# Mathematical Modeling Unveils Optimization Strategies for Targeted Radionuclide Therapy of Blood Cancers

**DOI:** 10.1158/2767-9764.CRC-24-0306

**Published:** 2024-11-14

**Authors:** Maxim Kuznetsov, Vikram Adhikarla, Enrico Caserta, Xiuli Wang, John E. Shively, Flavia Pichiorri, Russell C. Rockne

**Affiliations:** 1 Department of Computational and Quantitative Medicine, Beckman Research Institute, City of Hope National Medical Center, Duarte, California.; 2 Department of Hematologic Malignancies Translational Science, Beckman Research Institute, City of Hope National Medical Center, Duarte, California.; 3 Department of Hematology and Hematopoietic Cell Transplantation, City of Hope National Medical Center, Duarte, California.; 4 Department of Immunology and Theranostics, Beckman Research Institute, City of Hope National Medical Center, Duarte, California.

## Abstract

Targeted radionuclide therapy (TRT) is based on injections of cancer-specific molecules conjugated with radioactive nuclides. Despite the specificity of this treatment, it is not devoid of side effects limiting its use and is especially harmful for rapidly proliferating organs well perfused by blood, like bone marrow. Optimization of radioconjugate administration accounting for toxicity constraints can increase treatment efficacy. Based on our experiments on a disseminated multiple myeloma mouse model treated by ^225^Ac-DOTA-daratumumab, we developed a mathematical model, investigation of which highlighted the following principles for optimization of TRT: (i) Nuclide-to-antibody ratio importance. The density of radioconjugates on cancer cells determines the density of radiation energy deposited in them. A low labeling ratio as well as accumulation of unlabeled antibodies and antibodies attached to decay products in the bloodstream can mitigate cancer radiation damage due to excessive occupation of specific receptors by antibodies devoid of radioactive nuclides. (ii) Cancer-binding capacity–based dosing. The total number of specific receptors on cancer cells is a critical factor for treatment optimization, and its estimation may allow increasing treatment efficacy close to its theoretical limit. Injection of doses significantly exceeding cancer-binding capacity should be avoided because radioconjugates remaining in the bloodstream have a negligible efficacy-to-toxicity ratio. (iii) Particle range–guided multi-dosing. The use of short-range particle emitters and high-affinity antibodies can allow for robust treatment optimization via initial saturation of cancer-binding capacity, enabling redistribution of further injected radioconjugates and deposited doses toward still viable cells that continue expressing specific receptors.

**Significance:**

Mathematical modeling yields general principles for optimization of TRT in mouse models of multiple myeloma that can be extrapolated to other cancer models and clinical settings.

## Introduction

### Biological background

Targeted therapy is based on suppressing survival and proliferation of cancer cells through interactions with molecules specific to the cancer type under consideration. A large class of targeted agents consists of mAbs that specifically bind to receptors on the surfaces of cancer cells (1). One such example is daratumumab, which targets CD38. These receptors are overexpressed in multiple myeloma, which is a white blood cell cancer resulting in about 100,000 deaths worldwide annually (2). The main mode of action of daratumumab is induction of cancer cell killing by the immune system (3). Clinical trials have shown favorable safety profile of daratumumab (4). However, its action results in highly heterogeneous outcomes, including frequent cancer relapse after initial response. Relevant studies suggest that the mechanisms of multiple myeloma resistance to daratumumab are associated with immune escape (5), as relapsing patients show stable expression of unmutated CD38 on cancer cells, which preserves their ability to be targeted by daratumumab (6).

A way to enhance the efficacy of targeted therapy is to attach additional therapeutic payload to antibodies, ensuring its selective delivery to malignant cells. Conjugation of antibodies with cytotoxic agents has already led to more than a dozen clinically approved drugs (1), and conjugation of antibodies with radioactive nuclides is gaining growing clinical interest (7). Two main types of radionuclides are being investigated in trials: emitters of *β*-particles (electrons), which deposit comparably low energy over long distance, and emitters of *α*-particles (two protons and two neutrons bound together), which deposit higher energy over short range, corresponding to only a few cell diameters.

Toxicity of targeted radionuclide therapy (TRT) is a more complicated matter compared with that of external beam radiotherapy (8). Radioconjugates attach to specific receptors, expressed in some types of healthy cells, and thus affect them. The clearance of a part of radionuclides through the liver and kidneys is toxic for these organs. Radionuclides circulating in the bloodstream pose a significant threat to well-perfused rapidly proliferating organs such as bone marrow and thus suppress hematopoiesis. Therefore, bone marrow is often regarded as the dose-limiting organ for TRT (9).

In light of stable expression of CD38 in multiple myeloma cells, it is encouraging to use daratumumab to guide delivery of radionuclides to them. Previously, we have compared the efficacy and toxicity of *α*-emitter ^225^Ac-DOTA-daratumumab and *β*-emitter ^177^Lu-DOTA-daratumumab in a mouse model of disseminated multiple myeloma. We concluded that the actinium-based *α*-emitter shows more promise for clinical translation (10), and now we are conducting the phase I clinical trial for assessing its safety for patients (NCT#05363111).

Although radiolabeling of daratumumab increases its efficacy (11), achieving long-term response represents an extremely challenging task. Formally, cancer cure implies elimination of every single clonogenic malignant cell, whereas even a technically undetectable residual number of them can promote cancer recurrence (12). Treatment efficacy can be compromised not only by dose-limiting side effects but also by inherent restrictions in cancer cells’ exposure to drug. The choice of proper therapy protocol is further complicated by interpatient variability (7). Mathematical modeling provides a tool to help navigate this complexity and facilitate treatment optimization.

### Mathematical background

In this work, we focus on mechanistic mathematical modeling, which implies representation of cancer, its environment, and treatment as a system of equations, in which the terms correspond to specific natural laws (13). This approach allows formulating the tasks of treatment optimization as optimal control problems, which can be solved analytically or numerically. Quantitative agreement of modeling predictions with experimental data cannot be accurate on an individual level due to significant heterogeneity of biological objects and unavoidable uncertainty in individual quantitative characteristics. This issue can be addressed with *in silico* trials simulating treatment outcomes for heterogeneous virtual populations (14). Robust conclusions gained by mathematical modeling can be verified in clinical trials and eventually be implemented into clinical decision-making pipeline (15).

Mathematical modeling of TRT relies on modeling of drug pharmacokinetics, which is an extensive research area (16), and on modeling of continuous exposure of cells to irradiation, which has a well-established mathematical foundation (17). Yet, to date, there exist only a few studies on mathematical modeling of TRT. The works by Kletting and colleagues (18–20) present detailed pharmacokinetic models, designed with the goal of predicting the biologically effective doses received by tumors and healthy organs as well as the tumor response for varying amounts of injected *β*-emitters. These works consider data of patients with prostate cancer, neuroendocrine cancer, and meningioma. Other works of this and other research groups focus on constructing mathematical models using data from mouse tumor experiments, which include xenografted neuroendocrine tumor model treated by *α*-emitting ^212^Pb-DOTAMTATE (21), thyroid cancer xenograft model treated by *α*-emitting ^212^At which naturally accumulates in thyroid (22), and transgenic murine model of metastatic breast cancer treated by *α*-emitters ^225^Ac and ^213^Bi, conjugated with anti-HER2/neu antibodies (23). To the best of our knowledge, the latter work is the only study providing simulations of multidose TRT. Its results suggest that dose fractionation can affect mice survival; however, an explicit optimization problem is not considered.

Previously, based on our experiments on a disseminated multiple myeloma mouse model and its treatment by ^225^Ac-DOTA-daratumumab, we have developed the first mathematical model of TRT of blood cancer (10). Furthermore, we incorporated the experimental data on chimeric antigen receptor T-cell treatment within it in order to provide ground for optimization of scheduling of such combination therapy (24, 25). The current study is a continuation of this research, aimed at suggesting ways for optimization of TRT in single-dose and multidose setting.

## Materials and Methods

### Animal studies

Briefly, the previously reported animal studies (10) were performed as follows: Daratumumab (Janssen Biotech Inc., Thermo Fisher Scientific, Cat. # MA5-41886, RRID: AB 2911029) or control trastuzumab antibodies were reacted with a 30 M excess of chelator DOTA-mono-N-hydroxysuccinimide ester (Macrocyclics, Inc.). DOTA-conjugated antibody (50 μg) was incubated with 225Ac (Oak Ridge National Laboratory) at a labeling ratio of 1.85 kBq/μg for 45 minutes at 43°C and chased with 1 mmol/L diethylenetriamine pentaacetic acid.

Animal studies were conducted on 6- to 10-week-old NOD.Cg-PrkdcscidIl2rgtm1Wjl/SzJ mice (Jackson Laboratory, RRID: BCBC 1262). GFP luciferase–positive MM.1S cells (Dana-Farber Cancer Institute, RRID: CVCL B7F7) were injected intravenously, at 5 to 106 cells/200 μL of PBS per mouse. Tumor distribution and growth were followed by serial whole-body imaging on the Lago X (Spectral Instruments Imaging). Before imaging, the animals were anesthetized with 4% isoflurane and injected intraperitoneally with 200 μL of D-luciferin (15 mg/mL) in sterile PBS. After 9 days, mice were randomized and treated with saline; 22.2 kBq of 225Ac-DOTA-trastuzumab; and 0.925, 1.85, 3.7, 11.1, or 22.2 kBq of 225Ac-DOTA-daratumumab. Mice were given intravenous immunoglobulin by i.p. injection 2 hours before the injection of radioconjugates. All therapy doses were made up to 30 μg of antibody.

The animal studies were performed in accordance with Institutional Animal Care and Use Committee protocol #14043 approved by the City of Hope Institutional Animal Care and Use Committee, and in accordance with the NIH Office of Laboratory Animal Welfare guidelines (assurance number D16-00001).

The experimental data are reproduced in Supplementary Fig. S1. We assume that the total radiance signal over the mouse body is proportional to the current number of alive, both viable and damaged, cancer cells in it.

### Mathematical model

The presented mathematical model is parameterized using the data from our preclinical studies investigating the therapeutic efficacy and toxicity of ^225^Ac-DOTA-daratumumab in a disseminated human MM.1S xenograft mouse model, reported previously (10). The details of animal studies are summarized in Supplementary Section S.1.1, with experimental data shown in Supplementary Fig. S1. The mathematical model is supplemented by relevant literature data for quantifying aspects not directly measured in our experiments.

**Figure 1:**
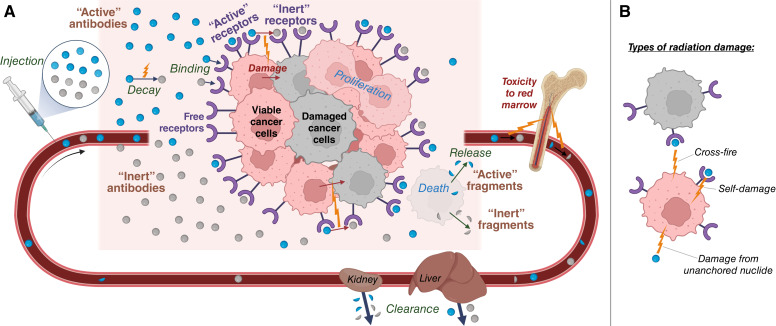
**A,** Schematic view of the main processes considered in the mathematical model of TRT of blood cancer. Note that antibody–receptor binding is considered irreversible. **B,** Illustration of three types of radiation damage, considered in the model. (**B,** Created with BioRender.com.)

For brevity, radioconjugates with yet undecayed radionuclides are referred to as *active antibodies*. Antibodies conjugated with final decay products and unlabeled antibodies are together termed *inert antibodies*. Likewise, receptors bound to active/inert antibodies are denoted as *active/inert receptors*. Radionuclides attached/unattached to cancer cells are referred to as *anchored/unanchored nuclides*. The schematic view of the model is shown in Fig. 1. The model is built on the following assumptions, illustrated in Fig. 1A.

*One-step nuclear reaction is considered*. Active antibodies transform directly into inert antibodies upon radionuclide decay. Such approximation is justified for ^225^Ac, as its half-life significantly exceeds the half-lives of its daughter nuclides (26).

*Antibody*–*receptor binding is irreversible*. We have shown previously that daratumumab is rapidly internalized by multiple myeloma cells (27). For simplicity, we do not consider internalized antibodies as a separate variable and assume sufficiently high affinity of antibodies that allows neglecting their unbinding.

*Blood plasma and cancer cell microenvironment represent a well-mixed medium*. Multiple myeloma cells are predominately confined to bone marrow. The rapid exchange of substances in it takes place through the discontinuous endothelium of venous sinuses. Experimental data suggest that equilibration of intravenously administered high-molecular-weight substances in blood plasma and bone marrow is achieved within only minutes (28).

*Cancer cell population is homogeneous*. Viable cells proliferate with a constant rate. This process can be ceased by their irradiation damage, with cancer cells having equal radiosensitivity. Damaged cells immediately stop proliferating and die at a constant rate. All cancer cells are reachable by antibodies and have an equal number of specific receptors. The relevant cancer-specific parameters vary in physiologically justified ranges for different virtual mice.

*Damage of cancer cells is irrepairable*, *with its rate being proportional to the delivered dose*. This approach is justified for modeling of the effect of *α*-particles manifested in DNA double-strand breaks (29).

*Antibodies are degraded upon cancer cell death and are released back in bloodstream in fragmented form*. Therefore, some of the radionuclides return back to the bloodstream being attached to the fragments of antibodies. The clearance rate of these fragments is higher than that of intact antibodies.

*Only decays of nuclides unattached to cancer cells are toxic to normal cells*. Decays of unanchored nuclides are attributed to treatment toxicity, as, in general, a part of them leads to the damage of red marrow, implied as the crucial organ at risk. We assume that the particles emitted from nuclides anchored to cancer cells effectively deposit all their energy in them, resulting in negligible toxicity to normal cells.

*Antibodies do not elicit therapeutic action*, *other than that due to the radionuclides*. Our experiments involved immunodeficient mice, with cancer dynamics not affected by unlabeled daratumumab.

*Conjugation of nuclides to antibodies and their decays do not alter antibody biodistribution*. Therefore, active and inert antibodies have equal rates of binding and equal rates of clearance.

*Antibodies do not bind to normal cells*. Our experiments used human cancer xenografts in mice and human antibodies, unable to attach to receptors of mouse cells. Immunoglobulin was preinjected to suppress antibodies recycling.

The following equations govern the model dynamics. All the used variables and parameters values are non-negative. The injection terms represent the external control of the otherwise autonomous system of equations. Initially, the number of viable cancer cells is *N*(0) = *N*_0_; damaged cancer cells are absent: *D*(*t*) = 0; all receptors are free: *f*_*FN*_(0) = *f*_*FD*_(0) = 1, *f*_*AN*_(0) = *f*_*AD*_(0) = 0; and antibodies are absent: *a*(0) = *b*(0) = *p*(0) = 0.

$${\text{Concentration}\;\text{of}\;\text{active}\;\text{antibodies}\;\text{in}\;\text{plasma}:\;a}^{\prime }=\overset{\text{injections}}{\overset{⏞}{\overset{I}{\underset{i=1}{\sum }}\frac{A_{i}}{V}\cdot \delta \left(t-t_{i}\right)}}\overset{\text{decay}}{\overset{⏞}{-\lambda a}}\overset{\text{binding}\;\text{with}\;\text{free}\;\text{receptors}}{\overset{⏞}{-k_{on}a\cdot \frac{\gamma }{V}\left[f_{FN}N+f_{FD}D\right]}}\overset{\text{clearance}}{\overset{⏞}{-\kappa_{c}a}},$$

$${\text{Concentration}\;\text{of}\;\text{inert}\;\text{antibodies}\;\text{in}\;\text{plasma}:\;b}^{\prime }=\overset{\text{injections}}{\overset{⏞}{\overset{I}{\underset{i=1}{\sum }}\frac{{\eta \cdot A}_{i}}{V}\cdot \delta \left(t-t_{i}\right)}}\overset{\text{decay}\;\text{of}\;a}{\overset{⏞}{+\lambda a}}\overset{\text{binding}\;\text{with}\;\text{free}\;\text{receptors}}{\overset{⏞}{-k_{on}b\cdot \frac{\gamma }{V}\left[f_{FN}N+f_{FD}D\right]}}\overset{\text{clearance}}{\overset{⏞}{-\kappa_{c}b}},$$

$${\text{Number}\;\text{of}\;\text{viable}\;\text{cancer}\;\text{cells}:\;N}^{\prime }=\overset{\text{proliferation}}{\overset{⏞}{\rho N}}\overset{\text{radiation}\;\text{damage}}{\overset{⏞}{-R_{D}\left(N,D,f_{AN},f_{AD},a,p\right)\cdot N}},$$

$${\text{Number}\;\text{of}\;\text{damaged}\;\text{cancer}\;\text{cells}:\;D}^{\prime }=\overset{\text{radiation}\;\text{damage}}{\overset{⏞}{R_{D}\left(N,D,f_{AN},f_{AD},a,p\right)\cdot N}}\overset{\text{death}}{\overset{⏞}{-\omega D}},$$

$${\text{Concentration}\;\text{of}\;\text{active}\;\text{fragments}\;\text{in}\;\text{plasma}:\;p}^{\prime }=\overset{\text{release}}{\overset{⏞}{\omega D\frac{\gamma f_{AD}}{V}}}\overset{\text{decay}}{\overset{⏞}{-\lambda p}}\overset{\text{clearance}}{\overset{⏞}{-\kappa_{p}p}},$$

$${\text{Fraction}\;\text{of}\;\text{free}\;\text{receptors}\;\text{of}\;\text{viable}\;\text{cancer}\;\text{cells}:\;f}_{FN}^{\prime }=\overset{\text{cell}\;\text{proliferation}}{\overset{⏞}{\left[1-f_{FN}\right]\rho }}\overset{\text{binding}}{\overset{⏞}{-k_{on}\left[a+b\right]f_{FN}},}$$

$${\text{Fraction}\;\text{of}\;\text{active}\;\text{receptors}\;\text{of}\;\text{viable}\;\text{cancer}\;\text{cells}:\;f}_{AN}^{\prime }=\overset{\text{binding}}{\overset{⏞}{k_{on}af_{FN}}}\overset{\text{decay}\;\text{and}\;\text{cell}\;\text{proliferation}}{\overset{⏞}{-\left[\lambda +\rho \right]f_{AN}}},$$

$${\text{Fraction}\;\text{of}\;\text{free}\;\text{receptors}\;\text{of}\;\text{damaged}\;\text{cancer}\;\text{cells}:\;f}_{FD}^{\prime }=\overset{\text{damage}\;\text{of}\;\text{viable}\;\text{cells}}{\overset{⏞}{\left[f_{FN}-f_{FD}\right]\cdot R_{D}\left(N,D,f_{AN},f_{AD},a,p\right)\cdot \frac{N}{D}}}\overset{\text{binding}}{\overset{⏞}{-k_{on}\left[a+b\right]f_{FD}}},$$

$${\text{Fraction}\;\text{of}\;\text{active}\;\text{receptors}\;\text{of}\;\text{damaged}\;\text{cancer}\;\text{cells}:\;f}_{AD}^{\prime }=\overset{\text{damage}\;\text{of}\;\text{viable}\;\text{cells}}{\overset{⏞}{\left[f_{AN}-f_{AD}\right]\cdot R_{D}\left(N,D,f_{AN},f_{AD},a,p\right)\cdot \frac{N}{D}}}\overset{\text{binding}}{\overset{⏞}{+k_{on}af_{FD}}}\overset{\text{decay}}{\overset{⏞}{-\lambda f_{AD}},}$$

$${\text{Radiation}\;\text{damage}:\;R}_{D}\left(N,D,f_{AN},f_{AD},a,p\right)=\alpha \cdot \left\{\overset{\text{self}‐\text{damage}}{\overset{⏞}{k_{S}\cdot \frac{\lambda \gamma f_{AN}}{\nu }}}\overset{\text{cross}‐\text{fire}}{\overset{⏞}{+\left[1-k_{S}\right]\cdot \frac{\lambda \gamma \left[f_{AN}N+f_{AD}D\right]}{\nu \left[N+D\right]}}}\overset{\text{unanchored}\;\text{nuclides}}{\overset{⏞}{+k_{f}\cdot \lambda \left[a+p\right]}}\right\}.$$

Injections of active antibodies result in instantaneous increase in their plasma concentration. Simultaneous injections of inert antibodies can be accounted for, with *η* taking positive values for such cases. This reflects the presence of impurities that accompany the production of radioconjugates, which are unlabeled antibodies and antibodies conjugated with nonradioactive ions (30). This can also reflect deliberate dilution of drug within unlabeled antibodies (see Supplementary Table S1).

The function of radiation damage accounts for three sources of radiation that can affect viable cancer cells (see Fig. 1B). The terms of self-damage and cross-fire stand for the damage of a cancer cell due to the decays taking place on its own receptors and on the receptors of neighboring cells, respectively. The numerator in each of these terms denotes the rate of decays within a certain volume, provided in the denominator. The relative significance of self-damage *k*_*s*_ for *α*-particles in dense cancer tissue can be small, because their range is generally greater than a cell diameter and their ionization density increases toward its end (31). The value of *k*_*s*_ should, however, increase with the decrease in cancer cell density, because cross-fire irradiation should be mitigated by increased intercellular distance. The third term of radiation damage corresponds to decays of unanchored nuclides. For brevity, we will refer to them as *decays in blood*.

Derivation of equations for receptors is performed in Supplementary Section S.1.2. Supplementary Section S.1.3 describes the process of estimation of model parameters and fitting of experimental data, illustrated in Supplementary Figs. S2–S5. The fitting process suggested significant heterogeneity of cancer cell response to treatment, in particular manifested in the decrease in cancer cell radiosensitivity under the increase in injected dose. Nevertheless, the presented model holds value for theoretical investigation, which is performed below. An augmentation of the model with account of cancer cell heterogeneity is also introduced during this study for verification and generalization of obtained qualitative conclusions.

Table 1 (32–37) lists the model parameters, formalizes their meaning, and provides their basic values as well as the ranges of their variation during parameter sweep. The following normalization parameters are used: *day* for time, mL for volume, pmol for amounts of antibodies and receptors, and nmol/L = pmol/mL for their concentrations.

**Table 1:** Model parameters.

	Parameter	Basic value	Range	Based on/comments
Estimated from literature				
λ	Radionuclide decay rate	0.07	Fixed	^225^Ac half-life is ≈ 9.9 days (26)
$k_{on}$	Antibody–receptor binding rate	11.15	Fixed	1.3·10^5^ M^−1^ s^−1^ (32)
$\kappa_{c}$	Antibody clearance rate	0.1	0.04–0.28	Half-life ≈ 6.9 (2.5–17.3) days (33), see Supplementary Section S.1.3.1
$\kappa_{p}$	Antibody fragment clearance rate	1	0.4–4	Half-life ≈ 16.6 (4.2–42) hours, see Supplementary Section S.1.3.1
γ	Amount of receptors on 10^7^ cancer cells	2.1	0.13–10	≈126,000 (8,000–600,000) per cell (34–36), see Supplementary Section S.1.3.1
V	Volume of drug distribution	1	0.75–1.5	See Supplementary Section S.1.3.1
ν	Volume of lesion with 10^7^ cancer cells	0.015	fixed	Ref. 37, see Supplementary Section S.1.3.1
$k_{s}$	Relative significance of self-damage	0.3	0–1	See description of radiation damage function
Estimated from our experimental data (10)				
ρ	Cancer cell proliferation rate	0.34	0.15–0.7	See Supplementary Section S.1.3.3, doubling time ≈2 (1–4.6) days
ω	Damaged cancer cell death rate	0.05	0.005–0.5	See Supplementary Section S.1.3.5, half of cells die in ≈14 (1.4–140) days
α	Cancer cell radiosensitivity	500	50–5,000	See Supplementary Section S.1.3.5, ≈0.2 (0.02–2) Gy^−1^
$k_{f}$	Significance of unanchored nuclide decays	0.05	0.01–0.25	See Supplementary Section S.1.3.4
Initial conditions and treatment				
$N_{0}$	Initial number of cancer cells × 10^7^	3	1–10	Ref. 10, see Supplementary Section S.1.3.3
$t_{1}$	Moment of the first drug injection	0	–	–
$A_{i}$	Injected doses	–	No limits	–
η	Coefficient of drug impurity	1,780	Mostly fixed	See Supplementary Section S.1.3.2, labeling ratio of 1.85 kBq/µg
$N_{cur}$	Viable cell number, corresponding to cure	0.01	–	–
$C_{d}$	Number of cancer cells leading to death	1,011	–	–
$A_{bl}^{cr}$	Lethal amount of decays in blood	0.0175	–	230 nCi·day (10), see Supplementary Section S.4.1

### Data availability

The data generated in this study are available within the article and its supplementary data files. For the sake of conciseness of this article, a significant part of mathematical reasoning and simulation results is provided in supplementary material (38–41). All the computational codes were implemented in Wolfram Mathematica (RRID: SCR_014448).

## Results

### Single-dose treatment by pure radioconjugates

**Figure 2:**
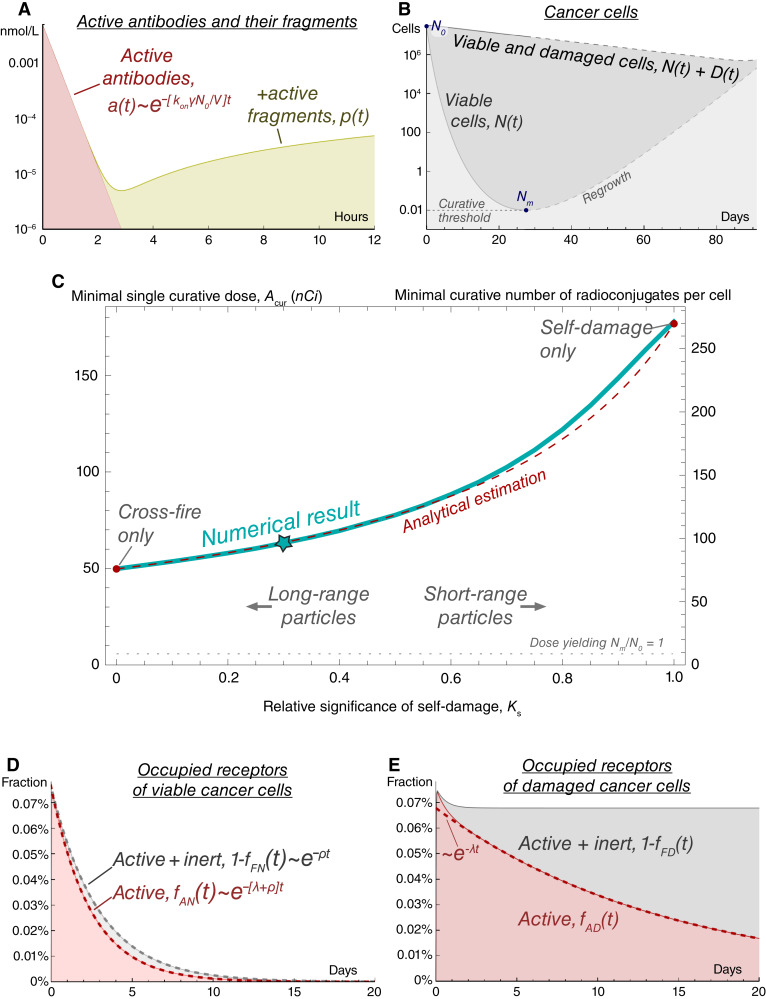
Model simulations of treatment by pure radioconjugates (coefficient of drug impurity *η* = 0) for the basic set of parameters with the dose *A*_*cur*_ leading to minimal number of viable cancer cells *N*_*m*_ = 0.01, which is considered as the curative threshold. **A,** Dynamics of active antibodies and their fragments in plasma. **B,** Dynamics of cancer cells. **C,** Dependence of minimal single curative dose, *A*_*cur*_, on relative significance of self-damage, *k*_*s*_. Star marks the case with basic value of *k*_*s*_ = 0.3. See Supplementary Section S.2.1.6 for analytical estimation of *A*_*cur*_. **D,** Dynamics of occupied receptors on viable cancer cells. The density of radionuclides on them decreases due to the decay of nuclides, rate of which is *λ*, and due the to the redistribution of nuclides among the newborn cells, characterized by cell proliferation rate *ρ*. **E,** Dynamics of occupied receptors on damaged cancer cells. After a transient period, the density of radionuclides on them decreases only due to the decay of nuclides.

In this section, we keep the coefficient of drug impurity *η* = 0. Thus, no inert antibodies are injected simultaneously with active antibodies. Understanding the model behavior in this setting, illustrated in Fig. 2, serves as an important steppingstone toward further investigation of the model with account of drug impurities in single-dose and multidose settings.

If the amount of injected radioconjugates is notably lower than the amount of free receptors on cancer cells, then the binding of drug to receptors is effectively performed within several hours, as illustrated in Fig. 2A, and irradiation from unanchored nuclides plays a minor role in the overall damage of cancer cells. These features facilitate analytical investigation of the model, which is performed in Supplementary Section S.2.1.

Figure 2B shows an example of cancer cell dynamics under prolonged irradiation. The minimal number of viable cancer cells *N*_*m*_ is achieved when the rate of nuclear decays decreases to the level at which they are not able to compensate for ongoing cell proliferation. The ratio *N*_*m*_*/N*_0_ can be regarded as *minimal surviving fraction* of cancer cells. For injected dose *A*, it can be estimated as follows for self-damage–only and cross-fire–only settings (see Supplementary Sections S.2.1.3 and S.2.1.4):

$$\frac{N_{m}}{N_{0}}{|}_{k_{s}=1}={\left[\frac{\alpha \cdot \frac{A}{\nu N_{0}}}{\rho /\lambda }\right]}^{\frac{\rho /\lambda }{1+\rho /\lambda }}\cdot e^{\frac{\rho /\lambda -\alpha \cdot \frac{A}{\nu N_{0}}}{1+\rho /\lambda }},$$

$$\frac{N_{m}}{N_{0}}{|}_{k_{s}=0}={\left[\frac{\alpha \cdot \frac{A}{\nu N_{0}}}{\rho /\lambda }\right]}^{\rho /\lambda }\cdot e^{2\rho /\lambda -\alpha \cdot \frac{A}{\nu N_{0}}}.$$

In the limit of *λ* → ∞ (immediate nuclide decay) or *ρ* → 0 (absence of cancer growth), analytical estimation of minimal surviving fraction in both settings tends to $e^{-\alpha \cdot A/\left[\nu N_{0}\;\right]}$. This expression corresponds to the classical formula for surviving fraction of cells after instantaneous irradiation, widely used in external beam setting, because its exponent corresponds to the energy deposited per unit of cancer mass. With the increase in the cancer cell proliferation rate, the minimal surviving fraction grows, highlighting the negative influence of cancer cell repopulation on the treatment outcome. These formulas are accurate unless the injected dose is sufficiently small, or the decay rate of nuclides is so fast that a large fraction of them decays in the bloodstream before anchoring to cancer cells (see Supplementary Fig. S6).

Given the continuous nature of our modeling approach, the numbers of cells are expressed as real numbers. We regard the cases, in which viable cancer cell number decreases to *N*_*m*_*< N*_*cur*_ = 0.01 cell, as curative. This approach allows introducing *minimal single curative dose A*_*cur*_ as the dose, resulting in *N*_*m*_ = *N*_*cur*_. Figure 2C illustrates the influence of particle range on *A*_*cur*_, emphasizing the qualitative difference of extreme opposite cases, in which irradiation of cells is provided mainly by either self-damage or cross-fire. Self-damage–only case demands greater dose for cure, because the density of radionuclides on viable cells decreases not only due to decay of nuclides but also due to their redistribution among the newborn cells. Therefore, as Fig. 2D shows, after binding of antibodies, the fraction of active receptors of viable cells decreases as $e^{-\left[\lambda +\rho \right]t}$. Figure 2E shows that the fraction of active receptors of damaged cells initially quickly decreases due to the transition of viable cells into damaged state, and then it decreases only due to decay of nuclides, decreasing as $e^{‐\text{λt}}$. Note that in case of introduction of receptor–antibody unbinding, the effect of particle range on *A*_*cur*_ will weaken with the decrease of antibody affinity (see Supplementary Fig. S7).

The ratio *A*_*cur*_*/N*_0_ can be regarded as the minimal activity of radioconjugates, initially residing on each cancer cell, that eventually will lead to cure. It can be converted into the *minimal curative number of radioconjugates per cell*. For the depicted variation in the particle range, it spans from 76 to 272, which is significantly smaller than the average number of CD38 receptors on a MM.1S cell, estimated as *≈*126,000 (38). However, in practice, the binding of radioconjugates to cancer cells is accompanied by binding of unlabeled antibodies, which are inevitable impurities of the drug and previously injected antibodies remaining in the bloodstream. This highlights the importance of the *nuclide*-*to*-*antibody ratio* for achieving the curative density of radioconjugates on cancer cells.

The fraction of activity spent via decays in blood in considered curative setting can be estimated as (see Supplementary Section S.2.1.1)

$$A_{bl}/A_{cur}=\overset{\text{due to intact radioconjugates}}{\overset{⏞}{\frac{\lambda }{\lambda +\kappa_{c}+k_{on}\gamma N_{0}/V}}}+\overset{\text{due to their fragments}}{\overset{⏞}{\frac{\omega \cdot \lambda }{\left[\lambda +\omega \right]\left[\lambda +\kappa_{p}\right]}}}.$$

In the used parameter range, ≈97% of decays in blood are due to the fragments of radioconjugates, released back to the bloodstream from dead cancer cells.

The global parameter sweep, performed in Supplementary Section S.2.2, confirms the validity of the analytical investigation (see Supplementary Figs. S8–S11).

The majority of radiation released in cancer is deposited in already damaged cells and thus formally does not contribute to the overall therapeutic effect. During the parameter sweep, the fraction of injected activity spent on viable cancer cells varies in the range 0.4% to 3.3% (see Supplementary Fig. S12). Analytical estimations show that in the single-dose curative setting, this measure cannot exceed 1*/*ln(*N*_0_*/N*_*cur*_), which is *<*5% if the initial number of cancer cells is greater than 10^7^ (see Supplementary Fig. S13).

Ongoing cancer cell proliferation hampers treatment efficacy. On the other hand, newborn cells express specific receptors, increasing the cancer capacity for drug binding. During the parameter sweep, the number of cancer cells born during treatment by minimal single curative doses constitutes 3% to 25% of their initial number for the vast majority of cases and is always less than 50% (see Supplementary Fig. S14).

### Variation by radioconjugate impurity

**Figure 3:**
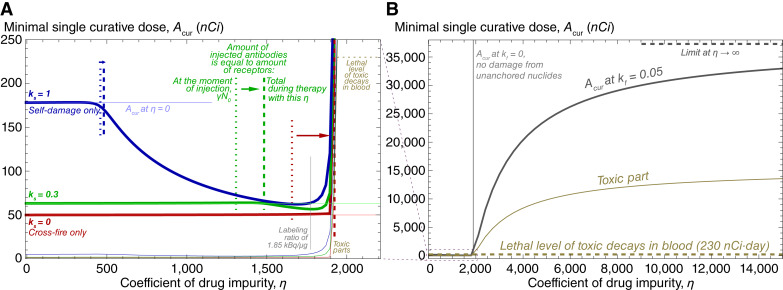
Dependence of minimal single curative dose, *A*_*cur*_, on the coefficient of drug impurity, *η*, i.e., the ratio of unlabeled antibodies to radioconjugates. **A,** Moderate variation in *η*. Under significant self-damage, saturation of specific receptors leads to redirection of radioconjugates and deposited doses toward still viable cells, which continue generating specific receptors. This effect yields the decrease in *A*_*cur*_ with an increase in *η*. **B,** Extended variation of *η*. The curative treatment becomes lethally toxic shortly after the amount of injected antibodies, (*η* + 1)*A*_*cur*_, exceeds the total amount of specific receptors expressed on cancer cells during treatment.

The influence of dilution of radioconjugates by unlabeled antibodies on the minimal single curative dose, *A*_*cur*_, is illustrated in Fig. 3. As Fig. 3A shows, an initial increase in the coefficient of drug impurity, *η*, affects the value of *A*_*cur*_ only slightly until the amount of injected antibodies, (*η* + 1)*A*_*cur*_, approaches the total amount of specific receptors expressed on cancer cells. The latter measure accounts for both $\gamma N_{0}$ of receptors at the moment of drug injection and the new receptors expressed during treatment.

For cross-fire–only case, *A*_*cur*_ remains almost constant until achieving the corresponding threshold, after which it rapidly grows, soon becoming lethal, because the anchored nuclides can no longer guarantee elimination of cancer cells. Self-damage–only case shows qualitatively different behavior: as the amount of injected antibodies approaches the total amount of specific receptors, the minimal single curative dose starts decreasing. This effect arises from the combination of dynamic processes taking place when cancer cells are nearly saturated, yet a substantial amount of radioconjugates remains in the bloodstream. New free receptors are produced only by still viable cancer cells, and therefore, they attract radioconjugates at a faster rate than damaged cells. In the case of significant self-damage, the radiation energy is therefore redirected toward viable cells, contributing to an increase of treatment efficacy and overall to reduced curative dose.

With the decrease in self-damage significance, *k*_*s*_, this effect weakens. However, under any value of *η*, the cross-fire–only case demands lower minimal single curative dose than the cases with *k*_*s*_*>* 0 and, as Supplementary Fig. S15 shows, remains less toxic and delivers greater fraction of injected dose to viable cells. This is explained by the fact that, independently of the value of *k*_*s*_, during single-dose treatment, the efficacy of radiation damage cannot decrease with time slower than $e^{-\lambda t}$, with the slowest decrease accompanying cross-fire–only case.

Notably, the decrease in *A*_*cur*_ under moderate drug impurity does not guarantee the accompanying decrease in treatment toxicity. When specific receptors are close to saturation, radioconjugates spend considerable time in the bloodstream before binding, thus amplifying the toxic effect. At *k*_*s*_ = 0.3, the amount of toxic decays in blood grows monotonically with the increase in drug impurity, whereas at *k*_*s*_ = 1, it decreases no more than twice compared with the pure radioconjugate case. It should be noted that the choice of optimal coefficient of drug impurity for increasing efficacy-to-toxicity ratio demands precise knowledge of values of model parameters. In real-life conditions, due to the inherent uncertainty and variability of characteristics, using the lowest possible coefficient of drug impurity may represent a reasonable strategy for single-dose treatment (see Supplementary Fig. S16).

Figure 3B corresponds to the behavior of *A*_*cur*_ upon further increase in drug impurity, where the curves for different values of *k*_*s*_ become indiscernible from each other. Under neglect of damage from unanchored nuclides, *k*_*f*_ = 0, the graph for *A*_*cur*_ skyrockets when the amount of injected antibodies exceeds the total amount of cancer receptors, implying that achieving cure becomes impossible. Under the value of *k*_*f*_ , estimated by our experimental data, *A*_*cur*_ tends to a certain limit that leads to the amount of toxic decays in blood exceeding their estimated lethal level by a factor of *≈*70 (see Supplementary Section S.3.1). For mathematical convenience, we will refer to all the doses that allow decreasing the viable cancer cell number to *N*_*cur*_ as curative, implying that they can be curative but lethally toxic simultaneously.

### Parameter sweep for the labeling ratio of 1.85 kBq/μg

**Figure 4:**
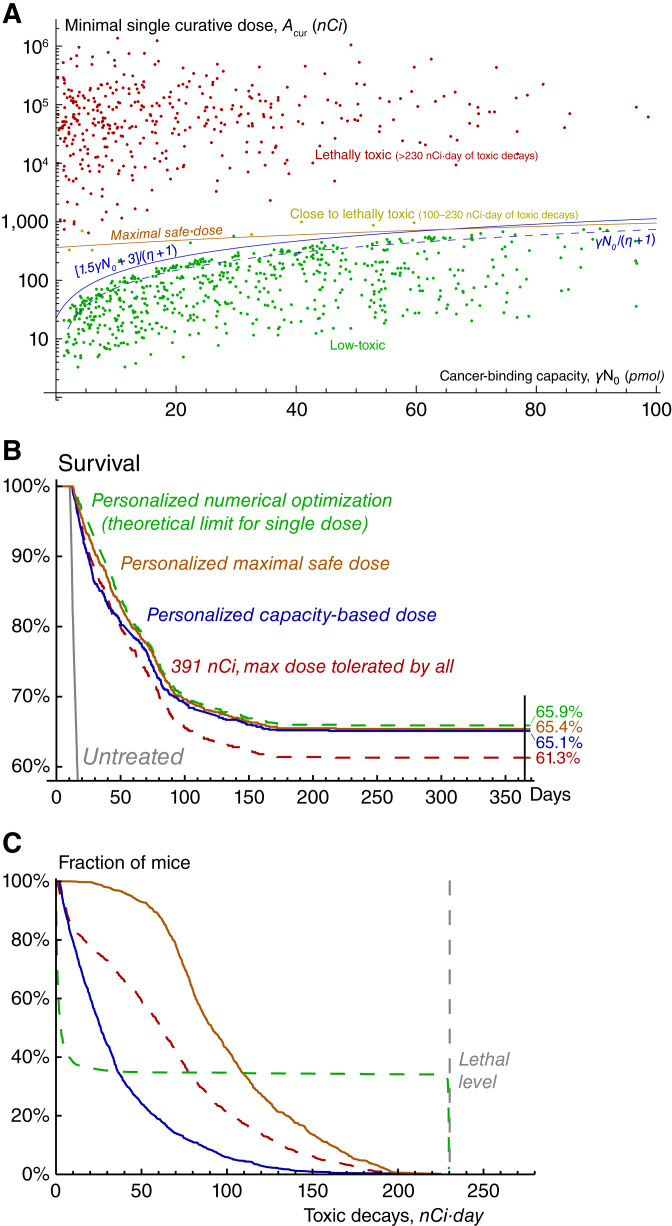
**A,** Scatter plot of cancer-binding capacity, i.e., total amount of specific receptors on cancer cells at the moment of drug injection, and minimal single curative dose produced by the global parameter sweep within the training set of 1,000 virtual mice. Cancer-binding capacity represents the product of the number of specific receptors on each cancer cell, γ, and the initial number of cancer cells, *N*_0_. Maximal safe dose is based on the exact value of only one parameter, cancer-binding capacity, and on physiologic ranges of other parameters. Coefficient of drug impurity, *η*, corresponds to the labeling ratio of 1.85 kBq/μg. **B,** Survival curves and (**C**) toxicity curves for a single test set of 1,000 virtual mice, treated by different approaches.

The above-discussed results show that *cancer*-*binding capacity*, i.e., the total number of specific receptors on cancer cells, plays a decisive role in determining whether cancer can be cured by radionuclides without exceeding lethal toxicity. This is confirmed by the global parameter sweep, the results of which are shown in Fig. 4. The used coefficient of drug impurity *η* = 1,780 corresponds to the labeling ratio of 1.85 kBq/μg (this value is chosen based on our experiments, see Supplementary Section S.1.3.2). Figure 4A shows the dependence of minimal single curative dose, *A*_*cur*_, on cancer-binding capacity at the moment of drug injection, which is the product of the number of specific receptors on each cancer cell, *γ*, and the initial number of cancer cells, *N*_0_. All the dots in the region *A*_*cur*_*< γN*_0_*/*(*η* + 1) correspond to low-toxic cases. In this region, the amount of injected antibodies is lower than cancer-binding capacity, and the analytical estimation of *A*_*cur*_, obtained under assumption of pure radioconjugates, still remains accurate (see Supplementary Fig. S17). As outlined above, during treatment with *A*_*cur*_, the number of newborn cancer cells is unlikely to exceed half of their initial number. This justifies low toxicity of cases, for which the amount of injected antibodies (*η* + 1)*A*_*cur*_ is lower than 1.5*γN*_0_ + 3 pmol. The latter coefficient compensates for slow drug binding under a small amount of specific receptors.

This provides a basis for a personalized dosing strategy based on only one parameter, cancer-binding capacity, $\gamma N_{0}$. In theory, it can be estimated before treatment. As Fig. 2A shows, the concentration of drug in blood after its injection follows exponential decrease, the rate of which depends on drug binding rate, *k*_*on*_; on volume of drug distribution, *V*, which by itself can be pre-estimated; and on cancer-binding capacity, $\gamma N_{0}$. The latter therefore can be assessed from the pharmacokinetic curve of a preliminary small diagnostic dose, which by itself would occupy a negligible fraction of receptors.

The personalized strategy was tested in *in silico* trial within a set of another 1,000 virtual mice. Its results are illustrated in Fig. 4B and C. We consider that a virtual mouse dies at the moment when the number of cancer cells reaches 10^11^, or at the moment when the total amount of decays in blood reaches 230 nCi*·*day. Direct numerical simulations identified 391 nCi as the maximal one-size-fits-all dose not leading to toxicity-related deaths and thus tolerated by all virtual mice. For determining the theoretical limit of single-dose treatment efficacy, we performed personalized numerical optimization, assuming precise knowledge of all model parameters for each virtual mouse, with the goal of curing a virtual mouse if it is feasible, otherwise prolonging its overall survival as much as possible. The treatment with personalized doses adjusted to cancer-binding capacity showed only marginally less efficiency than such explicit numerical optimization and was accompanied by less toxicity for non-cured mice.

The acceptable toxicity levels within the considered parameter ranges are ensured by moderate amount and fast clearance of radioactive fragments of antibodies, released to the bloodstream from dead cancer cells. In a more general case, injection of higher doses guided by greater cancer-binding capacities can exacerbate treatment-associated toxicity. Its prediction in real-life scenario is complicated by uncertainties in relevant parameters. However, the use of reasonable physiologic ranges of corresponding parameters allows estimating *maximal safe doses*, which will definitely not result in unacceptable toxicity for any specific parameter set (see Supplementary Section S.3.2). For negligible cancer-binding capacity, the maximal safe dose is ≈362 nCi, being determined only by the decays of nuclides attached to intact antibodies. With the increase in cancer-binding capacity, maximal safe dose tends to ≈1,763 nCi, being defined by the decays of nuclides attached to fragmented antibodies. Notably, these border values are independent of the coefficient of drug impurity, but its lower values would allow using greater doses safely under lower cancer-binding capacity (see Supplementary Fig. S18).

As noted above, our experimental data suggest significant intrapopulation heterogeneity of cancer cells, not accounted for herein. Supplementary Section S.4 presents the augmentation of the mathematical model accounting for heterogeneity of cancer cells (see Supplementary Table S2 for its newly introduced parameters). The study of the heterogeneous cancer model, illustrated in Supplementary Figs. S19–S22, confirms the optimization potential of the personalized dosing strategy based on cancer-binding capacity. Further improvement of treatment efficacy can be achieved by the use of multiple doses of radioconjugates.

### Optimization of multidose treatment

**Figure 5:**
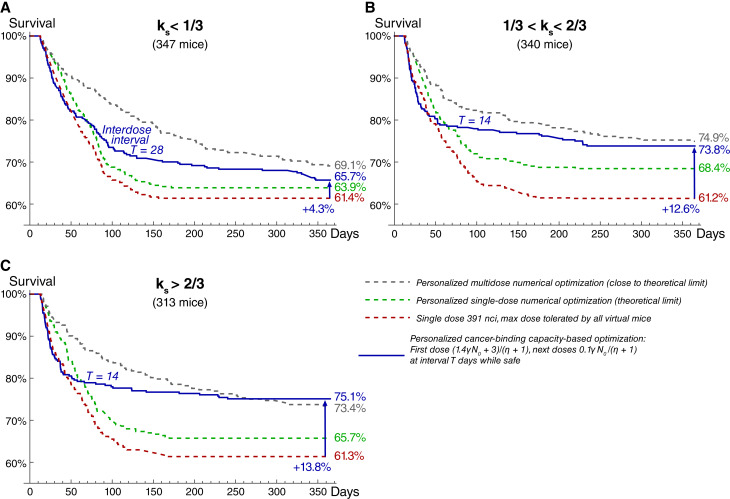
Survival curves for the test set of virtual mice, grouped by relative significance of self-damage, *k*_*s*_: (**A**) low *k*_*s*_; (**B**) intermediate *k*_*s*_; and (**C**) high *k*_*s*_. Personalized schedules based on cancer-binding capacity, *γN*_0_, yield robust treatment optimization via initial saturation of cancer-binding capacity with the first dose, enabling redistribution of further injected small doses toward still viable cells that continue expressing specific receptors. Coefficient of drug impurity, *η*, corresponds to the labeling ratio of 1.85 kBq/μg.

The extension of above-discussed results that includes multidose treatments is presented in Fig. 5, in which virtual mice are stratified into three groups, corresponding to low (Fig. 5A), intermediate (Fig. 5B), and high (Fig. 5C) values of relative significance of self-damage, *k*_*s*_. The administration of one-size-fits-all maximum tolerated dose results in comparable 1-year overall survival rates for these groups. Personalized single-dose numerical optimization assuming precise knowledge of all model parameters yields the greatest increase in treatment efficacy for the group with intermediate values of *k*_*s*_. These mice benefit from both slow decrease in cross-fire irradiation rate and redistribution of a part of radioconjugates to newborn viable cells that promote their prolonged self-damage irradiation.

The algorithm of similar personalized multidose numerical optimization, described in Supplementary Sections S.5.1 and S.5.2, is based on the auxiliary solution of two-dose treatment optimization task (see Supplementary Figs. S23 and S24) and on the analysis and simulations of idealized version of the model wherein injected antibodies immediately bind to cancer receptors and ensure their constant saturation, with further discretization of the corresponding total administered dose over variable time intervals (see Supplementary Section S.5.2.1). The analytical study of this version of the model shows that the necessary, but not sufficient, condition for cancer curability in the long term is the requirement of maximal possible rate of cancer cell damage due to irradiation from anchored nuclides to be greater than the cell proliferation rate: αλγ/{ν[η + 1]}> ρ (see Supplementary Figs. S25–S27). In particular, this formula shows that the treatment outcome crucially depends on the level of expression of specific receptors, characterized by *γ*. During the prolonged treatment, however, the irradiation rate of cancer cells is impaired by inevitable accumulation of inert antibodies on their receptors. The account for this effect allows deriving more strict conditions that indicate a positive impact of self-damage irradiation efficiency on increasing the likelihood of cancer cure in the long term. Consistent with this finding, personalized multidose numerical optimization yields greater increase in the 1-year survival rate for the groups with intermediate and high values of *k*_*s*_. The full algorithm of personalized multidose numerical optimization is summarized in Supplementary Fig. S28.

The simulations of curative multidose treatments, applied to virtual mice that cannot be cured by a single dose, suggest that the crucial factor enabling treatment optimization is redistribution of activity toward viable cancer cells. For some of the curative multidose treatments, the fraction of injected activity spent on viable cancer cells exceeds the theoretical threshold for a single-dose setting, estimated above to be <5% (see Supplementary Fig. S29). Redistribution of activity toward viable cells can be achieved via two methods, relevance of which depends on the value of *k*_*s*_. Under its high values, treatment efficacy benefits from maintaining near-saturation levels of cancer-binding capacity, enabling redirection of further injected radioconjugates and deposited dose to newborn cancer cells. Under low values of *k*_*s*_, when cross-fire dominates, optimal treatments aim to keep a considerable fraction of cancer receptors free but initiate shrinkage of cancer mass with the first dose in order to distribute further injected radioconjugates over fewer cancer cells (see Supplementary Section S.5.3.1).

From the practical point of view, the crucial question is whether these methods can be used in a robust way, given the uncertainty of parameters. For the study of this question, we followed our assumptions introduced in the previous section. The only parameter assumed to be known is cancer-binding capacity at the moment of first drug injection, $\gamma N_{0}$. Based on it, the upper limit on injected doses can be set, guaranteeing that they cannot lead to lethal toxicity for any specific parameter set. Also, we assume that the range of emitted particles and approximate knowledge of cancer cell density enable stratification of mice into three groups, corresponding to low, intermediate, and high values of *k*_*s*_. Using these assumptions, within the training set of virtual mice, we found optimized universal forms of schedules with doses tailored to $\gamma N_{0}$, as described in Supplementary Section S.5.3.1 (see Supplementary Figs. S30 and S31).

The administration of corresponding universal schedules in the test set of virtual mice yields an increase in treatment efficacy comparable to that in the training set, which confirms the universality of these schedules (see Supplementary Fig. S32). The greatest increase in the 1-year survival rate, compared with the outcome of administration of maximum tolerated dose, is achieved for the group with high values of *k*_*s*_. This shows that saturation of cancer receptors enabling redistribution of further injected radioconjugates toward viable cancer cells represents a robust method of increasing treatment efficacy, valid for the use of short-range particle emitters.

The universal schedules for the group with low values of *k*_*s*_ also initially saturate cancer receptors, as their undersaturation turns out to be beneficial only for a subset of virtual mice, hampering treatment efficacy for the others. The optimal interdose intervals are longer for this group, as they allow taking advantage of the relatively slow process of cancer mass shrinkage to facilitate cross-fire irradiation of viable cells. This, however, allows gaining only a moderate increase in the 1-year survival rate, suggesting that this approach, valid for emitters of long-range particles, is less robust.

## Discussion

### Optimization of TRT in experimental setting

This study highlights the general principles that can allow increasing the efficacy of TRT in mouse models of multiple myeloma and can be extrapolated on other cancer models. In particular, the general form of optimal multidose strategy is suggested for the use of high-affinity or rapidly internalizing antibodies and short-range particle emitters which provide high efficiency of self-damage irradiation of cancer cells. Namely, the first dose should aim at saturating cancer-binding capacity in order to enable redistribution of significantly lower subsequent doses toward still viable cells that continue producing specific receptors. It is noteworthy that eradication of cancer cells via self-damage irradiation may be a necessary condition for cure of disseminated multiple myeloma, because during prolonged treatment, the distance between its remaining cells should gradually increase, hindering the efficiency of cross-fire (32).

The crucial factor governing the rate of cancer radiation damage and overall TRT efficacy is the density of radionuclides attached to cancer cells, the maximum value of which is achieved upon saturation of specific receptors and is largely determined by the labeling ratio of antibodies. The simulations performed herein considered daratumumab incubated with ^225^Ac at the labeling ratio of 1.85 kBq/μg. It is technically feasible to achieve at least 20 times higher labeling ratios in this setting, resulting in a proportional increase of maximal radiation damage rate (11). However, for the first dose saturating cancer receptors, the use of higher labeling ratios implies injecting greater amount of radioconjugates, which may result in unacceptable toxicity. Such risk can be prevented by dilution of radioconjugates for the first dose. The effect of the following low doses, distributed over lower number of remaining viable cells, should on contrary benefit from high labeling ratio. Such deliberate regulation of nuclide-to-antibody ratio should have additional advantage in case of sufficiently heterogeneous cancer cell composition, providing initial damage of relatively radiosensitive cells by low radiation energy density, with its further increase for eradication of remaining radioresistant cells.

The used mathematical model does not account for the heterogeneity of receptor expression within the cancer cell population, and we are unaware of literature data quantitatively addressing the heterogeneity of CD38 expression in multiple myeloma lesions. Current findings suggest that the impact of receptor expression heterogeneity on treatment outcome should vary with the significance of self-damage and cross-fire. In the cross-fire–only case, radiation damage to each cell population will be primarily determined by the total number of radionuclides attached to all cancer cells, so the receptor heterogeneity should not affect the treatment outcome as long as the total number of specific receptors is maintained. In the self-damage–only case, the relevant model accounting for receptor heterogeneity should behave similarly to the model considering variable cell radiosensitivity, explored in Supplementary Section S.4, because the actual levels of heterogeneity of the parameters affecting cancer cell damage are structurally unidentifiable from experimental data. The long-term cancer curability should depend on the cell population with the lowest associated radiation damage rate, whereas an optimal multidose strategy should presumably also aim to initially saturate cancer-binding capacity in order to facilitate the redistribution of small subsequent doses to viable cells. A gradual increase in the nuclide-to-antibody ratio should as well improve the likelihood of eradicating cancer cells with relatively low expression of specific receptors.

Another factor that determines the rate of cancer radiation damage is the rate of nuclide decay. From this perspective, the use of radium isotope ^224^Ra, which decay chain yields ≈2.5 faster release of *α*-particle energy, comparable to that of ^225^Ac (30), represents a potentially more effective option for radiopharmaceutical therapy of blood cancers, that should mitigate the negative impact of cancer cell proliferation on treatment efficacy (see Supplementary Section S.2.1.5).

### Application in clinical setting

Multiple myeloma is currently considered a not curable disease (42), spurring the debates on curative versus control doctrines toward its treatment (43). High and uniform expression of CD38 in its cells, however, provides great potential for its effective treatment with targeted radioconjugates, which can be optimized based on the principles derived herein. Labeling of daratumumab with ^225^Ac leads to significant increase in its anticancer potency, suggesting that radiation damage should be regarded as the major factor for the effect of ^225^Ac-DOTA-daratumumab (11). Clinical trials with daratumumab did not identify its maximum tolerated dose, which suggests feasibility of using this antibody for saturation of CD38 (4). However, injections of the amounts of antibodies significantly exceeding cancer-binding capacity should be avoided as impeding the binding of further injected radioconjugates to cancer cells. Importantly, prolonged saturation of CD38 was witnessed in clinical setting several weeks after termination of daratumumab treatment, underscoring the necessity of tailoring the first dose to cancer-binding capacity for efficient prolonged TRT (44).

In this work, we suggest that estimation of cancer-binding capacity can be performed via preliminary injection of a small dose of diagnostic radioconjugates and measurement of their pharmacokinetic curve. In clinical setting, such estimation will be complicated by binding of radioconjugates to CD38 expressed in healthy cells and, to some extent, by FcRn-mediated recycling of antibodies. Preliminary cold dosing by unlabeled daratumumab allows redistributing further injected radioconjugates toward cancer cells (12); however, it should as well be associated with the risk of prolonged saturation of CD38 by antibodies devoid of radionuclides. Another complication accompanying clinical setting is on-target off-site toxicity of radioconjugates binding to CD38 expressed in healthy cells. However, low levels of CD38 expression can protect them from excessive irradiation (31). Differential expression of receptors in healthy and cancer cells may guide the optimal ratio of radioconjugates and antibodies during TRT, whereas the rate of healthy cell repopulation may be utilized to adjust the timing of injections, allowing for toxicity recovery. The mentioned complications can be addressed via quantitative systems pharmacology modeling incorporating clinical data (45). With that approach, the solutions of treatment optimization tasks, based on the collection of patient data and leveraging on the mechanistic insights gained in this study, can facilitate dynamic estimation of cancer-binding capacity and its saturation level and allow for treatment optimization in clinical setting.

In particular, the rapid exchange of substances between the bloodstream and the microenvironment of blood cancers can be leveraged for real-time optimization of TRT. A lateral flow or microfluidic chip-based device inserted in a patient’s vein could deliver a slow continuous infusion of radioconjugates while estimating the degree of saturation of blood cancer receptors by dynamically monitoring the blood radioactivity level. This would allow halting the drug inflow when treatment shifts from beneficial to harmful. Relevant devices are developed outside of TRT area, for antibiotics (46) and mAbs (47). This approach should further enable robust optimization of multidose TRT via the use of α-emitters and high-affinity or rapidly internalizing antibodies–initial saturation of cancer receptors will enable redistribution of small and low-toxic subsequent doses to still viable cells that continue producing target receptors.

The principles derived in this study can be adapted to the use of antibody–drug conjugates with cytotoxic payloads, as well as to treatment of extramedullary lesions of multiple myeloma and solid cancers. The penetration of drug to solid tumor sites is impeded by lower permeability of associated capillaries, compared with the bone marrow site, resulting in only a moderate fraction of injected activity reaching cancer cells (48) and in perivascular localization of the administered payload, which can be overcome by coadministration of nonconjugated antibodies (49, 50). The denser arrangement of cells in solid tumors, compared with blood cancers, should however promote effective cross-fire irradiation, as implied by complete remission of solid tumors reported for experimental setting using *β*-particle emitters (51). In light of this, the combination of initial administration of *β*-particle emitters or external beam irradiation to solid tumors with subsequent injections of *α*-particle emitters for redirection of deposited dose toward still viable cells represents an intriguing concept for further investigation.

## Supplementary Material

Supplementary Material Additional mathematical reasoning and simulation results.

Supplementary Figure S.1 Supplementary Figure S.1

Supplementary Figure S.2 Supplementary Figure S.2

Supplementary Figure S.3 Supplementary Figure S.3

Supplementary Figure S.4 Supplementary Figure S.4

Supplementary Figure S.5 Supplementary Figure S.5

Supplementary Figure S.6 Supplementary Figure S.6

Supplementary Figure S.7 Supplementary Figure S.7

Supplementary Figure S.8 Supplementary Figure S.8

Supplementary Figure S.9 Supplementary Figure S.9

Supplementary Figure S.10 Supplementary Figure S.10

Supplementary Figure S.11 Supplementary Figure S.11

Supplementary Figure S.12 Supplementary Figure S.12

Supplementary Figure S.13 Supplementary Figure S.13

Supplementary Figure S.14 Supplementary Figure S.14

Supplementary Figure S.15 Supplementary Figure S.15

Supplementary Figure S.16 Supplementary Figure S.16

Supplementary Figure S.17 Supplementary Figure S.17

Supplementary Figure S.18 Supplementary Figure S.18

Supplementary Figure S.19 Supplementary Figure S.19

Supplementary Figure S.20 Supplementary Figure S.20

Supplementary Figure S.21 Supplementary Figure S.21

Supplementary Figure S.22 Supplementary Figure S.22

Supplementary Figure S.23 Supplementary Figure S.23

Supplementary Figure S.24 Supplementary Figure S.24

Supplementary Figure S.25 Supplementary Figure S.25

Supplementary Figure S.26 Supplementary Figure S.26

Supplementary Figure S.27 Supplementary Figure S.27

Supplementary Figure S.28 Supplementary Figure S.28

Supplementary Figure S.29 Supplementary Figure S.29

Supplementary Figure S.30 Supplementary Figure S.30

Supplementary Figure S.31 Supplementary Figure S.31

Supplementary Figure S.32 Supplementary Figure S.32

Computational codes Designed in Wolfram Mathematica, version 13.3.1.0.
