# Supplementary figures and images for: Mathematical Modeling Unveils Optimization Strategies for Targeted Radionuclide Therapy of Blood Cancers
Source: Cancer Res Commun. 2024 Nov 14;4(11):2955–67. doi: 10.1158/2767-9764.CRC-24-0306 (PMC11562018; doi:10.1158/2767-9764.CRC-24-0306)

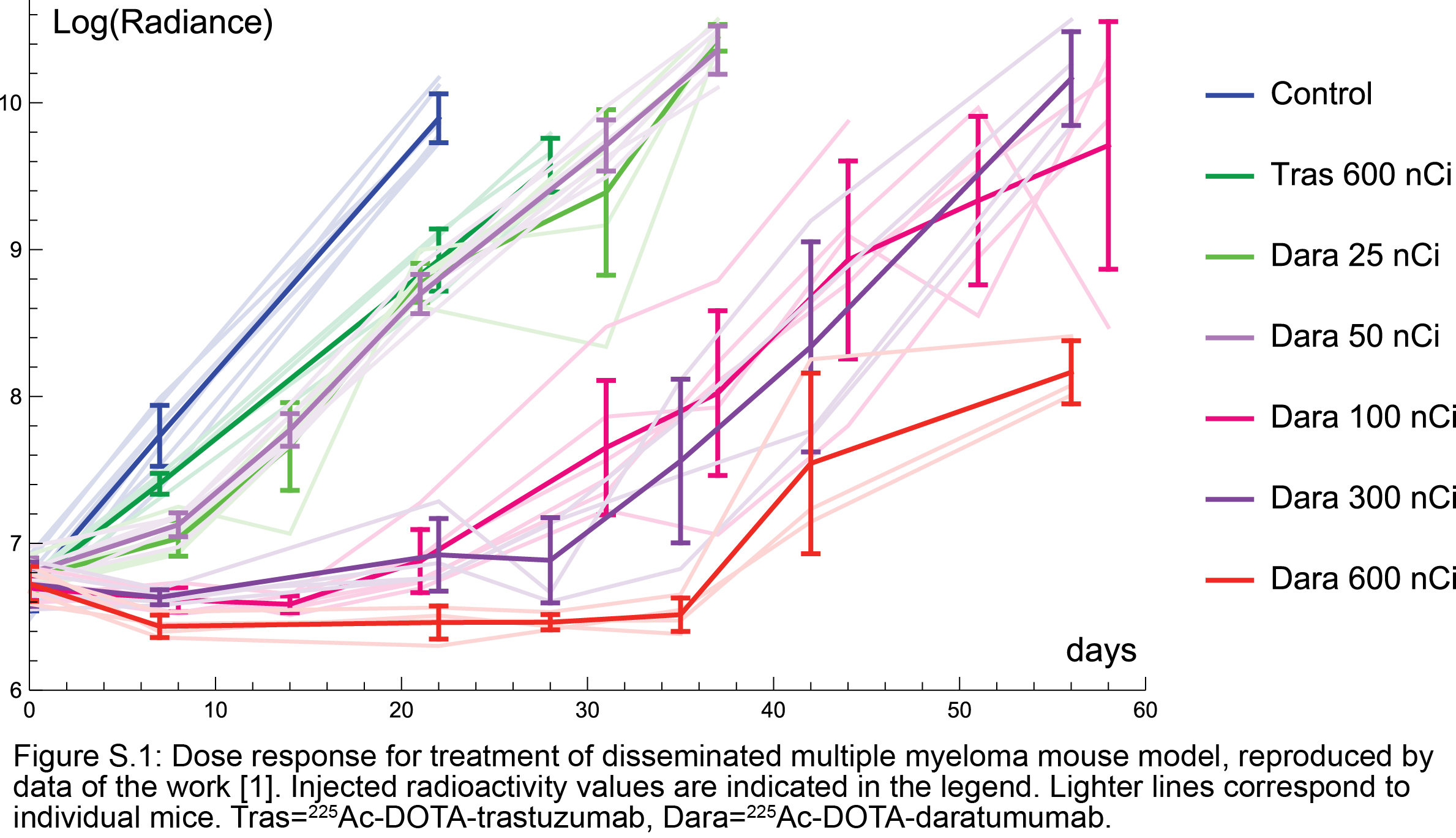

Supplement: Supplementary Figure S.1 [file crc-24-0306_supplementary_figure_s.1_suppsf1.jpeg]

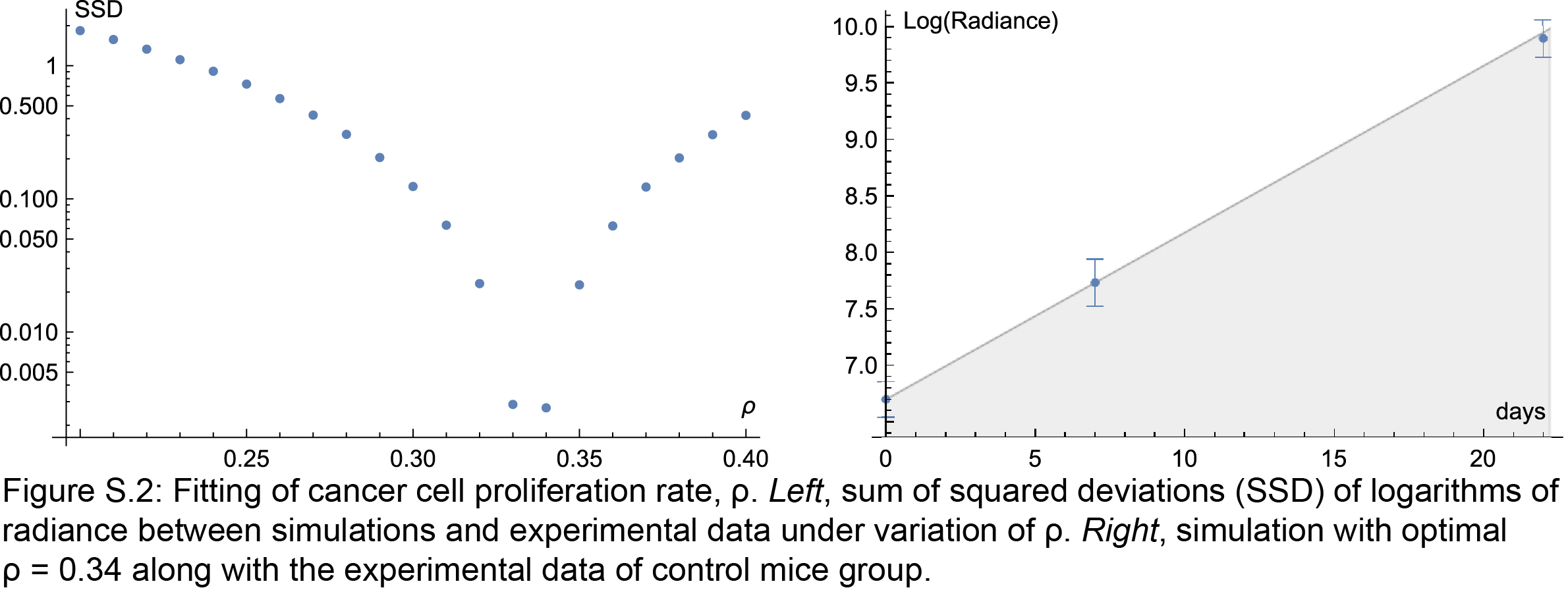

Supplement: Supplementary Figure S.2 [file crc-24-0306_supplementary_figure_s.2_suppsf2.jpeg]

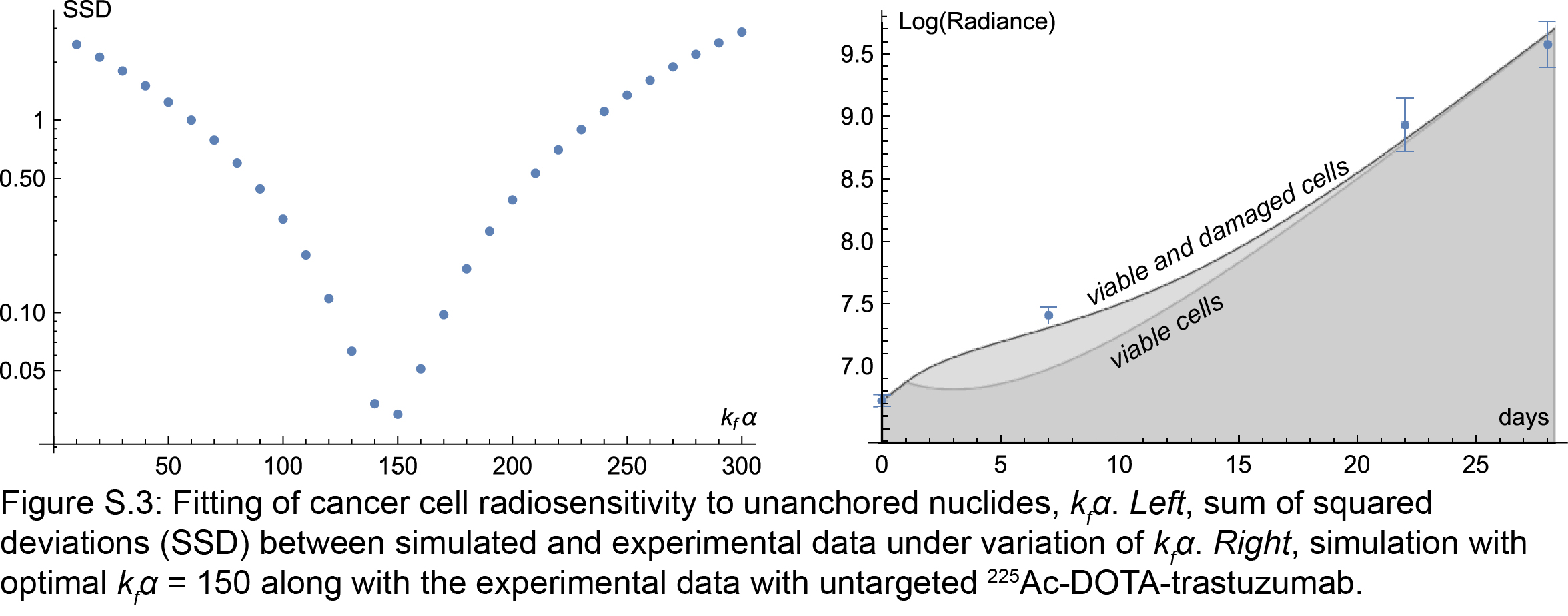

Supplement: Supplementary Figure S.3 [file crc-24-0306_supplementary_figure_s.3_suppsf3.jpeg]

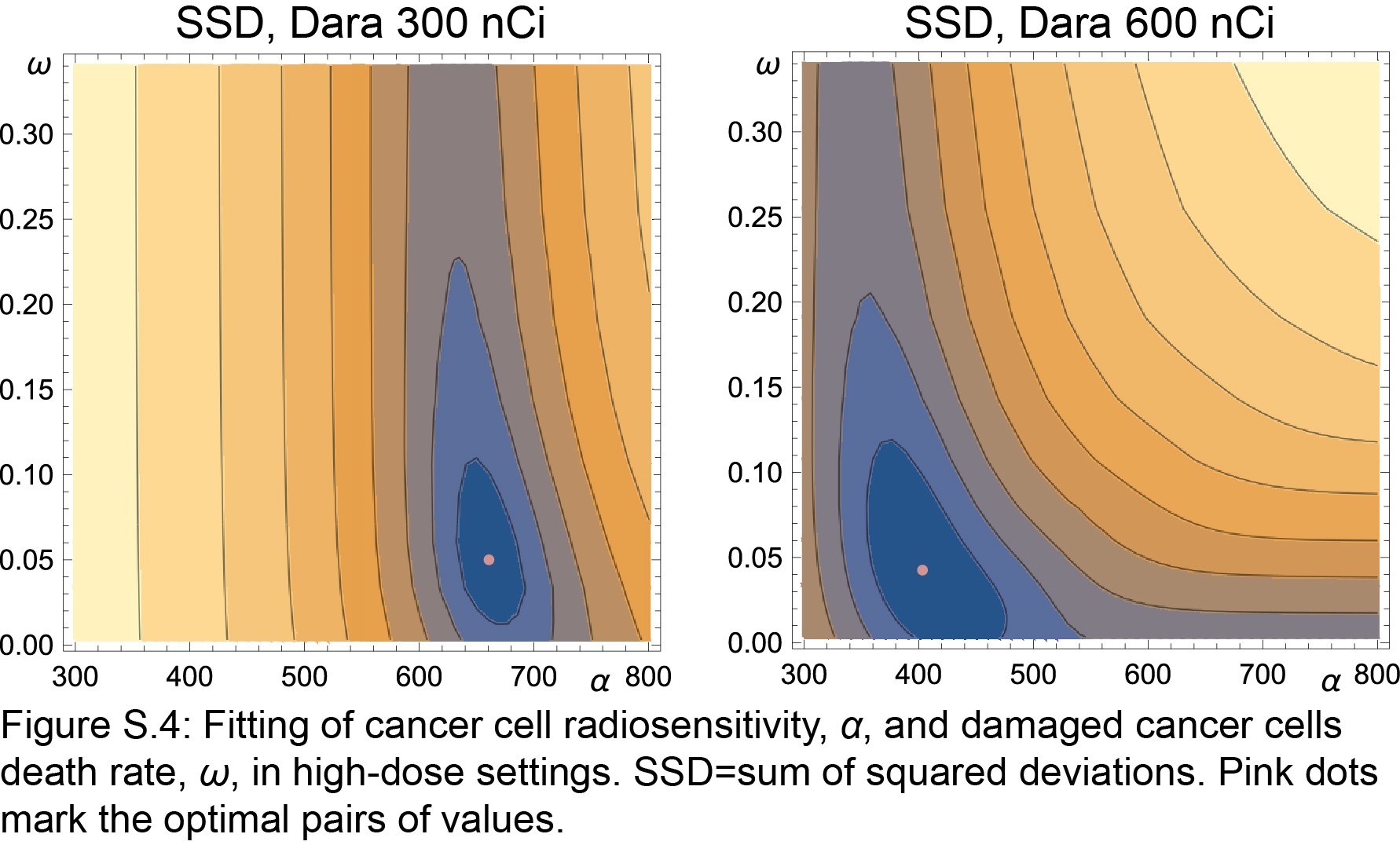

Supplement: Supplementary Figure S.4 [file crc-24-0306_supplementary_figure_s.4_suppsf4.jpeg]

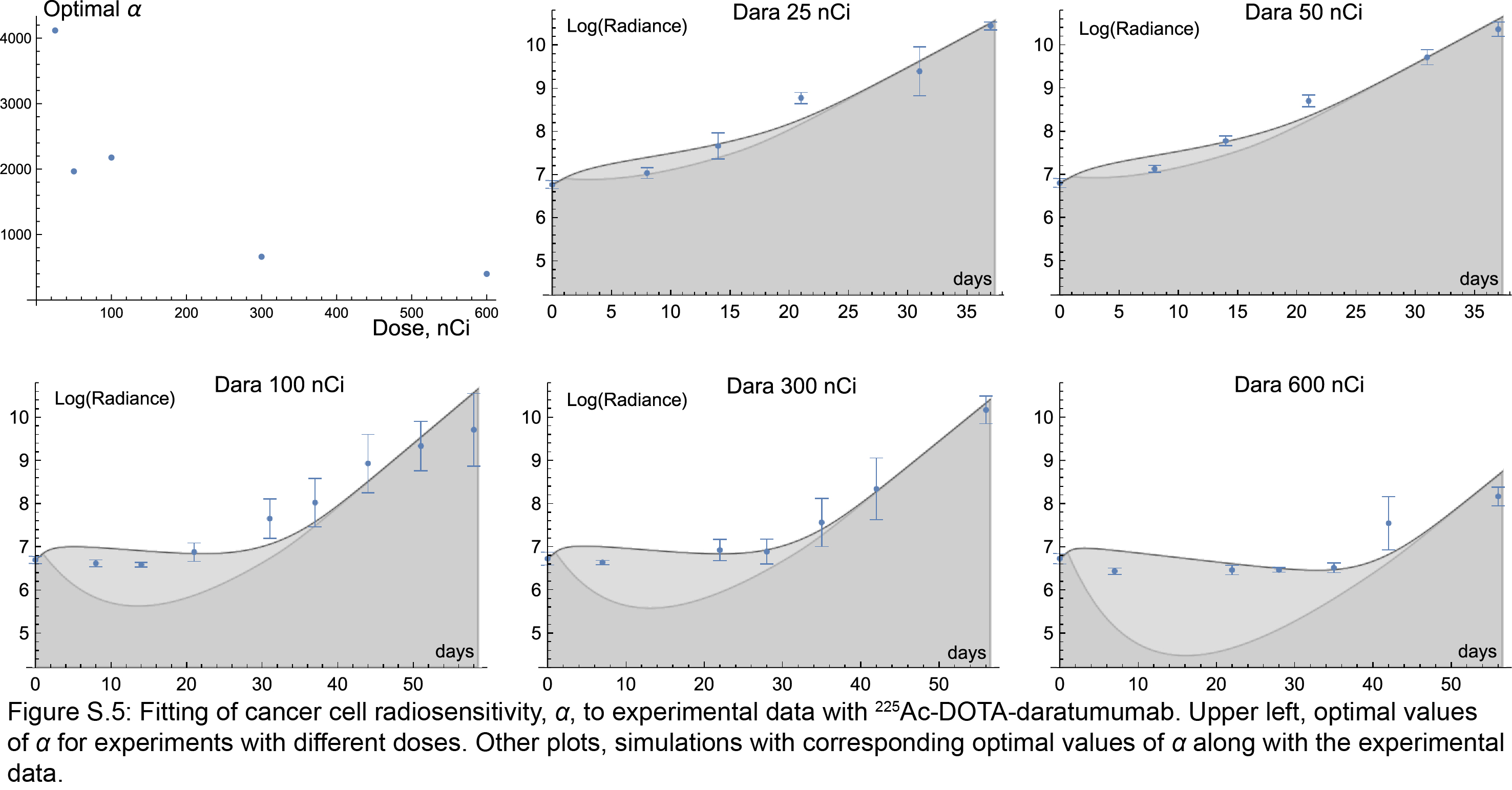

Supplement: Supplementary Figure S.5 [file crc-24-0306_supplementary_figure_s.5_suppsf5.jpeg]

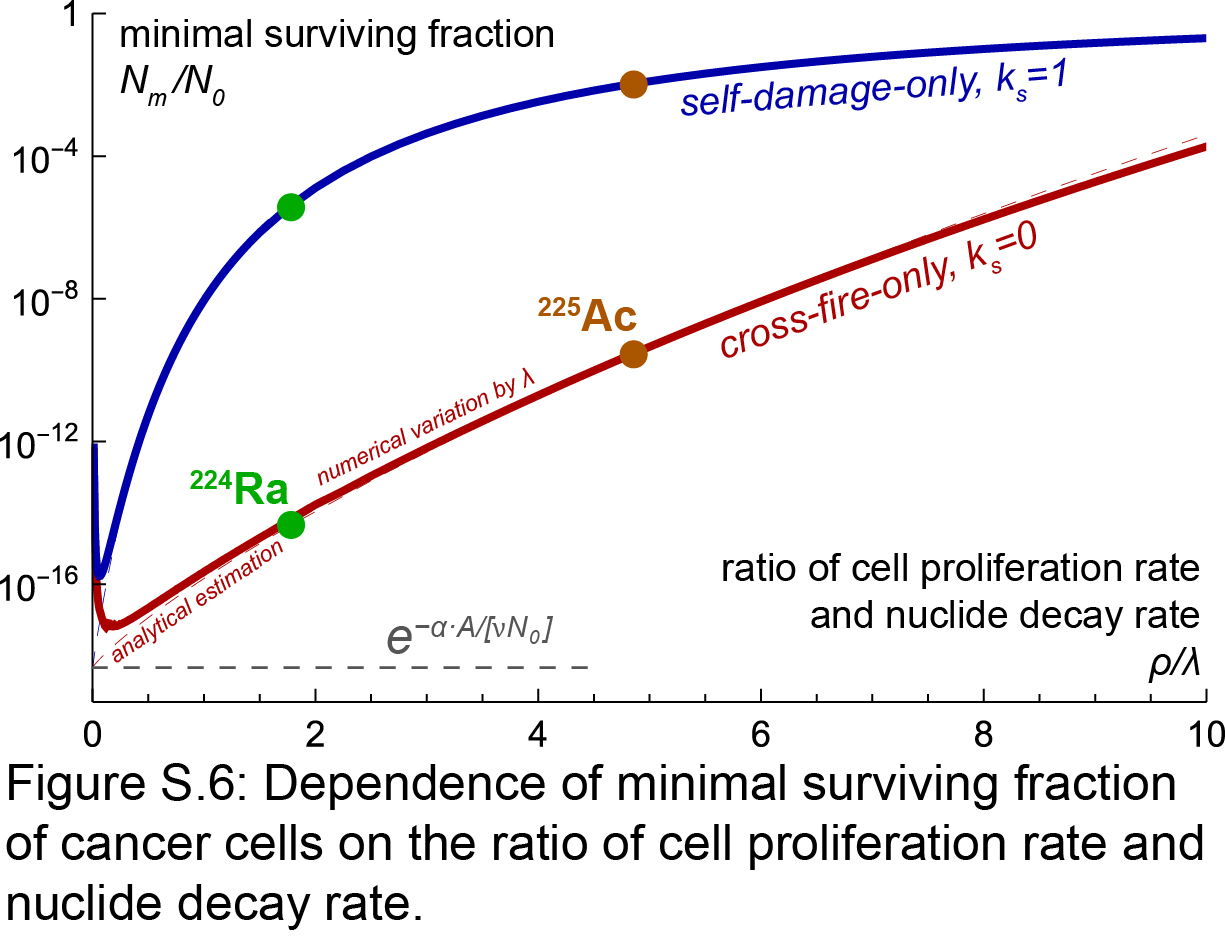

Supplement: Supplementary Figure S.6 [file crc-24-0306_supplementary_figure_s.6_suppsf6.jpeg]

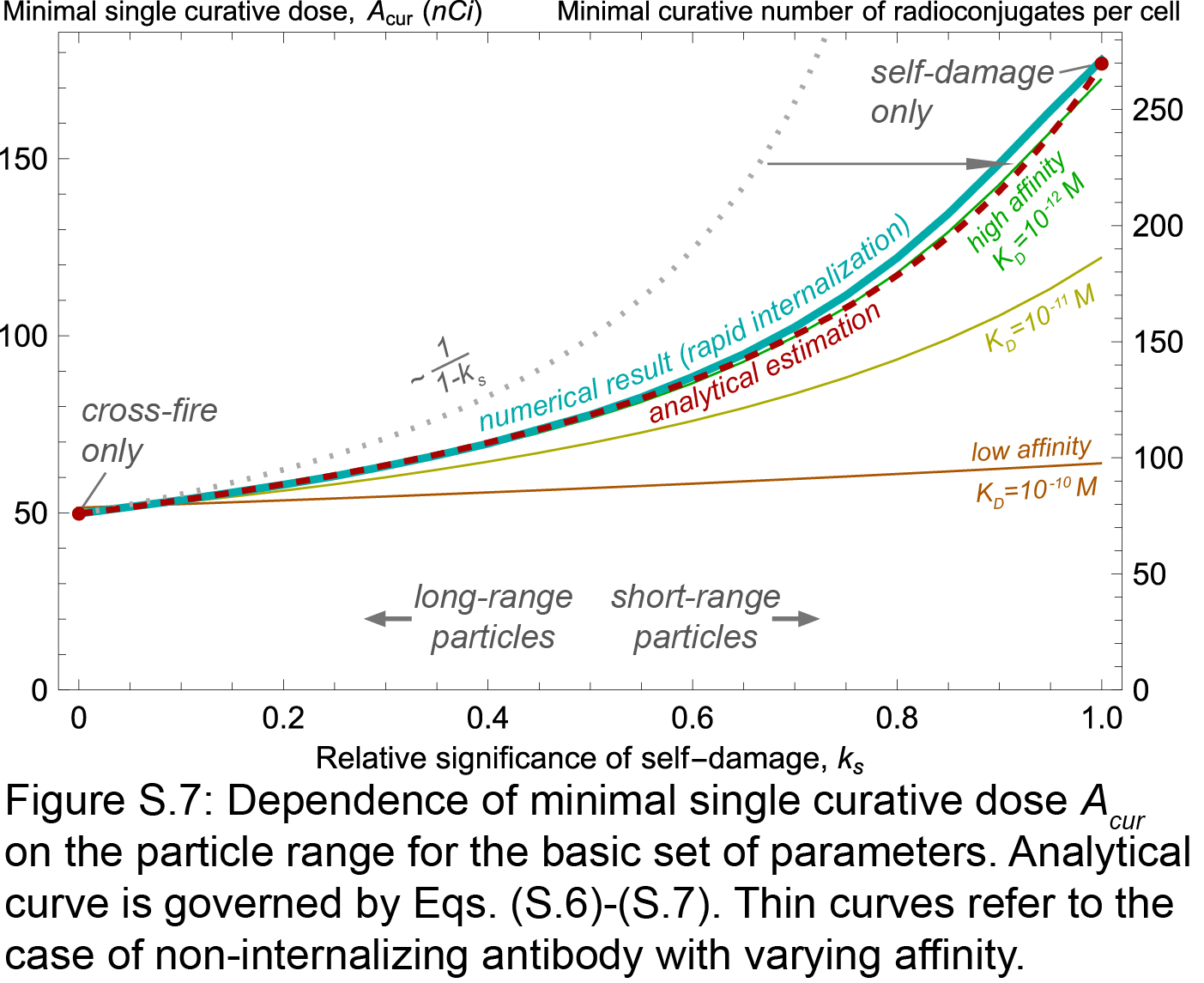

Supplement: Supplementary Figure S.7 [file crc-24-0306_supplementary_figure_s.7_suppsf7.jpeg]

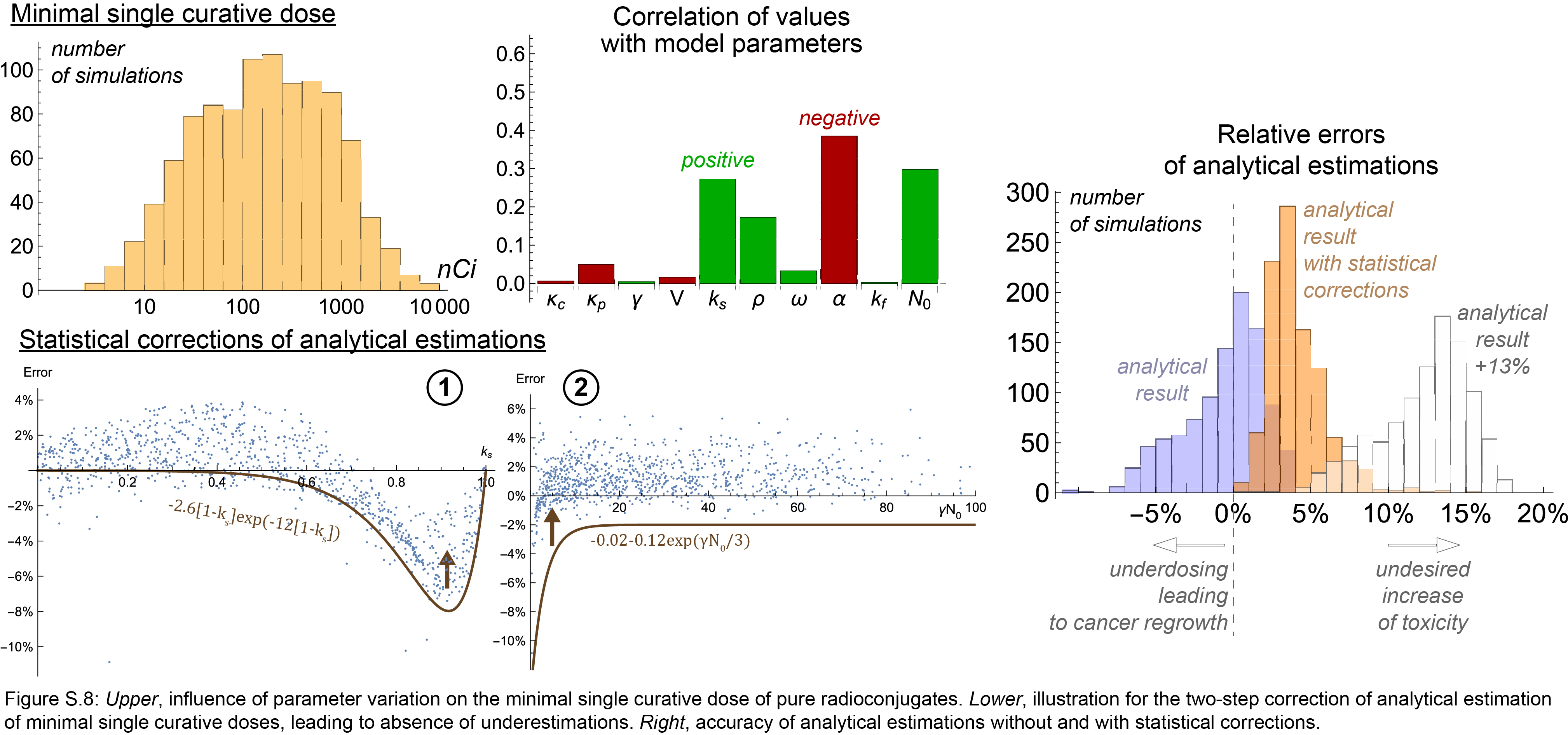

Supplement: Supplementary Figure S.8 [file crc-24-0306_supplementary_figure_s.8_suppsf8.jpeg]

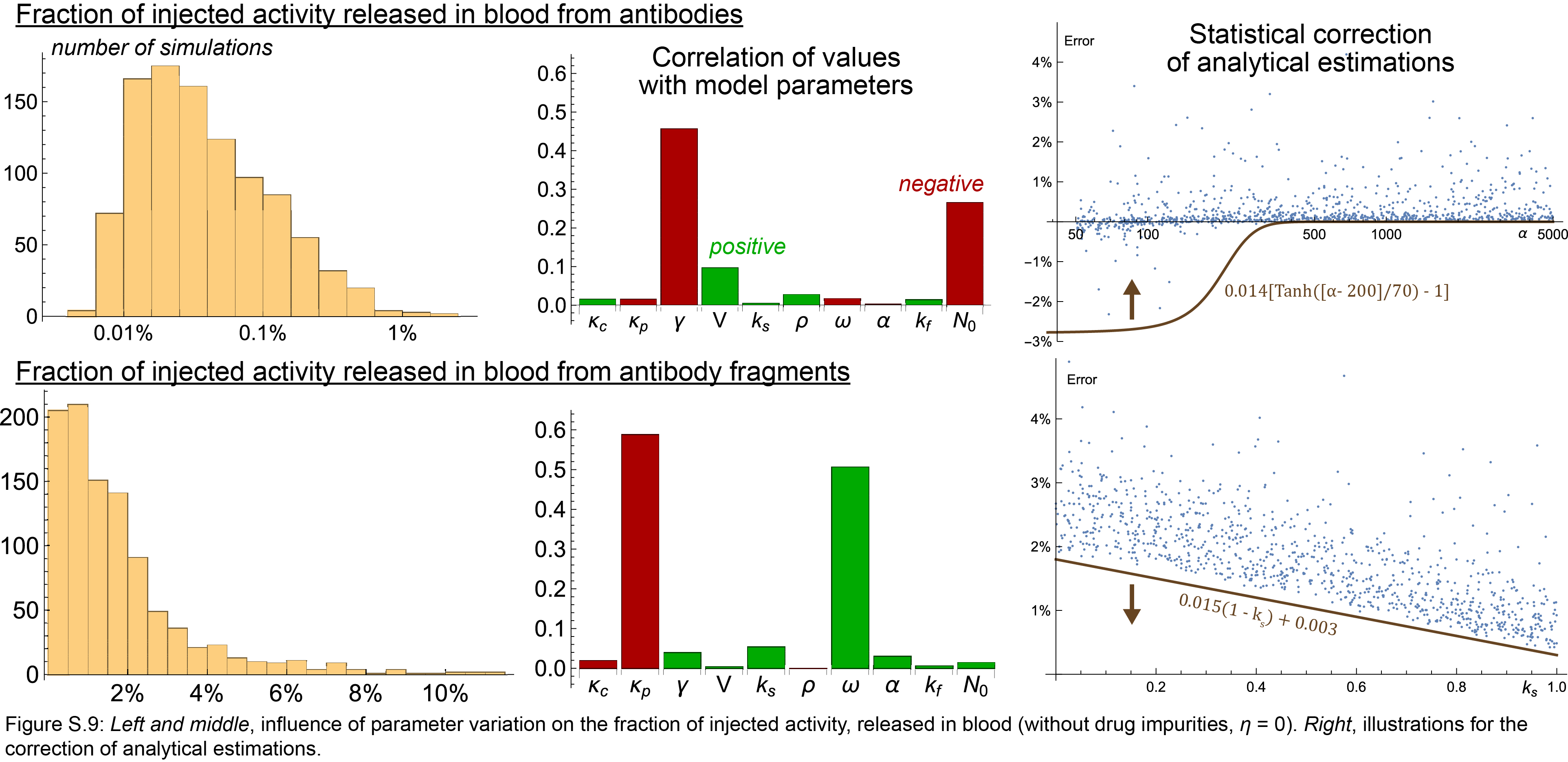

Supplement: Supplementary Figure S.9 [file crc-24-0306_supplementary_figure_s.9_suppsf9.jpeg]

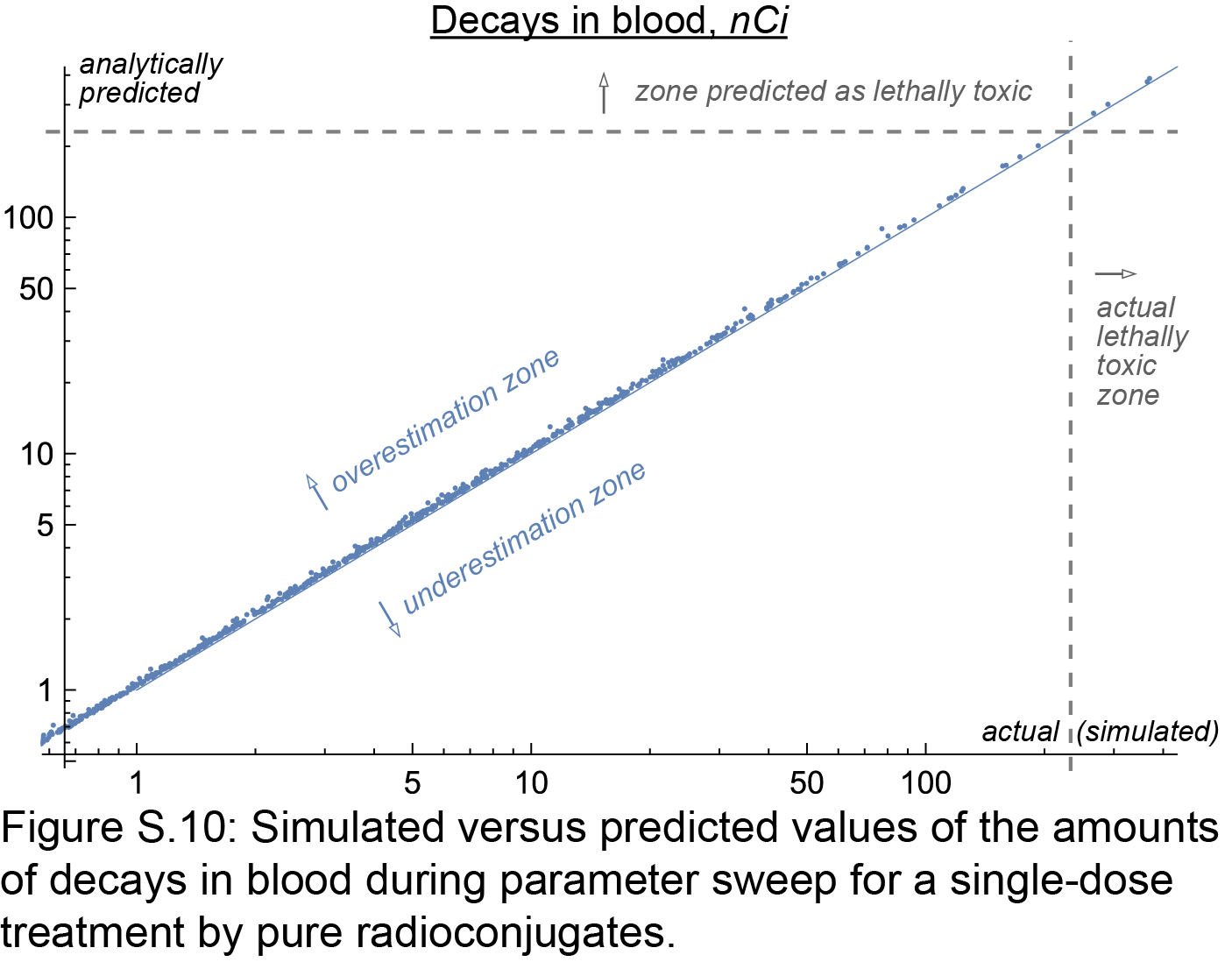

Supplement: Supplementary Figure S.10 [file crc-24-0306_supplementary_figure_s.10_suppsf10.jpeg]

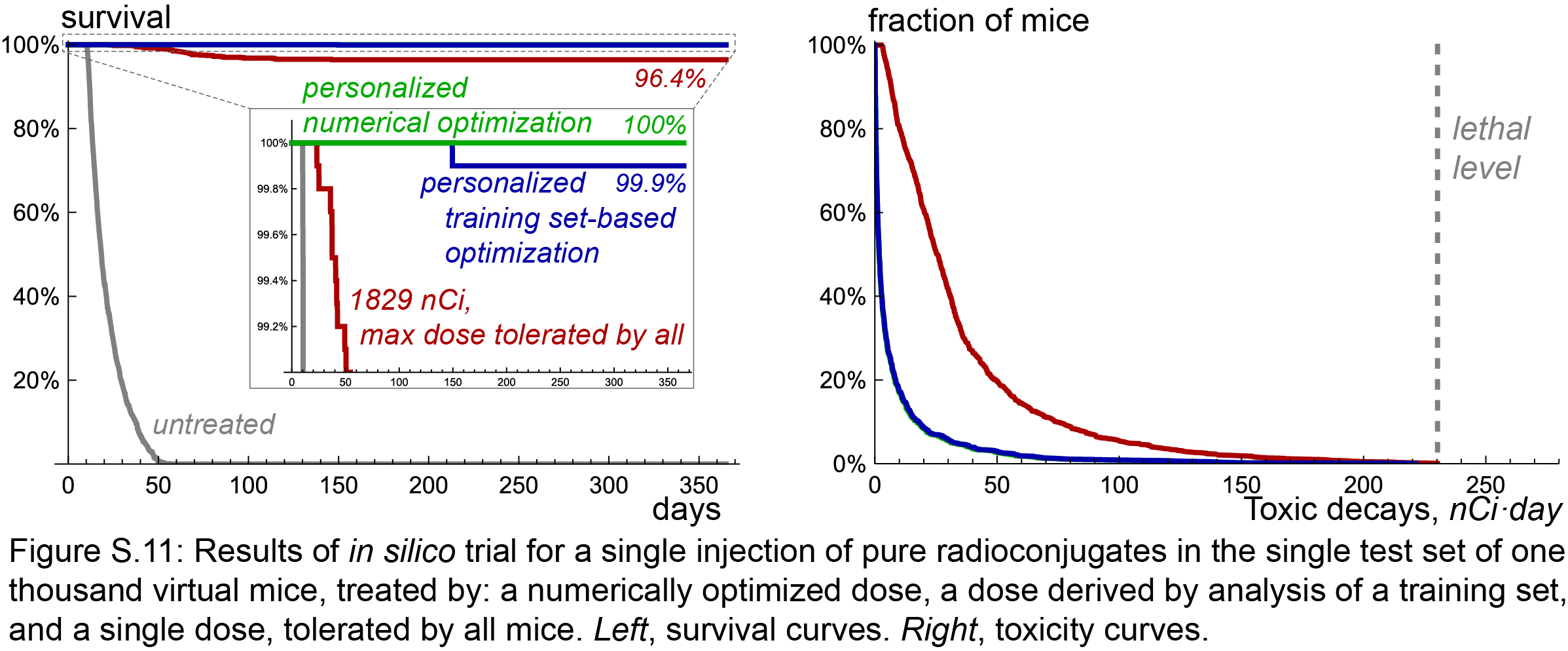

Supplement: Supplementary Figure S.11 [file crc-24-0306_supplementary_figure_s.11_suppsf11.jpeg]

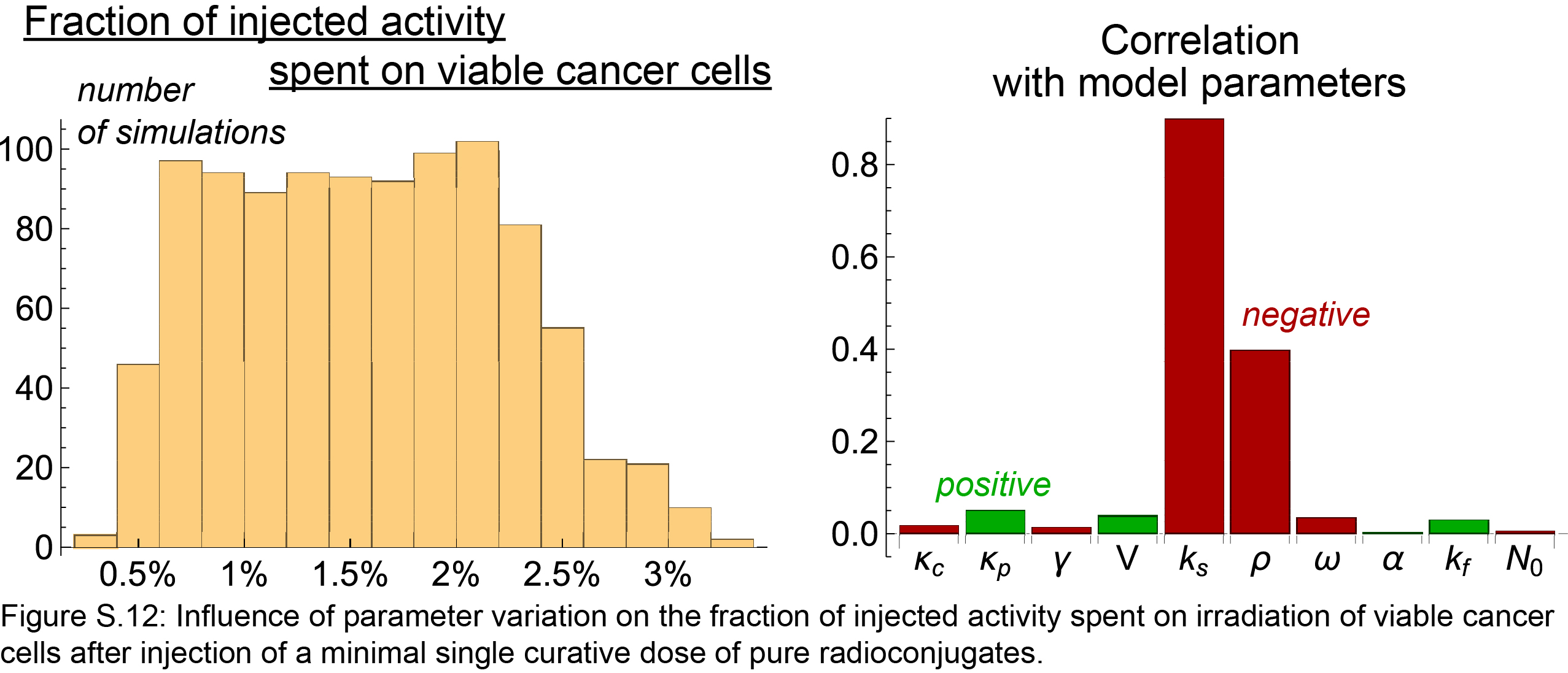

Supplement: Supplementary Figure S.12 [file crc-24-0306_supplementary_figure_s.12_suppsf12.jpeg]

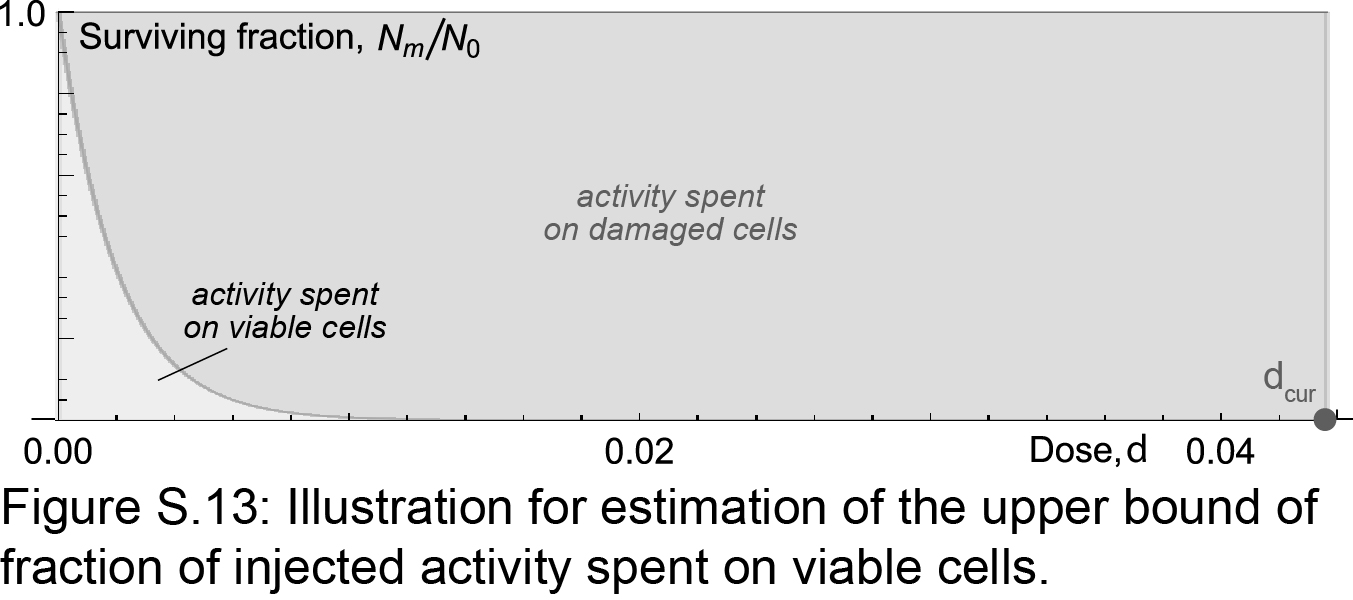

Supplement: Supplementary Figure S.13 [file crc-24-0306_supplementary_figure_s.13_suppsf13.jpeg]

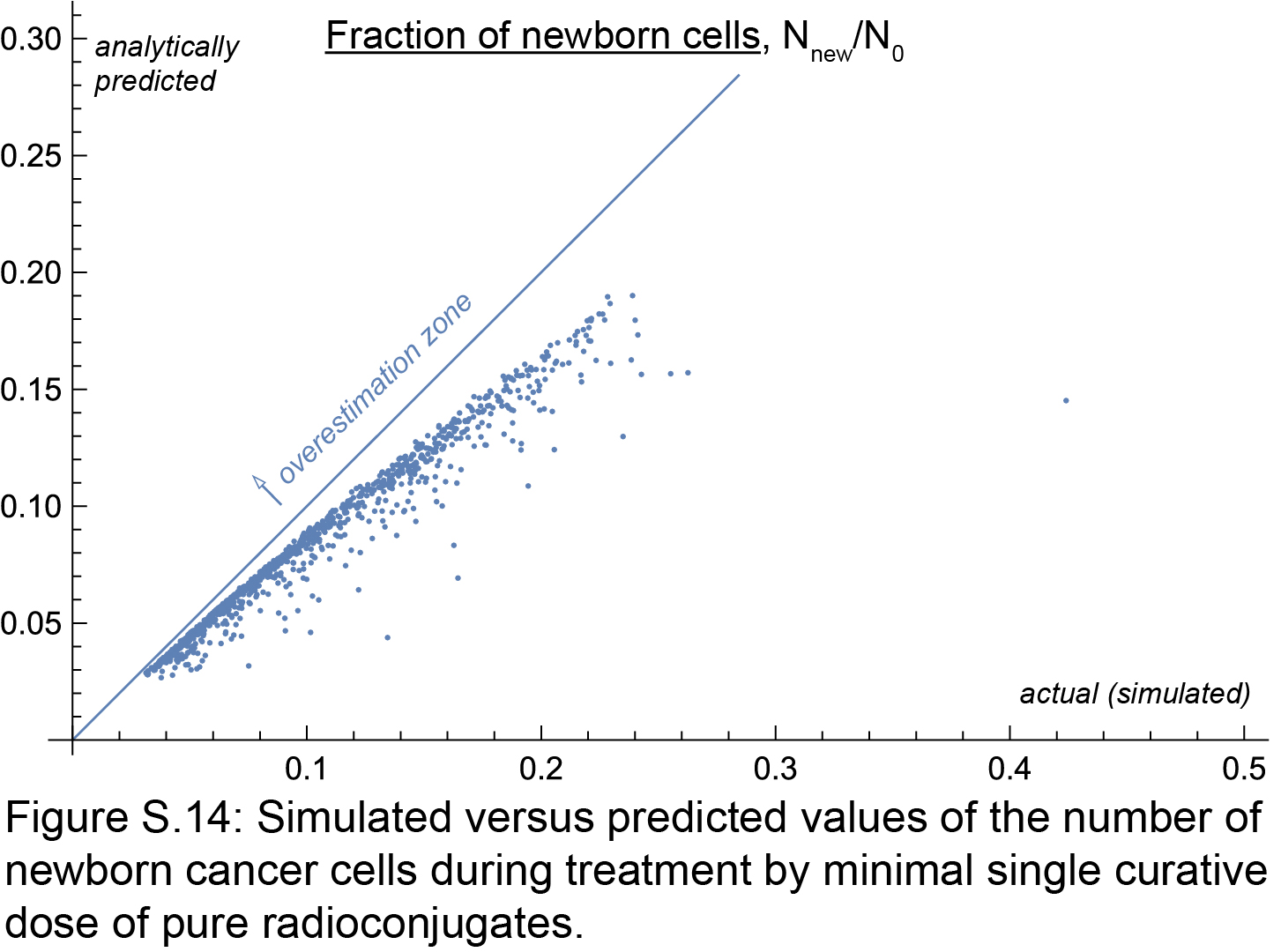

Supplement: Supplementary Figure S.14 [file crc-24-0306_supplementary_figure_s.14_suppsf14.jpeg]

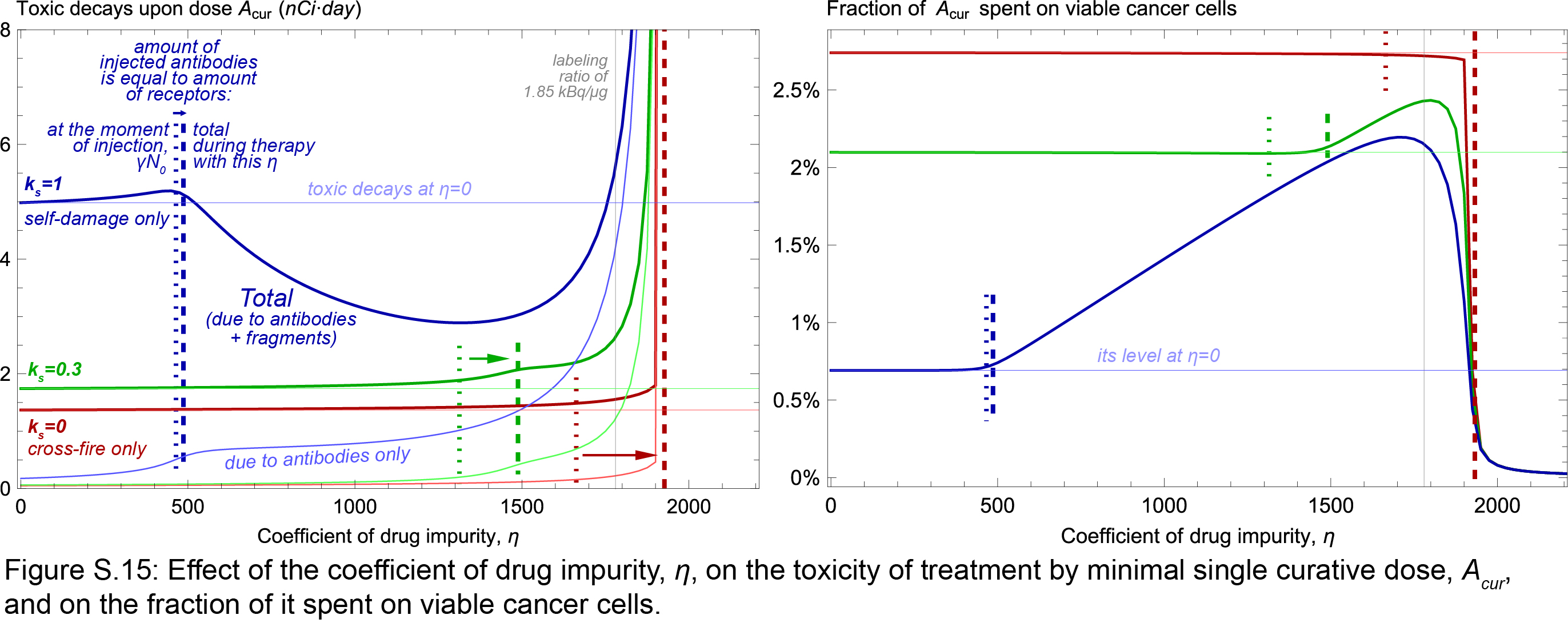

Supplement: Supplementary Figure S.15 [file crc-24-0306_supplementary_figure_s.15_suppsf15.jpeg]

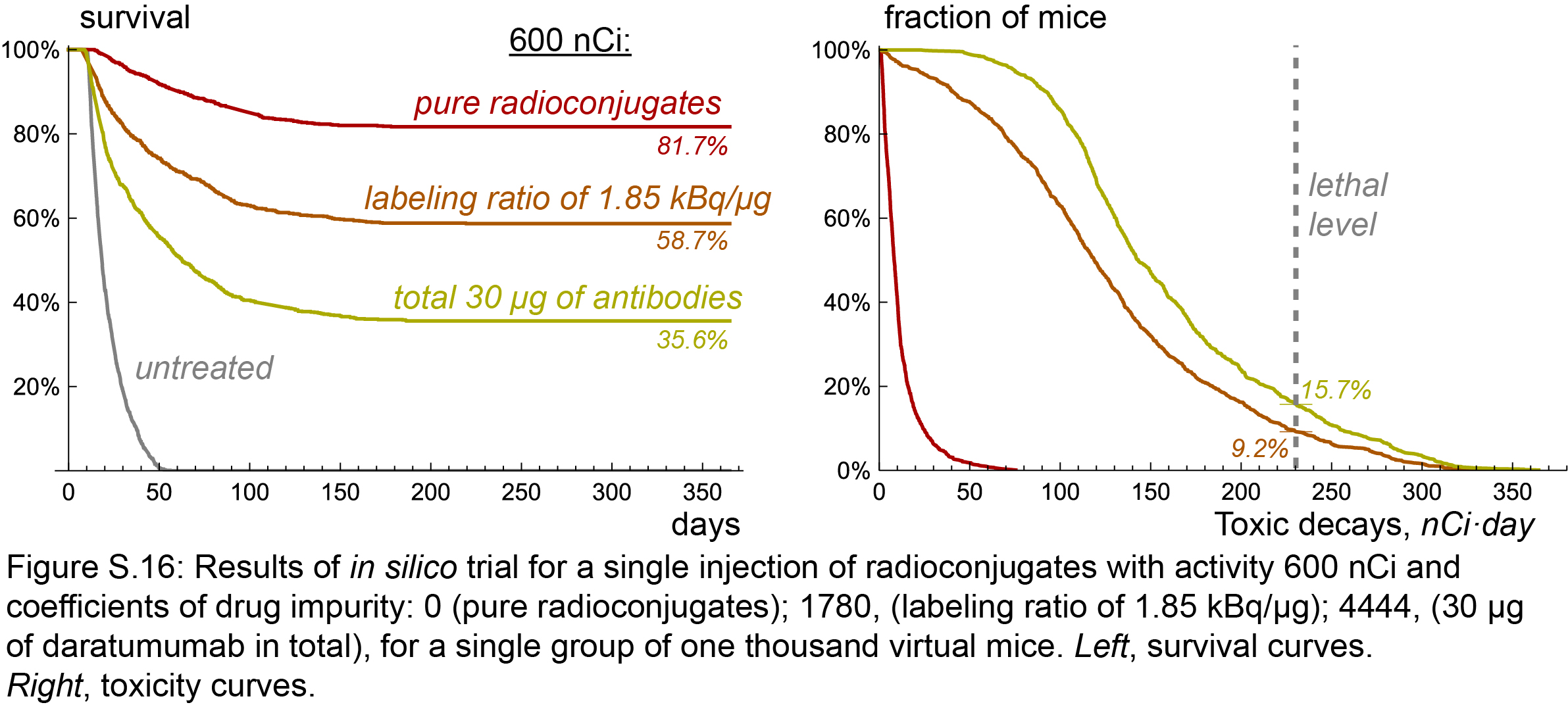

Supplement: Supplementary Figure S.16 [file crc-24-0306_supplementary_figure_s.16_suppsf16.jpeg]

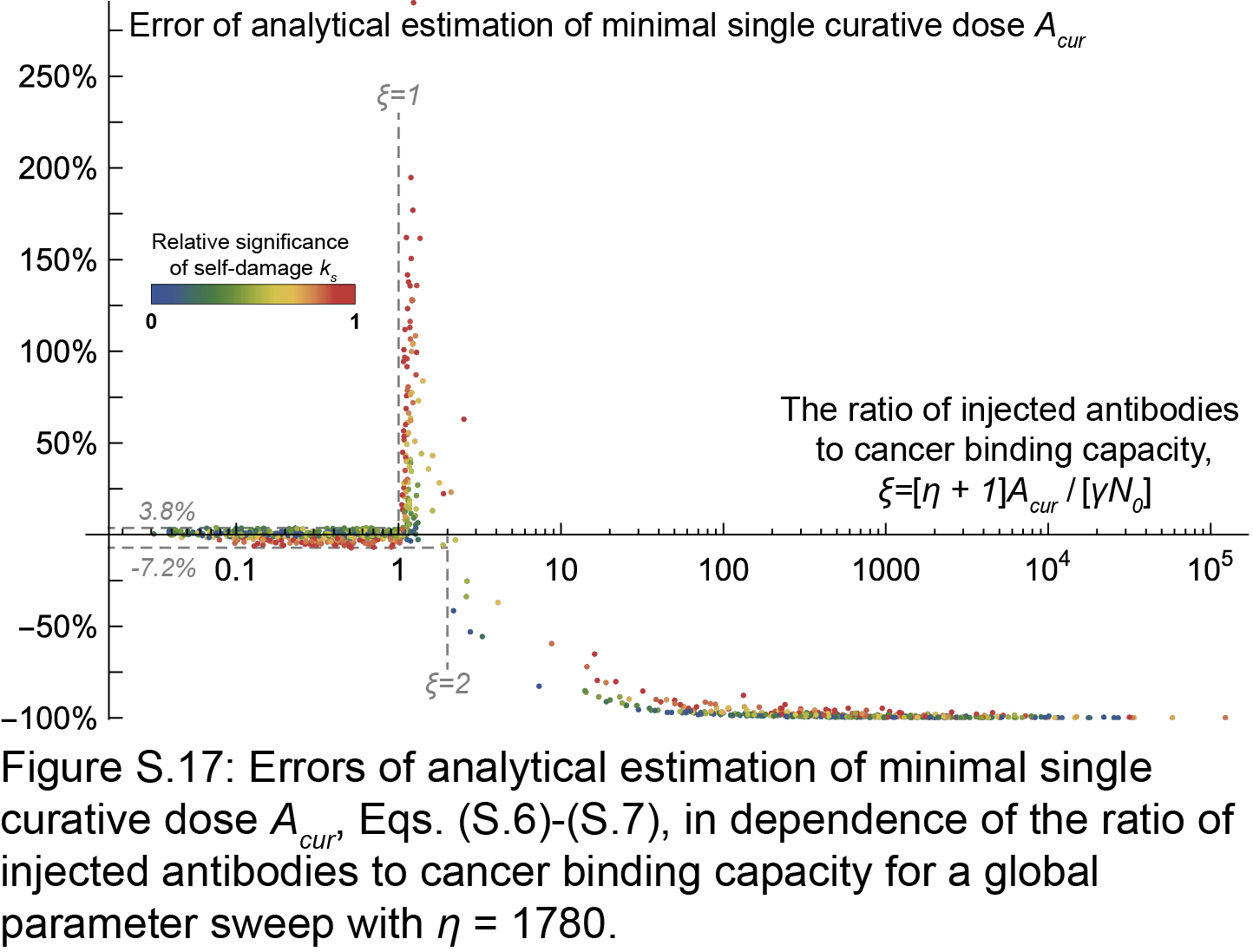

Supplement: Supplementary Figure S.17 [file crc-24-0306_supplementary_figure_s.17_suppsf17.jpeg]

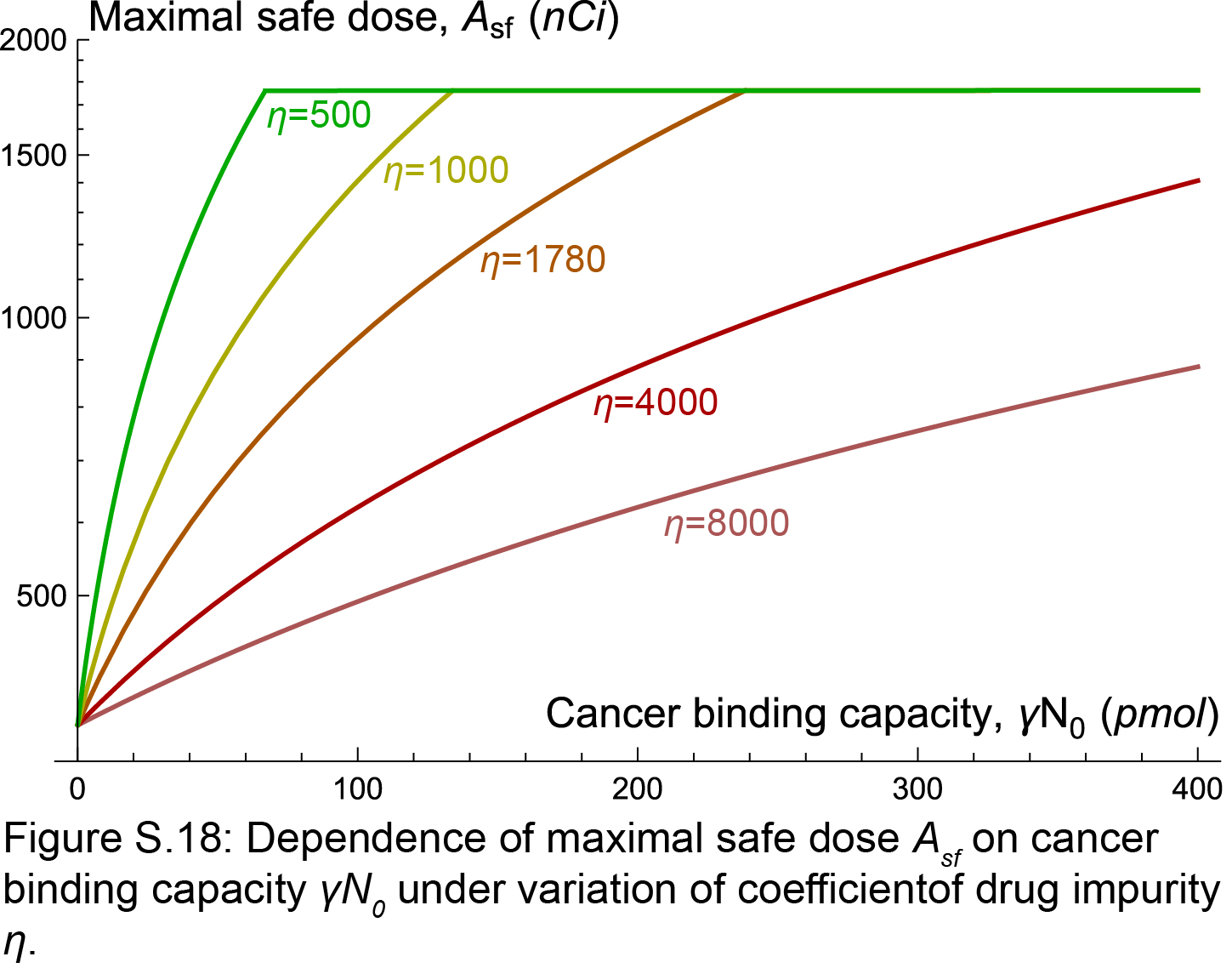

Supplement: Supplementary Figure S.18 [file crc-24-0306_supplementary_figure_s.18_suppsf18.jpeg]

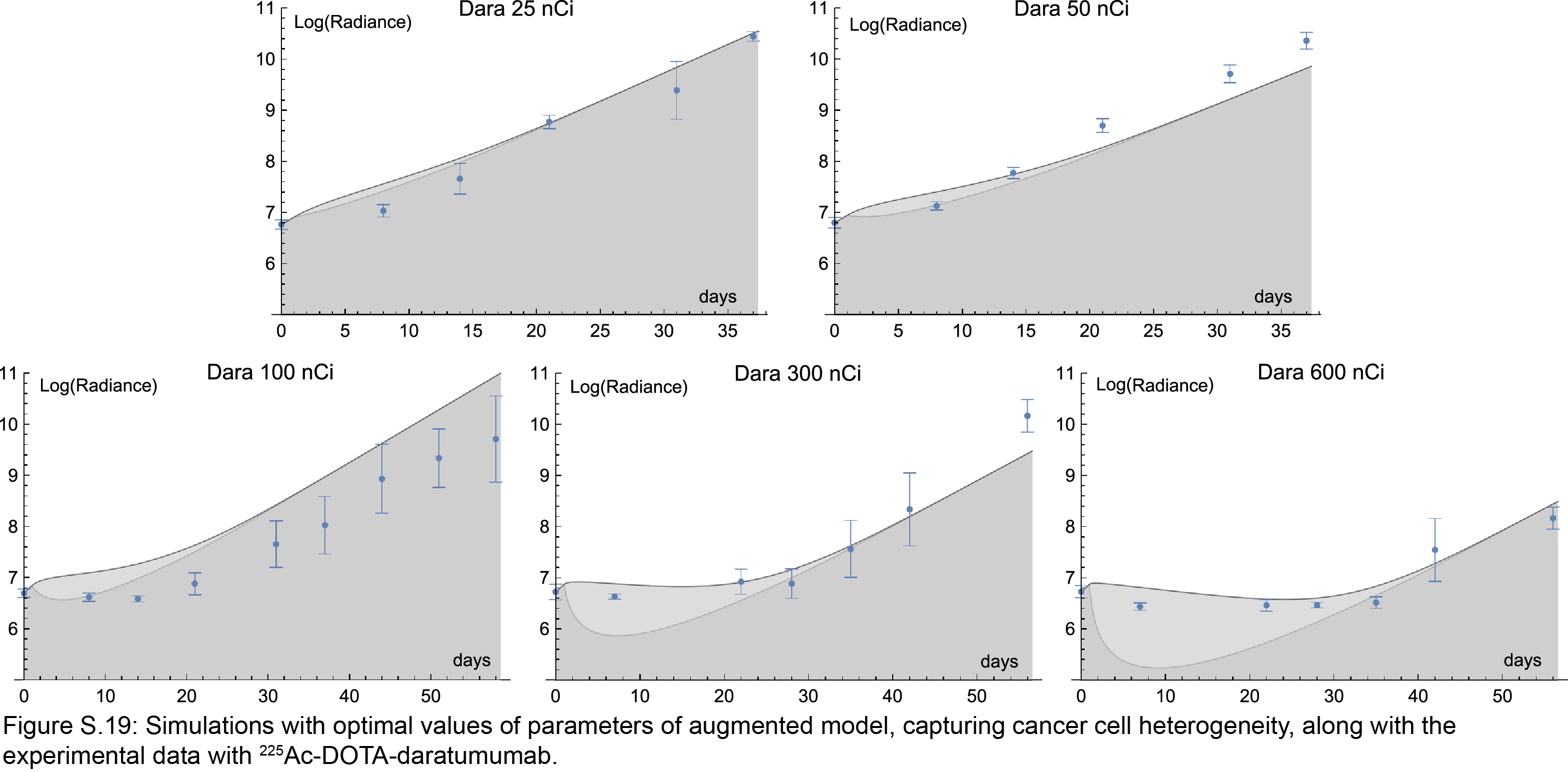

Supplement: Supplementary Figure S.19 [file crc-24-0306_supplementary_figure_s.19_suppsf19.jpeg]

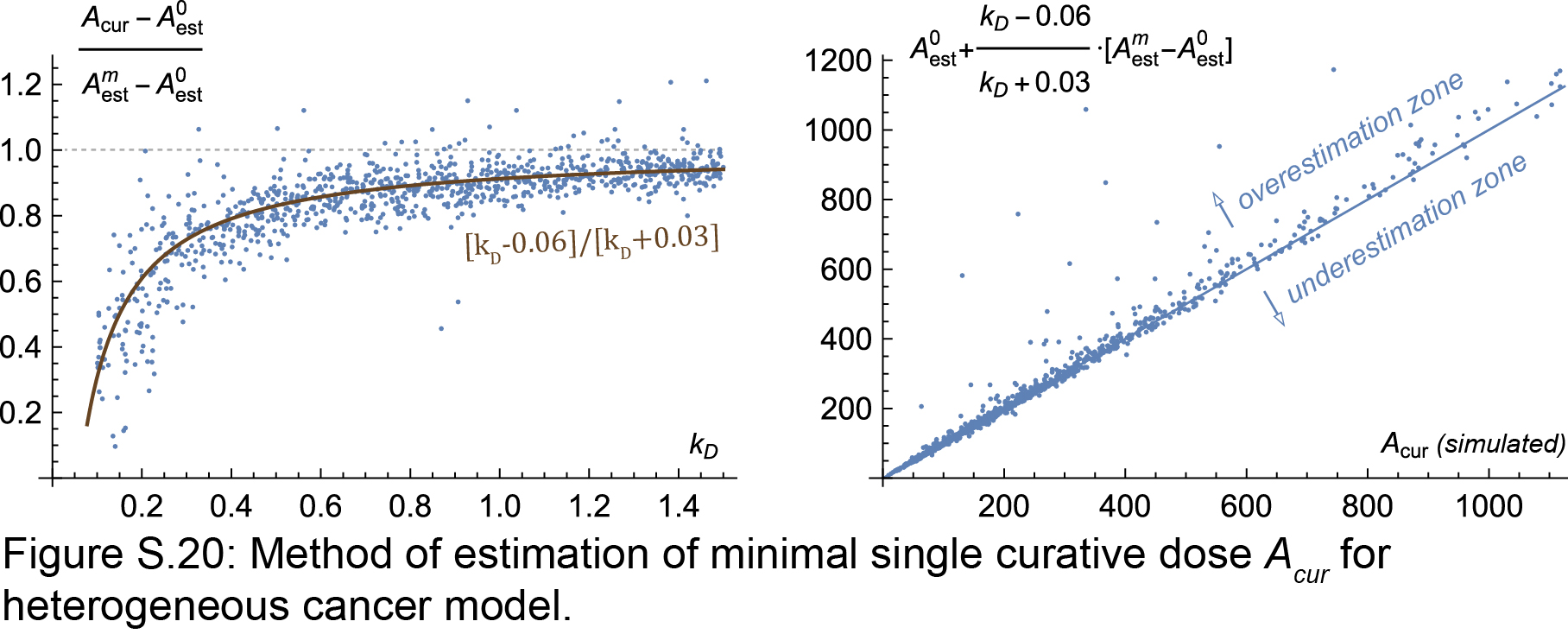

Supplement: Supplementary Figure S.20 [file crc-24-0306_supplementary_figure_s.20_suppsf20.jpeg]

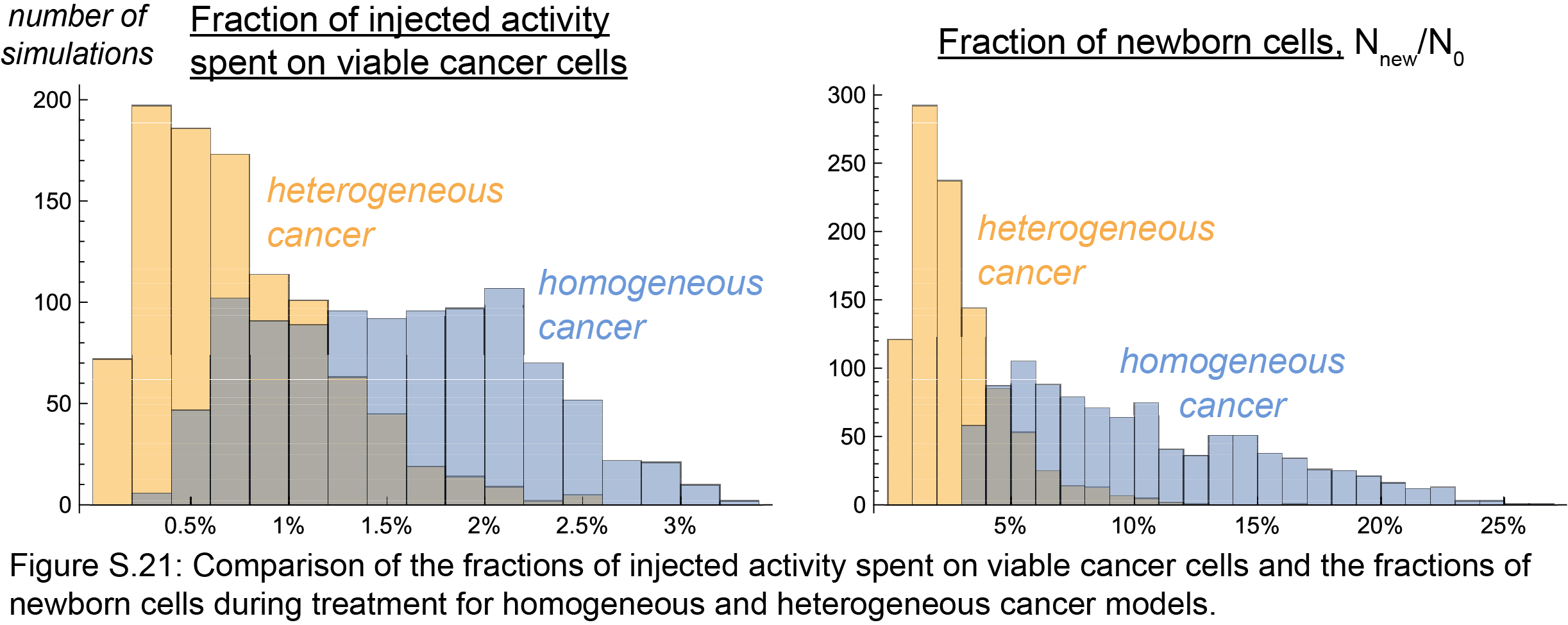

Supplement: Supplementary Figure S.21 [file crc-24-0306_supplementary_figure_s.21_suppsf21.jpeg]

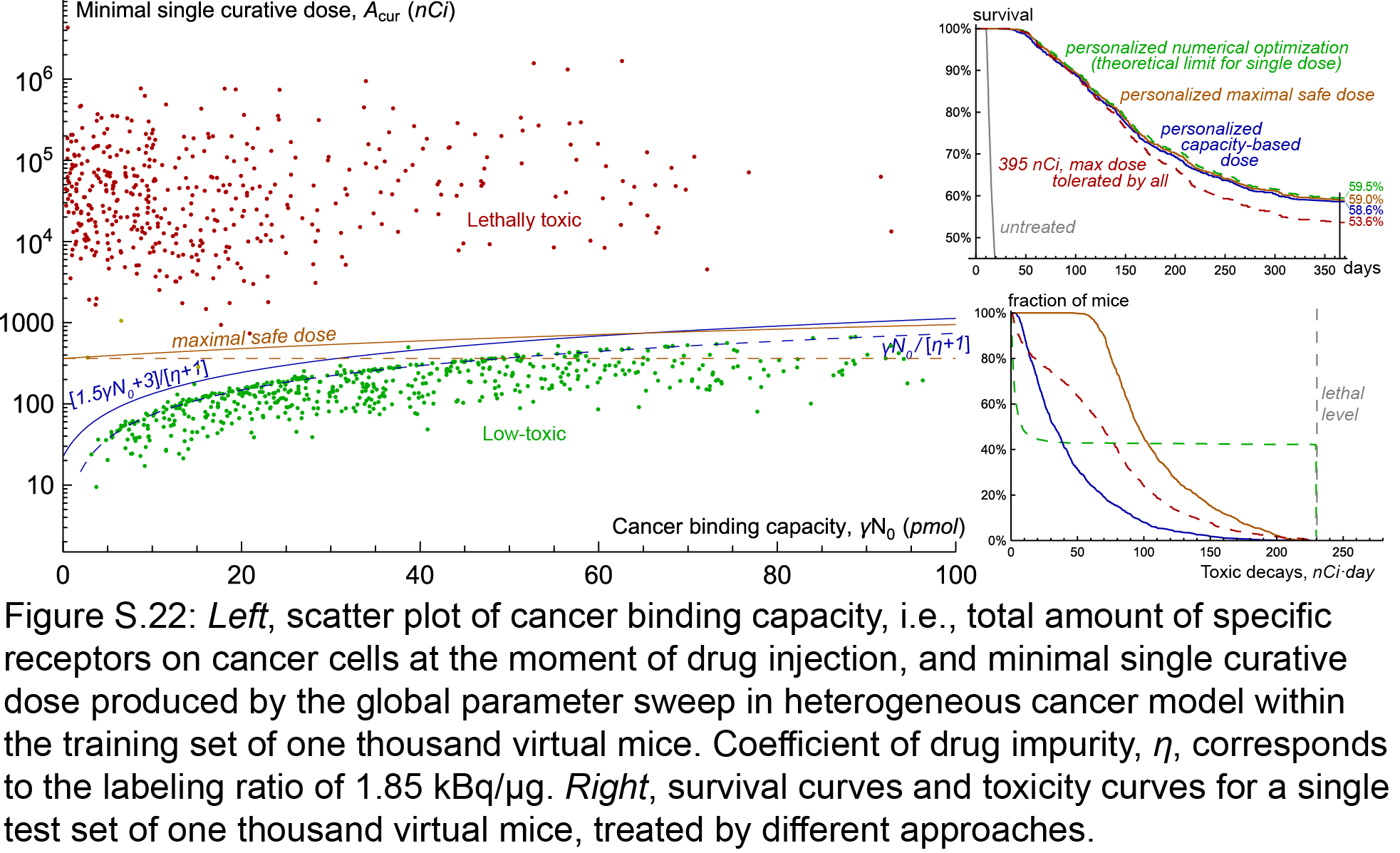

Supplement: Supplementary Figure S.22 [file crc-24-0306_supplementary_figure_s.22_suppsf22.jpeg]

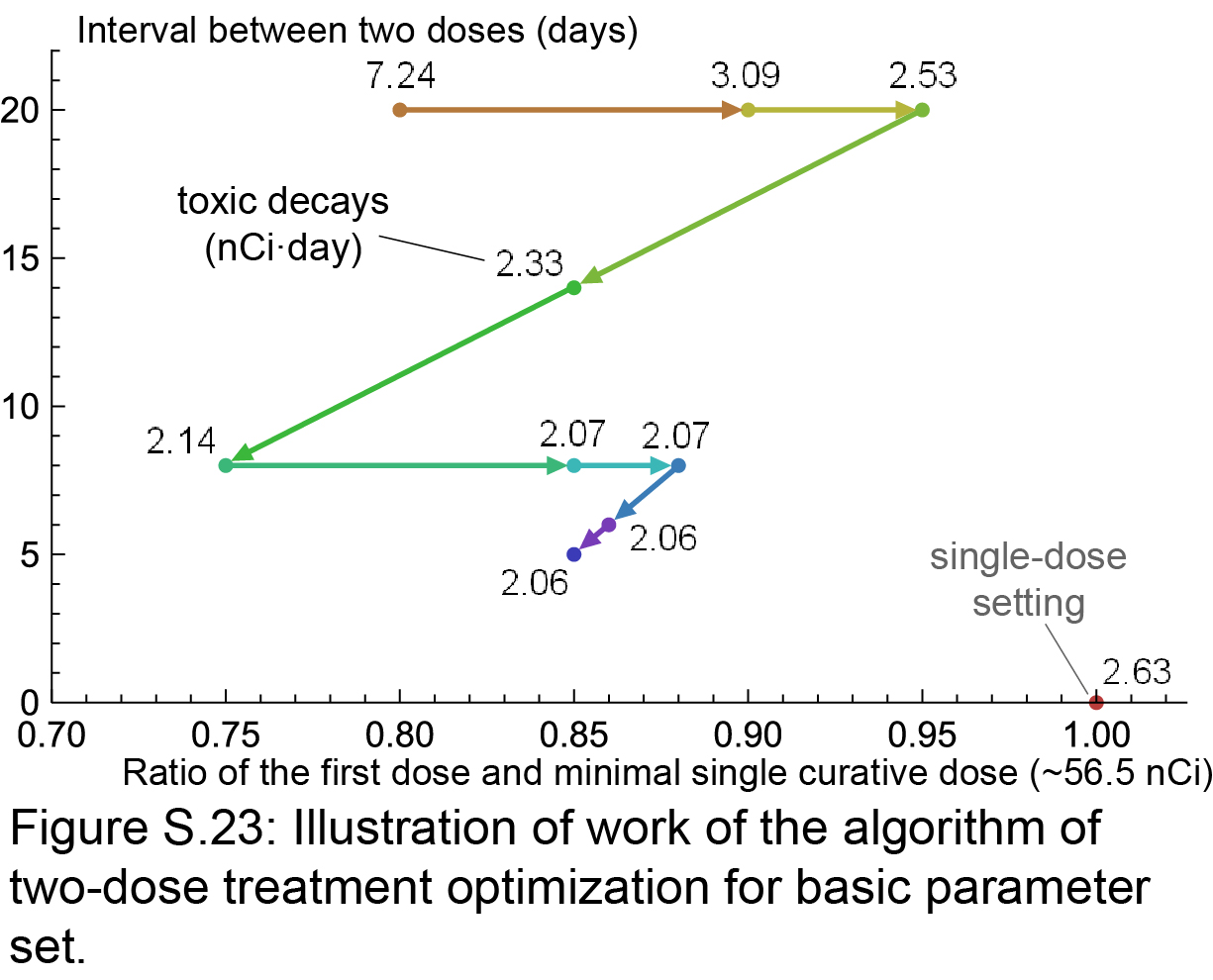

Supplement: Supplementary Figure S.23 [file crc-24-0306_supplementary_figure_s.23_suppsf23.jpeg]

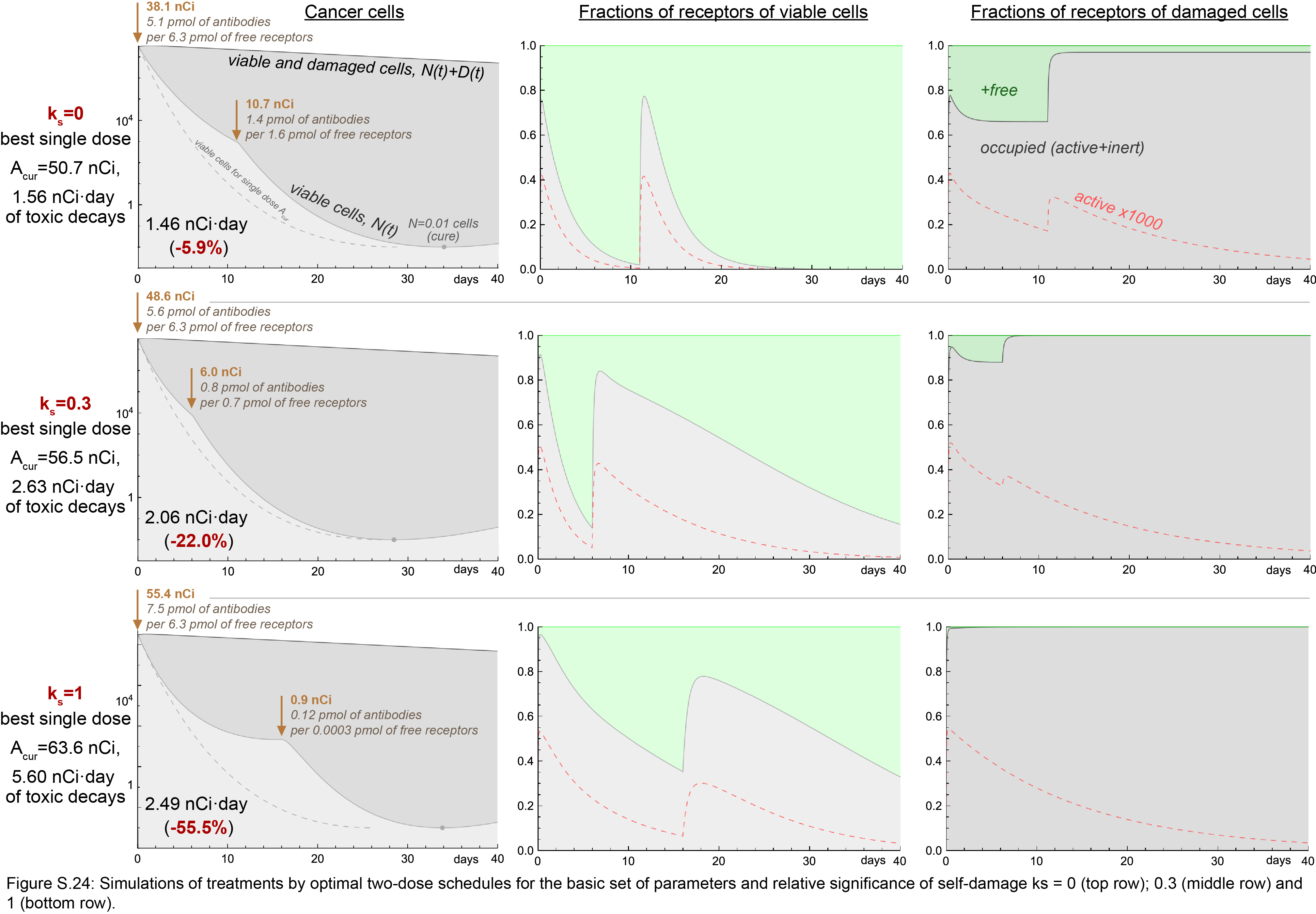

Supplement: Supplementary Figure S.24 [file crc-24-0306_supplementary_figure_s.24_suppsf24.jpeg]

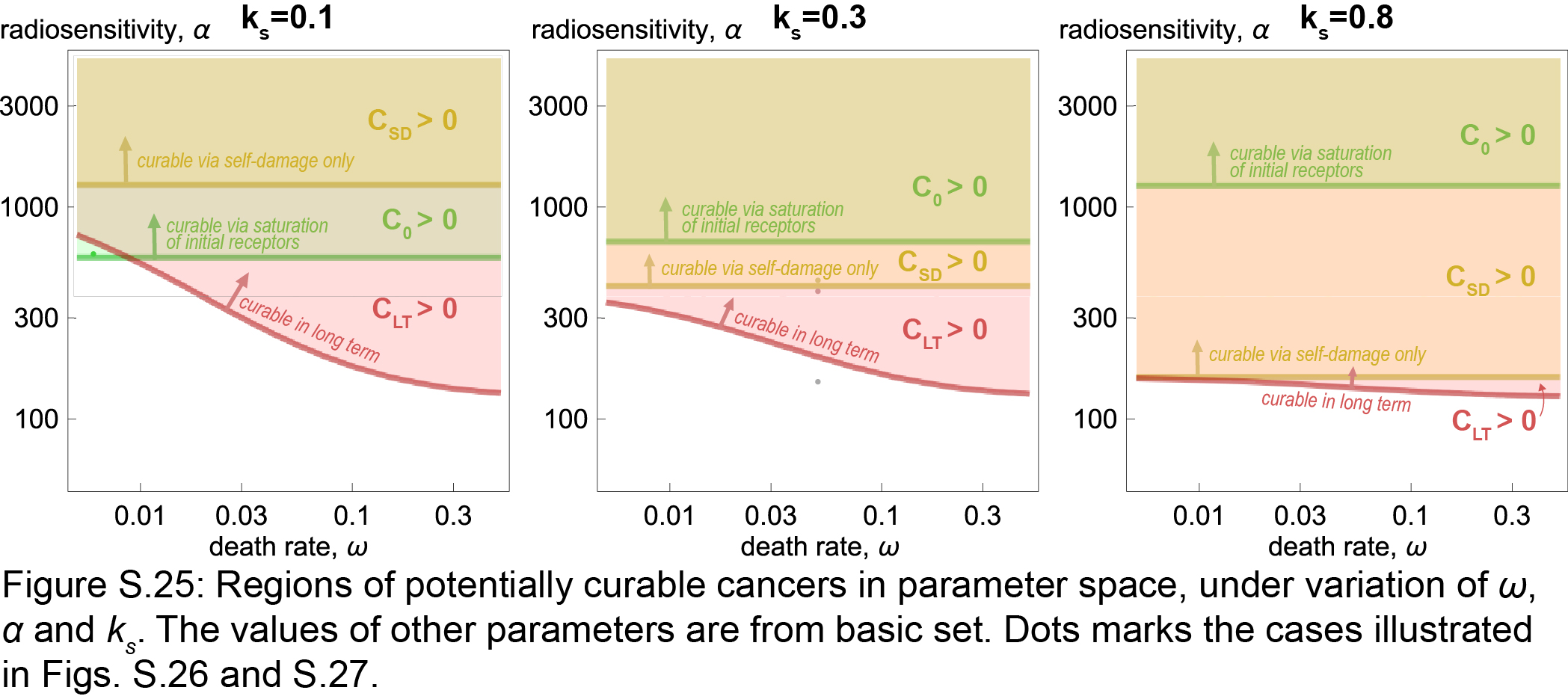

Supplement: Supplementary Figure S.25 [file crc-24-0306_supplementary_figure_s.25_suppsf25.jpeg]

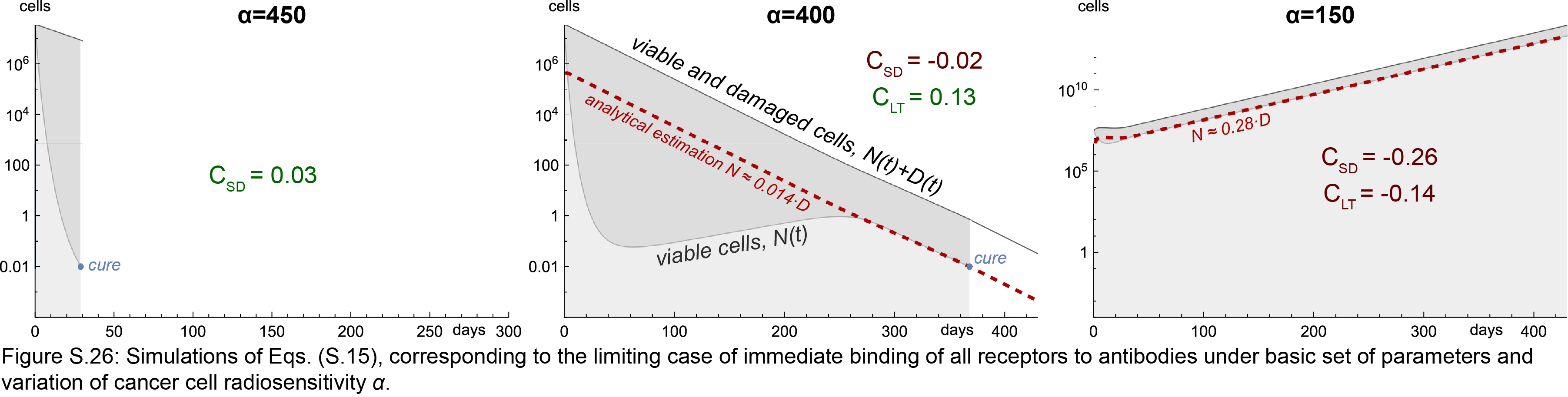

Supplement: Supplementary Figure S.26 [file crc-24-0306_supplementary_figure_s.26_suppsf26.jpeg]

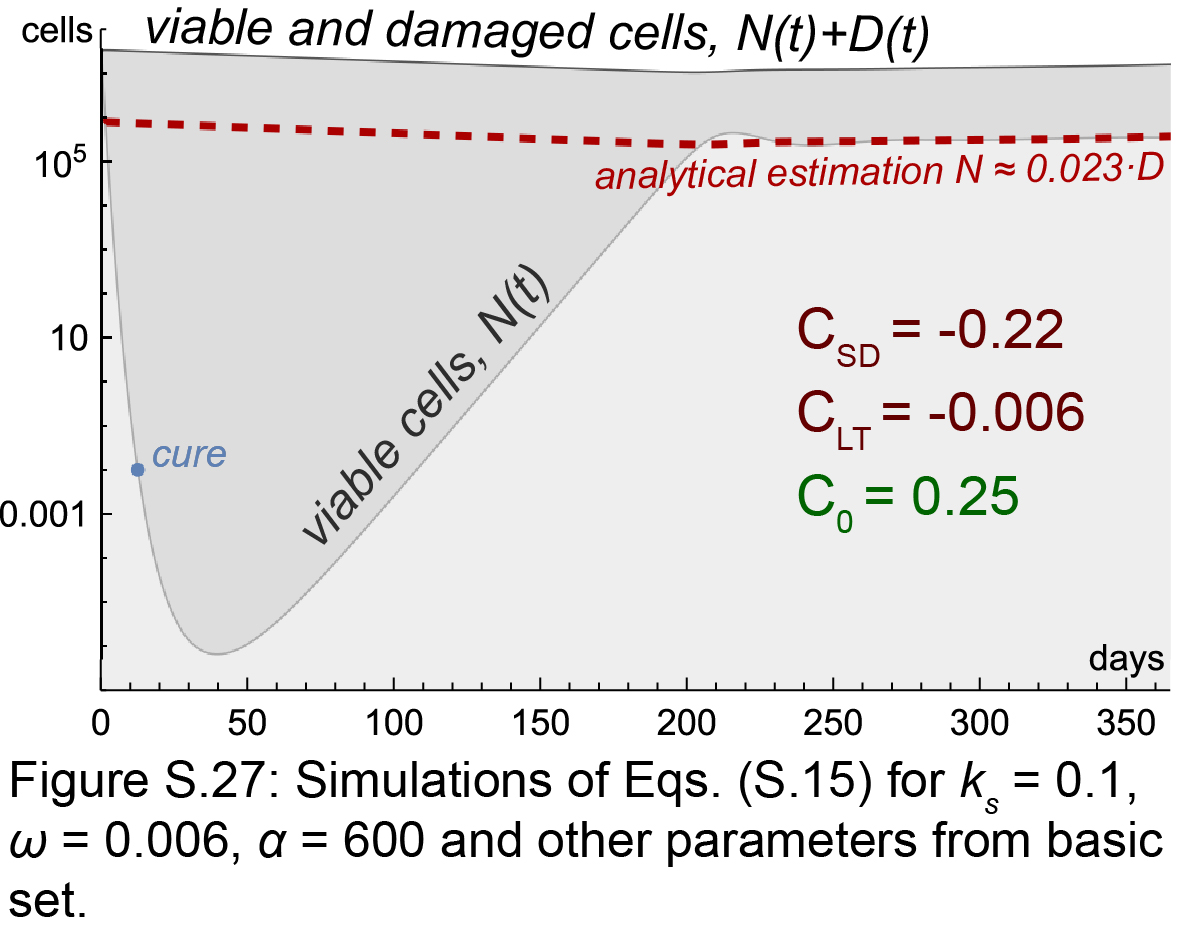

Supplement: Supplementary Figure S.27 [file crc-24-0306_supplementary_figure_s.27_suppsf27.jpeg]

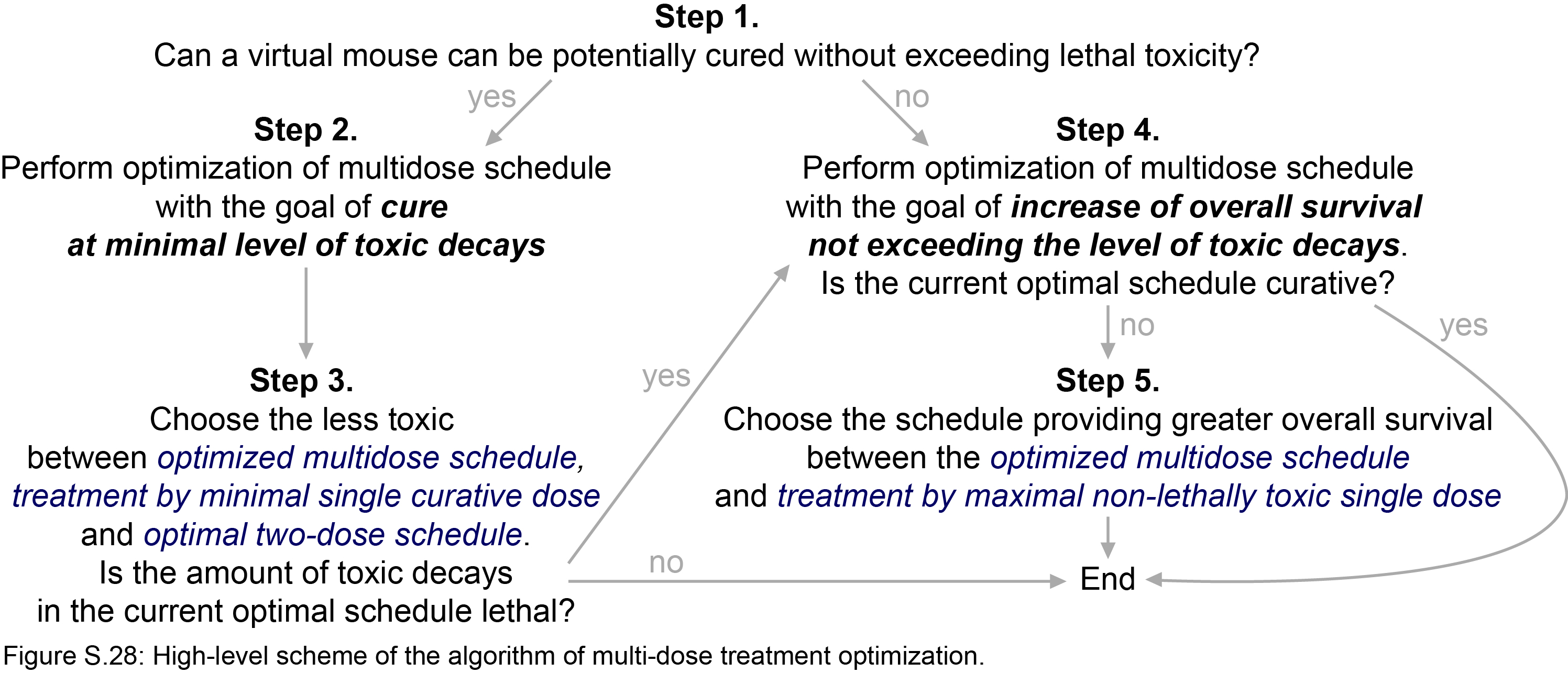

Supplement: Supplementary Figure S.28 [file crc-24-0306_supplementary_figure_s.28_suppsf28.jpeg]

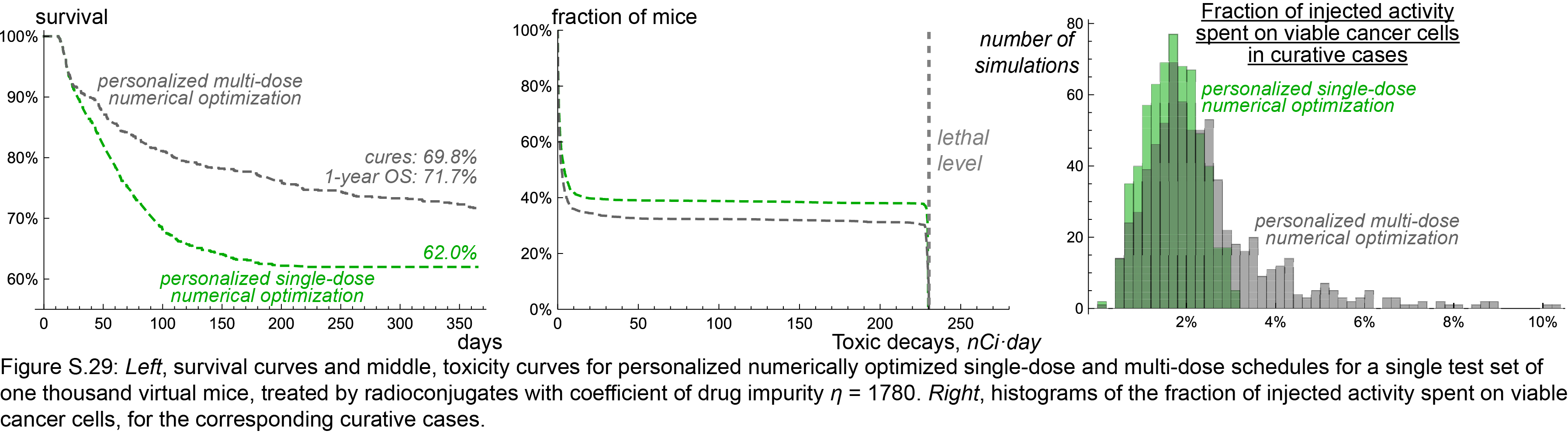

Supplement: Supplementary Figure S.29 [file crc-24-0306_supplementary_figure_s.29_suppsf29.jpeg]

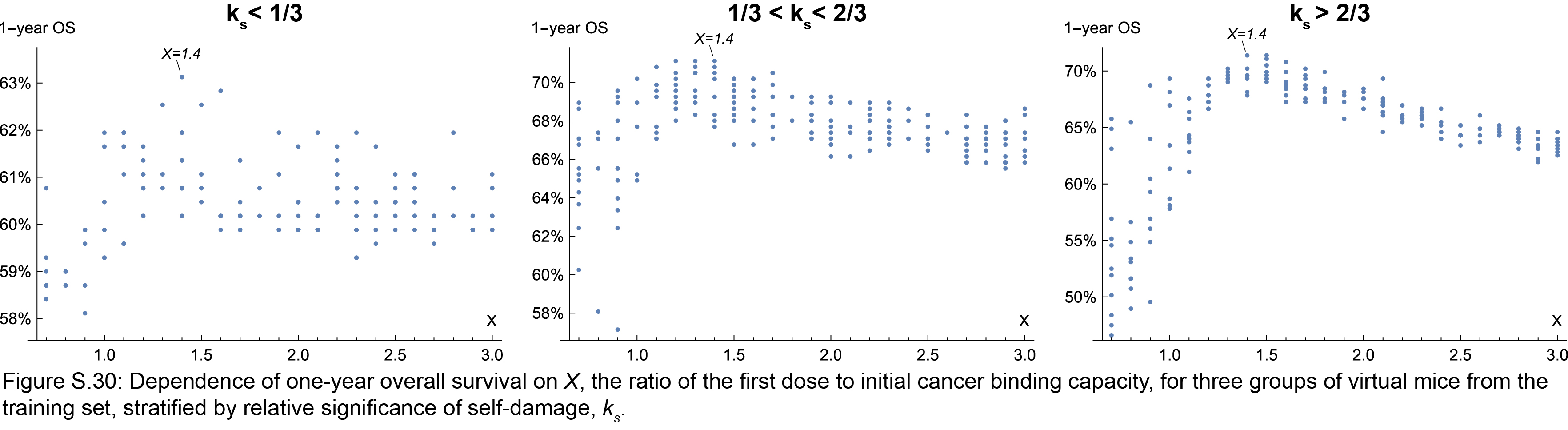

Supplement: Supplementary Figure S.30 [file crc-24-0306_supplementary_figure_s.30_suppsf30.jpeg]

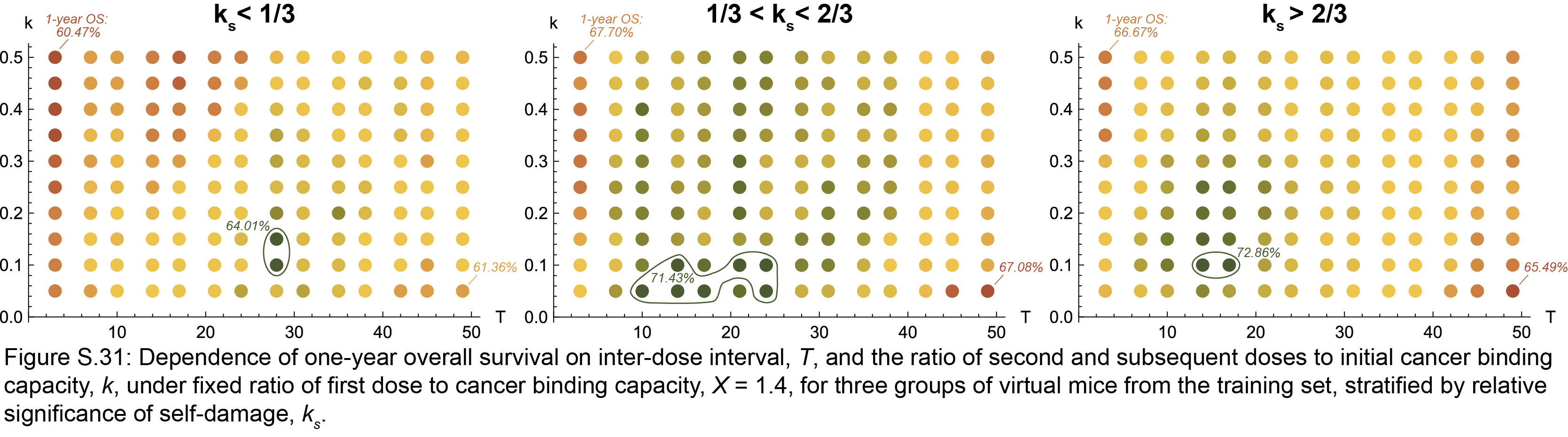

Supplement: Supplementary Figure S.31 [file crc-24-0306_supplementary_figure_s.31_suppsf31.jpeg]

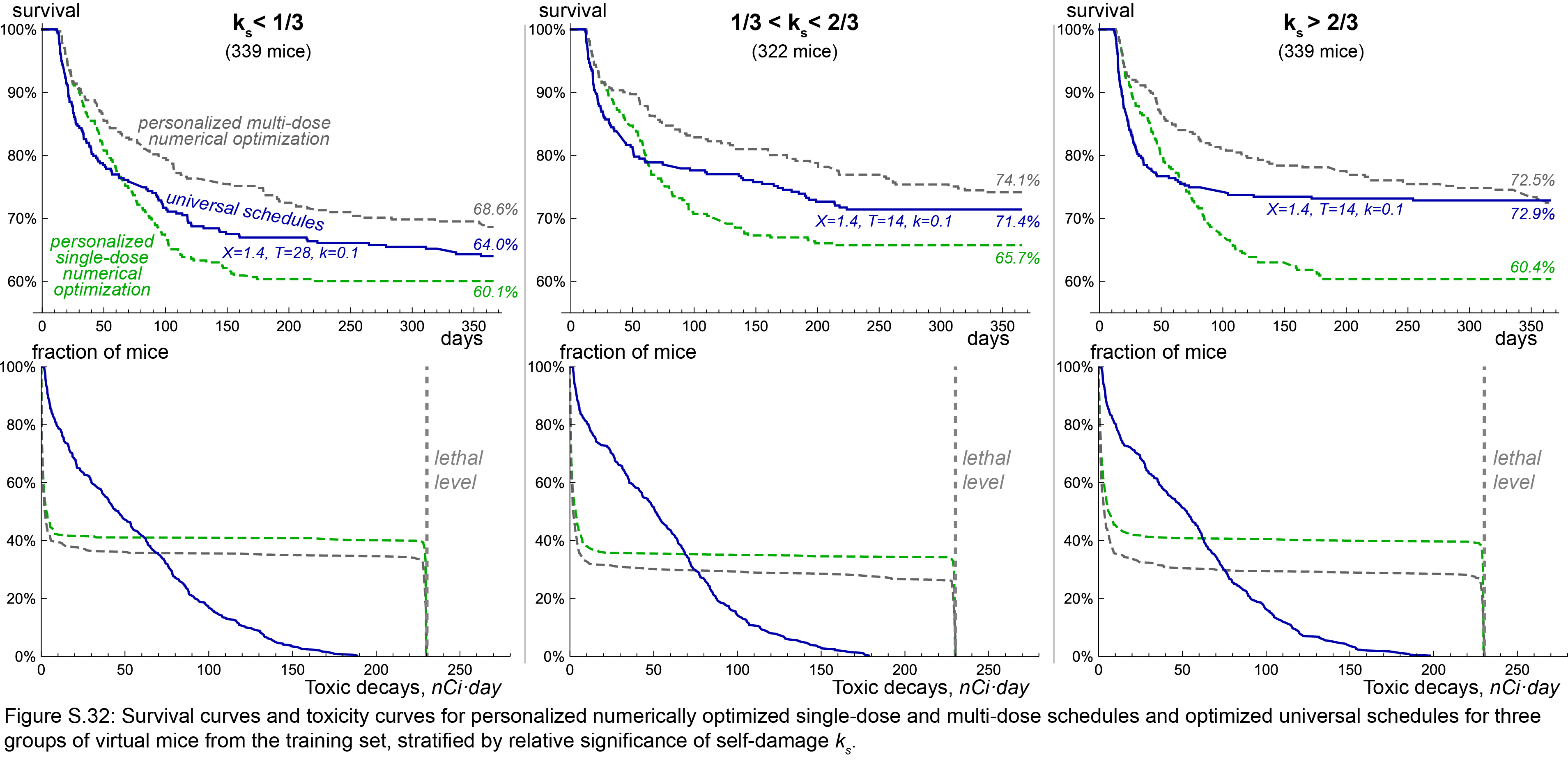

Supplement: Supplementary Figure S.32 [file crc-24-0306_supplementary_figure_s.32_suppsf32.jpeg]
