# Supplementary material for: Mathematical Modeling Unveils Optimization Strategies for Targeted Radionuclide Therapy of Blood Cancers
Source: Cancer Res Commun. 2024 Nov 14;4(11):2955–67. doi: 10.1158/2767-9764.CRC-24-0306 (PMC11562018; doi:10.1158/2767-9764.CRC-24-0306)
Supplement: Computational codes — Designed in Wolfram Mathematica, version 13.3.1.0. [file crc-24-0306_computational_codes_suppscc.zip › CodesPDF/01-Fitting-data.pdf]

```
(* This file contains simulations for the paper
  "Mathematical Modeling Unveils Optimization Strategies
for Targeted Radionuclide Therapy of Blood Cancers"
  by Maxim Kuznetsov, Vikram Adhikarla, Enrico Caserta,
 Flavia Pichiorri, John E.Shively, Xiuli Wang and Russell C.Rockne *)

(**************************************************************************************)

(* Supplementary S.1.1 Animal studies *)
(* Published in the work by M. Minnix, V. Adhikarla, E. Caserta, E. Poku, R. Rockne, J.E. Shively & F. Pichiorri
   Comparison of CD38-targeted α-versus β-radionuclide therapy of disseminated multiple myeloma in an animal model.
     Journal of Nuclear Medicine, 62 (6) 795-801. (2021) *)
```

In[∘]:=

```
(* Raw experimental data is hidden in this cell -- initialize it to plot the following figure *)
```

In[∘]:=

```
(* 1 -- Vehicle control (no treatment) *)
ControlData = (* {days, BLI signal} *)
   {{{0, 6210000}, {7, 64900000}, {22, 7360000000}},
     {{0, 5140000}, {7, 62500000}, {22, 7870000000}},
     {{0, 8320000}, {7, 89100000}, {22, 14800000000}},
     {{0, 4800000}, {7, 34000000}, {22, 5300000000}},
     {{0, 3460000}, {7, 23400000}, {22, 5990000000}},
     {{0, 3030000}, {7, 45500000}, {22, 13100000000}},
     {{0, 7420000}, {7, 97600000}, {22, 7190000000}},
     {{0, 3840000}, {7, 56100000}, {22, 5550000000}}};
(* Day 0 here corresponds to day 8 after injection of cancer cells, when the first BLI measurement was performed
  note that injection of antibodies were performed on day 1 in current notation (day 9 of experiment) *)

(* Logarithms of BLI will be used for statistical processing --
  to avoid giving much weight to greater values due to the exponential nature of tumor cell proliferation --
  e.g., the average of 10^7 and 10^9 is 5.05·10^8, but for fitting purposes let's assume that on average tumors grew to 10^8 cells *)
```

```
ControlData[[All, All, {2}]] = Log10[ControlData[[All, All, {2}]]];

(* Find means and errors *)
daysControl = ControlData[[1, All, 1]];
meansControl = Mean /@ Table[ControlData[[All, k, 2]], {k, 1, Length[daysControl]}];
errorsControl = N[StandardDeviation /@ Table[ControlData[[All, k, 2]], {k, 1, Length[daysControl]}]];
ControlErr = Table[{daysControl[[k]], Around[meansControl[[k]], errorsControl[[k]]]}, {k, 1, Length[daysControl]}];
ControlMean = Table[{daysControl[[k]], meansControl[[k]]}, {k, 1, Length[daysControl]}];

(* 2 -- Treatment by 600 nCi of of 225Ac-DOTA-trastumumab, which does not bind to multiple myeloma cells *)
TrasData =
  {{{0, 4620000}, {7, 20200000}, {22, 488000000}, {28, 2440000000}},
   {{0, 5540000}, {7, 27500000}, {22, 651000000}, {28, 2920000000}},
   {{0, 5050000}, {7, 25700000}, {22, 1240000000}, {28, 6180000000}},
   {{0, 5980000}, {7, 29300000}, {22, 1320000000}, {28, 4550000000}
    (*,{35,9590000000},{42,23500000000} -- this mouse survived for a longer time, let's neglect these data points for processing *)}};

TrasData[[All, All, {2}]] = Log10[TrasData[[All, All, {2}]]];
daysTras = TrasData[[1, All, 1]];
meansTras = Mean /@ Table[TrasData[[All, k, 2]], {k, 1, Length[daysTras]}];
errorsTras = N[StandardDeviation /@ Table[TrasData[[All, k, 2]], {k, 1, Length[daysTras]}]];
TrasErr = Table[{daysTras[[k]], Around[meansTras[[k]], errorsTras[[k]]]}, {k, 1, Length[daysTras]}];
TrasMean = Table[{daysTras[[k]], meansTras[[k]]}, {k, 1, Length[daysTras]}];

(* 3 -- Treatment by 25 nCi of 225Ac-DOTA-daratumumab *)
Dara25Data =
  {{{0, 6280000}, {8, 11235000}, {14, 54750000}, {21, 481000000}, {31, 6695000000}, {37, 37000000000}},
   {{0, 5705000}, {8, 11805000}, {14, 73850000}, {21, 608000000}, {31, 5400000000}, {37, 22650000000}},
   {{0, 5515000}, {8, 8595000}, {14, 46550000}, {21, 408000000}, {31, 217500000}, {37, 24050000000}},
   {{0, 8370000}, {8, 17750000}, {14, 11630000}, {21, 994000000}, {31, 1465000000}, {37, 32150000000}},
   {{0, 4665000}, {8, 9320000}, {14, 65100000}, {21, 559000000}, {31, 3985000000}, {37, 25200000000}},
```

```
    {{0, 4875000}, {8, 8445000}, {14, 63500000}, {21, 659500000}, {31, 4735000000}, {37, ""}}};

Dara25Data[[All, All, {2}]] = Log10[Dara25Data[[All, All, {2}]]];
daysDara25 = Dara25Data[[1, All, 1]];
meansDara25 = Join[N[Mean /@ Table[Dara25Data[[All, k, 2]], {k, 1, 5}]], N[Mean /@ Table[Dara25Data[[1 ;; 5, k, 2]], {k, {6}}]]];
errorsDara25 = Join[N[StandardDeviation /@ Table[Dara25Data[[All, k, 2]], {k, 1, 5}]],
   N[StandardDeviation /@ Table[Dara25Data[[1 ;; 5, k, 2]], {k, {6}}]]];
Dara25Err = Table[{daysDara25[[k]], Around[meansDara25[[k]], errorsDara25[[k]]]}, {k, 1, Length[daysDara25]}];
Dara25Mean = Table[{daysDara25[[k]], meansDara25[[k]]}, {k, 1, Length[daysDara25]}];

(* 4 -- Treatment by 50 nCi of 225Ac-DOTA-daratumumab *)
Dara50Data =
  {{{0, 9490000}, {8, 14970000}, {14, 89000000}, {21, 787000000}, {31, 6710000000}, {37, 31050000000}},
    {{0, 5590000}, {8, 9435000}, {14, 46550000}, {21, 406000000}, {31, 4265000000}, {37, 12670000000}},
    {{0, 6155000}, {8, 13645000}, {14, 51700000}, {21, 316500000}, {31, 3875000000}, {37, 27900000000}},
    {{0, 6650000}, {8, 15530000}, {14, 73350000}, {21, 527000000}, {31, 3120000000}, {37, 18050000000}},
    {{0, 6010000}, {8, 15030000}, {14, 55800000}, {21, 554000000}, {31, 5510000000}, {37, 20800000000}},
    {{0, 4670000}, {8, 12650000}, {14, 49000000}, {21, 519500000}, {31, 9415000000}, {37, 34350000000}}};

Dara50Data[[All, All, {2}]] = Log10[Dara50Data[[All, All, {2}]]];
daysDara50 = Dara50Data[[1, All, 1]];
meansDara50 = Mean /@ Table[Dara50Data[[All, k, 2]], {k, 1, Length[daysDara50]}];
errorsDara50 = N[StandardDeviation /@ Table[Dara50Data[[All, k, 2]], {k, 1, Length[daysDara50]}]];
Dara50Err = Table[{daysDara50[[k]], Around[meansDara50[[k]], errorsDara50[[k]]]}, {k, 1, Length[daysDara50]}];
Dara50Mean = Table[{daysDara50[[k]], meansDara50[[k]]}, {k, 1, Length[daysDara50]}];

(* 5 -- Treatment by 100 nCi of 225Ac-DOTA-daratumumab *)
Dara100Data =
  {{{0, 4420000}, {8, 5405000}, {14, 4515000}, {21, 8010000}, {31, 36790000},
      {37, 108000000}, {44, 766000000}, {51, 3450000000}, {58, 14930000000} (*,{65,128250000000}*)},
    {{0, 4545000}, {8, 3295000}, {14, 3910000}, {21, 5695000}, {31, 22150000},
```

```
      {37, 169 850 000}, {44, 1 431 000 000}, {51, 9 245 000 000}, {58, 297 000 000} (*,{65,117450000000}*) },
    {{0, 3 960 000}, {8, 4 275 000}, {14, 3 535 000}, {21, 8 120 000},
      {31, 72 850 000}, {37, 84 200 000}, {44, 1 252 000 000}, {51, 354 500 000}, {58, 20 300 000 000}},
    {{0, 6 855 000}, {8, 4 545 000}, {14, 3 235 000}, {21, 4 925 000},
      {31, 16 815 000}, {37, 11 445 000}, {44, 62 950 000}, {51, 884 000 000}, {58, 7 670 000 000}},
    {{0, 5 425 000}, {8, 4 215 000}, {14, 3 595 000},
      {21, 5 430 000}, {31, 27 350 000}, {37, 126 500 000}, {44, 588 500 000}, {51, 4 660 000 000}, {58, ""}},
    {{0, 4 920 000}, {8, 3 370 000}, {14, 4 420 000},
      {21, 18 880 000}, {31, 297 300 000}, {37, 612 000 000}, {44, 7 435 000 000}, {51, ""}, {58, ""}}};

Dara100Data[[All, All, {2}]] = Log10[Dara100Data[[All, All, {2}]]];
daysDara100 = Dara100Data[[1, All, 1]];
meansDara100 = Join[N[Mean /@ Table[Dara100Data[[All, k, 2]], {k, 1, 7}]],
    N[Mean /@ Table[Dara100Data[[1 ;; 5, k, 2]], {k, {8}}]], N[Mean /@ Table[Dara100Data[[1 ;; 4, k, 2]], {k, {9}}]]];
errorsDara100 = Join[N[StandardDeviation /@ Table[Dara100Data[[All ;;, k, 2]], {k, 1, 7}]],
    N[StandardDeviation /@ Table[Dara100Data[[1 ;; 5, k, 2]], {k, {8}}]],
    N[StandardDeviation /@ Table[Dara100Data[[1 ;; 4, k, 2]], {k, {9}}]]];
Dara100Err = Table[{daysDara100[[k]], Around[meansDara100[[k]], errorsDara100[[k]]]}, {k, 1, Length[daysDara100]}];
Dara100Mean = Table[{daysDara100[[k]], meansDara100[[k]]}, {k, 1, Length[daysDara100]}];

(* 6 -- Treatment by 300 nCi of 225Ac-DOTA-daratumumab *)
Dara300Data =
  {{{0, 3 540 000}, {7, 3 780 000}, {22, 5 850 000}, {28, 13 500 000}, {35, 69 400 000}, {42, 455 000 000}, {56, 18 400 000 000}},
    {{0, 4 470 000}, {7, 4 010 000}, {22, 7 340 000}, {28, 4 010 000}, {35, 6 690 000}, {42, 53 900 000}, {56, 6 890 000 000}},
    {{0, 7 660 000}, {7, 4 890 000}, {22, 19 300 000}, {28, 4 610 000}, {35, 129 000 000}, {42, 1 570 000 000}, {56, 36 700 000 000}},
    {{0, 6 340 000}, {7, 4 570 000}, {22, 5 840 000}, {28, 13 800 000}, {35, 29 000 000}, {42, 58 300 000}, {56, 9 860 000 000}}};

Dara300Data[[All, All, {2}]] = Log10[Dara300Data[[All, All, {2}]]];
daysDara300 = Dara300Data[[1, All, 1]];
meansDara300 = Mean /@ Table[Dara300Data[[All, k, 2]], {k, 1, Length[daysDara300]}];
errorsDara300 = N[StandardDeviation /@ Table[Dara300Data[[All, k, 2]], {k, 1, Length[daysDara300]}]];
```

```
Dara300Err = Table[{daysDara300[[k]], Around[meansDara300[[k]], errorsDara300[[k]]]}, {k, 1, Length[daysDara300]}];
Dara300Mean = Table[{daysDara300[[k]], meansDara300[[k]]}, {k, 1, Length[daysDara300]}];

(* 7 -- Treatment by 600 nCi of 225Ac-DOTA-daratumumab *)
Dara600Data =
  {{{0, 7080000}, {7, 2490000}, {22, 3220000}, {28, 2720000}, {35, 2410000}, {42, ""}, {56, ""}}, (* this mouse died due to toxicity *)
   {{0, 4670000}, {7, 2280000}, {22, 2000000}, {28, 2580000}, {35, 3520000}, {42, 14000000}, {56, 102000000}},
   {{0, 3860000}, {7, 2820000}, {22, 2960000}, {28, 2960000}, {35, 2980000}, {42, 17100000}, {56, 119000000}},
   {{0, 6170000}, {7, 3410000}, {22, 3650000}, {28, 3400000}, {35, 4480000}, {42, 179000000}, {56, 257000000}}};

Dara600Data[[All, All, {2}]] = Log10[Dara600Data[[All, All, {2}]]];
daysDara600 = Dara600Data[[1, All, 1]];
meansDara600 = Join[N[Mean /@ Table[Dara600Data[[All, k, 2]], {k, 1, 5}]], N[Mean /@ Table[Dara600Data[[2 ;;, k, 2]], {k, 6, 7}]]];
errorsDara600 = Join[N[StandardDeviation /@ Table[Dara600Data[[All, k, 2]], {k, 1, 5}]],
    N[StandardDeviation /@ Table[Dara600Data[[2 ;;, k, 2]], {k, 6, 7}]]];
Dara600Err = Table[{daysDara600[[k]], Around[meansDara600[[k]], errorsDara600[[k]]]}, {k, 1, Length[daysDara600]}];
Dara600Mean = Table[{daysDara600[[k]], meansDara600[[k]]}, {k, 1, Length[daysDara600]}];

(* Setting colorcode corresponding to the figures in experimental paper *)
ControlColor = RGBColor[51/255, 75/255, 160/255];
TrasColor = RGBColor[13/255, 156/255, 72/255];
Dara25Color = RGBColor[98/255, 186/255, 70/255];
Dara50Color = RGBColor[170/255, 121/255, 181/255];
Dara100Color = RGBColor[235/255, 11/255, 124/255];
Dara300Color = RGBColor[127/255, 70/255, 154/255];
Dara600Color = RGBColor[238/255, 43/255, 36/255];
```

In[ ]:=

```
(* Supplementary Figure S.1 *)

Show[
  (* 1 -- Vehicle control (no treatment) *)
  ListPlot[ControlData, Joined → True, PlotStyle → Lighter[Lighter[Lighter[Lighter[ControlColor]]]],
    PlotRange → {{0, 60}, {6, 10.7}}, AxesLabel → {"days", "Log(Radiance)"}],
  ListPlot[ControlErr, Joined → True, PlotStyle → Directive[Thick, ControlColor], PlotLegends → {"Control"}]
  (* 2 -- 225Ac-DOTA-trastumumab, 600 nCi *)
  , ListPlot[TrasData, Joined → True, PlotStyle → Lighter[Lighter[Lighter[Lighter[TrasColor]]]]],
  ListPlot[TrasErr, Joined → True, PlotStyle → Directive[Thick, TrasColor], PlotLegends → {"Tras 600 nCi"}]
  (* 3 -- 225Ac-DOTA-daratumumab, 25 nCi *)
  , ListPlot[Dara25Data, Joined → True, PlotStyle → Lighter[Lighter[Lighter[Lighter[Dara25Color]]]]],
  ListPlot[Dara25Err, Joined → True, PlotStyle → Directive[Thick, Dara25Color], PlotLegends → {"Dara 25 nCi"}]
  (* 4 -- 225Ac-DOTA-daratumumab, 50 nCi *)
  , ListPlot[Dara50Data, Joined → True, PlotStyle → Lighter[Lighter[Lighter[Lighter[Dara50Color]]]]],
  ListPlot[Dara50Err, Joined → True, PlotStyle → Directive[Thick, Dara50Color], PlotLegends → {"Dara 50 nCi"}]
  (* 5 -- 225Ac-DOTA-daratumumab, 100 nCi *)
  , ListPlot[Dara100Data, Joined → True, PlotStyle → Lighter[Lighter[Lighter[Lighter[Dara100Color]]]]],
  ListPlot[Dara100Err, Joined → True, PlotStyle → Directive[Thick, Dara100Color], PlotLegends → {"Dara 100 nCi"}]
  (* 6 -- 225Ac-DOTA-daratumumab, 300 nCi *)
  , ListPlot[Dara300Data, Joined → True, PlotStyle → Lighter[Lighter[Lighter[Lighter[Dara300Color]]]]],
  ListPlot[Dara300Err, Joined → True, PlotStyle → Directive[Thick, Dara300Color], PlotLegends → {"Dara 300 nCi"}]
  (* 7 -- 225Ac-DOTA-daratumumab, 600 nCi *)
  , ListPlot[Dara600Data, Joined → True, PlotStyle → Lighter[Lighter[Lighter[Lighter[Dara600Color]]]]],
  ListPlot[Dara600Err, Joined → True, PlotStyle → Directive[Thick, Dara600Color], PlotLegends → {"Dara 600 nCi"}]
  , ImageSize → 500]
```

Out[ ]=

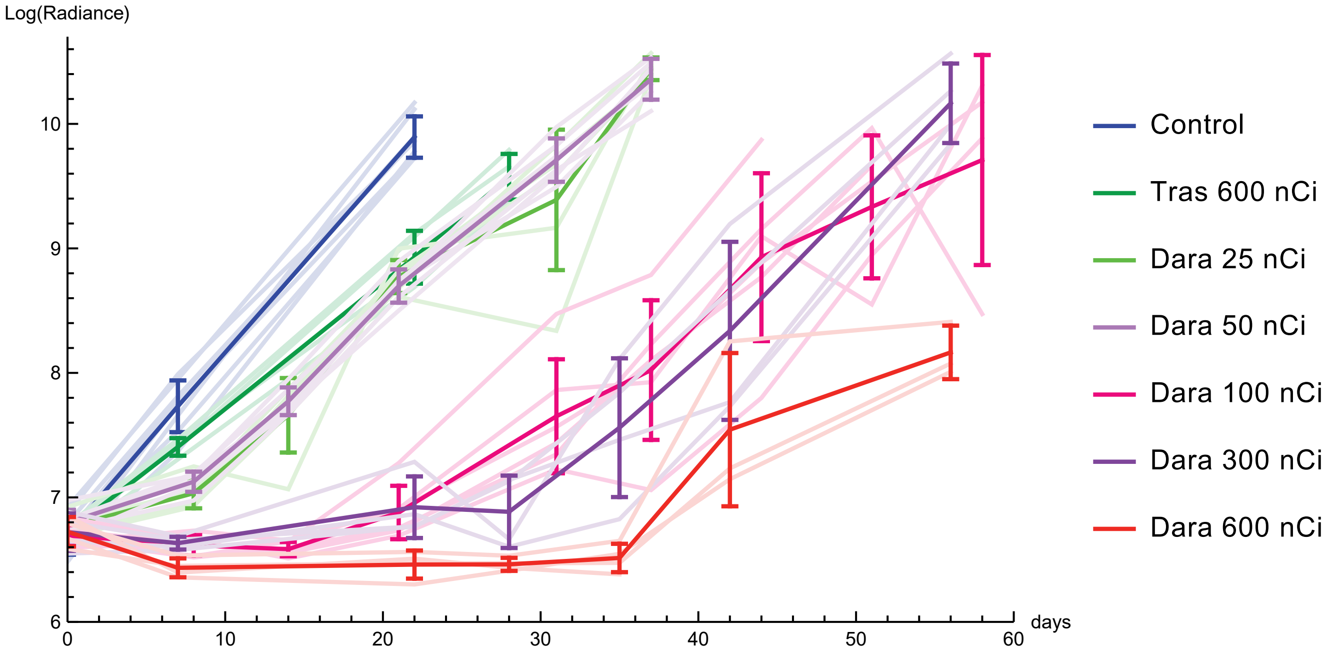


```
(* Supplementary S.1.3
Fitting of experimental data and estimation of corresponding parameters *)

(* S.1.3.3 Cancer cells proliferation rate *)
```

In[ ]:=

```
(* Fitting cancer cells proliferation rate *)

(* Setting boundaries and step for variation*)
rhomin = 0.2; rhomax = 0.4; rhostep = 0.01;

(* Table which will contain the deviations of simulations from the experimental data *)
result = Array[f, {Round[(rhomax - rhomin) / rhostep, 1] + 2, 2}];
result[[1, 1]] = "rho";
result[[1, 2]] = "SSD"; (* sum of squared deviations *)

N0 = 10^ControlMean[[1, 2]]; (* Initial "number of cancer cells"
```

```
(coefficient of proportionality between them and radiance can be set to 1 for now, since it does not affect the dynamics) *)

ii = 2;
For[rho = rhomin, rho ≤ rhomax, rho = rho + rhostep,

  NotebookDelete[pr]; (* To see the code running *)
  pr = PrintTemporary["Current rho is " <> ToString[rho] <> "; max value is " <> ToString[rhomax]];

  (* The day of the last experimental value *)
  tEnd = ControlData[[1, All, 1]][[Length[daysControl]]];
  Clear[NN];

  (* Straightforward solution of highly reduced model, which is now boiled down to one exponential equation *)
  sol = NDSolve[{
      (* Initial conditions *)
      NN[0] == N0,
      (* We need only the equation for the number of viable cancer cells, there are no damaged cells now *)
      NN'[t] == rho * NN[t]}
     , {NN}, {t, 0, tEnd}];
  NN = First[NN /. sol]; (* Save solution *)

  (* Save sum of squared deviations *)
  result[[ii, 1]] = rho;
  result[[ii, 2]] = Sum[(Log10[NN[ControlMean[[kk, 1]]]] - ControlMean[[kk, 2]])^2, {kk, 1, Length[daysControl]}];

  ii++;
 ];

(* Find optimal rho *)
DevMin = Min[result[[All, 2]][[2 ;;]]];
PosOpt = Position[result[[All, 2]], DevMin][[1, 1]];
```

```
rhoOpt = result⟦PosOpt, 1⟧;

IS = 300;
(* Visualize results *)
PicVar = ListLogPlot[result, AxesLabel → {"ρ", "SSD"}, PlotLabel → "Optimal ρ is " <> ToString[rhoOpt], ImageSize → IS];

(* Run simulation with it *)
Clear[NN];
sol = NDSolve[{
      NN[0] == N0,
      NN'[t] == rhoOpt * NN[t]}
    , {NN}, {t, 0, tEnd}];
NN = First[NN /. sol];

(* Plot simulation with optimal rho along with experimental data *)
PicOptSim =
   Show[Plot[{{Log10[NN[t]]}}, {t, 0, 1.01 * tEnd}, Filling → {1 → Axis}, PlotStyle → {Directive[Lighter[Gray], Thickness[0.002]]},
      PlotRange → {0.95 * Log10[N0], 1.02 * NMaximize[{Log10[NN[t]], t > 0, t < tEnd}, t]⟦1⟧}, AxesLabel → {"days", "Log(Radiance)"},
      (*PlotLegends→Placed[{"N"},Above],*) ImageSize → 280], ListPlot[ControlErr], ImageSize → IS];

(* Supplementary Figure S.2 *)

GraphicsGrid[{{PicVar, PicOptSim}}, ImageSize → {2.2 * IS, IS}]
```

Out[ ]=

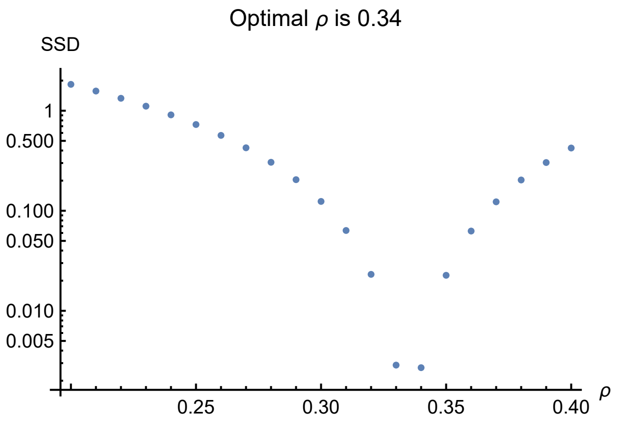

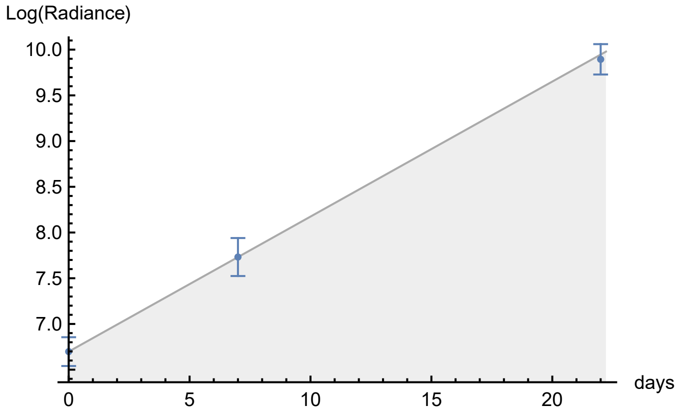

(* S.1.3.4 Cancer cells radiosensitivity to unanchored nuclides *)

In[ ]:=

```
(* Fitting cancer cells radiosensitivity to unanchored nuclides under basic values of other parameters *)

(* Setting parameters *)
rho = 0.34; (* we've just estimated it *)
lambda = 0.07; (* decay rate of 225Ac *)
kappac = 0.1 ; (* antibody clearance rate *)
V = 1.; (* volume of drug distribution *)
omega = 0.05; (* damaged cells outflow rate *)
DA1 = 0.0456; (* injected amount of radionuclides *)
t1 = 1; (* moment of their injection *)

(* As the injection takes place not at t=0, we cannot set drug concentration as initial value.
    Let's build close-to-delta function of drug injection
   (it is a smooth function, that will allow to avoid numerical difficulties) *)
```

```
kk = 250; (* Technical parameter, the larger it is, the sharper the function *)
AInjFunc[t_] := DA1/2 * kk * Sech[kk * (t - t1)]^2 / V;
(* To visualize the function: Plot[AInjFunc[t],{t,0,2},PlotRange→{0,1}]
    To check the total amount of injected drug: V*NIntegrate[AInjFunc[t],{t,0,2}] *)

(* Setting boundaries and step of variation *)
kfamin = 10; kfamax = 300; kfastep = 10;

(* Table which will contain the deviations of simulations from the experimental data *)
result = Array[f, {Round[(kfamax - kfamin) / kfastep, 1] + 2, 2}];
result[[1, 1]] = "kfa";
result[[1, 2]] = "SSD"; (* sum of squared deviations *)

N0 = 10^TrasMean[[1, 2]]; (* Initial "number of cancer cells"
  (coefficient of proportionality between them and radiance can be set to 1, since it does not affect the dynamics) *)

ii = 2;
For[kfa = kfamin, kfa ≤ kfamax, kfa = kfa + kfastep,

   NotebookDelete[pr]; (* To see the code running *)
   pr = PrintTemporary["Current kfa is " <> ToString[kfa] <> "; max value is " <> ToString[kfamax]];

   (* The day of the last experimental value *)
   tEnd = TrasData[[1, All, 1]][[Length[daysTras]]];

   Clear[a, NN, DD];

   (* Straightforward solution of the reduced model *)
   sol = NDSolve[{
```

```
            (* Initial conditions *)
            a[0] == 0, NN[0] == N0, DD[0] == 0,
            (* Active antibodies *)
            a'[t] == AInjFunc[t] (*injection*) - lambda*a[t] (*decay*) - kappac*a[t] (*clearance*),
            (* Viable cells *) NN'[t] == rho*NN[t] (*proliferation*) - kfa*lambda*a[t]*NN[t] (*damage*),
            (* Damaged cells *) DD'[t] == kfa*lambda*a[t]*NN[t] (*damage*) - omega*DD[t] (*death*)
          }
          , {a, NN, DD}, {t, 0, tEnd}];
      a = First[a /. sol]; NN = First[NN /. sol]; DD = First[DD /. sol]; (* Save solution *)

      (* Save sum of squared deviations *)
      result[[ii, 1]] = kfa;
      result[[ii, 2]] = Sum[(Log10[NN[TrasMean[[kk, 1]]] + DD[TrasMean[[kk, 1]]]] - TrasMean[[kk, 2]])^2, {kk, 1, Length[daysTras]}];

      ii++;
    ];

  (* Find optimal kfa *)
  DevMin = Min[result[[All, 2]][[2 ;;]]];
  PosOpt = Position[result[[All, 2]], DevMin][[1, 1]];
  kfaOpt = result[[PosOpt, 1]];

  IS = 300;
  (* Visualize results *)
  PicVar = ListLogPlot[result, AxesLabel → {"kfα", "SSD"}, PlotLabel → "Optimal kfα is " <> ToString[kfaOpt], ImageSize → IS];

  (* Run simulation with it *)
  Clear[a, NN, DD];
  sol = NDSolve[{
          (* Initial conditions *)
          a[0] == 0, NN[0] == N0, DD[0] == 0,
```

```
        (* Active antibodies *)
        a'[t] == AInjFunc[t] (*injection*) - lambda*a[t] (*decay*) - kappac*a[t] (*clearance*),
        (* Viable cells *) NN'[t] == rho*NN[t] (*proliferation*) - kfaOpt*lambda*a[t] *NN[t] (*damage*),
        DD'[t] == kfaOpt*lambda*a[t] *NN[t] (*damage*) - omega*DD[t] (*death*)
      }
     , {a, NN, DD}, {t, 0, tEnd}];
 a = First[a /. sol]; NN = First[NN /. sol]; DD = First[DD /. sol];(* Save solution *)

(* Plot simulation with optimal rho along with experimental data *)
PicOptSim = Show[Plot[{{Log10[NN[t]], Log10[NN[t] + DD[t]]}}, {t, 0, 1.01*tEnd}, Filling → {1 → Axis, 2 → Axis},
     PlotStyle → {Directive[Lighter[Gray], Thickness[0.002]], Directive[Darker[Gray], Thickness[0.002]]}, PlotRange →
       {0.95*Log10[N0], 1.02*NMaximize[{Log10[NN[t] + DD[t]], t > 0, t < tEnd}, t]〚1〛}, AxesLabel → {"days", "Log(Radiance)"},
     PlotLegends → Placed[{"N", "N+D"}, Above], ImageSize → 280], ListPlot[TrasErr], ImageSize → IS];

(* Supplementary Figure S.3 *)

GraphicsGrid[{{PicVar, PicOptSim}}, ImageSize → {2.2*IS, IS}]
```

Out[ ]=

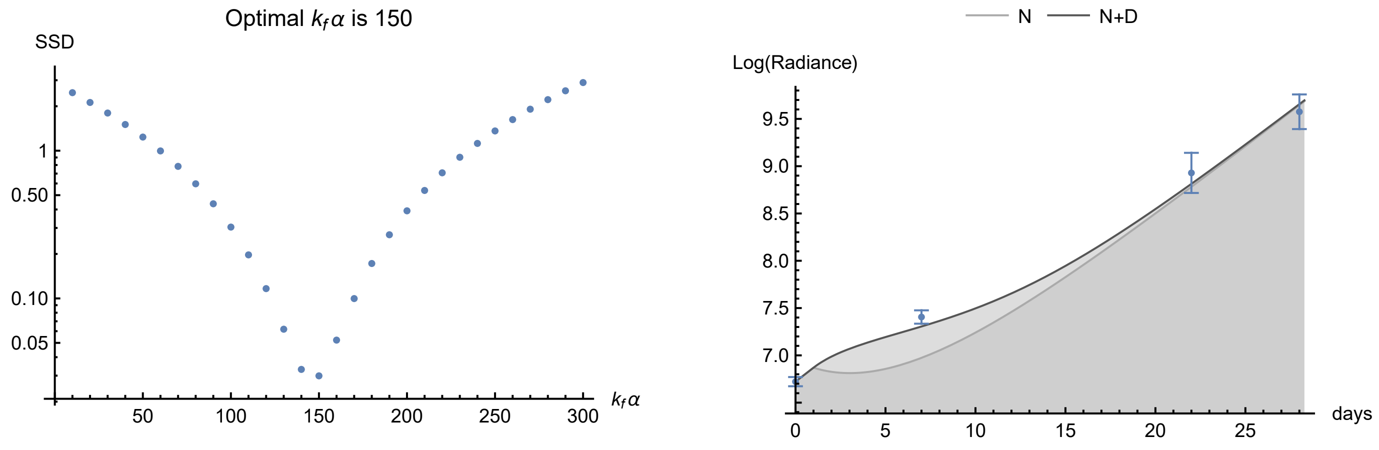

In[ ]:=

```
(* Fitting cancer cells radiosensitivity to unanchored nuclides under variation of kappac, V, omega *)

(* Setting parameters *)
rho = 0.34; (* we've just estimated it *)
lambda = 0.07; (* -225Ac decay rate *)
DA1 = 0.0456; (* injected amount of radionuclides *)
t1 = 1; (* moment of their injection *)

kk = 250;
tEnd = TrasData[[1, All, 1]][[Length[daysTras]]];

(* Setting boundaries and step of variation *)
kappacmin = 0.04; kappacmax = 0.28; kappacstep = 0.02;
Vmin = 0.75; Vmax = 1.5; Vstep = 0.125;
omegamin = 0.0034; omegamax = 0.34; omegak = 10^(1/8);
```

```
(* Table which will contain optimal kfa for each set (kappac, V, omega) *)
outcome = Array[f, {Round[(Round[(kappacmax - kappacmin) / kappacstep, 1] + 1) *
        ((Vmax - Vmin) / Vstep + 1) * (Log[omegak, omegamax / omegamin] + 1) + 1], 5}];
outcome[[1, 1]] = "kappac";
outcome[[1, 2]] = "V";
outcome[[1, 3]] = "omega";
outcome[[1, 4]] = "kfa";
outcome[[1, 5]] = "SSD";

jj = 2;
For[kappac = kappacmin, kappac ≤ kappacmax, kappac = kappac + kappacstep,
  For[V = Vmin, V ≤ Vmax, V = V + Vstep,
   AInjFunc[t_] := DA1/2 * kk * Sech[kk * (t - t1)]^2 / V;
   For[omega = omegamin, omega ≤ omegamax, omega = N[omega * omegak],

    (* Setting boundaries and step of variation of kfa *)
    kfamin = 10; kfamax = 300; kfastep = 10;

    (* Table which will contain the deviations of simulations from the experimental data *)
    result = Array[f, {Round[(kfamax - kfamin) / kfastep, 1] + 2, 2}];
    result[[1, 1]] = "kfa";
    result[[1, 2]] = "SSD";

    N0 = 10^TrasMean[[1, 2]];

    NotebookDelete[pr]; (* To see the code running, it's better not to ouput every kfa -- it takes a lot of time *)
    pr = PrintTemporary["Current kappac is " <> ToString[kappac] <> "; max value is " <> ToString[kappacmax] <> "
" <> "Current V is " <> ToString[V] <> "; max value is " <> ToString[Vmax] <> "
" <> "Current omega is " <> ToString[omega] <> "; max value is " <> ToString[omegamax] (*<>"
```

```
"<>"Current kfa is "<>ToString[kfa]<>"; max value is "<>ToString[kfamax] *) ];


    ii = 2;
    For[kfa = kfamin, kfa ≤ kfamax, kfa = kfa + kfastep,

      Clear[a, NN, DD];

      (* Straightforward solution of the reduced model *)
      sol = NDSolve[{
            (* Initial conditions *)
            a[0] == 0, NN[0] == N0, DD[0] == 0,
            (* Active antibodies *)
            a'[t] == AInjFunc[t] (*injection*) - lambda * a[t] (*decay*) - kappac * a[t] (*clearance*),
            (* Viable cells *) NN'[t] == rho * NN[t] (*proliferation*) - kfa * lambda * a[t] * NN[t] (*damage*),
            (* Damaged cells *) DD'[t] == kfa * lambda * a[t] * NN[t] (*damage*) - omega * DD[t] (*death*)
          }
        , {a, NN, DD}, {t, 0, tEnd}];
      a = First[a /. sol]; NN = First[NN /. sol]; DD = First[DD /. sol]; (* Save solution *)

      (* Save sum of squared deviations *)
      result⟦ii, 1⟧ = kfa;
      result⟦ii, 2⟧ = Sum[ (Log10[NN[TrasMean⟦kk, 1⟧] + DD[TrasMean⟦kk, 1⟧]] - TrasMean⟦kk, 2⟧) ^2, {kk, 1, Length[daysTras]}];

      ii++;
    ];

    (* Find optimal kfa *)
    DevMin = Min[result⟦All, 2⟧⟦2 ;;⟧];
    PosOpt = Position[result⟦All, 2⟧, DevMin]⟦1, 1⟧;
    kfaOpt = result⟦PosOpt, 1⟧;
```

```
        outcome[[jj, 1]] = kappac;
        outcome[[jj, 2]] = V;
        outcome[[jj, 3]] = omega;
        outcome[[jj, 4]] = kfaOpt;
        outcome[[jj, 5]] = DevMin;

        jj++;]]];
```

(* "Under variation of κ_c, V and ω the most probable optimal values of $k_f\alpha$ lie in the range [80,160]" *)

```
(* Let's now order the sets by ascending deviation and plot the ordered set of kfa
  the most probable values will be on the left side *)
outcomeSort = MatrixForm[Sort[outcome, #1[[5]] < #2[[5]] &]];
ListPlot[outcomeSort[[1, All, 4]][[2 ;; 600]]]
```

Out[ ]=

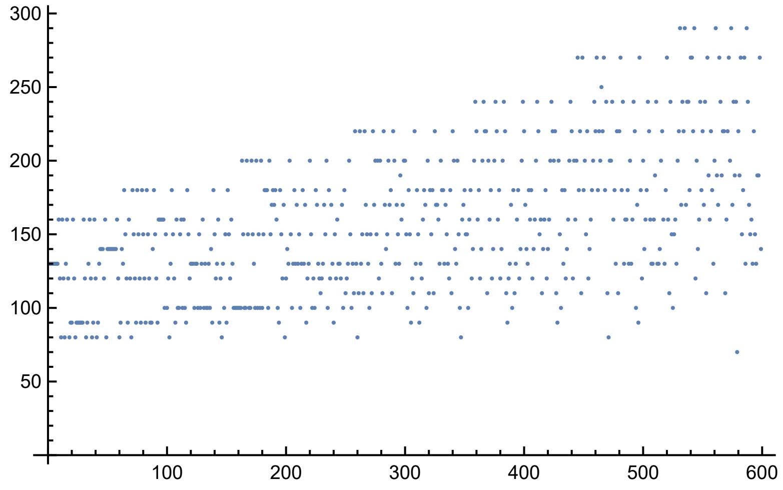

(* S.1.3.5 Cancer cells radiosensitivity and damaged cells death rate *)

In[ ]:=

(* The scripts for the solution of full system and finding optimal parameters are hidden here and have to be initialized *)

In[ ]:=

(* Solution of full system *)

```
nCpm = 0.000076; (* factor of conversion from nCi to pmol *)

FullSystemSolution[] := (Clear[a, b, NN, DD, p, fFN, fAN, fFD, fAD];

  sol = NDSolve[{
      (* Initial conditions *)
      a[0] == 0, b[0] == 0, NN[0] == N0, DD[0] == 0.0000000000000001
      (*to avoid division by zero*), p[0] == 0, fFN[0] == 1, fAN[0] == 0, fFD[0] == 1, fAD[0] == 0,
      (* Active antibodies *)
      a'[t] == AInjFunc[t] (*injection*) - lambda * a[t] (*decay*) -
        kon * gamma/V * (fFN[t] * NN[t] + fFD[t] * DD[t]) * a[t] (*binding*) - kappac * a[t] (*clearance*),
      (* Inert antibodies *)
      b'[t] == eta * AInjFunc[t] (*injection*) + lambda * a[t] (*decay of a*) -
        kon * gamma/V * (fFN[t] * NN[t] + fFD[t] * DD[t]) * b[t] (*binding*) - kappac * b[t] (*clearance*),
      (* Viable cells *) NN'[t] == rho * NN[t] (*proliferation*) - RD[NN[t], DD[t], fAN[t], fAD[t], a[t], p[t]] * NN[t] (*damage*),
      (* Damaged cells *) DD'[t] == RD[NN[t], DD[t], fAN[t], fAD[t], a[t], p[t]] * NN[t] (*damage*) - omega * DD[t] (*death*),
      (* Active fragments *)
      p'[t] == omega * DD[t] * (gamma * fAD[t])/V (*release*) - lambda * p[t] (*decay*) - kappap * p[t] (*clearance*),
      (* Free receptors on viable cells *) fFN'[t] == (1 - fFN[t]) * rho - kon * (a[t] + b[t]) * fFN[t],
      (* Active receptors on viable cells *) fAN'[t] == kon * a[t] * fFN[t] - (lambda + rho) * fAN[t],
      (* Free receptors on damaged cells *)
      fFD'[t] == (fFN[t] - fFD[t]) * RD[NN[t], DD[t], fAN[t], fAD[t], a[t], p[t]] * NN[t]/DD[t] - kon * (a[t] + b[t]) * fFD[t],
      (* Active receptors on damaged cells *)
      fAD'[t] == (fAN[t] - fAD[t]) * RD[NN[t], DD[t], fAN[t], fAD[t], a[t], p[t]] * NN[t]/DD[t] + kon * a[t] * fFD[t] - lambda * fAD[t]}
    , {a, b, NN, DD, p, fFN, fAN, fFD, fAD}, {t, 0, tEnd}];
```

```
a = First[a /. sol]; b = First[b /. sol]; NN = First[NN /. sol]; DD = First[DD /. sol];
p = First[p /. sol]; fFN = First[fFN /. sol]; fAN = First[fAN /. sol]; fFD = First[fFD /. sol]; fAD = First[fAD /. sol];)

(* Optimizing alpha and omega simultaneously *)
AlphaOmegaOptimize[DnCi_] (* injected dose in nCi *) :=
 (If[DnCi == 25, DaraMean = Dara25Mean; DaraData = Dara25Data; daysDara = daysDara25; DaraErr = Dara25Err];
  If[DnCi == 50, DaraMean = Dara50Mean; DaraData = Dara50Data; daysDara = daysDara50; DaraErr = Dara50Err];
  If[DnCi == 100, DaraMean = Dara100Mean; DaraData = Dara100Data; daysDara = daysDara100; DaraErr = Dara100Err];
  If[DnCi == 300, DaraMean = Dara300Mean; DaraData = Dara300Data; daysDara = daysDara300; DaraErr = Dara300Err];
  If[DnCi == 600, DaraMean = Dara600Mean; DaraData = Dara600Data; daysDara = daysDara600; DaraErr = Dara600Err];

DA1 = nCpm * DnCi; (* injected amount of radionuclides *)
  t1 = 1; (* moment of their injection *)
  eta = Round[202.7 / DA1 - 1]; (* coefficient of drug impurity *)

  (* the coeffient of transition of cell number to radiance *)
  kNR = 10^DaraMean[[1, 2]] / N0;

  (* Radiation damage function *)
  RD[NN_, DD_, fAN_, fAD_, a_, p_] :=
   ks * alpha * (lambda * gamma * fAN)/nu (*self-dose*) + (1 - ks) * alpha * (lambda * gamma * (fAN * NN + fAD * DD))/(nu * (NN + DD))
     (*cross-fire*) + kfa * lambda * (a + p) (*dose from unanchored nuclides*);


  (* Close-to-delta function of drug injection (it is a smooth function, that will allow to avoid numerical difficulties) *)
  kk = 250; (* Technical parameter, the larger it is, the sharper the function *)
  AInjFunc[t_] := DA1/2 * kk * Sech[kk * (t - t1)]^2 / V;
```

```
(* Setting boundaries and step of variation *)
alphamin = 300; alphamax = 800; alphastep = 10;
omegamin = 0.0034; omegamax = 0.34; omegak = 10^(1/8);

(* Table which will contain the deviations of simulations from the experimental data *)
result = Array[f, {Round[((alphamax - alphamin) / alphastep + 1) * (Log[omegak, omegamax / omegamin] + 1) + 1], 3}];
result[[1, 1]] = "alpha";
result[[1, 2]] = "omega";
result[[1, 3]] = "SSD"; (* sum of squared deviations *)

ii = 2;
For[alpha = alphamin, alpha ≤ alphamax, alpha = alpha + alphastep,
  For[omega = omegamin, omega ≤ omegamax, omega = N[omega * omegak],

    NotebookDelete[pr]; (* To see the code running *)
    pr = PrintTemporary["Current alpha is " <> ToString[alpha] <> "; max value is " <> ToString[alphamax] <> "
Current omega is " <> ToString[omega] <> "; max value is " <> ToString[omegamax]];

    (* The day of the last experimental value *)
    tEnd = DaraData[[1, All, 1]][[Length[daysDara]]];

    (* Straightforward solution of the reduced model *)
    FullSystemSolution[];

    (* Save sum of squared deviations *)
    result[[ii, 1]] = alpha;
    result[[ii, 2]] = omega;
    result[[ii, 3]] =
      Log10[Sum[(Log10[kNR * (NN[DaraMean[[kk, 1]]] + DD[DaraMean[[kk, 1]]])] - DaraMean[[kk, 2]])^2, {kk, 1, Length[daysDara]}]];
```

```
      ii++;
    ];];

(* Find optimal alpha and omega *)
DevMin = Min[result[[All, 3]][[2 ;;]]];
PosOpt = Position[result[[All, 3]], DevMin][[1, 1]];

(* Interpolate optimal alpha and omega*)
FitInt = Interpolation[Thread[{result[[2 ;;, {1, 2}]], result[[2 ;;, 3]]}]];
OptVal = NMinimize[{FitInt[al, om], alphamin ≤ al ≤ alphamax, omegamin ≤ om ≤ omegamax}, {al, om}];
alphaOpt = al /. OptVal[[2]]; omegaOpt = om /. OptVal[[2]];

IS = 300;
(* Visualize results *)
PicVar =
  Show[ListContourPlot[result], ListPlot[{{alphaOpt, omegaOpt}}, PlotStyle → Darker[Red]], AxesLabel → {"α", "ω"}, PlotLabel →
     "Optimal α is " <> ToString[Round[alphaOpt, 1]] <> "; Optimal ω is " <> ToString[Round[omegaOpt, 0.001]], ImageSize → IS / 1.4];

(* Run simulation with it *)
alpha = alphaOpt;
omega = omegaOpt;
FullSystemSolution[];

(* Plot simulation with optimal rho along with experimental data *)
PicOptSim = Show[Plot[{{Log10[kNR * NN[t]], Log10[kNR * (NN[t] + DD[t])]}}, {t, 0, 1.01 * tEnd}, Filling → {1 → Axis, 2 → Axis},
     PlotStyle → {Directive[Lighter[Gray], Thickness[0.002]], Directive[Darker[Gray], Thickness[0.002]]},
     PlotRange → {0.95 * NMinimize[{Log10[kNR * NN[t]], 0 ≤ t ≤ tEnd}, t][[1]],
        1.02 * NMaximize[{Log10[kNR * (NN[t] + DD[t])], t > 0, t < tEnd}, t][[1]]}, AxesLabel → {"days", "Log(Radiance)"},
     PlotLegends → Placed[{"N", "N+D"}, Above], ImageSize → 280], ListPlot[DaraErr], ImageSize → IS];
```

In[ ]:=

```
(* Fitting cancer cells radiosensitivity and damaged cells death rate -- 300 nCi *)

(* Setting parameters *)
rho = 0.34; (* cancer cells proliferation rate -- already estimated *)
lambda = 0.07; (* decay rate of 225Ac *)
kon = 11.15; (* daratumumab-CD38 association rate *)
kappac = 0.1 ; (* antibody clearance rate (0.04-0.28) *)
kappap = 1; (* antibody fragments clearance rate (0.4-4.) *)
gamma = 2.1; (* number of receptors on 10^7 cancer cells (0.13-10) *)
V = 1.; (* volume of drug distribution (0.75-1.5) *)
nu = 0.015; (* volume of lesion with 10^7 cancer cells *)
ks = 0.3; (* relative significance of self-dose (0.1-0.5) *)
kfa = 150; (* relative sensitivity to unanchored nuclides decays (80-160) *)
N0 = 3.; (* initial number of cancer cells, x10^7 (1-10) *)

AlphaOmegaOptimize[300];

(* Supplementary Figure S.4, left part *)

PicVar
```

Out[ ]=

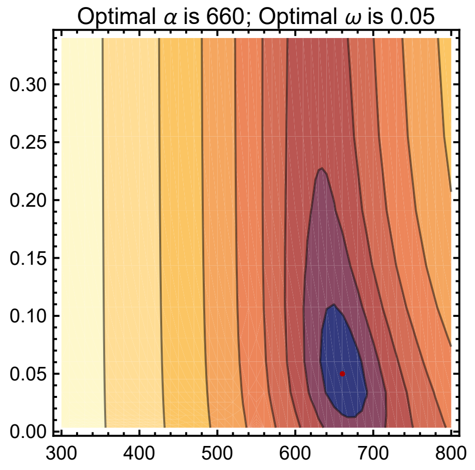
Optimal α is 660; Optimal ω is 0.05
0.30
0.25
0.20
0.15
0.10
0.05
0.00
300
400
500
600
700
800

In[ ]:=

```
(* Fitting cancer cells radiosensitivity and damaged cells death rate -- 600 nCi *)

(* Setting parameters *)
rho = 0.34; (* cancer cells proliferation rate -- already estimated *)
lambda = 0.07; (* decay rate of 225Ac *)
kon = 11.15; (* daratumumab-CD38 association rate *)
kappac = 0.1 ; (* antibody clearance rate (0.04-0.28) *)
kappap = 1; (* antibody fragments clearance rate (0.4-4.) *)
gamma = 2.1; (* number of receptors on 10^7 cancer cells (0.13-10) *)
V = 1.; (* volume of drug distribution (0.75-1.5) *)
nu = 0.015; (* volume of lesion with 10^7 cancer cells *)
ks = 0.3; (* relative significance of self-dose (0.1-0.5) *)
kfa = 150; (* relative sensitivity to unanchored nuclides decays (80-160) *)
N0 = 3.; (* initial number of cancer cells, x10^7 (1-10) *)

AlphaOmegaOptimize[600];

(* Supplementary Figure S.4, right part *)

PicVar
```

Out[ ]=

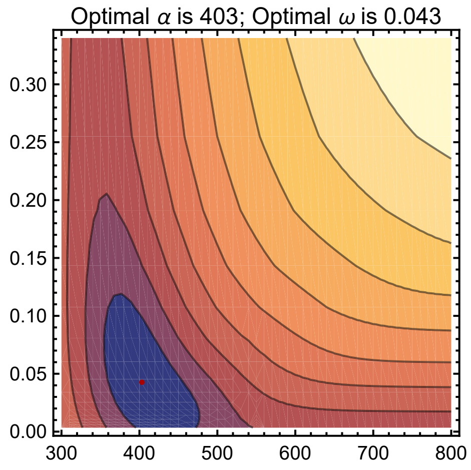

In[ ]:=

```
(* The script for optimizing alpha, once the value for ω is chosen is hidden here ans has to be initialized *)
```

In[ ]:=

```
AlphaOptimize[DnCi_] (* argument is injected dose in nCi *) :=
 (If[DnCi == 25, DaraMean = Dara25Mean; DaraData = Dara25Data; daysDara = daysDara25; DaraErr = Dara25Err];
  If[DnCi == 50, DaraMean = Dara50Mean; DaraData = Dara50Data; daysDara = daysDara50; DaraErr = Dara50Err];
  If[DnCi == 100, DaraMean = Dara100Mean; DaraData = Dara100Data; daysDara = daysDara100; DaraErr = Dara100Err];
  If[DnCi == 300, DaraMean = Dara300Mean; DaraData = Dara300Data; daysDara = daysDara300; DaraErr = Dara300Err];
  If[DnCi == 600, DaraMean = Dara600Mean; DaraData = Dara600Data; daysDara = daysDara600; DaraErr = Dara600Err];

  DA1 = nCpm * DnCi; (* injected amount of radionuclides *)
  t1 = 1; (* moment of their injection *)
  eta = Round[202.7 / DA1 - 1]; (* coefficient of drug impurity *)

  kNR = 10^DaraMean[[1, 2]] / N0;

  (* Radiation damage function *)
  RD[NN_, DD_, fAN_, fAD_, a_, p_] :=
```

```
ks * alpha * (lambda * gamma * fAN)/nu (*self-dose*) + (1 - ks) * alpha * (lambda * gamma * (fAN * NN + fAD * DD))/(nu * (NN + DD))
   (*cross-fire*) + kfa * lambda * (a + p) (*dose from unanchored nuclides*);

(* Close-to-delta function of drug injection *)
If[VarParFlg == 0,
  kk = 250, (* For all except fitting alpha under varied other parameters *)
  kk = 100]; (* For fitting alpha under varied other parameters -- there could be some bizzare numerical errors otherwise *)
AInjFunc[t_] := DA1/2 * kk * Sech[kk * (t - t1)]^2/V;

(* Setting boundaries and step of variation *)
If[VarParFlg == 0,
  alphamin = 300; alphamax = 5000; alphastep = 25, (* For all except fitting alpha under varied other parameters *)
  alphamin = 50; alphamax = 100 000; alphak = 10^(1/64) (* For fitting alpha under varied other parameters *) ];

(* Table which will contain the deviations of simulations from the experimental data *)
If[VarParFlg == 0,
  result = Array[f, {Round[((alphamax - alphamin) / alphastep + 1) + 1], 2}],
   (* For all except fitting alpha under varied other parameters *)
  result = Array[f, {Round[(Log[alphak, alphamax / alphamin] + 1) + 1], 2}] (* For fitting alpha under varied other parameters *) ];
result[[1, 1]] = "alpha";
result[[1, 2]] = "SSD"; (* sum of squared deviations *)

ii = 2;

If[VarParFlg == 0,
  Nextalpha[alpha_] := alpha + alphastep, (* For all except fitting alpha under varied other parameters *)
  Nextalpha[alpha_] := N[alpha * alphak] (* For fitting alpha under varied other parameters *)
];
```

```
For[alpha = alphamin, alpha ≤ alphamax, alpha = Nextalpha[alpha],

  (* The day of the last experimental value *)
  tEnd = DaraData[[1, All, 1]][[Length[daysDara]]];

  (* Straightforward solution of reduced model *)
  FullSystemSolution[];

  (* Save sum of squared deviations *)
  result[[ii, 1]] = alpha;
  result[[ii, 2]] =
    Sum[(Log10[kNR * (NN[DaraMean[[kk, 1]]] + DD[DaraMean[[kk, 1]]])] - DaraMean[[kk, 2]])^2, {kk, 1, Length[daysDara]}];

  ii++;
];

(* Find optimal alpha *)
DevMin = Min[result[[All, 2]][[2 ;;]]];
PosOpt = Position[result[[All, 2]], DevMin][[1, 1]];

(* Interpolate optimal alpha *)
FitInt = Interpolation[result[[2 ;;, {1, 2}]]];
OptVal = NMinimize[{FitInt[al], alphamin ≤ al ≤ alphamax}, al];
alphaOpt = al /. OptVal[[2]];

IS = 300; (* Figure size *)
(* Visualize results *)
PicVar = ListLogPlot[result, AxesLabel → {"α", "SSD"},
    PlotLabel → "DnCi=" <> ToString[DnCi] <> "; Optimal α is " <> ToString[Round[alphaOpt, 1]], ImageSize → IS];

(* Run simulation with it *)
```

```
alpha = alphaOpt;
FullSystemSolution[];

(* Plot simulation with optimal rho along with experimental data *)
PicOptSim = Show[Plot[{{Log10[kNR*NN[t]], Log10[kNR*(NN[t] + DD[t])]}}, {t, 0, 1.01*tEnd}, Filling → {1 → Axis, 2 → Axis},
    PlotStyle → {Directive[Lighter[Gray], Thickness[0.002]], Directive[Darker[Gray], Thickness[0.002]]},
    PlotRange → {4, 11 (*0.95*NMinimize[{Log10[kNR*NN[t]],0≤t≤tEnd},t][[1]],
      1.02*NMaximize[{Log10[kNR*(NN[t]+DD[t])],t>0,t<tEnd},t][[1]]*)}, AxesLabel → {"days", "Log(Radiance)"},
    PlotLabel → "Dara " <> ToString[DnCi] <> " nCi", ImageSize → 280], ListPlot[DaraErr], ImageSize → IS];)
```

In[ ]:=

```
(* Fitting cancer cells radiosensitivity under different doses *)

rho = 0.34; (* cancer cells proliferation rate -- already estimated *)
omega = 0.05; (* damaged cells death rate -- already estimated *)
lambda = 0.07; (* decay rate of 225Ac *)
kon = 11.15; (* daratumumab-CD38 association rate *)
kappac = 0.1; (* antibody clearance rate (0.04-0.28) *)
kappap = 1; (* antibody fragments clearance rate (0.4-4.) *)
gamma = 2.1; (* number of receptors on 10^7 cancer cells (0.13-10) *)
V = 1.; (* volume of drug distribution (0.75-1.5) *)
nu = 0.015; (* volume of lesion with 10^7 cancer cells *)
ks = 0.3; (* relative significance of self-dose (0.1-0.5) *)
kfa = 150; (* relative sensitivity to unanchored nuclides decays (80-160) *)
N0 = 3.; (* initial number of cancer cells, x10^7 (1-10) *)

VarParFlg = 0;

(* Supplementary Figure S.5 *)

AlphaOptimize[25];
```

```
PicAlpha25 = GraphicsGrid[{{PicVar, PicOptSim}}, ImageSize → {2.2 * IS, IS}]
alphaOpt25 = alphaOpt;

AlphaOptimize[50];
PicAlpha50 = GraphicsGrid[{{PicVar, PicOptSim}}, ImageSize → {2.2 * IS, IS}]
alphaOpt50 = alphaOpt;

AlphaOptimize[100];
PicAlpha100 = GraphicsGrid[{{PicVar, PicOptSim}}, ImageSize → {2.2 * IS, IS}]
alphaOpt100 = alphaOpt;

AlphaOptimize[300];
PicAlpha300 = GraphicsGrid[{{PicVar, PicOptSim}}, ImageSize → {2.2 * IS, IS}]
alphaOpt300 = alphaOpt;

AlphaOptimize[600];
PicAlpha600 = GraphicsGrid[{{PicVar, PicOptSim}}, ImageSize → {2.2 * IS, IS}]
alphaOpt600 = alphaOpt;

PicAlpha = ListPlot[{{25, alphaOpt25}, {50, alphaOpt50}, {100, alphaOpt100}, {300, alphaOpt300}, {600, alphaOpt600}},
   AxesLabel → {"Dose, nCi", "Optimal α"}, ImageSize → 400]
```

Out[ ]=

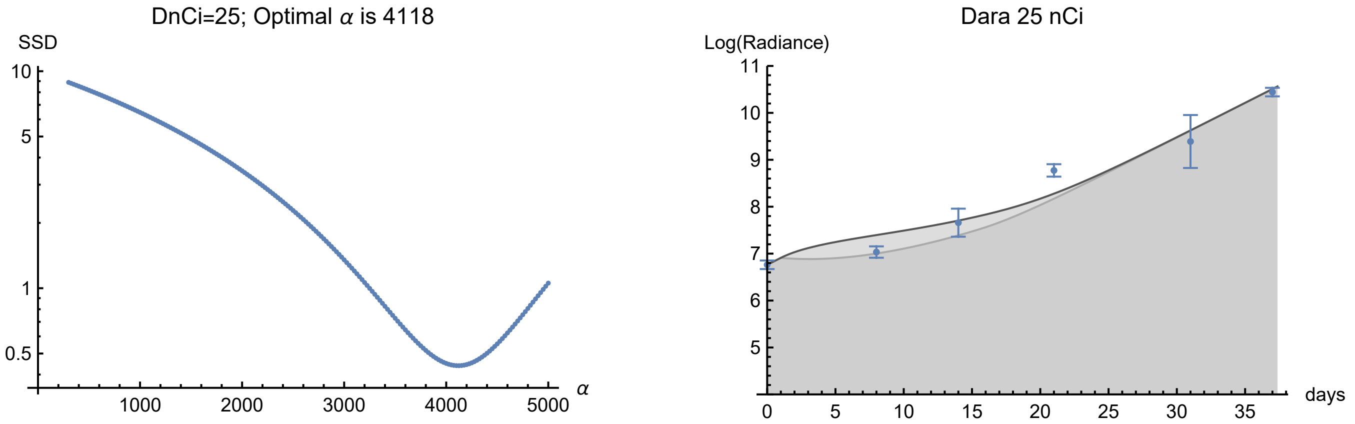
DnCi=25; Optimal α is 4118
SSD
α
1000 2000 3000 4000 5000
Dara 25 nCi
Log(Radiance)
days
0 5 10 15 20 25 30 35

Out[ ]=

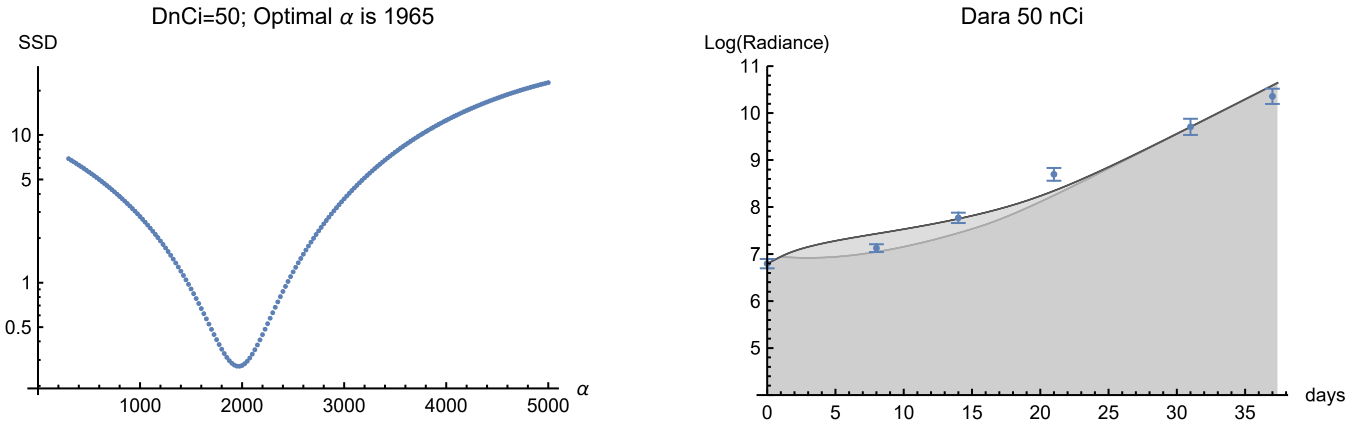
DnCi=50; Optimal α is 1965
SSD
α
1000 2000 3000 4000 5000
Dara 50 nCi
Log(Radiance)
days
0 5 10 15 20 25 30 35

Out[ ]=

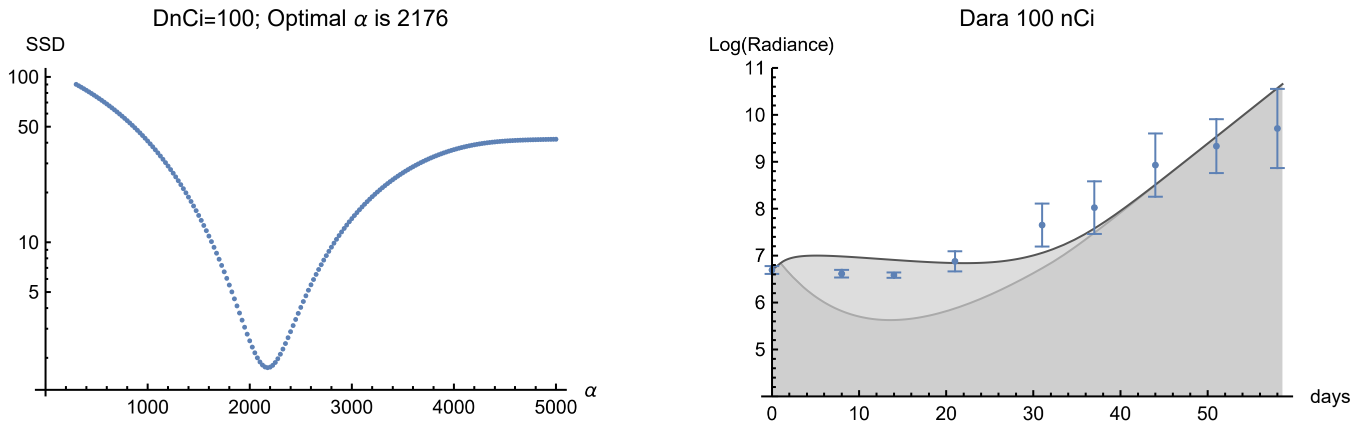
DnCi=100; Optimal α is 2176
SSD
100
50
10
5
1000
2000
3000
4000
5000
α
Dara 100 nCi
Log(Radiance)
11
10
9
8
7
6
5
0
10
20
30
40
50
days

Out[ ]=

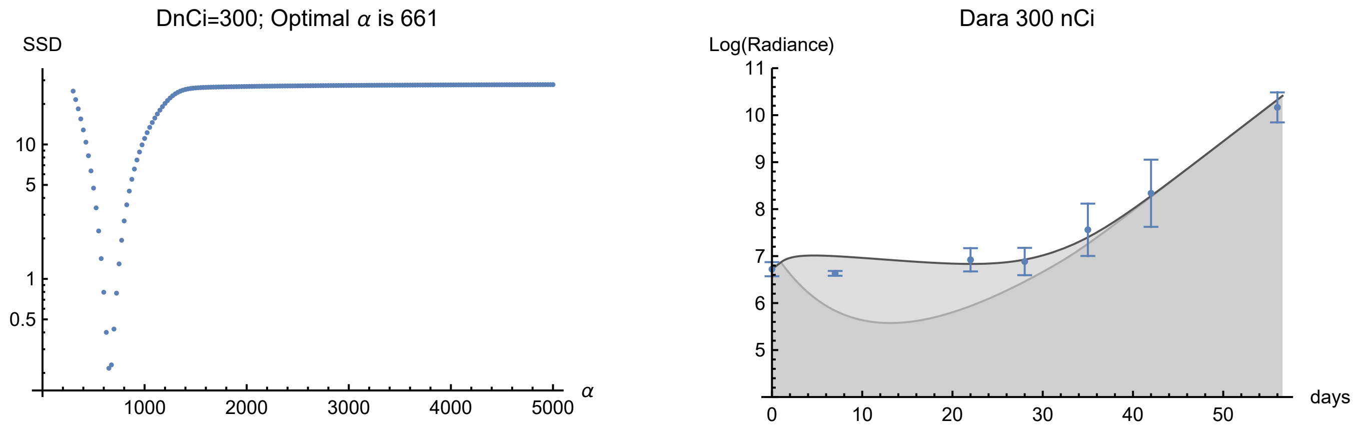
DnCi=300; Optimal α is 661
SSD
10
5
1
0.5
1000
2000
3000
4000
5000
α
Dara 300 nCi
Log(Radiance)
11
10
9
8
7
6
5
0
10
20
30
40
50
days

Out[ ]=

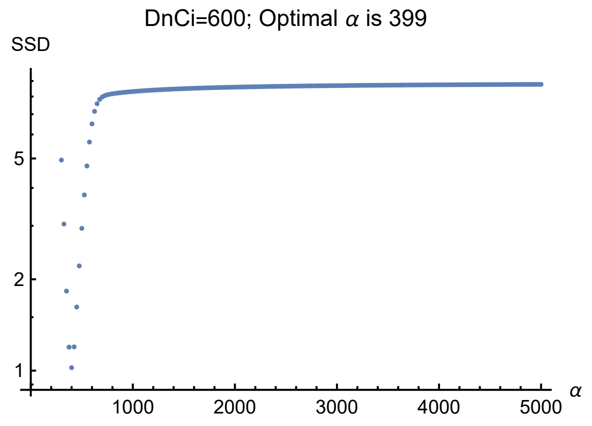

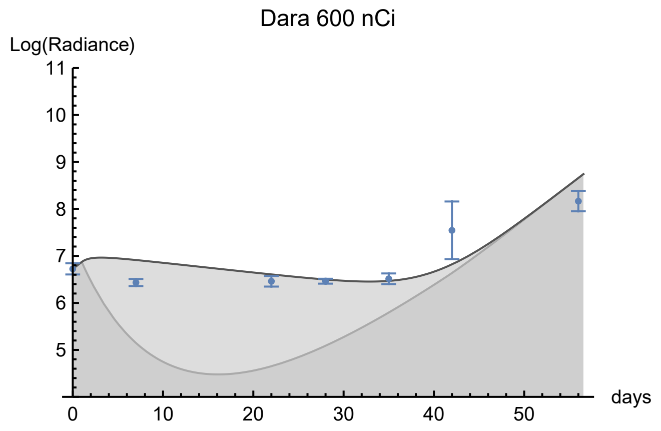

Out[ ]=

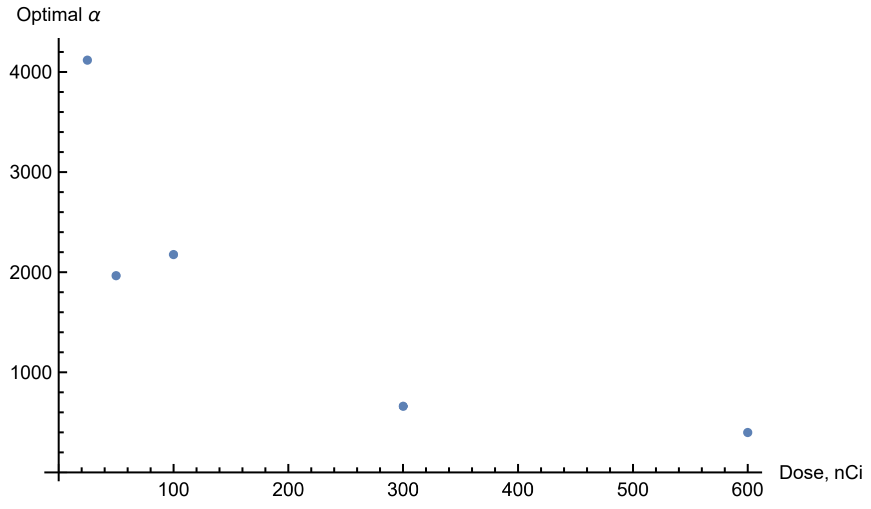

In[ ]:=

(* "Global variation of κ_c, κ_p, γ, V , k_s, N_0 and k_f α yielded ≈9.8 as the average ratio of optimal α in 25 nCi and 600 nCi settings, with its range being [8.4, 12.8]" *)

(* Fitting cancer cells radiosensitivity under different doses and varied parameters (long-running code) *)

```
rho = 0.34; (* cancer cells proliferation rate -- already estimated *)
omega = 0.05; (* damaged cells death rate -- already estimated *)
lambda = 0.07; (* -225Ac decay rate *)
kon = 11.15; (* daratumumab-CD38 association rate *)
nu = 0.015; (* volume of lesion with 10^7 cancer cells *)

(* Setting boundaries for variation *)
kappacmin = 0.04; kappacmax = 0.28;
kappapmin = 0.4; kappapmax = 4;
ksmin = 0.1; ksmax = 0.5;
Vmin = 0.75; Vmax = 1.5;
N0min = 1; N0max = 10;
gammamin = 0.13; gammamax = 10;
kfamin = 80; kfamax = 160;

(* How many parameters sets are going to be checked *)
JJ = 200;

outcome = Array[f, {JJ + 1, 13}];

outcome[[1, 1]] = "kappac";
outcome[[1, 2]] = "kappap";
outcome[[1, 3]] = "ks";
outcome[[1, 4]] = "V";
outcome[[1, 5]] = "N0";
outcome[[1, 6]] = "gamma";
outcome[[1, 7]] = "kfa";
outcome[[1, 8]] = "α-ratio"; (* ratio of optimal alphas *)
outcome[[1, 9]] = "α25";
outcome[[1, 10]] = "α50";
```

```
outcome[[1, 11]] = "α100";
outcome[[1, 12]] = "α300";
outcome[[1, 13]] = "α600";

VarParFlg = 1;

Quiet[jj = 2;
  While[jj ≤ JJ + 1,

    kappac = RandomReal[{kappacmin, kappacmax}];
    kappap = RandomReal[{kappapmin, kappapmax}];
    ks = RandomReal[{ksmin, ksmax}];
    V = RandomReal[{Vmin, Vmax}];
    N0 = 10^RandomReal[{Log10[N0min], Log10[N0max]}];
    gamma = 10^RandomReal[{Log10[gammamin], Log10[gammamax]}];
    kfa = RandomReal[{kfamin, kfamax}];

    NotebookDelete[pr]; (* To see the code running *)
    pr = PrintTemporary[ToString[jj - 1] <> "/" <> ToString[JJ]];

    Quiet[AlphaOptimize[25];
      alphaOpt25 = alphaOpt;

      AlphaOptimize[50];
      alphaOpt50 = alphaOpt;

      AlphaOptimize[100];
      alphaOpt100 = alphaOpt;

      AlphaOptimize[300];
      alphaOpt300 = alphaOpt;
```

```
  AlphaOptimize[600];
  alphaOpt600 = alphaOpt];

 outcome[[jj, 1]] = kappac;
 outcome[[jj, 2]] = kappap;
 outcome[[jj, 3]] = ks;
 outcome[[jj, 4]] = V;
 outcome[[jj, 5]] = N0;
 outcome[[jj, 6]] = gamma;
 outcome[[jj, 7]] = kfa;
 outcome[[jj, 8]] = Max[alphaOpt25, alphaOpt50, alphaOpt100, alphaOpt300, alphaOpt600] /
    Min[alphaOpt25, alphaOpt50, alphaOpt100, alphaOpt300, alphaOpt600];
 outcome[[jj, 9]] = alphaOpt25;
 outcome[[jj, 10]] = alphaOpt50;
 outcome[[jj, 11]] = alphaOpt100;
 outcome[[jj, 12]] = alphaOpt300;
 outcome[[jj, 13]] = alphaOpt600;

 If[outcome[[jj, 8]] == 1 || Not[NumericQ[alphaOpt25]] || Not[NumericQ[alphaOpt50]] || Not[NumericQ[alphaOpt100]] ||
    Not[NumericQ[alphaOpt300]] || Not[NumericQ[alphaOpt600]], jj--]; (* to overcome a bug *)

 jj++;];]

Histogram[outcome[[2;;, 8]]] (* Histogram of ratios of alphas *)
Mean[outcome[[2;;, 8]]] (* What is the average ratio*)
Min[outcome[[2;;, 8]]] (* What is the minimum ratio*)
Max[outcome[[2;;, 8]]] (* What is the maximum ratio*)
(* When the script is rerun, the results may a bit differ from the ones indicated in text, since this script uses randomization *)
```

Out[ ]=

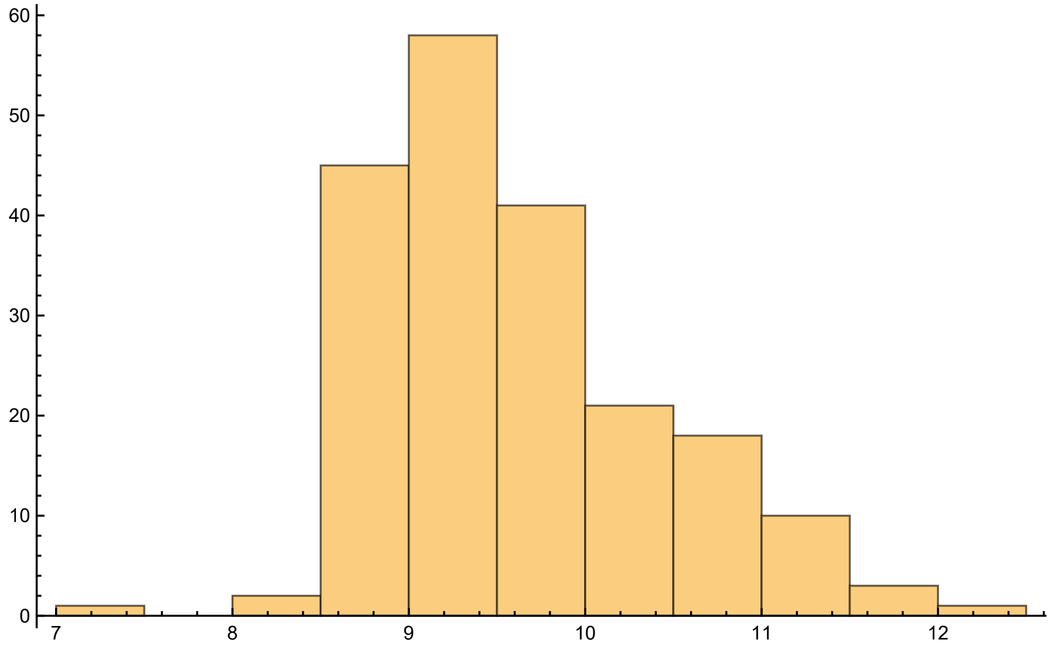

Out[ ]=

9.6161

Out[ ]=

7.04558

Out[ ]=

12.0399
