# Supplementary material for: Mathematical Modeling Unveils Optimization Strategies for Targeted Radionuclide Therapy of Blood Cancers
Source: Cancer Res Commun. 2024 Nov 14;4(11):2955–67. doi: 10.1158/2767-9764.CRC-24-0306 (PMC11562018; doi:10.1158/2767-9764.CRC-24-0306)
Supplement: Computational codes — Designed in Wolfram Mathematica, version 13.3.1.0. [file crc-24-0306_computational_codes_suppscc.zip › CodesPDF/02-Pure-drug.pdf]

```
(* This file contains simulations for the paper
  "Mathematical Modeling Unveils Optimization Strategies
for Targeted Radionuclide Therapy of Blood Cancers"
  by Maxim Kuznetsov, Vikram Adhikarla, Enrico Caserta,
 Flavia Pichiorri, John E.Shively, Xiuli Wang and Russell C.Rockne *)

(*****************************************************************************************************)

In[ ]:=
(* The script for the solution of full model and other scripts are hidden here
          and need to be initialized to run simulations *)

In[ ]:=
(* Solution of full model *)

(* To avoid numerical problems (linked with N / D and arriving under high rate of cell death)
   here we solve identical equations for concentrations of receptors of damaged cells instead of their fractions,
normalized by division by gamma / V, which thus provides their relative numbers (i.e., dF=fFD*D and dA=fAD*D).
        These equations are explicitly presented in supplemetary pdf-file, page 2 (such renormalization does not alter them).
      Original equations also work well if D is not getting too small (which includes working fine for the simulation for the basic set),
the corresponding parts of original equations are commented out and highlighted like this *)

FullSystemSolutionMD[] := (

   (* maximum time of simulation -- when little radioactivity of the last dose remains, namely 0.17% of the last dose *)
   tEnd = Ainj[[Length[Ainj], 1]] + (-Log[0.0017] / lambda);
   lAinj = Length[Ainj]; (* number of injections *)
   If[Ainj[[1, 1]] == 0, Npw = lAinj, Npw = lAinj + 1];
   (* the injections are treated as new initial conditions for a new system, which as well takes the actual values of other parameters *)
   (* therefore the number of injections has to me remembered
       and the logic differs whether the first injection is made at t=0 or t>0,
   although the latter case will never be considered in this study from now on *)

   apw = Array[ff, Npw]; bpw = Array[ff, Npw]; NNpw = Array[ff, Npw]; DDpw = Array[ff, Npw];
```

```
papw = Array[ff, Npw]; pbpw = Array[ff, Npw]; fFNpw = Array[ff, Npw]; fANpw = Array[ff, Npw];
(*fFDpw=Array[ff,Npw];fADpw=Array[ff,Npw];*) dFpw = Array[ff, Npw]; dApw = Array[ff, Npw];
(* decayed antibody fragments are as well accounted for as pb, just in case *)

(* times of beginnings and ends for solution of separate systems will be here *)
tB = Array[ff, Npw]; tE = Array[ff, Npw];

(* for monitoring the paths of activity *)
ActBloodpw = Array[ff, Npw]; ActBloodFragpw = Array[ff, Npw];
ActOutpw = Array[ff, Npw]; ActOutFragpw = Array[ff, Npw]; ActTumorpw = Array[ff, Npw];

(* for monitoring the influences of self-dose, cross-fire and decays in blood *)
SDpw = Array[ff, Npw]; CFNpw = Array[ff, Npw]; CFDpw = Array[ff, Npw]; UNpw = Array[ff, Npw];

(* for monitoring the number of new cancer cells appearing during treatment *)
NewCellspw = Array[ff, Npw];

Clear[a, b, NN, DD, pa, pb, fFN, fAN, dF, dA, ActBlood, ActBloodFrag, ActOut, ActOutFrag, ActTumor, SD, CFN, CFD, UN, NewCells];
(*fFD,fAD,*)

(* EQUATIONS *)

(* Radiation damage function *) (*fAD_,*)
RD[NN_, DD_, fAN_, dA_, a_, pa_] := alpha * (ks * (lambda * gamma * fAN)/nu (*self-dose*) +
    (1 - ks) * (lambda * gamma * (fAN * NN + dA))/(nu * (NN + DD)) (*cross-fire*) + kf * lambda * (a + pa) (*dose from unanchored nuclides*));


(* Active antibodies *)
```

```
Fa[t_] := (*injections are considered as initial conditions*)
  - lambda * a[t] (*decay*) - kon * gamma/V * (fFN[t] * NN[t] + dF[t]) * a[t] (*binding*) - kappac * a[t] (*clearance*);
(*fFD[t]*DD[t]*)

(* Inert antibodies *)
Fb[t_] := (*injections are considered as initial conditions*)
  + lambda * a[t] (*decay of a*) - kon * gamma/V * (fFN[t] * NN[t] + dF[t]) * b[t] (*binding*) - kappac * b[t] (*clearance*);
(*fFD[t]*DD[t]*)

(* Viable cells *) FNN[t_] := rho * NN[t] (*proliferation*) - RD[NN[t], DD[t], fAN[t], dA[t], a[t], pa[t]] * NN[t] (*damage*);
(*fAD[t],*)

(* Damaged cells *) FDD[t_] := RD[NN[t], DD[t], fAN[t], dA[t], a[t], pa[t]] * NN[t] (*damage*) - omega * DD[t] (*death*);
(*fAD[t],*)

(* Active fragments *) Fpa[t_] := omega * (gamma * dA[t])/V (*release*) - lambda * pa[t] (*decay*) - kappap * pa[t] (*clearance*);
(*fAD[t]*DD[t],*)

(* Inert fragments *)
Fpb[t_] := omega * (gamma * (DD[t] - dF[t] - dA[t]))/V (*release*) + lambda * pa[t] (*decay*) - kappap * pb[t] (*clearance*);
(*-fFD[t]*DD[t]-fAD[t]*DD[t]*)

(* Free receptors of viable cells *) FfFN[t_] := (1 - fFN[t]) * rho - kon * (a[t] + b[t]) * fFN[t];

(* Active receptors of viable cells *) FfAN[t_] := kon * a[t] * fFN[t] - (lambda + rho) * fAN[t];

(* Free receptors of damaged cells *)
```

```
(*FfFD[t_]:=(fFN[t]-fFD[t])*RD[NN[t],DD[t],fAN[t],fAD[t],a[t],pa[t]]*NN[t]/DD[t]-kon*(a[t]+b[t])*fFD[t];*)
FdF[t_] := RD[NN[t], DD[t], fAN[t], dA[t], a[t], pa[t]] * fFN[t] * NN[t] - kon * (a[t] + b[t]) * dF[t] - omega * dF[t];

(* Active receptors of damaged cells *)
(*FfAD[t_]:=(fAN[t]-fAD[t])*RD[NN[t],DD[t],fAN[t],fAD[t],a[t],pa[t]]*NN[t]/DD[t]+kon*a[t]*fFD[t]-lambda*fAD[t];*)
FdA[t_] := RD[NN[t], DD[t], fAN[t], dA[t], a[t], pa[t]] * fAN[t] * NN[t] + kon * a[t] * dF[t] - lambda * dA[t] - omega * dA[t];

(* Initial conditions *)

If[Ainj[[1, 1]] == 0
  , a0 = Ainj[[1, 2]] / V; b0 = eta * Ainj[[1, 2]] / V
  , a0 = 0; b0 = 0];
NN0 = N0;
DD0 = 0;
pa0 = 0; pb0 = 0; fFN0 = 1; fAN0 = 0;
(*fFD0=1;fAD0=0;*) dF0 = 0; dA0 = 0;
ActBlood0 = 0; ActBloodFrag0 = 0; ActOut0 = 0; ActOutFrag0 = 0; ActTumor0 = 0; NewCells0 = 0;
SD0 = 0; CFN0 = 0; CFD0 = 0; UN0 = 0;

(* SOLVER *)

tB[[1]] = 0; If[Ainj[[1, 1]] == 0, If[lAinj > 1, tE[[1]] = Ainj[[2, 1]], tE[[1]] = tEnd], tE[[1]] = Ainj[[1, 1]]];

For[npw = 1, npw ≤ Npw, npw++,

  Clear[a, b, NN, DD, pa, pb, fFN, fAN, dF, dA, ActBlood, ActBloodFrag, ActOut, ActOutFrag, ActTumor, SD, CFN, CFD, UN, NewCells];
  (*fFD,fAD,*)

  sol = NDSolve[{
```

```
(*INITIAL CONDITIONS*)
a[tB[[npw]]] == a0, b[tB[[npw]]] == b0, NN[tB[[npw]]] == NN0,
DD[tB[[npw]]] == DD0, pa[tB[[npw]]] == pa0, pb[tB[[npw]]] == pb0, fFN[tB[[npw]]] == fFN0, fAN[tB[[npw]]] == fAN0,
(*fFD[tB[[npw]]]==fFD0,fAD[tB[[npw]]]==fAD0,*) dF[tB[[npw]]] == dF0, dA[tB[[npw]]] == dA0,
ActBlood[tB[[npw]]] == ActBlood0, ActBloodFrag[tB[[npw]]] == ActBloodFrag0, ActOut[tB[[npw]]] == ActOut0,
ActOutFrag[tB[[npw]]] == ActOutFrag0, ActTumor[tB[[npw]]] == ActTumor0, SD[tB[[npw]]] == SD0,
CFN[tB[[npw]]] == CFN0, CFD[tB[[npw]]] == CFD0, UN[tB[[npw]]] == UN0, NewCells[tB[[npw]]] == NewCells0,

a'[t] == Fa[t], b'[t] == Fb[t], NN'[t] == FNN[t], DD'[t] == FDD[t], pa'[t] == Fpa[t], pb'[t] == Fpb[t],
fFN'[t] == FfFN[t], fAN'[t] == FfAN[t], (*fFD'[t]==FfFD[t],fAD'[t]==FfAD[t],*) dF'[t] == FdF[t], dA'[t] == FdA[t],

ActBlood'[t] == V*lambda*(a[t] + pa[t]),
ActBloodFrag'[t] == V*lambda*pa[t],
ActOut'[t] == V*(kappac*a[t] + kappap*pa[t]),
ActOutFrag'[t] == V*kappap*pa[t],
ActTumor'[t] == (lambda*gamma)*(fAN[t]*NN[t] + dA[t]), (*fAD[t]*DD[t]*)

SD'[t] == ks*(lambda*gamma)*(fAN[t]*NN[t]),
CFN'[t] == (1 - ks)*(lambda*gamma)*(fAN[t]*NN[t])*NN[t]/(NN[t] + DD[t]),
CFD'[t] == (1 - ks)*(lambda*gamma)*dA[t]*NN[t]/(NN[t] + DD[t]), (*(fAD[t]*DD[t])*)
UN'[t] == kf*lambda*(a[t] + pa[t])*nu*NN[t],
NewCells'[t] == If[t > Ainj[[1, 1]], rho*NN[t], 0] (* start counting new cells from the moment of the first injection *)

(* The simulations run until the injected activity decays to negligible amounts.
   If viable cancer cell number becomes too great (host death),
then it is just kept further at this level to allow assessment of activity paths *)
(* The cases of cancer cure will use additional script to virtually eliminate all cancer cells in case of cure *)
, WhenEvent[NN[t] > 10^9 / Nnor, NN[t] → 0.99*10^9 / Nnor]
```

```
        }(*fFD,fAD,*)
        , {a, b, NN, DD, pa, pb, fFN, fAN, dF, dA, ActBlood,
          ActBloodFrag, ActOut, ActOutFrag, ActTumor, SD, CFN, CFD, UN, NewCells}, {t, tB[[npw]], tE[[npw]]}
        , AccuracyGoal → 10, PrecisionGoal → 10];

apw[[npw]] = First[a /. sol]; bpw[[npw]] = First[b /. sol]; NNpw[[npw]] = First[NN /. sol]; DDpw[[npw]] = First[DD /. sol];
papw[[npw]] = First[pa /. sol]; pbpw[[npw]] = First[pb /. sol]; fFNpw[[npw]] = First[fFN /. sol]; fANpw[[npw]] = First[fAN /. sol];
(*fFDpw[[npw]]=First[fFD/.sol]; fADpw[[npw]]=First[fAD/.sol];*) dFpw[[npw]] = First[dF /. sol]; dApw[[npw]] = First[dA /. sol];
ActBloodpw[[npw]] = First[ActBlood /. sol];
ActBloodFragpw[[npw]] = First[ActBloodFrag /. sol];
ActOutpw[[npw]] = First[ActOut /. sol];
ActOutFragpw[[npw]] = First[ActOutFrag /. sol];
ActTumorpw[[npw]] = First[ActTumor /. sol];
SDpw[[npw]] = First[SD /. sol];
CFNpw[[npw]] = First[CFN /. sol];
CFDpw[[npw]] = First[CFD /. sol];
UNpw[[npw]] = First[UN /. sol];
NewCellspw[[npw]] = First[NewCells /. sol];

If[npw < Npw,
  (*renew initial conditions*)
  If[Ainj[[1, 1]] == 0
    , a0 = apw[[npw]][tE[[npw]]] + Ainj[[npw + 1, 2]] / V; b0 = bpw[[npw]][tE[[npw]]] + eta * Ainj[[npw + 1, 2]] / V
    , a0 = apw[[npw]][tE[[npw]]] + Ainj[[npw, 2]] / V; b0 = bpw[[npw]][tE[[npw]]] + eta * Ainj[[npw, 2]] / V];
  NN0 = NNpw[[npw]][tE[[npw]]];
  DD0 = DDpw[[npw]][tE[[npw]]];
  pa0 = papw[[npw]][tE[[npw]]];
  pb0 = pbpw[[npw]][tE[[npw]]];
  fFN0 = fFNpw[[npw]][tE[[npw]]]; fAN0 = fANpw[[npw]][tE[[npw]]];
  (*fFD0=fFDpw[[npw]][tE[[npw]]];fAD0=fADpw[[npw]][tE[[npw]]];*)
```

```
        dF0 = dFpw⟦npw⟧ [tE⟦npw⟧];
        dA0 = dApw⟦npw⟧ [tE⟦npw⟧];
        ActBlood0 = ActBloodpw⟦npw⟧ [tE⟦npw⟧];
        ActBloodFrag0 = ActBloodFragpw⟦npw⟧ [tE⟦npw⟧];
        ActOut0 = ActOutpw⟦npw⟧ [tE⟦npw⟧];
        ActOutFrag0 = ActOutFragpw⟦npw⟧ [tE⟦npw⟧];
        ActTumor0 = ActTumorpw⟦npw⟧ [tE⟦npw⟧];
        SD0 = SDpw⟦npw⟧ [tE⟦npw⟧];
        CFN0 = CFNpw⟦npw⟧ [tE⟦npw⟧];
        CFD0 = CFDpw⟦npw⟧ [tE⟦npw⟧];
        UN0 = UNpw⟦npw⟧ [tE⟦npw⟧];
        NewCells0 = NewCellspw⟦npw⟧ [tE⟦npw⟧];

        (* renew time frame *)
        If [Ainj⟦1, 1⟧ == 0, If [Npw > npw + 1, tE⟦npw + 1⟧ = Ainj⟦npw + 2, 1⟧, tE⟦npw + 1⟧ = tEnd],
          If [Npw > npw + 1, tE⟦npw + 1⟧ = Ainj⟦npw + 1, 1⟧, tE⟦npw + 1⟧ = tEnd] ];]
    ];
    npw--;
    (* It will be convenient to have estimation of minimal viable cell number here *)
    Nn = If [Ainj⟦1, 1⟧ == 0
        , Min [Table [NMinimize [{Nnor * (NNpw⟦nn⟧ [t]), t > tB⟦nn⟧, t < tE⟦nn⟧}, t] ⟦1⟧, {nn, 1, npw}] ]
        , Min [Table [NMinimize [{Nnor * (NNpw⟦nn⟧ [t]), t > tB⟦nn⟧, t < tE⟦nn⟧}, t] ⟦1⟧, {nn, 2, npw}] ] ];
    tminNn = If [Ainj⟦1, 1⟧ == 0
        , Min [Table [t /. NMinimize [{Nnor * (NNpw⟦nn⟧ [t]), t > tB⟦nn⟧, t < tE⟦nn⟧}, t] ⟦2⟧, {nn, 1, npw}] ]
        , Min [Table [t /. NMinimize [{Nnor * (NNpw⟦nn⟧ [t]), t > tB⟦nn⟧, t < tE⟦nn⟧}, t] ⟦2⟧, {nn, 2, npw}] ] ]; Return [Nn] )


(* The script for finding curative dose *)

FindCurDose [ ] := (DA1 = (nu * N0 * rho)/(alpha * lambda); (* at this dose N' (0) =0 for approximated system *)
```

```
  Ainj = {{t1, DA1}};

  While[FullSystemSolutionMD[] > Ncur, DA1 = 2 * DA1; Ainj = {{t1, DA1}}; ];
  Dmax = DA1; Dmin = DA1 / 2;

  While[Abs[Dmin - Dmax] / Dmax > 0.000001,
    DA1 = (Dmin + Dmax) / 2; Ainj = {{t1, DA1}};
    If[FullSystemSolutionMD[] > Ncur, Dmin = DA1, Dmax = DA1]];
  Dcur = Dmax;
  Return[Dcur / nCpm])

(* Set basic parameter values *)
SetBasicParameterValues[] := (
  Nnor = 10^5; (* due to numerical pecularities, it is better to use this normalization number of cells *)

  lambda = 0.07; (* radionuclide decay rate *)
  kon = 11.15; (* antibody-receptor association rate *)
  kappac = 0.1; (* antibody clearance rate (0.04-0.28) *)
  kappap = 1; (* antibody fragments clearance rate (0.4-4.) *)
  gamma = 2.1 * Nnor / 10^7; (* number of receptors of Nnor cancer cells (0.13-10) *Nnor/10^7 *)
  V = 1.; (* volume of drug distribution (0.75-1.5) *)
  nu = 0.015 * Nnor / 10^7; (* volume of lesion with Nnor cancer cells *)
  ks = 0.3; (* relative significance of self-dose (0.1-0.5) *)

  rho = 0.34; (* cancer cells proliferation rate (0.15-0.7) *)
  omega = 0.05; (* damaged cells death rate (0.005-0.5) *)
  alpha = 500; (* cancer cells radiosensitivity (50-5000) *)
  eta = 0; (* coefficient of drug impurity (0-10^5) *)
  kf = 0.05; (* significance of unanchored nuclides decays (0.01-0.25) *)
  N0 = 3. * 10^7 / Nnor; (* normalized initial number of viable cancer cells, (1-10) *Nnor/10^7 *)
  D0 = 0;
```

```
    (*0.1 / Nnor; *)
    (* initial number of damaged cancer cells -- use non-zero value if considering fraction of receptors of damaged cells, to avoid division by zero*)
    nCpm = 0.000076; (* parameter of conversion from nCi to pmol *)
    DA1 = 50 * nCpm;  (* injected amount of radionuclides *)
    t1 = 0;  (* moment of their injection *)
    Ncur = 0.01;  (* number of viable cancer cells (in cells), corresponding to cancer cure *)
    Abld = 0.0175;  (* critical amount of decays in blood, leading to death of toxicity (230 nCi) *)
    Cd = 10^11;  (* critical number of cancer cells, leading to death of tumor burden *)
    Ainj = {{t1, DA1}};  (* schedule of injections *)

    kGy = 2500; (* coefficient of conversion of nM of 225-Ac decay in bone marrow into Gy *)
  )
```

```
(* Main text: Single-dose treatment by pure radioconjugates *)
```

```
(* Let's find the minimal single curative dose for the basic set, since Figure 2 is plotted using this dose *)
SetBasicParameterValues[];
Acur = FindCurDose[]  (* nCi *)
```

Out[∘]=

63.0859

```
(* Figure 2 A,B,D,E  *)
DA1 = Acur * nCpm;
Ainj = {{t1, DA1}};
FullSystemSolutionMD[];

IS = 320; (* size of images *)

(* Antibodies in blood *)
Quiet[Show[LogPlot[{
        Piecewise[Table[{apw⟦nn⟧[t], tB⟦nn⟧ ≤ t < tE⟦nn⟧}, {nn, 1, npw}]],
        Piecewise[Table[{papw⟦nn⟧[t] + apw⟦nn⟧[t], tB⟦nn⟧ ≤ t < tE⟦nn⟧}, {nn, 1, npw}]],
        Max[10^(-6), Piecewise[Table[{apw⟦nn⟧[t], tB⟦nn⟧ ≤ t < tE⟦nn⟧}, {nn, 1, npw}]]]
```

```
    }, {t, Ainj⟦1, 1⟧, 0.5}, Filling → {1 → 10^(-6), 2 → {3}},
    PlotStyle → {Directive[Darker[Red], Thickness[0.002]], Directive[Darker[Yellow], Thickness[0.002]]}, PlotRange →
     {{0, 0.5}, {10^(-6), 1.05 * Max[Table[NMaximize[{papw⟦nn⟧[t] + pbpw⟦nn⟧[t] + apw⟦nn⟧[t] + bpw⟦nn⟧[t], t > tB⟦nn⟧, t < tE⟦nn⟧}, t]⟦
          1⟧, {nn, 1, npw}]]}}, AxesLabel → {"hours", "nM"},
    Ticks → {Table[{i / 24, i}, {i, 0, 12, 2}], Automatic}, ImageSize → IS],
  ListPlot[Ainj, PlotStyle → Red]]

(* Number of cancer cells *)
Nn = If[Ainj⟦1, 1⟧ == 0
    , Min[Table[NMinimize[{Nnor * (NNpw⟦nn⟧[t]), t > tB⟦nn⟧, t < tE⟦nn⟧}, t]⟦1⟧, {nn, 1, npw}]]
    , Min[Table[NMinimize[{Nnor * (NNpw⟦nn⟧[t]), t > tB⟦nn⟧, t < tE⟦nn⟧}, t]⟦1⟧, {nn, 2, npw}]]];
tminNn = If[Ainj⟦1, 1⟧ == 0
    , Min[Table[t /. NMinimize[{Nnor * (NNpw⟦nn⟧[t]), t > tB⟦nn⟧, t < tE⟦nn⟧}, t]⟦2⟧, {nn, 1, npw}]]
    , Min[Table[t /. NMinimize[{Nnor * (NNpw⟦nn⟧[t]), t > tB⟦nn⟧, t < tE⟦nn⟧}, t]⟦2⟧, {nn, 2, npw}]]];

Quiet[Show[LogPlot[{Piecewise[Table[{Nnor * NNpw⟦nn⟧[t], tB⟦nn⟧ ≤ t < tE⟦nn⟧}, {nn, 1, npw}]],
     Piecewise[Table[{Nnor * (NNpw⟦nn⟧[t] + DDpw⟦nn⟧[t]), tB⟦nn⟧ ≤ t < tE⟦nn⟧}, {nn, 1, npw}]]}, {t, 0, tminNn}, Filling → {1 → Axis, 2 → {1}
    PlotStyle → {Directive[Lighter[Gray], Thickness[0.002]], Directive[Darker[Gray], Thickness[0.002]]}, PlotRange →
     {{0, tE⟦npw⟧}, {Nn / 10, Max[Table[NMaximize[{Nnor * (NNpw⟦nn⟧[t] + DDpw⟦nn⟧[t]), t > tB⟦nn⟧, t < tE⟦nn⟧}, t]⟦1⟧, {nn, 1, npw}]]}},
    AxesLabel → {"days", "cells"}, Ticks → {Automatic, {{0.01, 0.01}, {1, 1}, {100, 100}, {10000, "10^4"}, {10^6, "10^6"}}},
    ImageSize → IS],
  LogPlot[{Piecewise[Table[{Nnor * NNpw⟦nn⟧[t], tB⟦nn⟧ ≤ t < tE⟦nn⟧}, {nn, 1, npw}]],
     Piecewise[Table[{Nnor * (NNpw⟦nn⟧[t] + DDpw⟦nn⟧[t]), tB⟦nn⟧ ≤ t < tE⟦nn⟧}, {nn, 1, npw}]]},
    {t, tminNn, tE⟦npw⟧}, Filling → {1 → Axis, 2 → {1}}, PlotStyle → {Directive[Lighter[Gray], Thickness[0.002], Dashed],
      Directive[Darker[Gray], Thickness[0.002], Dashed]}, PlotRange → {{tminNn, tE⟦npw⟧},
      {Nn / 10, Max[Table[NMaximize[{Nnor * (NNpw⟦nn⟧[t] + DDpw⟦nn⟧[t]), t > tB⟦nn⟧, t < tE⟦nn⟧}, t]⟦1⟧, {nn, 1, npw}]]}},
    AxesLabel → {"days", "cells"}, Ticks → {Automatic, {{0.01, 0.01}, {1, 1}, {100, 100}, {10000, "10^4"}, {10^6, "10^6"}}}, ImageSize → IS],
  ListLogPlot[{{tminNn, Nn}}, PlotStyle → Lighter[Gray]]]]

(* Receptors of viable cancer cells *)
```

```
MinRec = 1 - 1.05 * (1 - Min[Table[NMinimize[{fFNpw[[nn]][t], t > tB[[nn]], t < tE[[nn]]}, t][[1]], {nn, 1, npw}],
        Table[NMinimize[{dFpw[[nn]][t] / DDpw[[nn]][t], t > tB[[nn]], t < tE[[nn]]}, t][[1]], {nn, 1, npw}]]);

Quiet[Show[Plot[{Piecewise[Table[{fANpw[[nn]][t], tB[[nn]] ≤ t < tE[[nn]]}, {nn, 1, npw}]],
      Piecewise[Table[{1 - fFNpw[[nn]][t], tB[[nn]] ≤ t < tE[[nn]]}, {nn, 1, npw}], 1}, {t, 0, tE[[npw]]}, Filling → {1 → Axis, 2 → {1}, 3 → {2}}, PlotSty
      {Directive[Lighter[Red], Thickness[0.002]], Directive[Lighter[Gray], Thickness[0.002]], Directive[White, Thickness[0.002]]},
    PlotRange → {{0, 20}, {0, 1 - MinRec}}, AxesLabel → {"days", "fraction"},
    Ticks → {Automatic, Table[{i / 10 000, PercentForm[N[i / 10 000]]}, {i, 0, 8}]},
    ImageSize → IS]

   (* analytical estimations *)
   , Plot[(1 - fFNpw[[npw]][20]) * Exp[-(rho) * (t - 20)], {t, -60, 60}, PlotStyle → Directive[Dashed, Gray]]
   , Plot[fANpw[[npw]][20] * Exp[-(rho + lambda) * (t - 20)], {t, -60, 60}, PlotStyle → Directive[Dashed, Darker[Red]]]]]

(* Receptors of damaged cancer cells *)

Quiet[Show[Plot[{Piecewise[Table[{dApw[[nn]][t] / DDpw[[nn]][t], tB[[nn]] < t < tE[[nn]]}, {nn, 1, npw}]] (*fADpw[[nn]][t]*),
      Piecewise[Table[{1 - dFpw[[nn]][t] / DDpw[[nn]][t], tB[[nn]] < t < tE[[nn]]}, {nn, 1, npw}]] (*1-fFDpw[[nn]][t]*), 1}, {t, 0, tE[[npw]]},
    Filling → {1 → Axis, 2 → {1}, 3 → {2}}, PlotStyle → {Directive[Darker[Red], Thickness[0.002]], Directive[Darker[Gray], Thickness[0.002]],
       Directive[White, Thickness[0.002]]}, PlotRange → {{0, 20}, {0, 1 - MinRec}}, AxesLabel → {"days", "fraction"},
    Ticks → {Automatic, Table[{i / 10 000, PercentForm[N[i / 10 000]]}, {i, 0, 8}]}, ImageSize → IS]

   (* analytical estimations *)
   , Plot[(dApw[[npw]][10] / DDpw[[npw]][10]) * Exp[-(lambda) * (t - 10)], {t, 0, 60}, PlotStyle → Directive[Dashed, Darker[Red]]]]]

(* Somehow, this figure may cause Wolfram to glitch and freeze when being scrolled by.
   So, for safety it is hidden here. Expand this group of cells to see it, then it's better to collapse this cell group before scrolling further. *)
```

Out[ ]=

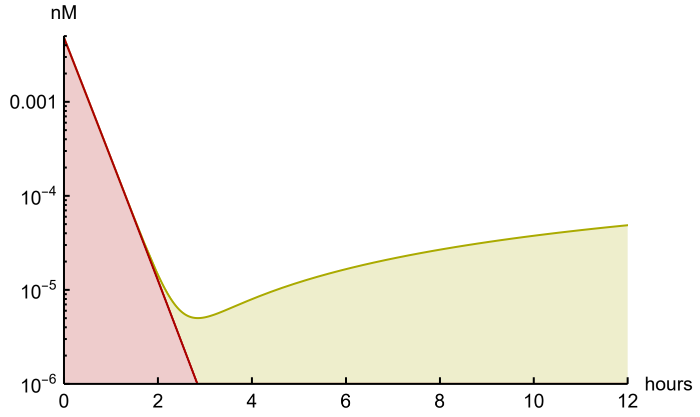
nM
0.001
10^{-4}
10^{-5}
10^{-6}
0
2
4
6
8
10
12
hours

Out[ ]=

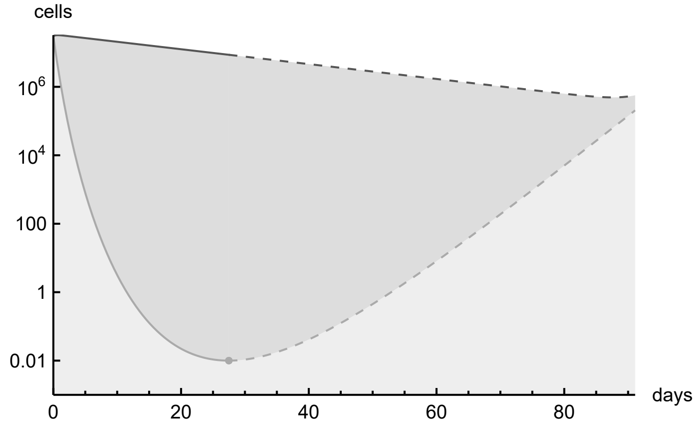
cells
10^6
10^4
100
1
0.01
0
20
40
60
80
days

Out[ ]=

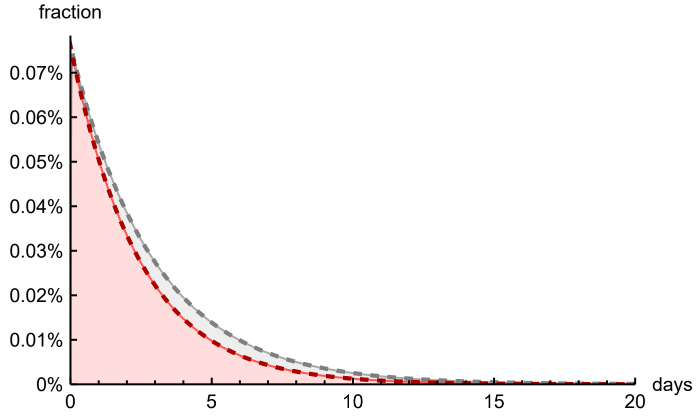
fraction
0.07%
0.06%
0.05%
0.04%
0.03%
0.02%
0.01%
0%
0
5
10
15
20
days

Out[ ]=

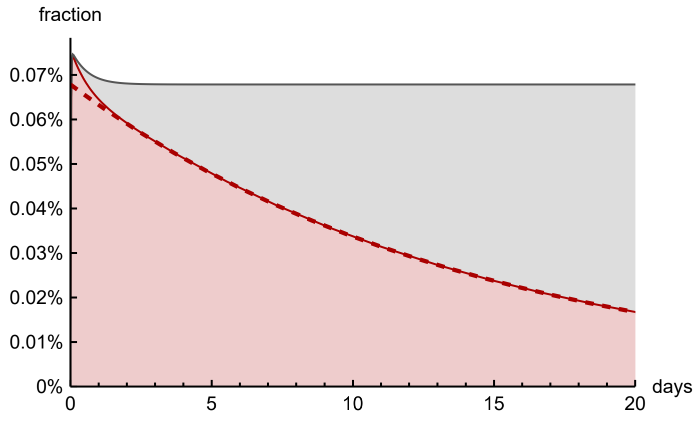

(* Supplementary S.2.1 Analytical investigation of the model in close–to–curative setting under low amount of injected radioconjugates *)

(* S.2.1.1 Estimation of total amount of toxic decays *)

```
(* "The amount of activity, that is lost due to clearance and decays in blood, can be estimated
from the relative rates of the three exponentially decaying processes comprising antibodies dynamics (...)
which is ≈0.12 nCi for basic parameter set. This estimation is only ≈0.4 % greater than the simulation result." *)

SetBasicParameterValues[];
FullSystemSolutionMD[];

(* estimation *)
DoseNotBoundEst = ((kappac + lambda) * DA1) / (kappac + lambda + kon * gamma * N0 / V) / nCpm

(* simulation result *)
DoseNotBoundSim =
  ((ActBloodpw[[Npw]][tEnd] - ActBloodFragpw[[Npw]][tEnd]) + (ActOutpw[[Npw]][tEnd] - ActOutFragpw[[Npw]][tEnd])) / nCpm

(* comparison *)
PercentForm[DoseNotBoundEst / DoseNotBoundSim - 1]
```

Out[ ]=

0.120713

Out[ ]=

0.120185

Out[ ]//PercentForm=

0.4389%

```
(* "So, we can make a reasonable approximation that at t = 0 all the injected drug is already on the cancer cell receptors (...)
   For basic set of parameters, this yields f0 ≈ 0.0006, \nwhich is ≈ 2 % greater than the maximum value of fAD attained in the simulation." *)

(* estimation *)
f0Est = DA1 / (gamma * N0)

(* simulation result *)
f0Sim = 1 - If[Ainj[[1, 1]] == 0
    , Min[Table[NMinimize[{dFpw[[nn]][t] / DDpw[[nn]][t], t > tB[[nn]], t < tE[[nn]]}, t][[1]], {nn, 1, npw}]]
    , Min[Table[NMinimize[{dFpw[[nn]][t] / DDpw[[nn]][t], t > tB[[nn]], t < tE[[nn]]}, t][[1]], {nn, 2, npw}]]]

(* comparison *)
PercentForm[f0Est / f0Sim - 1]
```

Out[ ]=

0.000603175

Out[ ]=

0.000591212

Out[ ]//PercentForm=

2.023%

```
(* "The amount of radioactivity that is lost due to decays in blood (...)
   for the basic parameter set this is ≈1.4 nCi,
   which is only ≈3 % greater than the simulation result." *)

(* decays in blood estimation *)
FrDecEst = (lambda * omega * DA1) / ((lambda + omega) * (lambda + kappap)) / nCpm

(* clearance simulation result *)
FrDecSim = ActBloodFragpw[[Npw]][tEnd] / nCpm

(* comparison *)
PercentForm[FrDecEst / FrDecSim - 1]
```

Out[∘]=

1.36293

Out[∘]=

1.32436

Out[∘]//PercentForm=

2.912%

```
(* S.2.1.2 Simplification of the system for the analysis of cancer cell dynamics *)
```

```
(* "Let's also notice that the radiation damage due to the unanchored nuclides is negligible in the corresponding simulation.
    In it ,after the antibodies are bound ,the maximum value of the term of dose from unanchored nuclides
    is more than four orders of magnitude smaller that the maximal sum of other terms of radiation damage function." *)
tMin = If[Ainj[[1, 1]] == 0, t /. Quiet[FindMinimum[papw[[1]][t] + pbpw[[1]][t] + apw[[1]][t] + bpw[[1]][t], {t, 0}]][[2]],
    t /. Quiet[FindMinimum[papw[[2]][t] + pbpw[[2]][t] + apw[[2]][t] + bpw[[2]][t], {t, 0}]][[2]]];

(* maximum value for the term of dose from anchored nuclides *)
MUN = alpha * kf * lambda * FindMaximum[papw[[1]][t], {t, 1.1 * tMin}][[1]]

(* maximum value for self-dose + cross-fire terms *)
MSC = alpha * lambda * DA1/(nu * N0)
(* their ratio *)
MSC / MUN
```

Out[∘]=

0.000225574

Out[∘]=

2.95556

Out[∘]=

13 102.4

```
(* S.2.1.3 The case of negligible cross-fire, ks=1 (self-damage-only) *)

(* "the border case for which (...) the number of viable cells does not decrease at all during treatment.
    For the basic parameter set the corresponding dose is ≈5.75 nCi" *)

(nu * N0 * rho)/(alpha * lambda) / nCpm
```

Out[∘]=

5.75188

In[ ]:=

```
(* We need to run the simulation with ks=1 to further compare the analytical estimations with its outcome *)
SetBasicParameterValues[];
PHI = (alpha * DA1)/(nu * N0); P = rho/lambda;
ks = 1;
FullSystemSolutionMD[];
```

```
(* "For the basic set of parameters with A =50 nCi and k_s =1, t_m^SD ≈5.3 days, less than 0.04 % greater that the actual simulation result,
    and N_m^SD ≈305772 cells, which is only ≈5 % less than the actual result." *)

(* Time, when minimal number of viable cancer cells is achieved *)
(* estimation *)
tSDminEst = Log[PHI/P] / (lambda + rho)

(* simulation result *)
tminNn = If[Ainj[[1, 1]] == 0
    , Min[Table[t /. NMinimize[{Nnor * (NNpw[[nn]][t]), t > tB[[nn]], t < tE[[nn]]}, t][[2]], {nn, 1, npw}]]
    , Min[Table[t /. NMinimize[{Nnor * (NNpw[[nn]][t]), t > tB[[nn]], t < tE[[nn]]}, t][[2]], {nn, 2, npw}]]]

(* comparison *)
PercentForm[tSDminEst / tminNn - 1]

(* The minimal number of viable cancer cells *)
(* estimation *)
NSDminEst = N0 * (PHI/P)^(P/(1+P)) * E^((P-PHI)/(1+P)) * Nnor

(* simulation result *)
Nn

(* comparison *)
PercentForm[NSDminEst / Nn - 1]
```

Out[ ]=

```
5.27438
```

Out[ ]=

5.27253

Out[ ]//PercentForm=

0.03502%

Out[ ]=

305 772.

Out[ ]=

321 080.

Out[ ]//PercentForm=

−4.768%

```
(* "For the basic set of parameters with k_s = 1, A_cur^SD ≈ 176.8 nCi,
    which is less than 0.7 % smaller than the full model simulation result." *)

(* estimation *)
```

$$ASDCurEst = -N0 * P * \frac{nu}{alpha} * ProductLog\left[-1, -E^{-1} * \left(\frac{Ncur}{N0 * Nnor}\right)^{\frac{1+P}{P}}\right] / nCpm$$

```
(* simulation result *)
SetBasicParameterValues[ ]; ks = 1;

ASDCurSim = FindCurDose[ ] (* This function has to be initiated in the first cell of this notebook *)

(*comparison*)
PercentForm[ASDCurEst / ASDCurSim - 1]
```

Out[ ]=

176.814

Out[ ]=

178.033

Out[ ]//PercentForm=

−0.685%

In[ ]:=

```
(* "we can obtain the minimal number of molecules of radioconjugates that should
   anchor on each cancer cell \nin order to achieve cure. (...) That is ≈272 molecules for the basic set." *)

ASDCurSim * nCpm * 6.02214076 * 10^11
-------------------------------------
            N0 * Nnor
```

Out[ ]=

271.609

```
(* S.2.1.4 The case of negligible self-damage, ks=0 (cross-fire-only) *)
```

```
(* "We can use the fact that during the initial period of active damaging of cancer cells\ntheir total number
   does not change drastically (in the basic set simulation with A =50 nCi it increases by only about 10% )" *)

SetBasicParameterValues[];
FullSystemSolutionMD[];
tminNn = If[Ainj[[1, 1]] == 0
    , Min[Table[t /. NMinimize[{Nnor * (NNpw[[nn]][t]), t > tB[[nn]], t < tE[[nn]]}, t][[2]], {nn, 1, npw}]]
    , Min[Table[t /. NMinimize[{Nnor * (NNpw[[nn]][t]), t > tB[[nn]], t < tE[[nn]]}, t][[2]], {nn, 2, npw}]]];
NDMax = If[Ainj[[1, 1]] == 0
   , Max[Table[NMaximize[{Nnor * (NNpw[[nn]][t] + DDpw[[nn]][t]), t > tB[[nn]], t < tminNn}, t][[1]], {nn, 1, npw}]]
   , Max[Table[NMaximize[{Nnor * (NNpw[[nn]][t] + DDpw[[nn]][t]), t > tB[[nn]], t < tminNn}, t][[1]], {nn, 2, npw}]]]

(* the percent of change from t=0 *)
PercentForm[NDMax / (N0 * Nnor) - 1]
```

Out[ ]=

3.30514×10^{7}

Out[ ]//PercentForm=

10.17%

```
In[ ]:=
(* We need to run the simulation with ks=0 to further compare the estimation with it *)
PHI = (alpha * DA1)/(nu * N0); P = rho/lambda;

SetBasicParameterValues[];
ks = 0;
FullSystemSolutionMD[];
```

(* "For the basic set of parameters with $k_s = 0$, $N_m^{CF} \approx 0.008$ cells,
with expanding of Lambert function resulting in negligible change.
This is ≈5% less than the corresponding simulation result." *)

(* estimation with Taylor expansion *)

$$NCFminEst = N0 * \left(\frac{PHI}{P}\right)^{P} * E^{2*P-PHI} * Nnor$$

(* Comparison to full formula with Lambert function *)

$$\frac{N0}{P} * ProductLog\left[P * \left(\frac{PHI}{P}\right)^{P} * E^{2*P-PHI}\right] * Nnor$$

(* simulation result *)

Nn

(*comparison*)

PercentForm[NCFminEst / Nn - 1]

Out[∘]=

0.00833181

Out[∘]=

0.00833181

Out[∘]=

0.00878688

Out[∘]//PercentForm=

−5.179%

```
(* "the moment of achieving minimum viable cancer cell number can be estimated (...)
   which is ≈30.9 days for the basic set, ≈7% greater than the simulation result." *)

(* estimation *)
tCFminEst = Log[PHI/P]/lambda

(* simulation result *)
tminNn = If[Ainj[[1, 1]] == 0
   , Min[Table[t /. NMinimize[{Nnor * (NNpw[[nn]][t]), t > tB[[nn]], t < tE[[nn]]}, t][[2]], {nn, 1, npw}]]
   , Min[Table[t /. NMinimize[{Nnor * (NNpw[[nn]][t]), t > tB[[nn]], t < tE[[nn]]}, t][[2]], {nn, 2, npw}]]]

(*comparison*)
PercentForm[tCFminEst / tminNn - 1]
```

Out[∘]=

30.8928

Out[∘]=

28.7993

Out[∘]//PercentForm=

7.269%

In[∘]:=

(* "For the basic set of parameters with $k_s = 0$, $A_{cur}^{CF} \approx 49.8$ nCi, which is only ≈0.1% smaller than the simulation result." *)

```
(* estimation *)
```

$$ACFCurEst = -N0 * P * \frac{nu}{alpha} * ProductLog\left[-1, -E^{-2} * \left(\frac{Ncur}{N0 * Nnor}\right)^{\frac{1}{P}}\right] / nCpm$$

```
(* simulation result *)
SetBasicParameterValues[]; ks = 0;

ACFCurSim = FindCurDose[] (* The function has to be initialized in the beginning of the notebook *)

(*comparison*)
PercentForm[ACFCurEst / ACFCurSim - 1]
```

Out[∘]=

49.7557

Out[∘]=

49.8243

Out[∘]//PercentForm=

−0.1377%

In[∘]:=

(* "The minimal number of radioconjugate molecules, that should anchor on each cancer cell in order to achieve cure, is about 76 in this case." *)

$$\frac{ACFCurSim * nCpm * 6.02214076 * 10^{11}}{N0 * Nnor}$$

Out[∘]=

76.0124

In[ ]:=

```
(* "The cancer cell proliferation,however,still affects the formula for A_cur^CF, yielding the factor
   e^-2 instead of e^-1 in self-damage-only case. Neglect of this alteration would lead to ≈13% error." *)

ACFCurEst = -N0 * P * nu/alpha * ProductLog[-1, -E^-1 * (Ncur/(N0 * Nnor))^(1/P)] / nCpm

PercentForm[ACFCurEst / ACFCurSim - 1]
```

Out[ ]=

43.1898

Out[ ]//PercentForm=

-13.32%

(* S.2.1.5 Influence of nuclide decay rate on minimal surviving fraction of cells *)

In[ ]:=

```
(* Supplementary Figure S.6 *)

NotebookDelete[pr];
pr = PrintTemporary["Processing cross-fire-only case"];

SetBasicParameterValues[];
ks = 0;
nn = 160;
Nnor = 10^3;
gamma = 2.1 * Nnor / 10^7;
nu = 0.015 * Nnor / 10^7;
N0 = 3. * 10^7 / Nnor;

Array0lambda1 = Array[f, {nn, 2}];

For[ii = 1, ii ≤ nn, ii++,

  lambda = rho / 0.01 / ii;
```

```
  Array0lambda1[[ii, 1]] = rho / lambda;
  Array0lambda1[[ii, 2]] = FullSystemSolutionMD[] / (N0 * Nnor);
]

SetBasicParameterValues[];
ks = 0;
nn = 40;

Array0lambda2 = Array[f, {nn - 1, 2}];

For[ii = 1, ii ≤ nn - 1, ii++,

  lambda = rho / 0.25 / (ii + 1);

  Array0lambda2[[ii, 1]] = rho / lambda;
  Array0lambda2[[ii, 2]] = FullSystemSolutionMD[] / (N0 * Nnor);
]
Array0lambda = Join[Array0lambda1, Array0lambda2];
SetBasicParameterValues[];
ks = 1;
nn = 160;
Nnor = 10^3;
gamma = 2.1 * Nnor / 10^7;
nu = 0.015 * Nnor / 10^7;
N0 = 3. * 10^7 / Nnor;

Array1lambda1 = Array[f, {nn, 2}];

For[ii = 1, ii ≤ nn, ii++,
```

```
    lambda = rho / 0.01 / ii;

    Array1lambda1[[ii, 1]] = rho / lambda;
    Array1lambda1[[ii, 2]] = FullSystemSolutionMD[] / (N0 * Nnor);
  ]

  NotebookDelete[pr];
  pr = PrintTemporary["Processing self-damage-only case"];

  SetBasicParameterValues[];
  ks = 1;
  nn = 40;

  Array1lambda2 = Array[f, {nn - 5, 2}];

  For[ii = 1, ii ≤ nn - 5, ii++,

    lambda = rho / 0.25 / (ii + 5);

    Array1lambda2[[ii, 1]] = rho / lambda;
    Array1lambda2[[ii, 2]] = FullSystemSolutionMD[] / (N0 * Nnor);
  ]
  Array1lambda = Join[Array1lambda1, Array1lambda2];

  NotebookDelete[pr];
  pr = PrintTemporary["Plotting"];

  SetBasicParameterValues[];
```

$$PHI = \frac{alpha * DA1}{nu * N0}; P = \frac{rho}{lambda};$$

$$PAc = \frac{rho}{lambda};$$

```
PRa = rho / N[Log[2] / 3.6319];
```

$$Show\left[LogPlot\left[\left\{\left(\frac{PHI}{P}\right)^{\frac{P}{1+P}} * Exp\left[\frac{P - PHI}{1 + P}\right], \left(\frac{PHI}{P}\right)^{P} * Exp[2 * P - PHI]\right\}, \{P, 0, PHI\}, AxesLabel \to \left\{"\rho / \lambda", "minimal surviving fraction, \frac{N_m}{N_0}"\right\},\right.\right.$$

```
PlotRange → {{0, 10}, {0, 1}}, PlotStyle → {Directive[Darker[Blue], Dashed, Thin], Directive[Darker[Red], Dashed, Thin]},
Ticks → {Automatic, {{10^-16, "10^-16"}, {10^-12, "10^-12"}, {10^-8, "10^-8"}, {10^-4, "10^-4"}, 1}}],

ListLogPlot[Array0lambda, Joined → True, PlotStyle → Darker[Red]],
ListLogPlot[Array1lambda, Joined → True, PlotStyle → Darker[Blue]],
```

$$ListLogPlot\left[\left\{\left\{PAc, \left(\frac{PHI}{PAc}\right)^{\frac{PAc}{1+PAc}} * Exp\left[\frac{PAc - PHI}{1 + PAc}\right]\right\}, \left\{PAc, \left(\frac{PHI}{PAc}\right)^{PAc} * Exp[2 * PAc - PHI]\right\} (*Actinium*)\right\}, PlotStyle \to Darker[Yellow]\right],$$

$$ListLogPlot\left[\left\{\left\{PRa, \left(\frac{PHI}{PRa}\right)^{\frac{PRa}{1+PRa}} * Exp\left[\frac{PRa - PHI}{1 + PRa}\right]\right\}, \left\{PRa, \left(\frac{PHI}{PRa}\right)^{PRa} * Exp[2 * PRa - PHI] (*Radium*)\right\}\right\}, PlotStyle \to Darker[Green]\right]$$

```
]

NotebookDelete[pr];
```

Out[ ]=

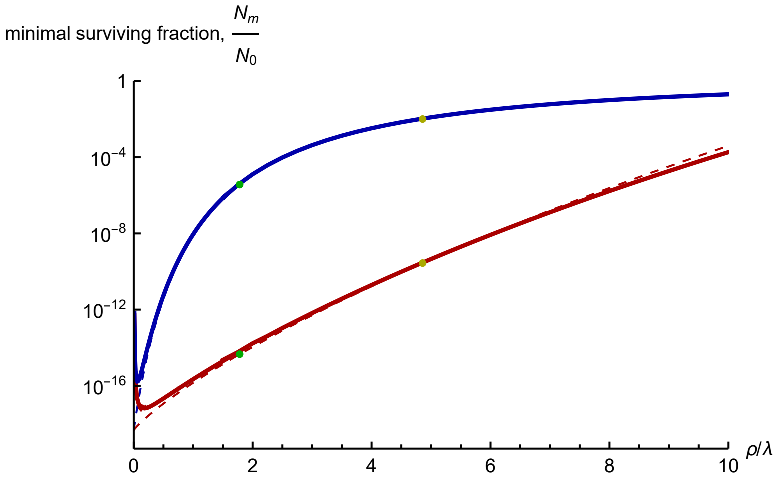

(* S.2.1.6 The case of non−negligible self−damage and cross−fire, 0<ks<1 *)

In[∘]:=

(* "It also follows from Eqs.(S.5),that the ratio $A_{cur}(1)/A_{cur}(0)$ grows with the increase of cancer cells proliferation rate ρ." *)

$$\text{Plot}\left[\frac{\text{ProductLog}\left[-1, -E^{-1} * \left(\frac{\text{Ncur}}{\text{N0}*\text{Nnor}}\right)^{\frac{\text{lambda}}{\text{rho}}} * \left(\frac{\text{Ncur}}{\text{N0}*\text{Nnor}}\right)\right]}{\text{ProductLog}\left[-1, -E^{-1} * \left(\frac{\text{Ncur}}{\text{N0}*\text{Nnor}}\right)^{\frac{\text{lambda}}{\text{rho}}} * E^{-1}\right]}, \{\text{rho}, 0.15, 0.7\}, \text{AxesLabel} \to \{\text{"rho"}, \text{"}A^{SD}_{cur} / A^{CF}_{cur}\text{"}\}\right]$$

(* more general case, x replaces $\left(\frac{\text{Ncur}}{\text{N0}*\text{Nnor}}\right)^{\frac{\text{lambda}}{\text{rho}}}$, the ratio of two Lambert functions of this kind changes monotonically with x and therefore with rho *)

$$\text{epsilon} = E^{-1} * \frac{\text{Ncur}}{\text{N0} * \text{Nnor}};$$

$$\text{LogLinearPlot}\left[\frac{\text{ProductLog}[-1, -x * \text{epsilon}]}{\text{ProductLog}\left[-1, -x * E^{-2}\right]}, \{x, 10^{\wedge}-100, 1\}, \text{PlotRange} \to \{1, 8\}\right]$$

Out[∘]=

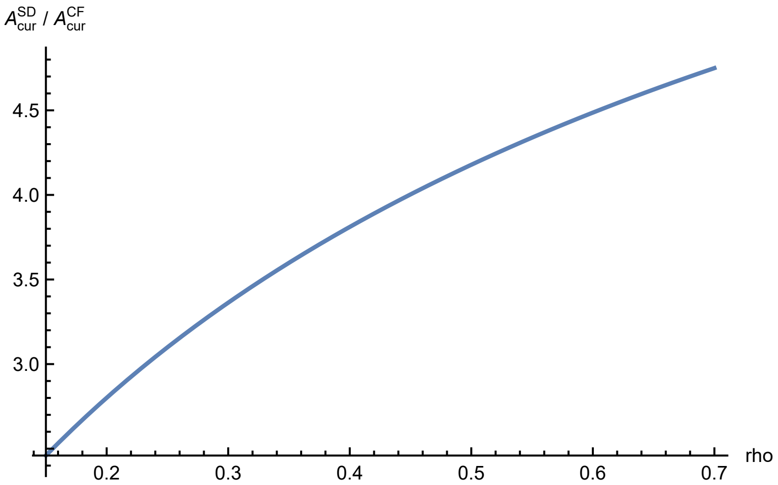

Out[ ]=

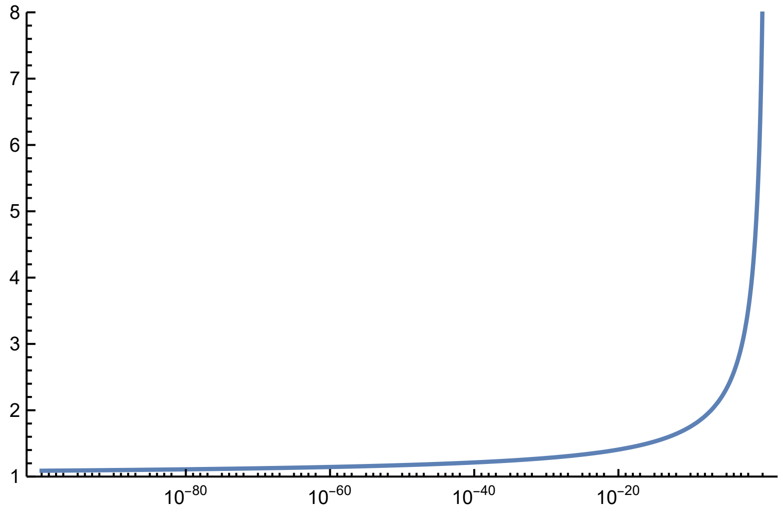

(* Supplementary Figure S.7 & Figure 2C of main paper *)

In[ ]:=

(* Numerical curve is plotted using the arrays saved here *)

In[ ]:=

```
result = {{0, 49.82433261727928`}, {0.05`, 51.580149715108085`}, {0.1`, 53.48635508601829`},
    {0.15000000000000002`, 55.563178958749404`}, {0.2`, 57.83510824791471`}, {0.25`, 60.331018204079555`}, {0.3`, 63.08592774814234`},
    {0.35`, 66.14196490524405`}, {0.39999999999999997`, 69.5514386399348`}, {0.44999999999999996`, 73.37806759024025`},
    {0.49999999999999994`, 77.70246548760205`}, {0.5499999999999999`, 82.6253446421229`},
    {0.6`, 88.27216757867569`}, {0.65`, 94.80727747866983`}, {0.7000000000000001`, 102.43648729826276`},
    {0.7500000000000001`, 111.41467158955737`}, {0.8000000000000002`, 122.03426906040734`},
    {0.8500000000000002`, 134.53163548519737`}, {0.9000000000000002`, 148.7406284647777`},
    {0.9500000000000003`, 163.58514025695342`}, {1.0000000000000002`, 178.02513524105674`}};
```

In[ ]:=

```
result10 = {{0, 51.35981136694887`}, {0.05`, 51.85858963127424`}, {0.1`, 52.36838261883959`},
    {0.15000000000000002`, 52.889234213004436`}, {0.2`, 53.4219343142402`}, {0.25`, 53.966790106063506`}, {0.3`, 54.52406488863149`},
    {0.35`, 55.09454856241555`}, {0.39999999999999997`, 55.67780229382049`}, {0.44999999999999996`, 56.27461598331766`},
    {0.49999999999999994`, 56.887622632478404`}, {0.5499999999999999`, 57.514803606764715`},
    {0.6`, 58.15712434008607`}, {0.65`, 58.81585744986856`}, {0.7000000000000001`, 59.49166118650509`},
    {0.7500000000000001`, 60.18440389991702`}, {0.8000000000000002`, 60.89487549057582`},
    {0.8500000000000002`, 61.62518235973847`}, {0.9000000000000002`, 62.374754023731214`},
    {0.9500000000000003`, 63.14512640013731`}, {1.0000000000000002`, 63.93559735520441`}};
```

In[ ]:=

```
result11 = {{0, 49.978450975920026`}, {0.05`, 51.36845638877468`}, {0.1`, 52.85109957357996`},
    {0.15000000000000002`, 54.43594710271163`}, {0.2`, 56.13423311620725`}, {0.25`, 57.95868378832823`}, {0.3`, 59.92426334467149`},
    {0.35`, 62.047559695136286`}, {0.39999999999999997`, 64.3498562690907`}, {0.44999999999999996`, 66.85480619731703`},
    {0.49999999999999994`, 69.58904667904503`}, {0.5499999999999999`, 72.58663120126367`},
    {0.6`, 75.88736197105922`}, {0.65`, 79.53942291718676`}, {0.7000000000000001`, 83.60095949101269`},
    {0.7500000000000001`, 88.14455476918614`}, {0.8000000000000002`, 93.25810712082944`},
    {0.8500000000000002`, 99.05588881413739`}, {0.9000000000000002`, 105.67578136472775`},
    {0.9500000000000003`, 113.29656557929245`}, {1.0000000000000002`, 122.1443285260882`}};
```

In[ ]:=

```
result12 = {{0, 49.839735676471456`}, {0.05`, 51.55035291399277`}, {0.1`, 53.40363495331959`},
    {0.15000000000000002`, 55.41731067169876`}, {0.2`, 57.615428150148325`}, {0.25`, 60.02304478695519`}, {0.3`, 62.67223931792985`},
    {0.35`, 65.6016290994515`}, {0.39999999999999997`, 68.85716000893957`}, {0.44999999999999996`, 72.49662643088433`},
    {0.49999999999999994`, 76.59107552435165`}, {0.5499999999999999`, 81.22871284198045`},
    {0.6`, 86.52174813406806`}, {0.65`, 92.61223183538682`}, {0.7000000000000001`, 99.68096338716667`},
    {0.7500000000000001`, 107.9560484922022`}, {0.8000000000000002`, 117.71553211642384`},
    {0.8500000000000002`, 129.25606577019943`}, {0.9000000000000002`, 142.73448858045995`},
    {0.9500000000000003`, 157.64403551144707`}, {1.0000000000000002`, 172.6459130308682`}};
```

```
(* The main numerical array for rapid-internalization-case is obtained by this code, but it runs about 10-15 minutes *)
SetBasicParameterValues[];
Var = "ks"; VarSymbol = "ks";
(* Setting boundaries and step of variation*)
Nx = 21; (* number of points *)
varmin = 0; varmax = 1; varstep = (varmax - varmin) / (Nx - 1);
result = Array[f, {Nx, 2}];
ii = 1;
Quiet[For[ks = varmin, ks ≤ varmax, ks = N[ks + varstep],
    NotebookDelete[pr]; (* To see the code running *)
    pr = PrintTemporary["Current " <> ToString[Var] <> " is " <> ToString[N[ks]] <> "; max value is " <> ToString[varmax]];

    result[[ii, 1]] = ks;
    result[[ii, 2]] = FindCurDose[]; (* The function has to be initialized in the beginning of the notebook *)
    ii++;]];

(* The arrays accounting for unbinding are obtained by the codes hidden here *)
```

In[∘]:=

```
(* Full system with unbinding *)
FullSystemSolutionMDUnb[] := (

   tEnd = Ainj[[Length[Ainj], 1]] + (-Log[0.0017] / lambda);
   lAinj = Length[Ainj]; (* number of injections *)
   If[Ainj[[1, 1]] == 0, Npw = lAinj, Npw = lAinj + 1];
   (* the injections are treated as new initial conditions for a new system, which as well takes the actual values of other parameters *)
   (* therefore the number of injections has to me remembered and logic differs whether the first injection is made at t=0 or t>0,
   although the letter case will never be considered in this study from now on *)

   apw = Array[ff, Npw]; bpw = Array[ff, Npw]; NNpw = Array[ff, Npw]; DDpw = Array[ff, Npw];
   papw = Array[ff, Npw]; pbpw = Array[ff, Npw]; fFNpw = Array[ff, Npw]; fANpw = Array[ff, Npw];
   (*fFDpw=Array[ff,Npw];fADpw=Array[ff,Npw];*) dFpw = Array[ff, Npw]; dApw = Array[ff, Npw];
```

```
(* decayed antibody fragments are as well accounted for as pb *)

(* times of beginnings and ends for solution of separate systems will be here *)
tB = Array[ff, Npw]; tE = Array[ff, Npw];

(* for monitoring the paths of activity *)
ActBloodpw = Array[ff, Npw]; ActBloodFragpw = Array[ff, Npw];
ActOutpw = Array[ff, Npw]; ActOutFragpw = Array[ff, Npw]; ActTumorpw = Array[ff, Npw];

(* for monitoring the influences of self-dose, cross-fire and decays in blood *)
SDpw = Array[ff, Npw]; CFNpw = Array[ff, Npw]; CFDpw = Array[ff, Npw]; UNpw = Array[ff, Npw];

(* for monitoring the number of new cancer cells appearing during treatment *)
NewCellspw = Array[ff, Npw];

Clear[a, b, NN, DD, pa, pb, fFN, fAN, dF, dA, ActBlood, ActBloodFrag, ActOut, ActOutFrag, ActTumor, SD, CFN, CFD, UN, NewCells];
(*fFD,fAD,*)

(* EQUATIONS *)

(* Radiation damage function *) (*fAD_,*)
RD[NN_, DD_, fAN_, dA_, a_, pa_] := alpha * (ks * (lambda * gamma * fAN)/nu (*self-dose*) +
    (1 - ks) * (lambda * gamma * (fAN * NN + dA))/(nu * (NN + DD)) (*cross-fire*) + kf * lambda * (a + pa) (*dose from unanchored nuclides*));


(* Active antibodies *)
Fa[t_] := (*injections are considered as initial conditions*)
  - lambda * a[t] (*decay*) - kon * gamma/V * (fFN[t] * NN[t] + dF[t]) * a[t] (*binding*) +
```

```
koff * gamma/V * (fAN[t] * NN[t] + dA[t]) (*unbinding*) - kappac * a[t] (*clearance*);
(*fFD[t]*DD[t]*)

(* Inert antibodies *)
Fb[t_] := (*injections are considered as initial conditions*)
  + lambda * a[t] (*decay of a*) - kon * gamma/V * (fFN[t] * NN[t] + dF[t]) * b[t] (*binding*) +
  koff * gamma/V * ((1 - fFN[t] - fAN[t]) * NN[t] + (DD[t] - dF[t] - dA[t])) (*unbinding*) - kappac * b[t] (*clearance*);
(*fFD[t]*DD[t]*)

(* Viable cells *) FNN[t_] := rho * NN[t] (*proliferation*) - RD[NN[t], DD[t], fAN[t], dA[t], a[t], pa[t]] * NN[t] (*damage*);
(*fAD[t],*)

(* Damaged cells *) FDD[t_] := RD[NN[t], DD[t], fAN[t], dA[t], a[t], pa[t]] * NN[t] (*damage*) - omega * DD[t] (*death*);
(*fAD[t],*)

(* Active fragments *) Fpa[t_] := omega * (gamma * dA[t])/V (*release*) - lambda * pa[t] (*decay*) - kappap * pa[t] (*clearance*);
(*fAD[t]*DD[t],*)

(* Inert fragments *)
Fpb[t_] := omega * (gamma * (DD[t] - dF[t] - dA[t]))/V (*release*) + lambda * pa[t] (*decay*) - kappap * pb[t] (*clearance*);
(*-fFD[t]*DD[t] - fAD[t]*DD[t]*)

(* Free receptors of viable cells *) FfFN[t_] := (1 - fFN[t]) * rho - kon * (a[t] + b[t]) * fFN[t] + koff * (1 - fFN[t]);

(* Active receptors of viable cells *) FfAN[t_] := kon * a[t] * fFN[t] - (koff + lambda + rho) * fAN[t];
```

```
(* Free receptors of damaged cells *)
(*FfFD[t_]:=(fFN[t]-fFD[t])*RD[NN[t],DD[t],fAN[t],fAD[t],a[t],pa[t]]*NN[t]/DD[t]-kon*(a[t]+b[t])*fFD[t];*)
FdF[t_] := RD[NN[t], DD[t], fAN[t], dA[t], a[t], pa[t]] * fFN[t] * NN[t]
   - kon * (a[t] + b[t]) * dF[t] - omega * dF[t] + koff * (DD[t] - dF[t]);

(* Active receptors of damaged cells *)
(*FfAD[t_]:=(fAN[t]-fAD[t])*RD[NN[t],DD[t],fAN[t],fAD[t],a[t],pa[t]]*NN[t]/DD[t]+kon*a[t]*fFD[t]-lambda*fAD[t];*)
FdA[t_] :=
  RD[NN[t], DD[t], fAN[t], dA[t], a[t], pa[t]] * fAN[t] * NN[t] + kon * a[t] * dF[t] - (koff + lambda) * dA[t] - omega * dA[t];

(* Initial conditions *)

If[Ainj[[1, 1]] == 0
  , a0 = Ainj[[1, 2]] / V; b0 = eta * Ainj[[1, 2]] / V
  , a0 = 0; b0 = 0]; (*complexes in blood*)
NN0 = N0;
DD0 = 0;
pa0 = 0; pb0 = 0; fFN0 = 1; fAN0 = 0;
(*fFD0=1;fAD0=0;*) dF0 = 0; dA0 = 0;
ActBlood0 = 0; ActBloodFrag0 = 0; ActOut0 = 0; ActOutFrag0 = 0; ActTumor0 = 0; NewCells0 = 0;
SD0 = 0; CFN0 = 0; CFD0 = 0; UN0 = 0;

(* SOLVER *)

tB[[1]] = 0; If[Ainj[[1, 1]] == 0, If[lAinj > 1, tE[[1]] = Ainj[[2, 1]], tE[[1]] = tEnd], tE[[1]] = Ainj[[1, 1]]];

For[npw = 1, npw ≤ Npw, npw++,

  Clear[a, b, NN, DD, pa, pb, fFN, fAN, dF, dA, ActBlood, ActBloodFrag, ActOut, ActOutFrag, ActTumor, SD, CFN, CFD, UN, NewCells];
  (*fFD,fAD,*)
```

```
sol = NDSolve[{

  (*INITIAL CONDITIONS*)
  a[tB[[npw]]] == a0, b[tB[[npw]]] == b0, NN[tB[[npw]]] == NN0,
  DD[tB[[npw]]] == DD0, pa[tB[[npw]]] == pa0, pb[tB[[npw]]] == pb0, fFN[tB[[npw]]] == fFN0, fAN[tB[[npw]]] == fAN0,
  (*fFD[tB[[npw]]]==fFD0,fAD[tB[[npw]]]==fAD0,*) dF[tB[[npw]]] == dF0, dA[tB[[npw]]] == dA0,
  ActBlood[tB[[npw]]] == ActBlood0, ActBloodFrag[tB[[npw]]] == ActBloodFrag0, ActOut[tB[[npw]]] == ActOut0,
  ActOutFrag[tB[[npw]]] == ActOutFrag0, ActTumor[tB[[npw]]] == ActTumor0, SD[tB[[npw]]] == SD0,
  CFN[tB[[npw]]] == CFN0, CFD[tB[[npw]]] == CFD0, UN[tB[[npw]]] == UN0, NewCells[tB[[npw]]] == NewCells0,

  a'[t] == Fa[t], b'[t] == Fb[t], NN'[t] == FNN[t], DD'[t] == FDD[t], pa'[t] == Fpa[t], pb'[t] == Fpb[t],
  fFN'[t] == FfFN[t], fAN'[t] == FfAN[t], (*fFD'[t]==FfFD[t],fAD'[t]==FfAD[t],*) dF'[t] == FdF[t], dA'[t] == FdA[t],

  ActBlood'[t] == V*lambda*(a[t] + pa[t]),
  ActBloodFrag'[t] == V*lambda*pa[t],
  ActOut'[t] == V*(kappac*a[t] + kappap*pa[t]),
  ActOutFrag'[t] == V*kappap*pa[t],
  ActTumor'[t] == (lambda*gamma)*(fAN[t]*NN[t] + dA[t]), (*fAD[t]*DD[t]*)

  SD'[t] == ks*(lambda*gamma)*(fAN[t]*NN[t]),
  CFN'[t] == (1 - ks)*(lambda*gamma)*(fAN[t]*NN[t])*NN[t]/(NN[t] + DD[t]),
  CFD'[t] == (1 - ks)*(lambda*gamma)*dA[t]*NN[t]/(NN[t] + DD[t]), (*(fAD[t]*DD[t])*)
  UN'[t] == kf*lambda*(a[t] + pa[t])*nu*NN[t],
  NewCells'[t] == If[t > Ainj[[1, 1]], rho*NN[t], 0] (* start counting new cells from the moment of the first injection *)

  (* The simulations run until the injected activity decays to negligible amounts.
     If viable cancer cell number becomes too great (host death), it is just kept further to allow assessment of activity paths *)
  (* The cases of cancer cure will use additional script to virtually eliminate all cancer cells in case of cure *)
```

```
        , WhenEvent[NN[t] > 10^9 / Nnor, NN[t] → 0.99 * 10^9 / Nnor]
      }(*fFD,fAD,*)
      , {a, b, NN, DD, pa, pb, fFN, fAN, dF, dA, ActBlood,
        ActBloodFrag, ActOut, ActOutFrag, ActTumor, SD, CFN, CFD, UN, NewCells}, {t, tB[[npw]], tE[[npw]]}
      , AccuracyGoal → 10, PrecisionGoal → 10];

apw[[npw]] = First[a /. sol]; bpw[[npw]] = First[b /. sol]; NNpw[[npw]] = First[NN /. sol]; DDpw[[npw]] = First[DD /. sol];
papw[[npw]] = First[pa /. sol]; pbpw[[npw]] = First[pb /. sol]; fFNpw[[npw]] = First[fFN /. sol]; fANpw[[npw]] = First[fAN /. sol];
(*fFDpw[[npw]]=First[fFD/.sol]; fADpw[[npw]]=First[fAD/.sol];*) dFpw[[npw]] = First[dF /. sol]; dApw[[npw]] = First[dA /. sol];
ActBloodpw[[npw]] = First[ActBlood /. sol];
ActBloodFragpw[[npw]] = First[ActBloodFrag /. sol];
ActOutpw[[npw]] = First[ActOut /. sol];
ActOutFragpw[[npw]] = First[ActOutFrag /. sol];
ActTumorpw[[npw]] = First[ActTumor /. sol];
SDpw[[npw]] = First[SD /. sol];
CFNpw[[npw]] = First[CFN /. sol];
CFDpw[[npw]] = First[CFD /. sol];
UNpw[[npw]] = First[UN /. sol];
NewCellspw[[npw]] = First[NewCells /. sol];

If[npw < Npw,
  (*renew initial conditions*)
  If[Ainj[[1, 1]] == 0
    , a0 = apw[[npw]][tE[[npw]]] + Ainj[[npw + 1, 2]] / V; b0 = bpw[[npw]][tE[[npw]]] + eta * Ainj[[npw + 1, 2]] / V
    , a0 = apw[[npw]][tE[[npw]]] + Ainj[[npw, 2]] / V; b0 = bpw[[npw]][tE[[npw]]] + eta * Ainj[[npw, 2]] / V];
  NN0 = NNpw[[npw]][tE[[npw]]];
  DD0 = DDpw[[npw]][tE[[npw]]];
  pa0 = papw[[npw]][tE[[npw]]];
  pb0 = pbpw[[npw]][tE[[npw]]];
  fFN0 = fFNpw[[npw]][tE[[npw]]]; fAN0 = fANpw[[npw]][tE[[npw]]];
```

```
      (*fFD0=fFDpw[[npw]][tE[[npw]]];fAD0=fADpw[[npw]][tE[[npw]]];*)
      dF0 = dFpw[[npw]][tE[[npw]]];
      dA0 = dApw[[npw]][tE[[npw]]];
      ActBlood0 = ActBloodpw[[npw]][tE[[npw]]];
      ActBloodFrag0 = ActBloodFragpw[[npw]][tE[[npw]]];
      ActOut0 = ActOutpw[[npw]][tE[[npw]]];
      ActOutFrag0 = ActOutFragpw[[npw]][tE[[npw]]];
      ActTumor0 = ActTumorpw[[npw]][tE[[npw]]];
      SD0 = SDpw[[npw]][tE[[npw]]];
      CFN0 = CFNpw[[npw]][tE[[npw]]];
      CFD0 = CFDpw[[npw]][tE[[npw]]];
      UN0 = UNpw[[npw]][tE[[npw]]];
      NewCells0 = NewCellspw[[npw]][tE[[npw]]];

      (* renew time frame *)
      If[Ainj[[1, 1]] == 0, If[Npw > npw + 1, tE[[npw + 1]] = Ainj[[npw + 2, 1]], tE[[npw + 1]] = tEnd],
        If[Npw > npw + 1, tE[[npw + 1]] = Ainj[[npw + 1, 1]], tE[[npw + 1]] = tEnd]];]
   ];
   npw--;
   (* It will be convenient to have estimation of minimal viable cell number here *)
   Nn = If[Ainj[[1, 1]] == 0
      , Min[Table[NMinimize[{Nnor* (NNpw[[nn]][t]), t > tB[[nn]], t < tE[[nn]]}, t][[1]], {nn, 1, npw}]]
      , Min[Table[NMinimize[{Nnor* (NNpw[[nn]][t]), t > tB[[nn]], t < tE[[nn]]}, t][[1]], {nn, 2, npw}]]];
   tminNn = If[Ainj[[1, 1]] == 0
      , Min[Table[t /. NMinimize[{Nnor* (NNpw[[nn]][t]), t > tB[[nn]], t < tE[[nn]]}, t][[2]], {nn, 1, npw}]]
      , Min[Table[t /. NMinimize[{Nnor* (NNpw[[nn]][t]), t > tB[[nn]], t < tE[[nn]]}, t][[2]], {nn, 2, npw}]]];Return[Nn])

SetBasicParameterValuesUnb[] := (
   Nnor = 10^5; (* due to numerical pecularities, it is better to use this normalization number of cells *)
```

```
lambda = 0.07; (* radionuclide decay rate *)
kon = 11.15; (* antibody–receptor association rate *)
koff = 4.1;
kappac = 0.1; (* antibody clearance rate (0.04–0.28) *)
kappap = 1; (* antibody fragments clearance rate (0.4–4.) *)
gamma = 2.1 * Nnor / 10 ^ 7; (* number of receptors of Nnor cancer cells (0.13–10) *Nnor / 10^7 *)
V = 1.; (* volume of drug distribution (0.75–1.5) *)
nu = 0.015 * Nnor / 10 ^ 7; (* volume of lesion with Nnor cancer cells *)
ks = 0.3; (* relative significance of self–dose (0.1–0.5) *)

rho = 0.34; (* cancer cells proliferation rate (0.15–0.7) *)
omega = 0.05; (* damaged cells death rate (0.005–0.5) *)
alpha = 500; (* cancer cells radiosensitivity (50–5000) *)
eta = 0; (* coefficient of drug impurity (0–10^5) *)
kf = 0.05; (* significance of unanchored nuclides decays (0.01–0.25) *)
N0 = 3. * 10 ^ 7 / Nnor; (* initial number of viable cancer cells, xNnor (1–10) *Nnor / 10^7 *)
D0 = 0;
(*0.1 / Nnor; *)
(* initial number of damaged cancer cells –– use non–zero value if considering fraction of receptors of damaged cells, to avoid division by zero*)
nCpm = 0.000076; (* parameter of conversion from nCi to pmol *)
DA1 = 50 * nCpm; (* injected amount of radionuclides *)
t1 = 0; (* moment of their injection *)
Ncur = 0.01; (* number of viable cancer cells (in cells), corresponding to cancer cure *)
Abld = 0.0175; (* critical amount of decays in blood, leading to death of toxicity (230 nCi) *)
Cd = 10 ^ 11; (* critical number of cancer cells, leading to death of tumor burden *)
Ainj = {{t1, DA1}}; (* schedule of injections *)

kGy = 2500; (* coefficient of conversion of nM of 225–Ac decay in bone marrow into Gy *)
)
```

FindCurDoseUnb[ ] := (DA1 = $\frac{\text{nu} * \text{N0} * \text{rho}}{\text{alpha} * \text{lambda}}$; (* at this dose N' (0) =0 for approximated system *)

```
    Ainj = {{t1, DA1}};

    While[FullSystemSolutionMDUnb[] > Ncur, DA1 = 2 * DA1; Ainj = {{t1, DA1}};];
    Dmax = DA1; Dmin = DA1 / 2;

    While[Abs[Dmin - Dmax] / Dmax > 0.000001,
      DA1 = (Dmin + Dmax) / 2; Ainj = {{t1, DA1}};
      If[FullSystemSolutionMDUnb[] > Ncur, Dmin = DA1, Dmax = DA1]];
    Dcur = Dmax;
    Return[Dcur / nCpm])

SetBasicParameterValuesUnb[];
koff = 1.113547684;
Var = "ks"; VarSymbol = "ks";
(* Setting boundaries and step of variation*)
Nx = 21; (* number of points *)
varmin = 0; varmax = 1; varstep = (varmax - varmin) / (Nx - 1);
result10 = Array[f, {Nx, 2}];
ii = 1;
Quiet[For[ks = varmin, ks ≤ varmax, ks = N[ks + varstep],
      NotebookDelete[pr]; (* To see the code running *)
      pr = PrintTemporary["Current " <> ToString[Var] <> " is " <> ToString[N[ks]] <> "; max value is " <> ToString[varmax]];

      result10[[ii, 1]] = ks;
      result10[[ii, 2]] = FindCurDoseUnb[];
      ii++;]];
```

```
SetBasicParameterValuesUnb[];
koff = 0.1113547684;
Var = "ks"; VarSymbol = "ks";
(* Setting boundaries and step of variation*)
Nx = 21; (* number of points *)
varmin = 0; varmax = 1; varstep = (varmax - varmin) / (Nx - 1);
result11 = Array[f, {Nx, 2}];
ii = 1;
Quiet[For[ks = varmin, ks ≤ varmax, ks = N[ks + varstep],
    NotebookDelete[pr]; (* To see the code running *)
    pr = PrintTemporary["Current " <> ToString[Var] <> " is " <> ToString[N[ks]] <> "; max value is " <> ToString[varmax]];

    result11[[ii, 1]] = ks;
    result11[[ii, 2]] = FindCurDoseUnb[];
    ii++;]];

SetBasicParameterValuesUnb[];
koff = 0.01113547684;
Var = "ks"; VarSymbol = "ks";
(* Setting boundaries and step of variation*)
Nx = 21; (* number of points *)
varmin = 0; varmax = 1; varstep = (varmax - varmin) / (Nx - 1);
result12 = Array[f, {Nx, 2}];
ii = 1;
Quiet[For[ks = varmin, ks ≤ varmax, ks = N[ks + varstep],
    NotebookDelete[pr]; (* To see the code running *)
    pr = PrintTemporary["Current " <> ToString[Var] <> " is " <> ToString[N[ks]] <> "; max value is " <> ToString[varmax]];

    result12[[ii, 1]] = ks;
    result12[[ii, 2]] = FindCurDoseUnb[];
    ii++;]];

(* Plotting the results and comparing with analytics *)
```

```
ASDCurEst = -(kappac + lambda + kon * gamma * N0 / V)/(kon * gamma * N0 / V) * N0 * rho/lambda * nu/alpha * ProductLog[-1, -E^-1 * (Ncur/(N0 * Nnor))^((lambda+rho)/rho)] / nCpm;
ACFCurEst = -(kappac + lambda + kon * gamma * N0 / V)/(kon * gamma * N0 / V) * N0 * rho/lambda * nu/alpha * ProductLog[-1, -E^-2 * (Ncur/(N0 * Nnor))^(lambda/rho)] / nCpm;

kk = 1 - ACFCurEst/ASDCurEst;
Thk = 0.0075; (* Thickness of graphs *)

AEst[ks_] := (* analytial approximation *)
  -(kappac + lambda + kon * gamma * N0 / V)/(kon * gamma * N0 / V) * N0 * 1/(1 - kk * ks) * rho/lambda * nu/alpha * ProductLog[-1, -E^-2 * (Ncur/(N0 * Nnor))^(lambda/rho)] / nCpm;
AEst1[ks_] := (* what does it look like with asymptote at ks=1 *)
  -(kappac + lambda + kon * gamma * N0 / V)/(kon * gamma * N0 / V) * N0 * 1/(1 - ks) * rho/lambda * nu/alpha * ProductLog[-1, -E^-2 * (Ncur/(N0 * Nnor))^(lambda/rho)] / nCpm;
AEstMol[ks_] := (AEst[ks] * nCpm * 6.02214076 * 10^11)/(N0 * Nnor); (* The dose in molecules per cancer cell *)

(* If the following does not work, remove the Show[] function from inside -- the results will be the same, just without the axis for molecules *)
AcurPlot = ResourceFunction["CombinePlots"][

    Show[(* Numerical result *) ListPlot[result, Joined → True, PlotStyle → Directive[Darker[Cyan], Thickness[Thk]],
      Frame → True, FrameLabel → {"Relative significance of self-damage, k_s", "Minimal single curative dose, A_cur (nCi)"},
      PlotRange → {{-0.02, 1.02}, {0, 1.05 * AEst[1]}}],
     ListPlot[{{(*point for cross-fire only*) 0, ACFCurEst}, {(*point for self-damage only*) 1, ASDCurEst}}, PlotStyle → Darker[Red]],

     (* analytical approximation, ks=0 *) Plot[AEst[ks], {ks, 0, 1}, PlotStyle → Directive[Darker[Red], Dashed, Thickness[Thk / 1.3]]],
     (* what it was with asymptote at ks=1 *)
     Plot[AEst1[ks], {ks, 0, 1}, PlotStyle → Directive[Lighter[Gray], Dotted]],
```

```
(* KD=10^{-12} *) ListPlot[result12, Joined → True, PlotStyle → Directive[Darker[Green], Thickness[Thk / 3]]],
(* KD=10^{-11} *) ListPlot[result11, Joined → True, PlotStyle → Directive[Darker[Yellow], Thickness[Thk / 3]]],
(* KD=10^{-10} *) ListPlot[result10, Joined → True, PlotStyle → Directive[Darker[Orange], Thickness[Thk / 3]]],

ImageSize → 400],

Plot[AEstMol[ks], {ks, 0, 1}, Frame → True, FrameLabel → {None, "Minimal curative number
of radioconjugates per cell, Mm"}, PlotStyle → Directive[Opacity[0, Darker[Cyan]], Thickness[0]],
PlotRange → {{-0.02, 1.02}, {0, 1.05 * AEstMol[1]}}], "AxesSides" → "TwoY"]

resInt = Interpolation[result];
```

Out[•]=

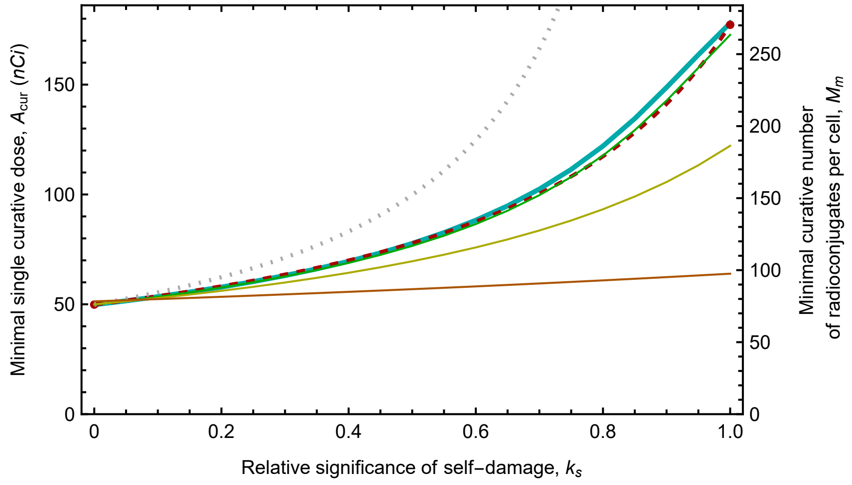

In[◦]:=

```
(* "The relative error, maximal by its absolute value, is ≈-5%, which is attained at ks≈0.89" *)
kMaxEr = kx /. NMaximize[{Abs[(AEst[kx] - resInt[kx])/resInt[kx]], kx > 0, kx < 1}, kx][[2]]
PercentForm[(AEst[kMaxEr] - resInt[kMaxEr])/resInt[kMaxEr]]
```

Out[◦]=

0.890548

Out[◦]//PercentForm=

−5.103%

In[◦]:=

```
(* For ks<0.5 the correspondence of analytical estimation to the numerical results is better.
   The maximum error in that region is only ≈0.8%, at ks≈0.29 *)
kMaxEr = kx /. NMaximize[{Abs[(AEst[kx] - resInt[kx])/resInt[kx]], kx > 0, kx < 0.5}, kx][[2]]
PercentForm[(AEst[kMaxEr] - resInt[kMaxEr])/resInt[kMaxEr]]
```

Out[◦]=

0.287528

Out[◦]//PercentForm=

0.7903%

```
(* Supplementary S.2.2 Global parameter sweep *)
(* S.2.2.1 Curative dose and toxicity: highly idealized in silico study *)
```

In[◦]:=

```
(* The outcome of the global parameter sweep, that was generated in this study
   is saved here in a closed cell -- open this group of cells to see it, and run it to upload it in the array "result" *)
```

In[◦]:=

```
Npar = 1000;
```

```
result = {{"\!\(\*SubscriptBox[\(κ\), \(c\)]\)", "\!\(\*SubscriptBox[\(κ\), \(p\)]\)", "γ", "V", "\!\(\*SubscriptBox[\(k\), \(s\)]\)",
    "ρ", "ω", "α", "\!\(\*SubscriptBox[\(k\), \(f\)]\)", "\!\(\*SubscriptBox[\(N\), \(0\)]\)", "\!\(\*SubsuperscriptBox[\(A\),
       \(cur\), \(sim\)]\)", "\!\(\*SubsuperscriptBox[\(A\), \(cur\), \(est\)]\)", "Err", "\!\(\*SubscriptBox[\(M\), \(m\)]\)",
    "Canc", "\!\(\*SubsuperscriptBox[\(Bl\), \(Frg\), \(sim\)]\)", "\!\(\*SubsuperscriptBox[\(Bl\), \(Frg\), \(est\)]\)",
    "Err", "\!\(\*SubsuperscriptBox[\(Bl\), \(Ab\), \(sim\)]\)", "\!\(\*SubsuperscriptBox[\(Bl\), \(Ab\), \(est\)]\)", "Err",
    "\!\(\*SubsuperscriptBox[\(Cld\), \(Frg\), \(sim\)]\)", "\!\(\*SubsuperscriptBox[\(Cld\), \(Ab\), \(sim\)]\)", "ToVbl",
    "\!\(\*SubsuperscriptBox[\(fD\), \(Max\), \(sim\)]\)", "\!\(\*SubsuperscriptBox[\(fD\), \(Max\), \(est\)]\)", "Err", "Nnew"},
  {0.172487255493398`, 1.2765963995094278`, 0.2324603897081641`, 0.9331633444796544`, 0.5595329534566904`,
    0.18253234530133355`, 0.2215389709063929`, 78.80610179612091`, 0.19190036455619353`, 1.548441048845115`, 214.41134334919198`,
    207.9605568694953`, -0.030086031731963336`, 633.7494225407845`, 51.88885724307789`, 7.852380699072512`, 8.46957984219302`,
    0.07860025726890818`, 3.30980874307499`, 3.303426382527135`, -0.001928317024724957`, 143.2045846859041`, 8.155711804300676`,
    4.230252889821418`, 0.040396489489872844`, 0.0452707524171753`, 0.12066055711411261`, 0.14063230077180636`},
  {0.14090572053825962`, 0.941193557649755`, 3.8052282638940014`, 1.32622706664442`, 0.616835667267962`, 0.48175991442091237`,
    0.08306143231635953`, 874.0744302472489`, 0.12491077781228116`, 5.511666182290989`, 116.14463035325602`, 116.78387911717152`,
    0.005503902866376364`, 96.44522362622601`, 53.77614310141242`, 4.307876641586207`, 4.363113642793668`, 0.012822326589909672`,
    0.04594024540252656`, 0.046052946941483264`, 0.0024532202205305165`, 57.922082031583`, 0.09247490543070082`,
    1.3572908059392432`, 0.00041580424949438920`, 0.00042087110303805835`, 0.012185670420228556`, 0.4992913039737422`},
  {0.19800522656535097`, 1.47676715957736`, 1.6845843495593211`, 1.0829526404841552`, 0.6930225215765629`, 0.2749384033948816`,
    0.06680029700083288`, 3207.8452923633004`, 0.18818451872370445`, 6.655704647717006`, 31.85234431205763`, 31.259021427744262`,
    -0.01862729093031834`, 21.90341616153816`, 16.470648610459886`, 0.6927716343278364`, 0.7038917127546827`, 0.016051578724979976`,
    0.019227788345414283`, 0.019269930028809307`, 0.0021917072644017654`, 14.615177123744067`, 0.05438860839548863`,
    0.4339106872017466`, 0.0002134108763558462`, 0.0002159077297038627`, 0.011699747410498285`, 0.3966816865306545`},
  {0.2711930593993829`, 3.0021543969280486`, 4.914701090552741`, 1.464602766667476`, 0.8471176050384048`, 0.28257943270869035`,
    0.04953192991909177`, 1933.8633413872876`, 0.19773521340575795`, 9.992985154010565`, 105.1122999806916`, 101.07977095724142`,
    -0.03836400710659871`, 48.141851790516995`, 61.93777924492877`, 0.9815171406865896`, 0.9924527949770666`, 0.01114158259409237`,
    0.019648061972327012`, 0.019661112085877182`, 0.0006641934236848446`, 42.095228565321285`, 0.07612025767908728`,
    1.064603912697659`, 0.0001619480204992918`, 0.00016265775718876`, 0.004382496848563289`, 0.43826047829541626`},
  {0.27990766836391096`, 3.477149477100016`, 9.931945787531824`, 0.8846854018858359`, 0.8967333599095253`, 0.5903168559967787`,
    0.24882968868487906`, 980.8551320933655`, 0.02004206946214948`, 1.1376503486553808`, 42.64261609820358`, 39.66545271571148`,
    -0.06981662137322575`, 171.5534794889944`, 9.527350710580022`, 0.6514468309869851`, 0.6567586955447084`, 0.008153949493737711`,
    0.02083203689588108`, 0.020909658018422456`, 0.003726045750078288`, 32.359685824640756`, 0.0833006696399474`,
```

0.24145334643973618`, 0.0002818082903783825`, 0.0002868232075518361`, 0.017795491987549594`, 0.0622921393617321`},
{0.11737985888146019`, 1.1225833279930164`, 5.309079794927442`, 0.8606501327539497`, 0.8514188342619913`,
0.5642039473140849`, 0.008986110654413243`, 2018.9822529242097`, 0.06040743860925685`, 4.536035630396375`, 73.50474481605842`,
69.69060371965857`, -0.05188972638357603`, 74.16575319677162`, 65.10738687516829`, 0.48704443478204285`, 0.49084651667432805`,
0.007806437402342325 5`, 0.016454725642484173`, 0.016481954909987596`, 0.0016547992409621237`, 7.810685178258629`,
0.027592191054970964`, 0.4684222906030412`, 0.00023006889958498977`, 0.0002319707659080231`, 0.008266507669937306`,
0.2556949147227681`}, {0.17121040978700802`, 0.4138167442315588`, 6.781135564610604`, 1.3665406840239216`, 0.9405996253397015`,
0.6832439228870748`, 0.04185895434111264`, 62.7113536738392`, 0.18142736225630413`, 8.46259785026923`, 6740.283132770514`,
6277.626993286135`, -0.06864046070038154`, 3645.347471845764`, 4226.033102805461`, 363.22448919749195`, 364.932805049157`,
0.004703195688813 5`, 1.0102067865637314`, 1.0071498148230658`, -0.003026085135563239`, 2147.2625077839557`, 2.4708273985334896`,
27.7966729416582`, 0.008865347188700468`, 0.00892659029265536`, 0.006908145011280675`, 0.3694482222514075`},
{0.07382619562964193`, 0.625700255424527`, 9.939999934198667`, 1.4556453073248168`, 0.9580351575689623`,
0.4163162212955882`, 0.006430521616962508`, 92.19524127790851`, 0.19149999891406427`, 4.303065608360628`,
1542.7367784040691`, 1481.9321176658373`, -0.039413503061185295`, 1640.8858124348885`, 1411.7852785554671`,
12.959492167531545`, 13.06011765141086`, 0.007764616281139336`, 0.32950973390717775`, 0.32946979988045305`,
-0.00012119225205031725`, 115.8393937056663`, 0.34752071538998874`, 9.579899884743671`, 0.0027248701251583185`,
0.0027412022277232446`, 0.005993717797459164`, 0.17192171118319985`}, {0.1627307868596377`, 1.5327585946689206`,
6.648641489637967`, 1.0898921981857375`, 0.08271984399349908`, 0.5169005126785295`, 0.006231976105310307`,
2017.0154068595314`, 0.2464092033815996`, 1.6313338993270357`, 8.430502121381775`, 8.556708305914556`, 0.014970185964688021`,
23.652392413209434`, 7.731976325744168`, 0.029384283256860613`, 0.030100379371032878`, 0.024370038496857704`,
0.005283453201692766`, 0.005307309752248053`, 0.004515332992377719`, 0.643414467287702`, 0.012282578526394135`,
0.19068651770554665`, 0.000057929124983369 11`, 0.000059073303464282906`, 0.019751351004219098`, 0.3173954984082079`},
{0.08926805346894312`, 1.228740834290055`, 5.100082063336529`, 1.0507919032716577`, 0.3229790419061651`, 0.45218339761418835`,
0.024533377777917375`, 814.1470590464116`, 0.1414263079606694`, 5.247711540674161`, 79.58707568747478`, 81.22694426914325`,
0.020604709590134496`, 69.41240429307831`, 59.24509739514081`, 1.0931975565816683`, 1.113243741586046`, 0.018337202533694308`,
0.019577491832015136`, 0.019606127675526504`, 0.0014626921444829133`, 19.189378253114434`, 0.024966351252112245`,
1.4396072219952472`, 0.0002243230579958011`, 0.0002260003028259440 8`, 0.007476916751796292`, 0.6906851892379211`},
{0.17431851399024534`, 2.020213173041662`, 9.224968841892476`, 1.3485881267881612`, 0.8890825947176819`, 0.5177484922558413`,
0.03701694525181502`, 131.5752033573906`, 0.228949740900746`, 4.795606274383001`, 1236.3276616941878`, 1160.1582463118507`,
-0.06160940804152126`, 1179.9254299547554`, 810.6993251070622`, 14.223944843418302`, 14.321518634203725`, 0.006859826290072535`,
0.2363712688106008`, 0.23644910101605468`, 0.0003292794671938548`, 410.50572493273785`, 0.5886269761295168`, 7.54658368668 7806`,

0.0021094927294597055`, 0.0021239229165676207`, 0.006840595801252736`, 0.23530636164600358`}, {0.25439204271280724`,
0.8534844729419486`, 2.8150623547133353`, 0.8024992452769948`, 0.43579385025887163`, 0.4594767236090769`, 0.057877631906885676`,
1195.6419878079198`, 0.17833211044546654`, 5.195270679096961`, 61.08964901322261`, 62.25545012434214`, 0.019083447522626917`,
53.81755271785605`, 33.805007870077105`, 2.0607629978147903`, 2.0958116027160334`, 0.017007586480545456`, 0.020967772909181 36`,
0.021010988171895704`, 0.0020610325618042324`, 25.126131729260376`, 0.0762004940214855 6`, 0.9572664243429916`,
0.0003140155015010082`, 0.00031745708452269217`, 0.010959914415794803`, 0.6037721695834154`}, {0.129274073243755`,
2.8469118022753515`, 4.121632588773114`, 1.2196787674185057`, 0.8228855513020883`, 0.4828002917324088`, 0.013782155470303842`,
388.66064646424104`, 0.2255512748456478`, 1.0022663108918284`, 67.88767209925422`, 64.35943027718949`, -0.051971760305852155`,
310.0075556105004`, 56.48727456551077`, 0.26433333867716624`, 0.26799782811430295`, 0.013863137565149009`,
0.12385539091423484`, 0.12517612226208427`, 0.010663495049351 38`, 10.750481451641033`, 0.22873272680971873`,
0.5453533505432343`, 0.0011978958328516809`, 0.0012489702711797005`, 0.042636794391740285`, 0.07165111136106354`},
{0.06343574194222029`, 3.9924293708982113`, 9.079106395846292`, 1.3161791897123671`, 0.10938228479468126`, 0.3240611741083924`,
0.010321331270591745`, 76.94152103093134`, 0.06038801289350482`, 9.425890207979073`, 1104.1714524295003`, 1114.925898737311`,
0.009739833686288168`, 536.1405214647216`, 964.7701094211801`, 2.3858252789190946`, 2.4448574963143876`, 0.024742892078852385`,
0.10659785823233735`, 0.10659315039545172`, -0.000044164460371920455`, 136.07484167697157`, 0.09660163180596562`,
27.225994957631908`, 0.0009782340589880034`, 0.0009805835977364844`, 0.0024018165457371232`, 1.1878110753660374`},
{0.110036074735397`, 2.490203037930516`, 9.729597307787852`, 1.4397421355398403`, 0.8533380591052817`, 0.37040757295392324`,
0.047916566872126407`, 665.4588326486067`, 0.16916230254726894`, 1.9013228378216027`, 67.24939749599474`, 64.01972861337667`,
-0.0480252463646299`, 161.8814283215872`, 40.11117782880465`, 0.7396587926471052`, 0.7471759717305696`, 0.010163036197490 26`,
0.032739962448475116`, 0.032817095105172`, 0.0023559176898346745`, 26.312865321169085`, 0.05146538506877642 4`,
0.5932887977122991`, 0.0002731559850721599`, 0.00027628115272842254`, 0.011440963504559809`, 0.09904413497395587`},
{0.21512225225566667`, 1.75750084110844`, 0.8044386006474246`, 1.3520587790631349`, 0.026316391204000755`, 0.16031186333306224`,
0.13761957869990635`, 493.8180531489964`, 0.06250485252841559`, 2.6863460670624115`, 36.24027706540802`, 35.867335292670376`,
-0.010290809092451059`, 61.74389807119533`, 12.773296223301598`, 0.8771675591715`, 0.9201187533682441`, 0.04896578053720124`,
0.13911291783098723`, 0.14010771409488182`, 0.0071509984795459225`, 22.02318175767066`, 0.42751834573800374`,
1.1705235531356009`, 0.00122247578354151`, 0.0012745305213387767`, 0.042581406108891606`, 0.24231728895489638`},
{0.15874286665935028`, 2.268211451796655`, 1.8981682437883747`, 1.375959688601855`, 0.24814706074480242`, 0.5134877163486878`,
0.3316652449509965`, 685.632474632321`, 0.05319219913715695`, 1.7896119638851753`, 32.2054930931124`, 32.06061117041807`,
-0.004498671151391687`, 82.36364786893252`, 6.0377510238372345`, 0.775572674813267`, 0.7961225385672352`, 0.026496374126275146`,
0.0799545529955229`, 0.0812215582909039`, 0.015846568430592622`, 25.130897467314497`, 0.18131735635681945`,
0.6060888515684539`, 0.0006778719873399286`, 0.0007205265152279122`, 0.06292416368371612`, 0.31586264613105997`},

{0.07432908705186819`, 3.0619407712521785`, 1.7905302646779082`, 0.9549392645174688`, 0.353226782755657`, 0.6119520610188782`, 0.0496137109124893`, 317.5186768305259`, 0.07812208014742161`, 6.620973402603631`, 311.7324920298368`, 321.0070772585055`, 0.02975174377325085`, 215.4888099619281`, 184.1076884282808`, 2.8450945439117112`, 2.889929967658103`, 0.015758851965864018`, 0.15695913276534207`, 0.15747981316233997`, 0.0033172991454810674`, 124.45015688671967`, 0.1666661291842049`, 4.753462884609781`, 0.001963181909530154`, 0.001998444787778835`, 0.017962104315193406`, 1.0262137088499657`},
{0.1228443083266203`, 2.0512729485932484`, 1.9256217622349043`, 1.4810122884336692`, 0.07741458536098089`, 0.4641958329015734`, 0.007197139507585793`, 240.76714600918612`, 0.03301937102418029`, 9.328377628444095`, 398.27706751086293`, 403.1457361693428`, 0.012224325866683428`, 195.4086015541345`, 361.0849145024699`, 1.196984110437254`, 1.2253086517522533`, 0.02366325590124352`, 0.20533979762786939`, 0.20585962486201534`, 0.002531546442292809`, 35.07630179479843`, 0.36035464873603024`, 8.951904908326195`, 0.0016602391149548312`, 0.0016850849076707984`, 0.014965189346622054`, 1.5919336595807714`}, {0.2606519449147342`, 3.818605783771848`, 8.595720359336667`, 1.112914365339814`, 0.820058745442926`, 0.4441576207168524`, 0.053270363092160024`, 1117.1606910609416`, 0.20810873381921946`, 6.830482236754131`, 158.16820447861517`, 151.84090893120717`, -0.040003587119581074`, 105.98204931432237`, 90.31244363490616`, 1.2198555375901772`, 1.2304112363527577`, 0.008653236745912896`, 0.01879950397872656`, 0.018811629060574715`, 0.0006449681790474671`, 66.54496301725663`, 0.07000181822119009`, 1.273111589634713`, 0.0002039001252606143`, 0.00020473831854525155`, 0.00411080318644208`, 0.3756868955034659`},
{0.24750930586460296`, 3.1846993652287674`, 3.026718658979185`, 1.4845570571034035`, 0.869864794311124`, 0.6669561203654737`, 0.2363643381944309`, 1924.496556700219`, 0.06437651322808385`, 2.548184450019704`, 51.50618468937827`, 47.98249780135377`, -0.06841288884577701`, 92.51092306044475`, 11.918878425454746`, 0.8454372020070509`, 0.8546539089928235`, 0.010901705015928354`, 0.061313207044787425`, 0.06190170021254959`, 0.00959814689406624`, 38.4637617224661`, 0.21679413308553142`, 0.2785273326412863`, 0.0004880360942673967`, 0.0005075397458116997`, 0.03996354321616402`, 0.16767500689654802`},
{0.24908529437358162`, 3.626300618072209`, 2.0230682918259397`, 1.4602193573319078`, 0.40130475942739396`, 0.6650543311625685`, 0.019582320105998748`, 125.14663606859848`, 0.03716084844005629`, 8.149858668846527`, 1079.857508207184`, 1116.7532325308846`, 0.0341672156217685`, 606.4302678547007`, 844.5208746633415`, 4.399325769583881`, 4.470324471036721`, 0.016138541488268876`, 0.5973034413425761`, 0.598890999032398`, 0.0026578746746435122`, 227.90396796204362`, 2.12542147881642`, 14.81868578019703`, 0.004869018477472675`, 0.004977593467102152`, 0.02229915333692345`, 1.2463186256571501`},
{0.2795730004018654`, 2.183215118244356`, 0.7910515142573491`, 1.2847699393543137`, 0.70345738565049`, 0.2632113724473232`, 0.1394636484917589`, 223.91272255356017`, 0.16150366940434352`, 9.859315316872873`, 682.0761628559404`, 667.1429568663535`, -0.02189375146473327`, 316.6289425386892`, 232.83024914179302`, 13.847758670848036`, 14.108492583935162`, 0.018828600301651566`, 0.7015258276695051`, 0.7017666134220021`, 0.00034323148628301325`, 431.8948011999251`, 2.801824007156012`, 9.195022375706763`, 0.006521139115730934`, 0.0066465295800241485`, 0.019228306905880288`, 0.5723559874932496`},
{0.08969576723701717`, 0.49356011207206274`, 0.2529879905685366`, 1.1041769014657603`, 0.7443718717514693`, 0.6180257213599576`,

0.03574805853679543`, 3205.3226496399375`, 0.061225880931137455`, 7.560768798013438`, 66.180506961155`, 64.00220933945363`,
-0.03291448980558487`, 40.061630998509536`, 43.62581294234702`, 2.735128522232697`, 2.778868145902911`, 0.01599179830661468`,
0.23226555007190855`, 0.23787573740443865`, 0.024154194760235503`, 19.285004856638075`, 0.29761766737756273`,
0.5132195713859414`, 0.0024285582393623883`, 0.0026295281138930576`, 0.08275275069517507`, 0.7740717394226644`},
{0.10298476113266081`, 3.435925289367793`, 4.954566778741102`, 0.9299871443466801`, 0.6352691398464292`, 0.5470174017743717`,
0.005507148986589441`, 973.5524715904572`, 0.1328513609688533`, 5.618259356059234`, 117.38562910913645`, 118.55990502554417`,
0.010003574758848499`, 95.6263639751378`, 108.7447298973425`, 0.16889579115007972`, 0.1709421130518381`, 0.012115884521598508`,
0.024572535214801915`, 0.02460835209787333`, 0.001457598198896593`, 8.29019028686201`, 0.03615138099314483`,
1.2439674214594985`, 0.00031807052841392736`, 0.00032049484914370066`, 0.007621959638518971`, 0.5178162708239497`},
{0.14277679649227548`, 3.0428582592918065`, 7.414614443890604`, 1.1046799505745009`, 0.2872163059276376`, 0.23677108379761724`,
0.04608733451503689`, 2695.430951291806`, 0.1274330436692639`, 8.066834406130887`, 27.508315273037844`, 27.636304624807984`,
0.00465275137716592`, 15.60721258320112`, 16.827710547630677`, 0.23994456259178587`, 0.24558364384430129`, 0.023501600501400377`,
0.0031872138763019277`, 0.0031884446620343686`, 0.00038616352093345796`, 10.43024705792251`, 0.006500859814211133`,
0.6414355968724725`, 0.00003487333650742386`, 0.00003495311377309303`, 0.0022876292795266373`, 0.6931554242928794`},
{0.18738335776550036`, 1.508373316422973`, 6.602438992729994`, 0.7532357689229783`, 0.8880958875806098`, 0.4853414284787141`,
0.10975729606650726`, 2118.598035369477`, 0.05093222572935868`, 5.0448517662608`, 76.68736023650659`, 72.14556157551598`,
-0.05922486635324953`, 69.57286267927743`, 30.14738993597934`, 2.0622492310016454`, 2.0766292696233455`, 0.00697298774829247`,
0.010872159221496066`, 0.010881742383852122`, 0.0008814405823922566`, 44.43773874080968`, 0.02910373858701669`,
0.4925197914865781`, 0.0001741063838482937`, 0.00017497845360066322`, 0.00500883272108732`, 0.24196897870233294`},
{0.061980185747317895`, 2.54906136842855`, 8.170240160100523`, 1.0602973698223939`, 0.7221801534586956`,
0.6067985789160708`, 0.38301062006579967`, 81.55317476370394`, 0.23749102694434165`, 4.188403663256118`,
1297.5314536717722`, 1289.906798998197`, -0.005876277335704505`, 1417.8616577831917`, 209.57069641184688`,
29.065359732061605`, 29.32058915180829`, 0.008781223494204404`, 0.25225150718561395`, 0.2523051681433652`,
0.00021272799655358554`, 1058.4197950353876`, 0.22335136100520261`, 10.958143339974784`,
0.0028597894753794595`, 0.0028816957989737935`, 0.007660117565621682 4`, 0.33953132412332687`},
{0.2562298689236346`, 3.2913375042385793`, 9.509882785943699`, 1.013989937500057`, 0.7792587778840148`, 0.49077497767429623`,
0.021163604915718004`, 145.96678185214677`, 0.05845136191000838`, 7.439112841768198`, 1289.8296968318164`, 1254.8338003361828`,
-0.02713218387016003`, 793.5526021092495`, 992.1983568332587`, 6.180226553711664`, 6.235717700420936`, 0.008978820796778919`,
0.11602168928245768`, 0.11601410446100098`, -0.000065374168430110 12`, 290.588734870319`, 0.4246888891022978`,
10.689921279105658`, 0.0013808493278488765`, 0.0013856375807360798`, 0.003467614308551914`, 0.4667790898738523`},
{0.2313093023364764`, 2.988629640967633`, 6.855539828479465`, 1.0799498768994156`, 0.4522471358822151`,

0.2285321965248518`, 0.04827992453162874`, 1204.6728692817303`, 0.13073517219860892`, 1.9378468299164582`,
16.252763217705784`, 16.229212911986043`, -0.0014490031882138377`, 38.38594671517339`, 9.733843290277406`,
0.14837039768106758`, 0.15182870897757766`, 0.023308634003556206`, 0.00826327507484866`,
0.008276369775760852`, 0.001584686555098358`, 6.334630976454205`, 0.02730531989396684`, 0.3416485583087768`,
0.00009214106542876355`, 0.00009297789196148047`, 0.009082014938972982`, 0.14746523280412052`},
{0.2663151909473739`, 2.2417238355718707`, 0.9398485531379794`, 1.0477480676389026`, 0.7226958973603346`, 0.5716146939488362`,
0.007506086251562624`, 76.16131590346896`, 0.024911856209502148`, 4.070747059506875`, 1324.6310152274689`, 1300.6080910345431`,
-0.01813555919857457`, 1489.3106540972783`, 1186.6234975841462`, 3.815891687687648`, 3.8844966552055604`, 0.017978751267829107`,
2.2593296064293655`, 2.2587610640935982`, -0.00025164205087624403`, 122.20250500357079`, 8.595625650704255`,
11.510541033884287`, 0.025044414995635367`, 0.026313372681478132`, 0.05066829015826135`, 0.37098656446073186`},
{0.19929196929676601`, 1.9362608032362312`, 0.5353176928827672`, 0.7753335837033051`, 0.8607728477687919`, 0.15482089410819944`,
0.02026432754563482`, 192.89182731397838`, 0.1831217833074511`, 5.333726168282188`, 382.3073291050847`, 373.0319275620772`,
-0.024261636743191928`, 328.0548049105959`, 295.7313265763829`, 2.9300426953946324`, 2.994603453317056`, 0.02203406729325086`,
0.6484894018569336`, 0.6475049935072078`, -0.0015180022170091512`, 81.04752604144655`, 1.8462675709166043`,
5.666334693501113`, 0.009984434004290055`, 0.010176159446451123`, 0.019202434717750627`, 0.20408807560809714`},
{0.10696417975467426`, 1.9440197080088568`, 2.0936739600677434`, 1.1829890119130342`, 0.8864433537218006`, 0.5974069860908109`,
0.31351620960128274`, 84.46268853549806`, 0.02353560759327844`, 4.656274919049821`, 2089.8152844089345`, 1944.627208129596`,
-0.0694741192499232`, 2054.1576986256314`, 390.0640636188494`, 58.93723716561315`, 59.377034097404994`, 0.007462123318675795`,
1.592572339002048`, 1.5890168278180437`, -0.0022325586706047895`, 1636.7878655077677`, 2.4335456277332037`,
11.49006407761115`, 0.015908728240008774`, 0.016291978473063757`, 0.024090563825909683`, 0.2608323793804504`},
{0.21459986298539707`, 1.6769788549349993`, 9.356964926187835`, 1.2601407238694713`, 0.895239496536256`, 0.38446102196425924`,
0.012005779035619358`, 146.09102757400478`, 0.02879957197754268`, 2.3826604675291154`, 434.85717436680454`,
411.7931442456674`, -0.05303817317656223`, 835.3124896174816`, 370.9398909229432`, 2.5260428682243026`, 2.5509557104260505`,
0.009862398819565854`, 0.15390869213608124`, 0.154086852851803`, 0.0011575740996112582`, 60.51600680959295`, 0.4718397749237969`,
3.3698958083090274`, 0.0014684968202234305`, 0.001482392029092695`, 0.00946219881303545`, 0.11171801016570031`},
{0.07383925157057758`, 2.6358268091067414`, 6.251616293116599`, 1.1087155777827833`, 0.9205185682116173`, 0.19659269091732745`,
0.29632431074176147`, 531.6045072192474`, 0.12599923353908937`, 2.5100327097656248`, 79.7718143526909`, 77.76483896547852`,
-0.02515895374197008`, 145.4569857042166`, 16.00989088526732`, 1.6476496318673675`, 1.669356360637861`, 0.013174359615455433`,
0.03531787112207444`, 0.03535293075883726`, 0.0009926882807187454`, 62.04170102415499`, 0.037254931010301466`,
0.9663725588435746`, 0.00038401942833876657`, 0.00038635929658886135`, 0.006093098623204618`, 0.0928558647678699`},
{0.0817054148315825`, 2.796991931647562`, 7.6919124752432`, 0.9355088496592321`, 0.8578766380044134`, 0.4976427721124693`,

0.008010560795693933`, 67.84344037448854`, 0.080482215315478`, 7.8347514420046025`, 3560.428141707568`, 3374.630578586424`,
-0.052184050829358064`, 2079.89541102839`, 3193.9786700253458`, 8.857298129726551`, 8.926543284597072`, 0.007817864303124589`,
0.34745482360293634`, 0.3469141572219432`, -0.001556076773914783`, 353.9113057863173`, 0.4055629282454775`,
23.962034067731224`, 0.004474336719659244`, 0.004490102554812349`, 0.0035236139211054507`, 0.40196049525728234`},
{0.23618801128357925`, 0.8353216061115365`, 4.620412867513723`, 1.4504705173961072`, 0.3108708881036355`, 0.3400688454787608`,
0.014061322572830477`, 2071.8308884477033`, 0.0364017192015193`, 3.610034284058532`, 18.548939790817297`, 18.75006111750761`,
0.010842739744612251`, 23.51647695296908`, 15.463659636664218`, 0.23445719697727962`, 0.2399077064809018`, 0.023247354203208115`,
0.01007664933124355`, 0.010102368589861075`, 0.002552362176361 4557`, 2.7978166049067164`, 0.033999768084976505`,
0.38290150977739595`, 0.00008343162740676036`, 0.00008451631882782727`, 0.0130009620425914 9`, 0.409388737904493`},
{0.1662441272521787`, 3.725772450959033`, 9.814227303865422`, 1.3148509050359762`, 0.25298305094984697`, 0.32303330970203903`,
0.055784478318004704`, 213.77275876328028`, 0.04990125708904308`, 9.833393949415544`, 466.4234481020703`, 471.2206746536005`,
0.010285131613881449`, 217.0908062099606`, 263.77122968554795`, 3.734649419736616`, 3.8147367120282465`, 0.021444393647336968`,
0.039875354570061125`, 0.03988367389810576`, 0.00020863333089660152`, 198.77791317206885`, 0.09470062170532795`,
10.074730768492309`, 0.0003664793436793934`, 0.0003673114078924226`, 0.002270425952730193`, 1.0787012018195048`},
{0.22831788422124638`, 2.590756298379799`, 9.795768388114066`, 1.125355926333821`, 0.3650031672541203`, 0.1680578019226482`,
0.4143448211959925`, 831.1374036828946`, 0.21412864715242685`, 3.6048538714465157`, 36.91532357978545`, 36.562140922848904`,
-0.009567372643319971`, 46.86876496855032`, 6.09401825798492`, 0.8100294279709689`, 0.8308198713386602`, 0.02566628155691686`,
0.007376250162619301`, 0.007379440590379793`, 0.0004325270550964433`, 29.97984060555386`, 0.02405899758022571`,
0.9109927971335643`, 0.00007920393173810325`, 0.00007945003615530621`, 0.003107224752638915`, 0.23142441634943425`},
{0.23359626472155715`, 2.56674003123695`, 1.1969232842409259`, 1.0442579072771299`, 0.8882810964774697`, 0.3176999965402193`,
0.007340467537817918 5`, 1908.0313626467632`, 0.2040761417499009`, 9.648978920291999`, 121.15401472121091`, 115.49344249505992`,
-0.04672211844714036`, 57.46732038707835`, 109.38942442049512`, 0.30163578982900824`, 0.30527094184505355`,
0.012051461194661384`, 0.06846242814397012`, 0.06860459087644473`, 0.002076507309610065`, 11.06029509439845`,
0.2284652498314241`, 1.0416079444975253`, 0.0007874836980574784`, 0.0007972668912448982`, 0.012423359634684994`,
0.42061891750711633`}, {0.2534162537452666`, 1.6838285982829593`, 0.8034274134000761`, 0.870316935261816`, 0.7856829676536639`,
0.46427070430809425`, 0.12261242146523575`, 210.18201198829036`, 0.1377493870208059`, 1.4006481974514937`, 163.84416314119767`,
155.13110013412629`, -0.05317896493854734 6`, 535.3852504514423`, 59.16646125319814`, 4.037036282105665`, 4.162862298510172`,
0.031167918148825358`, 0.7642446682001748`, 0.7780741395073665`, 0.01809560718265879`, 97.10967348736328`, 2.766743153715467`,
1.4344170856884972`, 0.010126775346289585`, 0.011065444239709706`, 0.09269178601498718`, 0.12689439365712055`},
{0.25695579183074796`, 3.423363059474246`, 4.134350766656727`, 1.2838485736639544`, 0.4149036036442244`, 0.6493708976181658`,
0.12875465899507466`, 1172.2742202486922`, 0.012611679323919878`, 3.4839902382375243`, 48.58822185763617`, 50.07292581143878`,

0.030556869484806404`, 63.8290779781692`, 17.485414600308406`, 0.6207122594623561`, 0.6307118019207926`, 0.01610978727421597`,
0.02698584482321726`, 0.02711752994031756`, 0.004879784863618841`, 30.356048851517453`, 0.09905955892530104`,
0.6753511114558601`, 0.00025084648810014265`, 0.0002563659534146057`, 0.02200335893185623`, 0.5272730304808976`},
{0.17682432929033887`, 2.45185381012344`, 3.9338825987195882`, 1.2879615271399696`, 0.02702036645869277`, 0.669439400464814 1`,
0.11330549796901213`, 154.96108716979026`, 0.11583322861965717`, 8.805457336592767`, 674.3830015713019`, 682.53277296402`,
0.01208478175418004`, 350.5251569387217`, 264.94200874970073`, 11.349627195437712`, 11.570716168221653`, 0.019479844489765474`,
0.15705943770572642`, 0.15729034075444898`, 0.0014701634750227122`, 397.537524037351`, 0.3967418533009242`,
13.73542721466785`, 0.0014645532418708918`, 0.001479608764413666`, 0.01027994210954164`, 1.9787234496346258`},
{0.2509750819824499`, 3.8883567883501247`, 6.384989889678234`, 1.4613334209290016`, 0.7183189010632474`, 0.4965470763778975`,
0.3859108436384297`, 748.6242458418322`, 0.020020198926280153`, 5.690775103224375`, 170.43768240674066`, 168.00523624843996`,
-0.014271762699141877`, 137.0751380417072`, 27.487696235928713`, 2.5244626500170324`, 2.5512662457452087`,
0.010617544976549986`, 0.042916648213998404`, 0.042983716177358654`, 0.001562749332749025`, 140.2287354590006`,
0.15387156148445985`, 1.6116952712617785`, 0.0003533890153339936`, 0.0003564901836797948 4`, 0.008775508607335558`,
0.4224326264499598`}, {0.1441623498715573`, 3.343288457669903`, 1.621696793707331`, 1.3375455077897458`, 0.06814809058713811`,
0.4010236514346772`, 0.3002310191555227`, 155.99799531014958`, 0.10001900063604485`, 4.2763953522223215`, 262.7661698799991`,
260.23408369886255`, -0.009636271603353341`, 281.2264058987037`, 54.243737103320036`, 4.256607043839301`, 4.369957936042617`,
0.02662940013863202`, 0.3153804684079007`, 0.31699164336113866`, 0.005108670683925176`, 203.30093140720476`, 0.649514134703628`,
6.2802143531640855`, 0.0028011412428842197`, 0.0028796223531287603`, 0.02801754836315639 7`, 0.68742228478323`},
{0.2657479684896014`, 3.193863728015164`, 2.3963245604206165`, 1.4113571594678591`, 0.26744798738201037`,
0.235299669124016`, 0.018584060295236755`, 466.949287196137`, 0.23679723903863448`, 8.690245518267893`, 168.58037369150787`,
169.33030615744755`, 0.004448515859337254`, 88.78497167695298`, 133.71172271379606`, 0.7393288779142638`, 0.7585043182859543`,
0.025936279434649823`, 0.0715032411534758`, 0.071582033325066`, 0.0011019384620774009`, 33.7330812320646`, 0.271454872527966 2`,
3.996588630440841`, 0.0006102968839836187`, 0.0006152376705667366`, 0.008095709994237144`, 0.7651891578818125`},
{0.18653936584035707`, 1.0683088955449334`, 5.185702152338592`, 0.9522710688882444`, 0.206752787597013`, 0.5240389817239608`,
0.06412446666117125`, 220.00685924183844`, 0.2055887216336732`, 7.885811409920794`, 430.59446346943605`, 440.29716253947225`,
0.022533264807583775`, 249.91167736179824`, 228.28075971586927`, 12.426915797552995`, 12.6596619720741`, 0.018729198645325518`,
0.06287341364399834`, 0.06291665500275857`, 0.0006877526804107692`, 189.65406701019577`, 0.16754809584844832`,
8.217487983858739`, 0.0007958474626336143`, 0.000800254413792479`, 0.005537431939886206`, 1.2742341168227242`},
{0.07587231017464335`, 3.3343312881276663`, 8.762311039358057`, 0.8284399428795388`, 0.17449762015817138`, 0.34311440561675743`,
0.03578987408308253`, 1364.0076205471958`, 0.21103062426335195`, 8.577574022027257`, 60.895569573004`, 61.60415779010116`,
0.011636121019406476`, 32.492693729289606`, 40.73300785129419`, 0.4143194357668188`, 0.4236117431298603`, 0.02242788187294042 5`,

0.004212020996461914`, 0.004213317015795703`, 0.0003076953640253066`, 19.73540368509581`, 0.004565368050096528`,
1.3950776401731306`, 0.00006146145978380702`, 0.00006157666292927 32`, 0.0018743965058984902`, 1.0637684100598852`},
{0.1141532151024588`, 1.4516849606811304`, 7.634928187688176`, 0.8394863515309923`, 0.0282734940185132`, 0.657649499282806 8`,
0.009900377337816624`, 932.466930942293`, 0.19488805826375527`, 9.750042758265863`, 122.34581736165055`, 124.69396284563918`,
0.01919269113260791`, 57.43109557366885`, 107.37235385998457`, 0.6838372335304949`, 0.6973733316294972`, 0.019794327414900392`,
0.008655114083883492`, 0.008660312840146279`, 0.0006006571620431611`, 14.18166039242869`, 0.01411441571077037`,
2.519407658800664`, 0.00012448278132726198`, 0.00012490828404182897`, 0.00341816522759375`, 2.152896501532221`},
{0.21903027892555205`, 2.3035966499415492`, 1.6904861150255108`, 0.7741880968122689`, 0.5538208564897962`, 0.4216122395122083`,
0.4439804577081556`, 61.55285726796139`, 0.16334632191055676`, 6.147879562247702`, 1578.5963229829147`, 1580.4759744211344`,
0.001190710640113446`, 1175.195799562293`, 232.76523840437866`, 39.60005653730803`, 40.21420192779929`, 0.01550869984017988 5`,
0.7390726212050569`, 0.736827768297474`, -0.003037391513600718`, 1303.1793939548425`, 2.3125611766964145`,
21.54025163072699`, 0.011393131807633017`, 0.011543771459621505`, 0.0132219704407847 7`, 0.5698315450723491`},
{0.19219532042344978`, 0.8633096904694177`, 0.1660296606087659`, 1.0595398890946446`, 0.3981268836303198`,
0.35447506211304225`, 0.06527455092271564`, 2352.6780619798324`, 0.04729158696423241`, 8.334762180963512`, 44.38283808517006`,
43.99726363837288`, -0.008687467125407133`, 24.371729664298186`, 22.93411976991881`, 1.550899102299569`, 1.6062573453277929`,
0.03569429045780104`, 0.20567132552786785`, 0.2095688993380075`, 0.018950496867447653`, 19.127231770793404`,
0.5647009473106667`, 0.7850694982342`, 0.0022554504024974165`, 0.0024375288785014938`, 0.0807282110049794`, 1.023451133870325`},
{0.059421230927129864`, 0.7565633878530198`, 4.881127251124937`, 0.9039794772130322`, 0.6691506700132113`, 0.3437226952926379`,
0.013854681683978944`, 1303.847591809871`, 0.1331369696675042`, 3.817043920573575`, 48.0046367438855`, 47.4413397178195`,
-0.011734221197658434`, 57.55996553120311`, 40.132172295366914`, 0.6624208218984294`, 0.6716983796510366`, 0.01400553461773546`,
0.01459406705740827`, 0.014614135510897856`, 0.001375110406896418 2`, 7.159476302855114`, 0.012388534696923626`,
0.6179831272102574`, 0.00019445941804718814`, 0.00019581659430762568`, 0.006979226175140463`, 0.26545219553420357`},
{0.27625614155976197`, 2.681313109654546`, 6.389652815751472`, 1.3504817156087858`, 0.7587234585548364`, 0.5970419449613528`,
0.3005699072645848`, 854.9062988397701`, 0.165124447314083`, 9.6053618622676`, 310.63951398933256`, 305.79004618169245`,
-0.015611239360252993`, 148.01559049221385`, 60.54727129173445`, 6.35755447805521`, 6.410472907316076`, 0.008323708344698977`,
0.0428411700765307 3`, 0.042882582243136624`, 0.0009666441540208126`, 243.52277381931955`, 0.16907337636065337`,
2.3757626653589567`, 0.0003823696255246256`, 0.0003846620116154474`, 0.005995209707561289`, 0.6853141056953311`},
{0.09922116949114601`, 0.5509823607034723`, 3.2884104382793122`, 1.3318058680427458`, 0.6719251293345294`,
0.6187329358641112`, 0.3259483616243979`, 1099.7736795288956`, 0.13645709285092156`, 2.0261792473532125`,
42.33828888251265`, 42.27683542351058`, -0.001451486600523632 7`, 95.63567635787079`, 7.787143119151758`,
3.8804686247746627`, 3.9288217918516186`, 0.012460651470868056`, 0.05246173525828720 7`,

0.052968544652899685`, 0.009660553394150728`, 30.543853764487157`, 0.0743616389408853`, 0.4011113824955628`,
0.00046513993157215605`, 0.0004829286969753557`, 0.038243900804375786`, 0.2088882415223282`},
{0.11800216420621701`, 3.285091391336728`, 1.6522276383131942`, 0.9670398406334181`, 0.4307591958110746`, 0.30003613158270603`,
0.29799743711056653`, 701.4335436984502`, 0.043710117716904806`, 7.7162513520402545`, 128.29865010730666`, 128.15523433073255`,
-0.001117827634617763`, 76.09922178669342`, 26.237794289628436`, 2.1259649429079777`, 2.167621785686193`, 0.019594322529719266`,
0.06092076622663797`, 0.06101804491859048`, 0.001596806770135073`, 99.77127331758095`, 0.10269688942640531`,
2.362249993667725`, 0.0007575139983534429`, 0.0007648203421130389`, 0.00964516005707794`, 0.6790196620370424`},
{0.19357111334803345`, 1.327860467490665`, 5.552252240997824`, 1.1813151963757291`, 0.7103742334207412`,
0.3776068940225674`, 0.2456309648230094`, 3934.634788049085`, 0.12355866435459295`, 9.745686147306856`,
47.12182648671423`, 46.36090634416943`, -0.016147933967698203`, 22.129631863609543`, 10.845988362686672`,
1.8153517615673316`, 1.8363685406155983`, 0.011577248824834463`, 0.006450639941798909`,
0.006455136082360078`, 0.0006970069019098801`, 34.43619769678286`, 0.017837965076296505`, 0.5204191096992588`,
0.00006592143650663296`, 0.00006618416573432697`, 0.0039854900259579384`, 0.6291201295268745`},
{0.11447070818541266`, 1.7335861954191927`, 8.736387174086214`, 1.0335531844510784`, 0.08646802454747982`, 0.4443642965951369`,
0.015374273787766714`, 123.60141703755143`, 0.21867059554150092`, 6.82672203062274`, 554.206633958985`, 562.2572548133888`,
0.01452638846434251`, 371.55575722730697`, 456.19927193244416`, 3.788521394114957`, 3.8734722216398034`, 0.022423214412041537`,
0.060257837446176105`, 0.060278041276155485`, 0.0003352896624846391`, 93.8246912841138`, 0.09853939037409645`,
12.676160745147644`, 0.0007036920789007528`, 0.0007062219636274741`, 0.003595158738568305`, 1.1116878859048496`},
{0.22987433852030326`, 3.2498283820092855`, 3.7918096781351274`, 0.808040368560279`, 0.9416246029763107`, 0.49895559050949523`,
0.02048494990466312`, 278.1506433463176`, 0.24538025309113942`, 1.1463070343242556`, 147.64594030819515`, 138.72157013652057`,
-0.0604443993044842`, 589.5016801794462`, 113.81870415806279`, 0.6970465458447466`, 0.7047944451342797`, 0.011115325562862433`,
0.17040784448587842`, 0.17146058071206263`, 0.006177450994719985`, 32.36116640382564`, 0.5596055789980313`,
0.8552307384722599`, 0.002503728713043829`, 0.0025815920449467995`, 0.03109894913826783`, 0.05896673259483973`},
{0.2774701327316953`, 3.217064080543545`, 9.848999257942918`, 1.1587780417232185`, 0.006285118350015439 5`,
0.6835477959342324`, 0.37074868705631564`, 2643.6047855840716`, 0.14999893314602414`, 6.1386041394007655`,
27.693060932993493`, 27.539831787753887`, -0.005533124186248739`, 20.647421708938847`, 4.820003015015584`,
0.48674386238325146`, 0.4960773805320262`, 0.019175420318758407`, 0.003326403373935983`,
0.003330223171966287`, 0.0011483267664511576`, 22.369802801402603`, 0.013185394081216774`, 0.5629533059844443`,
0.00003460477076133728`, 0.00003481150671877232`, 0.005974203928726052`, 1.4017412006542072`},
{0.07518728576930783`, 0.8073344777020095`, 0.2079911159294223`, 1.2717171333972301`, 0.7961690644438275`,
0.349516332980527`, 0.00930168975466583 7`, 660.9000723394831`, 0.24128632812714051`, 8.034216989913379`, 263.7111748764726`,

250.26261694398673`, -0.05099730012876946`, 150.22751078101564`, 230.65286080895135`, 2.4183911140310403`, 2.4679751682509066`,
0.02050290953030265`, 1.2313881386027192`, 1.2475885728213933`, 0.013156237022923678`, 27.89215038464899`, 1.3226390267150911`,
2.57229590433192`, 0.011270185361205165`, 0.011993716630704823`, 0.0641987018235064`, 0.5792711272943429`},
{0.19386888157639426`, 1.1721624963279647`, 7.488800420822528`, 1.2206737749782337`, 0.39882649538868`, 0.6168747383393647`,
0.005894727770540639`, 784.8706404426375`, 0.21917965839925868`, 7.12627169471982`, 142.8967798017912`, 147.87303991344814`,
0.03482415851889309`, 91.77503536577107`, 131.74749127278275`, 0.6158998057421508`, 0.6254523305178624`, 0.015509868141947214`,
0.020484943518129607`, 0.020508579283338088`, 0.0011538115878895194`, 10.313352196951818`, 0.05673418698593073`,
2.048524233893791`, 0.00020223517696882354`, 0.00020349858862894619`, 0.006247239867263277`, 1.020049378534369`},
{0.051543962131442966`, 1.0824196854643793`, 1.5789464925861978`, 0.9324716268311393`, 0.16692765658832065`, 0.33113273649509023`,
0.16543741477462187`, 443.37752852110856`, 0.07774169596012481`, 8.031423814374019`, 172.80619654175007`, 173.5060763026177`,
0.004050084863123304`, 98.4761955673701`, 54.01698575311122`, 7.207072537624216`, 7.37572876954845`, 0.023401489445786794`,
0.07958019637991515`, 0.07970951765168821`, 0.0016250433858655544`, 111.44395984705957`, 0.05859826612321683`,
4.016502602353098`, 0.0010256214645159023`, 0.0010356498506699271`, 0.00977786298452532`, 0.9914329720842712`},
{0.04886198458272409`, 2.4240564285395276`, 3.0585768323132374`, 1.3030342316587755`, 0.5506088802661759`, 0.40370465638646613`,
0.05636145780930632`, 1567.8212350001168`, 0.20179368484919008`, 9.505728478640599`, 93.67359522493639`, 94.32074935711191`,
0.006908607816551893`, 45.1020496406587`, 52.43535225512044`, 1.1561415807140005`, 1.1726719681957658`, 0.014297892020765035`,
0.026305302429273165`, 0.02634413502580835`, 0.001476226956127702`, 40.036463300451715`, 0.01836184688206557`,
1.3031595353785168`, 0.00024305785366529609`, 0.00024486458749929435`, 0.007433348919826388`, 0.8460855626322047`},
{0.26828907890664055`, 1.4916419764625646`, 9.30244345383333`, 0.8462433381711008`, 0.9150350508953056`, 0.16353772752060636`,
0.1124240025000419`, 2042.0256023360328`, 0.08362962700521354`, 9.175430964325553`, 69.61069965297537`, 68.30475947414355`,
-0.01876062423366265`, 34.72274265264087`, 27.266363254280026`, 1.8971301825522753`, 1.922957241218268`, 0.013613751393300122`,
0.004331012362802909`, 0.0043315198137115225`, 0.00011716681138373986`, 40.42627164441516`, 0.01659947596496887`,
0.915492520383118`, 0.00006191576779579488`, 0.00006198207060358323`, 0.0010708549719842253`, 0.29649547454651914`},
{0.11853219177887636`, 2.436539187916523`, 4.07490711978506`, 0.8391678158796785`, 0.8274679746994464`, 0.4783994312610165`,
0.02880518630114325`, 249.91551468654188`, 0.20172418525520536`, 8.758671893830364`, 977.9055353441262`, 936.8989095907302`,
-0.041933115491534445`, 511.0026371532502`, 694.5662514855044`, 7.898533150486966`, 7.961802358882124`, 0.008010247876373944`,
0.14429755120780452`, 0.14429135118758923`, -0.00004296691221294946`, 274.9297935459894`, 0.24434150018552483`,
7.298476310482959`, 0.002072019967288563`, 0.0020823538242181867`, 0.004987334626483486`, 0.48294058921602406`},
{0.15380346197105899`, 2.5002802569502807`, 7.104691399911929`, 0.895619363352984`, 0.7056556726651881`, 0.2904087856211036`,
0.27890663528622706`, 1570.4796825528501`, 0.2402855906015`, 2.015690994359673`, 19.89806018481054`, 19.471443184024665`,
-0.021440130184727235`, 45.18052564137176`, 4.199976786097584`, 0.426849260197647`, 0.4331894962904596`, 0.014853571703220991`,

0.00779081324278526`, 0.00780267264454762`, 0.00152222898852572`, 15.24632539951441`, 0.017117914975862913`,
0.2689596295439862`, 0.00010475252679287284`, 0.00010559787002694799`, 0.00806990780992467`, 0.12444688367755673`},
{0.07681933116098377`, 1.701376646381478`, 3.4749545233581878`, 0.9897106512386663`, 0.21660819673269338`, 0.4456181241675946`,
0.020856154503598144`, 170.86262737853005`, 0.11272605112949713`, 7.4521875824230435`, 488.82708949524687`, 497.7830012025341`,
0.018321226257192347`, 300.21748457599824`, 378.46254279421777`, 4.346598330826601`, 4.434271442707575`, 0.020170511560542836`,
0.11713556754039188`, 0.11722905791607578`, 0.0007981382397082015`, 105.64572701812997`, 0.1285467993374035`,
9.852884045787231`, 0.0014243893546389552`, 0.001434616937965955`, 0.00718032839384164`, 1.0774933388607104`},
{0.19680750576810457`, 2.9845730901840906`, 8.034687795284743`, 1.0668124507997105`, 0.6731645050394575`, 0.24725877139168673`,
0.05097134738484948`, 549.1786460489706`, 0.029127445194553014`, 6.0096318557240025`, 153.40060000906897`, 150.96974761642952`,
-0.015846433407012417`, 116.82712375015367`, 89.66885983247525`, 1.4585898248042353`, 1.4812152440050643`, 0.01551184494507618`,
0.021258520877004514`, 0.0212662977117418`, 0.0003658220053164296`, 62.189542010386354`, 0.05976909243038826`,
2.2819703589341005`, 0.00024075227177755565`, 0.0002414480911212663`, 0.002890188069974098`, 0.339962398295326`},
{0.2481156970490977`, 1.2202113043036036`, 9.306028403345813`, 0.8009108612869359`, 0.02681732734233533`, 0.15549305073805075`,
0.021015785720259927`, 53.28601671876581`, 0.06540227633756851`, 2.0322216725641393`, 243.07448189693466`, 243.45706886064008`,
0.0015739495183524355`, 547.4352829375808`, 188.48092771231975`, 2.9426483632895986`, 3.0451274565514574`, 0.034825463531530154`,
0.06454220270461014`, 0.06454860340344323`, 0.000099170752854371710`, 51.29503996394957`, 0.2287704801876694`,
8.137546620661489`, 0.00097302477169902 7`, 0.0009768266704842565`, 0.0039072990696742416`, 0.16477338451260923`},
{0.05969602287151182`, 2.177557565950017`, 1.7675183816518665`, 0.8372040785938111`, 0.9188853493106184`, 0.1878023064060833`,
0.1313271602899384`, 239.3049490553974`, 0.14060221077790058`, 6.359907705361997`, 449.37725078254476`, 438.8884012337126`,
-0.023340855663180227`, 323.3886433237487`, 159.64426613993135`, 9.011592612934558`, 9.129573400935312`, 0.013092112911475162`,
0.20999411086259745`, 0.2099301288180974`, -0.0003046849468170132`, 280.3323096507845`, 0.17908304635616398`,
5.43019628891052`, 0.003019841621200614`, 0.003038154973062123 4`, 0.0060643418293666596`, 0.22408805011592364`},
{0.1932263333480873`, 0.7780345756067408`, 9.679599745167298`, 1.1108495595363916`, 0.27977393862884203`, 0.2181645563751744`,
0.006193313005348089`, 412.49802062580375`, 0.21651680739670837`, 9.973248740719807`, 215.43066877546784`, 216.45596427487624`,
0.004759282906358297 5`, 98.86336160736413`, 198.08070225871262`, 1.4099372080153243`, 1.4454337197399527`, 0.025175952179171457`,
0.015556543711899812`, 0.015558722590973567`, 0.00014006190025916965`, 15.67114138957652`, 0.042941912871686035`,
5.157941720180926`, 0.00016935654669092415`, 0.0001696004778948902`, 0.001440341154400393`, 0.806797727342097`},
{0.05080144024762184`, 3.462591354860418`, 9.637683194306994`, 0.7734436451051352`, 0.6551811041034619`,
0.18020194036251547`, 0.05139770733113868`, 3391.683612113084`, 0.04739051831745644`, 1.6399754771823183`,
5.396051734835776`, 5.316314045179877`, -0.014777043211266827`, 15.059246614439521`, 3.151860555814629`,
0.04441138245546047`, 0.04527036595737394`, 0.019341516845933215`, 0.0016555962207532363`,

0.0016568674396051136`, 0.0007678314530694674`, 2.1968352706810994`, 0.0012015238926119827`, 0.10038440691939528`,
0.000025842019030219987`, 0.000025946554603759637`, 0.004045178258610615 5`, 0.08508340630938539`},
{0.17269587719558416`, 2.280528270589695`, 6.304702452448467`, 0.855238720891303`, 0.7302844321830138`, 0.25273349413775115`,
0.28098261768807636`, 2514.837947344666`, 0.04999285469609732`, 2.363210664893213`, 14.041982223196438`, 13.694419023912111`,
-0.02475171907782192`, 27.19508845014846`, 2.9524722165759014`, 0.3297304027061708`, 0.3347763926920347`, 0.015303381018 09936`,
0.005047754158023709`, 0.0050539348261895945`, 0.0012244392203730747`, 10.742278643490671`, 0.012453233316967332`,
0.19566708070468491`, 0.00007114261425156254`, 0.00007162670585537 66`, 0.006804523686777975`, 0.12994906742507387`},
{0.12416213981002666`, 0.8389921874064035`, 3.610730817659622`, 1.0777639636353653`, 0.18253050124358539`, 0.365469281758221 8`,
0.27799795600117 6`, 563.1802062075279`, 0.17509802310960038`, 1.6234053595359104`, 28.10779269199273`, 28.02836029534192`,
-0.0028259919774290543`, 79.24361165733531`, 6.126185470298745`, 1.685897131114977`, 1.7291375452869697`, 0.025648311141851954`,
0.032164757305706 21`, 0.03234172583962162`, 0.005501939039471937`, 20.206493168233344`, 0.05705207276495838`,
0.649106734701812`, 0.00035568515390060895`, 0.0003644334613297186`, 0.02459564964455674`, 0.22878590261106943`},
{0.05389681485101466`, 0.42474703837564753`, 9.702496123506663`, 1.2875902192251218`, 0.8398628787663107`, 0.4145702753216558`,
0.012323091180371398`, 1207.1917708220153`, 0.0487427592688896`, 1.997348446926472`, 41.045972757667585`, 39.110786457292406`,
-0.04714680077873601`, 94.05485343741995`, 34.908052191640216`, 0.8608451321473262`, 0.8693274272101589`, 0.009853450691733823`,
0.01706899496026219`, 0.017108564213106445`, 0.002318194652724203 7`, 5.22344886256671`, 0.013142349443804376`,
0.34461847027062004`, 0.00015927888269173796`, 0.00016097069929433703`, 0.010621725705304952`, 0.11048916641894123`},
{0.06391261825702438`, 1.7835225604286613`, 8.062142041499534`, 1.3025605866836996`, 0.9222349035188417`, 0.39598365567854665`,
0.09958099473376647`, 75.95083350193653`, 0.043061298974276296`, 9.739451519751867`, 3891.996296935089`, 3692.057315228333`,
-0.05137183246145582 4`, 1828.9524427948115`, 1621.665756722901`, 85.71179318166647`, 86.31213586088239`, 0.0070042016031970356`,
0.40577626430734837`, 0.4052491024209929`, -0.0012991442149907773`, 2183.841669061397`, 0.3704889068345102`,
26.34015790641488`, 0.003755637744505491`, 0.003767047228429806`, 0.003037961779196241`, 0.3967036822433033`},
{0.08850946488241984`, 0.9673836199236003`, 8.299835087678265`, 0.9900607622617293`, 0.0809660102577392`, 0.6707110673061827`,
0.0056133585030711565`, 223.20635429959376`, 0.07233923327715214`, 4.067688416849283`, 220.16565021723787`, 225.40772854810487`,
0.02380970113046521`, 247.72302698533753`, 203.82233717945553`, 1.0813710074989562`, 1.1028906813445607`, 0.01990036138972883 8`,
0.04045611956597`, 0.040516920104838455`, 0.0015028761908149058`, 14.944294281639563`, 0.05115356420004294`,
4.389550660832337`, 0.0004916287484149073`, 0.0004956167921437378`, 0.008111900985628973`, 0.8965079575767003`},
{0.05602702376588930 6`, 2.0857485685464603`, 9.366491292380005`, 1.196524731276077`, 0.9516747873190254`,
0.6119693625054259`, 0.014912996736033244`, 220.0557248806415`, 0.0820080753991021`, 2.040285256013867`, 411.46995123827054`,
386.7514678791356`, -0.06007360509497128`, 923.0213117249884`, 339.1529652864068`, 2.3329179992018036`, 2.346544601200308 4`,
0.005841011987205213 5`, 0.1612270959603389`, 0.1616247683979727 7`, 0.0024665360078908183`, 69.5125768195919`, 0.12904391910110 3`,

1.9143337448658824`, 0.001614129978631862`, 0.0016363789813164215`, 0.013783897814361845`, 0.0940992510969300 4`},
{0.04516470805711162`, 3.292123721374173`, 5.263943679637629`, 1.138536155221258`, 0.48426832878752823`,
0.5662939024082725`, 0.09160607397723192`, 3360.7159646360847`, 0.026694430856043894`, 4.180658858897127`,
20.553504041527464`, 21.067098041154125`, 0.024988147937644367`, 22.501197767255636`, 9.049109270743953`,
0.23929535195864685`, 0.24256984622758213`, 0.013683902516840929`, 0.006655257653162395`,
0.006672182225621616`, 0.0025430379019477645`, 11.254141494252046`, 0.0042940395564324526`, 0.2758741940324601`,
0.000070188388123115 79`, 0.00007098122667092265`, 0.011295864871781358`, 0.51682416989063`},
{0.2782184518736612`, 3.845021085593168`, 1.137061402050021`, 1.4385887178277776`, 0.42032329684699743`, 0.5854257024714923`,
0.03249096611383817`, 328.6688300716124`, 0.02617032692156479`, 8.869579059594898`, 430.54499271697676`, 441.1953338810919`,
0.024736883123191555`, 222.16724429621976`, 294.5791370192687`, 2.3964098640028655`, 2.4403915668466554`, 0.01835316383246921`,
0.38173822505575566`, 0.3838495600443357`, 0.005530845092266201`, 131.63209224020653`, 1.5172373999428304`,
6.073822156104434`, 0.0031478850318336082`, 0.003244480798795065`, 0.030685925942216086`, 1.2448861200938373`},
{0.09319840750005726`, 2.6994281444799046`, 8.85475415324603`, 1.038403514470577`, 0.3378909888092929`, 0.6279203689803818`,
0.23307552493819164`, 1846.0011275272348`, 0.034946033021005685`, 7.6621208156759835`, 62.20614530151405`, 63.93660734091749`,
0.02781818469888897`, 37.15769704466047`, 15.052889789539904`, 1.1914927018013826`, 1.209169082306737`, 0.014835492050123245`,
0.005970945230739712`, 0.005975851963543392 5`, 0.0008217681814295386`, 45.947841902643624`, 0.007949751239649723`,
0.9459239558454956`, 0.00006938405911194323`, 0.00006968212388624011`, 0.004295868216876553`, 1.1851744098227461`},
{0.06034484465157863`, 2.8968090048449424`, 3.142651522085412`, 1.307236858746047`, 0.3617717997572676`,
0.29303713371207274`, 0.18131081790115888`, 4578.414121282968`, 0.17962394399488657`, 3.3138716244433386`,
7.596768627013361`, 7.607813324098744`, 0.0014538677729514227`, 10.491986273167884`, 2.2225046138551057`,
0.12654058838131943`, 0.12931529186084054`, 0.021927379309789163`, 0.005959001802183251 5`,
0.005977485667094 58`, 0.0031459571835232847`, 5.236627370019772`, 0.005137071971875178`, 0.15685477140041795`,
0.00005466910550155912`, 0.00005543838690484465`, 0.0140715930181734`, 0.32473528509943117`},
{0.20102830374342656`, 1.9832552045818916`, 0.5122029117897267`, 0.8414910669557318`, 0.023679729667421823`, 0.562606520598186`,
0.21796137676857033`, 2537.351175593395`, 0.0373739667120736`, 9.865525950238812`, 42.92080812337952`, 42.751768039115404`,
-0.00393841802275019`, 19.911874289350123`, 11.02881373126372`, 1.081414014099687`, 1.1075626436122428`, 0.024180035741746098`,
0.04435203802429344`, 0.04469139034207035`, 0.00765133538149998`, 30.638856739585655`, 0.12737164245125368`,
0.9254635322740378`, 0.0006248171967424554`, 0.00064553411152208`, 0.033156761509821164`, 2.0321523574552125`},
{0.04583409051630541`, 3.6747726707008503`, 0.7420872691712788`, 1.2157743693615881`, 0.8915453306179026`,
0.4926202034522773`, 0.4217762472968932`, 73.4269782954483`, 0.10619296232023001`, 3.9517710932009376`, 1784.2615067651361`,
1654.7835367759387`, -0.07256670028371615`, 2066.480068470066`, 262.51616816382887`, 28.301114120556274`, 28.60524405384157`,

0.010746217692694904`, 4.669911938191502`, 4.624038505510868`, -0.009823190091759915`, 1485.7165817229431`, 3.057730949688227`, 11.295340192570384`, 0.044046717928946766`, 0.046240799805967604`, 0.04981260761721651`, 0.25043458354089254`},
{0.10119631213999619`, 1.851973173732965`, 1.6003758779141855`, 0.9328064269865228`, 0.39319494883808903`, 0.3867811485974866`, 0.006630825974655251`, 2020.948278622179`, 0.24307528382326266`, 8.411215463490773`, 52.05856114339918`, 52.77316385734188`, 0.01372690097934659`, 28.326824831718902`, 47.535344073120925`, 0.16095900431333288`, 0.16406162246745107`, 0.01927582844684128`, 0.0225737599375829`, 0.02262373349616466`, 0.002213789759434981`, 4.258453686558014`, 0.03263401795452689`, 0.9107920490910653`, 0.0002907655969103251`, 0.0002939171973984333`, 0.010838973116479744`, 0.9148135647305554`},
{0.19796477344593338`, 2.2434362012846876`, 1.2309355399833084`, 1.3299187291217778`, 0.059794768114105334`, 0.6215799505741797`, 0.02273921518352973`, 1004.7174087228818`, 0.138969497102004`, 8.58482448486276`, 100.10969788169231`, 101.46022806970909`, 0.013490503084054861`, 53.37147740087311`, 75.77558531823767`, 0.7265680276049937`, 0.7427261564299535`, 0.0222389758578041`, 0.0783552266984961`, 0.07885806746162871`, 0.00641745017301143`, 23.285843083215028`, 0.22159392430960576`, 2.0487260871677706`, 0.0007002600125622926`, 0.000719984275522158`, 0.02816705595924751`, 1.8443553945802242`},
{0.18841727652913837`, 3.2954166443999204`, 4.985214271603974`, 1.3919313698468052`, 0.8063258092579206`, 0.6389441864140679`, 0.008804142841395211`, 1247.8494355462315`, 0.09671339442963328`, 8.749674730462491`, 226.8921841491027`, 219.74992554964012`, -0.03147864535857714`, 118.68398556631944`, 201.40453321529262`, 0.5230477349724868`, 0.5272502181131189`, 0.008034607282742856`, 0.04534933148132502`, 0.04542173440908493`, 0.0015965599799354635`, 24.623717309200178`, 0.12206567900183153`, 1.4742753168856528`, 0.0003919828974616735`, 0.0003953278313498865 6`, 0.008533366914407647`, 0.571338288749412`},
{0.08822838789215687`, 3.6314565393637377`, 8.720964691808945`, 1.4591613734708808`, 0.09172379783619267`, 0.17510896742376914`, 0.2630216511777198`, 431.756995427934`, 0.16850915581941245`, 3.8927198296202143`, 63.849864672730305`, 63.410636702419914`, -0.006879074412478481`, 75.07084915966638`, 14.879135015099772`, 0.9253730705484062`, 0.9536837808013978`, 0.030593834156221744`, 0.01720861564982071`, 0.017218843747302624`, 0.0005943591099972778`, 48.006458405630205`, 0.02168983452342532`, 1.9260877129873002`, 0.0001424171977343347`, 0.0001429406977792542 3`, 0.003675820429328125`, 0.3207295137560808`},
{0.22004819658675462`, 1.2435615798896285`, 4.521096993231973`, 1.3090616735635763`, 0.5905184190150714`, 0.6892934240599866`, 0.09109967416767972`, 239.35075160225182`, 0.045734190683186515`, 5.8358773331232445`, 527.6900445317161`, 542.2782602107791`, 0.027645425245816257`, 413.8445504870073`, 231.94844298276072`, 15.723959316069264`, 15.901887075434018`, 0.011315709726043233`, 0.16378729842995862`, 0.1641542176059076`, 0.002240217522764043`, 279.3387384173054`, 0.5148728520466499`, 5.361894921524974`, 0.001499543169570905`, 0.0015199962417294134`, 0.013639535408881187`, 0.6665488039056203`},
{0.18183828174362843`, 3.2786199220563104`, 2.2240542093570888`, 0.9831747738881791`, 0.39067106215615355`, 0.5999409005287338`, 0.32534468961787655`, 53.98091103084368`, 0.10022266621367804`, 9.980846637487563`, 2894.6355483297284`,

2960.951428115113`, 0.022909923780784824`, 1327.366960850367`, 544.7297173498025`, 49.062116835878975`, 49.79593454927109`,
0.014956910967516057`, 0.8061773366495281`, 0.8040828470046012`, -0.0025980507634110195`, 2297.943338233817`,
2.0941985953901323`, 41.62557742769615`, 0.009802782218858552`, 0.0099104809842068`, 0.010986550852987165`, 1.403885428287714`},
{0.21252199352031792`, 1.818911285509734`, 0.45179175410691036`, 0.8473192883797733`, 0.5752582261250987`, 0.4244690624430477`,
0.010390335454685632`, 819.8846878599306`, 0.22518903335941576`, 6.3452240038645735`, 127.4122874127839`, 127.18453275932204`,
-0.0017875407316407799`, 91.9028223358843`, 110.2655616196575`, 0.5975077370172476`, 0.6102721053701391`, 0.021362682961414192`,
0.23266778338545732`, 0.23466909141661285`, 0.008601569164562894`, 15.52590808628647`, 0.7063860164718627`,
1.6780588768271756`, 0.0032402269774978176`, 0.0033778444490193244`, 0.04247155291194393`, 0.6255708745551045`},
{0.13408565703158576`, 3.9481177636984475`, 4.409802796026355`, 1.1882851460555317`, 0.7016940523769313`, 0.6437617694336766`,
0.472695817526219`, 268.88735252515323`, 0.02238688650951909`, 7.198883746978469`, 684.7135868164122`, 684.7364542320128`,
0.00003339705249150171`, 435.31965874375277`, 93.1878119153107`, 10.296868564336597`, 10.38985794686693`, 0.00903084097357576`,
0.1605883072718845`, 0.1607944338526815`, 0.0012835715395393965`, 580.7607098475044`, 0.30760840989091887`,
5.739059579822065`, 0.0016233959930257447`, 0.0016392236421641294`, 0.009749715538526438`, 0.6197963051274077`},
{0.11550511139863262`, 2.7266825017818803`, 9.715437717848832`, 1.4226217916614743`, 0.7434913376024772`, 0.6527594414247178`,
0.026462900292485443`, 517.125461393156`, 0.19263485762362964`, 9.20218742349427`, 501.92462167580794`, 498.92828017425677`,
-0.005969704159056954`, 249.6387047434431`, 365.104407705874`, 3.419299479671165`, 3.4464382386374988`, 0.007936935365761899`,
0.05009268286027127`, 0.05012827086218332`, 0.0007104431202322736`, 133.1906294224464`, 0.08265658448629766`,
3.7820017027532984`, 0.00042472213098454503`, 0.0004266764235888176`, 0.004601343941607938`, 0.7070440801707286`},
{0.1537432379066465`, 3.3010385217103515`, 7.8154151393713`, 1.099357680158021`, 0.2670513361076359`, 0.6099579487912872`,
0.01626886763741749`, 3071.882113963599`, 0.04550777584421739`, 6.952411393791335`, 30.857042972715615`, 31.830588209285622`,
0.03155017923884773`, 20.313433530789997`, 25.110189244061594`, 0.11882477572369933`, 0.12083475287876837`, 0.01691547190244913`,
0.0039136454553036016`, 0.00391789407613286`, 0.0010855916504908247`, 5.603502314250351`, 0.008595664633101678`,
0.5215709060566273`, 0.00004292438149378075`, 0.000043159895366773086`, 0.005486715586722202`, 1.1654239171025258`},
{0.08915960893105551`, 3.2848427178141772`, 0.6302374569507307`, 1.4630290564093034`, 0.8347103436436674`,
0.36812644478584344`, 0.040963141333673035`, 3229.889147199789`, 0.1995027638419964`, 3.7582834934312945`, 27.44455415099724`,
25.861034172501718`, -0.05769887788240802`, 33.42190019620044`, 17.253329965400322`, 0.20769326250047393`, 0.2113959031730457`,
0.017827447207457325`, 0.10384575407914388`, 0.10549362018498996`, 0.01586840136564649`, 9.746281440910709`, 0.1322692403263983`,
0.2452258645979959`, 0.0008326188703393633`, 0.0008805946133937052`, 0.05762029274545233`, 0.25478179153598196`},
{0.2095156599086041`, 1.856403794983298`, 1.6647511783688493`, 0.8963074241076089`, 0.5257557556911894`,
0.2962680347723602`, 0.005638948645619698`, 398.77430140016673`, 0.1712593265312492`, 6.243180864475704`,
199.6192482010315`, 199.48791869475178`, -0.0006579000144688596`, 146.33930687693385`,

```
  184.39525874999867`, 0.5301741319600903`, 0.5407615276895857`, 0.01996965730929401`, 0.10767943249658567`,
  0.10784237709714828`, 0.0015132379209723545`, 14.060246722466976`, 0.3222932479729393`, 3.333865542717488`,
  0.001443274543598605`, 0.0014596904287545753`, 0.011374055773920588`, 0.492749310504889`},
 {0.08606673974307905`, 3.001046395083865`, 6.871125535025986`, 1.29871337654279`, 0.0013879217267305233`, 0.4709543850320145`,
  0.028486772088755836`, 255.05656064251065`, 0.15733152617600732`, 4.419059383126843`, 166.38463825817024`, 167.92926200058616`,
  0.009283451637038809`, 172.32483997519353`, 119.24323981480677`, 1.0717633107999194`, 1.096958573190606`, 0.023508233708692483`,
  0.04458057929535302`, 0.04465109039562835`, 0.0015816550926397444`, 45.948734575132086`, 0.05481293022587648`,
  4.055869015007063`, 0.00041300198624472095`, 0.00041645597590178233`, 0.008363130861590351`, 0.822285691554453`},
 {0.2794012033077364`, 3.8964244114394253`, 7.589043584575673`, 0.9660750334911808`, 0.6823441866078543`, 0.315494435212184`,
  0.013950850602606538`, 1092.7384181067544`, 0.06831145072823913`, 6.790315469699049`, 100.9561130697813`, 99.50048411574802`,
  -0.014418433017791887`, 68.04671504781938`, 84.30399795314506`, 0.29197851607007125`, 0.2960786324869444`, 0.0140425277587517`,
  0.011869654438877275`, 0.011875026717163568`, 0.00045260612378883346`, 16.252460251875494`, 0.04737708190099557`,
  1.3035559284365381`, 0.00014842293529171435`, 0.00014889126339899444`, 0.003155362116775473`, 0.426875789945802`},
 {0.16053897675716755`, 1.21110484978606`, 6.017303762103152`, 1.2353982330406967`, 0.20086989110627473`, 0.3272106220983775`,
  0.02355461265150193`, 77.09093986775292`, 0.04848901873161415`, 3.5142859649597167`, 432.4464111120494`, 436.90334409952976`,
  0.010306324374433462`, 563.1961714210174`, 325.5305181475977`, 5.808613810311164`, 5.949170610121002`, 0.024197993600526324`,
  0.15827062529411387`, 0.15841603395778978`, 0.000918734372886254`, 100.4977193743161`, 0.36298006050623394`,
  10.097446852588794`, 0.0015411135132914477`, 0.001554199824541459`, 0.008491464864299303`, 0.42971715456905274`},
 {0.06315507597420161`, 1.4395182613839497`, 3.0858504661132873`, 1.1448617905347693`, 0.30610701790846395`,
  0.20244634599341438`, 0.2950980917537417`, 2179.6214593356067`, 0.03473000454292019`, 1.333335366603153`,
  5.162177229273032`, 5.1134156112022335`, -0.009445940328101754`, 17.719766982766362`, 1.0894222809805088`,
  0.18807422511532784`, 0.19348594263673746`, 0.028774370959609774`, 0.00894724413353735`,
  0.00898781509562655`, 0.004534464633319413`, 3.867661164987873`, 0.008072341185893481`, 0.13101166253574806`,
  0.0000934298941126510l`, 0.00009535253136605019`, 0.020578394866647365`, 0.11348084523334605`},
 {0.27116635506122466`, 2.640185082938654`, 3.1282641056246216`, 0.9199164829096633`, 0.32218318348593566`,
  0.5777637686875948`, 0.006117113694401221`, 324.8095638375007`, 0.04342122620091826`, 2.609798810681127`,
  110.65831133704218`, 113.6744910135902`, 0.02725669351090443`, 194.06244747688172`, 101.45829913832941`,
  0.22506647676175726`, 0.22969279299540998`, 0.020555332363201728`, 0.07761206613480863`,
  0.07801002733412997`, 0.005127568677660177`, 8.48881649451358`, 0.30065401546495746`, 1.8426951619395806`,
  0.0010051615979232986`, 0.001030118508623783`, 0.02482875465203449`, 0.42272791694989076`},
 {0.08076786811690995`, 2.5389877544798445`, 7.211847948605141`, 1.1500644274940979`, 0.7690232347129535`,
```

0.1924810447357974`, 0.3170033067881963`, 2456.45541532025`, 0.12977198817972552`, 1.000471570970415`,
5.33277724700941`, 5.188550962019851`, -0.027045248340429207`, 24.395694468329747`, 1.0311829009667102`,
0.11510623811646509`, 0.11720025572446115`, 0.01819204277944797`, 0.005311725531932204`,
0.005324912198413303`, 0.002482557956322351`, 4.1750475577420705`, 0.006128810674804736`, 0.084324178764209`,
0.000055489780309891934`, 0.000056171461217971199`, 0.012284800989895706`, 0.04903059690629938`},
{0.14196490482109098`, 3.5800560378690767`, 8.191314882292886`, 0.8521719014833709`, 0.20577614391452248`,
0.2973229046898421`, 0.032689134381726236`, 2192.8696238585753`, 0.20870222148197032`, 8.620659635124824`,
36.878689945575665`, 37.22335298098436`, 0.009345858974853405`, 19.579404614669333`, 25.392901671087944`,
0.22004739423481418`, 0.22514013414228054`, 0.02314383192391678`, 0.0027925528000668732`,
0.0027933935509724746`, 0.00030106893791992384`, 11.254028604967212`, 0.005663492749538841`, 0.8603038920924881`,
0.0000396172236752923`, 0.0000396912665937725`, 0.0018689577817736502`, 0.9377806786459013`},
{0.16225217210911858`, 2.1337475700719883`, 4.607583982995607`, 1.274907826520427`, 0.760916097212121`, 0.6512132808308`,
0.1988962707427588`, 88.66654318682235`, 0.17629703311441042`, 5.991536547797288`, 1962.1392833686511`, 1934.6599296796364`,
-0.014004792586302717`, 1498.842898526905`, 520.807226928439`, 45.72267684378615`, 46.100739023083804`, 0.008268592422734367`,
0.5683734631733084`, 0.56833283964031`, -0.000071473310473635373`, 1393.7235801802215`, 1.31742612813145`,
14.353056511653314`, 0.005338124561506619`, 0.0054017185530319265`, 0.011913171150760737`, 0.45648537213999973`},
{0.09998370398747453`, 1.3561107931887042`, 7.055733599099842`, 0.9997997594446719`, 0.4231050232131661`, 0.35289870383552235`,
0.01863838099230359`, 585.7262791471302`, 0.12303242645253354`, 2.5487735356675643`, 52.0514380377683`, 52.60038478619157`,
0.010546235975746887`, 93.46865168691279`, 41.251677878327826`, 0.5271314947934856`, 0.5372344770040892`, 0.01916596202350162 6`,
0.01812267756324611`, 0.018152129148845383`, 0.0016251233018129696`, 10.21212442170201`, 0.02588532041349921`,
0.9469171199163103`, 0.00021816478318914534`, 0.00021997478433990843`, 0.008296486372843415`, 0.2613732879663514`},
{0.0790420625297461`, 0.7951762600254768`, 1.0975467432818782`, 0.8295886125705915`, 0.21974762785224522`,
0.4003083061581494`, 0.12181590550696897`, 256.3718289629029`, 0.07504947990766975`, 1.4404680202816778`,
59.098677119194164`, 58.893481597787435`, -0.003472083156664918`, 187.77537298378036`,
22.190096527647324`, 2.953409088927535`, 3.0366209715749988`, 0.028174858322007834`, 0.1904232994971396`,
0.19333101416901005`, 0.015269742093267924`, 33.549725623694975`, 0.21502071922805246`, 1.285487903450367`,
0.0026774483423918083`, 0.0028409572949972058`, 0.06106894763068782`, 0.22931726429908042`},
{0.09310243320239603`, 1.7939569577957926`, 0.5112771848209494`, 1.3548866082012778`, 0.9609312900127882`,
0.31815651234043585`, 0.047992777894275945`, 1572.364241162158`, 0.10220690607690369`, 1.3696469849079624`, 23.448282714901687`,
22.07830369798928`, -0.05842555864621024`, 78.3550316910057`, 13.61388348384526`, 0.34577918339943975`, 0.35817367646202186`,
0.0358451105723856`, 0.26887589896970027`, 0.2769818110665452`, 0.03014741048902403`, 8.861613884576748`, 0.357614291765 1421`,

0.18484822590855857`, 0.002335980457573328`, 0.002544834682543584`, 0.08940752235025906`, 0.10297330109384302`},
{0.20315703606221613`, 1.4806269686939117`, 9.851530344798562`, 0.807291927894971`, 0.7692364729235686`, 0.5064599484352303`,
0.07616257600145751`, 550.4721115636684`, 0.18512304424700965`, 4.821016411964738`, 218.25976960641165`, 213.31168229520065`,
-0.022670633805459986`, 207.20468803637254`, 105.43721427561158`, 5.08903597714983`, 5.13416516163036`, 0.008867924039673403`,
0.02326740812246331`, 0.0232811461326495`, 0.0005904400745404548`, 107.64234160602292`, 0.0675276810144944`,
1.8431059328699775`, 0.0003478445428566346`, 0.00034925688430491385`, 0.004060266223182785`, 0.3195142235274357`},
{0.22897905358022103`, 2.219649931841208`, 8.024983925513396`, 1.3490395658771779`, 0.7992198564081268`,
0.5097552623833034`, 0.017934168491060075`, 2636.2345048980164`, 0.04871567850920905`, 1.38033814014487`,
13.589589992501551`, 13.07290615139485`, -0.03802056142913779`, 45.05939543601709`, 10.801750128414914`,
0.08374656144064192`, 0.08473423071001447`, 0.011793550115757334`, 0.010302796785369411`,
0.010356395798350052`, 0.005202375053806163`, 2.6555435627665207`, 0.03370178081633205`, 0.11049319652125557`,
0.0000911420633309401`, 0.0000932374284599636`, 0.022990099767822292`, 0.0955108121325542`},
{0.14728957561352346`, 3.8290117168547324`, 5.577517394641557`, 1.2037071314600827`, 0.7629976630105226`,
0.33391252098357616`, 0.00623505541513591`, 1551.1825745003182`, 0.05716501203669799`, 3.4524118308532703`,
41.84970574861765`, 40.64442531087087`, -0.028800212956971438`, 55.47972595944787`, 38.380983103751944`,
0.06063722946861787`, 0.0614499460399118`, 0.013402930483730868`, 0.016375164702076353`, 0.01640378794577127`,
0.0017479667664830334`, 3.3168666016135213`, 0.034455586565291296`, 0.46495606485828556`,
0.0001637070148537978`, 0.0001651742986588 7985`, 0.00896286458092499`, 0.2018290151098927`},
{0.1747499923357959`, 1.9364726507678487`, 4.539322141325334`, 1.0358217348301002`, 0.687467494258686`,
0.22118436656891216`, 0.06970356230137446`, 4108.781309212477`, 0.15897963252631786`, 3.216561469360766`,
10.346253999430653`, 10.156417516420506`, -0.01834833003525671`, 14.721625833286708`, 5.256592627685855`,
0.177001915764667 1`, 0.18009241637525356`, 0.017460266445338712`, 0.004594601626176822`,
0.004600793093937911`, 0.0013475526857029685`, 4.896562414454186`, 0.01147009427086241`, 0.16293442302067634`,
0.00005345534383538464`, 0.00005385349192545064`, 0.007448237379074518`, 0.17537888544157504`},
{0.061449808274615936`, 2.451738473864639`, 8.032153511575046`, 1.3988277328315561`, 0.653844119276888`,
0.6033333182307286`, 0.12509363159734996`, 3162.271766392656`, 0.18189469469951425`, 1.0210674887190017`,
6.9142449252502916`, 6.940073254802677`, 0.0037355242447461023`, 30.992371271482362`, 2.5202577283948817`,
0.12158915598498929`, 0.1230650869395774`, 0.012138672586643695`, 0.007326292718165662`,
0.007388790064727186`, 0.008530555489075775`, 4.25864016761609`, 0.006431418327072611`, 0.07049839049654791`,
0.0000620502356012409`, 0.00006407253447844167`, 0.032591316658277414`, 0.10847019948733619`},
{0.1951885853322612`, 0.6765436415231281`, 7.0581513804142055`, 1.4397439961417247`, 0.9899034195557828`, 0.3660842570171081`,

0.2551485659861665`, 53.30479560198744`, 0.15667683142074817`, 8.392793083340557`, 5040.925671351742`, 5013.044794160846`,
−0.005530904244302914`, 2748.959062331129`, 1109.85076859092`, 368.32526696433865`, 370.90648084540834`, 0.007007973963729208`,
0.7706107674163495`, 0.7687237253326382`, −0.002448761636225205`, 3559.8302482433087`, 2.148777507629248`,
32.18344091677435`, 0.006439885331277706`, 0.00646735054412299`, 0.004264860542141813`, 0.29923439950180547`},
{0.15955516943400022`, 0.8482855893464007`, 9.331572022970409`, 1.1667938097447013`, 0.29910958141522603`, 0.1963171731712634`,
0.010305064592809629`, 1013.0765277256257`, 0.20900313016467104`, 3.301983133021727`, 27.458321866585838`, 27.528929883671847`,
0.0025714614836651606`, 38.05954882743707`, 23.986610876969028`, 0.2616022294946104`, 0.2685975488521885`, 0.026740289526936856`,
0.006518542869531845`, 0.006522636400899435`, 0.0006279825798540806`, 3.170191448873832`, 0.014858103028723493`,
0.6929831533445153`, 0.00006746731564366559`, 0.00006772638902791463`, 0.0038399835798619275`, 0.25504469957675957`},
{0.17746707158856556`, 0.44139712602022296`, 2.8621670830493073`, 0.8100971992338867`, 0.0373958036205273`, 0.5075452637360592`,
0.09067013663296572`, 114.80525128927701`, 0.16719225120884412`, 9.820606806768879`, 889.3768626077475`, 900.2521256591069`,
0.012227958145292872`, 414.48803501946225`, 397.55738753863534`, 67.24233078513595`, 68.6996763598955`, 0.021673037768668847`,
0.16080165332015764`, 0.1608181693442379`, 0.00010271053648547301`, 424.00816507800437`, 0.407671407446875`,
19.998926185547358`, 0.0023891679755845274`, 0.002404728847250464`, 0.006513092350540761`, 1.812367375218305`},
{0.25772135278013303`, 3.070426405636976`, 1.2338255709842532`, 1.3135003325072887`, 0.5149580362176238`,
0.41732865316044054`, 0.04496826136197767`, 318.8324074107286`, 0.010846019130009599`, 7.957536959758443`, 374.9376677528621`,
377.69682844362916`, 0.007358985047577926`, 215.64773639980885`, 229.4546303668787`, 3.209946782590362`, 3.268862297010981`,
0.01835404709515953`, 0.31262258723746744`, 0.3136719251293102`, 0.003356564543577578`, 140.7986480279271`, 1.1509930870351386`,
5.417467530423034`, 0.002839157379154633`, 0.0029022861858328296`, 0.022235050139064017`, 0.7939350628651793`},
{0.08988753363169116`, 3.536295514824336`, 7.275260820034448`, 0.8993825689374235`, 0.025163868977214232`, 0.47083049518550046`,
0.018253871395130763`, 416.18519037331805`, 0.11499620973251179`, 2.265482484941062`, 52.295229716001764`, 52.87708431242897`,
0.011126341725374633`, 105.649114374068`, 41.673995267126`, 0.20504082449839323`, 0.2099520039701183`, 0.0239522030977961 1`,
0.017862626935146557`, 0.0179011421941074`, 0.0021561923171029562`, 10.35835640042218`, 0.022937535419760704`,
1.2754884287118493`, 0.0002386601025238244`, 0.00024113840887649067`, 0.010384250766920067`, 0.42410378164001555`},
{0.14192760186457132`, 2.3958894382433478`, 3.253512770748353`, 0.8655063905996925`, 0.8143036151546204`, 0.5834364577519011`,
0.17553594321370639`, 62.22207407824173`, 0.16384862691545943`, 3.0979894918131596`, 1502.8467783118874`, 1443.3649262227405`,
−0.03957945210885805`, 2220.236607113684`, 435.1897428740512`, 30.23831403833985`, 30.499317804140023`, 0.008631558144056584`,
0.8099616131841785`, 0.8088490128676338`, −0.0013736457363341392`, 1034.9665319249132`, 1.642227276595551`,
10.482677291729063`, 0.011127700901153892`, 0.01133172015683765`, 0.018334358327568134`, 0.2067637189738103`},
{0.14248286143558947`, 2.096310454690764`, 9.38011512102738`, 0.9675972750885355`, 0.0803295271719453`, 0.6033546111821413`,
0.13762578908199044`, 1262.8186365484712`, 0.18785001372865495`, 4.9706998985924695`, 45.473134703570096`, 46.131598125846885`,

0.01448027338711477`, 41.869892351833904`, 15.88876887756999`, 0.9553794975152033`, 0.973981625533766`, 0.019470930731655844`,
0.005915712784319727`, 0.005922093668302014`, 0.0010786331613663602`, 28.611028983404726`, 0.012041252641979437`,
0.9340626172237028`, 0.00007371279326096047`, 0.00007412125660667646`, 0.005541281609963367`, 1.0056952847897556`},
{0.10446764528219044`, 3.442117541556258`, 1.7911289531773882`, 0.8226507378787467`, 0.06660293233402026`, 0.33605831499521144`,
0.1966635974545483`, 110.27400709595466`, 0.23027159794407875`, 2.5346674177346316`, 199.765734930368`, 198.8557281848253`,
-0.004555369547534882`, 360.7152553554944`, 55.87910013282879`, 2.856586651513664`, 2.9363663645177907`, 0.027928336415719235`,
0.225808850703668`, 0.22661162647271443`, 0.003555112062900933 6`, 140.46724317358027`, 0.33699598452709784`,
5.192092426724734`, 0.0032705432182025262`, 0.0033441577312500015`, 0.022508344374648992`, 0.36626695794334785`},
{0.0959624592169791`, 0.7155878562202682`, 5.1612847015717165`, 1.2701047018452214`, 0.05672968500006248`, 0.6450160655606327`,
0.04218472167952095`, 1614.7615476312947`, 0.23305782159027644`, 5.6652476983851425`, 41.16843624031692`, 41.92333015469925`,
0.018336715778459745`, 33.25905938128649`, 25.956560554340815`, 1.3529498850976323`, 1.3793965097483225`, 0.019547379353805017`,
0.011192213623065082`, 0.011219400434862775`, 0.002429082638457203`, 13.830778683578172`, 0.015343319190732794`,
0.8434082203733578`, 0.00010582936038461277`, 0.00010700430345038512`, 0.011102241018015047`, 1.2350616629051725`},
{0.24311048293765425`, 1.2773072105524381`, 8.674158333323465`, 1.236248375623142`, 0.5430857314344402`, 0.29169516048698574`,
0.10383247735445839`, 522.6667880889086`, 0.04591098967785867`, 9.694591495258663`, 242.36667366332347`, 241.96339662525466`,
-0.001663912913328236`, 114.42156495567507`, 99.86260162861177`, 7.398671444328024`, 7.521537150821624`, 0.016606455282967225`,
0.0223535791058109 5`, 0.022359685829158125`, 0.0002731877216739509`, 135.00537691926593`, 0.07763413445429386`,
3.928981918749611`, 0.00021853264983440202`, 0.00021904312952893744`, 0.002335942454924966 3`, 0.7100535293980683`},
{0.08713889377857453`, 2.2859880795657075`, 9.600148060687761`, 1.2921363299986606`, 0.6294865567710719`, 0.5590186325118135`,
0.25992443653112657`, 633.6168560418654`, 0.16781120217455497`, 6.04666254822`, 195.3333133353014`, 197.3194594124301`,
0.010167984370998528`, 147.8512767940352`, 43.02214634860901`, 4.5235788053902946`, 4.5722913440335065`, 0.010768584065599995`,
0.027261816237646716`, 0.027288391923527635`, 0.0009748318178530724`, 147.7263889442611`, 0.03393663584773771`,
2.0590146032954486`, 0.00025436731957140246`, 0.0002557385778802673`, 0.005390858822490685`, 0.5634443408340508`},
{0.26758004713434824`, 2.448981429729897`, 4.582999027119474`, 1.4554483602004895`, 0.3942207099215642`, 0.5288182096517661`,
0.017684947331647518`, 53.19217741492592`, 0.1488296927599813`, 5.857737808697124`, 1589.5500016297913`, 1630.565544843584`,
0.025803241906035446`, 1241.963291511929`, 1271.2040483512783`, 8.759012378186805`, 8.908939164468196`, 0.017116859733497414`,
0.5399777028072834`, 0.5401348504633608`, 0.00029102619471221836`, 306.43798081362524`, 2.064103702409502`,
24.63644233247883`, 0.004445613488643141`, 0.004499954185780401`, 0.012223441663581358`, 0.7819566501732014`},
{0.13431037279286218`, 0.7397108148766112`, 9.99887072123514`, 1.436439642978205`, 0.2003363652340402`,
0.6779870054162531`, 0.009538236593206559`, 4558.309876478542`, 0.14371659865116293`, 1.1480275619139793`,
3.3041970113380166`, 3.396425327215184`, 0.027912474819357325`, 13.172800481884476`, 2.9059040157952682`,

0.033577390867176295`, 0.03425517311703606`, 0.020185673524810266`, 0.002571034564972271 5`,
0.0025898733918108072`, 0.007327333165876304`, 0.3548222737112779`, 0.004933094441210974`, 0.0602357366774054`,
0.000021254007531279484`, 0.000218764202819432`, 0.029284489042723516`, 0.24314308304814045`},
{0.27730778316543614`, 1.7365428506012295`, 3.277328223559843`, 1.1643406154035905`, 0.2621424262474452`, 0.5836632043576286`,
0.19147730163610321`, 594.1704278677969`, 0.14363610143643862`, 8.32222051121584`, 188.16133338898743`, 192.3193972710194`,
0.022098397195325914`, 103.47981835320181`, 52.51635158046463`, 5.246314337277064`, 5.33904349332531`, 0.017675104861592184`,
0.05026398577375303`, 0.05036134703403935`, 0.00193699840527084`, 130.14928077721706`, 0.19912277811404216`,
3.2154076610410898`, 0.0005187458757651697`, 0.0005243059874329588`, 0.010718372766988615`, 1.3576468948170541`},
{0.05614793436458493`, 1.997529172364887`, 6.840549262003957`, 1.2853232683568476`, 0.15723714782247722`, 0.6374125080636333`,
0.1308231427534364`, 1207.3035504224565`, 0.15724403465210185`, 1.842303075231806`, 18.939740702371978`, 19.281315954082512`,
0.018034843088836627`, 47.051930467226704`, 6.8110799000178`, 0.40990313772768633`, 0.4177257443762149`, 0.01908403700418959`,
0.012045714773525831`, 0.012113138387681806`, 0.005597311195194354`, 11.697049649356497`, 0.009662028606834235`,
0.363371666125854`, 0.00011168513310244599`, 0.00011421826776507777`, 0.022681037236246926`, 0.380443099127317`},
{0.0647068787712346`, 0.8380824418888393`, 1.0963289034750066`, 0.857562051048008`, 0.6907626550964823`, 0.2999007220652139`,
0.27885748113518277`, 1238.4520803651262`, 0.1379529162857775`, 8.96709675960637`, 117.02857234147464`, 115.07488447496439`,
-0.016694110057239975`, 59.731654670578855`, 24.656936700425643`, 7.111034608762892`, 7.211056991269819`, 0.014065798861908396`,
0.06390786842479802`, 0.0640221809159225`, 0.001788707618358254`, 85.13761784668613`, 0.05907540992423772`,
1.5172451226488288`, 0.0008954423189835303`, 0.00090471705902347`, 0.01035771913311856`, 0.5533491594728781`},
{0.20132622112515353`, 1.9086557669063273`, 0.7030652496772074`, 0.7755069309180247`, 0.32762519329281337`,
0.20767575421456186`, 0.011318073987463645`, 431.8155255411275`, 0.17436258525110954`, 5.81443415452358`,
121.53573591354466`, 121.52746827212849`, -0.00006802642329051256`, 95.66675278949099`,
104.46527266304263`, 0.5815749476589845`, 0.5984353430625919`, 0.028990924508484506`, 0.14375397950855737`,
0.1440819297032142`, 0.002281329503210916`, 15.857519681965242`, 0.4134492209451507`, 2.8690056362307144`,
0.0022205493457754244`, 0.002259511197709569`, 0.01754604193249243`, 0.4625638078627393`},
{0.045022613849116516`, 3.5058573906861508`, 8.47131465431961`, 1.2697383949459176`, 0.5022287267438617`,
0.23847623822456332`, 0.06077359284826833`, 243.2833936812314`, 0.1613793589669978`, 8.022277670460284`,
372.92725768898316`, 372.09299583320785`, -0.0022370632303607696`, 212.76046576625106`,
202.7530839852573`, 3.3298256640852677`, 3.3926326214564293`, 0.018861935640830385`, 0.04372672660602135`,
0.043735022168067496`, 0.0001897137675290761`, 166.76991305899668`, 0.028124164669895212`, 6.955623907465391`,
0.0004161524750425638`, 0.00041705104788524627`, 0.0021592394532570225`, 0.551473796724312`},
{0.17418182442148117`, 3.0892110646487794`, 3.4522019380421036`, 1.2965827481879664`, 0.41818871776887856`, 0.6556019601202148`,

0.075063644002762`, 2886.574837344593`, 0.1531514113867664 7`, 2.6062865811306466`, 14.820017850371201`, 15.261998266439404`,
0.02982320402921323 5`, 26.025018889775115`, 7.230677129479273`, 0.16713480008838255`, 0.1699179210610004`, 0.016651953818989984`,
0.013259701879905703`, 0.013365504467541115`, 0.007979258402162959`, 7.375923910298476`, 0.03299427235324203`,
0.20610918361726768`, 0.00012126094354103 5`, 0.00012518259755951115`, 0.0323406193614934 6`, 0.4081246412912823`},
{0.04583105977220422 5`, 2.899702133961444`, 5.925065562245003`, 0.8929739121804446`, 0.5933600691515191`, 0.4490635153302144`,
0.16447888961080792`, 1912.79886244215`, 0.11499785968845799`, 4.757985376452911`, 42.34937121651226`, 42.58205912795087`,
0.005494483265146677`, 40.736935768522216`, 13.006441144596327`, 0.6913256009310029`, 0.700226483117737`, 0.012875094130388565`,
0.008408687226400088`, 0.00841879167009823`, 0.0012016672075063628`, 28.63769028974007`, 0.005505414955408842 5`,
0.5349766819391214`, 0.00011348729481663877`, 0.00011416797436238335`, 0.005997848013245433`, 0.42674875368798076`},
{0.2429697761558342`, 1.8449858081251405`, 7.508754000794074`, 0.9127320360969664`, 0.44886430542069955`, 0.35316747155912975`,
0.02193269408167387`, 161.73721618998414`, 0.16775422018188862`, 9.221971574736251`, 727.0663473425552`, 734.7451436087945`,
0.010561341883454478`, 360.84007048326544`, 556.1298161060198`, 6.232318817400337`, 6.34057315485361`, 0.017369833062941487`,
0.06014238649975288`, 0.06014343158087113`, 0.000017376781652478357`, 164.26485385449826`, 0.20875403121898495`,
12.30687271611814`, 0.000796031241507178`, 0.0007979873011898653`, 0.002457264967369532 4`, 0.8631237140187292`},
{0.160442241636698`, 3.55050251528807`, 3.9052606138637995`, 1.2025377341211225`, 0.791383919489673`, 0.40672925070026333`,
0.35477124416027767`, 105.50559352550128`, 0.10792398319932495`, 9.162379917819035`, 2013.9089077686758`, 1945.1501623356062`,
-0.03414193420955236`, 1005.9954621155534`, 346.6782520566707`, 32.20773991554216`, 32.52088171680901`, 0.009722563647371452`,
0.42501986906562095`, 0.42462136282263385`, -0.0009376179138711471`, 1633.6237368839488`, 0.9741591504686485`,
18.11414034147711`, 0.0042515308023148846`, 0.004277550031647503`, 0.006119967264132553`, 0.5207452647019479`},
{0.20468313725879644`, 1.5880039947408164`, 1.8181726149942818`, 1.4361114186612707`, 0.6452252370101204`, 0.23877202353940497`,
0.026067763377395026`, 956.3204095901076`, 0.08759161290164996`, 6.530440575770296`, 91.25028387302363`, 89.88605786951616`,
-0.01495037544656641`, 63.9523101233412`, 66.66534457357844`, 1.025920516738457`, 1.0453766182256377`, 0.018964531042847455`,
0.06893137080787134`, 0.0690837349656595`, 0.0022103746959107884`, 23.27379826953184`, 0.20155841760723156`,
1.428368601049095`, 0.0005766015504934385`, 0.0005840771581314327`, 0.012964945431722663`, 0.39051485627373744`},
{0.07364401078716826`, 2.5859680523819737`, 7.8227327965838285`, 0.8181378301360093`, 0.36855652079172274`,
0.32445369334254925`, 0.005391697017678555`, 139.66827785366002`, 0.0644557011853657`, 2.5433237558513593`,
197.97876721741716`, 200.06966424811495`, 0.010561218559369978`, 356.27181191855385`, 183.80534427943076`,
0.36534445967053675`, 0.3731605883545561`, 0.021393861264703107`, 0.05104193465290674`,
0.05108320154818448`, 0.0008084900299796249`, 13.496701440325163`, 0.05369903980251842`, 3.9645457215585727`,
0.0007518757132010112`, 0.0007562616257095262`, 0.00583329456120163`, 0.2621973637191556`},
{0.2506656721970057`, 1.5258727034335298`, 1.8333712411976357`, 1.2495957919641723`, 0.1010075460664761`, 0.5024718466416268`,

```
0.012345305326089913`, 1052.1669408921373`, 0.03320454993831845`, 2.130087729732141`, 21.5975940949994`, 21.719906382444215`,
 0.005663236696958451`, 46.40581227031337`, 18.25025070294567`, 0.13777297401130378`, 0.1420260993580753`, 0.030870534495557767`,
 0.04243347617728269`, 0.04299044479190355`, 0.013125689073737457`, 3.0032002902100787`, 0.1519516547090588`,
 0.4781882235969407`, 0.00039870593637048035`, 0.00042031129586418524`, 0.05418870782407681`, 0.4191237292572324`},
{0.10121864512314288`, 0.9521305504715327`, 3.6368221202357223`, 1.491518490019038`, 0.3322534458031634`, 0.2564348282281962`,
 0.03507970829425946`, 4627.905596988043`, 0.07121256842175683`, 7.352385124666178`, 15.63452559293925`, 15.709695293981277`,
 0.004807929770249908`, 9.732422513570505`, 10.515941366312344`, 0.34954979411672976`, 0.35744851197101385`, 0.02259683165954418`,
 0.005463537880751665`, 0.005470340718603184`, 0.0012451341969248464`, 4.754529112708182`, 0.007900170026697304`,
 0.3412209442669575`, 0.000044154567577781 02`, 0.000044437333660351 32`, 0.006404005249789568`, 0.6485823530987753`},
{0.1942542932466596`, 3.1177323303054916`, 7.932133913996736`, 1.3085296181137158`, 0.8325654731630694`,
 0.19060678658466512`, 0.044940459115954866`, 2391.994107548152`, 0.24452644883302188`, 1.4227474861405387`,
 8.533858078755378`, 8.291418684634737`, -0.028409119519362758`, 27.452511607530045`, 5.230552226205812`,
 0.07201988196976293`, 0.07326999912633794`, 0.017357945089383264`, 0.006183402429240182`,
 0.006195016787078544`, 0.001878311814776934`, 3.207695920598793`, 0.017159320982168533`, 0.12248673977887296`,
 0.0000568746691120836 1`, 0.0000574699929560038 1`, 0.010467293317293569`, 0.06172281786562274 6`},
{0.12644890469661946`, 3.0196313965941393`, 8.688658870512302`, 1.3655010622962382`, 0.5847686349152417`,
 0.30770354208104556`, 0.013061738242062998`, 1213.9477293174991`, 0.0486468587160932`, 6.312801306430787`,
 72.47586287177245`, 72.23336044689572`, -0.0033459749945410344`, 52.54552904721668`, 61.19069383489821`,
 0.2541069186981418`, 0.25821693733669143`, 0.016174367308085857`, 0.011315374317976547`,
 0.011322525117971686`, 0.0006319543476152045`, 10.96156042561002`, 0.020440238410580108`, 1.1009959940982037`,
 0.00010005554637892988`, 0.00010042275233562804`, 0.003670021003208346`, 0.45906006882304506`},
{0.11280111274715143`, 3.2009360918012444`, 2.9222221030043283`, 0.9684721327188957`, 0.03329493891954294`, 0.1886848273472841`,
 0.39445850876705457`, 50.69043630972767`, 0.07560282132089569`, 3.828945975174229`, 531.8383316777534`, 523.3454148614499`,
 -0.01596898213318243`, 635.7185606500386`, 93.29256899107865`, 9.369008682585877`, 9.666294481096083`, 0.03173076347583814`,
 0.28870042532528944`, 0.28859023154965463`, -0.00038168899651136456`, 428.42282909268954`, 0.4652247032469864`,
 16.267586811906728`, 0.00358550418903969`, 0.0036124410413623915`, 0.0075127097620031336`, 0.34908346338218693`},
{0.21704875939335333`, 0.6532232080519234`, 2.918554590657344`, 1.428534955684801`, 0.6202310312510246`,
 0.3415578035649972`, 0.1858190740142067`, 1428.9901262383053`, 0.23526262585558444`, 9.378643714384769`,
 102.44187993740051`, 102.0586850921827`, -0.0037406073126730055`, 49.992170937726215`,
 28.987652261914253`, 7.096270881715948`, 7.202124003016933`, 0.014916725004639142`, 0.03347223801270195`,
 0.03351973737320003`, 0.001419067362034454`, 66.2206975794276`, 0.10378725335395625`, 1.389908399026109`,
```

0.00028214150020444695`, 0.00028443515554125786`, 0.008129450418137285`, 0.6845942047741643`},
{0.23408001184633387`, 2.4049572740765104`, 0.41973647277501946`, 0.9771854975111727`, 0.20272636213110817`,
0.5074761434023957`, 0.2724896408017817`, 4254.760257351377`, 0.16397942354351142`, 7.3871482427729305`,
21.04461020226337`, 21.039262638512433`, -0.0002541060966445574`, 13.038528068126848`,
4.563836605534726`, 0.4611218277169452`, 0.4735586527908623`, 0.026970801047291326`, 0.04076998493648963`,
0.04128295894134946`, 0.012582148501131929`, 15.842547054333158`, 0.13633483652726716`, 0.4014061058301745`,
0.0004899261844532043`, 0.000515823299615242`, 0.05285921835539553`, 1.2661446053587264`},
{0.1483945695425406`, 2.699298050205127`, 5.571982823617271`, 0.9775705576725262`, 0.05426683476548022`, 0.5307838109252759`,
0.3096243181655647`, 323.46321359362`, 0.06725520461051537`, 9.792268234153859`, 330.21241162188124`, 329.9903374078502`,
-0.0006725192821805948`, 154.33861059668715`, 66.16314292622062`, 6.671490291571488`, 6.807737029400675`, 0.020422234294684705`,
0.03710658125947784`, 0.03712951083123012`, 0.0006179381385724714`, 257.26201051430485`, 0.0786630736170591`,
7.073296958639292`, 0.00045795213985833083`, 0.0004599534861860053`, 0.0043702084857459855`, 1.8000186825596316`},
{0.11663741077922185`, 0.4431208542421814`, 9.780884572399291`, 1.0122403254522139`, 0.996318833483786`, 0.22155768960679945`,
0.39993089984673347`, 139.93164861672463`, 0.10490063957337303`, 5.3884722727325105`, 811.3966832881443`, 812.6481200164565`,
0.001542324185059174`, 689.179055147138`, 127.49984610158774`, 93.2616955290253`, 94.20252858384369`, 0.010088097256665707`,
0.09782454386045905`, 0.09780400779642863`, -0.0002099275214583196`, 590.3743170127984`, 0.16300002152283263`,
7.84605331060415`, 0.0011674930968604835`, 0.0011700462640967994`, 0.0021868799423154783`, 0.17427768103109334`},
{0.11314041374373823`, 3.32806970136921`, 5.495643049849155`, 1.495187492234928`, 0.8329498208958137`, 0.41734162088096116`,
0.32389371604169326`, 602.537083528763`, 0.025714962476490244`, 1.3686646556271693`, 55.473621944728635`, 52.66683962351665`,
-0.05059670205072486`, 185.50429312100178`, 10.21599056795776`, 0.9286080399457157`, 0.9396708387876294`, 0.011913313654446034`,
0.0685777099309538`, 0.06900397345522835`, 0.006215773677828107`, 44.14960403130267`, 0.11084157821693691`,
0.4760383401748952`, 0.0005455598077972468`, 0.0005605115235137899`, 0.027406189940773373`, 0.08461116126621974`},
{0.09294922428523011`, 0.8101676653238759`, 3.5463022431515436`, 0.9341830117643879`, 0.15981251792750606`, 0.6527933768073686`,
0.016252799814697117`, 738.0588634619963`, 0.024900612098687713`, 6.915149516601594`, 120.37792398395915`, 123.65131947629831`,
0.0271926561283391`, 79.67274296449726`, 97.98577414039039`, 1.7717459060404341`, 1.803991405719612`, 0.018199844328265602`,
0.028714848950292315`, 0.028772854880079465`, 0.0020200673835186755`, 20.505874917770186`, 0.03812889907712363 5`,
2.215067900356894`, 0.0003693739555129527`, 0.00037306380903359004`, 0.00998947940309769`, 1.3680930026791478`},
{0.2696435798732645`, 3.282053008859009`, 8.218787637608994`, 1.4011858805306123`, 0.38871702639887196`, 0.49514225831047376`,
0.01812451162452924`, 173.50575962443355`, 0.20495943261505556`, 3.6349041099248875`, 287.0768468790293`, 293.88747670914523`,
0.023724065190760202`, 361.4678730733935`, 228.55557644080702`, 1.2115733111503724`, 1.2329768415513094`, 0.0176658978899 2179`,
0.08426818805833798`, 0.08441047373535282`, 0.0016884862519690458`, 56.80639759020498`, 0.32460536996403266`,

4.67515453057286`, 0.0007229424317221067`, 0.0007303163814818147`, 0.010199912795466615`, 0.47500483123052323`},
{0.13023501639399904`, 3.4827106844829796`, 7.180310384726493`, 1.0417379014779535`, 0.3157954886073613`, 0.3090830033379006`,
0.027522996818882862`, 1326.2576636786057`, 0.24748434887668458`, 4.853044482161593`, 37.76231261896993`, 38.13568855652656`,
0.009887528375819521`, 35.613020191005056`, 27.30326815139869`, 0.2055736872349975`, 0.20998364140667006`, 0.02145193886915786`,
0.00707799664062057`, 0.007083528339707985`, 0.0007815345737334223`, 10.22790967116982`, 0.01316861440753903`,
0.7964878530194213`, 0.00008200229535204606`, 0.00008235968664113328`, 0.004358308356527063`, 0.49911932224803884`},
{0.0474731410927452`, 0.7132410347452449`, 0.7493200461382123`, 1.2237616503945397`, 0.9506868210145907`, 0.6554164129507942`,
0.030282495208288053`, 4252.97050681489`, 0.14327214907032687`, 7.932983145622682`, 93.05888839367078`, 86.4401640550493`,
-0.0711240425591807`, 53.68906287676082`, 64.92782798348847`, 2.495373026813049`, 2.5114642110691405`, 0.006448408347445422`,
0.11885034325500406`, 0.12001430965879661`, 0.009793546841468936`, 25.42574913885019`, 0.08060284448954132`,
0.38715875621789114`, 0.0011481239926370845`, 0.0011897824814147593`, 0.03628396326949934`, 0.4096623650696204`},
{0.16300334800486183`, 3.0919140294206544`, 3.354218048792589`, 0.9673340843818878`, 0.9052111330146941`, 0.3760205832601582`,
0.15433250667793078`, 4163.373290035795`, 0.2440496776808055`, 4.495712049614825`, 29.557678927069627`, 28.03471829670753`,
-0.05152504139854275`, 30.09098020862826`, 9.363455674304893`, 0.4461953323347096`, 0.4501772155810654`, 0.008924080907616538`,
0.011864011399246184`, 0.011887715983094028`, 0.001998024365464701`, 19.70853725582431`, 0.02762676541207154`,
0.2226567454973205`, 0.00014748869943725929`, 0.00014896839585002192`, 0.01003260872465761`, 0.20110389643773904`},
{0.2283283078987099`, 0.42840256075325867`, 9.446993924606975`, 1.3839314736668495`, 0.8927356525671915`,
0.3909626915031965`, 0.018767685887155043`, 53.5712236843488`, 0.10749676377570666`, 1.5496335657505222`,
766.687770109555`, 724.8129404127303`, -0.054617839659606204`, 2264.4045322636725`, 604.1446711961082`,
22.522971795601787`, 22.766283435903343`, 0.010802821337682733`, 0.4533350428108119`,
0.45387529186708875`, 0.0011917213655647796`, 137.84141132870317`, 1.4787031890881681`, 5.991721984546903`,
0.003921095375753136`, 0.0039802419899045115`, 0.01508420695837187`, 0.07668475393646046`},
{0.1380652378345807`, 3.317739091147252`, 0.7336293987943634`, 0.9484386065310282`, 0.5023326788118547`,
0.2130184622271264`, 0.06305960906666651`, 1932.031257216769`, 0.01203085244100871`, 4.258055262793977`,
23.512609527881533`, 23.298680309643302`, -0.009098488961191253`, 25.272839115378524`, 12.517097940891647`,
0.2244715682674012`, 0.23024698238863112`, 0.025728933805772458`, 0.04433912798428164`,
0.04456476424199118`, 0.005088744990547075`, 10.63911566988408`, 0.08745274643609918`, 0.4658564189727441`,
0.000558123651945408`, 0.0005720400076449035`, 0.024934180178511234`, 0.30219382988010707`},
{0.0926632627436077`, 3.578083963812018`, 1.8944161244777453`, 1.1381278842914715`, 0.7382269863407194`,
0.4777843546205588`, 0.04528998830707099`, 1233.5473532765689`, 0.08357615053600725`, 2.9903558138075255`,
54.443463629532914`, 53.133223137666015`, -0.024066075236920725`, 83.32731240312161`, 33.159909462405174`,

```
0.40533293836396195`, 0.4103832010524851`, 0.01245954180014941`, 0.06795438943838132`,
 0.06846848271773955`, 0.007565269640519512`, 20.71878981092707`, 0.08995536347299407`, 0.5159354515686989`,
 0.0007084541621078522`, 0.0007304005483279383`, 0.03097784922991975`, 0.23422696162944256`},
{0.2706310445313141`, 2.5166396966047158`, 0.5049158559505837`, 1.3168707464723388`, 0.5754269054535`, 0.5968444469720801`,
 0.1025449755078217`, 681.183394170123`, 0.05243619315919962`, 2.183793658787195`, 65.77432567179528`, 64.3566422857879`,
 -0.021553750213744838`, 137.85080241294733`, 26.207953976733624`, 1.0106149857924116`, 1.0578657940938057`,
 0.046754509843672354`, 0.4566479058853678`, 0.47580481246698786`, 0.04195115390812498`, 36.33362558898282`, 1.765472853611313`,
 0.7357066115407864`, 0.0038978580756028336`, 0.004533560199194159`, 0.16309011545860597`, 0.3553720511439756`},
{0.20804007909247985`, 0.9673945938397814`, 0.48619022305575754`, 1.3068640855075515`, 0.04779661652651645 6`,
 0.6050138674479171`, 0.4706922223098622`, 348.3137462309647`, 0.24685340199219946`, 5.551550038883448`, 190.42722682926214`,
 184.70134712080423`, -0.030068597877507153`, 156.992635014961`, 27.422707632361654`, 10.848649542744216`, 11.185876037373047`,
 0.031084651900694427`, 0.5610196775161438`, 0.5719393721960883`, 0.0194640136836024`, 149.92749883018826`, 1.667351115469956`,
 3.8681149152673378`, 0.004941293320219531`, 0.005361942534805161`, 0.0851293755147775`, 1.2748591201923212`},
{0.048451644236667674`, 3.3925416461261833`, 7.486002650281495`, 1.2431466818882033`, 0.1294136137122328`, 0.6416005560424389`,
 0.04082335443875802`, 58.92112932799084`, 0.09857305139503486`, 6.047753646247408`, 1271.2878313623348`, 1302.0139818510706`,
 0.024169310624022167`, 962.086881049471`, 810.9183138194043`, 9.298422360857463`, 9.46726035502372`, 0.018157703276310055`,
 0.21897766547748376`, 0.21908805591056296`, 0.0005041173164326285`, 450.6469300354263`, 0.15156897062139205`,
 24.339513858206303`, 0.0021186859523597557`, 0.002134093356135815`, 0.007272150815414102`, 1.2184120167724968`},
{0.09431215650771135`, 0.5526170445339527`, 7.677473256451986`, 0.7715239445968243`, 0.7622192109377914`, 0.1566986505563851`,
 0.018040482265180933`, 824.8087273379117`, 0.09073506826067207`, 9.996434684615583`, 151.7830998639991`, 148.99177950003994`,
 -0.0183901920995172`, 69.49327516443633`, 121.12996719154722`, 3.43794173269914`, 3.49676287670065`, 0.017109406899496582`,
 0.009577224181280918`, 0.009577854208587106`, 0.00006578391549183493`, 27.140931422916186`, 0.012903552369900515`,
 2.484781106789503`, 0.00015017043238807481`, 0.00015030504758767077`, 0.0008964161416815664`, 0.3875423045668269`},
{0.26113782676147984`, 1.4684069653405487`, 4.54225535313282`, 1.1193463339337828`, 0.6236318874525932`, 0.1751148412910668`,
 0.053183289402392066`, 554.4897241223483`, 0.22550063278101773`, 9.001504879035508`, 183.14485176813224`, 181.0793306310366`,
 -0.011278073705891845`, 93.12024040926305`, 105.4245188765496`, 3.529515226419025`, 3.5978654306788576`, 0.019365323528913958`,
 0.03144353580773747`, 0.03145160024699391`, 0.00025647367731629345`, 74.03949632498878`, 0.11730138009328107`,
 3.3618299275457626`, 0.0003394549620115983`, 0.0003404251525347489`, 0.0028580831972562226`, 0.4432692105905284`},
{0.23311836154945775`, 2.5262146716446363`, 6.3107841154355`, 1.4422657044398717`, 0.3118898255230633`, 0.2567889403043644`,
 0.03481026984978327`, 1604.0167510242688`, 0.16315782107376747`, 5.777943122601165`, 34.570870932400446`, 34.763770297899015`,
 0.005579823715629484`, 27.38429426617599`, 23.311756601292153`, 0.3025035116556812`, 0.3095788707143082`, 0.023389345200991896`,
```

0.008568004345769549`, 0.008575420828095351`, 0.0008656020733071035`, 10.916982990980102`, 0.028533701926216067`,
0.7752816251485339`, 0.00007168383099354081`, 0.00007205553164898902`, 0.00518527888781084`, 0.5227847601306089`},
{0.22640126428063923`, 3.7808015365334118`, 7.033092553948691`, 1.0460349638379132`, 0.8860375200295565`, 0.499376231890995`,
0.3693111113992884`, 210.995030019894`, 0.19589698445197923`, 2.861160479579169`, 436.77828668878715`, 410.0424834036011`,
-0.061211383669893316`, 698.6880533318347`, 71.8567375350229`, 6.622615001330922`, 6.674644359681307`, 0.007856316325187018`,
0.14214661313107413`, 0.14234497668367013`, 0.0013954856062106114`, 357.6970424700162`, 0.4597453275164303`,
2.8126232177799504`, 0.001632006990980539`, 0.001649628238823287`, 0.010797286984757903`, 0.14565042412271498`},
{0.19770244273120569`, 3.3508846225196347`, 6.757833537731408`, 0.8466672464019638`, 0.28373063232656337`, 0.6609960258126362`,
0.006803814757890119`, 1256.448281122042`, 0.0187925047862397`, 9.149585751239048`, 105.78070590085548`, 109.5878003583873`,
0.035990442917823495`, 52.91386972476258`, 96.42955861502112`, 0.18865108926371074`, 0.19175017256960963`, 0.016427592960074344`,
0.00908381112770119`, 0.009090561624236167`, 0.0007431348406605753`, 9.030686200504974`, 0.025655594989354543`,
1.6831359053800352`, 0.0001294769878548152`, 0.00013002029035301158`, 0.004196131738912312`, 1.565265206823182`},
{0.06481064744600706`, 2.3696983632913335`, 9.923821775061434`, 1.1006879887228784`, 0.22746584913410017`, 0.6483433064071293`,
0.08284957522863273`, 281.66193979187017`, 0.13350656503173153`, 1.0702403000648566`, 49.78853974666823`, 51.08487841422653`,
0.026036888692744675`, 212.9181006116588`, 23.22335139095791`, 0.7604312779876712`, 0.7743144145923657`, 0.018256924730178703`,
0.03217060071403114`, 0.03235286155687126`, 0.005665447296438808`, 25.742753640613195`, 0.029785678014315753`,
0.898708615690226`, 0.0003481314090114651`, 0.00035627284748563753`, 0.02338610726705314 7`, 0.21229522841688483`},
{0.1537084817657926`, 0.5942996643982652`, 0.8160795607483013`, 1.042756674430172`, 0.7722126796580249`,
0.49669902434825364`, 0.00611858149596996`, 1518.932458333751`, 0.21666908673557295`, 1.634333111202272`,
26.901375444556223`, 25.53568476102389`, -0.0507665746068271`, 75.33527897419738`, 24.361254898595657`,
0.22223206895819572`, 0.22786024247917216`, 0.025325658656560224`, 0.12670251555378143`,
0.13000139382913453`, 0.026036407098427583`, 1.8867492000055397`, 0.2782178757382672`, 0.23278344622193886`,
0.0014047532304344301`, 0.0015329041418264144`, 0.09122663583578494`, 0.15715070642575843`},
{0.13754450751212471`, 1.8598194811531465`, 9.485701362019345`, 1.4994328167980717`, 0.8397969618927028`, 0.642370724162252`,
0.08665379792235217`, 239.43302754758346`, 0.1633413998793405`, 9.552795266495377`, 1418.0820285280618`, 1355.6571922206913`,
-0.04402061026904491`, 679.4153861310649`, 638.0527968774545`, 28.277994714021276`, 28.45308626884489`, 0.006191795302118663`,
0.1472198966877591`, 0.14727121630800183`, 0.00034859160614408857`, 751.3137922440366`, 0.2892755455125675`,
8.333567417264398`, 0.0011837781539524306`, 0.0011893646536532579`, 0.00471921168858791`, 0.5568832045742859`},
{0.07858197291799773`, 2.783199788795301`, 0.9642830041255817`, 1.3105090636097434`, 0.9572373854271048`,
0.41778523295843695`, 0.00823451224525384`, 90.59081205787001`, 0.24323880940762027`, 7.348654585309644`, 2760.056769776339`,
2634.820754866145`, -0.045374434425253796`, 1718.9952442277684`, 2463.1969508199336`, 7.060737608498302`, 7.127272638300127`,

0.009423240671306399`, 3.2237021396469916`, 3.1966853602609495`, -0.008380668627468335`, 280.7349060101647`, 3.61892677476348`,
16.721053295908575`, 0.02888155422101968`, 0.029601877738317083`, 0.024940607828271233`, 0.3261993181560628`},
{0.09118935700706149`, 1.2770460819223013`, 0.666402509206284`, 1.1661439001587546`, 0.35202394044239993`,
0.23733544361366754`, 0.06689005296661862`, 1909.3731761645724`, 0.030837584446851807`,
5.2718694661097025`, 26.83154409726582`, 26.74565280618183`, -0.003201131130307955`, 23.294077303027592`,
13.937297300701449`, 0.6634336502871679`, 0.6813196292270243`, 0.026959710186714725`, 0.055300285433342665`,
0.055646864796055594`, 0.006267225566686907`, 12.103362053066265`, 0.07203996387104773`, 0.5933422044481431`,
0.0005646693152212556`, 0.0005804408726118909`, 0.02793060817277726`, 0.4682164281524639 5`},
{0.26692387934872025`, 2.295217680243936`, 3.3610155473383347`, 0.802490311252646`, 0.6241394164828811`, 0.45771836786665954`,
0.015176154328102385`, 1461.3995866509204`, 0.028435347409555445`, 8.762751383914615`, 109.83933729709884`, 110.26129061743359`,
0.003841550128743 4317`, 57.36961145170455`, 90.3807571308705`, 0.5718176825690405`, 0.5791999738755381`, 0.01291021864404529`,
0.01875570598405963`, 0.018773810616795998`, 0.0009652866573914842`, 18.749229355836796`, 0.07151922573127739`,
1.2830249140209693`, 0.0002817831873276333`, 0.0002834395727790716`, 0.005878226686081067`, 0.7373098174163232`},
{0.16789560992918506`, 3.010819021450227`, 9.549236085030234`, 1.3823009139889233`, 0.2184000924395355`, 0.5213598249132101`,
0.3210433649481226`, 558.1245473691072`, 0.20748907999743366`, 5.921770487462836`, 129.1220686043237`, 130.50736596502583`,
0.01072858710889446`, 99.79606069552106`, 24.970543392000433`, 2.364923107710534`, 2.4086356243850586`, 0.018483694684197527`,
0.019784534950968356`, 0.019805216531142575`, 0.0010453407282746685`, 101.71936395660053`, 0.04745337946797269`,
2.425910193544705`, 0.00017255062475962646`, 0.0001735377113513269`, 0.005720562258615391 5`, 0.9382936591037263`},
{0.05001315370963505`, 3.027404779172768`, 3.8220563025552714`, 0.9962642247459573`, 0.44362718458195727`, 0.3510033113401648`,
0.04839212642640241`, 1615.957133832461`, 0.048401134986770955`, 4.739296636982807`, 36.45952538654751`, 36.774237156456245`,
0.008631812031893782`, 35.20964230799384`, 21.795980134698596`, 0.33088550107885384`, 0.3367929345148687`, 0.01785340674267566`,
0.012561250488865463`, 0.012581740463343694`, 0.00163120499001223 6`, 14.310347818929964`, 0.008974682163992347`,
0.6352176006831113`, 0.0001517690984579323`, 0.00015297259371755523`, 0.007929778010485666`, 0.4615577250402279`},
{0.0729686004745061`, 0.5044823312287607`, 5.5024178276878075`, 1.2230366941116102`, 0.012758742281838265`, 0.5648708708999995`,
0.03232052448858605`, 634.5000769880222`, 0.22553164736796405`, 5.75812027698643`, 96.35976979326912`, 97.63326548519228`,
0.013216051622532143`, 76.59131326377452`, 66.50076808340052`, 3.631353226003601`, 3.7087944112732316`, 0.021325709852483055`,
0.02329801076205135`, 0.023340449199925718`, 0.0018215476981191525`, 26.170764870991082`, 0.024286046273531616`,
2.1619291897508073`, 0.00022910909325546402`, 0.00023113996767143368`, 0.00886422440555501`, 1.192050526534098`},
{0.25149333924897926`, 1.871859550111978`, 9.811462629766478`, 1.3386558973788294`, 0.5189637829018348`, 0.6377508608690272`,
0.024369302194970535`, 194.42061666753636`, 0.14619611626015583`, 6.325307886810732`, 604.8865328072178`, 624.4888962097375`,
0.0324066785080 2881`, 437.68003886604185`, 450.1235012742575`, 5.56125054717386`, 5.6307531704973774`, 0.012497660864935645`,

0.08179185736286954`, 0.08186166948120342`, 0.000853533842912535`, 148.71257067561234`, 0.2938586761651309`,
7.205253264342156`, 0.0007361821009821412`, 0.0007407507227865049`, 0.006205831136438578`, 0.7723310142101982`},
{0.1922243891646514`, 1.6850594067594091`, 0.2630953725218035`, 1.4711962401085972`, 0.15977146838462364`,
0.6883258791888056`, 0.09212778533128511`, 774.9062808250895`, 0.24229201708328568`, 1.1723783184073415`, 24.704865885799407`,
22.020026359336963`, -0.10867654731959964`, 96.44488889705877`, 9.939897897263483`, 0.501714392837837`, 0.5599147393817769`,
0.11600294385565157`, 0.5835023549499595`, 0.6651552064783347`, 0.1399357703284294`, 12.07740795939975`, 1.6023340536627404`,
0.42520968252225133`, 0.004063120465667369`, 0.006087165437679264`, 0.49815037213754976`, 0.49707646110693954`},
{0.2327924107307146`, 3.0114739814321467`, 6.475065538974626`, 1.3312648289930094`, 0.6507217418486848`,
0.3841095338158853`, 0.3176950069808959`, 737.689863101484`, 0.19812174358831114`, 7.321794649353192`,
171.82745207588`, 170.39967183857436`, -0.008309383745474519`, 107.40870999743643`, 32.69936726258799`,
3.1575176214838923`, 3.1985435379818044`, 0.012993091857594008`, 0.030243635431112276`,
0.030268257497067845`, 0.0008141238844003862`, 135.83974518588934`, 0.1005784114466853`, 2.102735769187523`,
0.0002740347896484918`, 0.0002754510483823355`, 0.005168171295550916`, 0.5374507843135516`},
{0.20811447549122308`, 0.49053077700921355`, 2.218461283385089`, 1.0634384889669943`, 0.30011342208125913`,
0.5589173637133283`, 0.16385051847701637`, 124.79731876547663`, 0.15435702825422665`, 9.111119072715418`, 994.3048692546382`,
1019.0250344849559`, 0.024861756182335437`, 499.4733702023049`, 308.2378306053539`, 85.51455522483825`, 87.0016773578219`,
0.01739028086006722`, 0.3277520048492066`, 0.3279910404397598`, 0.0007293184695031929`, 599.2503031433879`, 0.9744276654339367`,
16.608902412072077`, 0.003693731114298493`, 0.0037386049981830718`, 0.01214866012062199`, 1.3907964708490925`},
{0.19580212756702065`, 2.2297474474451375`, 8.070495694463702`, 1.2202670372174012`, 0.07482300382970508`, 0.34257881980750315`,
0.24674110677687536`, 1287.6149126957`, 0.12354587287124019`, 3.0573978018103922`, 20.974920596008555`, 20.911420931699155`,
-0.0030274090439933454`, 31.3987870273164`, 5.020491886157756`, 0.48487220927933566`, 0.4973421854993988`, 0.0257180675266937`,
0.00649500185263634`, 0.0065045090102019785`, 0.001463765181495713`, 15.444893871109791`, 0.018167645447121638`,
0.5342052962131939`, 0.00006410761343855764`, 0.0000646043495897001`, 0.007748473613333751`, 0.42771244213827997`},
{0.18591817623779283`, 1.5316284759435828`, 4.0240186618411435`, 1.0506796825025935`, 0.8560461598734517`, 0.5496710897949223`,
0.05348974137997375`, 3910.687147405355`, 0.18912146822955533`, 8.614502889074807`, 72.90049093362195`, 69.14576850182758`,
-0.05150476195301845`, 38.73153655893885`, 41.49180439898203`, 1.3704778206672765`, 1.3800865657919137`, 0.007011237234002765`,
0.01384302845069974`, 0.013862186257940801`, 0.0013839317970982812`, 29.98661222547287`, 0.03676672290217802`,
0.4581408556764876`, 0.0001586901217159875`, 0.00015982835814773127`, 0.007172698712657688`, 0.46316792896770387`},
{0.2552337642427184`, 2.0636949211593727`, 3.6788035742608756`, 1.2716726068122697`, 0.4767575621154567`, 0.33698161388823356`,
0.00790398094444615`, 742.5948236907665`, 0.22094334214516276`, 6.7847431915852425`, 116.91265727797185`, 117.69277763016758`,
0.006672676597717864`, 78.86650809663173`, 105.00265440446385`, 0.3818584465188585`, 0.3891468162866569`, 0.019086574709140436`,

0.03729010050916963`, 0.037340064942602336`, 0.0013398846543848375`, 11.257704809753935`, 0.13596703888492156`,
1.965166273066498`, 0.00035313321462504543`, 0.00035598779641925134`, 0.008083583407006545`, 0.6091564732409933`},
{0.2297403387592854`, 3.933034881515791`, 9.92502862171417`, 1.2059622632705849`, 0.1421363362803283`, 0.1853832242974326`,
0.0636701100922172`, 57.09955187350921`, 0.13182928467731042`, 9.7382206276645`, 1297.9825441940216`, 1300.5825095982962`,
0.002003081948909413`, 610.0335730579783`, 696.2139024425782`, 10.515232458509672`, 10.811322252825732`, 0.028158178669311473`,
0.10167179135847708`, 0.1016409504162580`, -0.0003033382397122919`, 590.8110863795088`, 0.3336873112705107`,
36.363764888514936`, 0.0010191980509540732`, 0.001020636432356675`, 0.001411287434523123`, 0.7819764164792986`},
{0.04776006202628119`, 3.695464511472898`, 0.268839094050918`, 1.3548340559244303`, 0.4311024896438589`,
0.6662260908047339`, 0.1893860365797585`, 146.80096070953766`, 0.0868075345269877`, 5.3982731410948785`, 658.1590785834825`,
652.3948615754017`, -0.00875809085622081`, 558.0081162403521`, 181.9632821760756`, 8.736061146361703`, 8.933295902857388`,
0.02257708058486152`, 3.7226333879789113`, 3.819722832958543`, 0.026080850532623545`, 461.197199091955`, 2.5399028787283373`,
8.669067321267084`, 0.03037696244873489`, 0.03446650049496496`, 0.13462629955617533`, 1.0497057434079562`},
{0.16250916383331093`, 2.090868942469317`, 5.046057727155544`, 1.3781211464947924`, 0.9692295654960448`, 0.507149644697747`,
0.012667564467634538`, 467.92228322579444`, 0.2269164868867607`, 2.233796179341983`, 187.5915196968694`, 179.90656765639656`,
-0.04096641496849662`, 384.35643146749715`, 158.5093964008242`, 0.9234372610625223`, 0.9311953413156078`, 0.008401307354826582`,
0.14297775694840723`, 0.14362240119863307`, 0.004508703059724617`, 27.582661278207993`, 0.33193136754926356`,
0.9848689677918584`, 0.001238158028993741`, 0.0012648267314434405`, 0.02153901345805842`, 0.10113731116882514`},
{0.12952636821033042`, 2.4189205073691173`, 1.5337482028150813`, 1.4058710509218408`, 0.9636406844740666`, 0.3882701702830901`,
0.27400686262252805`, 4688.706549038708`, 0.0765768774664311`, 6.843480813486918`, 46.997280302834966`, 45.29801271063936`,
-0.036156721862330876`, 31.431142458956693`, 9.77907967268889`, 1.0436005662009211`, 1.052819872632676`, 0.008834133221407248`,
0.039268581839208505`, 0.03942489775299135`, 0.00398068650461858`, 36.062668729791916`, 0.07266166843428526`,
0.30005485350910177`, 0.0003341187984804428`, 0.0003402947099348414`, 0.018484178329642997`, 0.2846562482218402`},
{0.08153578837307857`, 2.0915987061965353`, 5.850951169546045`, 1.037700922521303`, 0.7897818160030217`, 0.3237340771928561`,
0.17831164332408336`, 1464.5863160958056`, 0.2436610405225158`, 7.1217069723220146`, 96.09814366896528`, 93.08810398464354`,
-0.03132255805783912`, 61.75830852782157`, 27.801786264886104`, 2.2106186736245608`, 2.2347069782587186`, 0.010896634920152026`,
0.015010015777613227`, 0.015019408703058089`, 0.0006257771866482464`, 66.05324510924325`, 0.017483620998830364`,
1.0098978295158219`, 0.0001746270630991198`, 0.00017527420937437098`, 0.003705876189900037`, 0.3723112402808944`},
{0.2624813864915975`, 2.8946734437676342`, 8.625458110929756`, 1.37764442375384`, 0.19776637799413987`, 0.31049273319228277`,
0.046660769123637466`, 609.6700244678286`, 0.017643025422342112`, 4.098918143681717`, 62.55958952242308`, 63.1147102276820`,
0.008873471029729574`, 69.85365576894077`, 38.05802384525692`, 0.576766021505661`, 0.590802228947625`, 0.024336051221121036`,
0.015270717120078976`, 0.015286162116780482`, 0.0010114126651719069`, 23.85070408171352`, 0.05726112860572504`,

1.4819300528892982`, 0.00013367441944223124`, 0.0001344794941873952`, 0.006022653762202124`, 0.4787560707810575`},
{0.22181257821621975`, 3.747903553799671`, 8.070266666229205`, 0.9808557386469257`, 0.7823522438549146`, 0.2263615830500163`,
0.06976335847986787`, 287.144692837758`, 0.1821266272405107`, 3.59762935889718`, 191.92056030068417`, 186.6559259451605`,
-0.02743132026748729`, 244.15722419208308`, 97.32261158428138`, 1.7313042990492862`, 1.7564210825301987`, 0.014507434363043581`,
0.04065149635915873`, 0.04066883140517064`, 0.0004264306991001998`, 92.69659335879264`, 0.12881447451090244`,
2.588837037369191`, 0.0005003589872591974`, 0.0005023782064107603`, 0.004035540887600719`, 0.16878277600825561`},
{0.21058778737677059`, 1.3147483391864796`, 0.4651324593397846`, 0.8516412222118703`, 0.046686774964893374`, 0.4819914326166439`,
0.1058661660756647l`, 1877.0848108087591`, 0.011164572002709261`, 8.732010177522003`, 48.15623446621077 6`, 48.19667691733027`,
0.0008398175556660892`, 25.24078059505091`, 19.670276795399083`, 1.4273100664907024`, 1.4653946339266763`, 0.026682756837560717`,
0.0625521822673634`, 0.06306027842135323`, 0.008122756642096274`, 26.807907706039927`, 0.18818179513246314`,
1.0883592600357617`, 0.0008697860206482444`, 0.0009011046514263463`, 0.036007282290833276`, 1.6188991777733293`},
{0.19996482365829993`, 1.5454344843054368`, 8.94969050535013`, 1.027667483044064`, 0.3164796074442535`, 0.5223314119072894`,
0.012949156080520758`, 231.31648814801656`, 0.19929090705614294`, 9.341058456545078`, 537.9082248014286`, 552.5060846870274`,
0.027138197953726628`, 263.5582344545864`, 454.9109040327066`, 3.5771891955373`, 3.6387009916445905`, 0.01719556689482049`,
0.04148657118033683`, 0.04150024155273738`, 0.00032951318972895827`, 78.9758791381167`, 0.11851221271803003`,
9.098545692801212`, 0.0004874885266868967`, 0.0004890099210979173`, 0.003120882498220956`, 1.322469304814925`},
{0.10440009635624109`, 1.532425369535546`, 0.308138348960842`, 1.2734376519411275`, 0.20147673343032046`,
0.35081245482125123`, 0.05722019474177449`, 85.42879625764479`, 0.08356786949647793`, 8.411462154648635`, 1005.5958763563167`,
1003.1881020223896`, -0.0023943757035396196`, 547.1626990471976`, 558.5251437882024`, 19.19418552748589`, 19.75772731346903`,
0.029360025992046124`, 3.0792055361783675`, 3.0780976327627974`, -0.000359801709419183`, 420.1950978555908`, 4.592419352538066`,
22.12307107207821`, 0.027974246082664522`, 0.029486269428399516`, 0.05405054853907165`, 1.1642709911484193`},
{0.1446017732242294`, 0.797427468093673`, 5.032298069921831`, 1.3706636028120882`, 0.10121695844960277`, 0.606814192096909 7`,
0.25341251132298065`, 197.97452899503492`, 0.2275401984407136`, 3.68386367767849`, 219.81876431976445`, 221.39769915039446`,
0.007182893760303344`, 273.1025191886049`, 50.67363165062731`, 13.624619577897782`, 13.899554086759712`, 0.020179242971887223`,
0.10153705577712688`, 0.10188994439987734`, 0.0034754663708689293`, 155.2092270534646`, 0.2097491187617553`,
4.418095206424731`, 0.0008860249106481533`, 0.0009011735715076947`, 0.017097330647802833`, 0.747799265560408`},
{0.1841264162344997`, 3.4194642457460365`, 0.8344796094826652`, 1.4377258087185927`, 0.5867256025093719`,
0.6103981120475732`, 0.3542274534468216`, 522.9821129731852`, 0.2244081861480518`, 8.361737950667973`, 328.5809054659251`,
331.80243000799675`, 0.00980435712630201`, 179.8499260958718`, 56.82713949280829`, 5.420929927764418`, 5.503830572327649`,
0.015292698055113796`, 0.4195646043254922`, 0.42305278501027993`, 0.008313810671411348`, 264.8096580955017`, 1.1036132424755458`,
3.5170146194749887`, 0.0034391251899227315`, 0.0035788511201091024`, 0.04062833496023743`, 0.9575631180551527`},

```
{0.17849930658201318`, 3.2992561971358683`, 5.635580909426066`, 1.1878114797167083`, 0.48988207998590316`, 0.27890916833354473`,
  0.03715549210344845`, 144.67131391199885`, 0.0866110192158086`, 7.798176718489761`, 644.1891341620525`, 645.0586588509619`,
  0.0013497971989864244`, 378.0809687114478`, 424.43405956825393`, 4.556810148936168`, 4.640726582166619`, 0.01841560883330673`,
  0.10922970164681502`, 0.10924228990050969`, 0.00011524570244980659`, 214.77263032927507`, 0.27853465717305015`,
  11.461604209816882`, 0.0011098359549420733`, 0.001114025739653926`, 0.0037751387429787453`, 0.6031943414161709`},
{0.09510173640093084`, 0.7659692827065738`, 0.5464490010035838`, 1.4050402850247132`, 0.11782959453955089`,
  0.3589214817200733`, 0.08175234954735425`, 225.11260306454756`, 0.1010344837489014`, 6.4310165595390805`, 272.6778303966361`,
  272.0001720828581 7`, -0.0024851976883938365`, 194.05940550745646`, 128.3273961340222`, 11.953987041442815`,
  12.300488780689623`, 0.028986290351958433`, 0.6743883158659774`, 0.6804073335132297`, 0.00892515114162884`, 130.8055268516806`,
  0.9162214263906521`, 6.479041382764545`, 0.005638970526221865`, 0.00589704114392826`, 0.0457655553449583`, 0.9587870547153234`},
{0.06416093472533757`, 1.2573296539610634`, 9.92323628294643`, 1.30190007637989`, 0.030494015633026894`,
  0.4401773012522794`, 0.02586427825345657`, 3143.6595843648015`, 0.20592664064058636`, 7.935954028868563`,
  24.30276689714245`, 24.570839911027583`, 0.011030555286964061`, 14.015902657770804`, 17.884173335398746`,
  0.3380330127324157`, 0.34579485021595185`, 0.022961773528552776`, 0.002520231488832032`, 0.002521840933237827`,
  0.0006386097518926714`, 6.0716990132323385`, 0.0023100058292625904`, 0.5823529260758071`,
  0.00002337584386657987`, 0.000023453995512463613`, 0.0033432652241263128`, 1.3396907170025585`},
{0.25237835724682106`, 2.979406809596097`, 0.9138356130609361`, 0.7932538461611548`, 0.3933816374531405`,
  0.6211446249652819`, 0.0749645015318215`, 95.73291003138912`, 0.06307891905410445`, 9.368280708056208`, 1558.1224455762763`,
  1603.6612456016999`, 0.029226714597889503`, 761.2129765907953`, 761.2926390469207`, 18.195644729726286`, 18.496018413489555`,
  0.01650799893188437`, 0.9056284667031756`, 0.9039554818846559`, -0.0018473191601519057`, 774.4603973248263`,
  3.2651574957481797`, 22.16463059377348`, 0.01353282185890814`, 0.01383207075761469`, 0.02211282331405018`, 1.3826011979415427`},
{0.05665762575451494`, 1.7380994050720693`, 0.46391281319797173`, 1.3490794355447004`, 0.9653780951392605`, 0.17540498059526055`,
  0.4972377260445833`, 285.101267300382`, 0.1881031714219547`, 6.261558552266614`, 379.0013477147005`, 373.30730566539734`,
  -0.015023804225597237`, 277.027449042936`, 50.453379325292495`, 12.642587828669082`, 12.862205835395683`, 0.01737128582398162`,
  1.0998331491411697`, 1.0992525363186787`, -0.0005279099133758125`, 313.9153483368735`, 0.8901990708062217`,
  4.498781896619565`, 0.009678967452074416`, 0.009915977501121508`, 0.02448712119558727`, 0.23699077690693535`},
{0.25642346088610446`, 3.010945889122482`, 9.963520677018519`, 1.467899384826252`, 0.4946077322511526`, 0.6868780403912886`,
  0.012314696297102353`, 88.3173980921876`, 0.10491074891333174`, 1.4353196490865765`, 295.68274497057325`, 305.9005387329298`,
  0.034556611558017813`, 942.8483507860847`, 251.086452988837`, 0.9894859128019121`, 1.0050467791496265`, 0.01572621312379363`,
  0.18896612171700927`, 0.18996819541277785`, 0.005302927777018462`, 42.56126487850743`, 0.6922192417271373`,
  3.670718888503235`, 0.0015316798806553 9`, 0.0015713687677810148`, 0.02591199873216476 5`, 0.20723461480620792`},
```

{0.09174036922136902`, 1.4204870656163582`, 7.877654658381974`, 0.9368390881044215`, 0.3909649395372681`, 0.6267838157099552`,
0.0073779181496306 1`, 223.00641924802295`, 0.22589847441224364`, 3.7895604923910007`, 263.3866805924252`, 272.783670120552`,
0.03567754264183187`, 318.10424129562807`, 238.3073411248531`, 1.1614112585390735`, 1.1794524298288418`, 0.015533835372374627`,
0.05179475487667674 3`, 0.0518678859010176`, 0.0014119388056761384`, 23.56813815165673`, 0.06788099908737544`,
3.8555112646839986`, 0.0006651439283004157`, 0.0006705352582866089`, 0.008105508833207375`, 0.5667785669145375`},
{0.2260021855376967`, 2.1215536107061057`, 4.2648042233683015`, 1.1043329178413561`, 0.5641485347029902`, 0.48705118822040194`,
0.49950773055219905`, 4133.317514629252`, 0.08759305230550185`, 2.0466954881219834`, 8.356348251134007`, 8.370071383861253`,
0.0016422404039209137`, 18.68649260819168`, 1.1161921654137281`, 0.2303675198963158 2`, 0.234101966689920 9`, 0.016210821713433 8`,
0.006581556064255934 5`, 0.006615020287470515`, 0.005084545795533524`, 6.981957766077692`, 0.021249229353724395`,
0.10798685353196202`, 0.00007110226255824248`, 0.00007275750393027577`, 0.023279728555436918`, 0.2147805416817124`},
{0.1540246000020472`, 3.8655612430526176`, 1.2043257775886698`, 1.4535724207675174`, 0.7982285789403216`, 0.6724657214579106`,
0.417253246623641`, 114.93402398229541`, 0.10143088124264565`, 7.877832682736377`, 2264.882199133719`, 2187.8900356183904`,
-0.033993893167943545`, 1315.8408368836522`, 336.7298278753419`, 34.17142478825481`, 34.49705065893882`, 0.009529186233871334`,
2.1735603458025836`, 2.1718003969578077`, -0.0008097078363500376`, 1887.024789733791`, 4.782596612033127`,
14.547610313047855`, 0.017561254640779733`, 0.018142973906968122`, 0.03312515410132222`, 0.5937119741285953`},
{0.18967559668177286`, 1.3751140072639014`, 7.146984940201236`, 1.0524950668750344`, 0.5379883166421491`, 0.5342982154057455`,
0.18359081743318598`, 57.97987210615365`, 0.21651827946513758`, 4.096495164957771`, 1215.344603094822`, 1238.147553372664`,
0.01876253880568135`, 1357.8490252123336`, 346.0871550829435`, 42.056795172020216`, 42.6199364791488`, 0.013390019492099503`,
0.27402490730818685`, 0.27405890342179906`, 0.0001240621297755595 3`, 826.1841163096273`, 0.7425119685611433`,
15.465857930064937`, 0.0031289278958566147`, 0.003154842737958139`, 0.008282339179449094`, 0.4468277243475771`},
{0.11610573160351356`, 2.8105133752566074`, 0.4045001364193191`, 0.9020454264062931`, 0.06323256453719583`, 0.49478351534124754`,
0.0689529373461184 3`, 538.3487824750697`, 0.04516006162070901`, 5.298889101080752`, 104.03205652690329`, 103.61826085720948`,
-0.003977578484058886`, 89.8559516466248`, 53.109395708994`, 1.2201035460326035`, 1.2545280246455068`, 0.028214391085773727`,
0.2688658482596679`, 0.27294507673007545`, 0.015171984455488952`, 48.98739050460928`, 0.44595522879124777`,
2.330022204919758`, 0.003472299148664537`, 0.0036887334439024513`, 0.06233169608129985`, 1.0565582935218794`},
{0.06094054623730827`, 3.5785656752883286`, 2.8105466781089756`, 1.0666569400098407`, 0.8956485783622179`, 0.6416539064413922`,
0.4241071070816547`, 1544.2747045672372`, 0.05890410014619657`, 6.674766669452417`, 179.474632763705`, 166.74978111447882`,
-0.0709005582197213`, 123.06412818699557`, 26.17676020533756`, 2.9388235001408916`, 2.9555165452850103`, 0.00568017954916944`,
0.06384979723684296`, 0.06402273545622976`, 0.0027085163441522564`, 150.23961290478383`, 0.05558630743956596`,
0.8970952398815314`, 0.0007175681041589055`, 0.0007270926834764094`, 0.013273415111821452`, 0.344398196666992`},
{0.06400872221443815`, 3.352604720184938`, 3.3612644751655854`, 0.9279864636339992`, 0.6811335659068025`,

0.5445450422212498`, 0.04222211989978537`, 4877.834369436828`, 0.09303122695369598`, 6.110250683676178`,
27.63178788686365`, 27.640325295165788`, 0.0003089705355692196`, 20.697336130214442`, 17.325089358873083`,
0.2104682952021771`, 0.21262378640533988`, 0.01024140572380361`, 0.007817356355199628`, 0.007833869053305924`,
0.002112312315826869`, 10.080242856344231`, 0.007148271305591292`, 0.2680601082536514`,
0.00010126562843026932`, 0.0001022494227531939`, 0.00971498758438094`, 0.5146835501725464`},
{0.1838955211549798`, 0.6066893455768754`, 3.1997125487162617`, 1.2441392193895697`, 0.8430620231566095`, 0.17975264196625795`,
0.008385470075528607`, 217.99247821227752`, 0.10887259656335785`, 8.65519228817503`, 594.371531929783`, 579.1256605379922`,
-0.02565040647604899`, 314.30100816711735`, 530.6910338392473`, 6.475158196212689`, 6.57746503291299`, 0.015799897639588112`,
0.167479379292621`, 0.1674628301297533`, -0.00009881313710136119`, 56.12013555095737`, 0.4399815391103509`,
8.13168523490026`, 0.0016241916735457718`, 0.0016311124670340049`, 0.004261069429770226`, 0.3262152092434302`},
{0.10877154933938238`, 0.6652486439511347`, 7.420477343235575`, 1.1719120037873472`, 0.8277401239831172`, 0.4096056229223566`,
0.3960687102590752`, 4083.2161783729553`, 0.09124503977845877`, 8.628655226336182`, 52.906742425959614`, 50.78484465217116`,
-0.04010637730640754`, 28.06289041344439`, 8.314853761602583`, 4.24393358503002`, 4.280509020553468`, 0.008618286500161831`,
0.006073695452050628`, 0.00607752578117234`, 0.0006306422756869878`, 40.33244374942715`, 0.009437789493349785`,
0.43880354747371747`, 0.00006258041634177314`, 0.00006279855555629779`, 0.003485742461879937`, 0.44944214513590697`},
{0.27429237480005003`, 0.4974953244631517`, 8.109325428142139`, 1.1022725368505206`, 0.3620164170986986`, 0.5133215689169995`,
0.013475856804577652`, 252.124017519504`, 0.17376346726865255`, 1.283750502496817`, 67.4690937496721`, 69.06886369518107`,
0.023711152123141455`, 240.54079653884182`, 56.54253448818474`, 1.3165171937228484`, 1.3434952368716973`, 0.020491979350881318`,
0.044503584909379224`, 0.04470273428816462`, 0.004474906441602755`, 9.356587835035233`, 0.17438562845584352`,
1.1518716791888945`, 0.0004821843616328314`, 0.0004925531641826982`, 0.02150381342678709`, 0.18768304331832203`},
{0.2348606786833104`, 2.9248975834862243`, 4.089597609027345`, 1.0125076012045389`, 0.4800911130241774`, 0.44584481805285503`,
0.013867879702002976`, 69.31755646766315`, 0.08239556859020553`, 3.2481286029667444`, 676.3345449026027`, 686.6282603891304`,
0.015219857634227507`, 952.9998868165945`, 564.7017327874523`, 2.5687172250432657`, 2.6139184670106195`, 0.017596815066552418`,
0.32263115368267214`, 0.32297215806404106`, 0.0010569480890996186`, 107.33192577412139`, 1.082476738832733`,
10.265664331295463`, 0.003815343775317115`, 0.003869558069913836`, 0.014209543828646032`, 0.35726461731127884`},
{0.2276305764129738`, 0.759063573478838`, 9.75608531797917`, 0.9651630732227953`, 0.2616315835146603`, 0.25394525967231996`,
0.42721777606241185`, 115.33714629065052`, 0.15474162363619287`, 5.678602403105034`, 455.39945966185786`, 452.7492111157109`,
-0.005819612847399402`, 367.04181497637114`, 72.89197748168901`, 32.27823333801756`, 33.03736375927338`, 0.02351833860627428`,
0.049780810152600705`, 0.04978487421822184`, 0.000081639202107686`, 350.01758775913447`, 0.16188049299063684`,
10.61606032036913`, 0.000623210917126582`, 0.000624725238853185`, 0.0024298703456366866`, 0.52515651571228`},
{0.22740101487152548`, 3.662274246882813`, 5.673159128278421`, 0.7501649913658239`, 0.6663016096811909`,

0.46449661159333955`, 0.15875103883050143`, 79.3840483030526`, 0.23925351496597358`, 9.512843911813135`,
2373.968137339562`, 2370.391036458293`, -0.0015068023976420042`, 1142.1654255722597`, 743.6130376542975`,
30.561312746899578`, 30.899601494639317`, 0.011069182483794293`, 0.20731664663713145`, 0.20708971336573037`,
-0.0010946215611826737`, 1598.9129803414507`, 0.6734859406450078`, 25.485887056991523`,
0.0033324974461524937`, 0.003343129052883672`, 0.0031902820341103144`, 0.7378987276906037`},
{0.18907356960866467`, 0.8903250429215461`, 0.8912625465714132`, 1.4194566043771595`, 0.25430531352108066`,
0.6668345122251143`, 0.01430802337144644`, 354.71023908442675`, 0.05435127807039547`, 4.360013356436832`, 176.29873760349315`,
179.09233079134458`, 0.01584579235124406`, 185.06567581708305`, 145.5624318316673`, 2.128178702804182`, 2.180919932889226`,
0.024782331491030263`, 0.393791795664251l`, 0.4008948927332171`, 0.018037696943341563`, 27.06815421312646`, 1.0636517212692342`,
2.9226890224854873`, 0.0032168382134795115`, 0.0034480163658775855`, 0.07186502306189002`, 0.8926391023684579`},
{0.22841094141655494`, 2.082125305118022`, 3.0130589699894585`, 0.8668572537043583`, 0.35284672466002664`, 0.48298470604644195`,
0.055294539730325123`, 1324.8059697322965`, 0.2155561741975654`, 1.2621538558570524`, 12.226940196510242`, 12.41228281360483`,
0.015158544502204219`, 44.337375736883104`, 6.880586746478369`, 0.171501211762105`, 0.17550858962266674`, 0.023366469655739186`,
0.017234086173956685`, 0.017391107546952753`, 0.009111093643790147`, 5.101243040975474`, 0.0562350549635369`,
0.21579091969335187`, 0.00023524341819647354`, 0.00024434949757162 5`, 0.0387091781141613`, 0.18631037817006935`},
{0.1257670047049273`, 2.665283038938149`, 6.579106420165356`, 1.3213793494994266`, 0.07362490185046933`, 0.671746727583086`,
0.11039555694375124`, 54.48594040704261`, 0.2029696416579515`, 8.437004538119528`, 1900.4947643371315`, 1933.8645666824689`,
0.01755848159727824`, 1030.9625494339766`, 757.7134693434994`, 29.22516816817918`, 29.763788032575402`, 0.01843000051519561`,
0.28387416344832705`, 0.2839100006534596`, 0.00012624327870214 28`, 1112.7620718394724`, 0.5100286178580674`,
37.14730827881846`, 0.0025844472651759265`, 0.0026021065488179757`, 0.0068329053875459955`, 1.8179593236999383`},
{0.07898342764241062`, 2.336170261443863`, 9.558440542813312`, 0.8746675798923161`, 0.3314927429594914`, 0.5846930166673705`,
0.14173550706968557`, 4831.453144750826`, 0.22811466814427805`, 5.138718102637142`, 15.06360870463859`, 15.49155267258548 5`,
0.02840912667992468`, 13.416484290901057`, 5.132088842559341`, 0.28882236338559025`, 0.2933497209215322`, 0.015675231941433054`,
0.0016820959315533976`, 0.0016836432280725759`, 0.0009198622326787032`, 9.639117374019271`, 0.0018979671756719095`,
0.24178507453237094`, 0.000023201167758335473`, 0.0000233077730717444`, 0.004594825334626851`, 0.78002640912594`},
{0.2360815041275312`, 3.0069216843113518`, 1.9005978173203566`, 1.189378383663731`, 0.3594173887197991`, 0.5615883220885535`,
0.16515958276300607`, 91.7495984293873`, 0.10675594880460115`, 2.979677734696839`, 464.99981474004835`, 472.81623489754327`,
0.016809512412095495`, 714.2462662653643`, 142.5755044262006`, 7.274923863284408`, 7.4297717758554604`, 0.021285159196311243`,
0.6054946463657374`, 0.6095927862851601`, 0.0067682512868121325`, 312.5018045174859`, 2.0420869550740144`,
7.408059684219367`, 0.005996431557476645`, 0.006240319850460914`, 0.04067223825479638`, 0.46406720555466663`},
{0.19503604064380992`, 2.260639826798138`, 3.7301326124020027`, 1.0003156010412233`, 0.94917046396899`, 0.3935362271642996`,

```
0.08733555022372319`, 2234.7260448390443`, 0.16743068067899064`, 7.659400521016185`, 108.46227945995874`, 103.9560055503515`,
-0.0415469224143572`, 64.81095815818098`, 48.605338035920155`, 1.795075514825111`, 1.8082805094843493`, 0.00735623351228476`,
0.023795641459792936`, 0.023820924012654465`, 0.0010624867122934756`, 57.97170287034013`, 0.06630010992712154`,
0.7031141449877091`, 0.00028673433558512595`, 0.0002885182356380807`, 0.006221438563729809`, 0.3016881076309086`},
{0.09766567347807259`, 2.1751916900809825`, 2.9967711374909367`, 1.239918427231443`, 0.884426059472291`, 0.5758255537556012`,
0.011950287911566193`, 133.4222213549303`, 0.18860770237483848`, 7.181211491841234`, 1989.5589004628557`, 1860.9580701494772`,
-0.06463786032344188`, 1268.0126270487938`, 1698.611644721264`, 8.981633138135125`, 9.045431719893175`, 0.0071032273058635464`,
0.7194791089578688`, 0.7190278131595023`, -0.0006272535126422563`, 279.0967680775356`, 1.0038344532825283`,
11.172600242428572`, 0.006939034419573442`, 0.007026177202998475 4`, 0.012558344310733327`, 0.3715324319465174`},
{0.14345253817386028`, 1.510087082683639`, 7.446143698111681`, 1.3871400228991382`, 0.2507990125704793`, 0.592096235116642`,
0.03860344741119924`, 206.9907110306594`, 0.033941806565597976`, 5.68471806318026`, 362.12665162102354`, 372.39861564106803`,
0.028365667023023677`, 291.5520189438993`, 235.35652908226473`, 5.605205820812549`, 5.702430294670193`, 0.017345388727143973`,
0.07435053798726499`, 0.07445455178407111`, 0.0013989649519945413`, 120.91927008274502`, 0.1523681912699355`,
6.337680968835236`, 0.0006448962841194561`, 0.000650180196888396`, 0.00819343032214026`, 0.9619434606121184`},
{0.23351662183055255`, 2.7403756212365034`, 1.6128399905233286`, 1.1394774719326448`, 0.2292996808057013`,
0.38219770472020054`, 0.028922618161783233`, 3147.1184318469313`, 0.11734182225292555`, 5.055074464096112`,
16.938666847532307`, 17.124591104558913`, 0.010976321731818617`, 15.336143501543212`, 12.039236473933704`,
0.12038944296876186`, 0.12335447199513189`, 0.024628646443188407`, 0.014739113757490074`,
0.014806082844457287`, 0.004543630510564611`, 4.71303277951196`, 0.04916897219179129`, 0.35947526092647875`,
0.00015457454418432715`, 0.00015789703612094597`, 0.02149443140299212`, 0.6769023821410574`},
{0.05894445024535572`, 1.7190376171466255`, 3.4445271438587763`, 1.1005021934401353`, 0.8920369053637744`,
0.4553589712941485`, 0.02602138727445485`, 2996.975539830326`, 0.01616140506923186`, 6.007209949084906`,
62.51539088485999`, 58.79262707630034`, -0.05954955661104622`, 47.629786530204775`, 45.650783283732636`,
0.6579686400557373`, 0.6628694269385218`, 0.007448359366138524`, 0.020819325633357925`,
0.020860829799834727`, 0.001993540386836745`, 16.158183473694603`, 0.01753119577052641`, 0.414452914647519`,
0.0002274616607359503`, 0.00022961387148760518`, 0.009461861593252463`, 0.2861233064664137`},
{0.1969133083502746`, 1.0306356019031098`, 0.4532514022896326`, 0.7704659952544493`, 0.6922371279082573`,
0.35856337468106037`, 0.02342746544122668`, 625.7704338271294`, 0.24611682235411125`, 7.40783802504553`, 212.38353339282975`,
208.4260767058717`, -0.018633538220866885`, 131.21813435227472`, 158.84838996908587`, 3.3285668935637416`, 3.387084752888565`,
0.017580496711054883`, 0.3029423706672801`, 0.304290054178015`, 0.0044486464794160074`, 49.00770776889765`, 0.8521912063938906`,
2.5051078382187044`, 0.004668337924073063`, 0.004807328500538931`, 0.02977303244247609`, 0.5347822307307566`},
```

```
{0.23578418763137027`, 1.109904439772956`, 9.416464917532476`, 0.9968768007328397`, 0.9319056056422497`,
  0.20705868768300972`, 0.023381736885244357`, 73.41723955051782`, 0.19857821780967083`, 6.130556036938069`,
  1538.0502478281212`, 1501.8280382623007`, -0.023550732244911998`, 1148.2465578570852`, 1156.4234226385379`,
  22.578597112034295`, 22.847387646779012`, 0.011904660569077352`, 0.1667795402769706`, 0.16666400041527088`,
  -0.0006927699974939028`, 358.0012168355961`, 0.5617711202532057`, 17.052318632182228`,
  0.0020206871337120624`, 0.0020248663216697093`, 0.002068201399377134`, 0.21257705107601396`},
{0.06254010222908407`, 2.833030713124539`, 1.1523226094346608`, 0.8553536481119535`, 0.24975724964580737`, 0.6445417390978168`,
  0.0383494620604867 4`, 4173.282831948493`, 0.23424935668510183`, 4.880796947048751`, 16.162351733822625`, 16.570367674036373`,
  0.025244837319058133`, 15.155780549639136`, 10.51279503205999`, 0.13550818022303376`, 0.13793785881953516`, 0.017930124900964 46`,
  0.01527688590294284`, 0.015403669823257141`, 0.00829906835200478`, 5.484269092163861`, 0.013648828658742583`,
  0.2740490364352409`, 0.00021162404959373937`, 0.00021840034631045142`, 0.03202044724935882`, 0.9138693575451601`},
{0.1868109134318776`, 3.0098056229686154`, 1.9105557354746436`, 0.9827601516958456`, 0.5236893411586465`, 0.474044891890932`,
  0.006475636749568416`, 1404.5093361888864`, 0.13430982294875038`, 1.0667742763750176`, 11.870705302889279`, 11.874726467154034`,
  0.0003387468698912155`, 50.92939104045627`, 10.752004426094922`, 0.022261433838579734`, 0.022846024243660955`,
  0.026260231453200777`, 0.034921873349203`, 0.03554007518812782`, 0.01770242485971707`, 0.9571798391814427`, 0.09319695798738538`,
  0.16908226102306198`, 0.00041445784553817 42`, 0.0004426473508193224`, 0.0680153737819682`, 0.13644563652539607`},
{0.06031809352972184`, 0.6708570664744204`, 0.23575165127461165`, 0.8219834374256857`, 0.8758699419986131`, 0.1885257370258894 8`,
  0.186745146986022`, 107.40963970974032`, 0.12527667123697356`, 3.1788633087725113`, 470.65930898625004`, 453.21607308969226`,
  -0.037061278856098`, 677.6404058275282`, 131.12722891091653`, 31.511201544087044`, 32.34575899969487`, 0.026484469481121042`,
  3.2378410161469704`, 3.199884489664233`, -0.011722788825470287`, 301.99303184214966`, 2.7900056749471736`,
  6.151219676793096`, 0.045444302047121354`, 0.04773024330003197`, 0.050302043379174544`, 0.16868482447719862`},
{0.18801205905393248`, 2.0266227725882997`, 1.7626950623641149`, 1.1991174158264077`, 0.011000047841808414`, 0.3353078609658676`,
  0.05399718652686698`, 93.79336861840014`, 0.2334529633377767`, 8.960462961249508`, 815.8364315035797`, 818.8902542855299`,
  0.003743180206260410 3`, 416.71308783511927`, 468.4958353990287`, 11.548561526141995`, 11.861514309497734`, 0.027098854056180066`,
  0.38805238134761666`, 0.3881664964113976`, 0.00029407128848091 27`, 334.35111113594985`, 1.0422646748277256`,
  21.534401340385255`, 0.0038824594074537977`, 0.003925627165624432`, 0.011118663105081916`, 1.2731095237534742`},
{0.14122716980793654`, 1.0036898987844207`, 5.592205787770849`, 0.8071832042112255`, 0.9861798903854433`, 0.6717138208444808`,
  0.19154806886518252`, 64.00473270059831`, 0.09434989637003327`, 6.618037610942842`, 5584.458000369836`, 5466.411205656372`,
  -0.02113845152128402`, 3862.035777660914`, 1509.5114860609294`, 265.5180855522519`, 266.6407129487807`, 0.0042280637652001 3`,
  0.7677495771122961`, 0.7643363952735853`, -0.004445696800704946`, 3807.1117201910424`, 1.5489585699569364`,
  21.211483860223026`, 0.011396690091759143`, 0.011467856389786818`, 0.006244470759026344`, 0.26064818698426606`},
```

```
{0.14179341245484034`, 1.2991893501557028`, 6.1750864357305275`, 1.3220469832389021`, 0.954868801179809`, 0.5061480199077149`,
  0.3454995627472`, 2784.154142185204`, 0.062422742009026755`, 1.1334761108568099`, 15.14862480554901`, 14.34109081263816`,
  -0.05330741260520544`, 61.16814815900071`, 2.613163403546609`, 0.6381279969669009`, 0.6439982213238787`, 0.009199133065591392`,
  0.0177731698381989`, 0.017899153102907357`, 0.007088395927983937`, 11.8435585385084`, 0.03600169144996765`,
  0.08441002083170081`, 0.00015971646895140967`, 0.00016448692782173577`, 0.029868296623671187`, 0.05724380312181977`},
{0.17346773507380248`, 2.4886801471142252`, 4.186844167829875`, 0.9059139514582338`, 0.8823714489891827`, 0.3118060351580627`,
  0.18611640479783054`, 317.2758158326019`, 0.18366106179531033`, 4.660916164387663`, 333.2378388065451`, 318.59326113108165`,
  -0.043946322926326165`, 327.22578068667934`, 93.17845962741205`, 6.558283401211324`, 6.624965296431235`, 0.010167583670994462`,
  0.09696160847339512`, 0.09702113131507316`, 0.000613880510185405`, 233.1638528534767`, 0.24028158015665926`,
  3.0267718569353086`, 0.0012893015007094943`, 0.0012978061060685052`, 0.006596289040484926`, 0.20471282187042542`},
{0.17801738501831404`, 3.953347877342175`, 5.116473955634309`, 1.3026800881545206`, 0.2660443419983707`, 0.5551592463622865`,
  0.021882498869656555`, 368.98117336094424`, 0.13387054033002443`, 7.8289062285527375`, 276.6512403302413`, 284.1069513727965`,
  0.026949855831679193`, 161.73202528752455`, 211.64957807962037`, 1.1263269820543882`, 1.1463234933393998`, 0.01775373546369141`,
  0.05636704893408679`, 0.05644268419025657`, 0.0013418345930831244`, 63.61089119568482`, 0.14334735217777725`,
  4.849611694209912`, 0.0005208338473435825`, 0.0005248973193879735`, 0.007801858625578806`, 1.2374651997062023`},
{0.1502521637125906`, 3.604808817430471`, 9.516864148680163`, 1.170214417719616`, 0.46260070316916013`, 0.4333225308578431`,
  0.19319459383231874`, 1379.7428965277768`, 0.03462365036850307`, 7.002259791633362`, 72.20482045389672`, 73.14705605153169`,
  0.013049483285352048`, 47.19461716644947`, 19.985350879211445`, 0.9942316768685509`, 1.0095956894627565`, 0.015453151364676287`,
  0.007952354645686386`, 0.007957416652371646`, 0.0006365418685152502`, 51.20021593349203`, 0.017069407030361253`,
  1.10687841741525`, 0.00008205049090614924`, 0.00008234698356608492`, 0.0036135391350040624`, 0.7355922203595292`},
{0.23041123856532575`, 3.7235663376199373`, 7.422983674409959`, 1.0720190294684302`, 0.9567615568260381`,
  0.6795713708474191`, 0.42619421138851304`, 252.0789228698587`, 0.23264645009848384`, 7.716645878625323`,
  1577.6566034250452`, 1487.5028872302105`, -0.057144067979757796`, 935.7253679277107`, 227.84894943879746`,
  24.89237646820109`, 25.004529669955694`, 0.0045055240867772905`, 0.18526070039793535`,
  0.1852730016003512`, 0.00006639941654884574`, 1324.1202154336602`, 0.6098021062316799`, 6.293509426560573`,
  0.0020815253223813723`, 0.0020932398584496345`, 0.005627861425610892`, 0.32142358078938343`},
{0.17190873580866728`, 2.1300233762526144`, 7.263444695009253`, 1.2209987713639263`, 0.8761821538663868`,
  0.2004596237918942`, 0.1361627927608223`, 3975.405902408578`, 0.13971132873354192`, 8.98211109187212`,
  38.02477252596979`, 36.95751605476262`, -0.02806739923238888`, 19.375490119627166`, 13.210167781153444`,
  0.7890563004433245`, 0.7990712971346962`, 0.012692372756854864`, 0.004464497144462967`, 0.00446588630128873 1`,
  0.0003111563924924621`, 24.010119501766944`, 0.010964086573227116`, 0.4623905670706082`,
```

0.000044204196417818586`, 0.000044295454347423492`, 0.0020644630374531125`, 0.33138586951732085`},
{0.25963938649205515`, 2.4653900827356168`, 4.997042605235823`, 1.1014911256242188`, 0.41576920876875767`, 0.18114027248931974`,
0.22217255314368375`, 81.71471228407572`, 0.2378841388493836`, 9.715142052575505`, 1115.177933883837`, 1110.6431961884703`,
-0.004066380402249936`, 525.3630286621183`, 285.58950342385276`, 22.883593340684808`, 23.412533424497592`, 0.02311438050563419`,
0.1588235289658295`, 0.1587431210768498`, -0.0005062718949973011`, 805.9569154211313`, 0.5890977660169001`,
24.92860344411221`, 0.0017415128731961271`, 0.0017458042735680268`, 0.0024641795291606705`, 0.6058284826937145`},
{0.2762626642080059`, 0.4739284043667089`, 1.351212961630882`, 1.24608482511035`, 0.19517067645291641`, 0.24775742659101618`,
0.0850306691167671`, 2141.7873291111496`, 0.17338810608691535`, 4.319189194420735`, 17.35667055934416`, 17.374251841011947`,
0.001012940909817627`, 18.39198851486659`, 8.016620410721337`, 1.1873518926613862`, 1.2251258313520141`, 0.03181360043648040`,
0.0230113513254672`, 0.023112187093717332`, 0.004382001162116289`, 8.038854113011466`, 0.09081681748857608`,
0.4391036774060534`, 0.00022082578834325965`, 0.00022602370106845977`, 0.0235385222178867`, 0.4458420482468696`},
{0.1906362991887024`, 1.590121005227628`, 9.42412383550555`, 1.4957920989499214`, 0.709065663948995`, 0.3417182681188283`,
0.3753659226116254`, 505.4667159946151`, 0.11728445265057086`, 5.855972486658924`, 204.91499385030917`, 200.84376517520198`,
-0.01986789057554883`, 160.1545215769826`, 34.21499814633034`, 7.192200799005008`, 7.282322231548247`, 0.012530438882588868`,
0.03482250067457271`, 0.03484601075531095`, 0.000675140506362526`, 163.37813663304013`, 0.09483475224433184`,
2.430226908499417`, 0.0002809399291507919`, 0.0002821937025712376`, 0.004462781151242989`, 0.3679148414498811`},
{0.2658746234670645`, 3.614932865157483`, 8.460828570764`, 0.8159863776033582`, 0.2726846463148733`, 0.3456401667360370`,
0.1062304873288386`, 3778.1279822475276`, 0.23711150436988554`, 5.459482199196464`, 15.112974876103308`, 15.27567396521876`,
0.010765523693996082`, 12.66960283063495`, 6.183222996955401`, 0.1694793894025447`, 0.17305595648064317`, 0.0211032568072537`,
0.0016742496212792698`, 0.0016751768020429564`, 0.0005537888448068351`, 8.752237353115454`, 0.006359149823536761`,
0.31798264604883586`, 0.000024786524773112717`, 0.000024865614862523983`, 0.003190850275915258`, 0.6275054085283681`},
{0.26863157332159515`, 2.9396265733275415`, 6.862183762414347`, 0.9732277188028977`, 0.5002013826688112`, 0.5818631486183298`,
0.034332997185588514`, 711.1213518313073`, 0.15904422112946637`, 1.3044149622459462`, 30.589057855378964`, 31.342830826782997`,
0.02464191525504877`, 107.32844168761429`, 20.58440873687482`, 0.23031209527305`, 0.2341216425827737`, 0.01654080436030614`,
0.02070088892676765`, 0.020811046070384403`, 0.005321372623487086`, 9.671879363191454`, 0.07944160516504759`,
0.40934039274399836`, 0.0002536228830313236`, 0.00025971771758334373`, 0.024031090882550066`, 0.17044455222021487`},
{0.27209369466384475`, 3.5584095462332135`, 0.9720509149176574`, 0.9873292487031791`, 0.8069894772531618`, 0.17871886216847`,
0.1021878803564629`, 96.95116373019758`, 0.02386269594633994`, 2.5608838628408552`, 364.9664698661573`, 354.11743947309253`,
-0.0297261016800896`, 652.2702608426222`, 149.81197962969105`, 4.066156474068413`, 4.17860689899256`, 0.02765521337933996`,
0.8977927335583055`, 0.8978550558912406`, 0.000069417283751205`, 206.70071448290088`, 3.489767741660382`,
5.457632564604800`, 0.01080061096425744`, 0.01114262878479571`, 0.0316665253169576`, 0.12001020180044801`},

{0.14444262093504412`, 3.863449844229998`, 8.726684391435349`, 0.8553749492129846`, 0.451984917853794`, 0.5228822441273482`,
0.00811515726694 7242`, 403.9888714010655`, 0.1027695319041006`, 9.711355034259547`, 372.3972140813144`, 382.00440128556465`,
0.025798225230955873`, 175.50564362827828`, 333.90582818472063`, 0.6783116490296648`, 0.688481818275636`, 0.014993357788444017`,
0.02358524073676418`, 0.023592432772392744`, 0.0003049379783244266`, 37.43747192547209`, 0.04866734267722628`,
5.306025153460638`, 0.0003331034999426441`, 0.0003339572793995731`, 0.0025631056325616797`, 1.1494255411597163`},
{0.2538137652575522`, 0.7540411224449701`, 8.794714857317246`, 0.9184514413182489`, 0.11359389239638507`, 0.27911589396705727`,
0.11507441894157476`, 65.76953791858952`, 0.10155553687026442`, 2.859221903044178`, 360.72306490106195`, 361.9513676137458`,
0.0034051127643326407`, 577.4183015050256`, 141.94707587134832`, 18.551952295261593`, 19.05265324379356`, 0.026989124409286802`,
0.08259731392219505`, 0.08262723733886733`, 0.00036228074802124866`, 199.84192760378014`, 0.29949050352508677`,
9.588350982773713`, 0.0010845686204296046`, 0.001090229374466319`, 0.005219359964952819`, 0.33592659321645507`},
{0.07943689831455408`, 1.7727348695278575`, 3.5998215792473456`, 1.4458682286750966`, 0.6331999429153172`,
0.478090570864118`, 0.46084046549589247`, 472.0739644000896`, 0.10830302137693731`, 2.3782715249335666`, 93.57181295935698`,
92.95139716509517`, -0.006630370563956878`, 180.07279379392529`, 13.239300145112455`, 3.043614175744206`, 3.0857931838810435`,
0.01385819808337696`, 0.09838222849348355`, 0.098985739031479`, 0.006134345066552527`, 77.07887112472933`, 0.11164541543991852`,
1.0879538494030185`, 0.0008088671446160323`, 0.0008306464707966779`, 0.02692571496519891`, 0.22394155571133492`},
{0.055568082805285024`, 1.296674173505255`, 4.7380956025626375`, 1.4538691472242138`, 0.09550221320066177`, 0.19041481571096486`,
0.007131815235409 4295`, 86.20076273581975`, 0.04910104396213488`, 4.3238451034891945`, 363.3137560846476`, 364.3669758898248`,
0.002898926306913019`, 384.570714348569`, 330.12118604513944`, 1.6685900079246307`, 1.7206059698349692`, 0.03117360266050917`,
0.16165183144422035`, 0.16173702044148766`, 0.000526990610042688`, 30.908822420639957`, 0.12832403364739448`,
10.778831001148435`, 0.0013397874340926874`, 0.0013477876953798976`, 0.005971291477761875`, 0.3838150629215167`},
{0.1596260086552564`, 2.5471852815710525`, 0.22684585875990315`, 1.2716791760964523`, 0.8208442532787252`,
0.3888937433082962`, 0.04289548126799573`, 69.96603930346842`, 0.07461229317798801`, 1.378327695847128`, 511.98528429422777`,
459.63520063089135`, -0.10224919596175708`, 1700.0805166668597`, 294.81251893617906`, 4.760285879904997`, 5.203026462461665`,
0.0930071415302276`, 11.942647248626505`, 12.062614925163397`, 0.010045316925080261`, 173.21900184806438`, 27.23367304394169`,
4.410874390293924`, 0.1043539451691653`, 0.12444794587387084`, 0.19255621502504505`, 0.18530345398631262`},
{0.14264033419885946`, 0.8035889732308119`, 6.673404894657215`, 1.4157343373387477`, 0.18185291353504507`, 0.4765318357304136`,
0.03778101114485503`, 210.869852474353`, 0.22822304787397907`, 1.0663935058760412`, 54.595482135024675`, 55.298102970951305`,
0.01286957836893765`, 234.31695158851184`, 35.705788672426536`, 1.4969578892749218`, 1.5334828571712507`, 0.02439946250860814`,
0.0674021128430677 1`, 0.06792851462216584`, 0.007809870594468693`, 17.18484076017424`, 0.13734657002348769`,
1.1725169826131006`, 0.0005644979101586678`, 0.0005830493703826028`, 0.03286364730512581`, 0.1863321430702531`},
{0.2209992311467341`, 3.548991401043337`, 2.677760813541523`, 1.1645638964766105`, 0.17856966244183803`, 0.240969404618861 4`,

0.16792458477559433`, 53.365358865447796`, 0.019626895089088575`, 3.834874658630479`, 601.1962267705935`, 600.4590727330327`,
-0.0012261454825166496`, 717.512658027167`, 186.93010067864824`, 7.978592931360559`, 8.207326261826141`, 0.02866837955431034`,
0.4267008507750384`, 0.4267723496308721`, 0.00016756201845824492`, 404.5136815117678`, 1.3471508564416461`,
15.606272063171668`, 0.004393769660962499`, 0.00444945565645975`, 0.012673854069321644`, 0.3848191756645689`},
{0.08986394714808477`, 2.7800808748945913`, 9.536673819459821`, 0.8308776528106754`, 0.5315183679881144`, 0.4614339535602666`,
0.046951751845033245`, 1052.1471359283046`, 0.24213976319820152`, 2.8230201755788666`, 41.80442034179551`, 42.41455632259722`,
0.014595011145070336`, 67.77549819105595`, 25.238151394970227`, 0.40640082736233574`, 0.4122001368091441`, 0.014269925296283459`,
0.008086422375696395`, 0.008096166265275546`, 0.0012049691602107337`, 16.14038810987669`, 0.010381111899817474`,
0.5838819377582212`, 0.0001172925352885823`, 0.00011801165130946412`, 0.006130961523787 67`, 0.2895836819861905`},
{0.08237232438525194`, 0.904854561594771`, 7.190948808493694`, 0.9264355987892083`, 0.7428148600467364`, 0.6232177046085758`,
0.0730718251800762`, 62.25627183567972`, 0.1453325784006102`, 9.786938339680962`, 4296.592885078395`, 4273.0347315013605`,
-0.0054829848224273725`, 2009.28641273447`, 2118.64155156721`, 156.3327472230886`, 157.57171231515443`, 0.007925179554977069`,
0.3555833175535167`, 0.3550188751954968`, -0.0015873701890835035`, 2020.834277877912`, 0.4184317768524126`,
33.364160581677545`, 0.004622729552615667 5`, 0.00463985862553524`, 0.003705402343920472`, 0.7371781317460478`},
{0.14754836278222488`, 0.5816922789151606`, 9.91636448422577`, 0.7971447359984003`, 0.9834799375197849`, 0.654563746672 6528`,
0.017670578507760985`, 406.58618651198583`, 0.1328908282216244`, 3.7962615345853075`, 480.91203862828513`, 467.8107672977147`,
-0.027242552230423378`, 579.7944035715615`, 384.07812081040026`, 10.36175094059402`, 10.41161280395228`, 0.00481210788062069 7`,
0.06385852555889743`, 0.06390546596878227`, 0.0007350688020748564`, 86.10500740264902`, 0.1346031556557303`,
1.9169283619994912`, 0.0009651645060237968`, 0.0009708913580081125`, 0.005933550134275878`, 0.15268120186319745`},
{0.06758332214646862`, 3.0558788120173555`, 4.64800500980858`, 1.4828314301436276`, 0.7395538908104162`, 0.3075642133576061`,
0.04417074894369504`, 145.23097869725217`, 0.1743358052899487`, 7.770691560418564`, 943.1874189202263`, 920.5686943528164`,
-0.023981155933254783`, 555.5239955521387`, 582.2549996793435`, 8.071263730746628`, 8.17153820403508`, 0.012423639795893227`,
0.24294732987289264`, 0.24297788229844183`, 0.00012575740414688674`, 352.35434029989653`, 0.23455982370644154`,
11.15699506384369`, 0.0019737968773874126`, 0.001984656418011965`, 0.005501853178998983`, 0.4390377569565117`},
{0.16631267310517406`, 2.413572311283116`, 5.471184235465843`, 1.4579537524645423`, 0.9497208653345599`, 0.439033675828699`,
0.21085287275356013`, 3128.2027597762994`, 0.18409987492192065`, 5.612876798830806`, 61.65177066917617`, 58.81117334521899`,
-0.046074870082805086`, 50.27181200098163`, 15.627073636062642`, 1.2954719130430918`, 1.3045701490060762`, 0.007023105535042 04`,
0.01832581638060898`, 0.018357302044537598`, 0.0017181043002227891`, 44.66735913379687`, 0.04354022155849728`,
0.3687143204984228`, 0.00015125912225533167`, 0.00015257813338494038`, 0.008720208804214646`, 0.23108318726140734`},
{0.18539796406610926`, 1.3871953304353832`, 7.757698949197568`, 1.3585428901946988`, 0.4532718222049952`, 0.29058122288435795`,
0.010738744574908157`, 486.55354471220693`, 0.2025104802295493 2`, 4.51278311919668`, 106.67238750726494`, 107.1540460378 5166`,

0.004515306555353149`, 108.18624516463791`, 92.60070679013728`, 0.6681135097441767`, 0.6815595461248104`, 0.02012537717697449`,
0.025941824038713646`, 0.025964818892563808`, 0.0008864008103612875`, 13.24005629882736`, 0.0687080194419953`,
1.986291020865343`, 0.0002303239035293947`, 0.00023157316080860688`, 0.005423915017369385`, 0.38383069609791315`},
{0.04144083471615087`, 3.41458508027812`, 2.460004608784927`, 1.2787341279549589`, 0.569252466493285`, 0.673615157257989 8`,
0.4762197736813673`, 718.8382183063302`, 0.07598079948793823`, 4.331845758841565`, 124.91748470043039`, 127.1185550842855`,
0.017620194555898916`, 131.98201085159545`, 17.12127336468911`, 2.1624743248928042`, 2.1878130291447606`, 0.011717459005305297`,
0.09340405546315046`, 0.09399336577957336`, 0.006309258345376545`, 105.48503666090666`, 0.05529631463256872`,
1.3343478417036494`, 0.0008678199694431843`, 0.0008908978641772792`, 0.026592951933224418`, 0.5280007161562663`},
{0.13724293356211342`, 3.6916535362348286`, 5.489803233715362`, 1.3457045887873154`, 0.0754297582883523`, 0.6293482685634375`,
0.017573021108899616`, 778.5577282359714`, 0.17674997296512807`, 2.6916234726152264`, 40.07481252044195`, 40.791806463255426`,
0.01789138607816909`, 68.14306864682355`, 32.1191880209922`, 0.14653294388880608`, 0.1496465968685858`, 0.0212488256711913 5`,
0.022761997518790284`, 0.02287382556533446`, 0.004912927630883912`, 7.727840864028728`, 0.04462747590303184`,
0.8319060839235788`, 0.0002018668139047719`, 0.00020611709168414006`, 0.021054861357118204`, 0.589990209797913`},
{0.08176385784973572`, 2.023736863649704`, 9.823987283528833`, 1.0905304980935506`, 0.6417627833690904`, 0.2509575610218163`,
0.3071390754059633`, 168.49990583950182`, 0.0669392059366426`, 7.784078345121018`, 632.1602969186987`, 623.0325551517668`,
-0.01443896715978954`, 371.6931116088536`, 125.0851808739939`, 16.948963912645283`, 17.212212425032256`, 0.015531835087014834`,
0.05658572634439112`, 0.05658599074054368`, 4.672488446155754`*^-6, 490.0034724384108`, 0.06609524693063742`,
9.653954187692822`, 0.0006271310076216841`, 0.0006282692709311045`, 0.0018150327373176456`, 0.45781953867818415`},
{0.20636275093165019`, 3.041433098250965`, 7.709885134336993`, 1.0647262222072884`, 0.28984076250310165`, 0.17246518959666646`,
0.007377660819744997`, 464.8055142552055`, 0.19020267666710844`, 9.452984008311837`, 168.1821285953507`, 168.45404551549638`,
0.00161680032484246`, 81.42830905337138`, 152.35763275467028`, 0.3513444046779298`, 0.3607617675891829`, 0.02680379361636831`,
0.015417257048265576`, 0.015419379876554725`, 0.00013769169720045937`, 15.265578589610517`, 0.0454506796614484 1`,
4.285647013942409`, 0.0001751133079328504`, 0.00017537860292474606`, 0.0015149904654727653`, 0.6397773047965998`},
{0.2140607830978551`, 1.6917207171996171`, 1.9218182027339432`, 0.9997605714664014`, 0.5513493876922198`, 0.4464250883384161`,
0.2328414126003874`, 611.4769203082982`, 0.08477583973629349`, 2.188379571162489`, 56.81269889456728`, 56.845741971236045`,
0.0005816142748309439`, 118.81937502861766`, 13.64014951991352`, 1.70193322672085`, 1.7356068743279476`, 0.01978552805621958`,
0.08359979525129464`, 0.08427668005091085`, 0.008096727959459038`, 41.131367127629375`, 0.25564910911873534`,
0.781599911915468`, 0.000990062375939904`, 0.0010266538941691718`, 0.036958800898307054`, 0.23313398556413611`},
{0.17696168772261972`, 1.6101558302339125`, 0.7107550695951144`, 1.133554468522512`, 0.8128396324743541`,
0.15732538361458315`, 0.06392981831267872`, 277.86150200910555`, 0.15413566109625954`, 1.907309832357937`, 88.48324740292108`,
85.78491105755522`, -0.030495448851222684`, 212.32654858109325`, 46.08657615676338`, 1.699772021773594`, 1.7596881943661327`,

```
0.03524953454053237`, 0.4529143231954926`, 0.45605326875164415`, 0.006930550427297177`, 39.09854044181769`, 1.1449783289488664`,
  1.421525830895771`, 0.0047359220871577845`, 0.004960591123436351`, 0.04743934383713633`, 0.09395916806376833`},
 {0.04328176764500202`, 2.9602821723444555`, 0.6131528539518083`, 1.0058908218647606`, 0.19964040264767258`, 0.6669684114614443`,
  0.23851965705730427`, 2287.3379976105803`, 0.08593310169788088`, 8.660468891635798`, 52.16441012499537`, 52.776049773115126`,
  0.01172522888026828`, 27.567500388027437`, 12.453465370334454`, 0.9150380119499619`, 0.9316024162216223`, 0.01810242203639323`,
  0.06128778117261784`, 0.061916036422403654`, 0.010250905445839509`, 38.69672448275701`, 0.03789490720265887 4`,
  0.896022560792318`, 0.0007183579484026925`, 0.0007465823747474173`, 0.03929019844143622`, 1.723697122781661`},
 {0.08735803097627126`, 3.963951857586263`, 7.965851341925532`, 1.189504619761999`, 0.40822475538796854`,
  0.4952091412789442`, 0.01984161894285721`, 1116.2764923646498`, 0.2379409272833778`, 1.293491554272407`,
  15.835605825435906`, 16.167593467624545`, 0.020964631593404892`, 56.031929786556056`, 12.36982952137319`,
  0.05961850396112173`, 0.06068782143236914`, 0.017935999734993935`, 0.011402404810274595`,
  0.011458326702365131`, 0.004904394557202929`, 3.3760697074743278`, 0.014229880465998329`, 0.2596305028120002`,
  0.00011444672565752434`, 0.00011680259199668613`, 0.02058482954079066`, 0.17492687680949306`},
 {0.15281896372135534`, 0.55010519971598`, 2.817048022996838`, 1.3198176246010052`, 0.16238216376752557`, 0.6925657414172184`,
  0.11807724637466313`, 76.78768290686035`, 0.04193274426603899`, 1.2341700274455292`, 212.1227243267973`, 213.42264570130578`,
  0.006128157078096885`, 786.6412128565022`, 80.61421833284678`, 14.668275130954084`, 15.033144159382356`, 0.02487470579675022`,
  0.49242223054002543`, 0.5017332161374871`, 0.018908540313564925`, 115.27277743432056`, 1.0750207854927143`,
  3.928571053809228`, 0.004310959534241765`, 0.004636940709330271`, 0.0756168487547264`, 0.29427037322982086`},
 {0.19086658543964008`, 1.9905232804946325`, 4.078173781767919`, 0.8519485127716493`, 0.08904379736388202`, 0.23536266216215151`,
  0.24991927271295455`, 192.66677656391008`, 0.127316992739278`, 7.8449864029309175`, 323.1844564632654`, 321.83387849508483`,
  -0.00417896944351992`, 188.54836236930205`, 77.27173156311753`, 8.3473007554222`, 8.576899556146188`, 0.02750575394984378`,
  0.05398622423854539`, 0.05399562536915251`, 0.00017413943537869514`, 237.36423547083234`, 0.1472023754456265`,
  8.846755907927024`, 0.0007652066953981462`, 0.0007677257971830457`, 0.003292054029387259`, 0.7910250467977857`},
 {0.06664208120921677`, 2.003234095381499`, 6.199949723153024`, 0.888873086526756`, 0.04784068730663105`, 0.37023377411027647`,
  0.049828770496122325`, 349.42476257926376`, 0.05267583263583803`, 7.0912634633886915`, 183.41757026940232`, 184.87387159474466`,
  0.007939813635102322`, 118.38094693687886`, 108.89902634545501`, 2.51436932003137`, 2.575192516736402`, 0.02419023976329493`,
  0.02326283198226454`, 0.023274721206511317`, 0.0005110824105956091`, 71.95529071811453`, 0.02214690768778 4517`,
  4.596419538662869`, 0.00031596940539391216`, 0.0003170609375486215`, 0.003454550143386692`, 1.0463240880723947`},
 {0.22983292033798441`, 0.7814702114508636`, 3.2012106727008174`, 1.142026804432239`, 0.1064467335224153`,
  0.4404014420960507`, 0.006695571873889409`, 4217.329674971789`, 0.15347806495470107`, 2.7989551297088675`,
  6.6322538882955335`, 6.709674363270432`, 0.011673327993599436`, 10.84500362154817`, 6.039485450233456`,
```

0.04637071840186266`, 0.047600017290354554`, 0.026510240316710565`, 0.005263348777308475 6`,
0.005288888909480731`, 0.004852449125614555`, 0.5176762159233156`, 0.01728129743208801`, 0.15308579053247745`,
0.00005503865983624845`, 0.00005625544220127512`, 0.0221077760368229`, 0.47215199058538676`},
{0.13211782844464454`, 0.44983284117427225`, 1.0762780754680463`, 1.3187859174208114`, 0.8445651695343355`, 0.2965729998791703`,
0.0542223541794765`, 616.1259401245338`, 0.10515194159293045`, 5.24820204317008`, 175.65036379652005`, 167.99116842429544`,
-0.04360477944183083`, 153.18033052774268`, 99.33126493768849`, 10.17746210647784`, 10.324332778203658`, 0.014430972101810768`,
0.25523087112482856`, 0.25637597979420124`, 0.004486560204594703`, 65.40223850429167`, 0.48172212064345904`,
1.7671930579552024`, 0.0023067406057935003`, 0.002363347623273881`, 0.024539827901849387`, 0.26845294451520774`},
{0.17817224333688036`, 1.8900871904197354`, 9.98947566566499`, 0.792780936871982`, 0.9109839945193492`,
0.4314201285170226`, 0.03598067399007004`, 128.67700477089366`, 0.059019163579936806`,
2.366574414393561`, 555.0925130070793`, 523.7112384783147`, -0.056533413428266455`, 1073.5189111919826`,
367.62628800884943`, 6.6788453261944625`, 6.730241317000358`, 0.007695340780586646`, 0.11673162038020318`,
0.11677633702030008`, 0.0003830722125781616`, 180.3371428262127`, 0.2971190667353438`, 3.795842529474763`,
0.0017730969078854075`, 0.0017844981569065678`, 0.006430133046003483`, 0.10817633280209217`},
{0.2509149027719233`, 2.580879402706512`, 1.8893709427148198`, 0.8255744895957449`, 0.1652280455910582`, 0.6768016323195194`,
0.009322737452992792`, 2112.3599589856663`, 0.08696925005081763`, 4.203193192682905`, 26.0460256637396`, 26.711356940120876`,
0.02554444524361843`, 28.361330886381314`, 22.95106313821138`, 0.07918513176859804`, 0.08083422778492642`, 0.020825829035020504`,
0.01684748676981155`, 0.016948326012170226`, 0.005985417512798907`, 2.919532508316798`, 0.06038979292569325`,
0.4748511310889226`, 0.00024293648629525322`, 0.0002492633758102044`, 0.026043389411921414`, 0.8789598216601228`},
{0.08555200390318413`, 3.7277336286219658`, 7.5570643278825855`, 1.1117862222014783`, 0.20420751548200644`, 0.3245432768958507`,
0.00950175247006993`, 144.2017035489447`, 0.14385517513119161`, 5.452203080835416`, 362.1330374639905`, 366.2563246217706`,
0.011386111542474486`, 303.9909245441138`, 319.45336575276286`, 0.7793622657724315`, 0.7977534620704781`, 0.023597750501583636`,
0.0612956503481997`, 0.06132308704156065`, 0.0004476124032470885`, 41.50364181427001`, 0.07491379596907843`,
8.345866756611393`, 0.0006652835970040183`, 0.0006679694447079487`, 0.004037147039286149`, 0.6454704865705106`},
{0.07297367338130328`, 1.9451098216604938`, 8.396918429735464`, 1.2137718043700492`, 0.7314346040557373`, 0.4669601248496963`,
0.016599550382003152`, 192.8440550768832`, 0.21285961816083276`, 7.382641950639707`, 850.6062835570555`, 837.5850181225206`,
-0.015308216840443034`, 527.3285379311496`, 688.6620058988717`, 5.606943592309119`, 5.663808937292565`, 0.010141950609498851`,
0.10450113166139338`, 0.10453170661870287`, 0.0002925801550988094`, 155.8017292985267`, 0.10894044928324455`,
8.07609489466398`, 0.0010385775232661931`, 0.001042822609029353`, 0.0040874038461853335`, 0.5083209529671142`},
{0.07358197980029185`, 1.0291227850237563`, 2.532589152827489`, 1.0117020834715413`, 0.8098748594005145`,
0.5301759885595309`, 0.008133019998886755`, 830.3082383512614`, 0.02407211472876364`, 4.5236701681781`,

153.99313143748978`, 147.94232128815003`, -0.039292727492822976`, 155.80267617346266`, 137.81405865959468`,
1.011284732198387`, 1.0208699228192124`, 0.009478231318679642`, 0.08497769032992837`, 0.08527621399943991`,
0.0035129652071328454`, 14.867659429314436`, 0.08932609561903247`, 1.1444982473887444`,
0.0010046915983217275`, 0.0010215491536297102`, 0.016778835750335785`, 0.29111387232152547`},
{0.18797192534273177`, 0.513697145142666`, 2.3563599620417435`, 0.8181944603817968`, 0.26394673984920813`, 0.3209514271347109`,
0.025430853857263652`, 265.9518711391782`, 0.07720757078553087`, 7.134393115551401`, 271.5835460029277`, 274.4713869836533`,
0.01063334293711038`, 174.2251765338`, 200.45489039525017`, 8.487729577148373`, 8.679327135534882`, 0.022573475821184186`,
0.08282473815491001`, 0.08288877693972943`, 0.0007731842713423198`, 62.28746360748696`, 0.22241036424268124`,
5.879430987577477`, 0.0012189804171475194`, 0.0012277738409243513`, 0.007213753111316556 5`, 0.7880782921735906`},
{0.2528075291703835`, 2.1409422046846034`, 2.3989238084434295`, 1.1058748574151904`, 0.4698643832575349`, 0.20099009902051257`,
0.02857754945135185`, 57.682491948215834`, 0.240427409232489`, 4.4402688370357115`, 771.5014104450036`, 768.5749603616548`,
-0.003793188247913526`, 795.228531914394`, 550.7427657934055`, 6.912974681004453`, 7.0811589249415166`, 0.02432878054641252`,
0.5017999891781084`, 0.5013450714503769`, -0.0009065718165451875`, 211.43256077826453`, 1.8122687914548956`,
16.20958490011584`, 0.005447175271088844`, 0.005504585180768967`, 0.010539390936221249`, 0.29711136642085384`},
{0.07054141508315104`, 0.5799884401515247`, 2.99755260715291`, 1.1960365141205305`, 0.29917509086343896`, 0.44670601886119`,
0.13984458882947357`, 3612.976166750159`, 0.22678215214973713`, 7.656934137991781`, 25.72471156634488`, 26.18147720988362`,
0.0177559092299530 7`, 15.376592219024309`, 8.876224384313087`, 1.8126698762772402`, 1.846251294177831 3`, 0.01852594250066031`,
0.008393414739481518`, 0.008410295137919521`, 0.0020111479012945 3`, 15.018965343595623`, 0.008458333615752576`,
0.47382857483441654`, 0.00008438783673669903`, 0.00008518093073249857`, 0.009398202708692294`, 1.0168513595513893`},
{0.13968170289558773`, 2.9225254745826437`, 6.832387664740558`, 1.248628328449276`, 0.2500405375269379`, 0.6548966838897512`,
0.01310969508256717`, 2778.8012693248406`, 0.21649543926111503`, 9.334771326724606`, 47.06333142217389`, 48.64673984443831`,
0.033644206103063956`, 23.075093901021706`, 39.719226050799065`, 0.17081474129617255`, 0.1736535841355173`, 0.01661942533649663`,
0.005775893449242684 5`, 0.005782331368584714`, 0.001114618785579058`, 7.13157761246154`, 0.011525523324765885`,
0.7804613021562136`, 0.00005576790870742698`, 0.00005608155857298899`, 0.005624199881826275`, 1.6535460835329454`},
{0.14258976979051796`, 0.734197214461469`, 7.171919374129239`, 0.945223896823731`, 0.9937893052327855`, 0.2333579308191156`,
0.10311334331996398`, 86.76571756149787`, 0.17032024165228216`, 9.316237353631934`, 2397.3231448234947`, 2398.995745662609`,
0.0006976951950452559`, 1177.7429907338671`, 982.138339873582`, 123.12602740069347`, 124.29291640398034`, 0.009477192011478008`,
0.2130864582478431`, 0.2128586849398343`, -0.0010689243693934314`, 1291.4112335042205`, 0.43405641467245565`,
21.870503673464867`, 0.002722065500787463`, 0.0027268687016454326`, 0.0017645427182337858`, 0.2983197003099363`},
{0.06222401125326871`, 0.8031654209648247`, 7.912640972265759`, 1.0061100735669513`, 0.06612689335476452`, 0.2257508005074531`,
0.2688428052003874`, 1016.6634644345198`, 0.08792308855751246`, 8.5639571694838`, 65.07086771945971`, 64.71955709799926`,

-0.0053988925270685995`, 34.7757580917719`, 14.824890653444696`, 4.027205686205276`, 4.138931696646738`, 0.027742812050590482`,
0.006062737502405069`, 0.006064330494518883`, 0.00026275129232988803`, 46.20731928961425`, 0.005389254951073289`,
1.8267397793285127`, 0.00007285635544340696`, 0.00007298006456299348`, 0.0016979866592781168`, 0.8457527677234243`},
{0.045675945991483086`, 1.972555523342196`, 9.234608119853927`, 1.10067888297902`, 0.060950291683742996`, 0.5641176167348642`,
0.005654158004083115`, 79.62566643520867`, 0.10418567571675635`, 3.9217157633863025`, 535.7828788356711`, 545.4876655176232`,
0.018113282572675526`, 625.2838507281037`, 495.8730237551553`, 1.3432290165749308`, 1.3722970791736115`, 0.02164043676840821`,
0.10209907310483403`, 0.1021977041334794`, 0.0009660325568685568`, 37.8513402251198`, 0.06662102498452754`,
11.66991994293334`, 0.0011164355842609996`, 0.0011243663496392524`, 0.007103647975805316`, 0.7863583446352463`},
{0.19725457740267938`, 0.673691333497294`, 1.5958254230238804`, 0.9055402555445284`, 0.8526577157919533`, 0.18700866613724199`,
0.221308084367558`, 137.27532297707575`, 0.16306687928789393`, 6.444987143866487`, 721.5732463075416`, 703.6147238687225`,
-0.024888010372775105`, 512.416212211948`, 180.31036692308905`, 50.803200487814465`, 51.59771670082303`, 0.0156390976430536`,
0.3985026076726274`, 0.39800486649610234`, -0.0012490286561285258`, 488.938226893802`, 1.1229494781465643`,
9.627824230605396`, 0.005289108310573498`, 0.005331956282654428`, 0.008101171230559201`, 0.25143710663606694`},
{0.17905209129143224`, 1.6872990209581902`, 6.743325221637608`, 0.8692054788625995`, 0.5776472609105132`, 0.5432377341075647`,
0.12670077461750193`, 462.43790721394`, 0.057341353016641616`, 6.31060148160045`, 253.2972125640225`, 257.5730226906546`,
0.016880604738401583`, 183.70634228670815`, 91.96535006265714`, 6.421872575639784`, 6.499141362425684`, 0.01203212705885881`,
0.032441666204161605`, 0.032466321167517215`, 0.0007599783315830333`, 154.7945615656464`, 0.08298211684038595`,
2.9649712243676056`, 0.00045010274594059485`, 0.00045237556877111184`, 0.005049564462814793`, 0.6363816316598383`},
{0.21834559506325857`, 2.5253820753047362`, 6.659656299000567`, 1.4243771911650907`, 0.9747752944821462`, 0.1772255451247845`,
0.01986842431027196`, 102.11368670749582`, 0.09272454748376258`, 4.914431231863471`, 833.2594487594612`, 825.0287780513876`,
-0.009877680619556517`, 776.0174360963274`, 650.2944161558446`, 4.903155497706423`, 4.968591421926534`, 0.013345675912322275`,
0.22747729312190618`, 0.22741276381443468`, -0.00028367362115977457`, 176.89058580485212`, 0.709552356143904`,
9.801408597645528`, 0.001926806269920034`, 0.0019349456061860436`, 0.004224262912714938`, 0.15658246775233564`},
{0.242929202956864l`, 1.5636324867576725`, 8.233163527811005`, 0.9303215118662402`, 0.8597394087604655`, 0.6215210972087029`,
0.12468068919290341`, 61.92974770793832`, 0.07970211447796205`, 9.85507721294049`, 5844.759080543197`, 5537.399977063587`,
-0.05258712963940426`, 2714.382694260095`, 2121.9906224820434`, 159.4372626871239`, 160.39344383224957`, 0.005997225046456389`,
0.42147320989418763`, 0.4205874860737807`, -0.0021014949458573184`, 3561.4469076757778`, 1.4626878706744832`,
33.10107094747384`, 0.005455912374911609`, 0.005474613120283646`, 0.0034276110184667807`, 0.5312903827669399`},
{0.09149398523540997`, 2.310449584405898`, 9.73345399143432`, 1.4402850137921024`, 0.7807243420630963`,
0.37700223940662914`, 0.04452259966898517`, 785.475395176315`, 0.18361612690246515`, 4.855709318281368`,
130.8180163489932`, 126.76429235825827`, -0.030987505420663974`, 123.30462701463877`, 80.42088055089062`,

1.4801769421351632`, 1.4955327514797532`, 0.010374306549080003`, 0.024993782956804513`,
0.025016575992332618`, 0.000911948205979618`, 48.85534572576259`, 0.032668297268900005`, 1.2904350719903535`,
0.0002092953702805822`, 0.0002103591905825972`, 0.00508286590663154`, 0.27988933986467934`},
{0.11249424084619364`, 2.9716096914630903`, 4.4521238184847185`, 1.4266434477040648`, 0.9997833218301952`,
0.5867347171724102`, 0.06910846060462165`, 1820.2194505805683`, 0.24722705290784003`, 7.621221876581703`,
207.21154374916586`, 206.8524145465044`, -0.0017331524883391625`, 124.43823298406473`,
104.62363424307624`, 2.357682805546115`, 2.3691151908170047`, 0.004848992088331894`, 0.0545555820101648`,
0.054659034801152744`, 0.0018962824183357263`, 100.0873296336677`, 0.08767412545937364`, 0.8580925564524766`,
0.00045969546918789295`, 0.00046412578747742194`, 0.009637506972508536`, 0.292489716794196`},
{0.09866336490626898`, 1.7258477745644702`, 0.8848358466481533`, 0.910425763108387`, 0.9172877053292023`, 0.36334488527428765`,
0.3804604060347452`, 1006.1834168170927`, 0.013687075322010439`, 8.165252701558408`, 227.3451530982527`, 215.55649608133103`,
-0.05185356651006723`, 127.43260594103758`, 36.67129071283729`, 7.415421363923949`, 7.484573575074074`, 0.009325459438697559`,
0.17906917698819463`, 0.1795122242967652`, 0.0024741684527860297`, 182.8269779769428`, 0.25239382218070605`,
1.6723586408280031`, 0.0023526570372519595`, 0.0023914811023813144`, 0.016502220474388984`, 0.3604249250592713`},
{0.22803797815686577`, 0.698146546972529`, 1.035170425484365`, 0.9822431866703154`, 0.6417091531359376`, 0.21874316930074555`,
0.005531062831453447`, 1727.3746181384265`, 0.11140836748423671`, 9.157656749061658`, 68.21249178217899`, 67.236923907091`,
-0.01430189470578569`, 34.09133811768872`, 63.058208134740546`, 0.4461318209400213`, 0.4551985478644153`, 0.020322977422435384`,
0.044174248144425964`, 0.0442495367973203`, 0.0017043561816418862`, 4.449505575483468`, 0.14390580333506164`,
1.1050661269488884`, 0.0005410071120698356`, 0.0005468664618211706`, 0.010830448658831315`, 0.513916692489899`},
{0.12680960193351426`, 3.133735649340868`, 7.511849069758438`, 1.0897808931740574`, 0.04885314856347578`, 0.24060421848165847`,
0.3693592758602631`, 883.7388687037887`, 0.08961301811176398`, 2.076054864701238`, 17.748051170092666`, 17.490909590265012`,
-0.01448844030047447`, 39.126981071762295`, 3.235948561362099`, 0.3166050374455009`, 0.3260027388190762`, 0.029682728516892354`,
0.007765552067334285`, 0.007776640083562228`, 0.0014278464855810302`, 14.173664180055264`, 0.014067808092175466`,
0.5151445542257578`, 0.00008583935963801803`, 0.00008649252673592689`, 0.007609179526306464`, 0.2305616818554043`},
{0.11645318221252937`, 1.153640861264721`, 2.619716900319183`, 0.8529577052637052`, 0.5619295219152276`, 0.5231720769226155`,
0.04609815796088822`, 565.9088521347549`, 0.20476637224893374`, 8.831648882728363`, 279.3847026026529`, 284.47682400474406`,
0.018226199769188156`, 144.78558432064244`, 169.65750684836183`, 6.266827319637289`, 6.346071116468592`, 0.012644962560718787`,
0.06454277799386064`, 0.06462335188299337`, 0.001248379627235563`, 103.28097237748034`, 0.10737445551798097`,
3.392555376926471`, 0.0009104123763068461`, 0.0009177408409997552`, 0.00804961013671357`, 0.8957285220164396`},
{0.042446348872052314`, 3.5353553586920903`, 5.120248896798044`, 1.1724129507972807`, 0.07388809680757258`, 0.47979821691937163`,
0.43439767413472635`, 83.35178002891637`, 0.15043407845955598`, 2.47226753883535`, 306.7941439620821`, 302.7943791090315`,

-0.013037292046698679`, 567.9578332840254`, 48.02621343425431`, 5.018582583091212`, 5.129930065627934`, 0.022187038011863836`, 0.17769845645888024`, 0.17822098840155978`, 0.002940554201158463`, 253.4638975452901`, 0.10775215252644987`, 6.897129458736064`, 0.001812323107841629`, 0.001841934229535821З`, 0.016338765182692727`, 0.44049841737397083`},
{0.1455833195103322`, 2.7843188408171127`, 7.76885376753495`, 1.3211895319342402`, 0.9135586666939064`, 0.6453968687054246`, 0.1333226247130129`, 368.7414592704895`, 0.16919558147748776`, 3.2843355334407627`, 382.252621581877`, 353.9914916120266`, -0.0739331226896425 8`, 532.6812966887792`, 132.67784036380658`, 6.111277321740543`, 6.147013628069514`, 0.005847600173836120 5`, 0.12385184612468675`, 0.12413656540281397`, 0.0022988698758721338`, 243.08206554829317`, 0.2575823269471601`, 1.8456870279976623`, 0.0011242514452812769`, 0.0011385696397078712`, 0.012735758078578296`, 0.1639431963971469`},
{0.19543798537508206`, 1.2324134940956721`, 7.418056367639444`, 1.0725732268441615`, 0.3282068086166905`, 0.15859490968399015`, 0.32000646124266346`, 1339.7132456219883`, 0.07635813740352487`, 1.32927343177637`, 7.773007700491276`, 7.704380583589187`, -0.00882890118554136`, 26.763275704050997`, 1.5592578755724988`, 0.3328890570459299`, 0.34278767305987135`, 0.02973548034826412`, 0.005286689046004263`, 0.0052943375913051425`, 0.0014467552818642204`, 5.860813799145549`, 0.014760283663651588`, 0.20903549491520798`, 0.000059386929457394544`, 0.000059909850599131 23`, 0.008805323772663298`, 0.08963451736174742`},
{0.14288798158791094`, 1.8492145343896036`, 4.320087837799633`, 1.416387968755192`, 0.03040014321801321`, 0.26264512496919146`, 0.017396141813896236`, 476.32655268366966`, 0.1492295937607505`, 1.5210123574307455`, 24.05415755716876`, 24.07858169245523`, 0.0010153810304276423`, 72.38055410480358`, 19.314083145319536`, 0.16899685800417674`, 0.17463261048703838`, 0.03334826782828326`, 0.032264843579448435`, 0.032418022466773624`, 0.004747547805338259`, 4.46444922982141`, 0.065860833933101`, 0.7107813268893632`, 0.00027215668194213727`, 0.00027821364358168667`, 0.022255421385674934`, 0.19437867392486952`},
{0.08126639977214484`, 1.4848577063399988`, 1.4511591326569544`, 1.4690309046886825`, 0.09088454373979671`, 0.2634538760346167`, 0.006391735651833628`, 757.569692438266`, 0.1906091612906891`, 5.0187956659047614`, 53.87375409217286`, 54.016607131634046`, 0.002651625858795414 5`, 49.12948594196148`, 49.3009868086873`, 0.19696081072678867`, 0.20293504720337913`, 0.03033210746110071`, 0.06774175611209389`, 0.06803462550036517`, 0.004323321464927199`, 4.177982537923515`, 0.07864469476389084`, 1.4604733515878647`, 0.0005512170804569116`, 0.0005621811483033315`, 0.01989065331090889`, 0.5870167638302141`},
{0.15406825481222286`, 3.5722547377798985`, 5.518246507815627`, 1.3255687223204002`, 0.2843400333432804`, 0.5740150104722689`, 0.007989301755943026`, 636.0707307721444`, 0.19470033172956325`, 2.3727553064621407`, 49.11659480424569`, 50.42816979454327`, 0.02670329642201108`, 94.7413985486356`, 44.04249807525763`, 0.09483395849185296`, 0.0967007329482389`, 0.019684662393865127`, 0.03100168767989493`, 0.031154225132095058`, 0.004920295106999983`, 4.839586536070628`, 0.06823394167250323`, 0.8602127278300666`, 0.00027910315587242884`, 0.00028509381258317896`, 0.021463951892712663`, 0.39950246248374505`},
{0.1284209869933174`, 2.657038147126583`, 3.485135675760706`, 0.826438046300177`, 0.2940359222573743`, 0.4455502493814366`, 0.007792225179768616 5`, 642.5517685154364`, 0.18333530742444448`, 4.125506851960305`, 76.1176011004122`, 77.62052501425865`,

0.019744761948866874`, 84.44467617864329`, 68.50304053267712`, 0.19182442946747805`, 0.19571189391564775`, 0.020265742267351783`,
0.027383110821342108`, 0.027439586324074304`, 0.0020624209974047236`, 7.281211809226895`, 0.05023665883747914`,
1.4403532349104164`, 0.0003981542431785723`, 0.00040234784770287323`, 0.01053261291609564`, 0.563700842158425`},
{0.1519197321396482`, 1.414313316427302`, 9.693328908271443`, 1.0879341282517354`, 0.5935096502675883`, 0.32269331092414044`,
0.04853394307287227`, 323.7551755246228`, 0.012675975614693441`, 6.5738064876305415`, 293.5619158451159`, 292.58212804750974`,
-0.003337584832097562`, 204.38418723824455`, 175.03317202382672`, 5.584801167516416`, 5.668589584000881`, 0.015002936357307428`,
0.03144492922776196`, 0.03145488270541677`, 0.00031653681211096796`, 112.83798086881735`, 0.06824436036333349`,
4.3016122384432265`, 0.00034916238840021485`, 0.0003501252571055082`, 0.002757652992651849`, 0.4823842548694824`},
{0.10245812606330185`, 3.806669862078807 8`, 2.784482506819291`, 0.8153557037427375`, 0.41559745180116914`, 0.2970790998891699`,
0.019454402362339196`, 101.06708659388384`, 0.03906278599147861`, 1.0136093018296712`, 107.97655567558535`, 108.0350107557488`,
0.0005413682608943482`, 487.55473343849803`, 84.55635889924514`, 0.4137011041874447`, 0.4240185603761346`, 0.024939397270777208`,
0.19383688142659986`, 0.19496131530380884`, 0.00580092843494695`, 22.497478931701043`, 0.28371662332746156`,
2.1817225906058058`, 0.0028228896782102053`, 0.002907555282812552`, 0.029992530439952425`, 0.1035924521786034`},
{0.08994858347063645`, 1.3905867780774148`, 6.975873482712982`, 0.9748960320168515`, 0.19705721946727817`, 0.428438510479348`,
0.008778830773896138`, 53.85788205383214`, 0.2055785802889155`, 2.165498620130503`, 421.8567198178079`, 429.0082992361346 3`,
0.01695262652545959`, 891.6030691479411`, 375.1692727909161`, 2.2032871479438794`, 2.2530083079061285`, 0.022566808874026734`,
0.17052404930068368`, 0.17076075460312706`, 0.0013881050996271682`, 43.76945680340936`, 0.21911995260389802`,
9.133002400492853`, 0.002099060590043611`, 0.00212237468526517`, 0.011106918653107867`, 0.32671448902365685`},
{0.2555268425086469`, 0.6701554458004759`, 6.763443756394938`, 0.9242821290525386`, 0.9506231196396431`, 0.49497463337651615`,
0.1535425304369218`, 4370.366828894948`, 0.17262313668823565`, 9.906703002274519`, 87.38784696984796`, 83.73594047767244`,
-0.04178963801952407`, 40.372569505127224`, 27.672634692235356`, 5.644224657190934`, 5.676683375870749`, 0.005750784324018987`,
0.007560766272851207`, 0.007564964104136077`, 0.0005552124127872204`, 54.03582701911179`, 0.027599696180800642`,
0.4652916519636083`, 0.0000987909062365455 2`, 0.0000991214406744607`, 0.003345798216728202`, 0.39633935300979906`},
{0.13340663060001784`, 0.4096518827228479`, 7.81563732077705`, 1.2681845828171425`, 0.264399527619257`, 0.43786671579755854`,
0.11528071789135624`, 3397.5276395199044`, 0.15041338875391808`, 8.11261999427796`, 27.787571022021062`, 28.28796732701628`,
0.018007918165954928`, 15.676674709929046`, 10.810235588269142`, 2.4761800269636467`, 2.523183986781016`, 0.01898244849143982`,
0.0034853077675902158`, 0.0034879661421722023`, 0.0007627373991778708`, 14.491025857233845`, 0.006642330941135019`,
0.5346822830717722`, 0.00003317225095900156`, 0.00003330723999949536`, 0.004069336164755244`, 1.0914757045516614`},
{0.09831375749458693`, 3.5924209186692435`, 1.6118282365623244`, 0.8326891410736474`, 0.6715734708323489`,
0.5976781170151362`, 0.03815316295555065`, 115.73199628594814`, 0.0304507712440647`, 4.7148191409051226`, 930.4807282128774`,
933.570914825478`, 0.0033210646055354953`, 903.247647841703`, 604.2633344113362`, 6.204718787663843`, 6.273768498255751`,

0.011128580191126725`, 0.6383769229879979`, 0.639016269190773`, 0.001001518350291608`, 318.428022389481`, 0.8965890570973267`,
8.798773251870102`, 0.009097409511029886`, 0.00930544565823554`, 0.022867624784113305`, 0.4445654661802788`},
{0.09108239111305882`, 3.309852207951435`, 3.0566194803884894`, 1.4370488086380533`, 0.21629452377334557`, 0.2421124982368058`,
0.4670454509414495`, 208.22129409387404`, 0.23659860207487343`, 3.442603557657883`, 143.85381652535102`, 141.9034097581748`,
-0.013558255278076081`, 191.2488665270173`, 21.714137125190582`, 2.523777377568878`, 2.5910143751014796`, 0.026641413830791327`,
0.12281307049986549`, 0.12309252843082315`, 0.002275473854861154`, 119.33328750748899`, 0.15980154458681275`,
3.5802039831711383`, 0.0010253245369071795`, 0.0010389785724340982`, 0.01331679388860163`, 0.33446828958819713`},
{0.05787448179355209`, 1.5695297669455615`, 8.686108197389068`, 1.3362839901345183`, 0.8882645350301348`, 0.222001961378486`,
0.20455164120837535`, 127.50819012666335`, 0.2481722521034202`, 6.402394955038988`, 910.541969875601`, 882.9338938922734`,
-0.030320487024996012`, 650.9115856221524`, 239.7328007980173`, 28.629598798538346`, 28.963939179790465`, 0.011678137147670063`,
0.13734082134370654`, 0.13732023745455985`, -0.00014987451615111613`, 641.9286790002103`, 0.11355041234824208`,
10.28777205749892`, 0.0012411832436532988`, 0.001244359490624987`, 0.00255904757652003`, 0.24393270309691895`},
{0.2051260145065577`, 0.6712958473867032`, 3.046620158397806`, 1.125533167922526`, 0.5107051094055373`, 0.47305603590798795`,
0.03599405586564042`, 73.71677428550161`, 0.039721863923625866`, 8.822427071575206`, 1897.4909758031818`, 1928.7128440054398`,
0.016454290745199662`, 984.3649391887775`, 1260.3691418072378`, 59.96561134126086`, 60.846481168781466`, 0.014689583042981402`,
0.498776715863502`, 0.49831767830623125`, -0.0009203267567853279`, 575.0666554199046`, 1.4616011407683103`,
26.07721989436673`, 0.005319112311749863`, 0.005365212441641507`, 0.008666884094515215`, 0.9173708296863152`},
{0.04365375531995927`, 0.9346121184769336`, 9.554451222092258`, 0.75459846537052`, 0.7382972174511151`, 0.44670601219095474`,
0.02981461788584879`, 1443.346811017674`, 0.026466349135542522`, 4.5970910839514385`, 68.83146176467417`, 67.56781701392785`,
-0.01835853428576828`, 68.52805075191579`, 48.45612946344728`, 1.4183268568322756`, 1.4325893530316967`, 0.010055859924471333`,
0.00741805976720659`, 0.007422687548223772`, 0.0006238532935038954`, 18.936935262239185`, 0.004626088086083602`,
0.6710437862997775`, 0.00011868948882498565`, 0.00011909999101540179`, 0.0034586229537263424`, 0.31022338936013777`},
{0.18832502219499725`, 2.144648019015537`, 7.011391892939212`, 1.2684842496371584`, 0.8800953133738048`, 0.3019976503972389`,
0.01607749175819828`, 253.61560940445747`, 0.0505443969728126`, 8.205874646962478`, 728.1956193369529`, 696.9208588954659`,
-0.04294829522589505`, 406.1511415830851`, 592.8878910395226`, 4.255891820291069`, 4.299026101300688`, 0.010135192065729992`,
0.10072959411564096`, 0.10074080430388226`, 0.0001112899177220239`, 130.39128516473912`, 0.27099861496451755`,
6.654646047159114`, 0.0009586977966187238`, 0.0009619057785874878`, 0.003346186858964817`, 0.3452507246539287`},
{0.2789832227001883`, 3.864947879576305`, 9.015175334965505`, 1.1939022970453932`, 0.8102757584298972`, 0.657562608929255`,
0.013706864097109712`, 131.89661477643062`, 0.1558047394747739`, 7.390258992479394`, 1857.3204631795174`, 1798.6601708400856`,
-0.03158329082263611`, 1150.248510989819`, 1553.4732617973916`, 5.370526323459912`, 5.410319764745244`, 0.007409598033530473`,
0.2088366142382988`, 0.20883411778843985`, -0.000011954081271303707`, 296.5257760866451`, 0.8323130236851869`,

```
11.774921743545162`, 0.002107296344059928`, 0.002118686013847171`, 0.005404873319952541`, 0.478640749157751`},
{0.10740283086494345`, 3.8162968757181996`, 6.158389397925204`, 0.8038652030060122`, 0.9187954678523191`, 0.31270681581700654`,
 0.027540968538212463`, 2177.2729976761966`, 0.021577241346914133`, 9.805063749007854`, 112.656476488189`, 107.93017520872108`,
 -0.04195321411426722`, 52.58601208428416`, 81.07950874220413`, 0.5680513746470444`, 0.5729416255297667`, 0.008608817971369032`,
 0.009410716745727174`, 0.009413541017132395`, 0.0003001122530335021`, 30.96932409018498`, 0.014439108842275606`,
 0.9232607070684146`, 0.00014149761382720172`, 0.00014179215020448176`, 0.002081564270332903`, 0.3799019957327401`},
{0.14667176624268824`, 3.777226248423828`, 0.6973504720596608`, 0.7758342249413661`, 0.494379064234173`, 0.5228383904297224`,
 0.014800119213367281`, 81.50804211451084`, 0.12794308526059`, 9.842771813142875`, 1986.9979192662113`, 2024.6734164262446`,
 0.018961014903300155`, 923.9415333381411`, 1640.3628721040805`, 6.211256108334683`, 6.309812818389318`, 0.015867436205437624`,
 1.4151063512317883`, 1.4069158699010889`, -0.005787891011562518`, 335.16170868692234`, 2.965087827948965`,
 26.602717424863297`, 0.021529645285688792`, 0.022001003417421228`, 0.021893446244827697`, 1.1434609431364764`},
{0.15361999046758062`, 1.6832845721068805`, 5.017027003357642`, 1.2257765840301957`, 0.8758480691873987`, 0.30492603190517764`,
 0.17353190337485183`, 1218.2701158582597`, 0.01908888394356914`, 4.8706184705887665`, 88.35714979487148`, 84.46924615514638`,
 -0.04400213959765775`, 83.02752294948864`, 25.957015559380956`, 2.487788212198589`, 2.513684655403377`, 0.010409424354455687`,
 0.027764551897096194`, 0.027797705034637483`, 0.0011940814915423204`, 59.823650232331865`, 0.06093128853962366`,
 0.8239544596724205`, 0.0002729334850908316`, 0.00027480506964787973`, 0.006857291828539447`, 0.21529694531627233`},
{0.1285936813753743`, 1.2517058162042831`, 2.0098881407856393`, 1.434144339335301`, 0.06632650483661995`, 0.16162194262920127`,
 0.05011873598676769`, 354.0526180115314`, 0.1584833535532481`, 2.894273123117289`, 54.99921248949913`, 54.89213455738702`,
 -0.001946899369378418 7`, 86.97239989485732`, 32.613011846995065`, 1.1728642261031061`, 1.215371549786568`, 0.03624232262987026`,
 0.08449484527674596`, 0.08475362009794837`, 0.003062610746902505 5`, 20.972585334731306`, 0.1552214744482826`,
 1.7478538715396714`, 0.0007064589723777548`, 0.0007185527635126242`, 0.017118886740393302`, 0.2411435560917732`},
{0.05871769486491246`, 0.822876240174752`, 3.546171727568429 7`, 1.3634413021309426`, 0.14962847564878934`, 0.2425078078443763`,
 0.045889110192962865`, 1618.8495608622088`, 0.1824696213391762`, 5.484504386708753`, 27.87952476678952`, 28.0009968611173`,
 0.004357036045050444`, 23.26550442452689`, 17.105012103112266`, 0.8428776382787636`, 0.8654842090854338`, 0.02682070300599615`,
 0.012242426889843306`, 0.012260170415741666`, 0.001449347099069076`, 9.908342598774327`, 0.010269244093188403`,
 0.7364731956741485`, 0.00010816036533045637`, 0.00010894362141035761`, 0.007241618290657614`, 0.5561747819376455`},
{0.21414990207917795`, 3.2291184159683555`, 3.7200101298578847`, 0.893435725539721`, 0.18550851463991336`, 0.6299970912008048`,
 0.007788531351629846`, 1185.7534950662077`, 0.10596189074334122`, 9.85351453889281`, 107.86034412120155`, 110.93137469993229`,
 0.02847228611916774 5`, 50.09970105982152`, 97.09932324730326`, 0.22498515489947907`, 0.2291405429686185`, 0.01846961001047398`,
 0.016473984109320463`, 0.016494658313025937`, 0.00125496076530629`, 10.378624385791417`, 0.050398601198082184`,
 1.944235257080228`, 0.0002221280497820554 7`, 0.0002236351729148189 3`, 0.00678492938754105`, 1.8270964687626832`},
```

```
{0.14399581513021464`, 3.6650427925648215`, 8.960857802594514`, 0.9993788071734813`, 0.7678347002262149`,
  0.3917750513543251`, 0.06821121121423936`, 446.58045234253916`, 0.09781275073737189`, 4.158206599173292`,
  195.97347167086045`, 190.48444416864632`, -0.028009033342191403`, 215.7027676824539`, 100.15796962044392`,
  1.7938173024223303`, 1.8126428098953264`, 0.010494662665799126`, 0.03295747171265748`,
  0.032981579129435966`, 0.0007314704534580052`, 93.92024536315762`, 0.06779625719841369`, 1.9487221170085904`,
  0.0003978023901387573`, 0.0003997194024555676 6`, 0.004819006532720271`, 0.2511168379782778`},
{0.21762093156371237`, 1.4799568210517782`, 6.880501462590445`, 1.1700220578418317`, 0.49477438305707566`, 0.30056004401666714`,
  0.03776191240235276`, 139.28017196074`, 0.13055880095851463`, 4.30304009869343`, 377.413965710174`, 378.4194717998769 3`,
  0.002664199476060336`, 401.4274513755563`, 247.15979360680484`, 5.86430059319338`, 5.97289798742666`, 0.018518388085243753`,
  0.09348665377223545`, 0.09353996651616714`, 0.0005702711754085321`, 123.98445233706482`, 0.2906378954671122`,
  6.601653391403199`, 0.0009632398544674459`, 0.000968804336819627`, 0.005776839824861213`, 0.3556556281778914`},
{0.20676336326895633`, 3.023915070136316`, 2.1344025288266977`, 0.7647942269583019`, 0.38268253024987975`, 0.605820162116665 7`,
  0.06287157366362177`, 52.11423962493933`, 0.23050267576131828`, 4.129012828530946`, 1204.5928995184731`, 1240.1958675085789`,
  0.02955601681226727`, 1335.237624219998`, 641.3390647498643`, 12.68488002051129`, 12.895916226053176`, 0.016636831030379717`,
  0.6552343738672224`, 0.6548638659944698`, -0.0005654585405309165`, 547.9714265270734`, 1.9354066124312832`,
  17.99555666494538`, 0.010183376153252488`, 0.010387985438700368`, 0.020092480368853805`, 0.6226325837228727`},
{0.14056217319026204`, 2.827702131488536`, 4.822127390400377`, 0.9317030787852092`, 0.5881617813064273`, 0.663416624487535`,
  0.01875862506453023`, 1486.513173237306`, 0.1539754165327772`, 8.90838886614388`, 127.29547713662555`, 130.8783633404614`,
  0.02814621763812042`, 65.40008329671878`, 100.59060809085227`, 0.6428987035987094`, 0.6499017554409542`, 0.010892931970533315`,
  0.01730581136675221`, 0.01732598707759282 2`, 0.0011658344363658646`, 25.97037192138981`, 0.034750606493312`,
  1.312018571578753`, 0.00022382088650996224`, 0.0002252105440574193`, 0.006208792973372512`, 0.9732977784564557`},
{0.15432054500385795`, 3.142590945055387`, 0.6412922856028498`, 0.9338764110697222`, 0.4036132844000331`, 0.253764192888976`,
  0.009287914173605602`, 800.3088033129984`, 0.0773663480819351`, 1.9942494398991926`, 25.743416949458513`, 25.485976457477012`,
  -0.010000245596259671`, 59.0814583334383`, 22.44836687506701`, 0.0633774525237631`, 0.06570826506179996`, 0.03677668390289002`,
  0.11473711391055028`, 0.1163079801166659 7`, 0.013691003308139216`, 2.8452772631693657`, 0.2529470564406532`,
  0.5409694406456527`, 0.0014433753299256669`, 0.0015298339292519657`, 0.05990028895031174`, 0.19932642983906498`},
{0.1558313627948012`, 2.6183453593149384`, 1.5210778217010041`, 1.2213020905618333`, 0.8957140746385861`, 0.2067404945185849`,
  0.06533654021268133`, 3401.5830280519913`, 0.07866132934502523`, 1.2687008259414867`, 6.259139698511886`, 6.022943017224859`,
  -0.0377362852826536`, 22.57979095068198`, 3.2340002793526597`, 0.07672533063595208`, 0.07868078578013135`, 0.02548643489667768`,
  0.024325994429292404`, 0.02455380657217161`, 0.009364967320919959`, 2.8699059058935683`, 0.05415361233221919`,
  0.07724497637450074`, 0.0002363565726566 3745`, 0.0002465003727442170 3`, 0.0429173598752246`, 0.06259219162393986`},
```

{0.27749339228385056`, 3.960153029334033`, 7.7991060624230615`, 1.1159035815659704`, 0.47604804800745515`,
0.18775668040836846`, 0.02760276902429692`, 890.1551806101527`, 0.14097999756547475`, 3.493022856148759`,
38.064148995940734`, 37.92170771530289`, -0.00374213753348418`, 49.87457316442713`, 27.489578145650547`,
0.18273576750542314`, 0.18697482697181847`, 0.023197754464076237`, 0.009769931693561842`,
0.009776100367174594`, 0.0006313937298882966`, 10.33802290363256`, 0.03872987840041474`, 0.8233092279401115`,
0.00010572344406523104`, 0.00010618996143311984`, 0.004412619849964106`, 0.22020021562646766`},
{0.1388142019807993`, 3.4960143968183592`, 0.8496516336862268`, 1.4057148799798922`, 0.27289215927666`, 0.4161162296591887`,
0.1071416651953492`, 563.7099077527477`, 0.24491201094978954`, 8.47264522499269`, 174.27227490128496`, 175.8699614740291`,
0.009167761043167522`, 94.139908851974`, 70.55338371734227`, 2.0235858824225836`, 2.0690973153996035`, 0.0224904874917069`,
0.2115982036613823`, 0.21286462192054775`, 0.005985014226264962`, 101.06407683068208`, 0.4196119397404118`,
3.3679757432768302`, 0.0017883304302743763`, 0.001839848123997339`, 0.02880770401869115`, 1.1467320068247484`},
{0.0729917462714273`, 3.1893255155706086`, 3.1041281178047555`, 1.014074092539919`, 0.28719491349307913`, 0.2514291775131975`,
0.05245081359414425`, 423.2923229706584`, 0.11450677404966558`, 9.081189649788715`, 202.5631538961181`, 203.59340604182674`,
0.00508607871615685`, 102.08976391557508`, 117.65333929260967`, 1.8215248139917415`, 1.8634652909440939`, 0.023024927593735534`,
0.04570247528305931`, 0.04572677945182802`, 0.0005317910817341787`, 82.9919366644125`, 0.047655763997760496`,
4.6141495061658135`, 0.0005438583618900816`, 0.0005461244990759433`, 0.00416677823613143`, 0.8146896037388152`},
{0.11420284690084648`, 0.577787691297051`, 2.567084026939444`, 1.4491737131438955`, 0.8025258647238394`, 0.3690270257872911`,
0.13672661029124697`, 86.53603740064875`, 0.011299238359278513`, 6.563498155623039`, 1674.2038924999579`, 1610.6584187773474`,
-0.0379556361129485`, 1167.4478090059542`, 576.493257687339`, 118.36175153098388`, 119.65486444249359`, 0.010925091043209045`,
0.9036646321893258`, 0.9027341261398638`, -0.0010297028524927088`, 976.9709022137467`, 1.4743010519923923`,
15.80495098850239`, 0.007458181487003834`, 0.007551730583794514`, 0.012543151028664656`, 0.36617084998303545`},
{0.26373133325063186`, 3.0221428803953465`, 7.660698925394574`, 1.4577816176348994`, 0.5393449604126412`, 0.22813670918251328`,
0.05400872825934766`, 78.22373236540282`, 0.11174025693188905`, 6.564224566850031`, 963.6940441922061`, 958.6101552941893`,
-0.005275417990445508`, 671.9241328039872`, 551.0627189225809`, 9.32196207735246`, 9.501438441306778`, 0.01925306737627186`,
0.17526152482806287`, 0.1752372078088134`, -0.00013874704829441864`, 402.46144747694376`, 0.6603136515791307`,
17.632928909580396`, 0.0014511619607796566`, 0.0014564679849240524`, 0.0036563969341816627`, 0.42429722747862886`},
{0.21289488654178568`, 3.397179033451687`, 0.7632571166797604`, 1.0142699830327386`, 0.0835760348267307`,
0.5184383444209231`, 0.04973152926575851 6`, 2070.206130759874`, 0.06133590421436391`, 5.427522099525574`,
28.546297776800465`, 28.707693702110898`, 0.005653830369610979`, 24.072028336261152`, 16.822805729220963`,
0.23315719037056298`, 0.23938385067841214`, 0.02670584723530478`, 0.043157951444455625`,
0.04360784071556636`, 0.01042424990189228`, 11.315381694648297`, 0.13125867394490112`, 0.6179725233496736`,

0.0005019284813422287`, 0.0005237100657817879`, 0.04339579292514384`, 1.0544191343046163`},
{0.22999162513252186`, 2.379033562263336`, 2.5591649989178986`, 1.3870393830660817`, 0.8120421313192066`,
0.6084139253527632`, 0.017274035559015792`, 82.84440150538262`, 0.15048886650658344`, 7.465486944171833`, 2857.384334527993`,
2751.809049229709`, -0.03694822709795653`, 1751.761647356292`, 2290.2665539327445`, 16.021251748776578`, 16.165186279807084`,
0.008983975365189467`, 1.3021942893718634`, 1.299800564781924`, -0.0018382238422302022`, 544.5013659972805`, 4.278482583584034`,
18.953299958481864`, 0.011176060624180195`, 0.011366475600603738`, 0.017037754431249663`, 0.4900653343311172`},
{0.1769680798340355`, 1.1633029664887262`, 4.7984019847292405`, 1.1781683757559547`, 0.8107109545176789`, 0.3886685867136115`,
0.009172138674078711`, 82.52474973299203`, 0.0366989899628154`, 4.7780773811523165`, 1328.7042830170064`, 1277.213532675834`,
-0.0387526035697392`, 1272.7398747774785`, 1173.9599706933373`, 8.64116606132692`, 8.736849091899305`, 0.011072930423199345`,
0.428357461410629`, 0.42816880560106574`, -0.00044041677000805635`, 143.60420161518994`, 1.082937106120404`,
12.060542133591179`, 0.004366624610378622`, 0.004404454422834703`, 0.00866339926866333`, 0.26474160735570745`},
{0.06792516420354372`, 2.678804428347224`, 7.00137481313343`, 1.1889039591838686`, 0.130109850192629`, 0.17336690021836265`,
0.0065615912781447905`, 559.1859620197067`, 0.12479016185335001`, 7.679232726268854`, 100.6450421470949`, 100.94141817513415`,
0.002944765303054764`, 59.98450111881476`, 92.14983484970311`, 0.21322632380266265`, 0.21965687847209633`, 0.03015835265905098`,
0.013964476550171596`, 0.013968262750862628`, 0.0002711308710663385`, 8.159880292039604`, 0.013550562324095867`,
2.921514936061564`, 0.00014198945120824646`, 0.00014226721207080583`, 0.001956207733714077`, 0.5980293177853638`},
{0.04696109785508959`, 0.9059167168392257`, 1.3656449005023852`, 0.8785005151977332`, 0.7117341492461746`,
0.37541668809892415`, 0.06884837024201017`, 2425.2572297139236`, 0.025009444315193585`,
7.4432891311871785`, 58.004502065058546`, 56.91721347222889`, -0.018744900035692846`, 35.666566916133256`,
29.533517476395115`, 2.0383773408213957`, 2.063003018698031`, 0.012081020223032812`, 0.03136202934496742`,
0.03144348506111743`, 0.002597271855530492`, 26.380001546805566`, 0.02103993327148729`, 0.6463540325869581`,
0.00042842332753767476`, 0.0004336832198064218`, 0.012277324624169639`, 0.4990898082766359`},
{0.18481744149515084`, 2.8908169503663625`, 0.7719563125670259`, 1.477735031707543`, 0.05143123515478387`,
0.4060758128174351`, 0.19176878359926766`, 114.34865924476063`, 0.02384868002335655`, 4.461346915500394`, 374.74854531387257`,
370.3101746328675`, -0.011843596823805425`, 384.44875157539997`, 105.39759612097453`, 6.282841219768456`, 6.490624666879546`,
0.03307157380602188`, 0.9898210088186288`, 0.9996846640643838`, 0.009965089807022265`, 259.4649127795299`, 2.613374091257873 6`,
9.042540617905473`, 0.00782310043135026`, 0.008269796631292337`, 0.05709963765158732`, 0.7775985576734143`},
{0.11940413672862621`, 0.6385297463968325`, 0.6629513696361364`, 1.2353159995947312`, 0.3854086082897601`,
0.4772941268081121`, 0.0318040184551439`, 4110.432043833155`, 0.1129636900773972`, 2.562436110087784`,
8.58236989621461`, 8.559207296786242`, -0.002698858206820365`, 15.329171298398336`, 5.873055141319611`,
0.25750187122465673`, 0.2648892027557315`, 0.02868845766417638`, 0.03772297499758277`, 0.03870276137043 4845`,

0.025973199964076432`, 2.3488943504255677`, 0.06434684663459816`, 0.14244854742938876`,
0.0003518220220405377`, 0.0003839601058877273`, 0.09134756164719726`, 0.4042242655971549`},
{0.16756813285689298`, 1.9469615336157506`, 0.3903678609266308`, 1.2604526712473199`, 0.11212576188863221`,
0.45563235469719776`, 0.0065229352440185`, 190.16738409260836`, 0.045949495779464666`, 5.424569346408827`, 303.5542568754639`,
302.0807675654379`, -0.004854121715151982`, 256.11531963411954`, 274.52720156441103`, 0.867730295425211`, 0.8980245279336011`,
0.03491203737855564`, 1.1021586711470204`, 1.1201430070276193`, 0.016317374577185406`, 24.134821524941643`, 2.6383810090877406`,
6.687455410904872`, 0.010085066281032451`, 0.010894582687843383`, 0.08026882365001753`, 1.018892556678377`},
{0.07183551197561577`, 3.34536856927257`, 9.136834569708476`, 1.4150518758492396`, 0.289745011670542`, 0.5142172775325705`,
0.41137464999480466`, 1635.4988121892525`, 0.15390110584062433`, 5.743187227898911`, 45.39898241032576`, 45.857263482607`,
0.010094523003604028`, 36.17908997321375`, 7.242467994943019`, 0.781721649201364`, 0.7951716558072193`, 0.01720562123307756`,
0.007674052434116683`, 0.007683232320430311`, 0.0011962240800982293`, 37.35924335940228`, 0.00787527836475132`,
0.7936637302645405`, 0.00006536410217738986`, 0.00006575230388267375`, 0.005939065822863521`, 0.8335289529361642`},
{0.2384422062087977`, 2.1587544451320504`, 5.065398759698054`, 0.8914719118393273`, 0.7773656949632277`, 0.1997211683380481`,
0.4677952481930321`, 402.45383708693856`, 0.19717109492345014`, 2.7966746550751953`, 97.53504711130351`, 94.90704495109331`,
-0.02694418301978385`, 159.61850766960177`, 13.91353473186458`, 2.621038338368355`, 2.664620477565028`, 0.016627814465241197`,
0.03843516102009197`, 0.038466314872017175`, 0.0008105560403122158`, 80.83111662591749`, 0.13092235128023183`,
1.4340707992381387`, 0.000519867281275066`, 0.0005232614062652031`, 0.006528829784810073`, 0.12753831135059293`},
{0.049151474922052546`, 1.7029958107544303`, 5.984640650536118`, 1.1787547055685845`, 0.01700706976856159`, 0.4330742864041065`,
0.015091472025893045`, 551.6808300976855`, 0.14911096385124378`, 2.965493287771624`, 49.754091716768954`, 50.20468175525086`,
0.009056341356745934`, 76.78852963663479`, 41.08645947194751`, 0.3399624564441233`, 0.34838921265955225`, 0.024787314174540276`,
0.020681138690237723`, 0.020731572957702643`, 0.0024386600863872676`, 8.270780559116114`, 0.01452154956703681`,
1.2483885204137044`, 0.00021069066780809997`, 0.0002130626729627386`, 0.011258235494317592`, 0.5239809237060932`},
{0.05913727892151971`, 3.1040367182848003`, 9.835708161805986`, 1.4919208308945082`, 0.5501557876127892`, 0.5378623906029458`,
0.015011170514212647`, 280.8434087480442`, 0.0269165475419888`, 2.8649095039964387`, 177.19297569958835`, 180.6168778356671`,
0.019323012791904404`, 283.07406928275014`, 146.13594785755123`, 0.6808356060715631`, 0.6900350598281794`, 0.013512004475937145`,
0.05872835366533802`, 0.05886168355168425`, 0.002270281355169823`, 30.190553148025963`, 0.049614786161559`,
2.2343292342890226`, 0.00047266985507254944`, 0.0004779071757875066`, 0.01108029348339401`, 0.3144067983474527`},
{0.1028902787116574`, 3.248894721912677`, 2.8223976230891363`, 1.4617310236348016`, 0.21163710737184238`, 0.2736720564831455`,
0.052563491644450414`, 157.16268136885162`, 0.03718011423514023`, 9.1247890605319`, 532.2939873208295`, 535.4585552816059`,
0.005945150680180422`, 266.9889095542811`, 308.93824059662484`, 4.700845965479725`, 4.8148105747348815`, 0.02424342556468484`,
0.1893919963290655`, 0.18950432253767727`, 0.0005930884661915492`, 218.17933779673635`, 0.278379932686629`,

12.637632018396477`, 0.0015600697106964434`, 0.001570811678659874`, 0.006885569208721742`, 0.9384425535660269`},
{0.13701717915337458`, 3.8023949322113495`, 2.868993121081161`, 1.1378721602373125`, 0.9948229422967421`, 0.24001294614371227`,
0.1498271577324817`, 115.7171521166015`, 0.23683719126671393`, 3.874936558168838`, 745.2187344424568`, 741.6739840160454`,
-0.004756657693346l`, 880.2046580140563`, 241.50924573744857`, 9.0798266563168`, 9.181451774414702`, 0.011192407294163598`,
0.47816975579076837`, 0.4779492475456234`, -0.0004611505484705125`, 493.2155266190934`, 0.9359638727846467`,
6.841154783779438`, 0.005042072183864765`, 0.005094519784662071`, 0.010401993244988583`, 0.13603411658811249`},
{0.12767680767420947`, 3.2442504564252905`, 9.68431286349912`, 1.3341607174516774`, 0.1954172777492651`, 0.4247465971362525`,
0.2589115414709387`, 128.98950675665216`, 0.14024381160911475`, 6.508167926731167`, 546.4504200290061`, 550.3164500636733`,
0.007074804763553821`, 384.2877215932225`, 124.69526524883676`, 8.903526787836205`, 9.0852239765116`, 0.020407327680939336`,
0.07255824662722683`, 0.07259246947722361`, 0.0004716603775254402`, 412.6467263604618`, 0.13234293285426407`,
11.365282068888934`, 0.0006561914487357434`, 0.0006589262281538954`, 0.004167654765116158`, 0.9195761905642996`},
{0.15001338826271665`, 1.2298087626140388`, 4.614349177257099`, 1.406777099587714`, 0.7364706782176744`, 0.4119762663671258`,
0.007028619260401152`, 151.01374639678102`, 0.013332718449329678`, 9.705143720051169`, 1349.268860561551`, 1322.155919625336`,
-0.020094542851104458`, 636.2986782251547`, 1225.5138615489075`, 6.554170087176614`, 6.630327524218559`, 0.011619691895232931`,
0.26592765481756775`, 0.26592882784393307`, 4.411073252663655`*^-6, 115.14822578389467`, 0.5698958361704985`,
13.498779143067974`, 0.0022765814643015414`, 0.002289811136945693`, 0.005811201071256544`, 0.6232624125151748`},
{0.20002396942953493`, 1.8945790659495865`, 6.47237980815893`, 1.0186003524309086`, 0.9948715481686918`, 0.5599748214378268`,
0.06647091233379371`, 636.3545963390636`, 0.214859543893563`, 8.583745959022096`, 635.3482581160115`, 630.9838632713123`,
-0.006869295365097616`, 338.76573954989345`, 327.0869588256166`, 10.973535241681583`, 11.0263536189358`, 0.004813250797572888`,
0.07306349717305531`, 0.07309795054391`, 0.0004715538153488019`, 297.0032878335714`, 0.20877786749961336`,
2.7612639827374434`, 0.0008650942033665565`, 0.0008691296892582252`, 0.004664793586599325`, 0.31911204605636107`},
{0.0426995949672023`, 2.62335318941125`, 9.6559263809094`, 1.1610440596461276`, 0.9636197200326426`, 0.24951659449471042`,
0.04756766795277323`, 104.67802103676537`, 0.10920782191363448`, 6.683384921443938`, 1424.8382103818187`, 1396.2796812397198`,
-0.020043348735324873`, 975.7387965291556`, 853.4480342025097`, 14.842795344995638`, 14.982843458619476`, 0.009435427112525208`,
0.16097511941105283`, 0.1609044437477694`, -0.00043904712443765 26`, 556.2556358296107`, 0.09819389141051346`,
13.036123246254277`, 0.0016743757556524352`, 0.0016779875146907027`, 0.002157077959397568 6`, 0.23056603549637206`},
{0.2109009326123063`, 1.0923929642765158`, 8.593806993622852`, 1.1682615459081633`, 0.4499643089978256`, 0.499787017067712`,
0.035780106156421375`, 2693.2423996223015`, 0.018435796880638622`, 3.8866756448846873`, 21.334501116498608`, 21.816267438842065`,
0.02258156024894764`, 25.122837402740135`, 14.208886217447924`, 0.4278920762089266`, 0.4345754469051215`, 0.015619290629096838`,
0.004673441089904844`, 0.004680576388413783`, 0.0015267761744877095`, 6.6775184788614546`, 0.014080472633838478`,
0.3190612031531946`, 0.00004817145879665308`, 0.0000485436233516337`, 0.007725831109903547`, 0.46715743550674454`},

```
{0.1340526977662379`, 3.5323141681247634`, 7.368159769503098`, 1.0026662265470512`, 0.9539160554997537`, 0.16176440546396775`,
  0.3999369934637664`, 62.1244000754222`, 0.21257466381414752`, 3.7256546669386505`, 936.1978104457986`, 923.8931769965501`,
  -0.013143198277070556`, 1150.0838854555436`, 149.74307542691088`, 15.270189793262466`, 15.482319243139067`, 0.0138917363011557`,
  0.21470133751789178`, 0.2145336124440879`, -0.0007812018115159747`, 770.5586822385021`, 0.41116133583299425`,
  12.216441943065963`, 0.0025839068387364383`, 0.0025919079961193806`, 0.0030965347755551598`, 0.11963690969197034`},
{0.138783517590022`, 3.0993312847332284`, 6.04052192414912`, 1.482957943290502`, 0.5742828550914962`, 0.6307262387789596`,
  0.006158597883984412`, 223.4781142247734`, 0.1875211630254222`, 1.5541408452833192`, 135.63285896611043`, 138.3664271403334`,
  0.02015417351709714`, 399.42848799649465`, 124.30052132249305`, 0.23861888532625813`, 0.24224689945347483`,
  0.015204220413049807`, 0.1330626948924204`, 0.13411253603324993`, 0.00788982322715115`, 10.565128234569194`, 0.2638129836739618`,
  1.5420417867914427`, 0.0010628339806196818`, 0.0010980286425395073`, 0.03311397881662126`, 0.1918962812625605`},
{0.13089267345973798`, 3.445236909656975`, 9.841043091432308`, 1.0552044625838208`, 0.6603410104708771`, 0.19921054981100283`,
  0.006557758988700662`, 85.91513533777795`, 0.010838310480980468`, 8.608032245025406`, 1251.6604602543316`, 1233.5383325502896`,
  -0.0144784694248149`, 665.4985945943504`, 1144.8803196034046`, 2.097359298928695`, 2.1349918067037317`, 0.017942804456184014`,
  0.09789290397435464`, 0.09785969291852144`, -0.0003392590727711031`, 103.22713813544881`, 0.18304948448490305`,
  20.771820183879807`, 0.0011213936832121796`, 0.001122936263164324`, 0.001375591797276643`, 0.4340107170111282`},
{0.0796809200271959`, 1.3022197759162255`, 4.457724373908043`, 0.819757860237272`, 0.9902524891863433`,
  0.373286927571 5256`, 0.027666886465779635`, 3268.657024391763`, 0.02176038735057556`, 2.8954452621820828`,
  27.858450131059918`, 27.533037087968193`, -0.011680945693705924`, 44.03581990628602`, 19.998469481537754`,
  0.3995479859444179`, 0.4025726462012297`, 0.007570205239959549`, 0.011076759110909055`, 0.01109856980522729`,
  0.0019690501616806966`, 7.432846981775984`, 0.012608662241098802`, 0.18156151884284782`,
  0.0001625121147205144`, 0.00016403705831931754`, 0.009383568735326087`, 0.11143056403765421`},
{0.24784589915696786`, 0.6840125293527293`, 8.78766727614829`, 1.2765252846824708`, 0.05554020049431552`, 0.46845321895484016`,
  0.4169012658954577`, 507.69507152027995`, 0.17834533234287903`, 5.09521235383073`, 102.50740277650692`, 101.53892247432107`,
  -0.00944790596536138`, 92.07833037194197`, 16.56098693243239`, 7.971258266643243`, 8.148300137544847`, 0.022210028201251175`,
  0.018312295178810188`, 0.018332391289107224`, 0.001097410788806652`, 77.89200755843561`, 0.06483753234599711`,
  2.320085014439846`, 0.00017291834634070025`, 0.0001739934615387111`, 0.006217473280091257`, 0.8727926656877142`},
{0.1280056031133443`, 3.9743049299675812`, 6.787496113248967`, 1.4777257411519527`, 0.41697417826757177`,
  0.16856234271449722`, 0.016472885834375787`, 675.9035984651438`, 0.06538866409107441`, 1.0273997992144341`,
  12.898104435355876`, 12.838587797981816`, -0.004614370869173157`, 57.458053218207816`, 10.457870656714581`,
  0.041316536441057136`, 0.04252749204662446`, 0.029309223615461955`, 0.017043476159213696`,
  0.017094743844433824`, 0.003008053330271654`, 2.3457787780982646`, 0.031166577784400837`, 0.320387349187593`,
```

```
0.00013844310524524506`, 0.00014056927694786932`, 0.015357729074755078`, 0.06979174369314244`},
{0.2694840025057293`, 1.4398402455219186`, 1.997018914620883`, 1.3694289195380824`, 0.1761008172166878`, 0.6152080706831848`,
 0.020267602026759375`, 203.92916364743616`, 0.23224028188307977`, 5.1018397742421016`, 316.5715850674374`, 323.3464704979386 4`,
 0.021400800798524022`, 283.9942912737748`, 245.65315652087088`, 3.2244904072690974`, 3.295409851263064`, 0.021994000612961973`,
 0.26446667796373374`, 0.26604306202703387`, 0.005960615059097396`, 66.32501513836318`, 1.0181362701008374`,
 5.900229564717098`, 0.002291805344477371`, 0.002361437901573295`, 0.030383277211443405`, 0.9945198877588015`},
{0.05785779964873633`, 3.866745902394764`, 0.7980363800640653`, 1.2385898080722695`, 0.5722218009229685`,
 0.20306189134783081`, 0.040832709866995026`, 2800.195372434598`, 0.18949558604342587`, 8.332936748246446`,
 34.02351184782525`, 33.65059855503893`, -0.010960458592699673`, 18.68725655821028`, 21.668273997044807`,
 0.21837984987499115`, 0.22288459139844205`, 0.020628009067821917`, 0.03957873191671226`,
 0.03969926482213308`, 0.0030453958372003154`, 12.063134138138675`, 0.03271340487981448`, 0.6226872712075072`,
 0.00038331073838637764`, 0.0003888409143793384`, 0.014427396467526998`, 0.5015363969871174`},
{0.2528189510796667`, 3.3250798883848782`, 4.1415523484020405`, 0.9119401620778833`, 0.8401965817654944`, 0.5070162083757728`,
 0.10337827675659322`, 381.96079877048203`, 0.13245695994197593`, 7.95834379608233`, 623.3248578715389`, 594.3351606203837`,
 -0.04650816806848679`, 358.47283032928584`, 254.0336162693854`, 7.6037899446251185`, 7.662969467426645`, 0.007782898164271135`,
 0.1081297092342117`, 0.10818582316831785`, 0.0005189501988265288`, 361.1887002910879`, 0.3905319952743712`,
 4.337365265049108`, 0.001428172687513496`, 0.001437282664100172`, 0.006378764043259322`, 0.43562061098500393`},
{0.20018502874097505`, 2.5781656282009475`, 6.942068190225046`, 0.9929389842661138`, 0.15941471595895984`, 0.3421827829774996`,
 0.4839576109293149`, 375.34729484852807`, 0.1596957174670693`, 8.895509004013046`, 231.47323432549135`, 229.31477415562566`,
 -0.00932488015798194`, 119.09525840233542`, 33.595636632265894`, 5.228193865555507`, 5.345452395034273`, 0.022428114276957123`,
 0.023348915508570243`, 0.023357032001741825`, 0.00034761756573242764`, 192.55928173923198`, 0.06677290460227671`,
 5.251517883759616`, 0.00028407609579250703`, 0.0002848752329611318`, 0.002813109516995 4268`, 1.0916156900106764`},
{0.272440775669993`, 3.035804235517027`, 9.484814840956616`, 1.224288286053278`, 0.8025814347303761`, 0.5890268382574878`,
 0.3843203491912798`, 336.978314494752`, 0.04553466081406238`, 4.10287356038395`, 361.72893349084296`, 349.5526050928185`,
 -0.03366147208772463`, 403.5149309413368`, 57.8309908579744`, 6.841527393147404`, 6.896653405897242`, 0.008057559311251739`,
 0.07128605149038399`, 0.071376245807943`, 0.001265244962700418`, 296.70768339318124`, 0.27744610232189393`,
 2.560414960839749`, 0.0007007526078823823`, 0.0007064474301835361`, 0.008126722950575926`, 0.26866831803146074`},
{0.20623021381347656`, 2.305665489644068`, 6.160210330863352`, 1.4997848102690652`, 0.31029755609374554`, 0.2932546230651458`,
 0.047029701289319936`, 605.575343672007`, 0.11131413350838143`, 4.271934365443833`, 70.75929733309704`, 71.2856111484316`,
 0.00743808708072601 9`, 75.80946551281846`, 42.85730791253358`, 0.8191579413545201`, 0.8378617923593151`, 0.022833021643991147`,
 0.02524676911414679`, 0.02528149622710698`, 0.0013755072105732236`, 26.98148851355706`, 0.0743806656072810 9`,
```

1.532838790870543`, 0.00020276377670214174`, 0.00020435110553759415`, 0.007828463551377896`, 0.43119581527051365`},
{0.27799817940399246`, 2.8456068500128753`, 7.4650958706635215`, 1.2794446296629622`, 0.3185515090611555`,
0.5440861077528716`, 0.2050159519579307`, 96.61991147082354`, 0.24522478882121745`, 9.22867476531573`, 1308.816756952388`,
1338.367702429557`, 0.022578367307872105`, 649.0886280300828`, 348.197580327664`, 23.045040795645672`, 23.424897199189793`,
0.01648321679759812`, 0.15248224696911977`, 0.1525095812172366`, 0.000179261839723166`, 936.8160849559348`, 0.6055683864121457`,
21.5728475123643`, 0.0014369165039453957`, 0.0014438354758951324`, 0.004815152398026656`, 1.3321160942298673`},
{0.18731205279881885`, 3.3920364906001828`, 9.53367346846489`, 1.150711712471592`, 0.269617044263482`, 0.4407414224913243`,
0.0689482800442352`, 54.451263142554176`, 0.1525802464986004`, 2.2696676207606785`, 473.99120983259155`, 482.2794240258315 3`,
0.017486008224007588`, 955.8120918075226`, 242.91143436532622`, 4.66051006650787`, 4.755617847749078`, 0.020407161423100062`,
0.15785755795555134`, 0.15805374924313112`, 0.001242837467655633`, 225.83743157720218`, 0.4224089032929096`,
9.392433404539053`, 0.00164823056033514`, 0.0016647972311758983`, 0.010051185337438318`, 0.3215770187270674`},
{0.07598141369986516`, 3.9958506473293216`, 3.287957942109602`, 1.3569966945049934`, 0.38975760386481206`, 0.6382110342097327`,
0.02530287278972352`, 4836.578585308585`, 0.06124631354128618`, 7.15493409138095`, 23.528541101567065`, 24.32610285566832`,
0.03389762886948122`, 15.050601484497875`, 17.350631227569416`, 0.10598824328665372`, 0.1075487724808209`, 0.01472360656027294`,
0.008486907204306423`, 0.008514069861680943`, 0.003200536629025308`, 6.050188436375329`, 0.009212102961765075`,
0.3357888567414432`, 0.00007496616917490595`, 0.00007601104451305149`, 0.013937958276989182`, 1.0736831577032346`},
{0.21782025688272888`, 3.800225088388993`, 1.7719223148490801`, 1.3823014458300409`, 0.9034484926621247`,
0.21119490910082483`, 0.008011320454875006`, 549.8645476667474`, 0.16991323645757794`, 1.3522541901702763`, 42.54749815271274`,
40.956377463140775`, -0.03739633958878308`, 144.005867232847`, 37.64380190601362`, 0.07687043596231895`, 0.07902824165513711`,
0.02807068368749599`, 0.15054520541607713`, 0.1518368621722283`, 0.008579859800790679`, 4.173213704277197`, 0.4684542188027615`,
0.5088337168939392`, 0.0012927909016694494`, 0.001349536412733533`, 0.043893804474339326`, 0.06534656015756854`},
{0.12917865835123116`, 3.4713877505357775`, 5.802428296169513`, 1.4965547140183602`, 0.061568597458759644`, 0.5580721907461164`,
0.2777380762847274`, 381.024601401567`, 0.014984965589617844`, 6.51082587242481`, 190.2405511314148`, 190.5047218324733`,
0.0013886140441006223`, 133.73082059211632`, 41.209116079143584`, 2.9431400548904034`, 3.0033837769352263`, 0.020469199875391 64`,
0.04719540656256359 5`, 0.04727872839847219`, 0.0017654649462155447`, 145.95400478082453`, 0.08709484714415919`,
4.023443426637635`, 0.00037922813553348256`, 0.000382710894032112`, 0.009183808299798368`, 1.2565308299167253`},
{0.12408328322582662`, 2.9809522601952096`, 4.3074275514408615`, 1.1040930179447719`, 0.6796918144407815`, 0.5931470332615022`,
0.005369942238542098`, 502.88367879292525`, 0.06875474145528054`, 6.831128450148093`, 315.1512612005716`, 316.7693825577835`,
0.005134427674658992`, 211.15000322621228`, 292.40603318762834`, 0.5095939982688772`, 0.5151730183558411`, 0.010947970552863895`,
0.07407607094310245`, 0.07419145500481454`, 0.001557642842594964`, 21.701076870307478`, 0.13130860130127125`,
2.9153873498738703`, 0.000806654540246754`, 0.0008139958831663314`, 0.009100975142756651`, 0.5971151766140518`},

```
{0.2301374553102561`, 3.6285008777535515`, 6.8583997394674485`, 1.291013232269163`, 0.6431022470858925`, 0.6246309281545763`,
  0.18498728864660263`, 132.58216720365365`, 0.011374938240932164`, 6.052136677369937`, 1020.0640878953124`, 1033.9469726833836`,
  0.013609816238816519`, 771.4063784807073`, 286.8307434013871`, 13.861467261840719`, 14.006290449127334`, 0.0104478973654758`,
  0.1988595492492045`, 0.1990152474375886`, 0.0007829555531628785`, 718.5192303791592`, 0.6537861518340027`,
  9.879742675130178`, 0.0018521930328238012`, 0.0018677103650913387`, 0.008377815914726972`, 0.5845769797315333`},
{0.18974259222357154`, 1.3104397818774611`, 5.6865397453703626`, 1.3109561369338216`, 0.42925562092874125`, 0.6235284138933523`,
  0.06332638490149935`, 1635.1348002386078`, 0.08473820825835521`, 3.2599591654117823`, 32.2479792355681`, 33.263844598176085`,
  0.031501675041006205`, 45.27462273282477`, 17.10780234644931`, 0.7650467871072637`, 0.7766970789675944`, 0.015228208335312354`,
  0.014239581810240855`, 0.014293504958580694`, 0.003786849154590932`, 14.32211064032705`, 0.0385979309265159`,
  0.4502503880956216`, 0.00012999644829059243`, 0.00013220742871026254`, 0.01700800636281774`, 0.47022256612864227`},
{0.2309824769455583`, 1.2471345787782342`, 1.0215019422726535`, 1.0312798793045614`, 0.7898876098277747`,
  0.6152586670568927`, 0.006979047924720161`, 310.31208829749596`, 0.24081833704073502`, 8.344184598629436`, 818.1037057209577`,
  793.6411623107655`, -0.029901518889508538`, 448.7339735308864`, 741.3595662610882`, 3.898162696771640 5`, 3.941848181369828`,
  0.011206685814926676`, 0.6177574881697003`, 0.6193966763125427`, 0.0026534492486669503`, 69.45047846924747`, 2.038445068130042`,
  5.690199714684133`, 0.007107401715137129`, 0.007294555820015299`, 0.026332281806947133`, 0.6022200342568901`},
{0.1769680396277803`, 1.6046472005597128`, 8.695674701006173`, 0.7703180616996756`, 0.764260429383415`, 0.1992432648214062`,
  0.012440799720035065`, 274.53159119005693`, 0.16375790457626532`, 6.298702841855883`, 323.94081088354625`, 316.44969475345465`,
  -0.023124953319897057`, 235.38513875351947`, 275.5291769729575`, 2.0118953579928887`, 2.043367491382748`, 0.015643026991849496`,
  0.0285928913070621 3`, 0.028593626828917237`, 0.000025723941213362522`, 46.11974648603389`, 0.07228611317001793`,
  4.728393931587731`, 0.0004487807733457583`, 0.0004494949870734 3394`, 0.0015914534893084475`, 0.2783042755255124`},
{0.23450402912552432`, 3.0762297015464597`, 2.593937576427738`, 1.1039402177647473`, 0.4424612211889203`, 0.6666998367628838`,
  0.44528706596344714`, 463.5224063697932`, 0.20661121111075748`, 4.223015851637719`, 158.32159156448682`, 161.85171414311463`,
  0.022297164548083392`, 171.5860316100642`, 23.15432761395708`, 2.9976981729175844`, 3.043957605173457`, 0.015431651082753683`,
  0.0993794821074211`, 0.0998925306180781`, 0.005162519463548953 5`, 131.73725936858057`, 0.33292698523697223`,
  2.0611464272075004`, 0.0010719696883484398`, 0.0010984277781357615`, 0.024681751802222207`, 0.6176144119155416`},
{0.2755523363386182`, 3.945462181815995`, 5.876565708554855`, 1.4395306041819111`, 0.28572441613120825`,
  0.3101513953075886`, 0.39757529919045964`, 3302.499033894636`, 0.07285156297216239`, 3.794286848749824`,
  11.652019507003523`, 11.60631323378407`, -0.0039226052781650145`, 14.055151692272972`, 1.946336453242206`,
  0.16879051542642845`, 0.17271558862438907`, 0.023254109912778098`, 0.004705166897356323`,
  0.004713282249548538`, 0.0017247745657598124`, 9.51366564663147`, 0.018521710448992736`, 0.25098831368457747`,
  0.0000393406450208 6887`, 0.00003971559178875132`, 0.009530773267280024`, 0.4054157137917925`},
```

```
{0.1912671247263708`, 2.190434692724648`, 6.310276417856439`, 0.9527360176604049`, 0.6104292936966895`, 0.2522020081398627`,
  0.010518470607815496`, 284.3254609055096`, 0.1005439259073278`, 6.418961425083908`, 295.0193543766808`, 292.3574775379676`,
  -0.009022719354590203`, 210.35373958610086`, 256.7812883127491`, 1.1731076364082713`, 1.193475930234205`, 0.017362681133247015`,
  0.043529562657626496`, 0.04354054090856143`, 0.00025220218777022296`, 36.708795215555575`, 0.11893963271601651`,
  4.7342833357985725`, 0.0005518339086135748`, 0.0005535424748120454`, 0.0030961602246646347`, 0.40116351955834206`},
{0.2597914621776059`, 0.5051242650756058`, 1.0118962727972`, 0.8968589895031354`, 0.07276014875602654`, 0.6898744359309281`,
  0.03193322527994425`, 3177.5504656788207`, 0.10860322045075349`, 3.7076953543063436`, 14.479708329700177`, 14.629659111067838`,
  0.010355925544445421`, 17.873938762034452`, 9.954288375569904`, 0.5384199385965475`, 0.5521069083021943`, 0.025420621943019972`,
  0.021287331823804902`, 0.02157780424259871`, 0.013645318314105648`, 3.8852710826519123`, 0.07900381514808855`,
  0.28585560146155453`, 0.0002783125626965033`, 0.0002933143880111866`, 0.053902796084137394`, 0.8944623510350219`},
{0.058009311425788745`, 1.2821997162065424`, 2.0542232518025845`, 1.4132652568351216`, 0.6228832317992945`,
  0.16342794118309045`, 0.044680537911227475`, 201.38656694501756`, 0.06413530627116476`, 9.590029577588982`,
  521.9402103839819`, 515.4725975706392`, -0.012391482174911594`, 249.09516871169032`, 322.11683904927827`,
  10.321299575270487`, 10.527048588591725`, 0.019934409598400427`, 0.2348713319241669`, 0.2348780762080362`,
  0.000028714802330576106`, 189.05667694706463`, 0.19463891769393243`, 9.848456369668725`,
  0.002002991576011026`, 0.002013570135218065`, 0.005281379778993456`, 0.4589276345878671`},
{0.19641852899773504`, 2.1398264375306333`, 5.320272455292722`, 1.143748934609851`, 0.5956525384016933`, 0.542078693849333`,
  0.2264882318745723`, 868.9198508977777`, 0.040131295366706066`, 7.0792614425236255`, 156.0886620908733`, 158.0463055848333`,
  0.012541868626052333`, 100.91318216668833`, 38.14484042830619`, 3.7324901653141267`, 3.777021129123072`, 0.011930631250624657`,
  0.029697899960481517`, 0.029736400525953825`, 0.0012964070026344476`, 114.09830190803201`, 0.08333168320799172`,
  1.7648601737277791`, 0.00031270864118748687`, 0.0003149656014588399`, 0.007217454122093958`, 0.6905835561319676`},
{0.09296068013545083`, 1.2991493564864323`, 0.26387265122439096`, 0.9093728692378444`, 0.35146944109549483`, 0.5215346752572301`,
  0.009103351916553084`, 3690.6422305701226`, 0.14178477746644713`, 8.458943719263925`, 32.25461562368238`, 32.595012890128814`,
  0.01055344358828747`, 17.45180011097577`, 28.412491767031774`, 0.18560258813673697`, 0.18977785320497362`, 0.02249572654213572`,
  0.08066973598836158`, 0.0820102654127717`, 0.016617501073804553`, 3.4446497562865455`, 0.10713019319750497`,
  0.5223149761654488`, 0.0010350838470014612`, 0.0010982341573602778`, 0.06100985011190829`, 1.3079566724592817`},
{0.09381338426095137`, 2.303475789921615`, 0.696644313515133`, 0.9364121618865129`, 0.9663355677423924`, 0.5212308135336106`,
  0.24626013703252375`, 1385.562539836289`, 0.20934361709679128`, 7.7725134925150865`, 235.01676388651643`, 224.84143344026876`,
  -0.043296189931204654`, 138.38908947277974`, 52.78277908526712`, 5.3570815245382555`, 5.397115817373765`, 0.007473153554249823`,
  0.25318831302486355`, 0.25451561182267357`, 0.005242338328940388`, 176.28439423443206`, 0.3393207500028462`,
  1.157842741487179`, 0.0032107873511741936`, 0.0032986774611083614`, 0.027373382389221712`, 0.35575505858992423`},
```

```
{0.10039832860995906`, 2.055462616090492`, 1.0012272143748433`, 1.102551063992137`, 0.7631597204325553`,
 0.38648795662626834`, 0.021413905310258614`, 653.3550083645581`, 0.05519462287367316`, 4.402154187880063`,
 141.0612081662764`, 136.27928931814048`, -0.033899602238619764`, 146.65836667097068`, 107.91253221398878`,
 1.0730083464102969`, 1.0882678104463461`, 0.014221197893846016`, 0.2192132386041495`, 0.22068585432514304`,
 0.006717731695268236 4`, 31.507550611420566`, 0.31440918235766974`, 1.4202555883213714`,
 0.002358348471492633`, 0.0024323344780146894`, 0.031371956865742456`, 0.2993633955088252`},
{0.16980453046164407`, 1.1980162751260872`, 9.435065425338543`, 1.2054727390347812`, 0.15783339617816905`, 0.5987514193964574`,
 0.2639813183074236`, 624.8721767770594`, 0.14884913893972124`, 1.0597319526281854`, 20.31314073728813`, 20.47856510317336 3`,
 0.00814371189687923 5`, 87.72947753027218`, 4.53880759792242`, 0.8679291226684923`, 0.8863419576691282`, 0.021214675852821552`,
 0.015241850807997307`, 0.01533529163628772`, 0.006130543427270929`, 14.85418878018224`, 0.03697336171168856`,
 0.40203211223142915`, 0.0001504820272022478`, 0.0001544008633904518`, 0.026041888596683327`, 0.21310936596709032`},
{0.1106187483985549`, 0.9718711331456742`, 7.0205608914526`, 1.3199794157480607`, 0.2870266036717726`, 0.2842604073783652`,
 0.12440193317594331`, 1067.250206889352`, 0.20077332983402157`, 1.344770962154577`, 11.863269609161131`, 11.898091017421132`,
 0.002935228601153250 2`, 40.375747185050926`, 4.431623763205497`, 0.4975135442687174`, 0.5100526227068715`, 0.025203491608625228`,
 0.010354350940177807`, 0.010389439273996284`, 0.003388752614355006`, 6.907415028910856`, 0.016362647735456915`,
 0.27541836172904`, 0.00009401890354610032`, 0.00009549878792268315`, 0.015740285418848643`, 0.14452657676341454`},
{0.22322153760577584`, 3.532203149750069`, 4.201686494998453`, 0.809746094501941`, 0.17157339217602185`, 0.1641601493204358 8`,
 0.005086092661785311`, 81.42460105493491`, 0.09946867199032089`, 6.2584574953677805`, 566.6131076905876`, 567.965911237098`,
 0.0023875260352239636`, 414.3657057764615`, 528.3079901778902`, 0.7236420864225517`, 0.7458323531507285`, 0.030664698950662483`,
 0.10949020298585156`, 0.10945009669090376`, -0.000366300306822831`, 36.51501224219214`, 0.34915102090391004`,
 16.278390897215637`, 0.0016327813819794201`, 0.0016376053838332153`, 0.002954468924643683`, 0.4572140277177559`},
{0.11344753764961812`, 0.6515213816912864`, 6.9223785093110415`, 0.7705690533378804`, 0.053758347821018404`, 0.22122664443377937`,
 0.23238889135484875`, 1612.9665239639735`, 0.23659636795633343`, 7.8050806983048435`, 36.58081414650875`, 36.51366628616791`,
 -0.0018356032228235009`, 21.450650354540876`, 9.235197403556331`, 2.652163345136576`, 2.7274181906209534`, 0.02837489086876066`,
 0.003273725706333417`, 0.0032745638928962442`, 0.00025603445065836006`, 24.684873244205225`, 0.00530565886177534 7`,
 1.0493660179101036`, 0.0000513699359067088`, 0.000051455786305373 16`, 0.001671218722567147`, 0.7707200779519849`},
{0.05086592503936643`, 0.6345548618632657`, 4.1212243894392255`, 0.9757969272512688`, 0.4720155756440034`, 0.3491598005177542`,
 0.10619345643493772`, 163.13358888497802`, 0.24650090837908084`, 9.412849245254243`, 750.6217895191916`, 758.6602289634035`,
 0.01070904089975988`, 364.97621142481523`, 305.4250887708639`, 44.21151740447513`, 44.94820701849095`, 0.016662843920874915`,
 0.11849977893429538`, 0.1185053664672983`, 0.00004715226520390914`, 400.7804759908871`, 0.08610858389244457`,
 12.420767520117643`, 0.001464803502432388`, 0.001470575732816318 3`, 0.003940617546548175`, 0.853455625877078 3`},
```

{0.18344840265344153`, 3.595124583701854`, 4.199071914809606`, 1.0537057639813663`, 0.12507220232647875`, 0.4903855977737973`, 0.3568003256791643`, 83.2177314580599`, 0.1899680886834239`, 7.625578775230776`, 1012.332586604081`, 1012.7649361525209`, 0.0004270825163203984`, 607.5959909722299`, 182.2971420611776`, 15.8383501499296`, 16.16341096216662`, 0.020523653610377002`, 0.2089364413823793`, 0.20898488916121455`, 0.00023187807026237017`, 813.440599846994`, 0.5475579489676855`, 21.072708629864565`, 0.002385716056099274`, 0.0024027616114627617`, 0.007144838263509756`, 1.2594444655628991`},
{0.11456761033436458`, 3.133847301219358`, 1.1215010618012613`, 1.2799949994814193`, 0.7250649174618937`, 0.2748176967060689`, 0.05347527364447488`, 4312.248498236981`, 0.1363057898672484`, 6.512229405589068`, 24.37486650322244`, 23.76021414140445`, -0.025216645257799875`, 17.130776518960154`, 13.913907273375887`, 0.22701709234500256`, 0.23064364623995226`, 0.015974805498073952`, 0.02663650776813175`, 0.026741527623291128`, 0.003942703603398989`, 10.163384316800759`, 0.043595443466388516`, 0.3155029186998235`, 0.0002491020262007648`, 0.00025364508897628347`, 0.018237759221826355`, 0.3811073313305559`},
{0.2733102228285163`, 0.9906705811868095`, 1.8602414047947509`, 1.4451100284824412`, 0.2848844825831507`, 0.554129635350794`, 0.011612796259976983`, 302.8864567264163`, 0.1580490958422761`, 2.3921629765885086`, 104.05075640815696`, 105.62509746862344`, 0.015130510481738968`, 199.07603019911502`, 88.5666083406878`, 0.9507573908982361`, 0.9771051601751186`, 0.027712400165504025`, 0.20716114950962106`, 0.21003339504391577`, 0.013864788552746088`, 13.455533957268727`, 0.8088465704840873`, 1.8331845971231118`, 0.001677869233879914`, 0.0017770463802787182`, 0.05910898441678114`, 0.42108741262862426`},
{0.13611751569452646`, 1.612649572698647`, 9.438389550358693`, 0.8529700096304889`, 0.38386891649316235`, 0.16702474363312747`, 0.015283436679942941`, 2091.084776788581`, 0.04888707786432367`, 3.7550559614266916`, 15.364264337924686`, 15.347367230979733`, -0.0010997667427034008`, 18.72663955880811`, 12.666076504567734`, 0.1116893133396595`, 0.1145440303266921`, 0.02555944612490446`, 0.002319597164996795`, 0.002320391253520751 5`, 0.0003423389784826813 6`, 2.573081763317247`, 0.004510540050162204`, 0.37682701982389005`, 0.000032874515341396 74`, 0.000032946633976452376`, 0.002193755080696968 6`, 0.23736207906474466`},
{0.06405507608053002`, 1.3528358541887275`, 2.745226236199782`, 1.0639243234460365`, 0.36966523495796855`, 0.3262944894947615`, 0.12612633222162115`, 59.71942118372549`, 0.2416255104540047`, 1.4175051543133999`, 257.6066767311101`, 257.9706452479735`, 0.0014128846405767614`, 831.7579545196871`, 94.8780447664212`, 7.964527315470293`, 8.15023656339132`, 0.0233170457662073`, 0.438542587868711`, 0.44072024264240417`, 0.004965663162331602`, 153.92425877191005`, 0.4012982690068265`, 5.21083329346719`, 0.004885041777308641`, 0.00503115757710555`, 0.02991085981610775`, 0.15918045075171786`},
{0.20954125488723696`, 2.353113320872523`, 8.807677395871629`, 1.2953396401945112`, 0.754037675145256`, 0.6760987806408354`, 0.0257504108557459`, 115.6397082887867`, 0.08726278776335833`, 1.3938676616131378`, 340.58623007342686`, 335.8587829428483`, -0.01388032372758996`, 1118.3301606948253`, 248.98449275496952`, 2.618782658132704`, 2.6460309328431664`, 0.010404939343034991`, 0.22388885341663406`, 0.22501095281419686`, 0.005011859145460429`, 88.03274796174317`, 0.6701993042882863`,

2.5840867751020173`, 0.002056464172436767`, 0.0021084229568887236`, 0.02526607812981685`, 0.12016907977308147`},
{0.12907099769038483`, 2.604139615282878`, 9.661547795493739`, 1.0824039436985886`, 0.5892380332813292`, 0.2973399722037613`,
0.047844179127263436`, 189.25086236267023`, 0.09074457722059731`, 6.471849996577652`, 471.67700941520786`, 469.51896152472466`,
-0.004575266225417263`, 333.5652189940726`, 282.9736149868942`, 4.935530920196`, 5.01279151767355`, 0.015653958758804043`,
0.051235719389609594`, 0.051244574776682324`, 0.00017283620056929294`, 183.61159416765648`, 0.09447207741433394`,
7.225556263773162`, 0.0005718294412868641`, 0.0005733015965085863`, 0.002574465593113251`, 0.45652662453150816`},
{0.2477279255184573`, 2.8408395942947395`, 2.128090335748766`, 1.4989746812981888`, 0.49854461135120975`,
0.5556994499234343`, 0.04795352432490085`, 88.91629411642595`, 0.17336384521962678`, 2.1291108959892835`, 400.05661096680114`,
404.83461632418573`, 0.0119433230857946`, 859.9786385430717`, 237.32844038603253`, 3.824257774592706`, 3.911207230830743`,
0.022736295867842538`, 0.813763838824454`, 0.8231434779640793`, 0.011526242248826035`, 155.20147006932095`, 2.8798861093416437`,
5.370735291343376`, 0.006339026203089015`, 0.006710373625813666`, 0.05858114650854307`, 0.2904548908626055`},
{0.17138396941106926`, 2.571547212239655`, 8.060362653989774`, 1.424545143219825`, 0.3585647265624352`, 0.4155041928809974`,
0.0058496443892025185`, 1444.9053577030656`, 0.14315469959013916`, 8.6363710850225`, 74.42029622367988`, 75.77900703185838`,
0.018257261488112242`, 39.438881919770374`, 68.67948879248293`, 0.14928217442620215`, 0.1520922110335909`, 0.018823658070293625`,
0.009549947382547419`, 0.009556801108281738`, 0.0007176715703003378`, 5.484087992611056`, 0.023381541286972646`,
1.3174456652364768`, 0.00008092207766219328`, 0.00008124920305045877`, 0.004042473917081901`, 1.008456036899752`},
{0.046024454977220214`, 1.8018961949102152`, 1.8889771640586375`, 1.2101342547143499`, 0.33571961358008684`, 0.4325808303075249`,
0.005213559292121167`, 454.61857984803544`, 0.13129751322022398`, 8.467405558196546`, 231.27392570944016`, 235.38305322105828`,
0.017767361794085668`, 125.00886311956359`, 215.12046502209208`, 0.58809527302247`, 0.5994888731550743`, 0.019373731868388866`,
0.10950960448855059`, 0.10976516937746884`, 0.0023337212303142785`, 15.138380495769596`, 0.07200171230507767`,
4.137644548659023`, 0.0010853348862187584`, 0.0010989127073776496`, 0.012510259580980954`, 1.0632482251819955`},
{0.15552548139256261`, 1.3158122598456092`, 2.9803095987090753`, 0.8576161156930884`, 0.19622947720693906`, 0.41449590928153224`,
0.03892742585126438`, 2129.9557657820133`, 0.1528564450859799`, 8.904626117418289`, 44.77430620558069`, 45.478931981401686`,
0.015737279603746712`, 23.013234900921184`, 29.07003696497524`, 0.791661213044013`, 0.8082411380322307`, 0.020943207416296516`,
0.009067644567244694`, 0.009077873405077817`, 0.0011280589746618386`, 14.881107568107984`, 0.02014642552024444`,
0.9400821364821629`, 0.0001274538274066428`, 0.00012822283843121824`, 0.006033644027981344`, 1.2405296092535465`},
{0.20226341603934178`, 2.23359139800782`, 8.92211343859698`, 1.0176324123098368`, 0.17770526936314845`, 0.5466941503204505`,
0.02261985699694341`, 208.46398724865384`, 0.0356723302826476`, 4.800574849057675`, 272.0647752586762`, 278.46558224714255`,
0.023526775865712724`, 259.38422839152577`, 206.66634196871996`, 1.980676230575035`, 2.019066658669686`, 0.019382485386572057`,
0.04052059135772512`, 0.040557721511587895`, 0.0009163280351704994`, 63.20030558358492`, 0.11708333182790233`,
5.3364921292428695`, 0.0004799287619698944`, 0.00048275293473583477`, 0.005884566605986352`, 0.8359217163367221`},

{0.1612087208534252`, 3.1252528746197825`, 0.891851633192701`, 1.468881451365178`, 0.6554353298599058`,
0.19493335173588566`, 0.018954452797492544`, 551.070999166768`, 0.11474062580409572`, 7.406826770535428`,
165.61404379154953`, 162.7071253920935`, -0.017552366531879526`, 102.33624289822538`, 130.27883290082903`,
0.7563931327839611`, 0.7730961774408842`, 0.022082491145108962`, 0.22950879839378552`,
0.23013589897534495`, 0.0027323596565718145`, 33.770283036796215`, 0.5285545687667017`, 2.796196192010278`,
0.0018712644474365714`, 0.0019053990932545405`, 0.018241486853838085`, 0.4004132194115439`},
{0.2365245631846375`, 1.6915599352668007`, 9.677850457164503`, 0.7567859979545984`, 0.725618873672359`, 0.2424250415266589`,
0.36826031218846694`, 892.5435235500784`, 0.17475643237365474`, 6.493737892117458`, 108.86281477642893`, 106.4918582608181 3`,
-0.021779305637834367`, 76.72719099596509`, 18.64024824111204`, 3.5837885135803416`, 3.634987722900552`, 0.014286336687055368`,
0.008225725325254016`, 0.008227320384699303`, 0.00019391109989896904`, 86.60275808631253`, 0.027794086991871518`,
1.5095612543520913`, 0.0001314294032674912`, 0.00013164958347645166`, 0.0016752735954552733`, 0.3328309884846046`},
{0.22790261965108521`, 1.8196334440146895`, 4.716201039891137`, 1.2810805872690838`, 0.6237465626441163`, 0.5616811859186371`,
0.03042013590776336`, 637.6118251230974`, 0.08011306001688712`, 9.216145754556873`, 296.7317027267927`, 300.627681896006`,
0.013129635739664636`, 147.35982896946575`, 207.58548349916725`, 3.2925194315547417`, 3.3298507428173574`, 0.0113382204839374`,
0.054796914868677424`, 0.05486306517324325`, 0.0012071903085120184`, 85.58826389607484`, 0.1784051492481985`,
3.1204513245689074`, 0.0005150755991086831`, 0.000518842922229419`, 0.007314116854409525`, 0.8646800120593936`},
{0.10184647957852327`, 3.0801202872185955`, 8.412464860766345`, 0.9942252517730743`, 0.7984635108014702`,
0.4565736536432896`, 0.009191281945386102`, 103.82351882247931`, 0.22274711417837167`, 2.5201460619086546`, 591.688147865415`,
570.7648871115722`, -0.03536197375141936`, 1074.5624304908902`, 522.8380395339575`, 1.5107441890128588`, 1.5260279479536143`,
0.010116708739910552`, 0.17393234221007567`, 0.17407566938379448`, 0.0008240398070744615`, 66.4753403625159`, 0.25306281055631`,
5.087131467353969`, 0.002102649719220251`, 0.0021210821388023674`, 0.008766281617725635`, 0.15588431333331104`},
{0.18768732765242752`, 1.4966861207841546`, 1.129759551095228`, 1.2240080138702374`, 0.5362083693873154`,
0.686948995479657`, 0.005867661202712241`, 74.92355531972449`, 0.165025723744549`, 3.2590978632956187`, 870.490195206716`,
886.6336386954683`, 0.018545232993599337`, 1222.449639830594`, 797.0391422056507`, 2.9493742064966937`, 3.0080722377734563`,
0.019901859569893432`, 1.7857470523221757`, 1.8028685077952153`, 0.009587839134761689`, 63.06124914089119`, 4.788029887336377`,
9.834493474139014`, 0.016841471120195184`, 0.017967764756876094`, 0.06687620271665784`, 0.47420056468284566`},
{0.14949493319923252`, 0.867900380757324`, 9.54711835147964`, 0.8271983961134595`, 0.23831574478972195`, 0.375815029784779`,
0.04516845569069218`, 2094.687766706356`, 0.0279158592619283`, 5.743304871768913`, 28.82222318749643`, 29.234191605463575`,
0.014293429597265206`, 22.96836605128936`, 17.739879381413207`, 0.8264262649734856`, 0.8436659152362695`, 0.0208604820459537`,
0.0027275756204454115`, 0.0027289601837858397`, 0.000507616848475223`, 10.246509571976327`, 0.005825124788171205 5`,
0.6093496397208308`, 0.00003983756090419899`, 0.00003994909011618617`, 0.0027995994095970644`, 0.7237744581475678`},

```
{0.10785599050108607`, 1.35490623294936`, 7.749733046704847`, 1.2972878372630243`, 0.6070878493023009`, 0.6937073302769028`,
  0.28374543116039413`, 66.83074779041495`, 0.1939262594152683`, 9.425365274493942`, 3177.6609239452723`, 3263.6743014470067`,
  0.027068142121011052`, 1543.0281818079382`, 652.7601813937031`, 123.9941369314793`, 125.215284659993`, 0.009848431214038111`,
  0.3544058012708916`, 0.35420861671737175`, -0.0005563807161529377`, 2400.0061282519728`, 0.5460684105042611`,
  30.819328043744743`, 0.003288632728649543`, 0.0033062539860411987`, 0.005358232081723369`, 1.0103403402846947`},
{0.13969386881974227`, 3.165109549305347`, 4.62533417349867`, 1.0389272094346946`, 0.7061162924631639`,
  0.5973647953915502`, 0.19864442086151357`, 3440.0036010322765`, 0.16071761832429643`, 1.3360604448248414`,
  9.191933194312142`, 9.111498595296059`, -0.00875056392553586`, 31.488012374724676`, 2.4487676088607198`,
  0.14528413114753055`, 0.14706673606002899`, 0.012269784032285402`, 0.00959261683325849`,
  0.00967107983971871`, 0.00817952054419413`, 6.569145583679686`, 0.019143282250617143`, 0.08387509054885706`,
  0.00010944856663874791`, 0.00011304496655715827`, 0.032859269233564525`, 0.12582799803503655`},
{0.18701505616348296`, 1.1471835668231432`, 1.9652898147298057`, 1.007866470439728`, 0.8673024418570234`, 0.4964521059357`,
  0.11361709646832255`, 189.11029986824036`, 0.17527157137077032`, 1.608206453868661`, 258.9597560838956`, 241.9764243045456`,
  -0.06558289996939881`, 736.978759727253`, 99.10962309975461`, 9.085233414156239`, 9.215200904698284`, 0.014305355142503418`,
  0.5101311687641911`, 0.5146469777053958`, 0.008852250593007982`, 148.89186390675093`, 1.3628887025309742`,
  1.7879651100119405`, 0.00594838221607652`, 0.006226979924447838`, 0.046835878773620276`, 0.10494814554677627`},
{0.12078071855653999`, 3.7816423804380825`, 3.0527531818157634`, 1.3792123087306463`, 0.18684955879429088`,
  0.29398025647621684`, 0.011105429740027619`, 471.0240529628659`, 0.1734264666127333`, 9.864280424563177`,
  193.53315183398033`, 195.19533289033754`, 0.008588611514905198`, 89.79547541735087`, 167.39914898140037`,
  0.47002191762584694`, 0.48160711228880415`, 0.024648200921088437`, 0.05555189312504426`,
  0.05560480899779912`, 0.0009525485051562477`, 25.392211477552515`, 0.09585139384025797`, 4.589273487626514`,
  0.0004854805377267901`, 0.0004884407159876274`, 0.006097419012300653`, 1.0881211144746339`},
{0.12022941283008587`, 2.2264210098571606`, 6.2451953049416655`, 1.3438290154028227`, 0.5358944653344513`,
  0.32618163573980585`, 0.199782178892696`, 3338.755251465298`, 0.1788085018354036`, 4.512919273292992`,
  18.09326458894188`, 18.07935406327192`, -0.0007688234260644355`, 18.34948432904258`, 4.900288943646175`,
  0.40170344389260004`, 0.4084199521547449`, 0.01672006641780399`, 0.0054043573859994115`,
  0.005411621181734325`, 0.0013440628026806234`, 12.77658553163515`, 0.00928232450346558`, 0.2886889629049606`,
  0.000048457632112031135`, 0.00004878956398170497`, 0.006849939941481864`, 0.37064713513131314`},
{0.04681963491488872`, 2.902738250264786`, 8.529727895043578`, 1.3985345888875074`, 0.35876551940164103`, 0.2520539039703089`,
  0.05124631524713002`, 163.50426816944002`, 0.24665810410586203`, 8.118758344754049`, 498.1205543718326`, 500.3693224687036`,
  0.004514505729856566`, 280.80791354249084`, 291.88102956562267`, 4.85371244189814`, 4.957582229563156`, 0.021400070339642197`,
```

0.06312994827774192`, 0.06314139519892933`, 0.00018132315168473134`, 201.27223944119822`, 0.0422245875791506`,
10.620183001724588`, 0.0005453395931630522`, 0.0005466675098508516`, 0.0024350270995310286`, 0.6792309204931237`},
{0.1911559295611578`, 2.175586072104755`, 6.8117639016300915`, 1.0586708066957606`, 0.34517099105389626`,
0.196211531611845`, 0.1489787018319715`, 3704.576077864889`, 0.10408111144660032`, 4.8300445149655875`,
11.584929553161782`, 11.56950819460474`, -0.00133115686947205`, 10.977583736178905`, 3.8837355484573286`,
0.2397918306669244`, 0.2456880537679589`, 0.024588924003939416`, 0.0023372329778607375`,
0.002338511986102822`, 0.0005472318139438936`, 7.452682385763757`, 0.006382513464054624`, 0.2760229213369669`,
0.000026669930705880418`, 0.000026760626058597072`, 0.00340065948115312`, 0.35086490342610716`},
{0.17192712556630163`, 3.7260903741599414`, 4.118781130521407`, 1.2245874304759414`, 0.6062180312792598`, 0.5331783629983038`,
0.1203111914746069`, 226.51013650082294`, 0.017839169449302028`, 7.150760371208259`, 609.0762276936674`, 615.8922390440637`,
0.011190736135287649`, 389.8377746718134`, 228.0558464303901`, 7.015913435561706`, 7.1002697020243355`, 0.01202356147027861`,
0.15865896036088384`, 0.15884445475360282`, 0.0011691390911490185`, 373.45610740265585`, 0.38968255714474964`,
6.826100487746484`, 0.0015571744855087388`, 0.0015716806360640786`, 0.00931568728510257`, 0.6832231262472195`},
{0.1575695043509842`, 3.8758817205894927`, 3.3547063374262347`, 1.3334178116995439`, 0.4921394422884451`, 0.402330971011554`,
0.12204131883407823`, 201.57298534982124`, 0.22899817129952033`, 2.4884430439924596`, 171.47976959974272`, 172.48822024094045`,
0.005880872382506563`, 315.39127950364815`, 63.96752445681919`, 1.8974298686409263`, 1.9332102127216484`, 0.018857268282780115`,
0.17059848871879818`, 0.17139833011293557`, 0.004688443608992232`, 105.0601963423698`, 0.38401599014923776`,
2.693377635167728`, 0.001524888626118548`, 0.0015611486919703287`, 0.023778828978531674`, 0.2623119546899794`},
{0.06573776532431397`, 2.613579102769201`, 3.806271127858438`, 1.1314163600990936`, 0.4483602258230772`, 0.6823320235871475`,
0.18768855278686034`, 189.85674956999534`, 0.0631967400861071`, 1.3499467761035964`, 122.60348273844141`, 125.59922710335557`,
0.024434414895906365`, 415.6718896463756`, 34.3511538065577`, 2.293571239856638`, 2.3293197282807196`, 0.015586386767875604`,
0.1671101972021159`, 0.169032204440175`, 0.011501435999949683`, 85.63471233145349`, 0.15693501324247797`,
1.6297026706773823`, 0.0017348101343656053`, 0.0018134268124828494`, 0.04531716558480503`, 0.2177308207861667`},
{0.06757463956251891`, 2.3005441553217736`, 6.868151131987258`, 1.3860241166029201`, 0.025379963517129678`, 0.6856836713524472`,
0.11835507299142825`, 3863.192203814942`, 0.1108156231672533`, 2.823244077255117`, 8.591053953203659`, 8.680870278018249`,
0.010454634006936692`, 13.927158411775737`, 3.2902278807014262`, 0.1563059464568660 7`, 0.1594064878810817`, 0.01983636256008503`,
0.0038354050280604364`, 0.003851849218301135`, 0.004287471628260819`, 5.136981879476881`, 0.003702515890678237`,
0.17870787206219277`, 0.00003308039613270086`, 0.000033672221523728965`, 0.01789051704985689`, 0.6746957381740626`},
{0.06632069369203508`, 0.7568152865276287`, 0.22726671156421221`, 1.362987063673106`, 0.9228910129465389`,
0.1868567265073786`, 0.1296811990167267`, 123.29501155053357`, 0.19348527358813117`, 9.470702921306387`, 1336.569957639109`,
1300.1638755330707`, -0.027238441129071256`, 645.9129264640212`, 475.08005910037116`, 72.05293898052093`, 73.48878703578215`,

0.019927682001276814`, 5.353699649685919`, 5.272773835114139`, -0.015115867506039105`, 779.0109379957239`, 5.072301065513682`,
15.807233790277968`, 0.04560839231674063`, 0.04719404971383378`, 0.034766789982008106`, 0.40399264831546416`},
{0.1352416913789251`, 3.40347330811998`, 4.693938060979022`, 1.2778213334701187`, 0.7052646641955478`,
0.6043325609148968`, 0.013064813980624626`, 333.49561209931903`, 0.11153764285740131`,
8.005884287110216`, 594.2033976771594`, 594.3122039880631`, 0.00018311290599992702`, 339.69590905142246`,
500.9682922494878`, 1.8652380583563968`, 1.8834553913332317`, 0.009766760277713615`, 0.12661431478158863`,
0.12676811394308124`, 0.0012147059497806811`, 90.68982778436475`, 0.24462191548350595`, 5.130168499748635`,
0.0011915824135697584`, 0.0012017165983254 8`, 0.008504812290206054`, 0.6626183794708759`},
{0.11675242158451127`, 2.833886804623358`, 0.47584343623935504`, 1.0607616820427617`, 0.7666611109645796`,
0.4737758025893787`, 0.1681420982868212`, 120.33220123866614`, 0.17915094289032796`, 3.5814785888826783`, 722.7508505594438`,
690.8775746266773`, -0.04409994939209705`, 923.6145097144872`, 214.72414271012977`, 12.067356816698242`, 12.301193640610043`,
0.01937763401412229`, 2.782460863708234`, 2.7951107021998483`, 0.004546277238471452`, 488.53604642175554`, 4.640843482887171`,
6.391067191104353`, 0.030026830242623093`, 0.03223114704513977`, 0.07341157174118385`, 0.31504740785766583`},
{0.24323887781179143`, 3.4699698183729026`, 8.39097272098094`, 1.1938324247391086`, 0.7463001660827029`, 0.6306644967356683`,
0.2992521819376612`, 322.6100644812281`, 0.10289378547186823`, 7.076414822100832`, 606.0894189561442`, 599.8431189919705`,
-0.010305904984996395`, 392.0016665575945`, 118.56753382332995`, 9.633584786040766`, 9.712911650011137`, 0.008234407620029138`,
0.0764036348139217`, 0.07645966988711256`, 0.0007334084736585478`, 477.54640643282676`, 0.2654904913262156`,
4.657921990438285`, 0.0007713335204214689`, 0.0007757552114173055`, 0.005732527990512493`, 0.5372272482305175`},
{0.24390696598302103`, 0.8206355382610386`, 2.839705408503791`, 1.0872176805395473`, 0.8466198387041834`, 0.5785697848880403`,
0.1356337036504287`, 2343.8028799398503`, 0.22246042363346175`, 4.459217822696726`, 62.64064321486061`, 59.539001572637815`,
-0.04951484344731272`, 64.29275203843048`, 21.533437483193623`, 3.219019430317975`, 3.2473384244693566`, 0.00879739770585486`,
0.033548979448366475`, 0.0336833599734267`, 0.004005502619447654`, 37.73773918388189`, 0.11689756841534037`,
0.3981641950149825`, 0.00036903135024191 07`, 0.00037595669669194924`, 0.018766282174939253`, 0.2690886722598613`},
{0.06769491456810739`, 3.362305338073525`, 1.8871867183871915`, 1.4803795481758868`, 0.5363168854717104`,
0.37458837250617005`, 0.011183256466341666`, 407.888606379481`, 0.051902827430772014`,
9.719761204172809`, 348.82759282500564`, 350.39535508955447`, 0.004494375722551 5135`, 164.25542807444154`,
300.98145245535846`, 0.9643370985623779`, 0.9799970581853334`, 0.016239092788508547`, 0.17622444422687691`,
0.17656474295961863`, 0.0019310529491789463`, 46.319939631408864`, 0.17042140995356822`, 5.11817276192237`,
0.0014284180683821202`, 0.0014452865350073631`, 0.011809194379870158`, 0.8576368625137833`},
{0.21105892687940375`, 0.7047276416914965`, 5.768010388957135`, 0.810198720595394`, 0.17857267890229922`, 0.19525448649651245`,
0.036176279358565294`, 67.82203673017167`, 0.07478241594027568`, 5.650997388906681`, 650.0519947084391`, 652.2051495507056`,

0.0033122809556676014`, 526.4867954221254`, 434.13082614704877`, 19.46867054046608`, 20.012137573368246`, 0.027914953502991446`,
0.1014098605808796`, 0.10137705104393016`, -0.0003235339912853741`, 196.00157538359122`, 0.30576366213096484`,
17.812846812445674`, 0.0015115689155602574`, 0.0015156905643293579`, 0.0027267355968172424`, 0.47006060272497674`},
{0.13030085884608883`, 3.691040694570585`, 3.5546925177950874`, 1.392417125140739`, 0.5227226143537942`, 0.3153665172319682`,
0.008712610924073824`, 695.2883528301467`, 0.04802018879975545`, 9.078480195703946`, 172.35955351376717`, 172.6905156269788 2`,
0.0019201843266856145`, 86.89338274287356`, 153.34651530643035`, 0.3489106613049575`, 0.3550825825598893`, 0.0176891162678956`,
0.046605858433885844`, 0.046652637739554595`, 0.0010037215757994211`, 18.397763566373303`, 0.08675404830276925`,
2.7833644426062176`, 0.0004034351655610724`, 0.0004059137492233271`, 0.006143697609521936 4`, 0.7267402569976734`},
{0.21250061023000932`, 2.8149517595358526`, 2.295631419336827`, 1.0543549080196901`, 0.6519014079329188`, 0.6534060058028184`,
0.09088447710322001`, 650.6798329466749`, 0.04016188412840693`, 1.3861729584655489`, 48.79115942221823`, 48.81362755101295`,
0.00046049589845331695`, 161.0972810775264`, 21.315401809594352`, 0.6569690733107841`, 0.6687690903851393`,
0.0179612976527026`, 0.09903496856714007`, 0.100647025253115`, 0.016277651311435992`, 26.419089269669044`, 0.3006427322089596`,
0.4704667174658382`, 0.001095883301274081`, 0.0011652930282771514`, 0.06333678679324195`, 0.16485128184792214`},
{0.14347475236734758`, 3.69539554202769`, 4.673626861854084`, 0.8876695712812951`, 0.055199727721453984`, 0.3291461786147223`,
0.031312636638690675`, 836.9629099625735`, 0.07687709551472927`, 5.335882334514919`, 54.81422858509442`, 55.206737317354595`,
0.007160708859577092`, 47.016636502986174`, 38.25753794465625`, 0.3069993586893299`, 0.3149464829526289`, 0.0258864523275472 7`,
0.012229103371933638`, 0.012240852461395963`, 0.0009607482335367568`, 16.206915164370148`, 0.025065251113751193`,
1.4301147697465306`, 0.0001661488952467982`, 0.00016705005347677243`, 0.005423799108839278`, 0.7286796307718957`},
{0.0805761857877269`, 3.094619489710217`, 9.945877040804334`, 1.11372690786775`, 0.6430409751953907`, 0.42229007304684385`,
0.05506378209236767`, 302.8456666998151`, 0.050941087889112335`, 7.632080993355595`, 453.0392128181804`, 452.27432678171004`,
-0.0016883439994349603`, 271.67978077565823`, 255.84411431642866`, 4.359768777130652`, 4.412114915603321`, 0.01200663180746031`,
0.04170844515014224`, 0.04172204428657111`, 0.0003260523469506005`, 192.74036326198038`, 0.04801010607634015`,
5.3607508313214645`, 0.0004522731378607814`, 0.0004535898522456727 5`, 0.0029113256452049097`, 0.593551815894808`},
{0.26865716137877294`, 2.5539808428845117`, 7.725254140854183`, 1.421576189719117`, 0.2484256166411507`,
0.1545757521332899`, 0.008556741498662016`, 1177.1024036480305`, 0.08015420001442447`, 4.125195480316323`,
26.42240657107209`, 26.447305840484844`, 0.0009423543364901477`, 29.31516428363904`, 23.57245363475408`,
0.07452235809814851`, 0.07677763002269804`, 0.030263024172950237`, 0.007385296478279963`,
0.007389590298454349 5`, 0.0005814011918159334`, 2.7189810707035806`, 0.028344468397078916`, 0.7380441599908404`,
0.00006275195740756967`, 0.00006301278206003407`, 0.004156438511875526 5`, 0.27648919794782306`},
{0.05207035897383483`, 1.5461440288878858`, 9.695008671418819`, 0.9618476339297223`, 0.8208653770038725`, 0.48583250041037973`,
0.22651174945218666`, 56.560953214157614`, 0.1695178841867513`, 7.246656846935364`, 3529.9317214751736`, 3394.4323698820094`,

```
-0.03838582790959433`, 2229.42621310487`, 853.5026469935897`, 115.90115289686223`, 116.79738252606619`, 0.007732706766096609`,
 0.3037505816076209`, 0.3033505392319683`, -0.0013170094145511513`, 2559.9982213243607`, 0.2259485974680158`,
 26.713396094077577`, 0.003806908472748005`, 0.0038185105859222554`, 0.0030476469968492292`, 0.40874789718443566`},
{0.09188932948313594`, 3.928289836924776`, 1.4719659367359732`, 1.3955743442040527`, 0.5432280807299197`,
 0.32698938665455834`, 0.013007122973082902`, 2762.880112969401`, 0.13947463148509326`, 4.274432647648432`,
 20.988697522594453`, 20.91294882491946`, -0.003609023265662259`, 22.473540926874733`, 17.685050832074374`,
 0.05647410096950544`, 0.05758046138506891`, 0.01959058040004691`, 0.028959091535921046`,
 0.029133255145123323`, 0.006014125442652229 5`, 3.1692376698281732`, 0.0380147357668094 2`, 0.33189699445859056`,
 0.00024724854331070745`, 0.00025352619755289 63`, 0.025390055521176347`, 0.3716581547745721`},
{0.04429611977972214`, 3.507177302954225`, 7.234549676890296`, 0.7623401767303408`, 0.010148640019661537`,
 0.662687845406686`, 0.08775344792279212`, 3965.4644576584396`, 0.17480873042969614`, 5.118348118050843`,
 14.83985783839336`, 15.035708857056138`, 0.013197634424507276`, 13.269801141305033`, 6.739243630217131`,
 0.15845550852248996`, 0.16153723439980264`, 0.019448524737625483`, 0.001915337563942278`,
 0.00191764739267418 3`, 0.0012059643038331291`, 7.939022328830672`, 0.0012120288878525007`, 0.3131871438266558`,
 0.000030283090671434643`, 0.000030458043959740503`, 0.005777259996480044`, 1.1741098193122215`},
{0.14748247662042147`, 2.024427515527907`, 9.506929645646245`, 1.2214601071407813`, 0.2054881053340767`, 0.2020185622482833`,
 0.07806490597707526`, 302.23580657852216`, 0.2425913704635762`, 6.045088630499148`, 161.47224147319793`, 161.87784335873357`,
 0.0025118985271717076`, 122.25304806607139`, 78.48555644333071`, 2.771338978665118`, 2.8453407582321164`, 0.026702536260159393`,
 0.02153064379079933`, 0.021536611718985174`, 0.0002771829882948218`, 80.14821261806662`, 0.045362752421389534`,
 4.275692512975166`, 0.00021304515729048124`, 0.00021353472673247496`, 0.002297960902843732`, 0.502399405561023`},
{0.05191368478403385`, 1.8292747620439185`, 5.888828307542417`, 1.141375509610469`, 0.9869968200599144`, 0.28747330669734017`,
 0.24632331590197062`, 4082.2304223282904`, 0.0447948152020462`, 8.998917183247265`, 57.78999834240845`, 57.23017728004768`,
 -0.009687161765324914`, 29.391849937388933`, 13.13564459138007`, 1.645287384414426`, 1.6585831250576073`, 0.008081105324899474`,
 0.007808436063798547`, 0.007812373416104046`, 0.0005042433943658331`, 42.995466980265206`, 0.005790924121014807`,
 0.4498385095513972`, 0.0000826419286054092`, 0.00008287950085933788`, 0.002874718171970825`, 0.3052280251354012`},
{0.22928260338606776`, 2.222139650206395`, 4.336407604983492`, 1.4545262441104416`, 0.17670484975018264`, 0.15215120204412502`,
 0.00931582374755147`, 107.9598634817244`, 0.18384173269782222`, 2.0618261552271964`, 133.72068106965185`, 133.73819092118197`,
 0.0001309434815173205`, 296.83221305105087`, 117.87770215606176`, 0.4631186259036406`, 0.47964174054979675`,
 0.03567793157512522`, 0.13576162298572414`, 0.1359780782066122`, 0.0015943770863053874`, 14.701632305279743`, 0.444682547972629`,
 4.046477735473635`, 0.0011222628145830527`, 0.001136658568720541`, 0.012827435740029092`, 0.15158357755046387`},
{0.13057179539780978`, 1.32287055831243`, 2.838629919542937`, 0.8498204532586893`, 0.8949438289681946`, 0.46229782752490833`,
```

0.06831034580553909`, 355.6715560557054`, 0.046330937271765626`, 4.510601406855825`, 399.3800774852029`, 375.2547588267408`,
-0.0604069156638289 4`, 405.2438573206611`, 203.1806888113814`, 9.836210135692294`, 9.913008875138049`, 0.007807757092040735`,
0.16597405734534024`, 0.16621695188314234`, 0.0014634488165625736`, 185.8861827697378`, 0.3095932951002379`,
2.624471594035306`, 0.0023424697071411 37`, 0.00237059171461679 3`, 0.012005281173935511`, 0.21887806556267247`},
{0.16617324858328203`, 1.7665378156673919`, 1.5537022391595983`, 1.3738499573440288`, 0.09625830296842142`, 0.5757224393125404`,
0.011187699121044243`, 1140.5955024024072`, 0.09128758443232887`, 2.6133507581824666`, 26.19008115585315`, 26.34546496888898`,
0.005932925984885706`, 45.86734850051569`, 22.478244675736352`, 0.1338694520662141`, 0.1375579051758527`, 0.02755261228539463`,
0.05437538550661492`, 0.05523729384334865`, 0.015851075421412286`, 3.3783635633948585`, 0.12908192075146635`,
0.5503145304497479`, 0.0004624005955113919`, 0.0004902131320476287`, 0.06014814168973448`, 0.5669263863950961`},
{0.150610971367461`, 1.9051770945291775`, 1.9970914506574022`, 1.332029884280907`, 0.017627751946745374`,
0.6342635790195508`, 0.03224365795250487`, 84.16237057378729`, 0.025181518731154096`, 6.093120594420567`, 824.1342761336732`,
833.1324303485147`, 0.0109183108571278`, 619.0456810632252`, 567.9321574866569`, 9.014168100215377`, 9.210811682118727`,
0.021814945063943547`, 0.5631665446297395`, 0.565142270442214`, 0.003508244286374529`, 245.33695129665577`, 1.2117008618333929`,
17.53335618988513`, 0.005021419214132905`, 0.005147233179694131`, 0.025055459461962526`, 1.3835000999261238`},
{0.170193722625894`, 0.42908710938865147`, 8.009796665724636`, 0.9108642019460496`, 0.48256370773751156`, 0.1504864882770205`,
0.015501266854459406`, 231.18780020236528`, 0.059845835174358764`, 2.951447491236335`, 113.34685848069803`, 112.77890415115328`,
-0.0050107637490584`, 175.76763986519728`, 93.15628414683346`, 2.811807906346337`, 2.8822113093194455`, 0.02503848247037288`,
0.027385732602652316`, 0.02739488125320234`, 0.0003340663068160943`, 17.235864667004403`, 0.06658399683543738`,
2.6568183197326043`, 0.0003632190380471467`, 0.0003643900649688943`, 0.003224024071104 7807`, 0.16062068967091647`},
{0.14155286072340578`, 2.768731185287475`, 2.866796585793665`, 0.9268420036617817`, 0.38841014261523243`,
0.6739227770046636`, 0.08018336457681292`, 85.63532436040691`, 0.10664760400309609`, 4.590937221386067`, 869.9112353369064`,
900.1770672216628`, 0.0347918622674585 6`, 867.2375634893231`, 411.13271718876365`, 11.284373889703113`, 11.452786303057728`,
0.014924391463871034`, 0.3834475469900565`, 0.3840835411926363`, 0.0016586211271194529`, 446.3342556409238`, 0.7754013887691785`,
12.254042157312384`, 0.004935435154092671`, 0.005023313693866414`, 0.01780563152589898`, 0.725915745609645`},
{0.17254585725985527`, 2.4767354176461422`, 1.5265879737476418`, 1.3533294324013416`, 0.010476993931212819`,
0.3024537953894164`, 0.005382899126762437`, 1067.886819263142`, 0.025600178888030733`, 4.688973100729083`,
35.51315380947588`, 35.58280659174881`, 0.0019613234759889853`, 34.663786062153925`, 32.87444315951891`,
0.06753132743423404`, 0.06970226001018769`, 0.032147044319065454`, 0.04178027718691432`,
0.04197917705538841`, 0.00476061629711699 3`, 2.3893890065289405`, 0.102985910625294`, 0.98636178837421`,
0.00036870778002129345`, 0.00037705374313316923`, 0.02263571197600922`, 0.6478621812744261`},
{0.12080218784009655`, 2.1868434076988192`, 8.77616791832854`, 0.9616886370191489`, 0.26020855267635734`, 0.6201783502287406`,

```
0.03811904516021991`, 117.06014479510438`, 0.17593156125676795`, 4.349098586723594`, 502.5864919512881`, 518.3770138412615`,
 0.03141851630087955`, 528.9030287705898`, 328.06780345421157`, 5.405449869365983`, 5.496006496223341`, 0.01675283816256723`,
 0.07939442934563574`, 0.07946521855812037`, 0.0008916143496220386`, 168.8696058924207`, 0.13701458238954375`,
 8.60459810758487`, 0.0009940851411641694`, 0.0010007376228364222`, 0.006692064287835642`, 0.7543292828564867`},
{0.06629025039264891`, 1.530609759106781`, 1.900084106095834`, 1.2729265107514505`, 0.009336607275947317`,
 0.20692029297881853`, 0.07757288734346537`, 56.094679581136695`, 0.10338780030561462`, 4.34093728430811`, 546.7001309895655`,
 545.7271039866157`, -0.001779818492431251`, 576.4082142387616`, 267.25708513969465`, 12.175933366155204`, 12.567970120292854`,
 0.03219767572212362`, 0.5285408325870575`, 0.5286897105942924`, 0.00028167739946627357`, 266.2371776638712`, 0.5005300590712404`,
 16.834033072641677`, 0.004973501524652146`, 0.005037399951468784`, 0.012847774651302046`, 0.4435144857835872`},
{0.11054921486908309`, 1.9601623754484976`, 4.1405236153603`, 1.0982919546164496`, 0.2371613718862391`, 0.5811399077338627`,
 0.014126785714663546`, 63.169894546693705`, 0.15558848051416968`, 8.651696062244092`, 1781.7142493917843`, 1831.4724281805798`,
 0.027927137477730346`, 942.5432636933733`, 1486.043976450488`, 10.135081570468875`, 10.316072369498377`, 0.01785785321717226`,
 0.3428761500755158`, 0.34277327419121506`, -0.00030003802911948707`, 283.80579380763726`, 0.541495559831028`,
 31.560348634803`, 0.0037518180213146346`, 0.0037800289462379816`, 0.007519267929061657`, 1.4495343406832475`},
{0.09844925238529889`, 2.9547765664281824`, 3.97533240344332`, 0.8901779978265776`, 0.6485331039194935`, 0.172046787329591`,
 0.2843834249851245`, 265.56383421685405`, 0.07856132944640065`, 2.1823933339229455`, 90.29532280430436`, 88.7619002689460 9`,
 -0.016982303044440483`, 189.3636966985127`, 19.16364029918861`, 1.6429134184240293`, 1.6768757118781619`, 0.020671992250639093`,
 0.05801014935191304`, 0.05807458593448432`, 0.0011107811872779205`, 69.3491724204249`, 0.08158651192088168`,
 1.7048407880188647`, 0.0007847959842084418`, 0.0007909925117204678`, 0.007895717660018287`, 0.11150988614795235`},
{0.07379407071323735`, 1.0020834251379886`, 3.8959201685323244`, 1.3275435329440872`, 0.5495795554378471`, 0.5203295123605114`,
 0.006236204073249997`, 495.6126936444482`, 0.05339932410900344`, 4.300380134475608`, 149.76917839032532`, 152.23984495875848`,
 0.016496495440431413`, 159.39698227478078`, 137.39659740723377`, 0.7884139709042084`, 0.7999281134195154`, 0.014604183766685042`,
 0.07418545065335498`, 0.07442758492563899`, 0.003263905120903354 3`, 11.286522462719024`, 0.07820637702010666`,
 1.8969643415224537`, 0.000669115106250228 7`, 0.0006793899895454047`, 0.015355927850376982`, 0.463095208908063`},
{0.137830658132162`, 0.964244066849937`, 5.678313655870262`, 1.056362681888576`, 0.27898051740088525`, 0.6674603903550571`,
 0.022240144180942223`, 3206.233069292432`, 0.13749704640301397`, 7.378694929535063`, 33.320063915531485`, 34.5025438872988`,
 0.03548852651558465`, 20.66763416570333`, 25.388929824007192`, 0.5352197042929275`, 0.54374897707348`, 0.015936021622784624`,
 0.00526383993954674`, 0.005271558961467869`, 0.001466424133290456`, 7.372606061794757`, 0.010364550330989347`,
 0.5336874925797369`, 0.00006000986045429091`, 0.0000604394474101088`, 0.007158606145153623`, 1.291012607504407`},
{0.09412349267697989`, 2.412142947637907`, 8.593900106949853`, 1.0545990130363978`, 0.8443965196041454`, 0.4441557500447094`,
 0.020813982545198062`, 67.68562305269263`, 0.21037364591803787`, 1.9282456472076213`, 749.2217813574797`, 712.49275099315 8`,
```

-0.04902290787351926`, 1778.330715325724`, 578.1714670169473`, 4.798654581306001`, 4.842661465922547`, 0.00917067146028438 5`,
0.29883827147454795`, 0.2990622700827657`, 0.0007495646628943309`, 165.35772580639428`, 0.4018243122392151`,
5.937507270823116`, 0.003398455995647587`, 0.003436143765233560 6`, 0.01108967414444706`, 0.10796600741984742`},
{0.059604065321211375`, 2.060924190389435`, 4.151577648882975`, 1.0218595307135387`, 0.8259153199816331`, 0.67511248087556`,
0.012173840559063499`, 163.32402156550611`, 0.22896057989768043`, 4.41755081858776`, 940.4603143852846`, 902.5716181338538`,
-0.040287395089282585`, 974.3689014576614`, 801.0032154204682`, 4.543698777599931`, 4.576827442297895`, 0.007291122567650188`,
0.32832551118075637`, 0.3287599814814873`, 0.0013232913250280642`, 133.77455320855145`, 0.2795647887862164`,
5.689332668679504`, 0.003844174772881348`, 0.003897259701128423 7`, 0.013809186986388333`, 0.29041961920740506`},
{0.062303828390692484`, 3.237012523552033`, 1.9717708145626371`, 0.9726411613979222`, 0.1873520229034391`, 0.5560570337810927`,
0.026607710207102397`, 62.963998927305475`, 0.022649002664224704`, 8.659052494024476`, 1678.2937049346401`, 1715.5120279343114`,
0.022176287076713264`, 887.0785701301471`, 1223.2076962838603`, 9.603421270133591`, 9.784212564709305`, 0.018825717365744454`,
0.6003743030399891`, 0.5998235435523847`, -0.0009173601948245791`, 444.0913560052627`, 0.5343659649544724`,
31.83868106042453`, 0.007379783427235109`, 0.007470587405365161`, 0.0123044231616527`, 1.4980469529945941`},
{0.058207327156306`, 1.429925878818433`, 8.458349879741764`, 1.1245084153106533`, 0.19874114947102073`, 0.3371100826465334`,
0.04401493286992449`, 375.7204818983131`, 0.2159694260931322`, 8.593637256078832`, 224.34400261113166`, 226.9371167094659`,
0.011558651304037992`, 119.48185067819591`, 139.5646571706205`, 3.9543657879127982`, 4.041857647070449`, 0.02212538339904846`,
0.021777544889139848`, 0.02178514402741297`, 0.00034894375430316593`, 80.77785677786807`, 0.018108752571650604`,
5.055375244986755`, 0.00023398455277967578`, 0.00023456617861666604`, 0.002485744593310635`, 1.030030062585992`},
{0.0526423548412821`, 1.6513512325831732`, 4.083783641771872`, 1.373155154639566`, 0.8939370398042139`, 0.2364887781328291`,
0.0490949664593259`, 1618.3201075393572`, 0.07451928539997066`, 4.7651781050102695`, 55.8848688369167`, 53.94951629750541`,
-0.03463106525415738`, 53.675931874090296`, 33.06970580503671`, 0.9259892689757737`, 0.9368419779749005`, 0.011720123939590321`,
0.02470436817903139`, 0.02473763698853467`, 0.0013466772055121812`, 21.84476458117052`, 0.018578515940146208`,
0.6043498126230342`, 0.00021674707962349 16`, 0.00021825588636036728`, 0.006961139866320742 4`, 0.1901812616842048`},
{0.21322884859448837`, 1.5615738832323176`, 8.40996483053398`, 1.2929614343326012`, 0.3765546169235392`, 0.671739728089668`,
0.1608794324574438`, 485.1832614690647`, 0.0705178455433636`, 2.1513131242884285`, 69.85213890189914`, 72.10339317700169`,
0.032228852408717756`, 148.60744824164973`, 21.812176710118795`, 2.0556671614611024`, 2.0882682396611005`, 0.01585912292183833`,
0.031156808496979244`, 0.0312826101752621`, 0.00403769462764636`, 45.85823074222827`, 0.09490757716702199`,
1.0184228329634275`, 0.00028812014492407556`, 0.0002934239065808394`, 0.018408159756276188`, 0.3522368824138316`},
{0.11682922382752631`, 0.5721136495795571`, 9.687079392836907`, 0.91254998083727`, 0.43543762812336806`, 0.4740169657662334`,
0.10620954791167687`, 4372.038997558794`, 0.0729116423313943`, 5.645356891940546`, 18.442375732598265`, 18.820688243631256`,
0.0205132200166757 7`, 14.951678768882385`, 7.489732565789462`, 1.1934401955163036`, 1.211817241252478`, 0.015398380082400465`,

0.001930146786388270`, 0.0019314835947351673`, 0.0006925941365309995`, 9.754048940168143`, 0.003221393584666152`, 0.2844921185679904`, 0.000025536099634293485`, 0.000025629857781409194`, 0.0036715923127819128`, 0.6566170025714902`},
{0.26956767736567855`, 0.8444303847788461`, 1.0020073721955711`, 0.7502641425367418`, 0.966405616850982`, 0.6715103286393609`, 0.23328928938153737`, 246.8438332486948`, 0.10178810353613177`, 2.531792031152129`, 523.9275398326437`, 492.8971860424036`, -0.05922642241740528`, 947.1258575383188`, 121.49165748513728`, 30.45071934413862`, 30.850083630561556`, 0.013115101876889135`, 0.9584208872345833`, 0.9640855777365936`, 0.005910441411972167`, 367.33589503662387`, 3.69084703586524`, 2.139021721148948`, 0.014905554174649227`, 0.01569588739179399`, 0.053022732860810384`, 0.14342986317105225`},
{0.07372356616920961`, 2.107710372997479`, 6.08076458707294`, 0.8379230467338923`, 0.9571077593283461`, 0.37371168094765217`, 0.1939664465355383`, 131.4329681577719`, 0.026286944156709513`, 5.373906305884013`, 1241.669927614601`, 1197.247467780099`, -0.03577638376073333`, 1057.5004658638725`, 335.31647622119647`, 29.120495621087326`, 29.32796752283279`, 0.007124600640217915`, 0.1999410180196522`, 0.1998212482926006`, -0.0005990252937486673`, 876.8224383913346`, 0.21057664102763043`, 8.38121723215397`, 0.0028755562422962955`, 0.0028878290089614997`, 0.004267962658732083`, 0.20737286694104431`},
{0.2128059772734779`, 3.088861612111426`, 5.437928143232815`, 1.2368766361188581`, 0.5161736795080103`, 0.22159582314348258`, 0.17105793552921358`, 1293.4067318655482`, 0.22754002195492767`, 9.689789287743999`, 83.9348082028616`, 83.49817178337545`, -0.005202089917580421`, 39.645350702149145`, 25.4569762180326`, 1.2947555546000342`, 1.3198705410267915`, 0.019397473397606335`, 0.012356944364426227`, 0.01236190413249552`, 0.0004013749615616735`, 57.13315328102977`, 0.0375661660226276`, 1.5734213477714123`, 0.00012071946131841926`, 0.00012106201468412496`, 0.0028375985277313553`, 0.6212511138164449`},
{0.09487232790202699`, 3.2902977287485546`, 3.9754892015026773`, 1.2274952505878522`, 0.2657953162834845`, 0.47376077240276926`, 0.26204707729007887`, 80.73499841507218`, 0.18195262450329797`, 6.708419132160676`, 1008.3717820717515`, 1020.5640631804943`, 0.012091057411080408`, 687.962854543663`, 226.4387850426715`, 16.27454789502671`, 16.57756059191735`, 0.018618808881519566`, 0.2910339209506777`, 0.291177247681261`, 0.0004924743140426457`, 764.972971077369`, 0.3944437939867179`, 18.736065039674234`, 0.0028479844733321213`, 0.0028735814357196193`, 0.008987746466731839`, 0.9557055206721228`},
{0.05710457017724263`, 2.2468049124614753`, 4.035987116327698`, 0.7517191945594467`, 0.40903626729229714`, 0.6582116219387326`, 0.04046385674463665`, 815.0779000126262`, 0.186726177475597`, 7.464159626289564`, 151.15647510113305`, 156.82982566212948`, 0.03753296414990226`, 92.68518731099151`, 96.47886091146293`, 1.6505339777546`, 1.6729478486243354`, 0.013579769439358902`, 0.023642873948389063`, 0.02367289432617027`, 0.0012697431727943798`, 52.977540705766025`, 0.01928737363678521`, 2.0723180944958246`, 0.00037875717142499266`, 0.0003813376258204356`, 0.006812951912526177`, 1.093950489850027`},
{0.1356890149134694`, 1.6504364529320803`, 8.903330095009764`, 0.8652149931542472`, 0.7882939470826769`, 0.5853867881019097`, 0.12191584954894137`, 2002.2565687817998`, 0.19189449537551667`, 8.0829058846114`, 117.03153340286403`, 114.27482433280552`, -0.02355267455728557`, 66.26739033642419`, 43.21201212404337`, 3.002465289803741`, 3.024904207582138`, 0.007473497813479613`, 0.008826503130853182`, 0.008831486231732197`, 0.0005645611637066228`, 70.79111661364816`, 0.017109421642034044`,

0.8489961100263561`, 0.00012318990090176563`, 0.00012359374454168245`, 0.003278220348913674`, 0.5299615097854194`},
{0.21947794079961902`, 1.5730764365956098`, 2.118289006802259`, 0.973502067608673`, 0.7638917093126882`, 0.33981639014260245`,
0.009615294802656809`, 1765.6534921210653`, 0.16626255675053375`, 4.010994756897377`, 43.59862844558511`, 42.279620828130206`,
-0.030253419992354647`, 49.74909989921438`, 38.24962807436522`, 0.2210696616486402`, 0.2243257850543299`, 0.01472894734359742`,
0.031170165009323986`, 0.03126839590673847`, 0.003151439762513419 4`, 4.967992508366284`, 0.09773090900900899`,
0.47528085666647124`, 0.000383816392444003`, 0.00038998608628993173`, 0.016074597040116823`, 0.24085718594727912`},
{0.2345658346849942`, 2.081301191128982`, 3.6234436876627587`, 1.1134590239676974`, 0.8739003578896458`, 0.6696041669913848`,
0.06590896300137702`, 558.8606273551264`, 0.09113830356870467`, 1.2137876330314246`, 84.07057206444038`, 77.92531816944096`,
-0.07309637301253369`, 317.0047640754254`, 43.22806089792601`, 1.3103796096225657`, 1.3265914831704753`, 0.012371890884794068`,
0.13110148480656414`, 0.13270406109181795`, 0.012223936957070913`, 38.961352033408225`, 0.4393132744584995`,
0.45856564556061963`, 0.001384326180464135`, 0.0014527583166802764`, 0.04943353465524836`, 0.0846687420671654`},
{0.23053748957807368`, 3.8668800248110458`, 5.535000453565214`, 1.1361194466757834`, 0.24488877583427326`,
0.5300195072451362`, 0.08816752471869817`, 3094.9084693615328`, 0.16131439142801796`, 1.2619255962975515`,
4.930455432037856`, 5.015128559449715`, 0.01717349007186164`, 17.882069671470937`, 2.2218679669745973`,
0.047780309472686286`, 0.048867962013411086`, 0.022763614399495813`, 0.004976338511899394`,
0.005012829057254399`, 0.007332810110837418`, 2.6394389182745805`, 0.01638903696891528`, 0.09440357897208891`,
0.00005201685836897507`, 0.00005364746677170662`, 0.03134769099596557`, 0.2184046142694736`},
{0.05839671607004826`, 2.466861519533315`, 8.278076935325572`, 0.9179604454629582`, 0.7485509623778881`,
0.3739704677333707`, 0.3976055017684991`, 334.0137006202725`, 0.18218149392519362`, 2.0150765691128605`,
116.5735091550975`, 113.40578811217537`, -0.027173592575887584`, 264.77246063629275`, 18.507858133779568`,
2.703906203831691`, 2.7351044870095045`, 0.011538226856243172`, 0.04018777235200521`, 0.04024860893837443`,
0.0015138083752528342`, 95.28803095228287`, 0.03352619902180991`, 1.2670194796930778`,
0.0005267327599640659`, 0.0005311197494699556`, 0.008328681713643649`, 0.12914621541643742`},
{0.14569114559043927`, 3.1555485829295717`, 1.7894848948555018`, 1.1727674941432193`, 0.06666495623301039`,
0.21747003985461844`, 0.03219791632186156 5`, 1115.0145850241458`, 0.11404115123479613`,
9.530247146566754`, 64.98910863143358`, 65.16034366834462`, 0.0026348266735300996`, 31.210513332980625`,
45.00806633954164`, 0.43162129333748017`, 0.44434547201762714`, 0.02947996050370505`, 0.02798686504732558`,
0.028019870992971017`, 0.001179337006471659`, 19.45717086504735`, 0.0582491204318459`, 1.8515782473929294`,
0.000287592312801066`, 0.00028961561421924964`, 0.007035311196176508`, 0.9300723936257498`},
{0.24150266620923067`, 1.7188165992139313`, 4.322636691993072`, 1.2029528995785472`, 0.9471494725848191`, 0.6615414605337746`,
0.19861497081852103`, 2170.4118977268995`, 0.144713056075588`, 4.793357249969096`, 107.26065890713265`, 100.16442576989826`,

-0.0661587688304095`, 102.41537438597581`, 28.242890444830785`, 3.0853482516794704`, 3.1035193065416915`, 0.005889466400536891`,
0.03891344446467602`, 0.0390319163077991`, 0.0030444964395435203`, 75.75925413346233`, 0.1342528655657815`,
0.45573798922753417`, 0.00038773061022068234`, 0.00039342822995548925`, 0.01469478959003001`, 0.21828113163003895`},
{0.20918490948785712`, 3.1569386485962374`, 8.450247389660383`, 1.207442014040422`, 0.02962131605799323`, 0.6403349881706148`,
0.07844599973162336`, 325.7491474109025`, 0.10080604275363181`, 2.998503110312266`, 104.41475752590324`, 105.812684608349`,
0.01338821365455889`, 159.37561560653145`, 50.23496671207723`, 1.1725932622293949`, 1.1969376250000463`, 0.020761131378468578`,
0.031121245224568272`, 0.031200354350435857`, 0.0025419653133009756`, 52.882928408793205`, 0.0930013552207009`,
2.2277942415152148`, 0.00030932788733339933`, 0.00031318542980569896`, 0.012470723236608583`, 0.6783874029089245`},
{0.20782035028778872`, 3.1560227942933192`, 4.024085032034362`, 1.1076699632387197`, 0.7825797887689212`,
0.681476671590229`, 0.005917686238352286`, 2392.206038919967`, 0.17125268254719206`, 3.7066097570560648`,
48.30716355720533`, 47.22745072157395`, -0.022350988054862242`, 59.64845070595026`, 44.44104637760937`,
0.08092079923559423`, 0.08170546714096083`, 0.00969673943879501`, 0.02238548006742934`,
0.022480098948381492`, 0.004226797042866437`, 3.6483983845709855`, 0.06645940441390528`, 0.3290949690038813`,
0.00024160188473443256`, 0.00024613938797299757`, 0.018780909940138146`, 0.28218930323889124`},
{0.16569005768607342`, 0.9318724619299896`, 5.387727961962419`, 1.4204768501331082`, 0.162422581490264`, 0.6283948310518737`,
0.42435650593933405`, 403.29853257093646`, 0.17732127106821444`, 7.752705227642123`, 248.06175070488356`, 249.67682608788064`,
0.006510779587775017`, 146.44381275954862`, 38.67173797928553`, 14.61747744901038`, 14.877707489156164`, 0.0178026640406038`,
0.05283530411815176`, 0.05292320339760735`, 0.0016636467021942103`, 194.59463853736253`, 0.12506120838846907`,
4.50419480084044`, 0.0004473425518242147`, 0.0004513510592033461`, 0.008960711121231757`, 1.4510728848936771`},
{0.25387146604005345`, 2.177421517695631`, 2.9195785593706507`, 1.205984878491204`, 0.22933817566590875`, 0.5142965710820098`,
0.00715658453704801`, 291.5834795916249`, 0.18546405096886748`, 3.1957985304348036`, 131.0671822795807`, 133.67568667314936`,
0.01990204068020951`, 187.70639326052455`, 118.5816258389067`, 0.3700709130857451`, 0.37865202122147235`, 0.02318774005818458`,
0.10540694626650789`, 0.10595756739565892`, 0.005223765118466339`, 11.511433846088162`, 0.38228308542119416`,
2.5226292527964342`, 0.0010411266017464893`, 0.0010675986031168413`, 0.025426303896130653`, 0.5310236811642506`},
{0.27254750481249423`, 3.728211063877054`, 5.204302820921312`, 1.137745914342209`, 0.04515932299981973`, 0.21786268187051538`,
0.17709327429647695`, 87.47051100453898`, 0.05078893394192652`, 2.761931237968355`, 229.79889360136377`, 228.8289192029971`,
-0.004220970706888205`, 380.80230282308145`, 69.63901537888626`, 2.9414387343831896`, 3.035344732591002`, 0.03192519263111704`,
0.11381292671084016`, 0.11391637689996796`, 0.0009089493796308l9`, 156.6614919034845`, 0.443134702720979`,
6.876749340718641`, 0.001204303629749326`, 0.0012150274925692168`, 0.008904617203655762`, 0.28573350937110936`},
{0.24124989455736606`, 1.138551887899828`, 6.0414451617780465`, 1.0864227463934355`, 0.8606911125263128`,
0.6167713883354349`, 0.008616924398628963`, 4204.638992315412`, 0.042475052828396476`, 4.683417206387297`,

40.019182529369836`, 37.768398539037356`, -0.05624262786179213`, 39.10838307809127`, 35.592466554555145`,
0.2521101839735373`, 0.25406052136509477`, 0.007736051597829086`, 0.009617148037709996`,
0.009636543944414579`, 0.0020168044235702176`, 4.1005789417406255`, 0.03314479928628689`, 0.23225051145151687`,
0.00010643959741996056`, 0.0001074924893236281`, 0.00989191925927102`, 0.2643325472128737`},
{0.16291183507690532`, 0.9001170306894437`, 5.6406094859134015`, 1.3830538570390154`, 0.30535931966424634`,
0.523800888605104`, 0.2170932130058295`, 52.61233898698406`, 0.08477132140762966`, 2.26771827115102`, 556.5750626325263`,
565.4232426935394`, 0.01589755031273299`, 1123.3087434723561`, 142.42895260686052`, 29.793018668671372`, 30.3683345835056`,
0.019310427091404403`, 0.37573407473227394`, 0.3769563655873988`, 0.003253074281313939`, 383.1029071331384`, 0.8744503945081793`,
9.819598801912441`, 0.003238227434320784`, 0.0033069086907932936`, 0.021209522143065707`, 0.34988417165313884`},
{0.24307985322679526`, 3.5312268091205707`, 7.143117328957406`, 1.2474301004946668`, 0.43966589306292936`, 0.28635789146968393`,
0.04752738776816154`, 125.46273060513494`, 0.15229886979218882`, 3.763239253417467`, 336.3976962100848`, 337.7219201090041`,
0.003936483257282131`, 409.1246789102328`, 202.63011391296658`, 2.591496939330963`, 2.6442678209593975`, 0.020363088540655605`,
0.09780749499209059`, 0.09787623851230201`, 0.0007028451164912131`, 130.73090668456305`, 0.33964330753084276`,
6.431203433602037`, 0.0009448996224886708`, 0.0009510798736586852`, 0.0065406430724745945`, 0.324934223123631`},
{0.08579390683558069`, 2.8983719603389604`, 3.99564346069112`, 1.2420354947694623`, 0.9313337583971846`, 0.4737657288125191`,
0.010571420568870461`, 66.04207510362154`, 0.1244519483642591`, 8.73466885165751`, 4735.181254302265`, 4458.832146166972`,
-0.05836082998601366`, 2481.159352069068`, 4112.738200998983`, 14.54994628427358`, 14.65104685590322`, 0.006948518548066218`,
1.061304963933738`, 1.057413168238639`, -0.0036669909473276974`, 602.4450904968083`, 1.3007642742830636`,
27.207666450735566`, 0.010239477839712796`, 0.010311385579137218`, 0.00702259827601126`, 0.3693749550870399`},
{0.20336933942425872`, 1.2934536281273639`, 5.591224704743386`, 1.058733783245077`, 0.09184020179750241`,
0.3092776948325172`, 0.45477750215520385`, 95.84900263920687`, 0.14971414135963745`, 8.98848136080165`,
835.9684698772969`, 825.5427565762624`, -0.012471419289970087`, 425.6651253728774`, 128.48533012225957`,
36.300178038579844`, 37.193866641330665`, 0.024619399987543034`, 0.11051082640008002`, 0.11050509915755329`,
-0.00005182517146329335`, 670.7513855095748`, 0.3210644823457623`, 20.750783573304496`,
0.0012599948125541216`, 0.0012641838014763834`, 0.003324608070227164`, 1.0888243149136076`},
{0.12808268874911022`, 3.6824385947617753`, 2.1347222010883335`, 0.9228913029818868`, 0.1301948165557758`,
0.29194798379239884`, 0.27062051931711223`, 4508.281780436825`, 0.033597809391257005`, 7.5117374932700365`,
14.750468459398865`, 14.699178142567403`, -0.003477199179988122`, 8.987313792274946`, 3.3055693301238493`,
0.21321863288129353`, 0.21861510988491983`, 0.025309593869456526`, 0.0053165181931781`, 0.005324195009900596`,
0.0014439556949783494`, 11.216636040634498`, 0.00972791349950926`, 0.36683515544190504`,
0.00006939093091495874`, 0.00006990973183027926`, 0.007476494528605437`, 0.8674968294223778`},

```
{0.14553068051711526`, 1.8082756931498238`, 2.368533159601707`, 1.1602558844471123`, 0.9691057704573378`,
  0.4792380149128871`, 0.010639214796165341`, 788.9564435352116`, 0.18893878705870149`, 9.465562662789438`, 471.9584085477612`,
  454.34562662059227`, -0.03731850436008621`, 228.2032303307364`, 409.34106260482`, 2.304670254525218`, 2.3206290067341375`,
  0.00692452734944804`, 0.1529800436854424`, 0.15318651523741902`, 0.001349663309033744`, 59.535417171191`, 0.31804699804396536`,
  2.461479972118019`, 0.0015841302251091705`, 0.0015998947605326413`, 0.009951540077700605`, 0.3739064603614686`},
{0.24858998623664108`, 2.4028729322353692`, 5.907867944453356`, 0.899298954014168`, 0.6845394050547509`,
  0.4831163766793648`, 0.006208316855544933`, 932.6458115441258`, 0.22545041645445363`, 9.209981899602862`,
  206.44197002176813`, 205.67293952043224`, -0.0037251654847839655`, 102.58968888367316`, 189.5201073405555`,
  0.47059225064089877`, 0.47606443492409944`, 0.011628292382095395`, 0.021398556571831966`,
  0.021410689471033507`, 0.0005669961504559673`, 16.15390544549627`, 0.07599238405251205`, 2.0981330242745213`,
  0.00028723804242314355`, 0.00028835137799667314`, 0.003876003206739087`, 0.7045473189663884`},
{0.2519271524040908`, 1.2045387595495214`, 4.5140841978362705`, 1.0152142452788597`, 0.29419718036266773`, 0.20432622138273293`,
  0.02287101962327847`, 2390.149329793605`, 0.07799608049040802`, 9.888014794979117`, 36.46695154165181`, 36.57670032263964`,
  0.0030095408677766056`, 16.879316128391416`, 27.672897575504763`, 0.4812363814747132`, 0.4932303197842375`, 0.02492317449642889`,
  0.005201755687727579 5`, 0.005203750535725734`, 0.00038349513470237184`, 8.280969628452167`, 0.018720907113024834`,
  0.8788481346357344`, 0.00006192587539843863`, 0.00006209180562428705`, 0.002679497460161828`, 0.7549151579709839`},
{0.11736070154865536`, 2.38096944376945`, 5.3494850274310295`, 1.276899864236343`, 0.07495782665645989`, 0.3912082450614217`,
  0.41113333692039317`, 80.12914367323096`, 0.2186956786299285`, 8.297333470694408`, 1000.3106819408114`, 990.7239706208131`,
  -0.00958373382697264`, 551.7735223319887`, 164.1946916706263`, 23.865777649652525`, 24.41250207746451`, 0.022908301411244558`,
  0.180547705655564`, 0.18057370542762413`, 0.00014400499837829983`, 811.7669619374099`, 0.30270293426744166`,
  23.429583586931646`, 0.0017040818913161004`, 0.0017127657809417082`, 0.005095934455885187`, 1.2147883754744435`},
{0.16035901956271875`, 2.606522752147513`, 4.69883411815465`, 1.3021358666964324`, 0.6150695298380762`, 0.6434499333892203`,
  0.12329290515746044`, 1079.0196669558486`, 0.2419176548365854`, 8.070742396729585`, 163.0046822679274`, 166.48527560191096`,
  0.021352719968268152`, 92.43811666957397`, 60.005137790218974`, 2.6907649672826777`, 2.7192513486864796`, 0.010586722270496063`,
  0.03505046173113468`, 0.03511300360207632`, 0.0017843380044859636`, 100.19343011291065`, 0.08029510969190748`,
  1.628455942307690 5`, 0.0003237158275913865`, 0.0003266706169361087`, 0.009127725903016026`, 0.8320799461106768`},
{0.16449617597041116`, 3.191202853555228`, 6.979734889593845`, 0.755053893640484`, 0.8431581165413089`, 0.5308423930900061`,
  0.0662197921799213`, 915.9947229465104`, 0.1651520479769521`, 4.094114041787371`, 136.34383157209945`, 129.7920200739647`,
  -0.04805359672373677`, 152.41933180154047`, 70.47527972033973`, 1.4121978135702475`, 1.422667206838254`, 0.007413545869709548`,
  0.022580724735281554`, 0.022604582254230766`, 0.0010565435445006877`, 64.38013846356908`, 0.05306346956560490 5`,
  0.9318482982815859`, 0.00036040503886525244`, 0.0003626187227058404`, 0.006142211128784103`, 0.2306311198633892`},
```

{0.11360527105324508`, 0.4683409445122746`, 6.198135239531911`, 1.4397350406163711`, 0.8287446034488006`, 0.16487005319303316`,
0.2956243647100192`, 339.82112374821816`, 0.14870620145140367`, 8.492417686013223`, 346.9058390379178`, 339.8109113418568`,
-0.020452027315935428`, 186.9583034535206`, 70.50594091953 5`, 35.91973133479666`, 36.471811423911284`, 0.015369827907922184`,
0.05953406531129956`, 0.05954282007905261 5`, 0.00014705476112331084`, 240.32401285665483`, 0.09661976609412481`,
5.114511541503023`, 0.0004996937948673619`, 0.0005008789818541611`, 0.0023718265044971876`, 0.3135455495678625`},
{0.08657280459787114`, 2.8376273381057535`, 2.18201840556476`, 0.9732923844552535`, 0.8927467229995099`, 0.4599530868891304`,
0.04289399463935107`, 1129.687177458504`, 0.08547871234303578`, 2.1820677830816138`, 59.15552628530992`, 55.31880358150875`,
-0.0648582295641572 1`, 124.07708444069262`, 36.72628406857485`, 0.5358782302318115`, 0.5411034111385989`, 0.009750687025534166`,
0.07516441173455941`, 0.07569903013994525`, 0.007112653356136533 5`, 21.723181657164517`, 0.09295991328298873`,
0.40231352830606604`, 0.0009172990121323332`, 0.0009442397256107081`, 0.02936960917002307`, 0.12067361809616214`},
{0.14288327643386806`, 3.0416391272131467`, 7.259522365277451`, 0.8919048885163637`, 0.7100314935930478`, 0.32120037052790307`,
0.014548712666776912`, 690.6861027330066`, 0.2370448478751857`, 7.1501615854828415`, 177.62851648561568`, 174.40906433738718`,
-0.0181246356830842`, 113.70022572539288`, 147.31748673609465`, 0.6787545535963813`, 0.687605072976772`, 0.01303935175608939`,
0.019148325862573916`, 0.019155259844915992`, 0.0003621195080885098`, 29.49323439989775`, 0.0390853648209926`,
2.166380620906325`, 0.00025936675646087703`, 0.0002600772575156656`, 0.002739368238565021`, 0.4310359551750926`},
{0.05887079706837223`, 1.3439289639420835`, 4.010009055307599`, 1.087305166401462`, 0.6661788396152433`, 0.35567096782369767`,
0.008830796062004187`, 96.96443168903161`, 0.035453828625968 3`, 6.082802068311153`, 1058.5713828061027`, 1047.3100132405896`,
-0.01063827130453976`, 796.4911578797204`, 940.2533535408119`, 5.790458276715147`, 5.870762903001512`, 0.013868440535922621`,
0.2961606516868926`, 0.29608884172295175`, -0.0002424696310323604`, 111.17092275108071`, 0.24907448035844443`,
13.246302136679859`, 0.003276356734343788 3`, 0.0032982585976139497`, 0.006684822516601851 6`, 0.4249267365881108`},
{0.275808383383374`, 3.614253930768469`, 8.690573450897112`, 0.8084062901760818`, 0.7881641108236563`, 0.5461332704061045`,
0.16300280176413603`, 213.49162259077596`, 0.11620699031214832`, 3.4918574760984407`, 443.36925794709765`, 430.57994944349053`,
-0.02884572683912401`, 581.1303567515832`, 135.47889022084988`, 5.842899034242298`, 5.893156676806335`, 0.0086014908471807 6`,
0.07403522138403151`, 0.07408918609827524`, 0.0007289059617152649`, 301.6817257370489`, 0.2917076389052463`,
3.44204800335795`, 0.001103082017101742`, 0.0011103866343906965`, 0.006622007408068287`, 0.23137448548056555`},
{0.16345104545617634`, 3.976575006158881`, 6.050721111049688`, 0.8077170991659299`, 0.4737043917748365`, 0.42206087766889466`,
0.011122815850097148`, 819.7636995837653`, 0.013456085267181 7`, 8.084495823489597`, 140.3908847939162`, 142.53412542824952`,
0.015266237815079187`, 79.47864690176573`, 121.31058791073711`, 0.3277169401007776`, 0.3329822522032412`, 0.016066646114919703`,
0.014540804688423881`, 0.014548263780720777`, 0.0005129765825706567`, 18.617014187137418`, 0.03395299611565014`,
2.1443480617411845`, 0.0002173871305970465`, 0.0002181184618565273 3`, 0.003364188383517819`, 0.8219882589694497`},
{0.27078615466317746`, 3.6662669410444693`, 7.922278334055675`, 1.4466898496109064`, 0.8715287168554611`,

0.2219561421798496`, 0.02446106939657988`, 188.339766844502`, 0.03692414185938048`, 1.663076961237424`,
149.18099508472852`, 144.1499825141307`, -0.033724219145611856`, 410.5496130142856`, 110.62995544327586`,
0.7124155170165098`, 0.7237627447331295`, 0.015927822240790146`, 0.1023217662827714`,
0.10249309722259616`, 0.0016744329779381228`, 37.31293511892481`, 0.3958188232864837`, 1.8178440636568898`,
0.0008504450576076739`, 0.0008605272911111211`, 0.011855243808234883`, 0.07160949711384046`},
{0.24286662289220456`, 2.6814172670718373`, 2.6369699156223714`, 0.8218929956416956`, 0.8808977613854625`, 0.43575724866517207`,
0.02475179215943 2087`, 472.6370271533013`, 0.14270041872793765`, 9.280134575613399`, 584.164350548346`, 551.9109222570158`,
-0.0552129349575 2411`, 288.1013348629 3034`, 432.13707212720135`, 3.851124989470432`, 3.882360396155899`, 0.00811072265139 9227`,
0.12300947023314225`, 0.1230566142003597`, 0.0003832547780922013`, 147.52104349168357`, 0.4267842088470139`,
4.0569365988526584`, 0.0018021844941648046`, 0.001814216836569099`, 0.006676531977304778`, 0.4349882561037057`},
{0.042342161544045354`, 1.3013804906014936`, 7.13634807471312`, 1.1105942412761596`, 0.2407240140528013`, 0.6802475696307242`,
0.0062232822378432`, 4037.4772502763867`, 0.18461914595887624`, 5.716947148449252`, 19.850092309922772`, 20.542905524332767`,
0.034902266628940026`, 15.891425201743985`, 18.231942762095876`, 0.0813354772789983`, 0.08272455801886339`, 0.01707841136900501`,
0.0033859076164760565`, 0.003391421895 9820858`, 0.0016285971534475419`, 1.5121200474949934`, 0.002048094960999781`,
0.3316489977793768`, 0.00003670004998446 1636`, 0.00003697736167616 4775`, 0.007556166594338443`, 1.0674476228401164`},
{0.14720397501884225`, 0.5316247300014076`, 6.683828314747185`, 1.481587653959343`, 0.013428589995876372`, 0.17720084833285243`,
0.005004553323761107`, 364.08088787611194`, 0.035796657483315286`, 8.51611862140901`, 160.49119826275324`, 160.90869689080554`,
0.002601380216308069`, 86.25296083003457`, 149.8656071472552`, 1.2064923793531104`, 1.2459518860544108`, 0.03270597260005692`,
0.026204880655476343`, 0.026212871604244577`, 0.0003049412387445827`, 9.162874077462172`, 0.05510660853402671`,
5.012890364952759`, 0.00021375406852675027`, 0.00021428798642489583`, 0.002497813968292961`, 0.7310424257388889`},
{0.1926486177557311`, 2.6336525621782014`, 9.444109966651418`, 1.4788802142070587`, 0.6654095862145473`, 0.16653655494047714`,
0.03828733327035071`, 2433.4281263020134`, 0.12844440731237916`, 4.149230973672752`, 19.155597096615622`, 18.873506896335243`,
-0.01472625462195 6758`, 21.129663332823`, 12.494629279407986`, 0.17199177473088223`, 0.17535587174872766`, 0.0195596389601147`,
0.004532118174259642`, 0.004534582417471718`, 0.0005437288078833369`, 6.470951117050902`, 0.012472947168242716`,
0.3523734747277347`, 0.00003702308959363 5206`, 0.00003715186668122 1985`, 0.003478291223132146`, 0.19531168411089353`},
{0.05576285411897663 5`, 2.4026093631268655`, 6.857734870811047`, 0.7509249944976302`, 0.22671157472868875`, 0.4788061070906606`,
0.1564030997457896`, 55.02740807629177`, 0.03469990388231514`, 7.492235364197862`, 1598.515566560386`, 1624.5072407737396`,
0.01625988182853999`, 976.4948394584247`, 514.232996588241`, 30.688764564800874`, 31.26240520530449`, 0.018692203763769788`,
0.14672851301994266`, 0.14664690899017396`, -0.0005561565921247125`, 1053.3301869455229`, 0.11688572380873888`,
30.854149374515202`, 0.0023569122853961 4`, 0.00236449477905026 7`, 0.0032171301838890276`, 1.1100722370922511`},
{0.05296847355619744`, 1.6490117913903575`, 9.992115392915188`, 0.8066960527418563`, 0.1291629696144665`,

```
0.33931322884091664`, 0.26435083615790894`, 660.4050522121521`, 0.10186522527726305`, 2.8696181659920086`,
 39.67979988769313`, 39.64940251720767`, -0.0007660666276415595`, 63.286321763642306`, 9.029600930023092`,
 1.2476081083488064`, 1.277517718795704`, 0.023973562087924005`, 0.007000686272130637`, 0.007006260713789342`,
 0.0007962707428978977`, 29.39029259573429`, 0.005297366652573758 5`, 0.9710075041475178`,
 0.0001047249106727044`, 0.0001051723339592086`, 0.004272367325311332 6`, 0.37942369314765856`},
{0.21365538200916506`, 3.0547845209089406`, 0.35609106729359574`, 0.8256521864420123`, 0.9506371395328554`,
 0.271042805312945`, 0.12766968769895812`, 68.7970381277751`, 0.05673347586404265`, 3.4610065999125617`, 1177.885417192509`,
 1131.4414527495996`, -0.03942995113532233`, 1557.6329019610953`, 416.6852005673842`, 16.599393164998535`, 17.042320574844084`,
 0.026683349532289347`, 4.986639145338778`, 4.871026231833259`, -0.023184535743595358`, 724.3938470988462`, 15.220318450560693`,
 10.431344815147636`, 0.06879995063085342`, 0.07263620246584847`, 0.055759514357480944`, 0.1750645777439373`},
{0.22385277301638717`, 3.365536903395271`, 8.935305056074029`, 1.445762062116566`, 0.43390325179453604`, 0.6187135527847307`,
 0.01939946413637202`, 453.1242214832122`, 0.13325485950688953`, 8.825528492983036`, 321.5961748275638`, 332.6134505890837 5`,
 0.03425810573595056`, 166.77642024656362`, 252.52354925764755`, 1.4022918039543006`, 1.4219001978774166`, 0.013983105276535568`,
 0.036965746806125396`, 0.036997380636648994`, 0.0008557606231929249`, 67.42092593624197`, 0.11821264184527314`,
 4.364083051674295`, 0.0003083370550750475`, 0.0003099376681208719`, 0.005191114786495143 5`, 1.1936616334249217`},
{0.26129739321020845`, 3.355909888714172`, 1.041845959949887`, 1.206440545734901`, 0.09347792890054829`, 0.5333101115239811`,
 0.015503351594481202`, 579.4832207194656`, 0.24065061389170217`, 7.475999528371727`, 143.52535769501768`, 145.28993559905936`,
 0.012294537581235776`, 87.86660921731192`, 117.4223926451152`, 0.5187791940432775`, 0.5317324930603206`, 0.02496880978608096`,
 0.13804287426587622`, 0.13892831975727665`, 0.006414278868860812`, 24.87108896212863`, 0.5152891885273687`,
 3.0304017008619133`, 0.0013587719234094964`, 0.0014004559494808825`, 0.030677721075359266`, 1.4214062902527047`},
{0.14172561423935526`, 2.161658858177276`, 2.5726304321806133`, 1.1364876244324849`, 0.9181535957758225`,
 0.5861373014149901`, 0.010418800942482771`, 101.70999367656657`, 0.1500483081169689`, 9.64604938537483`, 3944.718665000919`,
 3665.997415616593`, -0.07065681308460592`, 1871.6776250795547`, 3430.928441353214`, 15.92622098367187`, 16.03047588856927`,
 0.006546116935353785`, 1.1373770862613632`, 1.133181620235433`, -0.003688720369531162`, 491.81509523775844`, 2.30279237388926`,
 19.580296404124166`, 0.011953678881284069`, 0.012080996083706304`, 0.010650880259262863`, 0.444277187765499`},
{0.06568219588241164`, 1.0305120784213448`, 8.720293141386708`, 1.4160313463775192`, 0.752508918071946`,
 0.31681781371940887`, 0.13417309715525413`, 209.13734568668048`, 0.22402731709711876`, 4.416848552940577`,
 379.90855976312633`, 370.1971593057519`, -0.02556246814609686`, 393.66886242622587`, 133.06489138219897`,
 15.690125078332184`, 15.879953759340472`, 0.012098608523550825`, 0.08758980608984179`,
 0.08764704420722542`, 0.0006534792110959309`, 230.98376293089976`, 0.0821870114411251`, 4.405867155554598`,
 0.0007458250473807437`, 0.0007496336590866968`, 0.005106575220728216`, 0.2517370173542297`},
```

```
{0.27044630440719625`, 2.4698840490073675`, 6.955264919348561`, 1.0249819835130385`, 0.106623120735865`, 0.6358905088319218`,
  0.012311596840628138`, 346.23093318344644`, 0.07401305589494472`, 1.2937187987733016`, 43.98998099397845`, 44.876248949853505`,
  0.020147041118212305`, 155.62464729449422`, 37.38274398012075`, 0.17724563286915787`, 0.18133902743807648`, 0.0230944734866349 6`,
  0.031159487314280202`, 0.031349580520384786`, 0.0061006525616826845`, 6.253945162567833`, 0.12038525987671497`,
  0.9005524092866529`, 0.0003617430296249946`, 0.000371547036157844`, 0.02710212977154769`, 0.2860633142028097`},
 {0.08403118541616988`, 3.378337092807227`, 2.524635889786053`, 0.8671019090417934`, 0.46085963082622694`, 0.5847463933658021`,
  0.0473316577589 3332`, 1030.6526695312073`, 0.10944158368617352`, 3.930070348109142`, 62.21184610375735`, 63.88330879656129`,
  0.026867273638146782`, 72.44981141647209`, 37.40102753496688`, 0.5021048124021786`, 0.5094457271560373`, 0.014620283599231287`,
  0.0339483164056804 7`, 0.03409128198283307`, 0.004211271494119817`, 24.232561603075673`, 0.040753103863561395`,
  0.8525804179019899`, 0.00046785173133 50117`, 0.00047652710140560036`, 0.01854299020297212`, 0.5220820236416713`},
 {0.11975601650409934`, 1.1540823534574054`, 5.101398975621729`, 0.9964575213162732`, 0.8596230582994027`, 0.5017711629445061`,
  0.09812020963113087`, 54.445260820005984`, 0.07947868464718594`, 4.273753987333478`, 2401.5975076656196`, 2272.535941141883`,
  -0.0537398819376631`, 2571.9066410930636`, 1008.6590425125149`, 79.54914708099956`, 80.15417927915867`, 0.007605765999515235`,
  0.6900650789495615`, 0.6885662055498684`, -0.0021720754250813545`, 1311.5180982681316`, 1.1805634997656032`,
  16.324931208334924`, 0.008294275177200494`, 0.008371726020779234`, 0.00933786761640576`, 0.22957604369919923`},
 {0.26353274820980593`, 3.2715371213501925`, 8.419438906869026`, 0.9426613525835754`, 0.1294979869931494`, 0.5387601140015186`,
  0.040849691062558216`, 744.2937861154428`, 0.1766059692159136`, 7.8822033078787435`, 120.79838093616839`, 123.2130336602 2315`,
  0.019989114964468868`, 70.14197263427936`, 77.08446115080189`, 0.9145578433373155`, 0.9325393201376391`, 0.01966138821215213`,
  0.010761131997331135`, 0.010767743768462158`, 0.0006144122321576351`, 42.742999058571534`, 0.04051300984431119`,
  2.4607282409579363`, 0.00013782421846086912`, 0.0001383387742297583`, 0.003733420545644295`, 1.3974207043778069`},
 {0.15837784563387913`, 1.590657770632209`, 9.4421417173358`, 0.8199017044479102`, 0.629826423052569`, 0.2661387442145513`,
  0.049173845467465206`, 187.50695430622352`, 0.06908425180315825`, 9.72292056298913 5`, 722.643836565428`, 715.449231953232`,
  -0.009955948211487375`, 340.1669061227229`, 428.8058391879212`, 12.379667365392477`, 12.568846425619927`, 0.015281433227867014`,
  0.04051353048173234`, 0.04051003110194197`, -0.00008637558239832366`, 281.3116298943357`, 0.09166350966733708`,
  10.862167747503829`, 0.0005974515150692916`, 0.0005982333816694936`, 0.001308669541345564`, 0.5965215282660009`},
 {0.18489841859634304`, 3.5401138358224227`, 7.262892362437768`, 0.8851173674042263`, 0.05104398233694862`, 0.6826543300991057`,
  0.13384148697112022`, 730.4506518091381`, 0.22533987085622137`, 8.188627124718394`, 136.4449308847219`, 138.5258195923069`,
  0.015250758632748829`, 76.26245903234222`, 48.43710301542611`, 1.705570045682828`, 1.7371316113991881`, 0.01850499532179839`,
  0.012733771601685437`, 0.012744212440209863`, 0.0008199329194065186`, 86.25588738122926`, 0.03363506045605698`,
  2.7024831814827985`, 0.00017355896560355966`, 0.00017436137872136107`, 0.004623288200704456`, 1.8131279234628175`},
 {0.22773890477301867`, 2.1437195677585157`, 6.152335866192775`, 1.1180903919170035`, 0.02705103803804154`,
```

0.3333465628704615`, 0.00917020779025847 5`, 2321.6700090659583`, 0.14789384405564093`, 7.237611958554762`,
26.591063213800286`, 26.792363890852588`, 0.007570237994388496`, 16.815310930857123`, 23.553769726019205`,
0.09485484951186883`, 0.0973931764186037`, 0.026760117377206516`, 0.004185960153839824`,
0.004188990352387559`, 0.0007238956980886169`, 2.9048885285054644`, 0.013618656869414185`, 0.7009986200144622`,
0.00004519570515970628`, 0.000045385169689448094`, 0.004192091462503322`, 1.0083245696945495`},
{0.17605039997850958`, 2.727076411741753`, 8.728585319721596`, 1.1320082850991182`, 0.7355770210071702`, 0.5402004706661186`,
0.09745706159326789`, 177.96581560566344`, 0.01922037387361028`, 4.980887347403458`, 678.7853001196466`, 670.0361409142529`,
-0.012889435295448504`, 623.7207663822047`, 286.9975246780879`, 9.7951792890919`, 9.886350069235345`, 0.009307719384470392`,
0.11082095397103628`, 0.11089326281144539`, 0.0006524834683159231`, 381.6028912580553`, 0.2787153324656682`,
5.932976292532321`, 0.0011792459195612004`, 0.0011865756725149335`, 0.006215627149645 4085`, 0.3688386089353362`},
{0.06947420594938447`, 0.8077718165326004`, 3.821443319804267`, 1.3891202366711977`, 0.6756008025235485`, 0.23550127195203518`,
0.0653335798785588`, 483.3383501610001`, 0.09612644227220152`, 2.381497911922425`, 65.71733472285908`, 64.47565792622012`,
-0.018894205035479783`, 126.29734796313699`, 34.419461403145526`, 2.485938349982277`, 2.530039109867275`, 0.01774008590571552`,
0.06267864075955476`, 0.0628545763483387`, 0.002806946459781301`, 28.686727667903416`, 0.062207839953649875`,
1.0305497809342345`, 0.0005412941097241442`, 0.0005488022791974318`, 0.01387077623496413 3`, 0.14223019218027946`},
{0.23569362997389992`, 1.2270876586626818`, 0.9845589085102358`, 1.1982217261670929`, 0.43286649637666286`,
0.634774301600445`, 0.09976341665112172`, 228.04937393006952`, 0.12724994607105566`, 3.0426000813607676`, 224.30270638905648`,
227.65403505998384`, 0.014941097790921898`, 337.40703684297324`, 93.2238351550279`, 6.94484090565037`, 7.113616257282839`,
0.02430226320881612`, 0.5477714021896914`, 0.5571493099440786`, 0.017120111997266374`, 121.74183666713363`, 1.8443747168282905`,
3.0651456305747535`, 0.005293214491106646`, 0.005690645439031113`, 0.07508309904920862`, 0.49902293272849985`},
{0.12274586224015488`, 2.0471402784080235`, 1.4612976870222134`, 0.9456822010625827`, 0.8327879461898879`,
0.22053943969717293`, 0.006767755909702022`, 174.3096643655477`, 0.1850505728558421`, 1.2537872990507584`, 114.258580896559`,
109.7953690642296`, -0.039062377611446375`, 417.0896896671247`, 103.30631963279372`, 0.3254650095144729`, 0.33304511849116963`,
0.023290088811712018`, 0.36456767841191773`, 0.3669752606183887`, 0.006603937619918954`, 9.518179002707546`, 0.6392739147365896`,
1.4954873878001438`, 0.004568206500384475`, 0.004739579966208132 4`, 0.037514386840707514`, 0.0669807520761631`},
{0.13491824188855928`, 1.669720674179887`, 1.3334401828831126`, 0.968437561771696`, 0.34828727193756603`,
0.3135712369052611`, 0.22891096239253717`, 1910.612731597179`, 0.10930115833371806`, 4.270908360205038`,
24.125953184975337`, 24.138049752596697`, 0.0005013923192429548`, 25.854058220066495`, 5.9928720819077475`,
0.7265976502349609`, 0.7434088804572325`, 0.023136917958426206`, 0.025564924305101648`,
0.025676175118912088`, 0.004351697367953378`, 17.331644548683478`, 0.049273923446552216`, 0.4872098898166279`,
0.00031551746953795146`, 0.0003219617517943875`, 0.020424486371144912`, 0.44549669424309524`},

```
{0.04199865921066254`, 2.754174936868967`, 5.53210979521042`, 1.3844956478579449`, 0.47136731805345633`, 0.24068198696313148`,
  0.022972136801125642`, 58.648848297948106`, 0.2240134714991286`, 4.479259216921056`, 824.5098359403563`, 823.9560726985757`,
  -0.0006716272112741173`, 842.4694079264048`, 624.2237552818326`, 4.948478417015255`, 5.049528964179758`, 0.020420529029093526`,
  0.28906821406345995`, 0.2890479911548488`, -0.00006995894957406712`, 194.69964616829202`, 0.1734353915869008`,
  16.191454250494893`, 0.002514229560421133`, 0.0025287881415003693`, 0.0057904740714278535`, 0.3320988473186464`},
{0.18074106663079648`, 0.9554759541683486`, 8.202625386518609`, 1.4769540911095849`, 0.5922233662884384`, 0.5544055394726339`,
  0.15090969809745627`, 4632.951795907658`, 0.16872394703920557`, 7.933658625009354`, 33.03910623547708`, 33.59942521349337`,
  0.016959265605515306`, 19.059841100000142`, 10.711893127745752`, 1.5229283830200389`, 1.5406472648653227`, 0.011634743985890 15`,
  0.004699961964663271`, 0.004705117511548924`, 0.0010969337463611062`, 20.787449284230455`, 0.012135373408820989`,
  0.37138729833770495`, 0.00003836840157644961`, 0.00003858473265485654`, 0.005638261421338830 5`, 0.784146593432866 3`},
{0.22308930012837075`, 1.8877770902637643`, 5.727481376105105`, 1.290937410786533`, 0.5385704303274257`,
  0.17837528465279628`, 0.11591468148922064`, 2665.0058694088225`, 0.1309938954283596`, 2.3264420617360244`,
  8.711740020890856`, 8.632861644598574`, -0.009054261961804588`, 17.13867171043912`, 3.3839499394911607`,
  0.1897041748361607`, 0.19420681404627096`, 0.023735055983870712`, 0.005277540105692553 5`,
  0.00528533763443844`, 0.0014774930345817783`, 5.115988502615644`, 0.01681946755111961`, 0.18243534591411725`,
  0.000049253927579595 57`, 0.00004968926490688096`, 0.008838631733111502`, 0.13692702868432075`},
{0.16528939075200133`, 0.44637388983991233`, 6.000554224390708`, 0.7620462868306999`, 0.45157540745748825`, 0.629355484702981 4`,
  0.08179042637541929`, 626.5470719450981`, 0.11922182895709121`, 4.5474321331423475`, 121.68240621952394`, 125.90750424026572`,
  0.034722341150284386`, 122.468967799627`, 56.92398985582869`, 8.768973448456752`, 8.888319269894453`, 0.01361001058324618`,
  0.02129193952022682 3`, 0.021321554216746537`, 0.0013908876874078047`, 55.91772554415077`, 0.05027616730303578`,
  1.6280601576853206`, 0.0003363811719181431`, 0.0003389095372575955`, 0.007516369971110270 5`, 0.6208532465822922`},
{0.12057640522465035`, 1.6899679349832946`, 5.872613897455656`, 0.8685022109314445`, 0.5819827862907108`, 0.45575489869095254`,
  0.00540859560413313 1`, 1249.3305456098487`, 0.22090492300977427`, 9.765554151588912`, 134.22075811612254`, 135.54928275900846`,
  0.009898056467066985`, 62.90530748834596`, 124.55025818181825`, 0.37768152773971897`, 0.3828922978413436`, 0.013796730099057575`,
  0.012751001324377004`, 0.012757719455721187`, 0.0005268708843546577`, 9.118138164508995`, 0.021963855752970555`,
  1.6807681208994718`, 0.00017729526620802183`, 0.00017787091479356146`, 0.003246835619763244`, 0.8736096139462535`},
{0.26839288378136816`, 1.20431418513886`, 3.4264235210106797`, 1.1464020290887187`, 0.9129873318113406`, 0.17958510842302844`,
  0.03476022275277427`, 85.14929883968256`, 0.03637248443077806`, 6.177611921122705`, 1179.8802642733738`, 1152.584120403935`,
  -0.02313467281042203`, 874.1416412707893`, 791.9429878720576`, 21.198682386018557`, 21.505304535215924`, 0.014464207898109649`,
  0.4010222533567292`, 0.4005190046414002`, -0.001254914686445896`, 364.7124843390777`, 1.5375931291270564`,
  14.81596796240296 5`, 0.00421335654801946 5`, 0.00423633078827761`, 0.005452716853252104`, 0.21433911693579405`},
```

{0.19312844950593372`, 3.9129912832786564`, 0.42277195731844414`, 0.8655327062249413`, 0.5847393470300801`,
0.16029168895981472`, 0.1525854675774217`, 2234.1231120850057`, 0.03649943574663328`, 5.234397199033289`,
24.30550146767184`, 23.92854371506977`, -0.015509153477184956`, 21.252127148830347`, 7.904806037113416`,
0.2843486010510042`, 0.2928260009777361`, 0.029813404727147885`, 0.05887115350319637`,
0.059135493499082745`, 0.004490144666046358`, 15.895051390357994`, 0.16242420852424488`, 0.4877718265692359`,
0.0008128797880341265`, 0.000834728670629336`, 0.02687836863067905`, 0.28696384518876417`},
{0.05831506452479063`, 1.4447664488109728`, 9.399667906542401`, 0.8838396243177019`, 0.4494233408794772`, 0.5909311609620335`,
0.2085880113243902`, 309.3416067223959`, 0.04816991376818669`, 2.147582543132131`, 110.74107221305343`, 113.64161889085416`,
0.026192149126210484`, 236.0061681698392`, 28.930414828888637`, 3.7780412705885404`, 3.831669193735803`, 0.014194636666557292`,
0.03036164789017302`, 0.03042455389175321`, 0.0020718902283480833`, 77.9769609995643`, 0.025293449368502998`,
1.5557861944926938`, 0.0004127185704178027`, 0.00041692692362806604`, 0.01019666550502718`, 0.2908490902137119`},
{0.23965113012644185`, 0.9128974489725401`, 8.261297062012524`, 0.788417407034967`, 0.8016641172773833`, 0.35686628470413617`,
0.030635478621033968`, 346.1316581378353`, 0.043365488693742626`, 1.9545026373639716`, 116.70546395082444`, 112.4722829314004`,
-0.03627234643621968`, 273.2872832363381`, 81.41593374801697`, 2.5011084835233244`, 2.530198388993655`, 0.011630805165776525`,
0.03568223257900966`, 0.03572708217777127`, 0.0012569168328326885`, 32.617936488743155`, 0.12216124804275667`,
1.1773327826066495`, 0.0005449505772771035`, 0.0005493135121147588`, 0.008006111048556086`, 0.11022558130430492`},
{0.20520507007680205`, 1.512442754504547`, 9.386452795492342`, 1.0402158263719963`, 0.9284696718653804`, 0.3818637796049891`,
0.053241178903524394`, 3017.2611079460976`, 0.15062298010038172`, 2.3864513615272553`, 22.463915137738805`, 21.371103971704514`,
-0.04864740448553406`, 43.0821488685705`, 12.814790660866914`, 0.4256832526504928`, 0.4292865689311132`, 0.008464782812536154`,
0.006531684174117444`, 0.0065415474997698635`, 0.0015100738782662937`, 9.197450731216646`, 0.019147638695279794`,
0.16284740259234867`, 0.00007562159195395601`, 0.00007621578976928998`, 0.007857515293988593`, 0.10307118695721539`},
{0.20853248950244857`, 2.7801356391066916`, 9.335557605849111`, 0.8182981643225358`, 0.24140252954455388`, 0.3825831953385048`,
0.013327430792399009`, 77.96529661289064`, 0.07323986754942219`, 3.7008101112034457`, 498.0006108044608`, 505.9028704823119`,
0.01586797185868094`, 615.8820803831932`, 419.4983572371741`, 1.914576959110768`, 1.9562341273408825`, 0.021757896976605462`,
0.07398729615941098`, 0.07400663865524908`, 0.00026142995949496317`, 76.03976625480514`, 0.22041078656684338`,
10.582610837266184`, 0.0010906892065341012`, 0.0010954849173742277`, 0.004396954523246732`, 0.47957694698755676`},
{0.13576335201419282`, 2.033104701519708`, 4.843203210944571`, 1.3428754702553487`, 0.2671111595179354`,
0.18962864690949777`, 0.12183777475102511`, 1231.483599580866`, 0.14101782654161166`, 2.1508880203421494`,
14.055910150216047`, 14.031142308825817`, -0.0017620944588814247`, 29.909259878623075`,
5.346323575104732`, 0.2887829449010606`, 0.2971282522742624`, 0.02889820025923262`, 0.011324978213673845`,
0.011348421870837157`, 0.002070084085018964`, 8.38751375710077`, 0.021964528625372882`, 0.37234540142904726`,

```
0.00010143557866237884`, 0.00010254679049286195`, 0.010954852775885593`, 0.1723674072032592`},
{0.25963449652183795`, 2.4481737185281203`, 5.334705083491999`, 0.8989699854247848`, 0.7130966522497681`, 0.695912989145927`,
 0.04220235704717508`, 140.4631283114693`, 0.11660454588716207`, 7.56536482611441`, 1473.7556693034473`, 1483.0165420576`,
 0.006283858951009025`, 891.5795682946958`, 923.0801227202641`, 15.279154160871284`, 15.40893307590966`, 0.00849385467755348`,
 0.2059549189715938`, 0.2059527847215907`, -0.000010362704681909918`, 534.371766542639`, 0.763900024190708`,
 11.546480486395385`, 0.0027563367251766113`, 0.002775228773618027`, 0.006854042276059458`, 0.652032676758423`},
{0.17509339161516935`, 3.0306683644318824`, 1.9789343166711684`, 0.8960196738550836`, 0.2152496986108292`,
 0.6250130758758401`, 0.02087454134280109`, 59.51435674676293`, 0.2018230747082378`, 3.546682887992379`, 778.929135996712`,
 798.0955218526343`, 0.024606071297355037`, 1005.1713096025944`, 601.1563193897927`, 3.959775686477655`, 4.039386230873065`,
 0.02010481166073963`, 0.6208378374937671`, 0.6225409329832551`, 0.0027432211547593344`, 171.4395271893478`, 1.5529228944261675`,
 14.00823490206896`, 0.00820124228843111`, 0.008434470081882754`, 0.028438105502704314`, 0.6773519657352577`},
{0.10593857456933181`, 2.955002391424368`, 8.212267239719637`, 0.932578721051657`, 0.3205115590912122`, 0.6246255275862143`,
 0.01773467921096997`, 102.27924186693049`, 0.015490300413002644`, 6.09864277306975`, 864.7624379591522`, 894.55644696043`,
 0.034453403262509896`, 648.9752232650285`, 692.1252095884371`, 3.9822047380014185`, 4.04502471694378`, 0.015775175581225653`,
 0.10102192271133299`, 0.10106054566145913`, 0.0003823224611998466`, 168.10606462765196`, 0.15288740703281328`,
 13.65380005286696`, 0.0013055549958493895`, 0.001312242624846972`, 0.005122441428238256`, 0.9769588391801256`},
{0.10583476133380088`, 3.0591620947997713`, 7.753926172578531`, 1.2866948671665401`, 0.7481949494801159`,
 0.21582592530952183`, 0.02844379415675546`, 101.39583001744663`, 0.09278999192622717`,
 1.6976300926057526`, 231.59476586590938`, 225.8821053951252`, -0.024666621671803002`, 624.3817053567102`,
 165.40167109085414`, 1.4721188554876543`, 1.4969176274504703`, 0.016845631635226344`, 0.14173723342141886`,
 0.14190372934986117`, 0.0011746802475485207`, 64.33500288211218`, 0.21429608958954974`, 3.4734393098397054`,
 0.001325070572815057`, 0.0013371422315575916`, 0.00911020061134482`, 0.08681070169747096`},
{0.23086787628538824`, 0.6622997055352342`, 7.183940408138384`, 1.353990838360309`, 0.25975387914696335`, 0.6772244715959861`,
 0.055054708296352145`, 683.0312933373987`, 0.1367743885435413`, 6.244797760097583`, 130.68275899970027`, 135.13511016954564`,
 0.03406992019395294`, 95.77770167655214`, 73.96405270158738`, 5.4113969192961875`, 5.499483377501676`, 0.016277951796769274`,
 0.02469923005677412`, 0.0247413019850880l`, 0.0017033700328787926`, 51.19952265977337`, 0.08146083984416409`,
 2.1322954004567785`, 0.00021946460342225915`, 0.00022138632333011028`, 0.008756400248078666`, 1.1344196323345872`},
{0.20388536464835005`, 2.2276602475557015`, 5.0708546023033385`, 0.8827779974574592`, 0.1554825605038257`, 0.2618026330626365`,
 0.006155066765134505`, 1063.8834649875878`, 0.12744889948308613`, 2.149276150894316`, 16.698275413695704`, 16.806112613077715`,
 0.006457984235519465`, 35.558537864479035`, 15.337478493595977`, 0.03995395104198202`, 0.04111665864160688`, 0.0291011919798152`,
 0.008459077986656474`, 0.008474414229577043`, 0.0018129922604757098`, 1.271483263843`, 0.024638317141404947`,
```

0.44296760521877376`, 0.00011530849469998383`, 0.00011644258399506713`, 0.009835262337211548`, 0.23847113107628393`},
{0.10738337044420926`, 3.7501964356372497`, 1.0776334746380822`, 0.9682422375974065`, 0.09998974406674432`,
0.504170653500009`, 0.22038799040453166`, 2051.555691372483`, 0.09521018879793502`, 2.016584418866474`,
10.628557350205678`, 10.51903903585091`, -0.01030415612826796`, 24.12250514200324`, 2.707346623509458`,
0.14379864555983135`, 0.14780731872876296`, 0.027876988363313204`, 0.029008882666982243`,
0.029520692325796294`, 0.01764320483107018`, 7.70390240039922`, 0.04450102276573248`, 0.23305275475224377`,
0.0003488122690517015`, 0.00037170673637296854`, 0.0656354989562411`, 0.40589382283699665`},
{0.2588804970943782`, 0.5492314132783744`, 9.644794872912858`, 0.9613308813426055`, 0.2817033035077452`, 0.232857138958534`,
0.012136672860729404`, 78.78277994386292`, 0.023082056754425706`, 4.302644367094656`, 486.8163799998133`, 489.41572993532355`,
0.005339487417229671`, 517.8383689250194`, 416.04204150431417`, 7.931213007912449`, 8.131539611736`, 0.02525800323654126`,
0.07074900615512797`, 0.0707515816069221`, 0.000036402656858444746`, 62.22959041925115`, 0.26165054117680964`,
11.722562570127636`, 0.000888875685065349`, 0.0008915594923605453`, 0.0030193280571051417`, 0.37660504176360204`},
{0.08494913534264076`, 3.4349155581389494`, 7.0407634051690415`, 1.119369452553694`, 0.9329533821244145`, 0.5630698272627359`,
0.4838120213175008`, 71.45974855722541`, 0.09271025542437755`, 2.443770975165119`, 1360.6962652097704`, 1270.9131225005497`,
-0.06598323593941768`, 2548.385850482922`, 177.9955720596822`, 23.596294091658113`, 23.74083283887592`, 0.006125485072204784`,
0.5552158906862869`, 0.5552455710596309`, 0.000053457355673636187`, 1157.8753955694103`, 0.6737872834612051`,
7.068275973030078`, 0.005930425325167188`, 0.0060102776069043936`, 0.013464849038454796`, 0.11538829291475704`},
{0.2312360951475228`, 2.6472972630846083`, 4.924168333426426`, 0.8758299082609615`, 0.33581096057577486`,
0.16094039113701897`, 0.009823651093858611`, 148.81624524939704`, 0.12101804849376735`, 7.0744188173362765`,
394.55333158258037`, 394.54053651975437`, -0.000032429235294118 01`, 255.25804715772384`,
346.72151668427404`, 1.2181817447969943`, 1.250857809923625`, 0.026823637167601833`, 0.06223861246802938`,
0.062234342776178585`, -0.0000686019768352 2529`, 46.06984569915312`, 0.20559733877864195`, 9.970721129446074`,
0.0008586984456444569`, 0.0008607869000712293`, 0.002432116230518089`, 0.443527940288998`},
{0.2359361250604497`, 3.266770315195857`, 7.411396165562348`, 1.2275925063587856`, 0.15344120088912327`, 0.536013355113413`,
0.3013372435966537`, 712.0245065436816`, 0.039861802547139946`, 9.042443794820379`, 149.7523390788011`, 150.93850243539916`,
0.007920833583600073`, 75.79704790060842`, 30.427847990175028`, 2.501656320146064`, 2.5493510542225635`, 0.01906526235934547`,
0.017199038867322214`, 0.017212618652418587`, 0.0007895665101480542`, 116.74766579250384`, 0.0579696369302001`,
2.931869749340665`, 0.0001690408677900157`, 0.00016982489723089524`, 0.004638105868300935`, 1.5345426379044371`},
{0.26580884355261647`, 3.957059703696273`, 5.486829732050921`, 1.3210508471658353`, 0.3282573265157698`, 0.6164824430541005`,
0.042206581416411024`, 114.97331259087073`, 0.22266675421103638`, 9.071401748075406`, 1157.954773290726`, 1195.8064006549514`,
0.03268834693487799`, 584.2270899711931`, 728.1837925284774`, 7.453641698632012`, 7.5711772783669895`, 0.015768879762029453`,

0.19268267258411703`, 0.19279306229030596`, 0.0005729093576940514`, 421.35007444924105`, 0.7316679767460789`,
17.998425691247633`, 0.0017552802314091398`, 0.0017681101006861942`, 0.007309299704671313`, 1.4132472768508495`},
{0.24965572729559277`, 1.088568489897936`, 8.460543344934337`, 1.1505850947024536`, 0.8975822933983539`, 0.5110185880609677`,
0.06613687599714729`, 1096.4114980052268`, 0.1003107894781316`, 4.101976455241168`, 127.75146274690617`, 119.7527871608091`,
-0.0626112250623978`, 142.5401504635222`, 66.00072273175408`, 3.7235885359421323`, 3.749817274657863`, 0.007043941204178949 5`,
0.026528948406132208`, 0.02656463146042157`, 0.0013450610157286214`, 57.90544499388272`, 0.09461577012457953`,
0.7739891988380548`, 0.000277679005918241`, 0.00027976156948269137`, 0.007499895635118881`, 0.19765087424609626`},
{0.1942611669549973`, 1.9123387865104826`, 2.4307010631404715`, 1.2953041584793583`, 0.36588469810004653`,
0.5550679926918506`, 0.01452356295191177`, 246.06581986793796`, 0.04070230178651557`, 1.4358727366404163`, 82.5136794819636`,
83.72056274363985`, 0.014626462778696503`, 263.01135514119403`, 67.93725798706079`, 0.48838399755681244`, 0.50065838151091`,
0.025132649749994407`, 0.18761303660774914`, 0.1905768111785942`, 0.015797274136346573`, 13.342223731984738`, 0.520656106105608`,
1.3426678863171115`, 0.0016899928713182133`, 0.0017967681708997059`, 0.06318091714683183`, 0.2380762805940968`},
{0.20522561065351508`, 3.886130079640968`, 5.862633336878224`, 1.1071510042707493`, 0.46587070079395265`, 0.3092363340879616`,
0.06237106286442892`, 3704.443052142306`, 0.23732711327647033`, 5.5948716983231535`, 18.23995650701205`, 18.32493562373855`,
0.004658953912189068`, 14.921007935993181`, 9.786341435610414`, 0.14930840382351404`, 0.1520692361099523`, 0.01849080303411199`,
0.0038584746104576096`, 0.003861973330205635`, 0.0009067624129346363`, 8.289026846310534`, 0.011312254401760683`,
0.32379439311849767`, 0.000042048803079675 07`, 0.00004226243596622584`, 0.005080593760206975`, 0.4824829448895981`},
{0.24537724051627174`, 3.881922170050893`, 2.190905545388226`, 1.4415825883984728`, 0.23029515887672103`,
0.5607538040686315`, 0.02143037658176177`, 612.4231659367224`, 0.18957440226421673`, 2.1066205948825285`, 43.160389193329856`,
43.75501464608105`, 0.013777110537341875`, 93.76991476472435`, 32.92686287505176`, 0.17447808758752112`, 0.1791902203268937 5`,
0.027007017354021334`, 0.08272773376390691`, 0.0838915310109844`, 0.014067800411392994`, 9.675862234201132`, 0.2899929003593286`,
0.8064057464127842`, 0.000672331796820469`, 0.0007107043346535282`, 0.05707381089891528`, 0.3956113113723902 5`},
{0.06117754469042569`, 1.77753347893454`, 8.494748688583062`, 1.4528026068281314`, 0.13374021962928717`, 0.2747744172973582`,
0.02769785108344557`, 761.9216006517844`, 0.1674213538626742`, 9.223974549251146`, 104.36832791047857`, 105.1197481057999`,
0.007199695639139136 5`, 51.78632879320572`, 75.46861904212413`, 1.0935472838769573`, 1.1210771826352024`, 0.025174859070239197`,
0.012141139769928427`, 0.01214605249453416`, 0.0004046345482242586`, 27.7688129698461`, 0.010610930298090286`,
2.6508538564802544`, 0.00010098289144089279`, 0.00010123103726682673`, 0.00245730561279478`, 1.0093890397606287`},
{0.2543463901897882`, 3.8184481545424687`, 6.435093510144283`, 1.0179149591357712`, 0.6456387658124723`, 0.39719113288117625`,
0.03560663844887868`, 836.6099873310699`, 0.05172688927212099`, 4.4940967912431145`, 92.44872290932476`, 92.02056915772759`,
-0.004631256529277428`, 94.15057768237892`, 61.59112124759425`, 0.5536873714018056`, 0.5611285929803662`, 0.01343939190760523 6`,
0.020384942047733064`, 0.02040769074183574`, 0.0011159557897888472`, 30.20323602175279`, 0.07406909177238344`,

1.1420084036039557`, 0.00024136328921808836`, 0.00024295017965491992`, 0.006574696765081489`, 0.34667576585033727`},
{0.25340300959745826`, 3.7481040027914663`, 6.564583042000866`, 0.9665595115932711`, 0.6900722172611409`, 0.5320596915161446`,
0.007953381223150922`, 557.3521763213881`, 0.16633866213427612`, 7.2685179596650595`, 289.52289864699577`, 289.14761110857484`,
-0.0012962274838181909`, 182.3062446173804`, 259.8965047719404`, 0.5356625889828519`, 0.541564322981782`, 0.011017633339182265`,
0.03677144264645645`, 0.036798200433933066`, 0.0007276779845557524`, 28.68170134160381`, 0.13311420340662836`,
2.7799572170997733`, 0.00045883591425177617`, 0.00046115136245521805`, 0.005046353459967623`, 0.5818524134913311`},
{0.21905085798311597`, 3.1393917093979224`, 6.344370247647537`, 0.9036844621009067`, 0.45657431516696256`, 0.6738956741194089`,
0.01713634873689236`, 595.7124001741428`, 0.1327223790362297`, 6.393427116779321`, 189.3653513086606`, 196.68153362507658`,
0.03863527443566395`, 135.55991687424978`, 152.4373736442643`, 0.8015685466230221`, 0.812259436493256`, 0.013337461799460826`,
0.02644020939987861`, 0.026470827030813052`, 0.0011579950245999004`, 35.94910928260657`, 0.08273929363274411`,
2.4130243460001464`, 0.0003524736938466111`, 0.0003548067485437154`, 0.006619088850697841`, 0.889746743381509`},
{0.26756748312672074`, 2.582420366138944`, 0.22100971269994044`, 1.2986139421663903`, 0.40694219440887625`,
0.4206092167171217`, 0.07540978051777308`, 286.7043124505423`, 0.12484831726628037`, 7.450517421109604`, 358.1443080616925`,
355.4653409680756`, -0.0074801331008601535`, 220.00680467726752`, 171.36685336060785`, 4.712344995483103`, 4.901712074734102`,
0.04018531738073339`, 1.70412206275012`, 1.7318727524198265`, 0.016284449498249254`, 173.84650983726414`, 6.513823589582315`,
5.838406486682021`, 0.015043574588178066`, 0.01653003796745748`, 0.09881051678020358`, 1.030557255583229`},
{0.1649279191778415`, 1.2798961157951103`, 8.443353077105307`, 1.1073246765756934`, 0.5543130008347743`, 0.4677559431287117`,
0.04549043803104127`, 88.19757502008439`, 0.08859698434873292`, 4.4693006714200685`, 830.9965005068252`, 841.841968152713`,
0.013051159227834397`, 850.9893339985038`, 507.57474356486324`, 16.74347739194009`, 16.97348329791075`, 0.013737045213880084`,
0.15293531651638823`, 0.1529939977160896`, 0.0003836994687560491`, 306.1415954121058`, 0.3603329074544892`,
10.982874483842584`, 0.001663418242994763`, 0.0016736254210243588`, 0.006136266734227291`, 0.4385913371189736`},
{0.24671033429293737`, 0.7439602453864245`, 2.8830481632165945`, 1.0433060628070765`, 0.2605457162416671`, 0.35613575939670206`,
0.02699949405773168`, 552.8129873973498`, 0.15941770646667952`, 8.472285283746757`, 162.34510267091554`, 164.49692857816075`,
0.013254640095963444`, 87.70071128399192`, 117.89720218402408`, 3.803468398860317`, 3.8861556854154027`, 0.021739969386852964`,
0.04343289139614997`, 0.04348055142191923`, 0.0010973256496915607`, 40.42327547622332`, 0.15307633079499566`,
3.382502114321693`, 0.0005015226521700722`, 0.0005051266890509524`, 0.007186189627299333`, 1.0018296173128451`},
{0.15869950794106824`, 3.1033559606377414`, 9.144330738839969`, 1.4178115100024233`, 0.3048291795319098`, 0.2869274730275907`,
0.17534043095376647`, 52.531250426358234`, 0.14088030152015352`, 4.046496400392222`, 762.7960983951896`, 765.6579789691419`,
0.003751829066736656`, 862.7675440919043`, 228.99351370419785`, 11.761761904493795`, 12.025431214541992`, 0.022417501067374612`,
0.1833133041186296`, 0.1833487118855228`, 0.00019315437612910102`, 521.4419130558967`, 0.41559615946668527`,
16.71246308716773`, 0.0015585653416529954`, 0.0015667184988936496`, 0.005231193728462591`, 0.4016649578857332`},

```
{0.2764621058346162`, 2.9381918751992178`, 0.9446276562373105`, 1.4121699137634485`, 0.5065538844804354`,
 0.6085910850890284`, 0.12806524333884275`, 90.5764532701865`, 0.169606806379912`, 4.122343563126204`, 825.9337216489414`,
 835.7787599341478`, 0.011919889002172335`, 916.9919200246716`, 295.0810348082261`, 12.140690049044485`, 12.426840133568216`,
 0.02356950744708719`, 1.841938860501637`, 1.859448011016202`, 0.009505826110751903`, 509.5953837344924`, 7.2746613741855946`,
 10.252444646310394`, 0.015088011261897516`, 0.016119588468847326`, 0.06837065462397285`, 0.582715476493102`},
{0.23050133288232844`, 1.3238077162346595`, 3.1108482993453865`, 1.1555015486294062`, 0.6747942262463269`, 0.6285668584610278`,
 0.027391537177886824`, 109.70658223761104`, 0.027210691652561736`, 1.9248854769177905`, 408.6075170194269`, 409.0361909384147`,
 0.0010491092335127572`, 971.5517777915757`, 293.1930941527146`, 5.6880367805154`, 5.771606474097563`, 0.014692185864274787`,
 0.4887995532146294`, 0.4924516114935641 5`, 0.007471484486670787 5`, 107.5695282896116`, 1.6095564075465254`,
 3.8211638544657416`, 0.004985752467569826`, 0.005186044215478461`, 0.040172822299431754`, 0.19909577907336307`},
{0.16511179970471734`, 1.257711050374506`, 7.3270141910702415`, 1.3219934358839476`, 0.5519839493462388`, 0.6927265248357952`,
 0.057669326830527015`, 282.0308127392517`, 0.18961708461999577`, 6.119113848125975`, 442.86170787168476`, 458.076884142214`,
 0.034356495493030215`, 331.2410035570577`, 244.7683831245201`, 10.429232128158095`, 10.546804946548022`, 0.011273391650042042`,
 0.08181118888963318`, 0.0819285179314242`, 0.0014341441969423219`, 187.38514992150388`, 0.19297132333642025`,
 4.802157419237953`, 0.0007443509998270592`, 0.0007506995519767412`, 0.008528976452180537`, 0.7429583761116817`},
{0.18051596911200918`, 3.1154638731836117`, 5.271301524292921`, 0.9821224785779399`, 0.579876513557519`,
 0.2578243252832043`, 0.0132470372825880 7`, 298.19257336110485`, 0.04626579120349833`, 3.252038865320179`,
 135.87857742329007`, 134.95470263162522`, -0.006799267472360815`, 191.2316440804886`, 114.41177619874017`,
 0.46636582573546453`, 0.4751451710102681`, 0.018825018451895437`, 0.048760874697044446`,
 0.048809859336172275`, 0.0010045890159309234`, 20.756369739518345`, 0.12574452215267032`, 2.299941881229796`,
 0.0005982355016240781`, 0.0006024083699483129`, 0.006975293697726537`, 0.22281078228211662`},
{0.2700603276661351`, 3.8874233694867435`, 9.935191315459868`, 0.9369398631417749`, 0.9680175802467335`,
 0.2817307366148821`, 0.03116132832319949`, 267.3428425281449`, 0.2310886914635361`, 4.719763737676903`,
 431.12192213130857`, 422.1325964721197`, -0.020851005707965165`, 418.06551207085346`, 299.22015691126535`,
 2.3277043866375067`, 2.3490219323009236`, 0.009158184254750346`, 0.05403913119174438`, 0.054047290650371976`,
 0.00015099166932652786`, 129.2681775695919`, 0.20848322109192052`, 3.6090002604897196`,
 0.0006966273900737008`, 0.0006987425741378977`, 0.003036320555775296`, 0.16876089750513132`},
{0.2505990837413898`, 3.9386610341401305`, 4.197732331385515`, 1.4863985983426793`, 0.6450186838764853`,
 0.5983146588433161`, 0.0058599277111039`, 4347.60789332823`, 0.1797443484893408`, 5.002549103667693`,
 25.067763174355747`, 25.33920199500803`, 0.010828202690615907`, 22.934470484330202`, 23.080392816883133`,
 0.033381292613262885`, 0.033813811130768916`, 0.012956913398080472`, 0.011076655405248905`,
```

0.01111693184321781`, 0.0036361551836141537`, 1.8782513783584045`, 0.03965428136392051`, 0.24915236548212405`,
0.00008923380798653291`, 0.00009072418443508954`, 0.016701925897654624`, 0.4857493041104974`},
{0.15385799276986173`, 3.328452062918277`, 1.1907303286310018`, 0.9499432416831306`, 0.24618506024325426`, 0.6220480490433855`,
0.08397939385457624`, 160.09510109398894`, 0.21511864519397922`, 7.34323009363505`, 625.6597203926133`, 640.846626530443`,
0.02427342793987064`, 389.9559527043332`, 289.2364477303063`, 6.901545900109782`, 7.028541572088081`, 0.018401047217012412`,
0.42446179975547194`, 0.4258064384366009`, 0.0031678673602750784`, 328.1637812649357`, 0.9329548645411438`,
10.668503709577744`, 0.005308841413530385`, 0.005438150674191897`, 0.02435734100701281`, 1.310510818890985`},
{0.10621732051626104`, 1.365898117434102`, 6.864395407461604`, 0.9825773772303991`, 0.8367483775504436`, 0.5624371522540671`,
0.07041397408927531`, 60.72834180417396`, 0.20758521960363197`, 6.929584578355666`, 3619.743151129345`, 3462.48657098948`,
-0.04344412671678133`, 2390.754874082628`, 1815.8147438276183`, 87.88325043662952`, 88.49132417251552`, 0.006919108395114115`,
0.47013703594058825`, 0.46926313700854494`, -0.0018588174622213849`, 1714.8509475054566`, 0.7133813747583017`,
23.809724958451188`, 0.005754083977474012`, 0.005783381685931383`, 0.005091637273989535`, 0.3964360080479024`},
{0.2268285366732684`, 3.377330494378361`, 0.792106071900994`, 1.4321394301198889`, 0.4771428999999012`, 0.6107585820948693`,
0.0427110994331284`, 2178.207920314213`, 0.06460930790004932`, 5.987666522418898`, 48.18564459381372`, 49.02494490095971`,
0.017418057062865255`, 36.83193733055787`, 29.916155422983167`, 0.3632470246936652`, 0.37077193775797335`, 0.02071569084606839`,
0.08932006354327866`, 0.09061666616270395`, 0.014516364722434894`, 17.525789335573165`, 0.28943341870123346`,
0.620221500062163`, 0.0007301344469975435`, 0.0007721298071899478`, 0.05751729748554868`, 0.8572551564229202`},
{0.27738294555080567`, 1.5184858354681863`, 2.5078605202307482`, 1.4658418288357904`, 0.7951974935096349`,
0.3102987260459962`, 0.028082255793227213`, 4738.743053997216`, 0.039514889508313555`, 1.270204126939964`,
5.035178324388556`, 4.816261691249143`, -0.04347743397270776`, 18.14286342117792`, 3.558863530248904`,
0.06192494437691002`, 0.06352886476140755`, 0.025901038759682526`, 0.014186440714080841`,
0.014340562203152926`, 0.010863999799407775`, 1.343316441407047`, 0.05621538160219519`, 0.0563629155621636`,
0.0001144018178771411`, 0.00012013002022334682`, 0.050070903177057646`, 0.08304870548416285`},
{0.12722141666194153`, 2.144800891916389`, 0.9267204145449971`, 1.474327309271881`, 0.07507296673368935`, 0.4110764361383803`,
0.01396212306727472`, 341.96044988390173`, 0.08796076167353961`, 8.378052186889676`, 238.93991798241555`, 240.40545798359213`,
0.006133508429865708`, 130.52994159653733`, 199.31649913764352`, 1.223548759785119`, 1.2557989357072825`, 0.026357900054450933`,
0.28232307335903595`, 0.28389500219610764`, 0.005567836940739923`, 37.48954958986166`, 0.5131077335583996`,
5.617359376698799`, 0.0022753839161886447`, 0.0023388938460300462`, 0.02791174244906469`, 1.3545824948280727`},
{0.14199823373416764`, 1.4769393574961134`, 9.82023825383516`, 0.8826223667346553`, 0.41403084809983204`, 0.6165765653923829`,
0.06076020685200448 5`, 76.65984772713675`, 0.1884299459763023`, 7.821991164404671`, 1636.9042138555903`, 1693.69650188925 6`,
0.03469493666944401`, 957.7903130106113`, 886.2772315783122`, 33.94964739372324`, 34.418478061183684`, 0.013809588713051824`,

0.11807653453800217`, 0.11805575210403735`, -0.00017600816323182222`, 716.3081486986456`, 0.23952370499815`,
22.935990698099516`, 0.0016142074259031247`, 0.001619561757876556`, 0.0033170036808842784`, 1.0865745788246686`},
{0.2704769900712498`, 1.6373927417773038`, 3.0886177841931914`, 1.4817168381054997`, 0.05399471731043759`,
0.4806448506103316`, 0.008090658829532001`, 56.76295821829396`, 0.22108849119145935`, 4.3771215003685064`, 779.5559450258826`,
788.0816867682037`, 0.010936664387875217`, 815.1230619123879`, 697.6088432512298`, 3.2272072389286897`, 3.311286040088929`,
0.026053114949057488`, 0.5333059579083823`, 0.5346035665953037`, 0.0024331411784910273`, 75.48865298904298`, 2.060671289744632`,
18.021478289822806`, 0.004291395822467292`, 0.004382360600871366`, 0.021197014250662782`, 0.805837424563105`},
{0.15635355369607173`, 1.2350233440589857`, 3.2938536959067304`, 0.7696047462951592`, 0.9490314349758091`,
0.5590905433465692`, 0.008743912631106248`, 94.37874467585033`, 0.17474749461052458`, 8.353315585312398`, 3793.839792252785`,
3575.664599969038`, -0.05750775051947943`, 2078.6654272233573`, 3370.0717537238406`, 22.45738975849383`, 22.59683249669039`,
0.006209213969037508`, 0.6684030770271004`, 0.6658251083936441`, -0.003856907189779157 4`, 396.2200085481584`,
1.492959948493735`, 18.1935655973344`, 0.01040943081511958`, 0.010479230312437469`, 0.00670540959996635`, 0.3465862191082777 5`},
{0.16339273559725687`, 2.0059391082075457`, 1.9960227683476044`, 0.8832597488866586`, 0.2201232888636404`, 0.41940637033719763`,
0.260701203173729`, 668.3313383270491`, 0.16081430237854888`, 8.573990161395482`, 142.19938842166056`, 143.2528471923 6257`,
0.007408321388684191`, 75.90654816199962`, 32.21477845030106`, 3.703479492030856`, 3.7799705699665185`, 0.020653841367356263`,
0.0459516850246602`, 0.046024803742914455`, 0.001591208640445263`, 106.12791927870482`, 0.10725959316388652`,
2.8783429961082247`, 0.0006256838327427516`, 0.0006314847239025223`, 0.00927128184588355`, 1.1726897618212566`},
{0.17131668518162624`, 1.9273467762184806`, 5.930228637778157`, 0.8979121960709446`, 0.761163722566164`, 0.6467717629026779`,
0.01019608706680365`, 260.442341207733`, 0.11039361670332914`, 7.204688135978646`, 802.6573156641324`, 792.3318933175954`,
-0.012864048137396833`, 509.8935008379228`, 700.5568825237677`, 3.5464752207483077`, 3.576474163917807`, 0.008458805236814637`,
0.10580083433217306`, 0.10585298198888289`, 0.0004928851179575311`, 97.64696548068542`, 0.2589349746748813`,
5.86384276765807`, 0.001419319857430934`, 0.0014277663307036316`, 0.005951071020725562`, 0.5330894220282161`},
{0.11942926109779373`, 3.6640895220352094`, 2.8978096019149504`, 1.1395608488550686`, 0.02531057599926112`,
0.3982329172386545`, 0.26228943719870584`, 89.66215495367044`, 0.22730124409898161`, 8.643250664580492`,
899.9512916464267`, 895.9059894825732`, -0.004495023454494706 5`, 476.54771450889626`, 205.55245452905896`,
13.004314223157994`, 13.316699020181774`, 0.02402162787388562`, 0.25679827881479494`, 0.2568604120494898`,
0.0002419534701774139 8`, 680.699592661808`, 0.43813183842905445`, 22.001094947158375`,
0.0027096532537571605`, 0.002730773259883954`, 0.00779435748744195`, 1.351588296885622`},
{0.06995397995088021`, 2.4465420953300523`, 1.0825256418482638`, 1.4045898231548657`, 0.8050454938859057`,
0.5373350569389552`, 0.3667462640330922`, 406.36701228872533`, 0.024784828878876453`, 8.741309230400322`, 621.407063619733`,
596.1152900300608`, -0.04070081444254292`, 325.36002764379316`, 103.19032666150366`, 14.382669964189112`, 14.51464859978620 2`,

0.009176226383953656`, 0.5757405570127144`, 0.5779972888319148`, 0.0039197027058675005`, 502.6829644375398`, 0.575362048317322`,
4.529194483423165`, 0.004865112550196837`, 0.004990856714274586`, 0.0258460956001234l`, 0.5839679374694695`},
{0.25135395656732756`, 1.8293827828852027`, 0.6280251816297326`, 0.776655925877336`, 0.712342015777852`, 0.6529270906121196`,
0.026921744082008515`, 2433.232128346219`, 0.014846384799260759`, 4.779446799164496`, 51.966090118808694`, 51.45791768695105`,
-0.00977892373076017`, 49.763040194888994`, 37.36845950956336`, 0.5237011888307953`, 0.5319705081226911`, 0.015790148023833384`,
0.08273116385560579`, 0.0837896794438324`, 0.01279464157030441`, 13.686427688908136`, 0.2970686480932349`,
0.42584335034786924`, 0.0012504472214309725`, 0.0013157668635280003`, 0.05223702446415768`, 0.47051019182779497`},
{0.22686431298761822`, 3.544454161776195`, 7.257561779040259`, 1.4835566223233323`, 0.41530778886586006`, 0.569342000607929 2`,
0.0281206594086524`, 300.82869963510313`, 0.2233455291441253`, 1.8496714258079727`, 92.09882975228088`, 94.54736795300296`,
0.026585986025098762`, 227.8893444177526`, 65.88401449659557`, 0.5022514740416353`, 0.5111807286118067`, 0.017778453686392304`,
0.06338344406292856`, 0.0637118314977597 5`, 0.005180965466394571 5`, 25.43153324893003`, 0.20542059274465216`,
1.3883680467995227`, 0.000509378120929771`, 0.0005214136247399008`, 0.023627838172871174`, 0.26647740946908505`},
{0.11505780978488611`, 2.035276516222745`, 0.3473218826840405`, 1.3302263404025527`, 0.8863987944255065`,
0.20525449010517516`, 0.166947674879067`, 1822.5924091420022`, 0.2368153750944742`, 8.635263957438244`,
83.16335311620116`, 80.35021178707962`, -0.033826694375716726`, 44.077897324961604`, 25.072492914844357`,
1.9114133276166452`, 1.948268501092122`, 0.019281634664247038`, 0.22861297719340387`,
0.22987286665604992`, 0.005511014633172939`, 55.57506654990479`, 0.37576726348961753`, 0.9759764431677309`,
0.0020455289921857966`, 0.0021073555729156355`, 0.030225228273969673`, 0.37735947811094805`},
{0.26450836027154073`, 2.000822664278666`, 6.699190267572199`, 1.2017845913999912`, 0.3438445280396565`, 0.6571515800951808`,
0.025420529665258214`, 670.8719376728277`, 0.0689542698910992`, 7.858447976519758`, 179.96524283824235`, 186.57460278879952`,
0.03672575796481903`, 104.81328894884135`, 132.58374317587536`, 1.5964394542818034`, 1.6206399472882633`, 0.015159042168214842`,
0.02574299828355005`, 0.02577401150966274`, 0.0012047247088737834`, 45.631317746795666`, 0.0972748323493335`,
2.6806195783911013`, 0.00025806582774223763`, 0.0002598023852240977`, 0.006729126041416622`, 1.253204441454277`},
{0.1895156330025849`, 2.3307833365396586`, 5.573081915828208`, 1.037167544233136`, 0.6437280244785568`, 0.6985848360999776`,
0.020283589241000377`, 257.16539910388553`, 0.09483519874959423`, 5.443513900146426`, 503.3968448634947`, 514.4863646190738`,
0.022029378747072226`, 423.24871439163417`, 390.9005087558659`, 3.264445083351863`, 3.2975512355281666`, 0.010141433331238847`,
0.10780039479450966`, 0.10795987565480542`, 0.0014794088704384034`, 108.69591719036204`, 0.2918551436770524`,
4.607344692839802`, 0.001248809083011615`, 0.0012610993988484533`, 0.009841629120120654`, 0.5597242603534988`},
{0.05605442916152037`, 2.091827585999483`, 5.134093728882359`, 0.7852275512421112`, 0.9528052242020535`, 0.26916872175155115`,
0.16382211865694157`, 830.5018741809612`, 0.0523186960509337`, 8.350099727317033`, 235.96375613757664`, 229.7808911145177`,
-0.026202604689230746`, 129.3356151535799`, 72.0646193698409`, 5.305474317611733`, 5.35314778691358`, 0.008985712953805791`,

0.027121867861935493`, 0.027128053046065286`, 0.0002280515546082551`, 158.5448219198857`, 0.021718583154117167`,
2.0547608607043832`, 0.00041732993506604643`, 0.00041831499984367013`, 0.002360398080401005`, 0.29607951911364233`},
{0.160097742020257`, 2.0916642582432887`, 7.779572730473025`, 1.4293729759208196`, 0.262481852877007`, 0.6066507214539851`,
0.010299825795425889`, 166.88640436294955`, 0.11119499215436468`, 1.1831617215189443`, 92.90831847007378`, 95.29698763613041`,
0.025709960156323763`, 359.39744388567726`, 80.87904536686874`, 0.3779880799703345`, 0.38590435029979375`, 0.020943174531007802`,
0.08959827596229532`, 0.09028881201391599`, 0.007707023870763718`, 11.294630813085071`, 0.2049211667210219`,
1.659720546213165`, 0.0007430114193562876`, 0.0007671288859822772`, 0.03245907935962`, 0.22022790345216353`},
{0.096645259209576`, 1.264318941007299`, 4.097121191163998`, 1.119171761441141`, 0.3848067085465605`, 0.38298305619587747`,
0.22103546881327146`, 186.61459180270506`, 0.035388155328102466`, 2.6745014933563738`, 172.59648176597855`, 173.79978678012495`,
0.006971781822169065`, 295.3612985252195`, 43.75831786161176`, 6.7452450284703`, 6.876799728217372`, 0.019503324073745`,
0.11019383092693066`, 0.11050139491668745`, 0.002791118043266261`, 121.8305864461473`, 0.15213873361742697`,
3.151304013275783`, 0.0011792989022809675`, 0.0011970819320406383`, 0.01507932359241182`, 0.3051003555342869`},
{0.08994789013179427`, 1.57009542864993`, 6.7950768142652755`, 1.2471941772808341`, 0.17502080201658954`, 0.4646155607499266`,
0.031711372215814826`, 510.77004725104206`, 0.22094423048581935`, 5.498912040996139`, 117.83016508104464`, 119.96732361506078`,
0.018137618092499475`, 98.07181389653461`, 81.76335551236332`, 1.5364538357712385`, 1.567945379759813`, 0.020496251338893545`,
0.024648346718195897`, 0.024679405486446603`, 0.0012600751119657971`, 34.462559198229634`, 0.031672382607682376`,
2.456999111234236`, 0.0002380820642816639`, 0.00023966186522264828`, 0.00663553109618297 8`, 0.859559824183392`},
{0.09559160148361812`, 1.3237772094663507`, 8.951678422145957`, 0.7806574543205589`, 0.8827653082752778`,
0.45488871998545977`, 0.011151733542884613`, 3151.7776331194414`, 0.22534056788365686`, 6.821338260162536`,
66.04616189235959`, 62.346854959035475`, -0.05601092973961397`, 44.31415713266297`, 56.98554901212675`,
0.4523628266572074`, 0.45582364362169947`, 0.007650533511045054 5`, 0.005297461158860481`,
0.005299986453587086`, 0.00047669905467473406`, 8.554680004836971`, 0.007234182799611685`, 0.447189251868076`,
0.00008197808045529165`, 0.00008220289612462089`, 0.0027423875758088023`, 0.3198298886618479`},
{0.20804533970301103`, 1.865488871682949`, 2.4561594295175926`, 0.8614398462681598`, 0.7232950760420711`,
0.3427980526897182`, 0.02113398228875948`, 356.75904569388376`, 0.23964513558905903`, 4.163032360670947`,
209.5986914034638 5`, 205.07945531435757`, -0.021561375497364366`, 230.43225758889437`,
161.17836659032378`, 1.7334277483983962`, 1.7579111440013353`, 0.014124266572725919`, 0.11043455189008475`,
0.11062643468711676`, 0.001737525020457209`, 46.19557392148055`, 0.32821991232752623`, 2.450445252125806`,
0.0015388963594118454`, 0.0015578865177273112`, 0.012340115173658406`, 0.26634142267468425`},
{0.23422673966465207`, 3.962742244147649`, 9.344278992351082`, 0.9436428211653483`, 0.6202800294740252`,
0.15583995298414144`, 0.0468134491479599`, 1145.0934679847246`, 0.11614669946806178`, 8.273469182440888`,

```
76.81031440192598`, 75.9401623927921`, -0.011328582833037504`, 42.490944410450695`, 46.60408567150128`,
0.5238406907624106`, 0.5343119792410351`, 0.019989452257678675`, 0.005883291350471605`, 0.005883994959572334`,
0.00011959446826859121`, 29.65493763553837`, 0.019686059307485484`, 1.4916821707130927`,
0.00007541714523784382`, 0.00007550916796514948`, 0.0012201831163913823`, 0.380982735856193`},
{0.23911413452544644`, 3.701379132317136`, 3.3596187018850023`, 1.2070387760959833`, 0.14815711868787962`,
0.5552188532365824`, 0.010060549211211993`, 324.2801725236705`, 0.014653386006449387`, 6.641284984634726`, 240.16784568748415`,
245.2566634936187`, 0.02118858913676691`, 165.5111439785637`, 210.0856353138165`, 0.5486472004257332`, 0.5601648240231768`,
0.020992768373749637`, 0.081262797812335`, 0.0814453508868006`, 0.002246453228046396`7`, 29.010732838000404`, 0.277586908114455`2`,
4.774386582171369`, 0.000807915888416666`, 0.0008180622416312762`, 0.012558675179039858`, 1.2031254661418018`},
{0.16379493488608782`, 3.773301948253276`, 6.66686177778092`, 1.3720180958436647`, 0.9115747518300852`, 0.36809943749279594`,
0.006089940921734969`, 188.41929546062013`, 0.11766502525697531`, 3.8904832664355844`, 560.5335389657265`, 532.1036746548102`,
-0.05071929212902038`, 659.4206547862593`, 514.9361731471528`, 0.8092059271680861`, 0.8171108360782545`, 0.009768723442044491`,
0.18577808832495663`, 0.1859422905013938`, 0.0008838619124444946`, 43.61969002173483`, 0.43470728400640785`,
4.25350630297008`, 0.0016287932608018973`, 0.0016424425696409345`, 0.008380013085464988`, 0.17015954949887857`},
{0.27173801863077446`, 2.1930338431922545`, 7.869758966833125`, 1.0214571559787395`, 0.457397629072652`, 0.6590886785471264`,
0.009120521470678952`, 4004.0778102470904`, 0.1596904202504179`, 8.692703920363371`, 38.135430179898115`, 39.572692393228934`,
0.03768837027799998`, 20.07882325784344`, 33.752806213078706`, 0.13414546384005285`, 0.13597722378975152`, 0.013655027141899811`,
0.003570140342037137`, 0.0035731969514181273`, 0.0008561594470108957`, 4.202650601599407`, 0.013859183753982958`,
0.4865177495686193`, 0.000042172368519666215`, 0.000042366825442869866`, 0.004611003129050273`, 1.1774410356920522`},
{0.2361473928644357`, 1.7414292069064263`, 7.944932185587515`, 1.1578150203530142`, 0.6270752096378571`,
0.30275674150027165`, 0.11839453780639704`, 3486.2324040163485`, 0.1006900893181421`, 6.697770970769804`,
28.185891662149604`, 27.940616848525956`, -0.008702042020299894`, 19.260430060989446`, 10.717426934649566`,
0.6743929733716072`, 0.6844974142679163`, 0.01498301627579579`, 0.0038456281313301007`, 0.0038478157443554784`,
0.0005688571413224874`, 16.777251725168366`, 0.012973357959135177`, 0.40472024113821586`,
0.0000401183375466907`, 0.000040255468290667935`, 0.0034181561939758964`, 0.45103638093140724`},
{0.08448906159522324`, 2.163318965633623`, 2.5852320656449397`, 0.9870547358990118`, 0.27911648709335135`, 0.1950863385031143`,
0.04472304557991584`, 1858.3083947017237`, 0.05117109151128901`, 9.531470241589851`, 43.93123088644959`, 44.008981881195744`,
0.0017698341971597031`, 21.09492424399985`, 27.202229629085405`, 0.5235405857065698`, 0.5367862227968139`, 0.025300115123582545`,
0.011034798543572172`, 0.011041736465825432`, 0.0006287312111648014`, 16.179789690542293`, 0.013318853911957213`,
1.0884385147995517`, 0.00013498994445504398`, 0.00013549633045223652`, 0.0037512868031528424`, 0.7166455338648993`},
{0.1142146683211071`, 3.422389653131356`, 1.8177299602895634`, 1.2969315528452774`, 0.1391750354375907`, 0.5170758706651055`,
```

0.01897326911835137`, 87.52693577556593`, 0.11000871045324723`, 3.4664980752399295`, 442.38517440621655`, 447.7609477503367 5`,
0.012151793629467056`, 584.0823669208339`, 348.59790516174166`, 1.847274688317379`, 1.8908563224686479`, 0.023592395016772683`,
0.5660371598320915`, 0.5696998956528795`, 0.006470839868312872`, 90.31562542555504`, 0.9235678066806701`,
9.267556311534747`, 0.005152036797230308`, 0.005335729783344225`, 0.03565443985428618`, 0.643383343212631`},
{0.14347242790835574`, 2.011431525472254`, 0.8946353742157331`, 1.3818589873326788`, 0.974530392330166`, 0.4363768778472087`,
0.006586014840232095`, 142.93650600689625`, 0.2189385420017279`, 6.889239014732425`, 1761.062890775304`, 1699.1087647897052`,
-0.03517996223196973`, 1169.9521718602623`, 1602.0729426523253`, 5.037738183669714`, 5.093121198535374`, 0.010993627069622036`,
2.475518125355858`, 2.4682219754978707`, -0.002947322333557212`, 144.75807713869443`, 5.073837082513563`,
9.940083961047785`, 0.021065769282163638`, 0.02171556529163555`, 0.03084606124600886`, 0.3094853983582026`},
{0.07350179944910268`, 2.001951652427108`, 6.856579719843172`, 1.3883617808830775`, 0.83650706130597`, 0.6246458892820506`,
0.03443555932639553`, 159.6804633773726`, 0.22561794329975743`, 6.769156049603524`, 1450.5677731348214`, 1386.2525862097975`,
-0.044337940023327804`, 980.7718522472919`, 974.7326732536071`, 16.053344021869094`, 16.159026451832233`, 0.006583203463351284`,
0.2721791264825443`, 0.27230437990090295`, 0.00046018745073284073`, 459.1145513080231`, 0.2857950795563741`,
8.866653021099928`, 0.002357339745116871`, 0.0023752512815213567`, 0.007598198962024405`, 0.4043890173419811`},
{0.2038713609791254`, 2.9048730428426977`, 6.578839990238183`, 1.1970767733981913`, 0.3047670438201835`, 0.4988995484689318`,
0.027219703475022124`, 126.68486054839573`, 0.1069997024432573`, 5.709865936189522`, 574.3778598226409`, 588.1721684885763`,
0.02401608702360991`, 460.40101703802213`, 415.87959937577`, 3.717013244477876`, 3.784045871924409`, 0.018034002850573216`,
0.11472709997711826`, 0.11482271867930657`, 0.0008334447764075659`, 154.24930819675765`, 0.33413671447880255`,
10.163360058050188`, 0.00115389453310355`, 0.0011620800574153026`, 0.00709382363545541`, 0.8136895798734217`},
{0.10518373092933642`, 1.253339618555911`, 0.9435760784539173`, 1.312750209379657`, 0.30938419913876847`, 0.5412461470886345`,
0.010398682812166098`, 560.2137041073427`, 0.038740677468453555`, 9.486718448691846`, 229.96781122572045`, 234.97140862671975`,
0.02175781634103613`, 110.94699269193946`, 200.10635996349257`, 1.5436690000983593`, 1.5733421390665658`, 0.019222475133150896`,
0.21000865891598255`, 0.2112418024564231`, 0.005871869982912781`, 27.639164510855153`, 0.31556420388927564`,
3.8540581892212096`, 0.0019003302514700726`, 0.0019524850190382856`, 0.027445107253261103`, 1.4527023700545607`},
{0.21800976245075693`, 1.265555352818649`, 7.66080710349685`, 0.9976040672991493`, 0.42066904207729006`, 0.6052137627060103`,
0.1434637338398527`, 296.0621032273413`, 0.0508815854649306`, 9.05489394101457`, 492.8645616832263`, 507.9712975264508`,
0.030650886709387537`, 249.1200710808149`, 165.97484755866606`, 17.1235694909768`, 17.361281067608612`, 0.0138821275994514`,
0.04446003104627356`, 0.0448256073655916`, 0.0005067403183356411`, 309.5832146952536`, 0.1384674400996869`,
6.852885245570723`, 0.0005377851494351438`, 0.0005399869816040576`, 0.004094259893986374`, 1.224762796147186`},
{0.14099839999887143`, 0.709118554874764`, 1.895334494340693`, 1.0269112445912816`, 0.6721979672345315`, 0.244304954855444`,
0.071658194148916 7`, 50.83934571528565`, 0.0295628703988986`, 3.2095234353654245`, 864.9946542511808`, 848.5840906059378`,

-0.018971867126103215`, 1233.4949249578667`, 432.68904517639925`, 38.5923981143075`, 39.312627783281876`, 0.018662475103027 1`,
0.9155013704593677`, 0.9138142371376159`, -0.0018428517708338754`, 390.95122257098984`, 1.8440604061641948`,
13.218911806437601`, 0.010628866511376733`, 0.010806886690286906`, 0.01674874538311566`, 0.19548342399180244`},
{0.16175267254619935`, 2.3816849613140647`, 5.017466007223192`, 1.1449585227321033`, 0.0709392075018902`, 0.21629140918390133`,
0.24748736884470657`, 967.6089395954832`, 0.2384053881049194`, 4.566103496741098`, 35.42090223683654`, 35.21388364659763`,
-0.005844531820638377`, 35.504088123317835`, 8.563701966772502`, 0.7657727182946159`, 0.7883509277287847`, 0.0294842175684328 9`,
0.01109181580468887`, 0.01110175064649188`, 0.0008956911995248173`, 26.054705242098162`, 0.025630440711399836`,
1.0228180570129153`, 0.0001168783601114276`, 0.0001175013947045754 7`, 0.005330624014179275`, 0.4483151750221562`},
{0.15219039437367055`, 3.36973706117323`, 6.4489465173864`, 1.4529455229052564`, 0.15905243014518256`,
0.19120675078037952`, 0.09107249936889779`, 1785.557342737041`, 0.07266026496560668`, 8.481021831493972`,
36.83026531274018`, 36.87130719182587`, 0.0011143519802854218`, 19.875641782874776`, 16.551166348667298`,
0.41229089624945175`, 0.42378296909955465`, 0.027873700231182852`, 0.006136562398943214`,
0.006139182603383123`, 0.00042698244873373703`, 19.847313043944375`, 0.013341797879877621`, 1.0141125407136913`,
0.000051039470782887 62`, 0.00005117778473838582`, 0.0027099410196975704`, 0.6934757095667906`},
{0.1147282820108807`, 1.275793939396646`, 2.8627986987042053`, 1.2289286563020643`, 0.9300053983658252`, 0.5333937455451996`,
0.40966617997380966`, 55.29536930365472`, 0.09207723861615985`, 9.133686457942215`, 6512.647310302873`, 6107.237475112454`,
-0.0622496222924691 1`, 3263.442427396805`, 978.6801274847853`, 287.5777447280656`, 289.3130643320633`, 0.006034262510955290 5`,
1.9342833826034669`, 1.9201380643106056`, -0.007312950325728429`, 5241.284911848886`, 3.1702429915476436`,
34.03676409455863`, 0.018741980556521876`, 0.01892928638893783`, 0.009993918831101123`, 0.402162441641722`},
{0.22214055647639447`, 3.77956922277194`, 8.844837651533133`, 1.3011494481281731`, 0.35962071678441987`, 0.36854620389209614`,
0.030699018263752917`, 153.2818434470183`, 0.18511332787655327`, 9.937679092387768`, 768.29514744178`, 779.0963116072494`,
0.01405861302317768`, 353.8405622560528`, 537.9733771898658`, 4.181249366629301`, 4.259054989604967`, 0.01860822356031555`,
0.07136210428765895`, 0.0713732051315912`, 0.00015555656665489082`, 225.76173455495427`, 0.22646310796787317`,
14.165751129111332`, 0.0006624493725649883`, 0.0006643039706614499`, 0.0027996072956950613`, 1.0638399728140262`},
{0.11158828176697494`, 2.207019844443386`, 8.337407964588238`, 0.903037166955644`, 0.0492331295750732`, 0.5332115600383506`,
0.10225753888572825`, 270.6598424430625`, 0.11536791182647016`, 7.224089837931905`, 285.1453668152983`, 288.42961260747984`,
0.011517794691396466`, 180.65403900784202`, 119.24444024459109`, 5.097975555736984`, 5.203728647218374`, 0.020744134671728975`,
0.026819018572694173`, 0.0268333499462711`, 0.000534373528176646`, 160.73333168569755`, 0.0427526885884367`,
6.265440329288738`, 0.0003584819299842223 5`, 0.0003598037791714142 5`, 0.0036873523506473216`, 1.3679791643673906`},
{0.11228066784233581`, 2.8383409912516155`, 8.777526606092515`, 0.8243326677721828`, 0.7215210925398621`,
0.47575335568293875`, 0.01179892034723288`, 1881.3977477117185`, 0.1350202375088484`, 6.891832227766253`,

80.57974948193227`, 79.5875429519073`, -0.012313348408305003`, 53.51255792201586`, 69.01171748777712`,
0.2768640078769646`, 0.27975208124241174`, 0.010431378883782427`, 0.006888410158673414`,
0.006892043458488472 5`, 0.0005274511434953943`, 11.226206607991422`, 0.011049075614114294`, 0.7742053992973389`,
0.00010092736253219936`, 0.00010123545846301717`, 0.003052650174223403 6`, 0.488215132056916`},
{0.18415971316226493`, 3.627641157385548`, 4.072786596426541`, 1.1318163513169097`, 0.431227341960456`, 0.35638232246082124`,
0.12315140656794354`, 98.11903649760767`, 0.20630818704214687`, 2.4221856850573946`, 300.597808223481`, 302.5086012930816`,
0.00635664338636821`, 567.9928531375411`, 111.98140838016654`, 3.5558866030254412`, 3.628278278980525`, 0.020358263363486007`,
0.21543476108127554`, 0.21594918230225227`, 0.002387828307719886`, 184.2782941733001`, 0.5667771972272608`,
5.379369502187245`, 0.002277965972542373`, 0.0023157960776485674`, 0.016606966724781014`, 0.25209498203998415`},
{0.2708373028347817`, 0.6873063866109219`, 4.963135106708849`, 1.480469510663712`, 0.1951402154410362`, 0.5673083531043152`,
0.10423026212852673`, 2550.63829867399`, 0.08286966503737198`, 2.6901867167370597`, 12.842561999478193`, 13.074087109963658`,
0.01802795349517261`, 21.84910952649589`, 5.279121018492048`, 0.6951219322253616`, 0.7101471224592312`, 0.021615186541112275`,
0.00886185542229803`, 0.008909764928940384`, 0.005406261370706211`, 6.825167764168789`, 0.03428744315267513`,
0.24623727823705724`, 0.00007137479330610041`, 0.00007310157626509894`, 0.024193176316364084`, 0.49199526192185344`},
{0.2248876472394919`, 0.40722939312005924`, 9.89488647226247`, 1.363579526182362`, 0.9039905300116995`, 0.6204673017769224`,
0.16140635189013572`, 124.79693831214993`, 0.1938029988590731`, 3.2922153328074497`, 1061.983866360389`, 990.3953472142992`,
-0.06741017581692321`, 1476.366494286799`, 324.69743535120136`, 107.97308152400957`, 108.6510982172682`, 0.006279497479266372`,
0.2784851963367189`, 0.27876740941468625`, 0.0010133862829322382`, 628.140178033218`, 0.8946840085028944`,
5.4698123895139625`, 0.002451590673821724`, 0.0024776072450169023`, 0.010612118683997807`, 0.16637158758212656`},
{0.23989116476268346`, 3.6056249656972357`, 9.989783431378257`, 1.4358686490541388`, 0.7020019529209951`, 0.17871498413227982`,
0.15143540034751335`, 1116.6227738463333`, 0.03611482374706981`, 5.098351562607867`, 55.84143155872808`, 54.81918623540333`,
-0.01830621627688156`, 50.1292559540677`, 18.27883760321687`, 0.7145240408148279`, 0.7272835238731256`, 0.017857318059931293`,
0.009871715198750342`, 0.009875712107261693`, 0.0004048849091449202 6`, 36.804367430754574`, 0.03383053224618412`,
0.9427823220494033`, 0.00008308356520414506`, 0.00008332671923459505`, 0.0029266200824740185`, 0.23549164373126633`},
{0.10260002089245757`, 1.5297774189947768`, 6.3178804183723365`, 1.026166177715942`, 0.11867434322817161`, 0.5793673959868294`,
0.011727656957059851`, 115.5782430006307`, 0.20678944231462698`, 5.80216317155719`, 583.8747062961285`, 596.8138967483573`,
0.022160902523608028`, 460.5684859767922`, 501.1345165794399`, 3.5942492714943906`, 3.6660665560472583`, 0.019981164111917193`,
0.10248167484542704`, 0.10256779932596645`, 0.0008403890809678405`, 78.54859105386504`, 0.15020888543193153`,
11.800136558101077`, 0.001202232429528327`, 0.001210519847526024`, 0.006893357552289814`, 1.1079546855770526`},
{0.15334449354379004`, 1.7605906069617427`, 2.299359928311352`, 0.8222860702278779`, 0.9712651951139977`,
0.6108690739001781`, 0.14744839931335676`, 2744.2788694525566`, 0.1958145599491105`, 2.932280984329149`, 50.22158855674389`,

48.01014166132958`, -0.04403379022779541`, 78.38795892782409`, 16.27723346021059`, 1.2933455243950647`, 1.3022102316394129`,
0.006854090478640273`, 0.0381414147221829`, 0.03835874171226288`, 0.00569792682476411`, 32.52931402579931`, 0.08355394176595539`,
0.2207037163457099`, 0.0005526156230863943`, 0.0005660978370239966`, 0.024397091530462323`, 0.13645991223613504`},
{0.19466496130115485`, 2.7024357312795884`, 5.257508792652555`, 1.2867770062917014`, 0.4348182682381361`, 0.22799121214772633`,
0.23891047775514818`, 1782.3644894856986`, 0.18533305313279363`, 5.119329477376578`, 29.470128760856426`, 29.35997092010539`,
-0.0037379490821009`, 26.34713802698103`, 7.131030077593813`, 0.563187319026841`, 0.5754679532746874`, 0.021805594396313133`,
0.0088272313152964`, 0.008835331252538088`, 0.0009176079058506037`, 21.742536204881333`, 0.0245478948912671`,
0.6086355676915958`, 0.00008276329746648159`, 0.00008321517609854605`, 0.005459891593220467`, 0.3770433329401852`},
{0.143202010216472 5`, 0.6405991610607145`, 3.208206356969493`, 1.3290628649451517`, 0.9481170196997712`, 0.4490165359974049`,
0.04841648328555665`, 413.10755212325614`, 0.04750626711264738`, 5.496555870600891`, 465.2295356354147`, 442.0704497662819`,
-0.04977991312933716`, 387.3835069813839`, 275.73019338628535`, 18.600426751943672`, 18.737934232624823`, 0.007392705689765133`,
0.2193995351945608`, 0.21981513939379208`, 0.0018942802174248818`, 170.22025389523387`, 0.44883506400599765`,
2.7379255891387615`, 0.001979212545708098`, 0.0020050625668887336`, 0.013060760572022101`, 0.2341366697217529`},
{0.2767015708016651`, 1.3448440647834508`, 8.399713367158164`, 0.7521061389207052`, 0.4173808724493866`, 0.513702172075632`,
0.342860965595718`, 77.17931109285855`, 0.021177395775830554`, 7.103551277505744`, 1357.9496590412748`, 1381.272017313927`,
0.01717468546596801`, 874.9286646812417`, 246.45333350258005`, 54.96541418360575`, 55.79398136643546`, 0.015074337110641434`,
0.10744845541421814`, 0.10741768936256804`, -0.0002863331216024223`, 1055.9987290455203`, 0.4247308056190192`,
20.401651535824872`, 0.0017240561274518562`, 0.0017296461976594628`, 0.0032423945593167414`, 0.8743481376646315`},
{0.2397400236888016`, 3.6266796947004982`, 2.6465237726626594`, 1.459013465973801`, 0.6743470274003898`, 0.6381986185617847`,
0.04750041433271958`, 90.95305912243636`, 0.11079011330617267`, 9.85823320912452`, 2630.6909049894393`, 2654.182988521078`,
0.008930005226795323`, 1221.3362017585987`, 1574.0321729729064`, 19.93033824405671`, 20.13789356676359`, 0.010414039148019638`,
0.9227916402128898`, 0.9221535932444886`, -0.0006914312403761835`, 1032.5850431176154`, 3.160429852635253`,
23.353917513063532`, 0.007555038735147401`, 0.007663171206406474`, 0.014312629630344853`, 0.9039253427604722`},
{0.10671756328879578`, 1.1279363098789865`, 5.068949642759208`, 1.0745694995138004`, 0.5897325137042213`, 0.3714383645112781`,
0.14529484820960328`, 91.27351773968599`, 0.0653436372538082`, 9.206661650127103`, 1574.9934444665064`, 1575.1598048786254`,
0.00010562609812980739`, 782.9626807321564`, 526.1445052105256`, 61.254662819968324`, 62.109729759375035`, 0.013959213879273236`,
0.22771092132665818`, 0.227592429163349`, -0.0005203622321618973`, 987.0194049148125`, 0.3471536379747409`,
21.483637030677396`, 0.0025547811506843043`, 0.0025649103090856456`, 0.003964785163155016`, 0.7284997874109452`},
{0.2358453438636856`, 3.7679680180462416`, 4.640233719273176`, 1.297156455219631`, 0.3876479327909328`, 0.3193535012332763`,
0.027047144947079417`, 401.3110605008155`, 0.0397014782942956`, 9.539243086069693`, 272.0658676495512`, 274.49066977263806`,
0.008912555419154033`, 130.5342983198531`, 197.44278739497412`, 1.3563257717366395`, 1.3829593472181982`, 0.019636562274752745`,

0.04998347228930189`, 0.050013455629103164`, 0.0005998650839569297`, 73.00845899936475`, 0.1684052744223774`,
5.136515443880958`, 0.00046495077423902575`, 0.0004671258283089096`, 0.0046780308591671815`, 0.9048819125813649`},
{0.07226742189042767`, 2.8421547517397343`, 4.74887948268691`, 0.940014135681887`, 0.26271764199006165`, 0.24510878200647013`,
0.011565327673117277`, 79.9069988164106`, 0.11613362579809078`, 3.4810370415732024`, 387.42444879404076`, 389.6317972761561`,
0.005697494024928806`, 509.3811550540169`, 333.3384786792194`, 1.2877620307424318`, 1.3204530706592135`, 0.025385932444315307`,
0.13815873199859058`, 0.13820692411474325`, 0.0003488170125443002`, 52.28598535406597`, 0.1426339339026295`,
9.405813165527245`, 0.001770273021336255`, 0.001781151345560689`, 0.006144998027605153`, 0.32702048695163266`},
{0.19794910251471837`, 2.250622511000005`, 5.810822485387694`, 0.8325726726239642`, 0.42888216834124404`, 0.3003167364214152`,
0.2221971352698835`, 492.4997381844193`, 0.06654773222193389`, 7.816458771254976`, 184.07673062667197`, 184.53653180583726`,
0.0024978778012838543`, 107.78376389303138`, 46.60047258004066`, 4.144433803627537`, 4.222356835255598`, 0.018801852151639098`,
0.021166995825950052`, 0.02117411657437241`, 0.00033640807986690113`, 133.25080019705027`, 0.05985696895254515`,
3.4041452548212674`, 0.0003071349417902436`, 0.0003080100126987084`, 0.0028491414990561292`, 0.6780138399004657`},
{0.23749910377351813`, 2.442156143626838`, 7.627688188064528`, 0.9684475529781722`, 0.7107198130457415`, 0.2572554624659992`,
0.4475955253238462`, 132.3529645103848`, 0.18259209603279808`, 1.6799945249508887`, 184.5124823233412`, 180.029510380224`,
-0.024296307147725682`, 502.66932079799363`, 27.174175046564176`, 4.373473269325103`, 4.446029961996811`, 0.01659017632063975`,
0.08726456837243511`, 0.08736148505815307`, 0.0011106075183267006`, 152.58149448100136`, 0.29607509685162153`,
2.651284114398659`, 0.001084289932214877`, 0.001094305410072893`, 0.009236900168903661`, 0.09792572503554253`},
{0.06924882755772316`, 2.8414116897854322`, 0.5206442594268132`, 0.9917884594202446`, 0.6526760474283868`,
0.5988068411525457`, 0.47108712727434415`, 720.0807499233221`, 0.11578003411625926`, 8.400733392823671`, 264.4737320963365`,
263.96338714216745`, -0.0019296621638899003`, 144.0886712294781`, 36.2848686470619`, 5.468608334970768`, 5.536190138939954`,
0.012358135713799845`, 0.3723564594399614`, 0.3754380983852985`, 0.008276045351736538`, 221.979537854914`, 0.3683606892820294`,
2.53694092915114`, 0.004416645225494453`, 0.004595553746758443`, 0.04050778636945207`, 0.8550650638328813`},
{0.06824086699485615`, 3.3015665983523297`, 9.09230580657946`, 1.0780523992371156`, 0.9189494010575767`, 0.47997916674377217`,
0.09047840499022508`, 3815.186560881826`, 0.15232905773407`, 1.5202594192596113`, 13.173152016188011`, 12.358327817838894`,
-0.06185491500802609`, 39.65851930526294`, 5.78692885731216`, 0.15308824115758488`, 0.15419998718988936`, 0.007262125581285472`,
0.006423523786252795`, 0.006443788328859453`, 0.003154739249199956`, 7.220443194377`, 0.006262097604797522`,
0.08246788442751894`, 0.00007144980806228496`, 0.00007242884484263394`, 0.013702441012871436`, 0.07386776122633167`},
{0.23257418904935773`, 1.8011483887717636`, 0.9612178256197179`, 0.8025101750942898`, 0.31352490932039023`,
0.2355458085864931`, 0.2680725991098069`, 2738.209295799684`, 0.0662676878204258`, 1.8613360761320532`,
6.253660393053786`, 6.190291429344035`, -0.010133099613170105`, 15.377084215309967`, 1.3925171131248926`,
0.17906510108332735`, 0.1855096712070095`, 0.03599009569532585`, 0.01726074635519734`,

```
0.017398311216558073`, 0.007969809562685004`, 4.607468832878394`, 0.05734862979923964`, 0.14713891602119167`,
  0.00025512431595797747`, 0.000265644772684922`, 0.04123658965019006`, 0.18000947282506116`},
{0.13474024315490335`, 3.3704964680175626`, 9.170639775134308`, 0.7502511885050491`, 0.6264710065934431`,
  0.21904625510680875`, 0.040564870634629845`, 2527.7664803980215`, 0.19403763861714918`, 6.378419170081072`,
  31.37808678982519`, 31.01930124068562`, -0.011434271042169097`, 22.51530830692031`, 20.05114290588379`,
  0.23027904099776503`, 0.23422665526879338`, 0.017142742361284657`, 0.0025254713094454494`,
  0.002526046622675588 3`, 0.0002278043025027987`, 11.087924204877686`, 0.00486118026166514`, 0.5260337840288271`,
  0.00004070657623411744`, 0.0000407687467503405`, 0.0015272843352260868`, 0.35867970283042366`},
{0.2693026298173556`, 1.772983767924213`, 1.344226083033309`, 1.4113484218782142`, 0.12593618066908396`, 0.656520191233777`,
  0.018659842469751033`, 117.56506476136576`, 0.1712196470744854`, 4.023445632685666`, 432.27447821458856`,
  437.8899240896687`, 0.012990463601444846`, 491.7291481579862`, 340.1142578831259`, 3.3673658231256827`, 3.4555496929855734`,
  0.026187790246690712`, 0.69473836915325`, 0.7026053229425416`, 0.011323620716212623`, 85.28978492949412`, 2.672783854971341`,
  8.263634641147485`, 0.005751250786920181`, 0.006074390843108043`, 0.056186048593597215`, 0.8988957265182721`},
{0.17866994815918202`, 2.442046867777872`, 1.8194825512463648`, 1.2285671890230836`, 0.7081083864311548`,
  0.17566313743095552`, 0.006280075202364708`, 2604.6083474637735`, 0.1295692924324935`, 1.862340891527415`,
  8.51207427976629`, 8.319287693798923`, -0.022648602400666573`, 20.918990382591808`, 7.75361676927164`,
  0.01901488011302544`, 0.019528075196854722`, 0.026989130658664262`, 0.019121370182440083`,
  0.019219973292024968`, 0.005156696860324272 4`, 0.6633604060169355`, 0.04880591741755837`, 0.148520445135913`,
  0.00018603236994818584`, 0.0001909158224811367`, 0.026250552709246255`, 0.09763623806768951`},
{0.26090114261485403`, 3.9086029270457496`, 1.920295645800332`, 1.1838686314265923`, 0.4729800090210985`, 0.36383922429133153`,
  0.014536748208445867`, 79.30674088085132`, 0.030053561477188684`, 7.670997166386538`, 1284.6285526585955`, 1294.8259937023915`,
  0.00793804638912321`, 766.4613202787403`, 1064.2133510724882`, 3.813917491889738`, 3.886572224336349`, 0.019049896229037744`,
  0.6470910343232816`, 0.646620893998955`, -0.0007265443336242905`, 212.95841531873114`, 2.4118112890111147`,
  20.909775958388543`, 0.006543114495052071`, 0.006627828212723194`, 0.01294700218606648`, 0.7309861659098355`},
{0.19544602681310375`, 0.41028512203974055`, 4.6003645589109095`, 1.2671666166106164`, 0.8219059973725353`, 0.22911191370711304`,
  0.03702179299429459`, 318.28074751099626`, 0.09079865705400514`, 1.4662081657512314`, 73.48337897560631`, 70.9829150533407`,
  -0.03402761219099171`, 229.38128374727825`, 48.17576741735044`, 3.6402791667838663`, 3.7048723586162073`, 0.017744021508495633`,
  0.08600550659389831`, 0.08628197817792585`, 0.0032145800307064043`, 21.336462602894894`, 0.2401347792546698`,
  0.9537381310860019`, 0.0008132231382184107`, 0.0008279704316107903`, 0.01813437505564286`, 0.07181136072581436`},
{0.26537062770714803`, 1.229239774996202`, 8.744773690909977`, 1.151653436583517`, 0.3537059210373261`, 0.2894924335103778`,
  0.13068544785366612`, 312.2140250313751`, 0.146894495801125`, 5.715731252046433`, 191.81236820366132`, 192.90097820548016`,
```

0.005675390028358151\`, 153.59224965616525\`, 69.3509991448894\`, 6.590778097168551\`, 6.729715245482963\`, 0.02108053802844645\`,
0.02771441465367882\`, 0.02772683756322971\`, 0.0004482472282429928\`, 115.73780836019134\`, 0.10506559447408394\`,
3.951161454328818\`, 0.00029062458154249793\`, 0.0002916552674591185\`, 0.003546451271086992\`, 0.5345493897246543\`},
{0.18712917295151626\`, 0.630329154174047\`, 6.232729389529142\`, 0.7582043174026267\`, 0.7607866373637777\`,
0.2530331438958473\`, 0.06090476948059027\`, 64.21018866863231\`, 0.08418997914362486\`, 2.694793972266142\`,
658.3920217587925\`, 641.0186879308368\`, -0.026387521801290226\`, 1118.2102966069144\`, 355.72677440341806\`,
30.183342877978657\`, 30.617958284973373\`, 0.014399180659071531\`, 0.1864856936956469\`, 0.18639672300613624\`,
-0.00047709123283135035\`, 271.79201409173606\`, 0.4985273375509113\`, 8.713332734455648\`,
0.0029626652348091476\`, 0.002979163327628624\`, 0.005568665884229995\`, 0.13992870538089344\`},
{0.19413873235593193\`, 0.8072205389170897\`, 9.684031161687852\`, 0.8518820755271745\`, 0.2120317281227424\`, 0.1532879757233685\`,
0.02372050181467318\`, 987.8329384880758\`, 0.08094704090521981\`, 8.693316755778081\`, 66.02812354236393\`, 66.13572837867821\`,
0.0016296818770753507\`, 34.76225537372002\`, 49.75115529808976\`, 1.2965253765366582\`, 1.3335443718099829\`, 0.028552464875166272\`,
0.004193131356343106\`, 0.00419358868069079\`, 0.00010906511359154436\`, 14.951170188108632\`, 0.011629274373174264\`,
1.851640608892224\`, 0.00005954925818629775\`, 0.00005960749418021826\`, 0.0009779465889956374\`, 0.5741819359992343\`},
{0.0816546741022599\`, 0.4036812381177306\`, 0.7902893788690406\`, 1.4273906025641667\`, 0.1897112398644374\`,
0.6362443390402239\`, 0.007446156154142883\`, 176.61944749055874\`, 0.10188842005948545\`, 9.51311671217389\`, 712.3396283018263\`,
726.5987051232179\`, 0.020017244941692214\`, 342.7115767278691\`, 642.7775323215486\`, 9.917911372577088\`, 10.121181994372664\`,
0.020495305327855373\`, 0.841190643912559\`, 0.846884310145202\`, 0.006768580076165209\`, 57.19535346319777\`, 0.9812449698077671\`,
12.720513217795142\`, 0.006932537334822464\`, 0.007200982034502042\`, 0.03872243115535312\`, 1.8701127048644224\`},
{0.2491419431624458\`, 3.7694376618574204\`, 4.74673147811253\`, 0.8416284883026488\`, 0.188731240527098\`, 0.3192559789027146\`,
0.07121130413051815\`, 671.9000833119555\`, 0.02760758509345429\`, 2.5790410082926702\`, 35.47881583469241\`, 35.74959607822846\`,
0.007632166890735537\`, 62.961543039991604\`, 17.960472618503204\`, 0.31812179146240105\`, 0.3261962851840927\`, 0.02538176867599451\`,
0.015255080194534197\`, 0.015282909725878457\`, 0.0018242795835470282\`, 17.13057516851321\`, 0.05429543318235528\`,
0.8507268588352741\`, 0.00021804164392980052\`, 0.00022025703882669705\`, 0.010160420995586561\`, 0.31708312583150766\`},
{0.11168227968802413\`, 2.173584861364678\`, 5.640951633995167\`, 0.9377704673639422\`, 0.3315982783471285\`, 0.5266083888319896\`,
0.005432939117039627\`, 621.842609802462\`, 0.09631877509870435\`, 9.43919460268938\`, 206.251006316924\`, 211.89342431830994\`,
0.02735704471044298\`, 100.00590194416503\`, 191.3765441208653\`, 0.455546588413072\`, 0.4634740510465008\`, 0.01740208978634805\`,
0.022782868117696115\`, 0.0227983737985314\`, 0.0006805851113733663\`, 14.145273831728275\`, 0.036349180703088464\`,
3.4153008554870183\`, 0.00029315564802012695\`, 0.0002943895248550986\`, 0.004208947851780609\`, 1.3212886909758321\`},
{0.15970197279447784\`, 3.9587696847183347\`, 4.0238996390694854\`, 0.8027865138486989\`, 0.03588289413016432\`,
0.35386710298208024\`, 0.016574217538633067\`, 2019.1576160982734\`, 0.07473946383041635\`, 3.5352801788382404\`,

15.125622566154323`, 15.240720866282377`, 0.007609491749820618`, 19.581858837711657`, 12.28186277738408`,
0.04900612190923616`, 0.05031335967136624`, 0.026674988985074366`, 0.005343311815574042`,
0.005352552268063926`, 0.0017293492891339302`, 2.771484996855645`, 0.012190534831472888`, 0.3971309846560025`,
0.00008010622345111518`, 0.00008080828182738421`, 0.00876409280107282`, 0.5275280869291428`},
{0.2064322311255224`, 0.43645383124552994`, 3.8213880668877356`, 0.8257710590869434`, 0.08868496943554072`, 0.17616539751645888`,
0.04042707492740236`, 2037.3062213977416`, 0.05324630165142419`, 4.6107951341494555`, 15.981092868758044`, 16.01087837764014`,
0.0018637967457360016`, 15.863358671379201`, 10.288744981989588`, 0.7841057234561072`, 0.8086515166500222`, 0.03130418827415826`,
0.004693368504508896`, 0.004696648016554904`, 0.0006987544325267603`, 4.888942101485219`, 0.01384089331256732`,
0.4838562105711181`, 0.00006862408174634904`, 0.0000689323521959646`, 0.004492161377910975`, 0.38373875834858273`},
{0.1394617695133732`, 3.387614469631334`, 8.352661875580207`, 1.2219251402369191`, 0.6325442693826859`, 0.17624447718949754`,
0.021853560156019736`, 288.68541357569393`, 0.164910064915528`, 5.7766720481305995`, 225.20904145757464`, 222.40867712464183`,
-0.012434511131562798`, 178.4319428131679`, 172.4829596404202`, 1.0642096182324763`, 1.0847595922442577`, 0.019310081077741348`,
0.035780681319715395`, 0.035788575970314526`, 0.00022064002998112286`, 51.5018843063597`, 0.07128624473199553`,
4.145680572282241`, 0.000353879994764994`, 0.0003547290867109514`, 0.0023993782031144217`, 0.28710200638494077`},
{0.10599692192016741`, 2.4119661618453145`, 1.4892081676893856`, 1.3031849980783952`, 0.42063329504650815`,
0.5860601104625098`, 0.3077592160131421`, 52.52666428206092`, 0.19647413521024198`, 3.2052596491028216`, 962.8990620376245`,
974.3797155965107`, 0.011923008352081599`, 1374.9346032147807`, 188.47442240973731`, 21.725538681723723`, 22.12477924042594`,
0.018376555101856873`, 1.6345337810708427`, 1.6433264718122083`, 0.00537932641294514`, 748.5894878311304`, 2.4750792795439596`,
13.85803194262978`, 0.01461550805621814`, 0.015331185209585313`, 0.04896697060508193`, 0.48284905529752137`},
{0.24388484229914548`, 3.2519594625966253`, 4.312340316913895`, 0.9914403572709836`, 0.29358995419342615`,
0.1835819110057716`, 0.13612159308161606`, 1157.8864188899545`, 0.08218618541111161`, 5.046821007659727`,
36.33647952493873`, 36.2902009980038`, -0.0012736106397751579`, 32.95258133948913`, 12.917823784353631`,
0.49249557843224806`, 0.5056500941902715`, 0.0267099164623934`, 0.010371733386255542`,
0.010378751003052012`, 0.0006766098332000592`, 22.879652236710683`, 0.03613583658965686`, 0.922756624066324`,
0.00012630133995650272`, 0.00012688944901641016`, 0.0046563960454415465`, 0.366645632272463`},
{0.23494551018662208`, 3.3331099682410468`, 0.9744758817090952`, 0.9989991912838305`, 0.20118476247466943`,
0.3741832660739852`, 0.07385095150097958`, 91.01998469145919`, 0.19994684540705993`, 6.360950891328324`, 719.2164475598951`,
724.8309440002016`, 0.007806407180140251`, 517.4901199777019`, 356.0552562706406`, 7.405116508444475`, 7.594951830354874`,
0.025635696844731326`, 0.7239020309256847`, 0.7245105547567378`, 0.000840616278248163`, 352.60096643260925`, 2.4296790282997525`,
15.65534394517365`, 0.008610919854886268`, 0.008818202453531637`, 0.024072062234761882`, 0.8587915518997824`},
{0.23944024111665796`, 0.7499363805955559`, 7.775454975750338`, 1.268235612825383`, 0.3786721827881383`, 0.6740341016595366`,

0.11977329505957622`, 117.53909816274609`, 0.2108164269301916`, 1.8777741577958924`, 251.84897784536446`, 260.2719771224575`,
0.03344464348894349`, 613.8486842510589`, 94.87053727817775`, 13.350158944110019`, 13.57009772444239`, 0.01647461886057955`,
0.1364047107243959`, 0.13700880298777726`, 0.004428675961213324`, 143.0252839845897`, 0.4665825260750276`,
3.6754146939657066`, 0.0012826508906362832`, 0.0013109454362621154`, 0.02205942851043119`, 0.31105081428516806`},
{0.24513403447625742`, 2.0133471925570054`, 9.940759001187548`, 1.083812287959604`, 0.5683273089881642`, 0.5900825394859205`,
0.005339720116331014`, 58.9416557608406`, 0.03145644437596545`, 6.28807827028038`, 2039.878948297754`, 2088.800631565277`,
0.023982640395572075`, 1484.7418559869777`, 1894.001923567171`, 4.795993047824554`, 4.85775128949621`, 0.012877049873887714`,
0.22201026153815515`, 0.22193785434053706`, -0.00032614347245230046`, 137.94284483372113`, 0.7774610157997823`,
23.314607256035913`, 0.00246835102756026 7`, 0.002480164599343018 3`, 0.004786017730398662`, 0.6745429431486223`},
{0.15958822823144275`, 2.509825654597928`, 3.728427574062339`, 0.9294074209823535`, 0.01651867581778177`, 0.5367648350921979`,
0.015785937236593408`, 685.9822133189093`, 0.020860236554209932`, 1.2753268301578835`, 18.83123140742199`, 18.95502249359305`,
0.006573711696956419`, 67.58054946295415`, 15.372985660954438`, 0.09159642379086876`, 0.0940244224390086`, 0.02650757035758744`,
0.022813660312046555`, 0.023015288511219718`, 0.008838046872587935`, 3.2841579185678063`, 0.05201130898104886`,
0.4469661641907754`, 0.0002905201212870523`, 0.00030098517494344003`, 0.036021786064338146`, 0.2781680863660519`},
{0.06806026871424425`, 1.205607754446362`, 0.7536813502622586`, 1.4624982795129167`, 0.7076540801030038`, 0.15470161284122685`,
0.268116863806763`, 324.06126360879364`, 0.036033823716353997`, 3.8267580932938614`, 135.22147105760928`, 132.10238540612022`,
-0.023066496963046745`, 161.7257379743389`, 29.746897704074208`, 5.74187370446558`, 5.884151476969225`, 0.02477897979417243`,
0.42620164102490615`, 0.4277865542205904`, 0.0037186933205441974`, 98.89210661650512`, 0.4143914030656733`,
2.4455037299919336`, 0.0034714769452334204`, 0.003563202220414007`, 0.02642255058226195`, 0.1885399208055789`},
{0.15319733389695195`, 3.558603911914262`, 4.579050417046558`, 0.9321187332487358`, 0.6642338797631109`, 0.569667348599446`,
0.2858050889038288`, 341.1212503566884`, 0.20343887540165378`, 9.501626804091558`, 619.0540281974038`, 622.6505384709761`,
0.005809687215903914`, 298.19137662045`, 126.45697393912847`, 9.497654928311691`, 9.59278766541139`, 0.01001644488221154`,
0.08318184730636036`, 0.08322683107267968`, 0.0005407882582080248`, 482.8341711700233`, 0.18204624622932059`,
5.97309392555974`, 0.001075535330110955`, 0.001081356144342214`, 0.00541201582904649`, 0.8219561567251431`},
{0.15071537712057487`, 1.2850575528534174`, 6.479674501252795`, 1.2297249014764047`, 0.051196968811160426`, 0.17856837950014437`,
0.01824538414392949`, 77.70419036862091`, 0.224107560352308`, 8.31176551046438`, 752.8113226583622`, 754.9154512745685`,
0.002795027854756382 3`, 414.5313250533497`, 601.2424948391449`, 7.799398461265308`, 8.040581435152086`, 0.03092327890215918`,
0.10788144963869198`, 0.10786351327457029`, -0.00016625994720831372`, 143.18108429088988`, 0.2322770480944676`,
22.888307366390716`, 0.0010599463792335673`, 0.0010623148735099913`, 0.002234541598355788`, 0.6993892807910213`},
{0.24584690511060947`, 2.473478204800995`, 6.676188280246508`, 1.4832363218386881`, 0.1442016176850626 8`, 0.523836460034543`,
0.04402629271492787`, 360.52310897688045`, 0.0850887320320231`, 2.528306265858081`, 78.16082518117435`, 79.4941615769920 6`,

0.01705888330537797`, 141.4894145188802`, 48.44610897801228`, 0.8124008109861455`, 0.8305509274059704`, 0.0223413322271222`,
0.04284645386536392`, 0.04301151258033589`, 0.0038523308251046107`, 28.706509993384064`, 0.15048097253947615`,
1.6359205405603705`, 0.00034555766380284236`, 0.00035192042305835016`, 0.018413017339815374`, 0.4599486757064223`},
{0.14137397451874373`, 3.652676161056461`, 8.39527364337399`, 1.0080745646578813`, 0.09190129083286003`, 0.15081167091134762`,
0.35462309792221747`, 1217.6311173423508`, 0.09724487810479643`, 6.223920941944753`, 35.2997246642143`, 34.93893906821083`,
-0.01022063484730884`, 25.958030902389034`, 6.691656736434515`, 0.5376942544575873`, 0.5543416429737594`, 0.030960696303079516`,
0.004272953360911057`, 0.004273949776619081`, 0.00023319133720001162`, 28.057471217060776`, 0.00862977713663938`,
1.0807738880082256`, 0.00005125826904384123`, 0.000051343560974147945`, 0.0016639643104952029`, 0.4437309044280341`},
{0.04933630800724648`, 1.8511857532828166`, 5.885062761777403`, 1.1138733840213622`, 0.4607664856084661`, 0.43439208322360623`,
0.01876107649607479`, 3507.5800668673905`, 0.21191062303011793`, 3.3573407631201917`, 13.303678316636786`, 13.516972340253732`,
0.016032710543685713`, 18.1359707330714`, 10.52510677078278`, 0.10079658668109202`, 0.10245557881659888`, 0.01645881264566728`,
0.004695651135818798`, 0.00470567846448581`, 0.0021354500956261013`, 2.665617217765344`, 0.0033095155818761`,
0.20973606351904206`, 0.0000506804469809774`, 0.000051172755741845485`, 0.009713978273571122`, 0.37003381877362584`},
{0.2339008817663396`, 3.669675022809618`, 9.160989469851096`, 1.2207724907901325`, 0.9659294978123167`, 0.3234992643572032`,
0.05037324961884807`, 93.86107259786391`, 0.14430488254373952`, 3.9894219201696473`, 1150.9116004218483`, 1120.832375395441`,
-0.026135130635039472`, 1320.3725670158021`, 672.3020585157832`, 8.938731852771824`, 9.015215804693769`, 0.00855646563536072`,
0.24118902480464913`, 0.24113059200078274`, -0.0002422697463689838`, 468.6034430815628`, 0.805918936774366`,
8.704061487381962`, 0.002380835074760279`, 0.0023933334253575943`, 0.005249565889637875`, 0.14990565312786142`},
{0.11569404259913263`, 3.2009969772206803`, 2.2264401820433353`, 0.7539773410821206`, 0.3237735314376855`,
0.22021748910844674`, 0.21956604535044472`, 1445.3927245947305`, 0.06533194774972478`, 1.3139082141801541`,
8.02075940301126`, 7.971503619916857`, -0.006141037353135204`, 27.93926365659165`, 2.0745553639550054`,
0.12651969611535033`, 0.13015202547966812`, 0.028709596022157058`, 0.01286384440058778`,
0.012922921279971685`, 0.004592474655648271`, 5.785559497487378`, 0.021261002315286957`, 0.19536614024904372`,
0.0002039177651200763`, 0.00020837855507648`, 0.021875435687412`, 0.1168211620769919`},
{0.1426983247121278`, 1.4581176174915065`, 6.531549332871599`, 0.9310047864665083`, 0.893336243428055`, 0.18377286371920176`,
0.08056837661087574`, 664.5727162634348`, 0.04347044784294363`, 7.864371974772275`, 191.66210545072016`, 186.8810641120699`,
-0.024945157142080787`, 111.54155698947253`, 90.36469182292035`, 4.637186693461259`, 4.697949640423377`, 0.01310340751382677`,
0.0217978636468196`, 0.021801227749109465`, 0.00015433174297641017`, 96.5937659047579`, 0.0444359803527621`,
2.4174232855712585`, 0.0002830583307505963`, 0.0002835760759889805`, 0.0018291114662172259`, 0.2754993930970519`},
{0.05831687901068116`, 0.732484500007835`, 9.716813224212896`, 0.9062398770267781`, 0.04581664783201011`, 0.2511270020278909`,
0.0489500536916562`, 2141.8470035057708`, 0.06373448098002471`, 7.679073247267311`, 27.969409780474987`, 28.085218278218846`,

0.004140541350454274`, 16.6701299894986`, 16.763464845753074`, 0.9770912384005372`, 1.0039988588761601`, 0.02753849325234925`, 0.002131788544251094 6`, 0.002132339146807869 3`, 0.0002582819756018484`, 10.22434553174076`, 0.0017759893516189084`, 0.7856266274749042`, 0.00002844413256852807`, 0.00002848814880320095`, 0.0015474627171996325`, 0.8465664125854829`},
{0.1624041267001165`, 2.3409738007594383`, 7.927758092789709`, 1.1108935833128895`, 0.2655878375527121`, 0.22381693809974978`, 0.10055816102060769`, 86.2207746546923`, 0.23778527923179793`, 1.5984483564142007`, 156.44493609717384`, 156.6598104222859`, 0.001373482136734605`, 447.94778709316836`, 66.36503292920031`, 2.6070969386248417`, 2.678008042223876`, 0.027199258511821034`, 0.08583620674304895`, 0.08594395032062281`, 0.0012552229608233656`, 87.187794705156`, 0.1991450599336265`, 3.9912921627147866`, 0.0009298416467284909`, 0.0009382662687434873`, 0.009060276063819206`, 0.1451553087744423 8`},
{0.05796853524365786`, 2.115435321939346`, 7.135826377560314`, 1.1217856002992745`, 0.9949497841824515`, 0.6493572179052653`, 0.16432568991023785`, 205.88622896743587`, 0.07308989915497993`, 5.091714109271411`, 1308.4739628718794`, 1295.5496737748254`, -0.009877375831527702`, 1176.1577347507703`, 394.29772309219373`, 29.26643670162799`, 29.390753950123663`, 0.00424777535314913`, 0.2534389593198482`, 0.25353386035451425`, 0.0003744532210860907`, 884.446484941797`, 0.20987836064893575`, 5.0731430243077265`, 0.0027154540466146626`, 0.0027369722361155 37`, 0.007924343086453955`, 0.20037000891672763`},
{0.09343457742759681`, 3.0751406184941388`, 6.999926605820257`, 1.4764339149831611`, 0.27064650694064607`, 0.6662559245780291`, 0.012835205507442616`, 1884.4110587763687`, 0.01681160009525748`, 8.661215052575166`, 66.17704216706454`, 68.48860350548834`, 0.034929958528340466`, 34.96979004094129`, 56.02779053915456`, 0.22457957165748785`, 0.22821973531815587`, 0.016208792428457075`, 0.01009970122777064`, 0.01011386690354096 7`, 0.001402583645878197`, 9.86591089839938`, 0.013480875948021484`, 1.0664418729522247`, 0.0000823923788757508`, 0.0000829561591196789 1`, 0.006842626121747353`, 1.5208335670211919`},
{0.23943678973010468`, 2.920807454124679`, 1.1099817114095707`, 1.3932739733707113`, 0.44392785132951373`, 0.17254202331513768`, 0.008938503858702969`, 2712.044809794402`, 0.1652368227234951`, 2.8738798121545877`, 9.742802512861669`, 9.659671775761543`, -0.008532528190978272`, 15.51600077000568`, 8.555101013336527`, 0.02491254381726308`, 0.025820784091396657`, 0.03645714708203407`, 0.02623608583070458`, 0.026395300299371628`, 0.006068529798782585`, 1.0394963383238522`, 0.08974120237696376`, 0.22336717938752468`, 0.0002246485514936225`, 0.0002321202699112486`, 0.03325958866838352`, 0.19541463982959673`},
{0.1765669287716667`, 3.4304813087836825`, 0.9837478487319995`, 1.1028599055297788`, 0.8616178142897495`, 0.24038775568738768`, 0.03874939820930588`, 2380.99712756261`, 0.18505877859540681`, 3.338709015344154`, 25.504000826271`, 24.472843412710375`, -0.04043120217038487`, 34.96183659071515`, 16.39828247295016`, 0.17832740809271913`, 0.18172584233338362`, 0.019057273792133733`, 0.053041892717734324`, 0.053367466584262564`, 0.006138051450403692`, 8.739269147227345`, 0.1337920584772239`, 0.29011030624599543`, 0.000573194031271651`, 0.0005901460988591859`, 0.029574745483525122`, 0.16110013528577727`},
{0.23886394100929725`, 3.974888040813318`, 7.275822510559582`, 1.2870566617016084`, 0.12273034821485096`, 0.6651538290269186`,

0.16897919530659566`, 150.6876048176701`, 0.1338282848735754`, 2.432758352876487`, 200.93783808927682`, 204.09327345667944`,
0.01570353994751672`, 378.0308541948781`, 61.23051470335077`, 2.410782485867311`, 2.458818110918054`, 0.019925325213842937`,
0.09119676178586006`, 0.09154351990649545`, 0.0038023073829047682`, 136.89414960108778`, 0.31119454182086725`,
3.8714422938070405`, 0.0008468505644743107`, 0.0008627684376256401`, 0.018796554928448783`, 0.5230253714378`},
{0.06973130535662914`, 1.4096929441217387`, 8.65071450995816`, 1.181254696978707`, 0.5406204719849208`,
0.2725259583108167`, 0.12003736669178822`, 4806.091595362366`, 0.20844978312325513`, 8.8784124085225`,
23.2295422616754`, 23.182078996595816`, -0.0020432285985201304`, 11.974843114798285`, 8.797200656271198`,
0.6825406752474092`, 0.6941359618323557`, 0.016988418427580587`, 0.0022417250240047335`, 0.0022425202865340886`,
0.0003547547182813915`, 13.745325342462346`, 0.002233120173892438`, 0.39042275613723393`,
0.000022938775615655l6`, 0.000022986187779645586`, 0.0020669003779814066`, 0.6287190621513985`},
{0.04409294567306185`, 1.3468266911815716`, 8.46560672493381`, 1.1221333539921368`, 0.8074609652543419`, 0.33934404303057597`,
0.1090535991677299`, 2100.6487939981484`, 0.1955313854386307`, 6.974828032327105`, 69.83200286061329`, 67.41831053599807`,
-0.034564271762804966`, 45.82320784268281`, 27.71235882511827`, 2.0803001212750014`, 2.101322722357823`, 0.010105561629221604`,
0.008325624489973085`, 0.008330021125900025`, 0.0005280848220137191`, 40.025767557163334`, 0.005244304404687321`,
0.6889183603292031`, 0.00008961688870312567`, 0.00008988278014603465`, 0.0029669791794468825`, 0.35752795080230554`},
{0.08301734541584582`, 0.4950033643125962`, 0.3447103403996028`, 1.3973171280993089`, 0.5174429014299606`,
0.6090072510200681`, 0.013328621450736109`, 78.08408450957157`, 0.15235476028821748`, 7.41363923833616`, 1779.6815484539895`,
1788.0964224805289`, 0.0047283032370872835`, 1098.6904353777547`, 1486.448074488274`, 34.57338879665067`, 35.26796007196658`,
0.02008976555353459`, 6.06278770323892`, 6.063565128014732`, 0.00012822892930874907`, 244.48491100042187`, 7.190236299181502`,
21.484285373229156`, 0.048713493111674944`, 0.052926128893031214`, 0.08647780137012284`, 1.0861032531994652`},
{0.07508111728102967`, 2.9328515590120503`, 2.9247828964602327`, 1.0997610261500241`, 0.15381049135387959`, 0.44875941041801015`,
0.005374403719665632`, 955.806314296473`, 0.04180941570568808`, 8.210271230243798`, 91.81897755107221`, 93.33694431988522`,
0.0165321680691628`, 51.18461518827526`, 85.2628807024933`, 0.14932074607752005`, 0.15261703989084127`, 0.022075256787224662`,
0.02633998668451728`, 0.026384209463154527`, 0.0016789218296471287`, 6.2562226132328185`, 0.028251937563444185`,
1.9573068681698742`, 0.00028815821083816306`, 0.0002905995288103482`, 0.00847214439971733`, 1.268480506394261`},
{0.1868411586163749`, 1.4148901359883457`, 3.2886483363036496`, 1.1024292265597047`, 0.35071605335062417`, 0.20216786873751635`,
0.033694215571138546`, 50.84055962824541`, 0.13756443231160648`, 9.762651891575405`, 1768.2349374080895`, 1770.9437098523865`,
0.0015319075463284815`, 828.9658848993573`, 1205.5098058754013`, 26.45493526405942`, 27.085976947667252`, 0.023853457863687977`,
0.3814385664787648`, 0.38087743545121516`, -0.0014710914859230595`, 534.7260993332555`, 1.0181203385978386`,
40.63372548912207`, 0.004170336589264911`, 0.004185702137969861`, 0.0036844864619567375`, 0.7042775839051814`},
{0.25728291653412544`, 1.1620186239014583`, 0.48798465750503445`, 1.2217305465551163`, 0.36537296293338284`,

0.2112385814144826`, 0.1783844678719564`, 175.70497466833672`, 0.14885286931859365`, 6.164090300210196`, 334.5159081628094`, 332.04691684460244`, -0.007380788948923023`, 248.37751531052405`, 98.40631671739405`, 13.191783278330103`, 13.649921233693366`, 0.03472903895531987`, 0.840706346898676`, 0.8429332544449673`, 0.0026488530204467065`, 218.9871121698774`, 3.089991155411927`, 7.4872654803577765`, 0.008156491895128015`, 0.008451916699377794`, 0.03621959146753273`, 0.5013619780341069`},
{0.13759840983832694`, 1.6824227355669867`, 8.881204796952819`, 1.07728991023014`, 0.8275568317133928`, 0.2913709338376398`, 0.47392581861361016`, 1028.758263171597`, 0.1629375199726112`, 7.630724789672264`, 147.11711077894086`, 141.76091626484666`, -0.0364076923869342`, 88.23926691615667`, 20.255476490965005`, 5.065711696125698`, 5.12027212842008`, 0.010770536415664944`, 0.014672723034590458`, 0.01467752263392678`, 0.0003271103342581405`, 121.7524075627067`, 0.028842047965079325`, 1.5315351854108517`, 0.00016459703139770632`, 0.00016498296495012944`, 0.002344717575680999`, 0.3522112297969869`},
{0.22999415874686646`, 3.253135821240728`, 9.26678168881324`, 0.8937302072810551`, 0.5038748821521797`, 0.2035592770142407`, 0.010501376095491947`, 229.44271937909937`, 0.1496315114369897`, 8.069758108498657`, 372.20727084594733`, 370.84183186210714`, -0.0036684908941645444`, 211.10029018992932`, 324.2829353351285`, 1.001828042560728`, 1.022769359900787`, 0.020903105573419456`, 0.027917271745005665`, 0.0279180696498135`, 0.000028581045279807427`, 46.5583241711112`, 0.0917258489928562`, 7.371552167001457`, 0.00037772750714049064`, 0.00037827618122835685`, 0.0014525658775019767`, 0.4997433670577949`},
{0.09925066924077935`, 1.4815445119155024`, 7.881183919866995`, 0.7663286317492166`, 0.2976933875506338`, 0.33062028037546887`, 0.05105854284999101`, 168.2571992844445`, 0.22488226539851913`, 5.574589576514775`, 347.18528453074236`, 351.2776832502037`, 0.01178736225814613`, 285.04465748660164`, 203.63412713037755`, 6.472089354490809`, 6.606448960607542`, 0.02075985029834393`, 0.03800028984727402`, 0.03800845363259813`, 0.0002148348172321679`, 136.9812637681797`, 0.053879345695441665`, 7.250528106601973`, 0.0005989103571646393`, 0.0006005795505577773`, 0.0027870504711928756`, 0.6065084240724694`},
{0.20983327896511272`, 1.8859920859197894`, 0.6151282764886545`, 0.8412663476815937`, 0.027122934645615437`, 0.6844735714100607`, 0.01254007978155685`, 549.5308557472557`, 0.10371695352022153`, 7.780558961111437`, 170.60489335950476`, 172.34439568419208`, 0.010196086937681148`, 100.35642456294848`, 144.44780660584954`, 0.9061831865169235`, 0.9275938179760752`, 0.023627266294188543`, 0.18577710489742755`, 0.18743902986608355`, 0.00894580077331697`, 24.415061688064156`, 0.556888843961082`, 3.4653293839030086`, 0.00260334847908239`, 0.002709122145708003`, 0.04062985323535906`, 1.867921843587196`},
{0.2225389666164696`, 1.2827559929647245`, 1.2157460525678818`, 1.1336603904726232`, 0.21910751958531538`, 0.6021275716391656`, 0.016887660465617623`, 151.92995003475355`, 0.056506174885467386`, 4.72149152068509`, 404.7345484538504`, 412.7749573420771`, 0.019865882265159396`, 392.3336496560202`, 325.5983638201975`, 3.9800842789563085`, 4.070616945617433`, 0.0227464195016656`, 0.4955429923362576`, 0.49924046681231843`, 0.007461460525612251`, 72.93538516194124`, 1.5753946489792652`, 7.287624574317465`, 0.005149055266400082`, 0.005358728915834522`, 0.04072079994997435`, 0.8883645633854179`},
{0.23629660338151143`, 1.375829581220887`, 0.7878883261410508`, 0.9498849347602389`, 0.6605330073956415`, 0.33733697402858687`, 0.028977316405205664`, 446.06430846982937`, 0.16303388369839966`, 6.718018447521894`, 247.4711424294480`,

243.92901886741885`, -0.014313279226239595`, 168.59623260879692`, 175.0630628467651`, 3.4455776242896565`, 3.507744768511214`,
0.018042589951626553`, 0.2765166552868099`, 0.27744515006076587`, 0.003357825853176566`, 67.72182314129287`, 0.9334278060382267`,
3.1876577400683987`, 0.0034702728204968336`, 0.003553303450965421`, 0.02392625443687746`, 0.4862766371528284`},
{0.2240884195389573`, 3.0168075094859823`, 9.22253837134286`, 1.0534395436787098`, 0.7253455428103461`,
0.1646055270291652`, 0.006115122680716056`, 258.28718729642685`, 0.11665954601209721`, 2.992281031623701`,
136.8007576070788`, 134.29902690148975`, -0.018287403881011688`, 209.24284562685236`, 125.75156943356743`,
0.24441514114844137`, 0.24923625498380597`, 0.019725103005940088`, 0.032739396771230604`,
0.03275151522261023`, 0.00037014889016750985`, 10.533620474981337`, 0.10480742398749024`, 2.3873275175806894`,
0.0003753981663271455`, 0.00037674651565523695`, 0.003591784534494602`, 0.13190725837743844`},
{0.04820076342563051`, 1.9965427255473651`, 1.4646173572921608`, 1.475676204663891`, 0.346581356777814`, 0.3525821474490592`,
0.16114708977216574`, 53.64321165141119`, 0.1945121008436112`, 8.557828905511698`, 1835.3942067227417`, 1847.670065883616`,
0.006688404657653191`, 981.5902856446647`, 578.5895451327791`, 42.49409504244117`, 43.34280807592495`, 0.019972493416700043`,
1.3575480573559588`, 1.354921465514312`, -0.0019348057900525628`, 1212.0182333672044`, 0.9347836107356423`,
34.92343046833078`, 0.010959060833486745`, 0.011128975149419561`, 0.015504459598729703`, 0.9340896213321267`},
{0.1395588171486265`, 3.3167971972296737`, 5.120579942113229`, 1.4414502422836475`, 0.46235265068089726`,
0.21590338795839814`, 0.018401804903068468`, 321.8138540224943`, 0.09243860589675884`, 1.0657915725386573`,
32.508543874085376`, 32.33094268905134`, -0.005463215631002494`, 139.60138589856464`, 25.750368503093124`,
0.13616218318921577`, 0.13986390679703745`, 0.027186135835363556`, 0.053392330721160064`,
0.053638715694589695`, 0.004614613561568781`, 6.451747822438058`, 0.10644815028933344`, 0.704532748173117`,
0.00044273345003364994`, 0.0004527095714484849`, 0.022533019391411946`, 0.08296564993291203`},
{0.2085065587668823`, 0.6294064238759702`, 7.1150002800734775`, 0.8698405330489325`, 0.07589384997930004`, 0.6271818704466325`,
0.014863365323890152`, 3717.018925299029`, 0.09558183779388002`, 3.993723504265942`, 12.490358870005473`, 12.754316693111443`,
0.02113292547100798`, 14.314013319921559`, 10.331117086247135`, 0.21458995468605646`, 0.21894762672643325`, 0.02030697125013159`,
0.0023945261286011954`, 0.002398572609903961`, 0.0016898881387981124`, 1.9294899425522456`, 0.007132491470740761`,
0.2570222852373466`, 0.000033132545583458577`, 0.000033406855081010764`, 0.008279155516778047`, 0.84675043555167`},
{0.11579616742063126`, 1.4637518716217537`, 6.091821149634162`, 0.8701973607843327`, 0.580668178059593`, 0.4892081276931839`,
0.43069272205186815`, 58.104542288448926`, 0.09639134486345691`, 1.0613512257562903`, 309.8072854486124`, 310.2650821877486`,
0.0014776822903737052`, 1335.9708902091782`, 46.56683139043719`, 11.982696012520417`, 12.162722975064915`, 0.015023911343189589`,
0.2602279798206908`, 0.26118809877310617`, 0.0036895300539048215`, 250.56705307716106`, 0.43047718169748456`,
3.984761370612228`, 0.0035585529177289965`, 0.0036416562491047313`, 0.023353125075562753`, 0.11174796878883375`},
{0.07650954660329923`, 2.451622188741575`, 3.5124331635719983`, 0.9208132913481966`, 0.22417245589301404`, 0.5962277485405788`,

0.1631058588288524`, 149.68967360762312`, 0.15724921240628176`, 3.991974201007654`, 344.02606153240106`, 351.1491436594416`,
0.02070506546891271`, 394.4283404991754`, 107.2593396847485`, 6.56440922009464`, 6.6822964491328385`, 0.017958543577284658`,
0.14136002475439682`, 0.14171496182589366`, 0.002510873014584636`, 229.90644714234026`, 0.1545055914547298`,
6.19989557741479`, 0.0018375368597937092`, 0.001864700730513996`, 0.014782762356852386`, 0.7101982166075185`},
{0.27715040216901043`, 3.1093705724520717`, 5.602120685872263`, 1.4185083740786066`, 0.20116695432127574`, 0.3482455682148583`,
0.014646070094414987`, 268.87083253973395`, 0.19497961893886057`, 8.247252623819538`, 305.4215226644244`, 309.1944495557074`,
0.012353179495566913`, 169.49419742920617`, 253.33860128158554`, 1.137252803162635`, 1.1635119185689362`, 0.02308995443517592`,
0.05877242476717188 5`, 0.05881363364927263`, 0.0007011601488962071`, 50.51629142275021`, 0.23269715943826394`,
6.799388989698797`, 0.0004997359273786195`, 0.0005024021299652725`, 0.005335222945923812`, 1.0154114278498307`},
{0.10373802184314534`, 1.9484823746249935`, 4.344672894707308`, 0.8659474233939041`, 0.34328087466446`, 0.6599529078817681`,
0.006604951530776707`, 74.02272771702236`, 0.12108236246583282`, 3.25882057231545`, 665.0328664790356`, 689.0231783053966`,
0.03607387399268824`, 934.0005982990581`, 607.3733490146402`, 1.9561135248465529`, 1.988515499207039`, 0.016564465174907284`,
0.2546565211298725`, 0.25510947301517917`, 0.0017786777393211661`, 54.44932465612967`, 0.3773937678781658`,
10.141302072593069`, 0.003515998678614296`, 0.003569761222136416 8`, 0.015290831549262363`, 0.5446594282304856`},
{0.08995654352927362`, 1.6673082382989595`, 4.04257302618344`, 1.1477945472560402`, 0.1500209798917984`, 0.1761016570773115`,
0.012888472951564884`, 1050.2473441342943`, 0.2022489804478793`, 4.465566609444316`, 31.23103757040664`, 31.304569092510476`,
0.0023544373746169356`, 32.00916429918918`, 26.47304236395421`, 0.1898948655372104`, 0.19566578942354795`, 0.030390099648095692`,
0.012443441885645223`, 0.012454988888355006`, 0.0009279589052528792`, 4.5230467674409125`, 0.015990986023431818`,
0.906292153247317`, 0.00013079419223105404`, 0.00013148177530034825`, 0.005256984714424817`, 0.3570434214749105`},
{0.18428208667431017`, 2.663013532673764`, 4.442362759588184`, 1.3723983485913218`, 0.7325310003414629`, 0.5418182340232052`,
0.027460635126683182`, 53.281000517524745`, 0.08334630855570291`, 6.274365305120075`, 2865.6144055153004`,
2829.8255603844996`, -0.012489065193809767`, 2090.318411626277`, 2062.5602989181625`, 20.475615673224283`, 20.68022930728153`,
0.009993039394894376`, 0.8861831937974894`, 0.8848097496543424`, -0.0015498422366388986`, 778.954880394619`, 2.332966973267988`,
24.99251744529525`, 0.007728621134007074`, 0.00781353431312302`, 0.010986847154703216`, 0.4725414595609441`},
{0.1326476876438774`, 2.8731759222440596`, 6.672951038295707`, 1.243076119232064`, 0.9225218753338189`, 0.23536778742386455`,
0.07924576221140303`, 2119.223051638056`, 0.014542489730864222`, 5.05029094558817`, 47.251096495395366`, 45.8017462716161`,
-0.030673366996266438`, 42.82131376019567`, 22.40572138447532`, 0.5901644024373527`, 0.596716089131823`, 0.011101460317518574`,
0.010926932359258417`, 0.010934713268570208`, 0.000712085428551168`, 24.223516446407935`, 0.020706175864247753`,
0.4878455879590754`, 0.00010611490614609309`, 0.00010655924792260239`, 0.004187364364225621`, 0.18790070800196879`},
{0.16449021574582717`, 1.5264131018768046`, 3.118813817198866`, 1.0873075186223824`, 0.9114527670749883`,
0.32623274128336255`, 0.005473446059082923`, 2600.919647538226`, 0.12372800503579795`, 1.4807994816594086`,

13.670639170019593`, 12.989179326075563`, -0.049848425919872486`, 42.252952495813965`, 12.612608367023173`,
0.04283805049091822`, 0.04347186171019029`, 0.01479551968422177`, 0.019983466000153556`,
0.020106370352926478`, 0.006150302093339333`, 0.9341223075456809`, 0.04695835191020866`, 0.11788376624043914`,
0.00021905850501935387`, 0.00022496590830726709`, 0.026967240041153806`, 0.07267195161707607`},
{0.1059475440552839`, 2.4614128361434053`, 6.764865578702089`, 0.897041430377437`, 0.5452264302150955`, 0.6377616415175531`,
0.031109305885935246`, 290.7153568287518`, 0.12025877488242465`, 1.854625189562679`, 119.76234634965553`, 123.11721945152065`,
0.02801275362516953`, 295.54841633690296`, 83.23477897948672`, 1.0060246157482136`, 1.0189539337090638`, 0.012851890260393128`,
0.053503466855179115`, 0.05369711193554908`, 0.003619299678170762`, 35.37488432398412`, 0.0809794415964362`,
1.4159322042853717`, 0.0007134146099532224`, 0.0007254685046719701`, 0.016896058127458424`, 0.2309832072423019`},
{0.09376889465512256`, 2.694562234128588`, 9.901749913963968`, 1.2671081853876889`, 0.9355728957697818`, 0.41426328356338693`,
0.1676359905682518`, 324.1423914220847`, 0.048114260882103244`, 6.992923371816153`, 690.732257831947`, 656.283359116043`,
-0.04987301277057954`, 452.0801764659932`, 206.53373248280903`, 12.255433318459703`, 12.337763931062376`, 0.006717886708963894`,
0.07931082172385191`, 0.0793339668268134`, 0.0002918278043073652`, 471.75753975432474`, 0.106241258388715`,
4.430248531394284`, 0.0007555114937300988`, 0.0007581455813764354`, 0.003486495795492761`, 0.2829688205563391 5`},
{0.07408607058446992`, 2.173441202450359`, 5.83110243938874`, 1.2671639264693393`, 0.15034130588796946`, 0.26151521374964837`,
0.00796428041791113`, 3349.7895146720416`, 0.06475249420724477`, 9.113413370060233`, 23.30944683909744`, 23.474497778761545`,
0.007080860425536350 5`, 11.706185245211708`, 20.962481028913988`, 0.07240392529089434`, 0.0742961284882554`, 0.026133986379313 3`,
0.0034864856863122 6`, 0.0034883688303670 5`, 0.0005401267133213761`, 2.2480810635195354`, 0.003690000352110271 6`,
0.5929371248005177`, 0.00003323685575262747`, 0.000033336025441675 82`, 0.0029837265530301416`, 0.9500737527527265`},
{0.2682300613650819`, 3.6108652762368214`, 9.881761872038165`, 0.9825580258076282`, 0.9296545706074566`,
0.25219322618587503`, 0.02446489800506527 3`, 1815.4892808719587`, 0.08994453092924143`, 7.425857575199714`,
87.58405352282145`, 84.83369812950119`, -0.031402467489171526`, 53.9812479449595`, 65.07928563921388`,
0.42699521999578766`, 0.43136619596004777`, 0.010236592260454813`, 0.007357631237178645 5`,
0.007359512670588677`, 0.00025571183841388567`, 22.026031614312735`, 0.02819339826072363`, 0.8366405659320559`,
0.00009053495944377765`, 0.00009071051525846462`, 0.0019390941992520894`, 0.2699631213885035`},
{0.04962174488386037`, 1.9694465660050104`, 4.880585401279637`, 1.2957318091391512`, 0.6289429156960646`,
0.36977064682826033`, 0.006935918860683708`, 414.38904513183815`, 0.2065379649808241`, 4.444261705692702`,
173.0676184166249`, 172.2383894475708`, -0.004791358294755632`, 178.22995074830575`, 157.42449180170442`,
0.5277282932377593`, 0.5355208509902037`, 0.014766230752258913`, 0.06475769267734238`,
0.0648640894804195`, 0.0016429986720996048`, 14.84760964143985`, 0.0459055672186322`, 2.280370762664378`,
0.0006010695562357338`, 0.0006063981506817508`, 0.008865187715358491`, 0.343538987267928`},

```
{0.2594824517719198`, 0.5971319663647825`, 9.453687965953478`, 0.8401327653868795`, 0.7997988949991743`,
  0.15899752713279824`, 0.005075100457461465`, 409.49724454586783`, 0.2091478816442816`, 4.600741868044251`,
  144.34953045979253`, 141.3117842430811`, -0.021044378925483054`, 143.59919425192948`, 134.52549410246309`,
  1.0057978305789943`, 1.0238831264097195`, 0.01798104477946061`, 0.017492232644409543`,
  0.017494821667142283`, 0.00014800985016449886`, 8.579914804843824`, 0.06484182019337377`, 2.302797808940957`,
  0.00025174486646806216`, 0.0002522317987739563`, 0.001934229335937454`, 0.17707087766053115`},
{0.1975048020318434`, 2.1175350807064808`, 9.527018017184652`, 0.8875453366861839`, 0.2770809005507884`, 0.4437813967164592`,
  0.08906290963263506`, 98.18685485298634`, 0.18494734685711195`, 2.236429925751402`, 261.5288084622031`, 266.1516029114938`,
  0.017676042943310488`, 535.2155648638229`, 117.69989015823177`, 4.594101475888334`, 4.685872909943449`, 0.019975926639141 05`,
  0.0682461295948304 5`, 0.06832618302579241`, 0.0011730105639282673`, 138.97387199312828`, 0.1925562616436424`,
  5.1278093094762225`, 0.000925309087019377`, 0.0009328693965024443`, 0.008170577366121812`, 0.31455298172415236`},
{0.18592803424857857`, 3.7511626605606008`, 0.2948073517622305`, 1.1421602142936857`, 0.24567142070681736`,
  0.41248813426514863`, 0.25834870652665815`, 931.542828982051`, 0.012674565140645466`, 9.754712584362995`, 120.58056737973124`,
  120.06366955327934`, -0.004286742363917462`, 56.57536180435881`, 27.24598347526525`, 1.6900678946282182`, 1.7380041915677034`,
  0.028363533259135876`, 0.2946005476279931`, 0.29794323404444967`, 0.011346504422244275`, 90.56742257345189`,
  0.7824928672717905`, 2.36539098562177`, 0.0030250983385314845`, 0.00318667760332525`, 0.05341289661089288`, 1.40950000263707`},
{0.05816505121822962 4`, 1.2475983837798745`, 4.326003024744654`, 1.2604302116068957`, 0.7278275017755875`, 0.49389546693051767`,
  0.021556729129120156`, 452.84942671713645`, 0.038214009527072046`, 8.73167788977904`, 442.69641402865375`, 436.6859383244124`,
  -0.01357696948467324`, 232.04530860956834`, 339.2042959246052`, 5.483489100212272`, 5.537498048927459`, 0.00984937650611828`,
  0.09260988235389392`, 0.09270366659680301`, 0.0010126807261314763`, 97.7313162699913`, 0.07695226500607649`,
  4.077391940847746`, 0.000884660048044994`, 0.0008907074222939172`, 0.006835817060222515 5`, 0.6236414326522628`},
{0.17310243018283944`, 0.49256885761049496`, 5.2474038118867075`, 1.2167083563026317`, 0.58861670692344 2`, 0.41089714710891945`,
  0.1821711177021871`, 4451.29023370465`, 0.12001647220652961`, 2.269196213787966`, 8.081093387198464`, 8.083891723590108`,
  0.0003462819024064867`, 16.299060433074942`, 2.3160036592777358`, 0.7151183790515176`, 0.7264013759102721`, 0.01577780293343789`,
  0.005154003794271372`, 0.005172455809553266`, 0.0035801322657935675`, 5.03207204322392`, 0.012745294028000143`,
  0.10977473561079625`, 0.000050738710338560367`, 0.00005157831868406769`, 0.016547687946834477`, 0.20657577710674`},
{0.1805022145737788`, 3.870623878161763`, 2.6853201865429206`, 1.4540056051610473`, 0.3465395939248448`, 0.3116685990581043`,
  0.3430495408207368`, 123.77189026179846`, 0.21554545019710575`, 7.5674497363872035`, 667.5216654759569`, 667.2203935368185`,
  -0.0004513290799685876`, 403.72004745437545`, 123.59489374472238`, 9.643126106982573`, 9.848115196439045`,
  0.021257534868079686`, 0.29916315900945656`, 0.2993721662668412`, 0.000698639692389591`, 533.2130595687388`, 0.7714230388578868`,
  13.25969232060591`, 0.002472117811554541 7`, 0.00249651015308656`, 0.009866981831533117`, 0.7478479908564255`},
```

{0.12919486377057743`, 2.6304051581320387`, 9.030615531469042`, 1.1852097760732927`, 0.8025656477879668`,
0.2709665069296936`, 0.01610528402445943`, 631.0560544240994`, 0.23139307171140971`, 4.580191033116536`,
129.2888274677662`, 125.09027476300521`, -0.03247421132199346`, 129.19386749991227`, 105.29077169023908`,
0.6190397511899655`, 0.6268573069011488`, 0.012628519729396714`, 0.02323316606616431`,
0.02324642558718362`, 0.000570715200052696`, 23.261790780269404`, 0.04288008178396441`, 1.5017082552570107`,
0.00023667460153786735`, 0.0002375601790102865`, 0.0037417511919946023`, 0.22129687260717393`},
{0.11792807938525018`, 2.9992967519426994`, 5.197929087471108`, 1.1316540273093707`, 0.8248866470317724`, 0.3561695895739371`,
0.01696020529134022`, 1283.8412020113915`, 0.1325959329361287`, 7.000673815586042`, 122.84876332894338`, 117.91162834438464`,
-0.04018872352291347`, 80.31477383175833`, 99.02080268099627`, 0.540942808279422`, 0.5464375023764498`, 0.010157624822677347`,
0.02395190024024396`, 0.02397230879581907`, 0.0008520641523390715`, 23.177828683703382`, 0.040351451327948915`,
1.130678487517111`, 0.00025529903352816685`, 0.0002565749116130237`, 0.004997582901997477`, 0.35415843649250583`},
{0.049303290582256715`, 1.1925070924339343`, 0.7957633600668377`, 1.4128683276782472`, 0.000022730879762677958`,
0.4285104885078085`, 0.00878130985829771`, 1257.460569712898`, 0.24085716752239034`, 7.587289884781604`,
56.654301083131934`, 56.7582502982993`, 0.0018347982973938581`, 34.17517157403495`, 50.31977971715527`,
0.3410152857914075`, 0.35013312854729195`, 0.026737343268130376`, 0.08234531130644157`,
0.08302350853617854`, 0.00823601512918093`, 5.809473527637695`, 0.057998497306112604`, 1.4045126775936223`,
0.0006905050025575177`, 0.0007131417500962557`, 0.032782887096972635`, 1.3610479623467813`},
{0.13128664635800402`, 2.4046868210274015`, 6.858470528577773`, 1.007040533285536`, 0.4840752461507001`, 0.3489790633820432`,
0.006590730128885638`, 3596.8950880067596`, 0.16587148750288666`, 3.462016809878005`, 12.368572077853594`, 12.463534577552196`,
0.007677253753193`, 16.35139789003659`, 11.30245739143819`, 0.029561686800876626`, 0.030106112356227604`, 0.01841659303875831`,
0.0032865474811251357`, 0.00329079514937072`, 0.001292440857763033`, 1.0155228379629668`, 0.006163997098474358`,
0.20759118359502582`, 0.00003933042766268713`, 0.00003958919832952938`, 0.006579401298698384`, 0.32118064795429496`},
{0.04846841194802434`, 1.828640157997615`, 8.053204702491172`, 0.7912000348817253`, 0.19137275290127498`, 0.26044015409759047`,
0.137444226583514`, 395.2194426771421`, 0.10623448877689068`, 6.754668978902`, 150.13266970261623`, 150.67600655491395`,
0.003619044764700207 8`, 101.72685812736012`, 53.01221881110682`, 3.57982950416081`, 3.6673784987232345`, 0.024456191128841898`,
0.013703872843609547`, 0.013707074014898735`, 0.00023359610277484677`, 93.5174284299018`, 0.009488642203761591`,
3.7129436873440165`, 0.00020936588010433965`, 0.00020975677179679636`, 0.0018670267202176571`, 0.6802971984368331`},
{0.22606436547954117`, 2.759207160262239`, 4.797641172785626`, 1.1197512107663665`, 0.8744312540778343`, 0.4079143329219078`,
0.021680363867763237`, 112.017364386689`, 0.04841847187742204`, 6.247097164626069`, 1535.293331907739`, 1454.3006392475324`,
-0.052753888118282743`, 1124.8059306318362`, 1173.5091319313146`, 8.904519209548818`, 8.982865430472927`, 0.008798478511910401`,
0.3599646543702072`, 0.3597488644365390 3`, -0.0005994753402821518`, 350.99161659542415`, 1.1625025883612352`,

```
11.530682948802912`, 0.003866298163084924`, 0.00389313023532879`, 0.00693999042806781`, 0.297195518124461`},
{0.21439187995731207`, 1.4871413304129364`, 0.5160724591468409`, 1.1806787917625174`, 0.8679222077666104`,
 0.6556391626343723`, 0.05962099501823598`, 325.40523317512117`, 0.12955288081431815`, 2.055442432220964`, 252.61864078071002`,
 228.36518105874237`, -0.09600819498914681`, 562.5026476273036`, 133.3999292922033 8`, 5.055516347148965`, 5.223471996655006 5`,
 0.03322254261082496`, 1.6632706968274746`, 1.7165113714032858`, 0.0320096269821637`, 107.40381866319208`, 5.094167593867909`,
 1.3733426874457304`, 0.01612216388516241`, 0.018099350298948807`, 0.1226377816197517`, 0.20881491579634393`},
{0.09778347249929609`, 1.5399588911598938`, 7.050045797549721`, 0.9082485710483456`, 0.7454636238668619`,
 0.6248569918637821`, 0.04029077002834171`, 1056.397985521477`, 0.2093818171480931 7`, 3.632048403677867 3`,
 92.44578580674406`, 91.54995376668423`, -0.009690350211663978`, 116.49304178250803`, 58.904600796760874`,
 1.4560298258216622`, 1.4683742849102075`, 0.008478163612877232`, 0.0205377360137715`, 0.020574993508728552`,
 0.0018140994183619696`, 32.031801086686535`, 0.028689302067209384`, 0.7318297345469675`,
 0.00027195313635719476`, 0.0002743829650028675`, 0.008934732940462542`, 0.28500911440934684`},
{0.12927232662770405`, 2.951438179906213`, 0.40510823994438105`, 1.0187470674786288`, 0.2032238445066854`,
 0.5456409554372916`, 0.01881027549673071`, 134.69705409101317`, 0.12921233603333943`, 7.23494261394152`, 667.6313108390607`,
 674.0663425712387`, 0.009638600868030922`, 422.343777634193`, 525.314085700534`, 3.1975924698488427`, 3.2760684132877227`,
 0.02454219672420921`, 1.437471502256223`, 1.4478733519537506`, 0.007236212809228482`, 134.82137856132118`, 2.654646936538337`,
 12.5534291725343`, 0.016363428370280153`, 0.017311875528269884`, 0.05796139638514464`, 1.3323343570133426`},
{0.20182779178318416`, 0.6881028184372244`, 1.057422534977741`, 1.0568090274497894`, 0.12452101430667706`,
 0.6254257177994904`, 0.38702446915906646`, 3365.5277260432094`, 0.12211162516890145`, 5.63708838473557`,
 20.98062217177111`, 20.916672179547568`, -0.003048050324722329`, 17.034445977929995`, 3.5092482686121893`,
 1.6049707875962953`, 1.6405416563726019`, 0.02216293844798245`, 0.02304140244622094`, 0.023252066180730523`,
 0.009142834729842342`, 15.77692746363461`, 0.06643421964724394`, 0.39827436609650135`,
 0.0002577484210000147`, 0.0002675029337691445`, 0.037845092246478806`, 1.154288952726082`},
{0.21480237208045305`, 2.4248480984277982`, 7.5563453916242835`, 0.9609967545999567`, 0.4216733331369118`, 0.3788510295397969`,
 0.02865689876021866`, 142.8377853769303`, 0.05340960656114529 5`, 2.2123406251771502`, 190.63892260980595`, 193.03675900242317`,
 0.01257789521568497 5`, 394.3883478211194`, 136.00061306090527`, 1.5245206054063931`, 1.5537002536031141`, 0.01914021240069940 8`,
 0.06859758714528653`, 0.06869982785959888`, 0.0014904418444894318`, 52.81044130048125`, 0.21049892054003266`,
 3.3872941039943854`, 0.0008583913631707851`, 0.000866685183449961`, 0.009662050010078804`, 0.23908288954591467`},
{0.19117013383013431`, 1.4594963084730592`, 8.271393984038092`, 0.8911461626518757`, 0.4023315908892062`, 0.6430024305607471`,
 0.425955961175158`, 811.9982424604128`, 0.08470327958141488`, 1.5517888018340713`, 30.272229147576326`, 30.92397173014905`,
 0.02152938851630281 6`, 89.28454366403611`, 4.621886647276803`, 1.1716905754785882`, 1.1899140564533877`, 0.01555315145157321`,
```

0.01312387073816355`, 0.01317344913344602`, 0.0037777265771370416`, 24.42968670833818`, 0.035841316076912516`,
0.4352566759689481`, 0.00017621798915079534`, 0.00017924484450266792`, 0.0171767670625409`, 0.23891132242272267`},
{0.16704561027767095`, 0.7136544792609025`, 7.482780404676742`, 1.2150283597546259`, 0.9046207606306917`, 0.271745131902447`,
0.2695234426940548`, 220.6020580904624`, 0.17006503868594403`, 3.14899802359108`, 300.0267629323425`, 288.87284735281145`,
-0.037176402099989625`, 436.065876289372`, 64.11235838760936`, 21.043747628169434`, 21.2745408318815`, 0.010967305243820835`,
0.09694866181307545`, 0.09701935042407041`, 0.0007291344684183798`, 214.5423536468443`, 0.23135497683075823`,
2.9334161480503695`, 0.0009614731193456905`, 0.0009676943385741137`, 0.006470507706608508`, 0.1297313508536759`},
{0.16595765366147208`, 2.092996799011412`, 4.948615364795604`, 0.8929763621996465`, 0.8391454185145462`,
0.5892008264762102`, 0.044299676614507844`, 130.42998143434642`, 0.19085435393011602`, 9.39386568753341`,
2400.490864656893`, 2293.579012162944`, -0.04453749608800539`, 1169.553803970645`, 1475.2696775789439`,
29.908486773154998`, 30.109100069207884`, 0.0067075709170598685`, 0.2896503534181655`, 0.28937342083760526`,
-0.0009560926727419927`, 894.2623868498373`, 0.6867099005065423`, 15.024782374934247`,
0.003904756196767445`, 0.003924511483497069`, 0.005059288143515417`, 0.5351902169703011`},
{0.27224148961405453`, 3.064354031176171`, 9.703480945324245`, 1.4628863651144104`, 0.3875440410966342`, 0.15502532019017645`,
0.01538110765065082`, 96.32001554152914`, 0.17185939304406467`, 6.478173160029735`, 572.6791633243092`, 571.7388234506207`,
-0.001642001200515275`, 404.597620264918`, 471.46441582006094`, 2.245906093080685`, 2.3040250665041833`, 0.025877739769509578`,
0.08361134369214993`, 0.08360936507772651`, -0.000023664425615521623`, 98.31787699964181`, 0.3251782393625612`,
14.050990100133463`, 0.0006907979590969893`, 0.0006923805577824168`, 0.002290971860276380`, 0.3790108707188194`},
{0.17789655356032075`, 0.42086448766877327`, 8.212577079102342`, 1.145177796821741`, 0.7675840121122328`, 0.5713332044931656`,
0.08476669830762888`, 407.4176887081235`, 0.039911846147414665`, 5.425104426537898`, 359.0334490062557`, 351.9321745619992`,
-0.019778865907651877`, 302.8944414847433`, 163.8224246664158`, 27.80891389232497`, 28.0426523114653`, 0.00840516174221539`,
0.05784481298481787`, 0.05790223581321401`, 0.000992704884553941`, 167.196918541691`, 0.14700561244778157`,
2.8122671767819534`, 0.0006085238243485058`, 0.0006124363259799014`, 0.006429496224875653`, 0.3801170333129755`},
{0.25890879804354505`, 0.827757285125033`, 6.146318214990161`, 1.0400613992204162`, 0.4812180568243971`,
0.29251954183586915`, 0.03603273141312643`, 1733.9600457555907`, 0.08508345454030858`, 2.745915615562448`,
18.582658993936747`, 18.625373468147195`, 0.0022986201395820416`, 30.973134977991744`, 12.357314976897815`,
0.48268047131042724`, 0.4923842978136806`, 0.020104037929913554`, 0.007165192382240595`,
0.007176274057041607`, 0.00154659836189186`, 5.7077468073541455`, 0.02650187639195828`, 0.3390105972500421`,
0.000082961885793486620`, 0.00008367952791374696`, 0.008650262869466685`, 0.2324272610820643`},
{0.13429919704858378`, 3.97301958069563`, 0.23275296336668916`, 1.4075463260369605`, 0.23061890929775308`,
0.5504417933404041`, 0.22771166040596233`, 272.6044063125219`, 0.22898591641919652`, 6.483734227937328`, 315.6135480519186`,

```
310.123946787966`, -0.0173934271764834`, 222.7896071057214`, 77.03243166802139`, 4.041436121583496`, 4.179624420677881`,
  0.03419286979605673`, 1.7673725370131008`, 1.8170288966525787`, 0.028096147586064824`, 229.38149778831198`,
  3.390810180093882`, 5.6593715446266915`, 0.0141161563946125`, 0.01589457083631308`, 0.1259843254768218`, 1.3329651811890244`},
{0.12030391986517214`, 0.7748656879950588`, 5.112522469071294`, 1.1531030157403883`, 0.37283988475313556`, 0.5692147153589893`,
  0.013539004333470354`, 948.0585012448981`, 0.020895787868669358`, 3.48210387762453`, 53.074120727516245`, 54.61572115989577`,
  0.02904617940434928`, 69.75985671162923`, 44.52971644444877`, 0.7008706836578088`, 0.7126734957469673`, 0.01684021370042199`,
  0.021491189461497882`, 0.02155843758922763`, 0.003129102176974818 4`, 7.758294921258202`, 0.036935347639770695`,
  0.8312726173903614`, 0.00022339261730497384`, 0.0002265788915176201`, 0.014263113307349862`, 0.51091115366912 7`},
{0.1321541010040706`, 0.831080421127889`, 5.684518793971316`, 1.0145204035612485`, 0.08667418093385804`, 0.2219847293666507`,
  0.02252392656695254 7`, 3181.1487538157703`, 0.21535243439086593`, 6.5479272590953945`, 15.758371838019794`, 15.829846222573646`,
  0.004535645261359189`, 11.014682738131862`, 12.017416057906782`, 0.28973276918632795`, 0.2980134589091203`, 0.02858043895430762`,
  0.002693740543800766`, 0.002695151544263959`, 0.0005238071151432155`, 3.4398747404274745`, 0.00508555514148851`,
  0.44613887444290007`, 0.000032077220997406286`, 0.00003217565402121818`, 0.003068626918143913`, 0.6428589725175897`},
{0.10292542985717545`, 0.9480363128916887`, 1.8187023134383153`, 1.095683835227761`, 0.3789693754557397`, 0.6741099730044744`,
  0.00792768211018952 4`, 599.0709679738629`, 0.23730403059109434`, 8.347995806673719`, 226.35519761084748`, 234.5920796790247 2`,
  0.036389189005229694`, 124.10027495666631`, 203.2403502675447`, 1.5590656155894709`, 1.5833601499283856`, 0.01558275296176625 5`,
  0.10206279257417775`, 0.1024399237563467 7`, 0.0036950897840162256`, 21.115011682280745`, 0.15006938283030388`,
  3.1839841313542596`, 0.0011132018684131273`, 0.0011330790822109433`, 0.017855893312639814`, 1.3163286359501376`},
{0.11768271904228267`, 0.8044018805503832`, 4.992994745146397`, 0.8839437991548327`, 0.9627780258088621`, 0.4036610370791115`,
  0.010677704183731065`, 75.90134138634313`, 0.09047277628943856`, 3.892249177500955`, 1660.7759031407054`, 1601.8081922372273`,
  -0.03550612144116716`, 1952.8770154748195`, 1440.332839110105`, 17.4589883357768`, 17.596340032042196`, 0.007867105105049843`,
  0.4746509047705061`, 0.4738760284305258`, -0.0016325184091979805`, 200.62918642580087`, 0.7979744152754192`,
  10.525305611725097`, 0.006445056807212968`, 0.006494756640259033`, 0.007711310316216968`, 0.15543388328461974`},
{0.23810204192481904`, 3.5212069305389937`, 6.24449843698252`, 1.12580298554529`, 0.9906238590423209`,
  0.651194539832239`, 0.008821192031195845`, 3794.027232128564`, 0.08728181755971864`, 9.039220988882679`,
  128.21948126806566`, 125.95857672132418`, -0.017633081372515 18`, 64.92134462066059`, 113.77520467094689`,
  0.2782663235530422`, 0.27970221330396133`, 0.005160127652476865`, 0.01602924601167521`, 0.016046182688800117`,
  0.0010566109667649037`, 13.997618671865403`, 0.05452280294135212`, 0.48815344351892664`,
  0.00017165073449709745`, 0.00017263905199751647`, 0.0057577236899952755`, 0.3445874668358851`},
{0.05170854445494355`, 0.9544694556169109`, 2.4941099826351163`, 0.9097774283707087`, 0.02554739328254807`, 0.2240892801663740 8`,
  0.36587983814629343`, 578.5087062785725`, 0.1431894567694563 2`, 8.489550606354296`, 110.70897756643201`, 109.38753992692125`,
```

-0.011936138049128187`, 59.68464747819577`, 20.321950513114555`, 6.172425441109756`, 6.349705202229808`, 0.028721247880829637`,
0.02983006066085373`, 0.02984951149057962`, 0.0006520546487327472`, 84.16273643731418`, 0.02203527168249715`,
3.189532143692178`, 0.0003956477214639431`, 0.0003973709641438153`, 0.0043554975458877 1`, 0.8593214393242319`},
{0.2758391104750425`, 2.3950700125014723`, 3.0825274454463827`, 1.2324094107661792`, 0.33578451389793784`,
0.4521239993250691`, 0.02669897011071422`, 4321.015062663416`, 0.06443072522161825`, 4.390191809873814`,
12.70458249377515`, 12.937766036958083`, 0.018354286203201342`, 13.244677776009537`, 9.229486672629607`,
0.09761415819171514`, 0.09960974756957724`, 0.020443646852362996`, 0.007217401865833887`,
0.007243077475243204`, 0.003557458748536968`, 3.3398963297221727`, 0.028440595865893224`, 0.22713445681164848`,
0.00007014665591631442`, 0.00007134828567928029`, 0.017130250149050896`, 0.5824953307296793`},
{0.04136033639560718`, 3.5342644851920078`, 2.1080294445485137`, 0.9950381948590621`, 0.26813425336643104`,
0.30392774702749736`, 0.047958977131448324`, 92.66270711931676`, 0.1731864034169307`, 4.57325203890367`, 485.27312290273034`,
488.76390838350073`, 0.00719344492002727`, 485.6524638291862`, 291.88634298753374`, 3.7459434163887986`, 3.8318327045909166`,
0.022928613343796256`, 0.3138566521515676`, 0.314123113381063`, 0.0008489902242592162`, 189.1307825725964`, 0.18544595304274258`,
10.802787460160289`, 0.0037797271091455364`, 0.0038255864563140254`, 0.012132978345851031`, 0.49855998326062584`},
{0.1958059683987035`, 3.200106783136203`, 7.203120771344906`, 1.1276577663917136`, 0.37865874140280775`,
0.19330414092595893`, 0.01322903896050458`, 235.27978791284824`, 0.1213328646575434`, 2.0980953870537427`,
78.78025844150503`, 78.76974539961955`, -0.00013344766942180453`, 171.85282154578442`, 66.4001021899335`,
0.2611533774054829`, 0.2680445135419194`, 0.026387313864744355`, 0.036798737946334335`,
0.03683834725678191`, 0.001076376872090945`, 11.938838492488797`, 0.10293446456330746`, 1.888342736525934`,
0.00039332668585589925`, 0.00039617317693196034`, 0.007236964026142711 5`, 0.15296826529933458`},
{0.2141293518430885`, 1.3445267096173987`, 9.440612710104876`, 1.3031822475396773`, 0.08631549676886618`, 0.2421378295292581`,
0.053475196461594834`, 50.8320364740117`, 0.167124755886029`, 6.69448094138391`, 1039.9467225900007`, 1044.355043042405`,
0.004238986821772261 5`, 710.9821145047109`, 601.0903279467381`, 21.68972994602299`, 22.28797649941296`, 0.027582019457077944`,
0.13457727633906563`, 0.1345532488070949`, -0.00017854078061574352`, 416.60601766880393`, 0.4116706422181631`,
28.711835141493093`, 0.0012471869882023645`, 0.001250568806761088`, 0.002711556960354189`, 0.7002230521002436`},
{0.14124099610833857`, 1.6304240145491873`, 4.431235349124869`, 0.7694928939095955`, 0.9477250896887188`, 0.5434618086833787`,
0.13057745213792676`, 1388.4740941330226`, 0.07045603839931946`, 8.981021929620123`, 269.8721338505538`, 254.917902533672`,
-0.05541228397135267`, 137.52979030549338`, 95.0127118852542`, 7.19423287421828`, 7.232441561515486`, 0.005311016193836782`,
0.032725457106280206`, 0.03274735254620417`, 0.0006690644489046882`, 167.56642920549567`, 0.06603108799721735`,
1.328931102269845`, 0.0005129695237795495`, 0.0005153722872056446`, 0.004684027636557397`, 0.3679225405533721`},
{0.08317638924124338`, 3.4814117090049965`, 4.537378351353009`, 1.1287962009049006`, 0.37603042712104306`, 0.6772825294518297`,

0.08278526718675269`, 52.354988641883`, 0.1936331782676135`, 8.933135500909255`, 2770.2075217218826`, 2873.020249012567`,
0.03711372757618503`, 1419.2956680973496`, 1288.699652497902`, 29.180266818459746`, 29.585635251145607`, 0.013891868611339175`,
0.4845890062747518`, 0.484145588059227`, -0.0009150397755277595`, 1451.2646081953696`, 0.5758051972525412`,
38.901220662105324`, 0.005154017875108385`, 0.00519417981540701`, 0.00779235564792846 2`, 1.386007527418358`},
{0.14458191325048447`, 2.576522592570102`, 7.664240990513161`, 1.1334692879511565`, 0.1460097548297743`, 0.5479004940247297`,
0.17641432391033549`, 874.6457178122505`, 0.14737345925317297`, 7.125853291619361`, 95.36151061132549`, 96.76209127673845`,
0.014687064586481391`, 61.24924504184095`, 28.33271691173981`, 1.7718922710020135`, 1.8057742319890202`, 0.01912190799717539`,
0.01240839685232247`, 0.01242014296456426 2`, 0.0009466260937320836`, 65.21886382624325`, 0.025628996532570406`,
1.891188873006625`, 0.00013202839525494614`, 0.00013270298305177787`, 0.005109414497761033`, 1.2550226394177149`},
{0.21964890100642098`, 3.948215647629967`, 1.3239539515383179`, 0.7541520677887659`, 0.2631388544858304`, 0.4680193255276971`,
0.07455346059859487`, 734.1983096733243`, 0.22794453971709777`, 5.810951908142973`, 94.60819788356025`, 96.20959270028948`,
0.016926596770188596`, 74.51539080349659`, 46.57064065082665`, 0.8326715438522138`, 0.8500273187670077`, 0.020843482694869575`,
0.05787025821991614`, 0.05807459667961083`, 0.0035309754264127857`, 46.965240268193014`, 0.18158769455659918`,
1.7899251566365193`, 0.0009178409745552729`, 0.0009345923050508128`, 0.01825079829722842`, 0.8474588833747576`},
{0.24357090033802437`, 2.585687351965424`, 7.1434831024545`, 1.2789397105658484`, 0.15335660895486725`, 0.5235228651937761`,
0.12790639986488742`, 56.5359989413313`, 0.05251799125371648`, 7.0717564062965526`, 1438.0668741551376`, 1460.857949049142`,
0.015848411018711595`, 930.7140810684383`, 525.6315460224113`, 24.023481980568555`, 24.49811188902719`, 0.01975691570616367`,
0.22838215608537465`, 0.22840515939102637`, 0.00010072286752182968`, 887.3887643903621`, 0.7946749625546481`,
28.894106507516394`, 0.0021503518407258726`, 0.002163492332545617`, 0.006110856637911111`, 1.2049332809630735`},
{0.09627432586752127`, 2.332762155680175`, 5.679853879558527`, 1.2640734140093113`, 0.8156330491360497`, 0.2895792785477985`,
0.02307681035132743`, 658.139699058721`, 0.029189068125338835`, 7.800914789874877`, 229.57205085482846`, 221.42610034897046`,
-0.035483197869801586`, 134.690813059399`, 173.1488948314327`, 1.6395331972468428`, 1.658213932716127`, 0.011393935481546436`,
0.04107846106285036`, 0.04110049562981109`, 0.0005364019583649071`, 54.637728507410806`, 0.05649715923571454`,
2.4503771782761743`, 0.00039226575316386736`, 0.0003937766037951765`, 0.00385159963398074`, 0.37005040449391324`},
{0.07523258003696548`, 2.5308231598738997`, 0.19036315944607196`, 0.7501464923569574`, 0.1153260012566284`,
0.48261384042066857`, 0.014254428894325608`, 71.6138994625911`, 0.24976914139283035`, 2.597638512857568`, 405.2289551569688`,
391.8124291340777`, -0.03310850780071761`, 713.9803344025833`, 331.51153425148794`, 1.7723811423317746`, 1.8452056203781775`,
0.04108849744959131`, 3.700167898943696`, 3.7845163978696816`, 0.022795857169093603`, 64.07976061624309`, 3.9767596801069285`,
8.840997200383567`, 0.053500672938327165`, 0.062280550602292546`, 0.16410779868295067`, 0.610262130406258`},
{0.20487865745542216`, 2.4577449266837927`, 0.3620012570001929`, 1.1621484663425825`, 0.45716579542364766`,
0.40055848752773193`, 0.09344580216885054`, 2051.8175124763634`, 0.1493227569977858`, 9.69569860745337`, 65.88008158615449`,

```
  66.05844948505614`, 0.002707463236340857`, 31.0985053163897`, 28.562705317485147`, 1.0187918505345865`, 1.0430495655041088`,
  0.02381027582503092 4`, 0.1344911186361278`, 0.135837307653097`, 0.01000950122670452`, 35.77043574283108`, 0.3936337117978318`,
  1.0363493531574957`, 0.0013652657211278285`, 0.0014265222015113373`, 0.044867808101785256`, 1.0780601362639362`},
{0.1890789829788747`, 2.8864588443110275`, 0.3020897698703884`, 0.7861583937331655`, 0.3631295895274096`,
  0.49565973888016235`, 0.15528595628066338`, 2271.132408212757`, 0.21092746640771154`, 4.683826377759731`,
  28.64142211119561`, 28.674373858185167`, 0.0011504926976608765`, 27.987124804153805`, 9.096801204543482`,
  0.45428394649933956`, 0.4674324312653339`, 0.028943318088422654`, 0.09669505439488184`,
  0.09863251017110906`, 0.020036761842183504`, 18.73245593145054`, 0.2611857506295796`, 0.47026682445941814`,
  0.001425202863972297`, 0.0015384074123101594`, 0.07943048052986712`, 0.727567299294831 1`},
{0.13636211858102731`, 3.2225156772879746`, 1.985405604082965`, 0.8074403105824717`, 0.51683495700684`, 0.17846947550569658`,
  0.2866421049071916`, 363.9511542941515`, 0.043491080354586376`, 7.48314403026421`, 209.2137241731765`, 207.16679860913504`,
  -0.009783897170852596`, 127.95891847161886`, 44.42039894492432`, 3.499093923971545`, 3.574931534491965`, 0.021673499531085216`,
  0.07128812997862086`, 0.07131055360896847`, 0.00031454928547480776`, 161.0840718043051`, 0.13887143476509894`,
  4.288413932579431`, 0.0010650752659149276`, 0.0010702134751633212`, 0.0048242686811243995`, 0.4247735217263845`},
{0.1760012024892597`, 3.3901909690471133`, 5.258617572133415`, 0.8627686224489919`, 0.7422533085216929`,
  0.41138487941188706`, 0.007592509582269234`, 424.62789093038504`, 0.09162929479721776`, 1.3722835015951313`,
  64.79000657779554`, 63.189675267236474`, -0.024700280106276784`, 216.08701819955493`, 58.31355061098681`,
  0.12646685402349936`, 0.12825418519298112`, 0.014132803281005524`, 0.0483153515027596 7`,
  0.048502713524997396`, 0.003877898357564913`, 6.124954091346672`, 0.12147942804538699`, 0.6832905215504892`,
  0.0006697328511239276`, 0.0006823484132615745`, 0.01883670797464365`, 0.09778051223786288`},
{0.1813951042683553`, 2.693386252451666`, 9.617741712202033`, 1.407583958403493`, 0.6407257633308476`, 0.4405499114150466`,
  0.008535253338252974`, 83.84476850180724`, 0.06822985382579094`, 7.163997237119144`, 1563.6019193563939`, 1564.1065748261265`,
  0.000322751886835837`, 998.9305146096426`, 1393.8860789039447`, 4.249802764779179`, 4.304615937359721`, 0.01289781564330772`,
  0.20045690468259691`, 0.20044497119071866`, -0.000059531458380779334`, 163.51943346124727`, 0.5194557303745992`,
  18.217827942214488`, 0.001717391827206649`, 0.001724690883304667`, 0.004250082003644850 6`, 0.5751819004126749`},
{0.17566594009418735`, 3.2466526484278377`, 9.344725381698979`, 1.0138972604548542`, 0.5838549167343343`, 0.6073333607184574`,
  0.10710975925951867`, 945.005957021721`, 0.208189974443157`, 1.4109192016259353`, 28.72138870034805`, 29.29115146081773`,
  0.019837577020179964`, 93.16821720648728`, 11.526099351409997`, 0.3618958451504986`, 0.3665979521118185`, 0.012992984098406879`,
  0.013787921166563412`, 0.013842631528655565`, 0.003967992087511307`, 16.785001487327108`, 0.03460097333812457`,
  0.3284324117146021`, 0.00016267661684621348`, 0.00016555806422444353`, 0.017712732377229257`, 0.16162509949056098`},
{0.22088610125498404`, 1.594941397589448`, 0.6585367344105002`, 1.4859166690390868`, 0.411769154740657`, 0.4715175396654723`,
```

```
0.02453817964421129`, 3156.4892991025154`, 0.15641735671760065`, 8.553654142069323`, 38.55673968673287`, 39.025938253147636`,
  0.012169041527549496`, 20.630659532821376`, 28.501339762112803`, 0.411495360488478`, 0.42076004947358003`, 0.02251468637241527`,
  0.06278601542955323`, 0.06341729121619626`, 0.010054401164401483`, 9.37587121941525`, 0.19812225945098022`,
  0.6038001533522392`, 0.00049899560742952`, 0.0005202142013608257`, 0.0425143472797602`, 1.1028487213848117`},
 {0.08792198919034061`, 0.7563929024204028`, 9.775416388823775`, 0.7563974438325178`, 0.8271569221820632`, 0.32401969363676375`,
  0.0333282008681181`, 102.10380045307154`, 0.1014972556908379`, 6.35713244492311`, 1315.392686514551`, 1265.6834373784666`,
  -0.037790425357921875`, 947.0189249534119`, 895.0258850583868`, 35.57859609051274`, 35.93849505780305`, 0.01011560339184614`,
  0.10054340299334648`, 0.10049796315647948`, -0.00045194249959901l8`, 384.4485365849446`, 0.12628537130211928`,
  12.881855803503678`, 0.0016055416181627313`, 0.0016086904738257758`, 0.001961242005453334`, 0.30698549050317336`},
 {0.13210283054552296`, 3.2703864704797683`, 0.8879166279114746`, 1.2876213813677582`, 0.37930173682375035`, 0.2978584700900475`,
  0.018740615394798876`, 111.76209976448992`, 0.22677958819730465`, 8.664567998658026`, 856.3146604768598`, 859.939215414434`,
  0.004232737222501504`, 452.32538309952514`, 676.6535142913308`, 3.7050669828363256`, 3.7896239395023725`, 0.022821977863762388`,
  0.8969313608179108`, 0.8970365147488283`, 0.0001172374336666504`, 173.10001332698891`, 1.6926738795582443`,
  16.763034452231402`, 0.00829828326196646`, 0.00845917224771269`, 0.019388225331331777`, 0.8276897749582444`},
 {0.19507506176309325`, 3.126733009164254`, 1.2939386659529877`, 0.8626822462482099`, 0.4036124839823858`,
  0.4778725494331981`, 0.06798274218289758`, 2442.14562500692`, 0.025650848833468687`, 3.9818096584261493`,
  22.565635484331786`, 22.916696717809067`, 0.015557338667507858`, 25.93770624691278`, 11.574827098647495`,
  0.23872165374076917`, 0.24345188963670217`, 0.019814858944759317`, 0.02347076355381329`,
  0.023626646615319533`, 0.00664158458879438`, 10.66312678219344`, 0.06540800928409969`, 0.363734344488398`,
  0.0003234894814369005`, 0.0003328641122580278`, 0.028979708333904286`, 0.5188710153964966`},
 {0.08607083224139878`, 0.4430857620402713`, 5.378081455692687`, 1.015604011959475`, 0.6736258822863768`,
  0.30157456176441344`, 0.06550214653497834`, 2724.6632477522535`, 0.20587133389175544`, 1.8687253311000447`,
  10.316136256307432`, 10.155378811198135`, -0.015583106030710558`, 25.265976725788597`, 5.390799935944584`,
  0.6699707527383373`, 0.6803533124701098`, 0.015497034294909673`, 0.006518513302932382`,
  0.006535486662707421`, 0.002603869776165668`, 4.240778593168005`, 0.008015055213717173`, 0.14453198934812367`,
  0.00007708092063085648`, 0.00007801135361954717`, 0.012070859832442604`, 0.12653306737347866`},
 {0.14471266481637884`, 3.5830029087352937`, 5.919067097135953`, 1.3383183012471727`, 0.9997339519904831`, 0.40166871745176214`,
  0.015827643464025123`, 2493.129738188907`, 0.10618580931649058`, 1.661604036688603`, 22.28214139846843`, 22.163864046958274`,
  -0.0053081680703401 8`, 61.37533589196511`, 18.143721703702624`, 0.0780183594050715`, 0.0787397475647466`, 0.009246389762307627`,
  0.018901344855592736`, 0.018985506727948572`, 0.004452692281889936`, 3.99342869547324`, 0.03907519975237666`,
  0.1370294530380975`, 0.00016888821883553895`, 0.00017218278038922587`, 0.0195073497512288l`, 0.0693324664811799`},
```

{0.15884129765285837`, 1.9554524456621927`, 0.3975714737116185`, 1.180621526159508`, 0.13357193827486524`, 0.6393982147520227`,
0.07953097836767822`, 3991.2803311425578`, 0.2365716915141447`, 8.584420428066604`, 27.54858357066705`, 27.721344184243357`,
0.0062711250882698`, 14.687666050076507`, 13.057944816655606`, 0.4942025264725811`, 0.5063845110465949`, 0.024649782065995884`,
0.05837457503153702`, 0.059406566753240436`, 0.01767878774527909`, 13.805564843474908`, 0.13246133211347091`,
0.5167049560728363`, 0.0005756572456753206`, 0.0006134605949984753`, 0.0656698922964245`, 1.8646388356988888`},
{0.04917531951043441`, 2.0521955455867618`, 5.720052321648424`, 1.1771597571169652`, 0.4322660150443707`, 0.1629991214278883`,
0.005091310116027936`, 189.05888707261903`, 0.1735533969163952`, 4.172239042736072`, 195.68761625080484`, 195.12319491825085`,
-0.002884297654434098`, 214.6637218205946`, 182.43567758881488`, 0.4267831361551723`, 0.437638835653652`, 0.025436102270293803`,
0.06054085011133044`, 0.060565295049969665`, 0.000403775939622264`, 12.512035013559917`, 0.04253022353798649`,
4.615129189567873`, 0.0006208461681012079`, 0.0006231717409978358`, 0.003745811790609066`, 0.24858564552858844`},
{0.27542629055144846`, 0.7843233583874856`, 9.783752583368699`, 1.3481471177954405`, 0.23017739141462767`, 0.5420497851091893`,
0.2059235624279975`, 3468.61907862177`, 0.15188055880627876`, 9.878186090289375`, 35.86804548500151`, 36.568662144054706`,
0.019533170809269862`, 16.61862174988949`, 9.55146475320813`, 2.155011703248164`, 2.1933135688388816`, 0.017773390990399873`,
0.003137698093426226 4`, 0.003139770228037290 5`, 0.000660399614419882`, 24.146085949370562`, 0.012345779239177913`,
0.6512154706438873`, 0.0000281016776556 5641`, 0.0000282058141016 22343`, 0.003705702102271946`, 1.5664938332737532`},
{0.242913589428772`, 2.017077890959616`, 6.7411875410249`, 1.4794325227990526`, 0.8119526659059784`,
0.6637027966074835`, 0.028536028992749693`, 2430.705278612085`, 0.11516348105007651`, 2.1124785829469186`,
28.285739131889102`, 27.221897987864867`, -0.03761051245872771`, 61.28295689492395`, 20.086464701574567`,
0.27213754035708326`, 0.2747422152393697`, 0.009571170808954754`, 0.01828980408819271`,
0.018394703420509566`, 0.005735399450482737`, 7.841751656491477`, 0.06346917087159863`, 0.18396422972265725`,
0.0001473265372176069`, 0.00015095670229711564`, 0.024640266092366137`, 0.15314639431727753`},
{0.27369802527373327`, 0.5072292829799574`, 6.3938654238644155`, 0.9166058122252008`, 0.8588715137395462`, 0.6363372429100727`,
0.4973592888199021`, 125.12883357781175`, 0.0982596068118865`, 5.59867632569453`, 1637.5317284359023`, 1554.5498262649037`,
-0.0506749889055641`, 1338.6555956066381`, 210.01748444099752`, 172.9565169020518`, 174.08103997027004`, 0.006501767544585091`,
0.2631114663987293`, 0.2630292234416006`, -0.0003125783845696617`, 1253.2658579276929`, 1.0287584111467067`,
9.329538549761413`, 0.003451765009853336`, 0.003476597905390687 4`, 0.007194260173118305`, 0.3161213431632557`},
{0.18540637687565897`, 1.1775430389730683`, 2.7268491301469933`, 1.4767982648221127`, 0.8902187555583527`, 0.5282942786301189`,
0.1497900469847022`, 304.29028125771407`, 0.023895791395609434`, 5.874748340307197`, 675.1036854218548`, 630.8827184887874`,
-0.06550248189718422`, 525.9515099423576`, 217.2819293010651`, 25.608886892045643`, 25.815938435361076`, 0.008085144199678096`,
0.38892619338494544`, 0.3898955911923727`, 0.0024924981241050137`, 430.7938070796719`, 1.0301342341076563`,
4.007275929259987`, 0.003146103555599189`, 0.003202828568851843`, 0.01803024352192706`, 0.3034189332911715`},

{0.2722155439547531`, 0.9139371325579537`, 7.770199974150746`, 0.8847133947824111`, 0.9018075103619831`,
0.19960287154002887`, 0.00858204856018103 4`, 1921.873640365849`, 0.18448070830995128`, 2.8786799978881135`,
25.038924135542548`, 24.350928499133843`, -0.027477044647940763`, 39.809504202517836`, 22.293428189370324`,
0.19176409824714566`, 0.19454226818041`, 0.014487435128153425`, 0.006205892132710286`, 0.006209962249564322`,
0.000655847179905411 1`, 2.503719001136541`, 0.024133432894718435`, 0.30669850838523743`,
0.00008470160868723209`, 0.00008507533550361085`, 0.004412275305877511`, 0.1077946157244639`},
{0.06964367539322192`, 3.707453741922901`, 4.6834761045808655`, 1.337177597600478`, 0.13817299390105364`, 0.15072857839162668`,
0.030325540973499845`, 324.16896723003435`, 0.11870351893768993`, 8.4259642987084`, 184.59909837651173`, 184.83204637468828`,
0.0012619129791273664`, 100.27079436115876`, 130.33974489466945`, 1.0036545807101427`, 1.0340118300068417`, 0.03024671025286363`,
0.03924179878011787 4`, 0.03925271066126346`, 0.00027806781250583334`, 53.157184726455334`, 0.03904204422988611 5`,
5.485202480282314`, 0.0003546022878763155 4`, 0.000355512802062561`, 0.00256770533461137`, 0.5832399792340747`},
{0.17261439973382386`, 3.0972705794401207`, 5.672236578564393`, 1.4223285072729144`, 0.7931550721000631`,
0.3896544494671119`, 0.01712882834694823`, 1350.492916562551`, 0.2427963854280547`, 3.05685280243325`,
49.55592464646186`, 47.78632408647853`, -0.035709162377816295`, 74.19686438329639`, 39.82107558119398`,
0.21281041330338524`, 0.21531497735000402`, 0.011768991976197185`, 0.025407459213178784`,
0.025475066664204963`, 0.002660929235738463`, 9.416163316043825`, 0.06265276172635546`, 0.47338432331536817`,
0.00021442750011235745`, 0.00021721024901511822`, 0.012977574710812068`, 0.1822782208425033`},
{0.11394032550346922`, 3.2108067304960466`, 9.096957387649113`, 1.399541009089664`, 0.15147624678363414`, 0.6188818627652795`,
0.08261238136824677`, 151.04115640404754`, 0.09860367843525403`, 6.06148281050898`, 497.73195386533973`, 509.04543069115323`,
0.022730059297889404`, 375.82108293946646`, 232.90314641880306`, 5.645994325284988`, 5.748682030791629`, 0.0181877096558111 8`,
0.07919342767757785`, 0.07927704992833795`, 0.0010559241241652195`, 258.9742368566796`, 0.12890464181851632`,
9.441377257905637`, 0.0006814400217796113`, 0.0006860157345969949`, 0.006714769709935675`, 1.1637689885010902`},
{0.20354065160019702`, 3.5209980508829766`, 0.4053576884306178`, 0.7576791600370161`, 0.6570403323895382`,
0.4683222917239164`, 0.005063986272754102`, 161.28160618619455`, 0.12637144234319758`, 8.874326085906798`, 1089.6333206396382`,
1081.535263812407`, -0.007431910050692547`, 561.9652838219748`, 1010.5816044597763`, 1.408711598200916`, 1.4329272502603727`,
0.01718992879052239`, 1.4378768570242306`, 1.4334309167893817`, -0.003092017381829182`, 70.85815416459529`, 4.180948462850711`,
11.812151854645654`, 0.022254000430734333`, 0.023020787191546044`, 0.03445613130090197`, 0.7780935548312717`},
{0.24345495866234085`, 1.8344199239977046`, 3.71003568577021`, 1.0568366445286865`, 0.9797132100793247`, 0.5295146440336447`,
0.23321770204647818`, 2779.737816031833`, 0.01572768263207122`, 9.835586906627377`, 155.21309272774684`, 150.79842698899552`,
-0.028442611774349036`, 72.22583429102885`, 36.36036967892911`, 4.3639860476724674`, 4.3880405874222985`, 0.00551205697888534 9`,
0.02816558845524764`, 0.02819860109983693 8`, 0.001172091420768683 9`, 114.36261362711998`, 0.09795788818674248`,

0.7250928867889541`, 0.00032108945206932304`, 0.00032326864121410583`, 0.006786859956745994`, 0.3766248899236249`},
{0.18352727434547023`, 0.4163243700753796`, 8.057415014521496`, 1.2522528089453002`, 0.16203828635471806`, 0.6604646928880291`,
0.2583073571896206 7`, 2461.4350174740794`, 0.12685187153570027`, 8.600866037504808`, 45.84706549708132`, 46.7140110580533 3`,
0.01890951038136124`, 24.39685553708622`, 10.36727429220653`, 5.104142084796967`, 5.192060104230099`, 0.017224837783219593`,
0.005193498198110483`, 0.005198887405916107`, 0.0010376835805170348`, 30.356839117547565`, 0.013616408123122526`,
0.8234415307660271`, 0.00005001136354287894`, 0.00005027906799204895`, 0.005352872431492184`, 1.668496244756305`},
{0.23920496896525717`, 1.1838719961494162`, 9.218551103388815`, 1.265080848919387`, 0.9542453823473309`, 0.22929853041342196`,
0.4982471338661781`, 90.20981971435951`, 0.0856312153630287`, 3.6772130334476696`, 821.6442825669218`, 804.168613189815`,
-0.02126914255705714`, 1022.6559310645391`, 108.40795873186842`, 39.77046288899211`, 40.21945948822653`, 0.011289700109542888`,
0.19234164400323409`, 0.19230678345959942`, -0.00018124282869336206`, 672.6162469739638`, 0.657272528348971`,
8.331601328962398`, 0.001833874436607985`, 0.0018421117448548438`, 0.004491751497498919`, 0.13085438689494638`},
{0.10156561975861944`, 0.5164671469302382`, 5.401464431301745`, 0.8562900606586841`, 0.5357330343495086`, 0.16718344164197263`,
0.02721990257094452`, 330.98847924494044`, 0.2190497368128987`, 7.446609110605853`, 225.51076075666256`, 224.26828730476`,
-0.005509597181676207`, 138.6031841123751`, 163.5871395398123`, 7.378235514750727`, 7.536210253508922`, 0.021410910297234187`,
0.0301268353654214`, 0.03012999284254787`, 0.00010480613340790867`, 54.43737493832383`, 0.0437121529322463`,
4.676291181943278`, 0.0004252946699659921`, 0.00042609926433935795`, 0.00189185153303284`, 0.3955699916048009`},
{0.2533884831264572`, 2.217775528299157`, 1.87005833593021`, 1.0852798553697922`, 0.3630118105229163`,
0.49869259108707253`, 0.006826900671537389`, 2180.4237702051914`, 0.21841135731377453`, 2.8539212819504436`,
17.589003776363175`, 17.903992482066336`, 0.017908274380295408`, 28.207444788999307`, 15.94331542376377`,
0.046733402336723566`, 0.04782292580865204`, 0.023313591937480505`, 0.022136197805165004`,
0.022323132085634908`, 0.00844473301671922`, 1.4806313722363451`, 0.08012939405768614`, 0.2955578269516511`,
0.00024167233816940747`, 0.0002504711385593413`, 0.036407974766917706`, 0.41124802991018483`},
{0.04019446235159935`, 2.9685609440334373`, 3.1967275735949383`, 1.2384363485833663`, 0.006837582253808039`, 0.6265805854044944`,
0.10123843723710083`, 64.97108080441517`, 0.1585540441972691`, 8.616308543170305`, 1497.5301359547873`, 1511.5958684136183`,
0.009392620636554438`, 795.4608741860367`, 628.8585692071628`, 19.9964563413857`, 20.39622697669098`, 0.01999207401952985`,
0.4223397307687243`, 0.42252520920431025`, 0.00043916880670535896`, 848.0099902015336`, 0.2425102629708438`,
31.923669073817422`, 0.004087167017623572`, 0.004132018846099978`, 0.010973818364409471`, 1.8925422248695833`},
{0.18466310913340017`, 2.870831467521074`, 5.7568671895307055`, 1.0469133934587485`, 0.5974897879057268`, 0.6447436436670198`,
0.1911914248698421`, 363.92035372259653`, 0.11435325769039895`, 2.2759611974712186`, 129.04954415343266`, 131.60494836516654`,
0.01980172985884887`, 259.51120598394215`, 35.504462701938245`, 2.221061229134115`, 2.2485050809681675`, 0.012356188777718 38`,
0.06435528079272809`, 0.06461710491736164`, 0.004068417096598775`, 91.08989239841793`, 0.16977208914767183`,

1.3746726073282884`, 0.0007344076610391603`, 0.0007485468775263931`, 0.019252544924744264`, 0.25874693385460945`},
{0.22919090442152185`, 1.3485088294529417`, 3.7111141569564556`, 1.1683364276022934`, 0.8698699225738455`,
0.45530089098812654`, 0.06782162054416256`, 403.6677278730082`, 0.1398561461848175`, 8.195868200542787`,
605.029732761786`, 572.6490328935768`, -0.05351918776024567`, 337.8673662627797`, 309.04022149437935`,
14.575540739028895`, 14.692426429305307`, 0.008019303871411587`, 0.14564079839008803`,
0.14575421453246915`, 0.0007787388124400607`, 280.78921972330704`, 0.4768506614815729`, 4.204886022318063`,
0.0015001786595930655`, 0.0015117884750346796`, 0.00773895520201795`, 0.40417418586711973`},
{0.12718220564190125`, 0.7794468104625536`, 3.4523481867286847`, 1.042660599955479`, 0.8212977484948716`,
0.42153700480377276`, 0.05083556365712203`, 907.9591078654141`, 0.03221255361915021`, 1.403008654938164`,
37.10118326200887`, 35.33333521340801`, -0.047649371075749736`, 121.02968563791774`, 21.54719374934459`,
1.2701836824242776`, 1.2862413934458465`, 0.012642038504951492`, 0.04959646583459106`,
0.04994923250557732`, 0.007112738076192171`, 14.143437428102635`, 0.090111255955525`, 0.32456602520906447`,
0.0005651962322511528`, 0.0005821386210756676`, 0.029976117776005173`, 0.09010091810255919`},
{0.24829534536640735`, 0.6361539074945153`, 1.4367506745172012`, 1.2157272327840285`, 0.5920355341003019`,
0.22834833308349511`, 0.008858258633323783`, 152.73617223659593`, 0.05098711045238086`, 6.308971873547048`, 505.67053619508823`,
500.2711245175664`, -0.010677726486003292`, 366.83735452032494`, 447.7769553991416`, 5.509291728286265`, 5.630766217378081`,
0.022049021014467485`, 0.42392279264301824`, 0.42415770171219286`, 0.000554131726935525`, 50.06796372109316`, 1.503686517256969`,
8.66491352400905`, 0.004179824531058807`, 0.0042397596833173385`, 0.014339155104041934`, 0.3964864645056275`},
{0.24634814165734203`, 1.0199939463339236`, 6.874973257490155`, 1.1669106806380989`, 0.9547234223304992`, 0.2467804242930165`,
0.010102520966714356`, 1373.9164154017546`, 0.22192762621195788`, 8.148592815397581`, 130.4392250968472`, 127.4325088057755`,
-0.023050706479123106`, 73.26390922766471`, 113.99625284906573`, 1.0452955311065661`, 1.056490850592743`, 0.01071019549306329`,
0.017041145999963862`, 0.01704742479868115`, 0.00036844932361379534`, 15.231358769408597`, 0.05997220926860141`,
1.2111931530225382`, 0.00017646108807389815`, 0.00017695717340263407`, 0.0028113015404742647`, 0.28273650508779946`},
{0.11968292559735771`, 0.4405556662519543`, 0.9343787484302961`, 1.4969299043499453`, 0.5720179190625709`, 0.5743610731089337`,
0.05837689913891526 4`, 4861.966479565238`, 0.13911792619470742`, 8.404040973858844`, 33.33261536867393`, 33.809111722886456`,
0.014295198529796105`, 18.152887846197878`, 18.28515555332324`, 2.0484040980129157`, 2.078157560596039`, 0.014525191885715438`,
0.03940926903632852`, 0.039762784258805715`, 0.00897035725659645`, 12.891943316475889`, 0.0673802373417405`,
0.3797868871318395`, 0.0003115656334738981`, 0.0003226055761785722`, 0.035433762644425215`, 0.9516153411825751`},
{0.0912885213280808`, 1.1352603856466574`, 9.64216394840902`, 1.0423303168159856`, 0.8496114679763396`,
0.3948758352214041`, 0.050073459411100744`, 1020.4170701645861`, 0.08585808288581304`, 1.5724283740137839`,
37.409677795718046`, 35.59516962613836`, -0.048503710175963666`, 108.88739060445447`, 21.920031251629304`,

0.8974248363771692`, 0.9060698901532807`, 0.00963317865261093`, 0.01609362629879642`, 0.01613004025671054З`,
0.002262632251927243 5`, 14.554440940491908`, 0.02098804782319544`, 0.3204863447656709`,
0.0001855328432149861`, 0.0001875219784570759`, 0.010721202821135645`, 0.0845222942174998 4` },
{0.23601424519793518`, 0.7493625485076763`, 8.159569145163797`, 1.0789985894921148`, 0.9135595458313224`, 0.43990663477596414`,
0.03553380189426036`, 63.68276264433202`, 0.11799293833856772`, 7.92015369426547`, 3998.1899714533124`, 3773.9061540394096`,
-0.05609633834691885`, 2310.437957304612`, 2659.184189608068`, 114.21357252777845`, 115.00995908142683`, 0.006972783847162045`,
0.41960909892146886`, 0.41889741080192056`, -0.001696074087472188`, 1222.676768336891`, 1.4147674965732602`,
25.600084290834687`, 0.004685036524366803 5`, 0.004701930239367599 5`, 0.003605887576955346 7`, 0.339952370679344 5` },
{0.09505824539478602`, 0.994707682773301`, 2.092747921993542`, 1.2334327544777528`, 0.1649122243213208`, 0.435178801412204`,
0.4947441968705133`, 1030.121381906519`, 0.19720224785958157`, 8.531247013968503`, 90.25968323555004`, 89.65267900640707`,
-0.006725087075243552`, 48.4223411355583`, 12.720770658925911`, 5.09180557077108`, 5.198647720667466`, 0.020983155859230163`,
0.03901277331842649`, 0.039107413430881935`, 0.002425875025161117`, 72.35511600619843`, 0.05297836828051942`,
1.8743650275187467`, 0.0003796879732030911`, 0.00038421822492131885`, 0.011931512288919865`, 1.251592014912129` },
{0.13338172675277454`, 1.0224437122231844`, 3.1176391252125253`, 1.1808523949493024`, 0.5954558720182712`,
0.3646371291004906`, 0.024821319877175146`, 118.84942434665426`, 0.15904005586434156`, 2.0945755856004613`, 264.619708738738`,
263.3571295988692`, -0.004771296687939852`, 578.2167185019077`, 195.6478871700804`, 4.360868026395415`, 4.43853892557123`,
0.0178108804728072 1`, 0.29820750636730514`, 0.29942561489348646`, 0.004084768156979113`, 63.69631562033039`, 0.568220458998617 2`,
3.7814779660488904`, 0.003007550314547358`, 0.00307973890435452`, 0.02400245457507033`, 0.18251857791844953` },
{0.2663723170815805`, 0.6117369492537685`, 4.996758576680092`, 1.4798016834758696`, 0.3454705507334743`, 0.5628818087325649`,
0.10659789380555212`, 347.2598858446038`, 0.128819557084905`, 4.579853140224845`, 185.8674897988866`, 190.67515693911798`,
0.025866100335423914`, 185.7446768541083`, 75.24559148375367`, 11.32148163202234`, 11.519874426982428`, 0.01752357168508234`,
0.07509202918593229`, 0.07530846851858955`, 0.002882321000027055`, 98.93955192294179`, 0.28574911155154664`,
2.9910958695119834`, 0.0006081760883809295`, 0.0006172727533311354`, 0.014957288068366603`, 0.68283075243991` },
{0.19858704302380392`, 3.77240735130054`, 4.552319154599175`, 1.490234534684174`, 0.9267739623702391`, 0.4663128979271708`,
0.016623264635311285`, 190.7315614324941`, 0.03981298108873932`, 1.7245962595124684`, 300.75131503802936`,
281.7995869223638`, -0.06301461429443511`, 798.1501320221192`, 242.2050157402678`, 1.039730771409348`, 1.051438661195322`,
0.011260501379702248`, 0.3547376018120006`, 0.3567663992454391`, 0.005719149656183697`, 56.03268579197131`, 1.0063755913315982`,
1.8764226963421138`, 0.0028273022873857023`, 0.002911394018483285`, 0.02974274504454888`, 0.08872843077613134` },
{0.16356962246540357`, 2.7590365436019733`, 9.315311335363422`, 1.411146654012818`, 0.2509435276133807`, 0.3090283790000361`,
0.2431141196841502`, 313.55839056266797`, 0.2467169299716112`, 8.038445579591425`, 254.95658034584537`, 255.8143850347918`,
0.00336451284286454 2`, 145.16390556976535`, 61.14741120349573`, 4.793010487235572`, 4.898163057230764`, 0.021938731466419226`,

0.03014057219693789`, 0.03015231666099009`, 0.00038965630696941034`, 188.91558697357783`, 0.07042974307349578`,
5.5999078699434675`, 0.0002580116052026682`, 0.00025876787974550965`, 0.002931164829765631`, 0.8581047896685918`},
{0.25383472083693576`, 3.9769524497483806`, 7.011565744250568`, 1.066285473960517`, 0.7734216029556533`, 0.6850552501893354`,
0.013843595247453381`, 297.6124430270534`, 0.10716201039628298`, 2.5950386184147853`, 263.8004356788171`, 259.1239774729543`,
-0.017727257325520496`, 465.2604944488193`, 220.06796848710297`, 0.746502976206939`, 0.753397972659981`, 0.00923639512875929`,
0.09660561477853989`, 0.09688907083403356`, 0.002934157151667227`, 42.41152628529493`, 0.3503122751227281`,
1.855400527437693`, 0.0010848453247417345`, 0.0011018697615128225`, 0.015692962289477697`, 0.20272548699037615`},
{0.17343366236722435`, 1.3936719133530833`, 0.599503921563171`, 0.8621470436054358`, 0.43567440743840313`, 0.22823781097601215`,
0.018669299661272933`, 3618.675535403083`, 0.22611694811187344`, 3.1306846738065635`, 8.856344344815527`, 8.793150178654933`,
-0.007135468507114551`, 12.94731342935989`, 6.958656237365039`, 0.0864528371929544`, 0.0891791940783169`, 0.03153577110809613`,
0.025077543413874637`, 0.02528679744296417`, 0.008344279407119348`, 1.7212413003643925`, 0.06213271710630322`,
0.18501219031418906`, 0.0003452720254454311`, 0.00035862183700713693`, 0.03866461971392954`, 0.2614440338547428`},
{0.27302715407014244`, 1.5486379048502288`, 9.868645627334566`, 1.0694698241775202`, 0.6816384789594119`, 0.4416154581373778`,
0.05961824305163796`, 87.63839207083008`, 0.05267766985704242`, 3.1260333504958955`, 683.7916450100372`, 678.6826155459381`,
-0.007471617270234487`, 1001.1396863193927`, 372.32551500620013`, 13.438018642835653`, 13.601448657929796`,
0.0121617642777474`, 0.14858603489265612`, 0.1486624882325664`, 0.0005145392025940332`, 297.2946433768475`, 0.5795431748755128`,
7.583137255023236`, 0.0016726713994968945`, 0.0016845589796302474`, 0.007106942903984947 4`, 0.2411413603090601 4`},
{0.12081145422022538`, 2.0244942048813837`, 8.652229532190159`, 0.835020699056165`, 0.9214042127932665`,
0.46285611589460973`, 0.11861503789539068`, 2689.091322411565`, 0.07096583948487911`, 5.509201411600676`,
69.05756059697832`, 65.05545866663405`, -0.05795313207920327`, 57.370294301087455`, 25.886140267031383`,
1.4421388506270822`, 1.4514214827068344`, 0.006436711746386825`, 0.007587470471273172`,
0.007592499801091234`, 0.0006628467072267163`, 41.70859636755457`, 0.013095047735557669`, 0.4217986942631338`,
0.00010969632314550282`, 0.00011010527937080975`, 0.00372807596080027`, 0.23769164604009124`},
{0.27038445563951496`, 3.3688963138915424`, 3.4133330216440854`, 1.1975198070883244`, 0.31492494684053174`, 0.45427916274833`,
0.007097071788183910 4`, 3067.136751023867`, 0.17569148508387616`, 1.2036801730392703`, 4.7142005202465525`, 4.772174222919766`,
0.0122976743191614`, 17.925093893007453`, 4.246902316499337`, 0.008589664037158606`, 0.00883340869895146`, 0.02837650701336191 8`,
0.008457619682377527`, 0.0085502131192223 6`, 0.010947931016307422`, 0.41339553589072`, 0.032668698483224116`,
0.08915298733489453`, 0.0000833138369880082`, 0.0000872030900687249`, 0.04668195849960055`, 0.18177147448072015`},
{0.1767050761987432`, 2.85608165701536`, 6.140634667151749`, 1.3554192221984884`, 0.6330624847112656`, 0.5906276578639604`,
0.04152902973081543`, 1510.3499876585004`, 0.15541682752363528`, 8.81551919212286`, 125.02409072492061`, 126.83535878980194`,
0.014487352432472456`, 64.90980494841759`, 78.88404039733447`, 1.1020260752581519`, 1.1137024206537618`, 0.010595344028383913`,

0.01961811839295022`, 0.01964214110273819`, 0.0012245165059561192`, 44.963949415677675`, 0.049523158650036966`,
1.2608099163978457`, 0.0001743994139387084`, 0.0001755278937795471`, 0.006470663033508295`, 0.8327529666785175`},
{0.16171803894826015`, 2.2156907324583015`, 1.0134587159780288`, 0.8322559746700846`, 0.34733770749260806`, 0.6849579573613365`,
0.018772440750507276`, 1617.8437562512436`, 0.21218330633531807`, 7.516753350682977`, 73.62344734943086`, 76.13952307942688`,
0.03417492416586043`, 44.82810121502415`, 58.11951548695335`, 0.4690354651005663`, 0.47680335559398473`, 0.01656141394713706`,
0.050076453129500625`, 0.05038194965726524`, 0.006100602352458573`, 14.846250474537063`, 0.11568951139409175`,
1.0718228789105702`, 0.0007156363547332623`, 0.0007345026709147644`, 0.02636299295964939`, 1.2690592094109394`},
{0.24108871826638628`, 1.1722622038849657`, 2.5945274526654174`, 1.1873482780450624`, 0.3399058776037083`, 0.3423735862363967`,
0.09173881369912114`, 1455.6792358614214`, 0.2278375557130481`, 4.818602627144232`, 36.70359382219128`, 37.03160868400174`,
0.008936859518430484`, 34.861973766829465`, 16.239160002542988`, 1.1476946950004832`, 1.1730924095834794`, 0.022129329946049436`,
0.021767080713736644`, 0.021826356664179383`, 0.0027231924768547078`, 19.219987322119295`, 0.07496853699536624`,
0.7247912385536086`, 0.00022001212485345256`, 0.00022312220801186256`, 0.014135962554252979`, 0.5270527257593858`},
{0.2202280085702742`, 2.180975046803316`, 5.6134680006393936`, 1.2506485034427453`, 0.4748208651226249`, 0.39757955628981323`,
0.017520218948128618`, 280.3947437474927`, 0.12359899846366301`, 9.521190146527957`, 472.57513124518033`, 478.57916033813115`,
0.012704919696326034`, 227.16641263745097`, 379.0442406905994`, 2.894526049897495`, 2.941909825356591`, 0.01637013267190124`,
0.06934709668317253`, 0.06938129040121963`, 0.0004930807442931062`, 90.18412981640856`, 0.21817390003807574`,
7.356017623996103`, 0.0006689801749714119`, 0.0006719886937829795`, 0.004497171850714743`, 0.9292591496619`},
{0.12315072752895034`, 2.8609501087269207`, 1.79674111361204`, 1.4514496090822089`, 0.3032919079034333`, 0.2763824218936657`,
0.028981582964232436`, 188.78817200372555`, 0.08856946510090113`, 4.546561013073182`, 235.7144068471969`, 236.62429088466408`,
0.0038601121146446538`, 237.28353213894684`, 167.6457087129226`, 1.6077726018195813`, 1.648327291580577`, 0.02522414532695616`,
0.2613007990232997`, 0.26212477478659196`, 0.0031533610550451563`, 65.71081714262708`, 0.4597054786230909`,
5.230960512942002`, 0.002151782019062409З`, 0.002192962425853413`, 0.019137815274126613`, 0.4569925847742799`},
{0.14162785770391484`, 1.92013166099325`, 8.540643644714823`, 1.2350689068338556`, 0.301574102817308`, 0.40858591883894513`,
0.080613435450821`, 585.1346359267128`, 0.15211712109726616`, 7.686797097032363`, 153.17622968985688`, 155.65831170640706`,
0.016204093948360976`, 91.20327901490326`, 72.65507032230538`, 2.83028691733575`, 2.8837082598889796`, 0.01887488587323749`,
0.018074048884248836`, 0.01808484004180465`, 0.0005970525821261674`, 77.6360502810195`, 0.036568411764358884`,
2.9129785967473274`, 0.00017668025809880472`, 0.00017732458367153946`, 0.003646845321984893`, 0.9492585686957938`},
{0.22524615675817938`, 2.031565502127312`, 0.245821316301889`, 1.298167355078078`, 0.689325845538592`, 0.661487626561178`,
0.00610096551453922`, 373.63064864758115`, 0.20592315239272913`, 2.994117316721619`, 220.3346227139022`, 208.6903090431616`,
-0.05284831556346192`, 336.8049206704033`, 194.04452233422646`, 0.5580800415709285`, 0.5883642262111846`,
0.0542649483665635 9`, 2.2111994512744353`, 2.3309044512577657`, 0.054135776813049485`, 16.19680228401817`, 7.115202546076603`,

```
1.8417660935776987`, 0.018945463063135604`, 0.022751392645817308`, 0.2008887072328862`, 0.4924600026280427`},
{0.06014178921319169`, 2.215935435785701`, 1.3525069683978295`, 1.0400202244696481`, 0.697929353581739`, 0.692932654029565`,
 0.014666911144259826`, 1018.9785490283374`, 0.16122419934759502`, 4.978072859014281`, 128.9391717351626`, 129.24837432632387`,
 0.002398050080516745`, 118.54633236198657`, 106.54545900995521`, 0.6769151671076052`, 0.6839807847563852`, 0.010437966221041872`,
 0.12379684652188679`, 0.12481499642893486`, 0.008224360600882274`, 21.428575797350593`, 0.10636234069679469`,
 1.0592317827007096`, 0.0014084623614459835`, 0.0014554514006714662`, 0.03336194172575690 5`, 0.4905550980924639`},
{0.11674773262981253`, 1.910335393882857`, 4.221858661435528`, 1.4404651869296854`, 0.4563596481936356`, 0.5439902833298367`,
 0.005611633628996954`, 451.6526207878965`, 0.24247834364497922`, 9.182241064841566`, 323.1663382650333`, 331.69397957468055`,
 0.026387777128735523`, 161.0801115739529`, 299.0131516114099 7`, 0.8349719904917438`, 0.8477857172468832`, 0.015346295326137849`,
 0.07522970688779498`, 0.07534067029828227`, 0.0014749945876140202`, 22.78680780481717`, 0.12546996722222184`,
 4.502437121123755`, 0.0006282817064148238`, 0.0006335593861930037`, 0.00840018056278602`, 1.1210157329789427`},
{0.12898119467887553`, 3.729766126068422`, 2.3744826976889595`, 1.2465399522360021`, 0.1415631230326866`, 0.3607064691421532`,
 0.03995461220531779`, 1238.1663657022145`, 0.14506115517150608`, 8.953141814946814`, 69.90164855158957`, 70.59463371820026`,
 0.009913717072055128`, 35.733573474232934`, 45.0073366868934`, 0.4572050929146387`, 0.46793126105334915`, 0.023460298900723542`,
 0.025660216378562913`, 0.025705029755932206`, 0.0017464146329930763`, 24.36097240312826`, 0.04728121948896874`,
 1.6182741503697289`, 0.000247658892518432`, 0.00024989440782803964`, 0.009026590108979438`, 1.1989008116508704`},
{0.2498624462217614`, 2.827129073229683`, 2.690968559152097`, 1.0446525535360744`, 0.06936419216205603`, 0.5607634545552321`,
 0.008891348377622028`, 196.01664126075448`, 0.10064633302983284`, 7.405206717033214`, 419.4170419088019`, 426.4593188000726`,
 0.016790631251464516`, 259.2229097208043`, 372.22450935965804`, 1.117370382695782`, 1.1421273107682905`, 0.022156420517232434`,
 0.13758144269255965`, 0.13782965456126334`, 0.0018041086344642299`, 45.127861349786`, 0.49109194036971004`,
 8.914003817511677`, 0.0015800672963041107`, 0.0015996090598453738`, 0.01236767800141969`, 1.447744932337894`},
{0.25890580889310233`, 3.9576703566675278`, 2.211552265086926`, 0.8006241502346895`, 0.26522568617457876`, 0.4892067360859801`,
 0.02025985822061832`, 94.72915936196773`, 0.06766512891079995`, 5.4984531961033785`, 706.606814123107`, 721.6572924041536`,
 0.02129965064053918`, 588.1685292377309`, 549.5816452727227`, 2.701945910667755`, 2.7565361822957986`, 0.020204057902311057`,
 0.2913197833622028`, 0.2915071983762719`, 0.0006433308850708475`, 152.76301765669754`, 1.0774912022563226`,
 13.1628154225018`, 0.00435685461832358`, 0.004416250234280791`, 0.013632682556680153`, 0.8191858332690953`},
{0.2032139210380186`, 1.0205320875718096`, 3.2697243543529257`, 1.0866214178514029`, 0.5111053959745286`, 0.4079750171889772`,
 0.04834589720060758 5`, 145.20906840025864`, 0.23728279570010086`, 2.517042827760779`, 247.6396922762206`, 249.57475028981793`,
 0.007814005888195696`, 450.2919107430016`, 147.50232060113672`, 6.376271489371716`, 6.493610942479263`, 0.01840251835310558`,
 0.20389130535260186`, 0.20460543675378715`, 0.0035025103201447028`, 92.95985219961621`, 0.5919078803751462`,
 3.772566894218131`, 0.0022398383293024127`, 0.0022868206257988595`, 0.020975753420149346`, 0.2585325224542857`},
```

```
{0.15288075349234037`, 1.8405591021591183`, 8.732063156275487`, 0.8366527628406224`, 0.7247685787231319`, 0.43308603413296065`,
  0.06703535093550567`, 1294.178726539122`, 0.16108367938164575`, 4.274431762914247`, 68.14089197965167`, 67.05642010388344`,
  -0.015915140589766175`, 72.96152798508342`, 35.12910889249919`, 1.208374707788055`, 1.221283946275656`, 0.010683141913183203`,
  0.00957728358653898`, 0.009584874445762078`, 0.0007925900026357624`, 31.772643817688177`, 0.02091689044457779`,
  0.6967755110588896`, 0.00013812872876572957`, 0.0001387478141782082`, 0.0044819453419324695`, 0.29444525892063683`},
{0.1609254870534111`, 1.384768951726243`, 6.196355509527487`, 0.9770497723863997`, 0.43819861370403546`, 0.17710326003602894`,
  0.2243750211158665`, 120.51310647409781`, 0.10277566384729958`, 6.21125463242891`, 481.95320812866595`, 479.34082128184997`,
  -0.005420415929918709`, 355.1321875273953`, 122.62550991706539`, 17.27780137911143`, 17.675936922713838`, 0.023043183265420986`,
  0.07677746252827396`, 0.07677163594695952`, -0.00007588921439405372`, 341.7966129126632`, 0.17650643645758668`,
  10.794447419893409`, 0.0009493641982898371`, 0.0009517059995886481`, 0.0024667048778850287`, 0.3798321508672612`},
{0.21184155187533854`, 1.0400436907537394`, 2.77253033361667`, 1.25783985819887`, 0.5916706672066951`, 0.18670557332607218`,
  0.05860949172916328`, 177.69964385957368`, 0.18251915101666627`, 3.5990512292385053`, 221.24814308632554`, 218.71684686132693`,
  -0.01144098291487572`, 281.35594786619663`, 121.94017696394745`, 6.218034195101131`, 6.3581758465336025`, 0.02253793514723368`,
  0.17432545184049805`, 0.17453487436559384`, 0.0012013307459395328`, 92.38610333579916`, 0.5275624892751248`,
  4.223956937028542`, 0.0016661148653526192`, 0.0016851124676572897`, 0.011402336477353225`, 0.20396669826136027`},
{0.15826860039724194`, 2.914200187526596`, 3.057619613104965`, 0.9127068016590583`, 0.36895217716385176`, 0.3294645201729436`,
  0.006405724718526674`, 799.6829736219844`, 0.2321605158663716`, 7.540832982633661`, 106.61077297380098`, 107.73544188039841`,
  0.0105492988675151`, 64.7062549950373`, 97.66689268922633`, 0.20539258655724807`, 0.209659019624217`, 0.020772088898056484`,
  0.026446872466291654`, 0.026472866180053928`, 0.0009828653197236914`, 8.550787346595765`, 0.05979584985893223`,
  2.0474261245188248`, 0.0003493136147530995`, 0.0003514082126149802`, 0.005996324716290191`, 0.7547788634341803`},
{0.2486148170283075`, 0.5446418582356878`, 7.121193243885838`, 1.048132429671941`, 0.7615900023009023`, 0.5400927540260018`,
  0.07717023450494316`, 388.33902457321517`, 0.04098404938849237`, 2.529691537294738`, 163.9360586308671`, 160.36937714211362`,
  -0.021756540437418592`, 296.6001841258279`, 78.56183876379649`, 9.692104319670328`, 9.789944605726538`, 0.010094844507362533`,
  0.05963979461661171`, 0.05978221809139727`, 0.0023880611209530223`, 75.41036724113414`, 0.2118190946600808`,
  1.3793689207249151`, 0.000682791661610338`, 0.0006916203172758461`, 0.01293023357181311`, 0.18433322178944914`},
{0.1973186549669898`, 3.31823274961833`, 5.404057922234477`, 1.0748992098641554`, 0.1837120164888255`, 0.5027766473922404`,
  0.30055211808677745`, 460.171808421071`, 0.10833443978919949`, 3.5562239100468123`, 88.80272054815109`, 89.40299163282324`,
  0.006759602419462718`, 114.28827243999427`, 18.0994816451485`, 1.4582591296112446`, 1.48806385548315`, 0.020438566278581094`,
  0.031069229341288942`, 0.031140538380421624`, 0.002295166009731453`, 69.12633144722817`, 0.08757912206410051`,
  1.7762933519806792`, 0.00034710911330315675`, 0.00035118080530851896`, 0.011730294162014987`, 0.5852700528940625`},
{0.16177424691159015`, 1.9234518490470327`, 6.336020872512709`, 1.465202064627133`, 0.3679027675625519`, 0.6088093981583469`,
```

0.02752967040620688`, 1523.605195139083`, 0.023927731291662258`, 6.9520681394676025`, 68.95854399771501`, 71.22133726144789`,
0.03281382019620160 4`, 45.3981920448968`, 49.73013067105637`, 0.6731929872517112`, 0.6835096155939573`, 0.015324919506906065`,
0.01436561051134644 5`, 0.014390569181632172`, 0.001737390155887475`, 18.497918515639988`, 0.0331997974556842`,
1.0344668885677715`, 0.00011797186699569195`, 0.00011897920317253 64`, 0.008538783037834285`, 1.0336020980138647`},
{0.12468946686374066`, 2.602287301847274`, 9.387923649086364`, 1.237964958422611`, 0.6279283704003791`, 0.5225362832845872`,
0.15386398985407668`, 4877.253164044286`, 0.10729269282887094`, 6.155025385024989`, 24.792262459999776`, 24.99339515091807`,
0.00811272029903698`, 18.435325374731345`, 7.932606014448452`, 0.44139250816158276`, 0.44635849341351164`, 0.011250723925089856`,
0.003330132875709142`, 0.003333878874904103`, 0.0009774420129033956`, 16.409001701705733`, 0.005931892755115951`,
0.2715558603197261`, 0.000032446529601770635`, 0.00003260846502919946`, 0.004990839680431902`, 0.5555044560347061`},
{0.1891437323001075`, 2.037539508644585`, 6.753647851102151`, 1.0264835122983769`, 0.08414675462879395`, 0.15050525073770327`,
0.01724785647741675`, 835.2003740810117`, 0.09305345804576048`, 5.34515441362371`, 43.22504151385589`, 43.30184218808783`,
0.0017767634579903824`, 37.01175322536206`, 34.90371349540363`, 0.27492225125593084`, 0.2838167992691468`, 0.03235295787293646`,
0.007708660077680718`, 0.007711253537176183`, 0.00033643453847131966`, 8.002356410563893`, 0.020829210544646155`,
1.355319389752894`, 0.00009077552834602098`, 0.00009100187562382534`, 0.0024934834522973848`, 0.39012611140239295`},
{0.11951180040558451`, 2.9137311414381974`, 6.217108794108201`, 1.4729889111648695`, 0.6540356010640074`, 0.40115742558392786`,
0.08157249569611574`, 391.2473393910366`, 0.0369147403900103`, 8.338813449127404`, 380.49980194865344`, 378.2894870521611`,
-0.005808977784410474`, 208.84047462559414`, 178.03829326570934`, 4.745545165075317`, 4.804145323594198`, 0.012348456600971058`,
0.0677886176817517`, 0.06783825753173106`, 0.0007322741144006883`, 197.53203900830593`, 0.11573628208781789`,
4.520034473003216`, 0.0005549515367506697`, 0.0005577958797368473`, 0.005125389872477459`, 0.6213406235651765`},
{0.17883917330505905`, 1.5543060950462326`, 9.905917854358197`, 1.2462903035005124`, 0.32028587133565645`, 0.3166477244063972`,
0.12099723319057126`, 400.41544183552224`, 0.027102889569123156`, 3.023770086067156`, 78.25331416510848`, 78.82841318062593`,
0.00734919692096425 2`, 118.44547342000604`, 29.658820346805367`, 2.091070195583774`, 2.1363936049477643`, 0.021674743133784258`,
0.020400220711257522`, 0.020422013062377092`, 0.00106824094837088`, 46.43090214521964`, 0.05211940867489373`,
1.6482803990569581`, 0.00019733440619729148`, 0.00019855134964327296`, 0.006166909610100246`, 0.3202143311694436`},
{0.25477439586299494`, 1.0781629016953422`, 9.675717925989822`, 1.4583691370753404`, 0.484439918991886`, 0.6784957922502513`,
0.09307366059264305`, 217.6033516010123`, 0.05467318278554256`, 4.774031073697932`, 400.953576908485`, 415.4168141224054`,
0.03607209923262911`, 384.39111920956105`, 174.6527643534126`, 13.774438547129304`, 13.951843525875454`, 0.012879289282039297`,
0.0792751580768874`, 0.07939937597691954`, 0.001566920874653599`, 212.15840904567295`, 0.28853257865708604`,
4.91641401732782`, 0.0006536900922885502`, 0.0006596890023092089`, 0.009176993947784373`, 0.6464316453140038`},
{0.26738035849024727`, 1.6336260676101997`, 9.900661691242231`, 0.846806505065853`, 0.2415106216321452`, 0.502680613934997`,
0.14320441138979886`, 77.88432317464672`, 0.12860121706274624`, 9.963544099078408`, 1565.834221302095`, 1596.97961887867 5`,

0.019890609844176455`, 719.2774212910442`, 532.3267921752044`, 42.448898654405944`, 43.21452600252277`, 0.01803644787936931`,
0.08438325198297388`, 0.08436499366139864`, -0.00021637376074246095`, 990.6518197597272`, 0.3223203452261914`,
29.014475456708436`, 0.001203679177379291`, 0.0012063721551523706`, 0.0022372886593775565`, 1.485971336868155`},
{0.0966033889391959`, 0.8191052864079662`, 6.963587929197736`, 1.39989556064551`, 0.208903818004208`, 0.4044327200024739`,
0.345646875315088`, 51.22809362991988`, 0.05669448608759026`, 3.5983266970468653`, 745.5771605044255`, 746.3315220387047`,
0.0010117819780972592`, 948.3234707064224`, 137.8823307552392`, 47.7954018416689`, 48.81413056180026`, 0.021314366672888108`,
0.2610610779241349`, 0.2612855314773191`, 0.0008597740994902292`, 559.2780902071995`, 0.3602769263948804`,
15.781425214651632`, 0.0022404170574971127`, 0.002261374721532854`, 0.009354358361810533`, 0.49783176840253257`},
{0.0726862650625391`, 2.0017174568948795`, 2.5547427204964173`, 0.8446935149578232`, 0.0630154383873418`, 0.49766282606547807`,
0.00568323780270915`, 197.50095618865632`, 0.04182941385906325`, 9.915201734242256`, 526.2828190994447`, 534.1129554343032`,
0.014878191061332924`, 242.93054935869128`, 486.8677222049884`, 1.3061018737469199`, 1.3353122464639657`, 0.0223645439181918 7`,
0.11005455264097962`, 0.11013047463887173`, 0.0006898578574916492`, 37.349241730890235`, 0.1142779197800404`,
11.67807815367689`, 0.001568039256622411`, 0.001579007015398117`, 0.006994569000351891`, 1.7707183825307238`},
{0.14445086415814334`, 1.1396367789378896`, 8.108045316850049`, 1.1452867101498132`, 0.43349891091368953`, 0.5412309783014353`,
0.17300117449492944`, 151.5195914284387`, 0.22841440999808194`, 1.401560283159844`, 137.05350855584413`, 139.9189659086689`,
0.020907581155846122`, 447.5513489287292`, 40.822000729918614`, 5.553504958227794`, 5.646429288378771`, 0.01673255553923747`,
0.08618150807361463`, 0.08654830937138962`, 0.0042561485169378965`, 90.41397860586183`, 0.17784276165264123`,
2.074842201486704`, 0.0008984220302045154`, 0.000916591447655143`, 0.0202236998201073`, 0.19129454725542847`},
{0.2737062633152032`, 1.87379900045931`, 2.9341815386065377`, 1.0257953180476898`, 0.5994241695021645`,
0.2666028641788709`, 0.16092630392754248`, 2757.7643970777704`, 0.13082540497870587`, 6.7306102844961035`,
32.459548132870914`, 32.16644146164344`, -0.009029906085804562`, 22.07255052598126`, 10.176414440189712`,
0.800592690249687 8`, 0.814596964663885 5`, 0.017492383561271474`, 0.010555764080946767`,
0.010567934566595813`, 0.001152970600301062`, 21.430711182355623`, 0.04127398204331102`, 0.5134953372198667`,
0.00012404977089408575`, 0.00012491501285215962`, 0.006974958130415487`, 0.44429212235920157`},
{0.14450675367477284`, 1.4907097867645147`, 8.628071674125838`, 0.8645742960679661`, 0.06677853457714877`, 0.6218432064874626`,
0.019848227343967184`, 449.15101395266043`, 0.20772413403212947`, 3.059722840773212`, 77.85256850400482`, 79.4214923242974`,
0.02015249914602224`, 116.45425234480962`, 60.92529312396964`, 0.7560254661533659`, 0.7713669803875604`, 0.020292324691457253`,
0.015969526008951358`, 0.015996650838201287`, 0.0016985369030191322`, 16.100208020543242`, 0.03296720516113002`,
1.632874989570446`, 0.00022222739215327358`, 0.00022412520764863728`, 0.008539971049359885`, 0.6562082768231047`},
{0.05092006104579416`, 0.9079056727720332`, 9.25200153286255`, 1.1365283513840736`, 0.03327106644526534`, 0.582893635885294`,
0.1249724078419535`, 82.97595681905067`, 0.15395748273911747`, 7.1070379565760815`, 945.3833534442603`, 955.8570385762621`,

0.011078770420320572`, 608.8128503962812`, 351.1797325017113`, 42.521334155316815`, 43.37605069090415`, 0.02010088706213531`,
0.10253025448614408`, 0.10256653092102933`, 0.0003538120047301696`, 551.5051499063918`, 0.07458352596323875`,
20.35241750369706`, 0.001087870101856181`, 0.0010926906130415473`, 0.004431145940256398`, 1.4493527119042076`},
{0.2364459713820966`, 3.884359137525399`, 0.954741626535867`, 0.8976843312448166`, 0.4180287949880024`,
0.15414803582881131`, 0.32241293356212986`, 4897.582666581565`, 0.05447310320870352`, 4.86045092756919`,
8.669082890523073`, 8.579765849280273`, -0.010302940042301478`, 8.16321222776748`, 1.7058703890181477`,
0.12245300627174482`, 0.12608523381542255`, 0.029662216177993805`, 0.010447890032861536`,
0.010472603594596749`, 0.0023654117393543395`, 6.795020768987238`, 0.035290878681617566`, 0.20750813938194143`,
0.00013987288392136588`, 0.00014197906995029206`, 0.015057858034229543`, 0.2887429575961709`},
{0.042914457849633436`, 3.519672647432988`, 3.392121169492343`, 1.4941116772804357`, 0.33812708289181614`,
0.3684690007615431`, 0.017148240098985187`, 3621.3180321373566`, 0.22166064632630456`, 1.0781698542512608`,
3.293799048807161`, 3.3047411232324793`, 0.0033220224619594507`, 13.982164578180337`, 2.6465083273296464`,
0.012337919980887702`, 0.01263866574906967`, 0.024375726917328366`, 0.00831021494516568 5`,
0.008413016952127785`, 0.012370559322524288`, 0.6203634211849637`, 0.0050946909855097726`, 0.06652641059232302`,
0.00006549143538681079`, 0.00006844664257437926`, 0.04512356722851152`, 0.1424574842249368`},
{0.1470328346746822`, 2.9686959435849998`, 9.643655350717612`, 1.0921756097932163`, 0.41500029005274564`, 0.5183995564120613`,
0.0407899820168490 5`, 4770.787596114335`, 0.08630863643009795`, 3.8345772490574745`, 11.611713167198674`, 11.907097656354242`,
0.02543849343350857`, 13.859364051891465`, 7.395347815621707`, 0.09696422267177161`, 0.09848273821545486`, 0.015660575641631258`,
0.002149030955526113`, 0.00215180629758349`, 0.0012914388460718573`, 4.112247064550874`, 0.004513973045637597`,
0.17905635183058569`, 0.000023706092759701036`, 0.000023864410970097614`, 0.0066783763989024525`, 0.493571185023175`},
{0.10223589016255652`, 0.4809064129783538`, 6.158635896611097`, 0.9490677758592585`, 0.9235714285317382`,
0.43663660593873277`, 0.45300992250929206`, 1331.4948553442105`, 0.21785131418517045`, 5.425736978481032`,
130.1447656249758`, 124.28791687321757`, -0.04500256866753505`, 109.78233494697974`, 18.135912761308745`,
14.224971879494264`, 14.323353289773069`, 0.006916105783001392`, 0.023174455944105346`,
0.023196012904321932`, 0.0009302035080598348`, 97.72686001836495`, 0.03384658760671568`, 0.8391177271435278`,
0.00029441818734199376`, 0.0002960036266559244`, 0.005384991084429913`, 0.2351531018989686`},
{0.08719902184880185`, 3.645892777856403`, 2.8891082836443758`, 1.428386787110956`, 0.151537003432892`, 0.34505159105650784`,
0.041961341889936235`, 290.12993265609543`, 0.15015043093723618`, 6.608503242578238`, 216.24896327580421`,
218.17800018994802`, 0.008920444680622808`, 149.7667554465174`, 136.85542373330972`, 1.491176147762598`, 1.5267578910464794`,
0.0238615292614956 5`, 0.1012735520955366`, 0.10146079682782343`, 0.001848900610400106`, 77.6666906805627`, 0.1261564954554358`,
5.081967442256913`, 0.0008518319592653034`, 0.0008607969102117104`, 0.010524318615773742`, 0.8619465087862593`},

{0.14456990131617375`, 0.9947424361823325`, 3.150441801621305`, 1.0015653051761273`, 0.8447006281771343`,
0.5703016332732033`, 0.00827173056980588`, 52.80371817926736`, 0.13650904531369146`, 3.8670961625783473`,
2368.001791138226`, 2246.0438273525215`, -0.051502479534478396`, 2802.6027864433745`, 2114.3127998145023`,
16.306120876124496`, 16.452312380401647`, 0.008965437297303813`, 1.2242886648416864`, 1.2202294736768127`,
-0.0033155507205473445`, 231.7198629285669`, 2.5285041636955286`, 15.326584534880467`,
0.014519856767073125`, 0.01477199615071293`, 0.0173651426239676`, 0.23336046726965695`},
{0.14643438827373756`, 2.4493630197222025`, 5.7044459384485755`, 0.7643715149180945`, 0.8231136724552808`,
0.4724466281569806`, 0.012687117803733845`, 1217.9307406282053`, 0.17452698640833697`, 5.969503699935295`,
132.6762642595534`, 127.20589312468667`, -0.041230970478374984`, 101.72308052290724`, 112.36586749589745`,
0.5607307463158338`, 0.5656211528902609`, 0.008721488176916647`, 0.018673944671855593`,
0.018688739869021396`, 0.0007922909393696109`, 19.620473629246217`, 0.03906439520975123`, 1.0217283851407915`,
0.0002946988404135542`, 0.00029611140339336434`, 0.004793242409192544`, 0.33542797249370493`},
{0.045156311345747796`, 1.2463805103118828`, 6.638333254697063`, 1.0774632518381033`, 0.7965921624862937`, 0.19987812094053103`,
0.1230725004501963`, 3016.4870397653995`, 0.18711874545495605`, 6.497285772809487`, 31.869703877545007`, 31.07130790622174`,
-0.02505187918880558`, 22.449700625034872`, 11.832720200879`, 1.0650519361459752`, 1.0802772434674663`, 0.014295366080067318`,
0.00499482622118367`, 0.004996899481952044`, 0.00041508166181669637`, 18.963713938318207`, 0.003222113256588206`,
0.44311839024875527`, 0.000056022242230000274`, 0.000056156573501112555`, 0.0023978203256895814`, 0.275129359943708`},
{0.21753427776894996`, 2.5707121359144134`, 7.635927852201033`, 1.3489791075447177`, 0.07755529833527275`, 0.4139475400754866`,
0.049678130502208456`, 727.9927710408293`, 0.24367647097486184`, 3.0823691783926943`, 40.24898373924204`, 40.649857556599194`,
0.00995984942015582`, 59.76332617457739`, 23.880528188538367`, 0.4323033134792808`, 0.44287620341870515`, 0.02445711057435851`,
0.014431571686641042`, 0.014460903688381955`, 0.0020324883787996573`, 15.876105347960015`, 0.04484802177032656`,
0.9708742862711737`, 0.00012862925708634343`, 0.00012996369245229792`, 0.010374275621126694`, 0.49869743004783895`},
{0.08480900190064672`, 0.6294563083477476`, 5.454864363444388`, 0.7943923084343845`, 0.9968128471314037`,
0.29575569030654725`, 0.01722905088446216`, 309.5962534203848`, 0.1957861782089137`, 7.847856100229725`,
686.4932779568235`, 685.3961634782964`, -0.0015981430754753445`, 400.35914450595914`, 551.5842644492996`,
13.458652972233013`, 13.569815542856494`, 0.008259561402825577`, 0.07996505285852079`, 0.07995536782539675`,
-0.00012111582219764827`, 121.02334307478908`, 0.09688223314078233`, 5.171593040458026`,
0.0012155502027917375`, 0.0012187507443955207`, 0.0026329982887030035`, 0.26441343711027293`},
{0.12061650440369748`, 3.919019990284525`, 1.2611661330191772`, 1.4204820377968523`, 0.21263599304474745`, 0.23829484446556248`,
0.00840490627656251 3`, 547.3513122366178`, 0.16414531089750106`, 3.5940701557610755`, 56.096797088029824`, 56.11786353256072`,
0.0003755373858125 2993`, 71.43581598091536`, 49.925521561329`, 0.10229959761687972`, 0.10552601246856343`, 0.03153888115735204`,

0.10913844878357777`, 0.10977858039467102`, 0.005865317110770318`, 5.727345257980208`, 0.18805568840453057`,
1.4279036603179875`, 0.0009151900352647457`, 0.0009405736494665926`, 0.02773589443038893`, 0.3658588858342448`},
{0.2541152986933387`, 3.9531330061598675`, 6.8389480578926864`, 1.4090336327472661`, 0.5067749551810137`, 0.5768128332044816`,
0.18877117253369932`, 176.17163988983094`, 0.11435366361110944`, 3.2749545355180913`, 318.65992848096846`, 325.76870276611584`,
0.022308340804049198`, 445.3348409983528`, 88.9504873905907`, 3.9867063336630446`, 4.044649413514406`, 0.014534072741225312`,
0.12529813536180376`, 0.12562726951442144`, 0.0026268080659563875`, 225.14257704957043`, 0.45485961561677946`,
4.1166507532666925`, 0.001065567900182307`, 0.0010813006550005613`, 0.014764666630406653`, 0.39958017670259344`},
{0.26839792559441034`, 1.5919359357927423`, 0.607262674381337`, 1.065158649344713`, 0.7342994823745972`, 0.6373140564402076`,
0.019311239061898532`, 131.67334362372645`, 0.026592393453336638`, 2.002632914376134`, 422.81325110068514`,
405.6051344203167`, -0.040699095015521825`, 966.299455198568`, 323.6366701869576`, 3.721869678377133`, 3.8506706677640503`,
0.034606528577614926`, 2.2110882213555723`, 2.264719597851467`, 0.024255647503297784`, 84.64254413351331`, 8.477878455972181`,
3.368219488944299`, 0.023411683018424223`, 0.0264231289627792`, 0.12863004944945944`, 0.2444576789411883`},
{0.0804335350475921`, 1.0990801178785707`, 6.42097149017635`, 1.215697698043305`, 0.5864093543250795`, 0.4707288466973014`,
0.04708688161958605`, 3199.4245739234016`, 0.17424214472199046`, 5.845755402883285`, 31.70412824121832`, 32.023801066041216`,
0.010083003146804437`, 24.822165731417474`, 19.100646684017185`, 0.7537730983785678`, 0.7634160240994385`, 0.012792875921963098`,
0.006435253620515871`, 0.006443672635832886`, 0.0013082647263775726`, 11.835100368851593`, 0.007394431394658493`,
0.39405442694827436`, 0.0000637802881449101`, 0.00006419305239393075`, 0.006471658580200268`, 0.5420315111256904`},
{0.04545514913582513`, 3.0084108739176516`, 7.583320976280454`, 0.7956411409554567`, 0.21703974982634144`,
0.40384085540761094`, 0.39906200752611665`, 3808.370230770943`, 0.07445295911170413`, 7.244769365152968`,
20.687835205388772`, 20.701815708607704`, 0.0006757837676165668`, 13.069379783377986`, 3.4299660718361022`,
0.3923562715999122`, 0.4002180104495528`, 0.020037245276041338`, 0.001879689488184358`, 0.0018806428528573833`,
0.0005071926395385784`, 16.86241248472801`, 0.0012205938002037848`, 0.4250158391722516`,
0.000028540475053850933`, 0.000028618353069733 76`, 0.00272868674175486`, 0.956226828029663`},
{0.21811178043156876`, 3.634131336571894`, 2.2696382083853486`, 1.2012104146171985`, 0.07478503245860835`, 0.304565647018059`,
0.049403646487048036`, 242.22074286172042`, 0.22363734402413937`, 5.499597099973035`, 193.01887669639555`, 193.8823594216119`,
0.004473566212772839`, 160.6324947066473`, 114.84599362932981`, 1.468195965436437`, 1.5092210791701084`, 0.027942532672385`,
0.11609490375381026`, 0.11632576883709343`, 0.001988589299085408`, 76.22309951744276`, 0.36173808795395146`,
5.056984652010224`, 0.001160187149334968`, 0.001175238114746684`, 0.012972877195152055`, 0.7065962348815189`},
{0.22643881069864102`, 1.8950339409059218`, 4.49788713237294`, 0.781319815193253`, 0.8852654232409718`, 0.1986674614673 6047`,
0.009828863939504507`, 672.8810907026934`, 0.061975672509268276`, 2.025268353742364`, 48.399087386185414`, 46.986655539037216`,
-0.029183026445894322`, 109.37525806443935`, 42.38994568377677`, 0.20894130517333506`, 0.21227973215545026`,

0.015977821998123698`, 0.025968749217761676`, 0.0260021252737332`, 0.001285239257834414`, 5.656440928009311`, 0.0840047526885 9`,
0.6176543009238274`, 0.0004003933574867613`, 0.00040379378951291067`, 0.008492728369655422`, 0.08047833898400024`},
{0.13526256211607002`, 2.462351571487578`, 7.058879409696349`, 0.9886974127254741`, 0.579411758510507`, 0.519067819756009`,
0.20147771873022094`, 163.35469430255884`, 0.08074145629003388`, 7.486187513545234`, 836.5673907875474`, 847.7592962635491`,
0.01337836688263172`, 511.4518165366756`, 222.83471292371775`, 16.957017502847496`, 17.161996383388516`, 0.0120881446579033 1`,
0.0982035780358617`, 0.0982293973955223`, 0.00026291668976829996`, 596.4876956554124`, 0.1897609653451416`,
9.925102313090095`, 0.001197848354663411`, 0.0012031452487622455`, 0.004422007241745351 5`, 0.7284602291221002`},
{0.20702996858127698`, 3.5459212947722705`, 9.044651158282573`, 1.2898647778788441`, 0.1120438185942787`, 0.3399243113730348`,
0.005774130262077124`, 68.71847405774774`, 0.13094328364017765`, 7.770082768301062`, 1035.5363032794862`, 1046.6645534884174`,
0.010746364153230159`, 609.963964408325`, 957.0401246070355`, 1.4902520162100157`, 1.5276012912309702`, 0.025062388518648504`,
0.11926636189810318`, 0.11926615528983266`, -1.732326426528985`*^-6, 75.49023369794861`, 0.35273873080814333`,
25.243396776554412`, 0.0011163134380507422`, 0.0011198541267572646`, 0.0031717693130211`, 1.0190016123952839`},
{0.10369509204579469`, 2.7722566029085502`, 8.491805279999415`, 1.0043154825901828`, 0.1196997608855428`, 0.46660526882559294`,
0.07137717728073698`, 124.06613686120853`, 0.02382042507485488`, 7.245869706736816`, 617.3223271701587`, 626.1601795871928`,
0.014316430862864493`, 389.92937980106296`, 311.86528602710416`, 7.518988889449216`, 7.675855729748659`, 0.020862757294343393`,
0.06322288391736466`, 0.06324181922172065`, 0.00029950080070273444`, 297.77952279959504`, 0.09365575381691336`,
13.401346026692124`, 0.0007598008761889874`, 0.0007624915400775396`, 0.003541275053600801`, 1.1782399943083706`},
{0.12495211269478451`, 3.2710300653782634`, 8.31741576802569`, 0.8301159562153473`, 0.45240597007929817`, 0.45987625913811714`,
0.011306277140564175`, 1464.8239131751898`, 0.06894180622066476`, 6.472250284276024`, 63.56005361670872`, 64.82847774873049`,
0.019956309975300845`, 44.94624826196673`, 54.80383955311929`, 0.18228529214369635`, 0.185181442091994`, 0.015888006729663173`,
0.006147945668633516`, 0.006151561880397521`, 0.000588198393238093`, 8.51800958683272`, 0.01097426857183902`,
0.9689807603556574`, 0.00008943437277530997`, 0.00008973340186841053`, 0.003343558900466892`, 0.7193064085480517`},
{0.2548094999525184`, 1.991565099208616`, 8.435964917534815`, 1.3597221193616629`, 0.25512389762112764`, 0.2087626588648025`,
0.2568272531538555`, 1822.5061165805678`, 0.026223328458720774`, 8.894025776493041`, 42.137410212447946`, 42.01370398082542`,
-0.0029357815537031096`, 21.68372800720322`, 9.81339111045622`, 1.096800417229064`, 1.124324488060192`, 0.02509487633188945`,
0.004790068730274824`, 0.004791580764688195`, 0.00031566027514684514`, 31.20499188215495`, 0.017436500255707667`,
1.040212676213916`, 0.000042588000672250104`, 0.00004268234615709454`, 0.002215306737935352`, 0.7113303135590948`},
{0.1329827943052238`, 0.6587055670228517`, 4.6853626661703505`, 1.028170006221078`, 0.5310002897488229`, 0.3139925260456507 5`,
0.010724910779551164`, 62.931190858884165`, 0.12157208629491184`, 4.054529845871336`, 836.8849722077925`, 837.9411628835317`,
0.001262049995894631 8`, 944.6909662902005`, 726.308874022308`, 10.49048227661873`, 10.680653753911212`, 0.018128001390015802`,
0.28412814604886455`, 0.2840811787410427`, -0.00016530325655861322`, 98.71627251947601`, 0.5397736400333648`,

13.753223607638962`, 0.003323109031715288`, 0.003348078617814417`, 0.007513923214923945 4`, 0.33070430333301404`},
{0.05388606525951978`, 3.0261209146571506`, 4.311810802135282`, 1.2867466894982391`, 0.14121116952696844`, 0.17229324040800142`,
0.239982541075217`, 290.98679707372355`, 0.1893837871763393`, 8.200911880146922`, 209.73683994134512`, 208.78873190020744`,
-0.0045204649855639 1`, 117.05152320487122`, 51.82832400912455`, 3.568227740121385`, 3.6711088416292386`, 0.02883254909742794`,
0.047879537897422136`, 0.04789537247832879`, 0.00033071707877763146`, 154.2555513234443`, 0.03685771291065172`,
5.987061297672305`, 0.0004494309136917618`, 0.0004507819013247058`, 0.003005996231648877 5`, 0.6260107947145644`},
{0.18877028807710305`, 3.0713928282220317`, 2.350050214349242`, 0.9003573816536171`, 0.8295355664150708`, 0.3697046533237569`,
0.07836282343745943`, 203.60143886304388`, 0.1119456128362123`, 8.250251130667593`, 951.1870811174654`, 911.4045474740751`,
-0.04182408953310557`, 527.6710520266001`, 452.71853940399376`, 11.084573005207632`, 11.195067615891812`, 0.009968236000400074`,
0.27710571397692213`, 0.2770077594290062`, -0.00035349162061715234`, 486.3582576014041`, 0.7472760779310307`,
8.405321167576117`, 0.0037002342927004994`, 0.003728509251235419`, 0.007641396813898416`, 0.4205153217095809`},
{0.1549731585118843`, 1.0794091822629426`, 5.872175042913531`, 1.1954059661912195`, 0.1582784842110334`, 0.47191465043849123`,
0.04704948286374014`, 132.48832982484745`, 0.0611350826684427`, 8.646340597834747`, 717.7660608703653`, 730.1536497532052`,
0.017258532491517542`, 379.9400490676759`, 434.6346696429162`, 17.22111388831871`, 17.570804714305545`, 0.02030593539155645`,
0.1059953098962315`, 0.10604346638476572`, 0.00045432659785960006`, 265.551835140672`, 0.2346632566017768`,
14.911206129061515`, 0.0010689755455272065`, 0.0010743979701732373`, 0.005072543210851821`, 1.3631674347798335`},
{0.0973630018497127`, 2.6509059407435345`, 2.661281949905481`, 1.0384603558144696`, 0.8313691963066465`, 0.6553290382276395`,
0.19978363792296042`, 613.3149478611653`, 0.10086553584387609`, 7.337446850354657`, 419.5251350409591`, 401.5841665626077`,
-0.04276494297916089`, 261.68420637267536`, 110.61338188784133`, 7.938697993757703`, 7.992576604051145`, 0.006786832089570094`,
0.13964967583668572`, 0.13995508625471448`, 0.0021869754884782378`, 300.63916676315733`, 0.1942387378117365`,
2.5096446875980694`, 0.001611722435922891`, 0.001632810262586585`, 0.013084031216342051`, 0.4611796609815446`},
{0.25760446309632484`, 3.1710442921339457`, 1.104410531583154`, 1.067160019670409`, 0.29416815684722497`,
0.2106415408174861`, 0.1640132441258384`, 295.52940308175954`, 0.22278260496644214`, 4.791464741488767`, 142.91714078908169`,
142.45611887402248`, -0.003225798616693476`, 136.5150451512427`, 44.85746274883892`, 2.0997554065978266`, 2.1633952637136766`,
0.0303082239559338`, 0.17941511270504412`, 0.1798757005737896`, 0.002567163165918318`, 95.1202485281349`, 0.6602590539964053`,
3.466589835379227`, 0.002012916559542499`, 0.002052575190546995 8`, 0.019702073996306302`, 0.3992299983910862`},
{0.08814664727699922`, 0.49325198699724604`, 7.4393784579313085`, 1.082856668773565`, 0.36684849391494345`, 0.47651248778047417`,
0.010016127241926027`, 222.2306387901171`, 0.10060123761752315`, 2.9576363340294876`, 173.67853589369457`, 177.62629435544488`,
0.022730261062119173`, 268.7607666170922`, 152.08431250820465`, 2.6535943054721978`, 2.7018657257134975`, 0.01819095712624774 2`,
0.05352849577190276 6`, 0.053623582688270506`, 0.00177637938441099 76`, 18.698438055124832`, 0.06740510622961399`,
2.973961511282933`, 0.0005942321803344974`, 0.0005998992459047107`, 0.009536786727072366`, 0.3909508566746293`},

```
{0.09405288750913654`, 1.8996899418118014`, 4.321657830772438`, 1.3285146456969388`, 0.4687194709250446`, 0.37672748824359026`,
  0.1617154754485949`, 124.22044587269936`, 0.08001764444851872`, 7.0616286930304`, 761.7345949828534`, 767.7063313617285`,
  0.007839654937832341`, 493.7001923550053`, 238.32382558088037`, 18.58396065159607`, 18.892976962357558`, 0.016628119083697435`,
  0.2079416915042554`, 0.20804623712847128`, 0.0005027641328663179`, 504.3394732694759`, 0.2793930931344824`,
  12.303899726821474`, 0.001883728767104298`, 0.0018969767829356346`, 0.007032868034234863`, 0.682243918832574`},
{0.26673333380428393`, 2.6775026015459398`, 7.186300359062184`, 1.3779081516185778`, 0.2197224262472608`, 0.44634915685926935`,
  0.2549448072449753`, 573.3906335364269`, 0.19327206597748986`, 8.09151224456528`, 160.4345057467781`, 162.00316950132856`,
  0.00977759583107618`, 90.74706332341783`, 36.892813699776056`, 3.1446353912424376`, 3.2069654258346416`, 0.019821068848168588`,
  0.0238304797214701`, 0.023850389360035452`, 0.0008354694826984144`, 120.28242058521525`, 0.0908054757466273`,
  3.180127494614615`, 0.0002086256661806285`, 0.0002096893204440167`, 0.00509838642033289`, 1.1473280386773947`},
{0.06695152315589192`, 3.739348796633019`, 8.531551488563299`, 1.3873826920360777`, 0.5359483678583272`, 0.39821450250245216`,
  0.041894311644931556`, 1681.9385322117223`, 0.03536815464018617`, 7.504735436115399`, 66.65244110150653`, 67.20013532431379`,
  0.00821716674972439 2`, 40.648560252801346`, 42.03582798106669`, 0.45197864781480046`, 0.4585750372201695`, 0.014594471303591261`,
  0.009058229923586373`, 0.009064764593039284`, 0.000721406887221443`, 24.14436875442991`, 0.008663747006856126`,
  0.9599999162753854`, 0.0000788087181733399`, 0.00007911635112866592`, 0.003903539639477094`, 0.676212872076415`},
{0.2432492145111665`, 1.524425096090667`, 0.7030547277601631`, 1.1448142947983793`, 0.5481222164790474`,
  0.4314400790527364`, 0.005980753513921293`, 1722.4066531232882`, 0.11386175120469438`, 3.8888258531503404`,
  35.91443038955693`, 35.81414327524097`, -0.002792390502317832`, 42.26831956460633`, 32.71361128351261`,
  0.12089399029284002`, 0.12411241013159006`, 0.02662183480712388`, 0.09203990772906785`,
  0.09331264357428962`, 0.013828086931249706`, 2.632769039556384`, 0.31983764655394264`, 0.4945133215674058`,
  0.0009431563515619468`, 0.0009983318960734 04`, 0.05850095206386707`, 0.4267228591529196`},
{0.24077340164047406`, 3.026302168091572`, 3.348461380748672`, 1.1251559242118545`, 0.33184191297746213`, 0.3141742813991918`,
  0.0703872676061783`, 173.09096476750136`, 0.24564160017502346`, 6.392098114270558`, 393.64905433707787`, 396.94334291839874`,
  0.008368592646230688`, 281.85794075569584`, 199.8783739858639`, 4.367656190195005`, 4.462007549441396`, 0.021602286246385827`,
  0.12962506471185375`, 0.12972405409478804`, 0.0007636592749584725`, 188.82639139808168`, 0.4458609681220022`,
  8.030742454293755`, 0.0013868975540561879`, 0.0013977647307174793`, 0.007835601576705375`, 0.6508166134088346`},
{0.09741803900657164`, 2.8226058170826276`, 6.285345102913118`, 0.8413213607246539`, 0.2506059249236361`, 0.26188997247349455`,
  0.05425664970500174`, 4955.988737338467`, 0.11641107051911387`, 2.1128007380367517`, 3.7978042932009797`, 3.8189056795083394`,
  0.005556206870674263`, 8.226943900501889`, 2.176349215932553`, 0.039150245721577215`, 0.04013051186401178`, 0.02503856934655979`,
  0.0015068743482220 2`, 0.0015090966967893243`, 0.0014748068211039023`, 1.5786530187705476`, 0.0020970963433279534`,
  0.09268191055526988`, 0.000021577760787927502`, 0.0000217349429928665 8`, 0.00728445395627042`, 0.21447382095568665`},
```

```
{0.14314599793313348`, 2.5518611864266925`, 6.295888200856282`, 1.4241645467251498`, 0.07884119203240614`, 0.5217209274223922`,
  0.00638149871598101З`, 359.4043872790348`, 0.24023808698930982`, 4.143010259943262`, 122.70687181765165`, 124.71106561227744`,
  0.016333183015244`, 135.55557095811403`, 112.43796881190094`, 0.26763176278193007`, 0.27371067831262663`, 0.022713729743841116`,
  0.04191892263647414`, 0.04201710830606932`, 0.0023422755982223l`, 9.756558681402332`, 0.08572180018684629`,
  2.703215293570549`, 0.00035343590286807114`, 0.0003575275071091484`, 0.011576651403761185`, 0.7794967514827961`},
{0.225655266424848`, 1.5867432872088365`, 3.0858574789264335`, 1.3244931698407223`, 0.6079478177713131`, 0.6933074034991797`,
  0.013957254253711016`, 59.76089956157712`, 0.13530140261382512`, 4.737681248427657`, 1766.6606164466834`, 1809.4596488040236`,
  0.024225950337548374`, 1706.6787623798266`, 1471.5657903161539`, 12.253585434558229`, 12.409026470856546`, 0.012685351330716133`,
  1.0015241367946845`, 1.0024028364578839`, 0.0008773624428179971`, 277.7613490360752`, 3.2285599417036517`,
  17.43533472410024`, 0.008976079553869587`, 0.009183854988123304`, 0.023147681903525896`, 0.5415386080708912`},
{0.1122606973400958`, 1.7187290694442563`, 9.979465273640976`, 1.2098446125137294`, 0.9761419756238592`, 0.37158902094365565`,
  0.0208086160906198l`, 179.99091662757314`, 0.030562090142117915`, 6.57677031568597`, 1152.0353653145621`, 1125.5596176980375`,
  -0.02298171428903606`, 801.7106096202494`, 889.2662165012093`, 10.257933740160658`, 10.330835041401908`, 0.007106821226173121`,
  0.1332886312315982`, 0.13328086133301914`, -0.000058293783252727494`, 251.86584159495968`, 0.21375820985094796`,
  7.475792301025691`, 0.0013297158928853303`, 0.0013340111432207779`, 0.003230201547886446S`, 0.24080820979238554`},
{0.24459409433338708`, 0.8193086201254509`, 4.65991702304226`, 0.8806460188595719`, 0.2108783317674836`, 0.532516488691834`,
  0.10515966693757768`, 2682.0355154099257`, 0.13211135275557007`, 8.414844442987327`, 38.239938037575726`, 39.07091456606537`,
  0.0217305929646931S`, 20.798670874775738`, 15.676412055128981`, 1.7741344931197764`, 1.8070817883911048`, 0.01857091184411330З`,
  0.005382661437331954`, 0.005388183125823362`, 0.0010258286826496654`, 20.76519547821324`, 0.018808102848133498`,
  0.7195422386703333`, 0.0000737060370364917`, 0.00007411506548738551`, 0.005549456562035759`, 1.3630006086996382`},
{0.06530542208683338`, 0.9596039403304868`, 4.254394351359874`, 1.2566656605190776`, 0.7704919647854709`, 0.333532652338865`,
  0.010126143082696715`, 1215.361514466369`, 0.2378411046297244`, 9.6572715838455`, 156.1055067889189`, 151.65796793849398`,
  -0.02849059550755450б`, 73.98237572800504`, 136.4419393337602`, 1.3256793749025735`, 1.3412690060113632`, 0.011759729693264331`,
  0.0299417854471514`, 0.02996455209629456S`, 0.0007603637793525131`, 18.173245025305228`, 0.02793372766656881`,
  1.6538511356114423`, 0.00028746200401119637`, 0.00028876172501361686`, 0.004521366247658420A`, 0.5268653229396743`},
{0.1695388010672208`, 1.173836844893498`, 5.733937529803059`, 0.8972303818101501`, 0.03459597061008757`, 0.4595594251326994З`,
  0.02896845637601857`, 512.9632938088884`, 0.14318523913512782`, 1.8784414403823462`, 34.94946265436272`, 35.288187541813336`,
  0.00969184822097202l`, 85.1544477726985`, 24.90211324449447`, 0.5616902614477906`, 0.5757098802874744`, 0.024959697188887464`,
  0.018185869736881036`, 0.018244859067906703`, 0.0032436903969479403`, 9.419038918646832`, 0.04404586502222455`,
  0.8564852128250628`, 0.00024288362610802583`, 0.00024660590830510404`, 0.01532537312919846`, 0.34757563835568694`},
{0.21476635225191587`, 0.5109603344620304`, 9.116157263242219`, 1.0856571152809298`, 0.22597102427026194`, 0.3946805779061345З`,
```

0.00812287667631087`, 1537.5523384133612`, 0.22468288668284858`, 1.5725594082493757`, 10.516928683399515`, 10.677467459556741`,
0.015264796500011446`, 30.6088041362219`, 9.420256443470876`, 0.12865279151092898`, 0.1317565214547231`, 0.024124855025244196`,
0.004977573458981238`, 0.004990530799886045`, 0.0026031440844791476`, 0.9390924768556884`, 0.015271647069303805`,
0.23024626858094677`, 0.000055052593374016 47`, 0.00005575497833128858`, 0.01275843542011268`, 0.2211200819998299`},
{0.06813064867410118`, 0.8082337111165119`, 3.599754755102856`, 0.8970806442820121`, 0.12954010392326953`, 0.2841840584225793`,
0.09462247566679993`, 471.57171291886044`, 0.0960155452146989`, 9.92783198210315`, 183.96962256219086`, 184.9915474345718`,
0.005554856601586389`, 84.81178298740232`, 80.72962172651901`, 8.224227446223054`, 8.428285371698598`, 0.02481180473300948`,
0.02896989768734624`, 0.02898267132542684`, 0.00044092796662442 7`, 94.95854099895905`, 0.028196256020841932`,
4.621601820885761`, 0.00038992801051851433`, 0.00039123020480107053`, 0.003339576145926504`, 1.1145459002239204`},
{0.12903273933048381`, 3.2486278014701124`, 1.90816116411313`, 1.4556940559632274`, 0.8634695831402321`,
0.3517692054558007`, 0.07017000005506284`, 82.66475099620249`, 0.22883036061603934`, 2.0030440452333433`, 569.7257980258842`,
537.9720355173645`, -0.05573516701990211`, 1301.786852088813`, 285.1268600214319`, 5.9222570152530105`, 6.015916250095906`,
0.015814787268041863`, 1.346723519708258`, 1.3530412827219889`, 0.004691210126856138`, 274.8458398171767`, 2.4824489266964522`,
5.062559842523355`, 0.010885204293454964`, 0.011328539568257221`, 0.040728245685643705`, 0.11733188294685475`},
{0.25243522618186365`, 1.7361695497710992`, 4.779805866927312`, 1.3770344402735035`, 0.053441804379047`,
0.21192069167526972`, 0.3494903419308112`, 922.4310224013639`, 0.09853668667446419`, 9.287299936208932`,
76.14621006845393`, 75.33254802252394`, -0.010685522565056393`, 37.52522594048549`, 14.429855232110938`,
2.3892386716146863`, 2.4586744528458264`, 0.029061885719526837`, 0.0148082075026111`, 0.014815869103799213`,
0.0005173888322920206`, 59.258906125614075`, 0.05340161728953936`, 2.175071988221757`,
0.00012988981881600914`, 0.00013036534414473787`, 0.003660990007248399`, 0.8782263794436402`},
{0.1954776887577972`, 2.556119761332786`, 4.179140500485959`, 0.8918497427192082`, 0.9985940433520333`, 0.19919828622889002`,
0.1034119468672016 8`, 185.89024422047143`, 0.23152045430083634`, 6.38817726005194`, 675.6379316906634`, 675.6902789725033`,
0.00007747830514603926`, 484.0626342961798`, 276.6819630401354`, 10.619968448982195`, 10.739627948106504`, 0.011267406273299807`,
0.141638665112431`, 0.14158578737174132`, -0.00037332843152471096`, 387.79873167391634`, 0.3955314127846472`,
6.995319908071766`, 0.0019165924067120477`, 0.0019233737538003933`, 0.0035382312194272014`, 0.20032215324841066`},
{0.21581636372683116`, 2.527066218755916`, 0.7074271020081202`, 1.2545848439034097`, 0.6959200155465519`,
0.5313070485281961`, 0.086636650755472`, 1874.5635156658839`, 0.10607902266846381`, 5.753646937249579`, 69.74948469331724`,
68.70074109101174`, -0.015035861654272398`, 55.48330071338634`, 31.28999301069213`, 1.0220563475855686`, 1.039834653472157`,
0.017394643581625013`, 0.1322988139766039`, 0.1339125846978168`, 0.012197922813565798`, 36.89720099497921`, 0.4078892708257452`,
0.6619419132216102`, 0.0012393321107608823`, 0.001302355933901936`, 0.05085305431355325`, 0.5333373411613205`},
{0.1492413868080179`, 2.613673396032418`, 3.0904979954224796`, 1.2072285951382535`, 0.18001792552689877`,

0.24732759197473053`, 0.00562211589804969`, 2528.5611678534033`, 0.0881107110472133`, 9.594393869960811`,
32.66762836471114`, 32.86948637483345`, 0.006179144928083025`, 15.583483940755878`, 30.242590539145528`,
0.0617017079658 7073`, 0.06334859029066484`, 0.026691033021404298`, 0.008336159286871998`,
0.008343278599426705`, 0.000854027893387288`, 2.303830180002259`, 0.01777285675178765`, 0.822094371332597`,
0.00008332473302541388`, 0.00008373079006887992`, 0.004873187452544281`, 0.9377667095958642`},
{0.07601227699098417`, 2.712563755157329`, 4.823883660189505`, 1.3951422907519642`, 0.1445858824844255`, 0.4724433117860949`,
0.00914361266793413`, 231.39818056782485`, 0.14481529205028987`, 4.810561016418756`, 223.06556459839388`, 226.8599513252022`,
0.01701018592286840 4`, 212.22732449354578`, 197.50717616732763`, 0.6343875536270089`, 0.6483169827075`, 0.021957286205966486`,
0.08394752531035453`, 0.08412786543625614`, 0.002148248268604 6023`, 24.583095495593568`, 0.09115775066570658`,
4.779938347725428`, 0.0007224301532764432`, 0.0007305561001640519`, 0.011248072703990752`, 0.7935221048233677`},
{0.12871665135347787`, 2.8014401685833805`, 7.283786002739568`, 0.7524429330430051`, 0.8818706538097647`, 0.37826653388239995`,
0.007972118927365966`, 65.78330734043783`, 0.1996049007295676`, 1.2990854064714608`, 494.58685954493967`, 468.6999904729794`,
-0.05234038990800993`, 1742.4862678429843`, 443.44262207131135`, 1.219798442097487`, 1.232750747365266`, 0.010618397942455893`,
0.24640876419615784`, 0.24656383470010979`, 0.0006293221933797444`, 48.81703361810465`, 0.4530987284496603`,
4.092592662791065`, 0.003925401816135987`, 0.00397247601821215`, 0.011992199596651876`, 0.0649842847011505`},
{0.09952335410600305`, 3.347751049949922`, 0.6305859630946937`, 1.1305375150416004`, 0.9039729545790072`,
0.47386245025326623`, 0.16157270551479597`, 562.0685567880952`, 0.16407167074796686`, 8.59213583452907`, 516.3825230267412`,
482.2728201479209`, -0.06605510713044371`, 275.0647228657877`, 157.80233520196086`, 7.310880543635071`, 7.379208429251762`,
0.009346054173485108`, 0.6715503671065706`, 0.6743085367027352`, 0.004107167133343026`, 349.6429716573247`, 0.9547849283647347`,
3.1741562526788867`, 0.007030009082037103`, 0.007243353722597813`, 0.030347704828126654`, 0.44727084788411925`},
{0.09189223639403032`, 1.6732139429664894`, 9.692701238874825`, 1.282228547207058`, 0.15855153018111912`, 0.4131843305852153`,
0.03830010741000983`, 3674.584524793622`, 0.10140311695331994`, 4.468652597668594`, 12.385243438825565`, 12.565270436264235`,
0.014535604271960878`, 12.685057756455327`, 8.092740116762588`, 0.17212835993826678`, 0.17588265020671306`, 0.02181099192365954`,
0.0022984008084439957`, 0.0023008330653950 78`, 0.0010582388163746792`, 4.114393883266604`, 0.0030172170059593498`,
0.2753837731274051`, 0.00002161499875119599`, 0.00002173185106486 9826`, 0.005406075430255175`, 0.6584135556407352`},
{0.15046133692934927`, 3.1811544489323884`, 8.294233740646352`, 1.3617577237735539`, 0.4847724596901488`, 0.37433619970600795`,
0.06464979105054385`, 793.4336208513492`, 0.16357169207826316`, 3.060886752086996`, 51.21461247573355`, 51.65330524257508`,
0.00856577343135001`, 76.57925268435571`, 26.987058027725038`, 0.5204673283514774`, 0.5294388069287217`, 0.01723735206523047`,
0.017198785760655658`, 0.017227927248836632`, 0.00169439218480405`, 23.652670815846527`, 0.03696788998728948`,
0.8356332208982302`, 0.0001519965258197642`, 0.0001533147543144 1214`, 0.008672754114203096`, 0.2984120091956064`},
{0.2766206750277398`, 2.8724354428398637`, 5.189326643025524`, 0.8952441889736982`, 0.9323758122824941`,

```
0.4464935529399745`, 0.01922331430839979`, 81.97107113159659`, 0.06878282991185858`, 7.425462926136905`,
3074.6195254184872`, 2906.5417901507467`, -0.05466619003691631`, 1895.1009155616373`, 2413.7353755772283`,
15.647660209270754`, 15.759135618849236`, 0.007124094470842168`, 0.44908130013357983`,
0.4481340736498793`, -0.0021092538999480936`, 642.0984826089187`, 1.774645319789638`, 18.61998045519261`,
0.006034052345722407`, 0.00606415691861767`, 0.004989113645426757`, 0.3077891028282203`},
{0.23204393359414766`, 1.3807564884192223`, 0.49288335289722696`, 0.9255887528096592`, 0.5628667572225596`,
0.43401537857521066`, 0.010225716982610468`, 1119.882052780943`, 0.015435359995479236`, 2.904839645045426`, 42.41796602370956`,
41.95705073816599`, -0.010866039293018881`, 66.833187010293`, 36.43342757844437`, 0.2528542990330039`, 0.26087563536092195`,
0.031723155819751714`, 0.16588771174124886`, 0.16919402980315845`, 0.019931060759140307`, 4.987574485921637`, 0.549903388105308`,
0.5716095484253089`, 0.0020805238925564185`, 0.002251630479341182`, 0.08224206768157716`, 0.3385124133185024`},
{0.06896766630182821`, 3.238145856087148`, 5.061610251787275`, 0.9979492539875935`, 0.9640249344246659`,
0.343776595701085`, 0.005657576047359979`, 3386.0339854064414`, 0.07870221044254616`, 4.290344560110242`,
36.062801320561576`, 35.00088655907136`, -0.029446263812144657`, 38.470849988685764`, 33.3325099646423`,
0.05656002906448834`, 0.05706250424385515`, 0.008883927177510742`, 0.010384555750391164`,
0.010398280888901221`, 0.0013216875945356588`, 2.6164231962191704`, 0.010231408224081378`, 0.2596942684968947`,
0.0001253816517973183`, 0.00012620953841920192`, 0.006602932805686024`, 0.16494577689473477`},
{0.14163054804702563`, 1.8677837362731973`, 0.4370922457889872`, 1.166715795330906`, 0.13182109027382305`,
0.4473367986720722`, 0.3453878303019619`, 2024.858554151485`, 0.17866806603084778`, 3.146330044100834`, 16.855845028720218`,
16.41311009375263`, -0.02626595903161344`, 24.519451289713185`, 3.0797613020812564`, 0.4882385549576865`, 0.5062867079896903`,
0.03696584968298544`, 0.0861176595400667`, 0.08835353176024377`, 0.02596299332933949`, 13.027486176735662`, 0.174241304531 2275`,
0.3653740803488827`, 0.0008456876325892981`, 0.0009315083295229843`, 0.10148037363503026`, 0.6021461726681335`},
{0.14346666218024207`, 3.6942323053195354`, 6.0819497997897844`, 0.8206348775106591`, 0.4021755195313119`, 0.4030089388647059`,
0.4636571249048107`, 744.6939263196219`, 0.08354527905403086`, 5.555269667846608`, 95.95280249076491`, 96.31417666979591`,
0.0037661659654577573`, 79.0527555409005`, 13.951274914033219`, 1.5240790106200397`, 1.5502934490900429`, 0.017200183381134826`,
0.014613399524821269`, 0.014624496684836015`, 0.0007593825102705232`, 80.43288452702814`, 0.029950509327604933`,
1.6325240370563268`, 0.000214860838066544`, 0.0002158356995119332`, 0.004537176035249946`, 0.6044327236510285`},
{0.09149267062932814`, 2.234522341263885`, 6.944559583117852`, 1.114683357002141`, 0.5085103250363343`, 0.6658564606426403`,
0.006987139923383322`, 1505.8338370139704`, 0.21635495075213407`, 1.8879751532316735`, 22.929089141274396`, 23.68236164183872`,
0.032852264471699755`, 55.584668883988435`, 20.82843101494257`, 0.06232637729788174`, 0.0632099103765913`, 0.014175909414513299`,
0.012164153984907234`, 0.012223232178749249`, 0.004856744983277483`, 1.989566893173699`, 0.015899013343 22227`,
0.281209998048472`, 0.0001302767570643404`, 0.00013291053682103443`, 0.020216804716694625`, 0.2575328682996467`},
```

{0.22068432056467163`, 3.569728941728428`, 4.540468856326312`, 1.2038410371742398`, 0.3148195694928344`, 0.5227498853534928`,
0.10369270254334745`, 4010.634501486441`, 0.16206884164220114`, 3.771999452670874`, 12.323519921310096`, 12.592969979701449`,
0.021864699380687025`, 14.952976304093992`, 5.078100939733653`, 0.138913178972306`, 0.14149151297632803`, 0.01856075876383212`,
0.005410982368863587`, 0.005428247612901865`, 0.0031907780993019053`, 7.084034219499189`, 0.017058842395145744`,
0.21322440225319714`, 0.000053882862769527584`, 0.000054685985322588016`, 0.014904971855256077`, 0.5608372791223145`},
{0.2097350071754976`, 1.1102722915344074`, 3.321403610485124`, 1.0935329934230222`, 0.7183578411763796`, 0.40562405317483374`,
0.006176942100465586`, 446.96867612346506`, 0.08354129713872654`, 7.284441181467236`, 325.39075225157944`, 319.8606836058835`,
-0.016995162300803`, 204.44357172592612`, 298.65349094926927`, 1.5451133936483932`, 1.564843930171778`, 0.012769636587510291`,
0.09212341481318889`, 0.0922253251084364`, 0.001106236622406609`, 24.507094117807203`, 0.27602150095539607`,
3.4331123345209864`, 0.001013856205302699`, 0.0010221176878707416`, 0.00814857425030624`, 0.49032677359585564`},
{0.15395695699718487`, 1.7653707587036722`, 8.640858401956272`, 1.0488086821127276`, 0.2563370285663329`, 0.39045184386637677`,
0.03142873441769787`, 98.7771934627432`, 0.2047745102794421`, 8.897923651735105`, 989.0570506261663`, 1005.0782529254774`,
0.016198461240601203`, 508.7414962038482`, 688.2349534329128`, 11.45909151226099`, 11.68857213798931`, 0.020026074971369212`,
0.0846801405628507`, 0.08467910295482263`, -0.000012253262939410092`, 288.99350110078575`, 0.1862442394163105`,
20.004056195612407`, 0.0009750883379029407`, 0.0009776634337020356`, 0.0026408846244976303`, 1.1135286288912647`},
{0.2495726529131425`, 0.6471319987804813`, 4.234303245586215`, 1.3697781718226154`, 0.5893758025334812`, 0.16113556190203493`,
0.016137549539750166`, 661.2554849181507`, 0.08255896742665592`, 6.142572860377916`, 96.2789779965973`, 95.31006301399012`,
-0.010063619315126338`, 71.73740285816858`, 78.44964343696829`, 1.7224042072345886`, 1.7606599526113096`, 0.022210666471921048`,
0.03176524987082118`, 0.03178465991274688`, 0.0006110464109247982`, 15.923183961937552`, 0.11325339543869672`,
1.9246921481523416`, 0.000279964170717073`, 0.0002813278905986475`, 0.004871051456625919`, 0.3058408886615471`},
{0.18703143603461198`, 3.5026925577041474`, 3.736145496532833`, 0.8654363814884891`, 0.7031489482980493`, 0.15906583747765657`,
0.008377691039792691`, 131.85234583624222`, 0.16088793676433022`, 8.514675246308464`, 758.387128372606`, 746.0678574384053`,
-0.016244040112648128`, 407.649918310765`, 677.7722009608865`, 1.5583523292720904`, 1.5882732549210745`, 0.019200359948741585`,
0.12951218029022607`, 0.12944495633768202`, -0.0005190550602530708`, 77.977558657461`, 0.34604070090936373`,
13.287305478351321`, 0.0018073284954649438`, 0.001811810101382393`, 0.002479685308284907`, 0.3619342336519853`},
{0.2617533772269884`, 3.6583043975291023`, 1.4938470678500337`, 1.229216471284039`, 0.8448795594977094`, 0.4744971024115313`,
0.027729197093825105`, 163.20786376161018`, 0.21172311156121143`, 7.676981105027913`, 1364.2473956749004`,
1294.6730225509723`, -0.050998355096371184`, 813.3306828847174`, 976.6878851923416`, 7.191591175082823`, 7.267629417421458`,
0.010573215368816946`, 0.91524523105242`, 0.9150930324291494`, -0.00016629272473289713`, 375.843280300528`, 3.422407574555612`,
9.80572792541681`, 0.008865647346624317`, 0.009040867720181105`, 0.019763968349531202`, 0.43156989313583277`},
{0.2277950960946843`, 2.381865480846251`, 9.991476447433318`, 0.832979598782657`, 0.5926733949589658`, 0.27276410945547525`,

```
0.04327492387940349`, 669.5530400360332`, 0.024018555255208573`, 9.681708943673282`, 194.42527896370262`, 193.19202307375122`,
 -0.006343084070778993`, 91.91051570187635`, 121.26175174459377`, 2.087331650617776`, 2.120589784848466`, 0.015933325315527602`,
 0.010506803843607963`, 0.010508209145304466`, 0.00013375158777306773`, 71.02490293834725`, 0.03419140558871177`,
 3.041202991784391`, 0.0001525459193313372`, 0.0001527512002955953`, 0.0013456994795921773`, 0.6352743804731232`},
{0.15963824410704142`, 2.654604852862681`, 7.469701098598773`, 1.491664105533315`, 0.25955793675814864`, 0.27946634303609463`,
 0.06966019051655106`, 86.7013584077103`, 0.017186465149371527`, 9.819694766966123`, 1090.6861216361706`, 1098.044073743162`,
 0.006746168270623576`, 508.35405621682327`, 558.2324837247143`, 13.667871951419276`, 13.976754244428651`, 0.022599150336442886`,
 0.13919887303802933`, 0.13919092743471206`, -0.000005708094572798306`, 518.3257030077627`, 0.31744948104955506`,
 24.557197031092894`, 0.0011268271174854627`, 0.0011300877493781857`, 0.0028936398868346647`, 0.9679597631936538`},
{0.13626056470386483`, 2.820076398995975`, 2.87943303050835`, 0.9523411928557022`, 0.8935766203190545`, 0.356125796963648`,
 0.009331421764352204`, 68.56313381348697`, 0.147099680293568`, 8.670054262776382`, 3313.431632229684`, 3148.447883065088`,
 -0.04979241085278563`, 1749.1243795244463`, 2922.5449179317193`, 9.352246202157458`, 9.439941698513381`, 0.009376944795967068`,
 0.7960284268688653`, 0.792973356939993`, -0.0038378904895258747`, 376.7721256043426`, 1.549532613792383`,
 26.037501853391007`, 0.010026208525505065`, 0.010087018294131965`, 0.00606508117921245`, 0.3750235385217479`},
{0.22962572899038486`, 1.387681996287987`, 7.569216994527352`, 0.873789488954491`, 0.15662470794012862`, 0.483300190980835`,
 0.02173528342644958`, 88.10021701991921`, 0.04718506749066664`, 8.783666403079792`, 1105.959326629311`, 1127.2365592524025`,
 0.019238711687471488`, 576.2723958267087`, 848.4652682451489`, 12.333748725734251`, 12.583553681936237`, 0.020253773751752302`,
 0.09122557319464666`, 0.0912197761797032`, -0.000063545941564999083`, 244.50458647773354`, 0.29925341067678574`,
 22.827561726344054`, 0.001260258171973283`, 0.0012642296122518206`, 0.0031512910345339318`, 1.4059735180271336`},
{0.16187203397799577`, 1.769575035055972`, 1.1614771267268384`, 0.8287067492253789`, 0.7900895875825826`, 0.2962983919698179`,
 0.014442973603799043`, 137.47659064397268`, 0.2186302306878819`, 9.341476742915006`, 1286.2803870802732`, 1244.5649883024462`,
 -0.03243103074323994`, 630.2090063877525`, 1066.4891444452016`, 8.263205449828517`, 8.371615622931515`, 0.01311962697299851`,
 0.6173559192149274`, 0.6158064454450541`, -0.0025098548854016745`, 208.89088676507146`, 1.4276094047384573`,
 14.097699757404191`, 0.008922349894279868`, 0.009009963704931342`, 0.009819589198989176`, 0.4782486641744434`},
{0.09685210682031803`, 1.7960713074535244`, 5.69551378974773`, 0.7869885608747933`, 0.9421312080797839`, 0.5036667090451988`,
 0.01697045527462802`, 52.99769310364423`, 0.21425647446502405`, 9.869621253243459`, 7212.950551597583`, 6812.491479879829`,
 -0.05551945335727515`, 3344.852434109134`, 5809.61668713339`, 52.485825156452556`, 52.79642420258769`, 0.005917770087624286`,
 0.6365461169528801`, 0.6338407778542878`, -0.004250028437126295`, 1346.6897801646694`, 0.8807261787893194`,
 38.246227895000914`, 0.009720297316106685`, 0.009751987872925095`, 0.0032602456270445224`, 0.40031877940310195`},
{0.16013522976515993`, 1.0007721789830901`, 6.380859482894074`, 1.440495462142086`, 0.5398127028451485`, 0.19075782788209084`,
 0.0465211016604952 6`, 84.8416541425776`, 0.16547472912452094`, 6.4422585881915015`, 805.5874704508226`, 800.2944951573206`,
```

-0.006570329712973222`, 572.3201602515785`, 489.8354414593446`, 20.60233688200135`, 21.026116808201653`, 0.02056950765476162`,
0.1771255377047683`, 0.17709913325452153`, -0.00014907195534263895`, 294.5463653363455`, 0.40520055253808684`,
15.855587991240643`, 0.001484199913409201`, 0.0014893918093270744`, 0.003498110915495012`, 0.373493929880729`},
{0.1912199176775608`, 2.2121001603415005`, 9.917361836602723`, 1.435922264503217`, 0.9790995032802718`, 0.6767636439858111`,
0.07238714810610002`, 269.421010262202`, 0.015607565070540702`, 2.5093255880098546`, 483.92110648171445`, 466.4187212793585`,
-0.03616784837017084`, 882.6368271054569`, 238.55796917786563`, 7.506123783472269`, 7.546204637582639`, 0.005339753948452541`,
0.17460990701674436`, 0.17506076184675642`, 0.002582069011518584`, 237.20425178517385`, 0.47698417207756355`,
1.9184334126793725`, 0.0014565814154406942`, 0.001477865744623321`, 0.014612522827079477`, 0.10946350502659938`},
{0.24179572266521915`, 2.5600514990256196`, 5.593167080478407`, 1.0641609773948193`, 0.6297315486340287`, 0.26623731831221253`,
0.08310036534342866`, 636.0105501064265`, 0.05032454414330245`, 1.4627567207550134`, 30.33101754551306`, 29.92352181295465`,
-0.013434950935851231`, 94.902875776028`, 14.077604421522564`, 0.4296737839031727`, 0.4381748774471793`, 0.019784994715717596`,
0.024608343843571735`, 0.024678048703607527`, 0.0028325701428300043`, 15.71410020819038`, 0.085002746903582`,
0.485178192158812 8`, 0.0002774775030026655`, 0.0002817543809182286`, 0.015413422238854668`, 0.10070742131078506`},
{0.20288510482307215`, 0.7288550347693712`, 9.019074578521877`, 1.2840716127441223`, 0.5874481378002983`, 0.6980893050148065`,
0.00822573595086472 5`, 1276.121996072157`, 0.1607915330045036`, 9.69181498105582`, 166.10171875774887`, 171.4304562333472`,
0.03208116999300903`, 78.43926332912469`, 148.6332907106119`, 1.5136122955441802`, 1.5304857714410938`, 0.01114781899340156`,
0.015300233646588378`, 0.015313107813534312`, 0.0008414359704111973`, 15.760056318517167`, 0.04434556438864697`,
1.6663842609637494`, 0.00014374672510653053`, 0.00014441776706063686`, 0.004668224292477152`, 1.0825003738526626`},
{0.1862496077639137`, 1.6905827743030857`, 2.538407600056317`, 0.9208769830081895`, 0.38109944279089136`, 0.3320011945053185`,
0.011054373793833769`, 1065.4164452217328`, 0.07943719416592698`, 4.231039339853934`, 44.96679068583393`, 45.37989215131294`,
0.00918681229365892`, 48.641764865774576`, 38.84777305279003`, 0.23866216309901012`, 0.24383226633189742`, 0.02166285248467492`,
0.024101175148466367`, 0.02415757382592136`, 0.002340079979817222 3`, 5.763973454472858`, 0.06412620597215932`,
0.8643101947253171`, 0.00031436047505994935`, 0.0003181977191295475 7`, 0.012206509322987946`, 0.43460215699291427`},
{0.05689751321611025`, 3.0250332600067678`, 2.529462845718742`, 1.466915953431471`, 0.22269501402715108`, 0.6695459886571014`,
0.14207393160159743`, 917.9363525294286`, 0.02546682059293326`, 3.574551553390743`, 53.50489111660605`, 54.55580532948984`,
0.0196414606394297 3`, 68.50723102978968`, 18.19985943576573`, 0.796279389369403`, 0.8106871986771339`, 0.01809391213697098`,
0.05390962665738938`, 0.054396488224812224`, 0.009031069172802608`, 34.411023387147495`, 0.0438189099316344`,
0.9217432313412577`, 0.00043456206694636457`, 0.00044973553852120635`, 0.0349166959773195`, 0.7123633209894129`},
{0.1967142498798619`, 3.0620830451218577`, 7.9498840213828466`, 1.4844762448061533`, 0.6042217303737281`, 0.6207898959804532`,
0.0334891282473782`, 576.7285784287807`, 0.16199601914875666`, 9.348325121692032`, 340.78920253103274`, 348.2636558055563`,
0.021932776094462536`, 166.8462741202257`, 231.49640535108952`, 2.4383659420588866`, 2.4646752936766054`, 0.010789747003890682`,

0.04267536189808204`, 0.042714978478021186`, 0.0009283244049285155`, 106.66398584258715`, 0.11992645434466068`,
3.525641475799647`, 0.00034657542993499213`, 0.0003485016330349421`, 0.005557817818508726`, 0.9539217410037093`},
{0.26434225760699476`, 2.569764850665571`, 5.652188171834926`, 0.8392687504503539`, 0.3834838466329171`,
0.17354269300981617`, 0.03414034179009787`, 716.202171206297`, 0.028057924329779993`, 1.7997126741152787`,
21.316670529855433`, 21.277599392329904`, -0.0018328911858354058`, 54.21016040886312`, 14.461911281819882`,
0.18033391904576948`, 0.18531093058714695`, 0.027598865303394726`, 0.011001153958426026`,
0.011014142691058819`, 0.0011806700171526074`, 6.620225236379832`, 0.04154385533789965`, 0.5295926694520834`,
0.00015802401546971545`, 0.00015926237142146435`, 0.00783650477472042`, 0.12205078678175806`},
{0.19233187457454282`, 2.1451040206615035`, 4.677354160755579`, 0.8253029687642099`, 0.9809911268657268`, 0.22566854695954142`,
0.015470928716805988`, 111.90002994139898`, 0.12242650367871766`, 3.4296984602053584`, 633.2077917842423`, 626.2614397257395`,
-0.010970098834269693`, 844.9962985058218`, 518.8811163483446`, 3.5802783445722888`, 3.6219973403673404`, 0.011652444804549411`,
0.20436548203700802`, 0.20426844324938856`, -0.0004748296368459748`, 109.71527817184743`, 0.5615142322645058`,
6.2573566674429735`, 0.0029828151541578807`, 0.002999878386501196`, 0.0057205128247823644`, 0.11797342622735922`},
{0.21830260718528288`, 1.0813728060954029`, 9.236753498274854`, 1.3292857287875703`, 0.7084275620345744`, 0.4530838967889582`,
0.022838882777727113`, 80.81382094438914`, 0.07293079158751065`, 5.841787640828064`, 1501.137114142784`, 1484.1228603976367`,
-0.011334243610959582`, 1176.08603110938`, 1134.60726591272`, 22.2059651061321`, 22.45160458023919`, 0.011061868868870586`,
0.23203201811541618`, 0.23201721451697566`, -0.00006379980901238813`, 343.0418114124956`, 0.7236170643581604`,
15.29554372755899`, 0.0021033344157139755`, 0.0021143108359222866`, 0.005218580614811508`, 0.4210751092053813`},
{0.27637635892023465`, 3.838779152844408`, 0.15938000205200176`, 1.3392032462951913`, 0.02123906289427646`,
0.6059474038465393`, 0.007164747514275051`, 100.75472745890974`, 0.10946913978259332`, 8.952098728322259`, 1062.8900340844698`,
1036.1989721798222`, -0.025111781133255295`, 543.4104258512448`, 938.3225509527417`, 1.6834221800926392`, 1.7673688352919146`,
0.04986666814301799`, 5.990399697056251`, 6.085792741135737`, 0.015924320396577762`, 92.31837100536458`, 23.65149795356105`,
22.141709567861028`, 0.04903449172446328`, 0.05661652842375751`, 0.15462659920897193`, 2.3510362984639004`},
{0.15064788979833238`, 0.9109936464272579`, 0.5800958535668599`, 1.4999646884179314`, 0.9779856120080317`, 0.34948818201364285`,
0.011067508252105101`, 2641.6705923333384`, 0.011474828296826944`, 3.667140614388831`, 42.0021008885658`, 40.484585634784644`,
-0.03612950832643391`, 52.421319135782085`, 35.79372839537999`, 0.4005433578178629`, 0.4091719833205884`, 0.021542300812910087`,
0.18048397133780078`, 0.18337069079111362`, 0.015994325877891624`, 5.212749344153037`, 0.38842184892088444`,
0.2888396122806677`, 0.0014159929468633425`, 0.0015005735318141052`, 0.059732349047445954`, 0.19882287556987366`},
{0.0419185722234717`, 1.709255049022068`, 2.3539768873704308`, 1.3792050115017793`, 0.22715212217109637`, 0.2926929451571887`,
0.00505223249889438`, 88.91455127714036`, 0.1107140298924858`, 2.282618721837757`, 236.1649508847856`, 237.1448201021905 2`,
0.00414908822724902`, 473.5289814423474`, 219.83175118317462`, 0.6088159158558925`, 0.625453447430866`, 0.027327688290776475`,

0.37778094100714416`, 0.37958966405278705`, 0.004787756208190164`, 14.866023972881113`, 0.22622910943228425`,
5.662264076779682`, 0.00325683203121252`, 0.003340361485037469 4`, 0.025647455264633967`, 0.2682334717999011`},
{0.24924491240080032`, 1.373280333190027`, 8.919496594562187`, 1.4037493221091317`, 0.24632944507518628`, 0.3548949637369072`,
0.18998328780525742`, 2947.236236962295`, 0.1153856006596437`, 7.477168052601716`, 26.530217308847327`, 26.738927371442475`,
0.00786688100461008 2`, 16.23933198324636`, 7.529718669338275`, 0.9207616142623067`, 0.9402819972068928`, 0.02120025709393336`,
0.0035013231522903793`, 0.003503599110359383`, 0.0006500279951353605`, 18.06376880603897`, 0.01246695689113499`,
0.5601547922831008`, 0.000030118472362117288`, 0.000030232698557703684`, 0.0037925627240664728`, 0.8874350258307818`},
{0.20687871564233656`, 0.4418165550312096 5`, 8.570924850704476`, 1.0055678634725929`, 0.5549096280819519`, 0.326726210951258`,
0.20129490353773463`, 306.54002765810475`, 0.1699967418388691`, 1.7003969931174137`, 73.90708599093448`, 73.7453632776144`,
-0.0021881895511332017`, 198.92998303823444`, 19.900124812619435`, 7.36915217420703`, 7.499994312155485`, 0.017755385538979596`,
0.03190284906477305`, 0.03195943968656982`, 0.0017738422572188828`, 46.511620387270135`, 0.09428600628358556`,
1.186386277695811`, 0.0003815767335976217`, 0.0003854088260265960 3`, 0.010042783250551413`, 0.1422224119314733`},
{0.20196179343132148`, 2.101313615300616`, 3.7695608295023266`, 0.8255643571631567`, 0.9698552359083252`, 0.3038610819528902`,
0.02519738143348337`, 88.57713238465558`, 0.03933307441687739`, 1.9858938348907018`, 566.8134786255362`, 553.1625545482643`,
-0.02408362643452644`, 1306.3171729839394`, 416.8137268123438`, 4.783582605224256`, 4.836665591931822`, 0.011096910221555234`,
0.39138192322700865`, 0.39138200121325856`, 1.9925869154668874`*^-7, 143.5972465467558`, 1.129202787593462`,
4.6019451583268065`, 0.005675167164211903`, 0.005754491830673847`, 0.013977503070248254`, 0.07941683405698047`},
{0.10010100507466935`, 2.4192735538683836`, 9.823330355933038`, 1.1133108806457672`, 0.011014651746178439`, 0.5998442212295849`,
0.013751847561218578`, 3832.28466694177`, 0.1060300423959204`, 2.751621101527464`, 7.71793051956826`, 7.833404248522687`,
0.014961747668193137`, 12.837389782188309`, 6.468985193726633`, 0.03487875021240865`, 0.03563632677227796`, 0.02172028972528328`,
0.001990095658391255`, 0.0019944369292289356`, 0.0021814382737712634`, 1.2054462568694522`, 0.002845865365709856`,
0.17306805906908093`, 0.000021485659694508996`, 0.000021700366862840087`, 0.009993045193113614`, 0.6088256496140791`},
{0.21311625184066546`, 1.4247782113917893`, 5.690068626185788`, 0.8615233428628426`, 0.12086241410351461`, 0.29764577871833753`,
0.005395932799405541`, 882.4049045614522`, 0.2481341739836153`, 1.9572384227218778`, 18.713969330433436`, 18.861109244487125`,
0.007862571080225367`, 43.760943324623085`, 17.35592565430593`, 0.06096685859589047`, 0.0627199651484833`, 0.02875507436282776`,
0.00905292005798997`, 0.009070739061159815`, 0.0019683155330769697`, 1.2409178820632705`, 0.02756177701388708`,
0.49287377572911545`, 0.00012638509331408532`, 0.00012770804849787505`, 0.010467652071134648`, 0.2463270459124536`},
{0.09607643000919447`, 2.545123365745101`, 1.6292494594570959`, 0.9689766494611143`, 0.9239031581606687`,
0.5340288721519064`, 0.06266594590032853`, 79.64776165873695`, 0.19537727971057256`, 3.908080115643834`, 1865.8234718623207`,
1736.820501985616`, -0.06913996518006293`, 2185.1013665956852`, 987.0317418154671`, 23.409210193225483`, 23.591119616614414`,
0.00777084839203912`, 1.7864684619737057`, 1.778577066722085`, -0.004417315737498151`, 851.13325480595`, 2.4519644592916583`,

```
10.175836619180062`, 0.02170178877959139`, 0.02227066066467304`, 0.02621313343610754`, 0.1983651079513249`},
{0.25410295729391263`, 0.5246172329407881`, 1.519575137014602`, 1.3035504391628365`, 0.641505202173666`, 0.18662868599370885`,
 0.02992528952684035`, 1308.2457641643925`, 0.1567967571574565`, 7.528281484333206`, 68.0792176898054`, 67.13460431424852`,
 -0.013875209022831347`, 41.38883499309038`, 47.882704402270974`, 2.35022381781166`, 2.4001481559559794`, 0.021242376051998857`,
 0.048462680972375445`, 0.04854130451265678`, 0.0016223522657805578`, 17.613827372741262`, 0.17592157933531902`,
 1.194594294265786`, 0.00044744102303562183`, 0.00045228285049374377`, 0.010821152305778847`, 0.3900213385320539`},
{0.20247384111644234`, 3.9381206949664582`, 3.525512124148932`, 0.8160399146038965`, 0.003770588899372651`, 0.4372678593782203`,
 0.005846407125549171`, 81.38331905868344`, 0.05740074457617078`, 6.792038617151225`, 783.4591456923383`, 791.0871599587367`,
 0.00973632678658376`, 527.935301309596`, 723.1116158070021`, 1.0288633789962391`, 1.0546968427529135`, 0.02510874065891766`,
 0.16746699282773997`, 0.16748157698084756`, 0.0000870867319067159`, 57.88268807311726`, 0.48439550425791605`,
 19.33156195869641`, 0.0024698518831678395`, 0.0024866094964186443`, 0.006784865669479556`, 1.179179202771082`},
{0.15622647656935051`, 2.478038073204126`, 4.93355773626655`, 1.0681326166577831`, 0.57283306272417`, 0.24473580012035412`,
 0.09417540555806864`, 340.729792091258`, 0.1606536419587833`, 9.486285960325056`, 346.9408340719854`, 344.6543695144871`,
 -0.006590358738296942`, 167.38776120104416`, 151.16132076237622`, 5.374063941355277`, 5.467350879088074`, 0.017358732376613784`,
 0.04967665409332505`, 0.049687401238164945`, 0.0002163419625587082`, 190.24478649302603`, 0.11086869481062961`,
 5.844643528145093`, 0.0005617827494165395`, 0.0005633944745348027`, 0.0028689473287264455`, 0.6036978565832033`},
{0.2472522705346833`, 1.1701552284997945`, 3.914120438755436`, 1.4973295978551664`, 0.163173719496931`,
 0.4864210576866177`, 0.39314364156002357`, 172.17497479862377`, 0.10619847023829598`, 4.473646521439088`,
 292.7200605180249`, 292.26024899910686`, -0.0015708233938743765`, 299.4713738430625`, 48.86943240833744`,
 13.724054717184671`, 14.025231153235431`, 0.021945149757647053`, 0.15629758798930107`,
 0.15676237683457356`, 0.002973742917288691`, 229.41820547901182`, 0.5520704787067243`, 5.966747025097044`,
 0.0012492877785436063`, 0.0012704870060788152`, 0.016969050605715852`, 0.7314643741851911`},
{0.1494891985884202`, 3.3979182405498527`, 4.585626936341594`, 1.2118171179612203`, 0.6586829775426448`, 0.36431745127605797`,
 0.33277789805879787`, 51.20287439819706`, 0.10641801334172246`, 7.126742388333813`, 2370.1658637460378`, 2342.756799405454`,
 -0.011564196734005772`, 1522.1314978828116`, 435.6043189928359`, 39.01421330998662`, 39.52726560557655`, 0.013150394486068029`,
 0.5522192768160721`, 0.5513562754990406`, -0.0015627873804174852`, 1893.8158149529593`, 1.1792973876593638`,
 29.41536425065522`, 0.005478465976095914`, 0.005511915497484267`, 0.006105636419812 21`, 0.504423698257343`},
{0.2014310029114028`, 3.9869430882246055`, 0.7136208449188892`, 1.3208947167282403`, 0.46844054682093383`,
 0.39516715199771224`, 0.14154388607116256`, 2748.591928872397`, 0.05354911946309293`, 6.211900336444712`,
 31.318540097643204`, 31.362363258604464`, 0.0013992721507654071`, 23.074990172810626`, 10.611027146059469`,
 0.3534393190648103 5`, 0.3615690689078102`, 0.023001826351722654`, 0.05763046151092948`,
```

0.058165035638580304`, 0.009275895310146831`, 20.130606432175096`, 0.1658365951484986`, 0.49472768499064385`,
0.0005159570588514351`, 0.0005369367070682999`, 0.040661616808901346`, 0.6777759091273408`},
{0.13228384635594964`, 2.6408616938406997`, 3.2993392931538836`, 0.952940068464383`, 0.8367144819205878`, 0.6111943245350302`,
0.13146835864980566`, 2246.7842161758563`, 0.024436379332296754`, 9.73655688789367`, 147.78990737212982`, 141.11053873505804`,
-0.04519502553211141`, 69.471050481799`, 51.909617898188976`, 2.4737755933271157`, 2.490289565277872`, 0.006675614390934115`,
0.027469612451770525`, 0.027508536856662727`, 0.0014169986912098143`, 93.32713147965197`, 0.05191122847175428`,
0.9053663497856034`, 0.0003470220092399723`, 0.0003496439229575571`, 0.00755546809070462`, 0.5698680908513771`},
{0.23113058156717692`, 2.2605125411397546`, 4.002625660970853`, 1.3839850516092966`, 0.507564008077771`, 0.4982541689482688`,
0.0228570122346136`, 138.3250229234806`, 0.07006503343759235`, 8.064244271550876`, 943.2451795482298`, 961.1811711253683`,
0.01901519558862641`, 535.3347243556718`, 713.1385076586868`, 6.872725422415488`, 6.973903685168476`, 0.014721708861377358`,
0.25341267039839044`, 0.2536108918897162`, 0.000782208289009878`, 221.9411715597173`, 0.8367345412235716`,
12.75015270823595`, 0.0022004236160432233`, 0.0022209027251169884`, 0.009306893874639677`, 0.8716592353822314`},
{0.17570245022422137`, 3.187809299248391`, 9.693328941627644`, 1.4591302419173326`, 0.2844948752823342`, 0.5066677812093952`,
0.2998214113578054`, 349.3700788988757`, 0.18995614940943706`, 5.5658132930392785`, 202.39605053060905`, 205.27011879352287`,
0.014200219101998535`, 166.43240717159415`, 41.052358409733955`, 3.464177282052593`, 3.5256971738446565`, 0.017758875133437657`,
0.034307811544910735`, 0.03434463692519686`, 0.001073381793470496`, 157.75909362817538`, 0.08611380786104328`,
3.6018615125060687`, 0.0002833735019204253`, 0.0002851110447365162`, 0.006131634765832228`, 0.8099920880498561`},
{0.2497541467888837`, 1.085030464715203`, 1.0043906551890043`, 0.864213346242746`, 0.8846254264305906`, 0.601718469375484`,
0.012654582561683364`, 62.423429857566624`, 0.12690014020771856`, 6.991109163451483`, 4308.56511692905`, 4013.3627399917723`,
-0.06851524090407268`, 2820.6621583021756`, 3637.4594432211966`, 39.59620819947328`, 39.977731656971066`, 0.009635353354437104`,
3.3746280332519953`, 3.3174005379172704`, -0.01695816391342453`, 613.7584597662062`, 12.040390645352856`,
23.33769064184784`, 0.04542372926318572`, 0.04663344571120359`, 0.026631817062151786`, 0.39010555796465474`},
{0.22558722869536918`, 1.12607467611575`, 0.7911165205665469`, 1.2733751455833207`, 0.6009341950827205`, 0.6641796486046712`,
0.175358669032309`, 3424.3638773504586`, 0.14969161390071423`, 8.91095466688921`, 57.237337816394835`, 58.21755736520024`,
0.017125526556629023`, 29.398128667119796`, 16.65193675864071`, 2.359437921923802`, 2.3941149797852144`, 0.014697168990628784`,
0.0639882977011776`, 0.06459807989236577`, 0.009529589207636358`, 37.955761339222306`, 0.2062134678192584`,
0.5732596116484949`, 0.0005935674718992034`, 0.0006170613289505874`, 0.03958076910146713`, 1.0351628335742782`},
{0.050627269144448594`, 3.276563036391985`, 7.788230793264948`, 0.8177022370463224`, 0.523932893406847`, 0.34959907144360225`,
0.11342446098213065`, 1447.0777642274236`, 0.2385428599947077`, 6.8056995790460695`, 64.71601993666037`, 65.02189070867793`,
0.004726353263332816 5`, 43.52146645452181`, 25.302432176829647`, 0.8241874480494701`, 0.8370671579219132`, 0.015627160912149662`,
0.00626410688636846 4`, 0.006266803744213348 5`, 0.00043052551525790683`, 38.57860181910201`, 0.0045304946469261545`,

```
  1.0070375034452068`, 0.00009255733030721913`, 0.0000927926986587581`, 0.002542946633807608`, 0.5777590887790477`},
 {0.060006217186970356`, 3.9647259629353027`, 0.34014645604634985`, 0.9146780185619144`, 0.0343140190064428 2`,
  0.5737721437004861`, 0.026766328146322068`, 169.17748604048344`, 0.08721098377832243`, 3.968142168145161`, 262.22698557961957`,
  258.54549957059066`, -0.014039310259740923`, 302.4507416821022`, 189.65000274547643`, 1.2236329837934796`, 1.2584208806050783`,
  0.02843000905692339`, 1.0815353460598829`, 1.106872437349181`, 0.023426965546343936`, 69.3052779992857`, 0.927126355300679`,
  5.753152215250934`, 0.013426756431344722`, 0.014765147880605869`, 0.09968092115953464`, 0.9632638976826579`},
 {0.15350947465095238`, 2.9043684969368213`, 4.5670292387828155`, 1.3486905179026798`, 0.8383532909017835`, 0.5087310077763043`,
  0.053857845014854355`, 402.8647291760491`, 0.025030223476360525`, 5.400937139619534`, 396.26124243963244`, 377.27283507788 7`,
  -0.0479189113849261`, 335.7971214422917`, 224.91529531799534`, 4.022213360631607`, 4.0551816873904745`, 0.008196563385113542`,
  0.13562912908358377`, 0.13587464146460373`, 0.001810174426974731`, 166.885568179669`, 0.29743366218536327`,
  2.796913848276764`, 0.001207462630818168`, 0.001220934243346460 7`, 0.011156960210987554`, 0.30807109078869555`},
 {0.2587691832069661`, 1.4843472048382624`, 4.771777259704052`, 1.1550607981645458`, 0.08010207781888057`, 0.5149452814008634`,
  0.01659115431008023`, 1531.656314816295`, 0.15778107864815993`, 1.195197590112899 3`, 8.112572636128597`, 8.192818148050538`,
  0.009891499961994121`, 31.0658602443012`, 6.549537120296323`, 0.06810277284406643`, 0.07000219778197196`, 0.027890566838660202`,
  0.010163972724565786`, 0.010253708361712433`, 0.008828795548591062`, 1.4441165787546435`, 0.03757318457248243`,
  0.18507385545656907`, 0.00010418286175362113`, 0.00010810662732867039`, 0.03766229405685228 6`, 0.24104807726885685`},
 {0.1547206763399963`, 3.3076918921708103`, 2.0706333626337727`, 1.4168143839445906`, 0.37770487420755305`,
  0.5386802863405199`, 0.00701518941055165`, 122.30089778268855`, 0.134914758355765`, 2.269217335741727`, 263.1164010142079`,
  267.43424716417223`, 0.016410402898948018`, 530.684400935631`, 237.87426489702472`, 0.4856636368087616`, 0.4966940326942812`,
  0.022712006931379536`, 0.4895257053132061`, 0.4950778011752018`, 0.011341786144699606`, 22.94893819706469`, 1.0819964030267635`,
  4.2289600395056945`, 0.004049110390208277`, 0.004255809821263966`, 0.05104810961823558`, 0.3512910868194726`},
 {0.1424711737630725`, 2.7520949793854754`, 4.596768650339829`, 0.8362726667059688`, 0.00009639230672808807`,
  0.36579283504807614`, 0.010146114868681232`, 3412.101956455065`, 0.24682488221918303`, 8.269337606653004`,
  21.12267497156553`, 21.285134963013444`, 0.0076912603004406 5`, 11.690758467904221`, 18.493074818111328`,
  0.06464997576752916`, 0.06632734762113889`, 0.025945436695000268`, 0.0029141501119565355`,
  0.0029161817749276875`, 0.000697171694353127`, 2.5417553389601393`, 0.005931176956750103`, 0.5508368459640721`,
  0.000042070711239849956`, 0.00004223175590838853`, 0.003827952126134626`, 1.2535205586737437`},
 {0.10882832688629612`, 2.4842578785622536`, 4.2235021418772565`, 1.4458161330640105`, 0.36880250968204553`, 0.4736058884190225`,
  0.023130853597073738`, 1306.5861668001219`, 0.20634842182153856`, 4.016571058814693`, 40.507446464382475`, 41.33134115477876`,
  0.020339339116839383`, 46.15767306419434`, 30.562065086705125`, 0.2708124847285259`, 0.27571871220644856`, 0.018116696070496374`,
  0.021570330298561435`, 0.0216446061414573`, 0.003443426311409814`, 9.610972125712284`, 0.03353518509681479`,
```

0.6865735554300405`, 0.00017871525847867886`, 0.00018147645436937052`, 0.015450252620825111`, 0.5285709929313601`},
{0.18639271747297592`, 2.4942906430499407`, 3.445345566170081`, 1.148752163319608`, 0.12885074455456724`,
0.17966201189701259`, 0.0741834712240346`, 1169.2351269821615`, 0.09106655178874168`, 4.423161126411237`,
27.63007201429066`, 27.640218284656402`, 0.00036721838294506703`, 28.589973409048955`, 13.792069029075051`,
0.37644453229817804`, 0.3880650074890565`, 0.030869023704331644`, 0.013038426228111471`,
0.013053105309239999`, 0.00112583228004004`, 13.413743921980805`, 0.0347181099461264 8`, 0.8082966695476125`,
0.00013685311653455212`, 0.00013779392186764354`, 0.00687456272034459`, 0.3652890938147585`},
{0.09766181155973352`, 3.6268090588227118`, 4.041820185573664`, 1.3253596170020208`, 0.4793934216010949`,
0.49032555462271876`, 0.006759043878711846`, 262.0748117860937`, 0.11178249583803845`, 2.0681460387230555`, 118.4399212610835 5`,
120.34155981324187`, 0.016055722867008848`, 262.10867932094646`, 107.80497268997564`, 0.1940099111471721`, 0.1974807826115991 8`,
0.01789017604257425 7`, 0.1168988386116336`, 0.1176149779615057`, 0.006126145976961261`, 10.05195576071362`, 0.16309360497200803`,
1.74784365840998`, 0.0010487594777680576`, 0.0010768457820347062`, 0.026780501022428016`, 0.2527206711760913`},
{0.07951891239009712`, 2.8897027315487103`, 1.5411848578052467`, 0.863635998632232`, 0.29085460372381067`, 0.43469992564602655`,
0.009145988318489378`, 180.1504573795744`, 0.0803419958474173`, 8.628584719983632`, 570.6536570727483`, 580.9736929095984`,
0.018084587225442927`, 302.68962261074114`, 505.074543076075`, 1.529255679150221`, 1.5596397208285613`, 0.0198685164898155 8`,
0.23226125568994055`, 0.23246317213859044`, 0.000869350542560765`, 63.129918761096704`, 0.2638451777546208`,
10.70073040519018`, 0.003225106751961415`, 0.003261308603103073 6`, 0.01122500863564957`, 1.1386387634216077`},
{0.07234637915492992`, 1.3584521644778373`, 7.973775020047323`, 0.7807691250526536`, 0.9100932527758132`,
0.18771974645581058`, 0.009648266694560257`, 1956.923094358971`, 0.1277442569760165`, 5.404137919151016`,
45.79239568405845`, 44.682124743129584`, -0.024245749197947752`, 38.782110129539014`, 40.27574179240898`,
0.268221896832218`, 0.27183080659535835`, 0.013454940874562116`, 0.0052063593635851505`,
0.005207728316305824`, 0.0002629385766661496`, 5.205237375886824`, 0.005380874979066864`, 0.5640898460945795`,
0.00008062315817358723`, 0.00008076376473581006`, 0.0017439972014008376`, 0.18885634723063333`},
{0.26334607729010473`, 1.8094502738990368`, 2.2460302448509974`, 1.2023222076508349`, 0.5513938303777435`,
0.6812297944975665`, 0.00739497505047526`, 1157.7965975362908`, 0.15524857884582421`, 1.7991882609973402`, 31.151116162007746`,
31.745236205426973`, 0.019072191196276345`, 79.24310752983465`, 27.9382914198103`, 0.10858894181097639`, 0.11085741849684944`,
0.020890494446679675`, 0.056766823482410786`, 0.05767391685411422`, 0.015979287126828 5`, 2.806947007175393`, 0.2135617183473295`,
0.35016191523729534`, 0.0005515192383225287`, 0.0005858615491007243`, 0.06226856361828714`, 0.2549208618467421`},
{0.22679920860671537`, 1.574644711817462`, 3.814781873556772`, 0.9990271813793515`, 0.3871644432916528`, 0.6894928310515707`,
0.05462293330962511 6`, 69.61156300085906`, 0.09745707491156125`, 5.357011938436007`, 1264.418589585801`, 1312.446590648646`,
0.0379842573166990 8`, 1080.271087371288`, 716.6017879436479`, 23.2458331432397`, 23.588149333931643`, 0.01472591619248953`,

0.3873107195087097`, 0.3875558090013368`, 0.0006327981134577332`, 522.9132604399073`, 1.2548823524205353`,
17.59634603034544`, 0.004639249950052604`, 0.004702319564200634`, 0.013594786835599448`, 0.8491944239640179`},
{0.054135826493752204`, 3.1195801041969027`, 7.2615169638158825`, 1.0984819310653413`, 0.10801447255588448`, 0.6366822484935735`,
0.01066160509264612`, 211.97043506851273`, 0.24501368432397974`, 8.92838638172081`, 514.0838891640927`, 526.5396726204798`,
0.02422908735117213`, 263.5272390868761`, 446.9659880903508`, 1.4635681198873456`, 1.4912630337645811`, 0.018922873148786046`,
0.05463618046241133`, 0.054672362037198566`, 0.0006622273826795144`, 65.22454268482043`, 0.04225392551136213`,
9.991024986214747`, 0.0005998067097017223`, 0.000602625151120305`, 0.00469891612246931`, 1.8018099790294195`},
{0.14590720393667378`, 1.5766246964498798`, 5.158062459855282`, 1.1478320866243528`, 0.3287329300123256`, 0.4460050972404973`,
0.032458645789603134`, 482.4725013461066`, 0.10279090509715616`, 8.020019312365584`, 207.31664646948747`, 211.48674349165788`,
0.020114627036397437`, 118.31049072464621`, 142.6700291030151`, 2.7426758821223576`, 2.7920254684945056`, 0.01799322577407869`,
0.03606159199790619`, 0.03609444916458332`, 0.0009111402147481407`, 61.77386471587944`, 0.07516637225606657`,
3.6982506398681014`, 0.00037876745024012504`, 0.0003808778847363862`, 0.005571847567480415`, 1.0204554885332786`},
{0.14965591009740292`, 1.460367057362724`, 7.337474105676788`, 0.8826427720923014`, 0.44478408083773613`, 0.631222883334907`,
0.365159753458356`, 157.97713196457462`, 0.16784289093699323`, 1.7957284623930878`, 187.2513741450132`, 191.74330634391688`,
0.023988780960427114`, 477.2531920764115`, 32.14436871831874`, 7.083444058957767`, 7.18723140818183`, 0.014652102615649643`,
0.07839962609150421`, 0.07864536986432431`, 0.003134501847410487`, 147.77754794819492`, 0.1676138199142123`,
2.564852276008406`, 0.0010629099971906086`, 0.0010800686434629776`, 0.016143084849818967`, 0.2572241047551177`},
{0.0810328468371882`, 1.4659479028658335`, 3.234238812997848`, 1.4263751048195057`, 0.08355223050311089`, 0.5582345497973803`,
0.12450549955799516`, 121.80826494117302`, 0.16010939682803116`, 9.339681585379608`, 866.7781632447746`, 877.4095169008085`,
0.012265368587777381`, 424.75684474547535`, 322.3055503661233`, 24.78882617573275`, 25.286351236647224`, 0.020070537321429605`,
0.2564730922968793`, 0.2567929121437504`, 0.0012469918150355142`, 519.1303963831592`, 0.29689635437080286`,
18.00242589826498`, 0.0021581114813811197`, 0.002180807836189391`, 0.010516766628638763`, 1.7707293501788561`},
{0.15633465648304357`, 2.148716979150252`, 1.5640986685796054`, 1.3949237088061017`, 0.3567264406830608`,
0.23012843132782612`, 0.08829396925964683`, 2593.62904544209`, 0.24948663022889017`, 4.657794257043932`, 17.186378573936935`,
17.167498804570332`, -0.001098530984022128`, 16.887624648831558`, 7.779182920455686`, 0.2947028603011137`, 0.3024455359951742`,
0.02627282166908529`, 0.020503943286857826`, 0.02057921715640812`, 0.0036711899022146`, 9.0461862819021`, 0.0457925275756935`,
0.38418962809800933`, 0.00017608327054408068`, 0.00017928894797039196`, 0.018205462770006564`, 0.39086232781187846`},
{0.24418896051468997`, 0.7850580710666257`, 9.594718039690303`, 1.3796447707658`, 0.3888924613344984`,
0.22950540339413805`, 0.26843428081063014`, 169.97373853516677`, 0.16535615066521753`, 5.752488029735448`,
334.38697970942934`, 333.6756888488899`, -0.0021271487937645217`, 266.04685516619554`,
74.68082113370129`, 21.241785650339157`, 21.712779294077674`, 0.02217297789797601`, 0.05242334471499508`,

0.05243799498782274`, 0.00027946085674823706`, 238.22907526665713`, 0.1828743150385664`, 7.192440097918599`,
0.000458989955128275`, 0.0004604420837326342`, 0.00316374811286102`, 0.4416433632741081`},
{0.161324838944309`, 0.8594526582032156`, 2.9743924517148237`, 1.2482811580906663`, 0.28485670242217753`, 0.27691573296739147`,
0.12631097975241765`, 80.56658004023716`, 0.24230111347476613`, 7.982613747504004`, 967.2887375353962`, 972.8237758702663`,
0.0057221935404027`, 554.5944385026663`, 357.8473180048104`, 45.819553803256234`, 46.873071254972494`, 0.022992747948614722`,
0.31899665636808056`, 0.31891496144940806`, -0.000256099608073046`, 562.5676759127682`, 0.7351726316050718`,
21.453377858358582`, 0.0030753892920917236`, 0.003096180973160015`, 0.006760666404658666`, 0.774741027581095`},
{0.26772168843838734`, 1.7983273472260084`, 5.340026177070573`, 1.4142901992580086`, 0.4537298076184495`, 0.2865003384437863`,
0.010579448173665554`, 2804.399167772796`, 0.15280115052346555`, 9.9218473198287`, 41.419036711978876`, 41.59375958663582`,
0.00421841956083968 1`, 19.106095719769915`, 36.02542824241342`, 0.19981327479462566`, 0.20374351294308152`, 0.01966955475053145`,
0.006930939035159501`, 0.006935452192445675`, 0.0006511610134325441`, 5.133281091456574`, 0.02650803858508846`,
0.7552424013767709`, 0.000059174425366403 94`, 0.000059412477227303424`, 0.004022884200826526`, 0.8093585680697615`},
{0.18205384870834052`, 1.3062696845921566`, 5.075170850216699`, 0.8732096494236974`, 0.8323770222253974`,
0.3345416157316672`, 0.015614629383397784`, 819.3644184370967`, 0.04873823503517111`, 3.447951588629733`,
90.13842023166414`, 86.42122963933532`, -0.041238692477361893`, 119.65015831283142`, 73.74395336594921`,
0.8267935132890389`, 0.8361575938140755`, 0.011325778897061767`, 0.028170750825743208`,
0.02820656420635755`, 0.0012712966308874307`, 15.428790026098778`, 0.07326562298328367`, 0.8725250627900408`,
0.00038856843971080135`, 0.00039148191880773584`, 0.007497981820404398`, 0.17428943163776467`},
{0.15466361671507556`, 2.9485738979940246`, 0.2621130142172472`, 1.4016672383633995`, 0.3670414989298545`, 0.40252781093021794`,
0.06082671195258038`, 80.30981146445014`, 0.15720245773080782`, 9.014567082907547`, 1434.7153063803532`, 1430.9542983656359`,
-0.002621431581577127`, 728.4258532911508`, 769.7529410229231`, 15.025676974174965`, 15.468915736410771`, 0.029498754897873347`,
5.2992948719932755`, 5.280075279759827`, -0.0036268206804311864`, 632.9188417963171`, 11.708687299174338`,
25.107522454037138`, 0.04304950919177 6525`, 0.04614725409730571`, 0.07195772875665973`, 1.2017602508498682`},
{0.07129651605023579`, 1.2229228380041173`, 7.261778729610821`, 1.077075564756948`, 0.8974689473036932`, 0.5583016490383189`,
0.012773781923945723`, 355.9690714870232`, 0.15455215759656415`, 2.741649121941279`, 278.74510663561955`, 260.0976679595555`,
-0.06689781535946493`, 465.3287372603535`, 235.6881005478547`, 2.3127236319233213`, 2.3289458144183426`, 0.007014319511030598`,
0.09440999606743229`, 0.0946071760159272 7`, 0.0020885494831939244`, 40.40403639244079`, 0.09615862571322421`,
1.5976153945608085`, 0.0010520180401021317`, 0.0010640596141393342`, 0.011446166869945928`, 0.13977266660775728`},
{0.11901300846945845`, 0.5315793015902206`, 2.6671039936143597`, 1.3880864279120932`, 0.8420811291146328`,
0.5566538571030129`, 0.2079188053795858`, 171.97845991450583`, 0.13095338568755877`, 2.9760745568663016`, 543.8175442825769`,
514.8047678466262`, -0.0533502030984038 8`, 836.3227331863164`, 139.27629800851193`, 46.8865071467361`, 47.340648859190644`,

0.009685978762147052`, 0.5922080067611922`, 0.5952831400521147`, 0.005192657403841006`, 356.0556674723828`, 1.0068636646335842`,
3.6380670068834187`, 0.005050654167744861`, 0.005206945715451876`, 0.030944812793785026`, 0.1939825260756924`},
{0.23041310070356935`, 3.6790901828404454`, 2.4540732736358937`, 1.23507526691096`, 0.821599531342406`,
0.33616875277136504`, 0.035023029642451846`, 3607.9630264867633`, 0.21590997641376625`, 4.226227139273737`,
24.922161731421134`, 23.894598744868432`, -0.04123089311539063`, 26.98965729799373`, 16.637773551482397`,
0.15316320113490317`, 0.1551768858491661`, 0.013147314102486929`, 0.018511072230101375`,
0.018572516800804628`, 0.003319341523789987`, 8.050017566683412`, 0.060931336426929134`, 0.24354052420719213`,
0.00017965876920933432`, 0.00018262445883106005`, 0.01650734687083477`, 0.22577790946917833`},
{0.07203443304476603`, 0.5285696054477942`, 8.334423467085383`, 0.8854719875782417`, 0.3459640631982801`, 0.2633909150103768`,
0.03987288196821301`, 568.5739309534505`, 0.056948467212013576`, 6.873946140568169`, 121.3149272711857`, 122.05475352084504`,
0.006098394206720759`, 80.77419004543601`, 78.18484649330108`, 5.040376955915777`, 5.148547607609389`, 0.021460825775472037`,
0.011765828594136564`, 0.011769148860019778`, 0.0002821956699987638`, 38.05985798423653`, 0.012107782744073467`,
2.5957254683819193`, 0.00016060821373431455`, 0.00016093338515792653`, 0.0020246251175539687`, 0.6037538154831483`},
{0.16979941581750402`, 2.100179291019132`, 5.184869462011486`, 1.1997264241396737`, 0.9307785088479614`,
0.3430074594655337`, 0.013577650230569586`, 4088.01556659017`, 0.029357726641530413`, 9.133680945256565`,
61.405867305792285`, 58.71573319441848`, -0.043809072803700166`, 30.770073068299123`, 51.45108094682305`,
0.3190329883916634`, 0.32177089225517896`, 0.008581883263289347`, 0.009753982372265243`,
0.00976101645728742`, 0.00072115006504192`, 9.571806791030273`, 0.023660292981501085`, 0.45821400186877925`,
0.00009813966185079348`, 0.00009854618096765375`, 0.004142251045029299`, 0.3571279829227824`},
{0.21619782984172958`, 1.7733623067535955`, 0.5980153626590834`, 0.9798687198034648`, 0.7538483940425484`,
0.5789929247394512`, 0.006264547120133815`, 61.29286164809138`, 0.15848957716004353`, 2.3238471342304834`, 1021.5933159128615`,
977.8571045834494`, -0.042811763397581415`, 2012.0324523644354`, 920.8236910527768`, 3.101485879868382`, 3.186635313733701`,
0.02745439997583765`, 4.432659866355082`, 4.441804810314639`, 0.0020630827167609223`, 78.57225934695859`, 13.690449193321326`,
8.269201804793903`, 0.050739811939116364`, 0.0558691080562995`, 0.10109016807823168`, 0.2441576137205167`},
{0.04816719755022911`, 1.0519290863610147`, 3.0181083809828984`, 0.7801564927089879`, 0.4325460609784135`, 0.2938523943485086`,
0.04249246462941157`, 626.2418769059092`, 0.2424260119832079`, 9.623530958234195`, 177.4029750081191`, 178.6150138023847`,
0.006832122145697506`, 84.37056268093933`, 111.56712035806208`, 4.104104228836826`, 4.181017841670048`, 0.01874065777686651`,
0.029892918001241622`, 0.029907003967643044`, 0.0004712141652023494`, 61.674665882438624`, 0.020569401238816638`,
3.29143651199255`, 0.00046254056107852826`, 0.0004642000808649337`, 0.003587836237617381`, 0.8200616296326847`},
{0.2279802782152826`, 2.528677960359964`, 2.539878173580604`, 0.7527299875368043`, 0.8436281080694634`,
0.27514078202436143`, 0.005327161585143342`, 920.5142066248217`, 0.04689927589992776`, 5.404864622946203`,

114.27343294427182`, 109.89088987851198`, -0.038351373130595334`, 96.76648116252247`, 106.01100953624224`,
0.2148367056159386`, 0.2176883153330412`, 0.013273382259921584`, 0.03924059020121395`,
0.03928022910428409 5`, 0.0010101505320612159`, 7.760754893819509`, 0.12780115244863482`, 1.2114573129183555`,
0.0006281082038068764`, 0.0006326465735038975`, 0.007225458399547469`, 0.24529795884124123`},
{0.18595171467721877`, 2.938499684754029`, 3.6306522502244434`, 1.1726699653227697`, 0.9888859131507246`,
0.3480894711653968`, 0.38543337220237905`, 80.27026890715871`, 0.03443629355490174`, 9.328937618149844`, 3593.3746935786753`,
3553.0445953329704`, -0.01122346030815391`, 1762.9289541178978`, 571.9524883896981`, 70.23407809709796`, 70.75791686856188`,
0.007458470099653169`, 0.782774535596237`, 0.7804401603810305`, -0.002982180831199832`, 2948.3259478187415`, 2.079403815712819`,
23.749962920028832`, 0.00801827851109671`, 0.008063048177593238`, 0.005583451165305675`, 0.33023632748731624`},
{0.2088003110428162`, 1.665360213541189`, 8.163801498355127`, 1.2665930603692175`, 0.5382347880039544`, 0.33255923205592086`,
0.03591580004997877`, 88.64295388071032`, 0.08847100949056225`, 8.845547783450662`, 1374.6815429276276`, 1378.4534169301235`,
0.002743816574755975`, 711.2820737966288`, 915.1436381973523`, 18.50858965841527`, 18.803353012382278`, 0.01592575984486122`,
0.15133609808842316`, 0.1513055235892789`, -0.0002020304443582921`, 440.3352689412101`, 0.4514146336125288`,
21.273999199200556`, 0.0014424566895426816`, 0.0014467667685914554`, 0.002988012797902506`, 0.7091222089059787`},
{0.21556335034608287`, 3.553132086531601`, 5.919169032409666`, 1.028261766481214`, 0.01873638959650825`, 0.38505243852661264`,
0.12008970777658311`, 72.3007614977653`, 0.1189730390969882`, 5.747570490343056`, 714.8640386150607`, 717.1005645461606`,
0.003128603217239511 7`, 569.2507856574742`, 273.6669725005647`, 8.513379063849328`, 8.725383176850759`, 0.024902463688204 7`,
0.13550811078107763`, 0.13554054057286954`, 0.00023931993151538045`, 432.1308616652779`, 0.41729403370052853`,
18.142337651356527`, 0.0015881707495978281`, 0.001596952483411053`, 0.005529464521021099`, 0.8984006368730754`},
{0.2061330284551588`, 3.7571620320677477`, 5.463889035312752`, 1.195880973038654`, 0.4606689625278244`, 0.6539206160067124`,
0.07294517832285098`, 79.70328938032284`, 0.04960591713533524`, 2.0731473047959454`, 445.1422109998676`, 459.08425823594456`,
0.03132043399065809`, 982.7275058842167`, 220.2973581814657`, 4.0913275144774595`, 4.154770588743907`, 0.015506720995068113`,
0.29295775121812495`, 0.2942685612559125`, 0.004474399575833621`, 219.5968628306968`, 0.8626895495432441`,
5.883163861436455`, 0.0029134381834953205`, 0.0029866225418570397`, 0.025119585092386654`, 0.30265075558505716`},
{0.13099744861910317`, 3.0069806711745155`, 9.6713740346189`, 0.7856145676036781`, 0.3887426527541791`,
0.16465925249784186`, 0.029006890169876405`, 3080.757337617379`, 0.22037025279153283`, 7.1587428033756435`,
20.289033969010653`, 20.26560233854502`, -0.001154891381295986`, 12.971467277050897`, 14.479842071898004`,
0.13200530317176642`, 0.13522917305951998`, 0.02442227554720744`, 0.0014448184016942485`, 0.0014450414496886453`,
0.00015437787484939847`, 5.670534216143344`, 0.0027038217762900187`, 0.48640080497040683`,
0.00002224747009182959`, 0.000022271528425028948`, 0.0010813963610267763`, 0.433570298895837`},
{0.20740821458680525`, 2.460110895835281`, 0.6082454159142117`, 0.8064214695794831`, 0.18139487742338534`,

```
  0.30057404623847583`, 0.2202140246268176`, 4056.9433443728003`, 0.07208592746284093`, 2.251460510548089`,
  5.111674598512508`, 5.051929785796079`, -0.0116879139242968`, 10.39114392350121`, 1.3049947056624145`,
  0.10330326539165358`, 0.10731199303619102`, 0.038805430102709426`, 0.018380398782209862`,
  0.018624672464828934`, 0.013289901134000637`, 3.6305355537910025`, 0.0544606527830242`, 0.12403469922933763`,
  0.0002675908525068271`, 0.00028368319737626346`, 0.060137873618178794`, 0.2980950111679363`},
 {0.20705709543566436`, 1.1783515457685674`, 5.677465659011105`, 1.0594933834891802`, 0.45712361519708566`, 0.4929063565541889`,
  0.07712216778297526`, 129.94158279886685`, 0.033100299542402456`, 5.957665662265306`, 686.9109098779845`, 701.207515052265`,
  0.020812895775407014`, 527.7020500579611`, 331.69063219218776`, 19.888602050076848`, 20.191224785964824`, 0.015215887729364441`,
  0.13489401288341307`, 0.13497498896488516`, 0.000600294110473909`, 334.79664241262844`, 0.39901089284699964`,
  10.09947674762608`, 0.001532811965096359`, 0.0015434172966914976`, 0.006918873179902363`, 0.6915337751376983`},
 {0.04662251177018917`, 1.087376542683649`, 6.170097112149202`, 0.7648803529340509`, 0.9868309112238562`, 0.551889656411283`,
  0.011192508637080182`, 2374.3826064993154`, 0.12804730636711914`, 7.114439815654695`, 136.41411963908118`, 133.7213940082184`,
  -0.019739346909154243`, 87.75727099635975`, 117.60019802836888`, 1.13127855144332`, 1.1373500908600858`, 0.005366971210600369`,
  0.014908917722335078`, 0.01491983946992605`, 0.0007325647504654587`, 17.573225144008603`, 0.0099298741712969`,
  0.6137876467636681`, 0.0002351972521376089`, 0.00023617841708046983`, 0.0041716683929915455`, 0.26955152897476703`},
 {0.061725502446144886`, 3.13849642385113`, 9.540008859499451`, 1.4660101376451562`, 0.45012796893408935`, 0.4212144239280121`,
  0.02511250628464842`, 120.07361261472948`, 0.1571099365418398`, 6.445559562130283`, 738.0098603086468`, 749.8244933861907`,
  0.016008774019093508`, 524.0419246516225`, 545.903841191113`, 4.183843195247373`, 4.251195500562687`, 0.016098190628134113`,
  0.11038240808986678`, 0.1104308973069647`, 0.00043928392156877827`, 187.58538437482537`, 0.0973344228650437`,
  11.71753265536368`, 0.0009081040583911459`, 0.0009121501893098345`, 0.004455580702785289`, 0.6830705882966607`},
 {0.047554055600075584`, 3.265459078802472`, 8.714860138547323`, 0.8369196863770172`, 0.4962846406178121`,
  0.46335229122842925`, 0.007763546254085084`, 1888.52152221599`, 0.22218896292653229`, 2.633616973587875`,
  20.753322792220196`, 21.11936622378476`, 0.017637822879225107`, 36.06612828777208`, 18.688130351212656`,
  0.04280782335233351`, 0.04348246454193145`, 0.015759763911499247`, 0.004742190856713481`,
  0.004749136290134819`, 0.0014646043635098138`, 1.9969599344235704`, 0.0032215772523755716`, 0.3052430474881168`,
  0.000068243224740083 26`, 0.00006872079880966655`, 0.0069981169764794515`, 0.2871038885045652`},
 {0.12567467788677716`, 3.0158399320010547`, 4.901658754742801`, 1.351978052591392`, 0.7221310556231506`, 0.5024953321675816`,
  0.09886516020555723`, 193.78321929587645`, 0.11023982026692764`, 3.349767411033996`, 387.109280800168`, 381.57357006751465`,
  -0.014300124040453999`, 528.9120055946362`, 162.36568490415084`, 5.0855036790740895`, 5.141163870699469`, 0.010944872944328221`,
  0.1992873767767348`, 0.19982120026845782`, 0.0026786618417937724`, 219.1009295669984 2`, 0.35779109833380546`,
  3.6760890077666426`, 0.0017641911060295445`, 0.0017917996587121664`, 0.015649411556527548`, 0.2595453591715719`},
```

```
{0.19691840575768232`, 1.6501723246031572`, 9.011299029553381`, 1.1479337267630148`, 0.646058584475099`,
  0.36485695404700413`, 0.027042804382441818`, 3550.5063392884595`, 0.2269993732427253`, 4.86925454673478`,
  22.581566135528416`, 22.43722741236239`, -0.006391882755152789`, 21.22540937089655`, 16.356039434789967`,
  0.25262857498488495`, 0.25607579578782186`, 0.013645411264910035`, 0.0037035018657715665`,
  0.003706570469487749`, 0.0008285681572199888`, 5.955438326342731`, 0.010418395473263552`, 0.2904928084923637`,
  0.00003893451429792805`, 0.00003911269769379569`, 0.004576489499886316`, 0.3564880151493411`},
{0.22383158609367865`, 3.396592529608882`, 7.0059346900996395`, 1.1116596498970173`, 0.7389255639756462`,
  0.40169511261271784`, 0.008944445083981989`, 1191.861359234809`, 0.06327031154858104`, 2.975039312230072`,
  50.070236491068556`, 48.965372209984444`, -0.02206628844825209`, 77.02849781011986`, 44.36061013807875`,
  0.1131329024642718`, 0.11455306441568913`, 0.012728603032634567`, 0.016712031925640445`,
  0.016741943953852288`, 0.0017898498725310574`, 5.48856795214994`, 0.05343829446806341`, 0.5254850763481181`,
  0.00018086807380657266`, 0.0001825721128690681`, 0.00942144750387408`, 0.19824116602957692`},
{0.22089456196581753`, 2.0637610394456862`, 7.594372198729954`, 1.2863006630967566`, 0.5843792508368972`, 0.43898418015861007`,
  0.0169422428941798`, 186.99955924932385`, 0.09805501720710208`, 5.47194276574208`, 487.09821632094565`, 490.68848203785547`,
  0.007370722364838711`, 407.41732007063655`, 392.9210159062332`, 3.0708191654604686`, 3.1139301197770353`, 0.014038910138852945`,
  0.09450780953656678`, 0.09457976005866962`, 0.0007613182704759858`, 90.5348136122919`, 0.2982323027132284`,
  6.293020548739438`, 0.0008852960517192754`, 0.0008908338423730447`, 0.006255298036193402`, 0.49047038466135257`},
{0.09910547662807062`, 2.346178866312991`, 7.421049922557871`, 1.3147434313884303`, 0.2750558853148206`,
  0.21685738835966661`, 0.006760522552087531`, 4988.988013498636`, 0.18492351030582366`, 2.8590709623133`,
  4.888150776073563`, 4.907208130103031`, 0.0038986837563910193`, 7.824996524188507`, 4.459828043265194`,
  0.012145604194385162`, 0.012472568429349556`, 0.026920376272062896`, 0.001897567604736568`,
  0.001899811583519495`, 0.0011825553815978385`, 0.4070822839924126`, 0.0026865620271633484`, 0.12281059399498544`,
  0.000017402576391267743`, 0.000017509264274610795`, 0.006130579802918534`, 0.2452786509672849`},
{0.20816183484471307`, 3.9485705583569546`, 6.550493120626773`, 0.7774564054459185`, 0.5499519895881679`,
  0.2003723071725072`, 0.34567679795977113`, 1808.1918719614162`, 0.23441319529101012`, 4.082991759791533`,
  24.4588746287839`, 24.192625322491004`, -0.010885591031222996`, 27.417159727128602`, 4.503033166347389`,
  0.34730478842771256`, 0.3543050637245442`, 0.02015599994610695`, 0.00445820619194945`, 0.00446034409150138 5`,
  0.0004795425469092951`, 19.590820890886455`, 0.013257548300465294`, 0.4722881106495243`,
  0.00006928135073158703`, 0.00006950204205921872`, 0.0031854362725505236`, 0.24387835723757958`},
{0.16112203008102222`, 2.4895492142589735`, 0.44352326574872514`, 1.2469876196885359`, 0.9780986797319231`,
  0.6188841396088389`, 0.029743069155058133`, 112.64100571320267`, 0.13296296295840093`, 7.528063016828725`, 3401.0279921314027`,
```

3234.835991727759`, -0.0488652256870995`, 2067.718698842278`, 2371.9668905446906`, 27.402372332123004`, 27.736254603276326`,
0.012184429402921415`, 8.088224902372994`, 7.913100750472747`, -0.021651741143952186`, 974.5650644038384`,
18.617017371744222`, 13.92116214480774`, 0.07330546634624235`, 0.07741482678608154`, 0.05605803556899169`, 0.41694508792232`},
{0.17781253097781763`, 1.7213960002432414`, 4.693599924413801`, 1.2010501070587627`, 0.4002263609998786`, 0.5127527245385753`,
0.08027502692564248`, 156.85332391803223`, 0.06371789077354856`, 6.562151621871855`, 600.3248351599984`, 614.4038492724043`,
0.023452326620226227`, 418.7015264514423`, 284.1719829213257`, 12.333564301636626`, 12.531019204074102`, 0.0160095571408562`,
0.14668782559133875`, 0.1468392136984243`, 0.0010320427511640862`, 303.29926082257305`, 0.37261333617218334`,
9.317991486010863`, 0.0014687128640139546`, 0.0014813155218907548`, 0.008580749978833513`, 0.8430752590196544`},
{0.11782384895680592`, 1.1169968183756591`, 9.741344822271138`, 0.8926206302886914`, 0.732042643272727`, 0.3854502756114396`,
0.005148133378435907`, 521.4771678665235`, 0.04124959681193141`, 8.98600497767481`, 343.6409439892825`, 336.9115011609798`,
-0.01958277366538863`, 175.0260707584259`, 319.94892573020377`, 1.3720265310514081`, 1.388305298424799`, 0.011864761362097243`,
0.02199110622638465`, 0.02199555022697533`, 0.00020208172089808407`, 21.893560998734504`, 0.03701538254873666`,
3.612713201045925`, 0.00029776820829452433`, 0.00029835472778238007`, 0.001969718296036538`, 0.5593287618613524`},
{0.1669047046062308`, 1.025805255669411`, 7.1552343684117155`, 0.8176510429563385`, 0.8282389242605148`, 0.29891546072913033`,
0.009889011750027802`, 94.42085507888534`, 0.16558325818795072`, 8.472484566593426`, 1818.7334408064787`, 1751.585645804869`,
-0.036920085975784445`, 982.477832980373`, 1594.3273717917223`, 14.220630372613943`, 14.38134673325977`, 0.01130163406506468`,
0.1540776956536384`, 0.15395759970132086`, -0.0007794505999592083`, 208.3942482165637`, 0.3673756039925108`,
18.530102992165695`, 0.002275284561883084`, 0.0022800690240120684`, 0.002102797254082578`, 0.39229815845527305`},
{0.1009959491070086`, 1.5581491170930981`, 4.3701504080039815`, 1.3889561652621691`, 0.5506040634320337`, 0.37952236600496847`,
0.006611879537937531`, 361.09151974058886`, 0.21353768992235467`, 8.563496620010564`, 353.99803128999145`, 355.9007652877697`,
0.0053749846880351`, 189.1969847736476`, 323.3910174407515`, 1.2930143438262467`, 1.3135104253945606`, 0.01585139535858704`,
0.08236282324587395`, 0.0824361918817384`, 0.0008907979713788095`, 28.7815594031654`, 0.11883302149784766`,
5.082818505214662`, 0.0007145044539033085`, 0.0007188974183422455`, 0.006148267396988816`, 0.7356741846313833`},
{0.23246941421316458`, 3.062147704374147`, 7.181406357346838`, 1.3012169805011569`, 0.348059834110821`, 0.639530677915989`,
0.09989941363865933`, 83.25264127475623`, 0.015153685907020209`, 6.841473199486905`, 1249.4007389945182`, 1290.5572182112453`,
0.03294097556709352`, 835.8274367708328`, 524.833275411064`, 16.173231410458524`, 16.41831650266256`, 0.015153749178754117`,
0.20747637616982612`, 0.20758865452233866`, 0.0005411621052249238`, 707.4974776549659`, 0.689027309017427`,
18.757246812440112`, 0.0019183704274609026`, 0.0019326633852906113`, 0.0074505724364333226`, 1.0705845250821489`},
{0.05240567594084544`, 2.6092929572780967`, 9.364433651132018`, 1.4357965076882815`, 0.3985784064329525`, 0.6640850131228939`,
0.14135898686373455`, 340.71602063464445`, 0.17921846525178464`, 4.7134535629319085`, 225.07169629237026`, 232.87437211084983`,
0.034667512383893095`, 218.54765252940192`, 76.51597395434966`, 3.8791151590660164`, 3.932796067522776`, 0.013838441566061732`,

0.04586852452722745`, 0.04594742077453943`, 0.0017200519991686924`, 144.59639807173767`, 0.0343395861750597`,
3.1444516117473182`, 0.0003842044940234324`, 0.00038753749408042113`, 0.008675067857966123`, 0.7147959752473131`},
{0.186251758126929`, 2.4933710973488976`, 3.5328550575209547`, 1.325175018028979`, 0.37317622556186136`, 0.25977278720819974`,
0.09748704597661054`, 108.88467391318876`, 0.2369656062609477`, 9.08676709988832`, 863.5393548704968`, 866.529826646155`,
0.0034630405189890467`, 434.9478942449361`, 370.67505258465746`, 13.436676956242666`, 13.72569611758976`, 0.021509720170269864`,
0.22359461407559564`, 0.22357925757870142`, -0.00006868008407845405`, 478.6088852444194`, 0.5949269997059409`,
17.858219955263674`, 0.0020336781797435366`, 0.0020443747587129283`, 0.005259720577196O365`, 0.7652976140394141`},
{0.2432348147394116`, 1.8751950266382789`, 2.5453519666394815`, 1.0050719073546701`, 0.45557496345664417`,
0.6934059109337196`, 0.0796473645979629`, 116.90111969840831`, 0.13836004707768368`, 5.732847922404471`, 880.2100758504521`,
912.1779534988016`, 0.036318463654784194`, 702.7169176010566`, 416.5880975808742`, 16.62285396459929`, 16.858679813292284`,
0.014186844761749029`, 0.3791640919609322`, 0.37988211093156493`, 0.00189368926503386`, 445.3013297564421`, 1.3175129666274565`,
11.0840266149253`, 0.0045012874187247975`, 0.004584391118141247`, 0.018462206850144325`, 0.8322100303243448`},
{0.10016679782659832`, 2.355527283731478`, 1.9202752019167662`, 1.4361517107451491`, 0.5493703103299528`, 0.5953848202767148`,
0.3151585002444098`, 3628.217577021284`, 0.16393396333788363`, 2.9174291385165`, 15.030229317922302`, 15.174746573997037`,
0.009615106530837192`, 23.579239033980805`, 2.8632614351972556`, 0.3494647588735111`, 0.3549335237298407`, 0.015648973801988042`,
0.0238086691046O971`, 0.02409514350757751`, 0.01203235685745807`, 11.759625346131385`, 0.034069116353177595`,
0.17878290051346052`, 0.00019493064596098897`, 0.00020389914711741808`, 0.04600867714881507`, 0.3691313905266742`},
{0.14701675506563494`, 1.5420515267654622`, 0.49939909194610793`, 1.0246394569387973`, 0.7670831420663697`,
0.6948893607838256`, 0.005182980912963712`, 1858.6105410956102`, 0.23965764027041103`, 6.073448972699818`, 102.57353210853087`,
99.68407164880875`, -0.028169649619405046`, 77.29731673918306`, 94.8356509891533`, 0.3026627560361835`, 0.3070538166221776`,
0.014508096878194099`, 0.21258262559749294`, 0.21612261553702797`, 0.0166523013326485`, 6.667450929151988`, 0.4464743971239511`,
0.7050016161872869`, 0.0024186123541393156`, 0.0025701931842314145`, 0.06267264360602365`, 0.565523100664794 2`},
{0.19166603750475464`, 2.2499911524034095`, 2.4198836269597095`, 0.7671801018390442`, 0.14979093568204394`, 0.302546021842622`,
0.008837119919208427`, 4450.048280356515`, 0.1035437468879743`, 7.585339524339819`, 15.356361758777375`, 15.492171315973327`,
0.008843862845203443`, 9.265690817112674`, 13.651314405410673`, 0.05063770897010863`, 0.05193738075370989`, 0.0256660858090647 1`,
0.004021051579722368`, 0.004025441916650644`, 0.0010918380033759778`, 1.6276342451531887`, 0.011009986041251031`,
0.37590956623913296`, 0.00006320125163361556`, 0.00006358174176127739`, 0.0060202941844818`, 0.8902601186384134`},
{0.14239569401382052`, 2.8387649854604886`, 1.4027026171382353`, 1.2048713691971291`, 0.21617980215068333`,
0.2290581611576099`, 0.32444520029725066`, 2595.236024076599`, 0.07657553404881084`, 7.455893283173221`,
24.894647373397476`, 24.726416475940617`, -0.006757713613434468`, 15.28166905950033`, 4.8994997190162515`,
0.47987813328170764`, 0.4927765846816168`, 0.026878597930064974`, 0.017927057404325878`,

0.017966045997328702`, 0.002174846218399251`, 19.460874886403577`, 0.036467654010205144`, 0.6185953610034998`,
0.00017884739059648158`, 0.00018090654551349816`, 0.011513474757160136`, 0.6781382748550664`},
{0.14874493002083228`, 3.1275671408493704`, 8.341714492380408`, 1.302209514279003`, 0.6258906648056601`, 0.45549735048188655`,
0.04029116024037646`, 765.0281459953038`, 0.12017823562381152`, 2.4241463017742984`, 56.06965724846459`, 56.176718488103454`,
0.001909432746564521`, 105.86040938689641`, 35.78450819991692`, 0.4424761184354501`, 0.44841004953042635`, 0.01341073754660016`,
0.02258400207760864`, 0.022639637169637666`, 0.002463473561410412`, 19.7696252661327`, 0.04798936869463615`,
0.6810375810829402`, 0.00020825606110730366`, 0.00021073047466983733`, 0.011881592062085167`, 0.21565248380280225`},
{0.06222670957040222`, 3.9065456744185374`, 1.0946055811329174`, 0.8246557540237`, 0.5907991824162977`,
0.2630274030573405`, 0.006980770676502568`, 3176.3819350381427`, 0.13217715504568522`, 5.816838705721813`,
23.838553394718883`, 23.62122517692009`, -0.009116669715660608`, 18.75674052584351`, 21.658642261093142`,
0.037352982589425945`, 0.03805336607111911`, 0.018750403130898485`, 0.01929936665973796`,
0.01935372422404599`, 0.002816546535769459 7`, 2.0845876080193255`, 0.01715622977183247`, 0.38621560059214405`,
0.00028085438063107926`, 0.00028454359225694455`, 0.013135674143930576`, 0.3987871294822133`},
{0.04971186392349858`, 0.9916432478771275`, 0.9981695561230755`, 0.8524884405534577`, 0.12034821743714219`, 0.2529703866830304`,
0.3237478827730366`, 360.7437887352434`, 0.010006209625245316`, 7.274213959868414`, 168.55557228229375`, 167.06749969804162`,
-0.00882837965012484`, 106.0526532079576`, 33.38808960587647`, 8.898369965059446`, 9.13800219156778`, 0.026929901481875795`,
0.1238757996144618`, 0.1240844298157975`, 0.0016841885338785278`, 126.05726418519696`, 0.08797281276959316`,
4.401714360996595`, 0.001743860083798121`, 0.0017642751449287784`, 0.011706822881221823`, 0.7711010264138985`},
{0.1940949007109463 7`, 1.638712293913553`, 0.39703017050116074`, 0.7643187357680644`, 0.5335846868412271`, 0.21830616448247264`,
0.0963055671350066`, 635.9574479204244`, 0.22613729069446092`, 5.479828782022301`, 97.08224634874256`, 96.04922537697776`,
-0.010640678503193501`, 81.0844027814601`, 41.56153451885019`, 2.2418174681899963`, 2.3031038330945743`, 0.027337803266409333`,
0.2113892157510834`, 0.21234802983049875`, 0.004535775753784721`, 52.481340654758604`, 0.5861366977510543`,
1.8194424640180695`, 0.0032915363173819934`, 0.0033912742295407134`, 0.030301325138666346`, 0.3827503483230187`},
{0.27029725962327555`, 2.240924310463286`, 1.2051421639323703`, 1.1356504301287609`, 0.773380004303778`, 0.6607812449412671`,
0.04949916223108672`, 204.2592199369304`, 0.19899441283798802`, 7.18276351221955`, 1074.5681191366805`, 1051.070353922555`,
-0.021867171373932015`, 684.7103275176466`, 630.0095831765242`, 13.33598093018902`, 13.482774104895254`, 0.011007302385528828`,
0.8792684198438285`, 0.8815287573831628`, 0.002570702516230261`, 426.9274838619338`, 3.3951977765295895`,
7.486473994421065`, 0.009156751296816035`, 0.009434474060972755`, 0.030329835894231216`, 0.5650886022186403`},
{0.14266269539818904`, 3.072017618366117`, 3.4103252419872643`, 0.7755852439360265`, 0.43949005760262017`, 0.1924018543570919`,
0.0671210280508097 7`, 656.0250616869308`, 0.04537130588160604`, 5.285505421294628`, 77.34877946877903`, 77.13759336015913`,
-0.00273030951581010 3`, 66.97788618868344`, 40.26745459447429`, 0.824703930525573`, 0.8435239699220581`, 0.022820358555210696`,

0.020865048777293557`, 0.020876975955773838`, 0.0005716343444766014`, 36.19292863586205`, 0.04252377283121002`,
1.694287617836488`, 0.0003247693788283046`, 0.0003261254829413945`, 0.0041755910547429664`, 0.34606995067208124`},
{0.10769494377900635`, 0.832286650700361`, 9.122967245453143`, 0.9815088864732582`, 0.8421400260693508`, 0.5448142839433918`,
0.07976087110962524`, 4710.110387525812`, 0.13361893143992154`, 5.1516712920505725`, 34.094735763084664`, 32.520379808776305`,
-0.0461759247893323`, 30.290307278614613`, 16.051803899791913`, 1.3988999945780496`, 1.4087449359310973`, 0.007037630560587038`,
0.004464274162765431`, 0.00446864607716945`, 0.000979311360508106`, 16.632654159316004`, 0.006868282213898099`,
0.2280293706228133`, 0.00005485985003539362`, 0.000055133639351723394`, 0.0049907047896256`, 0.2901491455889556`},
{0.21491444350519606`, 1.295137699490157`, 7.08600395884199`, 0.8977823469890502`, 0.4181882258302472`,
0.21987671758091132`, 0.14871331978208877`, 664.8642422386233`, 0.19734382780045878`, 6.556011365474522`,
98.5083915192579`, 98.46753453055675`, -0.0004147564290821748`, 68.76984170583265`, 32.899735874915876`,
3.3617146303038483`, 3.434546839594247`, 0.021665196871221593`, 0.011942149349882676`, 0.011945709875630948`,
0.00029814781610548735`, 62.19833360905911`, 0.036664862596865584`, 2.0865010727281885`,
0.00016077497668465224`, 0.00016115572254712436`, 0.002368191060098347`, 0.47267107241628464`},
{0.14904691011609433`, 3.9586033442731177`, 2.425615791755316`, 1.0187686401741827`, 0.6625310044158794`, 0.43390068486512956`,
0.00854220404180854`, 96.98019438566024`, 0.2008209408788944`, 6.643325956478872`, 1288.2491913030913`, 1281.4291603952768`,
-0.005294030808523864`, 887.5213547029459`, 1147.3803715758718`, 2.40239359402794`, 2.434502732222786`, 0.01336547777793995`,
0.5109991447156963`, 0.5106836764089487`, -0.0006173558410221824`, 135.85890450827605`, 1.0880406227403578`,
14.580234612087315`, 0.0060082618199126525`, 0.006075833969438322`, 0.011246538774612125`, 0.5209102446563978`},
{0.21251186737978806`, 3.785250570140386`, 5.706304857162573`, 0.8638958511126991`, 0.620802282746526`,
0.4325048599697877`, 0.008982590727965567`, 699.3542650472853`, 0.09331259312392975`, 7.885660147589962`,
198.5251317052007`, 199.03964077631105`, 0.0025916571201392014`, 115.22373035536526`, 175.99871723493806`,
0.40454865647546895`, 0.4099502543307182`, 0.013352158680514092`, 0.023902725928317655`,
0.023916400661908588`, 0.0005720993342743785`, 21.87597189390423`, 0.07256589889276252`, 2.4080855830860557`,
0.0003339636825057113`, 0.0003353019344566329`, 0.004007178088589747`, 0.6471381306033075`},
{0.24515641699904833`, 3.817676488470438`, 5.668256418323743`, 1.3090245110359662`, 0.8705848781731611`, 0.5675799494003652`,
0.13511363154659006`, 1804.7556662922755`, 0.20417175811210386`, 6.336735207604568`, 122.83848524739709`, 115.65896161662529`,
-0.05844685903047586`, 88.72242168066468`, 42.35712508926359`, 1.4468440115920642`, 1.4569579494988292`, 0.00699034438110302`,
0.028029582489060775`, 0.028076515925405908`, 0.0016744250958231621`, 78.9083195077039`, 0.09816617161439123`,
0.7295970878705395`, 0.00025763887825402954`, 0.0002599159890839663`, 0.008838382022807734`, 0.3347701508479694`},
{0.16393709899357184`, 1.7364683461504136`, 6.294465981846198`, 0.7908159510076651`, 0.7611075592144951`,
0.36561813704297086`, 0.008326625223173748`, 2834.6413370290525`, 0.20899318399811945`, 1.1709155007061636`,

7.917792232127606`, 7.682628901159168`, -0.029700619070834988`, 30.948744866028736`, 7.063759426746676`,
0.03216118915685585`, 0.032616051798479304`, 0.014143215893075656`, 0.005303867318301533`,
0.005321593059727214`, 0.003342040884868336`, 0.7978126706490861`, 0.012421437451559632`, 0.08682819039538602`,
0.00008039614356913916`, 0.00008164568698640154`, 0.01554233028836527`, 0.07612684071239292`},
{0.25878729889995966`, 3.1361195765858056`, 8.880297290408144`, 1.0867236136347516`, 0.0902130745148968`, 0.5352173770607672`,
0.11164972929109021`, 57.41366920823615`, 0.17534323232121918`, 1.541035034614442`, 287.31158807250375`, 290.57224792253567`,
0.011348862995421705`, 853.3066398430929`, 114.02355604919366`, 3.768828161391641`, 3.855621436198954`, 0.023029247047232193`,
0.1424845629087506`, 0.1429024357829953`, 0.002932758929907963`, 168.84993968183133`, 0.5267599310011523`,
6.335305489275143`, 0.0015685432578467218`, 0.0015956098826429889`, 0.01725589948563111`, 0.3018795104368248`},
{0.11203607225462164`, 0.6218658244264867`, 6.440483663444866`, 1.39680689202378`, 0.16534679246130501`,
0.5081267228820908`, 0.03214478110427463`, 1906.9275025607635`, 0.21932209633484323`, 1.251120855111619`,
7.226226495545671`, 7.332463087313262`, 0.014701530851970457`, 26.434846990150046`, 4.9825042401216395`,
0.22489161384257608`, 0.23008167044830752`, 0.023078035312443568`, 0.007783332154002354`,
0.007841984976573058`, 0.007535695690507493`, 1.9978915549830942`, 0.012457342336964931`, 0.15321734876390197`,
0.00006615253087760564`, 0.00006815652124242501`, 0.030293479905207477`, 0.22922931877350333`},
{0.24357827172423074`, 3.9240029450424014`, 2.804272198831022`, 0.75931848861878`, 0.2959071515485636`, 0.6138549707668646`,
0.10713705928623629`, 339.1202760730188`, 0.035220064551167996`, 7.297254233929856`, 304.4626203808729`, 313.1650779314956`,
0.028583008120130593`, 190.95850164446406`, 123.00139518390118`, 3.17478441764933`, 3.227408445913228`, 0.016575622574984683`,
0.07074862106901848`, 0.07085141212296958`, 0.0014529054050511636`, 177.96947721043813`, 0.2461832406694443`,
4.939022711559732`, 0.0011198321291311375`, 0.0011307534708000046`, 0.009752659693146093`, 1.1890210108103547`},
{0.24204908128005126`, 1.6039606621918692`, 7.422941916054803`, 1.4757185212808246`, 0.48127457855983957`, 0.4526414340963527`,
0.017845029630840916`, 286.8959659308558`, 0.1351657578784522`, 4.5748150305594475`, 233.96803542003354`, 237.9137277519639`,
0.016864236710142144`, 234.0709316668332`, 186.83779803725412`, 1.9556585334583403`, 1.987510355989846`, 0.016287006134542148`,
0.0636596395964155`, 0.0637537541180391`, 0.0014784017349178669`, 44.811419376385984`, 0.22012510398472623`,
3.4875327023880303`, 0.0005190752582938662`, 0.0005236251757297016`, 0.008765429218858145`, 0.4946842248338376`},
{0.0765658571842559`, 1.8453576022836256`, 0.8953985896867352`, 1.0948080634821278`, 0.11609992165592442`, 0.45437290836767896`,
0.20627344098654643`, 689.3327160072474`, 0.18998846953804188`, 8.643335566773064`, 132.7464808257968`, 133.1117969029543`,
0.0027519831402305073`, 70.29203829139261`, 35.57349614398671`, 3.5423911284316154`, 3.622224496519204`, 0.022536576338744174`,
0.11710201307326219`, 0.11767374412323432`, 0.004882333232089087`, 93.38540570161949`, 0.12808594298489467`,
2.880015003775221`, 0.0012745573540741129`, 0.0013035834212668804`, 0.022773449229244846`, 1.4154598286764815`},
{0.2258389851785662`, 0.888336357939937`, 0.32961763645801767`, 0.9210788231382718`, 0.8596897387491109`,

0.5585019604624183`, 0.04381971178906294`, 415.715019082411`, 0.24817184929202513`, 7.285653344604734`, 602.593619114097`, 563.6070404303182`, -0.06469796135759776`, 378.54761982786124`, 368.28211195500586`, 16.672842220764213`, 16.945587832575622`, 0.016358675275636836`, 1.4272132112375675`, 1.436376507854928`, 0.00642041185242026`, 211.58702764144206`, 4.604576903704745`, 3.697022767251288`, 0.018004644356442334`, 0.019070372516001532`, 0.05919184730676807 5`, 0.5013466335909396`},
{0.19210976086993148`, 0.9419570933782317`, 5.851922457612897`, 1.40657571618961`, 0.8492777725664675`, 0.5465312705852027`, 0.10025464283588155`, 725.5272924080149`, 0.05639485661927951`, 6.120948580338062`, 270.7207190953261`, 256.9789261210002`, -0.05076003425318598`, 202.4264498040151`, 112.23445176652679`, 10.9457064323163`, 11.02714197527482`, 0.00743995314163 4481`, 0.06657335707862519`, 0.0666791064940788`, 0.0015884645163486422`, 147.29122594222915`, 0.1827055958383373`, 1.7579622416763292`, 0.0005692814927235057`, 0.0005744044466815936`, 0.008998982091581897`, 0.3413293198142617`},
{0.04040676752094921`, 0.7630185090261814`, 4.034875094487921`, 0.9393317585048341`, 0.5565744536568444`, 0.3001585479016161`, 0.008819784570716005`, 3975.779851508773`, 0.23610962920225392`, 2.0975088246946054`, 6.788417447009773`, 6.765789493451768`, -0.0033333179249270817`, 14.812529864425485`, 6.029958147473185`, 0.06267011539844969`, 0.06383144317724108`, 0.01853080645229066`, 0.0047114261656 9492`, 0.004724978005293038`, 0.0028763773688724736`, 0.6831208287403405`, 0.0027196214538481424`, 0.11246592963431665`, 0.00006019881720353837`, 0.00006096046085084 4505`, 0.012652136415420623`, 0.16799937988008265`},
{0.2323130275502685`, 0.49681822670142894`, 1.2539687147478098`, 0.9468502150118296`, 0.13096405194857463`, 0.3219849827616931`, 0.04046745571036271`, 95.23490611137812`, 0.01783356001323902`, 6.936606555558118`, 664.4353243272811`, 668.6988730760784`, 0.006416800240285125`, 438.39959681940286`, 425.3930429729211`, 29.275585710301453`, 30.059274542667364`, 0.026769364757411207`, 0.4526480866945258`, 0.45273441766005157`, 0.00019072424707733404`, 207.7806368319662`, 1.5022292490696911`, 16.204666799341304`, 0.00571807128710855`, 0.005805405262429441`, 0.01527332747980692`, 0.8825263795292932`},
{0.1110467064554368`, 3.3883763096282147`, 5.159131249779884`, 1.2482205179674581`, 0.8201347270890553`, 0.46628896390017405`, 0.2764030660881924`, 2912.733505822841`, 0.03458542948115101`, 2.6312047625809307`, 23.560471794440954`, 22.51568479838448`, -0.04434490977820693`, 40.98206435595282`, 4.904025830860445`, 0.37691131743850315`, 0.38051423139877966`, 0.009559049552457122`, 0.013532674565231011`, 0.013580610934191798`, 0.0035422686572208484`, 18.244533983419764`, 0.02146798485717783`, 0.18914890279866828`, 0.0001298451979339088`, 0.00013190654709433088`, 0.015875436236550522`, 0.16022632872703785`},
{0.1632243866872038`, 2.5373481239797906`, 9.958152546995578`, 1.2439631103015851`, 0.6072525242529792`, 0.6395983432777625`, 0.3899581087433961`, 56.26697227139985`, 0.18832987989003086`, 4.752251898931052`, 1793.0383020175611`, 1825.9388683692819`, 0.01834905942315923`, 1726.849974932933`, 287.9917065829686`, 40.37983700809152`, 40.81203610776715`, 0.010703339381707 31`, 0.29572118083651755`, 0.29573538462284754`, 0.00004803100775485625`, 1463.681481129866`, 0.6895558338924255`, 18.421209344282012`, 0.0028589489271211 3`, 0.002879552038384321`, 0.00720653351577627`, 0.4993768573294861 7`},

{0.16346002078838484`, 3.218794874634134`, 9.025936619626364`, 1.1259348004332868`, 0.7415896232848811`, 0.4862549245509259`,
0.364081252118343`, 137.81289300851844`, 0.05819144446151425`, 8.24717117891944`, 1398.0445902970032`, 1371.9013689097633`,
-0.01869984803681124`, 775.8549032078441`, 235.8556518155578`, 24.727063559314303`, 24.957987527651703`, 0.009338915952695759`,
0.1327237826476209`, 0.13271579990534485`, -0.000060145530189181784`, 1137.0192207067637`, 0.30992903243820535`,
12.613579713349692`, 0.0014224104645627067`, 0.0014273725060661242`, 0.0034884737050517867`, 0.5602859736313021`},
{0.19514620721272602`, 2.87732243599938`, 0.26739185721478975`, 1.4891953183216857`, 0.05289250725423611`,
0.4217001319921505`, 0.03447210696714538`, 218.70480172461387`, 0.0713049851341147`, 6.944425315128572`, 317.87230714388363`,
313.1581644164324`, -0.014830303305743864`, 209.4984803996188`, 211.33972466953125`, 2.3910704130674016`, 2.491096543590857`,
0.04183320155558956`, 1.5401654532332552`, 1.5705020839397317`, 0.019696994659107014`, 98.28400779390202`, 4.293677809693392`,
7.467058325381253`, 0.01184254301342802`, 0.013010134820136915`, 0.09859299690826417`, 1.3298543391658537`},
{0.06305409129179324`, 2.891634576752894`, 4.887865973915014`, 1.4252999692587962`, 0.37958790888649707`, 0.5644849529569385`,
0.3706753829912659`, 1888.9507831786343`, 0.129560920022812`, 6.557119455026179`, 51.57550879450067`, 52.519613992881474`,
0.01830530072214165`, 35.99937162242446`, 8.81537431867243`, 1.0100161149670739`, 1.0253804752123727`, 0.015211995152968116`,
0.014360829976628613`, 0.01439163637847212`, 0.002145168621426663`, 41.72282173023452`, 0.0129358440624124`,
0.7803408680725418`, 0.00012109419080463812`, 0.00012229951330859615`, 0.009953594767420082`, 0.9112763816928784`},
{0.07134729442997761`, 1.925040188749243`, 1.1575324502817335`, 0.9987470940766925`, 0.48237853230783223`,
0.6023915002525595`, 0.11093155711801116`, 121.08543179928522`, 0.23351841944906743`, 5.375810156733433`, 766.1859686898816`,
783.7715638557264`, 0.022952123746033726`, 652.311095280429`, 301.69397131468304`, 16.243241519097683`, 16.48243508775197`,
0.014725728751435518`, 0.767727152807005`, 0.7704652489751043`, 0.003566496453965451`, 446.698467426047`, 0.7825036459033755`,
9.93352515180238`, 0.00907096886204306`, 0.009357733836321177`, 0.03161348899322847`, 0.718402767894861`},
{0.1360789534218197`, 0.4455509561284261`, 2.009215820725272`, 0.8936493225292423`, 0.96227260378033`, 0.5587588217121244`,
0.041826495849356216`, 90.5779416890061`, 0.15671264889903508`, 7.812316525319764`, 3790.6037160220135`, 3616.2623162987215`,
-0.04599304300430851`, 2220.7161336400477`, 2376.821635677763`, 191.39327453465472`, 192.50481328814087`, 0.005807616574765584`,
1.3621660389289516`, 1.3534291322340115`, -0.006413980708115274`, 1218.2208066495102`, 2.6480304137740354`,
17.736096247821035`, 0.01813064756873395`, 0.018353358852616634`, 0.012283691635303073`, 0.327278873757916`},
{0.15849386520968983`, 3.640724074855333`, 1.8861164484754733`, 1.1569149894265711`, 0.5885808127893892`, 0.1748767871381529`,
0.46502697264632115`, 193.59851086045592`, 0.1503277806325949`, 4.212693145809347`, 232.47848161244565`, 228.61635599735934`,
-0.016612830522201527`, 252.57329445268854`, 34.21625841725262`, 3.7270326545317123`, 3.81175172346607`, 0.02273097039580474`,
0.21167235984397564`, 0.2118776390084845`, 0.0009697967399246998`, 193.84425018750832`, 0.4792681495678639`,
4.528028797433691`, 0.0021981220922435707`, 0.002223658158023569`, 0.011617219020775282`, 0.22884632559387974`},
{0.2646664636377358`, 0.7835752608143567`, 7.19251403558616`, 1.494052846937745`, 0.8510899770532778`, 0.5885640033353514`,

```
0.48048609718479995`, 154.5393756420333`, 0.18357560397946`, 4.027566835631127`, 875.8787778104735`, 830.9401339460172`,
 -0.051306921691600005`, 995.3269015738416`, 115.93283899906226`, 62.21078428787122`, 62.69525816960831`, 0.007787618935894747`,
 0.28275704799348755`, 0.2831638965231836`, 0.0014388625591585935`, 696.383307483348`, 1.069090113729633`,
 5.443747345244324`, 0.0022697473613214614`, 0.002297915855978955`, 0.012410409694713298`, 0.23488463252985414`},
{0.12495672263621771`, 3.9102582538632182`, 1.1196234634441904`, 1.2648993449094799`, 0.05034343178950351`,
 0.5637162084123208`, 0.0377865951253564`, 87.92444212100203`, 0.05653305553347371`, 1.253921938823833`, 158.20803376928234`,
 154.91990124695994`, -0.02078360020021197`, 577.4608248002214`, 102.31905392717752`, 0.9410216568027295`, 0.9754125264029495`,
 0.03654631043994083`, 0.852027419111525`, 0.8810019251286076`, 0.03400654176986051`, 52.5662528653845`, 1.5209507698339113`,
 3.5332007530875034`, 0.007595357718841567`, 0.008564453056328768`, 0.12759048004851659`, 0.32006190143423147`},
{0.22032142146595135`, 2.68397072068061`, 2.6883455170430324`, 1.1162234719994681`, 0.40997309115380154`, 0.5558969738438672`,
 0.02165064984593792`, 1526.6454168541877`, 0.24054798278325668`, 4.966530798126495`, 48.92999711187832`, 50.225916449402206`,
 0.02648517093840752`, 45.090655811499715`, 37.44663328922119`, 0.2888940691021067`, 0.29379831391447214`, 0.016975927638833266`,
 0.02552528034442505`, 0.02562518169509528`, 0.003913819919789896`, 11.076903183547643`, 0.08033951498287091`,
 0.7300988472466615`, 0.0002735532955493447`, 0.0002785162661005014`, 0.01814260925349176`, 0.6807863018042949`},
{0.10964967041383433`, 0.7460930180313401`, 6.531951119775776`, 1.1455194328360876`, 0.1524629724176152`, 0.28792352560938195`,
 0.09608079533773954`, 1051.3203547256705`, 0.17626541392472717`, 8.294356744338359`, 70.11864714214212`, 70.57876004969503`,
 0.006561919351070644`, 38.69147732185844`, 30.497615904262634`, 3.396426417982862`, 3.4794378175492295`, 0.024440806115171965`,
 0.009299747303983997`, 0.009304340623876358`, 0.0004939187853409965`, 36.20071481020285`, 0.014567346097297575`,
 1.7311435682307603`, 0.00009806929358768812`, 0.00009836067123206265`, 0.0029711404427930077`, 0.9269080391776469`},
{0.07988018646299105`, 2.6019950195572488`, 9.606629629213668`, 1.0662798233306474`, 0.7693895670648874`,
 0.32124913017390155`, 0.04062772179058327`, 1822.0454711227774`, 0.21556365885863799`, 7.4522296585239225`,
 77.67696175470039`, 75.50036726293014`, -0.028021107450672544`, 47.705724378780566`, 49.448666024350885`,
 0.7390206351384017`, 0.7473312227107642`, 0.011245406660134849`, 0.007258969191476546`, 0.007261768097701905`,
 0.00038557901976576936`, 27.470400171145084`, 0.008283540179234642`, 0.8506999298898401`,
 0.000008226993008819683`, 0.000008246099008323803`, 0.0023223551404063514`, 0.4011743788141079`},
{0.1682679068810577`, 1.5454861727740674`, 1.469740171862469`, 0.9427424173065856`, 0.7350490973518766`,
 0.3805201167376455`, 0.012952191039290932`, 4650.232844583341`, 0.019462289964433777`, 1.394906478790226`,
 5.757320024557451`, 5.562664536924255`, -0.033810086429607256`, 18.890339967396443`, 4.819081416567948`,
 0.03812821872398815`, 0.038952070298189395`, 0.021607397402043427`, 0.01624083018080837`,
 0.016459027824821164`, 0.0134351287208605`, 0.841809069006128`, 0.039040150007647635`, 0.06394821213168589`,
 0.00020264851250384464`, 0.00021342648250749857`, 0.05318553721656083`, 0.11178884267255494`},
```

{0.1878353522665755`, 2.6490780379134238`, 9.484290061448991`, 1.1885927795104771`, 0.9576534469556572`, 0.2804046795618802`,
0.13562182147346188`, 68.8621810881709`, 0.06230339869012863`, 8.081807561887658`, 2865.207149060758`, 2791.485661910901`,
-0.02572989781001489`, 1622.6020325050586`, 990.8600667440596`, 48.226761088084416`, 48.651111298717495`, 0.008799060958251337`,
0.27912074195383485`, 0.2788324095366072`, -0.0010330024748763655`, 1825.0921948306052`, 0.7489820412743001`,
23.984845175144798`, 0.0028344010898936345`, 0.0028409022161841103`, 0.0022936507869886213`, 0.28654611501331756`},
{0.195165600718623`, 2.3826254740878197`, 7.662410402528582`, 0.9519993324167042`, 0.5246436284800724`, 0.22761517290878341`,
0.02889008666683709`, 101.1296763305189`, 0.18152629399038878`, 2.6385617746928904`, 285.91804681756184`, 284.68237155616373`,
-0.004321781276669712`, 495.9510300681243`, 203.7174575799793`, 2.3359324451844867`, 2.383990172842996`, 0.02057325234622276`,
0.08439229943615273`, 0.084427251603564 6`, 0.0004141629940810354`, 79.50931642349718`, 0.23529248307841505`,
5.501804930675953`, 0.0010690305712646175`, 0.0010747871982443834`, 0.00538490398170377`, 0.18051003579528854`},
{0.21914596002979703`, 3.4188550030486784`, 4.710500615641845`, 1.4527326168664971`, 0.03624911304432854`,
0.28384065291200533`, 0.4254237737178384`, 3505.5630456480862`, 0.12312118964896007`, 9.273808474100267`,
21.99317657378436`, 21.65050931560944`, -0.015580616880208953`, 10.854112864971842`, 3.5796025428755995`,
0.36906800002411433`, 0.3789204052379248`, 0.02669536565935471`, 0.004583982839302328 5`,
0.004587723811207525`, 0.0008160964027006 39`, 18.025571119251623`, 0.01435087600113193`, 0.5843250371722009`,
0.000038082069034661714`, 0.00003826276636489541`, 0.004744945188488447 5`, 1.1046920288193023`},
{0.1814951436259516`, 1.11858432682377`, 5.7029405020905966`, 0.816783193035002`, 0.6766309251156815`, 0.4321734260024682`,
0.2931263656368919`, 87.29527865588257`, 0.044724422492498916`, 2.4174522941648533`, 517.5925026400078`, 512.2382152503286`,
-0.010344599974631219`, 979.9288843069688`, 104.33816234224483`, 24.297385036366105`, 24.60668547694234`, 0.012729783065679623`,
0.19210233470027138`, 0.1922565867187113`, 0.0008029679528913647`, 388.2667726354505`, 0.4980805832469929`,
5.89507021413847`, 0.0028229350423908395`, 0.0028532827842887294`, 0.010750421615152606`, 0.18991414516716984`},
{0.22203693974513244`, 0.9431525918127432`, 4.196712511517475`, 1.3261224346879132`, 0.8036004422541301`, 0.6271444136150295`,
0.10733581071086257`, 369.9451385562586`, 0.16273465181192748`, 5.731309489564488`, 485.8052822292604`, 470.84370169781084`,
-0.03079748425705453`, 387.9474185813715`, 193.44765078858856`, 20.151064524012973`, 20.315785045964347`, 0.00817428388237662`,
0.16753779253151788`, 0.16791104120855074`, 0.0022278476479427045`, 271.50755333726704`, 0.5314225535050937`,
3.264564792040459`, 0.0015143058291664513`, 0.0015350154264693189`, 0.013675967498762986`, 0.3877538746431157`},
{0.18924733350115397`, 2.8150904404250037`, 2.4574844917932026`, 0.8737719488383665`, 0.6977173227044986`,
0.3482841124888072`, 0.48079795163551986`, 94.66261771766344`, 0.16471072416530214`, 1.3078470560939532`, 234.37297495517285`,
228.24486179453265`, -0.026146842065781106`, 820.191129839684`, 32.13124914877842`, 4.871361646559536`, 4.963824670545982`,
0.01898094017547436`, 0.3957683114013082`, 0.39750674085258425`, 0.004392543316873443`, 195.90462290118245`, 1.0699728230991388`,
2.933419449162295`, 0.005363751477149514`, 0.005542087521595888`, 0.03324837946092685`, 0.09909110877553828`},

{0.07907817004881895`, 1.0481324229512046`, 3.8137728667258273`, 1.2920968808020492`, 0.04181350128653394`, 0.5803710922764521`, 0.02851300126208627`, 266.99553906648964`, 0.22548933562284668`, 2.0140176417924156`, 81.64030486383005`, 82.42950631252553`, 0.009666811631972694`, 185.52645319815838`, 58.34926003975133`, 1.4460730550112981`, 1.4793086601970058`, 0.022983351408513597`, 0.08534217850931368`, 0.08602587167720456`, 0.008011198915156115`, 21.652515070192013`, 0.09641004720708582`, 1.8168549012161652`, 0.0007823795358298247`, 0.0008077930550547644`, 0.03248234144823958`, 0.44378260490365834`},
{0.22302087067747528`, 1.8201511620752422`, 1.875184623833773`, 0.7813352529024251`, 0.8705057770695381`, 0.22074020090969038`, 0.1367588752741694`, 187.45561858905666`, 0.07793294287132913`, 8.923384483271061`, 848.4445061877997`, 821.4750711921528`, -0.03178691688019164`, 435.1693813257464`, 293.33314693798343`, 20.519485702066422`, 20.78338600228203`, 0.01286096074956844`, 0.24866819434660065`, 0.24841498484578556`, -0.0010182625143533297`, 533.5509392257494`, 0.7922599601859693`, 9.762433026513174`, 0.003833248443349979`, 0.003853571266235657`, 0.005301723377970635`, 0.34873165545317103`},
{0.21429795289589076`, 2.896671823601787`, 8.636702505614757`, 0.9811380648364925`, 0.20610104158853804`, 0.1778381348410949`, 0.007936414694970003`, 185.13169573811163`, 0.014685687390926405`, 3.2353159752463547`, 132.47679948689233`, 132.84142613062733`, 0.002752381135015858`, 187.4077816314513`, 119.14830847713047`, 0.30910450590939487`, 0.3183114713543572`, 0.029785930871098598`, 0.029165960709066723`, 0.029176944988632967`, 0.0003766129864815 06`, 12.791061611658527`, 0.0892886524885022`, 3.716992996868302 4`, 0.0003590634512981028`, 0.0003603203221242123`, 0.003500414262620044`, 0.25122565630866567`},
{0.17507336986882482`, 0.7983306290353371`, 2.750450064120228`, 0.8050075785267179`, 0.7818959542274213`, 0.6526925477724106`, 0.0965325694706689 3`, 72.78219945862553`, 0.07700161447215975`, 3.655530763820291`, 1517.4183210196843`, 1481.9854223917985`, -0.02335077818493425`, 1899.850269811273`, 642.8310577257377`, 70.28918440496706`, 70.90762288865965`, 0.00879848712042941`, 0.7623238385259226`, 0.7613961053733861`, -0.0012169803771719945`, 801.6286971485465`, 1.9066086191723133`, 10.660148004821785`, 0.011253404392068234`, 0.011470034071720162`, 0.019250146187283157`, 0.2755389301298304`},
{0.1470766374450453`, 1.2533511161361544`, 4.593526904612924`, 1.479552314271993`, 0.7400477950348978`, 0.5768087033937906`, 0.10619660427114413`, 242.62879808709684`, 0.045931800134058565`, 3.771378090721337`, 394.84140365047256`, 389.07362025209494`, -0.014607848480559738`, 479.1672286973734`, 158.5783933230288`, 12.462816033413498`, 12.588057989439008`, 0.010049250160616197`, 0.21064443391908813`, 0.2113529586094471`, 0.0033636050911800552`, 223.14691979540527`, 0.44258392910493666`, 3.311038371978406`, 0.001701227612407874`, 0.0017321677209779218`, 0.01818693062843968`, 0.2989450973861311`},
{0.0788585810797619`, 2.548378832414274`, 3.111482597871378`, 0.9346713900878518`, 0.19963135196405957`, 0.5058944800586684`, 0.03749384602431879`, 1118.4164778142415`, 0.22872744797764172`, 1.4303214452036404`, 14.538205229017372`, 14.745160897849553`, 0.01423529696905845 3`, 46.52020713328444`, 9.537110960331592`, 0.1326004055965948`, 0.13556661235059772`, 0.022369514939696966`, 0.018945867407208578`, 0.019115069967104065`, 0.008930842608510403`, 4.827372382741561`, 0.021343488872249128`, 0.2972556361512708`, 0.00023991786034593954`, 0.00024826949509756366`, 0.03481039193823188`, 0.25412027054575803`},
{0.24182502946725698`, 1.8945200327623395`, 6.300131593939071`, 0.7669603268824658`, 0.6959163653444247`,

```
    0.5105300710554361`, 0.2329066367569749`, 178.13280663193103`, 0.21420184435482204`, 3.5903109736592462`,
    433.3873533845676`, 430.9635487074551`, -0.0055926982136963455`, 552.4699518436227`, 103.31690604930786`,
    11.746489530412255`, 11.873831659640212`, 0.010840866873312427`, 0.09209437439140745`,
    0.09216775092444572`, 0.0007967536944917253`, 317.9137104285599`, 0.31815321144254743`, 4.296022403680138`,
    0.0014450376098787965`, 0.0014561567878336944`, 0.0076947325653555578`, 0.2870191698215545`},
   {0.05758087297506348`, 2.9919516996565125`, 9.945704556467732`, 1.1770757830874041`, 0.6136122924727272`, 0.31603398268032157`,
    0.21009959511709828`, 118.22678602178958`, 0.2258513747270965`, 6.274152149731609`, 779.9904718139995`, 774.3914869512789`,
    -0.007178273408519065`, 568.9822861280111`, 203.17135553268463`, 13.182950215412868`, 13.37524470131632`, 0.014586604876853038`,
    0.0923439473964363`, 0.09234907889727316`, 0.000055569433422999725`, 563.4678614784522`, 0.07596064435788641`,
    11.211390189335555`, 0.000947319903974897`, 0.0009499752473855977`, 0.0028030060379382604`, 0.4416467711366696`},
   {0.12816326660379745`, 2.7095777054297967`, 7.985014815061977`, 0.8582103259977079`, 0.28527001685481057`, 0.4561351167228034`,
    0.41006088059385215`, 4928.696897400591`, 0.12257221770331383`, 5.545857987270676`, 13.652970545305315`, 13.7432291070492`,
    0.006610910163789896`, 11.267379016837012`, 2.1949079715833624`, 0.28843790610997727`, 0.2936962465445861`, 0.018230407041590 18`,
    0.0016592578251850036`, 0.0016605426467489277`, 0.00077433509393333205`, 11.164927425663535`, 0.003037941471620652`,
    0.25201218120011326`, 0.000023335956148895498`, 0.000023431294087044483`, 0.004085452404051004`, 0.7474064020426624`}};

(*MakeOutputForm[];*)
PatientsTrainingSet = result[[All, 1 ;; 10]];
```

In[•]:=

```
(* The scripts hidden in this group of cells have to be initialized to run the following codes *)

(* This is the script for the solution of full system for the curative case --
  where cell proliferation stops at the moment of achieving minimal viable tumor cell number *)

FullSystemSolutionMDStopNmin[TMIN_] := (

   (* maximum time of simulation-- where little radioactivity of the last dose remains, namely 0.17% of the last dose *)
   tEnd = Ainj[[Length[Ainj], 1]] + (-Log[0.0017] / lambda);

   krho = 1;  (*cells do proliferate yet*)
   lAinj = Length[Ainj]; (* number of injections*)
```

```
If[Ainj[[1, 1]] == 0, Npw = lAinj, Npw = lAinj + 1];

apw = Array[ff, Npw]; bpw = Array[ff, Npw]; NNpw = Array[ff, Npw]; DDpw = Array[ff, Npw];
papw = Array[ff, Npw]; pbpw = Array[ff, Npw]; fFNpw = Array[ff, Npw]; fANpw = Array[ff, Npw];
dFpw = Array[ff, Npw]; dApw = Array[ff, Npw];
(* decayed antibody fragments are as well accounted for as pb *)

(* times of beginning and end for solution of separate systems *)
tB = Array[ff, Npw]; tE = Array[ff, Npw];

(* for monitoring the paths of activity *)
ActBloodpw = Array[ff, Npw]; ActBloodFragpw = Array[ff, Npw];
ActOutpw = Array[ff, Npw]; ActOutFragpw = Array[ff, Npw]; ActTumorpw = Array[ff, Npw];

(* for monitoring influence of self-dose, croos-fire and decays in blood *)
SDpw = Array[ff, Npw]; CFNpw = Array[ff, Npw]; CFDpw = Array[ff, Npw]; UNpw = Array[ff, Npw];

(* for monitoring the number of new cancer cells appearing during treatment *)
NewCellspw = Array[ff, Npw];

Clear[a, b, NN, DD, pa, pb, fFN, fAN, dF, dA, ActBlood, ActBloodFrag, ActOut, ActOutFrag, ActTumor, SD, CFN, CFD, UN, NewCells];

(* EQUATIONS *)

(* Radiation damage function *)

RD[NN_, DD_, fAN_, dA_, a_, pa_] := alpha * (ks * (lambda * gamma * fAN)/nu (*self-dose*) +
    (1 - ks) * (lambda * gamma * (fAN * NN + dA))/(nu * (NN + DD)) (*cross-fire*) + kf * lambda * (a + pa) (*dose from unanchored nuclides*));
```

```
(* Active antibodies *)
Fa[t_] := (*injections are considered as initial conditions*)
  - lambda * a[t] (*decay*) - kon * gamma/V * (fFN[t] * NN[t] + dF[t]) * a[t] (*binding*) - kappac * a[t] (*clearance*);

(* Inert antibodies *)
Fb[t_] := (*injections are considered as initial conditions*)
  + lambda * a[t] (*decay of a*) - kon * gamma/V * (fFN[t] * NN[t] + dF[t]) * b[t] (*binding*) - kappac * b[t] (*clearance*);

(* Viable cells *)
FNN[t_] := krho * rho * NN[t] (*proliferation*) - RD[NN[t], DD[t], fAN[t], dA[t], a[t], pa[t]] * NN[t] (*damage*);

(* Damaged cells *) FDD[t_] := RD[NN[t], DD[t], fAN[t], dA[t], a[t], pa[t]] * NN[t] (*damage*) - omega * DD[t] (*death*);

(* Active fragments *) Fpa[t_] := omega * (gamma * dA[t])/V (*release*) - lambda * pa[t] (*decay*) - kappap * pa[t] (*clearance*);

(* Inert fragments *)
Fpb[t_] := omega * (gamma * (DD[t] - dF[t] - dA[t]))/V (*release*) + lambda * pa[t] (*decay*) - kappap * pb[t] (*clearance*);

(* Free receptors of viable cells *) FfFN[t_] := krho * (1 - fFN[t]) * rho - kon * (a[t] + b[t]) * fFN[t];

(* Active receptors of viable cells *) FfAN[t_] := kon * a[t] * fFN[t] - (lambda + krho * rho) * fAN[t];

(* Free receptors of damaged cells *)
FdF[t_] := RD[NN[t], DD[t], fAN[t], dA[t], a[t], pa[t]] * fFN[t] * NN[t] - kon * (a[t] + b[t]) * dF[t] - omega * dF[t];

(* Active receptors of damaged cells *)
FdA[t_] :=
```

```
RD[NN[t], DD[t], fAN[t], dA[t], a[t], pa[t]] * fAN[t] * NN[t] + kon * a[t] * dF[t] - lambda * dA[t] - omega * dA[t];

(* Initial conditions *)

If[Ainj[[1, 1]] == 0
  , a0 = Ainj[[1, 2]] / V; b0 = eta * Ainj[[1, 2]] / V
  , a0 = 0; b0 = 0]; (*complexes in blood*)
NN0 = N0;
DD0 = 0;
pa0 = 0; pb0 = 0; fFN0 = 1; fAN0 = 0;
dF0 = 0; dA0 = 0;
ActBlood0 = 0; ActBloodFrag0 = 0; ActOut0 = 0; ActOutFrag0 = 0; ActTumor0 = 0; NewCells0 = 0;
SD0 = 0; CFN0 = 0; CFD0 = 0; UN0 = 0;

(* SOLVER *)

tB[[1]] = 0; If[Ainj[[1, 1]] == 0, If[lAinj > 1, tE[[1]] = Ainj[[2, 1]], tE[[1]] = tEnd], tE[[1]] = Ainj[[1, 1]]];

For[npw = 1, npw ≤ Npw, npw ++,

  Clear[a, b, NN, DD, pa, pb, fFN, fAN, dF, dA, ActBlood, ActBloodFrag, ActOut, ActOutFrag, ActTumor, SD, CFN, CFD, UN, NewCells];
  (*fFD,fAD,*)

  sol = NDSolve[{
      (*INITIAL CONDITIONS*)
      a[tB[[npw]]] == a0, b[tB[[npw]]] == b0, NN[tB[[npw]]] == NN0, DD[tB[[npw]]] == DD0, pa[tB[[npw]]] == pa0,
      pb[tB[[npw]]] == pb0, fFN[tB[[npw]]] == fFN0, fAN[tB[[npw]]] == fAN0, dF[tB[[npw]]] == dF0, dA[tB[[npw]]] == dA0,
      ActBlood[tB[[npw]]] == ActBlood0, ActBloodFrag[tB[[npw]]] == ActBloodFrag0, ActOut[tB[[npw]]] == ActOut0,
      ActOutFrag[tB[[npw]]] == ActOutFrag0, ActTumor[tB[[npw]]] == ActTumor0, SD[tB[[npw]]] == SD0,
      CFN[tB[[npw]]] == CFN0, CFD[tB[[npw]]] == CFD0, UN[tB[[npw]]] == UN0, NewCells[tB[[npw]]] == NewCells0,
```

```
    a'[t] == Fa[t], b'[t] == Fb[t], NN'[t] == FNN[t], DD'[t] == FDD[t],
    pa'[t] == Fpa[t], pb'[t] == Fpb[t], fFN'[t] == FfFN[t], fAN'[t] == FfAN[t], dF'[t] == FdF[t], dA'[t] == FdA[t],

    ActBlood'[t] == V * lambda * (a[t] + pa[t]),
    ActBloodFrag'[t] == V * lambda * pa[t],
    ActOut'[t] == V * (kappac * a[t] + kappap * pa[t]),
    ActOutFrag'[t] == V * kappap * pa[t],
    ActTumor'[t] == (lambda * gamma) * (fAN[t] * NN[t] + dA[t]),

    SD'[t] == ks * (lambda * gamma) * (fAN[t] * NN[t]),
    CFN'[t] == (1 - ks) * (lambda * gamma) * (fAN[t] * NN[t]) * NN[t] / (NN[t] + DD[t]),
    CFD'[t] == (1 - ks) * (lambda * gamma) * dA[t] * NN[t] / (NN[t] + DD[t]),
    UN'[t] == kf * lambda * (a[t] + pa[t]) * nu * NN[t],
    NewCells'[t] == If[t > Ainj[[1, 1]], krho * rho * NN[t], 0] (* start counting new cells from the moment of the first injection *)

    , WhenEvent[t > TMIN, NN[t] -> 0]
    , WhenEvent[NN[t] > 10^9 / Nnor, NN[t] -> 0.99 * 10^9 / Nnor (*tE[[npw]]=t;
      tEnd=t;
      "StopIntegration"*) ] (* cancer wins *)
    (*,WhenEvent[NN[t]<0.01/Nnor,NN[t]->0 (*tE[[npw]]=t;
      tEnd=t;
      "StopIntegration"*) ] (* treatment wins *) *)
  }
  , {a, b, NN, DD, pa, pb, fFN, fAN, dF, dA, ActBlood,
    ActBloodFrag, ActOut, ActOutFrag, ActTumor, SD, CFN, CFD, UN, NewCells}, {t, tB[[npw]], tE[[npw]]}
  , AccuracyGoal -> 10, PrecisionGoal -> 10];
```

```
apw⟦npw⟧ = First[a /. sol]; bpw⟦npw⟧ = First[b /. sol]; NNpw⟦npw⟧ = First[NN /. sol]; DDpw⟦npw⟧ = First[DD /. sol];
papw⟦npw⟧ = First[pa /. sol]; pbpw⟦npw⟧ = First[pb /. sol]; fFNpw⟦npw⟧ = First[fFN /. sol];
fANpw⟦npw⟧ = First[fAN /. sol]; dFpw⟦npw⟧ = First[dF /. sol]; dApw⟦npw⟧ = First[dA /. sol];
ActBloodpw⟦npw⟧ = First[ActBlood /. sol];
ActBloodFragpw⟦npw⟧ = First[ActBloodFrag /. sol];
ActOutpw⟦npw⟧ = First[ActOut /. sol];
ActOutFragpw⟦npw⟧ = First[ActOutFrag /. sol];
ActTumorpw⟦npw⟧ = First[ActTumor /. sol];
SDpw⟦npw⟧ = First[SD /. sol];
CFNpw⟦npw⟧ = First[CFN /. sol];
CFDpw⟦npw⟧ = First[CFD /. sol];
UNpw⟦npw⟧ = First[UN /. sol];
NewCellspw⟦npw⟧ = First[NewCells /. sol];

If[npw < Npw,
  (*renew initial conditions*)
  If[Ainj⟦1, 1⟧ == 0
    , a0 = apw⟦npw⟧[tE⟦npw⟧] + Ainj⟦npw + 1, 2⟧/V; b0 = bpw⟦npw⟧[tE⟦npw⟧] + eta * Ainj⟦npw + 1, 2⟧/V
    , a0 = apw⟦npw⟧[tE⟦npw⟧] + Ainj⟦npw, 2⟧/V; b0 = bpw⟦npw⟧[tE⟦npw⟧] + eta * Ainj⟦npw, 2⟧/V];
  NN0 = NNpw⟦npw⟧[tE⟦npw⟧];
  DD0 = DDpw⟦npw⟧[tE⟦npw⟧];
  pa0 = papw⟦npw⟧[tE⟦npw⟧];
  pb0 = pbpw⟦npw⟧[tE⟦npw⟧];
  fFN0 = fFNpw⟦npw⟧[tE⟦npw⟧]; fAN0 = fANpw⟦npw⟧[tE⟦npw⟧];
  dF0 = dFpw⟦npw⟧[tE⟦npw⟧];
  dA0 = dApw⟦npw⟧[tE⟦npw⟧];
  ActBlood0 = ActBloodpw⟦npw⟧[tE⟦npw⟧];
  ActBloodFrag0 = ActBloodFragpw⟦npw⟧[tE⟦npw⟧];
  ActOut0 = ActOutpw⟦npw⟧[tE⟦npw⟧];
  ActOutFrag0 = ActOutFragpw⟦npw⟧[tE⟦npw⟧];
  ActTumor0 = ActTumorpw⟦npw⟧[tE⟦npw⟧];
```

```
        SD0 = SDpw[[npw]][tE[[npw]]];
        CFN0 = CFNpw[[npw]][tE[[npw]]];
        CFD0 = CFDpw[[npw]][tE[[npw]]];
        UN0 = UNpw[[npw]][tE[[npw]]];
        NewCells0 = NewCellspw[[npw]][tE[[npw]]];

        (*renew time frame*)

        If[Ainj[[1, 1]] == 0, If[Npw > npw + 1, tE[[npw + 1]] = Ainj[[npw + 2, 1]], tE[[npw + 1]] = tEnd],
          If[Npw > npw + 1, tE[[npw + 1]] = Ainj[[npw + 1, 1]], tE[[npw + 1]] = tEnd]];]
    ];
    npw--;
    (* It will be convenient to have estimation of minimal viable cell number here *)
    Nn = If[Ainj[[1, 1]] == 0
        , Min[Table[NMinimize[{Nnor * (NNpw[[nn]][t]), t > tB[[nn]], t < tE[[nn]]}, t][[1]], {nn, 1, npw}]]
        , Min[Table[NMinimize[{Nnor * (NNpw[[nn]][t]), t > tB[[nn]], t < tE[[nn]]}, t][[1]], {nn, 2, npw}]]];
    Return[Nn])
```

In[∘]:=

```
(* Here are the scripts to compactify the process of parameter variation *)

CreateResultArray[Njj_] := ( (* argument is how many random sets will be chosen *)
    result = {};
    (* Table that will contatin the results *)
    result = Array[f, {Njj + 1, 28}]; (* 10 variables and 17 measures *)

    result[[1, 1]] = "κc";
    result[[1, 2]] = "κp";
    result[[1, 3]] = "γ";
    result[[1, 4]] = "V";
    result[[1, 5]] = "ks";
```

```
result⟦1, 6⟧ = "ρ";
result⟦1, 7⟧ = "ω";
result⟦1, 8⟧ = "α";
result⟦1, 9⟧ = "k_f";
result⟦1, 10⟧ = "N_0";

(* minimal curative dose *)
result⟦1, 11⟧ = "A_cur^sim"; (* simulation result *)
result⟦1, 12⟧ = "A_cur^est"; (* analytical estimation *)
result⟦1, 13⟧ = "Err"; (* error in estimation *)
result⟦1, 14⟧ = "M_m"; (* minimal curative number of radioconjugate molecules of cancer cell *)

(* paths of activity *)
result⟦1, 15⟧ = "Canc"; (* released in cancer *)
result⟦1, 16⟧ = "Bl_Frg^sim"; (* released in blood from fragments, simulation result *)
result⟦1, 17⟧ = "Bl_Frg^est"; (* analytical estimation *)
result⟦1, 18⟧ = "Err"; (* error in estimation *)
result⟦1, 19⟧ = "Bl_Ab^sim"; (* released in blood from antibodies, simulation result *)
result⟦1, 20⟧ = "Bl_Ab^est"; (* analytical estimation *)
result⟦1, 21⟧ = "Err"; (* error in estimation *)
result⟦1, 22⟧ = "Cld_Frg^sim"; (* cleared in form of fragments,
simulation result *) (* no need to estimate -- error will be the same as for release *)
result⟦1, 23⟧ = "Cld_Ab^sim"; (* cleared in form of fragments, simulation result *)

result⟦1, 24⟧ = "ToVbl"; (* How much activity affects viable cells *)

(* receptors occupancy *)
result⟦1, 25⟧ = "fD_Max^sim"; (* maximum occupancy of receptors on damaged cancer cells, simulation result *)
result⟦1, 26⟧ = "fD_Max^est"; (* analytical estimation *)
result⟦1, 27⟧ = "Err"; (* error in estimation *)
result⟦1, 28⟧ = "Nnew"; (*number of newborn cells until Ncur is achieved*)
```

```
)

WriteDownMeasures[ ] := (

  result[[ij, 1]] = kappac;
  result[[ij, 2]] = kappap;
  result[[ij, 3]] = gamma * 10^7 / Nnor;
  result[[ij, 4]] = V;
  result[[ij, 5]] = ks;
  result[[ij, 6]] = rho;
  result[[ij, 7]] = omega;
  result[[ij, 8]] = alpha;
  result[[ij, 9]] = kf;
  result[[ij, 10]] = N0 * Nnor / 10^7;

  (* minimal curative dose *)
  result[[ij, 11]] = DA1 / nCpm; (* simulation result *)
```

$$\text{result}[[ij, 12]] = -\frac{\text{kappac} + \text{lambda} + \text{kon} * \text{gamma} * \text{N0} / \text{V}}{\text{kon} * \text{gamma} * \text{N0} / \text{V}} * \text{N0} * \frac{\frac{\text{rho}}{\text{lambda}} * \frac{\text{nu}}{\text{alpha}} * \text{ProductLog}\left[-1, -E^{-2} * \left(\frac{\text{Ncur}}{\text{N0} * \text{Nnor}}\right)^{\frac{\text{lambda}}{\text{rho}}}\right]}{1 - \left(1 - \frac{\text{ProductLog}\left[-1, -E^{-2} * \left(\frac{\text{Ncur}}{\text{N0} * \text{Nnor}}\right)^{\frac{\text{lambda}}{\text{rho}}}\right]}{\text{ProductLog}\left[-1, -E^{-1} * \left(\frac{\text{Ncur}}{\text{N0} * \text{Nnor}}\right)^{\frac{\text{lambda}+\text{rho}}{\text{rho}}}\right]}\right) * \text{ks}} \Big/ \text{nCpm};$$

```
  (* analytical estimation *)
  result[[ij, 13]] = result[[ij, 12]] / result[[ij, 11]] - 1; (* error in estimation *)
```

$$\text{result}[[ij, 14]] = \frac{\text{result}[[ij, 11]] * \text{nCpm} * 6.02214076 * 10^{11}}{\text{N0} * \text{Nnor}};$$

```
  (* minimal curative number of radioconjugate molecules of cancer cell *)
```

```
(* paths of activity *)
result[[ij, 15]] = ActTumorpw[[Npw]][tEnd] / nCpm; (* released in cancer *)
result[[ij, 16]] = ActBloodFragpw[[Npw]][tEnd] / nCpm; (* released in blood from fragments, simulation result *)
result[[ij, 17]] = (lambda * omega * DA1) / ((lambda + omega) * (lambda + kappap)) / nCpm; (* analytical estimation *)
result[[ij, 18]] = result[[ij, 17]] / result[[ij, 16]] - 1; (* error in estimation *)
result[[ij, 19]] = (ActBloodpw[[Npw]][tEnd] - ActBloodFragpw[[Npw]][tEnd]) / nCpm;
(* released in blood from antibodies, simulation result *)
result[[ij, 20]] = (lambda * DA1) / (lambda + kappac + kon * gamma * N0 / V) / nCpm; (* analytical estimation *)
result[[ij, 21]] = result[[ij, 20]] / result[[ij, 19]] - 1; (* error in estimation *)
result[[ij, 22]] = ActOutFragpw[[Npw]][tEnd] / nCpm; (* cleared in form of fragments,
simulation result *) (* no need to estimate -- error will be the same as for release *)
result[[ij, 23]] = (ActOutpw[[Npw]][tEnd] - ActOutFragpw[[Npw]][tEnd]) / nCpm; (* cleared in form of fragments, simulation result *)

result[[ij, 24]] = (SDpw[[Npw]][tEnd] + CFNpw[[Npw]][tEnd] + CFDpw[[Npw]][tEnd] + UNpw[[Npw]][tEnd]) / nCpm;
(* How much activity affects viable cells *)

(* receptors occupancy *)
result[[ij, 25]] = 1 - If[Ainj[[1, 1]] == 0
    , Min[Table[NMinimize[{dFpw[[nn]][t] / DDpw[[nn]][t], t > tB[[nn]], t < 10}, t][[1]], {nn, 1, npw}]]
    , Min[Table[NMinimize[{dFpw[[nn]][t] / DDpw[[nn]][t], t > tB[[nn]], t < 10}, t][[1]], {nn, 2, npw}]]];
(* maximum occupancy of receptors on damaged cancer cells, simulation result *)
result[[ij, 26]] = DA1 / (gamma * N0); (* analytical estimation *)
result[[ij, 27]] = result[[ij, 26]] / result[[ij, 25]] - 1; (* error in estimation *)

result[[ij, 28]] = NewCellspw[[npw]][tminNn] * Nnor / 10^7; (*number of newborn cells until Ncur is achieved*)
```

```
)

MakeOutputForm[] := (
  resultOutput = result;

  resultOutput[[2 ;;, 1]] = Round[resultOutput[[2 ;;, 1]], 0.01]; (*"κc"*)
  resultOutput[[2 ;;, 2]] = Round[resultOutput[[2 ;;, 2]], 0.01]; (*"κp"*)
  resultOutput[[2 ;;, 3]] = Round[resultOutput[[2 ;;, 3]], 0.01]; (*"γ"*)
  resultOutput[[2 ;;, 4]] = Round[resultOutput[[2 ;;, 4]], 0.01]; (*"V"*)
  resultOutput[[2 ;;, 5]] = Round[resultOutput[[2 ;;, 5]], 0.01]; (*"ks"*)
  resultOutput[[2 ;;, 6]] = Round[resultOutput[[2 ;;, 6]], 0.01]; (*"ρ"*)
  resultOutput[[2 ;;, 7]] = Round[resultOutput[[2 ;;, 7]], 0.001]; (*"ω"*)
  resultOutput[[2 ;;, 8]] = Round[resultOutput[[2 ;;, 8]], 1]; (*"α"*)
  resultOutput[[2 ;;, 9]] = Round[resultOutput[[2 ;;, 9]], 0.01]; (*"kf"*)
  resultOutput[[2 ;;, 10]] = Round[resultOutput[[2 ;;, 10]], 0.01]; (*"N0"*)

  resultOutput[[2 ;;, 11]] = Round[resultOutput[[2 ;;, 11]], 0.01]; (*"Acur^sim"*)
  resultOutput[[2 ;;, 12]] = Round[resultOutput[[2 ;;, 12]], 0.01]; (*"Acur^est"*)
  resultOutput[[2 ;;, 14]] = Round[resultOutput[[2 ;;, 14]], 0.01]; (*"Mm"*)
  resultOutput[[2 ;;, 15]] = Round[resultOutput[[2 ;;, 15]], 0.01]; (*"Canc"*)
  resultOutput[[2 ;;, 16]] = Round[resultOutput[[2 ;;, 16]], 0.01]; (*"BlFrg^sim"*)
  resultOutput[[2 ;;, 17]] = Round[resultOutput[[2 ;;, 17]], 0.01]; (*"BlFrg^est"*)
  resultOutput[[2 ;;, 19]] = Round[resultOutput[[2 ;;, 19]], 0.01]; (*"BlAb^sim"*)
  resultOutput[[2 ;;, 20]] = Round[resultOutput[[2 ;;, 20]], 0.01]; (*"BlAb^est"*)
  resultOutput[[2 ;;, 22]] = Round[resultOutput[[2 ;;, 22]], 0.01]; (*"CldFrg^sim"*)
  resultOutput[[2 ;;, 23]] = Round[resultOutput[[2 ;;, 23]], 0.01]; (*"CldAb^sim"*)
```

```
    resultOutput[[2 ;;, 24]] = Round[resultOutput[[2 ;;, 24]], 0.01]; (*"ToVbl"*)
    resultOutput[[2 ;;, 28]] = Round[resultOutput[[2 ;;, 28]], 0.01]; (*"Nnew"*)

    For[iii = 2, iii ≤ Npar + 1, iii++,
      resultOutput[[iii, 13]] = PercentForm[Round[resultOutput[[iii, 13]], 0.0001]]; (*"Err"*)
      resultOutput[[iii, 18]] = PercentForm[Round[resultOutput[[iii, 18]], 0.0001]]; (*"Err"*)
      resultOutput[[iii, 21]] = PercentForm[Round[resultOutput[[iii, 21]], 0.0001]]; (*"Err"*)
      resultOutput[[iii, 25]] = PercentForm[Round[resultOutput[[iii, 25]], 0.0001]]; (*"fD^sim_Max"*)
      resultOutput[[iii, 26]] = PercentForm[Round[resultOutput[[iii, 26]], 0.0001]]; (*"fD^est_Max"*)
      resultOutput[[iii, 27]] = PercentForm[Round[resultOutput[[iii, 27]], 0.0001]];(*"Err"*)
    ];
  )


(* The outcome of the global parameter sweep was obtained by this code.
   It uses randomization and will generate a new array of data.
   It takes a long time,at least half a day. *)


Npar = 1000; (*how many sets will be tested*)
SetBasicParameterValues[];
CreateResultArray[Npar];
Nnres = Array[f, {Npar + 1, 2}];
(* The minimal viable cell number achieved in simulations can be monitored with this array.
      Numerical acurracy can be adjusted if desired in the scripts
    for the solutions of the full system -- search for "AccuracyGoal->10,PrecisionGoal->10".
      With current setting N_min is kept within 99%-101.5% of desired N_cur=0.01 *)
Nnres[[1, 1]] = "#";
Nnres[[1, 2]] = "Nn";
Quiet[For[ij = 2, ij ≤ Npar + 1, ij++,
```

```
        NotebookDelete[pr]; (* To see the code running *)
        pr = PrintTemporary["Set " <> ToString[ij - 1] <> " of " <> ToString[Npar]];

        kappac = RandomReal[{0.04, 0.28}];
        kappap = RandomReal[{0.4, 4}];
        gamma = RandomReal[{0.13, 10}] * Nnor / 10^7;
        V = RandomReal[{0.75, 1.5}];
        ks = RandomReal[{0, 1}];
        rho = RandomReal[{0.15, 0.7}];
        omega = 0.05 * 10^RandomReal[{-1, 1}];
        alpha = 50 * 10^RandomReal[{0, 2}];
        kf = RandomReal[{0.01, 0.25}];
        N0 = RandomReal[{1, 10}] * 10^7 / Nnor;

        ACurSim = FindCurDose[]; (* find curative dose *)
        DA1 = ACurSim * nCpm; (* set curative dose *)
        Ainj = {{t1, DA1}}; (* set schedule *)
        FullSystemSolutionMD[]; (* find time of achieving of minimal N *)
        FullSystemSolutionMDStopNmin[tminNn]; (* solve it with cell proliferation stopping at this moment *)

        Nnres[[ij, 1]] = ij;
        Nnres[[ij, 2]] = Nn;

        WriteDownMeasures[];

        check = 11; OkFlg = 1; (* to avoid possible bugs *)
        While[check < 28, If[! NumericQ[result[[ij, check]]], OkFlg = 0]; check++];
        If[OkFlg == 0, ij--];
      ]];
   MakeOutputForm[];
```

```
(* The code hidden here can be used to check the simulation measures for the training parameter set used in this study *)

resultTrain = result;
Npar = 1000; (*how many sets will be tested*)
SetBasicParameterValues[];
CreateResultArray[Npar];
Nnres = Array[f, {Npar + 1, 2}];
(* The minimal viable cell number achieved in simulations can be monitored.
   Numerical acurracy can be adjusted if desired. *)
Nnres⟦1, 1⟧ = "#";
Nnres⟦1, 2⟧ = "Nn";
Quiet[For[ij = 2, ij ≤ Npar + 1, ij++,

    NotebookDelete[pr]; (* To see the code running *)
    pr = PrintTemporary["Set " <> ToString[ij - 1] <> " of " <> ToString[Npar]];

    kappac = resultTrain⟦ij, 1⟧;
    kappap = resultTrain⟦ij, 2⟧;
    gamma = resultTrain⟦ij, 3⟧ * Nnor / 10^7;
    V = resultTrain⟦ij, 4⟧;
    ks = resultTrain⟦ij, 5⟧;
    rho = resultTrain⟦ij, 6⟧;
    omega = resultTrain⟦ij, 7⟧;
    alpha = resultTrain⟦ij, 8⟧;
    kf = resultTrain⟦ij, 9⟧;
    N0 = resultTrain⟦ij, 10⟧ * 10^7 / Nnor;

    ACurSim = FindCurDose[]; (* find curative dose *)
    (*ACurSim=resultTrain⟦ij,11⟧;(*FindCurDose[];*) (* find curative dose *) *)
    DA1 = ACurSim * nCpm; (* set curative dose *)
```

```
        Ainj = {{t1, DA1}}; (* set schedule *)
        FullSystemSolutionMD[]; (*find time of achieving of minimal N *)

        Nnres[[ij, 1]] = ij;
        Nnres[[ij, 2]] = Nn;

        (*Print[Nn];*)

        FullSystemSolutionMDStopNmin[tminNn]; (* solve it with cell proliferation stopping at this moment*)

        WriteDownMeasures[];

        check = 11; OkFlg = 1; (* to avoid possible bugs *)
        While[check < 28, If[ ! NumericQ[result[[ij, check]]], OkFlg = 0]; check++];
        If[OkFlg == 0, ij--];
      ]];
    MakeOutputForm[];
```

In[•]:=

```
(* "In the performed simulations the maximum receptors occupancy of damaged cancer cells did not exceed 10.5%,
the average value being about only about 0.3%." *)
(* Max value *)
PercentForm[Max[result[[2 ;;, 25]]]]
(* average value *)
PercentForm[Mean[result[[2 ;;, 25]]]]
```

Out[•]//PercentForm=

10.44%

Out[•]//PercentForm=

0.2688%

In[ ]:=

```
(* Minimal single curative dose: Supplementary Figure S.8, upper plots *)

(* Correlations *)
Cor = Array[f, 10];
Cor[[1]] = Correlation[result[[2 ;;, 11]], result[[2 ;;, 1]]] ; (*"κc"*)
Cor[[2]] = Correlation[result[[2 ;;, 11]], result[[2 ;;, 2]]] ; (*"κp"*)
Cor[[3]] = Correlation[result[[2 ;;, 11]], result[[2 ;;, 3]]]; (*"γ"*)
Cor[[4]] = Correlation[result[[2 ;;, 11]], result[[2 ;;, 4]]]; (*"V"*)
Cor[[5]] = Correlation[result[[2 ;;, 11]], result[[2 ;;, 5]]] ; (*"ks"*)
Cor[[6]] = Correlation[result[[2 ;;, 11]], result[[2 ;;, 6]]]; (*"ρ"*)
Cor[[7]] = Correlation[result[[2 ;;, 11]], result[[2 ;;, 7]]] ; (*"ω"*)
Cor[[8]] = Correlation[result[[2 ;;, 11]], result[[2 ;;, 8]]]; (*"α"*)
Cor[[9]] = Correlation[result[[2 ;;, 11]], result[[2 ;;, 9]]]; (*"kf"*)
Cor[[10]] = Correlation[result[[2 ;;, 11]], result[[2 ;;, 10]]] ; (*"N0"*)

(* plots of distribution and correlations *)
MinCurDoseValuePlots = GraphicsGrid[{{Histogram[Log10[result[[2 ;;, 11]]], LabelStyle → 14, PlotRange → {{0, 4}, Automatic}, ImageSize → 300,
      Ticks → {{{1, 10}, {2, 100}, {3, 1000}, {4, 10 000}}, Automatic}], BarChart[{Table[Abs[Cor[[i]]], {i, 1, 10}]},
      PlotRange → {-0.05, 0.65}, ChartStyle → Table[If[Cor[[i]] > 0, Darker[Green], Darker[Red]], {i, 1, 10}],
      ChartLabels → {"κc", "κp", "γ", "V", "ks", "ρ", "ω", "α", "kf", "N0"}, LabelStyle → 14, ImageSize → 300]}}, ImageSize → 650]
```

Out[ ]=

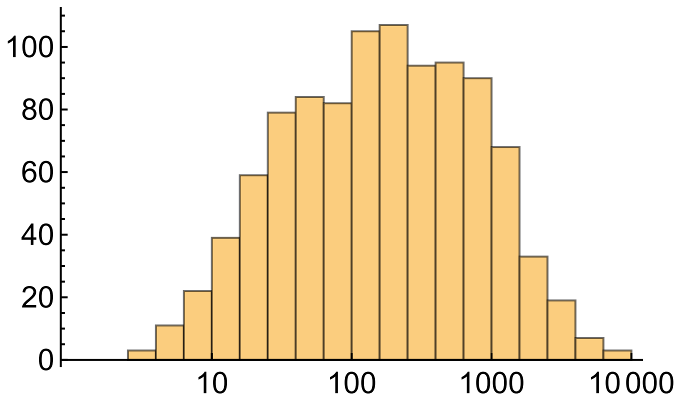

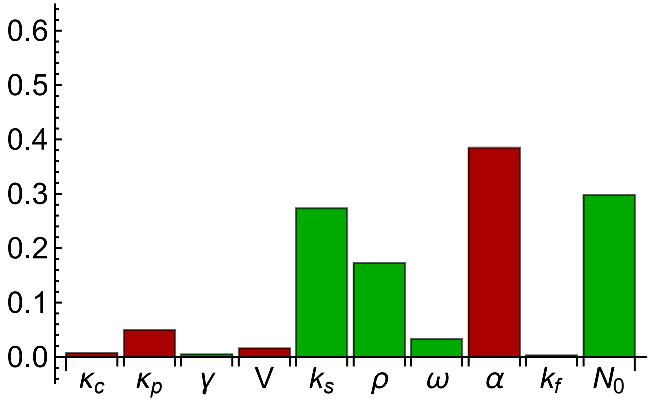

In[ ]:=

```
(* "The generated data suggests that adding 13 % to each estimated dose ensures cure
of all the considered virtual mice in the training set." *)
PercentForm[Max[Abs[result[[2 ;;, 13]]]]]
(* it is actually less than 11%, so 13% allows to play even safer *)
```

Out[ ]//PercentForm=

10.87%

In[ ]:=

```
(* "the values of errors correlate with some of the model parameters" *)
Table[ListPlot[Thread[{result[[2 ;;, ii]], result[[2 ;;, 13]]}], AxesLabel → {result[[1, ii]], "Error of A^est_cur"}], {ii, 1, 10}]
```

Out[ ]=

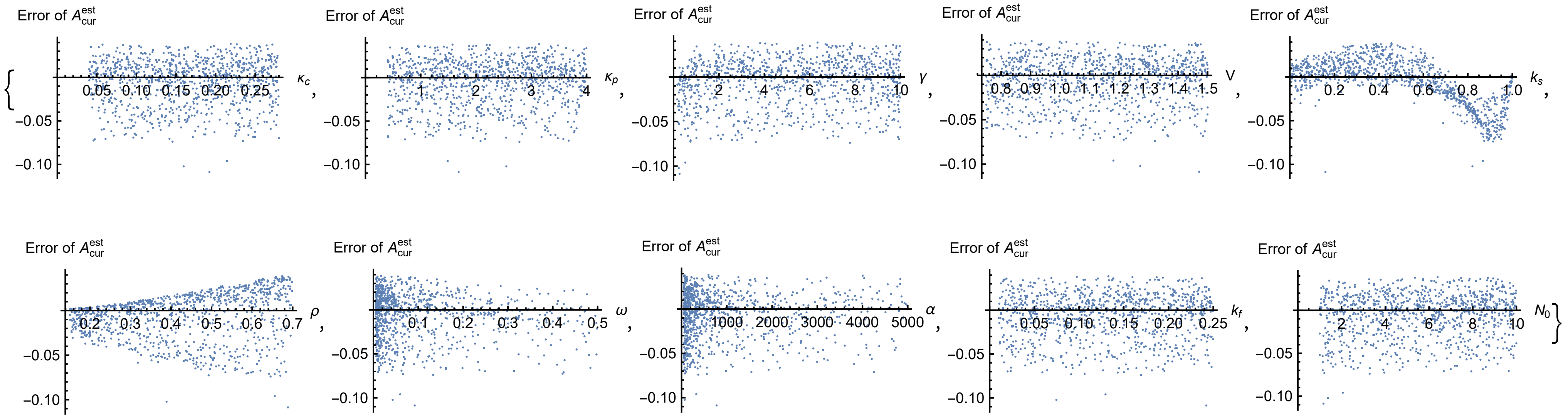

In[ ]:=

```
(* Statistical corrections of analytical estimations 1: Supplementary Figure S.8, lower left plot *)
ii = 5;
Show[ListPlot[Thread[{result[[2 ;;, ii]], result[[2 ;;, 13]]}],
   AxesLabel → {result[[1, ii]], "Error"}, Ticks → {Automatic, Table[{0.02 * i, PercentForm[0.02 * i]}, {i, -5, 4}]}],
  Plot[-2.6 * (-ks + 1) * Exp[-(12 * (-ks + 1))], {ks, 0, 1}, PlotStyle → Darker[Brown]]]
```

Out[ ]=

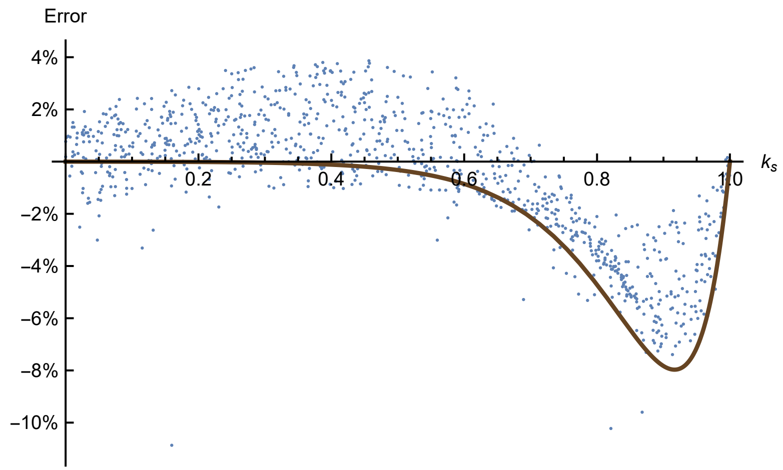

In[ ]:=

```
(* Statistical corrections of analytical estimations 2: Supplementary Figure S.8, lower middle plot *)
Show[ListPlot[ Thread[{result[[2 ;;, 3]] * result[[2 ;;, 10]],
    (1 + 2.6 * (-result[[2 ;;, 5]] + 1) * Exp[-(12 * (-result[[2 ;;, 5]] + 1))]) * result[[2 ;;, 12]] / result[[2 ;;, 11]] - 1}], AxesLabel → {"γ N0", "Error"},
  Ticks → {Automatic, Table[{0.02 * i, PercentForm[0.02 * i]}, {i, -5, 4}]}, PlotRange → {{0, 100}, {-0.12, 0.07}}]
 , Plot[-0.02 - 0.12 * Exp[-x / 3], {x, -100, 100}, PlotStyle → Darker[Brown]]]
```

Out[ ]=

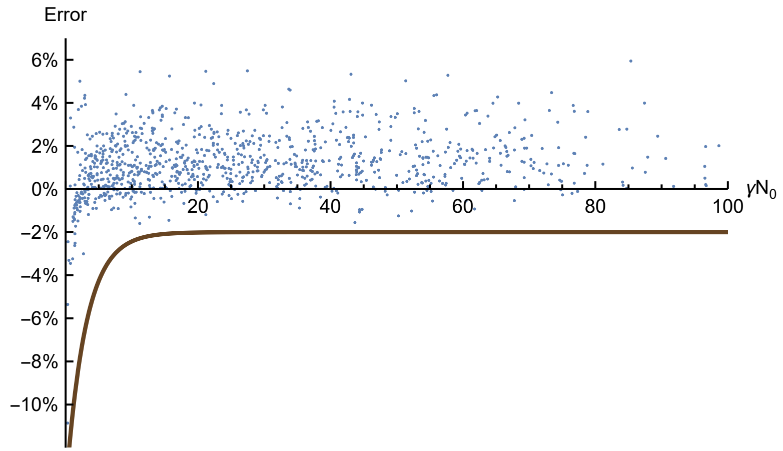

In[ ]:=

```
(* Relative errors of analytical estimations: Supplementary Figure S.8, right plot *)

AcurStatCor = (1 + 0.02 + 0.12 * Exp[-result[[2 ;;, 3]] * result[[2 ;;, 10]] / 3]) *
    (1 + 2.6 * (-result[[2 ;;, 5]] + 1) * Exp[-(12 * (-result[[2 ;;, 5]] + 1))]) * result[[2 ;;, 12]];
AcurStatCorErr = AcurStatCor / result[[2 ;;, 11]] - 1;
Pic1 = Histogram[{result[[2 ;;, 13]], (* error due to initial analytical formula *)
    AcurStatCorErr (* error with account for statistical corrections *),
    1.13 * result[[2 ;;, 12]] / result[[2 ;;, 11]] - 1 (* shift by 13% percent without statistical corrections *)
  }, Ticks → {Table[{0.05 * i, PercentForm[0.05 * i]}, {i, -5, 4}], Automatic}, PlotRange → {{Min[result[[2 ;;, 13]]], 0.2}, Automatic},
  LabelStyle → 14, ChartStyle → {Lighter[Lighter[Blue]], Orange, White}, ImageSize → 300]
```

Out[ ]=

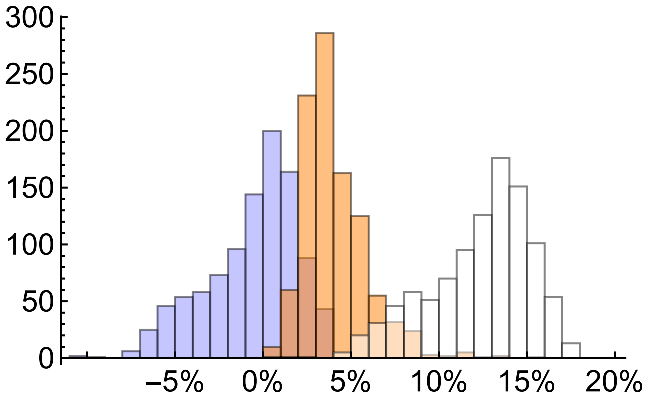

In[ ]:=

```
(* "It is however still needed to evaluate whether thus assessed minimal curative dose is expected to be lethally toxic by itself
for some mice. The training set contains four lethal cases." *)
Length[Select[(result[[2 ;;, 16]] + result[[2 ;;, 19]]) * nCpm, # > Abld &]]
```

Out[ ]=

4

In[ ]:=

```
(* Fraction of injected activity, released in blood from antibodies: Supplementary Figure S.9, upper row *)
FrAcBlAb = result[[2 ;;, 19]] / result[[2 ;;, 11]];

(* Correlations *)
Cor = Array[f, 10];
Cor[[1]] = Correlation[FrAcBlAb, result[[2 ;;, 1]]] ; (*"κc"*)
Cor[[2]] = Correlation[FrAcBlAb, result[[2 ;;, 2]]] ; (*"κp"*)
Cor[[3]] = Correlation[FrAcBlAb, result[[2 ;;, 3]]]; (*"γ"*)
Cor[[4]] = Correlation[FrAcBlAb, result[[2 ;;, 4]]]; (*"V"*)
Cor[[5]] = Correlation[FrAcBlAb, result[[2 ;;, 5]]] ; (*"ks"*)
Cor[[6]] = Correlation[FrAcBlAb, result[[2 ;;, 6]]]; (*"ρ"*)
Cor[[7]] = Correlation[FrAcBlAb, result[[2 ;;, 7]]] ; (*"ω"*)
Cor[[8]] = Correlation[FrAcBlAb, result[[2 ;;, 8]]]; (*"α"*)
Cor[[9]] = Correlation[FrAcBlAb, result[[2 ;;, 9]]]; (*"kf"*)
Cor[[10]] = Correlation[FrAcBlAb, result[[2 ;;, 10]]] ; (*"N0"*)

FrAcBlAbValuePlots = GraphicsGrid[
  {{Histogram[Log10[FrAcBlAb], Ticks → {Table[{Log10[0.01 * 10^i], PercentForm[0.01 * 10^i]}, {i, -2, 2}], Automatic}, LabelStyle → 14,
      ImageSize → 300, PlotRange → {{Log10[0.00003], Log10[0.03]}, Automatic}], BarChart[{Table[Abs[Cor[[i]]], {i, 1, 10}]},
      PlotRange → {-0.05, 0.65}, ChartStyle → Table[If[Cor[[i]] > 0, Darker[Green], Darker[Red]], {i, 1, 10}],
      ChartLabels → {"κc", "κp", "γ", "V", "ks", "ρ", "ω", "α", "kf", "N0"}, LabelStyle → 14, ImageSize → 300]}}, ImageSize → 650]
```

Out[•]=

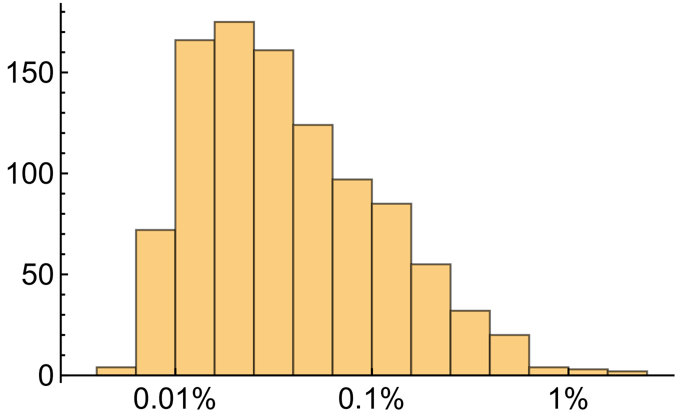

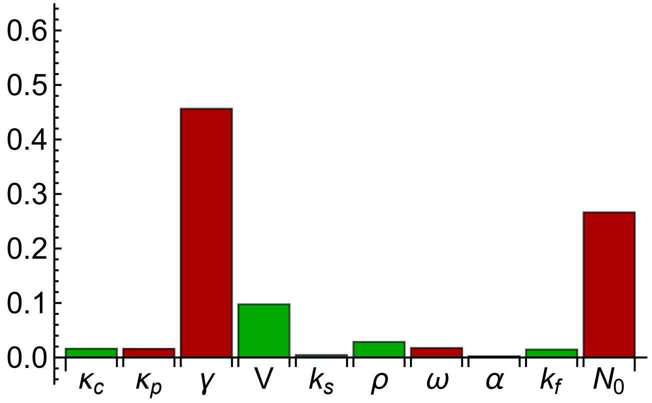

In[•]:=

```
(* Correlations of error of estimation of fraction of injected activity, released in blood from antibodies with model parameters *)
Table[ListPlot[Thread[{result[[2 ;;, ii]], result[[2 ;;, 21]]}], AxesLabel → {result[[1, ii]], "FrAcBlAb"}], {ii, 1, 10}]
```

Out[•]=

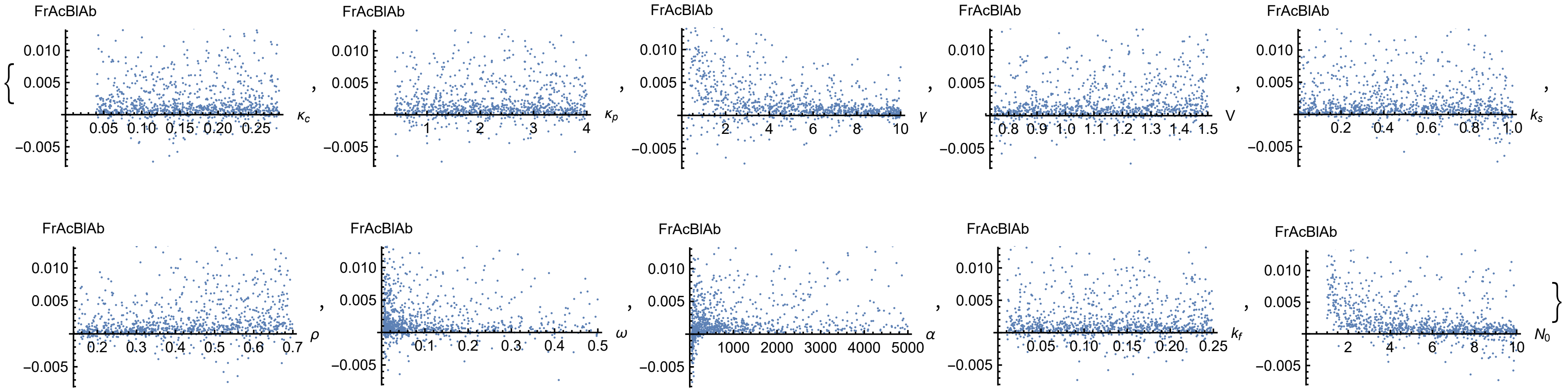

In[ ]:=

```
(* Statistical corrections of analytical estimations: Supplementary Figure S.9, upper right plot *)
Show[ListLogLinearPlot[Thread[{result⟦2 ;;, 8⟧, result⟦2 ;;, 21⟧}], AxesLabel → {"α", "FrAcBlAb"},
   PlotRange → {Automatic, {-0.03, 0.05}}, Ticks → {Automatic, Table[{0.01 * i, PercentForm[0.01 * i]}, {i, -5, 4}]}],
  LogLinearPlot[0.014 * (Tanh[(x - 200) / 70] - 1), {x, -5000, 5000}, PlotStyle → Darker[Brown]], ImageSize → 500]
```

Out[ ]=

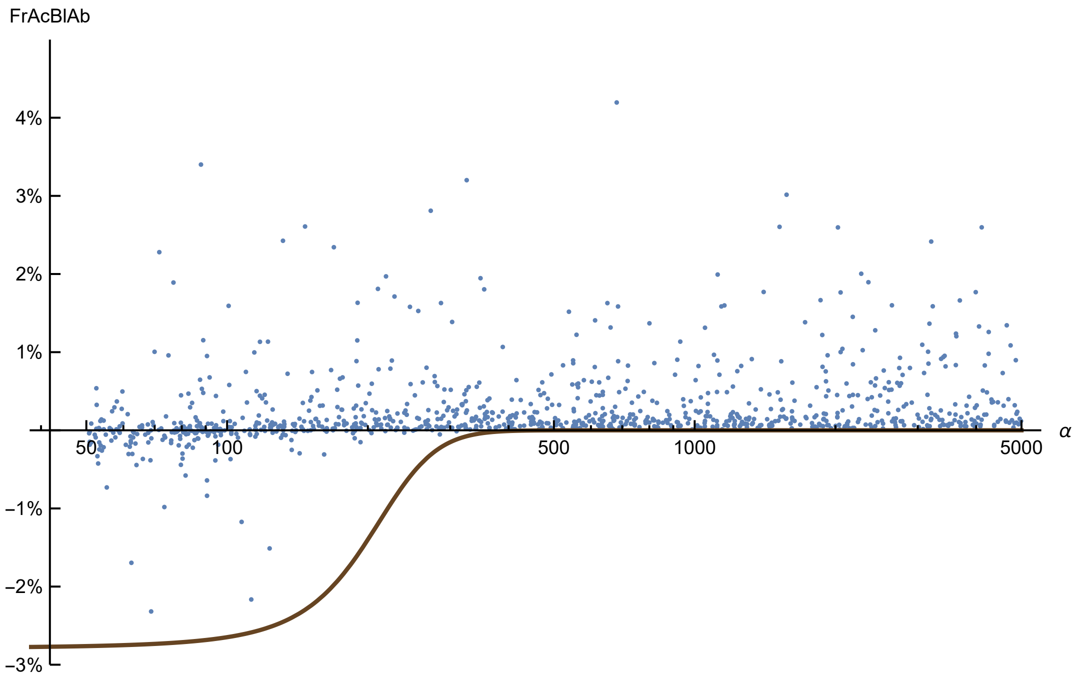

In[ ]:=

```
(* "Activity, released in blood from antibody fragments. This path of activity is generally more significant.
   Among all the decays in blood in all the simulations of this parameter sweep, ≈97.2 % of them happen via this path." *)
PercentForm[Total[result⟦2 ;;, 16⟧] / (Total[result⟦2 ;;, 16⟧] + Total[result⟦2 ;;, 19⟧])]
```

Out[ ]//PercentForm=

97.22%

In[ ]:=

```
(* Fraction of injected activity, released in blood from antibody fragments: Supplementary Figure S.9, lower row *)

FrAcBlFr = result[[2 ;;, 16]] / result[[2 ;;, 11]];

(* Correlations *)
Cor = Array[f, 10];
Cor[[1]] = Correlation[FrAcBlFr, result[[2 ;;, 1]]] ; (*"κc"*)
Cor[[2]] = Correlation[FrAcBlFr, result[[2 ;;, 2]]] ; (*"κp"*)
Cor[[3]] = Correlation[FrAcBlFr, result[[2 ;;, 3]]]; (*"γ"*)
Cor[[4]] = Correlation[FrAcBlFr, result[[2 ;;, 4]]]; (*"V"*)
Cor[[5]] = Correlation[FrAcBlFr, result[[2 ;;, 5]]] ; (*"ks"*)
Cor[[6]] = Correlation[FrAcBlFr, result[[2 ;;, 6]]]; (*"ρ"*)
Cor[[7]] = Correlation[FrAcBlFr, result[[2 ;;, 7]]] ; (*"ω"*)
Cor[[8]] = Correlation[FrAcBlFr, result[[2 ;;, 8]]]; (*"α"*)
Cor[[9]] = Correlation[FrAcBlFr, result[[2 ;;, 9]]]; (*"kf"*)
Cor[[10]] = Correlation[FrAcBlFr, result[[2 ;;, 10]]] ; (*"N0"*)

FrAcBlFrValuePlots = GraphicsGrid[{{Histogram[FrAcBlFr, Ticks → {Table[{0.02 * i, PercentForm[0.02 * i]}, {i, 1, 16}], Automatic}, PlotRange →
        {{Min[FrAcBlFr], Max[FrAcBlFr]}, Automatic}, LabelStyle → 14, ImageSize → 300], BarChart[{Table[Abs[Cor[[i]]], {i, 1, 10}]},
      PlotRange → {-0.05, 0.65}, ChartStyle → Table[If[Cor[[i]] > 0, Darker[Green], Darker[Red]], {i, 1, 10}],
      ChartLabels → {"κc", "κp", "γ", "V", "ks", "ρ", "ω", "α", "kf", "N0"}, LabelStyle → 14, ImageSize → 300]}}, ImageSize → 650]
```

Out[ ]=

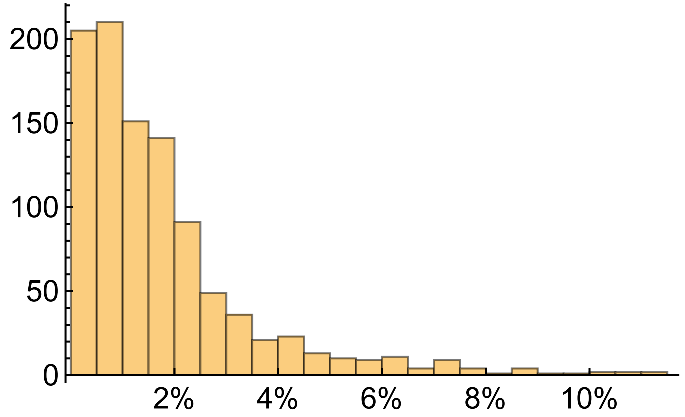

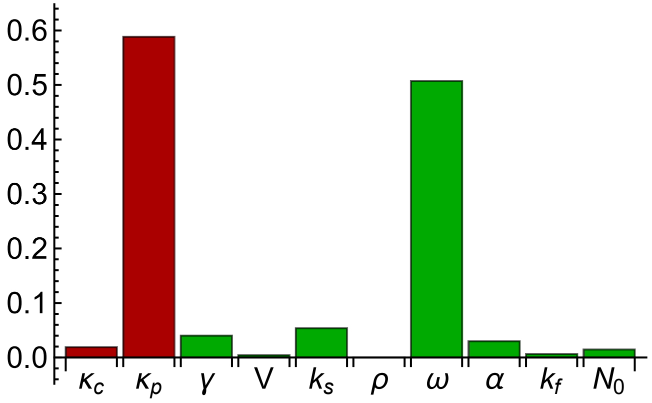

In[ ]:=

```
(* "the analytical error correlates with the values of parameters that determine the value
   of the curative dose, with the coefficient of self -damage significance ks being the strongest correlator" *)
Table[ListPlot[Thread[{result⟦2 ;;, ii⟧, result⟦2 ;;, 18⟧}], AxesLabel → {result⟦1, ii⟧, "FrAcBlFr"}], {ii, 1, 10}]
```

Out[ ]=

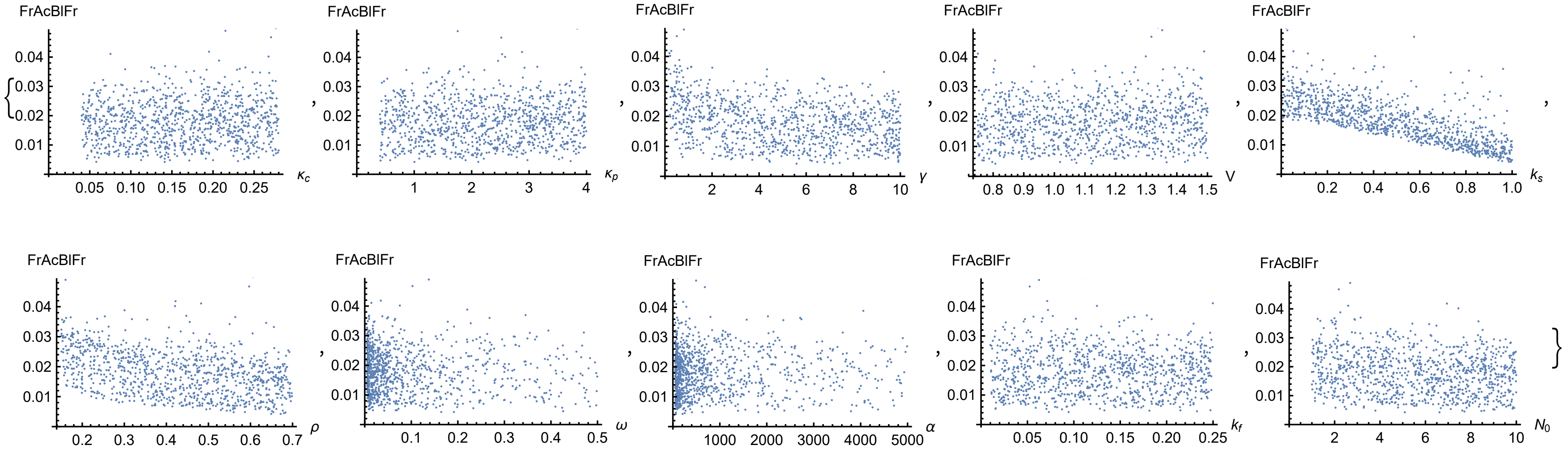

In[ ]:=

```
(* Statistical corrections of analytical estimations: Supplementary Figure S.9, lower right plot *)
Show[ListPlot[Thread[{result[[2 ;;, 5]], result[[2 ;;, 18]]}],
   AxesLabel → {"ks", "FrAcBlFr"}, Ticks → {Automatic, Table[{0.01 * i, PercentForm[0.01 * i]}, {i, -5, 5}]}],
  Plot[0.015 * (1 - x) + 0.003, {x, 0, 1}, PlotStyle → Darker[Brown]]]
```

Out[ ]=

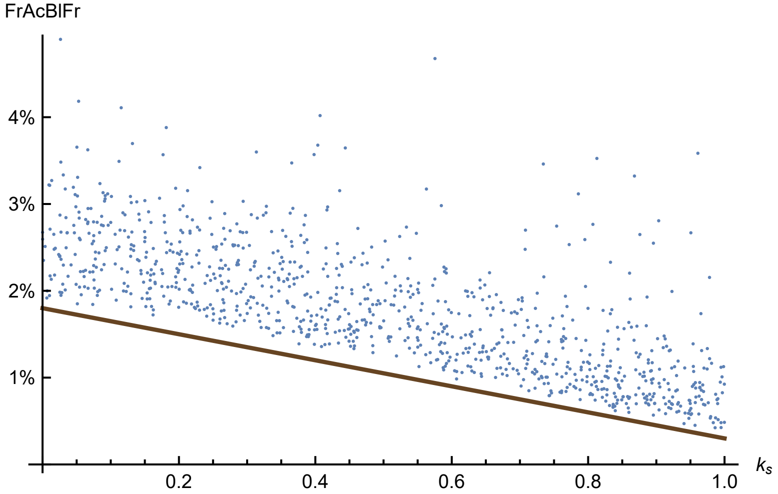

In[ ]:=

```
(* Supplementary Figure S.10 *)

AcurStatCor = (1 + 0.02 + 0.12 * Exp[-result[[2 ;;, 3]] * result[[2 ;;, 10]] / 3]) *
    (1 + 2.6 * (-result[[2 ;;, 5]] + 1) * Exp[- (12 * (-result[[2 ;;, 5]] + 1))]) * result[[2 ;;, 12]];
FrAcBlAbESTcorr = (result[[2 ;;, 20]] / result[[2 ;;, 11]]) * (1 - 0.014 * (Tanh[(result[[2 ;;, 8]] - 200) / 70] - 1));
FrAcBlFrESTcorr = (result[[2 ;;, 17]] / result[[2 ;;, 11]]) * (1 - 0.015 * (1 - result[[2 ;;, 5]]) - 0.003);
ToxEST = AcurStatCor * (FrAcBlAbESTcorr + FrAcBlFrESTcorr);

rr = 1.05 * Max[Max[result[[2 ;;, 16]] + result[[2 ;;, 19]]], Max[(result[[2 ;;, 17]] + result[[2 ;;, 20]]) * (1.12 * result[[2 ;;, 12]] / result[[2 ;;, 11]])]];

Show[ListLogLogPlot[Thread[{result[[2 ;;, 16]] + result[[2 ;;, 19]], ToxEST}],
  AxesLabel → {"A_bl^sim", "A_bl^est"}, PlotRange → {{0, rr}, {0, rr}}], Plot[x, {x, 0, rr}],
 LogLogPlot[{Abld / nCpm}, {x, 0, rr}, PlotStyle → Directive[Gray, Dashed]],
 LogLogPlot[{100 * (x - Abld / nCpm)}, {x, 100, rr}, PlotStyle → Directive[Gray, Dashed]], ImageSize → 600]
```

Out[∘]=

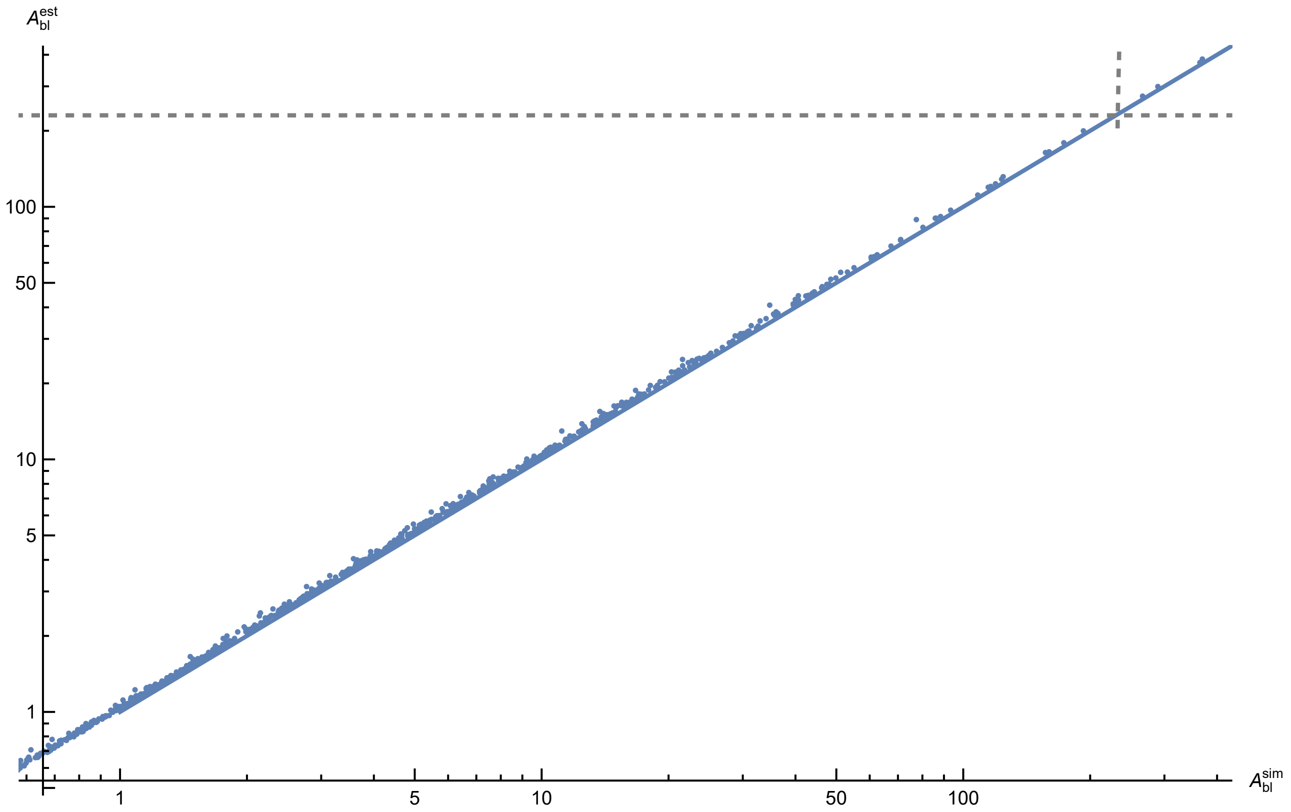

In[∘]:=

```
(* The outcome of the test, that was generated in this study, is saved here in a closed cell -- open this group of cells to see it,and run it to upload it *)
```

In[∘]:=

```
Npar = 1000;
Patients = {{"kappac", "kappap", "gamma", "V", "ks", "rho", "omega", "alpha", "kf", "N0"}, {0.2135521539372217`, 3.961577083383463`,
    0.017449556363494628`, 0.8968357850841964`, 0.5526193340404524`, 0.44754718012416395`, 0.060348499488465 74`, 106.16254136487882`,
    0.10190317263014587`, 883.5270672679858`}, {0.07152561216059383`, 2.5169021554225424`, 0.04134379025654024`,
    0.9380911271372574`, 0.93412718926926`, 0.17225303021845306`, 0.1175689330301931`, 2613.4682606921233`, 0.18822840660147933`,
    493.53347054969345`}, {0.08527801742841884`, 2.3342943915916523`, 0.007356128912919502`, 1.449543423692679`,
    0.2920284025196249`, 0.42703227038753633`, 0.03973423147454342`, 243.18803536822332`, 0.14706039466941528`, 772.0773970550337`},
   {0.08032499747193339`, 3.3228245803820906`, 0.058505885415950074`, 1.328072711539189`, 0.9755298339255658`,
    0.3834882995147877`, 0.016384649136306686`, 81.13118073476093`, 0.12842007570807257`, 778.1337523863579`},
   {0.12288971305592977`, 0.46847473663449035`, 0.035027886845599224`, 0.9755484915941668`, 0.875011150725298`, 0.508807503319318`,
    0.009673321455752245`, 67.20829764486504`, 0.24949007211501428`, 854.477971991049`}, {0.23358644138244972`, 1.7807941427787792`,
```

0.06034630860175783`, 1.045216035784662`, 0.6114168239067443`, 0.23619205669697263`, 0.3250660132336067`, 1792.5878930475694`, 0.0744857171754702`, 805.4037858164254`}, {0.2672425581653916`, 1.5294271887111437`, 0.07676744398776716`, 1.292359521032572`, 0.8529891799096945`, 0.393740102898689`, 0.1380993699197939`, 2546.257830379171`, 0.12485562910431564`, 269.9458351710083`},
{0.23183312051487565`, 3.3970641967209048`, 0.08395863347805453`, 1.1558870614014223`, 0.12544854381867032`, 0.6108675290264531`, 0.011088452522675474`, 482.67672135456445`, 0.02718649446571869`, 273.7693703287759`}, {0.08728636941166823`, 1.601719923640072`, 0.02854064232033517`, 1.12476800155736`, 0.3992080927357673`, 0.5147003606496414`, 0.06898789718338541`, 4299.502344187501`, 0.1107223992523067`, 582.4485009262825`}, {0.06789465848570125`, 3.3425223793990293`, 0.05746465325158763`, 1.2855437262048381`, 0.9962937730263095`, 0.5148610577647594`, 0.34967229657185783`, 129.46101746656242`, 0.19161277365419005`, 109.49077359602803`}, {0.21068632540051146`, 0.7531370432799127`, 0.03477678881607343`, 0.8221096630790845`, 0.8211187339561399`, 0.3670710605309486`, 0.2800655063342806`, 102.20325958949621`, 0.2320804455102461`, 131.9450462497348 7`},
{0.15242131810791038`, 3.789625832521282`, 0.044532267365124396`, 1.387509696935342`, 0.3025280009740767`, 0.43327817796146684`, 0.05700485989023632`, 1418.28871606834`, 0.07523468846369646`, 480.71702187415434`}, {0.047127741426415765`, 2.196394146604325`, 0.0020860451850145266`, 1.253945549403835`, 0.04333275562133232`, 0.4635809712022565`, 0.09752500840122344`, 2979.919142064 5693`, 0.19800512345941224`, 326.8262854148691`}, {0.21354080576404838`, 0.499272990997762`, 0.02136565456468402`, 1.4494872415741291`, 0.4261365642523509`, 0.22487734950611293`, 0.3748843819820306`, 66.77722732862037`, 0.11979197850581236`, 963.8394274052501`},
{0.06484047241450586`, 3.036571492120566`, 0.07651087746736028`, 0.9711768655928728`, 0.4574624946507835`, 0.5217597270510685`, 0.12598093752406897`, 242.96341995255446`, 0.14545623834203425`, 981.9658120220585`}, {0.2281503022999219`, 2.470286745790686`, 0.06276936772487546`, 1.1988495043217857`, 0.7133186669750637`, 0.3930261678642514`, 0.3166839777189734`, 137.26966163233058`, 0.159523733253794`, 623.7392541692013`}, {0.2086500877706095`, 1.2720061208981175`, 0.06218340621581904`, 0.9797444420614967`, 0.04659359568256205`, 0.28748130863864596`, 0.3811626852138692`, 349.8696799206167`, 0.19707584717198728`, 104.08157372885026`},
{0.11607219052414597`, 2.714811676304598`, 0.010818112381176023`, 0.8421879055822536`, 0.6583674774374331`, 0.40051060979188435`, 0.25210876559776124`, 309.1166496608548`, 0.2109324247903891`, 983.8961564091211`},
{0.0729343665897485`, 1.3500826412359075`, 0.07266241705510545`, 1.1245658298901933`, 0.8437686840308298`, 0.25155713345223996`, 0.03143796678764096`, 51.35885900577598`, 0.018795747835889998`, 737.3104099861529`},
{0.13040192171577758`, 2.9737527300384095`, 0.07310292644734392`, 1.466567655421121`, 0.5616562924844843`, 0.2174201253491196`, 0.017973630894190788`, 381.0744410819025`, 0.2070882324093905`, 363.12098913994095`}, {0.12850488774735863`, 1.94264952671025`, 0.05360394961309327`, 1.3166582015996433`, 0.7420300946941296`, 0.6747046075237411`, 0.46225759842348835`, 653.3639045731352`, 0.1781000624097991`, 153.16234178224983`}, {0.14131707783478825`, 2.47648774559096`, 0.046912654007802104`, 1.102943262592814`, 0.2119600715047596`, 0.4901715753981759`, 0.0580109813149486`, 2619.9859648711445`, 0.01885924901675312`, 866.9553146407405`},
{0.25596962129585343`, 1.7441590604011816`, 0.07394014025769799`, 1.0817705534126583`, 0.7417269961287187`, 0.3670491724777639`, 0.009656798208801406`, 431.47684692132157`, 0.21109646038438556`, 541.8299411106649`},

{0.17137889910326493`, 2.547743818855161`, 0.051249488967469524`, 1.4387428259441999`, 0.22533128633818977`, 0.21356004296130449`,
0.010718966990308196`, 426.834678908343`, 0.10633739166308992`, 565.8122888467649`}, {0.1356919186397164`, 3.275909693087268`,
0.09755833451316673`, 0.830833686577428`, 0.23758326238226246`, 0.37684838867012627`, 0.34622549381973555`, 2800.980761749347`,
0.05589233942552713`, 397.59708636141903`}, {0.06319547070029213`, 3.0135506306602915`, 0.0865865693268 8455`,
0.8750884263433183`, 0.43559283695953344`, 0.17423436075008547`, 0.3556921476320783`, 692.9068977663358`, 0.042185132157772975`,
222.69074576769867`}, {0.051353934055559936`, 3.5419315477765503`, 0.04801539957297914`, 1.3650767438471239`,
0.35603852921000145`, 0.4618444822640493`, 0.00593767767296163`, 2613.749958603078`, 0.019287810526956883`, 756.2455097050401`},
{0.20935469703068038`, 3.6183197679011254`, 0.03566796922371117`, 1.1753688638954878`, 0.7004011433506241`,
0.18665090004926455`, 0.010273985816104142`, 1326.129340467039`, 0.14178606357406276`, 910.8324510297567`},
{0.25107423404415036`, 0.9233938441474328`, 0.08706047090121377`, 0.9278477911660168`, 0.6395373622443865`,
0.41454776302472407`, 0.011168650588331405`, 350.2039717436121`, 0.018063541364192554`, 440.5654385955753`},
{0.18503330381957606`, 1.3843983227309558`, 0.05599341470950066`, 1.2326838759285517`, 0.5366716060108483`,
0.5872039546866128`, 0.021673868633287827`, 440.55468856416707`, 0.05873091092641808`, 958.3232953637463`},
{0.06679430502398687`, 2.97961750490882`, 0.06874693539558713`, 0.8324720698932511`, 0.047831959104126964`,
0.23900392037978713`, 0.05029201371754466`, 1312.9498214501468`, 0.05149188289007395`, 357.1225656698418 3`},
{0.2633133297348904`, 1.167395880011603`, 0.02628362214068364`, 1.0744483713964295`, 0.2579472965293179`, 0.6802631987807441`,
0.10725370629344262`, 53.88085410525317`, 0.04507037997684166`, 433.7101304465891`}, {0.2010390664309742`, 0.9179437940816833`,
0.04118734602457437`, 1.4112901498922994`, 0.03943650490351103`, 0.5936392993401745`, 0.007904983488694988`, 372.8977436408934`,
0.1257212210729608`, 514.9151276266798`}, {0.2679869983047456`, 2.831371041491759`, 0.04323230583074366`, 1.0163736311152665`,
0.9719920644948712`, 0.3094609684087656`, 0.02204068430568152`, 76.16804088198147`, 0.16897723749861843`, 499.6307316546634`},
{0.05683769076526879`, 0.650647885401221`, 0.02840792532917323`, 1.1275069757067548`, 0.7854264529060555`,
0.22126398655563229`, 0.06980518344934367`, 574.9844145764067`, 0.1210354338257757`, 562.7623109630207`},
{0.04996310769523532`, 2.710365865528229`, 0.02845470152648966`, 1.3080682866189928`, 0.07861434689660651`,
0.6705317255564853`, 0.006348108900509377`, 549.0755816993258`, 0.061170873719453955`, 318.91974598994375`},
{0.2755006956503554`, 3.698123728642071`, 0.09922032193054331`, 0.8332466983901297`, 0.2785036755762762`, 0.43490103767019017`,
0.15490829410477724`, 4617.932809455717`, 0.11837182487721798`, 340.2748740925912`}, {0.2734635980899412`, 1.5929961417122707`,
0.06137119836965004`, 0.7652341524879904`, 0.954168112916443`, 0.43307045621476403`, 0.1582788386688816`, 71.48508495772737`,
0.24624735395364544`, 972.2197337262259`}, {0.2181683242113333`, 2.1761487384705624`, 0.08088096446343027`, 1.0959670952629994`,
0.6079403055392678`, 0.3741554091001601`, 0.02009386290007227`, 69.7071844679123`, 0.040607395764710064`, 305.00876855756474`},
{0.24133385636483384`, 2.7891523258501225`, 0.005829082609902692`, 0.8597961081451015`, 0.9570483419454672`, 0.29444398106251457`,
0.04229917912093678`, 1941.779238616586`, 0.19957823673360453`, 490.6542116348769`}, {0.1230694271594923 7`, 2.252880201646872`,

0.011446250013559797`, 1.0462863109436527`, 0.5663933692592433`, 0.47495024626336324`, 0.3233936229451822`, 86.1453410021763 5`,
0.0819767202093 1229`, 782.5197776099736`}, {0.10769046541924182`, 2.4605116390609316`, 0.05377772146881331`, 0.989407475316354`,
0.2930401465570722`, 0.29181294086362775`, 0.10863060011045422`, 4646.540814363835`, 0.22419809836059507`, 512.9803490302319`},
{0.21497939836377283`, 3.1512094394139076`, 0.05781466570999411`, 0.9235677861713979`, 0.797875992877098`,
0.30592437760429736`, 0.011963817124529342`, 496.30104999186307`, 0.12299294969111368`, 343.6950402862427`},
{0.2657260134641566`, 2.9019690638082407`, 0.03280205239027208`, 1.2552566977527801`, 0.09347633453726867`,
0.6906835420223192`, 0.05597654901690233`, 214.21462946437896`, 0.14839555678735822`, 189.76155409360942`},
{0.21292359448836545`, 3.6237126070126635`, 0.023273632947346867`, 1.0934162632306244`, 0.6322993827793542`, 0.5537399147284487`,
0.3715227242167489`, 758.3220767157304`, 0.23574243432919756`, 314.70942110857914`}, {0.23082110188218175`, 3.79416458010805 7`,
0.09951024223913159`, 1.1044050381914734`, 0.2931375323115071`, 0.40868926988441434`, 0.10698117113686255`, 1766.3231774314004`,
0.1575507952957782`, 782.0701995703548`}, {0.1731048010854444`, 0.4511019492052632 5`, 0.06558149662017904`, 1.268779063180271`,
0.15752730810346982`, 0.1844619681378331`, 0.4734368872326295`, 214.57885630606498`, 0.10442024095385904`, 238.91185603032366`},
{0.2554292953302569`, 1.1427932465434605`, 0.04899287050638531`, 1.3736804562756375`, 0.5882660335289671`,
0.17180037030824735`, 0.05309276876348573`, 276.71914948998887`, 0.05937534025119473`, 386.16358526001164`},
{0.16644797368483177`, 2.4992189221756043`, 0.004445377489754368`, 1.4133660089877695`, 0.32679827704181763`,
0.3418600753563352`, 0.38105352658785596`, 1798.4227174359469`, 0.0853854152424785`, 140.42796197604304`},
{0.05401664285302743`, 1.3436659136216367`, 0.007303531204713778`, 0.8064085762190545`, 0.4041746333332612`,
0.5906806798990734`, 0.005958103091852778`, 2587.5652974623863`, 0.09992354552595534`, 820.3533386597787`},
{0.20307037888791557`, 1.4293968611238235`, 0.007006708117720848`, 1.1406730143707504`, 0.6144629354618545`,
0.3719133121966949`, 0.00813248394206873`, 121.80260956296803`, 0.14105543387259395`, 126.00065959923121`},
{0.2740843330487453`, 3.308166539499317`, 0.0033332412164113734`, 0.9387759672621804`, 0.8792897541259657`,
0.2588278323921356`, 0.0906560451270411`, 289.10750886695166`, 0.0689222507314608`, 274.0455429116968`},
{0.0941844879899188`, 3.012435636197103`, 0.0977143251520188`, 1.4935685648224934`, 0.03172920895860054`,
0.19879535583488106`, 0.12277001567292761`, 281.6381134207748`, 0.12553563404062174`, 818.5606422688558`},
{0.13120359665298414`, 3.8066558838990874`, 0.05832666562127484`, 1.4436216710518677`, 0.7256118790918118`,
0.22190148447236047`, 0.055627038916505056`, 331.2421431929122`, 0.023623634114193437`, 105.97098539393085`},
{0.1599611237255819`, 1.5653869796304836`, 0.04073827294465206`, 1.420732828376658`, 0.4167553998465383`,
0.5683789027240399`, 0.006867177748429021`, 437.8657191219929`, 0.20093958315759247`, 853.3201855869486`},
{0.21575208583629502`, 0.9356056552129512`, 0.03505308262533445`, 1.1968736187898597`, 0.7561599337939984`,
0.4654145195769487`, 0.10760666835377902`, 1053.3658392946543`, 0.20711098924554222`, 210.78286348037096`},
{0.14749719864367028`, 1.1298646044981888`, 0.09689905730204273`, 0.831362255602855`, 0.5798187583416305`,

0.19996262727341718`, 0.02080299930415397`, 1905.6099187599903`, 0.03509497286958235`, 329.9767515874418`},
{0.05611993210971444`, 2.018253520390404`, 0.05603878791329802`, 1.1233601094750865`, 0.7854886288850511`,
0.27746521744215147`, 0.005322507239848602 5`, 101.97524414758992`, 0.19072350614781147`, 586.0302656773133`},
{0.07988047106873392`, 1.2467973601355782`, 0.005734825596729962`, 1.4870141755274235`, 0.786482359746921`,
0.40753461440319994`, 0.0616456048522424`, 430.36934645621176`, 0.11501201441136555`, 555.9632996241309`},
{0.21976948435818772`, 2.6949030865877113`, 0.004565631201115927`, 0.851615304193428`, 0.28689924805671385`,
0.2511775784606734 6`, 0.1420011334809148`, 365.7629364689339`, 0.17048089811131317`, 458.8930260541114`},
{0.24569357480220383`, 2.806982006055639`, 0.03541916579786691`, 1.1486472973181063`, 0.9842633355008354`,
0.6578328260657522`, 0.06186663500336862`, 676.0260667202342`, 0.15355635401466894`, 104.93495329528014`},
{0.2568863641209479`, 0.7034804925577145`, 0.0941603933918832`, 0.9631193622252813`, 0.4879437161835407`,
0.2995943026897797`, 0.00877818042938852`, 1278.8744528151467`, 0.17037713399806426`, 719.843140498881`},
{0.15208884515258675`, 3.3804365982678615`, 0.007505379740790073`, 0.8013128272354585`, 0.48161188241254527`,
0.28391647832046085`, 0.008016681423664398`, 51.406574561981365`, 0.07108676915203138`, 187.7624783373312`},
{0.10637517181381878`, 2.6673257763233797`, 0.07389800171077983`, 1.0335545955573995`, 0.6258721856651546`,
0.552024947614703`, 0.393437725623221 1`, 1365.910979824601`, 0.017092456029375425`, 449.7500884619777`},
{0.0649812277693595`, 2.884896944749496`, 0.07783207858895651`, 1.428219843208142`, 0.5851112039722763`,
0.4850552612198272`, 0.13145374596336928`, 233.36752929196032`, 0.020935430712967162`, 793.3352241051653`},
{0.22088364361500656`, 2.753665575599883`, 0.056531767859618255`, 1.38986306399133`, 0.7216745774063014`,
0.3584382836914882`, 0.15422309470836812`, 2154.2453486409945`, 0.20075088684393572`, 196.6876979035824`},
{0.17574945972757133`, 3.479082463657387`, 0.0406948089429684`, 1.0632324439925762`, 0.7336036718363608`,
0.6225865045970325`, 0.01654846847609865`, 2647.8857343385694`, 0.1742065007038966`, 538.059550515096`},
{0.04025957477335049`, 2.723445036422185`, 0.0624307243457759`, 1.2961681244376777`, 0.2134882654614516`,
0.39895923327159644`, 0.012423634234475615`, 68.80792227348101`, 0.09152513701065051`, 342.5597984149974`},
{0.23836095562622683`, 1.2444047973172507`, 0.05553779779641564`, 1.076392253001211`, 0.35117607296612463`,
0.40534329593724594`, 0.006015625989151594`, 1819.5202248158637`, 0.12753202451029555`, 623.0420974247954`},
{0.26944870454169506`, 1.2060790013825349`, 0.039462741842031746`, 1.0581301327520456`, 0.10601447465615688`,
0.354379020364461`, 0.033711267495647235`, 990.9089845688537`, 0.031312059666100456`, 967.6243986980542`},
{0.14445853797988234`, 3.535198432366248`, 0.06372334173632162`, 1.4863884210475007`, 0.7021222356246233`,
0.1986028344377223`, 0.033605257874194515`, 401.94568584516094`, 0.22631225203628458`, 497.0198172800991`},
{0.19199489014371673`, 2.559659501669885`, 0.04358276707150084`, 0.9357767944167577`, 0.5821038208204001`,
0.3881836320458101`, 0.08395917774758936`, 399.00745257526154`, 0.15489271985369857`, 774.2528196011518`},

```
{0.09844334988931319`, 1.3873519913225651`, 0.04614436554245893`, 1.1498948867492238`, 0.47347293312976735`,
  0.48947207082211097`, 0.0900112564683952`, 300.85371376045117`, 0.10865036330785854`, 289.3128973476921`},
{0.2588239052872208`, 1.9064804051930793`, 0.065214860918082`, 1.2358133969471479`, 0.9062007858068677`,
  0.6285986730260349`, 0.02508709888565183`, 60.40797732056926`, 0.24714947667059417`, 952.5811518915539`},
{0.15433829014876738`, 3.2630592980638813`, 0.03425127382548034`, 1.0548030673325137`, 0.6906919434954677`,
  0.3631290272789046`, 0.0347694820539026`, 173.0213690165559`, 0.20951393482972103`, 475.6784304680295`},
{0.07919142001495871`, 2.7255459074120383`, 0.05226476773837156`, 1.4738421429170918`, 0.07762084300215877`,
  0.32839016838251456`, 0.28010379475182423`, 1228.3196986901144`, 0.022535205722582274`, 511.1200103954101`},
{0.05563602603579554`, 2.285803588715188`, 0.011271259422675524`, 0.8886812633057506`, 0.8488682312030629`,
  0.2180988504645489`, 0.04124220143069666`, 325.42600392283333`, 0.015282318354637336`, 540.3914665193738`},
{0.26726930802972265`, 3.468951790252442`, 0.03848449069490482`, 0.8094106772420622`, 0.06275944440065384`,
  0.3246635527776901`, 0.054987775783502536`, 681.4969078295769`, 0.11090812120155558`, 354.5762000041195`},
{0.13377593551305073`, 1.1907304154621343`, 0.01146637788789656`, 0.9181303013739212`, 0.44430229039926394`,
  0.21249075461775713`, 0.11547143720610627`, 203.15849668645484`, 0.01100896387178596`, 295.5191145619232`},
{0.16315983268315204`, 1.1080296708247115`, 0.06583864001022185`, 0.865015106617715`, 0.12286793950783781`,
  0.37991054755624265`, 0.13902707267015119`, 715.7992806626531`, 0.019079624096566744`, 581.5012259249219`},
{0.1551702158696348 6`, 1.952115790572729`, 0.0731100150366914`, 0.863005494839794`, 0.11113391823909224`,
  0.23929002865779792`, 0.3427593945863136`, 157.02882367804486`, 0.1258544997368633`, 151.66501695475426`},
{0.16216149919315814`, 0.7994153119966949`, 0.07344189350605326`, 1.46448250423246`, 0.11568032132459538`,
  0.4524728031579279`, 0.007439586263343828`, 2935.686946515946`, 0.23568961866347637`, 848.5436646987821`},
{0.15863268122870933`, 2.604649964738848`, 0.09105452346714177`, 1.3281546343893726`, 0.6415701788553387`,
  0.5395588121712969`, 0.10672502596586574`, 4293.9755867585145`, 0.22612411051961012`, 801.1531009053148`},
{0.0470360951706533`, 3.5075246181417876`, 0.0689698582393753`, 1.3992260841209352`, 0.49274029849989587`,
  0.3265485661944224`, 0.0896880649482509`, 3553.0072655615363`, 0.24883204385112412`, 930.4206898584084`},
{0.1301565511337785`, 3.3013385458906184`, 0.060172545647574065`, 1.1841312709206266`, 0.7389603235116047`,
  0.6545296783352468`, 0.09213436798988452`, 632.9865742133833`, 0.01144874409169866`, 153.66076223065957`},
{0.19211271095347948`, 1.3267678786947181`, 0.011925004369487074`, 0.7708856579991501`, 0.5967808987867167`,
  0.3563983615639521`, 0.008295516898745303`, 651.3850149278022`, 0.065165960143103`, 674.6701400488206`},
{0.04092327463376352`, 0.7045023567934869`, 0.08460571137678446`, 1.4657750963462932`, 0.7181575655127523`,
  0.6488519412096585`, 0.15923360328476563`, 212.85100756959832`, 0.05159171716399186`, 462.3207119503768`},
{0.19270602889895483`, 1.9529553547869458`, 0.06583688116670341`, 1.3515290363153516`, 0.005091282852797141`,
```

0.36050263163910423`, 0.04479551947501828`, 203.77608216685013`, 0.1482624290923 4083`, 529.3883166966048`},
{0.1050601938980929`, 3.4490435237177026`, 0.08223909341236009`, 1.2367817503973777`, 0.9277363378878729`,
0.3699674586709595`, 0.4385914759613132`, 1519.1710373444578`, 0.23151573895912042`, 820.0175517477519`},
{0.05132348611758153`, 3.959642395183179`, 0.0488282767798439`, 1.2096431470311648`, 0.026920294658858257`,
0.43312132839092765`, 0.3519494697432887`, 129.77286276747867`, 0.014447876552552863`, 379.4279055426431`},
{0.13421887508919655`, 3.5366003696861075`, 0.04365656524319377`, 0.8302898645594913`, 0.33283176141556736`,
0.32180578132251225`, 0.12171969152254764`, 58.615799213607424`, 0.0646250311088258 4`, 898.0928768715434`},
{0.15819611106702364`, 2.020855817361644`, 0.06579688155882536`, 1.2380340703095258`, 0.6378555510612616`,
0.3675344788090784`, 0.006545785906113509`, 101.24936252099768`, 0.22028280764448066`, 149.80163079680509`},
{0.2499495815562608 5`, 1.6151536382110443`, 0.09538293813329457`, 1.3475056084534551`, 0.8251324191653782`,
0.518473471172061`, 0.013209857312129224`, 465.53610432365656`, 0.19186631952926742`, 938.6440248484497`},
{0.1956555609490701`, 2.03149296201667`, 0.09660508820628273`, 1.431718750027923`, 0.5308578462289135`,
0.43609275022077343`, 0.05994448278703194`, 319.5342769319219`, 0.05601191301657277`, 788.8827504958957`},
{0.23193438538805855`, 3.16433220464147`, 0.06263350952730523`, 1.484540865185697`, 0.945228356849932`,
0.5695514219689578`, 0.023879141173510178`, 147.89101930946188`, 0.24041444251942057`, 974.5971477651331`},
{0.2528274636845232`, 1.6765266145916016`, 0.05359196557993448`, 1.3463142085764375`, 0.9224467805077987`,
0.16620719793609418`, 0.013039811850177031`, 103.76980611708078`, 0.22032399406907538`, 401.8839359060937`},
{0.23077639135389166`, 2.5631050348732005`, 0.04014355344850944`, 1.1219336672611722`, 0.11159784718139387`,
0.5983828871337603`, 0.12763464721917558`, 1560.2077328425014`, 0.20349144783421902`, 903.744744090405`},
{0.06895016542438792`, 1.301044343226704`, 0.015962130237339344`, 1.182285615620497`, 0.5628258630936316`,
0.17632340412293257`, 0.3831530705725889`, 1497.278387537139`, 0.10171305044347567`, 505.35404235107535`},
{0.2787317780563141`, 2.375067420232342`, 0.0815658084141512`, 0.8993075085878712`, 0.2038087026634039`,
0.6041957560913382`, 0.324966388605867`, 215.9571285973821`, 0.13818574272027367`, 691.7030542595149`},
{0.04704135783034269`, 0.4824465321524163`, 0.021143667148849198`, 1.297225751526362`, 0.5221151518138136`,
0.4606696788974354`, 0.006779779197302622`, 185.59373538640742`, 0.01894971206264362`, 558.5730827937659`},
{0.21423251173707536`, 2.493227952439149`, 0.05289405397630384`, 1.406475234089612`, 0.7998464081533074`,
0.509476952192685`, 0.4272743578374108`, 100.59740971479944`, 0.1412992063677468`, 892.9324365177549`},
{0.07990270577478725`, 1.026053986899301`, 0.09098769304378568`, 0.75484093198576`, 0.3137514363776597`,
0.2597158546619064`, 0.021526708814218906`, 71.8454436542471`, 0.14857027641402232`, 192.33905427474235`},
{0.20790548900468359`, 1.6561792271072155`, 0.03308057679124518`, 1.2176117409116913`, 0.5649384654171494`,
0.5537564006068142`, 0.021311473455272706`, 493.7190855791356`, 0.0879661543265085`, 522.8214036804926`},

{0.23583327723859243`, 0.8244276223039284`, 0.009610054530140194`, 0.999911169587162`, 0.6818020653793861`,
0.4770396571968506`, 0.3762680384381647`, 1045.715543915765`, 0.20181855407690935`, 285.3335505694424`},
{0.2149596477715155`, 1.1258091287082204`, 0.04117204642206028`, 0.8287635491092544`, 0.19708791744131293`,
0.30242514760323325`, 0.009963068581429268`, 2766.449995395716`, 0.1599911935585479`, 542.0030818591285`},
{0.09778089450463257`, 1.5512303348716232`, 0.04607092381086256`, 0.9234013469057234`, 0.7159741715951657`,
0.15221755315547447`, 0.008899731124616716`, 1902.692936123654`, 0.11247025125228444`, 519.6608670719012`},
{0.2733616407663576`, 1.391826052520571`, 0.05242996857884462`, 1.2463936486263583`, 0.3747282584855436`,
0.40124237280450004`, 0.11539389962842635`, 292.43427931036985`, 0.183553339648669`, 592.2315796450872`},
{0.24918162326616394`, 3.2909828859462893`, 0.0031304822935452315`, 1.0930940447805135`, 0.3406640390037241`,
0.20243554875381864`, 0.028649077189173346`, 1674.4319759967557`, 0.19194057055819636`, 777.538166666535`},
{0.25645888779994136`, 2.282435807658568`, 0.08257899475169463`, 1.378184234375194`, 0.8190909920565956`,
0.23648999352950728`, 0.029414947617452787`, 2478.9160402790253`, 0.12086031199538894`, 421.4774938421854`},
{0.12282576626019515`, 1.0173035286087062`, 0.0379861701996793`, 1.401110687144116`, 0.7192159067178037`,
0.40286532167194544`, 0.36624490921162295`, 2426.5499538363206`, 0.01808445874874337`, 556.291828749522`},
{0.08314629308908505`, 1.3023656036359537`, 0.09511446512381881`, 0.821564112530474`, 0.4777933736422373`,
0.2692725743194774`, 0.006069954672804891 5`, 1469.9917989704652`, 0.23042452149338993`, 154.46286861175`},
{0.21957774039482975`, 2.751122632050148`, 0.013942756799365678`, 1.0532031012381704`, 0.8873864151539932`,
0.23272558697907686`, 0.007181177159222858`, 4013.7501241473833`, 0.14211730184734184`, 952.1784892015081`},
{0.26384054503883103`, 3.138964037644092`, 0.08426203609396872`, 1.3770189510815902`, 0.4475271713808333`,
0.3491883286754355`, 0.07774969397541334`, 618.3912182185799`, 0.06384891902606338`, 535.1175352858716`},
{0.20560343993895913`, 1.5779437043773905`, 0.07680738613512926`, 0.7894682023823474`, 0.9949259646898643`,
0.44397150101458005`, 0.018989097980746553`, 294.52516577442924`, 0.14255921229578516`, 385.97631580256467`},
{0.27238164345669874`, 3.1728219863305904`, 0.04998959318576006`, 1.2550483187184474`, 0.08966409063298642`,
0.2754392206527779`, 0.028155523330625355`, 413.44364879258364`, 0.10742347205803915`, 204.45184033599216`},
{0.2451942638086284`, 3.321324534398453`, 0.09231223940162195`, 1.0826750771187965`, 0.290821416328257`,
0.5533414871497576`, 0.3812913244405754`, 2830.6500293967956`, 0.03030872516300448`, 250.95228864955956`},
{0.10306075838040374`, 2.5636670031903526`, 0.062478631313391465`, 1.1237391323830406`, 0.020325627807727287`,
0.4138983427169105`, 0.015670410941822525`, 640.0409779587225`, 0.2299818577146827`, 855.7014985263506`},
{0.1543081177985079`, 3.6905447676223817`, 0.02801124131684004`, 1.4594516817243341`, 0.19245562145181472`,
0.22404862937840908`, 0.011760833299589 24`, 1165.1333013103292`, 0.08358867889715743`, 831.8544035344403`},
{0.06171989970085079`, 3.434249919377687`, 0.06572710058482858`, 1.3211715182855723`, 0.01504624767015783`,

0.4567422389972121`, 0.16959599175310128`, 4317.9045896509315`, 0.1155486280077034 9`, 591.0128116780224`},
{0.242854939016651`, 2.284046375461106`, 0.07510045340186228`, 1.3823454805694586`, 0.12288532968332899`,
0.2505129792578149`, 0.19202736337063933`, 949.9596745663976`, 0.23010030070393422`, 645.8973615583986`},
{0.22700129023591364`, 3.923952544058909`, 0.030515149632633083`, 0.939180364827058`, 0.4621492504536673`,
0.6517443382103951`, 0.014468218060931168`, 427.04247682874535`, 0.2370408654331208`, 259.4368577693423`},
{0.07805880992570535`, 2.501643518243867`, 0.009418707493693895`, 0.9986888477411795`, 0.32030485689176835`,
0.15640932443277744`, 0.05282555610968247 4`, 1053.3265205661667`, 0.23821781066142345`, 975.8018452897034`},
{0.11861919681642336`, 1.4687434978704887`, 0.0829781170205376`, 1.0362850735520839`, 0.6712083946301921`,
0.44248360772337636`, 0.31715757872607053`, 458.6280472607073`, 0.19463738072293596`, 242.98487605538276`},
{0.059281890431487294`, 3.297569244457442`, 0.09408483617597145`, 1.4708447962140283`, 0.8202767387462129`,
0.5182490075472995`, 0.15407063447248953`, 64.57603365703892`, 0.059976329837068354`, 643.9595265565569`},
{0.2572247154689537`, 1.909920813474347`, 0.05239854389036264`, 1.3414720605906971`, 0.4513315960556996`,
0.24795401762018465`, 0.018990463442733085`, 1296.597641454505`, 0.13705502920429874`, 197.81881626257612`},
{0.12947779232825135`, 1.9145873156534714`, 0.0288911518259296`, 1.3705880176351148`, 0.34333617932115823`,
0.684343564948868`, 0.30854336196404863`, 221.38025911589182`, 0.07491746507159502`, 703.3196235432932`},
{0.07972116217449848`, 3.4709822723848074`, 0.04576191227888536`, 0.8016369799963272`, 0.2857357367482012`,
0.3331460762626077`, 0.19358831260020504`, 243.63380400065088`, 0.06895044823250152`, 836.6535842859868`},
{0.21425814556096656`, 3.412718953326978`, 0.054294933223100375`, 0.843453127486217`, 0.5949443832779631`,
0.4312511536994379`, 0.006774834458589081`, 264.20975235216673`, 0.18238909307892875`, 619.340566973434`},
{0.1030949911235679`, 3.2379695384683664`, 0.01929347915744433`, 0.9201548487613123`, 0.33739940615248143`,
0.2622793831217315`, 0.23841206764006043`, 1617.5278268905984`, 0.22935132610715692`, 896.4339292297652`},
{0.11659113279351957`, 3.589133205154993`, 0.07913036370723393`, 1.232106957500204`, 0.8783988188812624`,
0.36486871630814866`, 0.12887619218337418`, 531.40225777078`, 0.19649941700330426`, 196.14313660000954`},
{0.17261096694115408`, 2.323826295719984`, 0.07573381776980444`, 0.8286032399217114`, 0.47983148793793484`,
0.26566773118764775`, 0.03472365295524612`, 927.3460129902165`, 0.1071272043003238`, 118.60052904781728`},
{0.15812900546790443`, 2.970437030179216`, 0.04641714570644767`, 1.3852443817278377`, 0.43447243659924073`,
0.33574441370838803`, 0.06435446224943432`, 738.6696158761797`, 0.13010058805108404`, 159.186393081969`},
{0.18592939740642223`, 3.685442589505847`, 0.07945757449204009`, 1.1464224447055114`, 0.32318739791637174`,
0.4649314153677717`, 0.013135441017131483`, 91.15430963381306`, 0.11860323377042997`, 381.3120573740848`},
{0.14376640925421846`, 3.1586224337701454`, 0.06046685266972789`, 1.2403049905427794`, 0.41520844208600294`,
0.6171055455360721`, 0.32907344516358666`, 110.43943865511335`, 0.09753160070705702`, 466.9944611056849`},

```
{0.08898564055719721`, 3.6481907193018275`, 0.035736135100176744`, 1.4644447392392914`, 0.3855084819966672`,
 0.5403543534096678`, 0.02564672554096082`, 3261.3971739494646`, 0.059327692271889`, 178.0636196825421`},
{0.12073346816538172`, 0.6796921643614748`, 0.004902893841691274`, 0.8582452939012792`, 0.29165716690801435`,
 0.6326916410656882`, 0.010914425076951347`, 71.33612718800642`, 0.018422948540203682`, 499.39101647732576`},
{0.08643478967247115`, 0.989294451274473`, 0.06406214590812752`, 1.4778521994730294`, 0.08864943219429122`,
 0.598523331628892`, 0.33924017802608397`, 243.33847401579249`, 0.1630923948211001`, 823.5357318052028`},
{0.1858290979683801`, 1.3708640419198979`, 0.03326172130438391`, 1.4020046543416833`, 0.11208251576177464`,
 0.2625003629250131`, 0.04524672886209799`, 85.06379115175038`, 0.18737298414395726`, 615.0030267833952`},
{0.1360682807160305`, 1.260103979250026`, 0.09631205400585557`, 1.492449539848565`, 0.646188927535122`,
 0.44017189812527846`, 0.4105272425293273`, 393.8607844518778`, 0.02987920633520591`, 916.2850221914006`},
{0.1722967578431442`, 1.2064516945248789`, 0.006692486646882791`, 0.9574846319241982`, 0.7639212221033438`,
 0.18533797387085427`, 0.023148480320789932`, 1419.1935015960435`, 0.20782666110883385`, 726.3055622381481`},
{0.10120560496305181`, 1.0077236027617351`, 0.0022035031006077867`, 0.7556532356144234`, 0.4541695517780062`,
 0.5272435056704864`, 0.48611131067523233`, 2530.0708561728975`, 0.016102479809594256`, 464.0343986269878`},
{0.12017322050098367`, 3.9721385392144386`, 0.07574165293605646`, 1.1837442643757194`, 0.5577757261385461`,
 0.3183184732454556`, 0.006181796792011399`, 369.4013122196196`, 0.18128847005418425`, 194.03504301481541`},
{0.04827662252574555`, 0.44476989943772693`, 0.04589418991887675`, 1.0976519464428027`, 0.5211218352826839`,
 0.35897962206032186`, 0.009662240534770522`, 2226.690418934838`, 0.1554674743129576`, 512.7162631562013`},
{0.05162633508079817`, 2.3550751217523676`, 0.0924989591884071`, 1.4157997348260007`, 0.18969184963171148`,
 0.23851541168228685`, 0.13394186077419723`, 885.0580489506601`, 0.09290724513295379`, 581.8072838231085`},
{0.0703807113832926`, 3.5028014587851954`, 0.09731342789234894`, 1.4652588215217102`, 0.02379062714059521`,
 0.45288235271478583`, 0.00942034539711149`, 1971.3577596359983`, 0.20635147200229786`, 588.3355736982174`},
{0.1722158503566622`, 3.9025180218674205`, 0.03287113170420332`, 0.7633959662136292`, 0.5426808864022441`,
 0.3669211940284537`, 0.015798227561105518`, 637.0091161622244`, 0.018607030959375853`, 729.5190439080948`},
{0.07584294958741444`, 2.9472829223076644`, 0.08633754726872958`, 1.4485030454804857`, 0.4347108871800047`,
 0.4606559371854728`, 0.488724952784251`, 373.5721326650099`, 0.07716317740414885`, 124.4741797765185`},
{0.24562593090753987`, 1.7880136046455286`, 0.061199201036337365`, 0.9504446252866968`, 0.8812906909113087`,
 0.4211899677669948`, 0.13371199909368486`, 62.37984939467348`, 0.029994323176434023`, 335.34030777918923`},
{0.20825483037627235`, 2.8176663305166816`, 0.03003135959790159`, 1.3809928219048555`, 0.5442546740990999`,
 0.2518879146968922`, 0.41623765779197747`, 1730.0044330431615`, 0.13532415983523782`, 593.0952047283174`},
{0.263098249275039`, 2.8719245261273185`, 0.057294037398088714`, 0.9272583089313478`, 0.5794976650588739`,
```

0.23557943476926713`, 0.18465618219605576`, 743.7540401729835`, 0.12098568685424893`, 886.4027693713879`},
{0.08125449990693445`, 1.4583301942945166`, 0.06189519777160188`, 1.4288277781740808`, 0.40197254124717063`,
0.569085386893778`, 0.039194281573835754`, 4748.401415793844`, 0.20401727261566`, 992.5067856915206`},
{0.13086541558871118`, 1.1155725899250655`, 0.08821908955004687`, 1.3478216191079166`, 0.17697042399948426`,
0.36922117198662885`, 0.013930530425628602`, 265.9989136069838`, 0.2270416561589264`, 365.77204194037876`},
{0.11728100988180301`, 2.8151532257585448`, 0.0822952483643148`, 1.4499657290535213`, 0.142235450676183`,
0.18155390727644505`, 0.00812157039144657`, 2435.595550489932`, 0.23995058381388307`, 812.4638732087483`},
{0.08049510800011694`, 3.603435994634494`, 0.007656876221115407`, 0.8287914836133501`, 0.8266092354769616`,
0.16324385237676353`, 0.08964145435399143`, 249.97635146943674`, 0.11550610667653838`, 765.8954747498603`},
{0.11820480579010878`, 2.60227134202185`, 0.04712951554705157`, 0.9880816634883703`, 0.8571809674721294`,
0.5649946552056018`, 0.017402816460863866`, 131.80950340449587`, 0.2293131996837257`, 460.13754914511065`},
{0.07353660759709546`, 3.5071493949530606`, 0.06008403863777037`, 0.7789529963855885`, 0.038880804706464156`,
0.24085809668436897`, 0.009207422134001322`, 57.365635753359946`, 0.19618331785464976`, 195.58478591654787`},
{0.14064741473086906`, 1.4253939258069908`, 0.09267725847840932`, 1.0975927496329574`, 0.22744648794341704`,
0.6063348207456416`, 0.03639182347931457`, 146.15301297855677`, 0.07935681769679304`, 259.4064208000535`},
{0.15191662908335968`, 1.3875044471862585`, 0.07598535142080483`, 1.4975086468472412`, 0.14071696059051497`,
0.2702248025276742`, 0.015378992010870783`, 1297.8058794576364`, 0.09563415627019778`, 267.1180590070449`},
{0.25783578932170337`, 2.5098960068891536`, 0.050196727090169`, 1.3457348584940565`, 0.6334273233006393`,
0.673911062730165`, 0.45542142980584244`, 1102.3077248576458`, 0.23127744020305874`, 280.7607833294188`},
{0.15728354284965573`, 1.1786483564465087`, 0.018710974043178813`, 1.0906308648763483`, 0.4692891978030753`,
0.5884245578610128`, 0.013999626118543194`, 1146.3540981218957`, 0.12976415437247035`, 943.3629249089528`},
{0.15744525017814853`, 3.9343441044396323`, 0.048424319387596705`, 1.0189964999962107`, 0.004699907891199784`,
0.35673641117911215`, 0.05845873245808589`, 1452.1810356944638`, 0.20019375268922035`, 991.5190215908439`},
{0.17711189820828616`, 0.9222841106349815`, 0.07731703618957698`, 0.8581707670014744`, 0.3028714201791183`,
0.3403814085412159`, 0.23552438227321898`, 185.6757896447749`, 0.15816694433359801`, 971.5707714133565`},
{0.12677717388625714`, 3.135520314905561`, 0.0958777837314775`, 0.8945786394252853`, 0.6294861294982774`,
0.5552940848485673`, 0.015606330011113338`, 857.9173420903631`, 0.2215517078689302`, 931.1017779440821`},
{0.21702108783814894`, 3.801779784508854`, 0.04805641449981236`, 0.9791693500326768`, 0.2245653714607041`,
0.5686073977459262`, 0.13790116867346938`, 202.46130862913807`, 0.1350408422039594`, 899.1324956275001`},
{0.1342014919902998`, 2.2832877374577247`, 0.03691753722884416`, 1.293132587653252`, 0.6577552625086529`,
0.43953980905115686`, 0.01957893390503574`, 205.45267528315344`, 0.11907718935756234`, 594.1955832844324`},

{0.1763744245545265`, 3.739113668312668`, 0.013915261017202703`, 1.4628218122817214`, 0.33832232520701955`,
0.3614238110077841`, 0.16749424131931667`, 750.7001786075303`, 0.16098306031842058`, 199.9116742658522`},
{0.15496422412126426`, 3.2726277520924976`, 0.04166420456847202`, 1.246251490612826`, 0.03723582004781312`,
0.3169293011472515`, 0.01364338217624883`, 794.7700944318913`, 0.13403305026443924`, 181.03718972044246`},
{0.1794519767096271`, 2.1595478222580216`, 0.024155004216522703`, 1.2232218292039836`, 0.12872104355462577`,
0.3105596069491494`, 0.08640881754488854`, 438.81545657103055`, 0.13579526038432777`, 696.2213922750994`},
{0.17257389690071828`, 0.774117551399768`, 0.008560234856989925`, 1.4940154641879095`, 0.5531402581000995`,
0.5441107289119099`, 0.07350653490141655`, 1314.8314542940839`, 0.07345603701745179`, 217.02216774233065`},
{0.058763034644712275`, 2.3597566945503834`, 0.09055618598093744`, 0.9263657689345207`, 0.5592210171940297`,
0.26262790696306815`, 0.011057611204984635`, 211.09574188287445`, 0.05571628830959996`, 675.171201042442`},
{0.23649888242497868`, 3.0694012769080574`, 0.0293881057277982`, 0.8469899032416341`, 0.5329226292412448`,
0.4016300524993841`, 0.08030522569307784`, 885.044604178621`, 0.10534611402615895`, 622.0478384674557`},
{0.10023410603180455`, 2.710596168246937`, 0.00298453338827164`, 1.151795972074526`, 0.37899349738211896`,
0.3392482218824491`, 0.04819504839896153`, 379.39038491273027`, 0.04469242387250283`, 827.1435707909849`},
{0.2438903560692493`, 1.1739940858261848`, 0.09102105800199446`, 1.392584698828661`, 0.6056955824388208`,
0.5844521424450305`, 0.36108528400570095`, 1701.2530207661314`, 0.11138522848439819`, 308.591402552778`},
{0.18793054675634024`, 3.139542677489672`, 0.08904425028360773`, 1.4068659558890193`, 0.46779742309438377`,
0.4804254450552986`, 0.31816862647583416`, 52.007157617767675`, 0.20815008924208545`, 645.3217048463795`},
{0.11103510208969514`, 1.4116670170359447`, 0.01094255418186231`, 1.3998271086778162`, 0.20796976919688226`,
0.21292073278793122`, 0.025573376903052888`, 86.81893641374597`, 0.03626690081608863`, 355.9231685657631`},
{0.059767794596915114`, 1.8804870041256674`, 0.07095681077704744`, 0.986817038205352`, 0.25655361425528533`,
0.168862562826992 3`, 0.1897075342910607`, 67.57112317559029`, 0.19542023600496528`, 963.6021600539973`},
{0.0563343868292458`, 0.5317396897533424`, 0.021545836837937156`, 1.1439759156456886`, 0.8881137136810024`,
0.4156539248732849`, 0.006243471710013326`, 260.74733013676007`, 0.24312372574340813`, 696.7530796966602`},
{0.25281786498427167`, 3.164006130077736`, 0.003838750721372204`, 1.0744832678586205`, 0.0068497919358050385`,
0.5152441133630022`, 0.32224630023151535`, 264.37961782996655`, 0.06028750963433477`, 143.50957591757913`},
{0.16077715085906397`, 2.873224638724322`, 0.05173801459016383`, 0.7810632519144793`, 0.3328755824267646`,
0.25599177499415204`, 0.29027099579570653`, 56.71390358897392`, 0.03391158089690982`, 522.8798210385845`},
{0.21945025832526777`, 1.6613676443983767`, 0.0033920248574508707`, 1.057752773749318`, 0.2127399240494794`,
0.2783903022229003`, 0.011232097563550716`, 1371.276504196438`, 0.04975311118637471`, 328.70478941546`},
{0.042964316768383276`, 3.463948962572940 7`, 0.050691741081661766`, 0.9465946216833707`, 0.05502079292147011`,

```
  0.21238865048524036`, 0.0776422991682389`, 52.80026867227022`, 0.04502021470199485`, 474.41563595375555`},
 {0.2433495999796823`, 1.8960270447479708`, 0.01120198462392917`, 0.844049298875671`, 0.4639208209806316`,
  0.20677684984563183`, 0.005733096581983471`, 1740.6658389561255`, 0.1399372816422193`, 628.7700453738331`},
 {0.09814127364381425`, 3.1310383407484847`, 0.017016069480737403`, 0.9217748908925876`, 0.27863388851936555`,
  0.32184170341904983`, 0.006207472689688149`, 1391.556177922241`, 0.16816866777593836`, 615.9674047842972`},
 {0.045179834179820105`, 2.124737466901916`, 0.0641039677828362`, 0.8087710768531244`, 0.5820240872072311`,
  0.6898665398618178`, 0.014125759687518747`, 92.19454081613313`, 0.015821689323895105`, 557.0872764229717`},
 {0.13473842282979165`, 2.4117633636086957`, 0.01984561256760543`, 1.1204070544446296`, 0.3860645674275691`,
  0.4170622324306399`, 0.019865666343063196`, 4743.148389421012`, 0.04365212493615206`, 322.93322910718985`},
 {0.2527515364737705`, 2.1426280202987984`, 0.015606989419899584`, 1.125943287101995`, 0.17776360180897943`,
  0.332914794116577`, 0.006519056311462601`, 78.48896188261014`, 0.12889711459762526`, 805.9196842114062`},
 {0.11933913407793822`, 3.785485787216923`, 0.023574857312977055`, 1.4812641194980447`, 0.6275581210964223`,
  0.5280696447262914`, 0.07256024376782456`, 241.814705594111`, 0.02783432907145139`, 151.3743596717724`},
 {0.1349803930463928`, 1.8072527206786377`, 0.06447251791280252`, 1.3994960916143184`, 0.7359286316821669`,
  0.20162137238693434`, 0.4265854166639518`, 227.4386051384146`, 0.13592337876958527`, 497.305076361482`},
 {0.2637152651333133`, 3.0391917107143414`, 0.0836047103384918`, 0.8164164023077424`, 0.5538018525602337`,
  0.5004598109403166`, 0.04356169162799880 5`, 3000.1047519173976`, 0.17835324975540034`, 767.1983419134932`},
 {0.17730450742771336`, 0.5526401983890268`, 0.003866546119779244`, 1.4224823875795298`, 0.9713446082448272`,
  0.47362133992508215`, 0.008799575820309609`, 460.0427974972538`, 0.1268858776449615`, 614.5975351416361`},
 {0.17661690011227865`, 2.968666144862362`, 0.08820580921653833`, 1.2618748536532438`, 0.014044591388426397`,
  0.6372850210418368`, 0.42461957500782593`, 908.6535515532695`, 0.16038310667129974`, 176.50654123832936`},
 {0.13672640919029144`, 3.614316511877404`, 0.006473169531331831`, 1.1642896824935516`, 0.29325294311380845`,
  0.16242724995588664`, 0.030111728610588202`, 3933.205934300977`, 0.0980103812613437`, 106.80306509175406`},
 {0.07145172009956824`, 3.410868367663567`, 0.08572451188759761`, 0.9584474542559545`, 0.040382356795113816`,
  0.6455580091740019`, 0.09117312647503195`, 1954.631204089039`, 0.0777204277630249`, 144.9524499282374`},
 {0.13883370155342772`, 3.715034522567122`, 0.016916922596204795`, 1.0000211488246231`, 0.12894118452278325`,
  0.24258423424654219`, 0.02087499948517953`, 3780.1181299010777`, 0.24420906754435595`, 798.6323972467644`},
 {0.21440197837823782`, 1.7615321043929084`, 0.028950668418736624`, 0.8631531769083587`, 0.523525808424337`,
  0.2748912735476704`, 0.38068084592210494`, 404.89859548140885`, 0.15408624036569346`, 803.1703020004433`},
 {0.27645780167060263`, 3.175276677600297`, 0.05299560517709523`, 0.8086008634045363`, 0.7913310844792734`,
  0.3862667201926391`, 0.00961380004706706`, 2836.236561313336`, 0.22859831535956182`, 465.6465289390621`},
```

{0.06752882943979077`, 1.4908646201280487`, 0.03739887788507268`, 1.1321270467820062`, 0.1590886090266126`,
0.33058750825147076`, 0.13664295377993954`, 324.6730016626649`, 0.23288398005137873`, 299.5733325408761`},
{0.10900083730464943`, 1.3165965495337897`, 0.04096974974497696`, 1.1178893614799246`, 0.02481354114149492`,
0.6533849288221782`, 0.0989115744507999`, 59.94210305630967`, 0.08210787534991087`, 838.1221078005716`},
{0.18821745287135477`, 3.8834155988951853`, 0.08623976994927297`, 0.9451777127739934`, 0.2536651767905649`,
0.4966204244968453`, 0.052355463971012595`, 1253.2350223323115`, 0.025530907055034302`, 107.88292349347337`},
{0.13433362908189822`, 3.976975346921523`, 0.023713728561632463`, 1.330972786055094`, 0.5425931686264049`,
0.5867946121348381`, 0.083015502101061`, 2146.731613991672`, 0.11433497831643785`, 956.3793106500469`},
{0.05370292234035079`, 0.8618436041219777`, 0.09065607433649935`, 1.1199215929503574`, 0.7033869517843656`,
0.5744405842935578`, 0.20721453360880282`, 163.81435947307054`, 0.15960680813292716`, 808.9619958368114`},
{0.1982510252132108`, 2.465970568324745`, 0.012757083454587495`, 1.214300872852428`, 0.49977561113114644`,
0.2038568209362135`, 0.04565388315282881`, 78.88460864136573`, 0.1487998824136843`, 164.5768910062601 5`},
{0.07006449306202894`, 1.594466036555235`, 0.0695487720666083 5`, 1.0929666985547377`, 0.8182673122674597`,
0.16380036850673574`, 0.04761505660877746`, 3094.547812800814`, 0.026912730925264194`, 465.941138242661`},
{0.271620743989626`, 1.798748508191406`, 0.039226132696162194`, 1.201815394348386`, 0.019033121630884553`,
0.500394949620919`, 0.022898921692070705`, 167.54130076556643`, 0.17172411650490582`, 118.02597321115923`},
{0.17015700880843931`, 1.1880680435779825`, 0.09612747942645765`, 1.220203731936928`, 0.8171639571524099`,
0.29942524478114674`, 0.005372366003640249`, 607.4487584881643`, 0.0444162424317765`, 783.4604894784995`},
{0.05414527078515874`, 1.462858717153524`, 0.057585439178684264`, 1.0235777810825535`, 0.21014799698731879`,
0.27005953264512594`, 0.08376210292871798`, 147.35977444766536`, 0.10744240382696624`, 846.2849324778499`},
{0.09237320793743342`, 0.8096523848475279`, 0.004358295040692077`, 0.792421334681352`, 0.9354401956315697`,
0.4996618434240512`, 0.3257079961489366`, 106.0381723510655`, 0.194703596109299`, 875.1849354846129`},
{0.050247694605344284`, 2.3552798474120182`, 0.045898586534112516`, 0.7657469521997533`, 0.41572162978856886`,
0.6250502955136628`, 0.036232245589174594`, 4925.729350966778`, 0.04833882006927181 5`, 396.9216964218738`},
{0.16604829560323886`, 3.2400306473910465`, 0.07805779128319201`, 0.9800901947770135`, 0.02662429037050673`,
0.6202147453627995`, 0.0521761817913341`, 319.666779674947`, 0.1526499768736515`, 641.0814189728534`},
{0.13199054420769823`, 1.1822647026678919`, 0.05315251799812888`, 1.3177578061426032`, 0.29009102787676366`,
0.41153300112684843`, 0.027654998358556677`, 862.9125744805588`, 0.22565453257412804`, 148.78642951801135`},
{0.15326781447665427`, 3.7132135007643896`, 0.08379527648859465`, 0.9155066607147588`, 0.9247491129828225`,
0.43619880658774446`, 0.03926748261529228`, 1156.2321575105877`, 0.17547647957302887`, 889.8562914260897`},
{0.25318856010923574`, 0.5926097353022888`, 0.00482353837495765`, 1.3099001178559129`, 0.39149861531549`,

0.647818541877643`, 0.25232461737348105`, 3385.571345294367`, 0.19387361798630304`, 420.02567016405266`},
{0.24993243970651624`, 1.7126281402055845`, 0.053130097502228`, 0.8352588223269972`, 0.62388332746021`,
0.5762423608317969`, 0.014465580674644738`, 799.5116049181332`, 0.0931416280797775`, 865.5405963251784`},
{0.19561758310618566`, 1.3596417600698452`, 0.00286504944841953`, 0.8159672694367253`, 0.6358724387725987`,
0.6648416656940517`, 0.24177467962143764`, 3682.5795096853144`, 0.15293398533505775`, 386.4064670424524`},
{0.2447874085020979`, 1.7744291418487776`, 0.060075050805309844`, 0.9864750267055266`, 0.7342694514660453`,
0.5397411982908532`, 0.4042131026855616`, 575.9477405967004`, 0.08605927470106772`, 780.0883449415986`},
{0.07734058301535968`, 1.385482226243342`, 0.07152447691997342`, 0.8194619571046995`, 0.4573258756273968`,
0.6888219817424488`, 0.1334830351653064`, 199.92831385353017`, 0.08814262925395056`, 749.6278229482897`},
{0.10474568576280202`, 1.8596472368495407`, 0.026805293435349233`, 0.8198649413737059`, 0.5254103672469626`,
0.2748607556216799`, 0.0523834433135859`, 134.70642314731086`, 0.10106748211794048`, 140.99421671530888`},
{0.27890810575443187`, 1.4911450307991796`, 0.07330732877841264`, 1.0850224852017472`, 0.511581631756513`,
0.6844047819742511`, 0.4182444515115468`, 898.6500980057638`, 0.1508853505547692`, 117.74931414924143`},
{0.06710090564388627`, 3.150840959910446`, 0.0014124858884598979`, 1.2404545757648113`, 0.6590308151409987`,
0.18742776248185322`, 0.07838269037439821`, 63.02543384043976`, 0.23165453423661014`, 134.9827251445024`},
{0.2709692633763827`, 3.0318490895249166`, 0.02057996898061427`, 0.8497637000182061`, 0.007946519407763919`,
0.4537529292092372`, 0.23156314091749142`, 3625.9461118472623`, 0.20365524494076642`, 575.3949074180657`},
{0.15487330171159508`, 2.506241955627659`, 0.02405679630252637`, 0.9599539152532017`, 0.4302913454357331`,
0.26259574333059643`, 0.008099203461346816`, 910.2082053871129`, 0.14622107038108184`, 559.5315182891957`},
{0.2128087852183892`, 1.9571603978537304`, 0.06326297053797199`, 0.9699003392267482`, 0.2686671251031061`,
0.689623844783678`, 0.35675989285485865`, 1924.6232853270492`, 0.23326907367518723`, 915.1337901802619`},
{0.21780708062769377`, 1.1747776713117952`, 0.05034010906267806`, 1.0913007058111903`, 0.12353581217661103`,
0.3210562571265647`, 0.0322536912509453`, 2197.1982196175004`, 0.1572434618024398`, 414.86282493944026`},
{0.10779474152210772`, 2.5545130403913596`, 0.09607448919858275`, 1.2919315212701343`, 0.16453105789719658`,
0.6141245226266829`, 0.11684667552500605`, 196.8514855477255`, 0.10491847566430007`, 112.73037991828173`},
{0.12432532516395484`, 2.1300608216646877`, 0.04913772379496075`, 1.114341717459199`, 0.2610859336877449`,
0.503197213507616`, 0.005797395507410687`, 442.94048846434436`, 0.08741076870037628`, 101.71296622490348`},
{0.27464475878650824`, 0.5510255383265457`, 0.031636220540726276`, 1.3762194055027865`, 0.1612567072728912`,
0.4273900785282383`, 0.24603471434298904`, 429.9496642319049`, 0.08771573488057915`, 617.3490635717783`},
{0.13029287084326696`, 1.6894675116680409`, 0.020566584774051967`, 1.2832846584058795`, 0.1828960091839662`,
0.4605785391689514`, 0.0113553124712164`, 936.7188945707877`, 0.1761057360191931`, 440.679523047495`},

{0.20510392052346132`, 2.0152643396686836`, 0.05752244878800122`, 1.0760055282794754`, 0.9860596725458286`,
0.33518476119672647`, 0.31856559936642365`, 321.34121932230744`, 0.10497410797788737`, 281.7881521533858`},
{0.22222058819666546`, 1.9181392247611697`, 0.06353852306281663`, 1.3758404515279568`, 0.6438678141184426`,
0.1693786566729748`, 0.06897920477033548`, 383.0298020915635`, 0.10314770849680055`, 941.5930224696303`},
{0.21276124668973173`, 3.586839401277566`, 0.039178902951713945`, 1.4021843911360103`, 0.5049572433506289`,
0.25133132700774996`, 0.12364866403599967`, 623.8499997024826`, 0.11712847066144477`, 770.7321547534711`},
{0.14968687983730605`, 2.3633590766978303`, 0.04173348230150333`, 1.0168117310979305`, 0.07526686867368393`,
0.37085554163771406`, 0.0076816551378203 5`, 1659.4326212173246`, 0.1437616377539393`, 942.572084587879`},
{0.2713741706327081`, 0.4242782972192547`, 0.07078081316399253`, 1.131720191226623`, 0.4549262787557089`,
0.34468168234433216`, 0.012942279609896707`, 2864.005301731598`, 0.09798425989157222`, 187.35732163896745`},
{0.1768611384179205`, 3.4923686338372777`, 0.0164618490651386 1`, 1.2055226780828572`, 0.7343581951493021`,
0.49378742542959486`, 0.12048169571136383`, 2844.3094803530207`, 0.12693060413099172`, 545.4728905288805`},
{0.2369345296836391`, 0.41785553179229007`, 0.012526518118659648`, 1.2179877794222598`, 0.01846774830289344`,
0.24685867084706536`, 0.0219438069009795`, 2091.108395341874`, 0.0637607114780574`, 657.8903568599761`},
{0.21219769453448084`, 2.488104916250064`, 0.038757338160515825`, 0.774789527802302`, 0.4580558695696113`,
0.4263775736158787`, 0.34953699783630143`, 407.33606250987975`, 0.16713719417824968`, 389.7058187031195`},
{0.11474454899039521`, 1.9841405956471387`, 0.009912438082135093`, 0.9199083705387736`, 0.923535949012403 1`,
0.6470781918812183`, 0.3331422211969604`, 337.2489956721694`, 0.03475601824362995`, 538.9648304299371`},
{0.24234974629560802`, 3.9617359783801245`, 0.024444176238852366`, 1.4313282134950815`, 0.9940513113675349`,
0.4143492563365402`, 0.1686590259483461`, 68.11457543267173`, 0.19793807681647463`, 199.02629821091972`},
{0.16236198209412894`, 2.018699278359029`, 0.03626856618687025`, 0.8436685495890566`, 0.1369133257491073`,
0.18228484143603907`, 0.040551804892385857`, 156.7528279727044`, 0.1739090814809508`, 704.2383268945476`},
{0.1621577456082553`, 0.6421908435706589`, 0.0682618183426171`, 1.3238181641882336`, 0.9244165647014204`,
0.6930553521066385`, 0.13666203325037238`, 72.36369505518103`, 0.2211105145097111`, 327.06990857144615`},
{0.2702619348258324`, 1.3531215117783164`, 0.06648966931523986`, 1.278915076565248`, 0.7821690760804008`,
0.4832187850250197`, 0.03589870905112195`, 3130.704914059961`, 0.14156174429321455`, 311.74724954691885`},
{0.19935819825153228`, 3.831285341751962`, 0.061322567547608904`, 1.470671933797358`, 0.8459029813533634`,
0.6098411086562021`, 0.020718424330298454`, 338.23739251616234`, 0.13481110564933563`, 328.9796748551533`},
{0.12820856017846227`, 0.9705044791290884`, 0.020813708364227884`, 0.8227896910361784`, 0.7793252261285137`,
0.1540874083560777`, 0.019838047780930596`, 1485.5571438891636`, 0.16001313078246715`, 526.6105313836905`},
{0.11895043241393044`, 3.4783832049993846`, 0.08948985928895077`, 1.359795719210997`, 0.29460393871375956`,

0.35020600424213755`, 0.2746425669615434`, 50.083789355993105`, 0.020545547787407692`, 916.6997924739111`},
{0.1324468409940383`, 2.674634590833133`, 0.04368586320677444`, 0.9656288911809869`, 0.20832826835990526`,
0.3737566616358602`, 0.005582642109220031`, 55.02582924504472`, 0.2065757664044986`, 615.3366029893464`},
{0.06870110651611899`, 2.6561693493128598`, 0.08012546640983625`, 0.8012667555503843`, 0.031201182173437925`,
0.47282927837851574`, 0.011587617652012341`, 3773.634663778097`, 0.09147096998858567`, 835.9545348732762`},
{0.062399865483643235`, 1.7222840522480984`, 0.0921920191456875`, 0.9286857558090922`, 0.3251018367521341`,
0.6108891276310624`, 0.011783888986275243`, 670.9821418853228`, 0.11450398298509856`, 971.5077469427929`},
{0.24591568234282185`, 0.8566394248947855`, 0.0781061151752332`, 1.3941060542854928`, 0.531302066365049`,
0.6676078161595655`, 0.1738149187521547`, 158.69973172683407`, 0.1485119105497385`, 936.3340802553399`},
{0.06567085889032587`, 3.9337642436409004`, 0.06099064297489713`, 1.4242284268139727`, 0.6539174667789966`,
0.3970266459135522`, 0.44668463659234353`, 1229.440509286354`, 0.19297859064513334`, 104.53761160348893`},
{0.13077961834437962`, 2.6471887027919063`, 0.08803755851714129`, 0.8915760567705249`, 0.43528239011744874`,
0.6770072844670658`, 0.13724713444300793`, 2266.2113033407786`, 0.18158499094944586`, 450.76741342966307`},
{0.0626945060366299`, 2.0207863261901435`, 0.04028779663279089`, 1.1608394772654784`, 0.8954638473430083`,
0.6803040308141024`, 0.0438755308716426`, 1072.86320924307`, 0.09417670420636176`, 203.3883701052643`},
{0.06633302157481619`, 0.6897085661220981`, 0.028600562511313363`, 1.222942456179899`, 0.45117275354230246`,
0.29367119707525824`, 0.1114281718553938`, 56.37596421013418`, 0.06329831527765895`, 495.6622356704312`},
{0.0758463119875773`, 3.621924471713986`, 0.052843607016089394`, 0.9710310359872816`, 0.3507881375218056`,
0.16711099920227068`, 0.1488874639582541`, 4335.730117904631`, 0.02658055782022295`, 793.4396666616456`},
{0.06315507988926289`, 2.3297312843414817`, 0.00212708414905217`, 1.395352700358994`, 0.0641701490182165`,
0.5894635107517013`, 0.17809044151421888`, 379.3821707157771`, 0.14230190042275537`, 759.6382607226183`},
{0.2099022158481964`, 0.8809344339859928`, 0.0666751548993475`, 1.4387478328331107`, 0.3531306367025422`,
0.6983916858003694`, 0.014622538997794657`, 343.9480456094322`, 0.08685603103174627`, 426.825757963735`},
{0.18558107573674792`, 3.0194943343339986`, 0.07461391639025422`, 1.1191398296135584`, 0.8305222019918481`,
0.4880407736474275`, 0.30782204523980083`, 408.3075491000427`, 0.12514760530359148`, 124.23432338200726`},
{0.20051301950717854`, 2.977167267300211`, 0.06936215087558335`, 1.0259008901103701`, 0.6147776109399516`,
0.6258821206893599`, 0.01703908364047091`, 2344.5258662598194`, 0.08418799298306856`, 794.1399968502964`},
{0.23048732313041992`, 3.462281492713984`, 0.028460833429753602`, 1.0414779858519587`, 0.11068368992785804`,
0.46918663455326926`, 0.3985525154764997`, 883.0495583098383`, 0.2409468662662823`, 546.4207520227328`},
{0.211465410867755`, 1.6738825157761648`, 0.0523685881403539`, 1.2569145647413262`, 0.6940459633746912`,
0.19386279088766634`, 0.02514509653095577 5`, 502.1390324130799`, 0.03396962103018908`, 864.65280998117`},

{0.1929466873888901`, 1.5728514227245611`, 0.08398743525565142`, 0.8305271019348486`, 0.30866536632560715`,
0.24782227733300222`, 0.06727104200994766`, 4073.498181247491`, 0.09367172319166167`, 761.8812104829784`},
{0.09217356026466877`, 0.8891340726836079`, 0.02261394061546632`, 0.76288584990135`, 0.2352883211999115`,
0.4087782246426179`, 0.05761668157743837`, 592.1949999543295`, 0.1566262893866785`, 939.2503062920734`},
{0.12967920647240816`, 0.8123733202889643`, 0.0013573621543738134`, 1.2244900945773618`, 0.8403401749152237`,
0.45714635607461884`, 0.04113284132792122`, 683.6853491881902`, 0.10112850721928329`, 691.2742096973634`},
{0.17585942228676693`, 3.1377953149568008`, 0.0015744621411661974`, 1.416873746788956`, 0.5827830119619737`,
0.6742879545649707`, 0.09162791382644989`, 517.589004535642`, 0.16103345930524726`, 547.8413219418244`},
{0.2427638279415646`, 1.6566162823863317`, 0.06521965313277005`, 0.889062176257591`, 0.014518981829873656`,
0.2748704572491185`, 0.05735800450312215`, 2387.1739807284794`, 0.23199018500208857`, 381.464796278728`},
{0.2519418117913656`, 3.063462870735645`, 0.007778137389172581`, 1.4021617024508317`, 0.9924604733729623`,
0.36233195131635443`, 0.18682309925698343`, 66.44214693634154`, 0.1722230669694152`, 118.3771627882729`},
{0.17349345626891455`, 3.174288570189952`, 0.09652146927294172`, 1.3390466263031546`, 0.7909502427935802`,
0.6793254105715787`, 0.08700743247214943`, 763.4035938655402`, 0.2387926278324622`, 592.4203788389427`},
{0.1727078461124784`, 2.8460728910005475`, 0.040914427699617326`, 1.4313337292763195`, 0.42218527760026237`,
0.5016244570171771`, 0.0887548918690675`, 1587.4849620109512`, 0.1328615275829737`, 768.9319503194112`},
{0.08930008677181278`, 2.849971715639322`, 0.0916977930872597 8`, 1.2438628382038357`, 0.4154968472190934`,
0.15405240023737177`, 0.04634668494948532`, 124.90941433976057`, 0.24982063081795847`, 463.43559716325797`},
{0.04995942432359332`, 1.4610302960771593`, 0.0433388762401086`, 0.9505438290907596`, 0.488211452413962`,
0.42119172502998126`, 0.012759652857857887`, 646.7948127587483`, 0.026663072682046873`, 245.49015570500714`},
{0.06795497506231873`, 1.985917542879199`, 0.04349282644250348`, 1.0631785554908315`, 0.8091824452378926`,
0.2147272979078938`, 0.03845431657393897`, 607.4631067901736`, 0.08409909386800546`, 814.8177301929959`},
{0.27140987444452636`, 1.6298377410728744`, 0.06029749698810518`, 0.8272061170373771`, 0.9095594755225849`,
0.2626530714660902`, 0.22141343547525327`, 1531.0082827603965`, 0.16934746708503456`, 553.9108700580455`},
{0.20249329091250423`, 2.9260026808453974`, 0.09022408237555009`, 0.9840606653871036`, 0.4430644155249066`,
0.26716691431663997`, 0.012442882013869866`, 1252.39191368304`, 0.1408105612614876`, 866.1715515451732`},
{0.13229111529623555`, 0.9037627200690848`, 0.04928384913936972`, 1.0937814374110013`, 0.9849443259560173`,
0.43115790526717`, 0.019499938095150635`, 129.0159606559662`, 0.1987553839173642`, 393.89165319051233`},
{0.1970263766379653`, 3.9709307503384244`, 0.027958813802574572`, 0.9741203705977242`, 0.07278184160194923`,
0.2945649201344811`, 0.013078030291277284`, 4971.688891574707`, 0.03180082765265402`, 755.4217432398528`},
{0.2049473596100468`, 1.974812376290127`, 0.018059141094917592`, 0.7718344152131382`, 0.3708285362372443`,

0.2734681930545607`, 0.057458033126770496`, 523.7031096038135`, 0.02427329957544444`, 932.0905826464876`},
{0.06537885284118145`, 3.340707691253673`, 0.054351682254287964`, 1.020827128283552`, 0.11666967592415722`,
0.4905291706504276`, 0.0525916907549581`, 117.00169938700564`, 0.09552258651198203`, 745.6309012306111`},
{0.11289673562266261`, 0.6794824966294883`, 0.03387634458174361`, 0.7927471128390904`, 0.07141719167345717`,
0.5226577421579536`, 0.03829715492190054`, 2920.8934693444294`, 0.12081443854156904`, 964.8985972362055`},
{0.23766500383642747`, 3.8767325971078446`, 0.0584809543600726`, 0.8270011297527531`, 0.6149673245232845`,
0.22395807159158476`, 0.013437587174666855`, 406.005827450584`, 0.2418694331681166`, 429.2383814891535`},
{0.2081776292468483`, 2.815887981403362`, 0.005125757826924850`, 0.810347956089487`, 0.4735170922919895`,
0.44544132586608753`, 0.4203259951948646`, 204.28043485055284`, 0.1992997883804286`, 827.7788091184511`},
{0.17107980313911997`, 1.611778555980452`, 0.08454567986455815`, 1.1369505371366053`, 0.2822681213890343`,
0.6771477606702629`, 0.13278544535359882`, 251.49910155434924`, 0.03764393371120245`, 453.2818524693168`},
{0.10511512871602496`, 3.5633360415567505`, 0.08943300281707153`, 0.8304995882196464`, 0.4228782437765999`,
0.48401108468440646`, 0.019832659219655203`, 164.88993273954887`, 0.1933397413498107`, 248.59325812442776`},
{0.1315672644477009`, 2.3773291392807145`, 0.05190617787787307`, 1.2351268314391448`, 0.45158718255427366`,
0.3842243141032361`, 0.03844922629193891`, 225.9216681882473`, 0.04341773379489022`, 914.0642920476225`},
{0.20624347857829856`, 2.4671372276614543`, 0.050734528297655734`, 1.1490024452760461`, 0.2202047098600366`,
0.3908877988906867`, 0.02383144777523708`, 157.0379664488479`, 0.14716356068566494`, 110.7973831044216`},
{0.27080390848623775`, 1.9772333839416323`, 0.024251112595875153`, 0.8820022792455461`, 0.651342077315098`,
0.5674047955018092`, 0.009713259722204017`, 54.31416433169486`, 0.012456849152343602`, 380.638174364041`},
{0.2662342547666148`, 2.0899914364276`, 0.0775212957879528`, 0.7629254448634359`, 0.7485227165858468`,
0.5717925034597559`, 0.384531789999449`, 531.2378339914305`, 0.010110683485557853`, 104.86289985839541`},
{0.08332863693803971`, 2.021121394734722`, 0.03701544455983157`, 0.913330989395187`, 0.7759249909409063`,
0.6241937322174744`, 0.0295724732082631`, 100.8666790089409`, 0.19088570031299712`, 262.59746879189373`},
{0.14639594099937192`, 2.488995500972994`, 0.08291914891669944`, 1.3150002115787267`, 0.6540711522150526`,
0.2055454195673022`, 0.1946827902708822`, 1690.1447445658273`, 0.18733657548952937`, 812.4826047698352`},
{0.1305321301981358`, 1.4867396329579305`, 0.03655804827713382`, 1.4456727825450792`, 0.670555897133192`,
0.6184895636508891`, 0.05124255420048995`, 394.1191568715212`, 0.03171421941058544`, 345.85608840747835`},
{0.250721605591452`, 1.7010887783606767`, 0.08806284896739065`, 1.4847636082147322`, 0.4308392570475905`,
0.16893566899927792`, 0.08647127635050468`, 2056.3372882750145`, 0.12615446084315202`, 207.40781829993898`},
{0.04392874140447878`, 1.9818443681166702`, 0.09434829100781027`, 1.4634488477570156`, 0.6811682982929017`,
0.2554902190221564`, 0.06882358130064935`, 420.2484502763152`, 0.11914991474138031`, 179.7692000111727`},

```
{0.14635546873540428`, 2.6921520503610896`, 0.005572522395533781`, 1.0650322427085313`, 0.0125854793096740 2`,
  0.3289894737792243`, 0.20755635390945587`, 2318.8984027990427`, 0.23607139988674775`, 598.8352065199724`},
{0.20440309232409604`, 3.6324112419808596`, 0.02933293982791259`, 1.0491273986291727`, 0.6481946292303631`,
  0.5970564598987798`, 0.367615520297974`, 2827.3491736750175`, 0.18901453458314488`, 331.1017138964795`},
{0.211339616570286`, 0.8062529435299846`, 0.03215637597034366`, 1.1329056004996207`, 0.6216719034637128`,
  0.26914056317845936`, 0.046266726096773195`, 367.3076671022254`, 0.062345072344042685`, 883.4219863160993`},
{0.0754121889664276`, 3.3553240739439776`, 0.032766043984085905`, 1.35951382107467`, 0.417421746626248`,
  0.2423604100007123`, 0.37190628043481266`, 360.0955624739355`, 0.09650587549325668`, 113.8474438254784`},
{0.2783033002921034`, 3.397814643841863`, 0.023780737803670213`, 0.7785779441011293`, 0.28908440577847827`,
  0.4851687293566612`, 0.2500599862274582`, 176.08406451146294`, 0.07757174693073099`, 972.6494889929199`},
{0.08302491117654731`, 1.417237397662472`, 0.08064924241577312`, 1.4920703552127885`, 0.33660997181727526`,
  0.6086848413902193`, 0.43663790825582516`, 432.2028054996635`, 0.054044182009652`, 768.4690422643392`},
{0.09729817063298457`, 3.4299522173089496`, 0.07735506084120583`, 1.4020158593772418`, 0.655176777497416`,
  0.2796434167254044`, 0.020513648578027708`, 361.54728331563473`, 0.18982251022644547`, 624.3692576556642`},
{0.05918716742286234`, 1.9820539956622945`, 0.05196458247655251`, 1.4930492496804253`, 0.4915276700726614`,
  0.5297919483627916`, 0.24251322382900306`, 1013.8284387296288`, 0.1901510038138538`, 731.270474360067`},
{0.05448705906655 3045`, 3.0521940188838643`, 0.06431699428389767`, 0.9108516151810719`, 0.22179534032337966`,
  0.15276001240612846`, 0.00618021185065336`, 1431.6290748192448`, 0.09019004900189659`, 615.4166559817393`},
{0.1805007029704227`, 0.41916969450246455`, 0.016124853476479205`, 1.3709380974041798`, 0.9243445784418525`,
  0.5673574690172947`, 0.01323585349172664`, 768.6156722427371`, 0.14720827739001074`, 303.65297547748116`},
{0.13586333024917552`, 2.4333711568454603`, 0.06594815024548176`, 1.1422919388448811`, 0.127316527773377`,
  0.5373692465409584`, 0.0174769508156497 6`, 1883.6141269851823`, 0.017752075188298855`, 680.6112691329253`},
{0.09049024561283486`, 2.3894866858591284`, 0.001345988925684072`, 0.8533171338756729`, 0.3021314683838838`,
  0.34607340060030345`, 0.30321966856095933`, 90.59478899907405`, 0.23877865208899252`, 565.9928476917297`},
{0.10259701094971274`, 3.6083949275799876`, 0.07320779035427538`, 0.9294410249753255`, 0.6943909777193686`,
  0.5424209490023467`, 0.02226818211415045`, 425.8826453817381`, 0.14811442642104045`, 767.5349856757164`},
{0.0818626476168649`, 0.8476840265643926`, 0.05039571888660504`, 1.2803278760732222`, 0.39203490461702195`,
  0.678685283379783`, 0.046408600947453554`, 625.4764903683038`, 0.18505631212115975`, 473.24089743900083`},
{0.07029108203608453`, 0.7073037290148494`, 0.03261231636893799`, 0.7728998119403233`, 0.4329865779673474`,
  0.39085105872031733`, 0.3956962494413241`, 72.63981932713905`, 0.24239129287515127`, 232.20167186552362`},
{0.04958285162353873`, 3.761311858085456`, 0.07854169509494732`, 1.403762798092309`, 0.6354348772329161`,
```

0.5539469242046658`, 0.014552601524129273`, 191.8255147151473`, 0.1545116444713645`, 509.99375854807204`},
{0.15497576240515054`, 3.7755891399441612`, 0.04102415780603357`, 1.3426436269065887`, 0.4633835643985551`,
0.2105276629358308`, 0.005292621332827327`, 393.4300921998384`, 0.05255466894451982`, 181.97601415353938`},
{0.1462216796835833`, 3.3115430587931307`, 0.09201451210593234`, 1.2778172268764427`, 0.906784021865821`,
0.3687077782146817`, 0.05157961805804173`, 924.6290700374847`, 0.029607911315220503`, 889.789386014198`},
{0.13283897685814167`, 3.038408092963987`, 0.03461781675567849`, 1.3222463428410316`, 0.47510806724254606`,
0.6210207278956918`, 0.005408113696778361 6`, 102.55379834175085`, 0.04224157239084064`, 436.06625132099504`},
{0.11923152550729099`, 3.907162388051315`, 0.06641684248516906`, 1.318540470917755`, 0.5804312953963475`,
0.6280211407721501`, 0.005256835397320809`, 1226.3954007569562`, 0.17176906355966298`, 506.996443873342`},
{0.10227819097193708`, 3.914724924869418`, 0.0664380714143547`, 0.7944397029062766`, 0.15335804466948`,
0.3668024996284803`, 0.06541187335723456`, 1449.384487729464`, 0.024262601947109108`, 554.3384286777302`},
{0.27621673974857747`, 3.975786747546901`, 0.0440045228345514`, 1.2561736736681604`, 0.12219205626085827`,
0.44109764605630186`, 0.27834299040512644`, 71.91340898917194`, 0.10015764539366545`, 482.1830368850472`},
{0.16141532638885775`, 3.5473613273302282`, 0.060131606026769265`, 1.1204941160194362`, 0.8786219204877892`,
0.42963549162688086`, 0.23724506417809585`, 4491.815167956783`, 0.0648001518482983 5`, 512.4293881945277`},
{0.2575375457582068`, 2.947834811130747`, 0.033810464285172745`, 1.0447722344490806`, 0.8627980313064527`,
0.34879266259466135`, 0.11166656744723742`, 94.36401066143432`, 0.11812196317533258`, 725.2748373314915`},
{0.20820386673509828`, 2.6786477995612135`, 0.010157905183889078`, 0.7567059297671783`, 0.6398946017563087`,
0.2916906367963521`, 0.04126212921113298`, 3268.442039148205`, 0.023820623550554088`, 259.60704797706916`},
{0.15593088724620752`, 2.5519577904336437`, 0.024361841648533833`, 1.1168597646554808`, 0.13563727928772762`,
0.3874274982263488`, 0.1882999430765413`, 616.7470197654534`, 0.0830157326232564`, 890.7479015168708`},
{0.24014955563677381`, 0.5220448196870295`, 0.02671701264219644`, 1.3551783563181985`, 0.799728403554941`,
0.47303019566294113`, 0.027399592270438356`, 644.7909772591187`, 0.07858290125002376`, 181.11983864094333`},
{0.1581702628188732`, 2.346334984324618`, 0.0581411782688245`, 1.4807167920720685`, 0.40458616499170885`,
0.6310195170221491`, 0.006522970079115585`, 337.28265576556936`, 0.12691983292355058`, 454.93623014073796`},
{0.23136224869656058`, 3.6088316121947086`, 0.03632040382309938`, 1.3419685001039396`, 0.40455118868914375`,
0.3221101170539603`, 0.25966887129973093`, 1676.6659884454966`, 0.19241943128863082`, 511.50532059075465`},
{0.17957415783469988`, 3.5968176444073308`, 0.048288185227319896`, 0.7994547395333207`, 0.9581588123096916`,
0.21642006235154376`, 0.14264678878084103`, 1476.5632047936012`, 0.020697112352618785`, 228.14672992784074`},
{0.059998032534718804`, 3.7773982431410413`, 0.06986223440078802`, 1.120487977215339`, 0.4567291173152259`,
0.612033275511431`, 0.07354302601323719`, 486.6146546384414`, 0.119884658675466`, 347.4528922941486`},

```
{0.19887637677541542`, 2.992660923165513`, 0.047995275724685205`, 0.9722118856000259`, 0.2651497498489779`,
  0.5318886674778154`, 0.19636633656100977`, 112.46333630768565`, 0.06776166614050899`, 129.50929186524326`},
{0.09067863132057552`, 1.9262440927144207`, 0.03464921977559642`, 0.7578597622362966`, 0.480840305150003`,
  0.24100415846854528`, 0.20408648616921088`, 520.809954661363`, 0.10723760431187324`, 547.2814452851367`},
{0.20782506825689806`, 0.8141429404339053`, 0.09514857720029408`, 1.3616807374452509`, 0.4328504524684271`,
  0.33813491058186396`, 0.24032169387315838`, 461.50385309819023`, 0.1829235837022425`, 223.3752348589885`},
{0.11510496723960911`, 0.6612811922775106`, 0.0679688489060159`, 0.8790789776515309`, 0.5367830167767558`,
  0.6858865509647121`, 0.06457419286375914`, 320.31094965266385`, 0.17862589858250755`, 915.319702346644`},
{0.17264723461047932`, 3.7903015192126244`, 0.06554920731341767`, 1.1764259933061223`, 0.4660090251563338`,
  0.6413383966201236`, 0.241827388783345`, 56.79713040044933`, 0.023743987090588337`, 456.3571078119333`},
{0.1547837566229352`, 3.4953794802806852`, 0.07246861713817378`, 1.0879160111375143`, 0.07311426293155776`,
  0.6990761768110039`, 0.005355312003055951`, 1173.1945274337409`, 0.08510663567245824`, 820.7115603554513`},
{0.12332890101103666`, 3.575694377588479`, 0.07546663843052578`, 1.1730448937718991`, 0.7299661739602221`,
  0.3544423810526146`, 0.3517297854582263`, 207.52630070713013`, 0.16381025124403453`, 211.24976747011442`},
{0.07543310039566975`, 1.1413407123857509`, 0.06791044220530608`, 1.3022569182103092`, 0.8228503547730448`,
  0.5416450068944677`, 0.18860915993442676`, 2038.110511149941`, 0.1613952093900407`, 419.7511258067653`},
{0.21429674136023663`, 2.8226782716380345`, 0.07008452435050433`, 1.2033565563578725`, 0.22607654151729606`,
  0.3670473114848468`, 0.04920492797100079`, 426.3575789205089`, 0.22829240712916699`, 151.89428282997392`},
{0.07895444632032361`, 2.201415239192807`, 0.005337930232592641`, 1.0054037946739751`, 0.7982316099945579`,
  0.22745190186265252`, 0.014751475681337079`, 999.912509637654`, 0.18790976944806215`, 156.9072222771119`},
{0.09184666059394636`, 2.067005962614928`, 0.04145877572545418`, 0.8296320077051444`, 0.11557680968156858`,
  0.31351325881175707`, 0.021527373087813387`, 871.3636268629474`, 0.16510736043882146`, 233.95931170378006`},
{0.12260917712356023`, 2.30639494589911`, 0.05876506714974223`, 1.4161472400002522`, 0.4228788165875499`,
  0.614217815805707`, 0.04726593691280875`, 1944.5171329959344`, 0.08163047191172412`, 744.531780752293`},
{0.17238170923293145`, 3.7452628271391264`, 0.012298914408557682`, 1.0114832888719023`, 0.7075400969976455`,
  0.4701095442473505`, 0.009792251350088488`, 109.73168851300552`, 0.11561190536083749`, 580.7510496473817`},
{0.06459740373368028`, 2.9106606918316986`, 0.09546811583014093`, 0.7816504226428446`, 0.9051718026272408`,
  0.6152354921314405`, 0.12920206403408327`, 192.77909888895545`, 0.1990333439617078`, 537.0263017540406`},
{0.07800640828878808`, 1.8505382879500134`, 0.012842566220368603`, 1.4649243339915694`, 0.6600118067779042`,
  0.4967332224237151`, 0.013562590855424642`, 1252.0890643038667`, 0.10833365543192358`, 267.9181792628814`},
{0.04846281102903915 4`, 1.800487790371883`, 0.005299468930184581`, 1.0419920592385326`, 0.9100782296151841`,
```

0.32988971122619926`, 0.11628748764501183`, 321.3445579524093`, 0.10904785586908877`, 676.7351327707524`},
{0.0408586986071619`, 3.397631267565795`, 0.034535155982847086`, 1.1499926979165747`, 0.6535863974222924`,
0.4932189245887587`, 0.383854582094014`, 103.0179671920324`, 0.1262724601642624`, 838.6520403111433`},
{0.056181027140717654`, 3.561092902333039`, 0.032415896850996564`, 0.8977612893413814`, 0.19606568802210056`,
0.5191107847286981`, 0.3780120623597924`, 2750.1468252074155`, 0.22792524388897922`, 419.0619039860675`},
{0.24051173539528659`, 3.363998136218836`, 0.07316535800080796`, 1.1407631035414798`, 0.3740167133471626`,
0.5883310500889865`, 0.1388691166946849`, 1845.7599241956455`, 0.06093536750612072`, 554.8160924336624`},
{0.10694470201355061`, 3.916523604921749`, 0.040570584968233535`, 1.141416795902756`, 0.9898088237744289`,
0.3296418758797267`, 0.04709726814380818`, 2933.808240863522`, 0.04460415393167344`, 879.0499414979915`},
{0.06862055370778036`, 1.8272394963651166`, 0.06696112608756692`, 0.9180601754047588`, 0.03279196554733499`,
0.20147163161780768`, 0.010370667030870775`, 671.676842322565`, 0.19673134057127845`, 347.1374473999164`},
{0.25063858582919535`, 1.0026414593770951`, 0.08049236416776281`, 1.1585705609095718`, 0.27877982032187476`,
0.2471176538176596`, 0.005549153441024165`, 1284.3138459700085`, 0.027491789825886737`, 964.5039545904644`},
{0.26413366443306313`, 3.5187238265686887`, 0.059384833236120135`, 1.2922578870185246`, 0.008326928227079256`,
0.6434100059643171`, 0.031067351563113396`, 133.02587790916047`, 0.1993086925023529`, 393.43546913290197`},
{0.13920351751612248`, 3.9485961604821114`, 0.08033096245429228`, 0.961725822180624`, 0.7322696656937997`,
0.5625362274736092`, 0.0233705450075019`, 857.2150128038464`, 0.13429548054404716`, 132.1146622970133`},
{0.12997797022210922`, 0.8438763321472402`, 0.09917403003828128`, 0.8801736269011186`, 0.10893971026850768`,
0.568805635817268`, 0.4569650004322221`, 173.3039538372586`, 0.1310412151491429`, 829.8180726295691`},
{0.04282165785099121`, 1.644759382359756`, 0.006131876320932417`, 0.8656795781593337`, 0.5309250697882764`,
0.28882829576135427`, 0.43822920515546965`, 171.56453795936363`, 0.17422534922512378`, 500.01112026736826`},
{0.22211928728847102`, 1.1859807267849432`, 0.08277265577838502`, 1.3176134726640667`, 0.7769092359720373`,
0.31677855442324954`, 0.01004066290735241`, 1412.8983237640775`, 0.051220047014877035`, 737.1658163615933`},
{0.09296167151413748`, 0.46354983597275057`, 0.024036152116512888`, 1.3763603395412263`, 0.7459540780275167`,
0.45155144834323624`, 0.3445673156480595`, 2335.90860073376`, 0.22065872238926132`, 474.2017592615175`},
{0.1962229794306719`, 1.6992229864288415`, 0.073818422425421`, 1.0867919112277424`, 0.7575363665855621`,
0.6670448748187716`, 0.24718082226975022`, 1605.1879390833765`, 0.059896123435389914`, 160.67342656698588`},
{0.21138025591123977`, 3.7198017071227776`, 0.012025339542637924`, 0.8791888222818957`, 0.9121961522822317`,
0.34954022634411386`, 0.48855663620210427`, 4481.4869986353115`, 0.09130130840750089`, 978.8438514579077`},
{0.174527181711994`, 0.849373235921072`, 0.0314016740925031`, 1.2229771378389438`, 0.5565629319212622`,
0.15780972618613898`, 0.03994648200280237 5`, 1015.8195322962273`, 0.11236134949951643`, 212.56048531719287`},

{0.27104366314172257`, 3.4411670504738847`, 0.05742812389939894`, 0.9577515288651626`, 0.0851317171213124`,
0.6274935825967685`, 0.018124860207485008`, 2870.4044184423146`, 0.2324535673532167`, 472.3817854782288`},
{0.11781804356750541`, 0.5626502822415125`, 0.0145982048993097`, 1.05752032760362`, 0.6292609081665201`,
0.6860764088162852`, 0.029170421001759508`, 352.2383589143192`, 0.10359473073293657`, 222.23677753012652`},
{0.15398221319678085`, 0.584996006414568 7`, 0.05386556842999246`, 0.7888135605497072`, 0.7266422422856504`,
0.40377533435456936`, 0.43018868620379314`, 1603.092147190326`, 0.20097603617156867`, 537.0534533002889`},
{0.09435165103191007`, 2.1871870158802187`, 0.09386196089682226`, 1.0117647368175615`, 0.23992524376119828`,
0.6944778195963781`, 0.011558368513568394`, 1224.4139617227247`, 0.05758577870260467`, 112.08810079007688`},
{0.15418547479730832`, 3.244024899467486`, 0.031613474871280495`, 1.1180817216823555`, 0.1378867782832267`,
0.24122334591588945`, 0.1784500939520896`, 1165.0611910719738`, 0.018111200873128563`, 519.7221352316303`},
{0.18001954973510415`, 1.9120553169228627`, 0.0184175105696649 6`, 1.0848164059222056`, 0.5445307300173845`,
0.21487607390213648`, 0.1420658743866174`, 59.88761221845187`, 0.10283589598798698`, 180.0642150440039`},
{0.17238975113628274`, 3.831887800749228`, 0.005776257990374099`, 1.3458283817066912`, 0.08939800815002275`,
0.20519935162452907`, 0.06363342671890825`, 2876.3582213396467`, 0.1796261387487414`, 336.8941601044835`},
{0.14352493778031827`, 3.7979256036341535`, 0.03771360392389294`, 1.3633466357184463`, 0.3018171788859503`,
0.19173241304281086`, 0.03417718814733039`, 2084.1653883747413`, 0.05483174208182884`, 581.7554610960195`},
{0.20940311206829182`, 3.2582854913524706`, 0.06591828409332467`, 1.3970633596535897`, 0.8615344587047382`,
0.38613005595364347`, 0.02066648198066755`, 4820.6850448541545`, 0.07989037484848699`, 539.3449932522163`},
{0.13522362333277543`, 1.144461624392597`, 0.008064525972274694`, 1.2137320027234764`, 0.41558764869922493`,
0.5044332234582337`, 0.194694572581245`, 1561.3725943749705`, 0.2352162319299269`, 635.4535165669378`},
{0.06564694091416517`, 2.517837422245419`, 0.08205855339446029`, 1.0554951116327027`, 0.7103060442920281`,
0.393784837384779`, 0.34842807990801816`, 201.46762707189424`, 0.22439022497167005`, 178.8572875798012`},
{0.24425093983878532`, 2.571873191821844`, 0.03797741011070798`, 1.0612508346456866`, 0.8063780340542086`,
0.4683983500560849`, 0.06605028609142427`, 2270.9148163281043`, 0.08295143441277542`, 860.3274939678396`},
{0.13421688107301388`, 1.6885106577171634`, 0.01327671934783954`, 1.4235239040662608`, 0.41175527118821376`,
0.35897461552598586`, 0.28058853074140605`, 344.729203987344`, 0.16559799427233646`, 938.0192541245116`},
{0.12355675427841095`, 2.0332534396069946`, 0.006925443967133322`, 0.9868311030648986`, 0.27448369335996525`,
0.49997436829086617`, 0.010689681959538098`, 661.0107417281803`, 0.049567549414286594`, 614.1468732990146`},
{0.24989261079843417`, 2.046336678398201`, 0.05343056582535697`, 0.9590211509682223`, 0.6962751749667404`,
0.5979718555935737`, 0.06212292589548939`, 836.0233579874354`, 0.028192514982996186`, 946.2272832575387`},
{0.18446589532009483`, 0.6770723958796063`, 0.017239459819183126`, 1.3829614064739535`, 0.15821669976510688`,

```
  0.18877757485488766`, 0.0071198331982843645`, 1580.0156398402103`, 0.12251896238349375`, 604.1234967411239`},
 {0.24207945515763046`, 0.8472072700897755`, 0.09830987943529323`, 1.3431438063557357`, 0.7063009601868007`,
  0.2722426879126155`, 0.34992016942373105`, 1219.3651744969027`, 0.1756849319581012`, 396.46752882152185`},
 {0.16929669277606063`, 3.384845133452008`, 0.04997850478346777`, 0.855944189945163`, 0.5854410422632743`,
  0.6313899885471745`, 0.27030175060519007`, 69.39208015599864`, 0.14109734367472376`, 825.1088816761363`},
 {0.22032907726938156`, 0.43680070868525345`, 0.02505576509377308`, 0.9013024202439682`, 0.3637877243645866`,
  0.6575693669120928`, 0.08327886814493145`, 86.53687085308205`, 0.24093186652306348`, 957.0475588354033`},
 {0.0915185159652725`, 0.4356928503469506`, 0.07385942693598586`, 0.894137684029105`, 0.07375739107747048`,
  0.37304243578619745`, 0.3884276894579204`, 86.6179555964664`, 0.023525347333101687`, 957.1153747111897`},
 {0.19293304608541778`, 2.881169086349173`, 0.06288677922536799`, 0.8471255106831246`, 0.5957102709253561`,
  0.464151719061209`, 0.03816221209756031`, 288.4038900208833`, 0.10298551466758471`, 155.08445353721268`},
 {0.18184235602556043`, 2.816880901418977`, 0.05545256089460814`, 0.876197436405354`, 0.954624174973548`,
  0.4655369772467929`, 0.007648944239440386`, 383.2702509437632`, 0.14043117229016888`, 779.9607071498034`},
 {0.1500634817243091`, 3.590063801011035`, 0.04690872877366834`, 0.8713971696364884`, 0.8016145774652992`,
  0.57680193310708`, 0.007900899994569407`, 1759.5867354003908`, 0.1650460276258336`, 468.72962630703444`},
 {0.07044808633799604`, 0.5092168059858677`, 0.023939619197820134`, 0.8363921850258171`, 0.11231870814099265`,
  0.415964567014653`, 0.010393952295787622`, 272.30094842939224`, 0.24251268430438633`, 533.0209640034411`},
 {0.17446312671000164`, 0.923278108173573`, 0.04622273024289145`, 1.361488933545279`, 0.05073013179716934`,
  0.423140548702129`, 0.4945547029315037`, 756.8418975914155`, 0.05582670682313107`, 453.1988863343269`},
 {0.25969186879026457`, 0.6876551199003744`, 0.016004864008093787`, 0.801399153017793`, 0.8167373300888658`,
  0.5699218117881594`, 0.010392376675716438`, 2453.002800663684`, 0.22138430515888585`, 604.6992574427161`},
 {0.08344350966636604`, 3.6673612041438233`, 0.0357399550994862`, 0.9043067215227492`, 0.3349583654565387`,
  0.39299105484116215`, 0.07044574168572774`, 531.2053684025772`, 0.2316097051950603`, 942.4468571449918`},
 {0.11177106931508435`, 2.9258562986192613`, 0.04535829204836597`, 1.397904475123252`, 0.8323395818238934`,
  0.6262291788570806`, 0.2126230418930395`, 59.43001557400602`, 0.03786935133626829`, 393.82943302003673`},
 {0.09014630069728341`, 2.5385294043084468`, 0.004644443169930969`, 1.1109052294542912`, 0.48602912154060585`,
  0.653448250732096`, 0.13848641693128835`, 233.22231224941473`, 0.13058754405383072`, 772.176206082497`},
 {0.1204210700620526`, 0.6547366279014759`, 0.07856424763514301`, 1.2422551703125984`, 0.769232712809691`,
  0.34959559963353615`, 0.3952520916617596`, 225.12460677270738`, 0.24941607930389664`, 764.7416993751449`},
 {0.12507255683757496`, 0.8093899840252368`, 0.022011166366603607`, 0.9039266843419957`, 0.9040126296575397`,
  0.40644665651439293`, 0.36973776842713274`, 4266.893817006702`, 0.22996060684055697`, 344.3317857496396`},
```

{0.18799072009935214`, 3.5387044337310227`, 0.010288586533272871`, 0.9709034820394874`, 0.16526258297595087`,
0.2720662467892546`, 0.16314642121657652`, 213.79313950957913`, 0.23266932286427316`, 908.785142248831`},
{0.10303653650946687`, 2.045785570411666`, 0.032527698921882384`, 1.4813578055387457`, 0.42199129008222913`,
0.6459634548526738`, 0.010175611270196772`, 575.847247144136`, 0.04915259766651514`, 563.8812676357885`},
{0.2398844305865619`, 2.1056225174808283`, 0.015327944846502356`, 1.2552986509569295`, 0.6918103031115113`,
0.1889744468357467`, 0.03454744519234309`, 720.0345748750987`, 0.20720302058084095`, 561.8805940371304`},
{0.19472671711158268`, 3.4537873437782753`, 0.05393741401653683`, 0.9791709575229651`, 0.34020645306003683`,
0.15465211878990004`, 0.005783246570334714`, 712.2087645951867`, 0.18659332879596663`, 390.1452748573064`},
{0.20571859545893295`, 1.5514943629000975`, 0.03978136161333113`, 1.2978001829388508`, 0.10072004838372872`,
0.22845349492624245`, 0.25892912790336436`, 119.6045968067387`, 0.07803724137740647`, 936.2227260158392`},
{0.275720564806804`, 0.720336275328604`, 0.09315930706476526`, 1.2814448950695845`, 0.9719953187330976`,
0.5030571263630652`, 0.09198284855367962`, 2926.101672056841`, 0.10631897378157318`, 340.03246937203465`},
{0.05630051102503303`, 3.7356157314175347`, 0.02800700712577493`, 1.0094636270338093`, 0.18952763541881779`,
0.23258714070277053`, 0.4551334614672762`, 3326.1734768299775`, 0.20084773313292642`, 389.1282332545071`},
{0.1566545086835321`, 3.6144342723158855`, 0.01851051241133261`, 0.8062001646255728`, 0.7860505516306966`,
0.5903319695657847`, 0.1423722018298642`, 1684.7441306012342`, 0.08281365756813702`, 745.921161780724`},
{0.26785050867880617`, 0.9336989210532032`, 0.047863988220149846`, 0.8870676821839705`, 0.894276639371691`,
0.24671850596175082`, 0.25434132792942304`, 2463.897338729517`, 0.07340938538359204`, 135.25070223351997`},
{0.20621368926339484`, 3.895401327519891`, 0.021945870300711868`, 1.2149002945024092`, 0.09567565478536721`,
0.35708592619490087`, 0.010140662222165558`, 72.14119724036794`, 0.09062375760852914`, 269.2376393484709`},
{0.22132018814331722`, 1.3624506083622139`, 0.02157572172797998`, 1.017162028467816`, 0.022930198784160805`,
0.174154916693125`, 0.0063722205185720695`, 81.57393238873418`, 0.23095499884940496`, 559.6417164883817`},
{0.2667728012376422`, 2.2597067694039596`, 0.052618617939097785`, 1.1876063798389778`, 0.9039352407220065`,
0.17021035842318066`, 0.061291229169270336`, 2589.9662235455107`, 0.0930933143809865`, 467.7152997091941`},
{0.16814767223163635`, 2.329148917787961`, 0.015181235666702158`, 1.3509190984659396`, 0.20135617569594788`,
0.6016277316250633`, 0.0960580261415236`, 75.18782439155149`, 0.06872828852909052`, 330.2905596330332`},
{0.24629497802526884`, 1.6185892731875686`, 0.005355789298525409`, 0.7573206700198012`, 0.6605818641858783`,
0.4413473573778042`, 0.3432973026299589`, 62.23416744978072`, 0.03461390771204009`, 861.2270986066914`},
{0.12348100274215157`, 1.3439669835932486`, 0.06660199654711046`, 1.157413128080917`, 0.4506243858381027`,
0.25274164958094525`, 0.1821863441956961`, 1340.7965217357605`, 0.03495643955461325`, 657.1175564125843`},
{0.20026891357076415`, 3.645369762770067`, 0.023665341602336538`, 1.1690889262253168`, 0.5114922593058153`,

0.6682127068245862`, 0.22249927148912355`, 3097.4126005392104`, 0.07667851305193468`, 720.3291394146138`},
{0.1167144215735465`, 2.9739284718729824`, 0.06770393749145168`, 0.8030036195270651`, 0.817910504731294`,
0.2533089674739153`, 0.13586578835473073`, 245.55576330732927`, 0.12297451593509273`, 949.2797411932297`},
{0.2610230648306139`, 3.241876732828336`, 0.005221929989860801`, 1.2708350659440502`, 0.2709097063186865`,
0.6295136971445925`, 0.2167769645618444`, 67.25197219006178`, 0.012919428781842102`, 446.6417150509489`},
{0.22755856549372416`, 1.1571207715998462`, 0.03935231641744066`, 1.338611381035573`, 0.1970208179920212`,
0.512487214253471`, 0.008778903977610756`, 69.93484330017829`, 0.0885116413292587`, 703.9913507831651`},
{0.08043529968845808`, 3.6346678770302887`, 0.06436593471662284`, 1.4919978389096693`, 0.6239584327672116`,
0.5363538338794006`, 0.0057143426753125245`, 2341.702974881963`, 0.06269329048310018`, 332.55107547613017`},
{0.17838278501630495`, 2.4576612999234007`, 0.06181741342015828`, 0.823346074642842`, 0.013205254550742174`,
0.5017391269032923`, 0.16663083212381372`, 304.8776737280077`, 0.14165654641577402`, 678.4897161214598`},
{0.1527608465983989`, 2.070934262024398`, 0.045813432940116104`, 0.7944089298621102`, 0.6769693136188586`,
0.16430192509774133`, 0.17450886910735772`, 3923.7643815879096`, 0.06708022916564621`, 420.0877116062573`},
{0.11624661761519267`, 2.9732886208480265`, 0.08686637218032857`, 0.8721582238868211`, 0.700158456128364`,
0.3183544106471198`, 0.06872076435482898`, 2515.039310773072`, 0.08635525493495505`, 866.8956807307524`},
{0.1310858151843764`, 1.596637100973001`, 0.08661007645244247`, 1.3109557164680856`, 0.9039286133369202`,
0.3437790347559597`, 0.06680016028420104`, 3498.912503889652`, 0.1695299313540355`, 339.123637513298`},
{0.13093035477925424`, 3.0078393597426825`, 0.0788110046495581`, 0.9368136862014702`, 0.15232997356507805`,
0.5208667948995476`, 0.027452102279722775`, 439.8539822509126`, 0.023447155859907365`, 960.4131412752828`},
{0.11884479435624312`, 1.7884452074083192`, 0.07215967343468577`, 0.9483398419798006`, 0.06052883609711657`,
0.6592909099232407`, 0.008348617368503395`, 734.1801319364669`, 0.05937372894090781`, 336.2025788281121`},
{0.09279332232126897`, 2.395881928323834`, 0.035821424791727154`, 1.1256250545946367`, 0.566531337666109`,
0.301586294875144`, 0.12926859185498135`, 3190.2362801672193`, 0.011195844366674756`, 937.8412253936428`},
{0.10470396306835666`, 3.0481563632657567`, 0.02918159941393267`, 1.1229103226446677`, 0.06432569110103459`,
0.1636910757663953`, 0.0677993574358691`, 108.21923708078778`, 0.037422575295802174`, 716.3638099124112`},
{0.17624356912801198`, 1.573989236801781`, 0.09366396594728997`, 0.9674991919084199`, 0.39632574484069294`,
0.18908139883550235`, 0.042591608468658185`, 545.1774692484181`, 0.10286957749970482`, 735.5542101216969`},
{0.11969608505483581`, 0.4187980837332148`, 0.0887749696374739`, 1.1942873584807199`, 0.9542625089733192`,
0.26224686727401303`, 0.2044328771862869`, 4071.5728832408004`, 0.012536086303640448`, 986.0101272937204`},
{0.0681782584958246`, 1.9712297226556181`, 0.0710699974540059`, 1.4798684393175123`, 0.28400354899281655`,
0.18343756245630538`, 0.025080565221411666`, 190.74276766833862`, 0.021217036684959`, 114.63276406832605`},

```
{0.26466011364476083`, 2.3627816128360744`, 0.02039742244272034`, 0.8195822231735724`, 0.29110662117910135`,
 0.47981634198411705`, 0.013151438435585994`, 123.59545033098055`, 0.04800415213314124`, 576.5536164907207`},
{0.27910166898523386`, 1.349450515188618`, 0.0885331584316003`, 1.336068232065382`, 0.5987418653774925`,
 0.1580872600090233`, 0.005729492652206841`, 454.22089835832855`, 0.0493218089029025`, 734.2743634707255`},
{0.1870945044053341`, 1.8787994994414792`, 0.052561657322085654`, 1.3540062415635468`, 0.28234410939143695`,
 0.681042030488356`, 0.009986864266329082`, 981.5333621075416`, 0.18733052504041348`, 853.4539224410559`},
{0.1396988967578765`, 1.913702915438618`, 0.01617810449961091`, 1.199136555704491`, 0.16595422293922835`,
 0.6068296527307018`, 0.17305426475375874`, 717.4973492774137`, 0.1983198566762196`, 283.9891726135136`},
{0.15506300074217488`, 2.83069948310355`, 0.0846226593104205`, 0.8890994038076738`, 0.47796520586519553`,
 0.5728084495295004`, 0.005405423164330928`, 1159.371736373684`, 0.08490828359258434`, 608.2480837068873`},
{0.08918867947883585`, 1.0389500531703932`, 0.03170283696550655`, 1.1764272925199706`, 0.8188250505498167`,
 0.512109016528521`, 0.044640995253867236`, 632.0761666245681`, 0.15046863407887012`, 841.6135553783089`},
{0.05692826445298971`, 1.923331897508854`, 0.07552634610068665`, 1.455886213864955`, 0.32041248603883954`,
 0.47523042425037754`, 0.22105687499350363`, 2027.7508727407876`, 0.017420396497442786`, 274.64042522521055`},
{0.24257218102715322`, 2.573176975490081`, 0.09661060539215438`, 0.8445932242005463`, 0.8955286624705445`,
 0.4789936248391016`, 0.26410377851960004`, 3510.537763377287`, 0.18852831639094753`, 237.77037147960095`},
{0.0534772370796234`, 0.9129899365885157`, 0.07428512647308946`, 1.466096589680298`, 0.263816129356184`,
 0.30123412254131576`, 0.022303163735904144`, 4746.406027391749`, 0.18205991120633613`, 895.9248957958035`},
{0.27274128807061915`, 2.426636979804435`, 0.06334129103904147`, 1.210981179335779`, 0.5970208082221518`,
 0.22184996611628427`, 0.009365743994866976`, 204.27799326594078`, 0.22060849091929458`, 828.6461515322391`},
{0.10281443317302225`, 0.4962771981673755`, 0.04898875008264165`, 1.262839945648412`, 0.8347432488590383`,
 0.4192167501942007`, 0.030355367117986`, 1308.2409868067423`, 0.042950873976760395`, 417.52929653085965`},
{0.25848551367088474`, 3.558870975613896`, 0.037906846258373254`, 0.946132081756833`, 0.5725842788655087`,
 0.6625769316332577`, 0.09924894324330936`, 52.004626921156905`, 0.12255979122726918`, 619.4850012984921`},
{0.05861384430265282`, 2.8809993577690376`, 0.0034389149229243545`, 0.778023331623658`, 0.955566931044247`,
 0.311264533883984`, 0.005123688327250641`, 510.7904162444792`, 0.203481852542521`, 418.9270384908555`},
{0.26171770992100346`, 0.5361270884859564`, 0.038524708394197374`, 1.0508503305310222`, 0.11056009208990747`,
 0.571340638565827`, 0.027815464969581257`, 333.39873468372707`, 0.030150481088034198`, 186.42906095487143`},
{0.09228406045428905`, 3.787322761562357`, 0.04412628551769787`, 1.2485522327984204`, 0.00453916468120652`,
 0.356730274900637`, 0.03212483930675613`, 3375.2895893320583`, 0.05502779271988756`, 653.0147468261181`},
{0.2582323539483842`, 0.7609188588793034`, 0.043891058231223086`, 1.3021658200784163`, 0.09072647662703637`,
```

```
0.4622306981965607`, 0.06168457257766234`, 192.16050980483587`, 0.03732931870687156`, 184.32898889722597`},
{0.2477921144844203`, 0.9405947985440335`, 0.09500951785333488`, 0.8564439359315013`, 0.8813246793351337`,
 0.5526752397451847`, 0.04122909919220205`, 2544.2880548964617`, 0.18357258099770601`, 309.1199502666939`},
{0.06005306680926792`, 1.310324956982524`, 0.01917002825340564`, 0.9233944849507227`, 0.37862876807380963`,
 0.4654284132589105`, 0.028904406737409957`, 740.7733482913402`, 0.14207137738578401`, 125.45920277021416`},
{0.13221174566753952`, 1.5344979786934942`, 0.07993475417223245`, 1.1279318472161384`, 0.5224757382486951`,
 0.681045123252989`, 0.09251417322960646`, 4007.8291314421654`, 0.1250986785892464`, 411.07839231550474`},
{0.23641095513812793`, 3.2390712982841716`, 0.0904220832128998`, 1.2764555680953642`, 0.4087688116784247`,
 0.23715766669095795`, 0.050286530096096176`, 142.72560445944592`, 0.13322047650855234`, 994.1833005928358`},
{0.11515945727299054`, 1.0363839446782226`, 0.04991967937770255`, 1.2110778925771657`, 0.7975055001930793`,
 0.15766796336173805`, 0.11336570441348018`, 702.6737236802497`, 0.07984121314940101`, 718.3669578221027`},
{0.22406410922088188`, 3.5768982916995915`, 0.0850321138351546`, 1.0822829393722269`, 0.3956856744827095`,
 0.5136072284730188`, 0.021536821023513928`, 1505.4866148851402`, 0.07683669026613277`, 536.6032367164931`},
{0.09816955174932002`, 1.3610473147845434`, 0.01200296321417639`, 1.4480047032959393`, 0.1823923291352767`,
 0.5244723013770171`, 0.03492258343756444`, 1533.0073499542846`, 0.0394156510269485`, 214.7356679901721`},
{0.2686714308482082`, 2.6024579475953065`, 0.09605319126050717`, 1.4115359358964699`, 0.40944065631503346`,
 0.20238890153994604`, 0.44356457168170405`, 943.2699426207273`, 0.20955602538220924`, 179.63082811814246`},
{0.2789448341874723`, 3.460339370316608`, 0.09560289059485072`, 0.7655082688097814`, 0.18036074766112598`,
 0.3208070646396465`, 0.06769953888545853`, 1642.3905460053934`, 0.12624689441090442`, 706.9723251095423`},
{0.08907118739874043`, 0.5288966486743032`, 0.07776144026324502`, 0.8401856534758337`, 0.6264494338356676`,
 0.6033856818349079`, 0.2509533302600997`, 881.1682049810406`, 0.2136549221518534`, 541.1576363993561`},
{0.21035514306399716`, 1.171180695658463`, 0.003072872162929503`, 1.0221763640609527`, 0.6635332430632568`,
 0.41088172812983936`, 0.025266995789722163`, 240.41303897513703`, 0.11383871532153805`, 586.1694984071029`},
{0.1757725171577343`, 2.8661042899257385`, 0.05038868584079925`, 1.0221414200627101`, 0.4033811300549006`,
 0.2808753785255522`, 0.23688381111182788`, 1187.3099819834174`, 0.12417956003379604`, 375.8746376350517`},
{0.15358915554374314`, 1.5283095551064108`, 0.067290924027347`, 1.1004906376784402`, 0.30400512125859813`,
 0.48412227232879435`, 0.05306566889779679`, 235.92000045587324`, 0.04951315982948612`, 369.40105642182`},
{0.2480667235282486`, 0.5649579963672635`, 0.053140067348230215`, 1.2818674385347062`, 0.7623809660000294`,
 0.3909970176312558`, 0.15838111333051053`, 2237.6541846573073`, 0.19996140314425598`, 705.3371940684284`},
{0.1514523903966063`, 2.013347200420334`, 0.015461991282007386`, 1.316890683212132`, 0.6869826935753494`,
 0.5587698316891556`, 0.155845847769785`, 1008.4357476155614`, 0.08368596216890972`, 432.47408201145043`},
```

{0.10270003263878408`, 1.5137093678767606`, 0.08953511397171234`, 1.327942781940753`, 0.7426665475116903`,
0.6088434442795674`, 0.0731377274448417`, 629.1021204327045`, 0.13932635233516538`, 654.4427386466705`},
{0.0793896888891446`, 1.0605932032187937`, 0.028244713409935986`, 0.9969099319950825`, 0.928004076538486`,
0.27677090692739703`, 0.2620559892525522`, 130.80681937144962`, 0.17810627837194376`, 310.3444008701523`},
{0.27502930950392923`, 2.268988901211176`, 0.016690280319337808`, 1.4275341924064249`, 0.010424245503220186`,
0.33306898602448776`, 0.08933605249123197`, 948.3096011028355`, 0.06701293400753894`, 811.6908300502881`},
{0.1401731309912823`, 3.510459405926335`, 0.08747923189250421`, 0.877306477766522`, 0.5918814561880961`,
0.26160677626581574`, 0.2368095664541691`, 545.7040461576285`, 0.1892727670923422 5`, 868.6345989081833`},
{0.22884950103664703`, 2.906687483673287`, 0.09738225028393067`, 1.211823583578368`, 0.3123283466662281`,
0.6689345381975826`, 0.26177215931622877`, 1241.3038445786822`, 0.0415314605513189`, 456.6453916426678`},
{0.05854425920454692`, 0.5238963150414171`, 0.029075123644520265`, 1.1426557901500585`, 0.18407746295956828`,
0.6998927262860721`, 0.018954418749017626`, 3086.173448022591`, 0.07606552279053064`, 322.105662191812`},
{0.21395159305342204`, 0.7435306425677184`, 0.0767057005018636`, 1.1181708530382222`, 0.44751085978070226`,
0.3928647127765148`, 0.07805162691492751`, 4351.207257923278`, 0.18002244556017466`, 250.73279447897897`},
{0.10532042949270481`, 2.282286106803773`, 0.03578707315111876`, 1.3216192797313202`, 0.0418889004272697 8`,
0.35518315207091156`, 0.030710322655757117`, 714.5224002509336`, 0.1899050019419828`, 291.00389901709906`},
{0.0829906933156388`, 3.8005179296933402`, 0.05100636383257797`, 1.2003099776527455`, 0.07859762059433151`,
0.5748450314701046`, 0.06614617870175625`, 1151.2833065752827`, 0.239776274024895`, 683.3664621517701`},
{0.07620756796620615`, 2.1362921732694753`, 0.08270811763119688`, 0.7737426183146745`, 0.16169731434372636`,
0.25627790943141393`, 0.10296042384053361`, 1230.0671635154563`, 0.22674061100539578`, 156.89063483163608`},
{0.08669389820930451`, 3.045091924598611`, 0.06550910340602879`, 1.1417010593363959`, 0.8553204857574712`,
0.40177204632489383`, 0.3920145317856002`, 704.3045188157349`, 0.14263547938719845`, 197.59576868603688`},
{0.16716391272679498`, 1.9707940745517059`, 0.07610260692323212`, 1.0373304984519585`, 0.065601176736233`,
0.3311381406088889`, 0.12567743444949903`, 64.49465686753402`, 0.10130675587326837`, 483.8093443390175`},
{0.19822566409398995`, 2.5240412947893924`, 0.07566408618441202`, 1.4422150828920155`, 0.5695379812663179`,
0.5359514511624127`, 0.4361070820215791`, 2999.4348713305167`, 0.17170169259485252`, 352.0476518165484`},
{0.2582881650757237`, 2.8338294335694956`, 0.056678410389419474`, 1.074319457568079`, 0.10001974339589292`,
0.2593067051666722`, 0.07903831577731309`, 123.40669984562356`, 0.2485795541321016`, 273.9914626473702`},
{0.19497013167937116`, 3.160806920721086`, 0.03009239591987214`, 1.1171831820583615`, 0.4032199037756403`,
0.6542147113806289`, 0.029465449867892138`, 135.26925165074772`, 0.15317415942522017`, 921.0689346313153`},
{0.1360564482424821`, 0.498585531259212`, 0.09708640926926869`, 1.2162038348005786`, 0.11915352104228649`,

0.5956143691814719`, 0.017832698276087463`, 73.16788682178698`, 0.17076027369704966`, 648.9946415099051`},
{0.13584499805886296`, 2.3834753645488007`, 0.08131332514134307`, 1.195938774683543`, 0.5088595280581578`,
0.5258764750728102`, 0.0188849029484269`, 313.8058177427212`, 0.2364862024741387`, 393.5561696485299 3`},
{0.11252539515708737`, 0.48050955320077815`, 0.05937623337381773`, 1.0236120368069983`, 0.7678895649223039`,
0.34416849853115417`, 0.008058107074703271`, 595.8657256124042`, 0.07836079702593357`, 415.53467926543846`},
{0.05491046591497972`, 2.8601951288119833`, 0.051921452801939785`, 1.1535422934918167`, 0.154204134629650 1`,
0.3257327805969301`, 0.13649263155602906`, 63.90737715933027`, 0.027455815347211365`, 562.8847739679362`},
{0.06606106797338895`, 3.6656724808413053`, 0.08751889535990849`, 0.8556123556717206`, 0.6825935559995051`,
0.5266173914314858`, 0.022251439911425613`, 1853.305468841329`, 0.16065122014174432`, 222.6798638040009`},
{0.08977217568251161`, 3.4528260544503624`, 0.05764478135197445`, 0.9515190566806713`, 0.12846915530471392`,
0.2168250694214009`, 0.007812714529548206`, 538.1854674164173`, 0.10544198251462877`, 265.77458817227784`},
{0.22154304122492052`, 1.5332296117725148`, 0.02877908996913961`, 1.4000488050416342`, 0.4501923011081377`,
0.49546155556198335`, 0.03515207628009833`, 79.99141751015185`, 0.06122494520176597`, 976.7821412391111`},
{0.25142928822507965`, 0.8204095084034169`, 0.04775248977653711`, 1.4286128295323546`, 0.29951010343614115`,
0.5861829342129077`, 0.023219447176916163`, 80.52367411150172`, 0.0258898929268112 1`, 373.5821652599455`},
{0.1883756473924924`, 2.484499408271515`, 0.061812669070377274`, 0.7895040235728823`, 0.6199124969015315`,
0.675547123744697`, 0.4413864963579275`, 224.0804292528027`, 0.08535968882727879`, 719.8375736166386`},
{0.04016858296637793`, 2.227608827443719`, 0.014781301743870988`, 0.9058498293617421`, 0.1749409962982329`,
0.4847952385558868`, 0.08097216295115642`, 156.97217485042802`, 0.02129075017123533`, 574.4487237391048`},
{0.10741359519637045`, 3.89617613092826`, 0.04687805603635281`, 0.9794147612026731`, 0.29389906665208465`,
0.504394704133666`, 0.007650336867825106`, 94.96376645284155`, 0.03904567887129218`, 298.40389093358954`},
{0.11745053279334533`, 2.766853076583562`, 0.01709341111594279`, 1.380078086463911`, 0.636455229677482 1`,
0.3050852260798198`, 0.23148607657693196`, 632.2606814192264`, 0.2194692090207866`, 944.8863492578521`},
{0.06051380978707749`, 3.721291677689673`, 0.02351362713647468`, 1.4742360491119153`, 0.26914934000385093`,
0.3113498131806878`, 0.014199987869299453`, 193.52537465732863`, 0.03624750856265596`, 654.4974389371708`},
{0.24099369076587246`, 0.9331253409733131`, 0.08217248655684913`, 1.3431483271602298`, 0.5958343219475497`,
0.5465723977337402`, 0.3117537417312618`, 3620.513648000344`, 0.017044351379539774`, 583.8913660600036`},
{0.1670999366894979`, 0.6892897125951207`, 0.018177278706139326`, 0.8073727173778276`, 0.4996777893876556`,
0.5006175381891131`, 0.2838330520493472`, 735.6587801659197`, 0.09848547093144494`, 522.0721253919525`},
{0.20400009973703231`, 2.742493766989547`, 0.008983552040910397`, 1.241362072408477`, 0.6278550642154503`,
0.39751636324962747`, 0.007299390369356264`, 91.83643299080828`, 0.13001522795856457`, 108.9539719977738`},

{0.22650061618533962`, 3.64567942204856`, 0.07707002972279017`, 0.9032908420697698`, 0.9136296860108464`,
0.4341422027661652`, 0.24929767745737103`, 136.4576870478556`, 0.07080767715199654`, 971.7774116990597`},
{0.10155767105386482`, 0.4181451151728903`, 0.012394687986228644`, 1.0711096932931712`, 0.20820538774593245`,
0.1626988912346673`, 0.0864577360395456`, 674.1888341417106`, 0.170056418389111`, 730.2391664075723`},
{0.21231295112704185`, 2.380633825425009`, 0.06236561820134972`, 0.830378492099762`, 0.7458626837014395`,
0.3778428196310881`, 0.06821741509586535`, 516.5455006559224`, 0.0981833249923344`, 365.58490612208846`},
{0.06947494567464263`, 2.895860587431992`, 0.09178966303831615`, 1.2688043406444924`, 0.2941408877223748`,
0.3751778179392746`, 0.03775382322774307`, 60.369496178615435`, 0.1179420875716954 4`, 673.3642442045926`},
{0.09985693159346487`, 3.9019559576117224`, 0.06462003972312033`, 1.0811937700231284`, 0.15667266604108`,
0.623010278974039`, 0.040053923820923376`, 112.7677826146989`, 0.1201138368644335`, 956.8700593079004`},
{0.21079578713436742`, 3.6465182996128265`, 0.07263502170252384`, 1.2463245485615204`, 0.04598824508235744`,
0.19490478821937018`, 0.006055825079153585`, 455.84209633704694`, 0.04307337339516104`, 393.98363704378045`},
{0.14050505626384868`, 3.3486141508967293`, 0.06576905440282896`, 1.0969622150757512`, 0.25298608583995974`,
0.6081914621324831`, 0.07984197613603365`, 4147.732881753063`, 0.01970187856601205`, 417.6295038180262`},
{0.09029343760417469`, 3.1277911065164137`, 0.04916145932038262`, 1.1046401865081483`, 0.21999281746629284`,
0.29609111953575007`, 0.08848537504901788`, 1978.4176945251475`, 0.1604567549403832`, 631.7829673381813`},
{0.18744474850618575`, 1.1949686342264414`, 0.06413249212557762`, 1.3584803763102893`, 0.320102170913821`,
0.4723006569832039`, 0.10067644299750714`, 61.82330782936513`, 0.17695515305422665`, 265.1926737070246`},
{0.2388654681009152`, 3.8576612834574524`, 0.02902564289553176`, 1.4380459539751635`, 0.624215346482978`,
0.3925897588304593`, 0.28422329981310057`, 1921.54220161346`, 0.09343473821304771`, 199.86167324974923`},
{0.1455526991456984`, 1.8705049552506363`, 0.03961322047797989`, 1.4283548335168212`, 0.9360504979535096`,
0.6211597747744511`, 0.1850342062284412`, 2722.415223545606`, 0.05486666811414997 5`, 162.6815751148559`},
{0.13798249053354167`, 2.899902544251761`, 0.0459763868363342`, 1.0421434347893428`, 0.7895156656158571`,
0.5540265258106729`, 0.047360361099740046`, 2648.770558815322`, 0.09753399188330308`, 720.6816664753013`},
{0.17745255413169525`, 2.520375966029923`, 0.04665876128100216`, 0.8123275453028636`, 0.944701273242313`,
0.24813120748859874`, 0.24862758503488186`, 106.62623807073865`, 0.1979751584484703`, 843.0950202672676`},
{0.16499863275860222`, 1.077452191253113`, 0.023014633917973006`, 1.2950348307619706`, 0.9833706900164221`,
0.290091826190232`, 0.22192674995545697`, 3510.2131809670686`, 0.1789639285862865`, 441.136883561353`},
{0.09494055003043922`, 3.273540527547083`, 0.060548822068484894`, 0.9817396325621766`, 0.5953487435300235`,
0.3030053710238173`, 0.020212568263132794`, 207.40154426384788`, 0.04862769785226101`, 884.4963622485988`},
{0.22556943514719452`, 2.763355506916427`, 0.0659847718412378`, 1.1318711218549837`, 0.7290768537995906`,

0.6910097128365145`, 0.02883814164041025`, 1935.4928886970383`, 0.060672213321555424`, 592.2254589353502`},
{0.17976151229453585`, 3.607368069246487`, 0.03562542831805498`, 1.1702627239429857`, 0.8807800807300026`,
0.5048328928685707`, 0.08802036843157425`, 258.70537367208925`, 0.2013896965979159 5`, 520.6789088604623`},
{0.23694985915625555`, 1.7115996463959204`, 0.09475499820714504`, 1.0033560670730772`, 0.6058641758111063`,
0.5075289726151094`, 0.04433733902402368`, 990.3350746839715`, 0.06868554455100367`, 684.4534604177855`},
{0.060659588298575706`, 3.185819049116902`, 0.031246099794861325`, 0.9329844374373137`, 0.7312648065631473`,
0.27616453553670284`, 0.06002230172665507`, 72.75197129693021`, 0.01701545753405373`, 816.2999501758868`},
{0.27783663000790365`, 2.7970847777258587`, 0.08231332350426997`, 1.022697264732825`, 0.9687024837581177`,
0.3645779308390613`, 0.041020574150516895`, 4813.902596935811`, 0.09489024372224386`, 126.14406706341742`},
{0.1853383438805617`, 2.710478464072388`, 0.01489054849244626`, 1.1482283702493803`, 0.5568912869451013`,
0.42437036955952745`, 0.009568366829966814`, 179.8278356132767`, 0.21625752953014726`, 454.80717115717636`},
{0.05021881637759101`, 1.4247717975270389`, 0.062006838284966344`, 1.2900546523204781`, 0.9617562903977206`,
0.5950392020832158`, 0.01053774359341417`, 3195.955497745086`, 0.10119630409828645`, 778.7070525025722`},
{0.1629749532929909`, 1.3827669190112752`, 0.02163316926061519`, 0.7859725102428954`, 0.8835314458481405`,
0.623882909407651`, 0.021916393496908437`, 149.5675827629461`, 0.09031773923128839`, 469.7973445453567`},
{0.2303174353412305`, 0.7381196545439321`, 0.017853274128905045`, 1.009576893018485`, 0.49320693638055335`,
0.6517404058254557`, 0.03129869768398024`, 1241.6665530134712`, 0.22978761854559276`, 819.6026061342192`},
{0.05000320218233145`, 2.1972018685344494`, 0.006726117027854865`, 0.9936552562980645`, 0.8328018981538441`,
0.5156429010358797`, 0.19438658089409308`, 89.43952173057976`, 0.03393498174078835`, 570.4514942090268`},
{0.22380766185105788`, 3.972867022032534`, 0.05857466369517589`, 1.320944264492121`, 0.8375716850380956`,
0.6387604619615563`, 0.14529791195160408`, 354.612762024331`, 0.0916149378074263`, 197.12301088359715`},
{0.1925638236278404`, 3.172077483377868`, 0.030644598758412977`, 0.9803085988716782`, 0.875326409629076`,
0.5489970615034699`, 0.4213559070823191`, 63.50782532256897`, 0.15909673801152152`, 757.7936027644167`},
{0.09418442467434818`, 0.6452011369781001`, 0.05468238280755824`, 1.1696403162883113`, 0.9269902181221603`,
0.5827207204052004`, 0.0480602677307661`, 155.50909202321085`, 0.17256127653904146`, 540.5740309875913`},
{0.05187445469191648`, 2.8967354683291404`, 0.05468134937388834`, 1.0272048159360176`, 0.32845734934833803`,
0.591187325659497`, 0.3107635049414343`, 481.6720966539763`, 0.2446670824913575`, 391.7749895365258`},
{0.08569179084998313`, 1.8355994610956383`, 0.09902879473840677`, 1.0426364128937085`, 0.046121645528780064`,
0.5970016999064142`, 0.04585616299575304`, 1326.7471633941773`, 0.1575248274172592`, 749.1145234256358`},
{0.24342030247616103`, 3.756945920144269`, 0.048201731449734615`, 0.8715075343316259`, 0.6190895848455991`,
0.19127133299748744`, 0.06855426679028909`, 1305.765466028692`, 0.23417008838782194`, 415.6502949437222`},

{0.19292203783708667`, 3.220432103715133`, 0.09761210999008452`, 1.3591272090173214`, 0.14186149659118774`,
0.4413360685039047`, 0.010167923556678914`, 131.42190006936238`, 0.24362820427309634`, 380.03821419833645`},
{0.1897036930032619`, 1.8964339581865879`, 0.059440108944421456`, 1.1142569288613073`, 0.15129145170580593`,
0.3833845599206046`, 0.05519372846185666`, 4558.979306456515`, 0.013937979989310956`, 207.93733592881284`},
{0.21229489496584697`, 2.877170935416972`, 0.0852716204718659`, 1.187802399588918`, 0.766747964989742`,
0.2056469011396459`, 0.2516666100392663`, 249.75546686829057`, 0.04381049946278048`, 453.1293949612376`},
{0.18727208406868906`, 0.5920853885729089`, 0.00310465950751313`, 1.4761651930790642`, 0.9879910194473076`,
0.4582425433815992`, 0.1348498167291504`, 1379.8554077407293`, 0.19832956659061096`, 601.2373424307469`},
{0.21303838080640486`, 3.716490082492431`, 0.06530590830455278`, 1.2455728623234372`, 0.6800061917252811`,
0.3355795794234222`, 0.014627505674034404`, 79.98307290173638`, 0.017560362469609708`, 213.93067315001346`},
{0.06263632603162816`, 3.088015470379359`, 0.06468478054731996`, 0.9840993814948887`, 0.7841919183884491`,
0.464604288151068`, 0.01258311092046284`, 1881.0148437864943`, 0.0609287237146911`, 341.9306317400494`},
{0.1229419022884577`, 0.42323948511603504`, 0.04401981826231521`, 0.7802588542080802`, 0.3378063219388938`,
0.28733129925219736`, 0.006237735142161048`, 2301.7896008425946`, 0.1904542796497251`, 972.0175866826189`},
{0.07173116327385964`, 1.689724351901349`, 0.033537565627478`, 1.2797681367134839`, 0.19114975037520088`,
0.541681437492502`, 0.35548681580819513`, 4889.525485193395`, 0.07624198202888299`, 255.7119735830849`},
{0.12377155914664595`, 1.5057831665831483`, 0.025847870859220036`, 1.3931245282333518`, 0.1751216551172583`,
0.31643702992235223`, 0.006127436167226222`, 3977.3695715719014`, 0.19191205388712534`, 489.96663345919205`},
{0.07922678243367776`, 1.1007105803460338`, 0.0670178962975939`, 1.3858977973939028`, 0.6913244964468961`,
0.30596530488881035`, 0.03670449008923856`, 722.5750280061836`, 0.1062965670986098`, 454.2241152370099`},
{0.13900795297527618`, 3.9726186574282156`, 0.014365558963868033`, 0.9387566245260975`, 0.7733911807172529`,
0.2901636804835216`, 0.022925433557643798`, 983.22576571694`, 0.10414701635541929`, 690.7271079642408`},
{0.06983492300698985`, 1.9811052508516296`, 0.015092410881193086`, 1.3035668919937708`, 0.40820708656243165`,
0.31349067656511853`, 0.03294127758239607`, 588.7480044888102`, 0.2032334347292173`, 447.9335749964651`},
{0.05248318477610414`, 2.804613750674261`, 0.003640346777431844`, 1.3628720468665754`, 0.1995299750560573`,
0.4294363076423442`, 0.007453829399752168`, 688.7688808168017`, 0.24854011257885444`, 913.0182165489159`},
{0.22482399322255875`, 3.3120245448192804`, 0.03927421614929747`, 1.1730261223962148`, 0.8102112515480973`,
0.5310384924975508`, 0.021360928541744394`, 935.9962133496834`, 0.022365336640988054`, 615.9503154198303`},
{0.14054031835314812`, 1.0862525764402395`, 0.0349595746798 6743`, 1.4989441459825807`, 0.9776070717717082`,
0.31123951873946865`, 0.005138607602622309`, 2625.434021720543`, 0.07843546140834678`, 537.9180477646557`},
{0.12045530385052439`, 0.5766797014120977`, 0.026639235327100757`, 0.9789792038223954`, 0.1966739314900421`,

```
  0.3703973027163936`, 0.058252148400224385`, 4908.720210074871`, 0.20113853341018156`, 829.8609278324832`},
 {0.2466711089217451`, 3.818653323688843`, 0.022826073218276496`, 0.9885944445220116`, 0.08478033040666122`,
  0.24831675766148897`, 0.05221202249805683 6`, 61.098852909743506`, 0.07219665114050788`, 854.0668809251908`},
 {0.2622407564991055`, 2.390608019262051`, 0.06092577106875352`, 1.4943502181005281`, 0.48817005604720776`,
  0.5989574618060204`, 0.0663716035556017`, 59.28691333172426`, 0.07923371737796253`, 663.2501758461839`},
 {0.04240892049220529`, 3.585324421001501`, 0.06559550484561946`, 1.4902471895373985`, 0.5553889156366423`,
  0.6483753523879463`, 0.007118840678651323`, 2218.8393261162782`, 0.011166606091999653`, 925.472456163056`},
 {0.139826903045498`, 1.6937551480709319`, 0.08421189687207545`, 1.1119195234296138`, 0.8823668491031473`,
  0.5196063483063849`, 0.09432450978517919`, 330.21686735744527`, 0.2294519472818627`, 332.35073177164196`},
 {0.14802078413209885`, 1.733603865107586`, 0.05101449210330218`, 0.9572479232347667`, 0.5675011406257902`,
  0.3885036693205801`, 0.29314426920759284`, 2048.9810499540877`, 0.14325997637273158`, 127.28328705485782`},
 {0.09351974409461145`, 1.9687875339139866`, 0.015572094311755826`, 0.9484499792232198`, 0.5844423119728932`,
  0.5819082431078818`, 0.2373776682556218`, 112.86193139612024`, 0.23719097877429007`, 229.2738627795444`},
 {0.1102903206501954`, 3.0454263679631577`, 0.04069331752982141`, 0.9534089355456694`, 0.7297525123381821`,
  0.6576008896160583`, 0.013329813923119783`, 1715.4779634820493`, 0.02214911406080483`, 227.88476662786474`},
 {0.05909859147151786`, 3.30435803161393`, 0.029336284768972404`, 0.9231466780747943`, 0.9623144707471962`,
  0.37803674309323987`, 0.04397572398478963`, 3067.5791243622793`, 0.057241291718842735`, 398.5949730494525`},
 {0.17398185963078006`, 0.47005124431733236`, 0.09389471888393235`, 1.232881569185857`, 0.49856219410777936`,
  0.4191945121229328`, 0.14050812071087648`, 190.8204082787259`, 0.22639026429089515`, 506.13435159524533`},
 {0.05155342181122552`, 1.5581409866760865`, 0.0691893121207933`, 1.3639899124520563`, 0.2080210727331211`,
  0.429465542144159`, 0.08260476334651806`, 246.4705132305552`, 0.16650171241851036`, 476.3403890303273`},
 {0.04071142279712078`, 2.1496023906791892`, 0.0853156754644768 7`, 0.9683458228368205`, 0.49038233136090126`,
  0.6235182579165484`, 0.07161574481597612`, 131.50858611951176`, 0.19699207630915766`, 596.6367875356493`},
 {0.06590056983751874`, 3.9638284260232943`, 0.016048742823094812`, 1.3783253378441898`, 0.24532119252948448`,
  0.3220387333596423`, 0.016945992465929767`, 2197.071659291998`, 0.010352607682042353`, 236.6421867279362`},
 {0.26325550998326586`, 0.661917211789294`, 0.026986149925021862`, 1.002112989283701`, 0.06554947180049209`,
  0.6994424736047835`, 0.005881514200787017`, 211.33538862145804`, 0.24482509012686327`, 303.2466877164689`},
 {0.26127356110524397`, 1.329573031379259`, 0.07977956036816294`, 1.2076717867972873`, 0.5921373287024636`,
  0.41816652735672233`, 0.022774959106689946`, 64.98766538013531`, 0.19432837274522574`, 259.57035925631845`},
 {0.2602155506169461`, 3.5506896347378634`, 0.022465629838975154`, 0.8509530374249318`, 0.7983914367554696`,
  0.257847432063023 4`, 0.011809304810800692`, 142.12037985462777`, 0.1698027140526227`, 698.002014936651`},
```

{0.06365047180104016`, 3.416188751455385`, 0.03523713957757545`, 1.0192452557682743`, 0.8469643727981293`,
0.5498369694104505`, 0.18294125259438154`, 1018.12440025711`, 0.11189348348403971`, 231.55349754949057`},
{0.20010483475430668`, 2.008655229618358`, 0.06224827434543773`, 0.7776282320212735`, 0.6157609854264054`,
0.25612002607302176`, 0.16950601491382145`, 2258.159167246791`, 0.12768932854616544`, 318.9473026654234`},
{0.04033357276115293`, 3.365035676090897`, 0.05992514630623122`, 1.1732918415280658`, 0.196311756799749`,
0.30038372703117333`, 0.16280533449388682`, 87.04220764531692`, 0.23496316330764122`, 283.48137514032834`},
{0.1509427023460006`, 2.3319882937767247`, 0.018731546174507247`, 1.0584311371273147`, 0.6567026163933902`,
0.6265947759038382`, 0.06164026490982475`, 1022.7891111424142`, 0.168828731973697 7`, 154.17413301109093`},
{0.12172292087124631`, 2.8233234444817974`, 0.034053562361951124`, 0.8588222796104166`, 0.9440480252161128`,
0.6422691510398222`, 0.05980616075075349`, 1021.745763820812`, 0.044168716180875034`, 436.5175383363466`},
{0.10332818067094146`, 3.3200182476314417`, 0.013797086337646878`, 1.2403231995339712`, 0.8068678048493421`,
0.31817454060495975`, 0.03466270708497138`, 224.98168772666384`, 0.18064139380974548`, 882.0488176741194`},
{0.06283595024068839`, 1.3516727681414702`, 0.052426747231404766`, 0.8261017998522725`, 0.2587281077553354`,
0.25782009716699184`, 0.4461332906525768`, 78.37105686956872`, 0.05053665990821049`, 454.91374396216366`},
{0.26061686002038464`, 0.9197100483832488`, 0.09424919710819697`, 1.2431276888944953`, 0.03523684950904893`,
0.6986561589310964`, 0.46987667183492504`, 257.0362567649576`, 0.16991196769780592`, 326.7794760540163`},
{0.2589743226453006`, 2.4967568849644275`, 0.07508840824492798`, 0.781034619960081`, 0.3845979096021525 7`,
0.18475383400796352`, 0.10416671513341536`, 3175.918867658944`, 0.2205445770390796`, 431.51407680934443`},
{0.1822125191728448`, 0.53967615378293`, 0.06140923587336875`, 0.9836246668356532`, 0.5663752339069781`,
0.6127333889317503`, 0.015376137369944434`, 4374.881958546335`, 0.1720894071809011`, 902.0125127914057`},
{0.20287638930589763`, 1.8511964408244932`, 0.020237031457082787`, 0.9096848496624212`, 0.22198223428443487`,
0.15744015403827616`, 0.17491779271673957`, 2922.9247864169292`, 0.14133268345189937`, 866.3466773010839`},
{0.17277183156190062`, 1.3838708180759518`, 0.01858178773758265`, 1.4265746785083109`, 0.6133308818390555`,
0.6532402234702124`, 0.07637848286373418`, 1061.2255229483176`, 0.23800513499817172`, 715.6629218234075`},
{0.22973167487200014`, 3.4630938101482327`, 0.05848037010032728 5`, 1.2645217042191328`, 0.6981417065741804`,
0.542170464886519`, 0.006546556460297805`, 74.23410585835131`, 0.017772563055006546`, 241.35868804813666`},
{0.17947546203746156`, 1.7068442534238697`, 0.04056609287799669`, 1.1830573633622712`, 0.6640874832688464`,
0.42001594980708057`, 0.21752981815857997`, 97.3204887238174`, 0.050533940181592796`, 409.66978902022663`},
{0.08473729051323187`, 1.7479721342100945`, 0.06355457932773143`, 0.8574963830627491`, 0.22245663004688998`,
0.4791593777006873`, 0.28265674645892613`, 4498.232223037125`, 0.17673010273241208`, 336.2131425839168`},
{0.20435672898605034`, 2.6117732980489388`, 0.03475265809490025`, 0.976265205498944`, 0.85216518519733`,

0.548618924239519`, 0.2709183052632252`, 155.3772460870891`, 0.021213370069483317`, 486.638902544934`},
{0.2230119008971858`, 0.6358954372186516`, 0.03489317261697556`, 0.8021667773970862`, 0.7799870633314419`,
0.372553704845368`, 0.3954953790403783`, 3708.9097099582787`, 0.06863756553832256`, 831.4592304552722`},
{0.13277530106525864`, 3.102992336174254`, 0.0810346296150594`, 1.417175031427167`, 0.04111047270929191`,
0.577719723059283`, 0.25840948246645284`, 1582.3635767727842`, 0.11281028363913381`, 572.0784651822191`},
{0.17721564666118195`, 2.3073681400537707`, 0.06977110969724436`, 1.4389124933186648`, 0.06511833232445241`,
0.5785856768063609`, 0.024382684167548917`, 50.72398143128576`, 0.15027785530459653`, 471.87731805197507`},
{0.2532195731000343`, 2.951680905930308`, 0.08243784959141458`, 1.063512245713839`, 0.13059994709602862`,
0.20280590336854443`, 0.2607392691737333`, 517.660101290023`, 0.077733893061395`, 364.603949151811`},
{0.11562259606165537`, 2.075988177277`, 0.03552939359394358`, 1.0857990592224895`, 0.7681961757441904`,
0.6605331614852874`, 0.15159919874130345`, 3296.1941609770115`, 0.07746882734639965`, 160.02380566717028`},
{0.16010263731321916`, 1.8512370146128028`, 0.08948994106153972`, 1.0065876584784161`, 0.31909081739901013`,
0.6055123683081534`, 0.027623148293597768`, 400.21848632042986`, 0.19838189450352378`, 329.78449858468684`},
{0.25703658745875196`, 2.3078283713322856`, 0.021362941537208063`, 1.2100220391878165`, 0.8787828002021987`,
0.2513644151003869`, 0.024289521781396636`, 68.1341971888039`, 0.24810809198178096`, 481.5176012623595 6`},
{0.2455904611014849`, 3.7571997349914916`, 0.09211738529180043`, 1.4019497765522448`, 0.6695611235062522`,
0.6602799476916219`, 0.04340955542197445`, 856.0993716100127`, 0.15292526713206545`, 529.9607802643166`},
{0.059696149748876604`, 1.688845353034914`, 0.009294581379800135`, 1.2433968456838194`, 0.3929448551213022`,
0.4749866039118191`, 0.025863060541790456`, 192.8417865287921`, 0.09839655966119532`, 364.5695918572345`},
{0.09761501672592626`, 1.3774481728994479`, 0.0397488421074619`, 1.4571532371767337`, 0.8600435797101407`,
0.37053809894631096`, 0.30839879803159215`, 1053.3603252171624`, 0.09829081378486954`, 314.7778202000868`},
{0.22092704028098814`, 0.8304263766325484`, 0.048201718626776184`, 1.203623675331191`, 0.293563656489940 2`,
0.4030249377909366`, 0.033061104251936244`, 4358.833221490219`, 0.15893421696064525`, 180.8709305853121`},
{0.13823492725556935`, 1.1413185260053824`, 0.06971384118788397`, 1.3157780297053645`, 0.6082014740714308`,
0.31531914337240985`, 0.005597926269123681`, 97.23206007061579`, 0.1162100716591018`, 522.6554437517659`},
{0.14416900464242904`, 2.543812431689566`, 0.010106655971553415`, 0.8969401222383744`, 0.4328515562362101`,
0.20360668938810977`, 0.2923006645347409`, 1178.8843718309924`, 0.20797847649312406`, 120.9186114906668`},
{0.2133877273723686`, 0.8490524254535732`, 0.08700555866439949`, 1.2062638954363991`, 0.6738638517009694`,
0.46997962516177505`, 0.06892379315565185`, 1349.9514731760883`, 0.09645636257209894`, 836.2941738998895`},
{0.0539089201271194`, 1.0364880092644801`, 0.016154417078585476`, 0.8886530805439552`, 0.3168467801118968`,
0.15642019482584735`, 0.006844524019875138`, 85.57714734356885`, 0.0199193393194675 7`, 258.3461272090961`},

{0.2715756156789369`, 1.5445757615325304`, 0.00421616582195627`, 1.159485102317175`, 0.4702357167765736`,
0.34737964168870006`, 0.496052684967637`, 300.35079175305486`, 0.1259406113194213`, 781.8522906262123`},
{0.04827287438408412`, 1.8515860963247066`, 0.07692111119618293`, 1.2774889475291031`, 0.32248502080707353`,
0.20234972534433104`, 0.01272417451099904`, 140.98466458635835`, 0.11366894324343613`, 273.5929992840893`},
{0.2554976820569969`, 2.4378755210159753`, 0.04254022762089836`, 0.8817958104635948`, 0.692204018420977`,
0.4234476557446073`, 0.007554290015778277`, 79.39858197716293`, 0.11447843867339091`, 117.82612509068677`},
{0.2760063677033659`, 1.2105198937059631`, 0.067224388891334`, 0.9988908925658994`, 0.9019281244861579`,
0.67284715505845`, 0.007365273847592193`, 2972.356277007087`, 0.06361067504455276`, 438.4208211463028`},
{0.23497331294207247`, 2.932797407682453`, 0.022597452058598203`, 0.9589743833470734`, 0.1667506323796748`,
0.3129991011881105`, 0.05276499482811941`, 99.75864289244342`, 0.09875437541358872`, 580.7218610046771`},
{0.0552957146577317`, 0.9263334676885089`, 0.05382222458972377`, 1.1846475829708125`, 0.6978145776046678`,
0.40095280794552435`, 0.14194113807951353`, 67.36725348197879`, 0.21539237947591866`, 912.2514050198081`},
{0.08596797992646615`, 2.89469532919738`, 0.0723374629314705`, 1.412708246795334`, 0.2707681400237405`,
0.659961029627586`, 0.1545447128013493`, 953.1432644930773`, 0.14443128680071893`, 294.4093465447068`},
{0.26579336742267545`, 2.150651278297752`, 0.09864009518921925`, 1.381049269309322`, 0.7824667408282995`,
0.6485863724693819`, 0.1346970994193899`, 71.41513724603877`, 0.04324969499825426`, 231.68375988200472`},
{0.07893352000042303`, 0.9269382255330783`, 0.07450960524320785`, 1.192225010506579`, 0.29981085040929156`,
0.20655660387273633`, 0.005195235752158478`, 3935.7899816629233`, 0.09905090934440974`, 552.2915075373634`},
{0.13874147069686438`, 0.4677020963588703`, 0.06081979667173851`, 1.4293968405810138`, 0.04489099618087722`,
0.600807000017457`, 0.1990035962891371`, 80.51094589303605`, 0.24671702535367518`, 538.9722201215866`},
{0.2700245759823924`, 1.083893289879918`, 0.062021204290361266`, 1.2005471238256362`, 0.792954755831037`,
0.39473782087530096`, 0.17457967683710685`, 279.6749633279759`, 0.14741868150066417`, 148.434091412925`},
{0.23474828628269123`, 1.725907115336116`, 0.012821406564911335`, 0.8070142233189552`, 0.4777555512453826`,
0.4076167331863103`, 0.015638133522482764`, 120.11394196884895`, 0.019548998977537424`, 892.9399386284366`},
{0.09165311999697207`, 1.4061679173562647`, 0.03309545344659714`, 0.9052080425177362`, 0.22786616586188102`,
0.16948939359836301`, 0.041478452818892154`, 78.37706796679595`, 0.1493801427638014`, 220.7559134640384`},
{0.04235508864011306`, 1.3060003486013567`, 0.08466697376486561`, 0.8479712158967934`, 0.3936959911899278`,
0.2088063088308738`, 0.017308803394347618`, 4694.788266429514`, 0.026439759379240313`, 433.9918956105633`},
{0.2651923586966664`, 3.8881876111766713`, 0.0350309847210708`, 0.9591171183376157`, 0.9954879310389926`,
0.5341580610877573`, 0.0817474157614525`, 113.37527000346847`, 0.1996829793433833`, 201.49628341235228`},
{0.2787517386498274`, 1.5890965688604197`, 0.014994826667312413`, 0.9487067103902393`, 0.03893198736235193`,

```
  0.2195801988959788`, 0.313478573421096`, 168.03485395038084`, 0.026460496970460712`, 626.9887607453275`},
{0.08753933430632788`, 0.6662218380744758`, 0.010717888122421044`, 0.8318793084446489`, 0.9074866505690673`,
  0.6370551722773192`, 0.05914667212913143`, 92.90178750054149`, 0.041267271127747585`, 531.582902194676`},
{0.254499813976234`, 2.1602436577244424`, 0.0772554574037915`, 1.3316064464046935`, 0.367293601649199`,
  0.4330751915150024`, 0.012004917841621048`, 4701.7880352079455`, 0.15735639832958048`, 710.0683407032234`},
{0.1501371731881197`, 3.5104518987365614`, 0.0358546336850628 9`, 1.1463465777386583`, 0.5443112287696148`,
  0.3949552428603267`, 0.04879115335558662`, 259.24478342917615`, 0.1285655725832237`, 162.3475385554764`},
{0.14482453276755702`, 3.3636104411839076`, 0.038458077364182566`, 1.197124661291601`, 0.4328455972778613`,
  0.3205266052041652`, 0.010337491367857544`, 172.8822485760027`, 0.1748531192873402`, 294.504606203127`},
{0.1729081128249474`, 3.4171958647256986`, 0.09736003964750388`, 1.3614201648251067`, 0.8114455381033425`,
  0.2740485642532573`, 0.10719777411327538`, 697.2636909321262`, 0.16794794102451832`, 169.68999987903217`},
{0.06017132640138534`, 2.551705655373457`, 0.098001989150616`, 1.2777770866301428`, 0.33103821819782686`,
  0.42457056361391154`, 0.3044012087125646`, 128.19341360646206`, 0.23285467527685833`, 861.6547556863932`},
{0.06838353005846232`, 1.5583268641110415`, 0.07074535929409585`, 1.1186421654705394`, 0.17808501168205293`,
  0.6526418380839745`, 0.30389354478023717`, 2253.855886651074`, 0.2140132420516081`, 589.9585678536141`},
{0.045919511842929334`, 2.3909808568887367`, 0.05197740517398504`, 1.4076343252717902`, 0.4269585056408616`,
  0.672767818437489`, 0.1429025608766 2156`, 365.93648706136383`, 0.2152208567065702`, 325.8336782379203`},
{0.2110207852916774`, 0.6269225337264532`, 0.060343077081531735`, 1.2521859520371945`, 0.29021239339379945`,
  0.47386146148275243`, 0.1626064474205758`, 445.43014573417435`, 0.07466666385442788`, 270.0446625362414`},
{0.13913288219717507`, 0.71598345975513`, 0.02918338476342825`, 1.084418293337738`, 0.1601393499115018`,
  0.351547594930286`, 0.041991397294508556`, 2091.889065193303`, 0.09601982280233712`, 646.6328833508262`},
{0.2080876337219731`, 2.734433706560215`, 0.0261030709279631`, 1.338847931530077`, 0.982472586795901`,
  0.3916223755715482`, 0.018379378513228514`, 98.65983909130968`, 0.11579954319108776`, 761.345713618144`},
{0.19884229924079655`, 3.7544266932443486`, 0.05306832500507259`, 1.4048543300387752`, 0.1752488991857195`,
  0.2724812216186141`, 0.12261739216762069`, 80.04459592601863`, 0.2028403340066654`, 224.71523563844968`},
{0.09229081102449843`, 0.41731444207519`, 0.09042236613704813`, 1.4795137951182236`, 0.6627370782775894`,
  0.6415172939653717`, 0.007608010716527551`, 62.09999691656457`, 0.17995756856061956`, 974.7890405043591`},
{0.22753750499438513`, 1.1094910684892056`, 0.06205079076485845`, 1.3683254796121973`, 0.59456895101486`,
  0.6649055203122181`, 0.06079543818004673`, 62.1436627548072`, 0.23424854868652234`, 576.7021529851472`},
{0.07611810878804481`, 2.417695097886692`, 0.008902516116383463`, 1.17651838899742`, 0.8956068934881507`,
  0.1734469831112303`, 0.007686752240077523`, 84.3504067614311`, 0.15979761325133707`, 962.8822438221003`},
```

```
{0.14505993431662217`, 3.1227268390818725`, 0.056275163136378696`, 0.9590973923566892`, 0.13969476213382426`,
  0.20892815360032824`, 0.0531610997371578`, 246.79786465887622`, 0.227047105813231`, 454.33945080770786`},
{0.23654885566478462`, 3.9241924925395733`, 0.03358528642939117`, 1.3191430750498658`, 0.39258636888736453`,
  0.3377325502420989`, 0.025557835669326675`, 93.73616135543377`, 0.1670083520090136`, 309.9129735942125`},
{0.11070282825339567`, 3.833445407868260 3`, 0.07681857609135982`, 0.8958554753360997`, 0.32396640927167897`,
  0.44194855743506667`, 0.2131540641524383`, 1741.909189584937`, 0.022902468906996543`, 670.3098695990176`},
{0.10170301352011679`, 1.8752371643597758`, 0.03510782937111476`, 1.3860933640706163`, 0.6474965074918984`,
  0.1594306933477596`, 0.37318794312281267`, 201.47970914729365`, 0.22517965099104725`, 283.6656714712788`},
{0.19274669213409934`, 0.9288413277333225`, 0.038837310625680195`, 1.3836847187363839`, 0.14968000832109252`,
  0.4232153388345993`, 0.01563322978668864`, 922.263283889537`, 0.12359553135969004`, 486.72903455727567`},
{0.06356535997519341`, 2.311362126443121`, 0.07268080427945918`, 1.2586315568490016`, 0.3763201304339143`,
  0.4384209659583461`, 0.006474182269401951`, 4348.973288945735`, 0.17106111863805346`, 281.3921070619326`},
{0.06831716410403144`, 1.0015662947000914`, 0.06390469368977933`, 0.8290508858928134`, 0.7249330951564277`,
  0.37450775838270167`, 0.3666941924999965`, 191.80408739918798`, 0.153067071236483`, 887.5069081464457`},
{0.1293750817503745`, 3.1014207000970364`, 0.023014106546543296`, 1.351810814205737`, 0.5054291062898795`,
  0.669566365984742`, 0.05376259966791126`, 207.8155819891332`, 0.12505633467039579`, 477.98997920694734`},
{0.22605177518456349`, 2.2930688004852557`, 0.04762600540866055`, 1.435285876756151`, 0.7544760534695125`,
  0.32613259549063545`, 0.008000490945700793`, 658.1451070175192`, 0.1664301859365524`, 397.6391494406913`},
{0.21478835126656415`, 2.1859458812100163`, 0.0858199459854308`, 0.993933836221133`, 0.013991965571371612`,
  0.25438763086955674`, 0.010673305721101622`, 175.38786745903064`, 0.249124195297232`, 141.44233499151147`},
{0.2524903538890303`, 3.207130817880457`, 0.0760687494889642`, 0.886811483661367`, 0.4861660798083616`,
  0.6820047923981643`, 0.01500874534183673`, 477.62249790514926`, 0.21800800344067622`, 582.3093284125088`},
{0.2673762141971479`, 2.9843296894323084`, 0.06575888816802714`, 1.4582777652181538`, 0.8424633965803163`,
  0.2888169272806542`, 0.017193087756165917`, 1905.2031126287002`, 0.22394556493184148`, 302.0798156952443`},
{0.0718648334935813`, 0.9687764001979549`, 0.03778553570658904`, 1.0685686917623896`, 0.8556706065609418`,
  0.5975957325406767`, 0.1005409517041212`, 952.0313425448139`, 0.1102822616742215`, 595.1450083064373`},
{0.15187564029414063`, 2.880478775690314`, 0.004382096934887443`, 1.4674879291008858`, 0.5009373487763229`,
  0.43891155488357636`, 0.3536287231064343`, 240.19671819847136`, 0.18432705417277784`, 919.7867146052535`},
{0.22049198023775118`, 3.737295065096858`, 0.0901755947592022`, 0.8260708286898752`, 0.9798021204432015`,
  0.3779284943750736`, 0.1710978537094571`, 184.8847972532019`, 0.07398065840012735`, 365.0355841401655`},
{0.07654850782489453`, 2.4131335758873655`, 0.08059562189671154`, 1.2322892164920185`, 0.6217255346992006`,
```

```
0.4763260824504111`, 0.019197847472858853`, 122.85370840033258`, 0.08071204068111693`, 671.7123766572973`},
{0.2186000058002156`, 1.674921415227379`, 0.04684927805332688`, 0.7958293763534576`, 0.5351292729987009`,
 0.3413988165541403`, 0.023772881602852104`, 4578.839908213088`, 0.025990133203813937`, 720.8700500740513`},
{0.08466466489793933`, 3.1894928322423937`, 0.04884056021911439`, 0.9766729535722316`, 0.2661879637637754`,
 0.5761935324118598`, 0.024926658702996464`, 4783.240631357024`, 0.1159466946528968`, 380.9240212400814`},
{0.21930175757669085`, 1.638318457274547`, 0.028552151697051935`, 0.9929755101949111`, 0.48282817858365856`,
 0.6345477159456419`, 0.037102957315214465`, 1113.33955069597`, 0.08280464755403139`, 979.2309221100506`},
{0.15074472135509004`, 0.9706350461652997`, 0.03261694957200774`, 1.3446365626585761`, 0.7522030233980499`,
 0.24992054076492587`, 0.19250628663410368`, 341.383303547674`, 0.21302570685657762`, 810.5220628187353`},
{0.15291759685661022`, 0.42291903800035024`, 0.0019904081657203853`, 1.0349558440923765`, 0.804656482695703`,
 0.5600791477495833`, 0.008901523926716821`, 129.71753742837356`, 0.2016179651576812`, 261.7757399110873`},
{0.17393186946863948`, 3.634473205144226`, 0.03501927778959045`, 1.259756868930488`, 0.2400906424310547`,
 0.1769239851738713`, 0.19287151186810095`, 68.7455559557316`, 0.22859728678934704`, 432.35938430583155`},
{0.08252103338424227`, 3.0363282382170365`, 0.014054309199089551`, 1.1671357119318402`, 0.48811181819525484`,
 0.5135417689953007`, 0.03600069536054404`, 697.7036897164247`, 0.15194091944355315`, 879.883721957997`},
{0.152738213225879`, 2.1423984829886598`, 0.013570505913453062`, 1.4096468218064773`, 0.2076797705558071`,
 0.6307063081946709`, 0.005224131132178296`, 944.5685551789895`, 0.1507676005368857`, 103.54095034743213`},
{0.13639700931586268`, 0.4456680831486439`, 0.03342224700364268`, 0.8649458036959383`, 0.5930285970230691`,
 0.512351265340247`, 0.245770577144212`, 2154.7761980173996`, 0.03788382651562655`, 910.303522512114`},
{0.15928993459575497`, 3.999012236456535`, 0.05909250114200807`, 1.3761236219477655`, 0.9503401787669128`,
 0.5184686893967728`, 0.024376742099233118`, 2487.723402011106`, 0.03811059136471645`, 242.2171516528934`},
{0.09086020561938618`, 3.6933841261170146`, 0.051558219365949225`, 1.0052639956256584`, 0.6404497588004767`,
 0.6310251056832639`, 0.009282302874928348`, 1342.6385109725754`, 0.17780049337096682`, 290.68187157092353`},
{0.1467805861006215`, 3.449583346443368`, 0.044526946458641366`, 0.9116880264848481`, 0.3816708687678134`,
 0.5427311042687007`, 0.008463344612022042`, 84.3419318420348`, 0.15666870327157162`, 901.9122564215138`},
{0.11923417673201614`, 0.8516005442952741`, 0.011417621987304472`, 1.406269973162172`, 0.8163346696622038`,
 0.4684095363963826`, 0.03909433657582696`, 694.2186659403512`, 0.15728990858282532`, 465.4699780055223`},
{0.20474637151455272`, 2.273526081644097`, 0.04289425593502317`, 0.93188673114289`, 0.463730044259554`,
 0.2201193444564068`, 0.08911251750722911`, 165.09098140801413`, 0.18954199559487928`, 728.9231766980553`},
{0.05526814093912169`, 3.3906431390874365`, 0.04679651060056909`, 1.3224994050291778`, 0.2130837384463229`,
 0.5620196653380606`, 0.45821421853915184`, 166.36956673134458`, 0.04079620395491479`, 150.90572670851293`},
```

```
{0.1424344572203114`, 1.5158405980214011`, 0.07134698803292082`, 1.3412182398127908`, 0.9032816245031063`,
  0.29383720975190164`, 0.11721475717276858`, 2259.2805795521476`, 0.2041616321098142`, 875.6348666465524`},
{0.21167201791316342`, 1.191857769132941`, 0.04032467834992808`, 1.3612666973498941`, 0.990843843386328`,
  0.16867005012046943`, 0.32551015426475394`, 61.09769889812229`, 0.07090867627674724`, 397.7456784438642`},
{0.08319100731021534`, 1.1769760609325015`, 0.07914999980937851`, 1.2654227958186712`, 0.5286106444848078`,
  0.6131113894479372`, 0.34203180569092556`, 3018.9895646255973`, 0.20716587538358655`, 115.93712250962636`},
{0.15910463203606118`, 3.3362654217234775`, 0.09431988145352659`, 0.9102504372858342`, 0.8811348699089658`,
  0.5322317508995823`, 0.49238248141112334`, 1657.8316991656131`, 0.10134572490386001`, 783.7355808530842`},
{0.10233951732223695`, 3.8860213079913546`, 0.08245580990451895`, 1.0255637903426433`, 0.4817258525533532`,
  0.6808372231333493`, 0.05183250404760466`, 4623.677166174953`, 0.1226398356738308`, 284.97228231241803`},
{0.14767115153525173`, 3.9914449333138045`, 0.0245556961746785`, 1.4796764004437446`, 0.4415017997340658`,
  0.38166398917825395`, 0.06906175497737103`, 324.69168049200084`, 0.11969109369698483`, 735.4456200401828`},
{0.17843716795843323`, 3.273844277118725`, 0.0030946336041298927`, 0.8383571948484874`, 0.5814391172481872`,
  0.33735131984206335`, 0.09800031855604995`, 299.74935856154275`, 0.23538286721353047`, 296.3741029308432`},
{0.15153478181902558`, 3.940884011459042`, 0.014665112834793917`, 1.0805125654131587`, 0.23054812649590795`,
  0.6258941160756026`, 0.07918188154195355`, 98.54552815956583`, 0.07490635587591205`, 443.4663115601323`},
{0.134722684681424`, 1.1454968245380153`, 0.01650061661779107`, 1.0638009891109292`, 0.999069379656865`,
  0.5940301687777447`, 0.005569610207767832 5`, 77.43260394254949`, 0.24107274439535475`, 940.3682518559112`},
{0.16433469628892827`, 2.875058335652863`, 0.047444839802376605`, 0.8943437881772829`, 0.6054569837144246`,
  0.6619217328059503`, 0.14605802692460343`, 150.35965445533094`, 0.21808470663788926`, 986.5914920483302`},
{0.18172474747658007`, 0.5398125898895043`, 0.06746996069377663`, 0.8516154049831974`, 0.9176550555688654`,
  0.6251776245678378`, 0.47714073816368646`, 219.82174949020737`, 0.1448459760500183`, 388.6902609510781`},
{0.1627140613056554`, 2.526426793480817`, 0.08166994036129233`, 1.3765323556161237`, 0.2395228888894274`,
  0.6556866863306616`, 0.04100839920318810 6`, 966.1247163249689`, 0.01569822406304719`, 290.26321585148054`},
{0.12926587974943277`, 3.1942443269472287`, 0.08210234540290166`, 1.2603468170155185`, 0.11771113616842777`,
  0.5855250995493423`, 0.010816984036854691`, 52.9364314659927`, 0.0872384103249817`, 113.10642700446624`},
{0.2469537827294621`, 1.6562160115050997`, 0.08971323759474036`, 0.8904234033118983`, 0.4552788372237486`,
  0.6086911537768385`, 0.45126702440163974`, 3127.8941282749056`, 0.06787458866390378`, 920.4041061473141`},
{0.04659393582426277`, 0.8257639781936223`, 0.07933970228984723`, 1.0123444750721238`, 0.23360835713895778`,
  0.5040801713850254`, 0.008240901014236341`, 3556.051928374705`, 0.0470528014171252`, 139.7711872268978`},
{0.2677990399223792`, 2.190165256838246`, 0.03877434139180814`, 1.1644520688476412`, 0.10209034886491564`,
```

```
0.2364288033256654`, 0.301326771326778`, 351.0852119703326`, 0.017030644812824763`, 859.704636269683`},
{0.27526155344317305`, 0.42206525583727394`, 0.010509190579989251`, 1.2469728015728827`, 0.7164324088803142`,
 0.23095486560068912`, 0.4019760074402924`, 1837.167141511333`, 0.14638705750272024`, 598.0758928204975`},
{0.20827027070080567`, 0.6628955977865401`, 0.05578571612958859`, 1.4550917307077555`, 0.8160716291341483`,
 0.4316400149470674`, 0.0992623271553592`, 1291.2580266081497`, 0.17638713235092185`, 712.744419351735`},
{0.26108671366598557`, 2.7581406885672397`, 0.09215954227601131`, 1.3356715726742072`, 0.5159534131307919`,
 0.6292106530069272`, 0.19102200652406912`, 1359.6101170600552`, 0.13195246712822323`, 655.8014880925608`},
{0.12411610080756336`, 3.5491378450736537`, 0.03048497342324529`, 1.052629142532319`, 0.9334052639776818`,
 0.36612480189397933`, 0.17487769367297745`, 297.20407438965395`, 0.10198438571360102`, 142.8358321881406`},
{0.05206075217376971`, 3.80544265452421`, 0.06452586960506253`, 1.1764286006641393`, 0.7578123360385443`,
 0.3632439318173788`, 0.03825456601997797`, 1732.1715020363104`, 0.09945789721416326`, 862.5286889178561`},
{0.15332434762984987`, 3.71381929627883`, 0.04943415023567301`, 0.9774785033821363`, 0.4718998580816507`,
 0.16669947597303947`, 0.00694639422020862`, 208.05288466197266`, 0.20610825162042906`, 633.4494218195209`},
{0.06033541900347844`, 0.973178216355266`, 0.030553995574257496`, 0.8504754985225583`, 0.27347786882322955`,
 0.4969493066691505`, 0.00830637169085789`, 57.15499757894147`, 0.0772124603177262 7`, 682.9655440833467`},
{0.20494675314374677`, 2.7031536940694387`, 0.02716633958654084`, 1.0975656700162824`, 0.4564303296261911`,
 0.4441209590579568`, 0.3554217278310708`, 4265.584586744801`, 0.21396541728692714`, 985.913598004549`},
{0.05327195450009359`, 2.536575411744539`, 0.009856300363339`, 1.2242648903686608`, 0.25909020435504493`,
 0.5282694072066187`, 0.04434669218741346`, 786.4053543279437`, 0.1778964533530039`, 544.6097455298984`},
{0.12477833238312225`, 0.7549035136230082`, 0.025172412892961696`, 0.9147089841492775`, 0.8930485640293313`,
 0.5901323959258005`, 0.0508108243850171`, 901.2908925357414`, 0.07250789031346216`, 103.12569950478974`},
{0.08995934799066885`, 3.666207694088575`, 0.03007591771494079`, 1.3783264230132162`, 0.4826387854777239`,
 0.6038418084491215`, 0.23708076068251083`, 167.7294214818222`, 0.01048197934630371`, 522.6073774456877`},
{0.15902513428717435`, 1.7183982375849336`, 0.033022213369310126`, 0.7628337434543904`, 0.7778819919683766`,
 0.27145222438301553`, 0.043862649375531554`, 1185.7733981174872`, 0.08799090639918955`, 695.0405493319087`},
{0.11065553243087889`, 2.46805164022211`, 0.0463748475768039`, 0.8209475953416414`, 0.5218398256116175`,
 0.3133447370054039`, 0.011285096069233967`, 4139.924962213234`, 0.13167847716735503`, 267.39995064825325`},
{0.08562689605991824`, 3.9571879577725095`, 0.023793442849937278`, 1.226116327613754`, 0.7555535622077838`,
 0.20305217942323717`, 0.12860386825121028`, 531.0020640269107`, 0.051948220660308075`, 415.78314995313474`},
{0.10669261879032438`, 2.055705808398714`, 0.04769389095504181`, 1.156038712774391`, 0.9820313641369269`,
 0.38163507374297045`, 0.03953536850205264 4`, 2902.0412683197437`, 0.2090115829764676 6`, 336.26891906262557`},
```

{0.07060338528242072`, 3.595376249521064`, 0.03262882113877765`, 1.2426013551392963`, 0.6002233277932603`,
0.6255684512538187`, 0.011346312056874001`, 3533.244097205098`, 0.061628460123131446`, 431.95845626027455`},
{0.2296701087879804`, 3.9087772869642077`, 0.005974703984365633`, 0.7959312177065372`, 0.876747425533583`,
0.6239039023797257`, 0.07860570649462724`, 228.33223863779693`, 0.09890789121587351`, 844.6853450307202`},
{0.23123324268179735`, 1.3892537621982006`, 0.09784040419751712`, 1.4899932847388562`, 0.04016727483231097`,
0.3908129571471922`, 0.009391139743151554`, 3403.3990186754204`, 0.07086662039842229`, 218.43172418092502`},
{0.07037189900847057`, 2.1828807010264386`, 0.09644892533086193`, 1.4657375782847808`, 0.7479730618322393`,
0.5037584189194234`, 0.030916267299058386`, 50.37706857442957`, 0.0504519506394871`, 421.25447735673964`},
{0.24256545257709167`, 3.690547309616945`, 0.0023677786666569567`, 0.8901250940229146`, 0.885419510645867l`,
0.44399073208272666`, 0.23636352272736572`, 89.54810713350285`, 0.186159802578101`, 387.72460570093335`},
{0.13839843978976302`, 1.4644738520575267`, 0.08648420456992728`, 1.2182260759194046`, 0.42213262989843314`,
0.3820758811935525`, 0.019878840421350977`, 139.8221226740942`, 0.07043032032389362`, 893.4669900375276`},
{0.11263641432345284`, 3.0669748383334596`, 0.07040331826586925`, 1.0832638028187807`, 0.4284903169815901`,
0.6222704043007405`, 0.007844284225620671`, 2131.889703000162`, 0.08148303022699405`, 953.4820565221385`},
{0.08536639269975432`, 1.9832711606259998`, 0.06847562880410717`, 1.259414576524179`, 0.8112350819234888`,
0.30170114251846125`, 0.062372919072375`, 772.9300404210154`, 0.12506634187920385`, 925.8799331095055`},
{0.11730626511183101`, 3.630410531262336`, 0.09596398943788596`, 1.1797596414840856`, 0.00512306280685948`,
0.2706014914642383`, 0.007107925916470569`, 167.23494696565012`, 0.11063186891436805`, 990.8331649712547`},
{0.11173802137314093`, 2.161180887445509`, 0.00830056555397336`, 1.063559562488714`, 0.1885103833536148`,
0.48139701696537807`, 0.010634768624873106`, 123.30116070184943`, 0.040023829860516`, 666.6470268689523`},
{0.10813918810135675`, 3.496374135712781`, 0.08929026043826785`, 0.7770714221575694`, 0.3733447131761056`,
0.4939165094284049`, 0.2800021673947185`, 161.9820586332278`, 0.08289673671409309`, 517.9231146523`},
{0.2516406246499894`, 3.0929818341741733`, 0.0922614386482984`, 1.4236100743284779`, 0.03336817153694982`,
0.531009903965389`, 0.28645389162750856`, 1114.6653530749377`, 0.10635783873890536`, 232.713586458166`},
{0.1065188593842617`, 3.0239491530160656`, 0.022787241302245534`, 0.9140212299263315`, 0.4098506422400856`,
0.631556223988371`, 0.008249935916109901`, 842.7098345476253`, 0.19464933826626446`, 205.3036767061707`},
{0.10588487940933416`, 2.859735718128807`, 0.07786116606871861`, 1.047717859923827`, 0.07660672309433547`,
0.158735212392627`, 0.05520993734392543`, 139.0602212904327`, 0.07978847169291192`, 577.575995655312`},
{0.24741184670460048`, 2.0853129323232675`, 0.002699004039505635`, 1.042019307006856`, 0.6924193332252813`,
0.4588072902902137`, 0.0350404016550923`, 391.00460391265096`, 0.13592475687567207`, 785.2080587147564`},
{0.14083189694803505`, 0.7118335450358293`, 0.018460964897572776`, 1.1767917671701966`, 0.48592079833913515`,

```
  0.6846823750216606`, 0.24127390726138886`, 71.14275489785842`, 0.0433294805997953`, 476.2086967233275`},
 {0.1009771753749294`, 3.4383291777667013`, 0.09534658166572815`, 1.3953306821932303`, 0.24681268758922426`,
  0.3480290288704033`, 0.2692140132359644`, 2330.189032037055`, 0.14685923933486766`, 555.9442327003733`},
 {0.16178386458202587`, 3.0596682411026226`, 0.016235234307709075`, 0.7897132419424195`, 0.7440577027037574`,
  0.20415575695989485`, 0.014269716719641092`, 102.02000238842407`, 0.02548443659481625`, 410.89583584040685`},
 {0.1639764064141605`, 0.8690658024831128`, 0.09689964069135186`, 0.9303160183239412`, 0.9259213439155836`,
  0.6251018503757462`, 0.13591794228358278`, 311.5677558935227`, 0.18168686534025869`, 702.447340545002`},
 {0.12955597509758354`, 2.3266058652149084`, 0.07612788670577046`, 1.2957091127204743`, 0.08408476825169409`,
  0.22843448121491383`, 0.01377311432994412`, 55.28011697543834`, 0.05462266813297323`, 936.2737831126054`},
 {0.22722659605137513`, 3.181545366826299`, 0.09535885824182891`, 1.455710256248994`, 0.794512142132684`,
  0.40024598250807375`, 0.3807443177879789`, 208.1153783523671`, 0.02759817321864938`, 937.8080702420884`},
 {0.09757928432270024`, 1.5076906365385394`, 0.06532569914948308`, 1.2393804160164015`, 0.6875999373585513`,
  0.4482433478851079`, 0.047668506035977345`, 978.2925685396615`, 0.1537809380520726`, 134.17889109795115`},
 {0.25247158502971473`, 3.209043445695394`, 0.055750773529977184`, 1.2307446634362496`, 0.9584321093779467`,
  0.17045215686099446`, 0.02394094843679724`, 733.412724821379`, 0.14871489313227643`, 731.1114504116057`},
 {0.10371347338421405`, 1.7327368937733922`, 0.06071844641560931`, 1.2089839863490672`, 0.14765621815567243`,
  0.4738799094972982`, 0.010611619577856298`, 2438.8343629553387`, 0.18500920326393544`, 108.28078770273848`},
 {0.1984535123182241`, 2.161947896960034`, 0.06138419655750843`, 0.778982059344484`, 0.4287275067738694`,
  0.20720196920301803`, 0.4492524632515773`, 121.20549296704452`, 0.18396106207724583`, 119.02585475241914`},
 {0.12381163862088174`, 3.895341333824428`, 0.06002344154256473`, 1.1343022539214913`, 0.8129713762085851`,
  0.4638592661411107`, 0.007042757871236177`, 1176.2351771701979`, 0.2462865126714932`, 108.58547580007442`},
 {0.23071761590484513`, 2.2648352286735998`, 0.07729558723199069`, 0.9210169155643355`, 0.6313373838804968`,
  0.6041276627548808`, 0.24562846674087035`, 1229.9254758581137`, 0.24585331069089628`, 430.22606831878835`},
 {0.05479322654019092`, 3.8246177185802415`, 0.08580605416623638`, 0.8811793306182123`, 0.8761776950953761`,
  0.29070846616035273`, 0.033348914503464826`, 623.2110193634621`, 0.14957649554329666`, 152.49175504634724`},
 {0.19953212896288414`, 3.4927590196799247`, 0.09411150882849728`, 1.197122533048502`, 0.4043090283096169`,
  0.6122442379199733`, 0.044927979287668965`, 1565.6926144346771`, 0.04491818835648648`, 171.98402040125399`},
 {0.1517228798855726`, 3.821661938472987`, 0.04482964683170573`, 0.9498760179602059`, 0.3819160293947341`,
  0.6937284136376201`, 0.007172788536686403`, 154.83311013460644`, 0.21825034747286304`, 849.9173689310978`},
 {0.10089572826433801`, 0.9689485600216026`, 0.09904537540593211`, 0.9565780146244569`, 0.8315614208813649`,
  0.23778331363990324`, 0.010609351956832196`, 166.5397570471634`, 0.23685724436722827`, 123.70685669376229`},
```

{0.2617448383586014`, 1.7419134501480498`, 0.045608467431404856`, 0.787836989709293`, 0.662878849064974`,
0.4338361091174493`, 0.3345623113002579`, 73.9957072876414`, 0.12486123360347584`, 607.2676663733008`},
{0.10370303958455385`, 2.4325217216628605`, 0.02176754760468306`, 1.4452363933770138`, 0.40438966394356823`,
0.43523988865263297`, 0.04976478629271485`, 282.2104614433738`, 0.15573083503468216`, 771.737205400766`},
{0.12568862947200987`, 3.409935111150106`, 0.04842578409514237`, 1.2928588459089652`, 0.8548940812962251`,
0.3585702743647413`, 0.03943961787244869`, 103.22445359993986`, 0.12312508088340224`, 106.12725365941351`},
{0.25023693340814834`, 3.9316478547515894`, 0.07082339724222557`, 1.158336750472774`, 0.7299281236811437`,
0.5200623075713134`, 0.16855609928966492`, 115.49612111354244`, 0.11648069433492153`, 380.1124291681772`},
{0.2327122870521875`, 1.8349316139343976`, 0.005921840861416321`, 1.0160702776423072`, 0.724126729587736`,
0.6641678286901775`, 0.014844066228330453`, 4846.196302582017`, 0.19133524452025602`, 312.0736607519881`},
{0.26060624730373366`, 2.429263865356268`, 0.02302054068506065`, 1.1376576887137433`, 0.6882649764401805`,
0.6080978782814359`, 0.05257452924408477`, 81.26248932759681`, 0.16061945366682562`, 580.3732149020416`},
{0.22406406307822446`, 1.3829714288487063`, 0.037525031212394956`, 0.7596012900803544`, 0.17941039146586824`,
0.520203193915476`, 0.056506779103220175`, 93.77318192698738`, 0.011157058120984026`, 243.36956207188064`},
{0.16129175454120598`, 2.241594788665445`, 0.02595808801699503`, 1.4515663695326364`, 0.44349403825461486`,
0.5944541929016426`, 0.13242981207036078`, 1862.045541199846`, 0.19478161615729406`, 241.6655690221253`},
{0.15431366769375426`, 2.6965576187541354`, 0.08956453107270243`, 0.8164255077330567`, 0.4910378956214103`,
0.374255897689989`, 0.007597691279617231`, 3560.421166649914`, 0.2337580506715733`, 428.017043104418`},
{0.2212577173064723`, 0.5158974882846561`, 0.013003028286676788`, 0.9387448220782406`, 0.24284514367000565`,
0.3081553043694423`, 0.038153400492409215`, 56.65345283557565`, 0.12156476174517056`, 401.2477783269693`},
{0.1683373271076925`, 1.6782737549041533`, 0.05896916410328887`, 1.1152338969749211`, 0.5823548078906295`,
0.17654711643520027`, 0.009562790707407565`, 617.6094195707465`, 0.1697152454569904`, 676.3594994410081`},
{0.07670427099110139`, 3.20755225941694`, 0.05882096511027358`, 1.447867595309567`, 0.29934068229086064`,
0.292514599413645`, 0.006615326955656567`, 67.05743705305659`, 0.05138097017561577`, 233.60263470026138`},
{0.09939447843258498`, 1.0806802687605517`, 0.08727314073863666`, 1.0427681271407077`, 0.2371764885575376`,
0.5091900756791798`, 0.016527080711814128`, 61.46338920331662`, 0.06725149887765097`, 222.62215508117382`},
{0.058754537918439986`, 3.7681443191246293`, 0.07934826392769118`, 1.166166391041544`, 0.7017545799004972`,
0.6192265616934043`, 0.1452002074780104`, 372.914499705161`, 0.050248583952100856`, 447.3871254600834`},
{0.1573899141991117`, 1.1557291134580199`, 0.009009591269829347`, 1.325752168244685`, 0.33932371792024996`,
0.619859177738425`, 0.009052475129474827`, 1177.0140495795238`, 0.2452005274360191`, 480.6283895441986`},
{0.2160610264367817`, 3.9191762808523007`, 0.059018604533116775`, 1.0411018057212353`, 0.008462501397731081`,

```
  0.4634420979655972`, 0.32322042810200047`, 481.531032168865`, 0.019909072809315087`, 488.3969282323367`},
 {0.11630471140404741`, 1.5367142722565337`, 0.05695013627172493`, 1.4595782498770793`, 0.4347006603611545`,
  0.15170509102823682`, 0.02764562799329766`, 1429.026434078033`, 0.014789325697006195`, 556.0517717070035`},
 {0.04574862224020773`, 1.667137614048353`, 0.08615274073435199`, 1.367803712499899`, 0.9144234427837643`,
  0.29317668075534076`, 0.06903839047309805`, 1609.2665529077706`, 0.23754263722827756`, 367.7320637842886`},
 {0.2785629651947983`, 1.9405997998909799`, 0.09745189735065271`, 0.9223797816210384`, 0.7945408892658388`,
  0.34974400234234904`, 0.06721335250349363`, 788.540217506401`, 0.22345836350921944`, 636.4117130237988`},
 {0.1558631861871686`, 2.071958791081494`, 0.0906148168421321`, 1.3439374199675207`, 0.8630947847986346`,
  0.6733061517037251`, 0.2071553134363624`, 104.16809345728986`, 0.11868827346586763`, 553.1180333659046`},
 {0.1292208707400178`, 1.8632605914435363`, 0.046415812114243664`, 1.1633279615140635`, 0.018954763914399875`,
  0.24694457378275914`, 0.007904270013564875`, 164.82469298269328`, 0.060610611106801215`, 743.9579215785636`},
 {0.0898239866436944`, 0.5041817537224471`, 0.004730845503889416`, 0.8004221139970495`, 0.71803886182858`,
  0.24861999546798397`, 0.03932650979479891`, 324.23821280210257`, 0.08133023973628611`, 182.23375283503634`},
 {0.12178660308868972`, 0.8029832921015938`, 0.06274577468035605`, 1.11373872869435`, 0.3507051586575438`,
  0.45809878988528197`, 0.10454823064111732`, 716.7349133355972`, 0.057611453097593246`, 948.7347206740686`},
 {0.0685108116980988`, 2.7433614317525636`, 0.03948625806531251`, 1.4383757105618995`, 0.12700514465157853`,
  0.29596094652330907`, 0.005824134698501406`, 171.22697089194668`, 0.06525718195551272`, 301.6896298904339`},
 {0.22222570024441013`, 3.2839931217970175`, 0.03747668952108825`, 1.1936997453676481`, 0.5468462118059447`,
  0.582123988821347`, 0.030193979255150022`, 4841.3857036739555`, 0.21121655285668073`, 468.9585544167855`},
 {0.05500096557985035`, 3.021764046557614`, 0.03167342594848885`, 1.471078294602109`, 0.4110285739228652`,
  0.6035837025270763`, 0.17164962839295506`, 1968.9667019111098`, 0.24541253876143565`, 510.7332508800703`},
 {0.21198399370055476`, 3.919285200069673`, 0.014045043011751641`, 1.0494239320976348`, 0.47271455772763615`,
  0.6026643614464045`, 0.05622966821105292`, 117.93228694573206`, 0.21791598368515847`, 664.3246551556151`},
 {0.18261393242527846`, 1.24104755829402`, 0.017562609584990004`, 0.8208770412391784`, 0.5702360150514367`,
  0.2445337365136635`, 0.06636250536989298`, 71.77227660714203`, 0.12457334089639138`, 863.0628087051591`},
 {0.1516561445800767`, 0.8718874600546336`, 0.04870269917216002`, 1.3587651378620147`, 0.3907653327903866`,
  0.2341643054755288`, 0.16161562738072457`, 398.11431612220986`, 0.21962329680899473`, 517.7069194001951`},
 {0.07481472761035168`, 1.4356710981047938`, 0.03195742895113495`, 0.7620294331090656`, 0.6058100988285502`,
  0.1748877988840174`, 0.036278078120143255`, 1067.1675037581192`, 0.20966766369542061`, 245.76795972204266`},
 {0.23415203477003`, 2.637838297244735`, 0.010100103036724857`, 0.7781763734410485`, 0.9270607447506427`,
  0.25091053524524765`, 0.006631362838358266`, 410.1408598520636`, 0.09672454943968684`, 810.2426002994349`},
```

{0.05419057280488459`, 3.205052996318111`, 0.07207331803839295`, 0.8907501512355283`, 0.1443118496038216`,
0.23964954198579969`, 0.007902398391995273`, 453.6925070614146`, 0.03286507467345462`, 128.45869723434936`},
{0.1942266150934147`, 1.489765634070828`, 0.04812058961143629`, 1.1947522848110315`, 0.4017009524635866`,
0.3855054941178302`, 0.01145165307821007`, 1319.8238490809574`, 0.16195059607983425`, 743.204251876079`},
{0.14289375184211878`, 3.302390049355761`, 0.06615684983106804`, 1.29788834802556`, 0.9156578751693429`,
0.3558474159491941`, 0.00509231061202701`, 4261.526717057486`, 0.16050896664278197`, 220.23477447344888`},
{0.2554917877832018`, 1.993631504608799`, 0.04368181856665183 4`, 1.3194492976944885`, 0.9399541819359079`,
0.20985624905157363`, 0.1180452152100559`, 2753.2515506454065`, 0.05426315355547984`, 171.05806084971232`},
{0.20201473782765333`, 2.779452027424738`, 0.0266194566183942`, 0.9103804881826009`, 0.744670698555542`,
0.5706914134089118`, 0.00905331146486097`, 947.9277353727819`, 0.11184880683500475`, 329.63826832088097`},
{0.24790417478919824`, 3.0551603250746844`, 0.002284082483761942`, 0.961052650753995`, 0.6989086220525811`,
0.17002095436609788`, 0.48755300390089085`, 499.7708205836441`, 0.047042546081792314`, 684.0826745551606`},
{0.17492926953535737`, 3.975314242289789`, 0.023210045448957432`, 0.9727254901105757`, 0.4411891051406147`,
0.5520848832843507`, 0.2621570799727775`, 227.0108426167968`, 0.23671821431578927`, 367.0798924947729`},
{0.17539856022464784`, 2.4073130870702855`, 0.07197140330954806`, 1.0342299427745458`, 0.017371005489709157`,
0.6584543473805056`, 0.4728358484415665`, 1058.3389525653213`, 0.15082970917224703`, 917.1303711003585`},
{0.25951131167180713`, 0.8532719891968794`, 0.04557480110843374`, 0.7947841840785437`, 0.09920524665409447`,
0.24836596104002728`, 0.00597151633514079`, 436.2925795444242`, 0.1419534062375405`, 794.0516002827078`},
{0.17151022740710664`, 1.3676826689672605`, 0.009249620913063498`, 0.9876118143041603`, 0.1005607415916352`,
0.34049413724762323`, 0.11299721785819453`, 604.8937675217858`, 0.18735181675894919`, 945.3802305578456`},
{0.08003627269054897`, 0.4223118257784404`, 0.09788308557086342`, 1.1088763125629295`, 0.1846532162494996`,
0.44877823183875776`, 0.1744050351437392`, 232.77104633127487`, 0.186893582460665`, 400.88339340412807`},
{0.09629954489292863`, 0.8024243073579225`, 0.09137673334437835`, 1.1050982833461225`, 0.42757767860422`,
0.28094623108518857`, 0.05836165382907772`, 735.7662354861307`, 0.19909783079594584`, 717.4225578982439`},
{0.18721691020827746`, 0.8832247086125689`, 0.007468716509783384`, 0.7687691323991039`, 0.5945515211149526`,
0.3249811314880464`, 0.2800532125976925`, 679.3592862851432`, 0.05953883596172632`, 967.7513494997147`},
{0.05639051201063694`, 2.8104432162704684`, 0.055705228249153096`, 1.1263048402686886`, 0.5873694688928046`,
0.5281479596797096`, 0.1725920753398321`, 2534.050261866886`, 0.23842584574122794`, 882.2272354030342`},
{0.11940415250329756`, 0.8265537347095737`, 0.0814676981972948 1`, 0.8399194412982254`, 0.9872938360285297`,
0.2770401739511047`, 0.07923813270856427`, 3244.586795954999`, 0.06527667418181682`, 865.3808868374238`},
{0.12362063330315853`, 0.9363044869527668`, 0.08770822250254312`, 0.7786855706091862`, 0.2025852940468913`,

0.4566487841344946`, 0.10394338643070074`, 1334.4445729465897`, 0.16877657565130982`, 938.8021455901892`},
{0.25510163512616657`, 0.9371168864708563`, 0.07693845729623781`, 1.115586607271032`, 0.44362559437168314`,
0.23833942272790432`, 0.005303052515546934`, 2412.9883309521724`, 0.03447937580899002`, 556.3992268583279`},
{0.26333930199077354`, 2.0534773984698482`, 0.0643643244795264`, 1.2527519466886172`, 0.9177593225481775`,
0.33938623608355467`, 0.007825944721954872`, 939.4147129019324`, 0.07035677568445653`, 544.5854367414727`},
{0.18557844748229363`, 1.3040038526378845`, 0.03298157347075899`, 1.0816274180852803`, 0.0720559521645785`,
0.4038816001150133`, 0.01911693092881156`, 163.22630477861273`, 0.023327766650460102`, 342.443720752801`},
{0.08287518809588085`, 1.4167280782763267`, 0.028751858328181383`, 1.3710623936056974`, 0.3420455264471254`,
0.6180297694222441`, 0.06931546639283913`, 2809.59627019281`, 0.14779417738205175`, 701.4197770536504`},
{0.09993506797522744`, 1.9333379215144753`, 0.0294160752239147`, 1.306899867333013`, 0.15174981363752638`,
0.6973160529857587`, 0.32517481775011875`, 1904.6938693333132`, 0.22395301180399813`, 246.61499786462963`},
{0.1791717975737977`, 3.6792864853182463`, 0.003697989603839975`, 1.4268593787039923`, 0.6861577534974679`,
0.6003986216720203`, 0.037512309221476955`, 1019.7481977593816`, 0.10587101285101158`, 387.91912641915013`},
{0.24888562729039826`, 3.026347119869615`, 0.09462972939910319`, 1.164624390493581`, 0.6459998640897591`,
0.3154263692820086`, 0.3531314716559603 7`, 156.8927746768367`, 0.0941784253310613`, 806.7916066548876`},
{0.07400216536531751`, 2.1937414927491288`, 0.051724338318571414`, 0.8475382671591798`, 0.5283653100185577`,
0.3375223430599671`, 0.006370262255851855`, 419.8990383214368`, 0.056433548922067256`, 105.83929536957157`},
{0.08649617896036566`, 2.9261562137270944`, 0.06277833160761251`, 1.2717586436565882`, 0.677517886873324`,
0.2240537731188419`, 0.01575962778087511`, 67.09139852540285`, 0.15266972777055898`, 497.65886074130015`},
{0.21956975330290024`, 2.946279278154277`, 0.01351442569268972`, 1.0817364176432047`, 0.11142356213890037`,
0.6113477082885477`, 0.45234016742673816`, 389.8324142721476`, 0.06262043870838019`, 252.04928334155613`},
{0.16962065420490857`, 3.2987764634142422`, 0.06541243051493663`, 1.4967196726725034`, 0.6967420255395558`,
0.17574917802163403`, 0.035158157434036455`, 63.90845452931862`, 0.08229945239139341`, 618.3420143317427`},
{0.19774968406774135`, 1.6989982601552214`, 0.021311063743135836`, 0.7648586795987937`, 0.41768823186273707`,
0.37949798185162253`, 0.005204291178783 6925`, 973.0476525534903`, 0.05448802148770515`, 784.8493656086185`},
{0.2518902561813237`, 2.7254296067200725`, 0.08582594724985977`, 0.8908613191536825`, 0.4232867475157469`,
0.2276322578613562`, 0.014796112855772266`, 4775.5807799067`, 0.21732003999216098`, 657.2426602250014`},
{0.09904156316806545`, 3.4999116420802006`, 0.05005174807173392`, 0.889946639423957`, 0.9586911880217555`,
0.5850413707726637`, 0.007125457878312364`, 2230.565285121922`, 0.055223893991493855`, 299.18976270067026`},
{0.1091854910155387`, 2.8633835129131198`, 0.02035355266995058`, 0.8677831415002069`, 0.010141336872096618`,
0.28824854332128513`, 0.17109333973146398`, 3619.61715038073`, 0.2333055604695853 4`, 323.4056364398427`},

{0.1891953550945305`, 2.389035398016863`, 0.08454968650044813`, 1.066070932458544`, 0.0345292329688216`,
0.3128135317446209`, 0.38423156758654614`, 3793.7981099794624`, 0.125293090042303`, 233.53803394535984`},
{0.1570227614874073`, 2.40105806706515`, 0.05143382258328971`, 1.1808996551918274`, 0.2223234227077866`,
0.26986116152499395`, 0.10425235900754858`, 216.82613723714533`, 0.18708364772996822`, 798.8941796704306`},
{0.21694584394948213`, 2.9530758173431613`, 0.09769681722844141`, 0.9640446069284444`, 0.42193435394716805`,
0.6603635658814309`, 0.3338306865733826`, 1314.2123391672299`, 0.21796673788414078`, 229.9659279906819 6`},
{0.12690264500941062`, 1.3993259739099067`, 0.022125995185778395`, 0.9178738286195887`, 0.25309924713953147`,
0.251012090694433`, 0.019625422672818624`, 283.1749611392459`, 0.18026742864340645`, 758.8108031110586`},
{0.06920814358208571`, 2.042285023379372`, 0.029660070385393613`, 0.992143189021462 3`, 0.03257273935914129`,
0.20517882048284686`, 0.027701026007507824`, 104.22947983733822`, 0.22115350693221852`, 739.7214992264616`},
{0.2577647175823676`, 2.0669085310717623`, 0.015137977599734764`, 1.2344138658842803`, 0.6169124929132423`,
0.4497973990302503`, 0.20323917961090476`, 273.8208174543164`, 0.23260089052488175`, 786.949837857692`},
{0.22404984477095807`, 1.7971972613143468`, 0.014556371051440885`, 1.4736916256467743`, 0.10711810372521668`,
0.4838216983187773`, 0.15033397484285083`, 3525.8267227086753`, 0.24709636291116127`, 542.7438703089757`},
{0.2560915912056091`, 2.8056995396679163`, 0.02230409169328258`, 1.1596232786630103`, 0.9731942543354852`,
0.32967621772461264`, 0.008273413274888125`, 659.2862632916124`, 0.2493316428820775`, 801.2464425394161`},
{0.069529383599776`, 2.849786616236517`, 0.001548829693283195 9`, 1.1307747790060854`, 0.8754257019481757`,
0.4462637970794401`, 0.00987625334070338`, 470.49802215652267`, 0.196535530652720 6`, 591.5879320045749`},
{0.16317921960456533`, 3.795960913866608`, 0.060277538996435585`, 0.9943285566083784`, 0.31878466621504153`,
0.5268930320337564`, 0.03217382490455373`, 160.19057295170512`, 0.13130693314062597`, 466.7290497736911`},
{0.23870751571987275`, 2.817892132050006`, 0.034904301600875376`, 1.2212602625610964`, 0.2634282445359748`,
0.29343287356938585`, 0.0412321304066401`, 2956.916383781115`, 0.1710028618436959`, 734.0158474610636`},
{0.25110587793218636`, 0.5330897224408169`, 0.03508654146335001`, 1.2737631195521142`, 0.14437163587185342`,
0.22915335883220245`, 0.1301100912805756`, 130.55788460482208`, 0.03440422815097299`, 153.71031149577092`},
{0.04796641395535989`, 3.6542681080130706`, 0.06056371338194985`, 1.0410004898170107`, 0.13233719731300564`,
0.36420880945840706`, 0.16862770789835824`, 456.10576346306766`, 0.12687150881465203`, 491.49936597120086`},
{0.23314959452407125`, 1.9850975451070108`, 0.09033499334189088`, 1.2112065225297546`, 0.7891619630024773`,
0.23016874225029837`, 0.025757539951395944`, 1618.2342010883456`, 0.11509681389516696`, 818.8391633081792`},
{0.2668656374936109`, 3.051749683327815`, 0.0258024333714593`, 1.3861879676031486`, 0.13705271113057615`,
0.3169491905155566`, 0.04957453327253996`, 127.00810430555478`, 0.22326078444976738`, 605.3130708988924`},
{0.12837714622951152`, 2.913045083809414`, 0.0837521188025789`, 1.2333880824573145`, 0.14831200507330466`,

0.5511854573030723`, 0.319487783122794`, 260.13632482567476`, 0.08285153464288336`, 293.457784508192`},
{0.2580675113282032`, 1.3794239963466088`, 0.09606459315739223`, 0.9738093032034381`, 0.551722273950022`,
0.6189616163620202`, 0.03080803973244329`, 452.05919890664615`, 0.0556923772463625`, 491.93043733941124`},
{0.08884487120981721`, 2.332961071695622`, 0.031598395011471735`, 1.1141910450192687`, 0.7184499049851998`,
0.6730388302232508`, 0.123997295097641`, 484.8896162348586`, 0.15163592409494436`, 272.73357673474675`},
{0.2759587426432519`, 1.9534358692391445`, 0.08397889547253015`, 1.0356223413014591`, 0.42065948992616775`,
0.6805267491095484`, 0.0964860490180039`, 69.9818047589248`, 0.07247191711576301`, 633.9862534370685`},
{0.07608942110432254`, 2.164297732879585`, 0.044623011426671744`, 1.2845300660264662`, 0.14123590728202684`,
0.20715073503154113`, 0.010177080583516988`, 365.6404789674773`, 0.09043375256261627`, 318.6735532350724`},
{0.12578906287533304`, 0.5873288516562982`, 0.00917874148360891`, 0.7860432924669931`, 0.8658472891163045`,
0.28929582442115676`, 0.04769704077854567`, 2092.327054664775`, 0.10790373294003436`, 499.81053764102194`},
{0.13400527850674449`, 3.167561049379361`, 0.09503778083239858`, 1.0695890919772384`, 0.44461403458537974`,
0.35428116459826664`, 0.04474774833241626`, 249.9292825838412`, 0.2392270171205957`, 993.872691687206`},
{0.14569029844361497`, 1.581782399146463`, 0.02853446038002714`, 1.4746522591423932`, 0.17909427610451467`,
0.395505345829425`, 0.04611739955881583`, 816.4722344402478`, 0.17778523558925585`, 271.44027790378294`},
{0.12249361616240895`, 3.3852221257689097`, 0.05543978239026112`, 1.0925731163264347`, 0.17776966626445145`,
0.29066583025906045`, 0.0687429771171289`, 314.1609292490537`, 0.1111159201808452`, 685.2380116739549`},
{0.07368028774821661`, 3.0677536525276716`, 0.07059619046896788`, 1.2388956643695934`, 0.6420156583576027`,
0.15354672355808818`, 0.023520960948583497`, 1566.5430005580442`, 0.0643629153664671`, 602.9382331885815`},
{0.10293015258903027`, 2.7627847871257094`, 0.048868500737193926`, 1.1335235205351295`, 0.26256073599306595`,
0.17258322470135423`, 0.35448680892520623`, 639.8812577909154`, 0.21355261514569296`, 292.3004754224651`},
{0.17989985519582302`, 2.2848444130728813`, 0.06499018234377278`, 1.3356780269154247`, 0.8760585579430653`,
0.25119519210597663`, 0.17680773834886487`, 2893.233426659574`, 0.08968766773643105`, 889.4972829137831`},
{0.0700064475969871`, 2.5832650492672506`, 0.009367713712463904`, 1.4593574063080232`, 0.8612459654760951`,
0.39256758588428897`, 0.048637831394936666`, 69.10482903899627`, 0.16784750403438659`, 808.9797947692787`},
{0.16902358696866238`, 1.5466078808636725`, 0.09114667029195474`, 1.1632289455971812`, 0.7011539680870855`,
0.43898684142995004`, 0.12814910976660654`, 201.32808946828865`, 0.1990055903635441`, 501.3434969043132`},
{0.07225780293824052`, 3.2069795251700226`, 0.08284178682925689`, 1.2105070206615947`, 0.6775214967569712`,
0.488074793019582`, 0.026308005494110893`, 313.1548266866826`, 0.21343764900902945`, 758.2569582586476`},
{0.13893262337344747`, 1.0427327070975592`, 0.09311200953995581`, 1.095872791509625`, 0.7537435467200488`,
0.549904286540486`, 0.2574747272316228`, 4239.365928479679`, 0.23905861617289415`, 330.34293410544217`},

```
{0.06788146233466685`, 1.022821493462117`, 0.06599019909830307`, 1.0274069325364188`, 0.42743374336783724`,
  0.47977647156275904`, 0.02909328473333213`, 114.54872183983547`, 0.08606336865176462`, 305.0489819660903`},
{0.04270280673685248`, 1.2934684868479742`, 0.0959118906786280 7`, 1.4128815880743355`, 0.13315269485352843`,
  0.23204805195972866`, 0.2572508527807455`, 90.11608912792754`, 0.03659032712805199`, 523.1103863627065`},
{0.046236418394459605`, 3.574151007923631`, 0.092837745021803`, 1.1374323179308754`, 0.4550831015183179`,
  0.3756839361198018`, 0.00586392775091683`, 1204.6242266607505`, 0.029043894879770327`, 942.0566673133433`},
{0.14802462884786977`, 2.8463045630582817`, 0.07086620031807243`, 1.4262141974100975`, 0.40895975433086873`,
  0.2365796035874227`, 0.3310600416149043`, 102.98362169854454`, 0.1764312785940091`, 773.2109911740185`},
{0.17261791412834615`, 2.66725952372021`, 0.011878378245691455`, 1.1959533166982588`, 0.37293218245137805`,
  0.2820216148069596 4`, 0.4651224858250725`, 72.55708905719771`, 0.17887121192017358`, 925.2830578188941`},
{0.10523638690298753`, 3.5642755326059987`, 0.09015549116400706`, 1.3647813438407541`, 0.8329295324191079`,
  0.5634168718886515`, 0.02622479781769635`, 1558.3726673845852`, 0.0866750472996437`, 735.9237964380025`},
{0.20934959845181905`, 0.5253713637677127`, 0.09562730622062596`, 1.3267328346184806`, 0.026713277643319655`,
  0.15125889150158744`, 0.009145743694901407`, 347.6644603704283`, 0.2158426941139397`, 839.7488318339839`},
{0.16181023020779767`, 1.305821903245473`, 0.04345833700918577`, 0.9871466940785586`, 0.990363037222695`,
  0.41737973540465045`, 0.04349028756064097`, 389.8084434973402`, 0.2301500945529913`, 142.75918439051142`},
{0.0585933257218722`, 2.1564084302719815`, 0.00824084879767166 7`, 1.3548876424139005`, 0.78543219520227`,
  0.5684638437310318`, 0.03933279743012852`, 1164.1502368017577`, 0.03651331026085236`, 908.8904797965278`},
{0.17629686442565196`, 2.478708866004733`, 0.029760826245713457`, 1.1409573325432285`, 0.08473376105442143`,
  0.48472391979554497`, 0.008616495281357169`, 315.1653370205564`, 0.21238301827033085`, 252.6645414580406`},
{0.05701931707649882`, 3.239761060927444`, 0.018658291098521504`, 1.1958711178433945`, 0.6010462815373658`,
  0.2222121897761763`, 0.00866105970369655 6`, 2427.987481600541`, 0.16195229268590294`, 598.9762630900484`},
{0.12429129251918081`, 3.165176790650899`, 0.05944857413724836`, 1.1750421651261647`, 0.7024367598760777`,
  0.26578928465801155`, 0.28623135703022834`, 57.36286473144533`, 0.18225946471633314`, 279.6228390464558`},
{0.17592117157303727`, 3.225697565005621`, 0.06410385713825643`, 1.4723665071047831`, 0.040075633612683514`,
  0.16766841616104544`, 0.01926077754515973`, 451.97528485070285`, 0.1967336572740045`, 903.2247661915143`},
{0.20267367580625623`, 2.351165901182144`, 0.008675643302528258`, 1.2736483392354083`, 0.1108762459426682`,
  0.4077401628201436`, 0.03171361327346612`, 3972.464308751051`, 0.13615309860632413`, 596.0671079273882`},
{0.12207280755595895`, 3.8644474094244794`, 0.009427505375928522`, 1.1042754889246225`, 0.6660399151614447`,
  0.25206523111087553`, 0.10009599160633623`, 402.11380186936043`, 0.20397090745892643`, 309.50158431622725`},
{0.24507742036233432`, 2.3831843355711397`, 0.005052828156007507`, 1.0721739375121355`, 0.49387052070387516`,
```

0.2147676922991728`, 0.011565289716111508`, 84.02018174384712`, 0.07100872729300844`, 676.9347914709565`},
{0.05872459234515515`, 2.1332438180615902`, 0.07824887308364058`, 0.7585647474793751`, 0.26510390251726434`,
0.2713237544705841`, 0.036979209154785794`, 52.71577024813928`, 0.17679977189893264`, 564.239792184463`},
{0.14683813473126478`, 3.4711809547760266`, 0.030295203568552493`, 1.3035360306310906`, 0.42027841877180494`,
0.5921843153064568`, 0.010793050374412331`, 254.4238250041397`, 0.22813578414007268`, 746.5344746241171`},
{0.27118296549826965`, 0.40016893385797836`, 0.03738933189542754`, 1.0762552685066569`, 0.6570337540705835`,
0.22274705980247678`, 0.006224989465699251`, 331.2301635317647`, 0.1631894969050383 7`, 803.619268726775`},
{0.2651341645020733`, 3.921256061000774`, 0.09127020329702631`, 1.0515436089401342`, 0.37820568835934876`,
0.30543268981111493`, 0.008197803117955519`, 249.67697255719887`, 0.20481437271886815`, 450.4819383009913`},
{0.10266981380053875`, 1.7259937776730974`, 0.05788500808078473`, 0.8498356975392536`, 0.5149250125601736`,
0.3750799043189028`, 0.006831336220999067`, 241.15442777469008`, 0.08411211628353799`, 814.8105177183215`},
{0.1701903238305883`, 1.5737398864052752`, 0.013362842515380927`, 0.9723904628120438`, 0.9778114455260909`,
0.6469978525912341`, 0.013362665151551732`, 1406.2278798102645`, 0.1754741948990562`, 281.6011633652904`},
{0.20205010610888874`, 2.4886025319973255`, 0.03416173840731352`, 0.9461759215151488`, 0.19917032732038176`,
0.20522267238789926`, 0.007087646061753528`, 151.73293313624973`, 0.015624468793102558`, 798.3652981187157`},
{0.2548714778924099`, 2.595325989353224`, 0.04783518749288423`, 1.260048561023251`, 0.11179251567641679`,
0.5271320321183793`, 0.20942249013272063`, 61.94848210458544`, 0.13000680045559498`, 257.7742889938444`},
{0.25697985123874933`, 0.6423673175461451`, 0.03920104245257182`, 1.0494619196955428`, 0.6883576503405942`,
0.5372602583905404`, 0.0298184492004487`, 1024.1709922100576`, 0.18196374947080113`, 448.03818305726395`},
{0.24108459248749042`, 0.6097105829562341`, 0.05914817527701839`, 1.4448529749350583`, 0.06017529583805459`,
0.5679483071298559`, 0.028892937973694452`, 184.61048198703293`, 0.046198153900852024`, 876.6239668930126`},
{0.11138935148613482`, 1.9270561111646902`, 0.0506158838526531`, 0.7861832578093317`, 0.2623170852799024`,
0.19880501636260794`, 0.1716591058201036`, 672.6474872708164`, 0.14359452836986147`, 488.74274292141473`},
{0.22711687131412428`, 0.5423663037563959`, 0.07370528658374681`, 0.9917310500559307`, 0.6268413827963848`,
0.35482337483246806`, 0.0492957290107415`, 298.86562808346656`, 0.0341131398928744`, 451.4330811913654`},
{0.2649900949630745`, 2.9147648354215034`, 0.09385410641202459`, 0.8515585607662866`, 0.6282724158295114`,
0.40674785391584145`, 0.01227961889947592`, 57.08712154139038`, 0.026923769884349857`, 165.58243018457918`},
{0.0915398024811338`, 3.642607843612522`, 0.045773601129209036`, 1.2779360684969243`, 0.2244141258099137`,
0.6005450159014489`, 0.4759115782164151`, 141.33543981734994`, 0.23290101078240694`, 255.60470768923676`},
{0.09997582603329147`, 1.8799516342297675`, 0.01460194430259186`, 0.8270986899357093`, 0.5545841854424991`,
0.5876382120219206`, 0.052778297224976234`, 1510.139172300266`, 0.05587104924786124`, 122.66534784854954`},

```
{0.11587596442377335`, 1.570443051624368`, 0.05003858184900709`, 0.7979390254239629`, 0.2695501577041357`,
  0.4402434745919389`, 0.1955710791573617`, 1393.738907723443`, 0.22482510146060541`, 822.0077092395296`},
{0.22552960854228582`, 1.1654101546280868`, 0.07708737421212913`, 0.8741738696212267`, 0.34763140639716794`,
  0.6720908373189787`, 0.049522394564493144`, 1483.0319583713147`, 0.08855956469300003`, 824.4137036635007`},
{0.0476102421762471`, 1.8287647324569782`, 0.03110183117006317`, 1.3863628762732523`, 0.6811850740698742`,
  0.21530814035187884`, 0.02495067854594185`, 78.5692803520234`, 0.13933019803203`, 251.51485099763934`},
{0.07293365735317825`, 0.9536666649351204`, 0.041006966482838275`, 0.7665287875498985`, 0.935767572935885`,
  0.220179659831362`, 0.20106612820967829`, 178.2780155183392`, 0.1430151800284700 2`, 953.0856220301953`},
{0.19096668838686354`, 3.9791109702558582`, 0.04575838938969985`, 0.88278575745365`, 0.1319776886877686`,
  0.6321229402668154`, 0.005732045216178792`, 174.70115248623193`, 0.20628116633253563`, 438.2211382365572`},
{0.24680488679812707`, 0.815637935503644`, 0.0843637406190441`, 0.9487689240355479`, 0.3250491534008515`,
  0.6707507630339515`, 0.10643040624255257`, 4909.4593594307935`, 0.030006121749521886`, 121.48162188773526`},
{0.14271805685583516`, 2.3484760469035573`, 0.07745189816615884`, 1.1886412637022417`, 0.22453965782263952`,
  0.6988851450501361`, 0.008392473480111172`, 241.2772419245499`, 0.05856292707558125`, 560.9416229791005`},
{0.237923538483648`, 1.3045678824163405`, 0.05160012685748933`, 1.1613123620254255`, 0.8558167677781581`,
  0.6442348560245077`, 0.280278439293096`, 960.8793815230854`, 0.10811725374257602`, 308.2410580836482`},
{0.26686684415084866`, 0.9101714257367162`, 0.05201112615814223`, 1.0067387685359694`, 0.3144283486587576`,
  0.5640584563511031`, 0.07480454987166867`, 4843.746054037886`, 0.14590156821291428`, 291.7993314288994`},
{0.27466728617562586`, 3.909730616037213`, 0.015651626558488035`, 0.8183399746276282`, 0.8624521285063864`,
  0.31078149750433326`, 0.1052382875316152`, 561.582629527527`, 0.23527086491760263`, 644.9709948771053`},
{0.07007388150970617`, 1.2788530615475953`, 0.041240822222090405`, 1.153764990829969`, 0.8716462413619626`,
  0.15860191259805845`, 0.35155378239777585`, 296.5267589380548`, 0.19912668804556583`, 616.7284928222024`},
{0.1878952467648845`, 0.7675716449344554`, 0.05741879370926064`, 0.9528631412514192`, 0.9302947624844955`,
  0.2534812971784569`, 0.1207735933533045`, 1834.0932588583464`, 0.2124476008021367`, 697.2076816469347`},
{0.14168118293015808`, 1.8332481297075178`, 0.0300583699414835`, 0.7581988115722615`, 0.852062289257528`,
  0.22721700466029904`, 0.12430348358114858`, 272.5636471822111`, 0.15659940706565478`, 639.1777488262896`},
{0.19834052588138612`, 2.8473206103303275`, 0.06787428338874751`, 1.2634740859082962`, 0.8483257098618575`,
  0.27810606990948006`, 0.052306771734647074`, 2133.625806620023`, 0.10206688811673287`, 552.214584726826`},
{0.13732318942612998`, 2.533545686945514`, 0.07677083355764772`, 0.9473176337866864`, 0.9203815554724639`,
  0.31737934755677866`, 0.05120312298025456`, 1347.6776516438235`, 0.2303275954656494`, 112.23809273053274`},
{0.21392312209610825`, 3.0478754499742005`, 0.06538726038868102`, 0.988290831541475`, 0.07401087304537768`,
```

```
  0.28494540960049397`, 0.2526680322768358`, 279.52916817401405`, 0.2297370624504932`, 425.1990754299824`},
 {0.14320126116520115`, 3.5785950094384216`, 0.025604653350218314`, 0.8268074039814586`, 0.976648397359879`,
  0.5001702346122724`, 0.38123679071943467`, 388.98067038422806`, 0.2240399781178949`, 927.7111417924974`},
 {0.20926436934918818`, 3.54928785755524`, 0.04655442449302829`, 1.4735046423674272`, 0.6436030426422834`,
  0.23716114021464474`, 0.016767557842678662`, 762.9533208051863`, 0.22515295037189986`, 823.9967654417107`},
 {0.09715661458363001`, 2.6162532629232897`, 0.05330552481177518`, 1.3217583375467599`, 0.056961332005489806`,
  0.41403944176397645`, 0.0787432744072381`, 132.53142675262785`, 0.16676071183902036`, 308.900351347671`},
 {0.044494746845228667`, 3.8073919623023302`, 0.00891629517230891`, 1.317697440879989`, 0.2104073212959853 8`,
  0.43509792721016394`, 0.11970345154140186`, 395.1110764732494`, 0.23530085829797331`, 437.748607476885`},
 {0.1200252023437956`, 2.8019007957477235`, 0.028007311257585012`, 0.92009516518451`, 0.01280900474487301`,
  0.4526896126410833`, 0.04417068363712719`, 126.12721650529039`, 0.11309971446701528`, 297.99616984883437`},
 {0.159307524381112`, 2.1388708024966263`, 0.07728990983733347`, 0.8933915824609264`, 0.17018256737475035`,
  0.39427249092340955`, 0.2374099439396035`, 296.4953500654782`, 0.22256932318418826`, 146.08391069826325`},
 {0.2515615397486592`, 1.2188920022514846`, 0.056806007322854075`, 1.0589873581328146`, 0.8389110983305801`,
  0.5160905955986007`, 0.005163127675966413`, 854.4257970577178`, 0.2370298510099471`, 574.6962402010942`},
 {0.23870656719030897`, 0.4607483912247612`, 0.017860308849642834`, 0.8081937138032418`, 0.1724194202814684`,
  0.3241110683425861`, 0.018037887307016776`, 469.5800690255617`, 0.1120148648028150 8`, 307.02179419088134`},
 {0.06627522358143922`, 2.000113059980986`, 0.03069667022234471`, 0.8345023822924422`, 0.7625117937694375`,
  0.4912663179641469`, 0.07709548204647149`, 185.51291986100534`, 0.0763658907530384`, 325.2314705034143`},
 {0.11831505888067828`, 0.8158614012599301`, 0.09695106021032068`, 1.4738361110893088`, 0.2645508678881856`,
  0.2913223690175213`, 0.010373880238463022`, 1209.9954370966966`, 0.23975714360436612`, 767.8374529336429`},
 {0.20577599681754594`, 2.9313768979273176`, 0.0038206720060969704`, 0.781758458231594`, 0.56664249884203`,
  0.4709509229113563`, 0.021927764931302315`, 4686.470122623354`, 0.2192041879935001`, 347.008418687607`},
 {0.1869209879787055`, 2.3095757377059725`, 0.05297142960646317`, 0.8815543572140244`, 0.041400679606300006`,
  0.31871458453434554`, 0.1289212733641052`, 101.4389842310655`, 0.13597402845170187`, 914.947493051789`},
 {0.06483245092897544`, 2.8384211668748724`, 0.0911657087741262`, 1.289023251198742`, 0.4466087032681607`,
  0.267574352599688`, 0.007946224566772193`, 1055.04661000947`, 0.08980662020688435`, 572.8761673725373`},
 {0.09228610076587707`, 1.6450889610515498`, 0.08913657400655176`, 1.3611199649465364`, 0.621424219326399`,
  0.6158200041827449`, 0.19662513724207695`, 1190.4261163432097`, 0.21638593744388268`, 965.0447414955308`},
 {0.24524928286716796`, 3.6904456707313154`, 0.08967706170457866`, 0.7837922940103521`, 0.15260266641265163`,
  0.4719521309469129`, 0.0219019946570777 4`, 50.30445277851058`, 0.16737611791533596`, 158.4281260415155`},
```

{0.2085289521726456`, 3.7433965844871855`, 0.07373717758801387`, 0.8272531686673292`, 0.9243484340355741`,
0.4736315139016828`, 0.04000550244322193`, 775.1117022858083`, 0.18128754644027817`, 792.6217255082997`},
{0.1942796430577805`, 3.7771592955896995`, 0.014808087701342974`, 0.8727503183069723`, 0.9499179373546311`,
0.4711663886743095`, 0.28504637709688535`, 219.95821433792653`, 0.17829093188760597`, 601.1866479309697`},
{0.19978804404047035`, 1.7412366971449815`, 0.05869679069766164`, 1.2250897389227589`, 0.9947242752248806`,
0.3288744578871605`, 0.029790954073398563`, 177.76139833706597`, 0.1704177380158945`, 342.62682125921594`},
{0.18813828973766622`, 3.040504339634855`, 0.03041458173591515`, 1.2993625717609874`, 0.3197300168421664`,
0.4882990032931548`, 0.05916461107129405`, 288.10788143037024`, 0.10847906504084376`, 408.965425001152`},
{0.13939828058518033`, 1.5857112352411011`, 0.08574772946752571`, 1.1093571913830735`, 0.14309585058744245`,
0.5634406137724309`, 0.2817673310248056`, 53.991544293777636`, 0.11459032439612055`, 248.27985633277424`},
{0.043536324184198144`, 3.326291464260996`, 0.05139708072028187`, 0.9030702944800504`, 0.7051237824883041`,
0.5197885611320734`, 0.019273613149624703`, 329.1019165250263`, 0.23460236315419714`, 678.1062885700973`},
{0.09801716906192193`, 2.9539869268188017`, 0.020040830336135844`, 1.2343926494145465`, 0.7600540430041269`,
0.3516902841570241`, 0.029540615021220525`, 3778.034634812808`, 0.12448155103112013`, 335.4861228637068`},
{0.2351399190035529`, 0.6800114559723247`, 0.03213357322891234`, 1.2136689663675575`, 0.0851758437090655`,
0.2574027192263152`, 0.12005990640730702`, 487.2802108209037`, 0.1525337459876398`, 478.6782242639763`},
{0.19840884489282196`, 2.435275307859949`, 0.06582510415417815`, 1.19484171596350 3`, 0.18931490770635162`,
0.6654697402710699`, 0.012855112113943912`, 384.7644348925394`, 0.20206490703703606`, 653.6112811972781`},
{0.153998794720146`, 2.296727093897146`, 0.048890456587564815`, 1.0717959146758531`, 0.20411298992327565`,
0.5326406872061232`, 0.0433093428704623`, 1797.551253413897`, 0.09526457101922897`, 474.50386549092957`},
{0.2762960805421906`, 2.0251009856011146`, 0.06438086916711981`, 1.2552601159611685`, 0.824619961217385`,
0.2219622549088328`, 0.07771120847969075`, 235.23851588627025`, 0.03242543857282393`, 995.444729973487`},
{0.22210233579747424`, 3.0942680698763763`, 0.09032486696922916`, 1.0647099914995546`, 0.516081148067818`,
0.4664841812624002`, 0.2575335143375922`, 247.27548003665564`, 0.1261056310024135`, 988.0783976396131`},
{0.04246581734245333`, 2.6243765146123685`, 0.039300994341565525`, 1.1707132411393175`, 0.760389410309404`,
0.6197176888843219`, 0.05613616961797109`, 579.7528852266169`, 0.18044598974464704`, 692.5186634415095`},
{0.13098562468211145`, 0.6918886972002496`, 0.09307771379161338`, 0.7572969971235853`, 0.8621959711022029`,
0.17247619508234702`, 0.13638646340531488`, 1721.8193108366343`, 0.15630991181601384`, 116.41676929938268`},
{0.2369558402004424`, 3.8904595389867698`, 0.07694340666447698`, 1.136418408023505`, 0.5724535764038741`,
0.4530887158124406`, 0.11198137985855583`, 117.36224574039397`, 0.1511561710016185`, 454.7116411649565`},
{0.22006023145078546`, 1.8699454187445346`, 0.04190713219713553`, 1.0916024021867943`, 0.9816282992661436`,

```
0.6829534497485226`, 0.009835956205983078`, 624.6290470462591`, 0.021070658550712773`, 768.5490473777757`},
{0.09828617329733175`, 2.463942931528096`, 0.007572202379780535`, 1.2472200527061044`, 0.41644064652613144`,
 0.4645936104828291`, 0.02312500549439911`, 54.07758231391304`, 0.09597622753113771`, 416.81598350845786`},
{0.08362876114696788`, 3.673753657595393`, 0.08998734313926576`, 1.3432025605848703`, 0.2747343812629557`,
 0.6253363571442772`, 0.03699414192084745 5`, 388.9552816546361`, 0.04955150722579804`, 133.7754196526289`},
{0.22105845206099273`, 3.6723501488527104`, 0.0598021179692625`, 0.9344989193429194`, 0.3577079276157791`,
 0.25286474043446827`, 0.02760454342543124`, 121.18794663333252`, 0.020589883661662256`, 454.33911379556343`},
{0.2404415985731178`, 3.9242340192232827`, 0.025726059183975813`, 1.0889915012585847`, 0.7185429349809043`,
 0.6376702062000428`, 0.268650042773469`, 355.68354138078996`, 0.22139947989242942`, 504.918029373391`},
{0.1005874500764159`, 2.23272409892634`, 0.060802667136885716`, 0.991684052120384`, 0.9801615674605653`,
 0.4858907364942624`, 0.29370050837355216`, 1056.4414347359782`, 0.08590239477227651`, 977.0843750733277`},
{0.2630219935361097`, 0.7161334798425658`, 0.06498611082218136`, 0.9675557624650479`, 0.11067589370665232`,
 0.632848587449107`, 0.011686165277954987`, 164.13933362107778`, 0.16701870855521322`, 106.59616859139227`},
{0.21827180155004183`, 3.007771469369752`, 0.03198650183307903`, 1.2597421711376162`, 0.05877944668022894`,
 0.21087223032471758`, 0.1491736628807538`, 409.73754661816`, 0.07595835910505389`, 682.1222641287044`},
{0.22045085365596306`, 0.8751625033990953`, 0.040943556123142544`, 1.1343752751378642`, 0.40735695900069624`,
 0.30497304960520855`, 0.05995678076716148`, 952.3200487692166`, 0.18118346106920746`, 347.9890739313543`},
{0.18946179909757177`, 3.562633004956865`, 0.0313023029411473`, 1.4739498657051078`, 0.8014019749280452`,
 0.35240031523303095`, 0.015200151831610186`, 167.2762376390573`, 0.22621173221438579`, 745.402744487609`},
{0.13542699057027358`, 2.86206448171706`, 0.09450221741270137`, 1.0246625231410813`, 0.5482685534092477`,
 0.5509095992989991`, 0.015559917921534123`, 99.38093814456799`, 0.05756251023205006`, 408.71843737795535`},
{0.22732356256701053`, 1.5620331345808607`, 0.005007450975166154 5`, 1.19717278691931 7`, 0.105827486838725`,
 0.386862672674428 3`, 0.406019958242878`, 3005.96971854734`, 0.19859762557886712`, 993.8820694295587`},
{0.21970737996259793`, 3.936399929325778`, 0.011750041974777936`, 1.121349683519117`, 0.5859217781105792`,
 0.5996889464289015`, 0.1151074043533795`, 67.42346044078403`, 0.08547629013313163`, 411.81079788833034`},
{0.2790786490274024`, 1.2584508796810594`, 0.07665707924292071`, 0.7658270386209375`, 0.7483296604910108`,
 0.41920065323621736`, 0.023176749691687894`, 3277.281084867628`, 0.1396918716126389`, 580.5780481474799`},
{0.16453791668796786`, 3.8862921855966857`, 0.013327775962263057`, 0.8725709784378354`, 0.6113118115742937`,
 0.2156639660199009`, 0.03762803482789101`, 2547.318776383711`, 0.17247938020774817`, 892.0466365080326`},
{0.06375537189690972`, 1.7690459528540705`, 0.015096892864035833`, 0.8299986088232871`, 0.6367083940240161`,
 0.28113916107145065`, 0.04428098157483091`, 4084.099508293162`, 0.10954682401240934`, 746.6104278313039`},
```

{0.08187579925483629`, 1.8655110961595254`, 0.0874648172698457`, 1.4510572222714657`, 0.15566591480992242`,
0.41910638528278665`, 0.07239970607129952`, 723.8241834225208`, 0.12901960068026558`, 180.2504060368367`},
{0.1043519915364962 7`, 0.5893233643311877`, 0.04811942984145233`, 1.1349604857206697`, 0.5895670318233859`,
0.15288875305518912`, 0.04910349811258292`, 449.5316302223964`, 0.12434959568189269`, 703.0287573582831`},
{0.15686440216485814`, 3.299158386840012`, 0.04362586030096008`, 0.953069845851497`, 0.4069322991233839`,
0.6538520347193495`, 0.028206943433383688`, 616.8360622195407`, 0.1803006375790399`, 266.5600115768661 5`},
{0.06547279088568253`, 0.8381089463723068`, 0.07137472004433824`, 1.2017236457315694`, 0.07858577799252853`,
0.56356916944079`, 0.03424982094712966`, 121.26145217466427`, 0.2454354766292936`, 829.242714338502`},
{0.17569742909738978`, 2.0195575491588675`, 0.02434222613969263`, 1.434279867729109`, 0.8915491260180508`,
0.2781339725257843`, 0.04682272639132616`, 59.94019211717092`, 0.2222051195324956`, 236.65018069241324`},
{0.09460907585719619`, 0.8356897954881948`, 0.060460918351022866`, 1.069146699286762`, 0.014736646088169492`,
0.5865094862125538`, 0.07297164593929295`, 179.1610024491539`, 0.037596776460407155`, 892.0322556004016`},
{0.09233956131689997`, 0.6404053871541118`, 0.05959897753518367`, 1.3305103741913034`, 0.7729857515912517`,
0.22480536379085048`, 0.13093485314958295`, 1027.106817790177`, 0.09580485239452469`, 776.0064233531045`},
{0.1760992852032619`, 2.0576043555645516`, 0.009895906281638124`, 1.218879855416122`, 0.22293753096138147`,
0.47733786176532955`, 0.12573960627564046`, 190.0926217184925`, 0.16755982960498023`, 296.35846579200165`},
{0.266626264443372`, 3.5900664324260045`, 0.04123634989318651`, 1.010061724366724`, 0.22320942065814342`,
0.29156885927164033`, 0.008778055090297143`, 315.7126293940718`, 0.03106814470993058`, 971.4718069354443`},
{0.20633623222753245`, 1.8062049689164192`, 0.024683456051737968`, 1.4736425858155604`, 0.9975963414746294`,
0.45321134080754555`, 0.06164119417640365`, 981.5961666351051`, 0.06084524543598019`, 894.6393430913585`},
{0.1952457396539687`, 2.396633432716655`, 0.027692355669681295`, 1.483739684147085`, 0.49946151037920083`,
0.6843034163274524`, 0.015772529376378245`, 165.6691863878366`, 0.13164676433224554`, 744.7639235090896`},
{0.0412147820800699`, 0.6603123172269081`, 0.022261526033749313`, 1.1649615926526864`, 0.2836869733691665`,
0.15807495679202033`, 0.11234412980270021`, 3908.1378298311292`, 0.06462235682420547`, 548.6113009858772`},
{0.17218500939622683`, 3.313865785682787`, 0.027957414977543033`, 1.3071462915775924`, 0.8795651108976117`,
0.6440187783428721`, 0.005956691356262271 5`, 104.65032511018502`, 0.027977308543697232`, 907.2104908091411`},
{0.15502202925070413`, 3.316526162282644`, 0.0223320056796846 7`, 1.2759230603694547`, 0.9221203428299127`,
0.2943110669636566`, 0.005350880253335643`, 3898.5440559966614`, 0.22820486785889155`, 684.4986442435719`},
{0.2268261659238296`, 0.7994964989328879`, 0.08934756409882037`, 1.2877518377572021`, 0.5411906669030329`,
0.3691399078289529`, 0.43421420694016527`, 139.17013379956958`, 0.04485647842506657`, 905.8781238041313`},
{0.177020423569270 1`, 1.2781960851633176`, 0.06497396695791541`, 1.3605134684748712`, 0.244014227954229`,

0.1870138591753292`, 0.03077127158598826`, 60.18019994982649`, 0.2131346317141143`, 683.027244020558`},
{0.2717375993536527`, 3.135824470079611`, 0.0691606222305435`, 0.7845225922631083`, 0.16212023726406954`,
0.4466944141001402`, 0.022381478813530132`, 1383.5815052684432`, 0.187615308884319`, 843.6072606940219`},
{0.13674247358699154`, 3.6254060485372106`, 0.06512400723657372`, 1.01887393296334`, 0.2910940805340305`,
0.4365596853256456`, 0.08138806498834827`, 2017.3312921087977`, 0.1583601508053748`, 997.8620700747402`},
{0.25040288289621143`, 2.7133813844480077`, 0.08448585223614441`, 1.2018013366302356`, 0.3702885119817223`,
0.5701876195896491`, 0.00869107124078448`, 443.903801201532`, 0.12402133852573172`, 106.45272333045864`},
{0.041511277768737365`, 3.4931610590794087`, 0.03976168853825012`, 1.2668832550579765`, 0.9486019052531109`,
0.46529858524653356`, 0.012928244696006381`, 74.20790580822272`, 0.10554204343742091`, 509.5020269334231`},
{0.1336668665495257`, 2.350867715598585`, 0.0771275101676191`, 1.4505612510666444`, 0.07698080749571079`,
0.6199281441330318`, 0.01101960174542534`, 2269.347129396604`, 0.18489372818520305`, 687.4473514223084`},
{0.16253001008083912`, 1.051184352668212`, 0.056229861688529344`, 1.004023329401581`, 0.3681252451367141`,
0.678555581878113`, 0.021351933171038215`, 801.2312670528647`, 0.04412464112257197`, 454.387044888035`},
{0.11892797719939147`, 1.7514715754101635`, 0.04183594418628097`, 0.8769916901819208`, 0.1544196171401151`,
0.36811304221318286`, 0.3010643636498307`, 695.4685634811971`, 0.1100461062891433`, 498.29553639594855`},
{0.07330210226600109`, 0.43499060410436075`, 0.027517449896700585`, 0.9126335864120539`, 0.9987805296414807`,
0.22966152781322624`, 0.006114379328547516`, 1310.3025866798243`, 0.17060413983046802`, 156.9492509406908`},
{0.04276868172332263`, 2.4648491140401436`, 0.05187906428248695`, 1.0339646047806086`, 0.22491069842200573`,
0.545977126956078 5`, 0.008402091672076594`, 289.6225761610719`, 0.14646027342084122`, 755.2264179389915`},
{0.04164393094890753`, 1.242303977240664`, 0.07972480756303409`, 0.7846124621006639`, 0.174070187582688`,
0.36672176044371163`, 0.2674133971106355`, 845.6949135526564`, 0.03358000499684233`, 498.1809415613497 5`},
{0.229458031958857`, 0.643951541612365 1`, 0.006015292830911623`, 0.915892012371688 7`, 0.2560144381841183`,
0.2684667797484167`, 0.34315524666163677`, 154.42595917415295`, 0.0855610041334563`, 198.13557848737526`},
{0.17047138799782602`, 2.2898957920477256`, 0.06696496462752731`, 1.0845008770247568`, 0.2872211606807551`,
0.6001028117008813`, 0.005754832757542004`, 635.7506300024877`, 0.22579833707749164`, 724.3665386038351`},
{0.17254913268987798`, 2.6495590376973945`, 0.034760594449157356`, 1.2124525447208163`, 0.84194692679041 4`,
0.5402918593560716`, 0.18936332779093834`, 107.26507780664734`, 0.037511987734769214`, 537.2669943782652`},
{0.25016487015444`, 0.777229893080774`, 0.04915901947556062`, 1.0998264093102956`, 0.5377812514235516`,
0.25267335702204063`, 0.00974787647189834`, 1244.8070691537305`, 0.07483034108642977`, 805.776090435819`},
{0.05399158420479849`, 3.019162845487374`, 0.03785073545081486`, 0.9636051990488629`, 0.15564774020522387`,
0.21918784710924044`, 0.006223290787557694`, 2623.658439173353`, 0.1090049048368558 8`, 947.3435866888657`},

```
{0.1747811216286701`, 3.881854804860919`, 0.08016222833139366`, 1.4944210252995196`, 0.11903432009706139`,
  0.5878228241833926`, 0.37145683618740644`, 66.72046507331578`, 0.12877597314607542`, 125.71709418119497`},
{0.141601654527025`, 1.3089955011167094`, 0.0656032917699483`, 1.3368116485682675`, 0.4133506282593844`,
  0.6351089997359891`, 0.016818978674610487`, 1705.797093405403`, 0.15388894760263466`, 511.61503546845904`},
{0.084639232238298`, 1.212736951496196`, 0.05997810159304612`, 0.8889893736120966`, 0.13958728158655265`,
  0.6603558899173929`, 0.011413642036874375`, 536.7545673384209`, 0.0425999565316516`, 944.3686479349165`},
{0.16529311034127547`, 3.851126737390418`, 0.08113860877333676`, 1.058819349237051`, 0.13571240412272645`,
  0.4517872186170928`, 0.35559705335185826`, 1001.8429736474003`, 0.09802300913580997`, 620.1784409572178`},
{0.23418982946474864`, 3.956604059014369`, 0.04230369921779414`, 0.95232561320253`, 0.8349528260558194`,
  0.6299543509496768`, 0.06275886449027793`, 701.9720478982712`, 0.21824826209863868`, 426.4774271646496`},
{0.2761598760040886`, 2.6641557087508057`, 0.004462535100939409`, 0.9313534046677239`, 0.750233715330811`,
  0.2049305849598677`, 0.3933840236336692`, 50.2496550267719`, 0.2374943907090124`, 344.2970123705422`},
{0.1351836628142749`, 3.5624660341291037`, 0.025452437146326938`, 0.7612568037032439`, 0.652970660668506`,
  0.6408529036315123`, 0.21928764621486097`, 534.7730348589814`, 0.24746341528536303`, 560.0699314591134`},
{0.26232668071537735`, 1.207274588572547`, 0.027172732680876114`, 0.878898400796649`, 0.9275146387246145`,
  0.31349252382451775`, 0.12237771890255045`, 296.36361267724436`, 0.017576594718109073`, 538.5261184010628`},
{0.06921463438282771`, 3.9571800779527315`, 0.026742802177079528`, 1.1077760750375103`, 0.012836546117421577`,
  0.44641175777925235`, 0.3069910959195543`, 680.1511030834043`, 0.08770647443596591`, 182.30226827470233`},
{0.23559239220203682`, 3.2108513477276697`, 0.01736528262118654`, 1.3561109299681864`, 0.09396152800742397`,
  0.29521017097752533`, 0.02869751203681663`, 1077.23513295276`, 0.19412010713150385`, 318.4056793084999`},
{0.09115428875490028`, 3.0706223452006016`, 0.03232145089994729`, 0.7642247265036182`, 0.18050339436720364`,
  0.641645625372536`, 0.010505945470625598`, 58.10983830816918`, 0.09406933636073506`, 174.5022624084388`},
{0.23356040634394826`, 2.492582964596944`, 0.09819476019997214`, 1.1503432849681279`, 0.06854649517894851`,
  0.5510839557713577`, 0.0071381077335303`, 2385.9838126952855`, 0.013315355756726904`, 961.7670735590306`},
{0.25739033381964765`, 3.83031296180729`, 0.07795330721270595`, 1.3914735727751597`, 0.315795024852483`,
  0.18909057130226337`, 0.046455878996427676`, 2457.8987531381417`, 0.21479863643047675`, 436.99040574834953`},
{0.11782083138332561`, 0.9397991607947134`, 0.03435330327997967`, 1.298219743186714`, 0.15161593487328484`,
  0.2427618760211594`, 0.04640184603844971`, 394.52569979799523`, 0.22637503918894109`, 120.48416627959249`},
{0.10065409907604789`, 3.109979529809686`, 0.05985471266539985`, 1.4037113657202065`, 0.2551527049282132`,
  0.39211616335234245`, 0.053944330615504715`, 1232.2013118971267`, 0.18318402883994528`, 982.5075567784879`},
{0.20373034722529687`, 1.8258929347032087`, 0.028710915990495637`, 1.2999358225942528`, 0.47437870101991586`,
```

```
0.4479020312750728`, 0.02085535990534196`, 155.89057435108427`, 0.24707674305197086`, 593.832502401464`},
{0.1667072912115688`, 1.1926716420341554`, 0.02793807367428192`, 1.1097053421712721`, 0.7879852596126686`,
 0.47927016034261305`, 0.00796414514983457 4`, 178.9414360093533 7`, 0.1062391944982713`, 376.6999080921696`},
{0.04399597163424873`, 1.5345814599820171`, 0.040779489311776336`, 1.1444095951288937`, 0.08884309651814792`,
 0.5567432378994842`, 0.011138214889458318`, 268.907723173268`, 0.042126942027993275`, 429.31770518007414`},
{0.12554904125704935`, 2.572105912307064`, 0.04382715819624885`, 1.0196689186185788`, 0.004628348183958852 5`,
 0.4286118484279966`, 0.12186483055559326`, 183.56311720218602`, 0.19168490195367482`, 312.7756329069644`},
{0.10389836812335534`, 1.5065271361516794`, 0.0782203910368041`, 1.3432006880431453`, 0.16858721631501128`,
 0.20272940646961235`, 0.005194801604326796`, 692.6428769431301`, 0.2497553227485374`, 646.8923759638144`},
{0.1873969648530403`, 2.487936537546788`, 0.08787631545159552`, 1.4862886220253502`, 0.4510967953396121`,
 0.6132699008580953`, 0.20943864253875633`, 362.75371797287244`, 0.05566418089330266`, 990.5206074435366`},
{0.2609977488784161`, 2.682129125412585`, 0.04099814383534014`, 1.1943200062659527`, 0.3320420087757965`,
 0.3562748569841966`, 0.03843736896556402`, 4461.432848364856`, 0.19491126930886082`, 448.46031606671596`},
{0.11177943644202659`, 3.004766476051927`, 0.02365721593278737`, 0.9739347355449255`, 0.11623805347832561`,
 0.5332851817458878`, 0.014219460200483265`, 2321.5504339611107`, 0.11273311280208292`, 154.13280416226715`},
{0.12452460259052345`, 0.6179927758495714`, 0.018240920352788487`, 1.419600762592073`, 0.04159334889643196`,
 0.4953333574077009`, 0.005771350364962132`, 173.11009258100464`, 0.03618579436828123`, 460.51456633143266`},
{0.056338015570984834`, 2.9056643066548693`, 0.09482015263628527`, 0.9910228668352266`, 0.5398932209933094`,
 0.30993462834373164`, 0.326754145425203`, 4152.751222173614`, 0.1627448112336103`, 934.4752403980212`},
{0.24888413407324067`, 0.9185195851359875`, 0.001862748847094693`, 0.8264811519169353`, 0.45711823663268425`,
 0.647903170967445`, 0.01273956161975329`, 3902.2467544998485`, 0.10661606869776508`, 764.1046199784935`},
{0.10302491503594513`, 3.897572343584968`, 0.0776490643225345`, 1.3728855867542038`, 0.399366063625185`,
 0.5139103382918121`, 0.006363477494426043`, 1471.3257431593884`, 0.18415616996412293`, 396.38746581742976`},
{0.2217716187206022`, 2.3669670966982617`, 0.05150497952731632`, 1.3669514270973737`, 0.27563093514117476`,
 0.6097112540241125`, 0.024210358214628785`, 245.19099830513036`, 0.2227447189207991`, 922.0556423723906`},
{0.200567099512532`, 0.414291053781334`, 0.0965175412941376`, 1.4122041619788255`, 0.2832458523243746`,
 0.4111934355701189`, 0.47068189330284727`, 727.924890435336`, 0.13700773707558722`, 457.2663597106665`},
{0.26282708876445304`, 0.7299461735692212`, 0.002590123247159504`, 1.314069772735592`, 0.44387331112790007`,
 0.6627601406571553`, 0.017941283900867265`, 4804.295508555627`, 0.13656101682983213`, 961.2364213744279`},
{0.2217181783558071`, 1.7819525199900896`, 0.09096582175868409`, 0.8016110058394221`, 0.34870116285618513`,
 0.41254310619778667`, 0.28848841693193716`, 118.09472925522707`, 0.0491516892009966 6`, 318.7068061045219`},
```

{0.04238719280368508`, 3.9934041772699773`, 0.012369382023231542`, 1.0320497019704051`, 0.441711735053806`, 0.3953172978504792`, 0.055840071594635055`, 4470.752428881338`, 0.11971425032923705`, 391.1155394727787`},
{0.11325951664984701`, 1.2613891355679572`, 0.040579133378454896`, 0.792778865736141`, 0.4125188071599031`, 0.6760512259522733`, 0.120226085162758`, 240.62393709029973`, 0.23563525899548954`, 669.3575607124823`},
{0.11479796420185318`, 1.3645131007055955`, 0.05813851484662918`, 1.4328455678258982`, 0.8783089057298354`, 0.6273688332789649`, 0.005594475300849291`, 336.8131271096774`, 0.042923286813940253`, 400.6985075519651`},
{0.18904612246826086`, 0.9499182666483561`, 0.01636431294508675`, 1.334133788223376`, 0.9399135492285866`, 0.44862152471700667`, 0.015021474832741414`, 104.29224521509512`, 0.20020013193412523`, 362.68249145721364`},
{0.07377471208830438`, 0.9399350889911195`, 0.0035046964205100294`, 0.95254283141522`, 0.8226600861406266`, 0.5781817355083377`, 0.45292305898377666`, 4955.013777151356`, 0.18926919322875674`, 784.6286355590761`},
{0.0943766935246651`, 1.541984748199737`, 0.08520914815438847`, 1.068673226642868`, 0.827147192543344`, 0.5185364408449342`, 0.02499096081051123`, 2458.353306813159`, 0.11939278701608641`, 406.52581078470075`},
{0.2691170241960745`, 1.3843069189189876`, 0.0032282878414418015`, 1.35893455567664`, 0.05684986848649287`, 0.6495874733323252`, 0.012010940694653572`, 818.6583817232862`, 0.11477976407459428`, 802.4857212031947`},
{0.13195654495183612`, 2.820234715819688`, 0.06459455777685377`, 1.3215682050752708`, 0.2555253012593035`, 0.5650143175076483`, 0.17650684935600558`, 2548.3703743447245`, 0.05197610429597477`, 647.2541591160882`},
{0.17938754879434415`, 2.956564495685738`, 0.02344859424499825`, 1.2271661955652247`, 0.1023158483495108`, 0.2141005377740559`, 0.03160934186798711`, 55.01949141750772`, 0.11404091283217083`, 153.96775969344887`},
{0.2559468893711437`, 2.6639064911815478`, 0.09405265387739238`, 0.9418787060193106`, 0.3579196254838115`, 0.25349572195403014`, 0.005922563586734462`, 686.8472909729428`, 0.21618019082855489`, 944.0817596146342`},
{0.16846533721603613`, 2.3770892262064063`, 0.060764583227921456`, 1.4122925060330735`, 0.8131908524160045`, 0.5745691401533295`, 0.13815925889996158`, 111.92799118209615`, 0.07704963831958889`, 569.2109515105052`},
{0.1434225400058592`, 2.4623089922223533`, 0.0902878213107297`, 0.9014701813026362`, 0.7982192946025746`, 0.24884754841188483`, 0.008733563377012872`, 84.02892029266614`, 0.10452691271186154`, 174.89969925150496`},
{0.27498382758967205`, 3.0621895303657007`, 0.01477217114526983`, 0.8499949546355164`, 0.9543379068261812`, 0.511253393062205`, 0.026007868557453007`, 1193.5403577036534`, 0.24471853485794082`, 173.30853286735942`},
{0.10079216159245852`, 2.802263371434325`, 0.07168578045446232`, 1.0754522486079452`, 0.19795232328567214`, 0.4796042882951459`, 0.2511967396816989`, 916.4127582717309`, 0.09231077895158313`, 746.8086196132365`},
{0.1990395876740651`, 1.2891521935040648`, 0.08843781786859424`, 1.4217044468375937`, 0.7248232910439498`, 0.5288906890390438`, 0.010145562070591906`, 273.266580044107`, 0.1443450012114269`, 262.99773077705277`},
{0.22527530235766147`, 1.598090720025331`, 0.07516157972031196`, 0.911906670636256`, 0.0015950083386944147`,

```
0.2733183222114466`, 0.46257295121139497`, 1061.4916967982135`, 0.16946126329202837`, 514.5508656659653`},
{0.22800375706146692`, 2.963114044328991`, 0.08247902929296419`, 0.900155769535574`, 0.863503432146618 2`,
0.41996725230963483`, 0.060440776646127806`, 82.14976842278587`, 0.17068417206912262`, 306.6961010018577`},
{0.2015284416951108`, 1.4828427267730095`, 0.09645095598799114`, 0.8813953916233646`, 0.6596036330203583`,
0.46790731355733717`, 0.04929088349718639`, 2147.9230273071194`, 0.21677576367436763`, 585.5137656428404`},
{0.24804752677268144`, 3.775861546538554`, 0.09828394004173842`, 0.8259108347253036`, 0.8501628381099438`,
0.43041433567991594`, 0.1645511915243919`, 472.6904522654539`, 0.10093712153827977`, 240.41573863346227`},
{0.2257734465493289`, 2.04598315320208`, 0.061072784217809206`, 0.9205146346019395`, 0.18360134495814817`,
0.2485501642439466`, 0.008261271045257068`, 4070.225349557058`, 0.14432673822154007`, 514.018907335898`},
{0.1884239042817073`, 3.884724587861638`, 0.046102203625386924`, 1.272869675104722`, 0.002436310787800044 5`,
0.6425585670884819`, 0.21347280812774377`, 105.72896030123064`, 0.013398853493480106`, 477.3170113191213`},
{0.10358133559917804`, 1.3204671153076744`, 0.03146944673924043`, 0.7598009709392282`, 0.8379948599836853`,
0.31448462367280716`, 0.09423762512957796`, 873.6401432656386`, 0.24643359443672203`, 257.57090703914133`},
{0.07213812112223295`, 3.2444866813138393`, 0.08948863559851084`, 1.1981049871186626`, 0.6100944668641342`,
0.3489877456164385`, 0.060436350562816356`, 810.8798894147244`, 0.1940599582028637`, 929.3745703520485`},
{0.18529645231815123`, 3.7388067772724316`, 0.04964299226107711`, 1.104153730745968`, 0.8562902999854938`,
0.20061955425285494`, 0.044683490812461285`, 1237.7065014840275`, 0.17461953898337046`, 513.2497687518729`},
{0.11937104462964826`, 2.2670408410877547`, 0.04212633055221726`, 1.2938710702028255`, 0.4062855702251942`,
0.22896688687327793`, 0.008005950997757192`, 187.47575366480618`, 0.12062380115724536`, 420.08829844146976`},
{0.054668692745937386`, 3.776137055200575`, 0.0067187515890975115`, 0.903291115804289`, 0.19988932148575245`,
0.5550342082463592`, 0.0455873571973524`, 57.62421691500666`, 0.11425393330613232`, 212.43967681879158`},
{0.1180225681670306`, 3.9649936920105127`, 0.09323036671246253`, 1.0524617108638712`, 0.5152474998332999`,
0.3810548965315216`, 0.2344220817835223`, 72.9591968642363`, 0.19446142099795322`, 521.0444198605804`},
{0.07585484942115545`, 1.5268041836953499`, 0.03996691736666499`, 1.2200843385635163`, 0.7382765513873244`,
0.15072535011998411`, 0.46165250685061965`, 2014.6215653623833`, 0.0639150426736455`, 907.8090815554127`},
{0.15171074425765668`, 3.139959028863476`, 0.0344935047970057`, 1.313524716160249`, 0.15932561521340416`,
0.344519114270757`, 0.023892122521195856`, 4372.432115235335`, 0.19509438620204334`, 395.2405886014027`},
{0.09759208826045196`, 2.787459031458204`, 0.08286510303958249`, 1.1498428365580842`, 0.3502413486308751`,
0.6023991214203646`, 0.04223365699052448`, 151.71289774079654`, 0.15665841498595628`, 996.6803923172566`},
{0.06870492988603288`, 1.4433926464019216`, 0.0548016124689776`, 1.4156603871152607`, 0.5046761258494916`,
0.49679280153517125`, 0.23820389677781276`, 574.8760772624919`, 0.07570136980088016`, 774.5924346494874`},
```

{0.12928812481552754`, 3.4467014297623226`, 0.07698339025243396`, 1.1617442922699581`, 0.6397961941354378`,
0.23683288560045057`, 0.014840572388870685`, 390.22473096383766`, 0.12473219055267015`, 308.51541332429787`},
{0.09947361882549755`, 1.0723085231871234`, 0.08651203729858636`, 1.1986754319537722`, 0.20293837184497532`,
0.6267394010553271`, 0.3996069768023198`, 125.45020568494616`, 0.0747353381238216`, 815.5264595512623`},
{0.07385189036800854`, 1.1097460679658449`, 0.08534569405097261`, 1.4790081203811583`, 0.825389044253078`,
0.6393014572933857`, 0.005393929802389671`, 1009.7951092503188`, 0.05876766510907333`, 713.7033793838743`},
{0.08886981390180526`, 3.1852329380949485`, 0.004843225884898494`, 1.0495521924526`, 0.8894920527442816`,
0.6575087708412799`, 0.03091556833752079`, 1958.934458296337`, 0.09234610293139284`, 932.1837545744593`},
{0.14789843862328383`, 2.02419875820041`, 0.0514487209494189`, 0.7738844537901749`, 0.7290609776680101`,
0.3165995990604955`, 0.08454509023565165`, 148.72732800342848`, 0.13774453289126964`, 330.25011651458095`},
{0.25024837610835304`, 2.332733791115757`, 0.0077143684034007625`, 1.3224531384329063`, 0.8138980190182421`,
0.44646733111462167`, 0.4678523507493547`, 2146.7439649709167`, 0.20938413594616256`, 223.81131941609436`},
{0.22717212093597267`, 3.1880507135942393`, 0.07302399796830696`, 1.4035606527233073`, 0.6815685781194232`,
0.3786677309260351`, 0.11250145446111069`, 222.14009806288436`, 0.22433932328259149`, 562.8866527417559`},
{0.26122096963316843`, 2.4993722773914913`, 0.09696053620814965`, 1.104804193531995`, 0.030204720323329992`,
0.320390588871465`, 0.02428210132726833`, 110.42992015103017`, 0.03440723905644305`, 957.9351268355158`},
{0.209997188604718`, 3.564852173259874`, 0.04768067199603367`, 1.4849701489343874`, 0.9167498847129119`,
0.5930768777567338`, 0.007556568646764511`, 303.63963198830606`, 0.1798334582091491`, 979.6939793583849`},
{0.12957791995272494`, 3.292789025445294`, 0.029334797113071912`, 1.000823124755441`, 0.3770034479959583`,
0.6497986144453649`, 0.4644164461032186`, 69.35773206793411`, 0.22822270741491651`, 883.2881905591939`},
{0.1734007680973328`, 2.5509297754695135`, 0.028940050324794433`, 0.7542700070247144`, 0.5607952150482101`,
0.188194520643177`, 0.10842093468725295`, 89.42321140726736`, 0.04230251478503749`, 585.538823967249`},
{0.08976599585154404`, 3.410901375584835`, 0.03980847090975558`, 0.973029097362308`, 0.18413384146487743`,
0.4306490501572334`, 0.25777469695398525`, 394.50697488236744`, 0.14943635401103217`, 467.5668758424882`},
{0.1540668065647836`, 3.156731285149597`, 0.025268011683832015`, 1.4768603918368393`, 0.5930914034327641`,
0.3246832969886583`, 0.024701654832061146`, 2039.571837718409`, 0.09825821190266026`, 517.5447194924812`},
{0.25448003730676694`, 3.184178274174914`, 0.07969865646175218`, 1.1996160734264`, 0.014800627784428588`,
0.41181348081367897`, 0.1736752616356111`, 514.7971371394015`, 0.0242520492159774`, 345.1422226775872`},
{0.1805495720684696`, 3.5988294042989537`, 0.08629129509807985`, 1.45590487849237`, 0.6500204371524467`,
0.6598493533910432`, 0.006501958768912599`, 98.298066958251`, 0.19720820935827937`, 552.7953947456965`},
{0.2584100436319957`, 2.6280813527891524`, 0.008925623022125535`, 1.4383962385176845`, 0.1768680490063801`,

```
0.5799402745315277`, 0.03668114755652737`, 1557.060885044531`, 0.15015889209697436`, 661.2136292315887`},
{0.12632549433481927`, 3.227079423540302`, 0.024599881217287293`, 1.083115156883892`, 0.862537661831063`,
 0.4466766069622442`, 0.0175594229299586 8`, 838.9788940765472`, 0.11679264941372974`, 962.5385414773948`},
{0.12581743503205006`, 2.6912266074159374`, 0.06825202848377941`, 1.130575915810954`, 0.7123031893599316`,
 0.38183459324469116`, 0.005640134906792506`, 195.97188584350954`, 0.08695147378297474`, 810.3829986474054`},
{0.25215230411705725`, 3.2162318690382756`, 0.033696261063115164`, 1.4320906763160213`, 0.4333561915057187`,
 0.6173099764463517`, 0.06281247244822827`, 350.692872561691`, 0.14670856975401297`, 661.831914027344`},
{0.06363146413241788`, 2.590325492453749`, 0.07759599585526651`, 0.8055036563134401`, 0.389060478218378 4`,
 0.6596279029376648`, 0.006263246097646333`, 3940.278955744855`, 0.14629462701990292`, 857.5168588182237`},
{0.09237029959020937`, 0.7372750141972166`, 0.08246784417666007`, 0.761354059113222`, 0.607934228395967`,
 0.617252844721303`, 0.005938986299855843 5`, 1735.7120546460667`, 0.12202837863790356`, 173.2417595974299`},
{0.17533432125136816`, 2.980549279621548`, 0.032407398618161984`, 1.2260204195085989`, 0.09654786970227502`,
 0.6394186143434559`, 0.017429477978672333`, 442.47136750346704`, 0.02322996341809752`, 762.5628352008919`},
{0.26705012685276763`, 3.7498343916107544`, 0.09027963548734444`, 0.9359855383901472`, 0.7308970561854626`,
 0.6045717653608473`, 0.013690168592867258`, 53.13894335207241`, 0.0962366983944411`, 806.6075956869515`},
{0.1678815958956808`, 3.549708737453586`, 0.0464906102104119`, 0.8245440899304926`, 0.5174765540911901`,
 0.24692645213758668`, 0.06597541194694605`, 190.2602468493688`, 0.13329765093762808`, 380.5016809755655`},
{0.26055153005517384`, 1.525876220559283`, 0.09894984049853037`, 0.7715055101942483`, 0.5126639262751305`,
 0.17386878262666106`, 0.09828079193823042`, 3373.049666117342`, 0.10907780218183427`, 438.55829206861597`},
{0.05352312148646443`, 2.717121314814369`, 0.009558938401920933`, 1.2972752707604394`, 0.3755720109619096`,
 0.4102380523871805`, 0.05724239658462567`, 409.7682683541758`, 0.12801779059907553`, 304.9029332641055`},
{0.06648599129298854`, 2.0041637987546954`, 0.08747673292965043`, 1.0596372061777766`, 0.3670136645832196`,
 0.20965175246372458`, 0.041210859023618776`, 258.8957674631027`, 0.10593279391689964`, 291.6186786637164`},
{0.27763207727232625`, 2.6532447231581857`, 0.005216127449356379`, 0.7883455682883888`, 0.4799446618915817`,
 0.19310494144291912`, 0.22065665818548827`, 81.96509789217254`, 0.19648280620732078`, 916.8547512970214`},
{0.04552100665001646`, 2.514367034016656`, 0.03037177853258429`, 0.7530148351569228`, 0.6998681178203525`,
 0.5299837160000372`, 0.11693739269557663`, 590.5283463527218`, 0.08466788522227797`, 566.1770520758637`}};
TestFreeGrowth = {{"TBUR", 15.711390754719442`}, {"TBUR", 44.2019502737212`}, {"TBUR", 16.78193020158727`},
 {"TBUR", 18.667093982125085`}, {"TBUR", 13.885447416359366`}, {"TBUR", 30.162601174755157`}, {"TBUR", 20.869830546775166`},
 {"TBUR", 13.428810876131172`}, {"TBUR", 14.471079396823363`}, {"TBUR", 17.712876379060795`}, {"TBUR", 24.336228038953468`},
 {"TBUR", 17.63354851297323`}, {"TBUR", 17.313225221748393`}, {"TBUR", 30.881659977935886`}, {"TBUR", 13.2742212625022 8`},
```

{"TBUR", 18.776811164075987`}, {"TBUR", 31.898893336838885`}, {"TBUR", 17.28790705464217`}, {"TBUR", 28.67142534485458`},
{"TBUR", 36.430732714305776`}, {"TBUR", 13.019048693178771`}, {"TBUR", 14.383786156569203`}, {"TBUR", 20.48923939651125`},
{"TBUR", 35.012393113888294`}, {"TBUR", 20.777776008147782`}, {"TBUR", 48.266751175561275`}, {"TBUR", 15.561828191043215`},
{"TBUR", 37.50933743832283`}, {"TBUR", 18.640678481908232`}, {"TBUR", 11.83630544424025`}, {"TBUR", 33.210465747981885`},
{"TBUR", 11.382556282125856`}, {"TBUR", 12.754392233655562`}, {"TBUR", 24.564135844473306`}, {"TBUR", 33.81776364120207`},
{"TBUR", 12.006249305459617`}, {"TBUR", 18.362239037123956`}, {"TBUR", 16.015705098077394`}, {"TBUR", 21.635849256627885`},
{"TBUR", 25.878508033408714`}, {"TBUR", 15.06050668469422`}, {"TBUR", 25.959345718956083`}, {"TBUR", 26.071004424283036`},
{"TBUR", 12.40762480700718`}, {"TBUR", 14.56254208955875`}, {"TBUR", 17.503679634098326`}, {"TBUR", 45.2094052716283`},
{"TBUR", 45.74640593438107`}, {"TBUR", 25.9486748045628`}, {"TBUR", 12.029808404620965`}, {"TBUR", 24.1433235073872 6`},
{"TBUR", 31.68985410836966`}, {"TBUR", 35.75517668565276`}, {"TBUR", 41.2450833432457`}, {"TBUR", 12.432508808409507`},
{"TBUR", 18.187404300362225`}, {"TBUR", 40.089933116105406`}, {"TBUR", 26.821881272495755`}, {"TBUR", 18.390605378139213`},
{"TBUR", 30.602625753839515`}, {"TBUR", 13.927808899569715`}, {"TBUR", 24.1542551473147`}, {"TBUR", 30.221327254485974`},
{"TBUR", 13.960996725501133`}, {"TBUR", 14.718456359925968`}, {"TBUR", 23.80854323464943`}, {"TBUR", 12.09075567378241`},
{"TBUR", 19.999698349123253`}, {"TBUR", 18.2090009652405`}, {"TBUR", 19.585433005037192`}, {"TBUR", 38.301974279561016`},
{"TBUR", 18.454183803164156`}, {"TBUR", 16.646510085022577`}, {"TBUR", 11.066417337683328`},
{"TBUR", 21.069008316964855`}, {"TBUR", 23.078967851849022`}, {"TBUR", 34.49452725010505`}, {"TBUR", 24.47021605580779`},
{"TBUR", 38.24532060036566`}, {"TBUR", 19.609609481476582`}, {"TBUR", 36.74969805999738`}, {"TBUR", 15.629644394196557`},
{"TBUR", 13.213496525127617`}, {"TBUR", 21.374687993953817`}, {"TBUR", 13.415378272305858`}, {"TBUR", 20.486308168185502`},
{"TBUR", 11.835137164430243`}, {"TBUR", 20.925751084537062`}, {"TBUR", 19.20759419404488`}, {"TBUR", 18.18623427800386`},
{"TBUR", 21.799599241705636`}, {"TBUR", 23.960197220535875`}, {"TBUR", 13.445382717487496`}, {"TBUR", 16.383883583281705`},
{"TBUR", 12.173591504764826`}, {"TBUR", 47.04577973955561`}, {"TBUR", 11.71317520726345`}, {"TBUR", 43.04732735707591`},
{"TBUR", 12.0430402417071`}, {"TBUR", 16.25921008760382`}, {"TBUR", 13.780799320894396`}, {"TBUR", 32.94466117223283`},
{"TBUR", 13.645477733405803`}, {"TBUR", 17.10937777091369`}, {"TBUR", 24.866446880146157`}, {"TBUR", 49.681091167586025`},
{"TBUR", 18.521505502047162`}, {"TBUR", 35.36620849444657`}, {"TBUR", 32.86288801866426`}, {"TBUR", 18.602289956387867`},
{"TBUR", 32.58986490722991`}, {"TBUR", 29.8925362710101`}, {"TBUR", 21.572955113264005`}, {"TBUR", 17.703241149394813`},
{"TBUR", 30.84229648532772`}, {"TBUR", 14.982154622046883`}, {"TBUR", 17.06599961414592`}, {"TBUR", 31.653186835701078`},
{"TBUR", 16.275422384450472`}, {"TBUR", 29.3193189802495`}, {"TBUR", 12.669073931157723`}, {"TBUR", 44.32121321762277`},
{"TBUR", 18.808632019596182`}, {"TBUR", 14.178270621846558`}, {"TBUR", 34.39411506496662`}, {"TBUR", 10.60826678656911`},
{"TBUR", 21.27025036051607`}, {"TBUR", 17.128893875251595`}, {"TBUR", 26.75424152875178`}, {"TBUR", 23.396540980904476`},
{"TBUR", 34.026524663570804`}, {"TBUR", 26.047893572349185`}, {"TBUR", 16.931298267133226`}, {"TBUR", 12.427684719498808`},
{"TBUR", 15.977237159890077`}, {"TBUR", 12.015523318328619`}, {"TBUR", 11.865708889876311`}, {"TBUR", 28.167135785515615`},

{"TBUR", 15.891934741275676`}, {"TBUR", 38.99654017919275`}, {"TBUR", 14.557887937138537`}, {"TBUR", 26.8519501593052`},
{"TBUR", 21.10367132602165`}, {"TBUR", 31.23224297185312`}, {"TBUR", 16.42416186520581`}, {"TBUR", 19.68576686273544`},
{"TBUR", 19.518715653499076`}, {"TBUR", 18.99467059371834`}, {"TBUR", 29.497864678679566`}, {"TBUR", 29.8342642786606`},
{"TBUR", 12.151562604837842`}, {"TBUR", 21.43295361353254`}, {"TBUR", 39.191880874485925`}, {"TBUR", 43.94937234554008`},
{"TBUR", 13.600102266619292`}, {"TBUR", 35.45455477652378`}, {"TBUR", 13.618077419834844`}, {"TBUR", 30.44805565275776`},
{"TBUR", 12.135143649888148`}, {"TBUR", 11.838492109374219`}, {"TBUR", 19.387626849034557`}, {"TBUR", 20.378893406187636`},
{"TBUR", 12.568370803609564`}, {"TBUR", 12.335541498182756`}, {"TBUR", 16.900180308391676`}, {"TBUR", 23.566889218337202`},
{"TBUR", 27.188423556215096`}, {"TBUR", 23.4088487070332`}, {"TBUR", 15.503298507467774`}, {"TBUR", 27.798052195552458`},
{"TBUR", 18.381327565539564`}, {"TBUR", 20.92135437607756`}, {"TBUR", 13.830888602052188`}, {"TBUR", 15.29011768969299`},
{"TBUR", 37.29460988856768`}, {"TBUR", 41.127126860961276`}, {"TBUR", 17.488297437451834`}, {"TBUR", 17.17459464616633`},
{"TBUR", 29.517194080940293`}, {"TBUR", 28.809733816287626`}, {"TBUR", 36.03500813957433`}, {"TBUR", 35.650726659604366`},
{"TBUR", 22.968796241435946`}, {"TBUR", 10.861214749311419`}, {"TBUR", 19.273058909571365`}, {"TBUR", 21.397446408131632`},
{"TBUR", 16.656429073810475`}, {"TBUR", 37.7256973902334`}, {"TBUR", 14.332350044067626`}, {"TBUR", 15.612773946318637`},
{"TBUR", 13.56089086265119`}, {"TBUR", 56.29919814610204`}, {"TBUR", 13.692192912801652`}, {"TBUR", 29.40261067063076`},
{"TBUR", 25.92640970414381`}, {"TBUR", 19.86214014774299`}, {"TBUR", 24.541614900945888`}, {"TBUR", 10.842531622673606`},
{"TBUR", 18.393250674136546`}, {"TBUR", 11.848022816238698`}, {"TBUR", 12.394247222735197`}, {"TBUR", 42.73652768351775`},
{"TBUR", 46.834151303573464`}, {"TBUR", 18.074934308481833`}, {"TBUR", 23.88505995433176`}, {"TBUR", 26.1966477685208`},
{"TBUR", 14.091681063105517`}, {"TBUR", 12.529826141736114`}, {"TBUR", 11.85452964978897`}, {"TBUR", 21.415047182220583`},
{"TBUR", 16.10378219825058`}, {"TBUR", 12.002118223003633`}, {"TBUR", 12.238177469377737`}, {"TBUR", 11.820289133688204`},
{"TBUR", 13.258397547509006`}, {"TBUR", 10.446724842053175`}, {"TBUR", 32.25921307596167`}, {"TBUR", 13.218716299423596`},
{"TBUR", 47.540255662146905`}, {"TBUR", 16.441665661679554`}, {"TBUR", 28.516877697345826`}, {"TBUR", 10.145299260427528`},
{"TBUR", 24.256068708797287`}, {"TBUR", 14.802391486987581`}, {"TBUR", 18.26988607751062`}, {"TBUR", 17.29117337945663`},
{"TBUR", 16.777144378744026`}, {"TBUR", 24.38760930220397`}, {"TBUR", 41.13822571897459`}, {"TBUR", 28.52079654241579`},
{"TBUR", 18.78601604914881`}, {"TBUR", 24.89976517570905`}, {"TBUR", 15.21678572837678`}, {"TBUR", 29.678812755534587`},
{"TBUR", 18.411189747761647`}, {"TBUR", 11.630526775456948`}, {"TBUR", 20.56736788402131`}, {"TBUR", 39.81896501701757`},
{"TBUR", 11.579647419833996`}, {"TBUR", 16.7073757138579`}, {"TBUR", 13.15017054642574`}, {"TBUR", 48.991993507602885`},
{"TBUR", 19.973188444818835`}, {"TBUR", 19.781162115684367`}, {"TBUR", 14.98836187254622`}, {"TBUR", 11.355024994053888`},
{"TBUR", 10.445561077742212`}, {"TBUR", 23.08652006894122`}, {"TBUR", 11.38031929474993`}, {"TBUR", 12.494991747371342`},
{"TBUR", 25.91202648565012`}, {"TBUR", 42.720904591500386`}, {"TBUR", 12.185093864964474`}, {"TBUR", 11.11000437764382`},
{"TBUR", 18.42743793854317`}, {"TBUR", 11.405104181149731`}, {"TBUR", 16.01094471858759`}, {"TBUR", 36.38234275331097`},
{"TBUR", 28.971244963128317`}, {"TBUR", 17.051858746531526`}, {"TBUR", 15.918258745132839`}, {"TBUR", 11.136970219111259`},

{"TBUR", 28.637097143580235`}, {"TBUR", 24.954008886644395`}, {"TBUR", 10.939225822736441`}, {"TBUR", 14.294574319421093`},
{"TBUR", 49.83267457923563`}, {"TBUR", 19.73508313170569`}, {"TBUR", 33.12362322236299`}, {"TBUR", 28.54909224679254`},
{"TBUR", 26.39334136273501`}, {"TBUR", 18.182282129491867`}, {"TBUR", 24.402886666984113`}, {"TBUR", 25.5169732034708 5`},
{"TBUR", 14.68063527980366`}, {"TBUR", 13.284960655014292`}, {"TBUR", 34.62031115029852`}, {"TBUR", 15.931985120694774`},
{"TBUR", 11.369743621488627`}, {"TBUR", 17.14773227042683`}, {"TBUR", 18.212303044244837`}, {"TBUR", 23.300311304242033`},
{"TBUR", 13.876621007775102`}, {"TBUR", 16.02479346669339`}, {"TBUR", 13.208860978559992`}, {"TBUR", 34.617244530505324`},
{"TBUR", 12.88540383655441`}, {"TBUR", 50.201497491149226`}, {"TBUR", 33.75407787514724`}, {"TBUR", 22.555506182171825`},
{"TBUR", 13.420983562939275`}, {"TBUR", 26.126524688871935`}, {"TBUR", 37.467551796005104`}, {"TBUR", 14.294999566793445`},
{"TBUR", 11.781318983556558`}, {"TBUR", 26.386348334785268`}, {"TBUR", 13.629363716693975`}, {"TBUR", 48.3975545130189`},
{"TBUR", 14.276052535452093`}, {"TBUR", 13.57077890673767`}, {"TBUR", 21.605038413907803`}, {"TBUR", 13.222805104676372`},
{"TBUR", 11.280495079659019`}, {"TBUR", 21.409445105028386`}, {"TBUR", 13.685628959811423`}, {"TBUR", 40.90500767021971`},
{"TBUR", 19.05174281927019`}, {"TBUR", 12.459674892628335`}, {"TBUR", 12.080813962339308`}, {"TBUR", 20.44079628896667`},
{"TBUR", 17.31405015943552`}, {"TBUR", 17.63436164079323`}, {"TBUR", 20.72566502090729`}, {"TBUR", 28.305130093017823`},
{"TBUR", 18.128421806240034`}, {"TBUR", 18.21522543397489`}, {"TBUR", 12.19511139100093`}, {"TBUR", 23.52658971955829`},
{"TBUR", 38.746507555243205`}, {"TBUR", 13.013804608663998`}, {"TBUR", 16.830134710533237`}, {"TBUR", 31.163559201344587`},
{"TBUR", 24.861844391920066`}, {"TBUR", 10.200283327118301`}, {"TBUR", 11.994034603135827`}, {"TBUR", 10.163897828210832`},
{"TBUR", 23.875444478765356`}, {"TBUR", 14.355986815184721`}, {"TBUR", 23.9542029287897`}, {"TBUR", 38.51300337985342`},
{"TBUR", 26.666697824394866`}, {"TBUR", 11.726711325822064`}, {"TBUR", 15.84989810942918`}, {"TBUR", 12.238343820235636`},
{"TBUR", 16.557839402597924`}, {"TBUR", 22.123244065017477`}, {"TBUR", 14.362211816349216`}, {"TBUR", 14.982335434433553`},
{"TBUR", 12.742611257109857`}, {"TBUR", 21.346404563089`}, {"TBUR", 39.538021744880815`}, {"TBUR", 28.099557097951184`},
{"TBUR", 12.185998695163024`}, {"TBUR", 15.877804698917146`}, {"TBUR", 12.472281605292798`}, {"TBUR", 26.31625892762327`},
{"TBUR", 22.76889517879618`}, {"TBUR", 16.950178546001784`}, {"TBUR", 13.096775023957145`}, {"TBUR", 19.82357938171404`},
{"TBUR", 53.585315525906424`}, {"TBUR", 12.20366748251806`}, {"TBUR", 12.26068564409993`}, {"TBUR", 18.64753065556537`},
{"TBUR", 13.09793505577459`}, {"TBUR", 31.349437648549987`}, {"TBUR", 40.126370801513914`}, {"TBUR", 38.96572613913505`},
{"TBUR", 38.85342205795237`}, {"TBUR", 19.48865417681404`}, {"TBUR", 14.59295555893629`}, {"TBUR", 21.912784658619394`},
{"TBUR", 15.068792260286681`}, {"TBUR", 19.421262057828905`}, {"TBUR", 14.791311150730058`}, {"TBUR", 11.644407162921675`},
{"TBUR", 39.26171802714754`}, {"TBUR", 28.7718155898307`}, {"TBUR", 11.245023407343798`}, {"TBUR", 10.57174774201496`},
{"TBUR", 18.634841364000923`}, {"TBUR", 18.89800331371781`}, {"TBUR", 15.372070023482934`}, {"TBUR", 13.289630292735579`},
{"TBUR", 18.119211100621463`}, {"TBUR", 18.19532424240082`}, {"TBUR", 13.003150893677239`}, {"TBUR", 17.7282178239352`},
{"TBUR", 12.518727832782371`}, {"TBUR", 10.96689413046786`}, {"TBUR", 20.526495282875`}, {"TBUR", 19.618576611833166`},
{"TBUR", 25.741531481345167`}, {"TBUR", 11.580634768845037`}, {"TBUR", 39.60440853116702`}, {"TBUR", 50.7525628981767`},

{"TBUR", 30.525499970009236`}, {"TBUR", 15.875869808240488`}, {"TBUR", 33.75767720491689`}, {"TBUR", 12.198036043438151`},
{"TBUR", 36.10746753826354`}, {"TBUR", 23.019434665476034`}, {"TBUR", 42.99742959154252`}, {"TBUR", 45.04808549688851`},
{"TBUR", 13.323085661147942`}, {"TBUR", 15.990018308084574`}, {"TBUR", 28.99263986073146`}, {"TBUR", 10.828591309594154`},
{"TBUR", 27.475565098910344`}, {"TBUR", 12.253512274090394`}, {"TBUR", 14.163757228575793`}, {"TBUR", 14.931779341911481`},
{"TBUR", 14.540706199919255`}, {"TBUR", 47.32170377355336`}, {"TBUR", 22.146989872057624`}, {"TBUR", 23.239188532259003`},
{"TBUR", 13.339585238476273`}, {"TBUR", 12.130906923683536`}, {"TBUR", 23.117528909403312`}, {"TBUR", 44.23773482755419`},
{"TBUR", 38.157567892445215`}, {"TBUR", 26.394381760015914`}, {"TBUR", 49.465206421225076`}, {"TBUR", 15.544368884888053`},
{"TBUR", 45.64964820274593`}, {"TBUR", 10.375598986949994`}, {"TBUR", 13.4577709014758`}, {"TBUR", 12.92740658866155`},
{"TBUR", 13.82555155001455`}, {"TBUR", 17.2548882383809`}, {"TBUR", 17.420284405698727`}, {"TBUR", 23.296344746475494`},
{"TBUR", 31.98430657291525`}, {"TBUR", 18.561175904260594`}, {"TBUR", 11.148323633274817`}, {"TBUR", 24.98779313594612`},
{"TBUR", 15.030367488401826`}, {"TBUR", 20.558700394866957`}, {"TBUR", 18.6028068766275`}, {"TBUR", 14.6230201465156`},
{"TBUR", 19.3016362362253`}, {"TBUR", 11.448179213739792`}, {"TBUR", 29.151867850742914`}, {"TBUR", 45.909960904742036`},
{"TBUR", 14.661498452826745`}, {"TBUR", 16.10400151271037`}, {"TBUR", 42.61403021443065`}, {"TBUR", 22.61333940022501`},
{"TBUR", 12.465990105470024`}, {"TBUR", 18.11202847916702`}, {"TBUR", 28.0774161110492`}, {"TBUR", 16.325684810661727`},
{"TBUR", 18.55982079277458`}, {"TBUR", 13.86257402896593`}, {"TBUR", 12.042055362310814`}, {"TBUR", 29.18597186973557`},
{"TBUR", 21.366117344635636`}, {"TBUR", 26.943445824101442`}, {"TBUR", 11.498290153855072`}, {"TBUR", 11.488376172892458`},
{"TBUR", 21.10426942757126`}, {"TBUR", 22.923874181921168`}, {"TBUR", 12.679033292772049`}, {"TBUR", 34.181493073312765`},
{"TBUR", 21.229170276246265`}, {"TBUR", 23.05327809438495`}, {"TBUR", 14.836687161538524`}, {"TBUR", 31.632092206844426`},
{"TBUR", 10.684528417301543`}, {"TBUR", 12.323554426038513`}, {"TBUR", 14.908989326903038`}, {"TBUR", 22.62247856502531`},
{"TBUR", 22.971085682806184`}, {"TBUR", 15.969422089771053`}, {"TBUR", 37.97006518058343`}, {"TBUR", 13.989474739922914`},
{"TBUR", 13.46400948773041`}, {"TBUR", 10.712035781062992`}, {"TBUR", 15.392271050722684`}, {"TBUR", 16.09268015882418`},
{"TBUR", 22.82787006896125`}, {"TBUR", 23.547927678469136`}, {"TBUR", 13.622706960699572`}, {"TBUR", 15.09676395522251`},
{"TBUR", 22.953986000725653`}, {"TBUR", 15.97721606819315`}, {"TBUR", 44.38959844892823`}, {"TBUR", 20.945249163902453`},
{"TBUR", 19.466033967193685`}, {"TBUR", 11.158472319678523`}, {"TBUR", 40.220670046310836`}, {"TBUR", 12.793530253211959`},
{"TBUR", 24.880734816219235`}, {"TBUR", 17.43604164254628`}, {"TBUR", 21.696656293887454`}, {"TBUR", 14.044238126710205`},
{"TBUR", 13.059506531769134`}, {"TBUR", 28.526967482011788`}, {"TBUR", 26.633481667404485`}, {"TBUR", 23.20253311428919`},
{"TBUR", 10.754730378470608`}, {"TBUR", 14.975999203711073`}, {"TBUR", 14.35758409286505`}, {"TBUR", 25.748160126748434`},
{"TBUR", 24.625971986527414`}, {"TBUR", 18.134246823300774`}, {"TBUR", 12.029250528991964`}, {"TBUR", 12.283088733640653`},
{"TBUR", 10.904174263410239`}, {"TBUR", 14.48499028734746`}, {"TBUR", 13.356622879290526`}, {"TBUR", 13.08768298202193`},
{"TBUR", 12.90992184913878`}, {"TBUR", 13.269606005256925`}, {"TBUR", 12.05460333402895`}, {"TBUR", 40.70482577774895`},
{"TBUR", 17.844085961158733`}, {"TBUR", 22.11428074306187`}, {"TBUR", 37.439576225558724`}, {"TBUR", 16.184705880426666`},

{"TBUR", 25.179894928284636`}, {"TBUR", 17.177849782689034`}, {"TBUR", 24.139857635768177`}, {"TBUR", 15.269969164410572`},
{"TBUR", 24.084328130080067`}, {"TBUR", 25.156185065552094`}, {"TBUR", 25.081587472097127`}, {"TBUR", 24.596794037781738`},
{"TBUR", 16.297538420609563`}, {"TBUR", 13.92054315571153`}, {"TBUR", 24.186531212314744`}, {"TBUR", 19.15308878818269`},
{"TBUR", 28.45358129148927`}, {"TBUR", 12.218494240750383`}, {"TBUR", 10.773398710428566`}, {"TBUR", 15.414206830182799`},
{"TBUR", 23.086256460655893`}, {"TBUR", 14.401915522233667`}, {"TBUR", 12.753435398541292`}, {"TBUR", 20.705830590117454`},
{"TBUR", 18.103071927473387`}, {"TBUR", 17.811389048194037`}, {"TBUR", 11.906952088761239`}, {"TBUR", 25.925332734218312`},
{"TBUR", 11.582030311234943`}, {"TBUR", 19.74448506851293`}, {"TBUR", 28.18445197624289`}, {"TBUR", 15.223966526805105`},
{"TBUR", 31.432468538394286`}, {"TBUR", 27.19309801194287`}, {"TBUR", 14.008140819298191`}, {"TBUR", 12.045856459211306`},
{"TBUR", 22.105046931525724`}, {"TBUR", 29.847955271378463`}, {"TBUR", 11.48809023519896`}, {"TBUR", 41.93802236462971`},
{"TBUR", 11.44197833872413`}, {"TBUR", 44.78670292317672`}, {"TBUR", 11.0867350462611`}, {"TBUR", 15.362744626619742`},
{"TBUR", 18.57110216060741`}, {"TBUR", 16.691242237560672`}, {"TBUR", 13.903982971980977`}, {"TBUR", 19.037062837946692`},
{"TBUR", 12.92362732078675`}, {"TBUR", 13.237091463120482`}, {"TBUR", 39.03584032971677`}, {"TBUR", 13.232019942363335`},
{"TBUR", 13.240144328016669`}, {"TBUR", 30.388421395742554`}, {"TBUR", 11.423499346803078`}, {"TBUR", 16.667402897010415`},
{"TBUR", 21.761982158062477`}, {"TBUR", 21.382614476203088`}, {"TBUR", 23.96488850963543`}, {"TBUR", 44.303028035478064`},
{"TBUR", 15.078377322238609`}, {"TBUR", 52.81421814180943`}, {"TBUR", 20.593736250381497`}, {"TBUR", 40.543019978884935`},
{"TBUR", 21.363444597177413`}, {"TBUR", 11.491958120679236`}, {"TBUR", 23.805942604091097`}, {"TBUR", 17.457403469003896`},
{"TBUR", 12.319726553927222`}, {"TBUR", 12.90520056034087`}, {"TBUR", 36.316604818573694`}, {"TBUR", 12.526229763228413`},
{"TBUR", 22.332214087824713`}, {"TBUR", 17.2244921848201`}, {"TBUR", 49.669497007104646`}, {"TBUR", 37.079745045406426`},
{"TBUR", 15.931126455903573`}, {"TBUR", 33.584913292338776`}, {"TBUR", 11.835162353735948`}, {"TBUR", 16.741086716203625`},
{"TBUR", 22.093063393428466`}, {"TBUR", 25.36518273199658`}, {"TBUR", 31.678827935351443`}, {"TBUR", 16.62069037647161`},
{"TBUR", 11.392861767709837`}, {"TBUR", 11.934464273787754`}, {"TBUR", 17.340349154883945`}, {"TBUR", 20.889722876209845`},
{"TBUR", 18.335068321726613`}, {"TBUR", 30.830295475520987`}, {"TBUR", 10.807642353824672`}, {"TBUR", 11.216908859826905`},
{"TBUR", 40.04439458958592`}, {"TBUR", 36.83881667098762`}, {"TBUR", 23.92194362638417`}, {"TBUR", 16.53534180686229`},
{"TBUR", 51.23050055254762`}, {"TBUR", 18.023455873735564`}, {"TBUR", 18.648199093095407`}, {"TBUR", 18.763547866061003`},
{"TBUR", 11.419212755826193`}, {"TBUR", 24.008534349430825`}, {"TBUR", 34.84296157475952`}, {"TBUR", 10.921490347290051`},
{"TBUR", 28.062133886083`}, {"TBUR", 12.427641403611485`}, {"TBUR", 15.92887830547401`}, {"TBUR", 20.94447979878765`},
{"TBUR", 15.337560941696069`}, {"TBUR", 21.19237478142351`}, {"TBUR", 13.663656699879482`}, {"TBUR", 10.91918368235662`},
{"TBUR", 28.48038009165704`}, {"TBUR", 14.72653000188838`}, {"TBUR", 43.78294665591238`}, {"TBUR", 13.700386640340682`},
{"TBUR", 14.548044383987587`}, {"TBUR", 13.665883046173738`}, {"TBUR", 16.05820386245968`}, {"TBUR", 12.904844890817078`},
{"TBUR", 12.917986940552879`}, {"TBUR", 16.379817998638547`}, {"TBUR", 32.81829785954663`}, {"TBUR", 15.65577822695791`},
{"TBUR", 23.960754924812576`}, {"TBUR", 46.42020173562314`}, {"TBUR", 14.781102935419684`}, {"TBUR", 13.436700928696643`},

{"TBUR", 11.989824230197081`}, {"TBUR", 18.904151712050382`}, {"TBUR", 24.081388036131735`}, {"TBUR", 12.335774564394683`},
{"TBUR", 11.732129723869265`}, {"TBUR", 10.456303438241472`}, {"TBUR", 12.560794668456198`}, {"TBUR", 12.421668111923303`},
{"TBUR", 15.519712741204295`}, {"TBUR", 11.484802585076057`}, {"TBUR", 17.607325878236882`}, {"TBUR", 29.8564370010684З`},
{"TBUR", 32.13525239881099`}, {"TBUR", 16.78803495216286`}, {"TBUR", 11.648964281069263`}, {"TBUR", 24.182504340114686`},
{"TBUR", 19.42397801276626`}, {"TBUR", 44.17728583357691`}, {"TBUR", 14.66762514297404`}, {"TBUR", 15.585713064661457`},
{"TBUR", 14.22653096705157`}, {"TBUR", 15.555088977904298`}, {"TBUR", 12.514337331506693`}, {"TBUR", 26.78755123112613`},
{"TBUR", 26.254677688178298`}, {"TBUR", 38.34160621879318`}, {"TBUR", 20.956143477051647`}, {"TBUR", 12.384226094606502`},
{"TBUR", 11.342365937291804`}, {"TBUR", 21.567957025331644`}, {"TBUR", 15.428572810460327`}, {"TBUR", 17.692295535412782`},
{"TBUR", 18.374363606827465`}, {"TBUR", 11.177439745546469`}, {"TBUR", 23.151274579710364`}, {"TBUR", 25.561442209495276`},
{"TBUR", 15.191722397878516`}, {"TBUR", 15.317738134370043`}, {"TBUR", 15.754324049386259`}, {"TBUR", 13.444599229541677`},
{"TBUR", 46.975528713564856`}, {"TBUR", 15.582929873652624`}, {"TBUR", 11.172559186556574`}, {"TBUR", 21.53510755015808`},
{"TBUR", 38.1922652097825`}, {"TBUR", 11.615611300801511`}, {"TBUR", 30.52780202756511`}, {"TBUR", 17.419201062358916`},
{"TBUR", 19.891732201574825`}, {"TBUR", 42.363468891181704`}, {"TBUR", 19.268136573185007`}, {"TBUR", 43.61044372946785`},
{"TBUR", 19.678323929404883`}, {"TBUR", 12.83040029879762`}, {"TBUR", 30.230973806479213`}, {"TBUR", 14.157926609120352`},
{"TBUR", 10.191843501040474`}, {"TBUR", 37.83947540069915`}, {"TBUR", 17.072209389919802`}, {"TBUR", 16.46647436967203`},
{"TBUR", 25.520441400196084`}, {"TBUR", 15.142499976685482`}, {"TBUR", 12.153963131932542`}, {"TBUR", 12.254341695995448`},
{"TBUR", 15.99553699974471`}, {"TBUR", 14.009415411087728`}, {"TBUR", 20.72471693373452`}, {"TBUR", 25.379837072629677`},
{"TBUR", 41.34185799496326`}, {"TBUR", 28.586227977857817`}, {"TBUR", 16.516493534325388`}, {"TBUR", 12.454385454931874`},
{"TBUR", 12.326051525095739`}, {"TBUR", 16.451639019541044`}, {"TBUR", 49.40275306734192`}, {"TBUR", 26.97402791257719`},
{"TBUR", 21.04300518348442`}, {"TBUR", 11.138973134291232`}, {"TBUR", 29.170619014428308`}, {"TBUR", 34.63205089264964`},
{"TBUR", 15.194061817314392`}, {"TBUR", 27.389126428015043`}, {"TBUR", 13.167291347644422`}, {"TBUR", 12.55776636072074`},
{"TBUR", 12.140654160542763`}, {"TBUR", 28.850919257426384`}, {"TBUR", 32.311078542727124`}, {"TBUR", 47.522598894000616`},
{"TBUR", 28.369382096388907`}, {"TBUR", 37.38752450894326`}, {"TBUR", 18.6885524515911`}, {"TBUR", 23.664121316574818`},
{"TBUR", 41.330708798869644`}, {"TBUR", 14.048773944038226`}, {"TBUR", 42.861962534556994`}, {"TBUR", 14.327382038298191`},
{"TBUR", 10.622241238403388`}, {"TBUR", 28.74130609102452`}, {"TBUR", 20.452403102098977`}, {"TBUR", 17.429187525332168`},
{"TBUR", 25.7695058559047`}, {"TBUR", 21.35673335905624`}, {"TBUR", 13.316459464757374`}, {"TBUR", 25.456022184650543`},
{"TBUR", 15.26535501368181`}, {"TBUR", 31.442656021163415`}, {"TBUR", 22.144344460330387`}, {"TBUR", 19.756788197085175`},
{"TBUR", 11.750896770936777`}, {"TBUR", 11.91379735527266`}, {"TBUR", 13.082497845804456`}, {"TBUR", 22.58037325536216`},
{"TBUR", 27.11994888874441`}, {"TBUR", 33.94540348960336`}, {"TBUR", 13.55347506137448`}, {"TBUR", 42.039848568278266`},
{"TBUR", 18.840729303537266`}, {"TBUR", 32.18988964188309`}, {"TBUR", 13.869843964601282`}, {"TBUR", 27.880811077585708`},
{"TBUR", 26.732109568747337`}, {"TBUR", 26.429449941613587`}, {"TBUR", 12.686313766165187`}, {"TBUR", 28.619171533903188`},

{"TBUR", 35.136359473108946`}, {"TBUR", 15.890145348087076`}, {"TBUR", 15.540586680598453`}, {"TBUR", 21.62528445822497`},
{"TBUR", 16.655395917309367`}, {"TBUR", 14.556582063467172`}, {"TBUR", 24.59499458597921`}, {"TBUR", 38.31687592592987`},
{"TBUR", 20.91670961312049`}, {"TBUR", 30.880052623523927`}, {"TBUR", 23.37839937953543`}, {"TBUR", 14.756878385331708`},
{"TBUR", 12.306374151885091`}, {"TBUR", 12.19396976451413`}, {"TBUR", 10.820270170682432`}, {"TBUR", 38.86707580054765`},
{"TBUR", 26.275116375269466`}, {"TBUR", 19.515294983292456`}, {"TBUR", 20.76272418963084`}, {"TBUR", 25.06570624066885`},
{"TBUR", 48.28299572199504`}, {"TBUR", 47.15248698386937`}, {"TBUR", 27.965718787972648`}, {"TBUR", 18.136333387094034`},
{"TBUR", 17.30853490271965`}, {"TBUR", 14.720055891444831`}, {"TBUR", 14.575953384252578`}, {"TBUR", 16.872520172913198`},
{"TBUR", 32.56100616317757`}, {"TBUR", 18.54602887377109`}, {"TBUR", 30.28561416396665 3`}, {"TBUR", 24.769061979356383`},
{"TBUR", 12.804699929538211`}, {"TBUR", 46.8230835758209`}, {"TBUR", 21.21413815436887`}, {"TBUR", 12.319668225861111`},
{"TBUR", 17.08900165913073`}, {"TBUR", 33.39280618827887`}, {"TBUR", 30.784043360357213`}, {"TBUR", 41.80596014055507`},
{"TBUR", 18.210512416441464`}, {"TBUR", 32.0573658992787`}, {"TBUR", 33.9806026181684`}, {"TBUR", 27.568655924125853`},
{"TBUR", 12.1584928408606`}, {"TBUR", 31.993171768030024`}, {"TBUR", 25.227137856905184`}, {"TBUR", 18.96277268186363`},
{"TBUR", 12.635310561055041`}, {"TBUR", 34.75709683033686`}, {"TBUR", 15.676198179360494`}, {"TBUR", 14.351763370381466`},
{"TBUR", 12.394494955044776`}, {"TBUR", 38.34749460297427`}, {"TBUR", 21.709627816230363`}, {"TBUR", 21.404025449569534`},
{"TBUR", 13.773952319506515`}, {"TBUR", 15.325842453057636`}, {"TBUR", 16.13598200107213`}, {"TBUR", 10.565295180343039`},
{"TBUR", 38.493707225491875`}, {"TBUR", 31.591500399349755`}, {"TBUR", 12.233042653846113`},
{"TBUR", 13.441278132842836`}, {"TBUR", 10.71119305839793`}, {"TBUR", 12.549194360866233`}, {"TBUR", 14.430143043814553`},
{"TBUR", 23.638167870560885`}, {"TBUR", 46.60146622174525`}, {"TBUR", 28.674412294553544`}, {"TBUR", 32.37137987887265`},
{"TBUR", 26.9737868248472`}, {"TBUR", 28.656206467813305`}, {"TBUR", 27.243650264504783`}, {"TBUR", 13.96082705027568`},
{"TBUR", 29.94311776735254`}, {"TBUR", 19.52106732513822`}, {"TBUR", 17.775000251237888`}, {"TBUR", 17.933766746197712`},
{"TBUR", 22.399050329429524`}, {"TBUR", 14.458060263011596`}, {"TBUR", 24.95623448114484`}, {"TBUR", 16.347494456653916`},
{"TBUR", 24.618543776758553`}, {"TBUR", 16.9150565948905`}, {"TBUR", 21.952694405429753`}, {"TBUR", 27.898193165891037`},
{"TBUR", 11.274944049057206`}, {"TBUR", 18.540459835720974`}, {"TBUR", 15.07535764694947`}, {"TBUR", 15.740946903924218`},
{"TBUR", 24.261138236301495`}, {"TBUR", 15.977669186060735`}, {"TBUR", 14.732615628830892`}, {"TBUR", 14.0368739479989`},
{"TBUR", 22.74708834092515`}, {"TBUR", 29.698528331382423`}, {"TBUR", 11.019280529800811`}, {"TBUR", 14.368487002503988`},
{"TBUR", 31.141875705872618`}, {"TBUR", 14.833833148835154`}, {"TBUR", 11.739499287603133`}, {"TBUR", 52.5193285933069`},
{"TBUR", 16.985298517961542`}, {"TBUR", 10.499992605933624`}, {"TBUR", 16.751986127639093`}, {"TBUR", 14.26328092366482`},
{"TBUR", 30.43788025347739`}, {"TBUR", 11.90445215079268`}, {"TBUR", 14.264395316837868`}, {"TBUR", 14.452845699757372`},
{"TBUR", 34.572127537796206`}, {"TBUR", 26.11161702071263`}, {"TBUR", 20.43581000209806`}, {"TBUR", 14.162911780156453`},
{"TBUR", 17.87169579027483`}, {"TBUR", 12.99831618789261`}, {"TBUR", 17.775464460897613`}, {"TBUR", 32.559876696628045`},
{"TBUR", 25.609975482620715`}, {"TBUR", 20.570346406615123`}, {"TBUR", 47.48624482192006`}, {"TBUR", 12.586809730426838`},

```
{"TBUR", 12.589399932667439`}, {"TBUR", 30.01764746535113`}, {"TBUR", 11.972539963913935`}, {"TBUR", 31.855777981949387`},
{"TBUR", 17.019267590986306`}, {"TBUR", 23.790943672497843`}, {"TBUR", 15.487454208123516`},
{"TBUR", 10.525218926133745`}, {"TBUR", 47.49721606768071`}, {"TBUR", 10.877223296166196`}, {"TBUR", 24.758919081039558`},
{"TBUR", 18.98089486237934`}, {"TBUR", 38.9755916501208`}, {"TBUR", 15.844889206178705`}, {"TBUR", 15.828065961395247`},
{"TBUR", 16.043507902292482`}, {"TBUR", 16.29507796729022`}, {"TBUR", 11.747370042394191`}, {"TBUR", 11.342565597386283`},
{"TBUR", 20.65755986837501`}, {"TBUR", 38.141294834970815`}, {"TBUR", 13.166289584683993`}, {"TBUR", 20.736558416184195`},
{"TBUR", 31.76020149311386`}, {"TBUR", 12.048290529008609`}, {"TBUR", 13.935089448273782`}, {"TBUR", 28.193335218325988`},
{"TBUR", 31.76201967547971`}, {"TBUR", 15.27922375145718`}, {"TBUR", 11.931712667218223`}, {"TBUR", 10.547333726231798`},
{"TBUR", 16.347304675056495`}, {"TBUR", 12.318275290985008`}, {"TBUR", 38.91076514574312`}, {"TBUR", 11.683568662416686`},
{"TBUR", 24.00910384152908`}, {"TBUR", 19.286777915296657`}, {"TBUR", 27.276107180948763`}, {"TBUR", 13.486529801608391`},
{"TBUR", 12.605589745478484`}, {"TBUR", 40.909492536943254`}, {"TBUR", 37.17219671673012`}, {"TBUR", 17.6616094107079`},
{"TBUR", 16.586022700802683`}, {"TBUR", 16.45014093748146`}, {"TBUR", 13.92619220408081`}, {"TBUR", 18.8282812976119`},
{"TBUR", 36.22232583851177`}, {"TBUR", 11.27934026300811`}, {"TBUR", 21.639726206576118`}, {"TBUR", 16.459665972699216`},
{"TBUR", 15.511101673101416`}, {"TBUR", 22.506440992370166`}, {"TBUR", 11.076972857018646`}, {"TBUR", 15.242188743714676`},
{"TBUR", 11.462647176542138`}, {"TBUR", 18.702255009934902`}, {"TBUR", 10.482359657660846`}, {"TBUR", 19.51611566728699`},
{"TBUR", 19.84863193299701`}, {"TBUR", 10.811595168120101`}, {"TBUR", 12.468425027733264`}, {"TBUR", 17.65849908369357`},
{"TBUR", 12.366872881786685`}, {"TBUR", 15.057501302422027`}, {"TBUR", 10.97280472054204`}, {"TBUR", 12.995726450926323`},
{"TBUR", 41.0030139043523`}, {"TBUR", 27.476983556131657`}, {"TBUR", 13.003238300319067`}, {"TBUR", 34.76545358004131`},
{"TBUR", 16.93961788742202`}, {"TBUR", 15.01175401839218`}, {"TBUR", 15.586141564209756`}, {"TBUR", 27.70475129393068`},
{"TBUR", 19.2625809574968`}, {"TBUR", 15.907041087628507`}, {"TBUR", 19.36074195370645`}, {"TBUR", 30.469706303174217`},
{"TBUR", 11.901373794002273`}, {"TBUR", 26.278599600233694`}, {"TBUR", 20.003563989282735`}, {"TBUR", 37.75677788969558`},
{"TBUR", 33.957074484169915`}, {"TBUR", 15.236632905420791`}, {"TBUR", 19.838808863077166`}, {"TBUR", 46.4717876345526`},
{"TBUR", 22.744792861590497`}, {"TBUR", 11.472593768678601`}, {"TBUR", 14.418835243486974`}, {"TBUR", 34.13267009209965`},
{"TBUR", 11.347103240094883`}, {"TBUR", 11.332749256343055`}, {"TBUR", 10.61275667865575`}, {"TBUR", 25.317973500286957`},
{"TBUR", 18.824909678232107`}, {"TBUR", 19.75988885226173`}, {"TBUR", 21.694552645662377`}, {"TBUR", 11.68190930846636`},
{"TBUR", 10.8215974542`}, {"TBUR", 39.549387730743824`}, {"TBUR", 17.80560798712406`}, {"TBUR", 23.303984805326564`},
{"TBUR", 19.35719532039265`}, {"TBUR", 11.367022753870474`}, {"TBUR", 12.624461342996618`}, {"TBUR", 15.55025792512026`},
{"TBUR", 18.641589075281406`}, {"TBUR", 11.85870829496253`}, {"TBUR", 10.705231972566915`}, {"TBUR", 14.031239476593514`},
{"TBUR", 11.227113948157005`}, {"TBUR", 11.781352793042377`}, {"TBUR", 31.888118474974192`}, {"TBUR", 44.4704201674232`},
{"TBUR", 19.733705914844947`}, {"TBUR", 38.82659412985412`}, {"TBUR", 36.22155621382699`}, {"TBUR", 14.107232897488702`}};
DosesToCheck = Table[1500 + 100 * i, {i, 0, 15}];
```

```
TestConstantDose = {{{"1500 Res", "1500 OS", "1500 Tox"}, {"CURE", 365, 12.447507768742463`},
    {"CURE", 365, 25.854026107894878`}, {"CURE", 365, 18.088142048494284`}, {"TBUR", 53.9538853141049`, 6.0048629909683`},
    {"TBUR", 35.82732409678449`, 21.904932068559127`}, {"CURE", 365, 46.82786006345507`},
    {"CURE", 365, 44.0648764313932`}, {"CURE", 365, 4.602960786893216`}, {"CURE", 365, 31.77843922036445`},
    {"CURE", 365, 27.47074572663702`}, {"CURE", 365, 103.11039806933469`}, {"CURE", 365, 12.799331006839155`},
    {"CURE", 365, 45.053200319890216`}, {"CURE", 365, 152.71211641041745`}, {"CURE", 365, 21.726208751813243`},
    {"CURE", 365, 33.903223675631835`}, {"CURE", 365, 67.25727320502305`}, {"CURE", 365, 30.09188049451465`},
    {"TBUR", 97.238063465876`, 22.640897468481914`}, {"CURE", 365, 7.550082080128763`}, {"CURE", 365, 46.67905006979911`},
    {"CURE", 365, 18.923180805433443`}, {"CURE", 365, 7.2483963207562185`}, {"CURE", 365, 5.775155428726488`},
    {"CURE", 365, 26.290553452002303`}, {"CURE", 365, 28.853082965246188`}, {"CURE", 365, 2.6229509470431167`},
    {"CURE", 365, 3.97528112574067`}, {"CURE", 365, 14.727513860978805`}, {"CURE", 365, 17.221655337513067`},
    {"CURE", 365, 14.70315459094553`}, {"CURE", 365, 51.498427095574826`}, {"CURE", 365, 11.362774334097754`},
    {"TBUR", 101.50930531793446`, 9.018223464163773`}, {"CURE", 365, 73.26220338274044`}, {"CURE", 365, 4.485361537798568`},
    {"CURE", 365, 19.408559490917593`}, {"TBUR", 36.56585374234291`, 42.96416686377358`}, {"CURE", 365, 10.767666145134855`},
    {"CURE", 365, 16.567047678079625`}, {"TBUR", 93.85174800613959`, 37.70510487225733`}, {"CURE", 365, 25.553531394378137`},
    {"CURE", 365, 5.183082219237905`}, {"CURE", 365, 17.47375374290452`}, {"CURE", 365, 25.2236521708785`},
    {"CURE", 365, 16.543084161079573`}, {"CURE", 365, 175.7367739790885`}, {"CURE", 365, 37.87782728975529`},
    {"CURE", 365, 56.78145831671306`}, {"CURE", 365, 7.08116821876973`}, {"CURE", 365, 19.385475977335847`},
    {"CURE", 365, 26.866561307674214`}, {"CURE", 365, 21.78088714525279`}, {"CURE", 365, 14.143914995844444`},
    {"CURE", 365, 6.0951351501520845`}, {"CURE", 365, 64.50946558294719`}, {"CURE", 365, 20.273336446254444`},
    {"CURE", 365, 3.842431220450029`}, {"CURE", 365, 41.44005141385796`}, {"CURE", 365, 28.99923931359367`},
    {"CURE", 365, 19.883602161282546`}, {"CURE", 365, 15.229943290233095`}, {"CURE", 365, 8.524119331687368`},
    {"CURE", 365, 32.82742837956947`}, {"CURE", 365, 23.2917644913383`}, {"CURE", 365, 26.66884879082122`},
    {"CURE", 365, 6.104195065469968`}, {"CURE", 365, 6.191658395395282`}, {"CURE", 365, 6.599782825435534`},
    {"CURE", 365, 26.94767748144779`}, {"CURE", 365, 9.864113725866618`}, {"CURE", 365, 21.965466408142845`},
    {"CURE", 365, 41.23605729905727`}, {"TBUR", 20.53590633360536`, 11.682635023404101`}, {"CURE", 365, 11.01159456562708`},
    {"CURE", 365, 30.531112866963593`}, {"CURE", 365, 17.84127744570012`}, {"CURE", 365, 13.579717114192347`},
    {"CURE", 365, 54.06695199309688`}, {"CURE", 365, 59.40417245501526`}, {"CURE", 365, 43.731568069504114`},
    {"CURE", 365, 11.802554831696353`}, {"CURE", 365, 23.86452726684249`}, {"CURE", 365, 16.68017886131852`},
    {"CURE", 365, 18.858157584180308`}, {"CURE", 365, 8.83347766247543`}, {"CURE", 365, 94.21364470640394`},
    {"CURE", 365, 20.53006501116571`}, {"CURE", 365, 25.87796329351859`}, {"CURE", 365, 22.248055531581596`},
```

{"TBUR", 122.3749584503879`, 18.276956505670906`}, {"CURE", 365, 5.453252989257252`}, {"CURE", 365, 9.997104075396974`},
{"CURE", 365, 23.139271191574206`}, {"TBUR", 41.874805947924095`, 8.236136366591914`}, {"CURE", 365, 9.958293470008336`},
{"CURE", 365, 26.002854975181286`}, {"CURE", 365, 65.99473523194831`}, {"CURE", 365, 35.312782593451466`},
{"CURE", 365, 17.71141868091165`}, {"TBUR", 53.00010640778016`, 35.00180403610315`}, {"CURE", 365, 22.839145475526276`},
{"CURE", 365, 14.810300677192012`}, {"CURE", 365, 101.41855464762617`}, {"CURE", 365, 11.269165690158754`},
{"CURE", 365, 7.656488697721052`}, {"CURE", 365, 44.90962718391093`}, {"CURE", 365, 13.209656983294682`},
{"CURE", 365, 13.55950135805733`}, {"CURE", 365, 81.58094182073366`}, {"CURE", 365, 6.621091720133708`},
{"CURE", 365, 4.199610845301095`}, {"CURE", 365, 17.470534553543178`}, {"CURE", 365, 13.809950848811269`},
{"CURE", 365, 10.4016818565877`}, {"CURE", 365, 26.561649429544563`}, {"CURE", 365, 7.47212396133615`},
{"CURE", 365, 4.595028351011081`}, {"CURE", 365, 21.517534265103496`}, {"CURE", 365, 32.90114052196557`},
{"CURE", 365, 5.599183644710364`}, {"CURE", 365, 18.540571520461405`}, {"CURE", 365, 56.296367132341686`},
{"TBUR", 47.807125686144914`, 21.34844837223633`}, {"CURE", 365, 12.487511501309704`}, {"CURE", 365, 43.519782928978934`},
{"CURE", 365, 21.869361385648517`}, {"CURE", 365, 2.884122931293628`}, {"CURE", 365, 25.000994760368815`},
{"CURE", 365, 19.309566075240255`}, {"CURE", 365, 15.38263090196216`}, {"CURE", 365, 18.241983191838845`},
{"CURE", 365, 4.7426163028423565`}, {"CURE", 365, 27.052642913880504`}, {"CURE", 365, 9.719840034498736`},
{"CURE", 365, 21.875420038354463`}, {"CURE", 365, 82.02499654580755`}, {"CURE", 365, 28.93173305697495`},
{"CURE", 365, 67.36977007405095`}, {"CURE", 365, 22.20184987859654`}, {"CURE", 365, 91.5362335395182`},
{"CURE", 365, 2.8604919187562827`}, {"CURE", 365, 25.139420881760884`}, {"CURE", 365, 28.65138515960526`},
{"CURE", 365, 3.72180547607031`}, {"CURE", 365, 5.154152255708788`}, {"CURE", 365, 31.64493081081346`},
{"TBUR", 98.38410024202898`, 37.206221973056564`}, {"CURE", 365, 31.78336894156921`}, {"CURE", 365, 25.99732430706206`},
{"CURE", 365, 24.863276262106602`}, {"CURE", 365, 15.047582864440361`}, {"CURE", 365, 3.9815320178070013`},
{"CURE", 365, 17.293152274839553`}, {"CURE", 365, 8.20628268579537`}, {"CURE", 365, 4.01704762256232`},
{"CURE", 365, 24.367943054984455`}, {"CURE", 365, 13.643087411911596`}, {"CURE", 365, 36.064796664253926`},
{"CURE", 365, 14.55620960549458`}, {"CURE", 365, 12.116743044323345`}, {"CURE", 365, 81.08506271262188`},
{"CURE", 365, 6.05372253341603`}, {"CURE", 365, 18.082707491605728`}, {"CURE", 365, 10.25547555465029`},
{"CURE", 365, 24.199281885849995`}, {"CURE", 365, 6.661567844976349`}, {"CURE", 365, 26.605735517600092`},
{"CURE", 365, 70.21110897065141`}, {"CURE", 365, 6.007928957537708`}, {"CURE", 365, 18.26663443819505`},
{"CURE", 365, 19.66303887302929`}, {"CURE", 365, 71.0508159999982`}, {"TBUR", 76.5709252682164`, 26.549537994533782`},
{"CURE", 365, 22.138985868956038`}, {"CURE", 365, 38.6923066998422`}, {"CURE", 365, 14.927517333387186`},
{"CURE", 365, 44.717735228091605`}, {"CURE", 365, 28.638557350660932`}, {"CURE", 365, 17.29065428342038`},
{"CURE", 365, 15.801239105236524`}, {"CURE", 365, 5.15545922571145`}, {"CURE", 365, 3.494206263284436`},

{"CURE", 365, 8.17224199100069`}, {"CURE", 365, 10.971816056663538`}, {"CURE", 365, 4.813969976382869`},
{"CURE", 365, 17.660674492408344`}, {"CURE", 365, 48.28170732802093`}, {"CURE", 365, 13.064472572266833`},
{"CURE", 365, 24.14523198411187`}, {"CURE", 365, 30.36710317287466`}, {"CURE", 365, 25.0533024856518 4`},
{"CURE", 365, 17.773224438239158`}, {"CURE", 365, 7.058503554196753`}, {"CURE", 365, 48.601346069112196`},
{"CURE", 365, 4.208077004063603`}, {"CURE", 365, 45.32615156791934`}, {"TBUR", 74.37155570456714`, 43.721910219014426`},
{"CURE", 365, 12.295125094509396`}, {"CURE", 365, 14.604867329870254`}, {"CURE", 365, 83.78155053614294`},
{"CURE", 365, 21.552398307181452`}, {"CURE", 365, 25.839963906220976`}, {"CURE", 365, 16.16169298374797`},
{"CURE", 365, 6.082664523817039`}, {"CURE", 365, 37.2170957797823`}, {"TBUR", 42.85790835402266`, 98.74432532841332`},
{"CURE", 365, 15.153268961716433`}, {"CURE", 365, 13.68935129457767`}, {"CURE", 365, 25.23604952671657`},
{"CURE", 365, 10.076566798216001`}, {"CURE", 365, 127.84255705080022`}, {"CURE", 365, 10.233956904348943`},
{"CURE", 365, 63.131293910548365`}, {"CURE", 365, 48.60662491432304`}, {"CURE", 365, 47.21579160999891`},
{"CURE", 365, 25.23774661917052`}, {"CURE", 365, 58.564296665123884`}, {"CURE", 365, 96.3271260936395`},
{"CURE", 365, 26.60760705406512`}, {"CURE", 365, 4.88619139123528`}, {"CURE", 365, 43.412802434324`},
{"CURE", 365, 27.05433798108127`}, {"CURE", 365, 26.074710539596342`}, {"CURE", 365, 5.737202779931013`},
{"CURE", 365, 131.7515627187698`}, {"CURE", 365, 9.631250364839921`}, {"CURE", 365, 41.79201762302986`},
{"CURE", 365, 26.3279934745018`}, {"CURE", 365, 18.71973431001584`}, {"CURE", 365, 4.501347503407702`},
{"CURE", 365, 33.841540516911664`}, {"CURE", 365, 19.84862298558383`}, {"CURE", 365, 52.51575731454615`},
{"CURE", 365, 34.59327557421951`}, {"CURE", 365, 43.65114542932369`}, {"CURE", 365, 20.93765569756334`},
{"CURE", 365, 18.627753713773085`}, {"TBUR", 47.05340285800928`, 97.30381808823941`}, {"CURE", 365, 25.53995241867607`},
{"CURE", 365, 6.81121210160184`}, {"CURE", 365, 22.94575765737099`}, {"TBUR", 92.07851679174955`, 23.109089151511686`},
{"CURE", 365, 3.115457429662972`}, {"CURE", 365, 5.5771785656779445`}, {"CURE", 365, 8.516381519583716`},
{"CURE", 365, 80.24873245937033`}, {"CURE", 365, 24.73275683395176`}, {"CURE", 365, 25.785357818845167`},
{"CURE", 365, 20.64722358269528`}, {"CURE", 365, 84.58741034702474`}, {"CURE", 365, 19.552980417504983`},
{"CURE", 365, 39.154204285977144`}, {"CURE", 365, 19.48980059665002`}, {"CURE", 365, 28.740196664128245`},
{"CURE", 365, 6.912436476069493`}, {"CURE", 365, 25.8493899474671`}, {"CURE", 365, 16.12364407416985`},
{"CURE", 365, 31.426199055040573`}, {"CURE", 365, 49.63390515966109`}, {"CURE", 365, 55.447478064166845`},
{"CURE", 365, 33.30774139850261`}, {"CURE", 365, 27.69138068037592`}, {"CURE", 365, 37.66443532246752`},
{"CURE", 365, 18.12788917009141`}, {"CURE", 365, 20.52588533844221`}, {"CURE", 365, 14.523320007973629`},
{"CURE", 365, 11.395325452606759`}, {"CURE", 365, 18.35137594145748`}, {"CURE", 365, 47.108231678988005`},
{"CURE", 365, 5.398675209047403`}, {"CURE", 365, 23.895534893141143`}, {"CURE", 365, 4.517573730492292`},
{"CURE", 365, 23.499622006150673`}, {"CURE", 365, 13.325170630376613`}, {"CURE", 365, 49.72672758111728`},

{"CURE", 365, 4.583032354867925`}, {"CURE", 365, 32.67276160930488`}, {"CURE", 365, 41.03655712749278`},
{"CURE", 365, 6.713715014727638`}, {"CURE", 365, 15.365321894805364`}, {"CURE", 365, 12.371573065965437`},
{"CURE", 365, 7.063343690949181`}, {"CURE", 365, 41.88375923149025`}, {"CURE", 365, 15.723550172295603`},
{"CURE", 365, 30.333507373282686`}, {"CURE", 365, 29.48857316042528`}, {"CURE", 365, 33.44809987992006`},
{"CURE", 365, 26.14662946203415`}, {"CURE", 365, 31.27758732458621`}, {"CURE", 365, 24.776217484571326`},
{"CURE", 365, 47.857784080863134`}, {"CURE", 365, 29.145312551238195`}, {"CURE", 365, 23.770327991089594`},
{"CURE", 365, 60.90809610029597`}, {"CURE", 365, 7.052189236664045`}, {"CURE", 365, 40.020953557371094`},
{"CURE", 365, 2.940239908689846`}, {"CURE", 365, 36.511884370271616`}, {"CURE", 365, 8.608506948312039`},
{"CURE", 365, 44.79018740343578`}, {"CURE", 365, 7.0249408473356025`}, {"CURE", 365, 46.039838987781586`},
{"CURE", 365, 115.10205987926152`}, {"CURE", 365, 5.025835271329208`}, {"CURE", 365, 3.6060544421199974`},
{"CURE", 365, 13.299181757770942`}, {"CURE", 365, 3.224813156503308`}, {"CURE", 365, 2.2083797620198844`},
{"CURE", 365, 12.921434743203376`}, {"CURE", 365, 21.066852414864726`}, {"CURE", 365, 22.735821808017523`},
{"TBUR", 97.59325428890845`, 21.53311837270632`}, {"CURE", 365, 16.800597990236135`}, {"CURE", 365, 29.592127863413594`},
{"CURE", 365, 52.110469864680546`}, {"CURE", 365, 4.2158191133525555`}, {"CURE", 365, 23.1087745093269`},
{"CURE", 365, 19.85923571444721`}, {"CURE", 365, 14.396146359375113`}, {"CURE", 365, 26.613118493569996`},
{"CURE", 365, 39.46549221460503`}, {"CURE", 365, 92.36808632128829`}, {"CURE", 365, 68.7469418917985 7`},
{"CURE", 365, 21.20410704701316`}, {"CURE", 365, 2.2601595446723843`}, {"CURE", 365, 24.65086787259306`},
{"CURE", 365, 63.59363585447687`}, {"CURE", 365, 15.997392518901863`}, {"CURE", 365, 19.79729147106484`},
{"CURE", 365, 12.343080331037978`}, {"CURE", 365, 18.09451119709029`}, {"CURE", 365, 4.672543321085662`},
{"CURE", 365, 22.901083126077296`}, {"CURE", 365, 12.844684526456051`}, {"CURE", 365, 37.588618656157344`},
{"TBUR", 85.71833915346598`, 25.67926997591548`}, {"CURE", 365, 24.999628566032168`}, {"CURE", 365, 20.569753759550352`},
{"CURE", 365, 10.88528921497538`}, {"CURE", 365, 7.500431126116225`}, {"CURE", 365, 7.31244475332808`},
{"CURE", 365, 9.46192039122101`}, {"CURE", 365, 7.380597969898925`}, {"CURE", 365, 99.06791500190548`},
{"CURE", 365, 55.13148056960705`}, {"CURE", 365, 10.669701242160249`}, {"CURE", 365, 164.37351518859717`},
{"CURE", 365, 47.00680785045585`}, {"CURE", 365, 24.886345810389642`}, {"CURE", 365, 43.045821341345416`},
{"CURE", 365, 6.4728805856709455`}, {"CURE", 365, 51.575255471709234`}, {"CURE", 365, 137.99275138265773`},
{"CURE", 365, 7.485201630331805`}, {"CURE", 365, 23.353858698310095`}, {"CURE", 365, 38.18187348703262`},
{"CURE", 365, 19.226253935398834`}, {"CURE", 365, 9.476860063317542`}, {"CURE", 365, 7.550911805060424`},
{"CURE", 365, 65.5192892578278`}, {"CURE", 365, 34.39534305805513`}, {"CURE", 365, 19.573762421127608`},
{"CURE", 365, 48.59005638168684`}, {"CURE", 365, 8.764162373307549`}, {"CURE", 365, 23.458616298499845`},
{"CURE", 365, 14.168792817239098`}, {"CURE", 365, 95.58639595268673`}, {"TBUR", 43.64849811753152`, 23.89293131816105`},

{"TBUR", 68.70416646748818`, 110.98188107588929`}, {"CURE", 365, 173.3406942312784`}, {"CURE", 365, 13.336681772333229`},
{"CURE", 365, 3.759088733064132`}, {"CURE", 365, 3.277155844461657`}, {"CURE", 365, 23.957144315765124`},
{"CURE", 365, 93.01015069450587`}, {"CURE", 365, 18.632650129230942`}, {"CURE", 365, 14.306473266663714`},
{"TBUR", 51.87244485774863`, 26.81877753329315`}, {"CURE", 365, 29.404915599550314`}, {"CURE", 365, 122.67204997793318`},
{"CURE", 365, 101.30313690657229`}, {"CURE", 365, 21.175422486048316`}, {"CURE", 365, 7.036939024873433`},
{"CURE", 365, 17.239642013065133`}, {"CURE", 365, 2.706103089869882`}, {"CURE", 365, 50.72323174914896`},
{"CURE", 365, 75.71698643523442`}, {"CURE", 365, 24.76099696746202`}, {"CURE", 365, 19.62133279804557`},
{"CURE", 365, 82.98475899247406`}, {"CURE", 365, 5.248054435948816`}, {"CURE", 365, 6.834740422932461 5`},
{"CURE", 365, 21.459065907855152`}, {"CURE", 365, 27.538385995981724`}, {"TBUR", 53.719749366506306`, 51.89708826357238`},
{"CURE", 365, 53.83671175762655`}, {"CURE", 365, 22.100835040725396`}, {"CURE", 365, 22.760833227595963`},
{"CURE", 365, 28.401250514447998`}, {"CURE", 365, 9.83483087385419`}, {"CURE", 365, 2.7907558580681373`},
{"CURE", 365, 29.337409026951903`}, {"CURE", 365, 35.358956350692125`}, {"CURE", 365, 17.189967866622`},
{"CURE", 365, 31.155447533792408`}, {"CURE", 365, 9.695145641813433`}, {"CURE", 365, 6.375760678124909`},
{"CURE", 365, 27.914500076782005`}, {"CURE", 365, 16.914372221785634`}, {"CURE", 365, 24.241489672218417`},
{"CURE", 365, 160.06036194706186`}, {"CURE", 365, 15.240571065828117`}, {"CURE", 365, 7.421440397335023`},
{"CURE", 365, 5.768567660822363`}, {"CURE", 365, 6.993871945834748`}, {"CURE", 365, 39.945041283484336`},
{"CURE", 365, 2.752043851196423`}, {"CURE", 365, 37.19516251867025`}, {"CURE", 365, 40.62940384238607 5`},
{"CURE", 365, 31.713337636994318`}, {"CURE", 365, 25.99858494323403`}, {"CURE", 365, 5.14615146140834`},
{"CURE", 365, 56.583004559571975`}, {"TBUR", 45.29430149814277`, 17.011198367576508`}, {"CURE", 365, 7.626547828692402`},
{"CURE", 365, 50.37844320343217`}, {"CURE", 365, 8.962566237917716`}, {"CURE", 365, 60.34953380398057`},
{"CURE", 365, 38.746937610467604`}, {"CURE", 365, 25.791849275392707`}, {"CURE", 365, 37.548508840660915`},
{"CURE", 365, 13.269239191509168`}, {"CURE", 365, 58.88037630801759`}, {"CURE", 365, 6.9883125006135`},
{"CURE", 365, 29.540864013941103`}, {"CURE", 365, 34.61287493918922`}, {"CURE", 365, 14.715808215785584`},
{"CURE", 365, 137.12234471537514`}, {"CURE", 365, 27.396037757839473`}, {"CURE", 365, 28.068878007206063`},
{"CURE", 365, 28.65949220599245`}, {"CURE", 365, 114.84268630379762`}, {"CURE", 365, 36.47729119688457`},
{"CURE", 365, 34.03116974063273`}, {"CURE", 365, 74.0236328705493`}, {"CURE", 365, 26.04997832985625`},
{"CURE", 365, 22.691176644919295`}, {"CURE", 365, 28.054279615334284`}, {"CURE", 365, 38.75143199931137`},
{"CURE", 365, 68.48501140086098`}, {"CURE", 365, 14.77190566248408`}, {"CURE", 365, 13.487711367333066`},
{"CURE", 365, 28.866516526353344`}, {"CURE", 365, 29.386258875506698`}, {"CURE", 365, 32.95566205038761`},
{"CURE", 365, 35.33811166098945`}, {"CURE", 365, 19.73210887935559`}, {"CURE", 365, 9.904288498037854`},
{"CURE", 365, 37.18188625477416`}, {"CURE", 365, 9.42017462954447`}, {"CURE", 365, 20.030368526736957`},

{"CURE", 365, 23.763735243085517`}, {"CURE", 365, 7.187268563752888`}, {"CURE", 365, 3.571408419265426`},
{"TBUR", 80.13724152853833`, 21.94201074496285`}, {"CURE", 365, 29.863812140581757`}, {"CURE", 365, 35.47465412927396`},
{"CURE", 365, 25.3696441664737`}, {"CURE", 365, 3.2513890714873224`}, {"CURE", 365, 29.126991492408294`},
{"CURE", 365, 5.54425372308684`}, {"CURE", 365, 85.65822349518304`}, {"CURE", 365, 111.42677770501062`},
{"CURE", 365, 15.427058008392601`}, {"TBUR", 64.66681546197626`, 21.950003367426135`}, {"CURE", 365, 119.59520710318742`},
{"CURE", 365, 21.45051625753308`}, {"CURE", 365, 12.401209744089849`}, {"CURE", 365, 9.66834541845133`},
{"CURE", 365, 2.6524789557307304`}, {"CURE", 365, 16.72739231074513`}, {"CURE", 365, 18.650557384538235`},
{"CURE", 365, 49.33883658498576`}, {"CURE", 365, 23.64467498186967`}, {"CURE", 365, 41.181741944918116`},
{"CURE", 365, 14.549606582979504`}, {"TBUR", 129.1398228033898`, 31.467735011256238`}, {"CURE", 365, 70.57078166542708`},
{"CURE", 365, 7.164708503430426`}, {"CURE", 365, 11.071124338744726`}, {"CURE", 365, 16.432709799112647`},
{"CURE", 365, 22.968221987422`}, {"TBUR", 123.41884076430178`, 14.993506906840839`}, {"CURE", 365, 14.418084513571257`},
{"CURE", 365, 6.099677604983223`}, {"CURE", 365, 9.430612004844352`}, {"CURE", 365, 17.85529034988159`},
{"CURE", 365, 40.68054077888196`}, {"TBUR", 70.5673718855491`, 36.08990469131709`}, {"CURE", 365, 18.536557990498594`},
{"TBUR", 30.297959434779486`, 27.634064291853896`}, {"CURE", 365, 59.81591148965233`},
{"CURE", 365, 29.29120574149217`}, {"CURE", 365, 21.92023899024807`}, {"CURE", 365, 13.963138978908962`},
{"CURE", 365, 4.3701745058078885`}, {"CURE", 365, 24.339812312401737`}, {"CURE", 365, 28.07513373701861`},
{"CURE", 365, 109.92005798080842`}, {"CURE", 365, 5.601847748836379`}, {"CURE", 365, 5.4786980042233155`},
{"CURE", 365, 17.554459450687098`}, {"CURE", 365, 51.17453849384985`}, {"CURE", 365, 6.383800925647323`},
{"CURE", 365, 31.23389113649161`}, {"CURE", 365, 7.281887088152546`}, {"CURE", 365, 18.144220173893338`},
{"CURE", 365, 7.360868350783599`}, {"CURE", 365, 7.697699778178663`}, {"CURE", 365, 6.943058140900752`},
{"CURE", 365, 74.09222494058078`}, {"CURE", 365, 11.776205229360082`}, {"TBUR", 65.63426902047404`, 20.749469003577897`},
{"CURE", 365, 2.8781393051512`}, {"CURE", 365, 34.474975172574915`}, {"CURE", 365, 48.252637476642604`},
{"CURE", 365, 42.01532188410829`}, {"CURE", 365, 6.348311762713861`}, {"CURE", 365, 12.738026220792996`},
{"CURE", 365, 129.48399533127014`}, {"CURE", 365, 35.19293189635063`}, {"CURE", 365, 23.948297975019855`},
{"CURE", 365, 8.493782663885787`}, {"CURE", 365, 12.194700690659902`}, {"CURE", 365, 18.83005749419362`},
{"CURE", 365, 4.660753041522341`}, {"CURE", 365, 22.924341454717222`}, {"CURE", 365, 36.0787826120329`},
{"CURE", 365, 21.920037516639916`}, {"CURE", 365, 23.79207528344444`}, {"CURE", 365, 17.238278885969695`},
{"CURE", 365, 11.146861684970961`}, {"CURE", 365, 63.64323833988983`}, {"CURE", 365, 92.44903589203061`},
{"CURE", 365, 24.671739167233625`}, {"CURE", 365, 31.14833694066774`}, {"CURE", 365, 39.45765836772317`},
{"CURE", 365, 38.57022299210221`}, {"CURE", 365, 3.3634777260754234`}, {"CURE", 365, 45.050287846436056`},
{"CURE", 365, 46.6405581414178`}, {"CURE", 365, 31.48034043989835`}, {"CURE", 365, 126.51050916413134`},

{"CURE", 365, 26.29917597075582`}, {"CURE", 365, 11.659308035017357`}, {"CURE", 365, 27.674173724739266`},
{"CURE", 365, 35.172647354196236`}, {"CURE", 365, 15.755299886732207`}, {"CURE", 365, 12.33158676463756`},
{"CURE", 365, 10.753949995091256`}, {"CURE", 365, 19.45317812848684`}, {"CURE", 365, 60.09364222470194`},
{"CURE", 365, 38.56990996988786`}, {"CURE", 365, 6.700763317771252`}, {"CURE", 365, 39.1761474330137`},
{"CURE", 365, 56.767120448001045`}, {"CURE", 365, 10.412745200360574`}, {"CURE", 365, 59.49335049047874`},
{"CURE", 365, 8.945336523449168`}, {"CURE", 365, 5.703620174346265`}, {"CURE", 365, 8.108363847388105`},
{"CURE", 365, 15.569612753876811`}, {"TBUR", 65.98055070147888`, 69.39604404728644`}, {"CURE", 365, 24.96754262091068`},
{"CURE", 365, 31.486734983610415`}, {"CURE", 365, 7.537234411161476`}, {"CURE", 365, 143.42386278396182`},
{"CURE", 365, 65.87648858303601`}, {"CURE", 365, 11.20586135688805`}, {"CURE", 365, 27.49007834398413`},
{"CURE", 365, 15.334450454090524`}, {"CURE", 365, 15.475616470921896`}, {"CURE", 365, 52.266423604473694`},
{"TBUR", 46.01291928136662`, 65.81437337478914`}, {"CURE", 365, 7.110379144118544`}, {"CURE", 365, 13.845505806829298`},
{"CURE", 365, 4.910997506392487`}, {"CURE", 365, 18.953442793167813`}, {"CURE", 365, 32.399985252465214`},
{"CURE", 365, 52.627801129343396`}, {"CURE", 365, 29.36075989128253`}, {"CURE", 365, 105.75721627222968`},
{"CURE", 365, 50.555071218563945`}, {"TBUR", 59.5112898688587`, 8.280560952092094`}, {"CURE", 365, 18.47137650436206`},
{"TBUR", 28.379763237186076`, 17.95256177707174`}, {"TBUR", 59.27470946772926`, 41.11235205296395`},
{"TBUR", 176.89254216949863`, 5.399576861036606`}, {"CURE", 365, 14.503978876196575`}, {"CURE", 365, 8.165883990254146`},
{"CURE", 365, 20.397280071776642`}, {"CURE", 365, 46.584530799348244`}, {"CURE", 365, 19.824787479149045`},
{"CURE", 365, 4.306194604252097`}, {"CURE", 365, 81.88750991664719`}, {"CURE", 365, 15.458901167637018`},
{"CURE", 365, 5.254066308026556`}, {"CURE", 365, 6.9062357586644305`}, {"CURE", 365, 5.8303473002131785`},
{"CURE", 365, 7.450790966952729`}, {"CURE", 365, 59.953163165697184`}, {"CURE", 365, 32.75545131917065`},
{"CURE", 365, 19.743479377265594`}, {"CURE", 365, 9.241409254920034`}, {"CURE", 365, 15.461620744110338`},
{"CURE", 365, 8.945206627366797`}, {"CURE", 365, 21.580998251970115`}, {"CURE", 365, 74.16516673645253`},
{"CURE", 365, 42.08678849912588`}, {"CURE", 365, 21.36806525871619`}, {"CURE", 365, 12.333733215271335`},
{"CURE", 365, 12.775380203335178`}, {"CURE", 365, 158.577065140423`}, {"CURE", 365, 7.555366109362288`},
{"CURE", 365, 3.8919281391572014`}, {"TBUR", 89.36604291473073`, 3.378026282189084`}, {"CURE", 365, 43.097614271101264`},
{"CURE", 365, 25.20768101177533`}, {"CURE", 365, 27.998825178659793`}, {"CURE", 365, 41.61865014826578`},
{"CURE", 365, 68.53608085397806`}, {"CURE", 365, 71.06743474586885`}, {"CURE", 365, 27.080961612238447`},
{"CURE", 365, 11.69448408131865`}, {"CURE", 365, 13.550693924523202`}, {"CURE", 365, 26.813478074506108`},
{"CURE", 365, 15.32316640276401`}, {"TBUR", 22.827657968601788`, 5.664596200591568`}, {"CURE", 365, 24.048317062734014`},
{"CURE", 365, 149.9939397492935`}, {"CURE", 365, 15.46078810644956`}, {"CURE", 365, 5.554625926795679`},
{"CURE", 365, 52.72336338581431`}, {"CURE", 365, 13.1851736683014`}, {"CURE", 365, 37.86583782127072`},

{"CURE", 365, 182.41655067250528`}, {"CURE", 365, 84.22436492056103`}, {"CURE", 365, 27.343133463396768`},
{"CURE", 365, 22.908435983188518`}, {"CURE", 365, 9.764332474653177`}, {"CURE", 365, 2.785324034848396`},
{"CURE", 365, 10.864787515459588`}, {"CURE", 365, 31.97750090087384`}, {"CURE", 365, 17.719519701273892`},
{"CURE", 365, 56.54974878657023`}, {"CURE", 365, 22.40221011140633`}, {"CURE", 365, 22.896856398779164`},
{"CURE", 365, 6.358466192568752`}, {"CURE", 365, 18.006661807145104`}, {"CURE", 365, 18.484302538345062`},
{"CURE", 365, 4.8187483993553455`}, {"CURE", 365, 15.312326829004597`}, {"CURE", 365, 9.144620598315305`},
{"TBUR", 72.27202150986693`, 14.431037001299462`}, {"CURE", 365, 30.055836200516186`}, {"CURE", 365, 15.142132249755761`},
{"CURE", 365, 3.519078935125058`}, {"CURE", 365, 24.237791170098056`}, {"CURE", 365, 2.708888274816001`},
{"CURE", 365, 7.946197199922228`}, {"CURE", 365, 23.604079807305283`}, {"CURE", 365, 27.243069234516707`},
{"CURE", 365, 5.409730146206506`}, {"CURE", 365, 15.929556925728848`}, {"CURE", 365, 20.604656800396434`},
{"CURE", 365, 104.09990644927099`}, {"CURE", 365, 23.983580624897787`}, {"CURE", 365, 6.753126109515102`},
{"CURE", 365, 73.6859635914459`}, {"CURE", 365, 7.200897327032274`}, {"CURE", 365, 27.251701926961754`},
{"CURE", 365, 28.232728999061937`}, {"CURE", 365, 8.425632236096465`}, {"CURE", 365, 9.378207651586035`},
{"CURE", 365, 41.5529224517499`}, {"CURE", 365, 4.055019018491744`}, {"CURE", 365, 35.21591288908402`},
{"CURE", 365, 9.325284789303094`}, {"CURE", 365, 12.194856541478927`}, {"CURE", 365, 2.7197428702385964`},
{"CURE", 365, 13.999884029035133`}, {"TBUR", 100.61873833012989`, 47.59995395975862`}, {"CURE", 365, 18.156091222882868`},
{"CURE", 365, 13.182221826981323`}, {"CURE", 365, 18.8388637501488`}, {"CURE", 365, 14.726456741018778`},
{"TBUR", 72.88442744284907`, 18.588023999978702`}, {"CURE", 365, 32.879015595827376`}, {"CURE", 365, 31.75069493159596`},
{"CURE", 365, 3.911292533049901`}, {"CURE", 365, 64.00879339423491`}, {"CURE", 365, 7.461937968253717`},
{"CURE", 365, 3.739250409297755`}, {"CURE", 365, 17.828115405995874`}, {"CURE", 365, 18.73101316245769`},
{"CURE", 365, 12.61449054257453`}, {"CURE", 365, 21.926552945084335`}, {"CURE", 365, 18.91008252696051`},
{"CURE", 365, 30.39737420935337`}, {"CURE", 365, 25.682118180195225`}, {"TBUR", 52.20944966885982`, 36.597063893247565`},
{"CURE", 365, 5.786784287121224`}, {"CURE", 365, 73.9921042471082`}, {"CURE", 365, 72.06829526491181`},
{"CURE", 365, 3.990399512628698`}, {"CURE", 365, 10.0546563373466`}, {"CURE", 365, 24.94702762749758`},
{"CURE", 365, 12.655944656292576`}, {"CURE", 365, 38.79218814069787`}, {"CURE", 365, 78.07755767029853`},
{"CURE", 365, 24.684122406490953`}, {"CURE", 365, 4.232474638750876`}, {"CURE", 365, 4.154207439634552`},
{"CURE", 365, 9.758557638101895`}, {"CURE", 365, 2.9452424400929456`}, {"CURE", 365, 33.44046976032655`},
{"CURE", 365, 5.181666852818029`}, {"CURE", 365, 34.73949255204514`}, {"CURE", 365, 21.465098733624547`},
{"CURE", 365, 37.01150998452794`}, {"CURE", 365, 9.109100373736608`}, {"CURE", 365, 45.95885075700383`},
{"CURE", 365, 152.05436461553154`}, {"CURE", 365, 54.795072983707236`}, {"CURE", 365, 88.74691527298698`},
{"CURE", 365, 26.131532418367406`}, {"CURE", 365, 62.26012138606244`}, {"CURE", 365, 62.37275610587894`},

{"CURE", 365, 7.571454849831485`}, {"CURE", 365, 5.295278283876111`}, {"CURE", 365, 17.207632942346912`},
{"CURE", 365, 35.7344950029524`}, {"CURE", 365, 44.70539689774737`}, {"CURE", 365, 18.943335397502015`},
{"CURE", 365, 28.235629057776762`}, {"CURE", 365, 5.322770122816306`}, {"CURE", 365, 6.752528980655802`},
{"CURE", 365, 32.87303451730982`}, {"CURE", 365, 10.623661180754004`}, {"CURE", 365, 4.525134149875423`},
{"CURE", 365, 6.695710391154674`}, {"CURE", 365, 3.272215539065453`}, {"CURE", 365, 26.594611794010632`},
{"CURE", 365, 36.58046241798529`}, {"CURE", 365, 25.553337502405945`}, {"CURE", 365, 29.0780589350001`},
{"CURE", 365, 16.088044079653002`}, {"CURE", 365, 14.376811312777082`}, {"CURE", 365, 37.27002797249682`},
{"CURE", 365, 39.92953338342712`}, {"CURE", 365, 4.4534830053355385`}, {"CURE", 365, 16.284022370290355`},
{"CURE", 365, 8.844667554441608`}, {"CURE", 365, 13.903835410102614`}, {"CURE", 365, 114.47989791601658`},
{"CURE", 365, 20.216840742019045`}, {"CURE", 365, 13.87979308193467`}, {"CURE", 365, 14.652729148081136`},
{"CURE", 365, 29.276128787843213`}, {"CURE", 365, 22.285298328581316`}, {"CURE", 365, 29.074109482454823`},
{"TBUR", 76.41750701166312`, 29.845061528277288`}, {"CURE", 365, 6.798958061988783`}, {"CURE", 365, 66.14179603282594`},
{"CURE", 365, 12.683477814664936`}, {"CURE", 365, 26.93551024526182`}, {"CURE", 365, 15.27658719006546`},
{"CURE", 365, 8.681322994215655`}, {"CURE", 365, 31.646041506298516`}, {"CURE", 365, 32.13069575532772`},
{"TBUR", 58.19443161316844`, 17.702124900362374`}, {"CURE", 365, 42.08224866330974`}, {"CURE", 365, 8.902149929567594`},
{"CURE", 365, 74.47029974917514`}, {"CURE", 365, 28.567425248790595`}, {"CURE", 365, 60.3154444870339`},
{"CURE", 365, 2.344747617097129`}, {"CURE", 365, 29.64940725386073`}, {"CURE", 365, 33.69832785986626`},
{"CURE", 365, 8.058933715563992`}, {"CURE", 365, 20.444286715714412`}, {"CURE", 365, 30.635755081884557`},
{"CURE", 365, 18.614629482628107`}, {"CURE", 365, 5.919770072297436`}, {"CURE", 365, 4.495250242764615`},
{"CURE", 365, 26.540961754748754`}, {"CURE", 365, 7.090145490139631`}, {"CURE", 365, 15.760487379577944`},
{"CURE", 365, 19.183956107215337`}, {"CURE", 365, 8.90323018971866`}, {"CURE", 365, 16.390463557863672`},
{"CURE", 365, 4.480306336317191`}, {"CURE", 365, 18.4756679555929`}, {"CURE", 365, 2.9877763646592403`},
{"CURE", 365, 5.3379643076838565`}, {"CURE", 365, 12.616655435851841`}, {"CURE", 365, 4.066917208067329`},
{"CURE", 365, 30.2314312082157`}, {"CURE", 365, 44.48518504781726`}, {"CURE", 365, 45.0630144977984`},
{"CURE", 365, 37.59224229433621`}, {"CURE", 365, 70.93431926748406`}, {"CURE", 365, 5.734124930973065`},
{"CURE", 365, 25.584090489786856`}, {"CURE", 365, 27.421421156057008`}, {"CURE", 365, 47.26689339654233`},
{"CURE", 365, 35.30529950651758`}, {"CURE", 365, 16.112236121579105`}, {"CURE", 365, 75.70281012046006`},
{"CURE", 365, 2.3664228462732133`}, {"CURE", 365, 72.20243576381812`}, {"CURE", 365, 4.884643164790597`},
{"CURE", 365, 61.66021370039836`}, {"CURE", 365, 55.8488297838373`}, {"CURE", 365, 16.54523300004234`},
{"CURE", 365, 65.12986136390037`}, {"CURE", 365, 79.50453149663171`}, {"CURE", 365, 35.52949698955642`},
{"CURE", 365, 15.692132983501548`}, {"CURE", 365, 18.039527019817502`}, {"CURE", 365, 26.628897790837605`},

{"CURE", 365, 24.54756902391946`}, {"CURE", 365, 5.95248014248995`}, {"CURE", 365, 21.359205149667563`},
{"CURE", 365, 20.194252418365146`}, {"CURE", 365, 15.11238141770622`}, {"CURE", 365, 37.37404351940852`},
{"CURE", 365, 5.884837142832794`}, {"CURE", 365, 41.70955057334827`}, {"CURE", 365, 27.282908850329637`},
{"CURE", 365, 15.455960041574997`}, {"CURE", 365, 13.91261158230809`}, {"CURE", 365, 28.407565395637594`},
{"CURE", 365, 3.9060590703773594`}, {"CURE", 365, 45.23830178512966`}, {"CURE", 365, 7.133978375543426`},
{"CURE", 365, 10.129143589213822`}, {"CURE", 365, 22.703684640168813`}, {"CURE", 365, 17.80787228581309`},
{"CURE", 365, 16.377778484037037`}, {"CURE", 365, 50.9206811815442`}, {"CURE", 365, 6.896454234102432`},
{"CURE", 365, 12.00956804351381`}, {"CURE", 365, 88.87365173475209`}, {"CURE", 365, 6.741315455136442`},
{"CURE", 365, 17.370142958210494`}, {"CURE", 365, 26.38566913100421`}, {"CURE", 365, 26.054340099231013`},
{"CURE", 365, 17.712950595579166`}, {"CURE", 365, 91.61017340656353`}, {"CURE", 365, 16.519686782487206`},
{"CURE", 365, 6.965483600899949`}, {"CURE", 365, 13.825579649988967`}, {"CURE", 365, 10.727493318594185`},
{"CURE", 365, 8.21127972844286`}, {"CURE", 365, 21.55390146987386`}, {"CURE", 365, 36.924686061196`},
{"CURE", 365, 20.312740546669446`}, {"CURE", 365, 23.67779208820569`}, {"CURE", 365, 51.87212607833158`},
{"CURE", 365, 5.710114870537599`}, {"CURE", 365, 6.719848852374222`}, {"CURE", 365, 56.77679684803689`},
{"CURE", 365, 18.22911871504598`}, {"CURE", 365, 19.802809558940798`}, {"CURE", 365, 9.952356805163479`},
{"CURE", 365, 22.792893824931`}, {"CURE", 365, 28.40513710443747`}, {"CURE", 365, 65.80129764389031`},
{"CURE", 365, 9.70158342429115`}, {"CURE", 365, 37.8091103762755`}, {"CURE", 365, 22.26444869739217`},
{"CURE", 365, 58.941437778526414`}, {"CURE", 365, 96.44729212556912`}, {"CURE", 365, 35.25734640666155`},
{"CURE", 365, 3.4160653584563594`}, {"CURE", 365, 26.757658664723014`}, {"CURE", 365, 8.437790271575473`},
{"CURE", 365, 89.3813254756964`}, {"TBUR", 33.75504210186413`, 2.6919744867152993`}, {"CURE", 365, 2.9818748752266435`},
{"CURE", 365, 103.17587476040491`}, {"CURE", 365, 23.687186866904714`}, {"CURE", 365, 8.050303577974143`},
{"CURE", 365, 15.408779062232764`}, {"CURE", 365, 5.402679230643508`}, {"TBUR", 56.33498794954662`, 5.091311799018941`},
{"CURE", 365, 6.147571501570305`}, {"CURE", 365, 22.218950119943035`}, {"CURE", 365, 47.09002444791333`},
{"CURE", 365, 18.643474474850983`}, {"CURE", 365, 4.667312434463032`}, {"CURE", 365, 63.53694933806465`},
{"CURE", 365, 126.79717266807087`}, {"CURE", 365, 3.580214111744387`}, {"TBUR", 81.41212074604002`, 28.563612939585838`},
{"CURE", 365, 15.373620384360434`}, {"CURE", 365, 3.0235041769982667`}, {"CURE", 365, 23.619166740975853`},
{"CURE", 365, 15.10267789816568`}, {"CURE", 365, 11.586686626282829`}, {"CURE", 365, 22.543642831704076`},
{"CURE", 365, 12.796128089673166`}, {"CURE", 365, 37.45446225434687`}, {"CURE", 365, 22.36261212280245`},
{"CURE", 365, 52.63982412663137`}, {"CURE", 365, 23.32019130450762`}, {"CURE", 365, 11.549639111436454`},
{"CURE", 365, 5.611005461594946`}, {"CURE", 365, 3.899556946727685`}, {"CURE", 365, 11.10816130844495`},
{"CURE", 365, 44.18446887884925`}, {"CURE", 365, 14.575103974405472`}, {"CURE", 365, 13.337252946788944`},

{"CURE", 365, 9.4361970514132`}, {"CURE", 365, 9.567314774401275`}, {"CURE", 365, 25.85262350837845`},
{"CURE", 365, 4.838544227157623`}, {"CURE", 365, 30.801237730605337`}, {"CURE", 365, 14.10696319205201`},
{"CURE", 365, 8.27348622564230 5`}, {"CURE", 365, 13.122839383633144`}, {"CURE", 365, 29.153724172540947`},
{"CURE", 365, 21.613122858090318`}, {"CURE", 365, 2.6207607463284486`}, {"CURE", 365, 11.276271937863136`},
{"CURE", 365, 188.7402677073796 2`}, {"CURE", 365, 31.302612431852012`}, {"CURE", 365, 45.68895805203333`},
{"CURE", 365, 13.465345636836268`}, {"CURE", 365, 49.907686315588826`}, {"CURE", 365, 5.97520624763138`},
{"CURE", 365, 20.10873928851162`}, {"CURE", 365, 92.95245266729451`}, {"CURE", 365, 17.412649938410432`},
{"CURE", 365, 15.272916385048983`}, {"CURE", 365, 26.28686185940545`}, {"CURE", 365, 13.879573008994898`},
{"CURE", 365, 3.0864989223816686`}, {"TBUR", 80.46755608449361`, 28.63656167659578`}, {"CURE", 365, 5.116332925743365`},
{"CURE", 365, 12.134436109250402`}, {"CURE", 365, 28.739176210429683`}, {"CURE", 365, 10.320329316496181`},
{"CURE", 365, 54.825888744375064`}, {"CURE", 365, 16.279032387924392`}, {"CURE", 365, 28.065454941645292`},
{"CURE", 365, 19.451267906832378`}, {"CURE", 365, 5.50514106193536`}, {"CURE", 365, 20.388494323123716`},
{"CURE", 365, 44.13540517279695`}, {"CURE", 365, 14.78880941552223`}, {"CURE", 365, 11.132566660414811`},
{"CURE", 365, 5.276715332074514`}, {"CURE", 365, 16.64843808284018`}, {"CURE", 365, 20.039116831701822`},
{"CURE", 365, 57.356532193324064`}, {"CURE", 365, 9.213395096408803`}, {"CURE", 365, 13.829039070988191`},
{"CURE", 365, 53.81829728943998`}, {"CURE", 365, 5.671313342511695`}, {"CURE", 365, 77.65398156618816`},
{"CURE", 365, 6.5809759074286855`}, {"CURE", 365, 12.034617211767369`}, {"CURE", 365, 27.752531723800555`},
{"CURE", 365, 44.59698339339112`}, {"CURE", 365, 20.102578396514918`}, {"CURE", 365, 10.507595904541297`},
{"CURE", 365, 3.096438708738538`}, {"TBUR", 48.04555580991841`, 26.962165925252922`}, {"CURE", 365, 24.52687126550808`},
{"CURE", 365, 24.160207235141556`}, {"CURE", 365, 9.527911165886275`}, {"CURE", 365, 23.358107027346506`},
{"CURE", 365, 2.6962502344943697`}, {"CURE", 365, 15.562996110154778`}, {"CURE", 365, 6.800178396747142`},
{"CURE", 365, 3.008119985611463`}, {"CURE", 365, 15.648087669409877`}, {"CURE", 365, 3.3508507735642725`},
{"CURE", 365, 10.654503750239599`}, {"CURE", 365, 7.302593680699419`}, {"TBUR", 29.578628143968597`, 4.117447528075185`},
{"CURE", 365, 14.462761552220758`}, {"CURE", 365, 38.56661501656055`}, {"CURE", 365, 21.067604521753733`},
{"CURE", 365, 19.112385348179767`}, {"CURE", 365, 30.23401779485619`}, {"CURE", 365, 25.78405496366647`}},
{{"1600 Res", "1600 OS", "1600 Tox"}, {"CURE", 365, 13.287398069146903`}, {"CURE", 365, 27.578138241228764`},
{"CURE", 365, 19.300465646398827`}, {"TBUR", 56.40709377511925`, 6.42267911858442`},
{"TBUR", 37.85402042952295`, 23.65708881127556`}, {"CURE", 365, 49.951579102629914`}, {"CURE", 365, 47.00305765281321`},
{"CURE", 365, 4.910125912134397`}, {"CURE", 365, 33.8975477677516`}, {"CURE", 365, 29.30563779295946`},
{"CURE", 365, 109.99745532249861`}, {"CURE", 365, 13.653116129080011`}, {"CURE", 365, 48.18942321930959`},
{"CURE", 365, 163.10933959344942`}, {"CURE", 365, 23.182801308847857`}, {"CURE", 365, 36.177793488606625`},

{"CURE", 365, 71.7437047220973`}, {"CURE", 365, 32.10688147080118`}, {"TBUR", 102.12287381323708`, 24.18418316339044`},
{"CURE", 365, 8.054096344039861`}, {"CURE", 365, 49.792894003654716`}, {"CURE", 365, 20.185298203690927`},
{"CURE", 365, 7.732433768156528`}, {"CURE", 365, 6.160856392549445`}, {"CURE", 365, 28.043605352698854`},
{"CURE", 365, 30.777521880782007`}, {"CURE", 365, 2.7979172206777045`}, {"CURE", 365, 4.240563782067327`},
{"CURE", 365, 15.711003855882492`}, {"CURE", 365, 18.373165289945625`}, {"CURE", 365, 15.683763547457625`},
{"CURE", 365, 54.97387198518113`}, {"CURE", 365, 12.121752137515557`}, {"TBUR", 106.68839837089723`, 9.6252486583859`},
{"CURE", 365, 78.15280961785224`}, {"CURE", 365, 4.785289999505768`}, {"CURE", 365, 20.7026171031924`},
{"TBUR", 38.12954316877558`, 45.90502850360896`}, {"CURE", 365, 11.489793001262377`}, {"CURE", 365, 17.676049014475744`},
{"CURE", 365, 40.25350040399302`}, {"CURE", 365, 27.257396492155713`}, {"CURE", 365, 5.529004733723853`},
{"CURE", 365, 18.64135392439115`}, {"CURE", 365, 26.907045540699993`}, {"CURE", 365, 17.646604306372378`},
{"CURE", 365, 187.46993230887747`}, {"CURE", 365, 40.40775872383222`}, {"CURE", 365, 60.76876912228631`},
{"CURE", 365, 7.554385549734075`}, {"CURE", 365, 20.741863531102553`}, {"CURE", 365, 28.706184670907255`},
{"CURE", 365, 23.238548729916992`}, {"CURE", 365, 15.088754883909528`}, {"CURE", 365, 6.5025357779661945`},
{"CURE", 365, 68.81203176544744`}, {"CURE", 365, 21.625228203634506`}, {"CURE", 365, 4.1005274153366`},
{"CURE", 365, 44.213428707730635`}, {"CURE", 365, 30.943155730178503`}, {"CURE", 365, 21.212973920435076`},
{"CURE", 365, 16.24603122071186`}, {"CURE", 365, 9.110322781424903`}, {"CURE", 365, 35.01691066404361`},
{"CURE", 365, 24.85183759149375`}, {"CURE", 365, 28.44744409062461`}, {"CURE", 365, 6.511341008097363`},
{"CURE", 365, 6.607140660747837`}, {"CURE", 365, 7.039995443941893`}, {"CURE", 365, 28.74652466881606`},
{"CURE", 365, 10.522812076852258`}, {"CURE", 365, 23.43365382751297`}, {"CURE", 365, 43.988860722088795`},
{"TBUR", 21.372286166607847`, 12.677589681692622`}, {"CURE", 365, 11.74846150512404`},
{"CURE", 365, 32.567712302449166`}, {"CURE", 365, 19.034086849110082`}, {"CURE", 365, 14.485799470890349`},
{"CURE", 365, 57.6811399470036`}, {"CURE", 365, 63.368725620604586`}, {"CURE", 365, 46.65094822300631`},
{"CURE", 365, 12.58970248758807`}, {"CURE", 365, 25.45589531051304`}, {"CURE", 365, 17.792584264591863`},
{"CURE", 365, 20.116384070815116`}, {"CURE", 365, 9.423581122963`}, {"CURE", 365, 100.5134192598864`},
{"CURE", 365, 21.903479987083323`}, {"CURE", 365, 27.604399059796588`}, {"CURE", 365, 23.73712512572029`},
{"TBUR", 130.09607374703072`, 19.52388335371185`}, {"CURE", 365, 5.817892521230592`}, {"CURE", 365, 10.665399152176933`},
{"CURE", 365, 24.68705005652834`}, {"TBUR", 44.130145686775016`, 8.824925534177098`}, {"CURE", 365, 10.625574484156582`},
{"CURE", 365, 27.73773122471066`}, {"CURE", 365, 70.39706797211521`}, {"CURE", 365, 37.677358572041385`},
{"CURE", 365, 18.89718058952798`}, {"TBUR", 56.93536538123542`, 37.36808342153412`}, {"CURE", 365, 24.367234658284442`},
{"CURE", 365, 15.799164752491993`}, {"CURE", 365, 108.18669954322725`}, {"CURE", 365, 12.020697197841178`},
{"CURE", 365, 8.167159531259792`}, {"CURE", 365, 47.91176539104846`}, {"CURE", 365, 14.098114110405975`},

{"CURE", 365, 14.463710873697071`}, {"CURE", 365, 87.02144105853534`}, {"CURE", 365, 7.062707968105781`},
{"CURE", 365, 4.479899266170174`}, {"CURE", 365, 18.636581471727617`}, {"CURE", 365, 14.732247046293264`},
{"CURE", 365, 11.096020891821274`}, {"CURE", 365, 28.332720707893923`}, {"CURE", 365, 7.97114559532949`},
{"CURE", 365, 4.901724374259266`}, {"CURE", 365, 22.952331105532824`}, {"CURE", 365, 35.0965286288294`},
{"CURE", 365, 5.973357344060474`}, {"CURE", 365, 19.778504223945927`}, {"CURE", 365, 60.052179919816375`},
{"TBUR", 50.96698931528078`, 22.795265621479725`}, {"CURE", 365, 13.320666812122372`}, {"CURE", 365, 46.433986532189934`},
{"CURE", 365, 23.334085810191656`}, {"CURE", 365, 3.0769908200071567`}, {"CURE", 365, 26.669047220397616`},
{"CURE", 365, 20.59769493898304`}, {"CURE", 365, 16.408710642466406`}, {"CURE", 365, 19.459502696050627`},
{"CURE", 365, 5.060543302820127`}, {"CURE", 365, 28.866920134817388`}, {"CURE", 365, 10.369432773764172`},
{"CURE", 365, 23.35260387059075`}, {"CURE", 365, 87.51881518364581`}, {"CURE", 365, 30.879549890267928`},
{"CURE", 365, 71.87530935934018`}, {"CURE", 365, 23.684469299353125`}, {"CURE", 365, 97.67274025028138`},
{"CURE", 365, 3.051516576531634`}, {"CURE", 365, 26.815964268492735`}, {"CURE", 365, 30.563142733505714`},
{"CURE", 365, 3.970036440808774`}, {"CURE", 365, 5.498309144277918`}, {"CURE", 365, 33.75612854147057`},
{"CURE", 365, 39.705797366326145`}, {"CURE", 365, 33.903388210969354`}, {"CURE", 365, 27.733220622725717`},
{"CURE", 365, 26.521297778835837`}, {"CURE", 365, 16.052600436860498`}, {"CURE", 365, 4.247091143792769`},
{"CURE", 365, 18.451361046828776`}, {"CURE", 365, 8.756029629203526`}, {"CURE", 365, 4.286107271047564`},
{"CURE", 365, 25.996386842721854`}, {"CURE", 365, 14.55301208233035`}, {"CURE", 365, 38.470237007091214`},
{"CURE", 365, 15.527794714982514`}, {"CURE", 365, 12.925261178213553`}, {"CURE", 365, 86.52985637366491`},
{"CURE", 365, 6.457887582339469`}, {"CURE", 365, 19.29568157579431`}, {"CURE", 365, 10.94185889755584`},
{"CURE", 365, 25.821273965882273`}, {"CURE", 365, 7.106697641367992`}, {"CURE", 365, 28.38327989327722`},
{"CURE", 365, 74.90936615507125`}, {"CURE", 365, 6.410162606373329`}, {"CURE", 365, 19.48561954416131`},
{"CURE", 365, 20.98491496962858`}, {"CURE", 365, 75.78873874806948`}, {"TBUR", 82.54743265426583`, 28.35478300869064`},
{"CURE", 365, 23.62555272703709`}, {"CURE", 365, 41.32396331683509`}, {"CURE", 365, 15.926378635059564`},
{"CURE", 365, 47.880446268089514`}, {"CURE", 365, 30.572561680006302`}, {"CURE", 365, 18.47891417879843`},
{"CURE", 365, 16.867521802399427`}, {"CURE", 365, 5.499963838200148`}, {"CURE", 365, 3.7276029602624168`},
{"CURE", 365, 8.721959213925123`}, {"CURE", 365, 11.704459254352804`}, {"CURE", 365, 5.139120536801402`},
{"CURE", 365, 18.84421763967782`}, {"CURE", 365, 51.50989110457465`}, {"CURE", 365, 13.935736757608861`},
{"CURE", 365, 25.768039967164352`}, {"CURE", 365, 32.39233824580311`}, {"CURE", 365, 26.831276559668055`},
{"CURE", 365, 18.95849118639526`}, {"CURE", 365, 7.529410620146203`}, {"CURE", 365, 51.85005208675428`},
{"CURE", 365, 4.488725921333263`}, {"CURE", 365, 48.35188747600273`}, {"TBUR", 80.33216359924084`, 46.70372802857821`},
{"CURE", 365, 13.115345181329452`}, {"CURE", 365, 15.57919504294929`}, {"CURE", 365, 89.40718678216662`},

{"CURE", 365, 23.003367337495742`}, {"CURE", 365, 27.563006650357796`}, {"CURE", 365, 17.242385735331375`},
{"CURE", 365, 6.488865888981477`}, {"CURE", 365, 39.717733166537144`}, {"TBUR", 45.16370014957899`, 105.41499174879762`},
{"CURE", 365, 16.163690352032443`}, {"CURE", 365, 14.604432495463712`}, {"CURE", 365, 26.919697558073075`},
{"CURE", 365, 10.749025913048705`}, {"CURE", 365, 136.37601325128466`}, {"CURE", 365, 10.917212744987541`},
{"CURE", 365, 67.3675527272915`}, {"CURE", 365, 51.85299039706506`}, {"CURE", 365, 50.380271834924315`},
{"CURE", 365, 26.92472849510917`}, {"CURE", 365, 62.4698392082179`}, {"CURE", 365, 105.89108080215702`},
{"CURE", 365, 28.382055091827528`}, {"CURE", 365, 5.2123786654384325`}, {"CURE", 365, 46.30882083860441`},
{"CURE", 365, 28.858499170842478`}, {"CURE", 365, 27.814795452418863`}, {"CURE", 365, 6.121709809202976`},
{"CURE", 365, 140.55192804928947`}, {"CURE", 365, 10.274363266992617`}, {"CURE", 365, 44.581523836919494`},
{"CURE", 365, 28.08893298993241`}, {"CURE", 365, 19.969781283971493`}, {"CURE", 365, 4.801676989973094`},
{"CURE", 365, 36.09807398358131`}, {"CURE", 365, 21.172835442688196`}, {"CURE", 365, 56.01886382190983`},
{"CURE", 365, 36.90252769482458`}, {"CURE", 365, 46.568776011673314`}, {"CURE", 365, 22.340986904349958`},
{"CURE", 365, 19.877048875227516`}, {"TBUR", 49.85525160935914`, 103.83874515403375`}, {"CURE", 365, 27.242957530768088`},
{"CURE", 365, 7.265985684382515`}, {"CURE", 365, 24.476429728131233`}, {"TBUR", 99.15876753692392`, 24.69507032750842`},
{"CURE", 365, 3.326271064767604`}, {"CURE", 365, 5.949104140366384`}, {"CURE", 365, 9.08524103592651`},
{"CURE", 365, 85.6459005166552`}, {"CURE", 365, 26.38343049909944`}, {"CURE", 365, 27.50485352009514`},
{"CURE", 365, 22.02482051840353`}, {"CURE", 365, 90.29737466895426`}, {"CURE", 365, 20.85684155548363`},
{"CURE", 365, 41.79635514267864`}, {"CURE", 365, 20.79132121997526`}, {"CURE", 365, 30.657535755016113`},
{"CURE", 365, 7.37348127233807`}, {"CURE", 365, 27.57424580386492`}, {"CURE", 365, 17.201006444479887`},
{"CURE", 365, 33.52180077030349`}, {"CURE", 365, 52.949879834803916`}, {"CURE", 365, 59.2073965455279`},
{"CURE", 365, 35.619161255948605`}, {"CURE", 365, 29.537910496289257`}, {"CURE", 365, 40.252119542745774`},
{"CURE", 365, 19.337675454755928`}, {"CURE", 365, 21.895200536308266`}, {"CURE", 365, 15.496301320298153`},
{"CURE", 365, 12.155721020938529`}, {"CURE", 365, 19.57698560969329`}, {"CURE", 365, 50.25030035626885`},
{"CURE", 365, 5.758915646105715`}, {"CURE", 365, 25.495311676686963`}, {"CURE", 365, 4.8188892457378225`},
{"CURE", 365, 25.070026085484752`}, {"CURE", 365, 14.221442898039623`}, {"CURE", 365, 53.04331138915985`},
{"CURE", 365, 4.889018674041055`}, {"CURE", 365, 34.86440699386168`}, {"CURE", 365, 43.7790222519909`},
{"CURE", 365, 7.16222301025635`}, {"CURE", 365, 16.395336023599086`}, {"CURE", 365, 13.19852646103792`},
{"CURE", 365, 7.539048102362237`}, {"CURE", 365, 44.677190603308325`}, {"CURE", 365, 16.7757873714313`},
{"CURE", 365, 32.35702873059056`}, {"CURE", 365, 31.457114844799367`}, {"CURE", 365, 35.678431121878425`},
{"CURE", 365, 27.890906736059463`}, {"CURE", 365, 33.367446088694344`}, {"CURE", 365, 26.428716034574457`},
{"CURE", 365, 51.058618786143114`}, {"CURE", 365, 31.093592464256677`}, {"CURE", 365, 25.36733609790897`},

{"CURE", 365, 64.97841212946393`}, {"CURE", 365, 7.523404511415811`}, {"CURE", 365, 42.69160306134192`},
{"CURE", 365, 3.1363815632631504`}, {"CURE", 365, 38.94963492039562`}, {"CURE", 365, 9.18269785422726`},
{"CURE", 365, 47.8655441315335`}, {"CURE", 365, 7.494405932504124`}, {"CURE", 365, 49.112329763830814`},
{"CURE", 365, 122.80939058392681`}, {"CURE", 365, 5.36207883231796`}, {"CURE", 365, 3.8475301206576833`},
{"CURE", 365, 14.186927710969838`}, {"CURE", 365, 3.4410096580203713`}, {"CURE", 365, 2.355723739983342`},
{"CURE", 365, 13.783315289775727`}, {"CURE", 365, 22.484451694582493`}, {"CURE", 365, 24.251819513012503`},
{"TBUR", 103.0426524224106`, 22.98470984121322`}, {"CURE", 365, 17.925067996550442`}, {"CURE", 365, 31.568774920304016`},
{"CURE", 365, 55.58794552466173`}, {"CURE", 365, 4.497411325472966`}, {"CURE", 365, 24.650129872743438`},
{"CURE", 365, 21.183706791956993`}, {"CURE", 365, 15.356857171301495`}, {"CURE", 365, 28.390973056867338`},
{"CURE", 365, 42.10024124834633`}, {"CURE", 365, 98.52998795978306`}, {"CURE", 365, 73.3483849903643`},
{"CURE", 365, 22.635190167796054`}, {"CURE", 365, 2.4109790588759124`}, {"CURE", 365, 26.29662496430086`},
{"CURE", 365, 67.83442802366946`}, {"CURE", 365, 17.06479792209315`}, {"CURE", 365, 21.18162634272888`},
{"CURE", 365, 13.16658380108334`}, {"CURE", 365, 19.30144639816032`}, {"CURE", 365, 4.986536800109058`},
{"CURE", 365, 24.433787175562966`}, {"CURE", 365, 13.706659576173417`}, {"CURE", 365, 40.104552056886675`},
{"TBUR", 91.75429759378524`, 27.41229802937949`}, {"CURE", 365, 26.666818451783104`}, {"CURE", 365, 21.94163839186013`},
{"CURE", 365, 11.611291349188692`}, {"CURE", 365, 8.000850981374457`}, {"CURE", 365, 7.8004242878882755`},
{"CURE", 365, 10.095233481724353`}, {"CURE", 365, 7.873098989789209`}, {"CURE", 365, 105.71680619906`},
{"CURE", 365, 58.82467725370108`}, {"CURE", 365, 11.381511272364145`}, {"CURE", 365, 175.33505884365684`},
{"CURE", 365, 50.14133085707891`}, {"CURE", 365, 26.546174955642805`}, {"CURE", 365, 45.91732065442352`},
{"CURE", 365, 6.904551225891035`}, {"CURE", 365, 55.020632112155326`}, {"CURE", 365, 147.19636531548662`},
{"CURE", 365, 7.984676765127638`}, {"CURE", 365, 24.911843578161786`}, {"CURE", 365, 40.74069319051273`},
{"CURE", 365, 20.52258570397938`}, {"CURE", 365, 10.108983934788021`}, {"CURE", 365, 8.054424374144157`},
{"CURE", 365, 69.89144854235373`}, {"CURE", 365, 36.69123618519446`}, {"CURE", 365, 20.87936484262286`},
{"CURE", 365, 51.84148512776957`}, {"CURE", 365, 9.351332669858486`}, {"CURE", 365, 25.02490619250396`},
{"CURE", 365, 15.114322413412026`}, {"CURE", 365, 101.9615795848726`}, {"TBUR", 48.11794911112639`, 25.51898065933844`},
{"TBUR", 73.8549811699981 8`, 118.51834623231201`}, {"CURE", 365, 185.08340369196654`}, {"CURE", 365, 14.22675543470056`},
{"CURE", 365, 4.010375625373254`}, {"CURE", 365, 3.495777798136847`}, {"CURE", 365, 25.55858584230092`},
{"CURE", 365, 99.21584227294609`}, {"CURE", 365, 19.875563315699722`}, {"CURE", 365, 15.262501462503677`},
{"TBUR", 55.35789743720239`, 28.6250278294759`}, {"CURE", 365, 31.377274274725426`}, {"CURE", 365, 130.88836873089983`},
{"CURE", 365, 108.05796148612595`}, {"CURE", 365, 22.59533542773263`}, {"CURE", 365, 7.50681418100786`},
{"CURE", 365, 18.390708239760613`}, {"CURE", 365, 2.8867060125761363`}, {"CURE", 365, 54.14117532932377`},

{"CURE", 365, 80.76560056650455`}, {"CURE", 365, 26.412353043593107`}, {"CURE", 365, 20.930346718391373`},
{"CURE", 365, 88.51812893104315`}, {"CURE", 365, 5.600444800270098`}, {"CURE", 365, 7.294424865949933`},
{"CURE", 365, 22.89007874561855`}, {"CURE", 365, 29.386935892738418`}, {"TBUR", 58.32692536543443`, 55.43787134961543`},
{"CURE", 365, 57.42816318561733`}, {"CURE", 365, 23.57485094392501`}, {"CURE", 365, 24.286214579358056`},
{"CURE", 365, 30.31985256943004`}, {"CURE", 365, 10.500100057095953`}, {"CURE", 365, 2.9769843290486357`},
{"CURE", 365, 31.299089516490163`}, {"CURE", 365, 37.716630446307434`}, {"CURE", 365, 18.336490187054917`},
{"CURE", 365, 33.23280156107092`}, {"CURE", 365, 10.343382017070564`}, {"CURE", 365, 6.801122856171552`},
{"CURE", 365, 29.77622158688847`}, {"CURE", 365, 18.051931170373802`}, {"CURE", 365, 25.860475238068204`},
{"CURE", 365, 170.73446523932853`}, {"CURE", 365, 16.258241016324234`}, {"CURE", 365, 7.919388482765977`},
{"CURE", 365, 6.153950652722899`}, {"CURE", 365, 7.46067882266197`}, {"CURE", 365, 42.61190305172233`},
{"CURE", 365, 2.935647296720304`}, {"CURE", 365, 39.679318536860684`}, {"CURE", 365, 43.33866515204771`},
{"CURE", 365, 33.827811540886735`}, {"CURE", 365, 27.732265045380018`}, {"CURE", 365, 5.491059949552371`},
{"CURE", 365, 60.35690436318362`}, {"TBUR", 49.27998790259729`, 18.17348236547072`}, {"CURE", 365, 8.151572700733766`},
{"CURE", 365, 53.740215576601564`}, {"CURE", 365, 9.560280030925083`}, {"CURE", 365, 64.37860486126611`},
{"CURE", 365, 41.330515902802574`}, {"CURE", 365, 27.51840694472641`}, {"CURE", 365, 40.05212435316224`},
{"CURE", 365, 14.162334727591228`}, {"CURE", 365, 62.811059944963844`}, {"CURE", 365, 7.454434222570831`},
{"CURE", 365, 31.519886303503046`}, {"CURE", 365, 36.92116977432564`}, {"CURE", 365, 15.69742223190428`},
{"CURE", 365, 146.27132294508607`}, {"CURE", 365, 29.24015768387327`}, {"CURE", 365, 29.94102024572654`},
{"CURE", 365, 30.57416207189458`}, {"CURE", 365, 122.50207466576167`}, {"CURE", 365, 38.91175131337052`},
{"CURE", 365, 36.303088269543366`}, {"CURE", 365, 78.97467874533059`}, {"CURE", 365, 27.788854161353154`},
{"CURE", 365, 24.207126993417898`}, {"CURE", 365, 29.92549528009355`}, {"CURE", 365, 41.335779042250664`},
{"CURE", 365, 73.05112856008562`}, {"CURE", 365, 15.757706846542684`}, {"CURE", 365, 14.387624866310555`},
{"CURE", 365, 30.79145053714867`}, {"CURE", 365, 31.346363265150476`}, {"CURE", 365, 35.17562780908711`},
{"CURE", 365, 37.69444345486228`}, {"CURE", 365, 21.051532059253915`}, {"CURE", 365, 10.571161612284977`},
{"CURE", 365, 39.693543273075534`}, {"CURE", 365, 10.04925313718295`}, {"CURE", 365, 21.36700710999457`},
{"CURE", 365, 25.36756943341746`}, {"CURE", 365, 7.666587912673414`}, {"CURE", 365, 3.809794318753977`},
{"TBUR", 85.47649057806747`, 23.43553442521614`}, {"CURE", 365, 31.867743885396745`}, {"CURE", 365, 37.85035549289645`},
{"CURE", 365, 27.069663244206634`}, {"CURE", 365, 3.4690942013914996`}, {"CURE", 365, 31.07278630173144`},
{"CURE", 365, 5.9155404434043195`}, {"CURE", 365, 91.37000660235705`}, {"CURE", 365, 118.86251478436073`},
{"CURE", 365, 16.512369652080604`}, {"TBUR", 68.39233702937075`, 23.428948288596285`},
{"CURE", 365, 127.57999034833983`}, {"CURE", 365, 22.88193664536509`}, {"CURE", 365, 13.241492124551787`},

{"CURE", 365, 10.321010553860255`}, {"CURE", 365, 2.829541761294569`}, {"CURE", 365, 17.84276150503059`},
{"CURE", 365, 19.89448870950073`}, {"CURE", 365, 52.64770626064185`}, {"CURE", 365, 25.22303749038908`},
{"CURE", 365, 43.92886456786882`}, {"CURE", 365, 15.519962621427876`}, {"TBUR", 136.05199753964447`, 33.58927127351332`},
{"CURE", 365, 75.27674109129586`}, {"CURE", 365, 7.6451070773568555`}, {"CURE", 365, 11.809523523334452`},
{"CURE", 365, 17.531275419164995`}, {"CURE", 365, 24.50084652876309`}, {"TBUR", 130.31266825617183`, 16.00949968459773`},
{"CURE", 365, 15.379693486027701`}, {"CURE", 365, 6.5083807030092915`}, {"CURE", 365, 10.059541322606915`},
{"CURE", 365, 19.05106108899553`}, {"CURE", 365, 43.39511435202188`}, {"TBUR", 74.95390958219672`, 38.52043747432328`},
{"CURE", 365, 19.773649112921067`}, {"TBUR", 31.952969194244186`, 29.51736532621389`}, {"CURE", 365, 63.82341687764535`},
{"CURE", 365, 31.246136061080506`}, {"CURE", 365, 23.382683456538494`}, {"CURE", 365, 14.894478101580829`},
{"CURE", 365, 4.662614157073673`}, {"CURE", 365, 25.9628515332641`}, {"CURE", 365, 29.951331236979527`},
{"CURE", 365, 117.26685610775601`}, {"CURE", 365, 5.97671380206868`}, {"CURE", 365, 5.844111514001606`},
{"CURE", 365, 18.725420034361466`}, {"CURE", 365, 54.587179615205706`}, {"CURE", 365, 6.809812981812497`},
{"CURE", 365, 33.31792260339761`}, {"CURE", 365, 7.768129182605218`}, {"CURE", 365, 19.356138791352432`},
{"CURE", 365, 7.857515264225963`}, {"CURE", 365, 8.211387376099847`}, {"CURE", 365, 7.406221289659148`},
{"CURE", 365, 79.03287060032139`}, {"CURE", 365, 12.57662745342657`}, {"TBUR", 70.34290341190561`, 22.15959410636834`},
{"CURE", 365, 3.0701307011192056`}, {"CURE", 365, 36.776438830026635`}, {"CURE", 365, 51.470638909735534`},
{"CURE", 365, 44.82691157196355`}, {"CURE", 365, 6.772087879820219`}, {"CURE", 365, 13.58764791867759l`},
{"CURE", 365, 138.1474546408907`}, {"CURE", 365, 37.54521360621865`}, {"CURE", 365, 25.555209137524702`},
{"CURE", 365, 9.064103929006436`}, {"CURE", 365, 13.009731726301428`}, {"CURE", 365, 20.092302316575694`},
{"CURE", 365, 4.973487850102817`}, {"CURE", 365, 24.453759609068996`}, {"CURE", 365, 38.48455246922478`},
{"CURE", 365, 23.387783265681218`}, {"CURE", 365, 25.383925546873037`}, {"CURE", 365, 18.388295229344198`},
{"CURE", 365, 11.894003928445306`}, {"CURE", 365, 67.91988857839536`}, {"CURE", 365, 98.62284488115029`},
{"CURE", 365, 26.31684066870875`}, {"CURE", 365, 33.225468255425945`}, {"CURE", 365, 42.089292197746815`},
{"CURE", 365, 41.14418582711481`}, {"CURE", 365, 3.588732503514153`}, {"CURE", 365, 48.07131080404325`},
{"CURE", 365, 49.75031048442546`}, {"CURE", 365, 33.58827522252481`}, {"CURE", 365, 134.94706163895373`},
{"CURE", 365, 28.053313375322197`}, {"CURE", 365, 12.447112008026897`}, {"CURE", 365, 29.520860911610786`},
{"CURE", 365, 37.51904481414021`}, {"CURE", 365, 16.80683218660601`}, {"CURE", 365, 13.16167218441427`},
{"CURE", 365, 11.471482738105276`}, {"CURE", 365, 20.75751749317782`}, {"CURE", 365, 64.10185573562204`},
{"CURE", 365, 41.141968791731465`}, {"CURE", 365, 7.150710841169644`}, {"CURE", 365, 41.81427450423834`},
{"CURE", 365, 60.554712694718035`}, {"CURE", 365, 11.111217845780274`}, {"CURE", 365, 63.47711175730156`},
{"CURE", 365, 9.54325047558774`}, {"CURE", 365, 6.085834233184449`}, {"CURE", 365, 8.649073124761712`},

{"CURE", 365, 16.615798313801708`}, {"TBUR", 70.34185296017533`, 74.12547232940345`}, {"CURE", 365, 26.632858814245274`},
{"CURE", 365, 33.595511258790744`}, {"CURE", 365, 8.03983463803771`}, {"CURE", 365, 153.07783827901397`},
{"CURE", 365, 70.27181302349518`}, {"CURE", 365, 11.960772097420332`}, {"CURE", 365, 29.330014024765095`},
{"CURE", 365, 16.356894734546202`}, {"CURE", 365, 16.510413649230312`}, {"CURE", 365, 55.768444483933465`},
{"TBUR", 48.74736126616614`, 70.3078617652878`}, {"CURE", 365, 7.584514667364541`}, {"CURE", 365, 14.770648782321246`},
{"CURE", 365, 5.23936962679491`}, {"CURE", 365, 20.217604273890377`}, {"CURE", 365, 34.580661817596656`},
{"CURE", 365, 56.13755714845964`}, {"CURE", 365, 31.320645821512752`}, {"CURE", 365, 112.8135038368594`},
{"CURE", 365, 53.92688702147822`}, {"TBUR", 62.32523311592774`, 8.848567052351894`}, {"CURE", 365, 19.707571958413222`},
{"TBUR", 31.049471563267574`, 19.751117259334606`}, {"TBUR", 63.51634763664903`, 43.90424003199148`},
{"TBUR", 186.12592135507657`, 5.764686970126604`}, {"CURE", 365, 15.473278473093602`}, {"CURE", 365, 8.71285872226276`},
{"CURE", 365, 21.757798872613353`}, {"CURE", 365, 49.69639625439019`}, {"CURE", 365, 21.147493862674978`},
{"CURE", 365, 4.59342633405321`}, {"CURE", 365, 87.382058485861`}, {"CURE", 365, 16.493011124682706`},
{"CURE", 365, 5.604746873686143`}, {"CURE", 365, 7.367356345763691`}, {"CURE", 365, 6.2196743830623635`},
{"CURE", 365, 7.947759431157098`}, {"CURE", 365, 63.95344088262247`}, {"CURE", 365, 34.953663555346544`},
{"CURE", 365, 21.063227428720857`}, {"CURE", 365, 9.862266199618315`}, {"CURE", 365, 16.492633788308474`},
{"CURE", 365, 9.541736288171919`}, {"CURE", 365, 23.02145151920475`}, {"CURE", 365, 79.12530123084595`},
{"CURE", 365, 45.0693015547349`}, {"CURE", 365, 22.80459553138008`}, {"CURE", 365, 13.157596173386203`},
{"CURE", 365, 13.658177840046996`}, {"CURE", 365, 169.1548130647192`}, {"CURE", 365, 8.059414683241457`},
{"CURE", 365, 4.151652362551196`}, {"CURE", 365, 3.6072176935206657`}, {"CURE", 365, 45.97533932708031`},
{"CURE", 365, 26.898212618226264`}, {"CURE", 365, 29.86887530258108`}, {"CURE", 365, 44.39465473676159`},
{"CURE", 365, 73.14616541974503`}, {"CURE", 365, 75.80613308472918`}, {"CURE", 365, 28.887494949876015`},
{"CURE", 365, 12.474261021352225`}, {"CURE", 365, 14.456870677698795`}, {"CURE", 365, 28.646748005661514`},
{"CURE", 365, 16.35163400472899`}, {"TBUR", 23.570437661423995`, 6.125046577505361`}, {"CURE", 365, 25.66757740942969`},
{"CURE", 365, 160.0178557738235`}, {"CURE", 365, 16.492015501155805`}, {"CURE", 365, 5.926377374715332`},
{"CURE", 365, 56.23962518501508`}, {"CURE", 365, 14.064573048768667`}, {"CURE", 365, 40.398463057760914`},
{"CURE", 365, 194.58343956238744`}, {"CURE", 365, 89.84345745677501`}, {"CURE", 365, 29.167184021069897`},
{"CURE", 365, 24.438951270407355`}, {"CURE", 365, 10.415723970976899`}, {"CURE", 365, 2.9717291571495785`},
{"CURE", 365, 11.602266213516645`}, {"CURE", 365, 34.11004103650202`}, {"CURE", 365, 18.903692196499296`},
{"CURE", 365, 60.3256301099037`}, {"CURE", 365, 23.902199330984036`}, {"CURE", 365, 24.424537513729245`},
{"CURE", 365, 6.782613284443042`}, {"CURE", 365, 19.208788381755056`}, {"CURE", 365, 19.716959381677242`},
{"CURE", 365, 5.140329223880321`}, {"CURE", 365, 16.339340665054618`}, {"CURE", 365, 9.75444916726226`},

{"TBUR", 76.62186519092721`, 15.409282012039112`}, {"CURE", 365, 32.11396925605385`}, {"CURE", 365, 16.16069018199527`},
{"CURE", 365, 3.7538273617899502`}, {"CURE", 365, 25.856219626970976`}, {"CURE", 365, 2.890887450926185`},
{"CURE", 365, 8.48046201096626`}, {"CURE", 365, 25.184585032212098`}, {"CURE", 365, 29.059906574789277`},
{"CURE", 365, 5.772306234512786`}, {"CURE", 365, 16.997382281362114`}, {"CURE", 365, 21.990816169670598`},
{"CURE", 365, 111.11127817614899`}, {"CURE", 365, 25.583005577842314`}, {"CURE", 365, 7.206086447090206`},
{"CURE", 365, 78.61375646822418`}, {"CURE", 365, 7.692594811913802`}, {"CURE", 365, 29.07997830471229`},
{"CURE", 365, 30.115920870855483`}, {"CURE", 365, 8.988087484133922`}, {"CURE", 365, 10.004621300714287`},
{"CURE", 365, 44.32720625641707`}, {"CURE", 365, 4.326515841067321`}, {"CURE", 365, 37.56475428229382`},
{"CURE", 365, 9.947368482636398`}, {"CURE", 365, 13.008163117675867`}, {"CURE", 365, 2.902425270332462`},
{"CURE", 365, 14.93433331985482`}, {"CURE", 365, 50.81229456641769`}, {"CURE", 365, 19.371027335497676`},
{"CURE", 365, 14.064121089620459`}, {"CURE", 365, 20.10052529716202`}, {"CURE", 365, 15.720655087373935`},
{"TBUR", 77.5691492324557`, 19.841868291717798`}, {"CURE", 365, 35.07887859419699`}, {"CURE", 365, 33.86937918500155`},
{"CURE", 365, 4.172107217709284`}, {"CURE", 365, 68.31873503414893`}, {"CURE", 365, 7.960124925956545`},
{"CURE", 365, 3.989704935417524`}, {"CURE", 365, 19.022556997488692`}, {"CURE", 365, 19.981776724723815`},
{"CURE", 365, 13.459037457543976`}, {"CURE", 365, 23.390316502856006`}, {"CURE", 365, 20.171444346120726`},
{"CURE", 365, 32.424525189649756`}, {"CURE", 365, 27.396096596971642`}, {"TBUR", 55.65777261791283`, 39.05657542651722`},
{"CURE", 365, 6.174837328358764`}, {"CURE", 365, 78.97297729314967`}, {"CURE", 365, 76.88135762890316`},
{"CURE", 365, 4.25728736217448`}, {"CURE", 365, 10.725218387551996`}, {"CURE", 365, 26.610988147356384`},
{"CURE", 365, 13.506568091618515`}, {"CURE", 365, 41.42359212299887`}, {"CURE", 365, 83.29175086436805`},
{"CURE", 365, 26.3306941778396`}, {"CURE", 365, 4.515685438258861`}, {"CURE", 365, 4.4316589477729265`},
{"CURE", 365, 10.409635835702224`}, {"CURE", 365, 3.141858763393374`}, {"CURE", 365, 35.67089475948561`},
{"CURE", 365, 5.527752007775771`}, {"CURE", 365, 37.07657582828771`}, {"CURE", 365, 22.899644552751955`},
{"CURE", 365, 39.481791436112864`}, {"CURE", 365, 9.71783743056256`}, {"CURE", 365, 49.02951364400831`},
{"CURE", 365, 162.21475388549007`}, {"CURE", 365, 58.452802712552476`}, {"CURE", 365, 94.67504615137852`},
{"CURE", 365, 27.874450305513818`}, {"CURE", 365, 66.41226106463833`}, {"CURE", 365, 66.53482007189301`},
{"CURE", 365, 8.076391278977244`}, {"CURE", 365, 5.648592430473966`}, {"CURE", 365, 18.358214229134077`},
{"CURE", 365, 38.11772026145056`}, {"CURE", 365, 47.68742269365314`}, {"CURE", 365, 20.237574191975185`},
{"CURE", 365, 30.13145190739118`}, {"CURE", 365, 5.678885609705639`}, {"CURE", 365, 7.207173141079952`},
{"CURE", 365, 35.0707019290636`}, {"CURE", 365, 11.341300046514677`}, {"CURE", 365, 4.827210874904988`},
{"CURE", 365, 7.142181118257957`}, {"CURE", 365, 3.4905685614176276`}, {"CURE", 365, 28.368597057021283`},
{"CURE", 365, 39.01946855229415`}, {"CURE", 365, 27.265328079834617`}, {"CURE", 365, 31.017122700087867`},

{"CURE", 365, 17.164443599590307`}, {"CURE", 365, 15.344375746120342`}, {"CURE", 365, 39.76463410659672`},
{"CURE", 365, 42.593007509235335`}, {"CURE", 365, 4.750943985457868`}, {"CURE", 365, 17.432896393063704`},
{"CURE", 365, 9.436634352406218`}, {"CURE", 365, 14.831126580215912`}, {"CURE", 365, 122.12491915449246`},
{"CURE", 365, 21.566576949334433`}, {"CURE", 365, 14.805733489858659`}, {"CURE", 365, 15.635825623163095`},
{"CURE", 365, 31.230855588037624`}, {"CURE", 365, 23.773158132413716`}, {"CURE", 365, 31.014460897814022`},
{"CURE", 365, 31.863579672941217`}, {"CURE", 365, 7.252935106126065`}, {"CURE", 365, 70.5539759808539`},
{"CURE", 365, 13.53363270384159`}, {"CURE", 365, 28.733000384716345`}, {"CURE", 365, 16.29797776964994`},
{"CURE", 365, 9.260389389625567`}, {"CURE", 365, 33.75725259126821`}, {"CURE", 365, 34.27362147319302`},
{"TBUR", 61.39007973847859`, 18.909457967169445`}, {"CURE", 365, 44.897263201788284`}, {"CURE", 365, 9.49756989731547`},
{"CURE", 365, 79.4355396395856`}, {"CURE", 365, 30.478850413237424`}, {"CURE", 365, 64.36823877288374`},
{"CURE", 365, 2.501223981759125`}, {"CURE", 365, 31.64659309307206`}, {"CURE", 365, 35.98658220852856`},
{"CURE", 365, 8.59653814175012`}, {"CURE", 365, 21.811595778849355`}, {"CURE", 365, 32.68012473219522`},
{"CURE", 365, 19.857886062289626`}, {"CURE", 365, 6.315609411835383`}, {"CURE", 365, 4.795403846993399`},
{"CURE", 365, 28.32216757895892`}, {"CURE", 365, 7.564051904998482`}, {"CURE", 365, 16.813352505317642`},
{"CURE", 365, 20.469439235452914`}, {"CURE", 365, 9.50483907616421`}, {"CURE", 365, 17.499789072991348`},
{"CURE", 365, 4.780165517247517`}, {"CURE", 365, 19.71137194189393`}, {"CURE", 365, 3.1874283279838096`},
{"CURE", 365, 5.6954415246365535`}, {"CURE", 365, 13.460937414612697`}, {"CURE", 365, 4.339878700145824`},
{"CURE", 365, 32.25850862317925`}, {"CURE", 365, 47.4526954768608`}, {"CURE", 365, 48.087114964584664`},
{"CURE", 365, 40.10082852601821`}, {"CURE", 365, 75.6728517563906`}, {"CURE", 365, 6.1179229116240865`},
{"CURE", 365, 27.294138345359436`}, {"CURE", 365, 29.260555533354378`}, {"CURE", 365, 50.42049106885163`},
{"CURE", 365, 37.660728371163174`}, {"CURE", 365, 17.191463370329807`}, {"CURE", 365, 80.78695319046938`},
{"CURE", 365, 2.5247178451557204`}, {"CURE", 365, 77.01639345225303`}, {"CURE", 365, 5.211296203635886`},
{"CURE", 365, 65.77281518210299`}, {"CURE", 365, 59.572535974267744`}, {"CURE", 365, 17.650174035499077`},
{"CURE", 365, 69.48390108584351`}, {"CURE", 365, 84.80750770889145`}, {"CURE", 365, 37.90560379647769`},
{"CURE", 365, 16.738661531370973`}, {"CURE", 365, 19.24280131485342`}, {"CURE", 365, 28.40775636157005`},
{"CURE", 365, 26.189455180980154`}, {"CURE", 365, 6.349886826603784`}, {"CURE", 365, 22.787667123117966`},
{"CURE", 365, 21.546166640990208`}, {"CURE", 365, 16.123396567481944`}, {"CURE", 365, 39.86752303345471`},
{"CURE", 365, 6.277533926427996`}, {"CURE", 365, 44.49350544043885`}, {"CURE", 365, 29.106318352319207`},
{"CURE", 365, 16.487223260146422`}, {"CURE", 365, 14.858888087218245`}, {"CURE", 365, 30.325398869536407`},
{"CURE", 365, 4.166657838927698`}, {"CURE", 365, 48.257456705070595`}, {"CURE", 365, 7.611627901538375`},
{"CURE", 365, 10.805343378681732`}, {"CURE", 365, 24.22322151481099`}, {"CURE", 365, 18.998180117796235`},

{"CURE", 365, 17.47193926424511`}, {"CURE", 365, 54.33722619995438`}, {"CURE", 365, 7.3574966811052285`},
{"CURE", 365, 12.81142600967759`}, {"CURE", 365, 94.80638821828848`}, {"CURE", 365, 7.191756803826736`},
{"CURE", 365, 18.52863105883997`}, {"CURE", 365, 28.154838005688696`}, {"CURE", 365, 27.800955802462855`},
{"CURE", 365, 18.895743873789584`}, {"CURE", 365, 97.71827502989646`}, {"CURE", 365, 17.626885781472104`},
{"CURE", 365, 7.430635194864994`}, {"CURE", 365, 14.760081549581441`}, {"CURE", 365, 11.44338732338001`},
{"CURE", 365, 8.761421178583824`}, {"CURE", 365, 22.993775716793756`}, {"CURE", 365, 39.38938132001792`},
{"CURE", 365, 21.668861653542812`}, {"CURE", 365, 25.2598278890648`}, {"CURE", 365, 55.332118845781004`},
{"CURE", 365, 6.093026711492463`}, {"CURE", 365, 7.170382394193145`}, {"CURE", 365, 60.565495250003856`},
{"CURE", 365, 19.456190673757096`}, {"CURE", 365, 21.123317731675005`}, {"CURE", 365, 10.616377516556954`},
{"CURE", 365, 24.31303690947111`}, {"CURE", 365, 30.29967397738247`}, {"CURE", 365, 70.19715904397526`},
{"CURE", 365, 10.349004703030655`}, {"CURE", 365, 40.35445770146006`}, {"CURE", 365, 23.75771880091885`},
{"CURE", 365, 62.89826124052087`}, {"CURE", 365, 102.88355410014901`}, {"CURE", 365, 37.61829729279286`},
{"CURE", 365, 3.644708245349713`}, {"CURE", 365, 28.543755331025046`}, {"CURE", 365, 9.003839959074899`},
{"CURE", 365, 95.34146579559275`}, {"TBUR", 35.883340332978634`, 2.9044544870003723`}, {"CURE", 365, 3.1809303457643656`},
{"CURE", 365, 110.11813528202364`}, {"CURE", 365, 25.291273782223975`}, {"CURE", 365, 8.587426329364607`},
{"CURE", 365, 16.436706825160474`}, {"CURE", 365, 5.763610098881688`}, {"TBUR", 59.20748663029966`, 5.44398857691755`},
{"CURE", 365, 6.557589835301857`}, {"CURE", 365, 23.701377461277097`}, {"CURE", 365, 50.23239529120643`},
{"CURE", 365, 19.888508790428105`}, {"CURE", 365, 4.979543076537408`}, {"CURE", 365, 67.77605853291405`},
{"CURE", 365, 135.2846631540103`}, {"CURE", 365, 3.81925695466581`}, {"CURE", 365, 30.481729016189394`},
{"CURE", 365, 16.399419458270756`}, {"CURE", 365, 3.225188790473438`}, {"CURE", 365, 25.198243384819484`},
{"CURE", 365, 16.109965940807044`}, {"CURE", 365, 12.360959961435404`}, {"CURE", 365, 24.04779170537454`},
{"CURE", 365, 13.64999059937311`}, {"CURE", 365, 39.98752938177398`}, {"CURE", 365, 23.85566138672767`},
{"CURE", 365, 56.1578037233421`}, {"CURE", 365, 24.877532896497975`}, {"CURE", 365, 12.321697601365514`},
{"CURE", 365, 5.9872440288154145`}, {"CURE", 365, 4.159667295624368`}, {"CURE", 365, 11.848920810175484`},
{"CURE", 365, 47.134198050757824`}, {"CURE", 365, 15.547806580531992`}, {"CURE", 365, 14.231042942336499`},
{"CURE", 365, 10.0673063682159`}, {"CURE", 365, 10.20657169695152`}, {"CURE", 365, 27.58017660348846`},
{"CURE", 365, 5.161512842589155`}, {"CURE", 365, 32.8623833432702`}, {"CURE", 365, 15.047691841653933`},
{"CURE", 365, 8.828175813552127`}, {"CURE", 365, 14.001320888369985`}, {"CURE", 365, 31.097885463389918`},
{"CURE", 365, 23.0713206512547`}, {"CURE", 365, 2.795594958290459`}, {"CURE", 365, 12.031913370967702`},
{"CURE", 365, 201.3334427341253`}, {"CURE", 365, 33.39862042317184`}, {"CURE", 365, 48.74604066109328`},
{"CURE", 365, 14.3649835334568`}, {"CURE", 365, 53.247713622290924`}, {"CURE", 365, 6.374246751654104`},

{"CURE", 365, 21.456749207990622`}, {"CURE", 365, 99.15593818742688`}, {"CURE", 365, 18.573780515929247`},
{"CURE", 365, 16.301115099748227`}, {"CURE", 365, 28.039934639546026`}, {"CURE", 365, 14.811324438162547`},
{"CURE", 365, 3.292634557328253`}, {"CURE", 365, 30.55989059441421`}, {"CURE", 365, 5.458417059655312`},
{"CURE", 365, 12.948883018461986`}, {"CURE", 365, 30.657198705789888`}, {"CURE", 365, 11.009301338476492`},
{"CURE", 365, 58.483300355518274`}, {"CURE", 365, 17.369919424720308`}, {"CURE", 365, 29.937166412028013`},
{"CURE", 365, 20.748938452426525`}, {"CURE", 365, 5.872245792858761`}, {"CURE", 365, 21.75623008674992`},
{"CURE", 365, 47.079338083211326`}, {"CURE", 365, 15.7762305542997`}, {"CURE", 365, 11.875196343962383`},
{"CURE", 365, 5.629577306778445`}, {"CURE", 365, 17.781362854567647`}, {"CURE", 365, 21.388692865298083`},
{"CURE", 365, 61.182599918321756`}, {"CURE", 365, 9.827985063937728`}, {"CURE", 365, 14.759839722202303`},
{"CURE", 365, 57.412680127518335`}, {"CURE", 365, 6.049846864540476`}, {"CURE", 365, 82.88012081130807`},
{"CURE", 365, 7.020125516376456`}, {"CURE", 365, 12.839684555870019`}, {"CURE", 365, 29.60827108262683`},
{"CURE", 365, 47.5897354078603`}, {"CURE", 365, 21.44733762642985`}, {"CURE", 365, 11.216570842938784`},
{"CURE", 365, 3.303752186304047`}, {"TBUR", 54.31287535022695`, 28.799068447989573`}, {"CURE", 365, 26.176604569053218`},
{"CURE", 365, 25.773497729212625`}, {"CURE", 365, 10.16366470717641`}, {"CURE", 365, 24.916737906533648`},
{"CURE", 365, 2.8773863912180224`}, {"CURE", 365, 16.60289152139422`}, {"CURE", 365, 7.254255793260703`},
{"CURE", 365, 3.2097595276429938`}, {"CURE", 365, 16.693997043707416`}, {"CURE", 365, 3.5743103851829274`},
{"CURE", 365, 11.365052285323832`}, {"CURE", 365, 7.790577335918708`}, {"TBUR", 31.95101224168553`, 4.475924366593911`},
{"CURE", 365, 15.42955102501367`}, {"CURE", 365, 41.138175313087686`}, {"CURE", 365, 22.480047454378365`},
{"CURE", 365, 20.388472766877555`}, {"CURE", 365, 32.28147862107787`}, {"CURE", 365, 27.505268226393596`}},
{{"1700 Res", "1700 OS", "1700 Tox"}, {"CURE", 365, 14.127233555450243`}, {"CURE", 365, 29.30226118054651`},
{"CURE", 365, 20.513010772998225`}, {"TBUR", 58.85781061004984`, 6.8395087776425205`},
{"TBUR", 39.88718592250468`, 25.400295219914657`}, {"CURE", 365, 53.075299713473726`}, {"CURE", 365, 49.94125222124623`},
{"CURE", 365, 5.217302280846427`}, {"CURE", 365, 36.01667642652865`}, {"CURE", 365, 31.14070778191107`},
{"CURE", 365, 116.8846522647262`}, {"CURE", 365, 14.506916742608118`}, {"CURE", 365, 51.344049612587575`},
{"CURE", 365, 173.50528765819087`}, {"CURE", 365, 24.639366213088863`}, {"CURE", 365, 38.4523158151016`},
{"CURE", 365, 76.23023887382256`}, {"CURE", 365, 34.12189738782866`}, {"TBUR", 106.98387814690008`, 25.72698531088065`},
{"CURE", 365, 8.558120595751792`}, {"CURE", 365, 52.90683996794148`}, {"CURE", 365, 21.447418775774047`},
{"CURE", 365, 8.216473748708157`}, {"CURE", 365, 6.546565443448562`}, {"CURE", 365, 29.796660018241933`},
{"CURE", 365, 32.70197240451944`}, {"CURE", 365, 2.972888886288103`}, {"CURE", 365, 4.50585179576708`},
{"CURE", 365, 16.69449529439039`}, {"CURE", 365, 19.5246699837665`}, {"CURE", 365, 16.664379200759985`},
{"CURE", 365, 58.4489814934534`}, {"CURE", 365, 12.880744530118754`}, {"TBUR", 111.860115354956`, 10.232168231750125`},

{"CURE", 365, 83.04343325002758`}, {"CURE", 365, 5.085304729637833`}, {"CURE", 365, 21.99667816107906`},
{"TBUR", 39.70455366936943`, 48.84264361478503`}, {"CURE", 365, 12.211915726734654`}, {"CURE", 365, 18.785573564384663`},
{"CURE", 365, 42.80164542997761`}, {"CURE", 365, 28.961267846889317`}, {"CURE", 365, 5.874938594125715`},
{"CURE", 365, 19.809121339808357`}, {"CURE", 365, 28.59054463392495`}, {"CURE", 365, 18.75012518183321`},
{"CURE", 365, 199.20310250065532`}, {"CURE", 365, 42.93770507021468`}, {"CURE", 365, 64.78518106821842`},
{"CURE", 365, 8.027719428142229`}, {"CURE", 365, 22.1065616587242`}, {"CURE", 365, 30.5520341480907`},
{"CURE", 365, 24.696207878575112`}, {"CURE", 365, 16.03378347188849`}, {"CURE", 365, 6.9099404795144554`},
{"CURE", 365, 73.1146921500744 8`}, {"CURE", 365, 22.977123809528155`}, {"CURE", 365, 4.358622321953 5025`},
{"CURE", 365, 46.98755804524709`}, {"CURE", 365, 32.888051980675705`}, {"CURE", 365, 22.54275841116322`},
{"CURE", 365, 17.262119909753093`}, {"CURE", 365, 9.698744845805916`}, {"CURE", 365, 37.20639745528829`},
{"CURE", 365, 26.41189099521679`}, {"CURE", 365, 30.226092937498233`}, {"CURE", 365, 6.918498474248771`},
{"CURE", 365, 7.022629724843216`}, {"CURE", 365, 7.480212490558602`}, {"CURE", 365, 30.54537418265161`},
{"CURE", 365, 11.181517237428377`}, {"CURE", 365, 24.90184015247168`}, {"CURE", 365, 46.74169311845958`},
{"TBUR", 22.238099406703437`, 13.682133281644596`}, {"CURE", 365, 12.485342708284506`},
{"CURE", 365, 34.604322312193574`}, {"CURE", 365, 20.227013511904584`}, {"CURE", 365, 15.391902642920344`},
{"CURE", 365, 61.29570317497215`}, {"CURE", 365, 67.33327981549941`}, {"CURE", 365, 49.57036025805628`},
{"CURE", 365, 13.376851986867266`}, {"CURE", 365, 27.047264502035226`}, {"CURE", 365, 18.904991150975416`},
{"CURE", 365, 21.374682284615304`}, {"CURE", 365, 10.013742955691495`}, {"CURE", 365, 106.81314675705566`},
{"CURE", 365, 23.27689539296974`}, {"CURE", 365, 29.33083565489238`}, {"CURE", 365, 25.226203929254876`},
{"CURE", 365, 20.770567235369604`}, {"CURE", 365, 6.182596000310836`}, {"CURE", 365, 11.333691168107066`},
{"CURE", 365, 26.23482079140674`}, {"TBUR", 46.380618536729656`, 9.410936556334486`}, {"CURE", 365, 11.29286465319325`},
{"CURE", 365, 29.472610934221134`}, {"CURE", 365, 74.7994892642245`}, {"CURE", 365, 40.04190887547157`},
{"CURE", 365, 20.08297770696998`}, {"TBUR", 60.84120955961276`, 39.73395954968497`}, {"CURE", 365, 25.89533017126264`},
{"CURE", 365, 16.78804778715602`}, {"CURE", 365, 114.9553688041129`}, {"CURE", 365, 12.77223671580307`},
{"CURE", 365, 8.677838165482639`}, {"CURE", 365, 50.913899298098244`}, {"CURE", 365, 14.987502289975335`},
{"CURE", 365, 15.367925876599436`}, {"CURE", 365, 92.46195480018774`}, {"CURE", 365, 7.504342954328176`},
{"CURE", 365, 4.760217215653066`}, {"CURE", 365, 19.802630945316167`}, {"CURE", 365, 15.654011994541785`},
{"CURE", 365, 11.790419092679187`}, {"CURE", 365, 30.10380201348286`}, {"CURE", 365, 8.470168604782263`},
{"CURE", 365, 5.2084337059268995`}, {"CURE", 365, 24.387132444564145`}, {"CURE", 365, 37.291919020158`},
{"CURE", 365, 6.347609029781708`}, {"CURE", 365, 21.01649418597582`}, {"CURE", 365, 63.80800376001906`},
{"TBUR", 54.08987102597456`, 24.241600451616403`}, {"CURE", 365, 14.153882329430232`}, {"CURE", 365, 49.3481635431858`},

{"CURE", 365, 24.798802045931613`}, {"CURE", 365, 3.2698612922592867`}, {"CURE", 365, 28.33711437872887`},
{"CURE", 365, 21.88584963053257`}, {"CURE", 365, 17.43484000781565`}, {"CURE", 365, 20.677147967807255`},
{"CURE", 365, 5.378471406940998 5`}, {"CURE", 365, 30.681158593568576`}, {"CURE", 365, 11.019207931268873`},
{"CURE", 365, 24.830407651010553`}, {"CURE", 365, 93.0125734687883`}, {"CURE", 365, 32.82733729720906`},
{"CURE", 365, 76.3808231032741`}, {"CURE", 365, 25.167284149470714`}, {"CURE", 365, 103.8134543971201`},
{"CURE", 365, 3.24256900826017`}, {"CURE", 365, 28.49251732648425`}, {"CURE", 365, 32.47490236169513`},
{"CURE", 365, 4.218269719488786`}, {"CURE", 365, 5.842472122961842`}, {"CURE", 365, 35.8673954708214`},
{"CURE", 365, 42.205281211492306`}, {"CURE", 365, 36.02342915578085`}, {"CURE", 365, 29.469117226873603`},
{"CURE", 365, 28.179321138847673`}, {"CURE", 365, 17.057623107009334`}, {"CURE", 365, 4.512651846735676`},
{"CURE", 365, 19.609683815148717`}, {"CURE", 365, 9.305775337588901`}, {"CURE", 365, 4.555193425419904`},
{"CURE", 365, 27.624834252423`}, {"CURE", 365, 15.46295472583094`}, {"CURE", 365, 40.875712093360136`},
{"CURE", 365, 16.49939661260563`}, {"CURE", 365, 13.733781254034495`}, {"CURE", 365, 91.97455015158252`},
{"CURE", 365, 6.862052560215088`}, {"CURE", 365, 20.50863197684025`}, {"CURE", 365, 11.628249187023863`},
{"CURE", 365, 27.444236628566813`}, {"CURE", 365, 7.551937577748605`}, {"CURE", 365, 30.160842460168155`},
{"CURE", 365, 79.6096184038097`}, {"CURE", 365, 6.81239502779223`}, {"CURE", 365, 20.704616206607906`},
{"CURE", 365, 22.307784077520544`}, {"CURE", 365, 80.52666913128952`}, {"TBUR", 88.34831673279885`, 30.1595839614238`},
{"CURE", 365, 25.11257061255761`}, {"CURE", 365, 43.95543334034119`}, {"CURE", 365, 16.92525639827505`},
{"CURE", 365, 51.068259012303265`}, {"CURE", 365, 32.50647904886161`}, {"CURE", 365, 19.671812486150014`},
{"CURE", 365, 17.933783235518646`}, {"CURE", 365, 5.8445524902740695`}, {"CURE", 365, 3.961041980223652`},
{"CURE", 365, 9.271638192115695`}, {"CURE", 365, 12.437237749570867`}, {"CURE", 365, 5.464286374393393`},
{"CURE", 365, 20.02837907349382`}, {"CURE", 365, 54.7380734351474`}, {"CURE", 365, 14.807001822404269`},
{"CURE", 365, 27.392144721254184`}, {"CURE", 365, 34.41760083755105`}, {"CURE", 365, 28.624492394242104`},
{"CURE", 365, 20.143789279970044`}, {"CURE", 365, 8.000344719559388`}, {"CURE", 365, 55.098757395820215`},
{"CURE", 365, 4.769381410025509`}, {"CURE", 365, 51.377666022422616`}, {"CURE", 365, 49.68456205213829`},
{"CURE", 365, 13.935619200385311`}, {"CURE", 365, 16.553536092208926`}, {"CURE", 365, 95.03258475236527`},
{"CURE", 365, 24.45573776163941`}, {"CURE", 365, 29.28605423076426`}, {"CURE", 365, 18.323360316617695`},
{"CURE", 365, 6.895067775381094`}, {"CURE", 365, 42.21833232285783`}, {"TBUR", 47.482715706796796`, 112.08476705198788`},
{"CURE", 365, 17.17412335374478`}, {"CURE", 365, 15.51951218111739`}, {"CURE", 365, 28.603448803044362`},
{"CURE", 365, 11.421485349591748`}, {"CURE", 365, 144.91055920599288`}, {"CURE", 365, 11.600469399562696`},
{"CURE", 365, 71.60727133332409`}, {"CURE", 365, 55.09934966701047`}, {"CURE", 365, 53.54468060322921`},
{"CURE", 365, 28.61199673643881`}, {"CURE", 365, 66.37544572306632`}, {"CURE", 365, 116.19547941535022`},

{"CURE", 365, 30.156531772667318`}, {"CURE", 365, 5.538592157794809`}, {"CURE", 365, 49.20483969952221`},
{"CURE", 365, 30.662672032233125`}, {"CURE", 365, 29.554937749691707`}, {"CURE", 365, 6.506446957890473`},
{"CURE", 365, 149.35229105350155`}, {"CURE", 365, 10.91755678561372`}, {"CURE", 365, 47.3710471698890 8`},
{"CURE", 365, 29.849870053264482`}, {"CURE", 365, 21.219834556538665`}, {"CURE", 365, 5.102009659883126`},
{"CURE", 365, 38.35463605161179`}, {"CURE", 365, 22.497122878610643`}, {"CURE", 365, 59.52204688863479`},
{"CURE", 365, 39.21179376537759`}, {"CURE", 365, 49.48655694465511`}, {"CURE", 365, 23.744611023658294`},
{"CURE", 365, 21.126342630351466`}, {"TBUR", 52.64383312927865`, 110.37197170621671`}, {"CURE", 365, 28.94597669728715`},
{"CURE", 365, 7.720777565825931`}, {"CURE", 365, 26.007134120908304`}, {"TBUR", 106.00652318782858`, 26.280493521974552`},
{"CURE", 365, 3.537072434370303`}, {"CURE", 365, 6.321030590164034`}, {"CURE", 365, 9.654099726254312`},
{"CURE", 365, 91.04271211230694`}, {"CURE", 365, 28.034287681510154`}, {"CURE", 365, 29.224351934448322`},
{"CURE", 365, 23.402506869205013`}, {"CURE", 365, 96.00705230272864`}, {"CURE", 365, 22.16071260190067`},
{"CURE", 365, 44.441602618402264`}, {"CURE", 365, 22.09284796147787`}, {"CURE", 365, 32.5749407292061 1`},
{"CURE", 365, 7.834527692317911`}, {"CURE", 365, 29.299122777434157`}, {"CURE", 365, 18.278370191087316`},
{"CURE", 365, 35.617403364999234`}, {"CURE", 365, 56.26585612579469`}, {"CURE", 365, 62.97492299165857`},
{"CURE", 365, 37.94159583368904`}, {"CURE", 365, 31.384446822727448`}, {"CURE", 365, 42.84899499759226`},
{"CURE", 365, 20.547462453715976`}, {"CURE", 365, 23.264522748915695`}, {"CURE", 365, 16.4692817956897`},
{"CURE", 365, 12.916159146758897`}, {"CURE", 365, 20.80259809397585`}, {"CURE", 365, 53.392371820431514`},
{"CURE", 365, 6.119156752512134`}, {"CURE", 365, 27.09508184836544`}, {"CURE", 365, 5.120215581981958`},
{"CURE", 365, 26.64044008938779`}, {"CURE", 365, 15.117686380956336`}, {"CURE", 365, 56.35989824331698`},
{"CURE", 365, 5.195011138960041`}, {"CURE", 365, 37.056233978395795`}, {"CURE", 365, 46.5214795842589`},
{"CURE", 365, 7.610738310808684`}, {"CURE", 365, 17.425338510274713`}, {"CURE", 365, 14.025663546536588`},
{"CURE", 365, 8.014763618819114`}, {"CURE", 365, 47.47067531807959`}, {"CURE", 365, 17.82806022076869 5`},
{"CURE", 365, 34.380551200370746`}, {"CURE", 365, 33.42570100181914`}, {"CURE", 365, 37.908782353260726`},
{"CURE", 365, 29.635209369600314`}, {"CURE", 365, 35.45778606628091`}, {"CURE", 365, 28.081270483449508`},
{"CURE", 365, 54.25944783521592`}, {"CURE", 365, 33.042394928190056`}, {"CURE", 365, 26.964301689176814`},
{"CURE", 365, 69.04871452818595`}, {"CURE", 365, 7.994621780626321`}, {"CURE", 365, 45.36225626494219`},
{"CURE", 365, 3.332526029801399`}, {"CURE", 365, 41.387663164965375`}, {"CURE", 365, 9.756891573887374`},
{"CURE", 365, 50.950363996519535`}, {"CURE", 365, 7.963870378941948`}, {"CURE", 365, 52.18482928746931`},
{"CURE", 365, 130.51671345446488`}, {"CURE", 365, 5.6983235282934235`}, {"CURE", 365, 4.089126961612961`},
{"CURE", 365, 15.074673781971018`}, {"CURE", 365, 3.6572304740298667`}, {"CURE", 365, 2.503073864523447`},
{"CURE", 365, 14.645198636488757`}, {"CURE", 365, 23.902012656230394`}, {"CURE", 365, 25.767823134164125`},

{"TBUR", 108.46541145484913`, 24.436205004524314`}, {"CURE", 365, 19.05008244520141`}, {"CURE", 365, 33.54543054621788`},
{"CURE", 365, 59.06567685825416`}, {"CURE", 365, 4.779013850899987`}, {"CURE", 365, 26.19150458581636`},
{"CURE", 365, 22.508209482586643`}, {"CURE", 365, 16.317577128551296`}, {"CURE", 365, 30.168951767147494`},
{"CURE", 365, 44.73499861550948`}, {"CURE", 365, 104.69190069414623`}, {"CURE", 365, 77.94976657213647`},
{"CURE", 365, 24.066104061809806`}, {"CURE", 365, 2.561800034402688`}, {"CURE", 365, 27.942404041372587`},
{"CURE", 365, 72.0752278647709`}, {"CURE", 365, 18.13225585334406`}, {"CURE", 365, 22.574777328738225`},
{"CURE", 365, 13.990130984461906`}, {"CURE", 365, 20.508385322458423`}, {"CURE", 365, 5.300619088405056`},
{"CURE", 365, 25.966469665113827`}, {"CURE", 365, 14.56927836491257`}, {"CURE", 365, 42.62089174932567`},
{"CURE", 365, 29.14514470657602`}, {"CURE", 365, 28.334033512837234`}, {"CURE", 365, 23.313526358633826`},
{"CURE", 365, 12.337297906455888`}, {"CURE", 365, 8.501279087050758`}, {"CURE", 365, 8.28840460958109 5`},
{"CURE", 365, 10.728552919369545`}, {"CURE", 365, 8.365643505395727`}, {"CURE", 365, 112.36554331265843`},
{"CURE", 365, 62.51833811097606`}, {"CURE", 365, 12.09332285655852`}, {"CURE", 365, 186.29664442969636`},
{"CURE", 365, 53.275890086473744`}, {"CURE", 365, 28.20603573302627`}, {"CURE", 365, 48.78894405646214`},
{"CURE", 365, 7.336228519169307`}, {"CURE", 365, 58.46650524810166`}, {"CURE", 365, 156.39998130468757`},
{"CURE", 365, 8.484198651015337`}, {"CURE", 365, 26.469848654627953`}, {"CURE", 365, 43.2999769828646`},
{"CURE", 365, 21.820745170630147`}, {"CURE", 365, 10.741121742812178`}, {"CURE", 365, 8.557942518427565`},
{"CURE", 365, 74.26382629114084`}, {"CURE", 365, 38.98715251251077`}, {"CURE", 365, 22.184971827073763`},
{"CURE", 365, 55.09293949182146`}, {"CURE", 365, 9.938787772195674`}, {"CURE", 365, 26.591194859010336`},
{"CURE", 365, 16.05991379297574`}, {"CURE", 365, 108.33676602810365`}, {"TBUR", 52.37486129764864`, 27.14437004555327`},
{"TBUR", 78.90882499849691`, 126.05176011442278`}, {"CURE", 365, 196.8250464276807`}, {"CURE", 365, 15.11687330269283`},
{"CURE", 365, 4.261663386042812`}, {"CURE", 365, 3.714408967400149`}, {"CURE", 365, 27.160047991757356`},
{"CURE", 365, 105.42154611037604`}, {"CURE", 365, 21.11851783099683`}, {"CURE", 365, 16.218531230264666`},
{"TBUR", 58.800116685251545`, 30.431090482978167`}, {"CURE", 365, 33.350052908220356`},
{"CURE", 365, 139.1045998333294`}, {"CURE", 365, 114.81285233744343`}, {"CURE", 365, 24.01528923172684`},
{"CURE", 365, 7.97671225694835`}, {"CURE", 365, 19.54185530891529`}, {"CURE", 365, 3.067319705701252`},
{"CURE", 365, 57.55904132539065`}, {"CURE", 365, 85.81421955976829`}, {"CURE", 365, 28.063752457949477`},
{"CURE", 365, 22.239381448840778`}, {"CURE", 365, 94.05157776280976`}, {"CURE", 365, 5.953007657146948`},
{"CURE", 365, 7.754137541981232`}, {"CURE", 365, 24.321100649820576`}, {"CURE", 365, 31.235714018809507`},
{"TBUR", 62.84780352927199`, 58.97744713406293`}, {"CURE", 365, 61.01961696366577`}, {"CURE", 365, 25.048887678222393`},
{"CURE", 365, 25.811580990115246`}, {"CURE", 365, 32.23950884691777`}, {"CURE", 365, 11.165304298712563`},
{"CURE", 365, 3.1632299577642846`}, {"CURE", 365, 33.260765019497576`}, {"CURE", 365, 40.074314419453415`},

```
{"CURE", 365, 19.48301321321537`}, {"CURE", 365, 35.31016290635207`}, {"CURE", 365, 10.991616535688026`},
{"CURE", 365, 7.226493229815277`}, {"CURE", 365, 31.63794786752281`}, {"CURE", 365, 19.189489817130646`},
{"CURE", 365, 27.479460616540802`}, {"CURE", 365, 181.4085686655473`}, {"CURE", 365, 17.276022199753747`},
{"CURE", 365, 8.417355215106538`}, {"CURE", 365, 6.539334830186669`}, {"CURE", 365, 7.927488683020494`},
{"CURE", 365, 45.279051769171154`}, {"CURE", 365, 3.1192523538546277`}, {"CURE", 365, 42.163476374388786`},
{"CURE", 365, 46.04794384523418`}, {"CURE", 365, 35.942292899376895`}, {"CURE", 365, 29.465946718254916`},
{"CURE", 365, 5.8359676601569666`}, {"CURE", 365, 64.13081799249667`}, {"TBUR", 53.087016332754025`, 19.33343175085222`},
{"CURE", 365, 8.678713871316065`}, {"CURE", 365, 57.10207752652373`}, {"CURE", 365, 10.15800143339549`},
{"CURE", 365, 68.407758935169`}, {"CURE", 365, 43.91409845619934`}, {"CURE", 365, 29.245795882106794`},
{"CURE", 365, 42.55574481899744`}, {"CURE", 365, 15.05540880775972`}, {"CURE", 365, 66.74174605760213`},
{"CURE", 365, 7.920558459289976`}, {"CURE", 365, 33.50002077184696`}, {"CURE", 365, 39.22948738925816`},
{"CURE", 365, 16.679036916506544`}, {"CURE", 365, 155.42029816585475`}, {"CURE", 365, 31.085874195533524`},
{"CURE", 365, 31.813176244501758`}, {"CURE", 365, 32.48883606658601`}, {"CURE", 365, 130.1614650949174`},
{"CURE", 365, 41.34635977010159`}, {"CURE", 365, 38.575004847117675`}, {"CURE", 365, 83.92576229399653`},
{"CURE", 365, 29.527766509759523`}, {"CURE", 365, 25.72307578770465`}, {"CURE", 365, 31.79671367434918`},
{"CURE", 365, 43.92019080048084`}, {"CURE", 365, 77.61725806911173`}, {"CURE", 365, 16.74356966462589`},
{"CURE", 365, 15.287543141996435`}, {"CURE", 365, 32.71640667010983`}, {"CURE", 365, 33.30650319640812`},
{"CURE", 365, 37.39552761247027`}, {"CURE", 365, 40.050785621459084`}, {"CURE", 365, 22.370973340636855`},
{"CURE", 365, 11.237990920067459`}, {"CURE", 365, 42.204962381145606`}, {"CURE", 365, 10.678336310601113`},
{"CURE", 365, 22.703653254773048`}, {"CURE", 365, 26.97133453214209`}, {"CURE", 365, 8.145918698526566`},
{"CURE", 365, 4.048200284837391`}, {"TBUR", 90.7533433824809`, 24.927924432553155`}, {"CURE", 365, 33.871643781293464`},
{"CURE", 365, 40.22601479159808`}, {"CURE", 365, 28.76972476195733`}, {"CURE", 365, 3.686822264802244`},
{"CURE", 365, 33.01860411144539`}, {"CURE", 365, 6.286855833693652`}, {"CURE", 365, 97.08179206605708`},
{"CURE", 365, 126.29828294168405`}, {"CURE", 365, 17.604951968721277`}, {"TBUR", 72.10283086212706`, 24.907766697214562`},
{"CURE", 365, 135.5648229883573`}, {"CURE", 365, 24.313363870412566`}, {"CURE", 365, 14.081686503736744`},
{"CURE", 365, 10.973615729184951`}, {"CURE", 365, 3.0066119719026196`}, {"CURE", 365, 18.958138199793375`},
{"CURE", 365, 21.138425594650005`}, {"CURE", 365, 55.9565378790829`}, {"CURE", 365, 26.80161025219137`},
{"CURE", 365, 46.67615221945562`}, {"CURE", 365, 16.49032335663759`}, {"TBUR", 142.94871463232417`, 35.710697932654604`},
{"CURE", 365, 79.98275509044916`}, {"CURE", 365, 8.125500991858914`}, {"CURE", 365, 12.547926372971832`},
{"CURE", 365, 18.629851250873916`}, {"CURE", 365, 26.033471338869923`}, {"TBUR", 137.1843966693259`, 17.025383733425322`},
{"CURE", 365, 16.341347257998795`}, {"CURE", 365, 6.917208352472013`}, {"CURE", 365, 10.688473485634711`},
```

{"CURE", 365, 20.246845221226753`}, {"CURE", 365, 46.109707823598576`}, {"TBUR", 79.29621808021884`, 40.95112666149958`},
{"CURE", 365, 21.010791812675436`}, {"TBUR", 33.66728354023728`, 31.399202660705257`}, {"CURE", 365, 67.83083400329079`},
{"CURE", 365, 33.20107640330114`}, {"CURE", 365, 24.845128454586177`}, {"CURE", 365, 15.825827511409923`},
{"CURE", 365, 4.955056902746032`}, {"CURE", 365, 27.585925075916123`}, {"CURE", 365, 31.827529209025673`},
{"CURE", 365, 124.61539634813768`}, {"CURE", 365, 6.351609192038072`}, {"CURE", 365, 6.20953524882059`},
{"CURE", 365, 19.8963825010124`}, {"CURE", 365, 57.999907156284955`}, {"CURE", 365, 7.2358684262105655`},
{"CURE", 365, 35.401960700324665`}, {"CURE", 365, 8.254418710815001`}, {"CURE", 365, 20.5682021550965`},
{"CURE", 365, 8.354826416138248`}, {"CURE", 365, 8.725084842674004`}, {"CURE", 365, 7.869405795944381`},
{"CURE", 365, 83.97352457163701`}, {"CURE", 365, 13.376993126918073`}, {"TBUR", 74.95873245109212`, 23.568792427950143`},
{"CURE", 365, 3.2621241966691477`}, {"CURE", 365, 39.077905846651504`}, {"CURE", 365, 54.68874605627033`},
{"CURE", 365, 47.63887131831477`}, {"CURE", 365, 7.1959209339128085`}, {"CURE", 365, 14.437303327055735`},
{"CURE", 365, 146.8108590219808`}, {"CURE", 365, 39.897494804446495`}, {"CURE", 365, 27.162070849021823`},
{"CURE", 365, 9.634913532626344`}, {"CURE", 365, 13.82483659754423`}, {"CURE", 365, 21.354539537112537`},
{"CURE", 365, 5.286234287119559`}, {"CURE", 365, 25.98325698312505`}, {"CURE", 365, 40.890331282506736`},
{"CURE", 365, 24.855540824781883`}, {"CURE", 365, 26.97642007734297`}, {"CURE", 365, 19.538330909207854`},
{"CURE", 365, 12.641177431556144`}, {"CURE", 365, 72.19647360569795`}, {"CURE", 365, 104.79664841606208`},
{"CURE", 365, 27.961945498397245`}, {"CURE", 365, 35.302600911730714`}, {"CURE", 365, 44.72093919176142`},
{"CURE", 365, 43.71818726742669`}, {"CURE", 365, 3.814016097822302`}, {"CURE", 365, 51.09229316251392`},
{"CURE", 365, 52.860071813024405`}, {"CURE", 365, 35.69619139157219`}, {"CURE", 365, 143.38361680463757`},
{"CURE", 365, 29.807454033606316`}, {"CURE", 365, 13.234847632394139`}, {"CURE", 365, 31.367553242083577`},
{"CURE", 365, 39.86560578703463`}, {"CURE", 365, 17.858369353976332`}, {"CURE", 365, 13.991780749834135`},
{"CURE", 365, 12.189017862469905`}, {"CURE", 365, 22.062427535660877`}, {"CURE", 365, 68.11011511168196`},
{"CURE", 365, 43.71409844347979`}, {"CURE", 365, 7.60065314881732`}, {"CURE", 365, 44.4558001374697`},
{"CURE", 365, 64.3423042917284`}, {"CURE", 365, 11.809945650552992`}, {"CURE", 365, 67.46134979159874`},
{"CURE", 365, 10.141177670102236`}, {"CURE", 365, 6.468225730709099`}, {"CURE", 365, 9.189788267261184`},
{"CURE", 365, 17.66199084401656`}, {"TBUR", 74.61236558646056`, 78.85340753650408`}, {"CURE", 365, 28.298191766474172`},
{"CURE", 365, 35.704256523288535`}, {"CURE", 365, 8.542438317178174`}, {"CURE", 365, 162.7313509928667`},
{"CURE", 365, 74.66719552015314`}, {"CURE", 365, 12.715670902641474`}, {"CURE", 365, 31.170026509038998`},
{"CURE", 365, 17.379342063563172`}, {"CURE", 365, 17.545300913984175`}, {"CURE", 365, 59.27049382166908`},
{"TBUR", 51.463913398900054`, 74.78805806208105`}, {"CURE", 365, 8.058652424225805`}, {"CURE", 365, 15.695964206498155`},
{"CURE", 365, 5.567787874331197`}, {"CURE", 365, 21.48179034706559`}, {"CURE", 365, 36.761247109296946`},

{"CURE", 365, 59.647316086632436`}, {"CURE", 365, 33.28055591450919`}, {"CURE", 365, 119.8698071298851`},
{"CURE", 365, 57.298716514624644`}, {"TBUR", 65.13641631857`, 9.415456352851615`}, {"CURE", 365, 20.943810817922987`},
{"TBUR", 33.806041021786896`, 21.520738261620938`}, {"TBUR", 67.6730427384016`, 46.69242812034705`},
{"TBUR", 195.35710544127207`, 6.129852374043267`}, {"CURE", 365, 16.442583927544405`}, {"CURE", 365, 9.259889394712147`},
{"CURE", 365, 23.118319157642087`}, {"CURE", 365, 52.80832886593088`}, {"CURE", 365, 22.470219204072077`},
{"CURE", 365, 4.880673279455257`}, {"CURE", 365, 92.87648674977525`}, {"CURE", 365, 17.527170358379514`},
{"CURE", 365, 5.955447580650268`}, {"CURE", 365, 7.828509994883275`}, {"CURE", 365, 6.60900282255754`},
{"CURE", 365, 8.44474618184308`}, {"CURE", 365, 67.95372620767951`}, {"CURE", 365, 37.1523250770626 7`},
{"CURE", 365, 22.382973275271883`}, {"CURE", 365, 10.483101148215816`}, {"CURE", 365, 17.523650152512168`},
{"CURE", 365, 10.138280238429799`}, {"CURE", 365, 24.46190933881668`}, {"CURE", 365, 84.08542607533397`},
{"CURE", 365, 48.075912191707346`}, {"CURE", 365, 24.241133478201373`}, {"CURE", 365, 13.981496742224572`},
{"CURE", 365, 14.544843301954481`}, {"CURE", 365, 179.7325620014352`}, {"CURE", 365, 8.563497409159242`},
{"CURE", 365, 4.411399974971853`}, {"CURE", 365, 3.835996066765029`}, {"CURE", 365, 48.85330918114649`},
{"CURE", 365, 28.588733086341925`}, {"CURE", 365, 31.73907562659959`}, {"CURE", 365, 47.17066059547818`},
{"CURE", 365, 77.75616779281259`}, {"CURE", 365, 80.54490206848683`}, {"CURE", 365, 30.69402851779356`},
{"CURE", 365, 13.254047447553114`}, {"CURE", 365, 15.363066231902067`}, {"CURE", 365, 30.48584420174471`},
{"CURE", 365, 17.380204274884857`}, {"TBUR", 24.313438427555752`, 6.5895266389758955`}, {"CURE", 365, 27.286692941740593`},
{"CURE", 365, 170.0417178246794`}, {"CURE", 365, 17.523255752796025`}, {"CURE", 365, 6.298203034379104`},
{"CURE", 365, 59.75588714947661`}, {"CURE", 365, 14.944013204686854`}, {"CURE", 365, 42.93108722842362`},
{"CURE", 365, 206.75039442215476`}, {"CURE", 365, 95.46255168494199`}, {"CURE", 365, 30.991235781801716`},
{"CURE", 365, 25.969751488664972`}, {"CURE", 365, 11.067117195191393`}, {"CURE", 365, 3.158138516852011`},
{"CURE", 365, 12.339626234550462`}, {"CURE", 365, 36.24258852628972`}, {"CURE", 365, 20.088088864243208`},
{"CURE", 365, 64.1021606330838`}, {"CURE", 365, 25.40219656047545`}, {"CURE", 365, 25.952225035797987`},
{"CURE", 365, 7.206786632518776`}, {"CURE", 365, 20.410977575534208`}, {"CURE", 365, 20.94963829995503`},
{"CURE", 365, 5.461942651186026`}, {"CURE", 365, 17.366479943877334`}, {"CURE", 365, 10.364293803061187`},
{"TBUR", 80.92825552030304`, 16.386403306547532`}, {"CURE", 365, 34.17810240827481`}, {"CURE", 365, 17.17921279194056`},
{"CURE", 365, 3.988576945214701`}, {"CURE", 365, 27.474647821874623`}, {"CURE", 365, 3.072884773720336`},
{"CURE", 365, 9.01488876105761`}, {"CURE", 365, 26.765075408899698`}, {"CURE", 365, 30.87675966043931`},
{"CURE", 365, 6.135100205235496`}, {"CURE", 365, 18.065205644238016`}, {"CURE", 365, 23.378157935858457`},
{"CURE", 365, 118.1220806255459`}, {"CURE", 365, 27.182433024333264`}, {"CURE", 365, 7.659130178438455`},
{"CURE", 365, 83.54150199890584`}, {"CURE", 365, 8.184234692522478`}, {"CURE", 365, 30.908211608823`},

{"CURE", 365, 31.9991921649822`}, {"CURE", 365, 9.550545992266946`}, {"CURE", 365, 10.631174253948863`},
{"CURE", 365, 47.10155775214643`}, {"CURE", 365, 4.598147828957626`}, {"CURE", 365, 39.91359916288756`},
{"CURE", 365, 10.569477820704291`}, {"CURE", 365, 13.821492940925818`}, {"CURE", 365, 3.085102357782822`},
{"CURE", 365, 15.86881344070445`}, {"CURE", 365, 54.02433800895304`}, {"CURE", 365, 20.585980598917224`},
{"CURE", 365, 14.946272042587283`}, {"CURE", 365, 21.362174760267227`}, {"CURE", 365, 16.716389568747008`},
{"TBUR", 82.204476011758`, 21.095204896746672`}, {"CURE", 365, 37.27876969722149`}, {"CURE", 365, 35.988245381843896`},
{"CURE", 365, 4.432924668726909`}, {"CURE", 365, 72.62868937843648`}, {"CURE", 365, 8.458315002054594`},
{"CURE", 365, 4.240196122791573`}, {"CURE", 365, 20.216997151308963`}, {"CURE", 365, 21.232542136113057`},
{"CURE", 365, 14.303953896091599`}, {"CURE", 365, 24.854085100635835`}, {"CURE", 365, 21.43281311385219`},
{"CURE", 365, 34.451682770637014`}, {"CURE", 365, 29.11007513208608`}, {"TBUR", 59.07085792294927`, 41.51589854749571`},
{"CURE", 365, 6.562892285184601`}, {"CURE", 365, 83.95991666181486`}, {"CURE", 365, 81.69441385031536`},
{"CURE", 365, 4.5242261439378675`}, {"CURE", 365, 11.395799959069146`}, {"CURE", 365, 28.274978883294047`},
{"CURE", 365, 14.357210265564444`}, {"CURE", 365, 44.05475323193523`}, {"CURE", 365, 88.50594896088246`},
{"CURE", 365, 27.977325604427868`}, {"CURE", 365, 4.7989526078390705`}, {"CURE", 365, 4.709162437524758`},
{"CURE", 365, 11.06071846628852`}, {"CURE", 365, 3.3385054816745385`}, {"CURE", 365, 37.90142618175074`},
{"CURE", 365, 5.873897979259211`}, {"CURE", 365, 39.41582688401131`}, {"CURE", 365, 24.334253775683674`},
{"CURE", 365, 41.952072269063905`}, {"CURE", 365, 10.32657657188692`}, {"CURE", 365, 52.10023123030046`},
{"CURE", 365, 172.37512325524747`}, {"CURE", 365, 62.11053164444874`}, {"CURE", 365, 100.60322740179959`},
{"CURE", 365, 29.617370219025045`}, {"CURE", 365, 70.56440115830797`}, {"CURE", 365, 70.69688289799596`},
{"CURE", 365, 8.581330653640672`}, {"CURE", 365, 6.001911511733183`}, {"CURE", 365, 19.50883338136703`},
{"CURE", 365, 40.50096267200142`}, {"CURE", 365, 50.66957485858113`}, {"CURE", 365, 21.53555312921181`},
{"CURE", 365, 32.02723452537361`}, {"CURE", 365, 6.035144814803607`}, {"CURE", 365, 7.661809201143475`},
{"CURE", 365, 37.2688638741528`}, {"CURE", 365, 12.058911374608456`}, {"CURE", 365, 5.129300906460959`},
{"CURE", 365, 7.5886531811427025`}, {"CURE", 365, 3.7089419354078883`}, {"CURE", 365, 30.142679890132182`},
{"CURE", 365, 41.458487584662414`}, {"CURE", 365, 28.977310846868765`}, {"CURE", 365, 32.95619588858407`},
{"CURE", 365, 18.240855178036576`}, {"CURE", 365, 16.311935046508935`}, {"CURE", 365, 42.259256987494794`},
{"CURE", 365, 45.256592177341496`}, {"CURE", 365, 5.048422513604398`}, {"CURE", 365, 18.589932133837323`},
{"CURE", 365, 10.028602121383942`}, {"CURE", 365, 15.758426778914847`}, {"CURE", 365, 129.77008529520162`},
{"CURE", 365, 22.916317965133743`}, {"CURE", 365, 15.73167479729448`}, {"CURE", 365, 16.618937296040798`},
{"CURE", 365, 33.18559039073408`}, {"CURE", 365, 25.26101745830048`}, {"CURE", 365, 32.95488995203779`},
{"CURE", 365, 33.881667409305415`}, {"CURE", 365, 7.706943621805887`}, {"CURE", 365, 74.96632359729499`},

{"CURE", 365, 14.383777891875614`}, {"CURE", 365, 30.530612145045705`}, {"CURE", 365, 17.31936959203489`},
{"CURE", 365, 9.839459083811363`}, {"CURE", 365, 35.86849130909153`}, {"CURE", 365, 36.41654889345046`},
{"TBUR", 64.55666974901119`, 20.115362976109534`}, {"CURE", 365, 47.71226060895389`}, {"CURE", 365, 10.092987086582452`},
{"CURE", 365, 84.40078462772556`}, {"CURE", 365, 32.39027394940653`}, {"CURE", 365, 68.42098172047443`},
{"CURE", 365, 2.657701001632154`}, {"CURE", 365, 33.6437221989624`}, {"CURE", 365, 38.27461483937403`},
{"CURE", 365, 9.134143972084512`}, {"CURE", 365, 23.178904019234313`}, {"CURE", 365, 34.72461824531153`},
{"CURE", 365, 21.10127011891997`}, {"CURE", 365, 6.711553274234677`}, {"CURE", 365, 5.095605350690397`},
{"CURE", 365, 30.10336426696023`}, {"CURE", 365, 8.037959950619259`}, {"CURE", 365, 17.86645255683227`},
{"CURE", 365, 21.755594355458662`}, {"CURE", 365, 10.106885254922009`}, {"CURE", 365, 18.60904530654404`},
{"CURE", 365, 5.080034325637878`}, {"CURE", 365, 20.947077540294902`}, {"CURE", 365, 3.387082912120266`},
{"CURE", 365, 6.052917186480784`}, {"CURE", 365, 14.305571124948498`}, {"CURE", 365, 4.612843753020756`},
{"CURE", 365, 34.285592662363534`}, {"CURE", 365, 50.420220245655585`}, {"CURE", 365, 51.11115346204759`},
{"CURE", 365, 42.609420152386214`}, {"CURE", 365, 80.41137894078635`}, {"CURE", 365, 6.501734906429114`},
{"CURE", 365, 29.004230399940663`}, {"CURE", 365, 31.101051868705806`}, {"CURE", 365, 53.574090000336454`},
{"CURE", 365, 40.01615727344645`}, {"CURE", 365, 18.27079752562346`}, {"CURE", 365, 85.87100173818084`},
{"CURE", 365, 2.6830227097457704`}, {"CURE", 365, 81.83038675820235`}, {"CURE", 365, 5.537950154929834`},
{"CURE", 365, 69.88543681585206`}, {"CURE", 365, 63.29626043774924`}, {"CURE", 365, 18.75515214515227`},
{"CURE", 365, 73.83794105047951`}, {"CURE", 365, 90.11048561644147`}, {"CURE", 365, 40.28171201223272`},
{"CURE", 365, 17.78519447554208`}, {"CURE", 365, 20.44613750927618`}, {"CURE", 365, 30.186619051691054`},
{"CURE", 365, 27.83133397062198`}, {"CURE", 365, 6.747298127814625`}, {"CURE", 365, 24.21614816664613`},
{"CURE", 365, 22.89854724860507`}, {"CURE", 365, 17.134473228909844`}, {"CURE", 365, 42.36103594604866`},
{"CURE", 365, 6.670235406069767`}, {"CURE", 365, 47.27758024571545`}, {"CURE", 365, 30.929761743921492`},
{"CURE", 365, 17.5184875507005`}, {"CURE", 365, 15.807589415564157`}, {"CURE", 365, 32.24315045144668`},
{"CURE", 365, 4.427258877903169`}, {"CURE", 365, 51.27660967824173`}, {"CURE", 365, 8.089292491157936`},
{"CURE", 365, 11.48154341486039`}, {"CURE", 365, 25.742795339910124`}, {"CURE", 365, 20.1884981457585`},
{"CURE", 365, 18.56614019908063`}, {"CURE", 365, 57.75370812455492`}, {"CURE", 365, 7.818540205825106`},
{"CURE", 365, 13.613419912762746`}, {"CURE", 365, 100.73914157149875`}, {"CURE", 365, 7.642199869375526`},
{"CURE", 365, 19.68712910525577`}, {"CURE", 365, 29.92398885285499`}, {"CURE", 365, 29.54754125292727`},
{"CURE", 365, 20.078542402557353`}, {"CURE", 365, 103.82640301690817`}, {"CURE", 365, 18.73407073309426`},
{"CURE", 365, 7.895790603203378`}, {"CURE", 365, 15.695168517852379`}, {"CURE", 365, 12.15933074108908`},
{"CURE", 365, 9.311564873327102`}, {"CURE", 365, 24.433677556927204`}, {"CURE", 365, 41.85407497580608`},

{"CURE", 365, 23.02508208119069`}, {"CURE", 365, 26.841874668772245`}, {"CURE", 365, 58.79213566225136`},
{"CURE", 365, 6.4759445782057`}, {"CURE", 365, 7.620909002981987`}, {"CURE", 365, 64.35441726176681`},
{"CURE", 365, 20.683421307664023`}, {"CURE", 365, 22.443827637992175`}, {"CURE", 365, 11.280428339914977`},
{"CURE", 365, 25.833211789519673`}, {"CURE", 365, 32.19424051863058`}, {"CURE", 365, 74.59302103455686`},
{"CURE", 365, 10.996462352767159`}, {"CURE", 365, 42.89968601249002`}, {"CURE", 365, 25.251181187198206`},
{"CURE", 365, 66.85499356687681`}, {"CURE", 365, 109.3198166143926`}, {"CURE", 365, 39.97996556970469`},
{"CURE", 365, 3.873353096490414`}, {"CURE", 365, 30.329864996701776`}, {"CURE", 365, 9.569887950652893`},
{"CURE", 365, 101.30164152614898`}, {"TBUR", 38.004664812537854`, 3.1157829517333164`}, {"CURE", 365, 3.3800132544507564`},
{"CURE", 365, 117.06011510669096`}, {"CURE", 365, 26.895288897159464`}, {"CURE", 365, 9.12455004776646`},
{"CURE", 365, 17.464635498413255`}, {"CURE", 365, 6.1246175755331365`}, {"TBUR", 62.07025469858645`, 5.795626493520078`},
{"CURE", 365, 6.967610862184681`}, {"CURE", 365, 25.183811792156398`}, {"CURE", 365, 53.37477466454707`},
{"CURE", 365, 21.133786087276576`}, {"CURE", 365, 5.291774991281479`}, {"CURE", 365, 72.01516915654358`},
{"CURE", 365, 143.77472003139377`}, {"CURE", 365, 4.058301738965217`}, {"CURE", 365, 32.39977326830315`},
{"CURE", 365, 17.425221566971384`}, {"CURE", 365, 3.426877071347242`}, {"CURE", 365, 26.777394112064812`},
{"CURE", 365, 17.117260061283254`}, {"CURE", 365, 13.135232041986967`}, {"CURE", 365, 25.55194218004281`},
{"CURE", 365, 14.503867270683894`}, {"CURE", 365, 42.522541484503364`}, {"CURE", 365, 25.34872655085742`},
{"CURE", 365, 59.675790117952126`}, {"CURE", 365, 26.435121203319383`}, {"CURE", 365, 13.093975952058386`},
{"CURE", 365, 6.363600955496968`}, {"CURE", 365, 4.41977824557648`}, {"CURE", 365, 12.589686099496666`},
{"CURE", 365, 50.08428870913466`}, {"CURE", 365, 16.520510767988593`}, {"CURE", 365, 15.12484167036215`},
{"CURE", 365, 10.698462209820947`}, {"CURE", 365, 10.845846086472084`}, {"CURE", 365, 29.307752881917278`},
{"CURE", 365, 5.484483906871354`}, {"CURE", 365, 34.92350895643646`}, {"CURE", 365, 15.98843756853509`},
{"CURE", 365, 9.383239516750276`}, {"CURE", 365, 14.879900229787319`}, {"CURE", 365, 33.0420472752981 5`},
{"CURE", 365, 24.53165420349732`}, {"CURE", 365, 2.9704368101068157`}, {"CURE", 365, 12.787544983716964`},
{"CURE", 365, 213.92661598505615`}, {"CURE", 365, 35.4956463638996`}, {"CURE", 365, 51.80310915725005`},
{"CURE", 365, 15.264845160885717`}, {"CURE", 365, 56.58770777411194`}, {"CURE", 365, 6.773300047367203`},
{"CURE", 365, 22.80492348462545`}, {"CURE", 365, 105.36004851353773`}, {"CURE", 365, 19.734915540706147`},
{"CURE", 365, 17.33037054922114`}, {"CURE", 365, 29.793011215270628`}, {"CURE", 365, 15.743546515193328`},
{"CURE", 365, 3.498770579626786`}, {"CURE", 365, 32.48312657542469`}, {"CURE", 365, 5.8005178622929385`},
{"CURE", 365, 13.763986987187586`}, {"CURE", 365, 32.57522196777177`}, {"CURE", 365, 11.698285894764828`},
{"CURE", 365, 62.14071421533974`}, {"CURE", 365, 18.460793926578656`}, {"CURE", 365, 31.808879027313225`},
{"CURE", 365, 22.046615640756613`}, {"CURE", 365, 6.239355081564544`}, {"CURE", 365, 23.12394990515366 2`},

{"CURE", 365, 50.023325335046856`}, {"CURE", 365, 16.763651463582327`}, {"CURE", 365, 12.617834176524534`},
{"CURE", 365, 5.98245872289737`}, {"CURE", 365, 18.916772732198403`}, {"CURE", 365, 22.738203737257184`},
{"CURE", 365, 65.00867177281138`}, {"CURE", 365, 10.442610001401253`}, {"CURE", 365, 15.690584250362399`},
{"CURE", 365, 61.007059696755036`}, {"CURE", 365, 6.428390370717435`}, {"CURE", 365, 88.10596525434687`},
{"CURE", 365, 7.459276811289841`}, {"CURE", 365, 13.645024682846795`}, {"CURE", 365, 31.464015581277536`},
{"CURE", 365, 50.584801997751185`}, {"CURE", 365, 22.7920926146893 4`}, {"CURE", 365, 11.92551865943323`},
{"CURE", 365, 3.5110651665504062`}, {"TBUR", 60.07364734365527`, 30.63523302331012`}, {"CURE", 365, 27.826318585529858`},
{"CURE", 365, 27.386800486405477`}, {"CURE", 365, 10.799461777347037`}, {"CURE", 365, 26.475375995460382`},
{"CURE", 365, 3.0585149486245378`}, {"CURE", 365, 17.64299791215784`}, {"CURE", 365, 7.70834207774765`},
{"CURE", 365, 3.4113974840553496`}, {"CURE", 365, 17.739915799031266`}, {"CURE", 365, 3.7977709065765914`},
{"CURE", 365, 12.075619565897197`}, {"CURE", 365, 8.278569578761418`}, {"TBUR", 34.39272206018607`, 4.830769502657211`},
{"CURE", 365, 16.396351058414567`}, {"CURE", 365, 43.70973735819984`}, {"CURE", 365, 23.89330313525233`},
{"CURE", 365, 21.664567314254825`}, {"CURE", 365, 34.32897750474376`}, {"CURE", 365, 29.226491941555274`}},
{{"1800 Res", "1800 OS", "1800 Tox"}, {"CURE", 365, 14.967027531885142`}, {"CURE", 365, 31.026394897427764`},
{"CURE", 365, 21.72577895233892`}, {"TBUR", 61.305766174216686`, 7.255432199516423`},
{"TBUR", 41.91790554907755`, 27.133032722739454`}, {"CURE", 365, 56.19902197704848`}, {"CURE", 365, 52.87946007990982`},
{"CURE", 365, 5.524489875623598`}, {"CURE", 365, 38.135825117930125`}, {"CURE", 365, 32.975954854580124`},
{"CURE", 365, 123.77199087238243`}, {"CURE", 365, 15.360732790223707`}, {"CURE", 365, 54.51756729889508`},
{"CURE", 365, 183.90019799925108`}, {"CURE", 365, 26.09590840236693`}, {"CURE", 365, 40.726799608176016`},
{"CURE", 365, 80.71687534987964`}, {"CURE", 365, 36.13693176293334`}, {"TBUR", 111.82592776442728`, 27.269423823915858`},
{"CURE", 365, 9.062154938112363`}, {"CURE", 365, 56.020887060365524`}, {"CURE", 365, 22.709542512067355`},
{"CURE", 365, 8.700516391063353`}, {"CURE", 365, 6.932282619488608`}, {"CURE", 365, 31.5497173248287 2`},
{"CURE", 365, 34.62643455250371`}, {"CURE", 365, 3.147865926686923`}, {"CURE", 365, 4.77114517489313`},
{"CURE", 365, 17.67798844345229`}, {"CURE", 365, 20.676170613447606`}, {"CURE", 365, 17.645001608207565`},
{"CURE", 365, 61.92382409480865`}, {"CURE", 365, 13.639751677001367`}, {"CURE", 365, 10.836787356629939`},
{"CURE", 365, 87.93407472085559`}, {"CURE", 365, 5.385405245501714`}, {"CURE", 365, 23.29074276412912`},
{"TBUR", 41.288530787714066`, 51.77787871751225`}, {"CURE", 365, 12.934036556239793`},
{"CURE", 365, 19.895623172258016`}, {"CURE", 365, 45.34959875604891`}, {"CURE", 365, 30.665145390878198`},
{"CURE", 365, 6.220883827433753`}, {"CURE", 365, 20.97705530569473`}, {"CURE", 365, 30.274148827201014`},
{"CURE", 365, 19.853646754005567`}, {"CURE", 365, 210.93628526075585`}, {"CURE", 365, 45.46766670194829`},
{"CURE", 365, 68.83172469945599`}, {"CURE", 365, 8.501169657742505`}, {"CURE", 365, 23.479702568226564`},

```
{"CURE", 365, 32.40420995334996`}, {"CURE", 365, 26.15386516182934`}, {"CURE", 365, 16.979000626968897`},
{"CURE", 365, 7.317349546623537`}, {"CURE", 365, 77.41744648221557`}, {"CURE", 365, 24.32902321477498`},
{"CURE", 365, 4.616717045071173`}, {"CURE", 365, 49.76243950374131`}, {"CURE", 365, 34.833933006423216`},
{"CURE", 365, 23.872955514804627`}, {"CURE", 365, 18.278209404962535`}, {"CURE", 365, 10.28940845670325`},
{"CURE", 365, 39.3958887866679`}, {"CURE", 365, 27.971928426350512`}, {"CURE", 365, 32.004795131313166`},
{"CURE", 365, 7.3256673913427885`}, {"CURE", 365, 7.438126783460691`}, {"CURE", 365, 7.920433969235369`},
{"CURE", 365, 32.34422617486587`}, {"CURE", 365, 11.840229137284728`}, {"CURE", 365, 26.370026202589855`},
{"CURE", 365, 49.4945548241298`}, {"TBUR", 23.131753401435322`, 14.695005676950462`}, {"CURE", 365, 13.222239018309647`},
{"CURE", 365, 36.64094286205347`}, {"CURE", 365, 21.420057893673057`}, {"CURE", 365, 16.298026628100267`},
{"CURE", 365, 64.91064312540277`}, {"CURE", 365, 71.29783515998216`}, {"CURE", 365, 52.48980433766882`},
{"CURE", 365, 14.164003332238286`}, {"CURE", 365, 28.63863487504413`}, {"CURE", 365, 20.017399740121643`},
{"CURE", 365, 22.633051835766995`}, {"CURE", 365, 10.603963268653455`}, {"CURE", 365, 113.11283596786717`},
{"CURE", 365, 24.650312008812865`}, {"CURE", 365, 31.057273166823514`}, {"CURE", 365, 26.715293324532322`},
{"CURE", 365, 22.01705521958571`}, {"CURE", 365, 6.547363481098481`}, {"CURE", 365, 12.001980764864623`},
{"CURE", 365, 27.78258491705422`}, {"TBUR", 48.62503445925164`, 9.99437530639531`}, {"CURE", 365, 11.960164752731254`},
{"CURE", 365, 31.20749427795403`}, {"CURE", 365, 79.2019987930558`}, {"CURE", 365, 42.40643817933515`},
{"CURE", 365, 21.268811910705697`}, {"TBUR", 64.71047698473649`, 42.09951467799886`}, {"CURE", 365, 27.423432926484754`},
{"CURE", 365, 17.776949961400668`}, {"CURE", 365, 121.72456291866786`}, {"CURE", 365, 13.523784244279492`},
{"CURE", 365, 9.188524597786117`}, {"CURE", 365, 53.91603052492871`}, {"CURE", 365, 15.877825779483612`},
{"CURE", 365, 16.27214633857934`}, {"CURE", 365, 97.90248300386133`}, {"CURE", 365, 7.945996650810743`},
{"CURE", 365, 5.0405646586792106`}, {"CURE", 365, 20.968683136346087`}, {"CURE", 365, 16.576846421070034`},
{"CURE", 365, 12.484876365514907`}, {"CURE", 365, 31.874893260720647`}, {"CURE", 365, 8.969193078240899`},
{"CURE", 365, 5.51515634129468`}, {"CURE", 365, 25.821938284073912`}, {"CURE", 365, 39.48731170400602`},
{"CURE", 365, 6.721938482787339`}, {"CURE", 365, 22.254541492803845`}, {"CURE", 365, 67.5638387489338`},
{"TBUR", 57.17487328235786`, 25.68758788143778`}, {"CURE", 365, 14.987157989542432`}, {"CURE", 365, 52.26232130614849`},
{"CURE", 365, 26.26351188722849`}, {"CURE", 365, 3.462734539638127`}, {"CURE", 365, 30.005196290318214`},
{"CURE", 365, 23.17403001787017`}, {"CURE", 365, 18.461018974362723`}, {"CURE", 365, 21.894918746544846`},
{"CURE", 365, 5.696401455530801`}, {"CURE", 365, 32.495366381392465`}, {"CURE", 365, 11.669165335545019`},
{"CURE", 365, 26.308852592693178`}, {"CURE", 365, 98.50628211227703`}, {"CURE", 365, 34.77510294868597`},
{"CURE", 365, 80.88631591368632`}, {"CURE", 365, 26.65029474400419`}, {"CURE", 365, 109.95844196401848`},
{"CURE", 365, 3.433649155171581`}, {"CURE", 365, 30.169080053222057`}, {"CURE", 365, 34.3866640742607`},
```

{"CURE", 365, 4.466505285124626`}, {"CURE", 365, 6.186641241872141`}, {"CURE", 365, 37.978730995613084`},
{"CURE", 365, 44.704691650950195`}, {"CURE", 365, 38.14349166511164`}, {"CURE", 365, 31.205014389203164`},
{"CURE", 365, 29.83734627573884`}, {"CURE", 365, 18.0626510803246`}, {"CURE", 365, 4.778214124086925`},
{"CURE", 365, 20.768121576488543`}, {"CURE", 365, 9.855521836536973`}, {"CURE", 365, 4.824306285143825`},
{"CURE", 365, 29.253286281076498`}, {"CURE", 365, 16.372915273148898`}, {"CURE", 365, 43.2812215741894`},
{"CURE", 365, 17.471015391802634`}, {"CURE", 365, 14.542303247140033`}, {"CURE", 365, 97.41916123699143`},
{"CURE", 365, 7.2662175640173805`}, {"CURE", 365, 21.721563365403703`}, {"CURE", 365, 12.31464747532755`},
{"CURE", 365, 29.06817135732666`}, {"CURE", 365, 7.997287496458954`}, {"CURE", 365, 31.93842357568817З`},
{"CURE", 365, 84.3118763329054`}, {"CURE", 365, 7.21462663229577`}, {"CURE", 365, 21.92362449798292`},
{"CURE", 365, 23.631649759593216`}, {"CURE", 365, 85.26460711512625`}, {"TBUR", 94.01503415778677`, 31.964029175297526`},
{"CURE", 365, 26.600042059521385`}, {"CURE", 365, 46.58675042815675`}, {"CURE", 365, 17.9241520666974`},
{"CURE", 365, 54.282004995071574`}, {"CURE", 365, 34.44032570585845`}, {"CURE", 365, 20.86941112900969`},
{"CURE", 365, 19.00002842750972`}, {"CURE", 365, 6.189225224660914`}, {"CURE", 365, 4.194523279664375`},
{"CURE", 365, 9.82128655797962`}, {"CURE", 365, 13.170151524977435`}, {"CURE", 365, 5.7894708413681535`},
{"CURE", 365, 21.213157261029206`}, {"CURE", 365, 57.96625560940933`}, {"CURE", 365, 15.678267770107537`},
{"CURE", 365, 29.017547932208767`}, {"CURE", 365, 36.44289048273593`}, {"CURE", 365, 30.433331437584815`},
{"CURE", 365, 21.329118588119858`}, {"CURE", 365, 8.471305820290059`}, {"CURE", 365, 58.34746327420138`},
{"CURE", 365, 5.05004346102756`}, {"CURE", 365, 54.40348729258407`}, {"CURE", 365, 52.66459411616127`},
{"CURE", 365, 14.755947036289783`}, {"CURE", 365, 17.527890480365706`}, {"CURE", 365, 100.65778783071012`},
{"CURE", 365, 25.90951695405789`}, {"CURE", 365, 31.009106810718322`}, {"CURE", 365, 19.40461616172909`},
{"CURE", 365, 7.301270262413399`}, {"CURE", 365, 44.718900062657106`}, {"TBUR", 49.81132203417368`, 118.75389730460871`},
{"CURE", 365, 18.184567917700814`}, {"CURE", 365, 16.43459090796302`}, {"CURE", 365, 30.287302973814025`},
{"CURE", 365, 12.093945174253138`}, {"CURE", 365, 153.44619936081614`}, {"CURE", 365, 12.283727037398345`},
{"CURE", 365, 75.85049647615814`}, {"CURE", 365, 58.345704101181404`}, {"CURE", 365, 56.70903065369607`},
{"CURE", 365, 30.299552115533867`}, {"CURE", 365, 70.28111585508712`}, {"CURE", 365, 127.37156562124963`},
{"CURE", 365, 31.93103704949619`}, {"CURE", 365, 5.8648318557015235`}, {"CURE", 365, 52.1008591584948`},
{"CURE", 365, 32.466856527177185`}, {"CURE", 365, 31.295137137857797`}, {"CURE", 365, 6.8914139762188595`},
{"CURE", 365, 158.15265390183092`}, {"CURE", 365, 11.5608306378781`}, {"CURE", 365, 50.1605878962004`},
{"CURE", 365, 31.61080533859755`}, {"CURE", 365, 22.469894337105742`}, {"CURE", 365, 5.402345511911276`},
{"CURE", 365, 40.61122665355442`}, {"CURE", 365, 23.82148497890974`}, {"CURE", 365, 63.025306482151706`},
{"CURE", 365, 41.52107406229719`}, {"CURE", 365, 52.404490366558896`}, {"CURE", 365, 25.14853026798282Ø`},

{"CURE", 365, 22.375636240890167`}, {"TBUR", 55.4181780761911`, 116.904106220449`}, {"CURE", 365, 30.649009870568044`},
{"CURE", 365, 8.175587823089405`}, {"CURE", 365, 27.5378708578783`}, {"TBUR", 112.68377485054434`, 27.865470857466914`},
{"CURE", 365, 3.7478648991499175`}, {"CURE", 365, 6.6929579024974135`}, {"CURE", 365, 10.222957839990787`},
{"CURE", 365, 96.43923285852044`}, {"CURE", 365, 29.685327781994545`}, {"CURE", 365, 30.943853119076586`},
{"CURE", 365, 24.78028226364411`}, {"CURE", 365, 101.71649882555658`}, {"CURE", 365, 23.464582682042707`},
{"CURE", 365, 47.089954418182565`}, {"CURE", 365, 23.394381230094165`}, {"CURE", 365, 34.49241120436912`},
{"CURE", 365, 8.29557575656417`}, {"CURE", 365, 31.024020840587248`}, {"CURE", 365, 19.35573556107929`},
{"CURE", 365, 37.71300682595671`}, {"CURE", 365, 59.58183505792945`}, {"CURE", 365, 66.75016674108801`},
{"CURE", 365, 40.27519292321728`}, {"CURE", 365, 33.230989664308325`}, {"CURE", 365, 45.45518152879855`},
{"CURE", 365, 21.757250352128477`}, {"CURE", 365, 24.633851971893595`}, {"CURE", 365, 17.442262090282128`},
{"CURE", 365, 13.676639768840076`}, {"CURE", 365, 22.028213608439568`}, {"CURE", 365, 56.53444611912077`},
{"CURE", 365, 6.479398552708675`}, {"CURE", 365, 28.69484901153839`}, {"CURE", 365, 5.421552713974783`},
{"CURE", 365, 28.210864472338443`}, {"CURE", 365, 16.01390661974982`}, {"CURE", 365, 59.67648817752894`},
{"CURE", 365, 5.501009776610921`}, {"CURE", 365, 39.24824928378085`}, {"CURE", 365, 49.2639310290776`},
{"CURE", 365, 8.059260974163154`}, {"CURE", 365, 18.455331911017755`}, {"CURE", 365, 14.852984087336374`},
{"CURE", 365, 8.490497491478498`}, {"CURE", 365, 50.26421317864033`}, {"CURE", 365, 18.88037087258168`},
{"CURE", 365, 36.40407481781112`}, {"CURE", 365, 35.394331795893436`}, {"CURE", 365, 40.13915356586636`},
{"CURE", 365, 31.379537219728924`}, {"CURE", 365, 37.54860775125849`}, {"CURE", 365, 29.733880504992438`},
{"CURE", 365, 57.46027318158708`}, {"CURE", 365, 34.99171930979304`}, {"CURE", 365, 28.561233390012486`},
{"CURE", 365, 73.11900619524077`}, {"CURE", 365, 8.465841207566381`}, {"CURE", 365, 48.03291339452987`},
{"CURE", 365, 3.5286733136090835`}, {"CURE", 365, 43.82596834367052`}, {"CURE", 365, 10.331088108052116`},
{"CURE", 365, 54.0448673701969`}, {"CURE", 365, 8.433334543120393`}, {"CURE", 365, 55.25733794836351`},
{"CURE", 365, 138.22403983065135`}, {"CURE", 365, 6.03457001345096`}, {"CURE", 365, 4.330844890308281`},
{"CURE", 365, 15.962420011887362`}, {"CURE", 365, 3.8734764067564567`}, {"CURE", 365, 2.6504301206206384`},
{"CURE", 365, 15.507084752118203`}, {"CURE", 365, 25.319544335598433`}, {"CURE", 365, 27.28383262977514`},
{"TBUR", 113.86771337294948`, 25.887623234438944`}, {"CURE", 365, 20.17564401404052`}, {"CURE", 365, 35.52209547016171`},
{"CURE", 365, 62.5436635024121`}, {"CURE", 365, 5.0606267831383365`}, {"CURE", 365, 27.732898541610997`},
{"CURE", 365, 23.832743764402373`}, {"CURE", 365, 17.27830637178633`}, {"CURE", 365, 31.947054728416592`},
{"CURE", 365, 47.36976460020438`}, {"CURE", 365, 110.85382459376966`}, {"CURE", 365, 82.55109761064352`},
{"CURE", 365, 25.496881862276773`}, {"CURE", 365, 2.7126224894620266`}, {"CURE", 365, 29.588205320213042`},
{"CURE", 365, 76.31603532162296`}, {"CURE", 365, 19.199766078399136`}, {"CURE", 365, 23.976921937618837`},

{"CURE", 365, 14.813721846918368`}, {"CURE", 365, 21.715327881375366`}, {"CURE", 365, 5.614791957740133`},
{"CURE", 365, 27.499134700480003`}, {"CURE", 365, 15.432541194769342`}, {"CURE", 365, 45.137640015386985`},
{"CURE", 365, 30.877845627567577`}, {"CURE", 365, 30.00127373684894`}, {"CURE", 365, 24.68541763988456`},
{"CURE", 365, 13.063308873215425`}, {"CURE", 365, 9.001715430437176`}, {"CURE", 365, 8.776385753740369`},
{"CURE", 365, 11.361879537885148`}, {"CURE", 365, 8.858231376167641`}, {"CURE", 365, 119.01415326549129`},
{"CURE", 365, 66.21246766516906`}, {"CURE", 365, 12.805136022080308`}, {"CURE", 365, 197.25827178821928`},
{"CURE", 365, 56.41048531861579`}, {"CURE", 365, 29.865928002774066`}, {"CURE", 365, 51.660691620701186`},
{"CURE", 365, 7.767912423958006`}, {"CURE", 365, 61.912874592591876`}, {"CURE", 365, 165.60359955672874`},
{"CURE", 365, 8.983767150690298`}, {"CURE", 365, 28.02787390793695`}, {"CURE", 365, 45.85973005063161`},
{"CURE", 365, 23.120745073563327`}, {"CURE", 365, 11.373273490070572`}, {"CURE", 365, 9.061466220277904`},
{"CURE", 365, 78.63642173545915`}, {"CURE", 365, 41.283092253251674`}, {"CURE", 365, 23.490583410208682`},
{"CURE", 365, 58.34442246265021`}, {"CURE", 365, 10.526527562980244`}, {"CURE", 365, 28.157482818251463`},
{"CURE", 365, 17.005566897540536`}, {"CURE", 365, 114.71195539247203`}, {"TBUR", 56.44870087494493`, 28.769244708929882`},
{"CURE", 365, 133.58345658242663`}, {"CURE", 365, 208.56581917175419`}, {"CURE", 365, 16.007035197276615`},
{"CURE", 365, 4.5129522659667805`}, {"CURE", 365, 3.9330493493297443`}, {"CURE", 365, 28.761531465545936`},
{"CURE", 365, 111.62726237718286`}, {"CURE", 365, 22.361513598158872`}, {"CURE", 365, 17.174562963876372`},
{"TBUR", 62.2022471608596`, 32.23700880948868`}, {"CURE", 365, 35.323257381344945`}, {"CURE", 365, 147.32075865700028`},
{"CURE", 365, 121.56780935956557`}, {"CURE", 365, 25.43528592118235`}, {"CURE", 365, 8.446633266175455`},
{"CURE", 365, 20.693083224318055`}, {"CURE", 365, 3.2479441704286613`}, {"CURE", 365, 60.97684392836816`},
{"CURE", 365, 90.86284339998807`}, {"CURE", 365, 29.71519504585474`}, {"CURE", 365, 23.548437004261913`},
{"CURE", 365, 99.58510552019962`}, {"CURE", 365, 6.305743570247036`}, {"CURE", 365, 8.213879345270339`},
{"CURE", 365, 25.75213153555513`}, {"CURE", 365, 33.08472730464832`}, {"TBUR", 67.25882016842417`, 62.51610468111538`},
{"CURE", 365, 64.61107306991615`}, {"CURE", 365, 26.522945101217356`}, {"CURE", 365, 27.336935285001008`},
{"CURE", 365, 34.16024384905265`}, {"CURE", 365, 11.830456968257295`}, {"CURE", 365, 3.3494926339146165`},
{"CURE", 365, 35.22243669966974`}, {"CURE", 365, 42.43200825498201`}, {"CURE", 365, 20.62953687338008`},
{"CURE", 365, 37.38753141823226`}, {"CURE", 365, 11.639849603236426`}, {"CURE", 365, 7.651871787466274`},
{"CURE", 365, 33.49967893619746`}, {"CURE", 365, 20.327050286556478`}, {"CURE", 365, 29.098446042802685`},
{"CURE", 365, 192.08267223126217`}, {"CURE", 365, 18.2939145329727`}, {"CURE", 365, 8.915342301540878`},
{"CURE", 365, 6.92472021016491`}, {"CURE", 365, 8.394301583924218`}, {"CURE", 365, 47.94648637911211`},
{"CURE", 365, 3.3028590310268107`}, {"CURE", 365, 44.64763706271994`}, {"CURE", 365, 48.7572398132621`},
{"CURE", 365, 38.05678167777831`}, {"CURE", 365, 31.199629944298085`}, {"CURE", 365, 6.180875080681433`},

{"CURE", 365, 67.90474539215555`}, {"TBUR", 56.761978812918926`, 20.49207040135967`}, {"CURE", 365, 9.207991521423903`},
{"CURE", 365, 60.46402893818754`}, {"CURE", 365, 10.755730418918773`}, {"CURE", 365, 72.4369961334791`},
{"CURE", 365, 46.49768557407345`}, {"CURE", 365, 30.974019677532883`}, {"CURE", 365, 45.05937062998849`},
{"CURE", 365, 15.948465307157152`}, {"CURE", 365, 70.67243496405698`}, {"CURE", 365, 8.386685268649023`},
{"CURE", 365, 35.48127068334741`}, {"CURE", 365, 41.537827552014534`}, {"CURE", 365, 17.660652264689467`},
{"CURE", 365, 164.569271301293`}, {"CURE", 365, 32.93319773101365`}, {"CURE", 365, 33.6853458193462`},
{"CURE", 365, 34.40351493067133`}, {"CURE", 365, 137.82085772008654`}, {"CURE", 365, 43.7811158518379`},
{"CURE", 365, 40.84692009561926`}, {"CURE", 365, 88.87688724510446`}, {"CURE", 365, 31.266715371099718`},
{"CURE", 365, 27.23902345372706`}, {"CURE", 365, 33.667934883802516`}, {"CURE", 365, 46.50466703484025`},
{"CURE", 365, 82.18339991460347`}, {"CURE", 365, 17.729493934088982`}, {"CURE", 365, 16.187466190806845`},
{"CURE", 365, 34.641384843206936`}, {"CURE", 365, 35.26667844591429`}, {"CURE", 365, 39.61537361108216`},
{"CURE", 365, 42.40713811172468`}, {"CURE", 365, 23.690433117887068`}, {"CURE", 365, 11.904785686650904`},
{"CURE", 365, 44.71618699156691`}, {"CURE", 365, 11.30742431697381`}, {"CURE", 365, 24.040307035339968`},
{"CURE", 365, 28.575043764351637`}, {"CURE", 365, 8.625260859852622`}, {"CURE", 365, 4.286626306832729`},
{"TBUR", 95.9828894044139`, 26.41956558795207`}, {"CURE", 365, 35.87552095330246`}, {"CURE", 365, 42.60163979068761`},
{"CURE", 365, 30.46983203063527`}, {"CURE", 365, 3.904573568017155`}, {"CURE", 365, 34.96444539410734`},
{"CURE", 365, 6.658200263334835`}, {"CURE", 365, 102.79357965360231`}, {"CURE", 365, 133.73408280112895`},
{"CURE", 365, 18.70490108582882`}, {"TBUR", 75.79738619415728`, 26.38648301896735`}, {"CURE", 365, 143.54970555311937`},
{"CURE", 365, 25.744798071594815`}, {"CURE", 365, 14.921809175267146`}, {"CURE", 365, 11.626172220848566`},
{"CURE", 365, 3.183689593161506`}, {"CURE", 365, 20.073522192111117`}, {"CURE", 365, 22.382368052007845`},
{"CURE", 365, 59.265341677581745`}, {"CURE", 365, 28.380392787996538`}, {"CURE", 365, 49.42360452240496`},
{"CURE", 365, 17.4606887825296`}, {"CURE", 365, 37.8320353974036`}, {"CURE", 365, 84.68882351459943`},
{"CURE", 365, 8.605891330148133`}, {"CURE", 365, 13.286332955565367`}, {"CURE", 365, 19.728438394071507`},
{"CURE", 365, 27.566096546352515`}, {"TBUR", 144.03951635773797`, 18.041180811667633`},
{"CURE", 365, 17.303045816524524`}, {"CURE", 365, 7.326160960688627`}, {"CURE", 365, 11.317408483839046`},
{"CURE", 365, 21.44264688950469`}, {"CURE", 365, 48.82432147341024`}, {"TBUR", 83.59987547051904`, 43.38200781270313`},
{"CURE", 365, 22.247985840009687`}, {"TBUR", 35.43241718193337`, 33.28004059813419`}, {"CURE", 365, 71.83817966035745`},
{"CURE", 365, 35.15602696194477`}, {"CURE", 365, 26.307574019540375`}, {"CURE", 365, 16.757187204311208`},
{"CURE", 365, 5.247503042026163`}, {"CURE", 365, 29.20903295031118`}, {"CURE", 365, 33.70372814370029`},
{"CURE", 365, 131.96568595659647`}, {"CURE", 365, 6.726534237516737`}, {"CURE", 365, 6.574969247906873`},
{"CURE", 365, 21.067346849126523`}, {"CURE", 365, 61.41272076983549`}, {"CURE", 365, 7.661967157758361`},

{"CURE", 365, 37.48600554459161`}, {"CURE", 365, 8.7407556511564`}, {"CURE", 365, 21.780410097680566`},
{"CURE", 365, 8.852801554231705`}, {"CURE", 365, 9.23879218733976`}, {"CURE", 365, 8.332611588763845`},
{"CURE", 365, 88.91418689152064`}, {"CURE", 365, 14.177314494471426`}, {"TBUR", 79.50294235910917`, 24.977393242900707`},
{"CURE", 365, 3.4541197925057396`}, {"CURE", 365, 41.37937684929508`}, {"CURE", 365, 57.90695883834582`},
{"CURE", 365, 50.45120378049644`}, {"CURE", 365, 7.619810744239343`}, {"CURE", 365, 15.286992382724236`},
{"CURE", 365, 155.47421828825327`}, {"CURE", 365, 42.249776611885096`}, {"CURE", 365, 28.768892237349082`},
{"CURE", 365, 10.20621248359214`}, {"CURE", 365, 14.640015501045298`}, {"CURE", 365, 22.61677274691235`},
{"CURE", 365, 5.598993219678602`}, {"CURE", 365, 27.51283324904825`}, {"CURE", 365, 43.29611903433865`},
{"CURE", 365, 26.323311524719607`}, {"CURE", 365, 28.569560068498753`}, {"CURE", 365, 20.688385942813497`},
{"CURE", 365, 13.388383876521404`}, {"CURE", 365, 76.47300587742671`}, {"CURE", 365, 110.97044815733311`},
{"CURE", 365, 29.607053653717323`}, {"CURE", 365, 37.3797349437863`}, {"CURE", 365, 47.3525993056778`},
{"CURE", 365, 46.29222740412012`}, {"CURE", 365, 4.0393289856237695`}, {"CURE", 365, 54.11324536495558`},
{"CURE", 365, 55.969842156701986`}, {"CURE", 365, 37.80409501055515`}, {"CURE", 365, 151.82017459858295`},
{"CURE", 365, 31.561597940548317`}, {"CURE", 365, 14.022528518325249`}, {"CURE", 365, 33.214250767168394`},
{"CURE", 365, 42.212330124046`}, {"CURE", 365, 18.90991146766443`}, {"CURE", 365, 14.821917992351182`},
{"CURE", 365, 12.90655570442`}, {"CURE", 365, 23.36790889783993`}, {"CURE", 365, 72.11842009490685`},
{"CURE", 365, 46.2862989056712`}, {"CURE", 365, 8.050591975814914`}, {"CURE", 365, 47.10076846791531`},
{"CURE", 365, 68.12989555499344`}, {"CURE", 365, 12.508929693298512`}, {"CURE", 365, 71.44606755919634`},
{"CURE", 365, 10.73911822373769`}, {"CURE", 365, 6.850794920152196`}, {"CURE", 365, 9.730509261767313`},
{"CURE", 365, 18.708193649332994`}, {"TBUR", 78.8087969808513`, 83.58016449449003`}, {"CURE", 365, 29.963541168433277`},
{"CURE", 365, 37.812978499345846`}, {"CURE", 365, 9.045045440796383`}, {"CURE", 365, 172.38448468365496`},
{"CURE", 365, 79.0626360379179`}, {"CURE", 365, 13.470565117023467`}, {"CURE", 365, 33.010116613488314`},
{"CURE", 365, 18.40179242111028`}, {"CURE", 365, 18.58027910791145`}, {"CURE", 365, 62.77257438805685`},
{"TBUR", 54.16264131281336`, 79.258179109529`}, {"CURE", 365, 8.532792409705442`}, {"CURE", 365, 16.621451799132423`},
{"CURE", 365, 5.8962523319432325`}, {"CURE", 365, 22.746000895697847`}, {"CURE", 365, 38.941757497248155`},
{"CURE", 365, 63.15707796494492`}, {"CURE", 365, 35.24049040250091`}, {"CURE", 365, 126.92612643828083`},
{"CURE", 365, 60.670559707818505`}, {"TBUR", 67.9448232259399`, 9.98137411910369`}, {"CURE", 365, 22.180093840800257`},
{"TBUR", 36.5616857944559l`, 23.24620815032659`}, {"TBUR", 71.76298054607075`, 49.47829089392614`},
{"TBUR", 204.5903136761541`, 6.495077615622351`}, {"CURE", 365, 17.41189541574622`}, {"CURE", 365, 9.806976649635596`},
{"CURE", 365, 24.478840935738095`}, {"CURE", 365, 55.92032882735525`}, {"CURE", 365, 23.792963462673967`},
{"CURE", 365, 5.167935367390384`}, {"CURE", 365, 98.37081606153868`}, {"CURE", 365, 18.561380171411507`},

{"CURE", 365, 6.306168401821122`}, {"CURE", 365, 8.289696723441713`}, {"CURE", 365, 6.998332763260913`},
{"CURE", 365, 8.941751168452798`}, {"CURE", 365, 71.9540195611987`}, {"CURE", 365, 39.3514407618141`},
{"CURE", 365, 23.702717989923286`}, {"CURE", 365, 11.103918397377809`}, {"CURE", 365, 18.554669831988818`},
{"CURE", 365, 10.734838446834559`}, {"CURE", 365, 25.902371991930025`}, {"CURE", 365, 89.04554426587131`},
{"CURE", 365, 51.10735224009065`}, {"CURE", 365, 25.677682190152723`}, {"CURE", 365, 14.805435083322221`},
{"CURE", 365, 15.435411561956872`}, {"CURE", 365, 190.31031235570543`}, {"CURE", 365, 9.067614137858271`},
{"CURE", 365, 4.671170866026057`}, {"CURE", 365, 4.064746664036301`}, {"CURE", 365, 51.73152333759503`},
{"CURE", 365, 30.279245008564992`}, {"CURE", 365, 33.609425204994814`}, {"CURE", 365, 49.94666767540547`},
{"CURE", 365, 82.36610605594242`}, {"CURE", 365, 85.28374141111615`}, {"CURE", 365, 32.500562408393975`},
{"CURE", 365, 14.033843277554443`}, {"CURE", 365, 16.269281166329765`}, {"CURE", 365, 32.33086147790432`},
{"CURE", 365, 18.40888152586832`}, {"TBUR", 25.056635733069893`, 7.057717142384273`}, {"CURE", 365, 28.90569118715868`},
{"CURE", 365, 180.06553590932828`}, {"CURE", 365, 18.554508812993177`}, {"CURE", 365, 6.670103066908118`},
{"CURE", 365, 63.27214937769993`}, {"CURE", 365, 15.82349408550756`}, {"CURE", 365, 45.46371127988283`},
{"CURE", 365, 218.91741525362445`}, {"CURE", 365, 101.08164794707288`}, {"CURE", 365, 32.81528882453588`},
{"CURE", 365, 27.50083632760642`}, {"CURE", 365, 11.718512179034292`}, {"CURE", 365, 3.344552205694230 3`},
{"CURE", 365, 13.076891553969768`}, {"CURE", 365, 38.37514332315794`}, {"CURE", 365, 21.272709075883853`},
{"CURE", 365, 67.8793426506058`}, {"CURE", 365, 26.902205238142383`}, {"CURE", 365, 27.479919028818465`},
{"CURE", 365, 7.630986210461929`}, {"CURE", 365, 21.613229337831104`}, {"CURE", 365, 22.182339419669702`},
{"CURE", 365, 5.7835885056455085`}, {"CURE", 365, 18.393749914121045`}, {"CURE", 365, 10.974154517310582`},
{"TBUR", 85.19955629158866`, 17.362741262561112`}, {"CURE", 365, 36.24833300927314`}, {"CURE", 365, 18.197706444612308`},
{"CURE", 365, 4.22332768838234`}, {"CURE", 365, 29.093075955651827`}, {"CURE", 365, 3.2548806685287026`},
{"CURE", 365, 9.549479755254668`}, {"CURE", 365, 28.34555378363185`}, {"CURE", 365, 32.69362828815392 4`},
{"CURE", 365, 6.498111791909782`}, {"CURE", 365, 19.13302775748624`}, {"CURE", 365, 24.766687039587907`},
{"CURE", 365, 125.1324312640379`}, {"CURE", 365, 28.781862904960445`}, {"CURE", 365, 8.112258052244778`},
{"CURE", 365, 88.46920870897782`}, {"CURE", 365, 8.675827510715454`}, {"CURE", 365, 32.73640958815329`},
{"CURE", 365, 33.88254263954896`}, {"CURE", 365, 10.113007801770594`}, {"CURE", 365, 11.257866499570538`},
{"CURE", 365, 49.875977055242146`}, {"CURE", 365, 4.869914880032021`}, {"CURE", 365, 42.26244759913659`},
{"CURE", 365, 11.19161277249882`}, {"CURE", 365, 14.634845814590573`}, {"CURE", 365, 3.2677756481627633`},
{"CURE", 365, 16.803324459132387`}, {"CURE", 365, 57.236140573547075`}, {"CURE", 365, 21.800952351972803`},
{"CURE", 365, 15.828674482226013`}, {"CURE", 365, 22.623815637606302`}, {"CURE", 365, 17.713670401818796`},
{"CURE", 365, 22.34844690061489`}, {"CURE", 365, 39.47869118569759`}, {"CURE", 365, 38.10729289657 4`},

{"CURE", 365, 4.693744860146013`}, {"CURE", 365, 76.93867909048551`}, {"CURE", 365, 8.956508232601513`},
{"CURE", 365, 4.490724179409093`}, {"CURE", 365, 21.411438347036412`}, {"CURE", 365, 22.48330991274553`},
{"CURE", 365, 15.149238805787956`}, {"CURE", 365, 26.317858941727316`}, {"CURE", 365, 22.69418882421725`},
{"CURE", 365, 36.47884706090045`}, {"CURE", 365, 30.824053915125717`}, {"TBUR", 62.45073903655984`, 43.97507330523435`},
{"CURE", 365, 6.95094963250177`}, {"CURE", 365, 88.95303825289989`}, {"CURE", 365, 86.50746526356404`},
{"CURE", 365, 4.7912158846186115`}, {"CURE", 365, 12.066400929133241`}, {"CURE", 365, 29.938999591233408`},
{"CURE", 365, 15.20787826891379`}, {"CURE", 365, 46.68571838824085`}, {"CURE", 365, 93.72015252825969`},
{"CURE", 365, 29.624016717128974`}, {"CURE", 365, 5.082276330401326`}, {"CURE", 365, 4.986717888090594`},
{"CURE", 365, 11.711805549120887`}, {"CURE", 365, 3.535182536192194`}, {"CURE", 365, 40.132064025807374`},
{"CURE", 365, 6.220104596853935`}, {"CURE", 365, 41.75725851359464`}, {"CURE", 365, 25.768927023077406`},
{"CURE", 365, 44.4223526968677`}, {"CURE", 365, 10.935317927230965`}, {"CURE", 365, 55.171004214771905`},
{"CURE", 365, 182.53547608702112`}, {"CURE", 365, 65.76826006245034`}, {"CURE", 365, 106.53146052796671`},
{"CURE", 365, 31.360292253916718`}, {"CURE", 365, 74.71654179669646`}, {"CURE", 365, 74.8589448503999`},
{"CURE", 365, 9.086272966730885`}, {"CURE", 365, 6.355235554349556`}, {"CURE", 365, 20.659490909671852`},
{"CURE", 365, 42.88422211964752`}, {"CURE", 365, 53.6518526448268`}, {"CURE", 365, 22.837298007790896`},
{"CURE", 365, 33.92298408266383`}, {"CURE", 365, 6.391547791355737`}, {"CURE", 365, 8.116439674986268`},
{"CURE", 365, 39.467518259745056`}, {"CURE", 365, 12.776500813013566`}, {"CURE", 365, 5.431404262910241`},
{"CURE", 365, 8.035126574337843`}, {"CURE", 365, 3.927335584230938`}, {"CURE", 365, 31.9168602799144`},
{"CURE", 365, 43.89751944534208`}, {"CURE", 365, 30.689287660022742`}, {"CURE", 365, 34.89527875241444`},
{"CURE", 365, 19.31727935436451`}, {"CURE", 365, 17.27949176076183`}, {"CURE", 365, 44.75390053674494`},
{"CURE", 365, 47.920286956444166`}, {"CURE", 365, 5.345918652465476`}, {"CURE", 365, 19.755253854301323`},
{"CURE", 365, 10.620571709929441`}, {"CURE", 365, 16.68573595482887`}, {"CURE", 365, 137.41539684528166`},
{"CURE", 365, 24.266064054567142`}, {"CURE", 365, 16.657617042324667`}, {"CURE", 365, 17.60206613255361`},
{"CURE", 365, 35.140333545898535`}, {"CURE", 365, 26.74887685417161`}, {"CURE", 365, 34.89539644131002`},
{"CURE", 365, 35.89948114187746`}, {"CURE", 365, 8.160983603003224`}, {"CURE", 365, 79.37883914510408`},
{"CURE", 365, 15.23391512071305`}, {"CURE", 365, 32.32834512815459`}, {"CURE", 365, 18.340763081919892`},
{"CURE", 365, 10.41853207315958`}, {"CURE", 365, 37.97975757751616`}, {"CURE", 365, 38.55947804922807`},
{"TBUR", 67.6965937311812`, 21.320240135820857`}, {"CURE", 365, 50.527244286014245`}, {"CURE", 365, 10.688402240430229`},
{"CURE", 365, 89.36603469649401`}, {"CURE", 365, 34.30169819713912`}, {"CURE", 365, 72.4736825908791`},
{"CURE", 365, 2.8141786992504376`}, {"CURE", 365, 35.64080485843308`}, {"CURE", 365, 40.562475470787156`},
{"CURE", 365, 9.671751291518438`}, {"CURE", 365, 24.5462118234694`}, {"CURE", 365, 36.769235527267426`},

{"CURE", 365, 22.344781221404727`}, {"CURE", 365, 7.107601423227278`}, {"CURE", 365, 5.395854697652611`},
{"CURE", 365, 31.884556852373446`}, {"CURE", 365, 8.511869720540924`}, {"CURE", 365, 18.919787441382834`},
{"CURE", 365, 23.04242288513392`}, {"CURE", 365, 10.709372996323083`}, {"CURE", 365, 19.71824471106067`},
{"CURE", 365, 5.3799132700880365`}, {"CURE", 365, 22.182785387170206`}, {"CURE", 365, 3.586740200899339`},
{"CURE", 365, 6.410391870554321`}, {"CURE", 365, 15.150556547702607`}, {"CURE", 365, 4.885812794834281`},
{"CURE", 365, 36.312688877454136`}, {"CURE", 365, 53.38775954109506`}, {"CURE", 365, 54.135141145822146`},
{"CURE", 365, 45.11801730299877`}, {"CURE", 365, 85.14990211794183`}, {"CURE", 365, 6.885561387333697`},
{"CURE", 365, 30.714367504994865`}, {"CURE", 365, 32.94292013639924`}, {"CURE", 365, 56.727690260845726`},
{"CURE", 365, 42.371586374175635`}, {"CURE", 365, 19.350239664271598`}, {"CURE", 365, 90.9549725831006`},
{"CURE", 365, 2.8413375989321037`}, {"CURE", 365, 86.6444156551884`}, {"CURE", 365, 5.864605402977753`},
{"CURE", 365, 73.99807854838852`}, {"CURE", 365, 67.02000305077095`}, {"CURE", 365, 19.860167519006026`},
{"CURE", 365, 78.19198257045308`}, {"CURE", 365, 95.41346529504604`}, {"CURE", 365, 42.65782295243966`},
{"CURE", 365, 18.831731709815998`}, {"CURE", 365, 21.649535484660493`}, {"CURE", 365, 31.965486146301217`},
{"CURE", 365, 29.473207857362688`}, {"CURE", 365, 7.144714087379666`}, {"CURE", 365, 25.644649070662048`},
{"CURE", 365, 24.25139340184036`}, {"CURE", 365, 18.14561205512611`}, {"CURE", 365, 44.854582208000544`},
{"CURE", 365, 7.062941619858489`}, {"CURE", 365, 50.06177515752136`}, {"CURE", 365, 32.75324022546522`},
{"CURE", 365, 18.54975291830676`}, {"CURE", 365, 16.758742806478672`}, {"CURE", 365, 34.16083482890396`},
{"CURE", 365, 4.687862214610618`}, {"CURE", 365, 54.29576118957404`}, {"CURE", 365, 8.566972762890876`},
{"CURE", 365, 12.157743804565929`}, {"CURE", 365, 27.26240895887569`}, {"CURE", 365, 21.378826972445054`},
{"CURE", 365, 19.660381606974326`}, {"CURE", 365, 61.17013995021584`}, {"CURE", 365, 8.27958523230761`},
{"CURE", 365, 14.415549716720378`}, {"CURE", 365, 106.67191216031719`}, {"CURE", 365, 8.092644878939899`},
{"CURE", 365, 20.845637048725095`}, {"CURE", 365, 31.693125056003044`}, {"CURE", 365, 31.294101768343992`},
{"CURE", 365, 21.261346593587366`}, {"CURE", 365, 109.93455747386832`}, {"CURE", 365, 19.841244824054016`},
{"CURE", 365, 8.32006480591775`}, {"CURE", 365, 16.6308519008074`}, {"CURE", 365, 12.875323232198092`},
{"CURE", 365, 9.86171145954018`}, {"CURE", 365, 25.873607726117353`}, {"CURE", 365, 44.31876753161479`},
{"CURE", 365, 24.381401648218127`}, {"CURE", 365, 28.423932661820885`}, {"CURE", 365, 62.252176498861104`},
{"CURE", 365, 6.8588696971773535`}, {"CURE", 365, 8.071430477050058`}, {"CURE", 365, 68.14356207205394`},
{"CURE", 365, 21.910824784144506`}, {"CURE", 365, 23.764339286017137`}, {"CURE", 365, 11.9445092875811`},
{"CURE", 365, 27.353418458390887`}, {"CURE", 365, 34.08883627217372`}, {"CURE", 365, 78.98888422757574`},
{"CURE", 365, 11.64395629124028`}, {"CURE", 365, 45.444816714038346`}, {"CURE", 365, 26.744839130681118`},
{"CURE", 365, 70.81165097116173`}, {"CURE", 365, 115.75607997725835`}, {"CURE", 365, 42.342351610472925`},

{"CURE", 365, 4.1020000925865325`}, {"CURE", 365, 32.1159876280824 9`}, {"CURE", 365, 10.135937587652952`},
{"CURE", 365, 107.2618526172433`}, {"TBUR", 40.11356837931496`, 3.3258487176427005`}, {"CURE", 365, 3.5791235329128694`},
{"CURE", 365, 124.00186417072761`}, {"CURE", 365, 28.49924526782436`}, {"CURE", 365, 9.661674729621794`},
{"CURE", 365, 18.492565220627238`}, {"CURE", 365, 6.485701332184488`}, {"TBUR", 64.92365343009472`, 6.146352535895947`},
{"CURE", 365, 7.377634595694683`}, {"CURE", 365, 26.666253179311997`}, {"CURE", 365, 56.5171626368863`},
{"CURE", 365, 22.379306933354734`}, {"CURE", 365, 5.604008512590162`}, {"CURE", 365, 76.25428136404547`},
{"CURE", 365, 152.26737113235617`}, {"CURE", 365, 4.297348518164501`}, {"CURE", 365, 34.31776190513554`},
{"CURE", 365, 18.451026760816458`}, {"CURE", 365, 3.628569021027941`}, {"CURE", 365, 28.356619321788198`},
{"CURE", 365, 18.12456023762954`}, {"CURE", 365, 13.909503296093204`}, {"CURE", 365, 27.056094344691708`},
{"CURE", 365, 15.35775816977216`}, {"CURE", 365, 45.05952727122506`}, {"CURE", 365, 26.841808024196975`},
{"CURE", 365, 63.19378501655513`}, {"CURE", 365, 27.992955509028185`}, {"CURE", 365, 13.866474013875756`},
{"CURE", 365, 6.74007698862081`}, {"CURE", 365, 4.679889815853637`}, {"CURE", 365, 13.330457163364445`},
{"CURE", 365, 53.034741255076945`}, {"CURE", 365, 17.493216544724607`}, {"CURE", 365, 16.018651270855564`},
{"CURE", 365, 11.329665151603546`}, {"CURE", 365, 11.485138144875197`}, {"CURE", 365, 31.035352829406627`},
{"CURE", 365, 5.807457435370834`}, {"CURE", 365, 36.98461816922849`}, {"CURE", 365, 16.929200309541752`},
{"CURE", 365, 9.938678131107332`}, {"CURE", 365, 15.758578077549538`}, {"CURE", 365, 34.98620962714027`},
{"CURE", 365, 25.994142464207783`}, {"CURE", 365, 3.1452862689869914`}, {"CURE", 365, 13.543168964611707`},
{"CURE", 365, 226.51978814154077`}, {"CURE", 365, 37.59369225075747`}, {"CURE", 365, 54.86016660625582`},
{"CURE", 365, 16.164930708578936`}, {"CURE", 365, 59.92767532412446`}, {"CURE", 365, 7.172366261602516`},
{"CURE", 365, 24.153266055121975`}, {"CURE", 365, 111.56478443414876`}, {"CURE", 365, 20.89605500474083`},
{"CURE", 365, 18.360681456961593`}, {"CURE", 365, 31.546091601384322`}, {"CURE", 365, 16.67624072218635`},
{"CURE", 365, 3.7049070205962114`}, {"CURE", 365, 34.40628905414463`}, {"CURE", 365, 6.142635487344506`},
{"CURE", 365, 14.579750481495246`}, {"CURE", 365, 34.493246180298485`}, {"CURE", 365, 12.38728309081684`},
{"CURE", 365, 65.79813037470446`}, {"CURE", 365, 19.55165943275162`}, {"CURE", 365, 33.6805927151802`},
{"CURE", 365, 23.344299648798177`}, {"CURE", 365, 6.606468920713108`}, {"CURE", 365, 24.491658731186188`},
{"CURE", 365, 52.96736691512822`}, {"CURE", 365, 17.75107241890741`}, {"CURE", 365, 13.360480181358826`},
{"CURE", 365, 6.335359739734584`}, {"CURE", 365, 20.054690409774622`}, {"CURE", 365, 24.087661508522114`},
{"CURE", 365, 68.83474780250296`}, {"CURE", 365, 11.057269851860974`}, {"CURE", 365, 16.62128290136084`},
{"CURE", 365, 64.6014372321501`}, {"CURE", 365, 6.8069438925337895`}, {"CURE", 365, 93.33156851794439`},
{"CURE", 365, 7.898429849317209`}, {"CURE", 365, 14.450636878629272`}, {"CURE", 365, 33.31976647643595`},
{"CURE", 365, 53.582198132623795`}, {"CURE", 365, 24.136844750564585`}, {"CURE", 365, 12.634444212521048`},

{"CURE", 365, 3.718378328336793`}, {"TBUR", 65.47010143458051`, 32.4708216653925`}, {"CURE", 365, 29.476018725390645`},
{"CURE", 365, 29.00011571394672`}, {"CURE", 365, 11.435302223571979`}, {"CURE", 365, 28.034021364535608`},
{"CURE", 365, 3.239637867793146`}, {"CURE", 365, 18.683314311039474`}, {"CURE", 365, 8.162437352791061`},
{"CURE", 365, 3.613034442736118`}, {"CURE", 365, 18.785844728168094`}, {"CURE", 365, 4.021232323135495`},
{"CURE", 365, 12.786205526798087`}, {"CURE", 365, 8.766570666474806`}, {"TBUR", 36.85027319036503`, 5.180455768900189`},
{"CURE", 365, 17.36316185184962`}, {"CURE", 365, 46.2813011212259`}, {"CURE", 365, 25.307372071980623`},
{"CURE", 365, 22.94066915073464`}, {"CURE", 365, 36.3765356898268`}, {"CURE", 365, 30.947726435950464`}},
{{"1900 Res", "1900 OS", "1900 Tox"}, {"CURE", 365, 15.806790244975312`}, {"CURE", 365, 32.75053938588186`},
{"CURE", 365, 22.938771421971076`}, {"TBUR", 63.750832123958354`, 7.6705346885139525`},
{"TBUR", 43.94044136435608`, 28.854755204997208`}, {"CURE", 365, 59.32274591362439`}, {"CURE", 365, 55.8176811773227`},
{"CURE", 365, 5.8316886733866795`}, {"CURE", 365, 40.25499375470711`}, {"CURE", 365, 34.81137825931925`},
{"CURE", 365, 130.6594726247018`}, {"CURE", 365, 16.21456422901087`}, {"CURE", 365, 57.710487724637154`},
{"CURE", 365, 194.2942525830505`}, {"CURE", 365, 27.552431754493067`}, {"CURE", 365, 43.00125184522315`},
{"CURE", 365, 85.20361391571396`}, {"CURE", 365, 38.15198735923755`}, {"TBUR", 116.65287417823687`, 28.811583277533586`},
{"CURE", 365, 9.566199320361502`}, {"CURE", 365, 59.13503449828507`}, {"CURE", 365, 23.971669585412197`},
{"CURE", 365, 9.184561791732836`}, {"CURE", 365, 7.318007955120345`}, {"CURE", 365, 33.30277733177212`},
{"CURE", 365, 36.55090829931284`}, {"CURE", 365, 3.3228483268974887`}, {"CURE", 365, 5.036443924604834`},
{"CURE", 365, 18.661483471157517`}, {"CURE", 365, 21.82766816959234`}, {"CURE", 365, 18.625630652342924`},
{"CURE", 365, 65.39845233567799`}, {"CURE", 365, 14.398773735490783`}, {"CURE", 365, 11.443373756807159`},
{"CURE", 365, 92.82473435406045`}, {"CURE", 365, 5.685591121840824`}, {"CURE", 365, 24.584810810704674`},
{"TBUR", 42.879530878110245`, 54.7113394321885`}, {"CURE", 365, 13.656157126497385`}, {"CURE", 365, 21.006199835118686`},
{"CURE", 365, 47.897405220374125`}, {"CURE", 365, 32.36902906049962`}, {"CURE", 365, 6.566840448890407`},
{"CURE", 365, 22.145155238485206`}, {"CURE", 365, 31.957857583115928`}, {"CURE", 365, 20.957169101433166`},
{"CURE", 365, 222.66948099573648`}, {"CURE", 365, 47.99764376855839`}, {"CURE", 365, 72.90949515915578`},
{"CURE", 365, 8.974736098620431`}, {"CURE", 365, 24.8614250056881`}, {"CURE", 365, 34.262816730033144`},
{"CURE", 365, 27.611521075589053`}, {"CURE", 365, 17.924406264684766`}, {"CURE", 365, 7.724763206675511`},
{"CURE", 365, 81.72029456667157`}, {"CURE", 365, 25.680926439244214`}, {"CURE", 365, 4.874812451778048`},
{"CURE", 365, 52.53807348105759`}, {"CURE", 365, 36.78080405613279`}, {"CURE", 365, 25.20356533090953`},
{"CURE", 365, 19.294299725898725`}, {"CURE", 365, 10.882337105545123`}, {"CURE", 365, 41.58538466755346`},
{"CURE", 365, 29.53195276452743`}, {"CURE", 365, 33.78355054671278`}, {"CURE", 365, 7.732847711286697`},
{"CURE", 365, 7.8536327878061565`}, {"CURE", 365, 8.360659868370453`}, {"CURE", 365, 34.14308078427898`},

```
{"CURE", 365, 12.498947875370426`}, {"CURE", 365, 27.838212626266905`}, {"CURE", 365, 52.24744614820453`},
{"TBUR", 24.05119685153525`, 15.714788296573708`}, {"CURE", 365, 13.959151099836996`}, {"CURE", 365, 38.67757391647356`},
{"CURE", 365, 22.61322040992366`}, {"CURE", 365, 17.20417144269064`}, {"CURE", 365, 68.52596117524212`},
{"CURE", 365, 75.26239199130063`}, {"CURE", 365, 55.40928057036346`}, {"CURE", 365, 14.951156514552167`},
{"CURE", 365, 30.230006415819872`}, {"CURE", 365, 21.12980995842986`}, {"CURE", 365, 23.891492388009144`},
{"CURE", 365, 11.194242150660655`}, {"CURE", 365, 119.41249402922796`}, {"CURE", 365, 26.023730485207846`},
{"CURE", 365, 32.783711659410805`}, {"CURE", 365, 28.204394448498434`}, {"CURE", 365, 23.26338177935165`},
{"CURE", 365, 6.912195002599875`}, {"CURE", 365, 12.670268494061533`}, {"CURE", 365, 29.330343756123444`},
{"TBUR", 50.862704745704946`, 10.575476912367659`}, {"CURE", 365, 12.627475408324946`}, {"CURE", 365, 32.94238124069087`},
{"CURE", 365, 83.60459637884243`}, {"CURE", 365, 44.770950099025455`}, {"CURE", 365, 22.454684666704345`},
{"TBUR", 68.5410026785762`, 44.46480997308759`}, {"CURE", 365, 28.951543619843026`}, {"CURE", 365, 18.765871437467837`},
{"CURE", 365, 128.49428270513474`}, {"CURE", 365, 14.275339773332718`}, {"CURE", 365, 9.699218828208133`},
{"CURE", 365, 56.91816040765462`}, {"CURE", 365, 16.76908910884318`}, {"CURE", 365, 17.17637225200861`},
{"CURE", 365, 103.3430255585655`}, {"CURE", 365, 8.387669067122006`}, {"CURE", 365, 5.320941567096536`},
{"CURE", 365, 22.13473809611773`}, {"CURE", 365, 17.499150215016666`}, {"CURE", 365, 13.179392621023684`},
{"CURE", 365, 33.64599439528049`}, {"CURE", 365, 9.468219083022845`}, {"CURE", 365, 5.821892270336646`},
{"CURE", 365, 27.256748621341266`}, {"CURE", 365, 41.68270676615315`}, {"CURE", 365, 7.096345512530903`},
{"CURE", 365, 23.49264621393072`}, {"CURE", 365, 71.3196850607452`}, {"TBUR", 60.223863209978035`, 27.13331435648122`},
{"CURE", 365, 15.820493699793548`}, {"CURE", 365, 55.17646554637826`}, {"CURE", 365, 27.72821679759118`},
{"CURE", 365, 3.655610675302701`}, {"CURE", 365, 31.673292937714223`}, {"CURE", 365, 24.462236029798326`},
{"CURE", 365, 19.487247545855606`}, {"CURE", 365, 23.112814825924094`}, {"CURE", 365, 6.014334113100649`},
{"CURE", 365, 34.309549858717965`}, {"CURE", 365, 12.31930491885412`}, {"CURE", 365, 27.787956057610817`},
{"CURE", 365, 103.99994973524693`}, {"CURE", 365, 36.72285286296385`}, {"CURE", 365, 85.39179115742778`},
{"CURE", 365, 28.133501435067885`}, {"CURE", 365, 116.10777243285311`}, {"CURE", 365, 3.624756993145801`},
{"CURE", 365, 31.845652450497735`}, {"CURE", 365, 36.29842786849828`}, {"CURE", 365, 4.714743151463982`},
{"CURE", 365, 6.530816569572988`}, {"CURE", 365, 40.09013454322826`}, {"CURE", 365, 47.204042991369384`},
{"CURE", 365, 40.26357562326638`}, {"CURE", 365, 32.940912259000854`}, {"CURE", 365, 31.495373282063944`},
{"CURE", 365, 19.06768448754476`}, {"CURE", 365, 5.043777982850452`}, {"CURE", 365, 21.92667518031086`},
{"CURE", 365, 10.405270718034835`}, {"CURE", 365, 5.093446002684523`}, {"CURE", 365, 30.88174345994691`},
{"CURE", 365, 17.282893633768428`}, {"CURE", 365, 45.686765146660754`}, {"CURE", 365, 18.442651125740724`},
{"CURE", 365, 15.350827230407967`}, {"CURE", 365, 102.86370331351787`}, {"CURE", 365, 7.670382682815512`},
```

{"CURE", 365, 22.934479149313784`}, {"CURE", 365, 13.001054587370032`}, {"CURE", 365, 30.693080194888473`},
{"CURE", 365, 8.442747319680928`}, {"CURE", 365, 33.71602352334301`}, {"CURE", 365, 89.01615157845401`},
{"CURE", 365, 7.616857741418521`}, {"CURE", 365, 23.142644492385035`}, {"CURE", 365, 24.95651581504717`},
{"CURE", 365, 90.00255270818325`}, {"CURE", 365, 33.76818482326058`}, {"CURE", 365, 28.087969346349453`},
{"CURE", 365, 49.217940316700115`}, {"CURE", 365, 18.92306677475784`}, {"CURE", 365, 57.52257263006412`},
{"CURE", 365, 36.37411425340373`}, {"CURE", 365, 22.071774114460858`}, {"CURE", 365, 20.066261308514264`},
{"CURE", 365, 6.5339821042162765`}, {"CURE", 365, 4.428046822823175`}, {"CURE", 365, 10.37091019745098`},
{"CURE", 365, 13.903200636263817`}, {"CURE", 365, 6.114676548116601`}, {"CURE", 365, 22.3985511945313`},
{"CURE", 365, 61.19443864542758`}, {"CURE", 365, 16.549534605775012`}, {"CURE", 365, 30.64425206223801`},
{"CURE", 365, 38.46820704681407`}, {"CURE", 365, 32.25819028262661`}, {"CURE", 365, 22.51447898195766`},
{"CURE", 365, 8.942293895249634`}, {"CURE", 365, 61.59617072254435`}, {"CURE", 365, 5.330712062383564`},
{"CURE", 365, 57.42935158758507`}, {"CURE", 365, 55.64402390653817`}, {"CURE", 365, 15.576328620511033`},
{"CURE", 365, 18.50225813953144`}, {"CURE", 365, 106.28282946579492`}, {"CURE", 365, 27.364712654587294`},
{"CURE", 365, 32.73216437245033`}, {"CURE", 365, 20.48615288589462`}, {"CURE", 365, 7.707473422977505`},
{"CURE", 365, 47.219441842879895`}, {"TBUR", 52.14648503865909`, 125.422558962417`}, {"CURE", 365, 19.195024015034193`},
{"CURE", 365, 17.349669153643273`}, {"CURE", 365, 31.971260003285263`}, {"CURE", 365, 12.766405459280735`},
{"CURE", 365, 161.98293880450726`}, {"CURE", 365, 12.966985805928992`}, {"CURE", 365, 80.09727698972861`},
{"CURE", 365, 61.592054819907474`}, {"CURE", 365, 59.8733319811244`}, {"CURE", 365, 31.987395299490256`},
{"CURE", 365, 74.18684924311172`}, {"CURE", 365, 139.58313120979741`}, {"CURE", 365, 33.70557084025243`},
{"CURE", 365, 6.191097756799897`}, {"CURE", 365, 54.99687935373768`}, {"CURE", 365, 34.271052636865456`},
{"CURE", 365, 33.03539343178476`}, {"CURE", 365, 7.276610751607021`}, {"CURE", 365, 166.95301829587112`},
{"CURE", 365, 12.204184580633862`}, {"CURE", 365, 52.95014628091791`}, {"CURE", 365, 33.3717394413237`},
{"CURE", 365, 23.719960698295285`}, {"CURE", 365, 5.702684561663555`}, {"CURE", 365, 42.8678457425534`},
{"CURE", 365, 25.14592149977878`}, {"CURE", 365, 66.52864255891483`}, {"CURE", 365, 43.830368825954544`},
{"CURE", 365, 55.32257792492617`}, {"CURE", 365, 26.55274642899921`}, {"CURE", 365, 23.624930680210245`},
{"TBUR", 58.17802122369731`, 123.43552620460187`}, {"CURE", 365, 32.35205699465079`}, {"CURE", 365, 8.630416476309229`},
{"CURE", 365, 29.068639971558927`}, {"TBUR", 119.23098962399703`, 29.450085938453327`},
{"CURE", 365, 3.9586510546480573`}, {"CURE", 365, 7.064886088345891`}, {"CURE", 365, 10.791815548034418`},
{"CURE", 365, 101.83551324325602`}, {"CURE", 365, 31.336550346786954`}, {"CURE", 365, 32.663356984247685`},
{"CURE", 365, 26.15814628567542`}, {"CURE", 365, 107.42575739255071`}, {"CURE", 365, 24.7684554599727`},
{"CURE", 365, 49.741421182334044`}, {"CURE", 365, 24.695921394131574`}, {"CURE", 365, 36.40994691128441`},

```
{"CURE", 365, 8.756625460072033`}, {"CURE", 365, 32.74893998952219`}, {"CURE", 365, 20.43310273992752`},
{"CURE", 365, 39.808611168744136`}, {"CURE", 365, 62.89781741741585`}, {"CURE", 365, 70.53324353626651`},
{"CURE", 365, 42.62011335590598`}, {"CURE", 365, 35.07753892115548`}, {"CURE", 365, 48.0708082749738`},
{"CURE", 365, 22.96703952062172`}, {"CURE", 365, 26.00318823374931`}, {"CURE", 365, 18.415242810574554`},
{"CURE", 365, 14.437162802347574`}, {"CURE", 365, 23.25383231162241`}, {"CURE", 365, 59.67652333245286`},
{"CURE", 365, 6.839641056168977`}, {"CURE", 365, 30.294615890735546`}, {"CURE", 365, 5.722900633139414`},
{"CURE", 365, 29.781299560448584`}, {"CURE", 365, 16.91010797139928`}, {"CURE", 365, 62.99308121924528`},
{"CURE", 365, 5.807014620050399`}, {"CURE", 365, 41.440458281366965`}, {"CURE", 365, 52.00637811254138`},
{"CURE", 365, 8.507791165774263`}, {"CURE", 365, 19.485318086029476`}, {"CURE", 365, 15.680487925862534`},
{"CURE", 365, 8.966255277449662`}, {"CURE", 365, 53.05780397668574`}, {"CURE", 365, 19.932721010478144`},
{"CURE", 365, 38.42759961751208`}, {"CURE", 365, 37.36300728001021`}, {"CURE", 365, 42.369544645397866`},
{"CURE", 365, 33.12389036606133`}, {"CURE", 365, 39.63991188123288`}, {"CURE", 365, 31.38654584578457`},
{"CURE", 365, 60.66109638782265`}, {"CURE", 365, 36.9415653532056`}, {"CURE", 365, 30.15813791245341`},
{"CURE", 365, 77.18928887861995`}, {"CURE", 365, 8.937062913013518`}, {"CURE", 365, 50.70357465217412`},
{"CURE", 365, 3.7248234068218853`}, {"CURE", 365, 46.26454989913328`}, {"CURE", 365, 10.90528746784972`},
{"CURE", 365, 57.14928172753664`}, {"CURE", 365, 8.902798700115905`}, {"CURE", 365, 58.32985599537236`},
{"CURE", 365, 145.93137867859747`}, {"CURE", 365, 6.370818584618665`}, {"CURE", 365, 4.572683868394139`},
{"CURE", 365, 16.850166726469165`}, {"CURE", 365, 4.08974808730055`}, {"CURE", 365, 2.79779248530849`},
{"CURE", 365, 16.36897367010182`}, {"CURE", 365, 26.73705375402617`}, {"CURE", 365, 28.79984798469505`},
{"CURE", 365, 27.33897753738979`}, {"CURE", 365, 21.30175548155397`}, {"CURE", 365, 37.4987696806915`},
{"CURE", 365, 66.02190523718988`}, {"CURE", 365, 5.342250176037861`}, {"CURE", 365, 29.274311650247682`},
{"CURE", 365, 25.157309611470538`}, {"CURE", 365, 18.239044872103918`}, {"CURE", 365, 33.7252820258640 9`},
{"CURE", 365, 50.00453937388943`}, {"CURE", 365, 117.01575976613769`}, {"CURE", 365, 87.15238653915678`},
{"CURE", 365, 26.92754885512021`}, {"CURE", 365, 2.863446432683603`}, {"CURE", 365, 31.234028902765658`},
{"CURE", 365, 80.55685039166598`}, {"CURE", 365, 20.267328525102755`}, {"CURE", 365, 25.388244012904483`},
{"CURE", 365, 15.637356326979884`}, {"CURE", 365, 22.92227405560718`}, {"CURE", 365, 5.929056821209545`},
{"CURE", 365, 29.031785375997433`}, {"CURE", 365, 16.296448725764016`}, {"CURE", 365, 47.654798908126686`},
{"CURE", 365, 32.610427902087295`}, {"CURE", 365, 31.66853887323795`}, {"CURE", 365, 26.057312282685942`},
{"CURE", 365, 13.789324252860284`}, {"CURE", 365, 9.50216003842118`}, {"CURE", 365, 9.264367720946673`},
{"CURE", 365, 11.995214026008817`}, {"CURE", 365, 9.350862467246763`}, {"CURE", 365, 125.66265778034999`},
{"CURE", 365, 69.90706979406936`}, {"CURE", 365, 13.516950781881594`}, {"CURE", 365, 208.21994074670022`},
```

{"CURE", 365, 59.54511636963034`}, {"CURE", 365, 31.52585163169789`}, {"CURE", 365, 54.53256345353621`},
{"CURE", 365, 8.199602907824689`}, {"CURE", 365, 65.3597401475194`}, {"CURE", 365, 174.80722021737884`},
{"CURE", 365, 9.48338210617189`}, {"CURE", 365, 29.585919285821394`}, {"CURE", 365, 48.4199530087306`},
{"CURE", 365, 24.42259861255073`}, {"CURE", 365, 12.005439115552836`}, {"CURE", 365, 9.564995461675766`},
{"CURE", 365, 83.00923441128263`}, {"CURE", 365, 43.57905559927209`}, {"CURE", 365, 24.796199606437625`},
{"CURE", 365, 61.595936385110754`}, {"CURE", 365, 11.114552037187591`}, {"CURE", 365, 29.723770440611137`},
{"CURE", 365, 17.95128168927946`}, {"CURE", 365, 121.08714767555192`}, {"TBUR", 60.37982156498649`, 30.39370806853766`},
{"CURE", 365, 141.11304993593595`}, {"CURE", 365, 220.3058730656922`}, {"CURE", 365, 16.89724109536901`},
{"CURE", 365, 4.764242441074192`}, {"CURE", 365, 4.151698909055313`}, {"CURE", 365, 30.363036810525777`},
{"CURE", 365, 117.8329911820964`}, {"CURE", 365, 23.604550543816945`}, {"CURE", 365, 18.130596953626167`},
{"TBUR", 65.56769207218657`, 34.04281423188461`}, {"CURE", 365, 37.29689246323871`}, {"CURE", 365, 155.53685718896634`},
{"CURE", 365, 128.32283245983243`}, {"CURE", 365, 26.855327095033605`}, {"CURE", 365, 8.916577204038179`},
{"CURE", 365, 21.844391997924735`}, {"CURE", 365, 3.428579409264821`}, {"CURE", 365, 64.39459416232043`},
{"CURE", 365, 95.91147226002775`}, {"CURE", 365, 31.36668066242514`}, {"CURE", 365, 24.857513341483543`},
{"CURE", 365, 105.11871220079554`}, {"CURE", 365, 6.658653046342054`}, {"CURE", 365, 8.673650987709326`},
{"CURE", 365, 27.18317160344577`}, {"CURE", 365, 34.933981215898264`}, {"TBUR", 71.56288539133212`, 66.05405505655405`},
{"CURE", 365, 68.20253162038368`}, {"CURE", 365, 27.997023072705996`}, {"CURE", 365, 28.862279502642355`},
{"CURE", 365, 36.08207764432347`}, {"CURE", 365, 12.495568371566003`}, {"CURE", 365, 3.535772266399047`},
{"CURE", 365, 37.184105562459294`}, {"CURE", 365, 44.789711945233115`}, {"CURE", 365, 21.776061174982097`},
{"CURE", 365, 39.46490717610595`}, {"CURE", 365, 12.288081599212287`}, {"CURE", 365, 8.07725852353563`},
{"CURE", 365, 35.361414791516175`}, {"CURE", 365, 21.46461425163429`}, {"CURE", 365, 30.717431719061686`},
{"CURE", 365, 202.75677604425377`}, {"CURE", 365, 19.311917957213254`}, {"CURE", 365, 9.413351080294754`},
{"CURE", 365, 7.310106834240493`}, {"CURE", 365, 8.861117591912105`}, {"CURE", 365, 50.6142060746927`},
{"CURE", 365, 3.4864673263954877`}, {"CURE", 365, 47.13180139147691`}, {"CURE", 365, 51.46655292724781`},
{"CURE", 365, 40.17127784896482`}, {"CURE", 365, 32.93331471945236`}, {"CURE", 365, 6.525782594529504`},
{"CURE", 365, 71.67868664617995`}, {"TBUR", 60.33754170879339`, 21.64991722573154`}, {"CURE", 365, 9.739426695172263`},
{"CURE", 365, 63.826069667272876`}, {"CURE", 365, 11.353466966564554`}, {"CURE", 365, 76.46631662993347`},
{"CURE", 365, 49.081276921156565`}, {"CURE", 365, 32.703082209190015`}, {"CURE", 365, 47.56300135554101`},
{"CURE", 365, 16.841507244667266`}, {"CURE", 365, 74.60312682006705`}, {"CURE", 365, 8.852814632550439`},
{"CURE", 365, 37.46363983173586`}, {"CURE", 365, 43.846190169789246`}, {"CURE", 365, 18.64226837012171`},
{"CURE", 365, 173.71824274673855`}, {"CURE", 365, 34.782139003969`}, {"CURE", 365, 35.5575291081901`},

{"CURE", 365, 36.318199205833515`}, {"CURE", 365, 145.48025268602933`}, {"CURE", 365, 46.21601893418218`},
{"CURE", 365, 43.11883460304139`}, {"CURE", 365, 93.82805656117898`}, {"CURE", 365, 33.00570071932653`},
{"CURE", 365, 28.754970252551786`}, {"CURE", 365, 35.53915891741468`}, {"CURE", 365, 49.0892075616376`},
{"CURE", 365, 86.74955404717959`}, {"CURE", 365, 18.715479464015978`}, {"CURE", 365, 17.087394057176986`},
{"CURE", 365, 36.5663850908003`}, {"CURE", 365, 37.22688883779866`}, {"CURE", 365, 41.83517518573689`},
{"CURE", 365, 44.76350080767627`}, {"CURE", 365, 25.00991184773909`}, {"CURE", 365, 12.57155303814193`},
{"CURE", 365, 47.227250921958195`}, {"CURE", 365, 11.936517317949896`}, {"CURE", 365, 25.376968535847542`},
{"CURE", 365, 30.17870750590142`}, {"CURE", 365, 9.104614377368792`}, {"CURE", 365, 4.525072384364894`},
{"CURE", 365, 27.909348849651504`}, {"CURE", 365, 37.87938249839296`}, {"CURE", 365, 44.97723649169334`},
{"CURE", 365, 32.169987691295766`}, {"CURE", 365, 4.1223483612868135`}, {"CURE", 365, 36.91031051085822`},
{"CURE", 365, 7.029574009212617`}, {"CURE", 365, 108.50536954063678`}, {"CURE", 365, 141.1699148422776`},
{"CURE", 365, 19.812318598245458`}, {"TBUR", 79.47584652178179`, 27.865115821308294`},
{"CURE", 365, 151.53463831633093`}, {"CURE", 365, 27.176239327685654`}, {"CURE", 365, 15.761872576557437`},
{"CURE", 365, 12.278688590517318`}, {"CURE", 365, 3.3607746278720927`}, {"CURE", 365, 21.1889135174619`},
{"CURE", 365, 23.626316098574076`}, {"CURE", 365, 62.57412550133969`}, {"CURE", 365, 29.959384781022678`},
{"CURE", 365, 52.17122119393094`}, {"CURE", 365, 18.431058916686997`}, {"CURE", 365, 39.95329921361515`},
{"CURE", 365, 89.39494624270895`}, {"CURE", 365, 9.086278939989255`}, {"CURE", 365, 14.02474323315769`},
{"CURE", 365, 20.827037683436963`}, {"CURE", 365, 29.09872220180448`}, {"CURE", 365, 19.056765062474263`},
{"CURE", 365, 18.264789162625455`}, {"CURE", 365, 7.735238860098619`}, {"CURE", 365, 11.946346313444584`},
{"CURE", 365, 22.638469292870326`}, {"CURE", 365, 51.53895548363158`}, {"CURE", 365, 45.813108202792996`},
{"CURE", 365, 23.48523095240801`}, {"TBUR", 37.23998725134437`, 35.16016077122718`}, {"CURE", 365, 75.84546699232264`},
{"CURE", 365, 37.11098791348579`}, {"CURE", 365, 27.770020204887256`}, {"CURE", 365, 17.688557125480727`},
{"CURE", 365, 5.539952800665249`}, {"CURE", 365, 30.832175064217555`}, {"CURE", 365, 35.579928507241995`},
{"CURE", 365, 139.31773329071245`}, {"CURE", 365, 7.101489222015178`}, {"CURE", 365, 6.940413419017551`},
{"CURE", 365, 22.23831309669998`}, {"CURE", 365, 64.82562026903356`}, {"CURE", 365, 8.08810909516839`},
{"CURE", 365, 39.57005716774898`}, {"CURE", 365, 9.227140016473502`}, {"CURE", 365, 22.99276249014145`},
{"CURE", 365, 9.351440788208931`}, {"CURE", 365, 9.752509467947517`}, {"CURE", 365, 8.795838604648113`},
{"CURE", 365, 93.85485755544116`}, {"CURE", 365, 14.977601035372262`}, {"TBUR", 83.99123189070636`, 26.385588561362184`},
{"CURE", 365, 3.6461174884372998`}, {"CURE", 365, 43.68085224980146`}, {"CURE", 365, 61.125277181039976`},
{"CURE", 365, 53.26391117426467`}, {"CURE", 365, 8.043757143261804`}, {"CURE", 365, 16.136715027297058`},
{"CURE", 365, 164.1375400679853`}, {"CURE", 365, 44.60205985936204`}, {"CURE", 365, 30.37568028626558`},

{"CURE", 365, 10.778001925101494`}, {"CURE", 365, 15.455268569518363`}, {"CURE", 365, 23.879004736144445`},
{"CURE", 365, 5.9117653300198`}, {"CURE", 365, 29.042488136808057`}, {"CURE", 365, 45.70191573543682`},
{"CURE", 365, 27.79109646407325`}, {"CURE", 365, 30.163347030410893`}, {"CURE", 365, 21.838460378932137`},
{"CURE", 365, 14.135624579669502`}, {"CURE", 365, 80.74949508138195`}, {"CURE", 365, 117.1442455068796`},
{"CURE", 365, 31.252165078731934`}, {"CURE", 365, 39.45687037162049`}, {"CURE", 365, 49.98427254274449`},
{"CURE", 365, 48.86630625632756`}, {"CURE", 365, 4.264671540249123`}, {"CURE", 365, 57.134175539514295`},
{"CURE", 365, 59.07962143089955`}, {"CURE", 365, 39.91199077726759`}, {"CURE", 365, 160.25673521782045`},
{"CURE", 365, 33.31574513339377`}, {"CURE", 365, 14.810165140387822`}, {"CURE", 365, 35.06095350702972`},
{"CURE", 365, 44.55921777958651`}, {"CURE", 365, 19.96145871874978`}, {"CURE", 365, 15.652088223152381`},
{"CURE", 365, 13.624096236323064`}, {"CURE", 365, 24.673962332674822`}, {"CURE", 365, 76.12677044282633`},
{"CURE", 365, 48.858570109131605`}, {"CURE", 365, 8.500528673646047`}, {"CURE", 365, 49.74922522066716`},
{"CURE", 365, 71.91748653326158`}, {"CURE", 365, 13.208171013162529`}, {"CURE", 365, 75.43126751257287`},
{"CURE", 365, 11.337072253619487`}, {"CURE", 365, 7.233542059035378`}, {"CURE", 365, 10.271236076659884`},
{"CURE", 365, 19.75440929775077`}, {"TBUR", 82.9445894078767`, 88.30597233447148`}, {"CURE", 365, 31.628906933416946`},
{"CURE", 365, 39.921682913336625`}, {"CURE", 365, 9.547655988327215`}, {"CURE", 365, 182.03730417640773`},
{"CURE", 365, 83.45813452812028`}, {"CURE", 365, 14.225460425279563`}, {"CURE", 365, 34.85028500315019`},
{"CURE", 365, 19.424245807078037`}, {"CURE", 365, 19.615348906933185`}, {"CURE", 365, 66.2746883749253`},
{"TBUR", 56.84416677055886`, 83.72070618034105`}, {"CURE", 365, 9.006934610723645`}, {"CURE", 365, 17.547111355675028`},
{"CURE", 365, 6.224763068946863`}, {"CURE", 365, 24.010235874862285`}, {"CURE", 365, 41.122205726669186`},
{"CURE", 365, 66.66684282614693`}, {"CURE", 365, 37.20044944416966`}, {"CURE", 365, 133.98246211580238`},
{"CURE", 365, 64.04241662258421`}, {"TBUR", 70.750569569633`, 10.546456288707347`}, {"CURE", 365, 23.4164215675047`},
{"TBUR", 39.25979797379313`, 24.92497013576102`}, {"TBUR", 75.79964329706793`, 52.26264964649301`},
{"CURE", 365, 6.855755645732277`}, {"CURE", 365, 18.38121305726385`}, {"CURE", 365, 10.35412102358592`},
{"CURE", 365, 25.839364266893487`}, {"CURE", 365, 59.032396288830675`}, {"CURE", 365, 25.115726608806323`},
{"CURE", 365, 5.455212545296006`}, {"CURE", 365, 103.86506302883996`}, {"CURE", 365, 19.595641573104313`},
{"CURE", 365, 6.656909293168582`}, {"CURE", 365, 8.750916482878393`}, {"CURE", 365, 7.3876643099793755`},
{"CURE", 365, 9.438774333665052`}, {"CURE", 365, 75.9543212028082`}, {"CURE", 365, 41.551014459121305`},
{"CURE", 365, 25.022462219274708`}, {"CURE", 365, 11.724721260165579`}, {"CURE", 365, 19.58569282587242`},
{"CURE", 365, 11.331410865512247`}, {"CURE", 365, 27.342839658025735`}, {"CURE", 365, 94.00565812157292`},
{"CURE", 365, 54.16440101091573`}, {"CURE", 365, 27.114244088859863`}, {"CURE", 365, 15.62941135647735`},
{"CURE", 365, 16.329919434346984`}, {"CURE", 365, 200.88806434845577`}, {"CURE", 365, 9.571764743336256`},

{"CURE", 365, 4.930964943084611`}, {"CURE", 365, 4.293474900596667`}, {"CURE", 365, 54.609981360479374`},
{"CURE", 365, 31.96975047593769`}, {"CURE", 365, 35.479923164613965`}, {"CURE", 365, 52.722676067864576`},
{"CURE", 365, 86.97599432233824`}, {"CURE", 365, 90.02265082611505`}, {"CURE", 365, 34.30709669412268`},
{"CURE", 365, 14.81364849512263`}, {"CURE", 365, 17.175515951281017`}, {"CURE", 365, 34.181899137678535`},
{"CURE", 365, 19.437669168080827`}, {"TBUR", 25.80000812982083`, 7.529299538440954`}, {"CURE", 365, 30.524593182376204`},
{"CURE", 365, 190.08931787525728`}, {"CURE", 365, 19.585774496231558`}, {"CURE", 365, 7.042077591487915`},
{"CURE", 365, 66.7884118804502`}, {"CURE", 365, 16.70301561110062`}, {"CURE", 365, 47.996335947507795`},
{"TOX", 16.22662238774153`, 231.08450202455336`}, {"CURE", 365, 106.70074641031546`}, {"CURE", 365, 34.639343197650746`},
{"CURE", 365, 29.03220562897018`}, {"CURE", 365, 12.369908856676725`}, {"CURE", 365, 3.530970298383821`},
{"CURE", 365, 13.814080481545147`}, {"CURE", 365, 40.50770541680771`}, {"CURE", 365, 22.457552321718072`},
{"CURE", 365, 71.65717868559105`}, {"CURE", 365, 28.40222804560689`}, {"CURE", 365, 29.007619545216233`},
{"CURE", 365, 8.055212019594041`}, {"CURE", 365, 22.815543620079488`}, {"CURE", 365, 23.415062500210404`},
{"CURE", 365, 6.105266642323991`}, {"CURE", 365, 19.421154710761684`}, {"CURE", 365, 11.584031194081177`},
{"TBUR", 89.4431761378137`, 18.338529405602554`}, {"CURE", 365, 38.32476116803864`}, {"CURE", 365, 19.21617609682826`},
{"CURE", 365, 4.458079598577676`}, {"CURE", 365, 30.711504341953123`}, {"CURE", 365, 3.4368754607899894`},
{"CURE", 365, 10.08423688896973`}, {"CURE", 365, 29.926022387674205`}, {"CURE", 365, 34.51051247284433`},
{"CURE", 365, 6.861340854348437`}, {"CURE", 365, 20.20084923052312`}, {"CURE", 365, 26.156408740673847`},
{"CURE", 365, 132.14242000865838`}, {"CURE", 365, 30.381295332974947`}, {"CURE", 365, 8.56547068994784`},
{"CURE", 365, 93.3968829602031`}, {"CURE", 365, 9.167381424002269`}, {"CURE", 365, 34.56457831025515`},
{"CURE", 365, 35.76597216263031`}, {"CURE", 365, 10.675472952618758`}, {"CURE", 365, 11.884698088526026`},
{"CURE", 365, 52.65046427846716`}, {"CURE", 365, 5.141816958417322`}, {"CURE", 365, 44.611299610404636`},
{"CURE", 365, 11.813773310031308`}, {"CURE", 365, 15.448221584038347`}, {"CURE", 365, 3.4504463178189706`},
{"CURE", 365, 17.737866370674855`}, {"CURE", 365, 60.44774528729891`}, {"CURE", 365, 23.01594367123633`},
{"CURE", 365, 16.71132825914361`}, {"CURE", 365, 23.885450560763413`}, {"CURE", 365, 18.712508340327847`},
{"CURE", 365, 23.60102663944228`}, {"CURE", 365, 41.67864487317227`}, {"CURE", 365, 40.22652123316862`},
{"CURE", 365, 4.954567812034228`}, {"CURE", 365, 81.24872193100613`}, {"CURE", 365, 9.454704656738707`},
{"CURE", 365, 4.741289316252511`}, {"CURE", 365, 22.605882592753733`}, {"CURE", 365, 23.734080362659796`},
{"CURE", 365, 15.994891430502351`}, {"CURE", 365, 27.781638110850924`}, {"CURE", 365, 23.95557148774235`},
{"CURE", 365, 38.506017938781056`}, {"CURE", 365, 32.53803304813103`}, {"TBUR", 65.80001987594689`, 46.43412834621016`},
{"CURE", 365, 7.339009727614522`}, {"CURE", 365, 93.95246247276911`}, {"CURE", 365, 91.3205128483349`},
{"CURE", 365, 5.0582566035450744`}, {"CURE", 365, 12.73702123108621`}, {"CURE", 365, 31.603050056261328`},

{"CURE", 365, 16.05857759499537`}, {"CURE", 365, 49.31652376891657`}, {"CURE", 365, 98.9343621124286`},
{"CURE", 365, 31.27076755764231`}, {"CURE", 365, 5.365656767420696`}, {"CURE", 365, 5.264325291186228`},
{"CURE", 365, 12.36289710988211`}, {"CURE", 365, 3.7318898861781262`}, {"CURE", 365, 42.36280831549552`},
{"CURE", 365, 6.56637171028782`}, {"CURE", 365, 44.10088502082873`}, {"CURE", 365, 27.203664794606425`},
{"CURE", 365, 46.892633017253026`}, {"CURE", 365, 11.54406159749475`}, {"CURE", 365, 58.24183315049291`},
{"CURE", 365, 192.69581609347782`}, {"CURE", 365, 69.42598830348312`}, {"CURE", 365, 112.45974671299867`},
{"CURE", 365, 33.10321643277852`}, {"CURE", 365, 78.86868295935193`}, {"CURE", 365, 79.02100632795812`},
{"CURE", 365, 9.591218219099071`}, {"CURE", 365, 6.708564548304358`}, {"CURE", 365, 21.810187269153513`},
{"CURE", 365, 45.26749855175464`}, {"CURE", 365, 56.6342554403885`}, {"CURE", 365, 24.142837199492305`},
{"CURE", 365, 35.818706198379274`}, {"CURE", 365, 6.748094635284341`}, {"CURE", 365, 8.571066518615682`},
{"CURE", 365, 41.666663496751745`}, {"CURE", 365, 13.494072705675356`}, {"CURE", 365, 5.733520966940124`},
{"CURE", 365, 8.481601311211888`}, {"CURE", 365, 4.145749450321472`}, {"CURE", 365, 33.69113824243912`},
{"CURE", 365, 46.336564094155676`}, {"CURE", 365, 32.40126001202914`}, {"CURE", 365, 36.8343709004138`},
{"CURE", 365, 20.39371659497986`}, {"CURE", 365, 18.247047893846695`}, {"CURE", 365, 47.24856770632419`},
{"CURE", 365, 50.584091456693876`}, {"CURE", 365, 5.643432443025446`}, {"CURE", 365, 20.928992905673653`},
{"CURE", 365, 11.212543816442263`}, {"CURE", 365, 17.613054119597216`}, {"CURE", 365, 145.06085433769854`},
{"CURE", 365, 25.615815219575985`}, {"CURE", 365, 17.583560212582636`}, {"CURE", 365, 18.585213665383957`},
{"CURE", 365, 37.09508532392265`}, {"CURE", 365, 28.236736550535724`}, {"CURE", 365, 36.83598007758677`},
{"CURE", 365, 37.91707048828749`}, {"CURE", 365, 8.615055050585825`}, {"CURE", 365, 83.7915229394352`},
{"CURE", 365, 16.084045779905004`}, {"CURE", 365, 34.12619900893104`}, {"CURE", 365, 19.362158521423762`},
{"CURE", 365, 10.99760836467518`}, {"CURE", 365, 40.091051330757765`}, {"CURE", 365, 40.702408997240994`},
{"TBUR", 70.81259381301703`, 22.52438347625109`}, {"CURE", 365, 53.34221687629987`}, {"CURE", 365, 11.283815924603589`},
{"CURE", 365, 94.3312898269517`}, {"CURE", 365, 36.21312499210259`}, {"CURE", 365, 76.52634867260409`},
{"CURE", 365, 2.970657048911439`}, {"CURE", 365, 37.63784911603635`}, {"CURE", 365, 42.850202161888625`},
{"CURE", 365, 10.209360027094524`}, {"CURE", 365, 25.91351933198355`}, {"CURE", 365, 38.81397646773445`},
{"CURE", 365, 23.588418985596054`}, {"CURE", 365, 7.503753658818484`}, {"CURE", 365, 5.6961518473504`},
{"CURE", 365, 33.66574932532501`}, {"CURE", 365, 8.985781260018756`}, {"CURE", 365, 19.973357173298414`},
{"CURE", 365, 24.329926580611865`}, {"CURE", 365, 11.312306067872592`}, {"CURE", 365, 20.82739711311161`},
{"CURE", 365, 5.679802755017555`}, {"CURE", 365, 23.418496002157568`}, {"CURE", 365, 3.786400226260683`},
{"CURE", 365, 6.767866031554531`}, {"CURE", 365, 15.995893863200553`}, {"CURE", 365, 5.15878616031832`},
{"CURE", 365, 38.339801505430614`}, {"CURE", 365, 56.3553133962561`}, {"CURE", 365, 57.15908675267485`},

{"CURE", 365, 47.626620030643416`}, {"CURE", 365, 89.88842256262906`}, {"CURE", 365, 7.269402770058261`},
{"CURE", 365, 32.424550308652`}, {"CURE", 365, 34.78617078082345`}, {"CURE", 365, 59.88129198364691`},
{"CURE", 365, 44.7270157848216`}, {"CURE", 365, 20.429790541616732`}, {"CURE", 365, 96.0388789800815`},
{"CURE", 365, 2.999662629223308`}, {"CURE", 365, 91.45848016520645`}, {"CURE", 365, 6.191262214838957`},
{"CURE", 365, 78.11074033817066`}, {"CURE", 365, 70.74376380973648`}, {"CURE", 365, 20.96522034480437`},
{"CURE", 365, 82.54602668453178`}, {"CURE", 365, 100.71644683072847`}, {"CURE", 365, 45.03393768194337`},
{"CURE", 365, 19.878273218995826`}, {"CURE", 365, 22.852995444492052`}, {"CURE", 365, 33.74435787529182`},
{"CURE", 365, 31.115078794582484`}, {"CURE", 365, 7.542134736628583`}, {"CURE", 365, 27.073170429918417`},
{"CURE", 365, 25.604704541072707`}, {"CURE", 365, 19.156813569966513`}, {"CURE", 365, 47.348161808619345`},
{"CURE", 365, 7.455652592083191`}, {"CURE", 365, 52.846090317446695`}, {"CURE", 365, 34.57675477695532`},
{"CURE", 365, 19.58101940666398`}, {"CURE", 365, 17.712376366168545`}, {"CURE", 365, 36.07846349938784`},
{"CURE", 365, 4.9484678692324096`}, {"CURE", 365, 57.31491155737675`}, {"CURE", 365, 9.04466920168794`},
{"CURE", 365, 12.833944717914747`}, {"CURE", 365, 28.782064592290627`}, {"CURE", 365, 22.569167277345194`},
{"CURE", 365, 20.754663621284205`}, {"CURE", 365, 64.58653167473645`}, {"CURE", 365, 8.740632105912528`},
{"CURE", 365, 15.217815424423861`}, {"CURE", 365, 112.60470022722342`}, {"CURE", 365, 8.543092025984764`},
{"CURE", 365, 22.004154933821972`}, {"CURE", 365, 33.462249204134956`}, {"CURE", 365, 33.04064156422667`},
{"CURE", 365, 22.44415686773085`}, {"CURE", 365, 116.04273828103787`}, {"CURE", 365, 20.948410538911048`},
{"CURE", 365, 8.826113762401441`}, {"CURE", 365, 17.567141085516436`}, {"CURE", 365, 13.591364619270607`},
{"CURE", 365, 10.411861434798908`}, {"CURE", 365, 27.313566793343526`}, {"CURE", 365, 46.78345938492998`},
{"CURE", 365, 25.737820171397935`}, {"CURE", 365, 30.006002020992778`}, {"CURE", 365, 65.71224136538189`},
{"CURE", 365, 7.241803080930964`}, {"CURE", 365, 8.521948206848041`}, {"CURE", 365, 71.9329290426836`},
{"CURE", 365, 23.13841215209663`}, {"CURE", 365, 25.084852697891694`}, {"CURE", 365, 12.608620331787119`},
{"CURE", 365, 28.873656886979497`}, {"CURE", 365, 35.9834612848756`}, {"CURE", 365, 83.38474914708762`},
{"CURE", 365, 12.291486374013276`}, {"CURE", 365, 47.98986640103001`}, {"CURE", 365, 28.23869530063189`},
{"CURE", 365, 74.76824606818434`}, {"CURE", 365, 122.19234454704919`}, {"CURE", 365, 44.70545607154454`},
{"CURE", 365, 4.330649367068731`}, {"CURE", 365, 33.902123553150666`}, {"CURE", 365, 10.701991460958249`},
{"CURE", 365, 113.22209912002128`}, {"TBUR", 42.20741509272445`, 3.534633755814158`}, {"CURE", 365, 3.7782611237050117`},
{"CURE", 365, 130.9434214995076`}, {"CURE", 365, 30.103153072842247`}, {"CURE", 365, 10.198800397763526`},
{"CURE", 365, 19.520495925457855`}, {"CURE", 365, 6.846861109833317`}, {"TBUR", 67.76831056847956`, 6.496287510509178`},
{"CURE", 365, 7.787661004864277`}, {"CURE", 365, 28.14870173653664`}, {"CURE", 365, 59.659559340951674`},
{"CURE", 365, 23.62507194970534`}, {"CURE", 365, 5.916243920682204`}, {"CURE", 365, 80.49339523337609`},

{"CURE", 365, 160.76264597883014`}, {"CURE", 365, 4.53639733493026`}, {"CURE", 365, 36.23570736010759`},
{"CURE", 365, 19.4768350782676`}, {"CURE", 365, 3.8302646395849993`}, {"CURE", 365, 29.935919472881306`},
{"CURE", 365, 19.131866448431687`}, {"CURE", 365, 14.683774067758817`}, {"CURE", 365, 28.560248231249737`},
{"CURE", 365, 16.211663335814343`}, {"CURE", 365, 47.598513534745706`}, {"CURE", 365, 28.33490612422835`},
{"CURE", 365, 66.71178979332402`}, {"CURE", 365, 29.551035265839026`}, {"CURE", 365, 14.639191752701436`},
{"CURE", 365, 7.1166727669646175`}, {"CURE", 365, 4.940001996555754`}, {"CURE", 365, 14.071233995621151`},
{"CURE", 365, 55.985556220535116`}, {"CURE", 365, 18.465924074050573`}, {"CURE", 365, 16.912473473160915`},
{"CURE", 365, 11.960915636002829`}, {"CURE", 365, 12.124448051481593`}, {"CURE", 365, 32.76297684522556`},
{"CURE", 365, 6.130433440866384`}, {"CURE", 365, 39.04571378850391`}, {"CURE", 365, 17.869980051167687`},
{"CURE", 365, 10.4944925600922`7}, {"CURE", 365, 16.637354955540538`}, {"CURE", 365, 36.93037252772329`},
{"CURE", 365, 27.458805410355783`}, {"CURE", 365, 3.3201433040415202`}, {"CURE", 365, 14.298787056487457`},
{"TOX", 10.012103525346905`, 239.11295948820683`}, {"CURE", 365, 39.6927606881557`}, {"CURE", 365, 57.91721548871878`},
{"CURE", 365, 17.065240440465153`}, {"CURE", 365, 63.26762141686562`}, {"CURE", 365, 7.571445490492477`},
{"CURE", 365, 25.501780027612014`}, {"CURE", 365, 117.77014714190682`}, {"CURE", 365, 22.057198906096684`},
{"CURE", 365, 19.39204744803261`}, {"CURE", 365, 33.29917575375837`}, {"CURE", 365, 17.609408571160365`},
{"CURE", 365, 3.9110439063908653`}, {"CURE", 365, 36.329391786182306`}, {"CURE", 365, 6.48477006010939`},
{"CURE", 365, 15.396176206452319`}, {"CURE", 365, 36.411271465877114`}, {"CURE", 365, 13.076293015871178`},
{"CURE", 365, 69.45554891367412`}, {"CURE", 365, 20.642518316881958`}, {"CURE", 365, 35.55230757697739`},
{"CURE", 365, 24.641990443248943`}, {"CURE", 365, 6.973587296153023`}, {"CURE", 365, 25.85936045715534`},
{"CURE", 365, 55.91146282050689`}, {"CURE", 365, 18.738493444702055`}, {"CURE", 365, 14.103134360814437`},
{"CURE", 365, 6.688280428107966`}, {"CURE", 365, 21.195139180380163`}, {"CURE", 365, 25.437075460154997`},
{"CURE", 365, 72.66082806337954`}, {"CURE", 365, 11.67196456086894`}, {"CURE", 365, 17.551943584630706`},
{"CURE", 365, 68.19581357072946`}, {"CURE", 365, 7.185507440364899`}, {"CURE", 365, 98.55697206277091`},
{"CURE", 365, 8.337584665747443`}, {"CURE", 365, 15.256520642253852`}, {"CURE", 365, 35.1755246779477`},
{"CURE", 365, 56.581940208786584`}, {"CURE", 365, 25.481595121031738`}, {"CURE", 365, 13.34335125508689`},
{"CURE", 365, 3.925692203760095`}, {"TBUR", 70.61457148493119`, 34.305951268725856`}, {"CURE", 365, 31.125709233702207`},
{"CURE", 365, 30.613443616135758`}, {"CURE", 365, 12.07118591950617`}, {"CURE", 365, 29.59267404400465`},
{"CURE", 365, 3.420756679263383`}, {"CURE", 365, 19.72383994051451`}, {"CURE", 365, 8.616541714885383`},
{"CURE", 365, 3.8146708608412827`}, {"CURE", 365, 19.83178444812207`}, {"CURE", 365, 4.244694649213495`},
{"CURE", 365, 13.496810123966753`}, {"CURE", 365, 9.25458075687511`}, {"TBUR", 39.28502315143694`, 5.524578369637387`},
{"CURE", 365, 18.329983707905136`}, {"CURE", 365, 48.85286667335718`}, {"CURE", 365, 26.722255251322853`},

{"CURE", 365, 24.216778360092572`}, {"CURE", 365, 38.424169767415435`}, {"CURE", 365, 32.66897198068088`}},
{{"2000 Res", "2000 OS", "2000 Tox"}, {"CURE", 365, 16.64652972248509`}, {"CURE", 365, 34.47469465831783`},
{"CURE", 365, 24.15198920714789`}, {"TBUR", 66.19299325542853`, 8.084901896993218`},
{"TBUR", 45.951499445510755`, 30.56554537109182`}, {"CURE", 365, 62.446471569176886`}, {"CURE", 365, 58.7559154600388l`},
{"CURE", 365, 6.138898658518724`}, {"CURE", 365, 42.37418226501093`}, {"CURE", 365, 36.6469771937098`},
{"CURE", 365, 137.54709885722048`}, {"CURE", 365, 17.068410933869057`}, {"CURE", 365, 60.92334681620439`},
{"CURE", 365, 204.68759341627634`}, {"CURE", 365, 29.008939335215032`}, {"CURE", 365, 45.2756780450424`},
{"CURE", 365, 89.69045440188275`}, {"CURE", 365, 40.16706638083666`}, {"TBUR", 121.46776716290864`, 30.353524352150107`},
{"CURE", 365, 10.070253782848262`}, {"CURE", 365, 62.249281631091804`}, {"CURE", 365, 25.233799807244154`},
{"CURE", 365, 9.668610013920535`}, {"CURE", 365, 7.703741475438966`}, {"CURE", 365, 35.05583999129617`},
{"CURE", 365, 38.47539362313341`}, {"CURE", 365, 3.497836068358981`}, {"CURE", 365, 5.3017480495673315`},
{"CURE", 365, 19.644980575326105`}, {"CURE", 365, 22.979163451530496`}, {"CURE", 365, 19.60626643551154`},
{"CURE", 365, 68.87290715218862`}, {"CURE", 365, 15.157810805716487`}, {"CURE", 365, 12.049954896664916`},
{"CURE", 365, 97.71541246519935`}, {"CURE", 365, 5.985861993550818`}, {"CURE", 365, 25.87888236835108`},
{"TBUR", 44.475959430583345`, 57.643455987436184`}, {"CURE", 365, 14.378278879482833`}, {"CURE", 365, 22.117305672319898`},
{"CURE", 365, 50.445099629764464`}, {"CURE", 365, 34.07291904657432`}, {"CURE", 365, 6.9128084685124715`},
{"CURE", 365, 23.313420605758626`}, {"CURE", 365, 33.6416704390505`}, {"CURE", 365, 22.06069218724469`},
{"TOX", 11.254756140857827`, 234.4026902914182`}, {"CURE", 365, 50.52763651954393`}, {"CURE", 365, 77.01965672289462`},
{"CURE", 365, 9.44841866358967`}, {"CURE", 365, 26.25187398743424`}, {"CURE", 365, 36.127963411201044`},
{"CURE", 365, 29.06917595078085`}, {"CURE", 365, 18.870000394670058`}, {"CURE", 365, 8.13218165477114`},
{"CURE", 365, 86.02323624819307`}, {"CURE", 365, 27.03283346641954`}, {"CURE", 365, 5.132909221322505`},
{"CURE", 365, 55.31446069809802`}, {"CURE", 365, 38.72867067349522`}, {"CURE", 365, 26.534588199770802`},
{"CURE", 365, 20.310390902652205`}, {"CURE", 365, 11.477554853684167`}, {"CURE", 365, 43.77488511639663`},
{"CURE", 365, 31.091966352463544`}, {"CURE", 365, 35.562359075931205`}, {"CURE", 365, 8.140039393329454`},
{"CURE", 365, 8.269148507317425`}, {"CURE", 365, 8.80089018910584`}, {"CURE", 365, 35.94193810810909`},
{"CURE", 365, 13.157673498436374`}, {"CURE", 365, 29.30639996987681`}, {"CURE", 365, 55.0003673054161`},
{"TBUR", 24.99403388594637`, 16.73998196975679`}, {"CURE", 365, 14.696079480882705`}, {"CURE", 365, 40.71421544276265`},
{"CURE", 365, 23.806501430357688`}, {"CURE", 365, 18.110337070463263`}, {"CURE", 365, 72.14165871113832`},
{"CURE", 365, 79.22695041343256`}, {"CURE", 365, 58.32878901932819`}, {"CURE", 365, 15.738311552068856`},
{"CURE", 365, 31.82137911273613`}, {"CURE", 365, 22.242221840538413`}, {"CURE", 365, 25.15000365397982`},
{"CURE", 365, 11.78457969171792`}, {"CURE", 365, 125.71212644402269`}, {"CURE", 365, 27.397151294307122`},

{"CURE", 365, 34.51015115983706`}, {"CURE", 365, 29.69350814762639`}, {"CURE", 365, 24.509574197878088`},
{"CURE", 365, 7.277090596517974`}, {"CURE", 365, 13.338554757300889`}, {"CURE", 365, 30.87809827122704`},
{"TBUR", 53.093330121324286`, 11.154484762147355`}, {"CURE", 365, 13.294797079482949`}, {"CURE", 365, 34.677271905667254`},
{"CURE", 365, 88.00728176148756`}, {"CURE", 365, 47.135447511281676`}, {"CURE", 365, 23.64059715843765`},
{"TBUR", 72.33306786567339`, 46.82989196020378`}, {"CURE", 365, 30.47966283308969`}, {"CURE", 365, 19.75481230736379`},
{"CURE", 365, 135.26452929097056`}, {"CURE", 365, 15.026903290409434`}, {"CURE", 365, 10.209920849313244`},
{"CURE", 365, 59.920289935644234`}, {"CURE", 365, 17.66129703093127`}, {"CURE", 365, 18.080603576721998`},
{"CURE", 365, 108.78358243608535`}, {"CURE", 365, 8.82936015610643`}, {"CURE", 365, 5.601347920122179`},
{"CURE", 365, 23.300795878636503`}, {"CURE", 365, 18.421457029757125`}, {"CURE", 365, 13.873967790120895`},
{"CURE", 365, 35.41710537189453`}, {"CURE", 365, 9.967246676205908`}, {"CURE", 365, 6.128641491218672`},
{"CURE", 365, 28.691563389730362`}, {"CURE", 365, 43.87810417316485`}, {"CURE", 365, 7.470829965239579`},
{"CURE", 365, 24.73080842153886`}, {"CURE", 365, 75.07554273864261`}, {"TBUR", 63.23968949762178`, 28.57883779705335`},
{"CURE", 365, 16.653889462523207`}, {"CURE", 365, 58.09060082083446`}, {"CURE", 365, 29.192917905880524`},
{"CURE", 365, 3.848489820311039`}, {"CURE", 365, 33.341404359833675`}, {"CURE", 365, 25.750467681472433`},
{"CURE", 365, 20.513525722771675`}, {"CURE", 365, 24.330836064398426`}, {"CURE", 365, 6.332269909808996`},
{"CURE", 365, 36.12371402603714`}, {"CURE", 365, 12.969626666455925`}, {"CURE", 365, 29.267732606609993`},
{"CURE", 365, 109.49358296754369`}, {"CURE", 365, 38.67059182308568`}, {"CURE", 365, 89.89725170963483`},
{"CURE", 365, 29.616904612804273`}, {"CURE", 365, 122.26151841508046`}, {"CURE", 365, 3.815892464605988 6`},
{"CURE", 365, 33.522234507864795`}, {"CURE", 365, 38.210193920875376`}, {"CURE", 365, 4.962983293649066`},
{"CURE", 365, 6.874998131317803`}, {"CURE", 365, 42.20160554370963`}, {"CURE", 365, 49.703346327285004`},
{"CURE", 365, 42.383680928618155`}, {"CURE", 365, 34.676810957702145`}, {"CURE", 365, 33.15340210857211`},
{"CURE", 365, 20.07272347120856`}, {"CURE", 365, 5.309343418962835`}, {"CURE", 365, 23.085345348696055`},
{"CURE", 365, 10.955023211146957`}, {"CURE", 365, 5.36261271222094`}, {"CURE", 365, 32.51020645326177`},
{"CURE", 365, 18.192889780315884`}, {"CURE", 365, 48.09234253016018`}, {"CURE", 365, 19.414303872122634`},
{"CURE", 365, 16.1593532173073`}, {"CURE", 365, 108.3081871995786`}, {"CURE", 365, 8.074547983341196`},
{"CURE", 365, 24.1473821976608`}, {"CURE", 365, 13.687471168168383`}, {"CURE", 365, 32.318965655194994`},
{"CURE", 365, 8.88831696480681`}, {"CURE", 365, 35.4936425197761`}, {"CURE", 365, 93.72245665810641`},
{"CURE", 365, 8.019088613383282`}, {"CURE", 365, 24.361676228352014`}, {"CURE", 365, 26.282386272075573`},
{"CURE", 365, 94.74050581410907`}, {"CURE", 365, 35.57210162441369`}, {"CURE", 365, 29.576354603411982`},
{"CURE", 365, 51.84902343782873`}, {"CURE", 365, 19.922001429005586`}, {"CURE", 365, 60.79090907892684`},
{"CURE", 365, 38.30785464892135`}, {"CURE", 365, 23.278967470961774`}, {"CURE", 365, 21.13248498896843`},

{"CURE", 365, 6.878823198558835`}, {"CURE", 365, 4.661612582074668`}, {"CURE", 365, 10.920513669178012`},
{"CURE", 365, 14.636385137272395`}, {"CURE", 365, 6.439905565865024`}, {"CURE", 365, 23.584560306850495`},
{"CURE", 365, 64.42262334783453`}, {"CURE", 365, 17.420802355218182`}, {"CURE", 365, 32.2722602404627`},
{"CURE", 365, 40.493550324438175`}, {"CURE", 365, 34.09948117539354`}, {"CURE", 365, 23.699870423832525`},
{"CURE", 365, 9.41330892532419`}, {"CURE", 365, 64.84488053550069`}, {"CURE", 365, 5.61138720576896`},
{"CURE", 365, 60.45525883095179`}, {"CURE", 365, 58.62295872624812`}, {"CURE", 365, 16.396763891879676`},
{"CURE", 365, 19.476639061382027`}, {"CURE", 365, 111.90773585729498`}, {"CURE", 365, 28.821332960191054`},
{"CURE", 365, 34.455226892461106`}, {"CURE", 365, 21.567970209873717`}, {"CURE", 365, 8.113677312374216`},
{"CURE", 365, 49.71996203925844`}, {"TBUR", 54.48568147661058`, 132.0908830273062`}, {"CURE", 365, 20.20549158893268`},
{"CURE", 365, 18.264747263177362`}, {"CURE", 365, 33.65531964358609`}, {"CURE", 365, 13.43886624261084`},
{"CURE", 365, 170.52078320086196`}, {"CURE", 365, 13.650245828068368`}, {"CURE", 365, 84.347663667724`},
{"CURE", 365, 64.83840265861443`}, {"CURE", 365, 63.0375924460045`}, {"CURE", 365, 33.67552716981297`},
{"CURE", 365, 78.0926456243526`}, {"CURE", 365, 153.03411786430277`}, {"CURE", 365, 35.48013308092727`},
{"CURE", 365, 6.5173898589476185`}, {"CURE", 365, 57.892900305510075`}, {"CURE", 365, 36.0752603613592`},
{"CURE", 365, 34.77570640134088`}, {"CURE", 365, 7.662037264095918`}, {"CURE", 365, 175.75338556309185`},
{"CURE", 365, 12.847618414198282`}, {"CURE", 365, 55.73972245290509`}, {"CURE", 365, 35.132672831258496`},
{"CURE", 365, 24.970033768680086`}, {"CURE", 365, 6.0030268022632285`}, {"CURE", 365, 45.12449333185492`},
{"CURE", 365, 26.470432228558796`}, {"CURE", 365, 70.03205512777168`}, {"CURE", 365, 46.13967823673493`},
{"CURE", 365, 58.24082096364294`}, {"CURE", 365, 27.957260878331184`}, {"CURE", 365, 24.87422673820766`},
{"TBUR", 60.92347796366693`, 129.96646724285816`}, {"CURE", 365, 34.05511800186836`}, {"CURE", 365, 9.085263531780011`},
{"CURE", 365, 30.599441481882234`}, {"TBUR", 125.6761880889246`, 31.034402486107847`}, {"CURE", 365, 4.169432944458191`},
{"CURE", 365, 7.436815144581335`}, {"CURE", 365, 11.36067299570176`}, {"CURE", 365, 107.23159255051841`},
{"CURE", 365, 32.987955032306566`}, {"CURE", 365, 34.382863582754204`}, {"CURE", 365, 27.53609873978694`},
{"CURE", 365, 113.13486198927897`}, {"CURE", 365, 26.07233087899498`}, {"CURE", 365, 52.396015977905435`},
{"CURE", 365, 25.997468703196546`}, {"CURE", 365, 38.32754754354459`}, {"CURE", 365, 9.217676812464317`},
{"CURE", 365, 34.47388020380449`}, {"CURE", 365, 21.51047193948051`}, {"CURE", 365, 41.90421642612114`},
{"CURE", 365, 66.21380383334505`}, {"CURE", 365, 74.32427514555641`}, {"CURE", 365, 44.97652994415828`},
{"CURE", 365, 36.92409485099311`}, {"CURE", 365, 50.696012852893055`}, {"CURE", 365, 24.176829886659327`},
{"CURE", 365, 27.37253154216837`}, {"CURE", 365, 19.38822433407224`}, {"CURE", 365, 15.197728206013418`},
{"CURE", 365, 24.479454323128046`}, {"CURE", 365, 62.81860341651219`}, {"CURE", 365, 7.199884275943277`},
{"CURE", 365, 31.89438467603238`}, {"CURE", 365, 6.024259307416578`}, {"CURE", 365, 31.351745654515938`},

{"CURE", 365, 17.8062938667609`}, {"CURE", 365, 66.30967739808366`}, {"CURE", 365, 6.113025676452071`},
{"CURE", 365, 43.63286535638733`}, {"CURE", 365, 54.748822011772084`}, {"CURE", 365, 8.956328986598324`},
{"CURE", 365, 20.515298551703836`}, {"CURE", 365, 16.508174963143258`}, {"CURE", 365, 9.442041326814254`},
{"CURE", 365, 55.85144758071887`}, {"CURE", 365, 20.98511198447443`}, {"CURE", 365, 40.451125618445815`},
{"CURE", 365, 39.33172755025644`}, {"CURE", 365, 44.599955588675726`}, {"CURE", 365, 34.86826866184485`},
{"CURE", 365, 41.73169939526689`}, {"CURE", 365, 33.03926628672887`}, {"CURE", 365, 63.86191866770564`},
{"CURE", 365, 38.891933116352234`}, {"CURE", 365, 31.755020562940167`}, {"CURE", 365, 81.25956441655312`},
{"CURE", 365, 9.408286991689673`}, {"CURE", 365, 53.374240205733074`}, {"CURE", 365, 3.920976317976329`},
{"CURE", 365, 48.703407431875156`}, {"CURE", 365, 11.47948964593143`}, {"CURE", 365, 60.2638426975117`},
{"CURE", 365, 9.372263061540716`}, {"CURE", 365, 61.40238366048616`}, {"CURE", 365, 153.63873709112823`},
{"CURE", 365, 6.70706961750208`}, {"CURE", 365, 4.814643888211139`}, {"CURE", 365, 17.737913770372774`},
{"CURE", 365, 4.306046005745152`}, {"CURE", 365, 2.9451609426784895`}, {"CURE", 365, 17.230865389982775`},
{"CURE", 365, 28.1545464249352`}, {"CURE", 365, 30.315869135901515`}, {"CURE", 365, 28.790283062889557`},
{"CURE", 365, 22.428419726699538`}, {"CURE", 365, 39.475453681306185`}, {"CURE", 365, 69.50040196648844`},
{"CURE", 365, 5.623884086708989`}, {"CURE", 365, 30.815743858983115`}, {"CURE", 365, 26.48190700497999`},
{"CURE", 365, 19.19979272943772`}, {"CURE", 365, 35.50363367965149`}, {"CURE", 365, 52.63932310915356`},
{"CURE", 365, 123.17770630933046`}, {"CURE", 365, 91.75364012100542`}, {"CURE", 365, 28.358124631427664`},
{"CURE", 365, 3.0142718708139884`}, {"CURE", 365, 32.87987491296187`}, {"CURE", 365, 84.79767302893727`},
{"CURE", 365, 21.33494317996882`}, {"CURE", 365, 26.808933906471495`}, {"CURE", 365, 16.46103447923631`},
{"CURE", 365, 24.12922388813542`}, {"CURE", 365, 6.243414827783747`}, {"CURE", 365, 30.564424231234707`},
{"CURE", 365, 17.16100191296786`}, {"CURE", 365, 50.17237026396987`}, {"CURE", 365, 34.34291254716546`},
{"CURE", 365, 33.33582886106822`}, {"CURE", 365, 27.429210199454783`}, {"CURE", 365, 14.515344047089858`},
{"CURE", 365, 10.002612901591323`}, {"CURE", 365, 9.75235053252843`}, {"CURE", 365, 12.628556934298075`},
{"CURE", 365, 9.843536699901762`}, {"CURE", 365, 132.31107325258992`}, {"CURE", 365, 73.60214793748645`},
{"CURE", 365, 14.228767163223916`}, {"CURE", 365, 219.18165120590635`}, {"CURE", 365, 62.679783136911055`},
{"CURE", 365, 33.185806507607694`}, {"CURE", 365, 57.404559664380365`}, {"CURE", 365, 8.631299988754021`},
{"CURE", 365, 68.80710217470235`}, {"CURE", 365, 184.0108434443882`}, {"CURE", 365, 9.983043417734944`},
{"CURE", 365, 31.14398476385343`}, {"CURE", 365, 50.98064909341776`}, {"CURE", 365, 25.72631940248178`},
{"CURE", 365, 12.637618616061301`}, {"CURE", 365, 10.068530206638357`}, {"CURE", 365, 87.38226395774639`},
{"CURE", 365, 45.87504265066382`}, {"CURE", 365, 26.10182046517411`}, {"CURE", 365, 64.84748308917649`},
{"CURE", 365, 11.702861287673475`}, {"CURE", 365, 31.29005805950653`}, {"CURE", 365, 18.897058140960567`},

{"CURE", 365, 127.46234294140302`}, {"TBUR", 64.20041574880871`, 32.01783685163985`}, {"CURE", 365, 148.64163110837265`},
{"TOX", 14.622889602663713`, 232.0453257554585`}, {"CURE", 365, 17.787491113066352`}, {"CURE", 365, 5.015534062162831`},
{"CURE", 365, 4.370357628749811`}, {"CURE", 365, 31.96456447490268`}, {"CURE", 365, 124.0387326000555`},
{"CURE", 365, 24.847628613031198`}, {"CURE", 365, 19.086633408116384`}, {"TBUR", 68.89980962509341`, 35.848530340739494`},
{"CURE", 365, 39.27096211735812`}, {"CURE", 365, 163.75290500026733`}, {"CURE", 365, 135.07792159636062`},
{"CURE", 365, 28.27541404159787`}, {"CURE", 365, 9.386544051299548`}, {"CURE", 365, 22.9957816891649`},
{"CURE", 365, 3.6092254204006635`}, {"CURE", 365, 67.81230081262447`}, {"CURE", 365, 100.96010586497107`},
{"CURE", 365, 33.01820921143332`}, {"CURE", 365, 26.166610516198848`}, {"CURE", 365, 110.65239789319749`},
{"CURE", 365, 7.011736551971635`}, {"CURE", 365, 9.133453042980761`}, {"CURE", 365, 28.614220623634672`},
{"CURE", 365, 36.7834801604668`}, {"TBUR", 75.77255529744066`, 69.59145651870351`}, {"CURE", 365, 71.7939926552258`},
{"CURE", 365, 29.471121473111985`}, {"CURE", 365, 30.38761541781134`}, {"CURE", 365, 38.00502712465684`},
{"CURE", 365, 13.16064654710139`}, {"CURE", 365, 3.7220687723345995`}, {"CURE", 365, 39.14577233701912`},
{"CURE", 365, 47.147425474570255`}, {"CURE", 365, 22.922586078479615`}, {"CURE", 365, 41.542290219228995`},
{"CURE", 365, 12.93631278451663`}, {"CURE", 365, 8.502653428630241`}, {"CURE", 365, 37.223155463822486`},
{"CURE", 365, 22.602183029488824`}, {"CURE", 365, 32.33641754473682`}, {"CURE", 365, 213.43088016081464`},
{"CURE", 365, 20.330032435621494`}, {"CURE", 365, 9.911382625387223`}, {"CURE", 365, 7.695494750913067`},
{"CURE", 365, 9.327936738164436`}, {"CURE", 365, 53.282210268470756`}, {"CURE", 365, 3.67007724002278`},
{"CURE", 365, 49.615969994614886`}, {"CURE", 365, 54.17588310644078`}, {"CURE", 365, 42.28578142164158`},
{"CURE", 365, 34.66700107922556`}, {"CURE", 365, 6.8706905067637045`}, {"CURE", 365, 75.45264173143659`},
{"TBUR", 63.835652816957364`, 22.807253783379448`}, {"CURE", 365, 10.273041230516835`}, {"CURE", 365, 67.1881996668853`},
{"CURE", 365, 11.951211056083485`}, {"CURE", 365, 80.49572048410586`}, {"CURE", 365, 51.66487273266271`},
{"CURE", 365, 34.43298762969639`}, {"CURE", 365, 50.066637184415775`}, {"CURE", 365, 17.734536973010183`},
{"CURE", 365, 78.53382185636073`}, {"CURE", 365, 9.318946532895064`}, {"CURE", 365, 39.447132434501185`},
{"CURE", 365, 46.1545751321011`}, {"CURE", 365, 19.623885169022458`}, {"CURE", 365, 182.86721310768942`},
{"CURE", 365, 36.632709043230776`}, {"CURE", 365, 37.42972598190026`}, {"CURE", 365, 38.232889334052096`},
{"CURE", 365, 153.13964998069852`}, {"CURE", 365, 48.65106850773648`}, {"CURE", 365, 45.39074864122443`},
{"CURE", 365, 98.77927259072464`}, {"CURE", 365, 34.74472257399626`}, {"CURE", 365, 30.27091641990789`},
{"CURE", 365, 37.41038581103252`}, {"CURE", 365, 51.673812201204434`}, {"CURE", 365, 91.315720460618`},
{"CURE", 365, 19.701526253030526`}, {"CURE", 365, 17.98732672611807`}, {"CURE", 365, 38.491407375866494`},
{"CURE", 365, 39.18713419590219`}, {"CURE", 365, 44.05493985973411`}, {"CURE", 365, 47.11987362719853`},
{"CURE", 365, 26.32940969921565`}, {"CURE", 365, 13.238298559171199`}, {"CURE", 365, 49.738180466701685`},

{"CURE", 365, 12.565615401219413`}, {"CURE", 365, 26.713637784608956`}, {"CURE", 365, 31.782333922771674`},
{"CURE", 365, 9.58397921052693`}, {"CURE", 365, 4.763538506683258`}, {"CURE", 365, 29.39973810859497`},
{"CURE", 365, 39.8832340585448`}, {"CURE", 365, 47.352809650096205`}, {"CURE", 365, 33.87019385178369`},
{"CURE", 365, 4.3401468377017425`}, {"CURE", 365, 38.856199753223585`}, {"CURE", 365, 7.400977311654937`},
{"CURE", 365, 114.21716180664275`}, {"CURE", 365, 148.60577946642005`}, {"CURE", 365, 20.927311200121938`},
{"TBUR", 83.13845626894569`, 29.343679280056623`}, {"CURE", 365, 159.519621726256`}, {"CURE", 365, 28.60768760553399`},
{"CURE", 365, 16.601886610581044`}, {"CURE", 365, 12.931171628092608`}, {"CURE", 365, 3.537867077416594`},
{"CURE", 365, 22.304312121574696`}, {"CURE", 365, 24.870269650966907`}, {"CURE", 365, 65.88289567119322`},
{"CURE", 365, 31.53858597620793`}, {"CURE", 365, 54.919002039798784`}, {"CURE", 365, 19.40143373802384`},
{"CURE", 365, 42.0745018254846`}, {"CURE", 365, 94.10112318627272`}, {"CURE", 365, 9.566664495735411`},
{"CURE", 365, 14.763157209323007`}, {"CURE", 365, 21.92564977385035`}, {"CURE", 365, 30.631348509846962`},
{"CURE", 365, 20.07242650609147`}, {"CURE", 365, 19.22657729083854`}, {"CURE", 365, 8.144442350821203`},
{"CURE", 365, 12.575286986329496`}, {"CURE", 365, 23.83431496998906`}, {"CURE", 365, 54.2536099627474`},
{"CURE", 365, 48.24444918131604`}, {"CURE", 365, 24.722526940351056`}, {"TBUR", 39.08216284309023`, 37.03974122806336`},
{"CURE", 365, 79.8527064071524`}, {"CURE", 365, 39.06595937861719`}, {"CURE", 365, 29.232467024574497`},
{"CURE", 365, 18.619937339010143`}, {"CURE", 365, 5.832406352941815`}, {"CURE", 365, 32.455351425602444`},
{"CURE", 365, 37.456130614454736`}, {"CURE", 365, 146.6715476622098`}, {"CURE", 365, 7.476474360997409`},
{"CURE", 365, 7.305867753433681`}, {"CURE", 365, 23.40928126468281`}, {"CURE", 365, 68.23860544918634`},
{"CURE", 365, 8.514294180457492`}, {"CURE", 365, 41.65411562652033`}, {"CURE", 365, 9.713571812012095`},
{"CURE", 365, 24.205259249067364`}, {"CURE", 365, 9.85074454472896`}, {"CURE", 365, 10.266236658163038`},
{"CURE", 365, 9.25908678260803`}, {"CURE", 365, 98.79553656206303`}, {"CURE", 365, 15.777860227590157`},
{"CURE", 365, 27.7935472952644`}, {"CURE", 365, 3.838117281275411`}, {"CURE", 365, 45.98233233949412`},
{"CURE", 365, 64.3437010834727`}, {"CURE", 365, 56.07699539811901`}, {"CURE", 365, 8.467760013324078`},
{"CURE", 365, 16.9864712247217`}, {"CURE", 365, 172.80083033666895`}, {"CURE", 365, 46.95434525407977`},
{"CURE", 365, 31.98244059751563`}, {"CURE", 365, 11.350283127908591`}, {"CURE", 365, 16.27059591088416`},
{"CURE", 365, 25.14123766691867`}, {"CURE", 365, 6.224551159412966`}, {"CURE", 365, 30.572221420319437`},
{"CURE", 365, 48.107721353397736`}, {"CURE", 365, 29.258896459371286`}, {"CURE", 365, 31.757782748704017`},
{"CURE", 365, 22.988554117886974`}, {"CURE", 365, 14.882900602609554`}, {"CURE", 365, 85.02594891367298`},
{"CURE", 365, 123.31804153872422`}, {"CURE", 365, 32.89727998612693`}, {"CURE", 365, 41.5340071931484`},
{"CURE", 365, 52.61595892777994`}, {"CURE", 365, 51.4404238160677`}, {"CURE", 365, 4.490044073523375`},
{"CURE", 365, 60.15509012951319`}, {"CURE", 365, 62.18940960136198`}, {"CURE", 365, 42.01988242916209`},

{"CURE", 365, 168.69329853057283`}, {"CURE", 365, 35.06989559331978`}, {"CURE", 365, 15.59776575716024`},
{"CURE", 365, 36.90766146471127`}, {"CURE", 365, 46.90626877048765`}, {"CURE", 365, 21.01301103439878`},
{"CURE", 365, 16.482294842963768`}, {"CURE", 365, 14.341639460367787`}, {"CURE", 365, 25.980588708044316`},
{"CURE", 365, 80.13516593433225`}, {"CURE", 365, 51.43091210032065`}, {"CURE", 365, 8.950464307453512`},
{"CURE", 365, 52.401217561716685`}, {"CURE", 365, 75.70507761350429`}, {"CURE", 365, 13.90767062451245`},
{"CURE", 365, 79.41695175598687`}, {"CURE", 365, 11.935039849238889`}, {"CURE", 365, 7.616467412944183`},
{"CURE", 365, 10.811968688177313`}, {"CURE", 365, 20.800639844667742`}, {"TBUR", 87.03021543062391`, 93.03100262005132`},
{"CURE", 365, 33.29428919743813`}, {"CURE", 365, 42.030374685804425`}, {"CURE", 365, 10.050270025591102`},
{"CURE", 365, 191.6898605573356`}, {"CURE", 365, 87.85369093067314`}, {"CURE", 365, 14.980361328294837`},
{"CURE", 365, 36.690532314128774`}, {"CURE", 365, 20.4467022244203`}, {"CURE", 365, 20.650510841763236`},
{"CURE", 365, 69.77683754174296`}, {"TBUR", 59.50944851581399`, 88.17753357986199`}, {"CURE", 365, 9.481079030500288`},
{"CURE", 365, 18.47294273171158`}, {"CURE", 365, 6.55332014098587`}, {"CURE", 365, 25.274495218841516`},
{"CURE", 365, 43.302601678441206`}, {"CURE", 365, 70.17661067139389`}, {"CURE", 365, 39.16043317584802`},
{"CURE", 365, 141.0388140661281`}, {"CURE", 365, 67.41428719228126`}, {"TBUR", 73.55376910350516`, 11.110826314090355`},
{"CURE", 365, 24.652794472510408`}, {"TBUR", 41.87742167144052`, 26.561749731905046`}, {"CURE", 365, 55.047425034631516`},
{"CURE", 365, 7.220867519888588`}, {"CURE", 365, 19.350537029065293`}, {"CURE", 365, 10.901322927403887`},
{"CURE", 365, 27.19988915607279`}, {"CURE", 365, 62.1445313543713`}, {"CURE", 365, 26.43850862403523`},
{"CURE", 365, 5.742504789963812`}, {"CURE", 365, 109.35924078982946`}, {"CURE", 365, 20.62995534234457`},
{"CURE", 365, 7.007670237157664`}, {"CURE", 365, 9.2121693540464`}, {"CURE", 365, 7.776997545875242`},
{"CURE", 365, 9.935815639628991`}, {"CURE", 365, 79.95463142223818`}, {"CURE", 365, 43.75104926818628`},
{"CURE", 365, 26.342206641039134`}, {"CURE", 365, 12.345512346816957`}, {"CURE", 365, 20.61671913276699`},
{"CURE", 365, 11.92799741083298`}, {"CURE", 365, 28.783312457007227`}, {"CURE", 365, 98.96576953256631`},
{"CURE", 365, 57.24788673654648`}, {"CURE", 365, 28.550821127241235`}, {"CURE", 365, 16.45342563851851`},
{"CURE", 365, 17.22840538343854`}, {"CURE", 365, 211.46581831757626`}, {"CURE", 365, 10.075949119157155`},
{"CURE", 365, 5.190782122001531`}, {"CURE", 365, 4.522184962877557`}, {"CURE", 365, 57.48868295647781`},
{"CURE", 365, 33.660251139242284`}, {"CURE", 365, 37.35056873160794`}, {"CURE", 365, 55.49868578977494`},
{"CURE", 365, 91.58584376985598`}, {"CURE", 365, 94.7616302589531`}, {"CURE", 365, 36.113631432050255`},
{"CURE", 365, 15.593463132353799`}, {"CURE", 365, 18.081770955550823`}, {"CURE", 365, 36.039060829566104`},
{"CURE", 365, 20.466569939226105`}, {"TBUR", 26.543536634139798`, 8.003960857508005`}, {"CURE", 365, 32.14341526768607`},
{"CURE", 365, 200.11306991334365`}, {"CURE", 365, 20.617052861827773`}, {"CURE", 365, 7.414126701832436`},
{"CURE", 365, 70.30467474329753`}, {"CURE", 365, 17.582577792569996`}, {"CURE", 365, 50.528961823959186`},

{"TOX", 9.678072878971198`, 243.25165476061306`}, {"CURE", 365, 112.31984731284619`}, {"CURE", 365, 36.46339898421554`},
{"CURE", 365, 30.563859370776502`}, {"CURE", 365, 13.021307450853731`}, {"CURE", 365, 3.7173928559467275`},
{"CURE", 365, 14.551207260083972`}, {"CURE", 365, 42.64027479012931`}, {"CURE", 365, 23.642618211365`},
{"CURE", 365, 75.43567148517918`}, {"CURE", 365, 29.902267164753948`}, {"CURE", 365, 30.535326601419595`},
{"CURE", 365, 8.47946404394467`}, {"CURE", 365, 24.017920393556803`}, {"CURE", 365, 24.647807579308996`},
{"CURE", 365, 6.426976932593282`}, {"CURE", 365, 20.44869765149408`}, {"CURE", 365, 12.19392382400409`},
{"CURE", 365, 19.313465272030346`}, {"CURE", 365, 40.40749017782163`}, {"CURE", 365, 20.234625675567756`},
{"CURE", 365, 4.692832679737042`}, {"CURE", 365, 32.32993314441251`}, {"CURE", 365, 3.618869420071871`},
{"CURE", 365, 10.619161744278248`}, {"CURE", 365, 31.50648298872184`}, {"CURE", 365, 36.327412050505146`},
{"CURE", 365, 7.22478735771219`}, {"CURE", 365, 21.26867047590744`}, {"CURE", 365, 27.5473286214594`},
{"CURE", 365, 139.15211690252556`}, {"CURE", 365, 31.98073022299051`}, {"CURE", 365, 9.018768629849607`},
{"CURE", 365, 98.3245300524201`}, {"CURE", 365, 9.658902855951391`}, {"CURE", 365, 36.39272251544171`},
{"CURE", 365, 37.64948058574509`}, {"CURE", 365, 11.237941477420557`}, {"CURE", 365, 12.511669089802677`},
{"CURE", 365, 55.425019473734764`}, {"CURE", 365, 5.4138540639293335`}, {"CURE", 365, 46.960155236617666`},
{"CURE", 365, 12.435959412164289`}, {"CURE", 365, 16.261620301682182`}, {"CURE", 365, 3.633115286918786`},
{"CURE", 365, 18.672439247757165`}, {"CURE", 365, 63.65918565605454`}, {"CURE", 365, 24.230955390074293`},
{"CURE", 365, 17.59423332261577`}, {"CURE", 365, 25.147081656607654`}, {"CURE", 365, 19.7129146079781 7`},
{"CURE", 365, 24.85356219873219`}, {"CURE", 365, 43.87863220794846`}, {"CURE", 365, 42.34593001929279`},
{"CURE", 365, 5.2153934796784895`}, {"CURE", 365, 85.55883207583577`}, {"CURE", 365, 9.952904279140153`},
{"CURE", 365, 4.991891674506959`}, {"CURE", 365, 23.80033146002533`}, {"CURE", 365, 24.98485379618756`},
{"CURE", 365, 16.840911263677697`}, {"CURE", 365, 29.24542274860013`}, {"CURE", 365, 25.216961069549118`},
{"CURE", 365, 40.53319544809537`}, {"CURE", 365, 34.25201264044671`}, {"TBUR", 69.12088436737785`, 48.893084935554846`},
{"CURE", 365, 7.727072864321762`}, {"CURE", 365, 98.95831423550172`}, {"CURE", 365, 96.13355740082142`},
{"CURE", 365, 5.325348321155816`}, {"CURE", 365, 13.407660801114552`}, {"CURE", 365, 33.267130091813875`},
{"CURE", 365, 16.90931260592738`}, {"CURE", 365, 51.947197783385626`}, {"CURE", 365, 104.14857815587789`},
{"CURE", 365, 32.91757820298946`}, {"CURE", 365, 5.649094067041848`}, {"CURE", 365, 5.5419846505995`},
{"CURE", 365, 13.013993143087156`}, {"CURE", 365, 3.928627490066251`}, {"CURE", 365, 44.59365910332447`},
{"CURE", 365, 6.912699196884415`}, {"CURE", 365, 46.44672205083243`}, {"CURE", 365, 28.63846747635649`},
{"CURE", 365, 49.3629133985993`}, {"CURE", 365, 12.152807659681546`}, {"CURE", 365, 61.312718435324435`},
{"CURE", 365, 202.85614506591767`}, {"CURE", 365, 73.08371642390495`}, {"CURE", 365, 118.38808695229598`},
{"CURE", 365, 34.846142724891536`}, {"CURE", 365, 83.02082466106567`}, {"CURE", 365, 83.18306725499927`},

{"CURE", 365, 10.09616640580989`}, {"CURE", 365, 7.061898502731232`}, {"CURE", 365, 22.960922748827848`},
{"CURE", 365, 47.65079185085328`}, {"CURE", 365, 59.61678275083502`}, {"CURE", 365, 25.452201345330838`},
{"CURE", 365, 37.71440526544891`}, {"CURE", 365, 7.104785471344385`}, {"CURE", 365, 9.025691281992934`},
{"CURE", 365, 43.86629842846809`}, {"CURE", 365, 14.211630463459624`}, {"CURE", 365, 6.0356510284748985`},
{"CURE", 365, 8.928077382059977`}, {"CURE", 365, 4.364183467070768`}, {"CURE", 365, 35.46551383214053`},
{"CURE", 365, 48.775621475627354`}, {"CURE", 365, 34.1132290492092`}, {"CURE", 365, 38.77347242542957`},
{"CURE", 365, 21.4701672693681`}, {"CURE", 365, 19.214605044662225`}, {"CURE", 365, 49.7432609636499`},
{"CURE", 365, 53.248005397638416`}, {"CURE", 365, 5.940963920401771`}, {"CURE", 365, 22.11128736213302`},
{"CURE", 365, 11.80451896888209`}, {"CURE", 365, 18.54038123259923`}, {"CURE", 365, 152.7064581357267`},
{"CURE", 365, 26.96557158526358`}, {"CURE", 365, 18.509504341318817`}, {"CURE", 365, 19.568381174536526`},
{"CURE", 365, 39.04984597080405`}, {"CURE", 365, 29.72459679005379`}, {"CURE", 365, 38.776640658061794`},
{"CURE", 365, 39.934473772864294`}, {"CURE", 365, 9.069157961340727`}, {"CURE", 365, 88.20437532834504`},
{"CURE", 365, 16.93417096727324`}, {"CURE", 365, 35.92417353205564`}, {"CURE", 365, 20.38355616750516`},
{"CURE", 365, 11.576687956046012`}, {"CURE", 365, 42.20237252393087`}, {"CURE", 365, 42.84534172151391`},
{"TBUR", 73.90735529086334`, 23.728008981539187`}, {"CURE", 365, 56.15718040149771`}, {"CURE", 365, 11.879228586224244`},
{"CURE", 365, 99.29655000375412`}, {"CURE", 365, 38.12455579749321`}, {"CURE", 365, 80.57898567899842`},
{"CURE", 365, 3.12713609292737`}, {"CURE", 365, 39.63486132112674`}, {"CURE", 365, 45.137824560653954`},
{"CURE", 365, 10.746970229899345`}, {"CURE", 365, 27.280826727428277`}, {"CURE", 365, 40.85884101557701`},
{"CURE", 365, 24.832183074513114`}, {"CURE", 365, 7.900009824366487`}, {"CURE", 365, 5.996496767850501`},
{"CURE", 365, 35.44694479157257`}, {"CURE", 365, 9.459694629798769`}, {"CURE", 365, 21.027161845410177`},
{"CURE", 365, 25.618107497689373`}, {"CURE", 365, 11.915687870766764`}, {"CURE", 365, 21.936510231901757`},
{"CURE", 365, 5.979703086983813`}, {"CURE", 365, 24.65420978620559`}, {"CURE", 365, 3.9860630230269893`},
{"CURE", 365, 7.125340027505481`}, {"CURE", 365, 16.84158339971718`}, {"CURE", 365, 5.43176412245592`},
{"CURE", 365, 40.36693392252279`}, {"CURE", 365, 59.3228818148436`}, {"CURE", 365, 60.1829971662808`},
{"CURE", 365, 50.13522838091182`}, {"CURE", 365, 94.62694125947564`}, {"CURE", 365, 7.653259421773561`},
{"CURE", 365, 34.134779332663776`}, {"CURE", 365, 36.63081464927141`}, {"CURE", 365, 63.03489532668804`},
{"CURE", 365, 47.082445581220135`}, {"CURE", 365, 21.509450897308803`}, {"CURE", 365, 101.1227312759898`},
{"CURE", 365, 3.157997900202965`}, {"CURE", 365, 96.27258023231317`}, {"CURE", 365, 6.517920854166877`},
{"CURE", 365, 82.22342216306545`}, {"CURE", 365, 74.46754266884568`}, {"CURE", 365, 22.070310744706347`},
{"CURE", 365, 86.90007421097893`}, {"CURE", 365, 106.01943027672293`}, {"CURE", 365, 47.41005704292401`},
{"CURE", 365, 20.924819117568124`}, {"CURE", 365, 24.056517178854246`}, {"CURE", 365, 35.52323444676254`},

{"CURE", 365, 32.75694831116959`}, {"CURE", 365, 7.939560096240632`}, {"CURE", 365, 28.5017127767480l`},
{"CURE", 365, 26.95848033542165`}, {"CURE", 365, 20.168078219809086`}, {"CURE", 365, 49.84177469469061`},
{"CURE", 365, 7.848368340919668`}, {"CURE", 365, 55.63052595768192`}, {"CURE", 365, 36.40030614519973`},
{"CURE", 365, 20.612287027728723`}, {"CURE", 365, 18.66851901653633`}, {"CURE", 365, 37.99604549488249`},
{"CURE", 365, 5.209075819049455`}, {"CURE", 365, 60.334061108329365`}, {"CURE", 365, 9.52238220661553`},
{"CURE", 365, 13.510146245470004`}, {"CURE", 365, 30.301764023479716`}, {"CURE", 365, 23.759519456895152`},
{"CURE", 365, 21.848986418488224`}, {"CURE", 365, 68.00289135980064`}, {"CURE", 365, 9.20168109686473`},
{"CURE", 365, 16.02021708755634`}, {"CURE", 365, 118.53750606313523`}, {"CURE", 365, 8.993541465350226`},
{"CURE", 365, 23.162682726213326`}, {"CURE", 365, 35.23136336178927`}, {"CURE", 365, 34.787163969252674`},
{"CURE", 365, 23.626973455036495`}, {"CURE", 365, 122.150945530079`}, {"CURE", 365, 22.055569874302122`},
{"CURE", 365, 9.291281843133705`}, {"CURE", 365, 18.5040440231036`}, {"CURE", 365, 14.307454553157752`},
{"CURE", 365, 10.962015180093656`}, {"CURE", 365, 28.753555187652925`}, {"CURE", 365, 49.24815081156049`},
{"CURE", 365, 27.094337529338432`}, {"CURE", 365, 31.588082888445246`}, {"CURE", 365, 69.17233025497892`},
{"CURE", 365, 7.6247454435488455`}, {"CURE", 365, 8.972463310650735`}, {"CURE", 365, 75.72251770707817`},
{"CURE", 365, 24.36619214809556`}, {"CURE", 365, 26.405367862478556`}, {"CURE", 365, 13.272761478028912`},
{"CURE", 365, 30.393927079666717`}, {"CURE", 365, 37.878115415900275`}, {"CURE", 365, 87.78061615594456`},
{"CURE", 365, 12.939052521025472`}, {"CURE", 365, 50.53484818431479`}, {"CURE", 365, 29.732751826102973`},
{"CURE", 365, 78.72478885703951`}, {"CURE", 365, 128.62861042935842`}, {"CURE", 365, 47.06927987214893`},
{"CURE", 365, 4.559301031311625`}, {"CURE", 365, 35.68827292812726`}, {"CURE", 365, 11.268051607033108`},
{"CURE", 365, 119.18238093901432`}, {"TBUR", 44.28528503704429`, 3.7421730935985678`}, {"CURE", 365, 3.977425979324936`},
{"CURE", 365, 137.8848175858451`}, {"CURE", 365, 31.70702035413914`}, {"CURE", 365, 10.73592707509149`},
{"CURE", 365, 20.548427659463215`}, {"CURE", 365, 7.208096677999962`}, {"TBUR", 70.60473920710687`, 6.845542081581158`},
{"CURE", 365, 8.197690100367428`}, {"CURE", 365, 29.631157409712397`}, {"CURE", 365, 62.801964900638936`},
{"CURE", 365, 24.87108177827845`}, {"CURE", 365, 6.2284814173897045`}, {"CURE", 365, 84.73251090636442`},
{"CURE", 365, 169.26057560829236`}, {"CURE", 365, 4.775448220636285`}, {"CURE", 365, 38.1536193143216`},
{"CURE", 365, 20.502646529683236`}, {"CURE", 365, 4.031963923455849`}, {"CURE", 365, 31.515294842921733`},
{"CURE", 365, 20.139178702819684`}, {"CURE", 365, 15.458044653445011`}, {"CURE", 365, 30.06440390877175`},
{"CURE", 365, 17.06558280025165`}, {"CURE", 365, 50.13952580330372`}, {"CURE", 365, 29.828021099215736`},
{"CURE", 365, 70.22980555083069`}, {"CURE", 365, 31.109360115081255`}, {"CURE", 365, 15.412129196656968`},
{"CURE", 365, 7.4933888488187375`}, {"CURE", 365, 5.200114795077158`}, {"CURE", 365, 14.81201650907174`},
{"CURE", 365, 58.936734245834806`}, {"CURE", 365, 19.438633281246833`}, {"CURE", 365, 17.80630964657389`},

```
{"CURE", 365, 12.592214024384683`}, {"CURE", 365, 12.763775933813283`}, {"CURE", 365, 34.4906252551066`},
{"CURE", 365, 6.453411930284412`}, {"CURE", 365, 41.1067980669541`}, {"CURE", 365, 18.810776733612823`},
{"CURE", 365, 11.050683830475842`}, {"CURE", 365, 17.516231284736712`}, {"CURE", 365, 38.8745360018677`},
{"CURE", 365, 28.92566394334004`}, {"CURE", 365, 3.495007868977385`}, {"CURE", 365, 15.054400598392222`},
{"TOX", 8.017549040965166`, 251.70613050011193`}, {"CURE", 365, 41.79285479009148`}, {"CURE", 365, 60.974257730151464`},
{"CURE", 365, 17.965774673943855`}, {"CURE", 365, 66.60755012027045`}, {"CURE", 365, 7.970537804440376`},
{"CURE", 365, 26.850467899254976`}, {"CURE", 365, 123.97613816799586`}, {"CURE", 365, 23.218347214112146`},
{"CURE", 365, 20.42446891434163`}, {"CURE", 365, 35.0522636858426`}, {"CURE", 365, 18.543051596385702`},
{"CURE", 365, 4.1171812417353415`}, {"CURE", 365, 38.252445386711095`}, {"CURE", 365, 6.826921693221917`},
{"CURE", 365, 16.213267059603567`}, {"CURE", 365, 38.32929794530954`}, {"CURE", 365, 13.765315732798374`},
{"CURE", 365, 73.11296982783114`}, {"CURE", 365, 21.73337271047618`}, {"CURE", 365, 37.42402357719926`},
{"CURE", 365, 25.939688070678777`}, {"CURE", 365, 7.340710215824168`}, {"CURE", 365, 27.22705812725247`},
{"CURE", 365, 58.855613074665314`}, {"CURE", 365, 19.725914723823827`}, {"CURE", 365, 14.845796680647277`},
{"CURE", 365, 7.0412208833328815`}, {"CURE", 365, 22.33814297400949`}, {"CURE", 365, 26.786452896774556`},
{"CURE", 365, 76.48691256328628`}, {"CURE", 365, 12.286694044113423`}, {"CURE", 365, 18.48257255019724`},
{"CURE", 365, 71.79018948962496`}, {"CURE", 365, 7.56408102525533`}, {"CURE", 365, 103.78220835270481`},
{"CURE", 365, 8.776741297758061`}, {"CURE", 365, 16.062675624279585`}, {"CURE", 365, 37.03129104634607`},
{"CURE", 365, 59.58404561342479`}, {"CURE", 365, 26.8263445644734`}, {"CURE", 365, 14.052242779950824`},
{"CURE", 365, 4.1330072044680275`}, {"TBUR", 75.57572030460827`, 36.1407087977902`}, {"CURE", 365, 32.77539343428148`},
{"CURE", 365, 32.226784439916855`}, {"CURE", 365, 12.707112762566299`}, {"CURE", 365, 31.151334094412906`},
{"CURE", 365, 3.601872559659342`}, {"CURE", 365, 20.76457415652332`}, {"CURE", 365, 9.070655222742424`},
{"CURE", 365, 4.016307093458275`}, {"CURE", 365, 20.877735455219305`}, {"CURE", 365, 4.468157877272408`},
{"CURE", 365, 14.207433323029406`}, {"CURE", 365, 9.742600000650674`}, {"TBUR", 41.675816070642924`, 5.863460193470617`},
{"CURE", 365, 19.296816867187584`}, {"CURE", 365, 51.42443395722719`}, {"CURE", 365, 28.137954083674554`},
{"CURE", 365, 25.49289489709005`}, {"CURE", 365, 40.4718929207687`}, {"CURE", 365, 34.39022879561623`}},
{{"2100 Res", "2100 OS", "2100 Tox"}, {"CURE", 365, 17.486252301504365`}, {"CURE", 365, 36.19886068685395`},
{"CURE", 365, 25.36543317265238`}, {"TBUR", 68.6323037979064`, 8.498616518624859`},
{"TBUR", 47.94949496152919`, 32.26585531693702`}, {"CURE", 365, 65.5701989601351`}, {"CURE", 365, 61.69416292955935`},
{"CURE", 365, 6.4461198154582355`}, {"CURE", 365, 44.49339063531808`}, {"CURE", 365, 38.482751495343905`},
{"CURE", 365, 144.43487057133865`}, {"CURE", 365, 17.922272966037305`}, {"CURE", 365, 64.1567058904639`},
{"CURE", 365, 215.08033296853546`}, {"CURE", 365, 30.46543361144711`}, {"CURE", 365, 47.5500825816881`},
```

{"CURE", 365, 94.17739668331193`}, {"CURE", 365, 42.1821705923275`}, {"TBUR", 126.27308491835883`, 31.895291264781232`},
{"CURE", 365, 10.574318338031409`}, {"CURE", 365, 65.36362789172624`}, {"CURE", 365, 26.495933239014697`},
{"CURE", 365, 10.152661123686858`}, {"CURE", 365, 8.089483192501989`}, {"CURE", 365, 36.80890534574567`},
{"CURE", 365, 40.399890508622654`}, {"CURE", 365, 3.672829133977953`}, {"CURE", 365, 5.567057554523484`},
{"CURE", 365, 20.628479869481293`}, {"CURE", 365, 24.130657079005168`}, {"CURE", 365, 20.58690892607208`},
{"CURE", 365, 72.34722096103422`}, {"CURE", 365, 15.916862971979006`}, {"CURE", 365, 12.656533171825822`},
{"CURE", 365, 102.60610925211465`}, {"CURE", 365, 6.286217540298512`}, {"CURE", 365, 27.172957332981696`},
{"TBUR", 46.076502015482276`, 60.574537241600105`}, {"CURE", 365, 15.10040284953998`}, {"CURE", 365, 23.228942924453012`},
{"CURE", 365, 52.99270941611851`}, {"CURE", 365, 35.776815183742706`}, {"CURE", 365, 7.25878788385697`},
{"CURE", 365, 24.481850972495092`}, {"CURE", 365, 35.325586997442755`}, {"CURE", 365, 23.164216071428918`},
{"TOX", 112.1449087466743`, 246.13591336222072`}, {"CURE", 365, 53.057645103632545`}, {"CURE", 365, 81.16344668504262`},
{"CURE", 365, 9.922217303258654`}, {"CURE", 365, 27.651200628225023`}, {"CURE", 365, 37.99976312090947`},
{"CURE", 365, 30.526830099072995`}, {"CURE", 365, 19.815783113403192`}, {"CURE", 365, 8.539605019247025`},
{"CURE", 365, 90.32627141940107`}, {"CURE", 365, 28.38474432282579`}, {"CURE", 365, 5.391007898866923`},
{"CURE", 365, 58.09160214558864`}, {"CURE", 365, 40.67753865226451`}, {"CURE", 365, 27.86602461447534`},
{"CURE", 365, 21.32648293609478`}, {"CURE", 365, 12.07508633533613`}, {"CURE", 365, 45.96439013943725`},
{"CURE", 365, 32.65197099005003`}, {"CURE", 365, 37.34122061940098`}, {"CURE", 365, 8.547242402601944`},
{"CURE", 365, 8.684674527783368`}, {"CURE", 365, 9.241124926035384`}, {"CURE", 365, 37.74079821928552`},
{"CURE", 365, 13.816406020074016`}, {"CURE", 365, 30.774588644841273`}, {"CURE", 365, 57.75331845006186`},
{"TBUR", 25.957647671956195`, 17.7690885668455`}, {"CURE", 365, 15.43302459117117`}, {"CURE", 365, 42.750867415275344`},
{"CURE", 365, 24.999901300826963`}, {"CURE", 365, 19.016523518942073`}, {"CURE", 365, 75.75773712584956`},
{"CURE", 365, 83.19151058238988`}, {"CURE", 365, 61.248329769882126`}, {"CURE", 365, 16.52546843108013`},
{"CURE", 365, 33.412752965370125`}, {"CURE", 365, 23.354635323918757`}, {"CURE", 365, 26.408585397246846`},
{"CURE", 365, 12.374975972525812`}, {"CURE", 365, 132.01173745636174`}, {"CURE", 365, 28.770574886266736`},
{"CURE", 365, 36.236591724666084`}, {"CURE", 365, 31.182635141353323`}, {"CURE", 365, 25.755653945748964`},
{"CURE", 365, 7.642050289437045`}, {"CURE", 365, 14.00683987802945`}, {"CURE", 365, 32.42584921504713`},
{"TBUR", 55.31681874433878`, 11.731636176729394`}, {"CURE", 365, 13.962130169046121`}, {"CURE", 365, 36.412166342884255`},
{"CURE", 365, 92.4100547959933`}, {"CURE", 365, 49.499932761381274`}, {"CURE", 365, 24.826550343248524`},
{"TBUR", 76.08807314376189`, 49.194796655336845`}, {"CURE", 365, 32.00779101476266`}, {"CURE", 365, 20.743772678925897`},
{"CURE", 365, 142.03530403036623`}, {"CURE", 365, 15.778474781621327`}, {"CURE", 365, 10.720630675125735`},
{"CURE", 365, 62.922419989318065`}, {"CURE", 365, 18.554454505824427`}, {"CURE", 365, 18.984840379947084`},

{"CURE", 365, 114.22415354347827`}, {"CURE", 365, 9.271069934242707`}, {"CURE", 365, 5.88178370243548`},
{"CURE", 365, 24.466856574097882`}, {"CURE", 365, 19.339320484466242`}, {"CURE", 365, 14.568601827178242`},
{"CURE", 365, 37.18822612663525`}, {"CURE", 365, 10.466275874963875`}, {"CURE", 365, 6.435403990168186`},
{"CURE", 365, 30.12638262680009`}, {"CURE", 365, 46.073504035545874`}, {"CURE", 365, 7.845391700730271`},
{"CURE", 365, 25.969028195868503`}, {"CURE", 365, 78.83141190372207`}, {"TBUR", 66.2253776948965`, 30.02419874623618`},
{"CURE", 365, 17.48734521893323`}, {"CURE", 365, 61.00473075915147`}, {"CURE", 365, 30.657616142704242`},
{"CURE", 365, 4.0413720531000585`}, {"CURE", 365, 35.00953055303482`}, {"CURE", 365, 27.038724817170515`},
{"CURE", 365, 21.53985352898224`}, {"CURE", 365, 25.5489823648577l`}, {"CURE", 365, 6.650209266359541`},
{"CURE", 365, 37.93786280562853`}, {"CURE", 365, 13.620130616626142`}, {"CURE", 365, 30.748194728820796`},
{"CURE", 365, 114.98718714998783`}, {"CURE", 365, 40.61832366247049`}, {"CURE", 365, 94.40269990564306`},
{"CURE", 365, 31.100504676476394`}, {"CURE", 365, 128.41975580486528`}, {"CURE", 365, 4.007055544387019`},
{"CURE", 365, 35.19882619607307`}, {"CURE", 365, 40.121962118879836`}, {"CURE", 365, 5.211225736820286`},
{"CURE", 365, 7.219185955415307`}, {"CURE", 365, 44.31314363441315`}, {"CURE", 365, 52.202610537117096`},
{"CURE", 365, 44.50380749154224`}, {"CURE", 365, 36.41271061808163`}, {"CURE", 365, 34.81143279657141`},
{"CURE", 365, 21.077768101088683`}, {"CURE", 365, 5.574910429181835`}, {"CURE", 365, 24.24413273551877`},
{"CURE", 365, 11.50478030652622`}, {"CURE", 365, 5.631806532558651`}, {"CURE", 365, 34.13867574359672`},
{"CURE", 365, 19.102903685685614`}, {"CURE", 365, 50.49795346693697`}, {"CURE", 365, 20.38597366847384`},
{"CURE", 365, 16.96788121818357`}, {"CURE", 365, 113.75262155133956`}, {"CURE", 365, 8.478713504609622`},
{"CURE", 365, 25.360274742079817`}, {"CURE", 365, 14.373897756109576`}, {"CURE", 365, 33.94583064701149`},
{"CURE", 365, 9.3339964113361`}, {"CURE", 365, 37.27128076504838`}, {"CURE", 365, 98.43080485149602`},
{"CURE", 365, 8.421319452852147`}, {"CURE", 365, 25.58071974936959`}, {"CURE", 365, 27.609265375958046`},
{"CURE", 365, 99.47846615245133`}, {"CURE", 365, 37.37581908498947`}, {"CURE", 365, 31.065199810184968`},
{"CURE", 365, 54.48001600501347`}, {"CURE", 365, 20.92095675456312`}, {"CURE", 365, 64.08802347940323`},
{"CURE", 365, 40.24155483870892`}, {"CURE", 365, 24.49105919902233`}, {"CURE", 365, 22.198701978087282`},
{"CURE", 365, 7.223748597481764`}, {"CURE", 365, 4.895220537011571`}, {"CURE", 365, 11.470100598076991`},
{"CURE", 365, 15.369705139122345`}, {"CURE", 365, 6.765159554288864`}, {"CURE", 365, 24.77118439067901`},
{"CURE", 365, 67.65081035780284`}, {"CURE", 365, 18.292070996659927`}, {"CURE", 365, 33.90157619416705`},
{"CURE", 365, 42.518920135184345`}, {"CURE", 365, 35.957632709655165`}, {"CURE", 365, 24.885292820825065`},
{"CURE", 365, 9.884350894706177`}, {"CURE", 365, 68.09359335682448`}, {"CURE", 365, 5.8920688692565895`},
{"CURE", 365, 63.481209164529616`}, {"CURE", 365, 61.601481467167496`}, {"CURE", 365, 17.21725281063419`},
{"CURE", 365, 20.451033206177904`}, {"CURE", 365, 117.53252791148222`}, {"CURE", 365, 30.27938628924236`},

{"CURE", 365, 36.17829433056932`}, {"CURE", 365, 22.650068070618506`}, {"CURE", 365, 8.51988196667419`},
{"CURE", 365, 52.22046398416059`}, {"TBUR", 56.82683132356206`, 138.75896935739237`}, {"CURE", 365, 21.215970632647675`},
{"CURE", 365, 19.179825504251422`}, {"CURE", 365, 35.33948188206426`}, {"CURE", 365, 14.111327576845182`},
{"CURE", 365, 179.05973862270238`}, {"CURE", 365, 14.333507178001227`}, {"CURE", 365, 88.60170922640543`},
{"CURE", 365, 68.08474831189848`}, {"CURE", 365, 66.20181836597479`}, {"CURE", 365, 35.36394852220385`},
{"CURE", 365, 81.9985047148303`}, {"CURE", 365, 167.97533924030694`}, {"CURE", 365, 37.25472370786365`},
{"CURE", 365, 6.8437081603864875`}, {"CURE", 365, 60.78892212786992`}, {"CURE", 365, 37.87947956584182`},
{"CURE", 365, 36.5160758283418`}, {"CURE", 365, 8.04769357936393`}, {"CURE", 365, 184.55375683751592`},
{"CURE", 365, 13.491131963701722`}, {"CURE", 365, 58.52931657187232`}, {"CURE", 365, 36.893605728196704`},
{"CURE", 365, 26.22011362816441`}, {"CURE", 365, 6.303372250370553`}, {"CURE", 365, 47.38116940067469`},
{"CURE", 365, 27.795016996938017`}, {"CURE", 365, 73.53554416571814`}, {"CURE", 365, 48.44900244373884`},
{"CURE", 365, 61.15922053912105`}, {"CURE", 365, 29.36207483489041`}, {"CURE", 365, 26.123525052893314`},
{"TBUR", 63.65499684810571`, 136.49707777396532`}, {"CURE", 365, 35.75819289033846`}, {"CURE", 365, 9.540129002318617`},
{"CURE", 365, 32.130275410264765`}, {"CURE", 365, 32.61847002909351`}, {"CURE", 365, 4.38021219505198`},
{"CURE", 365, 7.808745074627897`}, {"CURE", 365, 11.929530289065852`}, {"CURE", 365, 112.6275022251806`},
{"CURE", 365, 34.639541584311914`}, {"CURE", 365, 36.10237287396964`}, {"CURE", 365, 28.91413937468613`},
{"CURE", 365, 118.84383984745878`}, {"CURE", 365, 27.376208963702194`}, {"CURE", 365, 55.05375413747642`},
{"CURE", 365, 27.299023407093305`}, {"CURE", 365, 40.245212883868724`}, {"CURE", 365, 9.678729821633329`},
{"CURE", 365, 36.19884144940851`}, {"CURE", 365, 22.587843223320355`}, {"CURE", 365, 43.99982244807109`},
{"CURE", 365, 69.5297948057522`}, {"CURE", 365, 78.12338914155202`}, {"CURE", 365, 47.34462667534947`},
{"CURE", 365, 38.77065715494438`}, {"CURE", 365, 53.330940963539106`}, {"CURE", 365, 25.386621596858845`},
{"CURE", 365, 28.741881904198195`}, {"CURE", 365, 20.361206996803812`}, {"CURE", 365, 15.95833593771941`},
{"CURE", 365, 25.7050797505065`}, {"CURE", 365, 65.96068642913936`}, {"CURE", 365, 7.5601282178490585`},
{"CURE", 365, 33.49415712571833`}, {"CURE", 365, 6.32562873225791`}, {"CURE", 365, 32.92220299048159`},
{"CURE", 365, 18.702467051667075`}, {"CURE", 365, 69.62627675792986`}, {"CURE", 365, 6.41904296766951`},
{"CURE", 365, 45.82547414608137`}, {"CURE", 365, 57.49126373527747`}, {"CURE", 365, 9.404874518882918`},
{"CURE", 365, 21.545274526553463`}, {"CURE", 365, 17.336045157110814`}, {"CURE", 365, 9.917859079147648`},
{"CURE", 365, 58.6451438718529`}, {"CURE", 365, 22.03754487213599`}, {"CURE", 365, 42.47465282077105`},
{"CURE", 365, 41.300492537903516`}, {"CURE", 365, 46.83038635562676`}, {"CURE", 365, 36.61267209468512`},
{"CURE", 365, 43.82397140230111`}, {"CURE", 365, 34.69204163742623`}, {"CURE", 365, 67.06274102742479`},
{"CURE", 365, 40.842822924009006`}, {"CURE", 365, 33.35188560423706`}, {"CURE", 365, 85.32983400307606`},

{"CURE", 365, 9.879513518736935`}, {"CURE", 365, 56.04491012085837`}, {"CURE", 365, 4.117132044771294`},
{"CURE", 365, 51.14254068805383`}, {"CURE", 365, 12.053694638999387`}, {"CURE", 365, 63.38879493858909`},
{"CURE", 365, 9.841727835109538`}, {"CURE", 365, 64.47492110122894`}, {"CURE", 365, 161.34612090472177`},
{"CURE", 365, 7.043323384491579`}, {"CURE", 365, 5.056724972006163`}, {"CURE", 365, 18.62566132346743`},
{"CURE", 365, 4.522370569779738`}, {"CURE", 365, 3.0925354726488963`}, {"CURE", 365, 18.09275996719243`},
{"CURE", 365, 29.57202680300907`}, {"CURE", 365, 31.831896102475135`}, {"CURE", 365, 30.241547194523985`},
{"CURE", 365, 23.55563969191731`}, {"CURE", 365, 41.45214754836863`}, {"CURE", 365, 72.97915370287026`},
{"CURE", 365, 5.905528546520528`}, {"CURE", 365, 32.35719510036816`}, {"CURE", 365, 27.80653592836542`},
{"CURE", 365, 20.160549966358225`}, {"CURE", 365, 37.28210975945027`}, {"CURE", 365, 55.274115891267876`},
{"CURE", 365, 129.33966417856522`}, {"CURE", 365, 96.35486368384689`}, {"CURE", 365, 29.78862464930246`},
{"CURE", 365, 3.1650988101147735`}, {"CURE", 365, 34.5257434440976`}, {"CURE", 365, 89.03850327208092`},
{"CURE", 365, 22.40260983228389`}, {"CURE", 365, 28.239188581160818`}, {"CURE", 365, 17.284756265461052`},
{"CURE", 365, 25.336177315590252`}, {"CURE", 365, 6.557866929965696`}, {"CURE", 365, 32.097053185847265`},
{"CURE", 365, 18.026201961572117`}, {"CURE", 365, 52.6903558274206`}, {"CURE", 365, 36.07531610425241`},
{"CURE", 365, 35.003143612136974`}, {"CURE", 365, 28.80111148524019`}, {"CURE", 365, 15.241368247178732`},
{"CURE", 365, 10.503074024803775`}, {"CURE", 365, 10.240334189940754`}, {"CURE", 365, 13.261908673126015`},
{"CURE", 365, 10.336253979312058`}, {"CURE", 365, 138.95941315259608`}, {"CURE", 365, 77.29770523789547`},
{"CURE", 365, 14.940585164520442`}, {"CURE", 365, 230.14340307438763`}, {"CURE", 365, 65.81448544439532`},
{"CURE", 365, 34.84579253573191`}, {"CURE", 365, 60.27668038650493`}, {"CURE", 365, 9.063003603934293`},
{"CURE", 365, 72.25496115138567`}, {"CURE", 365, 193.21446928088872`}, {"CURE", 365, 10.482750969980792`},
{"CURE", 365, 32.70207033007035`}, {"CURE", 365, 53.5418207201783`}, {"CURE", 365, 27.031921430976986`},
{"CURE", 365, 13.269811972386629`}, {"CURE", 365, 10.572070453134895`}, {"CURE", 365, 91.75551010395078`},
{"CURE", 365, 48.17105352270123`}, {"CURE", 365, 27.4074459127163`}, {"CURE", 365, 68.09906410272207`},
{"CURE", 365, 12.291455496269549`}, {"CURE", 365, 32.85634589497368`}, {"CURE", 365, 19.84289623642153`},
{"CURE", 365, 133.83754121704166`}, {"TBUR", 67.93327597991463`, 33.64168986795642`}, {"CURE", 365, 156.1693661409981`},
{"TOX", 9.766439154820398`, 243.78427071932774`}, {"CURE", 365, 18.677785068203693`}, {"CURE", 365, 5.266827243800069`},
{"CURE", 365, 4.589025488668419`}, {"CURE", 365, 33.566114787408246`}, {"CURE", 365, 130.2444866731698`},
{"CURE", 365, 26.090747759318834`}, {"CURE", 365, 20.04267252679106`}, {"TBUR", 72.20177600862387`, 37.65417539049495`},
{"CURE", 365, 41.245469710735385`}, {"CURE", 365, 171.96890963739193`}, {"CURE", 365, 141.8330767306018`},
{"CURE", 365, 29.69554780366881`}, {"CURE", 365, 9.85653378586805`}, {"CURE", 365, 24.147252291569554`},
{"CURE", 365, 3.7898822073310705`}, {"CURE", 365, 71.22997091189426`}, {"CURE", 365, 106.00874438096649`},

{"CURE", 365, 34.669780573940734`}, {"CURE", 365, 27.47572847106243`}, {"CURE", 365, 116.18616267753828`},
{"CURE", 365, 7.364994526949936`}, {"CURE", 365, 9.593285985680438`}, {"CURE", 365, 30.04527875152492`},
{"CURE", 365, 38.633227728688404`}, {"TBUR", 79.90158581432611`, 73.12843048682736`}, {"CURE", 365, 75.38545616155511`},
{"CURE", 365, 30.945240214672896`}, {"CURE", 365, 31.91294418687064`}, {"CURE", 365, 39.929106930504354`},
{"CURE", 365, 13.825697906463324`}, {"CURE", 365, 3.9083820787661754`}, {"CURE", 365, 41.10743761474062`},
{"CURE", 365, 49.50514886581307`}, {"CURE", 365, 24.069111796344075`}, {"CURE", 365, 43.619680311407514`},
{"CURE", 365, 13.584543425588047`}, {"CURE", 365, 8.928056496440005`}, {"CURE", 365, 39.08490092844575`},
{"CURE", 365, 23.739757693889388`}, {"CURE", 365, 33.955403758393935`}, {"CURE", 365, 224.1049846028001`},
{"CURE", 365, 21.348257969494657`}, {"CURE", 365, 10.409437796058386`}, {"CURE", 365, 8.080883986125228`},
{"CURE", 365, 9.794759053233424`}, {"CURE", 365, 55.95049851518702`}, {"CURE", 365, 3.8536887919143843`},
{"CURE", 365, 52.10014338700083`}, {"CURE", 365, 56.88523026426883`}, {"CURE", 365, 44.400292332348926`},
{"CURE", 365, 36.40068899391372`}, {"CURE", 365, 7.21559906133162`}, {"CURE", 365, 79.2266106193198`},
{"TBUR", 67.27159562383233`, 23.964242393726344`}, {"CURE", 365, 10.808857711370866`}, {"CURE", 365, 70.55041878916842`},
{"CURE", 365, 12.548962661832432`}, {"CURE", 365, 84.52520776855098`}, {"CURE", 365, 54.248472923749745`},
{"CURE", 365, 36.163740290755726`}, {"CURE", 365, 52.57027805366975`}, {"CURE", 365, 18.627556435753405`},
{"CURE", 365, 82.46452019662553`}, {"CURE", 365, 9.785081022979632`}, {"CURE", 365, 41.43175308215168`},
{"CURE", 365, 48.462982339196856`}, {"CURE", 365, 20.605502618058445`}, {"CURE", 365, 192.0161828627418`},
{"CURE", 365, 38.48491921135592`}, {"CURE", 365, 39.301936475765395`}, {"CURE", 365, 40.147585712302075`},
{"CURE", 365, 160.7990497067144`}, {"CURE", 365, 51.08626413044794`}, {"CURE", 365, 47.662662702007424`},
{"CURE", 365, 103.73053723247997`}, {"CURE", 365, 36.48378087549817`}, {"CURE", 365, 31.7868621364311`},
{"CURE", 365, 39.28161556962767`}, {"CURE", 365, 54.25848081979209`}, {"CURE", 365, 95.88189919380149`},
{"CURE", 365, 20.687634070742956`}, {"CURE", 365, 18.887264245429318`}, {"CURE", 365, 40.41645167462926`},
{"CURE", 365, 41.14741438686654`}, {"CURE", 365, 46.274673622863276`}, {"CURE", 365, 49.47625650244628`},
{"CURE", 365, 27.648926951081076`}, {"CURE", 365, 13.905026684593787`}, {"CURE", 365, 52.248996490021696`},
{"CURE", 365, 13.194718675539319`}, {"CURE", 365, 28.05031484726224`}, {"CURE", 365, 33.38592959767433`},
{"CURE", 365, 10.063355347694312`}, {"CURE", 365, 5.00202467323795`}, {"CURE", 365, 30.88997810514042`},
{"CURE", 365, 41.887080147165975`}, {"CURE", 365, 49.72836304013409`}, {"CURE", 365, 35.5704522256442`},
{"CURE", 365, 4.557969167509573`}, {"CURE", 365, 40.80211335044506`}, {"CURE", 365, 7.772410364920729`},
{"CURE", 365, 119.92895617331807`}, {"CURE", 365, 156.0416768444001`}, {"CURE", 365, 22.04999042272521`},
{"TBUR", 86.7858930362004`, 30.82218471155586`}, {"CURE", 365, 167.50465605671081`}, {"CURE", 365, 30.039143087102286`},
{"CURE", 365, 17.441859037740148`}, {"CURE", 365, 13.583626676566503`}, {"CURE", 365, 3.7149669430668903`},

{"CURE", 365, 23.41971798998999`}, {"CURE", 365, 26.11422877731236`}, {"CURE", 365, 69.1916572309041`},
{"CURE", 365, 33.117996327828244`}, {"CURE", 365, 57.66694693150595`}, {"CURE", 365, 20.37181323603445`},
{"CURE", 365, 44.19565290102631`}, {"CURE", 365, 98.80735426564061`}, {"CURE", 365, 10.04704853868002`},
{"CURE", 365, 15.501574878431153`}, {"CURE", 365, 23.024275193662287`}, {"CURE", 365, 32.163975490918986`},
{"CURE", 365, 21.088040410299584`}, {"CURE", 365, 20.18841021557536`}, {"CURE", 365, 8.553771707075397`},
{"CURE", 365, 13.204230474140898`}, {"CURE", 365, 25.030185954391143`}, {"CURE", 365, 56.96828504688382`},
{"CURE", 365, 50.676047835692955`}, {"CURE", 365, 25.959873609738754`}, {"TBUR", 40.95190972023353`, 38.91890076745566`},
{"CURE", 365, 83.8599060541895`}, {"CURE", 365, 41.02094145702822`}, {"CURE", 365, 30.694914574441807`},
{"CURE", 365, 19.551327810274596`}, {"CURE", 365, 6.1248638428780025`}, {"CURE", 365, 34.07856200214388`},
{"CURE", 365, 39.33233473743141`}, {"CURE", 365, 154.02713921060663`}, {"CURE", 365, 7.851489833901142`},
{"CURE", 365, 7.671332229040625`}, {"CURE", 365, 24.580251361334607`}, {"CURE", 365, 71.65167612867918`},
{"CURE", 365, 8.940522359116967`}, {"CURE", 365, 43.738180997906525`}, {"CURE", 365, 10.200051048484942`},
{"CURE", 365, 25.417900311029378`}, {"CURE", 365, 10.350713527306166`}, {"CURE", 365, 10.779973778791161`},
{"CURE", 365, 9.722356083076969`}, {"CURE", 365, 103.73622396430248`}, {"CURE", 365, 16.578098039973064`},
{"CURE", 365, 29.201160336630412`}, {"CURE", 365, 4.030119172644813`}, {"CURE", 365, 48.2838175440177`},
{"CURE", 365, 67.56223061221868`}, {"CURE", 365, 58.89045811512033`}, {"CURE", 365, 8.891819240592225`},
{"CURE", 365, 17.836260949272695`}, {"CURE", 365, 181.46409408533256`}, {"CURE", 365, 49.30663336477434`},
{"CURE", 365, 33.589177607856506`}, {"CURE", 365, 11.923057458787577`}, {"CURE", 365, 17.08599761259099`},
{"CURE", 365, 26.403473432393266`}, {"CURE", 365, 6.537351147328791`}, {"CURE", 365, 32.10203291180683`},
{"CURE", 365, 50.513535901720175`}, {"CURE", 365, 30.72671222459203`}, {"CURE", 365, 33.35286923585469`},
{"CURE", 365, 24.138667226736096`}, {"CURE", 365, 15.630212789094454`}, {"CURE", 365, 89.30237357849103`},
{"CURE", 365, 129.49183724478303`}, {"CURE", 365, 34.54239820504613`}, {"CURE", 365, 43.611145423260254`},
{"CURE", 365, 55.24765842958888`}, {"CURE", 365, 54.01458004779182`}, {"CURE", 365, 4.71544681889864`},
{"CURE", 365, 63.17599430666641`}, {"CURE", 365, 65.29920669726702`}, {"CURE", 365, 44.12777292365569`},
{"CURE", 365, 177.12986477401802`}, {"CURE", 365, 36.824049343872026`}, {"CURE", 365, 16.38533686194257`},
{"CURE", 365, 38.75437470905281`}, {"CURE", 365, 49.25348311001633`}, {"CURE", 365, 22.064568749287584`},
{"CURE", 365, 17.31254058548963`}, {"CURE", 365, 15.059185353064095`}, {"CURE", 365, 27.2877890093053`},
{"CURE", 365, 84.14360639707938`}, {"CURE", 365, 54.00332487231005`}, {"CURE", 365, 9.400399745629782`},
{"CURE", 365, 55.05679410297652`}, {"CURE", 365, 79.49266871462952`}, {"CURE", 365, 14.607429512610132`},
{"CURE", 365, 83.4031221603542`}, {"CURE", 365, 12.533021081732626`}, {"CURE", 365, 7.9995712646651995`},
{"CURE", 365, 11.352707077012345`}, {"CURE", 365, 21.84688694628135`}, {"TBUR", 91.07388482236561`, 97.75538729463055`},

{"CURE", 365, 34.95968762918286`}, {"CURE", 365, 44.139057408626535`}, {"CURE", 365, 10.552887463702074`},
{"CURE", 365, 201.34219437865627`}, {"CURE", 365, 92.24930524434383`}, {"CURE", 365, 15.735271418046443`},
{"CURE", 365, 38.530858975268984`}, {"CURE", 365, 21.469161670721263`}, {"CURE", 365, 21.685765374447442`},
{"CURE", 365, 73.27902329895878`}, {"TBUR", 62.159744044993055`, 92.6300981730854`}, {"CURE", 365, 9.955225655343936`},
{"CURE", 365, 19.398945862683163`}, {"CURE", 365, 6.881923604774987`}, {"CURE", 365, 26.538778873689623`},
{"CURE", 365, 45.48295345450103`}, {"CURE", 365, 73.6863815024613`}, {"CURE", 365, 41.12044163933732`},
{"CURE", 365, 148.0951826576053`}, {"CURE", 365, 70.78617148587024`}, {"TBUR", 76.35459609711506`, 11.674594219741829`},
{"CURE", 365, 25.889212931090256`}, {"TBUR", 44.413871095399465`, 28.16353456861402`}, {"CURE", 365, 57.8293896208642`},
{"CURE", 365, 7.586047426165906`}, {"CURE", 365, 20.319867373895594`}, {"CURE", 365, 11.448582718916951`},
{"CURE", 365, 28.56041556881699`}, {"CURE", 365, 65.25673409373977`}, {"CURE", 365, 27.76130947625502`},
{"CURE", 365, 6.02981205462384`}, {"CURE", 365, 114.8533598122376`}, {"CURE", 365, 21.664322177879953`},
{"CURE", 365, 7.358451190611129`}, {"CURE", 365, 9.67345529810801`}, {"CURE", 365, 8.166332559169488`},
{"CURE", 365, 10.432875044670386`}, {"CURE", 365, 83.95495038976219`}, {"CURE", 365, 45.95154775692036`},
{"CURE", 365, 27.66195174342453`}, {"CURE", 365, 12.966293761708892`}, {"CURE", 365, 21.64774874869814`},
{"CURE", 365, 12.524598070251779`}, {"CURE", 365, 30.22379054488376`}, {"CURE", 365, 103.92587999756009`},
{"CURE", 365, 60.35868848873031`}, {"CURE", 365, 29.98741483693031`}, {"CURE", 365, 17.27747801888714`},
{"CURE", 365, 18.130909388727527`}, {"CURE", 365, 222.0435743789829`}, {"CURE", 365, 10.580167167608725`},
{"CURE", 365, 5.450622327996478`}, {"CURE", 365, 4.750880162073389`}, {"CURE", 365, 60.36762791330353`},
{"CURE", 365, 35.35074830080134`}, {"CURE", 365, 39.22136121856258`}, {"CURE", 365, 58.27469687884135`},
{"CURE", 365, 96.19566336673135`}, {"CURE", 365, 99.50067957677116`}, {"CURE", 365, 37.920166672097395`},
{"CURE", 365, 16.37328689801242`}, {"CURE", 365, 18.988046482451775`}, {"CURE", 365, 37.90245442848875`},
{"CURE", 365, 21.49558606963067`}, {"TBUR", 27.28720459113871`, 8.481397871946658`}, {"CURE", 365, 33.76217035753116`},
{"CURE", 365, 210.1367970097723`}, {"CURE", 365, 21.648343723789853`}, {"CURE", 365, 7.786250473385137`},
{"CURE", 365, 73.82093794879886`}, {"CURE", 365, 18.462180624114076`}, {"CURE", 365, 53.061589387304025`},
{"TOX", 8.108619594910593`, 255.41887348565373`}, {"CURE", 365, 117.93895077921033`}, {"CURE", 365, 38.28745622154836`},
{"CURE", 365, 32.09579764739382`}, {"CURE", 365, 13.672707796546911`}, {"CURE", 365, 3.903819923135242`},
{"CURE", 365, 15.288283147065387`}, {"CURE", 365, 44.77285141196599`}, {"CURE", 365, 24.827906450826536`},
{"CURE", 365, 79.21482399043366`}, {"CURE", 365, 31.4023242737829`}, {"CURE", 365, 32.06304023735246`},
{"CURE", 365, 8.903742281796617`}, {"CURE", 365, 25.220359661016367`}, {"CURE", 365, 25.880574574123667`},
{"CURE", 365, 6.748719277540121`}, {"CURE", 365, 21.47638145744844`}, {"CURE", 365, 12.803832356043696`},
{"CURE", 365, 20.287994837391913`}, {"CURE", 365, 42.496626832164075`}, {"CURE", 365, 21.25305829927088`},

{"CURE", 365, 4.927586928906077`}, {"CURE", 365, 33.94836243943559`}, {"CURE", 365, 3.8008627599977767`},
{"CURE", 365, 11.154255670404531`}, {"CURE", 365, 33.08693700858695`}, {"CURE", 365, 38.144327025591956`},
{"CURE", 365, 7.588451354482105`}, {"CURE", 365, 22.336491919069093`}, {"CURE", 365, 28.93945257338915`},
{"CURE", 365, 146.1615773056322`}, {"CURE", 365, 33.5801675618619`}, {"CURE", 365, 9.472152341453718`},
{"CURE", 365, 103.2521524729816`}, {"CURE", 365, 10.150396900641864`}, {"CURE", 365, 38.22084600384383`},
{"CURE", 365, 39.53306783761987`}, {"CURE", 365, 11.80041334505119`}, {"CURE", 365, 13.138779621333917`},
{"CURE", 365, 58.199642712479246`}, {"CURE", 365, 5.686026237969035`}, {"CURE", 365, 49.309014483648305`},
{"CURE", 365, 13.058171025168134`}, {"CURE", 365, 17.075041753572805`}, {"CURE", 365, 3.815783300279283`},
{"CURE", 365, 19.607043115743274`}, {"CURE", 365, 66.87048813149084`}, {"CURE", 365, 25.44598818672086`},
{"CURE", 365, 18.47738961909055`}, {"CURE", 365, 26.408710569781448`}, {"CURE", 365, 20.7149008457435`},
{"CURE", 365, 26.106065350268114`}, {"CURE", 365, 46.07865434590691`}, {"CURE", 365, 44.465518975883676`},
{"CURE", 365, 5.47622189901706`}, {"CURE", 365, 89.86902097555941`}, {"CURE", 365, 10.451107135994862`},
{"CURE", 365, 5.24253138503651`}, {"CURE", 365, 24.99478617819141`}, {"CURE", 365, 26.23563042258299`},
{"CURE", 365, 17.68729800375685`}, {"CURE", 365, 30.709212906033684`}, {"CURE", 365, 26.478357613453166`},
{"CURE", 365, 42.56037953019934`}, {"CURE", 365, 35.9659927595086`}, {"CURE", 365, 51.351959649990704`},
{"CURE", 365, 8.115139282902428`}, {"CURE", 365, 103.97072302554487`}, {"CURE", 365, 100.9465995258818`},
{"CURE", 365, 5.592491051717338`}, {"CURE", 365, 14.07831955400364`}, {"CURE", 365, 34.931239536748045`},
{"CURE", 365, 17.760086756368132`}, {"CURE", 365, 54.5777629839212`}, {"CURE", 365, 109.36280099711706`},
{"CURE", 365, 34.564448700430205`}, {"CURE", 365, 5.932588365070626`}, {"CURE", 365, 5.819695969113759`},
{"CURE", 365, 13.665093662945605`}, {"CURE", 365, 4.125395328378337`}, {"CURE", 365, 46.82461646170803`},
{"CURE", 365, 7.259086958433569`}, {"CURE", 365, 48.79478643803336`}, {"CURE", 365, 30.073335393394757`},
{"CURE", 365, 51.83319396473326`}, {"CURE", 365, 12.761556184358774`}, {"CURE", 365, 64.38366045287643`},
{"CURE", 365, 213.0164652033152`}, {"CURE", 365, 76.7414446998431`}, {"CURE", 365, 124.3164820277852`},
{"CURE", 365, 36.58907116183173`}, {"CURE", 365, 87.17296704926527`}, {"CURE", 365, 87.34512784914472`},
{"CURE", 365, 10.60111753587587`}, {"CURE", 365, 7.415237428738971`}, {"CURE", 365, 24.111697649583963`},
{"CURE", 365, 50.03410196466361`}, {"CURE", 365, 62.599434142688516`}, {"CURE", 365, 26.765423067067086`},
{"CURE", 365, 39.61008489128478`}, {"CURE", 365, 7.461620468298492`}, {"CURE", 365, 9.480315202931394`},
{"CURE", 365, 46.066422270526935`}, {"CURE", 365, 14.929176905105294`}, {"CURE", 365, 6.3377944600341145`},
{"CURE", 365, 9.374554788316189`}, {"CURE", 365, 4.582637592444332`}, {"CURE", 365, 37.23998711650321`},
{"CURE", 365, 51.21469157531656`}, {"CURE", 365, 35.82519572484831`}, {"CURE", 365, 40.71258329575587`},
{"CURE", 365, 22.546631674661192`}, {"CURE", 365, 20.182164495904566`}, {"CURE", 365, 52.23798223732832`},

{"CURE", 365, 55.91202854125458`}, {"CURE", 365, 6.238513111185574`}, {"CURE", 365, 23.302281772662425`},
{"CURE", 365, 12.396497595192425`}, {"CURE", 365, 19.467717297369187`}, {"CURE", 365, 160.35220858524923`},
{"CURE", 365, 28.315333259822815`}, {"CURE", 365, 19.435449431970486`}, {"CURE", 365, 20.55156961531197`},
{"CURE", 365, 41.00461562721009`}, {"CURE", 365, 31.212457803908812`}, {"CURE", 365, 40.71737797573448`},
{"CURE", 365, 41.951721049759044`}, {"CURE", 365, 9.523292345423373`}, {"CURE", 365, 92.61739667717306`},
{"CURE", 365, 17.78429156630983`}, {"CURE", 365, 37.72226846716632`}, {"CURE", 365, 21.40495621092793`},
{"CURE", 365, 12.155770838876789`}, {"CURE", 365, 44.31372106283637`}, {"CURE", 365, 44.98827621619409`},
{"TBUR", 76.98336340887681`, 24.9312755555499`}, {"CURE", 365, 58.97213659219607`}, {"CURE", 365, 12.474640591204476`},
{"CURE", 365, 104.2618151997710８`}, {"CURE", 365, 40.03599178085226`}, {"CURE", 365, 84.63159826214022`},
{"CURE", 365, 3.2836158276484175`}, {"CURE", 365, 41.63184655100494`}, {"CURE", 365, 47.42536609533011`},
{"CURE", 365, 11.284581910006903`}, {"CURE", 365, 28.64813420185616`}, {"CURE", 365, 42.90382920732487`},
{"CURE", 365, 26.076073196176434`}, {"CURE", 365, 8.296369783073485`}, {"CURE", 365, 6.296889440472473`},
{"CURE", 365, 37.22814584595364`}, {"CURE", 365, 9.93360985128124`}, {"CURE", 365, 22.081201638416548`},
{"CURE", 365, 26.906967531859845`}, {"CURE", 365, 12.519521535219678`}, {"CURE", 365, 23.045590233668953`},
{"CURE", 365, 6.279614541846342`}, {"CURE", 365, 25.889927062212166`}, {"CURE", 365, 4.185728637856453`},
{"CURE", 365, 7.482814160152746`}, {"CURE", 365, 17.687625643541107`}, {"CURE", 365, 5.704746895520055`},
{"CURE", 365, 42.394088854311995`}, {"CURE", 365, 62.29046487974936`}, {"CURE", 365, 63.20687793948507`},
{"CURE", 365, 52.64384239212992`}, {"CURE", 365, 99.36545895660676`}, {"CURE", 365, 8.03713157064501`},
{"CURE", 365, 35.845054940078285`}, {"CURE", 365, 38.47686296805901`}, {"CURE", 365, 66.18850026988423`},
{"CURE", 365, 49.43787590573761`}, {"CURE", 365, 22.589221300827976`}, {"CURE", 365, 106.20653784253234`},
{"CURE", 365, 3.31634349727757`}, {"CURE", 365, 101.08671571957005`}, {"CURE", 365, 6.844581480058099`},
{"CURE", 365, 86.33612394771086`}, {"CURE", 365, 78.19133960542034`}, {"CURE", 365, 23.175438848734345`},
{"CURE", 365, 91.25412581437024`}, {"CURE", 365, 111.32241569333488`}, {"CURE", 365, 49.78618171166516`},
{"CURE", 365, 21.971369284875227`}, {"CURE", 365, 25.26010075818647`}, {"CURE", 365, 37.302115975266695`},
{"CURE", 365, 34.39881763832442`}, {"CURE", 365, 8.336990193599423`}, {"CURE", 365, 29.930276540514654`},
{"CURE", 365, 28.312720680547244`}, {"CURE", 365, 21.179406333273747`}, {"CURE", 365, 52.335420848776`},
{"CURE", 365, 8.24108888489525`}, {"CURE", 365, 58.41508223127888`}, {"CURE", 365, 38.22389500583504`},
{"CURE", 365, 21.643555814556155`}, {"CURE", 365, 19.62720046787468`}, {"CURE", 365, 39.91358805785663`},
{"CURE", 365, 5.469686072330257`}, {"CURE", 365, 63.353210027674905`}, {"CURE", 365, 10.000112103072798`},
{"CURE", 365, 14.186348435494104`}, {"CURE", 365, 31.82150868220152`}, {"CURE", 365, 24.94988383836938`},
{"CURE", 365, 22.943350108929298`}, {"CURE", 365, 71.41922535142923`}, {"CURE", 365, 9.662732419041799`},

{"CURE", 365, 16.822754767804412`}, {"CURE", 365, 124.47032983245781`}, {"CURE", 365, 9.443993323953913`},
{"CURE", 365, 24.321220330663007`}, {"CURE", 365, 37.00046919308147`}, {"CURE", 365, 36.53367164499846`},
{"CURE", 365, 24.80979657074588`}, {"CURE", 365, 128.2591791949400б`}, {"CURE", 365, 23.16272440028145`},
{"CURE", 365, 9.756454425298312`}, {"CURE", 365, 19.44156761472394`}, {"CURE", 365, 15.02359294221344`},
{"CURE", 365, 11.512173051243305`}, {"CURE", 365, 30.19357326234082`}, {"CURE", 365, 51.71284207452731`},
{"CURE", 365, 28.45095359782269`}, {"CURE", 365, 33.17017540265889`}, {"CURE", 365, 72.63244313099536`},
{"CURE", 365, 8.007697415223683`}, {"CURE", 365, 9.422976644820093`}, {"CURE", 365, 79.51232772697283`},
{"CURE", 365, 25.59417181683647`}, {"CURE", 365, 27.725884779649554`}, {"CURE", 365, 13.936932727623493`},
{"CURE", 365, 31.914229028767874`}, {"CURE", 365, 39.772798568241896`}, {"CURE", 365, 92.17648558267155`},
{"CURE", 365, 13.586654588547736`}, {"CURE", 365, 53.07977252336573`}, {"CURE", 365, 31.227010526967394`},
{"CURE", 365, 82.68128734096942`}, {"CURE", 365, 135.06487784897044`}, {"CURE", 365, 49.43382418084173`},
{"CURE", 365, 4.7879551778177385`}, {"CURE", 365, 37.47443587844072`}, {"CURE", 365, 11.834119660129478`},
{"CURE", 365, 125.142698067253`}, {"TBUR", 46.34722425544823`, 3.9485300138383956`}, {"CURE", 365, 4.1766180605327925`},
{"CURE", 365, 144.82607697990744`}, {"CURE", 365, 33.31085367173763`}, {"CURE", 365, 11.273054779532202`},
{"CURE", 365, 21.57636045500424`}, {"CURE", 365, 7.569407855690536`}, {"TBUR", 73.43382143629769`, 7.194216230843825`},
{"CURE", 365, 8.607721860641375`}, {"CURE", 365, 31.113620336264145`}, {"CURE", 365, 65.94437930312557`},
{"CURE", 365, 26.1173371145824`}, {"CURE", 365, 6.540721180052661`}, {"CURE", 365, 88.97162847218999`},
{"CURE", 365, 177.76119258890142`}, {"CURE", 365, 5.014501210351446`}, {"CURE", 365, 40.071505496811206`},
{"CURE", 365, 21.52846113862944`}, {"CURE", 365, 4.233666873911501`}, {"CURE", 365, 33.09474562019494`},
{"CURE", 365, 21.146496992415177`}, {"CURE", 365, 16.23231524402325`}, {"CURE", 365, 31.568561415465794`},
{"CURE", 365, 17.919516527516805`}, {"CURE", 365, 52.682588758467936`}, {"CURE", 365, 31.321153165948683`},
{"CURE", 365, 73.74783316842218`}, {"CURE", 365, 32.667929808943676`}, {"CURE", 365, 16.185286449566096`},
{"CURE", 365, 7.870225733165011`}, {"CURE", 365, 5.460228213416316`}, {"CURE", 365, 15.552804915071205`},
{"CURE", 365, 61.88827606354491`}, {"CURE", 365, 20.41134427648243`}, {"CURE", 365, 18.700160889778928`},
{"CURE", 365, 13.223560619665566`}, {"CURE", 365, 13.403121896628877`}, {"CURE", 365, 36.218298322730675`},
{"CURE", 365, 6.776392912186876`}, {"CURE", 365, 43.16787280751545`}, {"CURE", 365, 19.7515903198775`},
{"CURE", 365, 11.60725301518298`}, {"CURE", 365, 18.395207421145788`}, {"CURE", 365, 40.81870000425124`},
{"CURE", 365, 30.394739805765166`}, {"CURE", 365, 3.669879952667902`}, {"CURE", 365, 15.810010694357553`},
{"TOX", 6.994449666110623`, 264.29930157389316`}, {"CURE", 365, 43.893978104401306`}, {"CURE", 365, 64.03129489045635`},
{"CURE", 365, 18.866533784420714`}, {"CURE", 365, 69.94746469174189`}, {"CURE", 365, 8.369643267994803`},
{"CURE", 365, 28.199331706149522`}, {"CURE", 365, 130.18275933602732`}, {"CURE", 365, 24.379499940999825`},

{"CURE", 365, 21.457946910959915`}, {"CURE", 365, 36.80535539607767`}, {"CURE", 365, 19.47717134557 4174`},
{"CURE", 365, 4.323319058346551`}, {"CURE", 365, 40.175458394035076`}, {"CURE", 365, 7.169090452358874`},
{"CURE", 365, 17.031026111821202`}, {"CURE", 365, 40.24732567631641`}, {"CURE", 365, 14.454351265274092`},
{"CURE", 365, 76.77039313156232`}, {"CURE", 365, 22.824224254700773`}, {"CURE", 365, 39.295740799946635`},
{"CURE", 365, 27.2373925230797`}, {"CURE", 365, 7.707837661261814`}, {"CURE", 365, 28.594754205936557`},
{"CURE", 365, 61.799817687201624`}, {"CURE", 365, 20.713336243734087`}, {"CURE", 365, 15.58846720729743`},
{"CURE", 365, 7.394181176769053`}, {"CURE", 365, 23.483726422838796`}, {"CURE", 365, 28.135799706330474`},
{"CURE", 365, 80.31300130862685`}, {"CURE", 365, 12.90145826542253`}, {"CURE", 365, 19.4131747384077`},
{"CURE", 365, 75.38456557726148`}, {"CURE", 365, 7.942664651341859`}, {"CURE", 365, 109.00730308904716`},
{"CURE", 365, 9.215899784215607`}, {"CURE", 365, 16.869101625514837`}, {"CURE", 365, 38.8870661475464`},
{"CURE", 365, 62.588532856696474`}, {"CURE", 365, 28.171093825410253`}, {"CURE", 365, 14.761121179691834`},
{"CURE", 365, 4.340323655297842`}, {"CURE", 365, 37.97516046385822`}, {"CURE", 365, 34.42507407358733`},
{"CURE", 365, 33.84013824244076`}, {"CURE", 365, 13.343082664366342`}, {"CURE", 365, 32.71000155060449`},
{"CURE", 365, 3.7829864454113293`}, {"CURE", 365, 21.805516457380307`}, {"CURE", 365, 9.524777936116518`},
{"CURE", 365, 4.217943441959111`}, {"CURE", 365, 21.923698148060865`}, {"CURE", 365, 4.691622013344959`},
{"CURE", 365, 14.918075104880662`}, {"CURE", 365, 10.230628521272253`}, {"TBUR", 44.01536247676874`, 6.197746352204346`},
{"CURE", 365, 20.263661573351985`}, {"CURE", 365, 53.99600290590578`}, {"CURE", 365, 29.55447032818199`},
{"CURE", 365, 26.769019045613664`}, {"CURE", 365, 42.519715786648945`}, {"CURE", 365, 36.11149702248889`}},
{{"2200 Res", "2200 OS", "2200 Tox"}, {"CURE", 365, 18.325963113003755`}, {"CURE", 365, 37.92303748207161`},
{"CURE", 365, 26.5791040854015`}, {"TBUR", 71.068879758523`, 8.911756354987363`},
{"TBUR", 49.93394548458135`, 33.95633139629969`}, {"CURE", 365, 68.69392811457212`}, {"CURE", 365, 64.63242340526591`},
{"CURE", 365, 6.7533521274154165`}, {"CURE", 365, 46.61261878649464`}, {"CURE", 365, 40.31870025730493`},
{"CURE", 365, 151.32278868109356`}, {"CURE", 365, 18.77615017812886`}, {"CURE", 365, 67.41115279564892`},
{"CURE", 365, 225.47256146448487`}, {"CURE", 365, 31.92191657020034`}, {"CURE", 365, 49.82446904996033`},
{"CURE", 365, 98.66444061982294`}, {"CURE", 365, 44.19730144838401`}, {"TBUR", 131.07065362634563`, 33.43691690349638`},
{"CURE", 365, 11.078393006815988`}, {"CURE", 365, 68.47807279475732`}, {"CURE", 365, 27.758069924224554`},
{"CURE", 365, 10.636715160574216`}, {"CURE", 365, 8.475233123515128`}, {"CURE", 365, 38.56197328457419`},
{"CURE", 365, 42.324398948692284`}, {"CURE", 365, 3.8478275091684644`}, {"CURE", 365, 5.832372439914235`},
{"CURE", 365, 21.611981455983482`}, {"CURE", 365, 25.28214954795271`}, {"CURE", 365, 21.567558112573238`},
{"CURE", 365, 75.82141972842568`}, {"CURE", 365, 16.675930307369708`}, {"CURE", 365, 13.263110499848136`},
{"CURE", 365, 107.49682490060961`}, {"CURE", 365, 6.586657483532051`}, {"CURE", 365, 28.467035712360826`},

{"TBUR", 47.68006970494`, 63.50480941743713`}, {"CURE", 365, 15.822529934786745`}, {"CURE", 365, 24.341113916226487`},
{"CURE", 365, 55.540256510343944`}, {"CURE", 365, 37.48071748351581`}, {"CURE", 365, 7.604778720075689`},
{"CURE", 365, 25.65044595493344`}, {"CURE", 365, 37.0096069198132`}, {"CURE", 365, 24.26774072716465`},
{"TOX", 7.161302424156135`, 257.86915055708585`}, {"CURE", 365, 55.58766965075313 5`}, {"CURE", 365, 85.34218145812014`},
{"CURE", 365, 10.396132005977462`}, {"CURE", 365, 29.05956204186345`}, {"CURE", 365, 39.8783331363218`},
{"CURE", 365, 31.984483856118697`}, {"CURE", 365, 20.761754474119055`}, {"CURE", 365, 8.947033431708947`},
{"CURE", 365, 94.62939999519348`}, {"CURE", 365, 29.736658999533883`}, {"CURE", 365, 5.649108923395905`},
{"CURE", 365, 60.8694990647636`}, {"CURE", 365, 42.62741405985248`}, {"CURE", 365, 29.19787521955853`},
{"CURE", 365, 22.342575857204682`}, {"CURE", 365, 12.674956771270438`}, {"CURE", 365, 48.153899764484215`},
{"CURE", 365, 34.211968318972595`}, {"CURE", 365, 39.120135091701236`}, {"CURE", 365, 8.954456693921635`},
{"CURE", 365, 9.100211343327686`}, {"CURE", 365, 9.681364080302709`}, {"CURE", 365, 39.53966114713836`},
{"CURE", 365, 14.475145545554174`}, {"CURE", 365, 32.2427789499485`}, {"CURE", 365, 60.50629971064295`},
{"TBUR", 26.93932030391917`, 18.800681031666493`}, {"CURE", 365, 16.16998678367488`}, {"CURE", 365, 44.787529805800915`},
{"CURE", 365, 26.193420353477954`}, {"CURE", 365, 19.92273078195219`}, {"CURE", 365, 79.37419782504097`},
{"CURE", 365, 87.15607260069655`}, {"CURE", 365, 64.16790287597472`}, {"CURE", 365, 17.312627162207708`},
{"CURE", 365, 35.004128050556346`}, {"CURE", 365, 24.467050419077527`}, {"CURE", 365, 27.667237413121278`},
{"CURE", 365, 12.965431070692047`}, {"CURE", 365, 138.31133092443937`}, {"CURE", 365, 30.144001573536446`},
{"CURE", 365, 37.963033378030204`}, {"CURE", 365, 32.67177600525711`}, {"CURE", 365, 27.001638222710994`},
{"CURE", 365, 8.007074113603267`}, {"CURE", 365, 14.675124128469761`}, {"CURE", 365, 33.973597310448156`},
{"TBUR", 57.53331049819486`, 12.307155235546892`}, {"CURE", 365, 14.62947499894628`}, {"CURE", 365, 38.14706453641266`},
{"CURE", 365, 96.8129153540308`}, {"CURE", 365, 51.86440765858702`}, {"CURE", 365, 26.012544999227718`},
{"TBUR", 79.8079957301939`, 51.55955245011545`}, {"CURE", 365, 33.53592854568996`}, {"CURE", 365, 21.732752614326753`},
{"CURE", 365, 148.80660849869875`}, {"CURE", 365, 16.530054275681405`}, {"CURE", 365, 11.231348290961717`},
{"CURE", 365, 65.92455121412641`}, {"CURE", 365, 19.44856666651076`}, {"CURE", 365, 19.889082588609135`},
{"CURE", 365, 119.6647388563028`}, {"CURE", 365, 9.71279840493772`}, {"CURE", 365, 6.162248902929999`},
{"CURE", 365, 25.63292013285799`}, {"CURE", 365, 20.26608047055103`}, {"CURE", 365, 15.263294681889558`},
{"CURE", 365, 38.95935658489927`}, {"CURE", 365, 10.96530679092553`}, {"CURE", 365, 6.742179766424801`},
{"CURE", 365, 31.56120626660105`}, {"CURE", 365, 48.26890636722616`}, {"CURE", 365, 8.220030612932806`},
{"CURE", 365, 27.207305584551435`}, {"CURE", 365, 82.5872924850075`}, {"TBUR", 69.18380331151081`, 31.469426782880305`},
{"CURE", 365, 18.32086096684773`}, {"CURE", 365, 63.918858322779414`}, {"CURE", 365, 32.122312306343474`},
{"CURE", 365, 4.23425744659357`}, {"CURE", 365, 36.67767152526878`}, {"CURE", 365, 28.327007434398567`},

{"CURE", 365, 22.566230974204714`}, {"CURE", 365, 26.767253671067646`}, {"CURE", 365, 6.9681525292708795`},
{"CURE", 365, 39.751999491144765`}, {"CURE", 365, 14.270816880382089`}, {"CURE", 365, 32.22935336694269`},
{"CURE", 365, 120.48076685785428`}, {"CURE", 365, 42.56605151082696 6`}, {"CURE", 365, 98.90813754729162`},
{"CURE", 365, 32.58430207015181`}, {"CURE", 365, 134.5825634669076`}, {"CURE", 365, 4.198246201241389`},
{"CURE", 365, 36.87542751873688`}, {"CURE", 365, 42.03373251463396`}, {"CURE", 365, 5.459470448822157`},
{"CURE", 365, 7.5633800686044665`}, {"CURE", 365, 46.424748408673295`}, {"CURE", 365, 54.70184276490842`},
{"CURE", 365, 46.62395523322866`}, {"CURE", 365, 38.148611325378866`}, {"CURE", 365, 36.46946529943796`},
{"CURE", 365, 22.08281847090786`}, {"CURE", 365, 5.840479017357603`}, {"CURE", 365, 25.403037910048873`},
{"CURE", 365, 12.05454281395683`}, {"CURE", 365, 5.901027555198431`}, {"CURE", 365, 35.76715169806562`},
{"CURE", 365, 20.01293533022392`}, {"CURE", 365, 52.90359776370276`}, {"CURE", 365, 21.357660546413058`},
{"CURE", 365, 17.77641125989534`}, {"CURE", 365, 119.19701338577526`}, {"CURE", 365, 8.882879293798181`},
{"CURE", 365, 26.573158619097697`}, {"CURE", 365, 15.060334775054105`}, {"CURE", 365, 35.57367841839086`},
{"CURE", 365, 9.779785653954526`}, {"CURE", 365, 39.04893839447068`}, {"CURE", 365, 103.14121012984799`},
{"CURE", 365, 8.823550429791688`}, {"CURE", 365, 26.799775088047667`}, {"CURE", 365, 28.9371575902249`},
{"CURE", 365, 104.21643448063654`}, {"CURE", 365, 39.17936842868101`}, {"CURE", 365, 32.55450687908826`},
{"CURE", 365, 57.11093120573443`}, {"CURE", 365, 21.919933350463634`}, {"CURE", 365, 67.41499006720883`},
{"CURE", 365, 42.17522128061169`}, {"CURE", 365, 25.708119230377534`}, {"CURE", 365, 23.2649143078334 2`},
{"CURE", 365, 7.568758390620212`}, {"CURE", 365, 5.128870677952873`}, {"CURE", 365, 12.019673914111767`},
{"CURE", 365, 16.10316077620981`}, {"CURE", 365, 7.090439866185739`}, {"CURE", 365, 25.958423564530538`},
{"CURE", 365, 70.87900022910782`}, {"CURE", 365, 19.163340548290787`}, {"CURE", 365, 35.532204177375164`},
{"CURE", 365, 44.54431631635361`}, {"CURE", 365, 37.833090568568934`}, {"CURE", 365, 26.070746135008`},
{"CURE", 365, 10.355419797645066`}, {"CURE", 365, 71.34230971864605`}, {"CURE", 365, 6.172757070529812`},
{"CURE", 365, 66.50720259941372`}, {"CURE", 365, 64.57965755535767`}, {"CURE", 365, 18.03779533589233`},
{"CURE", 365, 21.425440552264412`}, {"CURE", 365, 123.15722246510565`}, {"CURE", 365, 31.73888137680544`},
{"CURE", 365, 37.90136667226502`}, {"CURE", 365, 23.732446376156236`}, {"CURE", 365, 8.926087421794092`},
{"CURE", 365, 54.720950622153346`}, {"TBUR", 59.16824375175578`, 145.42689584321957`}, {"CURE", 365, 22.226461121033292`},
{"CURE", 365, 20.094904149827858`}, {"CURE", 365, 37.02374659890876`}, {"CURE", 365, 14.783789433909945`},
{"CURE", 365, 187.59981156555935`}, {"CURE", 365, 15.016769933049192`}, {"CURE", 365, 92.85946820354772`},
{"CURE", 365, 71.3310923562753`}, {"CURE", 365, 69.36601485213983`}, {"CURE", 365, 37.05266020344883`},
{"CURE", 365, 85.90442634114032`}, {"CURE", 365, 184.70654301975514`}, {"CURE", 365, 39.02934267722885`},
{"CURE", 365, 7.1700526616573095`}, {"CURE", 365, 63.684944850349495`}, {"CURE", 365, 39.68371050728444`},

{"CURE", 365, 38.25650156694978` }, {"CURE", 365, 8.433579852753336` }, {"CURE", 365, 193.3541329165057` },
{"CURE", 365, 14.134725090271926` }, {"CURE", 365, 61.318928772328015` }, {"CURE", 365, 38.654538569160216` },
{"CURE", 365, 27.470200332881515` }, {"CURE", 365, 6.603720897647854` }, {"CURE", 365, 49.63787391692777` },
{"CURE", 365, 29.11967566665179` }, {"CURE", 365, 77.03910973979464` }, {"CURE", 365, 50.75834158851061` },
{"CURE", 365, 64.07777752145014` }, {"CURE", 365, 30.767189301789834` }, {"CURE", 365, 27.37282613990768` },
{"CURE", 365, 143.02767562687853` }, {"CURE", 365, 37.46128159831751` }, {"CURE", 365, 9.995012866704014` },
{"CURE", 365, 33.66114179450751` }, {"CURE", 365, 34.20232762614449` }, {"CURE", 365, 4.590990119046648` },
{"CURE", 365, 8.180675876513352` }, {"CURE", 365, 12.498387558564113` }, {"CURE", 365, 118.02326722353378` },
{"CURE", 365, 36.29130985209441` }, {"CURE", 365, 37.82188495041011` }, {"CURE", 365, 30.292268007910167` },
{"CURE", 365, 124.55271292911036` }, {"CURE", 365, 28.680089733810668` }, {"CURE", 365, 57.714652954032374` },
{"CURE", 365, 28.600585656262812` }, {"CURE", 365, 42.162942730631585` }, {"CURE", 365, 10.13978447918738` },
{"CURE", 365, 37.92382370194272` }, {"CURE", 365, 23.665216707604547` }, {"CURE", 365, 46.09542948462655` },
{"CURE", 365, 72.84579076438446` }, {"CURE", 365, 81.93071868129394` }, {"CURE", 365, 49.724598209791246` },
{"CURE", 365, 40.61722599658104` }, {"CURE", 365, 55.97574600269376` }, {"CURE", 365, 26.59641475308168` },
{"CURE", 365, 30.111239323941067` }, {"CURE", 365, 21.334191269509752` }, {"CURE", 365, 16.718985955131103` },
{"CURE", 365, 26.930708689223938` }, {"CURE", 365, 69.10277238808531` }, {"CURE", 365, 7.920372890093578` },
{"CURE", 365, 35.093934645325135` }, {"CURE", 365, 6.627008891038856` }, {"CURE", 365, 34.492671734354865` },
{"CURE", 365, 19.59862975527617` }, {"CURE", 365, 72.942879273461` }, {"CURE", 365, 6.725066512212752` },
{"CURE", 365, 48.018287718360696` }, {"CURE", 365, 60.23370404904681` }, {"CURE", 365, 9.853427835157383` },
{"CURE", 365, 22.57524700418449` }, {"CURE", 365, 18.164098536706412` }, {"CURE", 365, 10.393711312308312` },
{"CURE", 365, 61.43889275143498` }, {"CURE", 365, 23.09002055583793` }, {"CURE", 365, 44.498181280242456` },
{"CURE", 365, 43.26930231156544` }, {"CURE", 365, 49.06083708747712` }, {"CURE", 365, 38.35710064936998` },
{"CURE", 365, 45.91672915661067` }, {"CURE", 365, 36.344871746622296` }, {"CURE", 365, 70.26356427925083` },
{"CURE", 365, 42.79423532269906` }, {"CURE", 365, 34.948736448517685` }, {"CURE", 365, 89.4000987464189` },
{"CURE", 365, 10.350742539827658` }, {"CURE", 365, 58.715584559461035` }, {"CURE", 365, 4.313290589925094` },
{"CURE", 365, 53.581949532680134` }, {"CURE", 365, 12.627902453897494` }, {"CURE", 365, 66.52439290030199` },
{"CURE", 365, 10.311193100885541` }, {"CURE", 365, 67.54746844885177` }, {"CURE", 365, 169.05353457522705` },
{"CURE", 365, 7.37958010321746` }, {"CURE", 365, 5.298927163021157` }, {"CURE", 365, 19.513409430155804` },
{"CURE", 365, 4.738722101682438` }, {"CURE", 365, 3.2399160642584333` }, {"CURE", 365, 18.954657256764087` },
{"CURE", 365, 30.989498474944106` }, {"CURE", 365, 33.347928854685996` }, {"CURE", 365, 31.692777209056114` },
{"CURE", 365, 24.683418404409398` }, {"CURE", 365, 43.42885150419818` }, {"CURE", 365, 76.45816054291836` },

{"CURE", 365, 6.187183585893464`}, {"CURE", 365, 33.898665230621155`}, {"CURE", 365, 29.131196377005804`},
{"CURE", 365, 21.12131661408167`}, {"CURE", 365, 39.06071028105285`}, {"CURE", 365, 57.908917828853994`},
{"CURE", 365, 135.5016334692519 7`}, {"CURE", 365, 100.95606161492185`}, {"CURE", 365, 31.21906119087307`},
{"CURE", 365, 3.315927251678024`}, {"CURE", 365, 36.171634522683014`}, {"CURE", 365, 93.27934108935055`},
{"CURE", 365, 23.470328446304023`}, {"CURE", 365, 29.67921185991003`}, {"CURE", 365, 18.10852163621687`},
{"CURE", 365, 26.543134526339276`}, {"CURE", 365, 6.872413930836243`}, {"CURE", 365, 33.62967392908493`},
{"CURE", 365, 18.892050290479297`}, {"CURE", 365, 55.20875725209129`}, {"CURE", 365, 37.80765176673656`},
{"CURE", 365, 36.67048304162316`}, {"CURE", 365, 30.173016083206257`}, {"CURE", 365, 15.967396852519169`},
{"CURE", 365, 11.003543409099942`}, {"CURE", 365, 10.728318715006521`}, {"CURE", 365, 13.895269696199474`},
{"CURE", 365, 10.829014247369292`}, {"CURE", 365, 145.60768842107387`}, {"CURE", 365, 80.99374458553692`},
{"CURE", 365, 15.65240480372124`}, {"TOX", 10.48830325891453`, 241.10519623379005`}, {"CURE", 365, 68.94922321879594`},
{"CURE", 365, 36.5058096303346`}, {"CURE", 365, 63.1489257742266 9`}, {"CURE", 365, 9.494713737636332`},
{"CURE", 365, 75.70331775847252`}, {"CURE", 365, 202.41809783389783`}, {"CURE", 365, 10.98250471246412`},
{"CURE", 365, 34.260175924092785`}, {"CURE", 365, 56.103470140697794`}, {"CURE", 365, 28.339419007313555`},
{"CURE", 365, 13.90201917308701`}, {"CURE", 365, 11.075616186838039`}, {"CURE", 365, 96.1289726908858`},
{"CURE", 365, 50.4670882841202`}, {"CURE", 365, 28.713076075762782`}, {"CURE", 365, 71.35068062771072`},
{"CURE", 365, 12.880334923142524`}, {"CURE", 365, 34.42263415391619`}, {"CURE", 365, 20.788795972100107`},
{"CURE", 365, 140.2127425323485`}, {"TBUR", 71.5944963131579`, 35.26531274017178`}, {"CURE", 365, 163.69638660939427`},
{"TOX", 8.270897871853265`, 255.52278290960203`}, {"CURE", 365, 19.56812306605549`}, {"CURE", 365, 5.518122090338235`},
{"CURE", 365, 4.807702469674075`}, {"CURE", 365, 35.167688062356376`}, {"CURE", 365, 136.450253465314`},
{"CURE", 365, 27.33390795320964`}, {"CURE", 365, 20.998714516372324`}, {"TBUR", 75.47673574104718`, 39.45976370004169`},
{"CURE", 365, 43.22041817348148`}, {"CURE", 365, 180.184877520682`}, {"CURE", 365, 148.58829785750635`},
{"CURE", 365, 31.115729260751287`}, {"CURE", 365, 10.326546387147669`}, {"CURE", 365, 25.2988038583602`},
{"CURE", 365, 3.9705497754248715`}, {"CURE", 365, 74.64761017143285`}, {"CURE", 365, 111.0573877930448`},
{"CURE", 365, 36.321394692372735`}, {"CURE", 365, 28.784867205694646`}, {"CURE", 365, 121.72000662747459`},
{"CURE", 365, 7.718427384364837`}, {"CURE", 365, 10.053150205803545`}, {"CURE", 365, 31.47634583410732`},
{"CURE", 365, 40.48322692103207`}, {"TBUR", 83.9619715128238`, 76.66507170632971`}, {"CURE", 365, 78.97692225401376`},
{"CURE", 365, 32.41937918948996`}, {"CURE", 365, 33.43826715054869`}, {"CURE", 365, 41.854329982244`},
{"CURE", 365, 14.4907275795423`}, {"CURE", 365, 4.094712125371532`}, {"CURE", 365, 43.06910196656874`},
{"CURE", 365, 51.8628820552666`}, {"CURE", 365, 25.215638122654212`}, {"CURE", 365, 45.69707762664208`},
{"CURE", 365, 14.232773680862472`}, {"CURE", 365, 9.353467703300643`}, {"CURE", 365, 40.94665114443777`},

{"CURE", 365, 24.877339114995646`}, {"CURE", 365, 35.5743905040306`}, {"TOX", 17.352041044003553`, 234.7790894612729`},
{"CURE", 365, 22.36659455881909`}, {"CURE", 365, 10.90751730403601`}, {"CURE", 365, 8.466274565490542`},
{"CURE", 365, 10.261584567177263`}, {"CURE", 365, 58.61907054851625`}, {"CURE", 365, 4.037301974368727`},
{"CURE", 365, 54.584321947457276`}, {"CURE", 365, 59.594594299241784`}, {"CURE", 365, 46.51481058872397`},
{"CURE", 365, 38.13437849358357`}, {"CURE", 365, 7.560508457214146`}, {"CURE", 365, 83.00059334684616`},
{"TBUR", 70.65644989926341`, 25.120981674835544`}, {"CURE", 365, 11.346899427448234`}, {"CURE", 365, 73.91272701859064`},
{"CURE", 365, 13.146721771160461`}, {"CURE", 365, 88.55477851257027`}, {"CURE", 365, 56.8320774948253`},
{"CURE", 365, 37.89534475374619`}, {"CURE", 365, 55.07392392132106`}, {"CURE", 365, 19.52056711145586`},
{"CURE", 365, 86.39522191145726`}, {"CURE", 365, 10.251218056278097`}, {"CURE", 365, 43.41750667741619`},
{"CURE", 365, 50.77141171490898`}, {"CURE", 365, 21.587120937161004`}, {"CURE", 365, 201.16515219551584`},
{"CURE", 365, 40.33878121455308`}, {"CURE", 365, 41.17416056616093`}, {"CURE", 365, 42.062288574934264`},
{"CURE", 365, 168.45845194541175`}, {"CURE", 365, 53.521605462434195`}, {"CURE", 365, 49.93457689865633`},
{"CURE", 365, 108.68185204078416`}, {"CURE", 365, 38.222875628153986`}, {"CURE", 365, 33.30280761744852`},
{"CURE", 365, 41.152848204338454`}, {"CURE", 365, 56.84321332680055`}, {"CURE", 365, 100.44809015739108`},
{"CURE", 365, 21.67380285667062`}, {"CURE", 365, 19.78720657823027`}, {"CURE", 365, 42.341518073819174`},
{"CURE", 365, 43.10772926193316`}, {"CURE", 365, 48.49438125235776`}, {"CURE", 365, 51.8326493641157`},
{"CURE", 365, 28.96846378415541`}, {"CURE", 365, 14.571740967922176`}, {"CURE", 365, 54.7597158212694‌5`},
{"CURE", 365, 13.823827220179258`}, {"CURE", 365, 29.386999742844377`}, {"CURE", 365, 34.989499768896316`},
{"CURE", 365, 10.542742757133633`}, {"CURE", 365, 5.240530888922568`}, {"CURE", 365, 32.38009285420545`},
{"CURE", 365, 43.890924385636836`}, {"CURE", 365, 52.103899750368555`}, {"CURE", 365, 37.27076421696788`},
{"CURE", 365, 4.775815483699097`}, {"CURE", 365, 42.74805151284268`}, {"CURE", 365, 8.143873331776764`},
{"CURE", 365, 125.6407529485729`}, {"CURE", 365, 163.47760742069846`}, {"CURE", 365, 23.180472445728995`},
{"TBUR", 90.41909579932108`, 32.300640902079174`}, {"CURE", 365, 175.48974150371978`}, {"CURE", 365, 31.47060570548573`},
{"CURE", 365, 18.281796164907377`}, {"CURE", 365, 14.236058040697275`}, {"CURE", 365, 3.8920742270003985`},
{"CURE", 365, 24.535131122434986`}, {"CURE", 365, 27.358193458261624`}, {"CURE", 365, 72.50041428534166`},
{"CURE", 365, 34.69761572066733`}, {"CURE", 365, 60.41505581641439`}, {"CURE", 365, 21.34219742724948`},
{"CURE", 365, 46.316760314222755`}, {"CURE", 365, 103.51363941714202`}, {"CURE", 365, 10.527431507973276`},
{"CURE", 365, 16.23999625945733`}, {"CURE", 365, 24.12291442659684`}, {"CURE", 365, 33.69660320922548`},
{"CURE", 365, 22.103614827845174`}, {"CURE", 365, 21.150288066163792`}, {"CURE", 365, 8.963227174160034`},
{"CURE", 365, 13.833176779266429`}, {"CURE", 365, 26.22608389122782`}, {"CURE", 365, 59.68298074313614`},
{"CURE", 365, 53.10791799370584`}, {"CURE", 365, 27.197270746224575`}, {"TBUR", 42.84308030116525`, 40.79772302358207`},

{"CURE", 365, 87.8670725538096`}, {"CURE", 365, 42.97593423192149`}, {"CURE", 365, 32.15736285674944`},
{"CURE", 365, 20.48272850956407`}, {"CURE", 365, 6.417325399124397`}, {"CURE", 365, 35.701806749519086`},
{"CURE", 365, 41.20854109777197`}, {"CURE", 365, 161.38451880137814`}, {"CURE", 365, 8.226535801424975`},
{"CURE", 365, 8.036806823253512`}, {"CURE", 365, 25.751223392633`}, {"CURE", 365, 75.06483223460879`},
{"CURE", 365, 9.366793596630218`}, {"CURE", 365, 45.82225326110293`}, {"CURE", 365, 10.686577738935277`},
{"CURE", 365, 26.630685669092497`}, {"CURE", 365, 10.851348681900847`}, {"CURE", 365, 11.29372083595043`},
{"CURE", 365, 10.185646460936923`}, {"CURE", 365, 108.67691976364767`}, {"CURE", 365, 17.378319298075613`},
{"CURE", 365, 30.60863876717589`}, {"CURE", 365, 4.22212315526784`}, {"CURE", 365, 50.58530787353372`},
{"CURE", 365, 70.78086566904136`}, {"CURE", 365, 61.704300878892596`}, {"CURE", 365, 9.315934754683484`},
{"CURE", 365, 18.686084150018345`}, {"CURE", 365, 190.12733490659173`}, {"CURE", 365, 51.65892468425207`},
{"CURE", 365, 35.19589492794251`}, {"CURE", 365, 12.496326372744292`}, {"CURE", 365, 17.901473742336282`},
{"CURE", 365, 27.665713385352948`}, {"CURE", 365, 6.850165651037013`}, {"CURE", 365, 33.63192245668811`},
{"CURE", 365, 52.91935931085938`}, {"CURE", 365, 32.194544322911355`}, {"CURE", 365, 34.94860871861886`},
{"CURE", 365, 25.288799625777184`}, {"CURE", 365, 16.377561840771516`}, {"CURE", 365, 93.57877404569756`},
{"CURE", 365, 135.66563332917704`}, {"CURE", 365, 36.187519700753725`}, {"CURE", 365, 45.688285067225735`},
{"CURE", 365, 57.87937106367939`}, {"CURE", 365, 56.58877493531253`}, {"CURE", 365, 4.940879980356165`},
{"CURE", 365, 66.19689225995384`}, {"CURE", 365, 68.40901262197514`}, {"CURE", 365, 46.23566467705151`},
{"CURE", 365, 185.56643382314337`}, {"CURE", 365, 38.57820639966703`}, {"CURE", 365, 17.17288370071038`},
{"CURE", 365, 40.601093197338194`}, {"CURE", 365, 51.60086096480845`}, {"CURE", 365, 23.11613161332242`},
{"CURE", 365, 18.1428276652915`}, {"CURE", 365, 15.776734169906632`}, {"CURE", 365, 28.59556433721082`},
{"CURE", 365, 88.15209162720703`}, {"CURE", 365, 56.575808494810005`}, {"CURE", 365, 9.850335682954725`},
{"CURE", 365, 57.71600486039346`}, {"CURE", 365, 83.28026010605299`}, {"CURE", 365, 15.307448663858601`},
{"CURE", 365, 87.38978044390558`}, {"CURE", 365, 13.131016016053579`}, {"CURE", 365, 8.382853908535047`},
{"CURE", 365, 11.893451230746049`}, {"CURE", 365, 22.8931519562502`}, {"TBUR", 95.082157416211`, 102.47922969631877`},
{"CURE", 365, 36.62510223854361`}, {"CURE", 365, 46.24773408736768`}, {"CURE", 365, 11.055508340817674`},
{"CURE", 365, 210.99433836823604`}, {"CURE", 365, 96.64497738898889`}, {"CURE", 365, 16.49019359993779`},
{"CURE", 365, 40.37126547187494`}, {"CURE", 365, 22.49162408910053`}, {"CURE", 365, 22.721112878785927`},
{"CURE", 365, 76.78124682789037`}, {"TBUR", 64.79597842039179`, 97.07948268196658`}, {"CURE", 365, 10.429374475780262`},
{"CURE", 365, 20.325120712584827`}, {"CURE", 365, 7.210573493697722`}, {"CURE", 365, 27.80308677811565`},
{"CURE", 365, 47.663267500286935`}, {"CURE", 365, 77.19615533962725`}, {"CURE", 365, 43.08047491448946`},
{"CURE", 365, 155.1515679873091`}, {"CURE", 365, 74.15806943949387`}, {"TBUR", 79.15305381815051`, 12.237856312344922`},

{"CURE", 365, 27.12567726028179`}, {"TBUR", 46.8784016744189`, 29.736918359491913`}, {"CURE", 365, 60.61112972246623`},
{"CURE", 365, 7.951297008952307`}, {"CURE", 365, 21.28920418583412`}, {"CURE", 365, 11.995900688618805`},
{"CURE", 365, 29.920943604095058`}, {"CURE", 365, 68.36900456969134`}, {"CURE", 365, 29.08412914331881`},
{"CURE", 365, 6.317134332824813`}, {"CURE", 365, 120.3474286329465`}, {"CURE", 365, 22.698742525698222`},
{"CURE", 365, 7.709252140693608`}, {"CURE", 365, 10.134774344557488`}, {"CURE", 365, 8.555669387575177`},
{"CURE", 365, 10.929952518467767`}, {"CURE", 365, 87.95527826761145`}, {"CURE", 365, 48.15251210201867`},
{"CURE", 365, 28.981697854866177`}, {"CURE", 365, 13.58706717202279`}, {"CURE", 365, 22.678781656262807`},
{"CURE", 365, 13.121212810294761`}, {"CURE", 365, 31.664273990268054`}, {"CURE", 365, 108.88599076067048`},
{"CURE", 365, 63.49773858686226`}, {"CURE", 365, 31.424026527137393`}, {"CURE", 365, 18.101568551826453`},
{"CURE", 365, 19.03747283419862`}, {"TOX", 17.48492683278415`, 232.62133269141742`}, {"CURE", 365, 11.084418830240283`},
{"CURE", 365, 5.710485499445885`}, {"CURE", 365, 4.979563129815141`}, {"CURE", 365, 63.2468161089244`},
{"CURE", 365, 37.041243074258695`}, {"CURE", 365, 41.092300013340775`}, {"CURE", 365, 61.05070931997567`},
{"CURE", 365, 100.80546037601336`}, {"CURE", 365, 104.23979868177935`}, {"CURE", 365, 39.72670245120556`},
{"CURE", 365, 17.153120057065752`}, {"CURE", 365, 19.894342753990596`}, {"CURE", 365, 39.772192035719804`},
{"CURE", 365, 22.524719416969916`}, {"TBUR", 28.03099743879816`, 8.961320207150422`}, {"CURE", 365, 35.38086873733036`},
{"CURE", 365, 220.16050322755962`}, {"CURE", 365, 22.67964730109807`}, {"CURE", 365, 8.15844896583149`},
{"CURE", 365, 77.33720149446042`}, {"CURE", 365, 19.341824073035074`}, {"CURE", 365, 55.594219017991016`},
{"TOX", 7.1891248887326835`, 267.5861582393934`}, {"CURE", 365, 123.55805697113095`}, {"CURE", 365, 40.11151494109877`},
{"CURE", 365, 33.62802065208624`}, {"CURE", 365, 14.324109896333638`}, {"CURE", 365, 4.090251545514937`},
{"CURE", 365, 16.02531713526169`}, {"CURE", 365, 46.90543529023048`}, {"CURE", 365, 26.01341685975548`},
{"CURE", 365, 82.99463927605403`}, {"CURE", 365, 32.9024007356102`}, {"CURE", 365, 33.59076050938231`},
{"CURE", 365, 9.328046740993754`}, {"CURE", 365, 26.42286136272377`}, {"CURE", 365, 27.11363482286278`},
{"CURE", 365, 7.07049358503072`}, {"CURE", 365, 22.504208385324695`}, {"CURE", 365, 13.413756766670161`},
{"CURE", 365, 21.262479204680627`}, {"CURE", 365, 44.59228162010028`}, {"CURE", 365, 22.271476522206108`},
{"CURE", 365, 5.162342375812518`}, {"CURE", 365, 35.56679251069298`}, {"CURE", 365, 3.982855649538015`},
{"CURE", 365, 11.689519839390915`}, {"CURE", 365, 34.667385608930246`}, {"CURE", 365, 39.96125710237452`},
{"CURE", 365, 7.9523329776602845`}, {"CURE", 365, 23.404313862730387`}, {"CURE", 365, 30.332786784484107`},
{"CURE", 365, 153.17084559045134`}, {"CURE", 365, 35.17960738516859`}, {"CURE", 365, 9.925622244674285`},
{"CURE", 365, 108.17975869007789`}, {"CURE", 365, 10.641867705909295`}, {"CURE", 365, 40.04895186886365`},
{"CURE", 365, 41.41673385034562`}, {"CURE", 365, 12.362888635390753`}, {"CURE", 365, 13.766029814522062`},
{"CURE", 365, 60.97433409915222`}, {"CURE", 365, 5.958333566019331`}, {"CURE", 365, 51.65787740463597`},

{"CURE", 365, 13.680408242954796`}, {"CURE", 365, 17.888485913308685`}, {"CURE", 365, 3.9984509460206756`},
{"CURE", 365, 20.541677972399082`}, {"CURE", 365, 70.08167387282845`}, {"CURE", 365, 26.661042634625485`},
{"CURE", 365, 19.36079739304615`}, {"CURE", 365, 27.670338666800657`}, {"CURE", 365, 21.718479083129466`},
{"CURE", 365, 27.358545516069256`}, {"CURE", 365, 48.27871233440385`}, {"CURE", 365, 46.585287917219006`},
{"CURE", 365, 5.737053051107604`}, {"CURE", 365, 94.17929802325676`}, {"CURE", 365, 10.94931323961022`},
{"CURE", 365, 5.493208569638685`}, {"CURE", 365, 26.189247805170314`}, {"CURE", 365, 27.486410497389233`},
{"CURE", 365, 18.53405152798331`}, {"CURE", 365, 32.17300865396835`}, {"CURE", 365, 27.739761069025374`},
{"CURE", 365, 44.58757018201894`}, {"CURE", 365, 37.679973581252575`}, {"CURE", 365, 53.81076523465456`},
{"CURE", 365, 8.503209172086317`}, {"CURE", 365, 108.9898229786985`}, {"CURE", 365, 105.75963970519317`},
{"CURE", 365, 5.8596848144502305`}, {"CURE", 365, 14.748997458090637`}, {"CURE", 365, 36.59537824349185`},
{"CURE", 365, 18.61090286975248`}, {"CURE", 365, 57.20823755131299`}, {"CURE", 365, 114.57703091381788`},
{"CURE", 365, 36.21137912143188`}, {"CURE", 365, 6.216139789132536`}, {"CURE", 365, 6.0974592712817355`},
{"CURE", 365, 14.316198682797195`}, {"CURE", 365, 4.322193383073727`}, {"CURE", 365, 49.055680479682714`},
{"CURE", 365, 7.605534916045706`}, {"CURE", 365, 51.14509612252975`}, {"CURE", 365, 31.508268787369012`},
{"CURE", 365, 54.303474841670386`}, {"CURE", 365, 13.370307228418232`}, {"CURE", 365, 67.45465950219631`},
{"CURE", 365, 223.17677807030017`}, {"CURE", 365, 80.39917320742383`}, {"CURE", 365, 130.24493261101688`},
{"CURE", 365, 38.33200179388828`}, {"CURE", 365, 91.32511000222443`}, {"CURE", 365, 91.50718822659499`},
{"CURE", 365, 11.106071586616531`}, {"CURE", 365, 7.768581322737759`}, {"CURE", 365, 25.26251217879704`},
{"CURE", 365, 52.41742885808344`}, {"CURE", 365, 65.58220928297065`}, {"CURE", 365, 28.082536791250085`},
{"CURE", 365, 41.505747899496136`}, {"CURE", 365, 7.818599817199064`}, {"CURE", 365, 9.93493927764212`},
{"CURE", 365, 48.26703455110316`}, {"CURE", 365, 15.646714275078299`}, {"CURE", 365, 6.639951277041929`},
{"CURE", 365, 9.821033532609057`}, {"CURE", 365, 4.8011117858831245`}, {"CURE", 365, 39.01455818729943 6`},
{"CURE", 365, 53.65377435458408`}, {"CURE", 365, 37.53716080116957`}, {"CURE", 365, 42.65170347332029`},
{"CURE", 365, 23.62311006900119`}, {"CURE", 365, 21.149727296170475`}, {"CURE", 365, 54.7327331185697`},
{"CURE", 365, 58.57616072016911`}, {"CURE", 365, 6.53608003295295`}, {"CURE", 365, 24.502127004618206`},
{"CURE", 365, 12.988480080498523`}, {"CURE", 365, 20.39506228147349`}, {"CURE", 365, 167.99810608346374`},
{"CURE", 365, 29.665100240813178`}, {"CURE", 365, 20.3613954963471`}, {"CURE", 365, 21.534779805115463`},
{"CURE", 365, 42.95939443136879`}, {"CURE", 365, 32.70031978284833`}, {"CURE", 365, 42.65819187455391`},
{"CURE", 365, 43.96883635526502`}, {"CURE", 365, 9.977458194229836`}, {"CURE", 365, 97.0305873768489`},
{"CURE", 365, 18.63440829513189`}, {"CURE", 365, 39.52048368814862`}, {"CURE", 365, 22.426358825289945`},
{"CURE", 365, 12.734857030618292`}, {"CURE", 365, 46.42509692513745`}, {"CURE", 365, 47.13121252332725`},

{"TBUR", 80.04284150915413`, 26.134300491296006`}, {"CURE", 365, 61.78708680203187`}, {"CURE", 365, 13.070052230843213`},
{"CURE", 365, 109.22708541025455`}, {"CURE", 365, 41.947433913525565`}, {"CURE", 365, 88.68419021708908`},
{"CURE", 365, 3.44009625205081`}, {"CURE", 365, 43.62880894871208`}, {"CURE", 365, 49.71284557107178`},
{"CURE", 365, 11.822195076265118`}, {"CURE", 365, 30.015441812767502`}, {"CURE", 365, 44.94894095689577`},
{"CURE", 365, 27.320089099417537`}, {"CURE", 365, 8.692833436055134`}, {"CURE", 365, 6.597329853824806`},
{"CURE", 365, 39.00935448942694`}, {"CURE", 365, 10.40752695895295`}, {"CURE", 365, 23.13547678575989`},
{"CURE", 365, 28.196509311968153`}, {"CURE", 365, 13.1238099877741`}, {"CURE", 365, 24.15464211023745`},
{"CURE", 365, 6.579537316789629`}, {"CURE", 365, 27.125648107655152`}, {"CURE", 365, 4.385397083036699`},
{"CURE", 365, 7.840288657814234`}, {"CURE", 365, 18.534021185716785`}, {"CURE", 365, 5.977734656868834`},
{"CURE", 365, 44.42126849433267`}, {"CURE", 365, 65.25806262016788`}, {"CURE", 365, 66.23073354289038`},
{"CURE", 365, 55.15246209858167`}, {"CURE", 365, 104.10397625812038`}, {"CURE", 365, 8.421019475766167`},
{"CURE", 365, 37.555377443874825`}, {"CURE", 365, 40.32432730600616`}, {"CURE", 365, 69.34210695867239`},
{"CURE", 365, 51.793306753856754`}, {"CURE", 365, 23.669102268503682`}, {"CURE", 365, 111.29030545907648`},
{"CURE", 365, 3.474699478990019`}, {"CURE", 365, 105.90088684631336`}, {"CURE", 365, 7.1712442497320446`},
{"CURE", 365, 90.44884568147272`}, {"CURE", 365, 81.9151545693164`}, {"CURE", 365, 24.280604744767707`},
{"CURE", 365, 95.60818206274256`}, {"CURE", 365, 116.62540307399699`}, {"CURE", 365, 52.1623122585909`},
{"CURE", 365, 23.017923776810317`}, {"CURE", 365, 26.463746244724497`}, {"CURE", 365, 39.08100263289629`},
{"CURE", 365, 36.04068777263032`}, {"CURE", 365, 8.734425037869954`}, {"CURE", 365, 31.358862020964192`},
{"CURE", 365, 29.66742564277615`}, {"CURE", 365, 22.190798252075194`}, {"CURE", 365, 54.82910024426008`},
{"CURE", 365, 8.633814230693536`}, {"CURE", 365, 61.199759370453`}, {"CURE", 365, 40.047521832482005`},
{"CURE", 365, 22.67482576180063`}, {"CURE", 365, 20.588451200035564`}, {"CURE", 365, 41.831096961441354`},
{"CURE", 365, 5.730298625194352`}, {"CURE", 365, 66.3723586338058`}, {"CURE", 365, 10.477859155858976`},
{"CURE", 365, 14.862551369493973`}, {"CURE", 365, 33.34129973666896`}, {"CURE", 365, 26.14026078411328`},
{"TBUR", 365, 24.037754782231165`}, {"CURE", 365, 74.83553888032341`}, {"CURE", 365, 10.123786243538644`},
{"CURE", 365, 17.625428589579457`}, {"CURE", 365, 130.40317166112303`}, {"CURE", 365, 9.894447693646521`},
{"CURE", 365, 25.479767777250817`}, {"CURE", 365, 38.76956805274417`}, {"CURE", 365, 38.28016676643475`},
{"CURE", 365, 25.992626412219437`}, {"CURE", 365, 134.36743929353457`}, {"CURE", 365, 24.26987541862749`},
{"CURE", 365, 10.213229973266852`}, {"CURE", 365, 20.379717968506906`}, {"CURE", 365, 15.739779514228191`},
{"CURE", 365, 12.06233531257049`}, {"CURE", 365, 31.633621284942258`}, {"CURE", 365, 54.17753336258672`},
{"CURE", 365, 29.807668301838035`}, {"CURE", 365, 34.752279624946055`}, {"CURE", 365, 76.09258004398121`},
{"CURE", 365, 8.390659492077214`}, {"CURE", 365, 9.87348897453165`}, {"CURE", 365, 83.30235887655529`},

{"CURE", 365, 26.82235696083383`}, {"CURE", 365, 29.04640347195499`}, {"CURE", 365, 14.601134085007002`},
{"CURE", 365, 33.43456270860353`}, {"CURE", 365, 41.66751065067344`}, {"CURE", 365, 96.57235761008157`},
{"CURE", 365, 14.234292625975362`}, {"CURE", 365, 55.624647878572276`}, {"CURE", 365, 32.7214729293608`},
{"CURE", 365, 86.63774805117275`}, {"CURE", 365, 141.50114696107673`}, {"CURE", 365, 51.7990903734218`},
{"CURE", 365, 5.0166118773058095`}, {"CURE", 365, 39.26061258840539`}, {"CURE", 365, 12.400196931046258`},
{"CURE", 365, 131.1030505588579`}, {"TBUR", 48.3937855800099`, 4.153781479363103`}, {"CURE", 365, 4.375837334414813`},
{"CURE", 365, 151.76721938809516`}, {"CURE", 365, 34.91465815613697`}, {"CURE", 365, 11.810183508748798`},
{"CURE", 365, 22.60429431389518`}, {"CURE", 365, 7.9307944914513575`}, {"TBUR", 76.25684497482152`, 7.542398698539663`},
{"CURE", 365, 9.017756283487955`}, {"CURE", 365, 32.59609051550436`}, {"CURE", 365, 69.08680270865685`},
{"CURE", 365, 27.363838659822154`}, {"CURE", 365, 6.852963343640367`}, {"CURE", 365, 93.2107479508283 8`},
{"CURE", 365, 186.26453084595644`}, {"CURE", 365, 5.25355631061008`}, {"CURE", 365, 41.989372072285605`},
{"CURE", 365, 22.554278922219908`}, {"CURE", 365, 4.435373489311176`}, {"CURE", 365, 34.67427192286861`},
{"CURE", 365, 22.153821238773755`}, {"CURE", 365, 17.00658604600838`}, {"CURE", 365, 33.072720744948576`},
{"CURE", 365, 18.77346460281679`}, {"CURE", 365, 55.22772658047203`}, {"CURE", 365, 32.81430246767759`},
{"CURE", 365, 77.26587336664497`}, {"CURE", 365, 34.22674425152203`}, {"CURE", 365, 16.95866367167444`},
{"CURE", 365, 8.247183877328377`}, {"CURE", 365, 5.720342256679218`}, {"CURE", 365, 16.293598972955557`},
{"CURE", 365, 64.84018249573943`}, {"CURE", 365, 21.384056992443384`}, {"CURE", 365, 19.59402809458856`},
{"CURE", 365, 13.85495566092533`}, {"CURE", 365, 14.042486037141517`}, {"CURE", 365, 37.94599624015939`},
{"CURE", 365, 7.09937639313547`}, {"CURE", 365, 45.22893947293864`}, {"CURE", 365, 20.692420812327487`},
{"CURE", 365, 12.164201283109193`}, {"CURE", 365, 19.274283648486985`}, {"CURE", 365, 42.762864587532285`},
{"CURE", 365, 31.86605552330795`}, {"CURE", 365, 3.844759521357522`}, {"CURE", 365, 16.56561822832058`},
{"TOX", 6.312890324183046`, 276.89247288833553`}, {"CURE", 365, 45.99613454843577`}, {"CURE", 365, 67.08832822962866`},
{"CURE", 365, 19.767518189614815`}, {"CURE", 365, 73.28736776907535`}, {"CURE", 365, 8.768761918399012`},
{"CURE", 365, 29.54837317116669`}, {"CURE", 365, 136.3900127073669`}, {"CURE", 365, 25.54065704458941`},
{"CURE", 365, 22.492483063528216`}, {"CURE", 365, 38.558450863418926`}, {"CURE", 365, 20.41176940964585`},
{"CURE", 365, 4.529457364613968`}, {"CURE", 365, 42.09843760968876`}, {"CURE", 365, 7.511276455378164`},
{"CURE", 365, 17.849456583234538`}, {"CURE", 365, 42.1653547683919`}, {"CURE", 365, 15.143399663791861`},
{"CURE", 365, 80.42781888124769`}, {"CURE", 365, 23.915074349365277`}, {"CURE", 365, 41.1674591942243`},
{"CURE", 365, 28.535103893424377`}, {"CURE", 365, 8.074969640798276`}, {"CURE", 365, 29.962450688551346`},
{"CURE", 365, 64.74407672595531`}, {"CURE", 365, 21.700758166514508`}, {"CURE", 365, 16.331145883013658`},
{"CURE", 365, 7.74716136518427 5`}, {"CURE", 365, 24.63191486939873`}, {"CURE", 365, 29.48512056480917`},

{"CURE", 365, 84.1390943255354`}, {"CURE", 365, 13.516257195513063`}, {"CURE", 365, 20.34375414611559`},
{"CURE", 365, 78.97894232723503`}, {"CURE", 365, 8.321258330552928`}, {"CURE", 365, 114.232277085923`},
{"CURE", 365, 9.655060141963775`}, {"CURE", 365, 17.67579855408332`}, {"CURE", 365, 40.742850533800386`},
{"CURE", 365, 65.59542132407275`}, {"CURE", 365, 29.51584343690201`}, {"CURE", 365, 15.469988396493083`},
{"CURE", 365, 4.547641832436976`}, {"CURE", 365, 39.809357658917314`}, {"CURE", 365, 36.07475332525858`},
{"CURE", 365, 35.45350512225883`}, {"CURE", 365, 13.979095550097096`}, {"CURE", 365, 34.26867634391632`},
{"CURE", 365, 3.964099092536069`}, {"CURE", 365, 22.84666645208679`}, {"CURE", 365, 9.978909907486019`},
{"CURE", 365, 4.4195801315891785`}, {"CURE", 365, 22.969672809793312`}, {"CURE", 365, 4.915087048624137`},
{"CURE", 365, 15.628735410661887`}, {"CURE", 365, 10.718666404623809`}, {"TBUR", 46.30468885976529`, 6.528138433858012`},
{"CURE", 365, 21.230517832502102`}, {"CURE", 365, 56.56757362888904`}, {"CURE", 365, 30.97180605336894`},
{"CURE", 365, 28.04515069218989`}, {"CURE", 365, 44.56764704287106`}, {"CURE", 365, 37.832776830876625`}},
{{"2300 Res", "2300 OS", "2300 Tox"}, {"CURE", 365, 19.1656629613258`}, {"CURE", 365, 39.64722502716651`},
{"CURE", 365, 27.793002617711814`}, {"TBUR", 73.50281604640418`, 9.32439303303108`},
{"TBUR", 51.90502799172881`, 35.63770472866631`}, {"CURE", 365, 71.8176590512792`}, {"CURE", 365, 67.57069714254976`},
{"CURE", 365, 7.060595560366115`}, {"CURE", 365, 48.73186662206401`}, {"CURE", 365, 42.15482317248501`},
{"CURE", 365, 158.21085408126362`}, {"CURE", 365, 19.63004260069019`}, {"CURE", 365, 70.68730322679501`},
{"TOX", 11.537516800626404`, 235.86435208712226`}, {"CURE", 365, 33.37838986578214`}, {"CURE", 365, 52.098840432008046`},
{"CURE", 365, 103.15158626476158`}, {"CURE", 365, 46.21246015345424`}, {"TBUR", 135.86223180609306`, 34.97842605935983`},
{"CURE", 365, 11.582477769093588`}, {"CURE", 365, 71.59261592144799`}, {"CURE", 365, 29.020209792265998`},
{"CURE", 365, 11.120772163950756`}, {"CURE", 365, 8.86099129147268`}, {"CURE", 365, 40.31504406541055`},
{"CURE", 365, 44.24891891988464`}, {"CURE", 365, 4.022831186117945`}, {"CURE", 365, 6.097692709071474`},
{"CURE", 365, 22.595485419973873`}, {"CURE", 365, 26.433641297187204`}, {"CURE", 365, 22.548214039511105`},
{"CURE", 365, 79.29552455131515`}, {"CURE", 365, 17.435012864949027`}, {"CURE", 365, 13.869688449008637`},
{"CURE", 365, 112.387559574128`}, {"CURE", 365, 6.887181581475877`}, {"CURE", 365, 29.76111751993181`},
{"TBUR", 49.28575363707901`, 66.43443981468346`}, {"CURE", 365, 16.544660904535178`}, {"CURE", 365, 25.453821068812037`},
{"CURE", 365, 58.087758668585046`}, {"CURE", 365, 39.18462593209455`}, {"CURE", 365, 7.9507809571363905`},
{"CURE", 365, 26.819205245075455`}, {"CURE", 365, 38.69372994148653`}, {"CURE", 365, 25.371266202459577`},
{"TOX", 6.416550756974049`, 269.60240212211966`}, {"CURE", 365, 58.11771028930585`}, {"CURE", 365, 89.5572622352784`},
{"CURE", 365, 10.87016278969623`}, {"CURE", 365, 30.477121309056457`}, {"CURE", 365, 41.76379492943352`},
{"CURE", 365, 33.44213728770199`}, {"CURE", 365, 21.70791463701801`}, {"CURE", 365, 9.354466988614542`},
{"CURE", 365, 98.93262192211576`}, {"CURE", 365, 31.088577464521254`}, {"CURE", 365, 5.90721266841826`},

{"CURE", 365, 63.6481529378338`}, {"CURE", 365, 44.578303161012904`}, {"CURE", 365, 30.530140786369415`},
{"CURE", 365, 23.358669656012083`}, {"CURE", 365, 13.277191952588705`}, {"CURE", 365, 50.34341394544866`},
{"CURE", 365, 35.771959447230905`}, {"CURE", 365, 40.89910248767413`}, {"CURE", 365, 9.361682238991849`},
{"CURE", 365, 9.515759376531122`}, {"CURE", 365, 10.121607639471069`}, {"CURE", 365, 41.33852698962866`},
{"CURE", 365, 15.133892031993048`}, {"CURE", 365, 33.71097121607076`}, {"CURE", 365, 63.259311253221746`},
{"TBUR", 27.9363418632438`, 19.833457527915474`}, {"CURE", 365, 16.906966350674377`}, {"CURE", 365, 46.82420257867046`},
{"CURE", 365, 27.38705889689063`}, {"CURE", 365, 20.82895888271258`}, {"CURE", 365, 82.99104223340092`},
{"CURE", 365, 91.12063655770511`}, {"CURE", 365, 67.08750837788665`}, {"CURE", 365, 18.099787728591547`},
{"CURE", 365, 36.5955042587808`}, {"CURE", 365, 25.57946723375978`}, {"CURE", 365, 28.925959530068805`},
{"CURE", 365, 13.55594506558776`}, {"CURE", 365, 144.6109096582529`}, {"CURE", 365, 31.51743160289602 5`},
{"CURE", 365, 39.68947616979207`}, {"CURE", 365, 34.160931217294014`}, {"CURE", 365, 28.24754100003604`},
{"CURE", 365, 8.372162091351484`}, {"CURE", 365, 15.343407735335505`}, {"CURE", 365, 35.52134303300407`},
{"TBUR", 59.743012570980234`, 12.881248001690992`}, {"CURE", 365, 15.296831835077894`}, {"CURE", 365, 39.88196654829867`},
{"CURE", 365, 101.21586333327018`}, {"CURE", 365, 54.22887380591978`}, {"CURE", 365, 27.19858177712721`},
{"TBUR", 83.49500600571554`, 53.92418199547387`}, {"CURE", 365, 35.06407574420582`}, {"CURE", 365, 22.721752162432168`},
{"CURE", 365, 155.5784444454572`}, {"CURE", 365, 17.28164170619571`}, {"CURE", 365, 11.742073702581175`},
{"CURE", 365, 68.9266841905845`}, {"CURE", 365, 20.343638815434677`}, {"CURE", 365, 20.793330206922192`},
{"CURE", 365, 125.10533830112972`}, {"CURE", 365, 10.15454553911298`}, {"CURE", 365, 6.442743515976498`},
{"CURE", 365, 26.798986769559335`}, {"CURE", 365, 21.188397328829453`}, {"CURE", 365, 15.958046322012638`},
{"CURE", 365, 40.730496765836435`}, {"CURE", 365, 11.464339377762718`}, {"CURE", 365, 7.048968811319625`},
{"CURE", 365, 32.99603435470214`}, {"CURE", 365, 50.46431116565615`}, {"CURE", 365, 8.594746607819465`},
{"CURE", 365, 28.44564067970998`}, {"CURE", 365, 86.34318462712669`}, {"TBUR", 72.11761617603383`, 32.91454408216192`},
{"CURE", 365, 19.154436716772718`}, {"CURE", 365, 66.83298595639633`}, {"CURE", 365, 33.587006962531454`},
{"CURE", 365, 4.427146060161151`}, {"CURE", 365, 38.345827283858995`}, {"CURE", 365, 29.615315438929823`},
{"CURE", 365, 23.59265810901935`}, {"CURE", 365, 27.985649958344027`}, {"CURE", 365, 7.286099980521129`},
{"CURE", 365, 41.56612670812372`}, {"CURE", 365, 14.921685569628984`}, {"CURE", 365, 33.71121829399228`},
{"CURE", 365, 125.97432557926496`}, {"CURE", 365, 44.513777935493835`}, {"CURE", 365, 103.4135661317486`},
{"CURE", 365, 34.068297236450775`}, {"CURE", 365, 140.7500233872977`}, {"CURE", 365, 4.389464420826453`},
{"CURE", 365, 38.552038444757294`}, {"CURE", 365, 43.945505162844434`}, {"CURE", 365, 5.707717433009095`},
{"CURE", 365, 7.907580498135072`}, {"CURE", 365, 48.53641954333423`}, {"CURE", 365, 57.20104881576971`},
{"CURE", 365, 48.74412407560256`}, {"CURE", 365, 39.88451313935731`}, {"CURE", 365, 38.12749962216826 6`},

{"CURE", 365, 23.087874667028597`}, {"CURE", 365, 6.106049182679988`}, {"CURE", 365, 26.562061405694156`},
{"CURE", 365, 12.604311388155027`}, {"CURE", 365, 6.170275874239743`}, {"CURE", 365, 37.39563462922754`},
{"CURE", 365, 20.92298466046751`}, {"CURE", 365, 55.309275214435345`}, {"CURE", 365, 22.329364525100374`},
{"CURE", 365, 18.584943320649263`}, {"CURE", 365, 124.64136854483696`}, {"CURE", 365, 9.287045402549369`},
{"CURE", 365, 27.786035262295584`}, {"CURE", 365, 15.746782573303038`}, {"CURE", 365, 37.20251251756111`},
{"CURE", 365, 10.225684726923031`}, {"CURE", 365, 40.826615539602024`}, {"CURE", 365, 107.85368706518416`},
{"CURE", 365, 9.225781682855617`}, {"CURE", 365, 28.01884225820975`}, {"CURE", 365, 30.26606756494914`},
{"CURE", 365, 108.95440998398011`}, {"CURE", 365, 40.982774826138986`}, {"CURE", 365, 34.04427765317942`},
{"CURE", 365, 59.74177980098167`}, {"CURE", 365, 22.91893169738482`}, {"CURE", 365, 70.77295315259249`},
{"CURE", 365, 44.10885924170271`}, {"CURE", 365, 26.930219434662845`}, {"CURE", 365, 24.33112366898943`},
{"CURE", 365, 7.913852684007248`}, {"CURE", 365, 5.362562998866757`}, {"CURE", 365, 12.569235947373182`},
{"CURE", 365, 16.836752201081122`}, {"CURE", 365, 7.415747613262247`}, {"CURE", 365, 27.146278218932785`},
{"CURE", 365, 74.10719339544225`}, {"CURE", 365, 20.034611013702943`}, {"CURE", 365, 37.1641489155807`},
{"CURE", 365, 46.56973871867749`}, {"CURE", 365, 39.726318594680386`}, {"CURE", 365, 27.25623029592633`},
{"CURE", 365, 10.826515623728255`}, {"CURE", 365, 74.59103004585911`}, {"CURE", 365, 6.453451779838999`},
{"CURE", 365, 69.53323922606057`}, {"CURE", 365, 67.55753950550192`}, {"CURE", 365, 18.858391481591543`},
{"CURE", 365, 22.39986104547211`}, {"CURE", 365, 128.7818331630033`}, {"CURE", 365, 33.199827228262464`},
{"CURE", 365, 39.62444406660918`}, {"CURE", 365, 24.815105212571975`}, {"CURE", 365, 9.33229371700498`},
{"CURE", 365, 57.22142416343605`}, {"TBUR", 61.50856910416345`, 152.09472437391298`}, {"CURE", 365, 23.23696304255323`},
{"CURE", 365, 21.009983349785053`}, {"CURE", 365, 38.70811379723694`}, {"CURE", 365, 15.456251989191824`},
{"CURE", 365, 196.1410089127191`}, {"CURE", 365, 15.700034157335251`}, {"CURE", 365, 97.12099694759908`},
{"CURE", 365, 74.57743522366735`}, {"CURE", 365, 72.530186064012`}, {"CURE", 365, 38.74166309585558`},
{"CURE", 365, 89.81041031748245`}, {"CURE", 365, 203.5670173301667`}, {"CURE", 365, 40.803989954600354`},
{"CURE", 365, 7.496423365561246`}, {"CURE", 365, 66.58096852542154`}, {"CURE", 365, 41.48795291766026`},
{"CURE", 365, 39.996983453866605`}, {"CURE", 365, 8.81969627448353`}, {"CURE", 365, 202.1545145684815`},
{"CURE", 365, 14.778397678365277`}, {"CURE", 365, 64.10855911841104`}, {"CURE", 365, 40.41547154151375`},
{"CURE", 365, 28.720293965756884`}, {"CURE", 365, 6.9040727421247485`}, {"CURE", 365, 51.894606870836206`},
{"CURE", 365, 30.444408125418164`}, {"CURE", 365, 80.54275183598641`}, {"CURE", 365, 53.06769574455288`},
{"CURE", 365, 66.9964926542544`}, {"CURE", 365, 32.17260512581218`}, {"CURE", 365, 28.622130423836342`},
{"CURE", 365, 149.55778680599988`}, {"CURE", 365, 39.16438410007913`}, {"CURE", 365, 10.449915175285486`},
{"CURE", 365, 35.19204065986673`}, {"CURE", 365, 35.78600656071235`}, {"CURE", 365, 4.801767788357176`},

{"CURE", 365, 8.5526075512413`}, {"CURE", 365, 13.067244846434003`}, {"CURE", 365, 123.41890809649436`},
{"CURE", 365, 37.943259731885476`}, {"CURE", 365, 39.54139972647221`}, {"CURE", 365, 31.67048449241845`},
{"CURE", 365, 130.26149922434664`}, {"CURE", 365, 29.983973122895236`}, {"CURE", 365, 60.378731518958865`},
{"CURE", 365, 29.90215559334774`}, {"CURE", 365, 44.080736916568306`}, {"CURE", 365, 10.600840797605233`},
{"CURE", 365, 39.64882693538946`}, {"CURE", 365, 24.74259247800242`}, {"CURE", 365, 48.19103735111714`},
{"CURE", 365, 76.16179210214389`}, {"CURE", 365, 85.74640240553921`}, {"CURE", 365, 52.11664943788952`},
{"CURE", 365, 42.463801322781876`}, {"CURE", 365, 58.6305888276176`}, {"CURE", 365, 27.80620939088812 4`},
{"CURE", 365, 31.480603805775647`}, {"CURE", 365, 22.307177031015396`}, {"CURE", 365, 17.479678222625626`},
{"CURE", 365, 28.156341199890633`}, {"CURE", 365, 72.24486127901467`}, {"CURE", 365, 8.28061828916898`},
{"CURE", 365, 36.69371841559924`}, {"CURE", 365, 6.928399782770558`}, {"CURE", 365, 36.06315206581071`},
{"CURE", 365, 20.494783801253835`}, {"CURE", 365, 76.259485024798`}, {"CURE", 365, 7.031096314054189`},
{"CURE", 365, 50.211308705836345`}, {"CURE", 365, 62.97614358732373`}, {"CURE", 365, 10.30198901356518`},
{"CURE", 365, 23.60521678657449`}, {"CURE", 365, 18.992335152746122`}, {"CURE", 365, 10.869600287538177`},
{"CURE", 365, 64.23269413468769`}, {"CURE", 365, 24.142539783304166`}, {"CURE", 365, 46.521710960059934`},
{"CURE", 365, 45.23815682603733`}, {"CURE", 365, 51.291307427767364`}, {"CURE", 365, 40.101554281441736`},
{"CURE", 365, 48.009974041092526`}, {"CURE", 365, 37.997756479272894`}, {"CURE", 365, 73.46438909211614`},
{"CURE", 365, 44.746171050587265`}, {"CURE", 365, 36.545575937424`}, {"CURE", 365, 93.47035954876162`},
{"CURE", 365, 10.82197413693379`}, {"CURE", 365, 61.38626353555728`}, {"CURE", 365, 4.509451950020141`},
{"CURE", 365, 56.021633932496634`}, {"CURE", 365, 13.202113087601889`}, {"CURE", 365, 69.67090153282477`},
{"CURE", 365, 10.780659035133608`}, {"CURE", 365, 70.62002592076269`}, {"CURE", 365, 176.76098215120356`},
{"CURE", 365, 7.715839943686667`}, {"CURE", 365, 5.541250526972897`}, {"CURE", 365, 20.40115813843888`},
{"CURE", 365, 4.955100879743299`}, {"CURE", 365, 3.3873026931488477`}, {"CURE", 365, 19.816557417607434`},
{"CURE", 365, 32.40696434839022`}, {"CURE", 365, 34.86396735820231`}, {"CURE", 365, 33.1439790404377`},
{"CURE", 365, 25.81175895257025`}, {"CURE", 365, 45.40556568136928`}, {"CURE", 365, 79.93742266681039`},
{"CURE", 365, 6.468849221848062`}, {"CURE", 365, 35.440154300544265`}, {"CURE", 365, 30.455888352934846`},
{"CURE", 365, 22.08209267642103`}, {"CURE", 365, 40.83943531722763`}, {"CURE", 365, 60.54372902977434`},
{"CURE", 365, 141.66361424954428`}, {"CURE", 365, 105.5572375364904`}, {"CURE", 365, 32.649444303324515`},
{"CURE", 365, 3.4667572032986493`}, {"CURE", 365, 37.81754821391022`}, {"CURE", 365, 97.52018643732859`},
{"CURE", 365, 24.53809896301758`}, {"CURE", 365, 31.129214568723842`}, {"CURE", 365, 18.93233065624479`},
{"CURE", 365, 27.750095359706663`}, {"CURE", 365, 7.187056523537966`}, {"CURE", 365, 35.1622877240325`},
{"CURE", 365, 19.758548497216275`}, {"CURE", 365, 57.72757614260976`}, {"CURE", 365, 39.53993021783212`},

{"CURE", 365, 38.337847093313805`}, {"CURE", 365, 31.544924009316567`}, {"CURE", 365, 16.693429857661712`},
{"CURE", 365, 11.504021060299763`}, {"CURE", 365, 11.216304103982543`}, {"CURE", 365, 14.528640224657913`},
{"CURE", 365, 11.3218174642042`}, {"CURE", 365, 152.25590758606444`}, {"CURE", 365, 84.69026870875679`},
{"CURE", 365, 16.364226086999498`}, {"TOX", 8.744612307319342`, 252.06703062967853`}, {"CURE", 365, 72.08399633863786`},
{"CURE", 365, 38.165857718830594`}, {"CURE", 365, 66.02129600987364`}, {"CURE", 365, 9.926430383624211`},
{"CURE", 365, 79.15217284202993`}, {"CURE", 365, 211.62172911209294`}, {"CURE", 365, 11.482304590218508`},
{"CURE", 365, 35.81830156345046`}, {"CURE", 365, 58.66559959994495`}, {"CURE", 365, 29.648826809290473`},
{"CURE", 365, 14.534240202664277`}, {"CURE", 365, 11.579167371051032`}, {"CURE", 365, 100.50265164392987`},
{"CURE", 365, 52.76314699031484`}, {"CURE", 365, 30.018710878100546`}, {"CURE", 365, 74.60233366605055`},
{"CURE", 365, 13.469499899246397`}, {"CURE", 365, 35.988923049914064`}, {"CURE", 365, 21.734757353697294`},
{"CURE", 365, 146.58794689354653`}, {"TBUR", 75.19588065154592`, 36.88874154565616`}, {"CURE", 365, 171.22279822409487`},
{"TOX", 7.372066668306286`, 267.26092319748136`}, {"CURE", 365, 20.45850496491834`}, {"CURE", 365, 5.769418668748494`},
{"CURE", 365, 5.026388554717434`}, {"CURE", 365, 36.76928454626436`}, {"CURE", 365, 142.65603299432374`},
{"CURE", 365, 28.577109164757417`}, {"CURE", 365, 21.954759445806534`}, {"CURE", 365, 41.26530675622341`},
{"CURE", 365, 45.19581010916953`}, {"CURE", 365, 188.40081359254233`}, {"CURE", 365, 155.34358497708786`},
{"CURE", 365, 32.53595913578763`}, {"CURE", 365, 10.796581824231502`}, {"CURE", 365, 26.450436433079144`},
{"CURE", 365, 4.151228120635965`}, {"CURE", 365, 78.06522327266916`}, {"CURE", 365, 116.10603597533296`},
{"CURE", 365, 37.97305149932471`}, {"CURE", 365, 30.094026717267965`}, {"CURE", 365, 127.25392984817772`},
{"CURE", 365, 8.072035527119088`}, {"CURE", 365, 10.513046033935979`}, {"CURE", 365, 32.90742197617607`},
{"CURE", 365, 42.33348028139501`}, {"TBUR", 87.96347071367656`, 80.20145538477378`}, {"CURE", 365, 82.56839086701432`},
{"CURE", 365, 33.893538313468945`}, {"CURE", 365, 34.96358512271478`}, {"CURE", 365, 43.78070793568646`},
{"CURE", 365, 15.155739751938764`}, {"CURE", 365, 4.28105885575452`}, {"CURE", 365, 45.0307656949346`},
{"CURE", 365, 54.220625085405885`}, {"CURE", 365, 26.36216513087084`}, {"CURE", 365, 47.774482213280955`},
{"CURE", 365, 14.881003704545865`}, {"CURE", 365, 9.778887067407302`}, {"CURE", 365, 42.80840619289133`},
{"CURE", 365, 26.01492801420209`}, {"CURE", 365, 37.193377720644506`}, {"TOX", 13.015796796956435`, 245.4531946345628`},
{"CURE", 365, 23.385042240916846`}, {"CURE", 365, 11.405621732550102`}, {"CURE", 365, 8.851666508724382`},
{"CURE", 365, 10.728413307908243`}, {"CURE", 365, 61.28792621337054`}, {"CURE", 365, 4.220916792949896`},
{"CURE", 365, 57.068506077900615`}, {"CURE", 365, 62.303975124676576`}, {"CURE", 365, 48.62933618666542`},
{"CURE", 365, 39.8680695396056`}, {"CURE", 365, 7.905418860603161`}, {"CURE", 365, 86.77458988289366`},
{"TBUR", 73.99879966222485`, 26.277534494309844`}, {"CURE", 365, 11.88719034545588`}, {"CURE", 365, 77.27512432320881`},
{"CURE", 365, 13.744488372974674`}, {"CURE", 365, 92.58443279774984`}, {"CURE", 365, 59.41568643988936`},

{"CURE", 365, 39.62780574595003`}, {"CURE", 365, 57.577574721384785`}, {"CURE", 365, 20.413570336635967`},
{"CURE", 365, 90.32592716779361`}, {"CURE", 365, 10.717357648604542`}, {"CURE", 365, 45.40439842706087`},
{"CURE", 365, 53.07986312873103`}, {"CURE", 365, 22.568740002538032`}, {"CURE", 365, 210.3141215161497`},
{"CURE", 365, 42.19430710123771`}, {"CURE", 365, 43.04639819950321`}, {"CURE", 365, 43.976998223211005`},
{"CURE", 365, 176.1178566892968 4`}, {"CURE", 365, 55.95709221187889`}, {"CURE", 365, 52.20649151984988`},
{"CURE", 365, 113.63321833942754`}, {"CURE", 365, 39.962006783243694`}, {"CURE", 365, 34.818752921684066`},
{"CURE", 365, 43.0240837379187`}, {"CURE", 365, 59.428009626991965`}, {"CURE", 365, 105.01429331772871`},
{"CURE", 365, 22.660032563810038`}, {"CURE", 365, 20.68715377829871`}, {"CURE", 365, 44.26660644511443`},
{"CURE", 365, 45.0680787396264`}, {"CURE", 365, 50.714066811836624`}, {"CURE", 365, 54.18905216699133`},
{"CURE", 365, 30.28802036531974`}, {"CURE", 365, 15.238444323076163`}, {"CURE", 365, 57.270352223118365`},
{"CURE", 365, 14.452941090783765`}, {"CURE", 365, 30.723692503596304`}, {"CURE", 365, 36.59304878792787`},
{"CURE", 365, 11.022141411298476`}, {"CURE", 365, 5.479057144577264`}, {"CURE", 365, 33.87010175908041`},
{"CURE", 365, 45.894769815660396`}, {"CURE", 365, 54.47942228801261`}, {"CURE", 365, 38.9711310150706`},
{"CURE", 365, 4.993685902278103`}, {"CURE", 365, 44.69401436704536`}, {"CURE", 365, 8.515366336579385`},
{"CURE", 365, 131.3525518517642`}, {"CURE", 365, 170.9135712423993`}, {"CURE", 365, 24.31887796977382`},
{"CURE", 365, 33.77905507689187`}, {"CURE", 365, 183.4748783568898`}, {"CURE", 365, 32.90207567946393`},
{"CURE", 365, 19.121703144245615`}, {"CURE", 365, 14.888469197498441`}, {"CURE", 365, 4.069188930636574`},
{"CURE", 365, 25.650551426303263`}, {"CURE", 365, 28.60216368810004`}, {"CURE", 365, 75.8091702252184`},
{"CURE", 365, 36.27744420410575`}, {"CURE", 365, 63.163328676605026`}, {"CURE", 365, 22.312586296646128`},
{"CURE", 365, 48.437830447491905`}, {"CURE", 365, 108.21997861698071`}, {"CURE", 365, 11.007813766010488`},
{"CURE", 365, 16.978421310723128`}, {"CURE", 365, 25.221567790296316`}, {"CURE", 365, 35.22923167617938`},
{"CURE", 365, 23.119156327075014`}, {"CURE", 365, 22.112210583988613`}, {"CURE", 365, 9.37280897971457`},
{"CURE", 365, 14.462125910579765`}, {"CURE", 365, 27.42201013766695`}, {"CURE", 365, 62.39769719185646`},
{"CURE", 365, 55.54007108935333`}, {"CURE", 365, 28.434718254362583`}, {"TBUR", 44.75040822139048`, 42.676269574849634`},
{"CURE", 365, 91.87421129940451`}, {"CURE", 365, 44.930937779061416`}, {"CURE", 365, 33.61981185595176`},
{"CURE", 365, 21.414139522054644`}, {"CURE", 365, 6.709791091946668`}, {"CURE", 365, 37.32508569046268`},
{"CURE", 365, 43.08474987736067`}, {"CURE", 365, 168.74369795807124`}, {"CURE", 365, 8.60161238471794`},
{"CURE", 365, 8.402291511792047`}, {"CURE", 365, 26.92219736570465`}, {"CURE", 365, 78.4780736867235`},
{"CURE", 365, 9.793107862296399`}, {"CURE", 365, 47.9063324997714`}, {"CURE", 365, 11.173151894189925`},
{"CURE", 365, 27.84361532945481`}, {"CURE", 365, 11.352651169689768`}, {"CURE", 365, 11.807477818034325`},
{"CURE", 365, 10.648957885524343`}, {"CURE", 365, 113.61762392945242`}, {"CURE", 365, 18.178527940020548`},

```
{"CURE", 365, 32.01600310540946`}, {"CURE", 365, 4.414129229241449`}, {"CURE", 365, 52.88680373902243`},
{"CURE", 365, 73.99960646053185`}, {"CURE", 365, 64.51852510308824`}, {"CURE", 365, 9.740106492240118`},
{"CURE", 365, 19.53594084087267`}, {"CURE", 365, 198.79055640729277`}, {"CURE", 365, 54.0112194144256`},
{"CURE", 365, 36.80259543916646`}, {"CURE", 365, 13.070091408351814`}, {"CURE", 365, 18.71702436174739`},
{"CURE", 365, 28.927958743067563`}, {"CURE", 365, 7.162994968271601`}, {"CURE", 365, 35.16188994975612`},
{"CURE", 365, 55.3251916691171`}, {"CURE", 365, 33.6623931821443`}, {"CURE", 365, 36.545003570200834`},
{"CURE", 365, 26.438951329732863`}, {"CURE", 365, 17.124948347052573`}, {"CURE", 365, 97.85515447355002`},
{"CURE", 365, 141.83943017970145`}, {"CURE", 365, 37.83264456339977`}, {"CURE", 365, 47.76542612798052 5`},
{"CURE", 365, 60.5110968268425`}, {"CURE", 365, 59.16300842619169`}, {"CURE", 365, 5.166343730215957`},
{"CURE", 365, 69.21778741959544`}, {"CURE", 365, 71.5188274467705`}, {"CURE", 365, 48.343559656143086`},
{"CURE", 365, 194.00300567919945`}, {"CURE", 365, 40.332366716472514`}, {"CURE", 365, 17.960410540445437`},
{"CURE", 365, 42.447817019925594`}, {"CURE", 365, 53.94840245474684`}, {"CURE", 365, 24.16769987596456`},
{"CURE", 365, 18.973157921697855`}, {"CURE", 365, 16.494285709132573`}, {"CURE", 365, 29.903915899514008`},
{"CURE", 365, 92.1606215200022`}, {"CURE", 365, 59.14836294177479`}, {"CURE", 365, 10.300272690487143`},
{"CURE", 365, 60.37890126711172`}, {"CURE", 365, 87.06785182503927`}, {"CURE", 365, 16.007729044461726`},
{"CURE", 365, 91.37692823109656`}, {"CURE", 365, 13.729024702132262`}, {"CURE", 365, 8.76631564379394`},
{"CURE", 365, 12.434201120484737`}, {"CURE", 365, 23.939435983643722`}, {"TBUR", 99.06037659797221`, 107.20261192337566`},
{"CURE", 365, 38.29053293921838`}, {"CURE", 365, 48.35640728778491`}, {"CURE", 365, 11.558132642181846`},
{"CURE", 365, 220.6463194526113`}, {"CURE", 365, 101.04070734402661`}, {"CURE", 365, 17.24513025921181`},
{"CURE", 365, 42.21175216700478`}, {"CURE", 365, 23.514089573828535`}, {"CURE", 365, 23.756553679995747`},
{"CURE", 365, 80.28350910920507`}, {"TBUR", 67.41906206484036`, 101.52650295241465`}, {"CURE", 365, 10.903525493290822`},
{"CURE", 365, 21.25146728024514`}, {"CURE", 365, 7.539269868709306`}, {"CURE", 365, 29.067418896599193`},
{"CURE", 365, 49.843549071104896`}, {"CURE", 365, 80.70593219738016`}, {"CURE", 365, 45.04053305056466`},
{"CURE", 365, 162.20797005230287`}, {"CURE", 365, 77.52998106565015`}, {"TBUR", 81.9489112400813`, 12.800695976199664`},
{"CURE", 365, 28.36218772352258`}, {"TBUR", 49.282556271532926`, 31.28727444520961`}, {"CURE", 365, 63.39267944095844`},
{"CURE", 365, 8.3166176291732`}, {"CURE", 365, 22.25854752721942`}, {"CURE", 365, 12.543277092489836`},
{"CURE", 365, 31.28147315809153`}, {"CURE", 365, 71.48134283242024`}, {"CURE", 365, 30.40696759270225`},
{"CURE", 365, 6.604471583039872`}, {"CURE", 365, 125.84145423179937`}, {"CURE", 365, 23.733216822216665`},
{"CURE", 365, 8.060073061452941`}, {"CURE", 365, 10.596126488542152`}, {"CURE", 365, 8.945008085706728`},
{"CURE", 365, 11.4270480383035`}, {"CURE", 365, 91.9556151858688`}, {"CURE", 365, 50.35394419893642`},
{"CURE", 365, 30.301445387416283`}, {"CURE", 365, 14.207833954987015`}, {"CURE", 365, 23.709817874018764`},
```

{"CURE", 365, 13.71784160854605`}, {"CURE", 365, 33.10476292357081`}, {"CURE", 365, 113.84610278029969`},
{"CURE", 365, 66.66602539645604`}, {"CURE", 365, 32.860657219099494`}, {"CURE", 365, 18.92569727172108`},
{"CURE", 365, 19.948138432831325`}, {"TOX", 12.117447886037407`, 243.1990933664786`}, {"CURE", 365, 11.588704030849188`},
{"CURE", 365, 5.970371573183133`}, {"CURE", 365, 5.2082360051947765`}, {"CURE", 365, 66.12624748978544`},
{"CURE", 365, 38.73173633945249`}, {"CURE", 365, 42.96338455098197`}, {"CURE", 365, 63.826723241283304`},
{"CURE", 365, 105.41524077042921`}, {"CURE", 365, 108.97898748340451`}, {"CURE", 365, 41.53323882937528`},
{"CURE", 365, 17.932962444401717`}, {"CURE", 365, 20.800660024286955`}, {"CURE", 365, 41.64838994951592`},
{"CURE", 365, 23.55397153197184`}, {"TBUR", 28.77490219020127`, 9.443452489719359`}, {"CURE", 365, 36.99951881173894`},
{"CURE", 365, 230.18419188118116`}, {"CURE", 365, 23.710963270373714`}, {"CURE", 365, 8.530722235303198`},
{"CURE", 365, 80.85346548124099`}, {"CURE", 365, 20.22150811512249`}, {"CURE", 365, 58.12685106616987`},
{"TOX", 6.545749809355645`, 279.7535090621244`}, {"CURE", 365, 129.17716600404756`}, {"CURE", 365, 41.93557520860617`},
{"CURE", 365, 35.16052864952482`}, {"CURE", 365, 14.975513858648913`}, {"CURE", 365, 4.276687750000017`},
{"CURE", 365, 16.762316518137954`}, {"CURE", 365, 49.038026395272986`}, {"CURE", 365, 27.199149296300547`},
{"CURE", 365, 86.77512057269377`}, {"CURE", 365, 34.40249775168773`}, {"CURE", 365, 35.11848738237825`},
{"CURE", 365, 9.752377416431877`}, {"CURE", 365, 27.625425533131086`}, {"CURE", 365, 28.34617426158333`},
{"CURE", 365, 7.392299781760045`}, {"CURE", 365, 23.532180340524764`}, {"CURE", 365, 14.02369702590977`},
{"CURE", 365, 22.23692556658658`}, {"CURE", 365, 46.694568935233946`}, {"CURE", 365, 23.289882433316546`},
{"CURE", 365, 5.397098993549552`}, {"CURE", 365, 37.18522332899609`}, {"CURE", 365, 4.164848233843`},
{"CURE", 365, 12.224955290488973`}, {"CURE", 365, 36.24782973917509`}, {"CURE", 365, 41.778202373473064`},
{"CURE", 365, 8.316432417533427`}, {"CURE", 365, 24.472136515782047`}, {"CURE", 365, 31.727337724973403`},
{"CURE", 365, 160.17995773453774`}, {"CURE", 365, 36.77904965523205`}, {"CURE", 365, 10.379178716225546`},
{"CURE", 365, 113.10734631001101`}, {"CURE", 365, 11.133318631487368`}, {"CURE", 365, 41.87704260775139`},
{"CURE", 365, 43.30047851923226`}, {"CURE", 365, 12.92536734668424`}, {"CURE", 365, 14.393419821518723`},
{"CURE", 365, 63.74909368111058`}, {"CURE", 365, 6.230776138574548`}, {"CURE", 365, 54.00674401966258`},
{"CURE", 365, 14.302670950148036`}, {"CURE", 365, 18.70195274488136`}, {"CURE", 365, 4.181118712213571`},
{"CURE", 365, 21.476343879441178`}, {"CURE", 365, 73.29276006512545`}, {"CURE", 365, 27.876119164361015`},
{"CURE", 365, 20.24445659787371`}, {"CURE", 365, 28.931967089921056`}, {"CURE", 365, 22.723661686912163`},
{"CURE", 365, 28.611010284025763`}, {"CURE", 365, 50.47880693275618`}, {"CURE", 365, 48.70523672560105`},
{"CURE", 365, 5.997886927524665`}, {"CURE", 365, 98.48967093742073`}, {"CURE", 365, 11.447522600299846`},
{"CURE", 365, 5.743923314615411`}, {"CURE", 365, 27.383717181942014`}, {"CURE", 365, 28.737194092913025`},
{"CURE", 365, 19.38117187743925`}, {"CURE", 365, 33.636810027849165`}, {"CURE", 365, 29.00117148459879`},

{"CURE", 365, 46.61476751085379`}, {"CURE", 365, 39.3939550823933`}, {"CURE", 365, 56.26951217688144`},
{"CURE", 365, 8.891282692618494`}, {"CURE", 365, 114.01575299436342`}, {"CURE", 365, 110.57267843315891`},
{"CURE", 365, 6.126929621605374`}, {"CURE", 365, 15.41969446266377`}, {"CURE", 365, 38.25954607204723`},
{"CURE", 365, 19.46176326018913`}, {"CURE", 365, 59.83863628604978`}, {"CURE", 365, 119.79126812727078`},
{"CURE", 365, 37.858369540535925`}, {"CURE", 365, 6.499748460800625`}, {"CURE", 365, 6.3752745794144365`},
{"CURE", 365, 14.967308188987658`}, {"CURE", 365, 4.51902163701685`}, {"CURE", 365, 51.286851259419095`},
{"CURE", 365, 7.952043004912898`}, {"CURE", 365, 53.497670023137886`}, {"CURE", 365, 32.94326789320595`},
{"CURE", 365, 56.77375615455608`}, {"CURE", 365, 13.979060830881973`}, {"CURE", 365, 70.5257158440881`},
{"TOX", 20.52566340637362`, 233.33708523207886`}, {"CURE", 365, 84.05690217449184`}, {"CURE", 365, 136.17343925474455`},
{"CURE", 365, 40.07493459422862`}, {"CURE", 365, 95.47725360224248`}, {"CURE", 365, 95.66924848620593`},
{"CURE", 365, 11.611028571849841`}, {"CURE", 365, 8.121930182565162`}, {"CURE", 365, 26.413366547595356`},
{"CURE", 365, 54.800772425126446`}, {"CURE", 365, 68.56510790772512`}, {"CURE", 365, 29.403578578330556`},
{"CURE", 365, 43.401396722679735`}, {"CURE", 365, 8.175723731979202`}, {"CURE", 365, 10.389564361562815`},
{"CURE", 365, 50.468135099408144`}, {"CURE", 365, 16.364244327838236`}, {"CURE", 365, 6.942121484906069`},
{"CURE", 365, 10.267513611662684`}, {"CURE", 365, 5.019606013925998`}, {"CURE", 365, 40.789227138638864`},
{"CURE", 365, 56.09286977389236`}, {"CURE", 365, 39.24912496077972`}, {"CURE", 365, 44.59083288091404`},
{"CURE", 365, 24.69960264170933`}, {"CURE", 365, 22.11729430827451`}, {"CURE", 365, 57.22751486725011`},
{"CURE", 365, 61.24040179635277`}, {"CURE", 365, 6.833664716728362`}, {"CURE", 365, 25.710980146648485`},
{"CURE", 365, 13.580466690656758`}, {"CURE", 365, 21.322416192779386`}, {"CURE", 365, 175.6441509656949`},
{"CURE", 365, 31.014872647521596`}, {"CURE", 365, 21.28734254147767`}, {"CURE", 365, 22.51801243440629`},
{"CURE", 365, 44.914182517538265`}, {"CURE", 365, 34.188182845828436`}, {"CURE", 365, 44.599082194626135`},
{"CURE", 365, 45.98583901914746`}, {"CURE", 365, 10.431655507920158`}, {"CURE", 365, 101.443947844235`},
{"CURE", 365, 19.484521747267657`}, {"CURE", 365, 41.31881905699173`}, {"CURE", 365, 23.447764134373628`},
{"CURE", 365, 13.313946539547604`}, {"CURE", 365, 48.53650008582679`}, {"CURE", 365, 49.27415063468597`},
{"TBUR", 83.08780171287928`, 27.337170668509344`}, {"CURE", 365, 64.60203216714514`}, {"CURE", 365, 13.665463768636288`},
{"CURE", 365, 114.19236064149274`}, {"CURE", 365, 43.858883000447044`}, {"CURE", 365, 92.73676459399218`},
{"CURE", 365, 3.596577376257571`}, {"CURE", 365, 45.62575189381513`}, {"CURE", 365, 52.0002782194753`},
{"CURE", 365, 12.35980973507447`}, {"CURE", 365, 31.382749758424172`}, {"CURE", 365, 46.994176407836`},
{"CURE", 365, 28.5642305679367`}, {"CURE", 365, 9.089400692053706`}, {"CURE", 365, 6.897818005945023`},
{"CURE", 365, 40.79057243186013`}, {"CURE", 365, 10.881445991128317`}, {"CURE", 365, 24.189987574730495`},
{"CURE", 365, 29.48673526070668`}, {"CURE", 365, 13.728555999493961`}, {"CURE", 365, 25.263669954185374`},

{"CURE", 365, 6.879471581374736`}, {"CURE", 365, 28.36137313195932`}, {"CURE", 365, 4.585068387788905`},
{"CURE", 365, 8.197763719213304`}, {"CURE", 365, 19.380770719036622`}, {"CURE", 365, 6.250727560128875`},
{"CURE", 365, 46.44847470063585`}, {"CURE", 365, 68.22567498468`}, {"CURE", 365, 69.2545677707349`},
{"CURE", 365, 57.661087535872134`}, {"CURE", 365, 108.84249368371505`}, {"CURE", 365, 8.804923282129892`},
{"CURE", 365, 39.2657470822302`}, {"CURE", 365, 42.17321954286903`}, {"CURE", 365, 72.49571531781937`},
{"CURE", 365, 54.14873823985676`}, {"CURE", 365, 24.74909422826229`}, {"CURE", 365, 116.37403967558883`},
{"CURE", 365, 3.6330659130012855`}, {"CURE", 365, 110.71509339599746`}, {"CURE", 365, 7.497909279667577`},
{"CURE", 365, 94.56158727225396`}, {"CURE", 365, 85.63898755848281`}, {"CURE", 365, 25.38580851207655`},
{"CURE", 365, 99.96224342264841`}, {"CURE", 365, 121.92839242735225`}, {"CURE", 365, 54.538449128551946`},
{"CURE", 365, 24.06448254697844`}, {"CURE", 365, 27.66745363327274`}, {"CURE", 365, 40.85989447982621`},
{"CURE", 365, 37.682559535824296`}, {"CURE", 365, 9.131864643384374`}, {"CURE", 365, 32.78746950034131`},
{"CURE", 365, 31.02259547075017`}, {"CURE", 365, 23.202254234216756`}, {"CURE", 365, 57.32281286258175`},
{"CURE", 365, 9.026544396366003`}, {"CURE", 365, 63.98455757464655`}, {"CURE", 365, 41.871187082555906`},
{"CURE", 365, 23.70609690964051`}, {"CURE", 365, 21.552302428777327`}, {"CURE", 365, 43.74857710972789`},
{"CURE", 365, 5.990913474309269`}, {"CURE", 365, 69.39150693937214`}, {"CURE", 365, 10.955623584954528`},
{"CURE", 365, 15.538755100949517`}, {"CURE", 365, 34.861138157042504`}, {"CURE", 365, 27.330650482162678`},
{"CURE", 365, 25.13220050910159`}, {"CURE", 365, 78.25183611322929`}, {"CURE", 365, 10.58484275418858 9`},
{"CURE", 365, 18.428238668041416`}, {"CURE", 365, 136.3360316923518`}, {"CURE", 365, 10.34490466116723`},
{"CURE", 365, 26.638325017583085`}, {"CURE", 365, 40.53866104606329`}, {"CURE", 365, 40.02665111222551`},
{"CURE", 365, 27.175463135811132`}, {"CURE", 365, 140.47572588930757`}, {"CURE", 365, 25.377023958251925`},
{"CURE", 365, -1.8933855828218095`*^24}, {"CURE", 365, 21.318500592897156`}, {"CURE", 365, 16.456014152889463`},
{"CURE", 365, 12.612502145257555`}, {"CURE", 365, 33.07369951172087`}, {"CURE", 365, 56.64222486756685`},
{"CURE", 365, 31.164481559145514`}, {"CURE", 365, 36.334395665562454`}, {"CURE", 365, 79.55274094504769`},
{"CURE", 365, 8.773632086193375`}, {"CURE", 365, 10.324000841661544`}, {"CURE", 365, 87.09261102507472`},
{"CURE", 365, 28.050752355416158`}, {"CURE", 365, 30.36692391020198`}, {"CURE", 365, 15.265365556916013`},
{"CURE", 365, 34.95492817632818`}, {"CURE", 365, 43.56225158914392`}, {"CURE", 365, 100.96823253804607`},
{"CURE", 365, 14.8819664609771`}, {"CURE", 365, 58.16948117352756`}, {"CURE", 365, 34.216140369607004`},
{"CURE", 365, 90.59417628711252`}, {"CURE", 365, 147.93741785282765`}, {"CURE", 365, 54.165080051361635`},
{"CURE", 365, 5.245271191825067`}, {"CURE", 365, 41.04680305450436`}, {"CURE", 365, 12.966284500616075`},
{"CURE", 365, 137.06343836787008`}, {"TBUR", 50.42576609697602`, 4.358009734452512`}, {"CURE", 365, 4.575083775187931`},
{"CURE", 365, 158.70826089795096`}, {"CURE", 365, 36.51843817016879`}, {"CURE", 365, 12.347313272706778`},

{"CURE", 365, 23.632229289123842`}, {"CURE", 365, 8.292256475283851`}, {"TBUR", 79.0710405720401`, 7.890163239429869`},
{"CURE", 365, 9.427793371381084`}, {"CURE", 365, 34.07856796939851`}, {"CURE", 365, 72.2292350879463`},
{"CURE", 365, 28.610587154857527`}, {"CURE", 365, 7.165208028619339`}, {"CURE", 365, 97.4498693732785`},
{"CURE", 365, 194.77062569310945`}, {"CURE", 365, 5.4926135609749815`}, {"CURE", 365, 43.907224037923825`},
{"CURE", 365, 23.580099892778676`}, {"CURE", 365, 4.637083770593364`}, {"CURE", 365, 36.2538738542448`},
{"CURE", 365, 23.16115148531067`}, {"CURE", 365, 17.780857185302363`}, {"CURE", 365, 34.576881937187416`},
{"CURE", 365, 19.62742696315478`}, {"CURE", 365, 57.77496309156359`}, {"CURE", 365, 34.30746917708174`},
{"CURE", 365, 80.78392671860979`}, {"CURE", 365, 35.78580340805272`}, {"CURE", 365, 17.732261072914557`},
{"CURE", 365, 8.624263696866905`}, {"CURE", 365, 5.980456924307184`}, {"CURE", 365, 17.03439877723446`},
{"CURE", 365, 67.79245439280275`}, {"CURE", 365, 22.356771534013696`}, {"CURE", 365, 20.48791199024529`},
{"CURE", 365, 14.486399356434292`}, {"CURE", 365, 14.681868423497962`}, {"CURE", 365, 39.67371919567702`},
{"CURE", 365, 7.4223623718051055`}, {"CURE", 365, 47.289999290126765`}, {"CURE", 365, 21.63326816208743`},
{"CURE", 365, 12.721529851423798`}, {"CURE", 365, 20.15346021064279`}, {"CURE", 365, 44.70702973469924`},
{"CURE", 365, 33.339634358548786`}, {"CURE", 365, 4.0196465589574295`}, {"CURE", 365, 17.321223925765022`},
{"TOX", 5.807584414309793`, 289.4856446373758`}, {"CURE", 365, 48.0993283646267`}, {"CURE", 365, 70.14535883345837`},
{"CURE", 365, 20.668728335574126`}, {"CURE", 365, 76.6272615302907`}, {"CURE", 365, 9.16789378794917`},
{"CURE", 365, 30.897593730149826`}, {"CURE", 365, 142.59790056501598`}, {"CURE", 365, 26.701818595315448`},
{"CURE", 365, 23.528079494337597`}, {"CURE", 365, 40.311550126306805`}, {"CURE", 365, 21.34684737173105`},
{"CURE", 365, 4.735596168022622`}, {"CURE", 365, 44.02138857205229`}, {"CURE", 365, 7.853479718139344`},
{"CURE", 365, 18.668561827052525`}, {"CURE", 365, 44.08338525868307`}, {"CURE", 365, 15.832460973263933`},
{"CURE", 365, 84.08524707087788`}, {"CURE", 365, 25.00592405087289`}, {"CURE", 365, 43.039178769500026`},
{"CURE", 365, 29.83282212921641`}, {"CURE", 365, 8.442106145995123`}, {"CURE", 365, 31.330149217544637`},
{"CURE", 365, 67.68839016688796`}, {"CURE", 365, 22.68818052316866`}, {"CURE", 365, 17.07383275292517`},
{"CURE", 365, 8.100161495316872`}, {"CURE", 365, 25.782734387513703`}, {"TBUR", 365, 30.834419379708827`},
{"CURE", 365, 87.96519159754904`}, {"CURE", 365, 14.131090808493207`}, {"CURE", 365, 21.274314048133565`},
{"CURE", 365, 82.57332013589543`}, {"CURE", 365, 8.699862060103625`}, {"CURE", 365, 119.4571472604731 2`},
{"CURE", 365, 10.094222381301227`}, {"CURE", 365, 18.4827664231213`}, {"CURE", 365, 42.59864460649916`},
{"CURE", 365, 68.60473116468272`}, {"CURE", 365, 30.860593851151698`}, {"CURE", 365, 16.17884601928847`},
{"CURE", 365, 4.754961952583565`}, {"CURE", 365, 41.643341013758025`}, {"CURE", 365, 37.72443300369744`},
{"CURE", 365, 37.06688517822606`}, {"CURE", 365, 14.615151359007113`}, {"CURE", 365, 35.82735854867231`},
{"CURE", 365, 4.145211106856886`}, {"CURE", 365, 23.88802383976457`}, {"CURE", 365, 10.433051157498916`},

```
{"CURE", 365, 4.621217358688416`}, {"CURE", 365, 24.015659806872275`}, {"CURE", 365, 5.138552995216621`},
{"CURE", 365, 16.33941422498465`}, {"CURE", 365, 11.206713727182134`}, {"TBUR", 48.54859315710254`, 6.855271318887848`},
{"CURE", 365, 22.197385866693278`}, {"CURE", 365, 59.13914608611736`}, {"CURE", 365, 32.38996359769131`},
{"CURE", 365, 29.321289865271048`}, {"CURE", 365, 46.61569389478797`}, {"CURE", 365, 39.55406830777304`}},
{{"2400 Res", "2400 OS", "2400 Tox"}, {"CURE", 365, 20.00536527030677`}, {"CURE", 365, 41.37142334619004`},
{"CURE", 365, 29.007129380566667`}, {"TBUR", 75.93431694549236`, 9.73659174072273`},
{"TBUR", 53.86328367646835`, 37.310726282885135`}, {"CURE", 365, 74.94139179371372`}, {"CURE", 365, 70.50898386982276`},
{"CURE", 365, 7.367850115399441`}, {"CURE", 365, 50.85113429295553`}, {"CURE", 365, 43.99111967570454`},
{"CURE", 365, 165.09906748961708`}, {"CURE", 365, 20.483950151134085`}, {"CURE", 365, 73.9858021618949`},
{"TOX", 9.069281135890709`, 246.25576497716025`}, {"CURE", 365, 34.83485483534424`}, {"CURE", 365, 54.373199049751896`},
{"CURE", 365, 107.63883348458373`}, {"CURE", 365, 48.22764769852487`}, {"TBUR", 140.64858695199052`, 36.51983782750597`},
{"CURE", 365, 12.086572656422636`}, {"CURE", 365, 74.70725691582639`}, {"CURE", 365, 30.282352900549746`},
{"CURE", 365, 11.604832171944256`}, {"CURE", 365, 9.246757696973702`}, {"CURE", 365, 42.068117314212884`},
{"CURE", 365, 46.17345042499729`}, {"CURE", 365, 4.197840142875898`}, {"CURE", 365, 6.363018363116571`},
{"CURE", 365, 23.578991828917122`}, {"CURE", 365, 27.585132665840714`}, {"CURE", 365, 23.528876560743225`},
{"CURE", 365, 82.76955273102519`}, {"CURE", 365, 18.19411067857054`}, {"CURE", 365, 14.476268301184563`},
{"CURE", 365, 117.27831342152828`}, {"CURE", 365, 7.187789625760128`}, {"CURE", 365, 31.055202769197287`},
{"TBUR", 50.89279558000401`, 69.36355409310922`}, {"CURE", 365, 17.266796314313883`}, {"CURE", 365, 26.567066877238734`},
{"CURE", 365, 60.63523040999006`}, {"CURE", 365, 40.888540563470244`}, {"CURE", 365, 8.296794627363882`},
{"CURE", 365, 27.988128564219252`}, {"CURE", 365, 40.37795580780302`}, {"CURE", 365, 26.474792456990354`},
{"TOX", 5.879289668381484`, 281.3356682030533`}, {"CURE", 365, 60.647767087248134`}, {"CURE", 365, 93.81018166590509`},
{"CURE", 365, 11.344309705768937`}, {"CURE", 365, 31.90404748510312`}, {"CURE", 365, 43.656274169377646`},
{"CURE", 365, 34.89979068909928`}, {"CURE", 365, 22.654263785755745`}, {"CURE", 365, 9.761905768860357`},
{"CURE", 365, 103.23593716834448`}, {"CURE", 365, 32.440499780267544`}, {"CURE", 365, 6.165319424131637`},
{"CURE", 365, 66.42756544211426`}, {"CURE", 365, 46.530212454419654`}, {"CURE", 365, 31.862822185281438`},
{"CURE", 365, 24.374764359302986`}, {"CURE", 365, 13.881818265168286`}, {"CURE", 365, 52.53293267388866`},
{"CURE", 365, 37.33194542014877`}, {"CURE", 365, 42.67812272205945`}, {"CURE", 365, 9.768919004190344`},
{"CURE", 365, 9.931318948775537`}, {"CURE", 365, 10.561855614680322`}, {"CURE", 365, 43.13739576987787`},
{"CURE", 365, 15.792645521831393`}, {"CURE", 365, 35.1791656751141`}, {"CURE", 365, 66.01235312607686`},
{"TBUR", 28.946101035240023`, 20.86627554820746`}, {"CURE", 365, 17.6439635378351`}, {"CURE", 365, 48.86088571867618`},
{"CURE", 365, 28.58081721088977`}, {"CURE", 365, 21.735207795294595`}, {"CURE", 365, 86.6082717884946`},
```

```
{"CURE", 365, 95.08520250153289`}, {"CURE", 365, 70.00714632878504`}, {"CURE", 365, 18.886950138939476`},
{"CURE", 365, 38.186881691652665`}, {"CURE", 365, 26.691885704221377`}, {"CURE", 365, 30.184751598476833`},
{"CURE", 365, 14.146518036810699`}, {"CURE", 365, 150.91047613086573`}, {"CURE", 365, 32.89086519858187`},
{"CURE", 365, 41.41592009962038`}, {"CURE", 365, 35.6501011591146`}, {"CURE", 365, 29.49337372339219`},
{"CURE", 365, 8.73731425078679`}, {"CURE", 365, 16.011690870408312`}, {"CURE", 365, 37.069086837448836`},
{"TBUR", 61.94604697053545`, 13.454100312827459`}, {"CURE", 365, 15.96420089928975`}, {"CURE", 365, 41.61687232383067`},
{"CURE", 365, 105.61889866459894`}, {"CURE", 365, 56.59333246219624`}, {"CURE", 365, 28.38466122219968`},
{"TBUR", 87.151177064171`, 56.288703631902465`}, {"CURE", 365, 36.59223285240922`}, {"CURE", 365, 23.710771364563442`},
{"CURE", 365, 162.35081379564966`}, {"CURE", 365, 18.033237170852296`}, {"CURE", 365, 12.252806912206474`},
{"CURE", 365, 71.92881937504805`}, {"CURE", 365, 21.239676402240914`}, {"CURE", 365, 21.6975832208598`},
{"CURE", 365, 130.54595183397532`}, {"CURE", 365, 10.596311369707585`}, {"CURE", 365, 6.723267536930212`},
{"CURE", 365, 27.965056340229953`}, {"CURE", 365, 22.110717779210738`}, {"CURE", 365, 16.652856731905032`},
{"CURE", 365, 42.50164656296702`}, {"CURE", 365, 11.963373694711953`}, {"CURE", 365, 7.355771119134464`},
{"CURE", 365, 34.4308667513432`}, {"CURE", 365, 52.65971845814965`}, {"CURE", 365, 8.969539614029776`},
{"CURE", 365, 29.684033537967604`}, {"CURE", 365, 90.09908830825006`}, {"TBUR", 75.02922424905609`, 34.35956791676712`},
{"CURE", 365, 19.988072452924847`}, {"CURE", 365, 69.74711563114394`}, {"CURE", 365, 35.051700645329426`},
{"CURE", 365, 4.620037941287858`}, {"CURE", 365, 40.013997822090445`}, {"CURE", 365, 30.90364879625357`},
{"CURE", 365, 24.619134944264292`}, {"CURE", 365, 29.204171236100695`}, {"CURE", 365, 7.604051857453652`},
{"CURE", 365, 43.380246632376696`}, {"CURE", 365, 15.572736864111011`}, {"CURE", 365, 35.193798337820525`},
{"CURE", 365, 131.46786629926743`}, {"CURE", 365, 46.4615050708567`}, {"CURE", 365, 107.91898695359558`},
{"CURE", 365, 35.552490656267196`}, {"CURE", 365, 146.9222206581176`}, {"CURE", 365, 4.580710173845903`},
{"CURE", 365, 40.22865900573712`}, {"CURE", 365, 45.85728004500681`}, {"CURE", 365, 5.955966685956751`},
{"CURE", 365, 8.251787259239444`}, {"CURE", 365, 50.648156705885604`}, {"CURE", 365, 59.700233504280604`},
{"CURE", 365, 50.86431394533363`}, {"CURE", 365, 41.62041612604954`}, {"CURE", 365, 39.78553581438631`},
{"CURE", 365, 24.092936705493628`}, {"CURE", 365, 6.371620920934314`}, {"CURE", 365, 27.721203704943907`},
{"CURE", 365, 13.154086573600042`}, {"CURE", 365, 6.439551568622704`}, {"CURE", 365, 39.02412479807417`},
{"CURE", 365, 21.833051673757687`}, {"CURE", 365, 57.7149856470686`}, {"CURE", 365, 23.301085636854285`},
{"CURE", 365, 19.39347743535752`}, {"CURE", 365, 130.08569174161542`}, {"CURE", 365, 9.691211834619926`},
{"CURE", 365, 28.99890605144381`}, {"CURE", 365, 16.433241452031663`}, {"CURE", 365, 38.832336747811766`},
{"CURE", 365, 10.671693657324486`}, {"CURE", 365, 42.60431228879664`}, {"CURE", 365, 112.5682507966592 7`},
{"CURE", 365, 9.628013326293658`}, {"CURE", 365, 29.23792130619795`}, {"CURE", 365, 31.59600014863155`},
```

{"CURE", 365, 113.69239287324785`}, {"CURE", 365, 42.78605854737411`}, {"CURE", 365, 35.53451393365621`},
{"CURE", 365, 62.37257061233751`}, {"CURE", 365, 23.917952212381152`}, {"CURE", 365, 74.16313086636393`},
{"CURE", 365, 46.04247304944631`}, {"CURE", 365, 28.15743361561173`}, {"CURE", 365, 25.397331430745716`},
{"CURE", 365, 8.259031591499502`}, {"CURE", 365, 5.596297495971073`}, {"CURE", 365, 13.118788639038344`},
{"CURE", 365, 17.57047958747945`}, {"CURE", 365, 7.7410837225594165`}, {"CURE", 365, 28.3347489832256`},
{"CURE", 365, 77.33539021577575`}, {"CURE", 365, 20.90588237185717`}, {"CURE", 365, 38.797415563360815`},
{"CURE", 365, 48.59518724026056`}, {"CURE", 365, 41.63779946770534`}, {"CURE", 365, 28.44174528183818`},
{"CURE", 365, 11.297638371958056`}, {"CURE", 365, 77.83975471033911`}, {"CURE", 365, 6.7341530052296585`},
{"CURE", 365, 72.55931907545781`}, {"CURE", 365, 70.53516963348197`}, {"CURE", 365, 19.679041223479345`},
{"CURE", 365, 23.37429467570344`}, {"CURE", 365, 134.40637128576594`}, {"CURE", 365, 34.662233138921984`},
{"CURE", 365, 41.34752633657847`}, {"CURE", 365, 25.898044731315423`}, {"CURE", 365, 9.738500870766549`},
{"CURE", 365, 59.721886576430194`}, {"TBUR", 63.84673142250649`, 158.762504778168`}, {"CURE", 365, 24.247476353771177`},
{"CURE", 365, 21.925063289100066`}, {"CURE", 365, 40.39258346206806`}, {"CURE", 365, 16.128715123979116`},
{"CURE", 365, 204.68333781535193`}, {"CURE", 365, 16.38329990834692`}, {"CURE", 365, 101.38635361308289`},
{"CURE", 365, 77.82377733817975`}, {"CURE", 365, 75.69433548661515`}, {"CURE", 365, 40.43095810558965`},
{"CURE", 365, 93.71645653705076`}, {"CURE", 365, 224.90599032039597`}, {"CURE", 365, 42.57866550204241`},
{"CURE", 365, 7.822820278444174`}, {"CURE", 365, 69.47699318904564`}, {"CURE", 365, 43.29220687624332`},
{"CURE", 365, 41.73752132967404`}, {"CURE", 365, 9.206043110529347`}, {"CURE", 365, 210.9549023515524`},
{"CURE", 365, 15.422149634057835`}, {"CURE", 365, 66.89820771490257`}, {"CURE", 365, 42.17640484035327`},
{"CURE", 365, 29.970394516484333`}, {"CURE", 365, 7.204427799436201`}, {"CURE", 365, 54.15136824726451`},
{"CURE", 365, 31.769214288771366`}, {"CURE", 365, 84.04647051084342`}, {"CURE", 365, 55.37706500451832`},
{"CURE", 365, 69.9153665729291`}, {"CURE", 365, 33.57832323979611`}, {"CURE", 365, 29.871438269137524`},
{"CURE", 365, 156.08780230883292`}, {"CURE", 365, 40.86750037031358`}, {"CURE", 365, 10.904835869798998`},
{"CURE", 365, 36.722972038077096`}, {"CURE", 365, 37.36953219563253`}, {"CURE", 365, 5.012546086464586`},
{"CURE", 365, 8.924540102656952`}, {"CURE", 365, 13.63610223907318`}, {"CURE", 365, 128.8144415470888`},
{"CURE", 365, 39.59539118547041`}, {"CURE", 365, 41.26091718741942`}, {"CURE", 365, 33.04878871435299`},
{"CURE", 365, 135.97021359743505`}, {"CURE", 365, 31.287859200356642`}, {"CURE", 365, 63.04601061564351`},
{"CURE", 365, 31.203733353437535`}, {"CURE", 365, 45.99859529828319`}, {"CURE", 365, 11.061898770237708`},
{"CURE", 365, 41.373851136101784`}, {"CURE", 365, 25.819970609775844`}, {"CURE", 365, 50.28664614239149`},
{"CURE", 365, 79.47779902421054`}, {"CURE", 365, 89.57058438476675`}, {"CURE", 365, 54.52099524834185`},
{"CURE", 365, 44.31038313751075`}, {"CURE", 365, 61.29563751177872`}, {"CURE", 365, 29.016005667876755`},

{"CURE", 365, 32.849975314491786`}, {"CURE", 365, 23.28016476956955`}, {"CURE", 365, 18.24041272833579`},
{"CURE", 365, 29.38197732979837`}, {"CURE", 365, 75.38695315648535`}, {"CURE", 365, 8.64086443272972`},
{"CURE", 365, 38.293509421170135`}, {"CURE", 365, 7.229801391872964`}, {"CURE", 365, 37.63364410935637`},
{"CURE", 365, 21.390930695656294`}, {"CURE", 365, 79.57609397760217`}, {"CURE", 365, 7.337132386882382`},
{"CURE", 365, 52.40453939753546`}, {"CURE", 365, 65.71858285429619`}, {"CURE", 365, 10.750558083814878`},
{"CURE", 365, 24.6351845641899`}, {"CURE", 365, 19.820755102408352`}, {"CURE", 365, 11.345527868079886`},
{"CURE", 365, 67.02654797345501`}, {"CURE", 365, 25.1951031349571`}, {"CURE", 365, 48.545241906373995`},
{"CURE", 365, 47.20705612693135`}, {"CURE", 365, 53.521797706787005`}, {"CURE", 365, 41.84603297849713`},
{"CURE", 365, 50.10370754771543`}, {"CURE", 365, 39.65069573294705`}, {"CURE", 365, 76.6521602760378`},
{"CURE", 365, 46.698631024058095`}, {"CURE", 365, 38.14240638364738`}, {"CURE", 365, 97.5406170589399`},
{"CURE", 365, 11.293208335203552`}, {"CURE", 365, 64.05694716357473`}, {"CURE", 365, 4.705616126445494`},
{"CURE", 365, 58.46159394897069`}, {"CURE", 365, 13.77632653151344`}, {"CURE", 365, 72.8285969656611`},
{"CURE", 365, 11.250125690504493`}, {"CURE", 365, 73.69259350763318`}, {"CURE", 365, 184.46846687157677`},
{"CURE", 365, 8.052103075066693`}, {"CURE", 365, 5.783695145320731`}, {"CURE", 365, 21.28890744745675`},
{"CURE", 365, 5.171507123225656`}, {"CURE", 365, 3.5346953490705326`}, {"CURE", 365, 20.678460373207884`},
{"CURE", 365, 33.824426871404725`}, {"CURE", 365, 36.38001160911058`}, {"CURE", 365, 34.59515752920376`},
{"CURE", 365, 26.94066448688953`}, {"CURE", 365, 47.38229020533851`}, {"CURE", 365, 83.41694031218034`},
{"CURE", 365, 6.750525477967049`}, {"CURE", 365, 36.98166222123428`}, {"CURE", 365, 31.780611837999274`},
{"CURE", 365, 23.042878179469355`}, {"CURE", 365, 42.61828491391447`}, {"CURE", 365, 63.17854956442322`},
{"CURE", 365, 147.82560647625124`}, {"CURE", 365, 110.15839435595962`}, {"CURE", 365, 34.07978204212928`},
{"CURE", 365, 3.617588662482301`}, {"CURE", 365, 39.463484560180184`}, {"CURE", 365, 101.76103932709526`},
{"CURE", 365, 25.605921335462273`}, {"CURE", 365, 32.589414929833836`}, {"CURE", 365, 19.75618331606259`},
{"CURE", 365, 28.95705986141706`}, {"CURE", 365, 7.501795306752679`}, {"CURE", 365, 36.694895690197995`},
{"CURE", 365, 20.62569834241233`}, {"CURE", 365, 60.246814077078`}, {"CURE", 365, 41.27216016791114`},
{"CURE", 365, 40.005235704115705`}, {"CURE", 365, 32.916835284703474`}, {"CURE", 365, 17.419467273397238`},
{"CURE", 365, 12.004506976842269`}, {"CURE", 365, 11.704290368744632`}, {"CURE", 365, 15.162020545772378`},
{"CURE", 365, 11.814663546118274`}, {"CURE", 365, 158.90407839299635`}, {"CURE", 365, 88.38728021595453`},
{"CURE", 365, 17.076049015427188`}, {"TOX", 7.746557839898171`, 263.02890617225836`}, {"CURE", 365, 75.21880478989317`},
{"CURE", 365, 39.82593674393398`}, {"CURE", 365, 68.89379125639756`}, {"CURE", 365, 10.358153533519065`},
{"CURE", 365, 82.60152740946782`}, {"CURE", 365, 220.82536321218527`}, {"CURE", 365, 11.982150522840993`},
{"CURE", 365, 37.3764472146678`}, {"CURE", 365, 61.2282112546849`}, {"CURE", 365, 30.960159776445526`},

{"CURE", 365, 15.166475036850336`}, {"CURE", 365, 12.082724008462653`}, {"CURE", 365, 104.87654692423119`},
{"CURE", 365, 55.05922969306308`}, {"CURE", 365, 31.32435033051262`}, {"CURE", 365, 77.85402406274936`},
{"CURE", 365, 14.058950815104097`}, {"CURE", 365, 37.555212679148475`}, {"CURE", 365, 22.68078039127388`},
{"CURE", 365, 152.96315438346943`}, {"TBUR", 78.74647852286124`, 38.512005181791714`}, {"CURE", 365, 178.74868689527688`},
{"TOX", 6.7355402525223775`, 278.99874157073623`}, {"CURE", 365, 21.348930916455345`}, {"CURE", 365, 6.020717066935285`},
{"CURE", 365, 5.245083735618978`}, {"CURE", 365, 38.37090444557646`}, {"CURE", 365, 148.86182524082335`},
{"CURE", 365, 29.820351369450115`}, {"CURE", 365, 22.910807501959948`}, {"CURE", 365, 43.07081380368693`},
{"CURE", 365, 47.17164789121615`}, {"CURE", 365, 196.6167223223178`}, {"CURE", 365, 162.09893807080795`},
{"CURE", 365, 33.95623805754515`}, {"CURE", 365, 11.266640068741388`}, {"CURE", 365, 27.602150078315056`},
{"CURE", 365, 4.331917250865628`}, {"CURE", 365, 81.482814097654`}, {"CURE", 365, 121.15468903372539`},
{"CURE", 365, 39.62475093534914`}, {"CURE", 365, 31.40320698740662`}, {"CURE", 365, 132.78793244082885`},
{"CURE", 365, 8.42581934916801`}, {"CURE", 365, 10.972973751162742`}, {"CURE", 365, 34.33850708515844`},
{"CURE", 365, 44.183989972610924`}, {"TBUR", 91.91392693828551`, 83.73764194315991`}, {"CURE", 365, 86.15986201384004`},
{"CURE", 365, 35.36771752353435`}, {"CURE", 365, 36.48889885910799`}, {"CURE", 365, 45.708251489229944`},
{"CURE", 365, 15.820737866759183`}, {"CURE", 365, 4.467422220428219`}, {"CURE", 365, 46.99242921893801`},
{"CURE", 365, 56.578377927744356`}, {"CURE", 365, 27.50869284418097`}, {"CURE", 365, 49.851893804737706`},
{"CURE", 365, 15.529233615358159`}, {"CURE", 365, 10.204314555301636`}, {"CURE", 365, 44.670165990786344`},
{"CURE", 365, 27.15252499205632`}, {"CURE", 365, 38.81236550556885`}, {"TOX", 11.167576516076998`, 256.12730017085147`},
{"CURE", 365, 24.403601057086863`}, {"CURE", 365, 11.903751582208644`}, {"CURE", 365, 9.237059826341095`},
{"CURE", 365, 11.195245273754725`}, {"CURE", 365, 63.95706551667594`}, {"CURE", 365, 4.404533250072687`},
{"CURE", 365, 59.55269601429331`}, {"CURE", 365, 65.01337276766355`}, {"CURE", 365, 50.743869062281846`},
{"CURE", 365, 41.6017621465084`}, {"CURE", 365, 8.250330406705261`}, {"CURE", 365, 90.54860020816662`},
{"TBUR", 77.30533502219801`, 27.433942708444253`}, {"CURE", 365, 12.429755087091547`}, {"CURE", 365, 80.63761065053147`},
{"CURE", 365, 14.342262445237372`}, {"CURE", 365, 96.61417061920125`}, {"CURE", 365, 61.99929975686498`},
{"CURE", 365, 41.36112816341677`}, {"CURE", 365, 60.0812305197392`}, {"CURE", 365, 21.30656713919755`},
{"CURE", 365, 94.25663599559091`}, {"CURE", 365, 11.183499800844858`}, {"CURE", 365, 47.39243376727128`},
{"CURE", 365, 55.38833655813367`}, {"CURE", 365, 23.550359644473684`}, {"CURE", 365, 219.46309096256638`},
{"CURE", 365, 44.05150926587202`}, {"CURE", 365, 44.918649369887895`}, {"CURE", 365, 45.89171477290248`},
{"CURE", 365, 183.77726397999615`}, {"CURE", 365, 58.392724153589015`}, {"CURE", 365, 54.47840672695372`},
{"CURE", 365, 118.58463720652718`}, {"CURE", 365, 41.7011743258205`}, {"CURE", 365, 36.33469819870846`},
{"CURE", 365, 44.895322164338765`}, {"CURE", 365, 62.01286964787721`}, {"CURE", 365, 109.58050874785351`},

{"CURE", 365, 23.64632312590637`}, {"CURE", 365, 21.587105898616617`}, {"CURE", 365, 46.19171684609505`},
{"CURE", 365, 47.02846270540442`}, {"CURE", 365, 52.93373361016767`}, {"CURE", 365, 56.54546483145954`},
{"CURE", 365, 31.607596808113932`}, {"CURE", 365, 15.905139123852777`}, {"CURE", 365, 59.78091689101781`},
{"CURE", 365, 15.08206030915471`}, {"CURE", 365, 32.060393156237886`}, {"CURE", 365, 38.196580254188035`},
{"CURE", 365, 11.501551329817646`}, {"CURE", 365, 5.717603451686399`}, {"CURE", 365, 35.36002054897514`},
{"CURE", 365, 47.89861890345991`}, {"CURE", 365, 56.85493272122795`}, {"CURE", 365, 40.6715535998388`},
{"CURE", 365, 5.211580518589272`}, {"CURE", 365, 46.64000206301106`}, {"CURE", 365, 8.886889500815254`},
{"CURE", 365, 137.06435308677962`}, {"CURE", 365, 178.34956846749057`}, {"CURE", 365, 25.465332139615082`},
{"CURE", 365, 35.25743311531247`}, {"CURE", 365, 191.46006673790777`}, {"CURE", 365, 34.3335292924585`},
{"CURE", 365, 19.961584125563007`}, {"CURE", 365, 15.540863065008363`}, {"CURE", 365, 4.2463110511313715`},
{"CURE", 365, 26.765978953888915`}, {"CURE", 365, 29.84613942291455`}, {"CURE", 365, 79.11792782505255`},
{"CURE", 365, 37.85748185654911`}, {"CURE", 365, 65.9117655511114`}, {"CURE", 365, 23.282979840734036`},
{"CURE", 365, 50.55886859563782`}, {"CURE", 365, 112.92637181530708`}, {"CURE", 365, 11.488195613312216`},
{"CURE", 365, 17.716850065675114`}, {"CURE", 365, 26.32023559490336`}, {"CURE", 365, 36.76186100957962`},
{"CURE", 365, 24.134670262319155`}, {"CURE", 365, 23.07417799469818`}, {"CURE", 365, 9.782517344541581`},
{"CURE", 365, 15.091077847899026`}, {"CURE", 365, 28.61796581416149`}, {"CURE", 365, 65.11243442951381`},
{"CURE", 365, 57.97251669112684`}, {"CURE", 365, 29.672215930567198`}, {"TBUR", 46.6694500699952`, 44.55458732879655`},
{"CURE", 365, 95.87786810438118`}, {"CURE", 365, 46.88595212192496`}, {"CURE", 365, 35.08226165807485`},
{"CURE", 365, 22.345560791155087`}, {"CURE", 365, 7.002261038870036`}, {"CURE", 365, 38.94839885813967`},
{"CURE", 365, 44.96096122714776`}, {"CURE", 365, 176.1046887886999`}, {"CURE", 365, 8.976719693527087`},
{"CURE", 365, 8.767786288299119`}, {"CURE", 365, 28.093173293828936`}, {"CURE", 365, 81.8914003934392`},
{"CURE", 365, 10.21946513802598`}, {"CURE", 365, 49.990418707727194`}, {"CURE", 365, 11.659773538842973`},
{"CURE", 365, 29.056689325104198`}, {"CURE", 365, 11.854622343973219`}, {"CURE", 365, 12.321244752698526`},
{"CURE", 365, 11.112290320701998`}, {"CURE", 365, 118.55833651998324`}, {"CURE", 365, 18.97872722079505`},
{"CURE", 365, 33.42326999980454`}, {"CURE", 365, 4.606137398814223`}, {"CURE", 365, 55.18830509720886`},
{"CURE", 365, 77.21845304661426`}, {"CURE", 365, 67.33313215902629`}, {"CURE", 365, 10.164334405159481`},
{"CURE", 365, 20.385830991146715`}, {"CURE", 365, 207.45376120645105`}, {"CURE", 365, 56.36351806951523`},
{"CURE", 365, 38.40928162332334`}, {"CURE", 365, 13.644354155503496`}, {"CURE", 365, 19.532649521403922`},
{"CURE", 365, 30.19021046570213`}, {"CURE", 365, 7.475839347670226`}, {"CURE", 365, 36.69193528169543`},
{"CURE", 365, 57.731032909115875`}, {"CURE", 365, 35.13025925773966`}, {"CURE", 365, 38.1420563346767`},
{"CURE", 365, 27.589122281459424`}, {"CURE", 365, 17.872372788098716`}, {"CURE", 365, 102.13151825926307`},

{"CURE", 365, 148.01322850403`}, {"CURE", 365, 39.47777278668786`}, {"CURE", 365, 49.842568622721366`},
{"CURE", 365, 63.14283570773857`}, {"CURE", 365, 61.73728048614922`}, {"CURE", 365, 5.391838207104186`},
{"CURE", 365, 72.23868263624017`}, {"CURE", 365, 74.62865107378188`}, {"CURE", 365, 50.45145949780015`},
{"CURE", 365, 202.43958049006847`}, {"CURE", 365, 42.08653030748201`}, {"CURE", 365, 18.747920885602955`},
{"CURE", 365, 44.29454612659036`}, {"CURE", 365, 56.29610774900024`}, {"CURE", 365, 25.219273459254136`},
{"CURE", 365, 19.803532862642985`}, {"CURE", 365, 17.211840047818004`}, {"CURE", 365, 31.212845018903728`},
{"CURE", 365, 96.16919593298176`}, {"CURE", 365, 61.72098830316202`}, {"CURE", 365, 10.75021124486406`},
{"CURE", 365, 63.045536145004476`}, {"CURE", 365, 90.85544395245894`}, {"CURE", 365, 16.70827162244822`},
{"CURE", 365, 95.3645670910978`}, {"CURE", 365, 14.327047177957287`}, {"CURE", 365, 9.149956798427892`},
{"CURE", 365, 12.974956736087268`}, {"CURE", 365, 24.98573996451987`}, {"TBUR", 103.01297262712853`, 111.92560054321308`},
{"CURE", 365, 39.955979684690625`}, {"CURE", 365, 50.46507898885823`}, {"CURE", 365, 12.060760362218023`},
{"TOX", 35.33635294088076`, 230.29815971809828`}, {"CURE", 365, 105.43649508553163`}, {"CURE", 365, 18.00008336068019`},
{"CURE", 365, 44.05231940994595`}, {"CURE", 365, 24.536558185282797`}, {"CURE", 365, 24.79208806258306`},
{"CURE", 365, 83.78581095819565`}, {"TBUR", 70.03041576638678`, 105.97177354789139`}, {"CURE", 365, 11.377678677893492`},
{"CURE", 365, 22.17798565243739`}, {"CURE", 365, 7.8680127567383416`}, {"CURE", 365, 30.33177519000553`},
{"CURE", 365, 52.02380254250713`}, {"CURE", 365, 84.2157120658412`}, {"CURE", 365, 47.00061603503274`},
{"CURE", 365, 169.26438900671573`}, {"CURE", 365, 80.90190636842314`}, {"TBUR", 84.74242156406085`, 13.363185238111091`},
{"CURE", 365, 29.59874452302109`}, {"TBUR", 51.63683414017236`, 32.81876820702699`}, {"CURE", 365, 66.17406626855876`},
{"CURE", 365, 8.682010433135325`}, {"CURE", 365, 23.227897452097192`}, {"CURE", 365, 13.090712152380519`},
{"CURE", 365, 32.64200434986083`}, {"CURE", 365, 74.59374888807916`}, {"CURE", 365, 31.729824823067545`},
{"CURE", 365, 6.891823792921294`}, {"CURE", 365, 131.33544234798407`}, {"CURE", 365, 24.76774541847372`},
{"CURE", 365, 8.410913925492254`}, {"CURE", 365, 11.057511767903184`}, {"CURE", 365, 9.33434869791468`},
{"CURE", 365, 11.924161580316769`}, {"CURE", 365, 95.95596122793692`}, {"CURE", 365, 52.55584573721548`},
{"CURE", 365, 31.62119458681797`}, {"CURE", 365, 14.828595247498043`}, {"CURE", 365, 24.74085739362775`},
{"CURE", 365, 14.314484441457536`}, {"CURE", 365, 34.54525737076547`}, {"CURE", 365, 118.80621696629967`},
{"CURE", 365, 69.86459658719826`}, {"CURE", 365, 34.29730782179384`}, {"CURE", 365, 19.74986423725503`},
{"CURE", 365, 20.86295014978253`}, {"TOX", 10.277575990549423`, 253.77685651218377`}, {"CURE", 365, 12.093022733812075`},
{"CURE", 365, 6.230280520896966`}, {"CURE", 365, 5.436900535189539`}, {"CURE", 365, 69.00592207011074`},
{"CURE", 365, 40.42222883716369`}, {"CURE", 365, 44.83461434747496`}, {"CURE", 365, 66.60273853222986`},
{"CURE", 365, 110.02500943294034`}, {"CURE", 365, 113.71824597335957`}, {"CURE", 365, 43.339775786711286`},
{"CURE", 365, 18.71281405989175`}, {"CURE", 365, 21.70699840918325`}, {"CURE", 365, 43.531168702958205`},

{"CURE", 365, 24.583343732238205`}, {"TBUR", 29.51890721946304`, 9.92753566188931`}, {"CURE", 365, 38.61812737609007`},
{"TOX", 8.647472299495584`, 240.2078657284017`}, {"CURE", 365, 24.742291686145034`}, {"CURE", 365, 8.903070322378424`},
{"CURE", 365, 84.36972987999866`}, {"CURE", 365, 21.10123277311633`}, {"CURE", 365, 60.65948579160736`},
{"TOX", 6.055941270848667`, 291.92092601097926`}, {"CURE", 365, 134.7962779455211`}, {"CURE", 365, 43.75963697774016`},
{"CURE", 365, 36.693322020543796`}, {"CURE", 365, 15.626919603213747`}, {"CURE", 365, 4.463128569469992`},
{"CURE", 365, 17.49928727211437`}, {"CURE", 365, 51.170624712419084`}, {"CURE", 365, 28.385103710122234`},
{"CURE", 365, 90.55627123249147`}, {"CURE", 365, 35.90261624672369`}, {"CURE", 365, 36.64622091390377`},
{"CURE", 365, 10.176734323410392`}, {"CURE", 365, 28.828052136401933`}, {"CURE", 365, 29.579006933053428`},
{"CURE", 365, 7.714137792796902`}, {"CURE", 365, 24.56029897522718`}, {"CURE", 365, 14.633653136096227`},
{"CURE", 365, 23.21133978262789`}, {"CURE", 365, 48.80360729347242`}, {"CURE", 365, 24.308277741292024`},
{"CURE", 365, 5.631856792921352`}, {"CURE", 365, 38.803655023940834`}, {"CURE", 365, 4.346840630700345`},
{"CURE", 365, 12.760562955932892`}, {"CURE", 365, 37.82827017448072`}, {"CURE", 365, 43.59516262931457`},
{"CURE", 365, 8.680749927543108`}, {"CURE", 365, 25.53996021975563`}, {"CURE", 365, 33.123112148114274`},
{"CURE", 365, 167.188943202788`}, {"CURE", 365, 38.378494375870915`}, {"CURE", 365, 10.83282210331939`},
{"CURE", 365, 118.03491920104783`}, {"CURE", 365, 11.624752447773535`}, {"CURE", 365, 43.705120327613244`},
{"CURE", 365, 45.184301933758775`}, {"CURE", 365, 13.48784948677947`}, {"CURE", 365, 15.020949823968985`},
{"CURE", 365, 66.52392151545865`}, {"CURE", 365, 6.503354086219174`}, {"CURE", 365, 56.3556143077686`},
{"CURE", 365, 14.924959178681922`}, {"CURE", 365, 19.51544214518659`}, {"CURE", 365, 4.363786996555112`},
{"CURE", 365, 22.411040831674736`}, {"CURE", 365, 76.5037607414021`}, {"CURE", 365, 29.091218166112544`},
{"CURE", 365, 21.128367494445392`}, {"CURE", 365, 30.19359675270733`}, {"CURE", 365, 23.7304613701881`},
{"CURE", 365, 29.86346587869833`}, {"CURE", 365, 52.67893880246122`}, {"CURE", 365, 50.82536532357481`},
{"CURE", 365, 6.258723543361627`}, {"CURE", 365, 102.8001462587219`}, {"CURE", 365, 11.945735238429174`},
{"CURE", 365, 5.994675715541559`}, {"CURE", 365, 28.578195015576313`}, {"CURE", 365, 29.987981368521996`},
{"CURE", 365, 20.228659206508258`}, {"CURE", 365, 35.10061710280116`}, {"CURE", 365, 30.262588812648666`},
{"CURE", 365, 48.64197142744853`}, {"CURE", 365, 41.10793726427145`}, {"CURE", 365, 58.72820896795648`},
{"CURE", 365, 9.279359980881123`}, {"CURE", 365, 119.04865686371723`}, {"CURE", 365, 115.38571602899563`},
{"CURE", 365, 6.394225490504178`}, {"CURE", 365, 16.090410513707184`}, {"CURE", 365, 39.923742925547664`},
{"CURE", 365, 20.312669859735834`}, {"CURE", 365, 62.468971359986675`}, {"CURE", 365, 125.00551282680614`},
{"CURE", 365, 39.50542002263141`}, {"CURE", 365, 6.783414496892035`}, {"CURE", 365, 6.653141923402785`},
{"CURE", 365, 15.618422200888215`}, {"CURE", 365, 4.715880078949111`}, {"CURE", 365, 53.51812893497083`},
{"CURE", 365, 8.298611180573053`}, {"CURE", 365, 55.85252803525386`}, {"CURE", 365, 34.37833285147115`},

{"CURE", 365, 59.24403794817226`}, {"CURE", 365, 14.587817026119566`}, {"CURE", 365, 73.59682971478928`},
{"TOX", 14.684741432115526`, 243.4973873814079`}, {"CURE", 365, 87.71463154737111`}, {"CURE", 365, 142.1020024677433`},
{"CURE", 365, 41.81786960601921`}, {"CURE", 365, 99.6293978312097`}, {"CURE", 365, 99.83130868084086`},
{"CURE", 365, 12.11598848438903`}, {"CURE", 365, 8.475284022994261`}, {"CURE", 365, 27.56426092311696`},
{"CURE", 365, 57.18413267214992`}, {"CURE", 365, 71.54812979611037`}, {"CURE", 365, 30.728586010101097`},
{"CURE", 365, 45.297033256560276`}, {"CURE", 365, 8.532992453302283`}, {"CURE", 365, 10.844191110983955`},
{"CURE", 365, 52.66972396554942`}, {"CURE", 365, 17.08176861258028`}, {"CURE", 365, 7.244305095760859`},
{"CURE", 365, 10.713995020947666`}, {"CURE", 365, 5.238120254305058`}, {"CURE", 365, 42.56399409959792`},
{"CURE", 365, 58.531977809402576`}, {"CURE", 365, 40.96108865871406`}, {"CURE", 365, 46.52997137380997`},
{"CURE", 365, 25.77610956913228`}, {"CURE", 365, 23.08486624887484`}, {"CURE", 365, 59.722328578727584`},
{"CURE", 365, 63.90475165757309`}, {"CURE", 365, 7.1312671669916865`}, {"CURE", 365, 26.929004491925465`},
{"CURE", 365, 14.172457676693822`}, {"CURE", 365, 22.249779012590484`}, {"CURE", 365, 183.29034343450226`},
{"CURE", 365, 32.364650477269805`}, {"CURE", 365, 22.21329057526255`}, {"CURE", 365, 23.501268059458017`},
{"CURE", 365, 46.868979961872064`}, {"CURE", 365, 35.67604709379668`}, {"CURE", 365, 46.54004880854162`},
{"CURE", 365, 48.00274482637394`}, {"CURE", 365, 10.885884294880631`}, {"CURE", 365, 105.85747851611465`},
{"CURE", 365, 20.334632415084844`}, {"CURE", 365, 43.117274490006004`}, {"CURE", 365, 24.46917225433142`},
{"CURE", 365, 13.893039325177424`}, {"CURE", 365, 50.647930451288154`}, {"CURE", 365, 51.41709050009406`},
{"TBUR", 86.11999526461358`, 28.539950701577087`}, {"CURE", 365, 67.41697357631125`}, {"CURE", 365, 14.26087539279416`},
{"CURE", 365, 119.15764086482015`}, {"CURE", 365, 45.77033965937578`}, {"CURE", 365, 96.78932396558616`},
{"CURE", 365, 3.7530591966109363`}, {"CURE", 365, 47.6226781750958`}, {"CURE", 365, 54.2876765539014`},
{"CURE", 365, 12.897425902210601`}, {"CURE", 365, 32.75005804554933`}, {"CURE", 365, 49.039535607121095`},
{"CURE", 365, 29.80849742387268`}, {"CURE", 365, 9.486071512951527`}, {"CURE", 365, 7.198353900722607`},
{"CURE", 365, 42.57180106452851`}, {"CURE", 365, 11.35536695636295`}, {"CURE", 365, 25.244734343963632`},
{"CURE", 365, 30.777648022031862`}, {"CURE", 365, 14.33376222437141`}, {"CURE", 365, 26.3726771107217`},
{"CURE", 365, 7.179417476339179`}, {"CURE", 365, 29.59710233369222`}, {"CURE", 365, 4.784742571765864`},
{"CURE", 365, 8.555239493656105`}, {"CURE", 365, 20.227875023453663`}, {"CURE", 365, 6.523725723586296`},
{"CURE", 365, 48.475708939022084`}, {"CURE", 365, 71.1933020300483`}, {"CURE", 365, 72.27838352364883`},
{"CURE", 365, 60.16971870149346`}, {"CURE", 365, 113.58101166037983`}, {"CURE", 365, 9.188843162539824`},
{"CURE", 365, 40.97616403879715`}, {"CURE", 365, 44.023551870265266`}, {"CURE", 365, 75.64932552629217`},
{"CURE", 365, 56.504170347363164`}, {"CURE", 365, 25.829197573027198`}, {"CURE", 365, 121.45774510770076`},
{"CURE", 365, 3.7914428359728243`}, {"CURE", 365, 115.52933544309234`}, {"CURE", 365, 7.824576671745383`},

{"CURE", 365, 98.67434869850814`}, {"CURE", 365, 89.36283853142012`}, {"CURE", 365, 26.491050255884282`},
{"CURE", 365, 104.31631016299575`}, {"CURE", 365, 127.23138388705891`}, {"CURE", 365, 56.914592706974055`},
{"CURE", 365, 25.111045659809637`}, {"CURE", 365, 28.87122298194375`}, {"CURE", 365, 42.638791613618565`},
{"CURE", 365, 39.32443360830997`}, {"CURE", 365, 9.52930901773866`}, {"CURE", 365, 34.21609919171865`},
{"CURE", 365, 32.378230549841405`}, {"CURE", 365, 24.21377452558075`}, {"CURE", 365, 59.81655867990125`},
{"CURE", 365, 9.419279387325954`}, {"CURE", 365, 66.76947707419791`}, {"CURE", 365, 43.69489108261107`},
{"CURE", 365, 24.737369236441914`}, {"CURE", 365, 22.51878613540874`}, {"CURE", 365, 45.666032449762206`},
{"CURE", 365, 6.2515306407681965`}, {"CURE", 365, 72.41065518727888`}, {"CURE", 365, 11.433405577610678`},
{"CURE", 365, 16.214959670301678`}, {"CURE", 365, 36.381024760937606`}, {"CURE", 365, 28.521053142429686`},
{"CURE", 365, 26.22668734863002`}, {"CURE", 365, 81.66812061241154`}, {"CURE", 365, 11.045902030313423`},
{"CURE", 365, 19.231185153064025`}, {"CURE", 365, 142.26891000918883`}, {"CURE", 365, 10.795364297826866`},
{"CURE", 365, 27.796892066604016`}, {"CURE", 365, 42.30774908427815`}, {"CURE", 365, 41.773126122295714`},
{"CURE", 365, 28.358306845919554`}, {"CURE", 365, 146.58403895970176`}, {"CURE", 365, 26.484170923031925`},
{"CURE", 365, -8.60071523707571`*^23}, {"CURE", 365, 22.25792054064943`}, {"CURE", 365, 17.172296723911035`},
{"CURE", 365, 13.162673750329667`}, {"CURE", 365, 34.51380809793236`}, {"CURE", 365, 59.10691674381686`},
{"CURE", 365, 32.521393327906125`}, {"CURE", 365, 37.91652357338949`}, {"CURE", 365, 83.01292581708863`},
{"CURE", 365, 9.15661553223488`}, {"CURE", 365, 10.774512752934854`}, {"CURE", 365, 90.88308412924664`},
{"CURE", 365, 29.27936208942811`}, {"CURE", 365, 31.687446093569296`}, {"CURE", 365, 15.929627153838428`},
{"CURE", 365, 36.475325385088496`}, {"CURE", 365, 45.45702132780554`}, {"CURE", 365, 105.36411055620535`},
{"CURE", 365, 15.529676068055254`}, {"CURE", 365, 60.71427811026858`}, {"CURE", 365, 35.711014007797054`},
{"CURE", 365, 94.55057645953`}, {"CURE", 365, 154.37369063322961`}, {"CURE", 365, 56.53179504501483`},
{"CURE", 365, 5.473933176257386`}, {"CURE", 365, 42.833007376089554`}, {"CURE", 365, 13.532383275431915`},
{"CURE", 365, 143.02386152182632`}, {"TBUR", 52.444067641703555`, 4.561297558303252`}, {"CURE", 365, 4.774357359209588`},
{"CURE", 365, 165.64921482255514`}, {"CURE", 365, 38.12219721442026`}, {"CURE", 365, 12.884444082199662`},
{"CURE", 365, 24.660165219846853`}, {"CURE", 365, 8.653793696051695`}, {"TBUR", 81.87999123874553`, 8.237579959710807`},
{"CURE", 365, 9.837833110179089`}, {"CURE", 365, 35.5610527194243`}, {"CURE", 365, 75.37167653043896`},
{"CURE", 365, 29.857583351495016`}, {"CURE", 365, 7.4774553286897545`}, {"CURE", 365, 101.68899279840079`},
{"CURE", 365, 203.27951376791407`}, {"CURE", 365, 5.731672958126588`}, {"CURE", 365, 45.82506554136296`},
{"CURE", 365, 24.605924055277537`}, {"CURE", 365, 4.838797714889123`}, {"CURE", 365, 37.83355145308404`},
{"CURE", 365, 24.168487707890602`}, {"CURE", 365, 18.555128804783497`}, {"CURE", 365, 36.08104500263705`},
{"CURE", 365, 20.481403637070812`}, {"CURE", 365, 60.32432206608574`}, {"CURE", 365, 35.80065336389335`},

{"CURE", 365, 84.30199377780524`}, {"CURE", 365, 37.345107341341`}, {"CURE", 365, 18.506078905600265`},
{"CURE", 365, 9.001465596561726`}, {"CURE", 365, 6.240572210942778`}, {"CURE", 365, 17.77520430312918`},
{"CURE", 365, 70.74509268850123`}, {"CURE", 365, 23.329487910516942`}, {"CURE", 365, 21.381813231357274`},
{"CURE", 365, 15.11789188304254`}, {"CURE", 365, 15.321269113532965`}, {"CURE", 365, 41.40146734798675`},
{"CURE", 365, 7.745350866569256`}, {"CURE", 365, 49.351053169020624`}, {"CURE", 365, 22.574132376735285`},
{"CURE", 365, 13.279239999148759`}, {"CURE", 365, 21.032737329588375`}, {"CURE", 365, 46.65119546023516`},
{"CURE", 365, 34.815500270628625`}, {"CURE", 365, 4.1945410360997295`}, {"CURE", 365, 18.076828397649837`},
{"TOX", 5.409895495771662`, 302.0788170832021`}, {"CURE", 365, 50.20356408184916`}, {"CURE", 365, 73.20238750633484`},
{"CURE", 365, 21.57016472382248`}, {"CURE", 365, 79.96714781765827`}, {"CURE", 365, 9.567038903926784`},
{"CURE", 365, 32.246994629977905`}, {"CURE", 365, 148.80642537607488`}, {"CURE", 365, 27.862984487347024`},
{"CURE", 365, 24.56473877018641`}, {"CURE", 365, 42.064653108641124`}, {"CURE", 365, 22.282406871392695`},
{"CURE", 365, 4.941735482384101`}, {"CURE", 365, 45.94431581591292`}, {"CURE", 365, 8.195700307585469`},
{"CURE", 365, 19.48834531419169`}, {"CURE", 365, 46.00141715784292`}, {"CURE", 365, 16.521535171470738`},
{"CURE", 365, 87.74267771392185`}, {"CURE", 365, 26.096774149258614`}, {"CURE", 365, 44.910899558972424`},
{"CURE", 365, 31.130547268832846`}, {"CURE", 365, 8.809247176819932`}, {"CURE", 365, 32.697851129538414`},
{"CURE", 365, 70.63275810081565`}, {"CURE", 365, 23.675603365136098`}, {"CURE", 365, 17.816527731674398`},
{"CURE", 365, 8.453181618629706`}, {"CURE", 365, 26.936211785749112`}, {"CURE", 365, 32.18369929770695`},
{"CURE", 365, 91.79129315188987`}, {"CURE", 365, 14.745959083891577`}, {"CURE", 365, 22.204857114042102`},
{"CURE", 365, 86.16769931769727`}, {"CURE", 365, 9.078475846579703`}, {"CURE", 365, 124.68192751575998`},
{"CURE", 365, 10.533386526403511`}, {"CURE", 365, 19.290005347365792`}, {"CURE", 365, 44.45444871231868`},
{"CURE", 365, 71.61648336808908`}, {"CURE", 365, 32.20534547668365`}, {"CURE", 365, 16.88769538842219`},
{"CURE", 365, 4.9622841958730515`}, {"CURE", 365, 43.477142997954154`}, {"CURE", 365, 39.3741146092316 5`},
{"CURE", 365, 38.6802785044123`}, {"CURE", 365, 15.251250038370339`}, {"CURE", 365, 37.38604817598842`},
{"CURE", 365, 4.326322996825757`}, {"CURE", 365, 24.929588407689423`}, {"CURE", 365, 10.887201731074914`},
{"CURE", 365, 4.822855283391139`}, {"CURE", 365, 25.061659259304523`}, {"CURE", 365, 5.362019839180301`},
{"CURE", 365, 17.050111541037115`}, {"CURE", 365, 11.694770551834614`}, {"TBUR", 50.75289286504347`, 7.179677570798457`},
{"CURE", 365, 23.164265758250007`}, {"CURE", 365, 61.710720222234485`}, {"CURE", 365, 33.80894553757239`},
{"CURE", 365, 30.59743665907693`}, {"CURE", 365, 48.66386236966391`}, {"CURE", 365, 41.27537159920662`}},
{{"2500 Res", "2500 OS", "2500 Tox"}, {"CURE", 365, 20.84506287798869`}, {"CURE", 365, 43.09563239149779`},
{"CURE", 365, 30.22148494136364`}, {"TBUR", 78.3636086993474`, 10.148410978280069`},
{"TBUR", 55.8094315004391`, 38.97613064730858`}, {"CURE", 365, 78.06512632707836`}, {"CURE", 365, 73.44728364135572`},

{"CURE", 365, 7.67511575490135`}, {"CURE", 365, 52.97042158604492`}, {"CURE", 365, 45.82759007456743`},
{"CURE", 365, 171.98742957225`}, {"CURE", 365, 21.337872874149518`}, {"CURE", 365, 77.307325452172`},
{"TOX", 7.903880665974006`, 256.64684987207795`}, {"CURE", 365, 36.29131262232342`}, {"CURE", 365, 56.64754697116556`},
{"CURE", 365, 112.12618231262722`}, {"CURE", 365, 50.24286493448898`}, {"TBUR", 145.43155022057422`, 38.06116720448752`},
{"CURE", 365, 12.59067766526198`}, {"CURE", 365, 77.82199548870652`}, {"CURE", 365, 31.544499231361637`},
{"CURE", 365, 12.08889519748813`}, {"CURE", 365, 9.63253235338464`}, {"CURE", 365, 43.82119342037942`},
{"CURE", 365, 48.097993439745494`}, {"CURE", 365, 4.372854371112147`}, {"CURE", 365, 6.628349406564268`},
{"CURE", 365, 24.56250076396669`}, {"CURE", 365, 28.736623946564116`}, {"CURE", 365, 24.509545848697467`},
{"CURE", 365, 86.24351860667494`}, {"CURE", 365, 18.95322379023273`}, {"CURE", 365, 15.082851128523412`},
{"CURE", 365, 122.16908641219369`}, {"CURE", 365, 7.488481435964767`}, {"CURE", 365, 32.349291422516536`},
{"TBUR", 52.50055778862698`, 72.29224781531659`}, {"CURE", 365, 17.988936686700576`}, {"CURE", 365, 27.68085390598283`},
{"CURE", 365, 63.18268375375777`}, {"CURE", 365, 42.59246134340658`}, {"CURE", 365, 8.642819701186612`},
{"CURE", 365, 29.157215762262346`}, {"CURE", 365, 42.06228432309957`}, {"CURE", 365, 27.57831955939994`},
{"TOX", 5.462991429312433`, 293.06894899941824`}, {"CURE", 365, 63.1778401030098`}, {"CURE", 365, 98.10253119567501`},
{"CURE", 365, 11.818572821527614`}, {"CURE", 365, 33.34051565525847`}, {"CURE", 365, 45.55590076018734`},
{"CURE", 365, 36.35744408574085`}, {"CURE", 365, 23.600802111765795`}, {"CURE", 365, 10.169349840048296`},
{"CURE", 365, 107.53934574415176`}, {"CURE", 365, 33.79242588576689`}, {"CURE", 365, 6.423429441136427`},
{"CURE", 365, 69.20773844598178`}, {"CURE", 365, 48.48314863086196`}, {"CURE", 365, 33.19592041717584`},
{"CURE", 365, 25.390859967414123`}, {"CURE", 365, 14.488862680639743`}, {"CURE", 365, 54.7224560126325`},
{"CURE", 365, 38.89192709490031`}, {"CURE", 365, 44.457195760731`}, {"CURE", 365, 10.17616697328269`},
{"CURE", 365, 10.346890362166901`}, {"CURE", 365, 11.002107991172556`}, {"CURE", 365, 44.936267548265214`},
{"CURE", 365, 16.451406025418464`}, {"CURE", 365, 36.647362488391856`}, {"CURE", 365, 68.76542539474771`},
{"TBUR", 29.966155483268082`, 21.898168910951615`}, {"CURE", 365, 18.38097855171566`}, {"CURE", 365, 50.89757919149924`},
{"CURE", 365, 29.774695614911113`}, {"CURE", 365, 22.641477538610854`}, {"CURE", 365, 90.22588798678808`},
{"CURE", 365, 99.04977054224747`}, {"CURE", 365, 72.92681673274349`}, {"CURE", 365, 19.674114403714263`},
{"CURE", 365, 39.778260286129516`}, {"CURE", 365, 27.804305734417255`}, {"CURE", 365, 31.443613499384167`},
{"CURE", 365, 14.737150055112693`}, {"CURE", 365, 157.2100323727059`}, {"CURE", 365, 34.26430267201847`},
{"CURE", 365, 43.14236518548276`}, {"CURE", 365, 37.139286181994066`}, {"CURE", 365, 30.73914568853315`},
{"CURE", 365, 9.102530624564643`}, {"CURE", 365, 16.679973703083586`}, {"CURE", 365, 38.61682909758191`},
{"TBUR", 64.14283326202087`, 14.025879366569782`}, {"CURE", 365, 16.631582379080225`}, {"CURE", 365, 43.35178199859234`},
{"CURE", 365, 110.02202128848776`}, {"CURE", 365, 58.957784702027936`}, {"CURE", 365, 29.570783787029523`},

{"CURE", 365, 58.65313234970572`}, {"CURE", 365, 38.12040008611253`}, {"CURE", 365, 24.699810267144382`},
{"CURE", 365, 169.1237186051225`}, {"CURE", 365, 18.784840572305665`}, {"CURE", 365, 12.763547919623749`},
{"CURE", 365, 74.93095719042027`}, {"CURE", 365, 22.136685011437663`}, {"CURE", 365, 22.601841654027968`},
{"CURE", 365, 135.9865794206542`}, {"CURE", 365, 11.038095874432107`}, {"CURE", 365, 7.003820970168324`},
{"CURE", 365, 29.131128923392847`}, {"CURE", 365, 23.033041862843834`}, {"CURE", 365, 17.347725897453113`},
{"CURE", 365, 44.27280601783915`}, {"CURE", 365, 12.462409747737901`}, {"CURE", 365, 7.662586687034466`},
{"CURE", 365, 35.865703569255174`}, {"CURE", 365, 54.855128267382575`}, {"CURE", 365, 9.344409576710257`},
{"CURE", 365, 30.922484232851485`}, {"CURE", 365, 93.85500359705814`}, {"TBUR", 77.92082651540203`, 35.80451201308113`},
{"CURE", 365, 20.821768204027492`}, {"CURE", 365, 72.6612490482558`}, {"CURE", 365, 36.51639378956379`},
{"CURE", 365, 4.8129331347549895`}, {"CURE", 365, 41.682183174003725`}, {"CURE", 365, 32.192007595593594`},
{"CURE", 365, 25.645661552135355`}, {"CURE", 365, 30.422817532109175`}, {"CURE", 365, 7.9220083476591885`},
{"CURE", 365, 45.19436109534276`}, {"CURE", 365, 16.22397095370519`}, {"CURE", 365, 36.677101675731045`},
{"CURE", 365, 136.96139148010175`}, {"CURE", 365, 48.409234697338945`}, {"CURE", 365, 112.42440101988747`},
{"CURE", 365, 37.036882815950484`}, {"CURE", 365, 153.0992434554726`}, {"CURE", 365, 4.771983449891155`},
{"CURE", 365, 41.90528915670303`}, {"CURE", 365, 47.76905713068228`}, {"CURE", 365, 6.204218221318528`},
{"CURE", 365, 8.596000365746708`}, {"CURE", 365, 52.75995967108252`}, {"CURE", 365, 62.19940074148636`},
{"CURE", 365, 52.984524808878476`}, {"CURE", 365, 43.35632032833694`}, {"CURE", 365, 41.44357384123908`},
{"CURE", 365, 25.098004658584014`}, {"CURE", 365, 6.63719423298161`}, {"CURE", 365, 28.880465264579602`},
{"CURE", 365, 13.703868812913246`}, {"CURE", 365, 6.708854707890867`}, {"CURE", 365, 40.652622431597386`},
{"CURE", 365, 22.743136344638287`}, {"CURE", 365, 60.12072890416691`}, {"CURE", 365, 24.272823885002982`},
{"CURE", 365, 20.202013608470278`}, {"CURE", 365, 135.52998707258692`}, {"CURE", 365, 10.095378636482685`},
{"CURE", 365, 30.2117718674137`}, {"CURE", 365, 17.119711650970018`}, {"CURE", 365, 40.46315514323562`},
{"CURE", 365, 11.117812515177166`}, {"CURE", 365, 44.38202876311518`}, {"CURE", 365, 117.28491697160085`},
{"CURE", 365, 10.030245457627764`}, {"CURE", 365, 30.457012218511267`}, {"CURE", 365, 32.9269603700089`},
{"CURE", 365, 118.4303831416017`}, {"CURE", 365, 44.589236365178955`}, {"CURE", 365, 37.02521749519917`},
{"CURE", 365, 65.0033110150713`}, {"CURE", 365, 24.91699523961999`}, {"CURE", 365, 77.58682146045719`},
{"CURE", 365, 47.976066344179245`}, {"CURE", 365, 29.389837513175912`}, {"CURE", 365, 26.463538759474687`},
{"CURE", 365, 8.604295228691354`}, {"CURE", 365, 5.830074175576483`}, {"CURE", 365, 13.66833358910956`},
{"CURE", 365, 18.304343116931904`}, {"CURE", 365, 8.0664489710996`}, {"CURE", 365, 29.52383669499449`},
{"CURE", 365, 80.56359099511779`}, {"CURE", 365, 21.777154693813788`}, {"CURE", 365, 40.432009649692816`},
{"CURE", 365, 50.620661776714215`}, {"CURE", 365, 43.5680359404545`}, {"CURE", 365, 29.627291059953418`},

{"CURE", 365, 11.76878803781719`}, {"CURE", 365, 81.08848402819649`}, {"CURE", 365, 7.014860730839197`},
{"CURE", 365, 75.58544216644746`}, {"CURE", 365, 73.51258255104263`}, {"CURE", 365, 20.49974456847287`},
{"CURE", 365, 24.3487414035667`}, {"CURE", 365, 140.03084611167975`}, {"CURE", 365, 36.12610864990822`},
{"CURE", 365, 43.070613515852806`}, {"CURE", 365, 26.981265153088444`}, {"CURE", 365, 10.144708900655637`},
{"CURE", 365, 62.22233941514013`}, {"TBUR", 66.18190563889269`, 165.43027796729606`}, {"CURE", 365, 25.258001080549043`},
{"CURE", 365, 22.840144124346413`}, {"CURE", 365, 42.07715560328036`}, {"CURE", 365, 16.801178901656986`},
{"CURE", 365, 213.2268057758634`}, {"CURE", 365, 17.0665672178469`}, {"CURE", 365, 105.65559813507966`},
{"CURE", 365, 81.07011900721439`}, {"CURE", 365, 78.85846592619568`}, {"CURE", 365, 42.12054620640756`},
{"CURE", 365, 97.62256487117257`}, {"TOX", 10.794589997816926`, 249.02814203298425`}, {"CURE", 365, 44.35336929308737`},
{"CURE", 365, 8.149243399597413`}, {"CURE", 365, 72.37301887941179`}, {"CURE", 365, 45.09647237527921`},
{"CURE", 365, 43.47811506638748`}, {"CURE", 365, 9.592620670428822`}, {"CURE", 365, 219.7552968705348`},
{"CURE", 365, 16.065980882397337`}, {"CURE", 365, 69.68787459867502`}, {"CURE", 365, 43.93733861826798`},
{"CURE", 365, 31.22050208327781`}, {"CURE", 365, 7.504786059706962`}, {"CURE", 365, 56.40815811670273`},
{"CURE", 365, 33.09409408784971`}, {"CURE", 365, 87.55026581693653`}, {"CURE", 365, 57.68644943698061`},
{"CURE", 365, 72.83439983062304`}, {"CURE", 365, 34.98434425439102`}, {"CURE", 365, 31.120749966888415`},
{"CURE", 365, 162.6177359998983`}, {"CURE", 365, 42.570630379807`}, {"CURE", 365, 11.359774956858415`},
{"CURE", 365, 38.253935959503565`}, {"CURE", 365, 38.952925270984224`}, {"CURE", 365, 5.223325747778451`},
{"CURE", 365, 9.29647351900668`}, {"CURE", 365, 14.20495978168233`}, {"CURE", 365, 134.20988150328873`},
{"CURE", 365, 41.24770423071768`}, {"CURE", 365, 42.980437368554284`}, {"CURE", 365, 34.42718058664681`},
{"CURE", 365, 141.67886839884153`}, {"CURE", 365, 32.5917479389623`}, {"CURE", 365, 65.71651250120561`},
{"CURE", 365, 32.505319044803215`}, {"CURE", 365, 47.916517753569686`}, {"CURE", 365, 11.52295840306566`},
{"CURE", 365, 43.09889624628948`}, {"CURE", 365, 26.897351141595`}, {"CURE", 365, 52.38225580660515`},
{"CURE", 365, 82.79381180998924`}, {"CURE", 365, 93.40341409604152`}, {"CURE", 365, 56.93786043436021`},
{"CURE", 365, 46.156971419058245`}, {"CURE", 365, 63.971067321449695`}, {"CURE", 365, 30.2258035467127`},
{"CURE", 365, 34.21935388954744`}, {"CURE", 365, 24.253154585605884`}, {"CURE", 365, 19.001189450786615`},
{"CURE", 365, 30.607617145755665`}, {"CURE", 365, 78.52904807765009`}, {"CURE", 365, 9.001111319604687`},
{"CURE", 365, 39.893308433207395`}, {"CURE", 365, 7.531213709813429`}, {"CURE", 365, 39.204147982106`},
{"CURE", 365, 22.28707168956855`}, {"CURE", 365, 82.89270614078279`}, {"CURE", 365, 7.64317473766884`},
{"CURE", 365, 54.59798182636748`}, {"CURE", 365, 68.4610223838633`}, {"CURE", 365, 11.199135073450337`},
{"CURE", 365, 25.665150895769028`}, {"CURE", 365, 20.649358523296833`}, {"CURE", 365, 11.821495613447924`},
{"CURE", 365, 69.82045421260457`}, {"CURE", 365, 26.24771117167816`}, {"CURE", 365, 50.568774133916186`},

{"CURE", 365, 49.17600007021248`}, {"CURE", 365, 55.75230780796327`}, {"CURE", 365, 43.59053671940951`},
{"CURE", 365, 52.197931265106284`}, {"CURE", 365, 41.303689419856504`}, {"CURE", 365, 79.86604555409444`},
{"CURE", 365, 48.65161629189816`}, {"CURE", 365, 39.739229728456124`}, {"CURE", 365, 101.61087195210143`},
{"CURE", 365, 11.764445171189898`}, {"CURE", 365, 66.72763544845489`}, {"CURE", 365, 4.9017831239747744`},
{"CURE", 365, 60.901829715764094`}, {"CURE", 365, 14.350542800818447`}, {"CURE", 365, 75.99776719502954`},
{"CURE", 365, 11.71959320027908`}, {"CURE", 365, 76.76517130332135`}, {"CURE", 365, 192.1759913991726`},
{"CURE", 365, 8.38836962035605`}, {"CURE", 365, 6.026261118305758`}, {"CURE", 365, 22.176657495607525`},
{"CURE", 365, 5.38794103668398`}, {"CURE", 365, 3.6820940193722036`}, {"CURE", 365, 21.540366181906982`},
{"CURE", 365, 35.241888054910625`}, {"CURE", 365, 37.89606160540083`}, {"CURE", 365, 36.046316711893745`},
{"CURE", 365, 28.07013821569267`}, {"CURE", 365, 49.35902517610151`}, {"CURE", 365, 86.89671377896249`},
{"CURE", 365, 7.03221235974488`}, {"CURE", 365, 38.523188934659316`}, {"CURE", 365, 33.10536689544247`},
{"CURE", 365, 24.003673129717743`}, {"CURE", 365, 44.39725909642958`}, {"CURE", 365, 65.8133794815833`},
{"CURE", 365, 153.98761019896386`}, {"CURE", 365, 114.7595345971291`}, {"CURE", 365, 35.510081173290686`},
{"CURE", 365, 3.768421635266429`}, {"CURE", 365, 41.10944357835674`}, {"CURE", 365, 106.0018997287726`},
{"CURE", 365, 26.67379553190295`}, {"CURE", 365, 34.060038633063314`}, {"CURE", 365, 20.580079620581408`},
{"CURE", 365, 30.16402796555383`}, {"CURE", 365, 7.816630812666903`}, {"CURE", 365, 38.227498708015546`},
{"CURE", 365, 21.49350172083726`}, {"CURE", 365, 62.76647259720253`}, {"CURE", 365, 43.00434881776445`},
{"CURE", 365, 41.67264882059501`}, {"CURE", 365, 34.28874988108877`}, {"CURE", 365, 18.145509053240023`},
{"CURE", 365, 12.505001154735046`}, {"CURE", 365, 12.192277510964065`}, {"CURE", 365, 15.795410791572307`},
{"CURE", 365, 12.30755250762169`}, {"CURE", 365, 165.55220671970133`}, {"CURE", 365, 92.08478163507189`},
{"CURE", 365, 17.78787359376063`}, {"TOX", 7.05408406809448`, 273.9908228270126`}, {"CURE", 365, 78.35364841903633`},
{"CURE", 365, 41.4860466503421`}, {"CURE", 365, 71.7664117183194`}, {"CURE", 365, 10.789883157848239`},
{"CURE", 365, 86.05138260844706`}, {"CURE", 365, 230.02900019666538`}, {"CURE", 365, 12.482042494865397`},
{"CURE", 365, 38.93461285024517`}, {"CURE", 365, 63.791307237343176`}, {"CURE", 365, 32.27343317346086`},
{"CURE", 365, 15.798723674903101`}, {"CURE", 365, 12.586286107165805`}, {"CURE", 365, 109.2506585985884`},
{"CURE", 365, 57.35533642621764`}, {"CURE", 365, 32.629994452959636`}, {"CURE", 365, 81.10575250678522`},
{"CURE", 365, 14.64868812589608`}, {"CURE", 365, 39.12150315929379`}, {"CURE", 365, 23.62686510147463`},
{"CURE", 365, 159.33836481894966`}, {"CURE", 365, 40.13512725185612`}, {"CURE", 365, 186.27412309658254`},
{"TOX", 6.247251420476217`, 290.7362792874093`}, {"CURE", 365, 22.239400807041662`}, {"CURE", 365, 6.272017320387238`},
{"CURE", 365, 5.4637879941517715`}, {"CURE", 365, 39.972547941706395`}, {"CURE", 365, 155.06763024645724`},
{"CURE", 365, 31.063634571868906`}, {"CURE", 365, 23.866858600488893`}, {"CURE", 365, 44.87629252801503`},

```
{"CURE", 365, 49.14793369109498`}, {"CURE", 365, 204.83260705615578`}, {"CURE", 365, 168.85435724412233`},
{"CURE", 365, 35.37656654519264`}, {"CURE", 365, 11.736721106205382`}, {"CURE", 365, 28.753944813458588`},
{"CURE", 365, 4.512617158530314`}, {"CURE", 365, 84.90038585192227`}, {"CURE", 365, 126.20334689380115`},
{"CURE", 365, 41.276492966189636`}, {"CURE", 365, 32.71240800332872`}, {"CURE", 365, 138.3220145361043`},
{"CURE", 365, 8.779779232357251`}, {"CURE", 365, 11.432933599463484`}, {"CURE", 365, 35.76960121843869`},
{"CURE", 365, 46.034757886754285`}, {"TBUR", 95.81974055885173`, 87.27368063193299`}, {"CURE", 365, 89.75133579877617`},
{"CURE", 365, 36.841916740627305`}, {"CURE", 365, 38.01420898382081`}, {"CURE", 365, 47.636970649537105`},
{"CURE", 365, 16.485724761564608`}, {"CURE", 365, 4.6538021789814605`}, {"CURE", 365, 48.95409278598966`},
{"CURE", 365, 58.93614060076104`}, {"CURE", 365, 28.655221232067124`}, {"CURE", 365, 51.92931258095191`},
{"CURE", 365, 16.17746354644346`}, {"CURE", 365, 10.629750172742682`}, {"CURE", 365, 46.53193058393775`},
{"CURE", 365, 28.290130550926033`}, {"CURE", 365, 40.43135389445719`}, {"TOX", 10.000871118446248`, 266.8014061436178`},
{"CURE", 365, 25.422271079852667`}, {"CURE", 365, 12.401907262310145`}, {"CURE", 365, 9.622454548843839`},
{"CURE", 365, 11.662080487784387`}, {"CURE", 365, 66.62648844274032`}, {"CURE", 365, 4.5881513488961145`},
{"CURE", 365, 62.03689201507834`}, {"CURE", 365, 67.7227870906767`}, {"CURE", 365, 52.85840925546671`},
{"CURE", 365, 43.33545634456122`}, {"CURE", 365, 8.595243215407722`}, {"CURE", 365, 94.32262434808906`},
{"CURE", 365, 28.59026535524451`}, {"CURE", 365, 12.974618914318519`}, {"CURE", 365, 84.00018600115578`},
{"CURE", 365, 14.940043979332993`}, {"CURE", 365, 100.64399204114653`}, {"CURE", 365, 64.5829174253586`},
{"CURE", 365, 43.09531703834689`}, {"CURE", 365, 62.584891326400076`}, {"CURE", 365, 22.199558410574646`},
{"CURE", 365, 98.18734846047411`}, {"CURE", 365, 11.64964450403939`}, {"CURE", 365, 49.38161835497974`},
{"CURE", 365, 57.696831933704175`}, {"CURE", 365, 24.53198010918656`}, {"CURE", 365, 228.61206078101583`},
{"CURE", 365, 45.91040043577718`}, {"CURE", 365, 46.79091409102546`}, {"CURE", 365, 47.8064384869155`},
{"CURE", 365, 191.4366738871855`}, {"CURE", 365, 60.82850110613945`}, {"CURE", 365, 56.75032257810831`},
{"CURE", 365, 123.53610956222228`}, {"CURE", 365, 43.44037825520062`}, {"CURE", 365, 37.85064349300393`},
{"CURE", 365, 46.766563480269284`}, {"CURE", 365, 64.59779333945389`}, {"CURE", 365, 114.14673638790757`},
{"CURE", 365, 24.632674493593694`}, {"CURE", 365, 22.48706284938802`}, {"CURE", 365, 48.11684929134426`},
{"CURE", 365, 48.988881070466014`}, {"CURE", 365, 55.15338435685553`}, {"CURE", 365, 58.9018873573738`},
{"CURE", 365, 32.927193254633174`}, {"CURE", 365, 16.571827353209613`}, {"CURE", 365, 62.291419154921996`},
{"CURE", 365, 15.711184977948847`}, {"CURE", 365, 33.39710171193598`}, {"CURE", 365, 39.80009717082444`},
{"CURE", 365, 11.980972462789842`}, {"CURE", 365, 5.95616980642459`}, {"CURE", 365, 36.84986226694533`},
{"CURE", 365, 49.90247372647734`}, {"CURE", 365, 59.23043277957712`}, {"CURE", 365, 42.37203281084172`},
{"CURE", 365, 5.42949943509814`}, {"CURE", 365, 48.586014709773686`}, {"CURE", 365, 9.258442921536538`},
```

{"CURE", 365, 142.7761565346588`}, {"CURE", 365, 185.78559940012673`}, {"CURE", 365, 26.619964503436375`},
{"CURE", 365, 36.73577987181329`}, {"CURE", 365, 199.44530676313457`}, {"CURE", 365, 35.76503737407373`},
{"CURE", 365, 20.801442677742088`}, {"CURE", 365, 16.19324199464258`}, {"CURE", 365, 4.423440600469969`},
{"CURE", 365, 27.881413662042625`}, {"CURE", 365, 31.090120720896547`}, {"CURE", 365, 82.42668944960144`},
{"CURE", 365, 39.437728812791235`}, {"CURE", 365, 68.66036650258613`}, {"CURE", 365, 24.25337803818438`},
{"CURE", 365, 52.67987908669782`}, {"CURE", 365, 117.63281900242389`}, {"CURE", 365, 11.968577302493307`},
{"CURE", 365, 18.455282502813276`}, {"CURE", 365, 27.41891808059228`}, {"CURE", 365, 38.29449117807156`},
{"CURE", 365, 25.150161074753747`}, {"CURE", 365, 24.036190449420026`}, {"CURE", 365, 10.192352485400303`},
{"CURE", 365, 15.720032584578345`}, {"CURE", 365, 29.813951879466085`}, {"CURE", 365, 67.82719249211381`},
{"CURE", 365, 60.405262898627846`}, {"CURE", 365, 30.909763668939398`}, {"TBUR", 48.596504454919106`, 46.43271297916165`},
{"CURE", 365, 99.88842262999961`}, {"CURE", 365, 48.84097730806258`}, {"CURE", 365, 36.54471221057648`},
{"CURE", 365, 23.27699228733021`}, {"CURE", 365, 7.294735278640424`}, {"CURE", 365, 40.571746194705696`},
{"CURE", 365, 46.83717526602996`}, {"CURE", 365, 183.46750394156413`}, {"CURE", 365, 9.351857825887608`},
{"CURE", 365, 9.133291139500706`}, {"CURE", 365, 29.264151162435525`}, {"CURE", 365, 85.30481235393302`},
{"CURE", 365, 10.64586540889469`}, {"CURE", 365, 52.074511892794725`}, {"CURE", 365, 12.146442692313727`},
{"CURE", 365, 30.26990773244146`}, {"CURE", 365, 12.35726373011037`}, {"CURE", 365, 12.835021604868503`},
{"CURE", 365, 11.57564374254703`}, {"CURE", 365, 123.4990574850029`}, {"CURE", 365, 19.778919859322215`},
{"CURE", 365, 34.83045310804257`}, {"CURE", 365, 4.79814764929983`}, {"CURE", 365, 57.48981218494133`},
{"CURE", 365, 80.43740552447541`}, {"CURE", 365, 70.14812335428832`}, {"CURE", 365, 10.588618461761879`},
{"CURE", 365, 21.235754605831904`}, {"CURE", 365, 216.11695159197578`}, {"CURE", 365, 58.71582080095195`},
{"CURE", 365, 40.01595546728646`}, {"CURE", 365, 14.219116275817742`}, {"CURE", 365, 20.348349271815376`},
{"CURE", 365, 31.452469405022978`}, {"CURE", 365, 7.788698999648336`}, {"CURE", 365, 38.222058386568115`},
{"CURE", 365, 60.136883045404744`}, {"CURE", 365, 36.59814282675455`}, {"CURE", 365, 39.739769680661986`},
{"CURE", 365, 28.73931258887847`}, {"CURE", 365, 18.61983557941518`}, {"CURE", 365, 106.40786825657395`},
{"CURE", 365, 154.18702863182142`}, {"CURE", 365, 41.122904359984865`}, {"CURE", 365, 51.91971253265609`},
{"CURE", 365, 65.77458776321359`}, {"CURE", 365, 64.31159107713918`}, {"CURE", 365, 5.617363529990245`},
{"CURE", 365, 75.25958026843578`}, {"CURE", 365, 77.7384835439443`}, {"CURE", 365, 52.55936556588428`},
{"CURE", 365, 210.87615820092924`}, {"CURE", 365, 43.84069718985934`}, {"CURE", 365, 19.535417644398418`},
{"CURE", 365, 46.14128052794997`}, {"CURE", 365, 58.643977070025876`}, {"CURE", 365, 26.27085242499077`},
{"CURE", 365, 20.633953768323785`}, {"CURE", 365, 17.929397204040242`}, {"CURE", 365, 32.52235311506022`},
{"CURE", 365, 100.17781476997413`}, {"CURE", 365, 64.29368461521435`}, {"CURE", 365, 11.200151728774323`},

{"CURE", 365, 65.71596368719102`}, {"CURE", 365, 94.64303660692384`}, {"CURE", 365, 17.409077360218646`},
{"CURE", 365, 99.35269855868994`}, {"CURE", 365, 14.925083492554798`}, {"CURE", 365, 9.533777703735318`},
{"CURE", 365, 13.515718066457135`}, {"CURE", 365, 26.032064682809118`}, {"TBUR", 106.94363487081003`, 116.6482494763257`},
{"CURE", 365, 41.62144239578279`}, {"CURE", 365, 52.57375087129112`}, {"CURE", 365, 12.563391520821124`},
{"TOX", 14.543844641606134`, 239.94987745729108`}, {"CURE", 365, 109.83234056088031`}, {"CURE", 365, 18.755054558675326`},
{"CURE", 365, 45.89296751102566`}, {"CURE", 365, 25.559029720731797`}, {"CURE", 365, 25.82771628485607`},
{"CURE", 365, 87.28815307569388`}, {"CURE", 365, 110.42590775018229`}, {"CURE", 365, 11.851834099357815`},
{"CURE", 365, 23.104675897479368`}, {"CURE", 365, 8.19680219891836`}, {"CURE", 365, 31.59615563506163`},
{"CURE", 365, 54.204031529921956`}, {"CURE", 365, 87.72549497016948`}, {"CURE", 365, 48.960723937219505`},
{"CURE", 365, 176.3208248086578`}, {"CURE", 365, 84.27384534957098`}, {"TBUR", 87.53492636324629`, 13.925385946355604`},
{"CURE", 365, 30.83534786754514`}, {"TBUR", 53.94978901940871`, 34.33461029595204`}, {"CURE", 365, 68.95531282870293`},
{"CURE", 365, 9.047476396089905`}, {"CURE", 365, 24.19725396627833`}, {"CURE", 365, 13.638206060418504`},
{"CURE", 365, 34.00253710198115`}, {"CURE", 365, 77.70622278716762`}, {"CURE", 365, 33.05270078908835`},
{"CURE", 365, 7.1791909455431036`}, {"CURE", 365, 136.82939780462587`}, {"CURE", 365, 25.80232860468173`},
{"CURE", 365, 8.76177471623141`}, {"CURE", 365, 11.518930169745845`}, {"CURE", 365, 9.723691252395618`},
{"CURE", 365, 12.421293120634637`}, {"CURE", 365, 99.95631649536466`}, {"CURE", 365, 54.75821825740391`},
{"CURE", 365, 32.94094568698757`}, {"CURE", 365, 15.449351993237745`}, {"CURE", 365, 25.77190020233469`},
{"CURE", 365, 14.911141262273299`}, {"CURE", 365, 35.985757402197635`}, {"CURE", 365, 123.7663340110347`},
{"CURE", 365, 73.09456284200367`}, {"CURE", 365, 35.733979052119935`}, {"CURE", 365, 20.574069481270296`},
{"CURE", 365, 21.781953176565988`}, {"TOX", 9.162131098877968`, 264.3546222143681`}, {"CURE", 365, 12.597374861347443`},
{"CURE", 365, 6.490212278919027`}, {"CURE", 365, 5.665558169090976`}, {"CURE", 365, 71.8858399247249`},
{"CURE", 365, 42.11272118726319`}, {"CURE", 365, 46.705988990525306`}, {"CURE", 365, 69.37875524497065`},
{"CURE", 365, 114.63477051790841`}, {"CURE", 365, 118.45757411248876`}, {"CURE", 365, 45.1463133557054`},
{"CURE", 365, 19.492674886740776`}, {"CURE", 365, 22.61335806389956`}, {"CURE", 365, 45.4206531163368`},
{"CURE", 365, 25.6128371680861`}, {"TBUR", 30.26300238390365`, 10.413327682864194`}, {"CURE", 365, 40.23670006102738`},
{"TOX", 7.255156669559721`, 250.2315270984031`}, {"CURE", 365, 25.773632498522687`}, {"CURE", 365, 9.27549327047053`},
{"CURE", 365, 87.88599470977482`}, {"CURE", 365, 21.980998086807745`}, {"CURE", 365, 63.19212341331997`},
{"TOX", 5.663872828799063`, 304.08840913824224`}, {"CURE", 365, 140.4153928668207`}, {"CURE", 365, 45.58370035498196`},
{"CURE", 365, 38.22640116033561`}, {"CURE", 365, 16.278327201901405`}, {"CURE", 365, 4.649574026370711`},
{"CURE", 365, 18.236234322041568`}, {"CURE", 365, 53.30323022794493`}, {"CURE", 365, 29.571280110900165`},
{"CURE", 365, 94.33809472021136`}, {"CURE", 365, 37.40275698233659`}, {"CURE", 365, 38.17396110854457`},

{"CURE", 365, 10.60111745799898`}, {"CURE", 365, 30.030741177562806`}, {"CURE", 365, 30.811861391399514`},
{"CURE", 365, 8.036007570088158`}, {"CURE", 365, 25.588565710941232`}, {"CURE", 365, 15.243625053658953`},
{"CURE", 365, 24.185726666369163`}, {"CURE", 365, -3.976873219036739`*^14}, {"CURE", 365, 25.326663894207567`},
{"CURE", 365, 5.866615777081387`}, {"CURE", 365, 40.42208766264416`}, {"CURE", 365, 4.528832939388274`},
{"CURE", 365, 13.296343681856364`}, {"CURE", 365, 39.40870761414258`}, {"CURE", 365, 45.41213792520061`},
{"CURE", 365, 9.04528580735289`}, {"CURE", 365, 26.60778500669771`}, {"CURE", 365, 34.5201170408585`},
{"CURE", 365, 174.19782641347925`}, {"CURE", 365, 39.97794158734425`}, {"CURE", 365, 11.28655273097176`},
{"CURE", 365, 122.96247992104556`}, {"CURE", 365, 12.11617148620223`}, {"CURE", 365, 45.53318672609862`},
{"CURE", 365, 47.068203984723425`}, {"CURE", 365, 14.050335056249875`}, {"CURE", 365, 15.64862001276049`},
{"CURE", 365, 69.29881766581688`}, {"CURE", 365, 6.776067564748058`}, {"CURE", 365, 58.70448831915124`},
{"CURE", 365, 15.547272925604078`}, {"CURE", 365, 20.32895413319338`}, {"CURE", 365, 4.546456132217565`},
{"CURE", 365, 23.34576887380701`}, {"CURE", 365, 79.71468753087281`}, {"CURE", 365, 30.306339967481907`},
{"CURE", 365, 22.012530309158308`}, {"CURE", 365, 31.455228423274907`}, {"CURE", 365, 24.73889114276266`},
{"CURE", 365, 31.11591741202078`}, {"CURE", 365, 54.879108558597956`}, {"CURE", 365, 52.94567371095415`},
{"CURE", 365, 6.519562871090307`}, {"CURE", 365, 107.11072952860464`}, {"CURE", 365, 12.44395115430986`},
{"CURE", 365, 6.245465829274031`}, {"CURE", 365, 29.7726818762413`}, {"CURE", 365, 31.238772399750903`},
{"CURE", 365, 21.076513797699377`}, {"CURE", 365, 36.56442988249521`}, {"CURE", 365, 31.524013081936122`},
{"CURE", 365, 50.669181948669056`}, {"CURE", 365, 42.82192020439847`}, {"CURE", 365, 61.18686249965288`},
{"CURE", 365, 9.667441137019164`}, {"CURE", 365, 124.08868343483732`}, {"CURE", 365, 120.19875276822198`},
{"CURE", 365, 6.6615724391771085`}, {"CURE", 365, 16.76114559133853`}, {"CURE", 365, 41.58796868127418`},
{"CURE", 365, 21.16362426390164`}, {"CURE", 365, 65.09925289703781`}, {"CURE", 365, 130.21976518173105`},
{"CURE", 365, 41.152530687855624`}, {"CURE", 365, 7.067138007980803`}, {"CURE", 365, 6.931061337046697`},
{"CURE", 365, 16.26954072636445`}, {"CURE", 365, 4.912768709416454`}, {"CURE", 365, 55.74951361254703`},
{"CURE", 365, 8.645239406982546`}, {"CURE", 365, 58.20969088881706`}, {"CURE", 365, 35.81346385326342`},
{"CURE", 365, 61.71432032969268`}, {"CURE", 365, 15.196575848317076`}, {"CURE", 365, 76.66800131948108`},
{"TOX", 12.480565488044103`, 253.65768559877097`}, {"CURE", 365, 91.37236136618189`}, {"CURE", 365, 148.03062265397102`},
{"CURE", 365, 43.56080676932175`}, {"CURE", 365, 103.78154271532945`}, {"CURE", 365, 103.99336892284013`},
{"CURE", 365, 12.620951322087537`}, {"CURE", 365, 8.828642825464465`}, {"CURE", 365, 28.715195431438463`},
{"CURE", 365, 59.56750951234632`}, {"CURE", 365, 74.5312747845549`}, {"CURE", 365, 32.05759806626462`},
{"CURE", 365, 47.192659203454156`}, {"CURE", 365, 8.890406240214523`}, {"CURE", 365, 11.298820100986383`},
{"CURE", 365, 54.87180142340403`}, {"CURE", 365, 17.7992883329715`}, {"CURE", 365, 7.546502116063526`},

{"CURE", 365, 11.160477766306816`}, {"CURE", 365, 5.456654467647584`}, {"CURE", 365, 44.338859186206726`},
{"CURE", 365, 60.97109844994777`}, {"CURE", 365, 42.67305238075804`}, {"CURE", 365, 48.46911933495074`},
{"CURE", 365, 26.85263101998767`}, {"CURE", 365, 24.052443723976452`}, {"CURE", 365, 62.21717517812338`},
{"CURE", 365, 66.56921023889562`}, {"CURE", 365, 7.428887405890989`}, {"CURE", 365, 28.15636954977015`},
{"CURE", 365, 14.764453244299863`}, {"CURE", 365, 23.177150740540633`}, {"CURE", 365, 190.9366839057814`},
{"CURE", 365, 33.7144337662239`}, {"CURE", 365, 23.139239604688644`}, {"CURE", 365, 24.48454715575362`},
{"CURE", 365, 48.82378685313013`}, {"CURE", 365, 37.16391267329369`}, {"CURE", 365, 48.481091585996566`},
{"CURE", 365, 50.01956670739646`}, {"CURE", 365, 11.340144558622589`}, {"CURE", 365, 110.2711798321222`},
{"CURE", 365, 21.184740704532068`}, {"CURE", 365, 44.91584995335717`}, {"CURE", 365, 25.490583272758954`},
{"CURE", 365, 14.472135418156531`}, {"CURE", 365, 52.75938803039689`}, {"CURE", 365, 53.560032232880964`},
{"TBUR", 89.1410196141231`, 29.742688876501614`}, {"CURE", 365, 70.23191191609605`}, {"CURE", 365, 14.856287284694771`},
{"CURE", 365, 124.1229260614988 7`}, {"CURE", 365, 47.681804480665875`}, {"CURE", 365, 100.84187047968855`},
{"CURE", 365, 3.9095417254923883`}, {"CURE", 365, 49.61959013105689`}, {"CURE", 365, 56.575050933729514`},
{"CURE", 365, 13.435043538537839`}, {"CURE", 365, 34.11736673426251`}, {"CURE", 365, 51.08501861949247`},
{"CURE", 365, 31.052889526746043`}, {"CURE", 365, 9.882845848083486`}, {"CURE", 365, 7.498937545327351`},
{"CURE", 365, 44.35304156441628`}, {"CURE", 365, 11.82928988017781`}, {"CURE", 365, 26.299717464840867`},
{"CURE", 365, 32.06925039520159`}, {"CURE", 365, 14.939431228241586`}, {"CURE", 365, 27.481666430844502`},
{"CURE", 365, 7.479375143398553`}, {"CURE", 365, 30.832835839583442`}, {"CURE", 365, 4.984419633073213`},
{"CURE", 365, 8.91271614088011`}, {"CURE", 365, 21.075334954298867`}, {"CURE", 365, 6.79672925149478`},
{"CURE", 365, 50.502972539809825`}, {"CURE", 365, 74.16094376925687`}, {"CURE", 365, 75.30218346906544`},
{"CURE", 365, 62.67835563842919`}, {"CURE", 365, 118.31953053904499`}, {"CURE", 365, 9.572779244617832`},
{"CURE", 365, 42.68662848166774`}, {"CURE", 365, 45.87533676163869`}, {"CURE", 365, 78.80293757621396`},
{"CURE", 365, 58.85960318355999`}, {"CURE", 365, 26.909412664980618`}, {"CURE", 365, 126.54142557374378`},
{"CURE", 365, 3.9498302869877486`}, {"CURE", 365, 120.34361304253186`}, {"CURE", 365, 8.151246525708011`},
{"CURE", 365, 102.7871299172264`}, {"CURE", 365, 93.08670749895204`}, {"CURE", 365, 27.596330011658615`},
{"CURE", 365, 108.6703827275784`}, {"CURE", 365, 132.53437740716413`}, {"CURE", 365, 59.29074332628585`},
{"CURE", 365, 26.157613006036954`}, {"CURE", 365, 30.07505439393598`}, {"CURE", 365, 44.41769409634414`},
{"CURE", 365, 40.966310554387064`}, {"CURE", 365, 9.926758179246258`}, {"CURE", 365, 35.644751332932984`},
{"CURE", 365, 33.73433139611112`}, {"CURE", 365, 25.22535932910438`}, {"CURE", 365, 62.31033763273118`},
{"CURE", 365, 9.812019201774431`}, {"CURE", 365, 69.55451808101543`}, {"CURE", 365, 45.51863419368304`},
{"CURE", 365, 25.768642762509582`}, {"CURE", 365, 23.487935043454417`}, {"CURE", 365, 47.58346626115207`},

{"CURE", 365, 6.512150133132229`}, {"CURE", 365, 75.42980345099363`}, {"CURE", 365, 11.911205296609376`},
{"CURE", 365, 16.891165112737312`}, {"CURE", 365, 37.90096022151318`}, {"CURE", 365, 29.7114689658006`},
{"CURE", 365, 27.32121533887834`}, {"CURE", 365, 85.08439536479403`}, {"CURE", 365, 11.50696416743437`},
{"CURE", 365, 20.034268210157336`}, {"CURE", 365, 148.20180673711656`}, {"CURE", 365, 11.245826654846494`},
{"CURE", 365, 28.95546887043967`}, {"CURE", 365, 44.07683297240574`}, {"CURE", 365, 43.51959308233678`},
{"CURE", 365, 29.54115764260962`}, {"CURE", 365, 152.69237855437783`}, {"CURE", 365, 27.59131701911506`},
{"CURE", 365, 11.617191389961912`}, {"CURE", 365, 23.197982508375727`}, {"CURE", 365, 17.88862708997382`},
{"CURE", 365, 13.71285027502744`}, {"CURE", 365, 35.953947208444774`}, {"CURE", 365, 61.571609004057876`},
{"CURE", 365, 33.87840360389797`}, {"CURE", 365, 39.49866341054735`}, {"CURE", 365, 86.4731347240641`},
{"CURE", 365, 9.539610117765331`}, {"CURE", 365, 11.225025110710172`}, {"CURE", 365, 94.67377821656538`},
{"CURE", 365, 30.508189617881524`}, {"CURE", 365, 33.0079700558267`}, {"CURE", 365, 16.593918887164882`},
{"CURE", 365, 37.99575437336611`}, {"CURE", 365, 47.35181979633802`}, {"CURE", 365, 109.75999172008298`},
{"CURE", 365, 16.177421409933828`}, {"CURE", 365, 63.25904343802012`}, {"CURE", 365, 37.20609491298012`},
{"CURE", 365, 98.50695224487913`}, {"CURE", 365, 160.80996539279408`}, {"CURE", 365, 58.899237309739775`},
{"CURE", 365, 5.702597867181652`}, {"CURE", 365, 44.6192256032066`}, {"CURE", 365, 14.098493971965866`},
{"CURE", 365, 148.98432003026744`}, {"TBUR", 54.44961788822482`, 4.763725501691263`}, {"CURE", 365, 4.973658075441061`},
{"CURE", 365, 172.59009231336307`}, {"CURE", 365, 39.725938471672585`}, {"CURE", 365, 13.421575941061027`},
{"CURE", 365, 25.688102350125085`}, {"CURE", 365, 9.01540609052341`}, {"CURE", 365, 8.587592319423605`},
{"CURE", 365, 10.247875495587815`}, {"CURE", 365, 37.0435447826549`}, {"CURE", 365, 78.5141270455565`},
{"CURE", 365, 31.10482803417448`}, {"CURE", 365, 7.789705320824126`}, {"CURE", 365, 105.92811822744062`},
{"CURE", 365, 211.79123300742773`}, {"CURE", 365, 5.970734528777704`}, {"CURE", 365, 47.74289998391033`},
{"CURE", 365, 25.631751427991244`}, {"CURE", 365, 5.040515323401375`}, {"CURE", 365, 39.41330474567907`},
{"CURE", 365, 25.175829885149025`}, {"CURE", 365, 19.329400994399652`}, {"CURE", 365, 37.585209954731255`},
{"CURE", 365, 21.335394662664264`}, {"CURE", 365, 62.875827224324226`}, {"CURE", 365, 37.29385516223816`},
{"CURE", 365, 87.82007490950491`}, {"CURE", 365, 38.9046561917277`}, {"CURE", 365, 19.28011745743185`},
{"CURE", 365, 9.378789941505472`}, {"CURE", 365, 6.500688132225994`}, {"CURE", 365, 18.51601554062131`},
{"CURE", 365, 73.69809837760482`}, {"CURE", 365, 24.3022061222128`}, {"CURE", 365, 22.27573228500939`},
{"CURE", 365, 15.749433390657792`}, {"CURE", 365, 15.960688160968331`}, {"CURE", 365, 43.129240792335274`},
{"CURE", 365, 8.068341871012564`}, {"CURE", 365, 51.4121020309569`}, {"CURE", 365, 23.51501340837357`},
{"CURE", 365, 13.837333047483913`}, {"CURE", 365, 21.9121151836992`}, {"CURE", 365, 48.595361762502634`},
{"CURE", 365, 36.29367789421601`}, {"CURE", 365, 4.369442934797933`}, {"CURE", 365, 18.83243214293042`},

{"TOX", 5.0846160868822405`, 314.6719901699796`}, {"CURE", 365, 52.308846478933134`}, {"CURE", 365, 76.2594150306586`},
{"CURE", 365, 22.471827858925863`}, {"CURE", 365, 83.30702811296406`}, {"CURE", 365, 9.966197286610129`},
{"CURE", 365, 33.596576944047406`}, {"CURE", 365, 155.01558977233574`}, {"CURE", 365, 29.02415476741546`},
{"CURE", 365, 25.602463844085324`}, {"CURE", 365, 43.81775982897027`}, {"CURE", 365, 23.21844957749546`},
{"CURE", 365, 5.147875312968701`}, {"CURE", 365, 47.86722301273954`}, {"CURE", 365, 8.537938279757475`},
{"CURE", 365, 20.308810618508883`}, {"CURE", 365, 47.91945057685817`}, {"CURE", 365, 17.210622293719105`},
{"CURE", 365, 91.40011085394575`}, {"CURE", 365, 27.1876255341906`}, {"CURE", 365, 46.78262153806422`},
{"CURE", 365, 32.428279338906385`}, {"CURE", 365, 9.176392716126704`}, {"CURE", 365, 34.0655756282556`},
{"CURE", 365, 73.57718055400296`}, {"CURE", 365, 24.663026713993997`}, {"CURE", 365, 18.55923089833556`},
{"CURE", 365, 8.806221765087187`}, {"CURE", 365, 28.092374622659495`}, {"CURE", 365, 33.53296292263572`},
{"CURE", 365, 95.6173989840883`}, {"CURE", 365, 15.360862007759415`}, {"CURE", 365, 23.13538557374353`},
{"CURE", 365, 89.76208017381076`}, {"CURE", 365, 9.457099688671978`}, {"CURE", 365, 129.90662943974027`},
{"CURE", 365, 10.972552587707748`}, {"CURE", 365, 20.09751551035686`}, {"CURE", 365, 46.31026317891602`},
{"CURE", 365, 74.63069962657923`}, {"CURE", 365, 33.55009862888924`}, {"CURE", 365, 17.596537541563155`},
{"CURE", 365, 5.169608709092338`}, {"CURE", 365, 45.31078999528635`}, {"CURE", 365, 41.02379939287154`},
{"CURE", 365, 40.29368513591761`}, {"CURE", 365, 15.887391549886656`}, {"CURE", 365, 38.94474521962029`},
{"CURE", 365, 4.507435170212789`}, {"CURE", 365, 25.97136001056102`}, {"CURE", 365, 11.34136164712458`},
{"CURE", 365, 5.024494036403203`}, {"CURE", 365, 26.107671369643604`}, {"CURE", 365, 5.58548758908899`},
{"CURE", 365, 17.76082732727863`}, {"CURE", 365, 12.182836935615985`}, {"TBUR", 52.92311050001385`, 7.501791437158879`},
{"CURE", 365, 24.13115764009088`}, {"CURE", 365, 64.28229622107418`}, {"CURE", 365, 35.228754654578374`},
{"CURE", 365, 31.873591077813433`}, {"CURE", 365, 50.71215757729511`}, {"CURE", 365, 42.99668675305175`}},
{{"2600 Res", "2600 OS", "2600 Tox"}, {"CURE", 365, 21.684761489257646`}, {"CURE", 365, 44.819852197717495`},
{"CURE", 365, 31.43606983345542`}, {"TBUR", 80.79078113956784`, 10.559902538339065`},
{"TBUR", 57.74426154550912`, 40.63461720947434`}, {"CURE", 365, 81.18886267030965`}, {"CURE", 365, 76.38559643409215`},
{"CURE", 365, 7.982392495146529`}, {"CURE", 365, 55.08972852145298`}, {"CURE", 365, 47.66423349538025`},
{"CURE", 365, 178.87594096351765`}, {"CURE", 365, 22.19181066400879`}, {"CURE", 365, 80.6525817021533`},
{"TOX", 7.143183683753749`, 267.0376483237765`}, {"CURE", 365, 37.747764164492665`}, {"CURE", 365, 58.92188586748716`},
{"CURE", 365, 116.61363276468595`}, {"CURE", 365, 52.25811257407556`}, {"TBUR", 150.21159230024338`, 39.602426066004554`},
{"CURE", 365, 13.094792803463102`}, {"CURE", 365, 80.93683134326508`}, {"CURE", 365, 32.806648785958686`},
{"CURE", 365, 12.572961304018381`}, {"CURE", 365, 10.018315264064775`}, {"CURE", 365, 45.5742719788741`},
{"CURE", 365, 50.02254797319245`}, {"CURE", 365, 4.547873868566261`}, {"CURE", 365, 6.893685838539213`},

{"CURE", 365, 25.54601226760215`}, {"CURE", 365, 29.888115374053235`}, {"CURE", 365, 25.49022181562563`},
{"CURE", 365, 89.71743411915828`}, {"CURE", 365, 19.712352240182227`}, {"CURE", 365, 15.689437829736587`},
{"CURE", 365, 127.05987878146811`}, {"CURE", 365, 7.789256857571751`}, {"CURE", 365, 33.64338347698851`},
{"TBUR", 54.10850690874384`, 75.22059453421055`}, {"CURE", 365, 18.711082447402323`}, {"CURE", 365, 28.79518478749629`},
{"CURE", 365, 65.73012871271035`}, {"CURE", 365, 44.29638826252164`}, {"CURE", 365, 8.98885623053441`},
{"CURE", 365, 30.326466613730712`}, {"CURE", 365, 43.746715318673324`}, {"CURE", 365, 28.681847421541068`},
{"TOX", 5.125851092763625`, 304.8022445533301`}, {"CURE", 365, 65.70792943932167`}, {"CURE", 365, 102.4360093565737`},
{"CURE", 365, 12.29295222907216`}, {"CURE", 365, 34.786707019329924`}, {"CURE", 365, 47.462808942127495`},
{"CURE", 365, 37.81509768618782`}, {"CURE", 365, 24.547529837107223`}, {"CURE", 365, 10.576799265233856`},
{"CURE", 365, 111.8428476452644`}, {"CURE", 365, 35.14435580738247`}, {"CURE", 365, 6.681542939799112`},
{"CURE", 365, 71.98867398913019`}, {"CURE", 365, 50.437118575008206`}, {"CURE", 365, 34.5294365139144`},
{"CURE", 365, 26.406956485860665`}, {"CURE", 365, 15.098352773743635`}, {"CURE", 365, 56.91198396422554`},
{"CURE", 365, 40.45190523303418`}, {"CURE", 365, 46.23632160011483`}, {"CURE", 365, 10.58342611361474`},
{"CURE", 365, 10.762473844183406`}, {"CURE", 365, 11.442364766919384`}, {"CURE", 365, 46.73514234097858`},
{"CURE", 365, 17.110173569026035`}, {"CURE", 365, 38.11556184465094`}, {"CURE", 365, 71.51852813083833`},
{"TBUR", 30.994279183408917`, 22.92834899552381`}, {"CURE", 365, 19.118011558001022`}, {"CURE", 365, 52.93428300470232`},
{"CURE", 365, 30.96869437489484`}, {"CURE", 365, 23.547768116284015`}, {"CURE", 365, 93.84389225181124`},
{"CURE", 365, 103.01434066459163`}, {"CURE", 365, 75.8465196557904`}, {"CURE", 365, 20.46128049693606`},
{"CURE", 365, 41.36963999758308`}, {"CURE", 365, 28.91672747878722`}, {"CURE", 365, 32.702545140959025`},
{"CURE", 365, 15.327841201283704`}, {"CURE", 365, 163.5095801485029`}, {"CURE", 365, 35.637744044484904`},
{"CURE", 365, 44.86881147778893`}, {"CURE", 365, 38.62848654167953`}, {"CURE", 365, 31.98486482108251`},
{"CURE", 365, 9.467811233259187`}, {"CURE", 365, 17.348256361865424`}, {"CURE", 365, 40.1645702412031`},
{"TBUR", 66.3338555907637`, 14.596733113363454`}, {"CURE", 365, 17.298976436603503`}, {"CURE", 365, 45.08669551406902`},
{"CURE", 365, 114.42523117230031`}, {"CURE", 365, 61.322231421455584`}, {"CURE", 365, 30.75694986701727`},
{"CURE", 365, 61.01748052529444`}, {"CURE", 365, 39.64857766214548`}, {"CURE", 365, 25.68886887870322`},
{"CURE", 365, 175.89716107028931`}, {"CURE", 365, 19.53645194954741`}, {"CURE", 365, 13.274296725145764`},
{"CURE", 365, 77.9330979141518`}, {"CURE", 365, 23.034670356588318`}, {"CURE", 365, 23.50610547383344`},
{"CURE", 365, 141.4272210067482`}, {"CURE", 365, 11.47989906860417`}, {"CURE", 365, 7.284403815793715`},
{"CURE", 365, 30.297204529661975`}, {"CURE", 365, 23.955369680980986`}, {"CURE", 365, 18.04265381086041`},
{"CURE", 365, 46.04397505944544`}, {"CURE", 365, 12.961447568022084`}, {"CURE", 365, 7.969415508633291`},
{"CURE", 365, 37.300544802945446`}, {"CURE", 365, 57.05054057286819`}, {"CURE", 365, 9.719356453850764`},

{"CURE", 365, 32.160992830052045`}, {"CURE", 365, 97.61093045662511`}, {"TBUR", 80.79432704323835`, 37.24938736956699`},
{"CURE", 365, 21.655524001976943`}, {"CURE", 365, 75.57538761329232`}, {"CURE", 365, 37.981086766501505`},
{"CURE", 365, 5.005831671040112`}, {"CURE", 365, 43.35038331931633`}, {"CURE", 365, 33.480391625500374`},
{"CURE", 365, 26.67223796094879`}, {"CURE", 365, 31.64158890349687`}, {"CURE", 365, 8.23996964384222`},
{"CURE", 365, 47.008471625785596`}, {"CURE", 365, 16.875388049355465`}, {"CURE", 365, 38.16113590483575`},
{"CURE", 365, 142.454903290868`}, {"CURE", 365, 50.356968295687416`}, {"CURE", 365, 116.92980924785098`},
{"CURE", 365, 38.52147422041736`}, {"CURE", 365, 159.2811830843088`}, {"CURE", 365, 4.963284228220238`},
{"CURE", 365, 43.581928919459514`}, {"CURE", 365, 49.68083654165487`}, {"CURE", 365, 6.452472009482585`},
{"CURE", 365, 8.940219832615844`}, {"CURE", 365, 54.871828194179834`}, {"CURE", 365, 64.69855385216408`},
{"CURE", 365, 55.10475657990658`}, {"CURE", 365, 45.09222580444649`}, {"CURE", 365, 43.10161365981491`},
{"CURE", 365, 26.10307854499104`}, {"CURE", 365, 6.902769127608689`}, {"CURE", 365, 30.039846521282865`},
{"CURE", 365, 14.25365850280769`}, {"CURE", 365, 6.978185358819775`}, {"CURE", 365, 42.28112770703678`},
{"CURE", 365, 23.653238665046835`}, {"CURE", 365, 62.52650484738896`}, {"CURE", 365, 25.244579279903054`},
{"CURE", 365, 21.01055186011373`}, {"CURE", 365, 140.97425785410977`}, {"CURE", 365, 10.499545816701659`},
{"CURE", 365, 31.42463366096199`}, {"CURE", 365, 17.8061933984787`}, {"CURE", 365, 42.094971939776364`},
{"CURE", 365, 11.56404137719151`}, {"CURE", 365, 46.15976499578061`}, {"CURE", 365, 122.00370172115392`},
{"CURE", 365, 10.432478159056952`}, {"CURE", 365, 31.676115042528817`}, {"CURE", 365, 34.25895341567038`},
{"CURE", 365, 123.16838075500033`}, {"CURE", 365, 46.392322068398705`}, {"CURE", 365, 38.51639009594361`},
{"CURE", 365, 67.63400722242653`}, {"CURE", 365, 25.916061069968364`}, {"CURE", 365, 81.045408985798`},
{"CURE", 365, 49.909642166246165`}, {"CURE", 365, 30.627508838076757`}, {"CURE", 365, 27.52974663582354`},
{"CURE", 365, 8.949643722052684`}, {"CURE", 365, 6.063893045415049`}, {"CURE", 365, 14.217872132448814`},
{"CURE", 365, 19.038343004713546`}, {"CURE", 365, 8.391844020151787`}, {"CURE", 365, 30.71354237693373`},
{"CURE", 365, 83.79179599159822`}, {"CURE", 365, 22.648427891748888`}, {"CURE", 365, 42.0679370612862`},
{"CURE", 365, 52.646162184511866`}, {"CURE", 365, 45.517552033128055`}, {"CURE", 365, 30.812867607007323`},
{"CURE", 365, 12.239964632845737`}, {"CURE", 365, 84.33721822792225`}, {"CURE", 365, 7.295574953290417`},
{"CURE", 365, 78.61160852986698`}, {"CURE", 365, 76.48980700579918`}, {"CURE", 365, 21.320501524770744`},
{"CURE", 365, 25.323201224922308`}, {"CURE", 365, 145.655265568906`}, {"CURE", 365, 37.59146354681157`},
{"CURE", 365, 44.7937057087774`}, {"CURE", 365, 28.064766759694862`}, {"CURE", 365, 10.550917837807214`},
{"CURE", 365, 64.72278415206792`}, {"TBUR", 68.51345942667228`, 172.09807742792586`}, {"CURE", 365, 26.26853718402704`},
{"CURE", 365, 23.755225937083402`}, {"CURE", 365, 43.76183025488663`}, {"CURE", 365, 17.473643318993243`},
{"CURE", 365, 221.77142053635257`}, {"CURE", 365, 17.749836128102285`}, {"CURE", 365, 109.92879226712391`},

{"CURE", 365, 84.31646050884865`}, {"CURE", 365, 82.02257989461575`}, {"CURE", 365, 43.81042830760912`},
{"CURE", 365, 101.52873522444463`}, {"TOX", 7.030547163303931`, 276.1244821445994`}, {"CURE", 365, 46.12810129501784`},
{"CURE", 365, 8.475692740722874`}, {"CURE", 365, 75.2690456157496`}, {"CURE", 365, 46.900749297355645`},
{"CURE", 365, 45.21876458747449`}, {"CURE", 365, 9.979429302124336`}, {"CURE", 365, 228.55569849834728`},
{"CURE", 365, 16.709891369087796`}, {"CURE", 365, 72.4775598205262`}, {"CURE", 365, 45.69827308324479`},
{"CURE", 365, 32.47061665561622`}, {"CURE", 365, 7.805147528207228`}, {"CURE", 365, 58.66497637959628`},
{"CURE", 365, 34.41904747689212`}, {"CURE", 365, 91.0541378165454`}, {"CURE", 365, 59.995849085755474`},
{"CURE", 365, 75.7535929268496`}, {"CURE", 365, 36.390668958112414`}, {"CURE", 365, 32.370065784515916`},
{"CURE", 365, 169.14759942599883`}, {"CURE", 365, 44.273774129183266`}, {"CURE", 365, 11.814732458974648`},
{"CURE", 365, 39.78493245241068`}, {"CURE", 365, 40.53620297271061`}, {"CURE", 365, 5.434107389337581`},
{"CURE", 365, 9.668407808888922`}, {"CURE", 365, 14.773817529095416`}, {"CURE", 365, 139.60523956013012`},
{"CURE", 365, 42.90019891732943`}, {"CURE", 365, 44.699960360557334`}, {"CURE", 365, 35.805660041974335`},
{"CURE", 365, 147.38747401821414`}, {"CURE", 365, 33.89563931523882`}, {"CURE", 365, 68.39026088820394`},
{"CURE", 365, 33.806912720247844`}, {"CURE", 365, 49.83450418106882`}, {"CURE", 365, 11.984019695253657`},
{"CURE", 365, 44.82396224328638`}, {"CURE", 365, 27.974734180301745`}, {"CURE", 365, 54.47786635051017`},
{"CURE", 365, 86.10983067054593`}, {"CURE", 365, 97.24504644601623`}, {"CURE", 365, 59.36747957702968`},
{"CURE", 365, 48.00356621378225`}, {"CURE", 365, 66.65706065998693`}, {"CURE", 365, 31.435603085682324`},
{"CURE", 365, 35.588739508188524`}, {"CURE", 365, 25.226146570960164`}, {"CURE", 365, 19.762008385116445`},
{"CURE", 365, 31.833260658019203`}, {"CURE", 365, 81.67114596035115`}, {"CURE", 365, 9.361358955370868`},
{"CURE", 365, 41.49311615257719`}, {"CURE", 365, 7.832636733012473`}, {"CURE", 365, 40.77466377899987`},
{"CURE", 365, 23.1832078558608`}, {"CURE", 365, 86.20932153875341`}, {"CURE", 365, 7.949223374241123`},
{"CURE", 365, 56.79163781184499`}, {"CURE", 365, 71.20346251701729`}, {"CURE", 365, 11.647720012872577`},
{"CURE", 365, 26.695116255022082`}, {"CURE", 365, 21.478145567674805`}, {"CURE", 365, 12.297504834827315`},
{"CURE", 365, 72.61441281946385`}, {"CURE", 365, 27.30036431908544`}, {"CURE", 365, 52.592307614845815`},
{"CURE", 365, 51.144988726521206`}, {"CURE", 365, 57.982837559672916`}, {"CURE", 365, 45.335065464970064`},
{"CURE", 365, 54.29264687090189`}, {"CURE", 365, 42.95673746440461`}, {"CURE", 365, 83.06687808669825`},
{"CURE", 365, 50.60512803733072`}, {"CURE", 365, 41.33604761490714`}, {"CURE", 365, 105.68112474194707`},
{"CURE", 365, 12.235684680137341`}, {"CURE", 365, 69.3983284765796`}, {"CURE", 365, 5.097952938194057`},
{"CURE", 365, 63.34234144081268`}, {"CURE", 365, 14.924761871110551`}, {"CURE", 365, 79.1787127629940`},
{"CURE", 365, 12.189061606901069`}, {"CURE", 365, 79.83775937784804`}, {"CURE", 365, 199.88355802660882`},
{"CURE", 365, 8.724639665748617`}, {"CURE", 365, 6.268948557143506`}, {"CURE", 365, 23.064408207349388`},

{"CURE", 365, 5.604402774673794`}, {"CURE", 365, 3.8294986939256375`}, {"CURE", 365, 22.40227478522343`},
{"CURE", 365, 36.65934960699336`}, {"CURE", 365, 39.41211730922017`}, {"CURE", 365, 37.49745995220309`},
{"CURE", 365, 29.200183395647116`}, {"CURE", 365, 51.33577068320318`}, {"CURE", 365, 90.37674341606557`},
{"CURE", 365, 7.313909882198253`}, {"CURE", 365, 40.06473441182898`}, {"CURE", 365, 34.43015348282185`},
{"CURE", 365, 24.96447750104975`}, {"CURE", 365, 46.1763580008099`}, {"CURE", 365, 68.44821882763209`},
{"CURE", 365, 160.1496254188241`}, {"CURE", 365, 119.36066031727441`}, {"CURE", 365, 36.94034729607424`},
{"CURE", 365, 3.9192561204987686`}, {"CURE", 365, 42.75542529483333`}, {"CURE", 365, 110.2427676382924`},
{"CURE", 365, 27.7417214964133`}, {"CURE", 365, 35.5413193794932`}, {"CURE", 365, 21.4040196099985`},
{"CURE", 365, 31.370999847260148`}, {"CURE", 365, 8.131563519434136`}, {"CURE", 365, 39.76009757581801`},
{"CURE", 365, 22.36196065828554`}, {"CURE", 365, 65.28655327788357`}, {"CURE", 365, 44.73650214822868`},
{"CURE", 365, 43.34008639166065`}, {"CURE", 365, 35.66066784956837`}, {"CURE", 365, 18.871555271944533`},
{"CURE", 365, 13.005503613242666`}, {"CURE", 365, 12.680265530978728`}, {"CURE", 365, 16.42881124039621`},
{"CURE", 365, 12.800484304200472`}, {"CURE", 365, 172.2002977709881`}, {"CURE", 365, 95.78277543601355`},
{"CURE", 365, 18.499699834873656`}, {"TOX", 6.528972372627936`, 284.952780523832`}, {"CURE", 365, 81.48852724065524`},
{"CURE", 365, 43.14618739263261`}, {"CURE", 365, 74.63915759566072`}, {"CURE", 365, 11.221619250250235`},
{"CURE", 365, 89.50173969276953`}, {"TOX", 9.03252188036621`, 239.23263999718614`}, {"CURE", 365, 12.981980491443323`},
{"CURE", 365, 40.492798458517285`}, {"CURE", 365, 66.3548896958585`}, {"CURE", 365, 33.58866254716140 5`},
{"CURE", 365, 16.43098615218984`}, {"CURE", 365, 13.089853626602611`}, {"CURE", 365, 113.62498676861837`},
{"CURE", 365, 59.651467254011216`}, {"CURE", 365, 33.93564324878981`}, {"CURE", 365, 84.35751959569795`},
{"CURE", 365, 15.238712328923572`}, {"CURE", 365, 40.68779463356695`}, {"CURE", 365, 24.5730115165467`},
{"CURE", 365, 165.7135783399767`}, {"CURE", 365, 41.75812696033071`}, {"CURE", 365, 193.7991654151535`},
{"TOX", 5.854254062390474`, 302.4735713260409`}, {"CURE", 365, 23.129914675036478`}, {"CURE", 365, 6.523319479756983`},
{"CURE", 365, 5.682501316299505`}, {"CURE", 365, 41.5742151966895`}, {"CURE", 365, 161.27344798194719`},
{"CURE", 365, 32.306958756863466`}, {"CURE", 365, 24.822912988250604`}, {"CURE", 365, 46.6817491754291`},
{"CURE", 365, 51.12466956931693`}, {"CURE", 365, 213.0484707825153`}, {"CURE", 365, 175.60984250013897`},
{"CURE", 365, 36.79694506755402`}, {"CURE", 365, 12.206824888446526`}, {"CURE", 365, 29.9058207728609`},
{"CURE", 365, 4.693327864560559`}, {"CURE", 365, 88.31794130399729`}, {"CURE", 365, 131.25200961846534`},
{"CURE", 365, 42.92827754696529`}, {"CURE", 365, 34.02162975498186`}, {"CURE", 365, 143.8561762453762`},
{"CURE", 365, 9.133915562459743`}, {"CURE", 365, 11.89292579341094`}, {"CURE", 365, 37.20070435293943`},
{"CURE", 365, 47.885785691623155`}, {"TBUR", 99.68623015982574`, 90.8096119669160 8`}, {"CURE", 365, 93.34281217481154`},
{"CURE", 365, 38.3161359047803`}, {"CURE", 365, 39.53951603374496`}, {"CURE", 365, 49.5668748846789`},

{"CURE", 365, 17.150702824241314`}, {"CURE", 365, 4.840198692618878`}, {"CURE", 365, 50.915756651538906`},
{"CURE", 365, 61.29391309641475`}, {"CURE", 365, 29.801750264545365`}, {"CURE", 365, 54.00673839126933`},
{"CURE", 365, 16.825693564430576`}, {"CURE", 365, 11.055193895683777`}, {"CURE", 365, 48.393699953640095`},
{"CURE", 365, 29.4277451174297`}, {"CURE", 365, 42.050342919709`}, {"TOX", 9.158610422963653`, 277.4755125310181`},
{"CURE", 365, 26.441052366721536`}, {"CURE", 365, 12.900089139482878`}, {"CURE", 365, 10.007850677333819`},
{"CURE", 365, 12.128918975994365`}, {"CURE", 365, 69.29619520643656`}, {"CURE", 365, 4.771771086184025`},
{"CURE", 365, 64.52109427499795`}, {"CURE", 365, 70.43221811552236`}, {"CURE", 365, 54.97295673171352`},
{"CURE", 365, 45.06915210095171`}, {"CURE", 365, 8.940157378495767`}, {"CURE", 365, 98.09666227962148`},
{"CURE", 365, 29.7464491851836`}, {"CURE", 365, 13.521807714748041`}, {"CURE", 365, 87.3628503766807`},
{"CURE", 365, 15.537832957648526`}, {"CURE", 365, 104.67389705974196`}, {"CURE", 365, 67.16653946096096`},
{"CURE", 365, 44.830377535284164`}, {"CURE", 365, 65.08855705916686`}, {"CURE", 365, 23.092544885190804`},
{"CURE", 365, 102.11806463135231`}, {"CURE", 365, 12.11579176419979`}, {"CURE", 365, 51.371958059505644`},
{"CURE", 365, 60.005349190970676`}, {"CURE", 365, 25.513601403169382`}, {"TOX", 13.1220312533919`, 237.76103112475485`},
{"CURE", 365, 47.770993675445`}, {"CURE", 365, 48.663192300501095`}, {"CURE", 365, 49.72116945812633`},
{"CURE", 365, 199.0960865361331`}, {"CURE", 365, 63.26442293737182`}, {"CURE", 365, 59.02223936041799`},
{"CURE", 365, 128.48763622284193`}, {"CURE", 365, 45.1796185290959`}, {"CURE", 365, 39.366588931865785`},
{"CURE", 365, 48.637807681454234`}, {"CURE", 365, 67.18278071420592`}, {"CURE", 365, 118.71297627614847`},
{"CURE", 365, 25.619086638557135`}, {"CURE", 365, 23.387024706433785`}, {"CURE", 365, 50.04200376111445`},
{"CURE", 365, 50.94933375907017`}, {"CURE", 365, 57.37302138148223`}, {"CURE", 365, 61.25831965887197`},
{"CURE", 365, 34.24680977539251`}, {"CURE", 365, 17.238510652117057`}, {"CURE", 365, 64.80186681809784`},
{"CURE", 365, 16.34031508240341`}, {"CURE", 365, 34.733818206556094`}, {"CURE", 365, 41.40360195568699`},
{"CURE", 365, 12.460404809370473`}, {"CURE", 365, 6.194756212435579`}, {"CURE", 365, 38.339637667167814`},
{"CURE", 365, 51.90633602853247`}, {"CURE", 365, 61.60592393629907`}, {"CURE", 365, 44.072569380726534`},
{"CURE", 365, 5.647442718902925`}, {"CURE", 365, 50.532052349096034`}, {"CURE", 365, 9.630026685399924`},
{"CURE", 365, 148.48796225355628`}, {"CURE", 365, 193.22166408063515`}, {"CURE", 365, 27.78290900070436`},
{"CURE", 365, 38.21409934045822`}, {"CURE", 365, 207.43059877182785`}, {"CURE", 365, 37.19652913210199`},
{"CURE", 365, 21.64128174240834`}, {"CURE", 365, 16.845607994781275`}, {"CURE", 365, 4.60057757035459`},
{"CURE", 365, 28.996855443028323`}, {"CURE", 365, 32.33410751376227`}, {"CURE", 365, 85.73545705663457`},
{"CURE", 365, 41.01818523982147`}, {"CURE", 365, 71.40913163459662`}, {"CURE", 365, 25.223780911929023`},
{"CURE", 365, 54.800865599188285`}, {"CURE", 365, 122.33932016055854`}, {"CURE", 365, 12.448959039717996`},
{"CURE", 365, 19.193718610341524`}, {"CURE", 365, 28.5176154861712`}, {"CURE", 365, 39.8271221668238`},

{"CURE", 365, 26.165632482923964`}, {"CURE", 365, 24.99824776512234`}, {"CURE", 365, 10.602314610540835`},
{"CURE", 365, 16.348990131261736`}, {"CURE", 365, 31.0099691099853`}, {"CURE", 365, 70.54197142237017`},
{"CURE", 365, 62.83831669435979`}, {"CURE", 365, 32.14736133461876`}, {"TBUR", 50.528516422601314`, 48.31067574509827`},
{"CURE", 365, 103.89550197620208`}, {"CURE", 365, 50.796013388464296`}, {"CURE", 365, 38.00716358198338`},
{"CURE", 365, 24.208434068200336`}, {"CURE", 365, 7.587213878147938`}, {"CURE", 365, 42.19512773404472`},
{"CURE", 365, 48.71339211840973`}, {"CURE", 365, 190.8321565568682`}, {"CURE", 365, 9.727026862849225`},
{"CURE", 365, 9.498806044280704`}, {"CURE", 365, 30.435131000561498`}, {"CURE", 365, 88.71830953872009`},
{"CURE", 365, 11.072308669170964`}, {"CURE", 365, 54.15861212003793`}, {"CURE", 365, 12.633159380245012`},
{"CURE", 365, 31.48327061197846`}, {"CURE", 365, 12.860577008220647`}, {"CURE", 365, 13.348808408350655`},
{"CURE", 365, 12.039018131168367`}, {"CURE", 365, 128.43978689081916`}, {"CURE", 365, 20.579108116859825`},
{"CURE", 365, 36.237563734750836`}, {"CURE", 365, 4.990159986505254`}, {"CURE", 365, 59.79132505103259`},
{"CURE", 365, 83.6564640063259`}, {"CURE", 365, 72.96349998958425`}, {"CURE", 365, 11.012958648202861`},
{"CURE", 365, 22.085711677914098`}, {"CURE", 365, 224.78012945314146`}, {"CURE", 365, 61.06812783673292`},
{"CURE", 365, 41.62261869292461`}, {"CURE", 365, 14.794379475092011`}, {"CURE", 365, 21.164123663741837`},
{"CURE", 365, 32.7147362222391`}, {"CURE", 365, 8.101574102174634`}, {"CURE", 365, 39.75225921247191`},
{"CURE", 365, 62.54274208700493`}, {"CURE", 365, 38.06604418367405`}, {"CURE", 365, 41.33814640010077`},
{"CURE", 365, 29.88952211012917`}, {"CURE", 365, 19.36733708267067`}, {"CURE", 365, 110.68420690204678`},
{"CURE", 365, 160.3608309206083`}, {"CURE", 365, 42.76803919710218`}, {"CURE", 365, 53.99685788208927`},
{"CURE", 365, 68.40635294816258`}, {"CURE", 365, 66.88594013644594`}, {"CURE", 365, 5.84291980576832`},
{"CURE", 365, 78.28048232867853`}, {"CURE", 365, 80.84832480167184`}, {"CURE", 365, 54.66727899531589`},
{"CURE", 365, 219.31273884341084`}, {"CURE", 365, 45.59486733664411`}, {"CURE", 365, 20.322903244415684`},
{"CURE", 365, 47.988020282543424`}, {"CURE", 365, 60.99201065199927`}, {"CURE", 365, 27.322436768543152`},
{"CURE", 365, 21.464421720702823`}, {"CURE", 365, 18.646957113916798`}, {"CURE", 365, 33.83244169895093`},
{"CURE", 365, 104.18647790768614`}, {"CURE", 365, 66.86645196584882`}, {"CURE", 365, 11.650094511728156`},
{"CURE", 365, 68.39023956243896`}, {"CURE", 365, 98.43062973048895`}, {"CURE", 365, 18.110147220189354`},
{"CURE", 365, 103.34132417129149`}, {"CURE", 365, 15.523133671506573`}, {"CURE", 365, 9.91777870956486`},
{"CURE", 365, 14.056485093487945`}, {"CURE", 365, 27.078410743919296`}, {"CURE", 365, 121.3706028937538`},
{"CURE", 365, 43.28692104800997`}, {"CURE", 365, 54.68242433652031`}, {"CURE", 365, 13.066026084673878`},
{"TOX", 12.081017669189686`, 249.60148804913683`}, {"CURE", 365, 114.2282437748494`}, {"CURE", 365, 19.510045232878706`},
{"CURE", 365, 47.73369677647743`}, {"CURE", 365, 26.58150429272904`}, {"CURE", 365, 26.863438575491593`},
{"CURE", 365, 90.79053605630548`}, {"CURE", 365, 114.86604281142921`}, {"CURE", 365, 12.325991644029484`},

{"CURE", 365, 24.031538134679067`}, {"CURE", 365, 8.52563823246738`}, {"CURE", 365, 32.86056019767147`},
{"CURE", 365, 56.38423908638881`}, {"CURE", 365, 91.23528087441376`}, {"CURE", 365, 50.92085671932793`},
{"CURE", 365, 183.37727762412996`}, {"CURE", 365, 87.64579798937285`}, {"CURE", 365, 14.487664531902633`},
{"CURE", 365, 32.07199791613729`}, {"TBUR", 56.228127671755225`, 35.837308954529384`}, {"CURE", 365, 71.73643789782543`},
{"CURE", 365, 9.413016357012099`}, {"CURE", 365, 25.166617234950486`}, {"CURE", 365, 14.185758987946466`},
{"CURE", 365, 35.36307146929393`}, {"CURE", 365, 80.81876452110953`}, {"CURE", 365, 34.37559548387582`},
{"CURE", 365, 7.466573030077713`}, {"CURE", 365, 142.32332461358354`}, {"CURE", 365, 26.836966620473977`},
{"CURE", 365, 9.112655428173646`}, {"CURE", 365, 11.980381719833773`}, {"CURE", 365, 10.113035774298478`},
{"CURE", 365, 12.918442645504042`}, {"CURE", 365, 103.95668107611438`}, {"CURE", 365, 56.96106319346673`},
{"CURE", 365, 34.2606988498925`}, {"CURE", 365, 16.070104970813475`}, {"CURE", 365, 26.802946312550482`},
{"CURE", 365, 15.507812125635985`}, {"CURE", 365, 37.426263073650226`}, {"CURE", 365, 128.72645448412376`},
{"CURE", 365, 76.35710200229204`}, {"CURE", 365, 37.170671551364904`}, {"CURE", 365, 21.39831299705531`},
{"CURE", 365, 22.70519390250651`}, {"TOX", 8.370719338808346`, 274.9323905532149`}, {"CURE", 365, 13.101760414408787`},
{"CURE", 365, 6.750166819082571`}, {"CURE", 365, 5.894210103182772`}, {"CURE", 365, 74.76600117792421`},
{"CURE", 365, 43.80321390461636`}, {"CURE", 365, 48.57750809115305`}, {"CURE", 365, 72.15477341737495`},
{"CURE", 365, 119.24452751435062`}, {"CURE", 365, 123.19697191288961`}, {"CURE", 365, 46.95285156754444`},
{"CURE", 365, 20.272544967916126`}, {"CURE", 365, 23.51973911177718`}, {"CURE", 365, 47.316972379255716`},
{"CURE", 365, 26.64245282408358`}, {"TBUR", 31.007177996888558`, 10.900603608316306`}, {"CURE", 365, 41.85524159774455`},
{"TOX", 6.453351254901527`, 260.2551778757235`}, {"CURE", 365, 26.804985679650912`}, {"CURE", 365, 9.6479911389358`},
{"CURE", 365, 91.4022600003668`}, {"CURE", 365, 22.860804027963567`}, {"CURE", 365, 65.72476412845847`},
{"TOX", 5.339351454658425`, 316.25595852556904`}, {"CURE", 365, 146.0345108493593`}, {"CURE", 365, 47.40776530655378`},
{"CURE", 365, 39.75976655374962`}, {"CURE", 365, 16.929736613082774`}, {"CURE", 365, 4.836024137410618`},
{"CURE", 365, 18.97316179603848`}, {"CURE", 365, 55.43584292485388`}, {"CURE", 365, 30.75767856385757`},
{"CURE", 365, 98.12059460047493`}, {"CURE", 365, 38.902920672393655`}, {"CURE", 365, 39.70170798033302`},
{"CURE", 365, 11.02552685349716`}, {"CURE", 365, 31.233492672510803`}, {"CURE", 365, 32.04473769667948`},
{"CURE", 365, 8.357909068664537`}, {"CURE", 365, 26.61698181569431`}, {"CURE", 365, 15.853612771628033`},
{"CURE", 365, 25.16009022852312`}, {"CURE", 365, 53.042432070506216`}, {"CURE", 365, 26.345042098228575`},
{"CURE", 365, 6.101375942947543`}, {"CURE", 365, 42.04052130699874`}, {"CURE", 365, 4.710825243160676`},
{"CURE", 365, 13.832298252486208`}, {"CURE", 365, 40.98914256670751`}, {"CURE", 365, 47.229128171749515`},
{"CURE", 365, 9.410040396020122`}, {"CURE", 365, 27.675611112667376`}, {"CURE", 365, 35.91835965761678`},
{"CURE", 365, 181.20662772403682`}, {"CURE", 365, 41.57739122953008`}, {"CURE", 365, 11.740370903734485`},

{"CURE", 365, 127.89002930752956`}, {"CURE", 365, 12.607577675710155`}, {"CURE", 365, 47.361243293911734`},
{"CURE", 365, 48.95218469317358`}, {"CURE", 365, 14.612824080322518`}, {"CURE", 365, 16.276430596341754`},
{"CURE", 365, 72.07378220148227`}, {"CURE", 365, 7.048916722719606`}, {"CURE", 365, 61.053366029709736`},
{"CURE", 365, 16.169612182845565`}, {"CURE", 365, 21.14248861002794`}, {"CURE", 365, 4.729126404707579`},
{"CURE", 365, 24.28052800103672`}, {"CURE", 365, 82.92555010386906`}, {"CURE", 365, 31.521484831537105`},
{"CURE", 365, 22.896945279314092`}, {"CURE", 365, 32.716862752394036`}, {"CURE", 365, 25.74896431392105`},
{"CURE", 365, 32.368369154391516`}, {"CURE", 365, 57.07931667129984`}, {"CURE", 365, 55.066161915779986`},
{"CURE", 365, 6.780404917950978`}, {"CURE", 365, 111.42142537241412`}, {"CURE", 365, 12.942170358639196`},
{"CURE", 365, 6.4962937414218045`}, {"CURE", 365, 30.967178313754978`}, {"CURE", 365, 32.489567362975926`},
{"CURE", 365, 21.924736029821997`}, {"CURE", 365, 38.028248418147875`}, {"CURE", 365, 32.78544428900311`},
{"CURE", 365, 52.69639899888141`}, {"CURE", 365, 44.535903999999576`}, {"CURE", 365, 63.64547856818089`},
{"CURE", 365, 10.055526274684839`}, {"CURE", 365, 129.13598675329624`}, {"CURE", 365, 125.01178899069711`},
{"CURE", 365, 6.928970478548112`}, {"CURE", 365, 17.43189967770116`}, {"CURE", 365, 43.252223245097355`},
{"CURE", 365, 22.01462784449362`}, {"CURE", 365, 67.72948933856749`}, {"CURE", 365, 135.43402533395033`},
{"CURE", 365, 42.79970157960726`}, {"CURE", 365, 7.350919105645497`}, {"CURE", 365, 7.2090328614427746`},
{"CURE", 365, 16.92066375482251`}, {"CURE", 365, 5.109687524125765`}, {"CURE", 365, 57.9810054215318`},
{"CURE", 365, 8.991927661242375`}, {"CURE", 365, 60.56918017972429`}, {"CURE", 365, 37.248661019657746`},
{"CURE", 365, 64.18460334157442`}, {"CURE", 365, 15.805337328734621`}, {"CURE", 365, 79.73923083631459`},
{"TOX", 11.126840298462774`, 263.8179805700173`}, {"CURE", 365, 95.03009196816865`}, {"CURE", 365, 153.9593001875078`},
{"CURE", 365, 45.30374620164415`}, {"CURE", 365, 107.93368825363217`}, {"CURE", 365, 108.15542920632859`},
{"CURE", 365, 13.125917089735243`}, {"CURE", 365, 9.182006611037849`}, {"CURE", 365, 29.866170216600334`},
{"CURE", 365, 61.95090291358816`}, {"CURE", 365, 77.51454272603118`}, {"CURE", 365, 33.39065516110427`},
{"CURE", 365, 49.08827585743341`}, {"CURE", 365, 9.24796534981814`}, {"CURE", 365, 11.753451812657817`},
{"CURE", 365, 57.07436793233978`}, {"CURE", 365, 18.51680468668584`}, {"CURE", 365, 7.848712556534922`},
{"CURE", 365, 11.606961841817306`}, {"CURE", 365, 5.675208655142488`}, {"CURE", 365, 46.113822567429324`},
{"CURE", 365, 63.410231678441235`}, {"CURE", 365, 44.38501644642524`}, {"CURE", 365, 50.40827632684395`},
{"CURE", 365, 27.929167085819465`}, {"CURE", 365, 25.020027239339758`}, {"CURE", 365, 64.71205543180578`},
{"CURE", 365, 69.23377750047455`}, {"CURE", 365, 7.726525441431269`}, {"CURE", 365, 29.393251109467887`},
{"CURE", 365, 15.356453537448232`}, {"CURE", 365, 24.10453135567809`}, {"CURE", 365, 198.5831726793481`},
{"CURE", 365, 35.064222542884174`}, {"CURE", 365, 24.06518963456693`}, {"CURE", 365, 25.467850125019336`},
{"CURE", 365, 50.7786032397398`}, {"CURE", 365, 38.651779615984736`}, {"CURE", 365, 50.422210436353204`},

{"CURE", 365, 52.036315506879`}, {"CURE", 365, 11.794436306094568`}, {"CURE", 365, 114.68505223864122`},
{"CURE", 365, 22.03484696991179`}, {"CURE", 365, 46.71454539496599`}, {"CURE", 365, 26.51199727029828`},
{"CURE", 365, 15.05123484315279`}, {"CURE", 365, 54.87087277100625`}, {"CURE", 365, 55.70297575900753`},
{"TBUR", 92.15225153193981`, 30.94542141747703`}, {"CURE", 365, 73.04684773667726`}, {"CURE", 365, 15.451699571801472`},
{"CURE", 365, 129.08821625228714`}, {"CURE", 365, 49.59327792983361`}, {"CURE", 365, 104.89440598985844`},
{"CURE", 365, 4.066024958249149`}, {"CURE", 365, 51.61648973348797`}, {"CURE", 365, 58.86241000707424`},
{"CURE", 365, 13.972662742961296`}, {"CURE", 365, 35.48467590855927`}, {"CURE", 365, 53.13062556732701`},
{"CURE", 365, 32.29740675311977`}, {"CURE", 365, 10.279723683578217`}, {"CURE", 365, 7.799568956278249`},
{"CURE", 365, 46.13429492094473`}, {"CURE", 365, 12.3032147746674`}, {"CURE", 365, 27.354937340958948`},
{"CURE", 365, 33.361545256533255`}, {"CURE", 365, 15.545565510202342`}, {"CURE", 365, 28.59064023984372`},
{"CURE", 365, 7.779344658197926`}, {"CURE", 365, 32.068573808258904`}, {"CURE", 365, 5.184099603666728`},
{"CURE", 365, 9.27019375824812`}, {"CURE", 365, 21.923151438476804`}, {"CURE", 365, 7.069738232217992`},
{"CURE", 365, 52.53026651819361`}, {"CURE", 365, 77.1286002071091`}, {"CURE", 365, 78.32596968989415`},
{"CURE", 365, 65.18699838372575`}, {"CURE", 365, 123.0580506255553`}, {"CURE", 365, 9.956731652744745`},
{"CURE", 365, 44.39714052007266`}, {"CURE", 365, 47.72858696294097`}, {"CURE", 365, 81.9565514224358`},
{"CURE", 365, 61.21503667469066`}, {"CURE", 365, 27.989739833736227`}, {"CURE", 365, 131.62508428808584`},
{"CURE", 365, 4.108228314033242`}, {"CURE", 365, 125.1579262505887`}, {"CURE", 365, 8.477918890984611`},
{"CURE", 365, 106.89993086521054`}, {"CURE", 365, 96.81059444394866`}, {"CURE", 365, 28.70164788533645`},
{"CURE", 365, 113.02446133675767`}, {"CURE", 365, 137.83737303223927`}, {"CURE", 365, 61.66690124455464`},
{"CURE", 365, 27.204184679199123`}, {"CURE", 365, 31.278947824829988`}, {"CURE", 365, 46.19660199978684`},
{"CURE", 365, 42.60819085952197`}, {"CURE", 365, 10.324212126957658`}, {"CURE", 365, 37.073426062146574`},
{"CURE", 365, 35.090898654574126`}, {"CURE", 365, 26.237008840340522`}, {"CURE", 365, 64.80414976681074`},
{"CURE", 365, 10.204763853875285`}, {"CURE", 365, 72.33968084152916`}, {"CURE", 365, 47.34241665341737`},
{"CURE", 365, 26.799917506031598`}, {"CURE", 365, 24.45978258941768`}, {"CURE", 365, 49.500881310729`},
{"CURE", 365, 6.772771929077`}, {"CURE", 365, 78.4489518825626`}, {"CURE", 365, 12.389022872939298`},
{"CURE", 365, 17.567371474913596`}, {"CURE", 365, 39.42094514691916`}, {"CURE", 365, 30.901898076277224`},
{"CURE", 365, 28.415784539537434`}, {"CURE", 365, 88.50066272022468`}, {"CURE", 365, 11.968029301344217`},
{"CURE", 365, 20.83748802959951`}, {"CURE", 365, 154.1347219202566`}, {"CURE", 365, 11.696291789775346`},
{"CURE", 365, 30.114055436849597`}, {"CURE", 365, 45.845913388381355`}, {"CURE", 365, 45.26605297204487`},
{"CURE", 365, 30.72401564140342`}, {"CURE", 365, 158.80074465974985`}, {"CURE", 365, 28.698462870500226`},
{"CURE", 365, 12.082387591122913`}, {"CURE", 365, 24.138690912993418`}, {"CURE", 365, 18.605005147495937`},

{"CURE", 365, 14.263031854108442`}, {"CURE", 365, 37.394116987150625`}, {"CURE", 365, 64.03630186322135`},
{"CURE", 365, 35.235512355332546`}, {"CURE", 365, 41.080815218986196`}, {"CURE", 365, 89.9333676077259`},
{"CURE", 365, 9.922616082628512`}, {"CURE", 365, 11.675538253504042`}, {"CURE", 365, 98.46469337948939`},
{"CURE", 365, 31.73723793944998`}, {"CURE", 365, 34.32849578744917`}, {"CURE", 365, 17.258240770635837`},
{"CURE", 365, 39.51621514904973`}, {"CURE", 365, 49.24664695895698`}, {"CURE", 365, 114.15587613710204`},
{"CURE", 365, 16.825202433558946`}, {"CURE", 365, 65.80378111677418`}, {"CURE", 365, 38.70138402170397`},
{"CURE", 365, 102.46330672010292`}, {"CURE", 365, 167.24624219561954`}, {"CURE", 365, 61.267408998248236`},
{"CURE", 365, 5.931265300915081`}, {"CURE", 365, 46.40545778791594`}, {"CURE", 365, 14.66461722054526`},
{"CURE", 365, 154.9448139403658`}, {"TBUR", 56.44333599445757`, 4.965370388417695`}, {"CURE", 365, 5.172985905805753`},
{"CURE", 365, 179.53090258756106`}, {"CURE", 365, 41.329664228146044`}, {"CURE", 365, 13.95870885279014`},
{"CURE", 365, 26.71604055550867`}, {"CURE", 365, 9.377093587945108`}, {"CURE", 365, 8.933084658717494`},
{"CURE", 365, 10.657920520457955`}, {"CURE", 365, 38.526044160351034`}, {"CURE", 365, 81.65658667228315`},
{"CURE", 365, 32.35232198259901`}, {"CURE", 365, 8.101958082935282`}, {"CURE", 365, 110.16724579259686`},
{"CURE", 365, 220.3058226376933`}, {"CURE", 365, 6.209798275057466`}, {"CURE", 365, 49.660730229820516`},
{"CURE", 365, 26.657582015877683`}, {"CURE", 365, 5.242236594379966`}, {"CURE", 365, 40.99313373183407`},
{"CURE", 365, 26.183178002233298`}, {"CURE", 365, 20.103673847210924`}, {"CURE", 365, 39.089376815288624`},
{"CURE", 365, 22.18940003437076`}, {"CURE", 365, 65.42950240969842`}, {"CURE", 365, 38.787074618535826`},
{"CURE", 365, 91.33817049349481`}, {"CURE", 365, 40.46445015823601`}, {"CURE", 365, 20.05437705253101`},
{"CURE", 365, 9.756237097134484`}, {"CURE", 365, 6.760804676433399`}, {"CURE", 365, 19.256832507629014`},
{"CURE", 365, 76.65147247691809`}, {"CURE", 365, 25.27492618344112`}, {"CURE", 365, 23.169669604579763`},
{"CURE", 365, 16.381024014484506`}, {"CURE", 365, 16.600125608303326`}, {"CURE", 365, 44.85703966684287`},
{"CURE", 365, 8.391335388975712`}, {"CURE", 365, 53.47314654282134`}, {"CURE", 365, 24.4559112754920З`},
{"CURE", 365, 14.395810360292552`}, {"CURE", 365, 22.791593945638876`}, {"CURE", 365, 50.53952863266144`},
{"CURE", 365, 37.77419252060095`}, {"CURE", 365, 4.5443522419461315`}, {"CURE", 365, 19.588035591677016`},
{"TOX", 4.811263968090389`, 327.2651642061127`}, {"CURE", 365, 54.41518056359164`}, {"CURE", 365, 79.31644203494348`},
{"CURE", 365, 23.37371827762992`}, {"CURE", 365, 86.64690371275309`}, {"CURE", 365, 10.365368945604954`},
{"CURE", 365, 34.946341642587264`}, {"CURE", 365, 161.22539653494803`}, {"CURE", 365, 30.185329413747965`},
{"CURE", 365, 26.641258018835934`}, {"CURE", 365, 45.570870306652964`}, {"CURE", 365, 24.15497718175817`},
{"CURE", 365, 5.354015658425121`}, {"CURE", 365, 49.79011337319388`}, {"CURE", 365, 8.880193671723633`},
{"CURE", 365, 21.129961437366042`}, {"CURE", 365, 49.83748551152805`}, {"CURE", 365, 17.899722347871148`},
{"CURE", 365, 95.05754647468167`}, {"CURE", 365, 28.278478801994776`}, {"CURE", 365, 48.6543445914908`},

{"CURE", 365, 33.72601827049152`}, {"CURE", 365, 9.543542782969716`}, {"CURE", 365, 35.43326946424243`},
{"CURE", 365, 76.52165757685395`}, {"CURE", 365, 25.650450637778913`}, {"CURE", 365, 19.301942236427976`},
{"CURE", 365, 9.15928197366052`}, {"CURE", 365, 29.25125119941879`}, {"CURE", 365, 34.882212546797014`},
{"CURE", 365, 99.44350910744151`}, {"CURE", 365, 15.975799573340048`}, {"CURE", 365, 24.065901304072888`},
{"CURE", 365, 93.35646292967091`}, {"CURE", 365, 9.835733607910239`}, {"CURE", 365, 135.13126273711055`},
{"CURE", 365, 11.411720570227912`}, {"CURE", 365, 20.90529715913356`}, {"CURE", 365, 48.16608824584045`},
{"CURE", 365, 77.64740222327033`}, {"CURE", 365, 34.894853578902044`}, {"CURE", 365, 18.30537347465204`},
{"CURE", 365, 5.376935622058052`}, {"CURE", 365, 47.14430363633314`}, {"CURE", 365, 42.673488445038956`},
{"CURE", 365, 41.9071051371236`}, {"CURE", 365, 16.523575854016634`}, {"CURE", 365, 40.50344961258993`},
{"CURE", 365, 4.688547974204491`}, {"CURE", 365, 27.013338564463023`}, {"CURE", 365, 11.795530929197929`},
{"CURE", 365, 5.226133738888878`}, {"CURE", 365, 27.153696307435116`}, {"CURE", 365, 5.808956241090621`},
{"CURE", 365, 18.471561596407728`}, {"CURE", 365, 12.670912928469107`}, {"TBUR", 55.06401538034641`, 7.821965515781727`},
{"CURE", 365, 25.09806146647374`}, {"CURE", 365, 66.85387392965997`}, {"CURE", 365, 36.64939392738073`},
{"CURE", 365, 33.149753113204156`}, {"CURE", 365, 52.76058388704318`}, {"CURE", 365, 44.71801381473272`}},
{{"2700 Res", "2700 OS", "2700 Tox"}, {"CURE", 365, 22.524463107630567`}, {"CURE", 365, 46.54408274473506`},
{"CURE", 365, 32.650884564630715`}, {"TBUR", 83.21542778241039`, 10.971111911837607`},
{"TBUR", 59.66857784291294`, 42.286840749157776`}, {"CURE", 365, 84.31260082817089`}, {"CURE", 365, 79.3239222410067`},
{"CURE", 365, 8.289680277945184`}, {"CURE", 365, 57.209055088273544`}, {"CURE", 365, 49.50104993426475`},
{"CURE", 365, 185.76460225446925`}, {"CURE", 365, 23.045763521118523`}, {"CURE", 365, 84.02231396617655`},
{"TOX", 6.583263964575193`, 277.42819542621976`}, {"CURE", 365, 39.20421026254302`}, {"CURE", 365, 61.196217177556875`},
{"CURE", 365, 121.10118485650733`}, {"CURE", 365, 54.27339123357124`}, {"CURE", 365, 41.13930237979889`},
{"CURE", 365, 13.598918069798266`}, {"CURE", 365, 84.05176426984379`}, {"CURE", 365, 34.06880154174427`},
{"CURE", 365, 13.057030469457798`}, {"CURE", 365, 10.404106446547866`}, {"CURE", 365, 47.32735339726838`},
{"CURE", 365, 51.947113991861684`}, {"CURE", 365, 4.722898616945009`}, {"CURE", 365, 7.159027663964525`},
{"CURE", 365, 26.529526369846014`}, {"CURE", 365, 31.03960718683451`}, {"CURE", 365, 26.470904440135378`},
{"CURE", 365, 93.1913093558001`}, {"CURE", 365, 20.47149603193243`}, {"CURE", 365, 16.29602913634114`},
{"CURE", 365, 131.9506906008321`}, {"CURE", 365, 8.090115759906041`}, {"CURE", 365, 34.937478976223545`},
{"TBUR", 55.716198161073955`, 78.14865156445624`}, {"CURE", 365, 19.43323395779361`}, {"CURE", 365, 29.91006220949019`},
{"CURE", 365, 68.27757370944015`}, {"CURE", 365, 46.0003213440046`}, {"CURE", 365, 9.334904177074737`},
{"CURE", 365, 31.495881018674144`}, {"CURE", 365, 45.43124867607198`}, {"CURE", 365, 29.785376116289495`},
{"TOX", 4.844440385584757`, 316.53555499450783`}, {"CURE", 365, 68.23803511610177`}, {"CURE", 365, 106.81243024500534`},

{"CURE", 365, 12.7674480392397`}, {"CURE", 365, 36.24280900841827`}, {"CURE", 365, 49.37713739002611`},
{"CURE", 365, 39.27275159198134`}, {"CURE", 365, 25.494447269470303`}, {"CURE", 365, 10.98425409145738`},
{"CURE", 365, 116.14644290547125`}, {"CURE", 365, 36.496289538800326`}, {"CURE", 365, 6.939660092512394`},
{"CURE", 365, 74.77037427110415`}, {"CURE", 365, 52.39212933124989`}, {"CURE", 365, 35.86337161738701`},
{"CURE", 365, 27.423053911065818`}, {"CURE", 365, 15.710316712944142`}, {"CURE", 365, 59.10151643123783`},
{"CURE", 365, 42.01188038166876`}, {"CURE", 365, 48.01550022344601`}, {"CURE", 365, 10.99069640245756`},
{"CURE", 365, 11.178069621968998`}, {"CURE", 365, 11.882625937499686`}, {"CURE", 365, 48.53402016923464`},
{"CURE", 365, 17.768948163213096`}, {"CURE", 365, 39.58376387880116`}, {"CURE", 365, 74.27166137198455`},
{"TBUR", 32.02849167015026`, 23.956196666676078`}, {"CURE", 365, 19.855062722351306`}, {"CURE", 365, 54.9709970672113`},
{"CURE", 365, 32.1628138036128`}, {"CURE", 365, 24.45407953388072`}, {"CURE", 365, 97.46228631398066`},
{"CURE", 365, 106.97891296933366`}, {"CURE", 365, 78.7662551125494`}, {"CURE", 365, 21.248448448001657`},
{"CURE", 365, 42.96102103686798`}, {"CURE", 365, 30.029150805893295`}, {"CURE", 365, 33.96154643980479`},
{"CURE", 365, 15.91859155318127`}, {"CURE", 365, 169.80912089367644`}, {"CURE", 365, 37.01118955053164`},
{"CURE", 365, 46.59525895387922`}, {"CURE", 365, 40.11770250212996`}, {"CURE", 365, 33.23053774313066`},
{"CURE", 365, 9.833156118413246`}, {"CURE", 365, 18.01653894871792`}, {"CURE", 365, 41.712310219362934`},
{"TBUR", 68.5191704205102`, 15.166790281563577`}, {"CURE", 365, 17.96638320501431`}, {"CURE", 365, 46.82161288937222`},
{"CURE", 365, 118.82852830053866`}, {"CURE", 365, 63.68667340091333`}, {"CURE", 365, 31.9431597965834`},
{"CURE", 365, 63.381758495204075`}, {"CURE", 365, 41.176765657184056`}, {"CURE", 365, 26.677947229060916`},
{"CURE", 365, 182.67114349168503`}, {"CURE", 365, 20.288071286383666`}, {"CURE", 365, 13.785053329280663`},
{"CURE", 365, 80.93524179657499`}, {"CURE", 365, 23.93363826916802`}, {"CURE", 365, 24.410374688571725`},
{"CURE", 365, 146.86787652546812`}, {"CURE", 365, 11.921720947338164`}, {"CURE", 365, 7.5650160815367355`},
{"CURE", 365, 31.463283208219604`}, {"CURE", 365, 24.877701272613066`}, {"CURE", 365, 18.73764048633208`},
{"CURE", 365, 47.81515366720591`}, {"CURE", 365, 13.460487150591245`}, {"CURE", 365, 8.276257582677681`},
{"CURE", 365, 38.73539040121157`}, {"CURE", 365, 59.24595542141472`}, {"CURE", 365, 10.09438021771643`},
{"CURE", 365, 33.39955941797368`}, {"CURE", 365, 101.36686891806193`}, {"TBUR", 83.65148173829928`, 38.69420315810023`},
{"CURE", 365, 22.489339855006403`}, {"CURE", 365, 78.48953252441645`}, {"CURE", 365, 39.44577987085816`},
{"CURE", 365, 5.19873358193081`}, {"CURE", 365, 45.018598245317364`}, {"CURE", 365, 34.76880096251361`},
{"CURE", 365, 27.698864199623937`}, {"CURE", 365, 32.860485420591296`}, {"CURE", 365, 8.557935860926788`},
{"CURE", 365, 48.82257952069312`}, {"CURE", 365, 17.52698842278809`}, {"CURE", 365, 39.645908217352414`},
{"CURE", 365, 147.94840356126127`}, {"CURE", 365, 52.30470721432986`}, {"CURE", 365, 121.43521238518741`},
{"CURE", 365, 40.00626538790294`}, {"CURE", 365, 165.4681340135507`}, {"CURE", 365, 5.154612505946363`},

{"CURE", 365, 45.25857825212767`}, {"CURE", 365, 51.592618257030864`}, {"CURE", 365, 6.700728065406684`},
{"CURE", 365, 9.284445676820404`}, {"CURE", 365, 56.98376207882534`}, {"CURE", 365, 67.19769572125801`},
{"CURE", 365, 57.22500923230861`}, {"CURE", 365, 46.828132592683744`}, {"CURE", 365, 44.7596553364977`},
{"CURE", 365, 27.108158416143684`}, {"CURE", 365, 7.168345587799761`}, {"CURE", 365, 31.199347865481716`},
{"CURE", 365, 14.803455948088086`}, {"CURE", 365, 7.247543582853822`}, {"CURE", 365, 43.909640775514376`},
{"CURE", 365, 24.56335859201515`}, {"CURE", 365, 64.9323133513262`}, {"CURE", 365, 26.216351836156477`},
{"CURE", 365, 21.8190921671642`}, {"CURE", 365, 146.41850694476048`}, {"CURE", 365, 10.903713398983479`},
{"CURE", 365, 32.63749222872421`}, {"CURE", 365, 18.49268684161025`}, {"CURE", 365, 43.72779156474782`},
{"CURE", 365, 12.01038034404677`}, {"CURE", 365, 47.937521087170396`}, {"CURE", 365, 126.72462162395`},
{"CURE", 365, 10.834711499983364`}, {"CURE", 365, 32.8952297531881`}, {"CURE", 365, 35.59198465099854`},
{"CURE", 365, 127.9063857089324`}, {"CURE", 365, 48.1953272145971`}, {"CURE", 365, 40.00803347874538`},
{"CURE", 365, 70.26466446830959`}, {"CURE", 365, 26.915149949721187`}, {"CURE", 365, 84.54037049083036`},
{"CURE", 365, 51.84320309379295`}, {"CURE", 365, 31.870527302489865`}, {"CURE", 365, 28.595955885714964`},
{"CURE", 365, 9.295077200539662`}, {"CURE", 365, 6.297754117932804`}, {"CURE", 365, 14.767405390331298`},
{"CURE", 365, 19.772479460713736`}, {"CURE", 365, 8.717269429371239`}, {"CURE", 365, 31.903867214220995`},
{"CURE", 365, 87.02000543023016`}, {"CURE", 365, 23.519702032935516`}, {"CURE", 365, 43.70520399040633`},
{"CURE", 365, 54.671688403130794`}, {"CURE", 365, 47.4868936624688`}, {"CURE", 365, 31.998474904304384`},
{"CURE", 365, 12.71116815974069`}, {"CURE", 365, 87.58595753780219`}, {"CURE", 365, 7.576295683856596`},
{"CURE", 365, 81.63781821196098`}, {"CURE", 365, 79.46686677693782`}, {"CURE", 365, 22.141312127041832`},
{"CURE", 365, 26.297674087926744`}, {"CURE", 365, 151.27963617907315`}, {"CURE", 365, 39.05830785873263`},
{"CURE", 365, 46.51680285664899`}, {"CURE", 365, 29.14854989390848`}, {"CURE", 365, 10.957127681606341`},
{"CURE", 365, 67.2232218241945`}, {"TBUR", 70.84096960452064`, 178.7659316725034`}, {"CURE", 365, 27.27908466370797`},
{"CURE", 365, 24.670308830548883`}, {"CURE", 365, 45.44660747480631`}, {"CURE", 365, 18.146108408040146`},
{"TOX", 27.901266804880063`, 230.31719009406862`}, {"CURE", 365, 18.433106671993293`}, {"CURE", 365, 114.2059995544826`},
{"CURE", 365, 87.56280207595104`}, {"CURE", 365, 85.18667940109108`}, {"CURE", 365, 45.50060542646011`},
{"CURE", 365, 105.43496752219737`}, {"TOX", 5.401542372632362`, 306.2183260357464`}, {"CURE", 365, 47.902861527096874`},
{"CURE", 365, 8.802168311308709`}, {"CURE", 365, 78.16507341709266`}, {"CURE", 365, 48.705037944833286`},
{"CURE", 365, 46.95946975640071`}, {"CURE", 365, 10.366469394188568`}, {"TOX", 13.335461408066388`, 237.35610759597165`},
{"CURE", 365, 17.353881049182537`}, {"CURE", 365, 75.2672634266186`}, {"CURE", 365, 47.45920828958676`},
{"CURE", 365, 33.7207383084634`}, {"CURE", 365, 8.105512197943748`}, {"CURE", 365, 60.9218231483009`},
{"CURE", 365, 35.74407442191802`}, {"CURE", 365, 94.5580865725993`}, {"CURE", 365, 62.30526403261588`},

{"CURE", 365, 78.67294631285414`}, {"CURE", 365, 37.79729795031442`}, {"CURE", 365, 33.61938592278524`},
{"CURE", 365, 175.6774022780711`}, {"CURE", 365, 45.976931576979496`}, {"CURE", 365, 12.269708277568485`},
{"CURE", 365, 41.31596155666177`}, {"CURE", 365, 42.11937961359324`}, {"CURE", 365, 5.644891531261941`},
{"CURE", 365, 10.040342971846554`}, {"CURE", 365, 15.342675521094252`}, {"CURE", 365, 145.00052543397229`},
{"CURE", 365, 44.55287534113426`}, {"CURE", 365, 46.4194859374081`}, {"CURE", 365, 37.184227010423186`},
{"CURE", 365, 153.09603924463153`}, {"CURE", 365, 35.1995333467918`}, {"CURE", 365, 71.06728077341067`},
{"CURE", 365, 35.10851447054501`}, {"CURE", 365, 51.7525545037872`}, {"CURE", 365, 12.445082646724408`},
{"CURE", 365, 46.5490491403005`}, {"CURE", 365, 29.05211972393425`}, {"CURE", 365, 56.57347775763062`},
{"CURE", 365, 89.42585573474317`}, {"CURE", 365, 101.09564189850528`}, {"CURE", 365, 61.81009710142062`},
{"CURE", 365, 49.850167369838154`}, {"CURE", 365, 69.35380704468541`}, {"CURE", 365, 32.645404343810455`},
{"CURE", 365, 36.95813214708871`}, {"CURE", 365, 26.199140905706148`}, {"CURE", 365, 20.522869524264156`},
{"CURE", 365, 33.05890793469639`}, {"CURE", 365, 84.81324684914921`}, {"CURE", 365, 9.721607326817939`},
{"CURE", 365, 43.092933126418956`}, {"CURE", 365, 8.13407045702446`}, {"CURE", 365, 42.34519157764641`},
{"CURE", 365, 24.07934008183382`}, {"CURE", 365, 89.52594018741485`}, {"CURE", 365, 8.255278309164375`},
{"CURE", 365, 58.985508976842546`}, {"CURE", 365, 73.94590359775269`}, {"CURE", 365, 12.096312996186644`},
{"CURE", 365, 27.725081043790592`}, {"CURE", 365, 22.3071164428876`}, {"CURE", 365, 12.773556641694451`},
{"CURE", 365, 75.40842377516469`}, {"CURE", 365, 28.353062977921233`}, {"CURE", 365, 54.61584235551975`},
{"CURE", 365, 53.11402203471664`}, {"CURE", 365, 60.21338719200725`}, {"CURE", 365, 47.07961922031178`},
{"CURE", 365, 56.3878561180721`}, {"CURE", 365, 44.60983981829137`}, {"CURE", 365, 86.26771392237328`},
{"CURE", 365, 52.55916755504792`}, {"CURE", 365, 42.9328614249991`}, {"CURE", 365, 109.75137587223453`},
{"CURE", 365, 12.70692688197911`}, {"CURE", 365, 72.0690262652997`}, {"CURE", 365, 5.294125574053063`},
{"CURE", 365, 65.78312939057798`}, {"CURE", 365, 15.49898375504746`}, {"CURE", 365, 82.37174748718415`},
{"CURE", 365, 12.658530957012715`}, {"CURE", 365, 82.9103577797284`}, {"CURE", 365, 207.59116872725173`},
{"CURE", 365, 9.060913295074513`}, {"CURE", 365, 6.511757586195005`}, {"CURE", 365, 23.952159639004204`},
{"CURE", 365, 5.82089248096871`}, {"CURE", 365, 3.9769093564667366`}, {"CURE", 365, 23.264186207770457`},
{"CURE", 365, 38.07681295165959`}, {"CURE", 365, 40.92817870054526`}, {"CURE", 365, 38.94859006459975`},
{"CURE", 365, 30.330803344121062`}, {"CURE", 365, 53.31252679704222`}, {"CURE", 365, 93.85702962752924`},
{"CURE", 365, 7.595618049779063`}, {"CURE", 365, 41.60629861909176`}, {"CURE", 365, 35.7549716278839`},
{"CURE", 365, 25.925291414383338`}, {"CURE", 365, 47.95558166700843`}, {"CURE", 365, 71.08306767692869`},
{"CURE", 365, 166.3116520687841`}, {"CURE", 365, 123.96177331432455`}, {"CURE", 365, 38.37058512508362`},
{"CURE", 365, 4.070092125662088 6`}, {"CURE", 365, 44.401429723084945`}, {"CURE", 365, 114.48364303401813`},

```
{"CURE", 365, 28.80969927055632`}, {"CURE", 365, 37.03349899308581`}, {"CURE", 365, 22.228003297612425`},
{"CURE", 365, 32.57797532262249`}, {"CURE", 365, 8.446593860430598`}, {"CURE", 365, 41.2926929071415`},
{"CURE", 365, 23.23107727994234`}, {"CURE", 365, 67.80705765734756`}, {"CURE", 365, 46.46862519644429`},
{"CURE", 365, 45.00754841952342`}, {"CURE", 365, 37.03258904254098`}, {"CURE", 365, 19.597605861649523`},
{"CURE", 365, 13.506014337469292`}, {"CURE", 365, 13.168254434341742`}, {"CURE", 365, 17.062221972053994`},
{"CURE", 365, 13.293458927378186`}, {"CURE", 365, 178.84835589532818`}, {"CURE", 365, 99.48126405091688`},
{"CURE", 365, 19.211527752840833`}, {"TOX", 6.109483355507412`, 295.9147792423826`}, {"CURE", 365, 84.62344119684623`},
{"CURE", 365, 44.80635893322739`}, {"CURE", 365, 77.51202909443502`}, {"CURE", 365, 11.653361793380471`},
{"CURE", 365, 92.95260004461517`}, {"TOX", 7.574748703984796`, 248.43628267998335`}, {"CURE", 365, 13.481964489487469`},
{"CURE", 365, 42.05100404736872`}, {"CURE", 365, 68.91896072823249`}, {"CURE", 365, 34.905863700291114`},
{"CURE", 365, 17.063262395992467`}, {"CURE", 365, 13.59342657138253`}, {"CURE", 365, 117.99953161078338`},
{"CURE", 365, 61.94762214490221`}, {"CURE", 365, 35.241296685883476`}, {"CURE", 365, 87.60932582928494`},
{"CURE", 365, 15.829023972549018`}, {"CURE", 365, 42.25408712622127`}, {"CURE", 365, 25.519219662153873`},
{"CURE", 365, 172.08879501053477`}, {"CURE", 365, 43.38102024442728`}, {"CURE", 365, 201.32386275781383`},
{"TOX", 5.52758089336598`, 314.2106464094261`}, {"CURE", 365, 24.020472526680066`}, {"CURE", 365, 6.774623593884857`},
{"CURE", 365, 5.901223691154401`}, {"CURE", 365, 43.17590633861999`}, {"CURE", 365, 167.47927851874363`},
{"CURE", 365, 33.550323937354584`}, {"CURE", 365, 25.778970671927922`}, {"CURE", 365, 48.4871890796624`},
{"CURE", 365, 53.101857464878606`}, {"CURE", 365, 221.26431608608232`}, {"CURE", 365, 182.3653939310553`},
{"CURE", 365, 38.21737400371329`}, {"CURE", 365, 12.676951404853678`}, {"CURE", 365, 31.05777797203691`},
{"CURE", 365, 4.874049357513701`}, {"CURE", 365, 91.73548274302817`}, {"CURE", 365, 136.30067717027163`},
{"CURE", 365, 44.580104685492756`}, {"CURE", 365, 35.330872254229455`}, {"CURE", 365, 149.39041771368184`},
{"CURE", 365, 9.48822871592625`}, {"CURE", 365, 12.352950514537195`}, {"CURE", 365, 38.631816490334636`},
{"CURE", 365, 49.7370748674619`}, {"CURE", 365, 94.34546964216322`}, {"CURE", 365, 96.93429111576253`},
{"CURE", 365, 39.79037496588288`}, {"CURE", 365, 41.064820458403304`}, {"CURE", 365, 51.497973225807876`},
{"CURE", 365, 17.815674077124406`}, {"CURE", 365, 5.0266117260712875`}, {"CURE", 365, 52.87742103438619`},
{"CURE", 365, 63.651695437870956`}, {"CURE", 365, 30.948280185589443`}, {"CURE", 365, 56.08417147678744`},
{"CURE", 365, 17.473923753474487`}, {"CURE", 365, 11.480645739650178`}, {"CURE", 365, 50.255474090972186`},
{"CURE", 365, 30.565369047381107`}, {"CURE", 365, 43.6693326452119`}, {"TOX", 8.506192306078676`, 288.1496193724554`},
{"CURE", 365, 27.4599450169397`}, {"CURE", 365, 13.398297510536645`}, {"CURE", 365, 10.3932482212908`},
{"CURE", 365, 12.595760725035186`}, {"CURE", 365, 71.96618600770874`}, {"CURE", 365, 4.955392466877036`},
{"CURE", 365, 67.00530296437196`}, {"CURE", 365, 73.1416657529635`}, {"CURE", 365, 57.08751149272828`},
```

{"CURE", 365, 46.80284940970292`}, {"CURE", 365, 9.285072980003545`}, {"CURE", 365, 101.87071401768293`},
{"CURE", 365, 30.902560247690136`}, {"CURE", 365, 14.071347998969333`}, {"CURE", 365, 90.72560379177962`},
{"CURE", 365, 16.13562938754117`}, {"CURE", 365, 108.70388572088343`}, {"CURE", 365, 69.75016583518843`},
{"CURE", 365, 46.56631494187509`}, {"CURE", 365, 67.5922277359092`}, {"CURE", 365, 23.98552715638553`},
{"CURE", 365, 106.04878455109053`}, {"CURE", 365, 12.581941588273583`}, {"CURE", 365, 53.36345893073425`},
{"CURE", 365, 62.31388829867572`}, {"CURE", 365, 26.49522329242519`}, {"TOX", 10.711832370264629`, 246.9100021389342`},
{"CURE", 365, 49.63330238065808`}, {"CURE", 365, 50.53548403330996`}, {"CURE", 365, 51.63590770704439`},
{"CURE", 365, 206.75550170510004`}, {"CURE", 365, 65.7004895577542`}, {"CURE", 365, 61.29415712227261`},
{"CURE", 365, 133.43921780882815`}, {"CURE", 365, 46.91889514809302`}, {"CURE", 365, 40.88253453768177`},
{"CURE", 365, 50.509054789007116`}, {"CURE", 365, 69.76783169964136`}, {"CURE", 365, 123.2792283643569`},
{"CURE", 365, 26.605559534362598`}, {"CURE", 365, 24.2869913670851`}, {"CURE", 365, 51.96718028343283`},
{"CURE", 365, 52.909820703842584`}, {"CURE", 365, 59.59264664922184`}, {"CURE", 365, 63.61476172095287`},
{"CURE", 365, 35.56644648705514`}, {"CURE", 365, 17.905190437792584`}, {"CURE", 365, 67.31226646228538`},
{"CURE", 365, 16.969450654043992`}, {"CURE", 365, 36.07054263848647`}, {"CURE", 365, 43.00709683213488`},
{"CURE", 365, 12.939848315442669`}, {"CURE", 365, 6.433362680620094`}, {"CURE", 365, 39.82935580519481`},
{"CURE", 365, 53.910207281286056`}, {"CURE", 365, 63.98140744216615`}, {"CURE", 365, 45.77316393393629`},
{"CURE", 365, 5.865410433600748`}, {"CURE", 365, 52.478115100117016`}, {"CURE", 365, 10.001640871681394`},
{"CURE", 365, 154.1997702704778`}, {"CURE", 365, 200.65776263435808`}, {"CURE", 365, 28.954303983196958`},
{"CURE", 365, 39.692394867661605`}, {"CURE", 365, 215.41594282011894`}, {"CURE", 365, 38.62802827094417`},
{"CURE", 365, 22.481103633465665`}, {"CURE", 365, 17.49796274709225`}, {"CURE", 365, 4.777721963519046`},
{"CURE", 365, 30.112304419363994`}, {"CURE", 365, 33.57809994111426`}, {"CURE", 365, 89.04423232156002`},
{"CURE", 365, 42.5988513719838`}, {"CURE", 365, 74.15806109166937`}, {"CURE", 365, 26.194188445225368`},
{"CURE", 365, 56.92183120749015`}, {"CURE", 365, 127.04587529972204`}, {"CURE", 365, 12.929341018246197`},
{"CURE", 365, 19.932158394395277`}, {"CURE", 365, 29.61632798914505`}, {"CURE", 365, 41.35975411043975`},
{"CURE", 365, 27.181087586155435`}, {"CURE", 365, 25.96035009945554`}, {"CURE", 365, 11.01240392315532`},
{"CURE", 365, 16.977950474627413`}, {"CURE", 365, 32.20601819508744`}, {"CURE", 365, 73.25677124799272`},
{"CURE", 365, 65.27168411718296`}, {"CURE", 365, 33.385008832122345`}, {"TBUR", 52.462994833801034`, 50.18849930158655`},
{"CURE", 365, 107.9025674351579`}, {"CURE", 365, 52.751060385172046`}, {"CURE", 365, 39.46961574466961`},
{"CURE", 365, 25.139886115591107`}, {"CURE", 365, 7.879696882878844`}, {"CURE", 365, 43.81854351367765`},
{"CURE", 365, 50.58961186847829`}, {"CURE", 365, 198.19866024786447`}, {"CURE", 365, 10.102226880534442`},
{"CURE", 365, 9.86433098786788`}, {"CURE", 365, 31.60611279889688`}, {"CURE", 365, 92.13189195039561`},

{"CURE", 365, 11.498794912557072`}, {"CURE", 365, 56.242719338932446`}, {"CURE", 365, 13.11992363039271`},
{"CURE", 365, 32.696778075683596`}, {"CURE", 365, 13.364563995476162`}, {"CURE", 365, 13.86260512977475`},
{"CURE", 365, 12.502413464884441`}, {"CURE", 365, 133.3805247065191`}, {"CURE", 365, 21.379293934371034`},
{"CURE", 365, 37.64461133899275`}, {"CURE", 365, 5.182174396310401`}, {"CURE", 365, 62.0928438423567`},
{"CURE", 365, 86.87562867571667`}, {"CURE", 365, 75.77926333620573`}, {"CURE", 365, 11.437354944482465`},
{"CURE", 365, 22.93570221359445`}, {"TOX", 22.774701215387317`, 233.44329630354287`}, {"CURE", 365, 63.42043937732426`},
{"CURE", 365, 43.22927264628306`}, {"CURE", 365, 15.370145507875051`}, {"CURE", 365, 21.97997273780748`},
{"CURE", 365, 33.97701157900862`}, {"CURE", 365, 8.414464816454327`}, {"CURE", 365, 41.28253772501384`},
{"CURE", 365, 64.94861000883722`}, {"CURE", 365, 39.53396356666046`}, {"CURE", 365, 42.93718938989778`},
{"CURE", 365, 31.039750918479093`}, {"CURE", 365, 20.114877608201567`}, {"CURE", 365, 114.96053622242967`},
{"CURE", 365, 166.53463568577308`}, {"CURE", 365, 44.41317743719062`}, {"CURE", 365, 56.07400464910379`},
{"CURE", 365, 71.03813127438558`}, {"CURE", 365, 69.4603276507459`}, {"CURE", 365, 6.068507129915145`},
{"CURE", 365, 81.30139049431654`}, {"CURE", 365, 83.95817488037942`}, {"CURE", 365, 56.77520075761472`},
{"CURE", 365, 227.74932240333834`}, {"CURE", 365, 47.34904075270622`}, {"CURE", 365, 21.110379742842692`},
{"CURE", 365, 49.83476535473211`}, {"CURE", 365, 63.340208748584594`}, {"CURE", 365, 28.374026612580323`},
{"CURE", 365, 22.294937659641608`}, {"CURE", 365, 19.364520007025718`}, {"CURE", 365, 35.14311237283821`},
{"CURE", 365, 108.19518528228143`}, {"CURE", 365, 69.43929041643845`}, {"CURE", 365, 12.100039841912185`},
{"CURE", 365, 71.06842082148927`}, {"CURE", 365, 102.21822343174087`}, {"CURE", 365, 18.81148216831501`},
{"CURE", 365, 107.33044547398757`}, {"CURE", 365, 16.12119774008372`}, {"CURE", 365, 10.301960176645165`},
{"CURE", 365, 14.59725780809133`}, {"CURE", 365, 28.124778872691675`}, {"CURE", 365, 126.09269902362999`},
{"CURE", 365, 44.95241545742332`}, {"CURE", 365, 56.79110062504427`}, {"CURE", 365, 13.568664049927294`},
{"TOX", 10.679559129081875`, 259.2530044903612`}, {"CURE", 365, 118.62420473112299`}, {"CURE", 365, 20.265056564027773`},
{"CURE", 365, 49.5745074635939`}, {"CURE", 365, 27.603981887014626`}, {"CURE", 365, 27.899255148624047`},
{"CURE", 365, 94.29296042054429`}, {"CURE", 365, 119.30622338514927`}, {"CURE", 365, 12.800151426125131`},
{"CURE", 365, 24.95857252153039`}, {"CURE", 365, 8.854520892282967`}, {"CURE", 365, 34.12498886576416`},
{"CURE", 365, 58.564427799412805`}, {"CURE", 365, 94.74506976087235`}, {"CURE", 365, 52.88101440375638`},
{"CURE", 365, 190.43374737267905`}, {"CURE", 365, 91.01776427249685`}, {"CURE", 365, 15.048366595157535`},
{"CURE", 365, 33.30869482112291`}, {"TBUR", 58.47708707979045`, 37.32886336391732`}, {"CURE", 365, 74.5174569973283`},
{"CURE", 365, 9.778631047082634`}, {"CURE", 365, 26.135987184750306`}, {"CURE", 365, 14.733371088061817`},
{"CURE", 365, 36.7236074473778`}, {"CURE", 365, 83.93137412186019`}, {"CURE", 365, 35.69850887683711`},
{"CURE", 365, 7.7539700308315345`}, {"CURE", 365, 147.8172262003046`}, {"CURE", 365, 27.871659672120423`},

{"CURE", 365, 9.463556013393704`}, {"CURE", 365, 12.44186642664851`}, {"CURE", 365, 10.502382299179711`},
{"CURE", 365, 13.415610141007512`}, {"CURE", 365, 107.95705498650807`}, {"CURE", 365, 59.16438190387596`},
{"CURE", 365, 35.580454267410275`}, {"CURE", 365, 16.69085488771866`}, {"CURE", 365, 27.833995716832106`},
{"CURE", 365, 16.10449696496581`}, {"CURE", 365, 38.86677440717309`}, {"CURE", 365, 133.68657891746625`},
{"CURE", 365, 79.65346371762519`}, {"CURE", 365, 38.607385845365016`}, {"CURE", 365, 22.22259487786772`},
{"CURE", 365, 23.63271978993737`}, {"TOX", 7.763479684381829`, 285.5101615522994`}, {"CURE", 365, 13.60617931720483`},
{"CURE", 365, 7.010144112247886`}, {"CURE", 365, 6.122857346772246`}, {"CURE", 365, 77.64640601360311`},
{"CURE", 365, 45.49370745674594`}, {"CURE", 365, 50.44917133267422`}, {"CURE", 365, 74.93079301264389`},
{"CURE", 365, 123.85428333016893`}, {"CURE", 365, 127.9364392902204`}, {"CURE", 365, 48.75939042838532`},
{"CURE", 365, 21.052424187557637`}, {"CURE", 365, 24.4261416516005`}, {"CURE", 365, 49.22026013747426`},
{"CURE", 365, 27.672191583539004`}, {"TBUR", 31.751425659765022`, 11.389155443909647`}, {"CURE", 365, 43.47375593478933`},
{"TOX", 5.89458478285802`, 270.2788197677718`}, {"CURE", 365, 27.836351191472225`}, {"CURE", 365, 10.020563950180074`},
{"CURE", 365, 94.91852574959984`}, {"CURE", 365, 23.740650619540823`}, {"CURE", 365, 68.25740808111824`},
{"TOX", 5.064190838734596`, 328.4235742457679`}, {"CURE", 365, 151.65363190377383`}, {"CURE", 365, 49.23183181916625`},
{"CURE", 365, 41.29341873171951`}, {"CURE", 365, 17.581147832173407`}, {"CURE", 365, 5.022478928407708`},
{"CURE", 365, 19.71007314327988`}, {"CURE", 365, 57.56846280409395`}, {"CURE", 365, 31.94429917601495`},
{"CURE", 365, 101.90377453825417`}, {"CURE", 365, 40.40310783941111`}, {"CURE", 365, 41.22946153669915`},
{"CURE", 365, 11.449962519770198`}, {"CURE", 365, 32.4363066304601`}, {"CURE", 365, 33.27763581423704`},
{"CURE", 365, 8.679842237138791`}, {"CURE", 365, 27.645548417539466`}, {"CURE", 365, 16.463616264259`},
{"CURE", 365, 26.134433809963003`}, {"CURE", 365, 55.17247705911593`}, {"CURE", 365, 27.3634133756997`},
{"CURE", 365, 6.336137302380885`}, {"CURE", 365, 43.65895600578394`}, {"CURE", 365, 4.892817614390056`},
{"CURE", 365, 14.36842739443566`}, {"CURE", 365, 42.56957553430528`}, {"CURE", 365, 49.0461333256616`},
{"CURE", 365, 9.77501407379062`}, {"CURE", 365, 28.74343862842287`}, {"CURE", 365, 37.317847478349655`},
{"CURE", 365, 188.21536427218388`}, {"CURE", 365, 43.176843308396435`}, {"CURE", 365, 12.194276908349593`},
{"CURE", 365, 132.81756906289496`}, {"CURE", 365, 13.098972610429136`}, {"CURE", 365, 49.1892912421849`},
{"CURE", 365, 50.836244102133044`}, {"CURE", 365, 15.175316559552256`}, {"CURE", 365, 16.904381803168377`},
{"CURE", 365, 74.8488151950622`}, {"CURE", 365, 7.321901757511827`}, {"CURE", 365, 63.40224746334759`},
{"CURE", 365, 16.79197696686451`}, {"CURE", 365, 21.95604557087597`}, {"CURE", 365, 4.911798042258994`},
{"CURE", 365, 25.21531826113739`}, {"CURE", 365, 86.13635667791459`}, {"CURE", 365, 32.736652997861675`},
{"CURE", 365, 23.781612748273975`}, {"CURE", 365, 33.97850032491483`}, {"CURE", 365, 26.76069447925929`},
{"CURE", 365, 33.62082466793963`}, {"CURE", 365, 59.27956360001784`}, {"CURE", 365, 57.1868300141053`},

{"CURE", 365, 7.041249695722379`}, {"CURE", 365, 115.7322378651711`}, {"CURE", 365, 13.44039286111478`},
{"CURE", 365, 6.747159506179947`}, {"CURE", 365, 32.16168470292816`}, {"CURE", 365, 33.740366282164956`},
{"CURE", 365, 22.773326373974044`}, {"CURE", 365, 39.492072746929175`}, {"CURE", 365, 34.04688243167966`},
{"CURE", 365, 54.723622642694735`}, {"CURE", 365, 46.24988847658214`}, {"CURE", 365, 66.10406197675827`},
{"CURE", 365, 10.443615459691461`}, {"CURE", 365, 134.19072630477314`}, {"CURE", 365, 129.82482549800625`},
{"CURE", 365, 7.196419644403686`}, {"CURE", 365, 18.10267272455648`}, {"CURE", 365, 44.91650654998076`},
{"CURE", 365, 22.86568175971068`}, {"CURE", 365, 70.359687876525`}, {"CURE", 365, 140.64829342492524`},
{"CURE", 365, 44.4469328053376`}, {"CURE", 365, 7.63475789408598`}, {"CURE", 365, 7.487056540569069`},
{"CURE", 365, 17.571791296650073`}, {"CURE", 365, 5.306636524646175`}, {"CURE", 365, 60.21260453369197`},
{"CURE", 365, 9.338675931278777`}, {"CURE", 365, 62.931018267252924`}, {"CURE", 365, 38.68392445262396`},
{"CURE", 365, 66.65488703917558`}, {"CURE", 365, 16.414101481562373`}, {"CURE", 365, 82.81051842829929`},
{"TOX", 10.162237833213409`, 273.9782731175583`}, {"CURE", 365, 98.68782324232593`}, {"CURE", 365, 159.8880353861527`},
{"CURE", 365, 47.04668787301474`}, {"CURE", 365, 112.08583450231363`}, {"CURE", 365, 112.31748961472032`},
{"CURE", 365, 13.630885779921394`}, {"CURE", 365, 9.535375362370115`}, {"CURE", 365, 31.017185382339424`},
{"CURE", 365, 64.33431283822577`}, {"CURE", 365, 80.4979335681186`}, {"CURE", 365, 34.72779886775108`},
{"CURE", 365, 50.9838844171255`}, {"CURE", 365, 9.60567006947515`}, {"CURE", 365, 12.208086651514872`},
{"CURE", 365, 59.277424123493795`}, {"CURE", 365, 19.23431842993423`}, {"CURE", 365, 8.150936421485381`},
{"CURE", 365, 12.053447254144862`}, {"CURE", 365, 5.893782782645829`}, {"CURE", 365, 47.88888437907507`},
{"CURE", 365, 65.84937746477974`}, {"CURE", 365, 46.09698128199396`}, {"CURE", 365, 52.34744244026257`},
{"CURE", 365, 29.005717894550653`}, {"CURE", 365, 25.98761722565782`}, {"CURE", 365, 67.20696998370796`},
{"CURE", 365, 71.89845339035338`}, {"CURE", 365, 8.024181285821314`}, {"CURE", 365, 30.639831320492508`},
{"CURE", 365, 15.948458787076952`}, {"CURE", 365, 25.031920861064005`}, {"CURE", 365, 206.22981004444657`},
{"CURE", 365, 36.41401688622071`}, {"CURE", 365, 24.991140658853467`}, {"CURE", 365, 26.451177318327254`},
{"CURE", 365, 52.73342920352721`}, {"CURE", 365, 40.1396480095315`}, {"CURE", 365, 52.363405267574365`},
{"CURE", 365, 54.053000277791035`}, {"CURE", 365, 12.248759538632887`}, {"CURE", 365, 119.09909627985662`},
{"CURE", 365, 22.884951493816178`}, {"CURE", 365, 48.513360826045414`}, {"CURE", 365, 27.53341432028596`},
{"CURE", 365, 15.630337592037229`}, {"CURE", 365, 56.982384653617835`}, {"CURE", 365, 57.845921087017764`},
{"TBUR", 95.15474597929826`, 32.14817568967243`}, {"CURE", 365, 75.86178163217345`}, {"CURE", 365, 16.047112397874706`},
{"CURE", 365, 134.05351135984753`}, {"CURE", 365, 51.50476038111495`}, {"CURE", 365, 108.94693201012747`},
{"CURE", 365, 4.222508900118128`}, {"CURE", 365, 53.61337864392262`}, {"CURE", 365, 61.14976105595759`},
{"CURE", 365, 14.510283449826321`}, {"CURE", 365, 36.851985647551764`}, {"CURE", 365, 55.1763566072203`},

{"CURE", 365, 33.542049007528995`}, {"CURE", 365, 10.676705018868898`}, {"CURE", 365, 8.100248150873963`},
{"CURE", 365, 47.91556197716037`}, {"CURE", 365, 12.777141657023234`}, {"CURE", 365, 28.410394417989128`},
{"CURE", 365, 34.65453561602293`}, {"CURE", 365, 16.15216751154427`}, {"CURE", 365, 29.69960055863428`},
{"CURE", 365, 8.079326119049357`}, {"CURE", 365, 33.30431634230023`}, {"CURE", 365, 5.383782477985249`},
{"CURE", 365, 9.627672443861357`}, {"CURE", 365, 22.77132545320622`}, {"CURE", 365, 7.342752739850787`},
{"CURE", 365, 54.557591782226474`}, {"CURE", 365, 80.09627134627071`}, {"CURE", 365, 81.34974408059138`},
{"CURE", 365, 67.69564690296157`}, {"CURE", 365, 127.79657218249541`}, {"CURE", 365, 10.340700456946465`},
{"CURE", 365, 46.10770025320538`}, {"CURE", 365, 49.58331549439713`}, {"CURE", 365, 85.11016716834293`},
{"CURE", 365, 63.57047090001998`}, {"CURE", 365, 29.070179378977056`}, {"CURE", 365, 136.70872400313291`},
{"CURE", 365, 4.266636938467108`}, {"CURE", 365, 129.97227485745387`}, {"CURE", 365, 8.80459385599631`},
{"CURE", 365, 111.01275152752554`}, {"CURE", 365, 100.53449936327141`}, {"CURE", 365, 29.807003897939143`},
{"CURE", 365, 117.37854618327499`}, {"CURE", 365, 143.1403707690984`}, {"CURE", 365, 64.04306669894922`},
{"CURE", 365, 28.250760638209524`}, {"CURE", 365, 32.48290338018618`}, {"CURE", 365, 47.97551536695639`},
{"CURE", 365, 44.25007491586353`}, {"CURE", 365, 10.721670872786696`}, {"CURE", 365, 38.50212351055483`},
{"CURE", 365, 36.4479330692127`}, {"CURE", 365, 27.248723235611443`}, {"CURE", 365, 67.29799505912585`},
{"CURE", 365, 10.59751333970166`}, {"CURE", 365, 75.12496559617288`}, {"CURE", 365, 49.16623870086613`},
{"CURE", 365, 27.83119345140482`}, {"CURE", 365, 25.4343629918461`}, {"CURE", 365, 51.418279954156425`},
{"CURE", 365, 7.033396024730766`}, {"CURE", 365, 81.46810045931309`}, {"CURE", 365, 12.866858425965525`},
{"CURE", 365, 18.24357876550558`}, {"CURE", 365, 40.940980044830035`}, {"CURE", 365, 32.09234059430524`},
{"CURE", 365, 29.510394952340437`}, {"CURE", 365, 91.9169248612922`}, {"CURE", 365, 12.429097476083975`},
{"CURE", 365, 21.640844798597794`}, {"CURE", 365, 160.06765565281106`}, {"CURE", 365, 12.146759737345905`},
{"CURE", 365, 31.27265172773633`}, {"CURE", 365, 47.61499075450225`}, {"CURE", 365, 47.01250668656578`},
{"CURE", 365, 31.90688088058415`}, {"CURE", 365, 164.90913733933604`}, {"CURE", 365, 29.80560901919672`},
{"CURE", 365, 12.522121789119687`}, {"CURE", 365, 25.08004996414713`}, {"CURE", 365, 19.321430818296797`},
{"CURE", 365, 14.813218595314243`}, {"CURE", 365, 38.834317508277515`}, {"CURE", 365, 66.50099535915055`},
{"CURE", 365, 36.59271961382198`}, {"CURE", 365, 42.66297903416758`}, {"CURE", 365, 93.39362447313644`},
{"CURE", 365, 10.305633631694457`}, {"CURE", 365, 12.1260524356726`}, {"CURE", 365, 102.25582974860667`},
{"CURE", 365, 32.96650968957483`}, {"CURE", 365, 35.64902326690583`}, {"CURE", 365, 17.922592820304043`},
{"CURE", 365, 41.03670772927198`}, {"CURE", 365, 51.141502763656916`}, {"CURE", 365, 118.55176403016928`},
{"CURE", 365, 17.473019104387546`}, {"CURE", 365, 68.34849464270775`}, {"CURE", 365, 40.19688220658966`},
{"CURE", 365, 106.41964255119146`}, {"CURE", 365, 173.68252108416664`}, {"CURE", 365, 63.63631240243893`},

{"CURE", 365, 6.159935507539666`}, {"CURE", 365, 48.19170395790361`}, {"CURE", 365, 15.230753553230377`},
{"CURE", 365, 160.90534319327847`}, {"TBUR", 58.42610051353593`, 5.166304420289982`}, {"CURE", 365, 5.372340843508147`},
{"CURE", 365, 186.47165356843513`}, {"CURE", 365, 42.93337668109594`}, {"CURE", 365, 14.495842822962922`},
{"CURE", 365, 27.743979872329636`}, {"CURE", 365, 9.738856175383344`}, {"CURE", 365, 9.278586705135455`},
{"CURE", 365, 11.067968181024664`}, {"CURE", 365, 40.00855086885412`}, {"CURE", 365, 84.79905543116419`},
{"CURE", 365, 33.600066017108304`}, {"CURE", 365, 8.41421366281945`}, {"CURE", 365, 114.40637542903988`},
{"CURE", 365, 228.82332315401044`}, {"CURE", 365, 6.448864206204012`}, {"CURE", 365, 51.57855869573694`},
{"CURE", 365, 27.683415818973646`}, {"CURE", 365, 5.443961528392193`}, {"CURE", 365, 42.5730384302245`},
{"CURE", 365, 27.190532048346554`}, {"CURE", 365, 20.8779474405818`}, {"CURE", 365, 40.59354559892985`},
{"CURE", 365, 23.043419708588488`}, {"CURE", 365, 67.98537162353156`}, {"CURE", 365, 40.28031183479326`},
{"CURE", 365, 94.85628082644911`}, {"CURE", 365, 42.02448949748539`}, {"CURE", 365, 20.82885804237003`},
{"CURE", 365, 10.133807409907165`}, {"CURE", 365, 7.0209218427922915`}, {"CURE", 365, 19.99765517728108`},
{"CURE", 365, 79.60521606088105`}, {"CURE", 365, 26.24764803604357`}, {"CURE", 365, 24.063625559471344`},
{"CURE", 365, 17.01266387348128`}, {"CURE", 365, 17.239581493910983`}, {"CURE", 365, 46.584864062133605`},
{"CURE", 365, 8.714331428083769`}, {"CURE", 365, 55.5341873052575`}, {"CURE", 365, 25.396825946556667`},
{"CURE", 365, 14.954673338874867`}, {"CURE", 365, 23.67117377238882`}, {"CURE", 365, 52.48369608674181`},
{"CURE", 365, 39.257070075514136`}, {"CURE", 365, 4.719268935436016`}, {"CURE", 365, 20.343639106494358`},
{"TOX", 4.57687337201812`, 339.8583393011259`}, {"CURE", 365, 56.522571548839394`}, {"CURE", 365, 82.37346900037919`},
{"CURE", 365, 24.275836540559833`}, {"CURE", 365, 89.98677568727507`}, {"CURE", 365, 10.76455389823429`},
{"CURE", 365, 36.29628958165931`}, {"CURE", 365, 167.4358485790709`}, {"CURE", 365, 31.346508409528706`},
{"CURE", 365, 27.681124910367547`}, {"CURE", 365, 47.32398447771892`}, {"CURE", 365, 25.091991414333343`},
{"CURE", 365, 5.560156534952509`}, {"CURE", 365, 51.712989501978214`}, {"CURE", 365, 9.22246652586783 7`},
{"CURE", 365, 21.951801520531408`}, {"CURE", 365, 51.755522004233114`}, {"CURE", 365, 18.588835353676473`},
{"CURE", 365, 98.71498458144353`}, {"CURE", 365, 29.369334526939408`}, {"CURE", 365, 50.52606913575367`},
{"CURE", 365, 35.023764180288545`}, {"CURE", 365, 9.91069735865246`}, {"CURE", 365, 36.80098761397884`},
{"CURE", 365, 79.4661892237769`}, {"CURE", 365, 26.637875146641225`}, {"CURE", 365, 20.04466173942501`},
{"CURE", 365, 9.512362274720639`}, {"CURE", 365, 30.41287057410348`}, {"CURE", 365, 36.23145001678122`},
{"CURE", 365, 103.26962349341632`}, {"CURE", 365, 16.590771772453387`}, {"CURE", 365, 24.996405896164337`},
{"CURE", 365, 96.95084777836355`}, {"CURE", 365, 10.214377585695386`}, {"CURE", 365, 140.35583549334186`},
{"CURE", 365, 11.850890491562035`}, {"CURE", 365, 21.713350627955354`}, {"CURE", 365, 50.021924131224516`},
{"CURE", 365, 80.6666141803148`}, {"CURE", 365, 36.23961055841928`}, {"CURE", 365, 19.014203943691633`},

```
{"CURE", 365, 5.584265037815096`}, {"CURE", 365, 48.97770180507848`}, {"CURE", 365, 44.32318262282104`},
{"CURE", 365, 43.520538541043294`}, {"CURE", 365, 17.15980292783858`}, {"CURE", 365, 42.06216152550553`},
{"CURE", 365, 4.869661704779689 5`}, {"CURE", 365, 28.05552404637311`}, {"CURE", 365, 12.24970959398078`},
{"CURE", 365, 5.427774471656869`}, {"CURE", 365, 28.19973419417706`}, {"CURE", 365, 6.032425796280313`},
{"CURE", 365, 19.18231431858682`}, {"CURE", 365, 13.15899856116135`}, {"TBUR", 57.17957059483661`, 8.140487910481141`},
{"CURE", 365, 26.06497741446221`}, {"CURE", 365, 69.4254532650577`}, {"CURE", 365, 38.07086650452545`},
{"CURE", 365, 34.425922832798214`}, {"CURE", 365, 54.80914507046814`}, {"CURE", 365, 46.43935287909575`}},
{{"2800 Res", "2800 OS", "2800 Tox"}, {"CURE", 365, 23.36416943252406`}, {"CURE", 365, 48.26832408302731`},
{"CURE", 365, 33.865929628784095`}, {"TBUR", 85.6397287399807`, 11.382080658026801`},
{"TBUR", 61.58315685647283`, 43.93340736220174`}, {"CURE", 365, 87.43634082718481`}, {"CURE", 365, 82.26226103469689`},
{"CURE", 365, 8.596979152758912`}, {"CURE", 365, 59.32840127351464`}, {"CURE", 365, 51.33803923733997`},
{"CURE", 365, 192.65341401102592`}, {"CURE", 365, 23.899731416630246`}, {"CURE", 365, 87.41730228723745`},
{"TOX", 6.143630092716685`, 287.81852080206414`}, {"CURE", 365, 40.660651661775624`}, {"CURE", 365, 63.47054213162558`},
{"CURE", 365, 125.58883869534208`}, {"CURE", 365, 56.28870144410882`}, {"CURE", 365, 42.680285405905316`},
{"CURE", 365, 14.103053473047677`}, {"CURE", 365, 87.1667940913288`}, {"CURE", 365, 35.33095755849709`},
{"CURE", 365, 13.54110271478407`}, {"CURE", 365, 10.789905885462058`}, {"CURE", 365, 49.08043735936972`},
{"CURE", 365, 53.87169148078242`}, {"CURE", 365, 4.8979286081048`}, {"CURE", 365, 7.424374880770613`},
{"CURE", 365, 27.513043116179894`}, {"CURE", 365, 32.19109954001983`}, {"CURE", 365, 27.451593844988952`},
{"CURE", 365, 96.66515286868307`}, {"CURE", 365, 21.23065519522753`}, {"CURE", 365, 16.902625752089797`},
{"CURE", 365, 136.84152191232567`}, {"CURE", 365, 8.391058031366835`}, {"CURE", 365, 36.23157789878952`},
{"TBUR", 57.323257338924904`, 81.07646400869821`}, {"CURE", 365, 20.15539152536468`}, {"CURE", 365, 31.02548891802149`},
{"CURE", 365, 70.82502589129513`}, {"CURE", 365, 47.70426057779752`}, {"CURE", 365, 9.680963558380228`},
{"CURE", 365, 32.6654588923911`}, {"CURE", 365, 47.115884277676074`}, {"CURE", 365, 30.888905640459846`},
{"TOX", 4.604303189937778`, 328.2688805589984`}, {"CURE", 365, 70.76815721795572`}, {"CURE", 365, 111.23373390153981`},
{"CURE", 365, 13.24206037913871`}, {"CURE", 365, 37.70901541963569`}, {"CURE", 365, 51.29902930053235`},
{"CURE", 365, 40.73040584007513`}, {"CURE", 365, 26.441554638540882`}, {"CURE", 365, 11.391714362025857`},
{"CURE", 365, 120.4501315697063`}, {"CURE", 365, 37.84822708036956`}, {"CURE", 365, 7.197781052387345`},
{"CURE", 365, 77.55284163550589`}, {"CURE", 365, 54.34818815016173`}, {"CURE", 365, 37.197726923253754`},
{"CURE", 365, 28.439152292939557`}, {"CURE", 365, 16.32478327848143`}, {"CURE", 365, 61.291053480782075`},
{"CURE", 365, 43.57185308491511`}, {"CURE", 365, 49.79473162745882`}, {"CURE", 365, 11.397977843097989`},
{"CURE", 365, 11.59367785093548`}, {"CURE", 365, 12.322891509452662`}, {"CURE", 365, 50.33290106703007`},
```

{"CURE", 365, 18.427729818479943`}, {"CURE", 365, 41.05196871297394`}, {"CURE", 365, 77.02482518409842`},
{"TBUR", 33.06706626577092`, 24.98124528159425`}, {"CURE", 365, 20.5921321684973`}, {"CURE", 365, 57.007721425641755`},
{"CURE", 365, 33.357054145198845`}, {"CURE", 365, 25.36041179797373`}, {"CURE", 365, 101.08107150104193`},
{"CURE", 365, 110.94348755195588`}, {"CURE", 365, 81.68602314125006`}, {"CURE", 365, 22.035618207643004`},
{"CURE", 365, 44.552403164928805`}, {"CURE", 365, 31.141575789759138`}, {"CURE", 365, 35.220617332681066`},
{"CURE", 365, 16.50940118885429`}, {"CURE", 365, 176.1086558492345`}, {"CURE", 365, 38.38463917207713`},
{"CURE", 365, 48.3217076360572`}, {"CURE", 365, 41.60693425343234`}, {"CURE", 365, 34.47617008717312`},
{"CURE", 365, 10.198565311574269`}, {"CURE", 365, 18.684821553989067`}, {"CURE", 365, 43.2600496882514`},
{"TBUR", 70.70043110427906`, 15.736166851585349`}, {"CURE", 365, 18.63380280453455`}, {"CURE", 365, 48.556534144674636`},
{"CURE", 365, 123.23191268385855`}, {"CURE", 365, 66.05111127420483`}, {"CURE", 365, 33.12941387442408`},
{"CURE", 365, 65.74597492806546`}, {"CURE", 365, 42.704964392009266`}, {"CURE", 365, 27.667045338948505`},
{"CURE", 365, 189.44566828220624`}, {"CURE", 365, 21.039698601970287`}, {"CURE", 365, 14.295817736387159`},
{"CURE", 365, 83.93738919174123`}, {"CURE", 365, 24.833594696009946`}, {"CURE", 365, 25.314649284014983`},
{"CURE", 365, 152.3085460116193`}, {"CURE", 365, 12.363561541647051`}, {"CURE", 365, 7.845657776062808`},
{"CURE", 365, 32.6293649191672`}, {"CURE", 365, 25.800036724200627`}, {"CURE", 365, 19.43268592894584`},
{"CURE", 365, 49.586341837240774`}, {"CURE", 365, 13.959528533922366`}, {"CURE", 365, 8.583112899565357`},
{"CURE", 365, 40.17024033047519`}, {"CURE", 365, 61.441372819070104`}, {"CURE", 365, 10.469480856056201`},
{"CURE", 365, 34.63818405269875`}, {"CURE", 365, 105.12281905556212`}, {"TBUR", 86.4940596846253`, 40.13896682188643`},
{"CURE", 365, 23.323215809003948`}, {"CURE", 365, 81.40368480629547`}, {"CURE", 365, 40.91047337621798`},
{"CURE", 365, 5.39163889504164`}, {"CURE", 365, 46.686827975054754`}, {"CURE", 365, 36.05723550780098`},
{"CURE", 365, 28.725540358647024`}, {"CURE", 365, 34.07950717880431`}, {"CURE", 365, 8.875907136359062`},
{"CURE", 365, 50.63668588851469`}, {"CURE", 365, 18.17877231268173`}, {"CURE", 365, 41.131425455292465`},
{"CURE", 365, 153.44189370257462`}, {"CURE", 365, 54.25245250030504`}, {"CURE", 365, 125.94061112047433`},
{"CURE", 365, 41.49125685319587`}, {"CURE", 365, 171.66019417881324`}, {"CURE", 365, 5.345968256641504`},
{"CURE", 365, 46.93523720130226`}, {"CURE", 365, 53.504402196910306`}, {"CURE", 365, 6.948986365513149`},
{"CURE", 365, 9.628677901600357`}, {"CURE", 365, 59.09576113557137`}, {"CURE", 365, 69.69682864355734`},
{"CURE", 365, 59.34528271039157`}, {"CURE", 365, 48.56404071502606`}, {"CURE", 365, 46.417698863271774`},
{"CURE", 365, 28.11324427851418`}, {"CURE", 365, 7.433923626309595`}, {"CURE", 365, 32.35896972882312`},
{"CURE", 365, 15.353261436488562`}, {"CURE", 365, 7.516929435946309`}, {"CURE", 365, 45.538161790037556`},
{"CURE", 365, 25.473496146430136`}, {"CURE", 365, 67.33815430137993`}, {"CURE", 365, 27.18814156150532`},
{"CURE", 365, 22.6276344654801`}, {"CURE", 365, 151.8627367881457`}, {"CURE", 365, 11.307881388703342`},

{"CURE", 365, 33.85034808101551`}, {"CURE", 365, 19.179192154168497`}, {"CURE", 365, 45.361618605928555`},
{"CURE", 365, 12.45682950412612`}, {"CURE", 365, 49.71529706799557`}, {"CURE", 365, 131.44769369325059`},
{"CURE", 365, 11.236945534989284`}, {"CURE", 365, 34.11435640550752`}, {"CURE", 365, 36.92605958694605`},
{"CURE", 365, 132.64439795229163`}, {"CURE", 365, 49.998261551717576`}, {"CURE", 365, 41.50014938569389`},
{"CURE", 365, 72.89528723941369`}, {"CURE", 365, 27.914262107531755`}, {"CURE", 365, 88.07328250674855`},
{"CURE", 365, 53.77675133295036`}, {"CURE", 365, 33.11897463764241`}, {"CURE", 365, 29.66216721995638`},
{"CURE", 365, 9.64059580311749`}, {"CURE", 365, 6.531657408455719`}, {"CURE", 365, 15.316934321647866`},
{"CURE", 365, 20.50675271800754`}, {"CURE", 365, 9.042725696658794`}, {"CURE", 365, 33.09481253833676`},
{"CURE", 365, 90.24821949614723`}, {"CURE", 365, 24.390977078697567`}, {"CURE", 365, 45.34381693415656`},
{"CURE", 365, 56.69724034690006`}, {"CURE", 365, 49.476630314159834`}, {"CURE", 365, 33.1841129320606`},
{"CURE", 365, 13.182398621149934`}, {"CURE", 365, 90.83470214900807`}, {"CURE", 365, 7.8570229042317905`},
{"CURE", 365, 84.66407126306012`}, {"CURE", 365, 82.44378202942913`}, {"CURE", 365, 22.962176397082615`},
{"CURE", 365, 27.27215998675661`}, {"CURE", 365, 156.9039635825382`}, {"CURE", 365, 40.526651837922714`},
{"CURE", 365, 48.23990480483005`}, {"CURE", 365, 30.232614943813672`}, {"CURE", 365, 11.363338454157427`},
{"CURE", 365, 69.72365349354924`}, {"TBUR", 73.16408366022871`, 185.43386429957826`}, {"CURE", 365, 28.289643520498153`},
{"CURE", 365, 25.585392922606733`}, {"CURE", 365, 47.13148728690269`}, {"CURE", 365, 18.818574240709268`},
{"TOX", 12.26067447519191`, 238.8641226768863`}, {"CURE", 365, 19.116378853278402`}, {"CURE", 365, 118.48728542309955`},
{"CURE", 365, 90.80914390110372`}, {"CURE", 365, 88.35076620550427`}, {"CURE", 365, 47.19107856613788`},
{"CURE", 365, 109.3412617183417`}, {"TOX", 4.446428691064732`, 339.1580716952916`}, {"CURE", 365, 49.677649957342396`},
{"CURE", 365, 9.128670113282118`}, {"CURE", 365, 81.06110231045507`}, {"CURE", 365, 50.50933797930535`},
{"CURE", 365, 48.70023041949852`}, {"CURE", 365, 10.753741357740788`}, {"TOX", 10.865603838569646`, 246.1565244904871`},
{"CURE", 365, 17.997949895044`}, {"CURE", 365, 78.05698543491826`}, {"CURE", 365, 49.220144347504316`},
{"CURE", 365, 34.97086701662596`}, {"CURE", 365, 8.40588007875123`}, {"CURE", 365, 63.178698307106814`},
{"CURE", 365, 37.069174905659835`}, {"CURE", 365, 98.06211218520782`}, {"CURE", 365, 64.61469432742072`},
{"CURE", 365, 81.59246040429096`}, {"CURE", 365, 39.20423192977709`}, {"CURE", 365, 34.86871057401661`},
{"CURE", 365, 182.20715274673827`}, {"CURE", 365, 47.68010270546375`}, {"CURE", 365, 12.724702463236095`},
{"CURE", 365, 42.84702330347343`}, {"CURE", 365, 43.70246730003185`}, {"CURE", 365, 5.855678616050086`},
{"CURE", 365, 10.412279009201775`}, {"CURE", 365, 15.911533785407437`}, {"CURE", 365, 150.3957475196229`},
{"CURE", 365, 46.20573363319217`}, {"CURE", 365, 48.139014341668215`}, {"CURE", 365, 38.56288157348502`},
{"CURE", 365, 158.8045715775595`}, {"CURE", 365, 36.50343000307626`}, {"CURE", 365, 73.74759842076637`},
{"CURE", 365, 36.41012432453218`}, {"CURE", 365, 53.67066862721066`}, {"CURE", 365, 12.906147254011707`},

{"CURE", 365, 48.27415687892768`}, {"CURE", 365, 30.12950779458682`}, {"CURE", 365, 58.669090123423686`},
{"CURE", 365, 92.74188727742604`}, {"CURE", 365, 104.95536628117756`}, {"CURE", 365, 64.26596734871666`},
{"CURE", 365, 51.69677522471577`}, {"CURE", 365, 72.06150320347676`}, {"CURE", 365, 33.855207291445154`},
{"CURE", 365, 38.32753183708958`}, {"CURE", 365, 27.172137657783498`}, {"CURE", 365, 21.283772877763703`},
{"CURE", 365, 34.28455897566613`}, {"CURE", 365, 87.9553507508955`}, {"CURE", 365, 10.081856459401187`},
{"CURE", 365, 44.69275988760211`}, {"CURE", 365, 8.435514880988084`}, {"CURE", 365, 43.91573146002526`},
{"CURE", 365, 24.975469136701996`}, {"CURE", 365, 92.84256208575952`}, {"CURE", 365, 8.561339542787525`},
{"CURE", 365, 61.17959683213918`}, {"CURE", 365, 76.68834583720572`}, {"CURE", 365, 12.544913983100884`},
{"CURE", 365, 28.75504561501237`}, {"CURE", 365, 23.13627136091669`}, {"CURE", 365, 13.249651997888972`},
{"CURE", 365, 78.2024870567358`}, {"CURE", 365, 29.40580747338938`}, {"CURE", 365, 56.63937839512106`},
{"CURE", 365, 55.08309994222141`}, {"CURE", 365, 62.443956578288926`}, {"CURE", 365, 48.824197927005876`},
{"CURE", 365, 58.48356083014142`}, {"CURE", 365, 46.26299642791187`}, {"CURE", 365, 89.46855336812223`},
{"CURE", 365, 54.513736241604896`}, {"CURE", 365, 44.529672336360164`}, {"CURE", 365, 113.82162567002585`},
{"CURE", 365, 13.178171813657118`}, {"CURE", 365, 74.73972883098597`}, {"CURE", 365, 5.490301029008146`},
{"CURE", 365, 68.22419389000547`}, {"CURE", 365, 16.073208448663475`}, {"CURE", 365, 85.57719922080676`},
{"CURE", 365, 13.128001312044983`}, {"CURE", 365, 85.98296655223288`}, {"CURE", 365, 215.29882527929934`},
{"CURE", 365, 9.397190641053884`}, {"CURE", 365, 6.75468834139848`}, {"CURE", 365, 24.839911825724318`},
{"CURE", 365, 6.037410282986698`}, {"CURE", 365, 4.1243259938341605`}, {"CURE", 365, 24.126100471043028`},
{"CURE", 365, 39.49427933247197`}, {"CURE", 365, 42.444245795752494`}, {"CURE", 365, 40.39970943025298`},
{"CURE", 365, 31.462001418075936`}, {"CURE", 365, 55.289293577431756`}, {"CURE", 365, 97.33757284512875`},
{"CURE", 365, 7.8773368762664`}, {"CURE", 365, 43.147881512218845`}, {"CURE", 365, 37.079821347200266`},
{"CURE", 365, 26.886114762319732`}, {"CURE", 365, 49.734930169108694`}, {"CURE", 365, 73.71792604244062`},
{"CURE", 365, 172.47369030834528`}, {"CURE", 365, 128.56287502907568`}, {"CURE", 365, 39.800798598623246`},
{"CURE", 365, 4.220929644350444`}, {"CURE", 365, 46.04745689028402`}, {"CURE", 365, 118.72452592185157`},
{"CURE", 365, 29.87772877806381`}, {"CURE", 365, 38.53682786189258`}, {"CURE", 365, 23.052030721759188`},
{"CURE", 365, 33.7849544839959`}, {"CURE", 365, 8.761722227537804`}, {"CURE", 365, 42.82528527236034`},
{"CURE", 365, 24.100853813017256`}, {"CURE", 365, 70.3279873115905`}, {"CURE", 365, 48.20072220678156`},
{"CURE", 365, 46.67503483631503`}, {"CURE", 365, 38.40451361491847`}, {"CURE", 365, 20.323660841149618`},
{"CURE", 365, 14.006533335544548`}, {"CURE", 365, 13.656244231622393`}, {"CURE", 365, 17.695643169246726`},
{"CURE", 365, 13.786476375831032`}, {"CURE", 365, 185.4963847656335`}, {"CURE", 365, 103.18024989120958`},
{"CURE", 365, 19.92335732208361`}, {"TOX", 5.762611001960802`, 306.87681893599313`}, {"CURE", 365, 87.75839022730624`},

```
{"CURE", 365, 46.46656124448964`}, {"CURE", 365, 80.38502645236709`}, {"CURE", 365, 12.085110781758782`},
{"CURE", 365, 96.40396514364528`}, {"TOX", 6.740914962911088`, 257.6399284153116`}, {"CURE", 365, 13.981994462571377`},
{"CURE", 365, 43.60922957650415`}, {"CURE", 365, 71.48352245541294`}, {"CURE", 365, 36.22505272819355`},
{"CURE", 365, 17.695552399928825`}, {"CURE", 365, 14.097004926714794`}, {"CURE", 365, 122.37429333661831`},
{"CURE", 365, 64.24380113222189`}, {"CURE", 365, 36.54695479983131`}, {"CURE", 365, 90.86117163216998`},
{"CURE", 365, 16.41962364840948`}, {"CURE", 365, 43.82038075159969`}, {"CURE", 365, 26.465489580248466`},
{"CURE", 365, 178.46401481607035`}, {"CURE", 365, 45.00382050302259`}, {"CURE", 365, 208.84825628467559`},
{"TOX", 105.16501125168256`, 325.94752937869845`}, {"CURE", 365, 24.9110744121002`}, {"CURE", 365, 7.025929687219036`},
{"CURE", 365, 6.119955123378498`}, {"CURE", 365, 44.77762150055388`}, {"CURE", 365, 173.68512177672133`},
{"CURE", 365, 34.79373010699212`}, {"CURE", 365, 26.735031710373633`}, {"CURE", 365, 50.29261665828965`},
{"CURE", 365, 55.07949925358834`}, {"CURE", 365, 229.4801451096885`}, {"CURE", 365, 189.12101146836034`},
{"CURE", 365, 39.637853749518285`}, {"CURE", 365, 13.147100623576842`}, {"CURE", 365, 32.20981649759851`},
{"CURE", 365, 5.054781644355494`}, {"CURE", 365, 95.15301216059727`}, {"CURE", 365, 141.34934948251097`},
{"CURE", 365, 46.231974363382356`}, {"CURE", 365, 36.64013545420111`}, {"CURE", 365, 154.92473907766234`},
{"CURE", 365, 9.84271907135554`}, {"CURE", 365, 12.81300792780519`}, {"CURE", 365, 40.06293762295264`},
{"CURE", 365, 51.588626768527035`}, {"CURE", 365, 97.88128192489395`}, {"CURE", 365, 100.5257727207824`},
{"CURE", 365, 41.264633867597006`}, {"CURE", 365, 42.590122661582356`}, {"CURE", 365, 53.430274477699975`},
{"CURE", 365, 18.48064021260578`}, {"CURE", 365, 5.213041251119516`}, {"CURE", 365, 54.83908608509139`},
{"CURE", 365, 66.00948755102667`}, {"CURE", 365, 32.094810616340276`}, {"CURE", 365, 58.16161159994082`},
{"CURE", 365, 18.12215418423697`}, {"CURE", 365, 11.906105679611748`}, {"CURE", 365, 52.11725302671011`},
{"CURE", 365, 31.70300266682095`}, {"CURE", 365, 45.28832305605701`}, {"TOX", 7.978143823196068`, 298.82372660609093`},
{"CURE", 365, 28.478949112669397`}, {"CURE", 365, 13.896532652348693`}, {"CURE", 365, 10.778647193462577`},
{"CURE", 365, 13.06260575389033`}, {"CURE", 365, 74.63646113780213`}, {"CURE", 365, 5.1390154910354395`},
{"CURE", 365, 69.48951822876128`}, {"CURE", 365, 75.8511299940017`}, {"CURE", 365, 59.202073534442384`},
{"CURE", 365, 48.53654832211858`}, {"CURE", 365, 9.629990089944064`}, {"CURE", 365, 105.64477951480485`},
{"CURE", 365, 32.058608440807916`}, {"CURE", 365, 14.623266893915638`}, {"CURE", 365, 94.08844626846587`},
{"CURE", 365, 16.733433249248012`}, {"CURE", 365, 112.733958089895`}, {"CURE", 365, 72.33379660362168`},
{"CURE", 365, 48.303134670656746`}, {"CURE", 365, 70.09590331740363`}, {"CURE", 365, 24.878505861088545`},
{"CURE", 365, 109.97950826138754`}, {"CURE", 365, 13.048093948626466`}, {"CURE", 365, 55.35612719398621`},
{"CURE", 365, 64.6224491937499`}, {"CURE", 365, 27.4768460737741`}, {"TOX", 9.42230670570818`, 256.0589739391349`},
{"CURE", 365, 51.497340254969785`}, {"CURE", 365, 52.407789245567436`}, {"CURE", 365, 53.55065363728583`},
```

{"CURE", 365, 214.4149196064434`}, {"CURE", 365, 68.13670086922444`}, {"CURE", 365, 63.56607589604665`},
{"CURE", 365, 138.39085493819022`}, {"CURE", 365, 48.65820808437022`}, {"CURE", 365, 42.39848039877116`},
{"CURE", 365, 52.38030481181294`}, {"CURE", 365, 72.35294631476033`}, {"CURE", 365, 127.84549268860957`},
{"CURE", 365, 27.59209316918037`}, {"CURE", 365, 25.186962935613078`}, {"CURE", 365, 53.89237882693893`},
{"CURE", 365, 54.87034185597954`}, {"CURE", 365, 61.81226182856486`}, {"CURE", 365, 65.97121350361876`},
{"CURE", 365, 36.88610343684692`}, {"CURE", 365, 18.571867883083133`}, {"CURE", 365, 69.82262369563587`},
{"CURE", 365, 17.59859175784815`}, {"CURE", 365, 37.40727503081938`}, {"CURE", 365, 44.61058357709568`},
{"CURE", 365, 13.419303080552005`}, {"CURE", 365, 6.671989212351195`}, {"CURE", 365, 41.31902429550447`},
{"CURE", 365, 55.9140887629357`}, {"CURE", 365, 66.3568842988725`}, {"CURE", 365, 47.473817026653045`},
{"CURE", 365, 6.083402645001721`}, {"CURE", 365, 54.42420301657152`}, {"CURE", 365, 10.373285547417913`},
{"CURE", 365, 159.9115804890625`}, {"CURE", 365, 208.09389514357227`}, {"CURE", 365, 30.134292255229795`},
{"CURE", 365, 41.17066934526573`}, {"CURE", 365, 223.4013390644778`}, {"CURE", 365, 40.059534765863305`},
{"CURE", 365, 23.32091062263602`}, {"CURE", 365, 18.15030766977187`}, {"CURE", 365, 4.954873779601248`},
{"CURE", 365, 31.227760498707053`}, {"CURE", 365, 34.82209781248771`}, {"CURE", 365, 92.35301667283038`},
{"CURE", 365, 44.17972743138622`}, {"CURE", 365, 76.9071550024698`}, {"CURE", 365, 27.164600621954985`},
{"CURE", 365, 59.042778535555996`}, {"CURE", 365, 131.75248441146604`}, {"CURE", 365, 13.40972336571625`},
{"CURE", 365, 20.67060186614598`}, {"CURE", 365, 30.71505569940358`}, {"CURE", 365, 42.892386957913764`},
{"CURE", 365, 28.19652904795931`}, {"CURE", 365, 26.92249744559856`}, {"CURE", 365, 11.422620633738054`},
{"CURE", 365, 17.606913600413645`}, {"CURE", 365, 33.402099731060815`}, {"CURE", 365, 75.9715920035349`},
{"CURE", 365, 67.70537050788947`}, {"CURE", 365, 34.62270605720973`}, {"TBUR", 54.397925643153684`, 52.06620301297557`},
{"CURE", 365, 111.90962122818053`}, {"CURE", 365, 54.70611831558407`}, {"CURE", 365, 40.93206874406079`},
{"CURE", 365, 26.071348431834803`}, {"CURE", 365, 8.172184340914308`}, {"CURE", 365, 45.44199344467213`},
{"CURE", 365, 52.4658345931336`}, {"CURE", 365, 205.56702904392233`}, {"CURE", 365, 10.477457943438262`},
{"CURE", 365, 10.229865972395794`}, {"CURE", 365, 32.777096557279776`}, {"CURE", 365, 95.5455596023869`},
{"CURE", 365, 11.925324141784284`}, {"CURE", 365, 58.326833582693716`}, {"CURE", 365, 13.606735476775476`},
{"CURE", 365, 33.9104302422278`}, {"CURE", 365, 13.869226638673661`}, {"CURE", 365, 14.376411765631095`},
{"CURE", 365, 12.96582972834996`}, {"CURE", 365, 138.3212709360346`}, {"CURE", 365, 22.179478944162266`},
{"CURE", 365, 39.05160386691367`}, {"CURE", 365, 5.374190897333201`}, {"CURE", 365, 64.39436861748878`},
{"CURE", 365, 90.09489963104694`}, {"CURE", 365, 78.59541467618723`}, {"CURE", 365, 11.861807360094838`},
{"CURE", 365, 23.78572619405587`}, {"TOX", 16.694162107797823`, 242.10645362228806`}, {"CURE", 365, 65.77275559675651`},
{"CURE", 365, 44.83591873614534`}, {"CURE", 365, 15.946416172360054`}, {"CURE", 365, 22.79589654562171`},

{"CURE", 365, 35.23929594473423`}, {"CURE", 365, 8.727371269734514`}, {"CURE", 365, 42.81289390637148`},
{"CURE", 365, 67.35448680916355`}, {"CURE", 365, 41.0019011807388`}, {"CURE", 365, 44.536901645884654`},
{"CURE", 365, 32.18999894378132`}, {"CURE", 365, 20.8624574052204`}, {"CURE", 365, 119.23685795219426`},
{"CURE", 365, 172.70844307982995`}, {"CURE", 365, 46.05831899818456`}, {"CURE", 365, 58.15115285598761`},
{"CURE", 365, 73.66992275523744`}, {"CURE", 365, 72.03475357275407`}, {"CURE", 365, 6.294125586229192`},
{"CURE", 365, 84.32230623188298`}, {"CURE", 365, 87.06803375578991`}, {"CURE", 365, 58.883131671039344`},
{"TOX", 10.103584379238649`, 236.18590890844047`}, {"CURE", 365, 49.1032174196893`}, {"CURE", 365, 21.89784888121379`},
{"CURE", 365, 51.68151576010787`}, {"CURE", 365, 65.68857162952978`}, {"CURE", 365, 29.425621865921567`},
{"CURE", 365, 23.12550237359989`}, {"CURE", 365, 20.082085665302564`}, {"CURE", 365, 36.45436683796737`},
{"CURE", 365, 112.2039367873687`}, {"CURE", 365, 72.01220003121314`}, {"CURE", 365, 12.549987970669653`},
{"CURE", 365, 73.75056600339953`}, {"CURE", 365, 106.00581773789818`}, {"CURE", 365, 19.513083174590083`},
{"CURE", 365, 111.32006403134336`}, {"CURE", 365, 16.719275726441985`}, {"CURE", 365, 10.686322466354245`},
{"CURE", 365, 15.138036200671447`}, {"CURE", 365, 29.171169418217726`}, {"CURE", 365, 130.8145685004113`},
{"CURE", 365, 46.61792588828162`}, {"CURE", 365, 58.899780722705316`}, {"CURE", 365, 14.071305456810391`},
{"TOX", 9.711474273296437`, 268.9044378581934`}, {"CURE", 365, 123.02022339630312`}, {"CURE", 365, 21.020089562274713`},
{"CURE", 365, 51.41539988316737`}, {"CURE", 365, 28.626462494590104`}, {"CURE", 365, 28.9351661852656`},
{"CURE", 365, 97.7954266159811`}, {"CURE", 365, 123.74645832429361`}, {"CURE", 365, 13.274313344386398`},
{"CURE", 365, 25.885779231440956`}, {"CURE", 365, 9.18345021111611`}, {"CURE", 365, 35.389441574168366`},
{"CURE", 365, 60.74459984103777`}, {"CURE", 365, 98.2548617121911`}, {"CURE", 365, 54.84119695576813`},
{"CURE", 365, 197.4902342383157`}, {"CURE", 365, 94.38974425270429`}, {"CURE", 365, 15.609076235106183`},
{"CURE", 365, 34.54543869688351`}, {"TBUR", 60.7008131545125`, 38.81089752267314`}, {"CURE", 365, 77.29838329779726`},
{"CURE", 365, 10.144321108158165`}, {"CURE", 365, 27.105363864541058`}, {"CURE", 365, 15.281042505899846`},
{"CURE", 365, 38.08414493617468`}, {"CURE", 365, 87.04405157928372`}, {"CURE", 365, 37.0214409640103`},
{"CURE", 365, 8.041381932532628`}, {"CURE", 365, 153.31110548327175`}, {"CURE", 365, 28.906407928060194`},
{"CURE", 365, 9.814476489604457`}, {"CURE", 365, 12.903384339380805`}, {"CURE", 365, 10.891730831153387`},
{"CURE", 365, 13.912795583533802`}, {"CURE", 365, 111.95743831700148`}, {"CURE", 365, 61.36817570941281`},
{"CURE", 365, 36.90021206814623`}, {"CURE", 365, 17.311602271431102`}, {"CURE", 365, 28.865048420518164`},
{"CURE", 365, 16.701195762957127`}, {"CURE", 365, 40.30729144447036`}, {"CURE", 365, 138.64670772940616`},
{"CURE", 365, 82.98497464126486`}, {"CURE", 365, 40.044122397047424`}, {"CURE", 365, 23.046915077784423`},
{"CURE", 365, 24.564579593893914`}, {"TOX", 7.274882470699797`, 296.0879352944907`}, {"CURE", 365, 14.110631612929676`},
{"CURE", 365, 7.270144124715197`}, {"CURE", 365, 6.351500754111439`}, {"CURE", 365, 80.5270546316146`},

{"CURE", 365, 47.18420220328686`}, {"CURE", 365, 52.32097843689781`}, {"CURE", 365, 77.70681408536466`},
{"CURE", 365, 128.46404049135816`}, {"CURE", 365, 132.67597636097838`}, {"CURE", 365, 50.56592995143109`},
{"CURE", 365, 21.832312578470887`}, {"CURE", 365, 25.332565774746893`}, {"CURE", 365, 51.13065461053916`},
{"CURE", 365, 28.702054227082687`}, {"TBUR", 32.49573713996359`, 11.878791596194116`}, {"CURE", 365, 45.092246395529926`},
{"TOX", 5.469326158731443`, 280.3024541472803`}, {"CURE", 365, 28.867729004653807`}, {"CURE", 365, 10.393211742888719`},
{"CURE", 365, 98.43479196958509`}, {"CURE", 365, 24.620537919636714`}, {"CURE", 365, 70.7900554521085`},
{"TOX", 4.8265901798496`, 340.59125638192586`}, {"CURE", 365, 157.27275614683268`}, {"CURE", 365, 51.05589994244481`},
{"CURE", 365, 42.82735827140272`}, {"CURE", 365, 18.232560912734943`}, {"CURE", 365, 5.208938406742326`},
{"CURE", 365, 20.44697130557339`}, {"CURE", 365, 59.70108984188564`}, {"CURE", 365, 33.13114209786472`},
{"CURE", 365, 105.68763828782498`}, {"CURE", 365, 41.90331897358831`}, {"CURE", 365, 42.75722179667312`},
{"CURE", 365, 11.87442445324133`}, {"CURE", 365, 33.639183068815406`}, {"CURE", 365, 34.51055571665468`},
{"CURE", 365, 9.00180705145334`}, {"CURE", 365, 28.674266550174497`}, {"CURE", 365, 17.07363556675087`},
{"CURE", 365, 27.108760210059106`}, {"CURE", 365, 57.30979045012373`}, {"CURE", 365, 28.381778594738243`},
{"CURE", 365, 6.57089983773675`}, {"CURE", 365, 45.277391796046466`}, {"CURE", 365, 5.074810111092317`},
{"CURE", 365, 14.90473179662361`}, {"CURE", 365, 44.150006913650316`}, {"CURE", 365, 50.86315331334356`},
{"CURE", 365, 10.140207259663299`}, {"CURE", 365, 29.81126767185297`}, {"CURE", 365, 38.71858820876195`},
{"CURE", 365, 195.22405059423818`}, {"CURE", 365, 44.77629783745543`}, {"CURE", 365, 12.64827101494094`},
{"CURE", 365, 137.7451002378971`}, {"CURE", 365, 13.590357748653663`}, {"CURE", 365, 51.017331609200355`},
{"CURE", 365, 52.72038225952658`}, {"CURE", 365, 15.737812505425655`}, {"CURE", 365, 17.53247385059722`},
{"CURE", 365, 77.62391671435485`}, {"CURE", 365, 7.5950228451678035`}, {"CURE", 365, 65.75113263281592`},
{"CURE", 365, 17.414367275369408`}, {"CURE", 365, 22.769625019224843`}, {"CURE", 365, 5.094471247887943`},
{"CURE", 365, 26.15013967691103`}, {"CURE", 365, 89.34711409360246`}, {"CURE", 365, 33.95184465950438`},
{"CURE", 365, 24.666533044945027`}, {"CURE", 365, 35.24014152965944`}, {"CURE", 365, 27.77409550123121`},
{"CURE", 365, 34.873287007274214`}, {"CURE", 365, 61.479849691262785`}, {"CURE", 365, 59.30767811242716`},
{"CURE", 365, 7.302097204996313`}, {"CURE", 365, 120.04317052648715`}, {"CURE", 365, 13.938618672495013`},
{"CURE", 365, 6.998063192939026`}, {"CURE", 365, 33.356201417953145`}, {"CURE", 365, 34.99116920234521`},
{"CURE", 365, 23.622285372089845`}, {"CURE", 365, 40.95590284545404`}, {"CURE", 365, 35.30832748916037`},
{"CURE", 365, 56.750852837492154`}, {"CURE", 365, 47.963874062018306`}, {"CURE", 365, 68.56261680172271`},
{"CURE", 365, 10.831708758807363`}, {"CURE", 365, 139.25306717973535`}, {"CURE", 365, 134.6378603940593`},
{"CURE", 365, 7.463919949959622`}, {"CURE", 365, 18.77346472325454`}, {"CURE", 365, 46.5808185078073`},
{"CURE", 365, 23.7167870065663`}, {"CURE", 365, 72.98985455381913`}, {"CURE", 365, 145.86256949831662`},

```
{"CURE", 365, 46.09422448141065`}, {"CURE", 365, 7.918654483816546`}, {"CURE", 365, 7.765132423087731`},
{"CURE", 365, 18.222923341522172`}, {"CURE", 365, 5.503615719711733`}, {"CURE", 365, 62.444311108760424`},
{"CURE", 365, 9.685484213444056`}, {"CURE", 365, 65.2952282836163`}, {"CURE", 365, 40.11925428036947`},
{"CURE", 365, 69.12517145489969`}, {"CURE", 365, 17.022868329496863`}, {"CURE", 365, 85.88186424804923`},
{"TOX", 9.420845214151836`, 284.1385637676594`}, {"CURE", 365, 102.34555517268504`}, {"CURE", 365, 165.8168285423009`},
{"CURE", 365, 48.789631713827006`}, {"CURE", 365, 116.23798135187405`}, {"CURE", 365, 116.4795501410808`},
{"CURE", 365, 14.135857387474363`}, {"CURE", 365, 9.888749096894422`}, {"CURE", 365, 32.168241025051735`},
{"CURE", 365, 66.71773925721581`}, {"CURE", 365, 83.48144723523723`}, {"CURE", 365, 36.0690720823045`},
{"CURE", 365, 52.87948586907836`}, {"CURE", 365, 9.963520714277191`}, {"CURE", 365, 12.662724965664916`},
{"CURE", 365, 61.480970777323265`}, {"CURE", 365, 19.951830374815344`}, {"CURE", 365, 8.453173720589131`},
{"CURE", 365, 12.499934000346098`}, {"CURE", 365, 6.112376840865511`}, {"CURE", 365, 49.664044773869044`},
{"CURE", 365, 68.28853579529837`}, {"CURE", 365, 47.808947082843325`}, {"CURE", 365, 54.28661772195761`},
{"CURE", 365, 30.082283542483033`}, {"CURE", 365, 26.955214052489232`}, {"CURE", 365, 69.70191942319971`},
{"CURE", 365, 74.56323794783661`}, {"CURE", 365, 8.321854957530913`}, {"CURE", 365, 31.89629883009067`},
{"CURE", 365, 16.54046903403744`}, {"CURE", 365, 25.959319253398967`}, {"CURE", 365, 213.87659633452014`},
{"CURE", 365, 37.76381674843572`}, {"CURE", 365, 25.91709268628244`}, {"CURE", 365, 27.434529038379598`},
{"CURE", 365, 54.68826474892674`}, {"CURE", 365, 41.627517938974435`}, {"CURE", 365, 54.30467603428254`},
{"CURE", 365, 56.06962859396514`}, {"CURE", 365, 12.703114277973537`}, {"CURE", 365, 123.51331238067098`},
{"CURE", 365, 23.73505452915095`}, {"CURE", 365, 50.31229624993782`}, {"CURE", 365, 28.55483451597107`},
{"CURE", 365, 16.209443581933208`}, {"CURE", 365, 59.09392364074716`}, {"CURE", 365, 59.988868223430664`},
{"TBUR", 98.14970187822911`, 33.350972515098604`}, {"CURE", 365, 78.67671408225895`}, {"CURE", 365, 16.642525817554688`},
{"CURE", 365, 139.0188114990914`}, {"CURE", 365, 53.41625217123521`}, {"CURE", 365, 112.99944984574672`},
{"CURE", 365, 4.37899354919498`}, {"CURE", 365, 55.61025827796096`}, {"CURE", 365, 63.43711027003941`},
{"CURE", 365, 15.047905664703752`}, {"CURE", 365, 38.21929592709252`}, {"CURE", 365, 57.222211822021386`},
{"CURE", 365, 34.78681622848113`}, {"CURE", 365, 11.073789867595373`}, {"CURE", 365, 8.40097515388803`},
{"CURE", 365, 49.69684344024951`}, {"CURE", 365, 13.251070529144068`}, {"CURE", 365, 29.466089159858495`},
{"CURE", 365, 35.94822456797444`}, {"CURE", 365, 16.759239635986788`}, {"CURE", 365, 30.80854909490329`},
{"CURE", 365, 8.379319610094207`}, {"CURE", 365, 34.540063537553166`}, {"CURE", 365, 5.583468283496355`},
{"CURE", 365, 9.985152281742137`}, {"CURE", 365, 23.6198580381463`}, {"CURE", 365, 7.615772842665408`},
{"CURE", 365, 56.58494918421717`}, {"CURE", 365, 83.06395718654127`}, {"CURE", 365, 84.37350814073184`},
{"CURE", 365, 70.2043012310499`}, {"CURE", 365, 132.5350954237829`}, {"CURE", 365, 10.724685794639278`},
```

{"CURE", 365, 47.81830775624762`}, {"CURE", 365, 51.43953563532517`}, {"CURE", 365, 88.26378480763192`},
{"CURE", 365, 65.9259059624274`}, {"CURE", 365, 30.15073160022318`}, {"CURE", 365, 141.79234704375278`},
{"CURE", 365, 4.4250561867710125`}, {"CURE", 365, 134.786659073252`}, {"CURE", 365, 9.131271469226956`},
{"CURE", 365, 115.12559187834415`}, {"CURE", 365, 104.25842222561933`}, {"CURE", 365, 30.912398147187194`},
{"CURE", 365, 121.73263750220575`}, {"CURE", 365, 148.4433706422665`}, {"CURE", 365, 66.41923989327036`},
{"CURE", 365, 29.297340849907627`}, {"CURE", 365, 33.686921137171325`}, {"CURE", 365, 49.75443426128841`},
{"CURE", 365, 45.89196308533691`}, {"CURE", 365, 11.119134416102984`}, {"CURE", 365, 39.93084381429362`},
{"CURE", 365, 37.805435483202615`}, {"CURE", 365, 28.260502678697055`}, {"CURE", 365, 69.79187350701304`},
{"CURE", 365, 10.990267665970354`}, {"CURE", 365, 77.91037260499292`}, {"CURE", 365, 50.990100543473346`},
{"CURE", 365, 28.86247060856237`}, {"CURE", 365, 26.4117111973814`}, {"CURE", 365, 53.335664256044645`},
{"CURE", 365, 7.294022435372457`}, {"CURE", 365, 84.48724929367877`}, {"CURE", 365, 13.344712050369868`},
{"CURE", 365, 18.919787009211042`}, {"CURE", 365, 42.46106534249607`}, {"CURE", 365, 33.28279665369574`},
{"CURE", 365, 30.605046651306687`}, {"CURE", 365, 95.33318355050791`}, {"CURE", 365, 12.890168740089646`},
{"CURE", 365, 22.444338721894123`}, {"CURE", 365, 166.0006079659664`}, {"CURE", 365, 12.597230535936415`},
{"CURE", 365, 32.43125779912982`}, {"CURE", 365, 49.38406564404649`}, {"CURE", 365, 48.758954969251796`},
{"CURE", 365, 33.08975343952214`}, {"CURE", 365, 171.0175565860939`}, {"CURE", 365, 30.91275585631225`},
{"CURE", 365, 13.011358999704782`}, {"CURE", 365, 26.022063694213998`}, {"CURE", 365, 20.03790401529592`},
{"CURE", 365, 15.3634105907153`}, {"CURE", 365, 40.274548871821516`}, {"CURE", 365, 68.96568956556638`},
{"CURE", 365, 37.950025363429674`}, {"CURE", 365, 44.24515491131071`}, {"CURE", 365, 96.85390534861845`},
{"CURE", 365, 10.688662935990807`}, {"CURE", 365, 12.576568013203007`}, {"CURE", 365, 106.04718755646331`},
{"CURE", 365, 34.196007192153274`}, {"CURE", 365, 36.969552514961634`}, {"CURE", 365, 18.58697505344552`},
{"CURE", 365, 42.55723212078321`}, {"CURE", 365, 53.03638717117583`}, {"CURE", 365, 122.94765550472155`},
{"CURE", 365, 18.120871385910494`}, {"CURE", 365, 70.89318676032782`}, {"CURE", 365, 41.69259026758785`},
{"CURE", 365, 110.3759619826974`}, {"CURE", 365, 180.11880214167164`}, {"CURE", 365, 66.0059499690873`},
{"CURE", 365, 6.388608524287398`}, {"CURE", 365, 49.97796415786748`}, {"CURE", 365, 15.796903419776758`},
{"CURE", 365, 166.86590780407445`}, {"TBUR", 60.39879965910556`, 5.36659503280882`}, {"CURE", 365, 5.571722880508561`},
{"CURE", 365, 193.4123519236982`}, {"CURE", 365, 44.53707763965401`}, {"CURE", 365, 15.032977859647103`},
{"CURE", 365, 28.77192028445921`}, {"CURE", 365, 10.10069380146061`}, {"CURE", 365, 9.62409908718819`},
{"CURE", 365, 11.47801848140385`}, {"CURE", 365, 41.491064907731904`}, {"CURE", 365, 87.9415333477409`},
{"CURE", 365, 34.84806095482435`}, {"CURE", 365, 8.726472112502243`}, {"CURE", 365, 118.64550714910479`},
{"TOX", 10.46527501095405`, 237.34377632718596`}, {"CURE", 365, 6.687932330772533`}, {"CURE", 365, 53.496387419022305`},

```
{"CURE", 365, 28.70925284666645`}, {"CURE", 365, 5.645690124694787`}, {"CURE", 365, 44.153018800498906`},
{"CURE", 365, 28.197892005451727`}, {"CURE", 365, 21.652221838116184`}, {"CURE", 365, 42.09771629958645`},
{"CURE", 365, 23.89745370121494`}, {"CURE", 365, 70.54345913044337`}, {"CURE", 365, 41.77356685916993`},
{"CURE", 365, 98.37440617536498`}, {"CURE", 365, 43.58477451776359`}, {"CURE", 365, 21.603560797085983`},
{"CURE", 365, 10.511501223038595`}, {"CURE", 365, 7.2810396459407905`}, {"CURE", 365, 20.738483543963877`},
{"CURE", 365, 82.5593302330505`}, {"CURE", 365, 27.220371923916154`}, {"CURE", 365, 24.957600459742153`},
{"CURE", 365, 17.644353080673874`}, {"CURE", 365, 17.87905585508846`}, {"CURE", 365, 48.31271402138496`},
{"CURE", 365, 9.037329982148464`}, {"CURE", 365, 57.5952248414799`}, {"CURE", 365, 26.337757432477144`},
{"CURE", 365, 15.513923432530744`}, {"CURE", 365, 24.550854807737885`}, {"CURE", 365, 54.427864113777694`},
{"CURE", 365, 40.74233711942611`}, {"CURE", 365, 4.894193006859157`}, {"CURE", 365, 21.09924299178807`},
{"TOX", 4.372721956657599`, 352.45151541549853`}, {"CURE", 365, 58.63102483969169`}, {"CURE", 365, 85.43049636624163`},
{"CURE", 365, 25.178183237499084`}, {"CURE", 365, 93.32664495808339`}, {"CURE", 365, 11.163752145591106`},
{"CURE", 365, 37.64642154923635`}, {"CURE", 365, 173.6469489455633`}, {"CURE", 365, 32.50769174483692`},
{"CURE", 365, 28.72206841345118`}, {"CURE", 365, 49.07710237349493`}, {"CURE", 365, 26.029494030324926`},
{"CURE", 365, 5.766297945546137`}, {"CURE", 365, 53.635853613921995`}, {"CURE", 365, 9.564756868737879`},
{"CURE", 365, 22.774334728746155`}, {"CURE", 365, 53.67356006373537`}, {"CURE", 365, 19.2779613147284`},
{"CURE", 365, 102.37242518142074`}, {"CURE", 365, 30.46019310075757`}, {"CURE", 365, 52.39779474037665`},
{"CURE", 365, 36.32151701263233`}, {"CURE", 365, 10.277856450867166`}, {"CURE", 365, 38.168712765690586`},
{"CURE", 365, 82.41077555158945`}, {"CURE", 365, 27.62530026571028`}, {"CURE", 365, 20.787389404765495`},
{"CURE", 365, 9.86546269520752`}, {"CURE", 365, 31.5772625612514`}, {"CURE", 365, 37.58067685184653`},
{"CURE", 365, 107.0957422312471`}, {"CURE", 365, 17.205778607421742`}, {"CURE", 365, 25.926900673518187`},
{"CURE", 365, 100.54523490135684`}, {"CURE", 365, 10.59303162657959`}, {"CURE", 365, 145.58035466526053`},
{"CURE", 365, 12.290062345554047`}, {"CURE", 365, 22.521676272255583`}, {"CURE", 365, 51.877770982776106`},
{"CURE", 365, 83.68835907800835`}, {"CURE", 365, 37.58436976456488`}, {"CURE", 365, 19.7230295996429`},
{"CURE", 365, 5.791597043469758`}, {"CURE", 365, 50.810999497831965`}, {"CURE", 365, 45.972882727242556`},
{"CURE", 365, 45.13398538776328`}, {"CURE", 365, 17.79607275143017`}, {"CURE", 365, 43.6208808305826`},
{"CURE", 365, 5.0507766134219825`}, {"CURE", 365, 29.097916478417815`}, {"CURE", 365, 12.703897656577308`},
{"CURE", 365, 5.629416333924346`}, {"CURE", 365, 29.24578513639904`}, {"CURE", 365, 6.2558962458549345`},
{"CURE", 365, 19.893085507083406`}, {"CURE", 365, 13.647093866368557`}, {"TBUR", 59.27303654990987`, 8.457596424564464`},
{"CURE", 365, 27.031905514451754`}, {"CURE", 365, 71.99703448336601`}, {"CURE", 365, 39.493175676206384`},
{"CURE", 365, 35.70210023593089`}, {"CURE", 365, 56.857844430119634`}, {"CURE", 365, 48.16070398404372`}},
```

{{"2900 Res", "2900 OS", "2900 Tox"}, {"CURE", 365, 24.203881908163595`}, {"CURE", 365, 49.99257612998695`},
{"CURE", 365, 35.08120550510072`}, {"TBUR", 88.05856299639746`, 11.792840721419264`},
{"TBUR", 63.48874422931803`, 45.57487471327947`}, {"CURE", 365, 90.56008264248693`}, {"CURE", 365, 85.20061282426828`},
{"CURE", 365, 8.904289053576546`}, {"CURE", 365, 61.44776704202839`}, {"CURE", 365, 53.17520087623162`},
{"CURE", 365, 199.54237675934394`}, {"CURE", 365, 24.753714339090354`}, {"CURE", 365, 90.83836547295192`},
{"TOX", 5.784093800866374`, 298.20864982189306`}, {"CURE", 365, 42.1170888497697`}, {"CURE", 365, 65.7448617829867 2`},
{"CURE", 365, 130.07659431345226`}, {"CURE", 365, 58.30404366898312`}, {"CURE", 365, 44.221222780931875`},
{"CURE", 365, 14.607199016689675`}, {"CURE", 365, 90.28192065319962`}, {"CURE", 365, 36.593116770424196`},
{"CURE", 365, 14.025178070233705`}, {"CURE", 365, 11.17571362898182`}, {"CURE", 365, 50.833523967165064`},
{"CURE", 365, 55.79628050439489`}, {"CURE", 365, 5.0729638314374865`}, {"CURE", 365, 7.689727491305108`},
{"CURE", 365, 28.496562542717754`}, {"CURE", 365, 33.3425926071462`}, {"CURE", 365, 28.432289878664402`},
{"CURE", 365, 100.13897191409907`}, {"CURE", 365, 21.989829741620408`}, {"CURE", 365, 17.509228180707197`},
{"CURE", 365, 141.7323727550519`}, {"CURE", 365, 8.692083581127173`}, {"CURE", 365, 37.525680128144664`},
{"TBUR", 58.92938545064673`, 84.00406791883555`}, {"CURE", 365, 20.877555424804118`}, {"CURE", 365, 32.14146771015649`},
{"CURE", 365, 73.37249135979509`}, {"CURE", 365, 49.40820595384517`}, {"CURE", 365, 10.027034362617911`},
{"CURE", 365, 33.83520018642557`}, {"CURE", 365, 48.800622048364`}, {"CURE", 365, 31.99243599055924`},
{"TOX", 4.395893659453362`, 340.0022210748199`}, {"CURE", 365, 73.29829579913094`}, {"CURE", 365, 115.7019987459514`},
{"CURE", 365, 13.716789391892236`}, {"CURE", 365, 39.18552658881051`}, {"CURE", 365, 53.22863252845498`},
{"CURE", 365, 42.188060567321344`}, {"CURE", 365, 27.388852250242888`}, {"CURE", 365, 11.79918010101427`},
{"CURE", 365, 124.75391369307238`}, {"CURE", 365, 39.20016845951223`}, {"CURE", 365, 7.455905952272905`},
{"CURE", 365, 80.33607855090881`}, {"CURE", 365, 56.30530241895504`}, {"CURE", 365, 38.53250368990488`},
{"CURE", 365, 29.455251584065156`}, {"CURE", 365, 16.941781867484746`}, {"CURE", 365, 63.48059506409475`},
{"CURE", 365, 45.131823764206025`}, {"CURE", 365, 51.57401581608331`}, {"CURE", 365, 11.80527039459091`},
{"CURE", 365, 12.009298692695923`}, {"CURE", 365, 12.76316147112282`}, {"CURE", 365, 52.13178503338721`},
{"CURE", 365, 19.08651852085271`}, {"CURE", 365, 42.520176452614805`}, {"CURE", 365, 79.77801957260208`},
{"TBUR", 34.10852804746136`, 26.00316215333729`}, {"CURE", 365, 21.329219995401363`}, {"CURE", 365, 59.04445603223485`},
{"CURE", 365, 34.55141571275882`}, {"CURE", 365, 26.26676490560889`}, {"CURE", 365, 104.70024948403616`},
{"CURE", 365, 114.9080643028446`}, {"CURE", 365, 84.60582375789639`}, {"CURE", 365, 22.82278982993934`},
{"CURE", 365, 46.14378641790663`}, {"CURE", 365, 32.25400249728569`}, {"CURE", 365, 36.47975777092197`},
{"CURE", 365, 17.100270189697824`}, {"CURE", 365, 182.4081860991132`}, {"CURE", 365, 39.75809315072235`},
{"CURE", 365, 50.04815753441361`}, {"CURE", 365, 43.096181972057046`}, {"CURE", 365, 35.72176653437913`},

{"CURE", 365, 10.564038844551252`}, {"CURE", 365, 19.35310426661856`}, {"CURE", 365, 44.807788513964105`},
{"CURE", 365, 16.319366825783607`}, {"CURE", 365, 19.301235336017474`}, {"CURE", 365, 50.29145926349719`},
{"CURE", 365, 127.63538432391475`}, {"CURE", 365, 68.41554556709617`}, {"CURE", 365, 34.3157123484864`},
{"CURE", 365, 68.11013719037048`}, {"CURE", 365, 44.23317382844847`}, {"CURE", 365, 28.65616323275011`},
{"CURE", 365, 196.22073794567254`}, {"CURE", 365, 21.791333880373408`}, {"CURE", 365, 14.806589945349018`},
{"CURE", 365, 86.9395402358746`}, {"CURE", 365, 25.734545690677937`}, {"CURE", 365, 26.218929265749907`},
{"CURE", 365, 157.74922941092666`}, {"CURE", 365, 12.805420829590824`}, {"CURE", 365, 8.126328908223241`},
{"CURE", 365, 33.795449680018216`}, {"CURE", 365, 26.722376022713426`}, {"CURE", 365, 20.127790139904562`},
{"CURE", 365, 51.357539531321`}, {"CURE", 365, 14.458571707913107`}, {"CURE", 365, 8.889981463083858`},
{"CURE", 365, 41.60509468706234`}, {"CURE", 365, 63.63679277895624`}, {"CURE", 365, 10.844658372800556`},
{"CURE", 365, 35.876866812626936`}, {"CURE", 365, 108.87878077381706`}, {"CURE", 365, 41.583684780754645`},
{"CURE", 365, 24.157151892083785`}, {"CURE", 365, 84.31784533462526`}, {"CURE", 365, 42.375167511233`},
{"CURE", 365, 5.584547629884814`}, {"CURE", 365, 48.35507251785357`}, {"CURE", 365, 37.345695298194016`},
{"CURE", 365, 29.752266465950534`}, {"CURE", 365, 35.29865427436894`}, {"CURE", 365, 9.193883557667448`},
{"CURE", 365, 52.4507916671713`}, {"CURE", 365, 18.830740005460843`}, {"CURE", 365, 42.61769418946609`},
{"CURE", 365, 158.93537501236034`}, {"CURE", 365, 56.20020509011511`}, {"CURE", 365, 130.4460058929043`},
{"CURE", 365, 42.97644914897548`}, {"CURE", 365, 177.85746466541215`}, {"CURE", 365, 5.537351511431535`},
{"CURE", 365, 48.611905720921406`}, {"CURE", 365, 55.416188414876494`}, {"CURE", 365, 7.1972469184474805`},
{"CURE", 365, 9.97291652554367`}, {"CURE", 365, 61.20782521063771`}, {"CURE", 365, 72.19595470654357`},
{"CURE", 365, 61.46557697371674`}, {"CURE", 365, 50.29995021034822`}, {"CURE", 365, 48.07574417801296`},
{"CURE", 365, 29.118336163749436`}, {"CURE", 365, 7.699503237002752`}, {"CURE", 365, 33.51871246750867`},
{"CURE", 365, 15.903075201900197`}, {"CURE", 365, 7.78634297819675`}, {"CURE", 365, 47.16669085611713`},
{"CURE", 365, 26.383651314047448`}, {"CURE", 365, 69.74402760517398`}, {"CURE", 365, 28.159948453003675`},
{"CURE", 365, 23.436178925636238`}, {"CURE", 365, 157.30694950922404`}, {"CURE", 365, 11.712049804275264`},
{"CURE", 365, 35.06320183687389`}, {"CURE", 365, 19.865709469158386`}, {"CURE", 365, 46.996457812013126`},
{"CURE", 365, 12.903388980872139`}, {"CURE", 365, 51.4930930284399`}, {"CURE", 365, 136.17293534935598`},
{"CURE", 365, 11.639180322893372`}, {"CURE", 365, 35.333494986116`}, {"CURE", 365, 38.26118388314893`},
{"CURE", 365, 137.38241746588776`}, {"CURE", 365, 51.80113332370327`}, {"CURE", 365, 42.992739561365376`},
{"CURE", 365, 75.52587926060168`}, {"CURE", 365, 28.913397726958518`}, {"CURE", 365, 91.64583093511226`},
{"CURE", 365, 55.710288755903704`}, {"CURE", 365, 34.37293465150914`}, {"CURE", 365, 30.728381251387912`},
{"CURE", 365, 9.986199665288941`}, {"CURE", 365, 6.765602941066246`}, {"CURE", 365, 15.866459729212036`},

{"CURE", 365, 21.24116301359574`}, {"CURE", 365, 9.368213234746452`}, {"CURE", 365, 34.286379812249`},
{"CURE", 365, 93.47643840593474`}, {"CURE", 365, 25.262253041742913`}, {"CURE", 365, 46.983782667061995`},
{"CURE", 365, 58.72281794971654`}, {"CURE", 365, 51.4873562930067`}, {"CURE", 365, 34.36978165481719`},
{"CURE", 365, 13.6536560309058`}, {"CURE", 365, 94.08345222336366`}, {"CURE", 365, 8.137756611254863`},
{"CURE", 365, 87.69036766511948`}, {"CURE", 365, 85.4205697987463`}, {"CURE", 365, 23.783094365778254`},
{"CURE", 365, 28.246658883701908`}, {"CURE", 365, 162.52825256632804`}, {"CURE", 365, 41.99650595798315`},
{"CURE", 365, 49.9630117282518`}, {"CURE", 365, 31.316962349842242`}, {"CURE", 365, 11.769550146201793`},
{"CURE", 365, 72.22407995391112`}, {"TBUR", 75.48261532649578`, 192.1018956640698`}, {"CURE", 365, 29.300213736009905`},
{"CURE", 365, 26.500478231108662`}, {"CURE", 365, 48.81646979160617`}, {"CURE", 365, 19.491040737537478`},
{"TOX", 10.204216519290606`, 247.4122267433059`}, {"CURE", 365, 19.799652733132394`}, {"CURE", 365, 122.77271715163451`},
{"CURE", 365, 94.05548615024081`}, {"CURE", 365, 91.51484178435535`}, {"CURE", 365, 48.881848758695924`},
{"CURE", 365, 113.24761774858773`}, {"TOX", 3.805607978479`, 374.66123975885296`}, {"CURE", 365, 51.452466559272935`},
{"CURE", 365, 9.455198162074314`}, {"CURE", 365, 83.9571323017458`}, {"CURE", 365, 52.313649566004806`},
{"CURE", 365, 50.44104665155207`}, {"CURE", 365, 11.141245642709563`}, {"TOX", 9.552218348095648`, 254.95694945954554`},
{"CURE", 365, 18.642097894240038`}, {"CURE", 365, 80.84672591138529`}, {"CURE", 365, 50.9810813484774`},
{"CURE", 365, 36.22100282341222`}, {"CURE", 365, 8.706251169112548`}, {"CURE", 365, 65.43560197070629`},
{"CURE", 365, 38.39434891612486`}, {"CURE", 365, 101.56621467044543`}, {"CURE", 365, 66.92413998349063`},
{"CURE", 365, 84.51213560084365`}, {"CURE", 365, 40.61147153958986`}, {"CURE", 365, 36.11803989951278`},
{"CURE", 365, 188.7368578061469`}, {"CURE", 365, 49.383287532963436`}, {"CURE", 365, 13.179715015709899`},
{"CURE", 365, 44.37811772354594`}, {"CURE", 365, 45.28547621973521`}, {"CURE", 365, 6.066469030865065`},
{"CURE", 365, 10.784215905863954`}, {"CURE", 365, 16.48039236278983`}, {"CURE", 365, 155.79091286078153`},
{"CURE", 365, 47.85877393510509`}, {"CURE", 365, 49.85854540924768`}, {"CURE", 365, 39.941623575957465`},
{"CURE", 365, 164.5130774312482`}, {"CURE", 365, 37.807329339578736`}, {"CURE", 365, 76.43124124741051`},
{"CURE", 365, 37.711742367509004`}, {"CURE", 365, 55.58884652244566`}, {"CURE", 365, 13.36721352332829`},
{"CURE", 365, 49.999285453829344`}, {"CURE", 365, 31.206898459709606`}, {"CURE", 365, 60.76470330737256`},
{"CURE", 365, 96.05792531221839`}, {"CURE", 365, 108.82439139780315`}, {"CURE", 365, 66.73535476752367`},
{"CURE", 365, 53.543389295976304`}, {"CURE", 365, 74.7803531307885`}, {"CURE", 365, 35.06501201759148`},
{"CURE", 365, 39.696938554108584`}, {"CURE", 365, 28.145136748374625`}, {"CURE", 365, 22.04471844304589`},
{"CURE", 365, 35.510213864988714`}, {"CURE", 365, 91.09745768467876`}, {"CURE", 365, 10.442106340643319`},
{"CURE", 365, 46.29259682022179`}, {"CURE", 365, 8.736969995050972`}, {"CURE", 365, 45.48628348449874`},
{"CURE", 365, 25.871595666066874`}, {"CURE", 365, 96.1591872440679`}, {"CURE", 365, 8.867407083595532`},

{"CURE", 365, 63.37390276685236`}, {"CURE", 365, 79.43078961903669`}, {"CURE", 365, 12.993523023539266`},
{"CURE", 365, 29.785010257162796`}, {"CURE", 365, 23.965610564546857`}, {"CURE", 365, 13.725791724839455`},
{"CURE", 365, 80.99660266908404`}, {"CURE", 365, 30.45859809370672`}, {"CURE", 365, 58.66291574388489`},
{"CURE", 365, 57.0522224936486`}, {"CURE", 365, 64.67454575149495`}, {"CURE", 365, 50.56880161595646`},
{"CURE", 365, 60.57976290044777`}, {"CURE", 365, 47.91620726369017`}, {"CURE", 365, 92.66939667859813`},
{"CURE", 365, 56.4688355835524`}, {"CURE", 365, 46.12648136049041`}, {"CURE", 365, 117.8918745211349`},
{"CURE", 365, 13.649419478300361`}, {"CURE", 365, 77.41043625821116`}, {"CURE", 365, 5.686479301682946`},
{"CURE", 365, 70.66553530706582`}, {"CURE", 365, 16.64743593694275`}, {"CURE", 365, 88.79541063439969`},
{"CURE", 365, 13.597472711141991`}, {"CURE", 365, 89.05558577274002`}, {"CURE", 365, 223.0065290658213`},
{"CURE", 365, 9.733471695512316`}, {"CURE", 365, 6.997740968875488`}, {"CURE", 365, 25.72766474817734`},
{"CURE", 365, 6.253956286363334`}, {"CURE", 365, 4.271748596766082`}, {"CURE", 365, 24.988017547441885`},
{"CURE", 365, 40.911749790011285`}, {"CURE", 365, 43.96031856080705`}, {"CURE", 365, 41.85082013090797`},
{"CURE", 365, 32.59378102761073`}, {"CURE", 365, 57.26607107291269`}, {"CURE", 365, 100.81837352906689`},
{"CURE", 365, 8.15906635517305`}, {"CURE", 365, 44.68948306624379`}, {"CURE", 365, 38.40470265826104`},
{"CURE", 365, 27.846947578015183`}, {"CURE", 365, 51.51440362652711`}, {"CURE", 365, 76.35279399872867`},
{"CURE", 365, 178.6357400323611`}, {"CURE", 365, 133.16396685874858`}, {"CURE", 365, 41.23099114067623`},
{"CURE", 365, 4.371768683598646 5`}, {"CURE", 365, 47.693506774578374`}, {"CURE", 365, 122.96541626903165`},
{"CURE", 365, 30.945810049520198`}, {"CURE", 365, 40.051565353536176`}, {"CURE", 365, 23.876101904721008`},
{"CURE", 365, 34.991937286823706`}, {"CURE", 365, 9.07694899538469`}, {"CURE", 365, 44.357875088445034`},
{"CURE", 365, 24.971292575112837`}, {"CURE", 365, 72.84934380783454`}, {"CURE", 365, 49.93279681089813`},
{"CURE", 365, 48.342545608849484`}, {"CURE", 365, 39.77644147391368`}, {"CURE", 365, 21.049720203877392`},
{"CURE", 365, 14.507060616014398`}, {"CURE", 365, 14.144234917291936`}, {"CURE", 365, 18.329074848284193`},
{"CURE", 365, 14.27953662915241`}, {"CURE", 365, 192.144387623617`}, {"CURE", 365, 106.87973536034463`},
{"CURE", 365, 20.635188558101483`}, {"TOX", 5.468626013850296`, 317.8388995950442`}, {"CURE", 365, 90.89337430240785`},
{"CURE", 365, 48.12679429198529`}, {"CURE", 365, 83.25814987674391`}, {"CURE", 365, 12.516866191519282`},
{"CURE", 365, 99.85583655923926`}, {"TOX", 6.161193620496244`, 266.84357708206335`}, {"CURE", 365, 14.482070426490147`},
{"CURE", 365, 45.167475048444956`}, {"CURE", 365, 74.04857700809897`}, {"CURE", 365, 37.54624597912994`},
{"CURE", 365, 18.327856247225444`}, {"CURE", 365, 14.600588699719996`}, {"CURE", 365, 126.74927203987981`},
{"CURE", 365, 66.54000424143877`}, {"CURE", 365, 37.85261759304687`}, {"CURE", 365, 94.11305739138778`},
{"CURE", 365, 17.01051198566851`}, {"CURE", 365, 45.38667557697775`}, {"CURE", 365, 27.411821305541725`},
{"CURE", 365, 184.83923765333765`}, {"CURE", 365, 46.6265389560531`}, {"CURE", 365, 216.372381036619`},

{"TOX", 5.008953579421627`, 337.6842411833623`}, {"CURE", 365, 25.801720225924857`}, {"CURE", 365, 7.277237792861982`},
{"CURE", 365, 6.338695584934135`}, {"CURE", 365, 46.37936077419544`}, {"CURE", 365, 179.89097781194604`},
{"CURE", 365, 36.03717728682825`}, {"CURE", 365, 27.69109615054518`}, {"CURE", 365, 52.09803566952559`},
{"CURE", 365, 57.05759675558636`}, {"TOX", 9.567904316059057`, 237.69595965009805`}, {"CURE", 365, 195.87669534127025`},
{"CURE", 365, 41.05838458838351`}, {"CURE", 365, 13.61727251312021`}, {"CURE", 365, 33.36193644041495`},
{"CURE", 365, 5.235524729573093`}, {"CURE", 365, 98.57053121608118`}, {"CURE", 365, 146.39802660895327`},
{"CURE", 365, 47.88388653662581`}, {"CURE", 365, 37.94941937923523`}, {"CURE", 365, 160.45914049181474`},
{"CURE", 365, 10.19738700806102`}, {"CURE", 365, 13.273098184212055`}, {"CURE", 365, 41.4940677558236`},
{"CURE", 365, 53.4404426257024`}, {"CURE", 365, 101.41707274197485`}, {"CURE", 365, 104.11725694672405`},
{"CURE", 365, 42.73891258147181`}, {"CURE", 365, 44.11542295152672`}, {"CURE", 365, 55.36378713582221`},
{"CURE", 365, 19.14560269226437`}, {"CURE", 365, 5.399487240409574`}, {"CURE", 365, 56.80075195286622`},
{"CURE", 365, 68.367289503593`}, {"CURE", 365, 33.2413418488618`}, {"CURE", 365, 60.23905877861766`},
{"CURE", 365, 18.770384906388976`}, {"CURE", 365, 12.33157371255649`}, {"CURE", 365, 53.97903669236491`},
{"CURE", 365, 32.8406462458504`}, {"CURE", 365, 46.907314198549784`}, {"TOX", 7.537663539834714`, 309.49783435775186`},
{"CURE", 365, 29.49806477167242`}, {"CURE", 365, 14.394794794389256`}, {"CURE", 365, 11.164047593047668`},
{"CURE", 365, 13.529454064241625`}, {"CURE", 365, 77.30702094533582`}, {"CURE", 365, 5.322640157822279`},
{"CURE", 365, 71.97374020132806`}, {"CURE", 365, 78.56061074651525`}, {"CURE", 365, 61.31664283014382`},
{"CURE", 365, 50.2702487726966`}, {"CURE", 365, 9.974908767919493`}, {"CURE", 365, 109.41885878868035`},
{"CURE", 365, 33.21460213152582`}, {"CURE", 365, 15.177592140811745`}, {"CURE", 365, 97.45137784725854`},
{"CURE", 365, 17.331244514184494`}, {"CURE", 365, 116.76411417101713`}, {"CURE", 365, 74.91743163251361`},
{"CURE", 365, 50.04084222432308`}, {"CURE", 365, 72.59958388030729`}, {"CURE", 365, 25.771481364874912`},
{"CURE", 365, 113.91023578830183`}, {"CURE", 365, 13.51424887598681`}, {"CURE", 365, 57.3499692268147`},
{"CURE", 365, 66.93103183640343`}, {"CURE", 365, 28.458469502651138`}, {"TOX", 8.55069195214995`, 265.2079466499522`},
{"CURE", 365, 53.36312134352826`}, {"CURE", 365, 54.28010794305668`}, {"CURE", 365, 55.46540705419285`},
{"CURE", 365, 222.0743402066358`}, {"CURE", 365, 70.57305694753911`}, {"CURE", 365, 65.83799582691411`},
{"CURE", 365, 143.34254804148804`}, {"CURE", 365, 50.39755734288561`}, {"CURE", 365, 43.91442654707703`},
{"CURE", 365, 54.251557704015745`}, {"CURE", 365, 74.93812453243667`}, {"CURE", 365, 132.41176919056267`},
{"CURE", 365, 28.578687532723375`}, {"CURE", 365, 26.086939371178715`}, {"CURE", 365, 55.81759944299563`},
{"CURE", 365, 56.830897138537814`}, {"CURE", 365, 64.03186836739917`}, {"CURE", 365, 68.32767499710117`},
{"CURE", 365, 38.20578071111807`}, {"CURE", 365, 19.23854398603386`}, {"CURE", 365, 72.3329432171818`},
{"CURE", 365, 18.2277383697763`}, {"CURE", 365, 38.74401538545193`}, {"CURE", 365, 46.21406374914297`},

{"CURE", 365, 13.898769015140557`}, {"CURE", 365, 6.910635812950326`}, {"CURE", 365, 42.80864965773305`},
{"CURE", 365, 57.91798155162264`}, {"CURE", 365, 68.73235542180531`}, {"CURE", 365, 49.17452914728097`},
{"CURE", 365, 6.301419409352762`}, {"CURE", 365, 56.370316148076476`}, {"CURE", 365, 10.74496077430873`},
{"CURE", 365, 165.62339296603315`}, {"CURE", 365, 215.5300617075881`}, {"CURE", 365, 31.32302112774323`},
{"CURE", 365, 42.64892517223936`}, {"TOX", 36.480260833091016`, 231.3867876160799 4`}, {"CURE", 365, 41.49104856767045`},
{"CURE", 365, 24.160704458784934`}, {"CURE", 365, 18.802643974948964`}, {"CURE", 365, 5.132033030300595`},
{"CURE", 365, 32.34322375077886`}, {"CURE", 365, 36.066101245867415`}, {"CURE", 365, 95.66181139015568`},
{"CURE", 365, 45.76081370731578`}, {"CURE", 365, 79.6564135771812`}, {"CURE", 365, 28.13501747905533 2`},
{"CURE", 365, 61.163709819596725`}, {"CURE", 365, 136.459147529907`}, {"CURE", 365, 13.89010623553533`},
{"CURE", 365, 21.40904895504972`}, {"CURE", 365, 31.81379881432769`}, {"CURE", 365, 44.425020745096816`},
{"CURE", 365, 29.21195912999132`}, {"CURE", 365, 27.88468983941328`}, {"CURE", 365, 11.832964942670461`},
{"CURE", 365, 18.23587952451645`}, {"CURE", 365, 34.59821421367236`}, {"CURE", 365, 78.6864336899737`},
{"CURE", 365, 70.13938060853688`}, {"CURE", 365, 35.860452913777664`}, {"TBUR", 56.33168491700081`, 53.94380296437423`},
{"CURE", 365, 115.91666522987154`}, {"CURE", 365, 56.661187197911076`}, {"CURE", 365, 42.39452248735102`},
{"CURE", 365, 27.002821001069236`}, {"CURE", 365, 8.464676290256108`}, {"CURE", 365, 47.06547765657746`},
{"CURE", 365, 54.34206036076326`}, {"CURE", 365, 212.93727739945714`}, {"CURE", 365, 10.8527201137517`},
{"CURE", 365, 10.595410992683478`}, {"CURE", 365, 33.94808228390612`}, {"CURE", 365, 98.95931251571095`},
{"CURE", 365, 12.351896376274558`}, {"CURE", 365, 60.41095483111869`}, {"CURE", 365, 14.093594950265684`},
{"CURE", 365, 35.12422724372559`}, {"CURE", 365, 14.37456700000026`}, {"CURE", 365, 14.890228350770945`},
{"CURE", 365, 13.429266908859155`}, {"CURE", 365, 143.26202564107447`}, {"CURE", 365, 22.979664551183852`},
{"CURE", 365, 40.45854802871544`}, {"CURE", 365, 5.566209469615725`}, {"CURE", 365, 66.69589943178545`},
{"CURE", 365, 93.31427703325164`}, {"CURE", 365, 81.41195529452533`}, {"CURE", 365, 12.286315885395043`},
{"CURE", 365, 24.635783710305816`}, {"TOX", 14.2428585204536`, 250.76960257941715`}, {"CURE", 365, 68.1250766022908`},
{"CURE", 365, 46.44255789148104`}, {"CURE", 365, 16.523193308508336`}, {"CURE", 365, 23.611895136630974`},
{"CURE", 365, 36.50158970388883`}, {"CURE", 365, 9.040293581437414`}, {"CURE", 365, 44.343327748187065`},
{"CURE", 365, 69.76037248497222`}, {"CURE", 365, 42.46985720771811`}, {"CURE", 365, 46.137286259538456`},
{"CURE", 365, 33.340266196932234`}, {"CURE", 365, 21.610076729504154`}, {"CURE", 365, 123.51317359652377`},
{"CURE", 365, 178.88225352850662`}, {"TOX", 365, 47.70346391334339`}, {"CURE", 365, 60.22830250143867`},
{"CURE", 365, 76.3017273832073`}, {"CURE", 365, 74.60921785190725`}, {"CURE", 365, 6.5197752375376705`},
{"CURE", 365, 87.34323077297583`}, {"CURE", 365, 90.17790141855649`}, {"CURE", 365, 60.99107246183952`},
{"TOX", 8.197875420385197`, 244.62249835658537`}, {"CURE", 365, 50.85739736226981`}, {"CURE", 365, 22.6853121408537`},

{"CURE", 365, 53.528271485225616`}, {"CURE", 365, 68.03709958724951`}, {"CURE", 365, 30.477222589693564`},
{"CURE", 365, 23.95611656150953`}, {"CURE", 365, 20.79965410121032`}, {"CURE", 365, 37.766206851175596`},
{"CURE", 365, 116.2127323909298`}, {"CURE", 365, 74.58518087956917`}, {"CURE", 365, 12.999939120789982`},
{"CURE", 365, 76.4367351090557`}, {"CURE", 365, 109.79341266271851`}, {"CURE", 365, 20.214951211057606`},
{"CURE", 365, 115.31018143251919`}, {"CURE", 365, 17.3173676583682`}, {"CURE", 365, 11.070865990004837`},
{"CURE", 365, 15.678820262790808`}, {"CURE", 365, 30.21758283948457`}, {"CURE", 365, 135.53623777856427`},
{"CURE", 365, 48.283451985113835`}, {"CURE", 365, 61.008465609114545`}, {"CURE", 365, 14.573950306441672`},
{"TOX", 8.979717874837853`, 278.5557975988259 6`}, {"CURE", 365, 127.41629979143723`}, {"CURE", 365, 21.775145091109348`},
{"CURE", 365, 53.25637417683403`}, {"CURE", 365, 29.64894613343969`}, {"CURE", 365, 29.971171899516982`},
{"CURE", 365, 101.29793503935362`}, {"CURE", 365, 128.18675518956954`}, {"CURE", 365, 13.748477447154102`},
{"CURE", 365, 26.81315847086812`}, {"CURE", 365, 9.512426236852157`}, {"CURE", 365, 36.65391839499776`},
{"CURE", 365, 62.92475710517667`}, {"CURE", 365, 101.76465668148573`}, {"CURE", 365, 56.801404416976546`},
{"CURE", 365, 204.54673810347967`}, {"CURE", 365, 97.76173788999584`}, {"CURE", 365, 16.16979442608726`},
{"CURE", 365, 35.782229665122706`}, {"TBUR", 62.90264220731231`, 40.284752484689264`}, {"CURE", 365, 80.07922790480062`},
{"CURE", 365, 10.510087107683054`}, {"CURE", 365, 28.07474726292777`}, {"CURE", 365, 15.828773370164015`},
{"CURE", 365, 39.44468410156809`}, {"CURE", 365, 90.15679696992142`}, {"CURE", 365, 38.3443917290876`},
{"CURE", 365, 8.328808740561257`}, {"CURE", 365, 158.8049649505102`}, {"CURE", 365, 29.941211564951526`},
{"CURE", 365, 10.165416836098311`}, {"CURE", 365, 13.364935437483151`}, {"CURE", 365, 11.28108141267316`},
{"CURE", 365, 14.409998997864852`}, {"CURE", 365, 115.9578310979675`}, {"CURE", 365, 63.572445853714875`},
{"CURE", 365, 38.219972362594504`}, {"CURE", 365, 17.932347628730454`}, {"CURE", 365, 29.896104416321215`},
{"CURE", 365, 17.297908530108074`}, {"CURE", 365, 41.74781421122638`}, {"CURE", 365, 143.60684130031734`},
{"CURE", 365, 86.35304415737467`}, {"CURE", 365, 41.48088162033837`}, {"CURE", 365, 23.871273643687605`},
{"CURE", 365, 25.500823076757023`}, {"TOX", 6.868916725563336`, 306.66571184252024`}, {"CURE", 365, 14.615117218512838`},
{"CURE", 365, 7.5301668383606275`}, {"CURE", 365, 6.580141053763505`}, {"CURE", 365, 83.40794731216319`},
{"CURE", 365, 48.87469848041248`}, {"CURE", 365, 54.19292914385842`}, {"CURE", 365, 80.48283659602457`},
{"CURE", 365, 133.07380114977576`}, {"CURE", 365, 137.41558304627875`}, {"CURE", 365, 52.372470164824975`},
{"CURE", 365, 22.612210207191872`}, {"CURE", 365, 26.23901155277659`}, {"CURE", 365, 53.04829871638036`},
{"CURE", 365, 29.732041458186618`}, {"TBUR", 33.240105256812704`, 12.369336261691343`}, {"CURE", 365, 46.71071585898729`},
{"TOX", 5.128540369596856`, 290.32608222327815`}, {"CURE", 365, 29.899119024788735`}, {"CURE", 365, 10.76593456816025`},
{"CURE", 365, 101.95105869681592`}, {"CURE", 365, 25.500465918259597`}, {"CURE", 365, 73.32270630642248`},
{"TOX", 4.618461880341433`, 352.7590050301358`}, {"CURE", 365, 162.89188353810837`}, {"CURE", 365, 52.879969673824796`},

```
{"CURE", 365, 44.3615857912956`}, {"CURE", 365, 18.883975812219123`}, {"CURE", 365, 5.395402592511864`},
{"CURE", 365, 21.183858776310355`}, {"CURE", 365, 61.8337240274972`}, {"CURE", 365, 34.31820753021312`},
{"CURE", 365, 109.47218967654314`}, {"CURE", 365, 43.40355450538548`}, {"CURE", 365, 44.28498877105352`},
{"CURE", 365, 12.298912692921661`}, {"CURE", 365, 34.84212201266471`}, {"CURE", 365, 35.743497448619735`},
{"CURE", 365, 9.323803478359626`}, {"CURE", 365, 29.703137123494354`}, {"CURE", 365, 17.683670598659884`},
{"CURE", 365, 28.083071850215056`}, {"CURE", 365, 59.45451319335055`}, {"CURE", 365, 29.400138509245927`},
{"CURE", 365, 6.805663572901483`}, {"CURE", 365, 46.89582876138803`}, {"CURE", 365, 5.256802784147587`},
{"CURE", 365, 15.441212113312783`}, {"CURE", 365, 45.730437059364355`}, {"CURE", 365, 52.680188111087524`},
{"CURE", 365, 10.505620390753966`}, {"CURE", 365, 30.879098344164046`}, {"CURE", 365, 40.12058977383828`},
{"CURE", 365, 202.23269911312906`}, {"CURE", 365, 46.37575477652102`}, {"CURE", 365, 13.102353506174701`},
{"CURE", 365, 142.67262380285348`}, {"CURE", 365, 14.081734235289552`}, {"CURE", 365, 52.84536531448334`},
{"CURE", 365, 54.60459909183533`}, {"CURE", 365, 16.300311913090226`}, {"CURE", 365, 18.160706979036476`},
{"CURE", 365, 80.39908685981362`}, {"CURE", 365, 7.868280208883043`}, {"CURE", 365, 68.10002147488726`},
{"CURE", 365, 18.036783116010973`}, {"CURE", 365, 23.58322691575464`}, {"CURE", 365, 5.277146195109`},
{"CURE", 365, 27.084992264317002`}, {"CURE", 365, 92.55782826351373`}, {"CURE", 365, 35.16705999273242`},
{"CURE", 365, 25.551706569804825`}, {"CURE", 365, 36.50178684220956`}, {"CURE", 365, 28.789181519682298`},
{"CURE", 365, 36.125758728394175`}, {"CURE", 365, 63.6801753060139`}, {"CURE", 365, 61.428706343517725`},
{"CURE", 365, 7.562947422954852`}, {"CURE", 365, 124.35422643531297`}, {"CURE", 365, 14.436847786783154`},
{"CURE", 365, 7.24900483558035`}, {"CURE", 365, 34.55072879662955`}, {"CURE", 365, 36.24197619782559`},
{"CURE", 365, 24.47161365374203`}, {"CURE", 365, 42.41973876355066`}, {"CURE", 365, 36.569779474110234`},
{"CURE", 365, 58.778089524618835`}, {"CURE", 365, 49.677860395183274`}, {"CURE", 365, 71.02114652642275`},
{"CURE", 365, 11.219806242890408`}, {"CURE", 365, 144.32318029079298`}, {"CURE", 365, 139.45089598560517`},
{"CURE", 365, 7.731471410031704`}, {"CURE", 365, 19.4442756468519`}, {"CURE", 365, 48.24515905483358`},
{"CURE", 365, 24.567944474123657`}, {"CURE", 365, 75.61999456560932`}, {"CURE", 365, 151.0768536450217`},
{"CURE", 365, 47.741576651731485`}, {"CURE", 365, 8.202608976641422`}, {"CURE", 365, 8.043260566370705`},
{"CURE", 365, 18.87405990824661`}, {"CURE", 365, 5.700625111383346`}, {"CURE", 365, 64.67612527055155`},
{"CURE", 365, 10.032352513619957`}, {"CURE", 365, 67.66183409475634`}, {"CURE", 365, 41.55465059404942`},
{"CURE", 365, 71.59545661440416`}, {"CURE", 365, 17.631637899933935`}, {"CURE", 365, 88.95326843560389`},
{"TOX", 8.823902713065701`, 294.29885297664396`}, {"CURE", 365, 106.003287866804`}, {"CURE", 365, 171.7456799214639`},
{"CURE", 365, 50.532577778177554`}, {"CURE", 365, 120.39012892736835`}, {"CURE", 365, 120.6416108668977 8`},
{"CURE", 365, 14.640831910016757`}, {"CURE", 365, 10.242127797130024`}, {"CURE", 365, 33.319337233056956`},
```

{"CURE", 365, 69.10118213914141`}, {"CURE", 365, 86.46508369408009`}, {"CURE", 365, 37.41451888901892`},
{"CURE", 365, 54.77508106628829 4`}, {"CURE", 365, 10.3215175759826`}, {"CURE", 365, 13.117367052621528`},
{"CURE", 365, 63.685008810161314`}, {"CURE", 365, 20.669341174629427`}, {"CURE", 365, 8.755424457054355`},
{"CURE", 365, 12.946422077540666`}, {"CURE", 365, 6.330990823260463`}, {"CURE", 365, 51.43930392124078`},
{"CURE", 365, 70.72770667448178`}, {"CURE", 365, 49.52091404325584`}, {"CURE", 365, 56.22580208559258`},
{"CURE", 365, 31.15886410853291`}, {"CURE", 365, 27.922818048364444`}, {"CURE", 365, 72.19690422020965`},
{"CURE", 365, 77.22813116551795`}, {"CURE", 365, 8.619546452928914`}, {"CURE", 365, 33.16284892744817`},
{"CURE", 365, 17.13248441568518`}, {"CURE", 365, 26.88672651631238`}, {"CURE", 365, 221.52353182762846`},
{"CURE", 365, 39.11362215978248`}, {"CURE", 365, 26.84304572865167`}, {"CURE", 365, 28.41790554024204`},
{"CURE", 365, 56.64310994508304`}, {"CURE", 365, 43.11538939383526`}, {"CURE", 365, 56.24602266180733`},
{"CURE", 365, 58.08620707377284`}, {"CURE", 365, 13.157500524631732`}, {"CURE", 365, 127.92770104491758`},
{"CURE", 365, 24.58515630194622`}, {"CURE", 365, 52.11135172716239`}, {"CURE", 365, 29.57625785031083`},
{"CURE", 365, 16.788552900106207`}, {"CURE", 365, 61.20548975332591`}, {"CURE", 365, 62.13181715150786`},
{"TBUR", 101.13835091833688`, 34.553827808386494`}, {"CURE", 365, 81.49164547936677`}, {"CURE", 365, 17.237939993663083`},
{"CURE", 365, 143.98411655206078`}, {"CURE", 365, 55.32775355471542`}, {"CURE", 365, 117.05196064722584`},
{"CURE", 365, 4.535478910833118`}, {"CURE", 365, 57.60712986151376`}, {"CURE", 365, 65.72446293801076`},
{"CURE", 365, 15.585529393577453`}, {"CURE", 365, 39.58660684137783`}, {"CURE", 365, 59.26819148632822`},
{"CURE", 365, 36.031708373035`}, {"CURE", 365, 11.470978257734785`}, {"CURE", 365, 8.70174999031984`},
{"CURE", 365, 51.47813995900065`}, {"CURE", 365, 13.725001412361843`}, {"CURE", 365, 30.52202205582131`},
{"CURE", 365, 37.242615303184515`}, {"CURE", 365, 17.366784258213894`}, {"CURE", 365, 31.917487302843774`},
{"CURE", 365, 8.679325183220403`}, {"CURE", 365, 35.77581547489951`}, {"CURE", 365, 5.783157026516495`},
{"CURE", 365, 10.342633342648552`}, {"CURE", 365, 24.468750275536486`}, {"CURE", 365, 7.888798591881591`},
{"CURE", 365, 58.61233936304501`}, {"CURE", 365, 86.03165774293599`}, {"CURE", 365, 87.39726321518904`},
{"CURE", 365, 72.71296137760245`}, {"CURE", 365, 137.27362054702405`}, {"CURE", 365, 11.108687689632626`},
{"CURE", 365, 49.52896309419547`}, {"CURE", 365, 53.297260918586225`}, {"CURE", 365, 91.41740435537092`},
{"CURE", 365, 68.28134175598555`}, {"CURE", 365, 31.231396761537717`}, {"CURE", 365, 146.8759554314971`},
{"CURE", 365, 4.583486085607078`}, {"CURE", 365, 139.60107891372448`}, {"CURE", 365, 9.457951763947127`},
{"CURE", 365, 119.23845187669947`}, {"CURE", 365, 107.98236304258745`}, {"CURE", 365, 32.017830660913155`},
{"CURE", 365, 126.08673542463174`}, {"CURE", 365, 153.74637265093108`}, {"CURE", 365, 68.7954209991678`},
{"CURE", 365, 30.343925415478783`}, {"CURE", 365, 34.8910011583031`}, {"CURE", 365, 51.53335868403718`},
{"CURE", 365, 47.533855655311235`}, {"CURE", 365, 11.516602766814987`}, {"CURE", 365, 41.35958706939925`},

{"CURE", 365, 39.16340682557627`}, {"CURE", 365, 29.272347322678762`}, {"CURE", 365, 72.28578507323364`},
{"CURE", 365, 11.383026838556328`}, {"CURE", 365, 80.6959021254414`}, {"CURE", 365, 52.81400234713218`},
{"CURE", 365, 29.893748997316703`}, {"CURE", 365, 27.39186293134678`}, {"CURE", 365, 55.25303585592643`},
{"CURE", 365, 7.5546511557512375`}, {"CURE", 365, 87.50639845374168`}, {"CURE", 365, 13.82258388887914`},
{"CURE", 365, 19.59599625333107`}, {"CURE", 365, 43.98120144262231`}, {"CURE", 365, 34.473266297353156`},
{"CURE", 365, 31.699739604076385`}, {"CURE", 365, 98.74944032905576`}, {"CURE", 365, 13.35124316887049`},
{"CURE", 365, 23.247970025061502`}, {"CURE", 365, 171.93357893428242`}, {"CURE", 365, 13.047704212011567`},
{"CURE", 365, 33.58987354932866`}, {"CURE", 365, 51.15313847844313`}, {"CURE", 365, 50.505398447533956`},
{"CURE", 365, 34.27263336597443`}, {"CURE", 365, 177.1260024274936`}, {"CURE", 365, 32.01990376549916`},
{"CURE", 365, 13.478005547751183`}, {"CURE", 365, 26.964736012391153`}, {"CURE", 365, 20.754424672826755`},
{"CURE", 365, 15.913607928050569`}, {"CURE", 365, 41.7148111665498`}, {"CURE", 365, 71.43038456237352`},
{"CURE", 365, 39.30742969816518`}, {"CURE", 365, 45.82734285787732`}, {"CURE", 365, 100.31421020425908`},
{"CURE", 365, 11.07170414674818`}, {"CURE", 365, 13.027085081685318`}, {"CURE", 365, 109.83876704843321`},
{"CURE", 365, 35.42573253311908`}, {"CURE", 365, 38.29008351189664`}, {"CURE", 365, 19.251387490045033`},
{"CURE", 365, 44.07778837774654`}, {"CURE", 365, 54.9313001240238`}, {"CURE", 365, 127.34355045258724`},
{"CURE", 365, 18.76875927627715`}, {"CURE", 365, 73.437859978222`}, {"CURE", 365, 43.18850897534283`},
{"CURE", 365, 114.33226688487727`}, {"CURE", 365, 186.55508542212908`}, {"CURE", 365, 68.3763242766461`},
{"CURE", 365, 6.617284365067148`}, {"CURE", 365, 51.76423840732753`}, {"CURE", 365, 16.363067202194134`},
{"CURE", 365, 172.82650787137146`}, {"TBUR", 62.362221949284006`, 5.5663043477038405`}, {"CURE", 365, 5.771132017303976`},
{"CURE", 365, 200.3530033750397`}, {"CURE", 365, 46.140768640342614`}, {"CURE", 365, 15.570113968219271`},
{"CURE", 365, 29.799861798972977`}, {"CURE", 365, 10.462606462640787`}, {"CURE", 365, 9.969622348444915`},
{"CURE", 365, 11.88807139665703`}, {"CURE", 365, 42.973586287034045`}, {"CURE", 365, 91.08402044100859`},
{"CURE", 365, 36.09630763763924`}, {"CURE", 365, 9.038733476845293`}, {"CURE", 365, 122.8846410538695`},
{"TOX", 8.588796905588438`, 245.86722522446073`}, {"CURE", 365, 6.927002659578345`}, {"CURE", 365, 55.414218142990336`},
{"CURE", 365, 29.735093102953414`}, {"CURE", 365, 5.847422384315322`}, {"CURE", 365, 45.733074842277716`},
{"CURE", 365, 29.205257875972055`}, {"CURE", 365, 22.42649709300331`}, {"CURE", 365, 43.60188892824403`},
{"CURE", 365, 24.75150203118459`}, {"CURE", 365, 73.10378947616772`}, {"CURE", 365, 43.26683974464809`},
{"CURE", 365, 101.892546741324`}, {"CURE", 365, 45.145305571817026`}, {"CURE", 365, 22.37848572310347`},
{"CURE", 365, 10.889318865220856`}, {"CURE", 365, 7.541158075314827`}, {"CURE", 365, 21.479317581794007`},
{"CURE", 365, 85.51381612946916`}, {"CURE", 365, 28.193097614178708`}, {"CURE", 365, 25.851594614282163`},
{"CURE", 365, 18.276091731231407`}, {"CURE", 365, 18.518548709887334`}, {"CURE", 365, 50.04058971716675`},

```
{"CURE", 365, 9.36033105612811`}, {"CURE", 365, 59.65625953453409`}, {"CURE", 365, 27.278705703671047`},
{"CURE", 365, 16.07356209194607`}, {"CURE", 365, 25.43063718826883`}, {"CURE", 365, 56.37203271719506`},
{"CURE", 365, 42.230020833535896`}, {"CURE", 365, 5.069124437535794`}, {"CURE", 365, 21.854847509296064`},
{"TOX", 4.192665719891895`, 365.044692713356`}, {"CURE", 365, 60.74054600882872`}, {"CURE", 365, 88.48752453732384`},
{"CURE", 365, 26.08075895081218`}, {"CURE", 365, 96.66651231562054`}, {"CURE", 365, 11.562963701494365`},
{"CURE", 365, 38.9967382600718`}, {"CURE", 365, 179.8587007732596`}, {"CURE", 365, 33.66887950663011`},
{"CURE", 365, 29.764092686798456`}, {"CURE", 365, 50.830223946576375`}, {"CURE", 365, 26.96748682952458`},
{"CURE", 365, 5.972439892455765`}, {"CURE", 365, 55.55870767970774`}, {"CURE", 365, 9.907064746627057`},
{"CURE", 365, 23.597564996470588`}, {"CURE", 365, 55.59159970468754`}, {"CURE", 365, 19.967100224025764`},
{"CURE", 365, 106.02986827634544`}, {"CURE", 365, 31.55105498388779`}, {"CURE", 365, 54.26952154397976`},
{"CURE", 365, 37.61927676133227`}, {"CURE", 365, 10.64502005892071`}, {"CURE", 365, 39.53644545401184`},
{"CURE", 365, 85.35541662348149`}, {"CURE", 365, 28.6127260263001`}, {"CURE", 365, 21.530125241216865`},
{"CURE", 365, 10.218583263938507`}, {"CURE", 365, 32.74445772144788`}, {"CURE", 365, 38.92989450706182`},
{"CURE", 365, 110.9218651705742`}, {"CURE", 365, 17.82082009019796`}, {"CURE", 365, 26.857386787118827`},
{"CURE", 365, 104.13962443776165`}, {"CURE", 365, 10.971695757496509`}, {"CURE", 365, 150.80482616316738`},
{"CURE", 365, 12.729236149416876`}, {"CURE", 365, 23.330274528947008`}, {"CURE", 365, 53.73362908177579`},
{"CURE", 365, 86.71266110496136`}, {"CURE", 365, 38.92913137008047`}, {"CURE", 365, 20.43185104082615`},
{"CURE", 365, 5.998931731578576`}, {"CURE", 365, 52.64420929854474`}, {"CURE", 365, 47.622589432172944`},
{"CURE", 365, 46.747445710841895`}, {"CURE", 365, 18.432385310771807`}, {"CURE", 365, 45.17960756189207`},
{"CURE", 365, 5.2318929003596315`}, {"CURE", 365, 30.140515918140736`}, {"CURE", 365, 13.158095135979591`},
{"CURE", 365, 5.831059376986814`}, {"CURE", 365, 30.291849236414386`}, {"CURE", 365, 6.479367609025451`},
{"CURE", 365, 20.603875125971058`}, {"CURE", 365, 14.135198876114057`}, {"TBUR", 61.34711093396466`, 8.773489428750855`},
{"CURE", 365, 27.998845806071422`}, {"CURE", 365, 74.56861737165215`}, {"CURE", 365, 40.91632489443132`},
{"CURE", 365, 36.97828533488528`}, {"CURE", 365, 58.90668486706695`}, {"CURE", 365, 49.88206719072653`}},
{{"3000 Res", "3000 OS", "3000 Tox"}, {"CURE", 365, 25.043601799335637`}, {"CURE", 365, 51.71683895673711`},
{"CURE", 365, 36.29671266807018`}, {"TBUR", 90.4771566837272`, 12.203425642745689`},
{"TBUR", 65.38604232001155`, 47.21175258117261`}, {"CURE", 365, 93.68382631840265`}, {"CURE", 365, 88.13897756959555`},
{"CURE", 365, 9.211609986077768`}, {"CURE", 365, 63.567152403266086`}, {"CURE", 365, 55.01253599554563`},
{"CURE", 365, 206.43149104975026`}, {"CURE", 365, 25.607712254861966`}, {"CURE", 365, 94.2863641891522`},
{"TOX", 5.481659514927353`, 308.5986042624457`}, {"CURE", 365, 43.57352244107768`}, {"CURE", 365, 68.01917703200242`},
{"CURE", 365, 134.56445184035059`}, {"CURE", 365, 60.31941831094076`}, {"CURE", 365, 45.76211965377459`},
```

```
{"CURE", 365, 15.111354700742899`}, {"CURE", 365, 93.39714382309707`}, {"CURE", 365, 37.855279196411836`},
{"CURE", 365, 14.509256543857607`}, {"CURE", 365, 11.561529642984715`}, {"CURE", 365, 52.5866131880521`},
{"CURE", 365, 57.720880947149006`}, {"CURE", 365, 5.2480042936196485`}, {"CURE", 365, 7.9550854976925525`},
{"CURE", 365, 29.4800846701857`}, {"CURE", 365, 34.494086506379695`}, {"CURE", 365, 29.412992583195326`},
{"CURE", 365, 103.6127728107122`}, {"CURE", 365, 22.749019675306624`}, {"CURE", 365, 18.115836909672304`},
{"CURE", 365, 146.62324323061574`}, {"CURE", 365, 8.99319233369592`}, {"CURE", 365, 38.819785808747085`},
{"TBUR", 60.53433416606409`, 86.93149221687526`}, {"CURE", 365, 21.599725866550113`}, {"CURE", 365, 33.25800143199054`},
{"CURE", 365, 75.91997535129597`}, {"CURE", 365, 51.11215745267734`}, {"CURE", 365, 10.373116638237695`},
{"CURE", 365, 35.005104870389545`}, {"CURE", 365, 50.485461931385345`}, {"CURE", 365, 33.09596716271461`},
{"TOX", 4.212581205476185`, 351.7355768358927`}, {"CURE", 365, 75.82845085059783`}, {"CURE", 365, 120.21944804203484`},
{"CURE", 365, 14.19163523243938`}, {"CURE", 365, 40.67254958481815`}, {"CURE", 365, 55.1660997487228`},
{"CURE", 365, 43.64571569958495`}, {"CURE", 365, 28.33634041944814`}, {"CURE", 365, 12.206651357952328`},
{"CURE", 365, 129.05778934427423`}, {"CURE", 365, 40.55211363410946`}, {"CURE", 365, 7.71403490760743`},
{"CURE", 365, 83.12008762499546`}, {"CURE", 365, 58.26347970345509`}, {"CURE", 365, 39.8677032289155`},
{"CURE", 365, 30.4713518068718`}, {"CURE", 365, 17.561342499651303`}, {"CURE", 365, 65.67014127218125`},
{"CURE", 365, 46.69179290473338`}, {"CURE", 365, 53.35335279407638`}, {"CURE", 365, 12.212574037897488`},
{"CURE", 365, 12.424932270894196`}, {"CURE", 365, 13.203435816383353`}, {"CURE", 365, 53.930672113457184`},
{"CURE", 365, 19.74531434807564`}, {"CURE", 365, 43.98838719761605`}, {"CURE", 365, 82.53124463971416`},
{"TBUR", 35.15163849124447`, 27.021727282448264`}, {"CURE", 365, 22.066326356092347`}, {"CURE", 365, 61.08120088527536`},
{"CURE", 365, 35.74589876148139`}, {"CURE", 365, 27.173138877121342`}, {"CURE", 365, 108.31982186285691`},
{"CURE", 365, 118.8726433384895`}, {"CURE", 365, 87.525657003772`}, {"CURE", 365, 23.609963268418387`},
{"CURE", 365, 47.73517086980361`}, {"CURE", 365, 33.366430783376856`}, {"CURE", 365, 37.738967719413615`},
{"CURE", 365, 17.691198637124508`}, {"CURE", 365, 188.70771256145505`}, {"CURE", 365, 41.131551523117686`},
{"CURE", 365, 51.77460865295214`}, {"CURE", 365, 44.58544582571902`}, {"CURE", 365, 36.9673312304658`},
{"CURE", 365, 10.929576763524901`}, {"CURE", 365, 20.021387163323958`}, {"CURE", 365, 46.355526995403366`},
{"CURE", 365, 16.88479889924862`}, {"CURE", 365, 19.968680887608258`}, {"CURE", 365, 52.02638829841535`},
{"CURE", 365, 132.0389432473314`}, {"CURE", 365, 70.7799768517275`}, {"CURE", 365, 35.50205545022976`},
{"CURE", 365, 70.47425153632652`}, {"CURE", 365, 45.76139410113719`}, {"CURE", 365, 29.645300912007034`},
{"CURE", 365, 202.99635507870164`}, {"CURE", 365, 22.542977121024787`}, {"CURE", 365, 15.317369958292634`},
{"CURE", 365, 89.94169511519091`}, {"CURE", 365, 26.636497411294215`}, {"CURE", 365, 27.123214635176986`},
{"CURE", 365, 163.18992668703103`}, {"CURE", 365, 13.247298844176173`}, {"CURE", 365, 8.40702949057129`},
```

{"CURE", 365, 34.96153762613059`}, {"CURE", 365, 27.6447192622732`}, {"CURE", 365, 20.822953186166192`},
{"CURE", 365, 53.12874673734061`}, {"CURE", 365, 14.957616705787526`}, {"CURE", 365, 9.196863269566437`},
{"CURE", 365, 43.0399532779388`}, {"CURE", 365, 65.83221525618721`}, {"CURE", 365, 11.219912767692557`},
{"CURE", 365, 37.115607777063104`}, {"CURE", 365, 112.63475408835308`}, {"CURE", 365, 43.028362337615995`},
{"CURE", 365, 24.99114815189935`}, {"CURE", 365, 87.23201486349443`}, {"CURE", 365, 43.83986249357334`},
{"CURE", 365, 5.777459806650293`}, {"CURE", 365, 50.02333185890807`}, {"CURE", 365, 38.63418030137869`},
{"CURE", 365, 30.779042580258483`}, {"CURE", 365, 36.51792682608716`}, {"CURE", 365, 9.511865236275794`},
{"CURE", 365, 54.26489766991325`}, {"CURE", 365, 19.482891798864305`}, {"CURE", 365, 44.10472079659698 6`},
{"CURE", 365, 164.42884872705227`}, {"CURE", 365, 58.14796581234731`}, {"CURE", 365, 134.951397323033`},
{"CURE", 365, 44.46184284281052`}, {"CURE", 365, 184.06005027706914`}, {"CURE", 365, 5.728762230265135`},
{"CURE", 365, 50.288583840907116`}, {"CURE", 365, 57.327976897602554`}, {"CURE", 365, 7.445509722853827`},
{"CURE", 365, 10.31716154663144`}, {"CURE", 365, 63.319954177976115`}, {"CURE", 365, 74.69507566589506`},
{"CURE", 365, 63.58589198794318`}, {"CURE", 365, 52.035861079914774`}, {"CURE", 365, 49.73379136370082`},
{"CURE", 365, 30.12343409752329`}, {"CURE", 365, 7.965084422297792`}, {"CURE", 365, 34.67857646192394`},
{"CURE", 365, 16.452897446296312`}, {"CURE", 365, 8.05578425722102`}, {"CURE", 365, 48.7952280609601 1`},
{"CURE", 365, 27.29382405968014`}, {"CURE", 365, 72.14993315742718`}, {"CURE", 365, 29.131772517195927`},
{"CURE", 365, 24.244725422444446`}, {"CURE", 365, 162.75114689104228`}, {"CURE", 365, 12.116218655616322`},
{"CURE", 365, 36.276053886387835`}, {"CURE", 365, 20.552238888093648`}, {"CURE", 365, 48.63231406673567`},
{"CURE", 365, 13.350058893487203`}, {"CURE", 365, 53.27090897795934`}, {"CURE", 365, 140.9003644073232`},
{"CURE", 365, 12.04141590397325`}, {"CURE", 365, 36.552645480376135`}, {"CURE", 365, 39.59736334321488`},
{"CURE", 365, 142.12044425449082`}, {"CURE", 365, 53.603949594625696`}, {"CURE", 365, 44.485805752965035`},
{"CURE", 365, 78.1564439125794`}, {"CURE", 365, 29.912556968746024`}, {"CURE", 365, 95.25982070428239`},
{"CURE", 365, 57.64381697995718`}, {"CURE", 365, 35.63249326566008`}, {"CURE", 365, 31.794598507764608`},
{"CURE", 365, 10.331888935163228`}, {"CURE", 365, 6.999590733157609`}, {"CURE", 365, 16.415982325323608`},
{"CURE", 365, 21.9757105835997`}, {"CURE", 365, 9.693732418539971`}, {"CURE", 365, 35.47857061617078`},
{"CURE", 365, 96.70466219694029`}, {"CURE", 365, 26.133529942466996`}, {"CURE", 365, 48.62510821238392`},
{"CURE", 365, 60.74842113559642`}, {"CURE", 365, 53.51969232849626`}, {"CURE", 365, 35.5554812185471`},
{"CURE", 365, 14.124940396379255`}, {"CURE", 365, 97.33220790779747`}, {"CURE", 365, 8.418496811725271`},
{"CURE", 365, 90.716707415635`}, {"CURE", 365, 88.3972445452275`}, {"CURE", 365, 24.604066077146456`},
{"CURE", 365, 29.221170774493693`}, {"CURE", 365, 168.1525072723573`}, {"CURE", 365, 43.467880904272`},
{"CURE", 365, 51.686123662733635`}, {"CURE", 365, 32.401592591261426`}, {"CURE", 365, 12.175762797152412`},

{"CURE", 365, 74.72450195468453`}, {"TBUR", 77.79643097532666`, 198.77004318697715`}, {"CURE", 365, 30.310795315461924`},
{"CURE", 365, 27.41556484047991`}, {"CURE", 365, 50.501555071884276`}, {"CURE", 365, 20.163507955269285`},
{"TOX", 9.030502599135122`, 255.9615109608402`}, {"CURE", 365, 20.482928295771668`}, {"CURE", 365, 127.06236394840752`},
{"CURE", 365, 97.30182899416627`}, {"CURE", 365, 94.67890744213648`}, {"CURE", 365, 50.57291705689469`},
{"CURE", 365, 117.15403558782769`}, {"TOX", 3.340588118400479`, 412.3825588758041`}, {"CURE", 365, 53.22731136891924`},
{"CURE", 365, 9.781752466917439`}, {"CURE", 365, 86.85316342559189`}, {"CURE", 365, 54.11797265205452`},
{"CURE", 365, 52.18191844904652`}, {"CURE", 365, 11.528982718135042`}, {"TOX", 8.66642820417664`, 263.757382703559`},
{"CURE", 365, 19.28632504224213`}, {"CURE", 365, 83.6364848553285`}, {"CURE", 365, 52.74201940370197`},
{"CURE", 365, 37.471145733068646`}, {"CURE", 365, 9.006625459378837`}, {"CURE", 365, 67.69253411267162`},
{"CURE", 365, 39.71959645996777`}, {"CURE", 365, 105.07039417176132`}, {"CURE", 365, 69.23360104468243`},
{"CURE", 365, 87.43197228208322`}, {"CURE", 365, 42.01901739459992`}, {"CURE", 365, 37.367374023428326`},
{"CURE", 365, 195.26652343140674`}, {"CURE", 365, 51.08648601780262`}, {"CURE", 365, 13.634745886856434`},
{"CURE", 365, 45.90924486161036`}, {"CURE", 365, 46.868415121469894`}, {"CURE", 365, 6.277263092805521`},
{"CURE", 365, 11.156153694104477`}, {"CURE", 365, 17.049251259813243`}, {"CURE", 365, 161.18602757006005`},
{"CURE", 365, 49.51199646279429`}, {"CURE", 365, 51.578079176928966`}, {"CURE", 365, 41.320453110541386`},
{"CURE", 365, 170.22156236598764`}, {"CURE", 365, 39.11123133417743`}, {"CURE", 365, 79.11823782171616`},
{"CURE", 365, 39.013368626201135`}, {"CURE", 365, 57.50708813655379`}, {"CURE", 365, 13.828281425169935`},
{"CURE", 365, 51.72443482113688`}, {"CURE", 365, 32.284291734683556`}, {"CURE", 365, 62.860317459025254`},
{"CURE", 365, 99.3739699789543`}, {"CURE", 365, 112.70289458746676`}, {"CURE", 365, 69.21853402840829`},
{"CURE", 365, 55.3900101425665`}, {"CURE", 365, 77.51056823963677`}, {"CURE", 365, 36.27481850436322`},
{"CURE", 365, 41.06635228879561`}, {"CURE", 365, 29.118138737594485`}, {"CURE", 365, 22.80570623269677`},
{"CURE", 365, 36.73587256816821`}, {"CURE", 365, 94.23956760471422`}, {"CURE", 365, 10.80235698290327`},
{"CURE", 365, 47.89244431847332`}, {"CURE", 365, 9.03843580641033`}, {"CURE", 365, 47.05684771009002`},
{"CURE", 365, 26.767720238213535`}, {"CURE", 365, 99.47581566507421`}, {"CURE", 365, 9.173480936412405`},
{"CURE", 365, 65.568428091237`}, {"CURE", 365, 82.17323503804863`}, {"CURE", 365, 13.4421401292572`},
{"CURE", 365, 30.81497526559826`}, {"CURE", 365, 24.79513431433576`}, {"CURE", 365, 14.201976543428385`},
{"CURE", 365, 83.79077061448602`}, {"CURE", 365, 31.511435127198688`}, {"CURE", 365, 60.68645435356031`},
{"CURE", 365, 59.02138955959765`}, {"CURE", 365, 66.905154716871`}, {"CURE", 365, 52.31343025715706`},
{"CURE", 365, 62.67646426296449`}, {"CURE", 365, 49.5694723017663`}, {"CURE", 365, 95.87024405227305`},
{"CURE", 365, 58.424467150316325`}, {"CURE", 365, 47.72328937213809`}, {"CURE", 365, 121.96212265284368`},
{"CURE", 365, 14.120669898715756`}, {"CURE", 365, 80.0811484780805`}, {"CURE", 365, 5.882660397602294`},

{"CURE", 365, 73.10715404971486`}, {"CURE", 365, 17.2216662515025`}, {"CURE", 365, 92.0267400900915`},
{"CURE", 365, 14.066945198447195`}, {"CURE", 365, 92.12821534027975`}, {"TOX", 15.366892429788264`, 230.71428138643412`},
{"CURE", 365, 10.069756572935496`}, {"CURE", 365, 7.2409156228453515`}, {"CURE", 365, 26.61541845499798`},
{"CURE", 365, 6.4705305971574205`}, {"CURE", 365, 4.419177153926424`}, {"CURE", 365, 25.849937429787932`},
{"CURE", 365, 42.32922524794774`}, {"CURE", 365, 45.476397021571394`}, {"CURE", 365, 43.30192389936798`},
{"CURE", 365, 33.72614562596925`}, {"CURE", 365, 59.24285933776487`}, {"CURE", 365, 104.29943219361147`},
{"CURE", 365, 8.440806493272936`}, {"CURE", 365, 46.23110326900686`}, {"CURE", 365, 39.72961558573086`},
{"CURE", 365, 28.807789866987743`}, {"CURE", 365, 53.29400215165386`}, {"CURE", 365, 78.98767151877368`},
{"CURE", 365, 184.79780122279004`}, {"CURE", 365, 137.7650498490373`}, {"CURE", 365, 42.66116560962724`},
{"CURE", 365, 4.5226092376324125`}, {"CURE", 365, 49.339579389607735`}, {"CURE", 365, 127.20631403136903`},
{"CURE", 365, 32.013943075995314`}, {"CURE", 365, 41.57798011424902`}, {"CURE", 365, 24.700216885045084`},
{"CURE", 365, 36.19892374142468`}, {"CURE", 365, 9.392274502862394`}, {"CURE", 365, 45.89046282045563`},
{"CURE", 365, 25.842395960661232`}, {"CURE", 365, 75.37112874429215`}, {"CURE", 365, 51.66485210963741`},
{"CURE", 365, 50.01008076221296`}, {"CURE", 365, 41.148372662287045`}, {"CURE", 365, 21.77578395184806`},
{"CURE", 365, 15.007596178030811`}, {"CURE", 365, 14.632226488219857`}, {"CURE", 365, 18.96251726323854`},
{"CURE", 365, 14.772639737089206`}, {"CURE", 365, 198.79236714528844`}, {"CURE", 365, 110.57972285484439`},
{"CURE", 365, 21.347021464755617`}, {"TOX", 5.21479500874263`, 328.801021184171`}, {"CURE", 365, 94.02839340511969`},
{"CURE", 365, 49.78705805522302`}, {"CURE", 365, 86.13139960657027`}, {"CURE", 365, 12.948628058552325`},
{"CURE", 365, 103.30821594693911`}, {"TOX", 5.720401249246399`, 276.0472287469664`}, {"CURE", 365, 14.982192375189436`},
{"CURE", 365, 46.725740441958`}, {"CURE", 365, 76.61412652730681`}, {"CURE", 365, 38.86946007938985`},
{"CURE", 365, 18.960173841981895`}, {"CURE", 365, 15.104177860983846`}, {"CURE", 365, 131.12446818451505`},
{"CURE", 365, 68.83623145362255`}, {"CURE", 365, 39.158285040775745`}, {"CURE", 365, 97.3649834479672 5`},
{"CURE", 365, 17.60168964797155`}, {"CURE", 365, 46.952971615425604`}, {"CURE", 365, 28.358214885688035`},
{"CURE", 365, 191.21446361332568`}, {"CURE", 365, 48.24918511166481`}, {"CURE", 365, 223.89626685317066`},
{"TOX", 4.797605837254376`, 349.420800401135`}, {"CURE", 365, 26.692410106484722`}, {"CURE", 365, 7.528547933956029`},
{"CURE", 365, 6.557445080204765`}, {"CURE", 365, 47.98112426169998`}, {"CURE", 365, 186.09684657017254`},
{"CURE", 365, 37.28066548325652`}, {"CURE", 365, 28.647164029559377`}, {"CURE", 365, 53.90344935409032`},
{"CURE", 365, 59.03615176204738`}, {"TOX", 7.975855479260195`, 245.91176143360195`}, {"CURE", 365, 202.63244550041725`},
{"CURE", 365, 42.47896680882236`}, {"CURE", 365, 14.087467061863713`}, {"CURE", 365, 34.514137871953814`},
{"CURE", 365, 5.416278616428441`}, {"CURE", 365, 101.9880414106354`}, {"CURE", 365, 151.44670855802585`},
{"CURE", 365, 49.5358412551291`}, {"CURE", 365, 39.258724020998756`}, {"CURE", 365, 165.9936220599567 5`},

{"CURE", 365, 10.55223290080473`}, {"CURE", 365, 13.733221416910645`}, {"CURE", 365, 42.92520686188502`},
{"CURE", 365, 55.29252357957956`}, {"CURE", 365, 104.9528624828287`}, {"CURE", 365, 107.7087438072281`},
{"CURE", 365, 44.21321104826631`}, {"CURE", 365, 45.64072162053001`}, {"CURE", 365, 57.298519602831725`},
{"CURE", 365, 19.810562781514637`}, {"CURE", 365, 5.585949671060182`}, {"CURE", 365, 58.76241878738763`},
{"CURE", 365, 70.72510127760556`}, {"CURE", 365, 34.387873768488134`}, {"CURE", 365, 62.31651315230898`},
{"CURE", 365, 19.418615967545236`}, {"CURE", 365, 12.757049843486753`}, {"CURE", 365, 55.84082516255292`},
{"CURE", 365, 33.97830000883497`}, {"CURE", 365, 48.52630608401972`}, {"TOX", 7.162015099019468`, 320.17194258037546`},
{"CURE", 365, 30.517292107029157`}, {"CURE", 365, 14.893084147714301`}, {"CURE", 365, 11.549449439467598`},
{"CURE", 365, 13.996305669115722`}, {"CURE", 365, 79.9778658319763`}, {"CURE", 365, 5.506266472474199`},
{"CURE", 365, 74.4579689672491`}, {"CURE", 365, 81.27010808934607`}, {"CURE", 365, 63.43121941128337`},
{"CURE", 365, 52.0039507993725`}, {"CURE", 365, 10.319829071275755`}, {"CURE", 365, 113.19295178654144`},
{"CURE", 365, 34.37054836108515`}, {"CURE", 365, 15.734352101730671`}, {"CURE", 365, 100.81439858185277`},
{"CURE", 365, 17.929063221864876`}, {"CURE", 365, 120.79435401079787`}, {"CURE", 365, 77.50107112186456`},
{"CURE", 365, 51.7794432338128`}, {"CURE", 365, 75.10326929305477`}, {"CURE", 365, 26.664454096985267`},
{"CURE", 365, 117.84096718586565`}, {"CURE", 365, 13.980406345984036`}, {"CURE", 365, 59.34499154254888`},
{"CURE", 365, 69.239636237236`}, {"CURE", 365, 29.440093717472458`}, {"TOX", 7.898967076387563`, 274.3569202962754 6`},
{"CURE", 365, 55.23065999043203`}, {"CURE", 365, 56.15244010220124`}, {"CURE", 365, 57.38016810803131`},
{"CURE", 365, 229.73376349919073`}, {"CURE", 365, 73.00955769634878`}, {"CURE", 365, 68.10991695721714`},
{"CURE", 365, 148.29429783340032`}, {"CURE", 365, 52.13694290155406`}, {"CURE", 365, 45.43037302622191`},
{"CURE", 365, 56.12281353325566`}, {"CURE", 365, 77.52336641605399`}, {"CURE", 365, 136.9780579430245`},
{"CURE", 365, 29.565342631863246`}, {"CURE", 365, 26.986920704093446`}, {"CURE", 365, 57.742842122169634`},
{"CURE", 365, 58.79148651971576`}, {"CURE", 365, 66.25146749760349`}, {"CURE", 365, 70.68414613177693`},
{"CURE", 365, 39.52547837102537`}, {"CURE", 365, 19.905219637976483`}, {"CURE", 365, 74.84322924612215`},
{"CURE", 365, 18.856890554340072`}, {"CURE", 365, 40.080763715182044`}, {"CURE", 365, 47.81753867308241`},
{"CURE", 365, 14.378246115004641`}, {"CURE", 365, 7.149302495285656`}, {"CURE", 365, 44.298237409257005`},
{"CURE", 365, 59.92188661180375`}, {"CURE", 365, 71.10782158976076`}, {"CURE", 365, 50.87530072744245`},
{"CURE", 365, 6.519460774089955`}, {"CURE", 365, 58.3164545283582`}, {"CURE", 365, 11.11666660724461`},
{"CURE", 365, 171.33520768507066`}, {"CURE", 365, 222.96626240378933`}, {"CURE", 365, 32.52064250846448`},
{"CURE", 365, 44.12716442936162`}, {"TOX", 23.321360399485155`, 239.37228863005532`}, {"CURE", 365, 42.92256977947099`},
{"CURE", 365, 25.000486677840726`}, {"CURE", 365, 19.45497274490419`}, {"CURE", 365, 5.309199703425441`},
{"CURE", 365, 33.45869403593111`}, {"CURE", 365, 37.310110197896364`}, {"CURE", 365, 98.9706174490669`},

{"CURE", 365, 47.342110494308855`}, {"CURE", 365, 82.40583699862276`}, {"CURE", 365, 29.105438919533327`},
{"CURE", 365, 63.28462700741523`}, {"CURE", 365, 141.1658646579813`}, {"CURE", 365, 14.370489727223331`},
{"CURE", 365, 22.14749977556399`}, {"CURE", 365, 32.91255740315795`}, {"CURE", 365, 45.95765548836626`},
{"CURE", 365, 30.227379714129246`}, {"CURE", 365, 28.846927379203706`}, {"CURE", 365, 12.243437058999039`},
{"CURE", 365, 18.864848228333425`}, {"CURE", 365, 35.79436210397118`}, {"CURE", 365, 81.40129635609543`},
{"CURE", 365, 72.57371863209009`}, {"CURE", 365, 37.098249353471665`}, {"TBUR", 58.262982588692864`, 55.82131258159616`},
{"CURE", 365, 119.92370118738177`}, {"CURE", 365, 58.61626704969716`}, {"CURE", 365, 43.85697721469583`},
{"CURE", 365, 27.934303875288535`}, {"CURE", 365, 8.75717275311427`}, {"CURE", 365, 48.68899607770079`},
{"CURE", 365, 56.21828925657435`}, {"CURE", 365, 220.30942015802387`}, {"CURE", 365, 11.228013447192943`},
{"CURE", 365, 10.960966024907025`}, {"CURE", 365, 35.119069971401295`}, {"CURE", 365, 102.37315073502545`},
{"CURE", 365, 12.778511617187169`}, {"CURE", 365, 62.49508329331772`}, {"CURE", 365, 14.580502081600661`},
{"CURE", 365, 36.33816923676519`}, {"CURE", 365, 14.880587248230274`}, {"CURE", 365, 15.404054839524164`},
{"CURE", 365, 13.892724994792214`}, {"CURE", 365, 148.20278875192585`}, {"CURE", 365, 23.779851954930923`},
{"CURE", 365, 41.865449628916245`}, {"CURE", 365, 5.758230114667711`}, {"CURE", 365, 68.9974363852674`},
{"CURE", 365, 96.53376108074201`}, {"CURE", 365, 84.22888648709865`}, {"CURE", 365, 12.710880552827643`},
{"CURE", 365, 25.485874683827852`}, {"TOX", 12.718117557477468`, 259.4327442419024`}, {"CURE", 365, 70.47740253082776`},
{"CURE", 365, 48.04919097030639`}, {"CURE", 365, 17.100478785487752`}, {"CURE", 365, 24.42796855821981`},
{"CURE", 365, 37.76389326363677`}, {"CURE", 365, 9.353231855746508`}, {"CURE", 365, 45.873839268570904`},
{"CURE", 365, 72.16626706572069`}, {"CURE", 365, 43.93783177784983`}, {"CURE", 365, 47.73834640828416`},
{"CURE", 365, 34.490552697990616`}, {"CURE", 365, 22.357735785025778`}, {"CURE", 365, 127.78948444927539`},
{"CURE", 365, 185.0560670123054`}, {"CURE", 365, 49.34861216742992`}, {"CURE", 365, 62.30545358090392`},
{"CURE", 365, 78.93354516252518`}, {"CURE", 365, 77.1837204624714`}, {"CURE", 365, 6.7454561589490805`},
{"CURE", 365, 90.36416517412906`}, {"CURE", 365, 93.28777786343439`}, {"CURE", 365, 63.09902373679484`},
{"TOX", 7.219246505563817 5`, 253.05909076508377`}, {"CURE", 365, 52.61158054883558`}, {"CURE", 365, 23.47277080809276`},
{"CURE", 365, 55.37503253482793`}, {"CURE", 365, 70.38579292757517`}, {"CURE", 365, 31.5288287793717`},
{"CURE", 365, 24.786780850100815`}, {"CURE", 365, 21.517225521427378`}, {"CURE", 365, 39.078634262230985`},
{"CURE", 365, 120.22157202260229`}, {"CURE", 365, 77.15823306780318`}, {"CURE", 365, 13.449893430857166`},
{"CURE", 365, 79.12698967862225`}, {"CURE", 365, 113.58100828065919`}, {"CURE", 365, 20.91708725478498`},
{"CURE", 365, 119.30079930595754`}, {"CURE", 365, 17.915473553623475`}, {"CURE", 365, 11.455591119912247`},
{"CURE", 365, 16.219609987234644`}, {"CURE", 365, 31.264019515320225`}, {"CURE", 365, 140.25772920936024`},
{"CURE", 365, 49.94899382977593`}, {"CURE", 365, 63.11715592611748`}, {"CURE", 365, 15.07659853390689`},

{"TOX", 8.396545548386616`, 288.2070918036864`}, {"CURE", 365, 131.81243386149694`}, {"CURE", 365, 22.530223905831658`},
{"CURE", 365, 55.09743065120313`}, {"CURE", 365, 30.671432760816348`}, {"CURE", 365, 31.007272452148136`},
{"CURE", 365, 104.80048603379834`}, {"CURE", 365, 132.62712048674874`}, {"CURE", 365, 14.222643714006772`},
{"CURE", 365, 27.740710436455647`}, {"CURE", 365, 9.841448996196602`}, {"CURE", 365, 37.918419213548646`},
{"CURE", 365, 65.10490119227677`}, {"CURE", 365, 105.27445464294253`}, {"CURE", 365, 58.76163672423766`},
{"CURE", 365, 211.60325901564568`}, {"CURE", 365, 101.13374517545563`}, {"CURE", 365, 16.73052200514015`},
{"CURE", 365, 37.0190678378707`}, {"TBUR", 65.08533669554153`, 41.75155053556955`}, {"CURE", 365, 82.86000035117007`},
{"CURE", 365, 10.875929557471393`}, {"CURE", 365, 29.044137474915747`}, {"CURE", 365, 16.37656380222916`},
{"CURE", 365, 40.80522485499532`}, {"CURE", 365, 93.26961028569812`}, {"CURE", 365, 39.6673611519769`},
{"CURE", 365, 8.616250437285043`}, {"CURE", 365, 164.29880678223185`}, {"CURE", 365, 30.97607069367712`},
{"CURE", 365, 10.516377030763712`}, {"CURE", 365, 13.826519755526048`}, {"CURE", 365, 11.670434033354166`},
{"CURE", 365, 14.907220336454683`}, {"CURE", 365, 119.95823337325675`}, {"CURE", 365, 65.77719362678174`},
{"CURE", 365, 39.53973528340765`}, {"CURE", 365, 18.553091367446907`}, {"CURE", 365, 30.92716369898892`},
{"CURE", 365, 17.894635245113555`}, {"CURE", 365, 43.1883427319126`}, {"CURE", 365, 148.5669799786444`},
{"CURE", 365, 89.75917078773159`}, {"CURE", 365, 42.91766384051359`}, {"CURE", 365, 24.695670579672935`},
{"CURE", 365, 26.441501194108618`}, {"TOX", 6.523667760082286`, 317.243491212291`}, {"CURE", 365, 15.119636126353196`},
{"CURE", 365, 7.790212229581342`}, {"CURE", 365, 6.80877886822856`}, {"CURE", 365, 86.28908433282587`},
{"CURE", 365, 50.56519656608114`}, {"CURE", 365, 56.06502325610967`}, {"CURE", 365, 83.25886063354302`},
{"CURE", 365, 137.68356717519484`}, {"CURE", 365, 142.15525944762962`}, {"CURE", 365, 54.179011068236804`},
{"CURE", 365, 23.39211682488983`}, {"CURE", 365, 27.145479047456675`}, {"CURE", 365, 54.97334024705209`},
{"CURE", 365, 30.762153919905064`}, {"TBUR", 33.98452328140893`, 12.860628645311818`}, {"CURE", 365, 48.32916675205243`},
{"TOX", 4.845965984084976`, 300.34970505601467`}, {"CURE", 365, 30.93052135337265`}, {"CURE", 365, 11.138732466743209`},
{"CURE", 365, 105.46732587467714`}, {"CURE", 365, 26.380434648490354`}, {"CURE", 365, 75.85536079497673`},
{"TOX", 4.4340255090048535`, 364.92682027855585`}, {"CURE", 365, 168.51101421028582`}, {"CURE", 365, 54.70404103134775`},
{"CURE", 365, 45.89610195414754`}, {"CURE", 365, 19.535392544438054`}, {"CURE", 365, 5.581871494097549`},
{"CURE", 365, 21.92073769880347`}, {"CURE", 365, 63.96636535466074`}, {"CURE", 365, 35.50549569554818`},
{"CURE", 365, 113.25743262167337`}, {"CURE", 365, 44.90381478523087`}, {"CURE", 365, 45.81276245726658`},
{"CURE", 365, 12.72342725181442`}, {"CURE", 365, 36.04512348491807`}, {"CURE", 365, 36.97646093348152`},
{"CURE", 365, 9.645831496382147`}, {"CURE", 365, 30.73216102088153`}, {"CURE", 365, 18.293721404183668`},
{"CURE", 365, 29.05737081124882`}, {"CURE", 365, 61.60679118992369`}, {"CURE", 365, 30.418493758722562`},
{"CURE", 365, 7.040428488339084`}, {"CURE", 365, 48.514266882311304`}, {"CURE", 365, 5.43879568487732`},

{"CURE", 365, 15.977868968298026`}, {"CURE", 365, 47.31086625425588`}, {"CURE", 365, 54.49723770189546`},
{"CURE", 365, 10.871253945788927`}, {"CURE", 365, 31.946930721894248`}, {"CURE", 365, 41.52386029734946`},
{"CURE", 365, 209.24132043198324`}, {"CURE", 365, 47.97521418356506`}, {"CURE", 365, 13.556524621341792`},
{"CURE", 365, 147.60014084042058`}, {"CURE", 365, 14.573103114926193`}, {"CURE", 365, 54.67339313399745`},
{"CURE", 365, 56.48889477623597`}, {"CURE", 365, 16.862814794377`}, {"CURE", 365, 18.789081459138576`},
{"CURE", 365, 83.17432563830083`}, {"CURE", 365, 8.141674056139582`}, {"CURE", 365, 70.44891409163952`},
{"CURE", 365, 18.659224496247923`}, {"CURE", 365, 24.396851157556455`}, {"CURE", 365, 5.459823031148821`},
{"CURE", 365, 28.01987605606805`}, {"CURE", 365, 95.76850423064832`}, {"CURE", 365, 36.382299154226196`},
{"CURE", 365, 26.437133709811473`}, {"CURE", 365, 37.76343658167674`}, {"CURE", 365, 29.80596693039249`},
{"CURE", 365, 37.37824206432358`}, {"CURE", 365, 65.8805407175048`}, {"CURE", 365, 63.549914887385185`},
{"CURE", 365, 7.823800341079753`}, {"CURE", 365, 128.6654083109954`}, {"CURE", 365, 14.935080211375233`},
{"CURE", 365, 7.499984497448828`}, {"CURE", 365, 35.74526707421046`}, {"CURE", 365, 37.492787310917315`},
{"CURE", 365, 25.321311891164456`}, {"CURE", 365, 43.883580526201975`}, {"CURE", 365, 37.83123837056017`},
{"CURE", 365, 60.805332885265514`}, {"CURE", 365, 51.39184761267458`}, {"CURE", 365, 73.47965407657638`},
{"CURE", 365, 11.607907958568337`}, {"CURE", 365, 149.40124267008858`}, {"CURE", 365, 144.2639317311538`},
{"CURE", 365, 7.999074059078852`}, {"CURE", 365, 20.115105508208543`}, {"CURE", 365, 49.90952813315393`},
{"CURE", 365, 25.419154903925037`}, {"CURE", 365, 78.25011239218409`}, {"CURE", 365, 156.291145957156`},
{"CURE", 365, 49.38898941781665`}, {"CURE", 365, 8.486621481215913`}, {"CURE", 365, 8.321441013033741`},
{"CURE", 365, 19.525200982858944`}, {"CURE", 365, 5.897664704620232`}, {"CURE", 365, 66.90804721458571`},
{"CURE", 365, 10.379280844450417`}, {"CURE", 365, 70.03086028617642`}, {"CURE", 365, 42.990113478023645`},
{"CURE", 365, 74.06574259238656`}, {"CURE", 365, 18.240410196171478`}, {"CURE", 365, 92.02473112323389`},
{"TOX", 8.327816779508606`, 304.4591411561984`}, {"CURE", 365, 109.66102132365764`}, {"CURE", 365, 177.674589760491`},
{"CURE", 365, 52.27552605636357`}, {"CURE", 365, 124.54227711713692`}, {"CURE", 365, 124.80367175176191`},
{"CURE", 365, 15.145809367137543`}, {"CURE", 365, 10.595511470173662`}, {"CURE", 365, 34.47047411028924`},
{"CURE", 365, 71.4846414505275`}, {"CURE", 365, 89.44884294846389`}, {"CURE", 365, 38.76418455132329`},
{"CURE", 365, 56.67067074574929`}, {"CURE", 365, 10.679660979978015`}, {"CURE", 365, 13.572013173391166`},
{"CURE", 365, 65.88953927489902`}, {"CURE", 365, 21.38685139307508`}, {"CURE", 365, 9.057688644168403`},
{"CURE", 365, 13.392911490438005`}, {"CURE", 365, 6.549624717465688`}, {"CURE", 365, 53.2146619956758`},
{"CURE", 365, 73.16689008757459`}, {"CURE", 365, 51.23288244596723`}, {"CURE", 365, 58.164995529836375`},
{"CURE", 365, 32.23545967480674`}, {"CURE", 365, 28.89042948394739`}, {"CURE", 365, 74.6919247984138`},
{"CURE", 365, 79.89313310107433`}, {"CURE", 365, 8.917255786791097`}, {"CURE", 365, 34.43968372223227`},

{"CURE", 365, 17.724505020159388`}, {"CURE", 365, 27.814142650877326`}, {"CURE", 365, 229.17061685186735`},
{"CURE", 365, 40.46343318844236`}, {"CURE", 365, 27.768999787891982`}, {"CURE", 365, 29.40130704598362`},
{"CURE", 365, 58.597964795126224`}, {"CURE", 365, 44.603262459955666`}, {"CURE", 365, 58.18744511764032`},
{"CURE", 365, 60.1027412106937`}, {"CURE", 365, 13.611918291516952`}, {"CURE", 365, 132.34226281670186`},
{"CURE", 365, 25.43525698748124`}, {"CURE", 365, 53.91052729617573`}, {"CURE", 365, 30.59768439120724`},
{"CURE", 365, 17.367665519884977`}, {"CURE", 365, 63.317082933943894`}, {"CURE", 365, 64.27476793689564`},
{"TBUR", 104.1205198337638`, 35.75675383618591`}, {"CURE", 365, 84.30657621806115`}, {"CURE", 365, 17.833354926489477`},
{"CURE", 365, 148.9494265668183`}, {"CURE", 365, 57.2392649272617`}, {"CURE", 365, 121.10446537352438`},
{"CURE", 365, 4.691964982581021`}, {"CURE", 365, 59.60399444368169`}, {"CURE", 365, 68.01182360432102`},
{"CURE", 365, 16.123154623159998`}, {"CURE", 365, 40.95391838317199`}, {"CURE", 365, 61.31429566369168`},
{"CURE", 365, 37.27672541343407`}, {"CURE", 365, 11.868270226790488`}, {"CURE", 365, 9.002572691081966`},
{"CURE", 365, 53.259452050606114`}, {"CURE", 365, 14.198934300495253`}, {"CURE", 365, 31.578193619287273`},
{"CURE", 365, 38.53771109323509`}, {"CURE", 365, 17.974803737052103`}, {"CURE", 365, 33.026416408660694`},
{"CURE", 365, 8.979342894292126`}, {"CURE", 365, 37.0115722402275`}, {"CURE", 365, 5.982848691384186`},
{"CURE", 365, 10.700115693764849`}, {"CURE", 365, 25.318003293810946`}, {"CURE", 365, 8.161830042055811`},
{"CURE", 365, 60.63976289227351`}, {"CURE", 365, 88.99937299730324`}, {"CURE", 365, 90.42101045208454`},
{"CURE", 365, 75.22162736052607`}, {"CURE", 365, 142.01214771172414`}, {"CURE", 365, 11.492706241202368`},
{"CURE", 365, 51.23966631779251`}, {"CURE", 365, 55.1565051377188`}, {"CURE", 365, 94.57102586508081`},
{"CURE", 365, 70.63677836874734`}, {"CURE", 365, 32.3121751311864`}, {"CURE", 365, 151.9595508161725`},
{"CURE", 365, 4.7419266550029455`}, {"CURE", 365, 144.4155343415544`}, {"CURE", 365, 9.784634780075518`},
{"CURE", 365, 123.35133146039136`}, {"CURE", 365, 111.70632179708875`}, {"CURE", 365, 33.12330149430635`},
{"CURE", 365, 130.44084013438388`}, {"CURE", 365, 159.04937681929727`}, {"CURE", 365, 71.17161016620221`},
{"CURE", 365, 31.390514227818553`}, {"CURE", 365, 36.09514347535354`}, {"CURE", 365, 53.312288708896745`},
{"CURE", 365, 49.17575288592415`}, {"CURE", 365, 11.914075928877189`}, {"CURE", 365, 42.78835337361575`},
{"CURE", 365, 40.52184811106463`}, {"CURE", 365, 30.284257300508123`}, {"CURE", 365, 74.77972978241178`},
{"CURE", 365, 11.775790852077002`}, {"CURE", 365, 83.48155441756855`}, {"CURE", 365, 54.637944307812425`},
{"CURE", 365, 30.92502861264077`}, {"CURE", 365, 28.37485468812483`}, {"CURE", 365, 57.170396270183026`},
{"CURE", 365, 7.815282186573688`}, {"CURE", 365, 90.5255479907834`}, {"CURE", 365, 14.300473935874829`},
{"CURE", 365, 20.272206448396936`}, {"CURE", 365, 45.501388675673525`}, {"CURE", 365, 35.663749661231606`},
{"CURE", 365, 32.79447382789704`}, {"CURE", 365, 102.1656965136977`}, {"CURE", 365, 13.812320791221191`},
{"CURE", 365, 24.051738924256586`}, {"CURE", 365, 177.86656859291458`}, {"CURE", 365, 13.49818079537271`},

{"CURE", 365, 34.74849903742261`}, {"CURE", 365, 52.92220954837891`}, {"CURE", 365, 52.25183771111593`},
{"CURE", 365, 35.45552071605223`}, {"CURE", 365, 183.23447495198766`}, {"CURE", 365, 33.127053181072526`},
{"CURE", 365, 13.943221417042508`}, {"CURE", 365, 27.908070729708086`}, {"CURE", 365, 21.470992729672574`},
{"CURE", 365, 16.46381068533912`}, {"CURE", 365, 43.15510443470107`}, {"CURE", 365, 73.89508038476328`},
{"CURE", 365, 40.66493261216909`}, {"CURE", 365, 47.40954291935521`}, {"CURE", 365, 103.7745390481328`},
{"CURE", 365, 11.454757409051414`}, {"CURE", 365, 13.477603869114327`}, {"CURE", 365, 113.63056844477302`},
{"CURE", 365, 36.655687589997264`}, {"CURE", 365, 39.61061630103854`}, {"CURE", 365, 19.915830148386707`},
{"CURE", 365, 45.59837648805652`}, {"CURE", 365, 56.82624168996225`}, {"CURE", 365, 131.7394491234454`},
{"CURE", 365, 19.416682728191695`}, {"CURE", 365, 75.98251641762445`}, {"CURE", 365, 44.684639026093315`},
{"CURE", 365, 118.28855892907562`}, {"CURE", 365, 192.99137090975003`}, {"CURE", 365, 70.74743802726428`},
{"CURE", 365, 6.845963048264962`}, {"CURE", 365, 53.55052673897792`}, {"CURE", 365, 16.929245236150546`},
{"CURE", 365, 178.78714338014387`}, {"TBUR", 64.31700599404827`, 5.7654892086348255`}, {"CURE", 365, 5.9705682489424365`},
{"CURE", 365, 207.293612865604`}, {"CURE", 365, 47.7444510731628`}, {"CURE", 365, 16.107251136339638`},
{"CURE", 365, 30.82780445475895`}, {"CURE", 365, 10.824594161525383`}, {"CURE", 365, 10.315156947056577`},
{"CURE", 365, 12.29812693664771`}, {"CURE", 365, 44.45611503060693`}, {"CURE", 365, 94.2265166839465`},
{"CURE", 365, 37.344806903090145`}, {"CURE", 365, 9.35099779212546`}, {"CURE", 365, 127.12377706641085`},
{"TOX", 7.574477648355335`, 254.39371414009904`}, {"CURE", 365, 7.166075193877662`}, {"CURE", 365, 57.33205237164447`},
{"CURE", 365, 30.76093658751406`}, {"CURE", 365, 6.0491583040944175`}, {"CURE", 365, 47.313206525702505`},
{"CURE", 365, 30.212629644131194`}, {"CURE", 365, 23.200773249247522`}, {"CURE", 365, 45.10606350613319`},
{"CURE", 365, 25.60556466538193`}, {"CURE", 365, 75.66638756040945`}, {"CURE", 365, 44.76013054062504`},
{"CURE", 365, 105.41070278366868`}, {"CURE", 365, 46.70608304978972`}, {"CURE", 365, 23.15363323530053`},
{"CURE", 365, 11.267260662885183`}, {"CURE", 365, 7.8012771277180075`}, {"CURE", 365, 22.220157482580127`},
{"CURE", 365, 88.46867491890949`}, {"CURE", 365, 29.16582519595583`}, {"CURE", 365, 26.745608247320035`},
{"CURE", 365, 18.907879916647918`}, {"CURE", 365, 19.15806007765843`}, {"CURE", 365, 51.76849108780817`},
{"CURE", 365, 9.683334656081696`}, {"CURE", 365, 61.717291803890824`}, {"CURE", 365, 28.21967077819589`},
{"CURE", 365, 16.633590827970526`}, {"CURE", 365, 26.31052104744625`}, {"CURE", 365, 58.316201891839306`},
{"CURE", 365, 43.720149021239926`}, {"CURE", 365, 5.244063217785765`}, {"CURE", 365, 22.610452891717546`},
{"TOX", 4.032217973235975`, 377.63787125373534`}, {"CURE", 365, 62.851140794253766`}, {"CURE", 365, 91.54455385437264`},
{"CURE", 365, 26.983564303199472`}, {"CURE", 365, 100.0063784538343`}, {"CURE", 365, 11.96218856582457`},
{"CURE", 365, 40.347240381886124`}, {"CURE", 365, 186.0711073188145`}, {"CURE", 365, 34.830071556412676`},
{"CURE", 365, 30.80720212104974`}, {"CURE", 365, 52.58334927141516`}, {"CURE", 365, 27.90597161153885`},

```
{"CURE", 365, 6.178582376509427`}, {"CURE", 365, 57.48155333385253`}, {"CURE", 365, 10.249390171720165`},
{"CURE", 365, 24.42149633308349`}, {"CURE", 365, 57.50964096927451`}, {"CURE", 365, 20.656252084288145`},
{"CURE", 365, 109.68731388537383`}, {"CURE", 365, 32.6419204696695 6`}, {"CURE", 365, 56.141249622735664`},
{"CURE", 365, 38.91704352653898`}, {"CURE", 365, 11.012188180658704`}, {"CURE", 365, 40.90418620457739`},
{"CURE", 365, 88.30011250166642`}, {"CURE", 365, 29.600152426299257`}, {"CURE", 365, 22.272869216704976`},
{"CURE", 365, 10.571724001513418`}, {"CURE", 365, 33.91448738697751`}, {"CURE", 365, 40.2791041265877`},
{"CURE", 365, 114.74799243335775`}, {"CURE", 365, 18.435896197018074`}, {"CURE", 365, 27.787865229632157`},
{"CURE", 365, 107.7340165198880 3`}, {"CURE", 365, 11.350369952214994`}, {"CURE", 365, 156.02925514731407`},
{"CURE", 365, 13.168411905502476`}, {"CURE", 365, 24.13914586227425`}, {"CURE", 365, 55.58949838583647`},
{"CURE", 365, 89.7395450391533`}, {"CURE", 365, 40.27389554953445`}, {"CURE", 365, 21.140668749354916`},
{"CURE", 365, 6.20626915470403`}, {"CURE", 365, 54.47734190670819`}, {"CURE", 365, 49.27230328379527`},
{"CURE", 365, 48.36091952655859`}, {"CURE", 365, 19.068740596247196`}, {"CURE", 365, 46.7383417444237`},
{"CURE", 365, 5.413010754648071`}, {"CURE", 365, 31.183322501057052`}, {"CURE", 365, 13.612302042950056`},
{"CURE", 365, 6.032703674482828`}, {"CURE", 365, 31.337926556030894`}, {"CURE", 365, 6.702839870170125`},
{"CURE", 365, 21.314683192551893`}, {"CURE", 365, 14.623313614797055`}, {"TBUR", 63.404045355397926`, 9.088333909541559`},
{"CURE", 365, 28.96579832849162`}, {"CURE", 365, 77.14020197918445`}, {"CURE", 365, 42.34031772801003`},
{"CURE", 365, 38.254478225292495`}, {"CURE", 365, 60.955668954826095`}, {"CURE", 365, 51.603442507556416`}}};
```

In[ ]:=

```
TestConstantDose = {{"MTD Res", "MTD OS", "MTD Tox"}, {"CURE", 365, 15.210561548769954`},
  {"CURE", 365, 31.526395693712264`}, {"CURE", 365, 22.07752362804874`}, {"TBUR", 62.01513552454614`, 7.375892622622398`},
  {"TBUR", 42.50546893395557`, 27.63347190013832`}, {"CURE", 365, 57.10490175029676`}, {"CURE", 365, 53.731542851160214`},
  {"CURE", 365, 5.613576376406317`}, {"CURE", 365, 38.75038197002379`}, {"CURE", 365, 33.508209519391315`},
  {"CURE", 365, 125.76934578833927`}, {"CURE", 365, 15.60834232545655`}, {"CURE", 365, 55.44149344372122`},
  {"CURE", 365, 186.91455498824277`}, {"CURE", 365, 26.518302031200363`}, {"CURE", 365, 41.386393740354535`},
  {"CURE", 365, 82.01801902315916`}, {"CURE", 365, 36.721295594927625`}, {"TBUR", 113.22714649011873`, 27.716675761020905`},
  {"CURE", 365, 9.20832677239148`}, {"CURE", 365, 56.923979515298086`}, {"CURE", 365, 23.075559050564323`},
  {"CURE", 365, 8.840889255523143`}, {"CURE", 365, 7.044142125413225`}, {"CURE", 365, 32.05810444040313`},
  {"CURE", 365, 35.18453074191822`}, {"CURE", 365, 3.1986102702254606`}, {"CURE", 365, 4.848081259580153`},
  {"CURE", 365, 17.9632018040884`}, {"CURE", 365, 21.010105185459498`}, {"CURE", 365, 17.929383319782094`},
  {"CURE", 365, 62.93148635640521`}, {"CURE", 365, 13.85986653390077`}, {"CURE", 365, 11.0126981168015`},
  {"CURE", 365, 89.35236411244992`}, {"CURE", 365, 5.47245037845853`}, {"CURE", 365, 23.666022136739723`},
```

{"TBUR", 41.74927404140381`, 52.628743148580675`}, {"CURE", 365, 13.143451475224593`},
{"CURE", 365, 20.217636053127073`}, {"CURE", 365, 46.08847604603`}, {"CURE", 365, 31.159270995604622`},
{"CURE", 365, 6.321210075980161`}, {"CURE", 365, 21.315787218525397`}, {"CURE", 365, 30.76241362349144`},
{"CURE", 365, 20.173668124988357`}, {"CURE", 365, 214.33891062673231`}, {"CURE", 365, 46.20135843972243`},
{"CURE", 365, 70.0110131031643`}, {"CURE", 365, 8.638491966145454`}, {"CURE", 365, 23.879512303663983`},
{"CURE", 365, 32.94253910799946`}, {"CURE", 365, 26.576585500182304`}, {"CURE", 365, 17.253148851184108`},
{"CURE", 365, 7.43549902874229`}, {"CURE", 365, 78.66526278358926`}, {"CURE", 365, 24.721074757221363`},
{"CURE", 365, 4.691564610987446`}, {"CURE", 365, 50.56729585823729`}, {"CURE", 365, 35.39842344700272`},
{"CURE", 365, 24.258789856493596`}, {"CURE", 365, 18.572875510881833`}, {"CURE", 365, 10.461123524501199`},
{"CURE", 365, 40.03084212098693`}, {"CURE", 365, 28.42433669445916`}, {"CURE", 365, 32.52062873362034`},
{"CURE", 365, 7.443748511736405`}, {"CURE", 365, 7.55862256791846`}, {"CURE", 365, 8.048099020337276`},
{"CURE", 365, 32.86589373838746`}, {"CURE", 365, 12.031256865265588`}, {"CURE", 365, 26.79580019092096`},
{"CURE", 365, 50.29289024248331`}, {"TBUR", 23.395837597109637`, 14.99009681407572`}, {"CURE", 365, 13.435941872637704`},
{"CURE", 365, 37.23156478795063`}, {"CURE", 365, 21.766062846923596`}, {"CURE", 365, 16.56080648670261`},
{"CURE", 365, 65.95904637600637`}, {"CURE", 365, 72.44755646299892`}, {"CURE", 365, 53.33644913129737`},
{"CURE", 365, 14.39227756437501`}, {"CURE", 365, 29.100132526689162`}, {"CURE", 365, 20.339998526373666`},
{"CURE", 365, 22.99799229983218`}, {"CURE", 365, 10.775138111520212`}, {"CURE", 365, 114.93973975071444`},
{"CURE", 365, 25.048603151561238`}, {"CURE", 365, 31.557940236561972`}, {"CURE", 365, 27.1471313977765`},
{"CURE", 365, 22.378505187982977`}, {"CURE", 365, 6.653158025044022`}, {"CURE", 365, 12.195784370550752`},
{"CURE", 365, 28.231435477890866`}, {"TBUR", 49.274670404927555`, 10.163124576974933`},
{"CURE", 365, 12.153683748178713`}, {"CURE", 365, 31.710611122067505`}, {"CURE", 365, 80.47874307228365`},
{"CURE", 365, 43.09214828669824`}, {"CURE", 365, 21.612710984420534`}, {"TBUR", 65.82533698467066`, 42.78547476878576`},
{"CURE", 365, 27.86658418563191`}, {"CURE", 365, 18.063735204886246`}, {"CURE", 365, 123.68772749583081`},
{"CURE", 365, 13.741734521404613`}, {"CURE", 365, 9.33662512228513`}, {"CURE", 365, 54.786648286205306`},
{"CURE", 365, 16.136195182413577`}, {"CURE", 365, 16.534371295190493`}, {"CURE", 365, 99.48023887380658`},
{"CURE", 365, 8.074079725513558`}, {"CURE", 365, 5.121870929505919`}, {"CURE", 365, 21.306838792061118`},
{"CURE", 365, 16.844314215905502`}, {"CURE", 365, 12.686280010381983`}, {"CURE", 365, 32.38851156058404`},
{"CURE", 365, 9.113910458811127`}, {"CURE", 365, 5.604108392540221`}, {"CURE", 365, 26.23803283415052`},
{"CURE", 365, 40.12397601757817`}, {"CURE", 365, 6.830508543571534`}, {"CURE", 365, 22.613585947546433`},
{"CURE", 365, 68.65303301137291`}, {"TBUR", 58.06267042712447`, 26.10687242948653`}, {"CURE", 365, 15.228819159340372`},
{"CURE", 365, 53.107424309943454`}, {"CURE", 365, 26.68827676193899`}, {"CURE", 365, 3.5186683171305773`},

{"CURE", 365, 30.488942800713215`}, {"CURE", 365, 23.547607150721337`}, {"CURE", 365, 18.758620158139667`},
{"CURE", 365, 22.248095716346977`}, {"CURE", 365, 5.788601629721769`}, {"CURE", 365, 33.021481845113016`},
{"CURE", 365, 11.857687061753845`}, {"CURE", 365, 26.737724111553984`}, {"CURE", 365, 100.0994496460469`},
{"CURE", 365, 35.33995181319404`}, {"CURE", 365, 82.19290532688645`}, {"CURE", 365, 27.08040448108791`},
{"CURE", 365, 111.74129755140369`}, {"CURE", 365, 3.4890675800161604`}, {"CURE", 365, 30.655285060417494`},
{"CURE", 365, 34.94107537089953`}, {"CURE", 365, 4.5384940296273175`}, {"CURE", 365, 6.286451452318628`},
{"CURE", 365, 38.59103103955561`}, {"CURE", 365, 45.42950908297951`}, {"CURE", 365, 38.75831380919812`},
{"CURE", 365, 31.708424692879255`}, {"CURE", 365, 30.318173921726512`}, {"CURE", 365, 18.354110194768033`},
{"CURE", 365, 4.8552274823986785`}, {"CURE", 365, 21.10409016161034`}, {"CURE", 365, 10.014948707950055`},
{"CURE", 365, 4.9023540273700235`}, {"CURE", 365, 29.725538269566105`}, {"CURE", 365, 16.636807174116825`},
{"CURE", 365, 43.97882571625701`}, {"CURE", 365, 17.752788006976832`}, {"CURE", 365, 14.776774994686233`},
{"CURE", 365, 98.99808501738514`}, {"CURE", 365, 7.3834254331572575`}, {"CURE", 365, 22.073310410031212`},
{"CURE", 365, 12.513704598334774`}, {"CURE", 365, 29.539294533332413`}, {"CURE", 365, 8.126459539848183`},
{"CURE", 365, 32.453925610192854`}, {"CURE", 365, 85.67590793351798`}, {"CURE", 365, 7.331273704085365`},
{"CURE", 365, 22.277139090475906`}, {"CURE", 365, 24.015757753216498`}, {"CURE", 365, 86.63861057736925`},
{"TBUR", 95.637524431116`, 32.48726165529206`}, {"CURE", 365, 27.03149394763994`}, {"CURE", 365, 47.349807599441064`},
{"CURE", 365, 18.213835328794897`}, {"CURE", 365, 55.21896752912685`}, {"CURE", 365, 35.0011298792213`},
{"CURE", 365, 21.217603018720357`}, {"CURE", 365, 19.309237103907936`}, {"CURE", 365, 6.28919605514588`},
{"CURE", 365, 4.262240759859643`}, {"CURE", 365, 9.980679741004998`}, {"CURE", 365, 13.382721838031381`},
{"CURE", 365, 5.883778208318004`}, {"CURE", 365, 21.55685814418339`}, {"CURE", 365, 58.90242856233868`},
{"CURE", 365, 15.930935061910144`}, {"CURE", 365, 29.48915807392601`}, {"CURE", 365, 37.03022952200883`},
{"CURE", 365, 30.96087332273023`}, {"CURE", 365, 21.67286993286642`}, {"CURE", 365, 8.607889586437746`},
{"CURE", 365, 59.289588234284146`}, {"CURE", 365, 5.1314366769829265`}, {"CURE", 365, 55.28098356833441`},
{"CURE", 365, 53.52868549562665`}, {"CURE", 365, 14.993852162540286`}, {"CURE", 365, 17.810455720598423`},
{"CURE", 365, 102.28906523008803`}, {"CURE", 365, 26.331377524727362`}, {"CURE", 365, 31.508793000808815`},
{"CURE", 365, 19.718232908406826`}, {"CURE", 365, 7.4190691037782575`}, {"CURE", 365, 45.444059669295434`},
{"TBUR", 50.487965036965676`, 120.687850925023`}, {"CURE", 365, 18.477599007662242`}, {"CURE", 365, 16.69996366417339`},
{"CURE", 365, 30.775639955047282`}, {"CURE", 365, 12.288958597359672`}, {"CURE", 365, 155.92174038484768`},
{"CURE", 365, 12.481871955892219`}, {"CURE", 365, 77.08169458864991`}, {"CURE", 365, 59.28714615511867`},
{"CURE", 365, 57.62668267217162`}, {"CURE", 365, 30.788996948062316`}, {"CURE", 365, 71.41377204166486`},
{"CURE", 365, 130.79811502424636`}, {"CURE", 365, 32.44564891688502`}, {"CURE", 365, 5.9594462692889 7`},

{"CURE", 365, 52.94070495641816`}, {"CURE", 365, 32.99007220737944`}, {"CURE", 365, 31.799805609192166`},
{"CURE", 365, 7.003097392032606`}, {"CURE", 365, 160.70475935546273`}, {"CURE", 365, 11.747395046122863`},
{"CURE", 365, 50.96955801889979`}, {"CURE", 365, 32.12147633508227`}, {"CURE", 365, 22.83241289624049`},
{"CURE", 365, 5.489443508644746`}, {"CURE", 365, 41.26564326746882`}, {"CURE", 365, 24.205563914332856`},
{"CURE", 365, 64.04126607391221`}, {"CURE", 365, 42.19076804562427`}, {"CURE", 365, 53.25071981493031`},
{"CURE", 365, 25.55572232845524`}, {"CURE", 365, 22.7379315048907`}, {"TBUR", 56.21989647226661`, 118.79827900330264`},
{"CURE", 365, 31.14289212474762`}, {"CURE", 365, 8.307486252649749`}, {"CURE", 365, 27.9817905656976`},
{"TBUR", 114.59443072513793`, 28.325043444575154`}, {"CURE", 365, 3.808993433275031`}, {"CURE", 365, 6.800816987698817`},
{"CURE", 365, 10.387926607142612`}, {"CURE", 365, 98.00417693706481`}, {"CURE", 365, 30.164163557907173`},
{"CURE", 365, 31.442508968300846`}, {"CURE", 365, 25.17985369432542`}, {"CURE", 365, 103.37220149876516`},
{"CURE", 365, 23.842705486070127`}, {"CURE", 365, 47.858558557238986`}, {"CURE", 365, 23.771827152474135`},
{"CURE", 365, 35.04848985926758`}, {"CURE", 365, 8.42928000278868`}, {"CURE", 365, 31.524245223329228`},
{"CURE", 365, 19.668171839426396`}, {"CURE", 365, 38.32073199587956`}, {"CURE", 365, 60.54346955854773`},
{"CURE", 365, 67.8464473259862`}, {"CURE", 365, 40.95404668089398`}, {"CURE", 365, 33.766488298525154`},
{"CURE", 365, 46.21273548796791`}, {"CURE", 365, 22.108089067734937`}, {"CURE", 365, 25.030958760776468`},
{"CURE", 365, 17.724426400366895`}, {"CURE", 365, 13.897187079795165`}, {"CURE", 365, 22.383642698575873`},
{"CURE", 365, 57.44564821550332`}, {"CURE", 365, 6.5838688005141535`}, {"CURE", 365, 29.158781315168284`},
{"CURE", 365, 5.508942495728983`}, {"CURE", 365, 28.666289527388482`}, {"CURE", 365, 16.27380678989193`},
{"CURE", 365, 60.63829983839875`}, {"CURE", 365, 5.589750543298213`}, {"CURE", 365, 39.883969742827745`},
{"CURE", 365, 50.05924107366459`}, {"CURE", 365, 8.189333949766015`}, {"CURE", 365, 18.754028572586957`},
{"CURE", 365, 15.09294133906859`}, {"CURE", 365, 8.628464575915968`}, {"CURE", 365, 51.07434906503027`},
{"CURE", 365, 19.185548284025955`}, {"CURE", 365, 36.99089688781975`}, {"CURE", 365, 35.96524309249867`},
{"CURE", 365, 40.78596493223343`}, {"CURE", 365, 31.88539703954343`}, {"CURE", 365, 38.15503624331277`},
{"CURE", 365, 30.213147769414835`}, {"CURE", 365, 58.38851207452593`}, {"CURE", 365, 35.55712096013054`},
{"CURE", 365, 29.024338241332543`}, {"CURE", 365, 74.29938901485903`}, {"CURE", 365, 8.602495263112244`},
{"CURE", 365, 48.807404719249625`}, {"CURE", 365, 3.5855565509503755`}, {"CURE", 365, 44.53312856225348`},
{"CURE", 365, 10.497605640102018`}, {"CURE", 365, 54.94411692949642`}, {"CURE", 365, 8.569479143361281`},
{"CURE", 365, 56.14836719198234`}, {"CURE", 365, 140.45916647068728`}, {"CURE", 365, 6.132081863211975`},
{"CURE", 365, 4.4009657331077126`}, {"CURE", 365, 16.21986660898872`}, {"CURE", 365, 3.9361925232012163`},
{"CURE", 365, 2.6931645803817017`}, {"CURE", 365, 15.757032246538442`}, {"CURE", 365, 25.730624088579194`},
{"CURE", 365, 27.72347647819158`}, {"TBUR", 115.4292655976226`, 26.30852235589111`}, {"CURE", 365, 20.502159602294018`},

{"CURE", 365, 36.095330023289215`}, {"CURE", 365, 63.552327351354506`}, {"CURE", 365, 5.142296482869857`},
{"CURE", 365, 28.17990636887016`}, {"CURE", 365, 24.216864610322137`}, {"CURE", 365, 17.556919545161655`},
{"CURE", 365, 32.4627278515016`}, {"CURE", 365, 48.13384837270756`}, {"CURE", 365, 112.6407846285423б`},
{"CURE", 365, 83.88547541226245`}, {"CURE", 365, 25.91178573835663`}, {"CURE", 365, 2.7563612806705824`},
{"CURE", 365, 30.06549184638003`}, {"CURE", 365, 77.54587090974623`}, {"CURE", 365, 19.509353828020295`},
{"CURE", 365, 24.38525221291171`}, {"CURE", 365, 15.05257136221714`}, {"CURE", 365, 22.06534188394638`},
{"CURE", 365, 5.705919242006529`}, {"CURE", 365, 27.9436047641582`}, {"CURE", 365, 15.683007969620151`},
{"CURE", 365, 45.86757373907645`}, {"CURE", 365, 31.380305666085203`}, {"CURE", 365, 30.484778061773483`},
{"CURE", 365, 25.083266719321728`}, {"CURE", 365, 13.273852882680904`}, {"CURE", 365, 9.146843513938332`},
{"CURE", 365, 8.917900434806997`}, {"CURE", 365, 11.545545701881418`}, {"CURE", 365, 9.001089943860096`},
{"CURE", 365, 120.94222962122669`}, {"CURE", 365, 67.28385347096707`}, {"CURE", 365, 13.01156213853909`},
{"CURE", 365, 200.43715150907363`}, {"CURE", 365, 57.31952463350764`}, {"CURE", 365, 30.34730263175887`},
{"CURE", 365, 52.49352164509447`}, {"CURE", 365, 7.893101992354542`}, {"CURE", 365, 62.91241451065684`},
{"CURE", 365, 168.272649272526`}, {"CURE", 365, 9.128650709060842`}, {"CURE", 365, 28.479704993813755`},
{"CURE", 365, 46.602146145044294`}, {"CURE", 365, 23.498091170414067`}, {"CURE", 365, 11.55660009058909`},
{"CURE", 365, 9.207489132584568`}, {"CURE", 365, 79.90451506201674`}, {"CURE", 365, 41.9489191896147`},
{"CURE", 365, 23.869211643052004`}, {"CURE", 365, 59.28735822530528`}, {"CURE", 365, 10.697025347534202`},
{"CURE", 365, 28.611706245702727`}, {"CURE", 365, 17.279817838967688`}, {"CURE", 365, 116.56076083534008`},
{"TBUR", 57.60179578648953`, 29.240377580911385`}, {"CURE", 365, 135.76715435042382`}, {"CURE", 365, 211.97050312912117`},
{"CURE", 365, 16.265190311163213`}, {"CURE", 365, 4.585826277798649`}, {"CURE", 365, 3.9964567771898127`},
{"CURE", 365, 29.225965743901043`}, {"CURE", 365, 113.42692245028014`}, {"CURE", 365, 22.72199007593516`},
{"CURE", 365, 17.451812530612532`}, {"TBUR", 63.18188514812383`, 32.76070283306206`}, {"CURE", 365, 35.89556703368865`},
{"CURE", 365, 149.70343297137327`}, {"CURE", 365, 123.5267592580788`}, {"CURE", 365, 25.84709322273126`},
{"CURE", 365, 8.582914647099805`}, {"CURE", 365, 21.026954447409317`}, {"CURE", 365, 3.3003272801232773`},
{"CURE", 365, 61.96799644986186`}, {"CURE", 365, 92.32694533906347`}, {"CURE", 365, 30.194121444856734`},
{"CURE", 365, 23.928066996537492`}, {"CURE", 365, 101.18984331886183`}, {"CURE", 365, 6.408069426163062`},
{"CURE", 365, 8.347210022611476`}, {"CURE", 365, 26.167132288031745`}, {"CURE", 365, 33.62098595382028`},
{"TBUR", 68.51753421063128`, 63.54217524857744`}, {"CURE", 365, 65.65259581290888`}, {"CURE", 365, 26.950425602183742`},
{"CURE", 365, 27.779286053686388`}, {"CURE", 365, 34.71746172881802`}, {"CURE", 365, 12.023343129344077`},
{"CURE", 365, 3.403511983371907`}, {"CURE", 365, 35.791320926164964`}, {"CURE", 365, 43.115741314628316`},
{"CURE", 365, 20.96202884797612`}, {"CURE", 365, 37.9899696937188`}, {"CURE", 365, 11.827836979356203`},

{"CURE", 365, 7.775233099310377`}, {"CURE", 365, 34.039581844314014`}, {"CURE", 365, 20.65694341232148`},
{"CURE", 365, 29.567951848227622`}, {"CURE", 365, 195.17816230995675`}, {"CURE", 365, 18.589124092946665`},
{"CURE", 365, 9.05976256292158`}, {"CURE", 365, 7.036482200549118`}, {"CURE", 365, 8.529677907355113`},
{"CURE", 365, 48.72009577135727`}, {"CURE", 365, 3.356105268567127`}, {"CURE", 365, 45.3680443112054`},
{"CURE", 365, 49.542938857598394`}, {"CURE", 365, 38.66998479967061`}, {"CURE", 365, 31.70239836125447`},
{"CURE", 365, 6.280898235348955`}, {"CURE", 365, 68.99918695176832`}, {"TBUR", 57.80799112259705`, 20.827911915608272`},
{"CURE", 365, 9.361884652718404`}, {"CURE", 365, 61.439011553014275`}, {"CURE", 365, 10.929073242700905`},
{"CURE", 365, 73.60549049869505`}, {"CURE", 365, 47.24692659644876`}, {"CURE", 365, 31.47536128379474`},
{"CURE", 365, 45.785423041076186`}, {"CURE", 365, 16.207448849941592`}, {"CURE", 365, 71.81233526317482`},
{"CURE", 365, 8.521862518854595`}, {"CURE", 365, 36.0560423319086`}, {"CURE", 365, 42.2072504052745`},
{"CURE", 365, 17.945320900182278`}, {"CURE", 365, 167.22247315699104`}, {"CURE", 365, 33.46922367213915`},
{"CURE", 365, 34.22827756339945`}, {"CURE", 365, 34.95877279013516`}, {"CURE", 365, 140.04208203033983`},
{"CURE", 365, 44.487222636511696`}, {"CURE", 365, 41.505775394899466`}, {"CURE", 365, 90.31272166810975`},
{"CURE", 365, 31.771017375319946`}, {"CURE", 365, 27.67864810011505`}, {"CURE", 365, 34.21058956166587`},
{"CURE", 365, 47.254177180836905`}, {"CURE", 365, 83.50758335269128`}, {"CURE", 365, 18.015423449960693`},
{"CURE", 365, 16.448444789244633`}, {"CURE", 365, 35.199632641074345`}, {"CURE", 365, 35.83513584997267`},
{"CURE", 365, 40.25912027581017`}, {"CURE", 365, 43.09048223897889`}, {"CURE", 365, 24.073080014986544`},
{"CURE", 365, 12.098150769097087`}, {"CURE", 365, 45.44441082173214`}, {"CURE", 365, 11.489860774895687`},
{"CURE", 365, 24.42793807279627`}, {"CURE", 365, 29.040110528877392`}, {"CURE", 365, 8.764272206733786`},
{"CURE", 365, 4.355773605929377`}, {"TBUR", 97.49280119604799`, 26.85203828754719`}, {"CURE", 365, 36.456642138914475`},
{"CURE", 365, 43.29056552458055`}, {"CURE", 365, 30.96287208834992`}, {"CURE", 365, 3.96772582784457`},
{"CURE", 365, 35.52874380386567`}, {"CURE", 365, 6.765895617616395`}, {"CURE", 365, 104.44999849503863`},
{"CURE", 365, 135.89047077137718`}, {"CURE", 365, 19.025278664011207`}, {"TBUR", 76.8657977333707`, 26.81529442147545`},
{"CURE", 365, 145.8653308320932`}, {"CURE", 365, 26.159915297047718`}, {"CURE", 365, 15.165433161742618`},
{"CURE", 365, 11.815405755646731`}, {"CURE", 365, 3.2350434915099537`}, {"CURE", 365, 20.39698496479995`},
{"CURE", 365, 22.743112439303857`}, {"CURE", 365, 60.22489072928389`}, {"CURE", 365, 28.83827891315253`},
{"CURE", 365, 50.22039644562866`}, {"CURE", 365, 17.742095644623166`}, {"CURE", 365, 38.447208856292946`},
{"CURE", 365, 86.0535935187544`}, {"CURE", 365, 8.745203985710248`}, {"CURE", 365, 13.500471549781148`},
{"CURE", 365, 20.047030911695686`}, {"CURE", 365, 28.01055790360249`}, {"TBUR", 146.02642908923278`, 18.335748143862894`},
{"CURE", 365, 17.581946702979305`}, {"CURE", 365, 7.444780638287484`}, {"CURE", 365, 11.499800164717533`},
{"CURE", 365, 21.789433128011975`}, {"CURE", 365, 49.6115632332067`}, {"TBUR", 84.84155841827591`, 44.08700331677596`},

{"CURE", 365, 22.606781680153773`}, {"TBUR", 35.95260374378233`, 33.8253399865356`}, {"CURE", 365, 73.00029842765217`},
{"CURE", 365, 35.72296455740475`}, {"CURE", 365, 26.73168333607354`}, {"CURE", 365, 17.02728344760043`},
{"CURE", 365, 5.332313090804495`}, {"CURE", 365, 29.67974062487725`}, {"CURE", 365, 34.24782608789625`},
{"CURE", 365, 134.0975983298134`}, {"CURE", 365, 6.8352681049908535`}, {"CURE", 365, 6.680947010437565`},
{"CURE", 365, 21.406926860007403`}, {"CURE", 365, 62.402452811034884`}, {"CURE", 365, 7.785543873363599`},
{"CURE", 365, 38.090379807231656`}, {"CURE", 365, 8.881802231170825`}, {"CURE", 365, 22.131977428119402`},
{"CURE", 365, 8.997338550100155`}, {"CURE", 365, 9.387769188866935`}, {"CURE", 365, 8.46694524075249`},
{"CURE", 365, 90.34698052790894`}, {"CURE", 365, 14.409400808790972`}, {"TBUR", 80.80982004422401`, 25.38580544631476`},
{"CURE", 365, 3.509798907459082`}, {"CURE", 365, 42.046804247952934`}, {"CURE", 365, 58.84026029405357`},
{"CURE", 365, 51.26685025312149`}, {"CURE", 365, 7.742749379906367`}, {"CURE", 365, 15.533408490034121`},
{"CURE", 365, 157.98658517591073`}, {"CURE", 365, 42.93193856878427`}, {"CURE", 365, 29.234863945203816`},
{"CURE", 365, 10.371980871359264`}, {"CURE", 365, 14.876431249552434`}, {"CURE", 365, 22.982820049141726`},
{"CURE", 365, 5.689695748785952`}, {"CURE", 365, 27.95642508362146`}, {"CURE", 365, 43.99379916261593`},
{"CURE", 365, 26.748967656683664`}, {"CURE", 365, 29.03169160733212`}, {"CURE", 365, 21.021905554440483`},
{"CURE", 365, 13.605080099559435`}, {"CURE", 365, 77.71319180673991`}, {"CURE", 365, 112.76084957801554`},
{"CURE", 365, 30.084135594189817`}, {"CURE", 365, 37.98210407175666`}, {"CURE", 365, 48.11578318773988`},
{"CURE", 365, 47.038706282551956`}, {"CURE", 365, 4.104675258579959`}, {"CURE", 365, 54.989317071028914`},
{"CURE", 365, 56.87167723067157`}, {"CURE", 365, 38.415385417165986`}, {"CURE", 365, 154.26677688377328`},
{"CURE", 365, 32.070300287647434`}, {"CURE", 365, 14.250947281034458`}, {"CURE", 365, 33.74979402074801`},
{"CURE", 365, 42.89291073240286`}, {"CURE", 365, 19.21485967160521`}, {"CURE", 365, 15.062663798608245`},
{"CURE", 365, 13.114642177464003`}, {"CURE", 365, 23.746605463029667`}, {"CURE", 365, 73.28083703731608`},
{"CURE", 365, 47.032250258379996`}, {"CURE", 365, 8.181073785612815`}, {"CURE", 365, 47.86845973697343`},
{"CURE", 365, 69.22829690961055`}, {"CURE", 365, 12.711683144210363`}, {"CURE", 365, 72.60172580582464`},
{"CURE", 365, 10.912523499556507`}, {"CURE", 365, 6.96177326021846`}, {"CURE", 365, 9.887319439935036`},
{"CURE", 365, 19.011594766543087`}, {"TBUR", 80.0139056194057`, 84.95073800860203`}, {"CURE", 365, 30.446495572799208`},
{"CURE", 365, 38.4245042973501`}, {"CURE", 365, 9.190802150517172`}, {"CURE", 365, 175.18383218890585`},
{"CURE", 365, 80.33732463470902`}, {"CURE", 365, 13.689484422685391`}, {"CURE", 365, 33.54375736238338`},
{"CURE", 365, 18.698303578815885`}, {"CURE", 365, 18.880439896614604`}, {"CURE", 365, 63.78818391827578`},
{"TBUR", 54.94202757460631`, 80.55300010326339`}, {"CURE", 365, 8.670293412171217`}, {"CURE", 365, 16.889875374375322`},
{"CURE", 365, 5.9915156781682315`}, {"CURE", 365, 23.11262652962033`}, {"CURE", 365, 39.57409338224194`},
{"CURE", 365, 64.17490947006614`}, {"CURE", 365, 35.808875972924845`}, {"CURE", 365, 128.97246207005597`},

{"CURE", 365, 61.6483968151548`}, {"TBUR", 68.75876187944577`, 10.145328265165789`}, {"CURE", 365, 22.538624256317636`},
{"TBUR", 37.351642068626084`, 23.737734406616358`}, {"TBUR", 72.93858280119818`, 50.285882890552855`},
{"CURE", 365, 6.5965664306777105`}, {"CURE", 365, 17.692996896853465`}, {"CURE", 365, 9.965642618556307`},
{"CURE", 365, 24.873392562301966`}, {"CURE", 365, 56.82282143802722`}, {"CURE", 365, 24.176562830649253`},
{"CURE", 365, 5.251244189639986`}, {"CURE", 365, 99.96415552042576`}, {"CURE", 365, 18.86131062828312`},
{"CURE", 365, 6.407881189356162`}, {"CURE", 365, 8.423447039330659`}, {"CURE", 365, 7.1112387430777755`},
{"CURE", 365, 9.085886016205862`}, {"CURE", 365, 73.1141061583116`}, {"CURE", 365, 39.989269832471884`},
{"CURE", 365, 24.085443833132643`}, {"CURE", 365, 11.28395257573763`}, {"CURE", 365, 18.853666169088154`},
{"CURE", 365, 10.90784298515997`}, {"CURE", 365, 26.320107095812467`}, {"CURE", 365, 90.48397764171585`},
{"CURE", 365, 51.991224241889455`}, {"CURE", 365, 26.09428369691893`}, {"CURE", 365, 15.044384299338256`},
{"CURE", 365, 15.694411589534731`}, {"CURE", 365, 193.3778602635945`}, {"CURE", 365, 9.213814332367527`},
{"CURE", 365, 4.746508766866476`}, {"CURE", 365, 4.1310799498154935`}, {"CURE", 365, 52.56625106998021`},
{"CURE", 365, 30.769492176126093`}, {"CURE", 365, 34.151854373503944`}, {"CURE", 365, 50.75170997577344`},
{"CURE", 365, 83.70297825945376`}, {"CURE", 365, 86.6580179310476`}, {"CURE", 365, 33.024457334354615`},
{"CURE", 365, 14.259985827294907`}, {"CURE", 365, 16.53208719209052`}, {"CURE", 365, 32.86703809379291`},
{"CURE", 365, 18.707218444414842`}, {"TBUR", 25.272196611703137`, 7.194140844023779`}, {"CURE", 365, 29.375181864230473`},
{"CURE", 365, 182.97243610014115`}, {"CURE", 365, 18.853574540501324`}, {"CURE", 365, 6.777968005193573`},
{"CURE", 365, 64.29186547184102`}, {"CURE", 365, 16.07855113066641`}, {"CURE", 365, 46.19817234071419`},
{"CURE", 365, 222.44586362694574`}, {"CURE", 365, 102.71118626643842`}, {"CURE", 365, 33.34426444469916`},
{"CURE", 365, 27.944904142928845`}, {"CURE", 365, 11.907417047712993`}, {"CURE", 365, 3.398612996327049`},
{"CURE", 365, 13.290683511188957`}, {"CURE", 365, 38.99358557986683`}, {"CURE", 365, 21.616290675183617`},
{"CURE", 365, 68.97484765080087`}, {"CURE", 365, 27.33721029347651`}, {"CURE", 365, 27.922951486199217`},
{"CURE", 365, 7.754008996205316`}, {"CURE", 365, 21.96189404300115`}, {"CURE", 365, 22.539826855485348`},
{"CURE", 365, 5.876871846691833`}, {"CURE", 365, 18.691683264939833`}, {"CURE", 365, 11.151017098129953`},
{"TBUR", 86.43269752641925`, 17.64576811023718`}, {"CURE", 365, 36.849854630074574`}, {"CURE", 365, 18.49306492838079`},
{"CURE", 365, 4.291405618160649`}, {"CURE", 365, 29.562420185707936`}, {"CURE", 365, 3.307659255866784`},
{"CURE", 365, 9.704542145136946`}, {"CURE", 365, 28.80389059929571`}, {"CURE", 365, 33.2205230929955`},
{"CURE", 365, 6.603425834298351`}, {"CURE", 365, 19.442695999412294`}, {"CURE", 365, 25.16958331381663`},
{"CURE", 365, 127.16536183457264`}, {"CURE", 365, 29.245698077323578`}, {"CURE", 365, 8.243680965307863`},
{"CURE", 365, 89.89823731414111`}, {"CURE", 365, 8.818381842851267`}, {"CURE", 365, 33.266581296478414`},
{"CURE", 365, 34.42872907174285`}, {"CURE", 365, 10.276122348815624`}, {"CURE", 365, 11.439633316063798`},

{"CURE", 365, 50.68057135064747`}, {"CURE", 365, 4.948752582428316`}, {"CURE", 365, 42.943614311316`},
{"CURE", 365, 11.37203669534052`}, {"CURE", 365, 14.870722337740492`}, {"CURE", 365, 3.320750367260972`},
{"CURE", 365, 17.074338433759213`}, {"CURE", 365, 58.16752467157546`}, {"CURE", 365, 22.153297780916795`},
{"CURE", 365, 16.084618198681465`}, {"CURE", 365, 22.989690278462607`}, {"CURE", 365, 18.00317261150415`},
{"CURE", 365, 22.711700384446363`}, {"CURE", 365, 40.11667436984813`}, {"CURE", 365, 38.721850510514315`},
{"CURE", 365, 4.769383227130879`}, {"CURE", 365, 78.18858535992821`}, {"CURE", 365, 9.100984865729405`},
{"CURE", 365, 4.563384246299938`}, {"CURE", 365, 21.757826778878332`}, {"CURE", 365, 22.84603305303504`},
{"CURE", 365, 15.394440237358866`}, {"CURE", 365, 26.742354348139862`}, {"CURE", 365, 23.059989076416752`},
{"CURE", 365, 37.06672590958192`}, {"CURE", 365, 31.321107810524612`}, {"TBUR", 63.42508412623114`, 44.68821054068269`},
{"CURE", 365, 7.063486757452539`}, {"CURE", 365, 90.40221697514113`}, {"CURE", 365, 87.90324941873187`},
{"CURE", 365, 4.868652443139357`}, {"CURE", 365, 12.260878837533129`}, {"CURE", 365, 30.42157117084416`},
{"CURE", 365, 15.45457763902059`}, {"CURE", 365, 47.44866700757043`}, {"CURE", 365, 95.23227262477349`},
{"CURE", 365, 30.10156830959943`}, {"CURE", 365, 5.164450810641262`}, {"CURE", 365, 5.067218690532567`},
{"CURE", 365, 11.900621650031532`}, {"CURE", 365, 3.5922245498604295`}, {"CURE", 365, 40.77896890940016`},
{"CURE", 365, 6.3205158371325805`}, {"CURE", 365, 42.43668357065571`}, {"CURE", 365, 26.184994315674274`},
{"CURE", 365, 45.138733984192434`}, {"CURE", 365, 11.111853347437988`}, {"CURE", 365, 56.061538829549875`},
{"CURE", 365, 185.48197604484483`}, {"CURE", 365, 66.82900126130708`}, {"CURE", 365, 108.25065800990565`},
{"CURE", 365, 31.865740048984033`}, {"CURE", 365, 75.92066266774526`}, {"CURE", 365, 76.06594270286534`},
{"CURE", 365, 9.232706792232722`}, {"CURE", 365, 6.457700449408707`}, {"CURE", 365, 20.993188847855773`},
{"CURE", 365, 43.575370534614656`}, {"CURE", 365, 54.516736610561736`}, {"CURE", 365, 23.21551243136801`},
{"CURE", 365, 34.47274609839625`}, {"CURE", 365, 6.494931559694285`}, {"CURE", 365, 8.248281756241996`},
{"CURE", 365, 40.105219904187855`}, {"CURE", 365, 12.98459828979053`}, {"CURE", 365, 5.51901673224372`},
{"CURE", 365, 8.16460411012371`}, {"CURE", 365, 3.9906735293518683`}, {"CURE", 365, 32.43139084292039`},
{"CURE", 365, 44.604841083238874`}, {"CURE", 365, 31.185760033540515`}, {"CURE", 365, 35.4576144941071`},
{"CURE", 365, 19.62944479032715`}, {"CURE", 365, 17.560083022121745`}, {"CURE", 365, 45.477351467768926`},
{"CURE", 365, 48.692778977227036`}, {"CURE", 365, 5.432195831579261`}, {"CURE", 365, 20.09476562663583`},
{"CURE", 365, 10.792243340075823`}, {"CURE", 365, 16.954657299507563`}, {"CURE", 365, 139.63256448185214`},
{"CURE", 365, 24.657491362158616`}, {"CURE", 365, 16.926140465173162`}, {"CURE", 365, 17.8871769354121`},
{"CURE", 365, 35.70721066531645`}, {"CURE", 365, 27.18035611302727`}, {"CURE", 365, 35.4581577631552`},
{"CURE", 365, 36.48460330812107`}, {"CURE", 365, 8.29266108416739`}, {"CURE", 365, 80.65850011097659`},
{"CURE", 365, 15.480453633460998`}, {"CURE", 365, 32.849710318447634`}, {"CURE", 365, 18.636967548810343`},

{"CURE", 365, 10.586463855599742`}, {"CURE", 365, 38.59202993926946`}, {"CURE", 365, 39.18092786179578`},
{"TBUR", 68.60257495852203`, 21.669506416963042`}, {"CURE", 365, 51.343587399346085`}, {"CURE", 365, 10.861072323862698`},
{"CURE", 365, 90.80595816751106`}, {"CURE", 365, 34.85601163595224`}, {"CURE", 365, 73.6489590571669`},
{"CURE", 365, 2.8595573429683636`}, {"CURE", 365, 36.219951343156076`}, {"CURE", 365, 41.225928553287446`},
{"CURE", 365, 9.82765766820234`}, {"CURE", 365, 24.94273101498595`}, {"CURE", 365, 37.36219764592829`},
{"CURE", 365, 22.705423147928578`}, {"CURE", 365, 7.222474863283269`}, {"CURE", 365, 5.482935951528946`},
{"CURE", 365, 32.401102526030385`}, {"CURE", 365, 8.64930388261075`}, {"CURE", 365, 19.225298485978335`},
{"CURE", 365, 23.415729367815075`}, {"CURE", 365, 10.884177583601977`}, {"CURE", 365, 20.039903356190006`},
{"CURE", 365, 5.4668801214864775`}, {"CURE", 365, 22.541141161206053`}, {"CURE", 365, 3.64464132569011`},
{"CURE", 365, 6.514059413300289`}, {"CURE", 365, 15.395668129569646`}, {"CURE", 365, 4.96497461260704`},
{"CURE", 365, 36.900549684023375`}, {"CURE", 365, 54.248348669622665`}, {"CURE", 365, 55.01208933445623`},
{"CURE", 365, 45.845511526515494`}, {"CURE", 365, 86.52407328721551`}, {"CURE", 365, 6.996873830617309`},
{"CURE", 365, 31.21031578859957`}, {"CURE", 365, 33.47732003494078`}, {"CURE", 365, 57.64223462501194`},
{"CURE", 365, 43.05466086149769`}, {"CURE", 365, 19.663298174471613`}, {"CURE", 365, 92.42931158090575`},
{"CURE", 365, 2.887250808543559`}, {"CURE", 365, 88.04049070760922`}, {"CURE", 365, 5.959335705783929`},
{"CURE", 365, 75.19074840480583`}, {"CURE", 365, 68.09989179605768`}, {"CURE", 365, 20.180628981531576`},
{"CURE", 365, 79.4546550565459`}, {"CURE", 365, 96.95132974678776`}, {"CURE", 365, 43.346895793325416`},
{"CURE", 365, 19.13522825489412`}, {"CURE", 365, 21.998532548783892`}, {"CURE", 365, 32.4813584688702`},
{"CURE", 365, 29.94935065998836`}, {"CURE", 365, 7.259965591567997`}, {"CURE", 365, 26.058918124103556`},
{"CURE", 365, 24.643805780650965`}, {"CURE", 365, 18.438854027881305`}, {"CURE", 365, 45.57771687180028`},
{"CURE", 365, 7.1768273102551845`}, {"CURE", 365, 50.86921416863323`}, {"CURE", 365, 33.282055688100854`},
{"CURE", 365, 18.848820086731944`}, {"CURE", 365, 17.035039938049923`}, {"CURE", 365, 34.71695244078258`},
{"CURE", 365, 4.763437622144406`}, {"CURE", 365, 55.171314900001335`}, {"CURE", 365, 8.705503045713861`},
{"CURE", 365, 12.353842030160898`}, {"CURE", 365, 27.703104682420488`}, {"CURE", 365, 21.724024476596885`},
{"CURE", 365, 19.97771919758889`}, {"CURE", 365, 62.16089728629134`}, {"CURE", 365, 8.41328862262899`},
{"CURE", 365, 14.648192778933907`}, {"CURE", 365, 108.39241888147244`}, {"CURE", 365, 8.22327432357258`},
{"CURE", 365, 21.181606210995167`}, {"CURE", 365, 32.20617214859665`}, {"CURE", 365, 31.800600282587236`},
{"CURE", 365, 21.604360945671328`}, {"CURE", 365, 111.70592718865792`}, {"CURE", 365, 20.162323648927988`},
{"CURE", 365, 8.48838000631926`}, {"CURE", 365, 16.902313020569483`}, {"CURE", 365, 13.082970207533348`},
{"CURE", 365, 10.021254584095612`}, {"CURE", 365, 26.29119285201459`}, {"CURE", 365, 45.03352823826704`},
{"CURE", 365, 24.774752841177545`}, {"CURE", 365, 28.88273159992022`}, {"CURE", 365, 63.25559285160834`},

{"CURE", 365, 6.969919502754947`}, {"CURE", 365, 8.202080950164682`}, {"CURE", 365, 69.2424556452336`},
{"CURE", 365, 22.266805766602193`}, {"CURE", 365, 24.14728799391516`}, {"CURE", 365, 12.137098391881759`},
{"CURE", 365, 27.794284336452808`}, {"CURE", 365, 34.63827452177241`}, {"CURE", 365, 80.26368485930713`},
{"CURE", 365, 11.831736287243432`}, {"CURE", 365, 46.18288882004451`}, {"CURE", 365, 27.178036918485603`},
{"CURE", 365, 71.95906948001264`}, {"CURE", 365, 117.6225965814468`}, {"CURE", 365, 43.027577905666895`},
{"CURE", 365, 4.168308140193079`}, {"CURE", 365, 32.63396566515909`}, {"CURE", 365, 10.300092675909186`},
{"CURE", 365, 108.99032047898307`}, {"TBUR", 40.72239933753562`, 3.3865274209555163`}, {"CURE", 365, 3.6368706247288527`},
{"CURE", 365, 126.01493403302635`}, {"CURE", 365, 28.964383143573492`}, {"CURE", 365, 9.817441076995395`},
{"CURE", 365, 18.790665007553777`}, {"CURE", 365, 6.590429856397076`}, {"TBUR", 65.74946125678426`, 6.247909981849715`},
{"CURE", 365, 7.496541977656421`}, {"CURE", 365, 27.096162524557187`}, {"CURE", 365, 57.42845677464963`},
{"CURE", 365, 22.74055362585225`}, {"CURE", 365, 5.694556577847451`}, {"CURE", 365, 77.48362418225892`},
{"CURE", 365, 154.7307293786171`}, {"CURE", 365, 4.366672462946068`}, {"CURE", 365, 34.8739700546116`},
{"CURE", 365, 18.748510853486014`}, {"CURE", 365, 3.6870603737239738`}, {"CURE", 365, 28.814608636140644`},
{"CURE", 365, 18.416678418509225`}, {"CURE", 365, 14.134041857436324`}, {"CURE", 365, 27.49229877088486`},
{"CURE", 365, 15.605389197713423`}, {"CURE", 365, 45.7956261885951`}, {"CURE", 365, 27.27480474902103`},
{"CURE", 365, 64.21400533223739`}, {"CURE", 365, 28.444773387600428`}, {"CURE", 365, 14.090539546938466`},
{"CURE", 365, 6.849277410956974`}, {"CURE", 365, 4.755322284425117`}, {"CURE", 365, 13.54528185170931`},
{"CURE", 365, 53.89044025652488`}, {"CURE", 365, 17.77530158187598`}, {"CURE", 365, 16.277858345867713`},
{"CURE", 365, 11.51272287863541`}, {"CURE", 365, 11.670536177202335`}, {"CURE", 365, 31.53636129205189`},
{"CURE", 365, 5.901120222100183`}, {"CURE", 365, 37.58233718987743`}, {"CURE", 365, 17.202024696689676`},
{"CURE", 365, 10.099825584539445`}, {"CURE", 365, 16.013413155566038`}, {"CURE", 365, 35.55001681825244`},
{"CURE", 365, 26.4186699263175`}, {"CURE", 365, 3.1959940315537105`}, {"CURE", 365, 13.76229875218535`},
{"CURE", 365, 230.17180783145255`}, {"CURE", 365, 38.20231669706069`}, {"CURE", 365, 55.7467115710049`},
{"CURE", 365, 16.425997439438127`}, {"CURE", 365, 60.89626170916364`}, {"CURE", 365, 7.288097894288052`},
{"CURE", 365, 24.54431733997539`}, {"CURE", 365, 113.36427503054375`}, {"CURE", 365, 21.232786279893656`},
{"CURE", 365, 18.659668969744338`}, {"CURE", 365, 32.05448559231021`}, {"CURE", 365, 16.94681056036861`},
{"CURE", 365, 3.764686669233449`}, {"CURE", 365, 34.96399449834984`}, {"CURE", 365, 6.241852762919949`},
{"CURE", 365, 14.816445640402955`}, {"CURE", 365, 35.04947340828565`}, {"CURE", 365, 12.587094657911111`},
{"CURE", 365, 66.85878150325385`}, {"CURE", 365, 19.868009035454715`}, {"CURE", 365, 34.22338992012267`},
{"CURE", 365, 23.720629285634285`}, {"CURE", 365, 6.712932779049423`}, {"CURE", 365, 24.88829281661677`},
{"CURE", 365, 53.8211491284014`}, {"CURE", 365, 18.037424502777153`}, {"CURE", 365, 13.575849055099322`},

```
{"CURE", 365, 6.43770470813249`}, {"CURE", 365, 20.385158928972945`}, {"CURE", 365, 24.47899569392604`},
{"CURE", 365, 69.94431064562599`}, {"CURE", 365, 11.23552772866443`}, {"CURE", 365, 16.89117810654939`},
{"CURE", 365, 65.64380644641983`}, {"CURE", 365, 6.91672628963601`}, {"CURE", 365, 94.8469545188212`},
{"CURE", 365, 8.025784559640952`}, {"CURE", 365, 14.684315227916803`}, {"CURE", 365, 33.85793556501427`},
{"CURE", 365, 54.45188109071036`}, {"CURE", 365, 24.526822503927423`}, {"CURE", 365, 12.840029018580887`},
{"CURE", 365, 3.7784992594982594`}, {"TBUR", 66.98422000619843`, 33.00305219013285`}, {"CURE", 365, 29.954429790719324`},
{"CURE", 365, 29.467979502211378`}, {"CURE", 365, 11.61970404809846`}, {"CURE", 365, 28.486029896534184`},
{"CURE", 365, 3.2921626913105464`}, {"CURE", 365, 18.985045230119454`}, {"CURE", 365, 8.294126681446102`},
{"CURE", 365, 3.6715090431535464`}, {"CURE", 365, 19.08916609517501`}, {"CURE", 365, 4.086036305016201`},
{"CURE", 365, 12.992278943960839`}, {"CURE", 365, 8.908092657573638`}, {"TBUR", 37.559950661790545`, 5.280819670488001`},
{"CURE", 365, 17.643539039167987`}, {"CURE", 365, 47.02705498827553`}, {"CURE", 365, 25.71760431273683`},
{"CURE", 365, 23.310740021045536`}, {"CURE", 365, 36.97034111720024`}, {"CURE", 365, 31.44688649631935`}};
```

In[ ]:=

```
TestNumOpt = {{"NumOpt Res", "NumOpt OS", "NumOpt Tox", "NumOpt Acur"},
{"CURE", 365, 11.262308448509847`, 1358.9017170957736`}, {"CURE", 365, 0.5169934816070314`, 30.3675440652236`},
{"CURE", 365, 4.503352908939691`, 378.3853425203081`}, {"CURE", 365, 12.710223073882466`, 3123.6988642219208`},
{"CURE", 365, 66.44346333302205`, 4187.347522122973`}, {"CURE", 365, 1.7682429048516763`, 57.53208273566699`},
{"CURE", 365, 0.7686626610850422`, 26.380135010092808`}, {"CURE", 365, 0.20267360524737693`, 67.12699386866798`},
{"CURE", 365, 0.40529818291619396`, 19.413664747360265`}, {"CURE", 365, 6.286230676471531`, 344.7521342509133`},
{"CURE", 365, 19.133988839894126`, 280.5711505177873`}, {"CURE", 365, 0.3375131969283707`, 40.23196377789699`},
{"CURE", 365, 0.3235795158816863`, 11.55404717509055`}, {"CURE", 365, 152.03454613662663`, 1493.483654203616`},
{"CURE", 365, 9.06615766874718`, 631.0849113812225`}, {"CURE", 365, 19.779106115350377`, 879.141601325`},
{"CURE", 365, 1.0324482947126272`, 23.637947470945637`}, {"CURE", 365, 11.467896710281027`, 575.8026134512742`},
{"CURE", 365, 40.44628159684264`, 2655.02842745788`}, {"CURE", 365, 0.5331661563366434`, 107.6541956813907`},
{"CURE", 365, 2.0055420464591704`, 64.93733676117235`}, {"CURE", 365, 0.48089966211056534`, 38.802403894757575`},
{"CURE", 365, 1.1682516490496266`, 243.90247076115082`}, {"CURE", 365, 0.4162145897544651`, 110.47556598377516`},
{"CURE", 365, 0.2571736756715295`, 14.968753420372643`}, {"CURE", 365, 0.5432195693144362`, 28.883144442959377`},
{"CURE", 365, 0.06492346887297591`, 37.658818252269704`}, {"CURE", 365, 0.22915903226493775`, 87.69952762581045`},
{"CURE", 365, 2.1300674355431957`, 219.1963406286717`}, {"CURE", 365, 4.572961583963281`, 401.74079570715116`},
{"CURE", 365, 0.19502262199202633`, 20.443307356122514`}, {"CURE", 365, 39.50636762654783`, 1155.0688760101982`},
{"CURE", 365, 1.140291161503384`, 153.11969238945443`}, {"CURE", 365, 10.515627519342583`, 1747.055075045587`},
```

{"CURE", 365, 7.2823685064227615`, 150.9088630234329`}, {"CURE", 365, 0.20691531987241607`, 70.22456792872241`},
{"CURE", 365, 0.10796910865024963`, 8.49897881446788`}, {"CURE", 365, 137.23835315058133`, 4718.963629301181`},
{"CURE", 365, 4.85490361512274`, 681.3336169223076`}, {"CURE", 365, 0.697097325540217`, 63.775060952041365`},
{"CURE", 365, 39.37238242668741`, 1565.4233495175176`}, {"CURE", 365, 0.18252445870657305`, 10.941240423403732`},
{"CURE", 365, 0.4486249432642805`, 131.0664015282777`}, {"CURE", 365, 1.2605742648448814`, 109.97839572050614`},
{"CURE", 365, 1.4037515099223588`, 84.32238479361504`}, {"CURE", 365, 0.5574076290416683`, 51.442964951774336`},
{"CURE", 365, 9.512485394716718`, 83.37145795199443`}, {"CURE", 365, 3.6322982898871445`, 146.41022405072718`},
{"CURE", 365, 0.3127570853113713`, 9.019725592862386`}, {"CURE", 365, 0.2309590898798745`, 49.57391800418637`},
{"CURE", 365, 2.064680690096057`, 167.67899648167463`}, {"CURE", 365, 3.3447227070665453`, 192.06049202656192`},
{"CURE", 365, 2.963893265209053`, 209.28519685088855`}, {"CURE", 365, 0.4002838289940187`, 43.07266171936573`},
{"CURE", 365, 1.2130969152987419`, 301.75854050170415`}, {"CURE", 365, 1.9300516244244879`, 45.28857472160761`},
{"CURE", 365, 0.2538030456279162`, 19.127765782687018`}, {"CURE", 365, 2.5988824953010528`, 1018.2162155021305`},
{"CURE", 365, 8.151163671678116`, 297.5843917899356`}, {"CURE", 365, 2.2376784569294443`, 118.30383586685049`},
{"CURE", 365, 1.0283936250970946`, 78.10068996122509`}, {"CURE", 365, 0.6900364415758031`, 69.07791589143963`},
{"CURE", 365, 2.3662333909128455`, 426.68870941071106`}, {"CURE", 365, 1.4386583558585284`, 66.40182984618805`},
{"CURE", 365, 9.340036781357277`, 605.8822364582178`}, {"CURE", 365, 0.2890694242752465`, 16.445361452349868`},
{"CURE", 365, 0.21733422932489654`, 53.80267662313199`}, {"CURE", 365, 2.1152007504930146`, 519.003833646611`},
{"CURE", 365, 0.17959134861488152`, 41.497512876057`}, {"CURE", 365, 1.6044566089653842`, 91.23592483202093`},
{"CURE", 365, 1.0348405773829101`, 159.5598566903397`}, {"CURE", 365, 4.418841361395124`, 305.01589206144416`},
{"CURE", 365, 3.9104939716725045`, 144.09668764955808`}, {"CURE", 365, 63.08846152576158`, 6699.623485027649`},
{"CURE", 365, 3.5328279016395148`, 485.0537410719286`}, {"CURE", 365, 0.7311389387155368`, 36.76532025250913`},
{"CURE", 365, 3.293713900731684`, 279.67236777766175`}, {"CURE", 365, 0.3922524580940574`, 44.35870549480609`},
{"CURE", 365, 5.111037658200517`, 144.53476415694394`}, {"CURE", 365, 3.041227591417432`, 78.4249207413694`},
{"CURE", 365, 2.134369419408139`, 75.10812300515711`}, {"CURE", 365, 0.2319557500516319`, 30.07392552913352`},
{"CURE", 365, 0.6049247998063308`, 38.40454741033961`}, {"CURE", 365, 0.3712626386968593`, 33.91662828160966`},
{"CURE", 365, 0.8134617639551563`, 65.2046767903638`}, {"CURE", 365, 0.9249847253409209`, 158.93752163610057`},
{"CURE", 365, 35.51910680350309`, 568.4324904458653`}, {"CURE", 365, 3.0004252768687385`, 223.8711658698895`},
{"CURE", 365, 2.669374738526519`, 155.7185896147784`}, {"CURE", 365, 4.077454996928422`, 279.9548866662452`},
{"CURE", 365, 20.359370757976393`, 1667.0148950296127`}, {"CURE", 365, 0.8424589817122229`, 234.08443793532004`},
{"CURE", 365, 3.9342569970673047`, 592.8644704282113`}, {"CURE", 365, 5.864777716943824`, 384.1102605483269`},
{"CURE", 365, 16.101433100639234`, 2861.457405833577`}, {"CURE", 365, 3.919294918375409`, 594.9833613292074`},

{"CURE", 365, 1.1786386363108576`, 69.17784996943409`}, {"CURE", 365, 1.5275116178293924`, 35.391747944113014`},
{"CURE", 365, 9.665087292930973`, 415.67457441927456`}, {"CURE", 365, 5.536279092868568`, 473.2066725551486`},
{"CURE", 365, 56.963129671272306`, 2428.5234714852113`}, {"CURE", 365, 3.7303285935392494`, 249.64802371891506`},
{"CURE", 365, 1.9004259834422437`, 194.40472353768703`}, {"CURE", 365, 3.731699085192637`, 55.78456552349925`},
{"CURE", 365, 0.1337432403745121`, 18.197836178873217`}, {"CURE", 365, 0.15689978976695487`, 31.26401467988533`},
{"CURE", 365, 7.396934193557294`, 250.65839878269117`}, {"CURE", 365, 0.3690541803700627`, 42.910203004084224`},
{"CURE", 365, 0.25441378310396673`, 28.479383622349193`}, {"CURE", 365, 2.4460066466952233`, 45.43695246469321`},
{"CURE", 365, 0.05026919279171573`, 11.593320688749301`}, {"CURE", 365, 0.12589423994434568`, 45.39271865743663`},
{"CURE", 365, 1.240463125519124`, 108.17744630296411`}, {"CURE", 365, 4.47417073336891`, 487.7875003350981`},
{"CURE", 365, 0.269424704622017`, 39.78271973250605`}, {"CURE", 365, 0.20350180583046099`, 11.679907967982288`},
{"CURE", 365, 0.6079604441107459`, 124.59890666625671`}, {"CURE", 365, 0.17910266567058342`, 59.73049867779775`},
{"CURE", 365, 0.1863674663688693`, 13.277492570095113`}, {"CURE", 365, 1.1995372298732372`, 56.044686501231155`},
{"CURE", 365, 0.3858271494645126`, 104.52259276632596`}, {"CURE", 365, 0.9235195329501954`, 76.45338443878902`},
{"CURE", 365, 3.6912227578918793`, 99.39038657176916`}, {"CURE", 365, 41.095706388735366`, 2866.2229432498366`},
{"CURE", 365, 0.13069305126203173`, 15.994779534103953`}, {"CURE", 365, 14.473423216689932`, 503.55580233110834`},
{"CURE", 365, 5.212733059084754`, 363.0701296001111`}, {"CURE", 365, 0.75044064797399`, 393.7470576162805`},
{"CURE", 365, 0.9128745317045454`, 55.86694629958141`}, {"CURE", 365, 1.157943867210954`, 90.65681859745992`},
{"CURE", 365, 0.14065885961468527`, 13.972181055509228`}, {"CURE", 365, 0.3028899869326968`, 25.33814000411585`},
{"CURE", 365, 1.623781697106101`, 519.202596803415`}, {"CURE", 365, 12.145307973790526`, 678.583217315448`},
{"CURE", 365, 0.049320692832955274`, 7.73185553863715`}, {"CURE", 365, 14.666817367154296`, 1011.4825731313347`},
{"CURE", 365, 21.614988276557053`, 400.7674901507803`}, {"CURE", 365, 11.357387230969927`, 598.0217523317965`},
{"CURE", 365, 19.333651284225528`, 433.9982410221648`}, {"CURE", 365, 1.026513965144817`, 70.34553810281412`},
{"CURE", 365, 1.7650678129128925`, 29.48779727593221`}, {"CURE", 365, 0.13085974907177766`, 69.47311347828226`},
{"CURE", 365, 0.5238296555541851`, 31.72344360304976`}, {"CURE", 365, 1.0374956289142994`, 55.6252032485685`},
{"CURE", 365, 0.06986871795135641`, 28.74605132319932`}, {"CURE", 365, 0.5620680395005926`, 165.62921356469835`},
{"CURE", 365, 0.9524500489832699`, 45.83031514557432`}, {"CURE", 365, 37.42067866105935`, 1508.5795681804245`},
{"CURE", 365, 0.866710160499998`, 41.58647094547175`}, {"CURE", 365, 2.507461671106283`, 146.89839621040244`},
{"CURE", 365, 0.5218882249407955`, 31.92131031339462`}, {"CURE", 365, 1.3278570838882746`, 134.97321045997268`},
{"CURE", 365, 0.06477633029713245`, 25.046801990959537`}, {"CURE", 365, 4.818339199609526`, 422.381240167631`},
{"CURE", 365, 6.349106814053167`, 1162.1857955031635`}, {"CURE", 365, 0.6570572295216055`, 250.57446575182652`},
{"CURE", 365, 3.6487873683649434`, 227.87238029077884`}, {"CURE", 365, 0.15212782414763734`, 17.133891184290338`},

{"CURE", 365, 1.378696959192161`, 57.86624687898302`}, {"CURE", 365, 1.332228153708007`, 138.8553796645815`},
{"CURE", 365, 0.46969756906355997`, 59.51017991656429`}, {"CURE", 365, 30.368840094219895`, 568.826280695969`},
{"CURE", 365, 0.8927567835478935`, 223.15588398035254`}, {"CURE", 365, 6.773902941735524`, 568.0138483682161`},
{"CURE", 365, 3.677134381940434`, 541.6504963169508`}, {"CURE", 365, 0.47624400639173914`, 30.16866554119638`},
{"CURE", 365, 0.08073127862294133`, 18.585604461606252`}, {"CURE", 365, 2.4722216868762517`, 142.35761530676783`},
{"CURE", 365, 1.3880031829088226`, 30.126084051867483`}, {"CURE", 365, 1.5852803672634843`, 400.63916783833423`},
{"CURE", 365, 1.2584045311418717`, 104.69690843145362`}, {"CURE", 365, 3.322922298091437`, 257.7648417805108`},
{"CURE", 365, 1.6940929235865665`, 36.14156336705299`}, {"CURE", 365, 33.256541696346964`, 1871.6394007748945`},
{"CURE", 365, 4.858235345813528`, 335.5050602317977`}, {"CURE", 365, 29.384620702106833`, 1146.4058327980251`},
{"CURE", 365, 7.69340551803512`, 775.7474291114605`}, {"CURE", 365, 1.6684873168240462`, 60.61598551394992`},
{"CURE", 365, 17.097855074790328`, 903.4661863382355`}, {"CURE", 365, 0.2469533849319188`, 22.38730806300795`},
{"CURE", 365, 6.829049433311556`, 658.8227651038449`}, {"CURE", 365, 0.12446066821981357`, 36.853392470810626`},
{"CURE", 365, 0.10380336133745012`, 45.316138662867125`}, {"CURE", 365, 6.988350235283015`, 1284.6694719506872`},
{"CURE", 365, 0.06176242574371186`, 8.58365069005551`}, {"CURE", 365, 3.1457180296962237`, 986.9293365486691`},
{"CURE", 365, 1.412970643979275`, 121.57366337383282`}, {"CURE", 365, 9.466072822514393`, 297.7006696536064`},
{"CURE", 365, 0.378844320751192`, 44.01962982356288`}, {"CURE", 365, 8.461096205833321`, 529.3442312317381`},
{"CURE", 365, 0.43874051780787915`, 22.08332482662356`}, {"CURE", 365, 0.033396487489167015`, 2.1606566892535124`},
{"CURE", 365, 0.09764630975814192`, 8.397952787745245`}, {"CURE", 365, 0.07961143367642053`, 17.318519093999857`},
{"CURE", 365, 7.862381086917252`, 246.12578941959273`}, {"CURE", 365, 0.10014547262090011`, 36.01300608277443`},
{"CURE", 365, 2.5164808339953986`, 85.09785233655927`}, {"CURE", 365, 47.65203121659875`, 1631.8089427205907`},
{"CURE", 365, 0.0809271878408411`, 10.050225643881154`}, {"CURE", 365, 0.7992688424013238`, 82.96817584268148`},
{"CURE", 365, 65.66221727351288`, 1177.9583954994857`}, {"CURE", 365, 3.006945386577921`, 213.4641671233280 8`},
{"CURE", 365, 0.34196665668384985`, 20.151376844006926`}, {"CURE", 365, 0.732234995550705`, 69.36695213501204`},
{"CURE", 365, 1.0290644155160378`, 255.9421165346789`}, {"CURE", 365, 12.744166206390869`, 521.6269679477103`},
{"CURE", 365, 205.79828973849197`, 3105.3981516306258`}, {"CURE", 365, 0.12823141656297068`, 12.862908742961668`},
{"CURE", 365, 2.0295029929161723`, 226.07514996812154`}, {"CURE", 365, 0.3172285544001548`, 19.210283513587036`},
{"CURE", 365, 1.692399615177244`, 253.24030026186122`}, {"CURE", 365, 1.7376922609616978`, 20.688776542729702`},
{"CURE", 365, 1.5212938762641803`, 224.92272281532763`}, {"CURE", 365, 1.0294230930772097`, 24.868935988348245`},
{"CURE", 365, 10.684111048137655`, 331.94705974339024`}, {"CURE", 365, 21.119165868245894`, 675.5681420112626`},
{"CURE", 365, 2.043055439753597`, 123.4415202464278`}, {"CURE", 365, 0.9456328627665921`, 24.50129819009227`},
{"CURE", 365, 13.31176142146092`, 274.42829103564173`}, {"CURE", 365, 0.2667183984760891`, 15.36627023084441`},

{"CURE", 365, 0.21328462681620586`, 66.57651691191442`}, {"CURE", 365, 2.011714689939301`, 70.49304402157404`},
{"CURE", 365, 0.2960850892601901`, 16.803517528276224`}, {"CURE", 365, 1.185701162581132`, 69.37824722669832`},
{"CURE", 365, 0.10246685830372354`, 27.261023344354697`}, {"CURE", 365, 13.096370455161097`, 151.87120618481913`},
{"CURE", 365, 0.3257275783347632`, 51.62980904712751`}, {"CURE", 365, 6.917073851216613`, 249.76624252248152`},
{"CURE", 365, 4.837635651049944`, 279.7250193180668`}, {"CURE", 365, 1.763554798376024`, 143.6000698459758`},
{"CURE", 365, 0.1548983386638196`, 52.720302585641576`}, {"CURE", 365, 0.17656466953748426`, 7.961505811970977`},
{"CURE", 365, 0.5828701634167999`, 44.44185730884166`}, {"CURE", 365, 0.8175461850506357`, 23.987003814909933`},
{"CURE", 365, 3.023163467456621`, 132.9318908726596`}, {"CURE", 365, 21.03794113657084`, 724.7938472019458`},
{"CURE", 365, 14.08204155422618`, 1011.1851153025343`}, {"CURE", 365, 4.07587356144302`, 335.39428584776726`},
{"CURE", 365, 137.88005254436223`, 2121.1721731501393`}, {"CURE", 365, 0.41427265351534986`, 24.533326217129577`},
{"CURE", 365, 1.4901637505247682`, 329.69975479602175`}, {"CURE", 365, 0.6617588810694927`, 43.950296741258526`},
{"CURE", 365, 31.088436721404715`, 2003.4108636526307`}, {"CURE", 365, 2.3732098377333153`, 1147.99753568389`},
{"CURE", 365, 0.08026812496559793`, 22.045590933584357`}, {"CURE", 365, 1.1806298249092648`, 210.63508678279518`},
{"CURE", 365, 61.714766161657344`, 1156.6762246041878`}, {"CURE", 365, 0.23350990570166613`, 14.348671017297418`},
{"CURE", 365, 0.5785302624937771`, 34.07262232392555`}, {"CURE", 365, 1.092451437845935`, 79.76909253338049`},
{"CURE", 365, 57.15741069167925`, 1019.7388285372696`}, {"CURE", 365, 0.1994304660843152`, 15.667733161801229`},
{"CURE", 365, 6.115153693462912`, 239.4161790873653`}, {"CURE", 365, 2.5236762481250805`, 196.5628550995527`},
{"CURE", 365, 1.5611078082357812`, 82.08320567053856`}, {"CURE", 365, 0.330945269403695`, 72.48837621377623`},
{"CURE", 365, 1.116722474932149`, 66.05048444299644`}, {"CURE", 365, 2.346967919930907`, 221.33427428825985`},
{"CURE", 365, 0.3646520290170353`, 17.788356657589173`}, {"CURE", 365, 5.68652279239475`, 174.83869244423164`},
{"CURE", 365, 10.753817429330972`, 296.11934384658053`}, {"CURE", 365, 5.222239331318647`, 245.2701763772268`},
{"CURE", 365, 0.22234972041085768`, 12.372092209853097`}, {"CURE", 365, 13.873047489592071`, 563.9270122480958`},
{"CURE", 365, 2.98825783706832`, 248.62223928633185`}, {"CURE", 365, 0.9555232764774446`, 70.79995019134346`},
{"CURE", 365, 3.0297073584372396`, 318.8785728622766`}, {"CURE", 365, 0.39929730634686306`, 53.30691187546096`},
{"CURE", 365, 2.5774007628675957`, 213.01414745573302`}, {"CURE", 365, 2.380927880538883`, 76.51410562377555`},
{"CURE", 365, 0.273377523167228`, 77.29858665405337`}, {"CURE", 365, 17.346892521566147`, 1090.6650430168854`},
{"CURE", 365, 0.03766769614728915`, 12.796426075173045`}, {"CURE", 365, 2.891244708794527`, 187.78024502351917`},
{"CURE", 365, 6.043507706935825`, 687.9040905949414`}, {"CURE", 365, 1.1626440437713348`, 35.760790951675204`},
{"CURE", 365, 0.3897592363634432`, 129.4565159165434`}, {"CURE", 365, 13.030129807387317`, 603.5349470217193`},
{"CURE", 365, 7.083005561876669`, 262.17651195914175`}, {"CURE", 365, 0.9269443714183536`, 209.78396004373934`},
{"CURE", 365, 5.461789704906237`, 538.7322643231519`}, {"CURE", 365, 0.5806076623237179`, 71.7481181963682 7`},

{"CURE", 365, 7.031227908495171`, 1493.2487569737796`}, {"CURE", 365, 1.3586984474561143`, 49.065081392842494`},
{"CURE", 365, 7.788866570151953`, 745.8673745383564`}, {"CURE", 365, 1.2038645480042547`, 60.47700073992852`},
{"CURE", 365, 3.951904154352548`, 202.63412788465368`}, {"CURE", 365, 0.19323129557719462`, 8.868671812964113`},
{"CURE", 365, 1.0196248353933`, 59.32943617489331`}, {"CURE", 365, 0.44948516145748324`, 22.09660333747019`},
{"CURE", 365, 0.4170835558682474`, 25.497298982562647`}, {"CURE", 365, 10.498321549692829`, 332.9558293586796`},
{"CURE", 365, 0.6011264588177841`, 31.6056053138104`}, {"CURE", 365, 10.969491470653802`, 698.7298767395444`},
{"CURE", 365, 10.589310164851716`, 263.98868470935446`}, {"CURE", 365, 1.1716584520028996`, 252.1151893461383`},
{"CURE", 365, 3.111344745770356`, 118.02938233945783`}, {"CURE", 365, 0.06128745487937193`, 32.0706583113957`},
{"CURE", 365, 3.8350868085387373`, 158.4057250829463`}, {"CURE", 365, 0.23071874020302388`, 40.9399952769958`},
{"CURE", 365, 20.63412535062451`, 704.4016624670611`}, {"CURE", 365, 1.8995952324730487`, 408.36826853266007`},
{"CURE", 365, 3.74355097829473`, 123.47254738130208`}, {"CURE", 365, 30.98369612668278`, 408.80125029883595`},
{"CURE", 365, 1.809066589282704`, 543.4181674695252`}, {"CURE", 365, 0.10812875714011179`, 45.65775952982095`},
{"CURE", 365, 2.3465274182118443`, 266.283200220506`}, {"CURE", 365, 1.5681056965738527`, 733.4343960008703`},
{"CURE", 365, 0.1211613178407528`, 83.02284145291294`}, {"CURE", 365, 0.31268498060083644`, 37.076788376179806`},
{"CURE", 365, 9.646150640555078`, 694.6866615377274`}, {"CURE", 365, 0.4929444062260964`, 32.75034596680579`},
{"CURE", 365, 26.591642884339898`, 1848.5073559567559`}, {"CURE", 365, 0.1263133779963549`, 11.464898349902807`},
{"CURE", 365, 2.7885156361892904`, 144.09846779922634`}, {"CURE", 365, 2.4349370376494583`, 70.68020320069606`},
{"CURE", 365, 0.5964372913469385`, 214.48971966181026`}, {"CURE", 365, 0.5343071972849333`, 35.29559730993394`},
{"CURE", 365, 0.38601476049428984`, 29.46174126674526`}, {"CURE", 365, 1.1205307493667853`, 118.14605803597442`},
{"CURE", 365, 2.492627812138955`, 142.7279740141629`}, {"CURE", 365, 2.9899987689889644`, 115.65102688473769`},
{"CURE", 365, 3.4787654881287873`, 57.47232401010046`}, {"CURE", 365, 25.982525606999445`, 570.8429719353087`},
{"CURE", 365, 19.696044839697528`, 1394.6371528435657`}, {"CURE", 365, 0.12924469311141054`, 87.11560089441284`},
{"CURE", 365, 3.0064792475635955`, 184.78504802943021`}, {"CURE", 365, 2.5532263486766844`, 60.63445153464004`},
{"CURE", 365, 0.37167408927125595`, 35.55810090968587`}, {"CURE", 365, 0.30944070570769444`, 24.76318782720724`},
{"CURE", 365, 0.18618578496033128`, 23.157820029110475`}, {"CURE", 365, 0.7383867274777978`, 61.99206969240034`},
{"CURE", 365, 3.509493775366531`, 1129.362732392747`}, {"CURE", 365, 17.322942246954085`, 1136.0776574643014`},
{"CURE", 365, 0.36213136319903766`, 42.9436475681261`}, {"CURE", 365, 13.396777587902031`, 537.6777383988025`},
{"CURE", 365, 28.01054313874697`, 1634.5227674019852`}, {"CURE", 365, 0.29640049183952644`, 18.103334543545916`},
{"CURE", 365, 0.6060454980910399`, 44.81691243956344`}, {"CURE", 365, 0.6375127410672357`, 88.41523460145879`},
{"CURE", 365, 0.17639051148761786`, 36.27348547589925`}, {"CURE", 365, 0.33390108407364305`, 69.96042706529289`},
{"CURE", 365, 2.073432409993016`, 333.57851887699354`}, {"CURE", 365, 0.1793569876172841`, 36.76388434557834`},

{"CURE", 365, 36.832345149447086`, 564.3597653522113`}, {"CURE", 365, 13.465032244338447`, 371.0879607808598`},
{"CURE", 365, 0.7015746835944715`, 99.62421933772144`}, {"CURE", 365, 4.888754115889797`, 45.02539202468553`},
{"CURE", 365, 0.8826127578947062`, 28.37299779813403`}, {"CURE", 365, 0.9715075440094307`, 58.98676340234219`},
{"CURE", 365, 0.5657916171073015`, 20.13267424567961`}, {"CURE", 365, 0.08232744535002234`, 19.41554226880814`},
{"CURE", 365, 4.916182608994901`, 144.33658374242736`}, {"CURE", 365, 6.086229234529106`, 66.82595506370394`},
{"CURE", 365, 0.06177713296881779`, 12.56824368798674`}, {"CURE", 365, 0.5542283687941733`, 36.48531959491505`},
{"CURE", 365, 8.221567109773199`, 327.934643703918`}, {"CURE", 365, 0.10971119351207678`, 8.839055366395339`},
{"CURE", 365, 0.1472262204253477`, 23.840574559036654`}, {"CURE", 365, 0.1428767250079005`, 28.601245536316416`},
{"CURE", 365, 2.569463720169458`, 59.67283663921525`}, {"CURE", 365, 3.743541523166276`, 164.90410180343062`},
{"CURE", 365, 1.2864374581326201`, 99.31272499503518`}, {"CURE", 365, 10.846645243511235`, 339.29630720848763`},
{"CURE", 365, 0.670837050865314`, 116.64623439773091`}, {"CURE", 365, 4.2631002357378565`, 274.55528557396`},
{"CURE", 365, 0.26939942107525566`, 29.245345468773433`}, {"CURE", 365, 3.126854211520571`, 49.718605753720446`},
{"CURE", 365, 39.300408464778144`, 2448.5722469229436`}, {"CURE", 365, 129.11646398504902`, 1740.6816559651986`},
{"CURE", 365, 120.0957072772016`, 1046.7694262669763`}, {"CURE", 365, 0.7978065845507101`, 90.77697287528106`},
{"CURE", 365, 1.8781268222352165`, 751.481177946595`}, {"CURE", 365, 0.16942700576196768`, 78.04405089490304`},
{"CURE", 365, 3.045258294058763`, 194.26561804527893`}, {"CURE", 365, 3.553556619266842`, 58.53483416336265`},
{"CURE", 365, 0.9273142324674037`, 75.1439851011026`}, {"CURE", 365, 1.9823090681058295`, 211.04938426010656`},
{"CURE", 365, 39.824987720952436`, 2220.227747082859`}, {"CURE", 365, 11.910331304983828`, 612.2237138925813`},
{"CURE", 365, 55.10646974647723`, 677.7751870639568`}, {"CURE", 365, 1.55471345932558`, 23.18573653661004`},
{"CURE", 365, 5.231062164611453`, 377.1112424680296`}, {"CURE", 365, 0.7518561330827942`, 162.03080022695272`},
{"CURE", 365, 1.1069485425050172`, 97.75567648040469`}, {"CURE", 365, 0.07793769371627955`, 44.13851070007615`},
{"CURE", 365, 20.765791387205635`, 623.8221073783109`}, {"CURE", 365, 2.314784301191734`, 46.08838479433352`},
{"CURE", 365, 0.1585777357776277`, 9.842982654868056`}, {"CURE", 365, 1.6739801934997074`, 128.81543008344235`},
{"CURE", 365, 0.5629171721426681`, 10.287379459917902`}, {"CURE", 365, 1.166214067688521`, 338.2755534030456`},
{"CURE", 365, 2.0830079150202088`, 466.13206760423776`}, {"CURE", 365, 0.390847900077753`, 27.676400141123846`},
{"CURE", 365, 10.21581185865639`, 562.5522497166564`}, {"CURE", 365, 93.12690831119669`, 2665.536854198614`},
{"CURE", 365, 1.890239306226681`, 53.64658835856637`}, {"CURE", 365, 0.6469100178386853`, 44.38620722204272`},
{"CURE", 365, 10.455032573962821`, 693.3599196888216`}, {"CURE", 365, 18.06822328456774`, 960.5813380836081`},
{"CURE", 365, 7.730848448944072`, 1183.8404509547938`}, {"CURE", 365, 0.051061793867627665`, 27.698001032331838`},
{"CURE", 365, 4.33409514425881`, 225.68096042779626`}, {"CURE", 365, 0.2805379280305594`, 12.116842259788802`},
{"CURE", 365, 0.660044913080886`, 58.27989999889477`}, {"CURE", 365, 0.5029931133718111`, 24.411343330444307`},

{"CURE", 365, 1.5945364247653953`, 250.61620467429944`}, {"CURE", 365, 0.22485536186027313`, 53.81816848288783`},
{"CURE", 365, 0.7368243085919561`, 40.18479795598945`}, {"CURE", 365, 5.051195601481112`, 457.3316807520611`},
{"CURE", 365, 1.9772171380740644`, 124.89466556847823`}, {"CURE", 365, 5.943187787981251`, 56.1725282846741`},
{"CURE", 365, 0.46940899561154514`, 47.31565692942392`}, {"CURE", 365, 2.8267363914907273`, 577.299920414129`},
{"CURE", 365, 0.6372180809260248`, 168.56201802523788`}, {"CURE", 365, 0.5894903262422798`, 128.10902843546947`},
{"CURE", 365, 1.2917680163539103`, 49.35621442013609`}, {"CURE", 365, 0.1577983910778898`, 87.01455352879485`},
{"CURE", 365, 9.401018525975399`, 381.20094592641533`}, {"CURE", 365, 0.4511919229097131`, 16.941405143217427`},
{"CURE", 365, 0.45047134251043186`, 21.443993158337783`}, {"CURE", 365, 0.3175486713808148`, 18.708948221604384`},
{"CURE", 365, 1.6777360642631522`, 494.53085061718883`}, {"CURE", 365, 3.027185967673261`, 80.87388121767113`},
{"CURE", 365, 28.041228884513217`, 2452.515537585776`}, {"CURE", 365, 1.0643609891939356`, 215.45483794581807`},
{"CURE", 365, 2.082737066597158`, 63.16950700274097`}, {"CURE", 365, 0.09732096167452635`, 16.675271470830975`},
{"CURE", 365, 3.7998464550667426`, 96.36466012423459`}, {"CURE", 365, 1.0748224672638607`, 41.8531168859234`},
{"CURE", 365, 0.37094420271622414`, 21.985435171073632`}, {"CURE", 365, 0.4877481134152585`, 19.701524731703305`},
{"CURE", 365, 6.296498287873374`, 719.471637110601`}, {"CURE", 365, 5.1283162631570285`, 132.54706079025175`},
{"CURE", 365, 0.23217689943989397`, 50.55279316581811`}, {"CURE", 365, 0.31715537599896`, 16.467048287311492`},
{"CURE", 365, 0.3996838615495493`, 17.70526668494254`}, {"CURE", 365, 0.3896981785130443`, 40.59766333166595`},
{"CURE", 365, 11.740554996459714`, 129.6299139977325`}, {"CURE", 365, 8.247626962158769`, 456.6590946288247`},
{"CURE", 365, 0.6257165219791622`, 34.07718428814625`}, {"CURE", 365, 3.673984030517664`, 195.20566138665407`},
{"CURE", 365, 5.042532754433054`, 66.4884323769222`}, {"CURE", 365, 2.3438656511247498`, 97.2552847817593`},
{"CURE", 365, 6.261577164983947`, 277.78703131314586`}, {"CURE", 365, 25.930067148246568`, 528.6335924875472`},
{"CURE", 365, 1.238910280372101`, 73.01174971484024`}, {"CURE", 365, 3.1207404857489154`, 209.1454034672705`},
{"CURE", 365, 1.0150347020996167`, 55.037591317896435`}, {"CURE", 365, 0.35724223603955996`, 14.052872661563164`},
{"CURE", 365, 0.33507904158154544`, 7.459907679670742`}, {"CURE", 365, 0.3401239963860764`, 35.333449305253055`},
{"CURE", 365, 0.5926720863084607`, 67.1140105900065`}, {"CURE", 365, 0.19561684029863577`, 10.425778808017723`},
{"CURE", 365, 1.3704732809069458`, 70.50662401912099`}, {"CURE", 365, 14.128787107252492`, 652.2800520027203`},
{"CURE", 365, 0.49377418618267827`, 21.203677852595344`}, {"CURE", 365, 2.2796985008754205`, 177.31529602507698`},
{"CURE", 365, 7.369671621120531`, 1120.0269139900943`}, {"CURE", 365, 25.84000585686787`, 1048.6352645594811`},
{"CURE", 365, 1.2729011144591158`, 204.95059973220535`}, {"CURE", 365, 1.7963216688145591`, 135.82313261791884`},
{"CURE", 365, 12.801017364756927`, 816.738929945907`}, {"CURE", 365, 0.1205110349458912`, 25.391613519473612`},
{"CURE", 365, 0.08745807414932436`, 37.59426078338282`}, {"CURE", 365, 27.06528498010455`, 1843.3714034241852`},
{"CURE", 365, 12.494241082129928`, 633.5045910128265`}, {"CURE", 365, 17.08884662602862`, 726.2464439636018`},

{"CURE", 365, 6.863207338495257`, 411.4986868144822`}, {"CURE", 365, 0.8412303852119949`, 392.4213789076496`},
{"CURE", 365, 4.3401164725375265`, 226.1243417196343`}, {"CURE", 365, 1.2362263939862714`, 339.32066138486243`},
{"CURE", 365, 1.7471203887392492`, 30.92959449299805`}, {"CURE", 365, 8.315030207320202`, 113.33857040502501`},
{"CURE", 365, 1.9803591112288654`, 202.62479774227475`}, {"CURE", 365, 33.52546065575635`, 2282.8466786404965`},
{"CURE", 365, 6.396901082687634`, 82.33105655789551`}, {"CURE", 365, 1.9788961129727163`, 139.70797673054562`},
{"CURE", 365, 10.193738556644426`, 1237.3654922498422`}, {"CURE", 365, 6.8511580189895005`, 1068.5367920679976`},
{"CURE", 365, 0.10497200194595319`, 60.83487180531087`}, {"CURE", 365, 0.14729188556506467`, 13.420420806326971`},
{"CURE", 365, 0.3659214523799314`, 30.075899134302738`}, {"CURE", 365, 17.319158177477792`, 532.5972842135401`},
{"CURE", 365, 0.26537278366806494`, 17.06223312589189`}, {"CURE", 365, 0.7089766183447131`, 25.962477270739434`},
{"CURE", 365, 0.7348146011367359`, 76.28886356917735`}, {"CURE", 365, 36.08313231268866`, 1717.5563033012877`},
{"CURE", 365, 1.5037213068160298`, 32.214186736269006`}, {"CURE", 365, 2.8605190649908`, 604.1986101857764`},
{"CURE", 365, 0.6286735460534678`, 85.77821154019512`}, {"CURE", 365, 7.186215582774554`, 658.3144498435762`},
{"CURE", 365, 1.95744933725959`, 129.17230994967787`}, {"CURE", 365, 18.418239776191523`, 1837.1349076764338`},
{"CURE", 365, 0.0721149464309417`, 7.556532037204152`}, {"CURE", 365, 1.6075005215365243`, 398.9720292306999`},
{"CURE", 365, 0.7089768902704335`, 113.22321591039783`}, {"CURE", 365, 14.491388262354747`, 1218.681892609731`},
{"CURE", 365, 3.2460694586587313`, 120.99611847723574`}, {"CURE", 365, 45.80305580120355`, 1899.586527798367`},
{"CURE", 365, 2.3134885296492675`, 188.24191838012692`}, {"CURE", 365, 77.3315585925418`, 4145.885471770314`},
{"CURE", 365, 57.40683688249668`, 1439.887217753978`}, {"CURE", 365, 2.228064552485864`, 115.71203516220871`},
{"CURE", 365, 0.9105873125946479`, 63.47586605194777`}, {"CURE", 365, 0.3321787609867613`, 36.30954816684556`},
{"CURE", 365, 0.8544943983850697`, 297.9661017993744`}, {"CURE", 365, 0.06982668569048461`, 4.397734499631947`},
{"CURE", 365, 4.794150858939967`, 259.223074671056`}, {"CURE", 365, 12.617274797771774`, 173.30672520604963`},
{"CURE", 365, 1.6048907446307512`, 433.3821605258296`}, {"CURE", 365, 0.15896664857890194`, 43.873851873395814`},
{"CURE", 365, 0.5047460189006331`, 43.98098052864242`}, {"CURE", 365, 0.21021634749250967`, 6.274123160336462`},
{"CURE", 365, 0.04761091381171395`, 11.414590795630419`}, {"CURE", 365, 2.075460416154708`, 100.88197700376146`},
{"CURE", 365, 0.6076421578782659`, 126.42990404654543`}, {"CURE", 365, 1.0325063449795193`, 86.80627787285289`},
{"CURE", 365, 0.7084854413787142`, 147.1138967957469`}, {"CURE", 365, 0.9578432740127346`, 187.81185672879738`},
{"CURE", 365, 0.2554438778636214`, 55.570494134913474`}, {"CURE", 365, 0.8356657295507265`, 17.269931279624313`},
{"CURE", 365, 8.751878618225884`, 1122.2668504131204`}, {"CURE", 365, 26.879655502772568`, 1935.0810287514164`},
{"CURE", 365, 0.15868750640849177`, 83.48927749657256`}, {"CURE", 365, 7.599041599015267`, 332.2607939718365`},
{"CURE", 365, 0.2906714332987627`, 9.166621090263522`}, {"CURE", 365, 10.873561134611887`, 391.6343392345653`},
{"CURE", 365, 0.1485780047475988`, 35.38778541946305`}, {"CURE", 365, 0.3363580336410365`, 39.867411999707905`},

{"CURE", 365, 32.71127755532217`, 383.19440173414864`}, {"CURE", 365, 4.831571695912288`, 209.48247101224322`},
{"CURE", 365, 12.702111682860332`, 800.3387771787268`}, {"CURE", 365, 0.05836203252007357`, 10.535688346672897`},
{"CURE", 365, 1.406413367671904`, 175.66718510000106`}, {"CURE", 365, 8.003692431730055`, 642.4420525192533`},
{"CURE", 365, 2.646629458652507`, 855.9162425059945`}, {"CURE", 365, 1.078018568154247`, 71.00301501500577`},
{"CURE", 365, 0.4341909578054401`, 18.33915593657235`}, {"CURE", 365, 4.272337194920672`, 297.7942818597918`},
{"CURE", 365, 0.5331431648335739`, 34.03652261138117`}, {"CURE", 365, 2.290806798203227`, 200.10425820074647`},
{"CURE", 365, 5.9344438648127`, 802.2800708732283`}, {"CURE", 365, 22.428766688876802`, 536.5835096445707`},
{"CURE", 365, 9.456999698975105`, 155.94952196881263`}, {"CURE", 365, 0.1952381666361037`, 12.1459280888295`},
{"CURE", 365, 0.8314990233076966`, 40.469157716997444`}, {"CURE", 365, 0.5812494511411115`, 22.689944588917733`},
{"CURE", 365, 3.7744691582152488`, 148.08800956271122`}, {"CURE", 365, 1.6116499392537382`, 721.9165818437019`},
{"CURE", 365, 23.001048914296266`, 770.2622864497263`}, {"CURE", 365, 0.2635001440658714`, 8.63263307255151`},
{"CURE", 365, 21.162625227760227`, 1010.5602504338468`}, {"CURE", 365, 3.9929720032578975`, 47.793289299582256`},
{"CURE", 365, 0.6908740607876663`, 40.158505224856846`}, {"CURE", 365, 8.017172701404698`, 1037.8908149319632`},
{"CURE", 365, 0.9619384428514947`, 53.53391021171861`}, {"CURE", 365, 0.3277911285878348`, 14.089391815919642`},
{"CURE", 365, 1.2000092622914003`, 115.87600531226146`}, {"CURE", 365, 11.264279280293199`, 1371.4193109206683`},
{"CURE", 365, 1.0610123329369936`, 149.16190227312939`}, {"CURE", 365, 3.311827144834352`, 258.95524589540406`},
{"CURE", 365, 2.891635267033247`, 72.76603738752213`}, {"CURE", 365, 0.1164759251099423`, 4.616808615181819`},
{"CURE", 365, 3.4529712829131025`, 778.3050686763037`}, {"CURE", 365, 0.2481116616465356`, 9.793433933498637`},
{"CURE", 365, 4.674282598639216`, 124.70748840653467`}, {"CURE", 365, 1.6136843783192087`, 237.2701314394263`},
{"CURE", 365, 13.542038046026022`, 345.8167753913835`}, {"CURE", 365, 0.978755456684565`, 167.52000710808824`},
{"CURE", 365, 1.0421404304460256`, 276.7184965894145`}, {"CURE", 365, 0.3440639819336372`, 63.93744439557565`},
{"CURE", 365, 5.486496041187731`, 536.393867230651`}, {"CURE", 365, 121.25488471632346`, 2597.549499406887`},
{"CURE", 365, 0.7142070984002196`, 43.55131550160511`}, {"CURE", 365, 20.19492761133833`, 964.5878236818253`},
{"CURE", 365, 0.06030348509574797`, 12.27977939686655`}, {"CURE", 365, 71.58044588430258`, 756.1953961979005`},
{"CURE", 365, 4.997859904617854`, 114.82436941591291`}, {"CURE", 365, 7.825251638855262`, 1052.2499317521188`},
{"CURE", 365, 3.7853907152715176`, 211.44046708609739`}, {"CURE", 365, 0.08717226318300104`, 8.714924682108071`},
{"CURE", 365, 7.8880272691120386`, 766.5089109271086`}, {"CURE", 365, 9.542448142548613`, 280.16938315184814`},
{"CURE", 365, 107.78695660967549`, 2440.5655813573117`}, {"CURE", 365, 0.08981029747852234`, 19.253984756144455`},
{"CURE", 365, 0.8280212507897059`, 90.93198558454012`}, {"CURE", 365, 0.6405178342969554`, 198.33350450257495`},
{"CURE", 365, 0.5361852151505399`, 42.91110015136875`}, {"CURE", 365, 17.752727141342522`, 828.5577746606651`},
{"CURE", 365, 1.1891184985438066`, 34.44618022736061`}, {"CURE", 365, 2.902228147710747`, 149.99663082387514`},

{"CURE", 365, 5.091986224436674`, 73.4583115109068`}, {"CURE", 365, 0.9808436732909589`, 29.737500503656`},
{"CURE", 365, 14.45800739614469`, 2594.710680185111`}, {"CURE", 365, 2.9089480939653747`, 241.0020678082268`},
{"CURE", 365, 52.27826007863782`, 3722.5635084956953`}, {"CURE", 365, 54.3587700602587`, 1975.2471455245486`},
{"CURE", 365, 6.505099470167387`, 1803.942215840006`}, {"CURE", 365, 1.3401170171620234`, 141.99204009764497`},
{"CURE", 365, 2.039195751738362`, 379.37284918961`}, {"CURE", 365, 0.636435111136627`, 47.591467367804114`},
{"CURE", 365, 4.599627257494846`, 150.67512728344462`}, {"CURE", 365, 0.7019376862819263`, 54.16836191062148`},
{"CURE", 365, 0.023132084161411184`, 8.18103784250051`}, {"CURE", 365, 48.596976359192915`, 894.2115075640204`},
{"CURE", 365, 4.412442822657293`, 431.65432604487995`}, {"CURE", 365, 0.3849388143930022`, 110.95476309096284`},
{"CURE", 365, 0.2645814669729503`, 58.94187163641369`}, {"CURE", 365, 0.8637758675582875`, 224.42980603222875`},
{"CURE", 365, 0.15354410645579522`, 31.21302073300639`}, {"CURE", 365, 8.65071773025496`, 217.5516294909813`},
{"CURE", 365, 12.795291806279847`, 591.1758357926029`}, {"CURE", 365, 8.145309498589208`, 621.225095355659`},
{"CURE", 365, 6.207074104334999`, 1011.3605662223719`}, {"CURE", 365, 0.22094256398958265`, 21.75582423897724`},
{"CURE", 365, 0.061119196579973945`, 10.41297849684996`}, {"CURE", 365, 2.2370307784057664`, 157.1726214141531`},
{"CURE", 365, 18.59415830711943`, 379.75268325637967`}, {"CURE", 365, 17.103106155631835`, 631.309726353807`},
{"CURE", 365, 6.942465187158883`, 495.9597426104939`}, {"CURE", 365, 1.6510195105053798`, 203.0441220279238`},
{"CURE", 365, 0.11788677129130148`, 14.692153138138039`}, {"CURE", 365, 8.217658973091565`, 78.58119319210884`},
{"CURE", 365, 0.18840079059055725`, 37.604233612869926`}, {"CURE", 365, 0.12221268574331087`, 47.51608163721132`},
{"CURE", 365, 3.5031025071455537`, 1554.4956291283133`}, {"CURE", 365, 5.091229353803965`, 178.49913800074944`},
{"CURE", 365, 7.669067829272576`, 462.7364058768239`}, {"CURE", 365, 2.065011413699221`, 112.43794522675319`},
{"CURE", 365, 2.4574856342642883`, 89.31694978147985`}, {"CURE", 365, 50.14183352608762`, 1101.0307407921744`},
{"CURE", 365, 0.32176519992819913`, 6.874270860258676`}, {"CURE", 365, 2.9247003395689997`, 162.86860606159198`},
{"CURE", 365, 0.08554735675332883`, 11.10196047132094`}, {"CURE", 365, 2.644706701386894`, 296.48299692210634`},
{"CURE", 365, 2.543733143630054`, 146.74587235370853`}, {"CURE", 365, 6.098607228413265`, 602.8533631906172`},
{"CURE", 365, 28.536401466870526`, 6134.058424977911`}, {"CURE", 365, 23.003688493415098`, 1435.4930770304566`},
{"CURE", 365, 74.4021169915986`, 745.928816980436`}, {"CURE", 365, 0.4141340361209922`, 40.79975678571222`},
{"CURE", 365, 0.8840490245916194`, 242.11821167541933`}, {"CURE", 365, 1.735041831730886`, 49.97312609374847`},
{"CURE", 365, 0.0393250387326087`, 4.555175994851797`}, {"CURE", 365, 4.877577568890132`, 197.6465401317123`},
{"CURE", 365, 5.6453082185815076`, 47.073083235173264`}, {"CURE", 365, 7.738084942006664`, 138.85245997355034`},
{"CURE", 365, 1.60470801056626`, 89.00274995546643`}, {"CURE", 365, 1.995307462748964`, 131.67164014889994`},
{"CURE", 365, 0.6471530327153735`, 100.36506184872853`}, {"CURE", 365, 0.5225506230400836`, 286.0424566118915`},
{"CURE", 365, 10.72230754116792`, 1480.6823204764362`}, {"CURE", 365, 0.7037785394826124`, 33.48784045005932`},

```
{"CURE", 365, 1.020150729154369`, 87.74898294706678`}, {"CURE", 365, 1.5768562818482135`, 42.12016130935014`},
{"CURE", 365, 7.9687518440234095`, 537.9055702649293`}, {"CURE", 365, 1.5364885197706535`, 101.77523562019354`},
{"CURE", 365, 0.034177081386569434`, 8.18861592726607`}, {"CURE", 365, 1.29433890087769`, 109.26712907209031`},
{"CURE", 365, 0.45152222488477056`, 36.87076977643503`}, {"CURE", 365, 0.08035681051679905`, 25.25453426662042`},
{"CURE", 365, 14.722373440806118`, 1442.5511585682946`}, {"CURE", 365, 0.03415229584272132`, 5.730064000412201`},
{"CURE", 365, 19.135678601937794`, 1981.7572348463032`}, {"CURE", 365, 26.895505033186826`, 1345.8865249109897`},
{"CURE", 365, 8.210203326911785`, 819.6434201195755`}, {"CURE", 365, 0.17081577067989726`, 73.69196148351931`},
{"CURE", 365, 3.804634127747524`, 237.53478883401414`}, {"CURE", 365, 0.8232203849104209`, 464.2111208409248`},
{"CURE", 365, 3.2334493705648812`, 616.7769991686312`}, {"CURE", 365, 6.793160751280957`, 436.63311424339145`},
{"CURE", 365, 0.38779733815524586`, 21.789009994270582`}, {"CURE", 365, 0.13719033080921034`, 38.6032674315667`},
{"CURE", 365, 2.9459536835417444`, 284.30259440920844`}, {"CURE", 365, 5.761180281890824`, 423.87492901592475`},
{"CURE", 365, 86.23397163853454`, 1245.2405559077674`}, {"CURE", 365, 0.3860554527309174`, 24.635817532491846`},
{"CURE", 365, 2.476101242152288`, 554.9406352300782`}, {"CURE", 365, 48.665427619793526`, 992.2833814377833`},
{"CURE", 365, 6.317438455738639`, 1320.3636956569899`}, {"CURE", 365, 18.86615980088527`, 1041.3935290666361`},
{"CURE", 365, 0.4886980062981856`, 26.247567010463477`}, {"CURE", 365, 0.9272870329238787`, 166.84175300087628`},
{"CURE", 365, 0.02843580637272789`, 4.6383417307811445`}, {"CURE", 365, 2.5114428444919166`, 92.5554031346354`},
{"CURE", 365, 0.06158473953337022`, 23.013349788890014`}, {"CURE", 365, 1.7304376107320745`, 74.41205040257661`},
{"CURE", 365, 0.30838131714138994`, 50.07505782570754`}, {"CURE", 365, 0.13489696262865594`, 16.82010615464745`},
{"CURE", 365, 1.6343699156177642`, 906.0265417718443`}, {"CURE", 365, 1.1236580889292922`, 121.76035304892044`},
{"CURE", 365, 48.908735523796494`, 1540.7410528892483`}, {"CURE", 365, 4.375667004047235`, 365.8383771841079`},
{"CURE", 365, 2.0400839614091337`, 234.1017553924886`}, {"CURE", 365, 9.578303759925625`, 766.0970853224984`},
{"CURE", 365, 0.17134429675154558`, 17.84944007397093`}, {"CURE", 365, 21.21114517620046`, 1709.2073052711473`},
{"CURE", 365, 6.399643309179052`, 296.47552877686036`}, {"CURE", 365, 0.4368000810457781`, 20.919715325249225`},
{"CURE", 365, 0.041597239113397126`, 16.192057213345635`}, {"CURE", 365, 28.925618930733286`, 686.167384369676`},
{"CURE", 365, 0.5747627017858773`, 117.55543002514237`}, {"CURE", 365, 0.8349284775068903`, 339.4740942489791`},
{"CURE", 365, 5.015377948972019`, 427.5745864357007`}, {"CURE", 365, 3.6559259454835846`, 294.81054602975144`},
{"CURE", 365, 0.5053080140087646`, 61.022603368207584`}, {"CURE", 365, 1.4209512793673509`, 99.21687141503799`},
{"CURE", 365, 0.41927861881911505`, 34.023232083876884`}, {"CURE", 365, 1.045947284649168`, 52.062199950572214`},
{"CURE", 365, 2.858499505170747`, 168.4375322266244`}, {"CURE", 365, 50.35130848576334`, 2059.3041372471794`},
{"CURE", 365, 1.3015140917022157`, 344.34737375647967`}, {"CURE", 365, 4.018306873286116`, 83.1271894676612`},
{"CURE", 365, 8.138346736173384`, 171.8998343040002`}, {"CURE", 365, 0.394792997460212`, 151.00856482910635`},
```

{"CURE", 365, 0.1176560125485188`, 17.750102239381615`}, {"CURE", 365, 0.6719245806369956`, 40.9364397568429`},
{"CURE", 365, 8.090538610462552`, 963.3192793164995`}, {"CURE", 365, 38.528985733475615`, 1489.9982360816548`},
{"CURE", 365, 6.592117856555089`, 129.1162654977449`}, {"CURE", 365, 0.39387475425866797`, 24.391519241120893`},
{"CURE", 365, 1.184696480290610 1`, 422.6380180929969`}, {"CURE", 365, 0.05983103501785764`, 22.092535250287252`},
{"CURE", 365, 0.4523544477333447`, 70.63753436923471`}, {"CURE", 365, 0.02633217376305388`, 13.507910694463053`},
{"CURE", 365, 0.24705492929208145`, 11.208024816729667`}, {"CURE", 365, 0.30289525225099667`, 88.37268376388359`},
{"CURE", 365, 3.825048653240132`, 168.43213616979511`}, {"CURE", 365, 3.5557915243185745`, 251.35760955304826`},
{"CURE", 365, 2.5124588148707483`, 103.57902258936366`}, {"CURE", 365, 0.8712903094782568`, 146.845849516189`},
{"CURE", 365, 4.31964401322526`, 143.9435767376797`}, {"CURE", 365, 18.56420715908658`, 186.38465329859562`},
{"CURE", 365, 3.910485860445433`, 108.9387894634537`}, {"CURE", 365, 12.31356172368779`, 210.69568660369654`},
{"CURE", 365, 1.122508731377677`, 65.13659797144184`}, {"CURE", 365, 2.788610080222681`, 67.70402536294989`},
{"CURE", 365, 3.2358681369399998`, 79.2427329461955`}, {"CURE", 365, 0.12010916510570846`, 24.25306928723714`},
{"CURE", 365, 0.5303741032245651`, 151.26249900455173`}, {"CURE", 365, 2.218264566636428`, 197.14122890304353`},
{"CURE", 365, 0.8715937975638373`, 37.10739151713828`}, {"CURE", 365, 0.5039131065648861`, 17.190226107461186`},
{"CURE", 365, 1.148124917902916`, 94.00047251114138`}, {"CURE", 365, 14.921890719153161`, 797.8702616830432`},
{"CURE", 365, 0.11377789341359658`, 32.550759151913745`}, {"CURE", 365, 4.421058224315699`, 987.255108905101`},
{"CURE", 365, 1.7034770959611476`, 79.14623886110805`}, {"CURE", 365, 8.278450518625618`, 1173.2465261647546`},
{"CURE", 365, 0.30454623528129254`, 102.42885744360383`}, {"CURE", 365, 0.06120550917707549`, 13.988440983463239`},
{"CURE", 365, 0.12805613451517703`, 58.970578775503064`}, {"CURE", 365, 0.1218312524346371`, 7.0535567652303435`},
{"CURE", 365, 0.12141894708000094`, 5.106676034794442`}, {"CURE", 365, 5.650858908369164`, 337.71133331773405`},
{"CURE", 365, 0.5525227493134746`, 28.870895291681936`}, {"CURE", 365, 2.5712640512894342`, 244.33951094181936`},
{"CURE", 365, 4.840780020452553`, 514.6663220565682`}, {"CURE", 365, 12.750227821019141`, 517.1754583366474`},
{"CURE", 365, 0.43116091983632426`, 16.52046328101975`}, {"CURE", 365, 1.0288684042858411`, 348.3277876215215`},
{"CURE", 365, 4.033701352995854`, 388.067086351021`}, {"CURE", 365, 2.24240628028733`, 384.9066493584876`},
{"CURE", 365, 0.2202617710300091`, 24.26068501220349`}, {"CURE", 365, 6.854137045386818`, 92.1111400203984`},
{"CURE", 365, 1.354975380798829`, 102.63658160001755`}, {"CURE", 365, 0.7436921537177428`, 81.34641971207634`},
{"CURE", 365, 4.153517835489691`, 432.1684671040795`}, {"CURE", 365, 2.519646993981479`, 131.3098456094648`},
{"CURE", 365, 3.0492124898609467`, 207.2540122317371`}, {"CURE", 365, 2.8769162443225897`, 149.51755217511214`},
{"CURE", 365, 30.658326148560207`, 1540.28521947138`}, {"CURE", 365, 0.2937957876119368`, 66.3690885064685`},
{"CURE", 365, 2.1885359681718293`, 50.11429876353143`}, {"CURE", 365, 4.235485633484285`, 506.5352791277543`},
{"CURE", 365, 0.5917674681804367`, 33.62762828873359`}, {"CURE", 365, 1.9705691941442154`, 197.42921968243124`},

{"CURE", 365, 0.23357870661900276`, 41.10371905541906`}, {"CURE", 365, 0.7408537686963116`, 36.01984857627746`},
{"CURE", 365, 1.2807414233556467`, 60.39225369766557`}, {"CURE", 365, 36.79604223938127`, 3086.4411401690004`},
{"CURE", 365, 13.889852881960564`, 498.6363553947217`}, {"CURE", 365, 2.9474700949050052`, 500.05114006486235`},
{"CURE", 365, 0.9655774367777075`, 19.60990026610167`}, {"CURE", 365, 7.025707912318654`, 373.20857141288616`},
{"CURE", 365, 18.455730496447618`, 467.4462645945179`}, {"CURE", 365, 0.15934810646229136`, 103.40376799268027`},
{"CURE", 365, 15.139673404454637`, 773.6956294559353`}, {"CURE", 365, 31.021964759388798`, 1383.0553198408777`},
{"CURE", 365, 0.7963048150556623`, 149.08551696408995`}, {"CURE", 365, 2.119261343206719`, 159.90583608203042`},
{"CURE", 365, 2.5171850003523817`, 123.96962003174689`}, {"CURE", 365, 2.7612787387777327`, 223.96307454736709`},
{"CURE", 365, 0.31915915317065396`, 82.29013696985652`}, {"CURE", 365, 0.08857925084291687`, 29.985520008928972`},
{"CURE", 365, 12.71658182152053`, 723.9761552130266`}, {"CURE", 365, 0.6332006296259399`, 137.58697666422705`},
{"CURE", 365, 0.15365657014977765`, 14.931530653286885`}, {"CURE", 365, 1.3473385111506797`, 107.00232647703284`},
{"CURE", 365, 5.1259948342605375`, 870.5494983690157`}, {"CURE", 365, 10.90447696547097`, 1005.6308018240388`},
{"CURE", 365, 1.3770924627818804`, 465.0976461606579`}, {"CURE", 365, 3.87710772054443`, 318.70425274884985`},
{"CURE", 365, 0.3858170684040066`, 196.7710761767976`}, {"CURE", 365, 1.683181860742233`, 477.7986040152708`},
{"CURE", 365, 0.8472518840942839`, 101.43715526574174`}, {"CURE", 365, 1.141383954921467`, 428.32996798436807`},
{"CURE", 365, 9.10787070589324`, 458.2063420357379`}, {"CURE", 365, 2.82977841759492`, 96.27957516206895`},
{"CURE", 365, 15.6668189778117`, 528.3298004480257`}, {"CURE", 365, 1.4851388498090854`, 60.69495045299651`},
{"CURE", 365, 11.11440259051484`, 237.70636630915095`}, {"CURE", 365, 1.8127555532573505`, 478.19191139507507`},
{"CURE", 365, 3.974424296676927`, 236.30397058708851`}, {"CURE", 365, 0.2685723260932549`, 14.951419065402865`},
{"CURE", 365, 2.1481899231136397`, 69.36843645262107`}, {"CURE", 365, 2.016970240023925`, 86.82990088848067`},
{"CURE", 365, 4.366199361200346`, 411.095419709763`}, {"CURE", 365, 52.77004304395279`, 1048.9744915907852`},
{"CURE", 365, 0.4779264729618095`, 306.7236892598338`}, {"CURE", 365, 0.17264549670887025`, 3.6404643460939097`},
{"CURE", 365, 1.0483279659199498`, 325.7899584001048`}, {"CURE", 365, 4.751301662921344`, 116.2032263630501`},
{"CURE", 365, 0.29823566469487`, 8.138676931761058`}, {"CURE", 365, 2.775805896888534`, 253.6040973094693`},
{"CURE", 365, 12.644573902769283`, 294.63335097530853`}, {"CURE", 365, 4.2812182214778804`, 81.50780626150171`},
{"CURE", 365, 9.594950027548828`, 408.59801113338796`}, {"CURE", 365, 0.529700015934212`, 51.1397231966689`},
{"CURE", 365, 0.233139106292892 5`, 19.55486145763738`}, {"CURE", 365, 2.183720172352606`, 125.91318072843296`},
{"CURE", 365, 16.051669532172493`, 982.5706485284281`}, {"CURE", 365, 0.5641323712043985`, 144.07739727207178`},
{"CURE", 365, 3.0552469421761024`, 218.73570513782337`}, {"CURE", 365, 1.621514375101536`, 122.64859675039746`},
{"CURE", 365, 2.209898798873662`, 223.51037863420527`}, {"CURE", 365, 1.1894194629126293`, 48.74200619864712`},
{"CURE", 365, 0.7848998666840057`, 201.23787228778568`}, {"CURE", 365, 1.6443352827762343`, 60.445642648510486`},

{"CURE", 365, 7.578071737081565`, 419.30713020961844`}, {"CURE", 365, 0.6239254963813726`, 61.81495490536934`},
{"CURE", 365, 0.11443665082990893`, 12.730468475558332`}, {"CURE", 365, 14.443168896926524`, 772.1757529844604`},
{"CURE", 365, 0.15408289087470284`, 60.1994149124179`}, {"CURE", 365, 5.2219754536200025`, 174.67879418718496`},
{"CURE", 365, 1.554308291229197`, 331.8777522420732`}, {"CURE", 365, 2.4090844768052238`, 358.3536475663469`},
{"CURE", 365, 15.156606183495423`, 1003.306280709522`}, {"CURE", 365, 6.5471287614966815`, 553.9604153197181`},
{"CURE", 365, 1.9498544368924733`, 181.18067784320118`}, {"CURE", 365, 17.96558086906385`, 535.8100111185594`},
{"CURE", 365, 2.11567238376569`, 463.13290698651593`}, {"CURE", 365, 0.13638496982772413`, 17.221205440235565`},
{"CURE", 365, 4.556686889206556`, 78.84628359416314`}, {"CURE", 365, 1.0001851972183822`, 225.62345944321225`},
{"CURE", 365, 0.35908125159462`, 31.554228420327636`}, {"CURE", 365, 12.398683159670984`, 709.4978594638778`},
{"CURE", 365, 10.968354980366096`, 636.4863899876477`}, {"CURE", 365, 3.950749535471167`, 336.50818781125287`},
{"CURE", 365, 0.5638594839319394`, 9.369517044258984`}, {"CURE", 365, 7.019920434759783`, 642.1798197821915`},
{"CURE", 365, 3.1404123874334466`, 677.6593056336538`}, {"CURE", 365, 9.891616496942326`, 1078.3866169737037`},
{"CURE", 365, 0.3265337067119524`, 46.34713021258334`}, {"CURE", 365, 1.9929037118465722`, 369.8355782563341`},
{"CURE", 365, 5.291958880881603`, 370.55551370507806`}, {"CURE", 365, 9.19978108987603`, 375.15374401821776`},
{"CURE", 365, 1.0154925328112296`, 76.30907090631192`}, {"CURE", 365, 1.907991823526553`, 123.9891058768109`},
{"CURE", 365, 1.3824177694935078`, 40.7160931105519`}, {"CURE", 365, 3.6239604869071558`, 955.1869421491522`},
{"CURE", 365, 3.2392618026244233`, 727.6212705626751`}, {"CURE", 365, 1.2241237757980477`, 33.02765999264955`},
{"CURE", 365, 14.778024117323378`, 1218.6995873048365`}, {"CURE", 365, 0.49439741102441914`, 37.806714370211964`},
{"CURE", 365, 0.2810355151334751`, 43.02683429671546`}, {"CURE", 365, 0.39370128182244984`, 26.276080838391046`},
{"CURE", 365, 0.46337147294330633`, 24.981400956038453`}, {"CURE", 365, 6.868050174007312`, 159.42907223174208`},
{"CURE", 365, 0.4440338333609389`, 69.51379112197858`}, {"CURE", 365, 19.133065300401437`, 766.617207189513`},
{"CURE", 365, 12.18434428537619`, 824.6638881191531`}, {"CURE", 365, 21.15897061628364 3`, 545.54350050846`},
{"CURE", 365, 7.406840093116197`, 116.62822079166959`}, {"CURE", 365, 4.208982656917056`, 182.1786820171935`},
{"CURE", 365, 0.6550962870285124`, 292.57653505361117`}, {"CURE", 365, 6.390132618544438`, 359.6415748753217`},
{"CURE", 365, 4.773066461292536`, 852.6787761970288`}, {"CURE", 365, 0.6426759573036968`, 11.069758260059203`},
{"CURE", 365, 6.721411811964657`, 3474.7372899685597`}, {"CURE", 365, 0.0824117687325663`, 41.7642108926785`},
{"CURE", 365, 65.23065450416607`, 953.5523614518913`}, {"CURE", 365, 14.412053781334974`, 921.9533600784295`},
{"CURE", 365, 0.3447338046607711`, 65.46150269639102`}, {"CURE", 365, 0.5983872760138127`, 59.25456434144156`},
{"CURE", 365, 0.11993816493127633`, 33.76193209319631`}, {"CURE", 365, 8.476876730379441`, 2467.9536959814063`},
{"CURE", 365, 0.1426998410664864`, 35.43555796984833`}, {"CURE", 365, 1.3151039716724957`, 89.93114821408972`},
{"CURE", 365, 2.1596759669162617`, 70.23501498844759`}, {"CURE", 365, 0.3079248083778762`, 25.03175533215095`},

{"CURE", 365, 0.9948529212085347`, 324.0021526323604`}, {"CURE", 365, 2.422522007496859`, 58.389347727603514`},
{"CURE", 365, 9.986582277895286`, 120.8358105927978`}, {"CURE", 365, 0.3701925558176354`, 157.20407323280364`},
{"CURE", 365, 29.73833895504342`, 1561.243240363453`}, {"CURE", 365, 0.7882925336076709`, 78.17219522759852`},
{"CURE", 365, 0.057547587941686476`, 29.227381573028573`}, {"CURE", 365, 3.405362827624421`, 219.70707236641644`},
{"CURE", 365, 0.4828804878011229`, 48.575349002402156`}, {"CURE", 365, 1.7108322927472759`, 224.73893498191137`},
{"CURE", 365, 0.9731623479343817`, 66.00901944072925`}, {"CURE", 365, 1.7475628663375633`, 205.93895443741744`},
{"CURE", 365, 24.076025484983365`, 970.6297592081823`}, {"CURE", 365, 3.570896424202618`, 241.39268160813288`},
{"CURE", 365, 15.843165362630064`, 454.08026741542784`}, {"CURE", 365, 0.3869838760286834`, 25.451694349409923`},
{"CURE", 365, 0.18791431037190542`, 24.964091765373084`}, {"CURE", 365, 1.4029473683066294`, 379.637525325384`},
{"CURE", 365, 0.11382252540200415`, 44.601855600301725`}, {"CURE", 365, 0.10949018516395882`, 15.137395531445959`},
{"CURE", 365, 0.6965156098999018`, 24.263769197227642`}, {"CURE", 365, 0.8395014502333121`, 87.970311255717`},
{"CURE", 365, 4.9010096169120105`, 556.2414944989334`}, {"CURE", 365, 3.295270209421562`, 526.6452895976034`},
{"CURE", 365, 1.1104034250646093`, 177.00917269378266`}, {"CURE", 365, 2.6491207881386227`, 156.92041943197447`},
{"CURE", 365, 0.23140143760513388`, 73.48995274954011`}, {"CURE", 365, 9.391435775351333`, 461.5110747026781`},
{"CURE", 365, 0.10347430452720659`, 11.206405375949712`}, {"CURE", 365, 0.03928158583518111`, 7.275734769728744`},
{"CURE", 365, 2.3342005416433937`, 271.22338250419824`}, {"CURE", 365, 0.5777551577080509`, 30.184869444880853`},
{"CURE", 365, 0.4994598431695315`, 35.468103360103754`}, {"CURE", 365, 0.06571535003357797`, 38.10002472367065`},
{"CURE", 365, 3.8738321017979196`, 520.6781480483644`}, {"CURE", 365, 8.94092994894461`, 72.33150085133762`},
{"CURE", 365, 0.7432258288501183`, 36.186892829606585`}, {"CURE", 365, 9.78250003046555`, 325.7171921844514`},
{"CURE", 365, 0.10152473301040414`, 11.495988199688886`}, {"CURE", 365, 15.399097849615552`, 467.0798968883814`},
{"CURE", 365, 1.8009138910715852`, 453.7643727332815`}, {"CURE", 365, 15.883892826115405`, 1186.5123810114255`},
{"CURE", 365, 3.091085259904065`, 50.2525912522677`}, {"CURE", 365, 0.5495560529049436`, 47.67058976466687`},
{"CURE", 365, 1.205647920754033`, 120.89479082559522`}, {"CURE", 365, 0.5711872999125021`, 33.12427940811496`},
{"CURE", 365, 1.8752041823729517`, 207.27307757600278`}, {"CURE", 365, 0.27999360480079627`, 138.60504510524459`},
{"CURE", 365, 29.377330903285912`, 1538.5142707303933`}, {"CURE", 365, 1.1405994580297851`, 337.51606472206856`},
{"CURE", 365, 0.44830617910124126`, 56.04526637649103`}, {"CURE", 365, 1.766262043274433`, 93.81933253206319`},
{"CURE", 365, 1.5137799896470412`, 221.6977857268474`}, {"CURE", 365, 1.3648501224311642`, 38.32646957845204`},
{"CURE", 365, 10.817395777080787`, 999.3774283886669`}, {"CURE", 365, 0.9787205897100514`, 52.860485204129354`},
{"CURE", 365, 1.7174080574952324`, 133.39259772867248`}, {"CURE", 365, 0.03841972935212842`, 10.717798784341769`},
{"CURE", 365, 6.886348840268763`, 513.1872183738485`}, {"CURE", 365, 1.7658681125145181`, 60.5985567365628`},
{"CURE", 365, 1.736044761014094`, 178.18948361205128`}, {"CURE", 365, 0.4880319236585876`, 66.5459461113712`},

```
{"CURE", 365, 0.7678751017962907`, 221.8528570633963`}, {"CURE", 365, 4.9397386860544445`, 453.9116965992587 3`},
{"CURE", 365, 13.412329516665212`, 1009.0990311752558`}, {"CURE", 365, 2.0228264449514466`, 53.785340188808405`},
{"CURE", 365, 0.05122360964374249`, 8.515994427067652`}, {"CURE", 365, 8.947694288215374`, 975.7606464095044`},
{"CURE", 365, 7.704164667552754`, 217.19229252597182`}, {"CURE", 365, 0.37845243828915387`, 101.52416932614132`},
{"CURE", 365, 44.469071857006455`, 865.3067565891688`}, {"CURE", 365, 1.0432212787154618`, 238.9961258929074`},
{"CURE", 365, 1.6116825163015236`, 202.13578445097815`}, {"CURE", 365, 6.980262625728453`, 380.73045234101113`},
{"CURE", 365, 0.7838356326464628`, 26.822777280036917`}, {"CURE", 365, 6.0198060216096545`, 452.89611553759187`},
{"CURE", 365, 5.086928102129399`, 735.7390708208238`}, {"CURE", 365, 2.785551174804992`, 1350.0413185360944`},
{"CURE", 365, 37.056373172167675`, 2049.912988423125`}, {"CURE", 365, 11.40227666177556`, 704.5878091902853`},
{"CURE", 365, 2.0103662746262`, 127.10595511240042`}, {"CURE", 365, 0.23055697813413759`, 36.78131343913735`},
{"CURE", 365, 0.934502356038442`, 61.36329041104543`}, {"CURE", 365, 2.3551105654045656`, 1311.6836173637428`},
{"CURE", 365, 0.5403001639112364`, 52.95242756787014`}, {"CURE", 365, 1.502796609894814`, 333.27910436516646`},
{"CURE", 365, 1.5758706931444053`, 789.782377760367`}, {"CURE", 365, 3.2141305193896903`, 311.2912120192276`},
{"CURE", 365, 0.07767657066779862`, 35.21727233674405`}, {"CURE", 365, 0.14180966311164409`, 20.17076452929373`},
{"CURE", 365, 1.001842188911843`, 208.87307912218856`}, {"CURE", 365, 12.075129285367062`, 3956.921323976174`},
{"CURE", 365, 2.168525291598815`, 228.37849786666183`}, {"CURE", 365, 0.320997516971354`, 12.748964830927543`},
{"CURE", 365, 1.2820846642719688`, 92.95134283518355`}, {"CURE", 365, 1.2839954736870498`, 102.9159072082954 1`},
{"CURE", 365, 22.998142419655665`, 1146.6026661421076`}, {"CURE", 365, 3.667628124098002`, 215.09440321021663`}};
```

In[ ]:=

```
TestAnOpt = {{"AnOpt Res", "AnOpt OS", "AnOpt Tox", "AnOpt Acur"}, {"CURE", 365, 11.675852926364987`, 0.10701802215863025`},
{"CURE", 365, 0.5585534836593681`, 0.0024910813361694486`}, {"CURE", 365, 4.726569798332359`, 0.030158179616365716`},
{"CURE", 365, 13.249423553220959`, 0.24740845458223798`}, {"CURE", 365, 68.37470103898688`, 0.3274277248442162`},
{"CURE", 365, 1.7981777093259999`, 0.004445214258789645`}, {"CURE", 365, 0.7950023797464685`, 0.0020730011778467214`},
{"CURE", 365, 0.21173496842088518`, 0.005325618303902999`}, {"CURE", 365, 0.42380768326563073`, 0.0015417528281032 75`},
{"CURE", 365, 6.481806062503363`, 0.027012121011289265`}, {"CURE", 365, 20.18484581722107`, 0.02248285871026948`},
{"CURE", 365, 0.35049546343089805`, 0.003173073110063029`}, {"CURE", 365, 0.34471329416410634`, 0.00093437414891687 78`},
{"CURE", 365, 154.54143423314088`, 0.11533708937875499`}, {"CURE", 365, 9.489512590420778`, 0.050169364992260684`},
{"CURE", 365, 20.2970271124094`, 0.06854457336406651`}, {"CURE", 365, 1.0490951351383295`, 0.0018246439538828173`},
{"CURE", 365, 11.818456145852247`, 0.04508265357211628`}, {"CURE", 365, 42.30966783949289`, 0.21097212340972113`},
{"CURE", 365, 0.5432060282889755`, 0.008333030777180374`}, {"CURE", 365, 2.0920596665082454`, 0.005146432268818443 5`},
{"CURE", 365, 0.5006202276058571`, 0.0030675605782100597`}, {"CURE", 365, 1.2001276804734369`, 0.01903685805382329`},
```

{"CURE", 365, 0.4266049405668143`, 0.00860071209475544`}, {"CURE", 365, 0.2630053105317752`, 0.001162869461893124`},
{"CURE", 365, 0.5501040120648935`, 0.00222281469226443`}, {"CURE", 365, 0.06773353502582387`, 0.002984057491360146`},
{"CURE", 365, 0.23387831387661615`, 0.006800328298267486`}, {"CURE", 365, 2.196631117732192`, 0.017172939219805214`},
{"CURE", 365, 4.7965032466702375`, 0.03200645177039987`}, {"CURE", 365, 0.19968621270017503`, 0.0015897866870058788`},
{"CURE", 365, 41.44919627369796`, 0.0920309311033436`}, {"CURE", 365, 1.1820546150234634`, 0.012054686001431374`},
{"CURE", 365, 11.042158977021645`, 0.13937311828506269`}, {"CURE", 365, 7.541334967623138`, 0.011871320321506264`},
{"CURE", 365, 0.21597396787201334`, 0.005567415437595665`}, {"CURE", 365, 0.11189313257963536`, 0.0006689356896620057`},
{"CURE", 365, 144.23277797493643`, 0.3768079119815565`}, {"CURE", 365, 4.992975810930269`, 0.0532336609348789`},
{"CURE", 365, 0.7668280664146859`, 0.00532818622625858`}, {"CURE", 365, 40.85441993553533`, 0.12339221962987573`},
{"CURE", 365, 0.18757480655517633`, 0.0008540336312692079`}, {"CURE", 365, 0.46275911920882246`, 0.010271582728470022`},
{"CURE", 365, 1.3231728923185901`, 0.008766064675880727`}, {"CURE", 365, 1.4663571508150983`, 0.00669124736697018`},
{"CURE", 365, 0.5761099550314943`, 0.0040382984107514164`}, {"CURE", 365, 9.554325896629601`, 0.006363295025664947`},
{"CURE", 365, 3.695378231586881`, 0.011316539640551078`}, {"CURE", 365, 0.3377588651724285`, 0.000739149623816055`},
{"CURE", 365, 0.24619876408001262`, 0.004013128906350926`}, {"CURE", 365, 2.2389187412889546`, 0.013804847055688229`},
{"CURE", 365, 3.7741928389291446`, 0.01644949878231473`}, {"CURE", 365, 3.022162558991484`, 0.016209088945382175`},
{"CURE", 365, 0.41479733976244804`, 0.0033905855788939417`}, {"CURE", 365, 1.2764324179059614`, 0.024114120149674234`},
{"CURE", 365, 2.0002495041495414`, 0.0035659106565572403`}, {"CURE", 365, 0.2585242382205`, 0.0014802305123931092`},
{"CURE", 365, 2.680050638251067`, 0.07977412594153162`}, {"CURE", 365, 8.622658090577149`, 0.0239125998713818 7`},
{"CURE", 365, 2.4090385530256535`, 0.009665204342656525`}, {"CURE", 365, 1.0780469733794205`, 0.006221044129713216`},
{"CURE", 365, 0.7090635890627703`, 0.005392101466268142`}, {"CURE", 365, 2.576249875265279`, 0.035262507299748905`},
{"CURE", 365, 1.4900292022392492`, 0.005224723357848878`}, {"CURE", 365, 9.669175788858109`, 0.047649341882988756`},
{"CURE", 365, 0.296348729051815`, 0.0012809512076219492`}, {"CURE", 365, 0.22641464925538718`, 0.004258500331392996 5`},
{"CURE", 365, 2.192402925611245`, 0.0408551744950633 94`}, {"CURE", 365, 0.18661986129021085`, 0.003275025714526541`},
{"CURE", 365, 1.6537351704312218`, 0.007141817941063057`}, {"CURE", 365, 1.0587740109871235`, 0.012402560474207912`},
{"CURE", 365, 4.554634291761799`, 0.023883589453108783`}, {"CURE", 365, 4.076974774823822`, 0.011410543792373055`},
{"CURE", 365, 64.58200071804468`, 0.5211643982909725`}, {"CURE", 365, 3.6230887625170416`, 0.03779474893236289`},
{"CURE", 365, 0.7421400071828743`, 0.00283515294926836`}, {"CURE", 365, 3.5106457690426733`, 0.022638469388563034`},
{"CURE", 365, 0.40312979858208087`, 0.0034623861072583043`}, {"CURE", 365, 5.390471026561121`, 0.011572541032542401`},
{"CURE", 365, 3.126204894565366`, 0.006122933190655233`}, {"CURE", 365, 2.1609339893348674`, 0.005777274314032263 5`},
{"CURE", 365, 0.2406058606162077`, 0.002369006129855672`}, {"CURE", 365, 0.6305149579319941`, 0.003040872805730806 7`},
{"CURE", 365, 0.3817569947972099`, 0.0026492940549694677`}, {"CURE", 365, 0.8490940263281136`, 0.005170892495986323`},

{"CURE", 365, 0.9564692260005104`, 0.012485011957839373`}, {"CURE", 365, 37.118925210998036`, 0.04512993940539981`},
{"CURE", 365, 3.0796166764098576`, 0.017451720168978533`}, {"CURE", 365, 2.804018503179765`, 0.012427184424533543`},
{"CURE", 365, 4.114543429339093`, 0.021465545422252586`}, {"CURE", 365, 20.958981219202265`, 0.1303487137424499`},
{"CURE", 365, 0.8667904377551249`, 0.018298415500981585`}, {"CURE", 365, 4.064126213360373`, 0.046534161881362335`},
{"CURE", 365, 6.083591292974745`, 0.03026586991158542`}, {"CURE", 365, 16.5690889244684`, 0.22375651773057698`},
{"CURE", 365, 4.234737159813918`, 0.04881097119050002`}, {"CURE", 365, 1.2231216136881036`, 0.005452059162979865`},
{"CURE", 365, 1.5582094173495409`, 0.002742743630629526`}, {"CURE", 365, 10.01097015714535`, 0.03270128656114845`},
{"CURE", 365, 5.75480845496051`, 0.03736373477233357`}, {"CURE", 365, 58.78287415603297`, 0.1904170378544965`},
{"CURE", 365, 3.835018373554124`, 0.019493326187185855`}, {"CURE", 365, 1.987531707090375`, 0.015443839490873459`},
{"CURE", 365, 3.9889151702782497`, 0.004528672829636537`}, {"CURE", 365, 0.13745565547988248`, 0.0014205331701977939`},
{"CURE", 365, 0.1613156639198606`, 0.002441759642616844`}, {"CURE", 365, 7.662885416599118`, 0.019722507614119297`},
{"CURE", 365, 0.39477263864309714`, 0.0034846590599952555`}, {"CURE", 365, 0.2648522977468875`, 0.0022519097620408617`},
{"CURE", 365, 2.5058210295549106`, 0.0035367191484377276`}, {"CURE", 365, 0.0515572582979473 4`, 0.0009032523768284184`},
{"CURE", 365, 0.13320164672548243`, 0.0036482558132704106`}, {"CURE", 365, 1.279082742493718`, 0.008472938055157642`},
{"CURE", 365, 4.588394962896328`, 0.038012966294891655`}, {"CURE", 365, 0.27725842284297886`, 0.003109228393490276 7`},
{"CURE", 365, 0.21042027205114636`, 0.0009173196025089321`}, {"CURE", 365, 0.6266775053103508`, 0.009754117210351234`},
{"CURE", 365, 0.1836229060363542`, 0.0046514825185145605`}, {"CURE", 365, 0.1905410969269682 5`, 0.001031157699561616`},
{"CURE", 365, 1.2236194564725285`, 0.004342647449604589`}, {"CURE", 365, 0.41063096712559316`, 0.008448677939261401`},
{"CURE", 365, 0.9439870030588124`, 0.005936083811070825`}, {"CURE", 365, 3.7977390963825517`, 0.00776906401018819 5`},
{"CURE", 365, 42.406875260755484`, 0.22473052503499275`}, {"CURE", 365, 0.1340375002547923`, 0.0012461188051551093`},
{"CURE", 365, 15.173305235382095`, 0.04009335441845204`}, {"CURE", 365, 5.357424653925638`, 0.028343168107983152`},
{"CURE", 365, 0.7764488715369673`, 0.030949115675447027`}, {"CURE", 365, 0.9329931741253724`, 0.004337460286633252`},
{"CURE", 365, 1.2046052455207046`, 0.0071652122753007655`}, {"CURE", 365, 0.14474158787410002`, 0.0010921183776774924`},
{"CURE", 365, 0.31398952468249963`, 0.00199503753660329`}, {"CURE", 365, 1.6948988996695222`, 0.04115781098189813`},
{"CURE", 365, 12.717361274175108`, 0.053966313816662316`}, {"CURE", 365, 0.05208053577487069`, 0.0006200712352549124`},
{"CURE", 365, 16.02621808441055`, 0.08387830529244374`}, {"CURE", 365, 22.12910835842891`, 0.031168328950437744`},
{"CURE", 365, 11.6484969413985`, 0.046584211044267944`}, {"CURE", 365, 19.895208938843915`, 0.03393047945660899`},
{"CURE", 365, 1.080592946610801`, 0.005623885731708263`}, {"CURE", 365, 1.9343197588929264`, 0.0024528840171379636`},
{"CURE", 365, 0.13422174151058427`, 0.00541391960892696`}, {"CURE", 365, 0.5399143980250604`, 0.0024838330169821067`},
{"CURE", 365, 1.0611563925534349`, 0.00432145390963819`}, {"CURE", 365, 0.07213731766915465`, 0.00225405144670739`},
{"CURE", 365, 0.5792471297201878`, 0.012966959069489062`}, {"CURE", 365, 0.979429914346741`, 0.003580168643574747`},

{"CURE", 365, 38.75954305956427`, 0.11872286213971664`}, {"CURE", 365, 0.8795014931023295`, 0.003206394661965397`},
{"CURE", 365, 2.5542713093317757`, 0.011369056353852984`}, {"CURE", 365, 0.5494530455590539`, 0.002552224348906911`},
{"CURE", 365, 1.3693800590226377`, 0.01057152044882085`}, {"CURE", 365, 0.06633695490541701`, 0.0019481744221742498`},
{"CURE", 365, 5.1552963472529045`, 0.03431361670659124`}, {"CURE", 365, 6.53546288072817`, 0.09090223796032411`},
{"CURE", 365, 0.6751281852536264`, 0.019554118947136927`}, {"CURE", 365, 3.8311112960961395`, 0.018167941885822776`},
{"CURE", 365, 0.15630662374612864`, 0.0013370406771521198`}, {"CURE", 365, 1.4431907886381161`, 0.004601508041073364`},
{"CURE", 365, 1.4022753175047062`, 0.01110052491126327`}, {"CURE", 365, 0.4819269886555518`, 0.004637546491884598`},
{"CURE", 365, 31.26388803276261`, 0.044478720572338815`}, {"CURE", 365, 0.9331743984854004`, 0.01771934294315546`},
{"CURE", 365, 7.068816537781991`, 0.04501442776213072`}, {"CURE", 365, 3.792026705085477`, 0.04243706498764285`},
{"CURE", 365, 0.5069322958529124`, 0.00243808786527265`}, {"CURE", 365, 0.08350002530865051`, 0.0014599187433894075`},
{"CURE", 365, 2.537568098217128`, 0.01109821170585833`}, {"CURE", 365, 1.4996301391344855`, 0.002471444134175588 3`},
{"CURE", 365, 1.620540549421759`, 0.031114293602807385`}, {"CURE", 365, 1.3000854733822071`, 0.008216630499944871`},
{"CURE", 365, 3.5650934480524965`, 0.02099488016017455`}, {"CURE", 365, 1.7662785145063704`, 0.002862457190831398`},
{"CURE", 365, 34.50417147432099`, 0.14750064072441815`}, {"CURE", 365, 5.111771112992889`, 0.02679803741955006`},
{"CURE", 365, 29.946288448498425`, 0.08874807572788128`}, {"CURE", 365, 7.95553123833185`, 0.06095117054135843`},
{"CURE", 365, 1.7516334566342011`, 0.004831407708889974`}, {"CURE", 365, 17.42821963467653`, 0.0699605905638198`},
{"CURE", 365, 0.2696776283365377`, 0.0018558867034906114`}, {"CURE", 365, 6.978347271608495`, 0.05113351661536620 6`},
{"CURE", 365, 0.12829566701326786`, 0.0028857185344959148`}, {"CURE", 365, 0.10739303483300365`, 0.0035611290420323573`},
{"CURE", 365, 7.409239752972486`, 0.10345242137302467`}, {"CURE", 365, 0.06478043654529159`, 0.0006837394161386015`},
{"CURE", 365, 3.247672280786589`, 0.07738930285229384`}, {"CURE", 365, 1.499949113340129`, 0.00980264715636959`},
{"CURE", 365, 9.70323230547323`, 0.023183271670097795`}, {"CURE", 365, 0.3953273176705507`, 0.003489146665630105`},
{"CURE", 365, 9.085101166508915`, 0.04317636833989707`}, {"CURE", 365, 0.4414020791879803 6`, 0.0016883021215060086`},
{"CURE", 365, 0.03650377531935933`, 0.00017930351913186785`}, {"CURE", 365, 0.10110338485775204`, 0.0006603796794056766`},
{"CURE", 365, 0.0817427798379466 4`, 0.0013505955907205157`}, {"CURE", 365, 8.00972399166346`, 0.01904995406505628`},
{"CURE", 365, 0.1031312305085015`, 0.0028178376416841166`}, {"CURE", 365, 2.5866751341779`, 0.006643516411569152`},
{"CURE", 365, 49.25321277487635`, 0.12810007327162845`}, {"CURE", 365, 0.08471873203145223`, 0.0007989387398538756`},
{"CURE", 365, 0.8383658050850984`, 0.006610347794960522`}, {"CURE", 365, 68.52438043065817`, 0.09339052895888218`},
{"CURE", 365, 3.2179155365203576`, 0.017341730880047854`}, {"CURE", 365, 0.35931131251241677`, 0.001607973013739164`},
{"CURE", 365, 0.7688305924256124`, 0.005529916640140463`}, {"CURE", 365, 1.0659223489596388`, 0.020140941319180365`},
{"CURE", 365, 13.087545226442998`, 0.0406860294739212`}, {"CURE", 365, 221.398906162617`, 0.253789974533682`},
{"CURE", 365, 0.1355597666300411`, 0.0010326473346599194`}, {"CURE", 365, 2.103311853504219`, 0.017793614291583684`},

{"CURE", 365, 0.331025114402912`, 0.0015222419582246744`}, {"CURE", 365, 1.7612906423067125`, 0.02002468350062018`},
{"CURE", 365, 1.8870460270513971`, 0.0017055526123279739`}, {"CURE", 365, 1.5929847727769066`, 0.017891014615783244`},
{"CURE", 365, 1.1367479679063488`, 0.0020851443024508197`}, {"CURE", 365, 11.062393133969344`, 0.02611312243706006`},
{"CURE", 365, 22.35737512262075`, 0.0543142535547325`}, {"CURE", 365, 2.1521924003559234`, 0.009873977089262504`},
{"CURE", 365, 0.9971342650422818`, 0.001962244494330458`}, {"CURE", 365, 14.477982232292504`, 0.022568342430202632`},
{"CURE", 365, 0.2717783350114016`, 0.0011894747769698386`}, {"CURE", 365, 0.218833659122544`, 0.00518914312292359`},
{"CURE", 365, 2.1038924521083366`, 0.005599019446266498`}, {"CURE", 365, 0.3048185816054923`, 0.0013138049406485966`},
{"CURE", 365, 1.2357047958966656`, 0.005490879801161955`}, {"CURE", 365, 0.10877284373313552`, 0.002197697801106415`},
{"CURE", 365, 13.431879740474491`, 0.011831496800272581`}, {"CURE", 365, 0.3393060358513766`, 0.004084443095651758`},
{"CURE", 365, 7.179284853445973`, 0.019696513908106734`}, {"CURE", 365, 4.93407567823109`, 0.02167505277842642`},
{"CURE", 365, 1.7998606457390776`, 0.011134154827687752`}, {"CURE", 365, 0.159801842030147`, 0.004130662575284431`},
{"CURE", 365, 0.18221938287131373`, 0.0006241022771584267`}, {"CURE", 365, 0.6012572485091232`, 0.0034831640818474433`},
{"CURE", 365, 0.8421554540103321`, 0.0018763457681928606`}, {"CURE", 365, 3.1188994267718084`, 0.010417579924532742`},
{"CURE", 365, 21.741253155102847`, 0.05691692876415632`}, {"CURE", 365, 14.680288131231746`, 0.08009330603606846`},
{"CURE", 365, 4.1706576638363355`, 0.026065958599977224`}, {"CURE", 365, 140.47651900711756`, 0.1642308980358522`},
{"CURE", 365, 0.4268570451738967`, 0.001920675273275835`}, {"CURE", 365, 1.5326763690446856`, 0.02576780321313383`},
{"CURE", 365, 0.6908513752526765`, 0.0034846391898553618`}, {"CURE", 365, 31.89939429294488`, 0.15614990117586652`},
{"CURE", 365, 2.4500807108150795`, 0.09001770160487825`}, {"CURE", 365, 0.08299843434854993`, 0.001731162914325296 7`},
{"CURE", 365, 1.2468616890712645`, 0.016891990841734372`}, {"CURE", 365, 65.36361362186908`, 0.09304335357026285`},
{"CURE", 365, 0.24096196174842785`, 0.001124871944911661`}, {"CURE", 365, 0.6133215801366959`, 0.0027431179207856515`},
{"CURE", 365, 1.1173881558396712`, 0.006200151331195171`}, {"CURE", 365, 58.61406049248651`, 0.07943779563515152`},
{"CURE", 365, 0.2030740722624613`, 0.0012119651865030209`}, {"CURE", 365, 6.497668558707803`, 0.0193126421963842 8`},
{"CURE", 365, 2.6792049752870652`, 0.015845866783803897`}, {"CURE", 365, 1.6107472991433749`, 0.006435124687545020 4`},
{"CURE", 365, 0.3482279695717700 5`, 0.005793814200382373`}, {"CURE", 365, 1.1338504304104036`, 0.005095188765800666`},
{"CURE", 365, 2.4005463569806373`, 0.01719914044313496`}, {"CURE", 365, 0.3741751109365802`, 0.0013864112755775095`},
{"CURE", 365, 5.900712470502467`, 0.013777978474317918`}, {"CURE", 365, 11.663849661031183`, 0.024389765409552212`},
{"CURE", 365, 5.6532003710322485`, 0.02015200233322289`}, {"CURE", 365, 0.22770403306008918`, 0.0009622826828885527`},
{"CURE", 365, 15.179462920167783`, 0.04682571791019264`}, {"CURE", 365, 3.112624047899373`, 0.019676285900151858`},
{"CURE", 365, 0.9978325770906971`, 0.0056153748287288245`}, {"CURE", 365, 3.086918352770055`, 0.02468126626685473`},
{"CURE", 365, 0.4150864019150924`, 0.004209114489563558`}, {"CURE", 365, 2.6837849946382746`, 0.016848482003813208`},
{"CURE", 365, 2.5245759055480907`, 0.00616241261743693`}, {"CURE", 365, 0.2804703601081981`, 0.006024186269701683`},

{"CURE", 365, 17.936361915084337`, 0.08569071951397111`}, {"CURE", 365, 0.03870883507212293`, 0.0009987685497423854`},
{"CURE", 365, 2.968520126158353`, 0.014644908594729906`}, {"CURE", 365, 6.267208716762264`, 0.0541750779772649`},
{"CURE", 365, 1.2021128734553577`, 0.002808110189501305 2`}, {"CURE", 365, 0.3973281935271973`, 0.010026607221568007`},
{"CURE", 365, 13.761342700582228`, 0.04840446311537895`}, {"CURE", 365, 7.46398899788946`, 0.020979697683344026`},
{"CURE", 365, 0.9691709801566692`, 0.016658463942810002`}, {"CURE", 365, 5.652669186889103`, 0.04235078085271107`},
{"CURE", 365, 0.6097277842311666`, 0.00572105764373661`}, {"CURE", 365, 7.334310760147796`, 0.11832906698488838`},
{"CURE", 365, 1.4126800009298426`, 0.0038757802030117325`}, {"CURE", 365, 8.102615703591024`, 0.05895206583567592`},
{"CURE", 365, 1.2271613343443863`, 0.004683690873215353`}, {"CURE", 365, 4.126069379144341`, 0.016072389904883817`},
{"CURE", 365, 0.1966916965419304`, 0.0006858005979472919`}, {"CURE", 365, 1.0403214355924242`, 0.004599170200231354`},
{"CURE", 365, 0.47155602141987835`, 0.0017598160848276722`}, {"CURE", 365, 0.43530432502118044`, 0.0020215921894949523`},
{"CURE", 365, 10.719548909094382`, 0.02582955515078959`}, {"CURE", 365, 0.6273767706087355`, 0.0025048415067900703`},
{"CURE", 365, 11.364430570585785`, 0.054980878403533016`}, {"CURE", 365, 11.011221022147147`, 0.020849877377463518`},
{"CURE", 365, 1.19721705354876`, 0.019572730471730092`}, {"CURE", 365, 3.24597341725096`, 0.009352972506362677`},
{"CURE", 365, 0.06265550571366187`, 0.002490337972312227`}, {"CURE", 365, 3.987575035349543`, 0.012515001190565981`},
{"CURE", 365, 0.2403046722513744`, 0.003238122021512095`}, {"CURE", 365, 22.55967356657356`, 0.05840166651261356`},
{"CURE", 365, 1.9752904156862734`, 0.03226074570005758`}, {"CURE", 365, 3.969597202448211`, 0.0099423886458399`},
{"CURE", 365, 32.081856048234286`, 0.03215062989837209`}, {"CURE", 365, 1.8875791237798127`, 0.043073490708187355`},
{"CURE", 365, 0.11119072674013143`, 0.003567079162200001`}, {"CURE", 365, 2.453761067108228`, 0.021155308274467016`},
{"CURE", 365, 1.648940130241732`, 0.05858376771512718`}, {"CURE", 365, 0.12763869257950045`, 0.00664381615863988`},
{"CURE", 365, 0.3226423134912996`, 0.0029055088485719907`}, {"CURE", 365, 9.855869171452868`, 0.05391903199564725`},
{"CURE", 365, 0.5101847045643061`, 0.0025754343311021736`}, {"CURE", 365, 27.654989887958347`, 0.14605482991102878`},
{"CURE", 365, 0.13421175151409015`, 0.0009251084908572336`}, {"CURE", 365, 2.8598112554652753`, 0.011225205432712018`},
{"CURE", 365, 2.545413497673282`, 0.005613343106368492`}, {"CURE", 365, 0.6304936000183128`, 0.017219875488990084`},
{"CURE", 365, 0.5472332150786609`, 0.0027461411156310556`}, {"CURE", 365, 0.41300153487165225`, 0.002393953171644265`},
{"CURE", 365, 1.1816735938587686`, 0.009462324720781885`}, {"CURE", 365, 2.6181507883966773`, 0.011383658329152214`},
{"CURE", 365, 3.0488499354526315`, 0.00895907958365286`}, {"CURE", 365, 3.569684082574203`, 0.00447990443487 3941`},
{"CURE", 365, 27.651075508735637`, 0.046137723722766574`}, {"CURE", 365, 20.735562697335737`, 0.11151195187821646`},
{"CURE", 365, 0.13512573082198343`, 0.006916694524570524`}, {"CURE", 365, 3.0755318027957284`, 0.014362413398595454`},
{"CURE", 365, 2.6353035175784383`, 0.0047552593246437775`}, {"CURE", 365, 0.3848537316076298`, 0.0027961944953639587`},
{"CURE", 365, 0.34315988556166904`, 0.0020853200281497146`}, {"CURE", 365, 0.19239267834595825`, 0.0018172399941890631`},
{"CURE", 365, 0.7795323565769392`, 0.004970204725891684`}, {"CURE", 365, 3.6478318489700396`, 0.08918339111246754`},

{"CURE", 365, 17.76569040225202`, 0.08853706301831867`}, {"CURE", 365, 0.3836405913588133`, 0.003455905698925943`},
{"CURE", 365, 14.501492765705299`, 0.04420522824732079`}, {"CURE", 365, 28.936973070986603`, 0.12828695463108697`},
{"CURE", 365, 0.30417494798164196`, 0.001411245773557157`}, {"CURE", 365, 0.6367696753611434`, 0.0035760818974489043`},
{"CURE", 365, 0.6589853587875962`, 0.006944308701055185`}, {"CURE", 365, 0.18064995877024778`, 0.002821405577294564`},
{"CURE", 365, 0.3430674846791046`, 0.005459591284119224`}, {"CURE", 365, 2.139413242640293`, 0.026142528273722147`},
{"CURE", 365, 0.1862575496597255`, 0.0029006496487889426`}, {"CURE", 365, 37.52288285430278`, 0.04367939447557702`},
{"CURE", 365, 14.217960154505564`, 0.029753164070701586`}, {"CURE", 365, 0.722075247268289`, 0.007790224933207356`},
{"CURE", 365, 5.045382166830712`, 0.0035304743029419467`}, {"CURE", 365, 0.9185652460455721`, 0.002243495257179751`},
{"CURE", 365, 1.0203839435318527`, 0.004706799164835926`}, {"CURE", 365, 0.5819766770949885`, 0.0015729150979927413`},
{"CURE", 365, 0.0855689180130791`, 0.0015325677698399013`}, {"CURE", 365, 5.317738663320431`, 0.011856714904202864`},
{"CURE", 365, 6.276811525819406`, 0.005236057666537616`}, {"CURE", 365, 0.06538094440359558`, 0.0010100411870922136`},
{"CURE", 365, 0.5651850418590113`, 0.002826269386716843`}, {"CURE", 365, 8.653166306328398`, 0.026206754144636718`},
{"CURE", 365, 0.1175671941578087`, 0.0007189113917323972`}, {"CURE", 365, 0.15048816985459693`, 0.001851075159640714`},
{"CURE", 365, 0.14804515912784344`, 0.002251695355435125`}, {"CURE", 365, 2.7163414572293347`, 0.004790384818516976`},
{"CURE", 365, 3.8356800768743735`, 0.012837562509875897`}, {"CURE", 365, 1.3269980856462325`, 0.007783791811221205`},
{"CURE", 365, 11.144421897932826`, 0.02648192366999658`}, {"CURE", 365, 0.715349662579851`, 0.009444868318908095`},
{"CURE", 365, 4.450318104118964`, 0.021774121654011654`}, {"CURE", 365, 0.2765034508029542`, 0.0022797438941327204`},
{"CURE", 365, 3.1942812940550853`, 0.0038589431362018828`}, {"CURE", 365, 41.31132131888034`, 0.19550769650399605`},
{"CURE", 365, 137.13593099349015`, 0.14038555271463532`}, {"CURE", 365, 121.51254969188254`, 0.08047054682960522`},
{"CURE", 365, 0.8295912389914325`, 0.00717042169764921`}, {"CURE", 365, 1.952917732550996`, 0.05937437753662711`},
{"CURE", 365, 0.1752074006433577`, 0.006132352075547854`}, {"CURE", 365, 3.1452143372057204`, 0.01523790133781683`},
{"CURE", 365, 3.556080464119122`, 0.004451732531422425`}, {"CURE", 365, 0.9611188011437478`, 0.005917682204351315`},
{"CURE", 365, 2.055192933232784`, 0.016618471464634507`}, {"CURE", 365, 41.01649160118238`, 0.17375266256721983`},
{"CURE", 365, 12.853521365484264`, 0.050168109241946326`}, {"CURE", 365, 56.90897358525238`, 0.053177520591275396`},
{"CURE", 365, 1.6352650359724565`, 0.0018527362774043024`}, {"CURE", 365, 5.372144575762693`, 0.02941496792952326`},
{"CURE", 365, 0.7945641645432409`, 0.013004891740016712`}, {"CURE", 365, 1.1377981493627114`, 0.007633207743215023`},
{"CURE", 365, 0.07954107186779909`, 0.0034220020065863703`}, {"CURE", 365, 21.089924516774097`, 0.04813036218816992`},
{"CURE", 365, 2.3990037549060017`, 0.003629475015941648`}, {"CURE", 365, 0.16018874225256105`, 0.0007554744265386706`},
{"CURE", 365, 1.7345509951870635`, 0.010141581648999209`}, {"CURE", 365, 0.6041191119561087`, 0.0008384226579979914`},
{"CURE", 365, 1.2166892523588149`, 0.026802914799347204`}, {"CURE", 365, 2.1345014984800024`, 0.03627718276369946`},
{"CURE", 365, 0.42092502411170296`, 0.0022630947845312267`}, {"CURE", 365, 10.791861264230812`, 0.045122489923274715`},

```
{"CURE", 365, 97.81275019435247`, 0.21265269488797367`}, {"CURE", 365, 1.9302395441557407`, 0.004161701154873009`},
{"CURE", 365, 0.6819266448519344`, 0.003553769106582779`}, {"CURE", 365, 10.876130346441506`, 0.05479265890035875`},
{"CURE", 365, 19.439455802258724`, 0.07844980085646375`}, {"CURE", 365, 8.063521232518946`, 0.09377003922291274`},
{"CURE", 365, 0.053147373074882015`, 0.002190254849911554`}, {"CURE", 365, 4.441076333839299`, 0.0175654711420253`},
{"CURE", 365, 0.2865855632769368`, 0.0009403625724785995`}, {"CURE", 365, 0.6762604573412978`, 0.0045366952376289585`},
{"CURE", 365, 0.5272530318134292`, 0.0019439938593896926`}, {"CURE", 365, 1.6615022579855139`, 0.019830741390106908`},
{"CURE", 365, 0.2343848234281496`, 0.0042601933496990586`}, {"CURE", 365, 0.7535396524238314`, 0.0031222262728967717`},
{"CURE", 365, 5.1564910886164705`, 0.03546001716250254`}, {"CURE", 365, 2.0192525106255945`, 0.009689127919128878`},
{"CURE", 365, 6.320439848185638`, 0.004537639516439101`}, {"CURE", 365, 0.4827954696450767`, 0.0036960231155024137`},
{"CURE", 365, 2.954467520122949`, 0.045823169875183006`}, {"CURE", 365, 0.6490326763536838`, 0.013043550722300732`},
{"CURE", 365, 0.6238765603985259`, 0.010295417227659968`}, {"CURE", 365, 1.3621209440679267`, 0.003951635767064258`},
{"CURE", 365, 0.1662025445999456`, 0.006960681738526928`}, {"CURE", 365, 9.708748318131562`, 0.02991243945153136`},
{"CURE", 365, 0.4673973448470365`, 0.0013329472824564067`}, {"CURE", 365, 0.465435851215731`, 0.001683517777348303`},
{"CURE", 365, 0.3274544923756336`, 0.001465249775629895`}, {"CURE", 365, 1.710443057491526`, 0.038304593495218496`},
{"CURE", 365, 3.122576480821987`, 0.006338451936633577`}, {"CURE", 365, 29.609821094644605`, 0.19670186962525155`},
{"CURE", 365, 1.1928703067577047`, 0.018335568630522055`}, {"CURE", 365, 2.182168848041652`, 0.005025366202257951`},
{"CURE", 365, 0.09990518288573826`, 0.0013001336512698208`}, {"CURE", 365, 3.9409476256026026`, 0.007589485853038574`},
{"CURE", 365, 1.10616812706861`, 0.003273021237508188`}, {"CURE", 365, 0.4026681262290463`, 0.0018115403277911527`},
{"CURE", 365, 0.5171057874007756`, 0.0015863501287307902`}, {"CURE", 365, 6.446780358057298`, 0.05595767018612602`},
{"CURE", 365, 5.362845493486007`, 0.01052681231401106`}, {"CURE", 365, 0.2433843609440164`, 0.004024544834715997`},
{"CURE", 365, 0.3399200635026374`, 0.0013397714744903403`}, {"CURE", 365, 0.40398493726558277`, 0.0013597480496799284`},
{"CURE", 365, 0.40148792420922536`, 0.003176574066594078`}, {"CURE", 365, 12.34987663632247`, 0.010357653060342902`},
{"CURE", 365, 8.897368417359349`, 0.037407349052006454`}, {"CURE", 365, 0.6395694933171944`, 0.002646044622048406`},
{"CURE", 365, 3.822121302439955`, 0.015422860125115132`}, {"CURE", 365, 5.203140790679605`, 0.005212403140623898`},
{"CURE", 365, 2.4492896165313867`, 0.007720664144314298`}, {"CURE", 365, 6.523487942210288`, 0.021987536506388763`},
{"CURE", 365, 27.62763669880057`, 0.0427815282461806`}, {"CURE", 365, 1.2668889345789125`, 0.005671024182105451`},
{"CURE", 365, 3.1823027961242842`, 0.016203444070752585`}, {"CURE", 365, 1.065455020491336`, 0.004387353177204336`},
{"CURE", 365, 0.3763884284379456`, 0.0011242589886933016`}, {"CURE", 365, 0.34682395806085925`, 0.0005864798751230476`},
{"CURE", 365, 0.3502042213242307`, 0.0027630126065500793`}, {"CURE", 365, 0.6147338874091698`, 0.005286674767719244`},
{"CURE", 365, 0.2006313623402163`, 0.0008121317280037172`}, {"CURE", 365, 1.4167605680420783`, 0.005537963897968327`},
{"CURE", 365, 14.469960435021491`, 0.05073968472397686`}, {"CURE", 365, 0.5122172869538275`, 0.001670916527245348 2`},
```

{"CURE", 365, 2.3353857051295566`, 0.013796326260071181`}, {"CURE", 365, 7.803579704026223`, 0.09006448993658663`},
{"CURE", 365, 26.994373440719055`, 0.0831858872157397`}, {"CURE", 365, 1.3328077014005253`, 0.016299367599046127`},
{"CURE", 365, 1.840748941516157`, 0.010575057927855009`}, {"CURE", 365, 13.133485793821995`, 0.0636458871869862`},
{"CURE", 365, 0.12469543004525942`, 0.001996093452897163`}, {"CURE", 365, 0.08971944787978817`, 0.002929268533120451 5`},
{"CURE", 365, 28.26259978767797`, 0.14620126988378424`}, {"CURE", 365, 13.1220210689221`, 0.050524564226492506`},
{"CURE", 365, 17.968998654863327`, 0.05800870850215902`}, {"CURE", 365, 7.151662493427366`, 0.03256210322649286`},
{"CURE", 365, 0.8802216669547815`, 0.031185450212846095`}, {"CURE", 365, 4.430559622441085`, 0.017538524112760807`},
{"CURE", 365, 1.2737582435204962`, 0.02655657350614602`}, {"CURE", 365, 1.818595493760734`, 0.0024456746095821965`},
{"CURE", 365, 8.676979629848551`, 0.00898332324996776`}, {"CURE", 365, 2.142290964114945`, 0.016641735295135605`},
{"CURE", 365, 34.85537395409378`, 0.1803330897947196`}, {"CURE", 365, 6.570518379588628`, 0.00642224818897062`},
{"CURE", 365, 2.0266179695174382`, 0.010871059002651564`}, {"CURE", 365, 10.562426791748907`, 0.09737279935831235`},
{"CURE", 365, 7.172628663384794`, 0.0849489141029977`}, {"CURE", 365, 0.1074832914418079`, 0.004731186998054476`},
{"CURE", 365, 0.15455314630093572`, 0.0010693606896238065`}, {"CURE", 365, 0.3761357408566121`, 0.0023480940444703135`},
{"CURE", 365, 18.019717063218792`, 0.04208454128940476`}, {"CURE", 365, 0.27513085159854883`, 0.0013438140442402226`},
{"CURE", 365, 0.7299816416489934`, 0.0020313237199904043`}, {"CURE", 365, 0.7601990433407503`, 0.005996702439707503`},
{"CURE", 365, 38.58091547839663`, 0.13948303743038754`}, {"CURE", 365, 1.5737559746168541`, 0.0025613758733074254`},
{"CURE", 365, 2.928964614235372`, 0.0470012190759418 2`}, {"CURE", 365, 0.6592170647823273`, 0.0068333795861127 66`},
{"CURE", 365, 7.400825476931759`, 0.05151644285579798`}, {"CURE", 365, 2.0385013068948994`, 0.010218703543263938`},
{"CURE", 365, 18.868729311409364`, 0.1429930093179921`}, {"CURE", 365, 0.07566392988731668`, 0.0006023625188574764`},
{"CURE", 365, 1.6768069998755646`, 0.031614401326294624`}, {"CURE", 365, 0.7296482659585019`, 0.008854733613098308`},
{"CURE", 365, 14.885082315157186`, 0.09512203313796244`}, {"CURE", 365, 3.437529682042204`, 0.00973126488018959`},
{"CURE", 365, 48.33393050555151`, 0.15227968983309886`}, {"CURE", 365, 2.381960079176291`, 0.0147271676073411 4`},
{"CURE", 365, 79.60679225533065`, 0.3242998892083759`}, {"CURE", 365, 58.95515245965202`, 0.11236764648524508`},
{"CURE", 365, 2.322729819633075`, 0.0091616539742759`}, {"CURE", 365, 0.9450583067088253`, 0.005002981641283894`},
{"CURE", 365, 0.3383120213628503`, 0.002809545450007668`}, {"CURE", 365, 0.8866339631145157`, 0.02347960864922657`},
{"CURE", 365, 0.07214202190130967`, 0.0003450564498079601`}, {"CURE", 365, 4.943883421765422`, 0.020307161672240493`},
{"CURE", 365, 13.618700806693692`, 0.014210752143047223`}, {"CURE", 365, 1.6432150929202354`, 0.033714297282419696`},
{"CURE", 365, 0.1638128456506528`, 0.0034352073055020776`}, {"CURE", 365, 0.5200267656879463`, 0.00344162031605801`},
{"CURE", 365, 0.21641952831107003`, 0.0004906328328802681`}, {"CURE", 365, 0.049085994292630254`, 0.000893839803946439`},
{"CURE", 365, 2.116709852776723`, 0.00781736614630586`}, {"CURE", 365, 0.6270247826504344`, 0.009911916031548312`},
{"CURE", 365, 1.0721471133704177`, 0.006846071598314788`}, {"CURE", 365, 0.7544099968186723`, 0.01189714496747062`},

{"CURE", 365, 0.9881498565892703`, 0.014722045132800418`}, {"CURE", 365, 0.2669260565457473`, 0.004411867444944455`},
{"CURE", 365, 0.8617772248528257`, 0.001352624107005165З`}, {"CURE", 365, 8.966113729382393`, 0.08732521872556562`},
{"CURE", 365, 28.308653723769286`, 0.15478107622719656`}, {"CURE", 365, 0.16723693115624913`, 0.006683399833662371`},
{"CURE", 365, 7.829491356267621`, 0.026012636832559038`}, {"CURE", 365, 0.30156789903679404`, 0.0007223896882298272`},
{"CURE", 365, 11.661016987155168`, 0.03189451003080373`}, {"CURE", 365, 0.15555174933192664`, 0.0028147844833490746`},
{"CURE", 365, 0.35275117548366897`, 0.0031766259070732236`}, {"CURE", 365, 33.939783787694935`, 0.030199430442429086`},
{"CURE", 365, 5.006129798160904`, 0.016483819382460876`}, {"CURE", 365, 13.42519995747273`, 0.06424300069706422`},
{"CURE", 365, 0.06170583872218328`, 0.0008459385960854412`}, {"CURE", 365, 1.4732117276913315`, 0.01397333604675177`},
{"CURE", 365, 8.264858482811444`, 0.05039707967400028`}, {"CURE", 365, 2.7389090875911144`, 0.06729229623866874`},
{"CURE", 365, 1.1143136699338316`, 0.005576690060519603`}, {"CURE", 365, 0.442533274883293`, 0.0014201099736225198`},
{"CURE", 365, 4.363126224320248`, 0.02310188700874343`}, {"CURE", 365, 0.5748961020754084`, 0.0027874897885550687`},
{"CURE", 365, 2.3419830980699885`, 0.01554614916530957`}, {"CURE", 365, 6.112839452006774`, 0.06278811119062715`},
{"CURE", 365, 22.701337297350307`, 0.04126414375541988`}, {"CURE", 365, 9.548555981833921`, 0.011964639995088417`},
{"CURE", 365, 0.19915563156525584`, 0.0009411701938656627`}, {"CURE", 365, 0.8769132399273554`, 0.003241681366812644`},
{"CURE", 365, 0.5920688988673052`, 0.0017556509368139694`}, {"CURE", 365, 3.9695570536134563`, 0.01183042242756068`},
{"CURE", 365, 1.6712171593765275`, 0.056876930811887355`}, {"CURE", 365, 23.634006149798452`, 0.06013146788452806`},
{"CURE", 365, 0.2716503001965952`, 0.0006759676397369943`}, {"CURE", 365, 21.77315148720415`, 0.07900345342417876`},
{"CURE", 365, 4.114479984922489`, 0.0037416977301863623`}, {"CURE", 365, 0.7064470981417352`, 0.0031193926100840964`},
{"CURE", 365, 8.299065557283733`, 0.08159645638409763`}, {"CURE", 365, 0.9762840993880214`, 0.004127538111611102`},
{"CURE", 365, 0.3443297045158426`, 0.0011244162067100267`}, {"CURE", 365, 1.265529802461436`, 0.009279506242998087`},
{"CURE", 365, 11.916763870733035`, 0.11020195312348377`}, {"CURE", 365, 1.1152387700911703`, 0.011910331448193125`},
{"CURE", 365, 3.5454289842421742`, 0.02104830714936267`}, {"CURE", 365, 2.997373672005945`, 0.005730676539500836`},
{"CURE", 365, 0.12132391137999701`, 0.0003651897434151015`}, {"CURE", 365, 3.536572526379164`, 0.06056255821859172`},
{"CURE", 365, 0.2692780683118868`, 0.0008065459142350193`}, {"CURE", 365, 4.838845770335258`, 0.009807759216348888`},
{"CURE", 365, 1.6928999596169114`, 0.018897820769958745`}, {"CURE", 365, 14.262554677358768`, 0.027657468449024145`},
{"CURE", 365, 1.0015298177752376`, 0.013020781130733263`}, {"CURE", 365, 1.0915068767970488`, 0.02201757883874439`},
{"CURE", 365, 0.3509613373066074`, 0.004956188755237377`}, {"CURE", 365, 5.650081951560868`, 0.0419531083957699`},
{"CURE", 365, 125.01116330589335`, 0.203459283327384`}, {"CURE", 365, 0.7493450566238656`, 0.0034700445325281624`},
{"CURE", 365, 20.947541281158603`, 0.07602040902857153`}, {"CURE", 365, 0.061579893052957885`, 0.0009525451608020984`},
{"CURE", 365, 73.62217781417942`, 0.05907575412320965`}, {"CURE", 365, 5.155776480286214`, 0.008999628234973456`},
{"CURE", 365, 8.121065675340953`, 0.08294802053423432`}, {"CURE", 365, 3.9053246050099024`, 0.01656460411394805З`},

{"CURE", 365, 0.0891475578787835`, 0.000677002191441851`}, {"CURE", 365, 8.126461579687087`, 0.060006858492840695`},
{"CURE", 365, 9.67730549493788`, 0.021585169744209386`}, {"CURE", 365, 111.44896874617254`, 0.1917511382389895`},
{"CURE", 365, 0.09353579160414849`, 0.0015229521897423103`}, {"CURE", 365, 0.8651202836830401`, 0.007216227320500804`},
{"CURE", 365, 0.6605003079093795`, 0.015536301673698084`}, {"CURE", 365, 0.5549330564621343`, 0.003373934024240358`},
{"CURE", 365, 18.32492072897902`, 0.0649626477439428`}, {"CURE", 365, 1.2330646792306088`, 0.0027129121377852555`},
{"CURE", 365, 3.065169682686581`, 0.012030974090918887`}, {"CURE", 365, 5.283792312855001`, 0.005789119459311754`},
{"CURE", 365, 1.0117291364706895`, 0.002329565919591061`}, {"CURE", 365, 14.988727895524697`, 0.2043916357110308`},
{"CURE", 365, 2.981239924898935`, 0.018760208413445174`}, {"CURE", 365, 54.95851925637967`, 0.29728137111227215`},
{"CURE", 365, 57.43325422263189`, 0.1585177647748884`}, {"CURE", 365, 7.025188127443204`, 0.1479270018550003`},
{"CURE", 365, 1.3720797271617302`, 0.011041711250720312`}, {"CURE", 365, 2.107580063010642`, 0.029782770426390867`},
{"CURE", 365, 0.659179292233753`, 0.003743835524471304`}, {"CURE", 365, 4.693019876039921`, 0.01167930664128234`},
{"CURE", 365, 0.7267570176336506`, 0.004259213180433091`}, {"CURE", 365, 0.024085194704952424`, 0.000646974283163945`},
{"CURE", 365, 50.01056836649667`, 0.06991457615256241`}, {"CURE", 365, 4.667021614017515`, 0.03467620896776949`},
{"CURE", 365, 0.39482070689876103`, 0.008646803948704396`}, {"CURE", 365, 0.2716781095522168`, 0.004596502908259491`},
{"CURE", 365, 0.9182870949318811`, 0.01811978970573891`}, {"CURE", 365, 0.15973607598134715`, 0.00246690287579755`},
{"CURE", 365, 8.91454945113286`, 0.017035037911334365`}, {"CURE", 365, 13.469943077884343`, 0.04726495772418887`},
{"CURE", 365, 8.480324740413808`, 0.04914201424947204`}, {"CURE", 365, 6.439846851776259`, 0.07971157837662267`},
{"CURE", 365, 0.22729638605435887`, 0.0017002389003098685`}, {"CURE", 365, 0.06415561802751411`, 0.0008300332586884195`},
{"CURE", 365, 2.3648706367187997`, 0.012618979164758445`}, {"CURE", 365, 19.07050626199183`, 0.02959070531131967`},
{"CURE", 365, 18.357389207207234`, 0.051394675141443975`}, {"CURE", 365, 7.067809230183742`, 0.038355511859060235`},
{"CURE", 365, 1.7254719852290266`, 0.016117964712707503`}, {"CURE", 365, 0.12748779039542255`, 0.0012066899362951434`},
{"CURE", 365, 8.548255473916447`, 0.00620950873926234`}, {"CURE", 365, 0.1946272017156516`, 0.002951905414567305`},
{"CURE", 365, 0.12824918501713908`, 0.0037880193297312194`}, {"CURE", 365, 3.6812249786975646`, 0.12405840224325222`},
{"CURE", 365, 5.312457554664802`, 0.01415072589117009`}, {"CURE", 365, 7.82947678945722`, 0.035888492501187454`},
{"CURE", 365, 2.1237949287915185`, 0.008784108209645907`}, {"CURE", 365, 2.598316282071334`, 0.007173525831238945`},
{"CURE", 365, 52.07113038674211`, 0.08685838630417762`}, {"CURE", 365, 0.33741651690428903`, 0.0005475290040068464`},
{"CURE", 365, 3.0138346650098202`, 0.012752899186623982`}, {"CURE", 365, 0.09078669351560013`, 0.0008947726749182766`},
{"CURE", 365, 2.7313359972696394`, 0.023258750981562163`}, {"CURE", 365, 2.771294836492966`, 0.012136760780927892`},
{"CURE", 365, 6.441168604587973`, 0.048348034668721854`}, {"CURE", 365, 29.059260428630783`, 0.4746852353696326`},
{"CURE", 365, 24.29207530492168`, 0.1151440297925006`}, {"CURE", 365, 76.53982611849077`, 0.05831110044626773`},
{"CURE", 365, 0.43584882164213534`, 0.003260604599800026`}, {"CURE", 365, 0.923683212879046`, 0.01921226805699832`},

{"CURE", 365, 1.814942236816506`, 0.003970463574109058`}, {"CURE", 365, 0.04114086620046412`, 0.0003618761753399806`},
{"CURE", 365, 4.943032249080453`, 0.015217285651040623`}, {"CURE", 365, 5.849639974523004`, 0.0037051228892376717`},
{"CURE", 365, 7.998121999233274`, 0.010904356842377253`}, {"CURE", 365, 1.6911434572464863`, 0.007124000308274995`},
{"CURE", 365, 2.1417475927559266`, 0.01073612265506263`}, {"CURE", 365, 0.665277295677463`, 0.00783910815906626`},
{"CURE", 365, 0.5323351804105325`, 0.022137975328318`}, {"CURE", 365, 11.206428009783822`, 0.11752056530308458`},
{"CURE", 365, 0.7272565504163174`, 0.002628663189590855`}, {"CURE", 365, 1.078807345351774`, 0.007046052514310773`},
{"CURE", 365, 1.6741218392251107`, 0.003397437029355362`}, {"CURE", 365, 8.346669513129342`, 0.04279419215881864`},
{"CURE", 365, 1.583593732734206`, 0.007969155431063262`}, {"CURE", 365, 0.03513253309918244`, 0.0006394573935092862`},
{"CURE", 365, 1.3367095522407821`, 0.008572235938324025`}, {"CURE", 365, 0.4687144310600186`, 0.0029081865198616005`},
{"CURE", 365, 0.0844012115562126l`, 0.00201504688586453`}, {"CURE", 365, 15.356316782536114`, 0.1143255482400509`},
{"CURE", 365, 0.03518637794273624`, 0.000448357085129659`}, {"CURE", 365, 19.770974123325068`, 0.15556789638874413`},
{"CURE", 365, 29.641279442579787`, 0.11246652503902176`}, {"CURE", 365, 8.508112039770445`, 0.06451395252154508`},
{"CURE", 365, 0.180722709934068`, 0.005921002749869716`}, {"CURE", 365, 3.938158154514628`, 0.018679396603245697`},
{"CURE", 365, 0.8447210357967666`, 0.03617673879111153`}, {"CURE", 365, 3.407596826271402`, 0.04935708518525092`},
{"CURE", 365, 7.057783480351746`, 0.03445506529141721`}, {"CURE", 365, 0.39419020088575474`, 0.0016826582341084248`},
{"CURE", 365, 0.14762270366978988`, 0.003154662090031911`}, {"CURE", 365, 3.0097020445168643`, 0.022060213160936905`},
{"CURE", 365, 6.19788652087953`, 0.0346307698463686`}, {"CURE", 365, 91.22133922315912`, 0.10004244830008269`},
{"CURE", 365, 0.3956176936422127`, 0.0019176954770443206`}, {"CURE", 365, 2.5752342250415805`, 0.043841156836274715`},
{"CURE", 365, 50.064699797601236`, 0.07757133755397473`}, {"CURE", 365, 6.479818218402836`, 0.10285660070929786`},
{"CURE", 365, 19.4179994688527`, 0.08143929320572939`}, {"CURE", 365, 0.5052057968678525`, 0.0020614418259816488`},
{"CURE", 365, 0.9951105126746038`, 0.013596182192816081`}, {"CURE", 365, 0.029795260067432303`, 0.0003690566305899444`},
{"CURE", 365, 2.562853679383878`, 0.007174940612774837`}, {"CURE", 365, 0.06396390883332617`, 0.001816209891888491`},
{"CURE", 365, 1.8118059754011588`, 0.005918403061132334`}, {"CURE", 365, 0.32379339106791105`, 0.0039940433119724245`},
{"CURE", 365, 0.1422921560458103`, 0.0013473853524832429`}, {"CURE", 365, 1.737702366554335`, 0.07315402682231013`},
{"CURE", 365, 1.1748478844332542`, 0.009670115979161044`}, {"CURE", 365, 50.357734854463146`, 0.12052452175092446`},
{"CURE", 365, 4.543980203400435`, 0.028855721912047473`}, {"CURE", 365, 2.148169333221157`, 0.01872653082989021`},
{"CURE", 365, 9.902522650486985`, 0.06017565222384415`}, {"CURE", 365, 0.18559054141656092`, 0.0014685580165394241`},
{"CURE", 365, 22.13423711989332`, 0.13550032000732468`}, {"CURE", 365, 6.69097750767028`, 0.023537255300951146`},
{"CURE", 365, 0.46190793372367295`, 0.0016800275260205055`}, {"CURE", 365, 0.04300801967820587`, 0.0012716720000261954`},
{"CURE", 365, 30.33362689801819`, 0.05462952391298986`}, {"CURE", 365, 0.5852240471307814`, 0.009093698929001264`},
{"CURE", 365, 0.8598967243272747`, 0.02655834732078442`}, {"CURE", 365, 5.240828129293779`, 0.03392826122222727`},

{"CURE", 365, 3.815947278404606`, 0.023377423384557353`}, {"CURE", 365, 0.5411905086466499`, 0.0049628315903210134`},
{"CURE", 365, 1.4371370713090097`, 0.007624364569052619`}, {"CURE", 365, 0.4269703205987209`, 0.0026320726039569483`},
{"CURE", 365, 1.106513612196326`, 0.004183733471265366`}, {"CURE", 365, 2.9497887954434323`, 0.013205856837797425`},
{"CURE", 365, 51.82349596620693`, 0.16105747106208615`}, {"CURE", 365, 1.3302546214327227`, 0.02673262954948639`},
{"CURE", 365, 4.408747957320872`, 0.006922943530077237`}, {"CURE", 365, 8.475551491134404`, 0.013596182009635947`},
{"CURE", 365, 0.40647523039179856`, 0.011809855513415722`}, {"CURE", 365, 0.12346628590214502`, 0.0014148382889454304`},
{"CURE", 365, 0.7048859550124841`, 0.0032615903852663292`}, {"CURE", 365, 8.540692168139046`, 0.07723323437140664`},
{"CURE", 365, 39.34214531520507`, 0.11558831589360152`}, {"CURE", 365, 6.734788434487497`, 0.01002056560831721`},
{"CURE", 365, 0.4042465349963801`, 0.0019016176488468152`}, {"CURE", 365, 1.2706294863081344`, 0.03443109867092111`},
{"CURE", 365, 0.06170891614423457`, 0.0017305654045989866`}, {"CURE", 365, 0.46891917417464724`, 0.005561614730573752`},
{"CURE", 365, 0.027589615925784145`, 0.0010753343628032946`}, {"CURE", 365, 0.2670816997118559 7`, 0.0009200841223704001`},
{"CURE", 365, 0.31524937482213505`, 0.00698826101696735`}, {"CURE", 365, 4.158741540631813`, 0.013899868024266338`},
{"CURE", 365, 3.727283220466587`, 0.020011275676273747`}, {"CURE", 365, 2.533960614169115`, 0.00793802731769419`},
{"CURE", 365, 0.8949156793610196`, 0.011454868453682222`}, {"CURE", 365, 4.455086928399537`, 0.011274527855616149`},
{"CURE", 365, 19.194011861353`, 0.014635575681662615`}, {"CURE", 365, 4.01860044397007`, 0.008503750904379822`},
{"CURE", 365, 12.708344779940846`, 0.016518667470065168`}, {"CURE", 365, 1.1707813103333438`, 0.005160706603284954`},
{"CURE", 365, 2.9023851779096708`, 0.0053537032902349875`}, {"CURE", 365, 3.3629813781095863`, 0.006254210738130854`},
{"CURE", 365, 0.12293683803074895`, 0.0018857554164794033`}, {"CURE", 365, 0.5573307052431454`, 0.012075758096152798`},
{"CURE", 365, 2.291752037032748`, 0.015467590920865906`}, {"CURE", 365, 0.9174015665545933`, 0.002966067907413364`},
{"CURE", 365, 0.5220716358811215`, 0.0013526991531619256`}, {"CURE", 365, 1.239104503560244`, 0.007704574482041432`},
{"CURE", 365, 15.232604760112862`, 0.06188305572014538`}, {"CURE", 365, 0.11890876720849657`, 0.0025839941567414185`},
{"CURE", 365, 4.51370757115643`, 0.07657964301337528`}, {"CURE", 365, 1.7737536889999497`, 0.006258702884840157`},
{"CURE", 365, 8.474580596945891`, 0.09124328141939707`}, {"CURE", 365, 0.31544542218473576`, 0.008058704871246807`},
{"CURE", 365, 0.06264748051168867`, 0.001087643832063132`}, {"CURE", 365, 0.1318426585527464`, 0.00461374357143233`},
{"CURE", 365, 0.12537438597985992`, 0.0005512402043534228`}, {"CURE", 365, 0.12203412854201585`, 0.00039002101544234114`},
{"CURE", 365, 5.7985367469261755`, 0.026320839539059847`}, {"CURE", 365, 0.5808192191000424`, 0.0023049650990256795`},
{"CURE", 365, 2.6418381616304676`, 0.019067601466230932`}, {"CURE", 365, 4.9393621455232966`, 0.03988816506994697`},
{"CURE", 365, 13.175794512633805`, 0.04060117890941159`}, {"CURE", 365, 0.4455685332449309`, 0.0012966328524018484`},
{"CURE", 365, 1.077483388418443`, 0.027715647794217996`}, {"CURE", 365, 4.394634934966008`, 0.03208013987051674`},
{"CURE", 365, 2.350043252952275`, 0.030633217036391117`}, {"CURE", 365, 0.22669293888772127`, 0.0018964632772083833`},
{"CURE", 365, 7.11410655175386`, 0.007258590403500754`}, {"CURE", 365, 1.3899443103949298`, 0.007996980804062746`},

{"CURE", 365, 0.7699855830398662`, 0.006398036356835993`}, {"CURE", 365, 4.270774360536434`, 0.03375034973048316`},
{"CURE", 365, 2.585793181023302`, 0.010236290673575014`}, {"CURE", 365, 3.2166011100811467`, 0.016605572701985736`},
{"CURE", 365, 3.016709567839144`, 0.011910922189142529`}, {"CURE", 365, 32.530962557575684`, 0.12411319687461309`},
{"CURE", 365, 0.30118000180900006`, 0.005167654576000709`}, {"CURE", 365, 2.345032009545164`, 0.0040783120281562965`},
{"CURE", 365, 4.3761418597430355`, 0.03975289740141463`}, {"CURE", 365, 0.6147268912490405`, 0.002652746310996282`},
{"CURE", 365, 2.0254120052911926`, 0.015412159544809059`}, {"CURE", 365, 0.23832303845546762`, 0.003186112672798649`},
{"CURE", 365, 0.750250475138166`, 0.0027713020905898583`}, {"CURE", 365, 1.351569745338463`, 0.004840916810973516`},
{"CURE", 365, 38.30433250185106`, 0.24409788459707304`}, {"CURE", 365, 14.3221080072714 2`, 0.0390627141613787 5`},
{"CURE", 365, 3.0537560245059936`, 0.03935977818312076`}, {"CURE", 365, 1.0006044925157969`, 0.0015439396031647335`},
{"CURE", 365, 7.336417936130404`, 0.029597956084242906`}, {"CURE", 365, 18.7562578298795`, 0.03608874694854372`},
{"CURE", 365, 0.1649344238307663`, 0.0081297601782182`}, {"CURE", 365, 15.403796152052923`, 0.059805132582704486`},
{"CURE", 365, 31.57235323131459`, 0.10693983236063445`}, {"CURE", 365, 0.8221811032063783`, 0.011696200464034292`},
{"CURE", 365, 2.168132940443041`, 0.012424192283337418`}, {"CURE", 365, 2.621889310816165`, 0.009811209123714`},
{"CURE", 365, 2.8664776458723384`, 0.01766498502009525`}, {"CURE", 365, 0.3326291085993975`, 0.006513380075921685`},
{"CURE", 365, 0.09040565905024252`, 0.0023252361423324857`}, {"CURE", 365, 12.974898118397384`, 0.05612387387958085`},
{"CURE", 365, 0.6476792077838613`, 0.010688553632823405`}, {"CURE", 365, 0.160979739136866`, 0.0011878046072463053`},
{"CURE", 365, 1.4265311128286278`, 0.008603817098246147`}, {"CURE", 365, 5.402022419199702`, 0.0696642771963723`},
{"CURE", 365, 11.218783691378773`, 0.0785796367867445`}, {"CURE", 365, 1.4505691941479597`, 0.03720877127934376 5`},
{"CURE", 365, 3.9566692043100513`, 0.024710555961007376`}, {"CURE", 365, 0.39641198384183246`, 0.01535755755322931`},
{"CURE", 365, 1.738739033741758`, 0.03749290368529028`}, {"CURE", 365, 0.8901994944354501`, 0.008098447628756101`},
{"CURE", 365, 1.169809298698671`, 0.033343898072982894`}, {"CURE", 365, 9.366944523221747`, 0.03579359344047012`},
{"CURE", 365, 2.9391737281222214`, 0.007597260199614161`}, {"CURE", 365, 16.258325873006655`, 0.04163725583166865`},
{"CURE", 365, 1.5149523049791431`, 0.004703034882839716`}, {"CURE", 365, 11.409293178988316`, 0.01853829111666072`},
{"CURE", 365, 1.8685556269792916`, 0.037447285731866085`}, {"CURE", 365, 4.076241031759547`, 0.018411057888986435`},
{"CURE", 365, 0.29489487617895527`, 0.0012464192172566386`}, {"CURE", 365, 2.2264584418319515`, 0.005460361141264577`},
{"CURE", 365, 2.1389380805381273`, 0.00699210317977912`}, {"CURE", 365, 4.485890166427718`, 0.03208660182454340 5`},
{"CURE", 365, 56.818847485912414`, 0.08577335331443632`}, {"CURE", 365, 0.5001242444613128`, 0.024376158587816277`},
{"CURE", 365, 0.18264730043624897`, 0.0002924445187120201`}, {"CURE", 365, 1.1078502210776426`, 0.02614307386093222`},
{"CURE", 365, 4.8840252462475`, 0.009076661602668377`}, {"CURE", 365, 0.31286639446161835`, 0.0006483604217889136`},
{"CURE", 365, 2.9072460288106368`, 0.020178069195872082`}, {"CURE", 365, 13.543292316948317`, 0.02396022205922792`},
{"CURE", 365, 4.57060241891068`, 0.006609195926770696`}, {"CURE", 365, 10.1011913033767`, 0.032672091795033824`},

{"CURE", 365, 0.5529246142274672`, 0.004055220390117053`}, {"CURE", 365, 0.24726965213493687`, 0.0015754692924600875`},
{"CURE", 365, 2.218862058548677`, 0.009719310695886815`}, {"CURE", 365, 16.674656852481817`, 0.0775588206768595l`},
{"CURE", 365, 0.5753973373733201`, 0.011165205732884603`}, {"CURE", 365, 3.140505401822792`, 0.017076851045331524`},
{"CURE", 365, 1.713964868403847`, 0.00984314880030127`}, {"CURE", 365, 2.286003607569028`, 0.0175583885765479`},
{"CURE", 365, 1.2212158537240465`, 0.0038011627497244703`}, {"CURE", 365, 0.8094855841469403`, 0.015769819341747673`},
{"CURE", 365, 1.7240144315929529`, 0.0048112153602106016`}, {"CURE", 365, 7.837496137790548`, 0.032948394161079614`},
{"CURE", 365, 0.6431475933633914`, 0.004839418662074511`}, {"CURE", 365, 0.12550727096828998`, 0.0010602391149387733`},
{"CURE", 365, 14.777406216917344`, 0.0600082541701208`}, {"CURE", 365, 0.1580383883885814`, 0.004690414522800622`},
{"CURE", 365, 5.4863105081074295`, 0.013940504926539527`}, {"CURE", 365, 1.611797568517991`, 0.02613644100365685`},
{"CURE", 365, 2.497243992423225`, 0.0282255272640867`}, {"CURE", 365, 15.825994451967238`, 0.07959944277480258`},
{"CURE", 365, 6.702567290113387`, 0.043093354187901056`}, {"CURE", 365, 2.0337027171269133`, 0.014351694980357713`},
{"CURE", 365, 18.482164841987036`, 0.04186884714773157`}, {"CURE", 365, 2.194126791425151`, 0.03649079574633789`},
{"CURE", 365, 0.14123029994191638`, 0.0013548558057800528`}, {"CURE", 365, 4.658713295319581`, 0.006122829540208445`},
{"CURE", 365, 1.052516339493001`, 0.018029065748740608`}, {"CURE", 365, 0.37471779473513134`, 0.00250055647649856`},
{"CURE", 365, 12.911332242606509`, 0.05612329621969604`}, {"CURE", 365, 11.381533734793841`, 0.05016935662780392`},
{"CURE", 365, 4.108898903215312`, 0.026590443079862726`}, {"CURE", 365, 0.601928332412483`, 0.0007594295435276762`},
{"CURE", 365, 7.2736998920421385`, 0.05054641612318921`}, {"CURE", 365, 3.207742278702724`, 0.0526021768998485 7`},
{"CURE", 365, 10.600874497698092`, 0.08773982084847992`}, {"CURE", 365, 0.3436363983538392`, 0.003703905442142012`},
{"CURE", 365, 2.0449449336234378`, 0.028825759611157825`}, {"CURE", 365, 5.516803520643943`, 0.02934870953813452`},
{"CURE", 365, 9.536577745676666`, 0.029550039768281286`}, {"CURE", 365, 1.0691402344078578`, 0.0060999579724662955`},
{"CURE", 365, 1.9432936408680612`, 0.009592551611290268`}, {"CURE", 365, 1.4223269729182102`, 0.003181993838827292`},
{"CURE", 365, 3.7333192683875116`, 0.07476461701920017`}, {"CURE", 365, 3.3960083499556135`, 0.05794158554560718`},
{"CURE", 365, 1.2607840081558652`, 0.002583608469513596`}, {"CURE", 365, 15.717940326323278`, 0.09844421529287373`},
{"CURE", 365, 0.5093218613795584`, 0.002959161854798385`}, {"CURE", 365, 0.28681831204535163`, 0.0033362259512027996`},
{"CURE", 365, 0.4029927525297023`, 0.0020434174982316504`}, {"CURE", 365, 0.47866005700462744`, 0.001959832230908514`},
{"CURE", 365, 6.975272053352455`, 0.012301850327045916`}, {"CURE", 365, 0.47066040352351907`, 0.005595530570565933`},
{"CURE", 365, 19.893028436514236`, 0.0605289566862665`}, {"CURE", 365, 13.014929797769051`, 0.06690481012065404`},
{"CURE", 365, 21.853393867341552`, 0.04279289702049127`}, {"CURE", 365, 7.651759727826903`, 0.009152796458762102`},
{"CURE", 365, 4.498533867207945`, 0.01478049010050521`}, {"CURE", 365, 0.6728461649015873`, 0.022825214322934884`},
{"CURE", 365, 6.522093537031696`, 0.027894223840749327`}, {"CURE", 365, 5.057210458367973`, 0.0686168338970128`},
{"CURE", 365, 0.6560231274914545`, 0.0008583049266759935`}, {"CURE", 365, 6.885313125150396`, 0.2704847233413854`},

{"CURE", 365, 0.08714646892862721`, 0.003355224947397723`}, {"CURE", 365, 66.84142264209609`, 0.0742323106036188`},
{"CURE", 365, 14.750038075020619`, 0.07166861729207369`}, {"CURE", 365, 0.35811255372034473`, 0.005164092130645708`},
{"CURE", 365, 0.622060410512421`, 0.0046781435333339266`}, {"CURE", 365, 0.12655382038624124`, 0.00270559231555593`},
{"CURE", 365, 8.801194835884674`, 0.1946987657062933`}, {"CURE", 365, 0.1487053288465003`, 0.0028042403161857216`},
{"CURE", 365, 1.3953232040972934`, 0.007245532345587825`}, {"CURE", 365, 2.2035222501605207`, 0.005443740525269843`},
{"CURE", 365, 0.3223174405922327`, 0.0019905207764669367`}, {"CURE", 365, 1.0418911456103985`, 0.025767680452347886`},
{"CURE", 365, 2.479053251435686`, 0.004538782325242029`}, {"CURE", 365, 10.81926005158211`, 0.009931411853725675`},
{"CURE", 365, 0.39005052054169775`, 0.012578106600111516`}, {"CURE", 365, 30.60876876595251`, 0.12210337044478535`},
{"CURE", 365, 0.8055530908902195`, 0.006068863462937883`}, {"CURE", 365, 0.05903464145025151`, 0.0022772683060316573`},
{"CURE", 365, 3.4749541772299897`, 0.01703229433967867`}, {"CURE", 365, 0.5094595318651062`, 0.0038920498523180797`},
{"CURE", 365, 1.7946386219500627`, 0.017901461914993855`}, {"CURE", 365, 0.9928781471380851`, 0.005116136582586262`},
{"CURE", 365, 1.803484223797091`, 0.01614905996826185`}, {"CURE", 365, 26.319428714870828`, 0.08052467777124658`},
{"CURE", 365, 3.746707007423408`, 0.019240313480876157`}, {"CURE", 365, 16.73993593396719`, 0.03644699160764716`},
{"CURE", 365, 0.39793289293965917`, 0.0019878253207785985`}, {"CURE", 365, 0.19577593035634386`, 0.0019749440110602987`},
{"CURE", 365, 1.4932119712121719`, 0.03068024913798516`}, {"CURE", 365, 0.11794560373857654`, 0.0035100128364332754`},
{"CURE", 365, 0.11190058798793505`, 0.0011751482813469503`}, {"CURE", 365, 0.7320248798636514`, 0.0019355990578523582`},
{"CURE", 365, 0.8699087370960009`, 0.006923009667146042`}, {"CURE", 365, 5.094078538121836`, 0.04391495273533947`},
{"CURE", 365, 3.400434479386038`, 0.04129200460971668`}, {"CURE", 365, 1.153763476107041`, 0.01396754944183675`},
{"CURE", 365, 2.71207409478551`, 0.012202454235919369`}, {"CURE", 365, 0.2372223918541401`, 0.005722068831335145`},
{"CURE", 365, 9.881732041360497`, 0.03688087653530409`}, {"CURE", 365, 0.10695599581274078`, 0.0008797883639587936`},
{"CURE", 365, 0.0419038089648477`, 0.0005893011440281103`}, {"CURE", 365, 2.4191478859095685`, 0.02134783397835865`},
{"CURE", 365, 0.590629076611876`, 0.0023443314141769725`}, {"CURE", 365, 0.5533495554556314`, 0.002983179899050881`},
{"CURE", 365, 0.0688656869435126`, 0.0030325011940815737`}, {"CURE", 365, 4.078692211828647`, 0.04162944200831578`},
{"CURE", 365, 9.137594295962263`, 0.005615707673858904`}, {"CURE", 365, 0.808865535831001`, 0.0029902039663025665`},
{"CURE", 365, 10.08641748517285`, 0.02550908168496256`}, {"CURE", 365, 0.10700761183021569`, 0.0009201799938481725`},
{"CURE", 365, 16.3397337109707`, 0.03763610868920574`}, {"CURE", 365, 1.844684776606481`, 0.03531986700275626`},
{"CURE", 365, 16.689866235622436`, 0.09472066039094161`}, {"CURE", 365, 3.321227148205746`, 0.004101586315379794`},
{"CURE", 365, 0.5669166009409743`, 0.003736560363697149`}, {"CURE", 365, 1.285170071217267`, 0.009784993668226896`},
{"CURE", 365, 0.5955902142290431`, 0.0026230841911749165`}, {"CURE", 365, 1.9793103144246091`, 0.016606736428612632`},
{"CURE", 365, 0.2875607060120009`, 0.010812687762540334`}, {"CURE", 365, 30.344353687594857`, 0.12074828965052593`},
{"CURE", 365, 1.1808458586491333`, 0.026545492718556447`}, {"CURE", 365, 0.4827232561046604`, 0.004584845999656428`},

```
{"CURE", 365, 1.8220644447388166`, 0.007351038799886304`}, {"CURE", 365, 1.5664558219173723`, 0.01742992739742807`},
{"TBUR", 149.40520306523666`, 1.3637025719171123`, 0.002910431205594223`},
{"CURE", 365, 11.193155463646121`, 0.07857006673325696`}, {"CURE", 365, 1.0138852115217254`, 0.004160082925047552`},
{"CURE", 365, 1.773605651533599`, 0.010466870273971906`}, {"CURE", 365, 0.0394806383134889`, 0.0008364925413400646`},
{"CURE", 365, 7.037299778586796`, 0.03983958194356979`}, {"CURE", 365, 1.8466427414992719`, 0.00481398668522825`},
{"CURE", 365, 1.7830733223625819`, 0.013904098357611742`}, {"CURE", 365, 0.5158214261339943`, 0.005341811894073438`},
{"CURE", 365, 0.7857248553762565`, 0.017245202769627037`}, {"CURE", 365, 5.40229444117971`, 0.03766962891335013`},
{"CURE", 365, 13.810639422714887`, 0.07893325823259012`}, {"CURE", 365, 2.0835248680475216`, 0.004208187777154539`},
{"CURE", 365, 0.05283844394815509`, 0.0006671775839101874`}, {"CURE", 365, 9.439872781485661`, 0.07817327564231215`},
{"CURE", 365, 8.012521839319124`, 0.01715797603376372`}, {"CURE", 365, 0.3858745472744309`, 0.00786480571112456 3`},
{"CURE", 365, 45.92861974272674`, 0.06788332196320802`}, {"CURE", 365, 1.0794814869066276`, 0.01879106693774813`},
{"CURE", 365, 1.675827242462266 2`, 0.015970810776362 72`}, {"CURE", 365, 7.1548852253462485`, 0.029650305526278366`},
{"CURE", 365, 0.8459355872785026`, 0.002198618171009334`}, {"CURE", 365, 6.179916096246945`, 0.03532445142636075`},
{"CURE", 365, 5.227888563939536`, 0.05742537074572966`}, {"CURE", 365, 2.8511903317750384`, 0.10500939638715667`},
{"CURE", 365, 38.70201914357876`, 0.16261162459012252`}, {"CURE", 365, 11.600996332083525`, 0.05446358609018299 4`},
{"CURE", 365, 2.064102622139395`, 0.009912828459497584`}, {"CURE", 365, 0.23545084310640751`, 0.0028539075438499656`},
{"CURE", 365, 0.9535917593461456`, 0.004756527332781 92`}, {"CURE", 365, 2.4715652213587016`, 0.1045734161248200 7`},
{"CURE", 365, 0.568323205838485`, 0.004229662205033808`}, {"CURE", 365, 1.5521271644716734`, 0.026154842636332467`},
{"CURE", 365, 1.6192722443065364`, 0.06165871874078466`}, {"CURE", 365, 3.3774057401228745`, 0.024843482575402302`},
{"CURE", 365, 0.082443525131249`, 0.002838458376288959`}, {"CURE", 365, 0.1491428859110231`, 0.0016113860735856454`},
{"CURE", 365, 1.0445035782228704`, 0.016537881899911868`}, {"CURE", 365, 12.590218159701676`, 0.31344611958456203`},
{"CURE", 365, 2.2147249839338117`, 0.017719675473995385`}, {"CURE", 365, 0.3266958568495283`, 0.0009857474578130103`},
{"CURE", 365, 1.3719723619323285`, 0.007551971883062075`}, {"CURE", 365, 1.3090045077628505`, 0.007970404255386893`},
{"CURE", 365, 23.91640308392636`, 0.09054997044422351`}, {"CURE", 365, 3.803554402370392`, 0.01694705745999973 8`}};
```

In[ ]:=

```
(* Validation of parameter sweep results with a test set of virtual mice:
   the scripts hidden in this group of cells have to be initialized to run the following codes *)

(* This is the script for the solution of full system in case of death due to cancer burden:
   it runs until critical number of cancer cells is achieved (10^11) *)
```

```
FullSystemSolutionTBUR[] := (

  tEnd = 99 999; (* it will stop before this time*)
  lAinj = Length[Ainj]; (* number of injections*)
  If[Ainj[[1, 1]] == 0, Npw = lAinj, Npw = lAinj + 1];
  (* the injections are treated as new initial conditions for a new system, which as well takes the actual vaules of other parameters *)
  (* therefore the number of injections has to me remembered and logic differs whether the first injection is made at t=0 or t>0 *)

  apw = Array[ff, Npw]; bpw = Array[ff, Npw]; NNpw = Array[ff, Npw]; DDpw = Array[ff, Npw];
  papw = Array[ff, Npw]; pbpw = Array[ff, Npw]; fFNpw = Array[ff, Npw]; fANpw = Array[ff, Npw];
  dFpw = Array[ff, Npw]; dApw = Array[ff, Npw];
  (* decayed antibody fragments are as well accounted for as pb *)

  (* times of beginning and end for solution of separate systems *)
  tB = Array[ff, Npw]; tE = Array[ff, Npw];

  (* for monitoring the paths of activity *)
  ActBloodpw = Array[ff, Npw]; ActBloodFragpw = Array[ff, Npw];
  ActOutpw = Array[ff, Npw]; ActOutFragpw = Array[ff, Npw]; ActTumorpw = Array[ff, Npw];

  (* for monitoring influence of self-dose, croos-fire and decays in blood *)
  SDpw = Array[ff, Npw]; CFNpw = Array[ff, Npw]; CFDpw = Array[ff, Npw]; UNpw = Array[ff, Npw];

  (* for monitoring the number of new cancer cells appearing during treatment *)
  NewCellspw = Array[ff, Npw];

  Clear[a, b, NN, DD, pa, pb, fFN, fAN, dF, dA, ActBlood, ActBloodFrag, ActOut, ActOutFrag, ActTumor, SD, CFN, CFD, UN, NewCells];

  (* EQUATIONS *)

  (* Radiation damage function *)
```

```
RD[NN_, DD_, fAN_, dA_, a_, pa_] := alpha * (ks * (lambda * gamma * fAN)/nu (*self-dose*) +
    (1 - ks) * (lambda * gamma * (fAN * NN + dA))/(nu * (NN + DD)) (*cross-fire*) + kf * lambda * (a + pa) (*dose from unanchored nuclides*));


(* Active antibodies *)
Fa[t_] := (*injections are considered as initial conditions*)
  - lambda * a[t] (*decay*) - kon * gamma/V * (fFN[t] * NN[t] + dF[t]) * a[t] (*binding*) - kappac * a[t] (*clearance*);

(* Inert antibodies *)
Fb[t_] := (*injections are considered as initial conditions*)
  + lambda * a[t] (*decay of a*) - kon * gamma/V * (fFN[t] * NN[t] + dF[t]) * b[t] (*binding*) - kappac * b[t] (*clearance*);

(* Viable cells *) FNN[t_] := rho * NN[t] (*proliferation*) - RD[NN[t], DD[t], fAN[t], dA[t], a[t], pa[t]] * NN[t] (*damage*);

(* Damaged cells *) FDD[t_] := RD[NN[t], DD[t], fAN[t], dA[t], a[t], pa[t]] * NN[t] (*damage*) - omega * DD[t] (*death*);

(* Active fragments *) Fpa[t_] := omega * (gamma * dA[t])/V (*release*) - lambda * pa[t] (*decay*) - kappap * pa[t] (*clearance*);

(* Inert fragments *)
Fpb[t_] := omega * (gamma * (DD[t] - dF[t] - dA[t]))/V (*release*) + lambda * pa[t] (*decay*) - kappap * pb[t] (*clearance*);

(* Free receptors of viable cells *) FfFN[t_] := (1 - fFN[t]) * rho - kon * (a[t] + b[t]) * fFN[t];

(* Active receptors of viable cells *) FfAN[t_] := kon * a[t] * fFN[t] - (lambda + rho) * fAN[t];
```

```
(* Free receptors of damaged cells *)
FdF[t_] := RD[NN[t], DD[t], fAN[t], dA[t], a[t], pa[t]] * fFN[t] * NN[t] - kon * (a[t] + b[t]) * dF[t] - omega * dF[t];

(* Active receptors of damaged cells *)
FdA[t_] :=
  RD[NN[t], DD[t], fAN[t], dA[t], a[t], pa[t]] * fAN[t] * NN[t] + kon * a[t] * dF[t] - lambda * dA[t] - omega * dA[t];

(* Initial conditions *)

If[Ainj⟦1, 1⟧ == 0
  , a0 = Ainj⟦1, 2⟧ / V; b0 = eta * Ainj⟦1, 2⟧ / V
  , a0 = 0; b0 = 0]; (*complexes in blood*)
NN0 = N0;
DD0 = 0;
pa0 = 0; pb0 = 0; fFN0 = 1; fAN0 = 0;
dF0 = 0; dA0 = 0;
ActBlood0 = 0; ActBloodFrag0 = 0; ActOut0 = 0; ActOutFrag0 = 0; ActTumor0 = 0; NewCells0 = 0;
SD0 = 0; CFN0 = 0; CFD0 = 0; UN0 = 0;

(* SOLVER *)

tB⟦1⟧ = 0; If[Ainj⟦1, 1⟧ == 0, If[lAinj > 1, tE⟦1⟧ = Ainj⟦2, 1⟧, tE⟦1⟧ = tEnd], tE⟦1⟧ = Ainj⟦1, 1⟧];

For[npw = 1, npw ≤ Npw, npw++,

  Clear[a, b, NN, DD, pa, pb, fFN, fAN, dF, dA, ActBlood, ActBloodFrag, ActOut, ActOutFrag, ActTumor, SD, CFN, CFD, UN, NewCells];

  sol = NDSolve[{
        (*INITIAL CONDITIONS*)
        a[tB⟦npw⟧] == a0, b[tB⟦npw⟧] == b0, NN[tB⟦npw⟧] == NN0, DD[tB⟦npw⟧] == DD0, pa[tB⟦npw⟧] == pa0,
```

```
pb[tB[[npw]]] == pb0, fFN[tB[[npw]]] == fFN0, fAN[tB[[npw]]] == fAN0, dF[tB[[npw]]] == dF0, dA[tB[[npw]]] == dA0,
ActBlood[tB[[npw]]] == ActBlood0, ActBloodFrag[tB[[npw]]] == ActBloodFrag0, ActOut[tB[[npw]]] == ActOut0,
ActOutFrag[tB[[npw]]] == ActOutFrag0, ActTumor[tB[[npw]]] == ActTumor0, SD[tB[[npw]]] == SD0,
CFN[tB[[npw]]] == CFN0, CFD[tB[[npw]]] == CFD0, UN[tB[[npw]]] == UN0, NewCells[tB[[npw]]] == NewCells0,

a'[t] == Fa[t], b'[t] == Fb[t], NN'[t] == FNN[t], DD'[t] == FDD[t],
pa'[t] == Fpa[t], pb'[t] == Fpb[t], fFN'[t] == FfFN[t], fAN'[t] == FfAN[t], dF'[t] == FdF[t], dA'[t] == FdA[t],

ActBlood'[t] == V * lambda * (a[t] + pa[t]),
ActBloodFrag'[t] == V * lambda * pa[t],
ActOut'[t] == V * (kappac * a[t] + kappap * pa[t]),
ActOutFrag'[t] == V * kappap * pa[t],
ActTumor'[t] == (lambda * gamma) * (fAN[t] * NN[t] + dA[t]),

SD'[t] == ks * (lambda * gamma) * (fAN[t] * NN[t]),
CFN'[t] == (1 - ks) * (lambda * gamma) * (fAN[t] * NN[t]) * NN[t] / (NN[t] + DD[t]),
CFD'[t] == (1 - ks) * (lambda * gamma) * dA[t] * NN[t] / (NN[t] + DD[t]),
UN'[t] == kf * lambda * (a[t] + pa[t]) * nu * NN[t],
NewCells'[t] == If[t > Ainj[[1, 1]], rho * NN[t], 0] (* start counting new cells from the moment of the first injection *)

, WhenEvent[NN[t] + DD[t] > Cd / Nnor, "StopIntegration"; tEnd = t; tE[[npw]] = t; NN[t] -> 0]
}
, {a, b, NN, DD, pa, pb, fFN, fAN, dF, dA, ActBlood,
  ActBloodFrag, ActOut, ActOutFrag, ActTumor, SD, CFN, CFD, UN, NewCells}, {t, tB[[npw]], tE[[npw]]}
, AccuracyGoal -> 10, PrecisionGoal -> 10];

apw[[npw]] = First[a /. sol]; bpw[[npw]] = First[b /. sol]; NNpw[[npw]] = First[NN /. sol]; DDpw[[npw]] = First[DD /. sol];
papw[[npw]] = First[pa /. sol]; pbpw[[npw]] = First[pb /. sol]; fFNpw[[npw]] = First[fFN /. sol];
```

```
fANpw[[npw]] = First[fAN /. sol]; dFpw[[npw]] = First[dF /. sol]; dApw[[npw]] = First[dA /. sol];
ActBloodpw[[npw]] = First[ActBlood /. sol];
ActBloodFragpw[[npw]] = First[ActBloodFrag /. sol];
ActOutpw[[npw]] = First[ActOut /. sol];
ActOutFragpw[[npw]] = First[ActOutFrag /. sol];
ActTumorpw[[npw]] = First[ActTumor /. sol];
SDpw[[npw]] = First[SD /. sol];
CFNpw[[npw]] = First[CFN /. sol];
CFDpw[[npw]] = First[CFD /. sol];
UNpw[[npw]] = First[UN /. sol];
NewCellspw[[npw]] = First[NewCells /. sol];

If[npw < Npw,
 (*renew initial conditions*)
 If[Ainj[[1, 1]] == 0
   , a0 = apw[[npw]][tE[[npw]]] + Ainj[[npw + 1, 2]]/V; b0 = bpw[[npw]][tE[[npw]]] + eta*Ainj[[npw + 1, 2]]/V
   , a0 = apw[[npw]][tE[[npw]]] + Ainj[[npw, 2]]/V; b0 = bpw[[npw]][tE[[npw]]] + eta*Ainj[[npw, 2]]/V];
 NN0 = NNpw[[npw]][tE[[npw]]];
 DD0 = DDpw[[npw]][tE[[npw]]];
 pa0 = papw[[npw]][tE[[npw]]];
 pb0 = pbpw[[npw]][tE[[npw]]];
 fFN0 = fFNpw[[npw]][tE[[npw]]]; fAN0 = fANpw[[npw]][tE[[npw]]];
 dF0 = dFpw[[npw]][tE[[npw]]];
 dA0 = dApw[[npw]][tE[[npw]]];
 ActBlood0 = ActBloodpw[[npw]][tE[[npw]]];
 ActBloodFrag0 = ActBloodFragpw[[npw]][tE[[npw]]];
 ActOut0 = ActOutpw[[npw]][tE[[npw]]];
 ActOutFrag0 = ActOutFragpw[[npw]][tE[[npw]]];
 ActTumor0 = ActTumorpw[[npw]][tE[[npw]]];
 SD0 = SDpw[[npw]][tE[[npw]]];
 CFN0 = CFNpw[[npw]][tE[[npw]]];
```

```
        CFD0 = CFDpw⟦npw⟧[tE⟦npw⟧];
        UN0 = UNpw⟦npw⟧[tE⟦npw⟧];
        NewCells0 = NewCellspw⟦npw⟧[tE⟦npw⟧];

        (*renew time frame*)

        If[Ainj⟦1, 1⟧ == 0, If[Npw > npw + 1, tE⟦npw + 1⟧ = Ainj⟦npw + 2, 1⟧, tE⟦npw + 1⟧ = tEnd],
          If[Npw > npw + 1, tE⟦npw + 1⟧ = Ainj⟦npw + 1, 1⟧, tE⟦npw + 1⟧ = tEnd]];]
      ];

    npw--;)
```

In[∘]:=

```
(* This is the script for the solution of full system -- which saves the type of outcome (CURE, TOX, BUR) and time of it *)
TOC0 = 365; (* one year *)

PatientTEST[] := (
    Outcome = "";
    TOC = TOC0; (* maximum accountable overall survival *)

    FullSystemSolutionMD[]; (*find minimal N and time of achieving it *)

    If[Nn < Ncur, (* tumor cure is achieved, but toxicity can still be lethal, then it will be death of toxicity rather than cure *)
      FullSystemSolutionMDStopNmin[tminNn]; (* solve it with cell proliferation stopping at this moment*)

      If[Abs[ActBloodpw⟦npw⟧[tEnd]] > Ainj⟦1, 2⟧, (* it's a rare bug when activity in blood spikes *)

        ttt = t /. NMinimize[Abs[ActBloodpw⟦npw⟧[t] - Ainj⟦1, 2⟧], t]⟦2⟧;
        If[ActBloodpw⟦npw⟧[ttt - 1] > Abld, (*in that case that's a much more realistic actual toxicity, and yes, that's toxic*)
          Outcome = "TOX"; Tox = ActBloodpw⟦npw⟧[ttt - 1] / nCpm;
          TOC = t /. NMinimize[Abs[ActBloodpw⟦npw⟧[t] - Abld], {t, tB⟦npw⟧, tE⟦npw⟧}]⟦2⟧ (* time of death *),
```

```
        (* otherwise it is cure *)
        Outcome = "CURE"; Tox = ActBloodpw[[npw]][ttt - 1] / nCpm;],

      If[ActBloodpw[[npw]][tEnd] > Abld (*yes, that's toxic*),
        Outcome = "TOX"; Tox = ActBloodpw[[npw]][tEnd] / nCpm;
        TOC = t /. NMinimize[Abs[ActBloodpw[[npw]][t] - Abld], {t, tB[[npw]], tE[[npw]]}][[2]] (* time of death *),
        (* otherwise it is cure *)
        Outcome = "CURE"; Tox = ActBloodpw[[npw]][tEnd] / nCpm;]],

    (* tumor cure is not achieved, so what is the reason of death and when it happens? Need to run simulation until 10^11 cells *)
    Quiet[FullSystemSolutionTBUR[]]; TOC = tEnd; Tox = ActBloodpw[[npw]][tEnd] / nCpm;
    If[ActBloodpw[[npw]][tEnd] > Abld (*yes, that's toxic*),
      Outcome = "TOX";
      TOC = t /. NMinimize[Abs[ActBloodpw[[npw]][t] - Abld], {t, tB[[npw]], tE[[npw]]}][[2]] (* time of death *),
      (* otherwise it is death of tumor burden *)
      Outcome = "TBUR";]];
  Return[{Outcome, TOC, Tox}])

(* This is the script for finding minimal lethally toxic dose *)

FindToxDose[] := (DA1 = Abld;
  Ainj = {{t1, DA1}};

  While[FullSystemSolutionToxicity[] < Abld, DA1 = 2 * DA1; Ainj = {{t1, DA1}};];
  Dmax = DA1; Dmin = DA1 / 2;

  While[Abs[Dmin - Dmax] / Dmax > 0.000001,
    DA1 = (Dmin + Dmax) / 2; Ainj = {{t1, DA1}};
    If[FullSystemSolutionToxicity[] < Abld, Dmin = DA1, Dmax = DA1]];
  Dtox = Dmax;
  Return[Dtox / nCpm])
```

```
FullSystemSolutionToxicity[] := (

  (* maximum time of simulation-- where little radioactivity of the last dose remains, namely 0.17% of the last dose *)
  tEnd = Ainj[[Length[Ainj], 1]] + (-Log[0.0017] / lambda);
  lAinj = Length[Ainj]; (* number of injections*)
  If[Ainj[[1, 1]] == 0, Npw = lAinj, Npw = lAinj + 1];

  apw = Array[ff, Npw]; bpw = Array[ff, Npw]; NNpw = Array[ff, Npw]; DDpw = Array[ff, Npw];
  papw = Array[ff, Npw]; pbpw = Array[ff, Npw]; fFNpw = Array[ff, Npw]; fANpw = Array[ff, Npw];
  dFpw = Array[ff, Npw]; dApw = Array[ff, Npw];
  (* inert antibody fragments are as well accounted for as pb *)

  (* times of beginning and end for solution of separate systems *)
  tB = Array[ff, Npw]; tE = Array[ff, Npw];

  (* for monitoring the paths of activity *)
  ActBloodpw = Array[ff, Npw]; ActBloodFragpw = Array[ff, Npw];
  ActOutpw = Array[ff, Npw]; ActOutFragpw = Array[ff, Npw]; ActTumorpw = Array[ff, Npw];

  (* for monitoring influence of self-dose, croos-fire and decays in blood *)
  SDpw = Array[ff, Npw]; CFNpw = Array[ff, Npw]; CFDpw = Array[ff, Npw]; UNpw = Array[ff, Npw];

  (* for monitoring the number of new cancer cells appearing during treatment *)
  NewCellspw = Array[ff, Npw];

  Clear[a, b, NN, DD, pa, pb, fFN, fAN, dF, dA, ActBlood, ActBloodFrag, ActOut, ActOutFrag, ActTumor, SD, CFN, CFD, UN, NewCells];

  (* EQUATIONS *)

  (* Radiation damage function *)
```

```
RD[NN_, DD_, fAN_, dA_, a_, pa_] := alpha * (ks * (lambda * gamma * fAN)/nu (*self-dose*) +
    (1 - ks) * (lambda * gamma * (fAN * NN + dA))/(nu * (NN + DD)) (*cross-fire*) + kf * lambda * (a + pa) (*dose from unanchored nuclides*));



(* Active antibodies *)
Fa[t_] := (*injections are considered as initial conditions*)
  - lambda * a[t] (*decay*) - kon * gamma/V * (fFN[t] * NN[t] + dF[t]) * a[t] (*binding*) - kappac * a[t] (*clearance*);

(* Inert antibodies *)
Fb[t_] := (*injections are considered as initial conditions*)
  + lambda * a[t] (*decay of a*) - kon * gamma/V * (fFN[t] * NN[t] + dF[t]) * b[t] (*binding*) - kappac * b[t] (*clearance*);

(* Viable cells *) FNN[t_] := rho * NN[t] (*proliferation*) - RD[NN[t], DD[t], fAN[t], dA[t], a[t], pa[t]] * NN[t] (*damage*);

(* Damaged cells *) FDD[t_] := RD[NN[t], DD[t], fAN[t], dA[t], a[t], pa[t]] * NN[t] (*damage*) - omega * DD[t] (*death*);

(* Active fragments *) Fpa[t_] := omega * (gamma * dA[t])/V (*release*) - lambda * pa[t] (*decay*) - kappap * pa[t] (*clearance*);

(* Inert fragments *)
Fpb[t_] := omega * (gamma * (DD[t] - dF[t] - dA[t]))/V (*release*) + lambda * pa[t] (*decay*) - kappap * pb[t] (*clearance*);

(* Free receptors of viable cells *) FfFN[t_] := (1 - fFN[t]) * rho - kon * (a[t] + b[t]) * fFN[t];

(* Active receptors of viable cells *) FfAN[t_] := kon * a[t] * fFN[t] - (lambda + rho) * fAN[t];
```

```
(* Free receptors of damaged cells *)
FdF[t_] := RD[NN[t], DD[t], fAN[t], dA[t], a[t], pa[t]] * fFN[t] * NN[t] - kon * (a[t] + b[t]) * dF[t] - omega * dF[t];

(* Active receptors of damaged cells *)
FdA[t_] :=
  RD[NN[t], DD[t], fAN[t], dA[t], a[t], pa[t]] * fAN[t] * NN[t] + kon * a[t] * dF[t] - lambda * dA[t] - omega * dA[t];

(* Initial conditions *)

If[Ainj⟦1, 1⟧ == 0
  , a0 = Ainj⟦1, 2⟧ / V; b0 = 0
  , a0 = 0; b0 = 0]; (*complexes in blood*)
NN0 = N0;
DD0 = 0;
pa0 = 0; pb0 = 0; fFN0 = 1; fAN0 = 0;
dF0 = 0; dA0 = 0;
ActBlood0 = 0; ActBloodFrag0 = 0; ActOut0 = 0; ActOutFrag0 = 0; ActTumor0 = 0; NewCells0 = 0;
SD0 = 0; CFN0 = 0; CFD0 = 0; UN0 = 0;

(* SOLVER *)

tB⟦1⟧ = 0; If[Ainj⟦1, 1⟧ == 0, If[lAinj > 1, tE⟦1⟧ = Ainj⟦2, 1⟧, tE⟦1⟧ = tEnd], tE⟦1⟧ = Ainj⟦1, 1⟧];

For[npw = 1, npw ≤ Npw, npw++,

  Clear[a, b, NN, DD, pa, pb, fFN, fAN, dF, dA, ActBlood, ActBloodFrag, ActOut, ActOutFrag, ActTumor, SD, CFN, CFD, UN, NewCells];

  sol = NDSolve[{
        (*INITIAL CONDITIONS*)
        a[tB⟦npw⟧] == a0, b[tB⟦npw⟧] == b0, NN[tB⟦npw⟧] == NN0, DD[tB⟦npw⟧] == DD0, pa[tB⟦npw⟧] == pa0,
```

```
pb[tB[[npw]]] == pb0, fFN[tB[[npw]]] == fFN0, fAN[tB[[npw]]] == fAN0, dF[tB[[npw]]] == dF0, dA[tB[[npw]]] == dA0,
ActBlood[tB[[npw]]] == ActBlood0, ActBloodFrag[tB[[npw]]] == ActBloodFrag0, ActOut[tB[[npw]]] == ActOut0,
ActOutFrag[tB[[npw]]] == ActOutFrag0, ActTumor[tB[[npw]]] == ActTumor0, SD[tB[[npw]]] == SD0,
CFN[tB[[npw]]] == CFN0, CFD[tB[[npw]]] == CFD0, UN[tB[[npw]]] == UN0, NewCells[tB[[npw]]] == NewCells0,

a'[t] == Fa[t], b'[t] == Fb[t], NN'[t] == FNN[t], DD'[t] == FDD[t],
pa'[t] == Fpa[t], pb'[t] == Fpb[t], fFN'[t] == FfFN[t], fAN'[t] == FfAN[t], dF'[t] == FdF[t], dA'[t] == FdA[t],

ActBlood'[t] == V * lambda * (a[t] + pa[t]),
ActBloodFrag'[t] == V * lambda * pa[t],
ActOut'[t] == V * (kappac * a[t] + kappap * pa[t]),
ActOutFrag'[t] == V * kappap * pa[t],
ActTumor'[t] == (lambda * gamma) * (fAN[t] * NN[t] + dA[t]),

SD'[t] == ks * (lambda * gamma) * (fAN[t] * NN[t]),
CFN'[t] == (1 - ks) * (lambda * gamma) * (fAN[t] * NN[t]) * NN[t] / (NN[t] + DD[t]),
CFD'[t] == (1 - ks) * (lambda * gamma) * dA[t] * NN[t] / (NN[t] + DD[t]),
UN'[t] == kf * lambda * (a[t] + pa[t]) * nu * NN[t],
NewCells'[t] == If[t > Ainj[[1, 1]], rho * NN[t], 0] (* start counting new cells from the moment of the first injection *)
, WhenEvent[NN[t] < Ncur / Nnor, NN[t] -> 0] (* treatment wins *)
}
, {a, b, NN, DD, pa, pb, fFN, fAN, dF, dA, ActBlood,
ActBloodFrag, ActOut, ActOutFrag, ActTumor, SD, CFN, CFD, UN, NewCells}, {t, tB[[npw]], tE[[npw]]}
, AccuracyGoal -> 10, PrecisionGoal -> 10];

apw[[npw]] = First[a /. sol]; bpw[[npw]] = First[b /. sol]; NNpw[[npw]] = First[NN /. sol]; DDpw[[npw]] = First[DD /. sol];
papw[[npw]] = First[pa /. sol]; pbpw[[npw]] = First[pb /. sol]; fFNpw[[npw]] = First[fFN /. sol];
fANpw[[npw]] = First[fAN /. sol]; dFpw[[npw]] = First[dF /. sol]; dApw[[npw]] = First[dA /. sol];
```

```
ActBloodpw[[npw]] = First[ActBlood /. sol];
ActBloodFragpw[[npw]] = First[ActBloodFrag /. sol];
ActOutpw[[npw]] = First[ActOut /. sol];
ActOutFragpw[[npw]] = First[ActOutFrag /. sol];
ActTumorpw[[npw]] = First[ActTumor /. sol];
SDpw[[npw]] = First[SD /. sol];
CFNpw[[npw]] = First[CFN /. sol];
CFDpw[[npw]] = First[CFD /. sol];
UNpw[[npw]] = First[UN /. sol];
NewCellspw[[npw]] = First[NewCells /. sol];

If[npw < Npw,
  (*renew initial conditions*)
  If[Ainj[[1, 1]] == 0
    , a0 = apw[[npw]][tE[[npw]]] + Ainj[[npw + 1, 2]]/V; b0 = bpw[[npw]][tE[[npw]]]
    , a0 = apw[[npw]][tE[[npw]]] + Ainj[[npw, 2]]/V; b0 = bpw[[npw]][tE[[npw]]]];
  NN0 = NNpw[[npw]][tE[[npw]]];
  DD0 = DDpw[[npw]][tE[[npw]]];
  pa0 = papw[[npw]][tE[[npw]]];
  pb0 = pbpw[[npw]][tE[[npw]]];
  fFN0 = fFNpw[[npw]][tE[[npw]]]; fAN0 = fANpw[[npw]][tE[[npw]]];
  dF0 = dFpw[[npw]][tE[[npw]]];
  dA0 = dApw[[npw]][tE[[npw]]];
  ActBlood0 = ActBloodpw[[npw]][tE[[npw]]];
  ActBloodFrag0 = ActBloodFragpw[[npw]][tE[[npw]]];
  ActOut0 = ActOutpw[[npw]][tE[[npw]]];
  ActOutFrag0 = ActOutFragpw[[npw]][tE[[npw]]];
  ActTumor0 = ActTumorpw[[npw]][tE[[npw]]];
  SD0 = SDpw[[npw]][tE[[npw]]];
  CFN0 = CFNpw[[npw]][tE[[npw]]];
  CFD0 = CFDpw[[npw]][tE[[npw]]];
```

```
        UN0 = UNpw[[npw]][tE[[npw]]];
        NewCells0 = NewCellspw[[npw]][tE[[npw]]];

        (*renew time frame*)
        If[Ainj[[1, 1]] == 0, If[Npw > npw + 1, tE[[npw + 1]] = Ainj[[npw + 2, 1]], tE[[npw + 1]] = tEnd],
          If[Npw > npw + 1, tE[[npw + 1]] = Ainj[[npw + 1, 1]], tE[[npw + 1]] = tEnd]];]
    ];
    npw--;
    Return[ActBloodpw[[npw]][tEnd]])


(* The outcome of the test was obtained by the codes, hidden here.
    They use randomization and will generate new arrays of data.
    It takes a long time, at least a day. *)


(* generate 1000 "patients" *)

Npar = 1000; (*how many sets will be tested*)
SetBasicParameterValues[];

Patients = Array[f, {Npar + 1, 10}];

Patients[[1, 1]] = "kappac";
Patients[[1, 2]] = "kappap";
Patients[[1, 3]] = "gamma";
Patients[[1, 4]] = "V";
Patients[[1, 5]] = "ks";
Patients[[1, 6]] = "rho";
Patients[[1, 7]] = "omega";
```

```
Patients[[1, 8]] = "alpha";
Patients[[1, 9]] = "kf";
Patients[[1, 10]] = "N0";

Quiet[For[ij = 2, ij ≤ Npar + 1, ij++,

    NotebookDelete[pr]; (* To see the code running *)
    pr = PrintTemporary["Set " <> ToString[ij - 1] <> " of " <> ToString[Npar]];

    (*kappac*) Patients[[ij, 1]] = RandomReal[{0.04, 0.28}];
    (*kappap*) Patients[[ij, 2]] = RandomReal[{0.4, 4}];
    (*gamma*) Patients[[ij, 3]] = RandomReal[{0.13, 10}] * Nnor / 10^7;
    (*V*) Patients[[ij, 4]] = RandomReal[{0.75, 1.5}];
    (*ks*) Patients[[ij, 5]] = RandomReal[{0, 1}];
    (*rho*) Patients[[ij, 6]] = RandomReal[{0.15, 0.7}];
    (*omega*) Patients[[ij, 7]] = 0.05 * 10^RandomReal[{-1, 1}];
    (*alpha*) Patients[[ij, 8]] = 50 * 10^RandomReal[{0, 2}];
    (*kf*) Patients[[ij, 9]] = RandomReal[{0.01, 0.25}];
    (*N0*) Patients[[ij, 10]] = RandomReal[{1, 10}] * 10^7 / Nnor;
  ]];
```

```
(* TEST FOR NUMERICARILLY OPTIMAL PERSONALIZED DOSES *)

Npar = 1000; (*how many sets will be tested*)
SetBasicParameterValues[];
TestNumOpt = Array[f, {Npar + 1, 4}];

TestNumOpt[[1, 1]] = "NumOpt Res";
TestNumOpt[[1, 2]] = "NumOpt OS";
TestNumOpt[[1, 3]] = "NumOpt Tox";
TestNumOpt[[1, 4]] = "NumOpt Acur";
```

```
Quiet[For[ij = 2, ij ≤ Npar + 1, ij++,

   NotebookDelete[pr]; (* To see the code running *)
   pr = PrintTemporary["Set " <> ToString[ij - 1] <> " of " <> ToString[Npar]];

   kappac = Patients[[ij, 1]];
   kappap = Patients[[ij, 2]];
   gamma = Patients[[ij, 3]];
   V = Patients[[ij, 4]];
   ks = Patients[[ij, 5]];
   rho = Patients[[ij, 6]];
   omega = Patients[[ij, 7]];
   alpha = Patients[[ij, 8]];
   kf = Patients[[ij, 9]];
   N0 = Patients[[ij, 10]];

   AA = FindCurDose[];

   Ainj = {{0, AA * nCpm}};

   res = PatientTEST[];

   (* To avoid errors, TBUR cannot be achieved*)
   While[res[[1]] == "TBUR",
     (*Print["Failed at Acur="<>ToString[Acur]];*)
     AA = AA * 1.003;
     Ainj = {{0, AA * nCpm}};
     res = PatientTEST[];
   ];
```

```
    (* If the treatment is toxic, maximize OS *)
    While[res[[1]] == "TOX",
      AA = FindToxDose[];
      AA = AA * 0.998;
      Ainj = {{0, AA * nCpm}};
      res = PatientTEST[];];

    (*Print[ToString[ij-1]<>" -- "<>res];*)

    TestNumOpt[[ij, 1]] = res[[1]];
    TestNumOpt[[ij, 2]] = res[[2]];
    TestNumOpt[[ij, 3]] = res[[3]];
    TestNumOpt[[ij, 4]] = AA;

  ]];

(* TEST FOR ANALYTICAL OPTIMIZATION *)

Npar = 1000; (*how many sets will be tested*)
SetBasicParameterValues[];
TestAnOpt = Array[f, {Npar + 1, 4}];

TestAnOpt[[1, 1]] = "AnOpt Res";
TestAnOpt[[1, 2]] = "AnOpt OS";
TestAnOpt[[1, 3]] = "AnOpt Tox";
TestAnOpt[[1, 4]] = "AnOpt Acur";

Quiet[For[ij = 2, ij ≤ Npar + 1, ij++,

    NotebookDelete[pr]; (* To see the code running *)
```

```
pr = PrintTemporary["Set " <> ToString[ij - 1] <> " of " <> ToString[Npar]];

kappac = Patients[[ij, 1]];
kappap = Patients[[ij, 2]];
gamma = Patients[[ij, 3]];
V = Patients[[ij, 4]];
ks = Patients[[ij, 5]];
rho = Patients[[ij, 6]];
omega = Patients[[ij, 7]];
alpha = Patients[[ij, 8]];
kf = Patients[[ij, 9]];
N0 = Patients[[ij, 10]];
```

$$\text{Aan} = -\frac{\text{kappac} + \text{lambda} + \text{kon} * \text{gamma} * \text{N0} / \text{V}}{\text{kon} * \text{gamma} * \text{N0} / \text{V}} * \text{N0} * \frac{\frac{\text{rho}}{\text{lambda}} * \frac{\text{nu}}{\text{alpha}} * \text{ProductLog}\left[-1, -E^{-2} * \left(\frac{\text{Ncur}}{\text{N0} * \text{Nnor}}\right)^{\frac{\text{lambda}}{\text{rho}}}\right]}{1 - \left(1 - \frac{\text{ProductLog}\left[-1, -E^{-2} * \left(\frac{\text{Ncur}}{\text{N0} * \text{Nnor}}\right)^{\frac{\text{lambda}}{\text{rho}}}\right]}{\text{ProductLog}\left[-1, -E^{-1} * \left(\frac{\text{Ncur}}{\text{N0} * \text{Nnor}}\right)^{\frac{\text{lambda}+\text{rho}}{\text{rho}}}\right]}\right) * \text{ks}};$$

(* analytical estimation of curative dose *)

```
Aan = (1 + 0.02 + 0.12 * Exp[-gamma * N0 / 3]) * (1 + (2.6 * (-ks + 1) * Exp[-(12 * (-ks + 1))])) * Aan;
(*statistical correction based on training set *)
```

$$\text{ToxAnAb} = \text{Aan} * \left(\frac{\text{lambda}}{\text{lambda} + \text{kappac} + \text{kon} * \text{gamma} * \text{N0} / \text{V}}\right) * (1 + 0.014 * (\text{Tanh}[(\text{alpha} - 200) / 70] - 1));$$

(* analytical estimation of accompanying toxicity from antibodies, with statistical correction *)

$$\text{ToxAnFr} = \text{Aan} * \frac{\text{lambda} * \text{omega}}{(\text{lambda} + \text{omega}) * (\text{lambda} + \text{kappap})} * (1 - 0.015 * (1 - \text{ks}) - 0.003);$$

```
  (* analytical estimation of accompanying toxicity from antibody fragments, with statistical correction *)

  ToxAn = ToxAnAb + ToxAnFr;

  If[ToxAn > Abld, (* treatment is predicted to be lethally toxic *)
    (*Print["POTENTAILLY TOXIC -- "<>ToString[ij]];*)
    Aan = 0.99 * Aan * (Abld / ToxAn);
  ];

  Ainj = {{0, Aan}}; (* no nCpm*)

  res = PatientTEST[];

  TestAnOpt[[ij, 1]] = res[[1]];
  TestAnOpt[[ij, 2]] = res[[2]];
  TestAnOpt[[ij, 3]] = res[[3]];
  TestAnOpt[[ij, 4]] = Aan / nCpm;

]];
```

In[ ]:=

```
(* find max dose tolerated by all virtual mice *)
MinToxDoses = Array[f, Length[Patients]];
MinToxDoses[[1]] = "MinToxDoses";

Quiet[For[ij = 2, ij ≤ Length[Patients], ij++,

    NotebookDelete[pr]; (* To see the code running *)
    pr = PrintTemporary["Set " <> ToString[ij] <> " of " <> ToString[Npar + 1]];

    kappac = Patients[[ij, 1]];
    kappap = Patients[[ij, 2]];
    gamma = Patients[[ij, 3]];
    V = Patients[[ij, 4]];
    ks = Patients[[ij, 5]];
    rho = Patients[[ij, 6]];
    omega = Patients[[ij, 7]];
    alpha = Patients[[ij, 8]];
    kf = Patients[[ij, 9]];
    N0 = Patients[[ij, 10]];

    MinToxDoses[[ij]] = FindToxDose[];
  ]]
MTD = Min[MinToxDoses[[2 ;;]]]
```

Out[ ]=

```
1829.73
```

In[ ]:=

```
(* TEST FOR CONSTANT DOSES *)
Npar = Length[Patients] - 1; (*how many sets will be tested*)
SetBasicParameterValues[];
```

```
TestConstantDose = Array[f, {Npar + 1, 3}];

TestConstantDose[[1, 1]] = ToString[MTD] <> " Res";
TestConstantDose[[1, 2]] = ToString[MTD] <> " OS";
TestConstantDose[[1, 3]] = ToString[MTD] <> " Tox";

MTD = 1829;

Ainj = {{0, MTD * nCpm}};

Quiet[For[ij = 2, ij ≤ Npar + 1, ij++,

    NotebookDelete[pr]; (* To see the code running *)
    pr = PrintTemporary["Set " <> ToString[ij - 1] <> " of " <> ToString[Npar]];

    kappac = Patients[[ij, 1]];
    kappap = Patients[[ij, 2]];
    gamma = Patients[[ij, 3]];
    V = Patients[[ij, 4]];
    ks = Patients[[ij, 5]];
    rho = Patients[[ij, 6]];
    omega = Patients[[ij, 7]];
    alpha = Patients[[ij, 8]];
    kf = Patients[[ij, 9]];
    N0 = Patients[[ij, 10]];

    res = PatientTEST[];

    TestConstantDose[[ij, 1]] = res[[1]];
    TestConstantDose[[ij, 2]] = res[[2]];
    TestConstantDose[[ij, 3]] = res[[3]];
```

```
]];
```

In[ ]:=

```
(* Supplementary Figure S.11 *)

TestFreeSM = SurvivalModelFit[TestFreeGrowth[[All, 2]]];
TestMTDSM = SurvivalModelFit[TestConstantDose[[2 ;;, 2]]];
TestNumOptDoseSM = SurvivalModelFit[TestNumOpt[[2 ;;, 2]]];
TestAnOptDoseSM = SurvivalModelFit[TestAnOpt[[2 ;;, 2]]];

Pic01 = GraphicsGrid[{{
    Plot[{TestFreeSM[t], TestMTDSM[t], TestNumOptDoseSM[t], TestAnOptDoseSM[t]}, {t, 0, 365},
     PlotStyle → {Gray (*Free growth*),
        Darker[Red] (*MTD*),
        Darker[Green] (*Num Opt*),
        Darker[Blue] (*An Opt*)}, Exclusions → None,
     Ticks → {Automatic, Table[{0.2 * i, PercentForm[0.2 * i]}, {i, 0, 10}]},
     PlotRange → {0., 1.01} (*,AxesLabel→{"days","% survival"}*)],

    Show[ListPlot[Join[{{0, 1}}, Thread[{Sort[TestConstantDose[[2 ;;, 3]]], Table[N[1 - (i - 1) / Npar], {i, 1, Npar}]}]], Joined → True,
      PlotStyle → Darker[Red], PlotRange → {{0, 280}, {0, 1.01}} (*,AxesLabel→{"Toxic decays, nCi·day","% mice"}*),
      Ticks → {Automatic, Table[{0.2 * i, PercentForm[0.2 * i]}, {i, 0, 10}]}] (*MTD*),
     ListPlot[Join[{{0, 1}}, Thread[{Sort[TestNumOpt[[2 ;;, 3]]], Table[N[1 - (i - 1) / Npar], {i, 1, Npar}]}]],
      Joined → True, PlotStyle → Darker[Green]] (*numerical optimization*),
     ListPlot[Join[{{0, 1}}, Thread[{Sort[TestAnOpt[[2 ;;, 3]]], Table[N[1 - (i - 1) / Npar], {i, 1, Npar}]}]],
      Joined → True, PlotStyle → Darker[Blue]] (*analytical optimization*),
     Plot[1000 * (x - Abld / nCpm), {x, 0, 400}, PlotStyle → Directive[Gray, Dashed]]]}}]
```

Out[ ]=

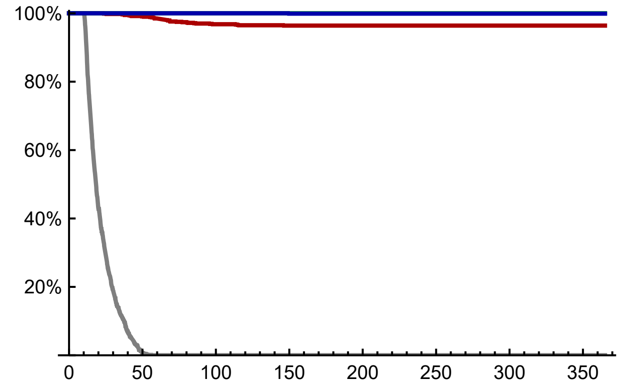

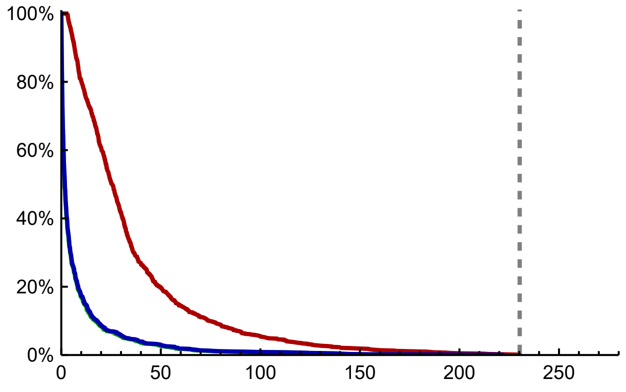

In[ ]:=

```
(* Inlet of survival plot *)
Plot[{TestFreeSM[t], TestMTDSM[t], TestNumOptDoseSM[t], TestAnOptDoseSM[t]}, {t, 0, 365},
 PlotStyle → {Gray (*Free growth*),
   Darker[Red] (*MTD*),
   Darker[Green] (*Num Opt*),
   Darker[Blue] (*An Opt*)}, Exclusions → None,
 Ticks → {Automatic, Table[{0.002 * i, PercentForm[0.002 * i]}, {i, 490, 500}]}, PlotRange → {0.99, 1.001}]
```

Out[ ]=

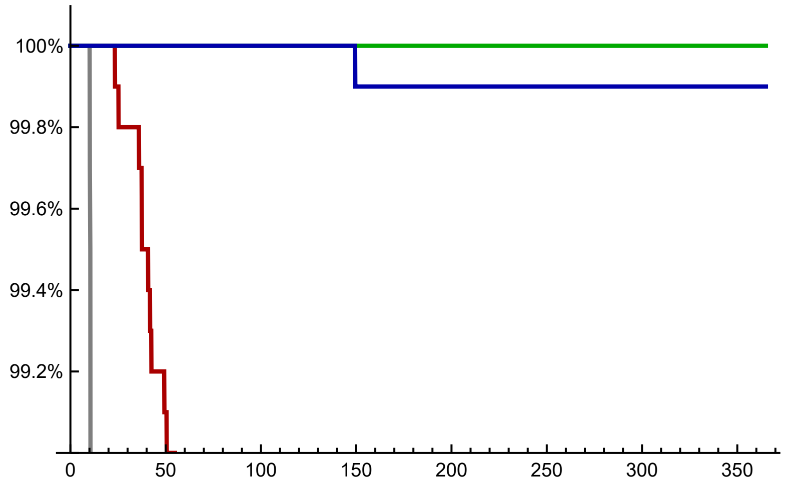


```
(* S.2.2.2 Other treatment-related measures *)
```

In[ ]:=

```
(* Activity, spent on viable cancer cells: Supplementary Figure S.12 *)
FrAcVC = result[[2 ;;, 24]] / result[[2 ;;, 11]];
(* Correlations *)
Cor = Array[f, 10];
Cor[[1]] = Correlation[FrAcVC, result[[2 ;;, 1]]] ; (*"κc"*)
Cor[[2]] = Correlation[FrAcVC, result[[2 ;;, 2]]] ; (*"κp"*)
Cor[[3]] = Correlation[FrAcVC, result[[2 ;;, 3]]]; (*"γ"*)
Cor[[4]] = Correlation[FrAcVC, result[[2 ;;, 4]]]; (*"V"*)
Cor[[5]] = Correlation[FrAcVC, result[[2 ;;, 5]]] ; (*"ks"*)
Cor[[6]] = Correlation[FrAcVC, result[[2 ;;, 6]]]; (*"ρ"*)
Cor[[7]] = Correlation[FrAcVC, result[[2 ;;, 7]]] ; (*"ω"*)
Cor[[8]] = Correlation[FrAcVC, result[[2 ;;, 8]]]; (*"α"*)
Cor[[9]] = Correlation[FrAcVC, result[[2 ;;, 9]]]; (*"kf"*)
Cor[[10]] = Correlation[FrAcVC, result[[2 ;;, 10]]] ; (*"N0"*)

(* plots of distribution and correlations *)
FrAcVCValuePlots = GraphicsGrid[
  {{Histogram[FrAcVC, Ticks → {Table[{0.005 * i, PercentForm[0.005 * i]}, {i, 1, 12}], Automatic}, LabelStyle → 14, ImageSize → 300],
    BarChart[{Table[Abs[Cor[[i]]], {i, 1, 10}]}, PlotRange → {-0.05, 0.9},
     ChartStyle → Table[If[Cor[[i]] > 0, Darker[Green], Darker[Red]], {i, 1, 10}],
     ChartLabels → {"κc", "κp", "γ", "V", "ks", "ρ", "ω", "α", "kf", "N0"}, LabelStyle → 14, ImageSize → 300]}}, ImageSize → 650]
```

Out[∘]=

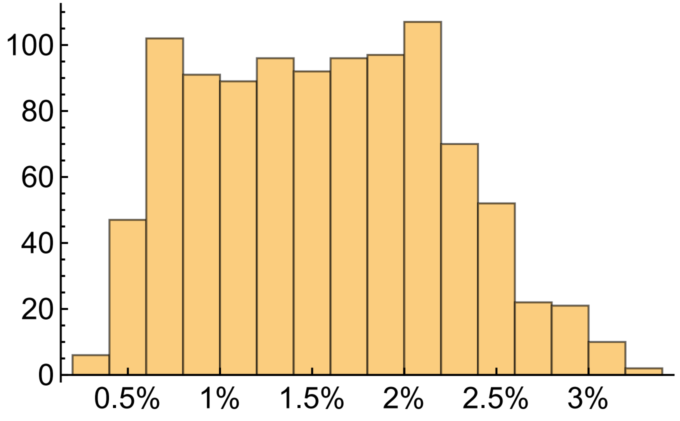

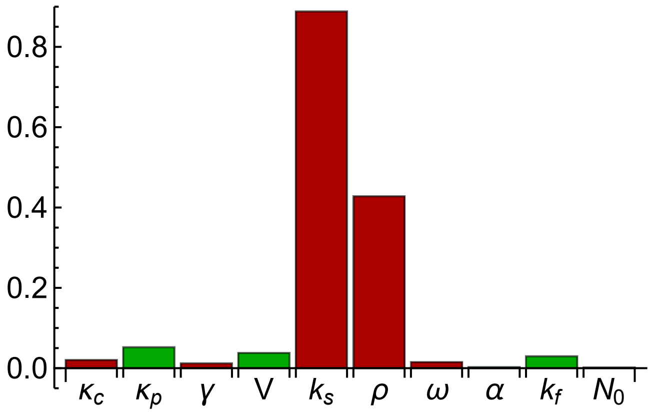


```
(* "During the parameter sweep the fraction of injected activity spent on viable cancer cells varies in the range 0.4%-3.3%." *)
PercentForm[Min[FrAcVC]]
PercentForm[Max[FrAcVC]]
```

Out[∘]//PercentForm=

0.3798%

Out[∘]//PercentForm=

3.348%

```
(* Supplementary Figure S.13: estimation for upper border of the fraction of injected activity, spent on viable cancer cells *)

SetBasicParameterValues[];
Gcur = Log[(N0 * Nnor)/Ncur] / alpha;
PicAcVUB = Show[Plot[{Exp[-alpha * G], 1}, {G, 0, Gcur}, PlotRange -> {0, 1}, Filling -> {1 -> Axis, 2 -> {1}},
    PlotStyle -> {Directive[Lighter[Gray], Thickness[0.002]], Directive[Darker[Gray], Thickness[0.002]]}],
   ListPlot[{{Gcur, Ncur/(N0 * Nnor)}}, PlotStyle -> Gray], AxesLabel -> {"Dose, d", "Surviving fraction, Nm/N0"}, ImageSize -> 410]
FrVCMaxEst = Integrate[Exp[-alpha * G], {G, 0, Gcur}] / Gcur;
PercentForm[FrVCMaxEst]
```

Out[ ]=

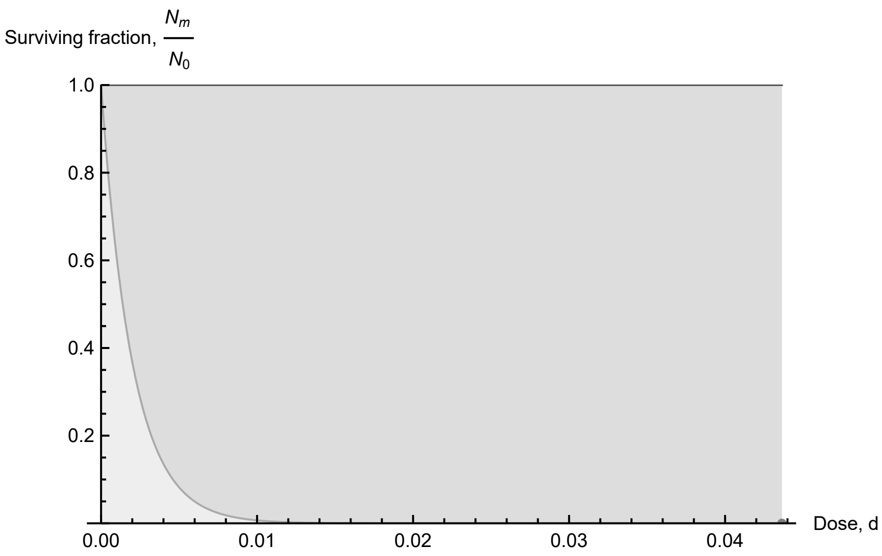

Out[ ]//PercentForm=

4.583%

In[ ]:=

```
(* "Simulations with very low cancer cell proliferation rate and negligible
   drug clearance yield very close values of the fraction of injected activity spent on viable cells." *)

resultSave = result;
SetBasicParameterValues[];
kappac = 0; gamma = 100 * Nnor / 10^7; ks = 0; rho = 0.00001; omega = 0;
Npar = 1; CreateResultArray[1];
Quiet[For[ij = 2, ij ≤ 2, ij++,
    ACurSim = FindCurDose[]; (* find curative dose *)
    DA1 = ACurSim * nCpm; (* set curative dose *)
    Ainj = {{t1, DA1}}; (* set schedule *)
    FullSystemSolutionMD[];
    WriteDownMeasures[];
  ];];
FrVCMaxSim = result[[2, 24]] / result[[2, 11]];
result = resultSave;
PercentForm[FrVCMaxSim]
PercentForm[N[FrVCMaxSim] / FrVCMaxEst - 1]
```

Out[ ]//PercentForm=

4.574%

Out[ ]//PercentForm=

−0.1892%

```
(* Supplementary Figure S.14: newborn cells during treatment *)

(* minimal single curative dose Acur with account of statistical corrections *)
AcurStatCor = (1 + 0.02 + 0.12 * Exp[-result[[2 ;;, 3]] * result[[2 ;;, 10]] / 3]) *
    (1 + 2.6 * (-result[[2 ;;, 5]] + 1) * Exp[- (12 * (-result[[2 ;;, 5]] + 1))]) * result[[2 ;;, 12]];
(* array of estimated Acur, implied as not injected, but situated on cancer cells *)
AcurCC = AcurStatCor / ((kappac + lambda + kon * result[[2 ;;, 3]] * result[[2 ;;, 10]] / result[[2 ;;, 4]]) /
    (kon * result[[2 ;;, 3]] * result[[2 ;;, 10]] / result[[2 ;;, 4]])) * nCpm;

(* estimated Nnew / N0 *)
NnewFrEst = 1 / (AcurCC / (result[[2 ;;, 10]] * result[[2;;,6]] / lambda * (nu*10^7 / Nnor) / result[[2;;,8]]) - 1);
NnewFrSim = result[[2 ;;, 28]] / result[[2 ;;, 10]];
Show[ListPlot[Thread[{NnewFrSim, NnewFrEst}]], Plot[x, {x, 0, 1}], PlotRange → {{0, 0.5}, {0, 0.3}}]
```

Out[ ]=

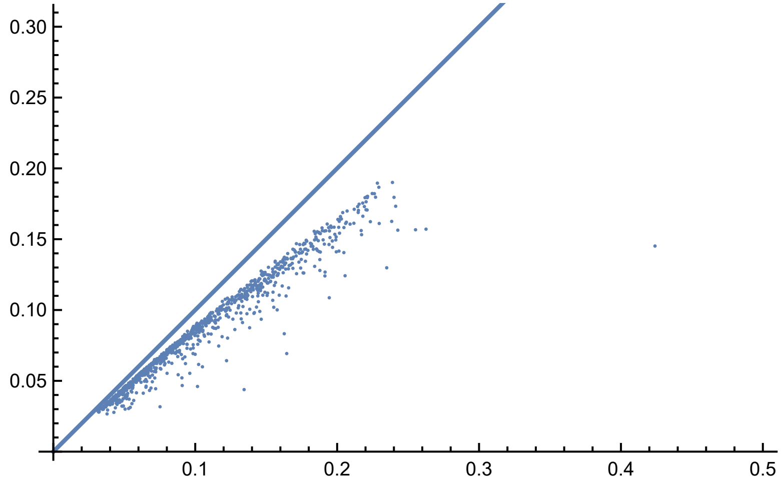

In[ ]:=

```
(* "The degree of underestimation can be corrected used the training set data,
analogically to the previously made corrections" *)
NnewFrEst2 = NnewFrEst * (1 + NnewFrEst^1.4 + 0.06);
NnewFrEst3 = NnewFrEst2 * (1 - (-0.3 / (result[[2 ;;, 3]] * result[[2 ;;, 10]] + 0.4)));
Pic1 = Show[ListPlot[{Thread[{NnewFrSim, NnewFrEst3}]}, AxesLabel → {"SIM", "EST"}, PlotStyle → {Gray}], Plot[x, {x, 0, 1}]]
```

Out[ ]=

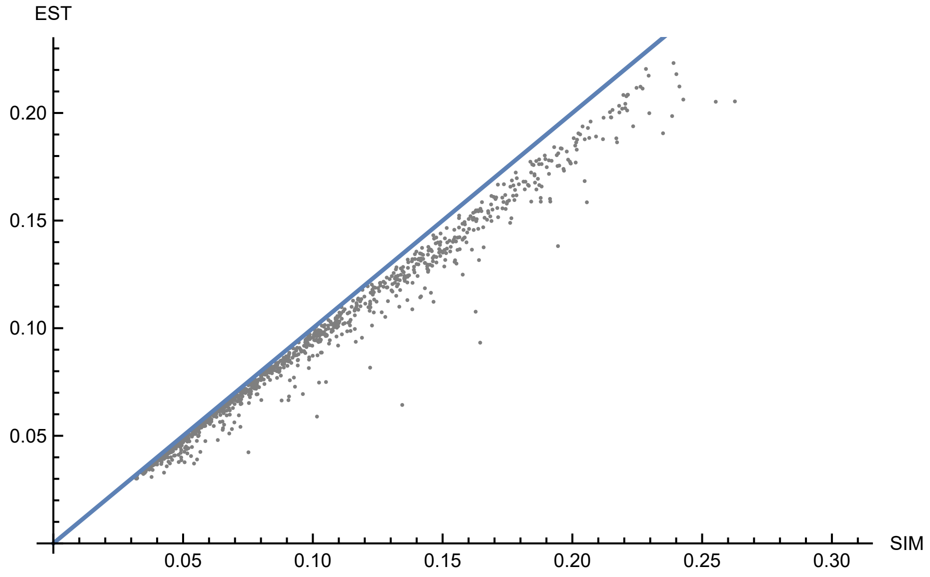

In[ ]:=

```
(* "It is important to notice, that the ratio N_new / N_0 varies in a moderate range for the training set, exceeding 0.25 in only three cases" *)
Length[Select[NnewFrSim, # > 0.25 &]]
```

Out[ ]=

3

In[ ]:=

```
(* "During the parameter sweep, the number of cancer cells
born during treatment by minimal single curative doses
constitutes 3-25 % of their initial number for the vast majority of cases" (main text of the paper) *)
PercentForm[Min[NnewFrSim]]
```

Out[ ]//PercentForm=

3.136%
