# Supplementary material for: Mathematical Modeling Unveils Optimization Strategies for Targeted Radionuclide Therapy of Blood Cancers
Source: Cancer Res Commun. 2024 Nov 14;4(11):2955–67. doi: 10.1158/2767-9764.CRC-24-0306 (PMC11562018; doi:10.1158/2767-9764.CRC-24-0306)
Supplement: Computational codes — Designed in Wolfram Mathematica, version 13.3.1.0. [file crc-24-0306_computational_codes_suppscc.zip › CodesPDF/03-Impure-drug.pdf]

```
(* This file contains simulations for the paper
  "Mathematical Modeling Unveils Optimization Strategies
for Targeted Radionuclide Therapy of Blood Cancers"
  by Maxim Kuznetsov, Vikram Adhikarla, Enrico Caserta,
 Flavia Pichiorri, John E.Shively, Xiuli Wang and Russell C.Rockne *)

(*****************************************************************************************************)
```

In[ ]:=

```
(* The script for the solution of full model and other scripts are hidden here
          and need to be initialized to run simulations *)

(* It allows consideration of multidose setting *)

(* To avoid numerical problems (linked with N / D multiplier and arriving under high rate of cell death)
   here we solve identical equations for concentrations of receptors of damaged cells instead of their fractions,
normalized by division by gamma / V, which thus provides their relative numbers (i.e., d_F=f_FD*D and d_A=f_AD*D) .
         These equations are explicitly presented in supplemetary pdf-file, page 2 (such renormalization does not alter them) .
      Original equations also work well if D is not getting too small (which includes working fine for simulation for the basic set) ,
the corresponding parts of original equations are commented out and highlighted like this *)

FullSystemSolutionMD[ ] := (

   lAinj = Length[Ainj]; (* number of injections"*)
   If[Ainj[[1, 1]] == 0, Npw = lAinj, Npw = lAinj + 1];
   (* the injections are treated as new initial conditions for a new system, which as well takes the actual vaules of other parameters *)
   (* therefore the number of injections has to me remembered and logic differs whether the first injection is made at t=0 or t>0 *)

   apw = Array[ff, Npw]; bpw = Array[ff, Npw]; NNpw = Array[ff, Npw]; DDpw = Array[ff, Npw];
   papw = Array[ff, Npw]; pbpw = Array[ff, Npw]; fFNpw = Array[ff, Npw]; fANpw = Array[ff, Npw];
   (*fFDpw=Array[ff,Npw];fADpw=Array[ff,Npw];*) dFpw = Array[ff, Npw]; dApw = Array[ff, Npw];
   (* inert antibody fragments are as well accounted for as pb *)
```

```
(* times of beginning and end for solution of separate systems *)
tB = Array[ff, Npw]; tE = Array[ff, Npw];

(* for monitoring the paths of activity *)
ActBloodpw = Array[ff, Npw]; ActBloodFragpw = Array[ff, Npw];
ActOutpw = Array[ff, Npw]; ActOutFragpw = Array[ff, Npw]; ActTumorpw = Array[ff, Npw];

(* for monitoring influence of self-dose, croos-fire and decays in blood *)
SDpw = Array[ff, Npw]; CFNpw = Array[ff, Npw]; CFDpw = Array[ff, Npw]; UNpw = Array[ff, Npw];

(* for monitoring the number of new cancer cells appearing during treatment *)
NewCellspw = Array[ff, Npw];

(*fFD,fAD,*)
Clear[a, b, NN, DD, pa, pb, fFN, fAN, dF, dA, ActBlood, ActBloodFrag, ActOut, ActOutFrag, ActTumor, SD, CFN, CFD, UN, NewCells];

(* EQUATIONS *)

(* Radiation damage function *) (*fAD_,*)
RD[NN_, DD_, fAN_, dA_, a_, pa_] := alpha * (ks * (lambda * gamma * fAN)/nu (*self-dose*) +
    (1 - ks) * (lambda * gamma * (fAN * NN + dA))/(nu * (NN + DD)) (*cross-fire*) + kf * lambda * (a + pa) (*dose from unanchored nuclides*));

(* Active antibodies *)
Fa[t_] := (*injections are considered as initial conditions*)
  - lambda * a[t] (*decay*) - kon * gamma/V * (fFN[t] * NN[t] + dF[t]) * a[t] (*binding*) - kappac * a[t] (*clearance*);
(*fFD[t] *DD[t] *)
```

```
(* Inert antibodies *)
Fb[t_] := (*injections are considered as initial conditions*)
  + lambda * a[t] (*decay of a*) - kon * gamma/V * (fFN[t] * NN[t] + dF[t]) * b[t] (*binding*) - kappac * b[t] (*clearance*);
(*fFD[t]*DD[t]*)

(* Viable cells *) FNN[t_] := rho * NN[t] (*proliferation*) - RD[NN[t], DD[t], fAN[t], dA[t], a[t], pa[t]] * NN[t] (*damage*);
(*fAD[t],*)

(* Damaged cells *) FDD[t_] := RD[NN[t], DD[t], fAN[t], dA[t], a[t], pa[t]] * NN[t] (*damage*) - omega * DD[t] (*death*);
(*fAD[t],*)

(* Active fragments *) Fpa[t_] := omega * (gamma * dA[t])/V (*release*) - lambda * pa[t] (*decay*) - kappap * pa[t] (*clearance*);
(*fAD[t]*DD[t],*)

(* Inert fragments *)
Fpb[t_] := omega * (gamma * (DD[t] - dF[t] - dA[t]))/V (*release*) + lambda * pa[t] (*decay*) - kappap * pb[t] (*clearance*);
(*-fFD[t]*DD[t]-fAD[t]*DD[t]*)

(* Free receptors of viable cells *) FfFN[t_] := (1 - fFN[t]) * rho - kon * (a[t] + b[t]) * fFN[t];

(* Active receptors of viable cells *) FfAN[t_] := kon * a[t] * fFN[t] - (lambda + rho) * fAN[t];

(* Free receptors of damaged cells *)
(*FfFD[t_]:=(fFN[t]-fFD[t])*RD[NN[t],DD[t],fAN[t],fAD[t],a[t],pa[t]]*NN[t]/DD[t]-kon*(a[t]+b[t])*fFD[t];*)
FdF[t_] := RD[NN[t], DD[t], fAN[t], dA[t], a[t], pa[t]] * fFN[t] * NN[t] - kon * (a[t] + b[t]) * dF[t] - omega * dF[t];

(* Active receptors of damaged cells *)
```

```
(*FfAD[t_]:=(fAN[t]-fAD[t])*RD[NN[t],DD[t],fAN[t],fAD[t],a[t],pa[t]]*NN[t]/DD[t]+kon*a[t]*fFD[t]-lambda*fAD[t];*)

FdA[t_] := RD[NN[t], DD[t], fAN[t], dA[t], a[t], pa[t]] * fAN[t] * NN[t] + kon * a[t] * dF[t] - lambda * dA[t] - omega * dA[t];

(* Initial conditions *)

If[Ainj⟦1, 1⟧ == 0
  , a0 = Ainj⟦1, 2⟧/V; b0 = Ainj⟦1, 3⟧/V (*eta*Ainj⟦1,2⟧/V*)
  , a0 = 0; b0 = 0]; (*complexes in blood*)
NN0 = N0;
DD0 = 0;
pa0 = 0; pb0 = 0; fFN0 = 1; fAN0 = 0;
(*fFD0=1;fAD0=0;*) dF0 = 0; dA0 = 0;
ActBlood0 = 0; ActBloodFrag0 = 0; ActOut0 = 0; ActOutFrag0 = 0; ActTumor0 = 0; NewCells0 = 0;
SD0 = 0; CFN0 = 0; CFD0 = 0; UN0 = 0;

(* SOLVER *)

tB⟦1⟧ = 0; If[Ainj⟦1, 1⟧ == 0, If[lAinj > 1, tE⟦1⟧ = Ainj⟦2, 1⟧, tE⟦1⟧ = tEnd], tE⟦1⟧ = Ainj⟦1, 1⟧];

For[npw = 1, npw ≤ Npw, npw++,

  Clear[a, b, NN, DD, pa, pb, fFN, fAN, dF, dA, ActBlood, ActBloodFrag, ActOut, ActOutFrag, ActTumor, SD, CFN, CFD, UN, NewCells];
  (*fFD,fAD,*)

  sol = NDSolve[{
        (*INITIAL CONDITIONS*)
        a[tB⟦npw⟧] == a0, b[tB⟦npw⟧] == b0, NN[tB⟦npw⟧] == NN0,
        DD[tB⟦npw⟧] == DD0, pa[tB⟦npw⟧] == pa0, pb[tB⟦npw⟧] == pb0, fFN[tB⟦npw⟧] == fFN0, fAN[tB⟦npw⟧] == fAN0,
        (*fFD[tB⟦npw⟧]==fFD0,fAD[tB⟦npw⟧]==fAD0,*) dF[tB⟦npw⟧] == dF0, dA[tB⟦npw⟧] == dA0,
        ActBlood[tB⟦npw⟧] == ActBlood0, ActBloodFrag[tB⟦npw⟧] == ActBloodFrag0, ActOut[tB⟦npw⟧] == ActOut0,
```

```
ActOutFrag[tB⟦npw⟧] == ActOutFrag0, ActTumor[tB⟦npw⟧] == ActTumor0, SD[tB⟦npw⟧] == SD0,
CFN[tB⟦npw⟧] == CFN0, CFD[tB⟦npw⟧] == CFD0, UN[tB⟦npw⟧] == UN0, NewCells[tB⟦npw⟧] == NewCells0,

a'[t] == Fa[t], b'[t] == Fb[t], NN'[t] == FNN[t], DD'[t] == FDD[t], pa'[t] == Fpa[t], pb'[t] == Fpb[t],
fFN'[t] == FfFN[t], fAN'[t] == FfAN[t], (*fFD'[t]==FfFD[t],fAD'[t]==FfAD[t],*) dF'[t] == FdF[t], dA'[t] == FdA[t],

ActBlood'[t] == V * lambda * (a[t] + pa[t]),
ActBloodFrag'[t] == V * lambda * pa[t],
ActOut'[t] == V * (kappac * a[t] + kappap * pa[t]),
ActOutFrag'[t] == V * kappap * pa[t],
ActTumor'[t] == (lambda * gamma) * (fAN[t] * NN[t] + dA[t]), (*fAD[t]*DD[t]*)

SD'[t] == ks * (lambda * gamma) * (fAN[t] * NN[t]),
CFN'[t] == (1 - ks) * (lambda * gamma) * (fAN[t] * NN[t]) * NN[t] / (NN[t] + DD[t]),
CFD'[t] == (1 - ks) * (lambda * gamma) * dA[t] * NN[t] / (NN[t] + DD[t]), (* (fAD[t]*DD[t]) *)
UN'[t] == kf * lambda * (a[t] + pa[t]) * nu * NN[t],
NewCells'[t] == If[t > Ainj⟦1, 1⟧, rho * NN[t], 0] (* start counting new cells from the moment of the first injection *)

 (* The simulations run until the injected activity decays to negligible amounts
  If viable cancer cell number becomes too small
  (cure) or too great (host death) it just stays there to allow assessment of activity paths *)
, WhenEvent[NN[t] > 10^9 / Nnor, NN[t] → 0.99 * 10^9 / Nnor]
}(*fFD,fAD,*)
, {a, b, NN, DD, pa, pb, fFN, fAN, dF, dA, ActBlood,
  ActBloodFrag, ActOut, ActOutFrag, ActTumor, SD, CFN, CFD, UN, NewCells}, {t, tB⟦npw⟧, tE⟦npw⟧}
, AccuracyGoal → 10, PrecisionGoal → 10];

apw⟦npw⟧ = First[a /. sol]; bpw⟦npw⟧ = First[b /. sol]; NNpw⟦npw⟧ = First[NN /. sol]; DDpw⟦npw⟧ = First[DD /. sol];
```

```
papw⟦npw⟧ = First[pa /. sol]; pbpw⟦npw⟧ = First[pb /. sol]; fFNpw⟦npw⟧ = First[fFN /. sol]; fANpw⟦npw⟧ = First[fAN /. sol];
(*fFDpw⟦npw⟧=First[fFD/.sol]; fADpw⟦npw⟧=First[fAD/.sol];*) dFpw⟦npw⟧ = First[dF /. sol]; dApw⟦npw⟧ = First[dA /. sol];
ActBloodpw⟦npw⟧ = First[ActBlood /. sol];
ActBloodFragpw⟦npw⟧ = First[ActBloodFrag /. sol];
ActOutpw⟦npw⟧ = First[ActOut /. sol];
ActOutFragpw⟦npw⟧ = First[ActOutFrag /. sol];
ActTumorpw⟦npw⟧ = First[ActTumor /. sol];
SDpw⟦npw⟧ = First[SD /. sol];
CFNpw⟦npw⟧ = First[CFN /. sol];
CFDpw⟦npw⟧ = First[CFD /. sol];
UNpw⟦npw⟧ = First[UN /. sol];
NewCellspw⟦npw⟧ = First[NewCells /. sol];

If[npw < Npw,
  (*renew initial conditions*)

  If[Ainj⟦1, 1⟧ == 0
    , a0 = apw⟦npw⟧[tE⟦npw⟧] + Ainj⟦npw + 1, 2⟧/V;
    b0 = bpw⟦npw⟧[tE⟦npw⟧] + Ainj⟦npw + 1, 3⟧/V (*eta*Ainj⟦npw+1,2⟧/V*)
    , a0 = apw⟦npw⟧[tE⟦npw⟧] + Ainj⟦npw, 2⟧/V;
    b0 = bpw⟦npw⟧[tE⟦npw⟧] + Ainj⟦npw, 3⟧/V (*eta*Ainj⟦npw,2⟧/V*) ];

  NN0 = NNpw⟦npw⟧[tE⟦npw⟧];
  DD0 = DDpw⟦npw⟧[tE⟦npw⟧];
  pa0 = papw⟦npw⟧[tE⟦npw⟧];
  pb0 = pbpw⟦npw⟧[tE⟦npw⟧];
  fFN0 = fFNpw⟦npw⟧[tE⟦npw⟧]; fAN0 = fANpw⟦npw⟧[tE⟦npw⟧];
  (*fFD0=fFDpw⟦npw⟧[tE⟦npw⟧];fAD0=fADpw⟦npw⟧[tE⟦npw⟧];*)
  dF0 = dFpw⟦npw⟧[tE⟦npw⟧];
  dA0 = dApw⟦npw⟧[tE⟦npw⟧];
```

```
    ActBlood0 = ActBloodpw[[npw]][tE[[npw]]];
    ActBloodFrag0 = ActBloodFragpw[[npw]][tE[[npw]]];
    ActOut0 = ActOutpw[[npw]][tE[[npw]]];
    ActOutFrag0 = ActOutFragpw[[npw]][tE[[npw]]];
    ActTumor0 = ActTumorpw[[npw]][tE[[npw]]];
    SD0 = SDpw[[npw]][tE[[npw]]];
    CFN0 = CFNpw[[npw]][tE[[npw]]];
    CFD0 = CFDpw[[npw]][tE[[npw]]];
    UN0 = UNpw[[npw]][tE[[npw]]];
    NewCells0 = NewCellspw[[npw]][tE[[npw]]];

    (*renew time frame*)
    tB[[npw + 1]] = tE[[npw]];

    If[Ainj[[1, 1]] == 0, If[Npw > npw + 1, tE[[npw + 1]] = Ainj[[npw + 2, 1]], tE[[npw + 1]] = tEnd],
      If[Npw > npw + 1, tE[[npw + 1]] = Ainj[[npw + 1, 1]], tE[[npw + 1]] = tEnd]];]
  ];
  npw--;
  (* It will be convenient to have estimation of minimal viable cell number here *)
  Nn = If[Ainj[[1, 1]] == 0
    , Min[Table[NMinimize[{Nnor* (NNpw[[nn]][t]), t > tB[[nn]], t < tE[[nn]]}, t][[1]], {nn, 1, npw}]]
    , Min[Table[NMinimize[{Nnor* (NNpw[[nn]][t]), t > tB[[nn]], t < tE[[nn]]}, t][[1]], {nn, 2, npw}]]];Return[Nn])

(* This is the script for finding minimal single curative dose, which we will need further, so let's initialize it as well *)
FindCurDose[] := (DA1 = (nu*N0*rho)/(alpha*lambda); (* at this dose N'(0) =0 for approximated system *)
  Ainj = {{t1, DA1, eta*DA1}};

  While[FullSystemSolutionMD[] > Ncur, DA1 = 2*DA1; Ainj = {{t1, DA1, eta*DA1}};];
```

```
    Dmax = DA1; Dmin = DA1 / 2;

    While[Abs[Dmin - Dmax] / Dmax > 0.000001,
      DA1 = (Dmin + Dmax) / 2; Ainj = {{t1, DA1, eta * DA1}};
      If[FullSystemSolutionMD[] > Ncur, Dmin = DA1, Dmax = DA1]];
    Dcur = Dmax;
    Return[Dcur / nCpm])

(* This is the script for finding minimal lethally toxic dose -- initialize it *)
FindToxDose[] := (DA1 = Abld;
    Ainj = {{t1, DA1, eta * DA1}};

    While[FullSystemSolutionToxicity[] < Abld, DA1 = 2 * DA1; Ainj = {{t1, DA1, eta * DA1}}; ];
    Dmax = DA1; Dmin = DA1 / 2;

    While[Abs[Dmin - Dmax] / Dmax > 0.000001,
      DA1 = (Dmin + Dmax) / 2; Ainj = {{t1, DA1, eta * DA1}};
      If[FullSystemSolutionToxicity[] < Abld, Dmin = DA1, Dmax = DA1]];
    Dtox = Dmax;
    Return[Dtox / nCpm])


FullSystemSolutionToxicity[] := (

    (* maximum time of simulation-- where little radioactivity of the last dose remains, namely 0.17% of the last dose *)
    tEnd = Ainj[[Length[Ainj], 1]] + (-Log[0.0017] / lambda);
    lAinj = Length[Ainj]; (* number of injections*)
    If[Ainj[[1, 1]] == 0, Npw = lAinj, Npw = lAinj + 1];

    apw = Array[ff, Npw]; bpw = Array[ff, Npw]; NNpw = Array[ff, Npw]; DDpw = Array[ff, Npw];
    papw = Array[ff, Npw]; pbpw = Array[ff, Npw]; fFNpw = Array[ff, Npw]; fANpw = Array[ff, Npw];
    dFpw = Array[ff, Npw]; dApw = Array[ff, Npw];
```

```
(* inert antibody fragments are as well accounted for as pb *)

(* times of beginning and end for solution of separate systems *)
tB = Array[ff, Npw]; tE = Array[ff, Npw];

(* for monitoring the paths of activity *)
ActBloodpw = Array[ff, Npw]; ActBloodFragpw = Array[ff, Npw];
ActOutpw = Array[ff, Npw]; ActOutFragpw = Array[ff, Npw]; ActTumorpw = Array[ff, Npw];

(* for monitoring influence of self-dose, croos-fire and decays in blood *)
SDpw = Array[ff, Npw]; CFNpw = Array[ff, Npw]; CFDpw = Array[ff, Npw]; UNpw = Array[ff, Npw];

(* for monitoring the number of new cancer cells appearing during treatment *)
NewCellspw = Array[ff, Npw];

Clear[a, b, NN, DD, pa, pb, fFN, fAN, dF, dA, ActBlood, ActBloodFrag, ActOut, ActOutFrag, ActTumor, SD, CFN, CFD, UN, NewCells];

(* EQUATIONS *)

(* Radiation damage function *)
RD[NN_, DD_, fAN_, dA_, a_, pa_] := alpha * (ks * (lambda * gamma * fAN)/nu (*self-dose*) +
    (1 - ks) * (lambda * gamma * (fAN * NN + dA))/(nu * (NN + DD)) (*cross-fire*) + kf * lambda * (a + pa) (*dose from unanchored nuclides*));


(* Active antibodies *)
Fa[t_] := (*injections are considered as initial conditions*)
  - lambda * a[t] (*decay*) - kon * gamma/V * (fFN[t] * NN[t] + dF[t]) * a[t] (*binding*) - kappac * a[t] (*clearance*);
```

```
(* Inert antibodies *)
Fb[t_] := (*injections are considered as initial conditions*)
  + lambda * a[t] (*decay of a*) - kon * gamma/V * (fFN[t] * NN[t] + dF[t]) * b[t] (*binding*) - kappac * b[t] (*clearance*);

(* Viable cells *) FNN[t_] := rho * NN[t] (*proliferation*) - RD[NN[t], DD[t], fAN[t], dA[t], a[t], pa[t]] * NN[t] (*damage*);

(* Damaged cells *) FDD[t_] := RD[NN[t], DD[t], fAN[t], dA[t], a[t], pa[t]] * NN[t] (*damage*) - omega * DD[t] (*death*);

(* Active fragments *) Fpa[t_] := omega * (gamma * dA[t])/V (*release*) - lambda * pa[t] (*decay*) - kappap * pa[t] (*clearance*);

(* Inert fragments *)
Fpb[t_] := omega * (gamma * (DD[t] - dF[t] - dA[t]))/V (*release*) + lambda * pa[t] (*decay*) - kappap * pb[t] (*clearance*);

(* Free receptors of viable cells *) FfFN[t_] := (1 - fFN[t]) * rho - kon * (a[t] + b[t]) * fFN[t];

(* Active receptors of viable cells *) FfAN[t_] := kon * a[t] * fFN[t] - (lambda + rho) * fAN[t];

(* Free receptors of damaged cells *)
FdF[t_] := RD[NN[t], DD[t], fAN[t], dA[t], a[t], pa[t]] * fFN[t] * NN[t] - kon * (a[t] + b[t]) * dF[t] - omega * dF[t];

(* Active receptors of damaged cells *)
FdA[t_] :=
  RD[NN[t], DD[t], fAN[t], dA[t], a[t], pa[t]] * fAN[t] * NN[t] + kon * a[t] * dF[t] - lambda * dA[t] - omega * dA[t];

(* Initial conditions *)

If[Ainj[[1, 1]] == 0
  , a0 = Ainj[[1, 2]]/V; b0 = Ainj[[1, 3]]/V
```

```
  , a0 = 0; b0 = 0]; (*complexes in blood*)
NN0 = N0;
DD0 = 0;
pa0 = 0; pb0 = 0; fFN0 = 1; fAN0 = 0;
dF0 = 0; dA0 = 0;
ActBlood0 = 0; ActBloodFrag0 = 0; ActOut0 = 0; ActOutFrag0 = 0; ActTumor0 = 0; NewCells0 = 0;
SD0 = 0; CFN0 = 0; CFD0 = 0; UN0 = 0;

(* SOLVER *)

tB[[1]] = 0; If[Ainj[[1, 1]] == 0, If[lAinj > 1, tE[[1]] = Ainj[[2, 1]], tE[[1]] = tEnd], tE[[1]] = Ainj[[1, 1]]];

For[npw = 1, npw ≤ Npw, npw++,

 Clear[a, b, NN, DD, pa, pb, fFN, fAN, dF, dA, ActBlood, ActBloodFrag, ActOut, ActOutFrag, ActTumor, SD, CFN, CFD, UN, NewCells];

 sol = NDSolve[{
      (*INITIAL CONDITIONS*)
      a[tB[[npw]]] == a0, b[tB[[npw]]] == b0, NN[tB[[npw]]] == NN0, DD[tB[[npw]]] == DD0, pa[tB[[npw]]] == pa0,
      pb[tB[[npw]]] == pb0, fFN[tB[[npw]]] == fFN0, fAN[tB[[npw]]] == fAN0, dF[tB[[npw]]] == dF0, dA[tB[[npw]]] == dA0,
      ActBlood[tB[[npw]]] == ActBlood0, ActBloodFrag[tB[[npw]]] == ActBloodFrag0, ActOut[tB[[npw]]] == ActOut0,
      ActOutFrag[tB[[npw]]] == ActOutFrag0, ActTumor[tB[[npw]]] == ActTumor0, SD[tB[[npw]]] == SD0,
      CFN[tB[[npw]]] == CFN0, CFD[tB[[npw]]] == CFD0, UN[tB[[npw]]] == UN0, NewCells[tB[[npw]]] == NewCells0,

      a'[t] == Fa[t], b'[t] == Fb[t], NN'[t] == FNN[t], DD'[t] == FDD[t],
      pa'[t] == Fpa[t], pb'[t] == Fpb[t], fFN'[t] == FfFN[t], fAN'[t] == FfAN[t], dF'[t] == FdF[t], dA'[t] == FdA[t],

      ActBlood'[t] == V*lambda*(a[t] + pa[t]),
      ActBloodFrag'[t] == V*lambda*pa[t],
      ActOut'[t] == V*(kappac*a[t] + kappap*pa[t]),
```

```
        ActOutFrag'[t] == V * kappap * pa[t],
        ActTumor'[t] == (lambda * gamma) * (fAN[t] * NN[t] + dA[t]),

        SD'[t] == ks * (lambda * gamma) * (fAN[t] * NN[t]),

        CFN'[t] == (1 - ks) * (lambda * gamma) * (fAN[t] * NN[t]) * NN[t] / (NN[t] + DD[t]),

        CFD'[t] == (1 - ks) * (lambda * gamma) * dA[t] * NN[t] / (NN[t] + DD[t]),

        UN'[t] == kf * lambda * (a[t] + pa[t]) * nu * NN[t],
        NewCells'[t] == If[t > Ainj[[1, 1]], rho * NN[t], 0] (* start counting new cells from the moment of the first injection *)
        , WhenEvent[NN[t] < Ncur / Nnor, NN[t] → 0] (* treatment wins *)
      }
      , {a, b, NN, DD, pa, pb, fFN, fAN, dF, dA, ActBlood,
        ActBloodFrag, ActOut, ActOutFrag, ActTumor, SD, CFN, CFD, UN, NewCells}, {t, tB[[npw]], tE[[npw]]}
      , AccuracyGoal → 10, PrecisionGoal → 10];

apw[[npw]] = First[a /. sol]; bpw[[npw]] = First[b /. sol]; NNpw[[npw]] = First[NN /. sol]; DDpw[[npw]] = First[DD /. sol];
papw[[npw]] = First[pa /. sol]; pbpw[[npw]] = First[pb /. sol]; fFNpw[[npw]] = First[fFN /. sol];
fANpw[[npw]] = First[fAN /. sol]; dFpw[[npw]] = First[dF /. sol]; dApw[[npw]] = First[dA /. sol];
ActBloodpw[[npw]] = First[ActBlood /. sol];
ActBloodFragpw[[npw]] = First[ActBloodFrag /. sol];
ActOutpw[[npw]] = First[ActOut /. sol];
ActOutFragpw[[npw]] = First[ActOutFrag /. sol];
ActTumorpw[[npw]] = First[ActTumor /. sol];
SDpw[[npw]] = First[SD /. sol];
CFNpw[[npw]] = First[CFN /. sol];
CFDpw[[npw]] = First[CFD /. sol];
UNpw[[npw]] = First[UN /. sol];
NewCellspw[[npw]] = First[NewCells /. sol];
```

```
If[npw < Npw,
  (*renew initial conditions*)
  If[Ainj⟦1, 1⟧ == 0
    , a0 = apw⟦npw⟧[tE⟦npw⟧] + Ainj⟦npw + 1, 2⟧ / V; b0 = bpw⟦npw⟧[tE⟦npw⟧] + Ainj⟦npw + 1, 3⟧ / V
    , a0 = apw⟦npw⟧[tE⟦npw⟧] + Ainj⟦npw, 2⟧ / V; b0 = bpw⟦npw⟧[tE⟦npw⟧] + Ainj⟦npw, 3⟧ / V];
  NN0 = NNpw⟦npw⟧[tE⟦npw⟧];
  DD0 = DDpw⟦npw⟧[tE⟦npw⟧];
  pa0 = papw⟦npw⟧[tE⟦npw⟧];
  pb0 = pbpw⟦npw⟧[tE⟦npw⟧];
  fFN0 = fFNpw⟦npw⟧[tE⟦npw⟧]; fAN0 = fANpw⟦npw⟧[tE⟦npw⟧];
  dF0 = dFpw⟦npw⟧[tE⟦npw⟧];
  dA0 = dApw⟦npw⟧[tE⟦npw⟧];
  ActBlood0 = ActBloodpw⟦npw⟧[tE⟦npw⟧];
  ActBloodFrag0 = ActBloodFragpw⟦npw⟧[tE⟦npw⟧];
  ActOut0 = ActOutpw⟦npw⟧[tE⟦npw⟧];
  ActOutFrag0 = ActOutFragpw⟦npw⟧[tE⟦npw⟧];
  ActTumor0 = ActTumorpw⟦npw⟧[tE⟦npw⟧];
  SD0 = SDpw⟦npw⟧[tE⟦npw⟧];
  CFN0 = CFNpw⟦npw⟧[tE⟦npw⟧];
  CFD0 = CFDpw⟦npw⟧[tE⟦npw⟧];
  UN0 = UNpw⟦npw⟧[tE⟦npw⟧];
  NewCells0 = NewCellspw⟦npw⟧[tE⟦npw⟧];

  (*renew time frame*)
  If[Ainj⟦1, 1⟧ == 0, If[Npw > npw + 1, tE⟦npw + 1⟧ = Ainj⟦npw + 2, 1⟧, tE⟦npw + 1⟧ = tEnd],
    If[Npw > npw + 1, tE⟦npw + 1⟧ = Ainj⟦npw + 1, 1⟧, tE⟦npw + 1⟧ = tEnd]];
];
npw--;
Return[ActBloodpw⟦npw⟧[tEnd]])
```

```
(* And this is the script for setting basic parameter values, with account for impure drug *)
SetBasicParameterValues[] := (
    Nnor = 10^5; (* due to numerical pecularities, it is better to use this normalization number of cells *)

    lambda = 0.07; (* radionuclide decay rate *)
    kon = 11.15; (* antibody-receptor association rate *)
    kappac = 0.1; (* antibody clearance rate (0.04-0.28) *)
    kappap = 1; (* antibody fragments clearance rate (0.4-4.) *)
    gamma = 2.1 * Nnor / 10^7; (* number of receptors of Nnor cancer cells (0.13-10) *Nnor/10^7 *)
    V = 1.; (* volume of drug distribution (0.75-1.5) *)
    nu = 0.015 * Nnor / 10^7; (* volume of lesion with Nnor cancer cells *)
    ks = 0.3; (* relative significance of self-dose (0.1-0.5) *)

    rho = 0.34; (* cancer cells proliferation rate (0.15-0.7) *)
    omega = 0.05; (* damaged cells death rate (0.005-0.5) *)
    alpha = 500; (* cancer cells radiosensitivity (50-5000) *)
    eta = 1780; (* coefficient of drug impurity (0-10^5) *)
    kf = 0.05; (* significance of unanchored nuclides decays (0.01-0.25) *)
    N0 = 3. * 10^7 / Nnor; (* initial number of viable cancer cells, xNnor (1-10) *Nnor/10^7 *)
    D0 = 0;
    (*0.1/Nnor; *)
    (* initial number of damaged cancer cells -- use non-zero value if considering fraction of receptors of damaged cells, to avoid division by zero*)
    nCpm = 0.000076; (* convenient parameter of conversion from nCi to pmol *)
    DA1 = 50 * nCpm; (* injected amount of radionuclides *)
    t1 = 0; (* moment of their injection *)
    Ncur = 0.01; (* number of viable cancer cells (in cells), corresponding to cancer cure *)
    Abld = 0.0175; (* lethal amount of decays in blood *)
    Ainj = {{t1, DA1, eta * DA1}}; (* Schedule of injections *)

    (* maximum time of simulation-- where little radioactivity of the last dose remains, namely 0.17% *)
```

```
    tEnd = -Log[0.0017] / lambda;

    kGy = 2500; (* coefficient of conversion of nM of 225-Ac decay in bone marrow into Gy *)
  )
SetBasicParameterValues[]
```

(* Main text: Variation by radioconjugates impurity & Supplementary S.3.1 Variation by radioconjugates impurity *)

(* Variation of coefficient of drug impurity η is performed with the pretty long-running codes hidden here *)

In[ ]:=

```
(* Minimal single curative dose depending on η, large-range variation *)
SetBasicParameterValues[];
Var = "eta"; VarSymbol = "η";
(* Setting boundaries and step of variation*)
Nx = 51; (* number of points *)
varmin = 0; varmax = 15 000; varstep = (varmax - varmin) / (Nx - 1);
AcurEtaDetBig = Array[f, {3, Nx, 2}];

Quiet[
  For[id = 1, id ≤ 3, id++,
   ii = 1;

   If[id == 1, ks = 0];
   If[id == 2, ks = 0.3];
   If[id == 3, ks = 1];

   NotebookDelete[pr2]; (* To see the code running *)
   pr2 = PrintTemporary["Current ks is " <> ToString[ks]];

   For[eta = varmin, eta ≤ varmax, eta = N[eta + varstep],
    NotebookDelete[pr]; (* To see the code running *)
    pr = PrintTemporary["Current " <> ToString[Var] <> " is " <> ToString[N[eta]] <> "; max value is " <> ToString[varmax]];

    AcurEtaDetBig[[id, ii, 1]] = eta;
    AcurEtaDetBig[[id, ii, 2]] = FindCurDose[]; (* The function has to be defined in script previously (S.2.1.1) *)
    ii++;]]
 ];
```

```
(* Minimal curative dose depending on η, in absense of damage from unanchored nuclides *)
SetBasicParameterValues[]; kf = 0;
Var = "eta"; VarSymbol = "η";
(* Setting boundaries and step of variation*)
Nx = 21; (* number of points *)
varmin = 0; varmax = 2100; varstep = (varmax - varmin) / (Nx - 1);
AcurEtaDetBigNokf = Array[f, {3, Nx, 2}];

Quiet[
  For[id = 1, id ≤ 3, id++,
   ii = 1;
   If[id == 1, ks = 0];
   If[id == 2, ks = 0.3];
   If[id == 3, ks = 1];

   NotebookDelete[pr2]; (* To see the code running *)
   pr2 = PrintTemporary["Current ks is " <> ToString[ks]];

   For[eta = varmin, eta ≤ varmax, eta = N[eta + varstep],
    NotebookDelete[pr]; (* To see the code running *)
    pr = PrintTemporary["Current " <> ToString[Var] <> " is " <> ToString[N[eta]] <> "; max value is " <> ToString[varmax]];

    AcurEtaDetBigNokf[[id, ii, 1]] = eta;
    AcurEtaDetBigNokf[[id, ii, 2]] = FindCurDose[]; (* The function has to be defined in script previously (S.2.1.1) *)
    ii++;];]
 ];
```

```
(* Toxic decays depending on η, large-range variation *)
SetBasicParameterValues[];
Var = "eta"; VarSymbol = "η";
varmax = 15000;

AcurEtaDetBigTox = Array[f, {3, Length[AcurEtaDetBig[[1]]], 2}];

Quiet[
  For[id = 1, id ≤ 3, id++,

    If[id == 1, ks = 0];
    If[id == 2, ks = 0.3];
    If[id == 3, ks = 1];

    NotebookDelete[pr2]; (* To see the code running *)
    pr2 = PrintTemporary["Current ks is " <> ToString[ks]];

    For[ii = 1, ii ≤ Length[AcurEtaDetBig[[1]]], ii++,
      NotebookDelete[pr]; (* To see the code running *)

      eta = AcurEtaDetBig[[id, ii, 1]];
      DA1 = AcurEtaDetBig[[id, ii, 2]] * nCpm;
      pr = PrintTemporary["Current " <> ToString[Var] <> " is " <> ToString[N[eta]] <> "; max value is " <> ToString[varmax]];

      AcurEtaDetBigTox[[id, ii, 1]] = eta;
      Ainj = {{t1, DA1, eta * DA1}}; (* Schedule of injections *)
      FullSystemSolutionMD[];
      AcurEtaDetBigTox[[id, ii, 2]] = ActBloodpw[[npw]][tEnd] / nCpm; (* The function has to be defined in script previously (S.2.1.1) *)
    ];]
];
```

```
(* Minimal curative dose depending on η, small–range variation *)
SetBasicParameterValues[];
Var = "eta"; VarSymbol = "η";
(* Setting boundaries and step of variation*)
Nx = 101; (* number of points *)
varmin = 0; varmax = 2500; varstep = (varmax − varmin) / (Nx − 1);
AcurEtaDet = Array[f, {3, Nx, 2}];

Quiet[
  For[id = 1, id ≤ 3, id++,
   ii = 1;
   If[id == 1, ks = 0];
   If[id == 2, ks = 0.3];
   If[id == 3, ks = 1];

   NotebookDelete[pr2]; (* To see the code running *)
   pr2 = PrintTemporary["Current ks is " <> ToString[ks]];

   For[eta = varmin, eta ≤ varmax, eta = N[eta + varstep],
    NotebookDelete[pr]; (* To see the code running *)
    pr = PrintTemporary["Current " <> ToString[Var] <> " is " <> ToString[N[eta]] <> "; max value is " <> ToString[varmax]];

    AcurEtaDet[[id, ii, 1]] = eta;
    AcurEtaDet[[id, ii, 2]] = FindCurDose[]; (* The function has to be defined in script previously (S.2.1.1) *)
    ii++;];]
 ];

(* Toxic decays depending on η, small–range variation *)
SetBasicParameterValues[];
Var = "eta"; VarSymbol = "η";
varmax = 2500;
```

```
AcurEtaDetTox = Array[f, {3, Length[AcurEtaDet⟦1⟧], 4}];

Quiet[
  For[id = 1, id ≤ 3, id++,

    If[id == 1, ks = 0];
    If[id == 2, ks = 0.3];
    If[id == 3, ks = 1];

    NotebookDelete[pr2]; (* To see the code running *)
    pr2 = PrintTemporary["Current ks is " <> ToString[ks]];

    For[ii = 1, ii ≤ Length[AcurEtaDet⟦1⟧], ii++,
      NotebookDelete[pr]; (* To see the code running *)

      eta = AcurEtaDet⟦id, ii, 1⟧;
      DA1 = AcurEtaDet⟦id, ii, 2⟧ * nCpm;
      pr = PrintTemporary["Current " <> ToString[Var] <> " is " <> ToString[N[eta]] <> "; max value is " <> ToString[varmax]];

      AcurEtaDetTox⟦id, ii, 1⟧ = eta;
      Ainj = {{t1, DA1, eta * DA1}}; (* Schedule of injections *)
      FullSystemSolutionMD[];
      AcurEtaDetTox⟦id, ii, 2⟧ = ActBloodpw⟦npw⟧[tEnd] / nCpm;
      AcurEtaDetTox⟦id, ii, 3⟧ = (ActBloodpw⟦npw⟧[tEnd] - ActBloodFragpw⟦npw⟧[tEnd]) / nCpm;
      AcurEtaDetTox⟦id, ii, 4⟧ = SDpw⟦npw⟧[tEnd] + CFNpw⟦npw⟧[tEnd] + CFDpw⟦npw⟧[tEnd] + UNpw⟦npw⟧[tEnd]; (*ActViable*)
    ];]
  ];
```

In[ ]:=

```
(* The resulting arrays are saved here, run the hidden cell to upload them *)
```

In[ ]:=

```
AcurEtaDetBig = {{{0, 49.82433261727928`}, {300.`, 49.85821057083015`}, {600.`, 49.904112564890006`}, {900.`, 49.9678312029157 4`},
    {1200.`, 50.067578079108905`}, {1500.`, 50.25412624043629`}, {1800.`, 50.81877342740396`}, {2100.`, 3545.219359433739`},
    {2400.`, 8098.416316670581`}, {2700.`, 11584.88112370771`}, {3000.`, 14335.58278019267`}, {3300.`, 16558.85279605263`},
    {3600.`, 18391.826318726507`}, {3900.`, 19928.398290648496`}, {4200.`, 21234.614221099626`}, {4500.`, 22358.5112928806 4`},
    {4800.`, 23335.55568609023`}, {5100.`, 24192.90031719925`}, {5400.`, 24951.025023496244`}, {5700.`, 25626.399054276317`},
    {6000.`, 26231.649582941733`}, {6300.`, 26777.224359727443`}, {6600.`, 27271.571457941733`}, {6900.`, 27721.566171287595`},
    {7200.`, 28132.870506343985`}, {7500.`, 28510.315143327065`}, {7800.`, 28857.89943609023`}, {8100.`, 29179.038563204882`},
    {8400.`, 29476.630932800745`}, {8700.`, 29753.19299224623`}, {9000.`, 30010.7693550282`}, {9300.`, 30251.472039473687`},
    {9600.`, 30476.716547227443`}, {9900.`, 30688.030721334584`}, {10200.`, 30886.62784891918`}, {10500.`, 31073.69874882519`},
    {10800.`, 31250.119683975572`}, {11100.`, 31416.78938557331`}, {11400.`, 31574.584116541355`}, {11700.`, 31723.998179041362`},
    {12000.`, 31865.97524083647`}, {12300.`, 32000.829857847755`}, {12600.`, 32129.146205357145`}, {12900.`, 32251.485990366542`},
    {13200.`, 32368.096363956767`}, {13500.`, 32479.44916000939`}, {13800.`, 32585.83646616540 5`},
    {14100.`, 32687.730116306375`}, {14400.`, 32785.242451832695`}, {14700.`, 32878.73296522555`}, {15000.`, 32968.4263392857`}},
   {{0, 63.08592774814234`}, {300.`, 63.14113301442082`}, {600.`, 63.223370430164756`}, {900.`, 63.35308964091136`},
    {1200.`, 63.603049256747816`}, {1500.`, 62.765184273397125`}, {1800.`, 56.47257381812072`}, {2100.`, 3547.2892497356665`},
    {2400.`, 8098.961172462405`}, {2700.`, 11585.184445488725`}, {3000.`, 14335.740058153197`}, {3300.`, 16558.98760573308`},
    {3600.`, 18391.93866012688`}, {3900.`, 19928.45446134868`}, {4200.`, 21234.68162593985`}, {4500.`, 22358.466356320492`},
    {4800.`, 23335.668027490603`}, {5100.`, 24192.967722039477`}, {5400.`, 24951.114896616546`}, {5700.`, 25626.33164943609`},
    {6000.`, 26231.672051221805`}, {6300.`, 26777.2692962876`}, {6600.`, 27271.54898966166`}, {6900.`, 27721.566171287595`},
    {7200.`, 28132.870506343985`}, {7500.`, 28510.315143327065`}, {7800.`, 28857.92190437031`}, {8100.`, 29179.016094924802`},
    {8400.`, 29476.630932800745`}, {8700.`, 29753.148055686084`}, {9000.`, 30010.83675986842`}, {9300.`, 30251.472039473687`},
    {9600.`, 30476.671610667298`}, {9900.`, 30688.053189614657`}, {10200.`, 30886.515507518805`}, {10500.`, 31073.69874882519`},
    {10800.`, 31250.0972156955`}, {11100.`, 31416.834322133454`}, {11400.`, 31574.494243421057`}, {11700.`, 31724.110520441736`},
    {12000.`, 31865.997709116542`}, {12300.`, 32000.85232612783`}, {12600.`, 32129.19114191729`}, {12900.`, 32251.46352208647`},
    {13200.`, 32368.096363956767`}, {13500.`, 32479.44916000939`}, {13800.`, 32585.858934445478`}, {14100.`, 32687.707648026302`},
    {14400.`, 32785.175046992474`}, {14700.`, 32878.73296522555`}, {15000.`, 32968.44880756578`}},
   {{0, 178.03338531264683`}, {300.`, 178.3883139244596`}, {600.`, 144.8420308048564`}, {900.`, 100.51773128652931`},
    {1200.`, 78.10689452895545`}, {1500.`, 65.59114097652578`}, {1800.`, 64.88729577315479`}, {2100.`, 3552.1789091870305`},
```

```
{2400.`, 8100.489015507519`}, {2700.`, 11585.970835291355`}, {3000.`, 14336.279296875`}, {3300.`, 16559.324629934206`},
{3600.`, 18392.197045347748`}, {3900.`, 19928.64544172932`}, {4200.`, 21234.8164356203`}, {4500.`, 22358.668570841164`},
{4800.`, 23335.78036889098`}, {5100.`, 24193.035126879706`}, {5400.`, 24951.18230145677`}, {5700.`, 25626.466459116546`},
{6000.`, 26231.716987781958`}, {6300.`, 26777.29176456767`}, {6600.`, 27271.593926221805`}, {6900.`, 27721.611107847748`},
{7200.`, 28132.915442904134`}, {7500.`, 28510.337611607138`}, {7800.`, 28857.94437265038`}, {8100.`, 29179.061031484955`},
{8400.`, 29476.653401080817`}, {8700.`, 29753.03571428571`}, {9000.`, 30010.814291588344`}, {9300.`, 30251.472039473687`},
{9600.`, 30476.69407894737`}, {9900.`, 30688.075657894733`}, {10200.`, 30886.695253759404`}, {10500.`, 31073.721217105263`},
{10800.`, 31250.14215225565`}, {11100.`, 31416.81185385338`}, {11400.`, 31574.606584821428`}, {11700.`, 31724.08805216166`},
{12000.`, 31865.930304276313`}, {12300.`, 32000.874794407904`}, {12600.`, 32129.168673637218`},
{12900.`, 32251.46352208647`}, {13200.`, 32368.073895676687`}, {13500.`, 32479.44916000939`}, {13800.`, 32585.858934445478`},
{14100.`, 32687.730116306375`}, {14400.`, 32785.13011043232`}, {14700.`, 32878.73296522555`}, {15000.`, 32968.44880756578`}}};
```

In[ ]:=

```
AcurEtaDetBigNokf = {{{0, 49.82836988635529`}, {105.`, 49.83885800928102`}, {210.`, 49.851277000025696`},
  {315.`, 49.86435424116321`}, {420.`, 49.87931846676016`}, {525.`, 49.89502870946898`}, {630.`, 49.91345972046816`},
  {735.`, 49.93399713272439`}, {840.`, 49.95734307999002`}, {945.`, 49.984462996174514`}, {1050.`, 50.01623454846834`},
  {1155.`, 50.05379870421905`}, {1260.`, 50.099612931559854`}, {1365.`, 50.156968482454914`},
  {1470.`, 50.2328428110682`}, {1575.`, 50.338513940796815`}, {1680.`, 50.49824936945637`}, {1785.`, 50.77054561528945`},
  {1890.`, 51.36626222079858`}, {1995.`, 1.204943154843718`*^52}, {2100.`, 9.661895008028567`*^50}},
 {{0, 63.092510252070625`}, {105.`, 63.109800295722216`}, {210.`, 63.128933440473745`}, {315.`, 63.15162113734654`},
  {420.`, 63.17742455274538`}, {525.`, 63.206563103467886`}, {630.`, 63.24017775686163`}, {735.`, 63.27993608058844`},
  {840.`, 63.32847107621961`}, {945.`, 63.38762584485506`}, {1050.`, 63.462227556042194`}, {1155.`, 63.560131331135445`},
  {1260.`, 63.6873930737488`}, {1365.`, 63.809125513062426`}, {1470.`, 63.34440073572604`},
  {1575.`, 60.737553646689975`}, {1680.`, 58.03157404849404`}, {1785.`, 56.471345084054114`},
  {1890.`, 67.19858843581122`}, {1995.`, 6.566884352312497`*^46}, {2100.`, 2.6269942494317217`*^47}},
 {{0, 178.08990707971097`}, {105.`, 178.1931207413064`}, {210.`, 178.34109542961406`}, {315.`, 178.4299153492863`},
  {420.`, 177.2810489970042`}, {525.`, 161.89282238035275`}, {630.`, 138.72302291984843`},
  {735.`, 120.72084010991836`}, {840.`, 106.9681463026463`}, {945.`, 96.22418339091136`}, {1050.`, 87.65025260753202`},
  {1155.`, 80.69337973917337`}, {1260.`, 74.98845523461362`}, {1365.`, 70.3023268047132`}, {1470.`, 66.51567559493215`},
  {1575.`, 63.65917607357626`}, {1680.`, 62.12299519015436`}, {1785.`, 63.95810951863913`},
  {1890.`, 104.15022025431013`}, {1995.`, 2.0523392573685326`*^45}, {2100.`, 5.434337811104703`*^48}}};
```

In[ ]:=

```
AcurEtaDetBigTox = {{{{0, 1.3695901568451236`}, {300.`, 1.3751648236021194`}, {600.`, 1.3826011908577944`}, {900.`, 1.393159340896395`},
```

{1200.`, 1.410047776152328`}, {1500.`, 1.4440823977484085`}, {1800.`, 1.574804553350108`}, {2100.`, 1442.4695069781733`},
{2400.`, 3319.6848366173544`}, {2700.`, 4757.063091579068`}, {3000.`, 5891.0924577118085`}, {3300.`, 6807.674238443873`},
{3600.`, 7563.349109677949`}, {3900.`, 8196.828139166866`}, {4200.`, 8735.340029331077`}, {4500.`, 9198.688785479533`},
{4800.`, 9601.495793426238`}, {5100.`, 9954.954991606559`}, {5400.`, 10267.50949239661`}, {5700.`, 10545.948769630992`},
{6000.`, 10795.4786047723`}, {6300.`, 11020.406131855252`}, {6600.`, 11224.21413387021`}, {6900.`, 11409.737037213818`},
{7200.`, 11579.309096863239`}, {7500.`, 11734.921795177834`}, {7800.`, 11878.223930361002`}, {8100.`, 12010.623456182237`},
{8400.`, 12133.315291378274`}, {8700.`, 12247.336873162432`}, {9000.`, 12353.5311838902`}, {9300.`, 12452.768822122487`},
{9600.`, 12545.633462690537`}, {9900.`, 12632.754940164383`}, {10200.`, 12714.633479333868`}, {10500.`, 12791.759980389712`},
{10800.`, 12864.49575651883`}, {11100.`, 12933.211318740996`}, {11400.`, 12998.267879515966`}, {11700.`, 13059.869330603402`},
{12000.`, 13118.404563366528`}, {12300.`, 13174.003388772417`}, {12600.`, 13226.906611388826`}, {12900.`, 13277.345760164182`},
{13200.`, 13325.422816797485`}, {13500.`, 13371.33226230331`}, {13800.`, 13415.19454713029`}, {14100.`, 13457.204120004448`},
{14400.`, 13497.407387789084`}, {14700.`, 13535.952514611146`}, {15000.`, 13572.93214414722`}},
{{0, 1.743311171196579`}, {300.`, 1.7525683168082304`}, {600.`, 1.7661918107655428`}, {900.`, 1.788999265735055`},
{1200.`, 1.8410700279106915`}, {1500.`, 2.0834618725153358`}, {1800.`, 2.830079930066934`}, {2100.`, 1443.3220321780911`},
{2400.`, 3319.9092278528465`}, {2700.`, 4757.1880050602995`}, {3000.`, 5891.157225659196`}, {3300.`, 6807.729752879494`},
{3600.`, 7563.395371115548`}, {3900.`, 8196.851269381308`}, {4200.`, 8735.36778555871`}, {4500.`, 9198.670280553488`},
{4800.`, 9601.54205382977`}, {5100.`, 9954.982747511162`}, {5400.`, 10267.546500384538`}, {5700.`, 10545.921013232997`},
{6000.`, 10795.487856472399`}, {6300.`, 11020.424635574445`}, {6600.`, 11224.204881657277`}, {6900.`, 11409.73703697102`},
{7200.`, 11579.309096644509`}, {7500.`, 11734.921795094004`}, {7800.`, 11878.233182269827`}, {8100.`, 12010.614204186837`},
{8400.`, 12133.31529118235`}, {8700.`, 12247.318369392287`}, {9000.`, 12353.55893915418`}, {9300.`, 12452.768822013453`},
{9600.`, 12545.614958813028`}, {9900.`, 12632.764191853672`}, {10200.`, 12714.58721993333`}, {10500.`, 12791.759980276907`},
{10800.`, 12864.486504562978`}, {11100.`, 12933.229822180007`}, {11400.`, 12998.230872183942`}, {11700.`, 13059.91558932181`},
{12000.`, 13118.413815171527`}, {12300.`, 13174.0126405297`}, {12600.`, 13226.925115036212`}, {12900.`, 13277.336508231423`},
{13200.`, 13325.422816437302`}, {13500.`, 13371.332262233938`}, {13800.`, 13415.203799087087`}, {14100.`, 13457.19486811929`},
{14400.`, 13497.37963256877`}, {14700.`, 13535.952514527167`}, {15000.`, 13572.941395875807`}},
{{0, 4.9839930464099105`}, {300.`, 5.08989481173881`}, {600.`, 4.547804028361946`}, {900.`, 3.407696460260839`},
{1200.`, 2.9337379766737426`}, {1500.`, 3.0467752345764967`}, {1800.`, 6.318433973857295`}, {2100.`, 1445.335935456297`},
{2400.`, 3320.538450983152`}, {2700.`, 4757.5118554535475`}, {3000.`, 5891.379288390297`}, {3300.`, 6807.868539128155`},
{3600.`, 7563.501772339206`}, {3900.`, 8196.929912766509`}, {4200.`, 8735.423297866922`}, {4500.`, 9198.753549257151`},
{4800.`, 9601.588313460312`}, {5100.`, 9955.010502900826`}, {5400.`, 10267.574255852705`}, {5700.`, 10545.9765247036`},
{6000.`, 10795.506359787405`}, {6300.`, 11020.433886921619`}, {6600.`, 11224.223384965739`}, {6900.`, 11409.755540326421`},

```
{7200.`, 11579.327600009996`}, {7500.`, 11734.93104645496`}, {7800.`, 11878.242433552827`}, {8100.`, 12010.632707548151`},
{8400.`, 12133.324542692488`}, {8700.`, 12247.272109611415`}, {9000.`, 12353.549687030214`}, {9300.`, 12452.768821812433`},
{9600.`, 12545.624210406477`}, {9900.`, 12632.773443479431`}, {10200.`, 12714.661234386129`}, {10500.`, 12791.7692318702 7`},
{10800.`, 12864.505008148604`}, {11100.`, 12933.220570218664`}, {11400.`, 12998.27713099452`}, {11700.`, 13059.906337451477`},
{12000.`, 13118.386059473249`}, {12300.`, 13174.021892076344`}, {12600.`, 13226.915863057226`}, {12900.`, 13277.336507979497`},
{13200.`, 13325.413564635583`}, {13500.`, 13371.332262083275`}, {13800.`, 13415.203798762846`}, {14100.`, 13457.204119795206`},
{14400.`, 13497.36112883217`}, {14700.`, 13535.952514761984`}, {15000.`, 13572.941395761949`}}};
```

In[∘]:=

```
AcurEtaDet = {{{0, 49.82433261727928`}, {25.`, 49.826790085412505`}, {50.`, 49.82933532026477`},
{75.`, 49.83188055511704`}, {100.`, 49.8345135566883 6`}, {125.`, 49.837190441619185`}, {150.`, 49.8400428599881 1`},
{175.`, 49.842807511637986`}, {200.`, 49.845791580085475`}, {225.`, 49.848863415252005`}, {250.`, 49.85184748369949`},
{275.`, 49.85505096894459`}, {300.`, 49.85821057083015`}, {325.`, 49.86154570615381`}, {350.`, 49.86474919139891`},
{375.`, 49.86865481039636`}, {400.`, 49.8719460623605`}, {425.`, 49.87519343096511`}, {450.`, 49.87905516660303`},
{475.`, 49.88282913552191`}, {500.`, 49.88677863787888`}, {525.`, 49.8912547405501`}, {550.`, 49.89555530978325`},
{575.`, 49.8996803455783`}, {600.`, 49.904112564890006`}, {625.`, 49.90863255092076`}, {650.`, 49.91310865359198`},
{675.`, 49.91789193977987`}, {700.`, 49.92276299268679`}, {725.`, 49.92802899582943`}, {750.`, 49.93290004873635`},
{775.`, 49.938253818598014`}, {800.`, 49.94409030541442`}, {825.`, 49.94948795863561`}, {850.`, 49.95571939568771`},
{875.`, 49.96146811578507`}, {900.`, 49.96783120291574`}, {925.`, 49.97450147356306`}, {950.`, 49.98117174421038`},
{975.`, 49.988544148610046`}, {1000.`, 49.99609208644781`}, {1025.`, 50.00337672412844`}, {1050.`, 50.01171456243759`},
{1075.`, 50.01983298394914`}, {1100.`, 50.028302472336854`}, {1125.`, 50.03729856103883`}, {1150.`, 50.046996783493164`},
{1175.`, 50.05713383954271`}, {1200.`, 50.067578079108905`}, {1225.`, 50.07815396875367`}, {1250.`, 50.090134125903134`},
{1275.`, 50.10228981649069`}, {1300.`, 50.11479657395442`}, {1325.`, 50.12844429876572`}, {1350.`, 50.14305745748649`},
{1375.`, 50.15841663331914`}, {1400.`, 50.17491677649935`}, {1425.`, 50.19260177038666`}, {1450.`, 50.21151549834058`},
{1475.`, 50.23200902723728`}, {1500.`, 50.25412624043629`}, {1525.`, 50.27826208817331`}, {1550.`, 50.30437268708882`},
{1575.`, 50.33316017093515`}, {1600.`, 50.36484395650992`}, {1625.`, 50.3999067607679`}, {1650.`, 50.43905071746138`},
{1675.`, 50.48297796034276`}, {1700.`, 50.532610039961966`}, {1725.`, 50.58926345710468`}, {1750.`, 50.65429859591607`},
{1775.`, 50.72995350773173`}, {1800.`, 50.81877342740396`}, {1825.`, 50.92479562400876`}, {1850.`, 51.054514834755345`},
{1875.`, 51.221447134376476`}, {1900.`, 51.487731359955056`}, {1925.`, 201.63016986129875`},
{1950.`, 710.289035883165`}, {1975.`, 1213.5111047271498`}, {2000.`, 1704.1263620476973`}, {2025.`, 2182.5560880962166`},
{2050.`, 2648.475194945371`}, {2075.`, 3102.4819005521617`}, {2100.`, 3545.219359433739`}, {2125.`, 3976.8659135632065`},
{2150.`, 4397.85969440202`}, {2175.`, 4808.605130991541`}, {2200.`, 5209.4673328829895`}, {2225.`, 5600.7580474624065`},
```

{2250.`, 5982.909789121241`}, {2275.`, 6355.916940789473`}, {2300.`, 6720.532189849626`}, {2325.`, 7076.598258341165`},
{2350.`, 7425.075665237313`}, {2375.`, 7765.503810796523`}, {2400.`, 8098.416316670581`}, {2425.`, 8423.99854617011`},
{2450.`, 8742.604374706298`}, {2475.`, 9054.059673108555`}, {2500.`, 9359.369896910246`}},
{{0, 63.08592774814234`}, {25.`, 63.08978948378026`}, {50.`, 63.093738986137225`}, {75.`, 63.09786402193228`},
{100.`, 63.10220847452495`}, {125.`, 63.106596810477136`}, {150.`, 63.1113362133055`}, {175.`, 63.11598784941481`},
{200.`, 63.12063948552412`}, {225.`, 63.125598305150085`}, {250.`, 63.1302499412594`}, {275.`, 63.13595477799724`},
{300.`, 63.14113301442082`}, {325.`, 63.14679396779914`}, {350.`, 63.15267433797506`}, {375.`, 63.158861891667655`},
{400.`, 63.165444395595934`}, {425.`, 63.17136864913137`}, {450.`, 63.17799503641917`}, {475.`, 63.1850163739426`},
{500.`, 63.19238877834234`}, {525.`, 63.19927846578727`}, {550.`, 63.20704582042263`}, {575.`, 63.21503259185562`},
{600.`, 63.223370430164756`}, {625.`, 63.23183991855248`}, {650.`, 63.240046106783055`}, {675.`, 63.249832095956435`},
{700.`, 63.25935478497267`}, {725.`, 63.26896524070798`}, {750.`, 63.27958501371226`}, {775.`, 63.29016090335704`},
{800.`, 63.301614460192226`}, {825.`, 63.31394568421787`}, {850.`, 63.32596972472686`}, {875.`, 63.33961744953815`},
{900.`, 63.35308964091136`}, {925.`, 63.36765891627262`}, {950.`, 63.383544692419534`}, {975.`, 63.39947435192594`},
{1000.`, 63.417203229172785`}, {1025.`, 63.43545870673386`}, {1050.`, 63.45476738492347`}, {1075.`, 63.47561198069635`},
{1100.`, 63.49786084397394`}, {1125.`, 63.521601741475244`}, {1150.`, 63.546966323278895`},
{1175.`, 63.574130122822915`}, {1200.`, 63.603049256747816`}, {1225.`, 63.633767608413116`},
{1250.`, 63.66593411094261`}, {1275.`, 63.69928546417923`}, {1300.`, 63.732768467494424`}, {1325.`, 63.764320602990615`},
{1350.`, 63.79021178510852`}, {1375.`, 63.80346455968411`}, {1400.`, 63.790782268782294`}, {1425.`, 63.727809647868455`},
{1450.`, 63.571014404296875`}, {1475.`, 63.26027633552263`}, {1500.`, 62.765184273397125`}, {1525.`, 62.13339554636103`},
{1550.`, 61.439643515680075`}, {1575.`, 60.73303366065923`}, {1600.`, 60.03809677927117`}, {1625.`, 59.36808564609155`},
{1650.`, 58.73208411654136`}, {1675.`, 58.13886886252495`}, {1700.`, 57.59914742376571`}, {1725.`, 57.12805955929864`},
{1750.`, 56.74956558342267`}, {1775.`, 56.505047504166896`}, {1800.`, 56.47257381812072`}, {1825.`, 56.81758479068153`},
{1850.`, 57.95569971988074`}, {1875.`, 61.27003433112812`}, {1900.`, 75.04949698770852`}, {1925.`, 245.77963807529076`},
{1950.`, 723.5347891212407`}, {1975.`, 1220.8350618979086`}, {2000.`, 1709.3895566553103`}, {2025.`, 2186.337780486372`},
{2050.`, 2651.4690932653903`}, {2075.`, 3104.9899223155553`}, {2100.`, 3547.2892497356665`},
{2125.`, 3978.680227179277`}, {2150.`, 4399.297664326832`}, {2175.`, 4809.781907160478`}, {2200.`, 5210.663768796993`},
{2225.`, 5601.915163886279`}, {2250.`, 5983.943330004699`}, {2275.`, 6357.001035303103`}, {2300.`, 6721.4253039826135`},
{2325.`, 7077.41273349389`}, {2350.`, 7425.845203829888`}, {2375.`, 7766.194710408837`}, {2400.`, 8098.961172462405`},
{2425.`, 8424.515316611842`}, {2450.`, 8743.14923049812`}, {2475.`, 9054.711253230733`}, {2500.`, 9359.909135632051`}},
{{0, 178.03338531264683`}, {25.`, 178.05286952427457`}, {50.`, 178.08797621189203`}, {75.`, 178.11255089322424`},
{100.`, 178.14520011270855`}, {125.`, 178.17890253282133`}, {150.`, 178.21348262012452`}, {175.`, 178.24911590805627`},

```
{200.`, 178.28703113068315`}, {225.`, 178.31950481672934`}, {250.`, 178.3514519024612`4}, {275.`, 178.37620211723157`},
{300.`, 178.3883139244596`}, {325.`, 178.37567551691728`}, {350.`, 178.31476541390097`}, {375.`, 178.15871618744129`},
{400.`, 177.80642057719987`}, {425.`, 177.0344245164914`}, {450.`, 175.38291816424606`}, {475.`, 172.23788555403405`},
{500.`, 167.51094559977827`}, {525.`, 161.8805350396866`}, {550.`, 156.0243884782146`}, {575.`, 150.29699569357962`},
{600.`, 144.8420308048564`}, {625.`, 139.70530803938559`}, {650.`, 134.8910401996813`}, {675.`, 130.3860622778871`},
{700.`, 126.1708900623752`}, {725.`, 122.22419624041794`}, {750.`, 118.52482903272589`}, {775.`, 115.05304092751409`},
{800.`, 111.79031314706442`}, {825.`, 108.71997001475859`}, {850.`, 105.82674012148291`}, {875.`, 103.0968440923476`},
{900.`, 100.51773128652931`}, {925.`, 98.0780797972715`}, {950.`, 95.76770868516509`}, {975.`, 93.5773146779914`},
{1000.`, 91.49847217072222`}, {1025.`, 89.52372099223889`}, {1050.`, 87.64595203829887`}, {1075.`, 85.85910940528815`},
{1100.`, 84.1574443731093`}, {1125.`, 82.53582258869831`}, {1150.`, 80.98959241594589`}, {1175.`, 79.5145410523378`6},
{1200.`, 78.10689452895545`}, {1225.`, 76.76322994375587`}, {1250.`, 75.48047546157262`}, {1275.`, 74.25599808083442`},
{1300.`, 73.08738421676753`}, {1325.`, 71.97265911819343`}, {1350.`, 70.91015521745037`}, {1375.`, 69.89864378047169`},
{1400.`, 68.93715937334792`}, {1425.`, 68.02530704584336`}, {1450.`, 67.16313068131755`}, {1475.`, 66.35128853016329`},
{1500.`, 65.59114097652578`}, {1525.`, 64.8850577218192`}, {1550.`, 64.23654943480526`}, {1575.`, 63.65075046854807`},
{1600.`, 63.134857694009206`}, {1625.`, 62.699359234114354`}, {1650.`, 62.35878048086526`}, {1675.`, 62.13510699738239`},
{1700.`, 62.060329752757134`}, {1725.`, 62.183905293170675`}, {1750.`, 62.586403466705086`},
{1775.`, 63.40377492115908`}, {1800.`, 64.88729577315479`}, {1825.`, 67.55549179880244`}, {1850.`, 72.65412380820827`},
{1875.`, 84.06506990131581`}, {1900.`, 120.45288831667794`}, {1925.`, 312.68333033511516`}, {1950.`, 752.424082361666`},
{1975.`, 1237.7262935781832`}, {2000.`, 1720.9593166265277`}, {2025.`, 2195.0161536654136`},
{2050.`, 2658.4721753113254`}, {2075.`, 3110.80639832002`}, {2100.`, 3552.1789091870305`}, {2125.`, 3982.752602942905`},
{2150.`, 4403.165017034776`}, {2175.`, 4813.286958852208`}, {2200.`, 5213.382430686091`}, {2225.`, 5604.572038005169`},
{2250.`, 5986.330584762688`}, {2275.`, 6359.129904840226`}, {2300.`, 6723.49238574953`}, {2325.`, 7079.311303160246`},
{2350.`, 7427.575261395677`}, {2375.`, 7767.818043644269`}, {2400.`, 8100.489015507519`}, {2425.`, 8426.003840166823`},
{2450.`, 8744.474859022557`}, {2475.`, 9055.95262570489`}, {2500.`, 9361.083103265979`}}};
```

In[ ]:=

```
AcurEtaDetTox =
  {{{0, 1.3695901568451236`, 0.04931418165121491`, 0.00010378540927814955`}, {25.`, 1.3700015638221714`, 0.049684288612400984`,
      0.00010378894283448495`}, {50.`, 1.3704223600056866`, 0.05006191107731042`, 0.00010379252717521823`},
    {75.`, 1.3708502840775891`, 0.050447197008943324`, 0.00010379620381319274`}, {100.`, 1.3712880684330317`,
      0.0508405053699751`, 0.00010379993860443602`}, {125.`, 1.3717347374255073`, 0.05124207701845251`, 0.00010380374920038966`},
    {150.`, 1.3721942841677628`, 0.05165233959168497`, 0.00010380759460281504`}, {175.`, 1.3726595475876917`,
```

0.05207132550942736`, 0.00010381157232450466`}, {200.`, 1.373139532424816`, 0.05249969540988496`, 0.00010381557471427816`},
{225.`, 1.3736308210331702`, 0.05293764148575756`, 0.0001038196534075009`}, {250.`, 1.3741287892063727`,
0.0533853460228617`, 0.00010382387467136007`}, {275.`, 1.3746424875071215`, 0.0538435198081051`, 0.00010382813150071896`},
{300.`, 1.3751648236021194`, 0.05431226895230641`, 0.00010383252318814127`}, {325.`, 1.3757024229259769`,
0.054792274447863006`, 0.00010383697723049111`}, {350.`, 1.3762469626017397`, 0.05528360804602051`, 0.00010384160353040265`},
{375.`, 1.3768226385435256`, 0.05578780068693047`, 0.00010384612051417689`}, {400.`, 1.3773924092092258`,
0.05630368593009534`, 0.0001038509863779329`}, {425.`, 1.3779730062732`, 0.05683249994925045`, 0.00010385601323256404`},
{450.`, 1.3785837352073755`, 0.05737567157311513`, 0.00010386097416664374`},
{475.`, 1.3792051609050755`, 0.057932922791141074`, 0.00010386612261123209`},
{500.`, 1.379845361282021`, 0.05850520691098954`, 0.00010387137083209892`},
{525.`, 1.3805150444795375`, 0.05909372904183075`, 0.0001038766048680569`},
{550.`, 1.3811947915093394`, 0.059698228495223014`, 0.00010388207926921583`},
{575.`, 1.381885299640337`, 0.060319455228350534`, 0.00010388779397937381`},
{600.`, 1.3826011908577944`, 0.06095895656423335`, 0.00010389359716066619`},
{625.`, 1.3833369766276224`, 0.06161727510766537`, 0.00010389957095733603`},
{650.`, 1.384089767719337`, 0.06229517071781376 4`, 0.00010390577124647802`},
{675.`, 1.3848706102402102`, 0.0629942120030026`, 0.00010391208591687488`},
{700.`, 1.38567420859728`, 0.06371514953862907`, 0.00010391860125371879`},
{725.`, 1.3865105372109`, 0.06445968820573099`, 0.00010392522504632513`},
{750.`, 1.3873579360998527`, 0.06522773629194438`, 0.00010393223809087261`},
{775.`, 1.3882430668715824`, 0.06602218898861803`, 0.00010393935433070694`},
{800.`, 1.3891673557842599`, 0.06684458051296732`, 0.00010394658716785048`},
{825.`, 1.390105529424572`, 0.06769480212367979`, 0.00010395427072021612`},
{850.`, 1.3910962203771298`, 0.06857702153390426`, 0.0001039619786382381`},
{875.`, 1.392102851224248`, 0.06949057438025409`, 0.00010397018760280782`},
{900.`, 1.3931593408963951`, 0.07043971589974976`, 0.00010397853710545688`},
{925.`, 1.3942588614345417`, 0.07142612863669628`, 0.00010398715417183986`},
{950.`, 1.39539470403616`, 0.07245162517569326`, 0.00010399616748434719`},
{975.`, 1.396590046752671`, 0.07352051168520957`, 0.00010400536010856895`},
{1000.`, 1.3978322186483723`, 0.07463463454451301`, 0.00010401494218450241`},
{1025.`, 1.3991112796607563`, 0.0757962005803053`, 0.00010402508732390041`},

```
{1050.`, 1.4004696059876103`, 0.07701211848440397`, 0.00010403538556192186`},
{1075.`, 1.401873249587111`, 0.07828326995003007`, 0.00010404630042029156`},
{1100.`, 1.4033433149760102`, 0.07961560404437255`, 0.00010405767773451934`},
{1125.`, 1.4048897588750255`, 0.08101468466542242`, 0.0001040695052765129`},
{1150.`, 1.4065231721308824`, 0.08248675962888372`, 0.00010408176472834882`},
{1175.`, 1.408241616321127`, 0.0840374961519107`, 0.00010409460458389792`},
{1200.`, 1.410047776152328`, 0.08567366911543438`, 0.00010410812328723288`},
{1225.`, 1.4119437936375974`, 0.0874027634601897`, 0.00010412244310606435`},
{1250.`, 1.413977152826574`, 0.08923849431613932`, 0.000104137218368872`},
{1275.`, 1.4161202116208709`, 0.09118726428319705`, 0.0001041529351016168`},
{1300.`, 1.4183895053441613`, 0.0932617458323706`, 0.00010416962605179578`},
{1325.`, 1.4208226231448522`, 0.09547921713059461`, 0.00010418713789872691`},
{1350.`, 1.4234294790693076`, 0.09785556564767421`, 0.00010420564551903851`},
{1375.`, 1.426220964684781`, 0.1004090351482493`, 0.00010422535623918745`},
{1400.`, 1.4292304911612872`, 0.10316406289347842`, 0.00010424629539876238`},
{1425.`, 1.4324843007993222`, 0.1061477146225212`, 0.00010426862471254978`},
{1450.`, 1.43601332603268`, 0.10939213390141034`, 0.00010429253473102335`},
{1475.`, 1.4398645403781272`, 0.11293773568734893`, 0.00010431814984992392`},
{1500.`, 1.4440823977484085`, 0.11683130224634011`, 0.00010434573076151021`},
{1525.`, 1.4487326800826337`, 0.12113231211487749`, 0.00010437547591472537`},
{1550.`, 1.4538784058897094`, 0.1259102463047641`, 0.00010440777110543815`},
{1575.`, 1.4596243073597275`, 0.13125764968712386`, 0.00010444284646737314`},
{1600.`, 1.466079215800579`, 0.13728578608870798`, 0.00010448115713687555`},
{1625.`, 1.4733886114408399`, 0.1441377634629093`, 0.00010452317539374491`},
{1650.`, 1.4817430071612383`, 0.1519988633421973`, 0.00010456942620326557`},
{1675.`, 1.491382627570652`, 0.1611080203059486`, 0.00010462057059530764`},
{1700.`, 1.5026240436527105`, 0.1717799910143117`, 0.00010467736471529425`},
{1725.`, 1.5158950902346893`, 0.18443764713122018`, 0.00010474063754268895`},
{1750.`, 1.5317593729802341`, 0.1996483628265039`, 0.00010481139608855624`},
{1775.`, 1.5510198807587419`, 0.21821047412146594`, 0.0001048906351217594`},
{1800.`, 1.574804553350108`, 0.2412610564773355`, 0.00010497947080450465`},
```

```
{1825.`, 1.6048583223246693`, 0.27055350909493625`, 0.0001050788853482085`},
{1850.`, 1.64417979325796`, 0.30911034360519407`, 0.00010518960159948475`},
{1875.`, 1.699320168623823 7`, 0.3635511920078508`, 0.00010531156144469343`},
{1900.`, 1.7994288149338662`, 0.4632800118889029`, 0.00010543985944031761`},
{1925.`, 63.875398989664205`, 62.55769799709286`, 0.00010444388640836152`},
{1950.`, 273.61939757636827`, 272.3216108735953`, 0.00010413066666461064`},
{1975.`, 481.1044365379804`, 479.8251785136055`, 0.00010390855588539332`},
{2000.`, 683.3885472550896`, 682.1271711683806`, 0.00010370599547462786`},
{2025.`, 880.6465312074582`, 879.4024800131241`, 0.00010351602448863068`},
{2050.`, 1072.7450672829566`, 1071.5178198248386`, 0.00010333697009716898`},
{2075.`, 1259.9309746367512`, 1258.7200410207975`, 0.00010316745224325155`},
{2100.`, 1442.4695069781733`, 1441.2744239959252`, 0.00010300643916234719`},
{2125.`, 1620.4344284398708`, 1619.2547538454232`, 0.00010285339002490182`},
{2150.`, 1794.006477106036`, 1792.8417894908985`, 0.00010270767604762377`},
{2175.`, 1963.3524857717923`, 1962.2023828523027`, 0.0001025687546459923`},
{2200.`, 2128.6230724969355`, 2127.4871696278437`, 0.00010243615624470038`},
{2225.`, 2289.946859920701`, 2288.8247887161465`, 0.00010230948155995909`},
{2250.`, 2447.502194497136`, 2446.3936025206217`, 0.00010218828250603882`},
{2275.`, 2601.286960063969`, 2600.191507650622`, 0.00010207241170038329`},
{2300.`, 2751.6113419060125`, 2750.5287053259194`, 0.00010196127163645955`},
{2325.`, 2898.410738296774`, 2897.340604373397`, 0.00010185483733640238`},
{2350.`, 3042.080905129308`, 3041.0229760732764`, 0.00010175243171152415`},
{2375.`, 3182.432298366147`, 3181.3862842969475`, 0.00010165424054709137`},
{2400.`, 3319.6848366173544`, 3318.6504597940307`, 0.00010155986614518157`},
{2425.`, 3453.9149999356905`, 3452.891992578189`, 0.00010146912120887234`},
{2450.`, 3585.2686610833216`, 3584.2567656219003`, 0.00010138175178651977`},
{2475.`, 3713.6742318238553`, 3712.673198203416`, 0.00010129778258001864`},
{2500.`, 3839.5459173117483`, 3838.555507502746`, 0.00010121661313868105`}},
{{0, 1.743311171196579`, 0.062446624728743766`, 0.00010059490669390659`}, {25.`, 1.743970632826435`, 0.06304168439389604`,
  0.00010059918265225572`}, {50.`, 1.7446467335679663`, 0.06365204311063219`, 0.00010060355924496785`},
 {75.`, 1.7453425655170378`, 0.0642784461201437`, 0.0001006080325625334`}, {100.`, 1.746060034284152`, 0.06492165349302095`,
```

0.00010061259222663517`}, {125.`, 1.7467948740909556`, 0.06558223207834715`, 0.00010061728096408834`},
{150.`, 1.747556540609693`, 0.06626133014361107`, 0.00010062204075864346`},
{175.`, 1.7483333939012868`, 0.06695931304581083`, 0.0001006269679652798`},
{200.`, 1.7491287704614567`, 0.067677191829573`, 0.00010063205450772244`}, {225.`, 1.749952301689055`, 0.06841630213542481`,
0.00010063724110276906`}, {250.`, 1.7507875009056009`, 0.06917695274659263`, 0.00010064266054269418`},
{275.`, 1.7516741115571572`, 0.06996194130939776`, 0.00010064804681961472`}, {300.`, 1.7525683168082304`,
0.07077052847269574`, 0.0001006537289430339`}, {325.`, 1.753500054798317`, 0.07160527490356802`, 0.00010065950910540025`},
{350.`, 1.7544631430716844`, 0.07246726091935982`, 0.00010066545299922862`}, {375.`, 1.7554615074290383`,
0.07335813005421152`, 0.00010067155220484858`}, {400.`, 1.7564991889156656`, 0.07427964957856548`, 0.00010067780062448 92`},
{425.`, 1.7575476832095145`, 0.07523208671564414`, 0.00010068442398459976`}, {450.`, 1.758647717174465`,
0.07621929567639035`, 0.00010069115934947833`}, {475.`, 1.7597924793077135`, 0.07724296864395583`, 0.00010069808359672842`},
{500.`, 1.7609828303859814`, 0.07830531782790567`, 0.00010070522054319013`}, {525.`, 1.7621970835832592`,
0.0794074993890539`, 0.0001007127558564130 8`}, {550.`, 1.7634769356742765`, 0.08055441000236607`, 0.0001007204313617224`},
{575.`, 1.7648062239147517`, 0.0817479928267485`, 0.00010072840112533343`}, {600.`, 1.7661918107655428`,
0.08299175960476542`, 0.00010073666065258053`}, {625.`, 1.7676307112882095`, 0.08428894046188207`, 0.00010074527361754694`},
{650.`, 1.7691151351869558`, 0.08564275146325256`, 0.00010075434355386352`}, {675.`, 1.7707028337386688`,
0.08706117940278812`, 0.00010076353637336088`}, {700.`, 1.7723447998512336`, 0.08854563577260245`, 0.00010077324381472597`},
{725.`, 1.774056316518934`, 0.09010224519807847`, 0.00010078342900685458`}, {750.`, 1.7758701674172301`,
0.09173920218188804`, 0.00010079394986173775`}, {775.`, 1.7777619171668189`, 0.09346115066961108`, 0.00010080505292295563`},
{800.`, 1.7797660035178737`, 0.09527806392902044`, 0.00010081660216668407`}, {825.`, 1.7818908428983424`,
0.09719899057686182`, 0.00010082864751163616`}, {850.`, 1.7841107219353816`, 0.09923106769444287`, 0.00010084147423872269`},
{875.`, 1.7864939299782467`, 0.10139103734260274`, 0.00010085477129533624`}, {900.`, 1.788999265735055`,
0.1036874224125041`, 0.00010086895464886747`}, {925.`, 1.7916792660508447`, 0.10613935531042497`, 0.00010088385926462326`},
{950.`, 1.7945576153587255`, 0.10876586722737326`, 0.00010089953174800449`}, {975.`, 1.7976154487259088`,
0.11158404725350812`, 0.00010091631384871723`}, {1000.`, 1.800929678812698`, 0.11462507487499721`, 0.00010093398927846196`},
{1025.`, 1.8044891806240826`, 0.11791437282625446`, 0.00010095292743946273`}, {1050.`, 1.8083434413206454`,
0.12148972119291691`, 0.00010097317805737442`}, {1075.`, 1.8125480927127473`, 0.1253968071561425`, 0.0001009948255699266`},
{1100.`, 1.8171486963404986`, 0.12968854277230504`, 0.00010101809371587409`}, {1125.`, 1.8222093761614362`,
0.13443137793594095`, 0.00010104319747141657`}, {1150.`, 1.827811094243893`, 0.13970860694811665`, 0.00010107038150832312`},
{1175.`, 1.8340568574235472`, 0.1456259748112241`, 0.00010109992785184717`}, {1200.`, 1.8410700279106915`,
0.1523176010572813`, 0.0001011322103118607`}, {1225.`, 1.849014589173527`, 0.15995875980856195`, 0.00010116764545839542`},

```
{1250.`, 1.858092825488994`, 0.16877756023234344`, 0.00010120678585273756`}, {1275.`, 1.8685831068312393`,
  0.17908138541532817`, 0.00010125024350451 96`}, {1300.`, 1.8808382292010475`, 0.19128081529710184`, 0.00010129883171501327`},
{1325.`, 1.895317659952096`, 0.20593056715721522`, 0.00010135358416448801`}, {1350.`, 1.9126174444537605`,
  0.22378059800877337`, 0.00010141581061917854`}, {1375.`, 1.9334703554885098`, 0.2458218681436289`, 0.00010148724322403523`},
{1400.`, 1.9586499173522454`, 0.2732720125195707`, 0.00010157028507462584`}, {1425.`, 1.9886400011701206`,
  0.30735773401601335`, 0.00010166829270067 23`}, {1450.`, 2.022555225146882`, 0.3483930722359539`, 0.00010178600330489567`},
{1475.`, 2.0563162212929065`, 0.3937504179328254`, 0.00010192854701264459`}, {1500.`, 2.0834618725153358`,
  0.43738806574236294`, 0.00010209662343337841`}, {1525.`, 2.101698094701096`, 0.47545999706731606`, 0.00010228321594934757`},
{1550.`, 2.114378653585607`, 0.5093832212223183`, 0.00010247980999087252`}, {1575.`, 2.125867242290668`,
  0.5424023001734237`, 0.00010268113814588967`}, {1600.`, 2.1393586586191446`, 0.5772249960009093`, 0.0001028846154665136`},
{1625.`, 2.1574071857096055`, 0.6162012419041204`, 0.00010308883624934086`}, {1650.`, 2.1825789158353945`,
  0.6618148619505975`, 0.00010329282120097991`}, {1675.`, 2.2181022701767383`, 0.7172678072138081`, 0.00010349563841523311`},
{1700.`, 2.268412251272027`, 0.7869977809199118`, 0.00010369624187972859`}, {1725.`, 2.3400069305341247`,
  0.8775224650493754`, 0.00010389333792049126`}, {1750.`, 2.443179729290901`, 0.9991665251759644`, 0.00010408517527062615`},
{1775.`, 2.5955550133206975`, 1.1695967229992397`, 0.00010426921287683491`}, {1800.`, 2.830079930066934`,
  1.4218177544406834`, 0.00010444152243269672`}, {1825.`, 3.216063259165128`, 1.8252225093405263`, 0.00010459552639850824`},
{1850.`, 3.926653544684304`, 2.5530998714846103`, 0.00010471895652865924`}, {1875.`, 5.535515822115184`,
  4.179399127168578`, 0.00010478506953851117`}, {1900.`, 11.467198864098282`, 10.129437128396074`, 0.00010471882546220747`},
{1925.`, 82.06448218261646`, 80.74738607769343`, 0.000104381395467418`}, {1950.`, 279.07537219020344`,
  277.7776890997097`, 0.00010411903541068126`}, {1975.`, 484.1211278925093`, 482.8419226064304`, 0.0001039022527059434`},
{2000.`, 685.5563943324291`, 684.2950544375097`, 0.00010370147377324273`}, {2025.`, 882.2041441410988`,
  880.9601177737773`, 0.00010351274899387474`}, {2050.`, 1073.978187865675`, 1072.7509592969998`, 0.0001033343612795 1456`},
{2075.`, 1260.9639632835942`, 1259.7530449309854`, 0.00010316526037673059`}, {2100.`, 1443.3220321780911`,
  1442.1269613215154`, 0.00010300460909159356`}, {2125.`, 1621.1816830118805`, 1620.0020186969434`, 0.00010285177934532602`},
{2150.`, 1794.598722132513`, 1793.4340423221654`, 0.00010270635653758495`}, {2175.`, 1963.8371503121539`,
  1962.6870535229027`, 0.0001025676382531608`}, {2200.`, 2129.1158319770916`, 2127.9799352228597`, 0.00010243505031128723`},
{2225.`, 2290.423422835052`, 2289.3013574002284`, 0.00010230842187703771`}, {2250.`, 2447.9278593812246`,
  2446.8192723965176`, 0.00010218732086733162`}, {2275.`, 2601.7334439115507`, 2600.637996641744`, 0.00010207143461466746`},
{2300.`, 2751.9791676420987`, 2750.8965351416637`, 0.0001019604269454354`}, {2325.`, 2898.7461748837377`,
  2897.67604457404`, 0.0001018540553604335`}, {2350.`, 3042.397833263208`, 3041.3399075343395`, 0.00010175169069498053`},
{2375.`, 3182.7168380496328`, 3181.670826878942`, 0.00010165355510931257`}, {2400.`, 3319.9092278528465`,
```

3318.874853217851`, 0.00010155927190388727`}, {2425.`, 3454.1278236936`, 3453.104818362434`, 0.00010146855239052076`},
{2450.`, 3585.4930508927996`, 3584.481157540288`, 0.00010138117496880145`},
{2475.`, 3713.9425742292847`, 3712.941543121675`, 0.0001012971546738009`},
{2500.`, 3839.767992441405`, 3838.7775846410686`, 0.00010121605174405154`}},
{{0, 4.9839930464099105`, 0.17638826862706058`, 0.00009358596047927139`},
{25.`, 4.989054262376066`, 0.1812431931736179`, 0.00009359618144792905`},
{50.`, 4.994904115848176`, 0.1864867612028478`, 0.00009360677338587879`},
{75.`, 5.000850730318898`, 0.19214174181068155`, 0.00009361834530340165`},
{100.`, 5.007477914168216`, 0.19829008001697784`, 0.00009363059409585457`},
{125.`, 5.014656432487222`, 0.20499842616921773`, 0.00009364375773057217`},
{150.`, 5.022466877217886`, 0.2123582534075303`, 0.00009365797131537049`},
{175.`, 5.031021230367809`, 0.22048440811809938`, 0.00009367336641923818`},
{200.`, 5.040490800040891`, 0.22952452573642507`, 0.00009369023950604199`},
{225.`, 5.050827694233763`, 0.2396529007679665`, 0.00009370859207185097`},
{250.`, 5.062404119637851`, 0.25112470817666704`, 0.00009372880473698238`},
{275.`, 5.0753366300228056`, 0.26425962782819995`, 0.00009375130893198068`},
{300.`, 5.08989481173881`, 0.27950536758877026`, 0.00009377653806741188`},
{325.`, 5.106332462552623`, 0.29748751115773286`, 0.00009380514859879593`},
{350.`, 5.124847691687086`, 0.3191051716869836`, 0.00009383823845111182`},
{375.`, 5.145391859738228`, 0.34567489204527657`, 0.00009387689326029027`},
{400.`, 5.166974241057835`, 0.3790880033478473`, 0.0000939238753795166`},
{425.`, 5.185708874398694`, 0.42170745698353684`, 0.00009398348032969186`},
{450.`, 5.190140415769147`, 0.4746908519375967`, 0.00009406313073673942`},
{475.`, 5.1588173628726555`, 0.5329930338444491`, 0.000094171941360113`},
{500.`, 5.078148941344394`, 0.5846402820709853`, 0.00009431129277205017`},
{525.`, 4.960156111270867`, 0.6226982576899597`, 0.0000944716779624643`},
{550.`, 4.8247090649707545`, 0.6486586510359093`, 0.00009464380060082242`},
{575.`, 4.684994408427543`, 0.6662930291268909`, 0.00009482222272863822`},
{600.`, 4.547804028361946`, 0.6786797993125693`, 0.0000950042995743541`},
{625.`, 4.416294761487661`, 0.6878617316370393`, 0.00009518873365792469`},
{650.`, 4.291798130863759`, 0.6951414962925505`, 0.00009537484468426562`},

{675.`, 4.1747038345310425`, 0.7013205603658872`, 0.00009556227467568029`},
{700.`, 4.064964127313507`, 0.7069164578330972`, 0.00009575080109180461`},
{725.`, 3.9622970107529842`, 0.7122496975429183`, 0.00009594029702634575`},
{750.`, 3.8662898754090778`, 0.7174958137189305`, 0.00009613067695194149`},
{775.`, 3.7765377861347416`, 0.7227975749582919`, 0.00009632187976047858`},
{800.`, 3.6925801338876627`, 0.7282022713995744`, 0.00009651386218042623`},
{825.`, 3.6140468199291855`, 0.7338003198319172`, 0.00009670657142941678`},
{850.`, 3.5405814679118652`, 0.7396558459839242`, 0.00009689996831851333`},
{875.`, 3.4718900376809025`, 0.7458561791297854`, 0.00009709401878653605`},
{900.`, 3.407696460260839`, 0.7524720292537277`, 0.0000972886972785186`},
{925.`, 3.3477717776620515`, 0.7595886899505604`, 0.00009748397953077355`},
{950.`, 3.2919408421110314`, 0.767315773149853`, 0.00009767983892985329`},
{975.`, 3.2400266012134606`, 0.7757352331578494`, 0.0000978762531879555`},
{1000.`, 3.1918770949133326`, 0.7849302754419637`, 0.00009807319905660324`},
{1025.`, 3.1474272838399546`, 0.7950490016687412`, 0.00009827064100871844`},
{1050.`, 3.1065057036757118`, 0.806115303713538`, 0.00009846856261564584`},
{1075.`, 3.0691115330044365`, 0.8183050589148606`, 0.00009866691956918347`},
{1100.`, 3.035166104183218`, 0.8317018184476536`, 0.00009886568139160804`},
{1125.`, 3.004644427923399`, 0.8464286799662049`, 0.00009906480876230294`},
{1150.`, 2.9775450195566457`, 0.8626198197406902`, 0.00009926425844348541`},
{1175.`, 2.9538895544781525`, 0.8804214046334838`, 0.00009946398328121148`},
{1200.`, 2.9337379766737426`, 0.9000077704506242`, 0.00009966392918059869`},
{1225.`, 2.917167509193767`, 0.9215615081728057`, 0.00009986403942959673`},
{1250.`, 2.9042843369595976`, 0.9452859741177829`, 0.00010006425280622434`},
{1275.`, 2.89527008734459`, 0.9714524998781999`, 0.00010026449391734542`},
{1300.`, 2.8903032938188717`, 1.0003227001452517`, 0.00010046469078075013`},
{1325.`, 2.8896568347451326`, 1.0322464062276868`, 0.00010066475459304838`},
{1350.`, 2.893652716733142`, 1.067617091188153`, 0.00010086459085140483`},
{1375.`, 2.902723338153445`, 1.1069336287084552`, 0.00010106408736414702`},
{1400.`, 2.917345432355068`, 1.1507346842496164`, 0.00010126313194175465`},
{1425.`, 2.938172949740312`, 1.1997320395253233`, 0.00010146158548875586`},

```
{1450.`, 2.9659878122623673`, 1.2547617062416068`, 0.00010165929822247479`},
{1475.`, 3.0017774382361435`, 1.3168618164050694`, 0.00010185609859475594`},
{1500.`, 3.0467752345764967`, 1.387313424676512`, 0.00010205179376077358`},
{1525.`, 3.102592555516409`, 1.4677728472868563`, 0.0001022461529707979`},
{1550.`, 3.1712760832820157`, 1.5603292700884994`, 0.00010243891453420402`},
{1575.`, 3.2555115943853905`, 1.6677088621609661`, 0.0001026297688961412`},
{1600.`, 3.358807615482002`, 1.793458640476408`, 0.00010281835989752594`},
{1625.`, 3.4860095159349695`, 1.9424611208487115`, 0.00010300423978860146`},
{1650.`, 3.643605763243531`, 2.121240517144014`, 0.0001031868999629645`},
{1675.`, 3.8411554254800295`, 2.3393914715667568`, 0.00010336564776232584`},
{1700.`, 4.092332387665085`, 2.610623014637425`, 0.00010353965898268366`},
{1725.`, 4.418016277991523`, 2.955850781595398`, 0.000103707850425625`},
{1750.`, 4.851936294163701`, 3.4088424659473175`, 0.00010386872442007922`},
{1775.`, 5.4506787973749695`, 4.026226402123051`, 0.00010402028304405405`},
{1800.`, 6.318433973857295`, 4.912242451067325`, 0.00010415960123600489`},
{1825.`, 7.669659202034765`, 6.281412336542003`, 0.0001042821258730239`},
{1850.`, 10.018683733801353`, 8.64815702426798`, 0.00010438039085722617`},
{1875.`, 14.96629842547616`, 13.613422340228894`, 0.00010444035632077887`},
{1900.`, 30.202842504511302`, 28.867849027373307`, 0.0001044327386975244`},
{1925.`, 109.62575951444923`, 108.30942029806069`, 0.00010430080656477562`},
{1950.`, 290.9749561473082`, 289.677497645981`, 0.0001040932607659 6382`},
{1975.`, 491.0785168081855`, 489.7994330436454`, 0.00010388748562305714`},
{2000.`, 690.3218328930735`, 689.0605721611038`, 0.00010369134811604236`},
{2025.`, 885.7786125366043`, 884.5346430549424`, 0.00010350511803513931`},
{2050.`, 1076.8626026631512`, 1075.6354182814887`, 0.00010332818638798904`},
{2075.`, 1263.3596173297199`, 1262.148734351065`, 0.00010316011230548652`},
{2100.`, 1445.335935456297`, 1444.1408932704671`, 0.00010300024974066952`},
{2125.`, 1622.8589556300637`, 1621.6793143061016`, 0.00010284809457750146`},
{2150.`, 1796.1915424250435`, 1795.026883874108`, 0.00010270289876961303`},
{2175.`, 1965.28074154141`, 1964.1306634348257`, 0.00010256449879724583`},
{2200.`, 2130.2355288553267`, 2129.0996459586418`, 0.00010243249179205002`},
```

```
{2225.`, 2291.5176663825796`, 2290.3956141740678`, 0.00010230595615878888`},
{2250.`, 2448.9110525196165`, 2447.8024770538987`, 0.00010218507171493215`},
{2275.`, 2602.610214902284`, 2601.5147775830483`, 0.00010206939175957378`},
{2300.`, 2752.8304873906036`, 2751.7478643258687`, 0.00010195845698283031`},
{2325.`, 2899.528089104312`, 2898.4579672174646`, 0.00010185221897306067`},
{2350.`, 3043.1103427673147`, 3042.0524244939324`, 0.00010174998319554466`},
{2375.`, 3183.3853907507437`, 3182.3393863962533`, 0.00010165193700400595`},
{2400.`, 3320.538450983152`, 3319.504082602659`, 0.00010155773178152405`},
{2425.`, 3454.7408511373997`, 3453.7178517660545`, 0.0001014670596877186`},
{2450.`, 3586.038989407297`, 3585.02710120624`, 0.00010137979016665753`},
{2475.`, 3714.45381105615`, 3713.452784653574`, 0.00010129583447583291`},
{2500.`, 3840.251467841934`, 3839.2610643860144`, 0.00010121478683244814`}}};

(* minimal single curative doses for pure drug *)
Acur01 = AcurEtaDet[[1, 1, 2]] * nCpm; (*ks=0*)
Acur02 = AcurEtaDet[[2, 1, 2]] * nCpm; (*ks=0.3*)
Acur03 = AcurEtaDet[[3, 1, 2]] * nCpm; (*ks=1*)
```

(* Figure 3A *)

```
(* For it, we need to find the values of η,
for which the total amount of injected antibodies (which by itself depends on η) equals
  the total amount of specific receptors, expressed on cancer cells during treatment (which also depends on η).
      Since both measures depend on η in non-trivial way, we found the sought values of η numerically via the code hidden here *)

SetBasicParameterValues[];
Nnew = Array[f, 3];

id = 3; (* set 1 or 2 or 3 for ks=0, 0.3, 1 correspondingly *)
```

```
step = 0.01; nnmax = 1.2;
iimax = Round[1 + (nnmax - 1) / step];
Arr01 = Array[f, {iimax, 2}];
Arr02 = Array[f, {iimax, 2}];
Arr03 = Array[f, {iimax, 2}];

If[id == 1, ks = 0; Acur = Acur01; Arr0 = Arr01];
If[id == 2, ks = 0.3; Acur = Acur02; Arr0 = Arr02];
If[id == 3, ks = 1; Acur = Acur03; Arr0 = Arr03];

etain = (gamma * N0 / Acur - 1); (* initial estimation, based on the amount of receptors at the moment of drug injection *)

For[ii = 1, ii ≤ iimax, ii++,
  eta = etain * (1 + (ii - 1) * step);
  DA1 = FindCurDose[] * nCpm; FullSystemSolutionMD[];
  TableNN = If[Ainj[[1, 1]] == 0, Table[NMinimize[{Nnor * (NNpw[[nn]][t]), t > tB[[nn]], t < tE[[nn]]}, t], {nn, 1, npw}],
     Table[NMinimize[{Nnor * (NNpw[[nn]][t]), t > tB[[nn]], t < tE[[nn]]}, t][[1]], {nn, 2, npw}]];
  Nn = Min[TableNN[[All, 1]]];
  tminNn = t /. TableNN[[Position[TableNN[[All, 1]], Nn][[1, 1]]]][[2]];
  Nnew[[id]] = NewCellspw[[npw]][tminNn];
  eta2 = (gamma * (N0 + Nnew[[id]]) / Acur - 1);
  (* is this equals to the value of η that we've set, then we found the desired η *)
  Arr0[[ii]] = {eta, eta2};
 ];

(* Plot the arrays with this code and find the sought value of η *)
Show[ListPlot[Arr0], Plot[x, {x, Arr0[[1, 1]], Arr0[[Length[Arr0], 1]]}]]
etacr = Quiet[x /. NMinimize[{Abs[Interpolation[Arr0][x] - x], etain < x < etain * (1 + (iimax - 1) * step)}, x][[2]]]
```

Out[ ]=

$Aborted

(* Resulting plots are hidden here *)

(* That's the result for ks=0 *)

Out[ ]=

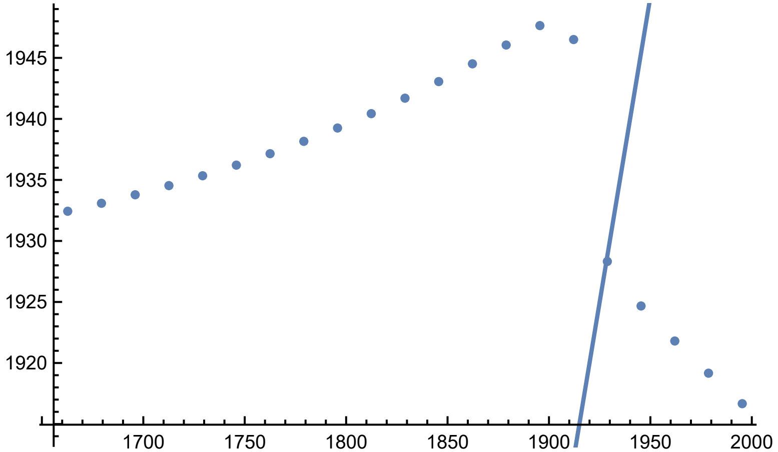

(* That's the result for ks=0.3 *)

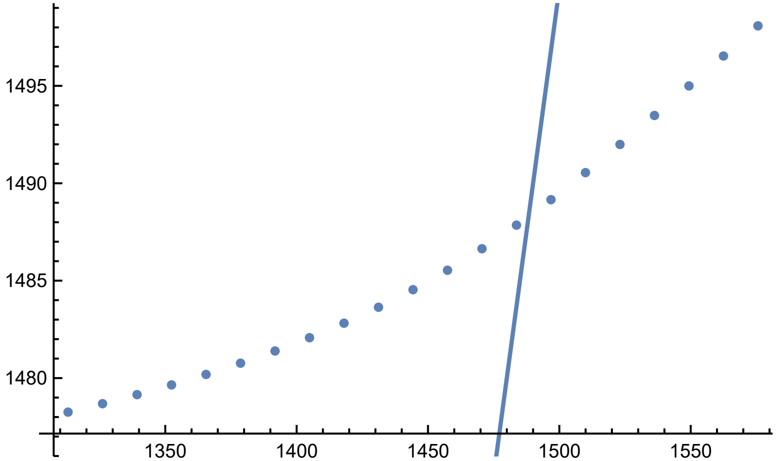

(* That's the result for ks=1 *)

Out[ ]=

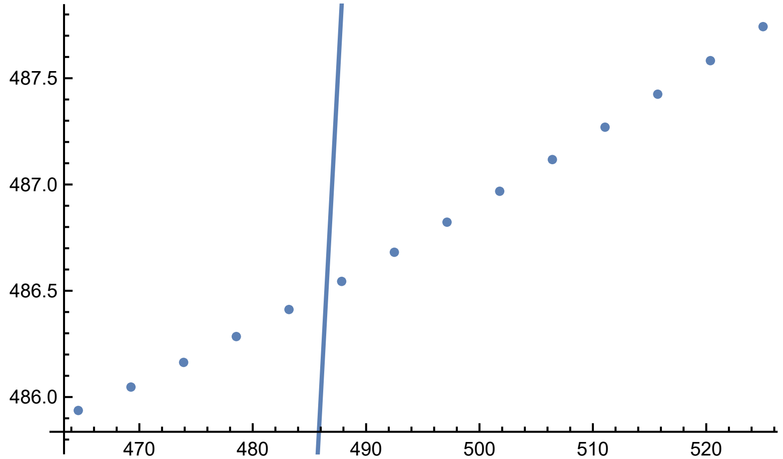

In[ ]:=

```
(* Here are the found values of η *)
etacr01 = 1928.5470657073904`; (* k_s=0 *)
etacr02 = 1488.29932122596`; (* k_s=0.3 *)
etacr03 = 486.50547128740044`; (* k_s=1 *)

Thk = 0.0075; (* Thickness of graphs *)

Show[ListPlot[{AcurEtaDet[[1]], AcurEtaDet[[2]], AcurEtaDet[[3]]}, Joined → True, PlotStyle →
    {Directive[Darker[Red], Thickness[Thk]], Directive[Darker[Green], Thickness[Thk]], Directive[Darker[Blue], Thickness[Thk]]},
   Frame → True, FrameLabel → {"Coefficient of drug impurity, η", "Minimal single curative dose, A_cur (nCi)"},
   PlotRange → {{0 - 10, 2200 + 10}, {0, 250}}]
 , Plot[{10 000 * (x - etacr01), 10 000 * (x - (gamma * N0/Acur01 - 1))},
   {x, 1600, 2200}, PlotStyle → {Directive[Darker[Red], Dashed], Directive[Darker[Red], Dotted]}],
 Plot[{10 000 * (x - etacr02), 10 000 * (x - (gamma * N0/Acur02 - 1))}, {x, 1300, 1500},
   PlotStyle → {Directive[Darker[Green], Dashed], Directive[Darker[Green], Dotted]}],
 Plot[{10 000 * (x - etacr03), 10 000 * (x - (gamma * N0/Acur03 - 1))}, {x, 400, 525},
   PlotStyle → {Directive[Darker[Blue], Dashed], Directive[Darker[Blue], Dotted]}],
 Plot[{10 000 * (x - 1780)}, {x, 1750, 1830}, PlotStyle → {Directive[Gray, Thin]}],
 Plot[{AcurEtaDet[[1, 1, 2]], AcurEtaDet[[2, 1, 2]], AcurEtaDet[[3, 1, 2]]}, {x, 0, 2500}, PlotStyle → {Directive[Lighter[Red], Thickness[Thk / 10]],
     Directive[Lighter[Green], Thickness[Thk / 10]], Directive[Lighter[Blue], Thickness[Thk / 10]]}]]
```

Out[ ]=

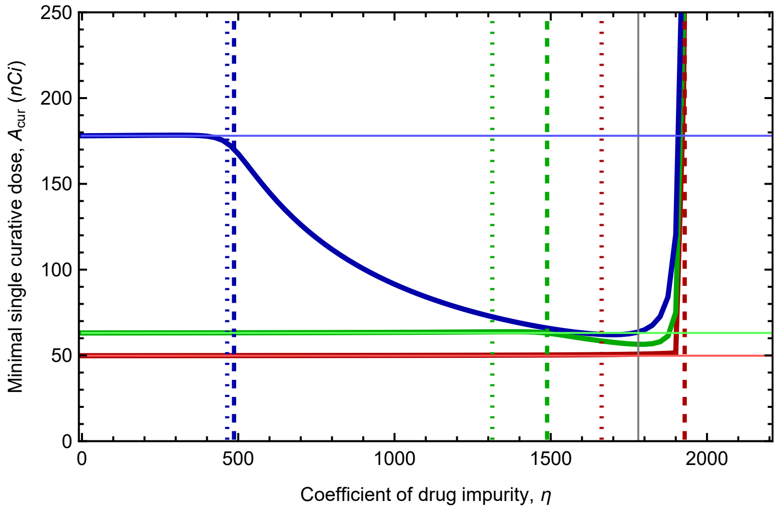

In[ ]:=

```
(* Supplementary Figure S.15:
  "under any value of η the cross-fire-only case demands lower minimal single curative dose
than the cases with ks > 0 and (...) remains less toxic and delivers greater fraction of injected dose to viable cells" *)

Thk = 0.0075; (* Thickness of graphs *)

FrAc1 = Thread[{AcurEtaDet[[1, All, 1]], AcurEtaDetTox[[1, All, 4]] / nCpm / AcurEtaDet[[1, All, 2]]}];
FrAc2 = Thread[{AcurEtaDet[[2, All, 1]], AcurEtaDetTox[[2, All, 4]] / nCpm / AcurEtaDet[[2, All, 2]]}];
FrAc3 = Thread[{AcurEtaDet[[3, All, 1]], AcurEtaDetTox[[3, All, 4]] / nCpm / AcurEtaDet[[3, All, 2]]}];

(* Toxicity *)
GraphicsRow[{Show[ListPlot[{AcurEtaDetTox[[1, All, {1, 2}]], AcurEtaDetTox[[2, All, {1, 2}]], AcurEtaDetTox[[3, All, {1, 2}]],
      AcurEtaDetTox[[1, All, {1, 3}]], AcurEtaDetTox[[2, All, {1, 3}]], AcurEtaDetTox[[3, All, {1, 3}]]}, Joined → True,
    PlotStyle → {Directive[Darker[Red], Thickness[Thk / 2]], Directive[Darker[Green], Thickness[Thk / 2]],
      Directive[Darker[Blue], Thickness[Thk / 2]], Directive[Lighter[Red], Thickness[Thk / 4]],
      Directive[Lighter[Green], Thickness[Thk / 4]], Directive[Lighter[Blue], Thickness[Thk / 4]]}, Frame → True,
    FrameLabel → {"Coefficient of drug impurity, η", "Toxic decays upon dose Acur (nCi · day)"}, PlotRange → {{0 - 10, 2200 + 10}, {0, 8}}],
   Plot[Abld / nCpm, {x, 0 - 10, 2500 + 10}]
```

```
, Plot[{10000 * (x - etacr01), 10000 * (x - (gamma * N0 / Acur01 - 1))},
  {x, 1600, 2200}, PlotStyle → {Directive[Darker[Red], Dashed], Directive[Darker[Red], Dotted]}],
 Plot[{10000 * (x - etacr02), 10000 * (x - (gamma * N0 / Acur02 - 1))}, {x, 1300, 1500},
  PlotStyle → {Directive[Darker[Green], Dashed], Directive[Darker[Green], Dotted]}],
 Plot[{10000 * (x - etacr03), 10000 * (x - (gamma * N0 / Acur03 - 1))}, {x, 400, 525},
  PlotStyle → {Directive[Darker[Blue], Dashed], Directive[Darker[Blue], Dotted]}],
 Plot[{10000 * (x - 1780)}, {x, 1750, 1830}, PlotStyle → {Directive[Gray, Thin]}],
 Plot[{AcurEtaDetTox[[1, 1, 2]], AcurEtaDetTox[[2, 1, 2]], AcurEtaDetTox[[3, 1, 2]]},
  {x, 0, 2500}, PlotStyle → {Directive[Lighter[Red], Thickness[Thk / 10]],
    Directive[Lighter[Green], Thickness[Thk / 10]], Directive[Lighter[Blue], Thickness[Thk / 10]]}]],

(* Fraction of activity deposited to viable cells *)
Show[ListPlot[{FrAc1, FrAc2, FrAc3}, Joined → True, PlotStyle → {Directive[Darker[Red], Thickness[Thk / 2]],
    Directive[Darker[Green], Thickness[Thk / 2]], Directive[Darker[Blue], Thickness[Thk / 2]]},
  Frame → True, FrameLabel → {"Coefficient of drug impurity, η", "Fraction of activity spent on viable cancer cells"},
  PlotRange → {{0 - 10, 2200 + 10}, Automatic},
  FrameTicks → {{Table[{0.005 * i, PercentForm[0.005 * i]}, {i, 0, 5}], Automatic}, {Automatic, Automatic}}],

 Plot[{10000 * (x - etacr01), 10000 * (x - gamma * N0 / Acur01 - 1)},
  {x, 1600, 2200}, PlotStyle → {Directive[Darker[Red], Dashed], Directive[Darker[Red], Dotted]}],
 Plot[{10000 * (x - etacr02), 10000 * (x - gamma * N0 / Acur02 - 1)}, {x, 1300, 1500},
  PlotStyle → {Directive[Darker[Green], Dashed], Directive[Darker[Green], Dotted]}],
 Plot[{10000 * (x - etacr03), 10000 * (x - gamma * N0 / Acur03 - 1)}, {x, 400, 525},
```

```
      PlotStyle → {Directive[Darker[Blue], Dashed], Directive[Darker[Blue], Dotted]}],
    Plot[{10000 * (x - 1780)}, {x, 1750, 1830}, PlotStyle → {Directive[Gray, Thin]}],
    Plot[{FrAc1[[1, 2]], FrAc2[[1, 2]], FrAc3[[1, 2]]}, {x, 0, 2500}, PlotStyle → {Directive[Lighter[Red], Thickness[Thk / 10]],
       Directive[Lighter[Green], Thickness[Thk / 10]], Directive[Lighter[Blue], Thickness[Thk / 10]]}]}]

(* Somehow, this figure may cause Wolfram to glitch and freeze when being scrolled by.
   So, for safety it is hidden here. Expand this group of cells to see it, then it's better to collapse this cell group before scrolling further. *)
```

Out[•]=

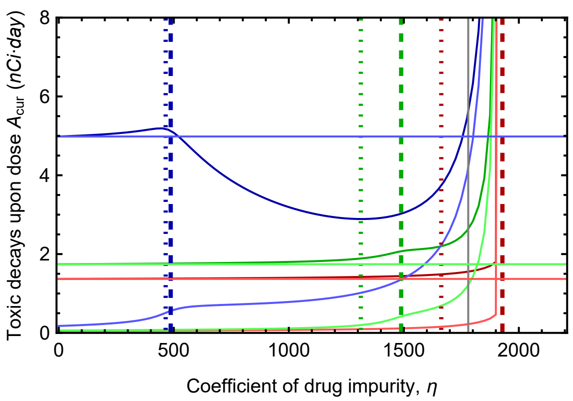

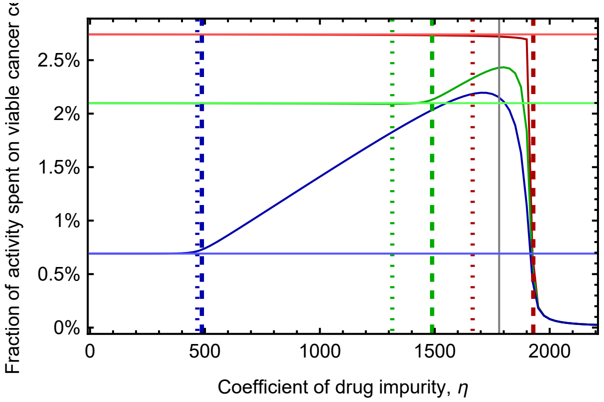

In[•]:=

```
(* "at ks=0.3 the amount of toxic decays in blood grows monotonically with the increase of drug impurity" *)
(* judged by the fact that all the differences of subsequent elements in the corresponding array are positive *)
Abs[AcurEtaDetTox[[2, 2 ;;, 2]] - AcurEtaDetTox[[2, 1 ;; Length[AcurEtaDetTox[[2]]] - 1, 2]]] ==
  AcurEtaDetTox[[2, 2 ;;, 2]] - AcurEtaDetTox[[2, 1 ;; Length[AcurEtaDetTox[[2]]] - 1, 2]]
```

Out[•]=

```
True
```

In[•]:=

```
(* "at ks=1 it falls no more than twice compared to the pure radioconjugates case" *)
PercentForm[1 - NMinimize[Interpolation[AcurEtaDetTox[[3, All, {1, 2}]]][x], {x, 500, 1780}][[1]] / AcurEtaDetTox[[3, All, {1, 2}]][[1, 2]]]
```

Out[•]//PercentForm=

```
42.03%
```

```
(* Supplementary Figure S.16:
  "In real-life conditions, due to the inherent uncertainty and variability of characteristics,
using the lowest possible coefficient of drug impurity may represent
a reasonable strategy for single-dose treatment" *)
(* in silico trial for a single injection of radioconjugates with activity 600 nCi
  and coefficients of drug impurity 0 (pure radioconjugates),1780 (labeling ratio 1.85 kBq/μg) and 4444 (corresponds to≈30 μg of daratumumab)
  in the same test set of 1000 virtual mice *)
```

In[•]:=

```
(* The results of the trial are saved here *)
```

In[•]:=

```
Npar = 1000;
TestFreeGrowth = {{"TBUR", 15.711390754719442`}, {"TBUR", 44.2019502737212`}, {"TBUR", 16.78193020158727`},
    {"TBUR", 18.667093982125085`}, {"TBUR", 13.885447416359366`}, {"TBUR", 30.162601174755157`},
    {"TBUR", 20.869830546775166`}, {"TBUR", 13.428810876131172`}, {"TBUR", 14.471079396823363`},
    {"TBUR", 17.712876379060795`}, {"TBUR", 24.336228038953468`}, {"TBUR", 17.63354851297323`}, {"TBUR", 17.313225221748393`},
    {"TBUR", 30.881659977935886`}, {"TBUR", 13.27422126250228`}, {"TBUR", 18.776811164075987`}, {"TBUR", 31.898893336838885`},
    {"TBUR", 17.28790705464217`}, {"TBUR", 28.67142534485458`}, {"TBUR", 36.430732714305776`}, {"TBUR", 13.019048693178771`},
    {"TBUR", 14.383786156569203`}, {"TBUR", 20.48923939651125`}, {"TBUR", 35.012393113888294`}, {"TBUR", 20.777776008147782`},
    {"TBUR", 48.266751175561275`}, {"TBUR", 15.561828191043215`}, {"TBUR", 37.50933743832283`}, {"TBUR", 18.640678481908232`},
    {"TBUR", 11.83630544424025`}, {"TBUR", 33.210465747981885`}, {"TBUR", 11.382556282125856`}, {"TBUR", 12.754392233655562`},
    {"TBUR", 24.564135844473306`}, {"TBUR", 33.81776364120207`}, {"TBUR", 12.006249305459617`}, {"TBUR", 18.362239037123956`},
    {"TBUR", 16.015705098077394`}, {"TBUR", 21.635849256627885`}, {"TBUR", 25.878508033408714`}, {"TBUR", 15.0605066846942`},
    {"TBUR", 25.959345718956083`}, {"TBUR", 26.071004424283036`}, {"TBUR", 12.40762480700718`}, {"TBUR", 14.5625420895587`},
    {"TBUR", 17.503679634098326`}, {"TBUR", 45.20940527162831`}, {"TBUR", 45.74640593438107`}, {"TBUR", 25.9486748045628`},
    {"TBUR", 12.029808404620965`}, {"TBUR", 24.14332350738726`}, {"TBUR", 31.68985410836966`}, {"TBUR", 35.7551766856527`},
    {"TBUR", 41.2450833432457`}, {"TBUR", 12.432508808409507`}, {"TBUR", 18.187404300362225`}, {"TBUR", 40.089933116105406`},
    {"TBUR", 26.821881272495755`}, {"TBUR", 18.390605378139213`}, {"TBUR", 30.602625753839515`},
    {"TBUR", 13.927808899569715`}, {"TBUR", 24.1542551473147`}, {"TBUR", 30.221327254485974`}, {"TBUR", 13.960996725501133`},
    {"TBUR", 14.718456359925968`}, {"TBUR", 23.80854323464943`}, {"TBUR", 12.09075567378241`}, {"TBUR", 19.999698349123253`},
    {"TBUR", 18.2090009652405`}, {"TBUR", 19.585433005037192`}, {"TBUR", 38.301974279561016`}, {"TBUR", 18.454183803164156`},
    {"TBUR", 16.646510085022577`}, {"TBUR", 11.066417337683328`}, {"TBUR", 21.069008316964855`}, {"TBUR", 23.078967851849022`},
```

```
{"TBUR", 34.49452725010505`}, {"TBUR", 24.47021605580779`}, {"TBUR", 38.24532060036566`}, {"TBUR", 19.609609481476582`},
{"TBUR", 36.74969805999738`}, {"TBUR", 15.629644394196557`}, {"TBUR", 13.213496525127617`}, {"TBUR", 21.374687993953817`},
{"TBUR", 13.415378272305858`}, {"TBUR", 20.486308168185502`}, {"TBUR", 11.835137164430243`}, {"TBUR", 20.925751084537062`},
{"TBUR", 19.20759419404488`}, {"TBUR", 18.18623427800386`}, {"TBUR", 21.799599241705636`}, {"TBUR", 23.960197220535875`},
{"TBUR", 13.445382717487496`}, {"TBUR", 16.383883583281705`}, {"TBUR", 12.173591504764826`}, {"TBUR", 47.04577973955561`},
{"TBUR", 11.71317520726345`}, {"TBUR", 43.04732735707591`}, {"TBUR", 12.0430402417071`}, {"TBUR", 16.25921008760382`},
{"TBUR", 13.780799320894396`}, {"TBUR", 32.94466117223283`}, {"TBUR", 13.645477733405803`}, {"TBUR", 17.10937777091369`},
{"TBUR", 24.866446880146157`}, {"TBUR", 49.681091167586025`}, {"TBUR", 18.521505502047162`}, {"TBUR", 35.36620849444657`},
{"TBUR", 32.86288801866426`}, {"TBUR", 18.602289956387867`}, {"TBUR", 32.58986490722991`}, {"TBUR", 29.8925362710101`},
{"TBUR", 21.572955113264005`}, {"TBUR", 17.703241149394813`}, {"TBUR", 30.84229648532772`}, {"TBUR", 14.982154622046883`},
{"TBUR", 17.06599961414592`}, {"TBUR", 31.653186835701078`}, {"TBUR", 16.275422384450472`}, {"TBUR", 29.3193189802495`},
{"TBUR", 12.669073931157723`}, {"TBUR", 44.32121321762277`}, {"TBUR", 18.808632019596182`}, {"TBUR", 14.178270621846558`},
{"TBUR", 34.39411506496662`}, {"TBUR", 10.60826678656911`}, {"TBUR", 21.27025036051607`}, {"TBUR", 17.128893875251595`},
{"TBUR", 26.75424152875178`}, {"TBUR", 23.396540980904476`}, {"TBUR", 34.026524663570804`}, {"TBUR", 26.047893572349185`},
{"TBUR", 16.931298267133226`}, {"TBUR", 12.427684719498808`}, {"TBUR", 15.977237159890077`}, {"TBUR", 12.015523318328619`},
{"TBUR", 11.865708889876311`}, {"TBUR", 28.167135785515615`}, {"TBUR", 15.891934741275676`}, {"TBUR", 38.99654017919275`},
{"TBUR", 14.557887937138537`}, {"TBUR", 26.85195015930527`}, {"TBUR", 21.10367132602165`}, {"TBUR", 31.23224297185312`},
{"TBUR", 16.42416186520581`}, {"TBUR", 19.68576686273544`}, {"TBUR", 19.518715653499076`}, {"TBUR", 18.99467059371834`},
{"TBUR", 29.497864678679566`}, {"TBUR", 29.8342642786606`}, {"TBUR", 12.151562604837842`}, {"TBUR", 21.43295361353254`},
{"TBUR", 39.191880874485925`}, {"TBUR", 43.94937234554008`}, {"TBUR", 13.600102266619292`}, {"TBUR", 35.4545547765237 8`},
{"TBUR", 13.618077419834844`}, {"TBUR", 30.44805565275776`}, {"TBUR", 12.135143649888148`}, {"TBUR", 11.838492109374219`},
{"TBUR", 19.387626849034557`}, {"TBUR", 20.378893406187636`}, {"TBUR", 12.568370803609564`}, {"TBUR", 12.335541498182756`},
{"TBUR", 16.900180308391676`}, {"TBUR", 23.566889218337202`}, {"TBUR", 27.188423556215096`}, {"TBUR", 23.4088487070332`},
{"TBUR", 15.503298507467774`}, {"TBUR", 27.798052195552458`}, {"TBUR", 18.381327565539564`}, {"TBUR", 20.92135437607756`},
{"TBUR", 13.830888602052188`}, {"TBUR", 15.29011768969299`}, {"TBUR", 37.29460988856768`}, {"TBUR", 41.127126860961276`},
{"TBUR", 17.488297437451834`}, {"TBUR", 17.174594646166334`}, {"TBUR", 29.517194080940293`}, {"TBUR", 28.809733816287626`},
{"TBUR", 36.03500813957433`}, {"TBUR", 35.650726659604366`}, {"TBUR", 22.968796241435946`}, {"TBUR", 10.861214749311419`},
{"TBUR", 19.273058909571365`}, {"TBUR", 21.397446408131632`}, {"TBUR", 16.656429073810475`}, {"TBUR", 37.7256973902334`},
{"TBUR", 14.332350044067626`}, {"TBUR", 15.612773946318637`}, {"TBUR", 13.56089086265119`}, {"TBUR", 56.29919814610204`},
{"TBUR", 13.692192912801652`}, {"TBUR", 29.40261067063076`}, {"TBUR", 25.92640970414381`}, {"TBUR", 19.86214014774299`},
{"TBUR", 24.541614900945888`}, {"TBUR", 10.842531622673606`}, {"TBUR", 18.393250674136546`}, {"TBUR", 11.848022816238698`},
```

{"TBUR", 12.394247222735197`}, {"TBUR", 42.73652768351775`}, {"TBUR", 46.834151303573464`}, {"TBUR", 18.074934308481833`},
{"TBUR", 23.88505995433176`}, {"TBUR", 26.19664776852087`}, {"TBUR", 14.091681063105517`}, {"TBUR", 12.529826141736114`},
{"TBUR", 11.85452964978897`}, {"TBUR", 21.415047182220583`}, {"TBUR", 16.10378219825058`}, {"TBUR", 12.002118223003633`},
{"TBUR", 12.238177469377737`}, {"TBUR", 11.820289133688204`}, {"TBUR", 13.258397547509006`}, {"TBUR", 10.446724842053175`},
{"TBUR", 32.25921307596167`}, {"TBUR", 13.218716299423596`}, {"TBUR", 47.540255662146905`}, {"TBUR", 16.441665661679554`},
{"TBUR", 28.516877697345826`}, {"TBUR", 10.145299260427528`}, {"TBUR", 24.256068708797287`}, {"TBUR", 14.802391486987581`},
{"TBUR", 18.26988607751062`}, {"TBUR", 17.29117337945663`}, {"TBUR", 16.777144378744026`}, {"TBUR", 24.38760930220397`},
{"TBUR", 41.13822571897459`}, {"TBUR", 28.52079654241579`}, {"TBUR", 18.78601604914881`}, {"TBUR", 24.89976517570905`},
{"TBUR", 15.21678572837678`}, {"TBUR", 29.678812755534587`}, {"TBUR", 18.411189747761647`}, {"TBUR", 11.630526775456948`},
{"TBUR", 20.56736788402131`}, {"TBUR", 39.81896501701757`}, {"TBUR", 11.579647419833996`}, {"TBUR", 16.7073757138579`},
{"TBUR", 13.15017054642574`}, {"TBUR", 48.991993507602885`}, {"TBUR", 19.973188444818835`}, {"TBUR", 19.781162115684367`},
{"TBUR", 14.98836187254622`}, {"TBUR", 11.355024994053888`}, {"TBUR", 10.445561077742212`}, {"TBUR", 23.08652006894122`},
{"TBUR", 11.38031929474993`}, {"TBUR", 12.494991747371342`}, {"TBUR", 25.91202648565012`}, {"TBUR", 42.720904591500386`},
{"TBUR", 12.185093864964474`}, {"TBUR", 11.11000437764382`}, {"TBUR", 18.42743793854317`}, {"TBUR", 11.405104181149731`},
{"TBUR", 16.01094471858759`}, {"TBUR", 36.38234275331097`}, {"TBUR", 28.971244963128317`}, {"TBUR", 17.051858746531526`},
{"TBUR", 15.918258745132839`}, {"TBUR", 11.136970219111259`}, {"TBUR", 28.637097143580235`}, {"TBUR", 24.954008886644395`},
{"TBUR", 10.939225822736441`}, {"TBUR", 14.294574319421093`}, {"TBUR", 49.83267457923563`}, {"TBUR", 19.73508313170569`},
{"TBUR", 33.12362322236299`}, {"TBUR", 28.54909224679254`}, {"TBUR", 26.39334136273501`}, {"TBUR", 18.182282129491867`},
{"TBUR", 24.402886666984113`}, {"TBUR", 25.51697320347085`}, {"TBUR", 14.68063527980366`}, {"TBUR", 13.284960655014292`},
{"TBUR", 34.62031115029852`}, {"TBUR", 15.931985120694774`}, {"TBUR", 11.369743621488627`}, {"TBUR", 17.14773227042683`},
{"TBUR", 18.212303044244837`}, {"TBUR", 23.300311304242033`}, {"TBUR", 13.876621007775102`}, {"TBUR", 16.02479346669339`},
{"TBUR", 13.208860978559992`}, {"TBUR", 34.617244530505324`}, {"TBUR", 12.88540383655441`}, {"TBUR", 50.20149749114922 6`},
{"TBUR", 33.75407787514724`}, {"TBUR", 22.555506182171825`}, {"TBUR", 13.420983562939275`}, {"TBUR", 26.126524688871935`},
{"TBUR", 37.467551796005104`}, {"TBUR", 14.294999566793445`}, {"TBUR", 11.781318983556558`}, {"TBUR", 26.386348334785268`},
{"TBUR", 13.629363716693975`}, {"TBUR", 48.3975545130189`}, {"TBUR", 14.276052535452093`}, {"TBUR", 13.57077890673767`},
{"TBUR", 21.605038413907803`}, {"TBUR", 13.222805104676372`}, {"TBUR", 11.280495079659019`}, {"TBUR", 21.409445105028386`},
{"TBUR", 13.685628959811423`}, {"TBUR", 40.90500767021971`}, {"TBUR", 19.05174281927019`}, {"TBUR", 12.459674892628335`},
{"TBUR", 12.080813962339308`}, {"TBUR", 20.44079628896667`}, {"TBUR", 17.31405015943552`}, {"TBUR", 17.63436164079323`},
{"TBUR", 20.72566502090729`}, {"TBUR", 28.305130093017823`}, {"TBUR", 18.128421806240034`}, {"TBUR", 18.21522543397489`},
{"TBUR", 12.19511139100093`}, {"TBUR", 23.52658971955829`}, {"TBUR", 38.746507555243205`}, {"TBUR", 13.013804608663998`},
{"TBUR", 16.830134710533237`}, {"TBUR", 31.163559201344587`}, {"TBUR", 24.861844391920066`}, {"TBUR", 10.200283327118301`},

{"TBUR", 11.994034603135827`}, {"TBUR", 10.163897828210832`}, {"TBUR", 23.875444478765356`}, {"TBUR", 14.355986815184721`},
{"TBUR", 23.9542029287897`}, {"TBUR", 38.51300337985342`}, {"TBUR", 26.666697824394866`}, {"TBUR", 11.726711325822064`},
{"TBUR", 15.84989810942918`}, {"TBUR", 12.238343820235636`}, {"TBUR", 16.557839402597924`}, {"TBUR", 22.123244065017477`},
{"TBUR", 14.362211816349216`}, {"TBUR", 14.982335434433553`}, {"TBUR", 12.742611257109857`}, {"TBUR", 21.346404563089`},
{"TBUR", 39.538021744880815`}, {"TBUR", 28.099557097951184`}, {"TBUR", 12.185998695163024`}, {"TBUR", 15.877804698917146`},
{"TBUR", 12.472281605292798`}, {"TBUR", 26.31625892762327`}, {"TBUR", 22.76889517879618`}, {"TBUR", 16.950178546001784`},
{"TBUR", 13.096775023957145`}, {"TBUR", 19.82357938171404`}, {"TBUR", 53.585315525906424`}, {"TBUR", 12.20366748251806`},
{"TBUR", 12.26068564409993`}, {"TBUR", 18.64753065556537`}, {"TBUR", 13.09793505577459`}, {"TBUR", 31.349437648549987`},
{"TBUR", 40.126370801513914`}, {"TBUR", 38.96572613913505`}, {"TBUR", 38.85342205795237`}, {"TBUR", 19.48865417681404`},
{"TBUR", 14.59295555893629`}, {"TBUR", 21.912784658619394`}, {"TBUR", 15.068792260286681`}, {"TBUR", 19.421262057828905`},
{"TBUR", 14.791311150730058`}, {"TBUR", 11.644407162921675`}, {"TBUR", 39.26171802714754`}, {"TBUR", 28.7718155898307`},
{"TBUR", 11.245023407343798`}, {"TBUR", 10.57174774201496`}, {"TBUR", 18.634841364000923`}, {"TBUR", 18.89800331371781`},
{"TBUR", 15.372070023482934`}, {"TBUR", 13.289630292735579`}, {"TBUR", 18.119211100621463`}, {"TBUR", 18.19532424240082`},
{"TBUR", 13.003150893677239`}, {"TBUR", 17.7282178239352`}, {"TBUR", 12.518727832782371`}, {"TBUR", 10.96689413046786`},
{"TBUR", 20.526495282875`}, {"TBUR", 19.618576611833166`}, {"TBUR", 25.741531481345167`}, {"TBUR", 11.580634768845037`},
{"TBUR", 39.60440853116702`}, {"TBUR", 50.7525628981767`}, {"TBUR", 30.525499970009236`}, {"TBUR", 15.875869808240488`},
{"TBUR", 33.7576772049169`}, {"TBUR", 12.198036043438151`}, {"TBUR", 36.10746753826354`}, {"TBUR", 23.019434665476034`},
{"TBUR", 42.99742959154252`}, {"TBUR", 45.04808549688851`}, {"TBUR", 13.323085661147942`}, {"TBUR", 15.990018308084574`},
{"TBUR", 28.99263986073146`}, {"TBUR", 10.828591309594154`}, {"TBUR", 27.475565098910344`}, {"TBUR", 12.253512274090394`},
{"TBUR", 14.163757228575793`}, {"TBUR", 14.931779341911481`}, {"TBUR", 14.540706199919255`}, {"TBUR", 47.32170377355336`},
{"TBUR", 22.146989872057624`}, {"TBUR", 23.239188532259003`}, {"TBUR", 13.339585238476273`}, {"TBUR", 12.130906923683536`},
{"TBUR", 23.117528909403312`}, {"TBUR", 44.23773482755419`}, {"TBUR", 38.157567892445215`}, {"TBUR", 26.394381760015914`},
{"TBUR", 49.465206421225076`}, {"TBUR", 15.544368884888053`}, {"TBUR", 45.64964820274593`}, {"TBUR", 10.375598986949994`},
{"TBUR", 13.4577709014758`}, {"TBUR", 12.92740658866155`}, {"TBUR", 13.82555155001455`}, {"TBUR", 17.2548882383809`},
{"TBUR", 17.420284405698727`}, {"TBUR", 23.296344746475494`}, {"TBUR", 31.98430657291525`}, {"TBUR", 18.561175904260594`},
{"TBUR", 11.148323633274817`}, {"TBUR", 24.98779313594612`}, {"TBUR", 15.030367488401826`}, {"TBUR", 20.558700394866957`},
{"TBUR", 18.6028068766275`}, {"TBUR", 14.6230201465156`}, {"TBUR", 19.3016362362253`}, {"TBUR", 11.448179213739792`},
{"TBUR", 29.151867850742914`}, {"TBUR", 45.909960904742036`}, {"TBUR", 14.661498452826745`}, {"TBUR", 16.10400151271037`},
{"TBUR", 42.61403021443065`}, {"TBUR", 22.61333940022501`}, {"TBUR", 12.465990105470024`}, {"TBUR", 18.11202847916702`},
{"TBUR", 28.0774161110492`}, {"TBUR", 16.325684810661727`}, {"TBUR", 18.55982079277458`}, {"TBUR", 13.86257402896593`},
{"TBUR", 12.042055362310814`}, {"TBUR", 29.18597186973557`}, {"TBUR", 21.366117344635636`}, {"TBUR", 26.943445824101442`},

{"TBUR", 11.498290153855072`}, {"TBUR", 11.488376172892458`}, {"TBUR", 21.10426942757126`}, {"TBUR", 22.923874181921168`},
{"TBUR", 12.679033292772049`}, {"TBUR", 34.181493073312765`}, {"TBUR", 21.229170276246265`}, {"TBUR", 23.05327809438495`},
{"TBUR", 14.836687161538524`}, {"TBUR", 31.632092206844426`}, {"TBUR", 10.684528417301543`}, {"TBUR", 12.323554426038513`},
{"TBUR", 14.908989326903038`}, {"TBUR", 22.62247856502531`}, {"TBUR", 22.971085682806184`}, {"TBUR", 15.969422089771053`},
{"TBUR", 37.97006518058343`}, {"TBUR", 13.989474739922914`}, {"TBUR", 13.46400948773041`}, {"TBUR", 10.712035781062992`},
{"TBUR", 15.392271050722684`}, {"TBUR", 16.09268015882418`}, {"TBUR", 22.82787006896125`}, {"TBUR", 23.547927678469136`},
{"TBUR", 13.622706960699572`}, {"TBUR", 15.09676395522251`}, {"TBUR", 22.953986000725653`}, {"TBUR", 15.97721606819315`},
{"TBUR", 44.38959844892823`}, {"TBUR", 20.945249163902453`}, {"TBUR", 19.466033967193685`}, {"TBUR", 11.15847231967852 3`},
{"TBUR", 40.220670046310836`}, {"TBUR", 12.793530253211959`}, {"TBUR", 24.880734816219235`}, {"TBUR", 17.43604164254628`},
{"TBUR", 21.696656293887454`}, {"TBUR", 14.044238126710205`}, {"TBUR", 13.059506531769134`}, {"TBUR", 28.526967482011788`},
{"TBUR", 26.633481667404485`}, {"TBUR", 23.20253311428919`}, {"TBUR", 10.754730378470608`}, {"TBUR", 14.975999203711073`},
{"TBUR", 14.35758409286505`}, {"TBUR", 25.748160126748434`}, {"TBUR", 24.625971986527414`}, {"TBUR", 18.134246823300774`},
{"TBUR", 12.029250528991964`}, {"TBUR", 12.283088733640653`}, {"TBUR", 10.904174263410239`}, {"TBUR", 14.48499028734746`},
{"TBUR", 13.356622879290526`}, {"TBUR", 13.087682982021931`}, {"TBUR", 12.90992184913878`}, {"TBUR", 13.269606005256925`},
{"TBUR", 12.05460333402895`}, {"TBUR", 40.70482577774895`}, {"TBUR", 17.844085961158733`}, {"TBUR", 22.11428074306187`},
{"TBUR", 37.439576225558724`}, {"TBUR", 16.184705880426666`}, {"TBUR", 25.179894928284636`}, {"TBUR", 17.177849782689034`},
{"TBUR", 24.139857635768177`}, {"TBUR", 15.269969164410572`}, {"TBUR", 24.084328130080067`}, {"TBUR", 25.156185065552094`},
{"TBUR", 25.081587472097127`}, {"TBUR", 24.596794037781738`}, {"TBUR", 16.297538420609563`}, {"TBUR", 13.92054315571153`},
{"TBUR", 24.186531212314744`}, {"TBUR", 19.15308878818269`}, {"TBUR", 28.45358129148927`}, {"TBUR", 12.218494240750383`},
{"TBUR", 10.773398710428566`}, {"TBUR", 15.414206830182799`}, {"TBUR", 23.086256460655893`}, {"TBUR", 14.401915522233667`},
{"TBUR", 12.753435398541292`}, {"TBUR", 20.705830590117454`}, {"TBUR", 18.103071927473387`}, {"TBUR", 17.811389048194037`},
{"TBUR", 11.906952088761239`}, {"TBUR", 25.925332734218312`}, {"TBUR", 11.582030311234943`}, {"TBUR", 19.7444850685129 3`},
{"TBUR", 28.18445197624289`}, {"TBUR", 15.223966526805105`}, {"TBUR", 31.432468538394286`}, {"TBUR", 27.19309801194287`},
{"TBUR", 14.008140819298191`}, {"TBUR", 12.045856459211306`}, {"TBUR", 22.105046931525724`}, {"TBUR", 29.847955271378463`},
{"TBUR", 11.48809023519896`}, {"TBUR", 41.93802236462971`}, {"TBUR", 11.44197833872413`}, {"TBUR", 44.78670292317672`},
{"TBUR", 11.0867350462611`}, {"TBUR", 15.362744626619742`}, {"TBUR", 18.57110216060741`}, {"TBUR", 16.691242237560672`},
{"TBUR", 13.903982971980977`}, {"TBUR", 19.037062837946692`}, {"TBUR", 12.92362732078675`}, {"TBUR", 13.237091463120482`},
{"TBUR", 39.03584032971677`}, {"TBUR", 13.232019942363335`}, {"TBUR", 13.240144328016669`}, {"TBUR", 30.38842139574255 4`},
{"TBUR", 11.423499346803078`}, {"TBUR", 16.667402897010415`}, {"TBUR", 21.761982158062477`}, {"TBUR", 21.382614476203088`},
{"TBUR", 23.96488850963543`}, {"TBUR", 44.303028035478064`}, {"TBUR", 15.078377322238609`}, {"TBUR", 52.81421814180943`},
{"TBUR", 20.593736250381497`}, {"TBUR", 40.543019978884935`}, {"TBUR", 21.363444597177413`}, {"TBUR", 11.491958120679236`},

{"TBUR", 23.805942604091097`}, {"TBUR", 17.457403469003896`}, {"TBUR", 12.319726553927222`}, {"TBUR", 12.9052005603408 7`},
{"TBUR", 36.316604818573694`}, {"TBUR", 12.526229763228413`}, {"TBUR", 22.332214087824713`}, {"TBUR", 17.2244921848201`},
{"TBUR", 49.669497007104646`}, {"TBUR", 37.079745045406426`}, {"TBUR", 15.931126455903573`}, {"TBUR", 33.584913292338776`},
{"TBUR", 11.835162353735948`}, {"TBUR", 16.741086716203625`}, {"TBUR", 22.093063393428466`}, {"TBUR", 25.3651827319965 8`},
{"TBUR", 31.678827935351443`}, {"TBUR", 16.62069037647161`}, {"TBUR", 11.392861767709837`}, {"TBUR", 11.934464273787754`},
{"TBUR", 17.340349154883945`}, {"TBUR", 20.889722876209845`}, {"TBUR", 18.335068321726613`}, {"TBUR", 30.830295475520987`},
{"TBUR", 10.807642353824672`}, {"TBUR", 11.216908859826905`}, {"TBUR", 40.04439458958592`}, {"TBUR", 36.83881667098762`},
{"TBUR", 23.92194362638417`}, {"TBUR", 16.53534180686229`}, {"TBUR", 51.23050055254762`}, {"TBUR", 18.023455873735564`},
{"TBUR", 18.648199093095407`}, {"TBUR", 18.763547866061003`}, {"TBUR", 11.419212755826193`}, {"TBUR", 24.008534349430825`},
{"TBUR", 34.84296157475952`}, {"TBUR", 10.921490347290051`}, {"TBUR", 28.062133886083`}, {"TBUR", 12.427641403611485`},
{"TBUR", 15.92887830547401`}, {"TBUR", 20.94447979878765`}, {"TBUR", 15.337560941696069`}, {"TBUR", 21.19237478142351`},
{"TBUR", 13.663656699879482`}, {"TBUR", 10.91918368235662`}, {"TBUR", 28.48038009165704`}, {"TBUR", 14.72653000188838`},
{"TBUR", 43.78294665591238`}, {"TBUR", 13.700386640340682`}, {"TBUR", 14.548044383987587`}, {"TBUR", 13.665883046173738`},
{"TBUR", 16.05820386245968`}, {"TBUR", 12.904844890817078`}, {"TBUR", 12.917986940552879`}, {"TBUR", 16.379817998638547`},
{"TBUR", 32.81829785954663`}, {"TBUR", 15.65577822695791`}, {"TBUR", 23.960754924812576`}, {"TBUR", 46.42020173562314`},
{"TBUR", 14.781102935419684`}, {"TBUR", 13.436700928696643`}, {"TBUR", 11.989824230197081`}, {"TBUR", 18.904151712050382`},
{"TBUR", 24.081388036131735`}, {"TBUR", 12.335774564394683`}, {"TBUR", 11.732129723869265`}, {"TBUR", 10.456303438241472`},
{"TBUR", 12.560794668456198`}, {"TBUR", 12.421668111923303`}, {"TBUR", 15.519712741204295`}, {"TBUR", 11.484802585076057`},
{"TBUR", 17.607325878236882`}, {"TBUR", 29.85643700106843`}, {"TBUR", 32.13525239881099`}, {"TBUR", 16.78803495216286`},
{"TBUR", 11.648964281069263`}, {"TBUR", 24.182504340114686`}, {"TBUR", 19.42397801276626`}, {"TBUR", 44.17728583357691`},
{"TBUR", 14.66762514297404`}, {"TBUR", 15.585713064661457`}, {"TBUR", 14.22653096705157`}, {"TBUR", 15.555088977904298`},
{"TBUR", 12.514337331506693`}, {"TBUR", 26.78755123112613`}, {"TBUR", 26.254677688178298`}, {"TBUR", 38.3416062187931 8`},
{"TBUR", 20.956143477051647`}, {"TBUR", 12.384226094606502`}, {"TBUR", 11.342365937291804`}, {"TBUR", 21.567957025331644`},
{"TBUR", 15.428572810460327`}, {"TBUR", 17.692295535412782`}, {"TBUR", 18.374363606827465`}, {"TBUR", 11.177439745546469`},
{"TBUR", 23.151274579710364`}, {"TBUR", 25.561442209495276`}, {"TBUR", 15.191722397878516`}, {"TBUR", 15.317738134370043`},
{"TBUR", 15.754324049386259`}, {"TBUR", 13.444599229541677`}, {"TBUR", 46.975528713564856`}, {"TBUR", 15.582929873652624`},
{"TBUR", 11.172559186556574`}, {"TBUR", 21.53510755015808`}, {"TBUR", 38.1922652097825`}, {"TBUR", 11.615611300801511`},
{"TBUR", 30.52780202756511`}, {"TBUR", 17.419201062358916`}, {"TBUR", 19.891732201574825`}, {"TBUR", 42.363468891181704`},
{"TBUR", 19.268136573185007`}, {"TBUR", 43.61044372946785`}, {"TBUR", 19.678323929404883`}, {"TBUR", 12.83040029879762`},
{"TBUR", 30.230973806479213`}, {"TBUR", 14.157926609120352`}, {"TBUR", 10.191843501040474`}, {"TBUR", 37.83947540069915`},
{"TBUR", 17.072209389919802`}, {"TBUR", 16.46647436967203`}, {"TBUR", 25.520441400196084`}, {"TBUR", 15.14249997668548 2`},

{"TBUR", 12.153963131932542`}, {"TBUR", 12.254341695995448`}, {"TBUR", 15.99553699974471`}, {"TBUR", 14.009415411087728`},
{"TBUR", 20.72471693373452`}, {"TBUR", 25.379837072629677`}, {"TBUR", 41.34185799496326`}, {"TBUR", 28.586227977857817`},
{"TBUR", 16.516493534325388`}, {"TBUR", 12.454385454931874`}, {"TBUR", 12.326051525095739`}, {"TBUR", 16.451639019541044`},
{"TBUR", 49.40275306734192`}, {"TBUR", 26.97402791257719`}, {"TBUR", 21.04300518348442`}, {"TBUR", 11.138973134291232`},
{"TBUR", 29.170619014428308`}, {"TBUR", 34.63205089264964`}, {"TBUR", 15.194061817314392`}, {"TBUR", 27.389126428015043`},
{"TBUR", 13.167291347644422`}, {"TBUR", 12.55776636072074`}, {"TBUR", 12.140654160542763`}, {"TBUR", 28.850919257426384`},
{"TBUR", 32.311078542727124`}, {"TBUR", 47.522598894000616`}, {"TBUR", 28.369382096388907`}, {"TBUR", 37.38752450894326`},
{"TBUR", 18.6885524515911`}, {"TBUR", 23.664121316574818`}, {"TBUR", 41.330708798869644`}, {"TBUR", 14.048773944038226`},
{"TBUR", 42.861962534556994`}, {"TBUR", 14.327382038298191`}, {"TBUR", 10.622241238403388`}, {"TBUR", 28.74130609102452`},
{"TBUR", 20.452403102098977`}, {"TBUR", 17.429187525332168`}, {"TBUR", 25.7695058559047`}, {"TBUR", 21.35673335905624`},
{"TBUR", 13.316459464757374`}, {"TBUR", 25.456022184650543`}, {"TBUR", 15.26535501368181`}, {"TBUR", 31.442656021163415`},
{"TBUR", 22.144344460330387`}, {"TBUR", 19.756788197085175`}, {"TBUR", 11.750896770936777`}, {"TBUR", 11.91379735527266`},
{"TBUR", 13.082497845804456`}, {"TBUR", 22.58037325536216`}, {"TBUR", 27.11994888874441`}, {"TBUR", 33.94540348960336`},
{"TBUR", 13.55347506137448`}, {"TBUR", 42.039848568278266`}, {"TBUR", 18.840729303537266`}, {"TBUR", 32.18988964188309`},
{"TBUR", 13.869843964601282`}, {"TBUR", 27.880811077585708`}, {"TBUR", 26.732109568747337`}, {"TBUR", 26.429449941613587`},
{"TBUR", 12.686313766165187`}, {"TBUR", 28.619171533903188`}, {"TBUR", 35.136359473108946`}, {"TBUR", 15.890145348087076`},
{"TBUR", 15.540586680598453`}, {"TBUR", 21.62528445822497`}, {"TBUR", 16.655395917309367`}, {"TBUR", 14.556582063467172`},
{"TBUR", 24.59499458597921`}, {"TBUR", 38.31687592592987`}, {"TBUR", 20.91670961312049`}, {"TBUR", 30.880052623523927`},
{"TBUR", 23.37839937953543`}, {"TBUR", 14.756878385331708`}, {"TBUR", 12.306374151885091`}, {"TBUR", 12.19396976451413`},
{"TBUR", 10.820270170682432`}, {"TBUR", 38.86707580054765`}, {"TBUR", 26.275116375269466`}, {"TBUR", 19.515294983292456`},
{"TBUR", 20.76272418963084`}, {"TBUR", 25.06570624066885`}, {"TBUR", 48.28299572199504`}, {"TBUR", 47.15248698386937`},
{"TBUR", 27.965718787972648`}, {"TBUR", 18.136333387094034`}, {"TBUR", 17.30853490271965`}, {"TBUR", 14.720055891444831`},
{"TBUR", 14.575953384252578`}, {"TBUR", 16.872520172913198`}, {"TBUR", 32.56100616317757`}, {"TBUR", 18.54602887377109`},
{"TBUR", 30.285614163966653`}, {"TBUR", 24.769061979356383`}, {"TBUR", 12.804699929538211`}, {"TBUR", 46.8230835758209`},
{"TBUR", 21.21413815436887`}, {"TBUR", 12.319668225861111`}, {"TBUR", 17.08900165913073`}, {"TBUR", 33.39280618827887`},
{"TBUR", 30.784043360357213`}, {"TBUR", 41.80596014055507`}, {"TBUR", 18.210512416441464`}, {"TBUR", 32.0573658992787`},
{"TBUR", 33.9806026181684`}, {"TBUR", 27.568655924125853`}, {"TBUR", 12.1584928408606`}, {"TBUR", 31.993171768030024`},
{"TBUR", 25.227137856905184`}, {"TBUR", 18.96277268186363`}, {"TBUR", 12.635310561055041`}, {"TBUR", 34.75709683033686`},
{"TBUR", 15.676198179360494`}, {"TBUR", 14.351763370381466`}, {"TBUR", 12.394494955044776`}, {"TBUR", 38.34749460297427`},
{"TBUR", 21.709627816230363`}, {"TBUR", 21.404025449569534`}, {"TBUR", 13.773952319506515`}, {"TBUR", 15.325842453057636`},
{"TBUR", 16.13598200107213`}, {"TBUR", 10.565295180343039`}, {"TBUR", 38.493707225491875`}, {"TBUR", 31.591500399349755`},

{"TBUR", 12.233042653846113`}, {"TBUR", 13.441278132842836`}, {"TBUR", 10.71119305839793`}, {"TBUR", 12.549194360866233`},
{"TBUR", 14.430143043814553`}, {"TBUR", 23.638167870560885`}, {"TBUR", 46.60146622174525`}, {"TBUR", 28.674412294553544`},
{"TBUR", 32.37137987887265`}, {"TBUR", 26.9737868248472`}, {"TBUR", 28.656206467813305`}, {"TBUR", 27.243650264504783`},
{"TBUR", 13.96082705027568`}, {"TBUR", 29.94311776735254`}, {"TBUR", 19.52106732513822`}, {"TBUR", 17.775000251237888`},
{"TBUR", 17.933766746197712`}, {"TBUR", 22.399050329429524`}, {"TBUR", 14.458060263011596`}, {"TBUR", 24.95623448114484`},
{"TBUR", 16.347494456653916`}, {"TBUR", 24.618543776758553`}, {"TBUR", 16.9150565948905`}, {"TBUR", 21.952694405429753`},
{"TBUR", 27.898193165891037`}, {"TBUR", 11.274944049057206`}, {"TBUR", 18.540459835720974`}, {"TBUR", 15.07535764694947`},
{"TBUR", 15.740946903924218`}, {"TBUR", 24.261138236301495`}, {"TBUR", 15.977669186060735`}, {"TBUR", 14.732615628830892`},
{"TBUR", 14.0368739479989`}, {"TBUR", 22.74708834092515`}, {"TBUR", 29.698528331382423`}, {"TBUR", 11.019280529800811`},
{"TBUR", 14.368487002503988`}, {"TBUR", 31.141875705872618`}, {"TBUR", 14.833833148835154`}, {"TBUR", 11.739499287603133`},
{"TBUR", 52.5193285933069`}, {"TBUR", 16.985298517961542`}, {"TBUR", 10.499992605933624`}, {"TBUR", 16.751986127639093`},
{"TBUR", 14.2632809236648`}, {"TBUR", 30.43788025347739`}, {"TBUR", 11.90445215079268`}, {"TBUR", 14.264395316837868`},
{"TBUR", 14.452845699757372`}, {"TBUR", 34.572127537796206`}, {"TBUR", 26.11161702071263`}, {"TBUR", 20.43581000209806`},
{"TBUR", 14.162911780156453`}, {"TBUR", 17.87169579027483`}, {"TBUR", 12.99831618789261`}, {"TBUR", 17.775464460897613`},
{"TBUR", 32.559876696628045`}, {"TBUR", 25.609975482620715`}, {"TBUR", 20.570346406615123`}, {"TBUR", 47.48624482192006`},
{"TBUR", 12.586809730426838`}, {"TBUR", 12.589399932667439`}, {"TBUR", 30.01764746535113`}, {"TBUR", 11.972539963913935`},
{"TBUR", 31.855777981949387`}, {"TBUR", 17.019267590986306`}, {"TBUR", 23.790943672497843`}, {"TBUR", 15.487454208123516`},
{"TBUR", 10.525218926133745`}, {"TBUR", 47.49721606768071`}, {"TBUR", 10.877223296166196`}, {"TBUR", 24.758919081039558`},
{"TBUR", 18.98089486237934`}, {"TBUR", 38.9755916501208`}, {"TBUR", 15.844889206178705`}, {"TBUR", 15.828065961395247`},
{"TBUR", 16.043507902292482`}, {"TBUR", 16.29507796729022`}, {"TBUR", 11.747370042394191`}, {"TBUR", 11.342565597386283`},
{"TBUR", 20.65755986837501`}, {"TBUR", 38.141294834970815`}, {"TBUR", 13.166289584683993`}, {"TBUR", 20.736558416184195`},
{"TBUR", 31.76020149311386`}, {"TBUR", 12.048290529008609`}, {"TBUR", 13.935089448273782`}, {"TBUR", 28.193335218325988`},
{"TBUR", 31.76201967547971`}, {"TBUR", 15.27922375145718`}, {"TBUR", 11.931712667218223`}, {"TBUR", 10.547333726231798`},
{"TBUR", 16.347304675056495`}, {"TBUR", 12.318275290985008`}, {"TBUR", 38.91076514574312`}, {"TBUR", 11.683568662416686`},
{"TBUR", 24.00910384152908`}, {"TBUR", 19.286777915296657`}, {"TBUR", 27.276107180948763`}, {"TBUR", 13.486529801608391`},
{"TBUR", 12.605589745478484`}, {"TBUR", 40.909492536943254`}, {"TBUR", 37.17219671673012`}, {"TBUR", 17.6616094107079`},
{"TBUR", 16.586022700802683`}, {"TBUR", 16.45014093748146`}, {"TBUR", 13.92619220408081`}, {"TBUR", 18.82828129761119`},
{"TBUR", 36.22232583851177`}, {"TBUR", 11.27934026300811`}, {"TBUR", 21.639726206576118`}, {"TBUR", 16.459665972699216`},
{"TBUR", 15.511101673101416`}, {"TBUR", 22.506440992370166`}, {"TBUR", 11.076972857018646`}, {"TBUR", 15.242188743714676`},
{"TBUR", 11.462647176542138`}, {"TBUR", 18.702255009934902`}, {"TBUR", 10.482359657660846`}, {"TBUR", 19.516115667286996`},
{"TBUR", 19.84863193299701`}, {"TBUR", 10.811595168120101`}, {"TBUR", 12.468425027733264`}, {"TBUR", 17.65849908369357`},

```
{"TBUR", 12.366872881786685`}, {"TBUR", 15.057501302422027`}, {"TBUR", 10.97280472054204`}, {"TBUR", 12.995726450926323`},
{"TBUR", 41.0030139043523`}, {"TBUR", 27.476983556131657`}, {"TBUR", 13.003238300319067`}, {"TBUR", 34.76545358004131`},
{"TBUR", 16.93961788742202`}, {"TBUR", 15.01175401839218`}, {"TBUR", 15.586141564209756`}, {"TBUR", 27.70475129393068`},
{"TBUR", 19.2625809574968`}, {"TBUR", 15.907041087628507`}, {"TBUR", 19.36074195370645`}, {"TBUR", 30.469706303174217`},
{"TBUR", 11.901373794002273`}, {"TBUR", 26.278599600233694`}, {"TBUR", 20.003563989282735`}, {"TBUR", 37.75677788969558`},
{"TBUR", 33.957074484169915`}, {"TBUR", 15.236632905420791`}, {"TBUR", 19.838808863077166`}, {"TBUR", 46.4717876345526`},
{"TBUR", 22.744792861590497`}, {"TBUR", 11.472593768678601`}, {"TBUR", 14.418835243486974`}, {"TBUR", 34.13267009209965`},
{"TBUR", 11.347103240094883`}, {"TBUR", 11.332749256343055`}, {"TBUR", 10.61275667865575`}, {"TBUR", 25.317973500286957`},
{"TBUR", 18.824909678232107`}, {"TBUR", 19.75988885226173`}, {"TBUR", 21.694552645662377`}, {"TBUR", 11.68190930846636`},
{"TBUR", 10.8215974542`}, {"TBUR", 39.549387730743824`}, {"TBUR", 17.80560798712406`}, {"TBUR", 23.303984805326564`},
{"TBUR", 19.35719532039265`}, {"TBUR", 11.367022753870474`}, {"TBUR", 12.624461342996618`}, {"TBUR", 15.55025792512026`},
{"TBUR", 18.641589075281406`}, {"TBUR", 11.85870829496253`}, {"TBUR", 10.705231972566915`}, {"TBUR", 14.031239476593514`},
{"TBUR", 11.227113948157005`}, {"TBUR", 11.781352793042377`}, {"TBUR", 31.888118474974192`}, {"TBUR", 44.4704201674232`},
{"TBUR", 19.733705914844947`}, {"TBUR", 38.82659412985412`}, {"TBUR", 36.22155621382699`}, {"TBUR", 14.107232897488702`}};

TestEta = {{{"0 Res", "0 OS", "0 Tox"}, {"TBUR", 44.120135788353586`, 4.861326196355177`},
   {"CURE", 365, 10.337504262653539`}, {"CURE", 365, 7.186426278637355`}, {"TBUR", 32.02493760570166`, 2.199892094193805`},
   {"TBUR", 20.365793999636534`, 6.621002536325695`}, {"CURE", 365, 18.714433026890628`}, {"CURE", 365, 17.621862548184076`},
   {"CURE", 365, 1.8389807944866148`}, {"CURE", 365, 12.70739113347037`}, {"CURE", 365, 10.964876830618918`},
   {"CURE", 365, 41.132243729315`}, {"CURE", 365, 5.11597429294633`}, {"CURE", 365, 17.586531949579708`},
   {"TBUR", 63.66503159539907`, 58.90263054368947`}, {"TBUR", 89.55274053173079`, 8.612908374401547`},
   {"TBUR", 75.86390234976218`, 13.4249132694345`}, {"CURE", 365, 26.884106488468888`}, {"CURE", 365, 11.955674679322415`},
   {"TBUR", 51.72152986771293`, 8.633617009204348`}, {"CURE", 365, 3.0143887915463874`},
   {"CURE", 365, 18.659311517030112`}, {"CURE", 365, 7.564260658677`}, {"CURE", 365, 2.892116339156526`},
   {"CURE", 365, 2.3041920986875613`}, {"CURE", 365, 10.513208718848723`}, {"CURE", 365, 11.53365822838632`},
   {"CURE", 365, 1.0485013198748447`}, {"CURE", 365, 1.5879744217061977`}, {"CURE", 365, 5.876041505839302`},
   {"CURE", 365, 6.857168675905919`}, {"CURE", 365, 5.877975821642121`}, {"TBUR", 28.23868913314177`, 19.988252340077025`},
   {"CURE", 365, 4.532530999713672`}, {"TBUR", 54.5083010756903`, 3.5301087055322626`},
   {"CURE", 365, 29.247315926814657`}, {"CURE", 365, 1.790013766718378`}, {"CURE", 365, 7.762200766314479`},
   {"TBUR", 23.51368313500588`, 15.842672525778097`}, {"TBUR", 105.65739019227388`, 4.268015744695488`},
   {"CURE", 365, 6.609410736002069`}, {"TBUR", 29.54011113356088`, 14.70734971479832`}, {"CURE", 365, 10.219028672758082`},
```

```
{"CURE", 365, 2.070282315612041`}, {"CURE", 365, 6.973031444027605`}, {"CURE", 365, 10.078039562462655`},
{"CURE", 365, 6.611421288034885`}, {"CURE", 365, 70.13852724106646`}, {"CURE", 365, 15.108960626004846`},
{"CURE", 365, 22.064056595430554`}, {"CURE", 365, 2.82753764133036`}, {"CURE", 365, 7.534375601252964`},
{"CURE", 365, 10.576306877161219`}, {"CURE", 365, 8.661519964452033`}, {"CURE", 365, 5.648940803891371`},
{"CURE", 365, 2.428561249704058`}, {"CURE", 365, 25.790697040215335`}, {"CURE", 365, 8.106481757059509`},
{"TBUR", 94.04834907767128`, 1.5191944501253916`}, {"CURE", 365, 16.51362646289519`}, {"CURE", 365, 11.54747104746299`},
{"CURE", 365, 7.938095180783986`}, {"CURE", 365, 6.085170045726911`}, {"CURE", 365, 3.3445663406400623`},
{"CURE", 365, 13.122270931026247`}, {"TBUR", 97.39075364697528`, 9.248201025416847`}, {"CURE", 365, 10.663959118286018`},
{"CURE", 365, 2.4404129965533796`}, {"CURE", 365, 2.451984065947791`}, {"CURE", 365, 2.638067321699513`},
{"CURE", 365, 10.758079870640973`}, {"CURE", 365, 3.9360914836608827`}, {"CURE", 365, 8.751305578026647`},
{"CURE", 365, 16.461926669005646`}, {"TBUR", 14.31295416407541`, 3.3292785429291745`}, {"CURE", 365, 4.3799952565221325`},
{"CURE", 365, 12.202192442516425`}, {"CURE", 365, 7.11109666628486`}, {"CURE", 365, 5.425908002655323`},
{"CURE", 365, 21.555794528284657`}, {"CURE", 365, 23.723108563332225`}, {"CURE", 365, 17.458506792504476`},
{"CURE", 365, 4.718306034057121`}, {"CURE", 365, 9.542262098776703`}, {"CURE", 365, 6.668600691071988`},
{"CURE", 365, 7.5374643803748995`}, {"CURE", 365, 3.5251334903483946`}, {"CURE", 365, 37.50874333521383`},
{"CURE", 365, 8.168939125775518`}, {"CURE", 365, 10.340037534338006`}, {"CURE", 365, 8.846112366140245`},
{"TBUR", 43.47909648202201`, 7.006626368627714`}, {"CURE", 365, 2.174340209580344`}, {"CURE", 365, 3.9819579671607173`},
{"CURE", 365, 9.2080702091512`}, {"TBUR", 22.2003322495203`, 2.865578128553233`}, {"CURE", 365, 3.9527754459933`},
{"CURE", 365, 10.389083186446884`}, {"CURE", 365, 26.377815315264396`}, {"CURE", 365, 14.027958549693158`},
{"CURE", 365, 7.040155110811144`}, {"TBUR", 23.357981090673793`, 13.561274150192743`}, {"CURE", 365, 9.086175469170527`},
{"CURE", 365, 5.911270942397595`}, {"CURE", 365, 40.529080366612604`}, {"CURE", 365, 4.505744493226987`},
{"CURE", 365, 3.0608020995842464`}, {"CURE", 365, 17.8893472369802`}, {"CURE", 365, 5.254965425629598`},
{"CURE", 365, 5.421864465186982`}, {"CURE", 365, 32.617111261891424`}, {"CURE", 365, 2.6473955309020303`},
{"CURE", 365, 1.6783557882979894`}, {"CURE", 365, 6.976179029002869`}, {"CURE", 365, 5.509206285124034`},
{"CURE", 365, 4.155323172050453`}, {"CURE", 365, 10.622480292109802`}, {"CURE", 365, 2.980947673907273`},
{"CURE", 365, 1.8353624346218074`}, {"CURE", 365, 8.604573213111255`}, {"CURE", 365, 13.142716473739288`},
{"CURE", 365, 2.2351842277911698`}, {"CURE", 365, 7.401728126513483`}, {"CURE", 365, 22.494466904409247`},
{"TBUR", 22.696834792885102`, 8.138771530110288`}, {"CURE", 365, 4.9918567354831245`},
{"CURE", 365, 17.286751613710535`}, {"CURE", 365, 8.685515863270085`}, {"CURE", 365, 1.1483398065659032`},
{"CURE", 365, 9.989168977388521`}, {"CURE", 365, 7.717581312674928`}, {"CURE", 365, 6.150160554209577`},
{"CURE", 365, 7.290074185635818`}, {"CURE", 365, 1.8808708811493502`}, {"TBUR", 70.79171625524961`, 10.717812743960504`},
```

{"CURE", 365, 3.8818229093266985`}, {"TBUR", 44.51025338021416`, 8.371501462611896`}, {"CURE", 365, 32.57208837945558`},
{"CURE", 365, 11.39596515019846`}, {"CURE", 365, 26.816398125877182`}, {"CURE", 365, 8.867040916321823`},
{"CURE", 365, 36.488391499088195`}, {"CURE", 365, 1.1425312648696282`}, {"CURE", 365, 10.050967169937838`},
{"CURE", 365, 11.445632481561514`}, {"CURE", 365, 1.487830831014959`}, {"CURE", 365, 2.0569851756012025`},
{"CURE", 365, 12.64744008880455`}, {"TBUR", 46.12370050071068`, 14.692640200565235`},
{"CURE", 365, 12.704196701888177`}, {"CURE", 365, 10.374165405004687`}, {"CURE", 365, 9.941159552015634`},
{"CURE", 365, 6.002559243013904`}, {"CURE", 365, 1.591570453819259`}, {"CURE", 365, 6.873982771725807`},
{"TBUR", 45.34192264010004`, 3.196775814730011`}, {"CURE", 365, 1.596616908788593`}, {"CURE", 365, 9.711634465157925`},
{"CURE", 365, 5.454587165942436`}, {"CURE", 365, 14.417483157240197`}, {"CURE", 365, 5.812639791644915`},
{"CURE", 365, 4.8401513596642705`}, {"CURE", 365, 32.06804232878863`}, {"CURE", 365, 2.416181418995692`},
{"CURE", 365, 7.16238940436839`}, {"CURE", 365, 4.077780599818743`}, {"CURE", 365, 9.645323456291797`},
{"CURE", 365, 2.660423868445219`}, {"CURE", 365, 10.608473350656842`}, {"CURE", 365, 28.01575185419393`},
{"CURE", 365, 2.387559011110273`}, {"CURE", 365, 7.296248053933448`}, {"CURE", 365, 7.810300911536647`},
{"CURE", 365, 28.40985859234573`}, {"TBUR", 24.44078158732981`, 10.135591274094782`}, {"CURE", 365, 8.779317366848558`},
{"TBUR", 124.15619171712581`, 14.979583160780994`}, {"TBUR", 80.89564742749732`, 5.930309055822927`},
{"CURE", 365, 17.273988651307956`}, {"TBUR", 105.00558822165186`, 11.219317560691273`},
{"CURE", 365, 6.796157619557559`}, {"TBUR", 173.57012320053138`, 6.201051561207254`}, {"CURE", 365, 2.058704581944865`},
{"CURE", 365, 1.3955548980436017`}, {"TBUR", 30.632085283114076`, 2.964426001108345`}, {"CURE", 365, 4.384162302934486`},
{"TBUR", 82.39321400837201`, 1.8849084503308646`}, {"CURE", 365, 7.037323760057046`}, {"CURE", 365, 19.22733082225396`},
{"CURE", 365, 5.223129441172221`}, {"CURE", 365, 9.598575211646565`}, {"CURE", 365, 12.141291670157841`},
{"CURE", 365, 9.68119998189347`}, {"CURE", 365, 7.1072726799537564`}, {"CURE", 365, 2.8215676281879536`},
{"CURE", 365, 19.362313585608828`}, {"CURE", 365, 1.6825356670020541`}, {"CURE", 365, 18.096351541583726`},
{"TBUR", 16.90583812843224`, 15.218205748793341`}, {"CURE", 365, 4.915613216614742`}, {"CURE", 365, 5.836514919309275`},
{"TBUR", 39.91930147699073`, 33.112638789844496`}, {"CURE", 365, 8.555722520043007`}, {"CURE", 365, 10.332804185879409`},
{"CURE", 365, 6.448358022730719`}, {"CURE", 365, 2.426831877058885`}, {"CURE", 365, 14.705990897827874`},
{"TBUR", 23.832626600254343`, 38.31709623191016`}, {"CURE", 365, 6.060009927745125`}, {"CURE", 365, 5.4532583728716695`},
{"CURE", 365, 10.087951903055423`}, {"CURE", 365, 4.024408847649084`}, {"CURE", 365, 51.09034218755481`},
{"CURE", 365, 4.084599213725688`}, {"CURE", 365, 25.154391263356125`}, {"CURE", 365, 19.388345330942478`},
{"TBUR", 68.36813973099964`, 18.725050668977254`}, {"CURE", 365, 10.067664705738057`},
{"CURE", 365, 23.41742027458658`}, {"CURE", 365, 31.160857950053376`}, {"CURE", 365, 10.638895461893794`},
{"CURE", 365, 1.9516854966865642`}, {"CURE", 365, 17.348592046802604`}, {"CURE", 365, 10.81741730490529`},

{"CURE", 365, 10.416571177878538`}, {"CURE", 365, 2.2871531503888147`}, {"CURE", 365, 52.547085489591055`},
{"CURE", 365, 3.846941898124723`}, {"CURE", 365, 16.68706946925232`}, {"CURE", 365, 10.479069794611497`},
{"CURE", 365, 7.46951294311448`}, {"CURE", 365, 1.7985214336957616`}, {"CURE", 365, 13.534042082873649`},
{"CURE", 365, 7.934184229876916`}, {"CURE", 365, 20.991267755506673`}, {"CURE", 365, 13.81048119966916`2},
{"TBUR", 56.14104386465955`, 17.39801604767651`}, {"TBUR", 57.169100443811374`, 8.319556643789157`},
{"CURE", 365, 7.38339561524173`}, {"TBUR", 22.5286562879871`, 37.903738965154574`}, {"CURE", 365, 10.21355740923217`},
{"CURE", 365, 2.719030191872994`}, {"CURE", 365, 9.171158235697309`}, {"TBUR", 31.028293807200598`, 8.62554851871458`},
{"TBUR", 63.45950524188225`, 1.2071323287857079`}, {"CURE", 365, 2.2298866820660366`}, {"CURE", 365, 3.3964902649230386`},
{"TBUR", 32.152426308349156`, 31.598150870415388`}, {"CURE", 365, 9.885192177330408`}, {"CURE", 365, 10.310013264332202`},
{"CURE", 365, 8.25300650717253`}, {"TBUR", 88.46000729752588`, 33.15296046870994`}, {"CURE", 365, 7.818317843530606`},
{"CURE", 365, 15.514851025062908`}, {"CURE", 365, 7.776160462941116`}, {"CURE", 365, 11.487207663244645`},
{"CURE", 365, 2.763101072205737`}, {"CURE", 365, 10.326617235066973`}, {"CURE", 365, 6.427321010325204`},
{"CURE", 365, 12.565819914812645`}, {"CURE", 365, 19.789700517921272`}, {"CURE", 365, 21.93690977895291`},
{"CURE", 365, 12.986032608462903`}, {"CURE", 365, 11.072906496649473`}, {"CURE", 365, 14.775574607477385`},
{"CURE", 365, 7.239737936550928`}, {"CURE", 365, 8.202343090171832`}, {"CURE", 365, 5.766097401051561`},
{"CURE", 365, 4.553701330898598`}, {"CURE", 365, 7.32091538056211`}, {"CURE", 365, 18.829715913923547`},
{"CURE", 365, 2.156534398312664`}, {"TBUR", 52.79504830280723`, 9.40622990992381`}, {"CURE", 365, 1.806227306529613`},
{"CURE", 365, 9.366210247383382`}, {"TBUR", 86.77294771410223`, 5.254253783371564`}, {"CURE", 365, 19.877586576769417`},
{"CURE", 365, 1.829418254261289`}, {"TBUR", 101.68801043839008`, 12.952657467046647`},
{"CURE", 365, 16.353024758513566`}, {"CURE", 365, 2.6773854891600597`}, {"CURE", 365, 6.093379230107219`},
{"CURE", 365, 4.937349944752054`}, {"TBUR", 33.15345235700245`, 2.594035703083765`}, {"CURE", 365, 16.745338556496556`},
{"TBUR", 64.65916136187622`, 6.2445133607217675`}, {"CURE", 365, 12.12184741979122`}, {"CURE", 365, 11.773563457139197`},
{"CURE", 365, 13.376030852072132`}, {"CURE", 365, 10.449278213739477`}, {"CURE", 365, 12.490659065850169`},
{"CURE", 365, 9.906346313860567`}, {"CURE", 365, 19.049006761394022`}, {"CURE", 365, 11.634775009662537`},
{"TBUR", 83.6432894220498`, 9.390492515029928`}, {"CURE", 365, 24.27329571671099`}, {"CURE", 365, 2.811265475130828`},
{"CURE", 365, 15.985147829208318`}, {"CURE", 365, 1.1750909148803466`}, {"CURE", 365, 14.584917881393421`},
{"CURE", 365, 3.4409116945060907`}, {"TBUR", 106.69849276548254`, 17.501107055372724`}, {"CURE", 365, 2.799544233731667`},
{"CURE", 365, 18.387624198074686`}, {"CURE", 365, 45.729771766475466`}, {"CURE", 365, 1.99940606586606`},
{"CURE", 365, 1.4382697499593085`}, {"CURE", 365, 5.309403040662869`}, {"TBUR", 68.42454070640743`, 1.2750221527758716`},
{"CURE", 365, 0.8825635595972613`}, {"CURE", 365, 5.164629301290836`}, {"TBUR", 89.68571302167639`, 8.30158126234952`},
{"CURE", 365, 9.092116075957343`}, {"TBUR", 46.45910828389855`, 8.448760573278983`}, {"CURE", 365, 6.70451610484366`},

{"CURE", 365, 11.802481753498217`}, {"CURE", 365, 20.82488866375496`}, {"CURE", 365, 1.6819064950517726`},
{"CURE", 365, 9.237467398055221`}, {"CURE", 365, 7.940433177130828`}, {"CURE", 365, 5.750114856633501`},
{"CURE", 365, 10.617903217630564`}, {"CURE", 365, 15.753006418475064`}, {"CURE", 365, 36.91140057683829`},
{"CURE", 365, 27.325264235378054`}, {"TBUR", 23.258564028088177`, 8.281289981772163`}, {"CURE", 365, 0.9028441934275048`},
{"CURE", 365, 9.839932631864743`}, {"CURE", 365, 25.426841546798734`}, {"CURE", 365, 6.393148592651098`},
{"CURE", 365, 7.708935402361587`}, {"CURE", 365, 4.933531295626072`}, {"CURE", 365, 7.232241942400319`},
{"TBUR", 55.27856715809899`, 1.8338092204047123`}, {"TBUR", 40.906641091824696`, 9.100539456638066`},
{"CURE", 365, 5.116175946584481`}, {"CURE", 365, 14.96258991224083`}, {"TBUR", 28.286869070970432`, 10.042082726394172`},
{"CURE", 365, 9.996092767164939`}, {"CURE", 365, 8.222932489893997`}, {"CURE", 365, 4.351465991443099`},
{"CURE", 365, 2.9970210524207728`}, {"CURE", 365, 2.920654902698162`}, {"CURE", 365, 3.761920658301864`},
{"CURE", 365, 2.950092921509175`}, {"CURE", 365, 39.205610031977415`}, {"CURE", 365, 21.911730249587997`},
{"CURE", 365, 4.263468822293343`}, {"CURE", 365, 65.72154002475048`}, {"CURE", 365, 18.797793626644474`},
{"CURE", 365, 9.949346902433083`}, {"CURE", 365, 17.207927226454114`}, {"CURE", 365, 2.5881507699445288`},
{"CURE", 365, 20.589453695095262`}, {"CURE", 365, 55.160238322627535`}, {"CURE", 365, 2.992088509159728`},
{"CURE", 365, 9.332907347307273`}, {"CURE", 365, 15.172338283583642`}, {"CURE", 365, 7.639782590554075`},
{"CURE", 365, 3.788378979872093`}, {"CURE", 365, 3.0195565606971018`}, {"CURE", 365, 26.17990728769913`},
{"CURE", 365, 13.733218402970781`}, {"CURE", 365, 7.823530521949697`}, {"CURE", 365, 19.326806449749206`},
{"CURE", 365, 3.492540754660476`}, {"CURE", 365, 9.361697433603736`}, {"CURE", 365, 5.661828416585101`},
{"CURE", 365, 38.209836929878016`}, {"TBUR", 16.409161783689644`, 8.853599201685054`},
{"TBUR", 17.91532411492415`, 38.19633724964021`}, {"TBUR", 48.045076926750085`, 67.47620416193949`},
{"CURE", 365, 5.328003688407721`}, {"TBUR", 68.87221718681718`, 1.491656307041244`}, {"CURE", 365, 1.3099789530361212`},
{"CURE", 365, 9.544756855105268`}, {"CURE", 365, 37.15938175343595`}, {"CURE", 365, 7.44831482156422`},
{"CURE", 365, 5.702100992458688`}, {"TBUR", 22.065153754899296`, 10.481827120681087`},
{"TBUR", 78.6166705944897 8`, 11.669540794202058`}, {"TBUR", 105.59003100798313`, 48.71296609153321`},
{"CURE", 365, 40.512746929036986`}, {"CURE", 365, 8.397020460967264`}, {"CURE", 365, 2.8090663025285894`},
{"CURE", 365, 6.883678285266469`}, {"CURE", 365, 1.0811608915037199`}, {"TBUR", 170.44400322406997`, 19.950534860975946`},
{"CURE", 365, 30.279679904508388`}, {"CURE", 365, 9.900797800242977`}, {"CURE", 365, 7.84112718309556`},
{"CURE", 365, 33.188000323388906`}, {"CURE", 365, 2.0841065223185815`}, {"CURE", 365, 2.698402541594715`},
{"CURE", 365, 8.580361417470845`}, {"CURE", 365, 10.907987333230052`}, {"TBUR", 24.029372725038854`, 19.45713517330503`},
{"CURE", 365, 21.513716694265824`}, {"CURE", 365, 8.835663681289333`}, {"TBUR", 135.8093629306811`, 9.030280730079095`},
{"TBUR", 41.2439503152036`, 11.168075983061522`}, {"TBUR", 42.502933680557625`, 3.695242418297541`},

```
{"CURE", 365, 1.1155024110209562`}, {"CURE", 365, 11.681438830185328`}, {"CURE", 365, 14.140337833085484`},
{"CURE", 365, 6.871286089610547`}, {"CURE", 365, 12.459595358827876`}, {"CURE", 365, 3.8607019615710954`},
{"CURE", 365, 2.54786741609022`}, {"CURE", 365, 11.159216165243159`}, {"CURE", 365, 6.675236119039037`},
{"CURE", 365, 9.670522484623055`}, {"CURE", 365, 63.9934005559939`}, {"CURE", 365, 6.086584048473344`},
{"CURE", 365, 2.9398377234178343`}, {"CURE", 365, 2.3001431984642227`}, {"CURE", 365, 2.792709329900106`},
{"CURE", 365, 15.956585599798636`}, {"CURE", 365, 1.0996805112867611`}, {"CURE", 365, 14.837318968269523`},
{"CURE", 365, 16.246869373002067`}, {"CURE", 365, 12.683417035080705`}, {"CURE", 365, 10.39553203910812`},
{"CURE", 365, 2.041686443492103`}, {"CURE", 365, 22.61850663318733`}, {"TBUR", 16.219335476751606`, 5.8801492759234835`},
{"CURE", 365, 2.993837150990726`}, {"CURE", 365, 20.12653131567057`}, {"CURE", 365, 3.583490920230909`},
{"CURE", 365, 24.091477630573802`}, {"CURE", 365, 15.49493087962409`}, {"CURE", 365, 10.28991801612104`},
{"CURE", 365, 15.016206280193058`}, {"TBUR", 145.69261152025473`, 5.22845860662873`}, {"CURE", 365, 23.504201894855168`},
{"CURE", 365, 2.7933281602749918`}, {"CURE", 365, 11.779667319378293`}, {"CURE", 365, 13.839286424817802`},
{"CURE", 365, 5.881304847526691`}, {"CURE", 365, 54.78100344000119`}, {"CURE", 365, 10.868914085950788`},
{"CURE", 365, 11.220217125021742`}, {"CURE", 365, 11.427290728481694`}, {"CURE", 365, 45.9082226943244`},
{"CURE", 365, 14.574035439737747`}, {"CURE", 365, 13.58348979899109`}, {"CURE", 365, 29.463982995818654`},
{"CURE", 365, 10.401714460307009`}, {"CURE", 365, 9.04736444863034`}, {"CURE", 365, 11.21344046628927`},
{"CURE", 365, 15.49530559060228`}, {"CURE", 365, 27.390522771943385`}, {"CURE", 365, 5.902519374162787`},
{"CURE", 365, 5.388682655810225`}, {"CURE", 365, 11.54311393533149`}, {"CURE", 365, 11.746970022421939`},
{"TBUR", 122.73657314011335`, 12.966347517250659`}, {"CURE", 365, 14.131621470255498`}, {"CURE", 365, 7.857896514084816`},
{"TBUR", 36.96840713017926`, 3.7934829634793767`}, {"TBUR", 42.53535688897206`, 14.117771945834448`},
{"CURE", 365, 3.758584619107748`}, {"CURE", 365, 8.000916318732367`}, {"TBUR", 98.94754292159011`, 9.318589136527503`},
{"CURE", 365, 2.873919250150761`}, {"CURE", 365, 1.4268401701362212`}, {"TBUR", 25.890975987661736`, 7.815480622943981`},
{"TBUR", 82.96585069454301`, 11.818893490975322`}, {"TBUR", 59.17332481062525`, 14.087078486449894`},
{"CURE", 365, 10.0696507806776`}, {"CURE", 365, 1.2929200907877099`}, {"CURE", 365, 11.615622871573187`},
{"CURE", 365, 2.203781244747429`}, {"CURE", 365, 34.25226547200853`}, {"CURE", 365, 44.50622505273443`},
{"CURE", 365, 5.9744163734934785`}, {"TBUR", 31.829276429285834`, 8.61035933221758`},
{"CURE", 365, 47.73416080593535`}, {"CURE", 365, 8.56798613049709`}, {"TBUR", 57.02599215120498`, 4.813712967589792`},
{"TBUR", 38.48014065102971`, 3.72620361157626`}, {"CURE", 365, 1.0592447331104762`}, {"CURE", 365, 6.689414054122399`},
{"CURE", 365, 7.455422706459424`}, {"CURE", 365, 19.551839755622726`}, {"CURE", 365, 9.449089164916812`},
{"CURE", 365, 16.46523503386716`}, {"CURE", 365, 5.816609107675788`}, {"TBUR", 66.24156555898772`, 12.355525889115903`},
{"CURE", 365, 28.219654110888726`}, {"TBUR", 138.39595123842324`, 2.8411076375265116`},
```

{"CURE", 365, 4.425692014271184`}, {"TBUR", 79.96964586453389`, 6.5454876764881655`},
{"CURE", 365, 9.174542546266`}, {"TBUR", 57.619566491475545`, 5.826581753188123`}, {"CURE", 365, 5.765633025403836`},
{"CURE", 365, 2.4267771164658405`}, {"CURE", 365, 3.770373758365172`}, {"TBUR", 39.38741363699935`, 6.895663938762562`},
{"CURE", 365, 16.250150670438398`}, {"TBUR", 29.78134615617619`, 14.188007687686087`}, {"CURE", 365, 7.405095824436832`},
{"TBUR", 18.657327825394994`, 10.225739480690939`}, {"TBUR", 35.15695057110269`, 23.279386881450144`},
{"CURE", 365, 11.697168783000699`}, {"CURE", 365, 8.758227556958404`}, {"CURE", 365, 5.581547443238992`},
{"CURE", 365, 1.7382114638436172`}, {"CURE", 365, 9.734026972091781`}, {"CURE", 365, 11.18910678822609`},
{"CURE", 365, 43.877050898445816`}, {"CURE", 365, 2.2291994272809883`}, {"CURE", 365, 2.190446411778079`},
{"CURE", 365, 7.015889189157286`}, {"CURE", 365, 20.4647673949063`}, {"CURE", 365, 2.5516794939215943`},
{"CURE", 365, 12.47785984775916`}, {"CURE", 365, 2.9078390342774942`}, {"CURE", 365, 7.243518475132522`},
{"CURE", 365, 2.921257261009896`}, {"CURE", 365, 3.07493564702548`}, {"CURE", 365, 2.775571573233364`},
{"CURE", 365, 29.62678111602246`}, {"TBUR", 92.50814388827804`, 4.562728396597595`},
{"TBUR", 20.191972630168983`, 7.375377207442529`}, {"CURE", 365, 1.1503088476695138`}, {"CURE", 365, 13.761673726979508`},
{"CURE", 365, 19.29544467112804`}, {"CURE", 365, 16.726343740829925`}, {"CURE", 365, 2.536954996218411`},
{"CURE", 365, 5.092971098398636`}, {"CURE", 365, 51.505347646375256`}, {"CURE", 365, 14.021818714632126`},
{"TBUR", 61.23461073928456`, 9.476921673508468`}, {"CURE", 365, 3.3828407891483505`}, {"CURE", 365, 4.862625389472053`},
{"TBUR", 102.48911648860985`, 7.468676716195933`}, {"TBUR", 114.021248042112`, 1.84703827558367`},
{"CURE", 365, 9.16324672957636`}, {"CURE", 365, 14.427259694348372`}, {"CURE", 365, 8.710164710992233`},
{"CURE", 365, 9.494533359271996`}, {"CURE", 365, 6.888987505395745`}, {"TBUR", 101.02163659672655`, 4.423288899954423`},
{"CURE", 365, 25.14375127291507`}, {"CURE", 365, 36.88361068336854`}, {"CURE", 365, 9.865975615514648`},
{"CURE", 365, 12.454206912979304`}, {"CURE", 365, 15.773542550596588`}, {"CURE", 365, 15.40620450056534`},
{"TBUR", 75.26265167565349`, 1.3349244080671117`}, {"TBUR", 84.1611548770158`, 17.8534575086973`},
{"CURE", 365, 18.653202789120485`}, {"TBUR", 49.9074884242514`, 12.504629720424589`}, {"CURE", 365, 50.58162397102399`},
{"CURE", 365, 10.512073568513312`}, {"TBUR", 44.124130228356854`, 4.483440107721088`}, {"CURE", 365, 11.054197327086973`},
{"CURE", 365, 14.062530513326232`}, {"CURE", 365, 6.291661886672615`}, {"TBUR", 80.77589132268031`, 4.857121303360887`},
{"CURE", 365, 4.29623697721033`}, {"CURE", 365, 7.739645129363739`}, {"CURE", 365, 24.02184871077165`},
{"CURE", 365, 15.42459703830445`}, {"TBUR", 107.61644935366587`, 2.6520489680384687`}, {"CURE", 365, 15.57965595745274`},
{"CURE", 365, 22.678625915879678`}, {"CURE", 365, 4.137715000416053`}, {"CURE", 365, 23.659403347814987`},
{"CURE", 365, 3.564638505261515`}, {"CURE", 365, 2.2716199356415046`}, {"CURE", 365, 3.2422510048698983`},
{"CURE", 365, 6.1525238221303145`}, {"TBUR", 28.455158067496708`, 26.28990906796901З`},
{"CURE", 365, 9.980464754817016`}, {"TBUR", 47.58835856672351`, 12.501069721520494`},

{"CURE", 365, 3.0139890834738607`}, {"TBUR", 63.83384829983235`, 56.46744332796567`}, {"CURE", 365, 26.321143208216235`},
{"TBUR", 65.78657938504644`, 4.3925983478124255`}, {"CURE", 365, 10.933740112506765`},
{"CURE", 365, 6.132589594323253`}, {"TBUR", 53.74030176356931`, 6.164332847141234`}, {"CURE", 365, 20.748103430038405`},
{"TBUR", 22.021020246504204`, 24.07638554077453`}, {"CURE", 365, 2.8432614603448343`}, {"CURE", 365, 5.5270796166788765`},
{"CURE", 365, 1.9576833771334394`}, {"CURE", 365, 7.5771180034788665`}, {"TBUR", 74.34594495850249`, 12.760333482907328`},
{"CURE", 365, 21.040110017336602`}, {"CURE", 365, 11.722721682716568`}, {"CURE", 365, 42.25116672551901`},
{"CURE", 365, 20.209338272935334`}, {"TBUR", 34.19578184207342`, 3.1036949094149775`}, {"CURE", 365, 7.347205573248782`},
{"TBUR", 14.466743305240845`, 4.25842028468955`}, {"TBUR", 18.853611582673274`, 14.261207568267055`},
{"TBUR", 93.15428833658079`, 2.1123377583879455`}, {"CURE", 365, 5.7804555686660235`}, {"CURE", 365, 3.245305929588845`},
{"CURE", 365, 8.15266478189856`}, {"CURE", 365, 18.580615796932282`}, {"CURE", 365, 7.921277571037943`},
{"CURE", 365, 1.72180952145439`}, {"TBUR", 76.4851093430011`, 32.419172618004595`}, {"CURE", 365, 6.153417288863416`},
{"CURE", 365, 2.0988519413175366`}, {"CURE", 365, 2.7576338265522686`}, {"CURE", 365, 2.326393195650498`},
{"CURE", 365, 2.9789148762160487`}, {"CURE", 365, 23.950801839376627`}, {"CURE", 365, 12.989011806201031`},
{"TBUR", 90.89140668176255`, 7.865137398611091`}, {"TBUR", 60.50159965591283`, 3.6341241548588257`},
{"CURE", 365, 6.182652470338151`}, {"CURE", 365, 3.5771004054423696`}, {"CURE", 365, 8.616999750322513`},
{"CURE", 365, 29.52198955558399`}, {"TBUR", 80.78853520336986`, 16.22090862223463`}, {"CURE", 365, 8.438024396041717`},
{"CURE", 365, 4.920556370583515`}, {"CURE", 365, 4.999975531623307`}, {"CURE", 365, 63.37720313402192`},
{"CURE", 365, 3.020513611062853`}, {"CURE", 365, 1.555492424813195`}, {"TBUR", 27.557371409427763`, 1.161011282633009`},
{"CURE", 365, 17.209249941510954`}, {"CURE", 365, 9.991021856687828`}, {"CURE", 365, 11.17531586723121`},
{"CURE", 365, 16.634636236678496`}, {"TBUR", 128.67357642671328`, 27.031515874303633`},
{"CURE", 365, 28.42241075441402`}, {"CURE", 365, 10.822124450968355`}, {"CURE", 365, 4.676932612623518`},
{"CURE", 365, 5.395642750505874`}, {"CURE", 365, 10.563351991605714`}, {"TBUR", 83.51034576876634`, 6.069240919782249`},
{"TBUR", 16.15679538609168`, 1.7596504191492135`}, {"TBUR", 24.839150143140323`, 9.397473467405632`},
{"TBUR", 55.50769024065771`, 59.77087067412596`}, {"CURE", 365, 6.1803327074665075`}, {"CURE", 365, 2.212106512409883`},
{"CURE", 365, 21.076984574790554`}, {"CURE", 365, 5.272438201674405`}, {"CURE", 365, 15.071681959570316`},
{"CURE", 365, 72.91751388380077`}, {"CURE", 365, 33.6524506351112`}, {"CURE", 365, 10.926685545024759`},
{"CURE", 365, 9.146801867593798`}, {"CURE", 365, 3.9018754493816488`}, {"CURE", 365, 1.1078219008262349`},
{"TBUR", 31.73298002647607`, 3.821528595210123`}, {"CURE", 365, 12.784970564264865`}, {"CURE", 365, 7.072256535008024`},
{"CURE", 365, 22.59588732838715`}, {"CURE", 365, 8.900791247861383`}, {"CURE", 365, 9.147986011377592`},
{"CURE", 365, 2.542333803003404`}, {"CURE", 365, 7.190339307271312`}, {"CURE", 365, 7.39140760210447`},
{"CURE", 365, 1.9260403163424613`}, {"TBUR", 32.89996114551276`, 6.0274122359228155`}, {"CURE", 365, 3.656905818643144`},

{"TBUR", 29.773766855293758`, 5.339076487796647`}, {"TBUR", 46.64886624367778`, 11.785491103100771`},
{"TBUR", 88.52516438061032`, 5.9695106864324625`}, {"CURE", 365, 1.4063905535608614`}, {"CURE", 365, 9.671768330968765`},
{"CURE", 365, 1.0705907277146425`}, {"TBUR", 98.6692339675076`, 3.1447445059904604`}, {"CURE", 365, 9.37735107067924`},
{"CURE", 365, 10.89227477389878`}, {"CURE", 365, 2.156511759473355`}, {"CURE", 365, 6.318659686762852`},
{"CURE", 365, 8.181655859945849`}, {"TBUR", 25.2379237335302`, 40.86015029184018`}, {"CURE", 365, 9.588866014205836`},
{"CURE", 365, 2.6799126543530645`}, {"TBUR", 44.39648719863112`, 29.324879867458712`},
{"TBUR", 86.73014292606014`, 2.7661037203678243`}, {"TBUR", 61.57533482245408`, 10.790814788117068`},
{"CURE", 365, 11.287651032817335`}, {"CURE", 365, 3.3636603929465188`}, {"CURE", 365, 3.7467997860740505`},
{"CURE", 365, 16.587342409453015`}, {"CURE", 365, 1.6177015397102799`}, {"CURE", 365, 14.076466500288136`},
{"CURE", 365, 3.727695530789436`}, {"CURE", 365, 4.876177143898577`}, {"TBUR", 48.691700396566645`, 1.05144324354354`},
{"CURE", 365, 5.591205942152514`}, {"TBUR", 35.755352373914846`, 18.634004573667006`}, {"CURE", 365, 7.221736604845174`},
{"CURE", 365, 5.2565355676937395`}, {"TBUR", 71.6720971932479`, 7.48147656362164`}, {"CURE", 365, 5.846572143773523`},
{"TBUR", 25.724631650883964`, 6.959832240799642`}, {"CURE", 365, 13.080341660590522`}, {"CURE", 365, 12.690969385766229`},
{"CURE", 365, 1.5640855592969707`}, {"TBUR", 123.98061137527796`, 25.209366638307845`}, {"CURE", 365, 2.9783767614501753`},
{"CURE", 365, 1.4866965899125448`}, {"CURE", 365, 7.076745744565183`}, {"CURE", 365, 7.473986776983484`},
{"CURE", 365, 5.030642829556795`}, {"CURE", 365, 8.752827272399216`}, {"CURE", 365, 7.5581360524867645`},
{"CURE", 365, 12.153312908353598`}, {"CURE", 365, 10.256232925536294`}, {"TBUR", 21.811925693612597`, 14.37686792584336`},
{"CURE", 365, 2.2941545678567303`}, {"CURE", 365, 29.4205340183568`}, {"CURE", 365, 28.74983163356199`},
{"CURE", 365, 1.5906831685807024`}, {"CURE", 365, 4.020504750951814`}, {"CURE", 365, 9.972801724464672`},
{"TBUR", 50.62787921462865`, 4.988802369223715`}, {"TBUR", 75.04822616197343`, 15.069211677383082`},
{"CURE", 365, 31.14970240971213`}, {"CURE", 365, 9.867660376274555`}, {"CURE", 365, 1.6860480616582907`},
{"CURE", 365, 1.6594963973698853`}, {"CURE", 365, 3.8990418355010292`}, {"CURE", 365, 1.177083732020691`},
{"CURE", 365, 13.371464477557117`}, {"CURE", 365, 2.0696891510389546`}, {"CURE", 365, 13.802304750515917`},
{"CURE", 365, 8.556692521374375`}, {"CURE", 365, 14.778800504484195`}, {"CURE", 365, 3.6304969433419667`},
{"CURE", 365, 18.32501826246433`}, {"CURE", 365, 60.6079966071353`}, {"CURE", 365, 21.875306199170634`},
{"CURE", 365, 35.39524877174882`}, {"CURE", 365, 10.445344602831396`}, {"CURE", 365, 24.89086841752309`},
{"CURE", 365, 24.914000576640248`}, {"CURE", 365, 3.027159304287652`}, {"CURE", 365, 2.1156658278827565`},
{"CURE", 365, 6.853847344014406`}, {"CURE", 365, 14.286255161125894`}, {"CURE", 365, 17.87310362492565`},
{"CURE", 365, 7.461505846338846`}, {"TBUR", 99.8108887984167`, 11.167435468461155`}, {"CURE", 365, 2.124230424051289`},
{"TBUR", 116.99963350255395`, 2.659930193641163`}, {"CURE", 365, 13.117052643585412`},
{"TBUR", 126.32373735841398`, 4.160781644535787`}, {"CURE", 365, 1.8070315103888062`}, {"CURE", 365, 2.677533991625318`},

{"CURE", 365, 1.3079764858758627`}, {"CURE", 365, 10.633167693014931`}, {"CURE", 365, 14.630006065105201`},
{"CURE", 365, 10.144107723404652`}, {"CURE", 365, 11.626924591779732`}, {"CURE", 365, 6.40070011236105`},
{"CURE", 365, 5.667167563516105`}, {"CURE", 365, 14.817270992795349`}, {"CURE", 365, 15.963397032478646`},
{"CURE", 365, 1.777094960535633`}, {"CURE", 365, 6.295659913520177`}, {"CURE", 365, 3.5165552715242643`},
{"CURE", 365, 5.558623839839809`}, {"CURE", 365, 45.68095266035558`}, {"CURE", 365, 8.069358885481419`},
{"CURE", 365, 5.546358835649372`}, {"CURE", 365, 5.804522823895884`}, {"CURE", 365, 11.683763315503974`},
{"CURE", 365, 8.894333476668294`}, {"CURE", 365, 11.614471373997196`}, {"TBUR", 20.10707389663781`, 11.11204038137411`},
{"CURE", 365, 2.7145782082780165`}, {"CURE", 365, 26.43970421920537`}, {"CURE", 365, 5.030713303886767`},
{"CURE", 365, 10.763705068009292`}, {"CURE", 365, 6.083930839645553`}, {"CURE", 365, 3.469871817462966`},
{"CURE", 365, 12.646398969101014`}, {"CURE", 365, 12.844432560240042`}, {"TBUR", 30.142104109173864`, 6.622477357046579`},
{"CURE", 365, 16.744573435853734`}, {"CURE", 365, 3.5428587875377553`}, {"CURE", 365, 29.783374807387684`},
{"CURE", 365, 11.363306265834783`}, {"CURE", 365, 23.83309357488771`}, {"CURE", 365, 0.9364839950799727`},
{"TBUR", 131.69995636380804`, 11.66648427463741`}, {"TBUR", 49.77724314950829`, 13.053376563635586`},
{"CURE", 365, 3.220542939728663`}, {"CURE", 365, 8.1383254728857`}, {"CURE", 365, 12.24205932326135`},
{"CURE", 365, 7.431134406581337`}, {"CURE", 365, 2.361981828406251`}, {"CURE", 365, 1.7960449104179288`},
{"TBUR", 127.1549679531507`, 10.506952988260924`}, {"CURE", 365, 2.8250241830680185`},
{"CURE", 365, 6.295392156359409`}, {"CURE", 365, 7.644825945396809`}, {"TBUR", 134.67156360950605`, 3.5082771187964523`},
{"TBUR", 96.9959749407013`, 6.396479127293212`}, {"CURE", 365, 1.7817361786284447`}, {"CURE", 365, 7.354076224134236`},
{"CURE", 365, 1.1909965276287402`}, {"CURE", 365, 2.1202943378675734`}, {"CURE", 365, 5.03413606591065`},
{"CURE", 365, 1.6102038493504818`}, {"CURE", 365, 11.985104841327102`}, {"CURE", 365, 17.778190051530537`},
{"CURE", 365, 17.837246937035275`}, {"CURE", 365, 15.015173430456638`}, {"CURE", 365, 28.28651794564269`},
{"CURE", 365, 2.2803019684382044`}, {"CURE", 365, 10.195163037033051`}, {"CURE", 365, 10.929238931474325`},
{"CURE", 365, 18.88449901356387`}, {"CURE", 365, 14.106364310896755`}, {"CURE", 365, 6.403522984584057`},
{"TBUR", 104.60680236853628`, 29.931920500995364`}, {"CURE", 365, 0.9421331412128543`}, {"CURE", 365, 28.878446476110096`},
{"CURE", 365, 1.9446158566066674`}, {"CURE", 365, 24.64768638048082`}, {"CURE", 365, 22.336312827739068`},
{"CURE", 365, 6.6023339451808845`}, {"CURE", 365, 25.94284852594555`}, {"CURE", 365, 31.77777622622837`},
{"CURE", 365, 14.143911335554549`}, {"CURE", 365, 6.273568559621009`}, {"CURE", 365, 7.212867235082841`},
{"CURE", 365, 10.619208960352431`}, {"TBUR", 48.7582516112508`, 9.768896350439844`}, {"CURE", 365, 2.3760081572405585`},
{"CURE", 365, 8.503495299262525`}, {"CURE", 365, 8.048337528168352`}, {"CURE", 365, 6.015700613274827`},
{"CURE", 365, 14.934222870978715`}, {"CURE", 365, 2.3507591818187232`}, {"CURE", 365, 16.65931155956648`},
{"CURE", 365, 10.873118152309601`}, {"CURE", 365, 6.174621147366489`}, {"CURE", 365, 5.501328752947062`},

{"TBUR", 106.58656920857463`, 11.135083645860709`}, {"CURE", 365, 1.560770422736008`}, {"CURE", 365, 18.065579802022683`},
{"CURE", 365, 2.835491791070619`}, {"CURE", 365, 4.043276190893801`}, {"TBUR", 53.40930963036612`, 9.027952685212682`},
{"CURE", 365, 7.095188344816248`}, {"CURE", 365, 6.531989765255555`}, {"CURE", 365, 20.16179110218173 8`},
{"CURE", 365, 2.746892785481342`}, {"CURE", 365, 4.79901830018591`}, {"CURE", 365, 35.47960099433656`},
{"CURE", 365, 2.687294839415666`}, {"CURE", 365, 6.944202582871782`}, {"TBUR", 148.9929975248523`, 10.460587686615728`},
{"TBUR", 94.74057436133957`, 10.330488064633439`}, {"CURE", 365, 7.067816545727889`}, {"CURE", 365, 36.638451715006326`},
{"TBUR", 96.53204267834084`, 6.552524035237083`}, {"TBUR", 49.3303156986524`, 2.7269074019666095`},
{"TBUR", 56.51578019548538`, 5.414303580336812`}, {"CURE", 365, 4.286751652669459`},
{"CURE", 365, 3.259781446074402`}, {"CURE", 365, 8.595878750796755`}, {"CURE", 365, 14.74210630901981`},
{"CURE", 365, 8.112159364327724`}, {"CURE", 365, 9.439851211682269`}, {"CURE", 365, 20.73326422581715`},
{"TBUR", 80.6205352090749`, 2.2621902703975247`}, {"TBUR", 74.84816677336987`, 2.660388574422594`},
{"CURE", 365, 22.68887690517388`}, {"TBUR", 36.3669814971452`, 7.17536682117124`},
{"CURE", 365, 7.9183114294591315`}, {"CURE", 365, 3.9775294308563307`}, {"CURE", 365, 9.113047154174142`},
{"CURE", 365, 11.355669489348855`}, {"CURE", 365, 26.23826729286757`}, {"CURE", 365, 3.8764533128159147`},
{"TBUR", 69.1975623388319`, 14.871797056203793`}, {"TBUR", 106.83947041104712`, 8.832172372767754`},
{"CURE", 365, 23.31700527251023`}, {"CURE", 365, 38.5207888744059`}, {"CURE", 365, 14.041082280190098`},
{"CURE", 365, 1.3582779399352236`}, {"CURE", 365, 10.683206705170814`}, {"TBUR", 53.60519872022429`, 3.3098803799249357`},
{"CURE", 365, 35.74166897206809`}, {"TBUR", 17.17714296126158`, 0.8466086573422319`}, {"CURE", 365, 1.1916317764856454`},
{"TBUR", 67.48544116156675`, 40.65366793050991`}, {"TBUR", 144.51849140699832`, 9.241107342060147`},
{"CURE", 365, 3.216229678261189`}, {"CURE", 365, 6.157456315602272`}, {"CURE", 365, 2.157851000038876`},
{"TBUR", 30.4542313355995`, 1.8671831406427077`}, {"CURE", 365, 2.4575276568180233`}, {"CURE", 365, 8.877369968751129`},
{"CURE", 365, 18.808990956032055`}, {"CURE", 365, 7.449047304424704`}, {"CURE", 365, 1.8571174887996533`},
{"CURE", 365, 25.38495813105903`}, {"CURE", 365, 50.52165095546137`}, {"CURE", 365, 1.4288885267596327`},
{"TBUR", 33.89368349758226`, 11.284502379201482`}, {"CURE", 365, 6.141547161953724`}, {"CURE", 365, 1.2085075318912044`},
{"CURE", 365, 9.410418788180644`}, {"CURE", 365, 6.037356989527773`}, {"CURE", 365, 4.6179450457302265`},
{"CURE", 365, 9.00634241774012`}, {"CURE", 365, 5.111968190941322`}, {"TBUR", 119.91623540541968`, 14.735650709400682`},
{"CURE", 365, 8.92567357109658`}, {"CURE", 365, 20.977417975241444`}, {"CURE", 365, 9.315519112835373`},
{"CURE", 365, 4.6111195527534`}, {"CURE", 365, 2.229872914063794`}, {"CURE", 365, 1.5585883887128755`},
{"CURE", 365, 4.441589024241569`}, {"CURE", 365, 17.653220980514067`}, {"CURE", 365, 5.820815288139443`},
{"CURE", 365, 5.292362732800186`}, {"CURE", 365, 3.7580100802459038`}, {"CURE", 365, 3.8146757076031426`},
{"CURE", 365, 10.305432289068312`}, {"CURE", 365, 1.9319296706698985`}, {"CURE", 365, 12.248098796797139`},

{"CURE", 365, 5.641189615841167`}, {"CURE", 365, 3.2980967411923268`}, {"CURE", 365, 5.220554039245501`},
{"CURE", 365, 11.656291259978063`}, {"CURE", 365, 8.583154616427597`}, {"CURE", 365, 1.047604987539647`},
{"CURE", 365, 4.473870696308807`}, {"CURE", 365, 75.40130855349732`}, {"CURE", 365, 12.48460411956053 8`},
{"CURE", 365, 18.17296735878511`}, {"CURE", 365, 5.378713622730304`}, {"CURE", 365, 19.84243222272627`},
{"CURE", 365, 2.3843427139537834`}, {"TBUR", 51.540807109160895`, 7.88612860298262`}, {"CURE", 365, 37.1494568081619`},
{"CURE", 365, 6.962676350229659`}, {"CURE", 365, 6.067655403226667`}, {"CURE", 365, 10.509373689962647`},
{"CURE", 365, 5.514671753259366`}, {"CURE", 365, 1.231280304808679`}, {"TBUR", 32.291352961529476`, 11.294859645033602`},
{"CURE", 365, 2.038249149027457`}, {"CURE", 365, 4.8337913080415325`}, {"CURE", 365, 11.476923456773463`},
{"CURE", 365, 4.1200828303323815`}, {"CURE", 365, 21.909260152827315`}, {"TBUR", 65.91456910625942`, 6.457393998784135`},
{"CURE", 365, 11.220088955751763`}, {"CURE", 365, 7.772506192691187`}, {"CURE", 365, 2.2014052331405165`},
{"CURE", 365, 8.07538984656962`}, {"CURE", 365, 17.642455638962147`}, {"CURE", 365, 5.901923798999419`},
{"CURE", 365, 4.449263487058253`}, {"CURE", 365, 2.1017715580672554`}, {"CURE", 365, 6.560077081073278`},
{"TBUR", 67.45730691697118`, 7.882947234605363`}, {"CURE", 365, 22.92208902368858`}, {"CURE", 365, 3.683689084590114`},
{"TBUR", 49.032195922303146`, 5.419564002623115`}, {"CURE", 365, 21.46810428272594`}, {"CURE", 365, 2.2649529842831404`},
{"TBUR", 43.27390277072805`, 30.57201969543038`}, {"CURE", 365, 2.6286752823046697`}, {"CURE", 365, 4.801561836815404`},
{"CURE", 365, 11.050473297340611`}, {"CURE", 365, 17.765067161008798`}, {"CURE", 365, 7.998832325152311`},
{"TBUR", 112.75559849962046`, 4.123653259597287`}, {"TBUR", 36.638570588832785`, 1.1640147473078952`},
{"TBUR", 15.388973498079778`, 9.910694667938738`}, {"TBUR", 178.04450753707553`, 9.675519935715565`},
{"CURE", 365, 9.641009947286978`}, {"CURE", 365, 3.808131746198342`}, {"CURE", 365, 9.33072459945241`},
{"TBUR", 32.94994021122148`, 0.9853186544894372`}, {"CURE", 365, 6.2137518375434455`}, {"CURE", 365, 2.713823956469185`},
{"TBUR", 90.36737551506155`, 1.1929036011303034`}, {"CURE", 365, 6.234909428659416`}, {"CURE", 365, 1.3397540720424257`},
{"CURE", 365, 4.260427755171511`}, {"CURE", 365, 2.9110086395045838`}, {"TBUR", 15.907170028239435`, 1.1472422854149447`},
{"CURE", 365, 5.761954340134697`}, {"CURE", 365, 15.422648789497028`}, {"CURE", 365, 8.392525751481458`},
{"CURE", 365, 7.627868259150544`}, {"TBUR", 110.5347533414981`, 11.796478976906`}, {"CURE", 365, 10.293433978172795`}},
{{"1780 Res", "1780 OS", "1780 Tox"}, {"TBUR", 22.41279465469096`, 67.79705363801799`}, {"CURE", 365, 224.32553046762246`},
{"TBUR", 27.842850637874715`, 174.29691765219852`}, {"TBUR", 58.779722248352215`, 50.50060813273945`},
{"TBUR", 19.268957811006228`, 40.17455834019597`}, {"CURE", 365, 66.0816391891566`}, {"CURE", 365, 96.97882405580108`},
{"CURE", 365, 97.82750936907597`}, {"CURE", 365, 214.0676858736456`}, {"TOX", 13.300876878665454`, 273.3532624994494`},
{"TBUR", 55.36623672569727`, 138.88015292007373`}, {"CURE", 365, 139.01697685194682`},
{"TOX", 8.922065924563764`, 355.1017283469213`}, {"TBUR", 44.49336844957738`, 97.6899308347876`},
{"TBUR", 89.63264957239915`, 9.097797975131197`}, {"TBUR", 91.31398908309396`, 66.30828638225307`},

{"CURE", 365, 140.28549314553902`}, {"TBUR", 46.39572100493293`, 175.06951500770003`},
{"TBUR", 73.45798740515419`, 36.836845056618`}, {"CURE", 365, 139.92593370036485`}, {"CURE", 365, 190.48096053501015`},
{"CURE", 365, 101.41031021075204`}, {"CURE", 365, 63.81401933698933`}, {"CURE", 365, 110.2558770296456 2`},
{"CURE", 365, 111.1290669511289`}, {"TOX", 22.432413324381145`, 242.3362840770888`}, {"CURE", 365, 189.4427765923874`},
{"CURE", 365, 89.88346199664356`}, {"CURE", 365, 68.49175325687257`}, {"CURE", 365, 49.77982986549906`},
{"CURE", 365, 215.0097259393117`}, {"TBUR", 13.617953998377716`, 56.55637086077508`}, {"CURE", 365, 108.85744813618068`},
{"TBUR", 51.87281866441041`, 72.93010998676534`}, {"TOX", 15.38184789502424`, 268.4412809924869`},
{"TOX", 11.840798041422016`, 303.561120020797`}, {"CURE", 365, 74.02829463150753`},
{"TBUR", 23.826271015975905`, 19.979311435942275`}, {"TBUR", 72.9330429625902`, 87.67056245466391`},
{"CURE", 365, 130.00537693920913`}, {"TBUR", 18.32786556020784`, 97.13795028822048`}, {"CURE", 365, 159.14930368712896`},
{"CURE", 365, 110.29143442509108`}, {"TBUR", 46.18160291626078`, 110.45899680874248`},
{"CURE", 365, 134.3658038816796`}, {"CURE", 365, 10.874627447998735`}, {"CURE", 365, 151.679211449738`},
{"CURE", 365, 101.05588489830922`}, {"TBUR", 114.03461521001024`, 176.15624728245945`},
{"TOX", 10.871594512323352`, 311.06130471176266`}, {"TBUR", 29.973031285631784`, 137.29562045952252`},
{"TBUR", 42.15703038067866`, 116.59876423950799`}, {"CURE", 365, 8.964745233600599`}, {"CURE", 365, 192.5026721685216`},
{"CURE", 365, 94.87988351263151`}, {"CURE", 365, 135.35815291029473`}, {"CURE", 365, 119.91859483798163`},
{"TBUR", 103.97397436414188`, 168.50490923164762`}, {"TOX", 15.639471158968218`, 239.75281601102913`},
{"TBUR", 53.78828566513054`, 137.60664178173664`}, {"CURE", 365, 126.60925620861292`},
{"CURE", 365, 25.26204518349607`}, {"TBUR", 33.75409158193514`, 158.62945752486644`}, {"CURE", 365, 143.96842902893712`},
{"CURE", 365, 55.04906292870267`}, {"CURE", 365, 125.80556782872313`}, {"CURE", 365, 124.41312776281269`},
{"TBUR", 57.790104797537886`, 215.17810161329402`}, {"CURE", 365, 78.34437926773707`}, {"CURE", 365, 68.62864647929746`},
{"CURE", 365, 118.65996470204473`}, {"CURE", 365, 91.51172394856242`}, {"CURE", 365, 205.08790507181646`},
{"TBUR", 14.28925466494035`, 5.696313773760521`}, {"TBUR", 80.31103368113685`, 139.65595093497427`},
{"CURE", 365, 191.0494558961641`}, {"TOX", 11.36756960111747`, 301.95760079575473`}, {"CURE", 365, 103.50922249356722`},
{"TBUR", 76.16471588732247`, 192.9469079608623`}, {"CURE", 365, 103.96310463131853`}, {"CURE", 365, 161.39131475749403`},
{"CURE", 365, 44.428125017041936`}, {"CURE", 365, 26.228967977231463`}, {"CURE", 365, 78.94186680497334`},
{"CURE", 365, 185.10653524773795`}, {"TBUR", 100.4393233483168`, 141.58724533706257`},
{"CURE", 365, 187.6212377689056`}, {"CURE", 365, 87.24405352073735`}, {"CURE", 365, 46.93531748920092`},
{"TOX", 25.314230950285747`, 240.35289934782026`}, {"TBUR", 36.684531561612964`, 40.18542209001306`},
{"TBUR", 89.81675903639893`, 156.08961212398123`}, {"CURE", 365, 4.0670259301933465`}, {"CURE", 365, 10.094562518822888`},
{"TBUR", 25.06078165360202`, 7.545676009191794`}, {"TBUR", 160.50503646293805`, 92.29788723044221`},

{"CURE", 365, 79.67106967656132`}, {"TOX", 13.133050860829739`, 274.033917841545`}, {"CURE", 365, 35.247335175385984`},
{"TOX", 18.09904071856587`, 243.76322674058002`}, {"TBUR", 25.777425165184905`, 27.71004895098603`},
{"TBUR", 127.70652386713452`, 211.32747749254622`}, {"CURE", 365, 115.58750725352384`},
{"TBUR", 92.82980320186519`, 133.4192767694357`}, {"CURE", 365, 107.69412031526285`}, {"CURE", 365, 176.9074692662527`},
{"CURE", 365, 77.88545128807736`}, {"TBUR", 143.00675212800786`, 127.35122600228303`},
{"CURE", 365, 75.52985956873908`}, {"CURE", 365, 168.70863384261025`}, {"CURE", 365, 224.71325446656414`},
{"CURE", 365, 121.20559928440498`}, {"CURE", 365, 57.99334044740452`}, {"CURE", 365, 94.42961253878228`},
{"CURE", 365, 106.90042978339211`}, {"CURE", 365, 97.91698014614425`}, {"CURE", 365, 78.31532757835207`},
{"CURE", 365, 132.55312432676217`}, {"CURE", 365, 169.3909593025718`}, {"CURE", 365, 60.84446973915097`},
{"CURE", 365, 124.86260589034114`}, {"TOX", 16.920228323754678`, 250.77934973978785`},
{"CURE", 365, 170.61369455549`}, {"TBUR", 23.480155311695192`, 12.699601894068056`},
{"CURE", 365, 112.3226409235953`}, {"TBUR", 39.897173937148175`, 125.44565784862492`},
{"CURE", 365, 135.87363380911498`}, {"CURE", 365, 79.30122841487463`}, {"CURE", 365, 191.57364647753886`},
{"CURE", 365, 182.07011711350572`}, {"CURE", 365, 154.34163379126974`}, {"CURE", 365, 167.33300499867067`},
{"TBUR", 72.50139935335282`, 83.62450900675054`}, {"TBUR", 47.423426400059796`, 94.78712288055652`},
{"TOX", 18.40656453592927`, 243.33274899967205`}, {"TBUR", 12.81806417661775`, 111.1673640561977`},
{"CURE", 365, 88.54012547938126`}, {"TBUR", 63.47495043586343`, 102.29705395643573`}, {"CURE", 365, 26.908084166097606`},
{"CURE", 365, 162.86654275763777`}, {"TOX", 18.00342887345899`, 240.87860111108267`}, {"CURE", 365, 179.25020980961887`},
{"TOX", 20.2865400417404`, 253.7030418289991`}, {"CURE", 365, 121.4348149567253`}, {"CURE", 365, 87.15674114530209`},
{"CURE", 365, 119.14033396740237`}, {"TOX", 17.571684289593538`, 249.35262253940738`},
{"TBUR", 40.6813544803152`, 78.33303075386297`}, {"CURE", 365, 119.98958710423656`}, {"CURE", 365, 52.11066976999106`},
{"CURE", 365, 73.36203134828541`}, {"CURE", 365, 123.18470358178072`}, {"CURE", 365, 40.19238090651988`},
{"TOX", 16.32207976033172`, 249.67275167175688`}, {"TBUR", 51.09266154562729`, 132.70017503146303`},
{"TOX", 26.99710882583056`, 234.33358296458997`}, {"CURE", 365, 131.52857747506968`}, {"CURE", 365, 142.75369404807506`},
{"CURE", 365, 107.3662324363143`}, {"CURE", 365, 141.5154059228703`}, {"CURE", 365, 76.19269343202633`},
{"CURE", 365, 32.61220507063635`}, {"CURE", 365, 2.4756002390965874`}, {"TBUR", 72.64919992694051`, 50.930824021018005`},
{"TBUR", 79.53983926568264`, 135.7463064557912`}, {"CURE", 365, 164.11531868937558`}, {"CURE", 365, 168.90728533080238`},
{"CURE", 365, 131.63613067361155`}, {"CURE", 365, 169.03290747764592`}, {"CURE", 365, 68.7882264956313`},
{"CURE", 365, 106.0071106292996`}, {"TBUR", 30.31003299504092`, 193.6160561481181`}, {"CURE", 365, 96.72834545634433`},
{"TBUR", 24.267233726692638`, 15.584724201699817`}, {"TBUR", 50.667891301453515`, 190.78337696530767`},
{"TBUR", 142.82454587691845`, 22.905471585659022`}, {"TOX", 20.011050344243042`, 249.38738745536625`},

{"TBUR", 21.422217300989477`, 116.3504425124137`}, {"TBUR", 63.6960820423708`, 93.24161574930096`},
{"TBUR", 91.96918190514613`, 142.65177522083133`}, {"TBUR", 81.08247758989083`, 207.22643004925973`},
{"CURE", 365, 122.13182253609243`}, {"CURE", 365, 216.10838976493048`}, {"TBUR", 35.48812581986378`, 59.53472081550466`},
{"CURE", 365, 188.9715083974559`}, {"TBUR", 28.56461467843985`, 74.02912590048471`},
{"TBUR", 53.86744597367437`, 206.37052835524838`}, {"CURE", 365, 127.40966159582979`}, {"CURE", 365, 29.7561846505839`},
{"TBUR", 25.025043277754474`, 140.53241043372137`}, {"CURE", 365, 138.96007471893446`},
{"CURE", 365, 201.42966230976373`}, {"TOX", 17.360578713474307`, 251.84853219028517`}, {"CURE", 365, 167.65390410208326`},
{"CURE", 365, 107.36908930022129`}, {"CURE", 365, 84.50937513576152`}, {"TOX", 15.441433679203481`, 261.3012474348742`},
{"TBUR", 14.44942952139253`, 32.00442018222035`}, {"CURE", 365, 144.20869327080754`}, {"CURE", 365, 147.17124679155398`},
{"TBUR", 40.51466464037646`, 33.717887675792554`}, {"TBUR", 57.62822690300832`, 144.51190881159846`},
{"CURE", 365, 183.9316731821572`}, {"TBUR", 65.40497759047275`, 113.53245822600529`}, {"CURE", 365, 11.575856507425392`},
{"TBUR", 144.10521744634227`, 117.12073308409293`}, {"TBUR", 16.174928206067772`, 135.13609626480877`},
{"TOX", 15.70211670585052`, 271.3957885293403`}, {"CURE", 365, 60.427500678096095`}, {"CURE", 365, 187.93069681415716`},
{"CURE", 365, 15.947173580084312`}, {"CURE", 365, 127.67821540979098`}, {"CURE", 365, 55.26537972834336`},
{"CURE", 365, 155.97621111314314`}, {"CURE", 365, 63.349473938444554`}, {"CURE", 365, 69.25387833821343`},
{"TBUR", 80.48709102536881`, 224.87092518845722`}, {"CURE", 365, 109.59401411963182`},
{"TOX", 10.29534507905134`, 289.35902257088446`}, {"CURE", 365, 106.37531123114084`},
{"CURE", 365, 154.7038784854651`}, {"CURE", 365, 52.825084431186006`}, {"CURE", 365, 110.74332633996003`},
{"CURE", 365, 201.7709518389068`}, {"CURE", 365, 201.65605116735568`}, {"CURE", 365, 102.69695510739494`},
{"CURE", 365, 184.49358601855985`}, {"CURE", 365, 123.44403790802482`}, {"CURE", 365, 42.788380421802806`},
{"CURE", 365, 93.8735209421059`}, {"CURE", 365, 97.48729748283107`}, {"CURE", 365, 104.98120541940854`},
{"CURE", 365, 151.42982592786493`}, {"CURE", 365, 124.59080930279363`}, {"CURE", 365, 120.9054537377653`},
{"TBUR", 23.581927107899276`, 157.63757479843753`}, {"TBUR", 28.6086362171242`, 102.85637669324377`},
{"TBUR", 157.44608278982886`, 118.77611103476261`}, {"TBUR", 25.61699499889744`, 88.83014422087986`},
{"CURE", 365, 94.20728242428693`}, {"CURE", 365, 112.98800539226768`}, {"CURE", 365, 184.00752386381706`},
{"TBUR", 30.99513618800816`, 8.785494092282422`}, {"TBUR", 34.134464830440486`, 60.77944091281233`},
{"CURE", 365, 53.23014279783072`}, {"CURE", 365, 3.456837816564479`}, {"TBUR", 31.968654163410445`, 32.03938378942114`},
{"TOX", 12.089467746438128`, 285.49069655369914`}, {"CURE", 365, 110.63156834285637`},
{"TOX", 12.624324844187218`, 283.38106004801523`}, {"TBUR", 39.959508485870536`, 160.6251459997111`},
{"CURE", 365, 142.87205531314234`}, {"TBUR", 15.701875680596677`, 178.42273226261287`}, {"CURE", 365, 93.50680454749657`},
{"CURE", 365, 145.6958057537231`}, {"CURE", 365, 50.142671648217004`}, {"CURE", 365, 113.15135771111736`},

{"CURE", 365, 67.21172179205234`}, {"CURE", 365, 43.39358695622453`}, {"CURE", 365, 188.68884686930406`},
{"TBUR", 22.3458358013067`, 175.7823767686753`}, {"TBUR", 14.076305745953185`, 128.1232345432547`},
{"CURE", 365, 96.24351381780839`}, {"TBUR", 28.18525488084027`, 116.07264072505406`},
{"CURE", 365, 51.6712808622439`}, {"CURE", 365, 107.1551417608636 1`}, {"CURE", 365, 121.37377421648472`},
{"TOX", 11.98810174310367`, 302.03607193210405`}, {"CURE", 365, 170.59354835948653`}, {"CURE", 365, 79.65711895061996`},
{"CURE", 365, 6.439151142823416`}, {"TBUR", 70.7949590605972`, 143.72697478384435`}, {"CURE", 365, 116.46997461774345`},
{"TBUR", 152.07006842746114`, 119.3047293916183`}, {"TBUR", 60.85787575131362`, 92.25639160128331`},
{"CURE", 365, 143.44380508625352`}, {"CURE", 365, 93.34004556781852`}, {"TBUR", 21.22370852065267`, 109.55591071704856`},
{"CURE", 365, 89.21452694151121`}, {"CURE", 365, 165.72942448298014`}, {"CURE", 365, 73.09652266265978`},
{"TBUR", 98.2002999751149`, 139.47973576379053`}, {"TBUR", 16.813764619948056`, 62.831396061731326`},
{"CURE", 365, 113.4331139774387`}, {"TBUR", 28.622493138411155`, 160.56896786601007`}, {"CURE", 365, 42.19133475382514`},
{"CURE", 365, 172.50302132260038`}, {"CURE", 365, 104.34798855094347`}, {"TOX", 13.63799130876277`, 291.8590132183014`},
{"CURE", 365, 186.0668483091596`}, {"CURE", 365, 135.42646723150312`}, {"CURE", 365, 99.267536752318`},
{"TOX", 12.479377552428666`, 274.96339280217074`}, {"TBUR", 34.114227004723745`, 62.037059849403676`},
{"CURE", 365, 69.45537985516728`}, {"CURE", 365, 97.46153725056324`}, {"CURE", 365, 174.5481955204585`},
{"CURE", 365, 172.42642237406707`}, {"CURE", 365, 156.52982966504226`}, {"CURE", 365, 91.15720035692895`},
{"TBUR", 23.6056521348697 1`, 204.57840789850127`}, {"CURE", 365, 64.95685167264355`}, {"CURE", 365, 193.94812277101315`},
{"TBUR", 39.72791424776915`, 217.55978216212452`}, {"CURE", 365, 150.68741473377713`}, {"CURE", 365, 168.70442124242544`},
{"CURE", 365, 5.479650980547399`}, {"TBUR", 23.91472806343157`, 87.4869442986277`}, {"CURE", 365, 127.3897583039924`},
{"CURE", 365, 133.90891435815578`}, {"TBUR", 30.984429965304486`, 59.62379734540504`}, {"CURE", 365, 115.60114687864184`},
{"TBUR", 40.769781700430215`, 64.72695600221073`}, {"CURE", 365, 146.09008294163377`}, {"CURE", 365, 134.31118891708118`},
{"CURE", 365, 127.7269230160377`}, {"CURE", 365, 116.27557657154706`}, {"CURE", 365, 108.92999108108128`},
{"CURE", 365, 146.23966529107742`}, {"CURE", 365, 221.1777985629759`}, {"TBUR", 49.51342812116183`, 137.64095689046871`},
{"CURE", 365, 200.98393955490874`}, {"CURE", 365, 120.27023479493495`}, {"CURE", 365, 52.90214344447161`},
{"TBUR", 18.73380523178496`, 37.720780227360585`}, {"CURE", 365, 46.03748956439162`}, {"CURE", 365, 172.6446148781123`},
{"CURE", 365, 194.83091620817478`}, {"CURE", 365, 128.25011177803952`}, {"TOX", 11.746823473283454`, 278.7090570646688`},
{"CURE", 365, 228.06109558051048`}, {"CURE", 365, 101.90930010861872`}, {"TBUR", 20.945371932524385`, 98.4558083475195`},
{"CURE", 365, 92.82684397340766`}, {"TOX", 12.892168785874413`, 270.40636364988234`},
{"TOX", 10.545178625457005`, 282.8650932619547`}, {"TBUR", 29.92074598268531`, 81.93943856731644`},
{"TOX", 13.964696252886663`, 277.64476103540585`}, {"CURE", 365, 70.5839122649999`}, {"CURE", 365, 133.85624696385383`},
{"CURE", 365, 215.61090066274127`}, {"CURE", 365, 7.317488874625764`}, {"TBUR", 55.70022433270279`, 74.58072095727809`},

{"CURE", 365, 174.0337106623718`}, {"CURE", 365, 39.290800407815595`}, {"TOX", 9.873933183077277`, 285.4372026142217`},
{"CURE", 365, 37.73853795275463`}, {"CURE", 365, 229.88486186921494`}, {"CURE", 365, 136.95918806439946`},
{"CURE", 365, 128.55254437919282`}, {"CURE", 365, 158.75707325456872`}, {"CURE", 365, 82.23357898063651`},
{"TBUR", 36.17673823974719`, 202.77324063281262`}, {"CURE", 365, 139.46430102543707`},
{"CURE", 365, 221.99177695542676`}, {"CURE", 365, 150.34260882456687`}, {"TBUR", 54.89345193953715`, 148.4614248334945`},
{"CURE", 365, 169.08745102890782`}, {"CURE", 365, 144.02231698310027`}, {"CURE", 365, 85.43764084846352`},
{"CURE", 365, 191.74757731540294`}, {"TOX", 18.35449982966913`, 250.82973099277098`}, {"CURE", 365, 81.89088856840836`},
{"TBUR", 65.03725322767554`, 165.09050720254717`}, {"TBUR", 46.445089595507255`, 196.53540010853862`},
{"CURE", 365, 51.179413780072565`}, {"CURE", 365, 144.0120362169998`}, {"CURE", 365, 87.84645057584633`},
{"TBUR", 15.789030802682605`, 22.336625630844072`}, {"TBUR", 13.98082914141123`, 52.27354712034864`},
{"TBUR", 47.69137235552186`, 68.397212084314`}, {"CURE", 365, 139.1671111053035`}, {"CURE", 365, 70.73778325267591`},
{"CURE", 365, 138.1099791276841`}, {"TOX", 22.616319453351167`, 240.37751574303567`}, {"CURE", 365, 135.27697631931153`},
{"CURE", 365, 112.34704083102203`}, {"CURE", 365, 153.38133717117503`}, {"TBUR", 18.121482048371828`, 67.98998165460199`},
{"TBUR", 13.941378235072467`, 125.32277617376772`}, {"CURE", 365, 82.84841871028607`},
{"CURE", 365, 198.7016471248684`}, {"TBUR", 51.42052301738066`, 129.75516260346208`}, {"CURE", 365, 180.23311713243967`},
{"CURE", 365, 120.96003628621176`}, {"CURE", 365, 117.15642471885728`}, {"TBUR", 102.83445272333907`, 77.4355203048517`},
{"CURE", 365, 85.58324260913719`}, {"TOX", 12.601572159044412`, 288.79815013291875`}, {"CURE", 365, 152.8487501890644`},
{"CURE", 365, 116.95697372484577`}, {"TBUR", 33.55984755490981`, 112.24531260174251`},
{"TBUR", 77.67345826908073`, 110.26339077993477`}, {"CURE", 365, 89.29444688082039`},
{"TBUR", 16.13581102520882`, 94.80533643921694`}, {"TBUR", 17.164871535209073`, 86.16923476819146`},
{"CURE", 365, 110.44281080340134`}, {"CURE", 365, 123.47204940893609`}, {"CURE", 365, 43.44907123129842`},
{"TBUR", 12.995132038912812`, 83.62094618559982`}, {"TBUR", 22.387651056974594`, 37.746340218136766`},
{"CURE", 365, 204.76255885872126`}, {"CURE", 365, 78.60513133067128`}, {"CURE", 365, 147.0302227805243`},
{"CURE", 365, 21.515064942898807`}, {"CURE", 365, 137.43856285978578`}, {"CURE", 365, 5.828375400366879`},
{"CURE", 365, 153.37866091297153`}, {"CURE", 365, 154.75932339069607`}, {"TBUR", 115.12831314215519`, 164.65106937379917`},
{"CURE", 365, 32.036950989327906`}, {"CURE", 365, 64.05917793258892`}, {"TOX", 13.524203872468757`, 272.3605825263528`},
{"TBUR", 25.90145895458383`, 74.3215020510553`}, {"CURE", 365, 24.322739732860086`}, {"CURE", 365, 69.83942742453203`},
{"CURE", 365, 187.4710180308734`}, {"CURE", 365, 66.23785700030196`}, {"CURE", 365, 173.30796733173375`},
{"TOX", 20.084878949701192`, 249.43999322082604`}, {"CURE", 365, 99.7163367750535`},
{"CURE", 365, 68.8311366023344`}, {"CURE", 365, 39.16660695978048`}, {"CURE", 365, 186.00492500965`},
{"TBUR", 13.85046222548245`, 33.30947183087658`}, {"TOX", 10.030778238624633`, 311.30935523990195`},

{"CURE", 365, 115.18114162266066`}, {"CURE", 365, 167.32118702444612`}, {"TBUR", 85.01143430318993`, 114.7068718339378`},
{"CURE", 365, 89.54499706271181`}, {"TOX", 10.27241073398416`, 312.42813893137406`}, {"CURE", 365, 128.98931933350875`},
{"TBUR", 145.56051303253784`, 5.308074023182152`}, {"CURE", 365, 135.38829762511457`}, {"CURE", 365, 62.82211236912057`},
{"TOX", 18.407506232559292`, 241.2152623666872`}, {"CURE", 365, 100.33856669479944`}, {"CURE", 365, 24.268589073588863`},
{"CURE", 365, 151.74906872946963`}, {"TBUR", 22.370283169029488`, 122.03047984593226`},
{"CURE", 365, 132.92525841097384`}, {"CURE", 365, 126.61969129710216`}, {"CURE", 365, 91.71057610336223`},
{"CURE", 365, 172.963032973639`}, {"CURE", 365, 70.1090109933447`}, {"TOX", 19.465519619196062`, 242.40422239577984`},
{"CURE", 365, 101.47962179204579`}, {"CURE", 365, 17.393942445887518`}, {"CURE", 365, 68.06200830928567`},
{"TOX", 12.367648867813031`, 289.6812907823246`}, {"CURE", 365, 119.18694484809002`}, {"CURE", 365, 207.90853963721125`},
{"CURE", 365, 154.49025693087634`}, {"TOX", 20.27618266290525`, 242.7501747814268`}, {"CURE", 365, 225.73176518869047`},
{"TBUR", 74.69967071553839`, 75.80793260916525`}, {"CURE", 365, 109.34053265719923`},
{"TBUR", 131.75972140187017`, 101.19484235015108`}, {"TBUR", 20.129206429040124`, 39.384260058306076`},
{"TBUR", 39.695586764641284`, 16.63489258200056`}, {"CURE", 365, 118.36144754852853`}, {"CURE", 365, 159.96864723043447`},
{"TBUR", 56.18476324873726`, 133.4271930683037`}, {"TOX", 29.865949530264537`, 234.32082050196783`},
{"CURE", 365, 212.17195042959474`}, {"TBUR", 19.852564237285154`, 38.45146937221198`},
{"TBUR", 26.907759309294924`, 66.2437819443897`}, {"CURE", 365, 62.475637032933825`},
{"TBUR", 27.054369895713336`, 182.3639137546674`}, {"TBUR", 44.470892014813124`, 157.59577606137012`},
{"CURE", 365, 177.0133966042965`}, {"TOX", 25.13229632331384`, 238.32656876398542`}, {"CURE", 365, 74.97469259706364`},
{"CURE", 365, 159.1024225307087`}, {"TBUR", 27.82346593147854`, 133.09297599908567`},
{"TBUR", 32.080416194838385`, 9.095236476003233`}, {"CURE", 365, 221.11985787926284`}, {"CURE", 365, 107.5293941947121`},
{"TBUR", 55.51633275698774`, 10.798593979450708`}, {"TBUR", 35.735132869193976`, 6.912036433444995`},
{"CURE", 365, 95.97266532893214`}, {"CURE", 365, 133.6451639647154`}, {"CURE", 365, 163.4577938763627`},
{"TBUR", 40.812933923905554`, 103.29590605838717`}, {"CURE", 365, 126.62848261534708`}, {"CURE", 365, 180.30070091623855`},
{"CURE", 365, 120.42384356995109`}, {"TBUR", 98.70817260177522`, 75.54839954986375`}, {"CURE", 365, 159.59757590519698`},
{"CURE", 365, 71.6142180773313`}, {"CURE", 365, 74.34047766336411`}, {"TBUR", 78.99017660390903`, 121.72684107189154`},
{"CURE", 365, 32.59667176970954`}, {"TBUR", 48.80921915541507`, 120.66778028009041`},
{"CURE", 365, 105.94407387785684`}, {"TBUR", 35.524509783362205`, 128.07503836480268`},
{"CURE", 365, 141.2468274524328`}, {"TBUR", 22.22203588735311`, 100.97815351060916`},
{"CURE", 365, 115.01163246466217`}, {"TBUR", 16.51081260645139`, 145.091174244369`}, {"CURE", 365, 121.04592504925606`},
{"TBUR", 16.91441073658512`, 45.588675259731`}, {"TBUR", 65.47480046671207`, 141.11004894691058`},
{"TOX", 21.212003996564572`, 248.750738480619`}, {"CURE", 365, 26.774218567025688`}, {"CURE", 365, 101.9229162982666`},

{"CURE", 365, 76.6045919062899`}, {"CURE", 365, 138.40727877445113`}, {"CURE", 365, 80.71402604211579`},
{"TBUR", 51.927951371267234`, 159.13874725250116`}, {"TBUR", 78.5952985464155`, 114.73229399394663`},
{"CURE", 365, 229.6675708045203`}, {"CURE", 365, 105.4477761895655`}, {"TOX", 13.952675566415683`, 266.92038620257756`},
{"CURE", 365, 182.89350712180604`}, {"CURE", 365, 178.03689343654824`}, {"CURE", 365, 174.79624282649942`},
{"TOX", 13.341259504527454`, 272.48224280745063`}, {"TOX", 10.441205327176283`, 303.0320655840821`},
{"CURE", 365, 98.43290740090744`}, {"CURE", 365, 153.25753430593375`}, {"CURE", 365, 167.96147651339137`},
{"TBUR", 41.00445887913375`, 68.96875129761675`}, {"TBUR", 18.125831906865105`, 21.26168454881142`},
{"CURE", 365, 89.86310852110202`}, {"CURE", 365, 130.95645515681034`}, {"CURE", 365, 178.5407849462983`},
{"TBUR", 20.948049983558995`, 157.14843408064542`}, {"CURE", 365, 205.6073995477535`},
{"TOX", 13.595574212415356`, 278.426939775561`}, {"CURE", 365, 94.39144340400068`}, {"CURE", 365, 196.62099925933651`},
{"TBUR", 78.44073592887449`, 84.63168493269117`}, {"TOX", 11.235841118082329`, 294.1685178555871`},
{"TBUR", 37.24153196128468`, 102.70868986962743`}, {"TBUR", 59.34766420276196`, 82.18593710127658`},
{"TBUR", 66.88801222389313`, 92.08548781867312`}, {"TOX", 12.705290928527276`, 281.6872832465607`},
{"CURE", 365, 120.72949055560707`}, {"TOX", 15.348897330445249`, 280.6008806220092`}, {"CURE", 365, 182.78463965323039`},
{"CURE", 365, 177.25481215042717`}, {"TBUR", 55.87198838575807`, 181.4743429537199`},
{"TBUR", 88.19778914790226`, 202.00042019435082`}, {"CURE", 365, 88.46647972329242`}, {"CURE", 365, 80.49296589973457`},
{"CURE", 365, 60.392804993163544`}, {"CURE", 365, 123.75699560322116`}, {"CURE", 365, 143.6684346301111`},
{"TBUR", 35.76907013740682`, 89.67942605263599`}, {"TBUR", 45.8094673922565`, 112.797600088664`},
{"CURE", 365, 204.43799507594713`}, {"TBUR", 43.10066975065773`, 103.22404138942481`}, {"CURE", 365, 109.7837050207541`},
{"CURE", 365, 92.75052672652185`}, {"TBUR", 23.224844275995274`, 31.72825843147534`}, {"CURE", 365, 85.06412455888697`},
{"CURE", 365, 211.02923091453883`}, {"CURE", 365, 114.03055403389136`}, {"TBUR", 43.86805314001531`, 92.64919881536925`},
{"CURE", 365, 52.94796653332929`}, {"TBUR", 26.48066856060099`, 209.20851708450462`}, {"CURE", 365, 214.3699061617775`},
{"CURE", 365, 130.36705640891083`}, {"TBUR", 102.11986351199137`, 92.88461017882346`},
{"CURE", 365, 193.19699675464307`}, {"CURE", 365, 33.81713354336671`}, {"TOX", 10.724803594816413`, 301.0583912823983`},
{"TBUR", 29.188249304885726`, 106.07016183589194`}, {"TOX", 19.15960559740068`, 256.9823742015277`},
{"TBUR", 43.7937651107952`, 112.4012073017152`}, {"CURE", 365, 77.87576571897038`},
{"TBUR", 42.7732846016037`, 92.3205132988347`}, {"TBUR", 29.46590339400415`, 42.003245462921`},
{"CURE", 365, 198.5952424861629`}, {"TBUR", 44.4130611510726`, 75.15369071841944`}, {"CURE", 365, 140.17561756302055`},
{"TBUR", 31.680063247249237`, 86.35770335293667`}, {"CURE", 365, 111.37841007776977`},
{"TBUR", 23.791762083146146`, 76.51547298285502`}, {"TBUR", 106.83146283882039`, 229.39207106115248`},
{"CURE", 365, 207.05285963006094`}, {"TBUR", 34.582983126919665`, 102.12521095346099`},

```
{"TBUR", 67.4446711421906`, 102.1793340368797`}, {"TBUR", 14.011232716615783`, 104.75560753350048`},
{"CURE", 365, 43.415262014290946`}, {"TBUR", 108.96536057954404`, 174.94165854628946`},
{"TBUR", 102.44855915355785`, 162.52246784750477`}, {"CURE", 365, 138.67558635503366`},
{"TBUR", 74.20589693274322`, 12.877958591044695`}, {"CURE", 365, 155.8832887808679`},
{"TOX", 15.138025899256387`, 277.91807293779226`}, {"CURE", 365, 126.2768386852015`},
{"CURE", 365, 157.86587788544983`}, {"TBUR", 30.465333414479147`, 70.10543554869702`},
{"TBUR", 85.60543357447614`, 127.03075436132164`}, {"TBUR", 14.458016785891248`, 4.354707947301743`},
{"TBUR", 16.84382996698321`, 30.631220649547124`}, {"TBUR", 53.77070759667485`, 197.44046242100515`},
{"CURE", 365, 132.0362369034449`}, {"TBUR", 52.28263463552337`, 106.37619406147071`}, {"CURE", 365, 88.29336809077348`},
{"TBUR", 219.4155258177875`, 214.25405209952464`}, {"CURE", 365, 122.75550835877742`},
{"TOX", 28.999937425076258`, 235.18490638549315`}, {"CURE", 365, 89.12737874892261`},
{"TBUR", 31.404703668918437`, 141.02147945913853`}, {"CURE", 365, 107.87015866600882`},
{"CURE", 365, 124.33902548593353`}, {"CURE", 365, 55.064277336658776`}, {"CURE", 365, 94.43192798230261`},
{"CURE", 365, 215.58767443264892`}, {"TBUR", 21.276275265812764`, 129.287904611164`}, {"CURE", 365, 83.85305529848051`},
{"TBUR", 91.58783109090187`, 54.945101652743986`}, {"CURE", 365, 87.17649678116274`}, {"CURE", 365, 209.51460583894755`},
{"CURE", 365, 94.27197034707577`}, {"CURE", 365, 132.98333976131562`}, {"TBUR", 16.07650384797061`, 143.5211693251683`},
{"TBUR", 89.56661152103686`, 128.80241791067485`}, {"TBUR", 87.41319212901324`, 224.74913490207635`},
{"CURE", 365, 184.6403204014424`}, {"CURE", 365, 149.43909716732057`}, {"CURE", 365, 150.90475630739797`},
{"CURE", 365, 211.45079047025658`}, {"TBUR", 22.68590331467407`, 21.77927172822773`},
{"TBUR", 80.44592068574956`, 204.93439396238387`}, {"TBUR", 149.83088300213487`, 90.50767906795089`},
{"TOX", 11.937948381862338`, 295.33337552366845`}, {"CURE", 365, 57.31479756910556`},
{"TBUR", 93.04998072822485`, 115.0710672864998`}, {"TOX", 17.841529270248518`, 246.0551056959686`},
{"CURE", 365, 24.072642655696846`}, {"CURE", 365, 173.9791395851371`}, {"TBUR", 95.63813590286287`, 141.99026199575772`},
{"TBUR", 34.47969037015264`, 157.44283353309504`}, {"TBUR", 15.739466799354316`, 90.56181856302328`},
{"TBUR", 14.213664700217203`, 56.234810916734304`}, {"TBUR", 33.11005599170677`, 26.338567691071773`},
{"CURE", 365, 125.43739421395018`}, {"CURE", 365, 127.81448159920892`}, {"TBUR", 37.620037945536176`, 151.42683597643241`},
{"CURE", 365, 21.172533460485564`}, {"TOX", 11.551732536414184`, 311.3477314542697`}, {"CURE", 365, 76.16653536366067`},
{"CURE", 365, 117.62402416048191`}, {"CURE", 365, 92.05853609861803`}, {"CURE", 365, 38.720474390159055`},
{"TBUR", 116.03938157424078`, 203.22097694935454`}, {"CURE", 365, 107.84807311501012`},
{"CURE", 365, 111.29910827720151`}, {"TBUR", 19.919751613205882`, 63.514247887792834`},
{"CURE", 365, 105.87275504107862`}, {"TOX", 10.847866278084547`, 311.79983658276126`},
```

{"CURE", 365, 208.75061991318353`}, {"TBUR", 37.85039039635162`, 159.89457377539452`}, {"CURE", 365, 132.7894661961327`},
{"CURE", 365, 197.11753255470356`}, {"TOX", 24.154594283448684`, 235.7420210697845`}, {"CURE", 365, 191.67999659108276`},
{"TOX", 19.55813913127035`, 246.03151110355256`}, {"TBUR", 14.692685449406468`, 84.58311448018792`},
{"CURE", 365, 103.45104553961625`}, {"TBUR", 39.97882883169914`, 46.01971543476174`},
{"TBUR", 18.939094893592426`, 111.85983667646424`}, {"TBUR", 88.30915781650206`, 6.322047622696538`},
{"CURE", 365, 37.723920321669276`}, {"CURE", 365, 62.14426696505775`}, {"CURE", 365, 1.1281704172554727`},
{"TBUR", 19.035254514614984`, 118.62073244763528`}, {"CURE", 365, 87.4188581845725`},
{"CURE", 365, 98.21394405257568`}, {"CURE", 365, 222.5511529671672`}, {"CURE", 365, 102.35113815469651`},
{"TBUR", 20.99505485827179`, 109.18882389560996`}, {"TBUR", 13.441736590664368`, 84.11683376268958`},
{"CURE", 365, 90.50502789585167`}, {"TBUR", 63.25166437279854`, 151.08915667867967`}, {"CURE", 365, 42.59407986531288`},
{"TBUR", 85.41795001663633`, 4.723988091531744`}, {"TBUR", 61.48664664048875`, 10.879057003450677`},
{"CURE", 365, 224.02482933862595`}, {"CURE", 365, 65.47446617064696`}, {"CURE", 365, 222.20934962126114`},
{"TBUR", 173.33009619306992`, 142.33759284554043`}, {"CURE", 365, 198.95477523348035`},
{"CURE", 365, 86.91023148958382`}, {"TOX", 13.624120999940054`, 281.7601662986853`}, {"CURE", 365, 125.17914030243838`},
{"TBUR", 42.0203107372185`, 44.896340684883505`}, {"CURE", 365, 207.40761005681176`},
{"TBUR", 30.081392994907763`, 55.97935596668332`}, {"TBUR", 68.07834786448478`, 175.4816080287859`},
{"TBUR", 75.5515167782958`, 196.12881387704374`}, {"TBUR", 65.84160332524934`, 76.78532616945226`},
{"CURE", 365, 135.4620978791695`}, {"TBUR", 15.97937087913138`, 55.69372331760065`},
{"TBUR", 33.13027519763997`, 104.31440129360068`}, {"CURE", 365, 167.85491965076366`},
{"CURE", 365, 99.1413878983415`}, {"TBUR", 30.51831847917492`, 107.90274819198545`}, {"CURE", 365, 89.4898614082314`},
{"TBUR", 77.3880601552817`, 217.07441562233194`}, {"TBUR", 52.50568321739721`, 153.86236206933856`},
{"CURE", 365, 174.59643579178785`}, {"TBUR", 79.19084274019002`, 172.8844735900649`}, {"CURE", 365, 94.4740300920973`},
{"CURE", 365, 139.78744914569373`}, {"CURE", 365, 224.82445639328503`}, {"CURE", 365, 34.74282285147943`},
{"TBUR", 55.186945185713135`, 39.391327461059554`}, {"TBUR", 145.2987256215585`, 109.60809077433412`},
{"TOX", 14.011481205080004`, 253.22120190202563`}, {"CURE", 365, 73.71364792238374`},
{"TOX", 18.307543623275333`, 250.00835169125259`}, {"CURE", 365, 113.05703899442146`},
{"TOX", 15.532539105417964`, 268.5530044021819`}, {"TBUR", 16.134992419688807`, 72.75849095301643`},
{"TBUR", 40.79234096017609`, 98.42911266112944`}, {"CURE", 365, 137.85273137179126`},
{"TOX", 14.523075273962776`, 262.3947200169618`}, {"TBUR", 86.2463720295187`, 120.53266643769108`},
{"TOX", 11.869807481300224`, 298.6729061115067`}, {"CURE", 365, 88.98903743588895`},
{"CURE", 365, 161.8577453801059`}, {"CURE", 365, 118.26325281244458`}, {"CURE", 365, 136.40914551925533`},

{"TBUR", 64.51849864972925`, 127.0793807838451`}, {"TBUR", 45.795220593325766`, 143.20046060363777`},
{"CURE", 365, 39.52305234523671`}, {"CURE", 365, 69.52720848001165`}, {"TBUR", 83.72622547860426`, 152.90317744445926`},
{"CURE", 365, 165.1321045955935`}, {"CURE", 365, 63.19423716703097`}, {"TBUR", 77.76935981445682`, 148.33418856435122`},
{"CURE", 365, 134.5571941432039`}, {"CURE", 365, 50.08054286449781`}, {"CURE", 365, 25.004227371484866`},
{"CURE", 365, 62.291887538564595`}, {"CURE", 365, 71.27946602244621`}, {"TBUR", 67.4306402205377`, 133.19142004312383`},
{"CURE", 365, 208.04872102413484`}, {"CURE", 365, 225.63108097551174`}, {"TBUR", 32.68279959911936`, 159.5925622679614 5`},
{"TBUR", 100.5208115253696`, 11.659615523158385`}, {"TOX", 13.179713889302924`, 270.88316431572866`},
{"TBUR", 103.18988583325067`, 139.5251727909518`}, {"TBUR", 39.88083001540981`, 134.41599164509648`},
{"TBUR", 126.65702315080301`, 73.3398519589420 8`}, {"CURE", 365, 122.55446100282728`},
{"CURE", 365, 41.46684916196683`}, {"CURE", 365, 201.9007643236652`}, {"CURE", 365, 215.97405880065588`},
{"CURE", 365, 126.01087128249542`}, {"CURE", 365, 87.51331230107208`}, {"CURE", 365, 108.28958769564524`},
{"TBUR", 124.1564436262856`, 163.28544193016776`}, {"TBUR", 91.80907105216937`, 194.0370888656742`},
{"TBUR", 42.541455095162604`, 99.64917864287541`}, {"CURE", 365, 130.0064426600305`}, {"CURE", 365, 98.37608043586435`},
{"TOX", 12.22032819825608`, 236.45288519842902`}, {"TBUR", 81.34769429733143`, 102.85112185753441`},
{"CURE", 365, 94.37640604588579`}, {"TBUR", 109.41257806407748`, 123.538883752757`}, {"CURE", 365, 221.71154503578444`},
{"CURE", 365, 16.572762270057034`}, {"TBUR", 56.26711000808186`, 87.56212491525912`},
{"CURE", 365, 144.60128478166277`}, {"CURE", 365, 54.6000610458529`}, {"TOX", 29.69594754232709`, 232.3306112191893`},
{"TBUR", 18.93644018220923`, 16.346463716089307`}, {"TOX", 26.71343408479304`, 235.0510012626465`},
{"CURE", 365, 203.2892666245099`}, {"CURE", 365, 5.083348243028305`}, {"CURE", 365, 175.9298595216051`},
{"CURE", 365, 112.18047313810908`}, {"CURE", 365, 140.04281383824386`}, {"CURE", 365, 201.4693325184659`},
{"CURE", 365, 56.91385837217756`}, {"TBUR", 21.294860752616792`, 128.12147626775712`}, {"CURE", 365, 77.16501112338793`},
{"CURE", 365, 51.892878113265404`}, {"CURE", 365, 135.7387393865833`}, {"TBUR", 74.85798639355428`, 208.1824664504581`},
{"TBUR", 171.92903524736852`, 132.1273965022229`}, {"CURE", 365, 1.023549136625972`},
{"TBUR", 140.63974674517948`, 53.06385436411212`}, {"TBUR", 31.118982560491844`, 102.14517548382344`},
{"CURE", 365, 43.17206455232444`}, {"CURE", 365, 8.409035800513658`}, {"CURE", 365, 166.94971410029362`},
{"TOX", 12.412501022743369`, 288.574839219013`}, {"TBUR", 90.0320674016518`, 151.29807617066015`},
{"TOX", 13.048543492141071`, 284.5332287892338`}, {"TBUR", 75.08012823647901`, 154.60512304589284`},
{"CURE", 365, 48.24876303703352`}, {"CURE", 365, 144.16888910619593`}, {"TBUR", 91.34799528958939`, 208.86962030407565`},
{"TBUR", 38.890657855066465`, 112.28144532591885`}, {"TBUR", 82.80921931637627`, 91.70165997913855`},
{"TBUR", 58.60693561739476`, 118.62150642044293`}, {"CURE", 365, 77.11030024899053`}, {"CURE", 365, 59.05127474184871`},
{"CURE", 365, 87.17549688174834`}, {"CURE", 365, 165.919228766348`}, {"TBUR", 127.86898945929259`, 93.77755292844442`},

{"TBUR", 26.269212042453198`, 78.2985586141211`}, {"CURE", 365, 103.20003282869197`},
{"TBUR", 82.7164022234523`, 41.223927576409494`}, {"CURE", 365, 164.0632800241329`}, {"CURE", 365, 91.85912819561594`},
{"TBUR", 64.47968649657577`, 91.74379438528312`}, {"TBUR", 51.43235570722226`, 203.1795299162793`},
{"TOX", 18.16565680185653`, 241.2820784119849`}, {"CURE", 365, 118.48127405712039`}, {"CURE", 365, 39.50076836804105`},
{"TOX", 11.673737861712684`, 300.28200838479654`}, {"TBUR", 157.10857845781234`, 150.3937808387508`},
{"TBUR", 57.59067589375217`, 104.75012729953909`}, {"CURE", 365, 119.36207161411474`},
{"CURE", 365, 75.22312632147262`}, {"CURE", 365, 113.30316528848067`}, {"CURE", 365, 105.33991578086425`},
{"TBUR", 121.51342383749581`, 105.59722538753074`}, {"CURE", 365, 209.56142766142588`}, {"CURE", 365, 97.58798529081011`},
{"TBUR", 167.34130137374802`, 149.8285355015755`}, {"CURE", 365, 86.58544230043047`}, {"CURE", 365, 181.5666121938852`},
{"CURE", 365, 97.93348905849088`}, {"TBUR", 87.36610712563508`, 130.23900050960793`}, {"CURE", 365, 78.58971478895461`},
{"TBUR", 82.674721770646`, 187.79623006056386`}, {"TOX", 9.916465554332747`, 327.6607856165573`},
{"TBUR", 42.56342994579733`, 170.55852161632703`}, {"CURE", 365, 158.11283027750696`}, {"CURE", 365, 76.84552607828445`},
{"TBUR", 121.93193462274468`, 126.6299797122129`}, {"TOX", 17.42462014062919`, 251.83357183403965`},
{"CURE", 365, 22.141058952907972`}, {"CURE", 365, 149.72584833845377`}, {"TBUR", 81.80564381517907`, 45.40221818709502`},
{"CURE", 365, 109.90552114639986`}, {"CURE", 365, 18.171081844708254`}, {"TBUR", 49.05137292327111`, 93.7029789030322`},
{"CURE", 365, 41.654988876896795`}, {"TBUR", 34.19246711163507`, 120.3539639116456`}, {"CURE", 365, 113.82468205488672`},
{"TBUR", 80.9458173364178`, 132.32551089564768`}, {"TBUR", 37.04673087140063`, 104.0690052657141`},
{"CURE", 365, 191.9488152893755`}, {"CURE", 365, 229.26459511802247`}, {"CURE", 365, 116.89971873716131`},
{"CURE", 365, 65.87081142478996`}, {"CURE", 365, 134.6365867296949`}, {"CURE", 365, 28.52012756845737`},
{"TBUR", 94.62405816080575`, 10.396516785834216`}, {"TOX", 32.02605518166218`, 236.67988597770517`},
{"CURE", 365, 185.84648985419537`}, {"TBUR", 84.51597432633277`, 67.27166613825686`}, {"CURE", 365, 82.13139535204564`},
{"TBUR", 18.388002818968555`, 135.13282287379494`}, {"TOX", 45.44594190885548`, 230.4812691828512`},
{"TBUR", 134.12480379484984`, 89.74123654142494`}, {"TBUR", 59.439019226733556`, 108.0949976748265`},
{"CURE", 365, 71.67903122571151`}, {"TBUR", 75.9877176101729`, 113.09031950609787`}, {"CURE", 365, 106.6245598760863`},
{"CURE", 365, 122.15469981397757`}, {"TBUR", 71.31702183411143`, 100.19652947025361`},
{"TBUR", 75.41935025735559`, 80.3648631994483`}, {"CURE", 365, 133.35947875753791`},
{"TBUR", 14.792610793216832`, 81.3984564161995`}, {"CURE", 365, 58.272802724011164`}, {"CURE", 365, 152.8445222377284 3`},
{"TOX", 14.188524137238538`, 270.95331829244253`}, {"CURE", 365, 223.63553702722075`}, {"CURE", 365, 148.89978494793297`},
{"CURE", 365, 156.57440968555696`}, {"TBUR", 66.84855314647628`, 22.523287497293126`},
{"TBUR", 43.68098912495509`, 135.43661869582775`}, {"TBUR", 80.13000784004643`, 60.37707273605915`},
{"CURE", 365, 131.41477629766493`}, {"TBUR", 26.513898989366183`, 137.19701827155728`},

{"CURE", 365, 58.13491372856274`}, {"CURE", 365, 110.52972414354524`}, {"TBUR", 23.8791407675012`, 62.67591846416471`},
{"TOX", 10.972503287741315`, 325.8003938235596`}, {"TBUR", 15.536177535135538`, 36.35592918679239`},
{"CURE", 365, 151.24797932983944`}, {"TBUR", 67.29828819156708`, 40.78348485423281`},
{"TBUR", 114.95134112460285`, 61.977764155350606`}, {"CURE", 365, 35.292411553846414`}, {"CURE", 365, 43.345245102921865`},
{"CURE", 365, 115.86906757697217`}, {"TBUR", 30.840582945475173`, 115.89549702863225`}, {"CURE", 365, 71.1415518702085`},
{"CURE", 365, 123.20932818159886`}, {"CURE", 365, 167.4018228816212`}, {"TOX", 12.371468893325634`, 277.4989983927675`},
{"CURE", 365, 165.05415583769388`}, {"CURE", 365, 200.87979315053042`}, {"TBUR", 41.34243809406419`, 132.26776934770695`},
{"CURE", 365, 65.23424596473023`}, {"TBUR", 27.807969989230294`, 83.34594571225725`},
{"CURE", 365, 69.1523948826294`}, {"CURE", 365, 188.39046882605518`}, {"TBUR", 43.08975748556391`, 134.596355331611`},
{"CURE", 365, 117.06413870076312`}, {"CURE", 365, 69.34709584314852`}, {"CURE", 365, 71.09234351559911`},
{"CURE", 365, 105.9874494603745`}, {"TBUR", 42.11422766726904`, 111.26667837212786`}, {"CURE", 365, 163.6570394859691`},
{"TBUR", 108.85142276502792`, 103.90845052855482`}, {"TOX", 12.130192589403086`, 282.316059169841`},
{"CURE", 365, 127.79432894789781`}, {"TBUR", 18.60356390878267`, 118.8694635955902`}, {"CURE", 365, 1.6157293951409457`},
{"CURE", 365, 76.0723208257119`}, {"CURE", 365, 212.31517871380217`}, {"CURE", 365, 68.32117139807556`},
{"TBUR", 48.40286599475252`, 102.19110470893148`}, {"TBUR", 50.828515383329055`, 141.02371468812288`},
{"TOX", 16.648063756533855`, 270.71445537743404`}, {"TBUR", 88.88028458210881`, 171.64538709451605`},
{"CURE", 365, 89.99685124831073`}, {"CURE", 365, 12.35184925555015`}, {"CURE", 365, 99.17625211518892`},
{"CURE", 365, 220.3999466837481`}, {"TBUR", 33.303068311114785`, 147.63281202143233`},
{"CURE", 365, 11.724863099035462`}, {"TBUR", 57.06150257239082`, 128.97658345620977`},
{"CURE", 365, 149.42154726018396`}, {"CURE", 365, 43.88143784101584`}, {"CURE", 365, 110.72524649301704`},
{"CURE", 365, 122.0604213417241`}, {"CURE", 365, 91.17457461814647`}, {"TOX", 9.487228089883114`, 351.1426630304253`},
{"TBUR", 63.70791745112217`, 135.06457986106878`}, {"CURE", 365, 153.63144564797352`},
{"TBUR", 25.267241912896946`, 110.63958601676929`}, {"TOX", 11.684395602530886`, 282.7168562532781`},
{"CURE", 365, 148.37695780426748`}, {"TBUR", 20.400344185780803`, 103.82886150320037`},
{"CURE", 365, 104.8107837993532`}, {"TBUR", 59.36358518219274`, 148.82897976019507`}, {"CURE", 365, 1.2968000838201108`},
{"TBUR", 50.676339275639194`, 70.98107954415867`}, {"TBUR", 146.78286798814386`, 153.47327197514466`},
{"CURE", 365, 117.64561356138395`}, {"CURE", 365, 86.7853043390391`}, {"CURE", 365, 108.52807890030122`},
{"CURE", 365, 84.12184920482237`}, {"TBUR", 73.03847785822062`, 85.93120994370364`}, {"CURE", 365, 53.96325715321024`},
{"CURE", 365, 94.52279991890109`}, {"CURE", 365, 87.70525439108374`}, {"TBUR", 29.96999802619394`, 71.32008466667119`},
{"CURE", 365, 218.60472975159493`}, {"CURE", 365, 6.03661302349555`}, {"CURE", 365, 113.61637157817984`},
{"TBUR", 163.0382750673003`, 167.96594541786675`}, {"TBUR", 16.66949863808461`, 170.40619238243357`},

{"TBUR", 83.55883376123087`, 58.917298537067325`}, {"CURE", 365, 168.62305282196633`},
{"CURE", 365, 157.91790291208855`}, {"TBUR", 48.885904664477586`, 5.545663148699298`}, {"CURE", 365, 146.12073974865137`},
{"CURE", 365, 147.83545122532902`}, {"TBUR", 42.77869389159647`, 31.407782634458588`}, {"CURE", 365, 68.19614432989478`},
{"TOX", 17.411134892951388`, 245.68022540412065`}, {"TBUR", 114.35280762472922`, 147.6714579427146`},
{"CURE", 365, 128.45260240103894`}, {"CURE", 365, 67.16685410456542`}, {"TBUR", 112.65370903309633`, 4.181924005990226`},
{"CURE", 365, 48.4499373360177`}, {"TBUR", 13.778455740285246`, 38.73311551036582`},
{"TBUR", 85.55436730369613`, 124.1335560174396`}, {"CURE", 365, 198.19330676260077`}, {"CURE", 365, 157.08069922463997`},
{"CURE", 365, 87.04850288531587`}, {"TBUR", 51.47666345265022`, 27.270027900260807`}, {"CURE", 365, 117.48974949044599`},
{"CURE", 365, 146.91060131618372`}, {"CURE", 365, 51.28920415554203`}, {"TBUR", 77.70478110286972`, 87.81340525795301`},
{"CURE", 365, 55.4955649799608`}, {"CURE", 365, 213.1201661357144`}, {"CURE", 365, 109.34642245500605`},
{"TBUR", 15.883765645184361`, 1.6969856955349598`}, {"TBUR", 164.2771945505306`, 134.96649838457748`},
{"CURE", 365, 67.25004873218855`}, {"TOX", 10.18920172133966`, 316.3897488125764`}, {"CURE", 365, 209.9514186169372`},
{"TBUR", 42.200460053212`, 101.07265133200673`}, {"TOX", 14.977345458656515`, 279.4883452002491`}},
{{"4444 Res", "4444 OS", "4444 Tox"}, {"TBUR", 18.496502146730386`, 88.36190689973456`},
{"TOX", 13.91814385565615`, 267.51606854957686`}, {"TBUR", 21.207500163021717`, 177.07975200472006`},
{"TBUR", 28.268654821114445`, 96.66157566194279`}, {"TBUR", 16.54090831030584`, 76.32630136600581`},
{"CURE", 365, 109.02643738680473`}, {"CURE", 365, 113.37311421504464`}, {"CURE", 365, 120.9470075818831`},
{"TOX", 17.7445749955454`, 245.35133750861792`}, {"TOX", 12.453854205448634`, 253.50302410074323`},
{"TBUR", 34.59167506397448`, 135.6227135844222`}, {"CURE", 365, 168.07870029320972`},
{"TOX", 8.837560436345061`, 356.23928546822816`}, {"TBUR", 36.56378614178611`, 110.66350879130198`},
{"TBUR", 65.73966395933739`, 158.66835892720107`}, {"TBUR", 41.92347664000635`, 95.17101027309197`},
{"CURE", 365, 146.2083064293913`}, {"TBUR", 27.988981887088837`, 169.89847601190795`},
{"TBUR", 42.73426202174447`, 113.48032622928666`}, {"CURE", 365, 180.41810823571228`},
{"CURE", 365, 202.09732300341784`}, {"CURE", 365, 158.8524526941544`}, {"CURE", 365, 101.032229906912`},
{"CURE", 365, 146.96218754711242`}, {"CURE", 365, 166.61703060199014`}, {"TOX", 12.294140632564595`, 285.5866686055502`},
{"TOX", 13.976170435240387`, 282.0040623604561`}, {"CURE", 365, 125.6190116572075`}, {"CURE", 365, 103.61183431371228`},
{"TBUR", 72.23452610292703`, 110.67393959640721`}, {"TOX", 14.075047581253497`, 269.60822546660296`},
{"TBUR", 12.360355737672494`, 70.56966597718743`}, {"TBUR", 48.77457355239346`, 130.00848602447985`},
{"TBUR", 33.19878487270823`, 87.37905264297345`}, {"TOX", 11.16867166132884`, 304.02948796998487`},
{"TOX", 10.303431173058495`, 316.93114541378173`}, {"CURE", 365, 102.42144647343558`},
{"TBUR", 21.15444689246695`, 48.67666550701139`}, {"TBUR", 35.48936428489114`, 99.5379523332495`},

```
{"TBUR", 94.73832264298066`, 132.82409403345503`}, {"TBUR", 16.563951549432478`, 120.8703673506056`},
{"CURE", 365, 205.25200362847517`}, {"CURE", 365, 131.5932638440116`}, {"TBUR", 20.76137527926037`, 102.21257182275524`},
{"TBUR", 61.5008164147857`, 141.78956083635453`}, {"CURE", 365, 87.38724437869972`},
{"TBUR", 157.59137728675955`, 163.44974092482857`}, {"TBUR", 158.9085284601243`, 116.88795801209135`},
{"TBUR", 62.797991158348395`, 176.8069553289115`}, {"TOX", 9.86550892724544`, 326.0003461960354`},
{"TBUR", 26.727505651724865`, 140.15100886183353`}, {"TBUR", 36.269320850976875`, 117.79364599654907`},
{"CURE", 365, 151.08321906979077`}, {"TBUR", 170.84624301599425`, 201.75680139500045`},
{"TBUR", 57.915490752158966`, 139.14878922927454`}, {"CURE", 365, 142.00277371819206`}, {"CURE", 365, 163.6013633181202`},
{"TBUR", 54.75217693021827`, 218.32709043580095`}, {"TBUR", 25.945293348279133`, 219.15467294351538`},
{"TBUR", 41.103422083844876`, 138.50945977102407`}, {"TBUR", 63.693035393944065`, 129.98982387371382`},
{"CURE", 365, 86.5675145467498`}, {"TBUR", 31.813519101086634`, 167.73672582891913`},
{"CURE", 365, 199.176579144812`}, {"TBUR", 69.3390473385897`, 186.8112000519141`}, {"CURE", 365, 136.82631549924042`},
{"CURE", 365, 151.7282002388115`}, {"TBUR", 30.80448877970218`, 180.3577292557993`},
{"CURE", 365, 112.51661794384988`}, {"CURE", 365, 100.41694200803394`}, {"CURE", 365, 163.55458810749673`},
{"TBUR", 84.45932598397606`, 128.75772262666155`}, {"TBUR", 57.58844488592582`, 225.7956800189627`},
{"TBUR", 13.223819873311292`, 29.91470568888606`}, {"TBUR", 40.454337350129514`, 149.3469830180304`},
{"TOX", 19.16135792097233`, 244.1692958882678`}, {"TOX", 10.305499970579662`, 299.8332446002732`},
{"CURE", 365, 115.51093460440919`}, {"TBUR", 51.88634475052333`, 191.3951076381498`}, {"CURE", 365, 148.07645792894556`},
{"TBUR", 107.9983413990038`, 174.98686116382132`}, {"CURE", 365, 125.6370607741592`}, {"CURE", 365, 120.16859186158855`},
{"TOX", 23.470071194593675`, 245.94641295457765`}, {"CURE", 365, 198.76741084765536`},
{"TBUR", 50.36715905573748`, 149.0740590730052`}, {"TOX", 15.60282566422951`, 272.4887148573374`},
{"TBUR", 75.40490146895382`, 124.83367891057259`}, {"CURE", 365, 161.36840165759412`},
{"TBUR", 34.47938236296976`, 217.51281076242853`}, {"TBUR", 27.680580069529444`, 87.05392399069807`},
{"TBUR", 46.42814662124807`, 161.9285149875601`}, {"CURE", 365, 68.10433778050943`}, {"CURE", 365, 93.34339939719212`},
{"TBUR", 21.62841974583358`, 48.06896117867282`}, {"TBUR", 90.40666768444164`, 111.13371621978945`},
{"CURE", 365, 114.38941400781152`}, {"TOX", 11.294103843158183`, 290.52524210928107`},
{"TBUR", 48.943039023921365`, 76.42108669038345`}, {"TBUR", 24.04317634485323`, 188.74150934719765`},
{"TBUR", 19.260862130401314`, 59.37183293019976`}, {"TOX", 20.089074051398995`, 238.4475656780725`},
{"TBUR", 54.55706374244257`, 132.94855677334039`}, {"TBUR", 48.779658001907855`, 135.30033115824287`},
{"CURE", 365, 131.27783086131416`}, {"CURE", 365, 220.64138556252297`}, {"TBUR", 71.8100924958276`, 101.12523555233848`},
{"TBUR", 86.17211214558012`, 129.7313785306117`}, {"CURE", 365, 107.23381880796927`}, {"CURE", 365, 197.6245727279293`},
```

{"TOX", 15.43796882670442`, 254.204246007049`}, {"CURE", 365, 135.32429754404467`}, {"CURE", 365, 97.54534089347625`},
{"TBUR", 90.49693679887511`, 126.19621081127502`}, {"TBUR", 143.33760911654946`, 115.82891545671133`},
{"CURE", 365, 118.92446400025483`}, {"CURE", 365, 172.99010106205756`}, {"CURE", 365, 164.47971968353943`},
{"TOX", 16.779526433306867`, 258.45965143269325`}, {"CURE", 365, 104.26210865836568`},
{"TBUR", 39.028432066700645`, 131.57458655383917`}, {"TOX", 12.959166189577948`, 269.8887558288359`},
{"CURE", 365, 200.36968756639368`}, {"TBUR", 20.6586186829388`, 62.15026307204398`}, {"CURE", 365, 121.80091679957509`},
{"TBUR", 16.68900595315819`, 104.15923269181687`}, {"TBUR", 70.32719488269045`, 208.18444765700087`},
{"TBUR", 64.41713682676699`, 114.3578228323475`}, {"CURE", 365, 221.50561712365666`}, {"CURE", 365, 206.9933465014018`},
{"CURE", 365, 165.43606258880945`}, {"CURE", 365, 177.0022391283313`}, {"TBUR", 32.00157978774296`, 98.6324430390275`},
{"TBUR", 19.51376640079562`, 91.29106816166792`}, {"TOX", 14.540300896325066`, 255.60688248255468`},
{"TBUR", 12.383447152705273`, 131.1994499755144`}, {"TBUR", 47.67885431629705`, 169.671802364967`},
{"TBUR", 39.093766410730204`, 117.2991882351674`}, {"CURE", 365, 117.00324208493493`},
{"TBUR", 135.59302004817746`, 168.92997450555484`}, {"TBUR", 28.62453405422124`, 228.8165513950365`},
{"CURE", 365, 203.06203707469194`}, {"TOX", 11.220594457707868`, 313.7258469508896`},
{"TOX", 19.36965512380815`, 254.12347688750765`}, {"CURE", 365, 213.17007215452088`},
{"TBUR", 103.98931858742672`, 149.09177968058302`}, {"TOX", 12.961488888547748`, 271.07106523329645`},
{"TBUR", 25.185211973851274`, 87.13904947958923`}, {"CURE", 365, 138.21606487792903`}, {"CURE", 365, 95.51297608015953`},
{"CURE", 365, 195.0205067329674`}, {"TBUR", 117.8723640069114`, 171.25946271092738`}, {"CURE", 365, 150.16189672425176`},
{"TOX", 13.517661150975444`, 254.74456025842213`}, {"TBUR", 22.692540544874067`, 119.89722129328271`},
{"TOX", 16.655748641493748`, 235.80430494537026`}, {"TBUR", 42.56039156721799`, 157.30921589774323`},
{"CURE", 365, 170.3387782048156`}, {"CURE", 365, 119.22760462530812`}, {"TBUR", 67.53883962565851`, 164.57064413456857`},
{"CURE", 365, 140.05781457269575`}, {"TBUR", 80.79675752389032`, 103.90013111780009`},
{"CURE", 365, 114.33621347789446`}, {"TBUR", 27.90665989010615`, 77.30496128853008`},
{"TBUR", 36.61599274385436`, 149.04769161811717`}, {"TBUR", 66.82678903494477`, 167.300792214531`},
{"CURE", 365, 179.18258057873584`}, {"TBUR", 73.42271268078834`, 150.53322409761654`},
{"TBUR", 44.06249486327119`, 170.3775874891945`}, {"TBUR", 137.60002241796616`, 213.87640695259907`},
{"CURE", 365, 123.49387392521807`}, {"TBUR", 24.910319425348792`, 197.829778584931`},
{"CURE", 365, 118.62630432609942`}, {"TBUR", 20.15435070721269`, 50.462266479095575`},
{"TBUR", 42.55017807766129`, 199.048076146686`}, {"TBUR", 80.45351429610541`, 159.59340653510455`},
{"TOX", 14.52601096001015`, 241.05549237392958`}, {"TBUR", 19.02257391226022`, 117.9195444726663`},
{"TBUR", 40.10850485111772`, 116.28340578976437`}, {"TBUR", 52.9940695116289`, 143.21300711207945`},

{"TBUR", 51.34559568400282`, 224.494811538134`}, {"TBUR", 186.34506424536124`, 129.07251881342683`},
{"TOX", 22.648449666691945`, 235.4964212829181`}, {"TBUR", 15.453139602495664`, 75.7652283775924`},
{"CURE", 365, 198.50500684304978`}, {"TBUR", 24.32075447571318`, 92.69994988515727`},
{"TBUR", 27.697088873756982`, 185.18421110662135`}, {"TBUR", 146.51161700644704`, 171.0768277300256`},
{"CURE", 365, 86.95627248566552`}, {"TBUR", 19.65123341588082`, 140.18035599005438`}, {"CURE", 365, 157.16566755806295`},
{"CURE", 365, 202.45410242136018`}, {"TOX", 12.396423253256222`, 278.47069229590187`},
{"CURE", 365, 187.52955871621376`}, {"TBUR", 88.20182924269496`, 129.134083673903`}, {"CURE", 365, 106.33597500189983`},
{"TOX", 11.998488134425665`, 284.45976797040925`}, {"TBUR", 12.48036047882625`, 60.089109604643824`},
{"CURE", 365, 155.06432846947567`}, {"CURE", 365, 180.66389500601372`}, {"TBUR", 47.83653685495313`, 158.59140667335245`},
{"TBUR", 48.831689081041205`, 147.37662510390336`}, {"TOX", 17.168943202944842`, 253.21109741799563`},
{"TBUR", 31.849384021974384`, 112.22633234469228`}, {"CURE", 365, 107.49715802410999`},
{"TBUR", 72.14227883527937`, 222.9491903918116`}, {"TBUR", 15.397756273574949`, 157.82254496376694`},
{"TOX", 10.738973724512459`, 317.5997521051276`}, {"TBUR", 71.61674765153602`, 122.58657968695654`},
{"CURE", 365, 199.52270763209594`}, {"CURE", 365, 117.45064570712059`}, {"TBUR", 53.11892391743893`, 128.92514120581413`},
{"CURE", 365, 98.30399319060405`}, {"TBUR", 45.5592973986146`, 157.13125632407235`}, {"CURE", 365, 102.50034806636388`},
{"TBUR", 36.12903593836887`, 144.2449882965731`}, {"TBUR", 49.78893600200978`, 220.6169998789011`},
{"CURE", 365, 115.78835157143683`}, {"TOX", 10.214905520179727`, 295.3606091612421`}, {"CURE", 365, 116.28290422568618`},
{"CURE", 365, 173.13927963126574`}, {"CURE", 365, 109.03203685376037`}, {"CURE", 365, 131.61619443744905`},
{"TBUR", 59.096268235417135`, 218.52383627352552`}, {"TBUR", 83.33719569674928`, 209.40156606272836`},
{"TBUR", 59.35966270977757`, 112.04948645514384`}, {"TBUR", 82.86148560657588`, 198.2249222898187`},
{"TBUR", 103.16006285640587`, 139.53939031134288`}, {"CURE", 365, 101.57546330260983`},
{"CURE", 365, 125.29464059167383`}, {"CURE", 365, 152.57878431665642`}, {"CURE", 365, 115.7315453619914`},
{"CURE", 365, 162.25145880357096`}, {"CURE", 365, 131.6710917188112`}, {"TBUR", 72.19149899776411`, 135.69814808984205`},
{"TBUR", 15.380401539501664`, 139.43803700535986`}, {"TBUR", 23.783831108503275`, 108.30929110559337`},
{"TBUR", 85.41737477987859`, 149.2924029731151`}, {"TBUR", 15.308413102548519`, 88.7583658325631`},
{"CURE", 365, 111.57772333663432`}, {"TBUR", 60.4534081305093`, 134.85265823168385`}, {"CURE", 365, 200.48240573138958`},
{"TBUR", 27.525675107111173`, 45.94102209899134`}, {"TBUR", 24.767742917380513`, 97.64458225142127`},
{"CURE", 365, 201.9879162939566`}, {"CURE", 365, 164.3916303173524`}, {"TBUR", 22.17649321905766`, 57.82754027306868`},
{"TOX", 10.775816210003505`, 299.6442563380801`}, {"CURE", 365, 168.986507749596`},
{"TOX", 10.837277633297923`, 301.9539923719726`}, {"TBUR", 31.17815936013599`, 180.70966107850222`},
{"CURE", 365, 229.59742813342862`}, {"TBUR", 14.21443176785192`, 192.80456715190732`},

{"TBUR", 57.4739817293357`, 121.88712645736992`}, {"CURE", 365, 156.09835479320742`}, {"CURE", 365, 112.24761944439591`},
{"TBUR", 96.62245675262466`, 128.07830088521925`}, {"CURE", 365, 114.87757361358402`},
{"CURE", 365, 112.93321939521161`}, {"TBUR", 69.77842516852294`, 225.66041629304104`},
{"TBUR", 19.475624122612192`, 178.79350900729293`}, {"TBUR", 12.881833826860294`, 134.4728333898155`},
{"CURE", 365, 118.9082278315714`}, {"TBUR", 26.424873181401914`, 120.14361817000098`}, {"CURE", 365, 121.499806230087`},
{"CURE", 365, 145.49878351069776`}, {"TBUR", 163.50716222575443`, 201.87880925515586`},
{"TOX", 10.035011975711319`, 328.9634043777007`}, {"TOX", 19.045784256912658`, 248.2751086507632`},
{"CURE", 365, 105.37875291918579`}, {"CURE", 365, 94.19786327094424`}, {"TBUR", 33.687392174894725`, 147.78704981096197`},
{"CURE", 365, 140.74245503375252`}, {"TBUR", 74.45485210173084`, 136.17138231660522`},
{"TBUR", 26.167265266430405`, 108.36847709141473`}, {"CURE", 365, 194.17264805360244`}, {"CURE", 365, 118.35258334616194`},
{"TBUR", 18.736647400851936`, 119.13869670088965`}, {"TBUR", 55.079685357753114`, 131.03716203694475`},
{"TBUR", 61.76952958928549`, 202.23985092890823`}, {"TBUR", 60.73328648952135`, 138.21783145323244`},
{"TBUR", 49.63700531262515`, 143.37990991713656`}, {"TBUR", 15.131117142776201`, 78.30973134498689`},
{"CURE", 365, 119.88826618298552`}, {"TBUR", 17.687528681519524`, 137.34067102312258`},
{"CURE", 365, 132.73802505925997`}, {"TBUR", 43.68972721375127`, 186.889807955598`}, {"CURE", 365, 120.23737126091895`},
{"TOX", 10.102656882184021`, 336.7075918066211`}, {"TBUR", 103.12739353560136`, 190.72037625663262`},
{"CURE", 365, 145.7503501238766`}, {"TBUR", 89.09101275146293`, 125.8814895985366`},
{"TOX", 11.595588286939991`, 282.49255593948675`}, {"TBUR", 19.870181526489695`, 73.95777250658591`},
{"CURE", 365, 182.60189483145126`}, {"CURE", 365, 185.85843082831843`}, {"TOX", 16.395040831058317`, 261.3036482645294`},
{"TOX", 15.28086580723485`, 270.68987044215925`}, {"TBUR", 44.32380187159064`, 161.14690364839282`},
{"CURE", 365, 157.66597464381886`}, {"TBUR", 23.197173830719024`, 220.1425593218037`}, {"CURE", 365, 163.9685240066697`},
{"TOX", 21.97710412726037`, 238.88145383433022`}, {"TBUR", 28.006315106009172`, 202.16019643083123`},
{"TOX", 23.4167726750507`, 240.85896109662804`}, {"TBUR", 155.62289950676757`, 178.88592153010043`},
{"CURE", 365, 114.60387709033701`}, {"TBUR", 15.871475351317574`, 96.02929603813034`},
{"CURE", 365, 182.41580510161364`}, {"CURE", 365, 198.79361232403755`}, {"TBUR", 21.877253103909382`, 75.11194827207596`},
{"CURE", 365, 154.86245756393694`}, {"TBUR", 27.433120433131997`, 82.50528106878733`}, {"CURE", 365, 148.9185332295011`},
{"TBUR", 75.32111482351178`, 161.50694981605335`}, {"TBUR", 68.65533225459689`, 131.70763966012237`},
{"TBUR", 56.564433713520536`, 149.87974435687804`}, {"CURE", 365, 126.83609513552855`},
{"CURE", 365, 159.29705142837764`}, {"TOX", 13.610763017987734`, 277.48286806250263`},
{"TBUR", 25.730441319719805`, 127.20497979679058`}, {"TOX", 23.82478831383749`, 235.3430236643479`},
{"CURE", 365, 138.20243875053237`}, {"TBUR", 56.16457414137852`, 140.38860649281955`},

```
{"TBUR", 14.672617945928062`, 63.19510616167015`}, {"CURE", 365, 127.71054791508233`},
{"TBUR", 89.8139607960312`, 196.5593980536096`}, {"TOX", 17.266874231156923`, 250.33851407777854`},
{"CURE", 365, 139.36556361223282`}, {"TOX", 11.544043066412037`, 280.384756872039`},
{"TOX", 16.875996743980824`, 246.343572660677`}, {"CURE", 365, 170.0990568936325`},
{"TBUR", 18.054645053088148`, 114.27687024245202`}, {"TBUR", 55.89860316677454`, 200.07529237830863`},
{"TOX", 11.976729765923292`, 277.1690709474683`}, {"TOX", 9.813426690124034`, 268.9502708088164`},
{"TBUR", 19.287990031848874`, 112.6339272157827`}, {"TOX", 10.731908686911714`, 310.15991789958696`},
{"CURE", 365, 108.701477261029`}, {"CURE", 365, 195.24888815773147`}, {"TOX", 14.295163061537714`, 267.1488672328198`},
{"CURE", 365, 80.76735866292631`}, {"TBUR", 21.879003748744683`, 76.89449834476558`}, {"CURE", 365, 189.55082186123283`},
{"TBUR", 54.05188146080306`, 108.05503261468256`}, {"TOX", 9.173138483924626`, 288.43536636705454`},
{"CURE", 365, 100.6402806684328`}, {"TOX", 16.73204960423236`, 246.1282528388427`}, {"CURE", 365, 149.1527119432986`},
{"CURE", 365, 140.7268424756944`}, {"CURE", 365, 166.40621429137397`}, {"CURE", 365, 106.44731826437034`},
{"TBUR", 19.167555051700944`, 166.83197832602113`}, {"CURE", 365, 167.78304712913317`},
{"TOX", 18.612537360449206`, 241.62944553344968`}, {"CURE", 365, 171.9324168113822`},
{"TBUR", 46.02270055184039`, 152.7007483868775`}, {"TBUR", 184.39827181992004`, 171.53482497733017`},
{"CURE", 365, 175.3043790923512`}, {"CURE", 365, 124.15539710513225`}, {"TBUR", 59.062014827205196`, 198.6400378524692`},
{"TOX", 12.421482783531747`, 282.16295239973795`}, {"CURE", 365, 112.28592845892186`},
{"TBUR", 34.41370510946053`, 165.12807328003973`}, {"TBUR", 24.788944512542965`, 176.64055668808115`},
{"CURE", 365, 96.4242812765654`}, {"CURE", 365, 156.32468471228447`}, {"CURE", 365, 115.59959269373655`},
{"TBUR", 13.582287750306538`, 51.42192227852724`}, {"TBUR", 12.18618928987528`, 65.72112507872484`},
{"TBUR", 32.19201462877462`, 110.94838918670465`}, {"TBUR", 75.7074196207953`, 149.98609370513978`},
{"TBUR", 81.58786839678001`, 122.74051150772793`}, {"CURE", 365, 168.95475877967482`},
{"TOX", 14.83706672263632`, 248.21285688099852`}, {"CURE", 365, 156.02030130887616`}, {"CURE", 365, 121.05123543236753`},
{"TBUR", 95.39133434212572`, 220.01635340097857`}, {"TBUR", 14.772830982523466`, 89.96216588672974`},
{"TBUR", 12.482590239143907`, 141.83783482202497`}, {"TBUR", 96.34831156872951`, 157.90137798122146`},
{"CURE", 365, 208.53773844951328`}, {"TBUR", 35.86171989386631`, 136.23127193971771`},
{"TBUR", 52.98321449388211`, 209.98828648635532`}, {"TBUR", 128.91225134480882`, 129.20478330982814`},
{"CURE", 365, 141.6784273104302`}, {"TBUR", 52.873735348950845`, 106.05579352528991`}, {"CURE", 365, 106.95063229509071`},
{"TOX", 10.395302227034268`, 314.8171422137489`}, {"CURE", 365, 170.9239480946267`}, {"CURE", 365, 121.33044257892347`},
{"TBUR", 26.88865012139672`, 120.0403978228815`}, {"TBUR", 55.40762194167891`, 122.42453251444012`},
{"CURE", 365, 110.42338332139339`}, {"TBUR", 14.521790501598392`, 109.03516505928225`},
```

{"TBUR", 16.544325558834636`, 100.27030082174174`}, {"CURE", 365, 173.64036062278228`},
{"CURE", 365, 141.95767372181416`}, {"TBUR", 126.53925074996732`, 145.20288044331505`},
{"TBUR", 12.586057832409626`, 94.72838480356386`}, {"TBUR", 17.125738516273326`, 64.3868984394819`},
{"TOX", 17.277728094182184`, 248.77315090036126`}, {"TBUR", 69.17305694825224`, 125.96287910913819`},
{"CURE", 365, 171.82644716874617`}, {"CURE", 365, 143.13076851122167`}, {"CURE", 365, 180.0750966393695`},
{"CURE", 365, 119.58447189867914`}, {"CURE", 365, 192.60143500762197`}, {"CURE", 365, 216.0017823537313`},
{"TBUR", 69.6708689810281`, 189.26001032071542`}, {"CURE", 365, 113.87032544846055`}, {"CURE", 365, 152.90167338889202`},
{"TOX", 11.417280199058856`, 290.16003843528665`}, {"TBUR", 19.054483114495465`, 84.98859446263636`},
{"CURE", 365, 80.96356066751831`}, {"CURE", 365, 122.97802295918261`}, {"TBUR", 41.105778661738626`, 191.91552310740872`},
{"CURE", 365, 136.77079306697783`}, {"TBUR", 73.9057592453993`, 221.45779200519956`},
{"TOX", 11.663724306624678`, 297.674234083031`}, {"CURE", 365, 120.38251486834793`},
{"TOX", 47.40228918324207`, 230.96010339189198`}, {"TBUR", 114.70992776217251`, 85.46465063758515`},
{"CURE", 365, 219.29226130930954`}, {"TBUR", 12.42103985695406`, 55.193739139533655`},
{"TOX", 9.751216287814207`, 306.6648140946206`}, {"TBUR", 43.898336372013105`, 119.57504504958541`},
{"CURE", 365, 221.6657502258108`}, {"TBUR", 39.484832667388346`, 117.54785144562663`},
{"CURE", 365, 114.87938519727312`}, {"TOX", 9.904670873427595`, 317.4879466640883`}, {"CURE", 365, 175.8082312408571`},
{"TBUR", 107.18608359740301`, 66.09554887705062`}, {"CURE", 365, 189.2118682296868`}, {"CURE", 365, 110.04379379800159`},
{"TOX", 16.48866018281823`, 245.5631712519523`}, {"CURE", 365, 114.40766819315812`}, {"CURE", 365, 81.43338146336094`},
{"CURE", 365, 216.80856617705876`}, {"TBUR", 20.24348325135131`, 128.64154402921113`},
{"CURE", 365, 155.2853287458902`}, {"TBUR", 63.04177603875173`, 157.10801483917353`}, {"CURE", 365, 115.58338391585566`},
{"TBUR", 54.11866134137321`, 181.05355781870816`}, {"CURE", 365, 169.29896145156687`},
{"TOX", 15.914558458321975`, 237.5680826069264`}, {"TBUR", 92.22947668356096`, 112.47826012259594`},
{"CURE", 365, 124.49611041539188`}, {"CURE", 365, 110.55613871727247`}, {"TOX", 10.488198037154074`, 311.24281206634475`},
{"CURE", 365, 136.33078570098763`}, {"CURE", 365, 225.8900356398911`}, {"CURE", 365, 223.89128474236898`},
{"TOX", 13.22024143280241`, 269.1982104378785`}, {"TOX", 16.112739456874184`, 250.2347135207027`},
{"TBUR", 36.98485184631904`, 101.39267812984941`}, {"CURE", 365, 137.41503424340138`},
{"TBUR", 67.59799195729872`, 113.49546410932652`}, {"TBUR", 13.868585496734381`, 63.096939805931996`},
{"TBUR", 19.426906591897833`, 49.54515552925255`}, {"TBUR", 87.87697165239649`, 165.09935615472335`},
{"CURE", 365, 200.35872390003038`}, {"TBUR", 32.42737294791514`, 150.02734356905017`},
{"TOX", 12.907486682960078`, 278.3729533906103`}, {"TOX", 19.074667816513518`, 241.7541531569905`},
{"TBUR", 16.45754106359419`, 65.23850311595501`}, {"TBUR", 17.22437136670526`, 75.14313883418728`},

```
{"TBUR", 33.25169318142822`, 98.51155098298727`}, {"TBUR", 19.300324238941204`, 177.3260365744984`},
{"TBUR", 23.394900644585903`, 134.73665015641026`}, {"TBUR", 79.21992514189573`, 202.11803580245768`},
{"TBUR", 41.50519838314786`, 230.1064991634658`}, {"CURE", 365, 110.72641852419983`},
{"TBUR", 53.876661917647866`, 167.82946448470184`}, {"TBUR", 25.062821119632684`, 136.87753891024028`},
{"TBUR", 38.42286641317286`, 58.141965124478574`}, {"TOX", 23.591510771510873`, 234.40393807962036`},
{"CURE", 365, 131.07507819851244`}, {"TBUR", 32.28727773656367`, 80.56949313410097`},
{"TBUR", 17.924195903778017`, 46.76556752165245`}, {"CURE", 365, 127.34644025598243`}, {"CURE", 365, 172.7969979356826`},
{"CURE", 365, 221.89913827942738`}, {"TBUR", 23.666309837458023`, 104.0392671212051`}, {"CURE", 365, 132.0859588466213`},
{"CURE", 365, 188.8257312322615`}, {"CURE", 365, 168.46875564251957`}, {"TBUR", 50.633182937045945`, 111.5738731553234`},
{"CURE", 365, 170.92759398143426`}, {"TBUR", 84.28339394787031`, 167.9585051311946`},
{"CURE", 365, 114.10745014546036`}, {"TBUR", 36.168942524016984`, 134.9833403782277`},
{"CURE", 365, 93.75476717163185`}, {"TBUR", 33.47344808496498`, 156.60880527943775`}, {"CURE", 365, 114.78628489679444`},
{"TBUR", 24.21063865608921`, 127.23690290564309`}, {"TOX", 17.00969739272384`, 264.6227371833964`},
{"TBUR", 15.630345325706065`, 103.17637824424786`}, {"TBUR", 62.83422874258217`, 128.45403883046936`},
{"TBUR", 15.412529834595759`, 173.1306158028027`}, {"TBUR", 57.51896936280741`, 132.03386642312645`},
{"TBUR", 14.937935203646491`, 71.16131863199952`}, {"TBUR", 26.82899711028191`, 141.80651143451638`},
{"TOX", 11.835393618738486`, 300.86470950990804`}, {"CURE", 365, 169.98241161310966`}, {"CURE", 365, 120.96183472672635`},
{"TBUR", 58.80483417016017`, 116.52986633558967`}, {"CURE", 365, 152.30634615770262`},
{"TBUR", 186.0346604078258`, 119.8290215615722`}, {"TBUR", 31.534418995888323`, 157.89655643913295`},
{"TBUR", 41.20372783610147`, 120.3197444513422`}, {"TOX", 12.904299891317407`, 281.05128825815353`},
{"CURE", 365, 172.09849024162597`}, {"TOX", 11.690366236572311`, 284.3375223909135`}, {"CURE", 365, 202.9876616944562`},
{"TOX", 22.666813576215727`, 238.4879913866679`}, {"TBUR", 102.25616306984165`, 189.31933132505208`},
{"TOX", 11.58505201601796`, 286.2320400216779`}, {"TOX", 9.834544850963525`, 283.76401167847837`},
{"CURE", 365, 123.15934576118886`}, {"CURE", 365, 180.61854647005632`}, {"CURE", 365, 199.19723146136505`},
{"TBUR", 33.25629800024363`, 92.5916497729912`}, {"TBUR", 14.81537853348408`, 46.5342569046594`},
{"TOX", 20.125316064520554`, 257.0495848240875`}, {"CURE", 365, 169.1254154340506`}, {"CURE", 365, 186.88080468575765`},
{"TBUR", 17.219569795290496`, 159.2267864792535`}, {"CURE", 365, 221.37030691349372`},
{"TOX", 10.808761038161164`, 306.10902461732303`}, {"TBUR", 80.15147987676472`, 135.5723351183725`},
{"TOX", 14.854808801298777`, 274.7038249665469`}, {"TBUR", 32.85725364349349`, 149.93246830923906`},
{"TOX", 10.518604658048083`, 302.7526727199742`}, {"TBUR", 17.894772101523344`, 93.61179986362056`},
{"TBUR", 29.865464722658675`, 88.78815970380778`}, {"TBUR", 40.841583818213934`, 102.33275774643452`},
```

{"TOX", 10.905428339884185`, 300.0313376624371`}, {"CURE", 365, 141.43004639011562`},
{"TOX", 11.266968348481353`, 286.59122511513016`}, {"TBUR", 61.33014751207109`, 186.5913923694669`},
{"CURE", 365, 200.29291438787092`}, {"TBUR", 33.68834969426245`, 178.56616801383066`},
{"TBUR", 49.19987052873624`, 214.62422838373698`}, {"TBUR", 57.85661431092267`, 108.08858419819794`},
{"CURE", 365, 108.69259168359501`}, {"CURE", 365, 123.50832651129535`}, {"CURE", 365, 141.68324787952605`},
{"TBUR", 56.586895386276076`, 158.94773822345059`}, {"TBUR", 20.604442616233154`, 89.27911083027718`},
{"TBUR", 25.942206717641536`, 112.79308863492444`}, {"TOX", 18.38461416619633`, 244.36034229766656`},
{"TBUR", 20.631863206337634`, 97.8868545765634`}, {"CURE", 365, 129.6685945097365`}, {"CURE", 365, 160.13529825064927`},
{"TBUR", 16.45882297198049`, 61.51431370590353`}, {"CURE", 365, 111.45248314269183`}, {"CURE", 365, 219.9711300302327`},
{"CURE", 365, 152.1738306465486`}, {"TBUR", 35.91195807346998`, 104.61079175855787`}, {"CURE", 365, 99.40052970452668`},
{"TBUR", 20.462911322526534`, 202.72610990242327`}, {"TOX", 22.88062971006604`, 235.3107796570377`},
{"CURE", 365, 138.70134268384902`}, {"TBUR", 50.08275417634064`, 131.70835927103545`},
{"TBUR", 150.78033488423057`, 194.87721601009343`}, {"CURE", 365, 101.32871476179803`},
{"TOX", 9.864673056831768`, 300.9382438928106`}, {"TBUR", 24.455697399672285`, 109.45876654460582`},
{"TOX", 11.390629493872588`, 310.70113183140745`}, {"TBUR", 28.27092456650898`, 109.73846455592941`},
{"CURE", 365, 103.62596255078765`}, {"TBUR", 30.162267022266654`, 103.39558992440486`},
{"TBUR", 23.619216204854155`, 94.67320703404964`}, {"TOX", 20.938804957827582`, 239.36508578609534`},
{"TBUR", 19.28944263640177`, 75.12026070679984`}, {"CURE", 365, 224.9633817087755`},
{"TBUR", 17.292473676327607`, 96.56002566744462`}, {"TBUR", 82.0910283329607`, 117.86178592639317`},
{"TBUR", 19.83105342644168`, 93.10780442299952`}, {"TOX", 19.28957630470633`, 234.4810961283769`},
{"TOX", 12.37132761000512`, 306.7960745019473`}, {"TBUR", 21.50397587843097`, 98.32176983663418`},
{"TBUR", 45.15833532606865`, 106.71546234997668`}, {"TBUR", 12.821105565164688`, 125.45221063714477`},
{"CURE", 365, 94.75635644840978`}, {"TBUR", 54.67082325951991`, 181.21924946724664`},
{"TBUR", 53.26973166982392`, 173.37013273018`}, {"CURE", 365, 158.8766519031925`},
{"TBUR", 58.24731971974854`, 129.33250312032882`}, {"TOX", 21.13153879652725`, 243.0779011612982`},
{"TOX", 10.962249892965936`, 316.45383777430584`}, {"TBUR", 100.30381446731717`, 139.0247467014154`},
{"CURE", 365, 183.07523149714964`}, {"TBUR", 22.937313964679877`, 90.44672127040933`},
{"TBUR", 48.52739875487494`, 133.0846923199382`}, {"TBUR", 13.7101439593809`, 22.99992601887103`},
{"TBUR", 13.695168403099547`, 52.62888163047681`}, {"TBUR", 46.28547881613365`, 225.1187897301429`},
{"TBUR", 139.5670976825695`, 167.72147646275667`}, {"TBUR", 32.57381991429373`, 109.50689809637923`},
{"CURE", 365, 173.57979570309493`}, {"TOX", 37.815374110093124`, 230.55546553563943`}, {"CURE", 365, 143.94939261013488`},

```
{"TOX", 12.645606754545186`, 282.4644867157058`}, {"TBUR", 67.05046194115931`, 196.40185377422057`},
{"TBUR", 16.43183096330078`, 116.50274772191375`}, {"CURE", 365, 127.42624766954927`},
{"TBUR", 125.09070447079074`, 137.2342926700608`}, {"CURE", 365, 96.60896732592134`}, {"CURE", 365, 112.30046186102345`},
{"TOX", 15.150011563193647`, 260.5383106966233`}, {"TBUR", 18.560804457955726`, 140.53589752121223`},
{"TBUR", 82.5295633964405`, 116.25330677005103`}, {"TBUR", 40.439793543367045`, 131.01030079315132`},
{"CURE", 365, 121.94168859699005`}, {"TOX", 17.6479563014166`, 246.3797503143163`}, {"CURE", 365, 122.19430898615188`},
{"TBUR", 95.56978739710834`, 163.92085726577642`}, {"TBUR", 15.616850275168863`, 153.41679679910752`},
{"TBUR", 60.03164186025423`, 142.18770780445755`}, {"TOX", 17.84901003007934`, 240.89081601470988`},
{"TBUR", 43.928347736062726`, 185.92526691118263`}, {"CURE", 365, 180.88956517871878`},
{"CURE", 365, 169.98556094918848`}, {"TOX", 19.79607412646082`, 240.22547406648533`},
{"TBUR", 16.67981391759897`, 59.47215837617255`}, {"TBUR", 40.39717560756262`, 207.59252700779376`},
{"TBUR", 77.1491745988620 5`, 121.56523068913249`}, {"TOX", 11.425300750221236`, 257.1233599190412`},
{"CURE", 365, 140.9083310517521`}, {"TBUR", 62.52639230184966`, 125.90045232084537`},
{"TOX", 13.614476340437113`, 262.7509180234636 7`}, {"CURE", 365, 118.30925995981835`}, {"CURE", 365, 215.50599220583675`},
{"TBUR", 45.790031141512365`, 159.51456816295033`}, {"TBUR", 30.006724999774434`, 158.9979553052028`},
{"TBUR", 13.770633700643137`, 106.42064674844592`}, {"TBUR", 13.019073703777815`, 85.85612835993018`},
{"TBUR", 16.227943055008247`, 58.612981798449155`}, {"TBUR", 43.103664253946`, 143.04433075502428`},
{"CURE", 365, 158.30596723366548`}, {"TBUR", 21.197861986740953`, 127.66955796384262`}, {"CURE", 365, 86.13375206617962`},
{"TOX", 9.679346257880816`, 340.4427093123599`}, {"TBUR", 110.05457367171991`, 102.63964189135778`},
{"TBUR", 155.3752988747879`, 119.91557594512314`}, {"CURE", 365, 126.5278623127152`}, {"CURE", 365, 90.54007256728627`},
{"TBUR", 59.561033244044395`, 208.52579361435048`}, {"TOX", 21.73633341893281`, 247.7351907250454`},
{"TBUR", 138.70367190830584`, 154.32569830517022`}, {"TBUR", 16.887130405546205`, 103.98614833987959`},
{"CURE", 365, 133.64445057486145`}, {"TOX", 10.031818845592529`, 307.0834787824754`},
{"TBUR", 73.69840608604613`, 212.38290023789506`}, {"TBUR", 18.346949099708052`, 125.4703234631275`},
{"CURE", 365, 162.3521935489715`}, {"CURE", 365, 218.19981210252453`}, {"TOX", 15.027549868695564`, 254.7851412521906`},
{"CURE", 365, 219.0077238220609`}, {"TOX", 12.663353148608127`, 276.9664007561368`},
{"TBUR", 12.939593962483391`, 95.69061921579946`}, {"CURE", 365, 124.92075068352472`},
{"TBUR", 21.47420019434713`, 83.95008227647953`}, {"TBUR", 18.456341973715183`, 118.88947857662285`},
{"TBUR", 62.39179496552794`, 93.28498591915874`}, {"CURE", 365, 151.17782674211006`}, {"CURE", 365, 184.19116711611156`},
{"TBUR", 114.71691534146537`, 99.55675106445283`}, {"TBUR", 16.816134091962343`, 138.63682879486828`},
{"TBUR", 56.227015143059354`, 158.32016484959132`}, {"CURE", 365, 117.22455522908506`},
```

{"TOX", 36.34939478795178`, 230.56297553655602`}, {"TBUR", 149.70933383140445`, 178.81504415913537`},
{"TBUR", 18.33855620487643`, 114.06738252109979`}, {"TBUR", 12.177587155536711`, 97.61828995468187`},
{"CURE", 365, 182.95887724535348`}, {"TBUR", 47.366526728461466`, 156.9773841264567`},
{"TBUR", 81.47003035698245`, 116.93734869230973`}, {"TBUR", 50.37218640633501`, 73.40323941945367`},
{"TBUR", 84.00106324084045`, 69.75941376978119`}, {"TOX", 19.41987351308486`, 239.54620410132037`},
{"CURE", 365, 103.6921557245003`}, {"TOX", 24.139785779259217`, 233.80604860531471`},
{"TBUR", 95.23469187431094`, 149.55359861129432`}, {"CURE", 365, 209.34956301000506`},
{"CURE", 365, 117.8097428733772`}, {"TOX", 10.596592120165482`, 313.93679125710327`}, {"CURE", 365, 143.25275265548873`},
{"TBUR", 16.14371703196571`, 63.12889329681522`}, {"TBUR", 144.44408617087572`, 229.05036489303123`},
{"TBUR", 21.880316364316805`, 74.15052069387237`}, {"TBUR", 32.722802197611394`, 173.70151319245699`},
{"TBUR", 42.151155439455025`, 191.11551144314083`}, {"TBUR", 28.140750707022526`, 87.51888001136035`},
{"CURE", 365, 137.3546665788594`}, {"TBUR", 13.893452782496343`, 72.34505238875562`},
{"TBUR", 20.810315484554675`, 101.7073878924164`}, {"CURE", 365, 175.73174417162897`},
{"CURE", 365, 151.7339931232569`}, {"TBUR", 27.61998678476481`, 118.56436821644014`}, {"CURE", 365, 140.54611657364245`},
{"TBUR", 44.62680881930091`, 210.24744989112781`}, {"TBUR", 25.54684215124419`, 132.12339875268705`},
{"TOX", 17.6419917779135`, 256.4685306316816`}, {"TBUR", 39.05520221885107`, 177.4965097220446`},
{"CURE", 365, 123.53411401869704`}, {"CURE", 365, 190.74779196901295`}, {"TOX", 11.974749212637322`, 306.9212120398389`},
{"CURE", 365, 85.27212049991114`}, {"TBUR", 19.22622130597871`, 66.82932066869506`},
{"TBUR", 73.80024178058561`, 157.93022358783418`}, {"TOX", 13.689028073206797`, 249.00859473652943`},
{"CURE", 365, 157.086833165734`}, {"TOX", 13.286409007970978`, 265.81576713973965`}, {"CURE", 365, 131.24799473375282`},
{"TOX", 11.02469290244509`, 307.6502017139393`}, {"TBUR", 13.947382952434921`, 88.76973988796996`},
{"TBUR", 33.85866257615373`, 120.23820510213923`}, {"TBUR", 158.42871197627895`, 167.28588197112865`},
{"TOX", 12.089419149735345`, 278.70625630692956`}, {"TBUR", 49.172105348121974`, 126.13620655631071`},
{"TOX", 10.131727408694337`, 321.6563393979879`}, {"CURE", 365, 129.99598742284127`}, {"CURE", 365, 182.99545367732006`},
{"CURE", 365, 124.67623818942805`}, {"TBUR", 79.1672631926526`, 146.20595898284083`},
{"TBUR", 52.13569228549262`, 128.45785032182548`}, {"TBUR", 23.48021313800436`, 132.32305485301717`},
{"CURE", 365, 115.78101244997947`}, {"TBUR", 150.19188321809267`, 102.58196045031026`},
{"TBUR", 44.87621200744856`, 160.0906352612085`}, {"TBUR", 94.45840296114734`, 227.6805795037884`},
{"CURE", 365, 174.90185976671228`}, {"TBUR", 41.287218246580885`, 150.98042749204356`},
{"TOX", 19.45847716656028`, 251.56903189524408`}, {"CURE", 365, 152.56509787901786`}, {"CURE", 365, 135.9803259394577`},
{"CURE", 365, 102.193254815117`}, {"CURE", 365, 103.35871077776925`}, {"TBUR", 33.64715467840537`, 131.60461788878342`},

{"TOX", 17.645924651641177`, 246.93798610715461`}, {"TOX", 20.15669309922031`, 237.98397088788832`},
{"TBUR", 20.11493751264752`, 148.47284866901015`}, {"TBUR", 85.89271238380871`, 74.59321547912197`},
{"TOX", 11.71909011405064`, 282.509496092706`}, {"TBUR", 57.60159319752015`, 177.26475166856153`},
{"TBUR", 20.952279339565663`, 123.54559915657678`}, {"TBUR", 70.42183938983636`, 116.68460167945571`},
{"TBUR", 100.84364541498155`, 141.57335958832695`}, {"CURE", 365, 94.71506932558499`}, {"CURE", 365, 229.2521483505474`},
{"CURE", 365, 226.9078477405702`}, {"CURE", 365, 147.5277085987518`}, {"TBUR", 87.17446707883475`, 139.265912119639`},
{"CURE", 365, 130.6840138359826`}, {"TBUR", 66.32642532431767`, 186.04709455254172`},
{"TBUR", 56.33591646972487`, 217.93772004188364`}, {"TBUR", 23.963541156899954`, 100.52064361142457`},
{"CURE", 365, 137.4950272285305`}, {"TBUR", 85.63524049159923`, 115.17044104087157`},
{"TOX", 11.237811469377574`, 243.18902737240927`}, {"TBUR", 35.516079056137386`, 123.23410130522558`},
{"CURE", 365, 119.11435511441549`}, {"TBUR", 62.622039784362464`, 125.5417974383747`},
{"TOX", 12.529451604298096`, 297.9067333479026`}, {"CURE", 365, 89.25204044340616`},
{"TBUR", 33.0841516442638`, 95.96249084292283`}, {"TBUR", 72.56711528646915`, 179.75011839034403`},
{"CURE", 365, 95.87936383739182`}, {"TOX", 16.985612899596806`, 244.76597919965653`},
{"TBUR", 14.268841841102565`, 39.335263194829906`}, {"TOX", 13.936670268164173`, 264.840294036208`},
{"TBUR", 140.77965955751282`, 209.75054033495164`}, {"TBUR", 119.99724146312606`, 97.20264110369028`},
{"TBUR", 111.95244685727431`, 186.45259781433057`}, {"TBUR", 115.71907176216072`, 171.1382840608287`},
{"TOX", 41.98142268937841`, 230.8295837015495`}, {"CURE", 365, 225.79909128195885`}, {"CURE", 365, 123.2014030375014`},
{"TBUR", 19.68017690473538`, 162.7300874090263`}, {"TBUR", 77.7655326942588`, 129.476757077463`},
{"TBUR", 95.47423156806303`, 185.82929023297788`}, {"CURE", 365, 174.67689619669926`},
{"TBUR", 35.122224943976185`, 202.30533007241084`}, {"TOX", 20.158474426296475`, 252.88008054019178`},
{"CURE", 365, 200.64961049006004`}, {"TBUR", 70.9366000884322`, 119.92925562582168`},
{"TBUR", 27.707579551255876`, 124.41579908184654`}, {"CURE", 365, 159.18805216526667`}, {"CURE", 365, 91.46193597984295`},
{"TBUR", 82.65607679024326`, 174.61633452875327`}, {"TOX", 11.184032613815647`, 280.4372134653786`},
{"TBUR", 43.198245157380185`, 157.54797406538398`}, {"TOX", 10.563014897864827`, 311.76664698387293`},
{"TBUR", 44.88832997299212`, 161.12529477345785`}, {"CURE", 365, 120.07953535392853`}, {"CURE", 365, 149.8894224768998`},
{"TBUR", 56.83390149114301`, 210.64559579869928`}, {"TBUR", 36.16725385026728`, 120.75533592371522`},
{"TBUR", 44.82993290024709`, 146.7849237976123`}, {"TBUR", 24.558690880481745`, 117.95190133206103`},
{"TBUR", 111.52638368557862`, 102.59431135749817`}, {"TBUR", 133.73359611357444`, 96.59087268782942`},
{"TBUR", 73.87931378852001`, 168.01125307153083`}, {"TBUR", 58.5605505407477`, 170.66254728753884`},
{"TBUR", 67.98274917663713`, 119.67191065749553`}, {"TBUR", 19.217849982153187`, 85.71854921837745`},

{"CURE", 365, 117.57312505272553`}, {"TBUR", 33.19110634554834`, 71.397930200247`}, {"CURE", 365, 203.65556221746132`},
{"TBUR", 110.33864329918386`, 119.29235494011878`}, {"TBUR", 32.81701781091962`, 95.56447442663111`},
{"TBUR", 23.905184154310003`, 167.1221931632789`}, {"TOX", 16.77936145159374`, 244.35424072821462`},
{"CURE", 365, 181.5356513113215`}, {"CURE", 365, 99.61942124352005`}, {"TOX", 10.15620152892786`, 289.45145530673085`},
{"TBUR", 81.90887553341057`, 221.9403420856218`}, {"TBUR", 23.899289061485284`, 103.20219888358966`},
{"CURE", 365, 127.2317502307123`}, {"TBUR", 47.56017521481762`, 131.48259858639773`}, {"CURE", 365, 126.40278229604066`},
{"CURE", 365, 116.83950984509363`}, {"TBUR", 64.80109421213723`, 114.13182272449713`},
{"TOX", 15.173635484912054`, 260.9278624811844`}, {"CURE", 365, 136.36767935707948`},
{"TBUR", 87.12707036761385`, 175.28830480574013`}, {"CURE", 365, 128.20141682133314`},
{"CURE", 365, 194.02838652824352`}, {"TBUR", 122.78300496668265`, 126.00607555442221`},
{"TBUR", 41.06072070938528`, 157.33475143502037`}, {"CURE", 365, 120.58134767113438`},
{"TBUR", 40.61917788190705`, 194.36546096009738`}, {"TOX", 9.446844611630457`, 288.9452722684168`},
{"TBUR", 25.326409525817937`, 153.5791473403241`}, {"TBUR", 110.39413862791997`, 172.06921138007579`},
{"CURE", 365, 107.8146576059578`}, {"TBUR", 62.245398893602854`, 131.0409399113759`},
{"TBUR", 28.856548894974363`, 212.83595141035275`}, {"CURE", 365, 141.46784867190726`},
{"CURE", 365, 151.23802087249484`}, {"TBUR", 38.143612976501686`, 85.15748861298547`}, {"CURE", 365, 229.46300633124895`},
{"CURE", 365, 150.74379346537572`}, {"TBUR", 26.219278450685543`, 93.9643179678251`}, {"CURE", 365, 105.03999622711184`},
{"TBUR", 21.947783153791498`, 118.28699243908459`}, {"TBUR", 67.58510355921139`, 134.53682632101442`},
{"TBUR", 37.108205793290594`, 139.15389288116845`}, {"TBUR", 20.1004679147163`, 101.80263043392418`},
{"TOX", 14.619805791232116`, 282.5427612648207`}, {"TOX", 18.27239673075214`, 241.4895086551825`},
{"TBUR", 121.58448956224264`, 128.4492670172433`}, {"TBUR", 67.04197500355738`, 113.48302142339486`},
{"CURE", 365, 165.59993137653382`}, {"TBUR", 123.86147119807138`, 80.40329210670666`},
{"TBUR", 81.52290183699306`, 70.48219490656801`}, {"TOX", 12.142385935412156`, 308.14816306800077`},
{"CURE", 365, 199.64843320191224`}, {"TBUR", 37.3861218984703`, 91.06246994365526`},
{"TBUR", 62.52125403098656`, 114.72178392886556`}, {"TBUR", 17.529798512575525`, 158.69477777871276`},
{"TOX", 15.404344554201812`, 254.11298045681994`}, {"TBUR", 68.29313900872127`, 114.80135089866401`},
{"TBUR", 26.00933143052579`, 110.9403486570958`}, {"CURE", 365, 173.18991225986008`},
{"TBUR", 33.68824599079541`, 115.19310867597447`}, {"TBUR", 126.6388799555898`, 128.39186904821958`},
{"CURE", 365, 135.22242167195017`}, {"TBUR", 35.338514225917265`, 113.64602893822189`},
{"TBUR", 31.039990113007786`, 105.32180440844873`}, {"TBUR", 85.73363662464881`, 137.87983345610627`},
{"TBUR", 13.819016041725327`, 95.95585423777395`}, {"CURE", 365, 95.2400159889996`}, {"CURE", 365, 168.3242563386353`},

```
{"TOX", 11.216783121256787`, 296.4179473087409`}, {"TOX", 15.4819127571998`, 254.5250391920728`},
{"CURE", 365, 202.48335343362746`}, {"TBUR", 75.09817773662198`, 171.95761066329064`},
{"TBUR", 26.86261123515795`, 84.50448679470239`}, {"TBUR", 35.39703697297477`, 142.07794198145436`},
{"TBUR", 32.43120971079026`, 111.1135645478656`}, {"CURE", 365, 206.6021225357931`},
{"TBUR", 20.79470398971064`, 136.6694298150217`}, {"TBUR", 88.62849597625771`, 93.69249759498366`},
{"TBUR", 90.41170393459673`, 133.32167010141245`}, {"TBUR", 14.157695391804694`, 73.3535505421278`},
{"TOX", 9.310435023383294`, 356.65868400852213`}, {"TBUR", 12.934191391576524`, 63.66283567141078`},
{"CURE", 365, 172.2572714494297`}, {"TBUR", 61.43982646584145`, 84.00931287017451`},
{"TBUR", 63.98283297593672`, 106.32617715133394`}, {"CURE", 365, 86.93531623951836`}, {"CURE", 365, 137.98963140074167`},
{"CURE", 365, 124.39934173920099`}, {"TBUR", 20.960517595301013`, 134.04060780497417`},
{"CURE", 365, 151.0741416656373`}, {"CURE", 365, 155.37331005871172`}, {"CURE", 365, 198.67475436693672`},
{"TOX", 11.351556038445645`, 286.65543565008994`}, {"TOX", 17.956555993278204`, 260.2612465113612`},
{"TOX", 12.57348870887599`, 303.6724417973424`}, {"TBUR", 35.876231647579004`, 133.2583130836959`},
{"CURE", 365, 127.18035783704158`}, {"TBUR", 18.135489211880973`, 92.19769688522605`}, {"CURE", 365, 105.7449636445272`},
{"TOX", 14.242483901834529`, 277.8037923738274`}, {"TBUR", 22.154458302034158`, 117.84136754606813`},
{"CURE", 365, 164.83514159033356`}, {"CURE", 365, 179.04658152374884`}, {"CURE", 365, 134.08768199196325`},
{"CURE", 365, 123.63070931785843`}, {"TBUR", 40.589284544954175`, 115.63476520824631`},
{"TBUR", 43.49980965292893`, 182.64341712251706`}, {"TBUR", 54.91092078198561`, 113.4739328308838`},
{"TOX", 11.090513678867017`, 292.58891737477336`}, {"TBUR", 126.70338127903737`, 133.26984599807346`},
{"TBUR", 15.457491169688055`, 130.52463072240224`}, {"CURE", 365, 73.11662703702576`},
{"CURE", 365, 107.25663824527945`}, {"TBUR", 125.11705197207979`, 218.61752718674774`}, {"CURE", 365, 172.7928685379782`},
{"TBUR", 25.1827213628769`, 104.03588591566653`}, {"TBUR", 25.86471159304995`, 130.97709630207405`},
{"TOX", 12.055564709035133`, 283.1420519778324`}, {"TBUR", 43.203820425508084`, 182.5825925173832`},
{"CURE", 365, 179.5780340994352`}, {"CURE", 365, 83.69366837337427`}, {"CURE", 365, 115.67898031130873`},
{"CURE", 365, 226.57033838805418`}, {"TBUR", 20.680515509173308`, 137.01800071920212`},
{"CURE", 365, 191.13922117477975`}, {"TBUR", 31.999111667101722`, 130.02511490206584`},
{"CURE", 365, 204.4291440715784`}, {"TBUR", 35.777626875441456`, 81.77508223327142`},
{"CURE", 365, 136.70181902695873`}, {"TBUR", 41.89792156303802`, 124.25691227762294`},
{"TBUR", 51.75900719202644`, 114.38436334515063`}, {"TOX", 8.892037434943228`, 364.3859341973283`},
{"TBUR", 26.17568232957693`, 142.37265082313462`}, {"TBUR", 56.79544611662532`, 189.73884056434147`},
{"TBUR", 20.77407442151379`, 118.59145092854958`}, {"TOX", 11.147940781096032`, 288.1709602132259`},
```

```
{"CURE", 365, 212.00911513881974`}, {"TBUR", 14.614223501442364`, 102.12777938027651`},
{"CURE", 365, 165.83535465518162`}, {"TBUR", 47.97574173448351`, 152.69400270629586`},
{"CURE", 365, 70.83469009019947`}, {"TBUR", 20.496002162488587`, 80.81692784388538`},
{"TBUR", 77.6182835350762`, 173.9653583120848`}, {"TBUR", 68.75007898577142`, 119.91612145479633`},
{"CURE", 365, 179.4295226503816`}, {"TBUR", 80.6971635054078`, 134.1342213531823`},
{"CURE", 365, 118.50679261476553`}, {"TBUR", 34.009094364289204`, 98.57437387864599`},
{"CURE", 365, 113.84851438860123`}, {"CURE", 365, 116.17972737401777`}, {"CURE", 365, 120.13419736445881`},
{"TBUR", 16.167100040564602`, 77.95175176233052`}, {"TOX", 27.781385102343023`, 232.13419789256483`},
{"CURE", 365, 171.08178455681633`}, {"CURE", 365, 143.77156908529125`}, {"TBUR", 84.87393163890881`, 194.9856749952104`},
{"TBUR", 15.970556135506131`, 194.00216307642`}, {"TBUR", 37.488891341366774`, 106.16796408561964`},
{"TOX", 22.106519150302567`, 239.63080417415827`}, {"CURE", 365, 176.66777719256535`},
{"TBUR", 34.2039135579832`, 58.29918536584245`}, {"TOX", 30.548383222892898`, 233.55434866867935`},
{"CURE", 365, 184.3768683600943`}, {"TBUR", 23.71366234854996`, 74.93847489467974`}, {"CURE", 365, 198.39182634250574`},
{"TOX", 15.078827620904805`, 245.9766085919063`}, {"TBUR", 58.00243496940045`, 166.35712574268794`},
{"TBUR", 76.13745881679215`, 129.95889868942052`}, {"TBUR", 79.78673485643397`, 106.74695086400496`},
{"TBUR", 68.9457828214394`, 47.01526697194482`}, {"TBUR", 41.512764945568655`, 93.42343099370257`},
{"TBUR", 12.32676238383649`, 67.62456759819663`}, {"TBUR", 55.07493274887786`, 138.14064873921845`},
{"TOX", 30.026621491316245`, 232.04891902698813`}, {"CURE", 365, 174.8604364355441`},
{"CURE", 365, 111.37761358395781`}, {"TBUR", 18.813492848626623`, 55.18474505621568`},
{"TBUR", 47.138155450671306`, 122.59742996966254`}, {"TBUR", 86.27321136356247`, 183.5239847840219`},
{"TBUR", 68.84205191606907`, 134.23858739475634`}, {"TBUR", 33.11371260873435`, 101.68875479182017`},
{"CURE", 365, 209.4616462962633`}, {"TOX", 19.781869196635434`, 239.98975737896959`},
{"TBUR", 40.29981725449257`, 133.49842747241064`}, {"TBUR", 14.549436508484176`, 24.665647066913056`},
{"TBUR", 84.05553825878035`, 156.37609172588967`}, {"CURE", 365, 103.02555739486026`},
{"TOX", 9.866139801721513`, 283.5988469822248`}, {"TOX", 14.654313433702859`, 266.35447146454555`},
{"TBUR", 39.277517818714166`, 109.61237863479224`}, {"TOX", 10.761568381819867`, 322.0423355826583`}}};
```

In[ ]:=

```
(* These scripts need to be initialized for running the following codes *)
```

In[ ]:=

```
(* The test set of mice is the same as was used for Fig S.10 *)
```

```
Npar = 1000;
Patients = {{"kappac", "kappap", "gamma", "V", "ks", "rho", "omega", "alpha", "kf", "N0"}, {0.2135521539372217`, 3.961577083383463`,
    0.017449556363494628`, 0.8968357850841964`, 0.5526193340404524`, 0.44754718012416395`, 0.06034849948846574`, 106.1625413648788 2`,
    0.10190317263014587`, 883.5270672679858`}, {0.07152561216059383`, 2.5169021554225424`, 0.04134379025654024`,
    0.9380911271372574`, 0.93412718926926`, 0.17225303021845306`, 0.1175689330301931`, 2613.4682606921233`, 0.18822840660147933`,
    493.53347054969345`}, {0.08527801742841884`, 2.3342943915916523`, 0.007356128912919502`, 1.449543423692679`,
    0.2920284025196249`, 0.42703227038753633`, 0.03973423147454342`, 243.18803536822332`, 0.14706039466941528`, 772.0773970550337`},
   {0.08032499747193339`, 3.3228245803820906`, 0.058505885415950074`, 1.328072711539189`, 0.9755298339255658`,
    0.3834882995147877`, 0.016384649136306686`, 81.13118073476093`, 0.12842007570807257`, 778.1337523863579`},
   {0.12288971305592977`, 0.46847473663449035`, 0.035027886845599224`, 0.9755484915941668`, 0.875011150725298`, 0.508807503319318`,
    0.009673321455752245`, 67.20829764486504`, 0.24949007211501428`, 854.477971991049`}, {0.23358644138244972`, 1.7807941427787792`,
    0.06034630860175783`, 1.045216035784662`, 0.6114168239067443`, 0.23619205669697263`, 0.3250660132336067`, 1792.5878930475694`,
    0.0744857171754702`, 805.4037858164254`}, {0.2672425581653916`, 1.5294271887111437`, 0.07676744398776716`, 1.292359521032572`,
    0.852989179909 6945`, 0.393740102898689`, 0.1380993699197939`, 2546.257830379171`, 0.12485562910431564`, 269.9458351710083`},
   {0.23183312051487565`, 3.3970641967209048`, 0.08395863347805453`, 1.1558870614014223`, 0.12544854381867032`, 0.610867529026453 1`,
    0.011088452522675474`, 482.67672135456445`, 0.02718649446571869`, 273.7693703287759`}, {0.08728636941166823`,
    1.601719923640072`, 0.02854064232033517`, 1.12476800155736`, 0.3992080927357673`, 0.5147003606496414`, 0.06898789718338541`,
    4299.502344187501`, 0.1107223992523067`, 582.4485009262825`}, {0.06789465848570125`, 3.3425223793990293`, 0.05746465325158763`,
    1.2855437262048381`, 0.9962937730263095`, 0.5148610577647594`, 0.34967229657185783`, 129.46101746656242`, 0.19161277365419005`,
    109.49077359602803`}, {0.21068632540051146`, 0.7531370432799127`, 0.03477678881607343`, 0.8221096630790845`,
    0.8211187339561399`, 0.3670710605309486`, 0.2800655063342806`, 102.20325958949621`, 0.2320804455102461`, 131.94504624973487`},
   {0.15242131810791038`, 3.789625832521282`, 0.044532267365124396`, 1.387509696935342`, 0.3025280009740767`, 0.43327817796146684`,
    0.05700485989023632`, 1418.28871606834`, 0.07523468846369646`, 480.71702187415434`}, {0.047127741426415765`, 2.196394146604325`,
    0.0020860451850145266`, 1.253945549403835`, 0.04333275562133232`, 0.4635809712022565`, 0.09752500840122344`, 2979.9191420645693`,
    0.19800512345941224`, 326.8262854148691`}, {0.21354080576404838`, 0.499272990997762`, 0.02136565456468402`, 1.4494872415741291`,
    0.4261365642523509`, 0.22487734950611293`, 0.3748843819820306`, 66.77722732862037`, 0.11979197850581236`, 963.8394274052501`},
   {0.06484047241450586`, 3.036571492120566`, 0.07651087746736028`, 0.9711768655928728`, 0.4574624946507835`, 0.5217597270510685`,
    0.12598093752406897`, 242.96341995255446`, 0.14545623834203425`, 981.9658120220585`}, {0.2281503022999219`, 2.470286745790686`,
    0.06276936772487546`, 1.1988495043217857`, 0.7133186669750637`, 0.3930261678642514`, 0.3166839777189734`, 137.26966163233058`,
    0.159523733253794`, 623.7392541692013`}, {0.2086500877706095`, 1.2720061208981175`, 0.06218340621581904`, 0.9797444420614967`,
    0.0465935956825620 5`, 0.28748130863864596`, 0.3811626852138692`, 349.8696799206167`, 0.19707584717198728`, 104.08157372885026`},
```

```
{0.11607219052414597`, 2.714811676304598`, 0.010818112381176023`, 0.8421879055822536`, 0.6583674774374331`,
  0.40051060979188435`, 0.25210876559776124`, 309.1166496608548`, 0.2109324247903891`, 983.8961564091211`},
{0.0729343665897485`, 1.3500826412359075`, 0.07266241705510545`, 1.1245658298901933`, 0.8437686840308298`,
  0.25155713345223996`, 0.03143796678764096`, 51.35885900577598`, 0.018795747835889998`, 737.3104099861529`},
{0.13040192171577758`, 2.9737527300384095`, 0.07310292644734392`, 1.466567655421121`, 0.5616562924844843`, 0.2174201253491196`,
  0.017973630894190788`, 381.0744410819025`, 0.2070882324093905`, 363.12098913994095`}, {0.12850488774735863`, 1.94264952671025`,
  0.05360394961309327`, 1.3166582015996433`, 0.7420300946941296`, 0.6747046075237411`, 0.46225759842348835`, 653.3639045731352`,
  0.1781000624097991`, 153.16234178224983`}, {0.14131707783478825`, 2.47648774559096`, 0.046912654007802104`, 1.102943262592814`,
  0.2119600715047596`, 0.4901715753981759`, 0.0580109813149486`, 2619.9859648711445`, 0.01885924901675312`, 866.9553146407405`},
{0.25596962129585343`, 1.7441590604011816`, 0.07394014025769799`, 1.0817705534126583`, 0.7417269961287187`,
  0.3670491724777639`, 0.009656798208801406`, 431.47684692132157`, 0.21109646038438556`, 541.8299411106649`},
{0.17137889910326493`, 2.547743818855161`, 0.051249488967469524`, 1.4387428259441999`, 0.22533128633818977`, 0.21356004296130449`,
  0.010718966990308196`, 426.834678908343`, 0.10633739166308992`, 565.8122888467649`}, {0.1356919186397164`, 3.275909693087268`,
  0.09755833451316673`, 0.830833686577428`, 0.23758326238226246`, 0.37684838867012627`, 0.34622549381973555`, 2800.980761749347`,
  0.05589233942552713`, 397.59708636141903`}, {0.06319547070029213`, 3.0135506306602915`, 0.08658656932688455`,
  0.8750884263433183`, 0.43559283695953344`, 0.17423436075008547`, 0.3556921476320783`, 692.9068977663358`, 0.042185132157772975`,
  222.69074576769867`}, {0.051353934055559936`, 3.5419315477765503`, 0.04801539957297914`, 1.3650767438471239`,
  0.35603852921000145`, 0.4618444822640493`, 0.00593767767296163`, 2613.749958603078`, 0.019287810526956883`, 756.2455097050401`},
{0.20935469703068038`, 3.6183197679011254`, 0.03566796922371117`, 1.1753688638954878`, 0.7004011433506241`,
  0.18665090004926455`, 0.010273985816104142`, 1326.129340467039`, 0.14178606357406276`, 910.8324510297567`},
{0.25107423404415036`, 0.9233938441474328`, 0.08706047090121377`, 0.9278477911660168`, 0.6395373622443865`,
  0.41454776302472407`, 0.011168650588331405`, 350.2039717436121`, 0.018063541364192554`, 440.5654385955753`},
{0.18503330381957606`, 1.3843983227309558`, 0.05599341470950066`, 1.2326838759285517`, 0.5366716060108483`,
  0.5872039546866128`, 0.021673868633287827`, 440.55468856416707`, 0.05873091092641808`, 958.3232953637463`},
{0.06679430502398687`, 2.97961750490882`, 0.06874693539558713`, 0.8324720698932511`, 0.04783195910412 6964`,
  0.23900392037978713`, 0.05029201371754466`, 1312.9498214501468`, 0.05149188289007395`, 357.12256566984183`},
{0.2633133297348904`, 1.167395880011603`, 0.02628362214068364`, 1.0744483713964295`, 0.2579472965293179`, 0.6802631987807441`,
  0.10725370629344262`, 53.88085410525317`, 0.04507037997684166`, 433.7101304465891`}, {0.2010390664309742`, 0.9179437940816833`,
  0.04118734602457437`, 1.4112901498922994`, 0.03943650490351103`, 0.5936392993401745`, 0.007904983488694988`, 372.8977436408934`,
  0.1257212210729608`, 514.9151276266798`}, {0.2679869983047456`, 2.831371041491759`, 0.04323230583074366`, 1.0163736311152665`,
  0.9719920644948712`, 0.3094609684087656`, 0.02204068430568152`, 76.16804088198147`, 0.16897723749861843`, 499.6307316546634`},
```

{0.05683769076526879`, 0.650647885401221`, 0.02840792532917323`, 1.1275069757067548`, 0.7854264529060555`,
0.22126398655563229`, 0.06980518344934367`, 574.9844145764067`, 0.1210354338257757`, 562.7623109630207`},
{0.04996310769523532`, 2.710365865528229`, 0.02845470152648966`, 1.3080682866189928`, 0.07861434689660651`,
0.6705317255564853`, 0.006348108900509377`, 549.0755816993258`, 0.061170873719453955`, 318.91974598994375`},
{0.2755006956503554`, 3.698123728642071`, 0.09922032193054331`, 0.8332466983901297`, 0.2785036755762762`, 0.43490103767019017`,
0.15490829410477724`, 4617.932809455717`, 0.11837182487721798`, 340.2748740925912`}, {0.2734635980899412`, 1.5929961417122707`,
0.06137119836965004`, 0.7652341524879904`, 0.954168112916443`, 0.43307045621476403`, 0.1582788386688816`, 71.48508495772737`,
0.24624735395364544`, 972.2197337262259`}, {0.2181683242113333`, 2.1761487384705624`, 0.08088096446343027`, 1.0959670952629994`,
0.6079403055392678`, 0.3741554091001601`, 0.02009386290007227`, 69.7071844679123`, 0.040607395764710064`, 305.00876855756474`},
{0.24133385636483384`, 2.7891523258501225`, 0.005829082609902692`, 0.8597961081451015`, 0.9570483419454672`, 0.29444398106251457`,
0.04229917912093678`, 1941.779238616586`, 0.19957823673360453`, 490.6542116348769`}, {0.12306942715949237`, 2.252880201646872`,
0.011446250013559797`, 1.0462863109436527`, 0.5663933692592433`, 0.47495024626336324`, 0.3233936229451822`, 86.1453410021763 5`,
0.08197672020931229`, 782.5197776099736`}, {0.10769046541924182`, 2.4605116390609316`, 0.05377772146881331`, 0.989407475316354`,
0.2930401465570722`, 0.29181294086362775`, 0.10863060011045422`, 4646.540814363835`, 0.22419809836059507`, 512.9803490302319`},
{0.21497939836377283`, 3.1512094394139076`, 0.05781466570999411`, 0.9235677861713979`, 0.797875992877098`,
0.30592437760429736`, 0.011963817124529342`, 496.30104999186307`, 0.12299294969111368`, 343.6950402862427`},
{0.2657260134641566`, 2.9019690638082407`, 0.03280205239027208`, 1.2552566977527801`, 0.09347633453726867`,
0.6906835420223192`, 0.05597654901690233`, 214.21462946437896`, 0.14839555678735822`, 189.76155409360942`},
{0.21292359448836545`, 3.6237126070126635`, 0.023273632947346867`, 1.0934162632306244`, 0.6322993827793542`, 0.5537399147284487`,
0.3715227242167489`, 758.3220767157304`, 0.23574243432919756`, 314.70942110857914`}, {0.23082110188218175`, 3.794164580108057`,
0.09951024223913159`, 1.1044050381914734`, 0.2931375323115071`, 0.40868926988441434`, 0.10698117113686255`, 1766.3231774314004`,
0.1575507952957782`, 782.0701995703548`}, {0.1731048010854444`, 0.4511019492052632 5`, 0.06558149662017904`, 1.268779063180271`,
0.15752730810346982`, 0.1844619681378331`, 0.4734368872326295`, 214.57885630606498`, 0.10442024095385904`, 238.91185603032366`},
{0.2554292953302569`, 1.1427932465434605`, 0.04899287050638531`, 1.3736804562756375`, 0.5882660335289671`,
0.17180037030824735`, 0.05309276876348573`, 276.71914948998887`, 0.05937534025119473`, 386.16358526001164`},
{0.16644797368483177`, 2.4992189221756043`, 0.004445377489754368`, 1.4133660089877695`, 0.32679827704181763`,
0.3418600753563352`, 0.38105352658785596`, 1798.4227174359469`, 0.0853854152424785`, 140.42796197604304`},
{0.05401664285302743`, 1.3436659136216367`, 0.007303531204713778`, 0.8064085762190545`, 0.4041746333332612`,
0.5906806798990734`, 0.005958103091852778`, 2587.5652974623863`, 0.09992354552595534`, 820.3533386597787`},
{0.20307037888791557`, 1.4293968611238235`, 0.007006708117720848`, 1.1406730143707504`, 0.6144629354618545`,
0.3719133121966949`, 0.00813248394206873`, 121.80260956296803`, 0.14105543387259395`, 126.00065959923121`},

```
{0.2740843330487453`, 3.308166539499317`, 0.0033332412164113734`, 0.9387759672621804`, 0.8792897541259657`,
  0.2588278323921356`, 0.0906560451270411`, 289.10750886695166`, 0.0689222507314608`, 274.0455429116968`},
{0.0941844879899188`, 3.012435636197103`, 0.0977143251520188`, 1.4935685648224934`, 0.03172920895860054`,
  0.19879535583488106`, 0.12277001567292761`, 281.6381134207748`, 0.12553563404062174`, 818.5606422688558`},
{0.13120359665298414`, 3.8066558838990874`, 0.05832666562127484`, 1.4436216710518677`, 0.7256118790918118`,
  0.22190148447236047`, 0.055627038916505056`, 331.2421431929122`, 0.023623634114193437`, 105.97098539393085`},
{0.1599611237255819`, 1.5653869796304836`, 0.04073827294465206`, 1.420732828376658`, 0.4167553998465383`,
  0.5683789027240399`, 0.006867177748429021`, 437.8657191219929`, 0.20093958315759247`, 853.3201855869486`},
{0.21575208583629502`, 0.9356056552129512`, 0.03505308262533445`, 1.1968736187898597`, 0.7561599337939984`,
  0.4654145195769487`, 0.10760666835377902`, 1053.3658392946543`, 0.20711098924554222`, 210.78286348037096`},
{0.14749719864367028`, 1.1298646044981888`, 0.09689905730204273`, 0.831362255602855`, 0.5798187583416305`,
  0.19996262727341718`, 0.02080299930415397`, 1905.6099187599903`, 0.03509497286958235`, 329.9767515874418`},
{0.05611993210971444`, 2.018253520390404`, 0.05603878791329802`, 1.1233601094750865`, 0.7854886288850511`,
  0.27746521744215147`, 0.0053225072398486025`, 101.97524414758992`, 0.19072350614781147`, 586.0302656773133`},
{0.07988047106873392`, 1.2467973601355782`, 0.005734825596729962`, 1.4870141755274235`, 0.786482359746921`,
  0.40753461440319994`, 0.0616456048522424`, 430.36934645621176`, 0.11501201441136555`, 555.9632996241309`},
{0.21976948435818772`, 2.6949030865877113`, 0.004565631201115927`, 0.851615304193428`, 0.28689924805671385`,
  0.25117757846067346`, 0.1420011334809148`, 365.7629364689339`, 0.17048089811131317`, 458.8930260541114`},
{0.24569357480220383`, 2.806982006055639`, 0.03541916579786691`, 1.1486472973181063`, 0.9842633355008354`,
  0.6578328260657522`, 0.06186663500336862`, 676.0260667202342`, 0.15355635401466894`, 104.93495329528014`},
{0.2568863641209479`, 0.7034804925577145`, 0.0941603933918832`, 0.9631193622252813`, 0.4879437161835407`,
  0.2995943026897797`, 0.00877818042938852`, 1278.8744528151467`, 0.17037713399806426`, 719.843140498881`},
{0.15208884515258675`, 3.3804365982678615`, 0.007505379740790073`, 0.8013128272354585`, 0.48161188241254527`,
  0.28391647832046085`, 0.008016681423664398`, 51.406574561981365`, 0.07108676915203138`, 187.7624783373312`},
{0.10637517181381878`, 2.6673257763233797`, 0.07389800171077983`, 1.0335545955573995`, 0.6258721856651546`,
  0.552024947614703`, 0.3934377256232211`, 1365.910979824601`, 0.017092456029375425`, 449.7500884619777`},
{0.0649812277693595`, 2.884896944749496`, 0.07783207858895651`, 1.428219843208142`, 0.5851112039722763`,
  0.4850552612198272`, 0.13145374596336928`, 233.36752929196032`, 0.020935430712967162`, 793.3352241051653`},
{0.22088364361500656`, 2.753665575599883`, 0.056531767859618255`, 1.38986306399133`, 0.7216745774063014`,
  0.3584382836914882`, 0.15422309470836812`, 2154.2453486409945`, 0.20075088684393572`, 196.6876979035824`},
{0.17574945972757133`, 3.479082463657387`, 0.0406948089429684`, 1.0632324439925762`, 0.7336036718363608`,
```

0.6225865045970325`, 0.01654846847609865`, 2647.8857343385694`, 0.1742065007038966`, 538.059550515096`},
{0.04025957477335049`, 2.723445036422185`, 0.0624307243457759`, 1.2961681244376777`, 0.2134882654614516`,
0.39895923327159644`, 0.012423634234475615`, 68.80792227348101`, 0.09152513701065051`, 342.5597984149974`},
{0.23836095562622683`, 1.2444047973172507`, 0.05553779779641564`, 1.076392253001211`, 0.35117607296612463`,
0.40534329593724594`, 0.006015625989151594`, 1819.5202248158637`, 0.12753202451029555`, 623.0420974247954`},
{0.26944870454169506`, 1.2060790013825349`, 0.039462741842031746`, 1.0581301327520456`, 0.10601447465615688`,
0.354379020364461`, 0.033711267495647235`, 990.9089845688537`, 0.031312059666100456`, 967.6243986980542`},
{0.1444585379798823 4`, 3.535198432366248`, 0.06372334173632162`, 1.4863884210475007`, 0.7021222356246233`,
0.1986028344377223`, 0.033605257874194515`, 401.94568584516094`, 0.22631225203628458`, 497.0198172800991`},
{0.19199489014371673`, 2.559659501669885`, 0.0435827670715008 4`, 0.9357767944167577`, 0.5821038208204001`,
0.3881836320458101`, 0.08395917774758936`, 399.00745257526154`, 0.15489271985369857`, 774.2528196011518`},
{0.09844334988931319`, 1.3873519913225651`, 0.04614436554245893`, 1.1498948867492238`, 0.47347293312976735`,
0.48947207082211097`, 0.0900112564683952`, 300.85371376045117`, 0.10865036330785854`, 289.3128973476921`},
{0.2588239052872208`, 1.9064804051930793`, 0.065214860918082`, 1.2358133969471479`, 0.9062007858068677`,
0.6285986730260349`, 0.02508709888565183`, 60.40797732056926`, 0.24714947667059417`, 952.5811518915539`},
{0.15433829014876738`, 3.2630592980638813`, 0.03425127382548034`, 1.0548030673325137`, 0.6906919434954677`,
0.3631290272789046`, 0.0347694820539026`, 173.0213690165559`, 0.20951393482972103`, 475.6784304680295`},
{0.07919142001495871`, 2.7255459074120383`, 0.05226476773837156`, 1.4738421429170918`, 0.07762084300215877`,
0.32839016838251456`, 0.28010379475182423`, 1228.3196986901144`, 0.022535205722582274`, 511.1200103954101`},
{0.05563602603579554`, 2.285803588715188`, 0.011271259422675524`, 0.8886812633057506`, 0.8488682312030629`,
0.2180988504645489`, 0.04124220143069666`, 325.42600392283333`, 0.015282318354637336`, 540.3914665193738`},
{0.26726930802972265`, 3.468951790252442`, 0.03848449069490482`, 0.8094106772420622`, 0.06275944440065384`,
0.3246635527776901`, 0.054987775783502536`, 681.4969078295769`, 0.11090812120155558`, 354.5762000041195`},
{0.13377593551305073`, 1.1907304154621343`, 0.01146637788789656`, 0.9181303013739212`, 0.44430229039926394`,
0.21249075461775713`, 0.11547143720610627`, 203.15849668645484`, 0.01100896387178596`, 295.5191145619232`},
{0.16315983268315204`, 1.1080296708247115`, 0.06583864001022185`, 0.865015106617715`, 0.12286793950783781`,
0.37991054755624265`, 0.13902707267015119`, 715.7992806626531`, 0.019079624096566744`, 581.5012259249219`},
{0.15517021586963486`, 1.952115790572729`, 0.0731100150366914`, 0.863005494839794`, 0.11113391823909224`,
0.23929002865779792`, 0.3427593945863136`, 157.02882367804486`, 0.1258544997368633`, 151.66501695475426`},
{0.16216149919315814`, 0.7994153119966949`, 0.07344189350605326`, 1.46448250423246`, 0.11568032132459538`,
0.4524728031579279`, 0.007439586263343828`, 2935.686946515946`, 0.23568961866347637`, 848.5436646987821`},

{0.15863268122870933`, 2.604649964738848`, 0.09105452346714177`, 1.3281546343893726`, 0.6415701788553387`,
0.5395588121712969`, 0.10672502596586574`, 4293.9755867585145`, 0.22612411051961012`, 801.1531009053148`},
{0.0470360951706533`, 3.5075246181417876`, 0.0689698582393753`, 1.3992260841209352`, 0.49274029849989587`,
0.3265485661944224`, 0.0896880649482509`, 3553.0072655615363`, 0.24883204385112412`, 930.4206898584084`},
{0.1301565511337785`, 3.3013385458906184`, 0.060172545647574065`, 1.1841312709206266`, 0.7389603235116047`,
0.6545296783352468`, 0.09213436798988452`, 632.9865742133833`, 0.01144874409169866`, 153.66076223065957`},
{0.19211271095347948`, 1.3267678786947181`, 0.011925004369487074`, 0.7708856579991501`, 0.5967808987867167`,
0.3563983615639521`, 0.008295516898745303`, 651.3850149278022`, 0.065165960143103`, 674.6701400488206`},
{0.04092327463376352`, 0.7045023567934869`, 0.08460571137678446`, 1.4657750963462932`, 0.7181575655127523`,
0.6488519412096585`, 0.15923360328476563`, 212.85100756959832`, 0.0515917167399186`, 462.3207119503768`},
{0.19270602889895483`, 1.9529553547869458`, 0.06583688116670341`, 1.3515290363153516`, 0.005091282852797141`,
0.36050263163910423`, 0.04479551947501828`, 203.77608216685013`, 0.14826242909234083`, 529.3883166966048`},
{0.1050601938980929`, 3.4490435237177026`, 0.08223909341236009`, 1.2367817503973777`, 0.9277363378878729`,
0.3699674586709595`, 0.4385914759613132`, 1519.1710373444578`, 0.23151573895912042`, 820.0175517477519`},
{0.05132348611758153`, 3.959642395183179`, 0.0488828276779843 9`, 1.2096431470311648`, 0.02692029465885825 7`,
0.43312132839092765`, 0.3519494697432887`, 129.77286276747867`, 0.014447876552552863`, 379.4279055426431`},
{0.13421887508919655`, 3.5366003696861075`, 0.04365656524319377`, 0.8302898645594913`, 0.33283176141556736`,
0.32180578132251225`, 0.12171969152254764`, 58.615799213607424`, 0.06462503110882584`, 898.0928768715434`},
{0.15819611106702364`, 2.020855817361644`, 0.06579688155882536`, 1.2380340703095258`, 0.6378555510612616`,
0.3675344788090784`, 0.006545785906113509`, 101.24936252099768`, 0.22028280764448066`, 149.80163079680509`},
{0.2499495815562608 5`, 1.6151536382110443`, 0.09538293813329457`, 1.3475056084534551`, 0.8251324191653782`,
0.518473471172061`, 0.013209857312129224`, 465.53610432365656`, 0.19186631952926742`, 938.6440248484497`},
{0.1956555609490701`, 2.03149296201667`, 0.09660508820628273`, 1.431718750027923`, 0.5308578462289135`,
0.43609275022077343`, 0.05994448278703194`, 319.5342769319219`, 0.05601191301657277`, 788.8827504958957`},
{0.23193438538805855`, 3.16433220464147`, 0.06263350952730523`, 1.484540865185697`, 0.945228356849932`,
0.5695514219689578`, 0.023879141173510178`, 147.89101930946188`, 0.24041444251942057`, 974.5971477651331`},
{0.2528274636845232`, 1.6765266145916016`, 0.05359196557993448`, 1.3463142085764375`, 0.9224467805077987`,
0.16620719793609418`, 0.013039811850177031`, 103.76980611708078`, 0.22032399406907538`, 401.8839359060937`},
{0.23077639135389166`, 2.5631050348732005`, 0.04014355344850944`, 1.1219336672611722`, 0.11159784718139387`,
0.5983828871337603`, 0.12763464721917558`, 1560.2077328425014`, 0.20349144783421902`, 903.744744090405`},
{0.06895016542438792`, 1.301044343226704`, 0.015962130237339344`, 1.182285615620497`, 0.5628258630936316`,

```
0.17632340412293257`, 0.3831530705725889`, 1497.278387537139`, 0.10171305044347567`, 505.35404235107535`},
{0.2787317780563141`, 2.375067420232342`, 0.0815658084141512`, 0.8993075085878712`, 0.2038087026634039`,
 0.6041957560913382`, 0.324966388605867`, 215.9571285973821`, 0.13818574272027367`, 691.7030542595149`},
{0.04704135783034269`, 0.4824465321524163`, 0.021143667148849198`, 1.297225751526362`, 0.5221151518138136`,
 0.4606696788974354`, 0.006779779197302622`, 185.59373538640742`, 0.01894971206264362`, 558.5730827937659`},
{0.21423251173707536`, 2.493227952439149`, 0.05289405397630384`, 1.406475234089612`, 0.7998464081533074`,
 0.509476952192685`, 0.4272743578374108`, 100.59740971479944`, 0.1412992063677468`, 892.9324365177549`},
{0.07990270577478725`, 1.026053986899301`, 0.09098769304378568`, 0.75484093198576`, 0.3137514363776597`,
 0.2597158546619064`, 0.021526708814218906`, 71.8454436542471`, 0.14857027641402232`, 192.33905427474235`},
{0.20790548900468359`, 1.6561792271072155`, 0.03308057679124518`, 1.2176117409116913`, 0.5649384654171494`,
 0.5537564006068142`, 0.021311473455272706`, 493.7190855791356`, 0.0879661543265085`, 522.8214036804926`},
{0.23583327723859243`, 0.8244276223039284`, 0.009610054530140194`, 0.999911169587162`, 0.6818020653793861`,
 0.4770396571968506`, 0.3762680384381647`, 1045.715543915765`, 0.20181855407690935`, 285.3335505694424`},
{0.2149596477715155`, 1.1258091287082204`, 0.04117204642206028`, 0.8287635491092544`, 0.19708791744131293`,
 0.30242514760323325`, 0.009963068581429268`, 2766.449995395716`, 0.1599911935585479`, 542.0030818591285`},
{0.09778089450463257`, 1.5512303348716232`, 0.04607092381086256`, 0.9234013469057234`, 0.7159741715951657`,
 0.15221755315547447`, 0.008899731124616716`, 1902.692936123654`, 0.11247025125228444`, 519.6608670719012`},
{0.2733616407663576`, 1.391826052520571`, 0.05242996857884462`, 1.2463936486263583`, 0.3747282584855436`,
 0.40124237280450004`, 0.11539389962842635`, 292.43427931036985`, 0.1835533339648669`, 592.2315796450872`},
{0.24918162326616394`, 3.2909828859462893`, 0.0031304822935452315`, 1.0930940447805135`, 0.3406640390037241`,
 0.20243554875381864`, 0.028649077189173346`, 1674.4319759967557`, 0.19194057055819636`, 777.538166666535`},
{0.25645888779994136`, 2.282435807658568`, 0.08257899475169463`, 1.378184234375194`, 0.8190909920565956`,
 0.23648999352950728`, 0.029414947617452787`, 2478.9160402790253`, 0.12086031199538894`, 421.4774938421854`},
{0.12282576626019515`, 1.0173035286087062`, 0.0379861701996793`, 1.401110687144116`, 0.7192159067178037`,
 0.40286532167194544`, 0.36624490921162295`, 2426.5499538363206`, 0.01808445874874337`, 556.291828749522`},
{0.08314629308908505`, 1.3023656036359537`, 0.09511446512381881`, 0.821564112530474`, 0.4777933736422373`,
 0.2692725743194774`, 0.006069954672804891 5`, 1469.9917989704652`, 0.23042452149338993`, 154.46286861175`},
{0.21957774039482975`, 2.751122632050148`, 0.013942756799365678`, 1.0532031012381704`, 0.8873864151539932`,
 0.23272558697907686`, 0.007181177159222858`, 4013.7501241473833`, 0.14211730184734184`, 952.1784892015081`},
{0.26384054503883103`, 3.138964037644092`, 0.08426203609396872`, 1.3770189510815902`, 0.4475271713808333`,
 0.3491883286754355`, 0.07774969397541334`, 618.3912182185799`, 0.0638489190260 6338`, 535.1175352858716`},
```

{0.20560343993895913`, 1.5779437043773905`, 0.07680738613512926`, 0.7894682023823474`, 0.9949259646898643`,
0.44397150101458005`, 0.018989097980746553`, 294.52516577442924`, 0.14255921229578516`, 385.97631580256467`},
{0.2723816434566987 4`, 3.1728219863305904`, 0.04998959318576006`, 1.2550483187184474`, 0.08966409063298642`,
0.2754392206527779`, 0.028155523330625355`, 413.44364879258364`, 0.10742347205803915`, 204.45184033599216`},
{0.2451942638086284`, 3.321324534398453`, 0.09231223940162195`, 1.0826750771187965`, 0.290821416328257`,
0.5533414871497576`, 0.3812913244405754`, 2830.6500293967956`, 0.03030872516300448`, 250.95228864955956`},
{0.10306075838040374`, 2.5636670031903526`, 0.062478631313391465`, 1.1237391323830406`, 0.020325627807727287`,
0.4138983427169105`, 0.01567041094182252 5`, 640.0409779587225`, 0.2299818577146827`, 855.7014985263506`},
{0.1543081177985079`, 3.6905447676223817`, 0.02801124131684004`, 1.4594516817243341`, 0.19245562145181472`,
0.22404862937840908`, 0.01176083329958924`, 1165.1333013103292`, 0.08358867889715743`, 831.8544035344403`},
{0.06171989970085079`, 3.434249919377687`, 0.06572710058482858`, 1.3211715182855723`, 0.01504624767015783`,
0.4567422389972121`, 0.16959599175310128`, 4317.9045896509315`, 0.11554862800770349`, 591.0128116780224`},
{0.242854939016651`, 2.284046375461106`, 0.07510045340186228`, 1.3823454805694586`, 0.12288532968332899`,
0.2505129792578149`, 0.19202736337063933`, 949.9596745663976`, 0.23010030070393422`, 645.8973615583986`},
{0.22700129023591364`, 3.923952544058909`, 0.030515149632633083`, 0.939180364827058`, 0.4621492504536673`,
0.6517443382103951`, 0.014468218060931168`, 427.04247682874535`, 0.2370408654331208`, 259.4368577693423`},
{0.07805880992570535`, 2.501643518243867`, 0.009418707493693895`, 0.9986888477411795`, 0.32030485689176835`,
0.15640932443277744`, 0.052825556109682474`, 1053.3265205661667`, 0.23821781066142345`, 975.8018452897034`},
{0.11861919681642336`, 1.4687434978704887`, 0.0829781170205376`, 1.0362850735520839`, 0.6712083946301921`,
0.44248360772337636`, 0.31715757872607053`, 458.6280472607073`, 0.19463738072293596`, 242.98487605538276`},
{0.059281890431487294`, 3.297569244457442`, 0.09408483617597145`, 1.4708447962140283`, 0.8202767387462129`,
0.5182490075472995`, 0.15407063447248953`, 64.57603365703892`, 0.059976329837068354`, 643.9595265565569`},
{0.2572247154689537`, 1.909920813474347`, 0.05239854389036264`, 1.3414720605906971`, 0.4513315960556996`,
0.24795401762018465`, 0.018990463442733085`, 1296.597641454505`, 0.13705502920429874`, 197.81881626257612`},
{0.12947779232825135`, 1.9145873156534714`, 0.0288911518259296`, 1.3705880176351148`, 0.34333617932115823`,
0.684343564948868`, 0.30854336196404863`, 221.38025911589182`, 0.07491746507159502`, 703.3196235432932`},
{0.07972116217449848`, 3.4709822723848074`, 0.04576191227888536`, 0.8016369799963272`, 0.2857357367482012`,
0.3331460762626077`, 0.19358831260020504`, 243.63380400065088`, 0.06895044823250152`, 836.6535842859868`},
{0.21425814556096656`, 3.412718953326978`, 0.054294933223100375`, 0.8434533127486217`, 0.5949443832779631`,
0.4312511536994379`, 0.006774834458589081`, 264.20975235216673`, 0.18238909307892875`, 619.340566973434`},
{0.1030949911235679`, 3.2379695384683664`, 0.01929347915744433`, 0.9201548487613123`, 0.33739940615248143`,

0.2622793831217315`, 0.23841206764006043`, 1617.5278268905984`, 0.22935132610715692`, 896.4339292297652`},
{0.11659113279351957`, 3.589133205154993`, 0.07913036370723393`, 1.232106957500204`, 0.8783988188812624`,
0.36486871630814866`, 0.12887619218337418`, 531.40225777078`, 0.19649941700330426`, 196.1431366000954`},
{0.17261096694115408`, 2.323826295719984`, 0.07573381776980444`, 0.8286032399217114`, 0.47983148793793484`,
0.26566773118764775`, 0.03472365295524612`, 927.3460129902165`, 0.1071272043003238`, 118.60052904781728`},
{0.15812900546790443`, 2.970437030179216`, 0.04641714570644767`, 1.3852443817278377`, 0.43447243659924073`,
0.33574441370838803`, 0.06435446224943432`, 738.6696158761797`, 0.13010058805108404`, 159.186393081969`},
{0.18592939740642223`, 3.685442589505847`, 0.07945757449204009`, 1.1464224447055114`, 0.32318739791637174`,
0.4649314153677717`, 0.013135441017131483`, 91.15430963381306`, 0.11860323377042997`, 381.3120573740848`},
{0.14376640925421846`, 3.1586224337701454`, 0.06046685266972789`, 1.2403049905427794`, 0.41520844208600294`,
0.6171055455360721`, 0.32907344516358666`, 110.43943865511335`, 0.09753160070705702`, 466.9944611056849`},
{0.08898564055719721`, 3.6481907193018275`, 0.035736135100176744`, 1.4644447392392914`, 0.3855084819966672`,
0.540354353409667 8`, 0.02564672554096082`, 3261.3971739494646`, 0.059327692271889`, 178.0636196825421`},
{0.12073346816538172`, 0.6796921643614748`, 0.004902893841691274`, 0.8582452939012792`, 0.29165716690801435`,
0.6326916410656882`, 0.010914425076951347`, 71.33612718800642`, 0.018422948540203682`, 499.39101647732576`},
{0.08643478967247115`, 0.989294451274473`, 0.06406214590812752`, 1.4778521994730294`, 0.08864943219429122`,
0.598523331628892`, 0.3392401780260839 7`, 243.33847401579249`, 0.1630923948211001`, 823.5357318052028`},
{0.1858290979683801`, 1.3708640419198979`, 0.03326172130438391`, 1.4020046543416833`, 0.11208251576177464`,
0.2625003629250131`, 0.04524672886209799`, 85.06379115175038`, 0.18737298414395726`, 615.0030267833952`},
{0.1360682807160305`, 1.260103979250026`, 0.09631205400585557`, 1.492449539848565`, 0.646188927535122`,
0.44017189812527846`, 0.4105272425293273`, 393.8607844518778`, 0.02987920633520591`, 916.2850221914006`},
{0.1722967578431442`, 1.2064516945248789`, 0.006692486646882791`, 0.9574846319241982`, 0.7639212221033438`,
0.18533797387085427`, 0.023148480320789932`, 1419.1935015960435`, 0.20782666110883385`, 726.3055622381481`},
{0.10120560496305181`, 1.0077236027617351`, 0.0022035031006077867`, 0.755653235614423 4`, 0.4541695517780062`,
0.5272435056704864`, 0.4861113106752323 3`, 2530.0708561728975`, 0.016102479809594256`, 464.0343986269878`},
{0.12017322050098367`, 3.9721385392144386`, 0.07574165293605646`, 1.1837442643757194`, 0.5577757261385461`,
0.3183184732454556`, 0.006181796792011399`, 369.4013122196196`, 0.18128847005418425`, 194.03504301481541`},
{0.04827662252574555`, 0.44476989943772693`, 0.04589418991887675`, 1.0976519464428027`, 0.5211218352826839`,
0.35897962206032186`, 0.009662240534770522`, 2226.690418934838`, 0.1554674743129576`, 512.7162631562013`},
{0.05162633508079817`, 2.3550751217523676`, 0.0924989591884071`, 1.4157997348260007`, 0.18969184963171148`,
0.23851541168228685`, 0.13394186077419723`, 885.0580489506601`, 0.09290724513295379`, 581.8072838231085`},

{0.0703807113832926`, 3.5028014587851954`, 0.09731342789234894`, 1.4652588215217102`, 0.02379062714059521`,
0.45288235271478583`, 0.00942034539711149`, 1971.3577596359983`, 0.20635147200229786`, 588.3355736982174`},
{0.1722158503566622`, 3.9025180218674205`, 0.03287113170420332`, 0.7633959662136292`, 0.5426808864022441`,
0.3669211940284537`, 0.015798227561105518`, 637.0091161622244`, 0.018607030959375853`, 729.5190439080948`},
{0.07584294958741444`, 2.9472829223076644`, 0.08633754726872958`, 1.4485030454804857`, 0.4347108871800047`,
0.4606559371854728`, 0.488724952784251`, 373.5721326650099`, 0.07716317740414885`, 124.4741797765185`},
{0.24562593090753987`, 1.7880136046455286`, 0.061199201036337365`, 0.9504446252866968`, 0.8812906909113087`,
0.4211899677669948`, 0.13371199909368486`, 62.37984939467348`, 0.029994323176434023`, 335.34030777918923`},
{0.20825483037627235`, 2.8176663305166816`, 0.03003135959790159`, 1.3809928219048555`, 0.5442546740990999`,
0.2518879146968922`, 0.41623765779197747`, 1730.0044330431615`, 0.13532415983523782`, 593.0952047283174`},
{0.263098249275039`, 2.8719245261273185`, 0.057294037398088714`, 0.9272583089313478`, 0.5794976650588739`,
0.23557943476926713`, 0.18465618219605576`, 743.7540401729835`, 0.12098568685424893`, 886.4027693713879`},
{0.08125449990693445`, 1.4583301942945166`, 0.06189519777160188`, 1.4288277781740808`, 0.40197254124717063`,
0.569085386893778`, 0.039194281573835754`, 4748.401415793844`, 0.20401727261566`, 992.5067856915206`},
{0.13086541558871118`, 1.1155725899250655`, 0.08821908955004687`, 1.3478216191079166`, 0.17697042399948426`,
0.36922117198662885`, 0.013930530425628602`, 265.9989136069838`, 0.2270416561589264`, 365.77204194037876`},
{0.11728100988180301`, 2.8151532257585448`, 0.0822952483643148`, 1.4499657290535213`, 0.1422354506761183`,
0.18155390727644505`, 0.00812157039144657`, 2435.595550489932`, 0.23995058381388307`, 812.4638732087483`},
{0.08049510800011694`, 3.603435994634494`, 0.007656876221115407`, 0.8287914836133501`, 0.8266092354769616`,
0.16324385237676353`, 0.08964145435399143`, 249.97635146943674`, 0.11550610667653838`, 765.8954747498603`},
{0.11820480579010878`, 2.60227134202185`, 0.04712951554705157`, 0.9880816634883703`, 0.8571809674721294`,
0.5649946552056018`, 0.017402816460863866`, 131.80950340449587`, 0.2293131996837257`, 460.13754914511065`},
{0.07353660759709546`, 3.5071493949530606`, 0.06008403863777037`, 0.7789529963855885`, 0.038880804706464156`,
0.24085809668436897`, 0.009207422134001322`, 57.365635753359946`, 0.19618331785464976`, 195.58478591654787`},
{0.14064741473086906`, 1.4253939258069908`, 0.09267725847840932`, 1.0975927496329574`, 0.22744648794341704`,
0.6063348207456416`, 0.03639182347931457`, 146.15301297855677`, 0.07935681769679304`, 259.4064208000535`},
{0.15191662908335968`, 1.3875044471862585`, 0.07598535142080483`, 1.4975086468472412`, 0.14071696059051497`,
0.2702248025276742`, 0.015378992010870783`, 1297.8058794576364`, 0.09563415627019778`, 267.1180590070449`},
{0.25783578932170337`, 2.5098960068891536`, 0.050196727090169`, 1.3457348584940565`, 0.6334273233006393`,
0.673911062730165`, 0.45542142980584244`, 1102.3077248576458`, 0.23127744020305874`, 280.7607833294188`},
{0.15728354284965573`, 1.1786483564465087`, 0.018710974043178813`, 1.0906308648763483`, 0.4692891978030753`,

0.5884245578610128`, 0.013999626118543194`, 1146.3540981218957`, 0.12976415437247035`, 943.3629249089528`},
{0.15744525017814853`, 3.9343441044396323`, 0.048424319387596705`, 1.0189964999962107`, 0.004699907891199784`,
0.35673641117911215`, 0.05845873245808589`, 1452.1810356944638`, 0.20019375268922035`, 991.5190215908439`},
{0.17711189820828616`, 0.9222841106349815`, 0.07731703618957698`, 0.8581707670014744`, 0.3028714201791183`,
0.3403814085412159`, 0.23552438227321898`, 185.6757896447749`, 0.15816694433359801`, 971.5707714133565`},
{0.12677717388625714`, 3.135520314905561`, 0.0958777837314775`, 0.8945786394252853`, 0.6294861294982774`,
0.5552940848485673`, 0.015606330011113338`, 857.9173420903631`, 0.2215517078689302`, 931.1017779440821`},
{0.21702108783814894`, 3.801779784508854`, 0.04805641449981236`, 0.9791693500326768`, 0.2245653714607041`,
0.5686073977459262`, 0.13790116867346938`, 202.46130862913807`, 0.1350408422039594`, 899.1324956275001`},
{0.1342014919902998`, 2.2832877374577247`, 0.03691753722884416`, 1.293132587653252`, 0.6577552625086529`,
0.43953980905115686`, 0.01957893390503574`, 205.45267528315344`, 0.11907718935756234`, 594.1955832844324`},
{0.1763744245545265`, 3.739113668312668`, 0.013915261017202703`, 1.4628218122817214`, 0.33832232520701955`,
0.3614238110077841`, 0.16749424131931667`, 750.7001786075303`, 0.16098306031842058`, 199.9116742658522`},
{0.15496422412126426`, 3.2726277520924976`, 0.04166420456847202`, 1.246251490612826`, 0.03723582004781312`,
0.3169293011472515`, 0.01364338217624883`, 794.7700944318913`, 0.13403305026443924`, 181.03718972044246`},
{0.1794519767096271`, 2.1595478222580216`, 0.024155004216522703`, 1.2232218292039836`, 0.12872104355462577`,
0.3105596069491494`, 0.08640881754488854`, 438.81545657103055`, 0.13579526038432777`, 696.2213922750994`},
{0.17257389690071828`, 0.774117551399768`, 0.008560234856989925`, 1.4940154641879095`, 0.5531402581000995`,
0.5441107289119099`, 0.07350653490141655`, 1314.8314542940839`, 0.07345603701745179`, 217.02216774233065`},
{0.058763034644712275`, 2.3597566945503834`, 0.09055618598093744`, 0.9263657689345207`, 0.5592210171940297`,
0.26262790696306815`, 0.011057611204984635`, 211.09574188287445`, 0.05571628830959996`, 675.171201042442`},
{0.23649888242497868`, 3.0694012769080574`, 0.0293881057277982`, 0.8469899032416341`, 0.5329226292412448`,
0.4016300524993841`, 0.08030522569307784`, 885.044604178621`, 0.10534611402615895`, 622.0478384674557`},
{0.10023410603180455`, 2.710596168246937`, 0.00298453338827164`, 1.151795972074526`, 0.37899349738211896`,
0.3392482218824491`, 0.04819504839896153`, 379.39038491273027`, 0.04469242387250283`, 827.1435707909849`},
{0.2438903560692493`, 1.1739940858261848`, 0.09102105800199446`, 1.392584698828661`, 0.6056955824388208`,
0.5844521424450305`, 0.36108528400570095`, 1701.2530207661314`, 0.11138522848439819`, 308.591402552778`},
{0.18793054675634024`, 3.139542677489672`, 0.08904425028360773`, 1.4068659558890193`, 0.46779742309438377`,
0.4804254450552986`, 0.31816862647583416`, 52.007157617767675`, 0.20815008924208545`, 645.3217048463795`},
{0.11103510208969514`, 1.4116670170359447`, 0.01094255418186231`, 1.3998271086778162`, 0.20796976919688226`,
0.21292073278793122`, 0.025573376903052888`, 86.81893641374597`, 0.03626690081608863`, 355.9231685657631`},

{0.059767794596915114`, 1.8804870041256674`, 0.07095681077704744`, 0.986817038205352`, 0.25655361425528533`,
0.1688625628269923`, 0.1897075342910607`, 67.57112317559029`, 0.19542023600496528`, 963.6021600539973`},
{0.0563343868292458`, 0.5317396897533424`, 0.021545836837937156`, 1.1439759156456886`, 0.8881137136810024`,
0.4156539248732849`, 0.006243471710013326`, 260.74733013676007`, 0.24312372574340813`, 696.7530796966602`},
{0.25281786498427167`, 3.164006130077736`, 0.003838750721372204`, 1.0744832678586205`, 0.006849791935805038 5`,
0.5152441133630022`, 0.32224630023151535`, 264.37961782996655`, 0.06028750963433477`, 143.50957591757913`},
{0.16077715085906397`, 2.873224638724322`, 0.05173801459016383`, 0.7810632519144793`, 0.3328755824267646`,
0.25599177499415204`, 0.29027099579570653`, 56.71390358897392`, 0.0339115808969098 2`, 522.8798210385845`},
{0.21945025832526777`, 1.6613676443983767`, 0.0033920248574508707`, 1.057752773749318`, 0.2127399240494794`,
0.2783903022229003`, 0.011232097563550716`, 1371.276504196438`, 0.04975311118637471`, 328.70478941546`},
{0.042964316768383276`, 3.4639489625729407`, 0.050691741081661766`, 0.9465946216833707`, 0.05502079292147011`,
0.21238865048524036`, 0.0776422991682389`, 52.80026867227022`, 0.045020214701994854`, 474.41563595375555`},
{0.2433495999796823`, 1.8960270447479708`, 0.01120198462392917`, 0.844049298875671`, 0.4639208209806316`,
0.20677684984563183`, 0.005733096581983471`, 1740.6658389561255`, 0.1399372816422193`, 628.7700453738331`},
{0.09814127364381425`, 3.1310383407484847`, 0.017016069480737403`, 0.9217748908925876`, 0.27863388851936555`,
0.32184170341904983`, 0.006207472689688149`, 1391.556177922241`, 0.16816866777593836`, 615.9674047842972`},
{0.045179834179820105`, 2.124737466901916`, 0.0641039677828362`, 0.8087710768531244`, 0.5820240872072311`,
0.6898665398618178`, 0.014125759687518747`, 92.19454081613313`, 0.015821689323895105`, 557.0872764229717`},
{0.13473842282979165`, 2.4117633636086957`, 0.01984561256760543`, 1.1204070544446296`, 0.3860645674275691`,
0.4170622324306399`, 0.019865666343063196`, 4743.148389421012`, 0.04365212493615206`, 322.93322910718985`},
{0.2527515364737705`, 2.1426280202987984`, 0.015606989419899584`, 1.125943287101995`, 0.17776360180897943`,
0.332914794116577`, 0.006519056311462601`, 78.48896188261014`, 0.12889711459762526`, 805.9196842114062`},
{0.11933913407793822`, 3.785485787216923`, 0.023574857312977055`, 1.4812641194980447`, 0.6275581210964223`,
0.5280696447262914`, 0.07256024376782456`, 241.814705594111`, 0.02783432907145139`, 151.3743596717724`},
{0.1349803930463928`, 1.8072527206786377`, 0.06447251791280252`, 1.3994960916143184`, 0.7359286316821669`,
0.20162137238693434`, 0.4265854166639518`, 227.4386051384146`, 0.13592337876958527`, 497.305076361482`},
{0.2637152651333133`, 3.0391917107143414`, 0.0836047103384918`, 0.8164164023077424`, 0.5538018525602337`,
0.5004598109403166`, 0.043561691627998805`, 3000.1047519173976`, 0.17835324975540034`, 767.1983419134932`},
{0.17730450742771336`, 0.5526401983890268`, 0.003866546119779244`, 1.4224823875795298`, 0.9713446082448272`,
0.47362133992508215`, 0.008799575820309609`, 460.0427974972538`, 0.1268858776449615`, 614.5975351416361`},
{0.17661690011227865`, 2.968666144862362`, 0.08820580921653833`, 1.2618748536532438`, 0.014044591388426397`,

```
  0.6372850210418368`, 0.4246195750078259 3`, 908.6535515532695`, 0.16038310667129974`, 176.50654123832936`},
 {0.13672640919029144`, 3.614316511877404`, 0.006473169531331831`, 1.1642896824935516`, 0.29325294311380845`,
  0.1624724995588664`, 0.030111728610588202`, 3933.205934300977`, 0.0980103812613437`, 106.80306509175406`},
 {0.07145172009956824`, 3.410868367663567`, 0.08572451188759761`, 0.9584474542559545`, 0.040382356795113816`,
  0.6455580091740019`, 0.09117312647503195`, 1954.631204089039`, 0.0777204277630249`, 144.9524499282374`},
 {0.13883370155342772`, 3.715034522567122`, 0.016916922596204795`, 1.0000211488246231`, 0.12894118452278325`,
  0.24258423424654219`, 0.02087499948517953`, 3780.1181299010777`, 0.24420906754435595`, 798.6323972467644`},
 {0.21440197837823782`, 1.7615321043929084`, 0.028950668418736624`, 0.863153176908 3587`, 0.523525808424337`,
  0.2748912735476704`, 0.38068084592210494`, 404.89859548140885`, 0.1540862403656934 6`, 803.1703020004433`},
 {0.27645780167060263`, 3.175276677600297`, 0.05299560517709523`, 0.8086008634045363`, 0.7913310844792734`,
  0.3862667201926391`, 0.00961380004706706`, 2836.236561313336`, 0.22859831535956182`, 465.6465289390621`},
 {0.06752882943979077`, 1.4908646201280487`, 0.03739887788507268`, 1.1321270467820062`, 0.1590886090266126`,
  0.33058750825147076`, 0.13664295377993954`, 324.6730016626649`, 0.23288398005137873`, 299.5733325408761`},
 {0.10900083730464943`, 1.3165965495337897`, 0.04096974974497696`, 1.1178893614799246`, 0.02481354114149492`,
  0.6533849288221782`, 0.0989115744507999`, 59.94210305630967`, 0.08210787534991087`, 838.1221078005716`},
 {0.18821745287135477`, 3.8834155988951853`, 0.08623976994927297`, 0.9451777127739934`, 0.2536651767905649`,
  0.4966204244968453`, 0.052355463971012595`, 1253.2350223323115`, 0.025530907055034302`, 107.88292349347337`},
 {0.13433362908189822`, 3.976975346921523`, 0.023713728561632463`, 1.330972786055094`, 0.5425931686264049`,
  0.5867946121348381`, 0.083015502101061`, 2146.731613991672`, 0.11433497831643785`, 956.3793106500469`},
 {0.05370292234035079`, 0.8618436041219777`, 0.09065607433649935`, 1.1199215929503574`, 0.7033869517843656`,
  0.5744405842935578`, 0.20721453360880282`, 163.81435947307054`, 0.15960680813292716`, 808.9619958368114`},
 {0.1982510252132108`, 2.465970568324745`, 0.012757083454587495`, 1.214300872852428`, 0.49977561113114644`,
  0.2038568209362135`, 0.04565388315282881`, 78.88460864136573`, 0.1487998824136843`, 164.57689100626015`},
 {0.07006449306202894`, 1.594466036555235`, 0.06954877206660835`, 1.0929666985547377`, 0.8182673122674597`,
  0.16380036850673574`, 0.04761505660877746`, 3094.547812800814`, 0.026912730925264194`, 465.941138242661`},
 {0.271620743989626`, 1.798748508191406`, 0.039226132696162194`, 1.201815394348386`, 0.019033121630884553`,
  0.500394949620919`, 0.022898921692070705`, 167.54130076556643`, 0.17172411650490582`, 118.02597321115923`},
 {0.17015700880843931`, 1.1880680435779825`, 0.09612747942645765`, 1.220203731936928`, 0.8171639571524099`,
  0.29942524478114674`, 0.005372366003640249`, 607.4487584881643`, 0.0444162424317765`, 783.4604894784995`},
 {0.05414527078515874`, 1.462858717153524`, 0.057585439178684264`, 1.0235777810825535`, 0.21014799698731879`,
  0.27005953264512594`, 0.08376210292871798`, 147.35977444766536`, 0.10744240382696624`, 846.2849324778499`},
```

{0.09237320793743342`, 0.8096523848475279`, 0.004358295040692077`, 0.792421334681352`, 0.9354401956315697`,
0.4996618434240512`, 0.3257079961489366`, 106.0381723510655`, 0.194703596109299`, 875.1849354846129`},
{0.05024769460534284`, 2.3552798474120182`, 0.045898586534112516`, 0.7657469521997533`, 0.41572162978856886`,
0.6250502955136628`, 0.036232245589174594`, 4925.729350966778`, 0.048338820069271815`, 396.9216964218738`},
{0.16604829560323886`, 3.2400306473910465`, 0.07805779128319201`, 0.9800901947770135`, 0.02662429037050673`,
0.6202147453627995`, 0.0521761817913341`, 319.666779674947`, 0.1526499768736515`, 641.0814189728534`},
{0.13199054420769823`, 1.1822647026678919`, 0.05315251799812888`, 1.3177578061426032`, 0.29009102787676366`,
0.41153300112684843`, 0.027654998358556677`, 862.9125744805588`, 0.22565453257412804`, 148.78642951801135`},
{0.15326781447665427`, 3.7132135007643896`, 0.08379527648859465`, 0.9155066607147588`, 0.9247491129828225`,
0.43619880658774446`, 0.03926748261529228`, 1156.2321575105877`, 0.17547647957302887`, 889.8562914260897`},
{0.25318856010923574`, 0.5926097353022888`, 0.00482353837495765`, 1.3099001178559129`, 0.39149861531549`,
0.647818541877643`, 0.25232461737348105`, 3385.571345294367`, 0.19387361798630304`, 420.02567016405266`},
{0.24993243970651624`, 1.7126281402055845`, 0.053130097502228`, 0.8352588223269972`, 0.62388332746021`,
0.5762423608317969`, 0.014465580674644738`, 799.5116049181332`, 0.0931416280797775`, 865.5405963251784`},
{0.19561758310618566`, 1.3596417600698452`, 0.00286504944841953`, 0.8159672694367253`, 0.6358724387725987`,
0.6648416656940517`, 0.24177467962143764`, 3682.5795096853144`, 0.15293398533505775`, 386.4064670424524`},
{0.2447874085020979`, 1.7744291418487776`, 0.060075050805309844`, 0.9864750267055266`, 0.7342694514660453`,
0.5397411982908532`, 0.4042131026855616`, 575.9477405967004`, 0.08605927470106772`, 780.0883449415986`},
{0.07734058301535968`, 1.385482226243342`, 0.07152447691997342`, 0.8194619571046995`, 0.4573258756273968`,
0.6888219817424488`, 0.1334830351653064`, 199.92831385353017`, 0.08814262925395056`, 749.6278229482897`},
{0.10474568576280202`, 1.8596472368495407`, 0.026805293435349233`, 0.8198649413737059`, 0.5254103672469626`,
0.2748607556216799`, 0.0523834433135859`, 134.70642314731086`, 0.10106748211794048`, 140.99421671530888`},
{0.27890810575443187`, 1.4911450307991796`, 0.07330732877841264`, 1.0850224852017472`, 0.511581631756513`,
0.6844047819742511`, 0.4182444515115468`, 898.6500980057638`, 0.1508853505547692`, 117.74931414924143`},
{0.06710090564388627`, 3.150840959910446`, 0.0014124858884598979`, 1.2404545757648113`, 0.6590308151409987`,
0.18742776248185322`, 0.07838269037439821`, 63.02543384043976`, 0.23165453423661014`, 134.9827251445024`},
{0.2709692633763827`, 3.0318490895249166`, 0.02057996898061427`, 0.8497637000182061`, 0.007946519407763919`,
0.45375292920923727`, 0.23156314091749142`, 3625.9461118472623`, 0.20365524494076642`, 575.3949074180657`},
{0.15487330171159508`, 2.506241955627659`, 0.02405679630252637`, 0.9599539152532017`, 0.4302913454357331`,
0.26259574333059643`, 0.008099203461346816`, 910.2082053871129`, 0.14622107038108184`, 559.5315182891957`},
{0.2128087852183892`, 1.9571603978537304`, 0.06326297053797199`, 0.9699003392267482`, 0.2686671251031061`,

```
  0.689623844783678`, 0.35675989285485865`, 1924.6232853270492`, 0.23326907367518723`, 915.1337901802619`},
 {0.21780708062769377`, 1.1747776713117952`, 0.05034010906267806`, 1.0913007058111903`, 0.12353581217661103`,
  0.3210562571265647`, 0.0322536912509453`, 2197.1982196175004`, 0.1572434618024398`, 414.86282493944026`},
 {0.10779474152210772`, 2.5545130403913596`, 0.09607448919858275`, 1.2919315212701343`, 0.16453105789719658`,
  0.6141245226266829`, 0.11684667552500605`, 196.8514855477255`, 0.10491847566430007`, 112.73037991828173`},
 {0.12432532516395484`, 2.1300608216646877`, 0.04913772379496075`, 1.114341717459199`, 0.2610859336877449`,
  0.503197213507616`, 0.005797395507410687`, 442.94048846434436`, 0.08741076870037628`, 101.71296622490348`},
 {0.27464475878650824`, 0.5510255383265457`, 0.031636220540726276`, 1.3762194055027865`, 0.161256707272891 2`,
  0.4273900785282383`, 0.24603471434298904`, 429.9496642319049`, 0.08771573488057915`, 617.3490635717783`},
 {0.13029287084326696`, 1.6894675116680409`, 0.020566584774051967`, 1.2832846584058795`, 0.1828960091839662`,
  0.4605785391689514`, 0.01135531247121644`, 936.7188945707877`, 0.1761057360191931`, 440.679523047495`},
 {0.20510392052346132`, 2.0152643396686836`, 0.05752244878800122`, 1.0760055282794754`, 0.9860596725458286`,
  0.33518476119672647`, 0.31856559936642365`, 321.34121932230744`, 0.10497410797788737`, 281.7881521533858`},
 {0.22222058819666546`, 1.9181392247611697`, 0.06353852306281663`, 1.3758404515279568`, 0.6438678141184426`,
  0.1693786566729748`, 0.06897920477033548`, 383.0298020915635`, 0.10314770849680055`, 941.5930224696303`},
 {0.21276124668973173`, 3.586839401277566`, 0.039178902951713945`, 1.4021843911360103`, 0.5049572433506289`,
  0.25133132700774996`, 0.12364866403599967`, 623.8499997024826`, 0.11712847066144477`, 770.7321547534711`},
 {0.14968687983730605`, 2.3633590766978303`, 0.04173348230150333`, 1.0168117310979305`, 0.07526686867368393`,
  0.37085554163771406`, 0.00768165513782035`, 1659.4326212173246`, 0.1437616377539393`, 942.572084587879`},
 {0.2713741706327081`, 0.4242782972192547`, 0.07078081316399253`, 1.131720191226623`, 0.4549262787557089`,
  0.34468168234433216`, 0.012942279609896707`, 2864.005301731598`, 0.09798425989157222`, 187.35732163896745`},
 {0.1768611384179205`, 3.4923686338372777`, 0.01646184906513861`, 1.2055226780828572`, 0.7343581951493021`,
  0.49378742542959486`, 0.12048169571136383`, 2844.3094803530207`, 0.12693060413099172`, 545.4728905288805`},
 {0.2369345296836391`, 0.41785553179229007`, 0.012526518118659648`, 1.2179877794222598`, 0.01846774830289344`,
  0.24685867084706536`, 0.0219438069009795`, 2091.108395341874`, 0.0637607114780574`, 657.8903568599761`},
 {0.21219769453448084`, 2.488104916250064`, 0.038757338160515825`, 0.774789527802302`, 0.4580558695696113`,
  0.4263775736158787`, 0.34953699783630143`, 407.33606250987975`, 0.16713719417824968`, 389.7058187031195`},
 {0.11474454899039521`, 1.9841405956471387`, 0.009912438082135093`, 0.9199083705387736`, 0.9235359490124031`,
  0.6470781918812183`, 0.3331422211969604`, 337.2489956721694`, 0.03475601824362995`, 538.9648304299371`},
 {0.24234974629560802`, 3.9617359783801245`, 0.024444176238852366`, 1.4313282134950815`, 0.9940513113675349`,
  0.4143492563365402`, 0.1686590259483461`, 68.11457543267173`, 0.19793807681647463`, 199.02629821091972`},
```

{0.16236198209412894`, 2.018699278359029`, 0.03626856618687025`, 0.8436685495890566`, 0.1369133257491073`,
0.18228484143603907`, 0.040551804892385857`, 156.7528279727044`, 0.1739090814809508`, 704.2383268945476`},
{0.1621577456082553`, 0.6421908435706589`, 0.0682618183426171`, 1.3238181641882336`, 0.9244165647014204`,
0.6930553521066385`, 0.13666203325037238`, 72.36369505518103`, 0.2211105145097111`, 327.06990857144615`},
{0.2702619348258324`, 1.3531215117783164`, 0.06648966931523986`, 1.278915076565248`, 0.7821690760804008`,
0.4832187850250197`, 0.03589870905112195`, 3130.704914059961`, 0.14156174429321455`, 311.74724954691885`},
{0.19935819825153228`, 3.831285341751962`, 0.061322567547608904`, 1.470671933797358`, 0.8459029813533634`,
0.6098411086562021`, 0.020718424330298454`, 338.23739251616234`, 0.1348110564933563`, 328.9796748551533`},
{0.12820856017846227`, 0.9705044791290884`, 0.020813708364227884`, 0.8227896910361784`, 0.779325226128513 7`,
0.1540874083560777`, 0.019838047780930596`, 1485.5571438891636`, 0.16001313078246715`, 526.6105313836905`},
{0.11895043241393044`, 3.4783832049993846`, 0.08948985928895077`, 1.359795719210997`, 0.29460393871375956`,
0.35020600424213755`, 0.2746425669615434`, 50.083789355993105`, 0.020545547787407692`, 916.6997924739111`},
{0.1324468409940383`, 2.674634590833133`, 0.04368586320677444`, 0.9656288911809869`, 0.20832826835990526`,
0.3737566616358602`, 0.005582642109220031`, 55.02582924504472`, 0.2065757664044986`, 615.3366029893464`},
{0.06870110651611899`, 2.6561693493128598`, 0.08012546640983625`, 0.8012667555503843`, 0.031201182173437925`,
0.47282927837851574`, 0.011587617652012341`, 3773.634663778097`, 0.09147096998858567`, 835.9545348732762`},
{0.062399865483643235`, 1.7222840522480984`, 0.0921920191456875`, 0.9286857558090922`, 0.3251018367521341`,
0.6108891276310624`, 0.011783888986275243`, 670.9821418853228`, 0.11450398298509856`, 971.5077469427929`},
{0.24591568234282185`, 0.8566394248947855`, 0.0781061151752332`, 1.3941060542854928`, 0.531302066365049`,
0.6676078161595655`, 0.1738149187521547`, 158.69973172683407`, 0.1485119105497385`, 936.3340802553399`},
{0.06567085889032587`, 3.9337642436409004`, 0.06099064297489713`, 1.4242284268139727`, 0.6539174667789966`,
0.3970266459135522`, 0.44668463659234353`, 1229.440509286354`, 0.19297859064513334`, 104.53761160348893`},
{0.13077961834437962`, 2.6471887027919063`, 0.08803755851714129`, 0.8915760567705249`, 0.43528239011744874`,
0.6770072844670658`, 0.13724713444300793`, 2266.2113033407786`, 0.18158499094944586`, 450.76741342966307`},
{0.0626945060366299`, 2.0207863261901435`, 0.04028779663279089`, 1.1608394772654784`, 0.8954638473430083`,
0.6803040308141024`, 0.0438755308716426`, 1072.86320924307`, 0.09417670420636176`, 203.3883701052643`},
{0.06633302157481619`, 0.6897085661220981`, 0.028600562511313363`, 1.222942456179899`, 0.45117275354230246`,
0.29367119707525824`, 0.1114281718553938`, 56.37596421013418`, 0.06329831527765895`, 495.6622356704312`},
{0.0758463119875773`, 3.621924471713986`, 0.052843607016089394`, 0.9710310359872816`, 0.3507881375218056`,
0.16711099920227068`, 0.1488874639582541`, 4335.730117904631`, 0.02658055782022295`, 793.4396666616456`},
{0.06315507988926289`, 2.3297312843414817`, 0.00212708414905217`, 1.395352700358994`, 0.0641701490182165`,

```
0.5894635107517013`, 0.17809044151421888`, 379.3821707157771`, 0.14230190042275537`, 759.6382607226183`},
{0.2099022158481964`, 0.8809344339859928`, 0.0666751548993475`, 1.4387478328331107`, 0.3531306367025422`,
 0.6983916858003694`, 0.014622538997794657`, 343.9480456094322`, 0.08685603103174627`, 426.825757963735`},
{0.18558107573674792`, 3.0194943343339986`, 0.07461391639025422`, 1.1191398296135584`, 0.8305222019918481`,
 0.4880407736474275`, 0.30782204523980083`, 408.3075491000427`, 0.12514760530359148`, 124.23432338200726`},
{0.20051301950717854`, 2.977167267300211`, 0.06936215087558335`, 1.0259008901103701`, 0.6147776109399516`,
 0.6258821206893599`, 0.01703908364047091`, 2344.5258662598194`, 0.08418799298306856`, 794.1399968502964`},
{0.23048732313041992`, 3.462281492713984`, 0.028460833429753602`, 1.0414779858519587`, 0.11068368992785804`,
 0.46918663455326926`, 0.3985525154764997`, 883.0495583098383`, 0.2409468662662823`, 546.4207520227328`},
{0.211465410867755`, 1.6738825157761648`, 0.0523685881403539`, 1.2569145647413262`, 0.6940459633746912`,
 0.19386279088766634`, 0.025145096530955775`, 502.1390324130799`, 0.03396962103018908`, 864.65280998117`},
{0.1929466873888901`, 1.5728514227245611`, 0.08398743525565142`, 0.8305271019348486`, 0.30866536632560715`,
 0.24782227733300222`, 0.06727104200994766`, 4073.498181247491`, 0.09367172319166167`, 761.8812104829784`},
{0.09217356026466877`, 0.8891340726836079`, 0.02261394061546632`, 0.76288584990135`, 0.2352883211999115`,
 0.4087782246426179`, 0.05761668157743837`, 592.1949999543295`, 0.1566262893866785`, 939.2503062920734`},
{0.12967920647240816`, 0.8123733202889643`, 0.0013573621543738134`, 1.2244900945773618`, 0.8403401749152237`,
 0.45714635607461884`, 0.04113284132792122`, 683.6853491881902`, 0.10112850721928329`, 691.2742096973634`},
{0.17585942228676693`, 3.1377953149568008`, 0.0015744621411661974`, 1.416873746788956`, 0.5827830119619737`,
 0.6742879545649707`, 0.09162791382644989`, 517.589004535642`, 0.16103345930524726`, 547.8413219418244`},
{0.2427638279415646`, 1.6566162823863317`, 0.06521965313277005`, 0.889062176257591`, 0.014518981829873656`,
 0.2748704572491185`, 0.05735800450312215`, 2387.1739807284794`, 0.23199018500208857`, 381.464796278728`},
{0.2519418117913656`, 3.063462870735645`, 0.007778137389172581`, 1.4021617024508317`, 0.9924604733729623`,
 0.36233195131635443`, 0.18682309925698343`, 66.44214693634154`, 0.1722230669694152`, 118.3771627882729`},
{0.17349345626891455`, 3.174288570189952`, 0.09652146927294172`, 1.3390466263031546`, 0.7909502427935802`,
 0.6793254105715787`, 0.08700743247214943`, 763.4035938655402`, 0.2387926278324622`, 592.4203788389427`},
{0.1727078461124784`, 2.8460728910005475`, 0.040914427699617326`, 1.4313337292763195`, 0.42218527760026237`,
 0.5016244570171771`, 0.0887548918690675`, 1587.4849620109512`, 0.1328615275829737`, 768.9319503194112`},
{0.08930008677181278`, 2.849971715639322`, 0.09169779308725978`, 1.2438628382038357`, 0.4154968472190934`,
 0.15405240023737177`, 0.04634668494948532`, 124.90941433976057`, 0.24982063081795847`, 463.43559716325797`},
{0.04995942432359332`, 1.4610302960771593`, 0.0433388762401086`, 0.9505438290907596`, 0.488211452413962`,
 0.42119172502998126`, 0.012759652857857887`, 646.7948127587483`, 0.026663072682046873`, 245.49015570500714`},
```

```
{0.06795497506231873`, 1.985917542879199`, 0.04349282644250348`, 1.0631785554908315`, 0.8091824452378926`,
  0.2147272979078938`, 0.03845431657393897`, 607.4631067901736`, 0.08409909386800546`, 814.8177301929959`},
{0.27140987444452636`, 1.6298377410728744`, 0.06029749698810518`, 0.8272061170373771`, 0.9095594755225849`,
  0.2626530714660902`, 0.22141343547525327`, 1531.0082827603965`, 0.16934746708503456`, 553.9108700580455`},
{0.20249329091250423`, 2.9260026808453974`, 0.09022408237555009`, 0.9840606653871036`, 0.4430644155249066`,
  0.26716691431663997`, 0.012442882013869866`, 1252.39191368304`, 0.1408105612614876`, 866.1715515451732`},
{0.13229111529623555`, 0.9037627200690848`, 0.04928384913936972`, 1.0937814374110013`, 0.9849443259560173`,
  0.43115790526717`, 0.019499938095150635`, 129.0159606559662`, 0.1987553839173642`, 393.89165319051233`},
{0.1970263766379653`, 3.9709307503384244`, 0.027958813802574572`, 0.9741203705977242`, 0.07278184160194923`,
  0.2945649201344811`, 0.013078030291277284`, 4971.688891574707`, 0.03180082765265402`, 755.4217432398528`},
{0.2049473596100468`, 1.974812376290127`, 0.018059141094917592`, 0.7718344152131382`, 0.3708285362372443`,
  0.2734681930545607`, 0.057458033126770496`, 523.7031096038135`, 0.02427329957544444`, 932.0905826464876`},
{0.06537885284118145`, 3.340707691253673`, 0.054351682254287964`, 1.020827128283552`, 0.11666967592415722`,
  0.49052917065042767`, 0.0525916907549581 1`, 117.00169938700564`, 0.09552258651198203`, 745.6309012306111`},
{0.11289673562266261`, 0.6794824966294883`, 0.03387634458174361`, 0.7927471128390904`, 0.07141719167345717`,
  0.5226577421579536`, 0.03829715492190054`, 2920.8934693444294`, 0.12081443854156904`, 964.8985972362055`},
{0.23766500383642747`, 3.8767325971078446`, 0.0584809543600726`, 0.8270011297527531`, 0.6149673245232845`,
  0.22395807159158476`, 0.013437587174666855`, 406.005827450584`, 0.2418694331681166`, 429.2383814891535`},
{0.2081776292468483`, 2.815887981403362`, 0.005125757826924850 5`, 0.8103479560894872`, 0.47351709229198957`,
  0.44544132586608753`, 0.4203259951948646`, 204.28043485055284`, 0.1992997883804286`, 827.7788091184511`},
{0.17107980313911997`, 1.611778555980452`, 0.08454567986455815`, 1.1369505371366053`, 0.28226812138903434`,
  0.677147760670262 9`, 0.13278544535359882`, 251.49910155434924`, 0.03764393371120245`, 453.2818524693168`},
{0.10511512871602496`, 3.5633360415567505`, 0.08943300281707153`, 0.8304995882196464`, 0.4228782437765999`,
  0.48401108468440646`, 0.019832659219655203`, 164.88993273954887`, 0.1933397413498107`, 248.59325812442776`},
{0.1315672644477009`, 2.3773291392807145`, 0.05190617787787307`, 1.2351268314391448`, 0.45158718255427366`,
  0.3842243141032361`, 0.038449226291938914`, 225.9216681882473`, 0.04341773379489022`, 914.0642920476225`},
{0.20624347857829856`, 2.4671372276614543`, 0.050734528297655734`, 1.1490024452760461`, 0.2202047098600366`,
  0.3908877988906867`, 0.02383144777523708`, 157.0379664488479`, 0.14716356068566494`, 110.7973831044216`},
{0.27080390848623775`, 1.9772333839416323`, 0.024251112595875153`, 0.8820022792455461`, 0.651342077315098`,
  0.5674047955018092`, 0.009713259722204017`, 54.31416433169486`, 0.012456849152343602`, 380.638174364041`},
{0.2662342547666148`, 2.0899914364276`, 0.0775212957879528`, 0.7629254448634359`, 0.7485227165858468`,
```

```
  0.5717925034597559`, 0.384531789999449`, 531.2378339914305`, 0.010110683485557853`, 104.86289985839541`},
 {0.08332863693803971`, 2.021121394734722`, 0.03701544455983157`, 0.9133309893951871`, 0.7759249909409063`,
  0.6241937322174744`, 0.02957247320826314`, 100.8666790089409`, 0.19088570031299712`, 262.59746879189373`},
 {0.14639594099937192`, 2.488995500972994`, 0.08291914891669944`, 1.3150002115787267`, 0.6540711522150526`,
  0.2055454195673022`, 0.1946827902708822`, 1690.1447445658273`, 0.18733657548952937`, 812.4826047698352`},
 {0.1305321301981358`, 1.4867396329579305`, 0.03655804827713382`, 1.4456727825450792`, 0.670555897133192`,
  0.6184895636508891`, 0.05124255420048954`, 394.1191568715212`, 0.03171421941058544`, 345.85608840747835`},
 {0.250721605591452`, 1.7010887783606767`, 0.08806284896739065`, 1.4847636082147322`, 0.4308392570475905`,
  0.16893566899927792`, 0.0864712763505O468`, 2056.3372882750145`, 0.12615446084315202`, 207.40781829993898`},
 {0.04392874140447878`, 1.9818443681166702`, 0.09434829100781027`, 1.4634488477570156`, 0.6811682982929017`,
  0.2554902190221564`, 0.06882358130064935`, 420.2484502763152`, 0.11914991474138031`, 179.7692000111727`},
 {0.14635546873540428`, 2.6921520503610896`, 0.005572522395533781`, 1.0650322427085313`, 0.01258547930967402`,
  0.3289894737792243`, 0.20755635390945587`, 2318.8984027990427`, 0.23607139988674775`, 598.8352065199724`},
 {0.20440309232409604`, 3.6324112419808596`, 0.02933293982791259`, 1.0491273986291727`, 0.6481946292303631`,
  0.5970564598987798`, 0.367615520297974`, 2827.3491736750175`, 0.18901453458314488`, 331.1017138964795`},
 {0.211339616570286`, 0.8062529435299846`, 0.03215637597034366`, 1.1329056004996207`, 0.6216719034637128`,
  0.26914056317845936`, 0.04626672609677319 5`, 367.3076671022254`, 0.06234507234404268 5`, 883.4219863160993`},
 {0.0754121889664276`, 3.3553240739439776`, 0.032766043984085905`, 1.35951382107467`, 0.417421746626248`,
  0.2423604100007123`, 0.37190628043481266`, 360.0955624739355`, 0.09650587549325668`, 113.8474438254784`},
 {0.2783033002921034`, 3.397814643841863`, 0.023780737803670213`, 0.7785779441011293`, 0.28908440577847827`,
  0.4851687293566612`, 0.2500599862274582`, 176.08406451146294`, 0.07757174693073099`, 972.6494889929199`},
 {0.08302491117654731`, 1.417237397662472`, 0.08064924241577312`, 1.4920703552127885`, 0.33660997181727526`,
  0.6086848413902193`, 0.43663790825582516`, 432.2028054996635`, 0.054044182009652`, 768.4690422643392`},
 {0.09729817063298457`, 3.4299522173089496`, 0.07735506084120583`, 1.4020158593772418`, 0.655176777497416`,
  0.2796434167254044`, 0.020513648578027708`, 361.54728331563473`, 0.18982251022644547`, 624.3692576556642`},
 {0.05918716742286234`, 1.9820539956622945`, 0.05196458247655251`, 1.4930492496804253`, 0.4915276700726614`,
  0.5297919483627916`, 0.24251322382900306`, 1013.8284387296288`, 0.1901510038138538`, 731.270474360067`},
 {0.05448705906655304 5`, 3.0521940188838643`, 0.06431699428389767`, 0.9108516151810719`, 0.22179534032337966`,
  0.1527600124061284 6`, 0.00618021185065336`, 1431.6290748192448`, 0.09019004900189659`, 615.4166559817393`},
 {0.1805007029704227`, 0.41916969450246455`, 0.016124853476479205`, 1.3709380974041798`, 0.9243445784418525`,
  0.5673574690172947`, 0.01323585349172664`, 768.6156722427371`, 0.14720827739001074`, 303.65297547748116`},
```

{0.13586333024917552`, 2.4333711568454603`, 0.06594815024548176`, 1.1422919388448811`, 0.127316527773377`,
0.5373692465409584`, 0.01747695081564976`, 1883.6141269851823`, 0.017752075188298855`, 680.6112691329253`},
{0.09049024561283486`, 2.3894866858591284`, 0.0013459889256840721`, 0.8533171338756729`, 0.3021314683838838`,
0.34607340060030345`, 0.30321966856095933`, 90.59478899907405`, 0.23877865208899252`, 565.9928476917297`},
{0.10259701094971274`, 3.6083949275799876`, 0.07320779035427538`, 0.9294410249753255`, 0.6943909777193686`,
0.5424209490023467`, 0.02226818211415045`, 425.8826453817381`, 0.14811442642104045`, 767.5349856757164`},
{0.0818626476168649`, 0.8476840265643926`, 0.05039571888660504`, 1.2803278760732222`, 0.39203490461702195`,
0.678685283379783`, 0.046408600947453554`, 625.4764903683038`, 0.18505631212115975`, 473.24089743900083`},
{0.07029108203608453`, 0.7073037290148494`, 0.03261231636893799`, 0.7728998119403233`, 0.4329865779673474`,
0.39085105872031733`, 0.3956962494413241`, 72.63981932713905`, 0.24239129287515127`, 232.2016718655236`},
{0.04958285162353873`, 3.761311858085456`, 0.07854169509494732`, 1.403762798092309`, 0.6354348772329161`,
0.5539469242046658`, 0.014552601524129273`, 191.8255147151473`, 0.1545116444713645`, 509.99375854807204`},
{0.15497576240515054`, 3.7755891399441612`, 0.04102415780603357`, 1.3426436269065887`, 0.4633835643985551`,
0.2105276629358308`, 0.005292621332827327`, 393.4300921998384`, 0.05255466894451982`, 181.97601415353938`},
{0.1462216796835833`, 3.3115430587931307`, 0.09201451210593234`, 1.2778172268764427`, 0.906784021865821`,
0.3687077782146817`, 0.05157961805804173`, 924.6290700374847`, 0.029607911315220503`, 889.789386014198`},
{0.13283897685814167`, 3.038408092963987`, 0.03461781675567849`, 1.3222463428410316`, 0.47510806724254606`,
0.6210207278956918`, 0.005408113696778361 6`, 102.55379834175085`, 0.04224157239084064`, 436.06625132099504`},
{0.11923152550729099`, 3.907162388051315`, 0.06641684248516906`, 1.318540470917755`, 0.5804312953963475`,
0.6280211407721501`, 0.005256835397320809`, 1226.3954007569562`, 0.17176906355966298`, 506.996443873342`},
{0.10227819097193708`, 3.914724924869418`, 0.0664380714143547`, 0.7944397029062766`, 0.15335804466948`,
0.366802499628480 3`, 0.06541187335723456`, 1449.384487729464`, 0.024262601947109108`, 554.3384286777302`},
{0.27621673974857747`, 3.975786747546901`, 0.0440045228345514`, 1.2561736736681604`, 0.12219205626085827`,
0.44109764605630186`, 0.27834299040512644`, 71.91340898917194`, 0.10015764539366545`, 482.1830368850472`},
{0.16141532638885775`, 3.5473613273302282`, 0.060131606026769265`, 1.1204941160194362`, 0.8786219204877892`,
0.42963549162688086`, 0.23724506417809585`, 4491.815167956783`, 0.06480015184829835`, 512.4293881945277`},
{0.2575375457582068`, 2.947834811130747`, 0.033810464285172745`, 1.0447722344490806`, 0.8627980313064527`,
0.34879266259466135`, 0.11166656744723742`, 94.36401066143432`, 0.11812196317533258`, 725.2748373314915`},
{0.20820386673509828`, 2.6786477995612135`, 0.010157905183889078`, 0.7567059297671783`, 0.6398946017563087`,
0.2916906367963521`, 0.04126212921113298`, 3268.442039148205`, 0.023820623550554088`, 259.60704797706916`},
{0.15593088724620752`, 2.5519577904336437`, 0.024361841648533833`, 1.1168597646554808`, 0.13563727928772762`,

0.3874274982263488`, 0.1882999430765413`, 616.7470197654534`, 0.0830157326232564`, 890.7479015168708`},
{0.24014955563677381`, 0.5220448196870295`, 0.02671701264219644`, 1.3551783563181985`, 0.799728403554941`,
0.47303019566294113`, 0.027399592270438356`, 644.7909772591187`, 0.07858290125002376`, 181.11983864094333`},
{0.1581702628188732`, 2.346334984324618`, 0.0581411782688245`, 1.4807167920720685`, 0.40458616499170885`,
0.6310195170221491`, 0.006522970079115585`, 337.28265576556936`, 0.12691983292355058`, 454.93623014073796`},
{0.23136224869656058`, 3.6088316121947086`, 0.03632040382309938`, 1.3419685001039396`, 0.40455118868914375`,
0.3221101170539603`, 0.25966887129973093`, 1676.6659884454966`, 0.19241943128863082`, 511.50532059075465`},
{0.17957415783469988`, 3.5968176444073308`, 0.048288185227319896`, 0.7994547395333207`, 0.9581588123096916`,
0.21642006235154376`, 0.14264678878084103`, 1476.5632047936012`, 0.020697112352618785`, 228.14672992784074`},
{0.059998032534718804`, 3.7773982431410413`, 0.06986223440078802`, 1.120487977215339`, 0.4567291173152259`,
0.612033275511431`, 0.07354302601323719`, 486.6146546384414`, 0.119884658675466`, 347.4528922941486`},
{0.19887637677541542`, 2.992660923165513`, 0.047995275724685205`, 0.9722118856000259`, 0.2651497498489779`,
0.5318886674778154`, 0.19636633656100977`, 112.46333630768565`, 0.06776166614050899`, 129.50929186524326`},
{0.09067863132057552`, 1.9262440927144207`, 0.03464921977559642`, 0.7578597622362966`, 0.480840305150003`,
0.24100415846854528`, 0.20408648616921088`, 520.809954661363`, 0.10723760431187324`, 547.2814452851367`},
{0.20782506825689806`, 0.8141429404339053`, 0.09514857720029408`, 1.3616807374452509`, 0.4328504524684271`,
0.33813491058186396`, 0.24032169387315838`, 461.50385309819023`, 0.1829235837022425`, 223.3752348589885`},
{0.11510496723960911`, 0.6612811922775106`, 0.0679688489060159`, 0.8790789776515309`, 0.5367830167767558`,
0.6858865509647121`, 0.06457419286375914`, 320.31094965266385`, 0.17862589858250755`, 915.319702346644`},
{0.17264723461047932`, 3.7903015192126244`, 0.06554920731341767`, 1.1764259933061223`, 0.4660090251563338`,
0.6413383966201236`, 0.241827388783345`, 56.79713040044933`, 0.023743987090588337`, 456.3571078119333`},
{0.1547837566229352`, 3.4953794802806852`, 0.07246861713817378`, 1.0879160111375143`, 0.07311426293155776`,
0.6990761768110039`, 0.005355312003055951`, 1173.1945274337409`, 0.08510663567245824`, 820.7115603554513`},
{0.12332890101103666`, 3.575694377588479`, 0.07546663843052578`, 1.1730448937718991`, 0.7299661739602221`,
0.3544423810526146`, 0.3517297854582263`, 207.52630070713013`, 0.16381025124403453`, 211.24976747011442`},
{0.07543310039566975`, 1.1413407123857509`, 0.06791044220530608`, 1.3022569182103092`, 0.8228503547730448`,
0.5416450068944677`, 0.18860915993442676`, 2038.110511149941`, 0.1613952093900407`, 419.7511258067653`},
{0.21429674136023663`, 2.8226782716380345`, 0.07008452435050433`, 1.2033565563578725`, 0.22607654151729606`,
0.3670473114848468`, 0.04920492797100079`, 426.3575789205089`, 0.22829240712916699`, 151.89428282997392`},
{0.07895444632032361`, 2.201415239192807`, 0.005337930232592641`, 1.0054037946739751`, 0.7982316099945579`,
0.22745190186265252`, 0.014751475681337079`, 999.912509637654`, 0.18790976944806215`, 156.9072222771119`},

{0.09184666059394636`, 2.067005962614928`, 0.04145877572545418`, 0.8296320077051444`, 0.11557680968156858`,
0.31351325881175707`, 0.021527373087813387`, 871.3636268629474`, 0.16510736043882146`, 233.95931170378006`},
{0.12260917712356023`, 2.30639494589911`, 0.05876506714974223`, 1.4161472400002522`, 0.4228788165875499`,
0.614217815805707`, 0.04726593691280875`, 1944.5171329959344`, 0.08163047191172412`, 744.531780752293`},
{0.17238170923293145`, 3.7452628271391264`, 0.012298914408557682`, 1.0114832888719023`, 0.7075400969976455`,
0.4701095442473505`, 0.009792251350088488`, 109.73168851300552`, 0.11561190536083749`, 580.7510496473817`},
{0.06459740373368028`, 2.9106606918316986`, 0.09546811583014093`, 0.7816504226428446`, 0.9051718026272408`,
0.6152354921314405`, 0.1292020640340832 7`, 192.77909888895545`, 0.1990333439617078`, 537.0263017540406`},
{0.07800640828878808`, 1.8505382879500134`, 0.012842566220368603`, 1.4649243339915694`, 0.6600118067779042`,
0.4967332224237151`, 0.013562590855424642`, 1252.0890643038667`, 0.10833365543192358`, 267.9181792628814`},
{0.048462811029039154`, 1.800487790371883`, 0.005299468930184581`, 1.0419920592385326`, 0.9100782296151841`,
0.32988971122619926`, 0.11628748764501183`, 321.3445579524093`, 0.10904785586908877`, 676.7351327707524`},
{0.0408586986071619`, 3.397631267565795`, 0.034535155982847086`, 1.149992697916574 7`, 0.6535863974222924`,
0.4932189245887587`, 0.383854582094014`, 103.0179671920324`, 0.1262724601642624`, 838.6520403111433`},
{0.056181027140717654`, 3.561092902333039`, 0.032415896850996564`, 0.8977612893413814`, 0.19606568802210056`,
0.5191107847286981`, 0.3780120623597924`, 2750.1468252074155`, 0.22792524388897922`, 419.0619039860675`},
{0.24051173539528659`, 3.363998136218836`, 0.07316535800080796`, 1.1407631035414798`, 0.3740167133471626`,
0.5883310500889865`, 0.1388691166946849`, 1845.7599241956455`, 0.06093536750612072`, 554.8160924336624`},
{0.10694470201355061`, 3.916523604921749`, 0.040570584968233535`, 1.141416795902756`, 0.9898088237744289`,
0.3296418758797267`, 0.04709726814380818`, 2933.808240863522`, 0.04460415393167344`, 879.0499414979915`},
{0.06862055370778036`, 1.8272394963651166`, 0.06696112608756692`, 0.9180601754047588`, 0.03279196554733499`,
0.20147163161780768`, 0.010370667030870775`, 671.676842322565`, 0.19673134057127845`, 347.1374473999164`},
{0.25063858582919535`, 1.0026414593770951`, 0.08049236416776281`, 1.1585705609095718`, 0.27877982032187476`,
0.2471176538176596`, 0.0055491534410242165`, 1284.3138459700085`, 0.027491789825886737`, 964.5039545904644`},
{0.26413366443306313`, 3.5187238265686887`, 0.059384833236120135`, 1.2922578870185246`, 0.008326928227079256`,
0.6434100059643171`, 0.031067351563113396`, 133.02587790916047`, 0.1993086925023529`, 393.43546913290197`},
{0.13920351751612248`, 3.9485961604821114`, 0.08033096245429228`, 0.961725822180624`, 0.7322696656937997`,
0.5625362274736092`, 0.02337054500750719`, 857.2150128038464`, 0.13429548054404716`, 132.1146622970133`},
{0.12997797022210922`, 0.8438763321472402`, 0.09917403003828128`, 0.8801736269011186`, 0.10893971026850768`,
0.568805635817268`, 0.4569650004322221`, 173.3039538372586`, 0.1310412151491429`, 829.8180726295691`},
{0.04282165785099121`, 1.644759382359756`, 0.006131876320932417`, 0.8656795781593337`, 0.5309250697882764`,

```
  0.28882829576135427`, 0.43822920515546965`, 171.56453795936363`, 0.17422534922512378`, 500.01112026736826`},
 {0.22211928728847102`, 1.1859807267849432`, 0.08277265577838502`, 1.3176134726640667`, 0.7769092359720373`,
  0.31677855442324954`, 0.01004066290735241`, 1412.8983237640775`, 0.05122004701487703 5`, 737.1658163615933`},
 {0.09296167151413748`, 0.46354983597275057`, 0.024036152116512888`, 1.3763603395412263`, 0.745954078027516 7`,
  0.45155144834323624`, 0.3445673156480595`, 2335.90860073376`, 0.22065872238926132`, 474.2017592615175`},
 {0.1962229794306719`, 1.6992229864288415`, 0.073818422425421`, 1.0867919112277424`, 0.7575363665855621`,
  0.6670448748187716`, 0.24718082226975022`, 1605.1879390833765`, 0.059896123435389914`, 160.67342656698588`},
 {0.21138025591123977`, 3.7198017071227776`, 0.012025339542637924`, 0.8791888222818957`, 0.9121961522822317`,
  0.34954022634411386`, 0.4885566362021042 7`, 4481.4869986353115`, 0.09130130840750089`, 978.8438514579077`},
 {0.174527181711994`, 0.849373235921072`, 0.0314016740925031`, 1.2229771378389438`, 0.5565629319212622`,
  0.15780972618613898`, 0.039946482002802375`, 1015.8195322962273`, 0.11236134949951643`, 212.56048531719287`},
 {0.27104366314172257`, 3.4411670504738847`, 0.05742812389939894`, 0.9577515288651626`, 0.0851317171213124`,
  0.6274935825967685`, 0.018124860207485008`, 2870.4044184423146`, 0.2324535673532167`, 472.3817854782288`},
 {0.11781804356750541`, 0.5626502822415125`, 0.01459820489930097`, 1.05752032760362`, 0.6292609081665201`,
  0.6860764088162852`, 0.029170421001759508`, 352.2383589143192`, 0.10359473073293657`, 222.23677753012652`},
 {0.15398221319678085`, 0.5849960064145687`, 0.05386556842999246`, 0.7888135605497072`, 0.7266422422856504`,
  0.40377533435456936`, 0.43018868620379314`, 1603.092147190326`, 0.20097603617156867`, 537.0534533002889`},
 {0.09435165103191007`, 2.1871870158802187`, 0.09386196089682226`, 1.0117647368175615`, 0.23992524376119828`,
  0.6944778195963781`, 0.011558368513568394`, 1224.4139617227247`, 0.05758577870260467`, 112.08810079007688`},
 {0.15418547479730832`, 3.244024899467486`, 0.031613474871280495`, 1.1180817216823555`, 0.1378867782832267`,
  0.24122334591588945`, 0.1784500939520896`, 1165.0611910719738`, 0.018111200873128563`, 519.7221352316303`},
 {0.18001954973510415`, 1.9120553169228627`, 0.01841751056966496`, 1.0848164059222056`, 0.5445307300173845`,
  0.21487607390213648`, 0.1420658743866174`, 59.88761221845187`, 0.10283589598798698`, 180.0642150440039`},
 {0.17238975113628274`, 3.831887800749228`, 0.005776257990374099`, 1.3458283817066912`, 0.08939800815002275`,
  0.20519935162452907`, 0.06363342671890825`, 2876.3582213396467`, 0.1796261387487414`, 336.8941601044835`},
 {0.14352493778031827`, 3.7979256036341535`, 0.03771360392389294`, 1.3633466357184463`, 0.3018171788859503`,
  0.19173241304281086`, 0.03417718814733039`, 2084.1653883747413`, 0.05483174208182884`, 581.7554610960195`},
 {0.20940311206829182`, 3.2582854913524706`, 0.06591828409332467`, 1.3970633596535897`, 0.8615344587047382`,
  0.38613005595364347`, 0.02066648198066755`, 4820.6850448541545`, 0.07989037484848699`, 539.3449932522163`},
 {0.13522362333277543`, 1.144461624392597`, 0.008064525972274694`, 1.2137320027234764`, 0.41558764869922493`,
  0.5044332234582337`, 0.194694572581245`, 1561.3725943749705`, 0.2352162319299269`, 635.4535165669378`},
```

{0.06564694091416517`, 2.517837422245419`, 0.08205855339446029`, 1.0554951116327027`, 0.7103060442920281`,
0.393784837384779`, 0.34842807990801816`, 201.46762707189424`, 0.22439022497167005`, 178.8572875798012`},
{0.24425093983878532`, 2.571873191821844`, 0.03797741011070798`, 1.0612508346456866`, 0.8063780340542086`,
0.4683983500560849`, 0.06605028609142427`, 2270.9148163281043`, 0.08295143441277542`, 860.3274939678396`},
{0.13421688107301388`, 1.6885106577171634`, 0.01327671934783954`, 1.4235239040662608`, 0.41175527118821376`,
0.35897461552598586`, 0.28058853074140605`, 344.729203987344`, 0.16559799427233646`, 938.0192541245116`},
{0.12355675427841095`, 2.0332534396069946`, 0.006925443967133322`, 0.9868311030648986`, 0.27448369335996525`,
0.49997436829086617`, 0.010689681959538098`, 661.0107417281803`, 0.04956754941428659 4`, 614.1468732990146`},
{0.24989261079843417`, 2.046336678398201`, 0.05343056582535697`, 0.9590211509682223`, 0.6962751749667404`,
0.5979718555935737`, 0.0621229258954893 9`, 836.0233579874354`, 0.028192514982996186`, 946.2272832575387`},
{0.18446589532009483`, 0.6770723958796063`, 0.017239459819183126`, 1.3829614064739535`, 0.15821669976510688`,
0.18877757485488766`, 0.0071198331982843645`, 1580.0156398402103`, 0.12251896238349375`, 604.1234967411239`},
{0.24207945515763046`, 0.8472072700897755`, 0.09830987943529323`, 1.3431438063557357`, 0.7063009601868007`,
0.2722426879126155`, 0.34992016942373105`, 1219.3651744969027`, 0.1756849319581012`, 396.46752882152185`},
{0.16929669277606063`, 3.384845133452008`, 0.04997850478346777`, 0.855944189945163`, 0.5854410422632743`,
0.6313899885471745`, 0.27030175060519007`, 69.39208015599864`, 0.14109734367472376`, 825.1088816761363`},
{0.22032907726938156`, 0.43680070868525345`, 0.02505576509377308`, 0.9013024202439682`, 0.3637877243645866`,
0.6575693669120928`, 0.08327886814493145`, 86.53687085308205`, 0.24093186652306348`, 957.0475588354033`},
{0.0915185159652725`, 0.4356928503469506`, 0.07385942693598586`, 0.894137684029105`, 0.07375739107747048`,
0.37304243578619745`, 0.3884276894579204`, 86.6179555964664`, 0.023525347333101687`, 957.1153747111897`},
{0.19293304608541778`, 2.881169086349173`, 0.06288677922536799`, 0.8471255106831246`, 0.5957102709253561`,
0.464151719061209`, 0.0381622120975603 1`, 288.4038900208833`, 0.10298551466758471`, 155.08445353721268`},
{0.18184235602556043`, 2.816880901418977`, 0.05545256089460814`, 0.876197436405354`, 0.954624174973548`,
0.4655369772467929`, 0.007648944239440386`, 383.2702509437632`, 0.14043117229016888`, 779.9607071498034`},
{0.1500634817243091`, 3.590063801011035`, 0.04690872877366834`, 0.8713971696364884`, 0.8016145774652992`,
0.57680193310708`, 0.007900899994569407`, 1759.5867354003908`, 0.1650460276258336`, 468.72962630703444`},
{0.07044808633799604`, 0.5092168059858677`, 0.023939619197820134`, 0.8363921850258171`, 0.11231870814099265`,
0.415964567014653`, 0.010393952295787622`, 272.30094842939224`, 0.24251268430438633`, 533.0209640034411`},
{0.17446312671000164`, 0.923278108173573`, 0.04622273024289145`, 1.361488933545279`, 0.05073013179716934`,
0.423140548702129`, 0.4945547029315037`, 756.8418975914155`, 0.05582670682313107`, 453.1988863343269`},
{0.25969186879026457`, 0.6876551199003744`, 0.016004864008093787`, 0.801399153017793`, 0.8167373300888658`,

```
0.5699218117881594`, 0.010392376675716438`, 2453.002800663684`, 0.22138430515888585`, 604.6992574427161`},
{0.08344350966636604`, 3.6673612041438233`, 0.0357399550994862`, 0.9043067215227492`, 0.3349583654565387`,
 0.3929910548411621 5`, 0.07044574168572774`, 531.2053684025772`, 0.2316097051950603`, 942.4468571449918`},
{0.11177106931508435`, 2.9258562986192613`, 0.04535829204836597`, 1.397904475123252`, 0.8323395818238934`,
 0.6262291788570806`, 0.2126230418930395`, 59.43001557400602`, 0.03786935133626829`, 393.82943302003673`},
{0.09014630069728341`, 2.5385294043084468`, 0.004644443169930969`, 1.1109052294542912`, 0.48602912154060585`,
 0.653448250732096`, 0.13848641693128835`, 233.22231224941473`, 0.13058754405383072`, 772.176206082497`},
{0.1204210700620526`, 0.6547366279014759`, 0.07856424763514301`, 1.2422551703125984`, 0.769232712809691`,
 0.34959559963353615`, 0.3952520916617596`, 225.12460677270738`, 0.24941607930389664`, 764.7416993751449`},
{0.12507255683757496`, 0.8093899840252368`, 0.022011166366603607`, 0.9039266843419957`, 0.9040126296575397`,
 0.40644665651439293`, 0.36973776842713274`, 4266.893817006702`, 0.22996060684055697`, 344.3317857496396`},
{0.18799072009935214`, 3.5387044337310227`, 0.010288586533272871`, 0.9709034820394874`, 0.1652625829759508 7`,
 0.2720662467892546`, 0.16314642121657652`, 213.79313950957913`, 0.23266932286427316`, 908.785142248831`},
{0.10303653650946687`, 2.045785570411666`, 0.032527698921882384`, 1.4813578055387457`, 0.42199129008222913`,
 0.6459634548526738`, 0.010175611270196772`, 575.847247144136`, 0.04915259766651514`, 563.8812676357885`},
{0.2398844305865619`, 2.1056225174808283`, 0.015327944846502356`, 1.2552986509569295`, 0.6918103031115113`,
 0.1889744468357467`, 0.03454744519234309`, 720.0345748750987`, 0.20720302058084095`, 561.8805940371304`},
{0.19472671711158268`, 3.4537873437782753`, 0.0539374140165368 3`, 0.9791709575229651`, 0.34020645306003683`,
 0.15465211878990004`, 0.005783246570334714`, 712.2087645951867`, 0.18659332879596663`, 390.1452748573064`},
{0.20571859545893295`, 1.5514943629000975`, 0.03978136161333113`, 1.2978001829388508`, 0.10072004838372872`,
 0.22845349492624245`, 0.25892912790336436`, 119.6045968067387`, 0.07803724137740647`, 936.2227260158392`},
{0.275720564806804`, 0.720336275328604`, 0.09315930706476526`, 1.2814448950695845`, 0.9719953187330976`,
 0.5030571263630652`, 0.09198284855367962`, 2926.101672056841`, 0.10631897378157318`, 340.03246937203465`},
{0.05630051102503303`, 3.7356157314175347`, 0.02800700712577493`, 1.0094636270338093`, 0.18952763541881779`,
 0.23258714070277053`, 0.4551334614672762`, 3326.1734768299775`, 0.20084773313292642`, 389.1282332545071`},
{0.1566545086835321`, 3.6144342723158855`, 0.01851051241133261`, 0.8062001646255728`, 0.7860505516306966`,
 0.5903319695657847`, 0.1423722018298642`, 1684.7441306012342`, 0.08281365756813702`, 745.921161780724`},
{0.26785050867880617`, 0.9336989210532032`, 0.047863988220149846`, 0.8870676821839705`, 0.894276639371691`,
 0.24671850596175082`, 0.25434132792942304`, 2463.897338729517`, 0.07340938538359204`, 135.25070223351997`},
{0.20621368926339484`, 3.895401327519891`, 0.021945870300711868`, 1.2149002945024092`, 0.09567565478536721`,
 0.35708592619490087`, 0.010140662222165558`, 72.14119724036794`, 0.09062375760852914`, 269.2376393484709`},
```

{0.22132018814331722`, 1.3624506083622139`, 0.02157572172797998`, 1.017162028467816`, 0.022930198784160805`,
0.174154916693125`, 0.0063722205185720695`, 81.57393238873418`, 0.23095499884940496`, 559.6417164883817`},
{0.2667728012376422`, 2.2597067694039596`, 0.052618617939097785`, 1.1876063798389778`, 0.9039352407220065`,
0.17021035842318066`, 0.061291229169270336`, 2589.9662235455107`, 0.0930933143809865`, 467.7152997091941`},
{0.16814767223163635`, 2.329148917787961`, 0.015181235666702158`, 1.3509190984659396`, 0.20135617569594788`,
0.6016277316250633`, 0.0960580261415236`, 75.18782439155149`, 0.06872828852909052`, 330.2905596330332`},
{0.24629497802526884`, 1.6185892731875686`, 0.005355789298525409`, 0.7573206700198012`, 0.6605818641858783`,
0.4413473573778042`, 0.3432973026299589`, 62.23416744978072`, 0.03461390771204009`, 861.2270986066914`},
{0.12348100274215157`, 1.3439669835932486`, 0.06660199654711046`, 1.157413128080917`, 0.4506243858381027`,
0.25274164958094525`, 0.1821863441956961`, 1340.7965217357605`, 0.03495643955461325`, 657.1175564125843`},
{0.20026891357076415`, 3.645369762770067`, 0.023665341602336538`, 1.1690889262253168`, 0.5114922593058153`,
0.6682127068245862`, 0.22249927148912355`, 3097.4126005392104`, 0.07667851305193468`, 720.3291394146138`},
{0.1167144215735465`, 2.9739284718729824`, 0.06770393749145168`, 0.8030036195270651`, 0.817910504731294`,
0.2533089674739153`, 0.13586578835473073`, 245.55576330732927`, 0.12297451593509273`, 949.2797411932297`},
{0.2610230648306139`, 3.241876732828336`, 0.005221929989860801`, 1.2708350659440502`, 0.2709097063186865`,
0.6295136971445925`, 0.2167769645618444`, 67.25197219006178`, 0.012919428781842102`, 446.6417150509489`},
{0.22755856549372416`, 1.1571207715998462`, 0.03935231641744066`, 1.338611381035573`, 0.1970208179920212`,
0.512487214253471`, 0.008778903977610756`, 69.93484330017829`, 0.0885116413292587`, 703.9913507831651`},
{0.08043529968845808`, 3.6346678770302887`, 0.06436593471662284`, 1.4919978389096693`, 0.6239584327672116`,
0.5363538338794006`, 0.0057143426753125245`, 2341.702974881963`, 0.06269329048310018`, 332.55107547613017`},
{0.17838278501630495`, 2.4576612999234007`, 0.06181741342015828`, 0.823346074642842`, 0.013205254550742174`,
0.5017391269032923`, 0.16663083212381372`, 304.8776737280077`, 0.14165654641577402`, 678.4897161214598`},
{0.1527608465983989`, 2.070934262024398`, 0.045813432940116104`, 0.7944089298621102`, 0.6769693136188586`,
0.16430192509774133`, 0.17450886910735772`, 3923.7643815879096`, 0.06708022916564621`, 420.0877116062573`},
{0.11624661761519267`, 2.9732886208480265`, 0.08686637218032857`, 0.8721582238868211`, 0.700158456128364`,
0.3183544106471198`, 0.06872076435482898`, 2515.039310773072`, 0.08635525493495505`, 866.8956807307524`},
{0.1310858151843764`, 1.596637100973001`, 0.08661007645244247`, 1.3109557164680856`, 0.9039286133369202`,
0.3437790347559597`, 0.06680016028420104`, 3498.912503889652`, 0.1695299313540355`, 339.123637513298`},
{0.13093035477925424`, 3.0078393597426825`, 0.0788110046495581`, 0.9368136862014702`, 0.15232997356507805`,
0.5208667948995476`, 0.027452102279722775`, 439.8539822509126`, 0.023447155859907365`, 960.4131412752828`},
{0.11884479435624312`, 1.7884452074083192`, 0.07215967343468577`, 0.9483398419798006`, 0.06052883609711657`,

0.6592909099232407`, 0.00834861736850339 5`, 734.1801319364669`, 0.0593737289409078 1`, 336.2025788281121`},
{0.09279332232126897`, 2.395881928323834`, 0.035821424791727154`, 1.1256250545946367`, 0.566531337666109`,
0.301586294875144`, 0.12926859185498135`, 3190.2362801672193`, 0.011195844366674756`, 937.8412253936428`},
{0.10470396306835666`, 3.0481563632657567`, 0.029181599413932 67`, 1.1229103226446677`, 0.06432569110103459`,
0.1636910757663953`, 0.0677993574358691`, 108.21923708078778`, 0.037422575295802174`, 716.3638099124112`},
{0.17624356912801198`, 1.573989236801781`, 0.09366396594728997`, 0.9674991919084199`, 0.39632574484069294`,
0.18908139883550235`, 0.042591608468658185`, 545.1774692484181`, 0.10286957749970482`, 735.5542101216969`},
{0.11969608505483581`, 0.4187980837332148`, 0.0887749696374739`, 1.1942873584807199`, 0.9542625089733192`,
0.26224686727401303`, 0.2044328771862869`, 4071.5728832408004`, 0.012536086303640448`, 986.0101272937204`},
{0.0681782584958246`, 1.9712297226556181`, 0.0710699974540059`, 1.4798684393175123`, 0.28400354899281655`,
0.18343756245630538`, 0.025080565221411666`, 190.74276766833862`, 0.021217036684959`, 114.63276406832605`},
{0.26466011364476083`, 2.3627816128360744`, 0.02039742244272034`, 0.8195822231735724`, 0.29110662117910135`,
0.47981634198411705`, 0.013151438435585994`, 123.59545033098055`, 0.04800415213314124`, 576.5536164907207`},
{0.27910166898523386`, 1.349450515188618`, 0.0885331584316003`, 1.336068232065382`, 0.5987418653774925`,
0.1580872600090233`, 0.005729492652206841`, 454.22089835832855`, 0.0493218089029025`, 734.2743634707255`},
{0.1870945044053341`, 1.8787994994414792`, 0.052561657322085654`, 1.3540062415635468`, 0.28234410939143695`,
0.681042030488356`, 0.009986864266329082`, 981.5333621075416`, 0.18733052504041348`, 853.4539224410559`},
{0.1396988967578765`, 1.913702915438618`, 0.01617810449961091`, 1.199136555704491`, 0.16595422293922835`,
0.6068296527307018`, 0.17305426475375874`, 717.4973492774137`, 0.1983198566762196`, 283.9891726135136`},
{0.15506300074217488`, 2.83069948310355`, 0.08462265931042055`, 0.8890994038076738`, 0.47796520586519553`,
0.5728084495295004`, 0.005405423164330928`, 1159.371736373684`, 0.08490828359258434`, 608.2480837068873`},
{0.08918867947883585`, 1.0389500531703932`, 0.03170283696550655`, 1.1764272925199706`, 0.8188250505498167`,
0.512109016528521`, 0.044640995253867236`, 632.0761666245681`, 0.15046863407887012`, 841.6135553783089`},
{0.05692826445298971`, 1.923331897508854`, 0.07552634610068665`, 1.455886213864955`, 0.32041248603883954`,
0.47523042425037754`, 0.22105687499350363`, 2027.7508727407876`, 0.017420396497442786`, 274.64042522521055`},
{0.24257218102715322`, 2.573176975490081`, 0.09661060539215438`, 0.8445932242005463`, 0.8955286624705445`,
0.4789936248391016`, 0.26410377851960004`, 3510.537763377287`, 0.18852831639094753`, 237.77037147960095`},
{0.05347723707962343`, 0.9129899365885157`, 0.07428512647308946`, 1.466096589680298`, 0.2638161293561847`,
0.30123412254131576`, 0.022303163735904144`, 4746.406027391749`, 0.18205991120633613`, 895.9248957958035`},
{0.27274128807061915`, 2.426636979804435`, 0.06334129103904147`, 1.210981179335779`, 0.5970208082221518`,
0.22184996611628427`, 0.009365743994866976`, 204.27799326594078`, 0.22060849091929458`, 828.6461515322391`},

{0.10281443317302225`, 0.4962771981673755`, 0.04898875008264165`, 1.262839945648412`, 0.8347432488590383`,
0.4192167501942007`, 0.030355367117986`, 1308.2409868067423`, 0.042950873976760395`, 417.52929653085965`},
{0.25848551367088474`, 3.558870975613896`, 0.037906846258373254`, 0.946132081756833`, 0.5725842788655087`,
0.6625769316332577`, 0.09924894324330936`, 52.004626921156905`, 0.12255979122726918`, 619.4850012984921`},
{0.05861384430265282`, 2.8809993577690376`, 0.0034389149229243545`, 0.778023331623658`, 0.955566931044247`,
0.311264533883984`, 0.005123688327250641`, 510.7904162444792`, 0.203481852542521`, 418.9270384908555`},
{0.26171770992100346`, 0.5361270884859564`, 0.038524708394197374`, 1.0508503305310222`, 0.11056009208990747`,
0.571340638565827`, 0.027815464969581257`, 333.39873468372707`, 0.030150481088034198`, 186.42906095487143`},
{0.09228406045428905`, 3.787322761562357`, 0.04412628551769787`, 1.2485522327984204`, 0.00453916468120652`,
0.356730274900637`, 0.03212483930675613`, 3375.2895893320583`, 0.05502779271988756`, 653.0147468261181`},
{0.2582323539483842`, 0.7609188588793034`, 0.043891058231223086`, 1.3021658200784163`, 0.09072647662703637`,
0.4622306981965607`, 0.061684572577666234`, 192.16050980483587`, 0.03732931870687156`, 184.32898889722597`},
{0.2477921144844203`, 0.9405947985440335`, 0.09500951785333488`, 0.8564439359315013`, 0.8813246793351337`,
0.5526752397451847`, 0.04122909919220205`, 2544.2880548964617`, 0.18357258099770601`, 309.1199502666939`},
{0.06005306680926792`, 1.310324956982524`, 0.01917002825340564`, 0.9233944849507227`, 0.37862876807380963`,
0.4654284132589105`, 0.028904406737409957`, 740.7733482913402`, 0.14207137738578401`, 125.45920277021416`},
{0.13221174566753952`, 1.5344979786934942`, 0.07993475417223245`, 1.1279318472161384`, 0.5224757382486951`,
0.681045123252989`, 0.09251417322960646`, 4007.8291314421654`, 0.1250986785892464`, 411.07839231550474`},
{0.23641095513812793`, 3.2390712982841716`, 0.0904220832128998`, 1.2764555680953642`, 0.4087688116784247`,
0.23715766669095795`, 0.050286530096096176`, 142.72560445944592`, 0.13322047650855234`, 994.1833005928358`},
{0.11515945727299054`, 1.0363839446782226`, 0.04991967937770255`, 1.2110778925771657`, 0.7975055001930793`,
0.15766796336173805`, 0.11336570441348018`, 702.6737236802497`, 0.07984121314940101`, 718.3669578221027`},
{0.22406410922088188`, 3.576898291699591 5`, 0.0850321138351546`, 1.0822829393722269`, 0.3956856744827095`,
0.5136072284730188`, 0.021536821023513928`, 1505.4866148851402`, 0.07683669026613277`, 536.6032367164931`},
{0.09816955174932002`, 1.3610473147845434`, 0.01200296321417639`, 1.4480047032959393`, 0.1823923291352767`,
0.5244723013770171`, 0.03492258343756444`, 1533.0073499542846`, 0.03941565102699485`, 214.7356679901721`},
{0.2686714308482082`, 2.6024579475953065`, 0.09605319126050717`, 1.4115359358964699`, 0.40944065631503346`,
0.20238890153994604`, 0.44356457168170405`, 943.2699426207273`, 0.20955602538220924`, 179.63082811814246`},
{0.2789448341874723`, 3.460339370316608`, 0.09560289059485072`, 0.7655082688097814`, 0.18036074766112598`,
0.3208070646396465`, 0.06769953888545853`, 1642.3905460053934`, 0.12624689441090442`, 706.9723251095423`},
{0.08907118739874043`, 0.5288966486743032`, 0.07776144026324502`, 0.8401856534758337`, 0.6264494338356676`,

```
0.6033856818349079`, 0.2509533302600997`, 881.1682049810406`, 0.2136549221518534`, 541.1576363993561`},
{0.21035514306399716`, 1.171180695658463`, 0.003072872162929503`, 1.0221763640609527`, 0.6635332430632568`,
 0.41088172812983936`, 0.025266995789722163`, 240.41303897513703`, 0.11383871532153805`, 586.1694984071029`},
{0.1757725171577343`, 2.8661042899257385`, 0.05038868584079925`, 1.0221414200627101`, 0.4033811300549006`,
 0.2808753785255522`, 0.23688381111182788`, 1187.3099819834174`, 0.12417956003379604`, 375.8746376350517`},
{0.15358915554374314`, 1.5283095551064108`, 0.067290924027347`, 1.1004906376784402`, 0.30400512125859813`,
 0.48412227232879435`, 0.05306566889779679`, 235.92000045587324`, 0.04951315982948612`, 369.40105642182`},
{0.2480667235282486`, 0.5649579963672635`, 0.053140067348230215`, 1.2818674385347062`, 0.7623809660000294`,
 0.3909970176312558`, 0.15838111333051053`, 2237.6541846573073`, 0.19996140314425598`, 705.3371940684284`},
{0.15145239039660063`, 2.013347200420334`, 0.015461991282007386`, 1.316890683212132`, 0.6869826935753494`,
 0.5587698316891556`, 0.155845847769785`, 1008.4357476155614`, 0.08368596216890972`, 432.47408201145043`},
{0.10270003263878408`, 1.5137093678767606`, 0.08953511397171234`, 1.327942781940753`, 0.7426665475116903`,
 0.6088434442795674`, 0.0731377274448417`, 629.1021204327045`, 0.13932635233516538`, 654.4427386466705`},
{0.0793896888891446`, 1.0605932032187937`, 0.028244713409935986`, 0.9969099319950825`, 0.928004076538486`,
 0.27677090692739703`, 0.2620559892525522`, 130.80681937144962`, 0.17810627837194376`, 310.3444008701523`},
{0.27502930950392923`, 2.268988901211176`, 0.016690280319337808`, 1.4275341924064249`, 0.010424245503220186`,
 0.33306898602448776`, 0.08933605249123197`, 948.3096011028355`, 0.06701293400753894`, 811.6908300502881`},
{0.1401731309912823`, 3.510459405926335`, 0.08747923189250421`, 0.877306477766522`, 0.5918814561880961`,
 0.26160677626581574`, 0.2368095664541691`, 545.7040461576285`, 0.18927276709234225`, 868.6345989081833`},
{0.22884950103664703`, 2.906687483673287`, 0.09738225028393067`, 1.211823583578368`, 0.3123283466662281`,
 0.6689345381975826`, 0.26177215931622877`, 1241.3038445786822`, 0.0415314605513189`, 456.6453916426678`},
{0.05854425920454692`, 0.5238963150414171`, 0.029075123644520265`, 1.1426557901500585`, 0.18407746295956828`,
 0.6998927262860721`, 0.018954418749017626`, 3086.173448022591`, 0.07606552279053064`, 322.105662191812`},
{0.21395159305342204`, 0.7435306425677184`, 0.0767057005018636`, 1.1181708530382222`, 0.44751085978070226`,
 0.3928647127765148`, 0.07805162691492751`, 4351.207257923278`, 0.18002244556017466`, 250.73279447897897`},
{0.10532042949270481`, 2.282286106803773`, 0.03578707315111876`, 1.3216192797313202`, 0.04188890042726978`,
 0.35518315207091156`, 0.030710322655757117`, 714.5224002509336`, 0.1899050019419828`, 291.00389901709906`},
{0.0829906933156388`, 3.8005179296933402`, 0.05100636383257797`, 1.2003099776527455`, 0.07859762059433151`,
 0.5748450314701046`, 0.06614617870175625`, 1151.2833065752827`, 0.239776274024895`, 683.3664621517701`},
{0.07620756796620615`, 2.1362921732694753`, 0.08270811763119688`, 0.7737426183146745`, 0.16169731434372636`,
 0.25627790943141393`, 0.10296042384053361`, 1230.0671635154563`, 0.22674061100539578`, 156.89063483163608`},
```

```
{0.08669389820930451`, 3.045091924598611`, 0.06550910340602879`, 1.1417010593363959`, 0.8553204857574712`,
  0.40177204632489383`, 0.3920145317856002`, 704.3045188157349`, 0.14263547938719845`, 197.59576868603688`},
{0.16716391272679498`, 1.9707940745517059`, 0.07610260692323212`, 1.0373304984519585`, 0.065601176736233`,
  0.3311381406088889`, 0.12567743444949903`, 64.49465686753402`, 0.10130675587326837`, 483.8093443390175`},
{0.19822566409398995`, 2.5240412947893924`, 0.07566408618441202`, 1.4422150828920155`, 0.5695379812663179`,
  0.5359514511624127`, 0.4361070820215791`, 2999.4348713305167`, 0.17170169259485252`, 352.0476518165484`},
{0.2582881650757237`, 2.8338294335694956`, 0.056678410389419474`, 1.074319457568079`, 0.10001974339589292`,
  0.2593067051666722`, 0.07903831577731309`, 123.40669984562356`, 0.2485795541321016`, 273.9914626473702`},
{0.19497013167937116`, 3.160806920721086`, 0.03009239591987214`, 1.1171831820583615`, 0.4032199037756403`,
  0.6542147113806289`, 0.029465449867892138`, 135.26925165074772`, 0.15317415942522017`, 921.0689346313153`},
{0.1360564482424821`, 0.498585531259212`, 0.09708640926926869`, 1.2162038348005786`, 0.11915352104228649`,
  0.5956143691814719`, 0.017832698276087463`, 73.16788682178698`, 0.17076027369704966`, 648.9946415099051`},
{0.13584499805886296`, 2.3834753645488007`, 0.08131332514134307`, 1.195938774683543`, 0.5088595280581578`,
  0.5258764750728102`, 0.0188849029484269`, 313.8058177427212`, 0.2364862024741387`, 393.55616964852993`},
{0.11252539515708737`, 0.48050955320077815`, 0.05937623337381773`, 1.0236120368069983`, 0.7678895649223039`,
  0.34416849853115417`, 0.008058107074703271`, 595.8657256124042`, 0.07836079702593357`, 415.53467926543846`},
{0.05491046591497972`, 2.8601951288119833`, 0.051921452801939785`, 1.1535422934918167`, 0.154204134629650`,
  0.3257327805969301`, 0.13649263155602906`, 63.90737715933027`, 0.027455815347211365`, 562.8847739679362`},
{0.06606106797338895`, 3.6656724808413053`, 0.08751889535990849`, 0.8556123556717206`, 0.6825935559995051`,
  0.5266173914314858`, 0.022251439911425613`, 1853.305468841329`, 0.16065122014174432`, 222.6798638040009`},
{0.08977217568251161`, 3.4528260544503624`, 0.05764478135197445`, 0.9515190566806713`, 0.12846915530471392`,
  0.2168250694214009`, 0.007812714529548206`, 538.1854674164173`, 0.10544198251462877`, 265.77458817227784`},
{0.22154304122492052`, 1.5332296117725148`, 0.02877908996913961`, 1.4000488050416342`, 0.4501923011081377`,
  0.49546155556198335`, 0.03515207628009833`, 79.99141751015185`, 0.06122494520176597`, 976.7821412391111`},
{0.25142928822507965`, 0.8204095084034169`, 0.04775248977653711`, 1.4286128295323546`, 0.29951010343614115`,
  0.5861829342129077`, 0.023219447176916163`, 80.52367411150172`, 0.02588989292681121`, 373.5821652599455`},
{0.1883756473924924`, 2.484499408271515`, 0.061812669070377274`, 0.7895040235728823`, 0.6199124969015315`,
  0.675547123744697`, 0.4413864963579275`, 224.0804292528027`, 0.08535968882727879`, 719.8375736166386`},
{0.04016858296637793`, 2.227608827443719`, 0.014781301743870988`, 0.9058498293617421`, 0.174940996298232`,
  0.4847952385558868`, 0.08097216295115642`, 156.97217485042802`, 0.02129075017123533`, 574.4487237391048`},
{0.10741359519637045`, 3.89617613092826`, 0.04687805603635281`, 0.9794147612026731`, 0.29389906665208465`,
```

0.504394704133666`, 0.007650336867825106`, 94.96376645284155`, 0.03904567887129218`, 298.40389093358954`},
{0.11745053279334533`, 2.766853076583562`, 0.01709341111594279`, 1.380078086463911`, 0.6364552296774821`,
0.3050852260798198`, 0.23148607657693196`, 632.2606814192264`, 0.2194692090207866`, 944.8863492578521`},
{0.06051380978707749`, 3.721291677689673`, 0.02351362713647468`, 1.4742360491119153`, 0.26914934000385093`,
0.3113498131806878`, 0.014199987869299453`, 193.52537465732863`, 0.03624750856265596`, 654.4974389371708`},
{0.24099369076587246`, 0.9331253409733131`, 0.0821724865568491 3`, 1.3431483271602298`, 0.5958343219475497`,
0.5465723977337402`, 0.3117537417312618`, 3620.513648000344`, 0.017044351379539774`, 583.8913660600036`},
{0.1670999366894979`, 0.6892897125951207`, 0.018177278706139326`, 0.8073727173778276`, 0.4996777893876556`,
0.5006175381891131`, 0.2838330520493472`, 735.6587801659197`, 0.09848547093144494`, 522.0721253919525`},
{0.20400009973703231`, 2.742493766989547`, 0.008983552040910397`, 1.241362072408477`, 0.6278550642154503`,
0.39751636324962747`, 0.007299390369356264`, 91.83643299080828`, 0.13001522795856457`, 108.9539719977738`},
{0.22650061618533962`, 3.64567942204856`, 0.07707002972279017`, 0.9032908420697698`, 0.9136296860108464`,
0.4341422027661652`, 0.24929767745737103`, 136.4576870478556`, 0.07080767715199654`, 971.7774116990597`},
{0.10155767105386482`, 0.4181451151728903`, 0.012394687986228644`, 1.0711096932931712`, 0.20820538774593245`,
0.1626988912346673`, 0.0864577360395456`, 674.1888341417106`, 0.170056418389111`, 730.2391664075723`},
{0.21231295112704185`, 2.380633825425009`, 0.06236561820134972`, 0.830378492099762`, 0.7458626837014395`,
0.3778428196310881`, 0.0682174150958653 5`, 516.5455006559224`, 0.0981833249923344`, 365.58490612208846`},
{0.06947494567464263`, 2.895860587431992`, 0.09178966303831615`, 1.2688043406444924`, 0.2941408877223748`,
0.3751778179392746`, 0.03775382322774307`, 60.369496178615435`, 0.11794208757169544`, 673.3642442045926`},
{0.09985693159346487`, 3.9019559576117224`, 0.06462003972312033`, 1.0811937700231284`, 0.15667266604108`,
0.623010278974039`, 0.040053923820923376`, 112.7677826146989`, 0.1201138368644335`, 956.8700593079004`},
{0.21079578713436742`, 3.646518299612826 5`, 0.07263502170252384`, 1.2463245485615204`, 0.04598824508235744`,
0.19490478821937018`, 0.006055825079153585`, 455.84209633704694`, 0.04307337339516104`, 393.98363704378045`},
{0.14050505626384868`, 3.3486141508967293`, 0.06576905440282896`, 1.0969622150757512`, 0.25298608583995974`,
0.6081914621324831`, 0.07984197613603365`, 4147.732881753063`, 0.01970187856601205`, 417.6295038180262`},
{0.09029343760417469`, 3.1277911065164137`, 0.04916145932038262`, 1.1046401865081483`, 0.21999281746629284`,
0.29609111953575007`, 0.08848537504901788`, 1978.4176945251475`, 0.1604567549403832`, 631.7829673381813`},
{0.18744474850618575`, 1.1949686342264414`, 0.06413249212557762`, 1.3584803763102893`, 0.320102170913821`,
0.4723006569832039`, 0.10067644299750714`, 61.82330782936513`, 0.17695515305422665`, 265.1926737070246`},
{0.2388654681009152`, 3.8576612834574524`, 0.02902564289553176`, 1.4380459539751635`, 0.624215346482978`,
0.3925897588304593`, 0.28422329981310057`, 1921.54220161346`, 0.09343473821304771`, 199.86167324974923`},

{0.1455526991456984`, 1.8705049552506363`, 0.03961322047797989`, 1.4283548335168212`, 0.9360504979535096`,
0.6211597747744511`, 0.1850342062284412`, 2722.415223545606`, 0.05486666814149975`, 162.6815751148559`},
{0.13798249053354167`, 2.899902544251761`, 0.0459763868363342`, 1.0421434347893428`, 0.7895156656158571`,
0.5540265258106729`, 0.047360361099740046`, 2648.770558815322`, 0.09753399188330308`, 720.6816664753013`},
{0.17745255413169525`, 2.520375966029923`, 0.04665876128100216`, 0.8123275453028636`, 0.944701273242313`,
0.24813120748859874`, 0.24862758503488186`, 106.62623807073865`, 0.1979751584484703`, 843.0950202672676`},
{0.16499863275860222`, 1.077452191253113`, 0.023014633917973006`, 1.2950348307619706`, 0.9833706900164221`,
0.290091826190232`, 0.2219267499554569 7`, 3510.2131809670686`, 0.1789639285862865`, 441.136883561353`},
{0.09494055003043922`, 3.273540527547083`, 0.060548822068484894`, 0.9817396325621766`, 0.5953487435300235`,
0.3030053710238173`, 0.020212568263132794`, 207.40154426384788`, 0.04862769785226101`, 884.4963622485988`},
{0.22556943514719452`, 2.763355506916427`, 0.0659847718412378`, 1.1318711218549837`, 0.7290768537995906`,
0.6910097128365145`, 0.02883814164041025`, 1935.4928886970383`, 0.060672213321555424`, 592.2254589353502`},
{0.17976151229453585`, 3.607368069246487`, 0.03562542831805498`, 1.1702627239429857`, 0.8807800807300026`,
0.5048328928685707`, 0.08802036843157425`, 258.70537367208925`, 0.20138969659791595`, 520.6789088604623`},
{0.23694985915625555`, 1.7115996463959204`, 0.09475499820714504`, 1.0033560670730772`, 0.6058641758111063`,
0.5075289726151094`, 0.04433733902402368`, 990.3350746839715`, 0.06868554455100367`, 684.4534604177855`},
{0.060659588298575706`, 3.185819049116902`, 0.031246099794861325`, 0.9329844374373137`, 0.7312648065631473`,
0.27616453553670284`, 0.06002230172665507`, 72.75197129693021`, 0.01701545753405373`, 816.2999501758868`},
{0.27783663000790365`, 2.7970847777258587`, 0.08231332350426997`, 1.022697264732825`, 0.9687024837581177`,
0.3645779308390613`, 0.041020574150516895`, 4813.902596935811`, 0.09489024372224386`, 126.14406706341742`},
{0.1853383438805617`, 2.710478464072388`, 0.01489054849244626`, 1.1482283702493803`, 0.5568912869451013`,
0.42437036955952745`, 0.009568366829966814`, 179.8278356132767`, 0.21625752953014726`, 454.80717115717636`},
{0.05021881637759101`, 1.4247717975270389`, 0.062006838284966344`, 1.2900546523204781`, 0.9617562903977206`,
0.5950392020832158`, 0.01053774359341417`, 3195.955497745086`, 0.10119630409828645`, 778.7070525025722`},
{0.1629749532929909`, 1.3827669190112752`, 0.02163316926061519`, 0.7859725102428954`, 0.8835314458481405`,
0.623882909407651`, 0.021916393496908437`, 149.5675827629461`, 0.09031773923128839`, 469.7973445453567`},
{0.2303174353412305`, 0.7381196545439321`, 0.017853274128905045`, 1.009576893018485`, 0.49320693638055335`,
0.6517404058254557`, 0.03129869768398024`, 1241.6665530134712`, 0.22978761854559276`, 819.6026061342192`},
{0.05000320218233145`, 2.1972018685344494`, 0.006726117027854865`, 0.9936552562980645`, 0.8328018981538441`,
0.5156429010358797`, 0.19438658089409308`, 89.43952173057976`, 0.03393498174078835`, 570.4514942090268`},
{0.22380766185105788`, 3.972867022032534`, 0.05857466369517589`, 1.320944264492121`, 0.8375716850380956`,

```
0.6387604619615563`, 0.14529791195160408`, 354.612762024331`, 0.0916149378074263`, 197.12301088359715`},
{0.1925638236278404`, 3.172077483377868`, 0.030644598758412977`, 0.9803085988716782`, 0.875326409629076`,
 0.5489970615034699`, 0.4213559070823191`, 63.50782532256897`, 0.15909673801152152`, 757.7936027644167`},
{0.09418442467434818`, 0.6452011369781001`, 0.05468238280755824`, 1.1696403162883113`, 0.9269902181221603`,
 0.5827207204052004`, 0.0480602677307661`, 155.50909202321085`, 0.17256127653904146`, 540.5740309875913`},
{0.05187445469191648`, 2.8967354683291404`, 0.05468134937388834`, 1.0272048159360176`, 0.32845734934833803`,
 0.591187325659497`, 0.3107635049414343`, 481.6720966539763`, 0.2446670824913575`, 391.7749895365258`},
{0.08569179084998313`, 1.8355994610956383`, 0.09902879473840677`, 1.0426364128937085`, 0.046121645528780064`,
 0.5970016999064142`, 0.04585616299575304`, 1326.7471633941773`, 0.1575248274172592`, 749.1145234256358`},
{0.24342030247616103`, 3.756945920144269`, 0.048201731449734615`, 0.8715075343316259`, 0.6190895848455991`,
 0.19127133299748744`, 0.06855426679028909`, 1305.765466028692`, 0.23417008838782194`, 415.6502949437222`},
{0.19292203783708667`, 3.220432103715133`, 0.09761210999008452`, 1.3591272090173214`, 0.14186149659118774`,
 0.4413360685039047`, 0.010167923556678914`, 131.42190006936238`, 0.24362820427309634`, 380.03821419833645`},
{0.1897036930032619`, 1.8964339581865879`, 0.059440108944421456`, 1.1142569288613073`, 0.15129145170580593`,
 0.3833845599206046`, 0.05519372846185666`, 4558.979306456515`, 0.013937979989310956`, 207.93733592881284`},
{0.21229489496584697`, 2.877170935416972`, 0.0852716204718659`, 1.187802399588918`, 0.766747964989742`,
 0.2056469011396459`, 0.2516666100392663`, 249.75546686829057`, 0.0438104994627804 8`, 453.1293949612376`},
{0.18727208406868906`, 0.5920853885729089`, 0.00310465950751313`, 1.4761651930790642`, 0.9879910194473076`,
 0.4582425433815992`, 0.1348498167291504`, 1379.8554077407293`, 0.19832956659061096`, 601.2373424307469`},
{0.21303838080640486`, 3.716490082492431`, 0.06530590830455278`, 1.2455728623234372`, 0.6800061917252811`,
 0.3355795794234222`, 0.014627505674034404`, 79.98307290173638`, 0.017560362469609708`, 213.93067315001346`},
{0.06263632603162816`, 3.088015470379359`, 0.06468478054731996`, 0.9840993814948887`, 0.7841919183884491`,
 0.464604288151068`, 0.01258311092046284`, 1881.0148437864943`, 0.0609287237146911`, 341.9306317400494`},
{0.1229419022884577`, 0.42323948511603504`, 0.04401981826231521`, 0.7802588542080802`, 0.3378063219388938`,
 0.28733129925219736`, 0.006237735142161048`, 2301.7896008425946`, 0.1904542796497251`, 972.0175866826189`},
{0.07173116327385964`, 1.689724351901349`, 0.0333537565627478`, 1.2797681367134839`, 0.19114975037520088`,
 0.541681437492502`, 0.35548681580819513`, 4889.525485193395`, 0.07624198202888299`, 255.7119735830849`},
{0.12377155914664595`, 1.5057831665831483`, 0.02584787085922036`, 1.3931245282333518`, 0.1751216551172583`,
 0.31643702992235223`, 0.006127436167226222`, 3977.3695715719014`, 0.19191205388712534`, 489.96663345919205`},
{0.07922678243367776`, 1.1007105803460338`, 0.0670178962975939`, 1.3858977973939028`, 0.6913244964468961`,
 0.30596530488881035`, 0.03670449008923856`, 722.5750280061836`, 0.1062965670986098`, 454.2241152370099`},
```

{0.13900795297527618`, 3.9726186574282156`, 0.014365558963868033`, 0.9387566245260975`, 0.7733911807172529`,
0.2901636804835216`, 0.022925433557643798`, 983.22576571694`, 0.10414701635541929`, 690.7271079642408`},
{0.06983492300698985`, 1.9811052508516296`, 0.015092410881193086`, 1.3035668919937708`, 0.40820708656243165`,
0.31349067656511853`, 0.03294127758239607`, 588.7480044888102`, 0.2032334347292173`, 447.9335749964651`},
{0.05248318477610414`, 2.804613750674261`, 0.003640346777431844`, 1.3628720468665754`, 0.1995299750560573`,
0.4294363076423442`, 0.007453829399752168`, 688.7688808168017`, 0.24854011257885444`, 913.0182165489159`},
{0.22482399322255875`, 3.3120245448192804`, 0.03927421614929747`, 1.1730261223962148`, 0.8102112515480973`,
0.5310384924975508`, 0.021360928541744394`, 935.9962133496834`, 0.022365336640988054`, 615.9503154198303`},
{0.14054031835314812`, 1.0862525764402395`, 0.03495957467986743`, 1.4989441459825807`, 0.9776070717717082`,
0.31123951873946865`, 0.005138607602622309`, 2625.434021720543`, 0.07843546140834678`, 537.9180477646557`},
{0.12045530385052439`, 0.5766797014120977`, 0.026639235327100757`, 0.9789792038223954`, 0.1966739314900421`,
0.3703973027163936`, 0.05825214840022438 5`, 4908.720210074871`, 0.20113853341018156`, 829.8609278324832`},
{0.2466711089217451`, 3.818653323688843`, 0.022826073218276496`, 0.9885944445220116`, 0.08478033040666122`,
0.24831675766148897`, 0.05221202249805683 6`, 61.098852909743506`, 0.07219665114050788`, 854.0668809251908`},
{0.2622407564991055`, 2.390608019262051`, 0.06092577106875352`, 1.4943502181005281`, 0.48817005604720776`,
0.5989574618060204`, 0.0663716035556017`, 59.28691333172426`, 0.07923371737796253`, 663.2501758461839`},
{0.04240892049220529`, 3.585324421001501`, 0.06559550484561946`, 1.4902471895373985`, 0.5553889156366423`,
0.6483753523879463`, 0.007118840678651323`, 2218.8393261162782`, 0.011166606091999653`, 925.472456163056`},
{0.139826903045498`, 1.6937551480709319`, 0.08421189687207545`, 1.1119195234296138`, 0.8823668491031473`,
0.5196063483063849`, 0.09432450978517919`, 330.21686735744527`, 0.2294519472818627`, 332.35073177164196`},
{0.14802078413209885`, 1.733603865107586`, 0.05101449210330218`, 0.9572479232347667`, 0.5675011406257902`,
0.3885036693205801`, 0.29314426920759284`, 2048.9810499540877`, 0.14325997637273158`, 127.28328705485782`},
{0.09351974409461145`, 1.9687875339139866`, 0.015572094311755826`, 0.9484499792232198`, 0.5844423119728932`,
0.5819082431078818`, 0.2373776682556218`, 112.86193139612024`, 0.23719097877429007`, 229.2738627795444`},
{0.1102903206501954`, 3.0454263679631577`, 0.04069331752982141`, 0.9534089355456694`, 0.7297525123381821`,
0.6576008896160583`, 0.013329813923119783`, 1715.4779634820493`, 0.02214911406080483`, 227.88476662786474`},
{0.05909859147151786`, 3.30435803161393`, 0.029336284768972404`, 0.9231466780747943`, 0.9623144707471962`,
0.37803674309323987`, 0.04397572398478963`, 3067.5791243622793`, 0.057241291718842735`, 398.5949730494525`},
{0.17398185963078006`, 0.47005124431733236`, 0.09389471888393235`, 1.232881569185857`, 0.49856219410777936`,
0.4191945121229328`, 0.14050812071087648`, 190.8204082787259`, 0.22639026429089515`, 506.13435159524533`},
{0.05155342181122552`, 1.5581409866760865`, 0.0691893121207933`, 1.3639899124520563`, 0.2080210727331211`,

```
  0.429465542144159`, 0.08260476334651806`, 246.4705132305552`, 0.16650171241851036`, 476.3403890303273`},
 {0.04071142279712078`, 2.1496023906791892`, 0.08531567546447687`, 0.9683458228368205`, 0.49038233136090126`,
  0.6235182579165484`, 0.07161574481597612`, 131.50858611951176`, 0.19699207630915766`, 596.6367875356493`},
 {0.06590056983751874`, 3.9638284260232943`, 0.016048742823094812`, 1.3783253378441898`, 0.24532119252948448`,
  0.3220387333596423`, 0.016945992465929767`, 2197.071659291998`, 0.010352607682042353`, 236.6421867279362`},
 {0.26325550998326586`, 0.661917211789294`, 0.026986149925021862`, 1.002112989283701`, 0.06554947180049209`,
  0.6994424736047835`, 0.005881514200787017`, 211.33538862145804`, 0.24482509012686327`, 303.2466877164689`},
 {0.26127356110524397`, 1.329573031379259`, 0.07977956036816294`, 1.2076717867972873`, 0.5921373287024636`,
  0.41816652735672233`, 0.022774959106689946`, 64.98766538013531`, 0.19432837274522574`, 259.57035925631845`},
 {0.260215506169461`, 3.5506896347378634`, 0.022465629838975154`, 0.8509530374249318`, 0.7983914367554696`,
  0.2578474320630234`, 0.011809304810800692`, 142.12037985462777`, 0.1698027140526227`, 698.002014936651`},
 {0.06365047180104016`, 3.416188751455385`, 0.03523713957757545`, 1.0192452557682743`, 0.8469643727981293`,
  0.5498369694104505`, 0.18294125259438154`, 1018.12440025711`, 0.11189348348403971`, 231.55349754949057`},
 {0.20010483475430668`, 2.008655229618358`, 0.06224827434543773`, 0.7776282320212735`, 0.6157609854264054`,
  0.25612002607302176`, 0.16950601491382145`, 2258.159167246791`, 0.12768932854616544`, 318.9473026654234`},
 {0.04033357276115293`, 3.365035676090897`, 0.05992514630623122`, 1.1732918415280658`, 0.196311756799749`,
  0.30038372703117333`, 0.16280533449388682`, 87.04220764531692`, 0.23496316330764122`, 283.48137514032834`},
 {0.1509427023460006`, 2.3319882937767247`, 0.018731546174507247`, 1.0584311371273147`, 0.6567026163933902`,
  0.6265947759038382`, 0.06164026490982475`, 1022.7891111424142`, 0.1688287319736977`, 154.17413301109093`},
 {0.12172292087124631`, 2.8233234444817974`, 0.034053562361951124`, 0.8588222796104166`, 0.9440480252161128`,
  0.6422691510398222`, 0.05980616075075349`, 1021.745763820812`, 0.044168716180875034`, 436.5175383363466`},
 {0.10332818067094146`, 3.3200182476314417`, 0.013797086337646878`, 1.2403231995339712`, 0.8068678048493421`,
  0.31817454060495975`, 0.03466270708497138`, 224.98168772666384`, 0.18064139380974548`, 882.0488176741194`},
 {0.06283595024068839`, 1.3516727681414702`, 0.052426747231404766`, 0.8261017998522725`, 0.2587281077553354`,
  0.25782009716699184`, 0.4461332906525768`, 78.37105686956872`, 0.05053665990821049`, 454.91374396216366`},
 {0.26061686002038464`, 0.9197100483832488`, 0.09424919710819697`, 1.2431276888944953`, 0.03523684950904893`,
  0.6986561589310964`, 0.46987667183492504`, 257.0362567649576`, 0.16991196769780592`, 326.7794760540163`},
 {0.2589743226453006`, 2.4967568849644275`, 0.07508840824492798`, 0.781034619960081`, 0.38459790960215257`,
  0.18475383400796352`, 0.10416671513341536`, 3175.918867658944`, 0.2205445770390796`, 431.51407680934443`},
 {0.1822125191728448`, 0.53967615378293`, 0.06140923587336875`, 0.9836246668356532`, 0.5663752339069781`,
  0.6127333889317503`, 0.015376137369944434`, 4374.881958546335`, 0.1720894071809011`, 902.0125127914057`},
```

{0.20287638930589763`, 1.8511964408244932`, 0.020237031457082787`, 0.9096848496624212`, 0.22198223428443487`,
0.15744015403827616`, 0.17491779271673957`, 2922.9247864169292`, 0.14133268345189937`, 866.3466773010839`},
{0.17277183156190062`, 1.3838708180759518`, 0.01858178773758265`, 1.4265746785083109`, 0.6133308818390555`,
0.6532402234702124`, 0.07637848286373418`, 1061.2255229483176`, 0.23800513499817172`, 715.6629218234075`},
{0.22973167487200014`, 3.4630938101482327`, 0.058480370100327285`, 1.2645217042191328`, 0.6981417065741804`,
0.542170464886519`, 0.006546556460297805`, 74.23410585835131`, 0.017772563055006546`, 241.35868804813666`},
{0.17947546203746156`, 1.7068442534238697`, 0.04056609287799669`, 1.1830573633622712`, 0.6640874832688464`,
0.42001594980708057`, 0.21752981815857997`, 97.3204887238174`, 0.050533940181592796`, 409.66978902022663`},
{0.08473729051323187`, 1.7479721342100945`, 0.06355457932773143`, 0.8574963830627491`, 0.22245663004688998`,
0.4791593777006873`, 0.28265674645892613`, 4498.232223037125`, 0.17673010273241208`, 336.2131425839168`},
{0.20435672898605034`, 2.6117732980489388`, 0.03475265809490025`, 0.976265205498944`, 0.85216518519733`,
0.548618924239519`, 0.2709183052632252`, 155.3772460870891`, 0.021213370069483317`, 486.638902544934`},
{0.2230119008971858`, 0.6358954372186516`, 0.03489317261697556`, 0.8021667773970862`, 0.7799870633314419`,
0.372553704845368`, 0.3954953790403783`, 3708.9097099582787`, 0.06863756553832256`, 831.4592304552722`},
{0.13277530106525864`, 3.102992336174254`, 0.0810346296150594`, 1.417175031427167`, 0.04111047270929191`,
0.577719723059283`, 0.25840948246645284`, 1582.3635767727842`, 0.11281028363913381`, 572.0784651822191`},
{0.17721564666118195`, 2.3073681400537707`, 0.06977110969724436`, 1.4389124933186648`, 0.06511833232445241`,
0.5785856768063609`, 0.024382684167548917`, 50.72398143128576`, 0.15027785530459653`, 471.87731805197507`},
{0.2532195731000343`, 2.951680905930308`, 0.08243784959141458`, 1.063512245713839`, 0.13059994709602862`,
0.20280590336854443`, 0.2607392691737333`, 517.660101290023`, 0.077733893061395`, 364.603949151811`},
{0.11562259606165537`, 2.075988177277`, 0.03552939359394358`, 1.0857990592224895`, 0.7681961757441904`,
0.6605331614852874`, 0.15159919874130345`, 3296.1941609770115`, 0.07746882734639965`, 160.02380566717028`},
{0.16010263731321916`, 1.8512370146128028`, 0.08948994106153972`, 1.0065876584784161`, 0.31909081739901013`,
0.6055123683081534`, 0.027623148293597768`, 400.21848632042986`, 0.19838189450352378`, 329.78449858468684`},
{0.25703658745875196`, 2.3078283713322856`, 0.021362941537208063`, 1.2100220391878165`, 0.8787828002021987`,
0.2513644151003869`, 0.024289521781396636`, 68.1341971888039`, 0.24810809198178096`, 481.51760126235956`},
{0.2455904611014849`, 3.7571997349914916`, 0.09211738529180043`, 1.4019497765522448`, 0.6695611235062522`,
0.6602799476916219`, 0.04340955542197445`, 856.0993716100127`, 0.15292526713206545`, 529.9607802643166`},
{0.059696149748876604`, 1.688845353034914`, 0.009294581379800135`, 1.2433968456838194`, 0.3929448551213022`,
0.4749866039118191`, 0.025863060541790456`, 192.8417865287921`, 0.09839655966119532`, 364.5695918572345`},
{0.09761501672592626`, 1.3774481728994479`, 0.0397488421074619`, 1.4571532371767337`, 0.8600435797101407`,

```
   0.37053809894631096`, 0.30839879803159215`, 1053.3603252171624`, 0.09829081378486954`, 314.7778202000868`},
  {0.22092704028098814`, 0.8304263766325484`, 0.048201718626776184`, 1.203623675331191`, 0.2935636564899402`,
   0.4030249377909366`, 0.033061104251936244`, 4358.833221490219`, 0.15893421696064525`, 180.8709305853121`},
  {0.13823492725556935`, 1.1413185260053824`, 0.06971384118788397`, 1.3157780297053645`, 0.6082014740714308`,
   0.31531914337240985`, 0.005597926269123681`, 97.23206007061579`, 0.1162100716591018`, 522.6554437517659`},
  {0.14416900464242904`, 2.543812431689566`, 0.010106655971553415`, 0.8969401222383744`, 0.4328515562362101`,
   0.20360668938810977`, 0.2923006645347409`, 1178.8843718309924`, 0.20797847649312406`, 120.9186114906668`},
  {0.2133877273723686`, 0.8490524254535732`, 0.08700555866439949`, 1.2062638954363991`, 0.6738638517009694`,
   0.46997962516177505`, 0.06892379315565185`, 1349.9514731760883`, 0.09645636257209894`, 836.2941738998895`},
  {0.0539089201271194`, 1.0364880092644801`, 0.016154417078585476`, 0.8886530805439552`, 0.3168467801118968`,
   0.15642019482584735`, 0.006844524019875138`, 85.57714734356885`, 0.01991933931946757`, 258.3461272090961`},
  {0.2715756156789369`, 1.5445757615325304`, 0.00421616582195627`, 1.159485102317175`, 0.4702357167765736`,
   0.34737964168870006`, 0.496052684967637`, 300.35079175305486`, 0.1259406113194213`, 781.8522906262123`},
  {0.04827287438408412`, 1.8515860963247066`, 0.07692111119618293`, 1.2774889475291031`, 0.32248502080707353`,
   0.20234972534433104`, 0.01272417451099904`, 140.98466458635835`, 0.11366894324343613`, 273.5929992840893`},
  {0.2554976820569969`, 2.4378755210159753`, 0.04254022762089836`, 0.8817958104635948`, 0.692204018420977`,
   0.4234476557446073`, 0.007554290015778277`, 79.39858197716293`, 0.11447843867339091`, 117.8261250906867`},
  {0.2760063677033659`, 1.2105198937059631`, 0.067224388891334`, 0.9988908925658994`, 0.9019281244861579`,
   0.67284715505845`, 0.007365273847592193`, 2972.356277007087`, 0.06361067504455276`, 438.4208211463028`},
  {0.23497331294207247`, 2.932797407682453`, 0.022597452058598203`, 0.9589743833470734`, 0.1667506323796748`,
   0.3129991011881105`, 0.05276499482811941`, 99.75864289244342`, 0.09875437541358872`, 580.7218610046771`},
  {0.0552957146577317`, 0.9263334676885089`, 0.0538222458972377`, 1.1846475829708125`, 0.6978145776046678`,
   0.40095280794552435`, 0.14194113807951353`, 67.36725348197879`, 0.21539237947591866`, 912.2514050198081`},
  {0.0859679799264661 5`, 2.89469532919738`, 0.0723374629314705`, 1.412708246795334`, 0.2707681400237405`,
   0.659961029627586`, 0.1545447128013493`, 953.1432644930773`, 0.14443128680071893`, 294.4093465447068`},
  {0.26579336742267545`, 2.150651278297752`, 0.09864009518921925`, 1.381049269309322`, 0.7824667408282995`,
   0.6485863724693819`, 0.1346970994193899`, 71.41513724603877`, 0.04324969499825426`, 231.68375988200472`},
  {0.07893352000042303`, 0.9269382255330783`, 0.07450960524320785`, 1.192225010506579`, 0.29981085040929156`,
   0.20655660387273633`, 0.005195235752158478`, 3935.7899816629233`, 0.09905090934440974`, 552.2915075373634`},
  {0.13874147069686438`, 0.4677020963588703`, 0.06081979667173851`, 1.4293968405810138`, 0.04489099618087722`,
   0.600807000017457`, 0.1990035962891371`, 80.51094589303605`, 0.24671702535367518`, 538.9722201215866`},
```

{0.2700245759823924`, 1.083893289879918`, 0.062021204290361266`, 1.2005471238256362`, 0.792954755831037`,
0.39473782087530096`, 0.17457967683710685`, 279.6749633279759`, 0.14741868150066417`, 148.434091412925`},
{0.23474828628269123`, 1.725907115336116`, 0.012821406564911335`, 0.8070142233189552`, 0.4777555512453826`,
0.4076167331863103`, 0.015638133522482764`, 120.11394196884895`, 0.019548998977537424`, 892.9399386284366`},
{0.09165311999697207`, 1.4061679173562647`, 0.03309545344659714`, 0.9052080425177362`, 0.22786616586188102`,
0.16948939359836301`, 0.041478452818892154`, 78.37706796679595`, 0.1493801427638014`, 220.7559134640384`},
{0.04235508864011306`, 1.3060003486013567`, 0.08466697376486561`, 0.8479712158967934`, 0.3936959911899278`,
0.2088063088308738`, 0.017308803394347618`, 4694.788266429514`, 0.026439759379240313`, 433.9918956105633`},
{0.2651923586966664`, 3.8881876111766713`, 0.0350309847210708`, 0.9591171183376157`, 0.9954879310389926`,
0.5341580610877573`, 0.0817474157614525`, 113.37527000346847`, 0.1996829793433833`, 201.49628341235228`},
{0.2787517386498274`, 1.5890965688604197`, 0.014994826667312413`, 0.9487067103902393`, 0.03893198736235193`,
0.2195801988959788`, 0.313478573421096`, 168.03485395038084`, 0.026460496970460712`, 626.9887607453275`},
{0.08753933430632788`, 0.6662218380744758`, 0.010717888122421044`, 0.8318793084446489`, 0.9074866505690673`,
0.6370551722773192`, 0.05914667212913143`, 92.90178750054149`, 0.041267271127747585`, 531.582902194676`},
{0.254499813976234`, 2.1602436577244424`, 0.0772554574037915`, 1.3316064464046935`, 0.367293601649199`,
0.4330751915150024`, 0.012004917841621048`, 4701.7880352079455`, 0.15735639832958048`, 710.0683407032234`},
{0.1501371731881197`, 3.5104518987365614`, 0.03585463368506289`, 1.1463465777386583`, 0.5443112287696148`,
0.3949552428603267`, 0.04879115335558662`, 259.24478342917615`, 0.1285655725832237`, 162.3475385554764`},
{0.14482453276755702`, 3.3636104411839076`, 0.038458077364182566`, 1.197124661291601`, 0.4328455972778613`,
0.3205266052041652`, 0.010337491367857544`, 172.8822485760027`, 0.1748531192873402`, 294.504606203127`},
{0.1729081128249474`, 3.4171958647256986`, 0.09736003964750388`, 1.3614201648251067`, 0.8114455381033425`,
0.2740485642532573`, 0.10719777411327538`, 697.2636909321262`, 0.16794794102451832`, 169.68999987903217`},
{0.06017132640138534`, 2.551705655373457`, 0.098001989150616`, 1.2777770866301428`, 0.33103821819782686`,
0.42457056361391154`, 0.3044012087125646`, 128.19341360646206`, 0.23285467527685833`, 861.6547556863932`},
{0.06838353005846232`, 1.5583268641110415`, 0.07074535929409585`, 1.1186421654705394`, 0.17808501168205293`,
0.6526418380839745`, 0.30389354478023717`, 2253.855886651074`, 0.2140132420516081`, 589.9585678536141`},
{0.045919511842929334`, 2.3909808568887367`, 0.05197740517398504`, 1.4076343252717902`, 0.4269585056408616`,
0.672767818437489`, 0.14290256087662156`, 365.93648706136383`, 0.2152208567065702`, 325.8336782379203`},
{0.2110207852916774`, 0.6269225337264532`, 0.060343077081531735`, 1.2521859520371945`, 0.29021239339379945`,
0.47386146148275243`, 0.1626064474205758`, 445.43014573417435`, 0.07466666385442788`, 270.0446625362414`},
{0.13913288219717507`, 0.71598345975513`, 0.02918338476342825`, 1.084418293337738`, 0.1601393499115018`,

```
  0.351547594930286`, 0.041991397294508556`, 2091.889065193303`, 0.09601982280233712`, 646.6328833508262`},
 {0.2080876337219731`, 2.734433706560215`, 0.0261030709279631`, 1.338847931530077`, 0.982472586795901`,
  0.3916223755715482`, 0.018379378513228514`, 98.65983909130968`, 0.11579954319108776`, 761.345713618144`},
 {0.19884229924079655`, 3.7544266932443486`, 0.05306832500507259`, 1.4048543300387752`, 0.1752488991857195`,
  0.2724812216186141`, 0.12261739216762069`, 80.04459592601863`, 0.2028403340066654`, 224.71523563844968`},
 {0.09229081102449843`, 0.41731444207519`, 0.09042236613704813`, 1.4795137951182236`, 0.6627370782775894`,
  0.6415172939653717`, 0.007608010716527551`, 62.09999691656457`, 0.17995756856061956`, 974.7890405043591`},
 {0.22753750499438513`, 1.1094910684892056`, 0.06205079076485845`, 1.3683254796121973`, 0.59456895101486`,
  0.6649055203122181`, 0.06079543818004673`, 62.1436627548072`, 0.23424854868652234`, 576.7021529851472`},
 {0.07611810878804481`, 2.417695097886692`, 0.008902516116383463`, 1.17651838899742`, 0.8956068934881507`,
  0.1734469831112303`, 0.007686752240077523`, 84.3504067614311`, 0.15979761325133707`, 962.8822438221003`},
 {0.14505993431662217`, 3.1227268390818725`, 0.056275163136378696`, 0.9590973923566892`, 0.13969476213382426`,
  0.20892815360032824`, 0.0531610997371578`, 246.79786465887622`, 0.227047105813231`, 454.33945080770786`},
 {0.23654885566478462`, 3.9241924925395733`, 0.03358528642939117`, 1.3191430750498658`, 0.39258636888736453`,
  0.3377325502420989`, 0.025557835669326675`, 93.73616135543377`, 0.1670083520090136`, 309.9129735942125`},
 {0.11070282825339567`, 3.8334454078682603`, 0.07681857609135982`, 0.8958554753360997`, 0.32396640927167897`,
  0.44194855743506667`, 0.2131540641524383`, 1741.909189584937`, 0.022902468906996543`, 670.3098695990176`},
 {0.10170301352011679`, 1.8752371643597758`, 0.03510782937111476`, 1.3860933640706163`, 0.6474965074918984`,
  0.1594306933477596`, 0.37318794312281267`, 201.47970914729365`, 0.22517965099104725`, 283.6656714712788`},
 {0.19274669213409934`, 0.9288413277333225`, 0.038837310625680195`, 1.3836847187363839`, 0.14968000832109252`,
  0.4232153388345993`, 0.01563322978668864`, 922.263283889537`, 0.12359553135969004`, 486.72903455727567`},
 {0.06356535997519341`, 2.311362126443121`, 0.07268080427945918`, 1.2586315568490016`, 0.3763201304339143`,
  0.4384209659583461`, 0.006474182269401951`, 4348.973288945735`, 0.17106111863805346`, 281.3921070619326`},
 {0.06831716410403144`, 1.0015662947000914`, 0.06390469368977933`, 0.8290508858928134`, 0.7249330951564277`,
  0.37450775838270167`, 0.3666941924999965`, 191.80408739918798`, 0.153067071236483`, 887.5069081464457`},
 {0.1293750817503745`, 3.1014207000970364`, 0.023014106546543296`, 1.351810814205737`, 0.5054291062898795`,
  0.669566365984742`, 0.05376259966791126`, 207.8155819891332`, 0.12505633467039579`, 477.98997920694734`},
 {0.22605177518456349`, 2.2930688004852557`, 0.04762600540866055`, 1.435285876756151`, 0.7544760534695125`,
  0.32613259549063545`, 0.008000490945700793`, 658.1451070175192`, 0.1664301859365524`, 397.6391494406913`},
 {0.21478835126656415`, 2.1859458812100163`, 0.0858199459854308`, 0.993933836221133`, 0.013991965571371612`,
  0.25438763086955674`, 0.010673305721101622`, 175.38786745903064`, 0.249124195297232`, 141.44233499151147`},
```

{0.2524903538890303`, 3.207130817880457`, 0.0760687494889642`, 0.886811483661367`, 0.4861660798083616`,
0.6820047923981643`, 0.01500874534183673`, 477.62249790514926`, 0.21800800344067622`, 582.3093284125088`},
{0.2673762141971479`, 2.9843296894323084`, 0.06575888816802714`, 1.4582777652181538`, 0.8424633965803163`,
0.2888169272806542`, 0.017193087756165917`, 1905.2031126287002`, 0.22394556493184148`, 302.0798156952443`},
{0.0718648334935813`, 0.9687764001979549`, 0.03778553570658904`, 1.0685686917623896`, 0.8556706065609418`,
0.5975957325406767`, 0.1005409517041212`, 952.0313425448139`, 0.1102822616742215`, 595.1450083064373`},
{0.15187564029414063`, 2.880478775690314`, 0.004382096934887443`, 1.4674879291008858`, 0.5009373487763229`,
0.43891155488357636`, 0.3536287231064343`, 240.19671819847136`, 0.18432705417277784`, 919.7867146052535`},
{0.22049198023775118`, 3.737295065096858`, 0.0901755947592022`, 0.8260708286898752`, 0.9798021204432015`,
0.3779284943750736`, 0.1710978537094571`, 184.8847972532019`, 0.07398065840012735`, 365.0355841401655`},
{0.07654850782489453`, 2.4131335758873655`, 0.08059562189671154`, 1.2322892164920185`, 0.6217255346992006`,
0.4763260824504111`, 0.019197847472858853`, 122.85370840033258`, 0.08071204068111693`, 671.7123766572973`},
{0.2186000058002156`, 1.674921415227379`, 0.04684927805332688`, 0.7958293763534576`, 0.5351292729987009`,
0.3413988165541403`, 0.023772881602852104`, 4578.839908213088`, 0.025990133203813937`, 720.8700500740513`},
{0.08466466489793933`, 3.1894928322423937`, 0.04884056021911439`, 0.9766729535722316`, 0.2661879637637754`,
0.5761935324118598`, 0.024926658702996464`, 4783.240631357024`, 0.11594669465289681`, 380.9240212400814`},
{0.21930175757669085`, 1.638318457274547`, 0.028552151697051935`, 0.9929755101949111`, 0.48282817858365856`,
0.6345477159456419`, 0.037102957315214465`, 1113.33955069597`, 0.08280464755403139`, 979.2309221100506`},
{0.15074472135509004`, 0.9706350461652997`, 0.03261694957200774`, 1.3446365626585761`, 0.7522030233980499`,
0.24992054076492587`, 0.19250628663410368`, 341.383303547674`, 0.21302570685657762`, 810.5220628187353`},
{0.15291759685661022`, 0.42291903800035024`, 0.0019904081657203853`, 1.0349558440923765`, 0.804656482695703`,
0.5600791477495833`, 0.008901523926716821`, 129.71753742837356`, 0.2016179651576812`, 261.7757399110873`},
{0.17393186946863948`, 3.634473205144226`, 0.03501927778959045`, 1.259756868930488`, 0.2400906424310547`,
0.1769239851738713`, 0.19287151186810095`, 68.7455559557316`, 0.22859728678934704`, 432.35938430583155`},
{0.08252103338424227`, 3.0363282382170365`, 0.014054309199089551`, 1.1671357119318402`, 0.48811181819525484`,
0.5135417689953007`, 0.03600069536054404`, 697.7036897164247`, 0.15194091944355315`, 879.883721957997`},
{0.152738213225879`, 2.1423984829886598`, 0.013570505913453062`, 1.4096468218064773`, 0.2076797705558071`,
0.6307063081946709`, 0.005224131132178296`, 944.5685551789895`, 0.1507676005368857`, 103.54095034743213`},
{0.13639700931586268`, 0.4456680831486439`, 0.03342224700364268`, 0.8649458036959383`, 0.5930285970230691`,
0.512351265340247`, 0.245770577144212`, 2154.7761980173996`, 0.03788382651562655`, 910.303522512114`},
{0.15928993459575497`, 3.999012236456535`, 0.05909250114200807`, 1.3761236219477655`, 0.950340178766912 8`,

0.5184686893967728`, 0.024376742099233118`, 2487.723402011106`, 0.03811059136471645`, 242.2171516528934`},
{0.09086020561938618`, 3.6933841261170146`, 0.051558219365949225`, 1.0052639956256584`, 0.6404497588004767`,
0.6310251056832639`, 0.009282302874928348`, 1342.6385109725754`, 0.17780049337096682`, 290.68187157092353`},
{0.1467805861006215`, 3.449583346443368`, 0.044526946458641366`, 0.9116880264848481`, 0.3816708687678134`,
0.5427311042687007`, 0.008463344612022042`, 84.3419318420348`, 0.15666870327157162`, 901.9122564215138`},
{0.11923417673201614`, 0.8516005442952741`, 0.011417621987304472`, 1.406269973162172`, 0.8163346696622038`,
0.4684095363963826`, 0.03909433657582696`, 694.2186659403512`, 0.15728990858282532`, 465.4699780055223`},
{0.20474637151455272`, 2.273526081644097`, 0.04289425593502317`, 0.93188673114289`, 0.463730044259554`,
0.2201193444564068`, 0.08911251750722911`, 165.09098140801413`, 0.18954199559487928`, 728.9231766980553`},
{0.05526814093912169`, 3.3906431390874365`, 0.04679651060056909`, 1.3224994050291778`, 0.2130837384463229`,
0.5620196653380606`, 0.45821421853915184`, 166.36956673134458`, 0.04079620395491479`, 150.90572670851293`},
{0.1424344572203114`, 1.5158405980214011`, 0.07134698803292082`, 1.3412182398127908`, 0.9032816245031063`,
0.29383720975190164`, 0.11721475717276858`, 2259.2805795521476`, 0.2041616321098142`, 875.6348666465524`},
{0.21167201791316342`, 1.191857769132941`, 0.04032467834992808`, 1.3612666973498941`, 0.990843843386328`,
0.16867005012046943`, 0.32551015426475394`, 61.09769889812229`, 0.07090867627674724`, 397.7456784438642`},
{0.08319100731021534`, 1.1769760609325015`, 0.07914999980937851`, 1.2654227958186712`, 0.5286106444848078`,
0.6131113894479372`, 0.3420318056909255 6`, 3018.9895646255973`, 0.20716587538358655`, 115.93712250962636`},
{0.15910463203606118`, 3.3362654217234775`, 0.09431988145352659`, 0.9102504372858342`, 0.8811348699089658`,
0.5322317508995823`, 0.49238248141112334`, 1657.8316991656131`, 0.10134572490386001`, 783.7355808530842`},
{0.10233951732223695`, 3.8860213079913546`, 0.08245580990451895`, 1.0255637903426433`, 0.4817258525533532`,
0.6808372231333493`, 0.05183250404760466`, 4623.677166174953`, 0.1226398356738308`, 284.97228231241803`},
{0.14767115153525173`, 3.9914449333138045`, 0.024555696174678 5`, 1.4796764004437446`, 0.4415017997340658`,
0.38166398917825395`, 0.06906175497737103`, 324.69168049200084`, 0.11969109369698483`, 735.4456200401828`},
{0.17843716795843323`, 3.273844277118725`, 0.0030946336041298927`, 0.8383571948484874`, 0.5814391172481872`,
0.33735131984206335`, 0.09800031855604995`, 299.74935856154275`, 0.23538286721353047`, 296.3741029308432`},
{0.15153478181902558`, 3.940884011459042`, 0.014665112834793917`, 1.0805125654131587`, 0.23054812649590795`,
0.6258941160756026`, 0.07918188154195355`, 98.54552815956583`, 0.07490635587591205`, 443.4663115601323`},
{0.134722684681424`, 1.1454968245380153`, 0.01650061661779107`, 1.0638009891109292`, 0.999069379656865`,
0.5940301687777447`, 0.005569610207767832 5`, 77.43260394254949`, 0.24107274439535475`, 940.3682518559112`},
{0.16433469628892827`, 2.875058335652863`, 0.047444839802376605`, 0.8943437881772829`, 0.6054569837144246`,
0.6619217328059503`, 0.14605802692460343`, 150.35965445533094`, 0.21808470663788926`, 986.5914920483302`},

```
{0.18172474747658007`, 0.5398125898895043`, 0.06746996069377663`, 0.8516154049831974`, 0.9176550555688654`,
  0.6251776245678378`, 0.47714073816368646`, 219.82174949020737`, 0.1448459760500183`, 388.6902609510781`},
{0.1627140613056554`, 2.526426793480817`, 0.08166994036129233`, 1.3765323556161237`, 0.2395228888894274`,
  0.6556866863306616`, 0.041008399203188106`, 966.1247163249689`, 0.01569822406304719`, 290.26321585148054`},
{0.12926587974943277`, 3.1942443269472287`, 0.08210234540290166`, 1.2603468170155185`, 0.11771113616842777`,
  0.5855250995493423`, 0.010816984036854691`, 52.9364314659927`, 0.0872384103249817`, 113.1064270044662 4`},
{0.2469537827294621`, 1.6562160115050997`, 0.08971323759474036`, 0.8904234033118983`, 0.4552788372237486`,
  0.6086911537768385`, 0.45126702440163974`, 3127.8941282749056`, 0.06787458866390378`, 920.4041061473141`},
{0.04659393582426277`, 0.8257639781936223`, 0.07933970228984723`, 1.0123444750721238`, 0.23360835713895778`,
  0.5040801713850254`, 0.008240901014236341`, 3556.051928374705`, 0.0470528014171252`, 139.7711872268978`},
{0.2677990399223792`, 2.190165256838246`, 0.03877434139180814`, 1.1644520688476412`, 0.10209034886491564`,
  0.2364288033256654`, 0.301326771326778`, 351.0852119703326`, 0.017030644812824763`, 859.704636269683`},
{0.27526155344317305`, 0.42206525583727394`, 0.010509190579989251`, 1.2469728015728827`, 0.7164324088803142`,
  0.23095486560068912`, 0.4019760074402924`, 1837.167141511333`, 0.14638705750272024`, 598.0758928204975`},
{0.20827027070080567`, 0.6628955977865401`, 0.05578571612958859`, 1.4550917307077555`, 0.8160716291341483`,
  0.4316400149470674`, 0.0992623271553592`, 1291.2580266081497`, 0.17638713235092185`, 712.744419351735`},
{0.26108671366598557`, 2.7581406885672397`, 0.09215954227601131`, 1.3356715726742072`, 0.5159534131307919`,
  0.6292106530069272`, 0.19102200652406912`, 1359.6101170600552`, 0.13195246712822323`, 655.8014880925608`},
{0.12411610080756336`, 3.5491378450736537`, 0.03048497342324529`, 1.052629142532319`, 0.933405263977 6818`,
  0.36612480189397933`, 0.17487769367297745`, 297.20407438965395`, 0.10198438571360102`, 142.8358321881406`},
{0.05206075217376971`, 3.80544265452421`, 0.06452586960506253`, 1.1764286006641393`, 0.7578123360385443`,
  0.3632439318173788`, 0.0382545660199779 7`, 1732.1715020363104`, 0.09945789721416326`, 862.5286889178561`},
{0.15332434762984987`, 3.71381929627883`, 0.04943415023567301`, 0.9774785033821363`, 0.4718998580816507`,
  0.16669947597303947`, 0.00694639422020862`, 208.05288466197266`, 0.20610825162042906`, 633.4494218195209`},
{0.06033541900347844`, 0.973178216355266`, 0.030553995574257496`, 0.8504754985225583`, 0.27347786882322955`,
  0.4969493066691505`, 0.00830637169085789`, 57.15499757894147`, 0.07721246031772627`, 682.9655440833467`},
{0.20494675314374677`, 2.7031536940694387`, 0.02716633958654084`, 1.0975656700162824`, 0.4564303296261911`,
  0.4441209590579568`, 0.3554217278310708`, 4265.584586744801`, 0.21396541728692714`, 985.913598004549`},
{0.05327195450009359`, 2.536575411744539`, 0.009856300363339`, 1.2242648903686608`, 0.25909020435504493`,
  0.528269407206618 7`, 0.04434669218741346`, 786.4053543279437`, 0.1778964533530039`, 544.6097455298984`},
{0.12477833238312225`, 0.7549035136230082`, 0.025172412892961696`, 0.9147089841492775`, 0.8930485640293313`,
```

0.5901323959258005`, 0.0508108243850171`, 901.2908925357414`, 0.07250789031346216`, 103.12569950478974`},
{0.08995934799066885`, 3.666207694088575`, 0.03007591771494079`, 1.3783264230132162`, 0.4826387854777239`,
0.6038418084491215`, 0.23708076068251083`, 167.7294214818222`, 0.01048197934630371`, 522.6073774456877`},
{0.15902513428717435`, 1.7183982375849336`, 0.033022213369310126`, 0.7628337434543904`, 0.7778819919683766`,
0.27145222438301553`, 0.043862649375531554`, 1185.7733981174872`, 0.08799090639918955`, 695.0405493319087`},
{0.11065553243087889`, 2.46805164022211`, 0.0463748475768039`, 0.8209475953416414`, 0.5218398256116175`,
0.3133447370054039`, 0.011285096069233967`, 4139.924962213234`, 0.13167847716735503`, 267.39995064825325`},
{0.08562689605991824`, 3.9571879577725095`, 0.023793442849937278`, 1.226116327613754`, 0.7555535622077838`,
0.20305217942323717`, 0.12860386825121028`, 531.0020640269107`, 0.051948220660308075`, 415.78314995313474`},
{0.10669261879032438`, 2.055705808398714`, 0.04769389095504181`, 1.156038712774391`, 0.9820313641369269`,
0.38163507374297045`, 0.039535368502052644`, 2902.0412683197437`, 0.20901158297646766`, 336.26891906262557`},
{0.07060338528242072`, 3.595376249521064`, 0.03262882113877765`, 1.2426013551392963`, 0.6002233277932603`,
0.6255684512538187`, 0.011346312056874001`, 3533.244097205098`, 0.061628460123131446`, 431.95845626027455`},
{0.2296701087879804`, 3.9087772869642077`, 0.005974703984365633`, 0.7959312177065372`, 0.876747425533583`,
0.6239039023797257`, 0.07860570649462724`, 228.33223863779693`, 0.09890789121587351`, 844.6853450307202`},
{0.23123324268179735`, 1.3892537621982006`, 0.09784040419751712`, 1.4899932847388562`, 0.04016727483231097`,
0.3908129571471922`, 0.009391139743151554`, 3403.3990186754204`, 0.07086662039842229`, 218.43172418092502`},
{0.07037189900847057`, 2.1828807010264386`, 0.09644892533086193`, 1.4657375782847808`, 0.7479730618322393`,
0.5037584189194234`, 0.030916267299058386`, 50.37706857442957`, 0.0504519506394871`, 421.25447735673964`},
{0.24256545257709167`, 3.690547309616945`, 0.0023677786666569567`, 0.8901250940229146`, 0.8854195106458671`,
0.44399073208272666`, 0.23636352272736572`, 89.54810713350285`, 0.186159802578101`, 387.72460570093335`},
{0.13839843978976302`, 1.4644738520575267`, 0.08648420456992728`, 1.2182260759194046`, 0.42213262989843314`,
0.3820758811935525`, 0.019878840421350977`, 139.8221226740942`, 0.07043032032389362`, 893.4669900375276`},
{0.11263641432345284`, 3.0669748383334596`, 0.07040331826586925`, 1.0832638028187807`, 0.4284903169815901`,
0.6222704043007405`, 0.007844284225620671`, 2131.889703000162`, 0.08148303022699405`, 953.4820565221385`},
{0.08536639269975432`, 1.9832711606259998`, 0.06847562880410717`, 1.259414576524179`, 0.8112350819234888`,
0.30170114251846125`, 0.062372919072375`, 772.9300404210154`, 0.12506634187920385`, 925.8799331095055`},
{0.11730626511183101`, 3.630410531262336`, 0.09596398943788596`, 1.1797596414840856`, 0.00512306280685948`,
0.2706014914642383`, 0.007107925916470569`, 167.23494696565012`, 0.11063186891436805`, 990.8331649712547`},
{0.11173802137314093`, 2.161180887445509`, 0.00830056555397336`, 1.063559562488714`, 0.1885103833536148`,
0.4813970169653780 7`, 0.010634768624873106`, 123.30116070184943`, 0.040023829860516`, 666.6470268689523`},

```
{0.10813918810135675`, 3.496374135712781`, 0.08929026043826785`, 0.7770714221575694`, 0.3733447131761056`,
  0.4939165094284049`, 0.2800021673947185`, 161.9820586332278`, 0.08289673671409309`, 517.9231146523`},
{0.2516406246499894`, 3.0929818341741733`, 0.0922614386482984`, 1.4236100743284779`, 0.03336817153694982`,
  0.531009903965389`, 0.28645389162750856`, 1114.6653530749377`, 0.10635783873890536`, 232.713586458166`},
{0.1065188593842617`, 3.0239491530160656`, 0.022787241302245534`, 0.9140212299263315`, 0.4098506422400856`,
  0.631556223988371`, 0.008249935916109901`, 842.7098345476253`, 0.19464933826626446`, 205.3036767061707`},
{0.10588487940933416`, 2.859735718128807`, 0.07786116606871861`, 1.047717859923827`, 0.07660672309433547`,
  0.158735212392627`, 0.05520993734392543`, 139.0602212904327`, 0.07978847169291192`, 577.575995655312`},
{0.24741184670460048`, 2.0853129323232675`, 0.002699004039505635`, 1.042019307006856`, 0.6924193332252813`,
  0.4588072902902137`, 0.0350404016550923`, 391.00460391265096`, 0.13592475687567207`, 785.2080587147564`},
{0.14083189694803505`, 0.7118335450358293`, 0.018460964897572776`, 1.1767917671701966`, 0.48592079833913515`,
  0.6846823750216606`, 0.24127390726138886`, 71.14275489785842`, 0.0433294805997953`, 476.2086967233275`},
{0.1009771753749294`, 3.4383291777667013`, 0.09534658166572815`, 1.3953306821932303`, 0.24681268758922426`,
  0.3480290288704033`, 0.2692140132359644`, 2330.189032037055`, 0.14685923933486766`, 555.9442327003733`},
{0.16178386458202587`, 3.0596682411026226`, 0.016235234307709075`, 0.7897132419424195`, 0.7440577027037574`,
  0.20415575695989485`, 0.014269716719641092`, 102.02000238842407`, 0.02548443659481625`, 410.89583584040685`},
{0.1639764064141605`, 0.8690658024831128`, 0.09689964069135186`, 0.9303160183239412`, 0.925921343915583 6`,
  0.6251018503757462`, 0.13591794228358278`, 311.5677558935227`, 0.18168686534025869`, 702.447340545002`},
{0.12955597509758354`, 2.3266058652149084`, 0.07612788670577046`, 1.2957091127204743`, 0.08408476825169409`,
  0.22843448121491383`, 0.01377311432994412`, 55.28011697543834`, 0.05462266813297323`, 936.2737831126054`},
{0.22722659605137513`, 3.181545366826299`, 0.09535885824182891`, 1.455710256248994`, 0.794512142132684`,
  0.40024598250807375`, 0.3807443177879789`, 208.1153783523671`, 0.02759817321864938`, 937.8080702420884`},
{0.09757928432270024`, 1.5076906365385394`, 0.06532569914948308`, 1.2393804160164015`, 0.6875999373585513`,
  0.4482433478851079`, 0.047668506035977345`, 978.2925685396615`, 0.1537809380520726`, 134.17889109795115`},
{0.25247158502971473`, 3.209043445695394`, 0.055750773529977184`, 1.2307446634362496`, 0.9584321093779467`,
  0.17045215686099446`, 0.02394094843679724`, 733.412724821379`, 0.14871489313227643`, 731.1114504116057`},
{0.10371347338421405`, 1.7327368937733922`, 0.06071844641560931`, 1.2089839863490672`, 0.14765621815567243`,
  0.4738799094972982`, 0.010611619577856298`, 2438.8343629553387`, 0.18500920326393544`, 108.28078770273848`},
{0.1984535123182241`, 2.161947896960034`, 0.06138419655750843`, 0.778982059344484`, 0.4287275067738694`,
  0.20720196920301803`, 0.4492524632515773`, 121.20549296704452`, 0.18396106207724583`, 119.02585475241914`},
{0.12381163862088174`, 3.895341333824428`, 0.06002344154256473`, 1.1343022539214913`, 0.8129713762085851`,
```

0.4638592661411107`, 0.007042757871236177`, 1176.2351771701979`, 0.2462865126714932`, 108.58547580007442`},
{0.23071761590484513`, 2.2648352286735998`, 0.07729558723199069`, 0.9210169155643355`, 0.6313373838804968`,
0.6041276627548808`, 0.24562846674087035`, 1229.9254758581137`, 0.2458533106908962 8`, 430.22606831878835`},
{0.05479322654019092`, 3.8246177185802415`, 0.08580605416623638`, 0.8811793306182123`, 0.8761776950953761`,
0.29070846616035273`, 0.033348914503464826`, 623.2110193634621`, 0.14957649554329666`, 152.49175504634724`},
{0.19953212896288414`, 3.4927590196799247`, 0.09411150882849728`, 1.197122533048502`, 0.4043090283096169`,
0.6122442379199733`, 0.044927979287668965`, 1565.6926144346771`, 0.04491818835648648`, 171.98402040125399`},
{0.1517228798855726`, 3.821661938472987`, 0.04482964683170573`, 0.9498760179602059`, 0.3819160293947341`,
0.6937284136376201`, 0.007172788536686403`, 154.83311013460644`, 0.21825034747286304`, 849.9173689310978`},
{0.10089572826433801`, 0.9689485600216026`, 0.09904537540593211`, 0.9565780146244569`, 0.8315614208813649`,
0.23778331363990324`, 0.010609351956832196`, 166.5397570471634`, 0.23685724436722827`, 123.70685669376229`},
{0.2617448383586014`, 1.7419134501480498`, 0.045608467431404856`, 0.787836989709293`, 0.662878849064974`,
0.4338361091174493`, 0.3345623113002579`, 73.9957072876414`, 0.12486123360347584`, 607.2676663733008`},
{0.10370303958455385`, 2.4325217216628605`, 0.02176754760468306`, 1.4452363933770138`, 0.40438966394356823`,
0.43523988865263297`, 0.04976478629271485`, 282.2104614433738`, 0.15573083503468216`, 771.737205400766`},
{0.12568862947200987`, 3.409935111150106`, 0.04842578409514237`, 1.2928588459089652`, 0.8548940812962251`,
0.3585702743647413`, 0.03943961787244869`, 103.22445359993986`, 0.12312508088340224`, 106.12725365941351`},
{0.25023693340814834`, 3.9316478547515894`, 0.07082339724222557`, 1.158336750472774`, 0.7299281236811437`,
0.5200623075713134`, 0.16855609928966492`, 115.49612111354244`, 0.11648069433492153`, 380.1124291681772`},
{0.2327122870521875`, 1.8349316139343976`, 0.005921840861416321`, 1.0160702776423072`, 0.724126729587736`,
0.6641678286901775`, 0.014844066228330453`, 4846.196302582017`, 0.19133524452025602`, 312.0736607519881`},
{0.26060624730373366`, 2.429263865356268`, 0.02302054068506065`, 1.1376576887137433`, 0.6882649764401805`,
0.6080978782814359`, 0.05257452924408477`, 81.26248932759681`, 0.16061945366682562`, 580.3732149020416`},
{0.22406406307822446`, 1.3829714288487063`, 0.037525031212394956`, 0.7596012900803544`, 0.17941039146586824`,
0.520203193915476`, 0.056506779103220175`, 93.77318192698738`, 0.011157058120984026`, 243.36956207188064`},
{0.16129175454120598`, 2.241594788665445`, 0.02595808801699503`, 1.4515663695326364`, 0.44349403825461486`,
0.5944541929016426`, 0.13242981207036078`, 1862.045541199846`, 0.19478161615729406`, 241.6655690221253`},
{0.15431366769375426`, 2.6965576187541354`, 0.08956453107270243`, 0.8164255077330567`, 0.4910378956214103`,
0.374255897689989`, 0.007597691279617231`, 3560.421166649914`, 0.2337580506715733`, 428.017043104418`},
{0.2212577173064723`, 0.5158974882846561`, 0.013003028286676788`, 0.9387448220782406`, 0.24284514367000565`,
0.3081553043694423`, 0.03815340049240 9215`, 56.65345283557565`, 0.12156476174517056`, 401.2477783269693`},

{0.1683373271076925`, 1.6782737549041533`, 0.05896916410328887`, 1.1152338969749211`, 0.5823548078906295`,
0.17654711643520027`, 0.009562790707407565`, 617.6094195707465`, 0.1697152454569904`, 676.3594994410081`},
{0.07670427099110139`, 3.20755225941694`, 0.05882096511027358`, 1.447867595309567`, 0.29934068229086064`,
0.292514599413645`, 0.006615326955656567`, 67.05743705305659`, 0.05138097017561577`, 233.60263470026138`},
{0.09939447843258498`, 1.0806802687605517`, 0.08727314073863666`, 1.0427681271407077`, 0.2371764885575376`,
0.5091900756791798`, 0.016527080711814128`, 61.46338920331662`, 0.06725149887765097`, 222.62215508117382`},
{0.058754537918439986`, 3.7681443191246293`, 0.07934826392769118`, 1.166166391041544`, 0.7017545799004972`,
0.6192265616934043`, 0.1452002074780104`, 372.914499705161`, 0.050248583952100856`, 447.3871254600834`},
{0.1573899141991117`, 1.1557291134580199`, 0.009009591269829347`, 1.325752168244685`, 0.33932371792024996`,
0.619859177738425`, 0.009052475129474827`, 1177.0140495795238`, 0.2452005274360191`, 480.6283895441986`},
{0.2160610264367817`, 3.9191762808523007`, 0.059018604533116775`, 1.0411018057212353`, 0.008462501397731081`,
0.4634420979655972`, 0.32322042810200047`, 481.531032168865`, 0.019909072809315087`, 488.3969282323367`},
{0.11630471140404741`, 1.5367142722565337`, 0.05695013627172493`, 1.4595782498770793`, 0.4347006603611545`,
0.15170509102823682`, 0.02764562799329766`, 1429.026434078033`, 0.014789325697006195`, 556.0517717070035`},
{0.04574862224020773`, 1.667137614048353`, 0.08615274073435199`, 1.367803712499899`, 0.9144234427837643`,
0.29317668075534076`, 0.06903839047309805`, 1609.2665529077706`, 0.23754263722827756`, 367.7320637842886`},
{0.2785629651947983`, 1.9405997998909799`, 0.09745189735065271`, 0.9223797816210384`, 0.7945408892658388`,
0.34974400234234904`, 0.06721335250349363`, 788.540217506401`, 0.22345836350921944`, 636.4117130237988`},
{0.1558631861871686`, 2.071958791081494`, 0.0906148168421321`, 1.3439374199675207`, 0.8630947847986346`,
0.6733061517037251`, 0.2071553134363624`, 104.16809345728986`, 0.11868827346586763`, 553.1180333659046`},
{0.1292208707400178`, 1.8632605914435363`, 0.046415812114243664`, 1.1633279615140635`, 0.018954763914399875`,
0.24694457378275914`, 0.007904270013564875`, 164.82469298269328`, 0.060610611106801215`, 743.9579215785636`},
{0.0898239866436944`, 0.5041817537224471`, 0.004730845503889416`, 0.8004221139970495`, 0.71803886182858`,
0.24861999546798397`, 0.03932650979479891`, 324.23821280210257`, 0.08133023973628611`, 182.23375283503634`},
{0.12178660308868972`, 0.8029832921015938`, 0.06274577468035605`, 1.11373872869435`, 0.3507051586575438`,
0.45809878988528197`, 0.10454823064111732`, 716.7349133355972`, 0.057611453097593246`, 948.7347206740686`},
{0.0685108116980988`, 2.7433614317525636`, 0.03948625806531251`, 1.4383757105618995`, 0.12700514465157853`,
0.29596094652330907`, 0.005824134698501406`, 171.22697089194668`, 0.06525718195551272`, 301.6896298904339`},
{0.22222570024441013`, 3.2839931217970175`, 0.03747668952108825`, 1.1936997453676481`, 0.5468462118059447`,
0.582123988821347`, 0.030193979255150022`, 4841.3857036739555`, 0.21121655285668073`, 468.9585544167855`},
{0.05500096557985035`, 3.021764046557614`, 0.03167342594848885`, 1.471078294602109`, 0.4110285739228652`,

0.6035837025270763`, 0.17164962839295506`, 1968.9667019111098`, 0.24541253876143565`, 510.7332508800703`},
{0.21198399370055476`, 3.919285200069673`, 0.014045043011751641`, 1.0494239320976348`, 0.47271455772763615`,
0.6026643614464045`, 0.05622966821105292`, 117.93228694573206`, 0.21791598368515847`, 664.3246551556151`},
{0.18261393242527846`, 1.24104755829402`, 0.017562609584990004`, 0.8208770412391784`, 0.5702360150514367`,
0.2445337365136635`, 0.06636250536989298`, 71.77227660714203`, 0.12457334089639138`, 863.0628087051591`},
{0.1516561445800767`, 0.8718874600546336`, 0.04870269917216002`, 1.3587651378620147`, 0.3907653327903866`,
0.2341643054755288`, 0.16161562738072457`, 398.11431612220986`, 0.21962329680899473`, 517.7069194001951`},
{0.07481472761035168`, 1.4356710981047938`, 0.03195742895113495`, 0.7620294331090656`, 0.6058100988285502`,
0.1748877988840174`, 0.036278078120143255`, 1067.1675037581192`, 0.20966766369542061`, 245.76795972204266`},
{0.23415203477003`, 2.637838297244735`, 0.010100103036724857`, 0.7781763734410485`, 0.9270607447506427`,
0.25091053524524765`, 0.006631362838358266`, 410.1408598520636`, 0.09672454943968684`, 810.2426002994349`},
{0.054190572804884596`, 3.205052996318111`, 0.07207331803839295`, 0.8907501512355283`, 0.1443118496038216`,
0.23964954198579969`, 0.007902398391995273`, 453.6925070614146`, 0.03286507467345462`, 128.45869723434936`},
{0.1942266150934147`, 1.489765634070828`, 0.04812058961143629`, 1.1947522848110315`, 0.4017009524635866`,
0.3855054941178302`, 0.01145165307821007`, 1319.8238490809574`, 0.16195059607983425`, 743.204251876079`},
{0.14289375184211878`, 3.302390049355761`, 0.06615684983106804`, 1.29788834802556`, 0.9156578751693429`,
0.3558474159491941`, 0.00509231061202701`, 4261.526717057486`, 0.16050896664278197`, 220.23477447344888`},
{0.2554917877832018`, 1.993631504608799`, 0.043681818566651834`, 1.3194492976944885`, 0.9399541819359079`,
0.20985624905157363`, 0.1180452152100559`, 2753.2515506454065`, 0.05426315355547984`, 171.05806084971232`},
{0.20201473782765333`, 2.779452027424738`, 0.0266194566183942`, 0.9103804881826009`, 0.744670698555542`,
0.5706914134089118`, 0.00905331146486097`, 947.9277353727819`, 0.11184880683500475`, 329.63826832088097`},
{0.24790417478919824`, 3.0551603250746844`, 0.002284082483761942`, 0.961052650753995`, 0.6989086220525811`,
0.17002095436609788`, 0.48755300390089085`, 499.7708205836441`, 0.047042546081792314`, 684.0826745551606`},
{0.17492926953535737`, 3.975314242289789`, 0.023210045448957432`, 0.9727254901105757`, 0.4411891051406147`,
0.5520848832843507`, 0.2621570799727775`, 227.0108426167968`, 0.23671821431578927`, 367.0798924947729`},
{0.17539856022464784`, 2.4073130870702855`, 0.07197140330954806`, 1.0342299427745458`, 0.017371005489709157`,
0.6584543473805056`, 0.4728358484415665`, 1058.3389525653213`, 0.15082970917224703`, 917.1303711003585`},
{0.25951131167180713`, 0.8532719891968794`, 0.04557480110843374`, 0.7947841840785437`, 0.09920524665409447`,
0.24836596104002728`, 0.00597151633514079`, 436.2925795444242`, 0.1419534062375405`, 794.0516002827078`},
{0.17151022740710664`, 1.3676826689672605`, 0.009249620913063498`, 0.9876118143041603`, 0.1005607415916352`,
0.34049413724762323`, 0.11299721785819453`, 604.8937675217858`, 0.18735181675894919`, 945.3802305578456`},

```
{0.08003627269054897`, 0.4223118257784404`, 0.09788308557086342`, 1.1088763125629295`, 0.1846532162494996`,
  0.44877823183875776`, 0.1744050351437392`, 232.77104633127487`, 0.186893582460665`, 400.88339340412807`},
{0.09629954489292863`, 0.8024243073579225`, 0.09137673334437835`, 1.1050982833461225`, 0.42757767860422`,
  0.28094623108518857`, 0.05836165382907772`, 735.7662354861307`, 0.19909783079594584`, 717.4225578982439`},
{0.18721691020827746`, 0.8832247086125689`, 0.007468716509783384`, 0.7687691323991039`, 0.5945515211149526`,
  0.3249811314880464`, 0.2800532125976925`, 679.3592862851432`, 0.05953883596172632`, 967.7513494997147`},
{0.05639051201063694`, 2.8104432162704684`, 0.055705228249153096`, 1.1263048402686886`, 0.5873694688928046`,
  0.5281479596797096`, 0.1725920753398321`, 2534.050261866886`, 0.23842584574122794`, 882.2272354030342`},
{0.11940415250329756`, 0.8265537347095737`, 0.08146769819729481`, 0.8399194412982254`, 0.9872938360285297`,
  0.2770401739511047`, 0.07923813270856427`, 3244.586795954999`, 0.06527667418181682`, 865.3808868374238`},
{0.12362063330315853`, 0.9363044869527668`, 0.08770822250254312`, 0.7786855706091862`, 0.2025852940468913`,
  0.4566487841344946`, 0.10394338643070074`, 1334.4445729465897`, 0.16877657565130982`, 938.8021455901892`},
{0.25510163512616657`, 0.9371168864708563`, 0.07693845729623781`, 1.115586607271032`, 0.44362559437168314`,
  0.23833942272790432`, 0.005303052515546934`, 2412.9883309521724`, 0.03447937580899002`, 556.3992268583279`},
{0.26333930199077354`, 2.0534773984698482`, 0.0643643244795264`, 1.2527519466886172`, 0.9177593225481775`,
  0.33938623608355467`, 0.007825944721954872`, 939.4147129019324`, 0.07035677568445653`, 544.5854367414727`},
{0.18557844748229363`, 1.3040038526378845`, 0.03298157347075899`, 1.0816274180852803`, 0.0720559521645785`,
  0.4038816001150133`, 0.01911693092881156`, 163.22630477861273`, 0.023327766650460102`, 342.443720752801`},
{0.08287518809588085`, 1.4167280782763267`, 0.028751858328181383`, 1.3710623936056974`, 0.3420455264471254`,
  0.6180297694222441`, 0.06931546639283913`, 2809.59627019281`, 0.14779417738205175`, 701.4197770536504`},
{0.09993506797522744`, 1.9333379215144753`, 0.0294160752239147`, 1.306899867333013`, 0.15174981363752638`,
  0.6973160529857587`, 0.32517481775011875`, 1904.6938693333132`, 0.22395301180399813`, 246.61499786462963`},
{0.1791717975737977`, 3.6792864853182463`, 0.003697989603839975`, 1.4268593787039923`, 0.6861577534974679`,
  0.6003986216720203`, 0.037512309221476955`, 1019.7481977593816`, 0.10587101285101158`, 387.91912641915013`},
{0.24888562729039826`, 3.026347119869615`, 0.09462972939910319`, 1.164624390493581`, 0.6459998640897591`,
  0.315426369282 0086`, 0.35313147165596037`, 156.8927746768367`, 0.0941784253310613`, 806.7916066548876`},
{0.07400216536531751`, 2.1937414927491288`, 0.051724338318571414`, 0.8475382671591798`, 0.5283653100185577`,
  0.3375223430599671`, 0.006370262255851855`, 419.8990383214368`, 0.056433548922067256`, 105.83929536957157`},
{0.08649617896036566`, 2.9261562137270944`, 0.06277833160761251`, 1.2717586436565882`, 0.677517886873324`,
  0.2240537731188419`, 0.01575962778087511`, 67.09139852540285`, 0.15266972777055898`, 497.65886074130015`},
{0.21956975330290024`, 2.946279278154277`, 0.01351442569268972`, 1.0817364176432047`, 0.11142356213890037`,
```

```
0.6113477082885477`, 0.45234016742673816`, 389.8324142721476`, 0.06262043870838019`, 252.04928334155613`},
{0.16962065420490857`, 3.2987764634142422`, 0.06541243051493663`, 1.4967196726725034`, 0.6967420255395558`,
 0.17574917802163403`, 0.035158157434036455`, 63.90845452931862`, 0.08229945239139341`, 618.3420143317427`},
{0.19774968406774135`, 1.6989982601552214`, 0.021311063743135836`, 0.7648586795987937`, 0.41768823186273707`,
 0.37949798185162253`, 0.005204291178783692 5`, 973.0476525534903`, 0.05448802148770515`, 784.8493656086185`},
{0.2518902561813237`, 2.7254296067200725`, 0.08582594724985977`, 0.8908613191536825`, 0.4232867475157469`,
 0.2276322578613562`, 0.014796112855772266`, 4775.5807799067`, 0.21732003999216098`, 657.2426602250014`},
{0.09904156316806545`, 3.4999116420802006`, 0.05005174807173392`, 0.889946639423957`, 0.9586911880217555`,
 0.5850413707726637`, 0.007125457878312364`, 2230.565285121922`, 0.055223893991493855`, 299.18976270067026`},
{0.1091854910155387`, 2.8633835129131198`, 0.02035355266995058`, 0.8677831415002069`, 0.010141336872096618`,
 0.28824854332128513`, 0.17109333973146398`, 3619.61715038073`, 0.23330556046958534`, 323.4056364398427`},
{0.1891953550945305`, 2.389035398016863`, 0.08454968650044813`, 1.066070932458544`, 0.0345292329688216`,
 0.3128135317446209`, 0.38423156758654614`, 3793.7981099794624`, 0.125293090042303`, 233.53803394535984`},
{0.1570227614874073`, 2.40105806706515`, 0.05143382258328971`, 1.1808996551918274`, 0.2223234227077866`,
 0.26986116152499395`, 0.10425235900754858`, 216.82613723714533`, 0.18708364772996822`, 798.8941796704306`},
{0.21694584394948213`, 2.9530758173431613`, 0.09769681722844141`, 0.9640446069284444`, 0.42193435394716805`,
 0.6603635658814309`, 0.3338306865733826`, 1314.2123391672299`, 0.21796673788414078`, 229.9659279906 8196`},
{0.12690264500941062`, 1.3993259739099067`, 0.022125995185778395`, 0.9178738286195887`, 0.25309924713953147`,
 0.251012090694433`, 0.019625422672818624`, 283.1749611392459`, 0.18026742864340645`, 758.8108031110586`},
{0.06920814358208571`, 2.042285023379372`, 0.02966007038539361 3`, 0.9921431890214623`, 0.03257273935914129`,
 0.20517882048284686`, 0.027701026007507824`, 104.22947983733822`, 0.22115350693221852`, 739.7214992264616`},
{0.2577647175823676`, 2.0669085310717623`, 0.015137977599734764`, 1.2344138658842803`, 0.6169124929132423`,
 0.4497973990302503`, 0.20323917961090476`, 273.8208174543164`, 0.23260089052488175`, 786.949837857692`},
{0.22404984477095807`, 1.7971972613143468`, 0.014556371051440885`, 1.4736916256467743`, 0.10711810372521668`,
 0.4838216983187773`, 0.15033397484285083`, 3525.8267227086753`, 0.24709636291116127`, 542.7438703089757`},
{0.2560915912056091`, 2.8056995396679163`, 0.02230409169328258`, 1.1596232786630103`, 0.9731942543354852`,
 0.32967621772461264`, 0.008273413274888125`, 659.2862632916124`, 0.2493316428820775`, 801.2464425394161`},
{0.069529383599776`, 2.849786616236517`, 0.0015488296932831959`, 1.1307747790060854`, 0.8754257019481757`,
 0.4462637970794401`, 0.00987625334070338`, 470.49802215652267`, 0.1965355306527206`, 591.5879320045749`},
{0.16317921960456533`, 3.795960913866608`, 0.060277538996435585`, 0.9943285566083784`, 0.31878466621504153`,
 0.5268930320337564`, 0.03217382490455373`, 160.19057295170512`, 0.13130693314062597`, 466.7290497736911`},
```

{0.23870751571987275`, 2.817892132050006`, 0.034904301600875376`, 1.2212602625610964`, 0.2634282445359748`,
0.29343287356938585`, 0.0412321304066401`, 2956.916383781115`, 0.1710028618436959`, 734.0158474610636`},
{0.25110587793218636`, 0.5330897224408169`, 0.03508654146335001`, 1.2737631195521142`, 0.14437163587185342`,
0.22915335883220245`, 0.1301100912805756`, 130.55788460482208`, 0.03440422815097299`, 153.71031149577092`},
{0.04796641395535989`, 3.6542681080130706`, 0.06056371338194985`, 1.0410004898170107`, 0.13233719731300564`,
0.36420880945840706`, 0.16862770789835824`, 456.10576346306766`, 0.12687150881465203`, 491.49936597120086`},
{0.23314959452407125`, 1.9850975451070108`, 0.09033499334189088`, 1.2112065225297546`, 0.7891619630024773`,
0.23016874225029837`, 0.025757539951395944`, 1618.2342010883456`, 0.11509681389516696`, 818.8391633081792`},
{0.2668656374936109`, 3.051749683327815`, 0.0258024333714593`, 1.3861879676031486`, 0.13705271113057615`,
0.3169491905155566`, 0.04957453327253996`, 127.00810430555478`, 0.22326078444976738`, 605.3130708988924`},
{0.12837714622951152`, 2.913045083809414`, 0.0837521188025789`, 1.2333880824573145`, 0.14831200507330466`,
0.5511854573030723`, 0.319487783122794`, 260.13632482567476`, 0.08285153464288336`, 293.457784508192`},
{0.2580675113282032`, 1.3794239963466088`, 0.09606459315739223`, 0.9738093032034381`, 0.551722273950022`,
0.6189616163620202`, 0.03080803973244329`, 452.05919890664615`, 0.0556923772463625`, 491.93043733941124`},
{0.08884487120981721`, 2.332961071695622`, 0.031598395011471735`, 1.1141910450192687`, 0.7184499049851998`,
0.6730388302232508`, 0.123997295097641`, 484.8896162348586`, 0.15163592409494436`, 272.73357673474675`},
{0.2759587426432519`, 1.9534358692391445`, 0.08397889547253015`, 1.0356223413014591`, 0.42065948992616775`,
0.6805267491095484`, 0.0964860490180039`, 69.9818047589248`, 0.07247191711576301`, 633.9862534370685`},
{0.07608942110432254`, 2.164297732879585`, 0.044623011426671744`, 1.2845300660264662`, 0.14123590728202684`,
0.20715073503154113`, 0.010177080583516988`, 365.6404789674773`, 0.09043375256261627`, 318.6735532350724`},
{0.12578906287533304`, 0.5873288516562982`, 0.00917874148360891`, 0.7860432924669931`, 0.8658472891163045`,
0.28929582442115676`, 0.04769704077854567`, 2092.327054664775`, 0.10790373294003436`, 499.81053764102194`},
{0.13400527850674449`, 3.167561049379361`, 0.09503778083239858`, 1.0695890919772384`, 0.44461403458537974`,
0.35428116459826664`, 0.04474774833241626`, 249.9292825838412`, 0.2392270171205957`, 993.872691687206`},
{0.14569029844361497`, 1.581782399146463`, 0.02853446038002714`, 1.4746522591423932`, 0.17909427610451467`,
0.395505345829425`, 0.04611739955881583`, 816.4722344402478`, 0.17778523558925585`, 271.44027790378294`},
{0.12249361616240895`, 3.3852221257689097`, 0.05543978239026112`, 1.0925731163264347`, 0.17776966626445145`,
0.29066583025906045`, 0.06874297711712889`, 314.16092924905377`, 0.1111159201808452`, 685.2380116739549`},
{0.07368028774821661`, 3.0677536525276716`, 0.07059619046896788`, 1.2388956643695934`, 0.6420156583576027`,
0.15354672355808818`, 0.023520960948583497`, 1566.5430005580442`, 0.06436291536646671`, 602.9382331885815`},
{0.10293015258903027`, 2.7627847871257094`, 0.048868500737193926`, 1.1335235205351295`, 0.26256073599306595`,

```
0.17258322470135423`, 0.35448680892520623`, 639.8812577909154`, 0.21355261514569296`, 292.3004754224651`},
{0.17989985519582302`, 2.2848444130728813`, 0.06499018234377278`, 1.3356780269154247`, 0.8760585579430653`,
 0.25119519210597663`, 0.17680773834886487`, 2893.233426659574`, 0.08968766773643105`, 889.4972829137831`},
{0.0700064475969871`, 2.5832650492672506`, 0.009367713712463904`, 1.4593574063080232`, 0.8612459654760951`,
 0.39256758588428897`, 0.048637831394936666`, 69.10482903899627`, 0.16784750403438659`, 808.9797947692787`},
{0.16902358696866238`, 1.5466078808636725`, 0.09114667029195474`, 1.1632289455971812`, 0.7011539680870855`,
 0.43898684142995004`, 0.12814910976660654`, 201.32808946828865`, 0.1990055903635441`, 501.3434969043132`},
{0.07225780293824052`, 3.2069795251700226`, 0.08284178682925689`, 1.2105070206615947`, 0.6775214967569712`,
 0.488074793019582`, 0.026308005494110893`, 313.1548266866826`, 0.21343764900902945`, 758.2569582586476`},
{0.13893262337344747`, 1.0427327070975592`, 0.09311200953995581`, 1.095872791509625`, 0.7537435467200488`,
 0.549904286540486`, 0.2574747272316 2285`, 4239.365928479679`, 0.23905861617289415`, 330.3429341054421 7`},
{0.06788146233466685`, 1.022821493462117`, 0.06599019909830307`, 1.0274069325364188`, 0.42743374336783724`,
 0.47977647156275904`, 0.02909328473333213`, 114.54872183983547`, 0.08606336865176462`, 305.0489819660903`},
{0.04270280673685248`, 1.2934684868479742`, 0.09591189067862807`, 1.4128815880743355`, 0.13315269485352843`,
 0.23204805195972866`, 0.2572508527807455`, 90.11608912792754`, 0.03659032712805199`, 523.1103863627065`},
{0.046236418394459605`, 3.574151007923631`, 0.092837745021803`, 1.1374323179308754`, 0.4550831015183179`,
 0.3756839361198018`, 0.00586392775091683`, 1204.6242266607505`, 0.029043894879770327`, 942.0566673133433`},
{0.14802462884786977`, 2.8463045630582817`, 0.07086620031807243`, 1.4262141974100975`, 0.40895975433086873`,
 0.2365796035874227`, 0.3310600416149043`, 102.98362169854454`, 0.1764312785940091`, 773.2109911740185`},
{0.17261791412834615`, 2.66725952372021`, 0.011878378245691455`, 1.1959533166982588`, 0.37293218245137805`,
 0.28202161480695964`, 0.4651224858250725`, 72.55708905719771`, 0.17887121192017358`, 925.2830578188941`},
{0.10523638690298753`, 3.5642755326059987`, 0.09015549116400706`, 1.3647813438407541`, 0.8329295324191079`,
 0.5634168718886515`, 0.02622479781769635`, 1558.3726673845852`, 0.0866750472996437`, 735.9237964380025`},
{0.20934959845181905`, 0.5253713637677127`, 0.09562730622062596`, 1.3267328346184806`, 0.026713277643319655`,
 0.15125889150158744`, 0.009145743694901407`, 347.6644603704283`, 0.2158426941139397`, 839.7488318339839`},
{0.16181023020779767`, 1.305821903245473`, 0.04345833700918577`, 0.9871466940785586`, 0.990363037222695`,
 0.41737973540465045`, 0.04349028756064097`, 389.8084434973402`, 0.2301500945529913`, 142.75918439051142`},
{0.0585933257218722`, 2.1564084302719815`, 0.008240848797671667`, 1.3548876424139005`, 0.78543219520227`,
 0.5684638437310318`, 0.03933279743012852`, 1164.1502368017577`, 0.0365133102608523 6`, 908.8904797965278`},
{0.17629686442565196`, 2.478708866004733`, 0.029760826245713457`, 1.1409573325432285`, 0.08473376105442143`,
 0.48472391979554497`, 0.008616495281357169`, 315.1653370205564`, 0.21238301827033085`, 252.6645414580406`},
```

{0.05701931707649882`, 3.239761060927444`, 0.018658291098521504`, 1.1958711178433945`, 0.6010462815373658`,
0.2222121897761763`, 0.008661059703696556`, 2427.987481600541`, 0.16195229268590294`, 598.9762630900484`},
{0.12429129251918081`, 3.165176790650899`, 0.05944857413724836`, 1.1750421651261647`, 0.7024367598760777`,
0.26578928465801155`, 0.28623135703022834`, 57.36286473144533`, 0.18225946471633314`, 279.6228390464558`},
{0.17592117157303727`, 3.225697565005621`, 0.06410385713825643`, 1.4723665071047831`, 0.040075633612683514`,
0.16766841616104544`, 0.01926077754515973`, 451.97528485070285`, 0.1967336572740045`, 903.2247661915143`},
{0.20267367580625623`, 2.351165901182144`, 0.008675643302528258`, 1.2736483392354083`, 0.1108762459426682`,
0.4077401628201436`, 0.03171361327346612`, 3972.464308751051`, 0.13615309860632413`, 596.0671079273882`},
{0.12207280755595895`, 3.8644474094244794`, 0.009427505375928522`, 1.1042754889246225`, 0.6660399151614447`,
0.25206523111087553`, 0.10009599160633623`, 402.11380186936043`, 0.20397090745892643`, 309.50158431622725`},
{0.24507742036233432`, 2.3831843355711397`, 0.005052828156007507`, 1.0721739375121355`, 0.49387052070387516`,
0.2147676922991728`, 0.011565289716111508`, 84.02018174384712`, 0.07100872729300844`, 676.9347914709565`},
{0.05872459234515515`, 2.1332438180615902`, 0.07824887308364058`, 0.7585647474793751`, 0.26510390251726434`,
0.2713237544705841`, 0.03697920915478579 4`, 52.71577024813928`, 0.17679977189893264`, 564.239792184463`},
{0.14683813473126478`, 3.4711809547760266`, 0.030295203568552493`, 1.3035360306310906`, 0.42027841877180494`,
0.5921843153064568`, 0.010793050374412331`, 254.4238250041397`, 0.22813578414007268`, 746.5344746241171`},
{0.27118296549826965`, 0.40016893385797836`, 0.03738933189542754`, 1.0762552685066569`, 0.6570337540705835`,
0.22274705980247678`, 0.006224989465699251`, 331.2301635317647`, 0.16318949690503837`, 803.619268726775`},
{0.2651341645020733`, 3.921256061000774`, 0.09127020329702631`, 1.0515436089401342`, 0.37820568835934876`,
0.30543268981111493`, 0.008197803117955519`, 249.67697255719887`, 0.20481437271886815`, 450.4819383009913`},
{0.10266981380053875`, 1.7259937776730974`, 0.05788500808078473`, 0.8498356975392536`, 0.5149250125601736`,
0.3750799043189028`, 0.006831336220999067`, 241.15442777469008`, 0.08411211628353799`, 814.8105177183215`},
{0.1701903238305883`, 1.5737398864052752`, 0.013362842515380927`, 0.9723904628120438`, 0.9778114455260909`,
0.6469978525912341`, 0.013362665151551732`, 1406.2278798102645`, 0.1754741948990562`, 281.6011633652904`},
{0.20205010610888874`, 2.4886025319973255`, 0.03416173840731352`, 0.9461759215151488`, 0.19917032732038176`,
0.20522267238789926`, 0.007087646061753528`, 151.73293313624973`, 0.015624468793102558`, 798.3652981187157`},
{0.2548714778924099`, 2.595325989353224`, 0.04783518749288423`, 1.260048561023251`, 0.11179251567641679`,
0.5271320321183793`, 0.20942249013272063`, 61.94848210458544`, 0.13000680045559498`, 257.7742889938444`},
{0.25697985123874933`, 0.6423673175461451`, 0.03920104245257182`, 1.0494619196955428`, 0.6883576503405942`,
0.5372602583905404`, 0.0298184492004487`, 1024.1709922100576`, 0.18196374947080113`, 448.03818305726395`},
{0.24108459248749042`, 0.6097105829562341`, 0.05914817527701839`, 1.4448529749350583`, 0.06017529583805459`,

```
   0.5679483071298559`, 0.028892937973694452`, 184.61048198703293`, 0.046198153900852024`, 876.6239668930126`},
  {0.11138935148613482`, 1.9270561111646902`, 0.0506158838526531`, 0.7861832578093317`, 0.2623170852799024`,
   0.19880501636260794`, 0.1716591058201036`, 672.6474872708164`, 0.1435945283698614 7`, 488.7427429214147 3`},
  {0.22711687131412428`, 0.5423663037563959`, 0.07370528658374681`, 0.9917310500559307`, 0.6268413827963848`,
   0.35482337483246806`, 0.0492957290107415`, 298.86562808346656`, 0.0341131398928744`, 451.4330811913654`},
  {0.2649900949630745`, 2.9147648354215034`, 0.09385410641202459`, 0.8515585607662866`, 0.6282724158295114`,
   0.40674785391584145`, 0.012279618899 47592`, 57.08712154139038`, 0.026923769884349857`, 165.58243018457918`},
  {0.0915398024811338`, 3.642607843612522`, 0.045773601129209036`, 1.2779360684969243`, 0.224414125809913 7`,
   0.6005450159014489`, 0.4759115782164151`, 141.33543981734994`, 0.23290101078240694`, 255.60470768923676`},
  {0.09997582603329147`, 1.8799516342297675`, 0.01460194430259186`, 0.8270986899357093`, 0.5545841854424991`,
   0.5876382120219206`, 0.052778297224976234`, 1510.139172300266`, 0.05587104924786124`, 122.66534784854954`},
  {0.11587596442377335`, 1.570443051624368`, 0.05003858184900709`, 0.7979390254239629`, 0.2695501577041357`,
   0.4402434745919389`, 0.1955710791573617`, 1393.738907723443`, 0.22482510146060541`, 822.0077092395296`},
  {0.22552960854228582`, 1.1654101546280868`, 0.07708737421212913`, 0.8741738696212267`, 0.34763140639716794`,
   0.6720908373189787`, 0.049522394564493144`, 1483.0319583713147`, 0.08855956469300003`, 824.4137036635007`},
  {0.0476102421762471`, 1.8287647324569782`, 0.03110183117006317`, 1.3863628762732523`, 0.6811850740698742`,
   0.21530814035187884`, 0.02495067854594185`, 78.5692803520234`, 0.13933019803203`, 251.51485099763934`},
  {0.07293365735317825`, 0.9536666649351204`, 0.041006966482838275`, 0.7665287875498985`, 0.935767572935885`,
   0.220179659831362`, 0.20106612820967829`, 178.2780155183392`, 0.14301518002847002`, 953.0856220301953`},
  {0.19096668838686354`, 3.9791109702558582`, 0.04575838938969985`, 0.88278575745365`, 0.1319776886877686`,
   0.6321229402668154`, 0.005732045216178792`, 174.70115248623193`, 0.20628116633253563`, 438.2211382365572`},
  {0.24680488679812707`, 0.815637935503644`, 0.0843637406190441`, 0.9487689240355479`, 0.3250491534008515`,
   0.6707507630339515`, 0.10643040624255257`, 4909.4593594307935`, 0.030006121749521886`, 121.48162188773526`},
  {0.14271805685583516`, 2.3484760469035573`, 0.07745189816615884`, 1.1886412637022417`, 0.22453965782263952`,
   0.6988851450501361`, 0.008392473480111172`, 241.2772419245499`, 0.05856292707558125`, 560.9416229791005`},
  {0.237923538483648`, 1.3045678824163405`, 0.05160012685748933`, 1.1613123620254255`, 0.8558167677781581`,
   0.6442348560245077`, 0.280278439293096`, 960.8793815230854`, 0.10811725374257602`, 308.2410580836482`},
  {0.26686684415084866`, 0.9101714257367162`, 0.05201112615814223`, 1.0067387685359694`, 0.3144283486587576`,
   0.5640584563511031`, 0.07480454987166867`, 4843.746054037886`, 0.1459015682129142 8`, 291.7993314288994`},
  {0.27466728617562586`, 3.909730616037213`, 0.015651626558488035`, 0.8183399746276282`, 0.8624521285063864`,
   0.31078149750433326`, 0.1052382875316152`, 561.582629527527`, 0.23527086491760263`, 644.9709948771053`},
```

{0.07007388150970617`, 1.2788530615475953`, 0.041240822222090405`, 1.153764990829969`, 0.8716462413619626`,
0.15860191259805845`, 0.35155378239777585`, 296.5267589380548`, 0.19912668804556583`, 616.7284928222024`},
{0.1878952467648845`, 0.7675716449344554`, 0.05741879370926064`, 0.9528631412514192`, 0.9302947624844955`,
0.2534812971784569`, 0.1207735933533045`, 1834.0932588583464`, 0.2124476008021367`, 697.2076816469347`},
{0.14168118293015808`, 1.8332481297075178`, 0.0300583699414835`, 0.7581988115722615`, 0.852062289257528`,
0.22721700466029904`, 0.12430348358114858`, 272.5636471822111`, 0.1565994070656547 8`, 639.1777488262896`},
{0.19834052588138612`, 2.8473206103303275`, 0.06787428338874751`, 1.2634740859082962`, 0.8483257098618575`,
0.27810606990948006`, 0.052306771734647074`, 2133.625806620023`, 0.1020668881167 3287`, 552.214584726826`},
{0.13732318942612998`, 2.533545686945514`, 0.07677083355764772`, 0.9473176337866864`, 0.9203815554724639`,
0.31737934755677866`, 0.05120312298025456`, 1347.6776516438235`, 0.2303275954656494`, 112.23809273053274`},
{0.21392312209610825`, 3.0478754499742005`, 0.06538726038868102`, 0.988290831541475`, 0.07401087304537768`,
0.28494540960049397`, 0.2526680322768358`, 279.52916817401405`, 0.2297370624504932`, 425.1990754299824`},
{0.14320126116520115`, 3.5785950094384216`, 0.025604653350218314`, 0.8268074039814586`, 0.976648397359879`,
0.5001702346122724`, 0.38123679071943467`, 388.98067038422806`, 0.2240399781178949`, 927.7111417924974`},
{0.20926436934918818`, 3.54928785755524`, 0.04655442449302829`, 1.4735046423674272`, 0.6436030426422834`,
0.23716114021464474`, 0.016767557842678662`, 762.9533208051863`, 0.22515295037189986`, 823.9967654417107`},
{0.09715661458363001`, 2.6162532629232897`, 0.05330552481177518`, 1.3217583375467599`, 0.056961332005489806`,
0.41403944176397645`, 0.0787432744072381`, 132.53142675262785`, 0.16676071183902036`, 308.900351347671`},
{0.04449474684522866 7`, 3.8073919623023302`, 0.00891629517230891`, 1.317697440879989`, 0.21040732129598538`,
0.43509792721016394`, 0.11970345154140186`, 395.1110764732494`, 0.23530085829797331`, 437.748607476885`},
{0.1200252023437956`, 2.8019007957477235`, 0.028007311257585012`, 0.92009516518451`, 0.01280900474487301`,
0.4526896126410833`, 0.04417068363712719`, 126.12721650529039`, 0.11309971446701528`, 297.99616984883437`},
{0.159307524381112`, 2.1388708024966263`, 0.07728990983733347`, 0.8933915824609264`, 0.17018256737475035`,
0.39427249092340955`, 0.2374099439396035`, 296.4953500654782`, 0.22256932318418826`, 146.08391069826325`},
{0.2515615397486592`, 1.2188920022514846`, 0.056806007322854075`, 1.0589873581328146`, 0.8389110983305801`,
0.5160905955986007`, 0.005163127675966413`, 854.4257970577178`, 0.2370298510099471`, 574.6962402010942`},
{0.23870656719030897`, 0.4607483912247612`, 0.017860308849642834`, 0.8081937138032418`, 0.1724194202814684`,
0.3241110683425861`, 0.018037887307016776`, 469.5800690255617`, 0.11201486480281508`, 307.02179419088134`},
{0.06627522358143922`, 2.000113059980986`, 0.03069667022234471`, 0.8345023822924422`, 0.7625117937694375`,
0.4912663179641469`, 0.07709548204647149`, 185.51291986100534`, 0.0763658907530384`, 325.2314705034143`},
{0.11831505888067828`, 0.815861401259930 1`, 0.09695106021032068`, 1.4738361110893088`, 0.2645508678881856`,

```
  0.2913223690175213`, 0.010373880238463022`, 1209.9954370966966`, 0.23975714360436612`, 767.8374529336429`},
 {0.20577599681754594`, 2.9313768979273176`, 0.0038206720060969704`, 0.781758458231594`, 0.56664249884203`,
  0.4709509229113563`, 0.021927764931302315`, 4686.470122623354`, 0.2192041879935001`, 347.008418687607`},
 {0.1869209879787055`, 2.3095757377059725`, 0.05297142960646317`, 0.8815543572140244`, 0.041400679606300006`,
  0.31871458453434554`, 0.1289212733641052`, 101.4389842310655`, 0.13597402845170187`, 914.947493051789`},
 {0.06483245092897544`, 2.8384211668748724`, 0.0911657087741262`, 1.289023251198742`, 0.4466087032681607`,
  0.267574352599688`, 0.007946224566772193`, 1055.04661000947`, 0.08980662020688435`, 572.8761673725373`},
 {0.09228610076587707`, 1.6450889610515498`, 0.08913657400655176`, 1.3611199649465364`, 0.621424219326399`,
  0.6158200041827449`, 0.19662513724207695`, 1190.4261163432097`, 0.21638593744388268`, 965.0447414955308`},
 {0.24524928286716796`, 3.6904456707313154`, 0.08967706170457866`, 0.7837922940103521`, 0.15260266641265163`,
  0.4719521309469129`, 0.02190199465707774`, 50.30445277851058`, 0.16737611791533596`, 158.4281260415155`},
 {0.2085289521726456`, 3.7433965844871855`, 0.07373717758801387`, 0.8272531686673292`, 0.9243484340355741`,
  0.4736315139016828`, 0.04000550244322193`, 775.1117022858083`, 0.18128754644027817`, 792.6217255082997`},
 {0.1942796430577805`, 3.7771592955896995`, 0.014808087701342974`, 0.8727503183069723`, 0.9499179373546311`,
  0.4711663886743095`, 0.28504637709688535`, 219.95821433792653`, 0.17829093188760597`, 601.1866479309697`},
 {0.19978804404047035`, 1.7412366971449815`, 0.05869679069766164`, 1.2250897389227589`, 0.9947242752248806`,
  0.3288744578871605`, 0.029790954073398563`, 177.76139833706597`, 0.1704177380158945`, 342.62682125921594`},
 {0.18813828973766622`, 3.040504339634855`, 0.03041458173591515`, 1.2993625717609874`, 0.3197300168421664`,
  0.4882990032931548`, 0.05916461107129405`, 288.10788143037024`, 0.10847906504084376`, 408.965425001152`},
 {0.13939828058518033`, 1.5857112352411011`, 0.08574772946752571`, 1.1093571913830735`, 0.14309585058744245`,
  0.5634406137724309`, 0.2817673310248056`, 53.991544293777636`, 0.11459032439612055`, 248.27985633277424`},
 {0.043536324184198144`, 3.326291464260996`, 0.05139708072028187`, 0.9030702944800504`, 0.7051237824883041`,
  0.5197885611320734`, 0.019273613149624703`, 329.1019165250263`, 0.23460236315419714`, 678.1062885700973`},
 {0.09801716906192193`, 2.9539869268188017`, 0.020040830336135844`, 1.2343926494145465`, 0.7600540430041269`,
  0.3516902841570241`, 0.029540615021220525`, 3778.034634812808`, 0.12448155103112013`, 335.4861228637068`},
 {0.2351399190035529`, 0.6800114559723247`, 0.03213357322891234`, 1.2136689663675575`, 0.0851758437090655`,
  0.2574027192263152`, 0.12005990640730702`, 487.2802108209037`, 0.1525337459876398`, 478.6782242639763`},
 {0.19840884489282196`, 2.435275307859949`, 0.06582510415417815`, 1.194841715963503`, 0.18931490770635162`,
  0.6654697402710699`, 0.012855112113943912`, 384.7644348925394`, 0.20206490703703606`, 653.6112811972781`},
 {0.153998794720146`, 2.296727093897146`, 0.048890456587564815`, 1.0717959146758531`, 0.20411298992327565`,
  0.5326406872061232`, 0.0433093428704623`, 1797.551253413897`, 0.09526457101922897`, 474.50386549092957`},
```

{0.2762960805421906`, 2.0251009856011146`, 0.06438086916711981`, 1.2552601159611685`, 0.824619961217385`,
0.2219622549088328`, 0.0777112084796907 5`, 235.23851588627025`, 0.03242543857282393`, 995.444729973487`},
{0.22210233579747424`, 3.0942680698763763`, 0.09032486696922916`, 1.0647099914995546`, 0.516081148067818`,
0.4664841812624002`, 0.2575335143375922`, 247.27548003665564`, 0.1261056310024135`, 988.0783976396131`},
{0.04246581734245333`, 2.6243765146123685`, 0.039300994341565525`, 1.1707132411393175`, 0.760389410309404`,
0.6197176888843219`, 0.05613616961797109`, 579.7528852266169`, 0.18044598974464704`, 692.5186634415095`},
{0.13098562468211145`, 0.6918886972002496`, 0.09307771379161338`, 0.7572969971235853`, 0.8621959711022029`,
0.17247619508234702`, 0.13638646340531488`, 1721.8193108366343`, 0.15630991181601384`, 116.41676929938268`},
{0.2369558402004424`, 3.8904595389867698`, 0.07694340666447698`, 1.136418408023505`, 0.5724535764038741`,
0.4530887158124406`, 0.11198137985855583`, 117.36224574039397`, 0.1511561710016185`, 454.7116411649565`},
{0.22006023145078546`, 1.8699454187445346`, 0.04190713219713553`, 1.0916024021867943`, 0.9816282992661436`,
0.6829534497485226`, 0.009835956205983078`, 624.6290470462591`, 0.021070658550712773`, 768.5490473777757`},
{0.09828617329733175`, 2.463942931528096`, 0.007572202379780535`, 1.2472200527061044`, 0.41644064652613144`,
0.4645936104828291`, 0.02312500549439911`, 54.07758231391304`, 0.09597622753113771`, 416.81598350845786`},
{0.08362876114696788`, 3.673753657595393`, 0.08998734313926576`, 1.3432025605848703`, 0.2747343812629557`,
0.6253363571442772`, 0.036994141920847455`, 388.9552816546361`, 0.04955150722579804`, 133.7754196526289`},
{0.22105845206099273`, 3.6723501488527104`, 0.0598021179692625`, 0.9344989193429194`, 0.3577079276157791`,
0.25286474043446827`, 0.02760454342543124`, 121.18794663333252`, 0.020589883661662256`, 454.33911379556343`},
{0.2404415985731178`, 3.9242340192232827`, 0.025726059183975813`, 1.0889915012585847`, 0.7185429349809043`,
0.6376702062000428`, 0.268650042773469`, 355.68354138078996`, 0.22139947989242942`, 504.918029373391`},
{0.1005874500764159`, 2.23272409892634`, 0.060802667136885716`, 0.991684052120384`, 0.9801615674605653`,
0.4858907364942624`, 0.29370050837355216`, 1056.4414347359782`, 0.08590239477227651`, 977.0843750733277`},
{0.2630219935361097`, 0.7161334798425658`, 0.06498611082218136`, 0.9675557624650479`, 0.11067589370665232`,
0.632848587449107`, 0.011686165277954987`, 164.13933362107778`, 0.16701870855521322`, 106.59616859139227`},
{0.21827180155004183`, 3.007771469369752`, 0.03198650183307903`, 1.2597421711376162`, 0.05877944668022894`,
0.21087223032471758`, 0.1491736628807538`, 409.73754661816`, 0.07595835910505389`, 682.1222641287044`},
{0.22045085365596306`, 0.8751625033990953`, 0.040943556123142544`, 1.1343752751378642`, 0.40735695900069624`,
0.30497304960520855`, 0.05995678076716148`, 952.3200487692166`, 0.18118346106920746`, 347.9890739313543`},
{0.18946179909757177`, 3.562633004956865`, 0.0313023029411473`, 1.4739498657051078`, 0.8014019749280452`,
0.35240031523303095`, 0.015200151831610186`, 167.2762376390573`, 0.22621173221438579`, 745.402744487609`},
{0.13542699057027358`, 2.86206448171706`, 0.09450221741270137`, 1.0246625231410813`, 0.5482685534092477`,

0.5509095992989991`, 0.015559917921534123`, 99.38093814456799`, 0.05756251023205006`, 408.71843737795535`},
{0.22732356256701053`, 1.5620331345808607`, 0.005007450975166154 5`, 1.197172786919317`, 0.105827486838725`,
0.3868626726744283`, 0.406019958242878`, 3005.96971854734`, 0.19859762557886712`, 993.8820694295587`},
{0.21970737996259793`, 3.936399929325778`, 0.011750041974777936`, 1.121349683519117`, 0.5859217781105792`,
0.5996889464289015`, 0.1151074043533795`, 67.42346044078403`, 0.08547629013313163`, 411.81079788833034`},
{0.2790786490274024`, 1.2584508796810594`, 0.07665707924292071`, 0.7658270386209375`, 0.7483296604910108`,
0.41920065323621736`, 0.023176749691687894`, 3277.281084867628`, 0.1396918716126389`, 580.5780481474799`},
{0.16453791668796786`, 3.8862921855966857`, 0.013327775962263057`, 0.8725709784378354`, 0.6113118115742937`,
0.2156639660199009`, 0.03762803482789101`, 2547.318776383711`, 0.17247938020774817`, 892.0466365080326`},
{0.06375537189690972`, 1.7690459528540705`, 0.015096892864035833`, 0.8299986088232871`, 0.6367083940240161`,
0.28113916107145065`, 0.04428098157483091`, 4084.099508293162`, 0.10954682401240934`, 746.6104278313039`},
{0.08187579925483629`, 1.8655110961595254`, 0.0874648172698457`, 1.4510572222714657`, 0.15566591480992242`,
0.41910638528278665`, 0.07239970607129952`, 723.8241834225208`, 0.12901960068026558`, 180.2504060368367`},
{0.10435199153649627`, 0.5893233643311877`, 0.04811942984145233`, 1.1349604857206697`, 0.5895670318233859`,
0.15288875305518912`, 0.04910349811258292`, 449.5316302223964`, 0.1243495956818926 9`, 703.0287573582831`},
{0.15686440216485814`, 3.299158386840012`, 0.04362586030096008`, 0.953069845851497`, 0.4069322991233839`,
0.6538520347193495`, 0.028206943433383688`, 616.8360622195407`, 0.1803006375790399`, 266.56001157686615`},
{0.06547279088568253`, 0.8381089463723068`, 0.07137472004433824`, 1.2017236457315694`, 0.07858577799252853`,
0.56356916944079`, 0.03424982094712966`, 121.26145217466427`, 0.2454354766292936`, 829.242714338502`},
{0.17569742909738978`, 2.0195575491588675`, 0.02434222613969263`, 1.434279867729109`, 0.8915491260180508`,
0.2781339725257843`, 0.04682272639132616`, 59.94019211717092`, 0.2222051195324956`, 236.65018069241324`},
{0.09460907585719619`, 0.8356897954881948`, 0.060460918351022866`, 1.069146699286762`, 0.014736646088169492`,
0.5865094862125538`, 0.07297164593929295`, 179.1610024491539`, 0.037596776460407155`, 892.0322556004016`},
{0.09233956131689997`, 0.6404053871541118`, 0.05959897753518367`, 1.3305103741913034`, 0.7729857515912517`,
0.22480536379085048`, 0.13093485314958295`, 1027.106817790177`, 0.09580485239452469`, 776.0064233531045`},
{0.1760992852032619`, 2.0576043555645516`, 0.009895906281638124`, 1.218879855416122`, 0.22293753096138147`,
0.47733786176532955`, 0.12573960627564046`, 190.0926217184925`, 0.16755982960498023`, 296.35846579200165`},
{0.266626264443372`, 3.5900664324260045`, 0.04123634989318651`, 1.010061724366724`, 0.22320942065814342`,
0.29156885927164033`, 0.008778055090297143`, 315.7126293940718`, 0.03106814470993058`, 971.4718069354443`},
{0.20633623222753245`, 1.8062049689164192`, 0.024683456051737968`, 1.4736425858155604`, 0.9975963414746294`,
0.4532113408075455 5`, 0.0616411941764036 5`, 981.5961666351051`, 0.06084524543598019`, 894.6393430913585`},

{0.1952457396539687`, 2.396633432716655`, 0.027692355669681295`, 1.483739684147085`, 0.49946151037920083`,
0.6843034163274524`, 0.015772529376378245`, 165.6691863878366`, 0.13164676433224554`, 744.7639235090896`},
{0.0412147820800699`, 0.6603123172269081`, 0.022261526033749313`, 1.1649615926526864`, 0.2836869733691665`,
0.15807495679202033`, 0.11234412980270021`, 3908.1378298311292`, 0.06462235682420547`, 548.6113009858772`},
{0.17218500939622683`, 3.313865785682787`, 0.027957414977543033`, 1.3071462915775924`, 0.8795651108976117`,
0.6440187783428721`, 0.0059566913562622715`, 104.65032511018502`, 0.027977308543697232`, 907.2104908091411`},
{0.15502202925070413`, 3.316526162282644`, 0.0223320056796846`, 1.2759230603694547`, 0.9221203428299127`,
0.2943110669636566`, 0.005350880253335643`, 3898.5440559966614`, 0.22820486785889155`, 684.4986442435719`},
{0.2268261659238296`, 0.7994964989328879`, 0.08934756409882037`, 1.2877518377572021`, 0.5411906669030329`,
0.3691399078289529`, 0.43421420694016527`, 139.17013379956958`, 0.04485647842506657`, 905.8781238041313`},
{0.1770204235692701`, 1.2781960851633176`, 0.06497396695791541`, 1.3605134684748712`, 0.244014227954229`,
0.1870138591753292`, 0.03077127158598826`, 60.18019994982649`, 0.2131346317141143`, 683.027244020558`},
{0.2717375993536527`, 3.135824470079611`, 0.0691606222305435`, 0.7845225922631083`, 0.16212023726406954`,
0.4466944141001402`, 0.022381478813530132`, 1383.5815052684432`, 0.187615308884319`, 843.6072606940219`},
{0.13674247358699154`, 3.6254060485372106`, 0.06512400723657372`, 1.01887393296334`, 0.2910940805340305`,
0.4365596853256456`, 0.08138806498834827`, 2017.3312921087977`, 0.1583601508053748`, 997.8620700747402`},
{0.25040288289621143`, 2.7133813844480077`, 0.08448585223614441`, 1.2018013366302356`, 0.3702885119817223`,
0.5701876195896491`, 0.00869107124078448`, 443.903801201532`, 0.12402133852573172`, 106.45272333045864`},
{0.041511277768737365`, 3.4931610590794087`, 0.03976168853825012`, 1.2668832550579765`, 0.9486019052531109`,
0.46529858524653356`, 0.012928244696006381`, 74.20790580822272`, 0.10554204343742091`, 509.5020269334231`},
{0.1336668665495257`, 2.350867715598585`, 0.0771275101676191`, 1.4505612510666444`, 0.07698080749571079`,
0.6199281441330318`, 0.01101960174542534`, 2269.347129396604`, 0.18489372818520305`, 687.4473514223084`},
{0.16253001008083912`, 1.051184352668212`, 0.056229861688529344`, 1.004023329401581`, 0.3681252451367141`,
0.678555581878113`, 0.021351933171038215`, 801.2312670528647`, 0.04412464112257197`, 454.387044888035`},
{0.11892797719939147`, 1.7514715754101635`, 0.04183594418628097`, 0.8769916901819208`, 0.1544196171401151`,
0.36811304221318286`, 0.3010643636498307`, 695.4685634811971`, 0.1100461062891433`, 498.29553639594855`},
{0.07330210226600109`, 0.43499060410436075`, 0.027517449896700585`, 0.9126335864120539`, 0.9987805296414807`,
0.22966152781322624`, 0.006114379328547516`, 1310.3025866798243`, 0.17060413983046802`, 156.9492509406908`},
{0.04276868172332263`, 2.4648491140401436`, 0.05187906428248695`, 1.0339646047806086`, 0.22491069842200573`,
0.5459771269560785`, 0.008402091672076594`, 289.6225761610719`, 0.14646027342084122`, 755.2264179389915`},
{0.04164393094890753`, 1.242303977240664`, 0.07972480756303409`, 0.7846124621006639`, 0.174070187582688`,

0.36672176044371163`, 0.2674133971106355`, 845.6949135526564`, 0.03358000499684233`, 498.1809415613497`},
{0.229458031958857`, 0.6439515416123651`, 0.006015292830911623`, 0.9158920123716887`, 0.2560144381841183`,
0.2684667797484167`, 0.3431552466616367`, 154.42595917415295`, 0.0855610041334563`, 198.13557848737526`},
{0.17047138799782602`, 2.2898957920477256`, 0.06696496462752731`, 1.0845008770247568`, 0.2872211606807551`,
0.6001028117008813`, 0.005754832757542004`, 635.7506300024877`, 0.22579833707749164`, 724.3665386038351`},
{0.17254913268987798`, 2.6495590376973945`, 0.034760594449157356`, 1.2124525447208163`, 0.841946926790414`,
0.5402918593560716`, 0.18936332779093834`, 107.26507780664734`, 0.037511987734769214`, 537.2669943782652`},
{0.25016487015444`, 0.777229893080774`, 0.04915901947556062`, 1.0998264093102956`, 0.5377812514235516`,
0.25267335702204063`, 0.00974787647189834`, 1244.8070691537305`, 0.07483034108642977`, 805.776090435819`},
{0.05399158420479849`, 3.019162845487374`, 0.03785073545081486`, 0.9636051990488629`, 0.1556477402052238 7`,
0.21918784710924044`, 0.006223290787557694`, 2623.658439173353`, 0.10900490483685588`, 947.3435866888657`},
{0.1747811216286701`, 3.881854804860919`, 0.08016222833139366`, 1.4944210252995196`, 0.11903432009706139`,
0.5878228241833926`, 0.37145683618740644`, 66.72046507331578`, 0.12877597314607542`, 125.71709418119497`},
{0.141601654527025`, 1.3089955011167094`, 0.06560329176994835`, 1.3368116485682675`, 0.4133506282593844`,
0.6351089997359891`, 0.016818978674610487`, 1705.797093405403`, 0.1538894760263466`, 511.61503546845904`},
{0.084639232238298`, 1.212736951496196`, 0.05997810159304612`, 0.8889893736120966`, 0.13958728158655265`,
0.6603558899173929`, 0.011413642036874375`, 536.7545673384209`, 0.04259995653165516`, 944.3686479349165`},
{0.16529311034127547`, 3.851126737390418`, 0.08113860877333676`, 1.058819349237051`, 0.13571240412272645`,
0.4517872186170928`, 0.35559705335185826`, 1001.8429736474003`, 0.09802300913580997`, 620.1784409572178`},
{0.23418982946474864`, 3.956604059014369`, 0.04230369921779414`, 0.95232561320253`, 0.8349528260558194`,
0.6299543509496768`, 0.06275886449027793`, 701.9720478982712`, 0.21824826209863868`, 426.47742716464967`},
{0.2761598760040886`, 2.6641557087508057`, 0.004462535100939409`, 0.931353404667723 9`, 0.750233715330811`,
0.2049305849598677`, 0.3933840236336692`, 50.2496550267719`, 0.2374943907090124`, 344.2970123705422`},
{0.1351836628142749`, 3.5624660341291037`, 0.025452437146326938`, 0.7612568037032439`, 0.652970660668506`,
0.6408529036315123`, 0.21928764621486097`, 534.7730348589814`, 0.24746341528536303`, 560.0699314591134`},
{0.26232668071537735`, 1.207274588572547`, 0.027172732680876114`, 0.878898400796649`, 0.9275146387246145`,
0.31349252382451775`, 0.12237771890255045`, 296.36361267724436`, 0.017576594718109073`, 538.5261184010628`},
{0.06921463438282771`, 3.9571800779527315`, 0.026742802177079528`, 1.1077760750375103`, 0.012836546117421577`,
0.44641175777925235`, 0.3069910959195543`, 680.1511030834043`, 0.08770647443596591`, 182.30226827470233`},
{0.23559239220203682`, 3.2108513477276697`, 0.01736528262118654`, 1.3561109299681864`, 0.09396152800742397`,
0.29521017097752533`, 0.02869751203681663`, 1077.23513295276`, 0.19412010713150385`, 318.4056793084999`},

{0.09115428875490028`, 3.0706223452006016`, 0.03232145089994729`, 0.7642247265036182`, 0.18050339436720364`,
0.6416456253725367`, 0.010505945470625598`, 58.10983830816918`, 0.09406933636073506`, 174.5022624084388`},
{0.23356040634394826`, 2.492582964596944`, 0.09819476019997214`, 1.1503432849681279`, 0.06854649517894851`,
0.5510839557713577`, 0.0071381077335303`, 2385.9838126952855`, 0.013315355756726904`, 961.7670735590306`},
{0.25739033381964765`, 3.83031296180729`, 0.07795330721270595`, 1.3914735727751597`, 0.315795024852483`,
0.18909057130226337`, 0.046455878996427676`, 2457.8987531381417`, 0.21479863643047675`, 436.99040574834953`},
{0.11782083138332561`, 0.9397991607947134`, 0.03435330327997967`, 1.298219743186714`, 0.15161593487328484`,
0.2427618760211594`, 0.04640184603844971`, 394.52569979799523`, 0.22637503918894109`, 120.48416627959249`},
{0.10065409907604789`, 3.109979529809686`, 0.05985471266539985`, 1.4037113657202065`, 0.2551527049282132`,
0.39211616335234245`, 0.053944330615504715`, 1232.2013118971267`, 0.18318402883994528`, 982.5075567784879`},
{0.20373034722529687`, 1.8258929347032087`, 0.028710915990495637`, 1.2999358225942528`, 0.47437870101991586`,
0.4479020312750728`, 0.02085535990534196`, 155.89057435108427`, 0.24707674305197086`, 593.832502401464`},
{0.1667072912115688`, 1.1926716420341554`, 0.02793807367428192`, 1.1097053421712721`, 0.7879852596126686`,
0.47927016034261305`, 0.007964145149834574`, 178.94143600935337`, 0.1062391944982713`, 376.6999080921696`},
{0.04399597163424873`, 1.5345814599820171`, 0.040779489311776336`, 1.144409595128893 7`, 0.08884309651814792`,
0.5567432378994842`, 0.011138214889458318`, 268.907723173268`, 0.042126942027993275`, 429.31770518007414`},
{0.12554904125704935`, 2.572105912307064`, 0.04382715819624885`, 1.0196689186185788`, 0.004628348183958852 5`,
0.4286118484279966`, 0.12186483055559326`, 183.56311720218602`, 0.19168490195367482`, 312.7756329069644`},
{0.10389836812335534`, 1.5065271361516794`, 0.0782203910368041`, 1.3432006880431453`, 0.16858721631501128`,
0.20272940646961235`, 0.005194801604326796`, 692.6428769431301`, 0.2497553227485374`, 646.8923759638144`},
{0.1873969648530403`, 2.487936537546788`, 0.08787631545159552`, 1.4862886220253502`, 0.4510967953396121`,
0.6132699008580953`, 0.20943864253875633`, 362.75371797287244`, 0.05566418089330266`, 990.5206074435366`},
{0.2609977488784161`, 2.682129125412585`, 0.04099814383534014`, 1.1943200062659527`, 0.3320420087757965`,
0.3562748569841966`, 0.03843736896556402`, 4461.432848364856`, 0.19491126930886082`, 448.46031606671596`},
{0.11177943644202659`, 3.004766476051927`, 0.02365721593278737`, 0.9739347355449255`, 0.11623805347832561`,
0.5332851817458878`, 0.014219460200483265`, 2321.5504339611107`, 0.11273311280208292`, 154.13280416226715`},
{0.12452460259052345`, 0.6179927758495714`, 0.018240920352788487`, 1.419600762592073`, 0.04159334889643196`,
0.4953333574077009`, 0.005771350364962132`, 173.11009258100464`, 0.03618579436828123`, 460.51456633143266`},
{0.056338015570984834`, 2.9056643066548693`, 0.09482015263628527`, 0.9910228668352266`, 0.5398932209933094`,
0.30993462834373164`, 0.326754145425203`, 4152.751222173614`, 0.1627448112336103`, 934.4752403980212`},
{0.24888413407324067`, 0.9185195851359875`, 0.001862748847094693`, 0.8264811519169353`, 0.45711823663268425`,

```
0.647903170967445`, 0.01273956161975329`, 3902.2467544998485`, 0.10661606869776508`, 764.1046199784935`},
{0.10302491503594513`, 3.897572343584968`, 0.0776490643225345`, 1.3728855867542038`, 0.399366063625185`,
 0.5139103382918121`, 0.006363477494426043`, 1471.3257431593884`, 0.18415616996412293`, 396.38746581742976`},
{0.2217716187206022`, 2.3669670966982617`, 0.05150497952731632`, 1.3669514270973737`, 0.27563093514117476`,
 0.6097112540241125`, 0.024210358214628785`, 245.19099830513036`, 0.2227447189207991`, 922.0556423723906`},
{0.200567099512532`, 0.414291053781334`, 0.0965175412941376`, 1.4122041619788255`, 0.2832458523243746`,
 0.4111934355701189`, 0.47068189330284727`, 727.924890435336`, 0.13700773707558722`, 457.2663597106665`},
{0.26282708876445304`, 0.7299461735692212`, 0.002590123247159504`, 1.314069772735592`, 0.44387331112790007`,
 0.6627601406571553`, 0.017941283900867265`, 4804.295508555627`, 0.13656101682983213`, 961.2364213744279`},
{0.2217181783558071`, 1.7819525199900896`, 0.09096582175868409`, 0.8016110058394221`, 0.34870116285618513`,
 0.41254310619778667`, 0.28848841693193716`, 118.09472925522707`, 0.04915168920099666`, 318.7068061045219`},
{0.04238719280368508`, 3.9934041772699773`, 0.012369382023231542`, 1.0320497019704051`, 0.441711735053806`,
 0.3953172978504792`, 0.055840071594635055`, 4470.752428881338`, 0.11971425032923705`, 391.1155394727787`},
{0.11325951664984701`, 1.2613891355679572`, 0.040579133378454896`, 0.792778865736141`, 0.4125188071599031`,
 0.6760512259522733`, 0.120226085162758`, 240.62393709029973`, 0.23563525899548954`, 669.3575607124823`},
{0.11479796420185318`, 1.3645131007055955`, 0.05813851484662918`, 1.4328455678258982`, 0.8783089057298354`,
 0.6273688332789649`, 0.005594475300849291`, 336.8131271096774`, 0.042923286813940253`, 400.6985075519651`},
{0.18904612246826086`, 0.9499182666483561`, 0.01636431294508675`, 1.334133788223376`, 0.9399135492285866`,
 0.44862152471700667`, 0.015021474832741414`, 104.29224521509512`, 0.20020013193412523`, 362.68249145721364`},
{0.07377471208830438`, 0.9399350889911195`, 0.0035046964205100294`, 0.95254283141522`, 0.8226600861406266`,
 0.5781817355083377`, 0.45292305898377666`, 4955.013777151356`, 0.18926919322875674`, 784.6286355590761`},
{0.0943766935246651`, 1.541984748199737`, 0.08520914815438847`, 1.068673226642868`, 0.827147192543344`,
 0.5185364408449342`, 0.02499096081051123`, 2458.353306813159`, 0.11939278701608641`, 406.52581078470075`},
{0.2691170241960745`, 1.3843069189189876`, 0.0032282878414418015`, 1.35893455567664`, 0.05684986848649287`,
 0.6495874733323252`, 0.012010940694653572`, 818.6583817232862`, 0.11477976407459428`, 802.4857212031947`},
{0.13195654495183612`, 2.820234715819688`, 0.06459455777685377`, 1.3215682050752708`, 0.2555253012593035`,
 0.5650143175076483`, 0.17650684935600558`, 2548.3703743447245`, 0.05197610429597477`, 647.2541591160882`},
{0.17938754879434415`, 2.956564495685738`, 0.02344859424499825`, 1.2271661955652247`, 0.1023158483495108`,
 0.2141005377740559`, 0.03160934186798711`, 55.01949141750772`, 0.11404091283217083`, 153.96775969344887`},
{0.2559468893711437`, 2.6639064911815478`, 0.09405265387739238`, 0.9418787060193106`, 0.3579196254838115`,
 0.25349572195403014`, 0.005922563586734462`, 686.8472909729428`, 0.21618019082855489`, 944.0817596146342`},
```

{0.16846533721603613`, 2.3770892262064063`, 0.060764583227921456`, 1.4122925060330735`, 0.8131908524160045`,
0.5745691401533295`, 0.13815925889996158`, 111.92799118209615`, 0.07704963831958889`, 569.2109515105052`},
{0.1434225400058592`, 2.4623089922223533`, 0.0902878213107297`, 0.9014701813026362`, 0.7982192946025746`,
0.24884754841188483`, 0.008733563377012872`, 84.02892029266614`, 0.10452691271186154`, 174.89969925150496`},
{0.27498382758967205`, 3.0621895303657007`, 0.01477217114526983`, 0.8499949546355164`, 0.9543379068261812`,
0.511253393062205`, 0.026007868557453007`, 1193.5403577036534`, 0.24471853485794082`, 173.30853286735942`},
{0.10079216159245852`, 2.802263371434325`, 0.07168578045446232`, 1.0754522486079452`, 0.19795232328567214`,
0.4796042882951459`, 0.2511967396816989`, 916.4127582717309`, 0.09231077895158313`, 746.8086196132365`},
{0.1990395876740651`, 1.2891521935040648`, 0.08843781786859424`, 1.4217044468375937`, 0.7248232910439498`,
0.5288906890390438`, 0.010145562070591906`, 273.266580044107`, 0.1443450012114269`, 262.99773077705277`},
{0.22527530235766147`, 1.598090720025331`, 0.07516157972031196`, 0.911906670636256`, 0.0015950083386944147`,
0.2733183222114466`, 0.46257295121139497`, 1061.4916967982135`, 0.16946126329202837`, 514.5508656659653`},
{0.22800375706146692`, 2.963114044328991`, 0.08247902929296419`, 0.900155769535574`, 0.8635034321466182`,
0.41996725230963483`, 0.060440776646127806`, 82.14976842278587`, 0.17068417206912262`, 306.6961010018577`},
{0.2015284416951108`, 1.4828427267730095`, 0.09645095598799114`, 0.8813953916233646`, 0.6596036330203583`,
0.46790731355733717`, 0.04929088349718639`, 2147.9230273071194`, 0.21677576367436763`, 585.5137656428404`},
{0.24804752677268144`, 3.775861546538554`, 0.09828394004173842`, 0.8259108347253036`, 0.8501628381099438`,
0.43041433567991594`, 0.1645511915243919`, 472.6904522654539`, 0.10093712153827977`, 240.41573863346227`},
{0.2257734465493289`, 2.04598315320208`, 0.061072784217809206`, 0.9205146346019395`, 0.18360134495814817`,
0.2485501642439466`, 0.008261271045257068`, 4070.225349557058`, 0.14432673822154007`, 514.018907335898`},
{0.1884239042817073`, 3.884724587861638`, 0.046102203625386924`, 1.272869675104722`, 0.0024363107878000445`,
0.6425585670884819`, 0.21347280812774377`, 105.72896030123064`, 0.013398853493480106`, 477.3170113191213`},
{0.10358133559917804`, 1.3204671153076744`, 0.03146944673924043`, 0.7598009709392282`, 0.8379948599836853`,
0.31448462367280716`, 0.09423762512957796`, 873.6401432656386`, 0.24643359443672203`, 257.57090703914133`},
{0.07213812112223295`, 3.2444866813138393`, 0.08948863559851084`, 1.1981049871186626`, 0.6100944668641342`,
0.3489877456164385`, 0.060436350562816356`, 810.8798894147244`, 0.1940599582028637`, 929.3745703520485`},
{0.18529645231815123`, 3.7388067772724316`, 0.04964299226107711`, 1.104153730745968`, 0.8562902999854938`,
0.20061955425285494`, 0.044683490812461285`, 1237.7065014840275`, 0.17461953898337046`, 513.2497687518729`},
{0.11937104462964826`, 2.2670408410877547`, 0.04212633055221726`, 1.2938710702028255`, 0.4062855702251942`,
0.22896688687327793`, 0.008005950997757192`, 187.47575366480618`, 0.12062380115724536`, 420.08829844146976`},
{0.054668692745937386`, 3.776137055200575`, 0.0067187515890975115`, 0.903291115804289`, 0.19988932148575245`,

```
0.5550342082463592`, 0.0455873571973524`, 57.62421691500666`, 0.11425393330613232`, 212.43967681879158`},
{0.1180225681670306`, 3.9649936920105127`, 0.09323036671246253`, 1.0524617108638712`, 0.5152474998332999`,
 0.3810548965315216`, 0.2344220817835223`, 72.9591968642363`, 0.19446142099795322`, 521.0444198605804`},
{0.07585484942115545`, 1.5268041836953499`, 0.03996691736666499`, 1.2200843385635163`, 0.7382765513873244`,
 0.15072535011998411`, 0.46165250685061965`, 2014.6215653623833`, 0.0639150426736455`, 907.8090815554127`},
{0.15171074425765668`, 3.139959028863476`, 0.0344935047970057`, 1.313524716160249`, 0.15932561521340416`,
 0.344519114270757`, 0.023892122521195856`, 4372.432115235335`, 0.19509438620204334`, 395.2405886014027`},
{0.09759208826045196`, 2.787459031458204`, 0.08286510303958249`, 1.149842836558084`, 0.3502413486308751`,
 0.6023991214203646`, 0.0422336569905244`, 151.71289774079654`, 0.15665841498595628`, 996.6803923172566`},
{0.06870492988603288`, 1.4433926464019216`, 0.0548016124689776`, 1.4156603871152607`, 0.5046761258494916`,
 0.49679280153517125`, 0.23820389677781276`, 574.8760772624919`, 0.07570136980088016`, 774.5924346494874`},
{0.12928812481552754`, 3.4467014297623226`, 0.07698339025243396`, 1.1617442922699581`, 0.6397961941354378`,
 0.23683288560045057`, 0.014840572388870685`, 390.22473096383766`, 0.12473219055267015`, 308.51541332429787`},
{0.09947361882549755`, 1.0723085231871234`, 0.08651203729858636`, 1.1986754319537722`, 0.20293837184497532`,
 0.6267394010553271`, 0.3996069768023198`, 125.45020568494616`, 0.0747353381238216`, 815.5264595512623`},
{0.07385189036800854`, 1.1097460679658449`, 0.08534569405097261`, 1.4790081203811583`, 0.825389044253078`,
 0.6393014572933857`, 0.005393929802389671`, 1009.7951092503188`, 0.05876766510907333`, 713.7033793838743`},
{0.08886981390180526`, 3.1852329380949485`, 0.004843225884898494`, 1.0495521924526`, 0.8894920527442816`,
 0.6575087708412799`, 0.03091556833752079`, 1958.934458296337`, 0.09234610293139284`, 932.1837545744593`},
{0.14789843862328383`, 2.02419875820041`, 0.0514487209494189`, 0.7738844537901749`, 0.7290609776680101`,
 0.3165995990604955`, 0.08454509023565165`, 148.72732800342848`, 0.13774453289126964`, 330.25011651458095`},
{0.25024837610835304`, 2.332733791115757`, 0.0077143684034007625`, 1.3224531384329063`, 0.8138980190182421`,
 0.44646733111462167`, 0.4678523507493547`, 2146.7439649709167`, 0.20938413594616256`, 223.81131941609436`},
{0.22717212093597267`, 3.1880507135942393`, 0.07302399796830696`, 1.4035606527233073`, 0.6815685781194232`,
 0.3786677309260351`, 0.11250145446111069`, 222.14009806288436`, 0.22433932328259149`, 562.8866527417559`},
{0.26122096963316843`, 2.4993722773914913`, 0.09696053620814965`, 1.104804193531995`, 0.030204720323329992`,
 0.320390588871465`, 0.02428210132726833`, 110.42992015103017`, 0.03440723905644305`, 957.9351268355158`},
{0.209997188604718`, 3.564852173259874`, 0.04768067199603367`, 1.4849701489343874`, 0.9167498847129119`,
 0.5930768777567338`, 0.007556568646764511`, 303.63963198830606`, 0.1798334582091491`, 979.6939793583849`},
{0.12957791995272494`, 3.292789025445294`, 0.029334797113071912`, 1.000823124755441`, 0.3770034479959583`,
 0.6497986144453649`, 0.4644164461032186`, 69.35773206793411`, 0.22822270741491651`, 883.2881905591939`},
```

```
{0.1734007680973328`, 2.5509297754695135`, 0.028940050324794433`, 0.7542700070247144`, 0.5607952150482101`,
  0.188194520643177`, 0.10842093468725295`, 89.42321140726736`, 0.04230251478503749`, 585.538823967249`},
{0.08976599585154404`, 3.410901375584835`, 0.0398084709097558`, 0.973029097362308`, 0.18413384146487743`,
  0.4306490501572334`, 0.25777469695398525`, 394.50697488236744`, 0.14943635401103217`, 467.5668758424882`},
{0.1540668065647836`, 3.156731285149597`, 0.025268011683832015`, 1.4768603918368393`, 0.5930914034327641`,
  0.3246832969886583`, 0.024701654832061146`, 2039.571837718409`, 0.0982582119026626`, 517.5447194924812`},
{0.25448003730676694`, 3.184178274174914`, 0.07969865646175218`, 1.1996160734264`, 0.014800627784428588`,
  0.41181348081367897`, 0.1736752616356111`, 514.7971371394015`, 0.0242520492159774`, 345.1422226775872`},
{0.1805495720684696`, 3.598829404298953 7`, 0.08629129509807985`, 1.45590487849237`, 0.6500204371524467`,
  0.6598493533910432`, 0.006501958768912599`, 98.298066958251`, 0.19720820935827937`, 552.7953947456965`},
{0.2584100436319957`, 2.6280813527891524`, 0.008925623022125535`, 1.4383962385176845`, 0.1768680490063801`,
  0.5799402745315277`, 0.03668114755652737`, 1557.060885044531`, 0.15015889209697436`, 661.2136292315887`},
{0.12632549433481927`, 3.227079423540302`, 0.024599881217287293`, 1.083115156883892`, 0.862537661831063`,
  0.4466766069622442`, 0.01755942292995868`, 838.9788940765472`, 0.11679264941372974`, 962.5385414773948`},
{0.12581743503205006`, 2.6912266074159374`, 0.06825202848377941`, 1.130575915810954`, 0.7123031893599316`,
  0.38183459324469116`, 0.005640134906792506`, 195.97188584350954`, 0.08695147378297474`, 810.3829986474054`},
{0.25215230411705725`, 3.2162318690382756`, 0.033696261063115164`, 1.4320906763160213`, 0.4333561915057187`,
  0.6173099764463517`, 0.06281247244822827`, 350.692872561691`, 0.14670856975401297`, 661.831914027344`},
{0.06363146413241788`, 2.590325492453749`, 0.07759599585526651`, 0.8055036563134401`, 0.3890604782183784`,
  0.6596279029376648`, 0.006263246097646333`, 3940.278955744855`, 0.14629462701990292`, 857.5168588182237`},
{0.09237029959020937`, 0.7372750141972166`, 0.08246784417666007`, 0.761354059113222`, 0.607934228395967`,
  0.617252844721303`, 0.005938986299855843 5`, 1735.7120546460667`, 0.12202837863790356`, 173.2417595974299`},
{0.17533432125136816`, 2.980549279621548`, 0.032407398618161984`, 1.2260204195085989`, 0.09654786970227502`,
  0.6394186143434559`, 0.01742947797867 2333`, 442.47136750346704`, 0.02322996341809752`, 762.5628352008919`},
{0.26705012685276763`, 3.7498343916107544`, 0.09027963548734444`, 0.9359855383901472`, 0.7308970561854626`,
  0.6045717653608473`, 0.013690168592867258`, 53.13894335207241`, 0.0962366983944411`, 806.6075956869515`},
{0.1678815958956808`, 3.549708737453586`, 0.0464906102104119`, 0.8245440899304926`, 0.5174765540911901`,
  0.24692645213758668`, 0.06597541194694605`, 190.2602468493688`, 0.13329765093762808`, 380.5016809755655`},
{0.26055153005517384`, 1.525876220559283`, 0.09894984049853037`, 0.7715055101942483`, 0.5126639262751305`,
  0.17386878262666106`, 0.09828079193823042`, 3373.049666117342`, 0.10907780218183427`, 438.5582920686159 7`},
{0.05352312148646443`, 2.717121314814369`, 0.009558938401920933`, 1.2972752707604394`, 0.3755720109619096`,
```

```
   0.4102380523871805`, 0.05724239658462567`, 409.7682683541758`, 0.12801779059907553`, 304.9029332641055`},
  {0.06648599129298854`, 2.0041637987546954`, 0.08747673292965043`, 1.0596372061777766`, 0.3670136645832196`,
   0.20965175246372458`, 0.041210859023618776`, 258.8957674631027`, 0.10593279391689964`, 291.6186786637164`},
  {0.27763207727232625`, 2.6532447231581857`, 0.005216127449356379`, 0.7883455682883888`, 0.4799446618915817`,
   0.19310494144291912`, 0.22065665818548827`, 81.96509789217254`, 0.19648280620732078`, 916.8547512970214`},
  {0.04552100665001646`, 2.514367034016656`, 0.03037177853258429`, 0.7530148351569228`, 0.6998681178203525`,
   0.5299837160000372`, 0.11693739269557663`, 590.5283463527218`, 0.08466788522227797`, 566.1770520758637`}};
```

In[ ]:=

```
(* Here is the script for the solution of full system -- which yields outcome type (CURE, TOX, BUR) and time of it *)
TOC0 = 365; (* one year *)

PatientTEST[] := (
   Outcome = "";
   TOC = TOC0; (* maximum accountable overall survival *)

   FullSystemSolutionMD[]; (*find minimal N and time of achieving it *)

   If[Nn < Ncur, (* tumor cure is achieved, but toxicity can still be lethal, then it will be death of toxicity rather than cure *)
    FullSystemSolutionMDStopNmin[tminNn]; (* solve it with cell proliferation stopping at this moment*)

    If[Abs[ActBloodpw[[npw]][tEnd]] > Ainj[[1, 2]], (* it's a rare bug when activity in blood spikes *)

     ttt = t /. NMinimize[Abs[ActBloodpw[[npw]][t] - Ainj[[1, 2]]], t][[2]];
     If[ActBloodpw[[npw]][ttt - 1] > Abld, (*in that case that's a much more realistic actual toxicity, and yes, that's toxic*)
      Outcome = "TOX"; Tox = ActBloodpw[[npw]][ttt - 1] / nCpm;
      TOC = t /. NMinimize[Abs[ActBloodpw[[npw]][t] - Abld], {t, tB[[npw]], tE[[npw]]}][[2]] (* time of death *),
      (* otherwise it is cure *)
      Outcome = "CURE"; Tox = ActBloodpw[[npw]][ttt - 1] / nCpm;],

     If[ActBloodpw[[npw]][tEnd] > Abld (*yes, that's toxic*),
```

```
        Outcome = "TOX"; Tox = ActBloodpw[[npw]][tEnd] / nCpm;
        TOC = t /. NMinimize[Abs[ActBloodpw[[npw]][t] - Abld], {t, tB[[npw]], tE[[npw]]}][[2]] (* time of death *),
        (* otherwise it is cure *)
        Outcome = "CURE"; Tox = ActBloodpw[[npw]][tEnd] / nCpm;]],

    (* tumor cure is not achieved, so what is the reason of death and when it happens? Need to run simulation until 10^11 cells *)
    Quiet[FullSystemSolutionTBUR[]]; TOC = tEnd; Tox = ActBloodpw[[npw]][tEnd] / nCpm;
    If[ActBloodpw[[npw]][tEnd] > Abld (*yes, that's toxic*),
      Outcome = "TOX";
      TOC = t /. NMinimize[Abs[ActBloodpw[[npw]][t] - Abld], {t, tB[[npw]], tE[[npw]]}][[2]] (* time of death *),
      (* otherwise it is death of tumor burden *)
      Outcome = "TBUR";]];
  Return[{Outcome, TOC, Tox}])

FullSystemSolutionMDStopNmin[TMIN_] := (

  (* maximum time of simulation-- where little radioactivity of the last dose remains, namely 0.17% of the last dose *)
  tEnd = Ainj[[Length[Ainj], 1]] + (-Log[0.0017] / lambda);

  lAinj = Length[Ainj]; (* number of injections*)
  If[Ainj[[1, 1]] == 0, Npw = lAinj, Npw = lAinj + 1];
  (* the injections are treated as new initial conditions for a new system, which as well takes the actual vaules of other parameters *)
  (* therefore the number of injections has to me remembered and logic differs whether the first injection is made at t=0 or t>0 *)

  apw = Array[ff, Npw]; bpw = Array[ff, Npw]; NNpw = Array[ff, Npw]; DDpw = Array[ff, Npw];
  papw = Array[ff, Npw]; pbpw = Array[ff, Npw]; fFNpw = Array[ff, Npw]; fANpw = Array[ff, Npw];
  dFpw = Array[ff, Npw]; dApw = Array[ff, Npw];
  (* inert antibody fragments are as well accounted for as pb *)

  (* times of beginning and end for solution of separate systems *)
  tB = Array[ff, Npw]; tE = Array[ff, Npw];
```

```
(* for monitoring the paths of activity *)
ActBloodpw = Array[ff, Npw]; ActBloodFragpw = Array[ff, Npw];
ActOutpw = Array[ff, Npw]; ActOutFragpw = Array[ff, Npw]; ActTumorpw = Array[ff, Npw];

(* for monitoring influence of self-dose, croos-fire and decays in blood *)
SDpw = Array[ff, Npw]; CFNpw = Array[ff, Npw]; CFDpw = Array[ff, Npw]; UNpw = Array[ff, Npw];

(* for monitoring the number of new cancer cells appearing during treatment *)
NewCellspw = Array[ff, Npw];

Clear[a, b, NN, DD, pa, pb, fFN, fAN, dF, dA, ActBlood, ActBloodFrag, ActOut, ActOutFrag, ActTumor, SD, CFN, CFD, UN, NewCells];

(* EQUATIONS *)

(* Radiation damage function *)
RD[NN_, DD_, fAN_, dA_, a_, pa_] := alpha * (ks * (lambda * gamma * fAN)/nu (*self-dose*) +
    (1 - ks) * (lambda * gamma * (fAN * NN + dA))/(nu * (NN + DD)) (*cross-fire*) + kf * lambda * (a + pa) (*dose from unanchored nuclides*));

(* Active antibodies *)
Fa[t_] := (*injections are considered as initial conditions*)
  - lambda * a[t] (*decay*) - kon * gamma/V * (fFN[t] * NN[t] + dF[t]) * a[t] (*binding*) - kappac * a[t] (*clearance*);

(* Inert antibodies *)
Fb[t_] := (*injections are considered as initial conditions*)
  + lambda * a[t] (*decay of a*) - kon * gamma/V * (fFN[t] * NN[t] + dF[t]) * b[t] (*binding*) - kappac * b[t] (*clearance*);
```

```
(* Viable cells *) FNN[t_] := rho * NN[t] (*proliferation*) - RD[NN[t], DD[t], fAN[t], dA[t], a[t], pa[t]] * NN[t] (*damage*);

(* Damaged cells *) FDD[t_] := RD[NN[t], DD[t], fAN[t], dA[t], a[t], pa[t]] * NN[t] (*damage*) - omega * DD[t] (*death*);

(* Active fragments *) Fpa[t_] := omega * (gamma * dA[t])/V (*release*) - lambda * pa[t] (*decay*) - kappap * pa[t] (*clearance*);

(* Inert fragments *)
Fpb[t_] := omega * (gamma * (DD[t] - dF[t] - dA[t]))/V (*release*) + lambda * pa[t] (*decay*) - kappap * pb[t] (*clearance*);

(* Free receptors of viable cells *) FfFN[t_] := (1 - fFN[t]) * rho - kon * (a[t] + b[t]) * fFN[t];

(* Active receptors of viable cells *) FfAN[t_] := kon * a[t] * fFN[t] - (lambda + rho) * fAN[t];

(* Free receptors of damaged cells *)
FdF[t_] := RD[NN[t], DD[t], fAN[t], dA[t], a[t], pa[t]] * fFN[t] * NN[t] - kon * (a[t] + b[t]) * dF[t] - omega * dF[t];

(* Active receptors of damaged cells *)
FdA[t_] :=
  RD[NN[t], DD[t], fAN[t], dA[t], a[t], pa[t]] * fAN[t] * NN[t] + kon * a[t] * dF[t] - lambda * dA[t] - omega * dA[t];

(* Initial conditions *)

If[Ainj[[1, 1]] == 0
  , a0 = Ainj[[1, 2]]/V; b0 = Ainj[[1, 3]]/V
  , a0 = 0; b0 = 0]; (*complexes in blood*)
NN0 = N0;
DD0 = 0;
pa0 = 0; pb0 = 0; fFN0 = 1; fAN0 = 0;
dF0 = 0; dA0 = 0;
```

```
ActBlood0 = 0; ActBloodFrag0 = 0; ActOut0 = 0; ActOutFrag0 = 0; ActTumor0 = 0; NewCells0 = 0;
SD0 = 0; CFN0 = 0; CFD0 = 0; UN0 = 0;

(* SOLVER *)

tB[[1]] = 0; If[Ainj[[1, 1]] == 0, If[lAinj > 1, tE[[1]] = Ainj[[2, 1]], tE[[1]] = tEnd], tE[[1]] = Ainj[[1, 1]]];

For[npw = 1, npw ≤ Npw, npw++,

  Clear[a, b, NN, DD, pa, pb, fFN, fAN, dF, dA, ActBlood, ActBloodFrag, ActOut, ActOutFrag, ActTumor, SD, CFN, CFD, UN, NewCells];

  sol = NDSolve[{
      (*INITIAL CONDITIONS*)
      a[tB[[npw]]] == a0, b[tB[[npw]]] == b0, NN[tB[[npw]]] == NN0, DD[tB[[npw]]] == DD0, pa[tB[[npw]]] == pa0,
      pb[tB[[npw]]] == pb0, fFN[tB[[npw]]] == fFN0, fAN[tB[[npw]]] == fAN0, dF[tB[[npw]]] == dF0, dA[tB[[npw]]] == dA0,
      ActBlood[tB[[npw]]] == ActBlood0, ActBloodFrag[tB[[npw]]] == ActBloodFrag0, ActOut[tB[[npw]]] == ActOut0,
      ActOutFrag[tB[[npw]]] == ActOutFrag0, ActTumor[tB[[npw]]] == ActTumor0, SD[tB[[npw]]] == SD0,
      CFN[tB[[npw]]] == CFN0, CFD[tB[[npw]]] == CFD0, UN[tB[[npw]]] == UN0, NewCells[tB[[npw]]] == NewCells0,

      a'[t] == Fa[t], b'[t] == Fb[t], NN'[t] == FNN[t], DD'[t] == FDD[t],
      pa'[t] == Fpa[t], pb'[t] == Fpb[t], fFN'[t] == FfFN[t], fAN'[t] == FfAN[t], dF'[t] == FdF[t], dA'[t] == FdA[t],

      ActBlood'[t] == V * lambda * (a[t] + pa[t]),
      ActBloodFrag'[t] == V * lambda * pa[t],
      ActOut'[t] == V * (kappac * a[t] + kappap * pa[t]),
      ActOutFrag'[t] == V * kappap * pa[t],
      ActTumor'[t] == (lambda * gamma) * (fAN[t] * NN[t] + dA[t]),

      SD'[t] == ks * (lambda * gamma) * (fAN[t] * NN[t]),
```

```
        CFN'[t] == (1 - ks) * (lambda * gamma) * (fAN[t] * NN[t]) * NN[t] / (NN[t] + DD[t]),

        CFD'[t] == (1 - ks) * (lambda * gamma) * dA[t] * NN[t] / (NN[t] + DD[t]),

        UN'[t] == kf * lambda * (a[t] + pa[t]) * nu * NN[t],
        NewCells'[t] == If[t > Ainj[[1, 1]], rho * NN[t], 0] (* start counting new cells from the moment of the first injection *)

        , WhenEvent[t > TMIN, NN[t] → 0]
        , WhenEvent[NN[t] > 10^9 / Nnor, NN[t] → 0.99 * 10^9 / Nnor]
      }
    , {a, b, NN, DD, pa, pb, fFN, fAN, dF, dA, ActBlood,
       ActBloodFrag, ActOut, ActOutFrag, ActTumor, SD, CFN, CFD, UN, NewCells}, {t, tB[[npw]], tE[[npw]]}
    , AccuracyGoal → 10, PrecisionGoal → 10];

apw[[npw]] = First[a /. sol]; bpw[[npw]] = First[b /. sol]; NNpw[[npw]] = First[NN /. sol]; DDpw[[npw]] = First[DD /. sol];
papw[[npw]] = First[pa /. sol]; pbpw[[npw]] = First[pb /. sol]; fFNpw[[npw]] = First[fFN /. sol];
fANpw[[npw]] = First[fAN /. sol]; dFpw[[npw]] = First[dF /. sol]; dApw[[npw]] = First[dA /. sol];
ActBloodpw[[npw]] = First[ActBlood /. sol];
ActBloodFragpw[[npw]] = First[ActBloodFrag /. sol];
ActOutpw[[npw]] = First[ActOut /. sol];
ActOutFragpw[[npw]] = First[ActOutFrag /. sol];
ActTumorpw[[npw]] = First[ActTumor /. sol];
SDpw[[npw]] = First[SD /. sol];
CFNpw[[npw]] = First[CFN /. sol];
CFDpw[[npw]] = First[CFD /. sol];
UNpw[[npw]] = First[UN /. sol];
NewCellspw[[npw]] = First[NewCells /. sol];

If[npw < Npw,
  (*renew initial conditions*)
```

```
    If[Ainj[[1, 1]] == 0
      , a0 = apw[[npw]][tE[[npw]]] + Ainj[[npw + 1, 2]]/V; b0 = bpw[[npw]][tE[[npw]]] + Ainj[[npw + 1, 3]]/V
      , a0 = apw[[npw]][tE[[npw]]] + Ainj[[npw, 2]]/V; b0 = bpw[[npw]][tE[[npw]]] + Ainj[[npw, 3]]/V];
    NN0 = NNpw[[npw]][tE[[npw]]];
    DD0 = DDpw[[npw]][tE[[npw]]];
    pa0 = papw[[npw]][tE[[npw]]];
    pb0 = pbpw[[npw]][tE[[npw]]];
    fFN0 = fFNpw[[npw]][tE[[npw]]]; fAN0 = fANpw[[npw]][tE[[npw]]];
    dF0 = dFpw[[npw]][tE[[npw]]];
    dA0 = dApw[[npw]][tE[[npw]]];
    ActBlood0 = ActBloodpw[[npw]][tE[[npw]]];
    ActBloodFrag0 = ActBloodFragpw[[npw]][tE[[npw]]];
    ActOut0 = ActOutpw[[npw]][tE[[npw]]];
    ActOutFrag0 = ActOutFragpw[[npw]][tE[[npw]]];
    ActTumor0 = ActTumorpw[[npw]][tE[[npw]]];
    SD0 = SDpw[[npw]][tE[[npw]]];
    CFN0 = CFNpw[[npw]][tE[[npw]]];
    CFD0 = CFDpw[[npw]][tE[[npw]]];
    UN0 = UNpw[[npw]][tE[[npw]]];
    NewCells0 = NewCellspw[[npw]][tE[[npw]]];

    (*renew time frame*)
    If[Ainj[[1, 1]] == 0, If[Npw > npw + 1, tE[[npw + 1]] = Ainj[[npw + 2, 1]], tE[[npw + 1]] = tEnd],
      If[Npw > npw + 1, tE[[npw + 1]] = Ainj[[npw + 1, 1]], tE[[npw + 1]] = tEnd]];]
];
npw--;
(* It will be convenient to have estimation of minimal viable cell number here *)
Nn = If[Ainj[[1, 1]] == 0
    , Min[Table[NMinimize[{Nnor* (NNpw[[nn]][t]), t > tB[[nn]], t < tE[[nn]]}, t][[1]], {nn, 1, npw}]]
    , Min[Table[NMinimize[{Nnor* (NNpw[[nn]][t]), t > tB[[nn]], t < tE[[nn]]}, t][[1]], {nn, 2, npw}]]];
```

```
  Return[Nn])

(* Here is the script for the solution of full system until tumor burden is lethal (10^11 cells) *)

FullSystemSolutionTBUR[] := (Cd = 10^11; (*critical number of cancer cells,leading to death of tumor burden*)

  tEnd = 99 999; (* it will stop before this time*)
  lAinj = Length[Ainj]; (* number of injections*)
  If[Ainj[[1, 1]] == 0, Npw = lAinj, Npw = lAinj + 1];

  apw = Array[ff, Npw]; bpw = Array[ff, Npw]; NNpw = Array[ff, Npw]; DDpw = Array[ff, Npw];
  papw = Array[ff, Npw]; pbpw = Array[ff, Npw]; fFNpw = Array[ff, Npw]; fANpw = Array[ff, Npw];
  dFpw = Array[ff, Npw]; dApw = Array[ff, Npw];
  (* inert antibody fragments are as well accounted for as pb *)

  (* times of beginning and end for solution of separate systems *)
  tB = Array[ff, Npw]; tE = Array[ff, Npw];

  (* for monitoring the paths of activity *)
  ActBloodpw = Array[ff, Npw]; ActBloodFragpw = Array[ff, Npw];
  ActOutpw = Array[ff, Npw]; ActOutFragpw = Array[ff, Npw]; ActTumorpw = Array[ff, Npw];

  (* for monitoring influence of self-dose, croos-fire and decays in blood *)
  SDpw = Array[ff, Npw]; CFNpw = Array[ff, Npw]; CFDpw = Array[ff, Npw]; UNpw = Array[ff, Npw];

  (* for monitoring the number of new cancer cells appearing during treatment *)
  NewCellspw = Array[ff, Npw];

  Clear[a, b, NN, DD, pa, pb, fFN, fAN, dF, dA, ActBlood, ActBloodFrag, ActOut, ActOutFrag, ActTumor, SD, CFN, CFD, UN, NewCells];

  (* EQUATIONS *)
```

```
(* Radiation damage function *)
RD[NN_, DD_, fAN_, dA_, a_, pa_] := alpha * (ks * (lambda * gamma * fAN)/nu (*self-dose*) +
    (1 - ks) * (lambda * gamma * (fAN * NN + dA))/(nu * (NN + DD)) (*cross-fire*) + kf * lambda * (a + pa) (*dose from unanchored nuclides*));

(* Active antibodies *)
Fa[t_] := (*injections are considered as initial conditions*)
  - lambda * a[t] (*decay*) - kon * gamma/V * (fFN[t] * NN[t] + dF[t]) * a[t] (*binding*) - kappac * a[t] (*clearance*);

(* Inert antibodies *)
Fb[t_] := (*injections are considered as initial conditions*)
  + lambda * a[t] (*decay of a*) - kon * gamma/V * (fFN[t] * NN[t] + dF[t]) * b[t] (*binding*) - kappac * b[t] (*clearance*);

(* Viable cells *) FNN[t_] := rho * NN[t] (*proliferation*) - RD[NN[t], DD[t], fAN[t], dA[t], a[t], pa[t]] * NN[t] (*damage*);

(* Damaged cells *) FDD[t_] := RD[NN[t], DD[t], fAN[t], dA[t], a[t], pa[t]] * NN[t] (*damage*) - omega * DD[t] (*death*);

(* Active fragments *) Fpa[t_] := omega * (gamma * dA[t])/V (*release*) - lambda * pa[t] (*decay*) - kappap * pa[t] (*clearance*);

(* Inert fragments *)
Fpb[t_] := omega * (gamma * (DD[t] - dF[t] - dA[t]))/V (*release*) + lambda * pa[t] (*decay*) - kappap * pb[t] (*clearance*);

(* Free receptors of viable cells *) FfFN[t_] := (1 - fFN[t]) * rho - kon * (a[t] + b[t]) * fFN[t];

(* Active receptors of viable cells *) FfAN[t_] := kon * a[t] * fFN[t] - (lambda + rho) * fAN[t];
```

```
(* Free receptors of damaged cells *)
FdF[t_] := RD[NN[t], DD[t], fAN[t], dA[t], a[t], pa[t]] * fFN[t] * NN[t] - kon * (a[t] + b[t]) * dF[t] - omega * dF[t];

(* Active receptors of damaged cells *)
FdA[t_] :=
  RD[NN[t], DD[t], fAN[t], dA[t], a[t], pa[t]] * fAN[t] * NN[t] + kon * a[t] * dF[t] - lambda * dA[t] - omega * dA[t];

(* Initial conditions *)

If[Ainj[[1, 1]] == 0
  , a0 = Ainj[[1, 2]] / V; b0 = eta * Ainj[[1, 2]] / V
  , a0 = 0; b0 = 0]; (*complexes in blood*)
NN0 = N0;
DD0 = 0;
pa0 = 0; pb0 = 0; fFN0 = 1; fAN0 = 0;
dF0 = 0; dA0 = 0;
ActBlood0 = 0; ActBloodFrag0 = 0; ActOut0 = 0; ActOutFrag0 = 0; ActTumor0 = 0; NewCells0 = 0;
SD0 = 0; CFN0 = 0; CFD0 = 0; UN0 = 0;

(* SOLVER *)

tB[[1]] = 0; If[Ainj[[1, 1]] == 0, If[lAinj > 1, tE[[1]] = Ainj[[2, 1]], tE[[1]] = tEnd], tE[[1]] = Ainj[[1, 1]]];

For[npw = 1, npw ≤ Npw, npw++,

  Clear[a, b, NN, DD, pa, pb, fFN, fAN, dF, dA, ActBlood, ActBloodFrag, ActOut, ActOutFrag, ActTumor, SD, CFN, CFD, UN, NewCells];

  sol = NDSolve[{
        (*INITIAL CONDITIONS*)
```

```
a[tB[[npw]]] == a0, b[tB[[npw]]] == b0, NN[tB[[npw]]] == NN0, DD[tB[[npw]]] == DD0, pa[tB[[npw]]] == pa0,
pb[tB[[npw]]] == pb0, fFN[tB[[npw]]] == fFN0, fAN[tB[[npw]]] == fAN0, dF[tB[[npw]]] == dF0, dA[tB[[npw]]] == dA0,
ActBlood[tB[[npw]]] == ActBlood0, ActBloodFrag[tB[[npw]]] == ActBloodFrag0, ActOut[tB[[npw]]] == ActOut0,
ActOutFrag[tB[[npw]]] == ActOutFrag0, ActTumor[tB[[npw]]] == ActTumor0, SD[tB[[npw]]] == SD0,
CFN[tB[[npw]]] == CFN0, CFD[tB[[npw]]] == CFD0, UN[tB[[npw]]] == UN0, NewCells[tB[[npw]]] == NewCells0,

a'[t] == Fa[t], b'[t] == Fb[t], NN'[t] == FNN[t], DD'[t] == FDD[t],
pa'[t] == Fpa[t], pb'[t] == Fpb[t], fFN'[t] == FfFN[t], fAN'[t] == FfAN[t], dF'[t] == FdF[t], dA'[t] == FdA[t],

ActBlood'[t] == V * lambda * (a[t] + pa[t]),
ActBloodFrag'[t] == V * lambda * pa[t],
ActOut'[t] == V * (kappac * a[t] + kappap * pa[t]),
ActOutFrag'[t] == V * kappap * pa[t],
ActTumor'[t] == (lambda * gamma) * (fAN[t] * NN[t] + dA[t]),

SD'[t] == ks * (lambda * gamma) * (fAN[t] * NN[t]),
CFN'[t] == (1 - ks) * (lambda * gamma) * (fAN[t] * NN[t]) * NN[t] / (NN[t] + DD[t]),
CFD'[t] == (1 - ks) * (lambda * gamma) * dA[t] * NN[t] / (NN[t] + DD[t]),
UN'[t] == kf * lambda * (a[t] + pa[t]) * nu * NN[t],
NewCells'[t] == If[t > Ainj[[1, 1]], rho * NN[t], 0] (* start counting new cells from the moment of the first injection *)

, WhenEvent[NN[t] + DD[t] > Cd / Nnor, "StopIntegration"; tEnd = t; tE[[npw]] = t; NN[t] → 0]
}
, {a, b, NN, DD, pa, pb, fFN, fAN, dF, dA, ActBlood,
ActBloodFrag, ActOut, ActOutFrag, ActTumor, SD, CFN, CFD, UN, NewCells}, {t, tB[[npw]], tE[[npw]]}
, AccuracyGoal → 10, PrecisionGoal → 10];

apw[[npw]] = First[a /. sol]; bpw[[npw]] = First[b /. sol]; NNpw[[npw]] = First[NN /. sol]; DDpw[[npw]] = First[DD /. sol];
```

```
papw[[npw]] = First[pa /. sol]; pbpw[[npw]] = First[pb /. sol]; fFNpw[[npw]] = First[fFN /. sol];
fANpw[[npw]] = First[fAN /. sol]; dFpw[[npw]] = First[dF /. sol]; dApw[[npw]] = First[dA /. sol];
ActBloodpw[[npw]] = First[ActBlood /. sol];
ActBloodFragpw[[npw]] = First[ActBloodFrag /. sol];
ActOutpw[[npw]] = First[ActOut /. sol];
ActOutFragpw[[npw]] = First[ActOutFrag /. sol];
ActTumorpw[[npw]] = First[ActTumor /. sol];
SDpw[[npw]] = First[SD /. sol];
CFNpw[[npw]] = First[CFN /. sol];
CFDpw[[npw]] = First[CFD /. sol];
UNpw[[npw]] = First[UN /. sol];
NewCellspw[[npw]] = First[NewCells /. sol];

If[npw < Npw,
  (*renew initial conditions*)
  If[Ainj[[1, 1]] == 0
    , a0 = apw[[npw]][tE[[npw]]] + Ainj[[npw + 1, 2]]/V; b0 = bpw[[npw]][tE[[npw]]] + eta * Ainj[[npw + 1, 2]]/V
    , a0 = apw[[npw]][tE[[npw]]] + Ainj[[npw, 2]]/V; b0 = bpw[[npw]][tE[[npw]]] + eta * Ainj[[npw, 2]]/V];
  NN0 = NNpw[[npw]][tE[[npw]]];
  DD0 = DDpw[[npw]][tE[[npw]]];
  pa0 = papw[[npw]][tE[[npw]]];
  pb0 = pbpw[[npw]][tE[[npw]]];
  fFN0 = fFNpw[[npw]][tE[[npw]]]; fAN0 = fANpw[[npw]][tE[[npw]]];
  dF0 = dFpw[[npw]][tE[[npw]]];
  dA0 = dApw[[npw]][tE[[npw]]];
  ActBlood0 = ActBloodpw[[npw]][tE[[npw]]];
  ActBloodFrag0 = ActBloodFragpw[[npw]][tE[[npw]]];
  ActOut0 = ActOutpw[[npw]][tE[[npw]]];
  ActOutFrag0 = ActOutFragpw[[npw]][tE[[npw]]];
  ActTumor0 = ActTumorpw[[npw]][tE[[npw]]];
  SD0 = SDpw[[npw]][tE[[npw]]];
```

```
            CFN0 = CFNpw[[npw]][tE[[npw]]];
            CFD0 = CFDpw[[npw]][tE[[npw]]];
            UN0 = UNpw[[npw]][tE[[npw]]];
            NewCells0 = NewCellspw[[npw]][tE[[npw]]];

            (*renew time frame*)
            If[Ainj[[1, 1]] == 0, If[Npw > npw + 1, tE[[npw + 1]] = Ainj[[npw + 2, 1]], tE[[npw + 1]] = tEnd],
              If[Npw > npw + 1, tE[[npw + 1]] = Ainj[[npw + 1, 1]], tE[[npw + 1]] = tEnd]];]
        ];

        npw--;)


(* The trial was performed via this long-running code *)

Npar = 1000; (*how many sets will be tested*)
SetBasicParameterValues[];

EtaToCheck = {0, 1780, 100 000};
DOSE = 600; (*nCi*)

TestEta = Array[f, {Length[EtaToCheck], Npar + 1, 3}];

For[id = 1, id ≤ Length[EtaToCheck], id++,

    eta = EtaToCheck[[id]];

    TestEta[[id, 1, 1]] = ToString[EtaToCheck[[id]]] <> " Res";
    TestEta[[id, 1, 2]] = ToString[EtaToCheck[[id]]] <> " OS";
    TestEta[[id, 1, 3]] = ToString[EtaToCheck[[id]]] <> " Tox";
```

```
NotebookDelete[prd]; (* To see the code running *)
prd = PrintTemporary["Coef " <> ToString[id] <> " of " <> ToString[Length[EtaToCheck]]];

Quiet[For[ij = 2, ij ≤ Npar + 1, ij++,

    NotebookDelete[pr]; (* To see the code running *)
    pr = PrintTemporary["Set " <> ToString[ij - 1] <> " of " <> ToString[Npar]];

    kappac = Patients[[ij, 1]];
    kappap = Patients[[ij, 2]];
    gamma = Patients[[ij, 3]];
    V = Patients[[ij, 4]];
    ks = Patients[[ij, 5]];
    rho = Patients[[ij, 6]];
    omega = Patients[[ij, 7]];
    alpha = Patients[[ij, 8]];
    kf = Patients[[ij, 9]];
    N0 = Patients[[ij, 10]];

    Ainj = {{0, DOSE * nCpm, eta * DOSE * nCpm}};

    res = PatientTEST[];

    TestEta[[id, ij, 1]] = res[[1]];
    TestEta[[id, ij, 2]] = res[[2]];
    TestEta[[id, ij, 3]] = res[[3]];

  ]];];
```

In[ ]:=

```
(* Kaplan–Meier curves and toxicity plots *)
TestFreeSM = SurvivalModelFit[TestFreeGrowth[[All, 2]]];
TestEta0SM = SurvivalModelFit[TestEta[[1, 2 ;;, 2]]];
TestEtaTechSM = SurvivalModelFit[TestEta[[2, 2 ;;, 2]]];
TestEta30mugSM = SurvivalModelFit[TestEta[[3, 2 ;;, 2]]];

GraphicsGrid[{{Plot[{TestFreeSM[t], TestEta0SM[t], TestEtaTechSM[t], TestEta30mugSM[t]}, {t, 0, 365},
    PlotStyle → {Gray (*Free*),
       Darker[Red], Darker[Orange], Darker[Yellow]}, Exclusions → None,
    Ticks → {Automatic, Table[{0.2 * i, PercentForm[0.2 * i]}, {i, 0, 10}]}, PlotRange → {0., 1.01}],

      Show[ListPlot[Table[Join[{{0, 1}}, Thread[{Sort[TestEta[[jj, 2 ;;, 3]]], Table[N[1 - (i - 1) / Npar], {i, 1, Npar}]}]], {jj, 1, 3}],
        Joined → True, PlotStyle → {Darker[Red], Darker[Orange], Darker[Yellow], Darker[Green], Darker[Cyan]},
        PlotRange → {{0, 380}, {0, 1.01}}, Ticks → {Automatic, Table[{0.2 * i, PercentForm[0.2 * i]}, {i, 0, 10}]}],
     Plot[1000 * (x - Abld / nCpm), {x, 0, 400}, PlotStyle → Directive[Gray, Dashed]]]}}]
```

Out[ ]=

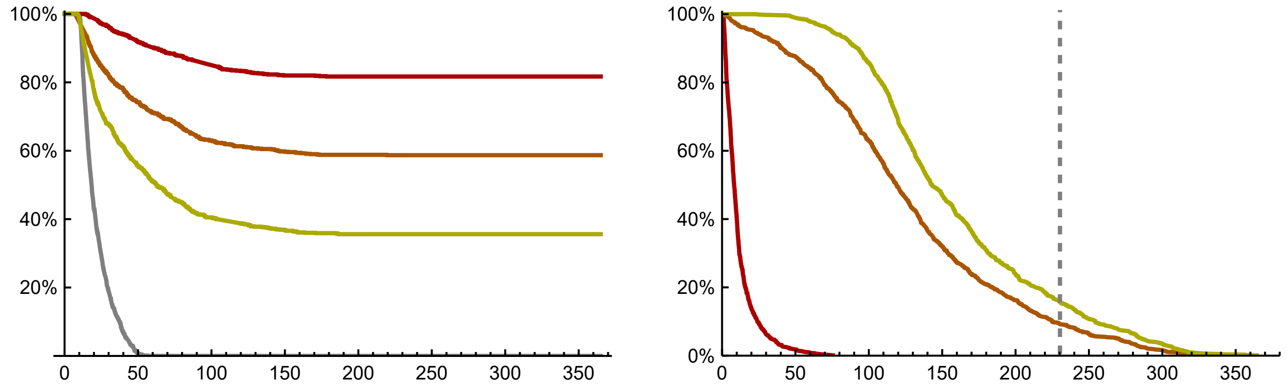

In[ ]:=

```
(* The use of pure radioconjugates results in 81.7% of cures and no deaths due to lethal toxicity *)
PercentForm[TestEta0SM[364]]
PercentForm[N[Length[Select[TestEta[[1, 2 ;;, 3]], # > Abld / nCpm &]] / Npar]]
```

Out[ ]//PercentForm=

81.7%

Out[ ]//PercentForm=

0%

In[ ]:=

```
(* The use of labeling ratio 1.85 kBq / μg yields 58.7% of cures and 9.2% of toxicity-associated deaths *)
PercentForm[TestEtaTechSM[364]]
PercentForm[N[Length[Select[TestEta[[2, 2 ;;, 3]], # > Abld / nCpm &]] / Npar]]
```

Out[ ]//PercentForm=

58.7%

Out[ ]//PercentForm=

9.2%

In[ ]:=

```
(* The use of total 30 μg of antibodies, which corresponds to our experimental setting,
leads to 35.6% of cures and 15.7% of toxicity-associated deaths *)
PercentForm[TestEta30mugSM[364]]
PercentForm[N[Length[Select[TestEta[[3, 2 ;;, 3]], # > Abld / nCpm &]] / Npar]]
```

Out[ ]//PercentForm=

35.6%

Out[ ]//PercentForm=

15.7%

In[ ]:=

(* Analytical estimation of minimal single curative dose in case of η→∞, radiation damage only from unanchored nuclides *)

(* "For the basic set of parameters, it is ≈37,300 nCi" *)

$$kfCur = -\frac{rho\ ProductLog\left[-1, -E^{-1}\left(\frac{Ncur}{N0\ Nnor}\right)^{\frac{kappac}{rho}+\frac{lambda}{rho}}\right]}{alpha\ kf\ lambda} * V / nCpm$$

Out[ ]=

37 301.2

In[ ]:=

```
(* Figure 3B, main text *)
Thk = 0.005; (* Thickness of graphs *)
Show[ListPlot[{AcurEtaDetBig[[1]], AcurEtaDetBig[[2]], AcurEtaDetBig[[3]], AcurEtaDetBigNokf[[1]], AcurEtaDetBigNokf[[2]],
    AcurEtaDetBigNokf[[3]], AcurEtaDetBigTox[[1]], AcurEtaDetBigTox[[2]], AcurEtaDetBigTox[[3]]}, Joined → True, PlotStyle →
    {Directive[Darker[Gray], Thickness[Thk]], Directive[Darker[Gray], Thickness[Thk]], Directive[Darker[Gray], Thickness[Thk]],
     Directive[Darker[Gray], Thickness[Thk / 2], Dotted], Directive[Darker[Gray], Thickness[Thk / 2], Dotted],
     Directive[Darker[Gray], Thickness[Thk / 2]], Directive[Darker[Red], Thickness[Thk / 2]],
     Directive[Darker[Red], Thickness[Thk / 2]], Directive[Darker[Red], Thickness[Thk / 2]]},
   Frame → True, FrameLabel → {"Coefficient of drug impurity, η", "Minimal single curative dose, A_cur (nCi)"},
   PlotRange → {{0 - 10, 15000 + 10}, {-500, 38000}}],
  Plot[Abld / nCpm, {x, 0, 15000}, PlotStyle → Directive[Darker[Red], Dashed]],
  Plot[kfCur, {x, 0, 15000}, PlotStyle → Directive[Darker[Gray], Dashed]]]
```

Out[ ]=

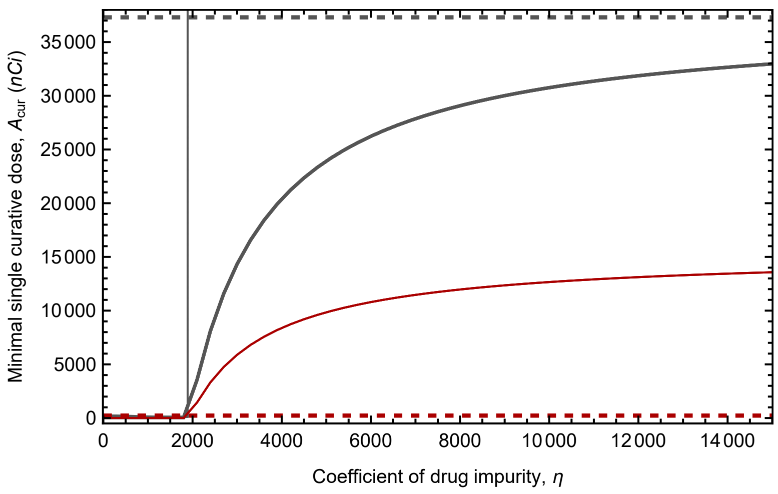


```
(* Main text: Parameter sweep for labeling ratio of 1.85 kBq / μg &
Supplementary S.3.2 Parameter sweep for labeling ratio of 1.85 kBq / μg *)
```

In[ ]:=

```
(* The outcome of the global parameter sweep, that was generated in this study
  is saved here in a closed cell -- open this group of cells to see it, and run it to upload it in the array "result" *)
```

In[ ]:=

```
PatientsTrainingSet = {{"\"\!\(\*SubscriptBox[\(κ\), \(c\)]\)\"",
```

```
"\"\!\(\*SubscriptBox[\(κ\), \(p\)]\)\"", "γ", "V", "\"\!\(\*SubscriptBox[\(k\), \(s\)]\)\"", "ρ",
"ω", "α", "\"\!\(\*SubscriptBox[\(k\), \(f\)]\)\"", "\"\!\(\*SubscriptBox[\(N\), \(0\)]\)\""},
{0.172487255493398`, 1.2765963995094278`, 0.2324603897081641`, 0.9331633444796544`, 0.5595329534566904`,
 0.18253234530133355`, 0.2215389709063929`, 78.80610179612091`, 0.19190036455619353`, 1.548441048845115`},
{0.14090572053825962`, 0.941193557649755`, 3.8052282638940014`, 1.32622706664442`, 0.616835667267962`,
 0.48175991442091237`, 0.08306143231635953`, 874.0744302472489`, 0.12491077781228116`, 5.511666182290989`},
{0.19800522656535097`, 1.47676715957736`, 1.6845843495593211`, 1.0829526404841552`, 0.6930225215765629`,
 0.2749384033948816`, 0.0668002970008328 8`, 3207.8452923633004`, 0.18818451872370445`, 6.655704647717006`},
{0.2711930593993829`, 3.0021543969280486`, 4.914701090552741`, 1.464602766667476`, 0.8471176050384048`,
 0.28257943270869035`, 0.04953192991909177`, 1933.8633413872876`, 0.19773521340575795`, 9.992985154010565`},
{0.27990766836391096`, 3.477149477100016`, 9.931945787531824`, 0.8846854018858359`, 0.8967333599095253`,
 0.5903168559967787`, 0.24882968868487906`, 980.8551320933655`, 0.02004206946214948`, 1.1376503486553808`},
{0.11737985888146019`, 1.1225833279930164`, 5.309079794927442`, 0.8606501327539497`, 0.8514188342619913`,
 0.5642039473140849`, 0.008986110654413243`, 2018.9822529242097`, 0.06040743860925685`, 4.536035630396375`},
{0.17121040978700802`, 0.4138167442315588`, 6.781135564610604`, 1.3665406840239216`, 0.9405996253397015`,
 0.6832439228870748`, 0.04185895434111264`, 62.7113536738392`, 0.18142736225630413`, 8.46259785026923`},
{0.07382619562964193`, 0.625700255424527`, 9.939999934198667`, 1.4556453073248168`, 0.9580351575689623`,
 0.4163162212955882`, 0.006430521616962508`, 92.19524127790851`, 0.19149999891406427`, 4.303065608360628`},
{0.1627307868596377`, 1.5327585946689206`, 6.648641489637967`, 1.0898921981857375`, 0.08271984399349908`,
 0.5169005126785295`, 0.006231976105310307`, 2017.0154068595314`, 0.2464092033815996`, 1.6313338993270357`},
{0.08926805346894312`, 1.228740834290055`, 5.100082063336529`, 1.0507919032716577`, 0.3229790419061651`,
 0.45218339761418835`, 0.024533377777917375`, 814.1470590464116`, 0.1414263079606694`, 5.247711540674161`},
{0.17431851399024534`, 2.020213173041662`, 9.224968841892476`, 1.3485881267881612`, 0.8890825947176819`,
 0.5177484922558413`, 0.03701694525181502`, 131.5752033573906`, 0.228949740900746`, 4.795606274383001`},
{0.25439204271280724`, 0.8534844729419486`, 2.8150623547133353`, 0.8024992452769948`, 0.43579385025887163`,
 0.4594767236090769`, 0.057877631906885676`, 1195.6419878079198`, 0.17833211044546654`, 5.195270679096961`},
{0.129274073243755`, 2.8469118022753515`, 4.121632588773114`, 1.2196787674185057`, 0.8228855513020883`,
 0.4828002917324088`, 0.013782155470303842`, 388.66064646424104`, 0.2255512748456478`, 1.0022663108918284`},
{0.06343574194222029`, 3.9924293708982113`, 9.079106395846292`, 1.3161791897123671`, 0.10938228479468126`,
 0.3240611741083924`, 0.010321331270591745`, 76.94152103093134`, 0.06038801289350482`, 9.425890207979073`},
{0.110036074735397`, 2.490203037930516`, 9.729597307787852`, 1.4397421355398403`, 0.8533380591052817`,
```

0.37040757295392324`, 0.047916566872126407`, 665.4588326486067`, 0.16916230254726894`, 1.9013228378216027`},
{0.21512225225566667`, 1.75750084110844`, 0.8044386006474246`, 1.3520587790631349`, 0.026316391204000755`,
0.16031186333306224`, 0.13761957869990635`, 493.8180531489964`, 0.06250485252841559`, 2.6863460670624115`},
{0.15874286665935028`, 2.268211451796655`, 1.8981682437883747`, 1.375959688601855`, 0.24814706074480242`,
0.5134877163486878`, 0.3316652449509965`, 685.632474632321`, 0.05319219913715695`, 1.7896119638851753`},
{0.07432908705186819`, 3.0619407712521785`, 1.7905302646779082`, 0.9549392645174688`, 0.353226782755657`,
0.6119520610188782`, 0.0496137109124893`, 317.5186768305259`, 0.07812208014742161`, 6.620973402603631`},
{0.1228443083266203`, 2.0512729485932484`, 1.9256217622349043`, 1.4810122884336692`, 0.0774145853609808 9`,
0.4641958329015734`, 0.007197139507585793`, 240.76714600918612`, 0.03301937102418029`, 9.328377628444095`},
{0.2606519449147342`, 3.818605783771848`, 8.595720359336667`, 1.112914365339814`, 0.820058745442926`,
0.4441576207168524`, 0.053270363092160024`, 1117.1606910609416`, 0.20810873381921946`, 6.830482236754131`},
{0.24750930586460296`, 3.1846993652287674`, 3.026718658979185`, 1.4845570571034035`, 0.869864794311124`,
0.6669561203654737`, 0.2363643381944309`, 1924.496556700219`, 0.06437651322808385`, 2.548184450019704`},
{0.24908529437358162`, 3.626300618072209`, 2.0230682918259397`, 1.4602193573319078`, 0.40130475942739396`,
0.6650543311625685`, 0.019582320105998748`, 125.14663606859848`, 0.03716084844005629`, 8.149858668846527`},
{0.2795730004018654`, 2.183215118244356`, 0.7910515142573491`, 1.2847699393543137`, 0.70345738565049`,
0.2632113724473232`, 0.1394636484917589`, 223.91272255356017`, 0.16150366940434352`, 9.859315316872873`},
{0.08969576723701717`, 0.49356011207206274`, 0.2529879905685366`, 1.1041769014657603`, 0.7443718717514693`,
0.6180257213599576`, 0.03574805853679543`, 3205.3226496399375`, 0.061225880931137455`, 7.560768798013438`},
{0.10298476113266081`, 3.435925289367793`, 4.954566778741102`, 0.9299871443466801`, 0.6352691398464292`,
0.5470174017743717`, 0.005507148986589441`, 973.5524715904572`, 0.1328513609688533`, 5.618259356059234`},
{0.14277679649227548`, 3.0428582592918065`, 7.414614443890604`, 1.1046799505745009`, 0.2872163059276376`,
0.23677108379761724`, 0.04608733451503689`, 2695.430951291806`, 0.1274330436692639`, 8.066834406130887`},
{0.18738335776550036`, 1.508373316422973`, 6.602438992729994`, 0.7532357689229783`, 0.8880958875806098`,
0.4853414284787141`, 0.10975729606650726`, 2118.598035369477`, 0.05093222572935868`, 5.0448517662608`},
{0.061980185747317895`, 2.54906136842855`, 8.170240160100523`, 1.0602973698223939`, 0.7221801534586956`,
0.6067985789160708`, 0.3830106200657996 7`, 81.55317476370394`, 0.23749102694434165`, 4.188403663256118`},
{0.2562298689236346`, 3.2913375042385793`, 9.509882785943699`, 1.013989937500057`, 0.7792587778840148`,
0.49077497767429623`, 0.021163604915718004`, 145.96678185214677`, 0.05845136191000838`, 7.439112841768198`},
{0.2313093023364764`, 2.988629640967633`, 6.855539828479465`, 1.0799498768994156`, 0.4522471358822151`,
0.2285321965248518`, 0.04827992453162874`, 1204.6728692817303`, 0.13073517219860892`, 1.9378468299164582`},

```
{0.2663151909473739`, 2.2417238355718707`, 0.9398485531379794`, 1.0477480676389026`, 0.7226958973603346`,
  0.5716146939488362`, 0.007506086251562624`, 76.16131590346896`, 0.024911856209502148`, 4.070747059506875`},
{0.19929196929676601`, 1.9362608032362312`, 0.5353176928827672`, 0.7753335837033051`, 0.8607728477687919`,
  0.15482089410819944`, 0.02026432754563482`, 192.89182731397838`, 0.1831217833074511`, 5.333726168282188`},
{0.10696417975467426`, 1.9440197080088568`, 2.0936739600677434`, 1.1829890119130342`, 0.8864433537218006`,
  0.5974069860908109`, 0.31351620960128274`, 84.46268853549806`, 0.02353560759327844`, 4.656274919049821`},
{0.21459986298539707`, 1.6769788549349993`, 9.356964926187835`, 1.2601407238694713`, 0.895239496536256`,
  0.38446102196425924`, 0.012005779035619358`, 146.09102757400478`, 0.02879957197754268`, 2.3826604675291154`},
{0.07383925157057758`, 2.6358268091067414`, 6.251616293116599`, 1.1087155777827833`, 0.9205185682116173`,
  0.19659269091732745`, 0.29632431074176147`, 531.6045072192474`, 0.12599923353908937`, 2.5100327097656248`},
{0.0817054148315825`, 2.796991931647562`, 7.6919124752432`, 0.9355088496592321`, 0.8578766380044134`,
  0.4976427721124693`, 0.008010560795693933`, 67.84344037448854`, 0.080482215315478`, 7.8347514420046025`},
{0.23618801128357925`, 0.8353216061115365`, 4.620412867513723`, 1.4504705173961072`, 0.3108708881036355`,
  0.3400688454787608`, 0.014061322572830477`, 2071.8308884477033`, 0.0364017192015193`, 3.610034284058532`},
{0.1662441272521787`, 3.725772450959033`, 9.814227303865422`, 1.3148509050359762`, 0.25298305094984697`,
  0.32303330970203903`, 0.055784478318004704`, 213.77275876328028`, 0.04990125708904308`, 9.833393949415544`},
{0.22831788422124638`, 2.590756298379799`, 9.795768388114066`, 1.125355926333821`, 0.3650031672541203`,
  0.1680578019226482`, 0.4143448211959925`, 831.1374036828946`, 0.21412864715242685`, 3.6048538714465157`},
{0.23359626472155715`, 2.56674003123695`, 1.1969232842409259`, 1.0442579072771299`, 0.8882810964774697`,
  0.3176999965402193`, 0.007340467537817918 5`, 1908.0313626467632`, 0.2040761417499009`, 9.648978920291999`},
{0.2534162537452666`, 1.6838285982829593`, 0.8034274134000761`, 0.870316935261816`, 0.7856829676536639`,
  0.46427070430809425`, 0.12261242146523575`, 210.18201198829036`, 0.1377493870208059`, 1.4006481974514937`},
{0.25695579183074796`, 3.423363059474246`, 4.134350766656727`, 1.2838485736639544`, 0.4149036036442244`,
  0.6493708976181658`, 0.12875465899507466`, 1172.2742202486922`, 0.012611679323919878`, 3.4839902382375243`},
{0.17682432929033887`, 2.45185381012344`, 3.9338825987195882`, 1.2879615271399696`, 0.02702036645869277`,
  0.6694394004648141`, 0.11330549796901213`, 154.96108716979026`, 0.11583322861965717`, 8.805457336592767`},
{0.2509750819824499`, 3.8883567883501247`, 6.384989889678234`, 1.4613334209290016`, 0.7183189010632474`,
  0.4965470763778975`, 0.3859108436384297`, 748.6242458418322`, 0.020020198926280153`, 5.690775103224375`},
{0.1441623498715573`, 3.343288457669903`, 1.621696793707331`, 1.3375455077897458`, 0.06814809058713811`,
  0.4010236514346772`, 0.3002310191555227`, 155.99799531014958`, 0.10001900063604485`, 4.2763953522223215`},
{0.2657479684896014`, 3.193863728015164`, 2.3963245604206165`, 1.4113571594678591`, 0.26744798738201037`,
```

0.235299669124016`, 0.018584060295236755`, 466.949287196137`, 0.23679723903863448`, 8.690245518267893`},
{0.18653936584035707`, 1.0683088955449334`, 5.185702152338592`, 0.9522710688882444`, 0.206752787597013`,
0.5240389817239608`, 0.06412446666117125`, 220.00685924183844`, 0.2055887216336732`, 7.885811409920794`},
{0.07587231017464335`, 3.3343312881276663`, 8.762311039358057`, 0.8284399428795388`, 0.17449762015817138`,
0.34311440561675743`, 0.03578987408308253`, 1364.0076205471958`, 0.21103062426335195`, 8.577574022027257`},
{0.1141532151024588`, 1.4516849606811304`, 7.634928187688176`, 0.8394863515309923`, 0.0282734940185132`,
0.6576494992828068`, 0.009900377337816624`, 932.466930942293`, 0.19488805826375527`, 9.750042758265863`},
{0.21903027892555205`, 2.3035966499415492`, 1.6904861150255108`, 0.7741880968122689`, 0.5538208564897962`,
0.4216122395122083`, 0.4439804577081556`, 61.55285726796139`, 0.16334632191055676`, 6.147879562247702`},
{0.19219532042344978`, 0.8633096904694177`, 0.1660296606087659`, 1.0595398890946446`, 0.3981268836303198`,
0.35447506211304225`, 0.06527455092271564`, 2352.6780619798324`, 0.04729158696423241`, 8.334762180963512`},
{0.059421230927129864`, 0.7565633878530198`, 4.881127251124937`, 0.9039794772130322`, 0.6691506700132113`,
0.3437226952926379`, 0.013854681683978944`, 1303.847591809871`, 0.133136969667504 2`, 3.817043920573575`},
{0.27625614155976197`, 2.681313109654546`, 6.389652815751472`, 1.3504817156087858`, 0.7587234585548364`,
0.5970419449613528`, 0.3005699072645848`, 854.9062988397701`, 0.165124447314083`, 9.6053618622676`},
{0.09922116949114601`, 0.5509823607034723`, 3.2884104382793122`, 1.3318058680427458`, 0.6719251293345294`,
0.6187329358641112`, 0.3259483616243979`, 1099.7736795288956`, 0.13645709285092156`, 2.0261792473532125`},
{0.11800216420621701`, 3.285091391336728`, 1.6522276383131942`, 0.9670398406334181`, 0.4307591958110746`,
0.30003613158270603`, 0.29799743711056653`, 701.4335436984502`, 0.043710117716904806`, 7.7162513520402545`},
{0.19357111334803345`, 1.327860467490665`, 5.552252240997824`, 1.1813151963757291`, 0.7103742334207412`,
0.3776068940225674`, 0.2456309648230094`, 3934.634788049085`, 0.12355866435459295`, 9.745686147306856`},
{0.11447070818541266`, 1.7335861954191927`, 8.736387174086214`, 1.0335531844510784`, 0.08646802454747982`,
0.4443642965951369`, 0.015374273787766714`, 123.60141703755143`, 0.21867059554150092`, 6.82672203062274`},
{0.22987433852030326`, 3.2498283820092855`, 3.7918096781351274`, 0.808040368560279`, 0.9416246029763107`,
0.49895559050949523`, 0.02048494990466312`, 278.1506433463176`, 0.24538025309113942`, 1.1463070343242556`},
{0.2774701327316953`, 3.217064080543545`, 9.848999257942918`, 1.1587780417232185`, 0.006285118350015 4395`,
0.6835477959342324`, 0.37074868705631564`, 2643.6047855840716`, 0.14999893314602414`, 6.1386041394007655`},
{0.07518728576930783`, 0.8073344777020095`, 0.2079911159294223`, 1.2717171333972301`, 0.7961690644438275`,
0.349516332980527`, 0.009301689754665837`, 660.9000723394831`, 0.24128632812714051`, 8.034216989913379`},
{0.19386888157639426`, 1.1721624963279647`, 7.488800420822528`, 1.2206737749782337`, 0.39882649538868`,
0.6168747383393647`, 0.005894727770540639`, 784.8706404426375`, 0.21917965839925868`, 7.12627169471982`},

{0.051543962131442966`, 1.0824196854643793`, 1.5789464925861978`, 0.9324716268311393`, 0.16692765658832065`, 0.33113273649509023`, 0.16543741477462187`, 443.37752852110856`, 0.07774169596012481`, 8.031423814374019`},
{0.04886198458272409`, 2.4240564285395276`, 3.0585768323132374`, 1.3030342316587755`, 0.5506088802661759`, 0.40370465638646613`, 0.05636145780930632`, 1567.8212350001168`, 0.20179368484919008`, 9.505728478640599`},
{0.26828907890664055`, 1.4916419764625646`, 9.30244345383333`, 0.8462433381711008`, 0.9150350508953056`, 0.16353772752060636`, 0.1124240025000419`, 2042.0256023360328`, 0.08362962700521354`, 9.175430964325553`},
{0.11853219177887636`, 2.436539187916523`, 4.07490711978506`, 0.8391678158796785`, 0.8274679746994464`, 0.4783994312610165`, 0.02880518630114325`, 249.91551468654188`, 0.20172418525520536`, 8.758671893830364`},
{0.15380346197105899`, 2.5002802569502807`, 7.104691399911929`, 0.895619363352984`, 0.7056556726651881`, 0.2904087856211036`, 0.27890663528622706`, 1570.4796825528501`, 0.2402855906015`, 2.015690994359673`},
{0.07681933116098377`, 1.701376646381478`, 3.4749545233581878`, 0.9897106512386663`, 0.21660819673269338`, 0.4456181241675946`, 0.020856154503598144`, 170.86262737853005`, 0.11272605112949713`, 7.4521875824230435`},
{0.19680750576810457`, 2.9845730901840906`, 8.034687795284743`, 1.0668124507997105`, 0.6731645050394575`, 0.24725877139168673`, 0.05097134738484948`, 549.1786460489706`, 0.029127445194553014`, 6.0096318557240025`},
{0.2481156970490977`, 1.2202113043036036`, 9.306028403345813`, 0.8009108612869359`, 0.02681732734233533`, 0.15549305073805075`, 0.021015785720259927`, 53.28601671876581`, 0.06540227633756851`, 2.0322216725641393`},
{0.05969602287151182`, 2.177557565950017`, 1.7675183816518665`, 0.8372040785938111`, 0.9188853493106184`, 0.1878023064060833`, 0.1313271602899384`, 239.3049490553974`, 0.14060221077790058`, 6.359907705361997`},
{0.1932263333480873`, 0.7780345756067408`, 9.679599745167298`, 1.1108495595363916`, 0.27977393862884203`, 0.2181645563751744`, 0.006193313005348089`, 412.49802062580375`, 0.21651680739670837`, 9.973248740719807`},
{0.05080144024762184`, 3.462591354860418`, 9.637683194306994`, 0.7734436451051352`, 0.6551811041034619`, 0.18020194036251547`, 0.05139770733113868`, 3391.683612113084`, 0.04739051831745644`, 1.6399754771823183`},
{0.17269587719558416`, 2.280528270589695`, 6.304702452448467`, 0.855238720891303`, 0.7302844321830138`, 0.25273349413775115`, 0.28098261768807636`, 2514.837947344666`, 0.04999285469609732`, 2.363210664893213`},
{0.12416213981002666`, 0.8389921874064035`, 3.610730817659622`, 1.0777639636353653`, 0.18253050124358539`, 0.3654692817582218`, 0.277997956001176`, 563.1802062075279`, 0.17509802310960038`, 1.6234053595359104`},
{0.05389681485101466`, 0.42474703837564753`, 9.702496123506663`, 1.2875902192251218`, 0.8398628787663107`, 0.4145702753216558`, 0.012323091180371398`, 1207.1917708220153`, 0.0487427592688896`, 1.997348446926472`},
{0.06391261825702438`, 1.7835225604286613`, 8.062142041499534`, 1.3025605866836996`, 0.9222349035188417`, 0.39598365567854665`, 0.09958099473376647`, 75.95083350193653`, 0.043061298974276296`, 9.739451519751867`},
{0.08850946488241984`, 0.9673836199236003`, 8.299835087678265`, 0.9900607622617293`, 0.0809660102577392`,

0.6707110673061827`, 0.0056133585030711565`, 223.20635429959376`, 0.07233923327715214`, 4.067688416849283`},
{0.056027023765889306`, 2.0857485685464603`, 9.366491292380005`, 1.196524731276077`, 0.9516747873190254`,
0.6119693625054259`, 0.014912996736033244`, 220.0557248806415`, 0.0820080753991021`, 2.040285256013867`},
{0.04516470805711162`, 3.292123721374173`, 5.263943679637629`, 1.138536155221258`, 0.48426832878752823`,
0.5662939024082725`, 0.09160607397723192`, 3360.7159646360847`, 0.026694430856043894`, 4.180658858897127`},
{0.2782184518736612`, 3.845021085593168`, 1.137061402050021`, 1.4385887178277776`, 0.42032329684699743`,
0.5854257024714923`, 0.03249096611383817`, 328.6688300716124`, 0.02617032692156479`, 8.869579059594898`},
{0.09319840750005726`, 2.6994281444799046`, 8.85475415324603`, 1.038403514470577`, 0.3378909888092929`,
0.6279203689803818`, 0.23307552493819164`, 1846.0011275272348`, 0.034946033021005685`, 7.6621208156759835`},
{0.06034484465157863`, 2.8968090048449424`, 3.142651522085412`, 1.307236858746047`, 0.3617717997572676`,
0.29303713371207274`, 0.18131081790115888`, 4578.414121282968`, 0.17962394399488657`, 3.3138716244433386`},
{0.20102830374342656`, 1.9832552045818916`, 0.5122029117897267`, 0.8414910669557318`, 0.023679729667421823`,
0.562606520598186`, 0.21796137676857033`, 2537.351175593395`, 0.0373739667120736`, 9.865525950238812`},
{0.04583409051630541`, 3.6747726707008503`, 0.7420872691712788`, 1.2157743693615881`, 0.8915453306179026`,
0.4926202034522773`, 0.4217762472968932`, 73.4269782954483`, 0.10619296232023001`, 3.9517710932009376`},
{0.10119631213999619`, 1.851973173732965`, 1.6003758779141855`, 0.9328064269865228`, 0.39319494883808903`,
0.3867811485974866`, 0.006630825974655251`, 2020.948278622179`, 0.24307528382326266`, 8.411215463490773`},
{0.19796477344593338`, 2.2434362012846876`, 1.2309355399833084`, 1.3299187291217778`, 0.059794768114105334`,
0.6215799505741797`, 0.02273921518352973`, 1004.7174087228818`, 0.138969497102004`, 8.58482448486276`},
{0.18841727652913837`, 3.2954166443999204`, 4.985214271603974`, 1.3919313698468052`, 0.8063258092579206`,
0.6389441864140679`, 0.008804142841395211`, 1247.8494355462315`, 0.09671339442963328`, 8.749674730462491`},
{0.08822838789215687`, 3.6314565393637377`, 8.720964691808945`, 1.4591613734708808`, 0.09172379783619267`,
0.17510896742376914`, 0.2630216511777198`, 431.756995427934`, 0.16850915581941245`, 3.8927198296202143`},
{0.22004819658675462`, 1.2435615798896285`, 4.521096993231973`, 1.3090616735635763`, 0.5905184190150714`,
0.6892934240599866`, 0.09109967416767972`, 239.35075160225182`, 0.045734190683186515`, 5.8358773331232445`},
{0.18183828174362843`, 3.2786199220563104`, 2.2240542093570888`, 0.9831747738881791`, 0.39067106215615355`,
0.5999409005287338`, 0.32534468961787655`, 53.98091103084368`, 0.10022266621367804`, 9.980846637487563`},
{0.21252199352031792`, 1.818911285509734`, 0.45179175410691036`, 0.8473192883797733`, 0.5752582261250987`,
0.4244690624430477`, 0.010390335454685632`, 819.8846878599306`, 0.22518903335941576`, 6.3452240038645735`},
{0.13408565703158576`, 3.9481177636984475`, 4.409802796026355`, 1.1882851460555317`, 0.7016940523769313`,
0.6437617694336766`, 0.472695817526219`, 268.88735252515323`, 0.02238688650951909`, 7.198883746978469`},

```
{0.11550511139863262`, 2.7266825017818803`, 9.715437717848832`, 1.4226217916614743`, 0.7434913376024772`,
  0.6527594414247178`, 0.026462900292485443`, 517.125461393156`, 0.19263485762362964`, 9.20218742349427`},
{0.1537432379066465`, 3.3010385217103515`, 7.8154151393713`, 1.099357680158021`, 0.2670513361076359`,
  0.6099579487912872`, 0.01626886763741749`, 3071.882113963599`, 0.04550777584421739`, 6.952411393791335`},
{0.08915960893105551`, 3.2848427178141772`, 0.6302374569507307`, 1.4630290564093034`, 0.8347103436436674`,
  0.36812644478584344`, 0.040963141333673035`, 3229.889147199789`, 0.1995027638419964`, 3.7582834934312945`},
{0.2095156599086041`, 1.856403794983298`, 1.6647511783688493`, 0.8963074241076089`, 0.5257557556911894`,
  0.2962680347723602`, 0.005638948645619698`, 398.77430140016673`, 0.1712593265312492`, 6.243180864475704`},
{0.08606673974307905`, 3.001046395083865`, 6.871125535025986`, 1.29871337654279`, 0.0013879217267305233`,
  0.4709543850320145`, 0.028486772088755836`, 255.05656064251065`, 0.15733152617600732`, 4.419059383126843`},
{0.2794012033077364`, 3.8964244114394253`, 7.589043584575673`, 0.9660750334911808`, 0.6823441866078543`,
  0.315494435212184`, 0.013950850602606538`, 1092.7384181067544`, 0.06831145072823913`, 6.790315469699049`},
{0.16053897675716755`, 1.21110484978606`, 6.017303762103152`, 1.2353982330406967`, 0.20086989110627473`,
  0.3272106220983775`, 0.02355461265150193`, 77.09093986775292`, 0.04848901873161415`, 3.5142859649597167`},
{0.06315507597420161`, 1.4395182613839497`, 3.0858504661132873`, 1.1448617905347693`, 0.30610701790846395`,
  0.20244634599341438`, 0.2950980917537417`, 2179.6214593356067`, 0.03473000454292019`, 1.333335366603153`},
{0.27116635506122466`, 2.640185082938654`, 3.1282641056246216`, 0.9199164829096633`, 0.3221831834859356 6`,
  0.5777637686875948`, 0.006117113694401221`, 324.8095638375007`, 0.04342122620091826`, 2.609798810681127`},
{0.08076786811690995`, 2.5389877544798445`, 7.211847948605141`, 1.1500644274940979`, 0.7690232347129535`,
  0.1924810447357974`, 0.3170033067881963`, 2456.45541532025`, 0.12977198817972552`, 1.000471570970415`},
{0.14196490482109098`, 3.5800560378690767`, 8.191314882292886`, 0.8521719014833709`, 0.20577614391452248`,
  0.2973229046898421`, 0.032689134381726236`, 2192.8696238585753`, 0.20870222148197032`, 8.620659635124824`},
{0.16225217210911858`, 2.1337475700719883`, 4.607583982995607`, 1.274907826520427`, 0.760916097212121`,
  0.6512132808308`, 0.1988962707427588`, 88.66654318682235`, 0.17629703311441042`, 5.991536547797288`},
{0.09998370398747453`, 1.3561107931887042`, 7.055733599099842`, 0.9997997594446719`, 0.4231050232131661`,
  0.35289870383552235`, 0.01863838099230359`, 585.7262791471302`, 0.12303242645253354`, 2.5487735356675643`},
{0.0790420625297461`, 0.7951762600254768`, 1.0975467432818782`, 0.8295886125705915`, 0.21974762785224522`,
  0.4003083061581494`, 0.12181590550696897`, 256.3718289629029`, 0.07504947990766975`, 1.4404680202816778`},
{0.09310243320239603`, 1.7939569577957926`, 0.5112771848209494`, 1.3548866082012778`, 0.9609312900127882`,
  0.31815651234043585`, 0.047992777894275945`, 1572.364241162158`, 0.10220690607690369`, 1.3696469849079624`},
{0.20315703606221613`, 1.4806269686939117`, 9.851530344798562`, 0.807291927894971`, 0.7692364729235686`,
```

0.5064599484352303`, 0.07616257600145751`, 550.4721115636684`, 0.18512304424700965`, 4.821016411964738`},
{0.22897905358022103`, 2.219649931841208`, 8.024983925513396`, 1.3490395658771779`, 0.7992198564081268`,
0.5097552623833034`, 0.017934168491060075`, 2636.2345048980164`, 0.04871567850920905`, 1.38033814014487`},
{0.14728957561352346`, 3.8290117168547324`, 5.577517394641557`, 1.2037071314600827`, 0.7629976630105226`,
0.33391252098357616`, 0.00623505541513591`, 1551.1825745003182`, 0.05716501203669799`, 3.4524118308532703`},
{0.1747499923357959`, 1.9364726507678487`, 4.539322141325334`, 1.0358217348301002`, 0.687467494258686`,
0.22118436656891216`, 0.06970356230137446`, 4108.781309212477`, 0.15897963252631786`, 3.216561469360766`},
{0.061449808274615936`, 2.451738473864639`, 8.032153511575046`, 1.3988277328315561`, 0.653844119276888`,
0.6033333182307286`, 0.12509363159734996`, 3162.271766392656`, 0.18189469469951425`, 1.0210674887190017`},
{0.1951885853322612`, 0.6765436415231281`, 7.0581513804142055`, 1.4397439961417247`, 0.9899034195557828`,
0.3660842570171081`, 0.2551485659861665`, 53.30479560198744`, 0.15667683142074817`, 8.392793083340557`},
{0.15955516943400022`, 0.8482855893464007`, 9.331572022970409`, 1.1667938097447013`, 0.29910958141522603`,
0.1963171731712634`, 0.010305064592809629`, 1013.0765277256257`, 0.20900313016467104`, 3.301983133021727`},
{0.17746707158856556`, 0.44139712602022296`, 2.8621670830493073`, 0.8100971992338867`, 0.0373958036205273`,
0.5075452637360592`, 0.09067013663296572`, 114.80525128927701`, 0.16719225120884412`, 9.820606806768879`},
{0.25772135278013303`, 3.070426405636976`, 1.2338255709842532`, 1.3135003325072887`, 0.5149580362176238`,
0.41732865316044054`, 0.04496826136197767`, 318.8324074107286`, 0.010846019130009599`, 7.957536959758443`},
{0.08988753363169116`, 3.536295514824336`, 7.275260820034448`, 0.8993825689374235`, 0.025163868977214232`,
0.47083049518550046`, 0.018253871395130763`, 416.18519037331805`, 0.11499620973251179`, 2.265482484941062`},
{0.14192760186457132`, 2.3958894382433478`, 3.253512770748353`, 0.8655063905996925`, 0.8143036151546204`,
0.5834364577519011`, 0.17553594321370639`, 62.22207407824173`, 0.16384862691545943`, 3.0979894918131596`},
{0.14248286143558947`, 2.096310454690764`, 9.38011512102738`, 0.9675972750885355`, 0.0803295271719453`,
0.6033546111821413`, 0.13762578908199044`, 1262.8186365484712`, 0.18785001372865495`, 4.9706998985924695`},
{0.10446764528219044`, 3.442117541556258`, 1.7911289531773882`, 0.8226507378787467`, 0.06660293233402026`,
0.33605831499521144`, 0.1966635974545483`, 110.27400709595466`, 0.23027159794407875`, 2.5346674177346316`},
{0.0959624592169791`, 0.7155878562202682`, 5.1612847015717165`, 1.2701047018452214`, 0.05672968500006248`,
0.6450160655606327`, 0.04218472167952095`, 1614.7615476312947`, 0.23305782159027644`, 5.6652476983851425`},
{0.24311048293765425`, 1.2773072105524381`, 8.674158333323465`, 1.236248375623142`, 0.5430857314344402`,
0.29169516048698574`, 0.10383247735445839`, 522.6667880889086`, 0.0459109896778586 7`, 9.694591495258663`},
{0.08713889377857453`, 2.2859880795657075`, 9.600148060687761`, 1.2921363299986606`, 0.6294865567710719`,
0.5590186325118135`, 0.25992443653112657`, 633.6168560418654`, 0.16781120217455497`, 6.04666254822`},

```
{0.26758004713434824`, 2.448981429729897`, 4.582999027119474`, 1.4554483602004895`, 0.3942207099215642`,
  0.5288182096517661`, 0.017684947331647518`, 53.19217741492592`, 0.1488296927599813`, 5.857737808697124`},
{0.13431037279286218`, 0.7397108148766112`, 9.99887072123514`, 1.436439642978205`, 0.2003363652340402`,
  0.6779870054162531`, 0.009538236593206559`, 4558.309876478542`, 0.14371659865116293`, 1.1480275619139793`},
{0.27730778316543614`, 1.7365428506012295`, 3.277328223559843`, 1.1643406154035905`, 0.2621424262474452`,
  0.5836632043576286`, 0.19147730163610321`, 594.1704278677969`, 0.14363610143643862`, 8.32222051121584`},
{0.05614793436458493`, 1.997529172364887`, 6.840549262003957`, 1.2853232683568476`, 0.15723714782247722`,
  0.6374125080636333`, 0.1308231427534364`, 1207.3035504224565`, 0.15724403465210185`, 1.842303075231806`},
{0.0647068787712346`, 0.8380824418888393`, 1.0963289034750066`, 0.857562051048008`, 0.6907626550964823`,
  0.2999007220652139`, 0.27885748113518277`, 1238.4520803651262`, 0.1379529162857775`, 8.96709675960637`},
{0.20132622112515353`, 1.9086557669063273`, 0.7030652496772074`, 0.7755069309180247`, 0.32762519329281337`,
  0.20767575421456186`, 0.011318073987463645`, 431.8155255411275`, 0.17436258525110954`, 5.81443415452358`},
{0.045022613849116516`, 3.5058573906861508`, 8.47131465431961`, 1.2697383949459176`, 0.5022287267438617`,
  0.23847623822456332`, 0.06077359284826833`, 243.2833936812314`, 0.1613793589669978`, 8.022277670460284`},
{0.17418182442148117`, 3.0892110646487794`, 3.4522019380421036`, 1.2965827481879664`, 0.41818871776887856`,
  0.6556019601202148`, 0.075063644002762`, 2886.574837344593`, 0.15315141138676647`, 2.6062865811306466`},
{0.04583105977220422 5`, 2.899702133961444`, 5.925065562245003`, 0.8929739121804446`, 0.5933600691515191`,
  0.4490635153302144`, 0.16447888961080792`, 1912.79886244215`, 0.11499785968845799`, 4.757985376452911`},
{0.2429697761558342`, 1.8449858081251405`, 7.508754000794074`, 0.9127320360969664`, 0.44886430542069955`,
  0.35316747155912975`, 0.02193269408167387`, 161.73721618998414`, 0.16775422018188862`, 9.221971574736251`},
{0.160442241636698`, 3.55050251528807`, 3.9052606138637995`, 1.2025377341211225`, 0.791383919489673`,
  0.40672925070026333`, 0.35477124416027767`, 105.50559352550128`, 0.10792398319932495`, 9.162379917819035`},
{0.20468313725879644`, 1.5880039947408164`, 1.8181726149942818`, 1.4361114186612707`, 0.6452252370101204`,
  0.23877202353940497`, 0.026067763377395026`, 956.3204095901076`, 0.08759161290164996`, 6.530440575770296`},
{0.07364401078716826`, 2.5859680523819737`, 7.8227327965838285`, 0.8181378301360093`, 0.36855652079172274`,
  0.32445369334254925`, 0.005391697017678555`, 139.66827785366002`, 0.0644557011853657`, 2.5433237558513593`},
{0.2506656721970057`, 1.5258727034335298`, 1.8333712411976357`, 1.2495957919641723`, 0.1010075460664761`,
  0.5024718466416268`, 0.012345305326089913`, 1052.1669408921373`, 0.03320454993831845`, 2.130087729732141`},
{0.10121864512314288`, 0.9521305504715327`, 3.6368221202357223`, 1.491518490019038`, 0.3322534458031634`,
  0.2564348282281962`, 0.03507970829425946`, 4627.905596988043`, 0.07121256842175683`, 7.352385124666178`},
{0.1942542932466596`, 3.1177323303054916`, 7.932133913996736`, 1.3085296181137158`, 0.8325654731630694`,
```

```
 0.19060678658466512`, 0.044940459115954866`, 2391.994107548152`, 0.24452644883302188`, 1.4227474861405387`},
{0.12644890469661946`, 3.0196313965941393`, 8.688658870512302`, 1.3655010622962382`, 0.5847686349152417`,
 0.30770354208104556`, 0.013061738242062998`, 1213.9477293174991`, 0.0486468587160932`, 6.312801306430787`},
{0.11280111274715143`, 3.2009360918012444`, 2.9222221030043283`, 0.9684721327188957`, 0.03329493891954294`,
 0.1886848273472841`, 0.39445850876705457`, 50.69043630972767`, 0.07560282132089569`, 3.828945975174229`},
{0.21704875939335333`, 0.6532232080519234`, 2.918554590657344`, 1.428534955684801`, 0.6202310312510246`,
 0.3415578035649972`, 0.1858190740142067`, 1428.9901262383053`, 0.23526262585558444`, 9.378643714384769`},
{0.23408001184633387`, 2.4049572740765104`, 0.41973647277501946`, 0.9771854975111727`, 0.20272636213110817`,
 0.5074761434023957`, 0.2724896408017817`, 4254.760257351377`, 0.16397942354351142`, 7.3871482427729305`},
{0.1483945695425406`, 2.699298050205127`, 5.571982823617271`, 0.9775705576725262`, 0.05426683476548022`,
 0.5307838109252759`, 0.3096243181655647`, 323.46321359362`, 0.06725520461051537`, 9.792268234153859`},
{0.11663741077922185`, 0.4431208542421814`, 9.780884572399291`, 1.0122403254522139`, 0.996318833483786`,
 0.22155768960679945`, 0.39993089984673347`, 139.93164861672463`, 0.10490063957337303`, 5.3884722727325105`},
{0.11314041374373823`, 3.32806970136921`, 5.495643049849155`, 1.495187492234928`, 0.8329498208958137`,
 0.41734162088096116`, 0.32389371604169326`, 602.537083528763`, 0.025714962476490244`, 1.3686646556271693`},
{0.09294922428523011`, 0.8101676653238759`, 3.5463022431515436`, 0.9341830117643879`, 0.15981251792750606`,
 0.6527933768073686`, 0.016252799814697117`, 738.0588634619963`, 0.024900612098687713`, 6.915149516601594`},
{0.2696435798732645`, 3.282053008859009`, 8.218787637608994`, 1.4011858805306123`, 0.38871702639887196`,
 0.49514225831047376`, 0.01812451162452924`, 173.50575962443355`, 0.20495943261505556`, 3.6349041099248875`},
{0.13023501639399904`, 3.4827106844829796`, 7.180310384726493`, 1.0417379014779535`, 0.3157954886073613`,
 0.3090830033379006`, 0.027522996818882862`, 1326.2576636786057`, 0.24748434887668458`, 4.853044482161593`},
{0.047473141092745`, 0.7132410347452449`, 0.7493200461382123`, 1.2237616503945397`, 0.9506868210145907`,
 0.6554164129507942`, 0.030282495208288053`, 4252.97050681489`, 0.14327214907032687`, 7.932983145622682`},
{0.16300334800486183`, 3.0919140294206544`, 3.354218048792589`, 0.9673340843818878`, 0.9052111330146941`,
 0.3760205832601582`, 0.15433250667793078`, 4163.373290035795`, 0.2440496776808055`, 4.495712049614825`},
{0.2283283078987099`, 0.42840256075325867`, 9.446993924606975`, 1.3839314736668495`, 0.8927356525671915`,
 0.3909626915031965`, 0.018767685887155043`, 53.5712236843488`, 0.10749676377570666`, 1.5496335657505222`},
{0.1380652378345807`, 3.317739091147252`, 0.7336293987943634`, 0.9484386065310282`, 0.5023326788118547`,
 0.2130184622271264`, 0.06305960906666651`, 1932.031257216769`, 0.01203085244100871`, 4.258055262793977`},
{0.0926632627436077`, 3.578083963812018`, 1.8944161244777453`, 1.1381278842914715`, 0.7382269863407194`,
 0.4777843546205588`, 0.04528998830707099`, 1233.5473532765689`, 0.08357615053600725`, 2.9903558138075255`},
```

```
{0.2706310445313141`, 2.5166396966047158`, 0.5049158559505837`, 1.3168707464723388`, 0.5754269054535`,
  0.5968444469720801`, 0.1025449755078217`, 681.183394170123`, 0.05243619315919962`, 2.183793658787195`},
{0.20804007909247985`, 0.9673945938397814`, 0.48619022305575754`, 1.3068640855075515`, 0.047796616526516456`,
  0.6050138674479171`, 0.4706922223098622`, 348.3137462309647`, 0.24685340199219946`, 5.551550038883448`},
{0.048451644236667674`, 3.3925416461261833`, 7.486002650281495`, 1.2431466818882033`, 0.1294136137122328`,
  0.6416005560424389`, 0.04082335443875802`, 58.92112932799084`, 0.09857305139503486`, 6.047753646247408`},
{0.09431215650771135`, 0.5526170445339527`, 7.677473256451986`, 0.7715239445968243`, 0.7622192109377914`,
  0.1566986505563851`, 0.018040482265180933`, 824.8087273379117`, 0.09073506826067207`, 9.996434684615583`},
{0.26113782676147984`, 1.4684069653405487`, 4.54225535313282`, 1.1193463339337828`, 0.6236318874525932`,
  0.1751148412910668`, 0.053183289402392066`, 554.4897241223483`, 0.22550063278101773`, 9.001504879035508`},
{0.23311836154945775`, 2.5262146716446363`, 6.3107841154355`, 1.4422657044398717`, 0.3118898255230633`,
  0.2567889403043644`, 0.03481026984978327`, 1604.0167510242688`, 0.16315782107376747`, 5.777943122601165`},
{0.22640126428063923`, 3.7808015365334118`, 7.033092553948691`, 1.0460349638379132`, 0.8860375200295565`,
  0.499376231890995`, 0.3693111113992884`, 210.995030019894`, 0.19589698445197923`, 2.861160479579169`},
{0.19770244273120569`, 3.3508846225196347`, 6.757833537731408`, 0.8466672464019638`, 0.28373063232656337`,
  0.6609960258126362`, 0.006803814757890119`, 1256.448281122042`, 0.0187925047862397`, 9.149585751239048`},
{0.06481064744600706`, 2.3696983632913335`, 9.923821775061434`, 1.1006879887228784`, 0.22746584913410017`,
  0.6483433064071293`, 0.08284957522863273`, 281.66193979187017`, 0.13350656503173153`, 1.0702403000648566`},
{0.1537084817657926`, 0.5942996643982652`, 0.8160795607483013`, 1.042756674430172`, 0.7722126796580249`,
  0.49669902434825364`, 0.00611858149596996`, 1518.932458333751`, 0.21666908673557295`, 1.634333111202272`},
{0.13754450751212471`, 1.8598194811531465`, 9.485701362019345`, 1.4994328167980717`, 0.8397969618927028`,
  0.642370724162252`, 0.08665379792235217`, 239.43302754758346`, 0.1633413998793405`, 9.552795266495377`},
{0.07858197291799773`, 2.783199788795301`, 0.9642830041255817`, 1.3105090636097434`, 0.9572373854271048`,
  0.41778523295843695`, 0.00823451224525384`, 90.59081205787001`, 0.24323880940762027`, 7.348654585309644`},
{0.09118935700706149`, 1.2770460819223013`, 0.666402509206284`, 1.1661439001587546`, 0.35202394044239993`,
  0.23733544361366754`, 0.06689005296661862`, 1909.3731761645724`, 0.030837584446851807`, 5.2718694661097025`},
{0.26692387934872025`, 2.295217680243936`, 3.3610155473383347`, 0.802490311252646`, 0.6241394164828811`,
  0.45771836786665954`, 0.015176154328102385`, 1461.3995866509204`, 0.028435347409555445`, 8.762751383914615`},
{0.16789560992918506`, 3.010819021450227`, 9.549236085030234`, 1.3823009139889233`, 0.2184000924395355`,
  0.5213598249132101`, 0.3210433649481226`, 558.1245473691072`, 0.20748907999743366`, 5.921770487462836`},
{0.05001315370963505`, 3.027404779172768`, 3.8220563025552714`, 0.9962642247459573`, 0.44362718458195727`,
```

0.3510033113401648`, 0.04839212642640241`, 1615.957133832461`, 0.048401134986770955`, 4.739296636982807`},
{0.0729686004745061`, 0.5044823312287607`, 5.5024178276878075`, 1.2230366941116102`, 0.012758742281838265`,
0.5648708708999995`, 0.03232052448858605`, 634.5000769880222`, 0.2255316473679640 5`, 5.7581202769864 3`},
{0.25149333924897926`, 1.871859550111978`, 9.811462629766478`, 1.3386558973788294`, 0.5189637829018348`,
0.6377508608690272`, 0.024369302194970535`, 194.42061666753636`, 0.14619611626015583`, 6.325307886810732`},
{0.1922243891646514`, 1.6850594067594091`, 0.2630953725218035`, 1.4711962401085972`, 0.15977146838462364`,
0.6883258791888056`, 0.09212778533128511`, 774.9062808250895`, 0.24229201708328568`, 1.1723783184073415`},
{0.2327924107307146`, 3.0114739814321467`, 6.475065538974626`, 1.3312648289930094`, 0.6507217418486848`,
0.3841095338158853`, 0.3176950069808959`, 737.689863101484`, 0.19812174358831114`, 7.321794649353192`},
{0.20811447549122308`, 0.49053077700921355`, 2.218461283385089`, 1.0634384889669943`, 0.30011342208125913`,
0.5589173637133283`, 0.16385051847701637`, 124.79731876547663`, 0.15435702825422665`, 9.111119072715418`},
{0.19580212756702065`, 2.2297474474451375`, 8.070495694463702`, 1.2202670372174012`, 0.07482300382970508`,
0.34257881980750315`, 0.24674110677687536`, 1287.6149126957`, 0.12354587287124019`, 3.0573978018103922`},
{0.18591817623779283`, 1.5316284759435828`, 4.0240186618411435`, 1.0506796825025935`, 0.8560461598734517`,
0.5496710897949223`, 0.05348974137997375`, 3910.687147405355`, 0.18912146822955533`, 8.614502889074807`},
{0.2552337642427184`, 2.0636949211593727`, 3.6788035742608756`, 1.2716726068122697`, 0.4767575621154567`,
0.33698161388823356`, 0.00790398094444615`, 742.5948236907665`, 0.22094334214516276`, 6.7847431915852425`},
{0.2297403387592854`, 3.933034881515791`, 9.92502862171417`, 1.2059622632705849`, 0.1421363362803283`,
0.1853832242974326`, 0.06367011009221722`, 57.09955187350921`, 0.13182928467731042`, 9.7382206276645`},
{0.04776006202628119`, 3.695464511472898`, 0.268839094050918`, 1.3548340559244303`, 0.4311024896438589`,
0.6662260908047339`, 0.1893860365797585`, 146.80096070953766`, 0.0868075345269877`, 5.3982731410948785`},
{0.16250916383331093`, 2.090868942469317`, 5.046057727155544`, 1.3781211464947924`, 0.9692295654960448`,
0.507149644697747`, 0.012667564467634538`, 467.92228322579444`, 0.2269164868867607`, 2.233796179341983`},
{0.12952636821033042`, 2.4189205073691173`, 1.5337482028150813`, 1.4058710509218408`, 0.9636406844740666`,
0.3882701702830901`, 0.27400686262252805`, 4688.706549038708`, 0.0765768774664311`, 6.843480813486918`},
{0.08153578837307857`, 2.0915987061965353`, 5.850951169546045`, 1.037700922521303`, 0.7897818160030217`,
0.3237340771928561`, 0.17831164332408336`, 1464.5863160958056`, 0.2436610405225158`, 7.1217069723220146`},
{0.2624813864915975`, 2.8946734437676342`, 8.625458110929756`, 1.37764442375384`, 0.19776637799413987`,
0.31049273319228277`, 0.046660769123637466`, 609.6700244678286`, 0.017643025422342112`, 4.098918143681717`},
{0.22181257821621975`, 3.747903553799671`, 8.070266666229205`, 0.9808557386469257`, 0.7823522438549146`,
0.2263615830500163`, 0.06976335847986787`, 287.144692837758`, 0.1821266272405107`, 3.59762935889718`},

```
{0.21058778737677059`, 1.3147483391864796`, 0.4651324593397846`, 0.8516412222118703`, 0.046686774964893374`,
  0.4819914326166439`, 0.10586616607566471`, 1877.0848108087591`, 0.011164572002709261`, 8.732010177522003`},
{0.19996482365829993`, 1.5454344843054368`, 8.94969050535013`, 1.027667483044064`, 0.3164796074442535`,
  0.5223314119072894`, 0.012949156080520758`, 231.31648814801656`, 0.19929090705614294`, 9.341058456545078`},
{0.10440009635624109`, 1.532425369535546`, 0.308138348960842`, 1.2734376519411275`, 0.20147673343032046`,
  0.35081245482125123`, 0.05722019474177449`, 85.42879625764479`, 0.08356786949647793`, 8.411462154648635`},
{0.1446017732242294`, 0.797427468093673`, 5.032298069921831`, 1.3706636028120882`, 0.10121695844960277`,
  0.6068141920969097`, 0.25341251132298065`, 197.97452899503492`, 0.2275401984407136`, 3.68386367767849`},
{0.1841264162344997`, 3.4194642457460365`, 0.8344796094826652`, 1.4377258087185927`, 0.5867256025093719`,
  0.6103981120475732`, 0.3542274534468216`, 522.9821129731852`, 0.2244081861480518`, 8.361737950667973`},
{0.17849930658201318`, 3.2992561971358683`, 5.635580909426066`, 1.1878114797167083`, 0.48988207998590316`,
  0.27890916833354473`, 0.03715549210344845`, 144.67131391199885`, 0.0866110192158086`, 7.798176718489761`},
{0.09510173640093084`, 0.7659692827065738`, 0.5464490010035838`, 1.4050402850247132`, 0.11782959453955089`,
  0.3589214817200733`, 0.08175234954735425`, 225.11260306454756`, 0.1010344837489014`, 6.4310165595390805`},
{0.06416093472533757`, 1.2573296539610634`, 9.92323628294643`, 1.30190007637989`, 0.030494015633026894`,
  0.4401773012522794`, 0.02586427825345657`, 3143.6595843648015`, 0.20592664064058636`, 7.935954028868563`},
{0.25237835724682106`, 2.979406809596097`, 0.9138356130609361`, 0.7932538461611548`, 0.3933816374531405`,
  0.6211446249652819`, 0.0749645015318215`, 95.73291003138912`, 0.06307891905410445`, 9.368280708056208`},
{0.05665762575451494`, 1.7380994050720693`, 0.46391281319797173`, 1.3490794355447004`, 0.9653780951392605`,
  0.17540498059526055`, 0.4972377260445833`, 285.101267300382`, 0.1881031714219547`, 6.261558552266614`},
{0.25642346088610446`, 3.010945889122482`, 9.963520677018519`, 1.467899384826252`, 0.4946077322511526`,
  0.6868780403912886`, 0.012314696297102353`, 88.3173980921876`, 0.10491074891333174`, 1.4353196490865765`},
{0.09174036922136902`, 1.4204870656163582`, 7.877654658381974`, 0.9368390881044215`, 0.3909649395372681`,
  0.6267838157099552`, 0.00737791814963061`, 223.00641924802295`, 0.22589847441224364`, 3.7895604923910007`},
{0.2260021855376967`, 2.1215536107061057`, 4.2648042233683015`, 1.1043329178413561`, 0.5641485347029902`,
  0.48705118822040194`, 0.49950773055219905`, 4133.317514629252`, 0.08759305230550185`, 2.0466954881219834`},
{0.1540246000020472`, 3.8655612430526176`, 1.2043257775886698`, 1.4535724207675174`, 0.7982285789403216`,
  0.6724657214579106`, 0.417253246623641`, 114.93402398229541`, 0.10143088124264565`, 7.877832682736377`},
{0.18967559668177286`, 1.3751140072639014`, 7.146984940201236`, 1.0524950668750344`, 0.5379883166421491`,
  0.5342982154057455`, 0.18359081743318598`, 57.97987210615365`, 0.21651827946513758`, 4.096495164957771`},
{0.11610573160351356`, 2.8105133752566074`, 0.4045001364193191`, 0.9020454264062931`, 0.06323256453719583`,
```

0.49478351534124754`, 0.06895293734611843`, 538.3487824750697`, 0.04516006162070901`, 5.298889101080752`},
{0.06094054623730827`, 3.5785656752883286`, 2.8105466781089756`, 1.0666569400098407`, 0.8956485783622179`,
0.6416539064413922`, 0.424107107081654`, 1544.2747045672372`, 0.0589041001461965`, 6.674766669452417`},
{0.06400872221443815`, 3.352604720184938`, 3.3612644751655854`, 0.9279864636339992`, 0.6811335659068025`,
0.5445450422212498`, 0.04222211989978537`, 4877.834369436828`, 0.09303122695369598`, 6.110250683676178`},
{0.1838955211549798`, 0.6066893455768754`, 3.1997125487162617`, 1.2441392193895697`, 0.8430620231566095`,
0.17975264196625795`, 0.008385470075528607`, 217.99247821227752`, 0.10887259656335785`, 8.65519228817503`},
{0.10877154933938238`, 0.6652486439511347`, 7.420477343235575`, 1.1719120037873472`, 0.8277401239831172`,
0.4096056229223566`, 0.3960687102590752`, 4083.2161783729553`, 0.09124503977845877`, 8.628655226336182`},
{0.27429237480005003`, 0.4974953244631517`, 8.109325428142139`, 1.1022725368505206`, 0.3620164170986986`,
0.5133215689169995`, 0.013475856804577652`, 252.124017519504`, 0.17376346726865255`, 1.283750502496817`},
{0.2348606786833104`, 2.9248975834862243`, 4.089597609027345`, 1.0125076012045389`, 0.4800911130241774`,
0.44584481805285503`, 0.013867879702002976`, 69.31755646766315`, 0.08239556859020553`, 3.2481286029667444`},
{0.2276305764129738`, 0.759063573478838`, 9.75608531797917`, 0.9651630732227953`, 0.2616315835146603`,
0.25394525967231996`, 0.42721777606241185`, 115.33714629065052`, 0.1547162363619287`, 5.678602403105034`},
{0.22740101487152548`, 3.662274246882813`, 5.673159128278421`, 0.7501649913658239`, 0.6663016096811909`,
0.46449661159333955`, 0.15875103883050143`, 79.3840483030526`, 0.2392535149659735 8`, 9.512843911813135`},
{0.18907356960866467`, 0.8903250429215461`, 0.8912625465714132`, 1.4194566043771595`, 0.25430531352108066`,
0.6668345122251143`, 0.01430802337144644`, 354.71023908442675`, 0.05435127807039547`, 4.360013356436832`},
{0.22841094141655494`, 2.082125305118022`, 3.0130589699894585`, 0.8668572537043583`, 0.35284672466002664`,
0.48298470604644195`, 0.055294539730325123`, 1324.8059697322965`, 0.2155561741975654`, 1.2621538558570524`},
{0.1257670047049273`, 2.665283038938149`, 6.579106420165356`, 1.3213793494994266`, 0.07362490185046933`,
0.671746727583086`, 0.11039555694375124`, 54.48594040704261`, 0.2029696416579515`, 8.437004538119528`},
{0.07898342764241062`, 2.336170261443863`, 9.558440542813312`, 0.8746675798923161`, 0.3314927429594914`,
0.5846930166673705`, 0.14173550706968557`, 4831.453144750826`, 0.22811466814427805`, 5.138718102637142`},
{0.2360815041275312`, 3.0069216843113518`, 1.9005978173203566`, 1.189378383663731`, 0.3594173887197991`,
0.5615883220885535`, 0.16515958276300607`, 91.7495984293873`, 0.10675594880460115`, 2.979677734696839`},
{0.19503604064380992`, 2.260639826798138`, 3.7301326124020027`, 1.0003156010412233`, 0.94917046396899`,
0.3935362271642996`, 0.08733555022372319`, 2234.7260448390443`, 0.16743068067899064`, 7.659400521016185`},
{0.09766567347807259`, 2.1751916900809825`, 2.9967711374909367`, 1.239918427231443`, 0.884426059472291`,
0.5758255537556012`, 0.011950287911566193`, 133.4222213549303`, 0.18860770237483848`, 7.181211491841234`},

```
{0.14345253817386028`, 1.510087082683639`, 7.446143698111681`, 1.3871400228991382`, 0.2507990125704793`,
  0.592096235116642`, 0.03860344741119924`, 206.9907110306594`, 0.033941806565597976`, 5.68471806318026`},
{0.23351662183055255`, 2.7403756212365034`, 1.6128399905233286`, 1.1394774719326448`, 0.2292996808057013`,
  0.38219770472020054`, 0.028922618161783233`, 3147.1184318469313`, 0.11734182225292555`, 5.055074464096112`},
{0.05894445024535572`, 1.7190376171466255`, 3.4445271438587763`, 1.1005021934401353`, 0.8920369053637744`,
  0.4553589712941485`, 0.02602138727445485`, 2996.975539830326`, 0.01616140506923186`, 6.007209949084906`},
{0.1969133083502746`, 1.0306356019031098`, 0.4532514022896326`, 0.7704659952544493`, 0.6922371279082573`,
  0.35856337468106037`, 0.02342746544122668`, 625.7704338271294`, 0.24611682235411125`, 7.40783802504553`},
{0.23578418763137027`, 1.109904439772956`, 9.416464917532476`, 0.9968768007328397`, 0.9319056056422497`,
  0.20705868768300972`, 0.023381736885244357`, 73.41723955051782`, 0.19857821780967083`, 6.130556036938069`},
{0.06254010222908407`, 2.833030713124539`, 1.1523226094346608`, 0.8553536481119535`, 0.24975724964580737`,
  0.6445417390978168`, 0.03834946206048674`, 4173.282831948493`, 0.23424935668510183`, 4.880796947048751`},
{0.1868109134318776`, 3.0098056229686154`, 1.9105557354746436`, 0.9827601516958456`, 0.5236893411586465`,
  0.474044891890932`, 0.006475636749568416`, 1404.5093361888864`, 0.13430982294875038`, 1.0667742763750176`},
{0.06031809352972184`, 0.6708570664744204`, 0.23575165127461165`, 0.8219834374256857`, 0.8758699419986131`,
  0.18852573702588948`, 0.186745146986022`, 107.40963970974032`, 0.12527667123697356`, 3.1788633087725113`},
{0.18801205905393248`, 2.0266227725882997`, 1.7626950623641149`, 1.1991174158264077`, 0.011000047841808414`,
  0.3353078609658676`, 0.05399718652686698`, 93.79336861840014`, 0.2334529633377767`, 8.960462961249508`},
{0.14122716980793654`, 1.0036898987844207`, 5.592205787770849`, 0.8071832042112255`, 0.9861798903854433`,
  0.6717138208444808`, 0.19154806886518252`, 64.00473270059831`, 0.09434989637003327`, 6.618037610942842`},
{0.14179341245484034`, 1.2991893501557028`, 6.1750864357305275`, 1.3220469832389021`, 0.954868801179809`,
  0.5061480199077149`, 0.3454995627472`, 2784.154142185204`, 0.062422742009026755`, 1.1334761108568099`},
{0.17346773507380248`, 2.4886801471142252`, 4.186844167829875`, 0.9059139514582338`, 0.8823714489891827`,
  0.3118060351580627`, 0.18611640479783054`, 317.2758158326019`, 0.18366106179531033`, 4.660916164387663`},
{0.17801738501831404`, 3.953347877342175`, 5.116473955634309`, 1.3026800881545206`, 0.2660443419983707`,
  0.5551592463622865`, 0.021882498869656555`, 368.98117336094424`, 0.13387054033002443`, 7.8289062285527375`},
{0.1502521637125906`, 3.604808817430471`, 9.516864148680163`, 1.170214417719616`, 0.46260070316916013`,
  0.4333225308578431`, 0.19319459383231874`, 1379.7428965277768`, 0.03462365036850307`, 7.002259791633362`},
{0.23041123856532575`, 3.7235663376199373`, 7.422983674409959`, 1.0720190294684302`, 0.9567615568260381`,
  0.6795713708474191`, 0.42619421138851304`, 252.0789228698587`, 0.23264645009848384`, 7.716645878625323`},
{0.17190873580866728`, 2.1300233762526144`, 7.263444695009253`, 1.2209987713639263`, 0.8761821538663868`,
```

0.2004596237918942`, 0.1361627927608223`, 3975.405902408578`, 0.13971132873354192`, 8.98211109187212`},
{0.25963938649205515`, 2.4653900827356168`, 4.997042605235823`, 1.1014911256242188`, 0.41576920876875767`,
0.18114027248931974`, 0.22217255314368375`, 81.71471228407572`, 0.2378841388493836`, 9.715142052575505`},
{0.2762626642080059`, 0.4739284043667089`, 1.351212961630882`, 1.24608482511035`, 0.19517067645291641`,
0.24775742659101618`, 0.08503066911676711`, 2141.7873291111496`, 0.17338810608691535`, 4.319189194420735`},
{0.1906362991887024`, 1.590121005227628`, 9.42412383550555`, 1.4957920989499214`, 0.709065663948995`,
0.3417182681188283`, 0.37536592261162544`, 505.4667159946151`, 0.11728445265057086`, 5.855972486658924`},
{0.2658746234670645`, 3.614932865157483`, 8.460828570764`, 0.8159863776033582`, 0.2726846463148733`,
0.34564016673603704`, 0.10623048732883861`, 3778.1279822475276`, 0.23711150436988554`, 5.459482199196464`},
{0.26863157332159515`, 2.9396265733275415`, 6.862183762414347`, 0.9732277188028977`, 0.5002013826688112`,
0.5818631486183298`, 0.034332997185588514`, 711.1213518313073`, 0.15904422112946637`, 1.3044149622459462`},
{0.27209369466384475`, 3.5584095462332135`, 0.9720509149176574`, 0.9873292487031791`, 0.8069894772531618`,
0.17871886216847`, 0.10218788035646292`, 96.95116373019758`, 0.02386269594633994`, 2.5608838628408552`},
{0.14444262093504412`, 3.863449844229998`, 8.726684391435349`, 0.8553749492129846`, 0.451984917853794`,
0.5228822441273482`, 0.008115157266947242`, 403.9888714010655`, 0.1027695319041006`, 9.711355034259547`},
{0.2538137652575522`, 0.7540411224449701`, 8.794714857317246`, 0.9184514413182489`, 0.11359389239638507`,
0.27911589396705727`, 0.11507441894157476`, 65.76953791858952`, 0.10155553687026442`, 2.859221903044178`},
{0.07943689831455408`, 1.7727348695278575`, 3.5998215792473456`, 1.4458682286750966`, 0.6331999429153172`,
0.478090570864118`, 0.46084046549589247`, 472.0739644000896`, 0.10830302137693731`, 2.3782715249335666`},
{0.055568082805285024`, 1.296674173505255`, 4.7380956025626375`, 1.4538691472242138`, 0.09550221320066177`,
0.19041481571096486`, 0.007131815235409429`, 86.20076273581975`, 0.04910104396213488`, 4.3238451034891945`},
{0.1596260086552564`, 2.5471852815710525`, 0.22684585875990315`, 1.271679176096452`, 0.8208442532787252`,
0.3888937433082962`, 0.04289548126799573`, 69.96603930346842`, 0.07461229317798801`, 1.378327695847128`},
{0.14264033419885946`, 0.8035889732308119`, 6.673404894657215`, 1.4157343373387477`, 0.18185291353504507`,
0.4765318357304136`, 0.03778101114485503`, 210.869852474353`, 0.22822304787397907`, 1.0663935058760412`},
{0.2209992311467341`, 3.548991401043337`, 2.677760813541523`, 1.1645638964766105`, 0.17856966244183803`,
0.2409694046188614`, 0.16792458477559433`, 53.365358865447796`, 0.019626895089088575`, 3.834874658630479`},
{0.08986394714808477`, 2.7800808748945913`, 9.536673819459821`, 0.8308776528106754`, 0.5315183679881144`,
0.4614339535602666`, 0.046951751845033245`, 1052.1471359283046`, 0.24213976319820152`, 2.8230201755788666`},
{0.08237232438525194`, 0.904854561594771`, 7.190948808493694`, 0.9264355987892083`, 0.7428148600467364`,
0.6232177046085758`, 0.0730718251800762`, 62.25627183567972`, 0.1453325784006102`, 9.786938339680962`},

```
{0.14754836278222488`, 0.5816922789151606`, 9.91636448422577`, 0.7971447359984003`, 0.9834799375197849`,
  0.6545637466726528`, 0.017670578507760985`, 406.58618651198583`, 0.1328908282216244`, 3.7962615345853075`},
{0.06758332214646862`, 3.0558788120173555`, 4.64800500980858`, 1.4828314301436276`, 0.7395538908104162`,
  0.3075642133576061`, 0.04417074894369504`, 145.23097869725217`, 0.174335805289948 7`, 7.770691560418564`},
{0.16631267310517406`, 2.413572311283116`, 5.471184235465843`, 1.4579537524645423`, 0.9497208653345599`,
  0.439033675828699`, 0.21085287275356013`, 3128.2027597762994`, 0.18409987492192065`, 5.612876798830806`},
{0.18539796406610926`, 1.3871953304353832`, 7.757698949197568`, 1.3585428901946988`, 0.4532718222049952`,
  0.29058122288435795`, 0.010738744574908157`, 486.55354471220693`, 0.20251048022954932`, 4.51278311919668`},
{0.04144083471615087`, 3.41458508027812`, 2.4600046087844927`, 1.2787341279549589`, 0.569252466493285`,
  0.6736151572579898`, 0.4762197736813673`, 718.8382183063302`, 0.07598079948793823`, 4.331845758841565`},
{0.13724293356211342`, 3.6916535362348286`, 5.489803233715362`, 1.3457045887873154`, 0.0754297582883523`,
  0.6293482685634375`, 0.017573021108899616`, 778.5577282359714`, 0.17674997296512807`, 2.6916234726152264`},
{0.08176385784973572`, 2.023736863649704`, 9.823987283528833`, 1.0905304980935506`, 0.6417627833690904`,
  0.2509575610218163`, 0.3071390754059633`, 168.49990583950182`, 0.0669392059366426`, 7.784078345121018`},
{0.20636275093165019`, 3.041433098250965`, 7.709885134336993`, 1.0647262222072884`, 0.28984076250310165`,
  0.17246518959666646`, 0.007377660819744997`, 464.8055142552055`, 0.19020267666710844`, 9.452984008311837`},
{0.2140607830978551`, 1.6917207171996171`, 1.9218182027339432`, 0.9997605714664014`, 0.5513493876922198`,
  0.4464250883384161`, 0.2328414126003874`, 611.4769203082982`, 0.08477583973629349`, 2.188379571162489`},
{0.17696168772261972`, 1.6101558302339125`, 0.7107550695951144`, 1.133554468522512`, 0.8128396324743541`,
  0.15732538361458315`, 0.06392981831267872`, 277.86150200910555`, 0.15413566109625954`, 1.907309832357937`},
{0.04328176764500202`, 2.9602821723444555`, 0.6131528539518083`, 1.0058908218647606`, 0.19964040264767258`,
  0.6669684114614443`, 0.23851965705730427`, 2287.3379976105803`, 0.0859331016978808 8`, 8.660468891635798`},
{0.08735803097627126`, 3.963951857586263`, 7.965851341925532`, 1.189504619761999`, 0.40822475538796854`,
  0.4952091412789442`, 0.01984161894285721`, 1116.2764923646498`, 0.2379409272833778`, 1.293491554272407`},
{0.15281896372135534`, 0.55010519971598`, 2.817048022996838`, 1.3198176246010052`, 0.16238216376752557`,
  0.6925657414172184`, 0.11807724637466313`, 76.78768290686035`, 0.04193274426603899`, 1.2341700274455292`},
{0.19086658543964008`, 1.9905232804946325`, 4.078173781767919`, 0.8519485127716493`, 0.08904379736388202`,
  0.23536266216215151`, 0.24991927271295455`, 192.66677656391008`, 0.127316992739278`, 7.8449864029309175`},
{0.06664208120921677`, 2.003234095381499`, 6.199949723153024`, 0.888873086526756`, 0.04784068730663105`,
  0.37023377411027647`, 0.049828770496122325`, 349.42476257926376`, 0.05267583263583803`, 7.0912634633886915`},
{0.22983292033798441`, 0.7814702114508636`, 3.2012106727008174`, 1.142026804432239`, 0.1064467335224153`,
```

```
0.4404014420960507`, 0.00669557187388940 9`, 4217.329674971789`, 0.15347806495470107`, 2.7989551297088675`},
{0.13211782844464454`, 0.44983284117427225`, 1.0762780754680463`, 1.3187859174208114`, 0.8445651695343355`,
 0.2965729998791703`, 0.054222354179476 5`, 616.1259401245338`, 0.10515194159293045`, 5.24820204317008`},
{0.17817224333688036`, 1.8900871904197354`, 9.98947566566499`, 0.792780936871982`, 0.9109839945193492`,
 0.4314201285170226`, 0.03598067399007004`, 128.67700477089366`, 0.059019163579936806`, 2.366574414393561`},
{0.2509149027719233`, 2.580879402706512`, 1.8893709427148198`, 0.8255744895957449`, 0.1652280455910582`,
 0.6768016323195194`, 0.009322737452992792`, 2112.3599589856663`, 0.08696925005081763`, 4.203193192682905`},
{0.08555200390318413`, 3.7277336286219658`, 7.5570643278825855`, 1.1117862222014783`, 0.20420751548200644`,
 0.3245432768958507`, 0.00950175247006993`, 144.2017035489447`, 0.14385517513119161`, 5.452203080835416`},
{0.07297367338130328`, 1.9451098216604938`, 8.396918429735464`, 1.2137718043700492`, 0.7314346040557373`,
 0.4669601248496963`, 0.016599550382003152`, 192.8440550768832`, 0.21285961816083276`, 7.382641950639707`},
{0.07358197980029185`, 1.0291227850237563`, 2.532589152827489`, 1.0117020834715413`, 0.8098748594005145`,
 0.5301759885595309`, 0.008133019998886755`, 830.3082383512614`, 0.02407211472876364`, 4.5236701681781`},
{0.18797192534273177`, 0.513697145142666`, 2.3563599620417435`, 0.8181944603817968`, 0.26394673984920813`,
 0.3209514271347109`, 0.025430853857263652`, 265.9518711391782`, 0.07720757078553087`, 7.134393115551401`},
{0.2528075291703835`, 2.1409422046846034`, 2.3989238084434295`, 1.1058748574151904`, 0.4698643832575349`,
 0.20099009902051257`, 0.02857754945135185`, 57.682491948215834`, 0.240427409232489`, 4.4402688370357115`},
{0.07054141508315104`, 0.5799884401515247`, 2.99755260715291`, 1.1960365141205305`, 0.29917509086343896`,
 0.44670601886119`, 0.13984458882947357`, 3612.976166750159`, 0.22678215214973713`, 7.656934137991781`},
{0.13968170289558773`, 2.9225254745826437`, 6.832387664740558`, 1.248628328449276`, 0.2500405375269379`,
 0.6548966838897512`, 0.01310969508256717`, 2778.8012693248406`, 0.21649543926111503`, 9.334771326724606`},
{0.14258976979051796`, 0.734197214461469`, 7.171919374129239`, 0.945223896823731`, 0.9937893052327855`,
 0.2333579308191156`, 0.10311334331996398`, 86.76571756149787`, 0.17032024165228216`, 9.316237353631934`},
{0.06222401125326871`, 0.8031654209648247`, 7.912640972265759`, 1.0061100735669513`, 0.06612689335476452`,
 0.2257508005074531`, 0.2688428052003874`, 1016.6634644345198`, 0.08792308855751246`, 8.5639571694838`},
{0.045675945991483086`, 1.972555523342196`, 9.234608119853927`, 1.10067888297902`, 0.060950291683742996`,
 0.5641176167348642`, 0.005654158004083115`, 79.62566643520867`, 0.10418567571675635`, 3.9217157633863025`},
{0.19725457740267938`, 0.673691333497294`, 1.5958254230238804`, 0.9055402555445284`, 0.8526577157919533`,
 0.18700866613724199`, 0.221308084367558`, 137.27532297707575`, 0.16306687928789393`, 6.444987143866487`},
{0.17905209129143224`, 1.6872990209581902`, 6.743325221637608`, 0.8692054788625995`, 0.5776472609105132`,
 0.5432377341075647`, 0.12670077461750193`, 462.43790721394`, 0.05734135301664161 6`, 6.31060148160045`},
```

{0.21834559506325857`, 2.5253820753047362`, 6.659656299000567`, 1.4243771911650907`, 0.9747752944821462`,
0.1772255451247845`, 0.01986842431027196`, 102.11368670749582`, 0.09272454748376258`, 4.914431231863471`},
{0.2429292029568641`, 1.5636324867576725`, 8.233163527811005`, 0.9303215118662402`, 0.8597394087604655`,
0.6215210972087029`, 0.12468068919290341`, 61.92974770793832`, 0.07970211447796205`, 9.85507721294049`},
{0.09149398523540997`, 2.310449584405898`, 9.73345399143432`, 1.4402850137921024`, 0.7807243420630963`,
0.37700223940662914`, 0.04452259966898517`, 785.475395176315`, 0.18361612690246515`, 4.855709318281368`},
{0.11249424084619364`, 2.9716096914630903`, 4.4521238184847185`, 1.4266434477040648`, 0.9997833218301952`,
0.5867347171724102`, 0.06910846060462165`, 1820.2194505805683`, 0.24722705290784003`, 7.621221876581703`},
{0.09866336490626898`, 1.7258477745644702`, 0.8848358466481533`, 0.910425763108387`, 0.9172877053292023`,
0.36334488527428765`, 0.3804604060347452`, 1006.1834168170927`, 0.013687075322010439`, 8.165252701558408`},
{0.22803797815686577`, 0.698146546972529`, 1.035170425484365`, 0.9822431866703154`, 0.6417091531359376`,
0.21874316930074555`, 0.0055310628314534475`, 1727.3746181384265`, 0.11140836748423671`, 9.157656749061658`},
{0.12680960193351426`, 3.133735649340868`, 7.511849069758438`, 1.0897808931740574`, 0.04885314856347578`,
0.24060421848165847`, 0.3693592758602631`, 883.7388687037887`, 0.08961301811176398`, 2.076054864701238`},
{0.11645318221252937`, 1.153640861264721`, 2.619716900319183`, 0.8529577052637052`, 0.5619295219152276`,
0.5231720769226155`, 0.04609815796088822`, 565.9088521347549`, 0.20476637224893374`, 8.831648882728363`},
{0.042446348872052314`, 3.5353553586920903`, 5.120248896798044`, 1.1724129507972807`, 0.07388809680757258`,
0.47979821691937163`, 0.43439767413472635`, 83.35178002891637`, 0.15043407845955598`, 2.47226753883535`},
{0.1455833195103322`, 2.7843188408171127`, 7.76885376753495`, 1.3211895319342402`, 0.9135586666939064`,
0.6453968687054246`, 0.1333226247130129`, 368.7414592704895`, 0.16919558147748776`, 3.2843355334407627`},
{0.19543798537508206`, 1.2324134940956721`, 7.418056367639444`, 1.0725732268441615`, 0.3282068086166905`,
0.15859490968399015`, 0.32000646124266346`, 1339.7132456219883`, 0.07635813740352487`, 1.32927343177637`},
{0.14288798158791094`, 1.8492145343896036`, 4.320087837799633`, 1.416387968755192`, 0.03040014321801321`,
0.26264512496919146`, 0.017396141813896236`, 476.32655268366966`, 0.1492295937607505`, 1.5210123574307455`},
{0.08126639977214484`, 1.4848577063399988`, 1.4511591326569544`, 1.4690309046886825`, 0.09088454373979671`,
0.2634538760346167`, 0.006391735651833628`, 757.569692438266`, 0.1906091612906891`, 5.0187956659047614`},
{0.15406825481222286`, 3.5722547377798985`, 5.518246507815627`, 1.3255687223204002`, 0.2843400333432804`,
0.5740150104722689`, 0.007989301755943026`, 636.0707307721444`, 0.19470033172956325`, 2.3727553064621407`},
{0.1284209869933174`, 2.657038147126583`, 3.485135675760706`, 0.826438046300177`, 0.2940359222573743`,
0.4455502493814366`, 0.0077922251797686165`, 642.5517685154364`, 0.18333530742444448`, 4.125506851960305`},
{0.1519197321396482`, 1.414313316427302`, 9.693328908271443`, 1.0879341282517354`, 0.5935096502675883`,

```
  0.32269331092414044`, 0.04853394307287227`, 323.7551755246228`, 0.012675975614693441`, 6.5738064876305415`},
 {0.10245812606330185`, 3.8066698620788078`, 2.784482506819291`, 0.8153557037427375`, 0.41559745180116914`,
  0.2970790998891699`, 0.019454402362339196`, 101.06708659388384`, 0.03906278599147861`, 1.0136093018296712`},
 {0.08994858347063645`, 1.3905867780774148`, 6.975873482712982`, 0.9748960320168515`, 0.19705721946727817`,
  0.428438510479348`, 0.008778830773896138`, 53.85788205383214`, 0.2055785802889155`, 2.165498620130503`},
 {0.2555268425086469`, 0.6701554458004759`, 6.763443756394938`, 0.9242821290525386`, 0.9506231196396431`,
  0.49497463337651615`, 0.1535425304369218`, 4370.366828894948`, 0.17262313668823565`, 9.906703002274519`},
 {0.13340663060001784`, 0.4096518827228479`, 7.81563732077705`, 1.2681845828171425`, 0.264399527619257`,
  0.43786671579755854`, 0.11528071789135624`, 3397.5276395199044`, 0.15041338875391808`, 8.11261999427796`},
 {0.09831375749458693`, 3.5924209186692435`, 1.6118282365623244`, 0.8326891410736474`, 0.6715734708323489`,
  0.5976781170151362`, 0.03815316295555065`, 115.73199628594814`, 0.0304507712440647`, 4.7148191409051226`},
 {0.09108239111305882`, 3.309852207951435`, 3.0566194803884894`, 1.4370488086380533`, 0.21629452377334557`,
  0.2421124982368058`, 0.4670454509414495`, 208.22129409387404`, 0.23659860207487343`, 3.442603557657883`},
 {0.05787448179355209`, 1.5695297669455615`, 8.686108197389068`, 1.3362839901345183`, 0.8882645350301348`,
  0.2220019613784866`, 0.20455164120837535`, 127.50819012666335`, 0.2481722521034202`, 6.402394955038988`},
 {0.2051260145065577`, 0.6712958473867032`, 3.046620158397806`, 1.125533167922526`, 0.5107051094055373`,
  0.47305603590798795`, 0.035994055865640424`, 73.71677428550161`, 0.039721863923625866`, 8.822427071575206`},
 {0.04365375531995927`, 0.9346121184769336`, 9.554451222092258`, 0.75459846537052`, 0.7382972174511151`,
  0.44670601219095474`, 0.02981461788584879`, 1443.346811017674`, 0.026466349135542522`, 4.5970910839514385`},
 {0.18832502219499725`, 2.1446480190155537`, 7.011391892939212`, 1.2684842496371584`, 0.8800953133738048`,
  0.3019976503972389`, 0.01607749175819828`, 253.61560940445747`, 0.0505443969728126`, 8.205874646962478`},
 {0.2789832227001883`, 3.864947879576305`, 9.015175334965505`, 1.1939022970453932`, 0.8102757584298972`,
  0.6575626089292559`, 0.013706864097109712`, 131.89661477643062`, 0.1558047394747739`, 7.390258992479394`},
 {0.10740283086494345`, 3.8162968757181996`, 6.158389397925204`, 0.8038652030060122`, 0.9187954678523191`,
  0.31270681581700654`, 0.027540968538212463`, 2177.2729976761966`, 0.021577241346914133`, 9.805063749007854`},
 {0.14667176624268824`, 3.777226248423828`, 0.6973504720596608`, 0.7758342249413661`, 0.494379064234173`,
  0.5228383904297224`, 0.014800119213367281`, 81.50804211451084`, 0.12794308526059`, 9.842771813142875`},
 {0.15361999046758062`, 1.6832845721068805`, 5.017027003357642`, 1.2257765840301957`, 0.8758480691873987`,
  0.30492603190517764`, 0.17353190337485183`, 1218.2701158582597`, 0.01908888394356914`, 4.8706184705887665`},
 {0.1285936813753743`, 1.2517058162042831`, 2.0098881407856393`, 1.434144339335301`, 0.06632650483661995`,
  0.16162194262920127`, 0.05011873598676769`, 354.0526180115314`, 0.1584833535532481`, 2.894273123117289`},
```

{0.05871769486491246`, 0.822876240174752`, 3.5461717275684297`, 1.3634413021309426`, 0.14962847564878934`,
0.2425078078443763`, 0.045889110192962865`, 1618.8495608622088`, 0.1824696213391762`, 5.484504386708753`},
{0.21414990207917795`, 3.2291184159683555`, 3.7200101298578847`, 0.893435725539721`, 0.18550851463991336`,
0.6299970912008048`, 0.007788531351629846`, 1185.7534950662077`, 0.10596189074334122`, 9.85351453889281`},
{0.14399581513021464`, 3.6650427925648215`, 8.960857802594514`, 0.9993788071734813`, 0.7678347002262149`,
0.3917750513543251`, 0.0682112112 1423936`, 446.58045234253916`, 0.09781275073737189`, 4.158206599173292`},
{0.21762093156371237`, 1.4799568210517782`, 6.880501462590445`, 1.1700220578418317`, 0.49477438305707566`,
0.30056004401666714`, 0.03776191240235276`, 139.28017196074`, 0.13055880095851463`, 4.30304009869343`},
{0.20676336326895633`, 3.023915070136316`, 2.1344025288266977`, 0.7647942269583019`, 0.38268253024987975`,
0.6058201621166657`, 0.06287157366362177`, 52.11423962493933`, 0.23050267576131828`, 4.129012828530946`},
{0.14056217319026204`, 2.827702131488536`, 4.822127390400377`, 0.9317030787852092`, 0.5881617813064273`,
0.663416624487535`, 0.01875862506453023`, 1486.513173237306`, 0.1539754165327772`, 8.90838886614388`},
{0.15432054500385795`, 3.142590945055387`, 0.6412922856028498`, 0.9338764110697222`, 0.4036132844000331`,
0.253764192888976`, 0.009287914173605602`, 800.3088033129984`, 0.0773663480819351`, 1.9942494398991926`},
{0.1558313627948012`, 2.6183453593149384`, 1.5210778217010041`, 1.2213020905618333`, 0.8957140746385861`,
0.2067404945185849`, 0.06533654021268133`, 3401.5830280519913`, 0.07866132934502523`, 1.2687008259414867`},
{0.27749339228385056`, 3.960153029334033`, 7.7991060624230615`, 1.1159035815659704`, 0.47604804800745515`,
0.18775668040836846`, 0.02760276902429692`, 890.1551806101527`, 0.14097999756547475`, 3.493022856148759`},
{0.1388142019807993`, 3.4960143968183592`, 0.8496516336862268`, 1.4057148799798922`, 0.27289215927666`,
0.4161162296591887`, 0.1071416651953492`, 563.7099077527477`, 0.24491201094978954`, 8.47264522499269`},
{0.0729917462714273`, 3.1893255155706086`, 3.1041281178047555`, 1.014074092539919`, 0.28719491349307913`,
0.2514291775131975`, 0.05245081359414425`, 423.2923229706584`, 0.11450677404966558`, 9.081189649788715`},
{0.11420284690084648`, 0.577787691297051`, 2.567084026939444`, 1.4491737131438955`, 0.8025258647238394`,
0.3690270257872911`, 0.13672661029124697`, 86.53603740064875`, 0.011299238359278513`, 6.563498155623039`},
{0.26373133325063186`, 3.0221428803953465`, 7.660698925394574`, 1.4577816176348994`, 0.5393449604126412`,
0.22813670918251328`, 0.05400872825934766`, 78.22373236540282`, 0.11174025693188905`, 6.564224566850031`},
{0.21289488654178568`, 3.397179033451687`, 0.7632571166797604`, 1.0142699830327386`, 0.0835760348267307`,
0.5184383444209231`, 0.049731529265758516`, 2070.206130759874`, 0.06133590421436391`, 5.427522099525574`},
{0.22999162513252186`, 2.379033562263336`, 2.5591649989178986`, 1.3870393830660817`, 0.8120421313192066`,
0.6084139253527632`, 0.017274035559015792`, 82.84440150538262`, 0.15048886650658344`, 7.465486944171833`},
{0.1769680798340355`, 1.1633029664887262`, 4.7984019847292405`, 1.1781683757559547`, 0.8107109545176789`,

```
  0.3886685867136115`, 0.009172138674078711`, 82.52474973299203`, 0.0366989899628154`, 4.7780773811523165`},
{0.06792516420354372`, 2.678804428347224`, 7.00137481313343`, 1.1889039591838686`, 0.130109850192629`,
  0.17336690021836265`, 0.0065615912781447905`, 559.1859620197067`, 0.12479016185335001`, 7.679232726268854`},
{0.04696109785508959`, 0.9059167168392257`, 1.3656449005023852`, 0.8785005151977332`, 0.7117341492461746`,
  0.37541668809892415`, 0.06884837024201017`, 2425.2572297139236`, 0.025009444315193585`, 7.4432891311871785`},
{0.18481744149515084`, 2.8908169503663625`, 0.7719563125670259`, 1.477735031707543`, 0.05143123515478387`,
  0.4060758128174351`, 0.19176878359926766`, 114.34865924476063`, 0.02384868002335655`, 4.461346915500394`},
{0.11940413672862621`, 0.6385297463968325`, 0.6629513696361364`, 1.2353159995947312`, 0.3854086082897601`,
  0.4772941268081121`, 0.0318040184551439`, 4110.432043833155`, 0.1129636900773972`, 2.562436110087784`},
{0.16756813285689298`, 1.9469615336157506`, 0.3903678609266308`, 1.2604526712473199`, 0.11212576188863221`,
  0.45563235469719776`, 0.0065229352440185`, 190.16738409260836`, 0.045949495779464666`, 5.424569346408827`},
{0.07183551197561577`, 3.34536856927257`, 9.136834569708476`, 1.4150518758492396`, 0.289745011670542`,
  0.5142172775325705`, 0.41137464999480466`, 1635.4988121892525`, 0.15390110584062433`, 5.743187227898911`},
{0.2384422062087977`, 2.1587544451320504`, 5.065398759698054`, 0.8914719118393273`, 0.7773656949632277`,
  0.1997211683380481`, 0.4677952481930321`, 402.45383708693856`, 0.19717109492345014`, 2.7966746550751953`},
{0.049151474922052546`, 1.7029958107544303`, 5.984640650536118`, 1.1787547055685845`, 0.01700706976856159`,
  0.4330742864041065`, 0.015091472025893045`, 551.6808300976855`, 0.1491109638512437`, 2.965493287771624`},
{0.05913727892151971`, 3.1040367182848003`, 9.835708161805986`, 1.4919208308945082`, 0.5501557876127892`,
  0.5378623906029458`, 0.015011170514212647`, 280.8434087480442`, 0.0269165475419888`, 2.8649095039964387`},
{0.1028902787116574`, 3.248894721912677`, 2.8223976230891363`, 1.4617310236348016`, 0.21163710737184238`,
  0.2736720564831455`, 0.052563491644450414`, 157.16268136885162`, 0.03718011423514023`, 9.1247890605319`},
{0.13701717915337458`, 3.8023949322113495`, 2.868993121081161`, 1.1378721602373125`, 0.9948229422967421`,
  0.24001294614371227`, 0.1498271577324817`, 115.7171521166015`, 0.23683719126671393`, 3.874936558168838`},
{0.12767680767420947`, 3.2442504564252905`, 9.68431286349912`, 1.3341607174516774`, 0.1954172777492651`,
  0.4247465971362525`, 0.25891115414709387`, 128.98950675665216`, 0.14024381160911475`, 6.508167926731167`},
{0.15001338826271665`, 1.2298087626140388`, 4.614349177257099`, 1.406777099587714`, 0.7364706782176744`,
  0.4119762663671258`, 0.007028619260401152`, 151.01374639678102`, 0.013332718449329678`, 9.705143720051169`},
{0.20002396942953493`, 1.8945790659495865`, 6.47237980815893`, 1.0186003524309086`, 0.9948715481686918`,
  0.5599748214378268`, 0.06647091233379371`, 636.3545963390636`, 0.214859543893563`, 8.583745959022096`},
{0.0426995949672023`, 2.62335318941125`, 9.6559263809094`, 1.1610440596461276`, 0.9636197200326426`,
  0.24951659449471042`, 0.04756766795277323`, 104.67802103676537`, 0.10920782191363448`, 6.683384921443938`},
```

```
{0.2109009326123063`, 1.0923929642765158`, 8.593806993622852`, 1.1682615459081633`, 0.4499643089978256`,
  0.499787017067712`, 0.035780106156421375`, 2693.2423996223015`, 0.018435796880638622`, 3.8866756448846873`},
{0.1340526977662379`, 3.5323141681247634`, 7.368159769503098`, 1.0026662265470512`, 0.9539160554997537`,
  0.16176440546396775`, 0.3999369934637664`, 62.1244000754222`, 0.21257466381414752`, 3.7256546669386505`},
{0.138783517590022`, 3.0993312847332284`, 6.04052192414912`, 1.482957943290502`, 0.5742828550914962`,
  0.6307262387789596`, 0.006158597883984412`, 223.4781142247734`, 0.1875211630254222`, 1.5541408452833192`},
{0.13089267345973798`, 3.445236909656975`, 9.841043091432308`, 1.0552044625838208`, 0.6603410104708771`,
  0.19921054981100283`, 0.006557758988700662`, 85.91513533777795`, 0.010838310480980468`, 8.608032245025406`},
{0.0796809200271959`, 1.3022197759162255`, 4.457724373908043`, 0.819757860237272`, 0.9902524891863433`,
  0.3732869275715256`, 0.027666886465779635`, 3268.657024391763`, 0.02176038735057556`, 2.8954452621820828`},
{0.24784589915696786`, 0.6840125293527293`, 8.78766727614829`, 1.2765252846824708`, 0.05554020049431552`,
  0.46845321895484016`, 0.4169012658954577`, 507.69507152027995`, 0.17834533234287903`, 5.09521235383073`},
{0.1280056031133443`, 3.9743049299675812`, 6.787496113248967`, 1.4777257411519527`, 0.41697417826757177`,
  0.16856234271449722`, 0.016472885834375787`, 675.9035984651438`, 0.06538866409107441`, 1.0273997992144341`},
{0.2694840025057293`, 1.4398402455219186`, 1.997018914620883`, 1.3694289195380824`, 0.1761008172166878`,
  0.6152080706831848`, 0.020267602026759375`, 203.92916364743616`, 0.23224028188307977`, 5.1018397742421016`},
{0.05785779964873633`, 3.866745902394764`, 0.7980363800640653`, 1.238589808072269 5`, 0.5722218009229685`,
  0.20306189134783081`, 0.040832709866995026`, 2800.195372434598`, 0.18949558604342587`, 8.332936748246446`},
{0.2528189510796667`, 3.3250798883848782`, 4.1415523484020405`, 0.9119401620778833`, 0.8401965817654944`,
  0.5070162083757728`, 0.10337827675659322`, 381.96079877048203`, 0.13245695994197593`, 7.95834379608233`},
{0.20018502874097505`, 2.5781656282009475`, 6.942068190225046`, 0.9929389842661138`, 0.15941471595895984`,
  0.3421827829774996`, 0.4839576109293149`, 375.34729484852807`, 0.1596957174670693`, 8.895509004013046`},
{0.272440775669993`, 3.035804235517027`, 9.484814840956616`, 1.224288286053278`, 0.8025814347303761`,
  0.5890268382574878`, 0.3843203491912798`, 336.978314494752`, 0.04553466081406238`, 4.10287356038395`},
{0.20623021381347656`, 2.305665489644068`, 6.160210330863352`, 1.4997848102690652`, 0.31029755609374554`,
  0.2932546230651458`, 0.047029701289319936`, 605.575343672007`, 0.11131413350838143`, 4.271934365443833`},
{0.27799817940399246`, 2.8456068500128753`, 7.4650958706635215`, 1.2794446296629622`, 0.3185515090611555`,
  0.5440861077528716`, 0.2050159519579307`, 96.61991147082354`, 0.24522478882121745`, 9.22867476531573`},
{0.18731205279881885`, 3.3920364906001828`, 9.53367346846489`, 1.150711712471592`, 0.269617044263482`,
  0.4407414224913243`, 0.0689482800442352`, 54.451263142554176`, 0.1525802464986004`, 2.2696676207606785`},
{0.07598141369986516`, 3.9958506473293216`, 3.287957942109602`, 1.3569966945049934`, 0.38975760386481206`,
```

```
  0.6382110342097327`, 0.02530287278972352`, 4836.578585308585`, 0.06124631354128618`, 7.15493409138095`},
{0.21782025688272888`, 3.800225088388993`, 1.7719223148490801`, 1.3823014458300409`, 0.9034484926621247`,
  0.21119490910082483`, 0.008011320454875006`, 549.8645476667474`, 0.16991323645757794`, 1.3522541901702763`},
{0.12917865835123116`, 3.4713877505357775`, 5.802428296169513`, 1.4965547140183602`, 0.061568597458759644`,
  0.5580721907461164`, 0.2777380762847274`, 381.024601401567`, 0.014984965589617844`, 6.51082587242481`},
{0.12408328322582662`, 2.9809522601952096`, 4.3074275514408615`, 1.1040930179447719`, 0.6796918144407815`,
  0.5931470332615022`, 0.005369942238542098`, 502.88367879292525`, 0.06875474145528054`, 6.831128450148093`},
{0.2301374553102561`, 3.6285008777535515`, 6.8583997394674485`, 1.291013232269163`, 0.6431022470858925`,
  0.6246309281545763`, 0.18498728864660263`, 132.58216720365365`, 0.011374938240932164`, 6.052136677369937`},
{0.18974259222357154`, 1.3104397818774611`, 5.6865397453703626`, 1.3109561369338216`, 0.42925562092874125`,
  0.6235284138933523`, 0.06332638490149935`, 1635.1348002386078`, 0.08473820825835521`, 3.2599591654117823`},
{0.2309824769455583`, 1.2471345787782342`, 1.0215019422726535`, 1.0312798793045614`, 0.7898876098277747`,
  0.6152586670568927`, 0.006979047924720161`, 310.31208829749596`, 0.24081833704073502`, 8.344184598629436`},
{0.1769680396277803`, 1.6046472005597128`, 8.695674701006173`, 0.7703180616996756`, 0.764260429383415`,
  0.1992432648214062`, 0.012440799720035065`, 274.53159119005693`, 0.16375790457626532`, 6.298702841855883`},
{0.23450402912552432`, 3.0762297015464597`, 2.593937576427738`, 1.1039402177647473`, 0.4424612211889203`,
  0.6666998367628838`, 0.44528706596344714`, 463.5224063697932`, 0.20661121111075748`, 4.223015851637719`},
{0.2755523363386182`, 3.945462181815995`, 5.876565708554855`, 1.4395306041819111`, 0.28572441613120825`,
  0.3101513953075886`, 0.39757529919045964`, 3302.499033894636`, 0.07285156297216239`, 3.794286848749824`},
{0.1912671247263708`, 2.190434692724648`, 6.310276417856439`, 0.9527360176604049`, 0.6104292936966895`,
  0.2522020081398627`, 0.010518470607815496`, 284.3254609055096`, 0.1005439259073278`, 6.418961425083908`},
{0.2597914621776059`, 0.5051242650756058`, 1.0118962727972`, 0.8968589895031354`, 0.07276014875602654`,
  0.6898744359309281`, 0.03193322527994425`, 3177.5504656788207`, 0.10860322045075349`, 3.7076953543063436`},
{0.058009311425788745`, 1.2821997162065424`, 2.0542232518025845`, 1.4132652568351216`, 0.6228832317992945`,
  0.16342794118309045`, 0.044680537911227475`, 201.38656694501756`, 0.06413530627116476`, 9.590029577588982`},
{0.19641852899773504`, 2.1398264375306333`, 5.320272455292722`, 1.1437489346098513`, 0.5956525384016933`,
  0.542078693849333`, 0.2264882318745723`, 868.9198508977777`, 0.040131295366706066`, 7.0792614425236255`},
{0.09296068013545083`, 1.2991493564864323`, 0.26387265122439096`, 0.9093728692378444`, 0.35146944109549483`,
  0.5215346752572301`, 0.009103351916553084`, 3690.6422305701226`, 0.14178477746644713`, 8.458943719263925`},
{0.09381338426095137`, 2.303475789921615`, 0.696644313515133`, 0.9364121618865129`, 0.9663355677423924`,
  0.5212308135336106`, 0.24626013703252375`, 1385.562539836289`, 0.20934361709679128`, 7.7725134925150865`},
```

```
{0.10039832860995906`, 2.055462616090492`, 1.0012272143748433`, 1.102551063992137`, 0.7631597204325553`,
  0.38648795662626834`, 0.021413905310258614`, 653.3550083645581`, 0.055519462287367316`, 4.402154187880063`},
{0.16980453046164407`, 1.1980162751260872`, 9.435065425338543`, 1.2054727390347812`, 0.15783339617816905`,
  0.5987514193964574`, 0.2639813183074236`, 624.8721767770594`, 0.14884913893972124`, 1.0597319526281854`},
{0.1106187483985549`, 0.9718711331456742`, 7.0205608914526`, 1.3199794157480607`, 0.2870266036717726`,
  0.2842604073783652`, 0.12440193317594331`, 1067.250206889352`, 0.20077332983402157`, 1.344770962154577`},
{0.22322153760577584`, 3.532203149750069`, 4.201686494998453`, 0.809746094501941`, 0.17157339217602185`,
  0.16416014932043588`, 0.005086092661785311`, 81.42460105493491`, 0.0994686719903208 9`, 6.2584574953677805`},
{0.11344753764961812`, 0.6515213816912864`, 6.9223785093110415`, 0.7705690533378804`, 0.053758347821018404`,
  0.22122664443377937`, 0.23238889135484875`, 1612.9665239639735`, 0.23659636795633343`, 7.8050806983048435`},
{0.05086592503936643`, 0.6345548618632657`, 4.1212243894392255`, 0.9757969272512688`, 0.4720155756440034`,
  0.3491598005177542`, 0.10619345643493772`, 163.13358888497802`, 0.24650090837908084`, 9.412849245254243`},
{0.18344840265344153`, 3.595124583701854`, 4.199071914809606`, 1.0537057639813663`, 0.12507220232647875`,
  0.4903855977737973`, 0.3568003256791643`, 83.2177314580599`, 0.1899680886834239`, 7.625578775230776`},
{0.11456761033436458`, 3.133847301219358`, 1.1215010618012613`, 1.2799949994814193`, 0.7250649174618937`,
  0.2748176967060689`, 0.05347527364447488`, 4312.248498236981`, 0.1363057898672484`, 6.512229405589068`},
{0.273310222828516 3`, 0.9906705811868095`, 1.8602414047947509`, 1.4451100284824412`, 0.2848844825831507`,
  0.554129635350794`, 0.011612796259976983`, 302.8864567264163`, 0.1580490958422761`, 2.3921629765885086`},
{0.13611751569452646`, 1.612649572698647`, 9.438389550358693`, 0.8529700096304889`, 0.38386891649316235`,
  0.16702474363312747`, 0.015283436679942941`, 2091.084776788581`, 0.04888707786432367`, 3.7550559614266916`},
{0.06405507608053002`, 1.3528358541887275`, 2.745226236199782`, 1.0639243234460365`, 0.36966523495796855`,
  0.3262944894947615`, 0.12612633222162115`, 59.71942118372549`, 0.2416255104540047`, 1.4175051543133999`},
{0.20954125488723696`, 2.353113320872523`, 8.807677395871629`, 1.2953396401945112`, 0.754037675145256`,
  0.6760987806408354`, 0.0257504108557459`, 115.6397082887867`, 0.08726278776335833`, 1.3938676616131378`},
{0.12907099769038483`, 2.604139615282878`, 9.661547795493739`, 1.0824039436985886`, 0.5892380332813292`,
  0.2973399722037613`, 0.047844179127263436`, 189.25086236267023`, 0.09074457722059731`, 6.471849996577652`},
{0.2477279255184573`, 2.8408395942947395`, 2.128090335748766`, 1.4989746812981888`, 0.49854461135120975`,
  0.5556994499234343`, 0.04795352432490085`, 88.91629411642595`, 0.17336384521962678`, 2.1291108959892835`},
{0.17138396941106926`, 2.571547212239655`, 8.060362653989774`, 1.424545143219825`, 0.3585647265624352`,
  0.4155041928809974`, 0.0058496443892025185`, 1444.9053577030656`, 0.14315469959013916`, 8.6363710850225`},
{0.046024454977220214`, 1.8018961949102152`, 1.8889771640586375`, 1.2101342547143499`, 0.33571961358008684`,
```

0.4325808303075249`, 0.005213559292121167`, 454.61857984803544`, 0.13129751322022398`, 8.467405558196546`},
{0.15552548139256261`, 1.3158122598456092`, 2.9803095987090753`, 0.857616156930884`, 0.19622947720693906`,
0.41449590928153224`, 0.03892742585126438`, 2129.9557657820133`, 0.1528564450859799`, 8.904626117418289`},
{0.20226341603934178`, 2.23359139800782`, 8.92211343859698`, 1.0176324123098368`, 0.17770526936314845`,
0.5466941503204505`, 0.02261985699694341`, 208.46398724865384`, 0.0356723302826476`, 4.800574849057675`},
{0.1612087208534252`, 3.1252528746197825`, 0.891851633192701`, 1.468881451365178`, 0.6554353298599058`,
0.19493335173588566`, 0.018954452797492544`, 551.070999166768`, 0.11474062580409572`, 7.406826770535428`},
{0.2365245631846375`, 1.6915599352668007`, 9.677850457164503`, 0.7567859979545984`, 0.725618873672359`,
0.2424250415266589`, 0.36826031218846694`, 892.5435235500784`, 0.17475643237365474`, 6.493737892117458`},
{0.22790261965108521`, 1.8196334440146895`, 4.716201039891137`, 1.2810805872690838`, 0.6237465626441163`,
0.5616811859186371`, 0.03042013590776336`, 637.6118251230974`, 0.08011306001688712`, 9.216145754556873`},
{0.10184647957852327`, 3.0801202872185955`, 8.412464860766345`, 0.9942252517730743`, 0.7984635108014702`,
0.4565736536432896`, 0.009191281945386102`, 103.82351882247931`, 0.22274711417837167`, 2.5201460619086546`},
{0.18768732765242752`, 1.4966861207841546`, 1.129759551095228`, 1.2240080138702374`, 0.5362083693873154`,
0.686948995479657`, 0.005867661202712241`, 74.92355531972449`, 0.165025723744549`, 3.2590978632956187`},
{0.14949493319923252`, 0.867900380757324`, 9.54711835147964`, 0.8271983961134595`, 0.23831574478972195`,
0.375815029784779`, 0.0451684556906921 8`, 2094.687766706356`, 0.0279158592619283`, 5.743304871768913`},
{0.10785599050108607`, 1.35490623294936`, 7.749733046704847`, 1.2972878372630243`, 0.6070878493023009`,
0.6937073302769028`, 0.28374543116039413`, 66.83074779041495`, 0.1939262594152683`, 9.425365274493942`},
{0.13969386881974227`, 3.165109549305347`, 4.62533417349867`, 1.0389272094346946`, 0.7061162924631639`,
0.5973647953915502`, 0.19864442086151357`, 3440.0036010322765`, 0.16071761832429643`, 1.3360604448248414`},
{0.18701505616348296`, 1.1471835668231432`, 1.9652898147298057`, 1.007866470439728`, 0.8673024418570234`,
0.4964521059357`, 0.11361709646832255`, 189.11029986824036`, 0.17527157137077032`, 1.608206453868661`},
{0.12078071855653999`, 3.7816423804380825`, 3.0527531818157634`, 1.3792123087306463`, 0.18684955879429088`,
0.29398025647621684`, 0.011105429740027619`, 471.0240529628659`, 0.1734264666127333`, 9.864280424563177`},
{0.12022941283008587`, 2.2264210098571606`, 6.2451953049416655`, 1.3438290154028227`, 0.5358944653344513`,
0.32618163573980585`, 0.199782178892696`, 3338.755251465298`, 0.1788085018354036`, 4.512919273292992`},
{0.04681963491488872`, 2.902738250264786`, 8.529727895043578`, 1.3985345888875074`, 0.35876551940164103`,
0.2520539039703089`, 0.05124631524713002`, 163.50426816944002`, 0.24665810410586203`, 8.118758344754049`},
{0.1911559295611578`, 2.175586072104755`, 6.8117639016300915`, 1.0586708066957606`, 0.34517099105389626`,
0.196211531611845`, 0.1489787018319715`, 3704.576077864889`, 0.10408111144660032`, 4.8300445149655875`},

{0.17192712556630163`, 3.7260903741599414`, 4.118781130521407`, 1.2245874304759414`, 0.6062180312792598`,
0.5331783629983038`, 0.1203111914746069`, 226.51013650082294`, 0.017839169449302028`, 7.150760371208259`},
{0.1575695043509842`, 3.8758817205894927`, 3.3547063374262347`, 1.3334178116995439`, 0.4921394422884451`,
0.402330971011554`, 0.12204131883407823`, 201.57298534982124`, 0.22899817129952033`, 2.4884430439924596`},
{0.06573776532431397`, 2.613579102769201`, 3.806271127858438`, 1.1314163600990936`, 0.4483602258230772`,
0.6823320235871475`, 0.18768855278686034`, 189.85674956999534`, 0.0631967400861071`, 1.3499467761035964`},
{0.06757463956251891`, 2.3005441553217736`, 6.868151131987258`, 1.3860241166029201`, 0.025379963517129678`,
0.6856836713524472`, 0.11835507299142825`, 3863.192203814942`, 0.1108156231672533`, 2.823244077255117`},
{0.06632069369203508`, 0.7568152865276287`, 0.22726671156421221`, 1.362987063673106`, 0.9228910129465389`,
0.1868567265073786`, 0.1296811990167267`, 123.29501155053357`, 0.19348527358813117`, 9.470702921306387`},
{0.1352416913789251`, 3.40347330811998`, 4.693938060979022`, 1.2778213334701187`, 0.7052646641955478`,
0.6043325609148968`, 0.013064813980624626`, 333.49561209931903`, 0.11153764285740131`, 8.005884287110216`},
{0.11675242158451127`, 2.833886804623358`, 0.47584343623935504`, 1.0607616820427617`, 0.7666611109645796`,
0.4737758025893787`, 0.1681420982868212`, 120.33220123866614`, 0.17915094289032796`, 3.5814785888826783`},
{0.24323887781179143`, 3.4699698183729026`, 8.39097272098094`, 1.1938324247391086`, 0.7463001660827029`,
0.6306644967356683`, 0.2992521819376612`, 322.6100644812281`, 0.10289378547186823`, 7.076414822100832`},
{0.24390696598302103`, 0.8206355382610386`, 2.839705408503791`, 1.0872176805395473`, 0.8466198387041834`,
0.5785697848880403`, 0.1356337036504287`, 2343.8028799398503`, 0.22246042363346175`, 4.459217822696726`},
{0.06769491456810739`, 3.362305338073525`, 1.8871867183871915`, 1.4803795481758868`, 0.5363168854717104`,
0.37458837250617005`, 0.011183256466341666`, 407.888606379481`, 0.051902827430772014`, 9.719761204172809`},
{0.21105892687940375`, 0.7047276416914965`, 5.768010388957135`, 0.810198720595394`, 0.17857267890229922`,
0.19525448649651245`, 0.036176279358565294`, 67.82203673017167`, 0.07478241594027568`, 5.650997388906681`},
{0.13030085884608883`, 3.691040694570585`, 3.5546925177950874`, 1.392417125140739`, 0.5227226143537942`,
0.3153665172319682`, 0.008712610924073824`, 695.2883528301467`, 0.04802018879975545`, 9.078480195703946`},
{0.21250061023000932`, 2.8149517595358526`, 2.295631419336827`, 1.0543549080196901`, 0.6519014079329188`,
0.6534060058028184`, 0.09088447710322001`, 650.6798329466749`, 0.04016188412840693`, 1.3861729584655489`},
{0.14347475236734758`, 3.69539554202769`, 4.673626861854084`, 0.8876695712812951`, 0.05519972772145398 4`,
0.3291461786147223`, 0.031312636638690675`, 836.9629099625735`, 0.07687709551472927`, 5.335882334514919`},
{0.0805761857877269`, 3.094619489710217`, 9.945877040804334`, 1.11372690786775`, 0.6430409751953907`,
0.42229007304684385`, 0.05506378209236767`, 302.8456666998151`, 0.050941087889112335`, 7.632080993355595`},
{0.26865716137877294`, 2.5539808428845117`, 7.725254140854183`, 1.421576189719117`, 0.2484256166411507`,

0.1545757521332899`, 0.00855674149866 2016`, 1177.1024036480305`, 0.08015420001442447`, 4.125195480316323`},
{0.05207035897383483`, 1.5461440288878858`, 9.695008671418819`, 0.9618476339297223`, 0.8208653770038725`,
0.48583250041037973`, 0.22651174945218666`, 56.560953214157614`, 0.1695178841867513`, 7.24665684693536 4`},
{0.09188932948313594`, 3.928289836924776`, 1.4719659367359732`, 1.3955743442040527`, 0.5432280807299197`,
0.32698938665455834`, 0.013007122973082902`, 2762.880112969401`, 0.13947463148509326`, 4.274432647648432`},
{0.04429611977972214`, 3.507177302954225`, 7.234549676890296`, 0.7623401767303408`, 0.010148640019661537`,
0.662687845406686`, 0.08775344792279212`, 3965.4644576584396`, 0.17480873042969614`, 5.118348118050843`},
{0.14748247662042147`, 2.024427515527907`, 9.506929645646245`, 1.2214601071407813`, 0.2054881053340767`,
0.2020185622482833`, 0.07806490597707526`, 302.23580657852216`, 0.2425913704635762`, 6.045088630499148`},
{0.05191368478403385`, 1.8292747620439185`, 5.888828307542417`, 1.141375509610469`, 0.9869968200599144`,
0.28747330669734017`, 0.24632331590197062`, 4082.2304223282904`, 0.0447948152020462`, 8.998917183247265`},
{0.22928260338606776`, 2.222139650206395`, 4.336407604983492`, 1.4545262441104416`, 0.17670484975018264`,
0.15215120204412502`, 0.00931582374755147`, 107.9598634817244`, 0.18384173269782222`, 2.0618261552271964`},
{0.13057179539780978`, 1.32287055831243`, 2.838629919542937`, 0.8498204532586893`, 0.8949438289681946`,
0.46229782752490833`, 0.06831034580553909`, 355.6715560557054`, 0.046330937271765626`, 4.510601406855825`},
{0.16617324858328203`, 1.7665378156673919`, 1.5537022391595983`, 1.3738499573440288`, 0.09625830296842142`,
0.5757224393125404`, 0.011187699121044243`, 1140.5955024024072`, 0.09128758443232887`, 2.6133507581824666`},
{0.150610971367461`, 1.9051770945291775`, 1.9970914506574022`, 1.332029884280907`, 0.017627751946745374`,
0.6342635790195508`, 0.03224365795250487`, 84.16237057378729`, 0.025181518731154096`, 6.093120594420567`},
{0.170193722625894`, 0.42908710938865147`, 8.009796665724636`, 0.9108642019460496`, 0.48256370773751156`,
0.1504864882770205`, 0.015501266854459406`, 231.18780020236528`, 0.059845835174358764`, 2.951447491236335`},
{0.14155286072340578`, 2.768731185287475`, 2.866796585793665`, 0.9268420036617817`, 0.38841014261523243`,
0.6739227770046636`, 0.08018336457681292`, 85.63532436040691`, 0.10664760400309609`, 4.590937221386067`},
{0.17254585725985527`, 2.4767354176461422`, 1.5265879737476418`, 1.3533294324013416`, 0.010476993931212819`,
0.3024537953894164`, 0.005382899126762437`, 1067.886819263142`, 0.025600178888030733`, 4.688973100729083`},
{0.12080218784009655`, 2.1868434076988192`, 8.77616791832854`, 0.9616886370191489`, 0.26020855267635734`,
0.6201783502287406`, 0.03811904516021991`, 117.06014479510438`, 0.17593156125676795`, 4.349098586723594`},
{0.06629025039264891`, 1.530609759106781`, 1.900084106095834`, 1.2729265107514505`, 0.009336607275947317`,
0.20692029297881853`, 0.07757288734346537`, 56.094679581136695`, 0.10338780030561462`, 4.34093728430811`},
{0.11054921486908309`, 1.9601623754484976`, 4.1405236153603`, 1.0982919546164496`, 0.2371613718862391`,
0.5811399077338627`, 0.014126785714663546`, 63.169894546693705`, 0.15558848051416968`, 8.651696062244092`},

{0.09844925238529889`, 2.9547765664281824`, 3.97533240344332`, 0.8901779978265776`, 0.6485331039194935`,
0.172046787329591`, 0.2843834249851245`, 265.56383421685405`, 0.07856132944640065`, 2.1823933339229455`},
{0.07379407071323735`, 1.0020834251379886`, 3.8959201685323244`, 1.3275435329440872`, 0.5495795554378471`,
0.5203295123605114`, 0.006236204073249997`, 495.6126936444482`, 0.05339932410900344`, 4.300380134475608`},
{0.137830658132162`, 0.964244066849937`, 5.678313655870262`, 1.056362681888576`, 0.27898051740088525`,
0.6674603903550571`, 0.022240144180942223`, 3206.233069292432`, 0.13749704640301397`, 7.378694929535063`},
{0.09412349267697989`, 2.412142947637907`, 8.593900106949853`, 1.0545990130363978`, 0.8443965196041454`,
0.4441557500447094`, 0.020813982545198062`, 67.68562305269263`, 0.21037364591803787`, 1.9282456472076213`},
{0.059604065321211375`, 2.060924190389435`, 4.151577648882975`, 1.0218595307135387`, 0.8259153199816331`,
0.67511248087556`, 0.012173840559063499`, 163.32402156550611`, 0.22896057989768043`, 4.41755081858776`},
{0.062303828390692484`, 3.237012523552033`, 1.9717708145626371`, 0.9726411613979222`, 0.1873520229034391`,
0.5560570337810927`, 0.026607710207102397`, 62.963998927305475`, 0.022649002664224704`, 8.659052494024476`},
{0.058207327156306`, 1.429925878818433`, 8.458349879741764`, 1.1245084153106533`, 0.19874114947102073`,
0.3371100826465334`, 0.04401493286992449`, 375.7204818983131`, 0.2159694260931322`, 8.593637256078832`},
{0.0526423548412821`, 1.6513512325831732`, 4.083783641771872`, 1.373155154639566`, 0.8939370398042139`,
0.2364887781328291`, 0.0490949664593259`, 1618.3201075393572`, 0.07451928539997066`, 4.7651781050102695`},
{0.21322884859448837`, 1.5615738832323176`, 8.40996483053398`, 1.2929614343326012`, 0.3765546169235392`,
0.671739728089668`, 0.1608794324574438`, 485.1832614690647`, 0.0705178455433636`, 2.1513131242884285`},
{0.11682922382752631`, 0.5721136495795571`, 9.687079392836907`, 0.91254998083727`, 0.43543762812336806`,
0.4740169657662334`, 0.10620954791167687`, 4372.038997558794`, 0.0729116423313943`, 5.645356891940546`},
{0.26956767736567855`, 0.8444303847788461`, 1.0020073721955711`, 0.7502641425367418`, 0.966405616850982`,
0.6715103286393609`, 0.23328928938153737`, 246.8438332486948`, 0.10178810353613177`, 2.531792031152129`},
{0.07372356616920961`, 2.107710372997479`, 6.08076458707294`, 0.8379230467338923`, 0.9571077593283461`,
0.37371168094765217`, 0.1939664465355383`, 131.4329681577719`, 0.026286944156709513`, 5.373906305884013`},
{0.2128059772734779`, 3.088861612111426`, 5.437928143232815`, 1.2368766361188581`, 0.5161736795080103`,
0.22159582314348258`, 0.17105793552921358`, 1293.4067318655482`, 0.22754002195492767`, 9.689789287743999`},
{0.09487232790202699`, 3.2902977287485546`, 3.9754892015026773`, 1.2274952505878522`, 0.2657953162834845`,
0.47376077240276926`, 0.26204707729007887`, 80.73499841507218`, 0.18195262450329797`, 6.708419132160676`},
{0.05710457017724263`, 2.2468049124614753`, 4.035987116327698`, 0.7517191945594467`, 0.40903626729229714`,
0.6582116219387326`, 0.04046385674463665`, 815.0779000126262`, 0.186726177475597`, 7.464159626289564`},
{0.1356890149134694`, 1.6504364529320803`, 8.903330095009764`, 0.8652149931542472`, 0.7882939470826769`,

```
  0.5853867881019097`, 0.12191584954894137`, 2002.2565687817998`, 0.1918944953755l667`, 8.0829058846114`},
{0.21947794079961902`, 1.5730764365956098`, 2.118289006802259`, 0.973502067608673`, 0.7638917093126882`,
  0.33981639014260245`, 0.009615294802656809`, 1765.6534921210653`, 0.16626255675053375`, 4.010994756897377`},
{0.2345658346849942`, 2.081301191128982`, 3.6234436876627587`, 1.1134590239676974`, 0.8739003578896458`,
  0.6696041669913848`, 0.06590896300137702`, 558.8606273551264`, 0.09113830356870467`, 1.2137876330314246`},
{0.23053748957807368`, 3.8668800248110458`, 5.535000453565214`, 1.1361194466757834`, 0.24488877583427326`,
  0.5300195072451362`, 0.08816752471869817`, 3094.9084693615328`, 0.16131439142801796`, 1.2619255962975515`},
{0.05839671607004826`, 2.466861519533315`, 8.278076935325572`, 0.9179604454629582`, 0.7485509623778881`,
  0.3739704677333707`, 0.3976055017684991`, 334.0137006202725`, 0.18218149392519362`, 2.0150765691128605`},
{0.14569114559043927`, 3.155548582929571 7`, 1.7894848948555018`, 1.1727674941432193`, 0.06666495623301039`,
  0.21747003985461844`, 0.032197916321861565`, 1115.0145850241458`, 0.11404115123479613`, 9.530247146566754`},
{0.24150266620923067`, 1.7188165992139313`, 4.322636691993072`, 1.2029528995785472`, 0.9471494725848191`,
  0.6615414605337746`, 0.19861497081852103`, 2170.4118977268995`, 0.144713056075588`, 4.793357249969096`},
{0.20918490948785712`, 3.1569386485962374`, 8.450247389660383`, 1.207442014040422`, 0.02962131605799323`,
  0.6403349881706148`, 0.07844599973162336`, 325.7491474109025`, 0.10080604275363181`, 2.998503110312266`},
{0.20782035028778872`, 3.1560227942933192`, 4.024085032034362`, 1.1076699632387197`, 0.7825797887689212`,
  0.681476671590229`, 0.005917686238352 2886`, 2392.206038919967`, 0.17125268254719206`, 3.7066097570560648`},
{0.16569005768607342`, 0.9318724619299896`, 5.387727961962419`, 1.4204768501331082`, 0.162422581490264`,
  0.6283948310518737`, 0.42435650593933405`, 403.29853257093646`, 0.17732127106821444`, 7.752705227642123`},
{0.25387146604005345`, 2.177421517695631`, 2.9195785593706507`, 1.205984878491204`, 0.22933817566590875`,
  0.5142965710820098`, 0.00715658453704801`, 291.5834795916249`, 0.18546405096886748`, 3.1957985304348036`},
{0.27254750481249423`, 3.728211063877054`, 5.204302820921312`, 1.137745914342209`, 0.04515932299981973`,
  0.21786268187051538`, 0.17709327429647695`, 87.47051100453898`, 0.05078893394192652`, 2.761931237968355`},
{0.24124989455736606`, 1.138551887899828`, 6.0414451617780465`, 1.0864227463934355`, 0.8606911125263128`,
  0.6167713883354349`, 0.008616924398628963`, 4204.638992315412`, 0.042475052828396476`, 4.683417206387297`},
{0.16291183507690532`, 0.9001170306894437`, 5.6406094859134015`, 1.3830538570390154`, 0.30535931966424634`,
  0.523800888605104`, 0.2170932130058295`, 52.61233898698406`, 0.08477132140762966`, 2.26771827115102`},
{0.24307985322679526`, 3.5312268091205707`, 7.143117328957406`, 1.2474301004946668`, 0.43966589306292936`,
  0.28635789146968393`, 0.04752738776816154`, 125.46273060513494`, 0.15229886979218882`, 3.763239253417467`},
{0.08579390683558069`, 2.8983719603389604`, 3.99564346069112`, 1.2420354947694623`, 0.9313337583971846`,
  0.4737657288125191`, 0.010571420568870461`, 66.04207510362154`, 0.1244519483642591`, 8.73466885165751`},
```

{0.20336933942425872`, 1.2934536281273639`, 5.591224704743386`, 1.058733783245077`, 0.09184020179750241`,
0.3092776948325172`, 0.45477750215520385`, 95.84900263920687`, 0.1497141413596374 5`, 8.98848136080165`},
{0.12808268874911022`, 3.6824385947617753`, 2.1347222010883335`, 0.9228913029818868`, 0.1301948165557758`,
0.29194798379239884`, 0.27062051931711223`, 4508.281780436825`, 0.033597809391257005`, 7.5117374932700365`},
{0.14553068051711526`, 1.8082756931498238`, 2.368533159601707`, 1.1602558844471123`, 0.9691057704573378`,
0.4792380149128871`, 0.010639214796165341`, 788.9564435352116`, 0.18893878705870149`, 9.465562662789438`},
{0.24858998623664108`, 2.4028729322353692`, 5.907867944453356`, 0.899298954014168`, 0.6845394050547509`,
0.4831163766793648`, 0.006208316855544933`, 932.6458115441258`, 0.22545041645445363`, 9.209981899602862`},
{0.2519271524040908`, 1.2045387595495214`, 4.5140841978362705`, 1.0152142452788597`, 0.29419718036266773`,
0.20432622138273293`, 0.02287101962327847`, 2390.149329793605`, 0.07799608049040802`, 9.888014794979117`},
{0.11736070154865536`, 2.38096944376945`, 5.3494850274310295`, 1.276899864236343`, 0.07495782665645989`,
0.3912082450614217`, 0.41113333692039317`, 80.12914367323096`, 0.2186956786299285`, 8.297333470694408`},
{0.16035901956271875`, 2.606522752147513`, 4.69883411815465`, 1.3021358666964324`, 0.6150695298380762`,
0.6434499333892203`, 0.12329290515746044`, 1079.0196669558486`, 0.2419176548365854`, 8.070742396729585`},
{0.16449617597041116`, 3.191202853555228`, 6.979734889593845`, 0.755053893640484`, 0.8431581165413089`,
0.5308423930900061`, 0.0662197921799213`, 915.9947229465104`, 0.1651520479769521`, 4.094114041787371`},
{0.11360527105324508`, 0.4683409445122746`, 6.198135239531911`, 1.4397350406163711`, 0.8287446034488006`,
0.16487005319303316`, 0.2956243647100192`, 339.82112374821816`, 0.14870620145140367`, 8.492417686013223`},
{0.08657280459787114`, 2.8376273381057535`, 2.18201840556476`, 0.9732923844552535`, 0.8927467229995099`,
0.4599530868891304`, 0.04289399463935107`, 1129.687177458504`, 0.08547871234303578`, 2.1820677830816138`},
{0.14288327643386806`, 3.0416391272131467`, 7.259522365277451`, 0.8919048885163637`, 0.7100314935930478`,
0.32120037052790307`, 0.014548712666776912`, 690.6861027330066`, 0.2370448478751857`, 7.1501615854828415`},
{0.05887079706837223`, 1.3439289639420835`, 4.010009055307599`, 1.087305166401462`, 0.6661788396152433`,
0.35567096782369767`, 0.008830796062004187`, 96.96443168903161`, 0.0354538286259683`, 6.082802068311153`},
{0.275808383383374`, 3.614253930768469`, 8.690573450897112`, 0.8084062901760818`, 0.7881641108236563`,
0.5461332704061045`, 0.16300280176413603`, 213.49162259077596`, 0.11620699031214832`, 3.4918574760984407`},
{0.16345104545617634`, 3.976575006158881`, 6.050721111049688`, 0.8077170991659299`, 0.4737043917748365`,
0.42206087766889466`, 0.011122815850097148`, 819.7636995837653`, 0.0134560852671817`, 8.084495823489597`},
{0.27078615466317746`, 3.6662669410444693`, 7.922278334055675`, 1.4466898496109064`, 0.8715287168554611`,
0.2219561421798496`, 0.02446106939657988`, 188.339766844502`, 0.03692414185938048`, 1.663076961237424`},
{0.24286662289220456`, 2.6814172670718373`, 2.6369699156223714`, 0.8218929956416956`, 0.8808977613854625`,

```
0.43575724866517207`, 0.024751792159432087`, 472.6370271533013`, 0.14270041872793765`, 9.280134575613399`},
{0.042342161544045354`, 1.3013804906014936`, 7.13634807471312`, 1.1105942412761596`, 0.2407240140528013`,
 0.6802475696307242`, 0.0062232822378432`, 4037.4772502763867`, 0.18461914595887624`, 5.716947148449252`},
{0.14720397501884225`, 0.5316247300014076`, 6.683828314747185`, 1.481587653959343`, 0.013428589995876372`,
 0.17720084833285243`, 0.005004553323761107`, 364.08088787611194`, 0.035796657483315286`, 8.51611862140901`},
{0.1926486177557311`, 2.6336525621782014`, 9.444109966651418`, 1.4788802142070587`, 0.6654095862145473`,
 0.16653655494047714`, 0.03828733327035071`, 2433.4281263020134`, 0.12844440731237916`, 4.149230973672752`},
{0.05576285411897663 5`, 2.4026093631268655`, 6.857734870811047`, 0.7509249944976302`, 0.22671157472868875`,
 0.4788061070906606`, 0.1564030997457896`, 55.02740807629177`, 0.03469990388231514`, 7.492235364197862`},
{0.05296847355619744`, 1.6490117913903575`, 9.992115392915188`, 0.8066960527418563`, 0.1291629696144665`,
 0.33931322884091664`, 0.26435083615790894`, 660.4050522121521`, 0.10186522527726305`, 2.8696181659920086`},
{0.21365538200916506`, 3.0547845209089406`, 0.35609106729359574`, 0.8256521864420123`, 0.9506371395328554`,
 0.271042805312945`, 0.12766968769895812`, 68.7970381277751`, 0.05673347586404265`, 3.4610065999125617`},
{0.22385277301638717`, 3.365536903395271`, 8.935305056074029`, 1.445762062116566`, 0.43390325179453604`,
 0.6187135527847307`, 0.01939946413637202`, 453.1242214832122`, 0.13325485950688953`, 8.825528492983036`},
{0.26129739321020845`, 3.355909888714172`, 1.041845959949887`, 1.206440545734901`, 0.09347792890054829`,
 0.5333101115239811`, 0.015503351594481202`, 579.4832207194656`, 0.24065061389170217`, 7.475999528371727`},
{0.14172561423935526`, 2.161658858177276`, 2.5726304321806133`, 1.1364876244324849`, 0.9181535957758225`,
 0.5861373014149901`, 0.010418800942482771`, 101.70999367656657`, 0.1500483081169689`, 9.64604938537483`},
{0.06568219588241164`, 1.0305120784213448`, 8.720293141386708`, 1.4160313463775192`, 0.752508918071946`,
 0.31681781371940887`, 0.13417309715525413`, 209.13734568668048`, 0.22402731709711876`, 4.416848552940577`},
{0.27044630440719625`, 2.4698840490073675`, 6.955264919348561`, 1.0249819835130385`, 0.106623120735865`,
 0.6358905088319218`, 0.012311596840628138`, 346.23093318344644`, 0.07401305589494472`, 1.2937187987733016`},
{0.08403118541616988`, 3.378337092807227`, 2.524635889786053`, 0.8671019090417934`, 0.46085963082622694`,
 0.5847463933658021`, 0.04733165775893332`, 1030.6526695312073`, 0.10944158368617352`, 3.930070348109142`},
{0.11975601650409934`, 1.1540823534574054`, 5.101398975621729`, 0.9964575213162732`, 0.8596230582994027`,
 0.5017711629445061`, 0.09812020963113087`, 54.445260820005984`, 0.07947868464718594`, 4.273753987333478`},
{0.26353274820980593`, 3.2715371213501925`, 8.419438906869026`, 0.9426613525835754`, 0.1294979869931494`,
 0.5387601140015186`, 0.040849691062558216`, 744.2937861154428`, 0.1766059692159136`, 7.8822033078787435`},
{0.15837784563387913`, 1.590657770632209`, 9.4421417173358`, 0.8199017044479102`, 0.629826423052569`,
 0.2661387442145513`, 0.049173845467465206`, 187.50695430622352`, 0.06908425180315825`, 9.722920562989135`},
```

```
{0.18489841859634304`, 3.5401138358224227`, 7.262892362437768`, 0.8851173674042263`, 0.05104398233694862`,
  0.6826543300991057`, 0.13384148697112022`, 730.4506518091381`, 0.22533987085622137`, 8.188627124718394`},
{0.22773890477301867`, 2.1437195677585157`, 6.152335866192775`, 1.1180903919170035`, 0.02705103803804154`,
  0.3333465628704615`, 0.009170207790258475`, 2321.6700090659583`, 0.14789384405564093`, 7.237611958554762`},
{0.17605039997850958`, 2.727076411741753`, 8.728585319721596`, 1.1320082850991182`, 0.7355770210071702`,
  0.5402004706661186`, 0.09745706159326789`, 177.96581560566344`, 0.01922037387361028`, 4.980887347403458`},
{0.06947420594938447`, 0.8077718165326004`, 3.821443319804267`, 1.3891202366711977`, 0.6756008025235485`,
  0.23550127195203518`, 0.0653335798785588`, 483.3383501610001`, 0.09612644227220152`, 2.381497911922425`},
{0.23569362997389992`, 1.2270876586626818`, 0.9845589085102358`, 1.1982217261670929`, 0.43286649637666286`,
  0.634774301600445`, 0.09976341665112172`, 228.04937393006952`, 0.12724994607105566`, 3.0426000813607676`},
{0.12274586224015488`, 2.0471402784080235`, 1.4612976870222134`, 0.9456822010625827`, 0.8327879461898879`,
  0.22053943969717293`, 0.006767755909702022`, 174.3096643655477`, 0.1850505728558421`, 1.2537872990507584`},
{0.13491824188855928`, 1.669720674179887`, 1.3334401828831126`, 0.968437561771696`, 0.34828727193756603`,
  0.3135712369052611`, 0.22891096239253717`, 1910.612731597179`, 0.10930115833371806`, 4.270908360205038`},
{0.04199865921066254`, 2.754174936868967`, 5.53210979521042`, 1.3844956478579449`, 0.47136731805345633`,
  0.24068198696313148`, 0.022972136801125642`, 58.648848297948106`, 0.2240134714991286`, 4.479259216921056`},
{0.18074106663079648`, 0.9554759541683486`, 8.202625386518609`, 1.4769540911095849`, 0.5922233662884384`,
  0.5544055394726339`, 0.15090969809745627`, 4632.951795907658`, 0.16872394703920557`, 7.933658625009354`},
{0.22308930012837075`, 1.8877770902637643`, 5.727481376105105`, 1.290937410786533`, 0.5385704303274257`,
  0.17837528465279628`, 0.11591468148922064`, 2665.0058694088225`, 0.1309938954283596`, 2.3264420617360244`},
{0.16528939075200133`, 0.44637388983991233`, 6.000554224390708`, 0.7620462868306999`, 0.45157540745748825`,
  0.6293554847029814`, 0.08179042637541929`, 626.5470719450981`, 0.11922182895709121`, 4.5474321331423475`},
{0.12057640522465035`, 1.6899679349832946`, 5.872613897455656`, 0.8685022109314445`, 0.5819827862907108`,
  0.45575489869095254`, 0.005408595604133131`, 1249.3305456098487`, 0.22090492300977427`, 9.765554151588912`},
{0.26839288378136816`, 1.20431418513886`, 3.4264235210106797`, 1.1464020290887187`, 0.9129873318113406`,
  0.17958510842302844`, 0.03476022275277427`, 85.14929883968256`, 0.03637248443077806`, 6.177611921122705`},
{0.19312844950593372`, 3.9129912832786564`, 0.42277195731844414`, 0.8655327062249413`, 0.5847393470300801`,
  0.16029168895981472`, 0.1525854675774217`, 2234.1231120850057`, 0.03649943574663328`, 5.234397199033289`},
{0.05831506452479063`, 1.4447664488109728`, 9.399667906542401`, 0.8838396243177019`, 0.4494233408794772`,
  0.5909311609620335`, 0.2085880113243902`, 309.3416067223959`, 0.04816991376818669`, 2.147582543132131`},
{0.23965113012644185`, 0.9128974489725401`, 8.261297062012524`, 0.788417407034967`, 0.8016641172773833`,
```

0.35686628470413617`, 0.030635478621033968`, 346.1316581378353`, 0.043365488693742626`, 1.9545026373639716`},
{0.20520507007680205`, 1.512442754504547`, 9.386452795492342`, 1.0402158263719963`, 0.9284696718653804`,
0.3818637796049891`, 0.053241178903524394`, 3017.2611079460976`, 0.15062298010038172`, 2.3864513615272553`},
{0.20853248950244857`, 2.7801356391066916`, 9.335557605849111`, 0.8182981643225358`, 0.24140252954455388`,
0.3825831953385048`, 0.013327430792399009`, 77.96529661289064`, 0.07323986754942219`, 3.7008101112034457`},
{0.13576335201419282`, 2.033104701519708`, 4.843203210944571`, 1.3428754702553487`, 0.2671111595179354`,
0.18962864690949777`, 0.12183777475102511`, 1231.483599580866`, 0.14101782654161166`, 2.1508880203421494`},
{0.25963449652183795`, 2.4481737185281203`, 5.334705083491999`, 0.8989699854247848`, 0.7130966522497681`,
0.695912989145927`, 0.04220235704717508`, 140.4631283114693`, 0.11660454588716207`, 7.56536482611441`},
{0.17509339161516935`, 3.0306683644318824`, 1.9789343166711684`, 0.8960196738550836`, 0.2152496986108292`,
0.6250130758758401`, 0.02087454134280109`, 59.51435674676293`, 0.2018230747082378`, 3.546682887992379`},
{0.10593857456933181`, 2.955002391424368`, 8.212267239719637`, 0.932578721051657`, 0.3205115590912122`,
0.6246255275862143`, 0.01773467921096997`, 102.27924186693049`, 0.015490300413002644`, 6.09864277306975`},
{0.10583476133380088`, 3.0591620947997713`, 7.753926172578531`, 1.2866948671665401`, 0.7481949494801159`,
0.21582592530952183`, 0.02844379415675546`, 101.39583001744663`, 0.09278999192622717`, 1.6976300926057526`},
{0.23086787628538824`, 0.6622997055352342`, 7.183940408138384`, 1.353990838360309`, 0.25975387914696335`,
0.6772244715959861`, 0.055054708296352145`, 683.0312933373987`, 0.1367743885435413`, 6.244797760097583`},
{0.20388536464835005`, 2.2276602475557015`, 5.0708546023033385`, 0.8827779974574592`, 0.1554825605038257`,
0.2618026330626365`, 0.006155066765134505`, 1063.8834649875878`, 0.12744889948308613`, 2.149276150894316`},
{0.10738337044420926`, 3.7501964356372497`, 1.0776334746380822`, 0.9682422375974065`, 0.09998974406674432`,
0.504170653500009`, 0.22038799040453166`, 2051.555691372483`, 0.09521018879793502`, 2.016584418866474`},
{0.2588804970943782`, 0.5492314132783744`, 9.644794872912858`, 0.9613308813426055`, 0.2817033035077452`,
0.232857138958534`, 0.012136672860729404`, 78.78277994386292`, 0.023082056754425706`, 4.302644367094656`},
{0.08494913534264076`, 3.4349155581389494`, 7.0407634051690415`, 1.119369452553694`, 0.9329533821244145`,
0.5630698272627359`, 0.4838120213175008`, 71.45974855722541`, 0.09271025542437755`, 2.443770975165119`},
{0.2312360951475228`, 2.6472972630846083`, 4.924168333426426`, 0.8758299082609615`, 0.33581096057577486`,
0.16094039113701897`, 0.009823651093858611`, 148.81624524939704`, 0.12101804849376735`, 7.0744188173362765`},
{0.2359361250604497`, 3.266770315195857`, 7.411396165562348`, 1.2275925063587856`, 0.15344120088912327`,
0.536013355113413`, 0.3013372435966537`, 712.0245065436816`, 0.039861802547139946`, 9.042443794820379`},
{0.26580884355261647`, 3.957059703696273`, 5.486829732050921`, 1.3210508471658353`, 0.3282573265157698`,
0.6164824430541005`, 0.042206581416411024`, 114.97331259087073`, 0.22266675421103638`, 9.071401748075406`},

```
{0.24965572729559277`, 1.088568489897936`, 8.460543344934337`, 1.1505850947024536`, 0.8975822933983539`,
  0.5110185880609677`, 0.06613687599714729`, 1096.4114980052268`, 0.1003107894781316`, 4.101976455241168`},
{0.1942611669549973`, 1.9123387865104826`, 2.4307010631404715`, 1.2953041584793583`, 0.36588469810004653`,
  0.5550679926918506`, 0.01452356295191177`, 246.06581986793796`, 0.04070230178651557`, 1.4358727366404163`},
{0.20522561065351508`, 3.886130079640968`, 5.862633336878224`, 1.1071510042707493`, 0.46587070079395265`,
  0.3092363340879616`, 0.06237106286442892`, 3704.443052142306`, 0.2373271132764703 3`, 5.5948716983231535`},
{0.24537724051627174`, 3.881922170050893`, 2.190905545388226`, 1.4415825883984728`, 0.23029515887672103`,
  0.5607538040686315`, 0.02143037658176177`, 612.4231659367224`, 0.1895744022642167 3`, 2.1066205948825285`},
{0.06117754469042569`, 1.77753347893454`, 8.494748688583062`, 1.4528026068281314`, 0.13374021962928717`,
  0.2747744172973582`, 0.02769785108344557`, 761.9216006517844`, 0.1674213538626742`, 9.223974549251146`},
{0.2543463901897882`, 3.8184481545424687`, 6.435093510144283`, 1.0179149591357712`, 0.6456387658124723`,
  0.39719113288117625`, 0.03560663844887868`, 836.6099873310699`, 0.05172688927212099`, 4.4940967912431145`},
{0.25340300959745826`, 3.7481040027914663`, 6.564583042000866`, 0.9665595115932711`, 0.6900722172611409`,
  0.5320596915161446`, 0.007953381223150922`, 557.3521763213881`, 0.16633866213427612`, 7.2685179596650595`},
{0.21905085798311597`, 3.1393917093979224`, 6.344370247647537`, 0.9036844621009067`, 0.45657431516696256`,
  0.6738956741194089`, 0.01713634873689236`, 595.7124001741428`, 0.1327223790362297`, 6.393427116779321`},
{0.26756748312672074`, 2.582420366138944`, 0.22100971269994044`, 1.2986139421663903`, 0.40694219440887625`,
  0.4206092167171217`, 0.07540978051777308`, 286.7043124505423`, 0.12484831726628037`, 7.450517421109604`},
{0.1649279191778415`, 1.2798961157951103`, 8.443353077105307`, 1.1073246765756934`, 0.5543130008347743`,
  0.4677559431287117`, 0.04549043803104127`, 88.19757502008439`, 0.08859698434873292`, 4.4693006714200685`},
{0.24671033429293737`, 0.7439602453864245`, 2.8830481632165945`, 1.0433060628070765`, 0.2605457162416671`,
  0.35613575939670206`, 0.02699949405773168`, 552.8129873973498`, 0.15941770646667952`, 8.472285283746757`},
{0.15869950794106824`, 3.1033559606377414`, 9.144330738839969`, 1.4178115100024233`, 0.3048291795319098`,
  0.2869274730275907`, 0.17534043095376647`, 52.531250426358234`, 0.14088030152015352`, 4.046496400392222`},
{0.2764621058346162`, 2.9381918751992178`, 0.9446276562373105`, 1.4121699137634485`, 0.5065538844804354`,
  0.6085910850890284`, 0.12806524333884275`, 90.5764532701865`, 0.169606806379912`, 4.122343563126204`},
{0.23050133288232844`, 1.3238077162346595`, 3.1108482993453865`, 1.1555015486294062`, 0.6747942262463269`,
  0.6285668584610278`, 0.027391537177886824`, 109.70658223761104`, 0.027210691652561736`, 1.9248854769177905`},
{0.16511179970471734`, 1.257711050374506`, 7.3270141910702415`, 1.3219934358839476`, 0.5519839493462388`,
  0.6927265248357952`, 0.057669326830527015`, 282.0308127392517`, 0.18961708461999577`, 6.119113848125975`},
{0.18051596911200918`, 3.1154638731836117`, 5.271301524292921`, 0.9821224785779399`, 0.579876513557519`,
```

0.2578243252832043`, 0.01324703728258807`, 298.19257336110485`, 0.04626579120349833`, 3.252038865320179`},
{0.2700603276661351`, 3.8874233694867435`, 9.935191315459868`, 0.9369398631417749`, 0.9680175802467335`,
0.2817307366148821`, 0.03116132832319949`, 267.3428425281449`, 0.2310886914635361`, 4.719763737676903`},
{0.2505990837413898`, 3.9386610341401305`, 4.197732331385515`, 1.4863985983426793`, 0.6450186838764853`,
0.5983146588433161`, 0.0058599277111039`, 4347.60789332823`, 0.1797443484893408`, 5.002549103667693`},
{0.15385799276986173`, 3.328452062918277`, 1.1907303286310018`, 0.9499432416831306`, 0.24618506024325426`,
0.6220480490433855`, 0.08397939385457624`, 160.09510109398894`, 0.21511864519397922`, 7.34323009363505`},
{0.10621732051626104`, 1.365898117434102`, 6.864395407461604`, 0.9825773772303991`, 0.8367483775504436`,
0.5624371522540671`, 0.07041397408927531`, 60.72834180417396`, 0.20758521960363197`, 6.929584578355666`},
{0.2268285366732684`, 3.377330494378361`, 0.792106071900994`, 1.4321394301198889`, 0.4771428999999012`,
0.6107585820948693`, 0.0427110994331284`, 2178.207920314213`, 0.06460930790004932`, 5.987666522418898`},
{0.27738294555080567`, 1.5184858354681863`, 2.5078605202307482`, 1.4658418288357904`, 0.7951974935096349`,
0.3102987260459962`, 0.028082255793227213`, 4738.743053997216`, 0.039514889508313555`, 1.270204126939964`},
{0.12722141666194153`, 2.144800891916389`, 0.9267204145449971`, 1.474327309271881`, 0.07507296673368935`,
0.4110764361383803`, 0.01396212306727472`, 341.96044988390173`, 0.08796076167353961`, 8.378052186889676`},
{0.14199823373416764`, 1.4769393574961134`, 9.82023825383516`, 0.8826223667346553`, 0.41403084809983204`,
0.6165765653923829`, 0.060760206852004485`, 76.65984772713675`, 0.1884299459763023`, 7.821991164404671`},
{0.2704769900712498`, 1.6373927417773038`, 3.0886177841931914`, 1.4817168381054997`, 0.05399471731043759`,
0.4806448506103316`, 0.008090658829532001`, 56.76295821829396`, 0.22108849119145935`, 4.3771215003685064`},
{0.15635355369607173`, 1.2350233440589857`, 3.2938536959067304`, 0.7696047462951592`, 0.9490314349758091`,
0.5590905433465692`, 0.008743912631106248`, 94.37874467585033`, 0.17474749461052458`, 8.353315585312398`},
{0.16339273559725687`, 2.0059391082075457`, 1.9960227683476044`, 0.8832597488866586`, 0.2201232888636404`,
0.41940637033719763`, 0.260701203173729`, 668.3313383270491`, 0.16081430237854888`, 8.573990161395482`},
{0.17131668518162624`, 1.9273467762184806`, 5.930228637778157`, 0.8979121960709446`, 0.761163722566164`,
0.6467717629026779`, 0.01019608706680365`, 260.442341207733`, 0.11039361670332914`, 7.204688135978646`},
{0.11942926109779373`, 3.6640895220352094`, 2.8978096019149504`, 1.1395608488550686`, 0.02531057599926112`,
0.3982329172386545`, 0.26228943719870584`, 89.66215495367044`, 0.22730124409898161`, 8.643250664580492`},
{0.06995397995088021`, 2.4465420953300523`, 1.0825256418482638`, 1.4045898231548657`, 0.8050454938859057`,
0.5373350569389552`, 0.3667462640330922`, 406.36701228872533`, 0.024784828878876453`, 8.741309230400322`},
{0.25135395656732756`, 1.8293827828852027`, 0.6280251816297326`, 0.776655925877336`, 0.712342015777852`,
0.6529270906121196`, 0.026921744082008515`, 2433.232128346219`, 0.014846384799260759`, 4.779446799164496`},

```
{0.22686431298761822`, 3.544454161776195`, 7.257561779040259`, 1.4835566223233323`, 0.41530778886586006`,
  0.5693420006079292`, 0.0281206594086524`, 300.82869963510313`, 0.2233455291441253`, 1.8496714258079727`},
{0.11505780978488611`, 2.035276516222745`, 0.3473218826840405`, 1.3302263404025527`, 0.8863987944255065`,
  0.20525449010517516`, 0.166947674879067`, 1822.5924091420022`, 0.2368153750944742`, 8.635263957438244`},
{0.26450836027154073`, 2.000822664278666`, 6.699190267572199`, 1.2017845913999912`, 0.3438445280396565`,
  0.6571515800951808`, 0.025420529665258214`, 670.8719376728277`, 0.0689542698910992`, 7.858447976519758`},
{0.1895156330025849`, 2.3307833365396586`, 5.573081915828208`, 1.037167544233136`, 0.6437280244785568`,
  0.6985848360999776`, 0.020283589241000377`, 257.1653991038853`, 0.09483519874959423`, 5.443513900146426`},
{0.05605442916152037`, 2.091827585999483`, 5.134093728882359`, 0.7852275512421112`, 0.9528052242020535`,
  0.26916872175155115`, 0.16382211865694157`, 830.5018741809612`, 0.0523186960509337`, 8.350099727317033`},
{0.160097742020257`, 2.0916642582432887`, 7.779572730473025`, 1.4293729759208196`, 0.262481852877007`,
  0.6066507214539851`, 0.010299825795425889`, 166.88640436294955`, 0.11119499215436468`, 1.1831617215189443`},
{0.096645259209576`, 1.264318941007299`, 4.097121191163998`, 1.119171761441141`, 0.3848067085465605`,
  0.38298305619587747`, 0.22103546881327146`, 186.61459180270506`, 0.035388155328102466`, 2.6745014933563738`},
{0.08994789013179427`, 1.57009542864993`, 6.7950768142652755`, 1.2471941772808341`, 0.17502080201658954`,
  0.4646155607499266`, 0.031711372215814826`, 510.77004725104206`, 0.22094423048581935`, 5.498912040996139`},
{0.09559160148361812`, 1.3237772094663507`, 8.951678422145957`, 0.7806574543205589`, 0.8827653082752778`,
  0.45488871998545977`, 0.011151733542884613`, 3151.7776331194414`, 0.22534056788365686`, 6.821338260162536`},
{0.20804533970301103`, 1.865488871682949`, 2.4561594295175926`, 0.8614398462681598`, 0.7232950760420711`,
  0.3427980526897182`, 0.02113398228875948`, 356.75904569388376`, 0.23964513558905903`, 4.163032360670947`},
{0.23422673966465207`, 3.962742244147649`, 9.344278992351082`, 0.9436428211653483`, 0.6202800294740252`,
  0.15583995298414144`, 0.0468134491479599`, 1145.0934679847246`, 0.11614669946806178`, 8.273469182440888`},
{0.23911413452544644`, 3.701379132317136`, 3.3596187018850023`, 1.2070387760959833`, 0.14815711868787962`,
  0.5552188532365824`, 0.010060549211211993`, 324.2801725236705`, 0.014653386006449387`, 6.641284984634726`},
{0.16379493488608782`, 3.773301948253276`, 6.66686177778092`, 1.3720180958436647`, 0.9115747518300852`,
  0.36809943749279594`, 0.006089940921734969`, 188.41929546062013`, 0.11766502525697531`, 3.8904832664355844`},
{0.27173801863077446`, 2.1930338431922545`, 7.869758966833125`, 1.0214571559787395`, 0.457397629072652`,
  0.6590886785471264`, 0.009120521470678952`, 4004.0778102470904`, 0.1596904202504179`, 8.692703920363371`},
{0.2361473928644357`, 1.7414292069064263`, 7.944932185587515`, 1.1578150203530142`, 0.6270752096378571`,
  0.30275674150027165`, 0.11839453780639704`, 3486.2324040163485`, 0.1006900893181421`, 6.697770970769804`},
{0.08448906159522324`, 2.163318965633623`, 2.5852320656449397`, 0.9870547358990118`, 0.27911648709335135`,
```

0.1950863385031143`, 0.04472304557991584`, 1858.3083947017237`, 0.05117109151128901`, 9.531470241589851`},
{0.1142146683211071`, 3.422389653131356`, 1.8177299602895634`, 1.2969315528452774`, 0.1391750354375907`,
0.5170758706651055`, 0.01897326911835137`, 87.52693577556593`, 0.11000871045324723`, 3.4664980752399295`},
{0.14347242790835574`, 2.011431525472254`, 0.8946353742157331`, 1.3818589873326788`, 0.974530392330166`,
0.4363768778472087`, 0.006586014840232095`, 142.93650600689625`, 0.2189385420017279`, 6.889239014732425`},
{0.07350179944910268`, 2.001951652427108`, 6.856579719843172`, 1.3883617808830775`, 0.83650706130597`,
0.6246458892820506`, 0.03443555932639553`, 159.6804633773726`, 0.22561794329975743`, 6.769156049603524`},
{0.2038713609791254`, 2.9048730428426977`, 6.578839990238183`, 1.1970767733981913`, 0.3047670438201835`,
0.4988995484689318`, 0.027219703475022124`, 126.68486054839573`, 0.1069997024432573`, 5.709865936189522`},
{0.10518373092933642`, 1.253339618555911`, 0.9435760784539173`, 1.312750209379657`, 0.30938419913876847`,
0.5412461470886345`, 0.010398682812166098`, 560.2137041073427`, 0.038740677468453555`, 9.486718448691846`},
{0.21800976245075693`, 1.265555352818649`, 7.66080710349685`, 0.9976040672991493`, 0.42066904207729006`,
0.6052137627060103`, 0.1434637338398527`, 296.0621032273413`, 0.0508815854649306`, 9.05489394101457`},
{0.14099839999887143`, 0.709118554874764`, 1.895334494340693`, 1.0269112445912816`, 0.6721979672345315`,
0.244304954855444`, 0.0716581941489167`, 50.83934571528565`, 0.0295628703988986`, 3.2095234353654245`},
{0.16175267254619935`, 2.3816849613140647`, 5.017466007223192`, 1.1449585227321033`, 0.0709392075018902`,
0.21629140918390133`, 0.24748736884470657`, 967.6089395954832`, 0.2384053881049194`, 4.566103496741098`},
{0.15219039437367055`, 3.36973706117323`, 6.4489465173864`, 1.4529455229052564`, 0.15905243014518256`,
0.19120675078037952`, 0.09107249936889779`, 1785.557342737041`, 0.07266026496560668`, 8.481021831493972`},
{0.1147282820108807`, 1.275793939396646`, 2.8627986987042053`, 1.2289286563020643`, 0.9300053983658252`,
0.5333937455451996`, 0.40966617997380966`, 55.29536930365472`, 0.09207723861615985`, 9.133686457942215`},
{0.22214055647639447`, 3.77956922277194`, 8.844837651533133`, 1.3011494481281731`, 0.35962071678441987`,
0.36854620389209614`, 0.030699018263752917`, 153.2818434470183`, 0.18511332787655327`, 9.937679092387768`},
{0.11158828176697494`, 2.207019844443386`, 8.337407964588238`, 0.903037166955644`, 0.0492331295750732`,
0.5332115600383506`, 0.10225753888572825`, 270.6598424430625`, 0.11536791182647016`, 7.224089837931905`},
{0.11228066784233581`, 2.8383409912516155`, 8.777526606092515`, 0.8243326677721828`, 0.7215210925398621`,
0.47575335568293875`, 0.01179892034723288`, 1881.3977477117185`, 0.1350202375088484`, 6.891832227766253`},
{0.18415971316226493`, 3.627641157385548`, 4.072786596426541`, 1.1318163513169097`, 0.431227341960456`,
0.35638232246082124`, 0.12315140656794354`, 98.11903649760767`, 0.20630818704214687`, 2.4221856850573946`},
{0.2708373028347817`, 0.6873063866109219`, 4.963135106708849`, 1.480469510663712`, 0.1951402154410362`,
0.5673083531043152`, 0.1042302621285267`, 2550.63829867399`, 0.08286966503737198`, 2.6901867167370597`},

```
{0.2248876472394919`, 0.40722939312005924`, 9.89488647226247`, 1.363579526182362`, 0.9039905300116995`,
  0.6204673017769224`, 0.16140635189013572`, 124.79693831214993`, 0.1938029988590731`, 3.2922153328074497`},
{0.23989116476268346`, 3.6056249656972357`, 9.989783431378257`, 1.4358686490541388`, 0.7020019529209951`,
  0.17871498413227982`, 0.15143540034751335`, 1116.6227738463333`, 0.03611482374706981`, 5.09835156260786`},
{0.10260002089245757`, 1.5297774189947768`, 6.3178804183723365`, 1.026166177715942`, 0.11867434322817161`,
  0.5793673959868294`, 0.011727656957059851`, 115.5782430006307`, 0.20678944231462698`, 5.80216317155719`},
{0.15334449354379004`, 1.7605906069617427`, 2.299359928311352`, 0.8222860702278779`, 0.9712651951139977`,
  0.6108690739001781`, 0.14744839931335676`, 2744.2788694525566`, 0.195814559949110 5`, 2.932280984329149`},
{0.19466496130115485`, 2.7024357312795884`, 5.257508792652555`, 1.2867770062917014`, 0.4348182682381361`,
  0.22799121214772633`, 0.23891047775514818`, 1782.3644894856986`, 0.18533305313279363`, 5.119329477376578`},
{0.1432020102164725`, 0.6405991610607145`, 3.208206356969493`, 1.3290628649451517`, 0.9481170196997712`,
  0.4490165359974049`, 0.04841648328555665`, 413.10755212325614`, 0.04750626711264738`, 5.496555870600891`},
{0.2767015708016651`, 1.3448440647834508`, 8.399713367158164`, 0.7521061389207052`, 0.4173808724493866`,
  0.513702172075632`, 0.342860965595718`, 77.17931109285855`, 0.021177395775830554`, 7.103551277505744`},
{0.2397400236888016`, 3.6266796947004982`, 2.6465237726626594`, 1.459013465973801`, 0.6743470274003898`,
  0.6381986185617847`, 0.04750041433271958`, 90.95305912243636`, 0.11079011330617267`, 9.85823320912452`},
{0.10671756328879578`, 1.1279363098789865`, 5.068949642759208`, 1.0745694995138004`, 0.5897325137042213`,
  0.3714383645112781`, 0.14529484820960328`, 91.27351773968599`, 0.0653436372538082`, 9.206661650127103`},
{0.2358453438636856`, 3.7679680180462416`, 4.640233719273176`, 1.297156455219631`, 0.3876479327909328`,
  0.3193535012332763`, 0.027047144947079417`, 401.3110605008155`, 0.0397014782942956`, 9.539243086069693`},
{0.07226742189042767`, 2.8421547517397343`, 4.74887948268691`, 0.9400141356818887`, 0.26271764199006165`,
  0.24510878200647013`, 0.011565327673117277`, 79.9069988164106`, 0.11613362579809078`, 3.4810370415732024`},
{0.19794910251471837`, 2.250622511000005`, 5.810822485387694`, 0.8325726726239642`, 0.42888216834124404`,
  0.3003167364214152`, 0.2221971352698835`, 492.4997381844193`, 0.06654773222193389`, 7.816458771254976`},
{0.23749910377351813`, 2.442156143626838`, 7.627688188064528`, 0.9684475529781722`, 0.7107198130457415`,
  0.2572554624659992`, 0.4475955253238462`, 132.3529645103848`, 0.18259209603279808`, 1.6799945249508887`},
{0.06924882755772316`, 2.8414116897854322`, 0.5206442594268132`, 0.9917884594202446`, 0.6526760474283868`,
  0.5988068411525457`, 0.47108712727434415`, 720.0807499233221`, 0.11578003411625926`, 8.400733392823671`},
{0.06824086699485615`, 3.3015665983523297`, 9.09230580657946`, 1.0780523992371156`, 0.9189494010575767`,
  0.47997916674377217`, 0.09047840499022508`, 3815.186560881826`, 0.152329057733407`, 1.5202594192596113`},
{0.23257418904935773`, 1.8011483887717636`, 0.9612178256197179`, 0.8025101750942898`, 0.31352490932039023`,
```

```
  0.2355458085864931`, 0.2680725991098069`, 2738.209295799684`, 0.0662676878204258`, 1.8613360761320532`},
{0.13474024315490335`, 3.3704964680175626`, 9.170639775134308`, 0.7502511885050491`, 0.6264710065934431`,
  0.21904625510680875`, 0.040564870634629845`, 2527.7664803980215`, 0.19403763861714918`, 6.378419170081072`},
{0.2693026298173556`, 1.772983767924213`, 1.344226083033309`, 1.4113484218782142`, 0.12593618066908396`,
  0.656520191233777`, 0.018659842469751033`, 117.56506476136576`, 0.1712196470744854`, 4.023445632685666`},
{0.17866994815918202`, 2.442046867777872`, 1.8194825512463648`, 1.2285671890230836`, 0.7081083864311548`,
  0.17566313743095552`, 0.006280075202364708`, 2604.6083474637735`, 0.1295692924324935`, 1.862340891527415`},
{0.26090114261485403`, 3.9086029270457496`, 1.920295645800332`, 1.1838686314265923`, 0.4729800090210985`,
  0.36383922429133153`, 0.014536748208445867`, 79.30674088085132`, 0.030053561477188684`, 7.670997166386538`},
{0.19544602681310375`, 0.41028512203974055`, 4.6003645589109095`, 1.2671666166106164`, 0.8219059973725353`,
  0.22911191370711304`, 0.03702179299429459`, 318.28074751099626`, 0.09079865705400514`, 1.4662081657512314`},
{0.26537062770714803`, 1.229239774996202`, 8.744773690909977`, 1.151653436583517`, 0.3537059210373261`,
  0.2894924335103778`, 0.13068544785366612`, 312.2140250313751`, 0.146894495801125`, 5.715731252046433`},
{0.18712917295151626`, 0.630329154174047`, 6.232729389529142`, 0.7582043174026267`, 0.7607866373637777`,
  0.2530331438958473`, 0.06090476948059027`, 64.21018866863231`, 0.08418997914362486`, 2.694793972266142`},
{0.19413873235593193`, 0.8072205389170897`, 9.684031161687852`, 0.8518820755271745`, 0.2120317281227424`,
  0.1532879757233685`, 0.02372050181467318`, 987.8329384880758`, 0.08094704090521981`, 8.693316755778081`},
{0.0816546741022599`, 0.4036812381177306`, 0.7902893788690406`, 1.4273906025641667`, 0.1897112398644374`,
  0.6362443390402239`, 0.007446156154142883`, 176.61944749055874`, 0.10188842005948545`, 9.51311671217389`},
{0.2491419431624458`, 3.7694376618574204`, 4.74673147811253`, 0.8416284883026488`, 0.188731240527098`,
  0.3192559789027146`, 0.07121130413051815`, 671.9000833119555`, 0.02760758509345429`, 2.5790410082926702`},
{0.11168227968802413`, 2.173584861364678`, 5.640951633995167`, 0.9377704673639422`, 0.3315982783471285`,
  0.5266083888319896`, 0.005432939117039627`, 621.842609802462`, 0.09631877509870435`, 9.43919460268938`},
{0.15970197279447784`, 3.9587696847183347`, 4.0238996390694854`, 0.8027865138486989`, 0.03588289413016432`,
  0.35386710298208024`, 0.016574217538633067`, 2019.1576160982734`, 0.07473946383041635`, 3.5352801788382404`},
{0.2064322311255224`, 0.43645383124552994`, 3.8213880668877356`, 0.8257710590869434`, 0.08868496943554072`,
  0.17616539751645888`, 0.04042707492740236`, 2037.3062213977416`, 0.05324630165142419`, 4.6107951341494555`},
{0.1394617695133732`, 3.387614469631334`, 8.352661875580207`, 1.2219251402369191`, 0.6325442693826859`,
  0.17624447718949754`, 0.021853560156019736`, 288.68541357569393`, 0.164910064915528`, 5.7766720481305995`},
{0.10599692192016741`, 2.4119661618453145`, 1.4892081676893856`, 1.3031849980783952`, 0.42063329504650815`,
  0.5860601104625098`, 0.3077592160131421`, 52.52666428206092`, 0.19647413521024198`, 3.2052596491028216`},
```

{0.24388484229914548`, 3.2519594625966253`, 4.312340316913895`, 0.9914403572709836`, 0.29358995419342615`,
0.1835819110057716`, 0.13612159308161606`, 1157.8864188899545`, 0.08218618541111161`, 5.046821007659727`},
{0.23494551018662208`, 3.3331099682410468`, 0.9744758817090952`, 0.9989991912838305`, 0.20118476247466943`,
0.3741832660739852`, 0.07385095150097958`, 91.01998469145919`, 0.19994684540705993`, 6.360950891328324`},
{0.23944024111665796`, 0.7499363805955559`, 7.775454975750338`, 1.268235612825383`, 0.3786721827881383`,
0.6740341016595366`, 0.11977329505957622`, 117.53909816274609`, 0.2108164269301916`, 1.8777741577958924`},
{0.24513403447625742`, 2.0133471925570054`, 9.940759001187548`, 1.083812287959604`, 0.5683273089881642`,
0.5900825394859205`, 0.005339720116331014`, 58.9416557608406`, 0.03145644437596545`, 6.28807827028038`},
{0.15958822823144275`, 2.509825654597928`, 3.728427574062339`, 0.9294074209823535`, 0.01651867581778177`,
0.5367648350921979`, 0.015785937236593408`, 685.9822133189093`, 0.020860236554209932`, 1.2753268301578835`},
{0.06806026871424425`, 1.205607754446362`, 0.7536813502622586`, 1.4624982795129167`, 0.7076540801030038`,
0.15470161284122685`, 0.268116863806763`, 324.06126360879364`, 0.036033823716353997`, 3.8267580932938614`},
{0.15319733389695195`, 3.558603911914262`, 4.579050417046558`, 0.9321187332487358`, 0.6642338797631109`,
0.569667348599446`, 0.2858050889038288`, 341.1212503566884`, 0.20343887540165378`, 9.501626804091558`},
{0.15071537712057487`, 1.2850575528534174`, 6.479674501252795`, 1.2297249014764047`, 0.051196968811160426`,
0.17856837950014437`, 0.01824538414392949`, 77.70419036862091`, 0.224107560352308`, 8.31176551046438`},
{0.24584690511060947`, 2.473478204800995`, 6.676188280246508`, 1.4832363218386881`, 0.14420161768506268`,
0.523836460034543`, 0.04402629271492787`, 360.52310897688045`, 0.0850887320320231`, 2.528306265858081`},
{0.14137397451874373`, 3.652676161056461`, 8.39527364337399`, 1.0080745646578813`, 0.09190129083286003`,
0.15081167091134762`, 0.35462309792221747`, 1217.6311173423508`, 0.09724487810479643`, 6.223920941944753`},
{0.04933630800724648`, 1.8511857532828166`, 5.885062761777403`, 1.1138733840213622`, 0.4607664856084661`,
0.43439208322360623`, 0.018761076496074794`, 3507.5800668673905`, 0.21191062303011793`, 3.3573407631201917`},
{0.2339008817663396`, 3.669675022809618`, 9.160989469851096`, 1.2207724907901325`, 0.9659294978123167`,
0.3234992643572032`, 0.05037324961884807`, 93.86107259786391`, 0.14430488254373952`, 3.9894219201696473`},
{0.11569404259913263`, 3.2009969772206803`, 2.2264401820433353`, 0.7539773410821206`, 0.3237735314376855`,
0.22021748910844674`, 0.21956604535044472`, 1445.3927245947305`, 0.06533194774972478`, 1.3139082141801541`},
{0.1426983247121278`, 1.4581176174915065`, 6.531549332871599`, 0.9310047864665083`, 0.893336243428055`,
0.18377286371920176`, 0.08056837661087574`, 664.5727162634348`, 0.04347044784294363`, 7.864371974772275`},
{0.05831687901068116`, 0.732484500007835`, 9.716813224212896`, 0.9062398770267781`, 0.045816647832010116`,
0.25112700202789096`, 0.0489500536916562`, 2141.8470035057708`, 0.06373448098002471`, 7.679073247267311`},
{0.1624041267001165`, 2.3409738007594383`, 7.927758092789709`, 1.1108935833128895`, 0.2655878375527121`,

0.22381693809974978`, 0.10055816102060769`, 86.2207746546923`, 0.23778527923179793`, 1.5984483564142007`},
{0.05796853524365786`, 2.115435321939346`, 7.135826377560314`, 1.1217856002992745`, 0.9949497841824515`,
0.6493572179052653`, 0.16432568991023785`, 205.88622896743587`, 0.07308989915497993`, 5.091714109271411`},
{0.09343457742759681`, 3.0751406184941388`, 6.999926605820257`, 1.4764339149831611`, 0.27064650694064607`,
0.6662559245780291`, 0.012835205507442616`, 1884.4110587763687`, 0.01681160009525748`, 8.661215052575166`},
{0.23943678973010468`, 2.920807454124679`, 1.1099817114095707`, 1.3932739733707113`, 0.44392785132951373`,
0.17254202331513768`, 0.008938503858702969`, 2712.044809794402`, 0.1652368227234951`, 2.8738798121545877`},
{0.176566928771 6667`, 3.4304813087836825`, 0.9837478487319995`, 1.1028599055297788`, 0.8616178142897495`,
0.24038775568738768`, 0.03874939820930588`, 2380.99712756261`, 0.18505877859540681`, 3.338709015344154`},
{0.23886394100929725`, 3.974888040813318`, 7.275822510559582`, 1.2870566617016084`, 0.12273034821485096`,
0.6651538290269186`, 0.16897919530659566`, 150.6876048176701`, 0.1338282848735754`, 2.432758352876487`},
{0.06973130535662914`, 1.4096929441217387`, 8.65071450995816`, 1.181254696978707`, 0.5406204719849208`,
0.2725259583108167`, 0.12003736669178822`, 4806.091595362366`, 0.20844978312325513`, 8.8784124085225`},
{0.04409294567306185`, 1.3468266911815716`, 8.46560672493381`, 1.1221333539921368`, 0.8074609652543419`,
0.33934404303057597`, 0.1090535991677299`, 2100.6487939981484`, 0.1955313854386307`, 6.974828032327105`},
{0.08301734541584582`, 0.4950033643125962`, 0.3447103403996028`, 1.3973171280993089`, 0.5174429014299606`,
0.6090072510200681`, 0.013328621450736109`, 78.08408450957157`, 0.15235476028821748`, 7.41363923833616`},
{0.07508111728102967`, 2.9328515590120503`, 2.9247828964602327`, 1.0997610261500241`, 0.15381049135387959`,
0.44875941041801015`, 0.005374403719665632`, 955.806314296473`, 0.04180941570568808`, 8.210271230243798`},
{0.1868411586163749`, 1.4148901359883457`, 3.2886483363036496`, 1.1024292265597047`, 0.35071605335062417`,
0.20216786873751635`, 0.033694215571138546`, 50.84055962824541`, 0.13756443231160648`, 9.762651891575405`},
{0.25728291653412544`, 1.1620186239014583`, 0.48798465750503445`, 1.2217305465551163`, 0.36537296293338284`,
0.2112385814144826`, 0.1783844678719564`, 175.70497466833672`, 0.14885286931859365`, 6.164090300210196`},
{0.13759840983832694`, 1.6824227355669867`, 8.881204796952819`, 1.07728991023014`, 0.8275568317133928`,
0.2913709338376398`, 0.47392581861361016`, 1028.758263171597`, 0.1629375199726112`, 7.630724789672264`},
{0.22999415874686646`, 3.253135821240728`, 9.26678168881324`, 0.8937302072810551`, 0.5038748821521797`,
0.2035592770142407`, 0.010501376095491947`, 229.44271937909937`, 0.14963151143698977`, 8.069758108498657`},
{0.09925066924077935`, 1.4815445119155024`, 7.881183919866995`, 0.7663286317492166`, 0.2976933875506338`,
0.33062028037546887`, 0.05105854284999101`, 168.2571992844445`, 0.22488226539851913`, 5.574589576514775`},
{0.20983327896511272`, 1.8859920859197894`, 0.6151282764886545`, 0.8412663476815937`, 0.027122934645615437`,
0.6844735714100607`, 0.0125400797815568 5`, 549.5308557472557`, 0.10371695352022153`, 7.780558961111437`},

{0.2225389666164696`, 1.2827559929647245`, 1.2157460525678818`, 1.1336603904726232`, 0.21910751958531538`,
0.6021275716391656`, 0.016887660465617623`, 151.92995003475355`, 0.056506174885467386`, 4.721491520685097`},
{0.23629660338151143`, 1.375829581220887`, 0.7878883261410508`, 0.9498849347602389`, 0.6605330073956415`,
0.33733697402858687`, 0.028977316405205664`, 446.06430846982937`, 0.16303388369839966`, 6.718018447521894`},
{0.2240884195389573`, 3.0168075094859823`, 9.22253837134286`, 1.0534395436787098`, 0.7253455428103461`,
0.1646055270291652`, 0.006115122680716056`, 258.28718729642685`, 0.11665954601209721`, 2.992281031623701`},
{0.04820076342563051`, 1.9965427255473651`, 1.4646173572921608`, 1.475676204663891`, 0.346581356777814`,
0.35258214744905925`, 0.16114708977216574`, 53.64321165141119`, 0.1945121008436112`, 8.557828905511698`},
{0.1395588171486265`, 3.3167971972296737`, 5.120579942113229`, 1.4414502422836475`, 0.46235265068089726`,
0.21590338795839814`, 0.018401804903068468`, 321.8138540224943`, 0.09243860589675884`, 1.0657915725386573`},
{0.2085065587668823`, 0.6294064238759702`, 7.1150002800734775`, 0.8698405330489325`, 0.07589384997930004`,
0.6271818704466325`, 0.014863365323890152`, 3717.018925299029`, 0.09558183779388002`, 3.993723504265942`},
{0.11579616742063126`, 1.4637518716217537`, 6.091821149634162`, 0.8701973607843327`, 0.580668178059593`,
0.4892081276931839`, 0.43069272205186815`, 58.104542288448926`, 0.09639134486345691`, 1.0613512257562903`},
{0.07650954660329923`, 2.451622188741575`, 3.5124331635719983`, 0.9208132913481966`, 0.22417245589301404`,
0.5962277485405788`, 0.1631058588288524`, 149.68967360762312`, 0.15724921240628176`, 3.991974201007654`},
{0.27715040216901043`, 3.1093705724520717`, 5.602120685872263`, 1.4185083740786066`, 0.20116695432127574`,
0.3482455682148583`, 0.014646070094414987`, 268.87083253973395`, 0.19497961893886057`, 8.247252623819538`},
{0.10373802184314534`, 1.9484823746249935`, 4.344672894707308`, 0.8659474233939041`, 0.34328087466446`,
0.6599529078817681`, 0.006604951530776707`, 74.02272771702236`, 0.12108236246583282`, 3.25882057231545`},
{0.08995654352927362`, 1.6673082382989595`, 4.04257302618344`, 1.1477945472560402`, 0.1500209798917984`,
0.1761016570773115`, 0.012888472951564884`, 1050.2473441342943`, 0.2022489804478793`, 4.465566609444316`},
{0.18428208667431017`, 2.663013532673764`, 4.442362759588184`, 1.3723983485913218`, 0.7325310003414629`,
0.5418182340232052`, 0.027460635126683182`, 53.281000517524745`, 0.08334630855570291`, 6.274365305120075`},
{0.1326476876438774`, 2.8731759222440596`, 6.672951038295707`, 1.243076119232064`, 0.9225218753338189`,
0.23536778742386455`, 0.07924576221140303`, 2119.223051638056`, 0.014542489730864222`, 5.05029094558817`},
{0.16449021574582717`, 1.5264131018768046`, 3.118813817198866`, 1.0873075186223824`, 0.9114527670749883`,
0.32623274128336255`, 0.005473446059082923`, 2600.919647538226`, 0.12372800503579795`, 1.4807994816594086`},
{0.1059475440552839`, 2.4614128361434053`, 6.764865578702089`, 0.897041430377437`, 0.5452264302150955`,
0.6377616415175531`, 0.031109305885935246`, 290.7153568287518`, 0.12025877488242465`, 1.854625189562679`},
{0.09376889465512256`, 2.694562234128588`, 9.901749913963968`, 1.2671081853876889`, 0.9355728957697818`,

0.41426328356338693`, 0.1676359905682518`, 324.1423914220847`, 0.048114260882103244`, 6.992923371816153`},
{0.07408607058446992`, 2.173441202450359`, 5.83110243938874`, 1.2671639264693393`, 0.15034130588796946`,
0.26151521374964837`, 0.00796428041791113`, 3349.7895146720416`, 0.06475249420724477`, 9.113413370060233`},
{0.2682300613650819`, 3.6108652762368214`, 9.881761872038165`, 0.9825580258076282`, 0.9296545706074566`,
0.25219322618587503`, 0.024464898005065273`, 1815.4892808719587`, 0.08994453092924143`, 7.425857575199714`},
{0.04962174488386037`, 1.9694465660050104`, 4.880585401279637`, 1.2957318091391512`, 0.6289429156960646`,
0.36977064682826033`, 0.006935918860683708`, 414.38904513183815`, 0.2065379649808241`, 4.444261705692702`},
{0.2594824517719198`, 0.5971319663647825`, 9.453687965953478`, 0.8401327653868795`, 0.7997988949991743`,
0.15899752713279824`, 0.005075100457461465`, 409.49724454586783`, 0.2091478816442816`, 4.600741868044251`},
{0.1975048020318434`, 2.1175350807064808`, 9.527018017184652`, 0.8875453366861839`, 0.2770809005507884`,
0.4437813967164592`, 0.08906290963263506`, 98.18685485298634`, 0.18494734685711195`, 2.236429925751402`},
{0.18592803424857857`, 3.7511626605606008`, 0.2948073517622305`, 1.1421602142936857`, 0.24567142070681736`,
0.41248813426514863`, 0.25834870652665815`, 931.542828982051`, 0.012674565140645466`, 9.754712584362995`},
{0.058165051218229624`, 1.2475983837798745`, 4.326003024744654`, 1.2604302116068957`, 0.7278275017755875`,
0.49389546693051767`, 0.021556729129120156`, 452.84942671713645`, 0.038214009527072046`, 8.73167788977904`},
{0.17310243018283944`, 0.49256885761049496`, 5.2474038118867075`, 1.2167083563026317`, 0.588616706923442`,
0.41089714710891945`, 0.1821711177021871`, 4451.29023370465`, 0.12001647220652961`, 2.269196213787966`},
{0.1805022145737788`, 3.870623878161763`, 2.6853201865429206`, 1.4540056051610473`, 0.3465395939248448`,
0.3116685990581043`, 0.3430495408207368`, 123.77189026179846`, 0.21554545019710575`, 7.5674497363872035`},
{0.12919486377057743`, 2.6304051581320387`, 9.030615531469042`, 1.1852097760732927`, 0.8025656477879668`,
0.2709665069296936`, 0.01610528402445943`, 631.0560544240994`, 0.23139307171140971`, 4.580191033116536`},
{0.11792807938525018`, 2.9992967519426994`, 5.197929087471108`, 1.1316540273093707`, 0.8248866470317724`,
0.3561695895739371`, 0.01696020529134022`, 1283.8412020113915`, 0.1325959329361287`, 7.000673815586042`},
{0.049303290582256715`, 1.1925070924339343`, 0.7957633600668377`, 1.4128683276782472`, 0.000022730879762677958`,
0.4285104885078085`, 0.00878130985829771`, 1257.460569712898`, 0.24085716752239034`, 7.587289884781604`},
{0.13128664635800402`, 2.4046868210274015`, 6.858470528577773`, 1.007040533285536`, 0.4840752461507001`,
0.3489790633820432`, 0.006590730128885638`, 3596.8950880067596`, 0.16587148750288666`, 3.462016809878005`},
{0.04846841194802434`, 1.828640157997615`, 8.053204702491172`, 0.7912000348817253`, 0.19137275290127498`,
0.26044015409759047`, 0.137444226583514`, 395.2194426771421`, 0.10623448877689068`, 6.754668978902`},
{0.22606436547954117`, 2.759207160262239`, 4.797641172785626`, 1.1197512107663665`, 0.8744312540778343`,
0.4079143329219078`, 0.021680363867763237`, 112.017364386689`, 0.04841847187742204`, 6.247097164626069`},

{0.21439187995731207`, 1.4871413304129364`, 0.5160724591468409`, 1.1806787917625174`, 0.8679222077666104`,
0.6556391626343723`, 0.05962099501823598`, 325.40523317512117`, 0.12955288081431815`, 2.055442432220964`},
{0.09778347249929609`, 1.5399588911598938`, 7.050045797549721`, 0.9082485710483456`, 0.7454636238668619`,
0.6248569918637821`, 0.04029077002834171`, 1056.397985521477`, 0.20938181714809317`, 3.632048403677867 3`},
{0.12927232662770405`, 2.951438179906213`, 0.40510823994438105`, 1.0187470674786288`, 0.2032238445066854`,
0.5456409554372916`, 0.01881027549673071`, 134.69705409101317`, 0.12921233603333943`, 7.23494261394152`},
{0.20182779178318416`, 0.6881028184372244`, 1.057422534977741`, 1.0568090274497894`, 0.12452101430667706`,
0.6254257177994904`, 0.3870244691590 6646`, 3365.5277260432094`, 0.1221116251689014 5`, 5.63708838473557`},
{0.21480237208045305`, 2.4248480984277982`, 7.5563453916242835`, 0.9609967545999567`, 0.4216733331369118`,
0.3788510295397969`, 0.02865689876021866`, 142.8377853769303`, 0.05340960656114529 5`, 2.2123406251771502`},
{0.19117013383013431`, 1.4594963084730592`, 8.271393984038092`, 0.8911461626518757`, 0.4023315908892062`,
0.6430024305607471`, 0.425955961175158`, 811.9982424604128`, 0.08470327958141488`, 1.5517888018340713`},
{0.16704561027767095`, 0.7136544792609025`, 7.482780404676742`, 1.2150283597546259`, 0.9046207606306917`,
0.271745131902447`, 0.2695234426940548`, 220.6020580904624`, 0.17006503868594403`, 3.14899802359108`},
{0.16595765366147208`, 2.092996799011412`, 4.948615364795604`, 0.8929763621996465`, 0.8391454185145462`,
0.5892008264762102`, 0.044299676614507844`, 130.42998143434642`, 0.19085435393011602`, 9.39386568753341`},
{0.27224148961405453`, 3.064354031176171`, 9.703480945324245`, 1.4628863651144104`, 0.3875440410966342`,
0.15502532019017645`, 0.01538110765065082`, 96.32001554152914`, 0.17185939304406467`, 6.478173160029735`},
{0.17789655356032075`, 0.42086448766877327`, 8.212577079102342`, 1.145177796821741`, 0.7675840121122328`,
0.5713332044931656`, 0.08476669830762888`, 407.4176887081235`, 0.039911846147414665`, 5.425104426537898`},
{0.25890879804354505`, 0.827757285125033`, 6.146318214990161`, 1.0400613992204162`, 0.4812180568243971`,
0.29251954183586915`, 0.03603273141312643`, 1733.9600457555907`, 0.08508345454030858`, 2.745915615562448`},
{0.13429919704858378`, 3.97301958069563`, 0.23275296336668916`, 1.4075463260369605`, 0.23061890929775308`,
0.5504417933404041`, 0.22771166040596233`, 272.6044063125219`, 0.22898591641919652`, 6.483734227937328`},
{0.12030391986517214`, 0.7748656879950588`, 5.112522469071294`, 1.1531030157403883`, 0.37283988475313556`,
0.5692147153589893`, 0.013539004333470354`, 948.0585012448981`, 0.020895787868669358`, 3.48210387762453`},
{0.1321541010040706`, 0.831080421127889`, 5.684518793971316`, 1.0145204035612485`, 0.08667418093385804`,
0.2219847293666507`, 0.022523926566952547`, 3181.1487538157703`, 0.21535243439086593`, 6.5479272590953945`},
{0.10292542985717545`, 0.9480363128916887`, 1.8187023134383153`, 1.095683835227761`, 0.3789693754557397`,
0.6741099730044744`, 0.007927682110189524`, 599.0709679738629`, 0.23730403059109434`, 8.347995806673719`},
{0.11768271904228267`, 0.8044018805503832`, 4.992994745146397`, 0.8839437991548327`, 0.9627780258088621`,

0.4036610370791115`, 0.010677704183731065`, 75.90134138634313`, 0.09047277628943856`, 3.892249177500955`},
{0.23810204192481904`, 3.5212069305389937`, 6.24449843698252`, 1.12580298554529`, 0.9906238590423209`,
0.651194539832239`, 0.008821920311958 45`, 3794.027232128564`, 0.08728181755971864`, 9.039220988882679`},
{0.05170854445494355`, 0.9544694556169109`, 2.4941099826351163`, 0.9097774283707087`, 0.02554739328254807`,
0.22408928016637408`, 0.36587983814629343`, 578.5087062785725`, 0.14318945676945632`, 8.489550606354296`},
{0.2758391104750425`, 2.3950700125014723`, 3.0825274454463827`, 1.2324094107661792`, 0.33578451389793784`,
0.4521239993250691`, 0.02669897011071422`, 4321.015062663416`, 0.06443072522161825`, 4.390191809873814`},
{0.04136033639560718`, 3.5342644851920078`, 2.1080294445485137`, 0.9950381948590621`, 0.26813425336643104`,
0.30392774702749736`, 0.047958977131448324`, 92.66270711931676`, 0.1731864034169307`, 4.57325203890367`},
{0.1958059683987035`, 3.200106783136203`, 7.203120771344906`, 1.1276577663917136`, 0.37865874140280775`,
0.19330414092595893`, 0.01322903896050458`, 235.27978791284824`, 0.1213328646575434`, 2.0980953870537427`},
{0.2141293518430885`, 1.3445267096173987`, 9.440612710104876`, 1.3031822475396773`, 0.08631549676886618`,
0.2421378295292581`, 0.053475196461594834`, 50.8320364740117`, 0.167124755886029`, 6.69448094138391`},
{0.14124099610833857`, 1.6304240145491873`, 4.431235349124869`, 0.7694928939095955`, 0.9477250896887188`,
0.5434618086833787`, 0.13057745213792676`, 1388.4740941330226`, 0.07045603839931946`, 8.981021929620123`},
{0.08317638924124338`, 3.4814117090049965`, 4.537378351353009`, 1.1287962009049006`, 0.37603042712104306`,
0.6772825294518297`, 0.08278526718675269`, 52.354988641883`, 0.1936331782676135`, 8.933135500909255`},
{0.14458191325048447`, 2.576522592570102`, 7.664240990513161`, 1.1334692879511565`, 0.1460097548297743`,
0.5479004940247297`, 0.17641432391033549`, 874.6457178122505`, 0.14737345925317297`, 7.125853291619361`},
{0.21964890100642098`, 3.948215647629967`, 1.3239539515383179`, 0.7541520677887659`, 0.2631388544858304`,
0.4680193255276971`, 0.07455346059859487`, 734.1983096733243`, 0.22794453971709777`, 5.810951908142973`},
{0.24357090033802437`, 2.585687351965424`, 7.1434831024545`, 1.2789397105658484`, 0.15335660895486725`,
0.5235228651937761`, 0.12790639986488742`, 56.5359989413313`, 0.05251799125371648`, 7.0717564062965526`},
{0.09627432586752127`, 2.332762155680175`, 5.679853879558527`, 1.2640734140093113`, 0.8156330491360497`,
0.2895792785477985`, 0.02307681035132743`, 658.139699058721`, 0.029189068125338835`, 7.800914789874877`},
{0.07523258003696548`, 2.5308231598738997`, 0.19036315944607196`, 0.7501464923569574`, 0.1153260012566284`,
0.48261384042066857`, 0.014254428894325608`, 71.6138994625911`, 0.24976914139283035`, 2.597638512857568`},
{0.20487865745542216`, 2.4577449266837927`, 0.3620012570001929`, 1.1621484663425825`, 0.45716579542364766`,
0.40055848752773193`, 0.09344580216885054`, 2051.8175124763634`, 0.1493227569977858`, 9.69569860745337`},
{0.1890789829788747`, 2.8864588443110275`, 0.3020897698703884`, 0.7861583937331655`, 0.3631295895274096`,
0.49565973888016235`, 0.1552859562806633 8`, 2271.132408212757`, 0.21092746640771154`, 4.683826377759731`},

{0.13636211858102731`, 3.2225156772879746`, 1.985405604082965`, 0.8074403105824717`, 0.51683495700684`,
0.17846947550569658`, 0.2866421049071916`, 363.9511542941515`, 0.043491080354586376`, 7.48314403026421`},
{0.1760012024892597`, 3.3901909690471133`, 5.258617572133415`, 0.8627686224489919`, 0.7422533085216929`,
0.41138487941188706`, 0.007592509582269234`, 424.62789093038504`, 0.09162929479721776`, 1.3722835015951313`},
{0.1813951042683553`, 2.693386252451666`, 9.617741712202033`, 1.407583958403493`, 0.6407257633308476`,
0.4405499114150466`, 0.008535253338252974`, 83.84476850180724`, 0.06822985382579094`, 7.163997237119144`},
{0.17566594009418735`, 3.2466526484278377`, 9.344725381698979`, 1.0138972604548542`, 0.5838549167343343`,
0.6073333607184574`, 0.10710975925951867`, 945.005957021721`, 0.208189974443157`, 1.4109192016259353`},
{0.22088610125498404`, 1.594941397589448`, 0.6585367344105002`, 1.4859166690390868`, 0.411769154740657`,
0.4715175396654723`, 0.024538179644211296`, 3156.4892991025154`, 0.15641735671760065`, 8.553654142069323`},
{0.08792198919034061`, 0.7563929024204028`, 9.775416388823775`, 0.7563974438325178`, 0.8271569221820632`,
0.32401969363676375`, 0.0333282008681181`, 102.10380045307154`, 0.1014972556908379`, 6.35713244492311`},
{0.13210283054552296`, 3.2703864704797683`, 0.8879166279114746`, 1.2876213813677582`, 0.37930173682375035`,
0.2978584700900475`, 0.018740615394798876`, 111.76209976448992`, 0.22677958819730465`, 8.664567998658026`},
{0.19507506176309325`, 3.126733009164254`, 1.2939386659529877`, 0.8626822462482099`, 0.4036124839823858`,
0.4778725494331981`, 0.06798274218289758`, 2442.14562500692`, 0.025650848833468687`, 3.9818096584261493`},
{0.08607083224139878`, 0.4430857620402713`, 5.378081455692687`, 1.015604011959475`, 0.6736258822863768`,
0.30157456176441344`, 0.06550214653497834`, 2724.6632477522535`, 0.20587133389175544`, 1.8687253311000447`},
{0.14471266481637884`, 3.5830029087352937`, 5.919067097135953`, 1.3383183012471727`, 0.9997339519904831`,
0.40166871745176214`, 0.015827643464025123`, 2493.129738188907`, 0.10618580931649058`, 1.661604036688603`},
{0.15884129765285837`, 1.9554524456621927`, 0.3975714737116185`, 1.180621526159508`, 0.13357193827486524`,
0.6393982147520227`, 0.07953097836767822`, 3991.2803311425578`, 0.2365716915141447`, 8.584420428066604`},
{0.04917531951043441`, 2.0521955455867618`, 5.720052321648424`, 1.1771597571169652`, 0.4322660150443707`,
0.1629991214278883`, 0.005091310116027936`, 189.05888707261903`, 0.1735533969163952`, 4.172239042736072`},
{0.27542629055144846`, 0.7843233583874856`, 9.783752583368699`, 1.3481471177954405`, 0.23017739141462767`,
0.5420497851091893`, 0.2059235624279975`, 3468.61907862177`, 0.15188055880627876`, 9.878186090289375`},
{0.242913589428772`, 2.017077890959616`, 6.7411875410249`, 1.4794325227990526`, 0.8119526659059784`,
0.6637027966074835`, 0.028536028992749693`, 2430.705278612085`, 0.11516348105007651`, 2.1124785829469186`},
{0.27369802527373327`, 0.5072292829799574`, 6.3938654238644155`, 0.9166058122252008`, 0.8588715137395462`,
0.6363372429100727`, 0.4973592888199021`, 125.12883357781175`, 0.0982596068118865`, 5.59867632569453`},
{0.18540637687565897`, 1.1775430389730683`, 2.7268491301469933`, 1.4767982648221127`, 0.8902187555583527`,

0.5282942786301189`, 0.1497900469847022`, 304.29028125771407`, 0.023895791395609434`, 5.874748340307197`},
{0.2722155439547531`, 0.9139371325579537`, 7.770199974150746`, 0.8847133947824111`, 0.9018075103619831`,
0.19960287154002887`, 0.008582048560181034`, 1921.873640365849`, 0.18448070830995128`, 2.8786799978881135`},
{0.06964367539322192`, 3.707453741922901`, 4.6834761045808655`, 1.337177597600478`, 0.13817299390105364`,
0.15072857839162668`, 0.030325540973499845`, 324.16896723003435`, 0.11870351893768993`, 8.4259642987084`},
{0.17261439973382386`, 3.0972705794401207`, 5.672236578564393`, 1.4223285072729144`, 0.7931550721000631`,
0.3896544494671119`, 0.01712882834694823`, 1350.492916562551`, 0.2427963854280547`, 3.05685280243325`},
{0.11394032550346922`, 3.2108067304960466`, 9.096957387649113`, 1.399541009089664`, 0.1514762467836341 4`,
0.6188818627652795`, 0.08261238136824677`, 151.04115640404754`, 0.09860367843525403`, 6.06148281050898`},
{0.20354065160019702`, 3.5209980508829766`, 0.4053576884306178`, 0.7576791600370161`, 0.6570403323895382`,
0.46832222917239164`, 0.005063986272754102`, 161.28160618619455`, 0.12637144234319758`, 8.874326085906798`},
{0.24345495866234085`, 1.8344199239977046`, 3.71003568577021`, 1.0568366445286865`, 0.9797132100793247`,
0.5295146440336447`, 0.23321770204647818`, 2779.737816031833`, 0.01572768263207122`, 9.835586906627377`},
{0.18352727434547023`, 0.4163243700753796`, 8.057415014521496`, 1.2522528089453002`, 0.16203828635471806`,
0.6604646928880291`, 0.25830735718962067`, 2461.4350174740794`, 0.12685187153570027`, 8.600866037504808`},
{0.23920496896525717`, 1.1838719961494162`, 9.218551103388815`, 1.265080848919387`, 0.9542453823473309`,
0.22929853041342196`, 0.4982471338661781`, 90.20981971435951`, 0.085631215363028 7`, 3.6772130334476696`},
{0.10156561975861944`, 0.5164671469302382`, 5.401464431301745`, 0.8562900606586841`, 0.5357330343495086`,
0.16718344164197263`, 0.02721990257094452`, 330.98847924494044`, 0.2190497368128987`, 7.446609110605853`},
{0.2533884831264572`, 2.217775528299157`, 1.87005833593021`, 1.0852798553697922`, 0.3630118105229163`,
0.49869259108707253`, 0.006826900671537389`, 2180.4237702051914`, 0.21841135731377453`, 2.8539212819504436`},
{0.04019446235159935`, 2.9685609440334373`, 3.1967275735949383`, 1.2384363485833663`, 0.006837582253808039`,
0.6265805854044944`, 0.10123843723710083`, 64.97108080441517`, 0.1585540441972691`, 8.616308543170305`},
{0.18466310913340017`, 2.870831467521074`, 5.7568671895307055`, 1.0469133934587485`, 0.5974897879057268`,
0.6447436436670198`, 0.1911914248698421`, 363.92035372259653`, 0.11435325769039895`, 2.2759611974712186`},
{0.22919090442152185`, 1.3485088294529417`, 3.7111141569564556`, 1.1683364276022934`, 0.8698699225738455`,
0.45530089098812654`, 0.06782162054416256`, 403.6677278730082`, 0.1398561461848175`, 8.195868200542787`},
{0.12718220564190125`, 0.7794468104625536`, 3.4523481867286847`, 1.042660599955479`, 0.8212977484948716`,
0.42153700480377276`, 0.05083556365712203`, 907.9591078654141`, 0.03221255361915021`, 1.403008654938164`},
{0.24829534536640735`, 0.6361539074945153`, 1.4367506745172012`, 1.2157272327840285`, 0.5920355341003019`,
0.22834833308349511`, 0.008858258633323783`, 152.73617223659593`, 0.05098711045238086`, 6.308971873547048`},

```
{0.24634814165734203`, 1.0199939463339236`, 6.874973257490155`, 1.1669106806380989`, 0.9547234223304992`,
  0.2467804242930165`, 0.010102520966714356`, 1373.9164154017546`, 0.22192762621195788`, 8.148592815397581`},
{0.11968292559735771`, 0.4405556662519543`, 0.9343787484302961`, 1.4969299043499453`, 0.5720179190625709`,
  0.5743610731089337`, 0.058376899138915264`, 4861.966479565238`, 0.13911792619470742`, 8.404040973858844`},
{0.0912885213280808`, 1.1352603856466574`, 9.64216394840902`, 1.0423303168159856`, 0.8496114679763396`,
  0.3948758352214041`, 0.050073459411100744`, 1020.4170701645861`, 0.08585808288581304`, 1.5724283740137839`},
{0.23601424519793518`, 0.7493625485076763`, 8.159569145163797`, 1.0789985894921148`, 0.9135595458313224`,
  0.43990663477596414`, 0.03553380189426036`, 63.68276264433202`, 0.11799293833856772`, 7.92015369426547`},
{0.09505824539478602`, 0.994707682773301`, 2.092747921993542`, 1.2334327544777528`, 0.1649122243213208`,
  0.435178801412204`, 0.4947441968705133`, 1030.121381906519`, 0.19720224785958157`, 8.531247013968503`},
{0.13338172675277454`, 1.0224437122231844`, 3.1176391252125253`, 1.1808523949493024`, 0.5954558720182712`,
  0.3646371291004906`, 0.024821319877175146`, 118.84942434665426`, 0.15904005586434156`, 2.0945755856004613`},
{0.2663723170815805`, 0.6117369492537685`, 4.996758576680092`, 1.4798016834758696`, 0.3454705507334743`,
  0.5628818087325649`, 0.10659789380555212`, 347.2598858446038`, 0.128819557084905`, 4.579853140224845`},
{0.19858704302380392`, 3.77240735130054`, 4.552319154599175`, 1.490234534684174`, 0.9267739623702391`,
  0.4663128979271708`, 0.016623264635311285`, 190.7315614324941`, 0.03981298108873932`, 1.7245962595124684`},
{0.16356962246540357`, 2.7590365436019733`, 9.315311335363422`, 1.411146654012818`, 0.2509435276133807`,
  0.3090283790000361`, 0.243114119684150`, 313.55839056266797`, 0.2467169299716112`, 8.038445579591425`},
{0.25383472083693576`, 3.9769524497483806`, 7.011565744250568`, 1.066285473960517`, 0.7734216029556533`,
  0.6850552501893354`, 0.013843595247453381`, 297.6124430270534`, 0.10716201039628298`, 2.5950386184147853`},
{0.17343366236722435`, 1.3936719133530833`, 0.599503921563171`, 0.8621470436054358`, 0.43567440743840313`,
  0.22823781097601215`, 0.018669299661272933`, 3618.675535403083`, 0.22611694811187344`, 3.1306846738065635`},
{0.27302715407014244`, 1.5486379048502288`, 9.868645627334566`, 1.0694698241775202`, 0.6816384789594119`,
  0.4416154581373778`, 0.05961824305163796`, 87.63839207083008`, 0.05267766985704242`, 3.1260333504958955`},
{0.12081145422022538`, 2.0244942048813837`, 8.652229532190159`, 0.835020699056165`, 0.9214042127932665`,
  0.46285611589460973`, 0.11861503789539068`, 2689.091322411565`, 0.07096583948487911`, 5.509201411600676`},
{0.27038445563951496`, 3.3688963138915424`, 3.4133330216440854`, 1.1975198070883244`, 0.31492494684053174`,
  0.45427916274833`, 0.0070970717881839104`, 3067.136751023867`, 0.17569148508387616`, 1.2036801730392703`},
{0.1767050761987432`, 2.85608165701536`, 6.140634667151749`, 1.3554192221984884`, 0.6330624847112656`,
  0.5906276578639604`, 0.04152902973081543`, 1510.3499876585004`, 0.15541682752363528`, 8.81551919212286`},
{0.16171803894826015`, 2.2156907324583015`, 1.0134587159780288`, 0.8322559746700846`, 0.34733770749260806`,
```

```
  0.6849579573613365`, 0.018772440750507276`, 1617.8437562512436`, 0.21218330633531807`, 7.516753350682977`},
 {0.24108871826638628`, 1.1722622038849657`, 2.5945274526654174`, 1.1873482780450624`, 0.3399058776037083`,
  0.3423735862363967`, 0.09173881369912114`, 1455.6792358614214`, 0.2278375557130481`, 4.818602627144232`},
 {0.2202280085702742`, 2.180975046803316`, 5.6134680006393936`, 1.2506485034427453`, 0.4748208651226249`,
  0.39757955628981323`, 0.017520218948128618`, 280.3947437474927`, 0.12359899846366301`, 9.521190146527957`},
 {0.12315072752895034`, 2.8609501087269207`, 1.79674111361204`, 1.4514496090822089`, 0.3032919079034333`,
  0.2763824218936657`, 0.028981582964232436`, 188.78817200372555`, 0.0885694651009113`, 4.546561013073182`},
 {0.14162785770391484`, 1.92013166099325`, 8.540643644714823`, 1.2350689068338556`, 0.301574102817308`,
  0.40858591883894513`, 0.080613435450821`, 585.1346359267128`, 0.1521171210972616`, 7.686797097032363`},
 {0.22524615675817938`, 2.031565502127312`, 0.245821316301889`, 1.298167355078078`, 0.689325845538592`,
  0.661487626561178`, 0.00610096551453922`, 373.63064864758115`, 0.20592315239272913`, 2.994117316721619`},
 {0.06014178921319169`, 2.215935435785701`, 1.3525069683978295`, 1.0400202244696481`, 0.697929353581739`,
  0.692932654029565`, 0.014666911144259826`, 1018.9785490283374`, 0.16122419934759502`, 4.978072859014281`},
 {0.11674773262981253`, 1.910335393882857`, 4.221858661435528`, 1.4404651869296854`, 0.4563596481936356`,
  0.5439902833298367`, 0.005611633628996954`, 451.6526207878965`, 0.24247834364497922`, 9.182241064841566`},
 {0.12898119467887553`, 3.729766126068422`, 2.3744826976889595`, 1.2465399522360021`, 0.1415631230326866`,
  0.3607064691421532`, 0.03995461220531779`, 1238.1663657022145`, 0.14506115517150608`, 8.953141814946814`},
 {0.2498624462217614`, 2.827129073229683`, 2.690968559152097`, 1.0446525535360744`, 0.06936419216205603`,
  0.5607634545552321`, 0.008891348377622028`, 196.01664126075448`, 0.10064633302983284`, 7.405206717033214`},
 {0.25890580889310233`, 3.9576703566675278`, 2.211552265086926`, 0.8006241502346895`, 0.26522568617457876`,
  0.4892067360859801`, 0.02025985822061832`, 94.72915936196773`, 0.06766512891079995`, 5.4984531961033785`},
 {0.2032139210380186`, 1.0205320875718096`, 3.2697243543529257`, 1.0866214178514029`, 0.5111053959745286`,
  0.4079750171889772`, 0.04834589720060758 5`, 145.20906840025864`, 0.23728279570010086`, 2.517042827760779`},
 {0.15288075349234037`, 1.8405591021591183`, 8.732063156275487`, 0.8366527628406224`, 0.7247685787231319`,
  0.43308603413296065`, 0.0670353509355 0567`, 1294.178726539122`, 0.16108367938164575`, 4.274431762914247`},
 {0.1609254870534111`, 1.384768951726243`, 6.196355509527487`, 0.9770497723863997`, 0.43819861370403546`,
  0.17710326003602894`, 0.2243750211158665`, 120.51310647409781`, 0.10277566384729958`, 6.21125463242891`},
 {0.21184155187533854`, 1.0400436907537394`, 2.77253033361667`, 1.25783985819887`, 0.5916706672066951`,
  0.18670557332607218`, 0.05860949172916328`, 177.69964385957368`, 0.18251915101666627`, 3.5990512292385053`},
 {0.15826860039724194`, 2.914200187526596`, 3.057619613104965`, 0.9127068016590583`, 0.36895217716385176`,
  0.3294645201729436`, 0.006405724718526674`, 799.6829736219844`, 0.2321605158663716`, 7.540832982633661`},
```

```
{0.2486148170283075`, 0.5446418582356878`, 7.121193243885838`, 1.048132429671941`, 0.7615900023009023`,
  0.5400927540260018`, 0.07717023450494316`, 388.33902457321517`, 0.04098404938849237`, 2.529691537294738`},
{0.1973186549669898`, 3.31823274961833`, 5.404057922234477`, 1.0748992098641554`, 0.1837120164888255`,
  0.5027766473922404`, 0.30055211808677745`, 460.171808421071`, 0.10833443978919949`, 3.5562239100468123`},
{0.16177424691159015`, 1.9234518490470327`, 6.336020872512709`, 1.465202064627133`, 0.3679027675625519`,
  0.6088093981583469`, 0.02752967040620688`, 1523.605195139083`, 0.023927731291662258`, 6.9520681394676025`},
{0.12468946686374066`, 2.602287301847274`, 9.387923649086364`, 1.237964958422611`, 0.6279283704003791`,
  0.5225362832845872`, 0.15386398985407668`, 4877.253164044286`, 0.10729269282887094`, 6.155025385024989`},
{0.1891437323001075`, 2.037539508644585`, 6.753647851102151`, 1.0264835122983769`, 0.08414675462879395`,
  0.15050525073770327`, 0.01724785647741675`, 835.2003740810117`, 0.09305345804576048`, 5.34515441362371`},
{0.11951180040558451`, 2.9137311414381974`, 6.217108794108201`, 1.4729889111648695`, 0.6540356010640074`,
  0.40115742558392786`, 0.08157249569611574`, 391.2473393910366`, 0.0369147403900103`, 8.338813449127404`},
{0.17883917330505905`, 1.5543060950462326`, 9.905917854358197`, 1.2462903035005124`, 0.32028587133565645`,
  0.3166477244063972`, 0.12099723319057126`, 400.41544183552224`, 0.027102889569123156`, 3.023770086067156`},
{0.25477439586299494`, 1.0781629016953422`, 9.675717925989822`, 1.4583691370753404`, 0.484439918991886`,
  0.6784957922502513`, 0.09307366059264305`, 217.6033516010123`, 0.05467318278554256`, 4.774031073697932`},
{0.26738035849024727`, 1.6336260676101997`, 9.900661691242231`, 0.846806505065853`, 0.2415106216321452`,
  0.502680613934997`, 0.14320441138979886`, 77.88432317464672`, 0.12860121706274624`, 9.963544099078408`},
{0.0966033889391959`, 0.8191052864079662`, 6.963587929197736`, 1.39989556064551`, 0.208903818004208`,
  0.4044327200024739`, 0.345646875315088`, 51.22809362991988`, 0.05669448608759026`, 3.5983266970468653`},
{0.07268626506253911`, 2.0017174568948795`, 2.5547427204964173`, 0.8446935149578232`, 0.0630154383873418`,
  0.49766282606547807`, 0.00568323780270915`, 197.50095618865632`, 0.04182941385906325`, 9.915201734242256`},
{0.14445086415814334`, 1.1396367789378896`, 8.108045316850049`, 1.1452867101498132`, 0.43349891091368953`,
  0.5412309783014353`, 0.17300117449492944`, 151.5195914284387`, 0.22841440999808194`, 1.401560283159844`},
{0.2737062633152032`, 1.87379900045931`, 2.9341815386065377`, 1.0257953180476898`, 0.5994241695021645`,
  0.2666028641788709`, 0.16092630392754248`, 2757.7643970777704`, 0.13082540497870587`, 6.7306102844961035`},
{0.14450675367477284`, 1.4907097867645147`, 8.628071674125838`, 0.8645742960679661`, 0.06677853457714877`,
  0.6218432064874626`, 0.019848227343967184`, 449.15101395266043`, 0.20772413403212947`, 3.059722840773212`},
{0.05092006104579416`, 0.9079056727720332`, 9.25200153286255`, 1.1365283513840736`, 0.03327106644526534`,
  0.582893635885294`, 0.1249724078419535`, 82.97595681905067`, 0.15395748273911747`, 7.1070379565760815`},
{0.2364459713820966`, 3.884359137525399`, 0.954741626535867`, 0.8976843312448166`, 0.4180287949880024`,
```

0.15414803582881131`, 0.32241293356212986`, 4897.582666581565`, 0.05447310320870352`, 4.86045092756919`},
{0.042914457849633436`, 3.519672647432988`, 3.392121169492343`, 1.4941116772804357`, 0.33812708289181614`,
0.3684690007615431`, 0.017148240098985187`, 3621.3180321373566`, 0.22166064632630456`, 1.0781698542512608`},
{0.1470328346746822`, 2.9686959435849998`, 9.643655350717612`, 1.0921756097932163`, 0.41500029005274564`,
0.5183995564120613`, 0.04078998201684905`, 4770.787596114335`, 0.08630863643009795`, 3.8345772490574745`},
{0.10223589016255652`, 0.4809064129783538`, 6.158635896611097`, 0.9490677758592585`, 0.9235714285317382`,
0.43663660593873277`, 0.45300992250929206`, 1331.4948553442105`, 0.21785131418517045`, 5.425736978481032`},
{0.08719902184880185`, 3.645892777856403`, 2.8891082836443758`, 1.428386787110956`, 0.151537003432892`,
0.34505159105650784`, 0.041961341889936235`, 290.12993265609543`, 0.15015043093723618`, 6.608503242578238`},
{0.14456990131617375`, 0.9947424361823325`, 3.150441801621305`, 1.0015653051761273`, 0.8447006281771343`,
0.5703016332732033`, 0.008271730569805881`, 52.80371817926736`, 0.13650904531369146`, 3.8670961625783473`},
{0.14643438827373756`, 2.4493630197222025`, 5.7044459384485755`, 0.7643715149180945`, 0.8231136724552808`,
0.4724466281569806`, 0.012687117803733845`, 1217.9307406282053`, 0.17452698640833697`, 5.969503699935295`},
{0.045156311345747796`, 1.2463805103118828`, 6.638333254697063`, 1.0774632518381033`, 0.7965921624862937`,
0.19987812094053103`, 0.1230725004501963`, 3016.4870397653995`, 0.18711874545495605`, 6.497285772809487`},
{0.21753427776894996`, 2.5707121359144134`, 7.635927852201033`, 1.3489791075447177`, 0.07755529833527275`,
0.4139475400754866`, 0.049678130502208456`, 727.9927710408293`, 0.24367647097486184`, 3.0823691783926943`},
{0.08480900190064672`, 0.6294563083477476`, 5.454864363444388`, 0.7943923084343845`, 0.9968128471314037`,
0.29575569030654725`, 0.01722905088446216`, 309.5962534203848`, 0.1957861782089137`, 7.847856100229725`},
{0.12061650440369748`, 3.919019990284525`, 1.2611661330191772`, 1.4204820377968523`, 0.21263599304474745`,
0.23829484446556248`, 0.008404906276562513`, 547.3513122366178`, 0.16414531089750106`, 3.5940701557610755`},
{0.2541152986933387`, 3.9531330061598675`, 6.8389480578926864`, 1.4090336327472661`, 0.5067749551810137`,
0.5768128332044816`, 0.18877117253369932`, 176.17163988983094`, 0.11435366361110944`, 3.2749545355180913`},
{0.26839792559441034`, 1.5919359357927423`, 0.607262674381337`, 1.065158649344713`, 0.7342994823745972`,
0.6373140564402076`, 0.019311239061898532`, 131.67334362372645`, 0.026592393453336638`, 2.002632914376134`},
{0.0804335350475921`, 1.0990801178785707`, 6.42097149017635`, 1.215697698043305`, 0.5864093543250795`,
0.4707288466973014`, 0.04708688161958605`, 3199.4245739234016`, 0.17424214472199046`, 5.845755402883285`},
{0.04545514913582513`, 3.0084108739176516`, 7.583320976280454`, 0.7956411409554567`, 0.21703974982634144`,
0.40384085540761094`, 0.39906200752611665`, 3808.370230770943`, 0.07445295911170413`, 7.244769365152968`},
{0.21811178043156876`, 3.634131336571894`, 2.2696382083853486`, 1.2012104146171985`, 0.07478503245860835`,
0.304565647018059`, 0.04940364648704803`, 242.22074286172042`, 0.22363734402413937`, 5.499597099973035`},

```
{0.22643881069864102`, 1.8950339409059218`, 4.49788713237294`, 0.781319815193253`, 0.8852654232409718`,
  0.19866746146736047`, 0.009828863939504507`, 672.8810907026934`, 0.061975672509268276`, 2.025268353742364`},
{0.13526256211607002`, 2.462351571487578`, 7.058879409696349`, 0.9886974127254741`, 0.579411758510507`,
  0.519067819756009`, 0.20147771873022094`, 163.35469430255884`, 0.08074145629003388`, 7.486187513545234`},
{0.20702996858127698`, 3.5459212947722705`, 9.044651158282573`, 1.2898647778788441`, 0.1120438185942787`,
  0.3399243113730348`, 0.005774130262077124`, 68.71847405774774`, 0.13094328364017765`, 7.770082768301062`},
{0.10369509204579469`, 2.7722566029085502`, 8.491805279999415`, 1.0043154825901828`, 0.1196997608855428`,
  0.46660526882559294`, 0.07137717728073698`, 124.06613686120853`, 0.02382042507485488`, 7.245869706736816`},
{0.12495211269478451`, 3.2710300653782634`, 8.31741576802569`, 0.830115956215347`, 0.45240597007929817`,
  0.45987625913811714`, 0.011306277140564175`, 1464.8239131751898`, 0.06894180622066476`, 6.472250284276024`},
{0.2548094999525184`, 1.991565099208616`, 8.435964917534815`, 1.3597221193616629`, 0.25512389762112764`,
  0.2087626588648025`, 0.2568272531538555`, 1822.5061165805678`, 0.026223328458720774`, 8.894025776493041`},
{0.1329827943052238`, 0.6587055670228517`, 4.6853626661703505`, 1.028170006221078`, 0.5310002897488229`,
  0.31399252604565075`, 0.010724910779551164`, 62.931190858884165`, 0.12157208629491184`, 4.054529845871336`},
{0.05388606525951978`, 3.0261209146571506`, 4.311810802135282`, 1.2867466894982391`, 0.1412111695269684`,
  0.17229324040800142`, 0.239982541075217`, 290.98679707372355`, 0.1893837871763393`, 8.200911880146922`},
{0.18877028807710305`, 3.0713928282220317`, 2.350050214349242`, 0.9003573816536171`, 0.8295355664150708`,
  0.3697046533237569`, 0.07836282343745943`, 203.60143886304388`, 0.1119456128362123`, 8.250251130667593`},
{0.1549731585118843`, 1.0794091822629426`, 5.872175042913531`, 1.1954059661912195`, 0.1582784842110334`,
  0.47191465043849123`, 0.04704948286374014`, 132.48832982484745`, 0.0611350826684427`, 8.646340597834747`},
{0.0973630018497127`, 2.6509059407435345`, 2.661281949905481`, 1.0384603558144696`, 0.8313691963066465`,
  0.6553290382276395`, 0.19978363792296042`, 613.3149478611653`, 0.10086553584387609`, 7.337446850354657`},
{0.25760446309632484`, 3.1710442921339457`, 1.104410531583154`, 1.067160019670409`, 0.29416815684722497`,
  0.2106415408174861`, 0.1640132441258384`, 295.52940308175954`, 0.22278260496644214`, 4.791464741488767`},
{0.08814664727699922`, 0.49325198699724604`, 7.4393784579313085`, 1.082856668773565`, 0.36684849391494345`,
  0.47651248778047417`, 0.010016127241926027`, 222.2306387901171`, 0.10060123761752315`, 2.9576363340294876`},
{0.09405288750913654`, 1.8996899418118014`, 4.321657830772438`, 1.3285146456969388`, 0.4687194709250446`,
  0.37672748824359026`, 0.1617154754485949`, 124.22044587269936`, 0.08001764444851872`, 7.0616286930304`},
{0.26673333380428393`, 2.6775026015459398`, 7.186300359062184`, 1.3779081516185778`, 0.2197224262472608`,
  0.44634915685926935`, 0.2549448072449753`, 573.3906335364269`, 0.19327206597748986`, 8.09151224456528`},
{0.06695152315589192`, 3.739348796633019`, 8.531551488563299`, 1.3873826920360777`, 0.5359483678583272`,
```

0.39821450250245216`, 0.041894311644931556`, 1681.9385322117223`, 0.03536815464018617`, 7.504735436115399`},
{0.2432492145111665`, 1.524425096090667`, 0.7030547277601631`, 1.1448142947983793`, 0.5481222164790474`,
0.4314400790527364`, 0.005980753513921293`, 1722.4066531232882`, 0.11386175120469438`, 3.8888258531503404`},
{0.24077340164047406`, 3.026302168091572`, 3.348461380748672`, 1.1251559242118545`, 0.33184191297746213`,
0.3141742813991918`, 0.0703872676061783`, 173.09096476750136`, 0.24564160017502346`, 6.392098114270558`},
{0.09741803900657164`, 2.8226058170826276`, 6.285345102913118`, 0.8413213607246539`, 0.2506059249236361`,
0.26188997247349455`, 0.05425664970500174`, 4955.988737338467`, 0.11641107051911387`, 2.1128007380367517`},
{0.14314599793313348`, 2.5518611864266925`, 6.295888200856282`, 1.4241645467251498`, 0.07884119203240614`,
0.5217209274223922`, 0.006381498715981013`, 359.4043872790348`, 0.24023808698930982`, 4.143010259943262`},
{0.225655266424848`, 1.5867432872088365`, 3.0858574789264335`, 1.3244931698407223`, 0.6079478177713131`,
0.6933074034991797`, 0.013957254253711016`, 59.76089956157712`, 0.13530140261382512`, 4.737681248427657`},
{0.1122606973400958`, 1.7187290694442563`, 9.979465273640976`, 1.2098446125137294`, 0.9761419756238592`,
0.37158902094365565`, 0.02080861609061981`, 179.99091662757314`, 0.030562090142117915`, 6.57677031568597`},
{0.24459409433338708`, 0.8193086201254509`, 4.65991702304226`, 0.8806460188595719`, 0.2108783317674836`,
0.532516488691834`, 0.10515966693757768`, 2682.0355154099257`, 0.13211135275557007`, 8.414844442987327`},
{0.06530542208683338`, 0.9596039403304868`, 4.254394351359874`, 1.2566656605190776`, 0.7704919647854709`,
0.333532652338865`, 0.010126143082696715`, 1215.361514466369`, 0.2378411046297244`, 9.6572715838455`},
{0.1695388010672208`, 1.173836844893498`, 5.733937529803059`, 0.8972303818101501`, 0.03459597061008757`,
0.45955942513269943`, 0.02896845637601857`, 512.9632938088884`, 0.14318523913512782`, 1.8784414403823462`},
{0.21476635225191587`, 0.5109603344620304`, 9.116157263242219`, 1.0856571152809298`, 0.22597102427026194`,
0.39468057790613453`, 0.008122876676631087`, 1537.5523384133612`, 0.22468288668284858`, 1.5725594082493757`},
{0.06813064867410118`, 0.8082337111165119`, 3.599754755102856`, 0.8970806442820121`, 0.12954010392326953`,
0.2841840584225793`, 0.09462247566679993`, 471.57171291886044`, 0.0960155452146989`, 9.92783198210315`},
{0.12903273933048381`, 3.2486278014701124`, 1.90816116411313`, 1.4556940559632274`, 0.8634695831402321`,
0.3517692054558007`, 0.07017000005506284`, 82.66475099620249`, 0.22883036061603934`, 2.0030440452333433`},
{0.25243522618186365`, 1.7361695497710992`, 4.779805866927312`, 1.3770344402735035`, 0.053441804379047`,
0.21192069167526972`, 0.3494903419308112`, 922.4310224013639`, 0.09853668667446419`, 9.287299936208932`},
{0.1954776887577972`, 2.556119761332786`, 4.179140500485959`, 0.8918497427192082`, 0.9985940433520333`,
0.19919828622889002`, 0.10341194686720168`, 185.89024422047143`, 0.23152045430083634`, 6.38817726005194`},
{0.21581636372683116`, 2.527066218755916`, 0.7074271020081202`, 1.2545848439034097`, 0.6959200155465519`,
0.5313070485281961`, 0.086636650755472`, 1874.5635156658839`, 0.10607902266846381`, 5.753646937249579`},

{0.1492413868080179`, 2.613673396032418`, 3.0904979954224796`, 1.2072285951382535`, 0.18001792552689877`,
0.24732759197473053`, 0.00562211589804969`, 2528.5611678534033`, 0.0881107110472133`, 9.594393869960811`},
{0.07601227699098417`, 2.712563755157329`, 4.823883660189505`, 1.3951422907519642`, 0.1445858824844255`,
0.4724433117860949`, 0.00914361266793413`, 231.39818056782485`, 0.1448152920502898 7`, 4.810561016418756`},
{0.12871665135347787`, 2.8014401685833805`, 7.283786002739568`, 0.7524429330430051`, 0.8818706538097647`,
0.37826653388239995`, 0.007972118927365966`, 65.78330734043783`, 0.1996049007295676`, 1.2990854064714608`},
{0.09952335410600305`, 3.347751049949922`, 0.6305859630946937`, 1.1305375150416004`, 0.9039729545790072`,
0.47386245025326623`, 0.16157270551479597`, 562.0685567880952`, 0.1640716707479668 6`, 8.5921358345290 7`},
{0.09189223639403032`, 1.6732139429664894`, 9.692701238874825`, 1.282228547207058`, 0.15855153018111912`,
0.4131843305852153`, 0.03830010741000983`, 3674.584524793622`, 0.10140311695331994`, 4.468652597668594`},
{0.15046133692934927`, 3.1811544489323884`, 8.294233740646352`, 1.3617577237735539`, 0.4847724596901488`,
0.37433619970600795`, 0.06464979105054385`, 793.4336208513492`, 0.16357169207826316`, 3.060886752086996`},
{0.2766206750277398`, 2.8724354428398637`, 5.189326643025524`, 0.8952441889736982`, 0.9323758122824941`,
0.4464935529399745`, 0.01922331430839979`, 81.97107113159659`, 0.06878282991185858`, 7.425462926136905`},
{0.23204393359414766`, 1.3807564884192223`, 0.49288335289722696`, 0.9255887528096592`, 0.5628667572225596`,
0.43401537857521066`, 0.010225716982610468`, 1119.882052780943`, 0.015435359995479236`, 2.904839645045426`},
{0.06896766630182821`, 3.238145856087148`, 5.061610251787275`, 0.9979492539875935`, 0.9640249344246659`,
0.343776595701085`, 0.005657576047359979`, 3386.0339854064414`, 0.07870221044254616`, 4.290344560110242`},
{0.14163054804702563`, 1.8677837362731973`, 0.4370922457889872`, 1.166715795330906`, 0.13182109027382305`,
0.4473367986720722`, 0.3453878303019619`, 2024.858554151485`, 0.17866806603084778`, 3.146330044100834`},
{0.14346666218024207`, 3.6942323053195354`, 6.0819497997897844`, 0.8206348775106591`, 0.4021755195313119`,
0.4030089388647059`, 0.4636571249048107`, 744.6939263196219`, 0.08354527905403086`, 5.555269667846608`},
{0.09149267062932814`, 2.234522341263885`, 6.944559583117852`, 1.114683357002141`, 0.5085103250363343`,
0.6658564606426403`, 0.006987139923383322`, 1505.8338370139704`, 0.21635495075213407`, 1.8879751532316735`},
{0.22068432056467163`, 3.569728941728428`, 4.540468856326312`, 1.2038410371742398`, 0.3148195694928344`,
0.5227498853534928`, 0.10369270254334745`, 4010.634501486441`, 0.16206884164220114`, 3.771999452670874`},
{0.2097350071754976`, 1.1102722915344074`, 3.321403610485124`, 1.0935329934230222`, 0.7183578411763796`,
0.40562405317483374`, 0.006176942100465586`, 446.96867612346506`, 0.08354129713872654`, 7.284441181467236`},
{0.15395695699718487`, 1.7653707587036722`, 8.640858401956272`, 1.0488086821127276`, 0.2563370285663329`,
0.39045184386637677`, 0.03142873441769787`, 98.7771934627432`, 0.2047745102794421`, 8.897923651735105`},
{0.2495726529131425`, 0.6471319987804813`, 4.234303245586215`, 1.3697781718226154`, 0.5893758025334812`,

0.16113556190203493`, 0.016137549539750166`, 661.2554849181507`, 0.08255896742665592`, 6.142572860377916`},
{0.18703143603461198`, 3.5026925577041474`, 3.736145496532833`, 0.8654363814884891`, 0.7031489482980493`,
0.15906583747765657`, 0.008377691039792691`, 131.85234583624222`, 0.16088793676433022`, 8.514675246308464`},
{0.2617533772269884`, 3.6583043975291023`, 1.4938470678500337`, 1.229216471284039`, 0.8448795594977094`,
0.4744971024115313`, 0.027729197093825105`, 163.20786376161018`, 0.21172311156121143`, 7.676981105027913`},
{0.2277950960946843`, 2.381865480846251`, 9.991476447433318`, 0.832979598782657`, 0.5926733949589658`,
0.27276410945547525`, 0.04327492387940349`, 669.5530400360332`, 0.024018555255208573`, 9.681708943673282`},
{0.15963824410704142`, 2.654604852862681`, 7.469701098598773`, 1.491664105533315`, 0.25955793675814864`,
0.27946634303609463`, 0.06966019051655106`, 86.7013584077103`, 0.017186465149371527`, 9.81969476696123`},
{0.13626056470386483`, 2.820076398995975`, 2.87943303050835`, 0.9523411928557022`, 0.8935766203190545`,
0.356125796963648`, 0.009331421764352204`, 68.56313381348697`, 0.147099680293568`, 8.670054262776382`},
{0.22962572899038486`, 1.387681996287987`, 7.569216994527352`, 0.873789488954491`, 0.15662470794012862`,
0.483300190980835`, 0.02173528342644958`, 88.10021701991921`, 0.04718506749066664`, 8.783666403079792`},
{0.16187203397799577`, 1.769575035055972`, 1.1614771267268384`, 0.8287067492253789`, 0.7900895875825826`,
0.2962983919698179`, 0.014442973603799043`, 137.47659064397268`, 0.2186302306878819`, 9.341476742915006`},
{0.09685210682031803`, 1.7960713074535244`, 5.69551378974773`, 0.7869885608747933`, 0.9421312080797839`,
0.5036667090451988`, 0.01697045527462802`, 52.99769310364423`, 0.21425647446502405`, 9.869621253243459`},
{0.16013522976515993`, 1.0007721789830901`, 6.380859482894074`, 1.440495462142086`, 0.5398127028451485`,
0.19075782788209084`, 0.04652110166049526`, 84.8416541425776`, 0.16547472912452094`, 6.4422585881915015`},
{0.1912199176775608`, 2.2121001603415005`, 9.917361836602723`, 1.435922264503217`, 0.9790995032802718`,
0.6767636439858111`, 0.07238714810610002`, 269.421010262202`, 0.015607565070540702`, 2.5093255880098546`},
{0.24179572266521915`, 2.5600514990256196`, 5.593167080478407`, 1.0641609773948193`, 0.6297315486340287`,
0.26623731831221253`, 0.08310036534342866`, 636.0105501064265`, 0.05032454414330245`, 1.4627567207550134`},
{0.20288510482307215`, 0.7288550347693712`, 9.019074578521877`, 1.2840716127441223`, 0.5874481378002983`,
0.6980893050148065`, 0.008225735950864725`, 1276.121996072157`, 0.1607915330045036`, 9.69181498105582`},
{0.1862496077639137`, 1.6905827743030857`, 2.538407600056317`, 0.9208769830081895`, 0.38109944279089136`,
0.3320011945053185`, 0.011054373793833769`, 1065.4164452217328`, 0.07943719416592698`, 4.231039339853934`},
{0.05689751321611025`, 3.0250332600067678`, 2.529462845718742`, 1.466915953431471`, 0.22269501402715108`,
0.6695459886571014`, 0.14207393160159743`, 917.9363525294286`, 0.02546682059293326`, 3.574551553390743`},
{0.1967142498798619`, 3.0620830451218577`, 7.9498840213828466`, 1.4844762448061533`, 0.6042217303737281`,
0.6207898959804532`, 0.0334891282473782`, 576.7285784287807`, 0.16199601914875666`, 9.348325121692032`},

```
{0.26434225760699476`, 2.569764850665571`, 5.652188171834926`, 0.8392687504503539`, 0.3834838466329171`,
  0.17354269300981617`, 0.03414034179009787`, 716.202171206297`, 0.028057924329779993`, 1.7997126741152787`},
{0.19233187457454282`, 2.145104020661503 5`, 4.677354160755579`, 0.8253029687642099`, 0.9809911268657268`,
  0.22566854695954142`, 0.015470928716805988`, 111.90002994139898`, 0.12242650367871766`, 3.4296984602053584`},
{0.21830260718528288`, 1.0813728060954029`, 9.236753498274854`, 1.3292857287875703`, 0.7084275620345744`,
  0.4530838967889582`, 0.022838882777727113`, 80.81382094438914`, 0.07293079158751065`, 5.841787640828064`},
{0.27637635892023465`, 3.838779152844408`, 0.15938000205200176`, 1.3392032462951913`, 0.02123906289427646`,
  0.6059474038465393`, 0.007164747514275051`, 100.75472745890974`, 0.10946913978259332`, 8.952098728322259`},
{0.15064788979833238`, 0.9109936464272579`, 0.5800958535668599`, 1.4999646884179314`, 0.9779856120080317`,
  0.34948818201364285`, 0.011067508252105101`, 2641.6705923333384`, 0.011474828296826944`, 3.667140614388831`},
{0.0419185722234717`, 1.709255049022068`, 2.3539768873704308`, 1.3792050115017793`, 0.22715212217109637`,
  0.2926929451571887`, 0.00505223249889438`, 88.91455127714036`, 0.1107140298924858`, 2.282618721837757`},
{0.24924491240080032`, 1.373280333190027`, 8.919496594562187`, 1.4037493221091317`, 0.24632944507518628`,
  0.3548949637369072`, 0.18998328780525742`, 2947.236236962295`, 0.1153856006596437`, 7.477168052601716`},
{0.20687871564233656`, 0.44181655503120965`, 8.570924850704476`, 1.0055678634725929`, 0.5549096280819519`,
  0.326726210951258`, 0.20129490353773463`, 306.54002765810475`, 0.1699967418388691`, 1.7003969931174137`},
{0.20196179343132148`, 2.101313615300616`, 3.7695608295023266`, 0.8255643571631567`, 0.9698552359083252`,
  0.3038610819528902`, 0.02519738143348337`, 88.57713238465558`, 0.03933307441687739`, 1.9858938348907018`},
{0.10010100507466935`, 2.4192735538683836`, 9.823330355933038`, 1.1133108806457672`, 0.011014651746178439`,
  0.5998442212295849`, 0.013751847561218578`, 3832.28466694177`, 0.1060300423959204`, 2.751621101527464`},
{0.21311625184066546`, 1.4247782113917893`, 5.690068626185788`, 0.8615233428628426`, 0.12086241410351461`,
  0.29764577871833753`, 0.005395932799405541`, 882.4049045614522`, 0.2481341739836153`, 1.9572384227218778`},
{0.09607643000919447`, 2.545123365745101`, 1.6292494594570959`, 0.9689766494611143`, 0.9239031581606687`,
  0.5340288721519064`, 0.06266594590032853`, 79.64776165873695`, 0.19537727971057256`, 3.908080115643834`},
{0.25410295729391263`, 0.5246172329407881`, 1.519575137014602`, 1.3035504391628365`, 0.641505202173666`,
  0.18662868599370885`, 0.02992528952684035`, 1308.2457641643925`, 0.1567967571574565`, 7.528281484333206`},
{0.20247384111644234`, 3.9381206949664582`, 3.525512124148932`, 0.8160399146038965`, 0.003770588899372651`,
  0.4372678593782203`, 0.005846407125549171`, 81.38331905868344`, 0.05740074457617078`, 6.792038617151225`},
{0.15622647656935051`, 2.478038073204126`, 4.93355773626655`, 1.0681326166577831`, 0.57283306272417`,
  0.24473580012035412`, 0.09417540555806864`, 340.729792091258`, 0.1606536419587833`, 9.486285960325056`},
{0.2472522705346833`, 1.1701552284997945`, 3.914120438755436`, 1.4973295978551664`, 0.1631737194969931`,
```

```
0.4864210576866177`, 0.39314364156002357`, 172.17497479862377`, 0.10619847023829598`, 4.473646521439088`},
{0.1494891985884202`, 3.3979182405498527`, 4.585626936341594`, 1.2118171179612203`, 0.6586829775426448`,
 0.3643174512760579 7`, 0.33277789805879787`, 51.20287439819706`, 0.10641801334172246`, 7.126742388333813`},
{0.2014310029114028`, 3.9869430882246055`, 0.7136208449188892`, 1.3208947167282403`, 0.46844054682093383`,
 0.39516715199771224`, 0.14154388607116256`, 2748.591928872397`, 0.05354911946309293`, 6.211900336444712`},
{0.13228384635594964`, 2.6408616938406997`, 3.2993392931538836`, 0.952940068464383`, 0.8367144819205878`,
 0.6111943245350302`, 0.13146835864980566`, 2246.7842161758563`, 0.024436379332296754`, 9.73655688789367`},
{0.23113058156717692`, 2.2605125411397546`, 4.002625660970853`, 1.3839850516092966`, 0.507564008077771`,
 0.4982541689482688`, 0.0228570122346136`, 138.3250229234806`, 0.07006503343759235`, 8.064244271550876`},
{0.17570245022422137`, 3.187809299248391`, 9.693328941627644`, 1.4591302419173326`, 0.2844948752823342`,
 0.5066677812093952`, 0.2998214113578054`, 349.3700788988757`, 0.18995614940943706`, 5.5658132930392785`},
{0.2497541467888837`, 1.085030464715203`, 1.0043906551890043`, 0.864213346242746`, 0.8846254264305906`,
 0.601718469375484`, 0.012654582561683364`, 62.423429857566624`, 0.12690014020771856`, 6.991109163451483`},
{0.22558722869536918`, 1.12607467611575`, 0.7911165205665469`, 1.2733751455833207`, 0.6009341950827205`,
 0.6641796486046712`, 0.175358669032309`, 3424.3638773504586`, 0.14969161390071423`, 8.91095466688921`},
{0.050627269144448594`, 3.276563036391985`, 7.788230793264948`, 0.8177022370463224`, 0.523932893406847`,
 0.34959907144360225`, 0.11342446098213065`, 1447.0777642274236`, 0.2385428599947077`, 6.8056995790460695`},
{0.060006217186970356`, 3.9647259629353027`, 0.34014645604634985`, 0.9146780185619144`, 0.03431401900644282`,
 0.5737721437004861`, 0.026766328146322068`, 169.17748604048344`, 0.08721098377832243`, 3.968142168145161`},
{0.15350947465095238`, 2.9043684969368213`, 4.5670292387828155`, 1.3486905179026798`, 0.8383532909017835`,
 0.5087310077763043`, 0.053857845014854355`, 402.8647291760491`, 0.025030223476360525`, 5.400937139619534`},
{0.2587691832069661`, 1.4843472048382624`, 4.771777259704052`, 1.1550607981645458`, 0.08010207781888057`,
 0.5149452814008634`, 0.01659115431008023`, 1531.656314816295`, 0.15778107864815993`, 1.1951975901128993`},
{0.1547206763399963`, 3.3076918921708103`, 2.0706333626337727`, 1.4168143839445906`, 0.37770487420755305`,
 0.5386802863405199`, 0.00701518941055165`, 122.30089778268855`, 0.134914758355765`, 2.269217335741727`},
{0.1424711737630725`, 2.7520949793854754`, 4.596768650339829`, 0.8362726667059688`, 0.00009639230672808807`,
 0.36579283504807614`, 0.010146114868681232`, 3412.101956455065`, 0.24682488221918303`, 8.269337606653004`},
{0.10882832688629612`, 2.4842578785622536`, 4.2235021418772565`, 1.4458161330640105`, 0.36880250968204553`,
 0.4736058884190225`, 0.023130853597073738`, 1306.5861668001219`, 0.20634842182153856`, 4.016571058814693`},
{0.18639271747297592`, 2.4942906430499407`, 3.445345566170081`, 1.148752163319608`, 0.12885074455456724`,
 0.17966201189701259`, 0.0741834712240346`, 1169.2351269821615`, 0.09106655178874168`, 4.423161126411237`},
```

```
{0.09766181155973352`, 3.6268090588227118`, 4.041820185573664`, 1.3253596170020208`, 0.4793934216010949`,
  0.49032555462271876`, 0.006759043878711846`, 262.0748117860937`, 0.11178249583803845`, 2.0681460387230555`},
{0.07951891239009712`, 2.8897027315487103`, 1.5411848578052467`, 0.863635998632232`, 0.29085460372381067`,
  0.43469992564602655`, 0.009145988318489378`, 180.1504573795744`, 0.0803419958474173`, 8.628584719983632`},
{0.07234637915492992`, 1.3584521644778373`, 7.973775020047323`, 0.7807691250526536`, 0.9100932527758132`,
  0.18771974645581058`, 0.009648266694560257`, 1956.923094358971`, 0.1277442569760165`, 5.404137919151016`},
{0.26334607729010473`, 1.8094502738990368`, 2.2460302448509974`, 1.2023222076508349`, 0.5513938303777435`,
  0.6812297944975665`, 0.00739497505047526`, 1157.7965975362908`, 0.15524857884582421`, 1.7991882609973402`},
{0.22679920860671537`, 1.574644711817462`, 3.814781873556772`, 0.9990271813793515`, 0.3871644432916528`,
  0.6894928310515707`, 0.05462293330962511 6`, 69.61156300085906`, 0.09745707491156125`, 5.357011938436007`},
{0.054135826493752204`, 3.1195801041969027`, 7.2615169638158825`, 1.0984819310653413`, 0.10801447255588448`,
  0.6366822484935735`, 0.01066160509264612`, 211.97043506851273`, 0.24501368432397974`, 8.92838638172081`},
{0.14590720393667378`, 1.5766246964498798`, 5.158062459855282`, 1.1478320866243528`, 0.3287329300123256`,
  0.4460050972404973`, 0.032458645789603134`, 482.4725013461066`, 0.10279090509715616`, 8.020019312365584`},
{0.14965591009740292`, 1.460367057362724`, 7.337474105676788`, 0.8826427720923014`, 0.44478408083773613`,
  0.631222883334907`, 0.365159753458356`, 157.97713196457462`, 0.16784289093699323`, 1.7957284623930878`},
{0.0810328468371882`, 1.4659479028658335`, 3.234238812997848`, 1.4263751048195057`, 0.08355223050311089`,
  0.5582345497973803`, 0.12450549955799516`, 121.80826494117302`, 0.16010939682803116`, 9.339681585379608`},
{0.15633465648304357`, 2.148716979150252`, 1.5640986685796054`, 1.3949237088061017`, 0.3567264406830608`,
  0.23012843132782612`, 0.08829396925964683`, 2593.62904544209`, 0.24948663022889017`, 4.657794257043932`},
{0.24418896051468997`, 0.7850580710666257`, 9.594718039690303`, 1.3796447707658`, 0.388924613344984`,
  0.22950540339413805`, 0.26843428081063014`, 169.97373853516677`, 0.16535615066521753`, 5.752488029735448`},
{0.161324838944309`, 0.8594526582032156`, 2.9743924517148237`, 1.2482811580906663`, 0.28485670242217753`,
  0.27691573296739147`, 0.12631097975241765`, 80.56658004023716`, 0.24230111347476613`, 7.982613747504004`},
{0.26772168843838734`, 1.7983273472260084`, 5.340026177070573`, 1.4142901992580086`, 0.4537298076184495`,
  0.2865003384437863`, 0.010579448173665554`, 2804.399167772796`, 0.15280115052346555`, 9.9218473198287`},
{0.18205384870834052`, 1.3062696845921566`, 5.075170850216699`, 0.8732096494236974`, 0.8323770222253974`,
  0.3345416157316672`, 0.015614629383397784`, 819.3644184370967`, 0.04873823503517111`, 3.447951588629733`},
{0.15466361671507556`, 2.9485738979940246`, 0.2621130142172472`, 1.4016672383633995`, 0.3670414989298545`,
  0.40252781093021794`, 0.06082671195258038`, 80.30981146445014`, 0.15720245773080782`, 9.014567082907547`},
{0.07129651605023579`, 1.2229228380041173`, 7.261778729610821`, 1.077075564756948`, 0.8974689473036932`,
```

0.5583016490383189`, 0.012773781923945723`, 355.9690714870232`, 0.1545521575965641 5`, 2.741649121941279`},
{0.11901300846945845`, 0.5315793015902206`, 2.6671039936143597`, 1.3880864279120932`, 0.8420811291146328`,
0.5566538571030129`, 0.2079188053795858`, 171.97845991450583`, 0.13095338568755877`, 2.9760745568663016`},
{0.23041310070356935`, 3.6790901828404454`, 2.4540732736358937`, 1.23507526691096`, 0.821599531342406`,
0.33616875277136504`, 0.035023029642451846`, 3607.9630264867633`, 0.21590997641376625`, 4.226227139273737`},
{0.07203443304476603`, 0.5285696054477942`, 8.334423467085383`, 0.8854719875782417`, 0.3459640631982801`,
0.2633909150103768`, 0.03987288196821301`, 568.5739309534505`, 0.056948467212013576`, 6.873946140568169`},
{0.16979941581750402`, 2.100179291019132`, 5.184869462011486`, 1.1997264241396737`, 0.9307785088479614`,
0.3430074594655337`, 0.013577650230569586`, 4088.01556659017`, 0.029357726641530413`, 9.133680945256565`},
{0.21619782984172958`, 1.7733623067535955`, 0.5980153626590834`, 0.9798687198034648`, 0.7538483940425484`,
0.5789929247394512`, 0.006264547120133815`, 61.29286164809138`, 0.15848957716004353`, 2.3238471342304834`},
{0.04816719755022911`, 1.0519290863610147`, 3.0181083809828984`, 0.7801564927089879`, 0.4325460609784135`,
0.2938523943485086`, 0.04249246462941157`, 626.2418769059092`, 0.2424260119832079`, 9.623530958234195`},
{0.2279802782152826`, 2.528677960359964`, 2.539878173580604`, 0.7527299875368043`, 0.8436281080694634`,
0.27514078202436143`, 0.005327161585143342`, 920.5142066248217`, 0.04689927589992776`, 5.404864622946203`},
{0.18595171467721877`, 2.938499684754029`, 3.6306522502244434`, 1.1726699653227697`, 0.9888859131507246`,
0.3480894711653968`, 0.38543337220237905`, 80.27026890715871`, 0.03443629355490174`, 9.328937618149844`},
{0.2088003110428162`, 1.665360213541189`, 8.163801498355127`, 1.2665930603692175`, 0.5382347880039544`,
0.33255923205592086`, 0.0359158004997877`, 88.64295388071032`, 0.08847100949056225`, 8.845547783450662`},
{0.21556335034608287`, 3.553132086531601`, 5.919169032409666`, 1.028261766481214`, 0.01873638959650825`,
0.38505243852661264`, 0.12008970777658311`, 72.3007614977653`, 0.1189730390969882`, 5.747570490343056`},
{0.2061330284551588`, 3.7571620320677477`, 5.463889035312752`, 1.195880973038654`, 0.4606689625278244`,
0.6539206160067124`, 0.07294517832285098`, 79.70328938032284`, 0.04960591713533524`, 2.0731473047959454`},
{0.13099744861910317`, 3.0069806711745155`, 9.6713740346189`, 0.7856145676036781`, 0.3887426527541791`,
0.16465925249784186`, 0.029006890169876405`, 3080.757337617379`, 0.22037025279153283`, 7.1587428033756435`},
{0.20740821458680525`, 2.460110895835281`, 0.6082454159142117`, 0.8064214695794831`, 0.18139487742338534`,
0.30057404623847583`, 0.2202140246268176`, 4056.9433443728003`, 0.07208592746284093`, 2.251460510548089`},
{0.20705709543566436`, 1.1783515457685674`, 5.677465659011105`, 1.0594933834891802`, 0.45712361519708566`,
0.4929063565541889`, 0.07712216778297526`, 129.94158279886685`, 0.033100299542402456`, 5.957665662265306`},
{0.04662251177018917`, 1.087376542683649`, 6.170097112149202`, 0.7648803529340509`, 0.9868309112238562`,
0.551889656411283`, 0.011192508637080182`, 2374.3826064993154`, 0.12804730636711914`, 7.114439815654695`},

```
{0.061725502446144886`, 3.13849642385113`, 9.540008859499451`, 1.4660101376451562`, 0.45012796893408935`,
  0.4212144239280121`, 0.02511250628464842`, 120.07361261472948`, 0.1571099365418398`, 6.445559562130283`},
{0.047554055600075584`, 3.265459078802472`, 8.714860138547323`, 0.8369196863770172`, 0.4962846406178121`,
  0.46335229122842925`, 0.007763546254085084`, 1888.52152221599`, 0.22218896292653229`, 2.633616973587875`},
{0.12567467788677716`, 3.0158399320010547`, 4.901658754742801`, 1.351978052591392`, 0.7221310556231506`,
  0.5024953321675816`, 0.09886516020555723`, 193.78321929587645`, 0.11023982026692764`, 3.349767411033996`},
{0.19691840575768232`, 1.6501723246031572`, 9.011299029553381`, 1.1479337267630148`, 0.646058584475099`,
  0.36485695404700413`, 0.027042804382441818`, 3550.5063392884595`, 0.2269993732427253`, 4.86925454673478`},
{0.22383158609367865`, 3.396592529608882`, 7.0059346900996395`, 1.1116596498970173`, 0.7389255639756462`,
  0.40169511261271784`, 0.008944445083981989`, 1191.861359234809`, 0.06327031154858104`, 2.975039312230072`},
{0.22089456196581753`, 2.0637610394456862`, 7.594372198729954`, 1.2863006630967566`, 0.5843792508368972`,
  0.43898418015861007`, 0.0169422428941798`, 186.99955924932385`, 0.09805501720710208`, 5.47194276574208`},
{0.09910547662807062`, 2.346178866312991`, 7.421049922557871`, 1.3147434313884303`, 0.2750558853148206`,
  0.21685738835966661`, 0.006760522552087531`, 4988.988013498636`, 0.18492351030582366`, 2.8590709623133`},
{0.20816183484471307`, 3.9485705583569546`, 6.550493120626773`, 0.7774564054459185`, 0.5499519895881679`,
  0.2003723071725072`, 0.34567679795977113`, 1808.1918719614162`, 0.23441319529101012`, 4.082991759791533`},
{0.16112203008102222`, 2.4895492142589735`, 0.44352326574872514`, 1.2469876196885359`, 0.9780986797319231`,
  0.6188841396088389`, 0.029743069155058133`, 112.64100571320267`, 0.13296296295840093`, 7.528063016828725`},
{0.17781253097781763`, 1.7213960002432414`, 4.693599924413801`, 1.2010501070587627`, 0.4002263609998786`,
  0.5127527245385753`, 0.08027502692564248`, 156.85332391803223`, 0.06371789077354856`, 6.562151621871855`},
{0.11782384895680592`, 1.1169968183756591`, 9.741344822271138`, 0.8926206302886914`, 0.732042643272727`,
  0.38545027561144396`, 0.005148133378435907`, 521.4771678665235`, 0.04124959681193141`, 8.98600497767481`},
{0.1669047046062308`, 1.025805255669411`, 7.1552343684117155`, 0.8176510429563385`, 0.8282389242605148`,
  0.29891546072913033`, 0.009889011750027802`, 94.42085507888534`, 0.16558325818795072`, 8.472484566593426`},
{0.1009959491070086`, 1.5581491170930981`, 4.3701504080039815`, 1.3889561652621691`, 0.5506040634320337`,
  0.37952236600496847`, 0.006611879537937531`, 361.09151974058886`, 0.21353768992235467`, 8.563496620010564`},
{0.23246941421316458`, 3.062147704374147`, 7.181406357346838`, 1.3012169805011569`, 0.348059834110821`,
  0.639530677915989`, 0.09989941363865933`, 83.25264127475623`, 0.015153685907020209`, 6.841473199486905`},
{0.05240567594084544`, 2.6092929572780967`, 9.364433651132018`, 1.4357965076882815`, 0.3985784064329525`,
  0.6640850131228939`, 0.14135898686373455`, 340.71602063464445`, 0.17921846525178464`, 4.7134535629319085`},
{0.186251758126929`, 2.4933710973488976`, 3.5328550575209547`, 1.325175018028979`, 0.37317622556186136`,
```

```
  0.25977278720819974`, 0.09748704597661054`, 108.88467391318876`, 0.2369656062609477`, 9.08676709988832`},
{0.2432348147394116`, 1.8751950266382789`, 2.5453519666394815`, 1.0050719073546701`, 0.45557496345664417`,
  0.6934059109337196`, 0.0796473645979629`, 116.90111969840831`, 0.13836004707768368`, 5.732847922404471`},
{0.10016679782659832`, 2.355527283731478`, 1.9202752019167662`, 1.4361517107451491`, 0.5493703103299528`,
  0.5953848202767148`, 0.3151585002444098`, 3628.217577021284`, 0.16393396333788363`, 2.9174291385165`},
{0.14701675506563494`, 1.5420515267654622`, 0.49939909194610793`, 1.0246394569387973`, 0.7670831420663697`,
  0.6948893607838256`, 0.005182980912963712`, 1858.6105410956102`, 0.23965764027041103`, 6.073448972699818`},
{0.19166603750475464`, 2.2499911524034095`, 2.4198836269597095`, 0.7671801018390442`, 0.14979093568204394`,
  0.302546021842622`, 0.008837119919208427`, 4450.048280356515`, 0.1035437468879743`, 7.585339524339819`},
{0.14239569401382052`, 2.8387649854604886`, 1.4027026171382353`, 1.2048713691971291`, 0.21617980215068333`,
  0.2290581611576099`, 0.32444520029725066`, 2595.236024076599`, 0.07657553404881084`, 7.455893283173221`},
{0.14874493002083228`, 3.1275671408493704`, 8.341714492380408`, 1.302209514279003`, 0.6258906648056601`,
  0.45549735048188655`, 0.04029116024037646`, 765.0281459953038`, 0.12017823562381152`, 2.4241463017742984`},
{0.06222670957040222`, 3.9065456744185374`, 1.0946055811329174`, 0.8246557540237`, 0.5907991824162977`,
  0.2630274030573405`, 0.006980770676502568`, 3176.3819350381427`, 0.13217715504568522`, 5.816838705721813`},
{0.04971186392349858`, 0.9916432478771275`, 0.9981695561230755`, 0.8524884405534577`, 0.12034821743714219`,
  0.2529703866830304`, 0.3237478827730366`, 360.7437887352434`, 0.010006209625245316`, 7.274213959868414`},
{0.19409490071094637`, 1.638712293913553`, 0.39703017050116074`, 0.7643187357680644`, 0.5335846868412271`,
  0.21830616448247264`, 0.0963055671350066`, 635.9574479204244`, 0.22613729069446092`, 5.479828782022301`},
{0.27029725962327555`, 2.240924310463286`, 1.2051421639323703`, 1.1356504301287609`, 0.773380004303778`,
  0.6607812449412671`, 0.04949916223108672`, 204.2592199369304`, 0.19899441283798802`, 7.18276351221955`},
{0.14266269539818904`, 3.072017618366117`, 3.4103252419872643`, 0.775852439360265`, 0.43949005760262017`,
  0.1924018543570919`, 0.06712102805080977`, 656.0250616869308`, 0.04537130588160604`, 5.285505421294628`},
{0.10769494377900635`, 0.832286650700361`, 9.122967245453143`, 0.9815088864732582`, 0.8421400260693508`,
  0.5448142839433918`, 0.07976087110962524`, 4710.110387525812`, 0.13361893143992154`, 5.1516712920505725`},
{0.21491444350519606`, 1.295137699490157`, 7.08600395884199`, 0.8977823469890502`, 0.4181882258302472`,
  0.21987671758091132`, 0.14871331978208877`, 664.8642422386233`, 0.19734382780045878`, 6.556011365474522`},
{0.14904691011609433`, 3.9586033442731177`, 2.425615791755316`, 1.0187686401741827`, 0.6625310044158794`,
  0.43390068486512956`, 0.00854220404180854`, 96.98019438566024`, 0.2008209408788944`, 6.643325956478872`},
{0.21251186737978806`, 3.785250570140386`, 5.706304857162573`, 0.8638958511126991`, 0.620802282746526`,
  0.4325048599697877`, 0.00898259072796556 7`, 699.3542650472853`, 0.09331259312392975`, 7.885660147589962`},
```

```
{0.24515641699904833`, 3.817676488470438`, 5.668256418323743`, 1.3090245110359662`, 0.8705848781731611`,
  0.5675799494003652`, 0.13511363154659006`, 1804.7556662922755`, 0.20417175811210386`, 6.336735207604568`},
{0.16393709899357184`, 1.7364683461504136`, 6.294465981846198`, 0.7908159510076651`, 0.7611075592144951`,
  0.36561813704297086`, 0.008326625223173748`, 2834.6413370290525`, 0.20899318399811945`, 1.1709155007061636`},
{0.25878729889995966`, 3.1361195765858056`, 8.880297290408144`, 1.0867236136347516`, 0.0902130745148968`,
  0.5352173770607672`, 0.11164972929109021`, 57.41366920823615`, 0.17534323232121918`, 1.541035034614442`},
{0.11203607225462164`, 0.6218658244264867`, 6.440483663444866`, 1.39680689202378`, 0.16534679246130501`,
  0.5081267228820908`, 0.03214478110427463`, 1906.9275025607635`, 0.21932209633484323`, 1.251120855111619`},
{0.24357827172423074`, 3.9240029450424014`, 2.804272198831022`, 0.75931848861878`, 0.2959071515485636`,
  0.6138549707668646`, 0.10713705928623629`, 339.1202760730188`, 0.035220064551167996`, 7.297254233929856`},
{0.24204908128005126`, 1.6039606621918692`, 7.422941916054803`, 1.4757185212808246`, 0.48127457855983957`,
  0.4526414340963527`, 0.017845029630840916`, 286.8959659308558`, 0.1351657578784522`, 4.5748150305594475`},
{0.0765658571842559`, 1.8453576022836256`, 0.8953985896867352`, 1.0948080634821278`, 0.11609992165592442`,
  0.45437290836767896`, 0.20627344098654643`, 689.3327160072474`, 0.18998846953804188`, 8.643335566773064`},
{0.2258389851785662`, 0.888336357939937`, 0.32961763645801767`, 0.9210788231382718`, 0.8596897387491109`,
  0.5585019604624183`, 0.04381971178906294`, 415.715019082411`, 0.24817184929202513`, 7.285653344604734`},
{0.19210976086993148`, 0.9419570933782317`, 5.851922457612897`, 1.40657571618961`, 0.8492777725664675`,
  0.5465312705852027`, 0.10025464283588155`, 725.5272924080149`, 0.05639485661927951`, 6.120948580338062`},
{0.04040676752094921`, 0.7630185090261814`, 4.034875094487921`, 0.9393317585048341`, 0.5565744536568444`,
  0.3001585479016161`, 0.008819784570716005`, 3975.779851508773`, 0.23610962920225392`, 2.0975088246946054`},
{0.2323130275502685`, 0.49681822670142894`, 1.2539687147478098`, 0.9468502150118296`, 0.13096405194857463`,
  0.3219849827616931`, 0.04046745571036271`, 95.23490611137812`, 0.01783356001323902`, 6.936606555558118`},
{0.1110467064554368`, 3.3883763096282147`, 5.159131249779884`, 1.2482205179674581`, 0.8201347270890553`,
  0.46628896390017405`, 0.2764030660881924`, 2912.733505822841`, 0.03458542948115101`, 2.6312047625809307`},
{0.1632243866872038`, 2.5373481239797906`, 9.958152546995578`, 1.2439631103015851`, 0.6072525242529792`,
  0.6395983432777625`, 0.3899581087433961`, 56.26697227139985`, 0.18832987989003086`, 4.752251898931052`},
{0.16346002078838484`, 3.218794874634134`, 9.025936619626364`, 1.1259348004332868`, 0.7415896232848811`,
  0.4862549245509259`, 0.364081252118343`, 137.81289300851844`, 0.05819144446151425`, 8.24717117891944`},
{0.19514620721272602`, 2.87732243599938`, 0.26739185721478975`, 1.4891953183216857`, 0.05289250725423611`,
  0.4217001319921505`, 0.03447210696714538`, 218.70480172461387`, 0.0713049851341147`, 6.944425315128572`},
{0.06305409129179324`, 2.891634576752894`, 4.887865973915014`, 1.4252999692587962`, 0.37958790888649707`,
```

0.5644849529569385`, 0.3706753829912659`, 1888.9507831786343`, 0.129560920022812`, 6.557119455026179`},
{0.07134729442997761`, 1.925040188749243`, 1.1575324502817335`, 0.9987470940766925`, 0.48237853230783223`,
0.6023915002525595`, 0.11093155711801116`, 121.08543179928522`, 0.23351841944906743`, 5.375810156733433`},
{0.1360789534218197`, 0.4455509561284261`, 2.009215820725272`, 0.8936493225292423`, 0.96227260378033`,
0.5587588217121244`, 0.041826495849356216`, 90.5779416890061`, 0.15671264889903508`, 7.812316525319764`},
{0.15849386520968983`, 3.640724074855333`, 1.8861164484754733`, 1.1569149894265711`, 0.5885808127893892`,
0.1748767871381529`, 0.46502697264632115`, 193.59851086045592`, 0.1503277806325949`, 4.212693145809347`},
{0.2646664636377358`, 0.7835752608143567`, 7.19251403558616`, 1.494052846937745`, 0.8510899770532778`,
0.5885640033353514`, 0.48048609718479995`, 154.5393756420333`, 0.18357560397946`, 4.027566835631127`},
{0.12495672263621771`, 3.9102582538632182`, 1.1196234634441904`, 1.2648993449094799`, 0.05034343178950351`,
0.5637162084123208`, 0.0377865951253564`, 87.92444212100203`, 0.05653305553347371`, 1.253921938823833`},
{0.22032142146595135`, 2.68397072068061`, 2.6883455170430324`, 1.1162234719994681`, 0.40997309115380154`,
0.5558969738438672`, 0.02165064984593792`, 1526.6454168541877`, 0.24054798278325668`, 4.966530798126495`},
{0.10964967041383433`, 0.7460930180313401`, 6.531951119775776`, 1.1455194328360876`, 0.1524629724176152`,
0.28792352560938195`, 0.09608079533773954`, 1051.3203547256705`, 0.17626541392472717`, 8.294356744338359`},
{0.07988018646299105`, 2.6019950195572488`, 9.606629629213668`, 1.0662798233306474`, 0.7693895670648874`,
0.32124913017390155`, 0.04062772179058327`, 1822.0454711227774`, 0.21556365885863799`, 7.4522296585239225`},
{0.1682679068810577`, 1.5454861727740674`, 1.469740171862469`, 0.9427424173065856`, 0.7350490973518766`,
0.3805201167376455`, 0.012952191039290932`, 4650.232844583341`, 0.019462289964433777`, 1.394906478790226`},
{0.1878353522665755`, 2.6490780379134238`, 9.484290061448991`, 1.1885927795104771`, 0.9576534469556572`,
0.2804046795618802`, 0.13562182147346188`, 68.8621810881709`, 0.06230339869012863`, 8.081807561887658`},
{0.195165600718623`, 2.3826254740878197`, 7.662410402528582`, 0.9519993324167042`, 0.5246436284800724`,
0.22761517290878341`, 0.02889008666683709`, 101.1296763305189`, 0.18152629399038878`, 2.6385617746928904`},
{0.21914596002979703`, 3.4188550030486784`, 4.710500615641845`, 1.4527326168664971`, 0.03624911304432854`,
0.28384065291200533`, 0.4254237737178384`, 3505.5630456480862`, 0.12312118964896007`, 9.273808474100267`},
{0.1814951436259516`, 1.11858432682377`, 5.7029405020905966`, 0.816783193035002`, 0.6766309251156815`,
0.4321734260024682`, 0.2931263656368919`, 87.29527865588257`, 0.044724422492498916`, 2.4174522941648533`},
{0.22203693974513244`, 0.9431525918127432`, 4.196712511517475`, 1.3261224346879132`, 0.8036004422541301`,
0.6271444136150295`, 0.10733581071086257`, 369.9451385562586`, 0.16273465181192748`, 5.731309489564488`},
{0.18924733350115397`, 2.8150904404250037`, 2.4574844917932026`, 0.8737719488383665`, 0.6977173227044986`,
0.3482841124888072`, 0.48079795163551986`, 94.66261771766344`, 0.1647107241653021 4`, 1.3078470560939532`},

```
{0.07907817004881895`, 1.0481324229512046`, 3.8137728667258273`, 1.2920968808020492`, 0.04181350128653394`,
  0.5803710922764521`, 0.02851300126208627`, 266.99553906648964`, 0.22548933562284668`, 2.0140176417924156`},
{0.22302087067747528`, 1.8201511620752422`, 1.875184623833773`, 0.7813352529024251`, 0.8705057770695381`,
  0.22074020090969038`, 0.1367588752741694`, 187.45561858905666`, 0.07793294287132913`, 8.923384483271061`},
{0.21429795289589076`, 2.896671823601787`, 8.636702505614757`, 0.9811380648364925`, 0.20610104158853804`,
  0.1778381348410949`, 0.007936414694970003`, 185.13169573811163`, 0.014685687390926405`, 3.2353159752463547`},
{0.17507336986882482`, 0.7983306290353371`, 2.750450064120228`, 0.8050075785267179`, 0.7818959542274213`,
  0.6526925477724106`, 0.0965325694706893`, 72.78219945862553`, 0.07700161447215975`, 3.655530763820291`},
{0.1470766374450453`, 1.2533511161361544`, 4.593526904612924`, 1.479552314271993`, 0.7400477950348978`,
  0.5768087033937906`, 0.10619660427114413`, 242.62879808709684`, 0.045931800134058565`, 3.771378090721337`},
{0.0788585810797619`, 2.548378832414274`, 3.111482597871378`, 0.9346713900878518`, 0.19963135196405957`,
  0.5058944800586684`, 0.03749384602431879`, 1118.4164778142415`, 0.22872744797764172`, 1.4303214452036404`},
{0.24182502946725698`, 1.8945200327623395`, 6.300131593939071`, 0.7669603268824658`, 0.6959163653444247`,
  0.5105300710554361`, 0.2329066367569749`, 178.13280663193103`, 0.21420184435482204`, 3.5903109736592462`},
{0.05758087297506348`, 2.9919516996565125`, 9.945704556467732`, 1.1770757830874041`, 0.6136122924727272`,
  0.31603398268032157`, 0.21009959511709828`, 118.22678602178958`, 0.2258513747270965`, 6.274152149731609`},
{0.12816326660379745`, 2.7095777054297967`, 7.985014815061977`, 0.8582103259977079`, 0.28527001685481057`,
  0.4561351167228034`, 0.41006088059385215`, 4928.696897400591`, 0.12257221770331383`, 5.545857987270676`}};
```

In[ ]:=

```
result = {{"\!\(\*SubscriptBox[\(κ\), \(c\)]\)", "\!\(\*SubscriptBox[\(κ\), \(p\)]\)",
    "γ", "V", "\!\(\*SubscriptBox[\(k\), \(s\)]\)", "ρ", "ω", "α", "\!\(\*SubscriptBox[\(k\), \(f\)]\)",
    "\!\(\*SubscriptBox[\(N\), \(0\)]\)", "\!\(\*SubsuperscriptBox[\(A\), \(cur\), \(sim\)]\)",
    "\!\(\*SubsuperscriptBox[\(A\), \(cur\), \(est\)]\)", "Err", "\!\(\*SubscriptBox[\(M\), \(m\)]\)", "Canc",
    "\!\(\*SubsuperscriptBox[\(Bl\), \(Frg\), \(sim\)]\)", "\!\(\*SubsuperscriptBox[\(Bl\), \(Frg\), \(est\)]\)", "Err",
    "\!\(\*SubsuperscriptBox[\(Bl\), \(Ab\), \(sim\)]\)", "\!\(\*SubsuperscriptBox[\(Bl\), \(Ab\), \(est\)]\)", "Err",
    "\!\(\*SubsuperscriptBox[\(Cld\), \(Frg\), \(sim\)]\)", "\!\(\*SubsuperscriptBox[\(Cld\), \(Ab\), \(sim\)]\)", "ToVbl",
    "\!\(\*SubsuperscriptBox[\(fD\), \(Max\), \(sim\)]\)", "\!\(\*SubsuperscriptBox[\(fD\), \(Max\), \(est\)]\)", "Err", "Nnew"},
  {0.172487255493398`, 1.2765963995094278`, 0.2324603897081641`, 0.9331633444796544`, 0.5595329534566904`,
    0.18253234530133355`, 0.2215389709063929`, 78.80610179612091`, 0.19190036455619353`, 1.548441048845115`,
    67743.09410144144`, 0, 0, 0, 0.6852674042781265`, 0.10718857420740713`, 0, 0, 19554.94224955615`, 0, 0,
    1.9548078271675062`, 48185.40457082622`, 4.008069782940419`, 1.0000000000003644`, 0, 0, 0.05191808718578425`},
```

{0.14090572053825962`, 0.941193557649755`, 3.8052282638940014`, 1.32622706664442`, 0.616835667267962`,
0.48175991442091237`, 0.08306143231635953`, 874.0744302472489`, 0.12491077781228116`, 5.511666182290989`,
116.46970588923584`, 0, 0, 0, 53.87426457434697`, 4.315726531299664`, 0, 0, 0.08363006331181534`, 0, 0,
58.02762868339093`, 0.16834220470863487`, 1.3599387416101831`, 0.7310244216325208`, 0, 0, 0.5110180678336491`},
{0.19800522656535097`, 1.47676715957736`, 1.6845843495593211`, 1.0829526404841552`, 0.6930225215765629`,
0.2749384033948816`, 0.06680029700083288`, 3207.8452923633004`, 0.18818451872370445`, 6.655704647717006`,
31.887329398445925`, 0, 0, 0, 16.478864419030586`, 0.6931194467673891`, 0, 0, 0.02421221660802215`, 0, 0,
14.622514809293001`, 0.06848779193029836`, 0.43414946439205965`, 0.3789034695609528`, 0, 0, 0.40056255200378343`},
{0.2711930593993829`, 3.0021543969280486`, 4.914701090552741`, 1.464602766667476`, 0.8471176050384048`,
0.28257943270869035`, 0.04953192991909177`, 1933.8633413872876`, 0.19773521340575795`, 9.992985154010565`,
105.13570106511645`, 0, 0, 0, 61.94141836278736`, 0.9815746806714228`, 0, 0, 0.02318432518635069`, 0, 0,
42.09769633558496`, 0.08982040110563455`, 1.0647436647159723`, 0.28815869775973646`, 0, 0, 0.43962980242854593`},
{0.27990766836391096`, 3.477149477100016`, 9.931945787531824`, 0.8846854018858359`, 0.8967333599095253`,
0.5903168559967787`, 0.24882968868487906`, 980.8551320933655`, 0.02004206946214948`, 1.1376503486553808`,
42.727467045547805`, 0, 0, 0, 9.53729452918055`, 0.6521216712025372`, 0, 0, 0.02897630434645414`, 0, 0,
32.39320754324975`, 0.11586699696319083`, 0.24177553336415214`, 0.49835109389883314`, 0, 0, 0.06389331109327692`},
{0.11737985888146019`, 1.1225833279930164`, 5.309079794927442`, 0.8606501327539497`, 0.8514188342619913`,
0.5642039473140849`, 0.008986110654413243`, 2018.9822529242097`, 0.06040743860925685`, 4.536035630396375`,
73.55385408728918`, 0, 0, 0, 65.13966916603187`, 0.4872861326413754`, 0, 0, 0.02119073517368962`, 0, 0,
7.814561263791457`, 0.03553379291831031`, 0.46864576046452`, 0.40857714960977276`, 0, 0, 0.25798090396825835`},
{0.17121040978700802`, 0.4138167442315588`, 6.781135564610604`, 1.3665406840239216`, 0.9405996253397015`,
0.6832439228870748`, 0.04185895434111264`, 62.7113536738392`, 0.18142736225630413`, 8.46259785026923`,
126972.27674519586`, 0, 0, 0, 298.1254945315542`, 25.354892784275624`, 0, 0, 36710.36451460944`, 0, 0,
149.88970260470268`, 89788.52217109627`, 29.778277025817353`, 1.000000000000286`, 0, 0, 0.9990782166212182`},
{0.07382619562964193`, 0.625700255424527`, 9.939999934198667`, 1.4556453073248168`, 0.9580351575689623`,
0.4163162212955882`, 0.006430521616962508`, 92.19524127790851`, 0.19149999891406427`, 4.303065608360628`,
10092.366546722247`, 0, 0, 0, 336.4548319381863`, 3.0304371986770398`, 0, 0, 4733.347998532603`, 0, 0,
27.08779041800309`, 4992.072504612018`, 10.739730236128622`, 0.9999999999999984`, 0, 0, 0.7137046293076434`},
{0.1627307868596377`, 1.5327585946689206`, 6.648641489637967`, 1.0898921981857375`, 0.08271984399349908`,
0.5169005126785295`, 0.006231976105310307`, 2017.0154068595314`, 0.2464092033815996`, 1.6313338993270357`,
8.432667214624587`, 0, 0, 0, 7.733066884282825`, 0.029388353758957216`, 0, 0, 0.005578197887301527`, 0, 0,

0.643503597245891`, 0.012967779020847576`, 0.190737104776319`, 0.10304664105276695`, 0, 0, 0.3179124752554388`},
{0.08926805346894312`, 1.228740834290055`, 5.100082063336529`, 1.0507919032716577`, 0.3229790419061651`,
0.45218339761418835`, 0.024533377777917375`, 814.1470590464116`, 0.1414263079606694`, 5.247711540674161`,
79.62930256895696`, 0, 0, 0, 59.267347626410526`, 1.0936076543521438`, 0, 0, 0.025010263836254435`, 0, 0,
19.196576879923473`, 0.031894536705833865`, 1.4402240475823056`, 0.39845435605455803`, 0, 0, 0.6932296020886753`},
{0.17431851399024534`, 2.020213173041662`, 9.224968841892476`, 1.3485881267881612`, 0.8890825947176819`,
0.5177484922558413`, 0.03701694525181502`, 131.5752033573906`, 0.228949740900746`, 4.795606274383001`,
443.45874279587423`, 0, 0, 0, 259.0825396937692`, 4.43540653733214`, 0, 0, 14.872912654837938`, 0, 0,
128.0066673501924`, 37.03748618140094`, 8.661545697586357`, 0.9999999995956493`, 0, 0, 0.9785932185552205`},
{0.25439204271280724`, 0.8534844729419486`, 2.8150623547133353`, 0.8024992452769948`, 0.43579385025887163`,
0.4594767236090769`, 0.057877631906885676`, 1195.6419878079198`, 0.17833211044546654`, 5.195270679096961`,
61.19257325377502`, 0, 0, 0, 33.83687275287343`, 2.06271165727129`, 0, 0, 0.03075389554574142`, 0, 0,
25.149891023391465`, 0.11176494727508911`, 0.9581936401948361`, 0.5558870737465285`, 0, 0, 0.6093891684973874`},
{0.129274073243755`, 2.8469118022753515`, 4.121632588773114`, 1.2196787674185057`, 0.8228855513020883`,
0.4828002917324088`, 0.013782155470303842`, 388.66064646424104`, 0.2255512748456478`, 1.0022663108918284`,
37.4176094379522`, 0, 0, 0, 29.4879073769192`, 0.13654781137290922`, 0, 0, 0.7806644759706806`, 0, 0,
5.5534225110343485`, 1.441709666363287`, 0.5920060413350425`, 0.9999924873036613`, 0, 0, 0.16352024139151572`},
{0.06343574194222029`, 3.9924293708982113`, 9.079106395846292`, 1.3161791897123671`, 0.10938228479468126`,
0.3240611741083924`, 0.010321331270591745`, 76.94152103093134`, 0.06038801289350482`, 9.425890207979073`,
70205.26592740085`, 0, 0, 0, 617.0349251028783`, 1.5271193117917539`, 0, 0, 36458.849768206324`, 0, 0,
87.09879990376076`, 33039.91693437305`, 27.081181575773556`, 1.000000000000078`, 0, 0, 1.1313574553864445`},
{0.110036074735397`, 2.490203037930516`, 9.729597307787852`, 1.4397421355398403`, 0.8533380591052817`,
0.37040757295392324`, 0.047916566872126407`, 665.4588326486067`, 0.16916230254726894`, 1.9013228378216027`,
67.32591358102374`, 0, 0, 0, 40.13822181606069`, 0.7401561002292918`, 0, 0, 0.04490276463618321`, 0, 0,
26.330556704768394`, 0.07058462807615701`, 0.5937917974429145`, 0.4840972551092325`, 0, 0, 0.1007400788020962`},
{0.21512225225566667`, 1.75750084110844`, 0.8044386006474246`, 1.3520587790631349`, 0.026316391204000755`,
0.16031186333306224`, 0.13761957869990635`, 493.8180531489964`, 0.06250485252841559`, 2.6863460670624115`,
31526.667301351725`, 0, 0, 0, 5.7374860567496535`, 0.41344711426966924`, 0, 0, 7736.013217670315`, 0, 0,
10.380480729754288`, 23774.122669497756`, 1.1123862754205833`, 1.0000000000000093`, 0, 0, 0.0968571533989443`},
{0.15874286665935028`, 2.268211451796655`, 1.8981682437883747`, 1.375959688601855`, 0.24814706074480242`,
0.5134877163486878`, 0.3316652449509965`, 685.632474632321`, 0.05319219913715695`, 1.7896119638851753`,

31.96808009735873`, 0, 0, 0, 5.723056881609258`, 0.7253203465303132`, 0, 0, 0.6172839032770692`, 0, 0,
23.50257023173107`, 1.3998488049839315`, 0.6308164743113699`, 0.9990402409674988`, 0, 0, 0.40176322274149523`},
{0.07432908705186819`, 3.0619407712521785`, 1.7905302646779082`, 0.9549392645174688`, 0.353226782755657`,
0.6119520610188782`, 0.0496137109124893`, 317.5186768305259`, 0.07812208014742161`, 6.620973402603631`,
20840.6317126216`, 0, 0, 0, 60.48673906551274`, 0.9317428431738233`, 0, 0, 10058.187504108044`, 0, 0,
40.75630571194792`, 10680.227065383699`, 4.815192177506494`, 1.0000000000000064`, 0, 0, 1.125521406931183`},
{0.1228443083266203`, 2.0512729485932484`, 1.9256217622349043`, 1.4810122884336692`, 0.07741458536098089`,
0.4641958329015734`, 0.007197139507585793`, 240.76714600918612`, 0.03301937102418029`, 9.328377628444095`,
131731.62537204823`, 0, 0, 0, 133.12817715678864`, 0.44449474733410377`, 0, 0, 47763.62158287618`,
0, 0, 13.025429299974743`, 83821.27223604081`, 8.468469846397717`, 1.`, 0, 0, 1.0021975742950489`},
{0.2606519449147342`, 3.818605783771848`, 8.595720359336667`, 1.112914365339814`, 0.820058745442926`,
0.4441576207168524`, 0.053270363092160024`, 1117.1606910609416`, 0.20810873381921946`, 6.830482236754131`,
158.217216712755`, 0, 0, 0, 90.32808338603802`, 1.2200679224930844`, 0, 0, 0.023366782525737022`, 0, 0,
66.55654893466564`, 0.08700853302474741`, 1.273332899934598`, 0.3626997465843119`, 0, 0, 0.37693957444604737`},
{0.24750930586460296`, 3.1846993652287674`, 3.026718658979185`, 1.4845570571034035`, 0.869864794311124`,
0.6669561203654737`, 0.2363643381944309`, 1924.496556700219`, 0.06437651322808385`, 2.548184450019704`,
51.48595308643028`, 0, 0, 0, 11.838152098414339`, 0.8394163885406494`, 0, 0, 0.13636790852796`, 0, 0,
38.18984056782889`, 0.48217609117090143`, 0.2806487892452064`, 0.8351464246350968`, 0, 0, 0.188360875388165`},
{0.24908529437358162`, 3.626300618072209`, 2.0230682918259397`, 1.4602193573319078`, 0.40130475942739396`,
0.6650543311625685`, 0.019582320105998748`, 125.14663606859848`, 0.03716084844005629`, 8.149858668846527`,
485275.3769879387`, 0, 0, 0, 103.34858566354733`, 0.541837019969642`, 0, 0, 106429.33765097226`, 0, 0,
28.069484577290076`, 378714.0414111101`, 13.938126391391329`, 1.000000000000063`, 0, 0, 0.6879769444359256`},
{0.2795730004018654`, 2.183215118244356`, 0.7910515142573491`, 1.2847699393543137`, 0.70345738565049`,
0.2632113724473232`, 0.1394636484917589`, 223.91272255356017`, 0.16150366940434352`, 9.859315316872873`,
53076.817736040095`, 0, 0, 0, 20.24333656208575`, 1.2211784841523583`, 0, 0, 10616.405231097064`, 0, 0,
38.08707612394481`, 42400.86091342664`, 8.984599844368203`, 1.000000000001575`, 0, 0, 0.33363349472311576`},
{0.08969576723701717`, 0.49356011207206274`, 0.2529879905685366`, 1.1041769014657603`, 0.7443718717514693`,
0.6180257213599576`, 0.03574805853679543`, 3205.3226496399375`, 0.061225880931137455`, 7.560768798013438`,
2171.9947059835604`, 0, 0, 0, 11.112362854328696`, 0.6868508337658673`, 0, 0, 944.7622127219953`, 0, 0,
4.842888207003865`, 1210.5881646663056`, 0.5500948044981915`, 0.9999999999999967`, 0, 0, 1.3729366406278294`},
{0.10298476113266081`, 3.435925289367793`, 4.954566778741102`, 0.9299871443466801`, 0.6352691398464292`,

```
0.5470174017743717`, 0.005507148986589441`, 973.5524715904572`, 0.1328513609688533`, 5.618259356059234`,
117.49696230705861`, 0, 0, 0, 108.82106480134435`, 0.16901447170440292`, 0, 0, 0.0362899394001093`, 0, 0,
8.29601567997564`, 0.05339015343770032`, 1.2448752933815868`, 0.5637226440483438`, 0, 0, 0.5222944997497027`},
{0.14277679649227548`, 3.0428582592918065`, 7.414614443890604`, 1.1046799505745009`, 0.2872163059276376`,
0.23677108379761724`, 0.04608733451503689`, 2695.430951291806`, 0.1274330436692639`, 8.066834406130887`,
27.508200949789227`, 0, 0, 0, 16.827455730939224`, 0.2399406828065002`, 0, 0, 0.00329055233667431 1`, 0, 0,
10.43007840596963`, 0.00671163601886364`, 0.6414452374245303`, 0.062102275311818`, 0, 0, 0.6932870847812999`},
{0.18738335776550036`, 1.508373316422973`, 6.602438992729994`, 0.7532357689229783`, 0.8880958875806098`,
0.4853414284787141`, 0.10975729606650726`, 2118.598035369477`, 0.05093222572935868`, 5.0448517662608`,
76.70717437665523`, 0, 0, 0, 30.152081431348936`, 2.062569830305099`, 0, 0, 0.013018988902814879`, 0, 0,
44.44464707558958`, 0.034850597933199036`, 0.4926114049094028`, 0.30970139788639983`, 0, 0, 0.24295238136663233`},
{0.061980185747317895`, 2.54906136842855`, 8.170240160100523`, 1.0602973698223939`, 0.7221801534586956`,
0.6067985789160708`, 0.38301062006579967`, 81.55317476370394`, 0.23749102694434165`, 4.188403663256118`,
22951.475716120684`, 0, 0, 0, 50.828471892691006`, 6.645904161706482`, 0, 0, 12014.150102668335`, 0, 0,
242.01167938406448`, 10637.703642279172`, 12.050133884605344`, 0.9999999999999992`, 0, 0, 0.7907448228697149`},
{0.2562298689236346`, 3.2913375042385793`, 9.509882785943699`, 1.013989937500057`, 0.7792587778840148`,
0.49077497767429623`, 0.021163604915718004`, 145.96678185214677`, 0.05845136191000838`, 7.439112841768198`,
627.823017294315`, 0, 0, 0, 467.54872550802116`, 2.870264203324775`, 0, 0, 4.783784735009045`, 0, 0,
134.95726027823258`, 17.510693365860277`, 11.695328836932138`, 0.9999999978600449`, 0, 0, 1.2103433598707598`},
{0.2313093023364764`, 2.988629640967633`, 6.855539828479465`, 1.0799498768994156`, 0.4522471358822151`,
0.2285321965248518`, 0.04827992453162874`, 1204.6728692817303`, 0.13073517219860892`, 1.9378468299164582`,
16.257879702552408`, 0, 0, 0, 9.734941782863014`, 0.14838698613263554`, 0, 0, 0.009028320800266611`, 0, 0,
6.33533921556925`, 0.029833351222570893`, 0.3416993786251959`, 0.16396532992320656`, 0, 0, 0.14777542398238708`},
{0.2663151909473739`, 2.2417238355718707`, 0.9398485531379794`, 1.0477480676389026`, 0.7226958973603346`,
0.5716146939488362`, 0.007506086251562624`, 76.16131590346896`, 0.024911856209502148`, 4.070747059506875`,
954570.6446215768`, 0, 0, 0, 27.240681578816293`, 0.0876924791752946`, 0, 0, 198676.22852994598`, 0, 0,
2.808318868109256`, 755864.2533950963`, 11.263422543948309`, 1.0000000000000069`, 0, 0, 0.2756279026641679`},
{0.19929196929676601`, 1.9362608032362312`, 0.5353176928827672`, 0.7753335837033051`, 0.8607728477687919`,
0.15482089410819944`, 0.02026432754563482`, 192.89182731397838`, 0.1831217833074511`, 5.333726168282188`,
25098.2949487175`, 0, 0, 0, 16.832505736651342`, 0.16752429125285953`, 0, 0, 6518.448603603513`, 0, 0,
4.633867410612032`, 18558.206556741323`, 5.600408897991077`, 1.000000000001345`, 0, 0, 0.13965757044260615`},
```

```
{0.10696417975467426`, 1.9440197080088568`, 2.0936739600677434`, 1.1829890119130342`, 0.8864433537218006`,
  0.5974069860908109`, 0.3135162096012827 4`, 84.46268853549806`, 0.02353560759327844`, 4.656274919049821`,
  572513.3395312632`, 0, 0, 0, 15.609951394631532`, 2.2684509853665826`, 0, 0, 226431.5209369653`, 0, 0,
  62.998763174353535`, 346000.8843946555`, 12.23863480062775`, 1.000000000000286`, 0, 0, 0.5811789555379678`},
{0.21459986298539707`, 1.6769788549349993`, 9.356964926187835`, 1.2601407238694713`, 0.895239496536256`,
  0.38446102196425924`, 0.012005779035619358`, 146.09102757400478`, 0.02879957197754268`, 2.3826604675291154`,
  193.3328311433502`, 0, 0, 0, 158.73957240461607`, 1.0659101113960512`, 0, 0, 1.9392171215859675`, 0, 0,
  25.535838829608362`, 5.9450818370183605`, 3.6383279149492043`, 0.9999999665113224`, 0, 0, 0.3104099907350998`},
{0.07383925157057758`, 2.6358268091067414`, 6.251616293116599`, 1.1087155777827833`, 0.9205185682116173`,
  0.19659269091732745`, 0.29632431074176147`, 531.6045072192474`, 0.12599923353908937`, 2.5100327097656248`,
  79.86268730333829`, 0, 0, 0, 16.01828676867377`, 1.6484978580661422`, 0, 0, 0.05949932473593963`, 0, 0,
  62.07364070065391`, 0.0627626515351894`, 0.9671079186658935`, 0.680115980217955`, 0, 0, 0.0948365295512122`},
{0.0817054148315825`, 2.796991931647562`, 7.6919124752432`, 0.9355088496592321`, 0.8578766380044134`,
  0.4976427721124693`, 0.008010560795693933`, 67.84344037448854`, 0.080482215315478`, 7.8347514420046025`,
  92958.46968521253`, 0, 0, 0, 454.23945465571876`, 1.2453927234059647`, 0, 0, 42659.58423111119`, 0, 0,
  49.762191415700954`, 49793.12894493963`, 25.94960471701653`, 1.0000000000000016`, 0, 0, 1.0851682251877146`},
{0.23618801128357925`, 0.8353216061115365`, 4.620412867513723`, 1.4504705173961072`, 0.3108708881036355`,
  0.3400688454787608`, 0.014061322572830477`, 2071.8308884477033`, 0.0364017192015193`, 3.610034284058532`,
  18.55464388397803`, 0, 0, 0, 15.46537846505077`, 0.2344826609461253`, 0, 0, 0.010912129974419132`, 0, 0,
  2.798120470668914`, 0.036818775393231105`, 0.38298554235939963`, 0.14841974544056524`, 0, 0, 0.4102708513984332`},
{0.1662441272521787`, 3.725772450959033`, 9.814227303865422`, 1.3148509050359762`, 0.25298305094984697`,
  0.32303330970203903`, 0.055784478318004704`, 213.77275876328028`, 0.04990125708904308`, 9.833393949415544`,
  466.6166735795884`, 0, 0, 0, 263.83326207866`, 3.7355284106241533`, 0, 0, 0.06462441888239463`, 0, 0,
  198.82469774397546`, 0.15347757308971366`, 10.077154442444469`, 0.6512817269992872`, 0, 0, 1.0813263206266797`},
{0.22831788422124638`, 2.590756298379799`, 9.795768388114066`, 1.125355926333821`, 0.3650031672541203`,
  0.1680578019226482`, 0.4143448211959925`, 831.1374036828946`, 0.21412864715242685`, 3.6048538714465157`,
  36.91913379485488`, 0, 0, 0, 6.0942373753121775`, 0.8100593739778663`, 0, 0, 0.007951813901167277`, 0, 0,
  29.980948931353613`, 0.02593630465196313`, 0.9110259063453554`, 0.14102749767699185`, 0, 0, 0.2315638436862914`},
{0.23359626472155715`, 2.56674003123695`, 1.1969232842409259`, 1.0442579072771299`, 0.8882810964774697`,
  0.3176999965402193`, 0.007340467537817918 5`, 1908.0313626467632`, 0.2040761417499009`, 9.648978920291999`,
  91.99309767384098`, 0, 0, 0, 81.80779411021146`, 0.22480149934642626`, 0, 0, 0.37772279339753806`, 0, 0,
```

8.24295724935086`, 1.2604947662550834`, 1.0623402223119554`, 0.9984625645942601`, 0, 0, 0.6210452897612784`},
{0.2534162537452666`, 1.6838285982829593`, 0.8034274134000761`, 0.870316935261816`, 0.7856829676536639`,
0.46427070430809425`, 0.12261242146523575`, 210.18201198829036`, 0.1377493870208059`, 1.4006481974514937`,
42825.83088445403`, 0, 0, 0, 3.2648574074127836`, 0.2226653540419275`, 0, 0, 9267.280386498287`, 0, 0,
5.35614701403706`, 33549.70682790549`, 1.3999479013669103`, 1.0000000000000007`, 0, 0, 0.09018742377791931`},
{0.25695579183074796`, 3.423363059474246`, 4.134350766656727`, 1.2838485736639544`, 0.4149036036442244`,
0.6493708976181658`, 0.12875465899507466`, 1172.2742202486922`, 0.012611679323919878`, 3.4839902382375243`,
48.702991447874965`, 0, 0, 0, 17.511948122187576`, 0.6216523980056863`, 0, 0, 0.03583194013995375`, 0, 0,
30.402026502374998`, 0.1315317793070353`, 0.6763770526054163`, 0.44344726123802747`, 0, 0, 0.5333666313252329`},
{0.17682432929033887`, 2.45185381012344`, 3.9338825987195882`, 1.2879615271399696`, 0.02702036645869277`,
0.6694394004648141`, 0.11330549796901213`, 154.96108716979026`, 0.11583322861965717`, 8.805457336592767`,
62075.72329942178`, 0, 0, 0, 112.03221704654264`, 4.883048713426663`, 0, 0, 17523.167392950818`, 0,
0, 171.03602275762168`, 44264.60459001253`, 12.638706545489008`, 1.`, 0, 0, 1.1176483827463262`},
{0.2509750819824499`, 3.8883567883501247`, 6.384989889678234`, 1.4613334209290016`, 0.7183189010632474`,
0.4965470763778975`, 0.3859108436384297`, 748.6242458418322`, 0.020020198926280153`, 5.690775103224375`,
170.7024644094458`, 0, 0, 0, 27.51216914856034`, 2.5266988536944233`, 0, 0, 0.06774702896279644`, 0, 0,
140.35295199827402`, 0.24289737354303692`, 1.6132276002711672`, 0.6253733053317605`, 0, 0, 0.4282447957611487`},
{0.1441623498715573`, 3.343288457669903`, 1.621696793707331`, 1.3375455077897458`, 0.06814809058713811`,
0.4010236514346772`, 0.3002310191555227`, 155.99799531014958`, 0.10001900063604485`, 4.2763953522223215`,
79261.33786662415`, 0, 0, 0, 11.038068702257304`, 0.9081687320150765`, 0, 0, 25888.86948936154`, 0, 0,
43.375286276610275`, 53317.1465872061`, 5.848386997227198`, 1.0000000000005629`, 0, 0, 0.34611033087063475`},
{0.2657479684896014`, 3.193863728015164`, 2.3963245604206165`, 1.4113571594678591`, 0.26744798738201037`,
0.235299669124016`, 0.018584060295236755`, 466.949287196137`, 0.23679723903863448`, 8.690245518267893`,
169.6572270508962 4`, 0, 0, 0, 132.32820284668716`, 0.730937257935614`, 0, 0, 0.6661125970682856`, 0, 0,
33.35019993679177`, 2.5288295636604`, 4.02174804841339`, 0.9953683071925011`, 0, 0, 0.8247132317345214`},
{0.18653936584035707`, 1.0683088955449334`, 5.185702152338592`, 0.9522710688882444`, 0.206752787597013`,
0.5240389817239608`, 0.06412446666117125`, 220.00685924183844`, 0.2055887216336732`, 7.885811409920794`,
991.1698661262881`, 0, 0, 0, 193.33399449579008`, 10.43390802795337`, 0, 0, 171.4020104812677`, 0, 0,
159.23766802229005`, 456.7603191276858`, 8.541748380285116`, 0.9999999999975049`, 0, 0, 1.6356876412557833`},
{0.07587231017464335`, 3.3343312881276663`, 8.762311039358057`, 0.8284399428795388`, 0.17449762015817138`,
0.3431144056167574 3`, 0.03578987408308253`, 1364.0076205471958`, 0.21103062426335195`, 8.577574022027257`,

60.89652107606923`, 0, 0, 0, 40.733300373690845`, 0.41432231599909114`, 0, 0, 0.004460976668674021`, 0, 0,
19.73554088007551`, 0.004835208649842515`, 1.3951068425481417`, 0.10944570623616079`, 0, 0, 1.0639702272693774`},
{0.1141532151024588`, 1.4516849606811304`, 7.634928187688176`, 0.8394863515309923`, 0.0282734940185132`,
0.6576494992828068`, 0.009900377337816624`, 932.466930942293`, 0.19488805826375527`, 9.750042758265863`,
122.34910866029524`, 0, 0, 0, 107.37265105301785`, 0.6838380596098153`, 0, 0, 0.009785880239781032`, 0, 0,
14.181677523956232`, 0.015958424171120936`, 2.5196697274032465`, 0.22155497504055477`, 0, 0, 2.15413464441722`},
{0.21903027892555205`, 2.3035966499415492`, 1.6904861150255108`, 0.7741880968122689`, 0.5538208564897962`,
0.4216122395122083`, 0.4439804577081556`, 61.55285726796139`, 0.1633463219105567 6`, 6.147879562247702`,
109561.838109268`, 0, 0, 0, 11.718227588417857`, 2.051820308996737`, 0, 0, 26515.0012184266`, 0, 0,
67.52237700124147`, 82965.5444654762`, 20.883070045574417`, 1.0000000000004252`, 0, 0, 0.3648777850749606`},
{0.19219532042344978`, 0.8633096904694177`, 0.1660296606087659`, 1.0595398890946446`, 0.3981268836303198`,
0.35447506211304225`, 0.06527455092271564`, 2352.6780619798324`, 0.04729158696423241`, 8.334762180963512`,
8886.551170717703`, 0, 0, 0, 5.6980565214198675`, 0.3893273780910939`, 0, 0, 2369.593592424266`, 0, 0,
4.801572832444199`, 6506.06856813336`, 0.7453522133390061`, 1.0000000000000784`, 0, 0, 0.5500117445726479`},
{0.059421230927129864`, 0.7565633878530198`, 4.881127251124937`, 0.9039794772130322`, 0.6691506700132113`,
0.3437226952926379`, 0.013854681683978944`, 1303.847591809871`, 0.1331369696675042`, 3.817043920573575`,
48.02279776226351`, 0, 0, 0, 40.14221392635678`, 0.662586202741585`, 0, 0, 0.0179251113764981`, 0, 0,
7.161263747012019`, 0.015216174035677002`, 0.6181848373081742`, 0.34563058142731384`, 0, 0, 0.2667847249431114`},
{0.27625614155976197`, 2.681313109654546`, 6.389652815751472`, 1.3504817156087858`, 0.7587234585548364`,
0.5970419449613528`, 0.3005699072645848`, 854.9062988397701`, 0.165124447314083`, 9.6053618622676`,
311.00243135268454`, 0, 0, 0, 60.58970821109794`, 6.362049740068461`, 0, 0, 0.07191140542513516`, 0, 0,
243.694962461712`, 0.2837995342410335`, 2.3775107428227904`, 0.6771290063903772`, 0, 0, 0.6928859478967527`},
{0.09922116949114601`, 0.5509823607034723`, 3.2884104382793122`, 1.3318058680427458`, 0.6719251293345294`,
0.6187329358641112`, 0.3259483616243979`, 1099.7736795288956`, 0.13645709285092156`, 2.0261792473532125`,
42.609141387369384`, 0, 0, 0, 7.813566728750504`, 3.8925148948596706`, 0, 0, 0.10936655832559596`, 0, 0,
30.63867208347441`, 0.15502111171840008`, 0.4042271621398462`, 0.7962636709403171`, 0, 0, 0.2262499476133617`},
{0.11800216420621701`, 3.285091391336728`, 1.6522276383131942`, 0.9670398406334181`, 0.4307591958110746`,
0.30003613158270603`, 0.29799743711056653`, 701.4335436984502`, 0.043710117716904806`, 7.7162513520402545`,
106.9250381098324`, 0, 0, 0, 21.872269761199796`, 1.7290395441317221`, 0, 0, 0.8117358276003599`, 0, 0,
81.14361316725574`, 1.3683797774365105`, 2.431710273119533`, 0.9999469609404865`, 0, 0, 0.9258766300280641`},
{0.19357111334803345`, 1.327860467490665`, 5.552252240997824`, 1.1813151963757291`, 0.7103742334207412`,

0.3776068940225674`, 0.2456309648230094`, 3934.634788049085`, 0.12355866435459295`, 9.745686147306856`,
47.12550835881893`, 0, 0, 0, 10.846474446758554`, 1.815434142335734`, 0, 0, 0.006862490885995592`, 0, 0,
34.43776041343489`, 0.018976857159180027`, 0.5204426963736446`, 0.11737129694225745`, 0, 0, 0.6295903412810214`},
{0.11447070818541266`, 1.7335861954191927`, 8.736387174086214`, 1.0335531844510784`, 0.08646802454747982`,
0.4443642965951369`, 0.015374273787766714`, 123.60141703755143`, 0.21867059554150092`, 6.82672203062274`,
961.1969955767904`, 0, 0, 0, 429.4682789069383`, 3.552858427494491`, 0, 0, 166.95074473642717`, 0, 0,
87.98837605975957`, 273.01385688658587`, 12.95781749730508`, 0.9999999999987393`, 0, 0, 1.2880746327407837`},
{0.22987433852030326`, 3.2498283820092855`, 3.7918096781351274`, 0.80804036856027 9`, 0.9416246029763107`,
0.49895559050949523`, 0.02048494990466312`, 278.1506433463176`, 0.24538025309113942`, 1.1463070343242556`,
698.8582853330865`, 0, 0, 0, 29.962424189665473`, 0.1793802431060896`, 0, 0, 154.15282369076837`, 0, 0,
8.327928645969925`, 506.225405385031`, 0.9795853002849119`, 0.9999999999937034`, 0, 0, 0.23421919287576345`},
{0.2774701327316953`, 3.217064080543545`, 9.848999257942918`, 1.1587780417232185`, 0.006285118350015439 5`,
0.6835477959342324`, 0.37074868705631564`, 2643.6047855840716`, 0.14999893314602414`, 6.1386041394007655`,
27.694623011240807`, 0, 0, 0, 4.820181153813061`, 0.4867619896063718`, 0, 0, 0.003433603078442154`, 0, 0,
22.37063589337957`, 0.013610318598898311`, 0.5629739621586601`, 0.061617378941259404`, 0, 0, 1.4020178749278014`},
{0.07518728576930783`, 0.8073344777020095`, 0.2079911159294223`, 1.2717171333972301`, 0.7961690644438275`,
0.349516332980527`, 0.009301689754665837`, 660.9000723394831`, 0.24128632812714051`, 8.034216989913379`,
5976.864073579396`, 0, 0, 0, 11.890254730517627`, 0.12370842595109272`, 0, 0, 2875.170388895669`, 0, 0,
1.4267725350366163`, 3088.2322523621597`, 2.618206837112195`, 1.0000000000001361`, 0, 0, 0.7330066576239155`},
{0.19386888157639426`, 1.1721624963279647`, 7.488800420822528`, 1.2206737749782337`, 0.39882649538868`,
0.6168747383393647`, 0.005894727770540639`, 784.8706404426375`, 0.21917965839925868`, 7.12627169471982`,
142.94961223057967`, 0, 0, 0, 131.77909561765668`, 0.6160474402646072`, 0, 0, 0.025411015884916784`, 0, 0,
10.315824363385909`, 0.07037721756184355`, 2.049136252502275`, 0.35948095808665914`, 0, 0, 1.0224831794428593`},
{0.051543962131442966`, 1.0824196854643793`, 1.5789464925861978`, 0.9324716268311393`, 0.16692765658832065`,
0.33113273649509023`, 0.16543741477462187`, 443.37752852110856`, 0.07774169596012481`, 8.031423814374019`,
6198.047736321502`, 0, 0, 0, 33.035885005246634`, 4.389950224756196`, 0, 0, 3508.8961654596783`, 0, 0,
67.88240773549808`, 2583.7487296516933`, 4.031117223316372`, 0.9999999999999973`, 0, 0, 1.0242639311582824`},
{0.04886198458272409`, 2.4240564285395276`, 3.0585768323132374`, 1.3030342316587755`, 0.5506088802661759`,
0.40370465638646613`, 0.05636145780930632`, 1567.8212350001168`, 0.20179368484919008`, 9.505728478640599`,
93.72953893448614`, 0, 0, 0, 52.458835543814736`, 1.1566623361110004`, 0, 0, 0.03449455826029714`, 0, 0,
40.05449673570605`, 0.02407817962712711`, 1.303773242633816`, 0.4315933904993403`, 0, 0, 0.8509606245398961`},

{0.26828907890664055`, 1.4916419764625646`, 9.30244345383333`, 0.8462433381711008`, 0.9150350508953056`,
0.16353772752060636`, 0.1124240025000419`, 2042.0256023360328`, 0.08362962700521354`, 9.175430964325553`,
69.61149000933557`, 0, 0, 0, 27.266190971873012`, 1.8971174428545485`, 0, 0, 0.004588893163660724`, 0, 0,
40.42600017201668`, 0.0175878560011244 8`, 0.915500733032626`, 0.11026287284073621`, 0, 0, 0.29658044489400576`},
{0.11853219177887636`, 2.436539187916523`, 4.07490711978506`, 0.8391678158796785`, 0.8274679746994464`,
0.4783994312610165`, 0.02880518630114325`, 249.91551468654188`, 0.20172418525520536`, 8.758671893830364`,
2309.3944678355565`, 0, 0, 0, 220.68233102569042`, 2.4602298994467726`, 0, 0, 742.7932856295268`, 0, 0,
85.63495087551426`, 1257.7845169185823`, 8.133091284430767`, 1.0000000000000067`, 0, 0, 1.5397622918616254`},
{0.15380346197105899`, 2.5002802569502807`, 7.104691399911929`, 0.895619363352984`, 0.7056556726651881`,
0.2904087856211036`, 0.27890663528622706`, 1570.4796825528501`, 0.2402855906015`, 2.015690994359673`,
19.903873302171796`, 0, 0, 0, 4.200637461128517`, 0.4269168838008991`, 0, 0, 0.008625743605087818`, 0, 0,
15.248740798944638`, 0.018952417550531554`, 0.2690035569380948`, 0.1863912047871622`, 0, 0, 0.12479641821661798`},
{0.07681933116098377`, 1.701376646381478`, 3.4749545233581878`, 0.9897106512386663`, 0.21660819673269338`,
0.4456181241675946`, 0.020856154503598144`, 170.86262737853005`, 0.11272605112949713`, 7.4521875824230435`,
22464.06171742289`, 0, 0, 0, 167.8768014369435`, 1.9294998962362573`, 0, 0, 10606.97322056454`, 0, 0,
46.89722946645518`, 11640.294120660501`, 9.77855768554486`, 1.00000000000004`, 0, 0, 1.0131487131480696`},
{0.19680750576810457`, 2.9845730901840906`, 8.034687795284743`, 1.0668124507997105`, 0.6731645050394575`,
0.24725877139168673`, 0.05097134738484948`, 549.1786460489706`, 0.029127445194553014`, 6.0096318557240025`,
153.44629044382324`, 0, 0, 0, 89.68107054491801`, 1.4587882745630063`, 0, 0, 0.02777023860583877`, 0, 0,
62.19800326481193`, 0.07807701992281582`, 2.282305451404751`, 0.42830304262427976`, 0, 0, 0.3408947948902159`},
{0.2481156970490977`, 1.2202113043036036`, 9.306028403345813`, 0.8009108612869359`, 0.02681732734233533`,
0.15549305073805075`, 0.021015785720259927`, 53.28601671876581`, 0.06540227633756851`, 2.0322216725641393`,
134925.1646269719`, 0, 0, 0, 112.09006009549734`, 1.7866195185451472`, 0, 0, 29657.786729442338`, 0, 0,
31.143619043117823`, 105122.3203901292`, 7.820222867439965`, 1.0000000000002116`, 0, 0, 0.07910152952873042`},
{0.05969602287151182`, 2.177557565950017`, 1.7675183816518665`, 0.8372040785938111`, 0.9188853493106184`,
0.1878023064060833`, 0.1313271602899384`, 239.3049490553974`, 0.14060221077790058`, 6.359907705361997`,
8945.507931814971`, 0, 0, 0, 32.20457042436641`, 1.759047425688187`, 0, 0, 4780.201276740189`, 0, 0,
54.720386152459994`, 4076.5572106673208`, 5.610960761035375`, 1.0000000000000235`, 0, 0, 0.44326106995985115`},
{0.1932263333480873`, 0.7780345756067408`, 9.679599745167298`, 1.1108495595363916`, 0.27977393862884203`,
0.2181645563751744`, 0.006193313005348089`, 412.49802062580375`, 0.21651680739670837`, 9.973248740719807`,
215.44831285761188`, 0, 0, 0, 198.0866882354231`, 1.409980010648914`, 0, 0, 0.018516310816165008`, 0, 0,

15.671617131417346`, 0.05111983516296866`, 5.158159519688026`, 0.3015175352037487`, 0, 0, 0.8072529272876969`},
{0.05080144024762184`, 3.462591354860418`, 9.637683194306994`, 0.7734436451051352`, 0.6551811041034619`,
0.18020194036251547`, 0.05139770733113868`, 3391.683612113084`, 0.04739051831745644`, 1.6399754771823183`,
5.39612108586466`, 0, 0, 0, 3.151862179612429`, 0.04441137925976248`, 0, 0, 0.0016949372238605162`, 0, 0,
2.196835112604007`, 0.0012300750300230639`, 0.10038633590585623`, 0.04601875058141702`, 0, 0, 0.0851165552354477`},
{0.17269587719558416`, 2.280528270589695`, 6.304702452448467`, 0.855238720891303`, 0.7302844321830138`,
0.25273349413775115`, 0.28098261768807636`, 2514.837947344666`, 0.04999285469609732`, 2.363210664893213`,
14.044368065944093`, 0, 0, 0, 2.952718466163068`, 0.32975791665742826`, 0, 0, 0.005398388136332847`, 0, 0,
10.743175019828943`, 0.0133182767807025`, 0.19568386875840552`, 0.12664251265206772`, 0, 0, 0.13016297821683165`},
{0.12416213981002666`, 0.8389921874064035`, 3.610730817659622`, 1.0777639636353653`, 0.18253050124358539`,
0.3654692817582218`, 0.277997956001176`, 563.1802062075279`, 0.17509802310960038`, 1.6234053595359104`,
28.236683392130647`, 0, 0, 0, 6.143203032394316`, 1.6904783065025228`, 0, 0, 0.05105038199128661`, 0, 0,
20.26140131622321`, 0.09055035237366182`, 0.6510595353265616`, 0.6239536701215939`, 0, 0, 0.2343581146888573`},
{0.05389681485101466`, 0.42474703837564753`, 9.702496123506663`, 1.2875902192251218`, 0.8398628787663107`,
0.4145702753216558`, 0.012323091180371398`, 1207.1917708220153`, 0.0487427592688896`, 1.997348446926472`,
41.061008317886824`, 0, 0, 0, 34.91631110925429`, 0.8610474066360961`, 0, 0, 0.020085745569764373`, 0, 0,
5.224676226710198`, 0.015465110144553222`, 0.3447579457197174`, 0.2830285743373264`, 0, 0, 0.11134226014408562`},
{0.06391261825702438`, 1.7835225604286613`, 8.062142041499534`, 1.3025605866836996`, 0.9222349035188417`,
0.39598365567854665`, 0.09958099473376647`, 75.95083350193653`, 0.043061298974276296`, 9.739451519751867`,
145277.60685188547`, 0, 0, 0, 277.85405077509415`, 14.221882690681829`, 0, 0, 75598.33640931966`,
0, 0, 362.35783758001156`, 69024.10879707066`, 28.63062760846588`, 1.`, 0, 0, 1.278116636017239`},
{0.08850946488241984`, 0.9673836199236003`, 8.299835087678265`, 0.9900607622617293`, 0.0809660102577392`,
0.6707110673061827`, 0.005613358503071565`, 223.20635429959376`, 0.07233923327715214`, 4.067688416849283`,
220.78650248063363`, 0, 0, 0, 204.2828868526055`, 1.0838069424294456`, 0, 0, 0.09505366196977594`, 0, 0,
14.977958332367512`, 0.12018783937226953`, 4.399246643194375`, 0.859670992152177`, 0, 0, 0.9074732622930282`},
{0.056027023765889306`, 2.0857485685464603`, 9.366491292380005`, 1.196524731276077`, 0.9516747873190254`,
0.6119693625054259`, 0.014912996736033244`, 220.0557248806415`, 0.0820080753991021`, 2.040285256013867`,
162.60731154020272`, 0, 0, 0, 132.85338966225322`, 0.9045827456389447`, 0, 0, 1.013519301981012`, 0, 0,
26.953316669260772`, 0.8112067145611457`, 2.092262077478548`, 0.9999906660420418`, 0, 0, 0.2924802700680639`},
{0.04516470805711162`, 3.292123721374173`, 5.263943679637629`, 1.138536155221258`, 0.48426832878752823`,
0.5662939024082725`, 0.09160607397723192`, 3360.7159646360847`, 0.026694430856043894`, 4.180658858897127`,

20.557542556718097`, 0, 0, 0, 9.050559156410635`, 0.23933360590074976`, 0, 0, 0.0071118697210037`, 0, 0,
11.255940590112516`, 0.004588650281279936`, 0.2759218443158702`, 0.12489322922752011`, 0, 0, 0.5176355411971597`},
{0.2782184518736612`, 3.845021085593168`, 1.137061402050021`, 1.4385887178277776`, 0.42032329684699743`,
0.5854257024714923`, 0.03249096611383817`, 328.6688300716124`, 0.02617032692156479`, 8.869579059594898`,
244319.42508432153`, 0, 0, 0, 54.87429782919783`, 0.44991090360616615`, 0, 0, 49097.79063161775`, 0, 0,
24.713098728628445`, 195141.58999922717`, 5.725676371215893`, 1.0000000000000016`, 0, 0, 0.6651344896556899`},
{0.09319840750005726`, 2.6994281444799046`, 8.85475415324603`, 1.038403514470577`, 0.3378909888092929`,
0.6279203689803818`, 0.23307552493819164`, 1846.0011275272348`, 0.034946033021005685`, 7.6621208156759835`,
62.210714593531755`, 0, 0, 0, 15.053766335273249`, 1.1915622822113954`, 0, 0, 0.0063741812626346706`, 0, 0,
45.950525150030565`, 0.00848662203992161`, 0.94597778630427`, 0.12352797921180092`, 0, 0, 1.185677890021089`},
{0.06034484465157863`, 2.8968090048449424`, 3.142651522085412`, 1.307236858746047`, 0.3617717997572676`,
0.29303713371207274`, 0.18131081790115888`, 4578.414121282968`, 0.17962394399488657`, 3.3138716244433386`,
7.5985320742277365`, 0, 0, 0, 2.2228502333596034`, 0.126560317559128`, 0, 0, 0.006271358614828696`, 0, 0,
5.2374438223045425`, 0.00540634516236525`, 0.1568797747276815`, 0.09729072250039572`, 0, 0, 0.3253152950828148`},
{0.20102830374342656`, 1.9832552045818916`, 0.5122029117897267`, 0.8414910669557318`, 0.023679729667421823`,
0.562606520598186`, 0.21796137676857033`, 2537.351175593395`, 0.0373739667120736`, 9.865525950238812`,
44.28800566628175`, 0, 0, 0, 11.202085414994313`, 1.0947180008851187`, 0, 0, 0.2519256601583918`, 0, 0,
31.01578818292707`, 0.7234884018726097`, 0.9450317378892474`, 0.9789355903716794`, 0, 0, 2.2837194990574226`},
{0.04583409051630541`, 3.6747726707008503`, 0.7420872691712788`, 1.2157743693615881`, 0.8915453306179026`,
0.4926202034522773`, 0.4217762472968932`, 73.4269782954483`, 0.10619296232023001`, 3.9517710932009376`,
114367.17200270195`, 0, 0, 0, 3.89224527544947`, 0.3894214933840609`, 0, 0, 69096.77595610723`, 0, 0,
20.443363732446596`, 45242.68405081549`, 12.1542648667526`, 1.0000000000000009`, 0, 0, 0.5699803508650577`},
{0.10119631213999619`, 1.851973173732965`, 1.6003758779141855`, 0.9328064269865228`, 0.39319494883808903`,
0.3867811485974866`, 0.006630825974655251`, 2020.948278622179`, 0.24307528382326266`, 8.411215463490773`,
52.12703977141673`, 0, 0, 0, 47.57719670408545`, 0.1611015653340528`, 0, 0, 0.03183893290136895`, 0, 0,
4.262225389215066`, 0.04602832274415613`, 0.911600990046119`, 0.5150454262856234`, 0, 0, 0.9230966925265243`},
{0.19796477344593338`, 2.2434362012846876`, 1.2309355399833084`, 1.3299187291217778`, 0.059794768114105334`,
0.6215799505741797`, 0.02273921518352973`, 1004.7174087228818`, 0.138969497102004`, 8.58482448486276`,
215.95651753218183`, 0, 0, 0, 73.37016952277021`, 0.7010189282799196`, 0, 0, 31.189911435346716`, 0, 0,
22.467017735556684`, 88.20719644424484`, 2.097845322381845`, 0.9999999999081356`, 0, 0, 2.09440021157553`},
{0.18841727652913837`, 3.2954166443999204`, 4.985214271603974`, 1.3919313698468052`, 0.8063258092579206`,

0.6389441864140679`, 0.00880414284139521l`, 1247.8494355462315`, 0.09671339442963328`, 8.749674730462491`,
227.245975107258`, 0, 0, 0, 201.612301031144`, 0.523587332669309`, 0, 0, 0.07782055596736777`, 0, 0,
24.649120155362876`, 0.20946767447652606`, 1.4760031678352414`, 0.6923198851548567`, 0, 0, 0.5822625190339311`},
{0.08822838789215687`, 3.6314565393637377`, 8.720964691808945`, 1.4591613734708808`, 0.09172379783619267`,
0.17510896742376914`, 0.2630216511777198`, 431.756995427934`, 0.16850915581941245`, 3.8927198296202143`,
63.86231170544817`, 0, 0, 0, 14.880630958346147`, 0.9254671524391951`, 0, 0, 0.019852375053510344`, 0, 0,
48.0113391813094`, 0.025022043525710358`, 1.9262817081036043`, 0.25347591193871777`, 0, 0, 0.3211538936786713`},
{0.22004819658675462`, 1.2435615798896285`, 4.521096993231973`, 1.3090616735635763`, 0.5905184190150714`,
0.6892934240599866`, 0.09109967416767972`, 239.35075160225182`, 0.045734190683186515`, 5.8358773331232445`,
37853.08680514885`, 0, 0, 0, 102.5230522980113`, 6.855128268692879`, 0, 0, 9079.645565672536`, 0, 0,
121.78248771659709`, 28542.280462473827`, 5.724965984041423`, 1.000000000001392`, 0, 0, 1.1068488847597557`},
{0.18183828174362843`, 3.2786199220563104`, 2.2240542093570888`, 0.9831747738881791`, 0.39067106215615355`,
0.5999409005287338`, 0.32534468961787655`, 53.98091103084368`, 0.10022266621367804`, 9.980846637487563`,
249893.91414954638`, 0, 0, 0, 32.976362884384315`, 3.040397266226465`, 0, 0, 69409.9578001431`, 0, 0,
142.4043864002231`, 180305.5351753684`, 39.73711627550715`, 1.0000000000012097`, 0, 0, 0.8873851930990582`},
{0.21252199352031792`, 1.818911285509734`, 0.45179175410691036`, 0.8473192883797733`, 0.5752582261250987`,
0.4244690624430477`, 0.010390335454685632`, 819.8846878599306`, 0.22518903335941576`, 6.3452240038645735`,
4949.901295132101`, 0, 0, 0, 19.75409747980972`, 0.10731120285943542`, 0, 0, 1220.8130872082522`, 0, 0,
2.78842225632359`, 3706.4233001313096`, 1.6359579812829674`, 1.0000000000006541`, 0, 0, 0.4506802867295168`},
{0.13408565703158576`, 3.9481177636984475`, 4.409802796026355`, 1.1882851460555317`, 0.7016940523769313`,
0.6437617694336766`, 0.472695817526219`, 268.88735252515323`, 0.02238688650951909`, 7.198883746978469`,
15489.633381362073`, 0, 0, 0, 42.155269566803945`, 4.278558158295536`, 0, 0, 5214.142550745218`, 0, 0,
241.3178781111923`, 9987.738996757467`, 6.514768190160185`, 1.000000000000201`, 0, 0, 1.676592693604853`},
{0.11550511139863262`, 2.7266825017818803`, 9.715437717848832`, 1.4226217916614743`, 0.7434913376024772`,
0.6527594414247178`, 0.026462900292485443`, 517.125461393156`, 0.19263485762362964`, 9.20218742349427`,
502.3896339203605`, 0, 0, 0, 365.35893319721896`, 3.421687781801913`, 0, 0, 0.09348953696429224`, 0, 0,
133.28366001714468`, 0.15426456259509833`, 3.7848400088157717`, 0.7515144416125528`, 0, 0, 0.7144807331574318`},
{0.1537432379066465`, 3.3010385217103515`, 7.8154151393713`, 1.099357680158021`, 0.2670513361076359`,
0.6099579487912872`, 0.01626886763741749`, 3071.882113963599`, 0.04550777584421739`, 6.952411393791335`,
30.85937412244917`, 0, 0, 0, 25.111673702980585`, 0.11883184273865889`, 0, 0, 0.004071812586004499`, 0, 0,
5.603835578373423`, 0.008943052158872993`, 0.5215921738342812`, 0.07642922341960157`, 0, 0, 1.1657549331107344`},

{0.08915960893105551`, 3.2848427178141772`, 0.6302374569507307`, 1.4630290564093034`, 0.8347103436436674`,
0.36812644478584344`, 0.040963141333673035`, 3229.889147199789`, 0.1995027638419964`, 3.7582834934312945`,
19.868013913202574`, 0, 0, 0, 12.077800309351087`, 0.14438483605991506`, 0, 0, 0.382445544670165`, 0, 0,
6.7754496756315`, 0.48712421714594945`, 0.2550380347307239`, 0.9957934256626366`, 0, 0, 0.4153557159184469`},
{0.2095156599086041`, 1.856403794983298`, 1.6647511783688493`, 0.8963074241076089`, 0.5257557556911894`,
0.2962680347723602`, 0.005638948645619698`, 398.77430140016673`, 0.1712593265312492`, 6.243180864475704`,
6593.453678424819`, 0, 0, 0, 76.43225147621729`, 0.2197271123677149`, 0, 0, 1630.5430053635562`, 0, 0,
5.827174932287811`, 4880.347053972915`, 3.3270199047394544`, 1.0000000000011917`, 0, 0, 0.47891799706932303`},
{0.08606673974307905`, 3.001046395083865`, 6.871125535025986`, 1.29871337654279`, 0.0013879217267305233`,
0.4709543850320145`, 0.028486772088755836`, 255.05656064251065`, 0.15733152617600732`, 4.419059383126843`,
166.6965921491207`, 0, 0, 0, 119.40940315014575`, 1.0732556166827885`, 0, 0, 0.08058980086013642`, 0, 0,
46.012712849276966`, 0.09908716309399489`, 4.061449217501498`, 0.7283190974727068`, 0, 0, 0.8294969124190619`},
{0.2794012033077364`, 3.8964244114394253`, 7.589043584575673`, 0.9660750334911808`, 0.6823441866078543`,
0.315494435212184`, 0.013950850602606538`, 1092.7384181067544`, 0.06831145072823913`, 6.790315469699049`,
100.97260273736335`, 0, 0, 0, 84.30978117087255`, 0.2919985211745414`, 0, 0, 0.013786662311285643`, 0, 0,
16.25357380012422`, 0.05502871484816052`, 1.3036658771644114`, 0.264181100867383`, 0, 0, 0.4274845256277511`},
{0.16053897675716755`, 1.21110484978606`, 6.017303762103152`, 1.2353982330406967`, 0.20086989110627473`,
0.3272106220983775`, 0.02355461265150193`, 77.09093986775292`, 0.04848901873161415`, 3.5142859649597167`,
223526.18527287157`, 0, 0, 0, 126.3090452450785`, 2.276012972571607`, 0, 0, 67819.65172155976`, 0, 0,
39.378433560820845`, 155538.535591524`, 9.686780526974268`, 1.000000000000008`, 0, 0, 0.2693133127647481`},
{0.06315507597420161`, 1.4395182613839497`, 3.0858504661132873`, 1.1448617905347693`, 0.30610701790846395`,
0.20244634599341438`, 0.2950980917537417`, 2179.6214593356067`, 0.03473000454292019`, 1.333335366603153`,
5.165641791158565`, 0, 0, 0, 1.0898227658467072`, 0.18814168177364365`, 0, 0, 0.009793320966624948`, 0, 0,
3.869048380580692`, 0.00883568470980081`, 0.13106667615880044`, 0.16609509668667566`, 0, 0, 0.11408838441204643`},
{0.27116635506122466`, 2.640185082938654`, 3.1282641056246216`, 0.9199164829096633`, 0.32218318348593566`,
0.5777637686875948`, 0.006117113694401221`, 324.8095638375007`, 0.04342122620091826`, 2.609798810681127`,
18981.386036116564`, 0, 0, 0, 64.66497911307027`, 0.14322591872378398`, 0, 0, 3880.152485095757`, 0, 0,
5.402041915781699`, 15030.954378073846`, 1.8534847415306202`, 1.0000000000000082`, 0, 0, 0.44048434744888754`},
{0.08076786811690995`, 2.5389877544798445`, 7.211847948605141`, 1.1500644274940979`, 0.7690232347129535`,
0.1924810447357974`, 0.3170033067881963`, 2456.45541532025`, 0.12977198817972552`, 1.000471570970415`,
5.333962766938453`, 0, 0, 0, 1.0312960187375153`, 0.11511868054806304`, 0, 0, 0.005594302665456636`, 0, 0,

4.175498860334408`, 0.006454855712706904`, 0.08433524697997714`, 0.09876437295911666`, 0, 0, 0.04916835237627691`},
{0.14196490482109098`, 3.5800560378690767`, 8.191314882292886`, 0.8521719014833709`, 0.20577614391452248`,
0.2973229046898421`, 0.032689134381726236`, 2192.8696238585753`, 0.20870222148197032`, 8.620659635124824`,
36.87870251732632`, 0, 0, 0, 25.39270028592754`, 0.22004552627608914`, 0, 0, 0.002895956841714297`, 0, 0,
11.253933070725603`, 0.00587320339143145`, 0.8603157758293198`, 0.07055087334483057`, 0, 0, 0.937912713988889`},
{0.16225217210911858`, 2.1337475700719883`, 4.607583982995607`, 1.274907826520427`, 0.760916097212121`,
0.6512132808308`, 0.1988962707427588`, 88.66654318682235`, 0.17629703311441042`, 5.991536547797288`,
81943.04021995429`, 0, 0, 0, 61.17626920176433`, 5.290666137242431`, 0, 0, 24628.7090805353`, 0, 0,
161.27065734861714`, 57086.59349372028`, 14.912828567794431`, 1.0000000000003275`, 0, 0, 0.7079595205338512`},
{0.09998370398747453`, 1.3561107931887042`, 7.055733599099842`, 0.9997997594446719`, 0.4231050232131661`,
0.35289870383552235`, 0.01863838099230359`, 585.7262791471302`, 0.12303242645253354`, 2.5487735356675643`,
52.08286953695101`, 0, 0, 0, 41.26730008165286`, 0.5273304248952306`, 0, 0, 0.02296070215683445`, 0, 0,
10.215978296817251`, 0.03279565782562263`, 0.9473463991230855`, 0.3874903477631825`, 0, 0, 0.2626472513160205`},
{0.0790420625297461`, 0.7951762600254768`, 1.0975467432818782`, 0.8295886125705915`, 0.21974762785224522`,
0.4003083061581494`, 0.12181590550696897`, 256.3718289629029`, 0.07504947990766975`, 1.4404680202816778`,
26896.800412169825`, 0, 0, 0, 4.885755968122807`, 0.6556713732704874`, 0, 0, 12626.397785247435`, 0, 0,
7.448204434328501`, 14257.378903813871`, 1.2358829528174902`, 1.0000000000001126`, 0, 0, 0.16488288439216547`},
{0.09310243320239603`, 1.7939569577957926`, 0.5112771848209494`, 1.3548866082012778`, 0.9609312900127882`,
0.31815651234043585`, 0.047992777894275945`, 1572.364241162158`, 0.10220690607690369`, 1.3696469849079624`,
1079.3676768922296`, 0, 0, 0, 3.532221426690215`, 0.08711057723517644`, 0, 0, 460.7293362592653`, 0, 0,
2.2324660875521816`, 612.7860321923214`, 0.19518149935578233`, 0.9999999999999161`, 0, 0, 0.18529482759872745`},
{0.20315703606221613`, 1.4806269686939117`, 9.851530344798562`, 0.807291927894971`, 0.7692364729235686`,
0.5064599484352303`, 0.07616257600145751`, 550.4721115636684`, 0.18512304424700965`, 4.821016411964738`,
218.39630892442125`, 0, 0, 0, 105.4785177847609`, 5.091037737463172`, 0, 0, 0.036309368680338586`, 0, 0,
107.68468246752009`, 0.10537862460550501`, 1.8438604772878473`, 0.6174815323864675`, 0, 0, 0.3216186925687856`},
{0.22897905358022103`, 2.219649931841208`, 8.024983925513396`, 1.3490395658771779`, 0.7992198564081268`,
0.5097552623833034`, 0.017934168491060075`, 2636.2345048980164`, 0.04871567850920905`, 1.38033814014487`,
13.596944980969587`, 0, 0, 0, 10.80439802079283`, 0.0837669196304792`, 0, 0, 0.011248634324142153`, 0, 0,
2.6561891064048724`, 0.03679573773731852`, 0.110542313086496`, 0.1619857979004531`, 0, 0, 0.09616685899135761`},
{0.14728957561352346`, 3.8290117168547324`, 5.577517394641557`, 1.2037071314600827`, 0.7629976630105226`,
0.33391252098357616`, 0.00623505541513591`, 1551.1825745003182`, 0.05716501203669799`, 3.4524118308532703`,

41.86753105286014`, 0, 0, 0, 38.38881982061887`, 0.060649530868368474`, 0, 0, 0.019368535642847098`, 0, 0,
3.3175394902389415`, 0.04075404850129067`, 0.46510681771226386`, 0.2910049940817847`, 0, 0, 0.20301383083622274`},
{0.1747499923357959`, 1.9364726507678487`, 4.539322141325334`, 1.0358217348301002`, 0.687467494258686`,
0.22118436656891216`, 0.06970356230137446`, 4108.781309212477`, 0.15897963252631786`, 3.216561469360766`,
10.347680597824837`, 0, 0, 0, 5.256901644327983`, 0.17701232425632085`, 0, 0, 0.0048289034452711896`, 0, 0,
4.896850353874509`, 0.01205501200073839`, 0.16294550377269001`, 0.09516713398030718`, 0, 0, 0.1756095626756004`},
{0.061449808274615936`, 2.451738473864639`, 8.032153511575046`, 1.3988277328315561`, 0.653844119276888`,
0.6033333182307286`, 0.12509363159734996`, 3162.271766392656`, 0.18189469469951425`, 1.0210674887190017`,
6.918144332950094`, 0, 0, 0, 2.521378800731132`, 0.12164317268642931`, 0, 0, 0.007769574944316761`, 0, 0,
4.260532093689695`, 0.006820555581478194`, 0.07053103876401944`, 0.1103103854102856`, 0, 0, 0.10899329614135286`},
{0.1951885853322612`, 0.6765436415231281`, 7.0581513804142055`, 1.4397439961417247`, 0.9899034195557828`,
0.3660842570171081`, 0.2551485659861665`, 53.30479560198744`, 0.15667683142074817`, 8.392793083340557`,
167811.20606479805`, 0, 0, 0, 104.67478435154212`, 33.904624031634974`, 0, 0, 44172.888863531196`,
0, 0, 327.6851115262117`, 123172.0526758837`, 33.13489491286899`, 1.`, 0, 0, 0.5562008416856223`},
{0.15955516943400022`, 0.8482855893464007`, 9.331572022970409`, 1.1667938097447013`, 0.29910958141522603`,
0.1963171731712634`, 0.010305064592809629`, 1013.0765277256257`, 0.20900313016467104`, 3.301983133021727`,
27.457654288105932`, 0, 0, 0, 23.984816466844165`, 0.26158176901850494`, 0, 0, 0.006944390332722704`, 0, 0,
3.1699435013448096`, 0.015828762516476767`, 0.6930193543426566`, 0.12011236804260406`, 0, 0, 0.25523051757683335`},
{0.17746707158856556`, 0.44139712602022296`, 2.8621670830493073`, 0.8100971992338867`, 0.0373958036205273`,
0.5075452637360592`, 0.09067013663296572`, 114.80525128927701`, 0.16719225120884412`, 9.820606806768879`,
45629.05944977913`, 0, 0, 0, 99.65064733179511`, 17.23092962768157`, 0, 0, 12843.109745784908`, 0, 0,
108.6526116616 4836`, 32560.415381071565`, 18.354586224025393`, 1.`, 0, 0, 0.8558878109151827`},
{0.25772135278013303`, 3.070426405636976`, 1.2338255709842532`, 1.3135003325072887`, 0.5149580362176238`,
0.41732865316044054`, 0.04496826136197767`, 318.8324074107286`, 0.010846019130009599`, 7.957536959758443`,
459343.15708679357`, 0, 0, 0, 47.168078434389265`, 0.6647065937046605`, 0, 0, 98097.45791651343`, 0, 0,
29.156181104454166`, 361168.7079790835`, 5.2318539397853865`, 1.0000000000004765`, 0, 0, 0.4940916165349102`},
{0.08988753363169116`, 3.536295514824336`, 7.275260820034448`, 0.8993825689374235`, 0.025163868977214232`,
0.47083049518550046`, 0.018253871395130763`, 416.18519037331805`, 0.11499620973251179`, 2.265482484941062`,
52.34014899122266`, 0, 0, 0, 41.69995458934051`, 0.2051682386651946`, 0, 0, 0.02328390873231041`, 0, 0,
10.364793173944816`, 0.02989904470361`, 1.2763544091891301`, 0.42339300346023445`, 0, 0, 0.42592985017809504`},
{0.14192760186457132`, 2.3958894382433478`, 3.253512770748353`, 0.8655063905996925`, 0.8143036151546204`,

0.5834364577519011`, 0.17553594321370639`, 62.22207407824173`, 0.16384862691545943`, 3.0979894918131596`,
90045.54264929114`, 0, 0, 0, 24.198372486978617`, 1.6542053842798152`, 0, 0, 29714.935338391857`, 0, 0,
56.61847441259001`, 60248.13588769615`, 10.897746739193282`, 1.0000000000080536`, 0, 0, 0.3376179128684148`},
{0.14248286143558947`, 2.096310454690764`, 9.38011512102738`, 0.9675972750885355`, 0.0803295271719453`,
0.6033546111821413`, 0.13762578908199044`, 1262.8186365484712`, 0.18785001372865495`, 4.9706998985924695`,
45.47802637953347`, 0, 0, 0, 15.890021577043804`, 0.9554551914470057`, 0, 0, 0.006342833643986323`, 0, 0,
28.613295811698936`, 0.012910644102888783`, 0.9341359328225532`, 0.1312185423988348`, 0, 0, 1.0061643785996222`},
{0.10446764528219044`, 3.442117541556258`, 1.7911289531773882`, 0.822650737878747`, 0.06660293233402026`,
0.33605831499521144`, 0.1966635974545483`, 110.27400709595466`, 0.23027159794407875`, 2.5346674177346316`,
24914.904403613527`, 0, 0, 0, 9.932913382555805`, 0.5245732241588901`, 0, 0, 9981.824543174687`, 0, 0,
25.794895667257563`, 14896.824366363391`, 4.909941662234955`, 1.0000000000000004`, 0, 0, 0.2086232219382652`},
{0.0959624592169791`, 0.7155878562202682`, 5.1612847015717165`, 1.2701047018452214`, 0.05672968500006248`,
0.6450160655606327`, 0.04218472167952095`, 1614.7615476312947`, 0.23305782159027644`, 5.6652476983851425`,
41.181567675359915`, 0, 0, 0, 25.963024646163475`, 1.3532871364464931`, 0, 0, 0.01240780575217576`, 0, 0,
13.834226297431567`, 0.01700976504951979`, 0.8436295375600448`, 0.1882151431782655`, 0, 0, 1.2368723602894067`},
{0.24311048293765425`, 1.2773072105524381`, 8.674158333323465`, 1.236248375623142`, 0.5430857314344402`,
0.29169516048698574`, 0.10383247735445839`, 522.6667880889086`, 0.0459109896778586 7`, 9.69459149525866 3`,
242.4184657661748`, 0, 0, 0, 99.87292501721628`, 7.399439451013098`, 0, 0, 0.02831971225563329`, 0, 0,
135.01939092607407`, 0.09835455604390633`, 3.929376215925806`, 0.3889029487845639`, 0, 0, 0.7110976988438057`},
{0.08713889377857453`, 2.2859880795657075`, 9.600148060687761`, 1.2921363299986606`, 0.6294865567710719`,
0.5590186325118135`, 0.25992443653112657`, 633.6168560418654`, 0.16781120217455497`, 6.04666254822`,
195.42311188862982`, 0, 0, 0, 43.037367386660655`, 4.525189693422276`, 0, 0, 0.03633180193400465`, 0, 0,
147.77899567052762`, 0.04522732899292192`, 2.0597426342625944`, 0.4519340572539954`, 0, 0, 0.5657815572465027`},
{0.26758004713434824`, 2.448981429729897`, 4.582999027119474`, 1.4554483602004895`, 0.3942207099215642`,
0.5288182096517661`, 0.017684947331647518`, 53.19217741492592`, 0.1488296927599813`, 5.857737808697124`,
280035.4092206887`, 0, 0, 0, 169.19417872092365`, 1.1755451280054454`, 0, 0, 58023.77529422624`, 0, 0,
41.12697411849718`, 221800.06468774052`, 23.198738608694565`, 1.0000000000003841`, 0, 0, 0.39448541983786584`},
{0.13431037279286218`, 0.7397108148766112`, 9.99887072123514`, 1.436439642978205`, 0.2003363652340402`,
0.6779870054162531`, 0.009538236593206559`, 4558.309876478542`, 0.14371659865116293`, 1.1480275619139793`,
3.3042548635671993`, 0, 0, 0, 2.9058272412855133`, 0.033576384391287004`, 0, 0, 0.0026211312609524524`, 0, 0,
0.35481163798127474`, 0.005029215954250556`, 0.0602462945966091 1`, 0.03782638590927245`, 0, 0, 0.24338692288349578`},

{0.27730778316543614`, 1.7365428506012295`, 3.277328223559843`, 1.1643406154035905`, 0.2621424262474452`, 0.5836632043576286`, 0.19147730163610321`, 594.1704278677969`, 0.14363610143643862`, 8.32222051121584`, 189.14428731602885`, 0, 0, 0, 52.67644380842103`, 5.2619975039848`, 0, 0, 0.1345350716031323`, 0, 0, 130.53834493466164`, 0.5329660352037449`, 3.225739805626592`, 0.8989569376745145`, 0, 0, 1.3887514389808886`},
{0.05614793436458493`, 1.997529172364887`, 6.840549262003957`, 1.2853232683568476`, 0.15723714782247722`, 0.6374125080636333`, 0.1308231427534364`, 1207.3035504224565`, 0.15724403465210185`, 1.842303075231806`, 18.954068087070414`, 0, 0, 0, 6.815332129818864`, 0.4101588765104715`, 0, 0, 0.013444988948635227`, 0, 0, 11.704347444772463`, 0.01078440510029886`, 0.3635979515998539`, 0.19836003372301791`, 0, 0, 0.3818272884502662`},
{0.0647068787712346`, 0.8380824418888393`, 1.0963289034750066`, 0.857562051048008`, 0.6907626550964823`, 0.2999007220652139`, 0.27885748113518277`, 1238.4520803651262`, 0.1379529162857775`, 8.96709675960637`, 80.42072928246569`, 0, 0, 0, 17.181032990215837`, 4.796910696398284`, 0, 0, 0.5254984684296022`, 0, 0, 57.43152328514522`, 0.4857623670163112`, 1.5756204122831978`, 0.9997765796433502`, 0, 0, 0.9196438205903477`},
{0.20132622112515353`, 1.9086557669063273`, 0.7030652496772074`, 0.7755069309180247`, 0.32762519329281337`, 0.20767575421456186`, 0.011318073987463645`, 431.8155255411275`, 0.17436258525110954`, 5.81443415452358`, 9566.819329129976`, 0, 0, 0, 27.051129901055535`, 0.15245891809875264`, 0, 0, 2460.067310171169`, 0, 0, 4.15702276064977`, 7075.3722181469075`, 2.762757576156191`, 1.0000000000001021`, 0, 0, 0.23010736097895226`},
{0.045022613849116516`, 3.5058573906861508`, 8.47131465431961`, 1.2697383949459176`, 0.5022287267438617`, 0.23847623822456332`, 0.06077359284826833`, 243.2833936812314`, 0.1613793589669978`, 8.022277670460284`, 373.1013242752542`, 0, 0, 0, 202.8153647572864`, 3.3308556035968717`, 0, 0, 0.07973717962268075`, 0, 0, 166.8214962168352`, 0.05128537496531917`, 6.9578220967984805`, 0.7388769358637977`, 0, 0, 0.5541833641433178`},
{0.17418182442148117`, 3.0892110646487794`, 3.4522019380421036`, 1.2965827481879664`, 0.41818871776887856`, 0.6556019601202148`, 0.075063644002762`, 2886.574837344593`, 0.15315141138676647`, 2.6062865811306466`, 14.839837611470706`, 0, 0, 0, 7.237474343856993`, 0.16729190847943057`, 0, 0, 0.014960224910682267`, 0, 0, 7.3828573528695705`, 0.037225703838533325`, 0.2063022009564729`, 0.21513548224898793`, 0, 0, 0.4109475802970207`},
{0.045831059772204225`, 2.899702133961444`, 5.925065562245003`, 0.8929739121804446`, 0.5933600691515191`, 0.4490635153302144`, 0.16447888961080792`, 1912.79886244215`, 0.11499785968845799`, 4.757985376452911`, 42.357168547527465`, 0, 0, 0, 13.008327945211114`, 0.6914263896813574`, 0, 0, 0.00939658032893252`, 0, 0, 28.64186539480406`, 0.006152217638704703 5`, 0.5350533783819423`, 0.20194493552601112`, 0, 0, 0.42749205835811055`},
{0.2429697761558342`, 1.8449858081251405`, 7.508754000794074`, 0.9127320360969664`, 0.44886430542069955`, 0.35316747155912975`, 0.02193269408167387`, 161.73721618998414`, 0.16775422018188862`, 9.221971574736251`, 588.9519172690613`, 0, 0, 0, 441.58024391633523`, 4.912626465773639`, 0, 0, 2.8721330230282875`, 0, 0,

129.48180157103303`, 9.969164538499692`, 12.71568676488441`, 0.9999997760947584`, 0, 0, 1.1981339747015711`},
{0.160442241636698`, 3.55050251528807`, 3.9052606138637995`, 1.2025377341211225`, 0.791383919489673`,
0.40672925070026333`, 0.3547712441602767`, 105.50559352550128`, 0.10792398319932495`, 9.162379917819035`,
89514.77688574418`, 0, 0, 0, 50.175942566805766`, 4.555296957208729`, 0, 0, 27104.534102772657`, 0, 0,
231.05133292076724`, 62124.46014238817`, 18.55023837225004`, 1.000000000000064`, 0, 0, 0.7538665588460479`},
{0.20468313725879644`, 1.5880039947408164`, 1.8181726149942818`, 1.4361114186612707`, 0.6452252370101204`,
0.23877202353940497`, 0.026067763377395026`, 956.3204095901076`, 0.08759161290164996`, 6.530440575770296`,
91.112966981299`, 0, 0, 0, 65.94955046636952`, 1.014495655327166`, 0, 0, 0.28533964351469066`, 0, 0,
23.01461647581059`, 0.8343459059841843`, 1.4358700521906775`, 0.9691988786357222`, 0, 0, 0.4268620632545388`},
{0.07364401078716826`, 2.5859680523819737`, 7.8227327965838285`, 0.8181378301360093`, 0.36855652079172274`,
0.32445369334254925`, 0.005391697017678555`, 139.66827785366002`, 0.0644557011853657`, 2.5433237558513593`,
168.49700533080954`, 0, 0, 0, 154.51058847848495`, 0.3054342988856176`, 0, 0, 1.0832796983189548`, 0, 0,
11.283476271712788`, 1.1396723112645946`, 4.085108711444888`, 0.9999857456835558`, 0, 0, 0.3475140781100951`},
{0.2506656721970057`, 1.5258727034335298`, 1.8333712411976357`, 1.2495957919641723`, 0.1010075460664761`,
0.5024718466416268`, 0.012345305326089913`, 1052.1669408921373`, 0.03320454993831845`, 2.130087729732141`,
21.908887159312037`, 0, 0, 0, 18.392971997680284`, 0.1388435726167236`, 0, 0, 0.07387912217471712`, 0, 0,
3.0265373929007087`, 0.2645565688749998`, 0.48192013005600964`, 0.6879687778596334`, 0, 0, 0.4390184448731985`},
{0.10121864512314288`, 0.9521305504715327`, 3.6368221202357223`, 1.491518490019038`, 0.3322534458031634`,
0.2564348282281962`, 0.03507970829425946`, 4627.905596988043`, 0.07121256842175683`, 7.352385124666178`,
15.635940940148682`, 0, 0, 0, 10.516518919511546`, 0.3495690673077044`, 0, 0, 0.005690970307999049`, 0, 0,
4.754791264050073`, 0.008229032914452606`, 0.34123838344021146`, 0.07861634866660128`, 0, 0, 0.6489912684494356`},
{0.1942542932466596`, 3.1177323303054916`, 7.932133913996736`, 1.3085296181137158`, 0.8325654731630694`,
0.19060678658466512`, 0.044940459115954866`, 2391.994107548152`, 0.24452644883302188`, 1.4227474861405387`,
8.535177468325813`, 0, 0, 0, 5.230586013405013`, 0.07202019927314394`, 0, 0, 0.0065201269012133915`, 0, 0,
3.2077100529846394`, 0.018093752043911296`, 0.12249927208082094`, 0.10123333433501236`, 0, 0, 0.061874702325715125`},
{0.12644890469661946`, 3.0196313965941393`, 8.688658870512302`, 1.3655010622962382`, 0.5847686349152417`,
0.30770354208104556`, 0.013061738242062998`, 1213.9477293174991`, 0.0486468587160932`, 6.312801306430787`,
72.48326340118683`, 0, 0, 0, 61.194223760578666`, 0.25412154163856593`, 0, 0, 0.012463623595405437`, 0, 0,
10.96219122403884`, 0.02251445074557796`, 1.1010717976956128`, 0.1781171472619033`, 0, 0, 0.459526311705594`},
{0.11280111274715143`, 3.2009360918012444`, 2.9222221030043283`, 0.9684721327188957`, 0.03329493891954294`,
0.1886848273472841`, 0.39445850876705457`, 50.69043630972767`, 0.07560282132089569`, 3.828945975174229`,

193146.62062500234`, 0, 0, 0, 14.210065098056779`, 1.5454248303912013`, 0, 0, 73928.50491493392`, 0, 0,
70.66865881092846`, 119131.68025911167`, 15.598834636997408`, 1.0000000000013987`, 0, 0, 0.1773268098610338`},
{0.21704875939335333`, 0.6532232080519234`, 2.918554590657344`, 1.428534955684801`, 0.6202310312510246`,
0.3415578035649972`, 0.1858190740142067`, 1428.9901262383053`, 0.23526262585558444`, 9.378643714384769`,
102.55197063756951`, 0, 0, 0, 29.003478163782347`, 7.100205082309487`, 0, 0, 0.0465474055251751`, 0, 0,
66.25741059561103`, 0.14432938031736794`, 1.3907016321762191`, 0.50062560456789`, 0, 0, 0.6903373846548709`},
{0.23408001184633387`, 2.4049572740765104`, 0.41973647277501946`, 0.9771854975111727`, 0.20272636213110817`,
0.5074761434023957`, 0.2724896408017817`, 4254.760257351377`, 0.16397942354351142`, 7.3871482427729305`,
21.437258244144928`, 0, 0, 0, 4.602951849244492`, 0.46480918345204486`, 0, 0, 0.09214212923548448`, 0, 0,
15.9692318114365`, 0.308123295756984`, 0.4060309503930818`, 0.8265741755072029`, 0, 0, 1.3658444699284693`},
{0.1483945695425406`, 2.699298050205127`, 5.571982823617271`, 0.9775705576725262`, 0.05426683476548022`,
0.5307838109252759`, 0.3096243181655647`, 323.46321359362`, 0.06725520461051537`, 9.792268234153859`,
330.6252128903389`, 0, 0, 0, 66.22111118081338`, 6.677331233677972`, 0, 0, 0.07677273983922155`, 0, 0,
257.4872454234386`, 0.1627522525862059`, 7.079466335526397`, 0.8090245549289123`, 0, 0, 1.8101295527336911`},
{0.11663741077922185`, 0.4431208542421814`, 9.780884572399291`, 1.0122403254522139`, 0.996318833483786`,
0.22155768960679945`, 0.39993089984673347`, 139.93164861672463`, 0.10490063957337303`, 5.3884722727325105`,
418.0089597902204`, 0, 0, 0, 68.74064004340796`, 47.24860992548619`, 0, 0, 1.0958965417555337`, 0, 0,
299.09777702767224`, 1.8260362158891166`, 8.12735928254823`, 0.9999397292701419`, 0, 0, 0.3737199637059701`},
{0.11314041374373823`, 3.32806970136921`, 5.495643049849155`, 1.495187492234928`, 0.8329498208958137`,
0.41734162088096116`, 0.32389371604169326`, 602.537083528763`, 0.025714962476490244`, 1.3686646556271693`,
54.67461567681306`, 0, 0, 0, 10.018687540369724`, 0.9093902182404022`, 0, 0, 0.19517058180971128`, 0, 0,
43.23591474353447`, 0.315452576807908`, 0.47997048008743576`, 0.9167141087138199`, 0, 0, 0.09661541331170055`},
{0.09294922428523011`, 0.8101676653238759`, 3.5463022431515436`, 0.9341830117643879`, 0.15981251792750606`,
0.6527933768073686`, 0.016252799814697117`, 738.0588634619963`, 0.024900612098687713`, 6.915149516601594`,
120.57807445002786`, 0, 0, 0, 98.11442412363415`, 1.7740689832765024`, 0, 0, 0.046851392258682595`, 0, 0,
20.532761804351775`, 0.06221143667327454`, 2.2180372146778127`, 0.6521487564441385`, 0, 0, 1.3791967483701655`},
{0.2696435798732645`, 3.282053008859009`, 8.218787637608994`, 1.4011858805306123`, 0.38871702639887196`,
0.49514225831047376`, 0.01812451162452924`, 173.50575962443355`, 0.20495943261505556`, 3.6349041099248875`,
263.54632725302497`, 0, 0, 0, 204.0059578879462`, 1.0773345536458587`, 0, 0, 1.6211471737613652`, 0, 0,
50.51241590487376`, 6.244741820491991`, 4.811886094534686`, 0.9999407587317961`, 0, 0, 0.5952049815402674`},
{0.13023501639399904`, 3.4827106844829796`, 7.180310384726493`, 1.0417379014779535`, 0.3157954886073613`,

0.3090830033379006`, 0.027522996818882862`, 1326.2576636786057`, 0.24748434887668458`, 4.853044482161593`,
37.76653376783778`, 0, 0, 0, 27.305129466395933`, 0.205587795719306`, 0, 0, 0.007652489365568896`, 0, 0,
10.22861161072758`, 0.014237458256860094`, 0.7965391564730171`, 0.1459855640397396`, 0, 0, 0.4994640667146315`},
{0.0474731410927452`, 0.7132410347452449`, 0.7493200461382123`, 1.2237616503945397`, 0.9506868210145907`,
0.6554164129507942`, 0.030282495208288053`, 4252.97050681489`, 0.14327214907032687`, 7.932983145622682`,
49.19049856623758`, 0, 0, 0, 33.93649674214015`, 1.2963265124262529`, 0, 0, 0.44313887501711247`, 0, 0,
13.208475187008498`, 0.300531347676694`, 0.4097127113454254`, 0.9991336353640207`, 0, 0, 0.895663659961093`},
{0.16300334800486183`, 3.0919140294206544`, 3.354218048792589`, 0.9673340843818878`, 0.9052111330146941`,
0.3760205832601582`, 0.15433250667793078`, 4163.373290035795`, 0.2440496776808055`, 4.495712049614825`,
29.572134404917954`, 0, 0, 0, 9.36601223745691`, 0.4463181954241059`, 0, 0, 0.01377141176146594`, 0, 0,
19.713964143107205`, 0.032068374626748664`, 0.22272556767703097`, 0.2622219260482481`, 0, 0, 0.20255566316874402`},
{0.2283283078987099`, 0.42840256075325867`, 9.446993924606975`, 1.3839314736668495`, 0.8927356525671915`,
0.3909626915031965`, 0.018767685887155043`, 53.5712236843488`, 0.10749676377570666`, 1.5496335657505222`,
198159.6868324399`, 0, 0, 0, 92.09012113511595`, 3.411762452567349`, 0, 0, 46469.03541460902`, 0, 0,
20.880111019452496`, 151574.23179861237`, 6.151915103242879`, 1.0000000000000002`, 0, 0, 0.12016557744657524`},
{0.1380652378345807`, 3.317739091147252`, 0.7336293987943634`, 0.9484386065310282`, 0.5023326788118547`,
0.2130184622271264`, 0.0630596090666651`, 1932.031257216769`, 0.01203085244100871`, 4.258055262793977`,
23.697070191819076`, 0, 0, 0, 12.455350344108883`, 0.22322489164024273`, 0, 0, 0.1474700649661753`, 0, 0,
10.580027844456323`, 0.29086413704335534`, 0.46939097175441147`, 0.9412860110318996`, 0, 0, 0.33679342038715754`},
{0.0926632627436077`, 3.578083963812018`, 1.8944161244777453`, 1.1381278842914715`, 0.7382269863407194`,
0.4777843546205588`, 0.04528998830707099`, 1233.5473532765689`, 0.08357615053600725`, 2.9903558138075255`,
46.48512135694232`, 0, 0, 0, 27.968871779570687`, 0.34070381507501224`, 0, 0, 0.32662985466384287`, 0, 0,
17.4152408161354`, 0.4323798291802552`, 0.529766776343631`, 0.9948824558579736`, 0, 0, 0.32067237225679696`},
{0.2706310445313141`, 2.5166396966047158`, 0.5049158559505837`, 1.3168707464723388`, 0.5754269054535`,
0.5968444469720801`, 0.1025449755078217`, 681.183394170123`, 0.05243619315919962`, 2.183793658787195`,
51157.04603679401`, 0, 0, 0, 3.6169021855438985`, 0.14033685249194672`, 0, 0, 10511.012122739663`, 0, 0,
5.045389912539885`, 40637.23128369062`, 0.6842433788530087`, 1.000000000001069`, 0, 0, 0.17775552395470823`},
{0.20804007909247985`, 0.9673945938397814`, 0.48619022305575754`, 1.3068640855075515`, 0.047796616526516456`,
0.6050138674479171`, 0.4706922223098622`, 348.3137462309647`, 0.24685340199219946`, 5.551550038883448`,
21419.69842270605`, 0, 0, 0, 2.972683060165869`, 1.2596076362932673`, 0, 0, 5387.223656718882`, 0, 0,
17.40768025299164`, 16010.834794752536`, 3.416581371681877`, 1.0000000000168994`, 0, 0, 0.4779974944021405`},

{0.048451644236667674`, 3.3925416461261833`, 7.486002650281495`, 1.2431466818882033`, 0.1294136137122328`, 0.6416005560424389`, 0.04082335443875802`, 58.92112932799084`, 0.09857305139503486`, 6.047753646247408`, 120598.42779870231`, 0, 0, 0, 253.0996638138065`, 2.9039282512539923`, 0, 0, 71032.72992777763`, 0, 0, 140.738536139165`, 49166.46513742825`, 24.11192991834449`, 1.0000000000000069`, 0, 0, 1.1504705369624977`},
{0.09431215650771135`, 0.5526170445339527`, 7.677473256451986`, 0.7715239445968243`, 0.7622192109377914`, 0.1566986505563851`, 0.018040482265180933`, 824.8087273379117`, 0.09073506826067207`, 9.996434684615583`, 151.78796140332793`, 0, 0, 0, 121.13091265837993`, 3.437968266246487`, 0, 0, 0.011144597155224957`, 0, 0, 27.141140892780708`, 0.015015299873121384`, 2.4848385843199945`, 0.2673985951106643`, 0, 0, 0.387782505054708`},
{0.26113782676147984`, 1.4684069653405487`, 4.54225535313282`, 1.1193463339337828`, 0.6236318874525932`, 0.1751148412910668`, 0.053183289402392066`, 554.4897241223483`, 0.22550063278101773`, 9.001504879035508`, 183.26146960123302`, 0, 0, 0, 105.44569117764044`, 3.530237184427758`, 0, 0, 0.04826571110374622`, 0, 0, 74.05464101311325`, 0.1800571843532451`, 3.3625616259722904`, 0.6034346837064544`, 0, 0, 0.44532485524450693`},
{0.23311836154945775`, 2.5262146716446363`, 6.3107841154355`, 1.4422657044398717`, 0.3118898255230633`, 0.2567889403043644`, 0.03481026984978327`, 1604.0167510242688`, 0.16315782107376747`, 5.777943122601165`, 34.574880388189655`, 0, 0, 0, 23.312714609764967`, 0.3025157966923146`, 0, 0, 0.009167428169442505`, 0, 0, 10.917426342976992`, 0.03052994049262022`, 0.7753309645965217`, 0.12761741945452254`, 0, 0, 0.5231857433840987`},
{0.22640126428063923`, 3.7808015365334118`, 7.033092553948691`, 1.0460349638379132`, 0.8860375200295565`, 0.499376231890995`, 0.3693111113992884`, 210.995030019894`, 0.19589698445197923`, 2.861160479579169`, 180.13242090714255`, 0, 0, 0, 29.92734128038333`, 2.5736081185385844`, 0, 0, 2.037445681304884`, 0, 0, 139.00430755722257`, 6.589718259293563`, 3.1017370774371567`, 0.9999998819133887`, 0, 0, 0.45472513705278866`},
{0.19770244273120569`, 3.3508846225196347`, 6.757833537731408`, 0.8466672464019638`, 0.28373063232656337`, 0.6609960258126362`, 0.006803814757890119`, 1256.448281122042`, 0.0187925047862397`, 9.149585751239048`, 105.78945537743523`, 0, 0, 0, 96.43320825096124`, 0.18865798749804952`, 0, 0, 0.010327924304054102`, 0, 0, 9.031016417467352`, 0.029169369475067874`, 1.6833439707750741`, 0.23041424358826057`, 0, 0, 1.5665897521176189`},
{0.06481064744600706`, 2.3696983632913335`, 9.923821775061434`, 1.1006879887228784`, 0.22746584913410017`, 0.6483433064071293`, 0.08284957522863273`, 281.66193979187017`, 0.13350656503173153`, 1.0702403000648566`, 49.97201871535207`, 0, 0, 0, 23.29286694258288`, 0.762699479064042`, 0, 0, 0.05029748191501782`, 0, 0, 25.819538674588813`, 0.04656874811165457`, 0.9013993123248479`, 0.6107874374737551`, 0, 0, 0.21613508709895082`},
{0.1537084817657926`, 0.5942996643982652`, 0.8160795607483013`, 1.042756674430172`, 0.7722126796580249`, 0.49669902434825364`, 0.00611858149596996`, 1518.932458333751`, 0.21666908673557295`, 1.634333111202272`, 20.73434981571264`, 0, 0, 0, 10.947282428171848`, 0.09846066038647738`, 0, 0, 2.766364054883044`, 0, 0,

0.8359305346302165`, 6.0744802698219305`, 0.2585379762415023`, 0.9999999955570568`, 0, 0, 0.3553556240625914`},
{0.13754450751212471`, 1.8598194811531465`, 9.485701362019345`, 1.4994328167980717`, 0.8397969618927028`,
0.642370724162252`, 0.08665379792235217`, 239.43302754758346`, 0.1633413998793405`, 9.552795266495377`,
758.8743360705054`, 0, 0, 0, 341.3179763565046`, 14.928916577178994`, 0, 0, 2.017858170415871`, 0, 0,
396.6441411821089`, 3.9649329754164824`, 8.87704512997872`, 0.999988009077999`, 0, 0, 1.2161908818259288`},
{0.07858197291799773`, 2.783199788795301`, 0.9642830041255817`, 1.3105090636097434`, 0.9572373854271048`,
0.41778523295843695`, 0.00823451224525384`, 90.59081205787001`, 0.24323880940762027`, 7.348654585309644`,
49788.50884527534`, 0, 0, 0, 51.58966451677703`, 0.14634945298953278`, 0, 0, 23429.2145132407`, 0, 0,
5.8188538092967965`, 26301.627148134878`, 17.64436861644863`, 1.000000000000011`, 0, 0, 0.7499950018352105`},
{0.09118935700706149`, 1.2770460819223013`, 0.666402509206284`, 1.1661439001587546`, 0.35202394044239993`,
0.23733544361366754`, 0.06689005296661862`, 1909.3731761645724`, 0.030837584446851807`, 5.2718694661097025`,
27.123683538190996`, 0, 0, 0, 13.929523775667976`, 0.6625859751579776`, 0, 0, 0.19262751850898308`, 0, 0,
12.087897478745202`, 0.2509368507814187`, 0.5989964379471928`, 0.944370426473294`, 0, 0, 0.5229163738441659`},
{0.26692387934872025`, 2.295217680243936`, 3.3610155473383347`, 0.802490311252646`, 0.6241394164828811`,
0.45771836786665954`, 0.015176154328102385`, 1461.3995866509204`, 0.028435347409555445`, 8.762751383914615`,
109.91228007536021`, 0, 0, 0, 90.41188715602163`, 0.5720141726090834`, 0, 0, 0.026059266559490025`, 0, 0,
18.75567203317822`, 0.09936915032915507`, 1.2835669431208339`, 0.5003832139050478`, 0, 0, 0.7413232310110921`},
{0.16789560992918506`, 3.010819021450227`, 9.549236085030234`, 1.3823009139889233`, 0.2184000924395355`,
0.5213598249132101`, 0.3210433649481226`, 558.1245473691072`, 0.20748907999743366`, 5.921770487462836`,
129.16341927127607`, 0, 0, 0, 24.975959523334478`, 2.365441180517958`, 0, 0, 0.02364899655918565`, 0, 0,
101.74164714893097`, 0.056722324307415174`, 2.4264307882846885`, 0.30688577555059493`, 0, 0, 0.9397658130838289`},
{0.05001315370963505`, 3.027404779172768`, 3.8220563025552714`, 0.9962642247459573`, 0.44362718458195727`,
0.3510033113401648`, 0.04839212642640241`, 1615.957133832461`, 0.048401134986770955`, 4.739296636982807`,
36.471937352834274`, 0, 0, 0, 21.801267891922844`, 0.3309655069022828`, 0, 0, 0.014651596364743662`, 0, 0,
14.313807961961519`, 0.010468179158308255`, 0.6353943783091293`, 0.26986387779801857`, 0, 0, 0.46300509483497754`},
{0.0729686004745061`, 0.5044823312287607`, 5.5024178276878075`, 1.2230366941116102`, 0.012758742281838265`,
0.5648708708999995`, 0.03232052448858605`, 634.5000769880222`, 0.22553164736796405`, 5.75812027698643`,
96.42441715249049`, 0, 0, 0, 66.53598103487538`, 3.6332799044768884`, 0, 0, 0.02996491210766959`, 0, 0,
26.184650231672965`, 0.031235681426263758`, 2.1630845313954663`, 0.40672546976271684`, 0, 0, 1.195764719316445`},
{0.25149333924897926`, 1.871859550111978`, 9.811462629766478`, 1.3386558973788294`, 0.5189637829018348`,
0.6377508608690272`, 0.02436930219497053 5`, 194.42061666753636`, 0.14619611626015583`, 6.325307886810732`,

535.7933383860712`, 0, 0, 0, 393.0388255897867`, 4.840151932106971`, 0, 0, 1.8257072432061032`, 0, 0,
129.42978025867703`, 6.559331586927809`, 7.409988490809702`, 0.9998173735714251`, 0, 0, 0.9740084095383307`},
{0.1922243891646514`, 1.6850594067594091`, 0.2630953725218035`, 1.4711962401085972`, 0.15977146838462364`,
0.6883258791888056`, 0.09212778533128511`, 774.9062808250895`, 0.24229201708328568`, 1.1723783184073415`,
9959.795383571882`, 0, 0, 0, 1.104007458284433`, 0.05664314016401417`, 0, 0, 2658.0631434225074`, 0, 0,
1.363529373739483`, 7299.2080586494785`, 0.3311366550878065`, 1.0000000000032747`, 0, 0, 0.12772787681008008`},
{0.2327924107307146`, 3.0114739814321467`, 6.475065538974626`, 1.3312648289930094`, 0.6507217418486848`,
0.3841095338158853`, 0.3176950069808959`, 737.689863101484`, 0.19812174358831114`, 7.321794649353192`,
171.9341475044876`, 0, 0, 0, 32.710268949662066`, 3.1585861898354866`, 0, 0, 0.04151470745448477`, 0, 0,
135.88571612572107`, 0.1380615547013875`, 2.103451330939696`, 0.48687732533321215`, 0, 0, 0.5401252884119456`},
{0.20811447549122308`, 0.49053077700921355`, 2.218461283385089`, 1.0634384889669943`, 0.30011342208125913`,
0.5589173637133283`, 0.16385051847701637`, 124.79731876547663`, 0.15435702825422665`, 9.111119072715418`,
71178.68437728196`, 0, 0, 0, 49.456052098965436`, 14.006844082770115`, 0, 0, 17874.634920457516`,
0, 0, 98.15411587668724`, 53142.43244383021`, 15.60793996680812`, 1.`, 0, 0, 0.7578815161476723`},
{0.19580212756702065`, 2.2297474474451375`, 8.070495694463702`, 1.2202670372174012`, 0.07482300382970508`,
0.34257881980750315`, 0.24674110677687536`, 1287.6149126957`, 0.12354587287124019`, 3.0573978018103922`,
20.978376512665147`, 0, 0, 0, 5.020952067600417`, 0.4849168071680647`, 0, 0, 0.006898002657615206 5`, 0, 0,
15.446314471517717`, 0.019294908518888747`, 0.5342538896590189`, 0.1141165002048583`, 0, 0, 0.42802946207427683`},
{0.18591817623779283`, 1.5316284759435828`, 4.0240186618411435`, 1.0506796825025935`, 0.8560461598734517`,
0.5496710897949223`, 0.05348974137997375`, 3910.687147405355`, 0.18912146822955533`, 8.614502889074807`,
72.92031233700214`, 0, 0, 0, 41.49802472664708`, 1.3706835905964023`, 0, 0, 0.016275402137891946`, 0, 0,
29.991114555229206`, 0.04322704404309801`, 0.4582535159371218`, 0.2821837688527753`, 0, 0, 0.4654001979332382`},
{0.2552337642427184`, 2.0636949211593727`, 3.6788035742608756`, 1.2716726068122697`, 0.4767575621154567`,
0.33698161388823356`, 0.00790398094444615`, 742.5948236907665`, 0.22094334214516276`, 6.7847431915852425`,
117.10515594649874`, 0, 0, 0, 105.08576355356632`, 0.3821615602392608`, 0, 0, 0.05882457103025551`, 0, 0,
11.266641013258624`, 0.21448595277163646`, 1.9667777381175904`, 0.625341391854903`, 0, 0, 0.615219579118226`},
{0.2297403387592854`, 3.933034881515791`, 9.92502862171417`, 1.2059622632705849`, 0.1421363362803283`,
0.1853832242974326`, 0.06367011009221722`, 57.09955187350921`, 0.13182928467731042`, 9.7382206276645`,
90501.93218328858`, 0, 0, 0, 396.5921028493311`, 6.114559400864283`, 0, 0, 20961.131755804516`, 0, 0,
343.5539344099945`, 68794.53586223634`, 35.2071389216039`, 1.0000000000008784`, 0, 0, 0.4473594399020187`},
{0.0477600620262811 9`, 3.695464511472898`, 0.268839094050918`, 1.3548340559244303`, 0.4311024896438589`,

0.6662260908047339`, 0.1893860365797585`, 146.80096070953766`, 0.0868075345269877`, 5.398273141094878 5`,
88985.20892200657`, 0, 0, 0, 3.5416873487739853`, 0.16824788063027388`, 0, 0, 52886.7474616363`, 0, 0,
8.882201028567225`, 36083.91913051457`, 8.548430795357481`, 1.0000000000000318`, 0, 0, 0.9599773198076297`},
{0.16250916383331093`, 2.090868942469317`, 5.046057727155544`, 1.3781211464947924`, 0.9692295654960448`,
0.507149644697747`, 0.012667564467634538`, 467.92228322579444`, 0.2269164868867607`, 2.233796179341983`,
93.88057303980034`, 0, 0, 0, 77.75460109631932`, 0.4497083624470351`, 0, 0, 0.6604876526039039`, 0, 0,
13.432589260131987`, 1.5333613735269624`, 1.04246345785151`, 0.9998447039015927`, 0, 0, 0.23768270212437162`},
{0.12952636821033042`, 2.4189205073691173`, 1.5337482028150813`, 1.4058710509218408`, 0.9636406844740666`,
0.3882701702830901`, 0.27400686262252805`, 4688.706549038708`, 0.0765768774664311`, 6.843480813486918`,
47.08218834930365`, 0, 0, 0, 9.784867407741704`, 1.044187252753108`, 0, 0, 0.059708394315838374`, 0, 0,
36.08294227454165`, 0.1104830209628453`, 0.3005961757784642`, 0.5892337231703326`, 0, 0, 0.2975159870004854`},
{0.08153578837307857`, 2.0915987061965353`, 5.850951169546045`, 1.037700922521303`, 0.7897818160030217`,
0.3237340771928561`, 0.17831164332408336`, 1464.5863160958056`, 0.2436610405225158`, 7.1217069723220146`,
96.11741179649037`, 0, 0, 0, 27.805448942445867`, 2.210915547262076`, 0, 0, 0.017983950328345953`, 0, 0,
66.06211568804503`, 0.020947650972525936`, 1.0100343715982412`, 0.3107172815679229`, 0, 0, 0.37332446051993295`},
{0.2624813864915975`, 2.8946734437676342`, 8.625458110929756`, 1.37764442375384`, 0.19776637799413987`,
0.31049273319228277`, 0.046660769123637466`, 609.6700244678286`, 0.017643025422342112`, 4.098918143681717`,
62.57851701665736`, 0, 0, 0, 38.06326729617098`, 0.5768451672782727`, 0, 0, 0.017445981720155074`, 0, 0,
23.853976955515883`, 0.06541779243733258`, 1.4821557032848367`, 0.23784890556697125`, 0, 0, 0.4794531041521771`},
{0.22181257821621975`, 3.747903553799671`, 8.070266666229205`, 0.9808557386469257`, 0.7823522438549146`,
0.2263615830500163`, 0.06976335847986787`, 287.144692837758`, 0.1821266272405107`, 3.59762935889718`,
192.2889886815009`, 0, 0, 0, 97.38339440838759`, 1.7323788485053793`, 0, 0, 0.10039054380613478`, 0, 0,
92.7541263262954`, 0.31811264785958154`, 2.5911718020083234`, 0.8823543991386094`, 0, 0, 0.17217954673598396`},
{0.21058778737677059`, 1.3147483391864796`, 0.4651324593397846`, 0.8516412222118703`, 0.046686774964893374`,
0.4819914326166439`, 0.10586616607566471`, 1877.0848108087591`, 0.011164572002709261`, 8.732010177522003`,
16818.565577651105`, 0, 0, 0, 13.755371371141498`, 1.010181814744984`, 0, 0, 4187.41627186414`, 0, 0,
18.97335518874785`, 12597.410393105127`, 1.0345747733561375`, 1.0000000000006053`, 0, 0, 1.107378426574178`},
{0.19996482365829993`, 1.5454344843054368`, 8.94969050535013`, 1.027667483044064`, 0.3164796074442535`,
0.5223314119072894`, 0.012949156080520758`, 231.31648814801656`, 0.19929090705614294`, 9.341058456545078`,
538.4992188076072`, 0, 0, 0, 455.231420556362`, 3.5797159644074874`, 0, 0, 0.09643893583726341`, 0, 0,
79.03166422020023`, 0.2754913542642124`, 9.10507114499417`, 0.8612913511533439`, 0, 0, 1.3301171578937585`},

{0.10440009635624109`, 1.532425369535546`, 0.308138348960842`, 1.2734376519411275`, 0.20147673343032046`,
0.35081245482125123`, 0.05722019474177449`, 85.42879625764479`, 0.08356786949647793`, 8.411462154648635`,
173222.50849639316`, 0, 0, 0, 11.438635560231702`, 0.398407193261451`, 0, 0, 69519.08391055709`, 0, 0,
8.721847005132823`, 103682.8436979968`, 20.886878485239617`, 1.000000000000095`, 0, 0, 0.6291983404868714`},
{0.1446017732242294`, 0.797427468093673`, 5.032298069921831`, 1.3706636028120882`, 0.10121695844960277`,
0.6068141920969097`, 0.25341251132298065`, 197.97452899503492`, 0.2275401984407136`, 3.68386367767849`,
7643.8237417228265`, 0, 0, 0, 37.50991816617386`, 10.094522771703554`, 0, 0, 2440.267346149572`, 0, 0,
114.99499622076387`, 5040.956934205909`, 4.376097820550947`, 0.999999999999969`, 0, 0, 0.7056729080551883`},
{0.1841264162344997`, 3.4194642457460365`, 0.8344796094826652`, 1.4377258087185927`, 0.5867256025093719`,
0.6103981120475732`, 0.3542274534468216`, 522.9821129731852`, 0.2244081861480518`, 8.361737950667973`,
12814.08333505623`, 0, 0, 0, 9.898660579326544`, 0.9426973968903923`, 0, 0, 3514.0125591989076`, 0, 0,
46.050286331779304`, 9243.179130404564`, 3.4867208508325045`, 1.0000000000000107`, 0, 0, 0.8772914240519025`},
{0.17849930658201318`, 3.2992561971358683`, 5.635580909426066`, 1.1878114797167083`, 0.48988207998590316`,
0.27890916833354473`, 0.03715549210344845`, 144.67131391199885`, 0.0866110192158086`, 7.798176718489761`,
19657.06600454017`, 0, 0, 0, 235.46408357564417`, 2.5092451491940118`, 0, 0, 5436.8617249152685`, 0, 0,
118.26632298016388`, 13863.943541138045`, 11.700968488881811`, 0.9999999999999984`, 0, 0, 0.7786486752925036`},
{0.09510173640093084`, 0.7659692827065738`, 0.5464490010035838`, 1.4050402850247132`, 0.11782959453955089`,
0.3589214817200733`, 0.08175234954735425`, 225.11260306454756`, 0.1010344837489014`, 6.4310165595390805`,
52018.17631537765`, 0, 0, 0, 13.193627452652244`, 1.249249204076487`, 0, 0, 22042.792903463233`, 0,
0, 13.669807382403246`, 29947.255432078142`, 6.113349086179084`, 1.`, 0, 0, 0.5416892021024865`},
{0.06416093472533757`, 1.2573296539610634`, 9.92323628294643`, 1.30190007637989`, 0.030494015633026894`,
0.4401773012522794`, 0.02586427825345657`, 3143.6595843648015`, 0.20592664064058636`, 7.935954028868563`,
24.301225123239742`, 0, 0, 0, 17.88297508105898`, 0.33800947148945903`, 0, 0, 0.002574050176195318`, 0, 0,
6.071276168905765`, 0.002359335219065146`, 0.582369920595796`, 0.04162524395206724`, 0, 0, 1.3399614037805696`},
{0.25237835724682106`, 2.979406809596097`, 0.9138356130609361`, 0.7932538461611548`, 0.3933816374531405`,
0.6211446249652819`, 0.0749645015318215`, 95.73291003138912`, 0.06307891905410445`, 9.368280708056208`,
227658.0785614993`, 0, 0, 0, 33.029373971486436`, 0.7992331873107785`, 0, 0, 49418.07006824128`, 0, 0,
34.017725724698906`, 178172.16203044305`, 20.7303843084989`, 1.0000000000000486`, 0, 0, 0.6869252432764058`},
{0.05665762575451494`, 1.7380994050720693`, 0.46391281319797173`, 1.3490794355447004`, 0.9653780951392605`,
0.17540498059526055`, 0.4972377260445833`, 285.101267300382`, 0.1881031714219547`, 6.261558552266614`,
14307.785124377497`, 0, 0, 0, 3.216975351542537`, 0.752818555879761`, 0, 0, 7894.896568253875`, 0, 0,

18.692478344311795`, 6390.087073353325`, 4.583280481933906`, 0.9999999999999957`, 0, 0, 0.35905008933353394`},
{0.25642346088610446`, 3.010945889122482`, 9.963520677018519`, 1.467899384826252`, 0.4946077322511526`,
0.6868780403912886`, 0.012314696297102353`, 88.3173980921876`, 0.10491074891333174`, 1.4353196490865765`,
96124.6612060573`, 0, 0, 0, 103.28809299713838`, 0.40648992930296063`, 0, 0, 20587.487754009624`, 0, 0,
17.484559737206283`, 75415.92658333486`, 3.6887068125091527`, 1.0000000000004474`, 0, 0, 0.21528375893815935`},
{0.09174036922136902`, 1.4204870656163582`, 7.877654658381974`, 0.9368390881044215`, 0.3909649395372681`,
0.6267838157099552`, 0.00737791814963061`, 223.00641924802295`, 0.22589847441224364`, 3.7895604923910007`,
257.84855811434767`, 0, 0, 0, 231.56215194067548`, 1.1270708698377105`, 0, 0, 0.8931377843274487`, 0, 0,
22.871279894820677`, 1.1705255728536461`, 3.9168445344992424`, 0.9981994870729389`, 0, 0, 0.6360789789437301`},
{0.2260021855376967`, 2.1215536107061057`, 4.2648042233683015`, 1.1043329178413561`, 0.5641485347029902`,
0.48705118822040194`, 0.49950773055219905`, 4133.317514629252`, 0.08759305230550185`, 2.0466954881219834`,
8.360890332626482`, 0, 0, 0, 1.116542878674277`, 0.23043926620057903`, 0, 0, 0.007041556460201166`, 0, 0,
6.9841322465186195`, 0.022734387851329182`, 0.10802373411491849`, 0.12644066280729738`, 0, 0, 0.2155529001093871`},
{0.1540246000020472`, 3.8655612430526176`, 1.2043257775886698`, 1.4535724207675174`, 0.7982285789403216`,
0.6724657214579106`, 0.417253246623641`, 114.93402398229541`, 0.10143088124264565`, 7.877832682736377`,
157235.21671098113`, 0, 0, 0, 11.83407280657064`, 1.171023658397862`, 0, 0, 49106.33981344825`, 0, 0,
64.6666238371495`, 108051.20496187091`, 15.008225850847673`, 1.0000000000020113`, 0, 0, 0.861943416869752`},
{0.18967559668177286`, 1.3751140072639014`, 7.146984940201236`, 1.0524950668750344`, 0.5379883166421491`,
0.5342982154057455`, 0.18359081743318598`, 57.97987210615365`, 0.21651827946513758`, 4.096495164957771`,
83944.59661895152`, 0, 0, 0, 67.25645226526096`, 8.205619702826532`, 0, 0, 22564.907254482987`, 0,
0, 161.19517988053374`, 61143.03210804171`, 15.27443884183193`, 1.`, 0, 0, 0.3905955607541936`},
{0.11610573160351356`, 2.8105133752566074`, 0.4045001364193191`, 0.9020454264062931`, 0.06323256453719583`,
0.49478351534124754`, 0.06895293734611843`, 538.3487824750697`, 0.04516006162070901`, 5.298889101080752`,
33913.91032449796`, 0, 0, 0, 8.912403155582608`, 0.20782549056893054`, 0, 0, 12749.478928451126`, 0, 0,
8.344233156617799`, 21146.96540787701`, 2.1456350018169905`, 1.0000000000006162`, 0, 0, 0.5536175243755074`},
{0.06094054623730827`, 3.5785656752883286`, 2.8105466781089756`, 1.0666569400098407`, 0.8956485783622179`,
0.6416539064413922`, 0.4241071070816547`, 1544.2747045672372`, 0.05890410014619657`, 6.674766669452417`,
146.73981626915102`, 0, 0, 0, 21.490677135964585`, 2.392255407237095`, 0, 0, 0.2989048569269657`, 0, 0,
122.29775838373718`, 0.2602203607725147`, 0.9130961011371358`, 0.9923308088130209`, 0, 0, 0.4695941553486991`},
{0.06400872221443815`, 3.352604720184938`, 3.3612644751655854`, 0.9279864636339992`, 0.6811335659068025`,
0.5445450422212498`, 0.04222211989978537`, 4877.834369436828`, 0.09303122695369598`, 6.110250683676178`,

27.638691796772214`, 0, 0, 0, 17.328451193523115`, 0.210509149333481`, 0, 0, 0.008624020557148046`, 0, 0,
10.082199538536349`, 0.007885893374482018`, 0.2681175936702911`, 0.1801409209337259`, 0, 0, 0.5161042656531725`},
{0.1838955211549798`, 0.6066893455768754`, 3.1997125487162617`, 1.2441392193895697`, 0.8430620231566095`,
0.17975264196625795`, 0.008385470075528607`, 217.99247821227752`, 0.10887259656335785`, 8.65519228817503`,
13476.826750153878`, 0, 0, 0, 194.39320467282874`, 2.3475642758867514`, 0, 0, 3655.7143835671523`, 0, 0,
20.346317631962638`, 9603.850025140517`, 8.33400229865434`, 0.9999999999999976`, 0, 0, 0.5496734408260051`},
{0.10877154933938238`, 0.6652486439511347`, 7.420477343235575`, 1.1719120037873472`, 0.8277401239831172`,
0.4096056229223566`, 0.3960687102590752`, 4083.2161783729553`, 0.09124503977845877`, 8.628655226336182`,
52.90970298330367`, 0, 0, 0, 8.315169314202365`, 4.244096288133143`, 0, 0, 0.006440153858308299`, 0, 0,
40.333990006837325`, 0.010007221616446018`, 0.4388207223872889`, 0.11142691863069842`, 0, 0, 0.449797465255998`},
{0.27429237480005003`, 0.4974953244631517`, 8.109325428142139`, 1.1022725368505206`, 0.3620164170986986`,
0.5133215689169995`, 0.013475856804577652`, 252.124017519504`, 0.17376346726865255`, 1.283750502496817`,
68.04699729848504`, 0, 0, 0, 56.80138748752961`, 1.322504345288549`, 0, 0, 0.09946400312171998`, 0, 0,
9.399138976617904`, 0.38974596604823875`, 1.1574505136285569`, 0.8332963415776767`, 0, 0, 0.19480960462239175`},
{0.2348606786833104`, 2.9248975834862243`, 4.089597609027345`, 1.0125076012045389`, 0.4800911130241774`,
0.44584481805285503`, 0.013867879702002976`, 69.31755646766315`, 0.08239556859020553`, 3.2481286029667444`,
221236.72662704941`, 0, 0, 0, 87.49473985025338`, 0.4003859484353412`, 0, 0, 50774.81174673964`, 0, 0,
16.729827043433843`, 170357.23924080888`, 9.871402377271412`, 1.0000000000001361`, 0, 0, 0.2187962959994254`},
{0.2276305764129738`, 0.759063573478838`, 9.75608531797917`, 0.9651630732227953`, 0.2616315835146603`,
0.25394525967231996`, 0.42721777606241185`, 115.33714629065052`, 0.1547416236361928 7`, 5.678602403105034`,
450.4943651944117`, 0, 0, 0, 71.80891222427346`, 31.561521418145315`, 0, 0, 1.14730811661039`, 0, 0,
342.2457318869473`, 3.7308915415322`, 10.677702148543808`, 0.9994255127085235`, 0, 0, 0.5611784520254767`},
{0.22740101487152548`, 3.662274246882813`, 5.673159128278421`, 0.7501649913658239`, 0.6663016096811909`,
0.46449661159333955`, 0.15875103883050143`, 79.3840483030526`, 0.23925351496597358`, 9.512843911813135`,
42809.697532988925`, 0, 0, 0, 134.25485084662975`, 5.519322280268204`, 0, 0, 9975.357227967039`, 0, 0,
288.76102638961123`, 32405.80510493875`, 25.4417178317641`, 1.0000000000003584`, 0, 0, 0.720133314631578`},
{0.18907356960866467`, 0.8903250429215461`, 0.8912625465714132`, 1.4194566043771595`, 0.25430531352108066`,
0.6668345122251143`, 0.01430802337144644`, 354.71023908442675`, 0.05435127807039547`, 4.360013356436832`,
88116.18412159599`, 0, 0, 0, 26.391195442974286`, 0.38845564458667997`, 0, 0, 23799.847372123335`,
0, 0, 4.940739834853598`, 64284.6014112676`, 2.684756127514098`, 1.`, 0, 0, 0.46502649181855615`},
{0.22841094141655494`, 2.082125305118022`, 3.0130589699894585`, 0.8668572537043583`, 0.35284672466002664`,

0.48298470604644195`, 0.055294539730325123`, 1324.8059697322965`, 0.2155561741975654`, 1.2621538558570524`,
12.277535646914766`, 0, 0, 0, 6.89677809527377`, 0.1719039615029102`, 0, 0, 0.022399754400557467`, 0, 0,
5.113222689932044`, 0.07309069985900915`, 0.21631121818460575`, 0.4148111905953411`, 0, 0, 0.18980280387838153`},
{0.1257670047049273`, 2.665283038938149`, 6.579106420165356`, 1.3213793494994266`, 0.07362490185046933`,
0.671746727583086`, 0.11039555694375124`, 54.48594040704261`, 0.2029696416579515`, 8.437004538119528`,
112676.07140636715`, 0, 0, 0, 183.38666864370958`, 7.175656712849308`, 0, 0, 40123.51492894714`, 0, 0,
273.21651614285537`, 72088.77558353017`, 34.621127010653645`, 1.0000000000000357`, 0, 0, 1.1205764339721322`},
{0.07898342764241062`, 2.336170261443863`, 9.558440542813312`, 0.8746675798923161`, 0.3314927429594914`,
0.5846930166673705`, 0.14173550706968557`, 4831.453144750826`, 0.22811466814427805`, 5.138718102637142`,
15.064003340588322`, 0, 0, 0, 5.132196552800319`, 0.2888284902461183`, 0, 0, 0.0017179134734818908`, 0, 0,
9.639321851010195`, 0.0019383813503999257`, 0.24178962811783522`, 0.04131636828392471`, 0, 0, 0.7801378780016732`},
{0.2360815041275312`, 3.0069216843113518`, 1.9005978173203566`, 1.189378383663731`, 0.3594173887197991`,
0.5615883220885535`, 0.16515958276300607`, 91.7495984293873`, 0.10675594880460115`, 2.979677734696839`,
176997.05695890484`, 0, 0, 0, 13.678774503264352`, 0.7129661159426484`, 0, 0, 40468.44570790517`, 0, 0,
30.626189631531346`, 136483.59332036562`, 6.901670403051124`, 1.0000000000063616`, 0, 0, 0.22866481863958865`},
{0.19503604064380992`, 2.260639826798138`, 3.7301326124020027`, 1.0003156010412233`, 0.94917046396899`,
0.3935362271642996`, 0.08733555022372319`, 2234.7260448390443`, 0.16743068067899064`, 7.659400521016185`,
108.52857909107487`, 0, 0, 0, 48.61887181320319`, 1.7955770959430126`, 0, 0, 0.03332102787862274`, 0, 0,
57.98790135964726`, 0.09284001925187925`, 0.7034320656853185`, 0.5090423085480824`, 0, 0, 0.30528781388373716`},
{0.09766567347807259`, 2.1751916900809825`, 2.9967711374909367`, 1.239918427231443`, 0.884426059472291`,
0.5758255537556012`, 0.011950287911566193`, 133.4222213549303`, 0.18860770237483848`, 7.181211491841234`,
36544.24702647398`, 0, 0, 0, 154.2889768634669`, 0.8074226286474061`, 0, 0, 15181.857455383579`, 0,
0, 25.089985603102495`, 21182.090471830536`, 12.070751786596034`, 1.`, 0, 0, 0.9786881964279124`},
{0.14345253817386028`, 1.510087082683639`, 7.446143698111681`, 1.3871400228991382`, 0.2507990125704793`,
0.592096235116642`, 0.03860344741119924`, 206.9907110306594`, 0.033941806565597976`, 5.68471806318026`,
365.65526130683713`, 0, 0, 0, 235.65634306470935`, 5.604308842293485`, 0, 0, 1.1398117299748707`, 0, 0,
120.89991985881568`, 2.3358412243603066`, 6.417555468705951`, 0.9980544716029628`, 0, 0, 1.0457120768565946`},
{0.23351662183055255`, 2.7403756212365034`, 1.6128399905233286`, 1.1394774719326448`, 0.2292996808057013`,
0.38219770472020054`, 0.028922618161783233`, 3147.1184318469313`, 0.11734182225292555`, 5.055074464096112`,
16.961401026417935`, 0, 0, 0, 12.047660307847641`, 0.1204736154468713`, 0, 0, 0.017259679965595177`, 0, 0,
4.716327982468962`, 0.05757745942060456`, 0.35974189430627834`, 0.27433617180483083`, 0, 0, 0.6811539582579005`},

{0.05894445024535572`, 1.7190376171466255`, 3.4445271438587763`, 1.1005021934401353`, 0.8920369053637744`,
0.4553589712941485`, 0.02602138727445485`, 2996.975539830326`, 0.01616140506923186`, 6.007209949084906`,
62.548788659013965`, 0, 0, 0, 45.66727535236215`, 0.6582052206564115`, 0, 0, 0.026711768146866675`, 0, 0,
16.16399334443806`, 0.022493006978555646`, 0.41468509797900915`, 0.40375450287784576`, 0, 0, 0.2896489379384584`},
{0.1969133083502746`, 1.0306356019031098`, 0.4532514022896326`, 0.7704659952544493`, 0.6922371279082573`,
0.35856337468106037`, 0.02342746544122668`, 625.7704338271294`, 0.24611682235411125`, 7.40783802504553`,
5445.851291335578`, 0, 0, 0, 19.774486855349103`, 0.4146926543015613`, 0, 0, 1421.317552132707`, 0, 0,
6.105671619584095`, 3998.2334486680697`, 2.478980409799793`, 1.0000000000000004`, 0, 0, 0.451943386583717`},
{0.23578418763137027`, 1.109904439772956`, 9.416464917532476`, 0.9968768007328397`, 0.9319056056422497`,
0.20705868768300972`, 0.023381736885244357`, 73.41723955051782`, 0.19857821780967083`, 6.130556036938069`,
24173.921788739648`, 0, 0, 0, 341.8477452892159`, 6.595777361811057`, 0, 0, 5430.157154842113`, 0, 0,
104.58117968040035`, 18290.645620930336`, 17.53365329854135`, 1.0000000000000513`, 0, 0, 0.39162424840818966`},
{0.06254010222908407`, 2.833030713124539`, 1.1523226094346608`, 0.8553536481119535`, 0.24975724964580737`,
0.6445417390978168`, 0.03834946206048674`, 4173.282831948493`, 0.23424935668510183`, 4.880796947048751`,
16.20045439658201`, 0, 0, 0, 10.532746464941145`, 0.13576569965935592`, 0, 0, 0.01922186490602511`, 0, 0,
5.494691384625678`, 0.017173391375092033`, 0.2745794414669904`, 0.37377940303342216`, 0, 0, 0.9250845773142755`},
{0.1868109134318776`, 3.0098056229686154`, 1.9105557354746436`, 0.9827601516958456`, 0.5236893411586465`,
0.474044891890932`, 0.006475636749568416`, 1404.5093361888864`, 0.13430982294875038`, 1.0667742763750176`,
12.064364390599708`, 0, 0, 0, 10.834631853286757`, 0.022431382115654343`, 0, 0, 0.0631171700801748`, 0, 0,
0.9644871431807739`, 0.16844251708446542`, 0.17066044061510274`, 0.7122700503846873`, 0, 0, 0.147676247587264`},
{0.06031809352972184`, 0.6708570664744204`, 0.23575165127461165`, 0.8219834374256857`, 0.8758699419986131`,
0.18852573702588948`, 0.186745146986022`, 107.40963970974032`, 0.12527667123697356`, 3.1788633087725113`,
40101.04512569063`, 0, 0, 0, 1.6812026979041739`, 0.3941713975163883`, 0, 0, 21536.87011968957`, 0, 0,
3.777609534656665`, 18558.042088813025`, 6.18255790553413`, 1.0000000000001872`, 0, 0, 0.18573936562784168`},
{0.18801205905393248`, 2.0266227725882997`, 1.7626950623641149`, 1.1991174158264077`, 0.011000047841808414`,
0.3353078609658676`, 0.05399718652686698`, 93.79336861840014`, 0.2334529633377767`, 8.960462961249508`,
64931.84912393902`, 0, 0, 0, 70.01211334794534`, 1.7691686934272184`, 0, 0, 17582.973512266195`, 0, 0,
51.22053660928405`, 47225.872204741885`, 19.887971272865396`, 1.0000000000005884`, 0, 0, 0.4906934676634336`},
{0.14122716980793654`, 1.0036898987844207`, 5.592205787770849`, 0.8071832042112255`, 0.9861798903854433`,
0.6717138208444808`, 0.19154806886518252`, 64.00473270059831`, 0.09434989637003327`, 6.618037610942842`,
132572.20946399213`, 0, 0, 0, 84.91643919137049`, 14.514772575389655`, 0, 0, 43832.07953204957`, 0,

0, 208.11900881531022`, 88432.57913011049`, 22.990056621251085`, 1.`, 0, 0, 0.837422711326799`},
{0.14179341245484034`, 1.2991893501557028`, 6.1750864357305275`, 1.3220469832389021`, 0.954868801179809`,
0.5061480199077149`, 0.3454995627472`, 2784.154142185204`, 0.062422742009026755`, 1.1334761108568099`,
15.166297898994495`, 0, 0, 0, 2.614599630672807`, 0.6384675112274864`, 0, 0, 0.02094475929756748`, 0, 0,
11.849859871530986`, 0.042426127054953994`, 0.08450220156369434`, 0.28316715757522837`, 0, 0, 0.05854423369861831`},
{0.17346773507380248`, 2.4886801471142252`, 4.186844167829875`, 0.9059139514582338`, 0.8823714489891827`,
0.3118060351580627`, 0.18611640479783054`, 317.2758158326019`, 0.18366106179531033`, 4.660916164387663`,
162.61028755708978`, 0, 0, 0, 45.7278074463258`, 3.0937273164090153`, 0, 0, 1.0921978698359556`, 0, 0,
109.98996789902978`, 2.7065870104696126`, 3.2065237690168535`, 0.9999993413330208`, 0, 0, 0.49366955711919047`},
{0.17801738501831404`, 3.953347877342175`, 5.116473955634309`, 1.3026800881545206`, 0.2660443419983707`,
0.5551592463622865`, 0.021882498869656555`, 368.98117336094424`, 0.13387054033002443`, 7.8289062285527375`,
277.6692222090391`, 0, 0, 0, 212.16490043682845`, 1.1290530614994065`, 0, 0, 0.15396131275258548`, 0, 0,
63.764850344076635`, 0.391539861288485`, 4.861625812655857`, 0.9063581285176536`, 0, 0, 1.2599256495474198`},
{0.1502521637125906`, 3.604808817430471`, 9.516864148680163`, 1.170214417719616`, 0.46260070316916013`,
0.4333225308578431`, 0.19319459383231874`, 1379.7428965277768`, 0.03462365036850307`, 7.002259791633362`,
72.21085203551476`, 0, 0, 0, 19.98645631083161`, 0.9942868170342185`, 0, 0, 0.008598023418422506`, 0, 0,
51.203055501426086`, 0.018455308889587296`, 1.1069384503358126`, 0.14607946503836033`, 0, 0, 0.7360118923636092`},
{0.23041123856532575`, 3.7235663376199373`, 7.422983674409959`, 1.0720190294684302`, 0.9567615568260381`,
0.6795713708474191`, 0.42619421138851304`, 252.0789228698587`, 0.23264645009848384`, 7.716645878625323`,
504.6156747600694`, 0, 0, 0, 75.58550755126718`, 7.701802013502826`, 0, 0, 2.7123314314737663`, 0, 0,
409.6881530927504`, 8.927880636078946`, 7.061694911598791`, 0.9999999626448`, 0, 0, 1.3025500217070463`},
{0.17190873580866728`, 2.1300233762526144`, 7.263444695009253`, 1.2209987713639263`, 0.8761821538663868`,
0.2004596237918942`, 0.1361627927608223`, 3975.405902408578`, 0.13971132873354192`, 8.98211109187212`,
38.025600682596114`, 0, 0, 0, 13.210233568722787`, 0.789060151511953`, 0, 0, 0.004650097860837522`, 0, 0,
24.010236685569897`, 0.011419892066288522`, 0.46239697544673436`, 0.07871941631301738`, 0, 0, 0.33151494749550514`},
{0.25963938649205515`, 2.4653900827356168`, 4.997042605235823`, 1.1014911256242188`, 0.41576920876875767`,
0.18114027248931974`, 0.22217255314368375`, 81.71471228407572`, 0.2378841388493836`, 9.715142052575505`,
52650.08568113075`, 0, 0, 0, 92.15729729999626`, 7.6802858203528235`, 0, 0, 11101.77550476024`, 0, 0,
270.49857848675396`, 41177.974014692285`, 24.239509655421934`, 1.000000000001409`, 0, 0, 0.32052903927949106`},
{0.2762626642080059`, 0.4739284043667089`, 1.351212961630882`, 1.24608482511035`, 0.19517067645291641`,
0.24775742659101618`, 0.08503066911676711`, 2141.7873291111496`, 0.17338810608691535`, 4.319189194420735`,

17.40648154681566`, 0, 0, 0, 8.025343503096861`, 1.1886445588186125`, 0, 0, 0.02928702841726609`, 0, 0,
8.047605987429652`, 0.11558446424698837`, 0.439595610400643`, 0.3911876956057405`, 0, 0, 0.45118036948652146`},
{0.1906362991887024`, 1.590121005227628`, 9.42412383550555`, 1.4957920989499214`, 0.709065663948995`,
0.3417182681188283`, 0.37536592261162544`, 505.4667159946151`, 0.11728445265057086`, 5.855972486658924`,
205.02681117245638`, 0, 0, 0, 34.22523152354299`, 7.194365850342365`, 0, 0, 0.04831525299171719`, 0, 0,
163.4273179703106`, 0.13158058606752904`, 2.430973522723896`, 0.49924207469114446`, 0, 0, 0.3698269438953232`},
{0.2658746234670645`, 3.614932865157483`, 8.460828570764`, 0.8159863776033582`, 0.2726846463148733`,
0.34564016673603704`, 0.10623048732883861`, 3778.1279822475276`, 0.23711150436988554`, 5.459482199196464`,
15.113367034537486`, 0, 0, 0, 6.183307702575298`, 0.16948175248232072`, 0, 0, 0.0017124029323062509`, 0, 0,
8.752359387040366`, 0.006504064069301846`, 0.3179863937494972`, 0.04414126941704333`, 0, 0, 0.6275771492296697`},
{0.26863157332159515`, 2.9396265733275415`, 6.862183762414347`, 0.9732277188028977`, 0.5002013826688112`,
0.5818631486183298`, 0.034332997185588514`, 711.1213518313073`, 0.15904422112946637`, 1.3044149622459462`,
30.670437836883494`, 0, 0, 0, 20.616838098286436`, 0.23067443037194965`, 0, 0, 0.02759849943969653`, 0, 0,
9.687095504408244`, 0.10591183322572847`, 0.41001738643907304`, 0.44811696955123526`, 0, 0, 0.17288377063682137`},
{0.27209369466384475`, 3.5584095462332135`, 0.9720509149176574`, 0.9873292487031791`, 0.8069894772531618`,
0.17871886216847`, 0.10218788035646292`, 96.95116373019758`, 0.02386269594633994`, 2.5608838628408552`,
607526.4899707471`, 0, 0, 0, 7.761980674233945`, 0.21367397770549934`, 0, 0, 124309.61554020383`, 0, 0,
10.861993172126759`, 483198.03677966207`, 5.341267617848818`, 1.0000000000000053`, 0, 0, 0.06284950370544327`},
{0.14444262093504412`, 3.863449844229998`, 8.726684391435349`, 0.8553749492129846`, 0.451984917853794`,
0.5228822441273482`, 0.008115157266947242`, 403.9888714010655`, 0.1027695319041006`, 9.711355034259547`,
372.50117745610584`, 0, 0, 0, 333.9655873736296`, 0.6784327923306188`, 0, 0, 0.03577123459481179`, 0, 0,
37.444158083575104`, 0.07381272684217945`, 5.307321387838847`, 0.5920163781553305`, 0, 0, 1.152078773428255`},
{0.2538137652575522`, 0.7540411224449701`, 8.794714857317246`, 0.9184514413182489`, 0.11359389239638507`,
0.27911589396705727`, 0.11507441894157476`, 65.76953791858952`, 0.10155553687026442`, 2.859221903044178`,
91049.2654400193`, 0, 0, 0, 76.35280168178367`, 10.26330912866354`, 0, 0, 19639.827510262498`, 0, 0,
110.55653050538653`, 71212.26527698334`, 9.115385281388017`, 1.0000000000012783`, 0, 0, 0.17831913269501568`},
{0.07943689831455408`, 1.7727348695278575`, 3.5998215792473456`, 1.4458682286750966`, 0.6331999429153172`,
0.478090570864118`, 0.46084046549589247`, 472.0739644000896`, 0.10830302137693731`, 2.3782715249335666`,
72.31348620505452`, 0, 0, 0, 10.332462955265331`, 2.303069693682795`, 0, 0, 0.6338779791643236`, 0, 0,
58.32474218491888`, 0.7193328653532274`, 1.1380746351948783`, 0.9987319883415517`, 0, 0, 0.3438225272557363`},
{0.055568082805285024`, 1.296674173505255`, 4.7380956025626375`, 1.4538691472242138`, 0.09550221320066177`,

0.19041481571096486`, 0.007131815235409429`, 86.20076273581975`, 0.04910104396213488`, 4.323845103489194`,
120296.3074315137`, 0, 0, 0, 147.29444538194028`, 0.7480883672879537`, 0, 0, 66970.1062076241`, 0, 0,
13.857526648028617`, 53162.86296034293`, 10.618865280747494`, 1.0000000000010396`, 0, 0, 0.3139499499033247`},
{0.1596260086552564`, 2.5471852815710525`, 0.22684585875990315`, 1.2716791760964523`, 0.8208442532787252`,
0.3888937433082962`, 0.04289548126799573`, 69.96603930346842`, 0.07461229317798801`, 1.378327695847128`,
284870.1840749884`, 0, 0, 0, 1.5408905589215078`, 0.024789390269905245`, 0, 0, 86840.07634335257`, 0, 0,
0.9020452862089052`, 198027.63968581587`, 4.157310378218687`, 1.000000000001114`, 0, 0, 0.09541639508677296`},
{0.14264033419885946`, 0.8035889732308119`, 6.673404894657215`, 1.4157343373387477`, 0.18185291353504507`,
0.4765318357304136`, 0.03778101114485503`, 210.869852474353`, 0.22822304787397907`, 1.0663935058760412`,
55.67351992410398`, 0, 0, 0, 36.104345828067714`, 1.513058629106873`, 0, 0, 0.22492073882351732`, 0, 0,
17.369674717171616`, 0.4583252764863029`, 1.187321608900792`, 0.9334175239895807`, 0, 0, 0.20214951318998842`},
{0.2209992311467341`, 3.548991401043337`, 2.677760813541523`, 1.1645638964766105`, 0.17856966244183803`,
0.2409694046188614`, 0.16792458477559433`, 53.365358865447796`, 0.019626895089088575`, 3.834874658630479`,
1.36730593164984`*^6, 0, 0, 0, 23.73654763818811`, 1.0631658604876244`, 0, 0, 328887.1441055133`, 0, 0,
53.902378525048384`, 1.0383400854480534`*^6, 14.719437257513663`, 1.`, 0, 0, 0.1450328050962076`},
{0.08986394714808477`, 2.7800808748945913`, 9.536673819459821`, 0.8308776528106754`, 0.5315183679881144`,
0.4614339535602666`, 0.046951751845033245`, 1052.1471359283046`, 0.24213976319820152`, 2.8230201755788666`,
41.81085216188932`, 0, 0, 0, 25.240682662126332`, 0.4064412749836607`, 0, 0, 0.009073161980929055`, 0, 0,
16.141994504997765`, 0.011647859267474288`, 0.5839724069515665`, 0.20869686132376963`, 0, 0, 0.2900659622935604`},
{0.08237232438525194`, 0.904854561594771`, 7.190948808493694`, 0.9264355987892083`, 0.7428148600467364`,
0.6232177046085758`, 0.0730718251800762`, 62.25627183567972`, 0.1453325784006102`, 9.786938339680962`,
70111.33250513591`, 0, 0, 0, 297.8249530014896`, 21.654976028603954`, 0, 0, 31933.820258429365`, 0, 0,
279.9229120101098`, 37578.04287410966`, 35.85536105202004`, 1.0000000000002305`, 0, 0, 1.5229819255557968`},
{0.14754836278222488`, 0.5816922789151606`, 9.91636448422577`, 0.7971447359984003`, 0.9834799375197849`,
0.6545637466726528`, 0.017670578507760985`, 406.58618651198583`, 0.1328908282216244`, 3.7962615345853075`,
298.7116857186342`, 0, 0, 0, 237.79234590030978`, 6.393896457161015`, 0, 0, 0.4145231932889547`, 0, 0,
53.132574304479334`, 0.8737459786435651`, 1.9746860794833625`, 0.9993786787050523`, 0, 0, 0.2716691106028291`},
{0.06758332214646862`, 3.0558788120173555`, 4.64800500980858`, 1.4828314301436276`, 0.7395538908104162`,
0.3075642133576061`, 0.04417074894369504`, 145.23097869725217`, 0.1743358052899487`, 7.770691560418564`,
15237.204425877404`, 0, 0, 0, 184.3143912870575`, 2.509473027088446`, 0, 0, 7601.601405435337`, 0, 0,
109.55207789726657`, 7339.163951608383`, 11.761375816465602`, 1.000000000000001`, 0, 0, 0.8837634520336762`},

{0.16631267310517406`, 2.413572311283116`, 5.471184235465843`, 1.4579537524645423`, 0.9497208653345599`,
0.439033675828699`, 0.21085287275356013`, 3128.2027597762994`, 0.18409987492192065`, 5.612876798830806`,
61.677985584717064`, 0, 0, 0, 15.631106174578136`, 1.2958080511211751`, 0, 0, 0.021363907468654995`, 0, 0,
44.67894904176853`, 0.05075840798692196`, 0.3688171121388909`, 0.2689619128817412`, 0, 0, 0.23271241439608115`},
{0.18539796406610926`, 1.3871953304353832`, 7.757698949197568`, 1.3585428901946988`, 0.4532718222049952`,
0.29058122288435795`, 0.010738744574908157`, 486.55354471220693`, 0.20251048022954932`, 4.51278311919668`,
106.72598666736846`, 0, 0, 0, 92.62360919403208`, 0.6682787533658657`, 0, 0, 0.03340615076036453`, 0, 0,
13.243330944261544`, 0.08847760483224389`, 1.9868498302723758`, 0.4094292875846528`, 0, 0, 0.385208275896955`},
{0.04144083471615087`, 3.41458508027812`, 2.4600046087844927`, 1.2787341279549589`, 0.569252466493285`,
0.6736151572579898`, 0.4762197736813673`, 718.8382183063302`, 0.07598079948793823`, 4.331845758841565`,
94.40191581040338`, 0, 0, 0, 13.134825083273519`, 1.6036667872693122`, 0, 0, 0.902554542365586`, 0, 0,
78.2265240792479`, 0.534323051606878`, 1.4243966133655426`, 0.9998637306805985`, 0, 0, 0.8559685758826732`},
{0.13724293356211342`, 3.6916535362348286`, 5.489803233715362`, 1.3457045887873154`, 0.0754297582883523`,
0.6293482685634375`, 0.017573021108899616`, 778.5577282359714`, 0.17674997296512807`, 2.6916234726152264`,
40.13379743236351`, 0, 0, 0, 32.15350230222615`, 0.14668913500733366`, 0, 0, 0.028254123308680688`, 0, 0,
7.736078056815016`, 0.05539541097299154`, 0.8328739550983658`, 0.35754090318291465`, 0, 0, 0.593808163959279`},
{0.08176385784973572`, 2.023736863649704`, 9.823987283528833`, 1.0905304980935506`, 0.6417627833690904`,
0.2509575610218163`, 0.3071390754059633`, 168.49990583950182`, 0.0669392059366426`, 7.784078345121018`,
600.1270112577715`, 0, 0, 0, 118.91723434942925`, 16.026942624528672`, 0, 0, 0.84660339981505`, 0, 0,
463.34735144082043`, 0.9888794291084954`, 9.703472982868652`, 0.9988644238651814`, 0, 0, 0.5000953611894512`},
{0.20636275093165019`, 3.041433098250965`, 7.709885134336993`, 1.0647262222072884`, 0.28984076250310165`,
0.17246518959666646`, 0.007377660819744997`, 464.8055142552055`, 0.19020267666710844`, 9.452984008311837`,
168.195936249229`, 0, 0, 0, 152.35919830019034`, 0.35134785365373683`, 0, 0, 0.01847792568260599`, 0, 0,
15.265728444313037`, 0.05447365107677613`, 4.285843041890438`, 0.3117558483111462`, 0, 0, 0.6402389552490766`},
{0.2140607830978551`, 1.6917207171996171`, 1.9218182027339432`, 0.9997605714664014`, 0.5513493876922198`,
0.4464250883384161`, 0.2328414126003874`, 611.4769203082982`, 0.08477583973629349`, 2.188379571162489`,
50.27417320276394`, 0, 0, 0, 9.121864531105887`, 1.0953497898928768`, 0, 0, 3.3477383984145823`, 0, 0,
26.47179903060029`, 10.237421473876893`, 0.8396101195026512`, 0.9999999545671305`, 0, 0, 0.4128582945988603`},
{0.17696168772261972`, 1.6101558302339125`, 0.7107550695951144`, 1.133554468522512`, 0.8128396324743541`,
0.15732538361458315`, 0.06392981831267872`, 277.86150200910555`, 0.15413566109625954`, 1.907309832357937`,
23455.448721662666`, 0, 0, 0, 5.471051765267195`, 0.20272701584411584`, 0, 0, 6645.394412924956`, 0, 0,

```
4.663172664390376`, 16799.71729848097`, 1.3989749076613207`, 1.0000000000003626`, 0, 0, 0.062211265570376845`},
{0.04328176764500202`, 2.9602821723444555`, 0.6131528539518083`, 1.0058908218647606`, 0.19964040264767258`,
 0.6669684114614443`, 0.23851965705730427`, 2287.3379976105803`, 0.08593310169788088`, 8.660468891635798`,
 50.06694733360331`, 0, 0, 0, 11.748199222905255`, 0.8533751044364636`, 0, 0, 0.850476358609902`, 0, 0,
 36.08901582836925`, 0.5258588591559813`, 0.9401618062871069`, 0.999978733694613`, 0, 0, 2.2352347480605093`},
{0.08735803097627126`, 3.963951857586263`, 7.965851341925532`, 1.189504619761999`, 0.40822475538796854`,
 0.4952091412789442`, 0.01984161894285721`, 1116.2764923646498`, 0.2379409272833778`, 1.293491554272407`,
 15.845252533906509`, 0, 0, 0, 12.374994498402648`, 0.059643218262354136`, 0, 0, 0.012761537633320138`, 0, 0,
 3.377469226049734`, 0.01592603999823442`, 0.259779674398017`, 0.20328661058968045`, 0, 0, 0.17577056159951326`},
{0.15281896372135534`, 0.55010519971598`, 2.817048022996838`, 1.3198176246010052`, 0.16238216376752557`,
 0.6925657414172184`, 0.11807724637466313`, 76.78768290686035`, 0.04193274426603899`, 1.2341700274455292`,
 454294.9909386107`, 0, 0, 0, 10.944462818426269`, 2.022821644362139`, 0, 0, 142710.60376230252`, 0, 0,
 15.896638638023441`, 311555.5225572018`, 3.5699321946107685`, 1.0000000000000147`, 0, 0, 0.15473927486259167`},
{0.19086658543964008`, 1.9905232804946325`, 4.078173781767919`, 0.8519485127716493`, 0.08904379736388202`,
 0.23536266216215151`, 0.24991927271295455`, 192.66677656391008`, 0.127316992739278`, 7.8449864029309175`,
 7092.509972562512`, 0, 0, 0, 60.69279583556107`, 6.643790057121158`, 0, 0, 1834.4147735632448`, 0, 0,
 188.92312542026292`, 5001.835487286369`, 8.731385021822419`, 0.9999999999999238`, 0, 0, 0.6784024881171183`},
{0.06664208120921677`, 2.003234095381499`, 6.199949723153024`, 0.888873086526756`, 0.04784068730663105`,
 0.37023377411027647`, 0.049828770496122325`, 349.42476257926376`, 0.05267583263583803`, 7.0912634633886915`,
 183.49474747556806`, 0, 0, 0, 108.93215738744479`, 2.515134803670689`, 0, 0, 0.03421204360599895`, 0, 0,
 71.97719704562552`, 0.032570882690229154`, 4.597842486210418`, 0.5613856038483607`, 0, 0, 1.0488433015379324`},
{0.22983292033798441`, 0.7814702114508636`, 3.2012106727008174`, 1.142026804432239`, 0.1064467335224153`,
 0.4404014420960507`, 0.006695571873889409`, 4217.329674971789`, 0.15347806495470107`, 2.7989551297088675`,
 6.6341998642552`, 0, 0, 0, 6.040177560275248`, 0.046375877536743555`, 0, 0, 0.00554119475661736`, 0, 0,
 0.51773381178369`, 0.018193556758212506`, 0.15312703442338224`, 0.09790367119486842`, 0, 0, 0.47303328205692763`},
{0.13211782844464454`, 0.44983284117427225`, 1.0762780754680463`, 1.3187859174208114`, 0.8445651695343355`,
 0.2965729998791703`, 0.0542223541794765`, 616.1259401245338`, 0.10515194159293045`, 5.24820204317008`,
 7264.328790209427`, 0, 0, 0, 26.05843805054559`, 2.624163748275537`, 0, 0, 2500.0998278442808`, 0, 0,
 16.86335763704736`, 4718.682287851659`, 1.838514004902742`, 1.0000000000000717`, 0, 0, 0.49109588439143365`},
{0.17817224333688036`, 1.8900871904197354`, 9.98947566566499`, 0.792780936871982`, 0.9109839945193492`,
 0.4314201285170226`, 0.03598067399007004`, 128.67700477089366`, 0.05901916357993680 6`, 2.366574414393561`,
```

208.9501876429785`, 0, 0, 0, 134.25215469954733`, 2.38745966340107`, 0, 0, 2.2093412762407003`, 0, 0,
64.46438467768824`, 5.623475592636604`, 4.197184645127444`, 0.9999999922172493`, 0, 0, 0.3698367400625289`},
{0.2509149027719233`, 2.580879402706512`, 1.8893709427148198`, 0.8255744895957449`, 0.1652280455910582`,
0.6768016323195194`, 0.009322737452992792`, 2112.3599589856663`, 0.08696925005081763`, 4.203193192682905`,
26.11496607326686`, 0, 0, 0, 22.99062886459954`, 0.07932150775683831`, 0, 0, 0.02212026486953407`, 0, 0,
2.9245606508749864`, 0.07929005870039847`, 0.4756950947512479`, 0.4291046464776467`, 0, 0, 0.8879924472674233`},
{0.08555200390318413`, 3.7277336286219658`, 7.5570643278825855`, 1.1117862222014783`, 0.20420751548200644`,
0.3245432768958507`, 0.00950175247006993`, 144.2017035489447`, 0.14385517513119161`, 5.452203080835416`,
347.82159403096705`, 0, 0, 0, 303.7020958110105`, 0.7388545679221763`, 0, 0, 1.7034427246476294`, 0, 0,
39.346471707206405`, 2.081899123255883`, 8.472313691387646`, 0.9998560459682716`, 0, 0, 0.7378553948090492`},
{0.07297367338130328`, 1.9451098216604938`, 8.396918429735464`, 1.2137718043700492`, 0.7314346040557373`,
0.4669601248496963`, 0.016599550382003152`, 192.8440550768832`, 0.21285961816083276`, 7.382641950639707`,
519.8715870231912`, 0, 0, 0, 418.5881191473881`, 3.3780919815434816`, 0, 0, 1.8804852844958806`, 0, 0,
93.86799845389687`, 1.9603702707021893`, 8.539009282811891`, 0.9999899670096274`, 0, 0, 0.9606237444246684`},
{0.07358197980029185`, 1.0291227850237563`, 2.532589152827489`, 1.0117020834715413`, 0.8098748594005145`,
0.5301759885595309`, 0.008133019998886755`, 830.3082383512614`, 0.02407211472876364`, 4.5236701681781`,
95.69019050420202`, 0, 0, 0, 84.7599321183816`, 0.6182647388006959`, 0, 0, 0.5580678106932286`, 0, 0,
9.089576141093662`, 0.5866247767660383`, 1.2066786581972506`, 0.9998101363542926`, 0, 0, 0.5527003372804556`},
{0.18797192534273177`, 0.513697145142666`, 2.3563599620417435`, 0.8181944603817968`, 0.26394673984920813`,
0.3209514271347109`, 0.025430853857263652`, 265.9518711391782`, 0.07720757078553087`, 7.134393115551401`,
22006.71583029791`, 0, 0, 0, 98.77975918658576`, 4.211206977815588`, 0, 0, 5935.125644386877`, 0, 0,
30.904071458696343`, 15937.67135037741`, 5.716528734236558`, 1.0000000000005265`, 0, 0, 0.5685692194572877`},
{0.2528075291703835`, 2.1409422046846034`, 2.3989238084434295`, 1.1058748574151904`, 0.4698643832575349`,
0.20099009902051257`, 0.02857754945135185`, 57.682491948215834`, 0.240427409232489`, 4.4402688370357115`,
106670.808020232`, 0, 0, 0, 57.70391870437626`, 0.7356931903117923`, 0, 0, 23113.742127655358`, 0, 0,
22.501094297679735`, 83476.11481677076`, 15.633138012423817`, 1.0000000000023601`, 0, 0, 0.1286401765036652`},
{0.07054141508315104`, 0.5799884401515247`, 2.99755260715291`, 1.1960365141205305`, 0.29917509086343896`,
0.44670601886119`, 0.13984458882947357`, 3612.976166750159`, 0.22678215214973713`, 7.656934137991781`,
25.730362813570387`, 0, 0, 0, 8.877677695843058`, 1.81296951242969`, 0, 0, 0.009098534466237384`, 0, 0,
15.021447993662413`, 0.00916890709186834`, 0.4739066492349142`, 0.15015708939027084`, 0, 0, 1.018280569757712`},
{0.13968170289558773`, 2.9225254745826437`, 6.832387664740558`, 1.248628328449276`, 0.2500405375269379`,

0.6548966838897512`, 0.0131096950825671 7`, 2778.8012693248406`, 0.21649543926111503`, 9.334771326724606`,
47.06514159535508`, 0, 0, 0, 39.719979516895776`, 0.17081787277185007`, 0, 0, 0.006083784864396233`, 0, 0,
7.131708352710705`, 0.012139906141558486`, 0.7805135882251287`, 0.09928244707312583`, 0, 0, 1.6542822326989568`},
{0.14258976979051796`, 0.734197214461469`, 7.171919374129239`, 0.945223896823731`, 0.9937893052327855`,
0.2333579308191156`, 0.10311334331996398`, 86.76571756149787`, 0.17032024165228216`, 9.316237353631934`,
24735.381002245496`, 0, 0, 0, 219.83002813840054`, 26.852061247322418`, 0, 0, 7970.723163752758`, 0, 0,
281.63869386189856`, 16236.336871192198`, 22.715317525872422`, 1.0000000000004348`, 0, 0, 0.6697108264379853`},
{0.0622240112532687 1`, 0.8031654209648247`, 7.912640972265759`, 1.0061100735669513`, 0.06612689335476452`,
0.2257508005074531`, 0.2688428052003874`, 1016.6634644345198`, 0.08792308855751246`, 8.5639571694838`,
65.07309714397097`, 0, 0, 0, 14.825208214017687`, 4.027293650722222`, 0, 0, 0.006494009052783631`, 0, 0,
46.20832857616125`, 0.005772618462559186`, 1.8267779452166237`, 0.12973715512977857`, 0, 0, 0.8459493654220915`},
{0.045675945991483086`, 1.972555523342196`, 9.234608119853927`, 1.10067888297902`, 0.060950291683742996`,
0.5641176167348642`, 0.005654158004083115`, 79.62566643520867`, 0.10418567571675635`, 3.9217157633863025`,
40611.54441421846`, 0, 0, 0, 295.2522704564025`, 0.7996396907627535`, 0, 0, 24381.987938731283`, 0, 0,
22.533338409967357`, 15909.576632206876`, 11.634072913337993`, 1.0000000000000002`, 0, 0, 0.7718963370398129`},
{0.19725457740267938`, 0.673691333497294`, 1.5958254230238804`, 0.9055402555445284`, 0.8526577157919533`,
0.18700866613724199`, 0.221308084367558`, 137.27532297707575`, 0.16306687928789393`, 6.444987143866487`,
41177.728449479386`, 0, 0, 0, 19.59093416776736`, 5.547098003118837`, 0, 0, 10764.808315885759`, 0, 0,
53.38616929659013`, 30334.395931012907`, 9.580071822118246`, 1.0000000000042728`, 0, 0, 0.2182239551681294`},
{0.17905209129143224`, 1.6872990209581902`, 6.743325221637608`, 0.8692054788625995`, 0.5776472609105132`,
0.5432377341075647`, 0.12670077461750193`, 462.43790721394`, 0.057341353016641616`, 6.31060148160045`,
253.65003828437148`, 0, 0, 0, 92.05097404757947`, 6.427850610123717`, 0, 0, 0.06536220023749234`, 0, 0,
154.93865773324632`, 0.16718912348473539`, 2.9677773808653445`, 0.7949588139241099`, 0, 0, 0.6429565089349959`},
{0.21834559506325857`, 2.5253820753047362`, 6.659656299000567`, 1.4243771911650907`, 0.9747752944821462`,
0.1772255451247845`, 0.01986842431027196`, 102.11368670749582`, 0.09272454748376258`, 4.914431231863471`,
42988.39881764499`, 0, 0, 0, 200.64256545594384`, 1.4928206051360497`, 0, 0, 10373.882872442024`, 0, 0,
53.85631996937347`, 32358.451841427155`, 10.073664898893513`, 1.000000000000125`, 0, 0, 0.29744134836411473`},
{0.2429292029568641`, 1.5636324867576725`, 8.233163527811005`, 0.9303215118662402`, 0.8597394087604655`,
0.6215210972087029`, 0.12468068919290341`, 61.92974770793832`, 0.07970211447796205`, 9.85507721294049`,
222607.84358287588`, 0, 0, 0, 239.38145025002552`, 17.795110265235756`, 0, 0, 49649.318539834814`, 0, 0,
397.5001788022503`, 172303.84828905124`, 34.333638267034566`, 1.0000000000007385`, 0, 0, 0.9180421450623234`},

{0.09149398523540997`, 2.310449584405898`, 9.73345399143432`, 1.4402850137921024`, 0.7807243420630963`,
0.37700223940662914`, 0.04452259966898517`, 785.475395176315`, 0.18361612690246515`, 4.855709318281368`,
130.85907622438398`, 0, 0, 0, 80.43720482414446`, 1.4804773164693015`, 0, 0, 0.03128741262913068`, 0, 0,
48.865260007983586`, 0.04089442955925364`, 1.29075587666227`, 0.3721330609862755`, 0, 0, 0.2811660598216434`},
{0.11249424084619364`, 2.9716096914630903`, 4.4521238184847185`, 1.4266434477040648`, 0.9997833218301952`,
0.5867347171724102`, 0.06910846060462165`, 1820.2194505805683`, 0.24722705290784003`, 7.621221876581703`,
207.3438173544296`, 0, 0, 0, 104.61399742519369`, 2.3574484833935996`, 0, 0, 0.1128938939196488`, 0, 0,
100.07738229110542`, 0.1814273270379867 8`, 0.859686294467838`, 0.8064175001367722`, 0, 0, 0.3072050590815158`},
{0.09866336490626898`, 1.7258477745644702`, 0.8848358466481533`, 0.910425763108387`, 0.9172877053292023`,
0.36334488527428765`, 0.3804604060347452`, 1006.1834168170927`, 0.013687075322010439`, 8.165252701558408`,
9257.29364849209`, 0, 0, 0, 10.603370033810643`, 1.9501860891169016`, 0, 0, 3816.8688610817944`, 0, 0,
48.08177602698558`, 5379.787503432669`, 1.8255941961394857`, 0.9999999999999732`, 0, 0, 1.1416191699944227`},
{0.22803797815686577`, 0.698146546972529`, 1.035170425484365`, 0.9822431866703154`, 0.6417091531359376`,
0.21874316930074555`, 0.005531062831453447 5`, 1727.3746181384265`, 0.11140836748423671`, 9.157656749061658`,
68.60746280959205`, 0, 0, 0, 63.05593122111219`, 0.4460889693692715`, 0, 0, 0.1375626728770324`, 0, 0,
4.449078194395586`, 0.44813591132477004`, 1.108479249915986`, 0.9345181075361613`, 0, 0, 0.5437884520623447`},
{0.12680960193351426`, 3.133735649340868`, 7.511849069758438`, 1.0897808931740574`, 0.04885314856347578`,
0.24060421848165847`, 0.3693592758602631`, 883.7388687037887`, 0.08961301811176398`, 2.076054864701238`,
17.752106102751814`, 0, 0, 0, 3.2363477476383697`, 0.31664411271944704`, 0, 0, 0.008429736026834661`, 0, 0,
14.175413488326283`, 0.015271020999561645`, 0.5152084613497765`, 0.15277261482881144`, 0, 0, 0.23084783414278584`},
{0.11645318221252937`, 1.153640861264721`, 2.619716900319183`, 0.8529577052637052`, 0.5619295219152276`,
0.5231720769226155`, 0.04609815796088822`, 565.9088521347549`, 0.20476637224893374`, 8.831648882728363`,
201.75684757988182`, 0, 0, 0, 121.03225856359961`, 4.425884341397462`, 0, 0, 1.2585050597463754`, 0, 0,
72.94115747811156`, 2.093670271971972`, 3.590680739169312`, 0.9999989204659508`, 0, 0, 1.4638077301961343`},
{0.042446348872052314`, 3.5353553586920903`, 5.120248896798044`, 1.1724129507972807`, 0.07388809680757258`,
0.47979821691937163`, 0.43439767413472635`, 83.35178002891637`, 0.15043407845955598`, 2.47226753883535`,
42440.865848042566`, 0, 0, 0, 16.932845710996283`, 1.7773363803811608`, 0, 0, 26351.784781619637`, 0, 0,
89.7645099511278`, 15979.100717740946`, 6.813498339632957`, 0.9999999999999994`, 0, 0, 0.40517968409536254`},
{0.1455833195103322`, 2.7843188408171127`, 7.76885376753495`, 1.3211895319342402`, 0.9135586666939064`,
0.6453968687054246`, 0.1333226247130129`, 368.7414592704895`, 0.16919558147748776`, 3.2843355334407627`,
209.76113366710553`, 0, 0, 0, 72.72623243809846`, 3.3064441658129824`, 0, 0, 0.7180381738289169`, 0, 0,

131.51706838546983`, 1.4933482983021409`, 1.9439028191082217`, 0.9997600974855981`, 0, 0, 0.3474388111136395`},
{0.19543798537508206`, 1.2324134940956721`, 7.418056367639444`, 1.0725732268441615`, 0.3282068086166905`,
0.15859490968399015`, 0.32000646124266346`, 1339.7132456219883`, 0.07635813740352487`, 1.32927343177637`,
7.774764526594433`, 0, 0, 0, 1.5593816481774654`, 0.3329152880753311`, 0, 0, 0.005588642238494854`, 0, 0,
5.861275620211226`, 0.015603328286769962`, 0.20905300170778526`, 0.10572036360969439`, 0, 0, 0.08975335270303055`},
{0.14288798158791094`, 1.8492145343896036`, 4.320087837799633`, 1.416387968755192`, 0.03040014321801321`,
0.26264512496919146`, 0.017396141813896236`, 476.32655268366966`, 0.1492295937607505`, 1.5210123574307455`,
24.129362571981456`, 0, 0, 0, 19.34541060822146`, 0.1692700180116515`, 0, 0, 0.044223479275774906`, 0, 0,
4.471665393479082`, 0.09027148132157664`, 0.7119632630914821`, 0.48085250573791394`, 0, 0, 0.1972311452823421`},
{0.08126639977214484`, 1.4848577063399988`, 1.4511591326569544`, 1.4690309046886825`, 0.09088454373979671`,
0.2634538760346167`, 0.006391735651833628`, 757.569692438266`, 0.1906091612906891`, 5.0187956659047614`,
54.5659135160035`, 0, 0, 0, 49.6376873683472`, 0.19826690112351747`, 0, 0, 0.21857445463209413`, 0, 0,
4.205687657791512`, 0.25375370014442983`, 1.471040573520449`, 0.9333098728755304`, 0, 0, 0.6275774939711962`},
{0.15406825481222286`, 3.5722547377798985`, 5.518246507815627`, 1.3255687223204002`, 0.2843400333432804`,
0.5740150104722689`, 0.007989301755943026`, 636.0707307721444`, 0.19470033172956325`, 2.3727553064621407`,
49.23941847690839`, 0, 0, 0, 44.11847846991745`, 0.09499733429187215`, 0, 0, 0.042954973749202985`, 0, 0,
4.847923964294297`, 0.09454282630063339`, 0.861771145071176`, 0.4926279495693625`, 0, 0, 0.404524864694873`},
{0.1284209869933174`, 2.657038147126583`, 3.485135675760706`, 0.826438046300177`, 0.2940359222573743`,
0.4455502493814366`, 0.007792225179768616 5`, 642.5517685154364`, 0.18333530742444448`, 4.125506851960305`,
76.29055054462059`, 0, 0, 0, 68.6069832167959`, 0.19211554008130477`, 0, 0, 0.047685869959401515`, 0, 0,
7.292261695026472`, 0.0874838069403041`, 1.4425994420496933`, 0.7017117720252724`, 0, 0, 0.5710135843551346`},
{0.1519197321396482`, 1.414313316427302`, 9.693328908271443`, 1.0879341282517354`, 0.5935096502675883`,
0.32269331092414044`, 0.04853394307287227`, 323.7551755246228`, 0.012675975614693441`, 6.5738064876305415`,
293.69172923458626`, 0, 0, 0, 175.07708225714228`, 5.586199257760622`, 0, 0, 0.049189479069000626`, 0, 0,
112.86622854953073`, 0.1067550354894853`, 4.302788707021305`, 0.6204183086822823`, 0, 0, 0.4843140864042932`},
{0.10245812606330185`, 3.8066698620788078`, 2.784482506819291`, 0.8153557037427375`, 0.41559745180116914`,
0.2970790998891699`, 0.019454402362339196`, 101.06708659388384`, 0.03906278599147861`, 1.0136093018296712`,
129608.872405188`, 0, 0, 0, 17.71607485126992`, 0.0869126942767891`, 0, 0, 52598.51996859304`, 0,
0, 4.726399056221741`, 76987.79699550293`, 2.142599016557059`, 1.`, 0, 0, 0.083558317113368`},
{0.08994858347063645`, 1.3905867780774148`, 6.975873482712982`, 0.9748960320168515`, 0.19705721946727817`,
0.428438510479348`, 0.008778830773896138`, 53.85788205383214`, 0.2055785802889155`, 2.165498620130503`,

53759.61969917746`, 0, 0, 0, 110.77461530327123`, 0.6531175596242664`, 0, 0, 23472.897637976705`, 0, 0,
12.974523470624176`, 30162.19846410374`, 8.871127221120604`, 1.00000000000024`, 0, 0, 0.2552540074158636`},
{0.2555268425086469`, 0.6701554458004759`, 6.763443756394938`, 0.9242821290525386`, 0.9506231196396431`,
0.49497463337651615`, 0.1535425304369218`, 4370.366828894948`, 0.17262313668823565`, 9.906703002274519`,
87.38994679506919`, 0, 0, 0, 27.67219742361427`, 5.644132332456548`, 0, 0, 0.008316248442089915`, 0, 0,
54.03494313448996`, 0.030357495798890292`, 0.46531991502527636`, 0.17586298846708914`, 0, 0, 0.396965231479573`},
{0.13340663060001784`, 0.4096518827228479`, 7.81563732077705`, 1.2681845828171425`, 0.264399527619257`,
0.43786671579755854`, 0.11528071789135624`, 3397.5276395199044`, 0.15041338875391808`, 8.11261999427796`,
27.78841443647035`, 0, 0, 0, 10.810443009228429`, 2.47622732225379`, 0, 0, 0.003592663228125407`, 0, 0,
14.491302637300299`, 0.006846929944943989`, 0.5346952025666641`, 0.059070602022710195`, 0, 0, 1.091698102642102`},
{0.09831375749458693`, 3.5924209186692435`, 1.6118282365623244`, 0.8326891410736474`, 0.6715734708323489`,
0.5976781170151362`, 0.03815316295555065`, 115.73199628594814`, 0.0304507712440647`, 4.7148191409051226`,
216972.37346790274`, 0, 0, 0, 41.29392194519502`, 0.4213925671086129`, 0, 0, 90210.26511593821`, 0, 0,
21.625992472181707`, 126698.71611615321`, 9.08823103305409`, 1.0000000000012792`, 0, 0, 0.6142964101707995`},
{0.09108239111305882`, 3.309852207951435`, 3.0566194803884894`, 1.4370488086380533`, 0.21629452377334557`,
0.2421124982368058`, 0.4670454509414495`, 208.22129409387404`, 0.23659860207487343`, 3.442603557657883`,
7466.42313325171`, 0, 0, 0, 12.774100120545762`, 1.4888313870709642`, 0, 0, 3207.8191782330055`, 0, 0,
70.3973121966317`, 4173.940585882697`, 3.561703129277759`, 1.0000000000514209`, 0, 0, 0.3149501130744804`},
{0.05787448179355209`, 1.5695297669455615`, 8.686108197389068`, 1.3362839901345183`, 0.8882645350301348`,
0.2220019613784866`, 0.20455164120837535`, 127.50819012666335`, 0.2481722521034202`, 6.402394955038988`,
448.8377815216411`, 0, 0, 0, 121.5453153777918`, 13.823049179231537`, 0, 0, 1.9329220196480417`, 0, 0,
309.9383879536612`, 1.5980980033494923`, 10.778788828533717`, 0.9999947975004293`, 0, 0, 0.5611499131644467`},
{0.2051260145065577`, 0.6712958473867032`, 3.046620158397806`, 1.125533167922526`, 0.5107051094055373`,
0.47305603590798795`, 0.035994055865640424`, 73.71677428550161`, 0.039721863923625866`, 8.822427071575206`,
491269.438664817`, 0, 0, 0, 141.10422104616686`, 6.75052543136971`, 0, 0, 124939.0332016028`, 0, 0,
64.73713842509723`, 366117.79909924744`, 25.30836598669515`, 1.0000000000005151`, 0, 0, 0.6302051961389624`},
{0.04365375531995927`, 0.9346121184769336`, 9.554451222092258`, 0.75459846537052`, 0.7382972174511151`,
0.44670601219095474`, 0.02981461788584879`, 1443.346811017674`, 0.026466349135542522`, 4.5970910839514385`,
68.83632808882238`, 0, 0, 0, 48.45851422789191`, 1.4183960108791893`, 0, 0, 0.008334817140057182`, 0, 0,
18.937858579529056`, 0.005197800972400321`, 0.671101132807143`, 0.2112609837489674`, 0, 0, 0.31064276204320623`},
{0.18832502219499725`, 2.1446480190155537`, 7.011391892939212`, 1.2684842496371584`, 0.8800953133738048`,

0.3019976503972389`, 0.01607749175819828`, 253.61560940445747`, 0.0505443969728126`, 8.205874646962478`,
459.3330175848634`, 0, 0, 0, 371.3653600555775`, 2.646249474138071`, 0, 0, 1.101450450282008`, 0, 0,
81.07533846473083`, 2.963295435657895`, 6.854008473153046`, 0.9999154103291084`, 0, 0, 0.6014286027580846`},
{0.2789832227001883`, 3.864947879576305`, 9.015175334965505`, 1.1939022970453932`, 0.8102757584298972`,
0.6575626089292559`, 0.013706864097109712`, 131.89661477643062`, 0.1558047394747739`, 7.390258992479394`,
9326.543438691775`, 0, 0, 0, 498.31187433571523`, 1.698031410079824`, 0, 0, 1751.5856868208812`, 0, 0,
93.75432711202822`, 6980.90028206876`, 13.401234184628784`, 1.0000000000001823`, 0, 0, 1.5654666173210223`},
{0.10740283086494345`, 3.8162968757181996`, 6.158389397925204`, 0.8038652030060122`, 0.9187954678523191`,
0.31270681581700654`, 0.027540968538212463`, 2177.2729976761966`, 0.021577241346914133`, 9.805063749007854`,
112.67195629309921`, 0, 0, 0, 81.08802088437051`, 0.5681113861480389`, 0, 0, 0.010844686850040584`, 0, 0,
30.972595828809855`, 0.016639286679127386`, 0.9233101057432938`, 0.25191613179752026`, 0, 0, 0.3804469398889166`},
{0.14667176624268824`, 3.777226248423828`, 0.6973504720596608`, 0.7758342249413661`, 0.494379064234173`,
0.5228383904297224`, 0.014800119213367281`, 81.50804211451084`, 0.12794308526059`, 9.842771813142875`,
91934.67769669769`, 0, 0, 0, 45.57292670276536`, 0.17304996882245732`, 0, 0, 29683.469841278893`, 0, 0,
9.3378412075016`, 62196.099283313684`, 25.926643445505526`, 1.0000000000000078`, 0, 0, 0.864259942503491`},
{0.15361999046758062`, 1.6832845721068805`, 5.017027003357642`, 1.2257765840301957`, 0.8758480691873987`,
0.30492603190517764`, 0.17353190337485183`, 1218.2701158582597`, 0.01908888394356914`, 4.8706184705887665`,
88.4270901347789`, 0, 0, 0, 25.96794117075366`, 2.4888321497265715`, 0, 0, 0.03805301639099156`, 0, 0,
59.84875371711911`, 0.08351005736062264`, 0.8243409445840376`, 0.4846190095228925`, 0, 0, 0.21768255053554647`},
{0.1285936813753743`, 1.2517058162042831`, 2.0098881407856393`, 1.434144339335301`, 0.06632650483661995`,
0.16162194262920127`, 0.05011873598676769`, 354.0526180115314`, 0.1584833535532481`, 2.894273123117289`,
3009.490192742772`, 0, 0, 0, 27.370525650810837`, 0.9878621827889326`, 0, 0, 1044.558156753674`, 0, 0,
17.664469140073848`, 1918.9082683947126`, 1.7316842912613932`, 0.9999999999998307`, 0, 0, 0.212137487924534`},
{0.05871769486491246`, 0.822876240174752`, 3.5461717275684297`, 1.3634413021309426`, 0.14962847564878934`,
0.2425078078443763`, 0.045889110192962865`, 1618.8495608622088`, 0.1824696213391762`, 5.484504386708753`,
27.885896118678033`, 0, 0, 0, 17.107389856656585`, 0.8429945785541966`, 0, 0, 0.01360299966270007`, 0, 0,
9.909717275548203`, 0.011410525477769011`, 0.7365941226932841`, 0.19245044943228562`, 0, 0, 0.5571666471303832`},
{0.21414990207917795`, 3.2291184159683555`, 3.7200101298578847`, 0.893435725539721`, 0.18550851463991336`,
0.6299970912008048`, 0.007788531351629846`, 1185.7534950662077`, 0.10596189074334122`, 9.85351453889281`,
107.90952971362401`, 0, 0, 0, 97.12715568321971`, 0.22504945458037526`, 0, 0, 0.02098035932911245`, 0, 0,
10.381590546987482`, 0.0641848842273687`, 1.9449681086712902`, 0.39465494154336356`, 0, 0, 1.8314993075330654`},

{0.14399581513021464`, 3.6650427925648215`, 8.960857802594514`, 0.9993788071734813`, 0.7678347002262149`,
0.3917750513543251`, 0.0682112112142393 6`, 446.58045234253916`, 0.09781275073737189`, 4.158206599173292`,
196.1648640455883`, 0, 0, 0, 100.21770377574656`, 1.7948871046332944`, 0, 0, 0.0573517222875094 7`, 0, 0,
93.97625780434001`, 0.1179772571415514`, 1.9499962161068367`, 0.7047383856138275`, 0, 0, 0.2539997093759647`},
{0.21762093156371237`, 1.4799568210517782`, 6.880501462590445`, 1.1700220578418317`, 0.49477438305707566`,
0.30056004401666714`, 0.03776191240235276`, 139.28017196074`, 0.13055880095851463`, 4.30304009869343`,
571.5957497424812`, 0, 0, 0, 163.74827429672212`, 3.821763543195586`, 0, 0, 78.66173228170787`, 0, 0,
80.80064320284755`, 244.54913510800776`, 6.953162845979232`, 0.999999999941123`, 0, 0, 0.6037110015464655`},
{0.20676336326895633`, 3.023915070136316`, 2.1344025288266977`, 0.7647942269583019`, 0.38268253024987975`,
0.6058201621166657`, 0.06287157366362177`, 52.11423962493933`, 0.23050267576131828`, 4.129012828530946`,
92316.29930931194`, 0, 0, 0, 37.50372427725364`, 0.7491832875300305`, 0, 0, 23331.114627698207`, 0, 0,
32.36380904937607`, 68914.56756052027`, 16.97394192424923`, 1.0000000000008284`, 0, 0, 0.3528800016323853`},
{0.14056217319026204`, 2.827702131488536`, 4.822127390400377`, 0.9317030787852092`, 0.5881617813064273`,
0.663416624487535`, 0.01875862506453023`, 1486.513173237306`, 0.1539754165327772`, 8.90838886614388`,
127.34888668402371`, 0, 0, 0, 100.62143918348326`, 0.6430961079209907`, 0, 0, 0.022090731131169255`, 0, 0,
25.978346216002386`, 0.04435887393088312`, 1.312465615972908`, 0.39772530066950007`, 0, 0, 0.9766647680453755`},
{0.15432054500385795`, 3.142590945055387`, 0.6412922856028498`, 0.9338764110697222`, 0.4036132844000331`,
0.253764192888976`, 0.009287914173605602`, 800.3088033129984`, 0.0773663480819351`, 1.9942494398991926`,
7595.197344877993`, 0, 0, 0, 8.936745842428042`, 0.02531232212004141`, 0, 0, 2366.953965352216`, 0, 0,
1.1363753470395328`, 5218.137513317069`, 0.526748637759796`, 1.0000000000005027`, 0, 0, 0.14166252556869594`},
{0.1558313627948012`, 2.6183453593149384`, 1.5210778217010041`, 1.2213020905618333`, 0.8957140746385861`,
0.2067404945185849`, 0.06533654021268133`, 3401.5830280519913`, 0.07866132934502523`, 1.2687008259414867`,
6.28614533852348`, 0, 0, 0, 3.235871917647214`, 0.07676698794608382`, 0, 0, 0.031620756064684355`, 0, 0,
2.871464094817349`, 0.07039293585945267`, 0.07740560265261512`, 0.4178958091816912`, 0, 0, 0.06536053561186543`},
{0.27749339228385056`, 3.960153029334033`, 7.7991060624230615`, 1.1159035815659704`, 0.47604804800745515`,
0.18775668040836846`, 0.02760276902429692`, 890.1551806101527`, 0.14097999756547475`, 3.493022856148759`,
38.071550460823495`, 0, 0, 0, 27.49114202214165`, 0.18274611856373807`, 0, 0, 0.01082580721438756`, 0, 0,
10.338608500417473`, 0.042915570973305464`, 0.8233694782474501`, 0.18820255665758512`, 0, 0, 0.22047195240840403`},
{0.1388142019807993`, 3.4960143968183592`, 0.8496516336862268`, 1.4057148799798922`, 0.27289215927666`,
0.416116229659188 7`, 0.1071416651953492`, 563.7099077527477`, 0.24491201094978954`, 8.47264522499269`,
7216.9781578825605`, 0, 0, 0, 23.484448851095387`, 0.68050297047411`, 0, 0, 2399.8265842172514`, 0, 0,

33.986402597930606`, 4759.000174006078`, 3.2482082982318086`, 1.0000000000000568`, 0, 0, 0.8046605905950109`},
{0.0729917462714273`, 3.1893255155706086`, 3.1041281178047555`, 1.014074092539919`, 0.28719491349307913`,
0.2514291775131975`, 0.05245081359414425`, 423.2923229706584`, 0.1145067740496655 8`, 9.081189649788715`,
203.0642495691459`, 0, 0, 0, 117.82037554138317`, 1.8240447851928925`, 0, 0, 0.15179434250972965`, 0, 0,
83.10675107084582`, 0.1582819161985703`, 4.621687301078058`, 0.9487384642673123`, 0, 0, 0.8308557708574072`},
{0.11420284690084648`, 0.577787691297051`, 2.567084026939444`, 1.4491737131438955`, 0.8025258647238394`,
0.3690270257872911`, 0.13672661029124697`, 86.53603740064875`, 0.011299238359278513`, 6.563498155623039`,
1.2392422190728874`*^6, 0, 0, 0, 46.844699996295624`, 9.482359965814343`, 0, 0, 470880.5028814399`, 0, 0,
78.2684410385064`, 768227.0568451777`, 16.20629520472433`, 1.0000000000004172`, 0, 0, 0.5421399241064936`},
{0.26373133325063186`, 3.0221428803953465`, 7.660698925394574`, 1.4577816176348994`, 0.5393449604126412`,
0.22813670918251328`, 0.05400872825934766`, 78.22373236540282`, 0.11174025693188905`, 6.564224566850031`,
102095.0625583668`, 0, 0, 0, 223.19343325927366`, 3.7948670748955826`, 0, 0, 21332.41775036452`, 0, 0,
163.83757874917694`, 80371.81392518697`, 17.438554599918582`, 1.0000000000006353`, 0, 0, 0.3472601621576046`},
{0.21289488654178568`, 3.397179033451687`, 0.7632571166797604`, 1.0142699830327386`, 0.0835760348267307`,
0.5184383444209231`, 0.049731529265758516`, 2070.206130759874`, 0.06133590421436391`, 5.427522099525574`,
29.086836908647165`, 0, 0, 0, 17.002229893389224`, 0.23559833954197962`, 0, 0, 0.10259209646355301`, 0, 0,
11.433853420114936`, 0.3120190390956047`, 0.624704428217243`, 0.84821873083187`, 0, 0, 1.1250303833672726`},
{0.22999162513252186`, 2.379033562263336`, 2.5591649989178986`, 1.3870393830660817`, 0.8120421313192066`,
0.6084139253527632`, 0.017274035559015792`, 82.84440150538262`, 0.15048886650658344`, 7.465486944171833`,
163958.07412768368`, 0, 0, 0, 122.57310065814526`, 0.8552947545961453`, 0, 0, 38222.3557716126`, 0, 0,
29.068213240171676`, 125583.16743295104`, 19.23656853077887`, 1.0000000000001046`, 0, 0, 0.60896598065567`},
{0.1769680798340355`, 1.1633029664887262`, 4.7984019847292405`, 1.1781683757559547`, 0.8107109545176789`,
0.3886685867136115`, 0.009172138674078711`, 82.52474973299203`, 0.0366989899628154`, 4.7780773811523165`,
340862.7226664043`, 0, 0, 0, 161.23356183885593`, 1.181885740320605`, 0, 0, 96561.6168829557`, 0, 0,
19.641302682366916`, 244118.9132206653`, 12.304064836429516`, 1.`, 0, 0, 0.3665646354493819`},
{0.06792516420354372`, 2.678804428347224`, 7.00137481313343`, 1.1889039591838686`, 0.130109850192629`,
0.17336690021836265`, 0.0065615912781447905`, 559.1859620197067`, 0.12479016185335001`, 7.679232726268854`,
100.64931860232065`, 0, 0, 0, 92.14990768111805`, 0.21322619563774586`, 0, 0, 0.01609918835343084`, 0, 0,
8.159875387343213`, 0.015622000177859596`, 2.9216775572623934`, 0.2527739675872085`, 0, 0, 0.5984900600379364`},
{0.04696109785508959`, 0.9059167168392257`, 1.3656449005023852`, 0.8785005151977332`, 0.7117341492461746`,
0.37541668809892415`, 0.06884837024201017`, 2425.2572297139236`, 0.025009444315193585`, 7.4432891311871785`,

58.154526164970484`, 0, 0, 0, 29.586516172274628`, 2.0420162168063456`, 0, 0, 0.05906779937711862`, 0, 0,
26.427094669452302`, 0.03962698152335503`, 0.6477431088653635`, 0.7522627709673673`, 0, 0, 0.5161587358744965`},
{0.18481744149515084`, 2.8908169503663625`, 0.7719563125670259`, 1.477735031707543`, 0.05143123515478387`,
0.4060758128174351`, 0.19176878359926766`, 114.34865924476063`, 0.02384868002335655`, 4.461346915500394`,
686710.1283072006`, 0, 0, 0, 7.443185993988482`, 0.4639587050561051`, 0, 0, 188636.28005863266`, 0, 0,
19.16028126923157`, 498046.78076570353`, 8.197513756815376`, 1.0000000000001192`, 0, 0, 0.28801828230870624`},
{0.11940413672862621`, 0.6385297463968325`, 0.6629513696361364`, 1.2353159995947312`, 0.3854086082897601`,
0.4772941268081121`, 0.0318040184551439`, 4110.432043833155`, 0.1129636900773972`, 2.562436110087784`,
8.7154545791285`, 0, 0, 0, 5.924453013748718`, 0.2597394660142647`, 0, 0, 0.059538460401905115`, 0, 0,
2.3693053623333844`, 0.10155912094916161`, 0.1438490604163631`, 0.6072809310888855`, 0, 0, 0.4333916138007292`},
{0.16756813285689298`, 1.9469615336157506`, 0.3903678609266308`, 1.2604526712473199`, 0.11212576188863221`,
0.45563235469719776`, 0.0065229352440185`, 190.16738409260836`, 0.045949495779464666`, 5.424569346408827`,
179836.78862903337`, 0, 0, 0, 15.366215348119287`, 0.04912031397985624`, 0, 0, 52984.37643818895`, 0, 0,
1.3662194548272593`, 126835.61471905855`, 6.049850066115853`, 1.0000000000003624`, 0, 0, 0.404550424563747`},
{0.07183551197561577`, 3.34536856927257`, 9.136834569708476`, 1.4150518758492396`, 0.289745011670542`,
0.5142172775325705`, 0.41137464999480466`, 1635.4988121892525`, 0.15390110584062433`, 5.743187227898911`,
45.403468842338704`, 0, 0, 0, 7.243022768729974`, 0.7817820466251569`, 0, 0, 0.008160093019026265`, 0, 0,
37.36212981144821`, 0.008374063711275748`, 0.7937241119503211`, 0.11636017107492314`, 0, 0, 0.8340293074342983`},
{0.2384422062087977`, 2.1587544451320504`, 5.065398759698054`, 0.8914719118393273`, 0.7773656949632277`,
0.1997211683380481`, 0.4677952481930321`, 402.45383708693856`, 0.19717109492345014`, 2.7966746550751953`,
97.80449009759113`, 0, 0, 0, 13.912185980322105`, 2.6203021949812744`, 0, 0, 0.10520973600488423`, 0, 0,
80.80841444293007`, 0.35837773668061224`, 1.436396598727791`, 0.909785788439908`, 0, 0, 0.132280847794653`},
{0.049151474922052546`, 1.7029958107544303`, 5.984640650536118`, 1.1787547055685845`, 0.01700706976856159`,
0.4330742864041065`, 0.015091472025893045`, 551.6808300976855`, 0.14911096385124378`, 2.965493287771624`,
49.79231323763358`, 0, 0, 0, 41.1106361419491`, 0.34016215005816713`, 0, 0, 0.02595168965460587`, 0, 0,
8.275638807518265`, 0.018222340332053208`, 1.2491790203797886`, 0.3739266237730092`, 0, 0, 0.5261905158243115`},
{0.05913727892151971`, 3.1040367182848003`, 9.835708161805986`, 1.4919208308945082`, 0.5501557876127892`,
0.5378623906029458`, 0.015011170514212647`, 280.8434087480442`, 0.0269165475419888`, 2.8649095039964387`,
177.74352047826127`, 0, 0, 0, 146.4857952566367`, 0.6824594161440132`, 0, 0, 0.12748656572769088`, 0, 0,
30.262558377860287`, 0.10770297994553507`, 2.2401464199504217`, 0.825890430329675`, 0, 0, 0.32268430419064315`},
{0.1028902787116574`, 3.248894721912677`, 2.8223976230891363`, 1.4617310236348016`, 0.21163710737184238`,

```
0.2736720564831455`, 0.052563491644450414`, 157.16268136885162`, 0.03718011423514023`, 9.1247890605319`,
132273.22917826028`, 0, 0, 0, 118.48503867324101`, 1.8181008262900569`, 0, 0, 53472.04324547977`,
0, 0, 84.3831168348405`, 78596.47761156005`, 12.345754966518049`, 1.`, 0, 0, 0.7060235077094906`},
{0.13701717915337458`, 3.8023949322113495`, 2.868993121081161`, 1.1378721602373125`, 0.9948229422967421`,
0.24001294614371227`, 0.1498271577324817`, 115.7171521166015`, 0.23683719126671393`, 3.874936558168838`,
28805.699328342518`, 0, 0, 0, 28.590752718562594`, 1.0530396152551995`, 0, 0, 9710.883948231896`, 0, 0,
57.20103566377387`, 19007.970366750324`, 6.995121739717677`, 1.0000000000001523`, 0, 0, 0.2263049794518096`},
{0.12767680767420947`, 3.2442504564252905`, 9.68431286349912`, 1.3341607174516774`, 0.1954172777492651`,
0.4247465971362525`, 0.25891115414709387`, 128.98950675665216`, 0.14024381160911475`, 6.508167926731167`,
541.0361674867996`, 0, 0, 0, 122.79114494641446`, 8.703493732480348`, 0, 0, 2.183328972932337`, 0, 0,
403.37590734420064`, 3.982292476665336`, 11.517310723644972`, 0.9994621695091643`, 0, 0, 1.0189340367057684`},
{0.15001338826271665`, 1.2298087626140388`, 4.614349177257099`, 1.406777099587714`, 0.7364706782176744`,
0.4119762663671258`, 0.007028619260401152`, 151.01374639678102`, 0.013332718449329678`, 9.705143720051169`,
337276.5667751779`, 0, 0, 0, 332.90751921478255`, 1.7677204003518183`, 0, 0, 107192.2732004795`, 0, 0,
31.056543402917974`, 229718.2299769535`, 14.06025857915104`, 1.000000000000174`, 0, 0, 1.0528707192618085`},
{0.20002396942953493`, 1.8945790659495865`, 6.47237980815893`, 1.0186003524309086`, 0.9948715481686918`,
0.5599748214378268`, 0.06647091233379371`, 636.3545963390636`, 0.214859543893563`, 8.583745959022096`,
435.5091992219396`, 0, 0, 0, 223.7750990181799`, 7.476216100890313`, 0, 0, 0.49492990966410005`, 0, 0,
202.3468931037431`, 1.4142549302916265`, 2.8188586656617667`, 0.9990490923393271`, 0, 0, 0.5048114078864476`},
{0.0426995949672023`, 2.62335318941125`, 9.6559263809094`, 1.1610440596461276`, 0.9636197200326426`,
0.24951659449471042`, 0.04756766795277323`, 104.67802103676537`, 0.10920782191363448`, 6.683384921443938`,
532.8192282341329`, 0, 0, 0, 319.5152868754587`, 5.396272893877545`, 0, 0, 3.5167956355763117`, 0, 0,
202.23328152981622`, 2.145224988879019`, 13.98366578549268`, 0.9999999696849449`, 0, 0, 0.7331128841026799`},
{0.2109009326123063`, 1.0923929642765158`, 8.593806993622852`, 1.1682615459081633`, 0.4499643089978256`,
0.499787017067712`, 0.035780106156421375`, 2693.2423996223015`, 0.018435796880638622`, 3.8866756448846873`,
21.33556390806201`, 0, 0, 0, 14.209031402753126`, 0.42789584099596756`, 0, 0, 0.004886607536086354`, 0, 0,
6.67757723067399`, 0.014722715523861868`, 0.31908587580155295`, 0.08575461212279667`, 0, 0, 0.46749411258398094`},
{0.1340526977662379`, 3.5323141681247634`, 7.368159769503098`, 1.0026662265470512`, 0.9539160554997537`,
0.16176440546396775`, 0.3999369934637664`, 62.1244000754222`, 0.21257466381414752`, 3.7256546669386505`,
34792.509861961975`, 0, 0, 0, 35.25959313580443`, 3.445521389276077`, 0, 0, 11862.600565203842`, 0, 0,
173.86662885595365`, 22717.337261269735`, 12.419086304555119`, 1.0000000000010818`, 0, 0, 0.18342198418845482`},
```

{0.138783517590022`, 3.0993312847332284`, 6.04052192414912`, 1.482957943290502`, 0.5742828550914962`,
0.6307262387789596`, 0.006158597883984412`, 223.4781142247734`, 0.1875211630254222`, 1.5541408452833192`,
95.9704023462689`, 0, 0, 0, 78.85662286688681`, 0.1499421270634252`, 0, 0, 3.4335079467266016`, 0, 0,
6.638861597186808`, 6.807347293142964`, 1.7086853677184761`, 0.9999999936590418`, 0, 0, 0.38058499399205803`},
{0.13089267345973798`, 3.445236909656975`, 9.841043091432308`, 1.0552044625838208`, 0.6603410104708771`,
0.19921054981100283`, 0.006557758988700662`, 85.91513533777795`, 0.010838310480980468`, 8.608032245025406`,
40476.67770227611`, 0, 0, 0, 625.533965574402`, 1.1295252334627681`, 0, 0, 13865.933304448203`, 0, 0,
55.592600353069216`, 25927.8440033377`, 21.64001286424822`, 1.000000000000019`, 0, 0, 0.8119379033413446`},
{0.0796809200271959`, 1.3022197759162255`, 4.457724373908043`, 0.819757860237272`, 0.9902524891863433`,
0.3732869275715256`, 0.027666886465779635`, 3268.657024391763`, 0.02176038735057556`, 2.8954452621820828`,
27.87668098845641`, 0, 0, 0, 20.008468586281868`, 0.3997483953763486`, 0, 0, 0.013088053303238634`, 0, 0,
7.4365752264265685`, 0.01489811612238699`, 0.18162266299817412`, 0.28893731394469435`, 0, 0, 0.11244327072180114`},
{0.24784589915696786`, 0.6840125293527293`, 8.78766727614829`, 1.2765252846824708`, 0.05554020049431552`,
0.46845321895484016`, 0.4169012658954577`, 507.69507152027995`, 0.17834533234287903`, 5.09521235383073`,
102.54709769645665`, 0, 0, 0, 16.5647415472258`, 7.973082777686043`, 0, 0, 0.021899341818264504`, 0, 0,
77.90983596433904`, 0.07753802948418609`, 2.3206097453311005`, 0.30752240064196834`, 0, 0, 0.8741424424367815`},
{0.1280056031133443`, 3.9743049299675812`, 6.787496113248967`, 1.4777257411519527`, 0.41697417826757177`,
0.16856234271449722`, 0.016472885834375787`, 675.9035984651438`, 0.06538866409107441`, 1.0273997992144341`,
12.90960733752032`, 0, 0, 0, 10.461392548266884`, 0.041330315794753276`, 0, 0, 0.01958254204596801`, 0, 0,
2.3465611117172154`, 0.035809644358376386`, 0.3205264052434416`, 0.24604180304524892`, 0, 0, 0.07026795290380472`},
{0.2694840025057293`, 1.4398402455219186`, 1.997018914620883`, 1.3694289195380824`, 0.1761008172166878`,
0.6152080706831848`, 0.020267602026759375`, 203.92916364743616`, 0.23224028188307977`, 5.1018397742421016`,
39622.275687718065`, 0, 0, 0, 63.329259377245364`, 0.8406115673772749`, 0, 0, 8153.1249865504815`, 0, 0,
17.29066236515808`, 31387.667918644107`, 5.349216653625247`, 1.000000000000169`, 0, 0, 0.42519072379818446`},
{0.05785779964873633`, 3.866745902394764`, 0.7980363800640653`, 1.2385898080722695`, 0.5722218009229685`,
0.20306189134783081`, 0.040832709866995026`, 2800.195372434598`, 0.18949558604342587`, 8.332936748246446`,
34.127750333283466`, 0, 0, 0, 21.70326270991887`, 0.21873410618963213`, 0, 0, 0.06658199332496435`, 0, 0,
12.082702983182028`, 0.05503268042869859`, 0.6238529560723276`, 0.6751761754936392`, 0, 0, 0.5180747339177854`},
{0.2528189510796667`, 3.3250798883848782`, 4.1415523484020405`, 0.9119401620778833`, 0.8401965817654944`,
0.5070162083757728`, 0.10337827675659322`, 381.96079877048203`, 0.13245695994197593`, 7.95834379608233`,
287.51497260134045`, 0, 0, 0, 115.2194257089084`, 3.365280716876808`, 0, 0, 1.967940619017245`, 0, 0,

159.85467472080717`, 7.107609758385419`, 4.717912899513644`, 0.9999999868683452`, 0, 0, 1.1720822916263298`},
{0.20018502874097505`, 2.5781656282009475`, 6.942068190225046`, 0.9929389842661138`, 0.15941471595895984`,
0.3421827829774996`, 0.4839576109293149`, 375.34729484852807`, 0.1596957174670693`, 8.895509004013046`,
231.55234637931534`, 0, 0, 0, 33.60180050731654`, 5.22918334056198`, 0, 0, 0.03255031215014048`, 0, 0,
192.59572503139864`, 0.09308693104716526`, 5.252500153148418`, 0.5051722856236773`, 0, 0, 1.0934837307023828`},
{0.272440775669993`, 3.035804235517027`, 9.484814840956616`, 1.224288286053278`, 0.8025814347303761`,
0.5890268382574878`, 0.3843203491912798`, 336.978314494752`, 0.04553466081406238`, 4.10287356038395`,
311.86392382124046`, 0, 0, 0, 49.83742101068079`, 5.8462854462366804`, 0, 0, 0.5385955961012368`, 0, 0,
253.5454017103833`, 2.09622002820353`, 2.602199314362214`, 0.9966059513995549`, 0, 0, 0.3400091204347524`},
{0.20623021381347656`, 2.305665489644068`, 6.160210330863352`, 1.4997848102690652`, 0.31029755609374554`,
0.2932546230651458`, 0.047029701289319936`, 605.575343672007`, 0.11131413350838143`, 4.271934365443833`,
70.80595675888081`, 0, 0, 0, 42.87102662673742`, 0.8194197054081458`, 0, 0, 0.03134288383742769`, 0, 0,
26.990110518483856`, 0.09234070907607139`, 1.5333724234143722`, 0.36035597585138723`, 0, 0, 0.43283313290464415`},
{0.27799817940399246`, 2.8456068500128753`, 7.4650958706635215`, 1.2794446296629622`, 0.3185515090611555`,
0.5440861077528716`, 0.2050159519579307`, 96.61991147082354`, 0.24522478882121745`, 9.22867476531573`,
47930.32433071013`, 0, 0, 0, 146.3294876622195`, 9.856276432073933`, 0, 0, 9529.195289482477`, 0, 0,
400.67268186757246`, 37844.27059516039`, 20.6482538457012`, 1.0000000000027915`, 0, 0, 0.8794896932498582`},
{0.18731205279881885`, 3.3920364906001828`, 9.53367346846489`, 1.150711712471592`, 0.269617044263482`,
0.4407414224913243`, 0.0689482800442352`, 54.451263142554176`, 0.1525802464986004`, 2.2696676207606785`,
105385.61397125013`, 0, 0, 0, 89.09724362518111`, 1.7279368148326135`, 0, 0, 28621.954819903043`, 0, 0,
83.73178184805226`, 76589.10160615832`, 8.99493436646208`, 1.000000000000081`, 0, 0, 0.2119124408473703`},
{0.07598141369986516`, 3.9958506473293216`, 3.287957942109602`, 1.3569966945049934`, 0.38975760386481206`,
0.6382110342097327`, 0.02530287278972352`, 4836.578585308585`, 0.06124631354128618`, 7.15493409138095`,
23.53519249599506`, 0, 0, 0, 17.354575053706704`, 0.10601233936017307`, 0, 0, 0.009113579726858247`, 0, 0,
6.051563926532053`, 0.009892323878763142`, 0.33586610040336695`, 0.13336152332993223`, 0, 0, 1.0755760392851568`},
{0.21782025688272888`, 3.800225088388993`, 1.7719223148490801`, 1.3823014458300409`, 0.9034484926621247`,
0.21119490910082483`, 0.008011320454875006`, 549.8645476667474`, 0.16991323645757794`, 1.3522541901702763`,
33.4745444670415`, 0, 0, 0, 17.4611858204885`, 0.03503862855065179`, 0, 0, 3.4194978656231076`, 0, 0,
1.9022096468704206`, 10.640512907142412`, 0.5355162831507864`, 0.9999999802528862`, 0, 0, 0.13968366611240923`},
{0.12917865835123116`, 3.4713877505357775`, 5.802428296169513`, 1.4965547140183602`, 0.061568597458759644`,
0.5580721907461164`, 0.2777380762847274`, 381.024601401567`, 0.014984965589617844`, 6.51082587242481`,

190.56984665272216`, 0, 0, 0, 41.26129040692625`, 2.946847067501354`, 0, 0, 0.07867725792027475`, 0, 0,
146.1378401832348`, 0.14519175172776075`, 4.028495239016292`, 0.6697089404146246`, 0, 0, 1.2662834782395656`},
{0.12408328322582662`, 2.9809522601952096`, 4.3074275514408615`, 1.1040930179447719`, 0.6796918144407815`,
0.5931470332615022`, 0.005369942238542098`, 502.88367879292525`, 0.06875474145528054`, 6.831128450148093`,
246.37891169750367`, 0, 0, 0, 226.66992812820945`, 0.39367117986740807`, 0, 0, 0.8276960675384191`, 0, 0,
16.76449990570666`, 1.467189222475327`, 3.0198730487402288`, 0.9997549305017908`, 0, 0, 0.8633388844216997`},
{0.2301374553102561`, 3.6285008777535515`, 6.8583997394674485`, 1.291013232269163`, 0.6431022470858925`,
0.6246309281545763`, 0.18498728864660263`, 132.58216720365365`, 0.011374938240932164`, 6.052136677369937`,
453621.9727944937`, 0, 0, 0, 99.61267405757036`, 4.739280898632772`, 0, 0, 105715.01960999363`, 0, 0,
245.66407000870942`, 347556.93715882447`, 10.30509003597132`, 1.000000000000469`, 0, 0, 0.8703039197089103`},
{0.18974259222357154`, 1.3104397818774611`, 5.6865397453703626`, 1.3109561369338216`, 0.42925562092874125`,
0.6235284138933523`, 0.06332638490149935`, 1635.1348002386078`, 0.08473820825835521`, 3.2599591654117823`,
32.271097197560664`, 0, 0, 0, 17.116202473802712`, 0.7654221094356638`, 0, 0, 0.01621126620375002`, 0, 0,
14.329136887615118`, 0.04394239532464371`, 0.450479782875668`, 0.23094526565212126`, 0, 0, 0.4721230292218956`},
{0.2309824769455583`, 1.2471345787782342`, 1.0215019422726535`, 1.0312798793045614`, 0.7898876098277747`,
0.6152586670568927`, 0.006979047924720161`, 310.31208829749596`, 0.24081833704073502`, 8.344184598629436`,
18896.563466806674`, 0, 0, 0, 62.13220203619788`, 0.3259709537517354`, 0, 0, 4378.914403541155`, 0, 0,
5.8075664014444195`, 14449.321360893158`, 5.763202513999704`, 1.0000000000002685`, 0, 0, 0.7169985686514875`},
{0.1769680396277803`, 1.6046472005597128`, 8.695674701006173`, 0.7703180616996756`, 0.764260429383415`,
0.1992432648214062`, 0.012440799720035065`, 274.53159119005693`, 0.16375790457626532`, 6.298702841855883`,
324.10454017424814`, 0, 0, 0, 275.58189049825705`, 2.012282495862444`, 0, 0, 0.05741885562716724`, 0, 0,
46.12862105315697`, 0.14516146168592725`, 4.729543503042238`, 0.797046192977441`, 0, 0, 0.2799032835304817`},
{0.23450402912552432`, 3.0762297015464597`, 2.593937576427738`, 1.1039402177647473`, 0.4424612211889203`,
0.6666998367628838`, 0.44528706596344714`, 463.5224063697932`, 0.20661121111075748`, 4.223015851637719`,
1155.1398207121988`, 0, 0, 0, 15.183794907417532`, 1.8745354388017086`, 0, 0, 242.68710973230955`, 0, 0,
82.37859419204685`, 813.0157864150681`, 2.220095410416822`, 1.0000000000681724`, 0, 0, 0.9909082489344998`},
{0.2755523363386182`, 3.945462181815995`, 5.876565708554855`, 1.4395306041819111`, 0.28572441613120825`,
0.3101513953075886`, 0.39757529919045964`, 3302.499033894636`, 0.07285156297216239`, 3.794286848749824`,
11.653610063142143`, 0, 0, 0, 1.9464590417096055`, 0.1688011653733941`, 0, 0, 0.004878785891588168`, 0, 0,
9.514265917531343`, 0.019205155013191477`, 0.2510041861396807`, 0.0700416206328971`, 0, 0, 0.4056813025834673`},
{0.1912671247263708`, 2.190434692724648`, 6.310276417856439`, 0.9527360176604049`, 0.6104292936966895`,

```
0.2522020081398627`, 0.010518470607815496`, 284.3254609055096`, 0.1005439259073278`, 6.418961425083908`,
 295.60125963965584`, 0, 0, 0, 256.90778739305284`, 1.1736607143279418`, 0, 0, 0.16071000761619517`, 0, 0,
 36.72610208788453`, 0.43912201530715933`, 4.74006816006764`, 0.9641058741362747`, 0, 0, 0.40949703536864224`},
{0.2597914621776059`, 0.5051242650756058`, 1.0118962727972`, 0.8968589895031354`, 0.07276014875602654`,
 0.6898744359309281`, 0.03193322527994425`, 3177.5504656788207`, 0.10860322045075349`, 3.7076953543063436`,
 14.58393856542346`, 0, 0, 0, 9.999643943644074`, 0.5408669738051731`, 0, 0, 0.02951477915563695`, 0, 0,
 3.902929037814356`, 0.10953839475274281`, 0.2871582036875228`, 0.4871921638461789`, 0, 0, 0.9154337115723002`},
{0.05800931142578874 5`, 1.2821997162065424`, 2.0542232518025845`, 1.4132652568351216`, 0.6228832317992945`,
 0.16342794118309045`, 0.044680537911227475`, 201.38656694501756`, 0.06413530627116476`, 9.590029577588982`,
 38611.37494832676`, 0, 0, 0, 95.56841612174067`, 3.0352448133882213`, 0, 0, 21029.553594539666`, 0, 0,
 55.597000547768026`, 17427.284623013817`, 9.973467375988829`, 1.0000000000000646`, 0, 0, 0.5864836912865944`},
{0.19641852899773504`, 2.1398264375306333`, 5.320272455292722`, 1.1437489346098513`, 0.5956525384016933`,
 0.542078693849333`, 0.2264882318745723`, 868.9198508977777`, 0.040131295366706066`, 7.0792614425236255`,
 156.24157045842674`, 0, 0, 0, 38.16942137925954`, 3.7348974122046164`, 0, 0, 0.04344815499546371`, 0, 0,
 114.17188891571686`, 0.12191460988384738`, 1.7659999268132076`, 0.5545161690463937`, 0, 0, 0.6956013501919427`},
{0.09296068013545083`, 1.2991493564864323`, 0.26387265122439096`, 0.9093728692378444`, 0.35146944109549483`,
 0.5215346752572301`, 0.009103351916553084`, 3690.6422305701226`, 0.1417847774664471 3`, 8.458943719263925`,
 330.9853641115859`, 0, 0, 0, 17.220687160908497`, 0.11189052149119676`, 0, 0, 133.83168100144565`, 0, 0,
 2.0766071284602856`, 177.72977270807232`, 0.5349567409842193`, 0.9999999999996935`, 0, 0, 1.5443482434126155`},
{0.09381338426095137`, 2.303475789921615`, 0.696644313515133`, 0.9364121618865129`, 0.9663355677423924`,
 0.5212308135336106`, 0.24626013703252375`, 1385.562539836289`, 0.20934361709679128`, 7.7725134925150865`,
 644.4886236714975`, 0, 0, 0, 11.157243471735109`, 1.0593559606970693`, 0, 0, 255.28333524142613`, 0, 0,
 34.86001154821206`, 342.1284803488745`, 1.3079983957754966`, 0.9999999999991838`, 0, 0, 1.4098753105391066`},
{0.10039832860995906`, 2.055462616090492`, 1.0012272143748433`, 1.102551063992137`, 0.7631597204325553`,
 0.38648795662626834`, 0.021413905310258614`, 653.3550083645581`, 0.055519462287367316`, 4.402154187880063`,
 11462.535829451072`, 0, 0, 0, 28.051611093764738`, 0.27556377341025895`, 0, 0, 4693.868935708436`, 0, 0,
 8.091586208480264`, 6732.237083704767`, 1.4913150440428338`, 0.9999999999999998`, 0, 0, 0.5345937887034802`},
{0.16980453046164407`, 1.1980162751260872`, 9.435065425338543`, 1.2054727390347812`, 0.15783339617816905`,
 0.5987514193964574`, 0.2639813183074236`, 624.8721767770594`, 0.14884913893972124`, 1.0597319526281854`,
 20.34133516162648`, 0, 0, 0, 4.543195001535352`, 0.8687651526828858`, 0, 0, 0.017770550507317986`, 0, 0,
 14.868497031092831`, 0.04310742835629348`, 0.40242251052055666`, 0.26686138964363315`, 0, 0, 0.21434537439221504`},
```

{0.1106187483985549`, 0.9718711331456742`, 7.0205608914526`, 1.3199794157480607`, 0.2870266036717726`,
0.2842604073783652`, 0.12440193317594331`, 1067.250206889352`, 0.20077332983402157`, 1.344770962154577`,
11.869506742128625`, 0, 0, 0, 4.433007669792648`, 0.49766896861431764`, 0, 0, 0.0113387054073921`, 0, 0,
6.909572920837651`, 0.017918191437512467`, 0.2755041481884452`, 0.16719802914414617`, 0, 0, 0.1449904589460031`},
{0.22322153760577584`, 3.532203149750069`, 4.201686494998453`, 0.809746094501941`, 0.17157339217602185`,
0.16416014932043588`, 0.005086092661785311`, 81.42460105493491`, 0.09946867199032089`, 6.2584574953677805`,
88862.3611754758`, 0, 0, 0, 186.75805318078113`, 0.2600243039333528`, 0, 0, 21166.045867864097`, 0, 0,
13.120838076642306`, 67495.96148084292`, 15.651746909591054`, 1.0000000000000053`, 0, 0, 0.19869122846706128`},
{0.11344753764961812`, 0.6515213816912864`, 6.9223785093110415`, 0.7705690533378804`, 0.053758347821018404`,
0.22122664443377937`, 0.23238889135484875`, 1612.9665239639735`, 0.23659636795633343`, 7.8050806983048435`,
36.58172375869865`, 0, 0, 0, 9.23531707623484`, 2.6521994671586975`, 0, 0, 0.003433355237720941`, 0, 0,
24.68520944805899`, 0.005564367108506654`, 1.0493798300273212`, 0.09148072588520051`, 0, 0, 0.7708329572476157`},
{0.05086592503936643`, 0.6345548618632657`, 4.1212243894392255`, 0.9757969272512688`, 0.4720155756440034`,
0.3491598005177542`, 0.10619345643493772`, 163.13358888497802`, 0.24650090837908084`, 9.412849245254243`,
6601.556914453258`, 0, 0, 0, 132.65832405728287`, 18.898796930826887`, 0, 0, 3636.2639134348788`, 0, 0,
171.31890679746854`, 2642.3132520590343`, 12.869139863976867`, 0.9999999999999989`, 0, 0, 1.224054879995296`},
{0.18344840265344153`, 3.595124583701854`, 4.199071914809606`, 1.0537057639813663`, 0.12507220232647875`,
0.4903855977737973`, 0.3568003256791643`, 83.2177314580599`, 0.1899680886834239`, 7.625578775230776`,
70364.39573615063`, 0, 0, 0, 44.42963655615566`, 4.049098427552748`, 0, 0, 19363.14107699567`, 0, 0,
207.957332838907`, 50744.81858468717`, 19.624285309079713`, 1.0000000000027942`, 0, 0, 0.6487363216170323`},
{0.11456761033436458`, 3.133847301219358`, 1.1215010618012613`, 1.2799949994814193`, 0.7250649174618937`,
0.2748176967060689`, 0.05347527364447488`, 4312.248498236981`, 0.1363057898672484`, 6.512229405589068`,
24.422370873582945`, 0, 0, 0, 13.92807428814374`, 0.2272485963022845`, 0, 0, 0.0352613886037789`, 0, 0,
10.173748574682868`, 0.057711614705801204`, 0.3158661537910846`, 0.44109972728729896`, 0, 0, 0.3890435552190303`},
{0.2733102228285163`, 0.9906705811868095`, 1.8602414047947509`, 1.4451100284824412`, 0.2848844825831507`,
0.554129635350794`, 0.011612796259976983`, 302.8864567264163`, 0.1580490958422761`, 2.3921629765885086`,
31773.060719865047`, 0, 0, 0, 30.72739190069384`, 0.33238103290326315`, 0, 0, 6471.142244619061`, 0, 0,
4.704001586310689`, 25266.133269026574`, 1.6983727755740867`, 1.0000000000010982`, 0, 0, 0.2142018621633714`},
{0.13611751569452646`, 1.612649572698647`, 9.438389550358693`, 0.8529700096304889`, 0.38386891649316235`,
0.16702474363312747`, 0.015283436679942941`, 2091.084776788581`, 0.04888707786432367`, 3.7550559614266916`,
15.362296473715713`, 0, 0, 0, 12.664293099919938`, 0.11167305858781539`, 0, 0, 0.0023899409320386313`, 0, 0,

2.5727072887655904`, 0.004647326033228428`, 0.3768350068312319`, 0.058536234373103735`, 0, 0, 0.23744670127518488`},
{0.06405507608053002`, 1.3528358541887275`, 2.745226236199782`, 1.0639243234460365`, 0.36966523495796855`,
0.3262944894947615`, 0.12612633222162115`, 59.71942118372549`, 0.2416255104540047`, 1.4175051543133999`,
45852.432199162926`, 0, 0, 0, 11.652011567390522`, 0.9794310356758635`, 0, 0, 23926.323458305073`, 0, 0,
18.928706025250122`, 21894.320992130044`, 5.153115884539112`, 1.0000000000000004`, 0, 0, 0.141716409655376`},
{0.20954125488723696`, 2.353113320872523`, 8.807677395871629`, 1.2953396401945112`, 0.754037675145256`,
0.6760987806408354`, 0.0257504108557459`, 115.6397082887867`, 0.08726278776335833`, 1.3938676616131378`,
39748.23335577215`, 0, 0, 0, 79.49913694701168`, 0.8256886661106985`, 0, 0, 9926.296567334206`, 0, 0,
27.756271415979374`, 29713.837701458255`, 2.845168467556828`, 1.0000000000017244`, 0, 0, 0.27313889461412155`},
{0.12907099769038483`, 2.604139615282878`, 9.661547795493739`, 1.0824039436985886`, 0.5892380332813292`,
0.2973399722037613`, 0.047844179127263436`, 189.25086236267023`, 0.09074457722059731`, 6.471849996577652`,
471.91695910904076`, 0, 0, 0, 282.76155621044995`, 4.931310740779148`, 0, 0, 0.26686706248161385`, 0, 0,
183.45459507618506`, 0.49206854293139074`, 7.236684017523637`, 0.9872253620888142`, 0, 0, 0.4671967053963582`},
{0.2477279255184573`, 2.8408395942947395`, 2.128090335748766`, 1.4989746812981888`, 0.49854461135120975`,
0.5556994499234343`, 0.04795352432490085`, 88.91629411642595`, 0.17336384521962678`, 2.1291108959892835`,
142269.56679491166`, 0, 0, 0, 21.47371149641406`, 0.34858182735582166`, 0, 0, 31336.092911040014`, 0, 0,
14.14664367148624`, 110897.50415293692`, 5.081844664215574`, 1.0000000000017248`, 0, 0, 0.16030707181555431`},
{0.17138396941106926`, 2.571547212239655`, 8.060362653989774`, 1.424545143219825`, 0.3585647265624352`,
0.4155041928809974`, 0.005849644389202518`, 1444.9053577030656`, 0.14315469959013916`, 8.6363710850225`,
74.42149826534792`, 0, 0, 0, 68.67817664860193`, 0.14927910684165038`, 0, 0, 0.010312743290212272`, 0, 0,
5.483975300632453`, 0.025249126865630384`, 1.3175371200479251`, 0.1440548940600534 5`, 0, 0, 1.0091255643037393`},
{0.046024454977220214`, 1.8018961949102152`, 1.8889771640586375`, 1.2101342547143499`, 0.33571961358008684`,
0.4325808303075249`, 0.005213559292121167`, 454.61857984803544`, 0.13129751322022398`, 8.467405558196546`,
3466.722922722436`, 0, 0, 0, 127.90781913024439`, 0.34759017560528727`, 0, 0, 2008.6297106975715`, 0, 0,
8.947448783019166`, 1320.6583955129654`, 4.28960643808751`, 0.9999999999999962`, 0, 0, 1.4132773636097318`},
{0.15552548139256261`, 1.3158122598456092`, 2.9803095987090753`, 0.8576161156930884`, 0.19622947720693906`,
0.41449590928153224`, 0.03892742585126438`, 2129.9557657820133`, 0.1528564450859799`, 8.904626117418289`,
44.784464050096815`, 0, 0, 0, 29.07407713059654`, 0.791771587248902`, 0, 0, 0.010288145544579375`, 0, 0,
14.883182307136016`, 0.022858125549429776`, 0.9402292107354331`, 0.2267718586017171`, 0, 0, 1.2421168018088982`},
{0.20226341603934178`, 2.23359139800782`, 8.92211343859698`, 1.0176324123098368`, 0.17770526936314845`,
0.5466941503204505`, 0.0226198569969 4341`, 208.46398724865384`, 0.0356723302826476`, 4.800574849057675`,

272.62929382791367`, 0, 0, 0, 206.94663050587897`, 1.9833540034646806`, 0, 0, 0.09091642369125472`, 0, 0,
63.28574916204422`, 0.26270094899813723`, 5.343825993576406`, 0.8442607161080874`, 0, 0, 0.8436678724539768`},
{0.1612087208534252`, 3.1252528746197825`, 0.891851633192701`, 1.468881451365178`, 0.6554353298599058`,
0.19493335173588566`, 0.018954452797492544`, 551.070999166768`, 0.11474062580409572`, 7.406826770535428`,
12565.975767021757`, 0, 0, 0, 40.53443895017065`, 0.23516582556795626`, 0, 0, 3788.9071665252795`, 0, 0,
10.499323890979825`, 8725.783967827332`, 2.790348587034135`, 0.9999999999999997`, 0, 0, 0.38408616225973197`},
{0.2365245631846375`, 1.6915599352668007`, 9.677850457164503`, 0.7567859979545984`, 0.725618873672359`,
0.2424250415266589`, 0.36826031218846694`, 892.5435235500784`, 0.17475643237365474`, 6.493737892117458`,
108.87173098858135`, 0, 0, 0, 18.64089377618501`, 3.583917772033314`, 0, 0, 0.009371665619386774`, 0, 0,
86.60588163517447`, 0.03166613024208092`, 1.5096143402310391`, 0.23401098756341276`, 0, 0, 0.33307105455648356`},
{0.22790261965108521`, 1.8196334440146895`, 4.716201039891137`, 1.2810805872690838`, 0.6237465626441163`,
0.5616811859186371`, 0.03042013590776336`, 637.6118251230974`, 0.08011306001688712`, 9.216145754556873`,
297.65408263271866`, 0, 0, 0, 207.9626080422809`, 3.2984764022637556`, 0, 0, 0.14511603332518916`, 0, 0,
85.7431139407485`, 0.47246177354544755`, 3.1270065061625925`, 0.8990107964062035`, 0, 0, 0.8858569627281917`},
{0.10184647957852327`, 3.0801202872185955`, 8.412464860766345`, 0.9942252517730743`, 0.7984635108014702`,
0.4565736536432896`, 0.009191281945386102`, 103.82351882247931`, 0.22274711417837167`, 2.5201460619086546`,
8657.49900539858`, 0, 0, 0, 161.10276100632785`, 0.45875492389757266`, 0, 0, 3452.459705735657`, 0, 0,
20.186004970833398`, 5023.155241655466`, 5.583815314642994`, 1.0000000000000049`, 0, 0, 0.4171594681587733`},
{0.18768732765242752`, 1.4966861207841546`, 1.129759551095228`, 1.2240080138702374`, 0.5362083693873154`,
0.686948995479657`, 0.005867661202712241`, 74.92355531972449`, 0.165025723744549`, 3.2590978632956187`,
141080.67716032866`, 0, 0, 0, 27.591686970782444`, 0.10229924736224338`, 0, 0, 38316.02315859303`, 0, 0,
2.1872837670533545`, 102734.74275578395`, 9.449466071537145`, 1.0000000000000004`, 0, 0, 0.3280397203672199`},
{0.14949493319923252`, 0.867900380757324`, 9.54711835147964`, 0.8271983961134595`, 0.23831574478972195`,
0.375815029784779`, 0.04516845569069218`, 2094.687766706356`, 0.0279158592619283`, 5.743304871768913`,
28.82201261093747`, 0, 0, 0, 17.73956249528244`, 0.8264104170606028`, 0, 0, 0.0028292266952608287`, 0, 0,
10.246313080410241`, 0.006042215083050599`, 0.6093642380555557`, 0.07093905797246414`, 0, 0, 0.7239293917339223`},
{0.10785599050108607`, 1.35490623294936`, 7.749733046704847`, 1.2972878372630243`, 0.6070878493023009`,
0.6937073302769028`, 0.28374543116039413`, 66.83074779041495`, 0.1939262594152683`, 9.425365274493942`,
75173.25433684074`, 0, 0, 0, 129.87317939297245`, 24.198238149793852`, 0, 0, 29341.468716293224`, 0, 0,
468.37633850783646`, 45209.33101646309`, 32.17684020950471`, 1.0000000000006573`, 0, 0, 1.4700063471580047`},
{0.13969386881974227`, 3.165109549305347`, 4.62533417349867`, 1.0389272094346946`, 0.7061162924631639`,

0.5973647953915502`, 0.19864442086151357`, 3440.0036010322765`, 0.16071761832429643`, 1.3360604448248414`,
9.20219692500833`, 0, 0, 0, 2.4506398422056974`, 0.14539493799792594`, 0, 0, 0.010684354524255302`, 0, 0,
6.574155809684221`, 0.02132198313335193`, 0.08394459582059134`, 0.19427593949172262`, 0, 0, 0.12703943031868117`},
{0.18701505616348296`, 1.1471835668231432`, 1.9652898147298057`, 1.007866470439728`, 0.8673024418570234`,
0.4964521059357`, 0.11361709646832255`, 189.11029986824036`, 0.17527157137077032`, 1.608206453868661`,
29410.57255772462`, 0, 0, 0, 9.901403178399516`, 0.8961572245259516`, 0, 0, 8003.251729392527`, 0, 0,
14.686526303800148`, 21381.836738040605`, 1.8339323610279963`, 1.000000000001476`, 0, 0, 0.1489920782748668`},
{0.12078071855653999`, 3.7816423804380825`, 3.0527531818157634`, 1.3792123087306463`, 0.18684955879429088`,
0.29398025647621684`, 0.011105429740027619`, 471.0240529628659`, 0.1734264666127333`, 9.864280424563177`,
194.00178108156413`, 0, 0, 0, 167.63488142568585`, 0.4706835810073993`, 0, 0, 0.12756547598810727`, 0, 0,
25.427956824484863`, 0.2201064264692886`, 4.595801171774258`, 0.8535193529426245`, 0, 0, 1.1036995761291348`},
{0.12022941283008587`, 2.2264210098571606`, 6.2451953049416655`, 1.3438290154028227`, 0.5358944653344513`,
0.32618163573980585`, 0.199782178892696`, 3338.755251465298`, 0.1788085018354036`, 4.512919273292992`,
18.095061850982948`, 0, 0, 0, 4.9005914963462445`, 0.401728437005387`, 0, 0, 0.005652658617219108`, 0, 0,
12.777380462941041`, 0.009708797521092152`, 0.2887065849709432`, 0.08627281655787244`, 0, 0, 0.3709452364340069`},
{0.04681963491488872`, 2.902738250264786`, 8.529727895043578`, 1.3985345888875074`, 0.35876551940164103`,
0.2520539039703089`, 0.05124631524713002`, 163.50426816944002`, 0.24665810410586203`, 8.118758344754049`,
498.79726428600173`, 0, 0, 0, 292.1282634787895`, 4.857756541844558`, 0, 0, 0.2175603426574267`, 0, 0,
201.43993892123146`, 0.14551565450261067`, 10.630769194416908`, 0.9563551728448765`, 0, 0, 0.6880007520947627`},
{0.1911559295611578`, 2.175586072104755`, 6.8117639016300915`, 1.0586708066957606`, 0.34517099105389626`,
0.196211531611845`, 0.1489787018319715`, 3704.576077864889`, 0.10408111144660032`, 4.8300445149655875`,
11.585309266571299`, 0, 0, 0, 3.8837907020579663`, 0.239795273425023`, 0, 0, 0.002394628659836233`, 0, 0,
7.452789386000446`, 0.006539249534643027`, 0.2760266485777854`, 0.04749500907942272`, 0, 0, 0.3509348642477312`},
{0.17192712556630163`, 3.7260903741599414`, 4.118781130521407`, 1.2245874304759414`, 0.6062180312792598`,
0.5331783629983038`, 0.1203111914746069`, 226.51013650082294`, 0.017839169449302028`, 7.150760371208259`,
96985.85234012778`, 0, 0, 0, 94.9118504195885`, 2.870771012165869`, 0, 0, 27989.701898238807`, 0, 0,
152.8107462121237`, 68745.55704031169`, 7.196003678634697`, 1.000000000000098`, 0, 0, 1.1077430257378866`},
{0.1575695043509842`, 3.8758817205894927`, 3.3547063374262347`, 1.3334178116995439`, 0.4921394422884451`,
0.402330971011554`, 0.12204131883407823`, 201.57298534982124`, 0.22899817129952033`, 2.4884430439924596`,
13254.481155256848`, 0, 0, 0, 25.678919192334924`, 0.7579965018288155`, 0, 0, 4056.014418848498`, 0, 0,
41.97006836727274`, 9130.059737405745`, 2.707978591595361`, 1.0000000000000036`, 0, 0, 0.27722395716804016`},

{0.06573776532431397`, 2.613579102769201`, 3.806271127858438`, 1.1314163600990936`, 0.4483602258230772`,
0.6823320235871475`, 0.18768855278686034`, 189.85674956999534`, 0.0631967400861071`, 1.3499467761035964`,
36717.984603400604`, 0, 0, 0, 13.097048208541445`, 0.858312699341934`, 0, 0, 18911.670117083493`, 0, 0,
32.046687637735744`, 17760.156172109626`, 1.7075283451989283`, 1.0000000000001865`, 0, 0, 0.292594518225327`},
{0.06757463956251891`, 2.3005441553217736`, 6.868151131987258`, 1.3860241166029201`, 0.025379963517129678`,
0.6856836713524472`, 0.1183550729914282 5`, 3863.192203814942`, 0.1108156231672533`, 2.823244077255117`,
8.59241485568045`, 0, 0, 0, 3.2906599403374575`, 0.15632649154012412`, 0, 0, 0.003954000954443936`, 0, 0,
5.137657091922740 4`, 0.003817002704663185 3`, 0.17873073971424458`, 0.05888130082232235`, 0, 0, 0.6751433383592685`},
{0.06632069369203508`, 0.7568152865276287`, 0.22726671156421221`, 1.362987063673106`, 0.9228910129465389`,
0.1868567265073786`, 0.129681199016726 7`, 123.29501155053357`, 0.19348527358813117`, 9.470702921306387`,
40466.119973540954`, 0, 0, 0, 6.067626277988171`, 0.9028770522232125`, 0, 0, 20770.476100509655`,
0, 0, 9.761587928250451`, 19678.748332851952`, 15.968892332303048`, 1.`, 0, 0, 0.504979886807201`},
{0.1352416913789251`, 3.40347330811998`, 4.693938060979022`, 1.2778213334701187`, 0.7052646641955478`,
0.6043325609148968`, 0.013064813980624626`, 333.49561209931903`, 0.11153764285740131`, 8.005884287110216`,
334.87069873308224`, 0, 0, 0, 276.6816484255834`, 1.0196898343797367`, 0, 0, 2.53054258075046`, 0, 0,
49.57838762675298`, 4.889069410386926`, 5.611612451201835`, 0.9999999875475801`, 0, 0, 1.47611956968482`},
{0.11675242158451127`, 2.833886804623358`, 0.47584343623935504`, 1.0607616820427617`, 0.7666611109645796`,
0.4737758025893787`, 0.1681420982868212`, 120.33220123866614`, 0.17915094289032796`, 3.5814785888826783`,
50878.84686836332`, 0, 0, 0, 4.172391937108239`, 0.2314646482954453`, 0, 0, 19065.642579468782`, 0, 0,
9.37063732201787`, 31799.427717396287`, 6.434804116296943`, 1.0000000000001792`, 0, 0, 0.3417130831629521`},
{0.24323887781179143`, 3.4699698183729026`, 8.39097272098094`, 1.1938324247391086`, 0.7463001660827029`,
0.6306644967356683`, 0.2992521819376612`, 322.6100644812281`, 0.10289378547186823`, 7.076414822100832`,
486.63939727132947`, 0, 0, 0, 95.31948256003494`, 7.655948565796998`, 0, 0, 0.9276216815272216`, 0, 0,
379.5130064904408`, 3.223337954979814`, 4.778077265952935`, 0.9992864250858389`, 0, 0, 0.7336279766231871`},
{0.24390696598302103`, 0.8206355382610386`, 2.839705408503791`, 1.0872176805395473`, 0.8466198387041834`,
0.5785697848880403`, 0.1356337036504287`, 2343.8028799398503`, 0.22246042363346175`, 4.459217822696726`,
62.83767603591975`, 0, 0, 0, 21.568665604801854`, 3.224282783827755`, 0, 0, 0.0546972837026407`, 0, 0,
37.7994433973184`, 0.19058640736334442`, 0.399036715841267`, 0.6490365577580033`, 0, 0, 0.2794500928886442`},
{0.06769491456810739`, 3.362305338073525`, 1.8871867183871915`, 1.4803795481758868`, 0.5363168854717104`,
0.37458837250617005`, 0.011183256466341666`, 407.888606379481`, 0.051902827430772014`, 9.719761204172809`,
16144.112966006198`, 0, 0, 0, 132.7533869186642`, 0.4215754866689822`, 0, 0, 8129.113135640726`, 0, 0,

20.249507274686604`, 7861.423131881135`, 5.3446267167496195`, 0.9999999999999998`, 0, 0, 1.3256346686714973`},
{0.21105892687940375`, 0.7047276416914965`, 5.768010388957135`, 0.810198720595394`, 0.17857267890229922`,
0.19525448649651245`, 0.036176279358565294`, 67.82203673017167`, 0.07478241594027568`, 5.650997388906681`,
128879.69321226129`, 0, 0, 0, 166.21041009393582`, 7.601388209212898`, 0, 0, 32036.177316214005`, 0, 0,
76.52726266085908`, 96593.16008112034`, 17.106822412961694`, 1.0000000000000009`, 0, 0, 0.22744827144041332`},
{0.13030085884608883`, 3.691040694570585`, 3.5546925177950874`, 1.392417125140739`, 0.5227226143537942`,
0.3153665172319682`, 0.008712610924073824`, 695.2883528301467`, 0.04802018879975545`, 9.078480195703946`,
172.6046088439054`, 0, 0, 0, 153.47391406433508`, 0.3492003336964452`, 0, 0, 0.08223521646574247`, 0, 0,
18.41303774616009`, 0.15307599046969142`, 2.785792982284983`, 0.7137214073388354`, 0, 0, 0.7352955029424042`},
{0.21250061023000932`, 2.8149517595358526`, 2.295631419336827`, 1.0543549080196901`, 0.6519014079329188`,
0.6534060058028184`, 0.09088447710322001`, 650.6798329466749`, 0.04016188412840693`, 1.3861729584655489`,
35.94675041263064`, 0, 0, 0, 12.86448296657865`, 0.3890069460211892`, 0, 0, 1.7468688170544804`, 0, 0,
15.643368388200162`, 5.303009851655024`, 0.5210382549318701`, 0.9999999803118867`, 0, 0, 0.3315744072654034`},
{0.14347475236734758`, 3.69539554202769`, 4.673626861854084`, 0.8876695712812951`, 0.055199727721453984`,
0.3291461786147223`, 0.031312636638690675`, 836.9629099625735`, 0.07687709551472927`, 5.335882334514919`,
54.829959766225926`, 0, 0, 0, 38.26368744020944`, 0.3070484562025946`, 0, 0, 0.014504634766559311`, 0, 0,
16.20950708910785`, 0.02972926973300662`, 1.4303929484425626`, 0.2955424915819511`, 0, 0, 0.7298592792533447`},
{0.0805761857877269`, 3.094619489710217`, 9.945877040804334`, 1.11372690786775`, 0.6430409751953907`,
0.42229007304684385`, 0.05506378209236767`, 302.8456666998151`, 0.050941087889112335`, 7.632080993355595`,
453.37242002750685`, 0, 0, 0, 255.98007983870508`, 4.362086054288794`, 0, 0, 0.08469997225835843`, 0, 0,
192.84280741993217`, 0.09749715286997629`, 5.363688034141614`, 0.8011129444596202`, 0, 0, 0.5980587129055255`},
{0.26865716137877294`, 2.5539808428845117`, 7.725254140854183`, 1.421576189719117`, 0.2484256166411507`,
0.1545757521332899`, 0.008556741498662016`, 1177.1024036480305`, 0.08015420001442447`, 4.125195480316323`,
26.423863208177067`, 0, 0, 0, 23.57183069912218`, 0.07452023945075942`, 0, 0, 0.007831593872572404`, 0, 0,
2.718903770920085`, 0.03005733969823982`, 0.738074439826507`, 0.11172825222410165`, 0, 0, 0.27666978853355695`},
{0.05207035897383483`, 1.5461440288878858`, 9.695008671418819`, 0.9618476339297223`, 0.8208653770038725`,
0.48583250041037973`, 0.22651174945218666`, 56.560953214157614`, 0.1695178841867513`, 7.246656846935364`,
43198.164833436254`, 0, 0, 0, 150.12924572576497`, 19.43175361598606`, 0, 0, 24427.828932178778`, 0, 0,
429.204140345391`, 18170.940306428245`, 29.262426872894498`, 1.000000000000123`, 0, 0, 1.1392376577518215`},
{0.09188932948313594`, 3.928289836924776`, 1.4719659367359732`, 1.3955743442040527`, 0.5432280807299197`,
0.32698938665455834`, 0.013007122973082902`, 2762.880112969401`, 0.13947463148509326`, 4.274432647648432`,

21.04301641057549`, 0, 0, 0, 17.71279560860649`, 0.05656260235784133`, 0, 0, 0.03825388979309847`, 0, 0,
3.174204228461792`, 0.050216061188703365`, 0.33246428912045956`, 0.4369244646708216`, 0, 0, 0.379599481699878`},
{0.04429611977972214`, 3.507177302954225`, 7.234549676890296`, 0.7623401767303408`, 0.010148640019661537`,
0.662687845406686`, 0.08775344792279212`, 3965.4644576584396`, 0.17480873042969614`, 5.118348118050843`,
14.840432833946723`, 0, 0, 0, 6.739464549255401`, 0.158460720923232`, 0, 0, 0.0019690858821729993`, 0, 0,
7.939283483310325`, 0.0012460409156287184`, 0.313197576799703`, 0.053923357346165934`, 0, 0, 1.1743194353465891`},
{0.14748247662042147`, 2.024427515527907`, 9.506929645646245`, 1.2214601071407813`, 0.2054881053340767`,
0.2020185622482833`, 0.07806490597707526`, 302.23580657852216`, 0.2425913704635762`, 6.045088630499148`,
161.50635741738552`, 0, 0, 0, 78.49369444112942`, 2.77163111762892`, 0, 0, 0.027078694322991405`, 0, 0,
80.1566613917336`, 0.05705189860558325`, 4.2761002307941`, 0.37915361502965805`, 0, 0, 0.5030237567882169`},
{0.05191368478403385`, 1.8292747620439185`, 5.888828307542417`, 1.141375509610469`, 0.9869968200599144`,
0.28747330669734017`, 0.24632331590197062`, 4082.2304223282904`, 0.0447948152020462`, 8.998917183247265`,
57.792533896672`, 0, 0, 0, 13.135972552796542`, 1.64532775379547`, 0, 0, 0.00844709161230426`, 0, 0,
42.99652193297801`, 0.006264566447198061`, 0.4498584994608277`, 0.1471392290177299`, 0, 0, 0.3056414954641059`},
{0.22928260338606776`, 2.222139650206395`, 4.336407604983492`, 1.4545262441104416`, 0.17670484975018264`,
0.15215120204412502`, 0.00931582374755147`, 107.9598634817244`, 0.18384173269782222`, 2.0618261552271964`,
43912.86751160214`, 0, 0, 0, 60.562614366910516`, 0.24152309583198886`, 0, 0, 10254.87010737125`, 0, 0,
7.667114966983765`, 33589.47593720064`, 3.9127625852013166`, 1.0000000000008717`, 0, 0, 0.07844183971651292`},
{0.13057179539780978`, 1.32287055831243`, 2.838629919542937`, 0.8498204532586893`, 0.8949438289681946`,
0.46229782752490833`, 0.06831034580553909`, 355.6715560557054`, 0.046330937271765626`, 4.510601406855825`,
5133.779070705771`, 0, 0, 0, 57.41302597358992`, 2.6937957107438972`, 0, 0, 1752.9557489552142`, 0, 0,
50.90775765502015`, 3269.8082770570754`, 2.9498096145602735`, 0.9999999999999893`, 0, 0, 0.8051596976277798`},
{0.16617324858328203`, 1.7665378156673919`, 1.5537022391595983`, 1.3738499573440288`, 0.09625830296842142`,
0.5757224393125404`, 0.011187699121044243`, 1140.5955024024072`, 0.09128758443232887`, 2.6133507581824666`,
26.706628066836902`, 0, 0, 0, 22.75723009031861`, 0.1355161731446444`, 0, 0, 0.11191033302040516`, 0, 0,
3.419920635636349`, 0.2656643369719646`, 0.5571413835871438`, 0.7834152280290758`, 0, 0, 0.6063788881816633`},
{0.150610971367461`, 1.9051770945291775`, 1.9970914506574022`, 1.332029884280907`, 0.017627751946745374`,
0.6342635790195508`, 0.03224365795250487`, 84.16237057378729`, 0.025181518731154096`, 6.093120594420567`,
745302.1534159952`, 0, 0, 0, 68.44502184959798`, 1.1000369788888436`, 0, 0, 236453.2722751199`, 0, 0,
29.93950364734405`, 508749.3860052945`, 15.83275989872226`, 1.0000000000001636`, 0, 0, 0.6583273234411555`},
{0.170193722625894`, 0.42908710938865147`, 8.009796665724636`, 0.9108642019460496`, 0.48256370773751156`,

```
0.1504864882770205`, 0.015501266854459406`, 231.18780020236528`, 0.059845835174358764`, 2.951447491236335`,
 113.43542295065046`, 0, 0, 0, 93.18201426414895`, 2.8125828358140397`, 0, 0, 0.04408105059819751`, 0, 0,
 17.240614841936864`, 0.10717597283669834`, 2.657633668119249`, 0.6452443111872204`, 0, 0, 0.1615757502316108`},
{0.14155286072340578`, 2.768731185287475`, 2.866796585793665`, 0.9268420036617817`, 0.38841014261523243`,
 0.6739227770046636`, 0.08018336457681292`, 85.63532436040691`, 0.10664760400309609`, 4.590937221386067`,
 110072.15334528571`, 0, 0, 0, 51.44947424047499`, 1.4187763073561648`, 0, 0, 36385.33498501213`, 0, 0,
 56.11728867320014`, 73577.83222154103`, 11.876741428061052`, 1.000000000000012`, 0, 0, 0.562210410217698`},
{0.17254585725985527`, 2.4767354176461422`, 1.5265879737476418`, 1.3533294324013416`, 0.010476993931212819`,
 0.3024537953894164`, 0.005382899126762437`, 1067.886819263142`, 0.025600178888030733`, 4.688973100729083`,
 35.70334589010506`, 0, 0, 0, 32.96645371893008`, 0.06771953558970577`, 0, 0, 0.06810145856353038`, 0, 0,
 2.3960481751653284`, 0.16786606497843357`, 0.9891132169028817`, 0.6472677490422285`, 0, 0, 0.6627491342713744`},
{0.12080218784009655`, 2.1868434076988192`, 8.77616791832854`, 0.9616886370191489`, 0.26020855267635734`,
 0.6201783502287406`, 0.03811904516021991`, 117.06014479510438`, 0.17593156125676795`, 4.349098586723594`,
 9884.244212671638`, 0, 0, 0, 221.26735494983524`, 3.6299212112516193`, 0, 0, 3502.1343933228977`, 0, 0,
 113.40098958988196`, 6043.792811763627`, 8.833052043478956`, 0.9999999999999991`, 0, 0, 0.889826393742482`},
{0.06629025039264891`, 1.530609759106781`, 1.900084106095834`, 1.2729265107514505`, 0.009336607275947317`,
 0.20692029297881853`, 0.07757288734346537`, 56.094679581136695`, 0.10338780030561462`, 4.34093728430811`,
 139732.2680952435`, 0, 0, 0, 31.333913547664746`, 1.457517852349525`, 0, 0, 71734.35241644784`, 0, 0,
 31.869872126836263`, 67932.68833486906`, 16.227797018482672`, 1.`, 0, 0, 0.27121429193054364`},
{0.11054921486908309`, 1.9601623754484976`, 4.1405236153603`, 1.0982919546164496`, 0.2371613718862391`,
 0.5811399077338627`, 0.014126785714663546`, 63.169894546693705`, 0.15558848051416968`, 8.651696062244092`,
 102818.72606315033`, 0, 0, 0, 247.0464506036112`, 1.6925238285493798`, 0, 0, 39748.558938570575`, 0, 0,
 47.394593261036135`, 62773.88546909241`, 30.310313057993852`, 1.0000000000000175`, 0, 0, 1.0494469612270647`},
{0.09844925238529889`, 2.9547765664281824`, 3.97533240344332`, 0.8901779978265776`, 0.6485331039194935`,
 0.172046787329591`, 0.2843834249851245`, 265.56383421685405`, 0.07856132944640065`, 2.1823933339229455`,
 69.3512138766139`, 0, 0, 0, 14.865826727383386`, 1.2348186256928588`, 0, 0, 0.4685485427665218`, 0, 0,
 52.123044842661606`, 0.6589750534511492`, 1.7420629555512792`, 0.999525598522091`, 0, 0, 0.1615931850007642`},
{0.07379407071323735`, 1.0020834251379886`, 3.8959201685323244`, 1.3275435329440872`, 0.5495795554378471`,
 0.5203295123605114`, 0.006236204073249997`, 495.6126936444482`, 0.05339932410900344`, 4.300380134475608`,
 139.97973413292246`, 0, 0, 0, 127.43094036165337`, 0.730091385289095`, 0, 0, 0.5996679973292796`, 0, 0,
 10.451606800489074`, 0.6321706085626099`, 1.9338999791045584`, 0.9968350766784568`, 0, 0, 0.5558444478979311`},
```

{0.137830658132162`, 0.964244066849937`, 5.678313655870262`, 1.056362681888576`, 0.27898051740088525`,
0.6674603903550571`, 0.022240144180942223`, 3206.233069292432`, 0.13749704640301397`, 7.378694929535063`,
33.32057608226024`, 0, 0, 0, 25.388644891537986`, 0.5352126553697917`, 0, 0, 0.00556736865967698`, 0, 0,
7.372508963476018`, 0.010962201234684988`, 0.5337426376831993`, 0.10681480727225168`, 0, 0, 1.291908435020394`},
{0.09412349267697989`, 2.412142947637907`, 8.593900106949853`, 1.0545990130363978`, 0.8443965196041454`,
0.4441557500447094`, 0.020813982545198062`, 67.68562305269263`, 0.21037364591803787`, 1.9282456472076213`,
33026.472906715855`, 0, 0, 0, 107.37599113448101`, 0.879041980594993`, 0, 0, 14026.947512079414`,
0, 0, 30.291070202426575`, 18860.93273476561`, 6.381233546593703`, 1.`, 0, 0, 0.2601377140780715`},
{0.059604065321211375`, 2.060924190389435`, 4.151577648882975`, 1.0218595307135387`, 0.8259153199816331`,
0.67511248087556`, 0.012173840559063499`, 163.32402156550611`, 0.22896057989768043`, 4.41755081858776`,
12073.803854849582`, 0, 0, 0, 138.91000642285542`, 0.7774714131251298`, 0, 0, 6433.233027132277`, 0, 0,
22.890137752083408`, 5477.812022510951`, 6.432896470909497`, 1.0000000000000506`, 0, 0, 0.9057248942928413`},
{0.062303828390692484`, 3.237012523552033`, 1.9717708145626371`, 0.9726411613979222`, 0.1873520229034391`,
0.5560570337810927`, 0.026607710207102397`, 62.963998927305475`, 0.022649002664224704`, 8.659052494024476`,
551979.9411245878`, 0, 0, 0, 104.7133167342808`, 0.8247189434873022`, 0, 0, 291966.6985908712`, 0, 0,
38.137507835414226`, 259866.32978289493`, 30.991413625726278`, 1.0000000000000002`, 0, 0, 1.2277537360017368`},
{0.058207327156306`, 1.429925878818433`, 8.458349879741764`, 1.1245084153106533`, 0.19874114947102073`,
0.3371100826465334`, 0.04401493286992449`, 375.7204818983131`, 0.2159694260931322`, 8.593637256078832`,
224.38273198488443`, 0, 0, 0, 139.5814254504947`, 3.9548434790934164`, 0, 0, 0.028178867273322677`, 0, 0,
80.78761482045729`, 0.023431664946639983`, 5.056053017153806`, 0.41628812663516135`, 0, 0, 1.031320304290618`},
{0.0526423548412821`, 1.6513512325831732`, 4.083783641771872`, 1.373155154639566`, 0.8939370398042139`,
0.2364887781328291`, 0.0490949664593259`, 1618.3201075393572`, 0.07451928539997066`, 4.7651781050102695`,
55.91013911539432`, 0, 0, 0, 33.07791563872257`, 0.9262177604831087`, 0, 0, 0.03123446304931371`, 0, 0,
21.850154863060066`, 0.02348936695886498`, 0.6045708521773702`, 0.3851496240014458`, 0, 0, 0.1919936733912312`},
{0.21322884859448837`, 1.5615738832323176`, 8.40996483053398`, 1.2929614343326012`, 0.3765546169235392`,
0.671739728089668`, 0.1608794324574438`, 485.1832614690647`, 0.0705178455433636`, 2.1513131242884285`,
70.01350433462389`, 0, 0, 0, 21.846711813760933`, 2.058915583098246`, 0, 0, 0.043789957015578126`, 0, 0,
45.930697176375105`, 0.1333897444918938`, 1.0200350816573338`, 0.5088971414437666`, 0, 0, 0.3562002119957909`},
{0.11682922382752631`, 0.5721136495795571`, 9.687079392836907`, 0.91254998083727`, 0.43543762812336806`,
0.4740169657662334`, 0.10620954791167687`, 4372.038997558794`, 0.0729116423313943`, 5.645356891940546`,
18.442711478826446`, 0, 0, 0, 7.489820480334162`, 1.193454026190729`, 0, 0, 0.0019754724293849207`, 0, 0,

9.754161978991371`, 0.0032970415802321316`, 0.28449693319519503`, 0.04547478639457747`, 0, 0, 0.6567236545839082`},
{0.26956767736567855`, 0.8444303847788461`, 1.0020073721955711`, 0.7502641425367418`, 0.966405616850982`,
0.6715103286393609`, 0.23328928938153737`, 246.8438332486948`, 0.10178810353613177`, 2.531792031152129`,
46725.70937092259`, 0, 0, 0, 4.814491318797165`, 1.1864800868138503`, 0, 0, 9628.059164731343`, 0, 0,
14.31285480343803`, 37077.336379656605`, 2.1956610508315406`, 1.0000000000090319`, 0, 0, 0.21426750454322224`},
{0.07372356616920961`, 2.107710372997479`, 6.08076458707294`, 0.8379230467338923`, 0.9571077593283461`,
0.37371168094765217`, 0.1939664465355383`, 131.4329681577719`, 0.026286944156709513`, 5.373906305884013`,
47586.938025175004`, 0, 0, 0, 77.38285970985962`, 6.339007807753926`, 0, 0, 23043.246206731226`, 0, 0,
190.86846444164263`, 24269.004092504798`, 9.209290301661785`, 1.0000000000000153`, 0, 0, 0.7588091139952156`},
{0.2128059772734779`, 3.088861612111426`, 5.437928143232815`, 1.2368766361188581`, 0.5161736795080103`,
0.22159582314348258`, 0.17105793552921358`, 1293.4067318655482`, 0.22754002195492767`, 9.689789287743999`,
83.94591454059119`, 0, 0, 0, 25.45840700517334`, 1.2948305306896501`, 0, 0, 0.013914356971754104`, 0, 0,
57.13646172053037`, 0.04230083333580931`, 1.5735096092818872`, 0.21491474920929676`, 0, 0, 0.6218295149899081`},
{0.09487232790202699`, 3.2902977287485546`, 3.9754892015026773`, 1.2274952505878522`, 0.2657953162834845`,
0.47376077240276926`, 0.26204707729007887`, 80.73499841507218`, 0.18195262450329797`, 6.708419132160676`,
61736.54428786671`, 0, 0, 0, 48.838017432236036`, 3.551613102868963`, 0, 0, 26118.413716366373`, 0, 0,
166.94092179661936`, 35398.78157685593`, 18.28888138114523`, 1.000000000000014`, 0, 0, 0.7727817705744372`},
{0.05710457017724263`, 2.2468049124614753`, 4.035987116327698`, 0.7517191945594467`, 0.40903626729229714`,
0.6582116219387326`, 0.04046385674463665`, 815.0779000126262`, 0.186726177475597`, 7.464159626289564`,
151.31900803907894`, 0, 0, 0, 96.56429622121492`, 1.6520006700260201`, 0, 0, 0.03936471273054896`, 0, 0,
53.02461744005885`, 0.032112928580334134`, 2.074210058089379`, 0.6700006913081767`, 0, 0, 1.101763784992707`},
{0.1356890149134694`, 1.6504364529320803`, 8.903330095009764`, 0.8652149931542472`, 0.7882939470826769`,
0.5853867881019097`, 0.12191584954894137`, 2002.2565687817998`, 0.19189449537551667`, 8.0829058846114`,
117.04286954479666`, 0, 0, 0, 43.21494823074433`, 3.0026708064874197`, 0, 0, 0.009966225066460556`, 0, 0,
70.79596221688303`, 0.019318675166615493`, 0.8490625335195947`, 0.21927900759277041`, 0, 0, 0.5306353613699298`},
{0.21947794079961902`, 1.5730764365956098`, 2.118289006802259`, 0.973502067608673`, 0.7638917093126882`,
0.33981639014260245`, 0.009615294802656809`, 1765.6534921210653`, 0.16626255675053375`, 4.010994756897377`,
43.75467344861621`, 0, 0, 0, 38.309366499549526`, 0.22141465726191958`, 0, 0, 0.052484453777358546`, 0, 0,
4.975745429365973`, 0.1645597119863978`, 0.47620107993397254`, 0.6756518774405146`, 0, 0, 0.24908948282009444`},
{0.2345658346849942`, 2.081301191128982`, 3.6234436876627587`, 1.1134590239676974`, 0.8739003578896458`,
0.6696041669913848`, 0.06590896300137702`, 558.8606273551264`, 0.09113830356870467`, 1.2137876330314246`,

41.63461180591715`, 0, 0, 0, 19.85727601983152`, 0.5929306412713616`, 0, 0, 0.8169966429984985`, 0, 0,
17.62953214192793`, 2.7377071357111866`, 0.5061750624869119`, 0.9999988242076193`, 0, 0, 0.21947771564398794`},
{0.23053748957807368`, 3.8668800248110458`, 5.535000453565214`, 1.1361194466757834`, 0.24488877583427326`,
0.5300195072451362`, 0.08816752471869817`, 3094.9084693615328`, 0.16131439142801796`, 1.2619255962975515`,
4.93320059035842`, 0, 0, 0, 2.222627288365176`, 0.0477966238532895`, 0, 0, 0.00522516626249449`, 0, 0,
2.6403401433098903`, 0.0172085244683358`, 0.09443597353094543`, 0.09251285560973999`, 0, 0, 0.21891258912196637`},
{0.05839671607004826`, 2.466861519533315`, 8.278076935325572`, 0.9179604454629582`, 0.7485509623778881`,
0.3739704677333707`, 0.3976055017684991`, 334.0137006202725`, 0.18218149392519362`, 2.015076569112860 5`,
116.7443927237742`, 0, 0, 0, 18.516523340505156`, 2.7046779233354408`, 0, 0, 0.11337914533604813`, 0, 0,
95.31522702582123`, 0.0945852822636001`, 1.270438374202759`, 0.9139132116197723`, 0, 0, 0.1349321340430081`},
{0.14569114559043927`, 3.1555485829295717`, 1.7894848948555018`, 1.1727674941432193`, 0.06666495623301039`,
0.21747003985461844`, 0.032197916321861565`, 1115.0145850241458`, 0.11404115123479613`, 9.530247146566754`,
65.05622278157867`, 0, 0, 0, 45.03050107674059`, 0.4318373715121046`, 0, 0, 0.03925422612029003`, 0, 0,
19.466911510443612`, 0.08169990246757111`, 1.8525231753413671`, 0.5104837081002765`, 0, 0, 0.9354106781918841`},
{0.24150266620923067`, 1.7188165992139313`, 4.322636691993072`, 1.2029528995785472`, 0.9471494725848191`,
0.6615414605337746`, 0.19861497081852103`, 2170.4118977268995`, 0.144713056075588`, 4.793357249969096`,
107.47949593117515`, 0, 0, 0, 28.26893340697244`, 3.088164096731122`, 0, 0, 0.06606742404243655`, 0, 0,
75.82839586511372`, 0.22793512936890128`, 0.4565971182447191`, 0.682385296622298`, 0, 0, 0.22772901119473937`},
{0.20918490948785712`, 3.1569386485962374`, 8.450247389660383`, 1.207442014040422`, 0.02962131605799323`,
0.6403349881706148`, 0.07844599973162336`, 325.7491474109025`, 0.10080604275363181`, 2.998503110312266`,
104.59496326856535`, 0, 0, 0, 50.294581157526`, 1.1739835277089419`, 0, 0, 0.04528776018130331`, 0, 0,
52.945628163424416`, 0.1353359430634095`, 2.230421343585763`, 0.5470981410156657`, 0, 0, 0.682723335965693`},
{0.20782035028778872`, 3.1560227942933192`, 4.024085032034362`, 1.1076699632387197`, 0.7825797887689212`,
0.681476671590229`, 0.005917686238352288 6`, 2392.206038919967`, 0.17125268254719206`, 3.7066097570560648`,
48.38473282362777`, 0, 0, 0, 44.487175177316985`, 0.08100477290852295`, 0, 0, 0.029317786285432832`, 0, 0,
3.652184424940749`, 0.0870404659357284`, 0.329490635377137`, 0.42764177304802853`, 0, 0, 0.2869849830791269`},
{0.16569005768607342`, 0.9318724619299896`, 5.387727961962419`, 1.4204768501331082`, 0.162422581490264`,
0.6283948310518737`, 0.42435650593933405`, 403.29853257093646`, 0.17732127106821444`, 7.752705227642123`,
248.6620777557737`, 0, 0, 0, 38.73811378442789`, 14.642433220362216`, 0, 0, 0.10533690108863092`, 0, 0,
194.92686133863432`, 0.24933253168333894`, 4.512113856617136`, 0.7861273539339508`, 0, 0, 1.4672545190065696`},
{0.25387146604005345`, 2.177421517695631`, 2.9195785593706507`, 1.205984878491204`, 0.22933817566590875`,

0.5142965710820098`, 0.00715658453704801`, 291.5834795916249`, 0.18546405096886748`, 3.1957985304348036`,
11155.114683384925`, 0, 0, 0, 70.25760311384663`, 0.22005258298296912`, 0, 0, 2394.285060077739`, 0, 0,
6.844960417308839`, 8683.437975996185`, 2.4334786419334304`, 1.0000000000007003`, 0, 0, 0.39931646496624223`},
{0.27254750481249423`, 3.728211063877054`, 5.204302820921312`, 1.137745914342209`, 0.04515932299981973`,
0.21786268187051538`, 0.17709327429647695`, 87.47051100453898`, 0.05078893394192652`, 2.761931237968355`,
216228.26157717768`, 0, 0, 0, 32.35781243075348`, 1.442369770094732`, 0, 0, 44163.90318472925`, 0, 0,
76.82084192955718`, 171953.73736826418`, 6.495627498322657`, 1.000000000001427`, 0, 0, 0.11682643634412401`},
{0.24124989455736606`, 1.138551887899828`, 6.0414451617780465`, 1.0864227463934355`, 0.8606911125263128`,
0.6167713883354349`, 0.008616924398628963`, 4204.638992315412`, 0.042475052828396476`, 4.683417206387297`,
40.026024938586524`, 0, 0, 0, 35.59440928144155`, 0.25212368497122845`, 0, 0, 0.01066602542292943`, 0, 0,
4.1007985358322045`, 0.036759678694687686`, 0.23230230364491455`, 0.18931702336638623`, 0, 0, 0.2654359057390758`},
{0.16291183507690532`, 0.9001170306894437`, 5.6406094859134015`, 1.3830538570390154`, 0.30535931966424634`,
0.523800888605104`, 0.2170932130058295`, 52.61233898698406`, 0.08477132140762966`, 2.26771827115102`,
309692.89217789605`, 0, 0, 0, 26.20586440063368`, 5.589977380177901`, 0, 0, 93044.84286653047`, 0, 0,
71.88048344381279`, 216544.37279755337`, 9.344408341695722`, 1.0000000000014535`, 0, 0, 0.22321304588624663`},
{0.24307985322679526`, 3.5312268091205707`, 7.143117328957406`, 1.2474301004946668`, 0.43966589306292936`,
0.28635789146968393`, 0.04752738776816154`, 125.46273060513494`, 0.15229886979218882`, 3.763239253417467`,
5018.292514045951`, 0, 0, 0, 134.69299746925606`, 1.7014033459723223`, 0, 0, 1072.3285503884101`, 0, 0,
85.82915869178385`, 3723.7352377045218`, 6.6468646002410585`, 0.9999999999995505`, 0, 0, 0.4620252472713346`},
{0.08579390683558069`, 2.8983719603389604`, 3.99564346069112`, 1.2420354947694623`, 0.9313337583971846`,
0.4737657288125191`, 0.010571420568870461`, 66.04207510362154`, 0.1244519483642591`, 8.73466885165751`,
114907.61684658368`, 0, 0, 0, 249.93135236172873`, 0.8746263278761671`, 0, 0, 51500.236941928415`, 0, 0,
36.21417749272142`, 63120.093288659285`, 29.129820703613195`, 1.0000000000000286`, 0, 0, 1.0125531043213005`},
{0.20336933942425872`, 1.2934536281273639`, 5.591224704743386`, 1.058733783245077`, 0.09184020179750241`,
0.3092776948325172`, 0.45477750215520385`, 95.84900263920687`, 0.14971414135963745`, 8.98848136080165`,
53395.708942902056`, 0, 0, 0, 57.49520095974479`, 17.351921744895332`, 0, 0, 13571.443048547631`, 0, 0,
320.6272305131`, 39428.7915402439`, 19.733292679774443`, 1.000000000000003`, 0, 0, 0.5946951790822039`},
{0.12808268874911022`, 3.6824385947617753`, 2.1347222010883335`, 0.9228913029818868`, 0.1301948165557758`,
0.29194798379239884`, 0.27062051931711223`, 4508.281780436825`, 0.033597809391257005`, 7.5117374932700365`,
14.752932769981127`, 0, 0, 0, 3.305893181408903`, 0.21323958300618914`, 0, 0, 0.0056760628299271З`, 0, 0,
11.217738148469923`, 0.0103857912681027`, 0.3668711584043515`, 0.12351643950964764`, 0, 0, 0.8683192084193201`},

{0.14553068051711526`, 1.8082756931498238`, 2.368533159601707`, 1.1602558844471123`, 0.9691057704573378`,
0.4792380149128871`, 0.010639214796165341`, 788.9564435352116`, 0.18893878705870149`, 9.465562662789438`,
188.00511743762038`, 0, 0, 0, 160.58781416128048`, 0.8946462835875092`, 0, 0, 1.068900365604254`, 0, 0,
23.110958979687386`, 2.222254251591198`, 2.6493946163246567`, 0.9999980343343114`, 0, 0, 1.1250725744804597`},
{0.24858998623664108`, 2.4028729322353692`, 5.907867944453356`, 0.899298954014168`, 0.6845394050547509`,
0.4831163766793648`, 0.006208316855544933`, 932.6458115441258`, 0.22545041645445363`, 9.209981899602862`,
206.52376409082336`, 0, 0, 0, 189.55932233240006`, 0.4706899927433083`, 0, 0, 0.029984081947482824`, 0, 0,
16.15726061481366`, 0.10648203598060986`, 2.0987087134326847`, 0.5104686165742491`, 0, 0, 0.7072676837125372`},
{0.2519271524040908`, 1.2045387595495214`, 4.5140841978362705`, 1.015214245278859`, 0.29419718036266773`,
0.20432622138273293`, 0.02287101962327847`, 2390.149329793605`, 0.07799608049040802`, 9.888014794979117`,
36.46856987462557`, 0, 0, 0, 27.673047257425146`, 0.4812387425510709`, 0, 0, 0.005511735210270012`, 0, 0,
8.281010257137709`, 0.019836510804686222`, 0.878870819077891`, 0.11026928930348223`, 0, 0, 0.7551898698837941`},
{0.11736070154865536`, 2.38096944376945`, 5.3494850274310295`, 1.276899864236343`, 0.07495782665645989`,
0.3912082450614217`, 0.41113333692039317`, 80.12914367323096`, 0.2186956786299285`, 8.297333470694408`,
49120.19641657008`, 0, 0, 0, 56.85487492792499`, 8.617128103237475`, 0, 0, 18217.872959090437`, 0, 0,
293.10169581222044`, 30543.74787433048`, 22.381710603597867`, 1.0000000000012923`, 0, 0, 0.789364873997676`},
{0.16035901956271875`, 2.606522752147513`, 4.69883411815465`, 1.3021358666964324`, 0.6150695298380762`,
0.6434499333892203`, 0.12329290515746044`, 1079.0196669558486`, 0.2419176548365854`, 8.070742396729585`,
163.20465155503854`, 0, 0, 0, 60.05769959740915`, 2.693135764488597`, 0, 0, 0.05229756007508371`, 0, 0,
100.28170921088166`, 0.11980550655947625`, 1.6299058646677747`, 0.5731747600102901`, 0, 0, 0.8400067196747673`},
{0.16449617597041116`, 3.191202853555228`, 6.979734889593845`, 0.755053893640484`, 0.8431581165413089`,
0.5308423930900061`, 0.0662197921799213`, 915.9947229465104`, 0.165152047976952l`, 4.094114041787371`,
136.4721946884852`, 0, 0, 0, 70.51812604110641`, 1.4130579962360044`, 0, 0, 0.036145607805766235`, 0, 0,
64.41935299753389`, 0.08494020374526691`, 0.9324955089514776`, 0.638583292006481`, 0, 0, 0.23363485189352354`},
{0.11360527105324508`, 0.4683409445122746`, 6.198135239531911`, 1.4397350406163711`, 0.8287446034488006`,
0.16487005319303316`, 0.2956243647100192`, 339.82112374821816`, 0.14870620145140367`, 8.492417686013223`,
347.2523030444991`, 0, 0, 0, 70.52987107076412`, 35.93185528131616`, 0, 0, 0.1469529000413674`, 0, 0,
240.40512915042828`, 0.238494629160585`, 5.117295463840134`, 0.8840239261869494`, 0, 0, 0.31833879669708753`},
{0.08657280459787114`, 2.8376273381057535`, 2.18201840556476`, 0.9732923844552535`, 0.8927467229995099`,
0.4599530868891304`, 0.04289399463935107`, 1129.687177458504`, 0.08547871234303578`, 2.1820677830816138`,
38.93171120702291 4`, 0, 0, 0, 23.862436046417827`, 0.3458705222240545`, 0, 0, 0.3135421770106559`, 0, 0,

```
  14.020737847255624`, 0.38777465176473414`, 0.41862005171926614`, 0.9980751094502535`, 0, 0, 0.21400805863886838`},
 {0.14288327643386806`, 3.0416391272131467`, 7.259522365277451`, 0.8919048885163637`, 0.7100314935930478`,
  0.32120037052790307`, 0.014548712666776912`, 690.6861027330066`, 0.2370448478751857`, 7.1501615854828415`,
  177.67594007321128`, 0, 0, 0, 147.34027328806292`, 0.6788603221831261`, 0, 0, 0.025698217347203043`, 0, 0,
  29.4978302552103`, 0.052454935615417905`, 2.1667295295262483`, 0.4613404294188883`, 0, 0, 0.43225695260395974`},
 {0.05887079706837223`, 1.3439289639420835`, 4.010009055307599`, 1.087305166401462`, 0.6661788396152433`,
  0.35567096782369767`, 0.008830796062004187`, 96.96443168903161`, 0.0354538286259683`, 6.082802068311153`,
  154167.19762556435`, 0, 0, 0, 178.1341783005994`, 1.0878488749984485`, 0, 0, 83631.04681256834`, 0,
  0, 20.885594450031792`, 70334.66265026089`, 13.791541253544137`, 1.`, 0, 0, 0.6927953288508171`},
 {0.275808383383374`, 3.614253930768469`, 8.690573450897112`, 0.8084062901760818`, 0.7881641108236563`,
  0.5461332704061045`, 0.16300280176413603`, 213.49162259077596`, 0.11620699031214832`, 3.4918574760984407`,
  258.9594454251696`, 0, 0, 0, 78.719045742071`, 3.3213781361140535`, 0, 0, 1.098954039418648`, 0, 0,
  171.49005691455196`, 4.33001052892405`, 3.6618776047914134`, 0.9999972841573727`, 0, 0, 0.4691621670628258`},
 {0.16345104545617634`, 3.976575006158881`, 6.050721111049688`, 0.8077170991659299`, 0.4737043917748365`,
  0.42206087766889466`, 0.011122815850097148`, 819.7636995837653`, 0.0134560852671817`, 8.084495823489597`,
  140.41931321801366`, 0, 0, 0, 121.32406389196018`, 0.3277530427130408`, 0, 0, 0.018394756264114583`, 0, 0,
  18.619065112074313`, 0.04295203060402728`, 2.144708962761351`, 0.3866908487528976`, 0, 0, 0.823487250352661`},
 {0.27078615466317746`, 3.6662669410444693`, 7.922278334055675`, 1.4466898496109064`, 0.8715287168554611`,
  0.2219561421798496`, 0.02446106939657988`, 188.339766844502`, 0.03692414185938048`, 1.663076961237424`,
  106.79079116375168`, 0, 0, 0, 77.15648129416783`, 0.4929714433127562`, 0, 0, 0.6783650872279261`, 0, 0,
  25.81949864995045`, 2.62416962040284`, 1.861741219255553`, 0.99967756660701`, 0, 0, 0.11349856965297846`},
 {0.24286662289220456`, 2.6814172670718373`, 2.6369699156223714`, 0.8218929956416956`, 0.8808977613854625`,
  0.43575724866517207`, 0.024751792159432087`, 472.6370271533013`, 0.14270041872793765`, 9.280134575613399`,
  233.98565963541557`, 0, 0, 0, 155.6198350286499`, 1.359698976289721`, 0, 0, 5.5673539188930015`, 0, 0,
  52.084575900616606`, 19.316063496103215`, 4.5001958457373945`, 0.999999999317221`, 0, 0, 1.4964586355645568`},
 {0.042342161544045354`, 1.3013804906014936`, 7.13634807471312`, 1.1105942412761596`, 0.2407240140528013`,
  0.6802475696307242`, 0.0062232822378432`, 4037.4772502763867`, 0.18461914595887624`, 5.716947148449252`,
  19.849475888450513`, 0, 0, 0, 18.231208207755326`, 0.08133206011704014`, 0, 0, 0.003501701676968845 4`, 0, 0,
  1.5120565185249113`, 0.0021181374012179837`, 0.3316740572033398`, 0.0653361200388406`, 0, 0, 1.0679602522873475`},
 {0.14720397501884225`, 0.5316247300014076`, 6.683828314747185`, 1.481587653959343`, 0.013428589995876372`,
  0.17720084833285243`, 0.005004553323761107`, 364.08088787611194`, 0.035796657483315286`, 8.51611862140901`,
```

160.522092861328`, 0, 0, 0, 149.87481767916253`, 1.2065648207600534`, 0, 0, 0.032986629692520704`, 0, 0,
9.163424243796566`, 0.06936804304592022`, 5.013438930470866`, 0.38037873517589815`, 0, 0, 0.7320543545577588`},
{0.1926486177557311`, 2.6336525621782014`, 9.444109966651418`, 1.4788802142070587`, 0.6654095862145473`,
0.16653655494047714`, 0.03828733327035071`, 2433.4281263020134`, 0.12844440731237916`, 4.149230973672752`,
19.155383302174474`, 0, 0, 0, 12.494114596367076`, 0.1719843920601078`, 0, 0, 0.004688342681947243`, 0, 0,
6.4706733543394686`, 0.012902896246324676`, 0.3523811932402043`, 0.06592613828015015`, 0, 0, 0.19540684877755854`},
{0.055762854118976635`, 2.4026093631268655`, 6.857734870811047`, 0.7509249944976302`, 0.22671157472868875`,
0.4788061070906606`, 0.1564030997457896`, 55.02740807629177`, 0.03469990388231514`, 7.492235364197862`,
219607.0192408077`, 0, 0, 0, 139.19080493791822`, 8.312296054817251`, 0, 0, 121991.76639370149`, 0,
0, 285.30286186266255`, 97180.1296161177`, 30.722267489891045`, 1.`, 0, 0, 1.0733028380482137`},
{0.05296847355619744`, 1.6490117913903575`, 9.992115392915188`, 0.8066960527418563`, 0.1291629696144665`,
0.33931322884091664`, 0.26435083615790894`, 660.4050522121521`, 0.10186522527726305`, 2.8696181659920086`,
39.684763508085595`, 0, 0, 0, 9.03042688716153`, 1.2477230354825688`, 0, 0, 0.00774957292669902`, 0, 0,
29.39299997000185`, 0.00586404355196638`, 0.9710940607810559`, 0.18640820724971063`, 0, 0, 0.3797133165338059`},
{0.21365538200916506`, 3.0547845209089406`, 0.35609106729359574`, 0.8256521864420123`, 0.9506371395328554`,
0.271042805312945`, 0.12766968769895812`, 68.7970381277751`, 0.05673347586404265`, 3.4610065999125617`,
288137.8627021797`, 0, 0, 0, 3.413262972618208`, 0.1356298075538366`, 0, 0, 71103.84264113051`, 0, 0,
5.918854809847409`, 217024.55231157603`, 10.328867006966568`, 1.0000000000001048`, 0, 0, 0.13934372488759988`},
{0.22385277301638717`, 3.365536903395271`, 8.935305056074029`, 1.445762062116566`, 0.43390325179453604`,
0.6187135527847307`, 0.01939946413637202`, 453.1242214832122`, 0.13325485950688953`, 8.825528492983036`,
321.8021318833832`, 0, 0, 0, 252.63027700486515`, 1.402884928477957`, 0, 0, 0.05365851455034574`, 0, 0,
67.44944282870857`, 0.17159438968623245`, 4.366020775831812`, 0.5473727380916782`, 0, 0, 1.1981103175274894`},
{0.26129739321020845`, 3.355909888714172`, 1.041845959949887`, 1.206440545734901`, 0.09347792890054829`,
0.5333101115239811`, 0.015503351594481202`, 579.4832207194656`, 0.24065061389170217`, 7.475999528371727`,
8747.97885270187`, 0, 0, 0, 51.288326879864684`, 0.229134290959011`, 0, 0, 1835.1533354710589`, 0, 0,
10.985057612469378`, 6850.296895708696`, 2.7695752133077964`, 1.0000000000003404`, 0, 0, 0.6556071215526034`},
{0.14172561423935526`, 2.161658858177276`, 2.5726304321806133`, 1.1364876244324849`, 0.9181535957758225`,
0.5861373014149901`, 0.010418800942482771`, 101.70999367656657`, 0.1500483081169689`, 9.64604938537483`,
78537.11429852412`, 0, 0, 0, 176.6420690322347`, 0.8138487974358283`, 0, 0, 25898.649094248194`, 0, 0,
25.132335174201177`, 52435.74215502642`, 20.72391833480457`, 1.0000000000000324`, 0, 0, 1.0336206151941896`},
{0.06568219588241164`, 1.0305120784213448`, 8.720293141386708`, 1.4160313463775192`, 0.752508918071946`,

0.31681781371940887`, 0.13417309715525413`, 209.13734568668048`, 0.22402731709711876`, 4.416848552940577`,
306.50031128237816`, 0, 0, 0, 107.49654833824754`, 12.561338987324715`, 0, 0, 0.783875178129728`, 0, 0,
184.9230221083295`, 0.7355234713895394`, 4.487528554212731`, 0.9988533989911941`, 0, 0, 0.3382678214604524`},
{0.27044630440719625`, 2.4698840490073675`, 6.955264919348561`, 1.0249819835130385`, 0.106623120735865`,
0.6358905088319218`, 0.012311596840628138`, 346.23093318344644`, 0.07401305589494472`, 1.2937187987733016`,
44.23096306646051`, 0, 0, 0, 37.509931434721466`, 0.17784682045980724`, 0, 0, 0.05005228576472407`, 0, 0,
6.275157500290781`, 0.1933779387457598`, 0.9036713139082971`, 0.6333491836326076`, 0, 0, 0.2915346112652073`},
{0.08403118541616988`, 3.378337092807227`, 2.524635889786053`, 0.8671019090417934`, 0.46085963082622694`,
0.5847463933658021`, 0.04733165775893332`, 1030.6526695312073`, 0.10944158368617352`, 3.930070348109142`,
62.54081465868531`, 0, 0, 0, 37.5488121118119`, 0.5040790110190088`, 0, 0, 0.07208778214840868`, 0, 0,
24.32784029473001`, 0.08653745411360485`, 0.8561619380460329`, 0.8116038857990568`, 0, 0, 0.540784627048789`},
{0.11975601650409934`, 1.1540823534574054`, 5.101398975621729`, 0.9964575213162732`, 0.8596230582994027`,
0.5017711629445061`, 0.09812020963113087`, 54.445260820005984`, 0.07947868464718594`, 4.273753987333478`,
200201.01107563608`, 0, 0, 0, 75.58911673300778`, 5.867610349450755`, 0, 0, 73787.36610668634`, 0, 0,
96.73865087521635`, 126235.44333237651`, 17.1656433809397`, 1.0000000000000033`, 0, 0, 0.4614915342077035`},
{0.26353274820980593`, 3.2715371213501925`, 8.419438906869026`, 0.9426613525835754`, 0.1294979869931494`,
0.5387601140015186`, 0.040849691062558216`, 744.2937861154428`, 0.1766059692159136`, 7.8822033078787435`,
120.81442853553429`, 0, 0, 0, 77.08988711707805`, 0.914621791906871`, 0, 0, 0.012349745213257298`, 0, 0,
42.745987774559524`, 0.046493747081979314`, 2.4609830141891633`, 0.2452900320628374`, 0, 0, 1.3983814768053315`},
{0.15837784563387913`, 1.590657770632209`, 9.4421417173358`, 0.8199017044479102`, 0.629826423052569`,
0.2661387442145513`, 0.049173845467465206`, 187.50695430622352`, 0.06908425180315825`, 9.722920562989135`,
713.5642589271415`, 0, 0, 0, 422.501346385137`, 12.190388376125343`, 0, 0, 0.5663325875181128`, 0, 0,
277.01051425011906`, 1.281350501762617`, 10.88510334272081`, 0.9990327945760411`, 0, 0, 0.6183111519610864`},
{0.18489841859634304`, 3.5401138358224227`, 7.262892362437768`, 0.8851173674042263`, 0.05104398233694862`,
0.6826543300991057`, 0.13384148697112022`, 730.4506518091381`, 0.22533987085622137`, 8.188627124718394`,
136.4777320365579`, 0, 0, 0, 48.44545983722825`, 1.7058671920142472`, 0, 0, 0.015238254072596432`, 0, 0,
86.27091497893132`, 0.04025041543134584`, 2.702943695165952`, 0.3087439051276356`, 0, 0, 1.8148322615798451`},
{0.22773890477301867`, 2.1437195677585157`, 6.152335866192775`, 1.1180903919170035`, 0.02705103803804154`,
0.3333465628704615`, 0.009170207790258475`, 2321.6700090659583`, 0.14789384405564093`, 7.237611958554762`,
26.590895556777824`, 0, 0, 0, 23.552954671219293`, 0.09485138678969592`, 0, 0, 0.004364039225486 91`, 0, 0,
2.904782484144325`, 0.014198021622844413`, 0.7010275881105487`, 0.08047210632316648`, 0, 0, 1.0086653256988045`},

{0.17605039997850958`, 2.727076411741753`, 8.728585319721596`, 1.1320082850991182`, 0.7355770210071702`,
0.5402004706661186`, 0.09745706159326789`, 177.96581560566344`, 0.01922037387361028`, 4.980887347403458`,
379.80400571840204`, 0, 0, 0, 159.1789846245606`, 5.323276605109517`, 0, 0, 2.2521135347366013`, 0, 0,
207.38545804244146`, 5.664078408391555`, 6.419737802060431`, 0.9999999301745343`, 0, 0, 0.8077482825940543`},
{0.06947420594938447`, 0.8077718165326004`, 3.821443319804267`, 1.3891202366711977`, 0.6756008025235485`,
0.23550127195203518`, 0.0653335798785588`, 483.3383501610001`, 0.09612644227220152`, 2.381497911922425`,
65.84106637907664`, 0, 0, 0, 34.35487077800581`, 2.4806897793547913`, 0, 0, 0.19022598505342425`, 0, 0,
28.62616127604673 4`, 0.1887971323218744`, 1.0352004261899022`, 0.9273736309072381`, 0, 0, 0.15361922394466737`},
{0.23569362997389992`, 1.2270876586626818`, 0.9845589085102358`, 1.1982217261670929`, 0.43286649637666286`,
0.634774301600445`, 0.09976341665112172`, 228.04937393006952`, 0.12724994607105566`, 3.0426000813607676`,
59653.73018148092`, 0, 0, 0, 10.033694085350785`, 0.7555851383189621`, 0, 0, 13654.451006858026`, 0,
0, 13.245274261430442`, 45975.244615816526`, 2.8619804171297307`, 1.`, 0, 0, 0.2642757759108095`},
{0.12274586224015488`, 2.0471402784080235`, 1.4612976870222134`, 0.9456822010625827`, 0.8327879461898879`,
0.22053943969717293`, 0.006767755909702022`, 174.3096643655477`, 0.1850505728558421`, 1.2537872990507584`,
19731.476101653963`, 0, 0, 0, 13.048767717499132`, 0.04093556089332934`, 0, 0, 7160.736288371719`, 0, 0,
1.1971547931994144`, 12556.439285579327`, 1.5013468104966172`, 1.0000000000007732`, 0, 0, 0.07215561243513452`},
{0.13491824188855928`, 1.669720674179887`, 1.3334401828831126`, 0.968437561771696`, 0.3482872719375660 3`,
0.3135712369052611`, 0.22891096239253717`, 1910.612731597179`, 0.10930115833371806`, 4.270908360205038`,
24.203493543643585`, 0, 0, 0, 6.0034635105778085`, 0.727870727981357`, 0, 0, 0.03762645344226842`, 0, 0,
17.362011466297684`, 0.07252135638474559`, 0.4881510498180058`, 0.556359446007006`, 0, 0, 0.45460753543937366`},
{0.04199865921066254`, 2.754174936868967`, 5.53210979521042`, 1.3844956478579449`, 0.47136731805345633`,
0.24068198696313148`, 0.022972136801125642`, 58.648848297948106`, 0.2240134714991286`, 4.479259216921056`,
43127.7408143082`, 0, 0, 0, 151.08597035688362`, 1.1908474661016089`, 0, 0, 26829.68161423397`, 0, 0,
46.854317782442386`, 16097.295069239897`, 16.43621562230337`, 1.000000000000079`, 0, 0, 0.4048307084464748`},
{0.18074106663079648`, 0.9554759541683486`, 8.202625386518609`, 1.4769540911095849`, 0.5922233662884384`,
0.5544055394726339`, 0.15090969809745627`, 4632.951795907658`, 0.16872394703920557`, 7.933658625009354`,
33.040934066417904`, 0, 0, 0, 10.712289054718704`, 1.5229848792360225`, 0, 0, 0.00486866201823349`, 0, 0,
20.788220438171454`, 0.012570959517735585`, 0.371401827893485`, 0.06831783768644684`, 0, 0, 0.7844876547957236`},
{0.22308930012837075`, 1.8877770902637643`, 5.727481376105105`, 1.290937410786533`, 0.5385704303274257`,
0.17837528465279628`, 0.11591468148922064`, 2665.0058694088225`, 0.1309938954283596`, 2.3264420617360244`,
8.71333110105202`, 0, 0, 0, 3.384167289328283`, 0.1897163558382017`, 0, 0, 0.005524262499664176`, 0, 0,

5.1163170028526626`, 0.01760576935392956`, 0.182447820449347`, 0.08768868794201012`, 0, 0, 0.1370954690716077`},
{0.16528939075200133`, 0.44637388983991233`, 6.000554224390708`, 0.7620462868306999`, 0.45157540745748825`,
0.6293554847029814`, 0.08179042637541929`, 626.5470719450981`, 0.11922182895709121`, 4.5474321331423475`,
121.81520506316748`, 0, 0, 0, 56.96845531464038`, 8.77583428321263`, 0, 0, 0.032514576824400516`, 0, 0,
55.96147550840125`, 0.07677592276950779`, 1.6293386138365742`, 0.5958716845127731`, 0, 0, 0.6249117123050363`},
{0.12057640522465035`, 1.6899679349832946`, 5.872613897455656`, 0.8685022109314445`, 0.5819827862907108`,
0.45575489869095254`, 0.005408595604133131`, 1249.3305456098487`, 0.22090492300977427`, 9.765554151588912`,
134.239724991178`, 0, 0, 0, 124.56135027301646`, 0.3777152917742554`, 0, 0, 0.015325913170547462`, 0, 0,
9.118953309305013`, 0.026399193098426074`, 1.6809714823287245`, 0.31547891981786536`, 0, 0, 0.8748968798843233`},
{0.26839288378136816`, 1.20431418513886`, 3.4264235210106797`, 1.1464020290887187`, 0.9129873318113406`,
0.17958510842302844`, 0.03476022275277427`, 85.14929883968256`, 0.03637248443077806`, 6.177611921122705`,
422073.38356184616`, 0, 0, 0, 107.99032373008933`, 2.8989640494591113`, 0, 0, 87276.90183825167`, 0, 0,
49.87519324244567`, 334635.70531245373`, 14.726337697245086`, 1.0000000000001266`, 0, 0, 0.17567054885659564`},
{0.19312844950593372`, 3.9129912832786564`, 0.42277195731844414`, 0.8655327062249413`, 0.5847393470300801`,
0.16029168895981472`, 0.1525854675774217`, 2234.1231120850057`, 0.03649943574663328`, 5.234397199033289`,
20.984268224598885`, 0, 0, 0, 5.854653886374717`, 0.20523903248764888`, 0, 0, 0.9182120923677841`, 0, 0,
11.472836358753073`, 2.53332682452272`, 0.5000619720221265`, 0.9999994424011686`, 0, 0, 0.4260828490366689`},
{0.05831506452479063`, 1.4447664488109728`, 9.399667906542401`, 0.8838396243177019`, 0.4494233408794772`,
0.5909311609620335`, 0.2085880113243902`, 309.3416067223959`, 0.04816991376818669`, 2.147582543132131`,
110.97250965331297`, 0, 0, 0, 28.97934176361099`, 3.7844020277743318`, 0, 0, 0.05483792199242731`, 0, 0,
78.10824397915084`, 0.04568395656261662`, 1.5584563011583141`, 0.7264990652800153`, 0, 0, 0.2950340307009241`},
{0.23965113012644185`, 0.9128974489725401`, 8.261297062012524`, 0.788417407034967`, 0.8016641172773833`,
0.35686628470413617`, 0.030635478621033968`, 346.1316581378353`, 0.043365488693742626`, 1.9545026373639716`,
116.95354517929417`, 0, 0, 0, 81.34456664304592`, 2.4987652523750543`, 0, 0, 0.1153346016635322`, 0, 0,
32.58737749249156`, 0.39485810901931506`, 1.1805178344034986`, 0.9418974839675771`, 0, 0, 0.11581131430142279`},
{0.20520507007680205`, 1.512442754504547`, 9.386452795492342`, 1.0402158263719963`, 0.9284696718653804`,
0.3818637796049891`, 0.053241178903524394`, 3017.2611079460976`, 0.15062298010038172`, 2.3864513615272553`,
22.46099795120003`, 0, 0, 0, 12.812062765049124`, 0.4255908782004235`, 0, 0, 0.007014680784464398`, 0, 0,
9.195454858820835`, 0.02056354374202737`, 0.16286751528836196`, 0.13455568328463563`, 0, 0, 0.1033786649093968`},
{0.20853248950244857`, 2.7801356391066916`, 9.335557605849111`, 0.8182981643225358`, 0.24140252954455388`,
0.3825831953385048`, 0.013327430792399009`, 77.96529661289064`, 0.07323986754942219`, 3.7008101112034457`,

68157.41842395031`, 0, 0, 0, 236.0548590551081`, 1.0823845957489935`, 0, 0, 17058.693981525594`, 0,
0, 42.988228426598084`, 50818.45605182816`, 10.304819183106764`, 1.`, 0, 0, 0.36984252950704843`},
{0.13576335201419282`, 2.033104701519708`, 4.843203210944571`, 1.3428754702553487`, 0.2671111595179354`,
0.18962864690949777`, 0.12183777475102511`, 1231.483599580866`, 0.14101782654161166`, 2.1508880203421494`,
14.062304512997493`, 0, 0, 0, 5.347448423874563`, 0.2888437794400696`, 0, 0, 0.0124958487831717`, 0, 0,
8.389280656918954`, 0.024235404529519552`, 0.3724242128799225`, 0.1804585200032418`, 0, 0, 0.1728591538078442`},
{0.25963449652183795`, 2.4481737185281203`, 5.334705083491999`, 0.8989699854247848`, 0.7130966522497681`,
0.695912989145927`, 0.04220235704717508`, 140.4631283114693`, 0.11660454588716207`, 7.56536482611441`,
51517.39459822761`, 0, 0, 0, 208.87380364420574`, 3.442295227607988`, 0, 0, 10869.394213417492`, 0, 0,
120.39052439492362`, 40315.28134425745`, 11.877411001165987`, 1.000000000000663`, 0, 0, 0.8875492589395017`},
{0.17509339161516935`, 3.0306683644318824`, 1.9789343166711684`, 0.8960196738550836`, 0.2152496986108292`,
0.6250130758758401`, 0.02087454134280109`, 59.51435674676293`, 0.2018230747082378`, 3.546682887992379`,
97708.20265429444`, 0, 0, 0, 43.93160268359486`, 0.2919679650464629`, 0, 0, 27889.746880935327`, 0, 0,
12.640829644198089`, 69761.57675245129`, 12.915017213597034`, 1.0000000000000742`, 0, 0, 0.34770386581547696`},
{0.10593857456933181`, 2.955002391424368`, 8.212267239719637`, 0.932578721051657`, 0.3205115590912122`,
0.6246255275862143`, 0.01773467921096997`, 102.27924186693049`, 0.015490300413002644`, 6.09864277306975`,
227762.3423639426`, 0, 0, 0, 348.4297661045804`, 2.000016737823238`, 0, 0, 90445.83706279755`, 0, 0,
84.42934633080581`, 136881.47220232585`, 13.879182444493322`, 1.`, 0, 0, 1.0937553363123036`},
{0.10583476133380088`, 3.0591620947997713`, 7.753926172578531`, 1.2866948671665401`, 0.7481949494801159`,
0.21582592530952183`, 0.02844379415675546`, 101.39583001744663`, 0.09278999192622717`, 1.6976300926057526`,
8561.006458364533`, 0, 0, 0, 76.9999401704853`, 0.6711330953265338`, 0, 0, 3365.5422993511315`, 0, 0,
29.330070368408165`, 5088.448085866253`, 3.6480650121362896`, 1.0000000000048883`, 0, 0, 0.1765227141574361`},
{0.23086787628538824`, 0.6622997055352342`, 7.183940408138384`, 1.353990838360309`, 0.25975387914696335`,
0.6772244715959861`, 0.055054708296352145`, 683.0312933373987`, 0.1367743885435413`, 6.244797760097583`,
130.76856833688336`, 0, 0, 0, 73.99648015698148`, 5.413766956174858`, 0, 0, 0.03135035642711215`, 0, 0,
51.22194658444273`, 0.10339700298737761`, 2.1333186171942065`, 0.38973108966027525`, 0, 0, 1.1379606702416871`},
{0.20388536464835005`, 2.2276602475557015`, 5.0708546023033385`, 0.8827779974574592`, 0.1554825605038257`,
0.2618026330626365`, 0.006155066765134505`, 1063.8834649875878`, 0.12744889948308613`, 2.149276150894316`,
16.705175788430296`, 0, 0, 0, 15.34018138969411`, 0.039960950176043446`, 0, 0, 0.009472157209855754`, 0, 0,
1.271706002310371`, 0.02758906038197066`, 0.4430659186650144`, 0.20511098268605288`, 0, 0, 0.2390010750085391`},
{0.10738337044420926`, 3.7501964356372497`, 1.0776334746380822`, 0.9682422375974065`, 0.09998974406674432`,

0.504170653500009`, 0.22038799040453166`, 2051.555691372483`, 0.09521018879793502`, 2.016584418866474`,
10.745437156692924`, 0, 0, 0, 2.7270488003687032`, 0.14481444957865908`, 0, 0, 0.04548081903495484`, 0, 0,
7.758323323409392`, 0.0697697662648037`, 0.23494318021597405`, 0.6032609361887338`, 0, 0, 0.425543942667319`},
{0.2588804970943782`, 0.5492314132783744`, 9.644794872912858`, 0.9613308813426055`, 0.2817033035077452`,
0.232857138958534`, 0.012136672860729404`, 78.78277994386292`, 0.023082056754425706`, 4.302644367094656`,
137772.13272659492`, 0, 0, 0, 282.3215864311667`, 5.393619556181917`, 0, 0, 29253.584769193883`, 0, 0,
42.319218450395255`, 108188.3223834497`, 11.63145159048731`, 1.0000000000005516`, 0, 0, 0.3402366293837788`},
{0.08494913534264076`, 3.4349155581389494`, 7.0407634051690415`, 1.119369452553694`, 0.9329533821244145`,
0.5630698272627359`, 0.4838120213175008`, 71.45974855722541`, 0.09271025542437755`, 2.443770975165119`,
95890.81008474117`, 0, 0, 0, 20.627754674663144`, 2.5143886386789522`, 0, 0, 43253.517140245785`, 0, 0,
123.38160934580272`, 52490.698308457`, 7.800929113162356`, 1.0000000000001543`, 0, 0, 0.38065468704163996`},
{0.2312360951475228`, 2.6472972630846083`, 4.924168333426426`, 0.8758299082609615`, 0.33581096057577486`,
0.16094039113701897`, 0.009823651093858611`, 148.81624524939704`, 0.12101804849376735`, 7.0744188173362765`,
9248.687097685755`, 0, 0, 0, 240.04361169803227`, 0.8432575827842137`, 0, 0, 2085.7403087632792`, 0, 0,
31.890764156856875`, 6889.977778431549`, 9.96795948882698`, 0.9999999999999839`, 0, 0, 0.4414456588569006`},
{0.2359361250604497`, 3.266770315195857`, 7.411396165562348`, 1.2275925063587856`, 0.15344120088912327`,
0.536013355113413`, 0.3013372435966537`, 712.0245065436816`, 0.039861802547139946`, 9.042443794820379`,
149.79165608831158`, 0, 0, 0, 30.432918836211947`, 2.502074871248999`, 0, 0, 0.020469798523094447`, 0, 0,
116.76719879705296`, 0.06899378491893797`, 2.9323484758205605`, 0.30073232914685155`, 0, 0, 1.5362696871862067`},
{0.26580884355261647`, 3.957059703696273`, 5.486829732050921`, 1.3210508471658353`, 0.3282573265157698`,
0.6164824430541005`, 0.042206581416411024`, 114.97331259087073`, 0.22266675421103638`, 9.071401748075406`,
55556.658656069565`, 0, 0, 0, 252.70336144734682`, 2.6069030186974453`, 0, 0, 11496.95056251396`, 0, 0,
147.3667269533114`, 43657.01619147745`, 17.070419450393373`, 1.0000000000003424`, 0, 0, 0.8726544584105114`},
{0.24965572729559277`, 1.088568489897936`, 8.460543344934337`, 1.1505850947024536`, 0.8975822933983539`,
0.5110185880609677`, 0.06613687599714729`, 1096.4114980052268`, 0.1003107894781316`, 4.101976455241168`,
127.8628755202702`, 0, 0, 0, 66.03446139642045`, 3.7254938768137342`, 0, 0, 0.03663259707346264`, 0, 0,
57.93507490867333`, 0.13065053807286173`, 0.7743986168113812`, 0.4928551527107755`, 0, 0, 0.19992525819466803`},
{0.1942611669549973`, 1.9123387865104826`, 2.4307010631404715`, 1.2953041584793583`, 0.36588469810004653`,
0.5550679926918506`, 0.01452356295191177`, 246.06581986793796`, 0.04070230178651557`, 1.4358727366404163`,
97040.48025496164`, 0, 0, 0, 23.86095189089513`, 0.17207659089975044`, 0, 0, 25697.386388061175`, 0, 0,
4.7009819861155515`, 71314.34667768893`, 1.2860653886072495`, 1.0000000000013012`, 0, 0, 0.16750805287136414`},

{0.20522561065351508`, 3.886130079640968`, 5.862633336878224`, 1.1071510042707493`, 0.46587070079395265`,
0.3092363340879616`, 0.06237106286442892`, 3704.443052142306`, 0.23732711327647033`, 5.5948716983231535`,
18.24073513341799`, 0, 0, 0, 9.786440843885856`, 0.14930982802442025`, 0, 0, 0.004010767270201246`, 0, 0,
8.289105912453135`, 0.01175874517453023`, 0.32380663667984183`, 0.0748705453448042`, 0, 0, 0.4827127466802505`},
{0.24537724051627174`, 3.881922170050893`, 2.190905545388226`, 1.4415825883984728`, 0.23029515887672103`,
0.5607538040686315`, 0.02143037658176177`, 612.4231659367224`, 0.18957440226421673`, 2.1066205948825285`,
44.96270739213868`, 0, 0, 0, 31.930028431004782`, 0.16876761013321223`, 0, 0, 0.7756196001635901`, 0, 0,
9.359181819466041`, 2.718848531121083`, 0.83486117459084`, 0.9996389277788165`, 0, 0, 0.48390691267912467`},
{0.06117754469042569`, 1.77753347893454`, 8.494748688583062`, 1.4528026068281314`, 0.13374021962928717`,
0.2747744172973582`, 0.02769785108344557`, 761.9216006517844`, 0.1674213538626742`, 9.223974549251146`,
104.37122598019772`, 0, 0, 0, 75.46905412178918`, 1.0935522749806401`, 0, 0, 0.013385042810014852`, 0, 0,
27.768939710615967`, 0.011698057924200568`, 2.6509876076003303`, 0.1797848348361325`, 0, 0, 1.0099053738560961`},
{0.2543463901897882`, 3.8184481545424687`, 6.435093510144283`, 1.0179149591357712`, 0.6456387658124723`,
0.39719113288117625`, 0.03560663844887868`, 836.6099873310699`, 0.05172688927212099`, 4.4940967912431145`,
92.50705857694102`, 0, 0, 0, 61.6106448630882`, 0.5538624651478468`, 0, 0, 0.026661635914319946`, 0, 0,
30.21278725591626`, 0.09687558359092514`, 1.142432810634279`, 0.42882045936404467`, 0, 0, 0.3484747525300642`},
{0.25340300959745826`, 3.7481040027914663`, 6.564583042000866`, 0.9665595115932711`, 0.6900722172611409`,
0.5320596915161446`, 0.007953381223150922`, 557.3521763213881`, 0.16633866213427612`, 7.2685179596650595`,
289.9526955762029`, 0, 0, 0, 260.1185272102084`, 0.5361207656716079`, 0, 0, 0.07628259979028275`, 0, 0,
28.706234111334048`, 0.27614629095393467`, 2.7826107197815406`, 0.8100390294346935`, 0, 0, 0.5893456868060367`},
{0.21905085798311597`, 3.1393917093979224`, 6.344370247647537`, 0.9036844621009067`, 0.45657431516696256`,
0.6738956741194089`, 0.01713634873689236`, 595.7124001741428`, 0.1327223790362297`, 6.393427116779321`,
189.5667522528146`, 0, 0, 0, 152.54896031016338`, 0.8021561218626119`, 0, 0, 0.04165305822734033`, 0, 0,
35.9754611231182`, 0.1303448306044758`, 2.4147933341625163`, 0.6244288343564561`, 0, 0, 0.8950860382912293`},
{0.26756748312672074`, 2.582420366138944`, 0.22100971269994044`, 1.2986139421663903`, 0.40694219440887625`,
0.4206092167171217`, 0.07540978051777308`, 286.7043124505423`, 0.12484831726628037`, 7.450517421109604`,
61364.44550581311`, 0, 0, 0, 6.200064112029521`, 0.1734064473586258`, 0, 0, 12722.247986401935`, 0, 0,
6.397262018267335`, 48629.426763365314`, 5.386502904980735`, 1.0000000000018558`, 0, 0, 0.37410626848781486`},
{0.1649279191778415`, 1.2798961157951103`, 8.443353077105307`, 1.1073246765756934`, 0.5543130008347743`,
0.4677559431287117`, 0.04549043803104127`, 88.19757502008439`, 0.08859698434873292`, 4.4693006714200685`,
66968.59336970124`, 0, 0, 0, 191.38166699056654`, 6.279472528682782`, 0, 0, 19861.102833137844`, 0,

```
0, 114.81532141004523`, 46795.00518352212`, 11.23371082872364`, 1.`, 0, 0, 0.5506820011070802`},
{0.24671033429293737`, 0.7439602453864245`, 2.8830481632165945`, 1.0433060628070765`, 0.2605457162416671`,
 0.3561357593967020 6`, 0.02699949405773168`, 552.8129873973498`, 0.15941770646667952`, 8.472285283746757`,
 162.91448578185557`, 0, 0, 0, 118.10149297053503`, 3.8100428607022123`, 0, 0, 0.10721732148495285`, 0, 0,
 40.49314887972586`, 0.3778803032222056`, 3.388528707302103`, 0.8785474011840592`, 0, 0, 1.0187096172125372`},
{0.15869950794106824`, 3.1033559606377414`, 9.144330738839969`, 1.4178115100024233`, 0.3048291795319098`,
 0.2869274730275907`, 0.17534043095376647`, 52.531250426358234`, 0.14088030152015352`, 4.046496400392222`,
 118882.02386303202`, 0, 0, 0, 86.72360529670013`, 4.535941489561502`, 0, 0, 36297.74689231964`, 0,
 0, 201.0948722676384`, 82291.9224454366`, 16.278288552748755`, 1.`, 0, 0, 0.28610578650798163`},
{0.2764621058346162`, 2.9381918751992178`, 0.9446276562373105`, 1.4121699137634485`, 0.5065538844804354`,
 0.6085910850890284`, 0.12806524333884275`, 90.5764532701865`, 0.169606806379912`, 4.122343563126204`,
 163538.67889342218`, 0, 0, 0, 11.02146588671046`, 0.4594339939708134`, 0, 0, 33035.51459589125`, 0, 0,
 19.28436040393394`, 130472.39903586148`, 9.611199253124855`, 1.0000000000021017`, 0, 0, 0.28843535938144205`},
{0.23050133288232844`, 1.3238077162346595`, 3.1108482993453865`, 1.1555015486294062`, 0.6747942262463269`,
 0.6285668584610278`, 0.027391537177886824`, 109.70658223761104`, 0.027210691652561736`, 1.9248854769177905`,
 486392.0716893193`, 0, 0, 0, 34.90003051867164`, 0.6767899247721898`, 0, 0, 113290.87258019214`, 0, 0,
 12.799138924047147`, 373052.8161876614`, 3.7909521704860194`, 1.0000000000003244`, 0, 0, 0.1823027494501815`},
{0.16511179970471734`, 1.257711050374506`, 7.3270141910702415`, 1.3219934358839476`, 0.5519839493462388`,
 0.6927265248357952`, 0.057669326830527015`, 282.0308127392517`, 0.18961708461999577`, 6.119113848125975`,
 386.1275084674433`, 0, 0, 0, 211.46831373242944`, 8.972245900066515`, 0, 0, 1.3327302751820447`, 0, 0,
 161.20704021701454`, 3.143564203660761`, 4.952134299030402`, 0.9996861392736609`, 0, 0, 0.957268756867683`},
{0.18051596911200918`, 3.1154638731836117`, 5.271301524292921`, 0.9821224785779399`, 0.579876513557519`,
 0.2578243252832043`, 0.01324703728258807`, 298.19257336110485`, 0.04626579120349833`, 3.252038865320179`,
 134.57577658022095`, 0, 0, 0, 112.5394751444557`, 0.458476026729155`, 0, 0, 0.30849162719865014`, 0, 0,
 20.40522139993495`, 0.7955380720957889`, 2.311269900478183`, 0.9900243657705059`, 0, 0, 0.2399256338833419`},
{0.2700603276661351`, 3.8874233694867435`, 9.935191315459868`, 0.9369398631417749`, 0.9680175802467335`,
 0.2817307366148821`, 0.03116132832319949`, 267.3428425281449`, 0.2310886914635361`, 4.719763737676903`,
 363.02233411688366`, 0, 0, 0, 250.8215835988255`, 1.9463754754179983`, 0, 0, 0.4377831251064967`, 0, 0,
 108.09122155622579`, 1.6889693458994506`, 3.6407035554075304`, 0.9982322319737682`, 0, 0, 0.21170419265869264`},
{0.2505990837413898`, 3.9386610341401305`, 4.197732331385515`, 1.4863985983426793`, 0.6450186838764853`,
 0.5983146588433161`, 0.0058599277111039`, 4347.60789332823`, 0.1797443484893408`, 5.002549103667693`,
```

25.07650818288479`, 0, 0, 0, 23.084273303790066`, 0.033386861391585934`, 0, 0, 0.012068040108825314`, 0, 0,
1.8785647145039597`, 0.04320342562608318`, 0.24923273258062129`, 0.15866413215865827`, 0, 0, 0.48751962110478475`},
{0.1538579927698617 3`, 3.328452062918277`, 1.1907303286310018`, 0.9499432416831306`, 0.24618506024325426`,
0.6220480490433855`, 0.08397939385457624`, 160.09510109398894`, 0.21511864519397922`, 7.34323009363505`,
32608.290024555277`, 0, 0, 0, 32.83289982363894`, 0.7926586587382974`, 0, 0, 10174.254449071743`, 0, 0,
37.69037639810738`, 22362.71953520019`, 10.005929227107977`, 1.0000000000007632`, 0, 0, 0.7730645504252401`},
{0.10621732051626104`, 1.365898117434102`, 6.864395407461604`, 0.9825773772303991`, 0.8367483775504436`,
0.5624371522540671`, 0.07041397408927531`, 60.72834180417396`, 0.20758521960363197`, 6.929584578355666`,
58409.78224203836`, 0, 0, 0, 200.48360499742128`, 9.556215528057246`, 0, 0, 23045.002754297864`, 0, 0,
186.46881142239891`, 34968.263483591145`, 25.45969464168525`, 1.0000000000002776`, 0, 0, 0.9041156194737352`},
{0.2268285366732684`, 3.377330494378361`, 0.792106071900994`, 1.4321394301198889`, 0.4771428999999012`,
0.6107585820948693`, 0.0427110994331284`, 2178.207920314213`, 0.06460930790004932`, 5.987666522418898`,
44.45312816329743`, 0, 0, 0, 26.001806075000907`, 0.3142910404330502`, 0, 0, 0.7008183984011627`, 0, 0,
15.163781642349205`, 2.270937311186252`, 0.6488609793179442`, 0.9997842411559313`, 0, 0, 1.1733277088559524`},
{0.27738294555080567`, 1.5184858354681863`, 2.5078605202307482`, 1.4658418288357904`, 0.7951974935096349`,
0.3102987260459962`, 0.028082255793227213`, 4738.743053997216`, 0.039514889508313555`, 1.270204126939964`,
5.0477582232509945`, 0, 0, 0, 3.561836097843187`, 0.061976471395522405`, 0, 0, 0.015886590095679956`, 0, 0,
1.3444341992342788`, 0.0629524165071266`, 0.05642032085155145`, 0.20302857666984309`, 0, 0, 0.08442698543933684`},
{0.12722141666194153`, 2.144800891916389`, 0.9267204145449971`, 1.474327309271881`, 0.07507296673368935`,
0.4110764361383803`, 0.01396212306727472`, 341.96044988390173`, 0.08796076167353961`, 8.378052186889676`,
40703.317562712655`, 0, 0, 0, 52.1193032669522`, 0.32317263433548166`, 0, 0, 14424.73142852852`, 0, 0,
9.902013633795866`, 26216.210961506204`, 5.264910612610111`, 1.0000000000000517`, 0, 0, 0.7439299038135906`},
{0.14199823373416764`, 1.4769393574961134`, 9.82023825383516`, 0.8826223667346553`, 0.41403084809983204`,
0.6165765653923829`, 0.060760206852004485`, 76.65984772713675`, 0.1884299459763023`, 7.821991164404671`,
34702.494111446664`, 0, 0, 0, 352.6424061133488`, 13.480607772427225`, 0, 0, 11243.657840386924`, 0, 0,
284.4291454580839`, 22808.279343518156`, 23.13840294977153`, 1.0000000000009193`, 0, 0, 1.1651935011061372`},
{0.2704769900712498`, 1.6373927417773038`, 3.0886177841931914`, 1.4817168381054997`, 0.05399471731043759`,
0.4806448506103316`, 0.008090658829532001`, 56.76295821829396`, 0.22108849119145935`, 4.3771215003685064`,
185341.5555474955`, 0, 0, 0, 94.8985104549962`, 0.4455467403882427`, 0, 0, 38083.33479382308`, 0, 0,
10.421928554774903`, 147152.36809869987`, 16.13816173716126`, 1.0000000000006162`, 0, 0, 0.2641975271580191`},
{0.15635355369607173`, 1.2350233440589857`, 3.2938536959067304`, 0.7696047462951592`, 0.9490314349758091`,

0.5590905433465692`, 0.008743912631106248`, 94.37874467585033`, 0.17474749461052458`, 8.353315585312398`,
48722.418553545765`, 0, 0, 0, 198.80115788512518`, 1.3149849045993227`, 0, 0, 14998.328776001294`, 0, 0,
23.200529346647773`, 33500.600051855`, 19.22882263139428`, 1.0000000000000067`, 0, 0, 0.8416312746893391`},
{0.16339273559725687`, 2.0059391082075457`, 1.9960227683476044`, 0.8832597488866586`, 0.2201232888636404`,
0.41940637033719763`, 0.260701203173729`, 668.3313383270491`, 0.16081430237854888`, 8.573990161395482`,
142.88418368381315`, 0, 0, 0, 32.15249716324679`, 3.6830814509823795`, 0, 0, 0.45145034900734454`, 0, 0,
105.54338744627644`, 1.0537672501521091`, 2.906524164501829`, 0.9954233907123432`, 0, 0, 1.2681171757710217`},
{0.17131668518162624`, 1.9273467762184806`, 5.930228637778157`, 0.8979121960709446`, 0.761163722566164`,
0.6467717629026779`, 0.01019608706680365`, 260.442341207733`, 0.11039361670332914`, 7.204688135978646`,
387.74531160173905`, 0, 0, 0, 329.8809395731619`, 1.6506287710266334`, 0, 0, 3.048573016883556`, 0, 0,
45.44762915102363`, 7.4610203398090915`, 6.551975816389322`, 0.9999999981344533`, 0, 0, 1.4411322673776423`},
{0.11942926109779373`, 3.6640895220352094`, 2.8978096019149504`, 1.1395608488550686`, 0.02531057599926112`,
0.3982329172386545`, 0.26228943719870584`, 89.66215495367044`, 0.22730124409898161`, 8.643250664580492`,
47645.08297986628`, 0, 0, 0, 44.48934368346081`, 2.9303698592837346`, 0, 0, 17532.133977629648`, 0,
0, 153.38767852984697`, 29912.140091654583`, 20.672779457443163`, 1.`, 0, 0, 0.7481501765293876`},
{0.06995397995088021`, 2.4465420953300523`, 1.0825256418482638`, 1.4045898231548657`, 0.8050454938859057`,
0.5373350569389552`, 0.3667462640330922`, 406.36701228872533`, 0.024784828878876453`, 8.741309230400322`,
82872.9610250695`, 0, 0, 0, 13.941054607322318`, 1.8390597231893664`, 0, 0, 41409.94425064903`, 0, 0,
64.27624326584012`, 41382.72014109954`, 4.874167506966916`, 1.0000000000001976`, 0, 0, 1.2942419330446062`},
{0.25135395656732756`, 1.8293827828852027`, 0.6280251816297326`, 0.776655925877336`, 0.712342015777852`,
0.6529270906121196`, 0.026921744082008515`, 2433.232128346219`, 0.014846384799260759`, 4.779446799164496`,
35.958359007037274`, 0, 0, 0, 19.5985766740256`, 0.27110396238749407`, 0, 0, 1.960361725435269`, 0, 0,
7.085041730909113`, 7.039209657018692`, 0.4742029272475706`, 0.999999997941037`, 0, 0, 1.0667052482680945`},
{0.22686431298761822`, 3.544454161776195`, 7.257561779040259`, 1.4835566223233323`, 0.41530778886586006`,
0.5693420006079292`, 0.0281206594086524`, 300.82869963510313`, 0.2233455291441253`, 1.8496714258079727`,
93.02073939466582`, 0, 0, 0, 66.25819263823034`, 0.505074282199308`, 0, 0, 0.15814807028218458`, 0, 0,
25.57446630782091`, 0.5125450473555149`, 1.397168661979499`, 0.8715748770394933`, 0, 0, 0.27989129610532903`},
{0.11505780978488611`, 2.035276516222745`, 0.3473218826840405`, 1.3302263404025527`, 0.8863987944255065`,
0.20525449010517516`, 0.166947674879067`, 1822.5924091420022`, 0.2368153750944742`, 8.635263957438244`,
911.616911200983`, 0, 0, 0, 7.379620742389669`, 0.5431642183314624`, 0, 0, 335.85773314574965`, 0, 0,
15.792705400321557`, 552.0436453580962`, 1.0026142414284305`, 0.9999999999995264`, 0, 0, 0.6233453797553505`},

{0.26450836027154073`, 2.000822664278666`, 6.699190267572199`, 1.2017845913999912`, 0.3438445280396565`,
0.6571515800951808`, 0.025420529665258214`, 670.8719376728277`, 0.0689542698910992`, 7.858447976519758`,
180.07809198342397`, 0, 0, 0, 132.63612120991124`, 1.597069022061446`, 0, 0, 0.03449699947507593`, 0, 0,
45.64931279654147`, 0.13035349664919996`, 2.681825195741119`, 0.4582561505051592`, 0, 0, 1.2573024367259216`},
{0.1895156330025849`, 2.3307833365396586`, 5.573081915828208`, 1.037167544233136`, 0.6437280244785568`,
0.6985848360999776`, 0.020283589241000377`, 257.16539910388553`, 0.09483519874959423`, 5.443513900146426`,
297.2872756457915`, 0, 0, 0, 215.75479832314957`, 1.7792133335823193`, 0, 0, 5.512060772430821`, 0, 0,
59.24229700089792`, 14.923166949084864`, 5.187641248734884`, 0.9999999991788738`, 0, 0, 1.3158340515296152`},
{0.05605442916152037`, 2.091827585999483`, 5.134093728882359`, 0.7852275512421112`, 0.9528052242020535`,
0.26916872175155115`, 0.16382211865694157`, 830.5018741809612`, 0.0523186960509337`, 8.350099727317033`,
236.07075787885512`, 0, 0, 0, 72.08490530847557`, 5.306970864413325`, 0, 0, 0.049610772082233995`, 0, 0,
158.58954360393372`, 0.03972719299100841`, 2.055474885950989`, 0.7407769595263233`, 0, 0, 0.2990840519882315`},
{0.160097742020257`, 2.0916642582432887`, 7.779572730473025`, 1.4293729759208196`, 0.262481852877007`,
0.6066507214539851`, 0.010299825795425889`, 166.88640436294955`, 0.11119499215436468`, 1.1831617215189443`,
89.48839484073358`, 0, 0, 0, 73.4242197789048`, 0.34171599536022135`, 0, 0, 1.6594539454847457`, 0, 0,
10.210787628071511`, 3.7953547094101823`, 1.7430679583449078`, 0.9999994370652392`, 0, 0, 0.29070295651371664`},
{0.096645259209576`, 1.264318941007299`, 4.097121191163998`, 1.119171761441141`, 0.3848067085465605`,
0.38298305619587747`, 0.22103546881327146`, 186.61459180270506`, 0.035388155328102466`, 2.6745014933563738`,
41233.10616888702`, 0, 0, 0, 23.586631923732092`, 3.578762721753753`, 0, 0, 17281.562258468384`, 0, 0,
64.63853563548703`, 23859.729485944317`, 3.217690631044048`, 1.0000000000031333`, 0, 0, 0.3678698275363588`},
{0.08994789013179427`, 1.57009542864993`, 6.7950768142652755`, 1.2471941772808341`, 0.17502080201658954`,
0.4646155607499266`, 0.031711372215814826`, 510.77004725104206`, 0.22094423048581935`, 5.498912040996139`,
117.8921968426536`, 0, 0, 0, 81.79460035858277`, 1.537041688784525`, 0, 0, 0.03209192139954003`, 0, 0,
34.47574470292791`, 0.04123715171665078`, 2.4579960392072966`, 0.42291179336679585`, 0, 0, 0.8621397757292277`},
{0.09559160148361812`, 1.3237772094663507`, 8.951678422145957`, 0.7806574543205589`, 0.8827653082752778`,
0.45488871998545977`, 0.011151733542884613`, 3151.7776331194414`, 0.22534056788365686`, 6.821338260162536`,
66.03533983615444`, 0, 0, 0, 56.97534787323443`, 0.452281129700052`, 0, 0, 0.005725595008703993`, 0, 0,
8.55313502526606`, 0.007818839947550634`, 0.4472120082534556`, 0.14592939415636963`, 0, 0, 0.3201932876358072`},
{0.20804533970301103`, 1.865488871682949`, 2.4561594295175926`, 0.8614398462681598`, 0.7232950760420711`,
0.3427980526897182`, 0.02113398228875948`, 356.75904569388376`, 0.23964513558905903`, 4.163032360670947`,
1984.185422153315`, 0, 0, 0, 65.34725459711909`, 0.6934759746045214`, 0, 0, 478.249185246399`, 0, 0,

18.48102447720318`, 1421.3930601039315`, 2.5848339324844942`, 0.9999999999998133`, 0, 0, 0.5092596014670594`},
{0.23422673966465207`, 3.962742244147649`, 9.344278992351082`, 0.9436428211653483`, 0.6202800294740252`,
0.15583995298414144`, 0.0468134491479599`, 1145.0934679847246`, 0.11614669946806178`, 8.27346918244088`,
76.81217943247714`, 0, 0, 0, 46.60407659757578`, 0.523840442245332`, 0, 0, 0.006317674776099342`, 0, 0,
29.654923566836562`, 0.02113954807246444`, 1.4917014446928603`, 0.13430322141815954`, 0, 0, 0.3810945602927282`},
{0.23911413452544644`, 3.701379132317136`, 3.3596187018850023`, 1.2070387760959833`, 0.14815711868787962`,
0.5552188532365824`, 0.010060549211211993`, 324.2801725236705`, 0.014653386006449387`, 6.641284984634726`,
40898.704017999575`, 0, 0, 0, 170.5122422821959`, 0.4448402950946854`, 0, 0, 9217.586941291758`, 0, 0,
23.521751221103845`, 31486.504626857717`, 4.790120164660258`, 0.9999999999999992`, 0, 0, 1.228976049617983`},
{0.16379493488608782`, 3.773301948253276`, 6.66686177778092`, 1.3720180958436647`, 0.9115747518300852`,
0.36809943749279594`, 0.006089940921734969`, 188.41929546062013`, 0.11766502525697531`, 3.8904832664355844`,
225.71959389388627`, 0, 0, 0, 199.575274552741`, 0.30891095199023727`, 0, 0, 2.6858382477627725`, 0, 0,
16.651632814021976`, 6.284667155812326`, 4.628680627825107`, 0.999999989715336`, 0, 0, 0.5283226316289775`},
{0.27173801863077446`, 2.1930338431922545`, 7.869758966833125`, 1.0214571559787395`, 0.457397629072652`,
0.6590886785471264`, 0.009120521470678952`, 4004.0778102470904`, 0.1596904202504179`, 8.692703920363371`,
38.1367075420029`, 0, 0, 0, 33.753326790727044`, 0.13414750630735928`, 0, 0, 0.003711532500213427`, 0, 0,
4.20271459016979`, 0.014408064109883373`, 0.4865355154945623`, 0.07509136504173985`, 0, 0, 1.177801458927541`},
{0.2361473928644357`, 1.7414292069064263`, 7.944932185587515`, 1.1578150203530142`, 0.6270752096378571`,
0.30275674150027165`, 0.11839453780639704`, 3486.2324040163485`, 0.1006900893181421`, 6.697770970769804`,
28.187080331557592`, 0, 0, 0, 10.7176384718845`, 0.6744063118203748`, 0, 0, 0.003990110264231114`, 0, 0,
16.777583553229217`, 0.013460773373488557`, 0.40472902945737355`, 0.07144044041278341`, 0, 0, 0.45119161414259945`},
{0.08448906159522324`, 2.163318965633623`, 2.5852320656449397`, 0.9870547358990118`, 0.27911648709335135`,
0.1950863385031143`, 0.04472304557991584`, 1858.3083947017237`, 0.05117109151128901`, 9.531470241589851`,
43.9374515470728`, 0, 0, 0, 27.20392295454003`, 0.5235725920666632`, 0, 0, 0.01262339163114912`, 0, 0,
16.18077883291099`, 0.015236264472349768`, 1.0885483474044169`, 0.24025321327724714`, 0, 0, 0.717679656428075`},
{0.1142146683211071`, 3.422389653131356`, 1.8177299602895634`, 1.2969315528452774`, 0.1391750354375907`,
0.5170758706651055`, 0.01897326911835137`, 87.52693577556593`, 0.11000871045324723`, 3.4664980752399295`,
128654.38482185059`, 0, 0, 0, 40.661462706846976`, 0.2172512043204479`, 0, 0, 48867.98878424787`, 0, 0,
10.621689625666033`, 79734.87329303497`, 8.663006262889986`, 1.0000000000004021`, 0, 0, 0.3752836561740276`},
{0.14347242790835574`, 2.011431525472254`, 0.8946353742157331`, 1.3818589873326788`, 0.974530392330166`,
0.4363768778472087`, 0.006586014840232095`, 142.93650600689625`, 0.2189385420017279`, 6.889239014732425`,

48131.69261379855`, 0, 0, 0, 44.90838995974941`, 0.14027046362465767`, 0, 0, 15766.812263808344`, 0, 0,
4.030634751817778`, 32315.754798054648`, 10.273017823465416`, 1.0000000000012867`, 0, 0, 0.5505999805201837`},
{0.07350179944910268`, 2.001951652427108`, 6.856579719843172`, 1.3883617808830775`, 0.83650706130597`,
0.6246458892820506`, 0.03443555932639553`, 159.6804633773726`, 0.22561794329975743`, 6.769156049603524`,
4954.5855265563205`, 0, 0, 0, 283.1279495553944`, 4.565203579291466`, 0, 0, 2212.7962654690145`, 0, 0,
130.5616692746957`6, 2323.4929618032425`, 10.254907506313257`, 0.999999999999996`, 0, 0, 1.5275530894221712`},
{0.2038713609791254`, 2.9048730428426977`, 6.578839990238183`, 1.1970767733981913`, 0.3047670438201835`,
0.4988995484689318`, 0.027219703475022124`, 126.68486054839573`, 0.1069997024432573`, 5.709865936189522`,
40974.57930763331`, 0, 0, 0, 226.06330895187114`, 2.0240191376584638`, 0, 0, 10393.097476077466`, 0, 0,
83.99312330259689`, 30269.356103380353`, 10.02578431383393`, 1.0000000000000617`, 0, 0, 0.7253838425013082`},
{0.10518373092933642`, 1.253339618555911`, 0.9435760784539173`, 1.312750209379657`, 0.30938419913876847`,
0.5412461470886345`, 0.010398682812166098`, 560.2137041073427`, 0.038740677468453555`, 9.486718448691846`,
38653.282609218615`, 0, 0, 0, 65.2084102817794`, 0.5035938328773518`, 0, 0, 15415.215452575325`, 0, 0,
9.016772891508706`, 23163.28391973476`, 3.7870910129018425`, 1.0000000000002616`, 0, 0, 1.2626216594838782`},
{0.21800976245075693`, 1.265555352818649`, 7.66080710349685`, 0.9976040672991493`, 0.42066904207729006`,
0.6052137627060103`, 0.1434637338398527`, 296.0621032273413`, 0.0508815854649306`, 9.05489394101457`,
493.94820755870967`, 0, 0, 0, 166.21017956480406`, 17.147494254154815`, 0, 0, 0.13969693281877602`, 0, 0,
310.0157591538936`, 0.43507564484206285`, 6.863216638882216`, 0.939856592738258`, 0, 0, 1.2402603819361724`},
{0.14099839999887143`, 0.709118554874764`, 1.895334494340693`, 1.0269112445912816`, 0.6721979672345315`,
0.244304954855444`, 0.0716581941489167`, 50.83934571528565`, 0.0295628703988986`, 3.2095234353654245`,
655663.7336615705`, 0, 0, 0, 23.621931809191572`, 2.1142737290270897`, 0, 0, 217504.7788812532`, 0,
0, 21.41815330481962`, 438111.7973480704`, 13.082745302398354`, 1.`, 0, 0, 0.16040883736982242`},
{0.16175267254619935`, 2.3816849613140647`, 5.017466007223192`, 1.1449585227321033`, 0.0709392075018902`,
0.21629140918390133`, 0.24748736884470657`, 967.6089395954832`, 0.2384053881049194`, 4.566103496741098`,
35.429388262638646`, 0, 0, 0, 8.564661952052175`, 0.7658602146194249`, 0, 0, 0.012439427936528372`, 0, 0,
26.057682223254886`, 0.028744438766845865`, 1.0229335383745985`, 0.2080131004415453`, 0, 0, 0.4488813299605002`},
{0.15219039437367055`, 3.36973706117323`, 6.4489465173864`, 1.4529455229052564`, 0.15905243014518256`,
0.19120675078037952`, 0.09107249936889779`, 1785.557342737041`, 0.07266026496560668`, 8.481021831493972`,
36.83199429513763`, 0, 0, 0, 16.55151862672847`, 0.4122997081041004`, 0, 0, 0.006433867291016219`, 0, 0,
19.84773723870415`, 0.013988182862385986`, 1.0141345217055322`, 0.09088795337634381`, 0, 0, 0.6936745696539156`},
{0.1147282820108807`, 1.275793939396646`, 2.8627986987042053`, 1.2289286563020643`, 0.9300053983658252`,

0.5333937455451996`, 0.40966617997380966`, 55.29536930365472`, 0.09207723861615985`, 9.133686457942215`,
242169.74999445686`, 0, 0, 0, 33.420501392465006`, 9.363771984092077`, 0, 0, 91685.69284942663`, 0,
0, 170.66062210281106`, 150270.60036559936`, 36.07134541671736`, 1.`, 0, 0, 0.9721772722707703`},
{0.22214055647639447`, 3.77956922277194`, 8.844837651533133`, 1.3011494481281731`, 0.35962071678441987`,
0.36854620389209614`, 0.030699018263752917`, 153.2818434470183`, 0.18511332787655327`, 9.937679092387768`,
735.0843607721722`, 0, 0, 0, 508.36947740236917`, 3.9405772854692014`, 0, 0, 2.3794677209933597`, 0, 0,
212.76692325876556`, 7.551089766558006`, 14.339139975146336`, 0.9996366044087849`, 0, 0, 1.1984986068070864`},
{0.11158828176697494`, 2.207019844443386`, 8.337407964588238`, 0.903037166955644`, 0.0492331295750732`,
0.5332115600383506`, 0.10225753888572825`, 270.6598424430625`, 0.11536791182647016`, 7.224089837931905`,
285.305710775736`, 0, 0, 0, 119.29410569337824`, 5.100107165789035`, 0, 0, 0.042755126488042486`, 0, 0,
160.80053890977572`, 0.06815673002179955`, 6.268021685978404`, 0.63629750307533`, 0, 0, 1.3715190903481698`},
{0.11228066784233581`, 2.8383409912516155`, 8.777526606092515`, 0.8243326677721828`, 0.7215210925398621`,
0.47575335568293875`, 0.01179892034723288`, 1881.3977477117185`, 0.1350202375088484`, 6.891832227766253`,
80.58248619015855`, 0, 0, 0, 69.0124915017005`, 0.27686700739819575`, 0, 0, 0.00759389932312848`, 0, 0,
11.226328231763752`, 0.01218068696468851`, 0.7742526611713688`, 0.1796712976070458`, 0, 0, 0.48866565198440703`},
{0.18415971316226493`, 3.627641157385548`, 4.072786596426541`, 1.1318163513169097`, 0.431227341960456`,
0.35638232246082124`, 0.12315140656794354`, 98.11903649760767`, 0.20630818704214687`, 2.4221856850573946`,
46780.71874464393`, 0, 0, 0, 28.96562433074999`, 0.930472962822017`, 0, 0, 12862.707949272182`, 0, 0,
48.22031451096596`, 33839.8943775422`, 5.227483232810534`, 1.0000000000000322`, 0, 0, 0.1765867816192133`},
{0.2708373028347817`, 0.6873063866109219`, 4.963135106708849`, 1.480469510663712`, 0.1951402154410362`,
0.5673083531043152`, 0.10423026212852673`, 2550.63829867399`, 0.08286966503737198`, 2.6901867167370597`,
12.85023651233897`, 0, 0, 0, 5.281037722028744`, 0.6953741438742828`, 0, 0, 0.009483985989084084`, 0, 0,
6.827644145269933`, 0.036694531220102376`, 0.24632656232512776`, 0.12691534682763816`, 0, 0, 0.49314910108862897`},
{0.2248876472394919`, 0.40722939312005924`, 9.89488647226247`, 1.363579526182362`, 0.9039905300116995`,
0.6204673017769224`, 0.16140635189013572`, 124.79693831214993`, 0.1938029988590731`, 3.2922153328074497`,
607.7271815578437`, 0, 0, 0, 94.60010477672807`, 29.82360613522872`, 0, 0, 73.54053007529187`, 0, 0,
173.50070038715603`, 236.262239791105`, 6.48167985222469`, 0.9999999998845347`, 0, 0, 0.8061799251175958`},
{0.23989116476268346`, 3.6056249656972357`, 9.989783431378257`, 1.4358686490541388`, 0.7020019529209951`,
0.17871498413227982`, 0.15143540034751335`, 1116.6227738463333`, 0.03611482374706981`, 5.098351562607867`,
55.847101211837916`, 0, 0, 0, 18.27951553422904`, 0.7145506304896654`, 0, 0, 0.010683923267340962`, 0, 0,
36.80573703640343`, 0.03661398281199109`, 0.9428165209860951`, 0.14793545244619133`, 0, 0, 0.23568512626486382`},

{0.10260002089245757`, 1.5297774189947768`, 6.3178804183723365`, 1.026166177715942`, 0.11867434322817161`,
0.5793673959868294`, 0.011727656957059851`, 115.5782430006307`, 0.20678944231462698`, 5.80216317155719`,
19681.456005117754`, 0, 0, 0, 269.3042838635489`, 1.9361477435117007`, 0, 0, 7854.807909856284`, 0, 0,
42.31250139802688`, 11512.906509392828`, 11.510384197310032`, 1.000000000000001`, 0, 0, 0.9382767375968091`},
{0.15334449354379004`, 1.7605906069617427`, 2.299359928311352`, 0.8222860702278779`, 0.9712651951139977`,
0.6108690739001781`, 0.14744839931335676`, 2744.2788694525566`, 0.195814559949105`, 2.932280984329149`,
48.72012410108711`, 0, 0, 0, 15.723860461195601`, 1.2486687801329581`, 0, 0, 0.10717570972362964`, 0, 0,
31.405636078692456`, 0.23478292753951266`, 0.2223144863322389`, 0.9245430295453997`, 0, 0, 0.15885810180664878`},
{0.19466496130115485`, 2.7024357312795884`, 5.257508792652555`, 1.2867770062917014`, 0.4348182682381361`,
0.22799121214772633`, 0.23891047775514818`, 1782.3644894856986`, 0.18533305313279363`, 5.119329477376578`,
29.475101309884298`, 0, 0, 0, 7.131566233544068`, 0.5632302123113518`, 0, 0, 0.009551284205452419`, 0, 0,
21.744192152662627`, 0.02656143386047796`, 0.6086823375567861`, 0.1473319050615819`, 0, 0, 0.37746569564297744`},
{0.143202010216472 5`, 0.6405991610607145`, 3.208206356969493`, 1.3290628649451517`, 0.9481170196997712`,
0.4490165359974049`, 0.04841648328555665`, 413.10755212325614`, 0.04750626711264738`, 5.496555870600891`,
159.61465056709488`, 0, 0, 0, 90.34063160931055`, 5.947569486258707`, 0, 0, 2.9202464481556722`, 0, 0,
54.42868604639467`, 5.974073738620095`, 3.0529416034413766`, 0.9999999950670907`, 0, 0, 0.8934898423739168`},
{0.2767015708016651`, 1.3448440647834508`, 8.399713367158164`, 0.7521061389207052`, 0.4173808724493866`,
0.513702172075632`, 0.342860965595718`, 77.17931109285855`, 0.021177395775830554`, 7.103551277505744`,
437287.0815572779`, 0, 0, 0, 85.42046867657348`, 19.45261763785817`, 0, 0, 88192.83334910797`, 0,
0, 373.7248196396493`, 348615.6503021081`, 19.741930901909846`, 1.`, 0, 0, 0.6163697660060206`},
{0.2397400236888016`, 3.6266796947004982`, 2.6465237726626594`, 1.459013465973801`, 0.6743470274003898`,
0.6381986185617847`, 0.04750041433271958`, 90.95305912243636`, 0.11079011330617267`, 9.85823320912452`,
219618.26699193037`, 0, 0, 0, 124.92514346083301`, 1.5829887360105777`, 0, 0, 49585.71904872256`, 0, 0,
82.01418722613113`, 169824.02084809987`, 23.159876470010182`, 1.0000000000000209`, 0, 0, 0.814505742434381`},
{0.10671756328879578`, 1.1279363098789865`, 5.068949642759208`, 1.0745694995138004`, 0.5897325137042213`,
0.3714383645112781`, 0.14529484820960328`, 91.27351773968599`, 0.0653436372538082`, 9.206661650127103`,
107108.53746142687`, 0, 0, 0, 126.96000441564674`, 14.647772843633108`, 0, 0, 42277.4195309786`, 0, 0,
236.02506927418943`, 64453.474206916515`, 21.841682006662396`, 1.0000000000000264`, 0, 0, 0.8940785781827851`},
{0.2358453438636856`, 3.7679680180462416`, 4.640233719273176`, 1.297156455219631`, 0.3876479327909328`,
0.3193535012332763`, 0.027047144947079417`, 401.3110605008155`, 0.0397014782942956`, 9.539243086069693`,
272.54522153681955`, 0, 0, 0, 197.61318104854269`, 1.3574941614147087`, 0, 0, 0.10602414161049617`, 0, 0,

73.07135121278748`, 0.3572185733712653`, 5.141070210655502`, 0.821251811127323`, 0, 0, 0.9141431628582797`},
{0.07226742189042767`, 2.8421547517397343`, 4.74887948268691`, 0.9400141356818887`, 0.26271764199006165`,
0.24510878200647013`, 0.011565327673117277`, 79.9069988164106`, 0.11613362579809078`, 3.4810370415732024`,
45756.333802778565`, 0, 0, 0, 113.47371635999882`, 0.43962673026437993`, 0, 0, 22448.617338908873`, 0, 0,
17.849817148753004`, 23175.767144109996`, 9.298519820330046`, 1.0000000000000004`, 0, 0, 0.2835814749894877`},
{0.19794910251471837`, 2.250622511000005`, 5.810822485387694`, 0.8325726726239642`, 0.42888216834124404`,
0.3003167364214152`, 0.2221971352698835`, 492.4997381844193`, 0.06654773222193389`, 7.816458771254976`,
184.15306461881525`, 0, 0, 0, 46.61054240736927`, 4.145337327955961`, 0, 0, 0.030652973014427026`, 0, 0,
133.27985008551875`, 0.0866818356802916`, 3.4048755082554574`, 0.5460542692018426`, 0, 0, 0.6798360649642274`},
{0.23749910377351813`, 2.442156143626838`, 7.627688188064528`, 0.9684475529781722`, 0.7107198130457415`,
0.2572554624659992`, 0.4475955253238462`, 132.3529645103848`, 0.18259209603279808`, 1.6799945249508887`,
119.28324170816055`, 0, 0, 0, 16.627877284230525`, 2.466119136762668`, 0, 0, 3.2214701001164188`, 0, 0,
86.03782858229536`, 10.929946594440343`, 2.7945497611718975`, 0.9999992541615398`, 0, 0, 0.19399799922562555`},
{0.06924882755772316`, 2.8414116897854322`, 0.5206442594268132`, 0.9917884594202446`, 0.6526760474283868`,
0.5988068411525457`, 0.47108712727434415`, 720.0807499233221`, 0.11578003411625926`, 8.400733392823671`,
8026.80557412179`, 0, 0, 0, 5.332485067283718`, 0.768568665266216`, 0, 0, 4016.2906613220925`, 0, 0,
31.197428427002937`, 3973.191706108386`, 2.6646309287199847`, 1.0000000000001283`, 0, 0, 1.320930425866904`},
{0.06824086699485615`, 3.3015665983523297`, 9.09230580657946`, 1.0780523992371156`, 0.9189494010575767`,
0.47997916674377217`, 0.09047840499022508`, 3815.186560881826`, 0.152329057733407`, 1.5202594192596113`,
13.174949957237954`, 0, 0, 0, 5.787335543593501`, 0.15309872078907125`, 0, 0, 0.006872411877080788`, 0, 0,
7.220937468680971`, 0.00669970492625373`, 0.08248532272884376`, 0.12710874738012135`, 0, 0, 0.0742048589969552`},
{0.23257418904935773`, 1.8011483887717636`, 0.9612178256197179`, 0.8025101750942898`, 0.31352490932039023`,
0.2355458085864931`, 0.2680725991098069`, 2738.209295799684`, 0.0662676878204258`, 1.8613360761320532`,
6.289638016238291`, 0, 0, 0, 1.3950524003995872`, 0.17937468933767398`, 0, 0, 0.023083040451530534`, 0, 0,
4.615434752671237`, 0.076693134482977`, 0.1474966472961464`, 0.4498127210230807`, 0, 0, 0.1849724918743638`},
{0.13474024315490335`, 3.3704964680175626`, 9.170639775134308`, 0.7502511885050491`, 0.6264710065934431`,
0.21904625510680875`, 0.040564870634629845`, 2527.7664803980215`, 0.19403763861714918`, 6.378419170081072`,
31.377266380232548`, 0, 0, 0, 20.050446815952935`, 0.2302707920316805`, 0, 0, 0.002621596071213253`, 0, 0,
11.087527017576951`, 0.005046207029843317`, 0.5260400685184886`, 0.07249011938201366`, 0, 0, 0.35876019070297793`},
{0.2693026298173556`, 1.772983767924213`, 1.344226083033309`, 1.4113484218782142`, 0.12593618066908396`,
0.656520191233777`, 0.018659842469751033`, 117.56506476136576`, 0.1712196470744854`, 4.023445632685666`,

```
  118587.99350201279`, 0, 0, 0, 34.055538749978965`, 0.3414516700750393 5`, 0, 0, 24456.472450990525`, 0, 0,
  8.648403836766564`, 94088.46210153509`, 7.2835317610097885`, 1.0000000000000433`, 0, 0, 0.3150852104471957`},
{0.17866994815918202`, 2.442046867777872`, 1.8194825512463648`, 1.2285671890230836`, 0.7081083864311548`,
  0.17566313743095552`, 0.006280075202364708`, 2604.6083474637735`, 0.1295692924324935`, 1.862340891527415`,
  8.533108465462853`, 0, 0, 0, 7.759442369725082`, 0.01902909987710558`, 0, 0, 0.023256004221967667`, 0, 0,
  0.6638564821645417`, 0.05935927241040969`, 0.14866371014559718`, 0.3299359212345786`, 0, 0, 0.09952686100136685`},
{0.26090114261485403`, 3.9086029270457496`, 1.920295645800332`, 1.1838686314265923`, 0.4729800090210985`,
  0.36383922429133153`, 0.014536748208445867`, 79.30674088085132`, 0.030053561477188684`, 7.670997166386538`,
  751917.9713923598`, 0, 0, 0, 94.43651665584603`, 0.341479354332147`, 0, 0, 159039.29731940423`, 0, 0,
  19.067245769547466`, 592764.7770185155`, 19.988165776266673`, 1.0000000000000033`, 0, 0, 0.3606640159881867`},
{0.19544602681310375`, 0.41028512203974055`, 4.6003645589109095`, 1.2671666166106164`, 0.8219059973725353`,
  0.22911191370711304`, 0.03702179299429459`, 318.28074751099626`, 0.09079865705400514`, 1.4662081657512314`,
  55.00755654891589`, 0, 0, 0, 35.112762094527525`, 2.6314481266734586`, 0, 0, 0.4842679657926547`, 0, 0,
  15.42348593990667`, 1.352117854671968`, 0.9777978065224117`, 0.9991813872096696`, 0, 0, 0.11061053367004757`},
{0.26537062770714803`, 1.229239774996202`, 8.744773690909977`, 1.151653436583517`, 0.3537059210373261`,
  0.2894924335103778`, 0.13068544785366612`, 312.2140250313751`, 0.146894495801125`, 5.715731252046433`,
  191.9126361577726`, 0, 0, 0, 69.36755138039021`, 6.592362913904827`, 0, 0, 0.039048260445709165`, 0, 0,
  115.76563864259505`, 0.14803230550499288`, 3.9520949796148996`, 0.5166560445132857`, 0, 0, 0.5360261192196613`},
{0.18712917295151626`, 0.630329154174047`, 6.232729389529142`, 0.7582043174026267`, 0.7607866373637777`,
  0.2530331438958473`, 0.06090476948059027`, 64.21018866863231`, 0.08418997914362486`, 2.694793972266142`,
  105602.6313301362`, 0, 0, 0, 70.85427819049693`, 5.998700718402652`, 0, 0, 28713.28513721277`, 0, 0,
  54.016513571056855`, 76758.47572068109`, 8.743410964880452`, 1.0000000000009288`, 0, 0, 0.1497141049342933`},
{0.19413873235593193`, 0.8072205389170897`, 9.684031161687852`, 0.8518820755271745`, 0.2120317281227424`,
  0.1532879757233685`, 0.02372050181467318`, 987.8329384880758`, 0.08094704090521981`, 8.693316755778081`,
  66.0282686364779`, 0, 0, 0, 49.75059239023731`, 1.2965098172749694`, 0, 0, 0.004432476171076335`, 0, 0,
  14.95099076302855`, 0.012293075786430543`, 1.8516576066540358`, 0.10604855253639223`, 0, 0, 0.5742670127337486`},
{0.0816546741022599`, 0.4036812381177306`, 0.7902893788690406`, 1.4273906025641667`, 0.1897112398644374`,
  0.6362443390402239`, 0.007446156154142883`, 176.61944749055874`, 0.10188842005948545`, 9.51311671217389`,
  73461.04814503502`, 0, 0, 0, 57.25246238249253`, 0.8864170176175891`, 0, 0, 33878.52570218356`, 0, 0,
  5.111855988007074`, 39519.14250395467`, 12.140761517949105`, 1.0000000000000064`, 0, 0, 1.3513092260928705`},
{0.2491419431624458`, 3.7694376618574204`, 4.74673147811253`, 0.8416284883026488`, 0.188731240527098`,
```

0.3192559789027146`, 0.07121130413051815`, 671.9000833119555`, 0.02760758509345429`, 2.5790410082926702`,
35.5148908973705`, 0, 0, 0, 17.969358898467316`, 0.3182789815223071`, 0, 0, 0.019327521222088105`, 0, 0,
17.139039713254384`, 0.06878994562547462`, 0.8511523607942953`, 0.38720459895078796`, 0, 0, 0.31853165989150006`},
{0.11168227968802413`, 2.173584861364678`, 5.640951633995167`, 0.9377704673639422`, 0.3315982783471285`,
0.5266083888319896`, 0.005432939117039627`, 621.842609802462`, 0.09631877509870435`, 9.43919460268938`,
206.3367253174613`, 0, 0, 0, 191.4333235520565`, 0.4556817186483322`, 0, 0, 0.03223574619918009`, 0, 0,
14.149469789352203`, 0.0514308803281233`, 3.4164579680323603`, 0.5207944496748775`, 0, 0, 1.3249343698668`},
{0.15970197279447784`, 3.9587696847183347`, 4.0238996390694854`, 0.8027865138486989`, 0.03588289413016432`,
0.35386710298208024`, 0.016574217538633067`, 2019.1576160982734`, 0.07473946383041635`, 3.5352801788382404`,
15.128961218806715`, 0, 0, 0, 12.283447768473755`, 0.04901243860734586`, 0, 0, 0.005766676235696886`, 0, 0,
2.7718422304697032`, 0.01315642244725667`, 0.3971858656542986`, 0.1425595473542709`, 0, 0, 0.5280895418121334`},
{0.2064322311255224`, 0.43645383124552994`, 3.8213880668877356`, 0.8257710590869434`, 0.08868496943554072`,
0.17616539751645888`, 0.04042707492740236`, 2037.3062213977416`, 0.05324630165142419`, 4.6107951341494555`,
15.98315551573246`, 0, 0, 0, 10.28927657296144`, 0.7841464650309774`, 0, 0, 0.005006422135475574`, 0, 0,
4.889196127434406`, 0.014764098448320669`, 0.48387933913581194`, 0.12218313808458448`, 0, 0, 0.3839775018519923`},
{0.1394617695133732`, 3.387614469631334`, 8.352661875580207`, 1.2219251402369191`, 0.6325442693826859`,
0.17624447718949754`, 0.021853560156019736`, 288.68541357569393`, 0.164910064915528`, 5.7766720481305995`,
225.3227622093112`, 0, 0, 0, 172.52252727715234`, 1.0644553355065969`, 0, 0, 0.056499664663467605`, 0, 0,
51.51377566912032`, 0.11256490301262276`, 4.1466022255087545`, 0.6290435451390459`, 0, 0, 0.28845008379986015`},
{0.10599692192016741`, 2.4119661618453145`, 1.4892081676893856`, 1.3031849980783952`, 0.42063329504650815`,
0.5860601104625098`, 0.3077592160131421`, 52.52666428206092`, 0.19647413521024198`, 3.2052596491028216`,
129035.76649976932`, 0, 0, 0, 7.720498582551073`, 0.8951847493056616`, 0, 0, 51306.24059079407`, 0, 0,
30.845076056074948`, 77690.05111313886`, 13.498367162978887`, 1.0000000000010505`, 0, 0, 0.38712965019929807`},
{0.24388484229914548`, 3.2519594625966253`, 4.312340316913895`, 0.9914403572709836`, 0.29358995419342615`,
0.1835819110057716`, 0.13612159308161606`, 1157.8864188899545`, 0.08218618541111161`, 5.046821007659727`,
36.346239182681884`, 0, 0, 0, 12.919089338988677`, 0.49254419134349264`, 0, 0, 0.011751544890092116`, 0, 0,
22.88191062552108`, 0.04094319531844243`, 0.9228455705924611`, 0.22480299646863522`, 0, 0, 0.36716744955080993`},
{0.23494551018662208`, 3.3331099682410468`, 0.9744758817090952`, 0.9989991912838305`, 0.20118476247466943`,
0.3741832660739852`, 0.07385095150097958`, 91.01998469145919`, 0.19994684540705993`, 6.360950891328324`,
81250.82668148632`, 0, 0, 0, 23.620189652633865`, 0.5032749682830447`, 0, 0, 18640.02438928343`, 0, 0,
23.963868765005948`, 62562.7148575896`, 14.490537505334727`, 1.000000000000303`, 0, 0, 0.32162020264735247`},

{0.23944024111665796`, 0.7499363805955559`, 7.775454975750338`, 1.268235612825383`, 0.3786721827881383`, 0.6740341016595366`, 0.11977329505957622`, 117.53909816274609`, 0.2108164269301916`, 1.8777741577958924`, 26931.64335491816`, 0, 0, 0, 47.06108450848899`, 6.620557741651935`, 0, 0, 6064.150918667003`, 0, 0, 70.92853014711945`, 20742.882259048933`, 3.6510383152917876`, 1.000000000000002`, 0, 0, 0.2965339153783451`},
{0.24513403447625742`, 2.0133471925570054`, 9.940759001187548`, 1.083812287959604`, 0.5683273089881642`, 0.5900825394859205`, 0.005339720116331014`, 58.9416557608406`, 0.03145644437596545`, 6.28807827028038`, 599847.2882128722`, 0, 0, 0, 469.9609134515975`, 1.1910664614842499`, 0, 0, 133130.31831094038`, 0, 0, 34.25757594825888`, 466211.0291238492`, 23.09250033229727`, 1.0000000000000018`, 0, 0, 0.6082134403641165`},
{0.15958822823144275`, 2.509825654597928`, 3.728427574062339`, 0.9294074209823535`, 0.01651867581778177`, 0.5367648350921979`, 0.015785937236593408`, 685.9822133189093`, 0.020860236554209932`, 1.2753268301578835`, 18.923628836260693`, 0, 0, 0, 15.42345318456745`, 0.09189605988777752`, 0, 0, 0.03222182091408246`, 0, 0, 3.2949012666115958`, 0.07346033300099397`, 0.44845843926197926`, 0.5101549205219732`, 0, 0, 0.2833547020988764`},
{0.06806026871424425`, 1.205607754446362`, 0.7536813502622586`, 1.4624982795129167`, 0.7076540801030038`, 0.15470161284122685`, 0.268116863806763`, 324.06126360879364`, 0.036033823716353997`, 3.8267580932938614`, 61566.02055294689`, 0, 0, 0, 5.025767250554875`, 0.9530842027643552`, 0, 0, 31204.04629622959`, 0, 0, 16.414938649900478`, 30339.368227044306`, 2.4523962948868805`, 1.0000000000000036`, 0, 0, 0.19985689494618974`},
{0.15319733389695195`, 3.558603911914262`, 4.579050417046558`, 0.9321187332487358`, 0.6642338797631109`, 0.569667348599446`, 0.2858050889038288`, 341.1212503566884`, 0.20343887540165378`, 9.501626804091558`, 381.0996592898564`, 0, 0, 0, 78.79705715365046`, 5.704073264340943`, 0, 0, 2.076008613551411`, 0, 0, 289.9791061761318`, 4.543414067760561`, 6.4303567910422`, 0.9999998158853786`, 0, 0, 1.612265364837121`},
{0.15071537712057487`, 1.2850575528534174`, 6.479674501252795`, 1.2297249014764047`, 0.051196968811160426`, 0.17856837950014437`, 0.01824538414392949`, 77.70419036862091`, 0.224107560352308`, 8.31176551046438`, 34586.80095434776`, 0, 0, 0, 332.75982968984044`, 4.37632232920313`, 0, 0, 10836.775910942757`, 0, 0, 80.34037232662202`, 23332.410974127135`, 22.180094645424653`, 1.`, 0, 0, 0.4205650608587388`},
{0.24584690511060947`, 2.473478204800995`, 6.676188280246508`, 1.4832363218386881`, 0.14420161768506268`, 0.523836460034543`, 0.04402629271492787`, 360.52310897688045`, 0.0850887320320231`, 2.528306265858081`, 78.43126692580904`, 0, 0, 0, 48.54753395264296`, 0.8140979146830841`, 0, 0, 0.06663702033100886`, 0, 0, 28.766477836322128`, 0.23403578877388082`, 1.6393182772190993`, 0.6084773889835442`, 0, 0, 0.46615515280872266`},
{0.14137397451874373`, 3.652676161056461`, 8.39527364337399`, 1.0080745646578813`, 0.09190129083286003`, 0.15081167091134762`, 0.35462309792221747`, 1217.6311173423508`, 0.09724487810479643`, 6.223920941944753`, 35.30083569932627`, 0, 0, 0, 6.691745722622908`, 0.5377016647434605`, 0, 0, 0.004480813802113485`, 0, 0,

```
  28.05785789384006`, 0.009049577946907262`, 1.0807872515535566`, 0.09128280821770851`, 0, 0, 0.44381335176285086`},
 {0.04933630800724648`, 1.8511857532828166`, 5.885062761777403`, 1.1138733840213622`, 0.4607664856084661`,
  0.43439208322360623`, 0.018761076496074794`, 3507.5800668673905`, 0.21191062303011793`, 3.3573407631201917`,
  13.305766777384259`, 0, 0, 0, 10.526452263209032`, 0.10080954876560111`, 0, 0, 0.004922409519083124`, 0, 0,
  2.665960006710717`, 0.003469335888164013`, 0.20975641139939036`, 0.0902160253868346`, 0, 0, 0.3703954360060874`},
 {0.2339008817663396`, 3.669675022809618`, 9.160989469851096`, 1.2207724907901325`, 0.9659294978123167`,
  0.3234992643572032`, 0.05037324961884807`, 93.86107259786391`, 0.14430488254373952`, 3.9894219201696473`,
  18463.93102232838`, 0, 0, 0, 177.18560333043416`, 2.29920195108497`, 0, 0, 4183.842864380277`, 0, 0,
  120.53319960416631`, 13980.06478786231`, 9.345938589766687`, 1.0000000000001037`, 0, 0, 0.4551585047962883`},
 {0.11569404259913263`, 3.2009969772206803`, 2.2264401820433353`, 0.7539773410821206`, 0.3237735314376855`,
  0.22021748910844674`, 0.21956604535044472`, 1445.3927245947305`, 0.06533194774972478`, 1.3139082141801541`,
  8.036716264969558`, 0, 0, 0, 2.0765556131152243`, 0.12663973561022038`, 0, 0, 0.016010493747584988`, 0, 0,
  5.791048726919161`, 0.026461696366667127`, 0.1955759043936973`, 0.36138722123021594`, 0, 0, 0.1183573333921082`},
 {0.1426983247121278`, 1.4581176174915065`, 6.531549332871599`, 0.9310047864665083`, 0.893336243428055`,
  0.18377286371920176`, 0.08056837661087574`, 664.5727162634348`, 0.04347044784294363`, 7.864371974772275`,
  191.70224383722768`, 0, 0, 0, 90.37142028860633`, 4.637529207844169`, 0, 0, 0.03033244126183768`, 0, 0,
  96.60090056555751`, 0.0618341221785563`, 2.4177014740680196`, 0.503627821132505`, 0, 0, 0.27643610073896374`},
 {0.058316879010681166`, 0.732484500007835`, 9.716813224212896`, 0.9062398770267781`, 0.045816647832010116`,
  0.25112700202789096`, 0.0489500536916562`, 2141.8470035057708`, 0.06373448098002471`, 7.679073247267311`,
  27.96932262473565`, 0, 0, 0, 16.763355141072694`, 0.9770842164410961`, 0, 0, 0.002187648736844265`, 0, 0,
  10.224272053505807`, 0.0018225263814781038`, 0.7856335098588071`, 0.05065566847948377`, 0, 0, 0.8466411016503986`},
 {0.1624041267001165`, 2.3409738007594383`, 7.927758092789709`, 1.1108935833128895`, 0.2655878375527121`,
  0.22381693809974978`, 0.10055816102060769`, 86.2207746546923`, 0.23778527923179793`, 1.5984483564142007`,
  13018.994692477769`, 0, 0, 0, 42.946919595295476`, 1.692059512715228`, 0, 0, 3890.8251924303718`, 0, 0,
  56.58667126560196`, 9026.943821706702`, 3.964513310999989`, 0.9999999999999974`, 0, 0, 0.1334569163178338`},
 {0.05796853524365786`, 2.115435321939346`, 7.135826377560314`, 1.1217856002992745`, 0.9949497841824515`,
  0.6493572179052653`, 0.16432568991023785`, 205.88622896743587`, 0.07308989915497993`, 5.091714109271411`,
  332.08050399628326`, 0, 0, 0, 101.25823649647742`, 7.1674751456162635`, 0, 0, 3.8564393618286825`, 0, 0,
  216.6047156022712`, 3.1936020151603746`, 5.9404639725448485`, 0.9999999926286909`, 0, 0, 1.1051218350872967`},
 {0.09343457742759681`, 3.0751406184941388`, 6.999926605820257`, 1.4764339149831611`, 0.27064650694064607`,
  0.6662559245780291`, 0.012835205507442616`, 1884.4110587763687`, 0.0168116000952574 8`, 8.661215052575166`,
```

66.18109341738075`, 0, 0, 0, 56.029605787779936`, 0.224586473136842`, 0, 0, 0.010923950523785 32`, 0, 0,
9.866214084392068`, 0.01458106715757844`, 1.0665839074693682`, 0.14662865995612395`, 0, 0, 1.5221896675528477`},
{0.23943678973010468`, 2.920807454124679`, 1.1099817114095707`, 1.3932739733707113`, 0.44392785132951373`,
0.17254202331513768`, 0.008938503858702969`, 2712.044809794402`, 0.1652368227234951`, 2.8738798121545877`,
9.787950706974414`, 0, 0, 0, 8.566402892630526`, 0.02494538644613259`, 0, 0, 0.03357171918675852`, 0, 0,
1.0408667239697842`, 0.11483292382568726`, 0.223691243093998`, 0.3976535306318262`, 0, 0, 0.1998676088931596`},
{0.1765669287716667`, 3.4304813087836825`, 0.9837478487319995`, 1.1028599055297788`, 0.8616178142897495`,
0.24038775568738768`, 0.03874939820930588`, 2380.99712756261`, 0.18505877859540681`, 3.338709015344154`,
24.805716106707905`, 0, 0, 0, 15.6914611999434`, 0.1704787055909662`, 0, 0, 0.1669058022078272`, 0, 0,
8.354628758220658`, 0.4210006412858177`, 0.2925356485496698`, 0.9516096995449252`, 0, 0, 0.190358783017881`},
{0.23886394100929725`, 3.974888040813318`, 7.275822510559582`, 1.2870566617016084`, 0.12273034821485096`,
0.6651538290269186`, 0.16897919530659566`, 150.6876048176701`, 0.1338282848735754`, 2.432758352876487`,
14917.350560072266`, 0, 0, 0, 47.87680507119167`, 1.8855419069742936`, 0, 0, 3345.2799752394258`, 0, 0,
107.06882823549186`, 11415.239409502452`, 3.8358742822286747`, 1.0000000000103144`, 0, 0, 0.4958697686548741`},
{0.06973130535662914`, 1.4096929441217387`, 8.65071450995816`, 1.181254696978707`, 0.5406204719849208`,
0.2725259583108167`, 0.12003736669178822`, 4806.091595362366`, 0.20844978312325513`, 8.8784124085225`,
23.229834666551877`, 0, 0, 0, 8.797274846909731`, 0.6825465497483377`, 0, 0, 0.002288838736324298`, 0, 0,
13.745443645926741`, 0.0022800530404710416`, 0.39042570562004664`, 0.04085177733975354`, 0, 0, 0.6287908848363538`},
{0.04409294567306185`, 1.3468266911815716`, 8.46560672493381`, 1.1221333539921368`, 0.8074609652543419`,
0.33934404303057597`, 0.1090535991677299`, 2100.6487939981484`, 0.1955313854386307`, 6.974828032327105`,
69.83561426610376`, 0, 0, 0, 27.71330858421421`, 2.0803715846721462`, 0, 0, 0.009071208657588313`, 0, 0,
40.02714254017356`, 0.005713947293302169 5`, 0.6889531565684387`, 0.15955078879193518`, 0, 0, 0.3578982973123104`},
{0.08301734541584582`, 0.4950033643125962`, 0.3447103403996028`, 1.3973171280993089`, 0.5174429014299606`,
0.6090072510200681`, 0.013328621450736109`, 78.08408450957157`, 0.15235476028821748`, 7.41363923833616`,
117207.01329950699`, 0, 0, 0, 17.967930437067338`, 0.41714676748455837`, 0, 0, 53608.23273805409`, 0, 0,
2.9498436188140094`, 63577.33106211575`, 21.225238399199775`, 1.0000000000000187`, 0, 0, 0.9836175383384149`},
{0.07508111728102967`, 2.9328515590120503`, 2.9247828964602327`, 1.0997610261500241`, 0.15381049135387959`,
0.44875941041801015`, 0.005374403719665632`, 955.806314296473`, 0.04180941570568808`, 8.210271230243798`,
91.90359966838545`, 0, 0, 0, 85.32099745530205`, 0.1494224207608528`, 0, 0, 0.03698674860558821`, 0, 0,
6.260482566854589`, 0.03967152014142433`, 1.95871108424927`, 0.5109077100055246`, 0, 0, 1.2752807767321597`},
{0.1868411586163749`, 1.4148901359883457`, 3.2886483363036496`, 1.1024292265597047`, 0.35071605335062417`,

0.20216786873751635`, 0.033694215571138546`, 50.84055962824541`, 0.13756443231160648`, 9.762651891575405`,
170456.0745443864`, 0, 0, 0, 166.63133851049434`, 3.7160067651892734`, 0, 0, 46389.53440110273`, 0, 0,
75.11059024760391`, 123821.06221680332`, 39.2399479544543`, 1.0000000000000002`, 0, 0, 0.3452514064088697`},
{0.25728291653412544`, 1.1620186239014583`, 0.48798465750503445`, 1.2217305465551163`, 0.36537296293338284`,
0.2112385814144826`, 0.1783844678719564`, 175.70497466833672`, 0.14885286931859365`, 6.164090300210196`,
68143.10139680833`, 0, 0, 0, 6.588168592351783`, 0.9238754078222671`, 0, 0, 14569.7115656451`, 0, 0,
15.336577572200326`, 53550.541209573785`, 7.121035852929336`, 1.0000000000011686`, 0, 0, 0.17533025216466067`},
{0.13759840983832694`, 1.6824227355669867`, 8.881204796952819`, 1.07728991023014`, 0.8275568317133928`,
0.2913709338376398`, 0.47392581861361016`, 1028.758263171597`, 0.1629375199726112`, 7.630724789672264`,
147.135753648158`, 0, 0, 0, 20.256905107375353`, 5.0660801274887675`, 0, 0, 0.017367186313950803`, 0, 0,
121.761262667017`, 0.03413853171663882`, 1.5316475770837685`, 0.29298599874487097`, 0, 0, 0.3527970574949527`},
{0.22999415874686646`, 3.253135821240728`, 9.26678168881324`, 0.8937302072810551`, 0.5038748821521797`,
0.2035592770142407`, 0.010501376095491947`, 229.44271937909937`, 0.14963151143698977`, 8.069758108498657`,
372.3238558562111`, 0, 0, 0, 324.3156632310892`, 1.0019293622537002`, 0, 0, 0.04635298639168399`, 0, 0,
46.56303283857702`, 0.15229880157939596`, 7.372596533453699`, 0.6717582822563974`, 0, 0, 0.5009574538719916`},
{0.09925066924077935`, 1.4815445119155024`, 7.881183919866995`, 0.7663286317492166`, 0.2976933875506338`,
0.3306202803754688 7`, 0.05105854284999101`, 168.2571992844445`, 0.22488226539851913`, 5.574589576514775`,
347.7521027408701`, 0, 0, 0, 203.4946333774482`, 6.463719180179649`, 0, 0, 0.4068501750316917`, 0, 0,
136.80410968511598`, 0.5768593164660593`, 7.274014436773202`, 0.9977976482091094`, 0, 0, 0.6265305946710429`},
{0.20983327896511272`, 1.8859920859197894`, 0.6151282764886545`, 0.8412663476815937`, 0.027122934645615437`,
0.6844735714100607`, 0.01254007978155685`, 549.5308557472557`, 0.10371695352022153`, 7.780558961111437`,
19165.672067231193`, 0, 0, 0, 33.009064603371236`, 0.20931210005051928`, 0, 0, 4784.5471136223405`, 0, 0,
5.639442345464739`, 14342.245845920552`, 3.0756587142349034`, 1.0000000000003735`, 0, 0, 0.7829658773852107`},
{0.2225389666164696`, 1.2827559929647245`, 1.2157460525678818`, 1.1336603904726232`, 0.21910751958531538`,
0.6021275716391656`, 0.016887660465617623`, 151.92995003475355`, 0.056506174885467386`, 4.721491520685097`,
190388.5285192307`, 0, 0, 0, 37.04579097617488`, 0.4573929166311305`, 0, 0, 45546.01396144325`, 0, 0,
8.38176435640278`, 144796.61257826933`, 6.644090290314435`, 1.0000000000002238`, 0, 0, 0.3929858632090125`},
{0.23629660338151143`, 1.375829581220887`, 0.7878883261410508`, 0.9498849347602389`, 0.6605330073956415`,
0.33733697402858687`, 0.028977316405205664`, 446.06430846982937`, 0.16303388369839966`, 6.718018447521894`,
14557.75897704643`, 0, 0, 0, 29.268939187275095`, 0.5778513283606073`, 0, 0, 3317.5636212119794`, 0, 0,
11.357499301518674`, 11198.985931349358`, 3.134404728247765`, 1.0000000000003124`, 0, 0, 0.36592168072365977`},

{0.2240884195389573`, 3.0168075094859823`, 9.22253837134286`, 1.0534395436787098`, 0.7253455428103461`,
0.1646055270291652`, 0.006115122680716056`, 258.28718729642685`, 0.11665954601209721`, 2.992281031623701`,
136.92517641644926`, 0, 0, 0, 125.7835193453226`, 0.24447694609430887`, 0, 0, 0.054087233880406635`, 0, 0,
10.536284098192988`, 0.17314746796421857`, 2.3881702267353617`, 0.6665827607579404`, 0, 0, 0.13301007412346386`},
{0.04820076342563051`, 1.9965427255473651`, 1.4646173572921608`, 1.475676204663891`, 0.346581356777814`,
0.35258214744905925`, 0.16114708977216574`, 53.64321165141119`, 0.1945121008436112`, 8.557828905511698`,
96135.59216903089`, 0, 0, 0, 32.272576739845086`, 2.3681800136294133`, 0, 0, 56871.010672096236`, 0, 0,
67.5453225571211`, 39160.373302603024`, 34.817674062913134`, 1.0000000000000762`, 0, 0, 0.9053458477045645`},
{0.1395588171486265`, 3.3167971972296737`, 5.120579942113229`, 1.4414502422836475`, 0.46235265068089726`,
0.21590338795839814`, 0.018401804903068468`, 321.8138540224943`, 0.09243860589675884`, 1.0657915725386573`,
32.76146072165554`, 0, 0, 0, 25.829661082200175`, 0.13657641227015474`, 0, 0, 0.10467687139524069`, 0, 0,
6.471375163219072`, 0.20869400506769054`, 0.7071376013769992`, 0.7722462791480589`, 0, 0, 0.08721292479858984`},
{0.2085065587668823`, 0.6294064238759702`, 7.1150002800734775`, 0.8698405330489325`, 0.07589384997930004`,
0.6271818704466325`, 0.014863365323890152`, 3717.018925299029`, 0.09558183779388002`, 3.993723504265942`,
12.489992847490445`, 0, 0, 0, 10.330576406600908`, 0.2145781642312358`, 0, 0, 0.0024680893671904995`, 0, 0,
1.9293839284378973`, 0.007351611724029095`, 0.25704215804423863`, 0.05898588024473084`, 0, 0, 0.8471246980566874`},
{0.11579616742063126`, 1.4637518716217537`, 6.091821149634162`, 0.8701973607843327`, 0.580668178059593`,
0.4892081276931839`, 0.43069272205186815`, 58.104542288448926`, 0.09639134486345691`, 1.0613512257562903`,
112608.65838155973`, 0, 0, 0, 8.149657742336753`, 2.063597394581422`, 0, 0, 42405.98889943247`, 0,
0, 43.15135069417614`, 70149.29986051611`, 4.035225452980323`, 1.`, 0, 0, 0.12660441227673397`},
{0.07650954660329923`, 2.451622188741575`, 3.5124331635719983`, 0.9208132913481966`, 0.22417245589301404`,
0.5962277485405788`, 0.163105858828852 4`, 149.68967360762312`, 0.15724921240628176`, 3.991974201007654`,
21862.27748609377`, 0, 0, 0, 37.55343022983086`, 2.3010771133082377`, 0, 0, 10387.8951331336`, 0, 0,
80.59102441417001`, 11353.902097123853`, 6.137372679041324`, 1.0000000000043676`, 0, 0, 0.6627791208363929`},
{0.27715040216901043`, 3.1093705724520717`, 5.602120685872263`, 1.4185083740786066`, 0.20116695432127574`,
0.3482455682148583`, 0.014646070094414987`, 268.87083253973395`, 0.19497961893886057`, 8.247252623819538`,
306.22565384499995`, 0, 0, 0, 253.6541386251208`, 1.1386666729645585`, 0, 0, 0.14430033035491266`, 0, 0,
50.57909492497004`, 0.5713270655855781`, 6.808118539478593`, 0.8785974021016731`, 0, 0, 1.0273035095609577`},
{0.10373802184314534`, 1.9484823746249935`, 4.344672894707308`, 0.8659474233939041`, 0.34328087466446`,
0.6599529078817681`, 0.006604951530776707`, 74.02272771702236`, 0.12108236246583282`, 3.25882057231545`,
81784.74233045777`, 0, 0, 0, 109.43739834180306`, 0.3528434402071802`, 0, 0, 32903.27846677182`, 0, 0,

9.821560346367704`, 48761.72886138687`, 9.965693721850466`, 1.0000000000001068`, 0, 0, 0.4787977901243346`},
{0.08995654352927362`, 1.6673082382989595`, 4.04257302618344`, 1.1477945472560402`, 0.1500209798917984`,
0.1761016570773115`, 0.012888472951564884`, 1050.2473441342943`, 0.2022489804478793`, 4.465566609444316`,
31.237520689619966`, 0, 0, 0, 26.475200729954675`, 0.18991012756568387`, 0, 0, 0.014169173924886718`, 0, 0,
4.523410288952453`, 0.018208713013523472`, 0.9064173078374559`, 0.23274062339121815`, 0, 0, 0.35770940281109737`},
{0.18428208667431017`, 2.663013532673764`, 4.442362759588184`, 1.3723983485913218`, 0.7325310003414629`,
0.5418182340232052`, 0.027460635126683182`, 53.281000517524745`, 0.08334630855570291`, 6.274365305120075`,
372683.15922425175`, 0, 0, 0, 161.25858862569763`, 1.597264083915644`, 0, 0, 102532.4505682869`, 0, 0,
60.76479815315875`, 269927.05632220715`, 25.26666000649568`, 1.0000000000005085`, 0, 0, 0.5465482001651858`},
{0.1326476876438774`, 2.8731759222440596`, 6.672951038295707`, 1.243076119232064`, 0.9225218753338189`,
0.23536778742386455`, 0.07924576221140303`, 2119.223051638056`, 0.014542489730864222`, 5.05029094558817`,
47.256296859171584`, 0, 0, 0, 22.406571200779076`, 0.5901862265277513`, 0, 0, 0.01211274928510822`, 0, 0,
24.22441222428019`, 0.022953259766856658`, 0.48788589167022295`, 0.18888552342481701`, 0, 0, 0.1883334657890097`},
{0.16449021574582717`, 1.5264131018768046`, 3.118813817198866`, 1.0873075186223824`, 0.9114527670749883`,
0.32623274128336255`, 0.005473446059082923`, 2600.919647538226`, 0.12372800503579795`, 1.4807994816594086`,
13.699394480882559`, 0, 0, 0, 12.622550885856187`, 0.042871614294049634`, 0, 0, 0.02536586899904758`, 0, 0,
0.9348541965292323`, 0.05960624663190951`, 0.11804461600631633`, 0.3877520648235925`, 0, 0, 0.07479163086644577`},
{0.1059475440552839`, 2.4614128361434053`, 6.764865578702089`, 0.897041430377437`, 0.5452264302150955`,
0.6377616415175531`, 0.031109305885935246`, 290.7153568287518`, 0.12025877488242465`, 1.854625189562679`,
107.9942079391424`, 0, 0, 0, 74.30036710395441`, 0.8954231460812143`, 0, 0, 0.5178696009505386`, 0, 0,
31.485800364917388`, 0.783814462308494`, 1.4579651073335045`, 0.9987988189675591`, 0, 0, 0.2928959005420834`},
{0.09376889465512256`, 2.694562234128588`, 9.901749913963968`, 1.2671081853876889`, 0.9355728957697818`,
0.41426328356338693`, 0.1676359905682518`, 324.1423914220847`, 0.048114260882103244`, 6.992923371816153`,
539.5368707617577`, 0, 0, 0, 161.6705190861599`, 9.53111457583444`, 0, 0, 0.6184643763736231`, 0, 0,
366.8883055027995`, 0.828467442230212`, 4.4934411217208075`, 0.9984173369459198`, 0, 0, 0.38675138256817326`},
{0.07408607058446992`, 2.173441202450359`, 5.83110243938874`, 1.2671639264693393`, 0.15034130588796946`,
0.26151521374964837`, 0.00796428041791113`, 3349.7895146720416`, 0.06475249420724477`, 9.113413370060233`,
23.308505588366984`, 0, 0, 0, 20.96143951678631`, 0.07240018871521363`, 0, 0, 0.0035938103802900146`, 0, 0,
2.2479650459832414`, 0.003803589850017114`, 0.5929516986788032`, 0.05918426606795424`, 0, 0, 0.9503211054995397`},
{0.2682300613650819`, 3.6108652762368214`, 9.881761872038165`, 0.9825580258076282`, 0.9296545706074566`,
0.25219322618587503`, 0.024464898005065273`, 1815.4892808719587`, 0.08994453092924143`, 7.425857575199714`,

87.5716937286244`, 0, 0, 0, 65.06775665335743`, 0.4269184477622557`, 0, 0, 0.008021967539908956`, 0, 0,
22.02207141156649`, 0.030739040649965233`, 0.8366693699996218`, 0.1611778359278765`, 0, 0, 0.2702280762585307`},
{0.04962174488386037`, 1.9694465660050104`, 4.880585401279637`, 1.2957318091391512`, 0.6289429156960646`,
0.36977064682826033`, 0.006935918860683708`, 414.38904513183815`, 0.2065379649808241`, 4.444261705692702`,
170.58419588601262`, 0, 0, 0, 154.6929284529902`, 0.5183506272848223`, 0, 0, 0.3712626697883502`, 0, 0,
14.583769469894841`, 0.26318144978770197`, 2.294972360502477`, 0.985966745021521`, 0, 0, 0.37419608100879637`},
{0.2594824517719198`, 0.5971319663647825`, 9.453687965953478`, 0.8401327653868795`, 0.7997988949991743`,
0.15899752713279824`, 0.005075100457461465`, 409.49724454586783`, 0.2091478816442816`, 4.600741868044251`,
144.37239454971117`, 0, 0, 0, 134.52173828750722`, 1.0057684011817902`, 0, 0, 0.02320686186429102`, 0, 0,
8.579663758646374`, 0.0860253344925436`, 2.303008290014094`, 0.4479837389201414`, 0, 0, 0.17750758088118113`},
{0.1975048020318434`, 2.1175350807064808`, 9.527018017184652`, 0.8875453366861839`, 0.2770809005507884`,
0.4437813967164592`, 0.08906290963263506`, 98.18685485298634`, 0.18494734685711195`, 2.236429925751402`,
9252.548489974435`, 0, 0, 0, 81.91996397719407`, 3.1815892316519747`, 0, 0, 2373.7299178561902`, 0, 0,
96.24466872030031`, 6697.472250046448`, 5.19689945275396`, 1.0000000000002145`, 0, 0, 0.348923950681847`},
{0.18592803424857857`, 3.7511626605606008`, 0.2948073517622305`, 1.1421602142936857`, 0.24567142070681736`,
0.41248813426514863`, 0.25834870652665815`, 931.542828982051`, 0.012674565140645466`, 9.754712584362995`,
107008.11656396952`, 0, 0, 0, 5.035692623044833`, 0.3237080395756408`, 0, 0, 29262.049159044513`, 0, 0,
17.346878728277446`, 77723.36111752044`, 2.2105784935090513`, 1.0000000000008644`, 0, 0, 0.6788133737550534`},
{0.058165051218229624`, 1.2475983837798745`, 4.326003024744654`, 1.2604302116068957`, 0.7278275017755875`,
0.49389546693051767`, 0.021556729129120156`, 452.84942671713645`, 0.038214009527072046`, 8.73167788977904`,
311.9395115847039`, 0, 0, 0, 237.9972229147099`, 3.8246808928361964`, 0, 0, 1.0241129258027049`, 0, 0,
68.16665286251737`, 0.8509654397509611`, 4.241499192854593`, 0.9998443136388088`, 0, 0, 1.0001751996514265`},
{0.17310243018283944`, 0.49256885761049496`, 5.2474038118867075`, 1.2167083563026317`, 0.588616706923442`,
0.41089714710891945`, 0.1821711177021871`, 4451.29023370465`, 0.12001647220652961`, 2.269196213787966`,
8.083272027047439`, 0, 0, 0, 2.3163803178288997`, 0.7152347127693163`, 0, 0, 0.005403666477170222`, 0, 0,
5.032890648459328`, 0.01336268284422703`, 0.10979295189601518`, 0.09029459825975683`, 0, 0, 0.20698661270069899`},
{0.1805022145737788`, 3.870623878161763`, 2.6853201865429206`, 1.4540056051610473`, 0.3465395939248448`,
0.3116685990581043`, 0.3430495408207368`, 123.77189026179846`, 0.21554545019710575`, 7.5674497363872035`,
50603.35160539281`, 0, 0, 0, 28.57963517275441`, 2.3157148154763387`, 0, 0, 14096.117602756778`, 0, 0,
128.0465865685096`, 36348.292059857435`, 12.785406941445546`, 0.9999999999999999`, 0, 0, 0.45041845533278396`},
{0.12919486377057743`, 2.6304051581320387`, 9.030615531469042`, 1.1852097760732927`, 0.8025656477879668`,

0.2709665069296936`, 0.01610528402445943`, 631.0560544240994`, 0.23139307171140971`, 4.580191033116536`,
129.3234760021952`, 0, 0, 0, 105.30290918390429`, 0.6191108680704439`, 0, 0, 0.030176478649678245`, 0, 0,
23.26446315468717 8`, 0.0556949435459957`, 1.502011776126406`, 0.42088431527835124`, 0, 0, 0.22226733530551915`},
{0.11792807938525018`, 2.9992967519426994`, 5.197929087471108`, 1.1316540273093707`, 0.8248866470317724`,
0.3561695895739371`, 0.01696020529134022`, 1283.8412020113915`, 0.1325959329361287`, 7.000673815586042`,
122.90846511155277`, 0, 0, 0, 99.05158720680863`, 0.5411114808104002`, 0, 0, 0.03196172426262296`, 0, 0,
23.185055811907674`, 0.053845496516179876`, 1.1310307815473633`, 0.453713416070862`, 0, 0, 0.3564500373476507`},
{0.049303290582256715`, 1.1925070924339343`, 0.7957633600668377`, 1.4128683276782472`, 0.000022730879762677958`,
0.4285104885078085`, 0.00878130985829771`, 1257.460569712898`, 0.24085716752239034`, 7.587289884781604`,
327.1525055128447`, 0, 0, 0, 46.601512089468855`, 0.3154072769727649`, 0, 0, 161.2458228989076 5`, 0, 0,
5.373220211361368`, 113.57070945085594`, 1.4036528399642243`, 0.9999999999986575`, 0, 0, 1.3555698221262484`},
{0.13128664635800402`, 2.4046868210274015`, 6.858470528577773`, 1.007040533285536`, 0.4840752461507001`,
0.3489790633820432`, 0.006590730128885638`, 3596.8950880067596`, 0.16587148750288666`, 3.462016809878005`,
12.368163834688406`, 0, 0, 0, 11.301768401220224`, 0.02955982512676542`, 0, 0, 0.003407286129207959`, 0, 0,
1.015458884488675`, 0.0063904452726567815`, 0.2076032080652111`, 0.07002086406109953`, 0, 0, 0.32139978522227547`},
{0.04846841194802434`, 1.828640157997615`, 8.053204702491172`, 0.7912000348817253`, 0.19137275290127498`,
0.26044015409759047`, 0.137444226583514`, 395.2194426771421`, 0.10623448877689068`, 6.754668978902`,
150.15138902721569`, 0, 0, 0, 53.01673350461782`, 3.580138073100756`, 0, 0, 0.017151301716950042`, 0, 0,
93.52548930926064`, 0.01187566224374669`, 3.7132526625558286`, 0.3726344190246683`, 0, 0, 0.680915905229137`},
{0.22606436547954117`, 2.759207160262239`, 4.797641172785626`, 1.1197512107663665`, 0.8744312540778343`,
0.4079143329219078`, 0.021680363867763237`, 112.017364386689`, 0.04841847187742204`, 6.247097164626069`,
179011.64375117325`, 0, 0, 0, 182.45224449733476`, 1.376164573490534`, 0, 0, 42268.32887362409`, 0, 0,
54.24461635534751`, 136505.1849528057`, 11.848911477775355`, 1.000000000001059`, 0, 0, 0.4778075987249561`},
{0.21439187995731207`, 1.4871413304129364`, 0.5160724591468409`, 1.1806787917625174`, 0.8679222077666104`,
0.6556391626343723`, 0.05962099501823598`, 325.40523317512117`, 0.12955288081431815`, 2.055442432220964`,
39489.35959638253`, 0, 0, 0, 4.6617489289387155`, 0.1755116702201063`, 0, 0, 9717.772351806689`, 0, 0,
3.728723696487519`, 29763.021192871664`, 1.3639250863187127`, 1.0000000000008105`, 0, 0, 0.19328814838030428`},
{0.09778347249929609`, 1.5399588911598938`, 7.050045797549721`, 0.9082485710483456`, 0.7454636238668619`,
0.6248569918637821`, 0.04029077002834171`, 1056.397985521477`, 0.20938181714809317`, 3.6320484036778673`,
92.52153069881075`, 0, 0, 0, 58.941298252668055`, 1.4569379816013033`, 0, 0, 0.02811300550940173 3`, 0, 0,
32.051779980506836`, 0.03927124715861237`, 0.732325049198392`, 0.48230875639429804`, 0, 0, 0.2876602453651809`},

{0.12927232662770405`, 2.951438179906213`, 0.40510823994438105`, 1.0187470674786288`, 0.2032238445066854`, 0.5456409554372916`, 0.01881027549673071`, 134.69705409101317`, 0.12921233603333943`, 7.23494261394152`, 67746.06365450741`, 0, 0, 0, 18.781520967460814`, 0.11524046831733357`, 0, 0, 23789.35931802493`, 0, 0, 4.858930258029305`, 43932.94040033616`, 11.647641841241272`, 1.0000000000000449`, 0, 0, 0.714166163244508`},
{0.20182779178318416`, 0.6881028184372244`, 1.057422534977741`, 1.0568090274497894`, 0.12452101430667706`, 0.6254257177994904`, 0.38702446915906646`, 3365.5277260432094`, 0.12211162516890145`, 5.63708838473557`, 21.067396734074475`, 0, 0, 0, 3.518761421252774`, 1.6092728464837467`, 0, 0, 0.030939408985170485`, 0, 0, 15.819216875713723`, 0.08920617992220255`, 0.3994165723752473`, 0.4535026004451209`, 0, 0, 1.173765862356064`},
{0.21480237208045305`, 2.4248480984277982`, 7.5563453916242835`, 0.9609967545999567`, 0.4216733331369118`, 0.3788510295397969`, 0.02865689876021866`, 142.8377853769303`, 0.053409606561145295`, 2.2123406251771502`, 159.10785880878555`, 0, 0, 0, 102.58668293562981`, 1.1376216675718818`, 0, 0, 3.9220169579647344`, 0, 0, 39.40799624774194`, 12.035122084436953`, 3.561158525373078`, 0.999999988273511`, 0, 0, 0.3649105613384747`},
{0.19117013383013431`, 1.4594963084730592`, 8.271393984038092`, 0.8911461626518757`, 0.4023315908892062`, 0.6430024305607471`, 0.425955961175158`, 811.9982424604128`, 0.08470327958141488`, 1.5517888018340713`, 30.304227280128735`, 0, 0, 0, 4.625278141582771`, 1.1725478777446567`, 0, 0, 0.01577052228880131`, 0, 0, 24.44756141537506`, 0.04306932652166065`, 0.43558099353041796`, 0.31267344909970585`, 0, 0, 0.24024560532698871`},
{0.16704561027767095`, 0.7136544792609025`, 7.482780404676742`, 1.2150283597546259`, 0.9046207606306917`, 0.271745131902447`, 0.2695234426940548`, 220.6020580904624`, 0.17006503868594403`, 3.14899802359108`, 188.19240292746207`, 0, 0, 0, 40.71278068257978`, 12.9647706933667`, 0, 0, 0.6904701972002221`, 0, 0, 132.17666682730876`, 1.647714506712291`, 3.0238056640481537`, 0.9996754334558481`, 0, 0, 0.2307612584358348`},
{0.16595765366147208`, 2.092996799011412`, 4.948615364795604`, 0.8929763621996465`, 0.8391454185145462`, 0.5892008264762102`, 0.044299676614507844`, 130.42998143434642`, 0.19085435393011602`, 9.39386568753341`, 26573.72469699617`, 0, 0, 0, 237.82303019615898`, 4.769167192238285`, 0, 0, 7769.176194246045`, 0, 0, 142.59788096149938`, 18419.34645828063`, 15.970104514385284`, 1.`, 0, 0, 1.1599018538288`},
{0.27224148961405453`, 3.064354031176171`, 9.703480945324245`, 1.4628863651144104`, 0.3875440410966342`, 0.15502532019017645`, 0.01538110765065082`, 96.32001554152914`, 0.17185939304406467`, 6.478173160029735`, 508.2653260559073`, 0, 0, 0, 409.08053665195206`, 1.9379518525833948`, 0, 0, 2.495170811902847`, 0, 0, 84.8367224527036`, 9.704128838199233`, 14.218417285847924`, 0.9999974563972014`, 0, 0, 0.4607189233507776`},
{0.17789655356032075`, 0.42086448766877327`, 8.212577079102342`, 1.145177796821741`, 0.7675840121122328`, 0.5713332044931656`, 0.08476669830762888`, 407.4176887081235`, 0.039911846147414665`, 5.425104426537898`, 350.12885558368856`, 0, 0, 0, 159.28670802717232`, 27.02110491540723`, 0, 0, 0.38413688065746515`, 0, 0,

162.4603353781003`, 0.9762375309197382`, 2.828516317260325`, 0.9909462018174414`, 0, 0, 0.413659316105187`},
{0.25890879804354505`, 0.827757285125033`, 6.146318214990161`, 1.0400613992204162`, 0.4812180568243971`,
0.29251954183586915`, 0.03603273141312643`, 1733.9600457555907`, 0.08508345454030858`, 2.745915615562448`,
18.586470565912578`, 0, 0, 0, 12.358016916321917`, 0.4827069567414854`, 0, 0, 0.0077546709381313824`, 0, 0,
5.708060000332828`, 0.0286821790259254`, 0.33905577222489813`, 0.14763958638999786`, 0, 0, 0.2328241413892715`},
{0.13429919704858378`, 3.97301958069563`, 0.23275296336668916`, 1.4075463260369605`, 0.23061890929775308`,
0.5504417933404041`, 0.22771166040596233`, 272.6044063125219`, 0.22898591641919652`, 6.483734227937328`,
26385.39698790132`, 0, 0, 0, 2.980489802199025`, 0.16013254043634617`, 0, 0, 9036.363075790885`, 0, 0,
9.088710266573386`, 17336.80436168836`, 5.155024561799034`, 1.000000000001482`, 0, 0, 0.6363633459151411`},
{0.12030391986517214`, 0.7748656879950588`, 5.112522469071294`, 1.1531030157403883`, 0.37283988475313556`,
0.5692147153589893`, 0.013539004333470354`, 948.0585012448981`, 0.020895787868669358`, 3.48210387762453`,
53.13242829537899`, 0, 0, 0, 44.56512270245526`, 0.7014268579029871`, 0, 0, 0.02743669089379966`, 0, 0,
7.764451497531577`, 0.047153449466466296`, 0.8319904517512042`, 0.3960387797166776 7`, 0, 0, 0.5143592684528563`},
{0.1321541010040706`, 0.831080421127889`, 5.684518793971316`, 1.0145204035612485`, 0.08667418093385804`,
0.219847293666507`, 0.022523926566952547`, 3181.1487538157703`, 0.21535243439086593`, 6.5479272590953945`,
15.757187434428145`, 0, 0, 0, 12.01634769376629`, 0.28970588100611155`, 0, 0, 0.002773527590048969`, 0, 0,
3.4395555084255034`, 0.005236186361040101`, 0.4461508780723974`, 0.0571175493678053 3`, 0, 0, 0.6430524464019814`},
{0.10292542985717545`, 0.9480363128916887`, 1.8187023134383153`, 1.095683835227761`, 0.3789693754557397`,
0.6741099730044744`, 0.007927682110189524`, 599.0709679738629`, 0.23730403059109434`, 8.347995806673719`,
1577.4614793503047`, 0, 0, 0, 122.60668025426332`, 0.9352006574482559`, 0, 0, 583.3714182714946`, 0, 0,
12.665774044301811`, 857.7679141711956`, 3.356830464462308`, 0.999999999999997`, 0, 0, 1.8409678845119857`},
{0.11768271904228267`, 0.8044018805503832`, 4.992994745146397`, 0.8839437991548327`, 0.9627780258088621`,
0.4036610370791115`, 0.010677704183731065`, 75.90134138634313`, 0.09047277628943856`, 3.892249177500955`,
90358.61297984415`, 0, 0, 0, 136.76330634924594`, 1.6407763187997662`, 0, 0, 33642.34794882681`, 0, 0,
18.854907948643824`, 56558.89973692154`, 11.11015996031324`, 1.0000000000000067`, 0, 0, 0.38034944707763585`},
{0.23810204192481904`, 3.5212069305389937`, 6.24449843698252`, 1.12580298554529`, 0.9906238590423209`,
0.651194539832239`, 0.008821192031195845`, 3794.027232128564`, 0.08728181755971864`, 9.039220988882679`,
128.21203881894698`, 0, 0, 0, 113.75649262887852`, 0.27822016872763866`, 0, 0, 0.01913060699263932`, 0, 0,
13.995296947706994`, 0.06507195126011893`, 0.4882626809160267`, 0.3051887356989189`, 0, 0, 0.3467097554612438`},
{0.05170854445494355`, 0.9544694556169109`, 2.4941099826351163`, 0.9097774283707087`, 0.02554739328254807`,
0.22408928016637408`, 0.36587983814629343`, 578.5087062785725`, 0.14318945676945632`, 8.489550606354296`,

110.81059570015968`, 0, 0, 0, 20.33343793956698`, 6.175992534439358`, 0, 0, 0.051642497781631436`, 0, 0,
84.21137474629195`, 0.038147977032861574`, 3.1914329859086092`, 0.7012068103906186`, 0, 0, 0.8648930201421581`},
{0.2758391104750425`, 2.3950700125014723`, 3.0825274454463827`, 1.2324094107661792`, 0.33578451389793784`,
0.4521239993250691`, 0.02669897011071422`, 4321.015062663416`, 0.06443072522161825`, 4.390191809873814`,
12.709093420348216`, 0, 0, 0, 9.230995290645087`, 0.09762993553985712`, 0, 0, 0.007712545701895708`, 0, 0,
3.340436156199478`, 0.03039173922727425`, 0.22719035037849017`, 0.12478063072199175`, 0, 0, 0.5837190166235212`},
{0.04136033639560718`, 3.5342644851920078`, 2.1080294445485137`, 0.9950381948590621`, 0.26813425336643104`,
0.30392774702749736`, 0.04795897713144824`, 92.66270711931676`, 0.1731864034169307`, 4.57325203890367`,
32961.012784380415`, 0, 0, 0, 47.25297300364927`, 0.606508126819986`, 0, 0, 20668.819262474222`, 0, 0,
30.622287608574418`, 12212.418822799145`, 10.758740270066053`, 1.0000000000000737`, 0, 0, 0.47788022176082323`},
{0.1958059683987035`, 3.200106783136203`, 7.203120771344906`, 1.1276577663917136`, 0.37865874140280775`,
0.19330414092595893`, 0.01322903896050458`, 235.27978791284824`, 0.1213328646575434`, 2.0980953870537427`,
78.94411939152164`, 0, 0, 0, 66.45333288588871`, 0.26136212076785414`, 0, 0, 0.06335239447437228`, 0, 0,
11.9483813646296`, 0.1772110992918677`, 1.8899825731810644`, 0.6960758182400872`, 0, 0, 0.15492308962211748`},
{0.2141293518430885`, 1.3445267096173987`, 9.440612710104876`, 1.3031822475396773`, 0.08631549676886618`,
0.2421378295292581`, 0.053475196461594834`, 50.8320364740117`, 0.167124755886029`, 6.69448094138391`,
113801.9853730022`, 0, 0, 0, 281.1255249589892`, 10.368359031053972`, 0, 0, 27916.135188146956`, 0, 0,
199.15050931649802`, 85395.19905431359`, 27.335245089536187`, 1.0000000000001608`, 0, 0, 0.3456836817320885`},
{0.14124099610833857`, 1.6304240145491873`, 4.431235349124869`, 0.7694928939095955`, 0.9477250896887188`,
0.5434618086833787`, 0.13057745213792676`, 1388.4740941330226`, 0.07045603839931946`, 8.981021929620123`,
270.06412862421405`, 0, 0, 0, 95.02431479727532`, 7.19502949786867`, 0, 0, 0.08609021713985418`, 0, 0,
167.5849839816406`, 0.1737066860573192`, 1.3306710992149524`, 0.9002848300622284`, 0, 0, 0.38016337207409434`},
{0.08317638924124338`, 3.4814117090049965`, 4.537378351353009`, 1.1287962009049006`, 0.37603042712104306`,
0.6772825294518297`, 0.08278526718675269`, 52.354988641883`, 0.1936331782676135`, 8.933135500909255`,
97609.87806211805`, 0, 0, 0, 160.39917969128118`, 3.6302763505867897`, 0, 0, 44449.18046894325`, 0, 0,
180.54980848366776`, 52816.03337341575`, 38.83461992112134`, 1.0000000000003122`, 0, 0, 1.3683406706881718`},
{0.14458191325048447`, 2.576522592570102`, 7.664240990513161`, 1.1334692879511565`, 0.1460097548297743`,
0.5479004940247297`, 0.17641432391033549`, 874.6457178122505`, 0.14737345925317297`, 7.125853291619361`,
95.38030714927152`, 0, 0, 0, 28.336698108603443`, 1.7721429349018143`, 0, 0, 0.01414991389174492`, 0, 0,
65.22809012911448`, 0.029226023182826067`, 1.8914501777516688`, 0.23493229381703784`, 0, 0, 1.2561806214165052`},
{0.21964890100642098`, 3.948215647629967`, 1.3239539515383179`, 0.7541520677887659`, 0.2631388544858304`,

0.4680193255276971`, 0.07455346059859487`, 734.1983096733243`, 0.22794453971709777`, 5.810951908142973`,
1081.510384640808`, 0, 0, 0, 32.20685495470932`, 0.5744434635542539`, 0, 0, 245.6180568685338`, 0, 0,
32.400381021195095`, 770.7105179786574`, 1.7969216403604862`, 0.9999999999995876`, 0, 0, 0.8734853697697074`},
{0.24357090033802437`, 2.585687351965424`, 7.1434831024545`, 1.2789397105658484`, 0.15335660895486725`,
0.5235228651937761`, 0.12790639986488742`, 56.5359989413313`, 0.05251799125371648`, 7.0717564062965526`,
528577.8097152971`, 0, 0, 0, 144.62179647282983`, 6.780873064319313`, 0, 0, 117907.35439788646`,
0, 0, 250.4745388241922`, 410268.5781023958`, 26.60427320508903`, 1.`, 0, 0, 0.5490126870635812`},
{0.09627432586752127`, 2.332762155680175`, 5.679853879558527`, 1.2640734140093113`, 0.8156330491360497`,
0.2895792785477985`, 0.02307681035132743`, 658.139699058721`, 0.029189068125338835`, 7.800914789874877`,
229.73736831393308`, 0, 0, 0, 173.2208752119352`, 1.64021322920117`, 0, 0, 0.07053374703797155`, 0, 0,
54.66039069037804`, 0.09700841352847822`, 2.451610902476585`, 0.6956868835533483`, 0, 0, 0.37416434980298635`},
{0.07523258003696548`, 2.5308231598738997`, 0.19036315944607196`, 0.7501464923569574`, 0.1153260012566284`,
0.48261384042066857`, 0.014254428894325608`, 71.6138994625911`, 0.24976914139283035`, 2.597638512857568`,
36879.97968610944`, 0, 0, 0, 3.3983333824590742`, 0.018286157970731857`, 0, 0, 17773.617804008507`, 0, 0,
0.6611290299634422`, 19102.21605695006`, 8.009699929426743`, 1.0000000000000036`, 0, 0, 0.3086311083813645`},
{0.20487865745542216`, 2.4577449266837927`, 0.3620012570001929`, 1.1621484663425825`, 0.45716579542364766`,
0.40055848752773193`, 0.09344580216885054`, 2051.8175124763634`, 0.1493227569977858`, 9.69569860745337`,
1986.8538965457685`, 0, 0, 0, 12.427268872178908`, 0.44326359366186296`, 0, 0, 498.72699168343627`, 0, 0,
15.563269264372448`, 1459.6930927554854`, 1.0243488474576605`, 0.9999999999999862`, 0, 0, 0.9533043743209016`},
{0.1890789829788747`, 2.8864588443110275`, 0.3020897698703884`, 0.7861583937331655`, 0.3631295895274096`,
0.49565973888016235`, 0.15528595628066338`, 2271.132408212757`, 0.21092746640771154`, 4.683826377759731`,
1042.0690091087292`, 0, 0, 0, 3.718471226732954`, 0.18677285776151895`, 0, 0, 278.41838249471317`, 0, 0,
7.7016023880426285`, 752.0437800674795`, 0.4527175609913666`, 1.0000000000006106`, 0, 0, 0.5256266812684689`},
{0.13636211858102731`, 3.2225156772879746`, 1.985405604082965`, 0.8074403105824717`, 0.51683495700684`,
0.17846947550569658`, 0.2866421049071916`, 363.9511542941515`, 0.043491080354586376`, 7.48314403026421`,
8549.383571778442`, 0, 0, 0, 25.484467054882078`, 1.9555899030677855`, 0, 0, 2860.186389505232`, 0, 0,
90.0274160140287`, 5571.729651565042`, 4.351443504723342`, 1.000000000001897`, 0, 0, 0.5410012632124653`},
{0.1760012024892597`, 3.3901909690471133`, 5.258617572133415`, 0.8627686224489919`, 0.7422533085216929`,
0.41138487941188706`, 0.007592509582269234`, 424.62789093038504`, 0.09162929479721776`, 1.3722835015951313`,
58.438503856773174`, 0, 0, 0, 51.9005049167311`, 0.11236509224947686`, 0, 0, 0.2658535214694121`, 0, 0,
5.441987442576034`, 0.6684362780660096`, 0.6949849042159959`, 0.9942589291505001`, 0, 0, 0.12294036857108155`},

{0.1813951042683553`, 2.693386252451666`, 9.617741712202033`, 1.407583958403493`, 0.6407257633308476`, 0.4405499114150466`, 0.008535253338252974`, 83.84476850180724`, 0.06822985382579094`, 7.163997237119144`, 101706.29625766253`, 0, 0, 0, 507.72808128870975`, 1.5387923109838262`, 0, 0, 28161.31271134242`, 0, 0, 59.208029368316964`, 72976.06079439593`, 18.88770460302187`, 1.000000000000163`, 0, 0, 0.8597545450760986`},
{0.17566594009418735`, 3.2466526484278377`, 9.344725381698979`, 1.0138972604548542`, 0.5838549167343343`, 0.6073333607184574`, 0.10710975925951867`, 945.005957021721`, 0.208189974443157`, 1.4109192016259353`, 28.749701412650325`, 0, 0, 0, 11.533936074182584`, 0.36214193280720125`, 0, 0, 0.01630004156209602`, 0, 0, 16.796415217932505`, 0.040905173208271056`, 0.328659339791929`, 0.28874284923544025`, 0, 0, 0.1627116381665004`},
{0.22088610125498404`, 1.594941397589448`, 0.6585367344105002`, 1.4859166690390868`, 0.411769154740657`, 0.4715175396654723`, 0.024538179644211296`, 3156.4892991025154`, 0.15641735671760065`, 8.553654142069323`, 39.198446573157874`, 0, 0, 0, 28.715936453755408`, 0.4145579169470008`, 0, 0, 0.148020532684469`, 0, 0, 9.445651191960271`, 0.4670811195765569`, 0.6094365177253301`, 0.8470116440783818`, 0, 0, 1.192990415473792`},
{0.08792198919034061`, 0.7563929024204028`, 9.775416388823775`, 0.7563974438325178`, 0.8271569221820632`, 0.32401969363676375`, 0.0333282008681181`, 102.10380045307154`, 0.1014972556908379`, 6.35713244492311`, 554.8359448931674`, 0, 0, 0, 359.9218592559756`, 13.947843007568355`, 0, 0, 13.388815266338074`, 0, 0, 150.71499221426754`, 16.81673244454918`, 14.103063731070058`, 0.9999999997631528`, 0, 0, 0.9387480084713695`},
{0.13210283054552296`, 3.2703864704797683`, 0.8879166279114746`, 1.2876213813677582`, 0.37930173682375035`, 0.2978584700900475`, 0.018740615394798876`, 111.76209976448992`, 0.22677958819730465`, 8.664567998658026`, 50581.820234339546`, 0, 0, 0, 47.5984003591553`, 0.2626713419276714`, 0, 0, 17498.600825094312`, 0, 0, 12.271954326043147`, 33023.06713687394`, 16.206351360554418`, 1.0000000000000409`, 0, 0, 0.5124616822293998`},
{0.19507506176309325`, 3.126733009164254`, 1.2939386659529877`, 0.8626822462482099`, 0.4036124839823858`, 0.4778725494331981`, 0.06798274218289758`, 2442.14562500692`, 0.025650848833468687`, 3.9818096584261493`, 22.673448332103852`, 0, 0, 0, 11.607816949515968`, 0.23939960480946418`, 0, 0, 0.03505373492013066`, 0, 0, 10.693409239123248`, 0.09768727863673615`, 0.36479898880883427`, 0.5682376625318122`, 0, 0, 0.5320444321820464`},
{0.08607083224139878`, 0.4430857620402713`, 5.378081455692687`, 1.015604011959475`, 0.6736258822863768`, 0.30157456176441344`, 0.06550214653497834`, 2724.6632477522535`, 0.20587133389175544`, 1.8687253311000447`, 10.318810318580342`, 0, 0, 0, 5.3916228342984915`, 0.6700725916949846`, 0, 0, 0.007013987535343537`, 0, 0, 4.241423213049599`, 0.008624282064257527`, 0.14455979334977806`, 0.13714482471883516`, 0, 0, 0.1269169005221545`},
{0.14471266481637884`, 3.5830029087352937`, 5.919067097135953`, 1.3383183012471727`, 0.9997339519904831`, 0.40166871745176214`, 0.015827643464025123`, 2493.129738188907`, 0.10618580931649058`, 1.661604036688603`, 22.298950885660187`, 0, 0, 0, 18.14844126164094`, 0.07803838962128709`, 0, 0, 0.0225007344196804`, 0, 0,

3.994453957229851`, 0.04651630340281911`, 0.13713169823774385`, 0.299666406309293`, 0, 0, 0.07062429700073451`},
{0.1588412976528583 7`, 1.9554524456621927`, 0.3975714737116185`, 1.180621526159508`, 0.13357193827486524`,
0.6393982147520227`, 0.07953097836767822`, 3991.2803311425578`, 0.2365716915141447`, 8.584420428066604`,
28.506128337321066`, 0, 0, 0, 13.30795609370322`, 0.5032991119871744`, 0, 0, 0.19428785427391315`, 0, 0,
14.059678277641874`, 0.4408704984436621`, 0.5284524182546331`, 0.9243864028096954`, 0, 0, 2.1022017370864705`},
{0.04917531951043441`, 2.0521955455867618`, 5.720052321648424`, 1.1771597571169652`, 0.4322660150443707`,
0.1629991214278883`, 0.005091310116027936`, 189.05888707261903`, 0.1735533969163952`, 4.172239042736072`,
187.91675973261079`, 0, 0, 0, 174.32167332886735`, 0.40714712410293846`, 0, 0, 0.6168800141772903`, 0, 0,
11.936364492607282`, 0.4333610256681225`, 4.642040709529168`, 0.9976480410290857`, 0, 0, 0.2743641473059501`},
{0.27542629055144846`, 0.7843233583874856`, 9.783752583368699`, 1.3481471177954405`, 0.23017739141462767`,
0.5420497851091893`, 0.2059235624279975`, 3468.61907862177`, 0.15188055880627876`, 9.878186090289375`,
35.8690582981044`, 0, 0, 0, 9.551626553766976`, 2.1550485005378857`, 0, 0, 0.003219056040381982`, 0, 0,
24.146498248996995`, 0.012665895203992434`, 0.6512265995433762`, 0.05004342769274028`, 0, 0, 1.5666894163102356`},
{0.242913589428772`, 2.017077890959616`, 6.7411875410249`, 1.4794325227990526`, 0.8119526659059784`,
0.6637027966074835`, 0.028536028992749693`, 2430.705278612085`, 0.11516348105007651`, 2.1124785829469186`,
28.31791319247922`, 0, 0, 0, 20.099992908699434`, 0.2723204769720097`, 0, 0, 0.021240104258927896`, 0, 0,
7.847023047940268`, 0.07370728521967662`, 0.18411731042493443`, 0.2613939988066486`, 0, 0, 0.1550316729767205 5`},
{0.27369802527373327`, 0.5072292829799574`, 6.3938654238644155`, 0.9166058122252008`, 0.8588715137395462`,
0.6363372429100727`, 0.4973592888199021`, 125.12883357781175`, 0.0982596068118865`, 5.59867632569453`,
60166.060284302344`, 0, 0, 0, 39.33952795771803`, 31.002922939288496`, 0, 0, 12193.769966013904`, 0, 0,
224.65129103968738`, 47677.29657628805`, 9.864159729400342`, 1.0000000000120837`, 0, 0, 0.6550637356796396`},
{0.18540637687565897`, 1.1775430389730683`, 2.7268491301469933`, 1.4767982648221127`, 0.8902187555583527`,
0.5282942786301189`, 0.1497900469847022`, 304.29028125771407`, 0.023895791395609434`, 5.874748340307197`,
94821.83147001214`, 0, 0, 0, 44.081064980288524`, 5.044130903510678`, 0, 0, 25951.387112928016`, 0, 0,
84.8525890442562`, 68736.46656436627`, 4.323054414527168`, 1.0000000000001628`, 0, 0, 0.7902664422089832`},
{0.2722155439547531`, 0.9139371325579537`, 7.770199974150746`, 0.8847133947824111`, 0.9018075103619831`,
0.19960287154002887`, 0.008582048560181034`, 1921.873640365849`, 0.18448070830995128`, 2.8786799978881135`,
25.034955966091786`, 0, 0, 0, 22.287636217222975`, 0.19171343975345823`, 0, 0, 0.006725569101591714`, 0, 0,
2.503057591444253`, 0.026154349305643667`, 0.30671968036813596`, 0.1507511159284114`, 0, 0, 0.10800077803892284`},
{0.06964367539322192`, 3.707453741922901`, 4.6834761045808655`, 1.337177597600478`, 0.13817299390105364`,
0.15072857839162668`, 0.030325540973499845`, 324.16896723003435`, 0.11870351893768993`, 8.4259642987084`,

184.69188829640746`, 0, 0, 0, 130.3730752099109`, 1.0039128503671308`, 0, 0, 0.062056759091877564`, 0, 0,
53.170863623687126`, 0.06174086837369313`, 5.4866135160238105`, 0.6301746432723709`, 0, 0, 0.5855575466092724`},
{0.17261439973382386`, 3.0972705794401207`, 5.672236578564393`, 1.4223285072729144`, 0.7931550721000631`,
0.3896544494671119`, 0.01712882834694823`, 1350.492916562551`, 0.2427963854280547`, 3.05685280243325`,
49.605842097918476`, 0, 0, 0, 39.84275544462721`, 0.21292618948008946`, 0, 0, 0.03203960917504181`, 0, 0,
9.421286032413906`, 0.07900711293509091`, 0.47369977021571125`, 0.3804935903856288`, 0, 0, 0.1844366656727454`},
{0.11394032550346922`, 3.2108067304960466`, 9.096957387649113`, 1.399541009089664`, 0.15147624678363414`,
0.6188818627652795`, 0.08261238136824677`, 151.04115640404754`, 0.09860367843525403`, 6.06148281050898`,
500.3812667100334`, 0, 0, 0, 231.4261452985217`, 5.587806721664983`, 0, 0, 2.687344225285333`, 0, 0,
256.30524900904294`, 4.374241082411589`, 9.65195816417009`, 0.9994282565464863`, 0, 0, 1.3249213320763602`},
{0.20354065160019702`, 3.5209980508829766`, 0.4053576884306178`, 0.7576791600370161`, 0.6570403323895382`,
0.46832222917239164`, 0.005063986272754102`, 161.28160618619455`, 0.12637144234319758`, 8.874326085906798`,
53801.02557794975`, 0, 0, 0, 26.390964160181678`, 0.036849636771129145`, 0, 0, 13760.623673189406`, 0, 0,
1.8535357035270212`, 40012.09012665813`, 11.576408172642804`, 1.0000000000001452`, 0, 0, 0.5854532593989298`},
{0.24345495866234085`, 1.8344199239977046`, 3.71003568577021`, 1.0568366445286865`, 0.9797132100793247`,
0.5295146440336447`, 0.23321770204647818`, 2779.737816031833`, 0.01572768263207122`, 9.835586906627377`,
155.3449258885264`, 0, 0, 0, 36.37701545868119`, 4.3659740951357415`, 0, 0, 0.04181039485735065`, 0, 0,
114.41471239678411`, 0.14541354216620625`, 0.7255311809278353`, 0.5694640953129739`, 0, 0, 0.382798509919777`},
{0.18352727434547023`, 0.4163243700753796`, 8.057415014521496`, 1.2522528089453002`, 0.16203828635471806`,
0.6604646928880291`, 0.25830735718962067`, 2461.4350174740794`, 0.12685187153570027`, 8.600866037504808`,
45.8502551707053`, 0, 0, 0, 10.367791013052232`, 5.104398274735264`, 0, 0, 0.00544010358723522`, 0, 0,
30.3583628049002`, 0.014262962621744734`, 0.8234820243969442`, 0.08904307529459743`, 0, 0, 1.6690012518064845`},
{0.23920496896525717`, 1.1838719961494162`, 9.218551103388815`, 1.265080848919387`, 0.9542453823473309`,
0.22929853041342196`, 0.4982471338661781`, 90.20981971435951`, 0.0856312153630287`, 3.6772130334476696`,
15280.30366185599`, 0, 0, 0, 40.26490004056679`, 13.078212214431058`, 0, 0, 3397.113289538133`, 0, 0,
221.18470286234566`, 11608.662557077618`, 8.843968393369343`, 1.00000000000102`, 0, 0, 0.3650384598913887`},
{0.10156561975861944`, 0.5164671469302382`, 5.401464431301745`, 0.8562900606586841`, 0.5357330343495086`,
0.16718344164197263`, 0.02721990257094452`, 330.98847924494044`, 0.2190497368128987`, 7.446609110605853`,
225.6291338421051`, 0, 0, 0, 163.62625890888182`, 7.380027002325344`, 0, 0, 0.056333526062077435`, 0, 0,
54.45059271655836`, 0.08173642125263166`, 4.677522590094146`, 0.755374277545537`, 0, 0, 0.3976350740360762`},
{0.2533884831264572`, 2.217775528299157`, 1.87005833593021`, 1.0852798553697922`, 0.3630118105229163`,

```
  0.4986925910870725`, 0.006826900671537389`, 2180.4237702051914`, 0.21841135731377453`, 2.8539212819504436`,
  17.65882355869093`, 0, 0, 0, 15.977957581466667`, 0.04683481065823965`, 0, 0, 0.029024283620812698`, 0, 0,
  1.483844242148122`, 0.10506313143585203`, 0.2962478099856194`, 0.4261442072144227`, 0, 0, 0.4188706039894201`},
 {0.04019446235159935`, 2.9685609440334373`, 3.1967275735949383`, 1.2384363485833663`, 0.006837582253808039`,
  0.626580585404494`, 0.10123843723710083`, 64.97108080441517`, 0.1585540441972691`, 8.616308543170305`,
  82754.54501528785`, 0, 0, 0, 100.41526749813409`, 3.2105641297246907`, 0, 0, 52414.44299781811`, 0, 0,
  136.15364691164908`, 30096.719367940746`, 30.94071411782546`, 1.0000000000000282`, 0, 0, 1.5689664200372246`},
 {0.18466310913340017`, 2.870831467521074`, 5.7568671895307055`, 1.0469133934587485`, 0.5974897879057268`,
  0.6447436436670198`, 0.1911914248698421`, 363.92035372259653`, 0.11435325769039895`, 2.2759611974712186`,
  112.51918288110635`, 0, 0, 0, 30.6425909987059`, 1.9009736670474748`, 0, 0, 0.553351649800174`, 0, 0,
  77.96250031841166`, 1.4597662299457475`, 1.4203160083692783`, 0.9987543809470352`, 0, 0, 0.34290711880219377`},
 {0.22919090442152185`, 1.3485088294529417`, 3.711141569564556`, 1.1683364276022934`, 0.869869922573845`,
  0.45530089098812654`, 0.06782162054416256`, 403.6677278730082`, 0.1398561461848175`, 8.19586820054278`,
  264.7668887685776`, 0, 0, 0, 131.88846145002162`, 6.088203301192917`, 0, 0, 2.2235027796832103`, 0, 0,
  117.28565581661694`, 7.280094472277014`, 4.568242137518486`, 0.999999989191112`, 0, 0, 1.1473282172917645`},
 {0.12718220564190125`, 0.7794468104625536`, 3.4523481867286847`, 1.042660599955479`, 0.821297748494871`,
  0.42153700480377276`, 0.05083556365712203`, 907.9591078654141`, 0.03221255361915021`, 1.403008654938164`,
  36.433778707561316`, 0, 0, 0, 20.990633597951664`, 1.2366676997873356`, 0, 0, 0.154635899784683`, 0, 0,
  13.77023848859002`, 0.28095621151481726`, 0.3277128232701847`, 0.9408696273065174`, 0, 0, 0.10457721619743707`},
 {0.24829534536640735`, 0.6361539074945153`, 1.4367506745172012`, 1.2157272327840285`, 0.5920355341003019`,
  0.22834833308349511`, 0.008858258633323783`, 152.73617223659593`, 0.05098711045238086`, 6.308971873547048`,
  194460.44354841363`, 0, 0, 0, 61.495969878706035`, 0.7626058633979217`, 0, 0, 42750.81076944352`, 0, 0,
  6.930495712554487`, 151640.3903527569`, 8.432621206318899`, 1.0000000000002724`, 0, 0, 0.21672397195787066`},
 {0.24634814165734203`, 1.0199939463339236`, 6.874973257490155`, 1.1669106806380989`, 0.9547234223304992`,
  0.2467804242930165`, 0.010102520966714356`, 1373.9164154017546`, 0.22192762621195788`, 8.148592815397581`,
  130.46850608034433`, 0, 0, 0, 114.008326888658`, 1.045407171815537`, 0, 0, 0.020461259406030788`, 0, 0,
  15.23298552437023`, 0.07200847472349382`, 1.2112985224193757`, 0.3140788603434711`, 0, 0, 0.2834700023117419`},
 {0.11968292559735771`, 0.4405556662519543`, 0.9343787484302961`, 1.4969299043499453`, 0.5720179190625709`,
  0.5743610731089337`, 0.058376899138915264`, 4861.966479565238`, 0.13911792619470742`, 8.404040973858844`,
  33.48884913923602`, 0, 0, 0, 18.343940845431863`, 2.054985851555301`, 0, 0, 0.05765523743579236`, 0, 0,
  12.933366585289802`, 0.09857639274751538`, 0.38109727202797605`, 0.5464929604647888`, 0, 0, 0.9838953149377347`},
```

{0.0912885213280808`, 1.1352603856466574`, 9.64216394840902`, 1.0423303168159856`, 0.8496114679763396`,
0.3948758352214041`, 0.050073459411100744`, 1020.4170701645861`, 0.08585808288581304`, 1.5724283740137839`,
37.42979637377136`, 0, 0, 0, 21.927178186410725`, 0.8977156114227023`, 0, 0, 0.01955042428498116`, 0, 0,
14.559156731782346`, 0.025496133204457847`, 0.32063974532357453`, 0.3295391528033339`, 0, 0, 0.08531539328667118`},
{0.23601424519793518`, 0.7493625485076763`, 8.159569145163797`, 1.0789985894921148`, 0.9135595458313224`,
0.43990663477596414`, 0.03553380189426036`, 63.68276264433202`, 0.11799293833856772`, 7.92015369426547`,
139480.46948593995`, 0, 0, 0, 342.37571951254813`, 14.600366994556234`, 0, 0, 31788.39281515719`, 0, 0,
156.2995460026859`, 107178.76480463958`, 26.414047573532336`, 1.0000000000006088`, 0, 0, 0.6025852195383089`},
{0.09505824539478602`, 0.994707682773301`, 2.092747921993542`, 1.2334327544777528`, 0.1649122243213208`,
0.435178801412204`, 0.4947441968705133`, 1030.121381906519`, 0.19720224785958157`, 8.531247013968503`,
90.45835009258096`, 0, 0, 0, 12.740088806256784`, 5.099552492760274`, 0, 0, 0.0651016981339128`, 0, 0,
72.46520061791965`, 0.08840647424030458`, 1.8774255151938661`, 0.6691537334855536`, 0, 0, 1.2675655622685091`},
{0.13338172675277454`, 1.0224437122231844`, 3.1176391252125253`, 1.1808523949493024`, 0.5954558720182712`,
0.3646371291004906`, 0.024821319877175146`, 118.84942434665426`, 0.15904005586434156`, 2.0945755856004613`,
44395.625014980316`, 0, 0, 0, 38.86754582382741`, 0.8650631723589228`, 0, 0, 15262.07561039796`, 0, 0,
12.635405732203093`, 29081.171410661183`, 3.781652835272766`, 1.0000000000005274`, 0, 0, 0.18262387950562123`},
{0.2663723170815805`, 0.6117369492537685`, 4.996758576680092`, 1.4798016834758696`, 0.3454705507334743`,
0.5628818087325649`, 0.10659789380555212`, 347.2598858446038`, 0.128819557084905`, 4.579853140224845`,
188.09878980109036`, 0, 0, 0, 75.46206065539009`, 11.343405823556367`, 0, 0, 0.44994928744018414`, 0, 0,
99.13114960928293`, 1.7122004894948653`, 3.0197858586805846`, 0.9856496709206579`, 0, 0, 0.7333093564182788`},
{0.19858704302380392`, 3.77240735130054`, 4.552319154599175`, 1.490234534684174`, 0.9267739623702391`,
0.4663128979271708`, 0.016623264635311285`, 190.7315614324941`, 0.03981298108873932`, 1.7245962595124684`,
59538.529850613384`, 0, 0, 0, 53.68049898485968`, 0.22693461879264332`, 0, 0, 15499.875493265816`, 0, 0,
12.229854631399334`, 43972.49202063981`, 2.041441918143971`, 1.0000000000011595`, 0, 0, 0.2481985915763493`},
{0.16356962246540357`, 2.7590365436019733`, 9.315311335363422`, 1.411146654012818`, 0.2509435276133807`,
0.3090283790000361`, 0.2431141196841502`, 313.55839056266797`, 0.2467169299716112`, 8.038445579591425`,
255.0347181232233`, 0, 0, 0, 61.1578029158479`, 4.793842183492308`, 0, 0, 0.04037059242109976`, 0, 0,
188.94836812165667`, 0.09433432229988671`, 5.600862075136447`, 0.45890709938303065`, 0, 0, 0.859620731978695`},
{0.25383472083693576`, 3.9769524497483806`, 7.011565744250568`, 1.066285473960517`, 0.7734216029556533`,
0.6850552501893354`, 0.013843595247453381`, 297.6124430270534`, 0.10716201039628298`, 2.5950386184147853`,
159.74436524213615`, 0, 0, 0, 129.7848271206461`, 0.437177468257577`, 0, 0, 0.9963309078275555`, 0, 0,

24.837628619453753`, 3.6129053978517573`, 1.9933286552524427`, 0.9999957921392091`, 0, 0, 0.41070774282119205`},
{0.17343366236722435`, 1.3936719133530833`, 0.599503921563171`, 0.8621470436054358`, 0.43567440743840313`,
0.22823781097601215`, 0.018669299661272933`, 3618.675535403083`, 0.22611694811187344`, 3.1306846738065635`,
8.932755013182055`, 0, 0, 0, 6.98086317638967`, 0.08672814155854232`, 0, 0, 0.03900456547960763`, 0, 0,
1.7267224998207276`, 0.09663863771672004`, 0.1856807031200472`, 0.6053454901077919`, 0, 0, 0.2737009644632621`},
{0.27302715407014244`, 1.5486379048502288`, 9.868645627334566`, 1.0694698241775202`, 0.6816384789594119`,
0.4416154581373778`, 0.05961824305163796`, 87.63839207083008`, 0.05267766985704242`, 3.1260333504958955`,
91650.78842242043`, 0, 0, 0, 139.47199081894604`, 4.980990699028664`, 0, 0, 18650.796339016943`, 0, 0,
110.19644286031756`, 72745.34065119475`, 7.882684135404812`, 1.000000000002026`, 0, 0, 0.3741505155050053`},
{0.12081145422022538`, 2.0244942048813837`, 8.652229532190159`, 0.835020699056165`, 0.9214042127932665`,
0.46285611589460973`, 0.11861503789539068`, 2689.091322411565`, 0.07096583948487911`, 5.509201411600676`,
69.05941686292778`, 0, 0, 0, 25.885982306069312`, 1.4421282691263906`, 0, 0, 0.008442564360954488`, 0, 0,
41.70829033631434`, 0.014570835397052683`, 0.42183349684365856`, 0.19525044163516647`, 0, 0, 0.2381658300652869`},
{0.27038445563951496`, 3.3688963138915424`, 3.4133330216440854`, 1.1975198070883244`, 0.31492494684053174`,
0.45427916274833`, 0.0070970717881839104`, 3067.136751023867`, 0.17569148508387616`, 1.2036801730392703`,
4.720745024840074`, 0, 0, 0, 4.249725916190107`, 0.00859534249416999`, 0, 0, 0.009164743755624196`, 0, 0,
0.41366882350349443`, 0.03540006073485821`, 0.08922750082613147`, 0.14791726775127545`, 0, 0, 0.18292942604020812`},
{0.1767050761987432`, 2.85608165701536`, 6.140634667151749`, 1.3554192221984884`, 0.6330624847112656`,
0.5906276578639604`, 0.04152902973081543`, 1510.3499876585004`, 0.15541682752363528`, 8.81551919212286`,
125.0655075646142`, 0, 0, 0, 78.90153610038297`, 1.1022707403464223`, 0, 0, 0.023503220852581476`, 0, 0,
44.973932036687906`, 0.05933054902386386`, 1.261127873658465`, 0.310108021728718`, 0, 0, 0.8351861156519741`},
{0.16171803894826015`, 2.2156907324583015`, 1.0134587159780288`, 0.8322559746700846`, 0.34733770749260806`,
0.6849579573613365`, 0.018772440750507276`, 1617.8437562512436`, 0.21218330633531807`, 7.516753350682977`,
69.77474506785634`, 0, 0, 0, 53.67414246720448`, 0.43193967532335276`, 0, 0, 0.5967239980912309`, 0, 0,
13.67206765135714`, 1.3785862109240026`, 1.1121787409691533`, 0.9998061050075341`, 0, 0, 1.5998592388966126`},
{0.24108871826638628`, 1.1722622038849657`, 2.5945274526654174`, 1.1873482780450624`, 0.3399058776037083`,
0.3423735862363967`, 0.09173881369912114`, 1455.6792358614214`, 0.2278375557130481`, 4.818602627144232`,
36.755851350699345`, 0, 0, 0, 16.25069512971041`, 1.148513712931908`, 0, 0, 0.02765969443689275`, 0, 0,
19.233703090195114`, 0.09526343256332741`, 0.7253125369770169`, 0.3903071419461721`, 0, 0, 0.5308975319968847`},
{0.2202280085702742`, 2.180975046803316`, 5.6134680006393936`, 1.2506485034427453`, 0.4748208651226249`,
0.39757955628981323`, 0.017520218948128618`, 280.3947437474927`, 0.12359899846366301`, 9.521190146527957`,

443.13414131294326`, 0, 0, 0, 351.39428838542267`, 2.6774415703015553`, 0, 0, 1.3238276064449392`, 0, 0,
83.42047505859398`, 4.164913106538831`, 7.45493695113794`, 0.9991506751179341`, 0, 0, 1.0697905438924589`},
{0.12315072752895034`, 2.8609501087269207`, 1.79674111361204`, 1.4514496090822089`, 0.3032919079034333`,
0.2763824218936657`, 0.028981582964232436`, 188.78817200372555`, 0.08856946510090113`, 4.546561013073182`,
58633.29990505694`, 0, 0, 0, 45.86430021621891`, 0.44328938231496273`, 0, 0, 21226.016774271637`,
0, 0, 18.117554379021193`, 37342.84868990359`, 5.080524084297705`, 1.`, 0, 0, 0.3130961184834628`},
{0.14162785770391484`, 1.92013166099325`, 8.540643644714823`, 1.2350689068338556`, 0.301574102817308`,
0.40858591883894513`, 0.080613435450821`, 585.1346359267128`, 0.1521171210972616`, 7.686797097032363`,
153.20803973682908`, 0, 0, 0, 72.66492137584426`, 2.8306727422462665`, 0, 0, 0.02170914075881159`, 0, 0,
77.64663363282335`, 0.043923129975259614`, 2.9133697817508404`, 0.3143702490925293`, 0, 0, 0.950418308024325`},
{0.22524615675817938`, 2.031565502127312`, 0.245821316301889`, 1.298167355078078`, 0.689325845538592`,
0.661487626561178`, 0.00610096551453922`, 373.63064864758115`, 0.20592315239272913`, 2.994117316721619`,
26349.97598308941`, 0, 0, 0, 5.4339895307532196`, 0.01562982934487665`, 0, 0, 6245.9227495607165`, 0, 0,
0.4536146014455515`, 20098.144210671828`, 1.7200689956793889`, 1.0000000000000422`, 0, 0, 0.2620804767691623`},
{0.06014178921319169`, 2.215935435785701`, 1.3525069683978295`, 1.0400202244696481`, 0.697929353581739`,
0.692932654029565`, 0.014666911144259826`, 1018.9785490283374`, 0.16122419934759502`, 4.978072859014281`,
70.28298129569727`, 0, 0, 0, 51.91608648711861`, 0.3253370616875185`, 0, 0, 4.149196366393443`, 0, 0,
10.298941765253865`, 3.5648584753110324`, 1.2217713060141497`, 0.9999999991299203`, 0, 0, 1.329718155499555`},
{0.11674773262981253`, 1.910335393882857`, 4.221858661435528`, 1.4404651869296854`, 0.4563596481936356`,
0.5439902833298367`, 0.005611633628996954`, 451.6526207878965`, 0.24247834364497922`, 9.182241064841566`,
320.8342668292945`, 0, 0, 0, 295.19591681894025`, 0.8238098441184336`, 0, 0, 0.751599409175239`, 0, 0,
22.48218718640802`, 1.2535360981016483`, 4.542903941439494`, 0.9956044256299839`, 0, 0, 1.2136189232695411`},
{0.12898119467887553`, 3.729766126068422`, 2.3744826976889595`, 1.2465399522360021`, 0.1415631230326866`,
0.3607064691421532`, 0.03995461220531779`, 1238.1663657022145`, 0.14506115517150608`, 8.953141814946814`,
69.96367527499407`, 0, 0, 0, 45.03223950605033`, 0.4574587477165292`, 0, 0, 0.033875449589081355`, 0, 0,
24.374487732954165`, 0.06241851368973307`, 1.6192034419924282`, 0.4395389726750034`, 0, 0, 1.2047306080235658`},
{0.2498624462217614`, 2.827129073229683`, 2.690968559152097`, 1.0446525535360744`, 0.06936419216205603`,
0.5607634545552321`, 0.008891348377622028`, 196.01664126075448`, 0.10064633302983284`, 7.405206717033214`,
58885.82980967864`, 0, 0, 0, 141.993093689474`, 0.43089469922865736`, 0, 0, 12851.810981722347`, 0, 0,
17.40278473842709`, 45874.07043246908`, 8.108321936456687`, 1.0000000000000375`, 0, 0, 0.647581184193504`},
{0.25890580889310233`, 3.9576703566675278`, 2.211552265086926`, 0.8006241502346895`, 0.26522568617457876`,

0.4892067360859801`, 0.02025985822061832`, 94.72915936196773`, 0.06766512891079995`, 5.4984531961033785`,
187331.1336026766`, 0, 0, 0, 74.22665409389836`, 0.3692943214246534`, 0, 0, 39848.77714700426`, 0, 0,
20.87921698268574`, 147386.85543780113`, 12.18508168897698`, 1.0000000000000089`, 0, 0, 0.34991172717359315`},
{0.2032139210380186`, 1.0205320875718096`, 3.2697243543529257`, 1.0866214178514029`, 0.5111053959745286`,
0.4079750171889772`, 0.048345897200607585`, 145.20906840025864`, 0.23728279570010086`, 2.517042827760779`,
26575.78611810111`, 0, 0, 0, 39.16650708934835`, 1.6995154689572323`, 0, 0, 6792.149859055458`, 0, 0,
24.777286705650017`, 19717.9915019498`, 3.6976022221948006`, 1.0000000000075226`, 0, 0, 0.20337906887486398`},
{0.15288075349234037`, 1.8405591021591183`, 8.732063156275487`, 0.8366527628406224`, 0.7247685787231319`,
0.43308603413296065`, 0.06703535093550567`, 1294.178726539122`, 0.16108367938164575`, 4.274431762914247`,
68.15273844370063`, 0, 0, 0, 35.132887270352306`, 1.2085048468673847`, 0, 0, 0.010995654514396071`, 0, 0,
31.776065655788315`, 0.02401462781856319`, 0.6968624896001716`, 0.245802846289621`, 0, 0, 0.2950155922816745`},
{0.1609254870534111`, 1.384768951726243`, 6.196355509527487`, 0.9770497723863997`, 0.43819861370403546`,
0.17710326003602894`, 0.2243750211158665`, 120.51310647409781`, 0.10277566384729958`, 6.21125463242891`,
11256.15117516689`, 0, 0, 0, 78.6196360060992`, 10.881244302821855`, 0, 0, 3319.6747599729456`, 0, 0,
215.2572752385117`, 7631.718251536588`, 10.925472507475028`, 1.0000000000007818`, 0, 0, 0.45983603687035696`},
{0.21184155187533854`, 1.0400436907537394`, 2.77253033361667`, 1.25783985819887`, 0.5916706672066951`,
0.18670557332607218`, 0.05860949172916328`, 177.69964385957368`, 0.1825191510166627`, 3.5990512292385053`,
23476.583376099206`, 0, 0, 0, 42.4984779751775`, 2.1781735697397084`, 0, 0, 5811.66267637715`, 0, 0,
32.362795408204676`, 17587.880576281685`, 4.180742713074167`, 1.0000000000000488`, 0, 0, 0.16505899389759981`},
{0.15826860039724194`, 2.914200187526596`, 3.057619613104965`, 0.9127068016590583`, 0.36895217716385176`,
0.3294645201729436`, 0.006405724718526674`, 799.6829736219844`, 0.2321605158663716`, 7.540832982633661`,
106.7202092065175`, 0, 0, 0, 97.72249819120857`, 0.20551011053949034`, 0, 0, 0.04139548743212558`, 0, 0,
8.55568003818276`, 0.09359436940920825`, 2.048692550041607`, 0.6192692417945318`, 0, 0, 0.7599100742754853`},
{0.2486148170283075`, 0.5446418582356878`, 7.121193243885838`, 1.048132429671941`, 0.7615900023009023`,
0.5400927540260018`, 0.07717023450494316`, 388.33902457321517`, 0.04098404938849237`, 2.529691537294738`,
145.99404495397135`, 0, 0, 0, 69.30859999182236`, 8.526752085903013`, 0, 0, 0.39880262737409977`, 0, 0,
66.34323001116063`, 1.4164034605003268`, 1.402697339474069`, 0.9961293151219387`, 0, 0, 0.23023385552895392`},
{0.1973186549669898`, 3.31823274961833`, 5.404057922234477`, 1.0748992098641554`, 0.1837120164888255`,
0.5027766473922404`, 0.30055211808677745`, 460.171808421071`, 0.10833443978919949`, 3.5562239100468123`,
88.9845536613686`, 0, 0, 0, 18.123103029081093`, 1.4601564893105565`, 0, 0, 0.048449700266410455`, 0, 0,
69.216272605686`, 0.13657185271596653`, 1.7786260472090378`, 0.6131787961064874`, 0, 0, 0.5907088185314855`},

{0.16177424691159015`, 1.9234518490470327`, 6.336020872512709`, 1.465202064627133`, 0.3679027675625519`,
0.6088093981583469`, 0.02752967040620688`, 1523.605195139083`, 0.023927731291662258`, 6.9520681394676025`,
68.97739749780388`, 0, 0, 0, 49.73952159093552`, 0.6733197124040651`, 0, 0, 0.016132878366665`, 0, 0,
18.5014006546202`, 0.03728406068978142`, 1.034694979242523`, 0.20984356706905294`, 0, 0, 1.0353628667243167`},
{0.12468946686374066`, 2.602287301847274`, 9.387923649086364`, 1.237964958422611`, 0.6279283704003791`,
0.5225362832845872`, 0.15386398985407668`, 4877.253164044286`, 0.10729269282887094`, 6.155025385024989`,
24.79324834329096`, 0, 0, 0, 7.932831964781891`, 0.4414051073451314`, 0, 0, 0.0034304435546571783`, 0, 0,
16.409470083068083`, 0.0061105739705188036`, 0.27156383047247096`, 0.057776850604039476`, 0, 0, 0.5557014098537689`},
{0.1891437323001075`, 2.037539508644585`, 6.753647851102151`, 1.0264835122983769`, 0.08414675462879395`,
0.15050525073770327`, 0.01724785647741675`, 835.2003740810117`, 0.09305345804576048`, 5.34515441362371`,
43.22750720053642`, 0, 0, 0, 34.903621306023695`, 0.2749211539464209`, 0, 0, 0.00840845345220087`, 0, 0,
8.002324470399909`, 0.022720089554583198`, 1.3553696582182078`, 0.16162662237745373`, 0, 0, 0.39033883476851244`},
{0.11951180040558451`, 2.9137311414381974`, 6.217108794108201`, 1.4729889111648695`, 0.6540356010640074`,
0.40115742558392786`, 0.08157249569611574`, 391.2473393910366`, 0.0369147403900103`, 8.338813449127404`,
381.22059843425484`, 0, 0, 0, 178.15304424797108`, 4.748289814215446`, 0, 0, 0.24843084441636978`, 0, 0,
197.64628428933335`, 0.42414882132141396`, 4.530011147261324`, 0.9603483043844986`, 0, 0, 0.6411175830137548`},
{0.17883917330505905`, 1.5543060950462326`, 9.905917854358197`, 1.2462903035005124`, 0.3202858713356564 5`,
0.3166477244063972`, 0.12099723319057126`, 400.41544183552224`, 0.027102889569123156`, 3.023770086067156`,
78.29135256141872`, 0, 0, 0, 29.66684422372291`, 2.091636304859099`, 0, 0, 0.025148028146053714`, 0, 0,
46.443472246606795`, 0.06424932234132459`, 1.6487180455104837`, 0.3508624167247224`, 0, 0, 0.3211022161499714`},
{0.25477439586299494`, 1.0781629016953422`, 9.675717925989822`, 1.4583691370753404`, 0.484439918991886`,
0.6784957922502513`, 0.09307366059264305`, 217.6033516010123`, 0.05467318278554256`, 4.774031073697932`,
393.88283325884083`, 0, 0, 0, 169.7770591487085`, 13.363882133780361`, 0, 0, 1.0575948394619668`, 0, 0,
205.83488484673114`, 3.8492583755959857`, 4.9821157218364736`, 0.9972392685356379`, 0, 0, 0.7188693284828338`},
{0.26738035849024727`, 1.6336260676101997`, 9.900661691242231`, 0.846806505065853`, 0.2415106216321452`,
0.502680613934997`, 0.14320441138979886`, 77.88432317464672`, 0.12860121706274624`, 9.963544099078408`,
61516.02042546346`, 0, 0, 0, 269.37810200237897`, 21.808366883288663`, 0, 0, 12597.38912811989`, 0, 0,
508.9530947506785`, 48118.491730254755`, 27.757174486568182`, 1.000000000000218`, 0, 0, 0.9898228926455391`},
{0.0966033889391959`, 0.8191052864079662`, 6.963587929197736`, 1.39989556064551`, 0.208903818004208`,
0.4044327200024739`, 0.345646875315088`, 51.22809362991988`, 0.05669448608759026`, 3.5983266970468653`,
313231.1273348109`, 0, 0, 0, 37.18778545383081`, 13.16211286421061`, 0, 0, 131521.14007521374`, 0, 0,

154.01651753390342`, 181505.54069160466`, 15.332350873656974`, 1.00000000000002`, 0, 0, 0.3812719754534076`},
{0.07268626506253911`, 2.0017174568948795`, 2.5547427204964173`, 0.8446935149578232`, 0.0630154383873418`,
0.49766282606547807`, 0.00568323780270915`, 197.50095618865632`, 0.04182941385906325`, 9.915201734242256`,
54749.04327797868`, 0, 0, 0, 197.13157167331536`, 0.5307609469389022`, 0, 0, 26754.575013408175`, 0, 0,
15.177620756080799`, 27781.287586573842`, 11.309149088071935`, 1.0000000000000007`, 0, 0, 1.4015415771737663`},
{0.14445086415814334`, 1.1396367789378896`, 8.108045316850049`, 1.1452867101498132`, 0.43349891091368953`,
0.5412309783014353`, 0.17300117449492944`, 151.5195914284387`, 0.22841440999808194`, 1.401560283159844`,
110.15771793878415`, 0, 0, 0, 30.91393956198547`, 4.1056446167251375`, 0, 0, 2.7079664851882006`, 0, 0,
66.84205152097633`, 5.5881156985244695`, 2.23051886079269`, 0.999999937715034`, 0, 0, 0.31080740399649354`},
{0.2737062633152032`, 1.87379900045931`, 2.9341815386065377`, 1.0257953180476898`, 0.5994241695021645`,
0.2666028641788709`, 0.16092630392754248`, 2757.7643970777704`, 0.13082540497870587`, 6.7306102844961035`,
32.471263536437455`, 0, 0, 0, 10.177959892055263`, 0.8007155805340579`, 0, 0, 0.011931987844734341`, 0, 0,
21.43400077795595`, 0.04665514009859763`, 0.5135737022803337`, 0.2207290294779013`, 0, 0, 0.4453870918623241`},
{0.14450675367477284`, 1.4907097867645147`, 8.628071674125838`, 0.8645742960679661`, 0.06677853457714877`,
0.6218432064874626`, 0.019848227343967184`, 449.15101395266043`, 0.20772413403212947`, 3.059722840773212`,
77.90011327461615`, 0, 0, 0, 60.9520306350899`, 0.7563567893063481`, 0, 0, 0.020345303897011217`, 0, 0,
16.107263830067982`, 0.04200048312403961`, 1.6336771716973972`, 0.3946127594529154`, 0, 0, 0.6580338014691158`},
{0.05092006104579416`, 0.9079056727720332`, 9.25200153286255`, 1.1365283513840736`, 0.03327106644526534`,
0.582893635885294`, 0.1249724078419535`, 82.97595681905067`, 0.15395748273911747`, 7.1070379565760815`,
28619.623157717273`, 0, 0, 0, 214.67789576773436`, 26.017656138801264`, 0, 0, 16232.799414382656`, 0, 0,
337.4511085807112`, 11808.216244635694`, 20.197265086659513`, 1.000000000000002`, 0, 0, 1.38412484938077`},
{0.2364459713820966`, 3.884359137525399`, 0.954741626535867`, 0.8976843312448166`, 0.4180287949880024`,
0.15414803582881131`, 0.32241293356212986`, 4897.582666581565`, 0.05447310320870352`, 4.86045092756919`,
8.678563707036247`, 0, 0, 0, 1.706388198902468`, 0.12248953215129474`, 0, 0, 0.012023930048381034`, 0, 0,
6.797047620901318`, 0.04061442600170851`, 0.20758305483707606`, 0.24866198113237914`, 0, 0, 0.29060194238737924`},
{0.042914457849633436`, 3.519672647432988`, 3.392121169492343`, 1.4941116772804357`, 0.33812708289181614`,
0.3684690007615431`, 0.017148240098985187`, 3621.3180321373566`, 0.22166064632630456`, 1.0781698542512608`,
3.2964433019883033`, 0, 0, 0, 2.647949240267482`, 0.01234458246926532`, 0, 0, 0.008843849164638028`, 0, 0,
0.6206984180150567`, 0.005421842745776806`, 0.06657366871535743`, 0.1163432902647008`, 0, 0, 0.1433245769072899 2`},
{0.1470328346746822`, 2.9686959435849998`, 9.643655350717612`, 1.0921756097932163`, 0.41500029005274564`,
0.5183995564120613`, 0.0407899820168 4905`, 4770.787596114335`, 0.08630863643009795`, 3.8345772490574745`,

11.611914838569161`, 0, 0, 0, 7.395385715289845`, 0.09696466183875081`, 0, 0, 0.0021956761649542106`, 0, 0,
4.112265689597008`, 0.004611949865154065`, 0.1790621782083117`, 0.042212457263193226`, 0, 0, 0.493712020172387`},
{0.10223589016255652`, 0.4809064129783538`, 6.158635896611097`, 0.9490677758592585`, 0.9235714285317382`,
0.43663660593873277`, 0.45300992250929206`, 1331.4948553442105`, 0.21785131418517045`, 5.425736978481032`,
130.2181377673123`, 0, 0, 0, 18.142705133465405`, 14.230402405701376`, 0, 0, 0.03286388047451553`, 0, 0,
97.76416823091942`, 0.047998115350071445`, 0.8394750717483217`, 0.5227791698604188`, 0, 0, 0.23756384221838825`},
{0.08719902184880185`, 3.645892777856403`, 2.8891082836443758`, 1.428386787110956`, 0.151537003432892`,
0.34505159105650784`, 0.041961341889936235`, 290.12993265609543`, 0.15015043093723618`, 6.608503242578238`,
5360.4407545880495`, 0, 0, 0, 101.02554303495141`, 1.0988913801069782`, 0, 0, 2316.0134875286003`, 0, 0,
57.23485923400981`, 2885.058724301796`, 5.095762145215109`, 1.0000000000001024`, 0, 0, 0.8822246072597989`},
{0.14456990131617375`, 0.9947424361823325`, 3.150441801621305`, 1.0015653051761273`, 0.8447006281771343`,
0.5703016332732033`, 0.008271730569805881`, 52.80371817926736`, 0.13650904531369146`, 3.8670961625783473`,
153936.2750796065`, 0, 0, 0, 88.7506952957576`, 0.6806885193864544`, 0, 0, 50186.891221064914`, 0, 0,
9.67299651508323`, 103650.19873135575`, 15.947648274902237`, 1.000000000000216`, 0, 0, 0.39951916117024533`},
{0.14643438827373756`, 2.4493630197222025`, 5.7044459384485755`, 0.7643715149180945`, 0.8231136724552808`,
0.4724466281569806`, 0.012687117803733845`, 1217.9307406282053`, 0.17452698640833697`, 5.969503699935295`,
132.7426642836786`, 0, 0, 0, 112.40163275387134`, 0.5609096906508442`, 0, 0, 0.02649001095230237`, 0, 0,
19.626735052628547`, 0.05541497927378731`, 1.0221107017333109`, 0.5234113864521879`, 0, 0, 0.3377871911494979`},
{0.045156311345747796`, 1.2463805103118828`, 6.638333254697063`, 1.0774632518381033`, 0.7965921624862937`,
0.19987812094053103`, 0.1230725004501963`, 3016.4870397653995`, 0.18711874545495605`, 6.497285772809487`,
31.870694821839166`, 0, 0, 0, 11.832923792060136`, 1.0650704233520012`, 0, 0, 0.005262120273223023`, 0, 0,
18.96404311107948`, 0.0033945420199473077`, 0.4431287262546541`, 0.09975923789638685`, 0, 0, 0.2752873306460161`},
{0.21753427776894996`, 2.5707121359144134`, 7.635927852201033`, 1.3489791075447177`, 0.07755529833527275`,
0.4139475400754866`, 0.049678130502208456`, 727.9927710408293`, 0.24367647097486184`, 3.0823691783926943`,
40.2670998924629`, 0, 0, 0, 23.886497922146752`, 0.43241109359009555`, 0, 0, 0.016398707390842614`, 0, 0,
15.8800635142297`, 0.050961156694467526`, 0.971147999221201`, 0.22873074370837254`, 0, 0, 0.4997070195356858`},
{0.08480900190064672`, 0.6294563083477476`, 5.454864363444388`, 0.7943923084343845`, 0.9968128471314037`,
0.29575569030654725`, 0.01722905088446216`, 309.5962534203848`, 0.1957861782089137`, 7.847856100229725`,
341.2031653600431`, 0, 0, 0, 273.33634169390234`, 6.6002299675293035`, 0, 0, 0.8100710193328942`, 0, 0,
59.35080556581665`, 0.9814473516895452`, 5.380142062382915`, 0.9999608966324525`, 0, 0, 0.5915478162872025`},
{0.12061650440369748`, 3.919019990284525`, 1.2611661330191772`, 1.4204820377968523`, 0.21263599304474745`,

0.2382948444656248`, 0.008404906276562513`, 547.3513122366178`, 0.16414531089750106`, 3.5940701557610755`,
3715.5206049358126`, 0, 0, 0, 32.556641202034974`, 0.06679912253209264`, 0, 0, 1351.0843607958775`, 0, 0,
3.739815664810516`, 2328.0438964814725`, 1.411527662845774`, 0.9999999999999819`, 0, 0, 0.3204442061326845`},
{0.2541152986933387`, 3.9531330061598675`, 6.8389480578926864`, 1.4090336327472661`, 0.5067749551810137`,
0.5768128332044816`, 0.18877117253369932`, 176.17163988983094`, 0.11435366361110944`, 3.2749545355180913`,
436.43178457413836`, 0, 0, 0, 58.135884950319976`, 2.5169902623987497`, 0, 0, 50.458976896266776`, 0, 0,
142.14281832102122`, 183.17711408221587`, 4.521670620470263`, 0.999999999452333`, 0, 0, 0.7611021153733701`},
{0.26839792559441034`, 1.5919359357927423`, 0.607262674381337`, 1.065158649344713`, 0.7342994823745972`,
0.6373140564402076`, 0.019311239061898532`, 131.67334362372645`, 0.026592393453336638`, 2.002632914376134`,
522742.8241948918`, 0, 0, 0, 7.589338503743456`, 0.08731591247029656`, 0, 0, 108131.04442384704`, 0, 0,
1.9857334118285486`, 414602.11451025127`, 3.231198490359931`, 1.000000000000285`, 0, 0, 0.1530458253701156`},
{0.0804335350475921`, 1.0990801178785707`, 6.42097149017635`, 1.215697698043305`, 0.5864093543250795`,
0.4707288466973014`, 0.04708688161958605`, 3199.4245739234016`, 0.17424214472199046`, 5.845755402883285`,
31.705913645576064`, 0, 0, 0, 19.10121589579792`, 0.7537949001462523`, 0, 0, 0.006831759172934952`, 0, 0,
11.835442681557252`, 0.007850036298207736`, 0.3940882141045094`, 0.1135329063930014`, 0, 0, 0.5425792150529647`},
{0.04545514913582513`, 3.0084108739176516`, 7.583320976280454`, 0.7956411409554567`, 0.21703974982634144`,
0.4038408554076 1094`, 0.39906200752611665`, 3808.370230770943`, 0.07445295911170413`, 7.244769365152968`,
20.688207038340703`, 0, 0, 0, 3.4300136092210907`, 0.3923617906128052`, 0, 0, 0.0019291983873497725`, 0, 0,
16.86264967699096`, 0.0012527428629896813`, 0.4250215594980936`, 0.05082593819317116`, 0, 0, 0.9563372061393004`},
{0.21811178043156876`, 3.634131336571894`, 2.2696382083853486`, 1.2012104146171985`, 0.07478503245860835`,
0.304565647018059`, 0.049403646487048036`, 242.22074286172042`, 0.22363734402413937`, 5.499597099973035`,
15686.16133302832`, 0, 0, 0, 58.135121905636204`, 0.7570453643442391`, 0, 0, 3787.2714007765026`, 0, 0,
39.30288973956982`, 11800.692974298934`, 4.776519189369514`, 1.0000000000004723`, 0, 0, 0.3623945011712749`},
{0.22643881069864102`, 1.8950339409059218`, 4.49788713237294`, 0.781319815193253`, 0.8852654232409718`,
0.19866746146736047`, 0.009828863939504507`, 672.8810907026934`, 0.061975672509268276`, 2.025268353742364`,
48.51080213629765`, 0, 0, 0, 42.41558729040904`, 0.20906659655604495`, 0, 0, 0.04545472735228421`, 0, 0,
5.6598328054769995`, 0.14703877717546313`, 0.6182741826480198`, 0.7078956060983954`, 0, 0, 0.08259167973104065`},
{0.13526256211607002`, 2.462351571487578`, 7.058879409696349`, 0.9886974127254741`, 0.579411758510507`,
0.519067819756009`, 0.20147771873022094`, 163.35469430255884`, 0.08074145629003388`, 7.486187513545234`,
873.2249300900974`, 0, 0, 0, 129.55235625178602`, 9.443263842462681`, 0, 0, 137.10934732246804`, 0, 0,
332.1805080351399`, 264.93945155570304`, 10.972788786912462`, 0.9999999999895204`, 0, 0, 1.5955923810279335`},

{0.20702996858127698`, 3.5459212947722705`, 9.044651158282573`, 1.2898647778788441`, 0.1120438185942787`,
0.3399243113730348`, 0.005774130262077124`, 68.71847405774774`, 0.13094328364017765`, 7.770082768301062`,
90101.20345696728`, 0, 0, 0, 516.3716874344561`, 0.8115193047036483`, 0, 0, 22625.5823592165`, 0, 0,
41.108336909534955`, 66916.76578516724`, 24.049782210728402`, 1.`, 0, 0, 0.6034165297477406`},
{0.10369509204579469`, 2.7722566029085502`, 8.491805279999415`, 1.0043154825901828`, 0.1196997608855428`,
0.46660526882559294`, 0.07137717728073698`, 124.06613686120853`, 0.02382042507485488`, 7.245869706736816`,
20549.77479733502`, 0, 0, 0, 271.9805668838826`, 6.520690717299001`, 0, 0, 8065.350688758405`, 0, 0,
258.24325566509464`, 11947.67545789167`, 13.67099544590684`, 1.0000000000002363`, 0, 0, 1.347742093915066`},
{0.12495211269478451`, 3.2710300653782634`, 8.31741576802569`, 0.8301159562153473`, 0.45240597007929817`,
0.45987625913811714`, 0.011306277140564175`, 1464.8239131751898`, 0.06894180622066476`, 6.472250284276024`,
63.56073112134365`, 0, 0, 0, 54.80311389634417`, 0.1822826569283911`, 0, 0, 0.006696958816451369 4`, 0, 0,
8.517886445854272`, 0.01195427361064159`, 0.9690429351109626`, 0.15921168759320858`, 0, 0, 0.7197678555720566`},
{0.2548094999525184`, 1.991565099208616`, 8.435964917534815`, 1.3597221193616629`, 0.25512389762112764`,
0.2087626588648025`, 0.2568272531538555`, 1822.5061165805678`, 0.026223328458720774`, 8.894025776493041`,
42.13888951048874`, 0, 0, 0, 9.813527909790823`, 1.096815834716801`, 0, 0, 0.004981580594226629`, 0, 0,
31.205430524019103`, 0.018133629431257518`, 1.0402265456018427`, 0.07584211330834767`, 0, 0, 0.7114583852315919`},
{0.1329827943052238`, 0.6587055670228517`, 4.6853626661703505`, 1.028170006221078`, 0.5310002897488229`,
0.31399252604565075`, 0.010724910779551164`, 62.931190858884165`, 0.12157208629491184`, 4.054529845871336`,
104722.53821266741`, 0, 0, 0, 130.46290351306445`, 1.88760669793842`, 0, 0, 36062.47935861134`, 0, 0,
17.762529146880848`, 68509.84678117985`, 13.624646693421196`, 1.0000000000000357`, 0, 0, 0.28970737890384785`},
{0.05388606525951978`, 3.0261209146571506`, 4.311810802135282`, 1.2867466894982391`, 0.14121116952696844`,
0.17229324040800142`, 0.239982541075217`, 290.98679707372355`, 0.1893837871763393`, 8.200911880146922`,
209.93122960746882`, 0, 0, 0, 51.85482077676696`, 3.5700850589807347`, 0, 0, 0.09632723709588271`, 0, 0,
154.33584377266502`, 0.07415279692039099`, 5.990275733843488`, 0.796253938336295`, 0, 0, 0.6307499467856054`},
{0.18877028807710305`, 3.0713928282220317`, 2.350050214349242`, 0.9003573816536171`, 0.8295355664150708`,
0.3697046533237569`, 0.07836282343745943`, 203.60143886304388`, 0.1119456128362123`, 8.250251130667593`,
32442.650373910943`, 0, 0, 0, 73.56303787726057`, 1.7856596241637053`, 0, 0, 8734.490565747023`, 0, 0,
78.34945947574326`, 23554.46143289725`, 8.59020352273772`, 1.0000000000000226`, 0, 0, 0.6112363772708249`},
{0.1549731585118843`, 1.0794091822629426`, 5.872175042913531`, 1.1954059661912195`, 0.1582784842110334`,
0.47191465043849123`, 0.04704948286374014`, 132.48832982484745`, 0.0611350826684427`, 8.646340597834747`,
65632.70263228439`, 0, 0, 0, 254.85550317068217`, 10.150241604995161`, 0, 0, 20290.33956883034`, 0,

0, 156.518057008844`, 44920.828718004246`, 14.506171383574891`, 1.`, 0, 0, 1.0912780582955397`},
{0.0973630018497127`, 2.6509059407435345`, 2.661281949905481`, 1.0384603558144696`, 0.8313691963066465`,
0.6553290382276395`, 0.19978363792296042`, 613.3149478611653`, 0.10086553584387609`, 7.337446850354657`,
176.24752619494032`, 0, 0, 0, 46.66588019074762`, 3.2199867285294985`, 0, 0, 1.8488803160397491`, 0, 0,
121.94117068248852`, 2.5716076804354686`, 2.827117152601293`, 0.9999999782970291`, 0, 0, 1.4477134106739136`},
{0.25760446309632484`, 3.1710442921339457`, 1.104410531583154`, 1.067160019670409`, 0.29416815684722497`,
0.2106415408174861`, 0.1640132441258384`, 295.52940308175954`, 0.22278260496644214`, 4.791464741488767`,
17326.968891317352`, 0, 0, 0, 12.415794054421802`, 0.6055029082248164`, 0, 0, 3693.6500914082426`, 0, 0,
27.42966487138294`, 13592.867838041591`, 3.312332845059602`, 1.0000000000062113`, 0, 0, 0.16825345202109734`},
{0.08814664727699922`, 0.49325198699724604`, 7.4393784579313085`, 1.082856668773565`, 0.36684849391494345`,
0.47651248778047417`, 0.010016127241926027`, 222.2306387901171`, 0.10060123761752315`, 2.9576363340294876`,
174.6579785741582`, 0, 0, 0, 152.46532560555116`, 2.659480111580967`, 0, 0, 0.29722712142435875`, 0, 0,
18.739912134528147`, 0.37427963190499064`, 2.992737840622153`, 0.9847424815463162`, 0, 0, 0.41209241338031916`},
{0.09405288750913654`, 1.8996899418118014`, 4.321657830772438`, 1.3285146456969388`, 0.4687194709250446`,
0.37672748824359026`, 0.1617154754485949`, 124.22044587269936`, 0.08001764444851872`, 7.0616286930304`,
64535.99375880696`, 0, 0, 0, 78.55498382665232`, 6.080543946380513`, 0, 0, 27430.437480711975`, 0, 0,
165.01640250976706`, 36855.883581425776`, 12.44925439829093`, 1.000000000000054`, 0, 0, 0.7737277641777537`},
{0.26673333380428393`, 2.6775026015459398`, 7.186300359062184`, 1.3779081516185778`, 0.2197224262472608`,
0.44634915685926935`, 0.2549448072449753`, 573.3906335364269`, 0.19327206597748986`, 8.09151224456528`,
160.50042410240889`, 0, 0, 0, 36.90128826719276`, 3.1453665833535247`, 0, 0, 0.02980585871929862`, 0, 0,
120.31038871063888`, 0.11357451518707325`, 3.180852683136688`, 0.3709825903773226`, 0, 0, 1.149434841394638`},
{0.06695152315589192`, 3.739348796633019`, 8.531551488563299`, 1.3873826920360777`, 0.5359483678583272`,
0.39821450250245216`, 0.041894311644931556`, 1681.9385322117223`, 0.03536815464018617`, 7.504735436115399`,
66.65528863153285`, 0, 0, 0, 42.03677029429763`, 0.4519883971636132`, 0, 0, 0.009761215552224778`, 0, 0,
24.144889557512098`, 0.00933611784390311`, 0.9600598190060812`, 0.14029993583719857`, 0, 0, 0.676723352504752`},
{0.2432492145111665`, 1.524425096090667`, 0.7030547277601631`, 1.1448142947983793`, 0.5481222164790474`,
0.4314400790527364`, 0.005980753513921293`, 1722.4066531232882`, 0.11386175120469438`, 3.8888258531503404`,
37.99101118815494`, 0, 0, 0, 21.810915697875622`, 0.07985967367945009`, 0, 0, 3.203912331378707`, 0, 0,
1.7391441531795004`, 11.133559399578742`, 0.5251823350138729`, 0.9999999936055809`, 0, 0, 0.6943670574735994`},
{0.24077340164047406`, 3.026302168091572`, 3.348461380748672`, 1.1251559242118545`, 0.33184191297746213`,
0.3141742813991918`, 0.0703872676061783`, 173.09096476750136`, 0.24564160017502346`, 6.392098114270558`,

```
  19415.596295889234`, 0, 0, 0, 85.06176206728789`, 1.8821065135428534`, 0, 0, 4335.344677461153`, 0, 0,
  81.3689003216286`, 14911.938361089275`, 7.765363217900851`, 1.0000000000001408`, 0, 0, 0.4180805565956372`},
 {0.09741803900657164`, 2.8226058170826276`, 6.285345102913118`, 0.8413213607246539`, 0.2506059249236361`,
  0.26188997247349455`, 0.05425664970500174`, 4955.988737338467`, 0.11641107051911387`, 2.1128007380367517`,
  3.7979111683690276`, 0, 0, 0, 2.1763703659193636`, 0.03915059493898787`, 0, 0, 0.001536682414195269`, 0, 0,
  1.578667100243323`, 0.0021385798195290655`, 0.09268473438796926`, 0.03842322633200457`, 0, 0, 0.21454472896756727`},
 {0.14314599793313348`, 2.5518611864266925`, 6.295888200856282`, 1.4241645467251498`, 0.07884119203240614`,
  0.5217209274223922`, 0.006381498715981013`, 359.4043872790348`, 0.24023808698930982`, 4.143010259943262`,
  122.94540160771717`, 0, 0, 0, 112.5889783816885`, 0.26799097100893765`, 0, 0, 0.06618326246708316`, 0, 0,
  9.769653674721567`, 0.13534098789028898`, 2.7069726361918436`, 0.6240483691659555`, 0, 0, 0.786338805655986`},
 {0.225655266424848`, 1.5867432872088365`, 3.0858574789264335`, 1.3244931698407223`, 0.6079478177713131`,
  0.6933074034991797`, 0.013957254253711016`, 59.76089956157712`, 0.13530140261382512`, 4.737681248427657`,
  247220.22263676152`, 0, 0, 0, 98.42544811278613`, 0.8207468506331641`, 0, 0, 58504.49500004633`, 0, 0,
  18.604493653428037`, 188597.82008981446`, 17.094656922225912`, 1.0000000000000417`, 0, 0, 0.43838560723796505`},
 {0.1122606973400958`, 1.7187290694442563`, 9.979465273640976`, 1.2098446125137294`, 0.9761419756238592`,
  0.37158902094365565`, 0.02080861609061981`, 179.99091662757314`, 0.030562090142117915`, 6.57677031568597`,
  531.2202130337728`, 0, 0, 0, 408.3868654022719`, 4.659335332735464`, 0, 0, 1.3959676701395354`, 0, 0,
  114.40192972373106`, 2.2387472016297063`, 7.863723528712906`, 0.999983487024757`, 0, 0, 0.5945836367901125`},
 {0.24459409433338708`, 0.8193086201254509`, 4.65991702304226`, 0.8806460188595719`, 0.2108783317674836`,
  0.532516488691834`, 0.10515966693757768`, 2682.0355154099257`, 0.13211135275557007`, 8.414844442987327`,
  38.24452045357361`, 0, 0, 0, 15.677571526373264`, 1.774266476741619`, 0, 0, 0.00577118973747996`, 0, 0,
  20.766740268486064`, 0.020165698958067763`, 0.7195959158535113`, 0.13120909501002143`, 0, 0, 1.3637300268808157`},
 {0.06530542208683338`, 0.9596039403304868`, 4.254394351359874`, 1.2566656605190776`, 0.7704919647854709`,
  0.333532652338865`, 0.010126143082696715`, 1215.361514466369`, 0.2378411046297244`, 9.6572715838455`,
  156.18001988518674`, 0, 0, 0, 136.48670396052668`, 1.3261171338977242`, 0, 0, 0.041977980427592106`, 0, 0,
  18.179246100400388`, 0.039162710431086746`, 1.654416075041823`, 0.5106920416564533`, 0, 0, 0.5303441248919486`},
 {0.1695388010672208`, 1.173836844893498`, 5.733937529803059`, 0.8972303818101501`, 0.03459597061008757`,
  0.45955942513269943`, 0.02896845637601857`, 512.9632938088884`, 0.14318523913512782`, 1.8784414403823462`,
  35.00221682664215`, 0, 0, 0, 24.92593118455567`, 0.5622262997322247`, 0, 0, 0.02385688338954254`, 0, 0,
  9.428027797054582`, 0.05778096295804812`, 0.857351470963138`, 0.43026116551087645`, 0, 0, 0.3498270554838933`},
 {0.21476635225191587`, 0.5109603344620304`, 9.116157263242219`, 1.0856571152809298`, 0.22597102427026194`,
```

```
0.39468057790613453`, 0.008122876676631087`, 1537.5523384133612`, 0.22468288668284858`, 1.5725594082493757`,
10.517775320361492`, 0, 0, 0, 9.420063981381597`, 0.12864966477355708`, 0, 0, 0.005239359616350735`, 0, 0,
0.9390696534446409`, 0.0160748307562795`, 0.23028272816036896`, 0.09796646344463233`, 0, 0, 0.22140411191316786`},
{0.06813064867410118`, 0.8082337111165119`, 3.599754755102856`, 0.8970806442820121`, 0.12954010392326953`,
0.2841840584225793`, 0.09462247566679993`, 471.57171291886044`, 0.0960155452146989`, 9.92783198210315`,
184.09040682748852`, 0, 0, 0, 80.76473218731564`, 8.227831154108255`, 0, 0, 0.04947420859408715`, 0, 0,
95.00015011607087`, 0.04815299891650146`, 4.623608434668151`, 0.6918601878782531`, 0, 0, 1.1193402967047292`},
{0.12903273933048381`, 3.2486278014701124`, 1.90816116411313`, 1.4556940559632274`, 0.8634695831402321`,
0.3517692054558007`, 0.07017000005506284`, 82.66475099620249`, 0.22883036061603934`, 2.0030440452333433`,
69303.4093640065`, 0, 0, 0, 15.364517957258593`, 0.31600134302788424`, 0, 0, 24363.40068326408`, 0, 0,
14.665296403746904`, 44909.66185096797`, 5.145152666505382`, 1.0000000000006786`, 0, 0, 0.15192462866503972`},
{0.25243522618186365`, 1.7361695497710992`, 4.779805866927312`, 1.3770344402735035`, 0.053441804379047`,
0.21192069167526972`, 0.3494903419308112`, 922.4310224013639`, 0.09853668667446419`, 9.287299936208932`,
76.1616549187417`, 0, 0, 0, 14.430988870255728`, 2.389429865360067`, 0, 0, 0.01684418477095051`, 0, 0,
59.26364819359689`, 0.06074379417868068`, 2.17524311314453`, 0.23120942670803402`, 0, 0, 0.8790261594157454`},
{0.1954776887577972`, 2.556119761332786`, 4.179140500485959`, 0.8918497427192082`, 0.9985940433520333`,
0.19919828622889002`, 0.10341194686720168`, 185.89024422047143`, 0.23152045430083634`, 6.38817726005194`,
3298.7595801783145`, 0, 0, 0, 89.00621002019965`, 3.2930762836467826`, 0, 0, 813.75848182193`, 0, 0,
120.24996234579832`, 2272.451817622927`, 7.324998891859227`, 0.9999999999998067`, 0, 0, 0.5108282957161648`},
{0.21581636372683116`, 2.527066218755916`, 0.7074271020081202`, 1.2545848439034097`, 0.6959200155465519`,
0.5313070485281961`, 0.086636650755472`, 1874.5635156658839`, 0.10607902266846381`, 5.753646937249579`,
53.559567860711624`, 0, 0, 0, 16.464245736439246`, 0.5251503983204138`, 0, 0, 4.313330600733108`, 0, 0,
18.95842616231043`, 13.298390368598415`, 0.7287770096600079`, 0.9999999969380268`, 0, 0, 1.1681229507010087`},
{0.1492413868080179`, 2.613673396032418`, 3.0904979954224796`, 1.2072285951382535`, 0.18001792552689877`,
0.24732759197473053`, 0.00562211589804969`, 2528.5611678534033`, 0.0881107110472133`, 9.594393869960811`,
32.67014173050535`, 0, 0, 0, 30.242924684209555`, 0.061702296670529705`, 0, 0, 0.009024386658346314`, 0, 0,
2.303852161169472`, 0.01924017114261856 7`, 0.8221591079959655`, 0.14832530444716785`, 0, 0, 0.9385960764533205`},
{0.07601227699098417`, 2.712563755157329`, 4.823883660189505`, 1.3951422907519642`, 0.1445858824844255`,
0.4724433117860949`, 0.00914361266793413`, 231.39818056782485`, 0.1448152920502898 7`, 4.810561016418756`,
218.95355784799887`, 0, 0, 0, 182.18486247231112`, 0.582561577881569`, 0, 0, 6.451630756274569`, 0, 0,
22.574791732983012`, 7.005759201278408`, 4.948174875201186`, 0.9999999977628957`, 0, 0, 0.9907655451519464`},
```

{0.12871665135347787`, 2.8014401685833805`, 7.283786002739568`, 0.7524429330430051`, 0.8818706538097647`,
0.37826653388239995`, 0.007972118927365966`, 65.78330734043783`, 0.1996049007295676`, 1.2990854064714608`,
32877.13968270576`, 0, 0, 0, 69.06298869753316`, 0.18817354573257253`, 0, 0, 11554.243385817254`, 0,
0, 7.530813281142713`, 21246.050250649536`, 4.292157424174587`, 1.`, 0, 0, 0.13149963710279522`},
{0.09952335410600305`, 3.347751049949922`, 0.6305859630946937`, 1.1305375150416004`, 0.9039729545790072`,
0.47386245025326623`, 0.16157270551479597`, 562.0685567880952`, 0.1640716707479668`, 8.59213583452907`,
9020.334132459173`, 0, 0, 0, 13.908811369912382`, 0.6263747683566765`, 0, 0, 3706.3262431444455`, 0, 0,
29.956382691831458`, 5269.514558983394`, 3.3594202001127123`, 1.0000000000004197`, 0, 0, 0.9748681160617912`},
{0.09189223639403032`, 1.6732139429664894`, 9.692701238874825`, 1.282228547207058`, 0.15855153018111912`,
0.4131843305852153`, 0.03830010741000983`, 3674.584524793622`, 0.10140311695331994`, 4.468652597668594`,
12.385367741288915`, 0, 0, 0, 8.092754585194593`, 0.17212855253955478`, 0, 0, 0.0023438214240436594`, 0, 0,
4.1143984870260395`, 0.0030768427480536422`, 0.2753905177711895`, 0.03849067822160057`, 0, 0, 0.658539126869065`},
{0.15046133692934927`, 3.1811544489323884`, 8.294233740646352`, 1.3617577237735539`, 0.4847724596901488`,
0.37433619970600795`, 0.06464979105054385`, 793.4336208513492`, 0.16357169207826316`, 3.060886752086996`,
51.2377712642987`, 0, 0, 0, 26.994507228780897`, 0.5206110672169495`, 0, 0, 0.02006708509306293`, 0, 0,
23.659203037723433`, 0.04313314930536999`, 0.8358720463045203`, 0.270260119377845`, 0, 0, 0.2993721025888442`},
{0.2766206750277398`, 2.8724354428398637`, 5.189326643025524`, 0.8952441889736982`, 0.9323758122824941`,
0.4464935529399745`, 0.01922331430839979`, 81.97107113159659`, 0.06878282991185858`, 7.425462926136905`,
198681.53433538997`, 0, 0, 0, 238.0351316654172`, 1.536626142531753`, 0, 0, 40062.57619930815`, 0, 0,
63.055134202892994`, 158316.24102289797`, 19.02367258825559`, 1.0000000000000315`, 0, 0, 0.4754505596290809`},
{0.23204393359414766`, 1.3807564884192223`, 0.49288335289722696`, 0.9255887528096592`, 0.5628667572225596`,
0.43401537857521066`, 0.010225716982610468`, 1119.882052780943`, 0.015435359995479236`, 2.904839645045426`,
45206.247438694016`, 0, 0, 0, 9.996568207598092`, 0.06947344680857712`, 0, 0, 10474.092931134615`, 0, 0,
1.3703701779112956`, 34720.71035101622`, 0.5544844393676486`, 1.0000000000003586`, 0, 0, 0.24134336230560097`},
{0.06896766630182821`, 3.238145856087148`, 5.061610251787275`, 0.9979492539875935`, 0.9640249344246659`,
0.343776595701085`, 0.005657576047359979`, 3386.0339854064414`, 0.07870221044254616`, 4.290344560110242`,
36.07799034501086`, 0, 0, 0, 33.344029279150526`, 0.056579666075244416`, 0, 0, 0.011758405670002367`, 0, 0,
2.617331589147821`, 0.011584997121287155`, 0.2597374055467094`, 0.2231176370715645`, 0, 0, 0.16568584631771738`},
{0.14163054804702563`, 1.8677837362731973`, 0.4370922457889872`, 1.166715795330906`, 0.13182109027382305`,
0.4473367986720722`, 0.3453878303019619`, 2024.858554151485`, 0.17866806603084778`, 3.146330044100834`,
741.4972791914795`, 0, 0, 0, 2.163443790390036`, 0.343533459157471`, 0, 0, 241.40028546387168`, 0, 0,

9.166374398285662`, 488.42363898509177`, 0.35352970502126907`, 0.9999999999964051`, 0, 0, 0.48063137018636065`},
{0.14346666218024207`, 3.6942323053195354`, 6.0819497997897844`, 0.8206348775106591`, 0.4021755195313119`,
0.4030089388647059`, 0.4636571249048107`, 744.6939263196219`, 0.08354527905403086`, 5.555269667846608`,
95.98575252739228`, 0, 0, 0, 13.954350785834414`, 1.524418228087694`, 0, 0, 0.018428078760942008`, 0, 0,
80.45078664313618`, 0.03776878500308928`, 1.6328854590147057`, 0.38208456836865523`, 0, 0, 0.60579641065929`},
{0.09149267062932814`, 2.234522341263885`, 6.944559583117852`, 1.114683357002141`, 0.5085103250363343`,
0.6658564606426403`, 0.006987139923383322`, 1505.8338370139704`, 0.21635495075213407`, 1.8879751532316735`,
22.94382407295265`, 0, 0, 0, 20.838291568192695`, 0.06235574681046822`, 0, 0, 0.013852938458385188`, 0, 0,
1.9905044193455068`, 0.018106319080306358`, 0.28139637149892627`, 0.23129667189769`, 0, 0, 0.25895481556777494`},
{0.22068432056467163`, 3.569728941728428`, 4.540468856326312`, 1.2038410371742398`, 0.3148195694928344`,
0.5227498853534928`, 0.10369270254334745`, 4010.634501486441`, 0.16206884164220114`, 3.771999452670874`,
12.326676862690043`, 0, 0, 0, 5.078924093600477`, 0.13893575517307366`, 0, 0, 0.005690317691304539`, 0, 0,
7.0851855183173855`, 0.017939484192903137`, 0.21325828581232759`, 0.0958899685134581`, 0, 0, 0.5615258116188699`},
{0.2097350071754976`, 1.1102722915344074`, 3.321403610485124`, 1.0935329934230222`, 0.7183578411763796`,
0.40562405317483374`, 0.006176942100465586`, 446.96867612346506`, 0.08354129713872654`, 7.284441181467236`,
205.63259620740783`, 0, 0, 0, 184.29012616068303`, 0.9455701100311007`, 0, 0, 1.301965601287317`, 0, 0,
14.997718469581024`, 3.900968067546396`, 3.6195028513121805`, 0.9999991114553949`, 0, 0, 0.9124344348098669`},
{0.15395695699718487`, 1.7653707587036722`, 8.640858401956272`, 1.0488086821127276`, 0.2563370285663329`,
0.39045184386637677`, 0.03142873441769787`, 98.7771934627432`, 0.2047745102794421`, 8.897923651735105`,
14362.54073416313`, 0, 0, 0, 443.9780791258702`, 7.388530740795001`, 0, 0, 4289.816383574525`, 0, 0,
186.33565885118063`, 9434.958235597227`, 20.013806786423853`, 0.9999999999999994`, 0, 0, 1.11840851797613`},
{0.2495726529131425`, 0.6471319987804813`, 4.234303245586215`, 1.3697781718226154`, 0.5893758025334812`,
0.16113556190203493`, 0.016137549539750166`, 661.2554849181507`, 0.08255896742665592`, 6.142572860377916`,
96.35706052546873`, 0, 0, 0, 78.46778549841127`, 1.7228016551819012`, 0, 0, 0.043999585606304285`, 0, 0,
15.92685826600269`, 0.15687276152633195`, 1.9252331395222115`, 0.4976113518082417`, 0, 0, 0.307653405691378`},
{0.18703143603461198`, 3.5026925577041474`, 3.736145496532833`, 0.8654363814884891`, 0.7031489482980493`,
0.15906583747765657`, 0.008377691039792691`, 131.85234583624222`, 0.16088793676433022`, 8.514675246308464`,
22834.21164833812`, 0, 0, 0, 218.66408445879256`, 0.5029357810874591`, 0, 0, 6152.079611988259`, 0, 0,
25.166134534545247`, 16437.604063277427`, 13.2708273584082`, 1.0000000000005123`, 0, 0, 0.35092599490052223`},
{0.2617533772269884`, 3.6583043975291023`, 1.4938470678500337`, 1.229216471284039`, 0.8448795594977094`,
0.4744971024115313`, 0.027729197093825105`, 163.20786376161018`, 0.21172311156121143`, 7.676981105027913`,

52647.37130250868`, 0, 0, 0, 64.60949989845876`, 0.47484058678741925`, 0, 0, 11089.629715163306`, 0, 0,
24.815877239567328`, 41467.82900201101`, 9.857539681722647`, 1.0000000000004021`, 0, 0, 0.47441407855301654`},
{0.2277950960946843`, 2.381865480846251`, 9.991476447433318`, 0.832979598782657`, 0.5926733949589658`,
0.27276410945547525`, 0.04327492387940349`, 669.5530400360332`, 0.024018555255208573`, 9.681708943673282`,
194.43401795257486`, 0, 0, 0, 121.26258138141593`, 2.0873445013092673`, 0, 0, 0.01226015282985287`, 0, 0,
71.02534020432522`, 0.039897181314510276`, 3.0413147034371812`, 0.2716001225143515`, 0, 0, 0.6356533514728432`},
{0.15963824410704142`, 2.654604852862681`, 7.469701098598773`, 1.491664105533315`, 0.25955793675814864`,
0.27946634303609463`, 0.06966019051655106`, 86.7013584077103`, 0.017186465149371527`, 9.81969476696123`,
432015.65701375157`, 0, 0, 0, 297.93417269011394`, 7.348088052837702`, 0, 0, 131512.15263502498`, 0, 0,
278.66100291893196`, 299919.5589341797`, 24.124187662842356`, 1.0000000000004412`, 0, 0, 0.7777444277594865`},
{0.13626056470386483`, 2.820076398995975`, 2.87943303050835`, 0.9523411928557022`, 0.8935766203190545`,
0.356125796963648`, 0.009331421764352204`, 68.56313381348697`, 0.147099680293568`, 8.670054262776382`,
89913.6527658248`, 0, 0, 0, 174.34072542995494`, 0.5546794277342522`, 0, 0, 30447.597397933245`, 0, 0,
22.346262330885054`, 59268.6687902614`, 26.735693028574303`, 1.000000000000001`, 0, 0, 0.617565759976627`},
{0.22962572899038486`, 1.387681996287987`, 7.569216994527352`, 0.873789488954491`, 0.15662470794012862`,
0.483300190980835`, 0.02173528342644958`, 88.10021701991921`, 0.04718506749066664`, 8.783666403079792`,
163469.5500264975`, 0, 0, 0, 411.1288288304322`, 6.031557456884641`, 0, 0, 38065.1168380581`, 0, 0,
119.56976703564796`, 124867.57432918876`, 21.53379113664193`, 1.0000000000000608`, 0, 0, 0.8284674051571311`},
{0.16187203397799577`, 1.769575035055972`, 1.1614771267268384`, 0.8287067492253789`, 0.7900895875825826`,
0.2962983919698179`, 0.014442973603799043`, 137.47659064397268`, 0.2186302306878819`, 9.341476742915006`,
29236.76291243101`, 0, 0, 0, 70.11789996064272`, 0.5426042686214169`, 0, 0, 8800.820983830052`, 0, 0,
13.716842395246612`, 20351.525618982752`, 14.133004590107186`, 1.0000000000002414`, 0, 0, 0.5028401783971442`},
{0.09685210682031803`, 1.7960713074535244`, 5.69551378974773`, 0.7869885608747933`, 0.9421312080797839`,
0.5036667090451988`, 0.0169704552746280 2`, 52.99769310364423`, 0.21425647446502405`, 9.869621253243459`,
55208.485035568345`, 0, 0, 0, 373.77954444001483`, 3.338928721786358`, 0, 0, 22967.56031258561`, 0, 0,
85.67077249761334`, 31777.951497094753`, 41.04548596216295`, 1.000000000000003`, 0, 0, 1.1519801623080321`},
{0.16013522976515993`, 1.0007721789830901`, 6.380859482894074`, 1.440495462142086`, 0.5398127028451485`,
0.19075782788209084`, 0.04652110166049526`, 84.8416541425776`, 0.16547472912452094`, 6.4422585881915015`,
47027.6331692644`, 0, 0, 0, 194.68541143606416`, 8.197104778590825`, 0, 0, 14206.988330026172`, 0, 0,
117.19192015175778`, 32500.562007138244`, 15.812702103507656`, 1.000000000000001`, 0, 0, 0.35505881457246236`},
{0.1912199176775608`, 2.2121001603415005`, 9.917361836602723`, 1.435922264503217`, 0.9790995032802718`,

0.6767636439858111`, 0.07238714810610002`, 269.421010262202`, 0.01560756507054070 2`, 2.5093255880098546`,
208.89881766280894`, 0, 0, 0, 101.99095719511912`, 3.1697903099670017`, 0, 0, 0.9560378368950885`, 0, 0,
100.16990932752765`, 2.611621092396032`, 2.0602893672280396`, 0.9999718786712853`, 0, 0, 0.30310644831137346`},
{0.24179572266521915`, 2.5600514990256196`, 5.593167080478407`, 1.0641609773948193`, 0.6297315486340287`,
0.26623731831221253`, 0.08310036534342866`, 636.0105501064265`, 0.05032454414330245`, 1.4627567207550134`,
30.402343053601534`, 0, 0, 0, 14.09138579167949`, 0.43009314564244494`, 0, 0, 0.033989965838497276`, 0, 0,
15.729437174608345`, 0.11740897647551443`, 0.4856820426414173`, 0.4914017623060243`, 0, 0, 0.10233105609565801`},
{0.20288510482307215`, 0.7288550347693712`, 9.019074578521877`, 1.2840716127441223`, 0.5874481378002983`,
0.6980893050148065`, 0.008225735950864725`, 1276.121996072157`, 0.1607915330045036`, 9.69181498105582`,
166.12595428875255`, 0, 0, 0, 148.64669031657846`, 1.5137483139833343`, 0, 0, 0.017678099402377915`, 0, 0,
15.76147257172002`, 0.051237472147459134`, 1.6666229904361092`, 0.2557661930715356`, 0, 0, 1.0840439236266957`},
{0.1862496077639137`, 1.6905827743030857`, 2.538407600056317`, 0.9208769830081895`, 0.38109944279089136`,
0.3320011945053185`, 0.011054373793833769`, 1065.4164452217328`, 0.07943719416592698`, 4.231039339853934`,
45.055776493899415`, 0, 0, 0, 38.88907297895234`, 0.23891577540419542`, 0, 0, 0.0353747161642397`, 0, 0,
5.770098491537104`, 0.09412181443356371`, 0.8652557027839695`, 0.5562533761787571`, 0, 0, 0.4397061137083344`},
{0.05689751321611025`, 3.0250332600067678`, 2.529462845718742`, 1.466915953431471`, 0.22269501402715108`,
0.6695459886571014`, 0.14207393160159743`, 917.9363525294286`, 0.02546682059293326`, 3.574551553390743`,
53.95020469379218`, 0, 0, 0, 18.32259539883518`, 0.8015716525159937`, 0, 0, 0.1027729251244113`, 0, 0,
34.639727273420995`, 0.08353605522185008`, 0.9281772354583665`, 0.7487557855632081`, 0, 0, 0.7422870861411103`},
{0.1967142498798619`, 3.0620830451218577`, 7.9498840213828466`, 1.4844762448061533`, 0.6042217303737281`,
0.6207898959804532`, 0.0334891282473782`, 576.7285784287807`, 0.16199601914875666`, 9.348325121692032`,
341.0713575055191`, 0, 0, 0, 231.6265489181281`, 2.439739120022417`, 0, 0, 0.06644094687830014`, 0, 0,
106.72405419915941`, 0.18671258609253605`, 3.527711677656057`, 0.6145430616184154`, 0, 0, 0.9599710603191095`},
{0.26434225760699476`, 2.569764850665571`, 5.652188171834926`, 0.8392687504503539`, 0.3834838466329171`,
0.17354269300981617`, 0.03414034179009787`, 716.202171206297`, 0.028057924329779993`, 1.7997126741152787`,
21.329250289769735`, 0, 0, 0, 14.46423143368313`, 0.18036247198599592`, 0, 0, 0.012923636836045893`, 0, 0,
6.621273441268088`, 0.04880376196763115`, 0.5297078431352612`, 0.2810720884802882`, 0, 0, 0.12246872518103848`},
{0.19233187457454282`, 2.145104020661503 5`, 4.677354160755579`, 0.8253029687642099`, 0.9809911268657268`,
0.22566854695954142`, 0.015470928716805988`, 111.90002994139898`, 0.12242650367871766`, 3.4296984602053584`,
34044.88615793034`, 0, 0, 0, 102.88466272570214`, 0.7040496990334464`, 0, 0, 9051.042235393568`, 0,
0, 21.575140573459542`, 24868.627428379958`, 6.397761857387997`, 1.`, 0, 0, 0.19757757407967005`},

{0.21830260718528288`, 1.0813728060954029`, 9.236753498274854`, 1.3292857287875703`, 0.7084275620345744`,
0.4530838967889582`, 0.022838882777727113`, 80.81382094438914`, 0.07293079158751065`, 5.841787640828064`,
128890.30204091553`, 0, 0, 0, 333.212858742036`, 6.48300512448861`, 0, 0, 31187.803852216497`, 0, 0,
100.15064919141612`, 97262.55561888675`, 15.770634688955711`, 1.0000000000001597`, 0, 0, 0.6156042831168366`},
{0.27637635892023465`, 3.838779152844408`, 0.1593800020520176`, 1.3392032462951913`, 0.02123906289427646`,
0.6059474038465393`, 0.007164747514275051`, 100.75472745890974`, 0.10946913978259332`, 8.952098728322259`,
231916.74584535134`, 0, 0, 0, 10.184564070349696`, 0.018522214618547757`, 0, 0, 46866.32264154167`, 0, 0,
1.015752733459872`, 185039.19439500396`, 18.689642474347835`, 1.0000000000000935`, 0, 0, 0.5887898962821706`},
{0.15064788979833238`, 0.9109936464272579`, 0.5800958535668599`, 1.4999646884179314`, 0.9779856120080317`,
0.34948818201364285`, 0.011067508252105101`, 2641.6705923333384`, 0.011474828296826944`, 3.667140614388831`,
20.30259376241613`, 0, 0, 0, 15.224711251918981`, 0.1682552867877102`, 0, 0, 0.8593262228152295`, 0, 0,
2.189707103448573`, 1.849366887364084`, 0.3104284428353334`, 0.9999946752072961`, 0, 0, 0.48777780870995335`},
{0.0419185722234717`, 1.709255049022068`, 2.3539768873704308`, 1.3792050115017793`, 0.22715212217109637`,
0.2926929451571887`, 0.00505223249889438`, 88.91455127714036`, 0.1107140298924858`, 2.282618721837757`,
70255.40262284165`, 0, 0, 0, 40.81629223213463`, 0.11314806457043461`, 0, 0, 43912.577666431214`, 0, 0,
2.762841437915573`, 26296.46512041589`, 5.596329491971364`, 1.0000000000000846`, 0, 0, 0.23852991542372498`},
{0.24924491240080032`, 1.373280333190027`, 8.919496594562187`, 1.4037493221091317`, 0.24632944507518628`,
0.3548949637369072`, 0.18998328780525742`, 2947.236236962295`, 0.1153856006596437`, 7.477168052601716`,
26.53115667188809`, 0, 0, 0, 7.529858291801952`, 0.920778822750132`, 0, 0, 0.0035988674005294373`, 0, 0,
18.064106407151744`, 0.012814276999812177`, 0.5601649695263842`, 0.05363466200215994`, 0, 0, 0.8875869994780539`},
{0.20687871564233656`, 0.44181655503120965`, 8.570924850704476`, 1.0055678634725929`, 0.5549096280819519`,
0.326726210951258`, 0.20129490353773463`, 306.54002765810475`, 0.1699967418388691`, 1.7003969931174137`,
74.07810128302826`, 0, 0, 0, 19.92331411155454`, 7.377705921149648`, 0, 0, 0.053464105220149334`, 0, 0,
46.565608773081294`, 0.1580083631559221`, 1.1878526509842307`, 0.673979387240365`, 0, 0, 0.1444998893281906`},
{0.20196179343132148`, 2.101313615300616`, 3.7695608295023266`, 0.8255643571631567`, 0.9698552359083252`,
0.3038610819528902`, 0.02519738143348337`, 88.57713238465558`, 0.03933307441687739`, 1.9858938348907018`,
218759.52169416408`, 0, 0, 0, 43.19072416120458`, 0.49271271675191297`, 0, 0, 56291.26219253943`, 0, 0,
14.790627716322149`, 162409.77524168516`, 4.682919920048927`, 1.000000000000478`, 0, 0, 0.11575756301601851`},
{0.10010100507466935`, 2.4192735538683836`, 9.823330355933038`, 1.1133108806457672`, 0.011014651746178439`,
0.5998442212295849`, 0.013751847561218578`, 3832.28466694177`, 0.1060300423959204`, 2.751621101527464`,
7.718266370888943`, 0, 0, 0, 6.469187209468058`, 0.03487982926791932`, 0, 0, 0.0020293271485728303`, 0, 0,

1.2054835501617376`, 0.002901966959964656`, 0.173075892169175`, 0.03825695374211602`, 0, 0, 0.6089777442105473`},
{0.21311625184066546`, 1.4247782113917893`, 5.690068626185788`, 0.8615233428628426`, 0.12086241410351461`,
0.29764577871833753`, 0.005395932799405541`, 882.4049045614522`, 0.2481341739836153`, 1.9572384227218778`,
18.721375600773758`, 0, 0, 0, 17.35827413912524`, 0.06097490765173577`, 0, 0, 0.01025996844119122`, 0, 0,
1.2410817123402813`, 0.031236657402714522`, 0.49301127510035087`, 0.2247398732313297`, 0, 0, 0.24694178572782563`},
{0.09607643000919447`, 2.545123365745101`, 1.6292494594570959`, 0.9689766494611143`, 0.9239031581606687`,
0.5340288721519064`, 0.06266594590032853`, 79.64776165873695`, 0.19537727971057256`, 3.908080115643834`,
57144.5511487094`, 0, 0, 0, 28.06069446946635`, 0.6551528323044373`, 0, 0, 24063.859819651312`, 0, 0,
23.82063973760139`, 33028.13919591121`, 10.834184689154943`, 1.0000000000002474`, 0, 0, 0.4640405945096687`},
{0.25410295729391263`, 0.5246172329407881`, 1.519575137014602`, 1.3035504391628365`, 0.641505202173666`,
0.18662868599370885`, 0.02992528952684035`, 1308.2457641643925`, 0.1567967571574565`, 7.528281484333206`,
68.38848774654484`, 0, 0, 0, 47.943377827407296`, 2.353200970285775`, 0, 0, 0.0966887550867633`, 0, 0,
17.636139736927166`, 0.3509842657801947`, 1.1964761275982982`, 0.7876247990839995`, 0, 0, 0.40249496795844947`},
{0.20247384111644234`, 3.9381206949664582`, 3.525512124148932`, 0.8160399146038965`, 0.003770588899372651`,
0.4372678593782203`, 0.005846407125549171`, 81.38331905868344`, 0.05740074457617078`, 6.792038617151225`,
201901.8125728869`, 0, 0, 0, 174.66950935384705`, 0.2518855596529265`, 0, 0, 51821.037581786135`, 0, 0,
14.170796217605705`, 149891.49328319664`, 17.63777785112771`, 1.0000000000000917`, 0, 0, 0.48076062694864474`},
{0.15622647656935051`, 2.478038073204126`, 4.93355773626655`, 1.0681326166577831`, 0.57283306272417`,
0.24473580012035412`, 0.09417540555806864`, 340.729792091258`, 0.1606536419587833`, 9.486285960325056`,
347.4636591568219`, 0, 0, 0, 151.16691938236076`, 5.373846588014266`, 0, 0, 0.2121671082459761`, 0, 0,
190.23709206653447`, 0.4735159966453461`, 5.852471491570005`, 0.9769046165582984`, 0, 0, 0.6172117732844979`},
{0.2472522705346833`, 1.1701552284997945`, 3.914120438755436`, 1.4973295978551664`, 0.163173719496993l`,
0.4864210576866177`, 0.39314364156002357`, 172.17497479862377`, 0.10619847023829598`, 4.473646521439088`,
63299.723146061595`, 0, 0, 0, 22.872887614750162`, 6.696724915020107`, 0, 0, 13935.51741085898`, 0, 0,
111.94582390193754`, 49222.690298721005`, 5.616904327480769`, 1.0000000000070322`, 0, 0, 0.42627797586870914`},
{0.1494891985884202`, 3.3979182405498527`, 4.585626936341594`, 1.2118171179612203`, 0.6586829775426448`,
0.36431745127605797`, 0.33277789805879787`, 51.20287439819706`, 0.10641801334172246`, 7.126742388333813`,
213930.34361840397`, 0, 0, 0, 47.57694881685964`, 4.254058174500717`, 0, 0, 68144.76979351569`, 0, 0,
206.49916953566088`, 145527.24320607205`, 29.420016490471124`, 1.0000000000331681`, 0, 0, 0.5056306207189001`},
{0.2014310029114028`, 3.9869430882246055`, 0.7136208449188892`, 1.3208947167282403`, 0.46844054682093383`,
0.39516715199771224`, 0.14154388607116256`, 2748.591928872397`, 0.05354911946309293`, 6.211900336444712`,

```
  31.788658869757267`, 0, 0, 0, 10.657059879675359`, 0.35482868777391824`, 0, 0, 0.14623283977864301`, 0, 0,
  20.209739774628986`, 0.420797536788503`, 0.49947122723703136`, 0.8724252628266254`, 0, 0, 0.7438354229766668`},
 {0.13228384635594964`, 2.640861693840699 7`, 3.2993392931538836`, 0.952940068464383`, 0.8367144819205878`,
  0.6111943245350302`, 0.13146835864980566`, 2246.7842161758563`, 0.024436379332296754`, 9.73655688789367`,
  147.94801617198212`, 0, 0, 0, 51.949607417873466`, 2.4756806508753795`, 0, 0, 0.042814355218300924`, 0, 0,
  93.3990028154198`, 0.08090925125033074`, 0.9061167900452572`, 0.6145463557655078`, 0, 0, 0.578410884412367`},
 {0.23113058156717692`, 2.2605125411397546`, 4.002625660970853`, 1.3839850516092966`, 0.507564008077771`,
  0.4982541689482688`, 0.0228570122346136`, 138.3250229234806`, 0.07006503343759235`, 8.064244271550876`,
  137295.50474742881`, 0, 0, 0, 195.74584601858857`, 1.8918096938838496`, 0, 0, 31855.184850235793`, 0, 0,
  61.09227912106`, 105181.53429092707`, 12.499286470668954`, 1.0000000000000084`, 0, 0, 0.6958404411750952`},
 {0.17570245022422137`, 3.187809299248391`, 9.693328941627644`, 1.4591302419173326`, 0.2844948752823342`,
  0.5066677812093952`, 0.2998214113578054`, 349.3700788988757`, 0.18995614940943706`, 5.5658132930392785`,
  202.53782306525252`, 0, 0, 0, 41.07142374538911`, 3.465796575574633`, 0, 0, 0.047796196554136174`, 0, 0,
  157.83283647028728`, 0.11997012637058864`, 3.6035267605028602`, 0.5030884043840982`, 0, 0, 0.812939813038693`},
 {0.2497541467888837`, 1.085030464715203`, 1.0043906551890043`, 0.864213346242746`, 0.8846254264305906`,
  0.601718469375484`, 0.012654582561683364`, 62.423429857566624`, 0.12690014020771856`, 6.991109163451483`,
  187560.33432596203`, 0, 0, 0, 47.1120916961373`, 0.5114201149434897`, 0, 0, 41048.20176668444`, 0,
  0, 7.92723435688339`, 146456.55156366044`, 23.696543146107683`, 1.`, 0, 0, 0.5036035005808313`},
 {0.22558722869536918`, 1.12607467611575`, 0.7911165205665469`, 1.2733751455833207`, 0.6009341950827205`,
  0.6641796486046712`, 0.175358669032309`, 3424.3638773504586`, 0.14969161390071423`, 8.91095466688921`,
  57.22783854061539`, 0, 0, 0, 16.43487670169562`, 2.325948836409714`, 0, 0, 0.24865359045226107`, 0, 0,
  37.41702975174107`, 0.8013296339325525`, 0.5821637459340423`, 0.9569931237793909`, 0, 0, 1.1896505459277373`},
 {0.050627269144448594`, 3.276563036391985`, 7.788230793264948`, 0.8177022370463224`, 0.523932893406847`,
  0.34959907144360225`, 0.11342446098213065`, 1447.0777642274236`, 0.2385428599947077`, 6.8056995790460695`,
  64.71978671862897`, 0, 0, 0, 25.303502878784084`, 0.8242228767588446`, 0, 0, 0.006845788212932835`, 0, 0,
  38.5802601676671`, 0.00495119374800163`, 1.0070808144581005`, 0.16479355125170647`, 0, 0, 0.5780766391722235`},
 {0.060006217186970356`, 3.9647259629353027`, 0.34014645604634985`, 0.9146780185619144`, 0.0343140190064428 2`,
  0.5737721437004861`, 0.026766328146322068`, 169.17748604048344`, 0.08721098377832243`, 3.968142168145161`,
  51646.57184929879`, 0, 0, 0, 8.315211731129057`, 0.05398393427736835`, 0, 0, 27802.00939124087`, 0, 0,
  3.0575929401553594`, 23832.763053785588`, 5.318433214578619`, 1.0000000000001288`, 0, 0, 0.5906375843184641`},
 {0.15350947465095238`, 2.9043684969368213`, 4.5670292387828155`, 1.3486905179026798`, 0.8383532909017835`,
```

0.5087310077763043`, 0.05385784501485435`, 402.8647291760491`, 0.025030223476360525`, 5.400937139619534`,
208.85862250688703`, 0, 0, 0, 117.19885746613353`, 2.068217992472714`, 0, 0, 1.182741236191996`, 0, 0,
85.81238831622383`, 2.5937426545121385`, 2.989216626187583`, 0.9999925367114894`, 0, 0, 0.7005959257596613`},
{0.2587691832069661`, 1.4843472048382624`, 4.771777259704052`, 1.1550607981645458`, 0.08010207781888057`,
0.5149452814008634`, 0.01659115431008023`, 1531.656314816295`, 0.15778107864815993`, 1.1951975901128993`,
8.1238810035257`, 0, 0, 0, 6.554559005034133`, 0.06815470789790856`, 0, 0, 0.011256328214981475`, 0, 0,
1.4452178594975522`, 0.041611297972863726`, 0.1852413343438131`, 0.18488799067050732`, 0, 0, 0.24234777909679678`},
{0.1547206763399963`, 3.3076918921708103`, 2.0706333626337727`, 1.4168143839445906`, 0.37770487420755305`,
0.5386802863405199`, 0.00701518941055165`, 122.30089778268855`, 0.134914758355765`, 2.269217335741727`,
87748.79668642554`, 0, 0, 0, 34.71290293126618`, 0.07115864719453859`, 0, 0, 27321.665258962224`, 0, 0,
3.3624411483316954`, 60388.95039430905`, 4.032860276088764`, 1.0000000000000004`, 0, 0, 0.22977633343481327`},
{0.1424711737630725`, 2.7520949793854754`, 4.596768650339829`, 0.8362726667059688`, 0.00009639230672808807`,
0.36579283504807614`, 0.010146114868681232`, 3412.101956455065`, 0.24682488221918303`, 8.269337606653004`,
21.123256610483015`, 0, 0, 0, 18.49327890745746`, 0.06465067228689285`, 0, 0, 0.0030292274750199043`, 0, 0,
2.5417827230664822`, 0.006165394199446895`, 0.5508532876716289`, 0.0749131696560195 7`, 0, 0, 1.2538048030851756`},
{0.10882832688629612`, 2.4842578785622536`, 4.2235021418772565`, 1.4458161330640105`, 0.36880250968204553`,
0.4736058884190225`, 0.023130853597073738`, 1306.5861668001219`, 0.20634842182153856`, 4.016571058814693`,
40.54404240459218`, 0, 0, 0, 30.581178343460607`, 0.27098167095373243`, 0, 0, 0.025994564648364176`, 0, 0,
9.616976443039643`, 0.040413499697425255`, 0.687047942331`, 0.3171667318637328`, 0, 0, 0.5317114515826485`},
{0.18639271747297592`, 2.4942906430499407`, 3.445345566170081`, 1.148752163319608`, 0.12885074455456724`,
0.17966201189701259`, 0.0741834712240346`, 1169.2351269821615`, 0.09106655178874168`, 4.423161126411237`,
27.641819441051528`, 0, 0, 0, 13.794438796240481`, 0.3765094501475352`, 0, 0, 0.014949435113730923`, 0, 0,
13.416057121755346`, 0.03980665479332093`, 0.808434380414435`, 0.24348933401805084`, 0, 0, 0.36610491222599256`},
{0.09766181155973352`, 3.6268090588227118`, 4.041820185573664`, 1.3253596170020208`, 0.4793934216010949`,
0.49032555462271876`, 0.006759043878711846`, 262.0748117860937`, 0.11178249583803845`, 2.0681460387230555`,
1470.4489382075433`, 0, 0, 0, 67.80350216838771`, 0.12071041243812188`, 0, 0, 582.9239965942276`, 0, 0,
6.254194533211518`, 813.277335843322`, 1.882401285500198`, 0.9999999999997817`, 0, 0, 0.4313924395028549`},
{0.07951891239009712`, 2.8897027315487103`, 1.5411848578052467`, 0.863635998632232`, 0.29085460372381067`,
0.43469992564602655`, 0.009145988318489378`, 180.1504573795744`, 0.0803419958474173`, 8.628584719983632`,
41939.599247497405`, 0, 0, 0, 96.61599165662427`, 0.29315710944773204`, 0, 0, 19583.689335845575`, 0, 0,
12.101955713486229`, 22246.766808171247`, 10.516603353420026`, 1.0000000000000193`, 0, 0, 0.9705745295865424`},

{0.07234637915492992`, 1.3584521644778373`, 7.973775020047323`, 0.7807691250526536`, 0.9100932527758132`,
0.18771974645581058`, 0.009648266694560257`, 1956.923094358971`, 0.1277442569760165`, 5.404137919151016`,
45.78956320934368`, 0, 0, 0, 40.27251661763253`, 0.26820004690719834`, 0, 0, 0.00561984951932333`, 0, 0,
5.204813346202008`, 0.005808225201693241`, 0.5641052555458007`, 0.14355025235689023`, 0, 0, 0.18900913336922961`},
{0.26334607729010473`, 1.8094502738990368`, 2.2460302448509974`, 1.2023222076508349`, 0.5513938303777435`,
0.6812297944975665`, 0.00739497505047526`, 1157.7965975362908`, 0.15524857884582421`, 1.7991882609973402`,
31.848849297753326`, 0, 0, 0, 28.09754757298095`, 0.10918509025011523`, 0, 0, 0.16642839473302196`, 0, 0,
2.8223570208394593`, 0.6261180700375718`, 0.35596149311936565`, 0.9063923399512008`, 0, 0, 0.2889421686136274`},
{0.22679920860671537`, 1.574644711817462`, 3.814781873556772`, 0.9990271813793515`, 0.3871644432916528`,
0.6894928310515707`, 0.054622933309625116`, 69.61156300085906`, 0.09745707491156125`, 5.357011938436007`,
210478.88519243314`, 0, 0, 0, 93.37590625530791`, 3.0535671509361575`, 0, 0, 49602.43507506206`, 0,
0, 68.68976237715918`, 160711.32885700068`, 16.625754689537448`, 1.`, 0, 0, 0.5068683718005674`},
{0.054135826493752204`, 3.1195801041969027`, 7.2615169638158825`, 1.0984819310653413`, 0.10801447255588448`,
0.6366822484935735`, 0.01066160509264612`, 211.97043506851273`, 0.24501368432397974`, 8.92838638172081`,
517.0449016496841`, 0, 0, 0, 448.98049096578944`, 1.4697413175523817`, 0, 0, 0.4287528333326963`, 0, 0,
65.49965389360777`, 0.3315841284857715`, 10.039874474639161`, 0.9948179346369059`, 0, 0, 1.8542735254217175`},
{0.14590720393667378`, 1.5766246964498798`, 5.158062459855282`, 1.1478320866243528`, 0.3287329300123256`,
0.4460050972404973`, 0.032458645789603134`, 482.4725013461066`, 0.10279090509715616`, 8.020019312365584`,
207.5348979371129`, 0, 0, 0, 142.7693337532617`, 2.744587346216255`, 0, 0, 0.060042583501342395`, 0, 0,
61.81691702297697`, 0.12515207822584565`, 3.7008519378375038`, 0.670950040392353`, 0, 0, 1.0268144326021957`},
{0.14965591009740292`, 1.460367057362724`, 7.337474105676788`, 0.8826427720923014`, 0.44478408083773613`,
0.631222883334907`, 0.365159753458356`, 157.97713196457462`, 0.16784289093699323`, 1.7957284623930878`,
1663.810184214276`, 0, 0, 0, 21.61101656584831`, 4.536729165085953`, 0, 0, 491.72854308884166`, 0, 0,
94.64699744097449`, 1051.2868948118705`, 2.799520016159948`, 1.0000000000294`, 0, 0, 0.44505626331488635`},
{0.0810328468371882`, 1.4659479028658335`, 3.234238812997848`, 1.4263751048195057`, 0.08355223050311089`,
0.5582345497973803`, 0.12450549955799516`, 121.80826494117302`, 0.16010939682803116`, 9.339681585379608`,
50113.962035273005`, 0, 0, 0, 93.96404491669944`, 7.301620699239646`, 0, 0, 23108.756349040952`, 0, 0,
152.9113650224588`, 26750.975911853486`, 17.275201949111285`, 1.000000000000055`, 0, 0, 1.321914454940688`},
{0.15633465648304357`, 2.148716979150252`, 1.5640986685796054`, 1.3949237088061017`, 0.3567264406830608`,
0.23012843132782612`, 0.08829396925964683`, 2593.62904544209`, 0.24948663022889017`, 4.657794257043932`,
17.211142276573653`, 0, 0, 0, 7.784357935792154`, 0.2948997926295885`, 0, 0, 0.02463174563507811 5`, 0, 0,

```
9.05223130815835`, 0.05501136417767677`, 0.3844577919524369`, 0.31254119319872775`, 0, 0, 0.39438628418909216`},
{0.24418896051468997`, 0.7850580710666257`, 9.594718039690303`, 1.3796447707658`, 0.3888924613344984`,
 0.22950540339413805`, 0.26843428081063014`, 169.97373853516677`, 0.16535615066521753`, 5.752488029735448`,
 334.8105392571657`, 0, 0, 0, 74.71862856394286`, 21.25259469548302`, 0, 0, 0.10895075464519142`, 0, 0,
 238.3502999542387`, 0.38006530748705736`, 7.19622826380652`, 0.81307792198309`, 0, 0, 0.44490573345677314`},
{0.161324838944309`, 0.8594526582032156`, 2.9743924517148237`, 1.2482811580906663`, 0.28485670242217753`,
 0.27691573296739147`, 0.12631097975241765`, 80.56658004023716`, 0.24230111347476613`, 7.982613747504004`,
 60463.398414618045`, 0, 0, 0, 67.3944071073658 4`, 8.838201347173214`, 0, 0, 18240.607479970007`, 0,
 0, 108.5145091651895`, 42038.04377075036`, 20.610770072639117`, 1.`, 0, 0, 0.4307851549407836`},
{0.26772168843838734`, 1.7983273472260084`, 5.340026177070573`, 1.4142901992580086`, 0.4537298076184495`,
 0.2865003384437863`, 0.010579448173665554`, 2804.399167772796`, 0.15280115052346555`, 9.9218473198287`,
 41.42050852053327`, 0, 0, 0, 36.02506154489168`, 0.19981106415955072`, 0, 0, 0.00732423496268786`, 0, 0,
 5.133224299377865`, 0.028012236439007115`, 0.7552739836201043`, 0.10535865963210866`, 0, 0, 0.8098073190982712`},
{0.18205384870834052`, 1.3062696845921566`, 5.075170850216699`, 0.8732096494236974`, 0.8323770222253974`,
 0.3345416157316672`, 0.015614629383397784`, 819.3644184370967`, 0.04873823503517111`, 3.447951588629733`,
 90.28689949452875`, 0, 0, 0, 73.80719436253649`, 0.8275020348292288`, 0, 0, 0.047962595162604406`, 0, 0,
 15.442011743367763`, 0.12473964347703752`, 0.8733481116044234`, 0.6873516025088179`, 0, 0, 0.1777062749558821`},
{0.15466361671507556`, 2.9485738979940246`, 0.2621130142172472`, 1.4016672383633995`, 0.3670414989298545`,
 0.40252781093021794`, 0.06082671195258038`, 80.30981146445014`, 0.15720245773080782`, 9.014567082907547`,
 137194.02505755573`, 0, 0, 0, 10.05548881632298`, 0.1985564589204616`, 0, 0, 42740.69220239166`, 0, 0,
 8.363691315014227`, 94434.71481325316`, 23.646104115945164`, 1.0000000000000278`, 0, 0, 0.607104691956466`},
{0.07129651605023579`, 1.2229228380041173`, 7.261778729610821`, 1.077075564756948`, 0.8974689473036932`,
 0.5583016490383189`, 0.012773781923945723`, 355.9690714870232`, 0.15455215759656415`, 2.741649121941279`,
 162.51193480243643`, 0, 0, 0, 136.64256135478362`, 1.3334672559830523`, 0, 0, 0.5710795278336147`, 0, 0,
 23.296108015319348`, 0.5816568674593063`, 1.6732530410986546`, 0.9996866757969151`, 0, 0, 0.27619096530157305`},
{0.11901300846945845`, 0.5315793015902206`, 2.6671039936143597`, 1.3880864279120932`, 0.8420811291146328`,
 0.5566538571030129`, 0.2079188053795858`, 171.97845991450583`, 0.13095338568755877`, 2.9760745568663016`,
 44126.950750833035`, 0, 0, 0, 17.37644146358779`, 5.678267687605614`, 0, 0, 16317.682025119371`, 0, 0,
 43.12070816599573`, 27743.09184367804`, 3.8617966118017297`, 1.0000000000009217`, 0, 0, 0.388931004135622`},
{0.23041310070356935`, 3.6790901828404454`, 2.4540732736358937`, 1.23507526691096`, 0.821599531342406`,
 0.33616875277136504`, 0.035023029642451846`, 3607.9630264867633`, 0.21590997641376625`, 4.226227139273737`,
```

24.95325756361989`, 0, 0, 0, 16.647604714070773`, 0.15325394185737695`, 0, 0, 0.022333242185996798`, 0, 0,
8.054786756701088`, 0.07351245115485214`, 0.24369361127118797`, 0.3189581637108555`, 0, 0, 0.22857639418276876`},
{0.07203443304476603`, 0.5285696054477942`, 8.334423467085383`, 0.8854719875782417`, 0.3459640631982801`,
0.2633909150103768`, 0.03987288196821301`, 568.5739309534505`, 0.056948467212013576`, 6.873946140568169`,
121.32092100878576`, 0, 0, 0, 78.18601442173417`, 5.040444419553298`, 0, 0, 0.013859775662848373`, 0, 0,
38.060367401783054`, 0.014262586885741294`, 2.595903677996945`, 0.2858913518481274`, 0, 0, 0.6042671977926947`},
{0.16979941581750402`, 2.100179291019132`, 5.184869462011486`, 1.1997264241396737`, 0.9307785088479614`,
0.3430074594655337`, 0.013577650230569586`, 4088.01556659017`, 0.029357726641530413`, 9.133680945256565`,
61.4034768838668`, 0, 0, 0, 51.44631265924629`, 0.3190029448264792`, 0, 0, 0.010720595780559873`, 0, 0,
9.570905407124151`, 0.026005012867924754`, 0.4582510414917612`, 0.1746742451163822`, 0, 0, 0.357895174020099`},
{0.21619782984172958`, 1.7733623067535955`, 0.5980153626590834`, 0.9798687198034648`, 0.7538483940425484`,
0.5789929247394512`, 0.006264547120133815`, 61.29286164809138`, 0.15848957716004353`, 2.3238471342304834`,
153388.4206672678`, 0, 0, 0, 10.163243997014836`, 0.03420582691301811`, 0, 0, 37513.96096594923`,
0, 0, 0.8665617731269122`, 115863.3849943653`, 8.077633797354013`, 1.`, 0, 0, 0.1846663980593181`},
{0.04816719755022911`, 1.0519290863610147`, 3.0181083809828984`, 0.7801564927089879`, 0.4325460609784135`,
0.2938523943485086`, 0.04249246462941157`, 626.2418769059092`, 0.2424260119832079`, 9.623530958234195`,
177.58049556924993`, 0, 0, 0, 111.64333174177102`, 4.10695093964694`, 0, 0, 0.06287498330920893`, 0, 0,
61.71744499531883`, 0.043264453457440094`, 3.293773275042274`, 0.8179156950240566`, 0, 0, 0.8274760829463038`},
{0.2279802782152826`, 2.528677960359964`, 2.539878173580604`, 0.7527299875368043`, 0.8436281080694634`,
0.27514078202436143`, 0.005327161585143342`, 920.5142066248217`, 0.04689927589992776`, 5.404864622946203`,
106.74849217596841`, 0, 0, 0, 98.27228643520625`, 0.19895377884495197`, 0, 0, 0.2299817977769493`, 0, 0,
7.187000509936569`, 0.7490187748805713`, 1.21969261815306`, 0.9929501622576247`, 0, 0, 0.2837069174699126`},
{0.18595171467721877`, 2.938499684754029`, 3.6306522502244434`, 1.1726699653227697`, 0.9888859131507246`,
0.3480894711653968`, 0.38543337220237905`, 80.27026890715871`, 0.03443629355490174`, 9.328937618149844`,
439769.12189084693`, 0, 0, 0, 43.37353810877927`, 5.165971824180637`, 0, 0, 120199.47042192846`, 0,
0, 216.86009395432828`, 319304.2518321612`, 24.346574113603307`, 1.`, 0, 0, 0.5728797095607162`},
{0.2088003110428162`, 1.665360213541189`, 8.163801498355127`, 1.2665930603692175`, 0.5382347880039544`,
0.33255923205592086`, 0.03591580004997877`, 88.64295388071032`, 0.08847100949056225`, 8.845547783450662`,
75417.26309473606`, 0, 0, 0, 385.9628193699226`, 7.789382385142075`, 0, 0, 18790.046948370713`, 0,
0, 185.31610731820322`, 56048.10924755633`, 21.41128498718074`, 1.`, 0, 0, 0.7707805923164529`},
{0.21556335034608287`, 3.553132086531601`, 5.919169032409666`, 1.028261766481214`, 0.01873638959650825`,

0.38505243852661264`, 0.12008970777658311`, 72.3007614977653`, 0.1189730390969882`, 5.747570490343056`,
115832.83713928123`, 0, 0, 0, 100.79158600590245`, 3.2380519640350864`, 0, 0, 28328.254617798597`, 0, 0,
164.3603761609962`, 87236.19249813685`, 16.76799906638972`, 1.000000000001483`, 0, 0, 0.39494762468019895`},
{0.2061330284551588`, 3.7571620320677477`, 5.463889035312752`, 1.195880973038654`, 0.4606689625278244`,
0.6539206160067124`, 0.07294517832285098`, 79.70328938032284`, 0.04960591713533524`, 2.0731473047959454`,
326312.9778560789`, 0, 0, 0, 45.93079411421758`, 0.8568811943128917`, 0, 0, 82697.14776171172`, 0, 0,
45.99202127521791`, 243523.0501817906`, 5.69846732734016`, 1.0000000000000213`, 0, 0, 0.2280647711096057`},
{0.13099744861910317`, 3.0069806711745155`, 9.6713740346189`, 0.785614567603678l`, 0.3887426527541791`,
0.16465925249784186`, 0.029006890169876405`, 3080.757337617379`, 0.22037025279153283`, 7.1587428033756435`,
20.28849897556103`, 0, 0, 0, 14.479404703592769`, 0.1320011672267636`, 0, 0, 0.001474164823042568`, 0, 0,
5.670356548905044`, 0.002758740438034927`, 0.48640347535316536`, 0.03962038146282865`, 0, 0, 0.4336119827347677`},
{0.20740821458680525`, 2.460110895835281`, 0.6082454159142117`, 0.8064214695794831`, 0.18139487742338534`,
0.30057404623847583`, 0.2202140246268176`, 4056.9433443728003`, 0.07208592746284093`, 2.251460510548089`,
5.154319536228488`, 0, 0, 0, 1.3092926534853337`, 0.10363027069279064`, 0, 0, 0.025074257172343697`, 0, 0,
3.6420279724242106`, 0.07429438446009008`, 0.12454185628995643`, 0.46944406974608177`, 0, 0, 0.30851971613096696`},
{0.20705709543566436`, 1.1783515457685674`, 5.677465659011105`, 1.0594933834891802`, 0.45712361519708566`,
0.4929063565541889`, 0.07712216778297526`, 129.94158279886685`, 0.033100299542402456`, 5.957665662265306`,
149139.72924688712`, 0, 0, 0, 133.96327144298098`, 8.035921990346752`, 0, 0, 37610.91884023905`, 0,
0, 135.2734442714388`, 111251.53731037713`, 10.057200863812247`, 1.`, 0, 0, 0.6637005727324241`},
{0.04662251177018917`, 1.087376542683649`, 6.170097112149202`, 0.7648803529340509`, 0.9868309112238562`,
0.551889656411283`, 0.011192508637080182`, 2374.3826064993154`, 0.12804730636711914`, 7.114439815654695`,
136.4379886532796`, 0, 0, 0, 117.61443152414277`, 1.1314154784376593`, 0, 0, 0.019325457124993433`, 0, 0,
17.575352161175807`, 0.012871447889642049`, 0.6139363437111349`, 0.4181363509438931`, 0, 0, 0.27134243175649775`},
{0.061725502446144886`, 3.13849642385113`, 9.540008859499451`, 1.4660101376451562`, 0.45012796893408935`,
0.4212144239280121`, 0.02511250628464842`, 120.07361261472948`, 0.1571099365418398`, 6.445559562130283`,
538.8634237080685`, 0, 0, 0, 393.20601754475183`, 2.980969711499458`, 0, 0, 4.744983020073123`, 0, 0,
133.65375398785125`, 4.1840923001777846`, 12.41903557016007`, 0.9999999859366913`, 0, 0, 1.1098460005447526`},
{0.047554055600075584`, 3.265459078802472`, 8.714860138547323`, 0.8369196863770172`, 0.4962846406178121`,
0.46335229122842925`, 0.007763546254085084`, 1888.52152221599`, 0.22218896292653229`, 2.633616973587875`,
20.753454841274323`, 0, 0, 0, 18.68777698167536`, 0.04280693493719975`, 0, 0, 0.005056494248324852`, 0, 0,
1.9969184903769368`, 0.003435097266091797`, 0.3052767349223153`, 0.12145934137856074`, 0, 0, 0.28742622701749027`},

```
{0.12567467788677716`, 3.0158399320010547`, 4.901658754742801`, 1.351978052591392`, 0.7221310556231506`,
  0.5024953321675816`, 0.09886516020555723`, 193.78321929587645`, 0.11023982026692764`, 3.349767411033996`,
  12011.429039086428`, 0, 0, 0, 60.609392735669736`, 1.8489569633778267`, 0, 0, 4246.0873410031`, 0, 0,
  79.65940346723234`, 7623.223697995529`, 4.012160186580301`, 1.0000000000001423`, 0, 0, 0.5895117975509551`},
{0.19691840575768232`, 1.6501723246031572`, 9.011299029553381`, 1.1479337267630148`, 0.646058584475099`,
  0.36485695404700413`, 0.027042804382441818`, 3550.5063392884595`, 0.2269993732427253`, 4.86925454673478`,
  22.58052204958477`, 0, 0, 0, 16.354919048714876`, 0.25261082887501735`, 0, 0, 0.00383802075051203`, 0, 0,
  5.95501998149454`, 0.01079681324936135`, 0.2905037808245897`, 0.06932242737702965`, 0, 0, 0.3566853989851077`},
{0.22383158609367865`, 3.396592529608882`, 7.0059346900996395`, 1.1116596498970173`, 0.7389255639756462`,
  0.40169511261271784`, 0.008944445083981989`, 1191.861359234809`, 0.06327031154858104`, 2.975039312230072`,
  50.09786658408003`, 0, 0, 0, 44.372202948996076`, 0.11314270896401002`, 0, 0, 0.020182446683471328`, 0, 0,
  5.489995429240973`, 0.06453527217732427`, 0.5256841562762193`, 0.3214045835549837`, 0, 0, 0.1994433628605833`},
{0.22089456196581753`, 2.0637610394456862`, 7.594372198729954`, 1.2863006630967566`, 0.5843792508368972`,
  0.43898418015861007`, 0.0169422428941798`, 186.99955924932385`, 0.09805501720710208`, 5.47194276574208`,
  357.82707975079154`, 0, 0, 0, 282.3981844529872`, 2.1901744231794797`, 0, 0, 2.0546209257488885`, 0, 0,
  64.57138063068908`, 6.483636991415819`, 6.594581984295816`, 0.9999991397368484`, 0, 0, 0.7761891195743341`},
{0.09910547662807062`, 2.346178866312991`, 7.421049922557871`, 1.3147434313884303`, 0.2750558853148206`,
  0.21685738835966661`, 0.006760522552087531`, 4988.988013498636`, 0.18492351030582366`, 2.8590709623133`,
  4.8884755885299205`, 0, 0, 0, 4.460058061569598`, 0.012146242782543755`, 0, 0, 0.0019277166020365089`, 0, 0,
  0.407103687450155`, 0.002729246752124255`, 0.12281248140309119`, 0.03099176083660571`, 0, 0, 0.24532636095835267`},
{0.20816183484471307`, 3.9485705583569546`, 6.550493120626773`, 0.7774564054459185`, 0.5499519895881679`,
  0.200372307172507`, 0.34567679795977113`, 1808.1918719614162`, 0.23441319529101012`, 4.082991759791533`,
  24.46088933504238`, 0, 0, 0, 4.503180104720139`, 0.34731653632355614`, 0, 0, 0.004758516036943434`, 0, 0,
  19.59148356796721`, 0.014150591848402418`, 0.4723040860547257`, 0.12336178499405681`, 0, 0, 0.24402471641989038`},
{0.16112203008102222`, 2.4895492142589735`, 0.44352326574872514`, 1.2469876196885359`, 0.9780986797319231`,
  0.6188841396088389`, 0.029743069155058133`, 112.64100571320267`, 0.13296296295840093`, 7.528063016828725`,
  112098.87372591226`, 0, 0, 0, 19.04086908848619`, 0.21816831846543355`, 0, 0, 33943.236225476845`, 0, 0,
  7.759153797311746`, 78128.61611669339`, 14.463714757787711`, 1.0000000000000018`, 0, 0, 0.7265878230892805`},
{0.17781253097781763`, 1.7213960002432414`, 4.693599924413801`, 1.2010501070587627`, 0.4002263609998786`,
  0.512752724538575`, 0.08027502692564248`, 156.85332391803223`, 0.06371789077354856`, 6.562151621871855`,
  68421.52213659421`, 0, 0, 0, 120.61289793872835`, 5.238852438796462`, 0, 0, 19255.19563588477`, 0, 0,
```

128.8305662001256`, 48911.64386413825`, 9.258953059410686`, 1.0000000000000129`, 0, 0, 0.7961560338308824`},
{0.11782384895680592`, 1.1169968183756591`, 9.741344822271138`, 0.8926206302886914`, 0.732042643272727`,
0.38545027561144396`, 0.005148133378435907`, 521.4771678665235`, 0.04124959681193141`, 8.98600497767481`,
343.69395222225114`, 0, 0, 0, 319.9749285794344`, 1.372137203622895`, 0, 0, 0.0313412791663986`, 0, 0,
21.895327011737844`, 0.05275357346590926`, 3.613258040826765`, 0.5296294827285093`, 0, 0, 0.5607683108553674`},
{0.1669047046062308`, 1.025805255669411`, 7.1552343684117155`, 0.8176510429563385`, 0.8282389242605148`,
0.29891546072913033`, 0.009889011750027802`, 94.42085507888534`, 0.16558325818795072`, 8.472484566593426`,
22505.774134398995`, 0, 0, 0, 425.7778382110164`, 3.763664847385679`, 0, 0, 6506.631939006286`, 0, 0,
55.15410258609217`, 15514.106882304386`, 19.210325169895096`, 0.9999999999999999`, 0, 0, 0.7177157674836364`},
{0.1009959491070086`, 1.5581491170930981`, 4.3701504080039815`, 1.3889561652621691`, 0.5506040634320337`,
0.37952236600496847`, 0.006611879537937531`, 361.09151974058886`, 0.21353768992235467`, 8.563496620010564`,
307.91252075697554`, 0, 0, 0, 279.1034006794144`, 1.112509482972122`, 0, 0, 1.0832355466279897`, 0, 0,
24.76365240929587`, 1.5628914591163634`, 5.1868348263164`, 0.999562585126199`, 0, 0, 0.9259752027204486`},
{0.23246941421316458`, 3.062147704374147`, 7.181406357346838`, 1.3012169805011569`, 0.348059834110821`,
0.639530677915989`, 0.09989941363865933`, 83.25264127475623`, 0.015153685907020209`, 6.841473199486905`,
1.0484676335373272`*^6, 0, 0, 0, 167.62545353424275`, 5.21392807374731`, 0, 0, 242552.3584200321`, 0, 0,
228.08311259710254`, 805514.352541805`, 17.93530935190787`, 1.0000000000001024`, 0, 0, 0.7238803093118942`},
{0.05240567594084544`, 2.6092929572780967`, 9.364433651132018`, 1.4357965076882815`, 0.3985784064329525`,
0.6640850131228939`, 0.14135898686373455`, 340.71602063464445`, 0.17921846525178464`, 4.7134535629319085`,
225.40676807168447`, 0, 0, 0, 76.61117490911195`, 3.883945125550361`, 0, 0, 0.07732181833285072`, 0, 0,
144.77643803647373`, 0.05788717363840836`, 3.148349305270226`, 0.678429105185048`, 0, 0, 0.7215245422381906`},
{0.186251758126929`, 2.4933710973488976`, 3.5328550575209547`, 1.325175018028979`, 0.37317622556186136`,
0.25977278720819974`, 0.09748704597661054`, 108.88467391318876`, 0.2369656062609477`, 9.08676709988832`,
43221.349195109644`, 0, 0, 0, 106.16372972632715`, 3.9119675352634515`, 0, 0, 11738.593231307543`, 0,
0, 139.34266837418696`, 31233.337532398396`, 17.334417287675652`, 1.`, 0, 0, 0.4763249286660489`},
{0.2432348147394116`, 1.8751950266382789`, 2.5453519666394815`, 1.0050719073546701`, 0.45557496345664417`,
0.6934059109337196`, 0.0796473645979629`, 116.90111969840831`, 0.13836004707768368`, 5.732847922404471`,
91128.56424834066`, 0, 0, 0, 55.5435613472234`, 2.23372192930428`, 0, 0, 20338.62812512636`, 0, 0,
59.83806075320364`, 70672.32062955583`, 10.568308096110375`, 1.0000000000000322`, 0, 0, 0.526750592915759`},
{0.10016679782659832`, 2.355527283731478`, 1.9202752019167662`, 1.4361517107451491`, 0.5493703103299528`,
0.5953848202767148`, 0.3151585002444098`, 3628.217577021284`, 0.16393396333788363`, 2.9174291385165`,

15.074600454037883`, 0, 0, 0, 2.869231081060213`, 0.35018517424205464`, 0, 0, 0.029336874105391708`, 0, 0,
11.783867604077447`, 0.04197972481969596`, 0.1792149104581407`, 0.3439826164832661`, 0, 0, 0.3770730160732839`},
{0.14701675506563494`, 1.5420515267654622`, 0.49939909194610793`, 1.0246394569387973`, 0.7670831420663697`,
0.6948893607838256`, 0.005182980912963712`, 1858.6105410956102`, 0.23965764027041103`, 6.073448972699818`,
1000.3214305472883`, 0, 0, 0, 24.592864341531794`, 0.07777240799842297`, 0, 0, 314.14003508377044`, 0, 0,
1.71327229277421`, 659.7692656317222`, 0.7624438247256285`, 0.9999999999999779`, 0, 0, 1.1064540858062712`},
{0.19166603750475464`, 2.2499911524034095`, 2.4198836269597095`, 0.7671801018390442`, 0.14979093568204394`,
0.302546021842622`, 0.008837119919208427`, 4450.048280356515`, 0.1035437468879743`, 7.585339524339819`,
15.357066200668918`, 0, 0, 0, 13.651128846968827`, 0.05063691779449218`, 0, 0, 0.004266152177056893`, 0, 0,
1.6276088146083723`, 0.011681092616697648`, 0.37593848350013465`, 0.11250716005659456`, 0, 0, 0.8909121142029123`},
{0.14239569401382052`, 2.8387649854604886`, 1.4027026171382353`, 1.2048713691971291`, 0.21617980215068333`,
0.2290581611576099`, 0.32444520029725066`, 2595.236024076599`, 0.07657553404881084`, 7.455893283173221`,
24.916355486371895`, 0, 0, 0, 4.901582087772632`, 0.4800821150157779`, 0, 0, 0.021601623870986162`, 0, 0,
19.469147117894384`, 0.043942546041893064`, 0.618873519742012`, 0.31774957006196847`, 0, 0, 0.6817958397275017`},
{0.14874493002083228`, 3.1275671408493704`, 8.341714492380408`, 1.302209514279003`, 0.6258906648056601`,
0.45549735048188655`, 0.04029116024037646`, 765.0281459953038`, 0.12017823562381152`, 2.4241463017742984`,
56.116400371856514`, 0, 0, 0, 35.80308866297329`, 0.4427051742036021`, 0, 0, 0.02824742647299284`, 0, 0,
19.77985937033116`, 0.06002373534278167`, 0.6814573933958684`, 0.36963063377596417`, 0, 0, 0.2172797362209177`},
{0.06222670957040222`, 3.9065456744185374`, 1.0946055811329174`, 0.8246557540237`, 0.5907991824162977`,
0.2630274030573405`, 0.006980770676502568`, 3176.3819350381427`, 0.13217715504568522`, 5.816838705721813`,
23.875326558918164`, 0, 0, 0, 21.679208704937462`, 0.037388526492123396`, 0, 0, 0.026795410933577186`, 0, 0,
2.086571234866963`, 0.023819860771187706`, 0.3866164256211813`, 0.4973208101601343`, 0, 0, 0.40523787477390716`},
{0.04971186392349858`, 0.9916432478771275`, 0.9981695561230755`, 0.8524884405534577`, 0.12034821743714219`,
0.2529703866830304`, 0.3237478827730366`, 360.7437887352434`, 0.010006209625245316`, 7.274213959868414`,
100108.64796647927`, 0, 0, 0, 11.434237231669137`, 3.091776412961654`, 0, 0, 58502.08880417312`, 0, 0,
43.799131483702745`, 41546.39825533525`, 4.336206779965715`, 1.0000000000000007`, 0, 0, 0.6513683314428592`},
{0.19409490071094637`, 1.638712293913553`, 0.39703017050116074`, 0.7643187357680644`, 0.5335846868412271`,
0.21830616448247264`, 0.0963055671350066`, 635.9574479204244`, 0.22613729069446092`, 5.479828782022301`,
5226.009218756005`, 0, 0, 0, 7.154763952661755`, 0.391660763886526`, 0, 0, 1380.7558379426055`, 0, 0,
9.168847268920347`, 3828.538103878989`, 1.769490141067366`, 1.0000000000011615`, 0, 0, 0.22179475266256654`},
{0.27029725962327555`, 2.240924310463286`, 1.2051421639323703`, 1.1356504301287609`, 0.773380004303778`,

```
0.6607812449412671`, 0.04949916223108672`, 204.2592199369304`, 0.19899441283798802`, 7.18276351221955`,
 45644.00349640439`, 0, 0, 0, 40.63839933517644`, 0.8590091506957326`, 0, 0, 9374.892886770454`, 0, 0,
 27.499635552921195`, 36200.1122365112`, 7.492991062991821`, 1.000000000000215`, 0, 0, 0.5718333929470829`},
{0.14266269539818904`, 3.072017618366117`, 3.4103252419872643`, 0.7755852439360265`, 0.43949005760262017`,
 0.1924018543570919`, 0.06712102805080977`, 656.0250616869308`, 0.04537130588160604`, 5.285505421294628`,
 77.40683502854972`, 0, 0, 0, 40.281376566182836`, 0.8249895124044744`, 0, 0, 0.03117264934782816`, 0, 0,
 36.2054616724831`, 0.06353105969525863`, 1.6948837400391388`, 0.5768867023958195`, 0, 0, 0.34805137842462436`},
{0.10769494377900635`, 0.832286650700361`, 9.122967245453143`, 0.9815088864732582`, 0.842140026069350 8`,
 0.5448142839433918`, 0.07976087110962524`, 4710.110387525812`, 0.13361893143992154`, 5.1516712920505725`,
 34.095338229861866`, 0, 0, 0, 16.051813465867586`, 1.3988999701173515`, 0, 0, 0.004697971214450261`, 0, 0,
 16.6326538684829`, 0.007227824940264355`, 0.22804169057407198`, 0.09766966655157483`, 0, 0, 0.29044315729841297`},
{0.21491444350519606`, 1.295137699490157`, 7.08600395884199`, 0.8977823469890502`, 0.4181882258302472`,
 0.21987671758091132`, 0.14871331978208877`, 664.8642422386233`, 0.19734382780045878`, 6.556011365474522`,
 98.52364269830204`, 0, 0, 0, 32.90190402783381`, 3.36194115265057`, 0, 0, 0.014071165181639967`, 0, 0,
 62.20252471807389`, 0.04320138049232362`, 2.0866375434861983`, 0.2861988332477583`, 0, 0, 0.47313079471238595`},
{0.14904691011609433`, 3.9586033442731177`, 2.425615791755316`, 1.0187686401741827`, 0.6625310044158794`,
 0.43390068486512956`, 0.00854220404180854`, 96.98019438566024`, 0.2008209408788944`, 6.643325956478872`,
 52483.96610627175`, 0, 0, 0, 114.78564282202817`, 0.24008429416116647`, 0, 0, 16730.974889144152`, 0, 0,
 13.5771212824835`, 35624.28729224145`, 14.63168268159128`, 1.0000000000002283`, 0, 0, 0.5461900967007979`},
{0.21251186737978806`, 3.785250570140386`, 5.706304857162573`, 0.8638958511126991`, 0.620802282746526`,
 0.4325048599697877`, 0.008982590727965567`, 699.3542650472853`, 0.09331259312392975`, 7.885660147589962`,
 198.6373289803386`, 0, 0, 0, 176.05378144752902`, 0.40467521826501923`, 0, 0, 0.03631771392434948`, 0, 0,
 21.882815723704994`, 0.11025636007184565`, 2.408988118185892`, 0.5930261941528048`, 0, 0, 0.6503361689121229`},
{0.24515641699904833`, 3.817676488470438`, 5.668256418323743`, 1.3090245110359662`, 0.8705848781731611`,
 0.5675799494003652`, 0.13511363154659006`, 1804.7556662922755`, 0.20417175811210386`, 6.336735207604568`,
 122.94450041578052`, 0, 0, 0, 42.37887253035392`, 1.4475898739438986`, 0, 0, 0.03754569164786958`, 0, 0,
 78.9489975243359`, 0.13149381768778998`, 0.7300113159985043`, 0.4572283927833528`, 0, 0, 0.33855789540392367`},
{0.16393709899357184`, 1.7364683461504136`, 6.294465981846198`, 0.7908159510076651`, 0.7611075592144951`,
 0.36561813704297086`, 0.008326625223173748`, 2834.6413370290525`, 0.20899318399811945`, 1.1709155007061636`,
 7.919504357196832`, 0, 0, 0, 7.064031553154856`, 0.03216234658749174`, 0, 0, 0.005725662301431857`, 0, 0,
 0.7978413826728311`, 0.01340926382162346`, 0.08685105087970829`, 0.1429835920922108`, 0, 0, 0.07645516728134889`},
```

{0.25878729889995966`, 3.1361195765858056`, 8.880297290408144`, 1.0867236136347516`, 0.0902130745148968`,
0.5352173770607672`, 0.11164972929109021`, 57.41366920823615`, 0.17534323232121918`, 1.541035034614442`,
112794.8560829351`, 0, 0, 0, 43.08512356784165`, 1.4600612640362003`, 0, 0, 23991.020523025254`, 0, 0,
65.41323875940788`, 88693.87712867449`, 5.767269864311419`, 1.000000000003577`, 0, 0, 0.13664026560732145`},
{0.11203607225462164`, 0.6218658244264867`, 6.440483663444866`, 1.39680689202378`, 0.16534679246130501`,
0.5081267228820908`, 0.03214478110427463`, 1906.9275025607635`, 0.21932209633484323`, 1.251120855111619`,
7.230030948388517`, 0, 0, 0, 4.9842356547041815`, 0.22496891340940184`, 0, 0, 0.008286587282042231`, 0, 0,
1.9985782686809808`, 0.013262809878214552`, 0.15329074286559266`, 0.11758722719969783`, 0, 0, 0.22993843541931833`},
{0.24357827172423074`, 3.9240029450424014`, 2.804272198831022`, 0.75931848861878`, 0.2959071515485636`,
0.6138549707668646`, 0.10713705928623629`, 339.1202760730188`, 0.035220064551167996`, 7.297254233929856`,
30837.35402677289`, 0, 0, 0, 69.78639293923663`, 1.8058014240124127`, 0, 0, 6845.236263028533`, 0, 0,
101.22814437123526`, 23819.29740703586`, 4.865557457489675`, 0.9999999999999998`, 0, 0, 1.0627923210308656`},
{0.24204908128005126`, 1.6039606621918692`, 7.422941916054803`, 1.4757185212808246`, 0.48127457855983957`,
0.4526414340963527`, 0.017845029630840916`, 286.8959659308558`, 0.1351657578784522`, 4.5748150305594475`,
234.98183715576994`, 0, 0, 0, 187.26312816812273`, 1.9600785885382555`, 0, 0, 0.1719059259250563`, 0, 0,
44.912699297427466`, 0.5944238776679516`, 3.4964399531920964`, 0.9031196227966126`, 0, 0, 0.5076318600774292`},
{0.0765658571842559`, 1.8453576022836256`, 0.8953985896867352`, 1.094808063482127 8`, 0.11609992165592442`,
0.45437290836767896`, 0.20627344098654643`, 689.3327160072474`, 0.18998846953804188`, 8.643335566773064`,
3592.048018278957`, 0, 0, 0, 17.345435709906592`, 1.739333284595913`, 0, 0, 1684.5488302460622`, 0, 0,
45.85274142334337`, 1842.5560736646466`, 2.8181815731141935`, 1.0000000000031193`, 0, 0, 1.199499208143396`},
{0.2258389851785662`, 0.888336357939937`, 0.32961763645801767`, 0.9210788231382718`, 0.8596897387491109`,
0.5585019604624183`, 0.04381971178906294`, 415.715019082411`, 0.24817184929202513`, 7.285653344604734`,
13479.357265858447`, 0, 0, 0, 11.778751967467588`, 0.5310834627150206`, 0, 0, 3184.913220594358`, 0, 0,
6.739724986148474`, 10275.393851726076`, 3.716559314200005`, 1.0000000000007299`, 0, 0, 0.5424963508098691`},
{0.19210976086993148`, 0.9419570933782317`, 5.851922457612897`, 1.40657571618961`, 0.8492777725664675`,
0.5465312705852027`, 0.10025464283588155`, 725.5272924080149`, 0.05639485661927951`, 6.120948580338062`,
268.9483672482861`, 0, 0, 0, 111.21428982042713`, 10.843810876877289`, 0, 0, 0.259090624224477`, 0, 0,
145.9200653532371`, 0.7110548266200625`, 1.7651244610569592`, 0.9644667140043115`, 0, 0, 0.36765767549999695`},
{0.04040676752094921`, 0.7630185090261814`, 4.034875094487921`, 0.9393317585048341`, 0.5565744536568444`,
0.3001585479016161`, 0.008819784570716005`, 3975.779851508773`, 0.23610962920225392`, 2.0975088246946054`,
6.788814298312004`, 0, 0, 0, 6.029930060408326`, 0.06266963828245461`, 0, 0, 0.0049843095127312794`, 0, 0,

```
0.6831156280498365`, 0.0028771405104775585`, 0.11248539374733864`, 0.1071123233232455`, 0, 0, 0.16839146411271355`},
{0.2323130275502685`, 0.49681822670142894`, 1.2539687147478098`, 0.9468502150118296`, 0.13096405194857463`,
 0.3219849827616931`, 0.04046745571036271`, 95.23490611137812`, 0.01783356001323902`, 6.936606555558118`,
 780592.5082509076`, 0, 0, 0, 42.771465308589285`, 3.008943985450297`, 0, 0, 180729.14777804067`, 0,
 0, 21.35568878707635`, 599796.2212413768`, 15.025906674358811`, 1.`, 0, 0, 0.3137468959580257`},
{0.1110467064554368`, 3.3883763096282147`, 5.159131249779884`, 1.2482205179674581`, 0.8201347270890553`,
 0.46628896390017405`, 0.2764030660881924`, 2912.733505822841`, 0.03458542948115101`, 2.6312047625809307`,
 23.575177885540555`, 0, 0, 0, 4.9060892178991695`, 0.3770692916092532`, 0, 0, 0.015403217017390449`, 0, 0,
 18.25218078252979`, 0.024435378837167555`, 0.18923684824037684`, 0.23072981895037614`, 0, 0, 0.16152421504556433`},
{0.1632243866872038`, 2.5373481239797906`, 9.958152546995578`, 1.2439631103015851`, 0.6072525242529792`,
 0.6395983432777625`, 0.3899581087433961`, 56.26697227139985`, 0.18832987989003086`, 4.752251898931052`,
 97167.93252372758`, 0, 0, 0, 64.02807892790582`, 8.853199293487346`, 0, 0, 29045.80447511475`, 0, 0,
 320.9092659792758`, 67728.33744695803`, 18.80636744414252`, 1.0000000000012554`, 0, 0, 0.6091800407089617`},
{0.16346002078838484`, 3.218794874634134`, 9.025936619626364`, 1.1259348004332868`, 0.7415896232848811`,
 0.4862549245509259`, 0.364081252118343`, 137.81289300851844`, 0.05819144446151425`, 8.24717117891944`,
 672.8629758010734`, 0, 0, 0, 116.17157154370891`, 11.316886329969748`, 0, 0, 7.493705058265925`, 0, 0,
 520.3819387960533`, 17.498874065801665`, 13.976733839643085`, 0.9999999740883458`, 0, 0, 1.5121110567605833`},
{0.19514620721272602`, 2.87732243599938`, 0.26739185721478975`, 1.4891953183216857`, 0.05289250725423611`,
 0.4217001319921505`, 0.03447210696714538`, 218.70480172461387`, 0.0713049851341147`, 6.944425315128572`,
 131416.31876315633`, 0, 0, 0, 9.80458899415031`, 0.11303184374317105`, 0, 0, 34690.75749174926`, 0, 0,
 4.646129428351463`, 96710.99642644738`, 6.657758326443616`, 1.0000000000000993`, 0, 0, 0.4330655310324824`},
{0.06305409129179324`, 2.891634576752894`, 4.887865973915014`, 1.4252999692587962`, 0.37958790888649707`,
 0.564484952956938`5, 0.3706753829912659`, 1888.9507831786343`, 0.129560920022812`, 6.557119455026179`,
 51.592938499520955`, 0, 0, 0, 8.817751936046072`, 1.0102899497160374`, 0, 0, 0.016184485675544348`, 0, 0,
 41.734133587783475`, 0.01457854338989386`, 0.7805522866618696`, 0.2153460291608431`, 0, 0, 0.9132998021993964`},
{0.07134729442997761`, 1.925040188749243`, 1.1575324502817335`, 0.9987470940766925`, 0.48237853230783223`,
 0.6023915002525595`, 0.11093155711801116`, 121.08543179928522`, 0.23351841944906743`, 5.375810156733433`,
 29915.823911628922`, 0, 0, 0, 20.825810736934756`, 1.1151253504108087`, 0, 0, 14789.245254759875`, 0, 0,
 30.666587357627026`, 15073.894794121446`, 10.049685362694934`, 1.0000000000000995`, 0, 0, 0.7896670057017313`},
{0.1360789534218197`, 0.4455509561284261`, 2.009215820725272`, 0.8936493225292423`, 0.96227260378033`,
 0.5587588217121244`, 0.041826495849356216`, 90.5779416890061`, 0.15671264889903508`, 7.812316525319764`,
```

68111.03518287199`, 0, 0, 0, 80.49776763498981`, 6.411209898539434`, 0, 0, 23092.275201082408`, 0, 0,
40.80743857477536`, 44891.037735599086`, 18.771917155196025`, 1.0000000000001383`, 0, 0, 0.8026463515983912`},
{0.1584938652096898`, 3.640724074855333`, 1.8861164484754733`, 1.1569149894265711`, 0.5885808127893892`,
0.1748767871381529`, 0.46502697264632115`, 193.59851086045592`, 0.1503277806325949`, 4.212693145809347`,
26164.56895608105`, 0, 0, 0, 8.868097048884618`, 0.9868829398639059`, 0, 0, 7996.875568213752`, 0, 0,
51.328121117521896`, 18106.510262959047`, 4.479586067441102`, 1.0000000000141984`, 0, 0, 0.18132889431597687`},
{0.2646664636377358`, 0.7835752608143567`, 7.19251403558616`, 1.494052846937745`, 0.8510899770532778`,
0.5885640033353514`, 0.48048609718479995`, 154.5393756420333`, 0.18357560397946`, 4.027566835631127`,
8249.340663343237`, 0, 0, 0, 36.74587992586362`, 17.922420843259424`, 0, 0, 1672.0632647379477`, 0, 0,
200.62236552402294`, 6321.986732239416`, 6.1604443985714115`, 1.0000000000304075`, 0, 0, 0.7960580692854555`},
{0.12495672263621771`, 3.9102582538632182`, 1.1196234634441904`, 1.2648993449094799`, 0.05034343178950351`,
0.5637162084123208`, 0.0377865951253564`, 87.92444212100203`, 0.05653305553347371`, 1.253921938823833`,
264111.2768128192`, 0, 0, 0, 7.534933223237838`, 0.07017288636376118`, 0, 0, 94826.08228671992`, 0, 0,
3.919915830018606`, 169273.6637568688`, 3.1324026814835038`, 1.000000000000137`, 0, 0, 0.1415128597843697`},
{0.22032142146595135`, 2.68397072068061`, 2.6883455170430324`, 1.1162234719994681`, 0.40997309115380154`,
0.5558969738438672`, 0.02165064984593792`, 1526.6454168541877`, 0.24054798278325668`, 4.966530798126495`,
49.03536488580379`, 0, 0, 0, 37.49712154398171`, 0.28928407597280986`, 0, 0, 0.03505329999561856`, 0, 0,
11.091856998145253`, 0.11032846974436966`, 0.7311122386497694`, 0.48362504035322307`, 0, 0, 0.688624887804471`},
{0.10964967041383433`, 0.7460930180313401`, 6.531951119775776`, 1.1455194328360876`, 0.1524629724176152`,
0.28792352560938195`, 0.09608079533773954`, 1051.3203547256705`, 0.17626541392472717`, 8.294356744338359`,
70.12448646374816`, 0, 0, 0, 30.49912016656039`, 3.396595300529472`, 0, 0, 0.010221911641800628`, 0, 0,
36.20251484004426`, 0.016011846321707466`, 1.7312349446859612`, 0.17459715725262526`, 0, 0, 0.927394773954033`},
{0.07988018646299105`, 2.6019950195572488`, 9.606629629213668`, 1.0662798233306474`, 0.7693895670648874`,
0.32124913017390155`, 0.04062772179058327`, 1822.0454711227774`, 0.21556365885863799`, 7.4522296585239225`,
77.67789447755827`, 0, 0, 0, 49.44846180450603`, 0.7390172740517948`, 0, 0, 0.007849358449318507`, 0, 0,
27.470275234993483`, 0.008957260236362246`, 0.8507303189082256`, 0.14648180752682116`, 0, 0, 0.40145492105319636`},
{0.1682679068810577`, 1.5454861727740674`, 1.469740171862469`, 0.9427424173065856`, 0.7350490973518766`,
0.3805201167376455`, 0.012952191039290932`, 4650.232844583341`, 0.019462289964433777`, 1.394906478790226`,
5.782774890399873`, 0, 0, 0, 4.829188176841602`, 0.038207958454593106`, 0, 0, 0.020206689360485416`, 0, 0,
0.8435695925928528`, 0.048573390338350474`, 0.06410459965505273`, 0.3578734872640926`, 0, 0, 0.11547351729422783`},
{0.1878353522665755`, 2.6490780379134238`, 9.484290061448991`, 1.1885927795104771`, 0.9576534469556572`,

0.2804046795618802`, 0.13562182147346188`, 68.8621810881709`, 0.06230339869012863`, 8.081807561887658`,
129865.38196915698`, 0, 0, 0, 213.9739256536155`, 10.15012215735319`, 0, 0, 35092.160591420135`, 0, 0,
384.12093841689665`, 94164.97637835183`, 24.91420723963223`, 1.000000000000055`, 0, 0, 0.6108021355796563`},
{0.195165600718623`, 2.3826254740878197`, 7.662410402528582`, 0.9519993324167042`, 0.5246436284800724`,
0.22761517290878341`, 0.02889008666683709`, 101.1296763305189`, 0.18152629399038878`, 2.6385617746928904`,
8075.8965636638795`, 0, 0, 0, 116.36193838402997`, 1.3223111672194472`, 0, 0, 2088.9695264407487`, 0, 0,
45.00817530982654`, 5824.214178724375`, 5.623471124146033`, 0.999999999999984`, 0, 0, 0.2428506212918482`},
{0.2191459600297970 3`, 3.4188550030486784`, 4.710500615641845`, 1.452732616866 4971`, 0.03624911304432854`,
0.28384065291200533`, 0.4254237737178384`, 3505.5630456480862`, 0.12312118964896007`, 9.273808474100267`,
21.994509170033602`, 0, 0, 0, 3.5797079400817586`, 0.36907910089500506`, 0, 0, 0.00474714065346806`, 0, 0,
18.026113294508555`, 0.014861667084298997`, 0.5843420313366317`, 0.06781182687726517`, 0, 0, 1.104993871951961`},
{0.1814951436259516`, 1.11858432682377`, 5.7029405020905966`, 0.816783193035002`, 0.6766309251156815`,
0.4321734260024682`, 0.2931263656368919`, 87.29527865588257`, 0.044724422492498916`, 2.4174522941648533`,
160375.63566669732`, 0, 0, 0, 22.610745555794903`, 5.208739455075934`, 0, 0, 44607.30555428446`, 0, 0,
83.23449024223619`, 115657.27611916566`, 5.967103799448903`, 1.000000000000898`, 0, 0, 0.2217743181979818`},
{0.22203693974513244`, 0.9431525918127432`, 4.196712511517475`, 1.3261224346879132`, 0.8036004422541301`,
0.6271444136150295`, 0.10733581071086257`, 369.9451385562586`, 0.16273465181192748`, 5.731309489564488`,
225.90071966344982`, 0, 0, 0, 85.73344751490892`, 8.697178506715423`, 0, 0, 3.424698367719071`, 0, 0,
117.18237785809609`, 10.86299350169288`, 3.657985047874977`, 0.9999999961675067`, 0, 0, 1.1251763612012213`},
{0.18924733350115397`, 2.8150904404250037`, 2.4574844917932026`, 0.8737719488383665`, 0.6977173227044986`,
0.3482841124888072`, 0.48079795163551986`, 94.66261771766344`, 0.16471072416530214`, 1.3078470560939532`,
57313.14013302701`, 0, 0, 0, 3.472129594665993`, 0.5283172748743727`, 0, 0, 15468.44267495709`, 0, 0,
21.246584428717583`, 41819.45042358689`, 2.9016667944648593`, 1.000000000002547`, 0, 0, 0.08386177806463371`},
{0.07907817004881895`, 1.0481324229512046`, 3.8137728667258273`, 1.2920968808020492`, 0.04181350128653394`,
0.5803710922764521`, 0.02851300126208627`, 266.99553906648964`, 0.22548933562284668`, 2.0140176417924156`,
2720.1059443241925`, 0, 0, 0, 49.37930452604416`, 1.2218238658840577`, 0, 0, 1244.8759718356946`, 0, 0,
18.294760128123777`, 1406.3216255787383`, 1.8166676355169908`, 0.9999999999999765`, 0, 0, 0.44352927612095394`},
{0.22302087067747528`, 1.8201511620752422`, 1.875184623833773`, 0.7813352529024251`, 0.8705057770695381`,
0.22074020090969038`, 0.1367588752741694`, 187.45561858905666`, 0.07793294287132913`, 8.923384483271061`,
51526.866471983536`, 0, 0, 0, 44.52287542734099`, 3.109676081754148`, 0, 0, 12278.600788756861`, 0, 0,
80.85829334117707`, 39119.774837281315`, 9.770238060739464`, 1.0000000000008369`, 0, 0, 0.3561446829275311`},

```
{0.21429795289589076`, 2.896671823601787`, 8.636702505614757`, 0.9811380648364925`, 0.20610104158853804`,
  0.1778381348410949`, 0.007936414694970003`, 185.13169573811163`, 0.014685687390926405`, 3.2353159752463547`,
  132.5848029001317`, 0, 0, 0, 119.18200137508019`, 0.3091914306230036`, 0, 0, 0.046544218469149856`, 0, 0,
  12.794658645496884`, 0.14249043910110062`, 3.7182116233445175`, 0.637771220332924`, 0, 0, 0.2523687303786486`},
 {0.17507336986882482`, 0.7983306290353371`, 2.750450064120228`, 0.8050075785267179`, 0.7818959542274213`,
  0.6526925477724106`, 0.09653256947066893`, 72.78219945862553`, 0.07700161447215975`, 3.655530763820291`,
  178673.84361342076`, 0, 0, 0, 34.94098801481757`, 3.792530410456041`, 0, 0, 51010.968739295444`, 0,
  0, 43.25275983164292`, 127580.88853516757`, 10.953387185997304`, 1.`, 0, 0, 0.38367480950420674`},
 {0.1470766374450453`, 1.2533511161361544`, 4.593526904612924`, 1.479552314271993`, 0.7400477950348978`,
  0.5768087033937906`, 0.10619660427114413`, 242.62879808709684`, 0.045931800134058565`, 3.771378090721337`,
  10744.69302168057`, 0, 0, 0, 63.5715783349201`, 4.838154824713873`, 0, 0, 3414.812063289552`, 0, 0,
  86.62723927992367`, 7174.843939648633`, 3.734095484894038`, 1.000000000000872`, 0, 0, 0.8190173474536607`},
 {0.0788585810797619`, 2.548378832414274`, 3.111482597871378`, 0.9346713900878518`, 0.19963135196405957`,
  0.5058944800586684`, 0.03749384602431879`, 1118.4164778142415`, 0.22872744797764172`, 1.4303214452036404`,
  14.58640832427198`, 0, 0, 0, 9.560662618618812`, 0.13292731229722263`, 0, 0, 0.024786684078228007`, 0, 0,
  4.839273555828064`, 0.027923467658307412`, 0.2979931039025121`, 0.4229115418020146`, 0, 0, 0.25829923738104343`},
 {0.24182502946725698`, 1.8945200327623395`, 6.300131593939071`, 0.7669603268824658`, 0.6959163653444247`,
  0.5105300710554361`, 0.2329066367569749`, 178.13280663193103`, 0.21420184435482204`, 3.5903109736592462`,
  684.3658492283569`, 0, 0, 0, 49.95781557132246`, 5.375957412063507`, 0, 0, 108.54608553203948`, 0, 0,
  145.49798589187859`, 374.9880047477282`, 4.804847584065826`, 0.9999999999402017`, 0, 0, 0.7462540209891684`},
 {0.05758087297506348`, 2.9919516996565125`, 9.945704556467732`, 1.1770757830874041`, 0.6136122924727272`,
  0.31603398268032157`, 0.21009959511709828`, 118.22678602178958`, 0.2258513747270965`, 6.274152149731609`,
  518.6124611616934`, 0, 0, 0, 137.30444178212255`, 8.62054807823717`, 0, 0, 2.318991480583124`, 0, 0,
  368.46090678074773`, 1.9075650553384622`, 11.732110604964895`, 0.9999946045542002`, 0, 0, 0.7535664838355827`},
 {0.12816326660379745`, 2.7095777054297967`, 7.985014815061977`, 0.8582103259977079`, 0.28527001685481057`,
  0.4561351167228034`, 0.41006088059385215`, 4928.696897400591`, 0.12257221770331383`, 5.545857987270676`,
  13.653307289162441`, 0, 0, 0, 2.194945443809597`, 0.2884429110447941`, 0, 0, 0.001694785956147883`, 0, 0,
  11.16512115794637`, 0.0031029900619198 7`, 0.2520163123611485`, 0.041556989900579766`, 0, 0, 0.7475095657139902`}};
```

In[ ]:=

```
AestcurPure = {"\!\(\*SubsuperscriptBox[\(A\), \(cur\), \(est\)]\)", 207.9605568694953`, 116.78387911717152`, 31.25902142774426 2`,
  101.07977095724142`, 39.66545271571148`, 69.69060371965857`, 6277.626993286135`, 1481.9321176658373`, 8.556708305914556`,
```

81.22694426914325`, 1160.1582463118507`, 62.25545012434214`, 64.35943027718949`, 1114.925898737311`, 64.01972861337667`,
35.867335292670376`, 32.06061117041807`, 321.0070772585055`, 403.1457361693428`, 151.84090893120717`, 47.98249780135377`,
1116.7532325308846`, 667.1429568663535`, 64.00220933945363`, 118.55990502554417`, 27.636304624807984`, 72.14556157551598`,
1289.906798998197`, 1254.8338003361828`, 16.22921291198604 3`, 1300.6080910345431`, 373.0319275620772`, 1944.627208129596`,
411.7931442456674`, 77.76483896547852`, 3374.630578586424`, 18.75006111750761`, 471.2206746536005`, 36.562140922848904`,
115.49344249505992`, 155.13110013412629`, 50.07292581143878`, 682.53277296402`, 168.00523624843996`, 260.23408369886255`,
169.33030615744755`, 440.29716253947225`, 61.60415779010116`, 124.69396284563918`, 1580.4759744211344`, 43.99726363837288`,
47.4413397178195`, 305.79004618169245`, 42.27683542351058`, 128.15523433073255`, 46.36090634416943`, 562.2572548133888`,
138.72157013652057`, 27.539831787753887`, 250.26261694398673`, 147.87303991344814`, 173.5060763026177`, 94.32074935711191`,
68.30475947414355`, 936.8989095907302`, 19.471443184024665`, 497.7830012025341`, 150.96974761642952`, 243.45706886064008`,
438.8884012337126`, 216.45596427487624`, 5.316314045179877`, 13.694419023912111`, 28.02836029534192`, 39.110786457292406`,
3692.057315228333`, 225.40772854810487`, 386.7514678791356`, 21.067098041154125`, 441.1953338810919`, 63.93660734091749`,
7.607813324098744`, 42.751768039115404`, 1654.7835367759387`, 52.77316385734188`, 101.46022806970909`, 219.74992554964012`,
63.410636702419914`, 542.2782602107791`, 2960.951428115113`, 127.18453275932204`, 684.7364542320128`, 498.92828017425677`,
31.830588209285622`, 25.861034172501718`, 199.48791869475178`, 167.92926200058616`, 99.50048411574802`, 436.90334409952976`,
5.1134156112022335`, 113.6744910135902`, 5.188550962019851`, 37.22335298098436`, 1934.6599296796364`, 52.60038478619157`,
58.893481597787435`, 22.07830369798928`, 213.31168229520065`, 13.07290615139485`, 40.64442531087087`, 10.156417516420506`,
6.940073254802677`, 5013.044794160846`, 27.528929883671847`, 900.2521256591069`, 377.69682844362916`, 52.87708431242897`,
1443.3649262227405`, 46.131598125846885`, 198.8557281848253`, 41.92333015469925`, 241.96339662525466`, 197.3194594124301`,
1630.565544843584`, 3.396425327215184`, 192.3193972710194`, 19.281315954082512`, 115.07488447496439`, 121.52746827212849`,
372.09299583320785`, 15.261998266439404`, 42.58205912795087`, 734.7451436087945`, 1945.1501623356062`, 89.88605786951616`,
200.06966424811495`, 21.719906382444215`, 15.709695293981277`, 8.291418684634737`, 72.23336044689572`, 523.3454148614499`,
102.0586850921827`, 21.039262638512433`, 329.9903374078502`, 812.6481200164565`, 52.66683962351665`, 123.65131947629831`,
293.88747670914523`, 38.13568855652656`, 86.4401640550493`, 28.03471829670753`, 724.8129404127303`, 23.298680309643302`,
53.133223137666015`, 64.3566422857879`, 184.70134712080423`, 1302.0139818510706`, 148.99177950003994`, 181.0793306310366`,
34.763770297899015`, 410.0424834036011`, 109.5878003583873`, 51.08487841422653`, 25.53568476102389`, 1355.6571922206913`,
2634.820754866145`, 26.74565280618183`, 110.26129061743359`, 130.50736596502583`, 36.774237156456245`, 97.63326548519228`,
624.4888962097375`, 22.020026359336963`, 170.39967183857436`, 1019.0250344849559`, 20.911420931699155`, 69.14576850182758`,
117.69277763016758`, 1300.5825095982962`, 652.3948615754017`, 179.90656765639656`, 45.29801271063936`, 93.08810398464354`,
63.11471022768208`, 186.6559259451605`, 48.19667691733027`, 552.5060846870274`, 1003.1881020223896`, 221.39769915039446`,
331.8024300079967 5`, 645.0586588509619`, 272.00017208285817`, 24.570839911027583`, 1603.6612456016999`, 373.30730566539734`,

305.9005387329298`, 272.783670120552`, 8.370071383861253`, 2187.8900356183904`, 1238.147553372664`, 103.61826085720948`,
166.74978111447882`, 27.640325295165788`, 579.1256605379922`, 50.78484465217116`, 69.06886369518107`, 686.6282603891304`,
452.7492111157109`, 2370.391036458293`, 179.09233079134458`, 12.41228281360483`, 1933.8645666824689`, 15.491552672585485`,
472.81623489754327`, 103.9560055503515`, 1860.9580701494772`, 372.39861564106803`, 17.124591104558913`, 58.79262707630034`,
208.4260767058717`, 1501.8280382623007`, 16.570367674036373`, 11.874726467154034`, 453.21607308969226`, 818.8902542855299`,
5466.411205656372`, 14.34109081263816`, 318.59326113108165`, 284.1069513727965`, 73.14705605153169`, 1487.5028872302105`,
36.95751605476262`, 1110.6431961884703`, 17.374251841011947`, 200.84376517520198`, 15.275673965218767`, 31.342830826782997`,
354.11743947309253`, 382.00440128556465`, 361.9513676137458`, 92.95139716509517`, 364.3669758898248`, 459.63520063089135`,
55.298102970951305`, 600.4590727330327`, 42.41455632259722`, 4273.0347315013605`, 467.8107672977147`, 920.5686943528164`,
58.81117334521899`, 107.15404603785166`, 127.1185550842855`, 40.791806463255426`, 623.0325551517668`, 168.45404551549638`,
56.845741971236045`, 85.78491105755522`, 52.776049773115126`, 16.167593467624545`, 213.42264570130578`, 321.83387849508483`,
184.87387159474466`, 6.709674363270432`, 167.99116842429544`, 523.7112384783147`, 26.711356940120876`, 366.2563246217706`,
837.5850181225206`, 147.94232128815003`, 274.4713869836533`, 768.5749603616548`, 26.18147720988362`, 48.64673984443831`,
2398.995745662609`, 64.71955709799926`, 545.4876655176232`, 703.6147238687225`, 257.5730226906546`, 825.0287780513876`,
5537.399977063587`, 126.76429235825827`, 206.8524145465044`, 215.55649608133103`, 67.236923907091`, 17.490909590265012`,
284.47682400474406`, 302.7943791090315`, 353.9914916120266`, 7.704380583589187`, 24.07858169245523`, 54.016607131634046`,
50.42816979454327`, 77.62052501425865`, 292.58212804750974`, 108.0350107557488`, 429.00829923613463`, 83.7359404776244`,
28.28796732701628`, 933.570914825478`, 141.9034097581748`, 882.9338938922734`, 1928.7128440054398`, 67.56781701392785`,
696.9208588954659`, 1798.6601708400856`, 107.93017520872108`, 2024.6734164262446`, 84.46924615514638`, 54.89213455738702`,
28.0009968611173`, 110.93137469993229`, 190.48444416864632`, 378.41947179987693`, 1240.1958675085789`, 130.8783633404614`,
25.485976457477012`, 6.022943017224859`, 37.92170771530289`, 175.8699614740291`, 203.59340604182674`, 1610.6584187773474`,
958.6101552941893`, 28.707693702110898`, 2751.809049229709`, 1277.213532675834`, 100.94141817513415`, 56.91721347222889`,
370.3101746328675`, 8.559207296786242`, 302.0807675654379`, 45.857263482607`, 94.90704495109331`, 50.20468175525086`,
180.6168778356671`, 535.4585552816059`, 741.6739840160454`, 550.3164500636733`, 1322.155919625336`, 630.9838632713123`,
1396.2796812397198`, 21.816267438842065`, 923.8931769965501`, 138.3664271403334`, 1233.5383325502896`, 27.533037087968193`,
101.53892247432107`, 12.838587797981816`, 323.34647049793864`, 33.65059855503893`, 594.3351606203837`, 229.31477415562566`,
349.5526050928185`, 71.2856111484316`, 1338.367702429557`, 482.27942402583153`, 24.32610285566832`, 40.956377463140775`,
190.5047218324733`, 316.7693825577835`, 1033.9469726833836`, 33.263844598176085`, 793.6411623107655`, 316.44969475345465`,
161.85171414311463`, 11.60631323378407`, 292.3574775379676`, 14.629659111067838`, 515.4725975706392`, 158.0463055848333`,
32.595012890128814`, 224.84143344026876`, 136.27928931814048`, 20.478565103173363`, 11.898091017421132`,
567.965911237098`, 36.51366628616791`, 758.6602289634035`, 1012.7649361525209`, 23.76021414140445`, 105.62509746862344`,

15.347367230979733`, 257.9706452479735`, 335.8587829428483`, 469.51896152472466`, 404.83461632418573`, 75.77900703185838`,
235.38305322105828`, 45.478931981401686`, 278.46558224714255`, 162.7071253920935`, 106.49185826081813`, 300.627681896006`,
570.7648871115722`, 886.6336386954683`, 29.234191605463575`, 3263.6743014470067`, 9.111498595296059`, 241.9764243045456`,
195.19533289033754`, 18.07935406327192`, 500.3693224687036`, 11.56950819460474`, 615.8922390440637`, 172.48822024094045`,
125.59922710335557`, 8.680870278018249`, 1300.1638755330707`, 594.3122039880631`, 690.8775746266773`, 599.8431189919705`,
59.539001572637815`, 350.39535508955447`, 652.2051495507056`, 172.69051562697882`, 48.81362755101295`, 55.206737317354595`,
452.27432678171004`, 26.447305840484844`, 3394.4323698820094`, 20.91294882491946`, 15.035708857056138`, 161.87784335873357`,
57.23017728004768`, 133.73819092118197`, 375.2547588267408`, 26.34546496888898`, 833.1324303485147`, 112.77890415115328`,
900.1770672216628`, 35.58280659174881`, 518.3770138412615`, 545.7271039866157`, 1831.4724281805798`, 88.76190026894609`,
152.23984495875848`, 34.5025438872988`, 712.492750993158`, 902.5716181338538`, 1715.5120279343114`, 226.9371167094659`,
53.94951629750541`, 72.10339317700169`, 18.820688243631256`, 492.8971860424036`, 1197.247467780099`, 83.49817178337545`,
1020.5640631804943`, 156.82982566212948`, 114.27482433280552`, 42.279620828130206`, 77.92531816944096`, 5.015128559449715`,
113.40578811217537`, 65.16034366834462`, 100.16442576989826`, 105.812684608349`, 47.22745072157395`, 249.67682608788064`,
133.67568667314936`, 228.8289192029971`, 37.768398539037356`, 565.4232426935394`, 337.7219201090041`, 4458.832146166972`,
825.5427565762624`, 14.699178142567403`, 454.34562662059227`, 205.67293952043224`, 36.57670032263964`, 990.7239706208131`,
166.48527560191096`, 129.7920200739647`, 339.8109113418568`, 55.31880358150875`, 174.40906433738718`, 1047.3100132405896`,
430.57994944349053`, 142.53412542824952`, 144.1499825141307`, 551.9109222570158`, 20.542905524332767`, 160.90869689080554`,
18.873506896335243`, 1624.5072407737396`, 39.64940251720767`, 1131.4414527495996`, 332.61345058908375`,
145.28993559905936`, 3665.997415616593`, 370.1971593057519`, 44.876248949853505`, 63.88330879656129`, 2272.535941141883`,
123.21303366022315`, 715.449231953232`, 138.5258195923069`, 26.792363890852588`, 670.0361409142529`, 64.47565792622012`,
227.65403505998384`, 109.7953690642296`, 24.138049752596697`, 823.9560726985757`, 33.59942521349337`, 8.632861644598574`,
125.90750424026572`, 135.54928275900846`, 1152.584120403935`, 23.92854371506977`, 113.64161889085416`, 112.4722829314004`,
21.371103971704514`, 505.9028704823119`, 14.031142308825817`, 1483.0165420576`, 798.0955218526343`, 894.55644696043`,
225.8821053951252`, 135.13511016954564`, 16.806112613077715`, 10.51903903585091`, 489.41572993532355`, 1270.9131225005497`,
394.54053651975437`, 150.93850243539916`, 1195.8064006549514`, 119.7527871608091`, 83.72056274363985`, 18.32493562373855`,
43.75501464608105`, 105.1197481057999`, 92.02056915772759`, 289.14761110857484`, 196.68153362507658`, 355.4653409680756`,
841.841968152713`, 164.49692857816075`, 765.6579789691419`, 835.7787599341478`, 409.0361909384147`, 458.076884142214`,
134.95470263162522`, 422.1325964721197`, 25.33920199500803`, 640.846626530443`, 3462.48657098948`, 49.02494490095971`,
4.816261691249143`, 240.40545798359213`, 1693.696501889256`, 788.0816867682037`, 3575.664599969038`, 143.25284719236257`,
792.3318933175954`, 895.9059894825732`, 596.1152900300608`, 51.45791768695105`, 94.54736795300296`, 80.35021178707962`,
186.57460278879952`, 514.4863646190738`, 229.7808911145177`, 95.29698763613041`, 173.79978678012495`, 119.96732361506078`,

62.346854959035475`, 205.07945531435757`, 75.9401623927921`, 245.2566634936187`, 532.1036746548102`, 39.572692393228934`,
27.940616848525956`, 44.008981881195744`, 447.76094775033675`, 1699.1087647897052`, 1386.2525862097975`, 588.1721684885763`,
234.97140862671975`, 507.9712975264508`, 848.5840906059378`, 35.21388364659763`, 36.87130719182587`, 6107.237475112454`,
779.0963116072494`, 288.42961260747984`, 79.5875429519073`, 302.5086012930816`, 13.074087109963658`, 990.3953472142992`,
54.81918623540333`, 596.8138967483573`, 48.01014166132958`, 29.35997092010539`, 442.0704497662819`, 1381.272017313927`,
2654.182988521078`, 1575.1598048786254`, 274.49066977263806`, 389.6317972761561`, 184.53653180583726`, 180.029510380224`,
263.96338714216745`, 12.358327817838894`, 6.190291429344035`, 31.01930124068562`, 437.8899240896687`, 8.319287693798923`,
1294.8259937023915`, 70.9829150533407`, 192.90097820548016`, 641.0186879308368`, 66.13572837867821`, 726.5987051232179`,
35.74959607822846`, 211.89342431830994`, 15.240720866282377`, 16.01087837764014`, 222.40867712464183`, 974.3797155965107`,
36.2902009980038`, 724.8309440002016`, 260.2719771224575`, 2088.800631565277`, 18.95502249359305`, 132.10238540612022`,
622.6505384709761`, 754.9154512745685`, 79.49416157699206`, 34.93893906821083`, 13.516972340253732`, 1120.832375395441`,
7.971503619916857`, 186.8810641120699`, 28.085218278218846`, 156.6598104222859`, 1295.5496737748254`, 68.48860350548834`,
9.659671775761543`, 24.472843412710375`, 204.09327345667944`, 23.182078996595816`, 67.41831053599807`, 1788.0964224805289`,
93.33694431988522`, 1770.9437098523865`, 332.04691684460244`, 141.76091626484666`, 370.84183186210714`, 351.2776832502037`,
172.34439568419208`, 412.7749573420771`, 243.92901886741885`, 134.29902690148975`, 1847.670065883616`, 32.33094268905134`,
12.754316693111443`, 310.2650821877486`, 351.1491436594416`, 309.1944495557074`, 689.0231783053966`, 31.304569092510476`,
2829.8255603844996`, 45.8017462716161`, 12.989179326075563`, 123.11721945152065`, 656.283359116043`, 23.474497778761545`,
84.83369812950119`, 172.2383894475708`, 141.3117842430811`, 266.1516029114938`, 120.06366955327934`, 436.6859383244124`,
8.083891723590108`, 667.2203935368185`, 125.09027476300521`, 117.91162834438464`, 56.7582502982993`, 12.463534577552196`,
150.67600655491395`, 1454.3006392475324`, 228.36518105874237`, 91.54995376668423`, 674.0663425712387`, 20.916672179547568`,
193.03675900242317`, 30.92397173014905`, 288.87284735281145`, 2293.579012162944`, 571.7388234506207`, 351.9321745619992`,
18.625373468147195`, 310.123946787966`, 54.61572115989577`, 15.829846222573646`, 234.59207967902472`, 1601.8081922372273`,
125.95857672132418`, 109.38753992692125`, 12.937766036958083`, 488.76390838350073`, 78.76974539961955`, 1044.355043042405`,
254.917902533672`, 2873.020249012567`, 96.76209127673845`, 96.20959270028948`, 1460.857949049142`, 221.42610034897046`,
391.8124291340777`, 66.05844948505614`, 28.674373858185167`, 207.16679860913504`, 63.189675267236474`, 1564.1065748261265`,
29.29115146081773`, 39.025938253147636`, 1265.6834373784666`, 859.939215414434`, 22.916696717809067`, 10.155378811198135`,
22.163864046958274`, 27.721344184243357`, 195.12319491825085`, 36.568662144054706`, 27.221897987864867`,
1554.5498262649037`, 630.8827184887874`, 24.350928499133843`, 184.83204637468828`, 47.78632408647853`, 509.04543069115323`,
1081.535263812407`, 150.79842698899552`, 46.71401105805333`, 804.168613189815`, 224.26828730476`, 17.903992482066336`,
1511.5958684136183`, 131.60494836516654`, 572.6490328935768`, 35.33333521340801`, 500.2711245175664`, 127.4325088057755`,
33.809111722886456`, 35.59516962613836`, 3773.9061540394096`, 89.65267900640707`, 263.3571295988692`, 190.67515693911798`,

281.7995869223638`, 255.8143850347918`, 259.1239774729543`, 8.793150178654933`, 678.6826155459381`, 65.05545866663405`,
4.772174222919766`, 126.83535878980194`, 76.13952307942688`, 37.03160868400174`, 478.57916033813115`, 236.62429088466408`,
155.65831170640706`, 208.6903090431616`, 129.24837432632387`, 331.69397957468055`, 70.59463371820026`, 426.4593188000726`,
721.6572924041536`, 249.57475028981793`, 67.05642010388344`, 479.34082128184997`, 218.71684686132693`, 107.73544188039841`,
160.36937714211362`, 89.40299163282324`, 71.22133726144789`, 24.99339515091807`, 43.30184218808783`, 378.2894870521611`,
78.82841318062593`, 415.4168141224054`, 1596.979618878675`, 746.3315220387047`, 534.1129554343032`, 139.9189659086689`,
32.16644146164344`, 79.4214923242974`, 955.8570385762621`, 8.579765849280273`, 3.3047411232324793`, 11.907097656354242`,
124.28791687321757`, 218.17800018994802`, 2246.0438273525215`, 127.20589312468667`, 31.07130790622174`, 40.649857556599194`,
685.3961634782964`, 56.11786353256072`, 325.76870276611584`, 405.6051344203167`, 32.023801066041216`, 20.701815708607704`,
193.8823594216119`, 46.986655539037216`, 847.7592962635491`, 1046.6645534884174`, 626.1601795871928`, 64.82847774873049`,
42.01370398082542`, 837.9411628835317`, 208.78873190020744`, 911.4045474740751`, 730.1536497532052`, 401.5841665626077`,
142.45611887402248`, 177.62629435544488`, 767.7063313617285`, 162.00316950132856`, 67.20013532431379`, 35.81414327524097`,
396.94334291839874`, 3.8189056795083394`, 124.71106561227744`, 1809.4596488040236`, 1125.5596176980375`,
39.07091456606537`, 151.65796793849398`, 35.288187541813336`, 10.677467459556741`, 184.9915474345718`, 537.9720355173645`,
75.33254802252394`, 675.6902789725033`, 68.70074109101174`, 32.86948637483345`, 226.8599513252022`, 468.6999904729794`,
482.2728201479209`, 12.565270436264235`, 51.65330524257508`, 2906.5417901507467`, 41.95705073816599`, 35.00088655907136`,
16.41311009375263`, 96.31417666979591`, 23.68236164183872`, 12.592969979701449`, 319.8606836058835`, 1005.0782529254774`,
95.31006301399012`, 746.0678574384053`, 1294.6730225509723`, 193.19202307375122`, 1098.044073743162`, 3148.447883065088`,
1127.2365592524025`, 1244.5649883024462`, 6812.491479879829`, 800.2944951573206`, 466.4187212793585`, 29.92352181295465`,
171.4304562333472`, 45.37989215131294`, 54.55580532948984`, 348.2636558055563`, 21.277599392329904`, 626.2614397257395`,
1484.1228603976367`, 1036.1989721798222`, 40.484585634784644`, 237.14482010219052`, 26.738927371442475`,
73.7453632776144`, 553.1625545482643`, 7.833404248522687`, 18.861109244487125`, 1736.820501985616`, 67.13460431424852`,
791.0871599587367`, 344.6543695144871`, 292.26024899910686`, 2342.756799405454`, 31.362363258604464`, 141.11053873505804`,
961.1811711253683`, 205.27011879352287`, 4013.3627399917723`, 58.21755736520024`, 65.02189070867793`, 258.54549957059066`,
377.272835077887`, 8.192818148050538`, 267.43424716417223`, 21.285134963013444`, 41.33134115477876`, 27.640218284656402`,
120.34155981324187`, 580.9736929095984`, 44.682124743129584`, 31.745236205426973`, 1312.446590648646`, 526.5396726204798`,
211.48674349165788`, 191.74330634391688`, 877.4095169008085`, 17.167498804570332`, 333.6756888488899`, 972.8237758702663`,
41.59375958663582`, 86.42122963933532`, 1430.9542983656359`, 260.0976679595555`, 514.8047678466262`, 23.894598744868432`,
122.05475352084504`, 58.71573319441848`, 977.8571045834494`, 178.6150138023847`, 109.89088987851198`, 3553.0445953329704`,
1378.4534169301235`, 717.1005645461606`, 459.08425823594456`, 20.26560233854502`, 5.051929785796079`, 701.207515052265`,
133.72139400821848`, 749.8244933861907`, 21.11936622378476`, 381.57357006751465`, 22.43722741236239`, 48.965372209984444`,

```
490.68848203785547`, 4.907208130103031`, 24.192625322491004`, 3234.835991727759`, 614.4038492724043`, 336.91150116097987`,
1751.585645804869`, 355.9007652877697`, 1290.5572182112453`, 232.87437211084983`, 866.529826646155`, 912.1779534988016`,
15.174746573997037`, 99.68407164880875`, 15.492171315973327`, 24.726416475940617`, 56.176718488103454`, 23.62122517692009`,
167.06749969804162`, 96.04922537697776`, 1051.070353922555`, 77.13759336015913`, 32.520379808776305`, 98.46753453055675`,
1281.4291603952768`, 199.03964077631105`, 115.65896161662529`, 7.682628901159168`, 290.57224792253567`, 7.332463087313262`,
313.16507793149566`, 237.9137277519639`, 133.11179690295432`, 563.6070404303182`, 256.9789261210002`, 6.765789493451768`,
668.6988730760784`, 22.51568479838448`, 1825.9388683692819`, 1371.9013689097633`, 313.15816441644324`, 52.519613992881474`,
783.7715638557264`, 3616.2623162987215`, 228.61635599735934`, 830.9401339460172`, 154.91990124695994`, 50.225916449402206`,
70.57876004969503`, 75.50036726293014`, 5.562664536924255`, 2791.485661910901`, 284.68237155616373`, 21.65050931560944`,
512.2382152503286`, 470.84370169781084`, 228.24486179453265`, 82.42950631252553`, 821.4750711921528`, 132.84142613062733`,
1481.9854223917985`, 389.07362025209494`, 14.745160897849553`, 430.9635487074551`, 774.3914869512789`, 13.7432291070492`};
result[[All, 12]] = AestcurPure;
```

In[∘]:=

```
result[[2 ;;, 13]] = (result[[2 ;;, 12]] - result[[2 ;;, 11]]) / result[[2 ;;, 11]];
```

In[∘]:=

```
(* The scripts hidden in this group of cells have to be initialized to run the following codes *)
```

In[∘]:=

```
CreateResultArray[Njj_] := ( (* argument is how many random sets will be chosen *)
   result = {};
   (* Table that will contatin the results *)
   result = Array[f, {Njj + 1, 28}]; (* 10 variables and 17 measures *)

   result[[1, 1]] = "κc";
   result[[1, 2]] = "κp";
   result[[1, 3]] = "γ";
   result[[1, 4]] = "V";
   result[[1, 5]] = "ks";
   result[[1, 6]] = "ρ";
   result[[1, 7]] = "ω";
   result[[1, 8]] = "α";
```

```
result[[1, 9]] = "kf";
result[[1, 10]] = "N0";

(* minimal curative dose *)
result[[1, 11]] = "Acur^sim"; (* simulation result *)
result[[1, 12]] = "Acur^est"; (* analytical estimation *)
result[[1, 13]] = "Err"; (* error in estimation *)
result[[1, 14]] = "Mm"; (* minimal curative number of radioconjugate molecules of cancer cell *)

(* paths of activity *)
result[[1, 15]] = "Canc"; (* released in cancer *)
result[[1, 16]] = "BlFrg^sim"; (* released in blood from fragments, simulation result *)
result[[1, 17]] = "BlFrg^est"; (* analytical estimation *)
result[[1, 18]] = "Err"; (* error in estimation *)
result[[1, 19]] = "BlAb^sim"; (* released in blood from antibodies, simulation result *)
result[[1, 20]] = "BlAb^est"; (* analytical estimation *)
result[[1, 21]] = "Err"; (* error in estimation *)
result[[1, 22]] = "CldFrg^sim"; (* cleared in form of fragments,
simulation result *) (* no need to estimate -- error will be the same as for release *)
result[[1, 23]] = "CldAb^sim"; (* cleared in form of fragments, simulation result *)

result[[1, 24]] = "ToVbl"; (* How much activity affects viable cells *)

(* receptors occupancy *)
result[[1, 25]] = "fDMax^sim"; (* maximum occupancy of receptors on damaged cancer cells, simulation result *)
result[[1, 26]] = "fDMax^est"; (* analytical estimation *)
result[[1, 27]] = "Err"; (* error in estimation *)
result[[1, 28]] = "Nnew"; (*number of newborn cells until Ncur is achieved*)
)
```

```
WriteDownMeasures[] := (

  result[[ij, 1]] = kappac;
  result[[ij, 2]] = kappap;
  result[[ij, 3]] = gamma * 10^7 / Nnor;
  result[[ij, 4]] = V;
  result[[ij, 5]] = ks;
  result[[ij, 6]] = rho;
  result[[ij, 7]] = omega;
  result[[ij, 8]] = alpha;
  result[[ij, 9]] = kf;
  result[[ij, 10]] = N0 * Nnor / 10^7;

  (* minimal curative dose *)
  result[[ij, 11]] = DA1 / nCpm; (* simulation result *)

  result[[ij, 12]] = 0 (* -(kappac+lambda+kon*gamma*N0/V)/(kon*gamma*N0/V) * N0 * (rho/lambda * nu/alpha * ProductLog[-1, -E^-2 * (Ncur/(N0*Nnor))^(lambda/rho)]) / (1 - (1 - ProductLog[-1, -E^-2 * (Ncur/(N0*Nnor))^(lambda/rho)] / ProductLog[-1, -E^-1 * (Ncur/(N0*Nnor))^((lambda+rho)/rho)]) * ks) / nCpm *);

  (* analytical estimation *)
  result[[ij, 13]] = 0 (* result[[ij,12]]/result[[ij,11]] - 1 *); (* error in estimation *)
  result[[ij, 14]] = 0 (* (result[[ij,11]] * nCpm * 6.02214076*10^11)/(N0*Nnor) *);
  (* minimal curative number of radioconjugate molecules of cancer cell *)

  (* paths of activity *)
  result[[ij, 15]] = ActTumorpw[[Npw]][tEnd] / nCpm; (* released in cancer *)
  result[[ij, 16]] = ActBloodFragpw[[Npw]][tEnd] / nCpm; (* released in blood from fragments, simulation result *)
  result[[ij, 17]] = 0 (* (lambda*omega*DA1)/((lambda+omega) * (lambda+kappap)) / nCpm *); (* analytical estimation *)
```

```
result[[ij, 18]] = 0 (*result[[ij,17]]/result[[ij,16]] -1*); (* error in estimation *)
result[[ij, 19]] = (ActBloodpw[[Npw]][tEnd] - ActBloodFragpw[[Npw]][tEnd]) / nCpm;
(* released in blood from antibodies, simulation result *)
result[[ij, 20]] = 0 (* lambda*DA1/(lambda+kappac+kon*gamma*N0/V) /nCpm*); (* analytical estimation *)
result[[ij, 21]] = 0 (*result[[ij,20]]/result[[ij,19]] -1*); (* error in estimation *)
result[[ij, 22]] = ActOutFragpw[[Npw]][tEnd] / nCpm; (* cleared in form of fragments,
simulation result *) (* no need to estimate -- error will be the same as for release *)
result[[ij, 23]] = (ActOutpw[[Npw]][tEnd] - ActOutFragpw[[Npw]][tEnd]) / nCpm; (* cleared in form of fragments, simulation result *)

result[[ij, 24]] = (SDpw[[Npw]][tEnd] + CFNpw[[Npw]][tEnd] + CFDpw[[Npw]][tEnd] + UNpw[[Npw]][tEnd]) / nCpm;
(* How much activity affects viable cells *)

(* receptors occupancy *)
result[[ij, 25]] = 1 - If[Ainj[[1, 1]] == 0
    , Min[Table[NMinimize[{dFpw[[nn]][t] / DDpw[[nn]][t], t > tB[[nn]], t < 10}, t][[1]], {nn, 1, npw}]]
    , Min[Table[NMinimize[{dFpw[[nn]][t] / DDpw[[nn]][t], t > tB[[nn]], t < 10}, t][[1]], {nn, 2, npw}]]];
(* maximum occupancy of receptors on damaged cancer cells, simulation result *)
result[[ij, 26]] = 0 (* DA1/(gamma*N0) *); (* analytical estimation *)
result[[ij, 27]] = 0 (*result[[ij,26]]/result[[ij,25]] -1*); (* error in estimation *)

result[[ij, 28]] = NewCellspw[[npw]][tminNn] * Nnor / 10^7; (*number of newborn cells until Ncur is achieved*)

)


MakeOutputForm[] := (
  resultOutput = result;
```

```
resultOutput[[2 ;;, 1]] = Round[resultOutput[[2 ;;, 1]], 0.01]; (*"κc"*)
resultOutput[[2 ;;, 2]] = Round[resultOutput[[2 ;;, 2]], 0.01]; (*"κp"*)
resultOutput[[2 ;;, 3]] = Round[resultOutput[[2 ;;, 3]], 0.01]; (*"γ"*)
resultOutput[[2 ;;, 4]] = Round[resultOutput[[2 ;;, 4]], 0.01]; (*"V"*)
resultOutput[[2 ;;, 5]] = Round[resultOutput[[2 ;;, 5]], 0.01]; (*"ks"*)
resultOutput[[2 ;;, 6]] = Round[resultOutput[[2 ;;, 6]], 0.01]; (*"ρ"*)
resultOutput[[2 ;;, 7]] = Round[resultOutput[[2 ;;, 7]], 0.001]; (*"ω"*)
resultOutput[[2 ;;, 8]] = Round[resultOutput[[2 ;;, 8]], 1]; (*"α"*)
resultOutput[[2 ;;, 9]] = Round[resultOutput[[2 ;;, 9]], 0.01]; (*"kf"*)
resultOutput[[2 ;;, 10]] = Round[resultOutput[[2 ;;, 10]], 0.01]; (*"N0"*)

resultOutput[[2 ;;, 11]] = Round[resultOutput[[2 ;;, 11]], 0.01]; (*"A^sim_cur"*)
resultOutput[[2 ;;, 12]] = Round[resultOutput[[2 ;;, 12]], 0.01]; (*"A^est_cur"*)
resultOutput[[2 ;;, 14]] = Round[resultOutput[[2 ;;, 14]], 0.01]; (*"Mm"*)
resultOutput[[2 ;;, 15]] = Round[resultOutput[[2 ;;, 15]], 0.01]; (*"Canc"*)
resultOutput[[2 ;;, 16]] = Round[resultOutput[[2 ;;, 16]], 0.01]; (*"Bl^sim_Frg"*)
resultOutput[[2 ;;, 17]] = Round[resultOutput[[2 ;;, 17]], 0.01]; (*"Bl^est_Frg"*)
resultOutput[[2 ;;, 19]] = Round[resultOutput[[2 ;;, 19]], 0.01]; (*"Bl^sim_Ab"*)
resultOutput[[2 ;;, 20]] = Round[resultOutput[[2 ;;, 20]], 0.01]; (*"Bl^est_Ab"*)
resultOutput[[2 ;;, 22]] = Round[resultOutput[[2 ;;, 22]], 0.01]; (*"Cld^sim_Frg"*)
resultOutput[[2 ;;, 23]] = Round[resultOutput[[2 ;;, 23]], 0.01]; (*"Cld^sim_Ab"*)
resultOutput[[2 ;;, 24]] = Round[resultOutput[[2 ;;, 24]], 0.01]; (*"ToVbl"*)
resultOutput[[2 ;;, 28]] = Round[resultOutput[[2 ;;, 28]], 0.01]; (*"Nnew"*)

For[iii = 2, iii ≤ Npar + 1, iii++,
  resultOutput[[iii, 13]] = PercentForm[Round[resultOutput[[iii, 13]], 0.0001]]; (*"Err"*)
  resultOutput[[iii, 18]] = PercentForm[Round[resultOutput[[iii, 18]], 0.0001]]; (*"Err"*)
  resultOutput[[iii, 21]] = PercentForm[Round[resultOutput[[iii, 21]], 0.0001]]; (*"Err"*)
  resultOutput[[iii, 25]] = PercentForm[Round[resultOutput[[iii, 25]], 0.0001]]; (*"fD^sim_Max"*)
  resultOutput[[iii, 26]] = PercentForm[Round[resultOutput[[iii, 26]], 0.0001]]; (*"fD^est_Max"*)
```

```
      resultOutput[[iii, 27]] = PercentForm[Round[resultOutput[[iii, 27]], 0.0001]]; (*"Err"*)
    ];
  )


(* The outcome of the global parameter sweep was obtained by this code.
   It uses the already generated test set of mice.
   It takes a long time, at least half a day *)

PatientsTrainingSet = {{"\"\!\(\*SubscriptBox[\(κ\), \(c\)]\)\"",
    "\"\!\(\*SubscriptBox[\(κ\), \(p\)]\)\"", "γ", "V", "\"\!\(\*SubscriptBox[\(k\), \(s\)]\)\"", "ρ",
    "ω", "α", "\"\!\(\*SubscriptBox[\(k\), \(f\)]\)\"", "\"\!\(\*SubscriptBox[\(N\), \(0\)]\)\""},
   {0.172487255493398`, 1.2765963995094278`, 0.2324603897081641`, 0.9331633444796544`, 0.5595329534566904`,
    0.18253234530133355`, 0.2215389709063929`, 78.80610179612091`, 0.19190036455619353`, 1.548441048845115`},
   {0.14090572053825962`, 0.941193557649755`, 3.8052282638940014`, 1.32622706664442`, 0.616835667267962`,
    0.48175991442091237`, 0.08306143231635953`, 874.0744302472489`, 0.12491077781228116`, 5.511666182290989`},
   {0.19800522656535097`, 1.47676715957736`, 1.6845843495593211`, 1.0829526404841552`, 0.6930225215765629`,
    0.2749384033948816`, 0.06680029700083288`, 3207.8452923633004`, 0.18818451872370445`, 6.655704647717006`},
   {0.2711930593993829`, 3.0021543969280486`, 4.914701090552741`, 1.464602766667476`, 0.8471176050384048`,
    0.28257943270869035`, 0.04953192991909177`, 1933.8633413872876`, 0.19773521340575795`, 9.992985154010565`},
   {0.27990766836391096`, 3.477149477100016`, 9.931945787531824`, 0.8846854018858359`, 0.8967333599095253`,
    0.5903168559967787`, 0.24882968868487906`, 980.8551320933655`, 0.02004206946214948`, 1.1376503486553808`},
   {0.11737985888146019`, 1.1225833279930164`, 5.309079794927442`, 0.8606501327539497`, 0.8514188342619913`,
    0.5642039473140849`, 0.008986110654413243`, 2018.9822529242097`, 0.06040743860925685`, 4.536035630396375`},
   {0.17121040978700802`, 0.4138167442315588`, 6.781135564610604`, 1.3665406840239216`, 0.9405996253397015`,
    0.6832439228870748`, 0.04185895434111264`, 62.7113536738392`, 0.18142736225630413`, 8.46259785026923`},
   {0.07382619562964193`, 0.625700255424527`, 9.939999934198667`, 1.4556453073248168`, 0.9580351575689623`,
    0.4163162212955882`, 0.006430521616962508`, 92.19524127790851`, 0.19149999891406427`, 4.303065608360628`},
   {0.1627307868596377`, 1.5327585946689206`, 6.648641489637967`, 1.0898921981857375`, 0.08271984399349908`,
    0.5169005126785295`, 0.006231976105310307`, 2017.0154068595314`, 0.2464092033815996`, 1.6313338993270357`},
   {0.08926805346894312`, 1.228740834290055`, 5.100082063336529`, 1.0507919032716577`, 0.3229790419061651`,
```

```
  0.45218339761418835`, 0.024533377777917375`, 814.1470590464116`, 0.1414263079606694`, 5.247711540674161`},
{0.17431851399024534`, 2.020213173041662`, 9.224968841892476`, 1.3485881267881612`, 0.8890825947176819`,
  0.5177484922558413`, 0.03701694525181502`, 131.5752033573906`, 0.228949740900746`, 4.795606274383001`},
{0.25439204271280724`, 0.8534844729419486`, 2.8150623547133353`, 0.8024992452769948`, 0.43579385025887163`,
  0.4594767236090769`, 0.057877631906885676`, 1195.6419878079198`, 0.17833211044546654`, 5.195270679096961`},
{0.129274073243755`, 2.8469118022753515`, 4.121632588773114`, 1.2196787674185057`, 0.8228855513020883`,
  0.4828002917324088`, 0.013782155470303842`, 388.66064646424104`, 0.2255512748456478`, 1.0022663108918284`},
{0.06343574194222029`, 3.9924293708982113`, 9.079106395846292`, 1.3161791897123671`, 0.10938228479468126`,
  0.3240611741083924`, 0.010321331270591745`, 76.94152103093134`, 0.06038801289350482`, 9.425890207979073`},
{0.110036074735397`, 2.490203037930516`, 9.729597307787852`, 1.4397421355398403`, 0.8533380591052817`,
  0.37040757295392324`, 0.047916566872126407`, 665.4588326486067`, 0.16916230254726894`, 1.9013228378216027`},
{0.21512225225566667`, 1.75750084110844`, 0.8044386006474246`, 1.3520587790631349`, 0.026316391204000755`,
  0.16031186333306224`, 0.13761957869990635`, 493.8180531489964`, 0.06250485252841559`, 2.6863460670624115`},
{0.15874286665935028`, 2.268211451796655`, 1.8981682437883747`, 1.375959688601855`, 0.24814706074480242`,
  0.5134877163486878`, 0.3316652449509965`, 685.632474632321`, 0.05319219913715695`, 1.7896119638851753`},
{0.07432908705186819`, 3.0619407712521785`, 1.7905302646779082`, 0.9549392645174688`, 0.353226782755657`,
  0.6119520610188782`, 0.0496137109124893`, 317.5186768305259`, 0.07812208014742161`, 6.620973402603631`},
{0.1228443083266203`, 2.0512729485932484`, 1.9256217622349043`, 1.4810122884336692`, 0.07741458536098089`,
  0.4641958329015734`, 0.007197139507585793`, 240.76714600918612`, 0.03301937102418029`, 9.328377628444095`},
{0.2606519449147342`, 3.818605783771848`, 8.595720359336667`, 1.112914365339814`, 0.820058745442926`,
  0.4441576207168524`, 0.053270363092160024`, 1117.1606910609416`, 0.20810873381921946`, 6.830482236754131`},
{0.24750930586460296`, 3.1846993652287674`, 3.026718658979185`, 1.4845570571034035`, 0.869864794311124`,
  0.6669561203654737`, 0.2363643381944309`, 1924.496556700219`, 0.06437651322808385`, 2.548184450019704`},
{0.24908529437358162`, 3.626300618072209`, 2.0230682918259397`, 1.4602193573319078`, 0.40130475942739396`,
  0.6650543311625685`, 0.019582320105998748`, 125.14663606859848`, 0.03716084844005629`, 8.149858668846527`},
{0.2795730004018654`, 2.183215118244356`, 0.7910515142573491`, 1.2847699393543137`, 0.70345738565049`,
  0.2632113724473232`, 0.1394636484917589`, 223.91272255356017`, 0.16150366940434352`, 9.859315316872873`},
{0.08969576723701717`, 0.49356011207206274`, 0.2529879905685366`, 1.1041769014657603`, 0.7443718717514693`,
  0.6180257213599576`, 0.03574805853679543`, 3205.3226496399375`, 0.061225880931137455`, 7.560768798013438`},
{0.10298476113266081`, 3.435925289367793`, 4.954566778741102`, 0.9299871443466801`, 0.6352691398464292`,
  0.5470174017743717`, 0.00550714898658944 1`, 973.5524715904572`, 0.1328513609688533`, 5.618259356059234`},
```

```
{0.14277679649227548`, 3.0428582592918065`, 7.414614443890604`, 1.1046799505745009`, 0.2872163059276376`,
  0.23677108379761724`, 0.04608733451503689`, 2695.430951291806`, 0.1274330436692639`, 8.066834406130887`},
{0.18738335776550036`, 1.508373316422973`, 6.602438992729994`, 0.7532357689229783`, 0.8880958875806098`,
  0.4853414284787141`, 0.10975729606650726`, 2118.598035369477`, 0.05093222572935868`, 5.0448517662608`},
{0.061980185747317895`, 2.54906136842855`, 8.170240160100523`, 1.0602973698223939`, 0.7221801534586956`,
  0.6067985789160708`, 0.38301062006579967`, 81.55317476370394`, 0.23749102694434165`, 4.188403663256118`},
{0.2562298689236346`, 3.2913375042385793`, 9.509882785943699`, 1.013989937500057`, 0.7792587778840148`,
  0.49077497767429623`, 0.021163604915718004`, 145.96678185214677`, 0.05845136191000838`, 7.439112841768198`},
{0.2313093023364764`, 2.988629640967633`, 6.855539828479465`, 1.0799498768994156`, 0.4522471358822151`,
  0.2285321965248518`, 0.04827992453162874`, 1204.6728692817303`, 0.13073517219860892`, 1.9378468299164582`},
{0.2663151909473739`, 2.2417238355718707`, 0.9398485531379794`, 1.0477480676389026`, 0.7226958973603346`,
  0.5716146939488362`, 0.007506086251562624`, 76.16131590346896`, 0.024911856209502148`, 4.070747059506875`},
{0.19929196929676601`, 1.9362608032362312`, 0.5353176928827672`, 0.7753335837033051`, 0.8607728477687919`,
  0.15482089410819944`, 0.02026432754563482`, 192.89182731397838`, 0.1831217833074511`, 5.333726168282188`},
{0.10696417975467426`, 1.9440197080088568`, 2.0936739600677434`, 1.1829890119130342`, 0.8864433537218006`,
  0.5974069860908109`, 0.31351620960128274`, 84.46268853549806`, 0.02353560759327844`, 4.656274919049821`},
{0.21459986298539707`, 1.6769788549349993`, 9.356964926187835`, 1.260140723869471 3`, 0.895239496536256`,
  0.38446102196425924`, 0.012005779035619358`, 146.09102757400478`, 0.02879957197754268`, 2.3826604675291154`},
{0.07383925157057758`, 2.6358268091067414`, 6.251616293116599`, 1.1087155777827833`, 0.9205185682116173`,
  0.19659269091732745`, 0.29632431074176147`, 531.6045072192474`, 0.12599923353908937`, 2.5100327097656248`},
{0.0817054148315825`, 2.796991931647562`, 7.6919124752432`, 0.9355088496592321`, 0.8578766380044134`,
  0.4976427721124693`, 0.008010560795693933`, 67.84344037448854`, 0.080482215315478`, 7.8347514420046025`},
{0.23618801128357925`, 0.8353216061115365`, 4.620412867513723`, 1.4504705173961072`, 0.3108708881036355`,
  0.3400688454787608`, 0.014061322572830477`, 2071.8308884477033`, 0.0364017192015193`, 3.610034284058532`},
{0.1662441272521787`, 3.725772450959033`, 9.814227303865422`, 1.3148509050359762`, 0.25298305094984697`,
  0.32303330970203903`, 0.055784478318004704`, 213.77275876328028`, 0.04990125708904308`, 9.833393949415544`},
{0.22831788422124638`, 2.590756298379799`, 9.795768388114066`, 1.125355926333821`, 0.3650031672541203`,
  0.1680578019226482`, 0.4143448211959925`, 831.1374036828946`, 0.21412864715242685`, 3.6048538714465157`},
{0.23359626472155715`, 2.56674003123695`, 1.1969232842409259`, 1.0442579072771299`, 0.8882810964774697`,
  0.3176999965402193`, 0.007340467537817918 5`, 1908.0313626467632`, 0.2040761417499009`, 9.648978920291999`},
{0.2534162537452666`, 1.6838285982829593`, 0.8034274134000761`, 0.870316935261816`, 0.7856829676536639`,
```

```
  0.46427070430809425`, 0.12261242146523575`, 210.18201198829036`, 0.1377493870208059`, 1.4006481974514937`},
{0.25695579183074796`, 3.423363059474246`, 4.134350766656727`, 1.2838485736639544`, 0.4149036036442244`,
  0.6493708976181658`, 0.12875465899507466`, 1172.2742202486922`, 0.012611679323919878`, 3.4839902382375243`},
{0.17682432929033887`, 2.45185381012344`, 3.9338825987195882`, 1.2879615271399696`, 0.02702036645869277`,
  0.6694394004648141`, 0.11330549796901213`, 154.96108716979026`, 0.11583322861965717`, 8.805457336592767`},
{0.2509750819824499`, 3.8883567883501247`, 6.384989889678234`, 1.4613334209290016`, 0.7183189010632474`,
  0.4965470763778975`, 0.3859108436384297`, 748.6242458418322`, 0.020020198926280153`, 5.690775103224375`},
{0.1441623498715573`, 3.343288457669903`, 1.621696793707331`, 1.3375455077897458`, 0.06814809058713811`,
  0.4010236514346772`, 0.3002310191555227`, 155.99799531014958`, 0.10001900063604485`, 4.2763953522223215`},
{0.2657479684896014`, 3.193863728015164`, 2.3963245604206165`, 1.4113571594678591`, 0.26744798738201037`,
  0.235299669124016`, 0.018584060295236755`, 466.949287196137`, 0.23679723903863448`, 8.690245518267893`},
{0.18653936584035707`, 1.0683088955449334`, 5.185702152338592`, 0.9522710688882444`, 0.206752787597013`,
  0.5240389817239608`, 0.06412446666117125`, 220.00685924183844`, 0.2055887216336732`, 7.885811409920794`},
{0.07587231017464335`, 3.3343312881276663`, 8.762311039358057`, 0.8284399428795388`, 0.17449762015817138`,
  0.34311440561675743`, 0.03578987408308253`, 1364.0076205471958`, 0.21103062426335195`, 8.577574022027257`},
{0.1141532151024588`, 1.4516849606811304`, 7.634928187688176`, 0.8394863515309923`, 0.0282734940185132`,
  0.6576494992828068`, 0.009900377337816624`, 932.466930942293`, 0.19488805826375527`, 9.750042758265863`},
{0.21903027892555205`, 2.3035966499415492`, 1.6904861150255108`, 0.7741880968122689`, 0.5538208564897962`,
  0.4216122395122083`, 0.4439804577081556`, 61.55285726796139`, 0.16334632191055676`, 6.147879562247702`},
{0.19219532042344978`, 0.8633096904694177`, 0.1660296606087659`, 1.0595398890946446`, 0.3981268836303198`,
  0.35447506211304225`, 0.06527455092271564`, 2352.6780619798324`, 0.04729158696423241`, 8.334762180963512`},
{0.059421230927129864`, 0.7565633878530198`, 4.881127251124937`, 0.9039794772130322`, 0.6691506700132113`,
  0.3437226952926379`, 0.013854681683978944`, 1303.847591809871`, 0.1331369696675042`, 3.817043920573575`},
{0.27625614155976197`, 2.681313109654546`, 6.389652815751472`, 1.3504817156087858`, 0.7587234585548364`,
  0.5970419449613528`, 0.3005699072645848`, 854.9062988397701`, 0.165124447314083`, 9.6053618622676`},
{0.09922116949114601`, 0.5509823607034723`, 3.2884104382793122`, 1.3318058680427458`, 0.6719251293345294`,
  0.6187329358641112`, 0.3259483616243979`, 1099.7736795288956`, 0.13645709285092156`, 2.0261792473532125`},
{0.11800216420621701`, 3.285091391336728`, 1.6522276383131942`, 0.9670398406334181`, 0.4307591958110746`,
  0.30003613158270603`, 0.29799743711056653`, 701.4335436984502`, 0.043710117716904806`, 7.7162513520402545`},
{0.19357111334803345`, 1.327860467490665`, 5.552252240997824`, 1.1813151963757291`, 0.7103742334207412`,
  0.3776068940225674`, 0.2456309648230094`, 3934.634788049085`, 0.12355866435459295`, 9.745686147306856`},
```

```
{0.11447070818541266`, 1.7335861954191927`, 8.736387174086214`, 1.0335531844510784`, 0.08646802454747982`,
  0.4443642965951369`, 0.015374273787766714`, 123.60141703755143`, 0.21867059554150092`, 6.82672203062274`},
{0.22987433852030326`, 3.2498283820092855`, 3.7918096781351274`, 0.808040368560279`, 0.941624602976310 7`,
  0.49895559050949523`, 0.02048494990466312`, 278.1506433463176`, 0.24538025309113942`, 1.1463070343242556`},
{0.2774701327316953`, 3.217064080543545`, 9.848999257942918`, 1.1587780417232185`, 0.006285118350015439 5`,
  0.6835477959342324`, 0.37074868705631564`, 2643.6047855840716`, 0.14999893314602414`, 6.1386041394007655`},
{0.07518728576930783`, 0.8073344777020095`, 0.2079911159294223`, 1.2717171333972301`, 0.7961690644438275`,
  0.349516332980527`, 0.009301689754665837`, 660.9000723394831`, 0.24128632812714051`, 8.034216989913379`},
{0.19386888157639426`, 1.1721624963279647`, 7.488800420822528`, 1.2206737749782337`, 0.39882649538868`,
  0.6168747383393647`, 0.005894727770540639`, 784.8706404426375`, 0.21917965839925868`, 7.12627169471982`},
{0.051543962131442966`, 1.0824196854643793`, 1.5789464925861978`, 0.9324716268311393`, 0.16692765658832065`,
  0.33113273649509023`, 0.16543741477462187`, 443.37752852110856`, 0.07774169596012481`, 8.031423814374019`},
{0.04886198458272409`, 2.4240564285395276`, 3.0585768323132374`, 1.3030342316587755`, 0.5506088802661759`,
  0.40370465638646613`, 0.05636145780930632`, 1567.8212350001168`, 0.20179368484919008`, 9.505728478640599`},
{0.26828907890664055`, 1.4916419764625646`, 9.30244345383333`, 0.8462433381711008`, 0.9150350508953056`,
  0.16353772752060636`, 0.1124240025000419`, 2042.0256023360328`, 0.08362962700521354`, 9.175430964325553`},
{0.11853219177887636`, 2.436539187916523`, 4.07490711978506`, 0.8391678158796785`, 0.8274679746994464`,
  0.4783994312610165`, 0.02880518630114325`, 249.91551468654188`, 0.20172418525520536`, 8.758671893830364`},
{0.15380346197105899`, 2.5002802569502807`, 7.104691399911929`, 0.895619363352984`, 0.7056556726651881`,
  0.2904087856211036`, 0.27890663528622706`, 1570.4796825528501`, 0.2402855906015`, 2.015690994359673`},
{0.07681933116098377`, 1.701376646381478`, 3.4749545233581878`, 0.9897106512386663`, 0.21660819673269338`,
  0.4456181241675946`, 0.020856154503598144`, 170.86262737853005`, 0.11272605112949713`, 7.4521875824230435`},
{0.19680750576810457`, 2.9845730901840906`, 8.034687795284743`, 1.0668124507997105`, 0.6731645050394575`,
  0.24725877139168673`, 0.05097134738484948`, 549.1786460489706`, 0.029127445194553014`, 6.0096318557240025`},
{0.2481156970490977`, 1.2202113043036036`, 9.306028403345813`, 0.8009108612869359`, 0.02681732734233533`,
  0.15549305073805075`, 0.021015785720259927`, 53.28601671876581`, 0.06540227633756851`, 2.0322216725641393`},
{0.05969602287151182`, 2.177557565950017`, 1.7675183816518665`, 0.8372040785938111`, 0.9188853493106184`,
  0.1878023064060833`, 0.1313271602899384`, 239.3049490553974`, 0.14060221077790058`, 6.359907705361997`},
{0.1932263333480873`, 0.7780345756067408`, 9.679599745167298`, 1.1108495595363916`, 0.27977393862884203`,
  0.2181645563751744`, 0.006193313005348089`, 412.49802062580375`, 0.21651680739670837`, 9.973248740719807`},
{0.05080144024762184`, 3.462591354860418`, 9.637683194306994`, 0.7734436451051352`, 0.6551811041034619`,
```

```
0.18020194036251547`, 0.05139770733113868`, 3391.683612113084`, 0.04739051831745644`, 1.6399754771823183`},
{0.17269587719558416`, 2.280528270589695`, 6.304702452448467`, 0.855238720891303`, 0.7302844321830138`,
 0.25273349413775115`, 0.28098261768807636`, 2514.837947344666`, 0.04999285469609732`, 2.363210664893213`},
{0.12416213981002666`, 0.8389921874064035`, 3.610730817659622`, 1.0777639636353653`, 0.18253050124358539`,
 0.3654692817582218`, 0.277997956001176`, 563.1802062075279`, 0.17509802310960038`, 1.6234053595359104`},
{0.05389681485101466`, 0.42474703837564753`, 9.702496123506663`, 1.2875902192251218`, 0.8398628787663107`,
 0.4145702753216558`, 0.012323091180371398`, 1207.1917708220153`, 0.0487427592688896`, 1.997348446926472`},
{0.06391261825702438`, 1.7835225604286613`, 8.062142041499534`, 1.3025605866836996`, 0.9222349035188417`,
 0.39598365567854665`, 0.09958099473376647`, 75.95083350193653`, 0.043061298974276296`, 9.73945151975186 7`},
{0.08850946488241984`, 0.9673836199236003`, 8.299835087678265`, 0.9900607622617293`, 0.0809660102577392`,
 0.6707110673061827`, 0.005613358503071156 5`, 223.20635429959376`, 0.07233923327715214`, 4.067688416849283`},
{0.056027023765889306`, 2.0857485685464603`, 9.366491292380005`, 1.196524731276077`, 0.9516747873190254`,
 0.6119693625054259`, 0.014912996736033244`, 220.0557248806415`, 0.0820080753991021`, 2.040285256013867`},
{0.04516470805711162`, 3.292123721374173`, 5.263943679637629`, 1.138536155221258`, 0.48426832878752823`,
 0.5662939024082725`, 0.09160607397723192`, 3360.7159646360847`, 0.026694430856043894`, 4.180658858897127`},
{0.2782184518736612`, 3.845021085593168`, 1.137061402050021`, 1.4385887178277776`, 0.42032329684699743`,
 0.5854257024714923`, 0.03249096611383817`, 328.6688300716124`, 0.02617032692156479`, 8.869579059594898`},
{0.09319840750005726`, 2.6994281444799046`, 8.85475415324603`, 1.038403514470577`, 0.3378909888092929`,
 0.6279203689803818`, 0.23307552493819164`, 1846.0011275272348`, 0.034946033021005685`, 7.6621208156759835`},
{0.06034484465157863`, 2.8968090048449424`, 3.142651522085412`, 1.307236858746047`, 0.3617717997572676`,
 0.29303713371207274`, 0.18131081790115888`, 4578.414121282968`, 0.17962394399488657`, 3.3138716244433386`},
{0.20102830374342656`, 1.9832552045818916`, 0.5122029117897267`, 0.8414910669557318`, 0.023679729667421823`,
 0.562606520598186`, 0.21796137676857033`, 2537.351175593395`, 0.0373739667120736`, 9.865525950238812`},
{0.04583409051630541`, 3.6747726707008503`, 0.7420872691712788`, 1.2157743693615881`, 0.8915453306179026`,
 0.4926202034522773`, 0.4217762472968932`, 73.4269782954483`, 0.10619296232023001`, 3.9517710932009376`},
{0.10119631213999619`, 1.851973173732965`, 1.6003758779141855`, 0.9328064269865228`, 0.39319494883808903`,
 0.3867811485974866`, 0.006630825974655251`, 2020.948278622179`, 0.24307528382326266`, 8.411215463490773`},
{0.19796477344593338`, 2.2434362012846876`, 1.2309355399833084`, 1.3299187291217778`, 0.059794768114105334`,
 0.6215799505741797`, 0.02273921518352973`, 1004.7174087228818`, 0.138969497102004`, 8.58482448486276`},
{0.18841727652913837`, 3.2954166443999204`, 4.985214271603974`, 1.3919313698468052`, 0.8063258092579206`,
 0.6389441864140679`, 0.00880414284139521 1`, 1247.8494355462315`, 0.09671339442963328`, 8.749674730462491`},
```

```
{0.08822838789215687`, 3.6314565393637377`, 8.720964691808945`, 1.4591613734708808`, 0.09172379783619267`,
  0.17510896742376914`, 0.2630216511777198`, 431.756995427934`, 0.16850915581941245`, 3.8927198296202143`},
{0.22004819658675462`, 1.2435615798896285`, 4.521096993231973`, 1.3090616735635763`, 0.5905184190150714`,
  0.6892934240599866`, 0.09109967416767972`, 239.35075160225182`, 0.045734190683186515`, 5.8358773331232445`},
{0.18183828174362843`, 3.2786199220563104`, 2.2240542093570888`, 0.9831747738881791`, 0.39067106215615355`,
  0.5999409005287338`, 0.32534468961787655`, 53.98091103084368`, 0.10022266621367804`, 9.980846637487563`},
{0.21252199352031792`, 1.818911285509734`, 0.45179175410691036`, 0.8473192883797733`, 0.5752582261250987`,
  0.4244690624430477`, 0.010390335454685632`, 819.8846878599306`, 0.22518903335941576`, 6.3452240038645735`},
{0.13408565703158576`, 3.9481177636984475`, 4.409802796026355`, 1.1882851460555317`, 0.7016940523769313`,
  0.6437617694336766`, 0.472695817526219`, 268.88735252515323`, 0.02238688650951909`, 7.198883746978469`},
{0.11550511139863262`, 2.7266825017818803`, 9.715437717848832`, 1.4226217916614743`, 0.7434913376024772`,
  0.6527594414247178`, 0.026462900292485443`, 517.125461393156`, 0.19263485762362964`, 9.20218742349427`},
{0.1537432379066465`, 3.3010385217103515`, 7.8154151393713`, 1.099357680158021`, 0.2670513361076359`,
  0.6099579487912872`, 0.01626886763741749`, 3071.882113963599`, 0.04550777584421739`, 6.952411393791335`},
{0.08915960893105551`, 3.2848427178141772`, 0.6302374569507307`, 1.4630290564093034`, 0.8347103436436674`,
  0.36812644478584344`, 0.040963141333673035`, 3229.889147199789`, 0.1995027638419964`, 3.7582834934312945`},
{0.2095156599086041`, 1.856403794983298`, 1.6647511783688493`, 0.8963074241076089`, 0.5257557556911894`,
  0.2962680347723602`, 0.005638948645619698`, 398.77430140016673`, 0.1712593265312492`, 6.243180864475704`},
{0.08606673974307905`, 3.001046395083865`, 6.871125535025986`, 1.29871337654279`, 0.0013879217267305233`,
  0.4709543850320145`, 0.028486772088755836`, 255.05656064251065`, 0.15733152617600732`, 4.419059383126843`},
{0.2794012033077364`, 3.8964244114394253`, 7.589043584575673`, 0.9660750334911808`, 0.6823441866078543`,
  0.315494435212184`, 0.013950850602606538`, 1092.7384181067544`, 0.06831145072823913`, 6.790315469699049`},
{0.16053897675716755`, 1.21110484978606`, 6.017303762103152`, 1.2353982330406967`, 0.20086989110627473`,
  0.3272106220983775`, 0.02355461265150193`, 77.09093986775292`, 0.04848901873161415`, 3.5142859649597167`},
{0.06315507597420161`, 1.4395182613839497`, 3.0858504661132873`, 1.1448617905347693`, 0.30610701790846395`,
  0.20244634599341438`, 0.2950980917537417`, 2179.6214593356067`, 0.03473000454292019`, 1.333335366603153`},
{0.27116635506122466`, 2.640185082938654`, 3.1282641056246216`, 0.9199164829096633`, 0.32218318348593566`,
  0.5777637686875948`, 0.006117113694401221`, 324.8095638375007`, 0.04342122620091826`, 2.609798810681127`},
{0.08076786811690995`, 2.5389877544798445`, 7.211847948605141`, 1.1500644274940979`, 0.7690232347129535`,
  0.1924810447357974`, 0.3170033067881963`, 2456.45541532025`, 0.12977198817972552`, 1.000471570970415`},
{0.14196490482109098`, 3.5800560378690767`, 8.191314882292886`, 0.8521719014833709`, 0.20577614391452248`,
```

0.2973229046898421`, 0.032689134381726236`, 2192.8696238585753`, 0.20870222148197032`, 8.620659635124824`},
{0.16225217210911858`, 2.1337475700719883`, 4.607583982995607`, 1.274907826520427`, 0.760916097212121`,
0.6512132808308`, 0.1988962707427588`, 88.66654318682235`, 0.17629703311441042`, 5.991536547797288`},
{0.09998370398747453`, 1.3561107931887042`, 7.055733599099842`, 0.9997997594446719`, 0.4231050232131661`,
0.35289870383552235`, 0.01863838099230359`, 585.7262791471302`, 0.12303242645253354`, 2.5487735356675643`},
{0.0790420625297461`, 0.7951762600254768`, 1.0975467432818782`, 0.8295886125705915`, 0.21974762785224522`,
0.4003083061581494`, 0.12181590550696897`, 256.3718289629029`, 0.07504947990766975`, 1.4404680202816778`},
{0.09310243320239603`, 1.7939569577957926`, 0.5112771848209494`, 1.3548866082012778`, 0.9609312900127882`,
0.31815651234043585`, 0.047992777894275945`, 1572.364241162158`, 0.10220690607690369`, 1.3696469849079624`},
{0.20315703606221613`, 1.4806269686939117`, 9.851530344798562`, 0.807291927894971`, 0.7692364729235686`,
0.5064599484352303`, 0.07616257600145751`, 550.4721115636684`, 0.18512304424700965`, 4.821016411964738`},
{0.22897905358022103`, 2.219649931841208`, 8.024983925513396`, 1.3490395658771779`, 0.7992198564081268`,
0.5097552623833034`, 0.017934168491060075`, 2636.2345048980164`, 0.04871567850920905`, 1.38033814014487`},
{0.14728957561352346`, 3.8290117168547324`, 5.577517394641557`, 1.2037071314600827`, 0.7629976630105226`,
0.33391252098357616`, 0.00623505541513591`, 1551.1825745003182`, 0.05716501203669799`, 3.4524118308532703`},
{0.1747499923357959`, 1.9364726507678487`, 4.539322141325334`, 1.0358217348301002`, 0.687467494258686`,
0.22118436656891216`, 0.06970356230137446`, 4108.781309212477`, 0.15897963252631786`, 3.216561469360766`},
{0.061449808274615936`, 2.451738473864639`, 8.032153511575046`, 1.3988277328315561`, 0.653844119276888`,
0.6033333182307286`, 0.12509363159734996`, 3162.271766392656`, 0.18189469469951425`, 1.0210674887190017`},
{0.1951885853322612`, 0.6765436415231281`, 7.0581513804142055`, 1.4397439961417247`, 0.9899034195557828`,
0.3660842570171081`, 0.2551485659861665`, 53.30479560198744`, 0.15667683142074817`, 8.392793083340557`},
{0.15955516943400022`, 0.8482855893464007`, 9.331572022970409`, 1.1667938097447013`, 0.29910958141522603`,
0.1963171731712634`, 0.010305064592809629`, 1013.0765277256257`, 0.20900313016467104`, 3.301983133021727`},
{0.17746707158856556`, 0.44139712602022296`, 2.8621670830493073`, 0.8100971992338867`, 0.0373958036205273`,
0.5075452637360592`, 0.09067013663296572`, 114.80525128927701`, 0.16719225120884412`, 9.820606806768879`},
{0.25772135278013303`, 3.070426405636976`, 1.2338255709842532`, 1.3135003325072887`, 0.5149580362176238`,
0.41732865316044054`, 0.04496826136197767`, 318.8324074107286`, 0.010846019130009599`, 7.957536959758443`},
{0.08988753363169116`, 3.536295514824336`, 7.275260820034448`, 0.8993825689374235`, 0.025163868977214232`,
0.47083049518550046`, 0.018253871395130763`, 416.18519037331805`, 0.11499620973251179`, 2.265482484941062`},
{0.14192760186457132`, 2.3958894382433478`, 3.253512770748353`, 0.8655063905996925`, 0.8143036151546204`,
0.5834364577519011`, 0.17553594321370639`, 62.22207407824173`, 0.16384862691545943`, 3.0979894918131596`},

{0.14248286143558947`, 2.096310454690764`, 9.38011512102738`, 0.9675972750885355`, 0.0803295271719453`,
0.6033546111821413`, 0.13762578908199044`, 1262.8186365484712`, 0.18785001372865495`, 4.9706998985924695`},
{0.10446764528219044`, 3.442117541556258`, 1.7911289531773882`, 0.822650737878746`7, 0.06660293233402026`,
0.33605831499521144`, 0.1966635974545483`, 110.27400709595466`, 0.23027159794407875`, 2.5346674177346316`},
{0.0959624592169791`, 0.7155878562202682`, 5.1612847015717165`, 1.2701047018452214`, 0.05672968500006248`,
0.6450160655606327`, 0.04218472167952095`, 1614.7615476312947`, 0.23305782159027644`, 5.6652476983851425`},
{0.24311048293765425`, 1.2773072105524381`, 8.674158333323465`, 1.236248375623142`, 0.5430857314344402`,
0.29169516048698574`, 0.10383247735445839`, 522.6667880889086`, 0.0459109896778586`7, 9.69459149525866`3},
{0.08713889377857453`, 2.2859880795657075`, 9.600148060687761`, 1.2921363299986606`, 0.6294865567710719`,
0.5590186325118135`, 0.25992443653112657`, 633.6168560418654`, 0.16781120217455497`, 6.04666254822`},
{0.26758004713434824`, 2.448981429729897`, 4.582999027119474`, 1.4554483602004895`, 0.3942207099215642`,
0.5288182096517661`, 0.017684947331647518`, 53.19217741492592`, 0.1488296927599813`, 5.857737808697124`},
{0.13431037279286218`, 0.7397108148766112`, 9.99887072123514`, 1.436439642978205`, 0.2003363652340402`,
0.6779870054162531`, 0.009538236593206559`, 4558.309876478542`, 0.14371659865116293`, 1.1480275619139793`},
{0.27730778316543614`, 1.7365428506012295`, 3.277328223559843`, 1.1643406154035905`, 0.2621424262474452`,
0.5836632043576286`, 0.19147730163610321`, 594.1704278677969`, 0.14363610143643862`, 8.32222051121584`},
{0.05614793436458493`, 1.997529172364887`, 6.840549262003957`, 1.2853232683568476`, 0.15723714782247722`,
0.6374125080636333`, 0.1308231427534364`, 1207.3035504224565`, 0.15724403465210185`, 1.842303075231806`},
{0.0647068787712346`, 0.8380824418888393`, 1.0963289034750066`, 0.857562051048008`, 0.6907626550964823`,
0.2999007220652139`, 0.27885748113518277`, 1238.4520803651262`, 0.1379529162857775`, 8.96709675960637`},
{0.20132622112515353`, 1.9086557669063273`, 0.7030652496772074`, 0.7755069309180247`, 0.32762519329281337`,
0.20767575421456186`, 0.011318073987463645`, 431.8155255411275`, 0.17436258525110954`, 5.81443415452358`},
{0.045022613849116516`, 3.5058573906861508`, 8.47131465431961`, 1.2697383949459176`, 0.5022287267438617`,
0.23847623822456332`, 0.06077359284826833`, 243.2833936812314`, 0.1613793589669978`, 8.022277670460284`},
{0.17418182442148117`, 3.0892110646487794`, 3.4522019380421036`, 1.2965827481879664`, 0.41818871776887856`,
0.6556019601202148`, 0.075063644002762`, 2886.574837344593`, 0.15315141138676647`, 2.6062865811306466`},
{0.045831059772204225`, 2.899702133961444`, 5.925065562245003`, 0.8929739121804446`, 0.5933600691515191`,
0.4490635153302144`, 0.16447888961080792`, 1912.79886244215`, 0.11499785968845799`, 4.757985376452911`},
{0.2429697761558342`, 1.8449858081251405`, 7.508754000794074`, 0.9127320360969664`, 0.44886430542069955`,
0.35316747155912975`, 0.02193269408167387`, 161.73721618998414`, 0.16775422018188862`, 9.221971574736251`},
{0.160442241636698`, 3.55050251528807`, 3.9052606138637995`, 1.2025377341211225`, 0.791383919489673`,

```
0.40672925070026333`, 0.35477124416027767`, 105.50559352550128`, 0.10792398319932495`, 9.162379917819035`},
{0.20468313725879644`, 1.5880039947408164`, 1.8181726149942818`, 1.4361114186612707`, 0.6452252370101204`,
0.23877202353940497`, 0.026067763377395026`, 956.3204095901076`, 0.08759161290164996`, 6.530440575770296`},
{0.07364401078716826`, 2.5859680523819737`, 7.8227327965838285`, 0.8181378301360093`, 0.36855652079172274`,
0.32445369334254925`, 0.005391697017678555`, 139.66827785366002`, 0.0644557011853657`, 2.5433237558513593`},
{0.2506656721970057`, 1.5258727034335298`, 1.8333712411976357`, 1.2495957919641723`, 0.1010075460664761`,
0.5024718466416268`, 0.012345305326089913`, 1052.1669408921373`, 0.03320454993831845`, 2.130087729732141`},
{0.10121864512314288`, 0.9521305504715327`, 3.6368221202357223`, 1.491518490019038`, 0.3322534458031634`,
0.2564348282281962`, 0.03507970829425946`, 4627.905596988043`, 0.07121256842175683`, 7.352385124666178`},
{0.1942542932466596`, 3.1177323303054916`, 7.932133913996736`, 1.3085296181137158`, 0.8325654731630694`,
0.19060678658466512`, 0.044940459115954866`, 2391.994107548152`, 0.24452644883302188`, 1.4227474861405387`},
{0.12644890469661946`, 3.0196313965941393`, 8.688658870512302`, 1.3655010622962382`, 0.5847686349152417`,
0.30770354208104556`, 0.013061738242062998`, 1213.9477293174991`, 0.0486468587160932`, 6.312801306430787`},
{0.11280111274715143`, 3.2009360918012444`, 2.9222221030043283`, 0.9684721327188957`, 0.03329493891954294`,
0.1886848273472841`, 0.39445850876705457`, 50.69043630972767`, 0.07560282132089569`, 3.828945975174229`},
{0.21704875939335333`, 0.6532232080519234`, 2.918554590657344`, 1.428534955684801`, 0.6202310312510246`,
0.3415578035649972`, 0.1858190740142067`, 1428.9901262383053`, 0.23526262585558444`, 9.378643714384769`},
{0.23408001184633387`, 2.4049572740765104`, 0.41973647277501946`, 0.9771854975111727`, 0.20272636213110817`,
0.5074761434023957`, 0.2724896408017817`, 4254.760257351377`, 0.16397942354351142`, 7.3871482427729305`},
{0.1483945695425406`, 2.699298050205127`, 5.571982823617271`, 0.9775705576725262`, 0.05426683476548022`,
0.5307838109252759`, 0.3096243181655647`, 323.46321359362`, 0.06725520461051537`, 9.792268234153859`},
{0.11663741077922185`, 0.4431208542421814`, 9.780884572399291`, 1.0122403254522139`, 0.996318833483786`,
0.22155768960679945`, 0.39993089984673347`, 139.93164861672463`, 0.10490063957337303`, 5.3884722727325105`},
{0.11314041374373823`, 3.32806970136921`, 5.495643049849155`, 1.495187492234928`, 0.8329498208958137`,
0.41734162088096116`, 0.32389371604169326`, 602.537083528763`, 0.025714962476490244`, 1.3686646556271693`},
{0.09294922428523011`, 0.8101676653238759`, 3.5463022431515436`, 0.9341830117643879`, 0.15981251792750606`,
0.6527933768073686`, 0.016252799814697117`, 738.0588634619963`, 0.024900612098687713`, 6.915149516601594`},
{0.2696435798732645`, 3.282053008859009`, 8.218787637608994`, 1.4011858805306123`, 0.38871702639887196`,
0.49514225831047376`, 0.01812451162452924`, 173.50575962443355`, 0.20495943261505556`, 3.6349041099248875`},
{0.13023501639399904`, 3.4827106844829796`, 7.180310384726493`, 1.0417379014779535`, 0.3157954886073613`,
0.3090830033379006`, 0.027522996818882862`, 1326.2576636786057`, 0.24748434887668458`, 4.853044482161593`},
```

```
{0.0474731410927452`, 0.7132410347452449`, 0.7493200461382123`, 1.2237616503945397`, 0.9506868210145907`,
  0.6554164129507942`, 0.030282495208288053`, 4252.97050681489`, 0.14327214907032687`, 7.932983145622682`},
{0.16300334800486183`, 3.0919140294206544`, 3.354218048792589`, 0.9673340843818878`, 0.9052111330146941`,
  0.3760205832601582`, 0.15433250667793078`, 4163.373290035795`, 0.2440496776808055`, 4.495712049614825`},
{0.2283283078987099`, 0.42840256075325867`, 9.446993924606975`, 1.3839314736668495`, 0.8927356525671915`,
  0.3909626915031965`, 0.018767685887155043`, 53.5712236843488`, 0.10749676377570666`, 1.5496335657505222`},
{0.1380652378345807`, 3.317739091147252`, 0.7336293987943634`, 0.9484386065310282`, 0.5023326788118547`,
  0.2130184622271264`, 0.0630596090666651`, 1932.031257216769`, 0.01203085244100871`, 4.258055262793977`},
{0.0926632627436077`, 3.578083963812018`, 1.8944161244777453`, 1.1381278842914715`, 0.7382269863407194`,
  0.4777843546205588`, 0.04528998830707099`, 1233.5473532765689`, 0.08357615053600725`, 2.9903558138075255`},
{0.2706310445313141`, 2.5166396966047158`, 0.5049158559505837`, 1.3168707464723388`, 0.5754269054535`,
  0.5968444469720801`, 0.1025449755078217`, 681.183394170123`, 0.05243619315919962`, 2.183793658787195`},
{0.20804007909247985`, 0.9673945938397814`, 0.48619022305575754`, 1.3068640855075515`, 0.047796616526516456`,
  0.6050138674479171`, 0.4706922223098622`, 348.3137462309647`, 0.24685340199219946`, 5.551550038883448`},
{0.04845164423667674`, 3.3925416461261833`, 7.486002650281495`, 1.2431466818882033`, 0.1294136137122328`,
  0.6416005560424389`, 0.04082335443875802`, 58.92112932799084`, 0.09857305139503486`, 6.047753646247408`},
{0.09431215650771135`, 0.5526170445339527`, 7.677473256451986`, 0.7715239445968243`, 0.7622192109377914`,
  0.1566986505563851`, 0.018040482265180933`, 824.8087273379117`, 0.09073506826067207`, 9.996434684615583`},
{0.26113782676147984`, 1.4684069653405487`, 4.54225535313282`, 1.1193463339337828`, 0.6236318874525932`,
  0.1751148412910668`, 0.053183289402392066`, 554.4897241223483`, 0.22550063278101773`, 9.001504879035508`},
{0.23311836154945775`, 2.5262146716446363`, 6.3107841154355`, 1.4422657044398717`, 0.3118898255230633`,
  0.2567889403043644`, 0.0348102698497832 7`, 1604.0167510242688`, 0.16315782107376747`, 5.777943122601165`},
{0.22640126428063923`, 3.7808015365334118`, 7.033092553948691`, 1.0460349638379132`, 0.8860375200295565`,
  0.499376231890995`, 0.3693111113992884`, 210.995030019894`, 0.19589698445197923`, 2.861160479579169`},
{0.19770244273120569`, 3.3508846225196347`, 6.757833537731408`, 0.8466672464019638`, 0.28373063232656337`,
  0.6609960258126362`, 0.00680381475789011 9`, 1256.448281122042`, 0.0187925047862397`, 9.149585751239048`},
{0.06481064744600706`, 2.3696983632913335`, 9.923821775061434`, 1.1006879887228784`, 0.22746584913410017`,
  0.6483433064071293`, 0.08284957522863273`, 281.66193979187017`, 0.13350656503173153`, 1.0702403000648566`},
{0.1537084817657926`, 0.5942996643982652`, 0.8160795607483013`, 1.042756674430172`, 0.7722126796580249`,
  0.49669902434825364`, 0.00611858149596996`, 1518.932458333751`, 0.21666908673557295`, 1.634333111202272`},
{0.13754450751212471`, 1.8598194811531465`, 9.485701362019345`, 1.4994328167980717`, 0.8397969618927028`,
```

0.642370724162252`, 0.08665379792235217`, 239.43302754758346`, 0.1633413998793405`, 9.552795266495377`},
{0.07858197291799773`, 2.783199788795301`, 0.9642830041255817`, 1.3105090636097434`, 0.9572373854271048`,
0.41778523295843695`, 0.00823451224525384`, 90.59081205787001`, 0.24323880940762027`, 7.348654585309644`},
{0.09118935700706149`, 1.2770460819223013`, 0.666402509206284`, 1.1661439001587546`, 0.35202394044239993`,
0.23733544361366754`, 0.06689005296661862`, 1909.3731761645724`, 0.030837584446851807`, 5.2718694661097025`},
{0.26692387934872025`, 2.295217680243936`, 3.3610155473383347`, 0.802490311252646`, 0.6241394164828811`,
0.45771836786665954`, 0.015176154328102385`, 1461.3995866509204`, 0.028435347409555445`, 8.762751383914615`},
{0.16789560992918506`, 3.010819021450227`, 9.549236085030234`, 1.3823009139889233`, 0.2184000924395355`,
0.5213598249132101`, 0.3210433649481226`, 558.1245473691072`, 0.20748907999743366`, 5.921770487462836`},
{0.05001315370963505`, 3.027404779172768`, 3.8220563025552714`, 0.9962642247459573`, 0.44362718458195727`,
0.3510033113401648`, 0.04839212642640241`, 1615.957133832461`, 0.048401134986770955`, 4.739296636982807`},
{0.0729686004745061`, 0.5044823312287607`, 5.5024178276878075`, 1.2230366941116102`, 0.012758742281838265`,
0.5648708708999995`, 0.03232052448858605`, 634.5000769880222`, 0.22553164736796405`, 5.75812027698643`},
{0.25149333924897926`, 1.871859550111978`, 9.811462629766478`, 1.3386558973788294`, 0.5189637829018348`,
0.6377508608690272`, 0.024369302194970535`, 194.42061666753636`, 0.14619611626015583`, 6.325307886810732`},
{0.1922243891646514`, 1.6850594067594091`, 0.2630953725218035`, 1.4711962401085972`, 0.15977146838462364`,
0.6883258791888056`, 0.09212778533128511`, 774.9062808250895`, 0.24229201708328568`, 1.1723783184073415`},
{0.2327924107307146`, 3.0114739814321467`, 6.475065538974626`, 1.3312648289930094`, 0.6507217418486848`,
0.3841095338158853`, 0.3176950069808959`, 737.689863101484`, 0.19812174358831114`, 7.321794649353192`},
{0.20811447549122308`, 0.49053077700921355`, 2.218461283385089`, 1.0634384889669943`, 0.30011342208125913`,
0.5589173637133283`, 0.16385051847701637`, 124.79731876547663`, 0.15435702825422665`, 9.111119072715418`},
{0.19580212756702065`, 2.2297474474451375`, 8.070495694463702`, 1.2202670372174012`, 0.07482300382970508`,
0.34257881980750315`, 0.24674110677687536`, 1287.6149126957`, 0.12354587287124019`, 3.0573978018103922`},
{0.18591817623779283`, 1.5316284759435828`, 4.0240186618411435`, 1.0506796825025935`, 0.8560461598734517`,
0.5496710897949223`, 0.05348974137997375`, 3910.687147405355`, 0.18912146822955533`, 8.614502889074807`},
{0.2552337642427184`, 2.0636949211593727`, 3.6788035742608756`, 1.2716726068122697`, 0.4767575621154567`,
0.33698161388823356`, 0.00790398094444615`, 742.5948236907665`, 0.22094334214516276`, 6.7847431915852425`},
{0.2297403387592854`, 3.933034881515791`, 9.92502862171417`, 1.2059622632705849`, 0.1421363362803283`,
0.1853832242974326`, 0.06367011009221722`, 57.09955187350921`, 0.13182928467731042`, 9.7382206276645`},
{0.04776006202628119`, 3.695464511472898`, 0.268839094050918`, 1.3548340559244303`, 0.4311024896438589`,
0.6662260908047339`, 0.189386036579758 5`, 146.80096070953766`, 0.0868075345269877`, 5.3982731410948785`},

```
{0.16250916383331093`, 2.090868942469317`, 5.046057727155544`, 1.3781211464947924`, 0.9692295654960448`,
  0.507149644697747`, 0.012667564467634538`, 467.92228322579444`, 0.2269164868867607`, 2.233796179341983`},
{0.12952636821033042`, 2.4189205073691173`, 1.5337482028150813`, 1.4058710509218408`, 0.9636406844740666`,
  0.3882701702830901`, 0.27400686262252805`, 4688.706549038708`, 0.0765768774664311`, 6.843480813486918`},
{0.08153578837307857`, 2.0915987061965353`, 5.850951169546045`, 1.037700922521303`, 0.7897818160030217`,
  0.3237340771928561`, 0.17831164332408336`, 1464.5863160958056`, 0.2436610405225158`, 7.1217069723220146`},
{0.2624813864915975`, 2.8946734437676342`, 8.625458110929756`, 1.37764442375384`, 0.19776637799413987`,
  0.31049273319228277`, 0.046660769123637466`, 609.6700244678286`, 0.017643025422342112`, 4.098918143681717`},
{0.22181257821621975`, 3.747903553799671`, 8.070266666229205`, 0.9808557386469257`, 0.7823522438549146`,
  0.2263615830500163`, 0.06976335847986787`, 287.144692837758`, 0.1821266272405107`, 3.59762935889718`},
{0.21058778737677059`, 1.3147483391864796`, 0.4651324593397846`, 0.8516412222118703`, 0.046686774964893374`,
  0.4819914326166439`, 0.10586616607566471`, 1877.0848108087591`, 0.011164572002709261`, 8.732010177522003`},
{0.19996482365829993`, 1.5454344843054368`, 8.94969050535013`, 1.027667483044064`, 0.3164796074442535`,
  0.5223314119072894`, 0.012949156080520758`, 231.31648814801656`, 0.19929090705614294`, 9.341058456545078`},
{0.10440009635624109`, 1.532425369535546`, 0.308138348960842`, 1.2734376519411275`, 0.20147673343032046`,
  0.35081245482125123`, 0.05722019474177449`, 85.42879625764479`, 0.08356786949647793`, 8.411462154648635`},
{0.1446017732242294`, 0.797427468093673`, 5.032298069921831`, 1.3706636028120882`, 0.10121695844960277`,
  0.6068141920969097`, 0.25341251132298065`, 197.97452899503492`, 0.2275401984407136`, 3.68386367767849`},
{0.1841264162344997`, 3.4194642457460365`, 0.8344796094826652`, 1.4377258087185927`, 0.5867256025093719`,
  0.6103981120475732`, 0.3542274534468216`, 522.9821129731852`, 0.2244081861480518`, 8.361737950667973`},
{0.17849930658201318`, 3.2992561971358683`, 5.635580909426066`, 1.1878114797167083`, 0.48988207998590316`,
  0.27890916833354473`, 0.03715549210344845`, 144.67131391199885`, 0.0866110192158086`, 7.798176718489761`},
{0.09510173640093084`, 0.7659692827065738`, 0.5464490010035838`, 1.4050402850247132`, 0.11782959453955089`,
  0.3589214817200733`, 0.08175234954735425`, 225.11260306454756`, 0.1010344837489014`, 6.4310165595390805`},
{0.06416093472533757`, 1.2573296539610634`, 9.92323628294643`, 1.30190007637989`, 0.030494015633026894`,
  0.4401773012522794`, 0.02586427825345657`, 3143.6595843648015`, 0.20592664064058636`, 7.935954028868563`},
{0.25237835724682106`, 2.979406809596097`, 0.9138356130609361`, 0.7932538461611548`, 0.3933816374531405`,
  0.6211446249652819`, 0.0749645015318215`, 95.73291003138912`, 0.06307891905410445`, 9.368280708056208`},
{0.05665762575451494`, 1.7380994050720693`, 0.46391281319797173`, 1.3490794355447004`, 0.9653780951392605`,
  0.17540498059526055`, 0.4972377260445833`, 285.101267300382`, 0.1881031714219547`, 6.261558552266614`},
{0.25642346088610446`, 3.010945889122482`, 9.963520677018519`, 1.467899384826252`, 0.4946077322511526`,
```

```
  0.6868780403912886`, 0.012314696297102353`, 88.3173980921876`, 0.10491074891333174`, 1.4353196490865765`},
 {0.09174036922136902`, 1.4204870656163582`, 7.877654658381974`, 0.9368390881044215`, 0.3909649395372681`,
  0.6267838157099552`, 0.0073779181496306 1`, 223.00641924802295`, 0.22589847441224364`, 3.7895604923910007`},
 {0.2260021855376967`, 2.1215536107061057`, 4.2648042233683015`, 1.1043329178413561`, 0.5641485347029902`,
  0.48705118822040194`, 0.49950773055219905`, 4133.317514629252`, 0.08759305230550185`, 2.0466954881219834`},
 {0.1540246000020472`, 3.8655612430526176`, 1.2043257775886698`, 1.4535724207675174`, 0.7982285789403216`,
  0.6724657214579106`, 0.417253246623641`, 114.93402398229541`, 0.10143088124264565`, 7.877832682736377`},
 {0.18967559668177286`, 1.3751140072639014`, 7.146984940201236`, 1.0524950668750344`, 0.5379883166421491`,
  0.5342982154057455`, 0.18359081743318598`, 57.97987210615365`, 0.21651827946513758`, 4.096495164957771`},
 {0.11610573160351356`, 2.8105133752566074`, 0.4045001364193191`, 0.9020454264062931`, 0.06323256453719583`,
  0.49478351534124754`, 0.06895293734611843`, 538.3487824750697`, 0.04516006162070901`, 5.298889101080752`},
 {0.06094054623730827`, 3.5785656752883286`, 2.8105466781089756`, 1.0666569400098407`, 0.8956485783622179`,
  0.6416539064413922`, 0.4241071070816547`, 1544.2747045672372`, 0.05890410014619657`, 6.674766669452417`},
 {0.06400872221443815`, 3.352604720184938`, 3.3612644751655854`, 0.9279864636339992`, 0.6811335659068025`,
  0.5445450422212498`, 0.04222211989978537`, 4877.834369436828`, 0.09303122695369598`, 6.110250683676178`},
 {0.1838955211549798`, 0.6066893455768754`, 3.1997125487162617`, 1.2441392193895697`, 0.8430620231566095`,
  0.17975264196625795`, 0.008385470075528607`, 217.99247821227752`, 0.10887259656335785`, 8.65519228817503`},
 {0.10877154933938238`, 0.6652486439511347`, 7.420477343235575`, 1.1719120037873472`, 0.8277401239831172`,
  0.4096056229223566`, 0.3960687102590752`, 4083.2161783729553`, 0.09124503977845877`, 8.628655226336182`},
 {0.27429237480005003`, 0.4974953244631517`, 8.109325428142139`, 1.1022725368505206`, 0.3620164170986986`,
  0.5133215689169995`, 0.013475856804577652`, 252.124017519504`, 0.17376346726865255`, 1.283750502496817`},
 {0.2348606786833104`, 2.9248975834862243`, 4.089597609027345`, 1.0125076012045389`, 0.4800911130241774`,
  0.44584481805285503`, 0.013867879702002976`, 69.31755646766315`, 0.08239556859020553`, 3.2481286029667444`},
 {0.2276305764129738`, 0.759063573478838`, 9.75608531797917`, 0.9651630732227953`, 0.2616315835146603`,
  0.25394525967231996`, 0.42721777606241185`, 115.33714629065052`, 0.1547416236361928 7`, 5.678602403105034`},
 {0.22740101487152548`, 3.662274246882813`, 5.673159128278421`, 0.7501649913658239`, 0.6663016096811909`,
  0.46449661159333955`, 0.15875103883050143`, 79.3840483030526`, 0.23925351496597358`, 9.512843911813135`},
 {0.18907356960866467`, 0.8903250429215461`, 0.8912625465714132`, 1.4194566043771595`, 0.25430531352108066`,
  0.6668345122251143`, 0.01430802337144644`, 354.71023908442675`, 0.0543512780703954 7`, 4.360013356436832`},
 {0.22841094141655494`, 2.082125305118022`, 3.0130589699894585`, 0.8668572537043583`, 0.35284672466002664`,
  0.48298470604644195`, 0.055294539730325123`, 1324.8059697322965`, 0.2155561741975654`, 1.2621538558570524`},
```

{0.1257670047049273`, 2.665283038938149`, 6.579106420165356`, 1.3213793494994266`, 0.07362490185046933`,
0.671746727583086`, 0.11039555694375124`, 54.48594040704261`, 0.2029696416579515`, 8.437004538119528`},
{0.07898342764241062`, 2.336170261443863`, 9.558440542813312`, 0.8746675798923161`, 0.3314927429594914`,
0.5846930166673705`, 0.14173550706968557`, 4831.453144750826`, 0.22811466814427805`, 5.138718102637142`},
{0.2360815041275312`, 3.0069216843113518`, 1.9005978173203566`, 1.189378383663731`, 0.3594173887197991`,
0.5615883220885535`, 0.16515958276300607`, 91.7495984293873`, 0.10675594880460115`, 2.979677734696839`},
{0.19503604064380992`, 2.260639826798138`, 3.7301326124020027`, 1.0003156010412233`, 0.94917046396899`,
0.3935362271642996`, 0.08733555022372319`, 2234.7260448390443`, 0.16743068067899064`, 7.659400521016185`},
{0.09766567347807259`, 2.1751916900809825`, 2.9967711374909367`, 1.239918427231443`, 0.884426059472291`,
0.5758255537556012`, 0.011950287911566193`, 133.4222213549303`, 0.18860770237483848`, 7.181211491841234`},
{0.14345253817386028`, 1.510087082683639`, 7.446143698111681`, 1.3871400228991382`, 0.2507990125704793`,
0.592096235116642`, 0.03860344741119924`, 206.9907110306594`, 0.033941806565597976`, 5.68471806318026`},
{0.23351662183055255`, 2.7403756212365034`, 1.6128399905233286`, 1.1394774719326448`, 0.2292996808057013`,
0.38219770472020054`, 0.028922618161783233`, 3147.1184318469313`, 0.11734182225292555`, 5.055074464096112`},
{0.05894445024535572`, 1.7190376171466255`, 3.4445271438587763`, 1.1005021934401353`, 0.8920369053637744`,
0.4553589712941485`, 0.02602138727445485`, 2996.975539830326`, 0.01616140506923186`, 6.007209949084906`},
{0.1969133083502746`, 1.0306356019031098`, 0.4532514022896326`, 0.7704659952544493`, 0.6922371279082573`,
0.35856337468106037`, 0.02342746544122668`, 625.7704338271294`, 0.24611682235411125`, 7.40783802504553`},
{0.23578418763137027`, 1.109904439772956`, 9.416464917532476`, 0.9968768007328397`, 0.9319056056422497`,
0.20705868768300972`, 0.023381736885244357`, 73.41723955051782`, 0.19857821780967083`, 6.130556036938069`},
{0.06254010222908407`, 2.833030713124539`, 1.1523226094346608`, 0.8553536481119535`, 0.24975724964580737`,
0.6445417390978168`, 0.03834946206048674`, 4173.282831948493`, 0.23424935668510183`, 4.880796947048751`},
{0.1868109134318776`, 3.0098056229686154`, 1.9105557354746436`, 0.9827601516958456`, 0.5236893411586465`,
0.474044891890932`, 0.006475636749568416`, 1404.5093361888864`, 0.13430982294875038`, 1.0667742763750176`},
{0.06031809352972184`, 0.6708570664744204`, 0.23575165127461165`, 0.8219834374256857`, 0.8758699419986131`,
0.18852573702588948`, 0.186745146986022`, 107.40963970974032`, 0.12527667123697356`, 3.1788633087725113`},
{0.18801205905393248`, 2.0266227725882997`, 1.7626950623641149`, 1.1991174158264077`, 0.011000047841808414`,
0.3353078609658676`, 0.05399718652686698`, 93.79336861840014`, 0.2334529633377767`, 8.960462961249508`},
{0.14122716980793654`, 1.0036898987844207`, 5.592205787770849`, 0.8071832042112255`, 0.9861798903854433`,
0.6717138208444808`, 0.19154806886518252`, 64.00473270059831`, 0.09434989637003327`, 6.618037610942842`},
{0.14179341245484034`, 1.2991893501557028`, 6.1750864357305275`, 1.3220469832389021`, 0.954868801179809`,

0.5061480199077149`, 0.3454995627472`, 2784.154142185204`, 0.062422742009026755`, 1.1334761108568099`},
{0.17346773507380248`, 2.4886801471142252`, 4.186844167829875`, 0.9059139514582338`, 0.8823714489891827`,
0.3118060351580627`, 0.18611640479783054`, 317.2758158326019`, 0.18366106179531033`, 4.660916164387663`},
{0.17801738501831404`, 3.953347877342175`, 5.116473955634309`, 1.3026800881545206`, 0.2660443419983707`,
0.5551592463622865`, 0.021882498869656555`, 368.98117336094424`, 0.13387054033002443`, 7.8289062285527375`},
{0.1502521637125906`, 3.604808817430471`, 9.516864148680163`, 1.170214417719616`, 0.46260070316916013`,
0.4333225308578431`, 0.19319459383231874`, 1379.7428965277768`, 0.03462365036850307`, 7.002259791633362`},
{0.23041123856532575`, 3.7235663376199373`, 7.422983674409959`, 1.0720190294684302`, 0.9567615568260381`,
0.6795713708474191`, 0.42619421138851304`, 252.0789228698587`, 0.23264645009848384`, 7.716645878625323`},
{0.17190873580866728`, 2.1300233762526144`, 7.263444695009253`, 1.2209987713639263`, 0.8761821538663868`,
0.2004596237918942`, 0.1361627927608223`, 3975.405902408578`, 0.13971132873354192`, 8.98211109187212`},
{0.25963938649205515`, 2.4653900827356168`, 4.997042605235823`, 1.1014911256242188`, 0.41576920876875767`,
0.18114027248931974`, 0.22217255314368375`, 81.71471228407572`, 0.2378841388493836`, 9.715142052575505`},
{0.2762626642080059`, 0.4739284043667089`, 1.351212961630882`, 1.24608482511035`, 0.19517067645291641`,
0.24775742659101618`, 0.08503066911676711`, 2141.7873291111496`, 0.17338810608691535`, 4.319189194420735`},
{0.1906362991887024`, 1.590121005227628`, 9.42412383550555`, 1.4957920989499214`, 0.709065663948995`,
0.3417182681188283`, 0.37536592261162544`, 505.4667159946151`, 0.11728445265057086`, 5.855972486658924`},
{0.2658746234670645`, 3.614932865157483`, 8.460828570764`, 0.8159863776033582`, 0.2726846463148733`,
0.34564016673603704`, 0.10623048732883861`, 3778.1279822475276`, 0.23711150436988554`, 5.459482199196464`},
{0.26863157332159515`, 2.9396265733275415`, 6.862183762414347`, 0.9732277188028977`, 0.5002013826688112`,
0.5818631486183298`, 0.034332997185588514`, 711.1213518313073`, 0.15904422112946637`, 1.3044149622459462`},
{0.27209369466384475`, 3.5584095462332135`, 0.9720509149176574`, 0.9873292487031791`, 0.8069894772531618`,
0.17871886216847`, 0.10218788035646292`, 96.95116373019758`, 0.02386269594633994`, 2.5608838628408552`},
{0.14444262093504412`, 3.863449844229998`, 8.726684391435349`, 0.8553749492129846`, 0.451984917853794`,
0.5228822441273482`, 0.008115157266947242`, 403.9888714010655`, 0.1027695319041006`, 9.711355034259547`},
{0.2538137652575522`, 0.7540411224449701`, 8.794714857317246`, 0.9184514413182489`, 0.11359389239638507`,
0.27911589396705727`, 0.11507441894157476`, 65.76953791858952`, 0.10155553687026442`, 2.859221903044178`},
{0.07943689831455408`, 1.7727348695278575`, 3.5998215792473456`, 1.4458682286750966`, 0.6331999429153172`,
0.478090570864118`, 0.46084046549589247`, 472.0739644000896`, 0.10830302137693731`, 2.3782715249335666`},
{0.05556808280528502 4`, 1.296674173505255`, 4.7380956025626375`, 1.4538691472242138`, 0.09550221320066177`,
0.19041481571096486`, 0.007131815235409429 5`, 86.20076273581975`, 0.04910104396213488`, 4.3238451034891945`},

{0.1596260086552564`, 2.5471852815710525`, 0.22684585875990315`, 1.2716791760964523`, 0.8208442532787252`,
0.3888937433082962`, 0.04289548126799573`, 69.96603930346842`, 0.07461229317798801`, 1.378327695847128`},
{0.14264033419885946`, 0.8035889732308119`, 6.673404894657215`, 1.4157343373387477`, 0.18185291353504507`,
0.4765318357304136`, 0.03778101114485503`, 210.869852474353`, 0.22822304787397907`, 1.0663935058760412`},
{0.2209992311467341`, 3.548991401043337`, 2.677760813541523`, 1.1645638964766105`, 0.17856966244183803`,
0.2409694046188614`, 0.16792458477559433`, 53.365358865447796`, 0.019626895089088575`, 3.834874658630479`},
{0.08986394714808477`, 2.7800808748945913`, 9.536673819459821`, 0.8308776528106754`, 0.5315183679881144`,
0.4614339535602666`, 0.046951751845033245`, 1052.1471359283046`, 0.24213976319820152`, 2.823020175578866`},
{0.08237232438525194`, 0.904854561594771`, 7.190948808493694`, 0.9264355987892083`, 0.7428148600467364`,
0.6232177046085758`, 0.0730718251800762`, 62.25627183567972`, 0.1453325784006102`, 9.786938339680962`},
{0.14754836278222488`, 0.5816922789151606`, 9.91636448422577`, 0.7971447359984003`, 0.9834799375197849`,
0.6545637466726528`, 0.017670578507760985`, 406.58618651198583`, 0.1328908282216244`, 3.7962615345853075`},
{0.06758332214646862`, 3.0558788120173555`, 4.64800500980858`, 1.4828314301436276`, 0.7395538908104162`,
0.3075642133576061`, 0.04417074894369504`, 145.23097869725217`, 0.1743358052899487`, 7.770691560418564`},
{0.16631267310517406`, 2.413572311283116`, 5.471184235465843`, 1.4579537524645423`, 0.9497208653345599`,
0.439033675828699`, 0.21085287275356013`, 3128.2027597762994`, 0.18409987492192065`, 5.612876798830806`},
{0.18539796406610926`, 1.3871953304353832`, 7.757698949197568`, 1.3585428901946988`, 0.4532718222049952`,
0.29058122288435795`, 0.010738744574908157`, 486.55354471220693`, 0.20251048022954932`, 4.51278311919668`},
{0.04144083471615087`, 3.41458508027812`, 2.4600046087844927`, 1.2787341279549589`, 0.569252466493285`,
0.6736151572579898`, 0.4762197736813673`, 718.8382183063302`, 0.07598079948793823`, 4.331845758841565`},
{0.13724293356211342`, 3.6916535362348286`, 5.489803233715362`, 1.3457045887873154`, 0.0754297582883523`,
0.6293482685634375`, 0.017573021108899616`, 778.5577282359714`, 0.17674997296512807`, 2.6916234726152264`},
{0.08176385784973572`, 2.023736863649704`, 9.823987283528833`, 1.0905304980935506`, 0.6417627833690904`,
0.2509575610218163`, 0.3071390754059633`, 168.49990583950182`, 0.0669392059366426`, 7.784078345121018`},
{0.20636275093165019`, 3.041433098250965`, 7.709885134336993`, 1.0647262222072884`, 0.28984076250310165`,
0.17246518959666646`, 0.007377660819744997`, 464.8055142552055`, 0.19020267666710844`, 9.452984008311837`},
{0.2140607830978551`, 1.6917207171996171`, 1.9218182027339432`, 0.9997605714664014`, 0.5513493876922198`,
0.4464250883384161`, 0.2328414126003874`, 611.4769203082982`, 0.08477583973629349`, 2.188379571162489`},
{0.17696168772261972`, 1.6101558302339125`, 0.7107550695951144`, 1.133554468522512`, 0.8128396324743541`,
0.15732538361458315`, 0.06392981831267872`, 277.86150200910555`, 0.15413566109625954`, 1.907309832357937`},
{0.04328176764500202`, 2.9602821723444555`, 0.6131528539518083`, 1.0058908218647606`, 0.19964040264767258`,

0.6669684114614443`, 0.23851965705730427`, 2287.3379976105803`, 0.0859331016978808`, 8.660468891635798`},
{0.08735803097627126`, 3.963951857586263`, 7.965851341925532`, 1.189504619761999`, 0.40822475538796854`,
0.4952091412789442`, 0.01984161894285721`, 1116.2764923646498`, 0.2379409272833778`, 1.293491554272407`},
{0.15281896372135534`, 0.55010519971598`, 2.817048022996838`, 1.3198176246010052`, 0.16238216376752557`,
0.6925657414172184`, 0.11807724637466313`, 76.78768290686035`, 0.04193274426603899`, 1.2341700274455292`},
{0.19086658543964008`, 1.9905232804946325`, 4.078173781767919`, 0.8519485127716493`, 0.08904379736388202`,
0.23536266216215151`, 0.24991927271295455`, 192.66677656391008`, 0.127316992739278`, 7.8449864029309175`},
{0.06664208120921677`, 2.003234095381499`, 6.199949723153024`, 0.888873086526756`, 0.04784068730663105`,
0.37023377411027647`, 0.049828770496122325`, 349.42476257926376`, 0.05267583263583803`, 7.0912634633886915`},
{0.22983292033798441`, 0.7814702114508636`, 3.2012106727008174`, 1.142026804432239`, 0.1064467335224153`,
0.4404014420960507`, 0.006695571873889409`, 4217.329674971789`, 0.15347806495470107`, 2.7989551297088675`},
{0.13211782844464454`, 0.44983284117427225`, 1.0762780754680463`, 1.3187859174208114`, 0.8445651695343355`,
0.2965729998791703`, 0.0542223541794765`, 616.1259401245338`, 0.10515194159293045`, 5.24820204317008`},
{0.17817224333688036`, 1.8900871904197354`, 9.98947566566499`, 0.792780936871982`, 0.9109839945193492`,
0.4314201285170226`, 0.03598067399007004`, 128.67700477089366`, 0.059019163579936806`, 2.366574414393561`},
{0.2509149027719233`, 2.580879402706512`, 1.8893709427148198`, 0.8255744895957449`, 0.1652280455910582`,
0.6768016323195194`, 0.009322737452992792`, 2112.3599589856663`, 0.08696925005081763`, 4.203193192682905`},
{0.08555200390318413`, 3.7277336286219658`, 7.5570643278825855`, 1.1117862222014783`, 0.20420751548200644`,
0.3245432768958507`, 0.00950175247006993`, 144.2017035489447`, 0.14385517513119161`, 5.452203080835416`},
{0.07297367338130328`, 1.9451098216604938`, 8.396918429735464`, 1.2137718043700492`, 0.7314346040557373`,
0.4669601248496963`, 0.016599550382003152`, 192.8440550768832`, 0.21285961816083276`, 7.382641950639707`},
{0.07358197980029185`, 1.0291227850237563`, 2.532589152827489`, 1.0117020834715413`, 0.8098748594005145`,
0.5301759885595309`, 0.008133019998886755`, 830.3082383512614`, 0.02407211472876364`, 4.5236701681781`},
{0.18797192534273177`, 0.513697145142666`, 2.3563599620417435`, 0.8181944603817968`, 0.26394673984920813`,
0.3209514271347109`, 0.025430853857263652`, 265.9518711391782`, 0.07720757078553087`, 7.134393115551401`},
{0.2528075291703835`, 2.1409422046846034`, 2.3989238084434295`, 1.1058748574151904`, 0.4698643832575349`,
0.20099009902051257`, 0.02857754945135185`, 57.682491948215834`, 0.240427409232489`, 4.4402688370357115`},
{0.07054141508315104`, 0.5799884401515247`, 2.99755260715291`, 1.1960365141205305`, 0.29917509086343896`,
0.44670601886119`, 0.13984458882947357`, 3612.976166750159`, 0.22678215214973713`, 7.656934137991781`},
{0.13968170289558773`, 2.9225254745826437`, 6.832387664740558`, 1.248628328449276`, 0.2500405375269379`,
0.6548966838897512`, 0.0131096950825671`, 2778.8012693248406`, 0.21649543926111503`, 9.334771326724606`},

```
{0.14258976979051796`, 0.734197214461469`, 7.171919374129239`, 0.945223896823731`, 0.9937893052327855`,
  0.2333579308191156`, 0.10311334331996398`, 86.7657175614978`, 0.17032024165228216`, 9.316237353631934`},
{0.06222401125326871`, 0.8031654209648247`, 7.912640972265759`, 1.0061100735669513`, 0.06612689335476452`,
  0.2257508005074531`, 0.2688428052003874`, 1016.6634644345198`, 0.08792308855751246`, 8.5639571694838`},
{0.045675945991483086`, 1.972555523342196`, 9.234608119853927`, 1.10067888297902`, 0.060950291683742996`,
  0.5641176167348642`, 0.005654158004083115`, 79.62566643520867`, 0.1041856757167563`, 3.9217157633863025`},
{0.19725457740267938`, 0.673691333497294`, 1.5958254230238804`, 0.9055402555445284`, 0.8526577157919533`,
  0.18700866613724199`, 0.221308084367558`, 137.27532297707575`, 0.16306687928789393`, 6.444987143866487`},
{0.17905209129143224`, 1.6872990209581902`, 6.743325221637608`, 0.8692054788625995`, 0.5776472609105132`,
  0.5432377341075647`, 0.12670077461750193`, 462.43790721394`, 0.057341353016641616`, 6.31060148160045`},
{0.21834559506325857`, 2.5253820753047362`, 6.659656299000567`, 1.4243771911650907`, 0.9747752944821462`,
  0.1772255451247845`, 0.01986842431027196`, 102.11368670749582`, 0.09272454748376258`, 4.914431231863471`},
{0.2429292029568641`, 1.5636324867576725`, 8.233163527811005`, 0.9303215118662402`, 0.8597394087604655`,
  0.6215210972087029`, 0.12468068919290341`, 61.92974770793832`, 0.07970211447796205`, 9.85507721294049`},
{0.09149398523540997`, 2.310449584405898`, 9.73345399143432`, 1.4402850137921024`, 0.7807243420630963`,
  0.37700223940662914`, 0.04452259966898517`, 785.475395176315`, 0.18361612690246515`, 4.855709318281368`},
{0.11249424084619364`, 2.9716096914630903`, 4.4521238184847185`, 1.4266434477040648`, 0.9997833218301952`,
  0.5867347171724102`, 0.06910846060462165`, 1820.2194505805683`, 0.24722705290784003`, 7.621221876581703`},
{0.09866336490626898`, 1.7258477745644702`, 0.8848358466481533`, 0.910425763108387`, 0.9172877053292023`,
  0.36334488527428765`, 0.3804604060347452`, 1006.1834168170927`, 0.013687075322010439`, 8.165252701558408`},
{0.22803797815686577`, 0.698146546972529`, 1.035170425484365`, 0.9822431866703154`, 0.6417091531359376`,
  0.21874316930074555`, 0.005531062831453447 5`, 1727.3746181384265`, 0.11140836748423671`, 9.157656749061658`},
{0.12680960193351426`, 3.133735649340868`, 7.511849069758438`, 1.0897808931740574`, 0.04885314856347578`,
  0.24060421848165847`, 0.3693592758602631`, 883.7388687037887`, 0.08961301811176398`, 2.076054864701238`},
{0.11645318221252937`, 1.153640861264721`, 2.619716900319183`, 0.8529577052637052`, 0.5619295219152276`,
  0.5231720769226155`, 0.04609815796088822`, 565.9088521347549`, 0.20476637224893374`, 8.831648882728363`},
{0.042446348872052314`, 3.5353553586920903`, 5.120248896798044`, 1.1724129507972807`, 0.07388809680757258`,
  0.47979821691937163`, 0.43439767413472635`, 83.35178002891637`, 0.15043407845955598`, 2.47226753883535`},
{0.1455833195103322`, 2.7843188408171127`, 7.76885376753495`, 1.3211895319342402`, 0.9135586666939064`,
  0.6453968687054246`, 0.1333226247130129`, 368.7414592704895`, 0.16919558147748776`, 3.2843355334407627`},
{0.19543798537508206`, 1.2324134940956721`, 7.418056367639444`, 1.0725732268441615`, 0.3282068086166905`,
```

```
  0.15859490968399015`, 0.32000646124266346`, 1339.7132456219883`, 0.07635813740352487`, 1.32927343177637`},
{0.14288798158791094`, 1.8492145343896036`, 4.320087837799633`, 1.416387968755192`, 0.03040014321801321`,
  0.26264512496919146`, 0.017396141813896236`, 476.32655268366966`, 0.1492295937607505`, 1.5210123574307455`},
{0.08126639977214484`, 1.4848577063399988`, 1.4511591326569544`, 1.4690309046886825`, 0.09088454373979671`,
  0.2634538760346167`, 0.006391735651833628`, 757.569692438266`, 0.1906091612906891`, 5.0187956659047614`},
{0.15406825481222286`, 3.5722547377798985`, 5.518246507815627`, 1.3255687223204002`, 0.2843400333432804`,
  0.5740150104722689`, 0.007989301755943026`, 636.0707307721444`, 0.19470033172956325`, 2.3727553064621407`},
{0.1284209869933174`, 2.657038147126583`, 3.485135675760706`, 0.826438046300177`, 0.2940359222573743`,
  0.4455502493814366`, 0.0077922251797686165`, 642.5517685154364`, 0.18333530742444448`, 4.125506851960305`},
{0.1519197321396482`, 1.414313316427302`, 9.693328908271443`, 1.0879341282517354`, 0.5935096502675883`,
  0.32269331092414044`, 0.04853394307287227`, 323.7551755246228`, 0.012675975614693441`, 6.5738064876305415`},
{0.10245812606330185`, 3.8066698620788078`, 2.784482506819291`, 0.8153557037427375`, 0.41559745180116914`,
  0.2970790998891699`, 0.019454402362339196`, 101.06708659388384`, 0.03906278599147861`, 1.0136093018296712`},
{0.08994858347063645`, 1.3905867780774148`, 6.975873482712982`, 0.9748960320168515`, 0.19705721946727817`,
  0.428438510479348`, 0.008778830773896138`, 53.85788205383214`, 0.2055785802889155`, 2.165498620130503`},
{0.2555268425086469`, 0.6701554458004759`, 6.763443756394938`, 0.9242821290525386`, 0.9506231196396431`,
  0.49497463337651615`, 0.1535425304369218`, 4370.366828894948`, 0.17262313668823565`, 9.906703002274519`},
{0.13340663060001784`, 0.4096518827228479`, 7.81563732077705`, 1.2681845828171425`, 0.264399527619257`,
  0.43786671579755854`, 0.11528071789135624`, 3397.5276395199044`, 0.15041338875391808`, 8.11261999427796`},
{0.09831375749458693`, 3.5924209186692435`, 1.6118282365623244`, 0.8326891410736474`, 0.6715734708323489`,
  0.5976781170151362`, 0.03815316295555065`, 115.73199628594814`, 0.0304507712440647`, 4.7148191409051226`},
{0.09108239111305882`, 3.309852207951435`, 3.0566194803884894`, 1.4370488086380533`, 0.21629452377334557`,
  0.2421124982368058`, 0.4670454509414495`, 208.22129409387404`, 0.23659860207487343`, 3.442603557657883`},
{0.05787448179355209`, 1.5695297669455615`, 8.686108197389068`, 1.3362839901345183`, 0.8882645350301348`,
  0.2220019613784866`, 0.20455164120837535`, 127.50819012666335`, 0.2481722521034202`, 6.402394955038988`},
{0.2051260145065577`, 0.6712958473867032`, 3.046620158397806`, 1.125533167922526`, 0.5107051094055373`,
  0.47305603590798795`, 0.035994055865640424`, 73.71677428550161`, 0.039721863923625866`, 8.822427071575206`},
{0.04365375531995927`, 0.9346121184769336`, 9.554451222092258`, 0.75459846537052`, 0.7382972174511151`,
  0.44670601219095474`, 0.02981461788584879`, 1443.346811017674`, 0.026466349135542522`, 4.5970910839514385`},
{0.18832502219499725`, 2.1446480190155537`, 7.011391892939212`, 1.2684842496371584`, 0.8800953133738048`,
  0.3019976503972389`, 0.01607749175819828`, 253.61560940445747`, 0.0505443969728126`, 8.205874646962478`},
```

```
{0.2789832227001883`, 3.864947879576305`, 9.015175334965505`, 1.1939022970453932`, 0.8102757584298972`,
  0.6575626089292559`, 0.013706864097109712`, 131.89661477643062`, 0.1558047394747739`, 7.390258992479394`},
{0.10740283086494345`, 3.8162968757181996`, 6.158389397925204`, 0.8038652030060122`, 0.9187954678523191`,
  0.31270681581700654`, 0.027540968538212463`, 2177.2729976761966`, 0.021577241346914133`, 9.805063749007854`},
{0.14667176624268824`, 3.777226248423828`, 0.6973504720596608`, 0.7758342249413661`, 0.494379064234173`,
  0.5228383904297224`, 0.014800119213367281`, 81.50804211451084`, 0.12794308526059`, 9.842771813142875`},
{0.1536199904675806 2`, 1.6832845721068805`, 5.017027003357642`, 1.2257765840301957`, 0.8758480691873987`,
  0.30492603190517764`, 0.17353190337485183`, 1218.2701158582597`, 0.01908888394356914`, 4.8706184705887665`},
{0.1285936813753743`, 1.2517058162042831`, 2.0098881407856393`, 1.434144339335301`, 0.06632650483661995`,
  0.16162194262920127`, 0.05011873598676769`, 354.0526180115314`, 0.1584833535532481`, 2.894273123117289`},
{0.05871769486491246`, 0.822876240174752`, 3.5461717275684297`, 1.3634413021309426`, 0.14962847564878934`,
  0.2425078078443763`, 0.045889110192962865`, 1618.8495608622088`, 0.1824696213391762`, 5.484504386708753`},
{0.21414990207917795`, 3.2291184159683555`, 3.7200101298578847`, 0.893435725539721`, 0.18550851463991336`,
  0.6299970912008048`, 0.007788531351629846`, 1185.7534950662077`, 0.10596189074334122`, 9.85351453889281`},
{0.14399581513021464`, 3.6650427925648215`, 8.960857802594514`, 0.9993788071734813`, 0.7678347002262149`,
  0.3917750513543251`, 0.06821121121423936`, 446.58045234253916`, 0.09781275073737189`, 4.158206599173292`},
{0.21762093156371237`, 1.4799568210517782`, 6.880501462590445`, 1.1700220578418317`, 0.49477438305707566`,
  0.30056004401666714`, 0.03776191240235276`, 139.28017196074`, 0.13055880095851463`, 4.30304009869343`},
{0.20676336326895633`, 3.023915070136316`, 2.1344025288266977`, 0.7647942269583019`, 0.38268253024987975`,
  0.605820162116665 7`, 0.06287157366362177`, 52.11423962493933`, 0.23050267576131828`, 4.129012828530946`},
{0.14056217319026204`, 2.827702131488536`, 4.822127390400377`, 0.9317030787852092`, 0.5881617813064273`,
  0.663416624487535`, 0.01875862506453023`, 1486.513173237306`, 0.1539754165327772`, 8.90838886614388`},
{0.15432054500385795`, 3.142590945055387`, 0.6412922856028498`, 0.9338764110697222`, 0.4036132844000331`,
  0.253764192888976`, 0.009287914173605602`, 800.3088033129984`, 0.0773663480819351`, 1.9942494398991926`},
{0.1558313627948012`, 2.6183453593149384`, 1.5210778217010041`, 1.2213020905618333`, 0.8957140746385861`,
  0.2067404945185849`, 0.06533654021268133`, 3401.5830280519913`, 0.07866132934502523`, 1.2687008259414867`},
{0.27749339228385056`, 3.960153029334033`, 7.7991060624230615`, 1.1159035815659704`, 0.47604804800745515`,
  0.18775668040836846`, 0.02760276902429692`, 890.1551806101527`, 0.14097999756547475`, 3.493022856148759`},
{0.1388142019807993`, 3.4960143968183592`, 0.8496516336862268`, 1.4057148799798922`, 0.27289215927666`,
  0.416116229659188 7`, 0.1071416651953492`, 563.7099077527477`, 0.24491201094978954`, 8.47264522499269`},
{0.0729917462714273`, 3.1893255155706086`, 3.1041281178047555`, 1.014074092539919`, 0.28719491349307913`,
```

```
  0.2514291775131975`, 0.05245081359414425`, 423.2923229706584`, 0.1145067740496658`, 9.081189649788715`},
{0.11420284690084648`, 0.577787691297051`, 2.567084026939444`, 1.4491737131438955`, 0.8025258647238394`,
  0.3690270257872911`, 0.13672661029124697`, 86.53603740064875`, 0.011299238359278513`, 6.563498155623039`},
{0.26373133325063186`, 3.0221428803953465`, 7.660698925394574`, 1.4577816176348994`, 0.5393449604126412`,
  0.22813670918251328`, 0.05400872825934766`, 78.22373236540282`, 0.11174025693188905`, 6.564224566850031`},
{0.21289488654178568`, 3.397179033451687`, 0.7632571166797604`, 1.0142699830327386`, 0.0835760348267307`,
  0.5184383444209231`, 0.049731529265758516`, 2070.206130759874`, 0.06133590421436391`, 5.427522099525574`},
{0.22999162513252186`, 2.379033562263336`, 2.5591649989178986`, 1.3870393830660817`, 0.8120421313192066`,
  0.6084139253527632`, 0.017274035559015792`, 82.84440150538262`, 0.15048886650658344`, 7.465486944171833`},
{0.1769680798340355`, 1.1633029664887262`, 4.7984019847292405`, 1.1781683757559547`, 0.8107109545176789`,
  0.3886685867136115`, 0.009172138674078711`, 82.52474973299203`, 0.0366989899628154`, 4.7780773811523165`},
{0.06792516420354372`, 2.678804428347224`, 7.00137481313343`, 1.1889039591838686`, 0.130109850192629`,
  0.17336690021836265`, 0.0065615912781447905`, 559.1859620197067`, 0.12479016185335001`, 7.679232726268854`},
{0.04696109785508959`, 0.9059167168392257`, 1.3656449005023852`, 0.8785005151977332`, 0.7117341492461746`,
  0.37541668809892415`, 0.06884837024201017`, 2425.2572297139236`, 0.025009444315193585`, 7.4432891311871785`},
{0.18481744149515084`, 2.8908169503663625`, 0.7719563125670259`, 1.477735031707543`, 0.05143123515478387`,
  0.4060758128174351`, 0.19176878359926766`, 114.34865924476063`, 0.02384868002335655`, 4.461346915500394`},
{0.11940413672862621`, 0.6385297463968325`, 0.6629513696361364`, 1.2353159995947312`, 0.3854086082897601`,
  0.4772941268081121`, 0.0318040184551439`, 4110.432043833155`, 0.1129636900773972`, 2.562436110087784`},
{0.16756813285689298`, 1.9469615336157506`, 0.3903678609266308`, 1.2604526712473199`, 0.11212576188863221`,
  0.45563235469719776`, 0.0065229352440185`, 190.16738409260836`, 0.045949495779464666`, 5.424569346408827`},
{0.07183551197561577`, 3.34536856927257`, 9.136834569708476`, 1.4150518758492396`, 0.289745011670542`,
  0.5142172775325705`, 0.41137464999480466`, 1635.4988121892525`, 0.15390110584062433`, 5.743187227898911`},
{0.2384422062087977`, 2.1587544451320504`, 5.065398759698054`, 0.8914719118393273`, 0.7773656949632277`,
  0.1997211683380481`, 0.4677952481930321`, 402.45383708693856`, 0.19717109492345014`, 2.7966746550751953`},
{0.049151474922052546`, 1.7029958107544303`, 5.984640650536118`, 1.1787547055685845`, 0.01700706976856159`,
  0.4330742864041065`, 0.015091472025893045`, 551.6808300976855`, 0.14911096385124378`, 2.965493287771624`},
{0.05913727892151971`, 3.1040367182848003`, 9.835708161805986`, 1.4919208308945082`, 0.5501557876127892`,
  0.5378623906029458`, 0.015011170514212647`, 280.8434087480442`, 0.0269165475419888`, 2.8649095039964387`},
{0.1028902787116574`, 3.248894721912677`, 2.8223976230891363`, 1.4617310236348016`, 0.21163710737184238`,
  0.2736720564831455`, 0.052563491644450414`, 157.16268136885162`, 0.03718011423514023`, 9.1247890605319`},
```

{0.13701717915337458`, 3.8023949322113495`, 2.868993121081161`, 1.1378721602373125`, 0.9948229422967421`, 0.24001294614371227`, 0.1498271577324817`, 115.7171521166015`, 0.23683719126671393`, 3.874936558168838`},
{0.12767680767420947`, 3.2442504564252905`, 9.68431286349912`, 1.3341607174516774`, 0.195417277749265 1`, 0.4247465971362525`, 0.2589111541470938 7`, 128.98950675665216`, 0.14024381160911475`, 6.508167926731167`},
{0.15001338826271665`, 1.2298087626140388`, 4.614349177257099`, 1.406777099587714`, 0.7364706782176744`, 0.4119762663671258`, 0.007028619260401152`, 151.01374639678102`, 0.013332718449329678`, 9.705143720051169`},
{0.20002396942953493`, 1.8945790659495865`, 6.47237980815893`, 1.0186003524309086`, 0.9948715481686918`, 0.5599748214378268`, 0.06647091233379371`, 636.3545963390636`, 0.214859543893563`, 8.583745959022096`},
{0.0426995949672023`, 2.62335318941125`, 9.6559263809094`, 1.1610440596461276`, 0.9636197200326426`, 0.24951659449471042`, 0.04756766795277323`, 104.67802103676537`, 0.10920782191363448`, 6.683384921443938`},
{0.2109009326123063`, 1.0923929642765158`, 8.593806993622852`, 1.1682615459081633`, 0.4499643089978256`, 0.499787017067712`, 0.035780106156421375`, 2693.2423996223015`, 0.018435796880638622`, 3.8866756448846873`},
{0.1340526977662379`, 3.5323141681247634`, 7.368159769503098`, 1.0026662265470512`, 0.9539160554997537`, 0.16176440546396775`, 0.3999369934637664`, 62.1244000754222`, 0.21257466381414752`, 3.7256546669386505`},
{0.138783517590022`, 3.0993312847332284`, 6.04052192414912`, 1.482957943290502`, 0.5742828550914962`, 0.6307262387789596`, 0.006158597883984412`, 223.4781142247734`, 0.1875211630254222`, 1.5541408452833192`},
{0.13089267345973798`, 3.445236909656975`, 9.841043091432308`, 1.0552044625838208`, 0.6603410104708771`, 0.19921054981100283`, 0.006557758988700662`, 85.91513533777795`, 0.010838310480980468`, 8.608032245025406`},
{0.0796809200271959`, 1.3022197759162255`, 4.457724373908043`, 0.819757860237272`, 0.9902524891863433`, 0.3732869275715256`, 0.027666886465779635`, 3268.657024391763`, 0.02176038735057556`, 2.8954452621820828`},
{0.24784589915696786`, 0.6840125293527293`, 8.78766727614829`, 1.2765252846824708`, 0.05554020049431552`, 0.46845321895484016`, 0.4169012658954577`, 507.69507152027995`, 0.17834533234287903`, 5.09521235383073`},
{0.1280056031133443`, 3.9743049299675812`, 6.787496113248967`, 1.4777257411519527`, 0.41697417826757177`, 0.16856234271449722`, 0.016472885834375787`, 675.9035984651438`, 0.06538866409107441`, 1.0273997992144341`},
{0.2694840025057293`, 1.4398402455219186`, 1.997018914620883`, 1.3694289195380824`, 0.1761008172166878`, 0.6152080706831848`, 0.020267602026759375`, 203.92916364743616`, 0.23224028188307977`, 5.1018397742421016`},
{0.05785779964873633`, 3.866745902394764`, 0.7980363800640653`, 1.2385898080722695`, 0.5722218009229685`, 0.20306189134783081`, 0.040832709866995026`, 2800.195372434598`, 0.18949558604342587`, 8.332936748246446`},
{0.2528189510796667`, 3.3250798883848782`, 4.1415523484020405`, 0.9119401620778833`, 0.8401965817654944`, 0.5070162083757728`, 0.10337827675659322`, 381.96079877048203`, 0.13245695994197593`, 7.95834379608233`},
{0.20018502874097505`, 2.5781656282009475`, 6.942068190225046`, 0.9929389842661138`, 0.1594147159589598 4`,

0.3421827829774996`, 0.4839576109293149`, 375.34729484852807`, 0.1596957174670693`, 8.895509004013046`},
{0.272440775669993`, 3.035804235517027`, 9.484814840956616`, 1.224288286053278`, 0.8025814347303761`,
0.5890268382574878`, 0.3843203491912798`, 336.978314494752`, 0.04553466081406238`, 4.10287356038395`},
{0.20623021381347656`, 2.305665489644068`, 6.160210330863352`, 1.4997848102690652`, 0.31029755609374554`,
0.2932546230651458`, 0.047029701289319936`, 605.575343672007`, 0.11131413350838143`, 4.271934365443833`},
{0.27799817940399246`, 2.8456068500128753`, 7.4650958706635215`, 1.2794446296629622`, 0.3185515090611555`,
0.5440861077528716`, 0.2050159519579307`, 96.61991147082354`, 0.24522478882121745`, 9.22867476531573`},
{0.18731205279881885`, 3.3920364906001828`, 9.53367346846489`, 1.150711712471592`, 0.269617044263482`,
0.4407414224913243`, 0.0689482800442352`, 54.451263142554176`, 0.1525802464986004`, 2.2696676207606785`},
{0.07598141369986516`, 3.9958506473293216`, 3.287957942109602`, 1.3569966945049934`, 0.38975760386481206`,
0.6382110342097327`, 0.02530287278972352`, 4836.578585308585`, 0.06124631354128618`, 7.15493409138095`},
{0.21782025688272888`, 3.800225088388993`, 1.7719223148490801`, 1.3823014458300409`, 0.9034484926621247`,
0.21119490910082483`, 0.008011320454875006`, 549.8645476667474`, 0.16991323645757794`, 1.3522541901702763`},
{0.12917865835123116`, 3.4713877505357775`, 5.802428296169513`, 1.4965547140183602`, 0.061568597458759644`,
0.5580721907461164`, 0.2777380762847274`, 381.024601401567`, 0.014984965589617844`, 6.51082587242481`},
{0.12408328322582662`, 2.9809522601952096`, 4.3074275514408615`, 1.1040930179447719`, 0.6796918144407815`,
0.5931470332615022`, 0.005369942238542098`, 502.88367879292525`, 0.06875474145528054`, 6.831128450148093`},
{0.2301374553102561`, 3.6285008777535515`, 6.8583997394674485`, 1.291013232269163`, 0.6431022470858925`,
0.6246309281545763`, 0.18498728864660263`, 132.58216720365365`, 0.011374938240932164`, 6.052136677369937`},
{0.18974259222357154`, 1.3104397818774611`, 5.6865397453703626`, 1.3109561369338216`, 0.42925562092874125`,
0.6235284138933523`, 0.06332638490149935`, 1635.1348002386078`, 0.08473820825835521`, 3.2599591654117823`},
{0.2309824769455583`, 1.2471345787782342`, 1.0215019422726535`, 1.0312798793045614`, 0.7898876098277747`,
0.6152586670568927`, 0.006979047924720161`, 310.31208829749596`, 0.24081833704073502`, 8.344184598629436`},
{0.1769680396277803`, 1.6046472005597128`, 8.695674701006173`, 0.7703180616996756`, 0.764260429383415`,
0.1992432648214062`, 0.012440799720035065`, 274.53159119005693`, 0.16375790457626532`, 6.298702841855883`},
{0.23450402912552432`, 3.0762297015464597`, 2.593937576427738`, 1.1039402177647473`, 0.4424612211889203`,
0.6666998367628838`, 0.44528706596344714`, 463.5224063697932`, 0.20661121111075748`, 4.223015851637719`},
{0.2755523363386182`, 3.945462181815995`, 5.876565708554855`, 1.4395306041819111`, 0.28572441613120825`,
0.3101513953075886`, 0.39757529919045964`, 3302.499033894636`, 0.07285156297216239`, 3.794286848749824`},
{0.1912671247263708`, 2.190434692724648`, 6.310276417856439`, 0.9527360176604049`, 0.6104292936966895`,
0.2522020081398627`, 0.010518470607815496`, 284.3254609055096`, 0.1005439259073278`, 6.418961425083908`},

{0.2597914621776059`, 0.5051242650756058`, 1.0118962727972`, 0.8968589895031354`, 0.07276014875602654`,
0.6898744359309281`, 0.03193322527994425`, 3177.5504656788207`, 0.10860322045075349`, 3.7076953543063436`},
{0.05800931142578874 5`, 1.2821997162065424`, 2.0542232518025845`, 1.4132652568351216`, 0.6228832317992945`,
0.16342794118309045`, 0.04468053791122747 5`, 201.38656694501756`, 0.06413530627116476`, 9.590029577588982`},
{0.19641852899773504`, 2.1398264375306333`, 5.320272455292722`, 1.1437489346098513`, 0.5956525384016933`,
0.542078693849333`, 0.2264882318745723`, 868.9198508977777`, 0.040131295366706066`, 7.0792614425236255`},
{0.09296068013545083`, 1.2991493564864323`, 0.26387265122439096`, 0.9093728692378444`, 0.35146944109549483`,
0.5215346752572301`, 0.009103351916553084`, 3690.6422305701226`, 0.1417847774664471 3`, 8.45894371926392 5`},
{0.09381338426095137`, 2.303475789921615`, 0.696644313515133`, 0.9364121618865129`, 0.9663355677423924`,
0.5212308135336106`, 0.24626013703252375`, 1385.562539836289`, 0.20934361709679128`, 7.7725134925150865`},
{0.10039832860995906`, 2.055462616090492`, 1.0012272143748433`, 1.102551063992137`, 0.7631597204325553`,
0.38648795662626834`, 0.021413905310258614`, 653.3550083645581`, 0.055519462287367316`, 4.402154187880063`},
{0.16980453046164407`, 1.1980162751260872`, 9.435065425338543`, 1.2054727390347812`, 0.15783339617816905`,
0.5987514193964574`, 0.2639813183074236`, 624.8721767770594`, 0.14884913893972124`, 1.0597319526281854`},
{0.1106187483985549`, 0.9718711331456742`, 7.0205608914526`, 1.3199794157480607`, 0.2870266036717726`,
0.2842604073783652`, 0.12440193317594331`, 1067.250206889352`, 0.20077332983402157`, 1.344770962154577`},
{0.22322153760577584`, 3.532203149750069`, 4.201686494998453`, 0.809746094501941`, 0.17157339217602185`,
0.16416014932043588`, 0.005086092661785311`, 81.42460105493491`, 0.09946867199032089`, 6.2584574953677805`},
{0.11344753764961812`, 0.6515213816912864`, 6.9223785093110415`, 0.7705690533378804`, 0.053758347821018404`,
0.22122664443377937`, 0.23238889135484875`, 1612.9665239639735`, 0.23659636795633343`, 7.8050806983048435`},
{0.05086592503936643`, 0.6345548618632657`, 4.1212243894392255`, 0.9757969272512688`, 0.4720155756440034`,
0.3491598005177542`, 0.10619345643493772`, 163.13358888497802`, 0.24650090837908084`, 9.412849245254243`},
{0.18344840265344153`, 3.595124583701854`, 4.199071914809606`, 1.0537057639813663`, 0.12507220232647875`,
0.4903855977737973`, 0.3568003256791643`, 83.2177314580599`, 0.1899680886834239`, 7.625578775230776`},
{0.11456761033436458`, 3.133847301219358`, 1.1215010618012613`, 1.2799949994814193`, 0.7250649174618937`,
0.2748176967060689`, 0.05347527364447488`, 4312.248498236981`, 0.1363057898672484`, 6.512229405589068`},
{0.2733102228285163`, 0.9906705811868095`, 1.8602414047947509`, 1.4451100284824412`, 0.2848844825831507`,
0.554129635350794`, 0.011612796259976983`, 302.8864567264163`, 0.1580490958422761`, 2.3921629765885086`},
{0.13611751569452646`, 1.612649572698647`, 9.438389550358693`, 0.8529700096304889`, 0.38386891649316235`,
0.16702474363312747`, 0.015283436679942941`, 2091.084776788581`, 0.04888707786432367`, 3.7550559614266916`},
{0.06405507608053002`, 1.3528358541887275`, 2.745226236199782`, 1.0639243234460365`, 0.36966523495796855`,

```
  0.3262944894947615`, 0.12612633222162115`, 59.71942118372549`, 0.2416255104540047`, 1.4175051543133999`},
 {0.20954125488723696`, 2.353113320872523`, 8.807677395871629`, 1.2953396401945112`, 0.754037675145256`,
  0.6760987806408354`, 0.0257504108557459`, 115.6397082887867`, 0.08726278776335833`, 1.393867616131378`},
 {0.12907099769038483`, 2.604139615282878`, 9.661547795493739`, 1.0824039436985886`, 0.5892380332813292`,
  0.2973399722037613`, 0.047844179127263436`, 189.25086236267023`, 0.09074457722059731`, 6.471849996577652`},
 {0.2477279255184573`, 2.8408395942947395`, 2.128090335748766`, 1.4989746812981888`, 0.49854461135120975`,
  0.5556994499234343`, 0.04795352432490085`, 88.91629411642595`, 0.17336384521962678`, 2.1291108959892835`},
 {0.17138396941106926`, 2.571547212239655`, 8.060362653989774`, 1.424545143219825`, 0.3585647265624352`,
  0.4155041928809974`, 0.0058496443892025185`, 1444.9053577030656`, 0.14315469959013916`, 8.6363710850225`},
 {0.046024454977220214`, 1.8018961949102152`, 1.8889771640586375`, 1.2101342547143499`, 0.33571961358008684`,
  0.4325808303075249`, 0.005213559292121167`, 454.61857984803544`, 0.13129751322022398`, 8.467405558196546`},
 {0.15552548139256261`, 1.3158122598456092`, 2.9803095987090753`, 0.857616156930884`, 0.19622947720693906`,
  0.41449590928153224`, 0.03892742585126438`, 2129.9557657820133`, 0.1528564450859799`, 8.904626117418289`},
 {0.20226341603934178`, 2.23359139800782`, 8.92211343859698`, 1.0176324123098368`, 0.17770526936314845`,
  0.5466941503204505`, 0.02261985699694341`, 208.46398724865384`, 0.0356723302826476`, 4.800574849057675`},
 {0.1612087208534252`, 3.1252528746197825`, 0.891851633192701`, 1.468881451365178`, 0.6554353298599058`,
  0.19493335173588566`, 0.018954452797492544`, 551.070999166768`, 0.1147406258040957 2`, 7.406826770535428`},
 {0.2365245631846375`, 1.6915599352668007`, 9.677850457164503`, 0.7567859979545984`, 0.725618873672359`,
  0.2424250415266589`, 0.36826031218846694`, 892.5435235500784`, 0.17475643237365474`, 6.493737892117458`},
 {0.22790261965108521`, 1.8196334440146895`, 4.716201039891137`, 1.2810805872690838`, 0.6237465626441163`,
  0.5616811859186371`, 0.03042013590776336`, 637.6118251230974`, 0.08011306001688712`, 9.216145754556873`},
 {0.10184647957852327`, 3.0801202872185955`, 8.412464860766345`, 0.9942252517730743`, 0.7984635108014702`,
  0.4565736536432896`, 0.009191281945386102`, 103.82351882247931`, 0.22274711417837167`, 2.5201460619086546`},
 {0.18768732765242752`, 1.4966861207841546`, 1.129759551095228`, 1.2240080138702374`, 0.5362083693873154`,
  0.686948995479657`, 0.005867661202712241`, 74.92355531972449`, 0.165025723744549`, 3.2590978632956187`},
 {0.14949493319923252`, 0.867900380757324`, 9.54711835147964`, 0.8271983961134595`, 0.23831574478972195`,
  0.375815029784779`, 0.04516845569069218`, 2094.687766706356`, 0.0279158592619283`, 5.743304871768913`},
 {0.10785599050108607`, 1.35490623294936`, 7.749733046704847`, 1.2972878372630243`, 0.6070878493023009`,
  0.6937073302769028`, 0.28374543116039413`, 66.83074779041495`, 0.1939262594152683`, 9.425365274493942`},
 {0.13969386881974227`, 3.165109549305347`, 4.62533417349867`, 1.0389272094346946`, 0.7061162924631639`,
  0.5973647953915502`, 0.19864442086151357`, 3440.0036010322765`, 0.16071761832429643`, 1.3360604448248414`},
```

```
{0.18701505616348296`, 1.1471835668231432`, 1.9652898147298057`, 1.007866470439728`, 0.8673024418570234`,
  0.4964521059357`, 0.11361709646832255`, 189.11029986824036`, 0.17527157137077032`, 1.608206453868661`},
{0.12078071855653999`, 3.7816423804380825`, 3.0527531818157634`, 1.3792123087306463`, 0.18684955879429088`,
  0.29398025647621684`, 0.011105429740027619`, 471.0240529628659`, 0.1734264666127333`, 9.864280424563177`},
{0.12022941283008587`, 2.2264210098571606`, 6.2451953049416655`, 1.3438290154028227`, 0.5358944653344513`,
  0.32618163573980585`, 0.199782178892696`, 3338.755251465298`, 0.1788085018354036`, 4.512919273292992`},
{0.04681963491488872`, 2.902738250264786`, 8.529727895043578`, 1.3985345888875074`, 0.35876551940164103`,
  0.2520539039703089`, 0.05124631524713002`, 163.50426816944002`, 0.24665810410586203`, 8.118758344754049`},
{0.1911559295611578`, 2.175586072104755`, 6.8117639016300915`, 1.0586708066957606`, 0.34517099105389626`,
  0.196211531611845`, 0.1489787018319715`, 3704.576077864889`, 0.10408111144660032`, 4.8300445149655875`},
{0.17192712556630163`, 3.7260903741599414`, 4.118781130521407`, 1.2245874304759414`, 0.6062180312792598`,
  0.5331783629983038`, 0.1203111914746069`, 226.51013650082294`, 0.017839169449302028`, 7.150760371208259`},
{0.1575695043509842`, 3.8758817205894927`, 3.3547063374262347`, 1.3334178116995439`, 0.4921394422884451`,
  0.402330971011554`, 0.12204131883407823`, 201.57298534982124`, 0.22899817129952033`, 2.4884430439924596`},
{0.06573776532431397`, 2.613579102769201`, 3.806271127858438`, 1.1314163600990936`, 0.4483602258230772`,
  0.6823320235871475`, 0.18768855278686034`, 189.85674956999534`, 0.0631967400861071`, 1.3499467761035964`},
{0.06757463956251891`, 2.3005441553217736`, 6.868151131987258`, 1.3860241166029201`, 0.025379963517129678`,
  0.6856836713524472`, 0.11835507299142825`, 3863.192203814942`, 0.1108156231672533`, 2.823244077255117`},
{0.06632069369203508`, 0.7568152865276287`, 0.22726671156421221`, 1.362987063673106`, 0.9228910129465389`,
  0.1868567265073786`, 0.1296811990167267`, 123.29501155053357`, 0.19348527358813117`, 9.470702921306387`},
{0.1352416913789251`, 3.40347330811998`, 4.693938060979022`, 1.2778213334701187`, 0.7052646641955478`,
  0.6043325609148968`, 0.013064813980624626`, 333.49561209931903`, 0.1115376428574013 1`, 8.005884287110216`},
{0.11675242158451127`, 2.833886804623358`, 0.47584343623935504`, 1.0607616820427617`, 0.7666611109645796`,
  0.4737758025893787`, 0.1681420982868212`, 120.33220123866614`, 0.17915094289032796`, 3.5814785888826783`},
{0.24323887781179143`, 3.4699698183729026`, 8.39097272098094`, 1.1938324247391086`, 0.7463001660827029`,
  0.6306644967356683`, 0.2992521819376612`, 322.6100644812281`, 0.10289378547186823`, 7.076414822100832`},
{0.24390696598302103`, 0.8206355382610386`, 2.839705408503791`, 1.0872176805395473`, 0.8466198387041834`,
  0.5785697848880403`, 0.1356337036504287`, 2343.8028799398503`, 0.22246042363346175`, 4.459217822696726`},
{0.06769491456810739`, 3.362305338073525`, 1.8871867183871915`, 1.4803795481758868`, 0.5363168854717104`,
  0.37458837250617005`, 0.011183256466341666`, 407.888606379481`, 0.051902827430772014`, 9.719761204172809`},
{0.21105892687940375`, 0.7047276416914965`, 5.768010388957135`, 0.810198720595394`, 0.17857267890229922`,
```

```
  0.19525448649651245`, 0.036176279358565294`, 67.82203673017167`, 0.0747824159402756 8`, 5.650997388906681`},
{0.13030085884608883`, 3.691040694570585`, 3.5546925177950874`, 1.392417125140739`, 0.5227226143537942`,
  0.3153665172319682`, 0.008712610924073824`, 695.2883528301467`, 0.04802018879975545`, 9.078480195703946`},
{0.21250061023000932`, 2.8149517595358526`, 2.295631419336827`, 1.0543549080196901`, 0.6519014079329188`,
  0.6534060058028184`, 0.09088447710322001`, 650.6798329466749`, 0.04016188412840693`, 1.3861729584655489`},
{0.14347475236734758`, 3.69539554202769`, 4.673626861854084`, 0.8876695712812951`, 0.055199727721453984`,
  0.3291461786147223`, 0.031312636638690675`, 836.9629099625735`, 0.07687709551472927`, 5.335882334514919`},
{0.0805761857877269`, 3.094619489710217`, 9.945877040804334`, 1.11372690786775`, 0.6430409751953907`,
  0.42229007304684385`, 0.05506378209236767`, 302.8456666998151`, 0.050941087889112335`, 7.632080993355595`},
{0.26865716137877294`, 2.5539808428845117`, 7.725254140854183`, 1.421576189719117`, 0.2484256166411507`,
  0.1545757521332899`, 0.008556741498662016`, 1177.1024036480305`, 0.08015420001442447`, 4.125195480316323`},
{0.05207035897383483`, 1.5461440288878858`, 9.695008671418819`, 0.9618476339297223`, 0.8208653770038725`,
  0.48583250041037973`, 0.22651174945218666`, 56.560953214157614`, 0.1695178841867513`, 7.246656846935364`},
{0.09188932948313594`, 3.928289836924776`, 1.4719659367359732`, 1.3955743442040527`, 0.5432280807299197`,
  0.32698938665455834`, 0.013007122973082902`, 2762.880112969401`, 0.13947463148509326`, 4.274432647648432`},
{0.04429611977972214`, 3.507177302954225`, 7.234549676890296`, 0.7623401767303408`, 0.010148640019661537`,
  0.662687845406686`, 0.08775344792279212`, 3965.4644576584396`, 0.17480873042969614`, 5.118348118050843`},
{0.14748247662042147`, 2.024427515527907`, 9.506929645646245`, 1.2214601071407813`, 0.2054881053340767`,
  0.2020185622482833`, 0.07806490597707526`, 302.23580657852216`, 0.2425913704635762`, 6.045088630499148`},
{0.05191368478403385`, 1.8292747620439185`, 5.888828307542417`, 1.141375509610469`, 0.9869968200599144`,
  0.28747330669734017`, 0.24632331590197062`, 4082.2304223282904`, 0.0447948152020462`, 8.998917183247265`},
{0.22928260338606776`, 2.222139650206395`, 4.336407604983492`, 1.4545262441104416`, 0.17670484975018264`,
  0.15215120204412502`, 0.00931582374755147`, 107.9598634817244`, 0.18384173269782222`, 2.0618261552271964`},
{0.13057179539780978`, 1.32287055831243`, 2.838629919542937`, 0.8498204532586893`, 0.8949438289681946`,
  0.46229782752490833`, 0.06831034580553909`, 355.6715560557054`, 0.046330937271765626`, 4.510601406855825`},
{0.16617324858328203`, 1.7665378156673919`, 1.5537022391595983`, 1.3738499573440288`, 0.09625830296842142`,
  0.5757224393125404`, 0.011187699121044243`, 1140.5955024024072`, 0.09128758443232887`, 2.6133507581824666`},
{0.150610971367461`, 1.9051770945291775`, 1.9970914506574022`, 1.332029884280907`, 0.017627751946745374`,
  0.6342635790195508`, 0.03224365795250487`, 84.16237057378729`, 0.025181518731154096`, 6.093120594420567`},
{0.170193722625894`, 0.42908710938865147`, 8.009796665724636`, 0.9108642019460496`, 0.48256370773751156`,
  0.150486488277020 5`, 0.015501266854459406`, 231.18780020236528`, 0.059845835174358764`, 2.951447491236335`},
```

{0.14155286072340578`, 2.768731185287475`, 2.866796585793665`, 0.9268420036617817`, 0.38841014261523243`,
0.6739227770046636`, 0.08018336457681292`, 85.63532436040691`, 0.10664760400309609`, 4.590937221386067`},
{0.17254585725985527`, 2.4767354176461422`, 1.5265879737476418`, 1.3533294324013416`, 0.010476993931212819`,
0.3024537953894164`, 0.005382899126762437`, 1067.886819263142`, 0.025600178888030733`, 4.688973100729083`},
{0.12080218784009655`, 2.186843407698192`, 8.77616791832854`, 0.9616886370191489`, 0.26020855267635734`,
0.6201783502287406`, 0.03811904516021991`, 117.06014479510438`, 0.17593156125676795`, 4.349098586723594`},
{0.06629025039264891`, 1.530609759106781`, 1.900084106095834`, 1.2729265107514505`, 0.009336607275947317`,
0.20692029297881853`, 0.07757288734346537`, 56.094679581136695`, 0.10338780030561462`, 4.3409372843081`},
{0.11054921486908309`, 1.9601623754484976`, 4.1405236153603`, 1.0982919546164496`, 0.2371613718862391`,
0.5811399077338627`, 0.014126785714663546`, 63.169894546693705`, 0.15558848051416968`, 8.651696062244092`},
{0.09844925238529889`, 2.9547765664281824`, 3.97533240344332`, 0.8901779978265776`, 0.6485331039194935`,
0.172046787329591`, 0.2843834249851245`, 265.56383421685405`, 0.07856132944640065`, 2.1823933339229455`},
{0.07379407071323735`, 1.0020834251379886`, 3.8959201685323244`, 1.3275435329440872`, 0.5495795554378471`,
0.5203295123605114`, 0.006236204073249997`, 495.6126936444482`, 0.05339932410900344`, 4.300380134475608`},
{0.137830658132162`, 0.964244066849937`, 5.678313655870262`, 1.056362681888576`, 0.27898051740088525`,
0.6674603903550571`, 0.022240144180942223`, 3206.233069292432`, 0.13749704640301397`, 7.378694929535063`},
{0.09412349267697989`, 2.412142947637907`, 8.593900106949853`, 1.0545990130363978`, 0.8443965196041454`,
0.4441557500447094`, 0.020813982545198062`, 67.68562305269263`, 0.21037364591803787`, 1.9282456472076213`},
{0.059604065321211375`, 2.060924190389435`, 4.151577648882975`, 1.0218595307135387`, 0.8259153199816331`,
0.67511248087556`, 0.012173840559063499`, 163.32402156550611`, 0.22896057989768043`, 4.41755081858776`},
{0.062303828390692484`, 3.237012523552033`, 1.9717708145626371`, 0.9726411613979222`, 0.1873520229034391`,
0.5560570337810927`, 0.026607710207102397`, 62.963998927305475`, 0.022649002664224704`, 8.659052494024476`},
{0.058207327156306`, 1.429925878818433`, 8.458349879741764`, 1.1245084153106533`, 0.19874114947102073`,
0.3371100826465334`, 0.04401493286992449`, 375.7204818983131`, 0.2159694260931322`, 8.593637256078832`},
{0.0526423548412821`, 1.6513512325831732`, 4.083783641771872`, 1.373155154639566`, 0.8939370398042139`,
0.2364887781328291`, 0.0490949664593259`, 1618.3201075393572`, 0.07451928539997066`, 4.7651781050102695`},
{0.21322884859448837`, 1.5615738832323176`, 8.40996483053398`, 1.2929614343326012`, 0.3765546169235392`,
0.671739728089668`, 0.1608794324574438`, 485.1832614690647`, 0.0705178455433636`, 2.1513131242884285`},
{0.11682922382752631`, 0.5721136495795571`, 9.687079392836907`, 0.91254998083727`, 0.43543762812336806`,
0.4740169657662334`, 0.10620954791167687`, 4372.038997558794`, 0.0729116423313943`, 5.645356891940546`},
{0.26956767736567855`, 0.8444303847788461`, 1.0020073721955711`, 0.7502641425367418`, 0.966405616850982`,

```
  0.6715103286393609`, 0.23328928938153737`, 246.8438332486948`, 0.10178810353613177`, 2.531792031152129`},
{0.07372356616920961`, 2.107710372997479`, 6.08076458707294`, 0.8379230467338923`, 0.9571077593283461`,
  0.37371168094765217`, 0.1939664465355383`, 131.4329681577719`, 0.026286944156709513`, 5.373906305884013`},
{0.2128059772734779`, 3.088861612111426`, 5.437928143232815`, 1.2368766361188581`, 0.5161736795080103`,
  0.22159582314348258`, 0.17105793552921358`, 1293.4067318655482`, 0.22754002195492767`, 9.689789287743999`},
{0.09487232790202699`, 3.2902977287485546`, 3.9754892015026773`, 1.2274952505878522`, 0.2657953162834845`,
  0.47376077240276926`, 0.26204707729007887`, 80.73499841507218`, 0.18195262450329797`, 6.708419132160676`},
{0.05710457017724263`, 2.2468049124614753`, 4.035987116327698`, 0.7517191945594467`, 0.40903626729229714`,
  0.6582116219387326`, 0.04046385674463665`, 815.0779000126262`, 0.186726177475597`, 7.464159626289564`},
{0.1356890149134694`, 1.6504364529320803`, 8.903330095009764`, 0.8652149931542472`, 0.7882939470826769`,
  0.5853867881019097`, 0.12191584954894137`, 2002.2565687817998`, 0.19189449537551667`, 8.0829058846114`},
{0.21947794079961902`, 1.5730764365956098`, 2.118289006802259`, 0.973502067608673`, 0.7638917093126882`,
  0.33981639014260245`, 0.009615294802656809`, 1765.6534921210653`, 0.16626255675053375`, 4.010994756897377`},
{0.2345658346849942`, 2.081301191128982`, 3.6234436876627587`, 1.1134590239676974`, 0.8739003578896458`,
  0.6696041669913848`, 0.0659089630013702`, 558.8606273551264`, 0.09113830356870467`, 1.2137876330314246`},
{0.23053748957807368`, 3.8668800248110458`, 5.535000453565214`, 1.1361194466757834`, 0.24488877583427326`,
  0.5300195072451362`, 0.08816752471869817`, 3094.9084693615328`, 0.16131439142801796`, 1.2619255962975515`},
{0.05839671607004826`, 2.466861519533315`, 8.278076935325572`, 0.9179604454629582`, 0.7485509623778881`,
  0.3739704677333707`, 0.3976055017684991`, 334.0137006202725`, 0.18218149392519362`, 2.0150765691128605`},
{0.14569114559043927`, 3.1555485829295717`, 1.7894848948555018`, 1.1727674941432193`, 0.06666495623301039`,
  0.21747003985461844`, 0.032197916321861565`, 1115.0145850241458`, 0.11404115123479613`, 9.530247146566754`},
{0.24150266620923067`, 1.7188165992139313`, 4.322636691993072`, 1.2029528995785472`, 0.9471494725848191`,
  0.6615414605337746`, 0.19861497081852103`, 2170.4118977268995`, 0.144713056075588`, 4.793357249969096`},
{0.20918490948785712`, 3.1569386485962374`, 8.450247389660383`, 1.207442014040422`, 0.02962131605799323`,
  0.6403349881706148`, 0.07844599973162336`, 325.7491474109025`, 0.10080604275363181`, 2.998503110312266`},
{0.20782035028778872`, 3.1560227942933192`, 4.024085032034362`, 1.1076699632387197`, 0.7825797887689212`,
  0.681476671590229`, 0.005917686238352288 6`, 2392.206038919967`, 0.17125268254719206`, 3.7066097570560648`},
{0.16569005768607342`, 0.9318724619299896`, 5.387727961962419`, 1.4204768501331082`, 0.162422581490264`,
  0.6283948310518737`, 0.42435650593933405`, 403.29853257093646`, 0.17732127106821444`, 7.752705227642123`},
{0.25387146604005345`, 2.177421517695631`, 2.9195785593706507`, 1.205984878491204`, 0.22933817566590875`,
  0.5142965710820098`, 0.00715658453704801`, 291.5834795916249`, 0.1854640509688 6748`, 3.1957985304348036`},
```

{0.27254750481249423`, 3.728211063877054`, 5.204302820921312`, 1.137745914342209`, 0.04515932299981973`,
0.21786268187051538`, 0.17709327429647695`, 87.47051100453898`, 0.05078893394192652`, 2.761931237968355`},
{0.24124989455736606`, 1.138551887899828`, 6.0414451617780465`, 1.0864227463934355`, 0.8606911125263128`,
0.6167713883354349`, 0.008616924398628963`, 4204.638992315412`, 0.042475052828396476`, 4.683417206387297`},
{0.16291183507690532`, 0.9001170306894437`, 5.6406094859134015`, 1.3830538570390154`, 0.30535931966424634`,
0.523800888605104`, 0.2170932130058295`, 52.61233898698406`, 0.08477132140762966`, 2.26771827115102`},
{0.24307985322679526`, 3.5312268091205707`, 7.143117328957406`, 1.2474301004946668`, 0.43966589306292936`,
0.28635789146968393`, 0.04752738776816154`, 125.46273060513494`, 0.15229886979218882`, 3.763239253417467`},
{0.08579390683558069`, 2.8983719603389604`, 3.99564346069112`, 1.2420354947694623`, 0.9313337583971846`,
0.4737657288125191`, 0.010571420568870461`, 66.04207510362154`, 0.1244519483642591`, 8.73466885165751`},
{0.20336933942425872`, 1.2934536281273639`, 5.591224704743386`, 1.058733783245077`, 0.09184020179750241`,
0.3092776948325172`, 0.45477750215520385`, 95.84900263920687`, 0.1497141413596374 5`, 8.98848136080165`},
{0.12808268874911022`, 3.6824385947617753`, 2.1347222010883335`, 0.9228913029818868`, 0.1301948165557758`,
0.29194798379239884`, 0.27062051931711223`, 4508.281780436825`, 0.033597809391257005`, 7.5117374932700365`},
{0.14553068051711526`, 1.8082756931498238`, 2.368533159601707`, 1.1602558844471123`, 0.9691057704573378`,
0.4792380149128871`, 0.010639214796165341`, 788.9564435352116`, 0.18893878705870149`, 9.465562662789438`},
{0.24858998623664108`, 2.4028729322353692`, 5.907867944453356`, 0.899298954014168`, 0.6845394050547509`,
0.4831163766793648`, 0.006208316855544933`, 932.6458115441258`, 0.22545041645445363`, 9.209981899602862`},
{0.2519271524040908`, 1.2045387595495214`, 4.5140841978362705`, 1.0152142452788597`, 0.29419718036266773`,
0.20432622138273293`, 0.02287101962327847`, 2390.149329793605`, 0.07799608049040802`, 9.888014794979117`},
{0.11736070154865536`, 2.38096944376945`, 5.3494850274310295`, 1.276899864236343`, 0.07495782665645989`,
0.3912082450614217`, 0.41113333692039317`, 80.12914367323096`, 0.2186956786299285`, 8.297333470694408`},
{0.16035901956271875`, 2.606522752147513`, 4.69883411815465`, 1.3021358666964324`, 0.6150695298380762`,
0.6434499333892203`, 0.12329290515746044`, 1079.0196669558486`, 0.2419176548365854`, 8.070742396729585`},
{0.16449617597041116`, 3.191202853555228`, 6.979734889593845`, 0.755053893640484`, 0.8431581165413089`,
0.5308423930900061`, 0.0662197921799213`, 915.9947229465104`, 0.1651520479769521`, 4.094114041787371`},
{0.11360527105324508`, 0.4683409445122746`, 6.198135239531911`, 1.4397350406163711`, 0.8287446034488006`,
0.16487005319303316`, 0.2956243647100192`, 339.82112374821816`, 0.14870620145140367`, 8.492417686013223`},
{0.08657280459787114`, 2.8376273381057535`, 2.18201840556476`, 0.9732923844552535`, 0.8927467229995099`,
0.4599530868891304`, 0.04289399463935107`, 1129.687177458504`, 0.08547871234303578`, 2.1820677830816138`},
{0.14288327643386806`, 3.0416391272131467`, 7.259522365277451`, 0.8919048885163637`, 0.7100314935930478`,

```
0.32120037052790307`, 0.014548712666776912`, 690.6861027330066`, 0.2370448478751857`, 7.1501615854828415`},
{0.05887079706837223`, 1.3439289639420835`, 4.010009055307599`, 1.087305166401462`, 0.6661788396152433`,
 0.35567096782369767`, 0.008830796062004187`, 96.96443168903161`, 0.0354538286259683`, 6.082802068311153`},
{0.275808383383374`, 3.614253930768469`, 8.690573450897112`, 0.8084062901760818`, 0.7881641108236563`,
 0.5461332704061045`, 0.16300280176413603`, 213.49162259077596`, 0.11620699031214832`, 3.4918574760984407`},
{0.16345104545617634`, 3.976575006158881`, 6.050721111049688`, 0.8077170991659299`, 0.4737043917748365`,
 0.42206087766889466`, 0.011122815850097148`, 819.7636995837653`, 0.0134560852671817`, 8.084495823489597`},
{0.27078615466317746`, 3.6662669410444693`, 7.922278334055675`, 1.4466898496109064`, 0.8715287168554611`,
 0.2219561421798496`, 0.02446106939657988`, 188.339766844502`, 0.03692414185938048`, 1.663076961237424`},
{0.24286662289220456`, 2.6814172670718373`, 2.6369699156223714`, 0.8218929956416956`, 0.8808977613854625`,
 0.43575724866517207`, 0.024751792159432087`, 472.6370271533013`, 0.14270041872793765`, 9.280134575613399`},
{0.042342161544045354`, 1.3013804906014936`, 7.13634807471312`, 1.1105942412761596`, 0.2407240140528013`,
 0.6802475696307242`, 0.0062232822378432`, 4037.4772502763867`, 0.18461914595887624`, 5.716947148449252`},
{0.14720397501884225`, 0.5316247300014076`, 6.683828314747185`, 1.481587653959343`, 0.013428589995876372`,
 0.17720084833285243`, 0.005004553323761107`, 364.08088787611194`, 0.035796657483315286`, 8.51611862140901`},
{0.1926486177557311`, 2.6336525621782014`, 9.444109966651418`, 1.4788802142070587`, 0.6654095862145473`,
 0.16653655494047714`, 0.03828733327035071`, 2433.4281263020134`, 0.12844440731237916`, 4.149230973672752`},
{0.055762854118976635`, 2.4026093631268655`, 6.857734870811047`, 0.7509249944976302`, 0.22671157472868875`,
 0.4788061070906606`, 0.1564030997457896`, 55.02740807629177`, 0.03469990388231514`, 7.492235364197862`},
{0.05296847355619744`, 1.6490117913903575`, 9.992115392915188`, 0.8066960527418563`, 0.1291629696144665`,
 0.33931322884091664`, 0.26435083615790894`, 660.4050522121521`, 0.10186522527726305`, 2.8696181659920086`},
{0.21365538200916506`, 3.0547845209089406`, 0.35609106729359574`, 0.8256521864420123`, 0.9506371395328554`,
 0.271042805312945`, 0.12766968769895812`, 68.7970381277751`, 0.05673347586404265`, 3.4610065999125617`},
{0.22385277301638717`, 3.365536903395271`, 8.935305056074029`, 1.445762062116566`, 0.43390325179453604`,
 0.6187135527847307`, 0.01939946413637202`, 453.1242214832122`, 0.13325485950688953`, 8.825528492983036`},
{0.26129739321020845`, 3.355909888714172`, 1.041845959949887`, 1.206440545734901`, 0.09347792890054829`,
 0.5333101115239811`, 0.015503351594481202`, 579.4832207194656`, 0.24065061389170217`, 7.475999528371727`},
{0.14172561423935526`, 2.161658858177276`, 2.5726304321806133`, 1.1364876244324849`, 0.9181535957758225`,
 0.5861373014149901`, 0.010418800942482771`, 101.70999367656657`, 0.1500483081169689`, 9.64604938537483`},
{0.06568219588241164`, 1.0305120784213448`, 8.720293141386708`, 1.4160313463775192`, 0.752508918071946`,
 0.31681781371940887`, 0.13417309715525413`, 209.13734568668048`, 0.22402731709711876`, 4.416848552940577`},
```

```
{0.27044630440719625`, 2.4698840490073675`, 6.955264919348561`, 1.0249819835130385`, 0.106623120735865`,
  0.6358905088319218`, 0.012311596840628138`, 346.23093318344644`, 0.07401305589494472`, 1.2937187987733016`},
{0.08403118541616988`, 3.378337092807227`, 2.524635889786053`, 0.8671019090417934`, 0.46085963082622694`,
  0.5847463933658021`, 0.04733165775893332`, 1030.6526695312073`, 0.10944158368617352`, 3.930070348109142`},
{0.11975601650409934`, 1.1540823534574054`, 5.101398975621729`, 0.9964575213162732`, 0.8596230582994027`,
  0.5017711629445061`, 0.09812020963113087`, 54.445260820005984`, 0.07947868464718594`, 4.273753987333478`},
{0.26353274820980593`, 3.2715371213501925`, 8.419438906869026`, 0.9426613525835754`, 0.1294979869931494`,
  0.5387601140015186`, 0.040849691062558216`, 744.2937861154428`, 0.1766059692159136`, 7.8822033078787435`},
{0.15837784563387913`, 1.590657770632209`, 9.4421417173358`, 0.8199017044479102`, 0.629826423052569`,
  0.2661387442145513`, 0.049173845467465206`, 187.50695430622352`, 0.06908425180315825`, 9.722920562989135`},
{0.18489841859634304`, 3.5401138358224227`, 7.262892362437768`, 0.8851173674042263`, 0.05104398233694862`,
  0.6826543300991057`, 0.13384148697112022`, 730.4506518091381`, 0.22533987085622137`, 8.188627124718394`},
{0.22773890477301867`, 2.1437195677585157`, 6.152335866192775`, 1.1180903919170035`, 0.02705103803804154`,
  0.3333465628704615`, 0.009170207790258475`, 2321.6700090659583`, 0.14789384405564093`, 7.237611958554762`},
{0.17605039997850958`, 2.727076411741753`, 8.728585319721596`, 1.1320082850991182`, 0.7355770210071702`,
  0.5402004706661186`, 0.09745706159326789`, 177.96581560566344`, 0.01922037387361028`, 4.980887347403458`},
{0.06947420594938447`, 0.8077718165326004`, 3.821443319804267`, 1.3891202366711977`, 0.6756008025235485`,
  0.23550127195203518`, 0.0653335798785588`, 483.3383501610001`, 0.09612644227220152`, 2.381497911922425`},
{0.23569362997389992`, 1.2270876586626818`, 0.9845589085102358`, 1.1982217261670929`, 0.43286649637666286`,
  0.634774301600445`, 0.09976341665112172`, 228.04937393006952`, 0.12724994607105566`, 3.0426000813607676`},
{0.12274586224015488`, 2.0471402784080235`, 1.4612976870222134`, 0.9456822010625827`, 0.8327879461898879`,
  0.22053943969717293`, 0.006767755909702022`, 174.3096643655477`, 0.1850505728558421`, 1.2537872990507584`},
{0.13491824188855928`, 1.669720674179887`, 1.3334401828831126`, 0.968437561771696`, 0.34828727193756603`,
  0.3135712369052611`, 0.22891096239253717`, 1910.612731597179`, 0.10930115833371806`, 4.270908360205038`},
{0.04199865921066254`, 2.754174936868967`, 5.53210979521042`, 1.3844956478579449`, 0.47136731805345633`,
  0.24068198696313148`, 0.022972136801125642`, 58.648848297948106`, 0.2240134714991286`, 4.479259216921056`},
{0.18074106663079648`, 0.9554759541683486`, 8.202625386518609`, 1.4769540911095849`, 0.5922233662884384`,
  0.5544055394726339`, 0.15090969809745627`, 4632.951795907658`, 0.16872394703920557`, 7.933658625009354`},
{0.22308930012837075`, 1.8877770902637643`, 5.727481376105105`, 1.290937410786533`, 0.5385704303274257`,
  0.17837528465279628`, 0.11591468148922064`, 2665.0058694088225`, 0.1309938954283596`, 2.3264420617360244`},
{0.16528939075200133`, 0.44637388983991233`, 6.000554224390708`, 0.7620462868306999`, 0.45157540745748825`,
```

```
  0.6293554847029814`, 0.08179042637541929`, 626.5470719450981`, 0.11922182895709121`, 4.5474321331423475`},
{0.12057640522465035`, 1.6899679349832946`, 5.872613897455656`, 0.8685022109314445`, 0.5819827862907108`,
  0.45575489869095254`, 0.005408595604133131`, 1249.3305456098487`, 0.22090492300977427`, 9.765554151588912`},
{0.26839288378136816`, 1.20431418513886`, 3.4264235210106797`, 1.1464020290887187`, 0.9129873318113406`,
  0.17958510842302844`, 0.03476022275277427`, 85.14929883968256`, 0.03637248443077806`, 6.177611921122705`},
{0.19312844950593372`, 3.9129912832786564`, 0.42277195731844414`, 0.8655327062249413`, 0.5847393470300801`,
  0.16029168895981472`, 0.1525854675774217`, 2234.1231120850057`, 0.03649943574663328`, 5.234397199033289`},
{0.05831506452479063`, 1.4447664488109728`, 9.399667906542401`, 0.8838396243177019`, 0.4494233408794772`,
  0.5909311609620335`, 0.2085880113243902`, 309.3416067223959`, 0.04816991376818669`, 2.147582543132131`},
{0.23965113012644185`, 0.9128974489725401`, 8.261297062012524`, 0.788417407034967`, 0.8016641172773833`,
  0.35686628470413617`, 0.030635478621033968`, 346.1316581378353`, 0.043365488693742626`, 1.9545026373639716`},
{0.20520507007680205`, 1.512442754504547`, 9.386452795492342`, 1.0402158263719963`, 0.9284696718653804`,
  0.3818637796049891`, 0.053241178903524394`, 3017.2611079460976`, 0.15062298010038172`, 2.3864513615272553`},
{0.20853248950244857`, 2.7801356391066916`, 9.335557605849111`, 0.8182981643225358`, 0.24140252954455388`,
  0.3825831953385048`, 0.013327430792399009`, 77.96529661289064`, 0.07323986754942219`, 3.7008101112034457`},
{0.13576335201419282`, 2.033104701519708`, 4.843203210944571`, 1.3428754702553487`, 0.2671111595179354`,
  0.18962864690949777`, 0.12183777475102511`, 1231.483599580866`, 0.14101782654161166`, 2.1508880203421494`},
{0.25963449652183795`, 2.4481737185281203`, 5.334705083491999`, 0.8989699854247848`, 0.7130966522497681`,
  0.695912989145927`, 0.04220235704717508`, 140.4631283114693`, 0.11660454588716207`, 7.56536482611441`},
{0.17509339161516935`, 3.0306683644318824`, 1.9789343166711684`, 0.8960196738550836`, 0.2152496986108292`,
  0.6250130758758401`, 0.02087454134280109`, 59.51435674676293`, 0.2018230747082378`, 3.546682887992379`},
{0.10593857456933181`, 2.955002391424368`, 8.212267239719637`, 0.932578721051657`, 0.3205115590912122`,
  0.6246255275862143`, 0.01773467921096997`, 102.27924186693049`, 0.015490300413002644`, 6.09864277306975`},
{0.10583476133380088`, 3.0591620947997713`, 7.753926172578531`, 1.2866948671665401`, 0.7481949494801159`,
  0.21582592530952183`, 0.02844379415675546`, 101.39583001744663`, 0.09278999192622717`, 1.6976300926057526`},
{0.23086787628538824`, 0.6622997055352342`, 7.183940408138384`, 1.353990838360309`, 0.25975387914696335`,
  0.6772244715959861`, 0.055054708296352145`, 683.0312933373987`, 0.1367743885435413`, 6.244797760097583`},
{0.20388536464835005`, 2.2276602475557015`, 5.0708546023033385`, 0.8827779974574592`, 0.1554825605038257`,
  0.2618026330626365`, 0.006155066765134505`, 1063.8834649875878`, 0.12744889948308613`, 2.149276150894316`},
{0.10738337044420926`, 3.7501964356372497`, 1.0776334746380822`, 0.9682422375974065`, 0.09998974406674432`,
  0.504170653500009`, 0.22038799040453166`, 2051.555691372483`, 0.09521018879793502`, 2.016584418866474`},
```

```
{0.2588804970943782`, 0.5492314132783744`, 9.644794872912858`, 0.9613308813426055`, 0.2817033035077452`,
  0.232857138958534`, 0.012136672860729404`, 78.78277994386292`, 0.023082056754425706`, 4.302644367094656`},
{0.08494913534264076`, 3.4349155581389494`, 7.0407634051690415`, 1.119369452553694`, 0.9329533821244145`,
  0.5630698272627359`, 0.4838120213175008`, 71.45974855722541`, 0.09271025542437755`, 2.443770975165119`},
{0.2312360951475228`, 2.6472972630846083`, 4.924168333426426`, 0.8758299082609615`, 0.33581096057577486`,
  0.16094039113701897`, 0.009823651093858611`, 148.81624524939704`, 0.12101804849376735`, 7.0744188173362765`},
{0.2359361250604497`, 3.266770315195857`, 7.411396165562348`, 1.2275925063587856`, 0.15344120088912327`,
  0.536013355113413`, 0.3013372435966537`, 712.0245065436816`, 0.039861802547139946`, 9.042443794820379`},
{0.26580884355261647`, 3.957059703696273`, 5.486829732050921`, 1.3210508471658353`, 0.3282573265157698`,
  0.6164824430541005`, 0.042206581416411024`, 114.97331259087073`, 0.22266675421103638`, 9.071401748075406`},
{0.24965572729559277`, 1.088568489897936`, 8.460543344934337`, 1.1505850947024536`, 0.8975822933983539`,
  0.5110185880609677`, 0.06613687599714729`, 1096.4114980052268`, 0.1003107894781316`, 4.101976455241168`},
{0.1942611669549973`, 1.9123387865104826`, 2.4307010631404715`, 1.2953041584793583`, 0.36588469810004653`,
  0.5550679926918506`, 0.01452356295191177`, 246.06581986793796`, 0.04070230178651557`, 1.4358727366404163`},
{0.20522561065351508`, 3.886130079640968`, 5.862633336878224`, 1.1071510042707493`, 0.46587070079395265`,
  0.3092363340879616`, 0.06237106286442892`, 3704.443052142306`, 0.23732711327647033`, 5.5948716983231535`},
{0.24537724051627174`, 3.881922170050893`, 2.190905545388226`, 1.4415825883984728`, 0.23029515887672103`,
  0.5607538040686315`, 0.02143037658176177`, 612.4231659367224`, 0.18957440226421673`, 2.1066205948825285`},
{0.06117754469042569`, 1.77753347893454`, 8.494748688583062`, 1.4528026068281314`, 0.13374021962928717`,
  0.2747744172973582`, 0.02769785108344557`, 761.9216006517844`, 0.1674213538626742`, 9.223974549251146`},
{0.2543463901897882`, 3.8184481545424687`, 6.435093510144283`, 1.0179149591357712`, 0.6456387658124723`,
  0.39719113288117625`, 0.03560663844887868`, 836.6099873310699`, 0.05172688927212099`, 4.4940967912431145`},
{0.25340300959745826`, 3.7481040027914663`, 6.564583042000866`, 0.9665595115932711`, 0.6900722172611409`,
  0.5320596915161446`, 0.007953381223150922`, 557.3521763213881`, 0.16633866213427612`, 7.2685179596650595`},
{0.21905085798311597`, 3.1393917093979224`, 6.344370247647537`, 0.9036844621009067`, 0.45657431516696256`,
  0.6738956741194089`, 0.01713634873689236`, 595.7124001741428`, 0.1327223790362297`, 6.393427116779321`},
{0.26756748312672074`, 2.582420366138944`, 0.22100971269994044`, 1.2986139421663903`, 0.40694219440887625`,
  0.4206092167171217`, 0.07540978051777308`, 286.7043124505423`, 0.12484831726628037`, 7.450517421109604`},
{0.1649279191778415`, 1.2798961157951103`, 8.443353077105307`, 1.1073246765756934`, 0.5543130008347743`,
  0.4677559431287117`, 0.04549043803104127`, 88.19757502008439`, 0.08859698434873292`, 4.4693006714200685`},
{0.24671033429293737`, 0.7439602453864245`, 2.8830481632165945`, 1.0433060628070765`, 0.2605457162416671`,
```

0.35613575939670206`, 0.02699949405773168`, 552.8129873973498`, 0.15941770646667952`, 8.472285283746757`},
{0.15869950794106824`, 3.1033559606377414`, 9.144330738839969`, 1.4178115100024233`, 0.3048291795319098`,
0.2869274730275907`, 0.17534043095376647`, 52.531250426358234`, 0.14088030152015352`, 4.046496400392222`},
{0.2764621058346162`, 2.9381918751992178`, 0.9446276562373105`, 1.4121699137634485`, 0.5065538844804354`,
0.6085910850890284`, 0.12806524333884275`, 90.5764532701865`, 0.169606806379912`, 4.122343563126204`},
{0.23050133288232844`, 1.3238077162346595`, 3.1108482993453865`, 1.1555015486294062`, 0.6747942262463269`,
0.6285668584610278`, 0.027391537177886824`, 109.70658223761104`, 0.027210691652561736`, 1.9248854769177905`},
{0.16511179970471734`, 1.257711050374506`, 7.3270141910702415`, 1.3219934358839476`, 0.5519839493462388`,
0.6927265248357952`, 0.057669326830527015`, 282.0308127392517`, 0.18961708461999577`, 6.119113848125975`},
{0.18051596911200918`, 3.1154638731836117`, 5.271301524292921`, 0.9821224785779399`, 0.579876513557519`,
0.2578243252832043`, 0.01324703728258807`, 298.19257336110485`, 0.04626579120349833`, 3.252038865320179`},
{0.2700603276661351`, 3.8874233694867435`, 9.935191315459868`, 0.9369398631417749`, 0.9680175802467335`,
0.2817307366148821`, 0.03116132832319949`, 267.3428425281449`, 0.2310886914635361`, 4.719763737676903`},
{0.2505990837413898`, 3.9386610341401305`, 4.197732331385515`, 1.4863985983426793`, 0.6450186838764853`,
0.5983146588433161`, 0.0058599277111039`, 4347.60789332823`, 0.1797443484893408`, 5.002549103667693`},
{0.15385799276986173`, 3.328452062918277`, 1.1907303286310018`, 0.9499432416831306`, 0.24618506024325426`,
0.6220480490433855`, 0.08397939385457624`, 160.09510109398894`, 0.21511864519397922`, 7.34323009363505`},
{0.10621732051626104`, 1.365898117434102`, 6.864395407461604`, 0.9825773772303991`, 0.8367483775504436`,
0.5624371522540671`, 0.07041397408927531`, 60.72834180417396`, 0.20758521960363197`, 6.929584578355666`},
{0.2268285366732684`, 3.377330494378361`, 0.792106071900994`, 1.4321394301198889`, 0.4771428999999012`,
0.6107585820948693`, 0.0427110994331284`, 2178.207920314213`, 0.06460930790004932`, 5.987666522418898`},
{0.27738294555080567`, 1.5184858354681863`, 2.5078605202307482`, 1.4658418288357904`, 0.7951974935096349`,
0.3102987260459962`, 0.028082255793227213`, 4738.743053997216`, 0.039514889508313555`, 1.270204126939964`},
{0.12722141666194153`, 2.144800891916389`, 0.9267204145449971`, 1.474327309271881`, 0.07507296673368935`,
0.4110764361383803`, 0.01396212306727472`, 341.96044988390173`, 0.08796076167353961`, 8.378052186889676`},
{0.14199823373416764`, 1.4769393574961134`, 9.82023825383516`, 0.8826223667346553`, 0.41403084809983204`,
0.6165765653923829`, 0.060760206852004485`, 76.65984772713675`, 0.1884299459763023`, 7.821991164404671`},
{0.2704769900712498`, 1.6373927417773038`, 3.0886177841931914`, 1.4817168381054997`, 0.05399471731043759`,
0.4806448506103316`, 0.008090658829532001`, 56.76295821829396`, 0.22108849119145935`, 4.3771215003685064`},
{0.15635355369607173`, 1.2350233440589857`, 3.2938536959067304`, 0.7696047462951592`, 0.9490314349758091`,
0.5590905433465692`, 0.008743912631106248`, 94.37874467585033`, 0.17474749461052458`, 8.353315585312398`},

{0.16339273559725687`, 2.0059391082075457`, 1.9960227683476044`, 0.8832597488866586`, 0.2201232888636404`,
0.41940637033719763`, 0.260701203173729`, 668.3313383270491`, 0.16081430237854888`, 8.573990161395482`},
{0.17131668518162624`, 1.9273467762184806`, 5.930228637778157`, 0.8979121960709446`, 0.761163722566164`,
0.6467717629026779`, 0.01019608706680365`, 260.442341207733`, 0.11039361670332914`, 7.204688135978646`},
{0.11942926109779373`, 3.6640895220352094`, 2.8978096019149504`, 1.1395608488550686`, 0.02531057599926112`,
0.3982329172386545`, 0.26228943719870584`, 89.66215495367044`, 0.22730124409898161`, 8.643250664580492`},
{0.06995397995088021`, 2.4465420953300523`, 1.0825256418482638`, 1.4045898231548657`, 0.8050454938859057`,
0.5373350569389552`, 0.3667462640330922`, 406.36701228872533`, 0.024784828878876453`, 8.741309230400322`},
{0.25135395656732756`, 1.8293827828852027`, 0.6280251816297326`, 0.776655925877336`, 0.712342015777852`,
0.6529270906121196`, 0.026921744082008515`, 2433.232128346219`, 0.014846384799260759`, 4.779446799164496`},
{0.22686431298761822`, 3.544454161776195`, 7.257561779040259`, 1.4835566223233323`, 0.41530778886586006`,
0.5693420006079292`, 0.0281206594086524`, 300.82869963510313`, 0.2233455291441253`, 1.8496714258079727`},
{0.11505780978488611`, 2.035276516222745`, 0.3473218826840405`, 1.3302263404025527`, 0.8863987944255065`,
0.20525449010517516`, 0.166947674879067`, 1822.5924091420022`, 0.2368153750944742`, 8.635263957438244`},
{0.26450836027154073`, 2.000822664278666`, 6.699190267572199`, 1.2017845913999912`, 0.3438445280396565`,
0.6571515800951808`, 0.025420529665258214`, 670.8719376728277`, 0.0689542698910992`, 7.858447976519758`},
{0.1895156330025849`, 2.3307833365396586`, 5.573081915828208`, 1.037167544233136`, 0.6437280244785568`,
0.6985848360999776`, 0.020283589241000377`, 257.16539910388553`, 0.09483519874959423`, 5.443513900146426`},
{0.05605442916152037`, 2.091827585999483`, 5.134093728882359`, 0.7852275512421112`, 0.9528052242020535`,
0.26916872175155115`, 0.16382211865694157`, 830.5018741809612`, 0.0523186960509337`, 8.350099727317033`},
{0.160097742020257`, 2.0916642582432887`, 7.779572730473025`, 1.4293729759208196`, 0.262481852877007`,
0.6066507214539851`, 0.010299825795425889`, 166.88640436294955`, 0.11119499215436468`, 1.1831617215189443`},
{0.096645259209576`, 1.264318941007299`, 4.097121191163998`, 1.119171761441141`, 0.3848067085465605`,
0.38298305619587747`, 0.22103546881327146`, 186.61459180270506`, 0.035388155328102466`, 2.6745014933563738`},
{0.08994789013179427`, 1.57009542864993`, 6.7950768142652755`, 1.2471941772808341`, 0.17502080201658954`,
0.4646155607499266`, 0.031711372215814826`, 510.77004725104206`, 0.22094423048581935`, 5.498912040996139`},
{0.09559160148361812`, 1.3237772094663507`, 8.951678422145957`, 0.7806574543205589`, 0.8827653082752778`,
0.45488871998545977`, 0.011151733542884613`, 3151.7776331194414`, 0.22534056788365686`, 6.821338260162536`},
{0.20804533970301103`, 1.865488871682949`, 2.4561594295175926`, 0.8614398462681598`, 0.7232950760420711`,
0.3427980526897182`, 0.02113398228875948`, 356.75904569388376`, 0.23964513558905903`, 4.163032360670947`},
{0.23422673966465207`, 3.962742244147649`, 9.344278992351082`, 0.9436428211653483`, 0.6202800294740252`,

0.15583995298414144`, 0.0468134491479599`, 1145.0934679847246`, 0.11614669946806178`, 8.273469182440888`},
{0.23911413452544644`, 3.701379132317136`, 3.3596187018850023`, 1.2070387760959833`, 0.14815711868787962`,
0.5552188532365824`, 0.010060549211211993`, 324.2801725236705`, 0.014653386006449387`, 6.641284984634726`},
{0.16379493488608782`, 3.773301948253276`, 6.66686177778092`, 1.3720180958436647`, 0.9115747518300852`,
0.36809943749279594`, 0.006089940921734969`, 188.41929546062013`, 0.11766502525697531`, 3.8904832664355844`},
{0.27173801863077446`, 2.1930338431922545`, 7.869758966833125`, 1.0214571559787395`, 0.457397629072652`,
0.6590886785471264`, 0.009120521470678952`, 4004.0778102470904`, 0.1596904202504179`, 8.692703920363371`},
{0.2361473928644357`, 1.7414292069064263`, 7.944932185587515`, 1.1578150203530142`, 0.6270752096378571`,
0.30275674150027165`, 0.11839453780639704`, 3486.2324040163485`, 0.1006900893181421`, 6.697770970769804`},
{0.08448906159522324`, 2.163318965633623`, 2.5852320656449397`, 0.9870547358990118`, 0.27911648709335135`,
0.1950863385031143`, 0.04472304557991584`, 1858.3083947017237`, 0.05117109151128901`, 9.531470241589851`},
{0.1142146683211071`, 3.422389653131356`, 1.8177299602895634`, 1.2969315528452774`, 0.1391750354375907`,
0.5170758706651055`, 0.01897326911835137`, 87.52693577556593`, 0.11000871045324723`, 3.4664980752399295`},
{0.14347242790835574`, 2.011431525472254`, 0.8946353742157331`, 1.3818589873326788`, 0.974530392330166`,
0.4363768778472087`, 0.006586014840232095`, 142.93650600689625`, 0.2189385420017279`, 6.889239014732425`},
{0.07350179944910268`, 2.001951652427108`, 6.856579719843172`, 1.3883617808830775`, 0.83650706130597`,
0.6246458892820506`, 0.03443555932639553`, 159.6804633773726`, 0.22561794329975743`, 6.769156049603524`},
{0.2038713609791254`, 2.9048730428426977`, 6.578839990238183`, 1.1970767733981913`, 0.3047670438201835`,
0.4988995484689318`, 0.027219703475022124`, 126.68486054839573`, 0.1069997024432573`, 5.709865936189522`},
{0.10518373092933642`, 1.253339618555911`, 0.9435760784539173`, 1.312750209379657`, 0.30938419913876847`,
0.5412461470886345`, 0.010398682812166098`, 560.2137041073427`, 0.038740677468453555`, 9.486718448691846`},
{0.21800976245075693`, 1.265555352818649`, 7.66080710349685`, 0.9976040672991493`, 0.42066904207729006`,
0.6052137627060103`, 0.1434637338398527`, 296.0621032273413`, 0.0508815854649306`, 9.05489394101457`},
{0.14099839999887143`, 0.709118554874764`, 1.895334494340693`, 1.0269112445912816`, 0.6721979672345315`,
0.244304954855444`, 0.0716581941489167`, 50.83934571528565`, 0.0295628703988986`, 3.2095234353654245`},
{0.16175267254619935`, 2.3816849613140647`, 5.017466007223192`, 1.1449585227321033`, 0.0709392075018902`,
0.21629140918390133`, 0.24748736884470657`, 967.6089395954832`, 0.2384053881049194`, 4.566103496741098`},
{0.15219039437367055`, 3.36973706117323`, 6.4489465173864`, 1.4529455229052564`, 0.15905243014518256`,
0.19120675078037952`, 0.09107249936889779`, 1785.557342737041`, 0.07266026496560668`, 8.481021831493972`},
{0.1147282820108807`, 1.275793939396646`, 2.8627986987042053`, 1.2289286563020643`, 0.9300053983658252`,
0.5333937455451996`, 0.40966617997380966`, 55.29536930365472`, 0.09207723861615985`, 9.133686457942215`},

```
{0.22214055647639447`, 3.77956922277194`, 8.844837651533133`, 1.3011494481281731`, 0.35962071678441987`,
  0.36854620389209614`, 0.030699018263752917`, 153.2818434470183`, 0.18511332787655327`, 9.937679092387768`},
{0.11158828176697494`, 2.207019844443386`, 8.337407964588238`, 0.903037166955644`, 0.0492331295750732`,
  0.5332115600383506`, 0.10225753888572825`, 270.6598424430625`, 0.11536791182647016`, 7.224089837931905`},
{0.11228066784233581`, 2.8383409912516155`, 8.777526606092515`, 0.8243326677721828`, 0.7215210925398621`,
  0.47575335568293875`, 0.01179892034723288`, 1881.3977477117185`, 0.1350202375088484`, 6.891832227766253`},
{0.18415971316226493`, 3.627641157385548`, 4.072786596426541`, 1.1318163513169097`, 0.431227341960456`,
  0.35638232246082124`, 0.12315140656794354`, 98.11903649760767`, 0.20630818704214687`, 2.4221856850573946`},
{0.2708373028347817`, 0.6873063866109219`, 4.963135106708849`, 1.480469510663712`, 0.1951402154410362`,
  0.5673083531043152`, 0.10423026212852673`, 2550.63829867399`, 0.08286966503737198`, 2.6901867167370597`},
{0.2248876472394919`, 0.40722939312005924`, 9.89488647226247`, 1.363579526182362`, 0.9039905300116995`,
  0.6204673017769224`, 0.16140635189013572`, 124.79693831214993`, 0.1938029988590731`, 3.2922153328074497`},
{0.23989116476268346`, 3.6056249656972357`, 9.989783431378257`, 1.4358686490541388`, 0.7020019529209951`,
  0.17871498413227982`, 0.15143540034751335`, 1116.6227738463333`, 0.03611482374706981`, 5.098351562607867`},
{0.10260002089245757`, 1.5297774189947768`, 6.3178804183723365`, 1.026166177715942`, 0.11867434322817161`,
  0.5793673959868294`, 0.011727656957059851`, 115.5782430006307`, 0.20678944231462698`, 5.80216317155719`},
{0.15334449354379004`, 1.7605906069617427`, 2.299359928311352`, 0.8222860702278779`, 0.9712651951139977`,
  0.6108690739001781`, 0.14744839931335676`, 2744.2788694525566`, 0.1958145599491105`, 2.932280984329149`},
{0.19466496130115485`, 2.7024357312795884`, 5.257508792652555`, 1.2867770062917014`, 0.4348182682381361`,
  0.22799121214772633`, 0.23891047775514818`, 1782.3644894856986`, 0.18533305313279363`, 5.119329477376578`},
{0.1432020102164725`, 0.6405991610607145`, 3.208206356969493`, 1.3290628649451517`, 0.9481170196997712`,
  0.4490165359974049`, 0.04841648328555665`, 413.10755212325614`, 0.0475062671126473 8`, 5.496555870600891`},
{0.2767015708016651`, 1.3448440647834508`, 8.399713367158164`, 0.7521061389207052`, 0.4173808724493866`,
  0.513702172075632`, 0.342860965595718`, 77.17931109285855`, 0.021177395775830554`, 7.103551277505744`},
{0.2397400236888016`, 3.6266796947004982`, 2.6465237726626594`, 1.459013465973801`, 0.6743470274003898`,
  0.6381986185617847`, 0.04750041433271958`, 90.95305912243636`, 0.11079011330617267`, 9.85823320912452`},
{0.10671756328879578`, 1.1279363098789865`, 5.068949642759208`, 1.0745694995138004`, 0.5897325137042213`,
  0.3714383645112781`, 0.14529484820960328`, 91.27351773968599`, 0.0653436372538082`, 9.206661650127103`},
{0.2358453438636856`, 3.7679680180462416`, 4.640233719273176`, 1.297156455219631`, 0.3876479327909328`,
  0.3193535012332763`, 0.027047144947079417`, 401.3110605008155`, 0.0397014782942956`, 9.539243086069693`},
{0.07226742189042767`, 2.8421547517397343`, 4.74887948268691`, 0.9400141356818887`, 0.26271764199006165`,
```

```
  0.24510878200647013`, 0.011565327673117277`, 79.9069988164106`, 0.11613362579809078`, 3.4810370415732024`},
 {0.19794910251471837`, 2.250622511000005`, 5.810822485387694`, 0.8325726726239642`, 0.42888216834124404`,
  0.3003167364214152`, 0.2221971352698835`, 492.4997381844193`, 0.06654773222193389`, 7.816458771254976`},
 {0.23749910377351813`, 2.442156143626838`, 7.627688188064528`, 0.9684475529781722`, 0.7107198130457415`,
  0.2572554624659992`, 0.4475955253238462`, 132.3529645103848`, 0.18259209603279808`, 1.6799945249508887`},
 {0.06924882755772316`, 2.8414116897854322`, 0.5206442594268132`, 0.9917884594202446`, 0.6526760474283868`,
  0.5988068411525457`, 0.47108712727434415`, 720.0807499233221`, 0.11578003411625926`, 8.400733392823671`},
 {0.06824086699485615`, 3.3015665983523297`, 9.09230580657946`, 1.0780523992371156`, 0.9189494010575767`,
  0.47997916674377217`, 0.09047840499022508`, 3815.186560881826`, 0.15232905773407`, 1.5202594192596113`},
 {0.23257418904935773`, 1.8011483887717636`, 0.9612178256197179`, 0.8025101750942898`, 0.31352490932039023`,
  0.2355458085864931`, 0.2680725991098069`, 2738.209295799684`, 0.0662676878204258`, 1.8613360761320532`},
 {0.13474024315490335`, 3.3704964680175626`, 9.170639775134308`, 0.7502511885050491`, 0.6264710065934431`,
  0.21904625510680875`, 0.040564870634629845`, 2527.7664803980215`, 0.19403763861714918`, 6.378419170081072`},
 {0.2693026298173556`, 1.772983767924213`, 1.344226083033309`, 1.4113484218782142`, 0.12593618066908396`,
  0.656520191233777`, 0.018659842469751033`, 117.56506476136576`, 0.1712196470744854`, 4.023445632685666`},
 {0.17866994815918202`, 2.442046867777872`, 1.8194825512463648`, 1.2285671890230836`, 0.7081083864311548`,
  0.17566313743095552`, 0.006280075202364708`, 2604.6083474637735`, 0.1295692924324935`, 1.862340891527415`},
 {0.26090114261485403`, 3.9086029270457496`, 1.920295645800332`, 1.1838686314265923`, 0.4729800090210985`,
  0.36383922429133153`, 0.014536748208445867`, 79.30674088085132`, 0.030053561477188684`, 7.670997166386538`},
 {0.19544602681310375`, 0.41028512203974055`, 4.6003645589109095`, 1.2671666166106164`, 0.8219059973725353`,
  0.22911191370711304`, 0.03702179299429459`, 318.28074751099626`, 0.09079865705400514`, 1.4662081657512314`},
 {0.26537062770714803`, 1.229239774996202`, 8.744773690909977`, 1.151653436583517`, 0.3537059210373261`,
  0.2894924335103778`, 0.13068544785366612`, 312.2140250313751`, 0.146894495801125`, 5.715731252046433`},
 {0.18712917295151626`, 0.630329154174047`, 6.232729389529142`, 0.7582043174026267`, 0.7607866373637777`,
  0.2530331438958473`, 0.06090476948059027`, 64.21018866863231`, 0.08418997914362486`, 2.694793972266142`},
 {0.19413873235593193`, 0.8072205389170897`, 9.684031161687852`, 0.8518820755271745`, 0.2120317281227424`,
  0.1532879757233685`, 0.02372050181467318`, 987.8329384880758`, 0.08094704090521981`, 8.693316755778081`},
 {0.0816546741022599`, 0.4036812381177306`, 0.7902893788690406`, 1.4273906025641667`, 0.1897112398644374`,
  0.6362443390402239`, 0.007446156154142883`, 176.61944749055874`, 0.10188842005948545`, 9.51311671217389`},
 {0.2491419431624458`, 3.7694376618574204`, 4.74673147811253`, 0.8416284883026488`, 0.188731240527098`,
  0.3192559789027146`, 0.07121130413051815`, 671.9000833119555`, 0.02760758509345429`, 2.5790410082926702`},
```

{0.11168227968802413`, 2.173584861364678`, 5.640951633995167`, 0.9377704673639422`, 0.3315982783471285`,
0.5266083888319896`, 0.005432939117039627`, 621.842609802462`, 0.09631877509870435`, 9.43919460268938`},
{0.15970197279447784`, 3.9587696847183347`, 4.0238996390694854`, 0.8027865138486989`, 0.03588289413016432`,
0.35386710298208024`, 0.016574217538633067`, 2019.1576160982734`, 0.07473946383041635`, 3.5352801788382404`},
{0.2064322311255224`, 0.43645383124552994`, 3.8213880668877356`, 0.8257710590869434`, 0.08868496943554072`,
0.17616539751645888`, 0.04042707492740236`, 2037.3062213977416`, 0.05324630165142419`, 4.6107951341494555`},
{0.1394617695133732`, 3.387614469631334`, 8.352661875580207`, 1.2219251402369191`, 0.6325442693826859`,
0.17624447718949754`, 0.021853560156019736`, 288.68541357569393`, 0.164910064915528`, 5.7766720481305995`},
{0.10599692192016741`, 2.4119661618453145`, 1.4892081676893856`, 1.3031849980783952`, 0.42063329504650815`,
0.5860601104625098`, 0.3077592160131421`, 52.52666428206092`, 0.19647413521024198`, 3.2052596491028216`},
{0.24388484229914548`, 3.2519594625966253`, 4.312340316913895`, 0.9914403572709836`, 0.29358995419342615`,
0.1835819110057716`, 0.13612159308161606`, 1157.8864188899545`, 0.08218618541111161`, 5.046821007659727`},
{0.23494551018662208`, 3.3331099682410468`, 0.9744758817090952`, 0.9989991912838305`, 0.20118476247466943`,
0.3741832660739852`, 0.07385095150097958`, 91.01998469145919`, 0.19994684540705993`, 6.360950891328324`},
{0.23944024111665796`, 0.7499363805955559`, 7.775454975750338`, 1.268235612825383`, 0.3786721827881383`,
0.6740341016595366`, 0.11977329505957622`, 117.53909816274609`, 0.2108164269301916`, 1.8777741577958924`},
{0.24513403447625742`, 2.0133471925570054`, 9.940759001187548`, 1.083812287959604`, 0.5683273089881642`,
0.5900825394859205`, 0.005339720116331014`, 58.9416557608406`, 0.03145644437596545`, 6.28807827028038`},
{0.15958822823144275`, 2.509825654597928`, 3.728427574062339`, 0.9294074209823535`, 0.01651867581778177`,
0.5367648350921979`, 0.015785937236593408`, 685.9822133189093`, 0.020860236554209932`, 1.2753268301578835`},
{0.06806026871424425`, 1.205607754446362`, 0.7536813502622586`, 1.4624982795129167`, 0.7076540801030038`,
0.15470161284122685`, 0.268116863806763`, 324.06126360879364`, 0.036033823716353997`, 3.8267580932938614`},
{0.15319733389695195`, 3.558603911914262`, 4.579050417046558`, 0.9321187332487358`, 0.6642338797631109`,
0.569667348599446`, 0.2858050889038288`, 341.1212503566884`, 0.20343887540165378`, 9.501626804091558`},
{0.15071537712057487`, 1.2850575528534174`, 6.479674501252795`, 1.2297249014764047`, 0.05119696881160426`,
0.17856837950014437`, 0.01824538414392949`, 77.70419036862091`, 0.224107560352308`, 8.31176551046438`},
{0.24584690511060947`, 2.473478204800995`, 6.676188280246508`, 1.4832363218386881`, 0.14420161768506268`,
0.523836460034543`, 0.04402629271492787`, 360.52310897688045`, 0.0850887320320231`, 2.528306265858081`},
{0.14137397451874373`, 3.652676161056461`, 8.39527364337399`, 1.0080745646578813`, 0.09190129083286003`,
0.15081167091134762`, 0.35462309792221747`, 1217.6311173423508`, 0.09724487810479643`, 6.223920941944753`},
{0.04933630800724648`, 1.8511857532828166`, 5.885062761777403`, 1.1138733840213622`, 0.4607664856084661`,

0.43439208322360623`, 0.018761076496074794`, 3507.5800668673905`, 0.21191062303011793`, 3.3573407631201917`},
{0.2339008817663396`, 3.669675022809618`, 9.160989469851096`, 1.2207724907901325`, 0.9659294978123167`,
0.3234992643572032`, 0.05037324961884807`, 93.86107259786391`, 0.14430488254373952`, 3.9894219201696473`},
{0.11569404259913263`, 3.2009969772206803`, 2.2264401820433353`, 0.7539773410821206`, 0.3237735314376855`,
0.22021748910844674`, 0.21956604535044472`, 1445.3927245947305`, 0.06533194774972478`, 1.3139082141801541`},
{0.1426983247121278`, 1.4581176174915065`, 6.531549332871599`, 0.9310047864665083`, 0.893336243428055`,
0.18377286371920176`, 0.08056837661087574`, 664.5727162634348`, 0.04347044784294363`, 7.864371974772275`},
{0.05831687901068116`, 0.732484500007835`, 9.716813224212896`, 0.9062398770267781`, 0.045816647832010116`,
0.25112700202789096`, 0.0489500536916562`, 2141.8470035057708`, 0.06373448098002471`, 7.679073247267311`},
{0.1624041267001165`, 2.3409738007594383`, 7.927758092789709`, 1.1108935833128895`, 0.2655878375527121`,
0.22381693809974978`, 0.10055816102060769`, 86.2207746546923`, 0.23778527923179793`, 1.5984483564142007`},
{0.05796853524365786`, 2.115435321939346`, 7.135826377560314`, 1.1217856002992745`, 0.9949497841824515`,
0.6493572179052653`, 0.16432568991023785`, 205.88622896743587`, 0.07308989915497993`, 5.091714109271411`},
{0.09343457742759681`, 3.0751406184941388`, 6.999926605820257`, 1.4764339149831611`, 0.27064650694064607`,
0.6662559245780291`, 0.012835205507442616`, 1884.4110587763687`, 0.01681160009525748`, 8.661215052575166`},
{0.23943678973010468`, 2.920807454124679`, 1.1099817114095707`, 1.3932739733707113`, 0.44392785132951373`,
0.17254202331513768`, 0.008938503858702969`, 2712.044809794402`, 0.1652368227234951`, 2.8738798121545877`},
{0.176566928771667`, 3.4304813087836825`, 0.9837478487319995`, 1.1028599055297788`, 0.8616178142897495`,
0.24038775568738768`, 0.03874939820930588`, 2380.99712756261`, 0.18505877859540681`, 3.338709015344154`},
{0.23886394100929725`, 3.974888040813318`, 7.275822510559582`, 1.2870566617016084`, 0.12273034821485096`,
0.6651538290269186`, 0.16897919530659566`, 150.6876048176701`, 0.1338282848735754`, 2.432758352876487`},
{0.06973130535662914`, 1.4096929441217387`, 8.65071450995816`, 1.181254696978707`, 0.5406204719849208`,
0.2725259583108167`, 0.12003736669178822`, 4806.091595362366`, 0.20844978312325513`, 8.8784124085225`},
{0.04409294567306185`, 1.3468266911815716`, 8.46560672493381`, 1.1221333539921368`, 0.8074609652543419`,
0.33934404303057597`, 0.1090535991677299`, 2100.6487939981484`, 0.1955313854386307`, 6.974828032327105`},
{0.08301734541584582`, 0.4950033643125962`, 0.3447103403996028`, 1.3973171280993089`, 0.5174429014299606`,
0.6090072510200681`, 0.013328621450736109`, 78.08408450957157`, 0.15235476028821748`, 7.41363923833616`},
{0.07508111728102967`, 2.9328515590120503`, 2.9247828964602327`, 1.0997610261500241`, 0.15381049135387959`,
0.44875941041801015`, 0.005374403719665632`, 955.806314296473`, 0.04180941570568808`, 8.210271230243798`},
{0.1868411586163749`, 1.4148901359883457`, 3.2886483363036496`, 1.1024292265597047`, 0.35071605335062417`,
0.20216786873751635`, 0.033694215571138546`, 50.84055962824541`, 0.13756443231160648`, 9.762651891575405`},

{0.25728291653412544`, 1.1620186239014583`, 0.48798465750503445`, 1.2217305465551163`, 0.36537296293338284`,
0.2112385814144826`, 0.1783844678719564`, 175.70497466833672`, 0.1488528693185936`, 6.164090300210196`},
{0.13759840983832694`, 1.6824227355669867`, 8.881204796952819`, 1.07728991023014`, 0.8275568317133928`,
0.2913709338376398`, 0.47392581861361016`, 1028.758263171597`, 0.1629375199726112`, 7.630724789672264`},
{0.22999415874686646`, 3.253135821240728`, 9.26678168881324`, 0.8937302072810551`, 0.5038748821521797`,
0.2035592770142407`, 0.010501376095491947`, 229.44271937909937`, 0.1496315114369897`, 8.069758108498657`},
{0.09925066924077935`, 1.4815445119155024`, 7.881183919866995`, 0.7663286317492166`, 0.2976933875506338`,
0.33062028037546887`, 0.0510585428499101`, 168.2571992844445`, 0.22488226539851913`, 5.574589576514775`},
{0.20983327896511272`, 1.8859920859197894`, 0.6151282764886545`, 0.8412663476815937`, 0.027122934645615437`,
0.6844735714100607`, 0.01254007978155685`, 549.5308557472557`, 0.10371695352022153`, 7.780558961111437`},
{0.2225389666164696`, 1.2827559929647245`, 1.2157460525678818`, 1.1336603904726232`, 0.21910751958531538`,
0.6021275716391656`, 0.016887660465617623`, 151.92995003475355`, 0.056506174885467386`, 4.721491520685097`},
{0.23629660338151143`, 1.375829581220887`, 0.7878883261410508`, 0.9498849347602389`, 0.6605330073956415`,
0.33733697402858687`, 0.028977316405205664`, 446.06430846982937`, 0.16303388369839966`, 6.718018447521894`},
{0.2240884195389573`, 3.0168075094859823`, 9.22253837134286`, 1.0534395436787098`, 0.7253455428103461`,
0.1646055270291652`, 0.006115122680716056`, 258.28718729642685`, 0.11665954601209721`, 2.992281031623701`},
{0.04820076342563051`, 1.9965427255473651`, 1.4646173572921608`, 1.47567620466389`, 0.346581356777814`,
0.35258214744905925`, 0.16114708977216574`, 53.64321165141119`, 0.194512100843611`, 8.557828905511698`},
{0.1395588171486265`, 3.3167971972296737`, 5.120579942113229`, 1.4414502422836475`, 0.46235265068089726`,
0.21590338795839814`, 0.018401804903068468`, 321.8138540224943`, 0.09243860589675884`, 1.0657915725386573`},
{0.2085065587668823`, 0.6294064238759702`, 7.1150002800734775`, 0.8698405330489325`, 0.07589384997930004`,
0.6271818704466325`, 0.014863365323890152`, 3717.018925299029`, 0.09558183779388002`, 3.993723504265942`},
{0.11579616742063126`, 1.4637518716217537`, 6.091821149634162`, 0.8701973607843327`, 0.580668178059593`,
0.4892081276931839`, 0.43069272205186815`, 58.104542288448926`, 0.09639134486345691`, 1.0613512257562903`},
{0.07650954660329923`, 2.451622188741575`, 3.5124331635719983`, 0.9208132913481966`, 0.22417245589301404`,
0.5962277485405788`, 0.1631058588288524`, 149.68967360762312`, 0.15724921240628176`, 3.991974201007654`},
{0.27715040216901043`, 3.1093705724520717`, 5.602120685872263`, 1.4185083740786066`, 0.20116695432127574`,
0.3482455682148583`, 0.014646070094414987`, 268.87083253973395`, 0.19497961893886057`, 8.247252623819538`},
{0.10373802184314534`, 1.9484823746249935`, 4.344672894707308`, 0.8659474233939041`, 0.34328087466446`,
0.6599529078817681`, 0.006604951530776707`, 74.02272771702236`, 0.12108236246583282`, 3.25882057231545`},
{0.08995654352927362`, 1.6673082382989595`, 4.04257302618344`, 1.1477945472560402`, 0.1500209798917984`,

0.1761016570773115`, 0.012888472951564884`, 1050.2473441342943`, 0.2022489804478793`, 4.465566609444316`},
{0.18428208667431017`, 2.663013532673764`, 4.442362759588184`, 1.3723983485913218`, 0.7325310003414629`,
0.5418182340232052`, 0.027460635126683182`, 53.281000517524745`, 0.08334630855570291`, 6.274365305120075`},
{0.1326476876438774`, 2.8731759222440596`, 6.672951038295707`, 1.243076119232064`, 0.9225218753338189`,
0.23536778742386455`, 0.07924576221140303`, 2119.223051638056`, 0.014542489730864222`, 5.05029094558817`},
{0.16449021574582717`, 1.5264131018768046`, 3.118813817198866`, 1.0873075186223824`, 0.9114527670749883`,
0.32623274128336255`, 0.005473446059082923`, 2600.919647538226`, 0.12372800503579795`, 1.4807994816594086`},
{0.1059475440552839`, 2.4614128361434053`, 6.764865578702089`, 0.897041430377437`, 0.5452264302150955`,
0.6377616415175531`, 0.031109305885935246`, 290.7153568287518`, 0.12025877488242465`, 1.854625189562679`},
{0.09376889465512256`, 2.694562234128588`, 9.901749913963968`, 1.2671081853876889`, 0.9355728957697818`,
0.41426328356338693`, 0.1676359905682518`, 324.1423914220847`, 0.048114260882103244`, 6.992923371816153`},
{0.07408607058446992`, 2.173441202450359`, 5.83110243938874`, 1.2671639264693393`, 0.15034130588796946`,
0.26151521374964837`, 0.00796428041791113`, 3349.7895146720416`, 0.06475249420724477`, 9.113413370060233`},
{0.2682300613650819`, 3.6108652762368214`, 9.881761872038165`, 0.9825580258076282`, 0.9296545706074566`,
0.25219322618587503`, 0.024464898005065273`, 1815.4892808719587`, 0.08994453092924143`, 7.425857575199714`},
{0.04962174488386037`, 1.9694465660050104`, 4.880585401279637`, 1.2957318091391512`, 0.6289429156960646`,
0.36977064682826033`, 0.006935918860683708`, 414.38904513183815`, 0.2065379649808241`, 4.444261705692702`},
{0.2594824517719198`, 0.5971319663647825`, 9.453687965953478`, 0.8401327653868795`, 0.7997988949991743`,
0.15899752713279824`, 0.005075100457461465`, 409.49724454586783`, 0.2091478816442816`, 4.600741868044251`},
{0.1975048020318434`, 2.1175350807064808`, 9.527018017184652`, 0.8875453366861839`, 0.2770809005507884`,
0.4437813967164592`, 0.08906290963263506`, 98.18685485298634`, 0.18494734685711195`, 2.236429925751402`},
{0.18592803424857857`, 3.7511626605606008`, 0.2948073517622305`, 1.142160214293685`, 0.24567142070681736`,
0.41248813426514863`, 0.25834870652665815`, 931.542828982051`, 0.012674565140645466`, 9.754712584362995`},
{0.05816505121822962`, 1.2475983837798745`, 4.326003024744654`, 1.2604302116068957`, 0.7278275017755875`,
0.49389546693051767`, 0.021556729129120156`, 452.84942671713645`, 0.038214009527072046`, 8.73167788977904`},
{0.17310243018283944`, 0.49256885761049496`, 5.2474038118867075`, 1.2167083563026317`, 0.588616706923442`,
0.41089714710891945`, 0.1821711177021871`, 4451.29023370465`, 0.12001647220652961`, 2.269196213787966`},
{0.1805022145737788`, 3.870623878161763`, 2.6853201865429206`, 1.4540056051610473`, 0.3465395939248448`,
0.3116685990581043`, 0.3430495408207368`, 123.77189026179846`, 0.21554545019710575`, 7.5674497363872035`},
{0.12919486377057743`, 2.6304051581320387`, 9.030615531469042`, 1.1852097760732927`, 0.8025656477879668`,
0.2709665069296936`, 0.01610528402445943`, 631.0560544240994`, 0.2313930717114097`, 4.580191033116536`},

{0.11792807938525018`, 2.9992967519426994`, 5.197929087471108`, 1.1316540273093707`, 0.8248866470317724`, 0.3561695895739371`, 0.01696020529134022`, 1283.8412020113915`, 0.1325959329361287`, 7.000673815586042`},
{0.049303290582256715`, 1.1925070924339343`, 0.7957633600668377`, 1.4128683276782472`, 0.000022730879762677958`, 0.4285104885078085`, 0.00878130985829771`, 1257.460569712898`, 0.24085716752239034`, 7.587289884781604`},
{0.13128664635800402`, 2.4046868210274015`, 6.858470528577773`, 1.007040533285536`, 0.4840752461507001`, 0.3489790633820432`, 0.006590730128885638`, 3596.8950880067596`, 0.16587148750288666`, 3.462016809878005`},
{0.04846841194802434`, 1.828640157997615`, 8.053204702491172`, 0.7912000348817253`, 0.19137275290127498`, 0.26044015409759047`, 0.137444226583514`, 395.2194426771421`, 0.10623448877689068`, 6.754668978902`},
{0.22606436547954117`, 2.759207160262239`, 4.797641172785626`, 1.1197512107663665`, 0.8744312540778343`, 0.4079143329219078`, 0.021680363867763237`, 112.017364386689`, 0.04841847187742204`, 6.247097164626069`},
{0.21439187995731207`, 1.4871413304129364`, 0.5160724591468409`, 1.1806787917625174`, 0.8679222077666104`, 0.6556391626343723`, 0.05962099501823598`, 325.40523317512117`, 0.12955288081431815`, 2.055442432220964`},
{0.09778347249929609`, 1.5399588911598938`, 7.050045797549721`, 0.9082485710483456`, 0.7454636238668619`, 0.6248569918637821`, 0.04029077002834171`, 1056.397985521477`, 0.20938181714809317`, 3.6320484036778673`},
{0.12927232662770405`, 2.951438179906213`, 0.40510823994438105`, 1.0187470674786288`, 0.2032238445066854`, 0.5456409554372916`, 0.01881027549673071`, 134.69705409101317`, 0.12921233603333943`, 7.23494261394152`},
{0.20182779178318416`, 0.6881028184372244`, 1.057422534977741`, 1.0568090274497894`, 0.12452101430667706`, 0.6254257177994904`, 0.38702446915906646`, 3365.5277260432094`, 0.12211162516890145`, 5.63708838473557`},
{0.21480237208045305`, 2.4248480984277982`, 7.5563453916242835`, 0.9609967545999567`, 0.4216733331369118`, 0.3788510295397969`, 0.02865689876021866`, 142.8377853769303`, 0.05340960656114529 5`, 2.2123406251771502`},
{0.19117013383013431`, 1.4594963084730592`, 8.271393984038092`, 0.8911461626518757`, 0.4023315908892062`, 0.6430024305607471`, 0.425955961175158`, 811.9982424604128`, 0.08470327958141488`, 1.5517888018340713`},
{0.16704561027767095`, 0.7136544792609025`, 7.482780404676742`, 1.2150283597546259`, 0.9046207606306917`, 0.271745131902447`, 0.2695234426940548`, 220.6020580904624`, 0.17006503868594403`, 3.14899802359108`},
{0.16595765366147208`, 2.092996799011412`, 4.948615364795604`, 0.8929763621996465`, 0.8391454185145462`, 0.5892008264762102`, 0.044299676614507844`, 130.42998143434642`, 0.19085435393011602`, 9.39386568753341`},
{0.27224148961405453`, 3.064354031176171`, 9.703480945324245`, 1.4628863651144104`, 0.3875440410966342`, 0.15502532019017645`, 0.01538110765065082`, 96.32001554152914`, 0.17185939304406467`, 6.478173160029735`},
{0.17789655356032075`, 0.42086448766877327`, 8.212577079102342`, 1.145177796821741`, 0.7675840121122328`, 0.5713332044931656`, 0.08476669830762888`, 407.4176887081235`, 0.039911846147414665`, 5.425104426537898`},
{0.25890879804354505`, 0.827757285125033`, 6.146318214990161`, 1.0400613992204162`, 0.4812180568243971`,

0.29251954183586915`, 0.03603273141312643`, 1733.9600457555907`, 0.08508345454030858`, 2.745915615562448`},
{0.13429919704858378`, 3.97301958069563`, 0.23275296336668916`, 1.4075463260369605`, 0.23061890929775308`,
0.5504417933404041`, 0.22771166040596233`, 272.6044063125219`, 0.22898591641919652`, 6.483734227937328`},
{0.12030391986517214`, 0.7748656879950588`, 5.112522469071294`, 1.153103015740388З`, 0.37283988475313556`,
0.5692147153589893`, 0.013539004333470354`, 948.0585012448981`, 0.020895787868669358`, 3.48210387762453`},
{0.1321541010040706`, 0.831080421127889`, 5.684518793971316`, 1.0145204035612485`, 0.08667418093385804`,
0.2219847293666507`, 0.022523926566952547`, 3181.1487538157703`, 0.21535243439086593`, 6.5479272590953945`},
{0.10292542985717545`, 0.9480363128916887`, 1.8187023134383153`, 1.095683835227761`, 0.3789693754557397`,
0.6741099730044744`, 0.007927682110189524`, 599.0709679738629`, 0.23730403059109434`, 8.347995806673719`},
{0.11768271904228267`, 0.8044018805503832`, 4.992994745146397`, 0.8839437991548327`, 0.9627780258088621`,
0.4036610370791115`, 0.010677704183731065`, 75.90134138634313`, 0.09047277628943856`, 3.892249177500955`},
{0.23810204192481904`, 3.5212069305389937`, 6.24449843698252`, 1.12580298554529`, 0.9906238590423209`,
0.651194539832239`, 0.008821192031195845`, 3794.027232128564`, 0.08728181755971864`, 9.039220988882679`},
{0.05170854445494355`, 0.9544694556169109`, 2.4941099826351163`, 0.9097774283707087`, 0.02554739328254807`,
0.22408928016637408`, 0.36587983814629343`, 578.5087062785725`, 0.14318945676945632`, 8.489550606354296`},
{0.2758391104750425`, 2.3950700125014723`, 3.0825274454463827`, 1.2324094107661792`, 0.33578451389793784`,
0.4521239993250691`, 0.02669897011071422`, 4321.015062663416`, 0.06443072522161825`, 4.390191809873814`},
{0.04136033639560718`, 3.5342644851920078`, 2.1080294445485137`, 0.9950381948590621`, 0.26813425336643104`,
0.30392774702749736`, 0.047958977131448324`, 92.66270711931676`, 0.1731864034169307`, 4.57325203890367`},
{0.1958059683987035`, 3.200106783136203`, 7.203120771344906`, 1.1276577663917136`, 0.37865874140280775`,
0.19330414092595893`, 0.01322903896050458`, 235.27978791284824`, 0.1213328646575434`, 2.0980953870537427`},
{0.2141293518430885`, 1.3445267096173987`, 9.440612710104876`, 1.3031822475396773`, 0.08631549676886618`,
0.2421378295292581`, 0.053475196461594834`, 50.8320364740117`, 0.167124755886029`, 6.69448094138391`},
{0.14124099610833857`, 1.6304240145491873`, 4.431235349124869`, 0.7694928939095955`, 0.9477250896887188`,
0.5434618086833787`, 0.13057745213792676`, 1388.4740941330226`, 0.07045603839931946`, 8.981021929620123`},
{0.08317638924124338`, 3.4814117090049965`, 4.537378351353009`, 1.1287962009049006`, 0.37603042712104306`,
0.6772825294518297`, 0.08278526718675269`, 52.354988641883`, 0.1936331782676135`, 8.933135500909255`},
{0.14458191325048447`, 2.576522592570102`, 7.664240990513161`, 1.1334692879511565`, 0.1460097548297743`,
0.5479004940247297`, 0.17641432391033549`, 874.6457178122505`, 0.14737345925317297`, 7.125853291619361`},
{0.21964890100642098`, 3.948215647629967`, 1.3239539515383179`, 0.7541520677887659`, 0.2631388544858304`,
0.4680193255276971`, 0.0745534605985948 7`, 734.1983096733243`, 0.22794453971709777`, 5.810951908142973`},

```
{0.24357090033802437`, 2.585687351965424`, 7.1434831024545`, 1.2789397105658484`, 0.15335660895486725`,
  0.5235228651937761`, 0.12790639986488742`, 56.5359989413313`, 0.05251799125371648`, 7.0717564062965526`},
{0.09627432586752127`, 2.332762155680175`, 5.679853879558527`, 1.2640734140093113`, 0.8156330491360497`,
  0.2895792785477985`, 0.02307681035132743`, 658.139699058721`, 0.029189068125338835`, 7.800914789874877`},
{0.07523258003696548`, 2.5308231598738997`, 0.19036315944607196`, 0.7501464923569574`, 0.1153260012566284`,
  0.48261384042066857`, 0.014254428894325608`, 71.6138994625911`, 0.24976914139283035`, 2.597638512857568`},
{0.20487865745542216`, 2.4577449266837927`, 0.3620012570001929`, 1.1621484663425825`, 0.45716579542364766`,
  0.40055848752773193`, 0.09344580216885054`, 2051.8175124763634`, 0.149322756997785 8`, 9.69569860745337`},
{0.1890789829788747`, 2.8864588443110275`, 0.3020897698703884`, 0.7861583937331655`, 0.3631295895274096`,
  0.49565973888016235`, 0.15528595628066338`, 2271.132408212757`, 0.21092746640771154`, 4.683826377759731`},
{0.13636211858102731`, 3.2225156772879746`, 1.985405604082965`, 0.8074403105824717`, 0.51683495700684`,
  0.17846947550569658`, 0.2866421049071916`, 363.9511542941515`, 0.043491080354586376`, 7.48314403026421`},
{0.1760012024892597`, 3.3901909690471133`, 5.258617572133415`, 0.8627686224489919`, 0.7422533085216929`,
  0.41138487941188706`, 0.007592509582269234`, 424.62789093038504`, 0.09162929479721776`, 1.3722835015951313`},
{0.1813951042683553`, 2.693386252451666`, 9.617741712202033`, 1.407583958403493`, 0.6407257633308476`,
  0.4405499114150466`, 0.008535253338252974`, 83.84476850180724`, 0.06822985382579094`, 7.163997237119144`},
{0.17566594009418735`, 3.2466526484278377`, 9.344725381698979`, 1.0138972604548542`, 0.5838549167343343`,
  0.6073333607184574`, 0.10710975925951867`, 945.005957021721`, 0.208189974443157`, 1.4109192016259353`},
{0.22088610125498404`, 1.594941397589448`, 0.6585367344105002`, 1.4859166690390868`, 0.411769154740657`,
  0.4715175396654723`, 0.024538179644211296`, 3156.4892991025154`, 0.15641735671760065`, 8.553654142069323`},
{0.08792198919034061`, 0.7563929024204028`, 9.775416388823775`, 0.7563974438325178`, 0.8271569221820632`,
  0.32401969363676375`, 0.0333282008681181`, 102.10380045307154`, 0.1014972556908379`, 6.35713244492311`},
{0.13210283054552296`, 3.2703864704797683`, 0.8879166279114746`, 1.2876213813677582`, 0.37930173682375035`,
  0.2978584700900475`, 0.018740615394798876`, 111.76209976448992`, 0.22677958819730465`, 8.664567998658026`},
{0.19507506176309325`, 3.126733009164254`, 1.2939386659529877`, 0.8626822462482099`, 0.4036124839823858`,
  0.4778725494331981`, 0.06798274218289758`, 2442.14562500692`, 0.025650848833468687`, 3.9818096584261493`},
{0.08607083224139878`, 0.4430857620402713`, 5.378081455692687`, 1.015604011959475`, 0.6736258822863768`,
  0.30157456176441344`, 0.06550214653497834`, 2724.6632477522535`, 0.20587133389175544`, 1.8687253311000447`},
{0.14471266481637884`, 3.5830029087352937`, 5.919067097135953`, 1.3383183012471727`, 0.9997339519904831`,
  0.40166871745176214`, 0.015827643464025123`, 2493.129738188907`, 0.10618580931649058`, 1.661604036688603`},
{0.15884129765285837`, 1.9554524456621927`, 0.3975714737116185`, 1.180621526159508`, 0.13357193827486524`,
```

```
  0.6393982147520227`, 0.07953097836767822`, 3991.2803311425578`, 0.2365716915141447`, 8.584420428066604`},
{0.04917531951043441`, 2.0521955455867618`, 5.720052321648424`, 1.1771597571169652`, 0.4322660150443707`,
  0.1629991214278883`, 0.005091310116027936`, 189.05888707261903`, 0.1735533969163952`, 4.172239042736072`},
{0.27542629055144846`, 0.7843233583874856`, 9.783752583368699`, 1.3481471177954405`, 0.23017739141462767`,
  0.5420497851091893`, 0.2059235624279975`, 3468.61907862177`, 0.15188055880627876`, 9.878186090289375`},
{0.242913589428772`, 2.017077890959616`, 6.7411875410249`, 1.4794325227990526`, 0.8119526659059784`,
  0.6637027966074835`, 0.028536028992749693`, 2430.705278612085`, 0.11516348105007651`, 2.1124785829469186`},
{0.27369802527373327`, 0.5072292829799574`, 6.3938654238644155`, 0.9166058122252008`, 0.858871513739546 2`,
  0.6363372429100727`, 0.4973592888199021`, 125.12883357781175`, 0.0982596068118865`, 5.59867632569453`},
{0.18540637687565897`, 1.1775430389730683`, 2.7268491301469933`, 1.4767982648221127`, 0.8902187555583527`,
  0.5282942786301189`, 0.1497900469847022`, 304.29028125771407`, 0.023895791395609434`, 5.874748340307197`},
{0.2722155439547531`, 0.9139371325579537`, 7.770199974150746`, 0.8847133947824111`, 0.9018075103619831`,
  0.19960287154002887`, 0.008582048560181034`, 1921.873640365849`, 0.18448070830995128`, 2.8786799978881135`},
{0.06964367539322192`, 3.707453741922901`, 4.6834761045808655`, 1.337177597600478`, 0.13817299390105364`,
  0.15072857839162668`, 0.030325540973499845`, 324.16896723003435`, 0.11870351893768993`, 8.4259642987084`},
{0.17261439973382386`, 3.0972705794401207`, 5.672236578564393`, 1.4223285072729144`, 0.7931550721000631`,
  0.3896544494671119`, 0.01712882834694823`, 1350.492916562551`, 0.2427963854280547`, 3.05685280243325`},
{0.11394032550346922`, 3.2108067304960466`, 9.096957387649113`, 1.399541009089664`, 0.15147624678363414`,
  0.6188818627652795`, 0.08261238136824677`, 151.04115640404754`, 0.09860367843525403`, 6.06148281050898`},
{0.20354065160019702`, 3.5209980508829766`, 0.4053576884306178`, 0.7576791600370161`, 0.6570403323895382`,
  0.46832222917239164`, 0.005063986272754102`, 161.28160618619455`, 0.12637144234319758`, 8.874326085906798`},
{0.24345495866234085`, 1.8344199239977046`, 3.71003568577021`, 1.0568366445286865`, 0.9797132100793247`,
  0.5295146440336447`, 0.23321770204647818`, 2779.737816031833`, 0.01572768263207122`, 9.835586906627377`},
{0.18352727434547023`, 0.4163243700753796`, 8.057415014521496`, 1.2522528089453002`, 0.16203828635471806`,
  0.6604646928880291`, 0.25830735718962067`, 2461.4350174740794`, 0.12685187153570027`, 8.600866037504808`},
{0.23920496896525717`, 1.1838719961494162`, 9.218551103388815`, 1.265080848919387`, 0.9542453823473309`,
  0.22929853041342196`, 0.4982471338661781`, 90.20981971435951`, 0.0856312153630287`, 3.6772130334476696`},
{0.10156561975861944`, 0.5164671469302382`, 5.401464431301745`, 0.8562900606586841`, 0.5357330343495086`,
  0.16718344164197263`, 0.02721990257094452`, 330.98847924494044`, 0.2190497368128987`, 7.446609110605853`},
{0.2533884831264572`, 2.217775528299157`, 1.87005833593021`, 1.0852798553697922`, 0.3630118105229163`,
  0.49869259108707253`, 0.006826900671537389`, 2180.4237702051914`, 0.21841135731377453`, 2.8539212819504436`},
```

```
{0.04019446235159935`, 2.9685609440334373`, 3.1967275735949383`, 1.2384363485833663`, 0.006837582253808039`,
  0.6265805854044944`, 0.10123843723710083`, 64.97108080441517`, 0.1585540441972691`, 8.616308543170305`},
{0.18466310913340017`, 2.870831467521074`, 5.7568671895307055`, 1.0469133934587485`, 0.5974897879057268`,
  0.6447436436670198`, 0.1911914248698421`, 363.92035372259653`, 0.11435325769039895`, 2.2759611974712186`},
{0.22919090442152185`, 1.3485088294529417`, 3.711141569564556`, 1.1683364276022934`, 0.8698699225738455`,
  0.45530089098812654`, 0.06782162054416256`, 403.6677278730082`, 0.1398561461848175`, 8.195868200542787`},
{0.12718220564190125`, 0.7794468104625536`, 3.4523481867286847`, 1.042660599955479`, 0.8212977484948716`,
  0.42153700480377276`, 0.05083556365712203`, 907.9591078654141`, 0.03221255361915021`, 1.403008654938164`},
{0.24829534536640735`, 0.6361539074945153`, 1.4367506745172012`, 1.2157272327840285`, 0.5920355341003019`,
  0.22834833308349511`, 0.008858258633323783`, 152.73617223659593`, 0.05098711045238086`, 6.308971873547048`},
{0.24634814165734203`, 1.0199939463339236`, 6.874973257490155`, 1.1669106806380989`, 0.9547234223304992`,
  0.2467804242930165`, 0.010102520966714356`, 1373.9164154017546`, 0.22192762621195788`, 8.148592815397581`},
{0.11968292559735771`, 0.4405556662519543`, 0.9343787484302961`, 1.4969299043499453`, 0.5720179190625709`,
  0.5743610731089337`, 0.058376899138915264`, 4861.966479565238`, 0.13911792619470742`, 8.404040973858844`},
{0.0912885213280808`, 1.1352603856466574`, 9.64216394840902`, 1.0423303168159856`, 0.8496114679763396`,
  0.3948758352214041`, 0.050073459411100744`, 1020.4170701645861`, 0.08585808288581304`, 1.5724283740137839`},
{0.23601424519793518`, 0.7493625485076763`, 8.159569145163797`, 1.0789985894921148`, 0.9135595458313224`,
  0.43990663477596414`, 0.03553380189426036`, 63.68276264433202`, 0.11799293833856772`, 7.92015369426547`},
{0.09505824539478602`, 0.994707682773301`, 2.092747921993542`, 1.2334327544777528`, 0.1649122243213208`,
  0.435178801412204`, 0.4947441968705133`, 1030.121381906519`, 0.19720224785958157`, 8.531247013968503`},
{0.13338172675277454`, 1.0224437122231844`, 3.1176391252125253`, 1.1808523949493024`, 0.5954558720182712`,
  0.3646371291004906`, 0.024821319877175146`, 118.84942434665426`, 0.15904005586434156`, 2.0945755856004613`},
{0.2663723170815805`, 0.6117369492537685`, 4.996758576680092`, 1.4798016834758696`, 0.3454705507334743`,
  0.5628818087325649`, 0.10659789380555212`, 347.2598858446038`, 0.128819557084905`, 4.579853140224845`},
{0.19858704302380392`, 3.77240735130054`, 4.552319154599175`, 1.490234534684174`, 0.9267739623702391`,
  0.4663128979271708`, 0.016623264635311285`, 190.7315614324941`, 0.03981298108873932`, 1.7245962595124684`},
{0.16356962246540357`, 2.7590365436019733`, 9.315311335363422`, 1.411146654012818`, 0.2509435276133807`,
  0.3090283790000361`, 0.2431141196841502`, 313.55839056266797`, 0.2467169299716112`, 8.038445579591425`},
{0.25383472083693576`, 3.9769524497483806`, 7.011565744250568`, 1.066285473960517`, 0.7734216029556533`,
  0.6850552501893354`, 0.013843595247453381`, 297.6124430270534`, 0.10716201039628298`, 2.5950386184147853`},
{0.17343366236722435`, 1.3936719133530833`, 0.599503921563171`, 0.8621470436054358`, 0.43567440743840313`,
```

```
  0.22823781097601215`, 0.018669299661272933`, 3618.675535403083`, 0.22611694811187344`, 3.1306846738065635`},
{0.27302715407014244`, 1.5486379048502288`, 9.868645627334566`, 1.0694698241775202`, 0.6816384789594119`,
  0.4416154581373778`, 0.05961824305163796`, 87.63839207083008`, 0.05267766985704242`, 3.1260333504958955`},
{0.12081145422022538`, 2.0244942048813837`, 8.652229532190159`, 0.835020699056165`, 0.9214042127932665`,
  0.46285611589460973`, 0.11861503789539068`, 2689.091322411565`, 0.07096583948487911`, 5.509201411600676`},
{0.27038445563951496`, 3.3688963138915424`, 3.4133330216440854`, 1.1975198070883244`, 0.31492494684053174`,
  0.45427916274833`, 0.0070970717881839104`, 3067.136751023867`, 0.17569148508387616`, 1.2036801730392703`},
{0.1767050761987432`, 2.85608165701536`, 6.140634667151749`, 1.3554192221984884`, 0.6330624847112656`,
  0.5906276578639604`, 0.04152902973081543`, 1510.3499876585004`, 0.15541682752363528`, 8.81551919212286`},
{0.16171803894826015`, 2.2156907324583015`, 1.0134587159780288`, 0.8322559746700846`, 0.34733770749260806`,
  0.6849579573613365`, 0.018772440750507276`, 1617.8437562512436`, 0.21218330633531807`, 7.516753350682977`},
{0.24108871826638628`, 1.1722622038849657`, 2.5945274526654174`, 1.1873482780450624`, 0.3399058776037083`,
  0.3423735862363967`, 0.09173881369912114`, 1455.6792358614214`, 0.2278375557130481`, 4.818602627144232`},
{0.2202280085702742`, 2.180975046803316`, 5.6134680006393936`, 1.2506485034427453`, 0.4748208651226249`,
  0.39757955628981323`, 0.017520218948128618`, 280.3947437474927`, 0.12359899846366301`, 9.521190146527957`},
{0.12315072752895034`, 2.8609501087269207`, 1.79674111361204`, 1.4514496090822089`, 0.3032919079034333`,
  0.2763824218936657`, 0.028981582964232436`, 188.78817200372555`, 0.08856946510090113`, 4.546561013073182`},
{0.14162785770391484`, 1.92013166099325`, 8.540643644714823`, 1.2350689068338556`, 0.301574102817308`,
  0.40858591883894513`, 0.080613435450821`, 585.1346359267128`, 0.15211712109726616`, 7.686797097032363`},
{0.22524615675817938`, 2.031565502127312`, 0.245821316301889`, 1.298167355078078`, 0.689325845538592`,
  0.661487626561178`, 0.00610096551453922`, 373.63064864758115`, 0.20592315239272913`, 2.994117316721619`},
{0.06014178921319169`, 2.215935435785701`, 1.3525069683978295`, 1.0400202244696481`, 0.697929353581739`,
  0.692932654029565`, 0.014666911144259826`, 1018.9785490283374`, 0.16122419934759502`, 4.978072859014281`},
{0.11674773262981253`, 1.910335393882857`, 4.221858661435528`, 1.4404651869296854`, 0.4563596481936356`,
  0.5439902833298367`, 0.005611633628996954`, 451.6526207878965`, 0.24247834364497922`, 9.182241064841566`},
{0.12898119467887553`, 3.729766126068422`, 2.3744826976889595`, 1.2465399522360021`, 0.1415631230326866`,
  0.3607064691421532`, 0.03995461220531779`, 1238.1663657022145`, 0.14506115517150608`, 8.953141814946814`},
{0.2498624462217614`, 2.827129073229683`, 2.690968559152097`, 1.0446525535360744`, 0.06936419216205603`,
  0.5607634545552321`, 0.008891348377622028`, 196.01664126075448`, 0.10064633302983284`, 7.405206717033214`},
{0.25890580889310233`, 3.9576703566675278`, 2.211552265086926`, 0.8006241502346895`, 0.26522568617457876`,
  0.4892067360859801`, 0.02025985822061832`, 94.72915936196773`, 0.06766512891079995`, 5.4984531961033785`},
```

```
{0.2032139210380186`, 1.0205320875718096`, 3.2697243543529257`, 1.0866214178514029`, 0.5111053959745286`,
  0.4079750171889772`, 0.048345897200607585`, 145.20906840025864`, 0.23728279570010086`, 2.517042827760779`},
{0.15288075349234037`, 1.8405591021591183`, 8.732063156275487`, 0.8366527628406224`, 0.7247685787231319`,
  0.43308603413296065`, 0.06703535093550567`, 1294.178726539122`, 0.16108367938164575`, 4.274431762914247`},
{0.1609254870534111`, 1.384768951726243`, 6.196355509527487`, 0.9770497723863997`, 0.43819861370403546`,
  0.17710326003602894`, 0.2243750211158665`, 120.51310647409781`, 0.10277566384729958`, 6.21125463242891`},
{0.21184155187533854`, 1.0400436907537394`, 2.77253033361667`, 1.25783985819887`, 0.5916706672066951`,
  0.18670557332607218`, 0.05860949172916328`, 177.69964385957368`, 0.1825191510166627`, 3.5990512292385053`},
{0.15826860039724194`, 2.914200187526596`, 3.057619613104965`, 0.9127068016590583`, 0.36895217716385176`,
  0.3294645201729436`, 0.006405724718526674`, 799.6829736219844`, 0.2321605158663716`, 7.540832982633661`},
{0.2486148170283075`, 0.5446418582356878`, 7.121193243885838`, 1.048132429671941`, 0.7615900023009023`,
  0.5400927540260018`, 0.07717023450494316`, 388.33902457321517`, 0.04098404938849237`, 2.529691537294738`},
{0.1973186549669898`, 3.31823274961833`, 5.404057922234477`, 1.0748992098641554`, 0.1837120164888255`,
  0.5027766473922404`, 0.30055211808677745`, 460.171808421071`, 0.10833443978919949`, 3.5562239100468123`},
{0.16177424691159015`, 1.9234518490470327`, 6.336020872512709`, 1.465202064627133`, 0.3679027675625519`,
  0.6088093981583469`, 0.02752967040620688`, 1523.605195139083`, 0.023927731291662258`, 6.9520681394676025`},
{0.12468946686374066`, 2.602287301847274`, 9.387923649086364`, 1.237964958422611`, 0.6279283704003791`,
  0.5225362832845872`, 0.15386398985407668`, 4877.253164044286`, 0.10729269282887094`, 6.155025385024989`},
{0.1891437323001075`, 2.037539508644585`, 6.753647851102151`, 1.0264835122983769`, 0.08414675462879395`,
  0.15050525073770327`, 0.01724785647741675`, 835.2003740810117`, 0.09305345804576048`, 5.34515441362371`},
{0.11951180040558451`, 2.9137311414381974`, 6.217108794108201`, 1.4729889111648695`, 0.6540356010640074`,
  0.40115742558392786`, 0.08157249569611574`, 391.2473393910366`, 0.0369147403900103`, 8.338813449127404`},
{0.17883917330505905`, 1.5543060950462326`, 9.905917854358197`, 1.2462903035005124`, 0.32028587133565645`,
  0.3166477244063972`, 0.12099723319057126`, 400.41544183552224`, 0.027102889569123156`, 3.023770086067156`},
{0.25477439586299494`, 1.0781629016953422`, 9.675717925989822`, 1.4583691370753404`, 0.484439918991886`,
  0.6784957922502513`, 0.09307366059264305`, 217.6033516010123`, 0.05467318278554256`, 4.774031073697932`},
{0.26738035849024727`, 1.6336260676101997`, 9.900661691242231`, 0.846806505065853`, 0.2415106216321452`,
  0.502680613934997`, 0.14320441138979886`, 77.88432317464672`, 0.12860121706274624`, 9.963544099078408`},
{0.0966033889391959`, 0.8191052864079662`, 6.963587929197736`, 1.39989556064551`, 0.208903818004208`,
  0.4044327200024739`, 0.345646875315088`, 51.22809362991988`, 0.05669448608759026`, 3.5983266970468653`},
{0.07268626506253911`, 2.0017174568948795`, 2.5547427204964173`, 0.8446935149578232`, 0.0630154383873418`,
```

0.49766282606547807`, 0.00568323780270915`, 197.50095618865632`, 0.04182941385906325`, 9.915201734242256`},
{0.14445086415814334`, 1.1396367789378896`, 8.108045316850049`, 1.1452867101498132`, 0.43349891091368953`,
0.5412309783014353`, 0.17300117449492944`, 151.5195914284387`, 0.22841440999808194`, 1.401560283159844`},
{0.2737062633152032`, 1.87379900045931`, 2.9341815386065377`, 1.0257953180476898`, 0.5994241695021645`,
0.2666028641788709`, 0.16092630392754248`, 2757.7643970777704`, 0.13082540497870587`, 6.7306102844961035`},
{0.14450675367477284`, 1.4907097867645147`, 8.628071674125838`, 0.864574296067966`, 0.06677853457714877`,
0.6218432064874626`, 0.019848227343967184`, 449.15101395266043`, 0.20772413403212947`, 3.059722840773212`},
{0.05092006104579416`, 0.9079056727720332`, 9.25200153286255`, 1.1365283513840736`, 0.03327106644526534`,
0.582893635885294`, 0.1249724078419535`, 82.97595681905067`, 0.15395748273911747`, 7.1070379565760815`},
{0.2364459713820966`, 3.884359137525399`, 0.954741626535867`, 0.8976843312448166`, 0.4180287949880024`,
0.15414803582881131`, 0.32241293356212986`, 4897.582666581565`, 0.05447310320870352`, 4.86045092756919`},
{0.042914457849633436`, 3.519672647432988`, 3.392121169492343`, 1.4941116772804357`, 0.33812708289181614`,
0.3684690007615431`, 0.017148240098985187`, 3621.3180321373566`, 0.22166064632630456`, 1.0781698542512608`},
{0.1470328346746822`, 2.9686959435849998`, 9.643655350717612`, 1.0921756097932163`, 0.41500029005274564`,
0.5183995564120613`, 0.04078998201684905`, 4770.787596114335`, 0.08630863643009795`, 3.8345772490574745`},
{0.10223589016255652`, 0.4809064129783538`, 6.158635896611097`, 0.9490677758592585`, 0.9235714285317382`,
0.43663660593873277`, 0.45300992250929206`, 1331.4948553442105`, 0.21785131418517045`, 5.425736978481032`},
{0.08719902184880185`, 3.645892777856403`, 2.8891082836443758`, 1.428386787110956`, 0.151537003432892`,
0.34505159105650784`, 0.041961341889936235`, 290.12993265609543`, 0.15015043093723618`, 6.608503242578238`},
{0.14456990131617375`, 0.9947424361823325`, 3.150441801621305`, 1.0015653051761273`, 0.8447006281771343`,
0.5703016332732033`, 0.008271730569805881`, 52.80371817926736`, 0.13650904531369146`, 3.8670961625783473`},
{0.14643438827373756`, 2.4493630197222025`, 5.7044459384485755`, 0.764371514918094 5`, 0.8231136724552808`,
0.4724466281569806`, 0.012687117803733845`, 1217.9307406282053`, 0.17452698640833697`, 5.969503699935295`},
{0.045156311345747796`, 1.2463805103118828`, 6.638333254697063`, 1.0774632518381033`, 0.7965921624862937`,
0.19987812094053103`, 0.1230725004501963`, 3016.4870397653995`, 0.18711874545495605`, 6.497285772809487`},
{0.21753427776894996`, 2.5707121359144134`, 7.635927852201033`, 1.3489791075447177`, 0.07755529833527275`,
0.4139475400754866`, 0.049678130502208456`, 727.9927710408293`, 0.24367647097486184`, 3.0823691783926943`},
{0.08480900190064672`, 0.6294563083477476`, 5.454864363444388`, 0.7943923084343845`, 0.9968128471314037`,
0.29575569030654725`, 0.01722905088446216`, 309.5962534203848`, 0.1957861782089137`, 7.847856100229725`},
{0.12061650440369748`, 3.919019990284525`, 1.2611661330191772`, 1.4204820377968523`, 0.21263599304474745`,
0.23829484446556248`, 0.00840490627656251 3`, 547.3513122366178`, 0.16414531089750106`, 3.5940701557610755`},

{0.2541152986933387`, 3.9531330061598675`, 6.8389480578926864`, 1.4090336327472661`, 0.5067749551810137`,
0.5768128332044816`, 0.18877117253369932`, 176.17163988983094`, 0.11435366361110944`, 3.2749545355180913`},
{0.26839792559441034`, 1.5919359357927423`, 0.607262674381337`, 1.065158649344713`, 0.7342994823745972`,
0.6373140564402076`, 0.019311239061898532`, 131.67334362372645`, 0.026592393453336638`, 2.002632914376134`},
{0.0804335350475921`, 1.0990801178785707`, 6.42097149017635`, 1.215697698043305`, 0.5864093543250795`,
0.4707288466973014`, 0.04708688161958605`, 3199.4245739234016`, 0.17424214472199046`, 5.845755402883285`},
{0.04545514913582513`, 3.0084108739176516`, 7.583320976280454`, 0.7956411409554567`, 0.21703974982634144`,
0.4038408554076109 4`, 0.39906200752611665`, 3808.370230770943`, 0.07445295911170413`, 7.244769365152968`},
{0.21811178043156876`, 3.634131336571894`, 2.2696382083853486`, 1.2012104146171985`, 0.07478503245860835`,
0.304565647018059`, 0.049403646487048036`, 242.22074286172042`, 0.22363734402413937`, 5.499597099973035`},
{0.22643881069864102`, 1.8950339409059218`, 4.49788713237294`, 0.781319815193253`, 0.8852654232409718`,
0.19866746146736047`, 0.009828863939504507`, 672.8810907026934`, 0.061975672509268276`, 2.025268353742364`},
{0.13526256211607002`, 2.462351571487578`, 7.058879409696349`, 0.9886974127254741`, 0.579411758510507`,
0.519067819756009`, 0.20147771873022094`, 163.35469430255884`, 0.08074145629003388`, 7.486187513545234`},
{0.20702996858127698`, 3.5459212947722705`, 9.044651158282573`, 1.2898647778788441`, 0.1120438185942787`,
0.3399243113730348`, 0.005774130262077124`, 68.71847405774774`, 0.13094328364017765`, 7.770082768301062`},
{0.10369509204579469`, 2.7722566029085502`, 8.491805279999415`, 1.0043154825901828`, 0.1196997608855428`,
0.46660526882559294`, 0.07137717728073698`, 124.06613686120853`, 0.02382042507485488`, 7.245869706736816`},
{0.12495211269478451`, 3.2710300653782634`, 8.31741576802569`, 0.8301159562153473`, 0.45240597007929817`,
0.45987625913811714`, 0.011306277140564175`, 1464.8239131751898`, 0.06894180622066476`, 6.472250284276024`},
{0.2548094999525184`, 1.991565099208616`, 8.435964917534815`, 1.3597221193616629`, 0.25512389762112764`,
0.2087626588648025`, 0.2568272531538555`, 1822.5061165805678`, 0.026223328458720774`, 8.894025776493041`},
{0.1329827943052238`, 0.6587055670228517`, 4.6853626661703505`, 1.028170006221078`, 0.5310002897488229`,
0.31399252604565075`, 0.010724910779551164`, 62.931190858884165`, 0.12157208629491184`, 4.054529845871336`},
{0.05388606525951978`, 3.0261209146571506`, 4.311810802135282`, 1.2867466894982391`, 0.14121116952696844`,
0.17229324040800142`, 0.239982541075217`, 290.98679707372355`, 0.1893837871763393`, 8.200911880146922`},
{0.18877028807710305`, 3.0713928282220317`, 2.350050214349242`, 0.9003573816536171`, 0.8295355664150708`,
0.3697046533237569`, 0.07836282343745943`, 203.60143886304388`, 0.1119456128362123`, 8.250251130667593`},
{0.1549731585118843`, 1.0794091822629426`, 5.872175042913531`, 1.1954059661912195`, 0.1582784842110334`,
0.47191465043849123`, 0.04704948286374014`, 132.48832982484745`, 0.0611350826684427`, 8.646340597834747`},
{0.0973630018497127`, 2.6509059407435345`, 2.661281949905481`, 1.0384603558144696`, 0.8313691963066465`,

0.6553290382276395`, 0.19978363792296042`, 613.3149478611653`, 0.10086553584387609`, 7.337446850354657`},
{0.25760446309632484`, 3.1710442921339457`, 1.104410531583154`, 1.067160019670409`, 0.29416815684722497`,
0.2106415408174861`, 0.1640132441258384`, 295.52940308175954`, 0.22278260496644214`, 4.791464741488767`},
{0.08814664727699922`, 0.49325198699724604`, 7.4393784579313085`, 1.082856668773565`, 0.36684849391494345`,
0.47651248778047417`, 0.010016127241926027`, 222.2306387901171`, 0.10060123761752315`, 2.9576363340294876`},
{0.09405288750913654`, 1.8996899418118014`, 4.321657830772438`, 1.3285146456969388`, 0.4687194709250446`,
0.37672748824359026`, 0.1617154754485949`, 124.22044587269936`, 0.08001764444851872`, 7.0616286930304`},
{0.26673333380428393`, 2.6775026015459398`, 7.186300359062184`, 1.3779081516185778`, 0.2197224262472608`,
0.44634915685926935`, 0.2549448072449753`, 573.3906335364269`, 0.19327206597748986`, 8.09151224456528`},
{0.06695152315589192`, 3.739348796633019`, 8.531551488563299`, 1.3873826920360777`, 0.5359483678583272`,
0.39821450250245216`, 0.041894311644931556`, 1681.9385322117223`, 0.03536815464018617`, 7.504735436115399`},
{0.2432492145111665`, 1.524425096090667`, 0.7030547277601631`, 1.1448142947983793`, 0.5481222164790474`,
0.4314400790527364`, 0.005980753513921293`, 1722.4066531232882`, 0.11386175120469438`, 3.8888258531503404`},
{0.24077340164047406`, 3.026302168091572`, 3.348461380748672`, 1.1251559242118545`, 0.33184191297746213`,
0.3141742813991918`, 0.0703872676061783`, 173.09096476750136`, 0.24564160017502346`, 6.392098114270558`},
{0.09741803900657164`, 2.8226058170826276`, 6.285345102913118`, 0.8413213607246539`, 0.2506059249236361`,
0.26188997247349455`, 0.05425664970500174`, 4955.988737338467`, 0.11641107051911387`, 2.1128007380367517`},
{0.14314599793313348`, 2.5518611864266925`, 6.295888200856282`, 1.4241645467251498`, 0.07884119203240614`,
0.5217209274223922`, 0.006381498715981013`, 359.4043872790348`, 0.24023808698930982`, 4.143010259943262`},
{0.225655266424848`, 1.5867432872088365`, 3.0858574789264335`, 1.3244931698407223`, 0.6079478177713131`,
0.6933074034991797`, 0.013957254253711016`, 59.76089956157712`, 0.13530140261382512`, 4.737681248427657`},
{0.1122606973400958`, 1.7187290694442563`, 9.979465273640976`, 1.2098446125137294`, 0.9761419756238592`,
0.37158902094365565`, 0.02080861609061981`, 179.99091662757314`, 0.030562090142117915`, 6.57677031568597`},
{0.24459409433338708`, 0.8193086201254509`, 4.65991702304226`, 0.8806460188595719`, 0.2108783317674836`,
0.532516488691834`, 0.10515966693757768`, 2682.0355154099257`, 0.13211135275557007`, 8.414844442987327`},
{0.06530542208683338`, 0.9596039403304868`, 4.254394351359874`, 1.2566656605190776`, 0.7704919647854709`,
0.333532652338865`, 0.010126143082696715`, 1215.361514466369`, 0.2378411046297244`, 9.6572715838455`},
{0.1695388010672208`, 1.173836844893498`, 5.733937529803059`, 0.8972303818101501`, 0.03459597061008757`,
0.45955942513269943`, 0.02896845637601857`, 512.9632938088884`, 0.14318523913512782`, 1.8784414403823462`},
{0.21476635225191587`, 0.5109603344620304`, 9.116157263242219`, 1.0856571152809298`, 0.22597102427026194`,
0.39468057790613453`, 0.008122876676631087`, 1537.5523384133612`, 0.22468288668284858`, 1.5725594082493757`},

```
{0.06813064867410118`, 0.8082337111165119`, 3.599754755102856`, 0.8970806442820121`, 0.12954010392326953`,
  0.2841840584225793`, 0.09462247566679993`, 471.57171291886044`, 0.0960155452146989`, 9.92783198210315`},
{0.12903273933048381`, 3.2486278014701124`, 1.90816116411313`, 1.4556940559632274`, 0.8634695831402321`,
  0.3517692054558007`, 0.07017000005506284`, 82.66475099620249`, 0.22883036061603934`, 2.0030440452333433`},
{0.25243522618186365`, 1.7361695497710992`, 4.779805866927312`, 1.3770344402735035`, 0.053441804379047`,
  0.21192069167526972`, 0.3494903419308112`, 922.4310224013639`, 0.09853668667446419`, 9.287299936208932`},
{0.1954776887577972`, 2.556119761332786`, 4.179140500485959`, 0.8918497427192082`, 0.9985940433520333`,
  0.19919828622889002`, 0.10341194686720168`, 185.89024422047143`, 0.23152045430083634`, 6.38817726005194`},
{0.21581636372683116`, 2.527066218755916`, 0.7074271020081202`, 1.2545848439034097`, 0.6959200155465519`,
  0.5313070485281961`, 0.086636650755472`, 1874.5635156658839`, 0.10607902266846381`, 5.753646937249579`},
{0.1492413868080179`, 2.613673396032418`, 3.0904979954224796`, 1.2072285951382535`, 0.18001792552689877`,
  0.24732759197473053`, 0.00562211589804969`, 2528.5611678534033`, 0.0881107110472133`, 9.594393869960811`},
{0.07601227699098417`, 2.712563755157329`, 4.823883660189505`, 1.3951422907519642`, 0.1445858824844255`,
  0.4724433117860949`, 0.00914361266793413`, 231.39818056782485`, 0.14481529205028987`, 4.810561016418756`},
{0.12871665135347787`, 2.8014401685833805`, 7.283786002739568`, 0.7524429330430051`, 0.8818706538097647`,
  0.37826653388239995`, 0.007972118927365966`, 65.78330734043783`, 0.1996049007295676`, 1.2990854064714608`},
{0.09952335410600305`, 3.347751049949922`, 0.6305859630946937`, 1.1305375150416004`, 0.9039729545790072`,
  0.47386245025326623`, 0.16157270551479597`, 562.0685567880952`, 0.16407167074796686`, 8.59213583452907`},
{0.09189223639403032`, 1.6732139429664894`, 9.692701238874825`, 1.282228547207058`, 0.15855153018111912`,
  0.4131843305852153`, 0.03830010741000983`, 3674.584524793622`, 0.10140311695331994`, 4.468652597668594`},
{0.15046133692934927`, 3.1811544489323884`, 8.294233740646352`, 1.3617577237735539`, 0.4847724596901488`,
  0.37433619970600795`, 0.06464979105054385`, 793.4336208513492`, 0.16357169207826316`, 3.060886752086996`},
{0.2766206750277398`, 2.8724354428398637`, 5.189326643025524`, 0.8952441889736982`, 0.9323758122824941`,
  0.4464935529399745`, 0.01922331430839979`, 81.97107113159659`, 0.06878282991185858`, 7.425462926136905`},
{0.23204393359414766`, 1.3807564884192223`, 0.49288335289722696`, 0.9255887528096592`, 0.5628667572225596`,
  0.43401537857521066`, 0.010225716982610468`, 1119.882052780943`, 0.015435359995479236`, 2.904839645045426`},
{0.06896766630182821`, 3.238145856087148`, 5.061610251787275`, 0.9979492539875935`, 0.9640249344246659`,
  0.343776595701085`, 0.005657576047359979`, 3386.0339854064414`, 0.07870221044254616`, 4.290344560110242`},
{0.14163054804702563`, 1.8677837362731973`, 0.4370922457889872`, 1.166715795330906`, 0.13182109027382305`,
  0.4473367986720722`, 0.3453878303019619`, 2024.858554151485`, 0.17866806603084778`, 3.146330044100834`},
{0.14346666218024207`, 3.6942323053195354`, 6.0819497997897844`, 0.8206348775106591`, 0.4021755195313119`,
```

0.4030089388647059`, 0.4636571249048107`, 744.6939263196219`, 0.08354527905403086`, 5.555269667846608`},
{0.09149267062932814`, 2.234522341263885`, 6.944559583117852`, 1.114683357002141`, 0.5085103250363343`,
0.6658564606426403`, 0.006987139923383322`, 1505.8338370139704`, 0.21635495075213407`, 1.8879751532316735`},
{0.22068432056467163`, 3.569728941728428`, 4.540468856326312`, 1.2038410371742398`, 0.3148195694928344`,
0.5227498853534928`, 0.10369270254334745`, 4010.634501486441`, 0.16206884164220114`, 3.771999452670874`},
{0.2097350071754976`, 1.1102722915344074`, 3.321403610485124`, 1.0935329934230222`, 0.7183578411763796`,
0.40562405317483374`, 0.006176942100465586`, 446.96867612346506`, 0.08354129713872654`, 7.284441181467236`},
{0.1539569569718487`, 1.7653707587036722`, 8.640858401956272`, 1.0488086821127276`, 0.2563370285663329`,
0.39045184386637677`, 0.03142873441769787`, 98.7771934627432`, 0.204774510279442 1`, 8.897923651735105`},
{0.2495726529131425`, 0.6471319987804813`, 4.234303245586215`, 1.3697781718226154`, 0.5893758025334812`,
0.16113556190203493`, 0.016137549539750166`, 661.2554849181507`, 0.08255896742665592`, 6.142572860377916`},
{0.18703143603461198`, 3.5026925577041474`, 3.736145496532833`, 0.8654363814884891`, 0.7031489482980493`,
0.15906583747765657`, 0.008377691039792691`, 131.85234583624222`, 0.16088793676433022`, 8.514675246308464`},
{0.2617533772269884`, 3.6583043975291023`, 1.4938470678500337`, 1.229216471284039`, 0.8448795594977094`,
0.4744971024115313`, 0.027729197093825105`, 163.20786376161018`, 0.21172311156121143`, 7.676981105027913`},
{0.2277950960946843`, 2.381865480846251`, 9.991476447433318`, 0.832979598782657`, 0.5926733949589658`,
0.27276410945547525`, 0.04327492387940349`, 669.5530400360332`, 0.024018555255208573`, 9.681708943673282`},
{0.15963824410704142`, 2.654604852862681`, 7.469701098598773`, 1.491664105533315`, 0.25955793675814864`,
0.27946634303609463`, 0.06966019051655106`, 86.7013584077103`, 0.017186465149371527`, 9.819694766966123`},
{0.13626056470386483`, 2.820076398995975`, 2.87943303050835`, 0.9523411928557022`, 0.8935766203190545`,
0.356125796963648`, 0.009331421764352204`, 68.56313381348697`, 0.147099680293568`, 8.670054262776382`},
{0.22962572899038486`, 1.387681996287987`, 7.569216994527352`, 0.873789488954491`, 0.15662470794012862`,
0.483300190980835`, 0.02173528342644958`, 88.10021701991921`, 0.04718506749066664`, 8.783666403079792`},
{0.16187203397799577`, 1.769575035055972`, 1.1614771267268384`, 0.8287067492253789`, 0.7900895875825826`,
0.2962983919698179`, 0.014442973603799043`, 137.47659064397268`, 0.2186302306878819`, 9.341476742915006`},
{0.09685210682031803`, 1.7960713074535244`, 5.69551378974773`, 0.7869885608747933`, 0.9421312080797839`,
0.5036667090451988`, 0.01697045527462802`, 52.99769310364423`, 0.21425647446502405`, 9.869621253243459`},
{0.16013522976515993`, 1.0007721789830901`, 6.380859482894074`, 1.440495462142086`, 0.5398127028451485`,
0.19075782788209084`, 0.04652110166049526`, 84.8416541425776`, 0.16547472912452094`, 6.4422585881915015`},
{0.1912199176775608`, 2.2121001603415005`, 9.917361836602723`, 1.435922264503217`, 0.9790995032802718`,
0.6767636439858111`, 0.07238714810610002`, 269.421010262202`, 0.015607565070540702`, 2.5093255880098546`},

```
{0.24179572266521915`, 2.5600514990256196`, 5.593167080478407`, 1.0641609773948193`, 0.6297315486340287`,
  0.26623731831221253`, 0.08310036534342866`, 636.0105501064265`, 0.05032454414330245`, 1.4627567207550134`},
{0.20288510482307215`, 0.7288550347693712`, 9.019074578521877`, 1.2840716127441223`, 0.5874481378002983`,
  0.6980893050148065`, 0.008225735950864725`, 1276.121996072157`, 0.1607915330045036`, 9.69181498105582`},
{0.1862496077639137`, 1.6905827743030857`, 2.538407600056317`, 0.9208769830081895`, 0.38109944279089136`,
  0.3320011945053185`, 0.011054373793833769`, 1065.4164452217328`, 0.07943719416592698`, 4.231039339853934`},
{0.05689751321611025`, 3.0250332600067678`, 2.529462845718742`, 1.466915953431471`, 0.22269501402715108`,
  0.6695459886571014`, 0.14207393160159743`, 917.9363525294286`, 0.02546682059293326`, 3.574551553390743`},
{0.1967142498798619`, 3.0620830451218577`, 7.9498840213828466`, 1.4844762448061533`, 0.6042217303737281`,
  0.6207898959804532`, 0.0334891282473782`, 576.7285784287807`, 0.16199601914875666`, 9.348325121692032`},
{0.26434225760699476`, 2.569764850665571`, 5.652188171834926`, 0.8392687504503539`, 0.3834838466329171`,
  0.17354269300981617`, 0.03414034179009787`, 716.202171206297`, 0.028057924329779993`, 1.7997126741152787`},
{0.19233187457454282`, 2.1451040206615035`, 4.677354160755579`, 0.8253029687642099`, 0.9809911268657268`,
  0.22566854695954142`, 0.015470928716805988`, 111.90002994139898`, 0.12242650367871766`, 3.4296984602053584`},
{0.21830260718528288`, 1.0813728060954029`, 9.236753498274854`, 1.3292857287875703`, 0.7084275620345744`,
  0.4530838967889582`, 0.022838882777727113`, 80.81382094438914`, 0.07293079158751065`, 5.841787640828064`},
{0.27637635892023465`, 3.838779152844408`, 0.15938000205200176`, 1.3392032462951913`, 0.02123906289427646`,
  0.6059474038465393`, 0.007164747514275051`, 100.75472745890974`, 0.10946913978259332`, 8.952098728322259`},
{0.15064788979833238`, 0.9109936464272579`, 0.5800958535668599`, 1.4999646884179314`, 0.9779856120080317`,
  0.34948818201364285`, 0.011067508252105101`, 2641.6705923333384`, 0.011474828296826944`, 3.667140614388831`},
{0.0419185722234717`, 1.709255049022068`, 2.3539768873704308`, 1.3792050115017793`, 0.22715212217109637`,
  0.2926929451571887`, 0.00505223249889438`, 88.91455127714036`, 0.1107140298924858`, 2.282618721837757`},
{0.24924491240080032`, 1.373280333190027`, 8.919496594562187`, 1.4037493221091317`, 0.24632944507518628`,
  0.3548949637369072`, 0.18998328780525742`, 2947.236236962295`, 0.1153856006596437`, 7.477168052601716`},
{0.20687871564233656`, 0.44181655503120965`, 8.570924850704476`, 1.0055678634725929`, 0.5549096280819519`,
  0.326726210951258`, 0.20129490353773463`, 306.54002765810475`, 0.1699967418388691`, 1.7003969931174137`},
{0.20196179343132148`, 2.101313615300616`, 3.7695608295023266`, 0.8255643571631567`, 0.9698552359083252`,
  0.3038610819528902`, 0.02519738143348337`, 88.57713238465558`, 0.03933307441687739`, 1.9858938348907018`},
{0.10010100507466935`, 2.4192735538683836`, 9.823330355933038`, 1.1133108806457672`, 0.011014651746178439`,
  0.5998442212295849`, 0.013751847561218578`, 3832.28466694177`, 0.1060300423959204`, 2.751621101527464`},
{0.21311625184066546`, 1.4247782113917893`, 5.690068626185788`, 0.8615233428628426`, 0.12086241410351461`,
```

```
  0.29764577871833753`, 0.005395932799405541`, 882.4049045614522`, 0.2481341739836153`, 1.9572384227218778`},
{0.09607643000919447`, 2.545123365745101`, 1.6292494594570959`, 0.9689766494611143`, 0.9239031581606687`,
  0.5340288721519064`, 0.06266594590032853`, 79.64776165873695`, 0.19537727971057256`, 3.908080115643834`},
{0.25410295729391263`, 0.5246172329407881`, 1.519575137014602`, 1.3035504391628365`, 0.641505202173666`,
  0.18662868599370885`, 0.02992528952684035`, 1308.2457641643925`, 0.1567967571574565`, 7.528281484333206`},
{0.20247384111644234`, 3.9381206949664582`, 3.525512124148932`, 0.8160399146038965`, 0.003770588899372651`,
  0.4372678593782203`, 0.005846407125549171`, 81.38331905868344`, 0.05740074457617078`, 6.792038617151225`},
{0.15622647656935051`, 2.478038073204126`, 4.93355773626655`, 1.0681326166577831`, 0.57283306272417`,
  0.24473580012035412`, 0.09417540555806864`, 340.729792091258`, 0.1606536419587833`, 9.486285960325056`},
{0.2472522705346833`, 1.1701552284997945`, 3.914120438755436`, 1.4973295978551664`, 0.1631737194969931`,
  0.4864210576866177`, 0.39314364156002357`, 172.17497479862377`, 0.10619847023829598`, 4.473646521439088`},
{0.1494891985884202`, 3.3979182405498527`, 4.585626936341594`, 1.2118171179612203`, 0.6586829775426448`,
  0.36431745127605797`, 0.33277789805879787`, 51.20287439819706`, 0.10641801334172246`, 7.126742388333813`},
{0.2014310029114028`, 3.9869430882246055`, 0.7136208449188892`, 1.3208947167282403`, 0.46844054682093383`,
  0.39516715199771224`, 0.14154388607116256`, 2748.591928872397`, 0.05354911946309293`, 6.211900336444712`},
{0.13228384635594964`, 2.6408616938406997`, 3.2993392931538836`, 0.952940068464383`, 0.8367144819205878`,
  0.6111943245350302`, 0.13146835864980566`, 2246.7842161758563`, 0.024436379332296754`, 9.73655688789367`},
{0.23113058156717692`, 2.2605125411397546`, 4.002625660970853`, 1.3839850516092966`, 0.507564008077771`,
  0.4982541689482688`, 0.0228570122346136`, 138.3250229234806`, 0.07006503343759235`, 8.064244271550876`},
{0.17570245022422137`, 3.187809299248391`, 9.693328941627644`, 1.4591302419173326`, 0.2844948752823342`,
  0.5066677812093952`, 0.2998214113578054`, 349.3700788988757`, 0.18995614940943706`, 5.5658132930392785`},
{0.2497541467888837`, 1.085030464715203`, 1.0043906551890043`, 0.864213346242746`, 0.8846254264305906`,
  0.601718469375484`, 0.012654582561683364`, 62.423429857566624`, 0.12690014020771856`, 6.991109163451483`},
{0.22558722869536918`, 1.12607467611575`, 0.7911165205665469`, 1.2733751455833207`, 0.6009341950827205`,
  0.6641796486046712`, 0.175358669032309`, 3424.3638773504586`, 0.14969161390071423`, 8.91095466688921`},
{0.050627269144448594`, 3.276563036391985`, 7.788230793264948`, 0.8177022370463224`, 0.523932893406847`,
  0.34959907144360225`, 0.11342446098213065`, 1447.0777642274236`, 0.2385428599947077`, 6.8056995790460695`},
{0.060006217186970356`, 3.9647259629353027`, 0.34014645604634985`, 0.9146780185619144`, 0.03431401900644282`,
  0.5737721437004861`, 0.026766328146322068`, 169.17748604048344`, 0.08721098377832243`, 3.968142168145161`},
{0.15350947465095238`, 2.9043684969368213`, 4.5670292387828155`, 1.3486905179026798`, 0.8383532909017835`,
  0.5087310077763043`, 0.05385784501485435 5`, 402.8647291760491`, 0.025030223476360525`, 5.400937139619534`},
```

{0.2587691832069661`, 1.4843472048382624`, 4.771777259704052`, 1.1550607981645458`, 0.08010207781888057`,
0.5149452814008634`, 0.01659115431008023`, 1531.656314816295`, 0.15778107864815993`, 1.1951975901128993`},
{0.1547206763399963`, 3.3076918921708103`, 2.0706333626337727`, 1.4168143839445906`, 0.37770487420755305`,
0.5386802863405199`, 0.00701518941055165`, 122.30089778268855`, 0.134914758355765`, 2.269217335741727`},
{0.1424711737630725`, 2.7520949793854754`, 4.596768650339829`, 0.8362726667059688`, 0.00009639230672808807`,
0.36579283504807614`, 0.010146114868681232`, 3412.101956455065`, 0.24682488221918303`, 8.269337606653004`},
{0.10882832688629612`, 2.4842578785622536`, 4.2235021418772565`, 1.4458161330640105`, 0.36880250968204553`,
0.4736058884190225`, 0.023130853597073738`, 1306.5861668001219`, 0.20634842182153856`, 4.016571058814693`},
{0.18639271747297592`, 2.4942906430499407`, 3.445345566170081`, 1.148752163319608`, 0.12885074455456724`,
0.17966201189701259`, 0.0741834712240346`, 1169.2351269821615`, 0.09106655178874168`, 4.423161126411237`},
{0.09766181155973352`, 3.6268090588227118`, 4.041820185573664`, 1.3253596170020208`, 0.4793934216010949`,
0.49032555462271876`, 0.006759043878711846`, 262.0748117860937`, 0.11178249583803845`, 2.0681460387230555`},
{0.07951891239009712`, 2.8897027315487103`, 1.5411848578052467`, 0.863635998632232`, 0.29085460372381067`,
0.43469992564602655`, 0.009145988318489378`, 180.1504573795744`, 0.0803419958474173`, 8.628584719983632`},
{0.07234637915492992`, 1.3584521644778373`, 7.973775020047323`, 0.7807691250526536`, 0.9100932527758132`,
0.18771974645581058`, 0.009648266694560257`, 1956.923094358971`, 0.1277442569760165`, 5.404137919151016`},
{0.26334607729010473`, 1.8094502738990368`, 2.2460302448509974`, 1.2023222076508349`, 0.551393830377435`,
0.6812297944975665`, 0.00739497505047526`, 1157.7965975362908`, 0.15524857884582421`, 1.7991882609973402`},
{0.22679920860671537`, 1.574644711817462`, 3.814781873556772`, 0.9990271813793515`, 0.3871644432916528`,
0.6894928310515707`, 0.054622933309625116`, 69.61156300085906`, 0.09745707491156125`, 5.357011938436007`},
{0.054135826493752204`, 3.1195801041969027`, 7.2615169638158825`, 1.0984819310653413`, 0.10801447255588448`,
0.6366822484935735`, 0.01066160509264612`, 211.97043506851273`, 0.24501368432397974`, 8.92838638172081`},
{0.14590720393667378`, 1.5766246964498798`, 5.158062459855282`, 1.1478320866243528`, 0.3287329300123256`,
0.4460050972404973`, 0.032458645789603134`, 482.4725013461066`, 0.10279090509715616`, 8.020019312365584`},
{0.14965591009740292`, 1.460367057362724`, 7.337474105676788`, 0.8826427720923014`, 0.44478408083773613`,
0.631222883334907`, 0.365159753458356`, 157.97713196457462`, 0.16784289093699323`, 1.7957284623930878`},
{0.0810328468371882`, 1.4659479028658335`, 3.234238812997848`, 1.4263751048195057`, 0.08355223050311089`,
0.5582345497973803`, 0.12450549955799516`, 121.80826494117302`, 0.16010939682803116`, 9.339681585379608`},
{0.15633465648304357`, 2.148716979150252`, 1.5640986685796054`, 1.3949237088061017`, 0.3567264406830608`,
0.23012843132782612`, 0.08829396925964683`, 2593.62904544209`, 0.24948663022889017`, 4.657794257043932`},
{0.24418896051468997`, 0.7850580710666257`, 9.594718039690303`, 1.3796447707658`, 0.3888924613344984`,

```
0.22950540339413805`, 0.26843428081063014`, 169.97373853516677`, 0.1653561506652175 3`, 5.752488029735448`},
{0.161324838944309`, 0.8594526582032156`, 2.9743924517148237`, 1.2482811580906663`, 0.28485670242217753`,
0.27691573296739147`, 0.12631097975241765`, 80.56658004023716`, 0.24230111347476613`, 7.982613747504004`},
{0.26772168843838734`, 1.7983273472260084`, 5.340026177070573`, 1.4142901992580086`, 0.4537298076184495`,
0.2865003384437863`, 0.010579448173665554`, 2804.399167772796`, 0.15280115052346555`, 9.9218473198287`},
{0.18205384870834052`, 1.3062696845921566`, 5.075170850216699`, 0.8732096494236974`, 0.8323770222253974`,
0.3345416157316672`, 0.015614629383397784`, 819.3644184370967`, 0.04873823503517111`, 3.447951588629733`},
{0.15466361671507556`, 2.9485738979940246`, 0.2621130142172472`, 1.4016672383633995`, 0.367041498929854 5`,
0.40252781093021794`, 0.06082671195258038`, 80.30981146445014`, 0.15720245773080782`, 9.014567082907547`},
{0.07129651605023579`, 1.2229228380041173`, 7.261778729610821`, 1.077075564756948`, 0.8974689473036932`,
0.5583016490383189`, 0.012773781923945723`, 355.9690714870232`, 0.1545521575965641 5`, 2.741649121941279`},
{0.11901300846945845`, 0.5315793015902206`, 2.6671039936143597`, 1.3880864279120932`, 0.842081129114632 8`,
0.5566538571030129`, 0.2079188053795858`, 171.97845991450583`, 0.13095338568755877`, 2.9760745568663016`},
{0.23041310070356935`, 3.6790901828404454`, 2.4540732736358937`, 1.23507526691096`, 0.821599531342406`,
0.33616875277136504`, 0.035023029642451846`, 3607.9630264867633`, 0.21590997641376625`, 4.226227139273737`},
{0.07203443304476603`, 0.5285696054477942`, 8.334423467085383`, 0.8854719875782417`, 0.3459640631982801`,
0.2633909150103768`, 0.03987288196821301`, 568.5739309534505`, 0.056948467212013576`, 6.873946140568169`},
{0.16979941581750402`, 2.100179291019132`, 5.184869462011486`, 1.1997264241396737`, 0.9307785088479614`,
0.3430074594655337`, 0.013577650230569586`, 4088.01556659017`, 0.029357726641530413`, 9.133680945256565`},
{0.21619782984172958`, 1.7733623067535955`, 0.5980153626590834`, 0.9798687198034648`, 0.7538483940425484`,
0.5789929247394512`, 0.006264547120133815`, 61.29286164809138`, 0.15848957716004353`, 2.3238471342304834`},
{0.04816719755022911`, 1.0519290863610147`, 3.0181083809828984`, 0.7801564927089879`, 0.4325460609784135`,
0.2938523943485086`, 0.04249246462941157`, 626.2418769059092`, 0.2424260119832079`, 9.623530958234195`},
{0.2279802782152826`, 2.528677960359964`, 2.539878173580604`, 0.7527299875368043`, 0.8436281080694634`,
0.27514078202436143`, 0.005327161585143342`, 920.5142066248217`, 0.04689927589992776`, 5.404864622946203`},
{0.18595171467721877`, 2.938499684754029`, 3.6306522502244434`, 1.1726699653227697`, 0.9888859131507246`,
0.3480894711653968`, 0.38543337220237905`, 80.27026890715871`, 0.03443629355490174`, 9.328937618149844`},
{0.2088003110428162`, 1.665360213541189`, 8.163801498355127`, 1.2665930603692175`, 0.5382347880039544`,
0.33255923205592086`, 0.03591580004997877`, 88.64295388071032`, 0.08847100949056225`, 8.845547783450662`},
{0.21556335034608287`, 3.553132086531601`, 5.919169032409666`, 1.028261766481214`, 0.01873638959650825`,
0.38505243852661264`, 0.12008970777658311`, 72.3007614977653`, 0.1189730390969882`, 5.747570490343056`},
```

```
{0.2061330284551588`, 3.7571620320677477`, 5.463889035312752`, 1.195880973038654`, 0.4606689625278244`,
  0.6539206160067124`, 0.07294517832285098`, 79.70328938032284`, 0.04960591713533524`, 2.0731473047959454`},
{0.13099744861910317`, 3.0069806711745155`, 9.6713740346189`, 0.785614567603678l`, 0.3887426527541791`,
  0.16465925249784186`, 0.029006890169876405`, 3080.757337617379`, 0.22037025279153283`, 7.1587428033756435`},
{0.20740821458680525`, 2.460110895835281`, 0.6082454159142117`, 0.8064214695794831`, 0.18139487742338534`,
  0.30057404623847583`, 0.2202140246268176`, 4056.9433443728003`, 0.07208592746284093`, 2.251460510548089`},
{0.20705709543566436`, 1.1783515457685674`, 5.677465659011105`, 1.0594933834891802`, 0.45712361519708566`,
  0.4929063565541889`, 0.07712216778297526`, 129.94158279886685`, 0.033100299542402456`, 5.957665662265306`},
{0.04662251177018917`, 1.087376542683649`, 6.170097112149202`, 0.7648803529340509`, 0.9868309112238562`,
  0.551889656411283`, 0.011192508637080182`, 2374.3826064993154`, 0.12804730636711914`, 7.114439815654695`},
{0.061725502446144886`, 3.13849642385113`, 9.540008859499451`, 1.4660101376451562`, 0.45012796893408935`,
  0.4212144239280121`, 0.02511250628464842`, 120.07361261472948`, 0.1571099365418398`, 6.445559562130283`},
{0.047554055600075584`, 3.265459078802472`, 8.714860138547323`, 0.8369196863770172`, 0.4962846406178121`,
  0.46335229122842925`, 0.007763546254085084`, 1888.52152221599`, 0.22218896292653229`, 2.633616973587875`},
{0.12567467788677716`, 3.0158399320010547`, 4.901658754742801`, 1.351978052591392`, 0.7221310556231506`,
  0.5024953321675816`, 0.09886516020555723`, 193.78321929587645`, 0.11023982026692764`, 3.349767411033996`},
{0.19691840575768232`, 1.6501723246031572`, 9.011299029553381`, 1.1479337267630148`, 0.646058584475099`,
  0.36485695404700413`, 0.027042804382441818`, 3550.5063392884595`, 0.2269993732427253`, 4.86925454673478`},
{0.22383158609367865`, 3.396592529608882`, 7.0059346900996395`, 1.1116596498970173`, 0.7389255639756462`,
  0.40169511261271784`, 0.008944445083981989`, 1191.861359234809`, 0.06327031154858104`, 2.975039312230072`},
{0.22089456196581753`, 2.0637610394456862`, 7.594372198729954`, 1.2863006630967566`, 0.5843792508368972`,
  0.43898418015861007`, 0.0169422428941798`, 186.99955924932385`, 0.09805501720710208`, 5.47194276574208`},
{0.09910547662807062`, 2.346178866312991`, 7.421049922557871`, 1.3147434313884303`, 0.2750558853148206`,
  0.21685738835966661`, 0.006760522552087531`, 4988.988013498636`, 0.18492351030582366`, 2.8590709623133`},
{0.20816183484471307`, 3.9485705583569546`, 6.550493120626773`, 0.7774564054459185`, 0.5499519895881679`,
  0.2003723071725072`, 0.34567679795977113`, 1808.1918719614162`, 0.23441319529101012`, 4.082991759791533`},
{0.16112203008102222`, 2.4895492142589735`, 0.44352326574872514`, 1.2469876196885359`, 0.9780986797319231`,
  0.6188841396088389`, 0.029743069155058133`, 112.64100571320267`, 0.13296296295840093`, 7.528063016828725`},
{0.17781253097781763`, 1.7213960002432414`, 4.693599924413801`, 1.2010501070587627`, 0.4002263609998786`,
  0.5127527245385753`, 0.08027502692564248`, 156.85332391803223`, 0.06371789077354856`, 6.562151621871855`},
{0.11782384895680592`, 1.1169968183756591`, 9.741344822271138`, 0.892620630288691 4`, 0.732042643272727`,
```

0.38545027561144396`, 0.005148133378435907`, 521.4771678665235`, 0.04124959681193141`, 8.98600497767481`},
{0.1669047046062308`, 1.025805255669411`, 7.1552343684117155`, 0.8176510429563385`, 0.8282389242605148`,
0.29891546072913033`, 0.009889011750027802`, 94.42085507888534`, 0.16558325818795072`, 8.472484566593426`},
{0.1009959491070086`, 1.5581491170930981`, 4.3701504080039815`, 1.3889561652621691`, 0.5506040634320337`,
0.37952236600496847`, 0.006611879537937531`, 361.09151974058886`, 0.21353768992235467`, 8.563496620010564`},
{0.23246941421316458`, 3.062147704374147`, 7.181406357346838`, 1.3012169805011569`, 0.348059834110821`,
0.639530677915989`, 0.09989941363865933`, 83.25264127475623`, 0.015153685907020209`, 6.841473199486905`},
{0.05240567594084544`, 2.6092929572780967`, 9.364433651132018`, 1.4357965076882815`, 0.3985784064329525`,
0.6640850131228939`, 0.14135898686373455`, 340.71602063464445`, 0.17921846525178464`, 4.7134535629319085`},
{0.186251758126929`, 2.4933710973488976`, 3.5328550575209547`, 1.325175018028979`, 0.37317622556186136`,
0.25977278720819974`, 0.09748704597661054`, 108.88467391318876`, 0.2369656062609477`, 9.08676709988832`},
{0.2432348147394116`, 1.8751950266382789`, 2.5453519666394815`, 1.0050719073546701`, 0.45557496345664417`,
0.6934059109337196`, 0.0796473645979629`, 116.90111969840831`, 0.13836004707768368`, 5.732847922404471`},
{0.10016679782659832`, 2.355527283731478`, 1.9202752019167662`, 1.4361517107451491`, 0.5493703103299528`,
0.5953848202767148`, 0.3151585002444098`, 3628.217577021284`, 0.16393396333788363`, 2.9174291385165`},
{0.14701675506563494`, 1.5420515267654622`, 0.49939909194610793`, 1.0246394569387973`, 0.7670831420663697`,
0.6948893607838256`, 0.005182980912963712`, 1858.6105410956102`, 0.23965764027041103`, 6.073448972699818`},
{0.19166603750475464`, 2.2499911524034095`, 2.4198836269597095`, 0.7671801018390442`, 0.14979093568204394`,
0.302546021842622`, 0.008837119919208427`, 4450.048280356515`, 0.1035437468879743`, 7.585339524339819`},
{0.14239569401382052`, 2.8387649854604886`, 1.4027026171382353`, 1.2048713691971291`, 0.21617980215068333`,
0.2290581611576099`, 0.32444520029725066`, 2595.236024076599`, 0.07657553404881084`, 7.455893283173221`},
{0.14874493002083228`, 3.1275671408493704`, 8.341714492380408`, 1.302209514279003`, 0.6258906648056601`,
0.45549735048188655`, 0.04029116024037646`, 765.0281459953038`, 0.12017823562381152`, 2.4241463017742984`},
{0.06222670957040222`, 3.9065456744185374`, 1.0946055811329174`, 0.8246557540237`, 0.5907991824162977`,
0.2630274030573405`, 0.006980770676502568`, 3176.3819350381427`, 0.13217715504568522`, 5.816838705721813`},
{0.04971186392349858`, 0.9916432478771275`, 0.9981695561230755`, 0.8524884405534577`, 0.12034821743714219`,
0.2529703866830304`, 0.3237478827730366`, 360.7437887352434`, 0.010006209625245316`, 7.274213959868414`},
{0.19409490071094637`, 1.638712293913553`, 0.39703017050116074`, 0.7643187357680644`, 0.5335846868412271`,
0.21830616448247264`, 0.0963055671350066`, 635.9574479204244`, 0.22613729069446092`, 5.479828782022301`},
{0.27029725962327555`, 2.240924310463286`, 1.2051421639323703`, 1.1356504301287609`, 0.773380004303778`,
0.6607812449412671`, 0.04949916223108672`, 204.2592199369304`, 0.19899441283798802`, 7.18276351221955`},

{0.14266269539818904`, 3.072017618366117`, 3.4103252419872643`, 0.7755852439360265`, 0.43949005760262017`,
0.1924018543570919`, 0.06712102805080977`, 656.0250616869308`, 0.04537130588160604`, 5.285505421294628`},
{0.10769494377900635`, 0.832286650700361`, 9.122967245453143`, 0.9815088864732582`, 0.8421400260693508`,
0.5448142839433918`, 0.07976087110962524`, 4710.110387525812`, 0.13361893143992154`, 5.1516712920505725`},
{0.21491444350519606`, 1.295137699490157`, 7.08600395884199`, 0.8977823469890502`, 0.4181882258302472`,
0.21987671758091132`, 0.14871331978208877`, 664.8642422386233`, 0.19734382780045878`, 6.556011365474522`},
{0.1490469101160943`, 3.9586033442731177`, 2.425615791755316`, 1.0187686401741827`, 0.6625310044158794`,
0.43390068486512956`, 0.00854220404180854`, 96.98019438566024`, 0.2008209408788944`, 6.643325956478872`},
{0.21251186737978806`, 3.785250570140386`, 5.706304857162573`, 0.8638958511126991`, 0.620802282746526`,
0.4325048599697877`, 0.008982590727965567`, 699.3542650472853`, 0.09331259312392975`, 7.885660147589962`},
{0.24515641699904833`, 3.817676488470438`, 5.668256418323743`, 1.3090245110359662`, 0.8705848781731611`,
0.5675799494003652`, 0.13511363154659006`, 1804.7556662922755`, 0.20417175811210386`, 6.336735207604568`},
{0.16393709899357184`, 1.7364683461504136`, 6.294465981846198`, 0.7908159510076651`, 0.7611075592144951`,
0.36561813704297086`, 0.008326625223173748`, 2834.6413370290525`, 0.20899318399811945`, 1.1709155007061636`},
{0.25878729889995966`, 3.1361195765858056`, 8.880297290408144`, 1.0867236136347516`, 0.0902130745148968`,
0.5352173770607672`, 0.11164972929109021`, 57.41366920823615`, 0.17534323232121918`, 1.541035034614442`},
{0.11203607225462164`, 0.6218658244264867`, 6.440483663444866`, 1.39680689202378`, 0.16534679246130501`,
0.5081267228820908`, 0.03214478110427463`, 1906.9275025607635`, 0.21932209633484323`, 1.251120855111619`},
{0.24357827172423074`, 3.9240029450424014`, 2.804272198831022`, 0.75931848861878`, 0.2959071515485636`,
0.6138549707668646`, 0.10713705928623629`, 339.1202760730188`, 0.035220064551167996`, 7.297254233929856`},
{0.24204908128005126`, 1.6039606621918692`, 7.422941916054803`, 1.4757185212808246`, 0.48127457855983957`,
0.4526414340963527`, 0.017845029630840916`, 286.8959659308558`, 0.1351657578784522`, 4.5748150305594475`},
{0.0765658571842559`, 1.8453576022836256`, 0.8953985896867352`, 1.0948080634821278`, 0.11609992165592442`,
0.45437290836767896`, 0.20627344098654643`, 689.3327160072474`, 0.18998846953804188`, 8.643335566773064`},
{0.2258389851785662`, 0.888336357939937`, 0.32961763645801767`, 0.9210788231382718`, 0.8596897387491109`,
0.5585019604624183`, 0.04381971178906294`, 415.715019082411`, 0.24817184929202513`, 7.285653344604734`},
{0.19210976086993148`, 0.9419570933782317`, 5.851922457612897`, 1.40657571618961`, 0.8492777725664675`,
0.5465312705852027`, 0.10025464283588155`, 725.5272924080149`, 0.05639485661927951`, 6.120948580338062`},
{0.04040676752094921`, 0.7630185090261814`, 4.034875094487921`, 0.9393317585048341`, 0.5565744536568444`,
0.3001585479016161`, 0.008819784570716005`, 3975.779851508773`, 0.23610962920225392`, 2.0975088246946054`},
{0.232313027550268 5`, 0.49681822670142894`, 1.2539687147478098`, 0.9468502150118296`, 0.13096405194857463`,

0.3219849827616931`, 0.04046745571036271`, 95.23490611137812`, 0.01783356001323902`, 6.936606555558118`},
{0.1110467064554368`, 3.3883763096282147`, 5.159131249779884`, 1.2482205179674581`, 0.8201347270890553`,
0.46628896390017405`, 0.2764030660881924`, 2912.733505822841`, 0.03458542948115101`, 2.6312047625809307`},
{0.1632243866872038`, 2.5373481239797906`, 9.958152546995578`, 1.2439631103015851`, 0.6072525242529792`,
0.6395983432777625`, 0.3899581087433961`, 56.26697227139985`, 0.18832987989003086`, 4.752251898931052`},
{0.16346002078838484`, 3.218794874634134`, 9.025936619626364`, 1.1259348004332868`, 0.7415896232848811`,
0.4862549245509259`, 0.364081252118343`, 137.81289300851844`, 0.05819144446151425`, 8.24717117891944`},
{0.19514620721272602`, 2.87732243599938`, 0.26739185721478975`, 1.4891953183216857`, 0.05289250725423611`,
0.4217001319921505`, 0.03447210696714538`, 218.70480172461387`, 0.0713049851341147`, 6.944425315128572`},
{0.06305409129179324`, 2.891634576752894`, 4.887865973915014`, 1.4252999692587962`, 0.37958790888649707`,
0.5644849529569385`, 0.3706753829912659`, 1888.9507831786343`, 0.129560920022812`, 6.557119455026179`},
{0.07134729442997761`, 1.925040188749243`, 1.1575324502817335`, 0.9987470940766925`, 0.48237853230783223`,
0.6023915002525595`, 0.11093155711801116`, 121.08543179928522`, 0.23351841944906743`, 5.375810156733433`},
{0.1360789534218197`, 0.4455509561284261`, 2.009215820725272`, 0.8936493225292423`, 0.96227260378033`,
0.5587588217121244`, 0.041826495849356216`, 90.5779416890061`, 0.15671264889903508`, 7.812316525319764`},
{0.15849386520968983`, 3.640724074855333`, 1.8861164484754733`, 1.1569149894265711`, 0.5885808127893892`,
0.1748767871381529`, 0.46502697264632115`, 193.59851086045592`, 0.1503277806325949`, 4.212693145809347`},
{0.2646664636377358`, 0.7835752608143567`, 7.19251403558616`, 1.494052846937745`, 0.8510899770532778`,
0.5885640033353514`, 0.48048609718479995`, 154.5393756420333`, 0.18357560397946`, 4.027566835631127`},
{0.12495672263621771`, 3.9102582538632182`, 1.1196234634441904`, 1.2648993449094799`, 0.05034343178950351`,
0.5637162084123208`, 0.0377865951253564`, 87.92444212100203`, 0.05653305553347371`, 1.253921938823833`},
{0.22032142146595135`, 2.68397072068061`, 2.6883455170430324`, 1.1162234719994681`, 0.40997309115380154`,
0.5558969738438672`, 0.02165064984593792`, 1526.6454168541877`, 0.24054798278325668`, 4.966530798126495`},
{0.10964967041383433`, 0.7460930180313401`, 6.531951119775776`, 1.1455194328360876`, 0.1524629724176152`,
0.28792352560938195`, 0.09608079533773954`, 1051.3203547256705`, 0.17626541392472717`, 8.294356744338359`},
{0.07988018646299105`, 2.6019950195572488`, 9.606629629213668`, 1.0662798233306474`, 0.7693895670648874`,
0.32124913017390155`, 0.04062772179058327`, 1822.0454711227774`, 0.21556365885863799`, 7.4522296585239225`},
{0.1682679068810577`, 1.5454861727740674`, 1.469740171862469`, 0.9427424173065856`, 0.7350490973518766`,
0.3805201167376455`, 0.012952191039290932`, 4650.232844583341`, 0.019462289964433777`, 1.394906478790226`},
{0.1878353522665755`, 2.6490780379134238`, 9.484290061448991`, 1.1885927795104771`, 0.9576534469556572`,
0.2804046795618802`, 0.13562182147346188`, 68.8621810881709`, 0.06230339869012863`, 8.081807561887658`},

```
{0.195165600718623`, 2.3826254740878197`, 7.662410402528582`, 0.9519993324167042`, 0.5246436284800724`,
  0.22761517290878341`, 0.02889008666683709`, 101.1296763305189`, 0.18152629399038878`, 2.6385617746928904`},
{0.21914596002979703`, 3.4188550030486784`, 4.710500615641845`, 1.452732616866497 1`, 0.03624911304432854`,
  0.28384065291200533`, 0.4254237737178384`, 3505.5630456480862`, 0.12312118964896007`, 9.273808474100267`},
{0.1814951436259516`, 1.11858432682377`, 5.7029405020905966`, 0.816783193035002`, 0.6766309251156815`,
  0.4321734260024682`, 0.2931263656368919`, 87.29527865588257`, 0.044724422492498916`, 2.4174522941648533`},
{0.22203693974513244`, 0.9431525918127432`, 4.196712511517475`, 1.3261224346879132`, 0.8036004422541301`,
  0.6271444136150295`, 0.10733581071086257`, 369.9451385562586`, 0.16273465181192748`, 5.73130948956448 8`},
{0.18924733350115397`, 2.8150904404250037`, 2.4574844917932026`, 0.8737719488383665`, 0.6977173227044986`,
  0.3482841124888072`, 0.48079795163551986`, 94.66261771766344`, 0.16471072416530214`, 1.3078470560939532`},
{0.07907817004881895`, 1.0481324229512046`, 3.8137728667258273`, 1.2920968808020492`, 0.04181350128653394`,
  0.5803710922764521`, 0.02851300126208627`, 266.99553906648964`, 0.22548933562284668`, 2.0140176417924156`},
{0.22302087067747528`, 1.8201511620752422`, 1.875184623833773`, 0.7813352529024251`, 0.8705057770695381`,
  0.22074020090969038`, 0.1367588752741694`, 187.45561858905666`, 0.07793294287132913`, 8.923384483271061`},
{0.21429795289589076`, 2.896671823601787`, 8.636702505614757`, 0.9811380648364925`, 0.20610104158853804`,
  0.1778381348410949`, 0.007936414694970003`, 185.13169573811163`, 0.014685687390926405`, 3.2353159752463547`},
{0.17507336986882482`, 0.7983306290353371`, 2.750450064120228`, 0.805007578526717 9`, 0.7818959542274213`,
  0.6526925477724106`, 0.09653256947066893`, 72.78219945862553`, 0.07700161447215975`, 3.655530763820291`},
{0.1470766374450453`, 1.2533511161361544`, 4.593526904612924`, 1.479552314271993`, 0.7400477950348978`,
  0.5768087033937906`, 0.10619660427114413`, 242.62879808709684`, 0.045931800134058565`, 3.771378090721337`},
{0.0788585810797619`, 2.548378832414274`, 3.111482597871378`, 0.9346713900878518`, 0.19963135196405957`,
  0.5058944800586684`, 0.03749384602431879`, 1118.4164778142415`, 0.22872744797764172`, 1.4303214452036404`},
{0.24182502946725698`, 1.8945200327623395`, 6.300131593939071`, 0.7669603268824658`, 0.6959163653444247`,
  0.5105300710554361`, 0.2329066367569749`, 178.13280663193103`, 0.21420184435482204`, 3.5903109736592462`},
{0.05758087297506348`, 2.9919516996565125`, 9.945704556467732`, 1.1770757830874041`, 0.6136122924727272`,
  0.31603398268032157`, 0.21009959511709828`, 118.22678602178958`, 0.2258513747270965`, 6.274152149731609`},
{0.12816326660379745`, 2.7095777054297967`, 7.985014815061977`, 0.8582103259977079`, 0.28527001685481057`,
  0.4561351167228034`, 0.41006088059385215`, 4928.696897400591`, 0.12257221770331383`, 5.545857987270676`}};

Npar = 1000; (*how many sets will be tested*)
SetBasicParameterValues[];
```

```
CreateResultArray[Npar];
Nnres = Array[f, {Npar + 1, 2}];
(* The minimal viable cell number achieved in simulations can be monitored.
   Numerical acurracy can be adjusted if desired. *)
Nnres⟦1, 1⟧ = "#";
Nnres⟦1, 2⟧ = "Nn";
Quiet[For[ij = 2, ij ≤ Npar + 1, ij++,

    NotebookDelete[pr]; (* To see the code running *)
    pr = PrintTemporary["Set " <> ToString[ij - 1] <> " of " <> ToString[Npar]];

    kappac = PatientsTrainingSet⟦ij, 1⟧;
    kappap = PatientsTrainingSet⟦ij, 2⟧;
    gamma = PatientsTrainingSet⟦ij, 3⟧ * Nnor / 10^7;
    V = PatientsTrainingSet⟦ij, 4⟧;
    ks = PatientsTrainingSet⟦ij, 5⟧;
    rho = PatientsTrainingSet⟦ij, 6⟧;
    omega = PatientsTrainingSet⟦ij, 7⟧;
    alpha = PatientsTrainingSet⟦ij, 8⟧;
    kf = PatientsTrainingSet⟦ij, 9⟧;
    N0 = PatientsTrainingSet⟦ij, 10⟧ * 10^7 / Nnor;

    ACurSim = FindCurDose[]; (* find curative dose *)
    (*ACurSim=resultTrain⟦ij,11⟧;(*FindCurDose[];*) (* find curative dose *) *)
    DA1 = ACurSim * nCpm; (* set curative dose *)
    Ainj = {{t1, DA1, eta * DA1}}; (* set schedule *)
    FullSystemSolutionMD[]; (*find time of achieving of minimal N *)

    Nnres⟦ij, 1⟧ = ij;
    Nnres⟦ij, 2⟧ = Nn;
```

```
    (*Print[Nn];*)

    FullSystemSolutionMDStopNmin[tminNn]; (* solve it with cell proliferation stopping at this moment*)

    WriteDownMeasures[];

    check = 11; OkFlg = 1; (* to avoid possible bugs *)
    While[check < 28, If[ ! NumericQ[result[[ij, check]]], OkFlg = 0]; check++];
    If[OkFlg == 0, ij--];
   ]];
MakeOutputForm[];
```

In[ ]:=

```
AestcurPure = {"\!\(\*SubsuperscriptBox[\(A\), \(cur\), \(est\)]\)", 207.9605568694953`, 116.78387911717152`, 31.259021427744262`,
    101.07977095724142`, 39.66545271571148`, 69.69060371965857`, 6277.626993286135`, 1481.9321176658373`, 8.556708305914556`,
    81.22694426914325`, 1160.1582463118507`, 62.25545012434214`, 64.35943027718949`, 1114.925898737311`, 64.01972861337667`,
    35.867335292670376`, 32.06061117041807`, 321.0070772585055`, 403.1457361693428`, 151.84090893120717`, 47.98249780135377`,
    1116.7532325308846`, 667.1429568663535`, 64.00220933945363`, 118.55990502554417`, 27.636304624807984`, 72.14556157551598`,
    1289.906798998197`, 1254.8338003361828`, 16.229212911986043`, 1300.6080910345431`, 373.0319275620772`, 1944.627208129596`,
    411.7931442456674`, 77.76483896547852`, 3374.630578586424`, 18.75006111750761`, 471.2206746536005`, 36.562140922848904`,
    115.49344249505992`, 155.13110013412629`, 50.07292581143878`, 682.53277296402`, 168.00523624843996`, 260.23408369886255`,
    169.33030615744755`, 440.29716253947225`, 61.60415779010116`, 124.69396284563918`, 1580.4759744211344`, 43.99726363837288`,
    47.4413397178195`, 305.79004618169245`, 42.27683542351058`, 128.15523433073255`, 46.36090634416943`, 562.2572548133888`,
    138.72157013652057`, 27.539831787753887`, 250.26261694398673`, 147.87303991344814`, 173.5060763026177`, 94.32074935711191`,
    68.30475947414355`, 936.8989095907302`, 19.471443184024665`, 497.7830012025341`, 150.96974761642952`, 243.45706886064008`,
    438.8884012337126`, 216.45596427487624`, 5.316314045179877`, 13.694419023912111`, 28.02836029534192`, 39.110786457292406`,
    3692.057315228333`, 225.40772854810487`, 386.7514678791356`, 21.067098041154125`, 441.1953338810919`, 63.93660734091749`,
    7.607813324098744`, 42.751768039115404`, 1654.7835367759387`, 52.77316385734188`, 101.46022806970909`, 219.74992554964012`,
    63.410636702419914`, 542.2782602107791`, 2960.951428115113`, 127.18453275932204`, 684.7364542320128`, 498.92828017425677`,
    31.830588209285622`, 25.861034172501718`, 199.48791869475178`, 167.92926200058616`, 99.50048411574802`, 436.90334409952976`,
    5.1134156112022335`, 113.6744910135902`, 5.188550962019851`, 37.22335298098436`, 1934.6599296796364`, 52.60038478619157`,
    58.893481597787435`, 22.07830369798928`, 213.31168229520065`, 13.07290615139485`, 40.64442531087087`, 10.156417516420506`,
```

6.940073254802677`, 5013.044794160846`, 27.528929883671847`, 900.2521256591069`, 377.69682844362916`, 52.87708431242897`,
1443.3649262227405`, 46.131598125846885`, 198.8557281848253`, 41.92333015469925`, 241.96339662525466`, 197.3194594124301`,
1630.565544843584`, 3.396425327215184`, 192.3193972710194`, 19.281315954082512`, 115.07488447496439`, 121.52746827212849`,
372.09299583320785`, 15.261998266439404`, 42.58205912795087`, 734.7451436087945`, 1945.1501623356062`, 89.88605786951616`,
200.06966424811495`, 21.719906382444215`, 15.709695293981277`, 8.291418684634737`, 72.23336044689572`, 523.3454148614499`,
102.0586850921827`, 21.039262638512433`, 329.9903374078502`, 812.6481200164565`, 52.66683962351665`, 123.6513194762983I`,
293.88747670914523`, 38.13568855652656`, 86.4401640550493`, 28.03471829670753`, 724.8129404127303`, 23.298680309643302`,
53.13322313766015`, 64.3566422857879`, 184.70134712080423`, 1302.0139818510706`, 148.99177950003994`, 181.0793306310366`,
34.763770297899015`, 410.0424834036011`, 109.5878003583873`, 51.08487841422653`, 25.53568476102389`, 1355.6571922206913`,
2634.820754866145`, 26.74565280618183`, 110.26129061743359`, 130.50736596502583`, 36.774237156456245`, 97.63326548519228`,
624.4888962097375`, 22.020026359336963`, 170.39967183857436`, 1019.0250344849559`, 20.911420931699155`, 69.14576850182758`,
117.69277763016758`, 1300.5825095982962`, 652.3948615754017`, 179.90656765639656`, 45.29801271063936`, 93.08810398464354`,
63.1147102276820 8`, 186.6559259451605`, 48.19667691733027`, 552.5060846870274`, 1003.1881020223896`, 221.39769915039446`,
331.8024300079967 5`, 645.0586588509619`, 272.00017208285817`, 24.570839911027583`, 1603.6612456016999`, 373.30730566539734`,
305.9005387329298`, 272.783670120552`, 8.370071383861253`, 2187.8900356183904`, 1238.147553372664`, 103.61826085720948`,
166.74978111447882`, 27.640325295165788`, 579.1256605379922`, 50.78484465217116`, 69.06886369518107`, 686.6282603891304`,
452.7492111157109`, 2370.391036458293`, 179.09233079134458`, 12.41228281360483`, 1933.8645666824689`, 15.491552672585485`,
472.81623489754327`, 103.9560055503515`, 1860.9580701494772`, 372.39861564106803`, 17.124591104558913`, 58.79262707630034`,
208.4260767058717`, 1501.8280382623007`, 16.570367674036373`, 11.874726467154034`, 453.21607308969226`, 818.8902542855299`,
5466.411205656372`, 14.34109081263816`, 318.59326113108165`, 284.1069513727965`, 73.14705605153169`, 1487.5028872302105`,
36.95751605476262`, 1110.6431961884703`, 17.374251841011947`, 200.84376517520198`, 15.275673965218767`, 31.342830826782997`,
354.11743947309253`, 382.00440128556465`, 361.9513676137458`, 92.95139716509517`, 364.3669758898248`, 459.63520063089135`,
55.298102970951305`, 600.4590727330327`, 42.41455632259722`, 4273.0347315013605`, 467.8107672977147`, 920.5686943528164`,
58.81117334521899`, 107.15404603785166`, 127.1185550842855`, 40.791806463255426`, 623.0325551517668`, 168.45404551549638`,
56.845741971236045`, 85.78491105755522`, 52.776049773115126`, 16.167593467624545`, 213.42264570130578`, 321.83387849508483`,
184.87387159474466`, 6.709674363270432`, 167.99116842429544`, 523.7112384783147`, 26.711356940120876`, 366.2563246217706`,
837.5850181225206`, 147.94232128815003`, 274.4713869836533`, 768.5749603616548`, 26.18147720988362`, 48.64673984443831`,
2398.995745662609`, 64.71955709799926`, 545.4876655176232`, 703.6147238687225`, 257.5730226906546`, 825.0287780513876`,
5537.399977063587`, 126.76429235825827`, 206.8524145465044`, 215.55649608133103`, 67.236923907091`, 17.490909590265012`,
284.47682400474406`, 302.7943791090315`, 353.9914916120266`, 7.704380583589187`, 24.07858169245523`, 54.016607131634046`,
50.42816979454327`, 77.62052501425865`, 292.58212804750974`, 108.0350107557488`, 429.00829923613463`, 83.73594047767244`,

28.28796732701628`, 933.570914825478`, 141.9034097581748`, 882.9338938922734`, 1928.7128440054398`, 67.5678170139278 5`,
696.9208588954659`, 1798.6601708400856`, 107.93017520872108`, 2024.6734164262446`, 84.46924615514638`, 54.89213455738702`,
28.0009968611173`, 110.93137469993229`, 190.48444416864632`, 378.41947179987693`, 1240.1958675085789`, 130.8783633404614`,
25.485976457477012`, 6.022943017224859`, 37.92170771530289`, 175.8699614740291`, 203.59340604182674`, 1610.6584187773474`,
958.6101552941893`, 28.707693702110898`, 2751.809049229709`, 1277.213532675834`, 100.94141817513415`, 56.91721347222889`,
370.3101746328675`, 8.559207296786242`, 302.0807675654379`, 45.857263482607`, 94.90704495109331`, 50.20468175525086`,
180.6168778356671`, 535.4585552816059`, 741.6739840160454`, 550.3164500636733`, 1322.155919625336`, 630.9838632713123`,
1396.2796812397198`, 21.816267438842065`, 923.8931769965501`, 138.3664271403334`, 1233.5383325502896`, 27.533037087968193`,
101.53892247432107`, 12.838587797981816`, 323.34647049793864`, 33.65059855503893`, 594.3351606203837`, 229.31477415562566`,
349.5526050928185`, 71.2856111484316`, 1338.367702429557`, 482.27942402583153`, 24.32610285566832`, 40.956377463140775`,
190.5047218324733`, 316.7693825577835`, 1033.9469726833836`, 33.263844598176085`, 793.6411623107655`, 316.44969475345465`,
161.85171414311463`, 11.60631323378407`, 292.3574775379676`, 14.629659111067838`, 515.4725975706392`, 158.0463055848333`,
32.595012890128814`, 224.84143344026876`, 136.27928931814048`, 20.478565103173363`, 11.898091017421132`,
567.965911237098`, 36.51366628616791`, 758.6602289634035`, 1012.7649361525209`, 23.76021414140445`, 105.62509746862344`,
15.347367230979733`, 257.9706452479735`, 335.8587829428483`, 469.51896152472466`, 404.83461632418573`, 75.77900703185838`,
235.38305322105828`, 45.478931981401686`, 278.46558224714255`, 162.7071253920935`, 106.49185826081813`, 300.627681896006`,
570.7648871115722`, 886.6336386954683`, 29.23419160546357 5`, 3263.6743014470067`, 9.111498595296059`, 241.9764243045456`,
195.19533289033754`, 18.07935406327192`, 500.3693224687036`, 11.56950819460474`, 615.8922390440637`, 172.48822024094045`,
125.59922710335557`, 8.680870278018249`, 1300.1638755330707`, 594.3122039880631`, 690.8775746266773`, 599.8431189919705`,
59.539001572637815`, 350.39535508955447`, 652.2051495507056`, 172.69051562697882`, 48.81362755101295`, 55.206737317354595`,
452.27432678171004`, 26.447305840484844`, 3394.4323698820094`, 20.91294882491946`, 15.035708857056138`, 161.87784335873357`,
57.23017728004768`, 133.73819092118197`, 375.2547588267408`, 26.34546496888898`, 833.1324303485147`, 112.7789041511532 8`,
900.1770672216628`, 35.58280659174881`, 518.3770138412615`, 545.7271039866157`, 1831.4724281805798`, 88.76190026894609`,
152.23984495875848`, 34.5025438872988`, 712.492750993158`, 902.5716181338538`, 1715.5120279343114`, 226.9371167094659`,
53.94951629750541`, 72.10339317700169`, 18.820688243631256`, 492.8971860424036`, 1197.247467780099`, 83.49817178337545`,
1020.5640631804943`, 156.82982566212948`, 114.27482433280552`, 42.279620828130206`, 77.92531816944096`, 5.015128559449715`,
113.40578811217537`, 65.16034366834462`, 100.16442576989826`, 105.812684608349`, 47.22745072157395`, 249.67682608788064`,
133.67568667314936`, 228.8289192029971`, 37.768398539037356`, 565.4232426935394`, 337.7219201090041`, 4458.832146166972`,
825.5427565762624`, 14.699178142567403`, 454.34562662059227`, 205.67293952043224`, 36.57670032263964`, 990.7239706208131`,
166.48527560191096`, 129.7920200739647`, 339.8109113418568`, 55.31880358150875`, 174.40906433738718`, 1047.3100132405896`,
430.57994944349053`, 142.53412542824952`, 144.1499825141307`, 551.9109222570158`, 20.542905524332767`, 160.90869689080554`,

18.873506896335243`, 1624.5072407737396`, 39.64940251720767`, 1131.4414527495996`, 332.61345058908375`,
145.28993559905936`, 3665.997415616593`, 370.1971593057519`, 44.876248949853505`, 63.88330879656129`, 2272.535941141883`,
123.21303366022315`, 715.449231953232`, 138.5258195923069`, 26.792363890852588`, 670.0361409142529`, 64.47565792622012`,
227.6540350599838 4`, 109.7953690642296`, 24.138049752596697`, 823.9560726985757`, 33.59942521349337`, 8.632861644598574`,
125.90750424026572`, 135.54928275900846`, 1152.584120403935`, 23.92854371506977`, 113.64161889085416`, 112.4722829314004`,
21.371103971704514`, 505.9028704823119`, 14.031142308825817`, 1483.0165420576`, 798.0955218526343`, 894.55644696043`,
225.8821053951252`, 135.13511016954564`, 16.806112613077715`, 10.51903903585091`, 489.41572993532355`, 1270.9131225005497`,
394.54053651975437`, 150.93850243539916`, 1195.8064006549514`, 119.7527871608091`, 83.72056274363985`, 18.32493562373855`,
43.75501464608105`, 105.1197481057999`, 92.02056915772759`, 289.14761110857484`, 196.68153362507658`, 355.4653409680756`,
841.841968152713`, 164.49692857816075`, 765.6579789691419`, 835.7787599341478`, 409.0361909384147`, 458.076884142214`,
134.95470263162522`, 422.1325964721197`, 25.33920199500803`, 640.846626530443`, 3462.48657098948`, 49.02494490095971`,
4.816261691249143`, 240.40545798359213`, 1693.696501889256`, 788.0816867682037`, 3575.664599969038`, 143.25284719236257`,
792.3318933175954`, 895.9059894825732`, 596.1152900300608`, 51.45791768695105`, 94.54736795300296`, 80.35021178707962`,
186.57460278879952`, 514.4863646190738`, 229.7808911145177`, 95.29698763613041`, 173.79978678012495`, 119.96732361506078`,
62.346854959035475`, 205.07945531435757`, 75.9401623927921`, 245.2566634936187`, 532.1036746548102`, 39.57269239322893 4`,
27.940616848525956`, 44.008981881195744`, 447.76094775033675`, 1699.1087647897052`, 1386.2525862097975`, 588.1721684885763`,
234.97140862671975`, 507.9712975264508`, 848.5840906059378`, 35.21388364659763`, 36.87130719182587`, 6107.237475112454`,
779.0963116072494`, 288.42961260747984`, 79.5875429519073`, 302.5086012930816`, 13.074087109963658`, 990.3953472142992`,
54.81918623540333`, 596.8138967483573`, 48.01014166132958`, 29.35997092010539`, 442.0704497662819`, 1381.272017313927`,
2654.182988521078`, 1575.1598048786254`, 274.49066977263806`, 389.6317972761561`, 184.53653180583726`, 180.029510380224`,
263.96338714216745`, 12.358327817838894`, 6.190291429344035`, 31.01930124068562`, 437.8899240896687`, 8.319287693798923`,
1294.8259937023915`, 70.9829150533407`, 192.90097820548016`, 641.0186879308368`, 66.13572837867821`, 726.5987051232179`,
35.74959607822846`, 211.89342431830994`, 15.240720866282377`, 16.01087837764014`, 222.40867712464183`, 974.3797155965107`,
36.2902009980038`, 724.8309440002016`, 260.2719771224575`, 2088.800631565277`, 18.95502249359305`, 132.10238540612022`,
622.6505384709761`, 754.9154512745685`, 79.49416157699206`, 34.93893906821083`, 13.516972340253732`, 1120.832375395441`,
7.971503619916857`, 186.8810641120699`, 28.085218278218846`, 156.6598104222859`, 1295.5496737748254`, 68.48860350548834`,
9.659671775761543`, 24.472843412710375`, 204.09327345667944`, 23.182078996595816`, 67.41831053599807`, 1788.0964224805289`,
93.33694431988522`, 1770.9437098523865`, 332.04691684460244`, 141.76091626484666`, 370.84183186210714`, 351.2776832502037`,
172.34439568419208`, 412.7749573420771`, 243.92901886741885`, 134.29902690148975`, 1847.670065883616`, 32.33094268905134`,
12.754316693111443`, 310.2650821877486`, 351.1491436594416`, 309.1944495557074`, 689.0231783053966`, 31.304569092510476`,
2829.8255603844996`, 45.8017462716161`, 12.989179326075563`, 123.11721945152065`, 656.283359116043`, 23.474497778761545`,

84.83369812950119`, 172.2383894475708`, 141.3117842430811`, 266.1516029114938`, 120.06366955327934`, 436.6859383244124`,
8.083891723590108`, 667.2203935368185`, 125.09027476300521`, 117.91162834438464`, 56.7582502982993`, 12.463534577552196`,
150.67600655491395`, 1454.3006392475324`, 228.36518105874237`, 91.54995376668423`, 674.0663425712387`, 20.916672179547568`,
193.03675900242317`, 30.92397173014905`, 288.87284735281145`, 2293.579012162944`, 571.7388234506207`, 351.9321745619992`,
18.625373468147195`, 310.123946787966`, 54.61572115989577`, 15.829846222573646`, 234.59207967902472`, 1601.8081922372273`,
125.95857672132418`, 109.38753992692125`, 12.937766036958083`, 488.76390838350073`, 78.76974539961955`, 1044.355043042405`,
254.917902533672`, 2873.020249012567`, 96.76209127673845`, 96.20959270028948`, 1460.857949049142`, 221.42610034897046`,
391.8124291340777`, 66.05844948505614`, 28.67437385818516 7`, 207.16679860913504`, 63.189675267236474`, 1564.1065748261265`,
29.29115146081773`, 39.025938253147636`, 1265.6834373784666`, 859.939215414434`, 22.916696717809067`, 10.155378811198135`,
22.163864046958274`, 27.721344184243357`, 195.12319491825085`, 36.568662144054706`, 27.221897987864867`,
1554.5498262649037`, 630.8827184887874`, 24.350928499133843`, 184.83204637468828`, 47.78632408647853`, 509.04543069115323`,
1081.535263812407`, 150.79842698899552`, 46.71401105805333`, 804.168613189815`, 224.26828730476`, 17.903992482066336`,
1511.5958684136183`, 131.60494836516654`, 572.6490328935768`, 35.33333521340801`, 500.2711245175664`, 127.4325088057755`,
33.809111722886456`, 35.59516962613836`, 3773.9061540394096`, 89.65267900640707`, 263.3571295988692`, 190.67515693911798`,
281.7995869223638`, 255.8143850347918`, 259.1239774729543`, 8.793150178654933`, 678.6826155459381`, 65.05545866663405`,
4.772174222919766`, 126.83535878980194`, 76.13952307942688`, 37.03160868400174`, 478.57916033813115`, 236.62429088466408`,
155.65831170640706`, 208.6903090431616`, 129.24837432632387`, 331.69397957468055`, 70.59463371820026`, 426.4593188000726`,
721.6572924041536`, 249.57475028981793`, 67.05642010388344`, 479.34082128184997`, 218.71684686132693`, 107.73544188039841`,
160.36937714211362`, 89.40299163282324`, 71.22133726144789`, 24.99339515091807`, 43.30184218808783`, 378.2894870521611`,
78.82841318062593`, 415.4168141224054`, 1596.979618878675`, 746.3315220387047`, 534.1129554343032`, 139.9189659086689`,
32.16644146164344`, 79.4214923242974`, 955.8570385762621`, 8.579765849280273`, 3.3047411232324793`, 11.907097656354242`,
124.28791687321757`, 218.17800018994802`, 2246.0438273525215`, 127.20589312468667`, 31.07130790622174`, 40.649857556599194`,
685.3961634782964`, 56.11786353256072`, 325.76870276611584`, 405.6051344203167`, 32.023801066041216`, 20.701815708607704`,
193.8823594216119`, 46.986655539037216`, 847.7592962635491`, 1046.6645534884174`, 626.1601795871928`, 64.82847774873049`,
42.01370398082542`, 837.9411628835317`, 208.78873190020744`, 911.4045474740751`, 730.1536497532052`, 401.5841665626077`,
142.45611887402248`, 177.62629435544488`, 767.7063313617285`, 162.00316950132856`, 67.20013532431379`, 35.81414327524097`,
396.94334291839874`, 3.8189056795083394`, 124.71106561227744`, 1809.4596488040236`, 1125.5596176980375`,
39.07091456606537`, 151.65796793849398`, 35.288187541813336`, 10.677467459556741`, 184.9915474345718`, 537.9720355173645`,
75.33254802252394`, 675.6902789725033`, 68.70074109101174`, 32.86948637483345`, 226.8599513252022`, 468.6999904729794`,
482.2728201479209`, 12.565270436264235`, 51.65330524257508`, 2906.5417901507467`, 41.95705073816599`, 35.00088655907136`,
16.41311009375263`, 96.31417666979591`, 23.68236164183872`, 12.592969979701449`, 319.8606836058835`, 1005.0782529254774`,

```
95.31006301399012`, 746.0678574384053`, 1294.6730225509723`, 193.19202307375122`, 1098.044073743162`, 3148.447883065088`,
1127.2365592524025`, 1244.5649883024462`, 6812.491479879829`, 800.2944951573206`, 466.4187212793585`, 29.92352181295465`,
171.4304562333472`, 45.37989215131294`, 54.55580532948984`, 348.2636558055563`, 21.277599392329904`, 626.2614397257395`,
1484.1228603976367`, 1036.1989721798222`, 40.484585634784644`, 237.14482010219052`, 26.738927371442475`,
73.7453632776144`, 553.1625545482643`, 7.833404248522687`, 18.861109244487125`, 1736.820501985616`, 67.13460431424852`,
791.0871599587367`, 344.6543695144871`, 292.26024899910686`, 2342.756799405454`, 31.362363258604464`, 141.11053873505804`,
961.1811711253683`, 205.27011879352287`, 4013.3627399917723`, 58.21755736520024`, 65.02189070867793`, 258.54549957059066`,
377.272835077887`, 8.192818148050538`, 267.43424716417223`, 21.285134963013444`, 41.33134115477876`, 27.640218284656402`,
120.34155981324187`, 580.9736929095984`, 44.682124743129584`, 31.745236205426973`, 1312.446590648646`, 526.5396726204798`,
211.48674349165788`, 191.74330634391688`, 877.4095169008085`, 17.167498804570332`, 333.6756888488899`, 972.8237758702663`,
41.59375958663582`, 86.42122963933532`, 1430.9542983656359`, 260.0976679595555`, 514.8047678466262`, 23.894598744868432`,
122.05475352084504`, 58.71573319441848`, 977.8571045834494`, 178.6150138023847`, 109.89088987851198`, 3553.0445953329704`,
1378.4534169301235`, 717.1005645461606`, 459.08425823594456`, 20.26560233854502`, 5.051929785796079`, 701.207515052265`,
133.72139400821848`, 749.8244933861907`, 21.11936622378476`, 381.57357006751465`, 22.43722741236239`, 48.965372209984444`,
490.68848203785547`, 4.907208130103031`, 24.192625322491004`, 3234.835991727759`, 614.4038492724043`, 336.91150116097987`,
1751.585645804869`, 355.9007652877697`, 1290.5572182112453`, 232.87437211084983`, 866.529826646155`, 912.1779534988016`,
15.174746573997037`, 99.68407164880875`, 15.492171315973327`, 24.726416475940617`, 56.176718488103454`, 23.62122517692009`,
167.06749969804162`, 96.04922537697776`, 1051.070353922555`, 77.13759336015913`, 32.520379808776305`, 98.46753453055675`,
1281.4291603952768`, 199.03964077631105`, 115.65896161662529`, 7.682628901159168`, 290.57224792253567`, 7.332463087313262`,
313.16507793149566`, 237.9137277519639`, 133.11179690295432`, 563.6070404303182`, 256.9789261210002`, 6.765789493451768`,
668.6988730760784`, 22.51568479838448`, 1825.9388683692819`, 1371.9013689097633`, 313.15816441644324`, 52.519613992881474`,
783.7715638557264`, 3616.2623162987215`, 228.61635599735934`, 830.9401339460172`, 154.91990124695994`, 50.225916449402206`,
70.57876004969503`, 75.50036726293014`, 5.562664536924255`, 2791.485661910901`, 284.68237155616373`, 21.65050931560944`,
512.2382152503286`, 470.84370169781084`, 228.24486179453265`, 82.42950631252553`, 821.4750711921528`, 132.84142613062733`,
1481.9854223917985`, 389.07362025209494`, 14.745160897849553`, 430.9635487074551`, 774.3914869512789`, 13.7432291070492`};
result[[All, 12]] = AestcurPure;
```

In[ ]:=

```
result[[2 ;;, 13]] = (result[[2 ;;, 12]] - result[[2 ;;, 11]]) / result[[2 ;;, 11]];
```

In[ ]:=

```
(* Figure 4A, main text: scatter plot of the minimal single curative dose with respect to cancer binding capacity *)
LethalTox = Select[result, #[[16]] + #[[19]] ≥ Abld / nCpm &];
Ill = Select[result, Abld / nCpm > #[[16]] + #[[19]] ≥ 100 &];
Cured = Select[result, #[[16]] + #[[19]] < 100 &];

Show[ListLogPlot[{Thread[{Cured[[1 ;;, 3]] * Cured[[1 ;;, 10]], Cured[[1 ;;, 11]]}], Thread[{Ill[[1 ;;, 3]] * Ill[[1 ;;, 10]], Ill[[1 ;;, 11]]}],
    Thread[{LethalTox[[1 ;;, 3]] * LethalTox[[1 ;;, 10]], LethalTox[[1 ;;, 11]]}]}, PlotStyle → {Darker[Green], Darker[Yellow], Darker[Red]},
  AxesLabel → {"γ N0" (*"Cancer binding capacity, γ N0 (pmol)"*), "Acur" (*"Minimal single curative dose, Acur (nCi)"*)}],
 LogPlot[{gN0/(eta + 1) / nCpm}, {gN0, 0, 100}, PlotStyle → Directive[Darker[Blue], Dashed]],
 LogPlot[{(gN0 + 2) * 1.5/(eta + 1) / nCpm}, {gN0, 0.1, 100}, PlotStyle → Darker[Blue]],
 LogPlot[(*non-toxic maximum dose*){((Abld - gN0/(eta + 1) * (lambda * 0.5)/((lambda + 0.5) * (lambda + 0.4))) * (lambda + 0.04)/lambda + gN0/(eta + 1)) / nCpm},
  {gN0, 0.1, 100}, PlotStyle → {Darker[Orange]}] (*,LogPlot[(*non-toxic maximum dose under eta=inf*)
  {((Abld) * (lambda+0.04)/lambda)/nCpm},{gN0,0.1,100},PlotStyle→{Directive[Dashed,Darker[Orange]]}]*) (*,ImageSize→800*)]
```

Out[ ]=

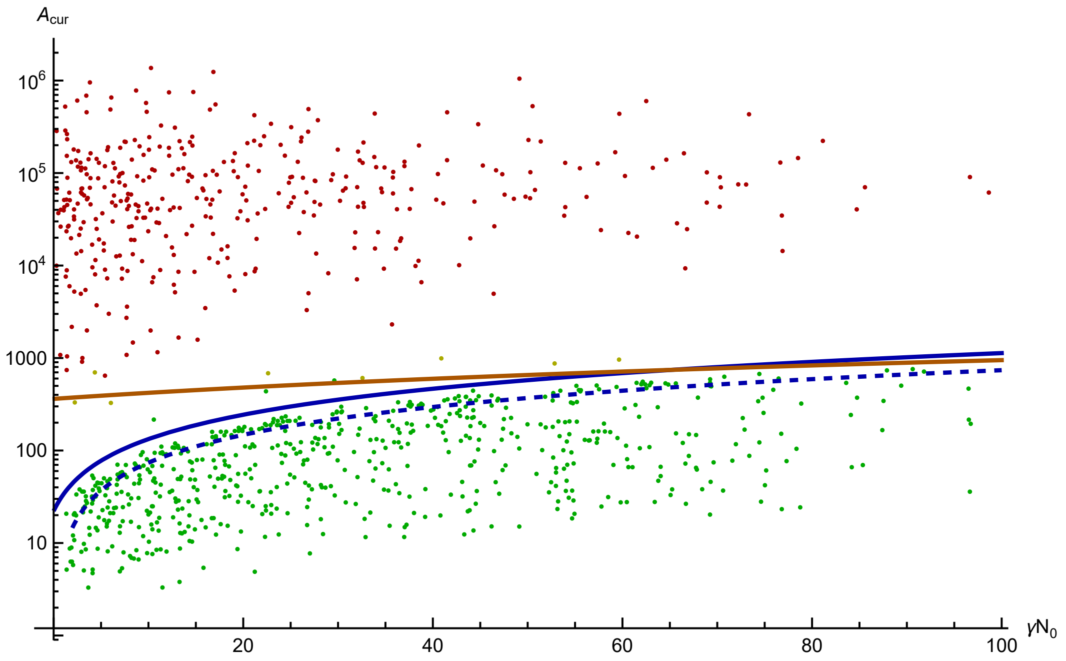


```
(* "In this region [Acur < γ N0 / [η + 1]]
the analytical estimation of Acur , obtained under assumption of pure radioconjugates, still remains accurate" *)
```

In[ ]:=

```
(* Supplementary Figure S.17 *)
Show[ListLogLinearPlot[
   Table[Style[{(result[[t, 11]] * (eta + 1)) / (result[[t, 3]] * result[[t, 10]] / nCpm), result[[t, 13]]}, ColorData["DarkRainbow"][result[[t, 5]]]], {t, 2, Length[result]}],
   PlotRange → {Min[result[[2 ;;, 13]]] - 0.01, Max[result[[2 ;;, 13]]] + 0.01},
   Ticks → {Automatic, Table[{0.25 * i, PercentForm[0.25 * i]}, {i, -10, 30}]}],
  LogLinearPlot[-7.2 / 100, {x, 0, 1}, PlotStyle → Directive[Gray, Dashed, Thickness[0.0025]]],
  LogLinearPlot[3.8 / 100, {x, 0, 1}, PlotStyle → Directive[Gray, Dashed, Thickness[0.0025]]]]
(* The range of errors for the cases where [η+1] Acur<N0 *)
resultFits = Join[{result[[1]]}, Select[result[[2 ;;]], #[[11]] * (eta + 1) < #[[3]] * #[[10]] / nCpm &]];
PercentForm[Min[resultFits[[2 ;;, 13]]]]
PercentForm[Max[resultFits[[2 ;;, 13]]]]
```

Out[ ]=

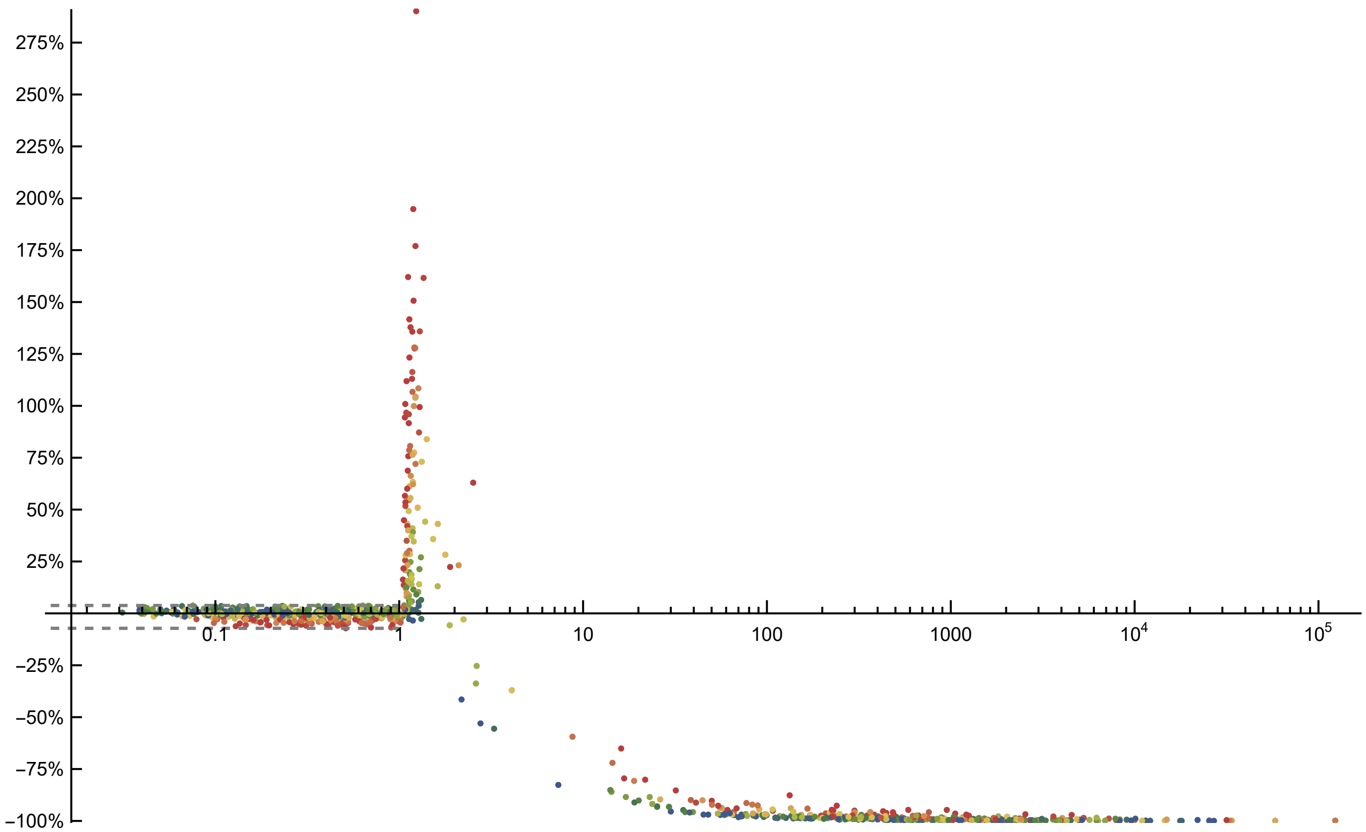

Out[ ]//PercentForm=

−7.166%

Out[ ]//PercentForm=

3.765%

(* "Until ξ ≈2 the analytical formulas for A_{cur} yield only moderate underestimations which can be corrected on statistical basis using the approach introduced in Section S.2.2.1" *)

In[ ]:=

```
(* "The absolute value of relative error in analytical estimation of Acur." *)
resultFits = Join[{result〚1〛}, Select[result〚2 ;;〛, #〚11〛 * (eta + 1) < 2 * #〚3〛 * #〚10〛 / nCpm &]];

(* Applying the same statistical correction of analytical estimations *)
AcurStatCor = (1 + 0.02 + 0.12 * Exp[-resultFits〚2 ;;, 3〛 * resultFits〚2 ;;, 10〛 / 3]) *
    (1 + 2.6 * (-resultFits〚2 ;;, 5〛 + 1) * Exp[- (12 * (-resultFits〚2 ;;, 5〛 + 1))]) * resultFits〚2 ;;, 12〛;
AcurStatCorErr = AcurStatCor / resultFits〚2 ;;, 11〛 - 1;

Histogram[{resultFits〚2 ;;, 13〛, (* Blue: error due to initial analytical formula *)
    AcurStatCorErr (* Orange: error with account for statistical corrections *),
    1.13 * resultFits〚2 ;;, 12〛 / resultFits〚2 ;;, 11〛 - 1 (* White: shift by 13% percent without statistical corrections *)
  }, Ticks → {Table[{0.05 * i, PercentForm[0.05 * i]}, {i, -5, 4}], Automatic}, PlotRange → {{Min[resultFits〚2 ;;, 13〛], 0.2}, Automatic},
  LabelStyle → 14, ChartStyle → {Lighter[Lighter[Blue]], Orange, White}, ImageSize → 300]
```

Out[ ]=

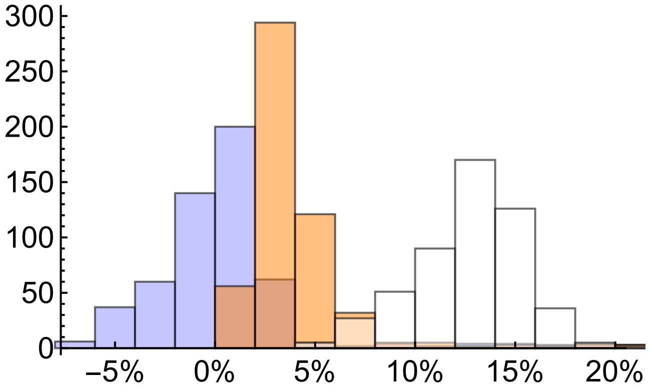

In[ ]:=

```
(* minimal error after statistical corrections, it is positive *)
PercentForm[Min[AcurStatCorErr]]
```

Out[ ]//PercentForm=

0.4071%

```
(* Figure 4B,C, main text: in silico trial *)
```

In[ ]:=

```
(* The results of the trial are saved here *)
```

In[ ]:=

```
Npar = 1000;
Patients = {{"kappac", "kappap", "gamma", "V", "ks", "rho", "omega", "alpha", "kf", "N0"}, {0.2135521539372217`, 3.961577083383463`,
    0.01744955636349462`8, 0.8968357850841964`, 0.5526193340404524`, 0.44754718012416395`, 0.06034849948846574`, 106.1625413648788`2,
    0.10190317263014587`, 883.5270672679858`}, {0.07152561216059383`, 2.5169021554225424`, 0.04134379025654024`,
    0.9380911271372574`, 0.93412718926926`, 0.17225303021845306`, 0.1175689330301931`, 2613.4682606921233`, 0.18822840660147933`,
    493.53347054969345`}, {0.08527801742841884`, 2.3342943915916523`, 0.007356128912919502`, 1.449543423692679`,
    0.292028402519624`9, 0.42703227038753633`, 0.03973423147454342`, 243.18803536822332`, 0.14706039466941528`, 772.0773970550337`},
   {0.08032499747193339`, 3.3228245803820906`, 0.058505885415950074`, 1.328072711539189`, 0.9755298339255658`,
    0.3834882995147877`, 0.016384649136306686`, 81.13118073476093`, 0.12842007570807257`, 778.1337523863579`},
   {0.12288971305592977`, 0.46847473663449035`, 0.035027886845599224`, 0.9755484915941668`, 0.875011150725298`, 0.508807503319318`,
    0.009673321455752245`, 67.20829764486504`, 0.24949007211501428`, 854.477971991049`}, {0.23358644138244972`, 1.7807941427787792`,
    0.06034630860175783`, 1.045216035784662`, 0.6114168239067443`, 0.23619205669697263`, 0.3250660132336067`, 1792.5878930475694`,
    0.0744857171754702`, 805.4037858164254`}, {0.2672425581653916`, 1.5294271887111437`, 0.07676744398776716`, 1.29235952103257`2,
    0.852989179909694`5, 0.393740102898689`, 0.1380993699197939`, 2546.257830379171`, 0.12485562910431564`, 269.9458351710083`},
   {0.23183312051487565`, 3.3970641967209048`, 0.08395863347805453`, 1.1558870614014223`, 0.12544854381867032`, 0.6108675290264531`,
    0.011088452522675474`, 482.67672135456445`, 0.02718649446571869`, 273.7693703287759`}, {0.08728636941166823`,
    1.601719923640072`, 0.02854064232033517`, 1.12476800155736`, 0.3992080927357673`, 0.5147003606496414`, 0.06898789718338541`,
    4299.502344187501`, 0.1107223992523067`, 582.4485009262825`}, {0.06789465848570125`, 3.3425223793990293`, 0.0574646532515876`3,
    1.2855437262048381`, 0.9962937730263095`, 0.5148610577647594`, 0.34967229657185783`, 129.46101746656242`, 0.19161277365419005`,
    109.49077359602803`}, {0.21068632540051146`, 0.7531370432799127`, 0.03477678881607343`, 0.8221096630790845`,
    0.8211187339561399`, 0.3670710605309486`, 0.2800655063342806`, 102.20325958949621`, 0.2320804455102461`, 131.94504624973487`},
   {0.15242131810791038`, 3.789625832521282`, 0.044532267365124396`, 1.387509696935342`, 0.3025280009740767`, 0.43327817796146684`,
    0.05700485989023632`, 1418.28871606834`, 0.07523468846369646`, 480.71702187415434`}, {0.047127741426415765`, 2.196394146604325`,
    0.0020860451850145266`, 1.253945549403835`, 0.04333275562133232`, 0.4635809712022565`, 0.09752500840122344`, 2979.9191420645693`,
    0.19800512345941224`, 326.8262854148691`}, {0.21354080576404838`, 0.499272990997762`, 0.02136565456468402`, 1.4494872415741291`,
    0.4261365642523509`, 0.22487734950611293`, 0.3748843819820306`, 66.77722732862037`, 0.11979197850581236`, 963.8394274052501`},
   {0.06484047241450586`, 3.036571492120566`, 0.07651087746736028`, 0.9711768655928728`, 0.4574624946507835`, 0.5217597270510685`,
    0.12598093752406897`, 242.96341995255446`, 0.14545623834203425`, 981.9658120220585`}, {0.2281503022999219`, 2.470286745790686`,
```

0.06276936772487546`, 1.1988495043217857`, 0.7133186669750637`, 0.3930261678642514`, 0.3166839777189734`, 137.2696616323305 8`,
0.159523733253794`, 623.7392541692013`}, {0.2086500877706095`, 1.2720061208981175`, 0.06218340621581904`, 0.9797444420614967`,
0.0465935956825620 5`, 0.28748130863864596`, 0.3811626852138692`, 349.8696799206167`, 0.19707584717198728`, 104.08157372885026`},
{0.11607219052414597`, 2.714811676304598`, 0.010818112381176023`, 0.842187905582253 6`, 0.6583674774374331`,
0.40051060979188435`, 0.25210876559776124`, 309.1166496608548`, 0.2109324247903891`, 983.8961564091211`},
{0.0729343665897485`, 1.3500826412359075`, 0.07266241705510545`, 1.1245658298901933`, 0.8437686840308298`,
0.25155713345223996`, 0.03143796678764096`, 51.35885900577598`, 0.018795747835889998`, 737.3104099861529`},
{0.13040192171577758`, 2.9737527300384095`, 0.07310292644734392`, 1.466567655421121`, 0.561656292484484 3`, 0.2174201253491196`,
0.017973630894190788`, 381.0744410819025`, 0.2070882324093905`, 363.12098913994095`}, {0.12850488774735863`, 1.9426495267102 5`,
0.05360394961309327`, 1.3166582015996433`, 0.7420300946941296`, 0.6747046075237411`, 0.46225759842348835`, 653.3639045731352`,
0.1781000624097991`, 153.16234178224983`}, {0.14131707783478825`, 2.47648774559096`, 0.046912654007802104`, 1.102943262592814`,
0.2119600715047596`, 0.4901715753981759`, 0.0580109813149486`, 2619.9859648711445`, 0.01885924901675312`, 866.9553146407405`},
{0.25596962129585343`, 1.7441590604011816`, 0.07394014025769799`, 1.0817705534126583`, 0.7417269961287187`,
0.3670491724777639`, 0.009656798208801406`, 431.47684692132157`, 0.21109646038438556`, 541.8299411106649`},
{0.17137889910326493`, 2.547743818855161`, 0.051249488967469524`, 1.4387428259441999`, 0.22533128633818977`, 0.21356004296130449`,
0.010718966990308196`, 426.834678908343`, 0.10633739166308992`, 565.8122888467649`}, {0.1356919186397164`, 3.275909693087268`,
0.09755833451316673`, 0.830833686577428`, 0.23758326238226246`, 0.37684838867012627`, 0.34622549381973555`, 2800.980761749347`,
0.05589233942552713`, 397.59708636141903`}, {0.06319547070029213`, 3.0135506306602915`, 0.08658656932688455`,
0.8750884263433183`, 0.43559283695953344`, 0.17423436075008547`, 0.3556921476320783`, 692.9068977663358`, 0.04218513215777297 5`,
222.69074576769867`}, {0.051353934055559936`, 3.5419315477765503`, 0.04801539957297914`, 1.3650767438471239`,
0.35603852921000145`, 0.4618444822640493`, 0.00593767767296163`, 2613.749958603078`, 0.019287810526956883`, 756.2455097050401`},
{0.20935469703068038`, 3.6183197679011254`, 0.03566796922371117`, 1.1753688638954878`, 0.7004011433506241`,
0.18665090004926455`, 0.010273985816104142`, 1326.129340467039`, 0.14178606357406276`, 910.8324510297567`},
{0.25107423404415036`, 0.9233938441474328`, 0.08706047090121377`, 0.9278477911660168`, 0.6395373622443865`,
0.41454776302472407`, 0.011168650588331405`, 350.2039717436121`, 0.018063541364192554`, 440.5654385955753`},
{0.18503330381957606`, 1.3843983227309558`, 0.05599341470950066`, 1.2326838759285517`, 0.5366716060108483`,
0.5872039546866128`, 0.021673868633287827`, 440.55468856416707`, 0.0587309109264180 8`, 958.3232953637463`},
{0.06679430502398687`, 2.97961750490882`, 0.06874693539558713`, 0.8324720698932511`, 0.047831959104126964`,
0.23900392037978713`, 0.05029201371754466`, 1312.9498214501468`, 0.05149188289007395`, 357.12256566984183`},
{0.2633133297348904`, 1.167395880011603`, 0.02628362214068364`, 1.0744483713964295`, 0.2579472965293179`, 0.6802631987807441`,
0.10725370629344262`, 53.88085410525317`, 0.04507037997684166`, 433.7101304465891`}, {0.2010390664309742`, 0.9179437940816833`,

0.04118734602457437`, 1.4112901498922994`, 0.03943650490351103`, 0.5936392993401745`, 0.007904983488694988`, 372.8977436408934`,
0.1257212210729608`, 514.9151276266798`}, {0.2679869983047456`, 2.831371041491759`, 0.04323230583074366`, 1.0163736311152665`,
0.9719920644948712`, 0.3094609684087656`, 0.02204068430568152`, 76.16804088198147`, 0.16897723749861843`, 499.6307316546634`},
{0.05683769076526879`, 0.650647885401221`, 0.02840792532917323`, 1.1275069757067548`, 0.7854264529060555`,
0.22126398655563229`, 0.06980518344934367`, 574.9844145764067`, 0.1210354338257757`, 562.7623109630207`},
{0.04996310769523532`, 2.710365865528229`, 0.02845470152648966`, 1.3080682866189928`, 0.07861434689660651`,
0.6705317255564853`, 0.006348108900509377`, 549.0755816993258`, 0.061170873719453955`, 318.91974598994375`},
{0.2755006956503554`, 3.698123728642071`, 0.09922032193054331`, 0.8332466983901297`, 0.2785036755762762`, 0.43490103767019017`,
0.15490829410477724`, 4617.932809455717`, 0.11837182487721798`, 340.2748740925912`}, {0.2734635980899412`, 1.5929961417122707`,
0.0613711983696500 4`, 0.7652341524879904`, 0.954168112916443`, 0.43307045621476403`, 0.1582788386688816`, 71.48508495772737`,
0.24624735395364544`, 972.2197337262259`}, {0.2181683242113333`, 2.1761487384705624`, 0.08088096446343027`, 1.0959670952629994`,
0.6079403055392678`, 0.3741554091001601`, 0.02009386290007227`, 69.7071844679123`, 0.040607395764710064`, 305.00876855756474`},
{0.24133385636483384`, 2.7891523258501225`, 0.005829082609902692`, 0.8597961081451015`, 0.9570483419454672`, 0.29444398106251457`,
0.04229917912093678`, 1941.779238616586`, 0.19957823673360453`, 490.6542116348769`}, {0.12306942715949237`, 2.252880201646872`,
0.011446250013559797`, 1.0462863109436527`, 0.5663933692592433`, 0.47495024626336324`, 0.3233936229451822`, 86.1453410021763 5`,
0.08197672020931229`, 782.5197776099736`}, {0.10769046541924182`, 2.4605116390609316`, 0.05377772146881331`, 0.989407475316354`,
0.2930401465570722`, 0.29181294086362775`, 0.10863060011045422`, 4646.540814363835`, 0.22419809836059507`, 512.9803490302319`},
{0.21497939836377283`, 3.1512094394139076`, 0.05781466570999411`, 0.9235677861713979`, 0.797875992877098`,
0.30592437760429736`, 0.011963817124529342`, 496.30104999186307`, 0.12299294969111368`, 343.6950402862427`},
{0.2657260134641566`, 2.9019690638082407`, 0.03280205239027208`, 1.2552566977527801`, 0.09347633453726867`,
0.6906835420223192`, 0.05597654901690233`, 214.21462946437896`, 0.14839555678735822`, 189.76155409360942`},
{0.21292359448836545`, 3.6237126070126635`, 0.023273632947346867`, 1.0934162632306244`, 0.6322993827793542`, 0.5537399147284487`,
0.3715227242167489`, 758.3220767157304`, 0.23574243432919756`, 314.70942110857914`}, {0.23082110188218175`, 3.79416458010805 7`,
0.09951024223913159`, 1.1044050381914734`, 0.2931375323115071`, 0.40868926988441434`, 0.10698117113686255`, 1766.3231774314004`,
0.1575507952957782`, 782.0701995703548`}, {0.1731048010854444`, 0.4511019492052632 5`, 0.06558149662017904`, 1.268779063180271`,
0.15752730810346982`, 0.1844619681378331`, 0.4734368872326295`, 214.57885630606498`, 0.10442024095385904`, 238.91185603032366`},
{0.2554292953302569`, 1.1427932465434605`, 0.04899287050638531`, 1.3736804562756375`, 0.5882660335289671`,
0.17180037030824735`, 0.05309276876348573`, 276.71914948998887`, 0.05937534025119473`, 386.16358526001164`},
{0.16644797368483177`, 2.4992189221756043`, 0.004445377489754368`, 1.4133660089877695`, 0.32679827704181763`,
0.3418600753563352`, 0.38105352658785596`, 1798.4227174359469`, 0.0853854152424785`, 140.42796197604304`},
{0.05401664285302743`, 1.3436659136216367`, 0.007303531204713778`, 0.8064085762190545`, 0.4041746333332612`,

```
0.5906806798990734`, 0.005958103091852778`, 2587.5652974623863`, 0.09992354552595534`, 820.3533386597787`},
{0.20307037888791557`, 1.4293968611238235`, 0.007006708117720848`, 1.1406730143707504`, 0.6144629354618545`,
 0.3719133121966949`, 0.00813248394206873`, 121.80260956296803`, 0.14105543387259395`, 126.00065959923121`},
{0.2740843330487453`, 3.308166539499317`, 0.0033332412164113734`, 0.9387759672621804`, 0.8792897541259657`,
 0.2588278323921356`, 0.0906560451270411`, 289.10750886695166`, 0.0689222507314608`, 274.0455429116968`},
{0.0941844879899188`, 3.012435636197103`, 0.0977143251520188`, 1.4935685648224934`, 0.03172920895860054`,
 0.19879535583488106`, 0.12277001567292761`, 281.6381134207748`, 0.12553563404062174`, 818.5606422688558`},
{0.13120359665298414`, 3.8066558838990874`, 0.05832666562127484`, 1.4436216710518677`, 0.7256118790918118`,
 0.22190148447236047`, 0.055627038916505056`, 331.2421431929122`, 0.023623634114193437`, 105.97098539393085`},
{0.1599611237255819`, 1.5653869796304836`, 0.04073827294465206`, 1.420732828376658`, 0.4167553998465383`,
 0.5683789027240399`, 0.006867177748429021`, 437.8657191219929`, 0.20093958315759247`, 853.3201855869486`},
{0.21575208583629502`, 0.9356056552129512`, 0.03505308262533445`, 1.1968736187898597`, 0.7561599337939984`,
 0.465414519576948`, 0.10760666835377902`, 1053.3658392946543`, 0.20711098924554222`, 210.78286348037096`},
{0.14749719864367028`, 1.1298646044981888`, 0.09689905730204273`, 0.831362255602855`, 0.5798187583416305`,
 0.19996262727341718`, 0.02080299930415397`, 1905.6099187599903`, 0.03509497286958235`, 329.9767515874418`},
{0.05611993210971444`, 2.018253520390404`, 0.05603878791329802`, 1.1233601094750865`, 0.7854886288850511`,
 0.27746521744215147`, 0.0053225072398486025`, 101.97524414758992`, 0.19072350614781147`, 586.0302656773133`},
{0.07988047106873392`, 1.2467973601355782`, 0.005734825596729962`, 1.4870141755274235`, 0.786482359746921`,
 0.40753461440319994`, 0.0616456048522424`, 430.36934645621176`, 0.11501201441136555`, 555.9632996241309`},
{0.21976948435818772`, 2.6949030865877113`, 0.004565631201115927`, 0.851615304193428`, 0.28689924805671385`,
 0.25117757846067346`, 0.1420011334809148`, 365.7629364689339`, 0.17048089811131317`, 458.8930260541114`},
{0.24569357480220383`, 2.806982006055639`, 0.0354191657978669`, 1.1486472973181063`, 0.9842633355008354`,
 0.6578328260657522`, 0.06186663500336862`, 676.0260667202342`, 0.15355635401466894`, 104.93495329528014`},
{0.2568863641209479`, 0.7034804925577145`, 0.0941603933918832`, 0.9631193622252813`, 0.4879437161835407`,
 0.2995943026897797`, 0.00877818042938852`, 1278.8744528151467`, 0.17037713399806426`, 719.843140498881`},
{0.15208884515258675`, 3.3804365982678615`, 0.007505379740790073`, 0.8013128272354585`, 0.48161188241254527`,
 0.28391647832046085`, 0.008016681423664398`, 51.406574561981365`, 0.07108676915203138`, 187.7624783373312`},
{0.10637517181381878`, 2.6673257763233797`, 0.07389800171077983`, 1.0335545955573995`, 0.6258721856651546`,
 0.552024947614703`, 0.3934377256232211`, 1365.910979824601`, 0.017092456029375425`, 449.7500884619777`},
{0.0649812277693595`, 2.884896944749496`, 0.07783207858895651`, 1.428219843208142`, 0.5851112039722763`,
 0.4850552612198272`, 0.13145374596336928`, 233.36752929196032`, 0.020935430712967162`, 793.3352241051653`},
```

{0.22088364361500656`, 2.753665575599883`, 0.056531767859618255`, 1.38986306399133`, 0.7216745774063014`,
0.3584382836914882`, 0.15422309470836812`, 2154.2453486409945`, 0.20075088684393572`, 196.6876979035824`},
{0.17574945972757133`, 3.479082463657387`, 0.0406948089429684`, 1.0632324439925762`, 0.7336036718363608`,
0.6225865045970325`, 0.01654846847609865`, 2647.8857343385694`, 0.1742065007038966`, 538.059550515096`},
{0.04025957477335049`, 2.723445036422185`, 0.0624307243457759`, 1.2961681244376777`, 0.2134882654614516`,
0.39895923327159644`, 0.012423634234475615`, 68.80792227348101`, 0.09152513701065051`, 342.5597984149974`},
{0.23836095562622683`, 1.2444047973172507`, 0.05553779779641564`, 1.076392253001211`, 0.35117607296612463`,
0.40534329593724594`, 0.006015625989151594`, 1819.5202248158637`, 0.12753202451029555`, 623.0420974247954`},
{0.26944870454169506`, 1.2060790013825349`, 0.039462741842031746`, 1.0581301327520456`, 0.10601447465615688`,
0.354379020364461`, 0.033711267495647235`, 990.9089845688537`, 0.031312059666100456`, 967.6243986980542`},
{0.14445853797988234`, 3.535198432366248`, 0.06372334173632162`, 1.4863884210475007`, 0.7021222356246233`,
0.1986028344377223`, 0.033605257874194515`, 401.94568584516094`, 0.22631225203628458`, 497.0198172800991`},
{0.19199489014371673`, 2.559659501669885`, 0.0435827670715008`, 0.9357767944167577`, 0.5821038208204001`,
0.3881836320458101`, 0.08395917774758936`, 399.00745257526154`, 0.15489271985369857`, 774.2528196011518`},
{0.09844334988931319`, 1.3873519913225651`, 0.04614436554245893`, 1.1498948867492238`, 0.47347293312976735`,
0.48947207082211097`, 0.0900112564683952`, 300.85371376045117`, 0.10865036330785854`, 289.3128973476921`},
{0.2588239052872208`, 1.9064804051930793`, 0.065214860918082`, 1.2358133969471479`, 0.9062007858068677`,
0.6285986730260349`, 0.02508709888565183`, 60.40797732056926`, 0.24714947667059417`, 952.5811518915539`},
{0.15433829014876738`, 3.2630592980638813`, 0.03425127382548034`, 1.0548030673325137`, 0.6906919434954677`,
0.3631290272789046`, 0.0347694820539026`, 173.0213690165559`, 0.20951393482972103`, 475.6784304680295`},
{0.07919142001495871`, 2.7255459074120383`, 0.05226476773837156`, 1.4738421429170918`, 0.07762084300215877`,
0.32839016838251456`, 0.28010379475182423`, 1228.3196986901144`, 0.022535205722582274`, 511.1200103954101`},
{0.05563602603579554`, 2.285803588715188`, 0.011271259422675524`, 0.8886812633057506`, 0.8488682312030629`,
0.2180988504645489`, 0.04124220143069666`, 325.42600392283333`, 0.015282318354637336`, 540.3914665193738`},
{0.26726930802972265`, 3.468951790252442`, 0.03848449069490482`, 0.8094106772420622`, 0.06275944440065384`,
0.3246635527776901`, 0.054987775783502536`, 681.4969078295769`, 0.11090812120155558`, 354.5762000041195`},
{0.13377593551305073`, 1.1907304154621343`, 0.01146637788789656`, 0.9181303013739212`, 0.44430229039926394`,
0.21249075461775713`, 0.11547143720610627`, 203.15849668645484`, 0.01100896387178596`, 295.5191145619232`},
{0.16315983268315204`, 1.1080296708247115`, 0.06583864001022185`, 0.865015106617715`, 0.12286793950783781`,
0.37991054755624265`, 0.13902707267015119`, 715.7992806626531`, 0.019079624096566744`, 581.5012259249219`},
{0.15517021586963486`, 1.952115790572729`, 0.0731100150366914`, 0.863005494839794`, 0.11113391823909224`,

```
  0.23929002865779792`, 0.3427593945863136`, 157.02882367804486`, 0.1258544997368633`, 151.66501695475426`},
 {0.16216149919315814`, 0.7994153119966949`, 0.07344189350605326`, 1.46448250423246`, 0.11568032132459538`,
  0.4524728031579279`, 0.007439586263343828`, 2935.686946515946`, 0.23568961866347637`, 848.5436646987821`},
 {0.15863268122870933`, 2.604649964738848`, 0.09105452346714177`, 1.3281546343893726`, 0.6415701788553387`,
  0.5395588121712969`, 0.10672502596586574`, 4293.9755867585145`, 0.22612411051961012`, 801.1531009053148`},
 {0.0470360951706533`, 3.5075246181417876`, 0.0689698582393753`, 1.3992260841209352`, 0.49274029849989587`,
  0.3265485661944224`, 0.0896880649482509`, 3553.0072655615363`, 0.24883204385112412`, 930.4206898584084`},
 {0.1301565511337785`, 3.3013385458906184`, 0.060172545647574065`, 1.1841312709206266`, 0.7389603235116047`,
  0.6545296783352468`, 0.09213436798988452`, 632.9865742133833`, 0.01144874409169866`, 153.66076223065957`},
 {0.19211271095347948`, 1.3267678786947181`, 0.011925004369487074`, 0.7708856579991501`, 0.5967808987867167`,
  0.3563983615639521`, 0.008295516898745303`, 651.3850149278022`, 0.065165960143103`, 674.6701400488206`},
 {0.04092327463376352`, 0.7045023567934869`, 0.08460571137678446`, 1.4657750963462932`, 0.7181575655127523`,
  0.6488519412096585`, 0.15923360328476563`, 212.85100756959832`, 0.0515917171639186`, 462.3207119503768`},
 {0.19270602889895483`, 1.9529553547869458`, 0.06583688116670341`, 1.3515290363153516`, 0.005091282852797141`,
  0.36050263163910423`, 0.04479551947501828`, 203.77608216685013`, 0.1482624290923408`, 529.3883166966048`},
 {0.1050601938980929`, 3.4490435237177026`, 0.08223909341236009`, 1.2367817503973777`, 0.9277363378878729`,
  0.3699674586709595`, 0.4385914759613132`, 1519.1710373444578`, 0.23151573895912042`, 820.0175517477519`},
 {0.05132348611758153`, 3.959642395183179`, 0.04888282767798439`, 1.2096431470311648`, 0.026920294658858257`,
  0.43312132839092765`, 0.3519494697432887`, 129.77286276747867`, 0.014447876552552863`, 379.4279055426431`},
 {0.13421887508919655`, 3.5366003696861075`, 0.04365656524319377`, 0.8302898645594913`, 0.33283176141556736`,
  0.32180578132251225`, 0.12171969152254764`, 58.615799213607424`, 0.06462503110882584`, 898.0928768715434`},
 {0.15819611106702364`, 2.020855817361644`, 0.06579688155882536`, 1.2380340703095258`, 0.6378555510612616`,
  0.3675344788090784`, 0.006545785906113509`, 101.24936252099768`, 0.22028280764448066`, 149.80163079680509`},
 {0.24994958155626085`, 1.6151536382110443`, 0.09538293813329457`, 1.3475056084534551`, 0.8251324191653782`,
  0.518473471172061`, 0.013209857312129224`, 465.53610432365656`, 0.19186631952926742`, 938.6440248484497`},
 {0.1956555609490701`, 2.03149296201667`, 0.09660508820628273`, 1.431718750027923`, 0.5308578462289135`,
  0.43609275022077343`, 0.05994448278703194`, 319.5342769319219`, 0.05601191301657277`, 788.8827504958957`},
 {0.23193438538805855`, 3.16433220464147`, 0.06263350952730523`, 1.484540865185697`, 0.945228356849932`,
  0.5695514219689578`, 0.023879141173510178`, 147.89101930946188`, 0.24041444251942057`, 974.5971477651331`},
 {0.2528274636845232`, 1.6765266145916016`, 0.05359196557993448`, 1.3463142085764375`, 0.9224467805077987`,
  0.16620719793609418`, 0.013039811850177031`, 103.76980611708078`, 0.22032399406907538`, 401.8839359060937`},
```

{0.23077639135389166`, 2.5631050348732005`, 0.04014355344850944`, 1.1219336672611722`, 0.11159784718139387`,
0.5983828871337603`, 0.12763464721917558`, 1560.2077328425014`, 0.20349144783421902`, 903.744744090405`},
{0.06895016542438792`, 1.301044343226704`, 0.015962130237339344`, 1.182285615620497`, 0.5628258630936316`,
0.17632340412293257`, 0.3831530705725889`, 1497.278387537139`, 0.10171305044347567`, 505.35404235107535`},
{0.2787317780563141`, 2.375067420232342`, 0.0815658084141512`, 0.8993075085878712`, 0.2038087026634039`,
0.6041957560913382`, 0.324966388605867`, 215.9571285973821`, 0.13818574272027367`, 691.7030542595149`},
{0.04704135783034269`, 0.4824465321524163`, 0.021143667148849198`, 1.297225751526362`, 0.5221151518138136`,
0.4606696788974354`, 0.006779779197302622`, 185.59373538640742`, 0.0189497120626436 2`, 558.5730827937659`},
{0.21423251173707536`, 2.493227952439149`, 0.05289405397630384`, 1.406475234089612`, 0.7998464081533074`,
0.509476952192685`, 0.4272743578374108`, 100.59740971479944`, 0.1412992063677468`, 892.9324365177549`},
{0.07990270577478725`, 1.026053986899301`, 0.09098769304378568`, 0.75484093198576`, 0.3137514363776597`,
0.2597158546619064`, 0.021526708814218906`, 71.8454436542471`, 0.14857027641402232`, 192.33905427474235`},
{0.20790548900468359`, 1.6561792271072155`, 0.03308057679124518`, 1.2176117409116913`, 0.5649384654171494`,
0.5537564006068142`, 0.021311473455272706`, 493.7190855791356`, 0.0879661543265085`, 522.8214036804926`},
{0.23583327723859243`, 0.8244276223039284`, 0.009610054530140194`, 0.999911169587162`, 0.6818020653793861`,
0.4770396571968506`, 0.3762680384381647`, 1045.715543915765`, 0.20181855407690935`, 285.3335505694424`},
{0.2149596477715155`, 1.1258091287082204`, 0.04117204642206028`, 0.8287635491092544`, 0.19708791744131293`,
0.30242514760323325`, 0.009963068581429268`, 2766.449995395716`, 0.1599911935585479`, 542.0030818591285`},
{0.09778089450463257`, 1.5512303348716232`, 0.04607092381086256`, 0.9234013469057234`, 0.7159741715951657`,
0.15221755315547447`, 0.008899731124616716`, 1902.692936123654`, 0.11247025125228444`, 519.6608670719012`},
{0.2733616407663576`, 1.391826052520571`, 0.05242996857884462`, 1.2463936486263583`, 0.3747282584855436`,
0.40124237280450004`, 0.11539389962842635`, 292.43427931036985`, 0.183553339648669`, 592.2315796450872`},
{0.24918162326616394`, 3.2909828859462893`, 0.0031304822935452315`, 1.0930940447805135`, 0.3406640390037241`,
0.20243554875381864`, 0.028649077189173346`, 1674.4319759967557`, 0.19194057055819636`, 777.538166666535`},
{0.25645888779994136`, 2.282435807658568`, 0.08257899475169463`, 1.378184234375194`, 0.8190909920565956`,
0.23648999352950728`, 0.029414947617452787`, 2478.9160402790253`, 0.12086031199538894`, 421.4774938421854`},
{0.12282576626019515`, 1.0173035286087062`, 0.0379861701996793`, 1.401110687144116`, 0.7192159067178037`,
0.40286532167194544`, 0.36624490921162295`, 2426.5499538363206`, 0.01808445874874337`, 556.291828749522`},
{0.08314629308908505`, 1.3023656036359537`, 0.09511446512381881`, 0.821564112530474`, 0.4777933736422373`,
0.2692725743194774`, 0.006069954672804891 5`, 1469.9917989704652`, 0.23042452149338993`, 154.46286861175`},
{0.21957774039482975`, 2.751122632050148`, 0.013942756799365678`, 1.0532031012381704`, 0.8873864151539932`,

```
  0.23272558697907686`, 0.007181177159222858`, 4013.7501241473833`, 0.14211730184734184`, 952.1784892015081`},
 {0.26384054503883103`, 3.138964037644092`, 0.08426203609396872`, 1.3770189510815902`, 0.4475271713808333`,
  0.3491883286754355`, 0.07774969397541334`, 618.3912182185799`, 0.0638489190260633 8`, 535.1175352858716`},
 {0.20560343993895913`, 1.5779437043773905`, 0.07680738613512926`, 0.7894682023823474`, 0.9949259646898643`,
  0.44397150101458005`, 0.018989097980746553`, 294.52516577442924`, 0.14255921229578516`, 385.97631580256467`},
 {0.27238164345669874`, 3.1728219863305904`, 0.04998959318576006`, 1.2550483187184474`, 0.08966409063298642`,
  0.2754392206527779`, 0.028155523330625355`, 413.44364879258364`, 0.10742347205803915`, 204.45184033599216`},
 {0.2451942638086284`, 3.321324534398453`, 0.09231223940162195`, 1.0826750771187965`, 0.290821416328257`,
  0.5533414871497576`, 0.3812913244405754`, 2830.6500293967956`, 0.03030872516300448`, 250.95228864955956`},
 {0.10306075838040374`, 2.5636670031903526`, 0.062478631313391465`, 1.1237391323830406`, 0.020325627807727287`,
  0.4138983427169105`, 0.01567041094182525`, 640.0409779587225`, 0.2299818577146827`, 855.7014985263506`},
 {0.1543081177985079`, 3.6905447676223817`, 0.02801124131684004`, 1.4594516817243341`, 0.19245562145181472`,
  0.22404862937840908`, 0.01176083329958924`, 1165.1333013103292`, 0.08358867889715743`, 831.8544035344403`},
 {0.06171989970085079`, 3.434249919377687`, 0.06572710058482858`, 1.3211715182855723`, 0.01504624767015783`,
  0.4567422389972121`, 0.16959599175310128`, 4317.9045896509315`, 0.11554862800770349`, 591.0128116780224`},
 {0.242854939016651`, 2.284046375461106`, 0.07510045340186228`, 1.3823454805694586`, 0.12288532968332899`,
  0.2505129792578149`, 0.19202736337063933`, 949.9596745663976`, 0.23010030070393422`, 645.8973615583986`},
 {0.22700129023591364`, 3.923952544058909`, 0.030515149632633083`, 0.939180364827058`, 0.4621492504536673`,
  0.6517443382103951`, 0.014468218060931168`, 427.04247682874535`, 0.2370408654331208`, 259.4368577693423`},
 {0.07805880992570535`, 2.501643518243867`, 0.009418707493693895`, 0.9986888477411795`, 0.32030485689176835`,
  0.15640932443277744`, 0.05282555610968247 4`, 1053.3265205661667`, 0.23821781066142345`, 975.8018452897034`},
 {0.11861919681642336`, 1.4687434978704887`, 0.0829781170205376`, 1.0362850735520839`, 0.6712083946301921`,
  0.44248360772337636`, 0.31715757872607053`, 458.6280472607073`, 0.19463738072293596`, 242.98487605538276`},
 {0.059281890431487294`, 3.297569244457442`, 0.09408483617597145`, 1.4708447962140283`, 0.8202767387462129`,
  0.5182490075472995`, 0.15407063447248953`, 64.57603365703892`, 0.059976329837068354`, 643.9595265565569`},
 {0.2572247154689537`, 1.909920813474347`, 0.05239854389036264`, 1.3414720605906971`, 0.4513315960556996`,
  0.24795401762018465`, 0.018990463442733085`, 1296.597641454505`, 0.13705502920429874`, 197.81881626257612`},
 {0.12947779232825135`, 1.9145873156534714`, 0.0288911518259296`, 1.3705880176351148`, 0.34333617932115823`,
  0.684343564948868`, 0.30854336196404863`, 221.38025911589182`, 0.07491746507159502`, 703.3196235432932`},
 {0.07972116217449848`, 3.4709822723848074`, 0.04576191227888536`, 0.8016369799963272`, 0.2857357367482012`,
  0.3331460762626077`, 0.19358831260020504`, 243.63380400065088`, 0.06895044823250152`, 836.6535842859868`},
```

{0.21425814556096656`, 3.412718953326978`, 0.054294933223100375`, 0.8434533127486217`, 0.5949443832779631`,
0.4312511536994379`, 0.006774834458589081`, 264.20975235216673`, 0.18238909307892875`, 619.340566973434`},
{0.1030949911235679`, 3.2379695384683664`, 0.01929347915744433`, 0.9201548487613123`, 0.33739940615248143`,
0.2622793831217315`, 0.23841206764006043`, 1617.5278268905984`, 0.22935132610715692`, 896.4339292297652`},
{0.11659113279351957`, 3.589133205154993`, 0.07913036370723393`, 1.232106957500204`, 0.8783988188812624`,
0.36486871630814866`, 0.12887619218337418`, 531.40225777078`, 0.19649941700330426`, 196.14313660000954`},
{0.17261096694115408`, 2.323826295719984`, 0.07573381776980444`, 0.8286032399217114`, 0.47983148793793484`,
0.26566773118764775`, 0.03472365295524612`, 927.3460129902165`, 0.1071272043003238`, 118.60052904781728`},
{0.15812900546790443`, 2.970437030179216`, 0.04641714570644767`, 1.3852443817278377`, 0.43447243659924073`,
0.33574441370838803`, 0.06435446224943432`, 738.6696158761797`, 0.13010058805108404`, 159.186393081969`},
{0.18592939740642223`, 3.685442589505847`, 0.07945757449204009`, 1.1464224447055114`, 0.32318739791637174`,
0.4649314153677717`, 0.013135441017131483`, 91.15430963381306`, 0.11860323377042997`, 381.3120573740848`},
{0.14376640925421846`, 3.1586224337701454`, 0.060466852669727789`, 1.2403049905427794`, 0.41520844208600294`,
0.6171055455360721`, 0.32907344516358666`, 110.43943865511335`, 0.09753160070705702`, 466.9944611056849`},
{0.08898564055719721`, 3.6481907193018275`, 0.035736135100176744`, 1.4644447392392914`, 0.3855084819966672`,
0.5403543534096678`, 0.02564672554096082`, 3261.3971739494646`, 0.059327692271889`, 178.0636196825421`},
{0.12073346816538172`, 0.6796921643614748`, 0.004902893841691274`, 0.8582452939012792`, 0.29165716690801435`,
0.6326916410656882`, 0.010914425076951347`, 71.33612718800642`, 0.018422948540203682`, 499.39101647732576`},
{0.08643478967247115`, 0.989294451274473`, 0.06406214590812752`, 1.4778521994730294`, 0.08864943219429122`,
0.598523331628892`, 0.33924017802608397`, 243.33847401579249`, 0.1630923948211001`, 823.5357318052028`},
{0.1858290979683801`, 1.3708640419198979`, 0.03326172130438391`, 1.4020046543416833`, 0.11208251576177464`,
0.2625003629250131`, 0.04524672886209799`, 85.06379115175038`, 0.18737298414395726`, 615.0030267833952`},
{0.1360682807160305`, 1.260103979250026`, 0.09631205400585557`, 1.492449539848565`, 0.646188927535122`,
0.44017189812527846`, 0.4105272425293273`, 393.8607844518778`, 0.02987920633520591`, 916.2850221914006`},
{0.1722967578431442`, 1.2064516945248789`, 0.006692486646882791`, 0.9574846319241982`, 0.7639212221033438`,
0.18533797387085427`, 0.023148480320789932`, 1419.1935015960435`, 0.20782666110883385`, 726.3055622381481`},
{0.10120560496305181`, 1.0077236027617351`, 0.0022035031006077867`, 0.7556532356144234`, 0.4541695517780062`,
0.5272435056704864`, 0.48611131067523233`, 2530.0708561728975`, 0.016102479809594256`, 464.0343986269878`},
{0.12017322050098367`, 3.9721385392144386`, 0.07574165293605646`, 1.1837442643757194`, 0.5577757261385461`,
0.3183184732454556`, 0.006181796792011399`, 369.4013122196196`, 0.18128847005418425`, 194.03504301481541`},
{0.04827662252574555`, 0.44476989943772693`, 0.04589418991887675`, 1.0976519464428027`, 0.5211218352826839`,

```
0.35897962206032186`, 0.009662240534770522`, 2226.690418934838`, 0.1554674743129576`, 512.7162631562013`},
{0.05162633508079817`, 2.3550751217523676`, 0.0924989591884071`, 1.4157997348260007`, 0.18969184963171148`,
 0.23851541168228685`, 0.13394186077419723`, 885.0580489506601`, 0.09290724513295379`, 581.8072838231085`},
{0.0703807113832926`, 3.5028014587851954`, 0.09731342789234894`, 1.4652588215217102`, 0.02379062714059521`,
 0.45288235271478583`, 0.00942034539711149`, 1971.3577596359983`, 0.20635147200229786`, 588.3355736982174`},
{0.1722158503566622`, 3.9025180218674205`, 0.03287113170420332`, 0.7633959662136292`, 0.5426808864022441`,
 0.3669211940284537`, 0.015798227561105518`, 637.0091161622244`, 0.018607030959375853`, 729.5190439080948`},
{0.07584294958741444`, 2.9472829223076644`, 0.08633754726872958`, 1.4485030454804857`, 0.4347108871800047`,
 0.4606559371854728`, 0.488724952784251`, 373.5721326650099`, 0.07716317740414885`, 124.4741797765185`},
{0.24562593090753987`, 1.7880136046455286`, 0.061199201036337365`, 0.9504446252866968`, 0.8812906909113087`,
 0.4211899677669948`, 0.13371199909368486`, 62.37984939467348`, 0.029994323176434023`, 335.34030777918923`},
{0.20825483037627235`, 2.8176663305166816`, 0.03003135959790159`, 1.3809928219048555`, 0.5442546740990999`,
 0.2518879146968922`, 0.41623765779197747`, 1730.0044330431615`, 0.13532415983523782`, 593.0952047283174`},
{0.263098249275039`, 2.8719245261273185`, 0.057294037398088714`, 0.9272583089313478`, 0.5794976650588739`,
 0.23557943476926713`, 0.18465618219605576`, 743.7540401729835`, 0.12098568685424893`, 886.4027693713879`},
{0.08125449990693445`, 1.4583301942945166`, 0.06189519777160188`, 1.4288277781740808`, 0.40197254124717063`,
 0.569085386893778`, 0.039194281573835754`, 4748.401415793844`, 0.20401727261566`, 992.5067856915206`},
{0.13086541558871118`, 1.1155725899250655`, 0.08821908955004687`, 1.3478216191079166`, 0.17697042399948426`,
 0.36922117198662885`, 0.013930530425628602`, 265.9989136069838`, 0.2270416561589264`, 365.77204194037876`},
{0.11728100988180301`, 2.8151532257585448`, 0.0822952483643148`, 1.4499657290535213`, 0.1422354506761183`,
 0.18155390727644505`, 0.00812157039144657`, 2435.595550489932`, 0.23995058381388307`, 812.4638732087483`},
{0.08049510800011694`, 3.603435994634494`, 0.007656876221115407`, 0.8287914836133501`, 0.8266092354769616`,
 0.16324385237676353`, 0.08964145435399143`, 249.97635146943674`, 0.11550610667653838`, 765.8954747498603`},
{0.11820480579010878`, 2.60227134202185`, 0.04712951554705157`, 0.9880816634883703`, 0.8571809674721294`,
 0.5649946552056018`, 0.017402816460863866`, 131.80950340449587`, 0.2293131996837257`, 460.13754914511065`},
{0.07353660759709546`, 3.5071493949530606`, 0.06008403863777037`, 0.7789529963855885`, 0.038880804706464156`,
 0.24085809668436897`, 0.009207422134001322`, 57.365635753359946`, 0.19618331785464976`, 195.58478591654787`},
{0.14064741473086906`, 1.4253939258069908`, 0.09267725847840932`, 1.0975927496329574`, 0.22744648794341704`,
 0.6063348207456416`, 0.03639182347931457`, 146.15301297855677`, 0.07935681769679304`, 259.4064208000535`},
{0.15191662908335968`, 1.3875044471862585`, 0.07598535142080483`, 1.4975086468472412`, 0.14071696059051497`,
 0.2702248025276742`, 0.015378992010870783`, 1297.8058794576364`, 0.09563415627019778`, 267.1180590070449`},
```

```
{0.25783578932170337`, 2.5098960068891536`, 0.050196727090169`, 1.3457348584940565`, 0.6334273233006393`,
  0.673911062730165`, 0.45542142980584244`, 1102.3077248576458`, 0.23127744020305874`, 280.7607833294188`},
{0.15728354284965573`, 1.1786483564465087`, 0.018710974043178813`, 1.0906308648763483`, 0.4692891978030753`,
  0.5884245578610128`, 0.013999626118543194`, 1146.3540981218957`, 0.12976415437247035`, 943.3629249089528`},
{0.15744525017814853`, 3.9343441044396323`, 0.048424319387596705`, 1.0189964999962107`, 0.004699907891199784`,
  0.35673641117911215`, 0.05845873245808589`, 1452.1810356944638`, 0.20019375268922035`, 991.5190215908439`},
{0.17711189820828616`, 0.9222841106349815`, 0.07731703618957698`, 0.8581707670014744`, 0.3028714201791183`,
  0.3403814085412159`, 0.23552438227321898`, 185.6757896447749`, 0.15816694433359801`, 971.5707714133565`},
{0.12677717388625714`, 3.135520314905561`, 0.0958777837314775`, 0.8945786394252853`, 0.6294861294982774`,
  0.5552940848485673`, 0.015606330011113338`, 857.9173420903631`, 0.2215517078689302`, 931.1017779440821`},
{0.21702108783814894`, 3.801779784508854`, 0.04805641449981236`, 0.9791693500326768`, 0.2245653714607041`,
  0.5686073977459262`, 0.13790116867346938`, 202.46130862913807`, 0.1350408422039594`, 899.1324956275001`},
{0.1342014919902998`, 2.2832877374577247`, 0.03691753722884416`, 1.293132587653252`, 0.6577552625086529`,
  0.43953980905115686`, 0.01957893390503574`, 205.45267528315344`, 0.11907718935756234`, 594.1955832844324`},
{0.1763744245545265`, 3.739113668312668`, 0.013915261017202703`, 1.4628218122817214`, 0.33832232520701955`,
  0.3614238110077841`, 0.16749424131931667`, 750.7001786075303`, 0.16098306031842058`, 199.9116742658522`},
{0.15496422412126426`, 3.2726277520924976`, 0.04166420456847202`, 1.246251490612826`, 0.03723582004781312`,
  0.3169293011472515`, 0.01364338217624883`, 794.7700944318913`, 0.13403305026443924`, 181.03718972044246`},
{0.1794519767096271`, 2.1595478222580216`, 0.024155004216522703`, 1.2232218292039836`, 0.12872104355462577`,
  0.3105596069491494`, 0.08640881754488854`, 438.81545657103055`, 0.13579526038432777`, 696.2213922750994`},
{0.17257389690071828`, 0.774117551399768`, 0.008560234856989925`, 1.4940154641879095`, 0.5531402581000995`,
  0.5441107289119099`, 0.07350653490141655`, 1314.8314542940839`, 0.07345603701745179`, 217.02216774233065`},
{0.058763034644712275`, 2.3597566945503834`, 0.09055618598093744`, 0.9263657689345207`, 0.5592210171940297`,
  0.26262790696306815`, 0.011057611204984635`, 211.09574188287445`, 0.05571628830959996`, 675.171201042442`},
{0.23649888242497868`, 3.0694012769080574`, 0.0293881057277982`, 0.8469899032416341`, 0.5329226292412448`,
  0.4016300524993841`, 0.08030522569307784`, 885.044604178621`, 0.10534611402615895`, 622.0478384674557`},
{0.10023410603180455`, 2.710596168246937`, 0.00298453338827164`, 1.151795972074526`, 0.37899349738211896`,
  0.3392482218824491`, 0.04819504839896153`, 379.39038491273027`, 0.04469242387250283`, 827.1435707909849`},
{0.2438903560692493`, 1.1739940858261848`, 0.09102105800199446`, 1.392584698828661`, 0.6056955824388208`,
  0.5844521424450305`, 0.36108528400570095`, 1701.2530207661314`, 0.11138522848439819`, 308.591402552778`},
{0.18793054675634024`, 3.139542677489672`, 0.08904425028360773`, 1.4068659558890193`, 0.46779742309438377`,
```

```
0.4804254450552986`, 0.31816862647583416`, 52.007157617767675`, 0.20815008924208545`, 645.3217048463795`},
{0.11103510208969514`, 1.4116670170359447`, 0.01094255418186231`, 1.3998271086778162`, 0.20796976919688226`,
0.21292073278793122`, 0.025573376903052888`, 86.81893641374597`, 0.03626690081608863`, 355.9231685657631`},
{0.059767794596915114`, 1.8804870041256674`, 0.07095681077704744`, 0.986817038205352`, 0.25655361425528533`,
0.1688625628269923`, 0.1897075342910607`, 67.57112317559029`, 0.19542023600496528`, 963.6021600539973`},
{0.0563343868292458`, 0.5317396897533424`, 0.021545836837937156`, 1.1439759156456886`, 0.8881137136810024`,
0.4156539248732849`, 0.006243471710013326`, 260.74733013676007`, 0.24312372574340813`, 696.7530796966602`},
{0.25281786498427167`, 3.164006130077736`, 0.003838750721372204`, 1.0744832678586205`, 0.006849791935805038 5`,
0.5152441133630022`, 0.32224630023151535`, 264.37961782996655`, 0.06028750963433477`, 143.50957591757913`},
{0.16077715085906397`, 2.873224638724322`, 0.05173801459016383`, 0.7810632519144793`, 0.3328755824267646`,
0.25599177499415204`, 0.29027099579570653`, 56.71390358897392`, 0.03391158089690982`, 522.8798210385845`},
{0.21945025832526777`, 1.6613676443983767`, 0.0033920248574508707`, 1.057752773749318`, 0.2127399240494794`,
0.2783903022229003`, 0.011232097563550716`, 1371.276504196438`, 0.04975311118637471`, 328.70478941546`},
{0.042964316768383276`, 3.4639489625729407`, 0.050691741081661766`, 0.9465946216833707`, 0.05502079292147011`,
0.21238865048524036`, 0.0776422991682389`, 52.80026867227022`, 0.045020214701994854`, 474.41563595375555`},
{0.2433495999796823`, 1.8960270447479708`, 0.01120198462392917`, 0.844049298875671`, 0.4639208209806316`,
0.20677684984563183`, 0.005733096581983471`, 1740.6658389561255`, 0.1399372816422193`, 628.7700453738331`},
{0.09814127364381425`, 3.1310383407484847`, 0.017016069480737403`, 0.9217748908925876`, 0.27863388851936555`,
0.32184170341904983`, 0.006207472689688149`, 1391.556177922241`, 0.16816866777593836`, 615.9674047842972`},
{0.045179834179820105`, 2.124737466901916`, 0.0641039677828362`, 0.8087710768531244`, 0.5820240872072311`,
0.6898665398618178`, 0.014125759687518747`, 92.19454081613313`, 0.015821689323895105`, 557.0872764229717`},
{0.13473842282979165`, 2.4117633636086957`, 0.01984561256760543`, 1.1204070544446296`, 0.3860645674275691`,
0.4170622324306399`, 0.019865666343063196`, 4743.148389421012`, 0.04365212493615206`, 322.93322910718985`},
{0.2527515364737705`, 2.1426280202987984`, 0.015606989419899584`, 1.125943287101995`, 0.17776360180897943`,
0.332914794116577`, 0.006519056311462601`, 78.48896188261014`, 0.12889711459762526`, 805.9196842114062`},
{0.11933913407793822`, 3.785485787216923`, 0.023574857312977055`, 1.4812641194980447`, 0.6275581210964223`,
0.5280696447262914`, 0.07256024376782456`, 241.814705594111`, 0.02783432907145139`, 151.3743596717724`},
{0.1349803930463928`, 1.8072527206786377`, 0.06447251791280252`, 1.3994960916143184`, 0.7359286316821669`,
0.20162137238693434`, 0.4265854166639518`, 227.4386051384146`, 0.13592337876958527`, 497.305076361482`},
{0.2637152651333133`, 3.0391917107143414`, 0.0836047103384918`, 0.8164164023077424`, 0.5538018525602337`,
0.5004598109403166`, 0.04356169162799880 5`, 3000.1047519173976`, 0.17835324975540034`, 767.1983419134932`},
```

{0.17730450742771336`, 0.5526401983890268`, 0.003866546119779244`, 1.4224823875795298`, 0.9713446082448272`,
0.47362133992508215`, 0.008799575820309609`, 460.0427974972538`, 0.1268858776449615`, 614.5975351416361`},
{0.17661690011227865`, 2.968666144862362`, 0.08820580921653833`, 1.2618748536532438`, 0.014044591388426397`,
0.6372850210418368`, 0.42461957500782593`, 908.6535515532695`, 0.16038310667129974`, 176.50654123832936`},
{0.13672640919029144`, 3.614316511877404`, 0.006473169531331831`, 1.1642896824935516`, 0.29325294311380845`,
0.16242724995588664`, 0.030111728610588202`, 3933.205934300977`, 0.0980103812613437`, 106.80306509175406`},
{0.07145172009956824`, 3.410868367663567`, 0.08572451188759761`, 0.9584474542559545`, 0.040382356795113816`,
0.6455580091740019`, 0.09117312647503195`, 1954.631204089039`, 0.0777204277630249`, 144.9524499282374`},
{0.13883370155342772`, 3.715034522567122`, 0.016916922596204795`, 1.0000211488246231`, 0.12894118452278325`,
0.24258423424654219`, 0.02087499948517953`, 3780.1181299010777`, 0.24420906754435595`, 798.6323972467644`},
{0.21440197837823782`, 1.7615321043929084`, 0.028950668418736624`, 0.8631531769083587`, 0.523525808424337`,
0.2748912735476704`, 0.38068084592210494`, 404.89859548140885`, 0.15408624036569346`, 803.1703020004433`},
{0.27645780167060263`, 3.175276677600297`, 0.05299560517709523`, 0.8086008634045363`, 0.7913310844792734`,
0.3862667201926391`, 0.00961380004706706`, 2836.236561313336`, 0.22859831535956182`, 465.6465289390621`},
{0.06752882943979077`, 1.4908646201280487`, 0.03739887788507268`, 1.1321270467820062`, 0.1590886090266126`,
0.33058750825147076`, 0.13664295377993954`, 324.6730016626649`, 0.23288398005137873`, 299.5733325408761`},
{0.10900083730464943`, 1.3165965495337897`, 0.04096974974497696`, 1.1178893614799246`, 0.02481354114149492`,
0.6533849288221782`, 0.0989115744507999`, 59.94210305630967`, 0.08210787534991087`, 838.1221078005716`},
{0.18821745287135477`, 3.8834155988951853`, 0.08623976994927297`, 0.9451777127739934`, 0.2536651767905649`,
0.4966204244968453`, 0.052355463971012595`, 1253.2350223323115`, 0.025530907055034302`, 107.88292349347337`},
{0.13433362908189822`, 3.976975346921523`, 0.023713728561632463`, 1.330972786055094`, 0.5425931686264049`,
0.5867946121348381`, 0.083015502101061`, 2146.731613991672`, 0.11433497831643785`, 956.3793106500469`},
{0.05370292234035079`, 0.8618436041219777`, 0.09065607433649935`, 1.1199215929503574`, 0.7033869517843656`,
0.5744405842935578`, 0.20721453360880282`, 163.81435947307054`, 0.15960680813292716`, 808.9619958368114`},
{0.1982510252132108`, 2.465970568324745`, 0.012757083454587495`, 1.214300872852428`, 0.49977561113114644`,
0.2038568209362135`, 0.04565388315282881`, 78.88460864136573`, 0.1487998824136843`, 164.57689100626015`},
{0.07006449306202894`, 1.594466036555235`, 0.06954877206660835`, 1.0929666985547377`, 0.8182673122674597`,
0.16380036850673574`, 0.04761505660877746`, 3094.547812800814`, 0.026912730925264194`, 465.941138242661`},
{0.271620743989626`, 1.798748508191406`, 0.039226132696162194`, 1.201815394348386`, 0.019033121630884553`,
0.500394949620919`, 0.022898921692070705`, 167.54130076556643`, 0.17172411650490582`, 118.02597321115923`},
{0.17015700880843931`, 1.1880680435779825`, 0.09612747942645765`, 1.220203731936928`, 0.8171639571524099`,

0.29942524478114674`, 0.005372366003640249`, 607.4487584881643`, 0.0444162424317765`, 783.4604894784995`},
{0.05414527078515874`, 1.462858717153524`, 0.057585439178684264`, 1.0235777810825535`, 0.21014799698731879`,
0.27005953264512594`, 0.08376210292871798`, 147.35977444766536`, 0.10744240382696624`, 846.2849324778499`},
{0.09237320793743342`, 0.8096523848475279`, 0.004358295040692077`, 0.792421334681352`, 0.9354401956315697`,
0.4996618434240512`, 0.3257079961489366`, 106.0381723510655`, 0.194703596109299`, 875.1849354846129`},
{0.050247694605344284`, 2.3552798474120182`, 0.045898586534112516`, 0.7657469521997533`, 0.41572162978856886`,
0.6250502955136628`, 0.036232245589174594`, 4925.729350966778`, 0.048338820069271815`, 396.9216964218738`},
{0.16604829560323886`, 3.2400306473910465`, 0.07805779128319201`, 0.9800901947770135`, 0.02662429037050673`,
0.6202147453627995`, 0.0521761817913341`, 319.666779674947`, 0.1526499768736515`, 641.0814189728534`},
{0.13199054420769823`, 1.1822647026678919`, 0.05315251799812888`, 1.3177578061426032`, 0.29009102787676366`,
0.41153300112684843`, 0.027654998358556677`, 862.9125744805588`, 0.22565453257412804`, 148.78642951801135`},
{0.15326781447665427`, 3.7132135007643896`, 0.08379527648859465`, 0.9155066607147588`, 0.9247491129828225`,
0.43619880658774446`, 0.0392674826152922 8`, 1156.2321575105877`, 0.17547647957302887`, 889.8562914260897`},
{0.25318856010923574`, 0.5926097353022888`, 0.00482353837495765`, 1.3099001178559129`, 0.39149861531549`,
0.647818541877643`, 0.25232461737348105`, 3385.571345294367`, 0.19387361798630304`, 420.02567016405266`},
{0.24993243970651624`, 1.7126281402055845`, 0.053130097502228`, 0.8352588223269972`, 0.62388332746021`,
0.5762423608317969`, 0.014465580674644738`, 799.5116049181332`, 0.0931416280797775`, 865.5405963251784`},
{0.19561758310618566`, 1.3596417600698452`, 0.00286504944841953`, 0.8159672694367253`, 0.6358724387725987`,
0.6648416656940517`, 0.24177467962143764`, 3682.5795096853144`, 0.15293398533505775`, 386.4064670424524`},
{0.2447874085020979`, 1.7744291418487776`, 0.060075050805309844`, 0.9864750267055266`, 0.7342694514660453`,
0.5397411982908532`, 0.4042131026855616`, 575.9477405967004`, 0.08605927470106772`, 780.0883449415986`},
{0.07734058301535968`, 1.385482226243342`, 0.07152447691997342`, 0.8194619571046995`, 0.4573258756273968`,
0.6888219817424488`, 0.1334830351653064`, 199.92831385353017`, 0.08814262925395056`, 749.6278229482897`},
{0.10474568576280202`, 1.8596472368495407`, 0.026805293435349233`, 0.8198649413737059`, 0.5254103672469626`,
0.2748607556216799`, 0.0523834433135859`, 134.70642314731086`, 0.10106748211794048`, 140.99421671530888`},
{0.27890810575443187`, 1.4911450307991796`, 0.07330732877841264`, 1.0850224852017472`, 0.511581631756513`,
0.6844047819742511`, 0.4182444515115468`, 898.6500980057638`, 0.1508853505547692`, 117.74931414924143`},
{0.06710090564388627`, 3.150840959910446`, 0.0014124858884598979`, 1.2404545757648113`, 0.6590308151409987`,
0.18742776248185322`, 0.07838269037439821`, 63.02543384043976`, 0.23165453423661014`, 134.9827251445024`},
{0.2709692633763827`, 3.0318490895249166`, 0.02057996898061427`, 0.8497637000182061`, 0.007946519407763919`,
0.4537529292092372 7`, 0.2315631409174914 2`, 3625.9461118472623`, 0.20365524494076642`, 575.3949074180657`},

{0.15487330171159508`, 2.506241955627659`, 0.02405679630252637`, 0.9599539152532017`, 0.4302913454357331`,
0.26259574333059643`, 0.008099203461346816`, 910.2082053871129`, 0.14622107038108184`, 559.5315182891957`},
{0.2128087852183892`, 1.9571603978537304`, 0.06326297053797199`, 0.9699003392267482`, 0.2686671251031061`,
0.689623844783678`, 0.35675989285485865`, 1924.6232853270492`, 0.23326907367518723`, 915.1337901802619`},
{0.21780708062769377`, 1.1747776713117952`, 0.05034010906267806`, 1.0913007058111903`, 0.12353581217661103`,
0.3210562571265647`, 0.0322536912509453`, 2197.1982196175004`, 0.1572434618024398`, 414.86282493944026`},
{0.10779474152210772`, 2.5545130403913596`, 0.09607448919858275`, 1.2919315212701343`, 0.16453105789719658`,
0.6141245226266829`, 0.11684667552500605`, 196.8514855477255`, 0.10491847566430007`, 112.73037991828173`},
{0.12432532516395484`, 2.130060821664687`, 0.04913772379496075`, 1.114341717459199`, 0.2610859336877449`,
0.503197213507616`, 0.005797395507410687`, 442.94048846434436`, 0.08741076870037628`, 101.71296622490348`},
{0.27464475878650824`, 0.5510255383265457`, 0.031636220540726276`, 1.3762194055027865`, 0.1612567072728912`,
0.4273900785282383`, 0.24603471434298904`, 429.9496642319049`, 0.08771573488057915`, 617.3490635717783`},
{0.13029287084326696`, 1.6894675116680409`, 0.020566584774051967`, 1.2832846584058795`, 0.1828960091839662`,
0.4605785391689514`, 0.01135531247121644`, 936.7188945707877`, 0.1761057360191931`, 440.679523047495`},
{0.20510392052346132`, 2.0152643396686836`, 0.05752244878800122`, 1.0760055282794754`, 0.9860596725458286`,
0.33518476119672647`, 0.31856559936642365`, 321.34121932230744`, 0.10497410797788737`, 281.7881521533858`},
{0.22222058819666546`, 1.9181392247611697`, 0.06353852306281663`, 1.3758404515279568`, 0.6438678141184426`,
0.1693786566729748`, 0.06897920477033548`, 383.0298020915635`, 0.10314770849680055`, 941.5930224696303`},
{0.21276124668973173`, 3.586839401277566`, 0.039178902951713945`, 1.4021843911360103`, 0.5049572433506289`,
0.25133132700774996`, 0.12364866403599967`, 623.8499997024826`, 0.11712847066144477`, 770.7321547534711`},
{0.14968687983730605`, 2.3633590766978303`, 0.04173348230150333`, 1.0168117310979305`, 0.07526686867368393`,
0.37085554163771406`, 0.00768165513782035`, 1659.4326212173246`, 0.1437616377539393`, 942.572084587879`},
{0.2713741706327081`, 0.4242782972192547`, 0.07078081316399253`, 1.131720191226623`, 0.4549262787557089`,
0.34468168234433216`, 0.012942279609896707`, 2864.005301731598`, 0.09798425989157222`, 187.35732163896745`},
{0.1768611384179205`, 3.4923686338372777`, 0.01646184906513861`, 1.2055226780828572`, 0.7343581951493021`,
0.49378742542959486`, 0.12048169571136383`, 2844.3094803530207`, 0.12693060413099172`, 545.4728905288805`},
{0.2369345296836391`, 0.41785553179229007`, 0.012526518118659648`, 1.2179877794222598`, 0.01846774830289344`,
0.24685867084706536`, 0.0219438069009795`, 2091.108395341874`, 0.0637607114780574`, 657.8903568599761`},
{0.21219769453448084`, 2.488104916250064`, 0.038757338160515825`, 0.774789527802302`, 0.4580558695696113`,
0.4263775736158787`, 0.34953699783630143`, 407.33606250987975`, 0.16713719417824968`, 389.7058187031195`},
{0.11474454899039521`, 1.9841405956471387`, 0.009912438082135093`, 0.9199083705387736`, 0.923535949012403`,

0.6470781918812183`, 0.3331422211969604`, 337.2489956721694`, 0.03475601824362995`, 538.9648304299371`},
{0.24234974629560802`, 3.9617359783801245`, 0.024444176238852366`, 1.4313282134950815`, 0.9940513113675349`,
0.4143492563365402`, 0.1686590259483461`, 68.11457543267173`, 0.19793807681647463`, 199.02629821091972`},
{0.16236198209412894`, 2.018699278359029`, 0.03626856618687025`, 0.8436685495890566`, 0.1369133257491073`,
0.18228484143603907`, 0.040551804892385857`, 156.7528279727044`, 0.1739090814809508`, 704.2383268945476`},
{0.1621577456082553`, 0.6421908435706589`, 0.0682618183426171`, 1.3238181641882336`, 0.9244165647014204`,
0.6930553521066385`, 0.13666203325037238`, 72.36369505518103`, 0.2211105145097111`, 327.06990857144615`},
{0.2702619348258324`, 1.3531215117783164`, 0.06648966931523986`, 1.278915076565248`, 0.7821690760804008`,
0.4832187850250197`, 0.03589870905112195`, 3130.704914059961`, 0.14156174429321455`, 311.74724954691885`},
{0.19935819825153228`, 3.831285341751962`, 0.061322567547608904`, 1.470671933797358`, 0.8459029813533634`,
0.6098411086562021`, 0.020718424330298454`, 338.23739251616234`, 0.13481110564933563`, 328.9796748551533`},
{0.12820856017846227`, 0.9705044791290884`, 0.020813708364227884`, 0.8227896910361784`, 0.7793252261285137`,
0.1540874083560777`, 0.019838047780930596`, 1485.5571438891636`, 0.16001313078246715`, 526.6105313836905`},
{0.11895043241393044`, 3.4783832049993846`, 0.08948985928895077`, 1.359795719210997`, 0.29460393871375956`,
0.35020600424213755`, 0.2746425669615434`, 50.083789355993105`, 0.020545547787407692`, 916.6997924739111`},
{0.1324468409940383`, 2.674634590833133`, 0.04368586320677444`, 0.9656288911809869`, 0.20832826835990526`,
0.3737566616358602`, 0.005582642109220031`, 55.02582924504472`, 0.2065757664044986`, 615.3366029893464`},
{0.06870110651611899`, 2.6561693493128598`, 0.08012546640983625`, 0.8012667555503843`, 0.031201182173437925`,
0.47282927837851574`, 0.011587617652012341`, 3773.634663778097`, 0.09147096998858567`, 835.9545348732762`},
{0.062399865483643235`, 1.7222840522480984`, 0.0921920191456875`, 0.9286857558090922`, 0.3251018367521341`,
0.6108891276310624`, 0.011783888986275243`, 670.9821418853228`, 0.11450398298509856`, 971.5077469427929`},
{0.24591568234282185`, 0.8566394248947855`, 0.0781061151752332`, 1.3941060542854928`, 0.531302066365049`,
0.6676078161595655`, 0.1738149187521547`, 158.69973172683407`, 0.1485119105497385`, 936.3340802553399`},
{0.06567085889032587`, 3.9337642436409004`, 0.06099064297489713`, 1.4242284268139727`, 0.6539174667789966`,
0.3970266459135522`, 0.44668463659234353`, 1229.440509286354`, 0.19297859064513334`, 104.53761160348893`},
{0.13077961834437962`, 2.6471887027919063`, 0.08803755851714129`, 0.8915760567705249`, 0.43528239011744874`,
0.6770072844670658`, 0.13724713444300793`, 2266.2113033407786`, 0.18158499094944586`, 450.76741342966307`},
{0.0626945060366299`, 2.0207863261901435`, 0.04028779663279089`, 1.1608394772654784`, 0.8954638473430083`,
0.6803040308141024`, 0.0438755308716426`, 1072.86320924307`, 0.09417670420636176`, 203.3883701052643`},
{0.06633302157481619`, 0.6897085661220981`, 0.028600562511313363`, 1.222942456179899`, 0.45117275354230246`,
0.29367119707525824`, 0.1114281718553938`, 56.37596421013418`, 0.0632983152776589 5`, 495.6622356704312`},

{0.0758463119875773`, 3.621924471713986`, 0.05284360701608939`, 0.9710310359872816`, 0.3507881375218056`, 0.16711099920227068`, 0.1488874639582541`, 4335.730117904631`, 0.02658055782022295`, 793.4396666616456`},
{0.06315507988926289`, 2.3297312843414817`, 0.00212708414905217`, 1.395352700358994`, 0.064170149018216`, 0.5894635107517013`, 0.17809044151421888`, 379.3821707157771`, 0.14230190042275537`, 759.6382607226183`},
{0.2099022158481964`, 0.8809344339859928`, 0.0666751548993475`, 1.4387478328331107`, 0.3531306367025422`, 0.6983916858003694`, 0.014622538997794657`, 343.9480456094322`, 0.08685603103174627`, 426.825757963735`},
{0.18558107573674792`, 3.0194943343339986`, 0.07461391639025422`, 1.1191398296135584`, 0.8305222019918481`, 0.4880407736474275`, 0.30782204523980083`, 408.3075491000427`, 0.12514760530359148`, 124.23432338200726`},
{0.20051301950717854`, 2.977167267300211`, 0.06936215087558335`, 1.0259008901103701`, 0.6147776109399516`, 0.6258821206893599`, 0.01703908364047091`, 2344.5258662598194`, 0.08418799298306856`, 794.1399968502964`},
{0.23048732313041992`, 3.462281492713984`, 0.028460833429753602`, 1.0414779858519587`, 0.11068368992785804`, 0.46918663455326926`, 0.3985525154764997`, 883.0495583098383`, 0.2409468662662823`, 546.4207520227328`},
{0.211465410867755`, 1.6738825157761648`, 0.0523685881403539`, 1.2569145647413262`, 0.6940459633746912`, 0.19386279088766634`, 0.025145096530955775`, 502.1390324130799`, 0.03396962103018908`, 864.65280998117`},
{0.1929466873888901`, 1.5728514227245611`, 0.08398743525565142`, 0.8305271019348486`, 0.30866536632560715`, 0.24782227733300222`, 0.06727104200994766`, 4073.498181247491`, 0.09367172319166167`, 761.8812104829784`},
{0.09217356026466877`, 0.8891340726836079`, 0.02261394061546632`, 0.76288584990135`, 0.2352883211999115`, 0.4087782246426179`, 0.05761668157743837`, 592.1949999543295`, 0.1566262893866785`, 939.2503062920734`},
{0.12967920647240816`, 0.8123733202889643`, 0.0013573621543738134`, 1.2244900945773618`, 0.8403401749152237`, 0.45714635607461884`, 0.04113284132792122`, 683.6853491881902`, 0.10112850721928329`, 691.2742096973634`},
{0.17585942228676693`, 3.1377953149568008`, 0.0015744621411661974`, 1.416873746788956`, 0.5827830119619737`, 0.6742879545649707`, 0.09162791382644989`, 517.589004535642`, 0.16103345930524726`, 547.8413219418244`},
{0.2427638279415646`, 1.6566162823863317`, 0.06521965313277005`, 0.889062176257591`, 0.014518981829873656`, 0.2748704572491185`, 0.05735800450312215`, 2387.1739807284794`, 0.23199018500208857`, 381.464796278728`},
{0.2519418117913656`, 3.063462870735645`, 0.007778137389172581`, 1.4021617024508317`, 0.9924604733729623`, 0.36233195131635443`, 0.18682309925698343`, 66.44214693634154`, 0.1722230669694152`, 118.3771627882729`},
{0.17349345626891455`, 3.174288570189952`, 0.09652146927294172`, 1.3390466263031546`, 0.7909502427935802`, 0.6793254105715787`, 0.08700743247214943`, 763.4035938655402`, 0.2387926278324622`, 592.4203788389427`},
{0.1727078461124784`, 2.8460728910005475`, 0.040914427699617326`, 1.4313337292763195`, 0.42218527760026237`, 0.5016244570171771`, 0.0887548918690675`, 1587.4849620109512`, 0.1328615275829737`, 768.9319503194112`},
{0.08930008677181278`, 2.849971715639322`, 0.09169779308725978`, 1.2438628382038357`, 0.4154968472190934`,

```
  0.15405240023737177`, 0.04634668494948532`, 124.90941433976057`, 0.24982063081795847`, 463.43559716325797`},
 {0.04995942432359332`, 1.4610302960771593`, 0.0433388762401086`, 0.9505438290907596`, 0.488211452413962`,
  0.42119172502998126`, 0.012759652857857887`, 646.7948127587483`, 0.026663072682046873`, 245.49015570500714`},
 {0.06795497506231873`, 1.985917542879199`, 0.04349282644250348`, 1.0631785554908315`, 0.8091824452378926`,
  0.2147272979078938`, 0.03845431657393897`, 607.4631067901736`, 0.08409909386800546`, 814.8177301929959`},
 {0.27140987444452636`, 1.6298377410728744`, 0.06029749698810518`, 0.8272061170373771`, 0.9095594755225849`,
  0.2626530714660902`, 0.22141343547525327`, 1531.0082827603965`, 0.16934746708503456`, 553.9108700580455`},
 {0.20249329091250423`, 2.9260026808453974`, 0.09022408237555009`, 0.9840606653871036`, 0.4430644155249066`,
  0.26716691431663997`, 0.012442882013869866`, 1252.39191368304`, 0.1408105612614876`, 866.1715515451732`},
 {0.13229111529623555`, 0.9037627200690848`, 0.04928384913936972`, 1.0937814374110013`, 0.9849443259560173`,
  0.43115790526717`, 0.019499938095150635`, 129.0159606559662`, 0.1987553839173642`, 393.89165319051233`},
 {0.1970263766379653`, 3.9709307503384244`, 0.027958813802574572`, 0.9741203705977242`, 0.07278184160194923`,
  0.2945649201344811`, 0.013078030291277284`, 4971.688891574707`, 0.03180082765265402`, 755.4217432398528`},
 {0.2049473596100468`, 1.974812376290127`, 0.018059141094917592`, 0.7718344152131382`, 0.3708285362372443`,
  0.2734681930545607`, 0.057458033126770496`, 523.7031096038135`, 0.02427329957544444`, 932.0905826464876`},
 {0.06537885284118145`, 3.340707691253673`, 0.05435168225428796`, 1.020827128283552`, 0.11666967592415722`,
  0.49052917065042767`, 0.0525916907549581`, 117.00169938700564`, 0.09552258651198203`, 745.6309012306111`},
 {0.11289673562266261`, 0.6794824966294883`, 0.03387634458174361`, 0.7927471128390904`, 0.07141719167345717`,
  0.5226577421579536`, 0.03829715492190054`, 2920.8934693444294`, 0.12081443854156904`, 964.8985972362055`},
 {0.23766500383642747`, 3.8767325971078446`, 0.0584809543600726`, 0.8270011297527531`, 0.6149673245232845`,
  0.22395807159158476`, 0.013437587174666855`, 406.005827450584`, 0.2418694331681166`, 429.2383814891535`},
 {0.2081776292468483`, 2.815887981403362`, 0.005125757826924850 5`, 0.8103479560894872`, 0.47351709229198957`,
  0.44544132586608753`, 0.4203259951948646`, 204.28043485055284`, 0.1992997883804286`, 827.7788091184511`},
 {0.17107980313911997`, 1.611778555980452`, 0.0845456798645581 5`, 1.1369505371366053`, 0.28226812138903434`,
  0.6771477606702629`, 0.13278544535359882`, 251.49910155434924`, 0.03764393371120245`, 453.2818524693168`},
 {0.10511512871602496`, 3.5633360415567505`, 0.08943300281707153`, 0.8304995882196464`, 0.4228782437765999`,
  0.48401108468440646`, 0.019832659219655203`, 164.88993273954887`, 0.1933397413498107`, 248.59325812442776`},
 {0.1315672644477009`, 2.3773291392807145`, 0.05190617787787307`, 1.2351268314391448`, 0.45158718255427366`,
  0.3842243141032361`, 0.038449226291938914`, 225.9216681882473`, 0.04341773379489022`, 914.0642920476225`},
 {0.20624347857829856`, 2.4671372276614543`, 0.050734528297655734`, 1.1490024452760461`, 0.2202047098600366`,
  0.3908877988906867`, 0.02383144777523708`, 157.0379664488479`, 0.14716356068566494`, 110.7973831044216`},
```

{0.27080390848623775`, 1.9772333839416323`, 0.024251112595875153`, 0.8820022792455461`, 0.651342077315098`,
0.5674047955018092`, 0.009713259722204017`, 54.31416433169486`, 0.012456849152343602`, 380.638174364041`},
{0.2662342547666148`, 2.0899914364276`, 0.0775212957879528`, 0.7629254448634359`, 0.7485227165858468`,
0.5717925034597559`, 0.384531789999449`, 531.2378339914305`, 0.010110683485557853`, 104.86289985839541`},
{0.08332863693803971`, 2.021121394734722`, 0.03701544455983157`, 0.9133309893951871`, 0.7759249909409063`,
0.6241937322174744`, 0.02957247320826314`, 100.8666790089409`, 0.19088570031299712`, 262.59746879189373`},
{0.14639594099937192`, 2.488995500972994`, 0.08291914891669944`, 1.3150002115787267`, 0.6540711522150526`,
0.2055454195673022`, 0.1946827902708822`, 1690.1447445658273`, 0.18733657548952937`, 812.4826047698352`},
{0.1305321301981358`, 1.4867396329579305`, 0.03655804827713382`, 1.4456727825450792`, 0.670555897133192`,
0.6184895636508891`, 0.051242554200489954`, 394.1191568715212`, 0.03171421941058544`, 345.85608840747835`},
{0.250721605591452`, 1.7010887783606767`, 0.08806284896739065`, 1.4847636082147322`, 0.4308392570475905`,
0.16893566899927792`, 0.08647127635050468`, 2056.3372882750145`, 0.12615446084315202`, 207.40781829993898`},
{0.04392874140447878`, 1.9818443681166702`, 0.09434829100781027`, 1.4634488477570156`, 0.6811682982929017`,
0.2554902190221564`, 0.06882358130064935`, 420.2484502763152`, 0.11914991474138031`, 179.7692000111727`},
{0.14635546873540428`, 2.6921520503610896`, 0.005572522395533781`, 1.0650322427085313`, 0.01258547930967402`,
0.3289894737792243`, 0.20755635390945587`, 2318.8984027990427`, 0.23607139988674775`, 598.8352065199724`},
{0.20440309232409604`, 3.6324112419808596`, 0.02933293982791259`, 1.0491273986291727`, 0.6481946292303631`,
0.5970564598987798`, 0.367615520297974`, 2827.3491736750175`, 0.18901453458314488`, 331.1017138964795`},
{0.211339616570286`, 0.8062529435299846`, 0.03215637597034366`, 1.1329056004996207`, 0.6216719034637128`,
0.26914056317845936`, 0.046266726096773195`, 367.3076671022254`, 0.062345072344042685`, 883.4219863160993`},
{0.0754121889664276`, 3.3553240739439776`, 0.032766043984085905`, 1.35951382107467`, 0.417421746626248`,
0.2423604100007123`, 0.37190628043481266`, 360.0955624739355`, 0.09650587549325668`, 113.8474438254784`},
{0.2783033002921034`, 3.397814643841863`, 0.023780737803670213`, 0.7785779441011293`, 0.28908440577847827`,
0.4851687293566612`, 0.2500599862274582`, 176.08406451146294`, 0.07757174693073099`, 972.6494889929199`},
{0.08302491117654731`, 1.417237397662472`, 0.08064924241577312`, 1.4920703552127885`, 0.33660997181727526`,
0.6086848413902193`, 0.43663790825582516`, 432.2028054996635`, 0.054044182009652`, 768.4690422643392`},
{0.09729817063298457`, 3.4299522173089496`, 0.07735506084120583`, 1.4020158593772418`, 0.655176777497416`,
0.2796434167254044`, 0.020513648578027708`, 361.54728331563473`, 0.18982251022644547`, 624.3692576556642`},
{0.05918716742286234`, 1.9820539956622945`, 0.05196458247655251`, 1.4930492496804253`, 0.4915276700726614`,
0.5297919483627916`, 0.24251322382900306`, 1013.8284387296288`, 0.1901510038138538`, 731.270474360067`},
{0.054487059066553045`, 3.0521940188838643`, 0.06431699428389767`, 0.9108516151810719`, 0.22179534032337966`,

```
0.15276001240612846`, 0.00618021185065336`, 1431.6290748192448`, 0.09019004900189659`, 615.4166559817393`},
{0.1805007029704227`, 0.41916969450246455`, 0.016124853476479205`, 1.3709380974041798`, 0.9243445784418525`,
 0.5673574690172947`, 0.01323585349172664`, 768.6156722427371`, 0.14720827739001074`, 303.65297547748116`},
{0.13586333024917552`, 2.4333711568454603`, 0.06594815024548176`, 1.1422919388448811`, 0.127316527773377`,
 0.5373692465409584`, 0.01747695081564976`, 1883.6141269851823`, 0.017752075188298855`, 680.6112691329253`},
{0.09049024561283486`, 2.3894866858591284`, 0.001345988925684072`, 0.8533171338756729`, 0.3021314683838838`,
 0.34607340060030345`, 0.30321966856095933`, 90.59478899907405`, 0.23877865208899252`, 565.9928476917297`},
{0.10259701094971274`, 3.6083949275799876`, 0.07320779035427538`, 0.9294410249753255`, 0.6943909777193686`,
 0.5424209490023467`, 0.02226818211415045`, 425.8826453817381`, 0.14811442642104045`, 767.5349856757164`},
{0.0818626476168649`, 0.8476840265643926`, 0.05039571888660504`, 1.2803278760732222`, 0.39203490461702195`,
 0.678685283379783`, 0.046408600947453554`, 625.4764903683038`, 0.18505631212115975`, 473.24089743900083`},
{0.07029108203608453`, 0.7073037290148494`, 0.03261231636893799`, 0.7728998119403233`, 0.4329865779673474`,
 0.39085105872031733`, 0.3956962494413241`, 72.63981932713905`, 0.24239129287515127`, 232.20167186552362`},
{0.04958285162353873`, 3.761311858085456`, 0.07854169509494732`, 1.403762798092309`, 0.6354348772329161`,
 0.5539469242046658`, 0.014552601524129273`, 191.8255147151473`, 0.1545116444713645`, 509.99375854807204`},
{0.15497576240515054`, 3.7755891399441612`, 0.04102415780603357`, 1.3426436269065887`, 0.4633835643985551`,
 0.2105276629358308`, 0.005292621332827327`, 393.4300921998384`, 0.05255466894451982`, 181.97601415353938`},
{0.1462216796835833`, 3.3115430587931307`, 0.09201451210593234`, 1.2778172268764427`, 0.906784021865821`,
 0.3687077782146817`, 0.05157961805804173`, 924.6290700374847`, 0.029607911315220503`, 889.789386014198`},
{0.13283897685814167`, 3.038408092963987`, 0.03461781675567849`, 1.3222463428410316`, 0.47510806724254606`,
 0.6210207278956918`, 0.005408113696778361 6`, 102.55379834175085`, 0.04224157239084064`, 436.06625132099504`},
{0.11923152550729099`, 3.907162388051315`, 0.06641684248516906`, 1.318540470917755`, 0.5804312953963475`,
 0.6280211407721501`, 0.005256835397320809`, 1226.3954007569562`, 0.17176906355966298`, 506.996443873342`},
{0.10227819097193708`, 3.914724924869418`, 0.0664380714143547`, 0.7944397029062766`, 0.15335804466948`,
 0.3668024996284803`, 0.06541187335723456`, 1449.384487729464`, 0.024262601947109108`, 554.3384286777302`},
{0.27621673974857747`, 3.975786747546901`, 0.0440045228345514`, 1.2561736736681604`, 0.12219205626085827`,
 0.44109764605630186`, 0.27834299040512644`, 71.91340898917194`, 0.10015764539366545`, 482.1830368850472`},
{0.16141532638885775`, 3.5473613273302282`, 0.060131606026769265`, 1.1204941160194362`, 0.8786219204877892`,
 0.42963549162688086`, 0.23724506417809585`, 4491.815167956783`, 0.06480015184829835`, 512.4293881945277`},
{0.2575375457582068`, 2.947834811130747`, 0.033810464285172745`, 1.0447722344490806`, 0.8627980313064527`,
 0.34879266259466135`, 0.11166656744723742`, 94.36401066143432`, 0.11812196317533258`, 725.2748373314915`},
```

{0.20820386673509828`, 2.6786477995612135`, 0.010157905183889078`, 0.7567059297671783`, 0.6398946017563087`,
0.2916906367963521`, 0.04126212921113298`, 3268.442039148205`, 0.02382062355054088`, 259.60704797706916`},
{0.15593088724620752`, 2.5519577904336437`, 0.024361841648533833`, 1.1168597646554808`, 0.13563727928772762`,
0.3874274982263488`, 0.1882999430765413`, 616.7470197654534`, 0.0830157326232564`, 890.7479015168708`},
{0.24014955563677381`, 0.5220448196870295`, 0.02671701264219644`, 1.3551783563181985`, 0.799728403554941`,
0.47303019566294113`, 0.027399592270438356`, 644.7909772591187`, 0.07858290125002376`, 181.11983864094333`},
{0.1581702628188732`, 2.346334984324618`, 0.0581411782688245`, 1.4807167920720685`, 0.40458616499170885`,
0.6310195170221491`, 0.006522970079115585`, 337.28265576556936`, 0.12691983292355058`, 454.93623014073796`},
{0.23136224869656058`, 3.6088316121947086`, 0.03632040382309938`, 1.3419685001039396`, 0.40455118868914375`,
0.3221101170539603`, 0.25966887129973093`, 1676.6659884454966`, 0.19241943128863082`, 511.50532059075465`},
{0.17957415783469988`, 3.5968176444073308`, 0.048288185227319896`, 0.7994547395333207`, 0.9581588123096916`,
0.21642006235154376`, 0.14264678878084103`, 1476.5632047936012`, 0.020697112352618785`, 228.14672992784074`},
{0.059998032534718804`, 3.7773982431410413`, 0.06986223440078802`, 1.120487977215339`, 0.4567291173152259`,
0.612033275511431`, 0.07354302601323719`, 486.6146546384414`, 0.119884658675466`, 347.4528922941486`},
{0.19887637677541542`, 2.992660923165513`, 0.047995275724685205`, 0.9722118856000259`, 0.2651497498489779`,
0.5318886674778154`, 0.19636633656100977`, 112.46333630768565`, 0.06776166614050899`, 129.50929186524326`},
{0.09067863132057552`, 1.9262440927144207`, 0.03464921977559642`, 0.7578597622362966`, 0.480840305150003`,
0.24100415846854528`, 0.20408648616921088`, 520.809954661363`, 0.10723760431187324`, 547.2814452851367`},
{0.20782506825689806`, 0.8141429404339053`, 0.09514857720029408`, 1.3616807374452509`, 0.4328504524684271`,
0.33813491058186396`, 0.24032169387315838`, 461.50385309819023`, 0.1829235837022425`, 223.3752348589885`},
{0.11510496723960911`, 0.6612811922775106`, 0.0679688489060159`, 0.8790789776515309`, 0.5367830167767558`,
0.6858865509647121`, 0.06457419286375914`, 320.31094965266385`, 0.17862589858250755`, 915.319702346644`},
{0.17264723461047932`, 3.7903015192126244`, 0.06554920731341767`, 1.1764259933061223`, 0.4660090251563338`,
0.6413383966201236`, 0.241827388783345`, 56.79713040044933`, 0.023743987090588337`, 456.3571078119333`},
{0.1547837566229352`, 3.4953794802806852`, 0.07246861713817378`, 1.0879160111375143`, 0.07311426293155776`,
0.6990761768110039`, 0.005355312003055951`, 1173.1945274337409`, 0.08510663567245824`, 820.7115603554513`},
{0.12332890101103666`, 3.575694377588479`, 0.07546663843052578`, 1.1730448937718991`, 0.7299661739602221`,
0.3544423810526146`, 0.3517297854582263`, 207.52630070713013`, 0.16381025124403453`, 211.24976747011442`},
{0.07543310039566975`, 1.1413407123857509`, 0.06791044220530608`, 1.3022569182103092`, 0.8228503547730448`,
0.5416450068944677`, 0.18860915993442676`, 2038.110511149941`, 0.1613952093900407`, 419.7511258067653`},
{0.21429674136023663`, 2.8226782716380345`, 0.07008452435050433`, 1.2033565563578725`, 0.22607654151729606`,

```
0.3670473114848468`, 0.04920492797100079`, 426.3575789205089`, 0.22829240712916699`, 151.89428282997392`},
{0.07895444632032361`, 2.201415239192807`, 0.005337930232592641`, 1.0054037946739751`, 0.7982316099945579`,
 0.22745190186265252`, 0.014751475681337079`, 999.912509637654`, 0.18790976944806215`, 156.9072222771119`},
{0.09184666059394636`, 2.067005962614928`, 0.04145877572545418`, 0.8296320077051444`, 0.11557680968156858`,
 0.31351325881175707`, 0.021527373087813387`, 871.3636268629474`, 0.16510736043882146`, 233.95931170378006`},
{0.12260917712356023`, 2.30639494589911`, 0.05876506714974223`, 1.4161472400002522`, 0.4228788165875499`,
 0.614217815805707`, 0.04726593691280875`, 1944.5171329959344`, 0.08163047191172412`, 744.531780752293`},
{0.17238170923293145`, 3.7452628271391264`, 0.012298914408557682`, 1.0114832888719023`, 0.7075400969976455`,
 0.4701095442473505`, 0.009792251350088488`, 109.73168851300552`, 0.11561190536083749`, 580.7510496473817`},
{0.06459740373368028`, 2.9106606918316986`, 0.09546811583014093`, 0.7816504226428446`, 0.9051718026272408`,
 0.6152354921314405`, 0.12920206403408327`, 192.77909888895545`, 0.1990333439617078`, 537.0263017540406`},
{0.07800640828878808`, 1.8505382879500134`, 0.012842566220368603`, 1.4649243339915694`, 0.6600118067779042`,
 0.4967332224237151`, 0.013562590855424642`, 1252.0890643038667`, 0.10833365543192358`, 267.9181792628814`},
{0.048462811029039154`, 1.800487790371883`, 0.005299468930184581`, 1.0419920592385326`, 0.9100782296151841`,
 0.32988971122619926`, 0.11628748764501183`, 321.3445579524093`, 0.10904785586908877`, 676.7351327707524`},
{0.0408586986071619`, 3.397631267565795`, 0.034535155982847086`, 1.1499926979165747`, 0.6535863974222924`,
 0.4932189245887587`, 0.383854582094014`, 103.0179671920324`, 0.1262724601642624`, 838.6520403111433`},
{0.056181027140717654`, 3.561092902333039`, 0.032415896850996564`, 0.8977612893413814`, 0.19606568802210056`,
 0.5191107847286981`, 0.3780120623597924`, 2750.1468252074155`, 0.22792524388897922`, 419.0619039860675`},
{0.24051173539528659`, 3.363998136218836`, 0.07316535800080796`, 1.1407631035414798`, 0.3740167133471626`,
 0.5883310500889865`, 0.1388691166946849`, 1845.7599241956455`, 0.06093536750612072`, 554.8160924336624`},
{0.10694470201355061`, 3.916523604921749`, 0.040570584968233535`, 1.141416795902756`, 0.9898088237744289`,
 0.3296418758797267`, 0.04709726814380818`, 2933.808240863522`, 0.04460415393167344`, 879.0499414979915`},
{0.06862055370778036`, 1.8272394963651166`, 0.06696112608756692`, 0.9180601754047588`, 0.03279196554733499`,
 0.20147163161780768`, 0.010370667030870775`, 671.676842322565`, 0.19673134057127845`, 347.1374473999164`},
{0.25063858582919535`, 1.0026414593770951`, 0.08049236416776281`, 1.1585705609095718`, 0.27877982032187476`,
 0.2471176538176596`, 0.005549153441024216 5`, 1284.3138459700085`, 0.027491789825886737`, 964.5039545904644`},
{0.26413366443306313`, 3.5187238265686887`, 0.059384833236120135`, 1.2922578870185246`, 0.008326928227079256`,
 0.6434100059643171`, 0.031067351563113396`, 133.02587790916047`, 0.1993086925023529`, 393.43546913290197`},
{0.13920351751612248`, 3.9485961604821114`, 0.08033096245429228`, 0.961725822180624`, 0.7322696656937997`,
 0.5625362274736092`, 0.0233705450075071 9`, 857.2150128038464`, 0.13429548054404716`, 132.1146622970133`},
```

```
{0.12997797022210922`, 0.8438763321472402`, 0.09917403003828128`, 0.8801736269011186`, 0.10893971026850768`,
  0.568805635817268`, 0.4569650004322221`, 173.3039538372586`, 0.1310412151491429`, 829.8180726295691`},
{0.04282165785099121`, 1.644759382359756`, 0.006131876320932417`, 0.8656795781593337`, 0.5309250697882764`,
  0.28882829576135427`, 0.43822920515546965`, 171.56453795936363`, 0.17422534922512378`, 500.0111202673682 6`},
{0.22211928728847102`, 1.1859807267849432`, 0.08277265577838502`, 1.3176134726640667`, 0.7769092359720373`,
  0.31677855442324954`, 0.01004066290735241`, 1412.8983237640775`, 0.05122004701487703 5`, 737.1658163615933`},
{0.09296167151413748`, 0.46354983597275057`, 0.024036152116512888`, 1.3763603395412263`, 0.745954078027516 7`,
  0.45155144834323624`, 0.3445673156480595`, 2335.90860073376`, 0.22065872238926132`, 474.2017592615175`},
{0.1962229794306719`, 1.6992229864288415`, 0.073818422425421`, 1.0867919112277424`, 0.7575363665855621`,
  0.6670448748187716`, 0.24718082226975022`, 1605.1879390833765`, 0.059896123435389914`, 160.67342656698588`},
{0.21138025591123977`, 3.7198017071227776`, 0.012025339542637924`, 0.8791888222818957`, 0.9121961522822317`,
  0.34954022634411386`, 0.48855663620210427`, 4481.4869986353115`, 0.09130130840750089`, 978.8438514579077`},
{0.174527181711994`, 0.849373235921072`, 0.0314016740925031`, 1.2229771378389438`, 0.5565629319212622`,
  0.15780972618613898`, 0.039946482002802375`, 1015.8195322962273`, 0.11236134949951643`, 212.56048531719287`},
{0.27104366314172257`, 3.4411670504738847`, 0.05742812389939894`, 0.9577515288651626`, 0.0851317171213124`,
  0.6274935825967685`, 0.018124860207485008`, 2870.4044184423146`, 0.2324535673532167`, 472.3817854782288`},
{0.11781804356750541`, 0.5626502822415125`, 0.01459820489930097`, 1.05752032760362`, 0.6292609081665201`,
  0.6860764088162852`, 0.029170421001759508`, 352.2383589143192`, 0.10359473073293657`, 222.23677753012652`},
{0.15398221319678085`, 0.5849960064145687`, 0.05386556842999246`, 0.7888135605497072`, 0.7266422422856504`,
  0.40377533435456936`, 0.43018868620379314`, 1603.092147190326`, 0.20097603617156867`, 537.0534533002889`},
{0.09435165103191007`, 2.1871870158802187`, 0.09386196089682226`, 1.0117647368175615`, 0.23992524376119828`,
  0.6944778195963781`, 0.011558368513568394`, 1224.4139617227247`, 0.05758577870260467`, 112.08810079007688`},
{0.15418547479730832`, 3.244024899467486`, 0.031613474871280495`, 1.1180817216823555`, 0.1378867782832267`,
  0.24122334591588945`, 0.1784500939520896`, 1165.0611910719738`, 0.018111200873128563`, 519.7221352316303`},
{0.18001954973510415`, 1.9120553169228627`, 0.01841751056966496`, 1.0848164059222056`, 0.5445307300173845`,
  0.21487607390213648`, 0.1420658743866174`, 59.88761221845187`, 0.10283589598798698`, 180.0642150440039`},
{0.17238975113628274`, 3.831887800749228`, 0.005776257990374099`, 1.3458283817066912`, 0.08939800815002275`,
  0.20519935162452907`, 0.06363342671890825`, 2876.3582213396467`, 0.1796261387487414`, 336.8941601044835`},
{0.14352493778031827`, 3.7979256036341535`, 0.03771360392389294`, 1.3633466357184463`, 0.3018171788859503`,
  0.19173241304281086`, 0.03417718814733039`, 2084.1653883747413`, 0.05483174208182884`, 581.7554610960195`},
{0.20940311206829182`, 3.2582854913524706`, 0.06591828409332467`, 1.3970633596535897`, 0.8615344587047382`,
```

0.38613005595364347`, 0.02066648198066755`, 4820.6850448541545`, 0.07989037484848699`, 539.3449932522163`},
{0.13522362333277543`, 1.144461624392597`, 0.008064525972274694`, 1.2137320027234764`, 0.41558764869922493`,
0.5044332234582337`, 0.194694572581245`, 1561.3725943749705`, 0.2352162319299269`, 635.4535165669378`},
{0.06564694091416517`, 2.517837422245419`, 0.08205855339446029`, 1.0554951116327027`, 0.7103060442920281`,
0.393784837384779`, 0.34842807990801816`, 201.46762707189424`, 0.22439022497167005`, 178.8572875798012`},
{0.24425093983878532`, 2.571873191821844`, 0.03797741011070798`, 1.0612508346456866`, 0.8063780340542086`,
0.4683983500560849`, 0.06605028609142427`, 2270.9148163281043`, 0.08295143441277542`, 860.3274939678396`},
{0.13421688107301388`, 1.6885106577171634`, 0.01327671934783954`, 1.4235239040662608`, 0.41175527118821376`,
0.35897461552598586`, 0.28058853074140605`, 344.729203987344`, 0.16559799427233646`, 938.0192541245116`},
{0.12355675427841095`, 2.0332534396069946`, 0.006925443967133322`, 0.9868311030648986`, 0.27448369335996525`,
0.49997436829086617`, 0.010689681959538098`, 661.0107417281803`, 0.049567549414286594`, 614.1468732990146`},
{0.24989261079843417`, 2.046336678398201`, 0.05343056582535697`, 0.9590211509682223`, 0.6962751749667404`,
0.5979718555935737`, 0.06212292589548939`, 836.0233579874354`, 0.028192514982996186`, 946.2272832575387`},
{0.18446589532009483`, 0.6770723958796063`, 0.017239459819183126`, 1.3829614064739535`, 0.15821669976510688`,
0.18877757485488766`, 0.007119833198284364 5`, 1580.0156398402103`, 0.12251896238349375`, 604.1234967411239`},
{0.24207945515763046`, 0.8472072700897755`, 0.09830987943529323`, 1.3431438063557357`, 0.7063009601868007`,
0.2722426879126155`, 0.34992016942373105`, 1219.3651744969027`, 0.1756849319581012`, 396.46752882152185`},
{0.16929669277606063`, 3.384845133452008`, 0.04997850478346777`, 0.855944189945163`, 0.5854410422632743`,
0.6313899885471745`, 0.27030175060519007`, 69.39208015599864`, 0.14109734367472376`, 825.1088816761363`},
{0.22032907726938156`, 0.43680070868525345`, 0.02505576509377308`, 0.9013024202439682`, 0.3637877243645866`,
0.6575693669120928`, 0.08327886814493145`, 86.53687085308205`, 0.24093186652306348`, 957.0475588354033`},
{0.0915185159652725`, 0.4356928503469506`, 0.07385942693598586`, 0.894137684029105`, 0.07375739107747048`,
0.37304243578619745`, 0.3884276894579204`, 86.6179555964664`, 0.023525347333101687`, 957.1153747111897`},
{0.19293304608541778`, 2.881169086349173`, 0.06288677922536799`, 0.8471255106831246`, 0.5957102709253561`,
0.464151719061209`, 0.03816221209756031`, 288.4038900208833`, 0.10298551466758471`, 155.08445353721268`},
{0.18184235602556043`, 2.816880901418977`, 0.05545256089460814`, 0.876197436405354`, 0.954624174973548`,
0.4655369772467929`, 0.007648944239440386`, 383.2702509437632`, 0.14043117229016888`, 779.9607071498034`},
{0.1500634817243091`, 3.590063801011035`, 0.04690872877366834`, 0.8713971696364884`, 0.8016145774652992`,
0.57680193310708`, 0.007900899994569407`, 1759.5867354003908`, 0.1650460276258336`, 468.72962630703444`},
{0.07044808633799604`, 0.5092168059858677`, 0.023939619197820134`, 0.8363921850258171`, 0.11231870814099265`,
0.415964567014653`, 0.010393952295787622`, 272.30094842939224`, 0.24251268430438633`, 533.0209640034411`},

{0.17446312671000164`, 0.923278108173573`, 0.04622273024289145`, 1.361488933545279`, 0.05073013179716934`,
0.423140548702129`, 0.4945547029315037`, 756.8418975914155`, 0.05582670682313107`, 453.1988863343269`},
{0.2596918687902645 7`, 0.6876551199003744`, 0.016004864008093787`, 0.801399153017793`, 0.8167373300888658`,
0.5699218117881594`, 0.010392376675716438`, 2453.002800663684`, 0.22138430515888585`, 604.6992574427161`},
{0.08344350966636604`, 3.6673612041438233`, 0.0357399550994862`, 0.9043067215227492`, 0.3349583654565387`,
0.39299105484116215`, 0.07044574168572774`, 531.2053684025772`, 0.2316097051950603`, 942.4468571449918`},
{0.1117106931508435`, 2.9258562986192613`, 0.04535829204836597`, 1.397904475123252`, 0.8323395818238934`,
0.6262291788570806`, 0.2126230418930395`, 59.43001557400602`, 0.0378693513362 6829`, 393.82943302003673`},
{0.09014630069728341`, 2.5385294043084468`, 0.004644443169930969`, 1.1109052294542912`, 0.48602912154060585`,
0.653448250732096`, 0.13848641693128835`, 233.22231224941473`, 0.13058754405383072`, 772.176206082497`},
{0.1204210700620526`, 0.6547366279014759`, 0.07856424763514301`, 1.2422551703125984`, 0.769232712809691`,
0.34959559963353615`, 0.3952520916617596`, 225.12460677270738`, 0.24941607930389664`, 764.7416993751449`},
{0.12507255683757496`, 0.8093899840252368`, 0.022011166366603607`, 0.9039266843419957`, 0.9040126296575397`,
0.40644665651439293`, 0.36973776842713274`, 4266.893817006702`, 0.22996060684055697`, 344.3317857496396`},
{0.18799072009935214`, 3.5387044337310227`, 0.010288586533272871`, 0.9709034820394874`, 0.16526258297595087`,
0.2720662467892546`, 0.16314642121657652`, 213.79313950957913`, 0.23266932286427316`, 908.785142248831`},
{0.10303653650946687`, 2.045785570411666`, 0.032527698921882384`, 1.4813578055387457`, 0.42199129008222913`,
0.6459634548526738`, 0.010175611270196772`, 575.847247144136`, 0.04915259766651514`, 563.8812676357885`},
{0.2398844305865619`, 2.1056225174808283`, 0.015327944846502356`, 1.2552986509569295`, 0.6918103031115113`,
0.1889744468357467`, 0.03454744519234309`, 720.0345748750987`, 0.20720302058084095`, 561.8805940371304`},
{0.19472671711158268`, 3.4537873437782753`, 0.05393741401653683`, 0.9791709575229651`, 0.34020645306003683`,
0.15465211878990004`, 0.005783246570334714`, 712.2087645951867`, 0.18659332879596663`, 390.1452748573064`},
{0.20571859545893295`, 1.551494362900097 5`, 0.03978136161333113`, 1.2978001829388508`, 0.10072004838372872`,
0.22845349492624245`, 0.25892912790336436`, 119.6045968067387`, 0.07803724137740647`, 936.2227260158392`},
{0.275720564806804`, 0.720336275328604`, 0.09315930706476526`, 1.2814448950695845`, 0.9719953187330976`,
0.5030571263630652`, 0.09198284855367962`, 2926.101672056841`, 0.10631897378157318`, 340.03246937203465`},
{0.05630051102503303`, 3.7356157314175347`, 0.02800700712577493`, 1.0094636270338093`, 0.18952763541881779`,
0.23258714070277053`, 0.4551334614672762`, 3326.1734768299775`, 0.20084773313292642`, 389.1282332545071`},
{0.1566545086835321`, 3.6144342723158855`, 0.01851051241133261`, 0.8062001646255728`, 0.7860505516306966`,
0.5903319695657847`, 0.1423722018298642`, 1684.7441306012342`, 0.08281365756813702`, 745.921161780724`},
{0.26785050867880617`, 0.9336989210532032`, 0.047863988220149846`, 0.8870676821839705`, 0.894276639371691`,

0.24671850596175082`, 0.25434132792942304`, 2463.897338729517`, 0.07340938538359204`, 135.25070223351997`},
{0.20621368926339484`, 3.895401327519891`, 0.021945870300711868`, 1.2149002945024092`, 0.09567565478536721`,
0.3570859261949008`, 0.010140662222165558`, 72.14119724036794`, 0.09062375760852914`, 269.2376393484709`},
{0.22132018814331722`, 1.3624506083622139`, 0.02157572172797998`, 1.017162028467816`, 0.022930198784160805`,
0.174154916693125`, 0.006372220518572069`, 81.57393238873418`, 0.23095499884940496`, 559.6417164883817`},
{0.2667728012376422`, 2.2597067694039596`, 0.052618617939097785`, 1.1876063798389778`, 0.9039352407220065`,
0.17021035842318066`, 0.061291229169270336`, 2589.9662235455107`, 0.0930933143809865`, 467.7152997091941`},
{0.16814767223163635`, 2.329148917787961`, 0.015181235666702158`, 1.3509190984659396`, 0.20135617569594788`,
0.6016277316250633`, 0.0960580261415236`, 75.18782439155149`, 0.06872828852909052`, 330.2905596330332`},
{0.24629497802526884`, 1.6185892731875686`, 0.005355789298525409`, 0.7573206700198012`, 0.6605818641858783`,
0.4413473573778042`, 0.3432973026299589`, 62.23416744978072`, 0.03461390771204009`, 861.2270986066914`},
{0.12348100274215157`, 1.3439669835932486`, 0.06660199654711046`, 1.157413128080917`, 0.4506243858381027`,
0.25274164958094525`, 0.1821863441956961`, 1340.7965217357605`, 0.03495643955461325`, 657.1175564125843`},
{0.20026891357076415`, 3.645369762770067`, 0.023665341602336538`, 1.1690889262253168`, 0.5114922593058153`,
0.6682127068245862`, 0.22249927148912355`, 3097.4126005392104`, 0.07667851305193468`, 720.3291394146138`},
{0.1167144215735465`, 2.9739284718729824`, 0.06770393749145168`, 0.8030036195270651`, 0.817910504731294`,
0.2533089674739153`, 0.13586578835473073`, 245.55576330732927`, 0.12297451593509273`, 949.2797411932297`},
{0.2610230648306139`, 3.241876732828336`, 0.005221929989860801`, 1.2708350659440502`, 0.2709097063186865`,
0.6295136971445925`, 0.2167769645618444`, 67.25197219006178`, 0.012919428781842102`, 446.6417150509489`},
{0.22755856549372416`, 1.1571207715998462`, 0.03935231641744066`, 1.338611381035573`, 0.1970208179920212`,
0.512487214253471`, 0.008778903977610756`, 69.93484330017829`, 0.0885116413292587`, 703.9913507831651`},
{0.08043529968845808`, 3.6346678770302887`, 0.06436593471662284`, 1.4919978389096693`, 0.6239584327672116`,
0.5363538338794006`, 0.005714342675312524 5`, 2341.702974881963`, 0.06269329048310018`, 332.55107547613017`},
{0.17838278501630495`, 2.4576612999234007`, 0.06181741342015828`, 0.823346074642842`, 0.013205254550742174`,
0.5017391269032923`, 0.16663083212381372`, 304.8776737280077`, 0.14165654641577402`, 678.4897161214598`},
{0.1527608465983989`, 2.070934262024398`, 0.045813432940116104`, 0.7944089298621102`, 0.6769693136188586`,
0.16430192509774133`, 0.17450886910735772`, 3923.7643815879096`, 0.06708022916564621`, 420.0877116062573`},
{0.11624661761519267`, 2.9732886208480265`, 0.08686637218032857`, 0.8721582238868211`, 0.700158456128364`,
0.3183544106471198`, 0.06872076435482898`, 2515.039310773072`, 0.08635525493495505`, 866.8956807307524`},
{0.1310858151843764`, 1.596637100973001`, 0.08661007645244247`, 1.3109557164680856`, 0.9039286133369202`,
0.3437790347559597`, 0.06680016028420104`, 3498.912503889652`, 0.1695299313540355`, 339.123637513298`},

{0.13093035477925424`, 3.0078393597426825`, 0.0788110046495581`, 0.9368136862014702`, 0.15232997356507805`,
0.5208667948995476`, 0.027452102279722775`, 439.8539822509126`, 0.023447155859907365`, 960.4131412752828`},
{0.11884479435624312`, 1.7884452074083192`, 0.0721596734346857`, 0.9483398419798006`, 0.06052883609711657`,
0.6592909099232407`, 0.008348617368503395`, 734.1801319364669`, 0.0593737289409078`, 336.2025788281121`},
{0.09279332232126897`, 2.395881928323834`, 0.035821424791727154`, 1.1256250545946367`, 0.566531337666109`,
0.301586294875144`, 0.12926859185498135`, 3190.2362801672193`, 0.011195844366674756`, 937.8412253936428`},
{0.10470396306835666`, 3.0481563632657567`, 0.02918159941393267`, 1.1229103226446677`, 0.06432569110103459`,
0.1636910757663953`, 0.0677993574358691`, 108.21923708078778`, 0.037422575295802174`, 716.3638099124112`},
{0.17624356912801198`, 1.573989236801781`, 0.09366396594728997`, 0.9674991919084199`, 0.39632574484069294`,
0.18908139883550235`, 0.042591608468658185`, 545.1774692484181`, 0.10286957749970482`, 735.5542101216969`},
{0.11969608505483581`, 0.4187980837332148`, 0.0887749696374739`, 1.1942873584807199`, 0.9542625089733192`,
0.26224686727401303`, 0.2044328771862869`, 4071.5728832408004`, 0.012536086303640448`, 986.0101272937204`},
{0.0681782584958246`, 1.9712297226556181`, 0.0710699974540059`, 1.4798684393175123`, 0.28400354899281655`,
0.18343756245630538`, 0.025080565221411666`, 190.74276766833862`, 0.021217036684959`, 114.63276406832605`},
{0.26466011364476083`, 2.3627816128360744`, 0.02039742244272034`, 0.8195822231735724`, 0.29110662117910135`,
0.47981634198411705`, 0.013151438435585994`, 123.59545033098055`, 0.04800415213314124`, 576.5536164907207`},
{0.27910166898523386`, 1.349450515188618`, 0.0885331584316003`, 1.336068232065382`, 0.5987418653774925`,
0.1580872600090233`, 0.005729492652206841`, 454.22089835832855`, 0.0493218089029025`, 734.2743634707255`},
{0.1870945044053341`, 1.8787994994414792`, 0.052561657322085654`, 1.3540062415635468`, 0.28234410939143695`,
0.681042030488356`, 0.009986864266329082`, 981.5333621075416`, 0.18733052504041348`, 853.4539224410559`},
{0.1396988967578765`, 1.913702915438618`, 0.01617810449961091`, 1.199136555704491`, 0.16595422293922835`,
0.6068296527307018`, 0.17305426475375874`, 717.4973492774137`, 0.198319856676219`, 283.9891726135136`},
{0.15506300074217488`, 2.83069948310355`, 0.08462265931042055`, 0.8890994038076738`, 0.47796520586519553`,
0.5728084495295004`, 0.005405423164330928`, 1159.371736373684`, 0.08490828359258434`, 608.2480837068873`},
{0.08918867947883585`, 1.0389500531703932`, 0.03170283696550655`, 1.1764272925199706`, 0.8188250505498167`,
0.512109016528521`, 0.044640995253867236`, 632.0761666245681`, 0.15046863407887012`, 841.6135553783089`},
{0.05692826445298971`, 1.923331897508854`, 0.07552634610068665`, 1.455886213864955`, 0.32041248603883954`,
0.47523042425037754`, 0.22105687499350363`, 2027.7508727407876`, 0.017420396497442786`, 274.64042522521055`},
{0.24257218102715322`, 2.573176975490081`, 0.09661060539215438`, 0.8445932242005463`, 0.8955286624705445`,
0.4789936248391016`, 0.26410377851960004`, 3510.537763377287`, 0.18852831639094753`, 237.77037147960095`},
{0.05347723707962343`, 0.9129899365885157`, 0.07428512647308946`, 1.466096589680298`, 0.263816129356184`,

```
  0.30123412254131576`, 0.022303163735904144`, 4746.406027391749`, 0.18205991120633613`, 895.9248957958035`},
 {0.27274128807061915`, 2.426636979804435`, 0.06334129103904147`, 1.210981179335779`, 0.5970208082221518`,
  0.22184996611628427`, 0.009365743994866976`, 204.27799326594078`, 0.22060849091929458`, 828.6461515322391`},
 {0.10281443317302225`, 0.4962771981673755`, 0.04898875008264165`, 1.262839945648412`, 0.8347432488590383`,
  0.4192167501942007`, 0.030355367117986`, 1308.2409868067423`, 0.042950873976760395`, 417.52929653085965`},
 {0.25848551367088474`, 3.558870975613896`, 0.037906846258373254`, 0.946132081756833`, 0.5725842788655087`,
  0.6625769316332577`, 0.09924894324330936`, 52.004626921156905`, 0.12255979122726918`, 619.4850012984921`},
 {0.05861384430265282`, 2.8809993577690376`, 0.0034389149229243545`, 0.778023331623658`, 0.955566931044247`,
  0.311264533883984`, 0.005123688327250641`, 510.7904162444792`, 0.203481852542521`, 418.9270384908555`},
 {0.26171770992100346`, 0.5361270884859564`, 0.038524708394197374`, 1.0508503305310222`, 0.11056009208990747`,
  0.571340638565827`, 0.027815464969581257`, 333.39873468372707`, 0.030150481088034198`, 186.42906095487143`},
 {0.09228406045428905`, 3.787322761562357`, 0.04412628551769787`, 1.2485522327984204`, 0.00453916468120652`,
  0.356730274900637`, 0.03212483930675613`, 3375.2895893320583`, 0.05502779271988756`, 653.0147468261181`},
 {0.2582323539483842`, 0.7609188588793034`, 0.043891058231223086`, 1.3021658200784163`, 0.09072647662703637`,
  0.4622306981965607`, 0.06168457257766234`, 192.16050980483587`, 0.03732931870687156`, 184.32898889722597`},
 {0.2477921144844203`, 0.9405947985440335`, 0.09500951785333488`, 0.8564439359315013`, 0.8813246793351337`,
  0.5526752397451847`, 0.04122909919220205`, 2544.2880548964617`, 0.18357258099770601`, 309.1199502666939`},
 {0.06005306680926792`, 1.310324956982524`, 0.01917002825340564`, 0.9233944849507227`, 0.37862876807380963`,
  0.4654284132589105`, 0.028904406737409957`, 740.7733482913402`, 0.14207137738578401`, 125.45920277021416`},
 {0.13221174566753952`, 1.5344979786934942`, 0.07993475417223245`, 1.1279318472161384`, 0.5224757382486951`,
  0.681045123252989`, 0.09251417322960646`, 4007.8291314421654`, 0.1250986785892464`, 411.07839231550474`},
 {0.23641095513812793`, 3.2390712982841716`, 0.0904220832128998`, 1.2764555680953642`, 0.4087688116784247`,
  0.23715766669095795`, 0.050286530096096176`, 142.72560445944592`, 0.13322047650855234`, 994.1833005928358`},
 {0.11515945727299054`, 1.0363839446782226`, 0.04991967937770255`, 1.2110778925771657`, 0.7975055001930793`,
  0.15766796336173805`, 0.11336570441348018`, 702.6737236802497`, 0.07984121314940101`, 718.3669578221027`},
 {0.22406410922088188`, 3.5768982916995915`, 0.0850321138351546`, 1.0822829393722269`, 0.3956856744827095`,
  0.5136072284730188`, 0.021536821023513928`, 1505.4866148851402`, 0.07683669026613277`, 536.6032367164931`},
 {0.09816955174932002`, 1.3610473147845434`, 0.01200296321417639`, 1.4480047032959393`, 0.1823923291352767`,
  0.5244723013770171`, 0.03492258343756444`, 1533.0073499542846`, 0.0394156510269485`, 214.7356679901721`},
 {0.2686714308482082`, 2.6024579475953065`, 0.09605319126050717`, 1.4115359358964699`, 0.40944065631503346`,
  0.20238890153994604`, 0.44356457168170405`, 943.2699426207273`, 0.20955602538220924`, 179.63082811814246`},
```

{0.2789448341874723`, 3.460339370316608`, 0.09560289059485072`, 0.7655082688097814`, 0.18036074766112598`,
0.3208070646396465`, 0.06769953888545853`, 1642.3905460053934`, 0.12624689441090442`, 706.9723251095423`},
{0.08907118739874043`, 0.5288966486743032`, 0.07776144026324502`, 0.8401856534758337`, 0.6264494338356676`,
0.6033856818349079`, 0.2509533302600997`, 881.1682049810406`, 0.2136549221518534`, 541.1576363993561`},
{0.21035514306399716`, 1.171180695658463`, 0.003072872162929503`, 1.0221763640609527`, 0.6635332430632568`,
0.41088172812983936`, 0.025266995789722163`, 240.41303897513703`, 0.11383871532153805`, 586.1694984071029`},
{0.1757725171577343`, 2.8661042899257385`, 0.05038868584079925`, 1.0221414200627101`, 0.4033811300549006`,
0.2808753785255522`, 0.23688381111182788`, 1187.3099819834174`, 0.12417956003379604`, 375.8746376350517`},
{0.15358915554374314`, 1.5283095551064108`, 0.067290924027347`, 1.1004906376784402`, 0.30400512125859813`,
0.48412227232879435`, 0.05306566889779679`, 235.92000045587324`, 0.04951315982948612`, 369.40105642182`},
{0.2480667235282486`, 0.5649579963672635`, 0.053140067348230215`, 1.2818674385347062`, 0.7623809660000294`,
0.3909970176312558`, 0.15838111333051053`, 2237.6541846573073`, 0.19996140314425598`, 705.3371940684284`},
{0.15145239039660063`, 2.013347200420334`, 0.015461991282007386`, 1.316890683212132`, 0.6869826935753494`,
0.5587698316891556`, 0.155845847769785`, 1008.4357476155614`, 0.08368596216890972`, 432.47408201145043`},
{0.10270003263878408`, 1.5137093678767606`, 0.08953511397171234`, 1.327942781940753`, 0.7426665475116903`,
0.6088434442795674`, 0.0731377274448417`, 629.1021204327045`, 0.13932635233516538`, 654.4427386466705`},
{0.0793896888891446`, 1.0605932032187937`, 0.028244713409935986`, 0.9969099319950825`, 0.928004076538486`,
0.27677090692739703`, 0.2620559892525522`, 130.80681937144962`, 0.17810627837194376`, 310.3444008701523`},
{0.27502930950392923`, 2.268988901211176`, 0.016690280319337808`, 1.4275341924064249`, 0.010424245503220186`,
0.33306898602448776`, 0.08933605249123197`, 948.3096011028355`, 0.06701293400753894`, 811.6908300502881`},
{0.1401731309912823`, 3.510459405926335`, 0.08747923189250421`, 0.877306477766522`, 0.5918814561880961`,
0.26160677626581574`, 0.2368095664541691`, 545.7040461576285`, 0.1892727670923422 5`, 868.6345989081833`},
{0.22884950103664703`, 2.906687483673287`, 0.09738225028393067`, 1.211823583578368`, 0.3123283466662281`,
0.6689345381975826`, 0.26177215931622877`, 1241.3038445786822`, 0.0415314605513189`, 456.6453916426678`},
{0.05854425920454692`, 0.5238963150414171`, 0.029075123644520265`, 1.1426557901500585`, 0.18407746295956828`,
0.6998927262860721`, 0.018954418749017626`, 3086.173448022591`, 0.07606552279053064`, 322.105662191812`},
{0.21395159305342204`, 0.7435306425677184`, 0.0767057005018636`, 1.1181708530382222`, 0.44751085978070226`,
0.3928647127765148`, 0.07805162691492751`, 4351.207257923278`, 0.18002244556017466`, 250.73279447897897`},
{0.10532042949270481`, 2.282286106803773`, 0.03578707315111876`, 1.3216192797313202`, 0.04188890042726978`,
0.35518315207091156`, 0.030710322655757117`, 714.5224002509336`, 0.1899050019419828`, 291.00389901709906`},
{0.0829906933156388`, 3.8005179296933402`, 0.05100636383257797`, 1.2003099776527455`, 0.07859762059433151`,

```
  0.5748450314701046`, 0.06614617870175625`, 1151.2833065752827`, 0.239776274024895`, 683.3664621517701`},
 {0.07620756796620615`, 2.1362921732694753`, 0.08270811763119688`, 0.7737426183146745`, 0.16169731434372636`,
  0.25627790943141393`, 0.10296042384053361`, 1230.0671635154563`, 0.22674061100539578`, 156.89063483163608`},
 {0.08669389820930451`, 3.045091924598611`, 0.06550910340602879`, 1.1417010593363959`, 0.8553204857574712`,
  0.40177204632489383`, 0.3920145317856002`, 704.3045188157349`, 0.14263547938719845`, 197.59576868603688`},
 {0.16716391272679498`, 1.9707940745517059`, 0.07610260692323212`, 1.0373304984519585`, 0.065601176736233`,
  0.3311381406088889`, 0.12567743444949903`, 64.49465686753402`, 0.10130675587326837`, 483.8093443390175`},
 {0.19822566409398995`, 2.5240412947893924`, 0.07566408618441202`, 1.4422150828920155`, 0.5695379812663179`,
  0.5359514511624127`, 0.4361070820215791`, 2999.4348713305167`, 0.17170169259485252`, 352.0476518165484`},
 {0.2582881650757237`, 2.8338294335694956`, 0.056678410389419474`, 1.074319457568079`, 0.10001974339589292`,
  0.2593067051666722`, 0.07903831577731309`, 123.40669984562356`, 0.2485795541321016`, 273.9914626473702`},
 {0.19497013167937116`, 3.160806920721086`, 0.03009239591987214`, 1.1171831820583615`, 0.4032199037756403`,
  0.6542147113806289`, 0.029465449867892138`, 135.26925165074772`, 0.15317415942522017`, 921.0689346313153`},
 {0.1360564482424821`, 0.498585531259212`, 0.09708640926926869`, 1.2162038348005786`, 0.11915352104228649`,
  0.5956143691814719`, 0.017832698276087463`, 73.16788682178698`, 0.17076027369704966`, 648.9946415099051`},
 {0.13584499805886296`, 2.3834753645488007`, 0.08131332514134307`, 1.195938774683543`, 0.5088595280581578`,
  0.5258764750728102`, 0.0188849029484269`, 313.8058177427212`, 0.2364862024741387`, 393.55616964852993`},
 {0.11252539515708737`, 0.48050955320077815`, 0.05937623337381773`, 1.0236120368069983`, 0.7678895649223039`,
  0.34416849853115417`, 0.008058107074703271`, 595.8657256124042`, 0.07836079702593357`, 415.53467926543846`},
 {0.05491046591497972`, 2.8601951288119833`, 0.051921452801939785`, 1.1535422934918167`, 0.1542041346296501`,
  0.3257327805969301`, 0.13649263155602906`, 63.90737715933027`, 0.027455815347211365`, 562.8847739679362`},
 {0.06606106797338895`, 3.6656724808413053`, 0.08751889535990849`, 0.856123556717206`, 0.6825935559995051`,
  0.5266173914314858`, 0.022251439911425613`, 1853.305468841329`, 0.16065122014174432`, 222.6798638040009`},
 {0.08977217568251161`, 3.4528260544503624`, 0.05764478135197445`, 0.9515190566806713`, 0.12846915530471392`,
  0.2168250694214009`, 0.007812714529548206`, 538.1854674164173`, 0.10544198251462877`, 265.77458817227784`},
 {0.22154304122492052`, 1.5332296117725148`, 0.02877908996913961`, 1.4000488050416342`, 0.4501923011081377`,
  0.49546155556198335`, 0.03515207628009833`, 79.99141751015185`, 0.06122494520176597`, 976.7821412391111`},
 {0.25142928822507965`, 0.8204095084034169`, 0.04775248977653711`, 1.4286128295323546`, 0.29951010343614115`,
  0.5861829342129077`, 0.023219447176916163`, 80.52367411150172`, 0.02588989292681121`, 373.5821652599455`},
 {0.1883756473924924`, 2.484499408271515`, 0.061812669070377274`, 0.7895040235728823`, 0.6199124969015315`,
  0.675547123744697`, 0.4413864963579275`, 224.0804292528027`, 0.08535968882727879`, 719.8375736166386`},
```

{0.04016858296637793`, 2.227608827443719`, 0.014781301743870988`, 0.9058498293617421`, 0.1749409962982329`,
0.4847952385558868`, 0.08097216295115642`, 156.97217485042802`, 0.02129075017123533`, 574.4487237391048`},
{0.10741359519637045`, 3.89617613092826`, 0.04687805603635281`, 0.9794147612026731`, 0.29389906665208465`,
0.504394704133666`, 0.007650336867825106`, 94.96376645284155`, 0.03904567887129218`, 298.40389093358954`},
{0.11745053279334533`, 2.766853076583562`, 0.01709341111594279`, 1.380078086463911`, 0.6364552296774821`,
0.3050852260798198`, 0.23148607657693196`, 632.2606814192264`, 0.2194692090207866`, 944.8863492578521`},
{0.06051380978707749`, 3.721291677689673`, 0.02351362713647468`, 1.4742360491119153`, 0.26914934000385093`,
0.3113498131806878`, 0.014199987869299453`, 193.52537465732863`, 0.03624750856265596`, 654.4974389371708`},
{0.24099369076587246`, 0.9331253409733131`, 0.08217248655684913`, 1.3431483271602298`, 0.5958343219475497`,
0.5465723977337402`, 0.3117537417312618`, 3620.513648000344`, 0.017044351379539774`, 583.8913660600036`},
{0.1670999366894979`, 0.6892897125951207`, 0.018177278706139326`, 0.8073727173778276`, 0.4996777893876556`,
0.5006175381891131`, 0.2838330520493472`, 735.6587801659197`, 0.09848547093144494`, 522.0721253919525`},
{0.20400009973703231`, 2.742493766989547`, 0.008983552040910397`, 1.241362072408477`, 0.6278550642154503`,
0.39751636324962747`, 0.007299390369356264`, 91.83643299080828`, 0.13001522795856457`, 108.9539719977738`},
{0.22650061618533962`, 3.64567942204856`, 0.07707002972279017`, 0.9032908420697698`, 0.9136296860108464`,
0.4341422027661652`, 0.24929767745737103`, 136.4576870478556`, 0.07080767715199654`, 971.7774116990597`},
{0.10155767105386482`, 0.4181451151728903`, 0.012394687986228644`, 1.0711096932931712`, 0.20820538774593245`,
0.1626988912346673`, 0.0864577360395456`, 674.1888341417106`, 0.170056418389111`, 730.2391664075723`},
{0.21231295112704185`, 2.380633825425009`, 0.06236561820134972`, 0.830378492099762`, 0.7458626837014395`,
0.3778428196310881`, 0.0682174150958653 5`, 516.5455006559224`, 0.0981833249923344`, 365.58490612208846`},
{0.06947494567464263`, 2.895860587431992`, 0.09178966303831615`, 1.2688043406444924`, 0.2941408877223748`,
0.3751778179392746`, 0.03775382322774307`, 60.369496178615435`, 0.11794208757169544`, 673.3642442045926`},
{0.09985693159346487`, 3.9019559576117224`, 0.06462003972312033`, 1.0811937700231284`, 0.15667266604108`,
0.623010278974039`, 0.040053923820923376`, 112.7677826146989`, 0.1201138368644335`, 956.8700593079004`},
{0.21079578713436742`, 3.6465182996128265`, 0.07263502170252384`, 1.2463245485615204`, 0.04598824508235744`,
0.19490478821937018`, 0.006055825079153585`, 455.84209633704694`, 0.04307337339516104`, 393.98363704378045`},
{0.14050505626384868`, 3.3486141508967293`, 0.06576905440282896`, 1.0969622150757512`, 0.25298608583995974`,
0.6081914621324831`, 0.07984197613603365`, 4147.732881753063`, 0.01970187856601205`, 417.6295038180262`},
{0.09029343760417469`, 3.1277911065164137`, 0.04916145932038262`, 1.1046401865081483`, 0.21999281746629284`,
0.29609111953575007`, 0.08848537504901788`, 1978.4176945251475`, 0.1604567549403832`, 631.7829673381813`},
{0.18744474850618575`, 1.1949686342264414`, 0.06413249212557762`, 1.3584803763102893`, 0.320102170913821`,

0.4723006569832039`, 0.10067644299750714`, 61.82330782936513`, 0.17695515305422665`, 265.1926737070246`},
{0.2388654681009152`, 3.8576612834574524`, 0.02902564289553176`, 1.4380459539751635`, 0.624215346482978`,
0.3925897588304593`, 0.28422329981310057`, 1921.54220161346`, 0.09343473821304771`, 199.86167324974923`},
{0.1455526991456984`, 1.8705049552506363`, 0.03961322047797989`, 1.4283548335168212`, 0.9360504979535096`,
0.6211597747744511`, 0.1850342062284412`, 2722.415223545606`, 0.054866668114149975`, 162.6815751148559`},
{0.13798249053354167`, 2.899902544251761`, 0.0459763868363342`, 1.0421434347893428`, 0.7895156656158571`,
0.5540265258106729`, 0.047360361099740046`, 2648.770558815322`, 0.09753399188330308`, 720.6816664753013`},
{0.17745255413169525`, 2.520375966029923`, 0.04665876128100216`, 0.8123275453028636`, 0.944701273242313`,
0.24813120748859874`, 0.24862758503488186`, 106.62623807073865`, 0.1979751584484703`, 843.0950202672676`},
{0.16499863275860222`, 1.077452191253113`, 0.023014633917973006`, 1.2950348307619706`, 0.9833706900164221`,
0.290091826190232`, 0.22192674995545697`, 3510.2131809670686`, 0.1789639285862865`, 441.136883561353`},
{0.09494055003043922`, 3.273540527547083`, 0.060548822068484894`, 0.9817396325621766`, 0.5953487435300235`,
0.3030053710238173`, 0.020212568263132794`, 207.40154426384788`, 0.04862769785226101`, 884.4963622485988`},
{0.22556943514719452`, 2.763355506916427`, 0.0659847718412378`, 1.1318711218549837`, 0.7290768537995906`,
0.6910097128365145`, 0.02883814164041025`, 1935.4928886970383`, 0.060672213321555424`, 592.2254589353502`},
{0.17976151229453585`, 3.607368069246487`, 0.03562542831805498`, 1.1702627239429857`, 0.8807800807300026`,
0.504832892868570 7`, 0.08802036843157425`, 258.70537367208925`, 0.2013896965979159 5`, 520.6789088604623`},
{0.23694985915625555`, 1.7115996463959204`, 0.09475499820714504`, 1.0033560670730772`, 0.6058641758111063`,
0.5075289726151094`, 0.04433733902402368`, 990.3350746839715`, 0.06868554455100367`, 684.4534604177855`},
{0.060659588298575706`, 3.185819049116902`, 0.031246099794861325`, 0.9329844374373137`, 0.7312648065631473`,
0.27616453553670284`, 0.06002230172665507`, 72.75197129693021`, 0.01701545753405373`, 816.2999501758868`},
{0.27783663000790365`, 2.7970847777258587`, 0.08231332350426997`, 1.022697264732825`, 0.9687024837581177`,
0.3645779308390613`, 0.041020574150516895`, 4813.902596935811`, 0.09489024372224386`, 126.14406706341742`},
{0.1853383438805617`, 2.710478464072388`, 0.01489054849244626`, 1.1482283702493803`, 0.5568912869451013`,
0.42437036955952745`, 0.009568366829966814`, 179.8278356132767`, 0.21625752953014726`, 454.80717115717636`},
{0.05021881637759101`, 1.4247717975270389`, 0.062006838284966344`, 1.2900546523204781`, 0.9617562903977206`,
0.5950392020832158`, 0.01053774359341417`, 3195.955497745086`, 0.10119630409828645`, 778.7070525025722`},
{0.1629749532929909`, 1.3827669190112752`, 0.02163316926061519`, 0.7859725102428954`, 0.8835314458481405`,
0.623882909407651`, 0.021916393496908437`, 149.5675827629461`, 0.09031773923128839`, 469.7973445453567`},
{0.2303174353412305`, 0.7381196545439321`, 0.017853274128905045`, 1.009576893018485`, 0.49320693638055335`,
0.6517404058254557`, 0.03129869768398024`, 1241.6665530134712`, 0.22978761854559276`, 819.6026061342192`},

```
{0.05000320218233145`, 2.1972018685344494`, 0.006726117027854865`, 0.9936552562980645`, 0.8328018981538441`,
  0.5156429010358797`, 0.1943865808940930 8`, 89.43952173057976`, 0.0339349817407883 5`, 570.4514942090268`},
{0.22380766185105788`, 3.972867022032534`, 0.05857466369517589`, 1.320944264492121`, 0.8375716850380956`,
  0.6387604619615563`, 0.14529791195160408`, 354.612762024331`, 0.0916149378074263`, 197.12301088359715`},
{0.1925638236278404`, 3.172077483377868`, 0.030644598758412977`, 0.9803085988716782`, 0.875326409629076`,
  0.5489970615034699`, 0.4213559070823191`, 63.50782532256897`, 0.15909673801152152`, 757.7936027644167`},
{0.09418442467434818`, 0.6452011369781001`, 0.05468238280755824`, 1.1696403162883113`, 0.9269902181221603`,
  0.5827207204052004`, 0.0480602677307661`, 155.50909202321085`, 0.17256127653904146`, 540.5740309875913`},
{0.05187445469191648`, 2.8967354683291404`, 0.05468134937388834`, 1.0272048159360176`, 0.32845734934833803`,
  0.591187325659497`, 0.3107635049414343`, 481.6720966539763`, 0.2446670824913575`, 391.7749895365258`},
{0.08569179084998313`, 1.8355994610956383`, 0.09902879473840677`, 1.0426364128937085`, 0.046121645528780064`,
  0.5970016999064142`, 0.04585616299575304`, 1326.7471633941773`, 0.1575248274172592`, 749.1145234256358`},
{0.24342030247616103`, 3.756945920144269`, 0.04820173144973461 5`, 0.8715075343316259`, 0.6190895848455991`,
  0.19127133299748744`, 0.06855426679028909`, 1305.765466028692`, 0.23417008838782194`, 415.6502949437222`},
{0.19292203783708667`, 3.220432103715133`, 0.09761210999008452`, 1.3591272090173214`, 0.14186149659118774`,
  0.4413360685039047`, 0.010167923556678914`, 131.42190006936238`, 0.24362820427309634`, 380.03821419833645`},
{0.1897036930032619`, 1.8964339581865879`, 0.059440108944421456`, 1.1142569288613073`, 0.15129145170580593`,
  0.3833845599206046`, 0.05519372846185666`, 4558.979306456515`, 0.013937979989310956`, 207.93733592881284`},
{0.21229489496584697`, 2.877170935416972`, 0.0852716204718659`, 1.187802399588918`, 0.766747964989742`,
  0.2056469011396459`, 0.2516666100392663`, 249.75546686829057`, 0.04381049946278048`, 453.1293949612376`},
{0.18727208406868906`, 0.5920853885729089`, 0.00310465950751313`, 1.4761651930790642`, 0.9879910194473076`,
  0.4582425433815992`, 0.1348498167291504`, 1379.8554077407293`, 0.19832956659061096`, 601.2373424307469`},
{0.21303838080640486`, 3.716490082492431`, 0.06530590830455278`, 1.2455728623234372`, 0.6800061917252811`,
  0.3355795794234222`, 0.014627505674034404`, 79.98307290173638`, 0.017560362469609708`, 213.93067315001346`},
{0.06263632603162816`, 3.088015470379359`, 0.06468478054731996`, 0.9840993814948887`, 0.7841919183884491`,
  0.464604288151068`, 0.01258311092046284`, 1881.0148437864943`, 0.0609287237146911`, 341.9306317400494`},
{0.1229419022884577`, 0.42323948511603504`, 0.04401981826231521`, 0.7802588542080802`, 0.3378063219388938`,
  0.28733129925219736`, 0.006237735142161048`, 2301.7896008425946`, 0.1904542796497251`, 972.0175866826189`},
{0.07173116327385964`, 1.689724351901349`, 0.0333537565627478`, 1.2797681367134839`, 0.19114975037520088`,
  0.541681437492502`, 0.35548681580819513`, 4889.525485193395`, 0.07624198202888299`, 255.7119735830849`},
{0.12377155914664595`, 1.5057831665831483`, 0.02584787085922036`, 1.3931245282333518`, 0.1751216551172583`,
```

0.31643702992235223`, 0.006127436167226222`, 3977.3695715719014`, 0.19191205388712534`, 489.96663345919205`},
{0.07922678243367776`, 1.1007105803460338`, 0.0670178962975939`, 1.3858977973939028`, 0.6913244964468961`,
0.30596530488881035`, 0.03670449008923856`, 722.5750280061836`, 0.1062965670986098`, 454.2241152370099`},
{0.13900795297527618`, 3.9726186574282156`, 0.014365558963868033`, 0.9387566245260975`, 0.7733911807172529`,
0.2901636804835216`, 0.022925433557643798`, 983.22576571694`, 0.10414701635541929`, 690.7271079642408`},
{0.06983492300698985`, 1.9811052508516296`, 0.015092410881193086`, 1.3035668919937708`, 0.40820708656243165`,
0.31349067656511853`, 0.03294127758239607`, 588.7480044888102`, 0.2032334347292173`, 447.9335749964651`},
{0.05248318477610414`, 2.804613750674261`, 0.003640346777431844`, 1.3628720468665754`, 0.1995299750560573`,
0.4294363076423442`, 0.007453829399752168`, 688.7688808168017`, 0.24854011257885444`, 913.0182165489159`},
{0.22482399322255875`, 3.3120245448192804`, 0.03927421614929747`, 1.1730261223962148`, 0.8102112515480973`,
0.5310384924975508`, 0.021360928541744394`, 935.9962133496834`, 0.022365336640988054`, 615.9503154198303`},
{0.14054031835314812`, 1.0862525764402395`, 0.03495957467986743`, 1.4989441459825807`, 0.9776070717717082`,
0.31123951873946865`, 0.005138607602622309`, 2625.434021720543`, 0.07843546140834678`, 537.9180477646557`},
{0.12045530385052439`, 0.5766797014120977`, 0.026639235327100757`, 0.9789792038223954`, 0.1966739314900421`,
0.3703973027163936`, 0.05825214840022438`, 4908.720210074871`, 0.20113853341018156`, 829.8609278324832`},
{0.2466711089217451`, 3.818653323688843`, 0.022826073218276496`, 0.9885944445220116`, 0.08478033040666122`,
0.24831675766148897`, 0.05221202249805683`, 61.098852909743506`, 0.07219665114050788`, 854.0668809251908`},
{0.2622407564991055`, 2.390608019262051`, 0.06092577106875352`, 1.4943502181005281`, 0.48817005604720776`,
0.5989574618060204`, 0.0663716035556017`, 59.28691333172426`, 0.07923371737796253`, 663.2501758461839`},
{0.04240892049220529`, 3.585324421001501`, 0.06559550484561946`, 1.4902471895373985`, 0.5553889156366423`,
0.6483753523879463`, 0.007118840678651323`, 2218.8393261162782`, 0.011166606091999653`, 925.472456163056`},
{0.139826903045498`, 1.6937551480709319`, 0.08421189687207545`, 1.1119195234296138`, 0.8823668491031473`,
0.5196063483063849`, 0.09432450978517919`, 330.21686735744527`, 0.2294519472818627`, 332.35073177164196`},
{0.14802078413209885`, 1.733603865107586`, 0.05101449210330218`, 0.9572479232347667`, 0.5675011406257902`,
0.3885036693205801`, 0.29314426920759284`, 2048.9810499540877`, 0.14325997637273158`, 127.28328705485782`},
{0.09351974409461145`, 1.9687875339139866`, 0.015572094311755826`, 0.9484499792232198`, 0.5844423119728932`,
0.5819082431078818`, 0.2373776682556218`, 112.86193139612024`, 0.23719097877429007`, 229.2738627795444`},
{0.1102903206501954`, 3.0454263679631577`, 0.04069331752982141`, 0.9534089355456694`, 0.7297525123381821`,
0.6576008896160583`, 0.013329813923119783`, 1715.4779634820493`, 0.02214911406080483`, 227.88476662786474`},
{0.05909859147151786`, 3.30435803161393`, 0.029336284768972404`, 0.9231466780747943`, 0.9623144707471962`,
0.37803674309323987`, 0.04397572398478963`, 3067.5791243622793`, 0.057241291718842735`, 398.5949730494525`},

{0.17398185963078006`, 0.47005124431733236`, 0.09389471888393235`, 1.232881569185857`, 0.49856219410777936`,
0.4191945121229328`, 0.14050812071087648`, 190.8204082787259`, 0.22639026429089515`, 506.13435159524533`},
{0.05155342181122552`, 1.5581409866760865`, 0.0691893121207933`, 1.3639899124520563`, 0.2080210727331211`,
0.429465542144159`, 0.08260476334651806`, 246.4705132305552`, 0.16650171241851036`, 476.3403890303273`},
{0.04071142279712078`, 2.1496023906791892`, 0.08531567546447687`, 0.9683458228368205`, 0.49038233136090126`,
0.6235182579165484`, 0.07161574481597612`, 131.50858611951176`, 0.19699207630915766`, 596.6367875356493`},
{0.06590056983751874`, 3.9638284260232943`, 0.016048742823094812`, 1.3783253378441898`, 0.24532119252948448`,
0.3220387333596423`, 0.016945992465929767`, 2197.071659291998`, 0.010352607682042353`, 236.6421867279362`},
{0.26325550998326586`, 0.661917211789294`, 0.026986149925021862`, 1.002112989283701`, 0.06554947180049209`,
0.6994424736047835`, 0.005881514200787017`, 211.33538862145804`, 0.24482509012686327`, 303.2466877164689`},
{0.26127356110524397`, 1.329573031379259`, 0.07977956036816294`, 1.2076717867972873`, 0.5921373287024636`,
0.41816652735672233`, 0.022774959106689946`, 64.98766538013531`, 0.19432837274522574`, 259.57035925631845`},
{0.260215550616946 1`, 3.5506896347378634`, 0.022465629838975154`, 0.8509530374249318`, 0.7983914367554696`,
0.2578474320630234`, 0.011809304810800692`, 142.12037985462777`, 0.1698027140526227`, 698.002014936651`},
{0.06365047180104016`, 3.416188751455385`, 0.03523713957757545`, 1.0192452557682743`, 0.8469643727981293`,
0.5498369694104505`, 0.18294125259438154`, 1018.12440025711`, 0.11189348348403971`, 231.55349754949057`},
{0.20010483475430668`, 2.008655229618358`, 0.06224827434543773`, 0.7776282320212735`, 0.6157609854264054`,
0.25612002607302176`, 0.16950601491382145`, 2258.159167246791`, 0.12768932854616544`, 318.9473026654234`},
{0.04033357276115293`, 3.365035676090897`, 0.05992514630623122`, 1.1732918415280658`, 0.196311756799749`,
0.30038372703117333`, 0.16280533449388682`, 87.04220764531692`, 0.23496316330764122`, 283.48137514032834`},
{0.1509427023460006`, 2.3319882937767247`, 0.018731546174507247`, 1.0584311371273147`, 0.6567026163933902`,
0.626594775903838 2`, 0.06164026490982475`, 1022.7891111424142`, 0.168828731973697 7`, 154.17413301109093`},
{0.12172292087124631`, 2.8233234444817974`, 0.034053562361951124`, 0.8588222796104166`, 0.9440480252161128`,
0.6422691510398222`, 0.05980616075075349`, 1021.745763820812`, 0.044168716180875034`, 436.5175383363466`},
{0.10332818067094146`, 3.3200182476314417`, 0.013797086337646878`, 1.2403231995339712`, 0.8068678048493421`,
0.31817454060495975`, 0.03466270708497138`, 224.98168772666384`, 0.18064139380974548`, 882.0488176741194`},
{0.06283595024068839`, 1.3516727681414702`, 0.05242674723140476 6`, 0.8261017998522725`, 0.2587281077553354`,
0.25782009716699184`, 0.4461332906525768`, 78.37105686956872`, 0.05053665990821049`, 454.91374396216366`},
{0.26061686002038464`, 0.9197100483832488`, 0.09424919710819697`, 1.2431276888944953`, 0.03523684950904893`,
0.6986561589310964`, 0.46987667183492504`, 257.0362567649576`, 0.16991196769780592`, 326.7794760540163`},
{0.2589743226453006`, 2.4967568849644275`, 0.07508840824492798`, 0.781034619960081`, 0.38459790960215257`,

0.18475383400796352`, 0.10416671513341536`, 3175.918867658944`, 0.2205445770390796`, 431.51407680934443`},
{0.1822125191728448`, 0.53967615378293`, 0.06140923587336875`, 0.9836246668356532`, 0.5663752339069781`,
0.6127333889317503`, 0.015376137369944434`, 4374.881958546335`, 0.1720894071809011`, 902.0125127914057`},
{0.20287638930589763`, 1.8511964408244932`, 0.020237031457082787`, 0.9096848496624212`, 0.22198223428443487`,
0.15744015403827616`, 0.17491779271673957`, 2922.9247864169292`, 0.14133268345189937`, 866.3466773010839`},
{0.17277183156190062`, 1.3838708180759518`, 0.01858178773758265`, 1.4265746785083109`, 0.6133308818390555`,
0.6532402234702124`, 0.07637848286373418`, 1061.2255229483176`, 0.23800513499817172`, 715.6629218234075`},
{0.22973167487200014`, 3.4630938101482327`, 0.05848037010032728 5`, 1.2645217042191328`, 0.6981417065741804`,
0.542170464886519`, 0.006546556460297805`, 74.23410585835131`, 0.017772563055006546`, 241.35868804813666`},
{0.17947546203746156`, 1.7068442534238697`, 0.04056609287799669`, 1.1830573633622712`, 0.6640874832688464`,
0.42001594980708057`, 0.21752981815857997`, 97.3204887238174`, 0.050533940181592796`, 409.66978902022663`},
{0.08473729051323187`, 1.7479721342100945`, 0.06355457932773143`, 0.8574963830627491`, 0.22245663004688998`,
0.4791593777006873`, 0.28265674645892613`, 4498.232223037125`, 0.17673010273241208`, 336.2131425839168`},
{0.20435672898605034`, 2.6117732980489388`, 0.03475265809490025`, 0.976265205498944`, 0.85216518519733`,
0.548618924239519`, 0.2709183052632252`, 155.3772460870891`, 0.021213370069483317`, 486.638902544934`},
{0.2230119008971858`, 0.6358954372186516`, 0.03489317261697556`, 0.8021667773970862`, 0.7799870633314419`,
0.372553704845368`, 0.3954953790403783`, 3708.9097099582787`, 0.06863756553832256`, 831.4592304552722`},
{0.13277530106525864`, 3.102992336174254`, 0.0810346296150594`, 1.417175031427167`, 0.04111047270929191`,
0.577719723059283`, 0.2584094824664528 4`, 1582.3635767727842`, 0.11281028363913381`, 572.0784651822191`},
{0.17721564666118195`, 2.3073681400537707`, 0.06977110969724436`, 1.4389124933186648`, 0.06511833232445241`,
0.5785856768063609`, 0.024382684167548917`, 50.72398143128576`, 0.15027785530459653`, 471.87731805197507`},
{0.2532195731000343`, 2.951680905930308`, 0.08243784959141458`, 1.063512245713839`, 0.13059994709602862`,
0.20280590336854443`, 0.2607392691737333`, 517.660101290023`, 0.077733893061395`, 364.603949151811`},
{0.11562259606165537`, 2.075988177277`, 0.03552939359394358`, 1.0857990592224895`, 0.7681961757441904`,
0.6605331614852874`, 0.15159919874130345`, 3296.1941609770115`, 0.07746882734639965`, 160.02380566717028`},
{0.16010263731321916`, 1.8512370146128028`, 0.08948994106153972`, 1.0065876584784161`, 0.31909081739901013`,
0.6055123683081534`, 0.027623148293597768`, 400.21848632042986`, 0.19838189450352378`, 329.78449858468684`},
{0.25703658745875196`, 2.3078283713322856`, 0.021362941537208063`, 1.2100220391878165`, 0.8787828002021987`,
0.2513644151003869`, 0.024289521781396636`, 68.1341971888039`, 0.24810809198178096`, 481.51760126235956`},
{0.2455904611014849`, 3.7571997349914916`, 0.09211738529180043`, 1.4019497765522448`, 0.6695611235062522`,
0.6602799476916219`, 0.04340955542197445`, 856.0993716100127`, 0.15292526713206545`, 529.9607802643166`},

{0.05969614974887660`, 1.688845353034914`, 0.009294581379800135`, 1.2433968456838194`, 0.3929448551213022`,
 0.4749866039118191`, 0.025863060541790456`, 192.8417865287921`, 0.09839655966119532`, 364.5695918572345`},
{0.09761501672592626`, 1.3774481728994479`, 0.0397488421074619`, 1.4571532371767337`, 0.8600435797101407`,
 0.37053809894631096`, 0.30839879803159215`, 1053.3603252171624`, 0.09829081378486954`, 314.7778202000868`},
{0.22092704028098814`, 0.8304263766325484`, 0.048201718626776184`, 1.203623675331191`, 0.2935636564899402`,
 0.4030249377909366`, 0.033061104251936244`, 4358.833221490219`, 0.1589342169606452`, 180.8709305853121`},
{0.13823492725556935`, 1.1413185260053824`, 0.06971384118788397`, 1.3157780297053645`, 0.6082014740714308`,
 0.31531914337240985`, 0.005597926269123681`, 97.23206007061579`, 0.1162100716591018`, 522.6554437517659`},
{0.14416900464242904`, 2.543812431689566`, 0.010106655971553415`, 0.8969401222383744`, 0.4328515562362101`,
 0.20360668938810977`, 0.2923006645347409`, 1178.8843718309924`, 0.20797847649312406`, 120.9186114906668`},
{0.2133877273723686`, 0.8490524254535732`, 0.08700555866439949`, 1.2062638954363991`, 0.6738638517009694`,
 0.46997962516177505`, 0.06892379315565185`, 1349.9514731760883`, 0.09645636257209894`, 836.2941738998895`},
{0.0539089201271194`, 1.0364880092644801`, 0.016154417078585476`, 0.8886530805439552`, 0.3168467801118968`,
 0.15642019482584735`, 0.006844524019875138`, 85.57714734356885`, 0.01991933931946757`, 258.3461272090961`},
{0.2715756156789369`, 1.5445757615325304`, 0.00421616582195627`, 1.159485102317175`, 0.4702357167765736`,
 0.34737964168870006`, 0.496052684967637`, 300.35079175305486`, 0.1259406113194213`, 781.8522906262123`},
{0.04827287438408412`, 1.8515860963247066`, 0.07692111119618293`, 1.2774889475291031`, 0.32248502080707353`,
 0.20234972534433104`, 0.01272417451099904`, 140.98466458635835`, 0.11366894324343613`, 273.5929992840893`},
{0.2554976820569969`, 2.4378755210159753`, 0.04254022762089836`, 0.8817958104635948`, 0.692204018420977`,
 0.4234476557446073`, 0.007554290015778277`, 79.39858197716293`, 0.11447843867339091`, 117.82612509068677`},
{0.2760063677033659`, 1.2105198937059631`, 0.067224388891334`, 0.9988908925658994`, 0.9019281244861579`,
 0.67284715505845`, 0.007365273847592193`, 2972.356277007087`, 0.06361067504455276`, 438.4208211463028`},
{0.23497331294207247`, 2.932797407682453`, 0.022597452058598203`, 0.9589743833470734`, 0.1667506323796748`,
 0.3129991011881105`, 0.05276499482811941`, 99.75864289244342`, 0.09875437541358872`, 580.7218610046771`},
{0.0552957146577317`, 0.9263334676885089`, 0.05382222458972377`, 1.1846475829708125`, 0.6978145776046678`,
 0.40095280794552435`, 0.14194113807951353`, 67.36725348197879`, 0.21539237947591866`, 912.2514050198081`},
{0.08596797992646615`, 2.89469532919738`, 0.0723374629314705`, 1.412708246795334`, 0.2707681400237405`,
 0.659961029627586`, 0.1545447128013493`, 953.1432644930773`, 0.14443128680071893`, 294.4093465447068`},
{0.26579336742267545`, 2.150651278297752`, 0.09864009518921925`, 1.381049269309322`, 0.7824667408282995`,
 0.6485863724693819`, 0.1346970994193899`, 71.41513724603877`, 0.04324969499825426`, 231.68375988200472`},
{0.07893352000042303`, 0.9269382255330783`, 0.07450960524320785`, 1.192225010506579`, 0.29981085040929156`,

0.20655660387273633`, 0.005195235752158478`, 3935.7899816629233`, 0.09905090934440974`, 552.2915075373634`},
{0.13874147069686438`, 0.4677020963588703`, 0.06081979667173851`, 1.4293968405810138`, 0.04489099618087722`,
0.600807000017457`, 0.1990035962891371`, 80.51094589303605`, 0.24671702535367518`, 538.9722201215866`},
{0.2700245759823924`, 1.083893289879918`, 0.062021204290361266`, 1.2005471238256362`, 0.792954755831037`,
0.39473782087530096`, 0.17457967683710685`, 279.6749633279759`, 0.14741868150066417`, 148.434091412925`},
{0.23474828628269123`, 1.725907115336116`, 0.012821406564911335`, 0.8070142233189552`, 0.4777555512453826`,
0.4076167331863103`, 0.015638133522482764`, 120.11394196884895`, 0.019548998977537424`, 892.9399386284366`},
{0.09165311999697207`, 1.4061679173562647`, 0.03309545344659714`, 0.9052080425177362`, 0.22786616586188102`,
0.16948939359836301`, 0.041478452818892154`, 78.37706796679595`, 0.1493801427638014`, 220.7559134640384`},
{0.04235508864011306`, 1.3060003486013567`, 0.08466697376486561`, 0.8479712158967934`, 0.3936959911899278`,
0.2088063088308738`, 0.017308803394347618`, 4694.788266429514`, 0.026439759379240313`, 433.9918956105633`},
{0.2651923586966664`, 3.8881876111766713`, 0.0350309847210708`, 0.9591171183376157`, 0.9954879310389926`,
0.5341580610877573`, 0.0817474157614525`, 113.37527000346847`, 0.1996829793433833`, 201.49628341235228`},
{0.2787517386498274`, 1.5890965688604197`, 0.014994826667312413`, 0.9487067103902393`, 0.03893198736235193`,
0.2195801988959788`, 0.313478573421096`, 168.03485395038084`, 0.026460496970460712`, 626.9887607453275`},
{0.08753933430632788`, 0.6662218380744758`, 0.010717888122421044`, 0.8318793084446489`, 0.9074866505690673`,
0.6370551722773192`, 0.05914667212913143`, 92.90178750054149`, 0.041267271127747585`, 531.582902194676`},
{0.254499813976234`, 2.1602436577244424`, 0.0772554574037915`, 1.3316064464046935`, 0.367293601649199`,
0.4330751915150024`, 0.012004917841621048`, 4701.7880352079455`, 0.15735639832958048`, 710.0683407032234`},
{0.1501371731881197`, 3.5104518987365614`, 0.03585463368506289`, 1.1463465777386583`, 0.5443112287696148`,
0.3949552428603267`, 0.04879115335558662`, 259.24478342917615`, 0.1285655725832237`, 162.3475385554764`},
{0.14482453276755702`, 3.3636104411839076`, 0.038458077364182566`, 1.197124661291601`, 0.4328455972778613`,
0.3205266052041652`, 0.010337491367857544`, 172.8822485760027`, 0.1748531192873402`, 294.504606203127`},
{0.1729081128249474`, 3.4171958647256986`, 0.09736003964750388`, 1.3614201648251067`, 0.8114455381033425`,
0.2740485642532573`, 0.10719777411327538`, 697.2636909321262`, 0.16794794102451832`, 169.68999987903217`},
{0.06017132640138534`, 2.551705655373457`, 0.098001989150616`, 1.2777770866301428`, 0.33103821819782686`,
0.42457056361391154`, 0.3044012087125646`, 128.19341360646206`, 0.23285467527685833`, 861.6547556863932`},
{0.06838353005846232`, 1.5583268641110415`, 0.07074535929409585`, 1.1186421654705394`, 0.17808501168205293`,
0.6526418380839745`, 0.30389354478023717`, 2253.855886651074`, 0.2140132420516081`, 589.9585678536141`},
{0.04591951184292933 4`, 2.3909808568887367`, 0.05197740517398504`, 1.4076343252717902`, 0.4269585056408616`,
0.672767818437489`, 0.1429025608766215 6`, 365.93648706136383`, 0.2152208567065702`, 325.8336782379203`},

```
{0.2110207852916774`, 0.6269225337264532`, 0.060343077081531735`, 1.2521859520371945`, 0.29021239339379945`,
  0.47386146148275243`, 0.1626064474205758`, 445.43014573417435`, 0.07466666385442788`, 270.0446625362414`},
{0.13913288219717507`, 0.71598345975513`, 0.02918338476342825`, 1.084418293337738`, 0.1601393499115018`,
  0.351547594930286`, 0.041991397294508556`, 2091.889065193303`, 0.09601982280233712`, 646.6328833508262`},
{0.2080876337219731`, 2.734433706560215`, 0.0261030709279631`, 1.338847931530077`, 0.982472586795901`,
  0.3916223755715482`, 0.018379378513228514`, 98.65983909130968`, 0.11579954319108776`, 761.345713618144`},
{0.19884229924079655`, 3.7544266932443486`, 0.05306832500507259`, 1.4048543300387752`, 0.1752488991857195`,
  0.2724812216186141`, 0.12261739216762069`, 80.04459592601863`, 0.2028403340066654`, 224.71523563844968`},
{0.09229081102449843`, 0.41731444207519`, 0.09042236613704813`, 1.4795137951182236`, 0.6627370782775894`,
  0.6415172939653717`, 0.007608010716527551`, 62.09999691656457`, 0.17995756856061956`, 974.7890405043591`},
{0.22753750499438513`, 1.1094910684892056`, 0.06205079076485845`, 1.3683254796121973`, 0.59456895101486`,
  0.6649055203122181`, 0.06079543818004673`, 62.1436627548072`, 0.23424854868652234`, 576.7021529851472`},
{0.07611810878804481`, 2.417695097886692`, 0.008902516116383463`, 1.17651838899742`, 0.8956068934881507`,
  0.1734469831112303`, 0.007686752240077523`, 84.3504067614311`, 0.15979761325133707`, 962.8822438221003`},
{0.14505993431662217`, 3.1227268390818725`, 0.056275163136378696`, 0.9590973923566892`, 0.13969476213382426`,
  0.20892815360032824`, 0.0531610997371578`, 246.79786465887622`, 0.227047105813231`, 454.33945080770786`},
{0.23654885566478462`, 3.9241924925395733`, 0.03358528642939117`, 1.3191430750498658`, 0.39258636888736453`,
  0.3377325502420989`, 0.025557835669326675`, 93.73616135543377`, 0.1670083520090136`, 309.9129735942125`},
{0.11070282825339567`, 3.8334454078682603`, 0.07681857609135982`, 0.8958554753360997`, 0.32396640927167897`,
  0.44194855743506667`, 0.2131540641524383`, 1741.909189584937`, 0.022902468906996543`, 670.3098695990176`},
{0.10170301352011679`, 1.8752371643597758`, 0.03510782937111476`, 1.3860933640706163`, 0.6474965074918984`,
  0.1594306933477596`, 0.37318794312281267`, 201.47970914729365`, 0.22517965099104725`, 283.6656714712788`},
{0.19274669213409934`, 0.9288413277333225`, 0.038837310625680195`, 1.3836847187363839`, 0.14968000832109252`,
  0.4232153388345993`, 0.01563322978668864`, 922.263283889537`, 0.12359553135969004`, 486.72903455727567`},
{0.06356535997519341`, 2.311362126443121`, 0.07268080427945918`, 1.2586315568490016`, 0.3763201304339143`,
  0.4384209659583461`, 0.006474182269401951`, 4348.973288945735`, 0.17106111863805346`, 281.3921070619326`},
{0.06831716410403144`, 1.0015662947000914`, 0.06390469368977933`, 0.8290508858928134`, 0.7249330951564277`,
  0.37450775838270167`, 0.3666941924999965`, 191.80408739918798`, 0.153067071236483`, 887.5069081464457`},
{0.1293750817503745`, 3.1014207000970364`, 0.023014106546543296`, 1.351810814205737`, 0.5054291062898795`,
  0.669566365984742`, 0.05376259966791126`, 207.8155819891332`, 0.12505633467039579`, 477.98997920694734`},
{0.22605177518456349`, 2.2930688004852557`, 0.04762600540866055`, 1.435285876756151`, 0.7544760534695125`,
```

```
0.32613259549063545`, 0.008000490945700793`, 658.1451070175192`, 0.1664301859365524`, 397.6391494406913`},
{0.21478835126656415`, 2.1859458812100163`, 0.0858199459854308`, 0.993933836221133`, 0.013991965571371612`,
  0.25438763086955674`, 0.010673305721101622`, 175.38786745903064`, 0.249124195297232`, 141.44233499151147`},
{0.2524903538890303`, 3.207130817880457`, 0.0760687494889642`, 0.886811483661367`, 0.4861660798083616`,
  0.6820047923981643`, 0.01500874534183673`, 477.62249790514926`, 0.21800800344067622`, 582.3093284125088`},
{0.2673762141971479`, 2.9843296894323084`, 0.06575888816802714`, 1.4582777652181538`, 0.8424633965803163`,
  0.2888169272806542`, 0.017193087756165917`, 1905.2031126287002`, 0.22394556493184148`, 302.0798156952443`},
{0.0718648334935813`, 0.9687764001979549`, 0.03778553570658904`, 1.0685686917623896`, 0.855670606560941 8`,
  0.5975957325406767`, 0.1005409517041212`, 952.0313425448139`, 0.1102822616742215`, 595.1450083064373`},
{0.15187564029414063`, 2.880478775690314`, 0.004382096934887443`, 1.4674879291008858`, 0.5009373487763229`,
  0.43891155488357636`, 0.3536287231064343`, 240.19671819847136`, 0.18432705417277784`, 919.7867146052535`},
{0.22049198023775118`, 3.737295065096858`, 0.0901755947592022`, 0.8260708286898752`, 0.9798021204432015`,
  0.3779284943750736`, 0.1710978537094571`, 184.8847972532019`, 0.07398065840012735`, 365.0355841401655`},
{0.07654850782489453`, 2.4131335758873655`, 0.08059562189671154`, 1.2322892164920185`, 0.6217255346992006`,
  0.4763260824504111`, 0.019197847472858853`, 122.85370840033258`, 0.08071204068111693`, 671.7123766572973`},
{0.2186000058002156`, 1.674921415227379`, 0.04684927805332688`, 0.7958293763534576`, 0.5351292729987009`,
  0.3413988165541403`, 0.023772881602852104`, 4578.839908213088`, 0.025990133203813937`, 720.8700500740513`},
{0.08466466489793933`, 3.1894928322423937`, 0.04884056021911439`, 0.9766729535722316`, 0.2661879637637754`,
  0.5761935324118598`, 0.024926658702996464`, 4783.240631357024`, 0.11594669465289681`, 380.9240212400814`},
{0.21930175757669085`, 1.638318457274547`, 0.028552151697051935`, 0.9929755101949111`, 0.48282817858365856`,
  0.6345477159456419`, 0.037102957315214465`, 1113.33955069597`, 0.08280464755403139`, 979.2309221100506`},
{0.15074472135509004`, 0.9706350461652997`, 0.03261694957200774`, 1.3446365626585761`, 0.7522030233980499`,
  0.24992054076492587`, 0.19250628663410368`, 341.383303547674`, 0.21302570685657762`, 810.5220628187353`},
{0.15291759685661022`, 0.42291903800035024`, 0.0019904081657203853`, 1.0349558440923765`, 0.804656482695703`,
  0.5600791477495833`, 0.008901523926716821`, 129.71753742837356`, 0.2016179651576812`, 261.7757399110873`},
{0.17393186946863948`, 3.634473205144226`, 0.03501927778959045`, 1.259756868930488`, 0.2400906424310547`,
  0.1769239851738713`, 0.19287151186810095`, 68.7455559557316`, 0.22859728678934704`, 432.35938430583155`},
{0.08252103338424227`, 3.0363282382170365`, 0.014054309199089551`, 1.1671357119318402`, 0.48811181819525484`,
  0.5135417689953007`, 0.03600069536054404`, 697.7036897164247`, 0.15194091944355315`, 879.883721957997`},
{0.152738213225879`, 2.1423984829886598`, 0.013570505913453062`, 1.4096468218064773`, 0.2076797705558071`,
  0.6307063081946709`, 0.005224131132178296`, 944.5685551789895`, 0.1507676005368857`, 103.54095034743213`},
```

```
{0.13639700931586268`, 0.4456680831486439`, 0.03342224700364268`, 0.8649458036959383`, 0.5930285970230691`,
  0.512351265340247`, 0.245770577144212`, 2154.7761980173996`, 0.03788382651562655`, 910.303522512114`},
{0.15928993459575497`, 3.999012236456535`, 0.05909250114200807`, 1.3761236219477655`, 0.9503401787669128`,
  0.5184686893967728`, 0.024376742099233118`, 2487.723402011106`, 0.03811059136471645`, 242.2171516528934`},
{0.09086020561938618`, 3.6933841261170146`, 0.051558219365949225`, 1.0052639956256584`, 0.6404497588004767`,
  0.6310251056832639`, 0.009282302874928348`, 1342.6385109725754`, 0.17780049337096682`, 290.68187157092353`},
{0.1467805861006215`, 3.449583346443368`, 0.044526946458641366`, 0.9116880264848481`, 0.3816708687678134`,
  0.542731104268707`, 0.008463344612022042`, 84.3419318420348`, 0.15666870327157162`, 901.9122564215138`},
{0.11923417673201614`, 0.8516005442952741`, 0.011417621987304472`, 1.406269973162172`, 0.816334669622038`,
  0.4684095363963826`, 0.03909433657582696`, 694.2186659403512`, 0.15728990858282532`, 465.4699780055223`},
{0.20474637151455272`, 2.273526081644097`, 0.04289425593502317`, 0.93188673114289`, 0.463730044259554`,
  0.2201193444564068`, 0.08911251750722911`, 165.09098140801413`, 0.18954199559487928`, 728.9231766980553`},
{0.05526814093912169`, 3.3906431390874365`, 0.04679651060056909`, 1.3224994050291778`, 0.2130837384463229`,
  0.5620196653380606`, 0.45821421853915184`, 166.36956673134458`, 0.04079620395491479`, 150.90572670851293`},
{0.1424344572203114`, 1.5158405980214011`, 0.07134698803292082`, 1.3412182398127908`, 0.9032816245031063`,
  0.29383720975190164`, 0.11721475717276858`, 2259.2805795521476`, 0.2041616321098142`, 875.6348666465524`},
{0.21167201791316342`, 1.191857769132941`, 0.04032467834992808`, 1.3612666973498941`, 0.990843843386328`,
  0.16867005012046943`, 0.32551015426475394`, 61.09769889812229`, 0.07090867627674724`, 397.7456784438642`},
{0.08319100731021534`, 1.1769760609325015`, 0.07914999980937851`, 1.2654227958186712`, 0.5286106444848078`,
  0.6131113894479372`, 0.34203180569092556`, 3018.9895646255973`, 0.20716587538358655`, 115.93712250962636`},
{0.15910463203606118`, 3.3362654217234775`, 0.09431988145352659`, 0.9102504372858342`, 0.8811348699089658`,
  0.5322317508995823`, 0.49238248141112334`, 1657.8316991656131`, 0.10134572490386001`, 783.7355808530842`},
{0.10233951732223695`, 3.8860213079913546`, 0.08245580990451895`, 1.0255637903426433`, 0.4817258525533532`,
  0.6808372231333493`, 0.05183250404760466`, 4623.677166174953`, 0.1226398356738308`, 284.97228231241803`},
{0.14767115153525173`, 3.9914449333138045`, 0.0245556961746785`, 1.4796764004437446`, 0.4415017997340658`,
  0.38166398917825395`, 0.06906175497737103`, 324.69168049200084`, 0.11969109369698483`, 735.4456200401828`},
{0.17843716795843323`, 3.273844277118725`, 0.0030946336041298927`, 0.8383571948484874`, 0.5814391172481872`,
  0.33735131984206335`, 0.09800031855604995`, 299.74935856154275`, 0.23538286721353047`, 296.3741029308432`},
{0.15153478181902558`, 3.940884011459042`, 0.014665112834793917`, 1.0805125654131587`, 0.23054812649590795`,
  0.6258941160756026`, 0.07918188154195355`, 98.54552815956583`, 0.07490635587591205`, 443.4663115601323`},
{0.134722684681424`, 1.1454968245380153`, 0.01650061661779107`, 1.0638009891109292`, 0.999069379656865`,
```

```
  0.5940301687777447`, 0.005569610207767832 5`, 77.43260394254949`, 0.24107274439535475`, 940.3682518559112`},
{0.16433469628892827`, 2.875058335652863`, 0.047444839802376605`, 0.8943437881772829`, 0.6054569837144246`,
  0.6619217328059503`, 0.1460580269246034 3`, 150.35965445533094`, 0.21808470663788926`, 986.5914920483302`},
{0.18172474747658007`, 0.5398125898895043`, 0.06746996069377663`, 0.8516154049831974`, 0.9176550555688654`,
  0.6251776245678378`, 0.47714073816368646`, 219.82174949020737`, 0.1448459760500183`, 388.6902609510781`},
{0.1627140613056554`, 2.526426793480817`, 0.08166994036129233`, 1.3765323556161237`, 0.2395228888894274`,
  0.6556866863306616`, 0.041008399203188106`, 966.1247163249689`, 0.01569822406304719`, 290.26321585148054`},
{0.12926587974943277`, 3.1942443269472287`, 0.08210234540290166`, 1.2603468170155185`, 0.1177111361684277 7`,
  0.5855250995493423`, 0.010816984036854691`, 52.9364314659927`, 0.0872384103249817`, 113.10642700446624`},
{0.2469537827294621`, 1.6562160115050997`, 0.08971323759474036`, 0.8904234033118983`, 0.4552788372237486`,
  0.6086911537768385`, 0.45126702440163974`, 3127.8941282749056`, 0.06787458866390378`, 920.4041061473141`},
{0.04659393582426277`, 0.8257639781936223`, 0.07933970228984723`, 1.0123444750721238`, 0.23360835713895778`,
  0.5040801713850254`, 0.008240901014236341`, 3556.051928374705`, 0.0470528014171252`, 139.7711872268978`},
{0.2677990399223792`, 2.190165256838246`, 0.03877434139180814`, 1.1644520688476412`, 0.10209034886491564`,
  0.2364288033256654`, 0.301326771326778`, 351.0852119703326`, 0.017030644812824763`, 859.704636269683`},
{0.27526155344317305`, 0.42206525583727394`, 0.010509190579989251`, 1.2469728015728827`, 0.7164324088803142`,
  0.23095486560068912`, 0.4019760074402924`, 1837.167141511333`, 0.14638705750272024`, 598.0758928204975`},
{0.20827027070080567`, 0.6628955977865401`, 0.05578571612958859`, 1.4550917307077555`, 0.8160716291341483`,
  0.4316400149470674`, 0.0992623271553592`, 1291.2580266081497`, 0.17638713235092185`, 712.744419351735`},
{0.26108671366598557`, 2.7581406885672397`, 0.09215954227601131`, 1.3356715726742072`, 0.5159534131307919`,
  0.6292106530069272`, 0.19102200652406912`, 1359.6101170600552`, 0.13195246712822323`, 655.8014880925608`},
{0.12411610080756336`, 3.5491378450736537`, 0.03048497342324529`, 1.052629142532319`, 0.933405263977681 8`,
  0.36612480189397933`, 0.17487769367297745`, 297.20407438965395`, 0.10198438571360102`, 142.8358321881406`},
{0.05206075217376971`, 3.80544265452421`, 0.06452586960506253`, 1.1764286006641393`, 0.7578123360385443`,
  0.3632439318173788`, 0.03825456601997797`, 1732.1715020363104`, 0.09945789721416326`, 862.5286889178561`},
{0.15332434762984987`, 3.71381929627883`, 0.04943415023567301`, 0.9774785033821363`, 0.4718998580816507`,
  0.16669947597303947`, 0.00694639422020862`, 208.05288466197266`, 0.20610825162042906`, 633.4494218195209`},
{0.06033541900347844`, 0.973178216355266`, 0.030553995574257496`, 0.8504754985225583`, 0.27347786882322955`,
  0.4969493066691505`, 0.00830637169085789`, 57.15499757894147`, 0.07721246031772627`, 682.9655440833467`},
{0.20494675314374677`, 2.7031536940694387`, 0.02716633958654084`, 1.0975656700162824`, 0.4564303296261911`,
  0.4441209590579568`, 0.3554217278310708`, 4265.584586744801`, 0.21396541728692714`, 985.913598004549`},
```

{0.05327195450009359`, 2.536575411744539`, 0.009856300363339`, 1.2242648903686608`, 0.25909020435504493`,
0.5282694072066187`, 0.04434669218741346`, 786.4053543279437`, 0.1778964533530039`, 544.6097455298984`},
{0.12477833238312225`, 0.7549035136230082`, 0.025172412892961696`, 0.9147089841492775`, 0.8930485640293313`,
0.5901323959258005`, 0.0508108243850171`, 901.2908925357414`, 0.07250789031346216`, 103.12569950478974`},
{0.08995934799066885`, 3.666207694088575`, 0.03007591771494079`, 1.3783264230132162`, 0.4826387854777239`,
0.6038418084491215`, 0.23708076068251083`, 167.7294214818222`, 0.01048197934630371`, 522.6073774456877`},
{0.15902513428717435`, 1.7183982375849336`, 0.033022213369310126`, 0.7628337434543904`, 0.7778819919683766`,
0.27145222438301553`, 0.043862649375531554`, 1185.7733981174872`, 0.08799090639918955`, 695.0405493319087`},
{0.11065553243087889`, 2.46805164022211`, 0.0463748475768039`, 0.8209475953416414`, 0.5218398256116175`,
0.3133447370054039`, 0.011285096069233967`, 4139.924962213234`, 0.13167847716735503`, 267.39995064825325`},
{0.08562689605991824`, 3.9571879577725095`, 0.023793442849937278`, 1.226116327613754`, 0.7555535622077838`,
0.20305217942323717`, 0.12860386825121028`, 531.0020640269107`, 0.051948220660308075`, 415.78314995313474`},
{0.10669261879032438`, 2.055705808398714`, 0.04769389095504181`, 1.156038712774391`, 0.9820313641369269`,
0.38163507374297045`, 0.039535368502052644`, 2902.0412683197437`, 0.20901158297646766`, 336.26891906262557`},
{0.07060338528242072`, 3.595376249521064`, 0.03262882113877765`, 1.2426013551392963`, 0.6002233277932603`,
0.6255684512538187`, 0.011346312056874001`, 3533.244097205098`, 0.061628460123131446`, 431.95845626027455`},
{0.2296701087879804`, 3.9087772869642077`, 0.005974703984365633`, 0.7959312177065372`, 0.876747425533583`,
0.6239039023797257`, 0.07860570649462724`, 228.33223863779693`, 0.09890789121587351`, 844.6853450307202`},
{0.23123324268179735`, 1.3892537621982006`, 0.09784040419751712`, 1.4899932847388562`, 0.04016727483231097`,
0.3908129571471922`, 0.009391139743151554`, 3403.3990186754204`, 0.07086662039842229`, 218.43172418092502`},
{0.07037189900847057`, 2.1828807010264386`, 0.09644892533086193`, 1.4657375782847808`, 0.7479730618322393`,
0.5037584189194234`, 0.030916267299058386`, 50.37706857442957`, 0.0504519506394871`, 421.25447735673964`},
{0.24256545257709167`, 3.690547309616945`, 0.0023677786666569567`, 0.8901250940229146`, 0.8854195106458671`,
0.44399073208272666`, 0.23636352272736572`, 89.54810713350285`, 0.186159802578101`, 387.72460570093335`},
{0.13839843978976302`, 1.4644738520575267`, 0.08648420456992728`, 1.2182260759194046`, 0.42213262989843314`,
0.3820758811935525`, 0.019878840421350977`, 139.8221226740942`, 0.07043032032389362`, 893.4669900375276`},
{0.11263641432345284`, 3.0669748383334596`, 0.07040331826586925`, 1.0832638028187807`, 0.4284903169815901`,
0.6222704043007405`, 0.007844284225620671`, 2131.889703000162`, 0.08148303022699405`, 953.4820565221385`},
{0.08536639269975432`, 1.9832711606259998`, 0.06847562880410717`, 1.259414576524179`, 0.8112350819234888`,
0.30170114251846125`, 0.062372919072375`, 772.9300404210154`, 0.12506634187920385`, 925.8799331095055`},
{0.11730626511183101`, 3.630410531262336`, 0.09596398943788596`, 1.1797596414840856`, 0.00512306280685948`,

0.2706014914642383`, 0.007107925916470569`, 167.23494696565012`, 0.11063186891436805`, 990.8331649712547`},
{0.11173802137314093`, 2.161180887445509`, 0.00830056555397336`, 1.063559562488714`, 0.1885103833536148`,
0.4813970169653780 7`, 0.010634768624873106`, 123.30116070184943`, 0.040023829860516`, 666.6470268689523`},
{0.10813918810135675`, 3.496374135712781`, 0.08929026043826785`, 0.7770714221575694`, 0.3733447131761056`,
0.4939165094284049`, 0.2800021673947185`, 161.9820586332278`, 0.08289673671409309`, 517.9231146523`},
{0.2516406246499894`, 3.0929818341741733`, 0.0922614386482984`, 1.4236100743284779`, 0.03336817153694982`,
0.531009903965389`, 0.28645389162750856`, 1114.6653530749377`, 0.10635783873890536`, 232.713586458166`},
{0.1065188593842617`, 3.0239491530160656`, 0.022787241302245534`, 0.914021229926331 5`, 0.4098506422400856`,
0.631556223988371`, 0.008249935916109901`, 842.7098345476253`, 0.19464933826626446`, 205.3036767061707`},
{0.10588487940933416`, 2.859735718128807`, 0.07786116606871861`, 1.047717859923827`, 0.07660672309433547`,
0.158735212392627`, 0.05520993734392543`, 139.0602212904327`, 0.07978847169291192`, 577.575995655312`},
{0.24741184670460048`, 2.0853129323232675`, 0.002699004039505635`, 1.042019307006856`, 0.6924193332252813`,
0.4588072902902137`, 0.0350404016550923`, 391.00460391265096`, 0.13592475687567207`, 785.2080587147564`},
{0.14083189694803505`, 0.7118335450358293`, 0.018460964897572776`, 1.1767917671701966`, 0.48592079833913515`,
0.6846823750216606`, 0.24127390726138886`, 71.14275489785842`, 0.0433294805997953`, 476.2086967233275`},
{0.1009771753749294`, 3.4383291777667013`, 0.09534658166572815`, 1.3953306821932303`, 0.24681268758922426`,
0.3480290288704033`, 0.2692140132359644`, 2330.189032037055`, 0.14685923933486766`, 555.9442327003733`},
{0.16178386458202587`, 3.0596682411026226`, 0.016235234307709075`, 0.7897132419424195`, 0.7440577027037574`,
0.20415575695989485`, 0.014269716719641092`, 102.02000238842407`, 0.02548443659481625`, 410.89583584040685`},
{0.1639764064141605`, 0.8690658024831128`, 0.09689964069135186`, 0.9303160183239412`, 0.9259213439155836`,
0.6251018503757462`, 0.13591794228358278`, 311.5677558935227`, 0.18168686534025869`, 702.447340545002`},
{0.12955597509758354`, 2.3266058652149084`, 0.07612788670577046`, 1.2957091127204743`, 0.08408476825169409`,
0.22843448121491383`, 0.01377311432994412`, 55.28011697543834`, 0.05462266813297323`, 936.2737831126054`},
{0.22722659605137513`, 3.181545366826299`, 0.09535885824182891`, 1.455710256248994`, 0.794512142132684`,
0.40024598250807375`, 0.3807443177879789`, 208.1153783523671`, 0.02759817321864938`, 937.8080702420884`},
{0.09757928432270024`, 1.5076906365385394`, 0.06532569914948308`, 1.2393804160164015`, 0.6875999373585513`,
0.4482433478851079`, 0.047668506035977345`, 978.2925685396615`, 0.1537809380520726`, 134.17889109795115`},
{0.25247158502971473`, 3.209043445695394`, 0.055750773529977184`, 1.2307446634362496`, 0.9584321093779467`,
0.17045215686099446`, 0.02394094843679724`, 733.412724821379`, 0.14871489313227643`, 731.1114504116057`},
{0.10371347338421405`, 1.7327368937733922`, 0.06071844641560931`, 1.2089839863490672`, 0.14765621815567243`,
0.4738799094972982`, 0.010611619577856298`, 2438.8343629553387`, 0.18500920326393544`, 108.28078770273848`},

```
{0.1984535123182241`, 2.161947896960034`, 0.06138419655750843`, 0.778982059344484`, 0.4287275067738694`,
  0.20720196920301803`, 0.4492524632515773`, 121.20549296704452`, 0.18396106207724583`, 119.02585475241914`},
{0.12381163862088174`, 3.895341333824428`, 0.06002344154256473`, 1.1343022539214913`, 0.8129713762085851`,
  0.4638592661411107`, 0.007042757871236177`, 1176.2351771701979`, 0.2462865126714932`, 108.58547580007442`},
{0.23071761590484513`, 2.2648352286735998`, 0.07729558723199069`, 0.9210169155643355`, 0.6313373838804968`,
  0.6041276627548808`, 0.24562846674087035`, 1229.9254758581137`, 0.2458533106908962 8`, 430.22606831878835`},
{0.05479322654019092`, 3.8246177185802415`, 0.08580605416623638`, 0.8811793306182123`, 0.8761776950953761`,
  0.29070846616035273`, 0.033348914503464826`, 623.2110193634621`, 0.1495764955432966 6`, 152.49175504634724`},
{0.19953212896288414`, 3.4927590196799247`, 0.09411150882849728`, 1.197122533048502`, 0.4043090283096169`,
  0.6122442379199733`, 0.044927979287668965`, 1565.6926144346771`, 0.04491818835648648`, 171.98402040125399`},
{0.1517228798855726`, 3.821661938472987`, 0.04482964683170573`, 0.9498760179602059`, 0.3819160293947341`,
  0.6937284136376201`, 0.007172788536686403`, 154.83311013460644`, 0.21825034747286304`, 849.9173689310978`},
{0.10089572826433801`, 0.9689485600216026`, 0.09904537540593211`, 0.9565780146244569`, 0.8315614208813649`,
  0.23778331363990324`, 0.010609351956832196`, 166.5397570471634`, 0.23685724436722827`, 123.70685669376229`},
{0.2617448383586014`, 1.7419134501480498`, 0.045608467431404856`, 0.787836989709293`, 0.662878849064974`,
  0.4338361091174493`, 0.3345623113002579`, 73.9957072876414`, 0.12486123360347584`, 607.2676663733008`},
{0.10370303958455385`, 2.4325217216628605`, 0.02176754760468306`, 1.4452363933770138`, 0.40438966394356823`,
  0.43523988865263297`, 0.04976478629271485`, 282.2104614433738`, 0.15573083503468216`, 771.737205400766`},
{0.12568862947200987`, 3.409935111150106`, 0.04842578409514237`, 1.2928588459089652`, 0.8548940812962251`,
  0.3585702743647413`, 0.03943961787244869`, 103.22445359993986`, 0.12312508088340224`, 106.12725365941351`},
{0.25023693340814834`, 3.9316478547515894`, 0.07082339724222557`, 1.158336750472774`, 0.7299281236811437`,
  0.5200623075713134`, 0.16855609928966492`, 115.49612111354244`, 0.11648069433492153`, 380.1124291681772`},
{0.2327122870521875`, 1.8349316139343976`, 0.005921840861416321`, 1.0160702776423072`, 0.724126729587736`,
  0.6641678286901775`, 0.014844066228330453`, 4846.196302582017`, 0.19133524452025602`, 312.0736607519881`},
{0.26060624730373366`, 2.429263865356268`, 0.02302054068506065`, 1.1376576887137433`, 0.6882649764401805`,
  0.6080978782814359`, 0.05257452924408477`, 81.26248932759681`, 0.16061945366682562`, 580.3732149020416`},
{0.22406406307822446`, 1.3829714288487063`, 0.037525031212394956`, 0.7596012900803544`, 0.17941039146586824`,
  0.520203193915476`, 0.056506779103220175`, 93.77318192698738`, 0.011157058120984026`, 243.36956207188064`},
{0.16129175454120598`, 2.241594788665445`, 0.02595808801699503`, 1.4515663695326364`, 0.44349403825461486`,
  0.5944541929016426`, 0.13242981207036078`, 1862.045541199846`, 0.19478161615729406`, 241.6655690221253`},
{0.15431366769375426`, 2.6965576187541354`, 0.08956453107270243`, 0.8164255077330567`, 0.4910378956214103`,
```

```
0.374255897689989`, 0.007597691279617231`, 3560.421166649914`, 0.2337580506715733`, 428.017043104418`},
{0.2212577173064723`, 0.5158974882846561`, 0.013003028286676788`, 0.9387448220782406`, 0.24284514367000565`,
 0.3081553043694423`, 0.03815340049240921 5`, 56.65345283557565`, 0.12156476174517056`, 401.2477783269693`},
{0.1683373271076925`, 1.6782737549041533`, 0.05896916410328887`, 1.1152338969749211`, 0.5823548078906295`,
 0.17654711643520027`, 0.009562790707407565`, 617.6094195707465`, 0.1697152454569904`, 676.3594994410081`},
{0.07670427099110139`, 3.20755225941694`, 0.05882096511027358`, 1.447867595309567`, 0.29934068229086064`,
 0.292514599413645`, 0.006615326955656567`, 67.05743705305659`, 0.05138097017561577`, 233.60263470026138`},
{0.09939447843258498`, 1.0806802687605517`, 0.08727314073863666`, 1.0427681271407077`, 0.2371764885575376`,
 0.5091900756791798`, 0.016527080711814128`, 61.46338920331662`, 0.06725149887765097`, 222.62215508117382`},
{0.058754537918439986`, 3.7681443191246293`, 0.07934826392769118`, 1.166166391041544`, 0.7017545799004972`,
 0.6192265616934043`, 0.1452002074780104`, 372.914499705161`, 0.050248583952100856`, 447.3871254600834`},
{0.1573899141991117`, 1.1557291134580199`, 0.009009591269829347`, 1.325752168244685`, 0.33932371792024996`,
 0.619859177738425`, 0.009052475129474827`, 1177.0140495795238`, 0.2452005274360191`, 480.6283895441986`},
{0.2160610264367817`, 3.9191762808523007`, 0.059018604533116775`, 1.0411018057212353`, 0.008462501397731081`,
 0.4634420979655972`, 0.32322042810200047`, 481.531032168865`, 0.019909072809315087`, 488.3969282323367`},
{0.11630471140404741`, 1.5367142722565337`, 0.05695013627172493`, 1.4595782498770793`, 0.4347006603611545`,
 0.15170509102823682`, 0.02764562799329766`, 1429.026434078033`, 0.014789325697006195`, 556.0517717070035`},
{0.04574862224020773`, 1.667137614048353`, 0.08615274073435199`, 1.367803712499899`, 0.9144234427837643`,
 0.29317668075534076`, 0.06903839047309805`, 1609.2665529077706`, 0.23754263722827756`, 367.7320637842886`},
{0.2785629651947983`, 1.9405997998909799`, 0.09745189735065271`, 0.9223797816210384`, 0.7945408892658388`,
 0.34974400234234904`, 0.06721335250349363`, 788.540217506401`, 0.22345836350921944`, 636.4117130237988`},
{0.1558631861871686`, 2.071958791081494`, 0.0906148168421321`, 1.3439374199675207`, 0.8630947847986346`,
 0.6733061517037251`, 0.2071553134363624`, 104.16809345728986`, 0.11868827346586763`, 553.1180333659046`},
{0.1292208707400178`, 1.8632605914435363`, 0.046415812114243664`, 1.1633279615140635`, 0.018954763914399875`,
 0.24694457378275914`, 0.007904270013564875`, 164.82469298269328`, 0.060610611106801215`, 743.9579215785636`},
{0.0898239866436944`, 0.5041817537224471`, 0.004730845503889416`, 0.8004221139970495`, 0.71803886182858`,
 0.24861999546798397`, 0.03932650979479891`, 324.23821280210257`, 0.0813302397362861 1`, 182.23375283503634`},
{0.12178660308868972`, 0.8029832921015938`, 0.06274577468035605`, 1.11373872869435`, 0.3507051586575438`,
 0.45809878988528197`, 0.10454823064111732`, 716.7349133355972`, 0.057611453097593246`, 948.7347206740686`},
{0.0685108116980988`, 2.7433614317525636`, 0.03948625806531251`, 1.4383757105618995`, 0.12700514465157853`,
 0.29596094652330907`, 0.005824134698501406`, 171.22697089194668`, 0.06525718195551272`, 301.6896298904339`},
```

{0.22222570024441013`, 3.2839931217970175`, 0.03747668952108825`, 1.1936997453676481`, 0.5468462118059447`,
0.582123988821347`, 0.030193979255150022`, 4841.3857036739555`, 0.21121655285668073`, 468.9585544167855`},
{0.05500096557985035`, 3.021764046557614`, 0.03167342594848885`, 1.471078294602109`, 0.4110285739228652`,
0.6035837025270763`, 0.17164962839295506`, 1968.9667019111098`, 0.24541253876143565`, 510.7332508800703`},
{0.21198399370055476`, 3.919285200069673`, 0.014045043011751641`, 1.0494239320976348`, 0.47271455772763615`,
0.6026643614464045`, 0.05622966821105292`, 117.93228694573206`, 0.21791598368515847`, 664.3246551556151`},
{0.18261393242527846`, 1.24104755829402`, 0.017562609584990004`, 0.8208770412391784`, 0.5702360150514367`,
0.2445337365136635`, 0.06636250536989298`, 71.77227660714203`, 0.12457334089639138`, 863.0628087051591`},
{0.1516561445800767`, 0.8718874600546336`, 0.04870269917216002`, 1.3587651378620147`, 0.3907653327903866`,
0.2341643054755288`, 0.16161562738072457`, 398.11431612220986`, 0.21962329680899473`, 517.7069194001951`},
{0.07481472761035168`, 1.4356710981047938`, 0.03195742895113495`, 0.7620294331090656`, 0.6058100988285502`,
0.1748877988840174`, 0.036278078120143255`, 1067.1675037581192`, 0.20966766369542061`, 245.76795972204266`},
{0.23415203477003`, 2.637838297244735`, 0.010100103036724857`, 0.7781763734410485`, 0.9270607447506427`,
0.25091053524524765`, 0.006631362838358266`, 410.1408598520636`, 0.09672454943968684`, 810.2426002994349`},
{0.054190572804884596`, 3.205052996318111`, 0.07207331803839295`, 0.8907501512355283`, 0.1443118496038216`,
0.23964954198579969`, 0.007902398391995273`, 453.6925070614146`, 0.03286507467345462`, 128.45869723434936`},
{0.1942266150934147`, 1.489765634070828`, 0.04812058961143629`, 1.1947522848110315`, 0.4017009524635866`,
0.3855054941178302`, 0.01145165307821007`, 1319.8238490809574`, 0.16195059607983425`, 743.204251876079`},
{0.14289375184211878`, 3.302390049355761`, 0.06615684983106804`, 1.29788834802556`, 0.9156578751693429`,
0.3558474159491941`, 0.00509231061202701`, 4261.526717057486`, 0.16050896664278197`, 220.23477447344888`},
{0.2554917877832018`, 1.993631504608799`, 0.043681818566651834`, 1.3194492976944885`, 0.9399541819359079`,
0.20985624905157363`, 0.1180452152100559`, 2753.2515506454065`, 0.05426315355547984`, 171.05806084971232`},
{0.20201473782765333`, 2.779452027424738`, 0.0266194566183942`, 0.9103804881826009`, 0.744670698555542`,
0.5706914134089118`, 0.00905331146486097`, 947.9277353727819`, 0.11184880683500475`, 329.63826832088097`},
{0.24790417478919824`, 3.0551603250746844`, 0.002284082483761942`, 0.961052650753995`, 0.6989086220525811`,
0.17002095436609788`, 0.48755300390089085`, 499.7708205836441`, 0.047042546081792314`, 684.0826745551606`},
{0.17492926953535737`, 3.975314242289789`, 0.023210045448957432`, 0.9727254901105757`, 0.4411891051406147`,
0.5520848832843507`, 0.2621570799727775`, 227.0108426167968`, 0.23671821431578927`, 367.0798924947729`},
{0.17539856022464784`, 2.4073130870702855`, 0.07197140330954806`, 1.0342299427745458`, 0.017371005489709157`,
0.6584543473805056`, 0.4728358484415665`, 1058.3389525653213`, 0.15082970917224703`, 917.1303711003585`},
{0.25951131167180713`, 0.8532719891968794`, 0.04557480110843374`, 0.7947841840785437`, 0.09920524665409447`,

```
0.24836596104002728`, 0.00597151633514079`, 436.2925795444242`, 0.1419534062375405`, 794.0516002827078`},
{0.17151022740710664`, 1.3676826689672605`, 0.009249620913063498`, 0.9876118143041603`, 0.1005607415916352`,
 0.34049413724762323`, 0.11299721785819453`, 604.8937675217858`, 0.18735181675894919`, 945.3802305578456`},
{0.08003627269054897`, 0.4223118257784404`, 0.09788308557086342`, 1.1088763125629295`, 0.1846532162494996`,
 0.44877823183875776`, 0.1744050351437392`, 232.77104633127487`, 0.186893582460665`, 400.88339340412807`},
{0.09629954489292863`, 0.8024243073579225`, 0.09137673334437835`, 1.1050982833461225`, 0.42757767860422`,
 0.28094623108518857`, 0.05836165382907772`, 735.7662354861307`, 0.19909783079594584`, 717.4225578982439`},
{0.18721691020827746`, 0.8832247086125689`, 0.007468716509783384`, 0.7687691323991039`, 0.5945515211149526`,
 0.3249811314880464`, 0.2800532125976925`, 679.3592862851432`, 0.05953883596172632`, 967.7513494997147`},
{0.05639051201063694`, 2.8104432162704684`, 0.055705228249153096`, 1.1263048402686886`, 0.5873694688928046`,
 0.5281479596797096`, 0.1725920753398321`, 2534.050261866886`, 0.23842584574122794`, 882.2272354030342`},
{0.11940415250329756`, 0.8265537347095737`, 0.08146769819729481`, 0.8399194412982254`, 0.9872938360285297`,
 0.2770401739511047`, 0.07923813270856427`, 3244.586795954999`, 0.06527667418181682`, 865.3808868374238`},
{0.12362063330315853`, 0.9363044869527668`, 0.08770822250254312`, 0.7786855706091862`, 0.2025852940468913`,
 0.4566487841344946`, 0.10394338643070074`, 1334.4445729465897`, 0.16877657565130982`, 938.8021455901892`},
{0.25510163512616657`, 0.9371168864708563`, 0.07693845729623781`, 1.115586607271032`, 0.44362559437168314`,
 0.23833942272790432`, 0.005303052515546934`, 2412.9883309521724`, 0.03447937580899002`, 556.3992268583279`},
{0.26333930199077354`, 2.0534773984698482`, 0.0643643244795264`, 1.2527519466886172`, 0.9177593225481775`,
 0.33938623608355467`, 0.007825944721954872`, 939.4147129019324`, 0.07035677568445653`, 544.5854367414727`},
{0.18557844748229363`, 1.3040038526378845`, 0.03298157347075899`, 1.0816274180852803`, 0.0720559521645785`,
 0.4038816001150133`, 0.01911693092881156`, 163.22630477861273`, 0.023327766650460102`, 342.443720752801`},
{0.08287518809588085`, 1.4167280782763267`, 0.028751858328181383`, 1.3710623936056974`, 0.3420455264471254`,
 0.6180297694222441`, 0.06931546639283913`, 2809.59627019281`, 0.14779417738205175`, 701.4197770536504`},
{0.09993506797522744`, 1.9333379215144753`, 0.0294160752239147`, 1.306899867333013`, 0.15174981363752638`,
 0.6973160529857587`, 0.32517481775011875`, 1904.6938693333132`, 0.22395301180399813`, 246.61499786462963`},
{0.1791717975737977`, 3.6792864853182463`, 0.003697989603839975`, 1.4268593787039923`, 0.6861577534974679`,
 0.6003986216720203`, 0.037512309221476955`, 1019.7481977593816`, 0.10587101285101158`, 387.91912641915013`},
{0.24888562729039826`, 3.026347119869615`, 0.09462972939910319`, 1.164624390493581`, 0.6459998640897591`,
 0.3154263692820086`, 0.35313147165596037`, 156.8927746768367`, 0.0941784253310613`, 806.7916066548876`},
{0.07400216536531751`, 2.1937414927491288`, 0.051724338318571414`, 0.8475382671591798`, 0.5283653100185577`,
 0.3375223430599671`, 0.006370262255851855`, 419.8990383214368`, 0.056433548922067256`, 105.83929536957157`},
```

```
{0.08649617896036566`, 2.9261562137270944`, 0.06277833160761251`, 1.2717586436565882`, 0.677517886873324`,
  0.2240537731188419`, 0.01575962778087511`, 67.09139852540285`, 0.15266972777055898`, 497.65886074130015`},
{0.21956975330290024`, 2.946279278154277`, 0.01351442569268972`, 1.0817364176432047`, 0.1114235621389037`,
  0.6113477082885477`, 0.45234016742673816`, 389.8324142721476`, 0.06262043870838019`, 252.04928334155613`},
{0.16962065420490857`, 3.2987764634142422`, 0.06541243051493663`, 1.4967196726725034`, 0.6967420255395558`,
  0.17574917802163403`, 0.035158157434036455`, 63.90845452931862`, 0.08229945239139341`, 618.3420143317427`},
{0.19774968406774135`, 1.6989982601552214`, 0.021311063743135836`, 0.7648586795987937`, 0.41768823186273707`,
  0.37949798185162253`, 0.005204291178783692 5`, 973.0476525534903`, 0.05448802148770515`, 784.849365608618 5`},
{0.2518902561813237`, 2.7254296067200725`, 0.08582594724985977`, 0.8908613191536825`, 0.4232867475157469`,
  0.2276322578613562`, 0.014796112855772266`, 4775.5807799067`, 0.21732003999216098`, 657.2426602250014`},
{0.09904156316806545`, 3.4999116420802006`, 0.05005174807173392`, 0.889946639423957`, 0.9586911880217555`,
  0.5850413707726637`, 0.007125457878312364`, 2230.565285121922`, 0.055223893991493855`, 299.18976270067026`},
{0.1091854910155387`, 2.8633835129131198`, 0.0203535526699505 8`, 0.8677831415002069`, 0.010141336872096618`,
  0.28824854332128513`, 0.17109333973146398`, 3619.61715038073`, 0.2333055604695853 4`, 323.4056364398427`},
{0.1891953550945305`, 2.389035398016863`, 0.08454968650044813`, 1.066070932458544`, 0.0345292329688216`,
  0.3128135317446209`, 0.38423156758654614`, 3793.7981099794624`, 0.125293090042303`, 233.53803394535984`},
{0.1570227614874073`, 2.40105806706515`, 0.05143382258328971`, 1.1808996551918274`, 0.2223234227077866`,
  0.26986116152499395`, 0.10425235900754858`, 216.82613723714533`, 0.18708364772996822`, 798.8941796704306`},
{0.21694584394948213`, 2.9530758173431613`, 0.09769681722844141`, 0.9640446069284444`, 0.42193435394716805`,
  0.6603635658814309`, 0.3338306865733826`, 1314.2123391672299`, 0.21796673788414078`, 229.96592799068196`},
{0.12690264500941062`, 1.3993259739099067`, 0.022125995185778395`, 0.9178738286195887`, 0.25309924713953147`,
  0.251012090694433`, 0.019625422672818624`, 283.1749611392459`, 0.18026742864340645`, 758.8108031110586`},
{0.06920814358208571`, 2.042285023379372`, 0.029660070385393613`, 0.9921431890214623`, 0.03257273935914129`,
  0.20517882048284686`, 0.027701026007507824`, 104.22947983733822`, 0.22115350693221852`, 739.7214992264616`},
{0.2577647175823676`, 2.0669085310717623`, 0.015137977599734764`, 1.2344138658842803`, 0.6169124929132423`,
  0.4497973990302503`, 0.20323917961090476`, 273.8208174543164`, 0.23260089052488175`, 786.949837857692`},
{0.22404984477095807`, 1.7971972613143468`, 0.014556371051440885`, 1.4736916256467743`, 0.10711810372521668`,
  0.4838216983187773`, 0.15033397484285083`, 3525.8267227086753`, 0.24709636291116127`, 542.7438703089757`},
{0.2560915912056091`, 2.8056995396679163`, 0.02230409169328258`, 1.1596232786630103`, 0.9731942543354852`,
  0.32967621772461264`, 0.008273413274888125`, 659.2862632916124`, 0.2493316428820775`, 801.2464425394161`},
{0.069529383599776`, 2.849786616236517`, 0.0015488296932831959`, 1.1307747790060854`, 0.8754257019481757`,
```

```
0.4462637970794401`, 0.00987625334070338`, 470.49802215652267`, 0.1965355306527206`, 591.5879320045749`},
{0.16317921960456533`, 3.795960913866608`, 0.060277538996435585`, 0.9943285566083784`, 0.31878466621504153`,
 0.5268930320337564`, 0.03217382490455373`, 160.19057295170512`, 0.13130693314062597`, 466.7290497736911`},
{0.23870751571987275`, 2.817892132050006`, 0.034904301600875376`, 1.2212602625610964`, 0.2634282445359748`,
 0.29343287356938585`, 0.0412321304066401`, 2956.916383781115`, 0.1710028618436959`, 734.0158474610636`},
{0.25110587793218636`, 0.5330897224408169`, 0.03508654146335001`, 1.2737631195521142`, 0.14437163587185342`,
 0.22915335883220245`, 0.1301100912805756`, 130.55788460482208`, 0.03440422815097299`, 153.71031149577092`},
{0.04796641395535989`, 3.6542681080130706`, 0.06056371338194985`, 1.0410004898170107`, 0.13233719731300564`,
 0.36420880945840706`, 0.16862770789835824`, 456.10576346306766`, 0.12687150881465203`, 491.49936597120086`},
{0.23314959452407125`, 1.9850975451070108`, 0.09033499334189088`, 1.2112065225297546`, 0.7891619630024773`,
 0.23016874225029837`, 0.025757539951395944`, 1618.2342010883456`, 0.11509681389516696`, 818.8391633081792`},
{0.2668656374936109`, 3.051749683327815`, 0.0258024333714593`, 1.3861879676031486`, 0.13705271113057615`,
 0.3169491905155566`, 0.04957453327253996`, 127.00810430555478`, 0.22326078444976738`, 605.3130708988924`},
{0.12837714622951152`, 2.913045083809414`, 0.0837521188025789`, 1.2333880824573145`, 0.14831200507330466`,
 0.5511854573030723`, 0.319487783122794`, 260.13632482567476`, 0.08285153464288336`, 293.457784508192`},
{0.2580675113282032`, 1.3794239963466088`, 0.09606459315739223`, 0.9738093032034381`, 0.551722273950022`,
 0.6189616163620202`, 0.03080803973244329`, 452.05919890664615`, 0.0556923772463625`, 491.93043733941124`},
{0.08884487120981721`, 2.332961071695622`, 0.031598395011471735`, 1.1141910450192687`, 0.7184499049851998`,
 0.6730388302232508`, 0.123997295097641`, 484.8896162348586`, 0.15163592409494436`, 272.73357673474675`},
{0.2759587426432519`, 1.9534358692391445`, 0.08397889547253015`, 1.0356223413014591`, 0.42065948992616775`,
 0.6805267491095484`, 0.0964860490180039`, 69.9818047589248`, 0.07247191711576301`, 633.9862534370685`},
{0.07608942110432254`, 2.164297732879585`, 0.044623011426671744`, 1.2845300660264662`, 0.14123590728202684`,
 0.20715073503154113`, 0.010177080583516988`, 365.6404789674773`, 0.09043375256261627`, 318.6735532350724`},
{0.12578906287533304`, 0.5873288516562982`, 0.00917874148360891`, 0.7860432924669931`, 0.8658472891163045`,
 0.28929582442115676`, 0.04769704077854567`, 2092.327054664775`, 0.10790373294003436`, 499.81053764102194`},
{0.13400527850674449`, 3.167561049379361`, 0.09503778083239858`, 1.0695890919772384`, 0.44461403458537974`,
 0.35428116459826664`, 0.04474774833241626`, 249.9292825838412`, 0.2392270171205957`, 993.872691687206`},
{0.14569029844361497`, 1.581782399146463`, 0.02853446038002714`, 1.4746522591423932`, 0.17909427610451467`,
 0.395505345829425`, 0.04611739955881583`, 816.4722344402478`, 0.17778523558925585`, 271.44027790378294`},
{0.12249361616240895`, 3.3852221257689097`, 0.05543978239026112`, 1.0925731163264347`, 0.17776966626445145`,
 0.29066583025906045`, 0.0687429771171289`, 314.16092924905377`, 0.1111159201808452`, 685.2380116739549`},
```

```
{0.07368028774821661`, 3.0677536525276716`, 0.07059619046896788`, 1.2388956643695934`, 0.6420156583576027`,
  0.15354672355808818`, 0.023520960948583497`, 1566.5430005580442`, 0.0643629153664667l`, 602.9382331885815`},
{0.10293015258903027`, 2.7627847871257094`, 0.048868500737193926`, 1.1335235205351295`, 0.26256073599306595`,
  0.17258322470135423`, 0.3544868089252062 3`, 639.8812577909154`, 0.21355261514569296`, 292.3004754224651`},
{0.17989985519582302`, 2.2848444130728813`, 0.06499018234377278`, 1.3356780269154247`, 0.8760585579430653`,
  0.25119519210597663`, 0.17680773834886487`, 2893.233426659574`, 0.0896876677364310 5`, 889.4972829137831`},
{0.0700064475969871`, 2.5832650492672506`, 0.009367713712463904`, 1.4593574063080232`, 0.8612459654760951`,
  0.39256758588428897`, 0.048637831394936666`, 69.10482903899627`, 0.16784750403438659`, 808.9797947692787`},
{0.16902358696866238`, 1.5466078808636725`, 0.09114667029195474`, 1.1632289455971812`, 0.7011539680870855`,
  0.43898684142995004`, 0.12814910976660654`, 201.32808946828865`, 0.1990055903635441`, 501.3434969043132`},
{0.07225780293824052`, 3.2069795251700226`, 0.08284178682925689`, 1.2105070206615947`, 0.6775214967569712`,
  0.488074793019582`, 0.026308005494110893`, 313.1548266866826`, 0.21343764900902945`, 758.2569582586476`},
{0.13893262337344747`, 1.0427327070975592`, 0.09311200953995581`, 1.095872791509625`, 0.7537435467200488`,
  0.549904286540486`, 0.25747472723162285`, 4239.365928479679`, 0.23905861617289415`, 330.34293410544217`},
{0.06788146233466685`, 1.022821493462117`, 0.06599019909830307`, 1.0274069325364188`, 0.42743374336783724`,
  0.47977647156275904`, 0.02909328473333213`, 114.54872183983547`, 0.08606336865176462`, 305.0489819660903`},
{0.04270280673685248`, 1.2934684868479742`, 0.09591189067862807`, 1.4128815880743355`, 0.13315269485352843`,
  0.23204805195972866`, 0.2572508527807455`, 90.11608912792754`, 0.03659032712805199`, 523.1103863627065`},
{0.046236418394459605`, 3.574151007923631`, 0.092837745021803`, 1.1374323179308754`, 0.4550831015183179`,
  0.3756839361198018`, 0.00586392775091683`, 1204.6242266607505`, 0.029043894879770327`, 942.0566673133433`},
{0.14802462884786977`, 2.8463045630582817`, 0.07086620031807243`, 1.4262141974100975`, 0.40895975433086873`,
  0.2365796035874227`, 0.3310600416149043`, 102.98362169854454`, 0.1764312785940091`, 773.2109911740185`},
{0.17261791412834615`, 2.66725952372021`, 0.011878378245691455`, 1.1959533166982588`, 0.37293218245137805`,
  0.28202161480695964`, 0.4651224858250725`, 72.55708905719771`, 0.17887121192017358`, 925.2830578188941`},
{0.10523638690298753`, 3.5642755326059987`, 0.09015549116400706`, 1.3647813438407541`, 0.8329295324191079`,
  0.5634168718886515`, 0.02622479781769635`, 1558.3726673845852`, 0.0866750472996437`, 735.9237964380025`},
{0.20934959845181905`, 0.5253713637677127`, 0.09562730622062596`, 1.3267328346184806`, 0.026713277643319655`,
  0.15125889150158744`, 0.009145743694901407`, 347.6644603704283`, 0.2158426941139397`, 839.7488318339839`},
{0.16181023020779767`, 1.305821903245473`, 0.04345833700918577`, 0.9871466940785586`, 0.990363037222695`,
  0.41737973540465045`, 0.04349028756064097`, 389.8084434973402`, 0.2301500945529913`, 142.75918439051142`},
{0.0585933257218722`, 2.1564084302719815`, 0.008240848797671667`, 1.3548876424139005`, 0.78543219520227`,
```

0.5684638437310318`, 0.03933279743012852`, 1164.1502368017577`, 0.0365133102608523б`, 908.8904797965278`},
{0.17629686442565196`, 2.478708866004733`, 0.029760826245713457`, 1.1409573325432285`, 0.08473376105442143`,
0.48472391979554497`, 0.008616495281357169`, 315.1653370205564`, 0.21238301827033085`, 252.6645414580406`},
{0.05701931707649882`, 3.239761060927444`, 0.018658291098521504`, 1.1958711178433945`, 0.6010462815373658`,
0.2222121897761763`, 0.008661059703696556`, 2427.987481600541`, 0.16195229268590294`, 598.9762630900484`},
{0.12429129251918081`, 3.165176790650899`, 0.05944857413724836`, 1.1750421651261647`, 0.7024367598760777`,
0.26578928465801155`, 0.28623135703022834`, 57.36286473144533`, 0.18225946471633314`, 279.6228390464558`},
{0.17592117157303727`, 3.225697565005621`, 0.06410385713825643`, 1.4723665071047831`, 0.040075633612683514`,
0.16766841616104544`, 0.01926077754515973`, 451.97528485070285`, 0.1967336572740045`, 903.2247661915143`},
{0.20267367580625623`, 2.351165901182144`, 0.008675643302528258`, 1.2736483392354083`, 0.1108762459426682`,
0.4077401628201436`, 0.03171361327346612`, 3972.464308751051`, 0.13615309860632413`, 596.0671079273882`},
{0.12207280755595895`, 3.8644474094244794`, 0.009427505375928522`, 1.1042754889246225`, 0.6660399151614447`,
0.25206523111087553`, 0.10009599160633623`, 402.11380186936043`, 0.20397090745892643`, 309.50158431622725`},
{0.24507742036233432`, 2.3831843355711397`, 0.005052828156007507`, 1.0721739375121355`, 0.49387052070387516`,
0.2147676922991728`, 0.011565289716111508`, 84.02018174384712`, 0.07100872729300844`, 676.9347914709565`},
{0.05872459234515515`, 2.1332438180615902`, 0.07824887308364058`, 0.7585647474793751`, 0.26510390251726434`,
0.2713237544705841`, 0.036979209154785794`, 52.71577024813928`, 0.17679977189893264`, 564.239792184463`},
{0.14683813473126478`, 3.4711809547760266`, 0.030295203568552493`, 1.3035360306310906`, 0.42027841877180494`,
0.5921843153064568`, 0.010793050374412331`, 254.4238250041397`, 0.22813578414007268`, 746.5344746241171`},
{0.27118296549826965`, 0.40016893385797836`, 0.03738933189542754`, 1.0762552685066569`, 0.6570337540705835`,
0.22274705980247678`, 0.006224989465699251`, 331.2301635317647`, 0.16318949690503837`, 803.619268726775`},
{0.2651341645020733`, 3.921256061000774`, 0.09127020329702631`, 1.0515436089401342`, 0.37820568835934876`,
0.30543268981111493`, 0.008197803117955519`, 249.67697255719887`, 0.20481437271886815`, 450.4819383009913`},
{0.10266981380053875`, 1.7259937776730974`, 0.05788500808078473`, 0.8498356975392536`, 0.5149250125601736`,
0.3750799043189028`, 0.006831336220999067`, 241.15442777469008`, 0.08411211628353799`, 814.8105177183215`},
{0.1701903238305883`, 1.5737398864052752`, 0.013362842515380927`, 0.9723904628120438`, 0.9778114455260909`,
0.6469978525912341`, 0.013362665151551732`, 1406.2278798102645`, 0.1754741948990562`, 281.6011633652904`},
{0.20205010610888874`, 2.4886025319973255`, 0.03416173840731352`, 0.9461759215151488`, 0.19917032732038176`,
0.20522267238789926`, 0.007087646061753528`, 151.73293313624973`, 0.015624468793102558`, 798.3652981187157`},
{0.2548714778924099`, 2.595325989353224`, 0.04783518749288423`, 1.260048561023251`, 0.11179251567641679`,
0.5271320321183793`, 0.20942249013272063`, 61.94848210458544`, 0.13000680045559498`, 257.7742889938444`},

```
{0.25697985123874933`, 0.6423673175461451`, 0.03920104245257182`, 1.0494619196955428`, 0.6883576503405942`,
  0.5372602583905404`, 0.0298184492004487`, 1024.1709922100576`, 0.18196374947080113`, 448.03818305726395`},
{0.24108459248749042`, 0.6097105829562341`, 0.05914817527701839`, 1.4448529749350583`, 0.06017529583805459`,
  0.5679483071298559`, 0.028892937973694452`, 184.61048198703293`, 0.046198153900852024`, 876.6239668930126`},
{0.11138935148613482`, 1.9270561111646902`, 0.0506158838526531`, 0.7861832578093317`, 0.2623170852799024`,
  0.19880501636260794`, 0.1716591058201036`, 672.6474872708164`, 0.14359452836986147`, 488.74274292141473`},
{0.22711687131412428`, 0.5423663037563959`, 0.07370528658374681`, 0.9917310500559307`, 0.6268413827963848`,
  0.35482337483246806`, 0.0492957290107415`, 298.86562808346656`, 0.034113139892874`, 451.4330811913654`},
{0.2649900949630745`, 2.9147648354215034`, 0.09385410641202459`, 0.851585607662866`, 0.6282724158295114`,
  0.40674785391584145`, 0.01227961889947592`, 57.08712154139038`, 0.026923769884349857`, 165.58243018457918`},
{0.0915398024811338`, 3.642607843612522`, 0.045773601129209036`, 1.2779360684969243`, 0.2244141258099137`,
  0.6005450159014489`, 0.4759115782164151`, 141.33543981734994`, 0.23290101078240694`, 255.60470768923676`},
{0.09997582603329147`, 1.8799516342297675`, 0.01460194430259186`, 0.8270986899357093`, 0.5545841854424991`,
  0.5876382120219206`, 0.052778297224976234`, 1510.139172300266`, 0.05587104924786124`, 122.66534784854954`},
{0.11587596442377335`, 1.570443051624368`, 0.05003858184900709`, 0.7979390254239629`, 0.2695501577041357`,
  0.4402434745919389`, 0.1955710791573617`, 1393.738907723443`, 0.22482510146060541`, 822.0077092395296`},
{0.22552960854228582`, 1.1654101546280868`, 0.07708737421212913`, 0.874173869621267`, 0.34763140639716794`,
  0.6720908373189787`, 0.049522394564493144`, 1483.0319583713147`, 0.08855956469300003`, 824.4137036635007`},
{0.0476102421762471`, 1.8287647324569782`, 0.03110183117006317`, 1.3863628762732523`, 0.6811850740698742`,
  0.21530814035187884`, 0.02495067854594185`, 78.5692803520234`, 0.13933019803203`, 251.51485099763934`},
{0.07293365735317825`, 0.9536666649351204`, 0.041006966482838275`, 0.7665287875498985`, 0.935767572935885`,
  0.220179659831362`, 0.20106612820967829`, 178.2780155183392`, 0.1430151800284700 2`, 953.0856220301953`},
{0.19096668838686354`, 3.9791109702558582`, 0.04575838938969985`, 0.88278575745365`, 0.1319776886877686`,
  0.6321229402668154`, 0.005732045216178792`, 174.70115248623193`, 0.20628116633253563`, 438.2211382365572`},
{0.24680488679812707`, 0.815637935503644`, 0.0843637406190441`, 0.9487689240355479`, 0.3250491534008515`,
  0.6707507630339515`, 0.10643040624255257`, 4909.4593594307935`, 0.030006121749521886`, 121.48162188773526`},
{0.14271805685583516`, 2.3484760469035573`, 0.07745189816615884`, 1.1886412637022417`, 0.22453965782263952`,
  0.6988851450501361`, 0.008392473480111172`, 241.2772419245499`, 0.05856292707558125`, 560.9416229791005`},
{0.237923538483648`, 1.3045678824163405`, 0.05160012685748933`, 1.1613123620254255`, 0.8558167677781581`,
  0.6442348560245077`, 0.280278439293096`, 960.8793815230854`, 0.10811725374257602`, 308.2410580836482`},
{0.26686684415084866`, 0.9101714257367162`, 0.05201112615814223`, 1.0067387685359694`, 0.3144283486587576`,
```

0.5640584563511031`, 0.07480454987166867`, 4843.746054037886`, 0.14590156821291428`, 291.7993314288994`},
{0.27466728617562586`, 3.909730616037213`, 0.015651626558488035`, 0.8183399746276282`, 0.8624521285063864`,
0.31078149750433326`, 0.1052382875316152`, 561.582629527527`, 0.23527086491760263`, 644.9709948771053`},
{0.07007388150970617`, 1.2788530615475953`, 0.041240822222090405`, 1.153764990829969`, 0.8716462413619626`,
0.15860191259805845`, 0.35155378239777585`, 296.5267589380548`, 0.19912668804556583`, 616.7284928222024`},
{0.1878952467648845`, 0.7675716449344554`, 0.05741879370926064`, 0.9528631412514192`, 0.9302947624844955`,
0.2534812971784569`, 0.1207735933533045`, 1834.0932588583464`, 0.2124476008021367`, 697.2076816469347`},
{0.14168118293015808`, 1.8332481297075178`, 0.0300583699414835`, 0.7581988115722615`, 0.852062289257528`,
0.22721700466029904`, 0.12430348358114858`, 272.5636471822111`, 0.15659940706565478`, 639.1777488262896`},
{0.19834052588138612`, 2.8473206103303275`, 0.06787428338874751`, 1.2634740859082962`, 0.8483257098618575`,
0.27810606990948006`, 0.052306771734647074`, 2133.625806620023`, 0.10206688811673287`, 552.214584726826`},
{0.13732318942612998`, 2.533545686945514`, 0.07677083355764772`, 0.9473176337866864`, 0.9203815554724639`,
0.31737934755677866`, 0.05120312298025456`, 1347.6776516438235`, 0.2303275954656494`, 112.23809273053274`},
{0.21392312209610825`, 3.0478754499742005`, 0.06538726038868102`, 0.988290831541475`, 0.07401087304537768`,
0.28494540960049397`, 0.2526680322768358`, 279.52916817401405`, 0.2297370624504932`, 425.1990754299824`},
{0.14320126116520115`, 3.5785950094384216`, 0.025604653350218314`, 0.8268074039814586`, 0.976648397359879`,
0.5001702346122724`, 0.38123679071943467`, 388.98067038422806`, 0.2240399781178949`, 927.7111417924974`},
{0.20926436934918818`, 3.54928785755524`, 0.04655442449302829`, 1.4735046423674272`, 0.6436030426422834`,
0.23716114021464474`, 0.016767557842678662`, 762.9533208051863`, 0.22515295037189986`, 823.9967654417107`},
{0.09715661458363001`, 2.6162532629232897`, 0.05330552481177518`, 1.3217583375467599`, 0.056961332005489806`,
0.41403944176397645`, 0.0787432744072381`, 132.53142675262785`, 0.16676071183902036`, 308.900351347671`},
{0.044494746845228667`, 3.8073919623023302`, 0.00891629517230891`, 1.317697440879989`, 0.21040732129598538`,
0.43509792721016394`, 0.11970345154140186`, 395.1110764732494`, 0.23530085829797331`, 437.748607476885`},
{0.1200252023437956`, 2.8019007957477235`, 0.028007311257585012`, 0.92009516518451`, 0.01280900474487301`,
0.4526896126410833`, 0.04417068363712719`, 126.12721650529039`, 0.11309971446701528`, 297.99616984883437`},
{0.159307524381112`, 2.1388708024966263`, 0.07728990983733347`, 0.8933915824609264`, 0.17018256737475035`,
0.39427249092340955`, 0.2374099439396035`, 296.4953500654782`, 0.22256932318418826`, 146.08391069826325`},
{0.2515615397486592`, 1.2188920022514846`, 0.056806007322854075`, 1.0589873581328146`, 0.8389110983305801`,
0.5160905955986007`, 0.005163127675966413`, 854.4257970577178`, 0.2370298510099471`, 574.6962402010942`},
{0.23870656719030897`, 0.4607483912247612`, 0.017860308849642834`, 0.8081937138032418`, 0.1724194202814684`,
0.3241110683425861`, 0.018037887307016776`, 469.5800690255617`, 0.11201486480281508`, 307.02179419088134`},

{0.06627522358143922`, 2.000113059980986`, 0.03069667022234471`, 0.8345023822924422`, 0.7625117937694375`,
0.4912663179641469`, 0.07709548204647149`, 185.51291986100534`, 0.0763658907530384`, 325.2314705034143`},
{0.11831505888067828`, 0.815861401259930l`, 0.09695106021032068`, 1.4738361110893088`, 0.2645508678881856`,
0.2913223690175213`, 0.010373880238463022`, 1209.9954370966966`, 0.23975714360436612`, 767.8374529336429`},
{0.20577599681754594`, 2.9313768979273176`, 0.0038206720060969704`, 0.781758458231594`, 0.56664249884203`,
0.4709509229113563`, 0.021927764931302315`, 4686.470122623354`, 0.2192041879935001`, 347.008418687607`},
{0.1869209879787055`, 2.3095757377059725`, 0.05297142960646317`, 0.8815543572140244`, 0.041400679606300006`,
0.31871458453434554`, 0.1289212733641052`, 101.4389842310655`, 0.13597402845170187`, 914.947493051789`},
{0.06483245092897544`, 2.8384211668748724`, 0.0911657087741262`, 1.289023251198742`, 0.4466087032681607`,
0.267574352599688`, 0.007946224566772193`, 1055.04661000947`, 0.08980662020688435`, 572.8761673725373`},
{0.09228610076587707`, 1.6450889610515498`, 0.08913657400655176`, 1.3611199649465364`, 0.621424219326399`,
0.6158200041827449`, 0.19662513724207695`, 1190.4261163432097`, 0.21638593744388268`, 965.0447414955308`},
{0.24524928286716796`, 3.6904456707313154`, 0.08967706170457866`, 0.7837922940103521`, 0.15260266641265163`,
0.4719521309469129`, 0.02190199465707774`, 50.30445277851058`, 0.16737611791533596`, 158.4281260415155`},
{0.2085289521726456`, 3.7433965844871855`, 0.07373717758801387`, 0.8272531686673292`, 0.9243484340355741`,
0.4736315139016828`, 0.04000550244322193`, 775.1117022858083`, 0.18128754644027817`, 792.6217255082997`},
{0.1942796430577805`, 3.7771592955896995`, 0.014808087701342974`, 0.8727503183069723`, 0.9499179373546311`,
0.4711663886743095`, 0.2850463770968853 5`, 219.95821433792653`, 0.17829093188760597`, 601.1866479309697`},
{0.19978804404047035`, 1.7412366971449815`, 0.05869679069766164`, 1.2250897389227589`, 0.9947242752248806`,
0.3288744578871605`, 0.029790954073398563`, 177.76139833706597`, 0.1704177380158945`, 342.62682125921594`},
{0.18813828973766622`, 3.040504339634855`, 0.03041458173591515`, 1.2993625717609874`, 0.3197300168421664`,
0.4882990032931548`, 0.05916461107129405`, 288.10788143037024`, 0.10847906504084376`, 408.965425001152`},
{0.13939828058518033`, 1.5857112352411011`, 0.08574772946752571`, 1.1093571913830735`, 0.14309585058744245`,
0.5634406137724309`, 0.2817673310248056`, 53.991544293777636`, 0.11459032439612055`, 248.27985633277424`},
{0.043536324184198144`, 3.326291464260996`, 0.05139708072028187`, 0.9030702944800504`, 0.7051237824883041`,
0.5197885611320734`, 0.019273613149624703`, 329.1019165250263`, 0.23460236315419714`, 678.1062885700973`},
{0.09801716906192193`, 2.9539869268188017`, 0.020040830336135844`, 1.2343926494145465`, 0.7600540430041269`,
0.3516902841570241`, 0.029540615021220525`, 3778.034634812808`, 0.12448155103112013`, 335.4861228637068`},
{0.2351399190035529`, 0.6800114559723247`, 0.03213357322891234`, 1.2136689663675575`, 0.0851758437090655`,
0.2574027192263152`, 0.12005990640730702`, 487.2802108209037`, 0.1525337459876398`, 478.6782242639763`},
{0.19840884489282196`, 2.435275307859949`, 0.06582510415417815`, 1.19484171596350 3`, 0.18931490770635162`,

0.6654697402710699`, 0.012855112113943912`, 384.7644348925394`, 0.20206490703703606`, 653.6112811972781`},
{0.153998794720146`, 2.296727093897146`, 0.048890456587564815`, 1.0717959146758531`, 0.20411298992327565`,
0.5326406872061232`, 0.0433093428704623`, 1797.551253413897`, 0.09526457101922897`, 474.50386549092957`},
{0.2762960805421906`, 2.0251009856011146`, 0.06438086916711981`, 1.2552601159611685`, 0.824619961217385`,
0.2219622549088328`, 0.0777112084796907 5`, 235.23851588627025`, 0.03242543857282393`, 995.444729973487`},
{0.22210233579747424`, 3.0942680698763763`, 0.09032486696922916`, 1.0647099914995546`, 0.516081148067818`,
0.4664841812624002`, 0.2575335143375922`, 247.27548003665564`, 0.1261056310024135`, 988.0783976396131`},
{0.04246581734245333`, 2.6243765146123685`, 0.03930099434156552 5`, 1.1707132411393175`, 0.760389410309404`,
0.6197176888843219`, 0.0561361696179710 9`, 579.7528852266169`, 0.18044598974464704`, 692.5186634415095`},
{0.13098562468211145`, 0.6918886972002496`, 0.09307771379161338`, 0.7572969971235853`, 0.8621959711022029`,
0.17247619508234702`, 0.13638646340531488`, 1721.8193108366343`, 0.15630991181601384`, 116.41676929938268`},
{0.2369558402004424`, 3.8904595389867698`, 0.07694340666447698`, 1.136418408023505`, 0.5724535764038741`,
0.4530887158124406`, 0.11198137985855583`, 117.36224574039397`, 0.1511561710016185`, 454.7116411649565`},
{0.22006023145078546`, 1.8699454187445346`, 0.04190713219713553`, 1.0916024021867943`, 0.9816282992661436`,
0.6829534497485226`, 0.009835956205983078`, 624.6290470462591`, 0.021070658550712773`, 768.5490473777757`},
{0.09828617329733175`, 2.463942931528096`, 0.007572202379780535`, 1.2472200527061044`, 0.41644064652613144`,
0.4645936104828291`, 0.02312500549439911`, 54.07758231391304`, 0.09597622753113771`, 416.81598350845786`},
{0.08362876114696788`, 3.673753657595393`, 0.08998734313926576`, 1.3432025605848703`, 0.2747343812629557`,
0.6253363571442772`, 0.036994141920847455`, 388.9552816546361`, 0.04955150722579804`, 133.7754196526289`},
{0.22105845206099273`, 3.6723501488527104`, 0.0598021179692625`, 0.9344989193429194`, 0.3577079276157791`,
0.25286474043446827`, 0.027604543425431 24`, 121.18794663333252`, 0.020589883661662256`, 454.33911379556343`},
{0.2404415985731178`, 3.9242340192232827`, 0.025726059183975813`, 1.0889915012585847`, 0.7185429349809043`,
0.6376702062000428`, 0.268650042773469`, 355.68354138078996`, 0.22139947989242942`, 504.918029373391`},
{0.1005874500764159`, 2.23272409892634`, 0.060802667136885716`, 0.991684052120384`, 0.9801615674605653`,
0.4858907364942624`, 0.29370050837355216`, 1056.4414347359782`, 0.08590239477227651`, 977.0843750733277`},
{0.2630219935361097`, 0.7161334798425658`, 0.06498611082218136`, 0.9675557624650479`, 0.11067589370665232`,
0.632848587449107`, 0.011686165277954987`, 164.13933362107778`, 0.16701870855521322`, 106.59616859139227`},
{0.21827180155004183`, 3.007771469369752`, 0.03198650183307903`, 1.2597421711376162`, 0.05877944668022894`,
0.21087223032471758`, 0.1491736628807538`, 409.73754661816`, 0.07595835910505389`, 682.1222641287044`},
{0.22045085365596306`, 0.8751625033990953`, 0.040943556123142544`, 1.1343752751378642`, 0.40735695900069624`,
0.30497304960520855`, 0.05995678076716148`, 952.3200487692166`, 0.18118346106920746`, 347.9890739313543`},

{0.18946179909757177`, 3.562633004956865`, 0.0313023029411473`, 1.4739498657051078`, 0.8014019749280452`,
0.35240031523303095`, 0.015200151831610186`, 167.2762376390573`, 0.22621173221438579`, 745.402744487609`},
{0.13542699057027358`, 2.86206448171706`, 0.09450221741270137`, 1.0246625231410813`, 0.5482685534092477`,
0.5509095992989991`, 0.015559917921534123`, 99.38093814456799`, 0.05756251023205006`, 408.71843737795535`},
{0.22732356256701053`, 1.5620331345808607`, 0.0050074509751661545`, 1.197172786919317`, 0.105827486838725`,
0.3868626726744283`, 0.406019958242878`, 3005.96971854734`, 0.19859762557886712`, 993.8820694295587`},
{0.21970737996259793`, 3.936399929325778`, 0.011750041974777936`, 1.121349683519117`, 0.5859217781105792`,
0.5996889464289015`, 0.1151074043533795`, 67.42346044078403`, 0.08547629013313163`, 411.81079788833034`},
{0.2790786490274024`, 1.2584508796810594`, 0.07665707924292071`, 0.7658270386209375`, 0.7483296604910108`,
0.41920065323621736`, 0.023176749691687894`, 3277.281084867628`, 0.1396918716126389`, 580.5780481474799`},
{0.16453791668796786`, 3.8862921855966857`, 0.013327775962263057`, 0.8725709784378354`, 0.6113118115742937`,
0.2156639660199009`, 0.03762803482789101`, 2547.318776383711`, 0.17247938020774817`, 892.0466365080326`},
{0.06375537189690972`, 1.7690459528540705`, 0.015096892864035833`, 0.8299986088232871`, 0.6367083940240161`,
0.28113916107145065`, 0.04428098157483091`, 4084.099508293162`, 0.10954682401240934`, 746.6104278313039`},
{0.08187579925483629`, 1.8655110961595254`, 0.0874648172698457`, 1.4510572222714657`, 0.15566591480992242`,
0.41910638528278665`, 0.07239970607129952`, 723.8241834225208`, 0.12901960068026558`, 180.2504060368367`},
{0.10435199153649627`, 0.5893233643311877`, 0.04811942984145233`, 1.1349604857206697`, 0.5895670318233859`,
0.15288875305518912`, 0.04910349811258292`, 449.5316302223964`, 0.12434959568189269`, 703.0287573582831`},
{0.15686440216485814`, 3.299158386840012`, 0.04362586030096008`, 0.953069845851497`, 0.4069322991233839`,
0.6538520347193495`, 0.028206943433383688`, 616.8360622195407`, 0.1803006375790399`, 266.56001157686615`},
{0.06547279088568253`, 0.8381089463723068`, 0.07137472004433824`, 1.2017236457315694`, 0.07858577799252853`,
0.5635691694407 9`, 0.03424982094712966`, 121.26145217466427`, 0.2454354766292936`, 829.242714338502`},
{0.17569742909738978`, 2.0195575491588675`, 0.024342226139692 63`, 1.434279867729109`, 0.8915491260180508`,
0.2781339725257843`, 0.04682272639132616`, 59.94019211717092`, 0.2222051195324956`, 236.65018069241324`},
{0.09460907585719619`, 0.8356897954881948`, 0.060460918351022866`, 1.069146699286762`, 0.014736646088169492`,
0.5865094862125538`, 0.07297164593929295`, 179.1610024491539`, 0.037596776460407155`, 892.0322556004016`},
{0.09233956131689997`, 0.6404053871541118`, 0.05959897753518367`, 1.3305103741913034`, 0.7729857515912517`,
0.22480536379085048`, 0.13093485314958295`, 1027.106817790177`, 0.09580485239452469`, 776.0064233531045`},
{0.1760992852032619`, 2.0576043555645516`, 0.009895906281638124`, 1.218879855416122`, 0.22293753096138147`,
0.47733786176532955`, 0.12573960627564046`, 190.0926217184925`, 0.16755982960498023`, 296.35846579200165`},
{0.266626264443372`, 3.5900664324260045`, 0.04123634989318651`, 1.010061724366724`, 0.22320942065814342`,

```
0.29156885927164033`, 0.008778055090297143`, 315.7126293940718`, 0.03106814470993058`, 971.4718069354443`},
{0.20633623222753245`, 1.8062049689164192`, 0.024683456051737968`, 1.4736425858155604`, 0.9975963414746294`,
 0.45321134080754555`, 0.06164119417640365`, 981.5961666351051`, 0.06084524543598019`, 894.6393430913585`},
{0.1952457396539687`, 2.396633432716655`, 0.027692355669681295`, 1.483739684147085`, 0.49946151037920083`,
 0.6843034163274524`, 0.015772529376378245`, 165.6691863878366`, 0.13164676433224554`, 744.7639235090896`},
{0.0412147820800699`, 0.6603123172269081`, 0.022261526033749313`, 1.1649615926526864`, 0.2836869733691665`,
 0.15807495679202033`, 0.11234412980270021`, 3908.1378298311292`, 0.06462235682420547`, 548.6113009858772`},
{0.17218500939622683`, 3.313865785682787`, 0.027957414977543033`, 1.3071462915775924`, 0.8795651108976117`,
 0.6440187783428721`, 0.0059566913562622715`, 104.65032511018502`, 0.027977308543697232`, 907.2104908091411`},
{0.15502202925070413`, 3.316526162282644`, 0.0223320056796846 7`, 1.2759230603694547`, 0.9221203428299127`,
 0.2943110669636566`, 0.005350880253335643`, 3898.5440559966614`, 0.22820486785889155`, 684.4986442435719`},
{0.2268261659238296`, 0.7994964989328879`, 0.08934756409882037`, 1.2877518377572021`, 0.5411906669030329`,
 0.3691399078289529`, 0.43421420694016527`, 139.17013379956958`, 0.04485647842506657`, 905.8781238041313`},
{0.1770204235692701`, 1.2781960851633176`, 0.06497396695791541`, 1.3605134684748712`, 0.244014227954229`,
 0.1870138591753292`, 0.0307712715859882 6`, 60.18019994982649`, 0.2131346317141143`, 683.027244020558`},
{0.2717375993536527`, 3.135824470079611`, 0.0691606222305435`, 0.7845225922631083`, 0.16212023726406954`,
 0.4466944141001402`, 0.022381478813530132`, 1383.5815052684432`, 0.187615308884319`, 843.6072606940219`},
{0.13674247358699154`, 3.6254060485372106`, 0.06512400723657372`, 1.01887393296334`, 0.2910940805340305`,
 0.4365596853256456`, 0.08138806498834827`, 2017.3312921087977`, 0.1583601508053748`, 997.8620700747402`},
{0.25040288289621143`, 2.7133813844480077`, 0.08448585223614441`, 1.2018013366302356`, 0.3702885119817223`,
 0.5701876195896491`, 0.00869107124078444 8`, 443.903801201532`, 0.12402133852573172`, 106.45272333045864`},
{0.041511277768737365`, 3.493161059079408 7`, 0.03976168853825012`, 1.2668832550579765`, 0.9486019052531109`,
 0.46529858524653356`, 0.012928244696006381`, 74.20790580822272`, 0.10554204343742091`, 509.5020269334231`},
{0.1336668665495257`, 2.350867715598585`, 0.0771275101676191`, 1.4505612510666444`, 0.07698080749571079`,
 0.6199281441330318`, 0.01101960174542534`, 2269.347129396604`, 0.18489372818520305`, 687.4473514223084`},
{0.16253001008083912`, 1.051184352668212`, 0.056229861688529344`, 1.004023329401581`, 0.3681252451367141`,
 0.678555581878113`, 0.021351933171038215`, 801.2312670528647`, 0.04412464112257197`, 454.387044888035`},
{0.11892797719939147`, 1.7514715754101635`, 0.04183594418628097`, 0.8769916901819208`, 0.1544196171401151`,
 0.36811304221318286`, 0.3010643636498307`, 695.4685634811971`, 0.1100461062891433`, 498.29553639594855`},
{0.07330210226600109`, 0.43499060410436075`, 0.027517449896700585`, 0.9126335864120539`, 0.9987805296414807`,
 0.22966152781322624`, 0.006114379328547516`, 1310.3025866798243`, 0.17060413983046802`, 156.9492509406908`},
```

{0.04276868172332263`, 2.4648491140401436`, 0.05187906428248695`, 1.0339646047806086`, 0.22491069842200573`,
0.5459771269560785`, 0.008402091672076594`, 289.6225761610719`, 0.14646027342084122`, 755.2264179389915`},
{0.04164393094890753`, 1.242303977240664`, 0.07972480756303409`, 0.7846124621006639`, 0.17407018758268 8`,
0.36672176044371163`, 0.2674133971106355`, 845.6949135526564`, 0.03358000499684233`, 498.1809415613 4975`},
{0.229458031958857`, 0.6439515416123651`, 0.006015292830911623`, 0.9158920123716887`, 0.2560144381841183`,
0.2684667797484167`, 0.3431552466616 3677`, 154.42595917415295`, 0.0855610041334563`, 198.13557848737526`},
{0.17047138799782602`, 2.2898957920477256`, 0.06696496462752731`, 1.0845008770247568`, 0.2872211606807551`,
0.6001028117008813`, 0.005754832757542004`, 635.7506300024877`, 0.22579833707749164`, 724.3665386038351`},
{0.17254913268987798`, 2.6495590376973945`, 0.034760594449157356`, 1.2124525447208163`, 0.84194692679041 4`,
0.5402918593560716`, 0.18936332779093834`, 107.26507780664734`, 0.037511987734769214`, 537.2669943782652`},
{0.25016487015444`, 0.777229893080774`, 0.04915901947556062`, 1.0998264093102956`, 0.5377812514235516`,
0.25267335702204063`, 0.00974787647189834`, 1244.8070691537305`, 0.07483034108642977`, 805.776090435819`},
{0.05399158420479849`, 3.019162845487374`, 0.03785073545081486`, 0.9636051990488629`, 0.15564774020522387`,
0.21918784710924044`, 0.006223290787557694`, 2623.658439173353`, 0.10900490483685588`, 947.3435866888657`},
{0.1747811216286701`, 3.881854804860919`, 0.08016222833139366`, 1.4944210252995196`, 0.11903432009706139`,
0.5878228241833926`, 0.37145683618740644`, 66.72046507331578`, 0.12877597314607542`, 125.71709418119497`},
{0.141601654527025`, 1.3089955011167094`, 0.0656032917699483 5`, 1.3368116485682675`, 0.4133506282593844`,
0.6351089997359891`, 0.016818978674610487`, 1705.797093405403`, 0.15388894760263466`, 511.61503546845904`},
{0.084639232238298`, 1.212736951496196`, 0.05997810159304612`, 0.8889893736120966`, 0.13958728158655265`,
0.6603558899173929`, 0.011413642036874375`, 536.7545673384209`, 0.04259995653165516`, 944.3686479349165`},
{0.16529311034127547`, 3.851126737390418`, 0.08113860877333676`, 1.058819349237051`, 0.13571240412272645`,
0.4517872186170928`, 0.35559705335185826`, 1001.8429736474003`, 0.09802300913580997`, 620.1784409572178`},
{0.23418982946474864`, 3.956604059014369`, 0.04230369921779414`, 0.95232561320253`, 0.8349528260558194`,
0.6299543509496768`, 0.06275886449027793`, 701.9720478982712`, 0.21824826209863868`, 426.47742716464967`},
{0.2761598760040886`, 2.6641557087508057`, 0.004462535100939409`, 0.9313534046677239`, 0.750233715330811`,
0.2049305849598677`, 0.3933840236336692`, 50.2496550267719`, 0.2374943907090124`, 344.2970123705422`},
{0.1351836628142749`, 3.5624660341291037`, 0.025452437146326938`, 0.7612568037032439`, 0.652970660668506`,
0.6408529036315123`, 0.21928764621486097`, 534.7730348589814`, 0.24746341528536303`, 560.0699314591134`},
{0.26232668071537735`, 1.207274588572547`, 0.027172732680876114`, 0.878898400796649`, 0.9275146387246145`,
0.31349252382451775`, 0.12237771890255045`, 296.36361267724436`, 0.017576594718109073`, 538.5261184010628`},
{0.06921463438282771`, 3.9571800779527315`, 0.026742802177079528`, 1.1077760750375103`, 0.012836546117421577`,

```
  0.4464117577792523`, 0.3069910959195543`, 680.1511030834043`, 0.0877064744359659`, 182.30226827470233`},
 {0.23559239220203682`, 3.2108513477276697`, 0.01736528262118654`, 1.3561109299681864`, 0.09396152800742397`,
  0.29521017097752533`, 0.02869751203681663`, 1077.23513295276`, 0.19412010713150385`, 318.4056793084999`},
 {0.09115428875490028`, 3.0706223452006016`, 0.03232145089994729`, 0.7642247265036182`, 0.18050339436720364`,
  0.6416456253725367`, 0.010505945470625598`, 58.10983830816918`, 0.09406933636073506`, 174.5022624084388`},
 {0.23356040634394826`, 2.492582964596944`, 0.09819476019997214`, 1.1503432849681279`, 0.06854649517894851`,
  0.5510839557713577`, 0.0071381077335303`, 2385.9838126952855`, 0.01331535575672690`, 961.7670735590306`},
 {0.25739033381964765`, 3.83031296180729`, 0.07795330721270595`, 1.3914735727751597`, 0.315795024852483`,
  0.18909057130226337`, 0.046455878996427676`, 2457.8987531381417`, 0.21479863643047675`, 436.99040574834953`},
 {0.11782083138332561`, 0.9397991607947134`, 0.03435330327997967`, 1.298219743186714`, 0.15161593487328484`,
  0.2427618760211594`, 0.04640184603844971`, 394.52569979799523`, 0.22637503918894109`, 120.48416627959249`},
 {0.10065409907604789`, 3.109979529809686`, 0.05985471266539985`, 1.4037113657202065`, 0.2551527049282132`,
  0.39211616335234245`, 0.053944330615504715`, 1232.2013118971267`, 0.18318402883994528`, 982.5075567784879`},
 {0.20373034722529687`, 1.8258929347032087`, 0.028710915990495637`, 1.2999358225942528`, 0.47437870101991586`,
  0.4479020312750728`, 0.02085535990534196`, 155.89057435108427`, 0.24707674305197086`, 593.832502401464`},
 {0.1667072912115688`, 1.1926716420341554`, 0.02793807367428192`, 1.1097053421712721`, 0.7879852596126686`,
  0.47927016034261305`, 0.007964145149834574`, 178.94143600935337`, 0.1062391944982713`, 376.6999080921696`},
 {0.04399597163424873`, 1.5345814599820171`, 0.040779489311776336`, 1.1444095951288937`, 0.08884309651814792`,
  0.5567432378994842`, 0.011138214889458318`, 268.907723173268`, 0.042126942027993275`, 429.31770518007414`},
 {0.12554904125704935`, 2.572105912307064`, 0.04382715819624885`, 1.0196689186185788`, 0.004628348183958852 5`,
  0.4286118484279966`, 0.12186483055559326`, 183.56311720218602`, 0.19168490195367482`, 312.7756329069644`},
 {0.10389836812335534`, 1.5065271361516794`, 0.0782203910368041`, 1.3432006880431453`, 0.16858721631501128`,
  0.20272940646961235`, 0.005194801604326796`, 692.6428769431301`, 0.2497553227485374`, 646.8923759638144`},
 {0.1873969648530403`, 2.487936537546788`, 0.08787631545159552`, 1.4862886220253502`, 0.4510967953396121`,
  0.6132699008580953`, 0.20943864253875633`, 362.75371797287244`, 0.05566418089330266`, 990.5206074435366`},
 {0.2609977488784161`, 2.682129125412585`, 0.04099814383534014`, 1.1943200062659527`, 0.3320420087757965`,
  0.3562748569841966`, 0.03843736896556402`, 4461.432848364856`, 0.19491126930886082`, 448.46031606671596`},
 {0.11177943644202659`, 3.004766476051927`, 0.02365721593278737`, 0.9739347355449255`, 0.11623805347832561`,
  0.5332851817458878`, 0.014219460200483265`, 2321.5504339611107`, 0.11273311280208292`, 154.13280416226715`},
 {0.12452460259052345`, 0.6179927758495714`, 0.018240920352788487`, 1.419600762592073`, 0.04159334889643196`,
  0.4953333574077009`, 0.005771350364962132`, 173.11009258100464`, 0.03618579436828123`, 460.51456633143266`},
```

```
{0.05633801557098483`, 2.9056643066548693`, 0.09482015263628527`, 0.9910228668352266`, 0.5398932209933094`,
  0.30993462834373164`, 0.326754145425203`, 4152.751222173614`, 0.1627448112336103`, 934.4752403980212`},
{0.24888413407324067`, 0.9185195851359875`, 0.001862748847094693`, 0.8264811519169353`, 0.45711823663268425`,
  0.647903170967445`, 0.01273956161975329`, 3902.2467544998485`, 0.10661606869776508`, 764.1046199784935`},
{0.10302491503594513`, 3.897572343584968`, 0.0776490643225345`, 1.3728855867542038`, 0.399366063625185`,
  0.5139103382918121`, 0.006363477494426043`, 1471.3257431593884`, 0.18415616996412293`, 396.38746581742976`},
{0.2217716187206022`, 2.3669670966982617`, 0.05150497952731632`, 1.3669514270973737`, 0.27563093514117476`,
  0.6097112540241125`, 0.02421035821462878 5`, 245.19099830513036`, 0.2227447189207991`, 922.0556423723906`},
{0.200567099512532`, 0.414291053781334`, 0.0965175412941376`, 1.4122041619788255`, 0.2832458523243746`,
  0.4111934355701189`, 0.47068189330284727`, 727.924890435336`, 0.13700773707558722`, 457.2663597106665`},
{0.26282708876445304`, 0.7299461735692212`, 0.002590123247159504`, 1.314069772735592`, 0.44387331112790007`,
  0.6627601406571553`, 0.017941283900867265`, 4804.295508555627`, 0.13656101682983213`, 961.2364213744279`},
{0.2217181783558071`, 1.7819525199900896`, 0.09096582175868409`, 0.8016110058394221`, 0.34870116285618513`,
  0.41254310619778667`, 0.28848841693193716`, 118.09472925522707`, 0.04915168920099666`, 318.7068061045219`},
{0.04238719280368508`, 3.9934041772699773`, 0.012369382023231542`, 1.0320497019704051`, 0.441711735053806`,
  0.3953172978504792`, 0.055840071594635055`, 4470.752428881338`, 0.11971425032923705`, 391.1155394727787`},
{0.11325951664984701`, 1.2613891355679572`, 0.040579133378454896`, 0.792778865736141`, 0.4125188071599031`,
  0.6760512259522733`, 0.120226085162758`, 240.62393709029973`, 0.23563525899548954`, 669.3575607124823`},
{0.11479796420185318`, 1.3645131007055955`, 0.05813851484662918`, 1.4328455678258982`, 0.8783089057298354`,
  0.6273688332789649`, 0.005594475300849291`, 336.8131271096774`, 0.042923286813940253`, 400.6985075519651`},
{0.18904612246826086`, 0.9499182666483561`, 0.01636431294508675`, 1.334133788223376`, 0.9399135492285866`,
  0.44862152471700667`, 0.015021474832741414`, 104.29224521509512`, 0.20020013193412523`, 362.68249145721364`},
{0.07377471208830438`, 0.9399350889911195`, 0.0035046964205100294`, 0.95254283141522`, 0.8226600861406266`,
  0.5781817355083377`, 0.45292305898377666`, 4955.013777151356`, 0.18926919322875674`, 784.6286355590761`},
{0.0943766935246651`, 1.541984748199737`, 0.08520914815438847`, 1.068673226642868`, 0.827147192543344`,
  0.5185364408449342`, 0.02499096081051123`, 2458.353306813159`, 0.11939278701608641`, 406.52581078470075`},
{0.2691170241960745`, 1.3843069189189876`, 0.0032282878414418015`, 1.35893455567664`, 0.05684986848649287`,
  0.6495874733323252`, 0.012010940694653572`, 818.6583817232862`, 0.11477976407459428`, 802.4857212031947`},
{0.13195654495183612`, 2.820234715819688`, 0.06459455777685377`, 1.3215682050752708`, 0.2555253012593035`,
  0.5650143175076483`, 0.17650684935600558`, 2548.3703743447245`, 0.05197610429597477`, 647.2541591160882`},
{0.17938754879434415`, 2.956564495685738`, 0.02344859424499825`, 1.2271661955652247`, 0.1023158483495108`,
```

0.2141005377740559`, 0.03160934186798711`, 55.01949141750772`, 0.11404091283217083`, 153.96775969344887`},
{0.2559468893711437`, 2.6639064911815478`, 0.09405265387739238`, 0.9418787060193106`, 0.3579196254838115`,
0.25349572195403014`, 0.005922563586734462`, 686.8472909729428`, 0.21618019082855489`, 944.0817596146342`},
{0.16846533721603613`, 2.3770892262064063`, 0.060764583227921456`, 1.4122925060330735`, 0.8131908524160045`,
0.5745691401533295`, 0.13815925889996158`, 111.92799118209615`, 0.07704963831958889`, 569.2109515105052`},
{0.1434225400058592`, 2.4623089922223533`, 0.0902878213107297`, 0.9014701813026362`, 0.7982192946025746`,
0.24884754841188483`, 0.008733563377012872`, 84.02892029266614`, 0.10452691271186154`, 174.89969925150496`},
{0.27498382758967205`, 3.0621895303657007`, 0.01477217114526983`, 0.8499949546355164`, 0.9543379068261812`,
0.511253393062205`, 0.026007868557453007`, 1193.5403577036534`, 0.24471853485794082`, 173.30853286735942`},
{0.10079216159245852`, 2.802263371434325`, 0.07168578045446232`, 1.0754522486079452`, 0.19795232328567214`,
0.4796042882951459`, 0.2511967396816989`, 916.4127582717309`, 0.09231077895158313`, 746.8086196132365`},
{0.1990395876740651`, 1.2891521935040648`, 0.08843781786859424`, 1.4217044468375937`, 0.7248232910439498`,
0.5288906890390438`, 0.010145562070591906`, 273.266580044107`, 0.1443450012114269`, 262.99773077705277`},
{0.22527530235766147`, 1.598090720025331`, 0.07516157972031196`, 0.911906670636256`, 0.0015950083386944147`,
0.2733183222114466`, 0.46257295121139497`, 1061.4916967982135`, 0.16946126329202837`, 514.5508656659653`},
{0.22800375706146692`, 2.963114044328991`, 0.08247902929296419`, 0.900155769535574`, 0.8635034321466182`,
0.41996725230963483`, 0.060440776646127806`, 82.14976842278587`, 0.17068417206912262`, 306.6961010018577`},
{0.2015284416951108`, 1.4828427267730095`, 0.09645095598799114`, 0.8813953916233646`, 0.6596036330203583`,
0.46790731355733717`, 0.04929088349718639`, 2147.9230273071194`, 0.21677576367436763`, 585.5137656428404`},
{0.24804752677268144`, 3.775861546538554`, 0.09828394004173842`, 0.8259108347253036`, 0.8501628381099438`,
0.43041433567991594`, 0.1645511915243919`, 472.6904522654539`, 0.10093712153827977`, 240.41573863346227`},
{0.2257734465493289`, 2.04598315320208`, 0.061072784217809206`, 0.9205146346019395`, 0.18360134495814817`,
0.2485501642439466`, 0.008261271045257068`, 4070.225349557058`, 0.14432673822154007`, 514.018907335898`},
{0.1884239042817073`, 3.884724587861638`, 0.046102203625386924`, 1.272869675104722`, 0.002436310787800445`,
0.6425585670884819`, 0.21347280812774377`, 105.72896030123064`, 0.013398853493480106`, 477.3170113191213`},
{0.10358133559917804`, 1.3204671153076744`, 0.03146944673924043`, 0.7598009709392282`, 0.8379948599836853`,
0.31448462367280716`, 0.09423762512957796`, 873.6401432656386`, 0.24643359443672203`, 257.57090703914133`},
{0.07213812112223295`, 3.2444866813138393`, 0.08948863559851084`, 1.1981049871186626`, 0.6100944668641342`,
0.3489877456164385`, 0.060436350562816356`, 810.8798894147244`, 0.1940599582028637`, 929.3745703520485`},
{0.18529645231815123`, 3.7388067772724316`, 0.04964299226107711`, 1.104153730745968`, 0.8562902999854938`,
0.20061955425285494`, 0.044683490812461285`, 1237.7065014840275`, 0.17461953898337046`, 513.2497687518729`},

```
{0.11937104462964826`, 2.2670408410877547`, 0.04212633055221726`, 1.2938710702028255`, 0.4062855702251942`,
  0.22896688687327793`, 0.008005950997757192`, 187.47575366480618`, 0.12062380115724536`, 420.08829844146976`},
{0.054668692745937386`, 3.776137055200575`, 0.006718751589097511`, 0.903291115804289`, 0.1998893214857524 5`,
  0.5550342082463592`, 0.0455873571973524`, 57.62421691500666`, 0.11425393330613232`, 212.43967681879158`},
{0.1180225681670306`, 3.9649936920105127`, 0.09323036671246253`, 1.0524617108638712`, 0.5152474998332999`,
  0.3810548965315216`, 0.2344220817835223`, 72.9591968642363`, 0.19446142099795322`, 521.0444198605804`},
{0.07585484942115545`, 1.5268041836953499`, 0.03996691736666499`, 1.2200843385635163`, 0.7382765513873244`,
  0.15072535011998411`, 0.46165250685061965`, 2014.6215653623833`, 0.0639150426736455`, 907.8090815554127`},
{0.15171074425765668`, 3.139959028863476`, 0.0344935047970057`, 1.313524716160249`, 0.15932561521340416`,
  0.344519114270757`, 0.023892122521195856`, 4372.432115235335`, 0.19509438620204334`, 395.2405886014027`},
{0.09759208826045196`, 2.787459031458204`, 0.08286510303958249`, 1.1498428365580842`, 0.3502413486308751`,
  0.6023991214203646`, 0.04223365699052448`, 151.71289774079654`, 0.15665841498595628`, 996.6803923172566`},
{0.06870492988603288`, 1.4433926464019216`, 0.054801612468977 6`, 1.4156603871152607`, 0.5046761258494916`,
  0.49679280153517125`, 0.23820389677781276`, 574.8760772624919`, 0.07570136980088016`, 774.5924346494874`},
{0.12928812481552754`, 3.4467014297623226`, 0.07698339025243396`, 1.1617442922699581`, 0.6397961941354378`,
  0.23683288560045057`, 0.014840572388870685`, 390.22473096383766`, 0.12473219055267015`, 308.51541332429787`},
{0.09947361882549755`, 1.0723085231871234`, 0.08651203729858636`, 1.1986754319537722`, 0.20293837184497532`,
  0.6267394010553271`, 0.3996069768023198`, 125.45020568494616`, 0.0747353381238216`, 815.5264595512623`},
{0.07385189036800854`, 1.1097460679658449`, 0.08534569405097261`, 1.4790081203811583`, 0.825389044253078`,
  0.6393014572933857`, 0.005393929802389671`, 1009.7951092503188`, 0.05876766510907333`, 713.7033793838743`},
{0.08886981390180526`, 3.1852329380949485`, 0.004843225884898494`, 1.0495521924526`, 0.8894920527442816`,
  0.6575087708412799`, 0.03091556833752079`, 1958.934458296337`, 0.09234610293139284`, 932.1837545744593`},
{0.14789843862328383`, 2.02419875820041`, 0.0514487209494189`, 0.7738844537901749`, 0.7290609776680101`,
  0.3165995990604955`, 0.08454509023565165`, 148.72732800342848`, 0.13774453289126964`, 330.25011651458095`},
{0.25024837610835304`, 2.332733791115757`, 0.007714368403400 7625`, 1.3224531384329063`, 0.8138980190182421`,
  0.44646733111462167`, 0.4678523507493547`, 2146.7439649709167`, 0.20938413594616256`, 223.81131941609436`},
{0.22717212093597267`, 3.1880507135942393`, 0.07302399796830696`, 1.4035606527233073`, 0.6815685781194232`,
  0.3786677309260351`, 0.11250145446111069`, 222.14009806288436`, 0.22433932328259149`, 562.8866527417559`},
{0.26122096963316843`, 2.4993722773914913`, 0.09696053620814965`, 1.104804193531995`, 0.030204720323329992`,
  0.320390588871465`, 0.02428210132726833`, 110.42992015103017`, 0.03440723905644305`, 957.9351268355158`},
{0.209997188604718`, 3.564852173259874`, 0.04768067199603367`, 1.4849701489343874`, 0.9167498847129119`,
```

0.5930768777567338`, 0.00755656864676451 1`, 303.63963198830606`, 0.1798334582091491`, 979.6939793583849`},
{0.12957791995272494`, 3.292789025445294`, 0.029334797113071912`, 1.000823124755441`, 0.3770034479959583`,
0.6497986144453649`, 0.4644164461032186`, 69.35773206793411`, 0.22822270741491651`, 883.2881905591939`},
{0.1734007680973328`, 2.5509297754695135`, 0.028940050324794433`, 0.7542700070247144`, 0.5607952150482101`,
0.188194520643177`, 0.10842093468725295`, 89.42321140726736`, 0.04230251478503749`, 585.538823967249`},
{0.08976599585154404`, 3.410901375584835`, 0.03980847090975558`, 0.973029097362308`, 0.18413384146487743`,
0.4306490501572334`, 0.25777469695398525`, 394.50697488236744`, 0.14943635401103217`, 467.5668758424882`},
{0.1540668065647836`, 3.156731285149597`, 0.025268011683832015`, 1.4768603918368393`, 0.5930914034327641`,
0.3246832969886583`, 0.024701654832061146`, 2039.571837718409`, 0.09825821190266026`, 517.5447194924812`},
{0.25448003730676694`, 3.184178274174914`, 0.07969865646175218`, 1.1996160734264`, 0.014800627784428588`,
0.41181348081367897`, 0.1736752616356111`, 514.7971371394015`, 0.0242520492159774`, 345.1422226775872`},
{0.1805495720684696`, 3.5988294042989537`, 0.08629129509807985`, 1.45590487849237`, 0.6500204371524467`,
0.6598493533910432`, 0.006501958768912599`, 98.298066958251`, 0.19720820935827937`, 552.7953947456965`},
{0.2584100436319957`, 2.6280813527891524`, 0.008925623022125535`, 1.4383962385176845`, 0.1768680490063801`,
0.5799402745315277`, 0.0366811475565 2737`, 1557.060885044531`, 0.15015889209697436`, 661.2136292315887`},
{0.12632549433481927`, 3.227079423540302`, 0.024599881217287293`, 1.083115156883892`, 0.862537661831063`,
0.4466766069622442`, 0.0175594229299 5868`, 838.9788940765472`, 0.11679264941372974`, 962.5385414773948`},
{0.12581743503205006`, 2.6912266074159374`, 0.06825202848377941`, 1.130575915810954`, 0.7123031893599316`,
0.38183459324469116`, 0.005640134906792506`, 195.97188584350954`, 0.08695147378297474`, 810.3829986474054`},
{0.25215230411705725`, 3.2162318690382756`, 0.033696261063115164`, 1.4320906763160213`, 0.4333561915057187`,
0.6173099764463517`, 0.06281247244822827`, 350.692872561691`, 0.14670856975401297`, 661.831914027344`},
{0.06363146413241788`, 2.590325492453749`, 0.07759599585526651`, 0.8055036563134401`, 0.3890604782183784`,
0.6596279029376648`, 0.006263246097646333`, 3940.278955744855`, 0.14629462701990292`, 857.5168588182237`},
{0.09237029959020937`, 0.7372750141972166`, 0.08246784417666007`, 0.761354059113222`, 0.607934228395967`,
0.617252844721303`, 0.005938986299855 8435`, 1735.7120546460667`, 0.12202837863790356`, 173.2417595974299`},
{0.17533432125136816`, 2.980549279621548`, 0.032407398618161984`, 1.2260204195085989`, 0.09654786970227502`,
0.6394186143434559`, 0.017429477978672333`, 442.47136750346704`, 0.02322996341809752`, 762.5628352008919`},
{0.26705012685276763`, 3.7498343916107544`, 0.09027963548734444`, 0.9359855383901472`, 0.7308970561854626`,
0.6045717653608473`, 0.013690168592867258`, 53.13894335207241`, 0.0962366983944411`, 806.6075956869515`},
{0.1678815958956808`, 3.549708737453586`, 0.0464906102104119`, 0.8245440899304926`, 0.5174765540911901`,
0.24692645213758668`, 0.06597541194694605`, 190.2602468493688`, 0.13329765093762808`, 380.5016809755655`},

```
{0.26055153005517384`, 1.525876220559283`, 0.09894984049853037`, 0.7715055101942483`, 0.5126639262751305`,
  0.17386878262666106`, 0.09828079193823042`, 3373.049666117342`, 0.10907780218183427`, 438.5582920686159`},
{0.05352312148646443`, 2.717121314814369`, 0.009558938401920933`, 1.2972752707604394`, 0.3755720109619096`,
  0.4102380523871805`, 0.05724239658462567`, 409.7682683541758`, 0.12801779059907553`, 304.9029332641055`},
{0.06648599129298854`, 2.0041637987546954`, 0.08747673292965043`, 1.0596372061777766`, 0.3670136645832196`,
  0.20965175246372458`, 0.041210859023618776`, 258.8957674631027`, 0.10593279391689964`, 291.6186786637164`},
{0.27763207727232625`, 2.6532447231581857`, 0.005216127449356379`, 0.7883455682883888`, 0.4799446618915817`,
  0.19310494144291912`, 0.22065665818548827`, 81.96509789217254`, 0.19648280620732078`, 916.8547512970214`},
{0.04552100665001646`, 2.514367034016656`, 0.03037177853258429`, 0.7530148351569228`, 0.6998681178203525`,
  0.5299837160000372`, 0.11693739269557663`, 590.5283463527218`, 0.08466788522227797`, 566.1770520758637`}};
```

In[ ]:=

```
TestFreeGrowth = {{"TBUR", 15.711390754719442`}, {"TBUR", 44.2019502737212`}, {"TBUR", 16.7819302015872`},
   {"TBUR", 18.667093982125085`}, {"TBUR", 13.885447416359366`}, {"TBUR", 30.162601174755157`}, {"TBUR", 20.869830546775166`},
   {"TBUR", 13.428810876131172`}, {"TBUR", 14.471079396823363`}, {"TBUR", 17.712876379060795`}, {"TBUR", 24.336228038953468`},
   {"TBUR", 17.63354851297323`}, {"TBUR", 17.313225221748393`}, {"TBUR", 30.881659977935886`}, {"TBUR", 13.2742212625022`},
   {"TBUR", 18.776811164075987`}, {"TBUR", 31.898893336838885`}, {"TBUR", 17.28790705464217`}, {"TBUR", 28.67142534485458`},
   {"TBUR", 36.430732714305776`}, {"TBUR", 13.019048693178771`}, {"TBUR", 14.383786156569203`}, {"TBUR", 20.48923939651125`},
   {"TBUR", 35.012393113888294`}, {"TBUR", 20.777776008147782`}, {"TBUR", 48.266751175561275`}, {"TBUR", 15.561828191043215`},
   {"TBUR", 37.50933743832283`}, {"TBUR", 18.640678481908232`}, {"TBUR", 11.83630544424025`}, {"TBUR", 33.210465747981885`},
   {"TBUR", 11.382556282125856`}, {"TBUR", 12.754392233655562`}, {"TBUR", 24.564135844473306`}, {"TBUR", 33.81776364120207`},
   {"TBUR", 12.006249305459617`}, {"TBUR", 18.362239037123956`}, {"TBUR", 16.015705098077394`}, {"TBUR", 21.635849256627885`},
   {"TBUR", 25.878508033408714`}, {"TBUR", 15.06050668469422`}, {"TBUR", 25.959345718956083`}, {"TBUR", 26.071004424283036`},
   {"TBUR", 12.40762480700718`}, {"TBUR", 14.56254208955875`}, {"TBUR", 17.503679634098326`}, {"TBUR", 45.2094052716283`},
   {"TBUR", 45.74640593438107`}, {"TBUR", 25.9486748045628`}, {"TBUR", 12.029808404620965`}, {"TBUR", 24.1433235073872`},
   {"TBUR", 31.68985410836966`}, {"TBUR", 35.75517668565276`}, {"TBUR", 41.2450833432457`}, {"TBUR", 12.432508808409507`},
   {"TBUR", 18.187404300362225`}, {"TBUR", 40.089933116105406`}, {"TBUR", 26.821881272495755`}, {"TBUR", 18.390605378139213`},
   {"TBUR", 30.602625753839515`}, {"TBUR", 13.927808899569715`}, {"TBUR", 24.1542551473147`}, {"TBUR", 30.221327254485974`},
   {"TBUR", 13.960996725501133`}, {"TBUR", 14.718456359925968`}, {"TBUR", 23.80854323464943`}, {"TBUR", 12.09075567378241`},
   {"TBUR", 19.999698349123253`}, {"TBUR", 18.2090009652405`}, {"TBUR", 19.585433005037192`}, {"TBUR", 38.301974279561016`},
   {"TBUR", 18.454183803164156`}, {"TBUR", 16.646510085022577`}, {"TBUR", 11.066417337683328`},
   {"TBUR", 21.069008316964855`}, {"TBUR", 23.078967851849022`}, {"TBUR", 34.49452725010505`}, {"TBUR", 24.47021605580779`},
```

{"TBUR", 38.24532060036566`}, {"TBUR", 19.609609481476582`}, {"TBUR", 36.74969805999738`}, {"TBUR", 15.629644394196557`},
{"TBUR", 13.213496525127617`}, {"TBUR", 21.374687993953817`}, {"TBUR", 13.415378272305858`}, {"TBUR", 20.486308168185502`},
{"TBUR", 11.835137164430243`}, {"TBUR", 20.925751084537062`}, {"TBUR", 19.20759419404488`}, {"TBUR", 18.18623427800386`},
{"TBUR", 21.799599241705636`}, {"TBUR", 23.960197220535875`}, {"TBUR", 13.445382717487496`}, {"TBUR", 16.383883583281705`},
{"TBUR", 12.173591504764826`}, {"TBUR", 47.04577973955561`}, {"TBUR", 11.71317520726345`}, {"TBUR", 43.04732735707591`},
{"TBUR", 12.0430402417071`}, {"TBUR", 16.25921008760382`}, {"TBUR", 13.780799320894396`}, {"TBUR", 32.94466117223283`},
{"TBUR", 13.645477733405803`}, {"TBUR", 17.10937777091369`}, {"TBUR", 24.866446880146157`}, {"TBUR", 49.681091167586025`},
{"TBUR", 18.521505502047162`}, {"TBUR", 35.36620849444657`}, {"TBUR", 32.86288801866426`}, {"TBUR", 18.602289956387867`},
{"TBUR", 32.58986490722991`}, {"TBUR", 29.8925362710101`}, {"TBUR", 21.572955113264005`}, {"TBUR", 17.703241149394813`},
{"TBUR", 30.84229648532772`}, {"TBUR", 14.982154622046883`}, {"TBUR", 17.06599961414592`}, {"TBUR", 31.653186835701078`},
{"TBUR", 16.275422384450472`}, {"TBUR", 29.3193189802495`}, {"TBUR", 12.669073931157723`}, {"TBUR", 44.32121321762277`},
{"TBUR", 18.808632019596182`}, {"TBUR", 14.178270621846558`}, {"TBUR", 34.39411506496662`}, {"TBUR", 10.60826678656911`},
{"TBUR", 21.27025036051607`}, {"TBUR", 17.128893875251595`}, {"TBUR", 26.75424152875178`}, {"TBUR", 23.396540980904476`},
{"TBUR", 34.026524663570804`}, {"TBUR", 26.047893572349185`}, {"TBUR", 16.931298267133226`}, {"TBUR", 12.427684719498808`},
{"TBUR", 15.977237159890077`}, {"TBUR", 12.015523318328619`}, {"TBUR", 11.865708889876311`}, {"TBUR", 28.167135785515615`},
{"TBUR", 15.891934741275676`}, {"TBUR", 38.99654017919275`}, {"TBUR", 14.557887937138537`}, {"TBUR", 26.85195015930527`},
{"TBUR", 21.10367132602165`}, {"TBUR", 31.23224297185312`}, {"TBUR", 16.42416186520581`}, {"TBUR", 19.68576686273544`},
{"TBUR", 19.518715653499076`}, {"TBUR", 18.99467059371834`}, {"TBUR", 29.497864678679566`}, {"TBUR", 29.8342642786606`},
{"TBUR", 12.151562604837842`}, {"TBUR", 21.43295361353254`}, {"TBUR", 39.191880874485925`}, {"TBUR", 43.94937234554008`},
{"TBUR", 13.600102266619292`}, {"TBUR", 35.45455477652378`}, {"TBUR", 13.618077419834844`}, {"TBUR", 30.44805565275776`},
{"TBUR", 12.135143649888148`}, {"TBUR", 11.838492109374219`}, {"TBUR", 19.387626849034557`}, {"TBUR", 20.378893406187636`},
{"TBUR", 12.568370803609564`}, {"TBUR", 12.335541498182756`}, {"TBUR", 16.900180308391676`}, {"TBUR", 23.566889218337202`},
{"TBUR", 27.188423556215096`}, {"TBUR", 23.4088487070332`}, {"TBUR", 15.503298507467774`}, {"TBUR", 27.798052195552458`},
{"TBUR", 18.381327565539564`}, {"TBUR", 20.92135437607756`}, {"TBUR", 13.830888602052188`}, {"TBUR", 15.29011768969299`},
{"TBUR", 37.29460988856768`}, {"TBUR", 41.127126860961276`}, {"TBUR", 17.488297437451834`}, {"TBUR", 17.174594646166334`},
{"TBUR", 29.517194080940293`}, {"TBUR", 28.809733816287626`}, {"TBUR", 36.03500813957433`}, {"TBUR", 35.650726659604366`},
{"TBUR", 22.968796241435946`}, {"TBUR", 10.861214749311419`}, {"TBUR", 19.273058909571365`}, {"TBUR", 21.397446408131632`},
{"TBUR", 16.656429073810475`}, {"TBUR", 37.7256973902334`}, {"TBUR", 14.332350044067626`}, {"TBUR", 15.612773946318637`},
{"TBUR", 13.56089086265119`}, {"TBUR", 56.29919814610204`}, {"TBUR", 13.692192912801652`}, {"TBUR", 29.40261067063076`},
{"TBUR", 25.92640970414381`}, {"TBUR", 19.86214014774299`}, {"TBUR", 24.541614900945888`}, {"TBUR", 10.842531622673606`},
{"TBUR", 18.393250674136546`}, {"TBUR", 11.848022816238698`}, {"TBUR", 12.394247222735197`}, {"TBUR", 42.73652768351775`},

{"TBUR", 46.834151303573464`}, {"TBUR", 18.074934308481833`}, {"TBUR", 23.88505995433176`}, {"TBUR", 26.19664776852087`},
{"TBUR", 14.091681063105517`}, {"TBUR", 12.529826141736114`}, {"TBUR", 11.85452964978897`}, {"TBUR", 21.415047182220583`},
{"TBUR", 16.10378219825058`}, {"TBUR", 12.002118223003633`}, {"TBUR", 12.238177469377737`}, {"TBUR", 11.820289133688204`},
{"TBUR", 13.258397547509006`}, {"TBUR", 10.446724842053175`}, {"TBUR", 32.25921307596167`}, {"TBUR", 13.218716299423596`},
{"TBUR", 47.540255662146905`}, {"TBUR", 16.441665661679554`}, {"TBUR", 28.516877697345826`}, {"TBUR", 10.145299260427528`},
{"TBUR", 24.256068708797287`}, {"TBUR", 14.802391486987581`}, {"TBUR", 18.26988607751062`}, {"TBUR", 17.29117337945663`},
{"TBUR", 16.777144378744026`}, {"TBUR", 24.38760930220397`}, {"TBUR", 41.13822571897459`}, {"TBUR", 28.52079654241579`},
{"TBUR", 18.78601604914881`}, {"TBUR", 24.89976517570905`}, {"TBUR", 15.21678572837678`}, {"TBUR", 29.678812755534587`},
{"TBUR", 18.411189747761647`}, {"TBUR", 11.630526775456948`}, {"TBUR", 20.56736788402131`}, {"TBUR", 39.81896501701757`},
{"TBUR", 11.579647419833996`}, {"TBUR", 16.7073757138579`}, {"TBUR", 13.15017054642574`}, {"TBUR", 48.991993507602885`},
{"TBUR", 19.973188444818835`}, {"TBUR", 19.781162115684367`}, {"TBUR", 14.98836187254622`}, {"TBUR", 11.355024994053888`},
{"TBUR", 10.445561077742212`}, {"TBUR", 23.08652006894122`}, {"TBUR", 11.38031929474993`}, {"TBUR", 12.494991747371342`},
{"TBUR", 25.91202648565012`}, {"TBUR", 42.720904591500386`}, {"TBUR", 12.185093864964474`}, {"TBUR", 11.11000437764382`},
{"TBUR", 18.42743793854317`}, {"TBUR", 11.405104181149731`}, {"TBUR", 16.01094471858759`}, {"TBUR", 36.38234275331097`},
{"TBUR", 28.971244963128317`}, {"TBUR", 17.051858746531526`}, {"TBUR", 15.918258745132839`}, {"TBUR", 11.136970219111259`},
{"TBUR", 28.637097143580235`}, {"TBUR", 24.954008886644395`}, {"TBUR", 10.939225822736441`}, {"TBUR", 14.294574319421093`},
{"TBUR", 49.83267457923563`}, {"TBUR", 19.73508313170569`}, {"TBUR", 33.12362322236299`}, {"TBUR", 28.5490922467925 4`},
{"TBUR", 26.39334136273501`}, {"TBUR", 18.182282129491867`}, {"TBUR", 24.402886666984113`}, {"TBUR", 25.51697320347085`},
{"TBUR", 14.68063527980366`}, {"TBUR", 13.284960655014292`}, {"TBUR", 34.62031115029852`}, {"TBUR", 15.931985120694774`},
{"TBUR", 11.369743621488627`}, {"TBUR", 17.14773227042683`}, {"TBUR", 18.212303044244837`}, {"TBUR", 23.300311304242033`},
{"TBUR", 13.876621007775102`}, {"TBUR", 16.02479346669339`}, {"TBUR", 13.208860978559992`}, {"TBUR", 34.617244530505324`},
{"TBUR", 12.88540383655441`}, {"TBUR", 50.201497491149226`}, {"TBUR", 33.75407787514724`}, {"TBUR", 22.555506182171825`},
{"TBUR", 13.420983562939275`}, {"TBUR", 26.126524688871935`}, {"TBUR", 37.467551796005104`}, {"TBUR", 14.294999566793445`},
{"TBUR", 11.781318983556558`}, {"TBUR", 26.386348334785268`}, {"TBUR", 13.629363716693975`}, {"TBUR", 48.3975545130189`},
{"TBUR", 14.276052535452093`}, {"TBUR", 13.57077890673767`}, {"TBUR", 21.605038413907803`}, {"TBUR", 13.222805104676372`},
{"TBUR", 11.280495079659019`}, {"TBUR", 21.409445105028386`}, {"TBUR", 13.685628959811423`}, {"TBUR", 40.90500767021971`},
{"TBUR", 19.05174281927019`}, {"TBUR", 12.459674892628335`}, {"TBUR", 12.080813962339308`}, {"TBUR", 20.44079628896667`},
{"TBUR", 17.31405015943552`}, {"TBUR", 17.63436164079323`}, {"TBUR", 20.72566502090729`}, {"TBUR", 28.305130093017823`},
{"TBUR", 18.128421806240034`}, {"TBUR", 18.21522543397489`}, {"TBUR", 12.19511139100093`}, {"TBUR", 23.52658971955829`},
{"TBUR", 38.746507555243205`}, {"TBUR", 13.013804608663998`}, {"TBUR", 16.830134710533237`}, {"TBUR", 31.163559201344587`},
{"TBUR", 24.861844391920066`}, {"TBUR", 10.200283327118301`}, {"TBUR", 11.994034603135827`}, {"TBUR", 10.163897828210832`},

{"TBUR", 23.875444478765356`}, {"TBUR", 14.355986815184721`}, {"TBUR", 23.9542029287897`}, {"TBUR", 38.51300337985342`},
{"TBUR", 26.666697824394866`}, {"TBUR", 11.726711325822064`}, {"TBUR", 15.84989810942918`}, {"TBUR", 12.238343820235636`},
{"TBUR", 16.557839402597924`}, {"TBUR", 22.123244065017477`}, {"TBUR", 14.362211816349216`}, {"TBUR", 14.982335434433553`},
{"TBUR", 12.742611257109857`}, {"TBUR", 21.346404563089`}, {"TBUR", 39.538021744880815`}, {"TBUR", 28.099557097951184`},
{"TBUR", 12.185998695163024`}, {"TBUR", 15.877804698917146`}, {"TBUR", 12.472281605292798`}, {"TBUR", 26.31625892762327`},
{"TBUR", 22.76889517879618`}, {"TBUR", 16.950178546001784`}, {"TBUR", 13.096775023957145`}, {"TBUR", 19.82357938171404`},
{"TBUR", 53.585315525906424`}, {"TBUR", 12.20366748251806`}, {"TBUR", 12.26068564409993`}, {"TBUR", 18.64753065556537`},
{"TBUR", 13.09793505577459`}, {"TBUR", 31.349437648549987`}, {"TBUR", 40.126370801513914`}, {"TBUR", 38.96572613913505`},
{"TBUR", 38.85342205795237`}, {"TBUR", 19.48865417681404`}, {"TBUR", 14.59295555893629`}, {"TBUR", 21.912784658619394`},
{"TBUR", 15.068792260286681`}, {"TBUR", 19.421262057828905`}, {"TBUR", 14.791311150730058`}, {"TBUR", 11.644407162921675`},
{"TBUR", 39.26171802714754`}, {"TBUR", 28.7718155898307`}, {"TBUR", 11.245023407343798`}, {"TBUR", 10.57174774201496`},
{"TBUR", 18.634841364000923`}, {"TBUR", 18.89800331371781`}, {"TBUR", 15.372070023482934`}, {"TBUR", 13.289630292735579`},
{"TBUR", 18.119211100621463`}, {"TBUR", 18.19532424240082`}, {"TBUR", 13.003150893677239`}, {"TBUR", 17.7282178239352`},
{"TBUR", 12.518727832782371`}, {"TBUR", 10.96689413046786`}, {"TBUR", 20.526495282875`}, {"TBUR", 19.618576611833166`},
{"TBUR", 25.741531481345167`}, {"TBUR", 11.580634768845037`}, {"TBUR", 39.60440853116702`}, {"TBUR", 50.7525628981767`},
{"TBUR", 30.525499970009236`}, {"TBUR", 15.875869808240488`}, {"TBUR", 33.75767720491689`}, {"TBUR", 12.198036043438151`},
{"TBUR", 36.10746753826354`}, {"TBUR", 23.019434665476034`}, {"TBUR", 42.99742959154252`}, {"TBUR", 45.04808549688851`},
{"TBUR", 13.323085661147942`}, {"TBUR", 15.990018308084574`}, {"TBUR", 28.99263986073146`}, {"TBUR", 10.828591309594154`},
{"TBUR", 27.475565098910344`}, {"TBUR", 12.253512274090394`}, {"TBUR", 14.163757228575793`}, {"TBUR", 14.931779341911481`},
{"TBUR", 14.540706199919255`}, {"TBUR", 47.32170377355336`}, {"TBUR", 22.146989872057624`}, {"TBUR", 23.239188532259003`},
{"TBUR", 13.339585238476273`}, {"TBUR", 12.130906923683536`}, {"TBUR", 23.117528909403312`}, {"TBUR", 44.23773482755419`},
{"TBUR", 38.157567892445215`}, {"TBUR", 26.394381760015914`}, {"TBUR", 49.465206421225076`}, {"TBUR", 15.544368884888053`},
{"TBUR", 45.64964820274593`}, {"TBUR", 10.375598986949994`}, {"TBUR", 13.4577709014758`}, {"TBUR", 12.92740658866155`},
{"TBUR", 13.82555155001455`}, {"TBUR", 17.2548882383809`}, {"TBUR", 17.420284405698727`}, {"TBUR", 23.296344746475494`},
{"TBUR", 31.98430657291525`}, {"TBUR", 18.561175904260594`}, {"TBUR", 11.148323633274817`}, {"TBUR", 24.98779313594612`},
{"TBUR", 15.030367488401826`}, {"TBUR", 20.558700394866957`}, {"TBUR", 18.6028068766275`}, {"TBUR", 14.6230201465156`},
{"TBUR", 19.3016362362253`}, {"TBUR", 11.448179213739792`}, {"TBUR", 29.151867850742914`}, {"TBUR", 45.909960904742036`},
{"TBUR", 14.661498452826745`}, {"TBUR", 16.10400151271037`}, {"TBUR", 42.61403021443065`}, {"TBUR", 22.61333940022501`},
{"TBUR", 12.465990105470024`}, {"TBUR", 18.11202847916702`}, {"TBUR", 28.0774161110492`}, {"TBUR", 16.325684810661727`},
{"TBUR", 18.55982079277458`}, {"TBUR", 13.86257402896593`}, {"TBUR", 12.042055362310814`}, {"TBUR", 29.1859718697357`},
{"TBUR", 21.366117344635636`}, {"TBUR", 26.943445824101442`}, {"TBUR", 11.498290153855072`}, {"TBUR", 11.488376172892458`},

{"TBUR", 21.10426942757126`}, {"TBUR", 22.923874181921168`}, {"TBUR", 12.679033292772049`}, {"TBUR", 34.181493073312765`},
{"TBUR", 21.229170276246265`}, {"TBUR", 23.05327809438495`}, {"TBUR", 14.836687161538524`}, {"TBUR", 31.632092206844426`},
{"TBUR", 10.684528417301543`}, {"TBUR", 12.323554426038513`}, {"TBUR", 14.908989326903038`}, {"TBUR", 22.62247856502531`},
{"TBUR", 22.971085682806184`}, {"TBUR", 15.969422089771053`}, {"TBUR", 37.97006518058343`}, {"TBUR", 13.989474739922914`},
{"TBUR", 13.46400948773041`}, {"TBUR", 10.712035781062992`}, {"TBUR", 15.392271050722684`}, {"TBUR", 16.09268015882418`},
{"TBUR", 22.82787006896125`}, {"TBUR", 23.547927678469136`}, {"TBUR", 13.622706960699572`}, {"TBUR", 15.09676395522251`},
{"TBUR", 22.953986000725653`}, {"TBUR", 15.97721606819315`}, {"TBUR", 44.38959844892823`}, {"TBUR", 20.945249163902453`},
{"TBUR", 19.466033967193685`}, {"TBUR", 11.158472319678523`}, {"TBUR", 40.220670046310836`}, {"TBUR", 12.793530253211959`},
{"TBUR", 24.880734816219235`}, {"TBUR", 17.43604164254628`}, {"TBUR", 21.696656293887454`}, {"TBUR", 14.044238126710205`},
{"TBUR", 13.059506531769134`}, {"TBUR", 28.526967482011788`}, {"TBUR", 26.633481667404485`}, {"TBUR", 23.20253311428919`},
{"TBUR", 10.754730378470608`}, {"TBUR", 14.975999203711073`}, {"TBUR", 14.35758409286505`}, {"TBUR", 25.748160126748434`},
{"TBUR", 24.625971986527414`}, {"TBUR", 18.134246823300774`}, {"TBUR", 12.029250528991964`}, {"TBUR", 12.283088733640653`},
{"TBUR", 10.904174263410239`}, {"TBUR", 14.48499028734746`}, {"TBUR", 13.356622879290526`}, {"TBUR", 13.08768298202193`},
{"TBUR", 12.90992184913878`}, {"TBUR", 13.269606005256925`}, {"TBUR", 12.05460333402895`}, {"TBUR", 40.70482577774895`},
{"TBUR", 17.844085961158733`}, {"TBUR", 22.11428074306187`}, {"TBUR", 37.439576225558724`}, {"TBUR", 16.184705880426666`},
{"TBUR", 25.179894928284636`}, {"TBUR", 17.177849782689034`}, {"TBUR", 24.139857635768177`}, {"TBUR", 15.269969164410572`},
{"TBUR", 24.084328130080067`}, {"TBUR", 25.156185065552094`}, {"TBUR", 25.081587472097127`}, {"TBUR", 24.596794037781738`},
{"TBUR", 16.297538420609563`}, {"TBUR", 13.92054315571153`}, {"TBUR", 24.186531212314744`}, {"TBUR", 19.15308878818269`},
{"TBUR", 28.45358129148927`}, {"TBUR", 12.218494240750383`}, {"TBUR", 10.773398710428566`}, {"TBUR", 15.414206830182799`},
{"TBUR", 23.086256460655893`}, {"TBUR", 14.401915522233667`}, {"TBUR", 12.753435398541292`}, {"TBUR", 20.705830590117454`},
{"TBUR", 18.103071927473387`}, {"TBUR", 17.811389048194037`}, {"TBUR", 11.906952088761239`}, {"TBUR", 25.925332734218312`},
{"TBUR", 11.582030311234943`}, {"TBUR", 19.74448506851293`}, {"TBUR", 28.18445197624289`}, {"TBUR", 15.223966526805105`},
{"TBUR", 31.432468538394286`}, {"TBUR", 27.19309801194287`}, {"TBUR", 14.008140819298191`}, {"TBUR", 12.045856459211306`},
{"TBUR", 22.105046931525724`}, {"TBUR", 29.847955271378463`}, {"TBUR", 11.48809023519896`}, {"TBUR", 41.93802236462971`},
{"TBUR", 11.44197833872413`}, {"TBUR", 44.78670292317672`}, {"TBUR", 11.0867350462611`}, {"TBUR", 15.362744626619742`},
{"TBUR", 18.57110216060741`}, {"TBUR", 16.691242237560672`}, {"TBUR", 13.903982971980977`}, {"TBUR", 19.037062837946692`},
{"TBUR", 12.92362732078675`}, {"TBUR", 13.237091463120482`}, {"TBUR", 39.03584032971677`}, {"TBUR", 13.232019942363335`},
{"TBUR", 13.240144328016669`}, {"TBUR", 30.388421395742554`}, {"TBUR", 11.423499346803078`}, {"TBUR", 16.667402897010415`},
{"TBUR", 21.761982158062477`}, {"TBUR", 21.382614476203088`}, {"TBUR", 23.96488850963543`}, {"TBUR", 44.303028035478064`},
{"TBUR", 15.078377322238609`}, {"TBUR", 52.81421814180943`}, {"TBUR", 20.593736250381497`}, {"TBUR", 40.543019978884935`},
{"TBUR", 21.363444597177413`}, {"TBUR", 11.491958120679236`}, {"TBUR", 23.805942604091097`}, {"TBUR", 17.457403469003896`},

{"TBUR", 12.319726553927222`}, {"TBUR", 12.90520056034087`}, {"TBUR", 36.316604818573694`}, {"TBUR", 12.526229763228413`},
{"TBUR", 22.332214087824713`}, {"TBUR", 17.2244921848201`}, {"TBUR", 49.669497007104646`}, {"TBUR", 37.079745045406426`},
{"TBUR", 15.931126455903573`}, {"TBUR", 33.584913292338776`}, {"TBUR", 11.835162353735948`}, {"TBUR", 16.741086716203625`},
{"TBUR", 22.093063393428466`}, {"TBUR", 25.36518273199658`}, {"TBUR", 31.678827935351443`}, {"TBUR", 16.6206903764716`},
{"TBUR", 11.392861767709837`}, {"TBUR", 11.934464273787754`}, {"TBUR", 17.340349154883945`}, {"TBUR", 20.889722876209845`},
{"TBUR", 18.335068321726613`}, {"TBUR", 30.830295475520987`}, {"TBUR", 10.807642353824672`}, {"TBUR", 11.216908859826905`},
{"TBUR", 40.04439458958592`}, {"TBUR", 36.83881667098762`}, {"TBUR", 23.92194362638417`}, {"TBUR", 16.53534180686229`},
{"TBUR", 51.23050055254762`}, {"TBUR", 18.023455873735564`}, {"TBUR", 18.648199093095407`}, {"TBUR", 18.763547866061003`},
{"TBUR", 11.419212755826193`}, {"TBUR", 24.008534349430825`}, {"TBUR", 34.84296157475952`}, {"TBUR", 10.921490347290051`},
{"TBUR", 28.062133886083`}, {"TBUR", 12.427641403611485`}, {"TBUR", 15.92887830547401`}, {"TBUR", 20.94447979878765`},
{"TBUR", 15.337560941696069`}, {"TBUR", 21.19237478142351`}, {"TBUR", 13.663656699879482`}, {"TBUR", 10.91918368235662`},
{"TBUR", 28.48038009165704`}, {"TBUR", 14.72653000188838`}, {"TBUR", 43.7829466559123 8`}, {"TBUR", 13.700386640340682`},
{"TBUR", 14.548044383987587`}, {"TBUR", 13.665883046173738`}, {"TBUR", 16.05820386245968`}, {"TBUR", 12.904844890817078`},
{"TBUR", 12.917986940552879`}, {"TBUR", 16.379817998638547`}, {"TBUR", 32.81829785954663`}, {"TBUR", 15.65577822695791`},
{"TBUR", 23.960754924812576`}, {"TBUR", 46.42020173562314`}, {"TBUR", 14.781102935419684`}, {"TBUR", 13.436700928696643`},
{"TBUR", 11.989824230197081`}, {"TBUR", 18.904151712050382`}, {"TBUR", 24.081388036131735`}, {"TBUR", 12.335774564394683`},
{"TBUR", 11.732129723869265`}, {"TBUR", 10.456303438241472`}, {"TBUR", 12.560794668456198`}, {"TBUR", 12.421668111923303`},
{"TBUR", 15.519712741204295`}, {"TBUR", 11.484802585076057`}, {"TBUR", 17.607325878236882`}, {"TBUR", 29.85643700106843`},
{"TBUR", 32.13525239881099`}, {"TBUR", 16.78803495216286`}, {"TBUR", 11.648964281069263`}, {"TBUR", 24.182504340114686`},
{"TBUR", 19.42397801276626`}, {"TBUR", 44.17728583357691`}, {"TBUR", 14.66762514297404`}, {"TBUR", 15.585713064661457`},
{"TBUR", 14.22653096705157`}, {"TBUR", 15.555088977904298`}, {"TBUR", 12.514337331506693`}, {"TBUR", 26.78755123112613`},
{"TBUR", 26.254677688178298`}, {"TBUR", 38.34160621879318`}, {"TBUR", 20.956143477051647`}, {"TBUR", 12.384226094606502`},
{"TBUR", 11.342365937291804`}, {"TBUR", 21.567957025331644`}, {"TBUR", 15.428572810460327`}, {"TBUR", 17.692295535412782`},
{"TBUR", 18.374363606827465`}, {"TBUR", 11.177439745546469`}, {"TBUR", 23.151274579710364`}, {"TBUR", 25.561442209495276`},
{"TBUR", 15.191722397878516`}, {"TBUR", 15.317738134370043`}, {"TBUR", 15.754324049386259`}, {"TBUR", 13.444599229541677`},
{"TBUR", 46.975528713564856`}, {"TBUR", 15.582929873652624`}, {"TBUR", 11.172559186556574`}, {"TBUR", 21.53510755015808`},
{"TBUR", 38.1922652097825`}, {"TBUR", 11.615611300801511`}, {"TBUR", 30.52780202756511`}, {"TBUR", 17.419201062358916`},
{"TBUR", 19.891732201574825`}, {"TBUR", 42.363468891181704`}, {"TBUR", 19.268136573185007`}, {"TBUR", 43.61044372946785`},
{"TBUR", 19.678323929404883`}, {"TBUR", 12.83040029879762`}, {"TBUR", 30.230973806479213`}, {"TBUR", 14.157926609120352`},
{"TBUR", 10.191843501040474`}, {"TBUR", 37.83947540069915`}, {"TBUR", 17.072209389919802`}, {"TBUR", 16.46647436967203`},
{"TBUR", 25.520441400196084`}, {"TBUR", 15.142499976685482`}, {"TBUR", 12.153963131932542`}, {"TBUR", 12.254341695995448`},

{"TBUR", 15.99553699974471`}, {"TBUR", 14.009415411087728`}, {"TBUR", 20.72471693373452`}, {"TBUR", 25.379837072629677`},
{"TBUR", 41.34185799496326`}, {"TBUR", 28.586227977857817`}, {"TBUR", 16.516493534325388`}, {"TBUR", 12.454385454931874`},
{"TBUR", 12.326051525095739`}, {"TBUR", 16.45163901954104`}, {"TBUR", 49.40275306734192`}, {"TBUR", 26.97402791257719`},
{"TBUR", 21.04300518348442`}, {"TBUR", 11.138973134291232`}, {"TBUR", 29.170619014428308`}, {"TBUR", 34.63205089264964`},
{"TBUR", 15.194061817314392`}, {"TBUR", 27.389126428015043`}, {"TBUR", 13.167291347644422`}, {"TBUR", 12.55776636072074`},
{"TBUR", 12.140654160542763`}, {"TBUR", 28.850919257426384`}, {"TBUR", 32.311078542727124`}, {"TBUR", 47.522598894000616`},
{"TBUR", 28.369382096388907`}, {"TBUR", 37.38752450894326`}, {"TBUR", 18.6885524515911`}, {"TBUR", 23.66412131657481`},
{"TBUR", 41.330708798869644`}, {"TBUR", 14.048773944038226`}, {"TBUR", 42.861962534556994`}, {"TBUR", 14.327382038298191`},
{"TBUR", 10.622241238403388`}, {"TBUR", 28.74130609102452`}, {"TBUR", 20.452403102098977`}, {"TBUR", 17.429187525332168`},
{"TBUR", 25.7695058559047`}, {"TBUR", 21.35673335905624`}, {"TBUR", 13.316459464757374`}, {"TBUR", 25.456022184650543`},
{"TBUR", 15.26535501368181`}, {"TBUR", 31.442656021163415`}, {"TBUR", 22.144344460330387`}, {"TBUR", 19.756788197085175`},
{"TBUR", 11.750896770936777`}, {"TBUR", 11.91379735527266`}, {"TBUR", 13.082497845804456`}, {"TBUR", 22.58037325536216`},
{"TBUR", 27.11994888874441`}, {"TBUR", 33.94540348960336`}, {"TBUR", 13.55347506137448`}, {"TBUR", 42.039848568278266`},
{"TBUR", 18.840729303537266`}, {"TBUR", 32.18988964188309`}, {"TBUR", 13.869843964601282`}, {"TBUR", 27.880811077585708`},
{"TBUR", 26.732109568747337`}, {"TBUR", 26.429449941613587`}, {"TBUR", 12.686313766165187`}, {"TBUR", 28.619171533903188`},
{"TBUR", 35.136359473108946`}, {"TBUR", 15.890145348087076`}, {"TBUR", 15.540586680598453`}, {"TBUR", 21.62528445822497`},
{"TBUR", 16.655395917309367`}, {"TBUR", 14.556582063467172`}, {"TBUR", 24.59499458597921`}, {"TBUR", 38.3168759259298`},
{"TBUR", 20.91670961312049`}, {"TBUR", 30.880052623523927`}, {"TBUR", 23.37839937953543`}, {"TBUR", 14.756878385331708`},
{"TBUR", 12.306374151885091`}, {"TBUR", 12.19396976451413`}, {"TBUR", 10.820270170682432`}, {"TBUR", 38.86707580054765`},
{"TBUR", 26.275116375269466`}, {"TBUR", 19.515294983292456`}, {"TBUR", 20.76272418963084`}, {"TBUR", 25.06570624066885`},
{"TBUR", 48.28299572199504`}, {"TBUR", 47.15248698386937`}, {"TBUR", 27.965718787972648`}, {"TBUR", 18.136333387094034`},
{"TBUR", 17.30853490271965`}, {"TBUR", 14.720055891444831`}, {"TBUR", 14.575953384252578`}, {"TBUR", 16.872520172913198`},
{"TBUR", 32.56100616317757`}, {"TBUR", 18.54602887377109`}, {"TBUR", 30.285614163966653`}, {"TBUR", 24.769061979356383`},
{"TBUR", 12.80469992953821`}, {"TBUR", 46.8230835758209`}, {"TBUR", 21.21413815436887`}, {"TBUR", 12.319668225861111`},
{"TBUR", 17.08900165913073`}, {"TBUR", 33.39280618827887`}, {"TBUR", 30.784043360357213`}, {"TBUR", 41.8059601405507`},
{"TBUR", 18.210512416441464`}, {"TBUR", 32.0573658992787`}, {"TBUR", 33.9806026181684`}, {"TBUR", 27.56865592412585`},
{"TBUR", 12.1584928408606`}, {"TBUR", 31.993171768030024`}, {"TBUR", 25.227137856905184`}, {"TBUR", 18.96277268186363`},
{"TBUR", 12.635310561055041`}, {"TBUR", 34.75709683033686`}, {"TBUR", 15.676198179360494`}, {"TBUR", 14.351763370381466`},
{"TBUR", 12.394494955044776`}, {"TBUR", 38.34749460297427`}, {"TBUR", 21.709627816230363`}, {"TBUR", 21.404025449569534`},
{"TBUR", 13.773952319506515`}, {"TBUR", 15.325842453057636`}, {"TBUR", 16.13598200107213`}, {"TBUR", 10.565295180343039`},
{"TBUR", 38.493707225491875`}, {"TBUR", 31.591500399349755`}, {"TBUR", 12.233042653846113`},

{"TBUR", 13.441278132842836`}, {"TBUR", 10.71119305839793`}, {"TBUR", 12.549194360866233`}, {"TBUR", 14.430143043814553`},
{"TBUR", 23.638167870560885`}, {"TBUR", 46.60146622174525`}, {"TBUR", 28.674412294553544`}, {"TBUR", 32.37137987887265`},
{"TBUR", 26.9737868248472`}, {"TBUR", 28.656206467813305`}, {"TBUR", 27.243650264504783`}, {"TBUR", 13.96082705027568`},
{"TBUR", 29.94311776735254`}, {"TBUR", 19.52106732513822`}, {"TBUR", 17.775000251237888`}, {"TBUR", 17.933766746197712`},
{"TBUR", 22.399050329429524`}, {"TBUR", 14.458060263011596`}, {"TBUR", 24.95623448114484`}, {"TBUR", 16.347494456653916`},
{"TBUR", 24.618543776758553`}, {"TBUR", 16.9150565948905`}, {"TBUR", 21.952694405429753`}, {"TBUR", 27.898193165891037`},
{"TBUR", 11.274944049057206`}, {"TBUR", 18.540459835720974`}, {"TBUR", 15.07535764694947`}, {"TBUR", 15.740946903924218`},
{"TBUR", 24.261138236301495`}, {"TBUR", 15.977669186060735`}, {"TBUR", 14.732615628830892`}, {"TBUR", 14.0368739479989`},
{"TBUR", 22.74708834092515`}, {"TBUR", 29.698528331382423`}, {"TBUR", 11.019280529800811`}, {"TBUR", 14.368487002503988`},
{"TBUR", 31.141875705872618`}, {"TBUR", 14.833833148835154`}, {"TBUR", 11.739499287603133`}, {"TBUR", 52.5193285933069`},
{"TBUR", 16.985298517961542`}, {"TBUR", 10.499992605933624`}, {"TBUR", 16.751986127639093`}, {"TBUR", 14.26328092366482`},
{"TBUR", 30.43788025347739`}, {"TBUR", 11.90445215079268`}, {"TBUR", 14.264395316837868`}, {"TBUR", 14.452845699757372`},
{"TBUR", 34.572127537796206`}, {"TBUR", 26.11161702071263`}, {"TBUR", 20.43581000209806`}, {"TBUR", 14.162911780156453`},
{"TBUR", 17.87169579027483`}, {"TBUR", 12.99831618789261`}, {"TBUR", 17.775464460897613`}, {"TBUR", 32.559876696628045`},
{"TBUR", 25.609975482620715`}, {"TBUR", 20.570346406615123`}, {"TBUR", 47.48624482192006`}, {"TBUR", 12.586809730426838`},
{"TBUR", 12.589399932667439`}, {"TBUR", 30.01764746535113`}, {"TBUR", 11.972539963913935`}, {"TBUR", 31.855777981949387`},
{"TBUR", 17.019267590986306`}, {"TBUR", 23.790943672497843`}, {"TBUR", 15.487454208123516`},
{"TBUR", 10.525218926133745`}, {"TBUR", 47.49721606768071`}, {"TBUR", 10.877223296166196`}, {"TBUR", 24.758919081039558`},
{"TBUR", 18.98089486237934`}, {"TBUR", 38.9755916501208`}, {"TBUR", 15.844889206178705`}, {"TBUR", 15.828065961395247`},
{"TBUR", 16.043507902292482`}, {"TBUR", 16.29507796729022`}, {"TBUR", 11.747370042394191`}, {"TBUR", 11.342565597386283`},
{"TBUR", 20.65755986837501`}, {"TBUR", 38.141294834970815`}, {"TBUR", 13.166289584683993`}, {"TBUR", 20.736558416184195`},
{"TBUR", 31.76020149311386`}, {"TBUR", 12.048290529008609`}, {"TBUR", 13.935089448273782`}, {"TBUR", 28.19333521832598`},
{"TBUR", 31.76201967547971`}, {"TBUR", 15.27922375145718`}, {"TBUR", 11.931712667218223`}, {"TBUR", 10.547333726231798`},
{"TBUR", 16.347304675056495`}, {"TBUR", 12.318275290985008`}, {"TBUR", 38.91076514574312`}, {"TBUR", 11.683568662416686`},
{"TBUR", 24.00910384152908`}, {"TBUR", 19.286777915296657`}, {"TBUR", 27.276107180948763`}, {"TBUR", 13.486529801608391`},
{"TBUR", 12.605589745478484`}, {"TBUR", 40.909492536943254`}, {"TBUR", 37.17219671673012`}, {"TBUR", 17.6616094107079`},
{"TBUR", 16.586022700802683`}, {"TBUR", 16.45014093748146`}, {"TBUR", 13.92619220408081`}, {"TBUR", 18.82828129761119`},
{"TBUR", 36.22232583851177`}, {"TBUR", 11.27934026300811`}, {"TBUR", 21.639726206576118`}, {"TBUR", 16.459665972699216`},
{"TBUR", 15.511101673101416`}, {"TBUR", 22.506440992370166`}, {"TBUR", 11.076972857018646`}, {"TBUR", 15.242188743714676`},
{"TBUR", 11.462647176542138`}, {"TBUR", 18.702255009934902`}, {"TBUR", 10.482359657660846`}, {"TBUR", 19.516115667286996`},
{"TBUR", 19.84863193299701`}, {"TBUR", 10.811595168120101`}, {"TBUR", 12.468425027733264`}, {"TBUR", 17.65849908369357`},

```
{"TBUR", 12.366872881786685`}, {"TBUR", 15.057501302422027`}, {"TBUR", 10.97280472054204`}, {"TBUR", 12.995726450926323`},
{"TBUR", 41.0030139043523`}, {"TBUR", 27.476983556131657`}, {"TBUR", 13.003238300319067`}, {"TBUR", 34.76545358004131`},
{"TBUR", 16.93961788742202`}, {"TBUR", 15.01175401839218`}, {"TBUR", 15.58614156420975 6`}, {"TBUR", 27.7047512939306 8`},
{"TBUR", 19.2625809574968`}, {"TBUR", 15.907041087628507`}, {"TBUR", 19.36074195370645`}, {"TBUR", 30.469706303174217`},
{"TBUR", 11.901373794002273`}, {"TBUR", 26.278599600233694`}, {"TBUR", 20.003563989282735`}, {"TBUR", 37.7567778896955 8`},
{"TBUR", 33.957074484169915`}, {"TBUR", 15.236632905420791`}, {"TBUR", 19.838808863077166`}, {"TBUR", 46.4717876345526`},
{"TBUR", 22.744792861590497`}, {"TBUR", 11.472593768678601`}, {"TBUR", 14.418835243486974`}, {"TBUR", 34.13267009209965`},
{"TBUR", 11.347103240094883`}, {"TBUR", 11.332749256343055`}, {"TBUR", 10.61275667865575`}, {"TBUR", 25.317973500286957`},
{"TBUR", 18.824909678232107`}, {"TBUR", 19.75988885226173`}, {"TBUR", 21.694552645662377`}, {"TBUR", 11.68190930846636`},
{"TBUR", 10.8215974542`}, {"TBUR", 39.549387730743824`}, {"TBUR", 17.80560798712406`}, {"TBUR", 23.303984805326564`},
{"TBUR", 19.35719532039265`}, {"TBUR", 11.367022753870474`}, {"TBUR", 12.624461342996618`}, {"TBUR", 15.55025792512026`},
{"TBUR", 18.641589075281406`}, {"TBUR", 11.85870829496253`}, {"TBUR", 10.705231972566915`}, {"TBUR", 14.031239476593514`},
{"TBUR", 11.227113948157005`}, {"TBUR", 11.781352793042377`}, {"TBUR", 31.888118474974192`}, {"TBUR", 44.4704201674232`},
{"TBUR", 19.733705914844947`}, {"TBUR", 38.82659412985412`}, {"TBUR", 36.22155621382699`}, {"TBUR", 14.107232897488702`}};
```

In[ ]:=

```
TestNumOpt =
 {{"NumOpt Res", "NumOpt OS", "NumOpt Tox", "NumOpt Acur"}, {"TBUR", 24.40132761838062`, 229.4284269373576`, 1352.054982687297 5`},
  {"CURE", 365, 0.5180606888084087`, 30.372470226331362`}, {"TBUR", 28.467231988903745`, 229.45787955639682`, 735.4330284971941`},
  {"TBUR", 62.01044756160214`, 229.33314671665616`, 983.0491685867308`},
  {"TBUR", 23.02801636877116`, 227.01443307713052`, 1352.2224185341283`}, {"CURE", 365, 1.7690085138719398`, 57.537378835718826`},
  {"CURE", 365, 0.7697847222770119`, 26.389330048791848`}, {"CURE", 365, 0.20847204434166325`, 67.1947065755882`},
  {"CURE", 365, 0.406114147384631`, 19.43933926712946`}, {"TBUR", 70.89032688160748`, 229.74008820261892`, 514.1275469880355`},
  {"TBUR", 56.71621908639164`, 229.78021917936084`, 963.385537800036`}, {"CURE", 365, 0.34011134542010246`, 40.253603513049214`},
  {"TBUR", 84.87818250787656`, 229.79457674191838`, 390.6199410087184`},
  {"TBUR", 46.986281499821935`, 229.70349445976106`, 1157.1842025455674`},
  {"CURE", 365, 9.950321040924281`, 620.2315108906364`}, {"TBUR", 94.98874583211311`, 229.65290553138558`, 1293.8179899516858`},
  {"CURE", 365, 1.041943862599815`, 23.699393429406346`}, {"TBUR", 47.767409102854614`, 229.687596134571`, 740.7151473195931`},
  {"TBUR", 81.73233642231551`, 229.28706530177408`, 994.9414968490603`},
  {"CURE", 365, 0.550022217804297`, 107.74529012029494`}, {"CURE", 365, 2.1053731372908606`, 63.54805119068841`},
  {"CURE", 365, 0.4813959722042533`, 38.84497831360089`}, {"CURE", 365, 1.213955236782113`, 244.293127819335`},
  {"CURE", 365, 0.4304538575741596`, 110.67107908492731`}, {"CURE", 365, 0.25723289396714466`, 14.969202200933932`},
```

{"CURE", 365, 0.5442261014156998`, 28.887288151823142`}, {"CURE", 365, 0.06561031745850048`, 37.661922725972545`},
{"CURE", 365, 0.23330308368929437`, 87.46793713652771`}, {"CURE", 365, 2.1616836400604726`, 219.48858177419163`},
{"CURE", 365, 4.740276960112816`, 401.88912808232936`}, {"CURE", 365, 0.1952921334864900 8`, 20.444384155051566`},
{"TBUR", 14.261218065698609`, 226.33497113180317`, 1665.1643993980008`},
{"CURE", 365, 1.273502779030596`, 154.51776352827443`}, {"TBUR", 56.495984332509494`, 229.6889592997096`, 1353.5601520538332`},
{"CURE", 365, 6.377528924381772`, 125.29092674108114`}, {"CURE", 365, 0.3607860844170827`, 71.48406862658959`},
{"CURE", 365, 0.10799365399122861`, 8.499226981974571`}, {"TBUR", 44.814656060639194`, 229.5392001568308`, 1726.0786124279623`},
{"TBUR", 74.15895306253545`, 229.6845256998884`, 1184.1685493368852`}, {"CURE", 365, 3.0219376983493293`, 36.07601350772998`},
{"TBUR", 18.96616720651472`, 229.5902139194432`, 1106.0961068303961`}, {"CURE", 365, 0.18259722677431425`, 10.95262563959985 9`},
{"CURE", 365, 0.5034176440009522`, 131.3997257031305`}, {"TBUR", 46.82224946549761`, 229.7703451538139`, 1170.8201428463583`},
{"CURE", 365, 1.6275545013041246`, 64.6738366190496`}, {"CURE", 365, 0.5559383711833238`, 51.34268568035498`},
{"CURE", 365, 9.552066232131494`, 83.54990163064704`}, {"CURE", 365, 3.8775375242755827`, 146.02424860387842`},
{"TBUR", 115.67191124662756`, 229.79970360770287`, 781.1649303687246`},
{"CURE", 365, 0.4029199119775586`, 49.88966364076135`}, {"TBUR", 30.526253325728188`, 229.74364035724926`, 970.8088192186859`},
{"TBUR", 43.40853384639492`, 229.79045994591368`, 1157.3604046671012`}, {"CURE", 365, 2.9698614749265975`, 209.31490210477264`},
{"CURE", 365, 0.5013210011271069`, 43.28157083578681`}, {"CURE", 365, 2.1089442928384896`, 296.1099901596531`},
{"CURE", 365, 1.980912637180696`, 45.58427521956962`}, {"CURE", 365, 0.2540044260607922`, 19.14710231457143`},
{"TBUR", 104.34759014471382`, 229.47157994955813`, 709.4072738446688`}, {"TBUR", 38.25786358208256`,
229.67173078571668`, 578.2020020484923`}, {"TBUR", 56.66707529109255`, 229.7858498879653`, 978.4275885632165`},
{"CURE", 365, 0.9805677708433517`, 34.27582636976438`}, {"CURE", 365, 0.6904649290261651`, 69.07631977016662`},
{"TBUR", 33.9505702099560 7`, 229.75152847786657`, 839.8209510351483`}, {"CURE", 365, 1.4367714229417965`, 66.22456881068253`},
{"CURE", 365, 9.193092768021721`, 511.8783178313361`}, {"CURE", 365, 0.29063992089495994`, 16.457723506297224`},
{"CURE", 365, 0.22091021346387663`, 53.83835484479421`}, {"TBUR", 57.83632580951616`, 229.47296920388945`, 622.9021133874592`},
{"CURE", 365, 0.18048027593028582`, 41.543433715383806`}, {"CURE", 365, 1.608118215660635`, 91.35704134889995`},
{"CURE", 365, 1.0670309887384657`, 159.8948740465079`}, {"CURE", 365, 4.641002631732996`, 275.66358437089764`},
{"CURE", 365, 4.07819780051215`, 117.3655617544463`}, {"TBUR", 21.9011568741557`, 227.8453897391601`, 2148.010810048957`},
{"TBUR", 81.3967157878454`, 229.70586657826502`, 887.28112396441`}, {"CURE", 365, 0.7326068023923876`, 36.77434447282472`},
{"TBUR", 97.02765976083073`, 229.73805343670116`, 470.38295959171506`}, {"CURE", 365, 0.3976219380125695`, 44.40587090561384`},
{"TBUR", 76.35706045455258`, 229.7766675432557`, 707.3055594845824`}, {"CURE", 365, 3.0435207009158347`, 78.44151853793775`},
{"CURE", 365, 2.195947308116813`, 75.43336819519433`}, {"CURE", 365, 0.23208839134322332`, 30.072153106922922`},
{"CURE", 365, 0.6051106466658821`, 38.40649638838458`}, {"CURE", 365, 0.371447271404944`, 33.91789753939548`},

{"CURE", 365, 0.8980194156197168`, 65.52770860101886`}, {"TBUR", 101.99929929946869`, 229.7652099731794`, 929.630120427985`},
{"CURE", 365, 22.695325406754613`, 340.0908127885077`}, {"CURE", 365, 3.075083759759301`, 224.4723369813427`},
{"CURE", 365, 2.67321545534397`, 155.89985110570385`}, {"TBUR", 71.56051420954918`, 229.59237436738394`, 581.3166594505311`},
{"TBUR", 43.59908005016137`, 229.44381238554982`, 1242.5519388600396`},
{"TBUR", 90.43182458614393`, 229.7463322356118`, 839.8227042900891`}, {"CURE", 365, 4.019382395107906`, 594.2406871815796`},
{"CURE", 365, 5.898070177312372`, 384.78833667548724`}, {"TBUR", 66.8239401608384`, 229.52737350197543`, 1559.403678241529`},
{"TBUR", 164.41257883111328`, 229.72750486441058`, 1233.0773491608468`},
{"CURE", 365, 1.181014178923279`, 69.2039090689223 6`}, {"CURE", 365, 1.5451345942156396`, 35.4507132809926`},
{"CURE", 365, 9.798990630513456`, 417.01816135018555`}, {"TBUR", 44.16819271402706`, 229.4813182863501`, 575.1272311963535`},
{"TBUR", 45.71499387965184`, 229.54755827070926`, 1437.499862219158`},
{"TBUR", 127.791848258988`, 229.66491092161388`, 639.2244785710385`}, {"CURE", 365, 2.5216419951068767`, 152.52587294195138`},
{"CURE", 365, 176.63914009547318`, 788.6466611430167`}, {"CURE", 365, 0.13358863341674032`, 18.145139387529724`},
{"CURE", 365, 0.1576698451391273`, 31.26837888914869`}, {"CURE", 365, 7.817456486836897`, 251.81836716039425`},
{"TBUR", 154.86358099684492`, 229.79403830687798`, 1066.6355978815177`}, {"CURE", 365, 0.2548400222787732`, 28.507049132350904`},
{"CURE", 365, 2.450312640418392`, 45.46329675807675`}, {"CURE", 365, 0.05050723234003253`, 11.595110133678935`},
{"CURE", 365, 0.1332224757003357`, 45.348330961192005`}, {"CURE", 365, 1.2450072636622713`, 108.2187214866056`},
{"CURE", 365, 2.756239359732879`, 239.98915624566445`}, {"CURE", 365, 0.2822417484290424`, 39.8880538534861`},
{"CURE", 365, 0.20363889349024583`, 11.681374807886977`}, {"CURE", 365, 0.6113229423334429`, 124.6209283047372`},
{"CURE", 365, 0.18445700029042708`, 59.76318810433488`}, {"CURE", 365, 0.18644074444306163`, 13.278050671129405`},
{"CURE", 365, 1.2004755647907457`, 56.051648146239714`}, {"CURE", 365, 7.232678541356566`, 102.55396372684298`},
{"CURE", 365, 1.2467296352013206`, 73.05669648881333`}, {"CURE", 365, 3.7155988721584596`, 99.55317331909733`},
{"TBUR", 54.77670944644364`, 229.09351850264383`, 1084.1856798372771`}, {"CURE", 365, 0.1322706799272399`, 16.005570162720883`},
{"TBUR", 40.75712481337362`, 229.63111251911866`, 896.3735040865446`}, {"CURE", 365, 6.105372787630634`, 323.0532005084489`},
{"CURE", 365, 1.9171804679166058`, 287.2050460241764`}, {"CURE", 365, 0.9190221132429647`, 55.901247317367506`},
{"CURE", 365, 1.2014428439415312`, 90.86552210790836`}, {"CURE", 365, 0.1416639375935008`, 13.978086664449753`},
{"CURE", 365, 0.31321758992548465`, 25.409736544066103`}, {"TBUR", 73.34766334743084`, 229.6394471514768`, 1132.51765878577`},
{"TBUR", 48.5972331614447`, 229.59814711209845`, 1010.347348012422`}, {"CURE", 365, 0.05033797873448218`, 7.7396297429639285`},
{"TBUR", 12.91235991604776`, 229.08736943091435`, 1079.6526391882644`}, {"CURE", 365, 21.9197998073702`, 402.6287322313`},
{"TBUR", 64.82020240788044`, 229.6730177717569`, 1064.010975235387`}, {"CURE", 365, 19.371068506395254`, 434.2621393734632`},
{"CURE", 365, 2.8303915505396597`, 46.92327766003299`}, {"TBUR", 53.604453686595825`, 229.7935717945862`, 572.8984058530708`},
{"CURE", 365, 0.1514514890761925 5`, 69.60558270460413`}, {"CURE", 365, 0.5248141443172577`, 31.726626406207927`},

{"CURE", 365, 1.0382479808821985`, 55.62884589735772`}, {"CURE", 365, 0.07002731863524772`, 28.744423141805015`},
{"CURE", 365, 0.6217042770749737`, 166.06499689760173`}, {"CURE", 365, 0.9725975689560512`, 45.958920377482066`},
{"TBUR", 44.31950448969148`, 229.69067221781836`, 1286.4359100241409`}, {"CURE", 365, 0.8710079241869999`, 41.615115271086914`},
{"CURE", 365, 2.5122277427793547`, 146.9347546581437`}, {"CURE", 365, 0.522089009414039`, 31.92348527762497`},
{"CURE", 365, 1.3410100840776622`, 134.6930254418906`}, {"CURE", 365, 0.06486298557755618`, 25.046072428944314`},
{"TBUR", 89.78059200783713`, 229.73963680133568`, 557.4719539441561`}, {"TBUR", 52.02841795255654`,
229.60247365081474`, 857.8334539815002`}, {"TBUR", 88.80040035662533`, 229.68457027246842`, 590.4914425548754`},
{"CURE", 365, 4.9707689077800685`, 220.26730088327645`}, {"CURE", 365, 0.1526851512446409`, 17.15321673753062`},
{"CURE", 365, 1.3965731362403155`, 58.04772714318349`}, {"CURE", 365, 1.5330835273970953`, 139.79161185650514`},
{"CURE", 365, 0.4704646724422774`, 59.5151575284871`}, {"CURE", 365, 30.580074612194437`, 569.7971511924982`},
{"CURE", 365, 0.8958932964721528`, 223.1768859231565`}, {"TBUR", 74.41722209158277`, 229.61046514088457`, 1328.2396442011782`},
{"TBUR", 80.26402930683129`, 229.66104600292235`, 873.1836393004966`}, {"CURE", 365, 4.001923968093943`, 36.98145979001912`},
{"CURE", 365, 0.08484634101960938`, 18.61391479894461`}, {"CURE", 365, 3.2775749641859515`, 143.36900733913023`},
{"CURE", 365, 106.49505161319316`, 383.3638130783425`}, {"CURE", 365, 1.640648163393721`, 400.90984824658534`},
{"CURE", 365, 1.2874257188042535`, 104.95401038868788`}, {"TBUR", 30.57993912298529`, 229.65390844811975`, 693.993971347809`},
{"CURE", 365, 1.6955872396255147`, 36.1549956200516`}, {"TBUR", 43.37054361991881`, 229.4287293544495`, 1523.622374785574`},
{"TBUR", 50.86627411337197`, 229.71905211844478`, 704.7444923300493`}, {"TBUR", 148.42574748143855`,
229.19846810981156`, 980.5665595907914`}, {"TBUR", 68.7927328631799`, 229.62269802393735`, 564.6502179848521`},
{"TBUR", 21.953035882107734`, 229.7732254720194`, 1140.917503206354`}, {"TBUR", 66.48284697462817`,
229.62508617652412`, 1051.3515979365302`}, {"TBUR", 93.75176709983424`, 229.797739956511`, 960.1595487092671`},
{"TBUR", 81.19167769952443`, 229.48044747163323`, 636.2715589372733`}, {"CURE", 365, 0.14444544447624555`, 36.99064097494317`},
{"CURE", 365, 0.11623747520609047`, 45.395572218543`}, {"TBUR", 37.910425327591796`, 229.02855531136586`, 900.8364145379317`},
{"CURE", 365, 0.06273398033643852`, 8.59067517405954`}, {"TBUR", 30.00665846134699`, 229.59880724206187`, 1382.3372020219501`},
{"TBUR", 53.95210833904238`, 229.77162568276304`, 663.3475132992394`}, {"CURE", 365, 8.493070700851478`, 251.11450784672803`},
{"CURE", 365, 0.3790156606317777`, 44.02020795879889`}, {"TBUR", 26.239857338608836`, 229.4980967610823`, 919.6050086774325`},
{"CURE", 365, 0.4401571565518183`, 22.097957833758986`}, {"CURE", 365, 0.039206089433327956`, 2.1775454857375474`},
{"CURE", 365, 0.09786039660074507`, 8.400058790906732`}, {"CURE", 365, 0.08040239950475085`, 17.322587274284576`},
{"CURE", 365, 7.0761721535125615`, 193.43101574625058`}, {"CURE", 365, 0.10103615714033579`, 36.05240563244399`},
{"CURE", 365, 2.6819122980796544`, 85.77954223785792`}, {"TBUR", 16.739758878324743`, 225.88613327943196`, 1587.0665346948724`},
{"CURE", 365, 0.08145942851580208`, 10.065145591989722`}, {"CURE", 365, 0.8114687869492913`, 83.08705705395849`},
{"CURE", 365, 38.52467147723949`, 643.3653173654284`}, {"TBUR", 58.374768792251196`, 229.77814588821153`, 928.542225737321`},

{"CURE", 365, 0.3421579262993968 4`, 20.151854330337812`}, {"TBUR", 65.93321984239702`, 229.78278708740783`, 1166.7052535006874`},
{"CURE", 365, 1.0379113587253466`, 255.9834874288147`}, {"TBUR", 144.66449598516618`, 229.3602175282617`, 798.6137618516623`},
{"TBUR", 16.691816462927612`, 229.32833829097788`, 896.8591557051005`}, {"CURE", 365, 0.12840514308707165`, 12.877355107310848`},
{"CURE", 365, 2.0456992853828098`, 226.25642449102014`}, {"CURE", 365, 0.3216793684351495`, 19.24230757249674`},
{"CURE", 365, 1.6989530325961562`, 253.2556098978866 7`}, {"CURE", 365, 2.0753161016778203`, 20.93856632579893`},
{"CURE", 365, 1.5383147349517399`, 225.11797708760537`}, {"CURE", 365, 110.23729793720346`, 426.6441959342883`},
{"CURE", 365, 10.783594136406016`, 332.6452904591382`}, {"CURE", 365, 18.70118544362438`, 494.56539991857755`},
{"TBUR", 80.52190377156921`, 229.7712422233559`, 612.220845975374`}, {"CURE", 365, 0.9519307801392465`, 24.563414602925334`},
{"TBUR", 50.20098382562729`, 229.7820661851423`, 480.88758656853133`}, {"CURE", 365, 0.2674685118199025`, 15.373712874097583`},
{"CURE", 365, 0.23273199446570023`, 66.76669870837075`}, {"CURE", 365, 2.0125838476233655`, 70.50228445029182`},
{"CURE", 365, 0.296409758737363`, 16.805082359008303`}, {"CURE", 365, 1.2525793917817274`, 69.9525321376173`},
{"CURE", 365, 0.13090100163859159`, 27.494127006140562`}, {"CURE", 365, 13.381888539460054`, 153.64529055687657`},
{"CURE", 365, 0.3656592952291538`, 51.95286882307834`}, {"CURE", 365, 4.112012421137454`, 131.3106388677318 5`},
{"CURE", 365, 4.861965479758639`, 279.8742417272052`}, {"CURE", 365, 1.7894989016724159`, 143.78335349832977`},
{"CURE", 365, 0.15579263194915394`, 52.727869971775085`}, {"CURE", 365, 0.1767365953609669`, 7.962029663856848`},
{"CURE", 365, 0.6053818145511386`, 44.49202240949365`}, {"CURE", 365, 0.8240546260621142`, 24.03132713781273`},
{"CURE", 365, 3.258222739719513`, 126.26840441674722`}, {"TBUR", 24.32222356201753`, 229.67409157993646`, 806.0681635455081`},
{"TBUR", 29.773480635714577`, 229.73595747845724`, 1190.8896521518107`}, {"TBUR", 159.10097324843122`,
229.67800223529807`, 967.1261072158815`}, {"TBUR", 29.94592470078291`, 229.4373778605452`, 1080.0690372366655`},
{"CURE", 365, 0.41515695428675126`, 24.5400335368879`}, {"CURE", 365, 1.8431204242866426`, 174.0041846758651`},
{"CURE", 365, 0.671036137661967`, 43.990810663126865`}, {"TBUR", 63.1359695183881`, 229.17825782251404`, 1463.6417700115007`},
{"TBUR", 37.65993923647646`, 229.4361219537363`, 1159.2696992974531`}, {"CURE", 365, 0.08030828616382658`, 22.04627815503393`},
{"CURE", 365, 1.183143560681389`, 210.87505255425773`}, {"TBUR", 74.25994832568341`, 229.57142079220188`, 1552.2442616914448`},
{"CURE", 365, 0.2374528136317283`, 14.369098937004306`}, {"CURE", 365, 0.5788789927910621`, 34.076301231477366`},
{"CURE", 365, 1.1003214216659416`, 65.086184035105`}, {"TBUR", 40.74589050168045`, 229.53184792243832`, 757.3079520777654`},
{"CURE", 365, 0.1994917214076044`, 15.667996301513222`}, {"TBUR", 16.06231462578092`, 229.30500008472518`, 729.3290714213723`},
{"CURE", 365, 2.635390189676918`, 197.77952735538886`}, {"CURE", 365, 1.5911751709313897`, 73.27611403500809`},
{"CURE", 365, 0.33180224705930106`, 72.49257179482004`}, {"CURE", 365, 1.1312751651294197`, 66.18663327916096`},
{"CURE", 365, 2.3720121837794554`, 221.49053177253631`}, {"CURE", 365, 0.3646816797473542`, 17.788546102691203`},
{"CURE", 365, 6.094913505301616`, 175.63979569491556`}, {"TBUR", 23.03394675334575`, 229.38342101621234`, 757.9904065634076`},
{"TBUR", 14.95883470550363`, 229.47536551783793`, 988.8708516171102`}, {"CURE", 365, 0.22245221418853936`, 12.385069618141154`},

{"TBUR", 28.587370093357972`, 229.7750639080896`, 1140.1986686806933`}, {"CURE", 365, 3.0085367220381944`, 248.84518897144034`},
{"CURE", 365, 0.9597183504059843`, 70.83909809471855`}, {"CURE", 365, 3.266350838355652`, 319.27595078875`},
{"CURE", 365, 0.41947288593072274`, 53.44298837662551`}, {"CURE", 365, 2.620770839061804`, 213.19667577954672`},
{"CURE", 365, 2.376274273554538`, 76.30368818233096`}, {"CURE", 365, 0.27381148690260587`, 77.29632101411612`},
{"TBUR", 71.60443912866225`, 229.6685268759301`, 847.2775442976703`}, {"CURE", 365, 0.0378287288233243`, 12.797542923637641`},
{"CURE", 365, 142.20289084778477`, 690.5785625584157`}, {"TBUR", 61.38525724390752`, 229.36213956979034`, 864.4629492257769`},
{"CURE", 365, 1.1596349121712144`, 35.692866499319365`}, {"CURE", 365, 0.40888656808587154`, 129.5942398407158`},
{"TBUR", 22.882677527228683`, 229.7118470105677`, 1096.5347310116417`},
{"CURE", 365, 7.18112492059235`, 263.1288529161455`}, {"CURE", 365, 1.6590845598576955`, 190.69876331482953`},
{"CURE", 365, 7.055539957558642`, 410.5282210560528`}, {"TBUR", 98.80684544264922`, 229.7775983979605`, 956.1577443072672`},
{"TBUR", 17.487898482646244`, 229.2875929000666`, 1589.9550222095693`}, {"CURE", 365, 1.390338322711289`, 49.31219887500403`},
{"TBUR", 29.518875765541004`, 229.36465690021504`, 761.5749362895364`}, {"CURE", 365, 1.2043820534009504`, 60.479933974279405`},
{"CURE", 365, 4.818096267421334`, 120.96819795846083`}, {"CURE", 365, 0.19339326657619954`, 8.878604514853837`},
{"CURE", 365, 1.0314639593868615`, 59.38247479780622`}, {"CURE", 365, 0.5063481674211577`, 22.406598106807856`},
{"CURE", 365, 0.4214559964247951`, 25.539337645686967`}, {"CURE", 365, 8.737204911224454`, 236.72432456773134`},
{"CURE", 365, 0.8081674688255254`, 29.839910806677022`}, {"TBUR", 38.578544558598615`, 229.6809627749718`, 1430.1914189991198`},
{"CURE", 365, 10.615174660120191`, 264.19332439351314`}, {"CURE", 365, 1.2056949449678562`, 252.31963402104324`},
{"CURE", 365, 3.121508680576783`, 118.10607790731788`}, {"CURE", 365, 0.061561085909169665`, 32.07113859420104`},
{"CURE", 365, 15.799792754599627`, 97.1655931120319`}, {"CURE", 365, 0.23117138679091084`, 40.9439841763354`},
{"TBUR", 23.756691150419833`, 229.73306981672386`, 664.177241199895`}, {"CURE", 365, 2.0083255807491955`, 409.4037110591186`},
{"CURE", 365, 3.780225048858378`, 123.78968123405082`}, {"TBUR", 39.88986317915796`, 229.66098719994008`, 625.2365723409149`},
{"CURE", 365, 3.2661765112238483`, 349.86391175800225`}, {"CURE", 365, 0.16237833171097352`, 45.94356277409684`},
{"CURE", 365, 2.3552315183114563`, 266.6213014706556`}, {"TBUR", 25.937665736882423`, 229.4685875722202`, 1074.6944342161478`},
{"CURE", 365, 0.1255731484361588`, 83.06640397008073`}, {"CURE", 365, 0.3130734293130817`, 37.0792931669654`},
{"TBUR", 34.6796767839432`, 229.67342021946362`, 1470.281346471686`}, {"CURE", 365, 0.49358475929185935`, 32.75557550909353`},
{"TBUR", 46.29197482050346`, 229.6696206056771`, 1374.8043421695108`}, {"CURE", 365, 0.135964180155496`, 11.495327241152369`},
{"CURE", 365, 2.8616914565468408`, 144.63313857281187`}, {"CURE", 365, 2.1533881112582516`, 44.28822221974125`},
{"CURE", 365, 0.9716653335871913`, 216.25142942264063`}, {"CURE", 365, 0.5369281420278708`, 35.31669370298456`},
{"CURE", 365, 0.38932464444647846`, 29.481163232720426`}, {"CURE", 365, 1.1432412044522602`, 118.35399926446277`},
{"TBUR", 50.100565888762276`, 229.76536028482107`, 953.6286740554009`}, {"CURE", 365, 3.024125247383397`, 115.81751338894503`},
{"CURE", 365, 3.485885890255409`, 57.519817201362685`}, {"CURE", 365, 24.98653835435324`, 526.6949578020378`},

{"TBUR", 23.323438974711113`, 229.48888409919854`, 1463.4769640470809`}, {"CURE", 365, 0.13038857377210966`, 87.12307007696452`},
{"CURE", 365, 2.7083797609934006`, 130.96824514628835`}, {"CURE", 365, 2.5571788118473417`, 60.66151962919879`},
{"CURE", 365, 0.38018534855155245`, 35.62909930592542`}, {"TBUR", 140.3735431836652`, 229.79600736999555`, 495.9677706266703 6`},
{"CURE", 365, 0.18880623427766696`, 23.173048718064653`}, {"CURE", 365, 0.7398923613956027`, 62.00679134500789`},
{"TBUR", 21.924288960484578`, 229.5501575349354`, 1132.5798993361623`}, {"CURE", 365, 8.17169111498529`, 438.66766446242684`},
{"CURE", 365, 0.8996312900144963`, 31.11205061415607`}, {"TBUR", 39.60171931840672`, 229.66718703352961`, 507.22322903181384`},
{"TBUR", 34.78984754076312`, 229.26690033330456`, 884.2024082886544`}, {"CURE", 365, 0.29724702094261674`, 18.109970700671383`},
{"CURE", 365, 0.6067697024544693`, 44.82516759093145`}, {"CURE", 365, 0.64148271348902`, 88.44107073243474`},
{"CURE", 365, 0.17750317843669225`, 36.277494860568986`}, {"CURE", 365, 0.3343249036680489`, 69.96030730093918`},
{"TBUR", 56.18832556883875`, 229.6941826274551`, 1336.6964694073324`}, {"CURE", 365, 0.1866453518736218`, 36.867053017237616`},
{"CURE", 365, 36.93162877141965`, 565.0490521190959`}, {"TBUR", 39.123620846590455`, 229.6763335890706`, 504.9702964330975`},
{"CURE", 365, 0.7033039966340572`, 99.62891237152546`}, {"CURE", 365, 4.909486143029813`, 45.11858388311339`},
{"CURE", 365, 0.8867019690623786`, 28.410973374211913`}, {"CURE", 365, 0.9902256503101814`, 59.09762009378818`},
{"CURE", 365, 0.5725665932738656`, 20.16665715929269`}, {"CURE", 365, 0.08227694310602642`, 19.378495053875973`},
{"TBUR", 36.41338155709064`, 229.64146668744846`, 672.1874247099224`}, {"CURE", 365, 6.0895913399905774`, 66.8452181780275`},
{"CURE", 365, 0.062469346279521866`, 12.574500461235536`}, {"CURE", 365, 0.5572967835153517`, 36.54087468334051`},
{"TBUR", 55.55140324418147`, 229.76629120858`, 893.9969670145134`}, {"CURE", 365, 0.1285120477652827 7`, 8.912436002964647`},
{"CURE", 365, 0.1479855302715676`, 23.843798087871807`}, {"CURE", 365, 0.14326460350058698`, 28.598308912718043`},
{"CURE", 365, 3.895815130756506`, 51.21542363533518`}, {"CURE", 365, 3.2229661245081394`, 120.53522953188383`},
{"CURE", 365, 1.2926271037029449`, 99.37028878195147`}, {"TBUR", 65.96268953162053`, 229.71865999510996`, 787.2000721881263`},
{"TBUR", 46.729345467942565`, 229.75263978333953`, 691.5670281962344`}, {"CURE", 365, 4.292217836566346`, 274.8752894118963`},
{"CURE", 365, 0.2757045031743216`, 29.282035507176182`}, {"CURE", 365, 3.128140847813005`, 49.7270982354219`},
{"TBUR", 21.247180046470312`, 229.40714195232826`, 1649.6594893304932`}, {"TBUR", 15.98572374533345`,
220.37095368762866`, 1521.025804218493`}, {"TBUR", 81.01377111656392`, 229.36915019238924`, 1028.7994796351384`},
{"CURE", 365, 1.0641981432072558`, 81.83311507062297`}, {"CURE", 365, 1.741465998730943`, 352.96252821400645`},
{"CURE", 365, 0.17647671909693777`, 78.11524660589825`}, {"TBUR", 79.21135729948293`, 229.6785058710371`, 578.6894069219888`},
{"CURE", 365, 3.5616490185791103`, 58.58999343539531`}, {"CURE", 365, 1.0176495406572945`, 74.18334159965205`},
{"CURE", 365, 2.026678266263203`, 211.35460299951365`}, {"TBUR", 20.071453791767564`, 229.41774443420337`, 1216.3968816556433`},
{"TBUR", 14.55564414719855`, 228.99223602168215`, 939.5114652734056`}, {"CURE", 365, 39.91766002669792`, 483.2296274426118`},
{"CURE", 365, 1.5606661539057822`, 23.214848249464037`}, {"TBUR", 54.12724505581692`, 229.73022777345662`, 961.6287763495199`},
{"CURE", 365, 1.3890729259351666`, 158.84570566739333`}, {"CURE", 365, 1.713904283429257`, 72.73262454599111`},

{"CURE", 365, 0.08020111848514148`, 44.14962736325824`}, {"TBUR", 104.60752281953685`, 229.65234529890063`, 1198.4426744360674`},
{"CURE", 365, 2.3160667261972527`, 46.094270029701775`}, {"CURE", 365, 0.15897341414736704`, 9.844797188157427`},
{"CURE", 365, 1.681740301077112`, 111.14021556151452`}, {"CURE", 365, 0.5641551833477436`, 10.295332826390252`},
{"TBUR", 34.43018393964749`, 229.69807401013236`, 1086.6297172245227`},
{"TBUR", 79.35307490712134`, 229.7318180217398`, 1095.0418344296909`}, {"CURE", 365, 0.39154527958089713`, 27.67917724710205`},
{"TBUR", 16.578299488552958`, 229.08711688490254`, 1196.8735112641984`},
{"TBUR", 17.389574407140394`, 229.68760686872415`, 1357.4830599835043`},
{"CURE", 365, 1.8911716837603787`, 53.651810910693044`}, {"CURE", 365, 0.6518019573703154`, 44.44059532440344`},
{"CURE", 365, 8.389163319709521`, 506.60018179466897`}, {"TBUR", 13.10416928346885`, 229.62119094979968`, 1433.0711402391137`},
{"TBUR", 25.766397769352128`, 228.92641450490405`, 1587.1489376770826`},
{"CURE", 365, 0.05225526031963367`, 27.705620078262985`}, {"CURE", 365, 4.358672499734148`, 225.90878609607012`},
{"CURE", 365, 0.2806864949963911`, 12.129724231389865`}, {"CURE", 365, 0.6602952491028158`, 58.28159955352719`},
{"CURE", 365, 0.5033638558785376`, 24.410511888256384`}, {"CURE", 365, 1.6011975516404862`, 250.92290184656045`},
{"CURE", 365, 0.22739653033635823`, 53.84320475040696`}, {"CURE", 365, 0.7376477028957817`, 40.189794722583294`},
{"TBUR", 115.36297421991864`, 229.65072663686084`, 762.0873250459371`}, {"CURE", 365, 1.9788972785617214`, 124.90421427491428`},
{"CURE", 365, 5.943391061823318`, 56.229138418918005`}, {"CURE", 365, 0.5198612558226128`, 47.51682920233118`},
{"TBUR", 27.766759445240517`, 229.52828060766583`, 1385.9164719832572`}, {"CURE", 365, 0.6422425146146977`, 168.5881856786367`},
{"CURE", 365, 0.5960692525228082`, 128.18533210972154`}, {"CURE", 365, 112.67420374295799`, 376.31325384493806`},
{"CURE", 365, 0.15904394876156205`, 87.11030392899205`}, {"CURE", 365, 6.234315205834106`, 221.65309943091134`},
{"CURE", 365, 0.4517071607844869`, 16.94504663681903`}, {"CURE", 365, 0.45084869549193035`, 21.447343003691433`},
{"CURE", 365, 0.3176163054573655`, 18.71003682727064`}, {"CURE", 365, 2.604363644367468`, 421.4509143809499`},
{"CURE", 365, 3.042743548152637`, 80.96753494757024`}, {"TBUR", 15.329643691474939`, 227.62830609172283`, 1887.790958504928`},
{"TBUR", 51.75791948403445`, 229.75313451060407`, 453.9049930321543`}, {"CURE", 365, 2.550614028374007`, 66.22683983265756`},
{"CURE", 365, 0.09750783605847067`, 16.67585048104511`}, {"TBUR", 85.30456620952975`, 229.77620518354374`, 1139.436879408987`},
{"CURE", 365, 1.0756663523765602`, 41.85701688252712`}, {"CURE", 365, 0.6051944690237497`, 21.24251395818681`},
{"CURE", 365, 0.48796989464544377`, 19.703718616888157`}, {"CURE", 365, 7.300913294302306`, 714.1269870083379`},
{"CURE", 365, 5.139949313248661`, 132.59678351784092`}, {"CURE", 365, 0.23280519877310427`, 50.556416138376036`},
{"CURE", 365, 0.3758500426222961`, 16.830480479043942`}, {"CURE", 365, 0.40041312975853915`, 17.71039045634086`},
{"CURE", 365, 0.3898273447620042`, 40.5988515591465`}, {"CURE", 365, 11.748819202829385`, 129.67949554306355`},
{"TBUR", 23.211815095101713`, 229.5465131649704`, 1051.2113375412794`},
{"CURE", 365, 0.6275048087793008`, 34.0894451303774`}, {"CURE", 365, 3.906590926600323 6`, 196.03246280819317`},

{"CURE", 365, 5.045334973544554`, 66.50778633612654`}, {"CURE", 365, 2.591551225393361`, 62.17294251396242`},
{"CURE", 365, 6.289071514275809`, 278.02628043396857`}, {"TBUR", 78.68549703503044`, 229.7234718199945`, 573.0237635813263`},
{"CURE", 365, 1.2781175763138206`, 73.3488585418234`}, {"CURE", 365, 3.124746238501188`, 209.17066430493446`},
{"CURE", 365, 1.0160694263006969`, 55.04966081420012`}, {"CURE", 365, 0.3587161521087868`, 14.06501937268971`},
{"CURE", 365, 0.33516622156154113`, 7.460516408830865`}, {"CURE", 365, 0.3497996397063357`, 35.399107278446934`},
{"CURE", 365, 0.5951040840427722`, 67.13639737742311`}, {"CURE", 365, 0.19585933907076628`, 10.4270655757386`},
{"CURE", 365, 1.4024520139226035`, 70.65089651623006`}, {"TBUR", 75.5376014898205`, 229.5993652144614`, 1119.6215920699274`},
{"CURE", 365, 0.4942552634530668`, 21.20818395856511`}, {"TBUR", 133.122350179586`, 229.7488435231202`, 1202.0561328687168`},
{"TBUR", 25.652043358898315`, 229.16749114817839`, 1411.974223538449`},
{"TBUR", 54.511719877940564`, 229.18458407411765`, 1313.2984055970846`},
{"CURE", 365, 1.3358482972354617`, 205.51582101668745`}, {"CURE", 365, 1.82117387572635`, 135.61317812994162`},
{"TBUR", 57.12253717633724`, 229.41200777109322`, 776.5398438353288`}, {"CURE", 365, 0.12084385954952048`, 25.344075035826183`},
{"CURE", 365, 0.09060493179434309`, 37.60854157794418`}, {"TBUR", 22.629056894096447`, 228.3486827317091`, 1614.321759374518`},
{"TBUR", 29.574803086916276`, 229.02510812609344`, 1385.366826559368`},
{"CURE", 365, 15.863009363009013`, 428.9946074553358`}, {"TBUR", 27.411421201025654`, 229.33063896153865`, 698.040483750795`},
{"TBUR", 44.798361321498916`, 229.63476721480924`, 784.524605148717`}, {"CURE", 365, 5.369358253084507`, 141.97986273500328`},
{"TBUR", 77.12738136584362`, 229.63550708682328`, 583.7891872305619`}, {"CURE", 365, 1.747479043378616`, 30.932628378896553`},
{"CURE", 365, 7.171287688873559`, 86.45298442582188`}, {"TBUR", 28.251039502894255`, 229.75031444960197`, 994.8371781800923`},
{"TBUR", 99.53055823094599`, 229.51053291885512`, 1587.6188100011727`}, {"CURE", 365, 10.933893794953143`, 85.52864781534132`},
{"CURE", 365, 2.009634212674844`, 139.57462762966327`}, {"TBUR", 73.19107171376524`, 229.18554037644031`, 1066.8074168657`},
{"TBUR", 52.337147042977165`, 229.22541071886906`, 1226.827871924953`}, {"CURE", 365, 0.10794118899548574`, 60.8512148326348`},
{"CURE", 365, 0.14741683374703013`, 13.421659084637161`}, {"CURE", 365, 0.3664243843972965`, 30.07886873368381`},
{"TBUR", 42.46646376870754`, 229.66828095315614`, 1066.5961496453538`}, {"CURE", 365, 0.27266832943200664`, 17.117563023577276`},
{"CURE", 365, 0.7242646810840956`, 26.02253796629614`}, {"CURE", 365, 0.7379457551799498`, 76.31551811028837`},
{"TBUR", 101.89099086013303`, 229.61237267776048`, 1140.8675354405455`},
{"CURE", 365, 1.5122405594580686`, 32.257068592503515`}, {"CURE", 365, 3.625492933975846`, 440.2776520301337`},
{"CURE", 365, 0.6317319887631629`, 85.81482537568496`}, {"TBUR", 80.81711028841647`, 229.7088108224421`, 983.8433930748388`},
{"CURE", 365, 1.9596923238694595`, 129.19321119061618`}, {"TBUR", 50.87049148534047`, 229.42808319950203`, 823.275922474108`},
{"CURE", 365, 0.0723306834672783`, 7.555052376626303`}, {"TBUR", 37.22536000840672`, 229.69712719504093`, 968.8057254490104`},
{"CURE", 365, 0.712936743772483`, 113.25140993629049`}, {"TBUR", 24.175037489839244`, 229.09835245433274`, 1062.100803977565`},
{"CURE", 365, 3.4957880505390118`, 121.3614775019012`}, {"TBUR", 16.677251373957063`, 229.39197629883483`, 847.9402746652303`},

{"CURE", 365, 2.0775145674005753`, 101.88060056226139`}, {"TBUR", 18.945704088718088`, 229.56961700703894`, 1546.267415598819`},
{"TBUR", 66.09377331414058`, 229.56487568497354`, 806.4161846512242`}, {"CURE", 365, 2.2588289706094717`, 115.96090694427696`},
{"CURE", 365, 0.9109620814854241`, 63.47836768964417`}, {"CURE", 365, 0.3336630394190144`, 36.31963310466172`},
{"CURE", 365, 1.3312829704865028`, 301.24954742640995`}, {"CURE", 365, 0.06989179909891358`, 4.398227265330866`},
{"CURE", 365, 4.875402030398364`, 259.6728516568361`}, {"TBUR", 54.75484833471953`, 229.79342088044382`, 858.7490913742469`},
{"TBUR", 79.46205064702637`, 229.73510280957277`, 1064.896368980408`}, {"CURE", 365, 0.16101401114422548`, 43.89198318582777`},
{"CURE", 365, 0.5051171181513945`, 43.98085002244357`}, {"CURE", 365, 0.21059173660449612`, 6.276834962574245`},
{"CURE", 365, 0.04812953046186834`, 11.416930342565022`}, {"CURE", 365, 2.079290700120494`, 100.73531936563394`},
{"CURE", 365, 0.9292098869474782`, 81.685909967108`}, {"TBUR", 135.53656256239682`, 229.74647064763226`, 514.757842139194`},
{"TBUR", 40.41503599044138`, 229.68046949444576`, 476.9901008354991`}, {"CURE", 365, 1.134532729225811`, 185.63211326559178`},
{"CURE", 365, 0.2630768604837308`, 55.604678497036275`}, {"CURE", 365, 0.8334404866858138`, 17.220270042217027`},
{"TBUR", 43.037759201538066`, 229.65275228328912`, 1373.8987859926726`},
{"TBUR", 23.764812817428226`, 228.90046188217227`, 1851.28293087608`}, {"CURE", 365, 0.15957057465633645`, 83.24764509761044`},
{"CURE", 365, 5.595498778489359`, 225.08813038541533`}, {"CURE", 365, 0.2917098746900783`, 9.173614127835867`},
{"TBUR", 21.6072030851409`, 229.58124530936072`, 802.5515724483291`}, {"CURE", 365, 0.1577852635294115`, 35.47821028310489`},
{"CURE", 365, 0.3431832924861169`, 39.90359241212177`}, {"CURE", 365, 32.88722605538609`, 381.0963079104884`},
{"CURE", 365, 4.907255753932145`, 209.97347289355562`}, {"TBUR", 79.18987977635403`, 229.20912036509569`, 827.2540579344097`},
{"CURE", 365, 0.06422175172458001`, 10.56789961360098`}, {"TBUR", 38.53612679831402`, 229.70157724078126`, 1197.5607872009277`},
{"TBUR", 61.149379785158246`, 229.70278417641316`, 1295.8947204288684`},
{"TBUR", 70.21583635406994`, 229.7293793345705`, 1244.0912966979176`},
{"CURE", 365, 1.1413502957515553`, 63.91908492351077`}, {"CURE", 365, 0.43452170132563595`, 18.341381518850962`},
{"TBUR", 92.4041515769388`, 229.60114145700274`, 519.7831091127898`}, {"CURE", 365, 0.8114340880068365`, 26.55056292185715`},
{"CURE", 365, 1.673758793547136`, 119.87782618894525`}, {"TBUR", 56.6187794220238`, 229.67030048442751`, 717.8044883828414`},
{"TBUR", 88.30181376471224`, 229.57404089139223`, 652.302007173237`}, {"CURE", 365, 9.49823120449729`, 156.31933886744653`},
{"CURE", 365, 0.19528819929204747`, 12.146273684879205`}, {"CURE", 365, 0.8314182047080083`, 40.51158551211618`},
{"CURE", 365, 0.5820200403000813`, 22.694361431864117`}, {"CURE", 365, 3.712496102703009`, 116.93424507147336`},
{"TBUR", 37.73482924120963`, 229.67728747502102`, 1204.5790667282909`},
{"TBUR", 47.39830368650364`, 229.69144949235664`, 1017.5225438569721`}, {"CURE", 365, 0.26357369536528336`, 8.633172360290432`},
{"TBUR", 44.497158579458`, 229.70269955696534`, 1095.6098890304565`}, {"CURE", 365, 3.9945061352096762`, 47.803206574735434`},
{"CURE", 365, 0.6914291939534357`, 40.16389682235493`}, {"TBUR", 27.97108355340901`, 228.99843937035246`, 1480.75178497716`},
{"CURE", 365, 0.96373212126103`, 53.546662116613234`}, {"CURE", 365, 0.33178039882651295`, 14.120281980657122`},

{"CURE", 365, 1.2112652759992841`, 115.99052765701884`}, {"TBUR", 46.117226764370734`, 229.70587534857657`, 1250.3127218547622`},
{"CURE", 365, 1.06922417159919`, 149.26583081587685`}, {"TBUR", 26.6303908752442`, 229.37082173310625`, 644.4215645288168`},
{"CURE", 365, 2.942231318772441`, 72.93725080850271`}, {"CURE", 365, 0.1166304240899345`, 4.617740366262071`},
{"TBUR", 103.22731851988488`, 229.58527519372154`, 1005.8844375610349`}, {"CURE", 365, 0.34223134384915016`, 9.62782409203975`},
{"CURE", 365, 4.676440387296385`, 124.7247745744432`}, {"TBUR", 84.3486675828888`, 229.73420899141362`, 472.4588134414272`},
{"TBUR", 30.942038160585216`, 229.76629415778044`, 1207.577132676777`}, {"CURE", 365, 1.422276194529762`, 166.7488752746063`},
{"TBUR", 45.089643766643945`, 229.76098624158425`, 1145.6854800174115`},
{"CURE", 365, 0.3465892653461862`, 63.96007427776753`}, {"TBUR", 44.67729210791056`, 229.69484143455836`, 1206.3586204930357`},
{"TBUR", 45.72574454279542`, 229.1351937416935`, 1028.4321727250754`},
{"CURE", 365, 0.7176864528473896`, 43.5852563241181`}, {"TBUR", 46.57236015810487`, 229.7021990825741`, 1340.60798117989`},
{"CURE", 365, 0.06022614606987295`, 12.254088713676737`}, {"TBUR", 36.2446635868832`, 229.54139618384656`, 1038.32579035508`},
{"CURE", 365, 3.841054578057157`, 78.09621846015094`}, {"TBUR", 25.01518437342969`, 229.34502294349173`, 1348.700129358392`},
{"TBUR", 106.8342373781494`, 229.7410758755012`, 600.8045264294275`}, {"CURE", 365, 0.08718772026381039`, 8.714451330586934`},
{"TBUR", 36.762362683830126`, 229.74688699057364`, 1208.1723627291228`},
{"TBUR", 69.35766069150343`, 229.76593326064616`, 1235.5652179216086`},
{"TBUR", 14.358925624297331`, 225.81104774496336`, 1055.6979169343647`},
{"CURE", 365, 0.08988066538624266`, 19.25406917148173`}, {"TBUR", 109.51780244685523`, 229.77085741878525`, 772.328963781658`},
{"TBUR", 103.0476034799822`, 229.7363019655026`, 805.9511337782207`}, {"CURE", 365, 0.5414743452425025`, 42.945417806517696`},
{"CURE", 365, 18.393103966314673`, 722.8602500158206`}, {"CURE", 365, 1.1895749424080537`, 34.45002044882974`},
{"CURE", 365, 3.3962260131071744`, 146.3310198531559`}, {"CURE", 365, 5.118674926729159`, 73.64889771111066`},
{"CURE", 365, 0.9823401867288387`, 29.746710706385628`}, {"TBUR", 34.198363728380514`, 229.5194186559324`, 1269.8518715406722`},
{"TBUR", 86.3618586168749`, 229.74388111940857`, 993.8237968244051`},
{"TBUR", 36.9159063857544`, 227.55386706505516`, 1700.6809613579208`},
{"TBUR", 22.368176526590993`, 227.81377627871328`, 1654.1408089587567`},
{"TBUR", 54.13135780890691`, 229.6446344934964`, 671.4370315953305`}, {"CURE", 365, 1.3690860925550197`, 142.3010818351615`},
{"TBUR", 53.72589376767883`, 229.73556354377644`, 1139.0178514781755`}, {"CURE", 365, 0.6368191700821981`, 47.59465017826948`},
{"TBUR", 219.71171787308128`, 229.74170546175662`, 637.9665181511327`}, {"CURE", 365, 0.7088616049750349`, 54.22697886710936`},
{"CURE", 365, 0.023234943280919518`, 8.189762518924251`}, {"CURE", 365, 27.88887876397486`, 479.69356606862175`},
{"TBUR", 32.357942888241176`, 229.5957345243698`, 854.454931710896`}, {"CURE", 365, 0.4354225034709798`, 111.32191819156917`},
{"CURE", 365, 0.2834913616790067`, 59.08114964720767`}, {"CURE", 365, 0.8835344051465868`, 224.6830792990371`},
{"CURE", 365, 0.15531280657707716`, 31.22253663357349`}, {"CURE", 365, 7.386412253498604`, 178.51275326343065`},

{"TBUR", 22.260791349627453`, 229.69048391866409`, 950.5122633984219`}, {"CURE", 365, 4.452564675556549`, 271.1390670424217`},
{"TBUR", 92.64547629828544`, 229.33869352752225`, 964.110508717989`}, {"CURE", 365, 0.22108483430144463`, 21.75585892499878`},
{"CURE", 365, 0.061255781105126035`, 10.413531264014788`}, {"CURE", 365, 2.2703841383457335`, 157.53819832980759`},
{"CURE", 365, 12.262560734644998`, 220.22603313223786`}, {"TBUR", 16.419759215984037`, 229.49428037386488`, 911.5356528131589`},
{"TBUR", 91.2022695145465`, 229.71447856295174`, 950.3632367284679`},
{"TBUR", 87.48758188406877`, 229.69909092419877`, 610.7384689230668`}, {"CURE", 365, 1.442409126994917`, 17.902334753981286`},
{"CURE", 365, 8.223227719421915`, 78.61322710612127`}, {"CURE", 365, 0.1936723502337337`, 37.633792164972064`},
{"CURE", 365, 0.1283107965676 2148`, 47.57356524757726`}, {"TBUR", 31.150146574555023`, 229.22485805736662`, 1406.0254295248735`},
{"TBUR", 80.85551920112957`, 229.76763566330212`, 666.9907770658793`},
{"TBUR", 152.67564155205804`, 229.67722456092773`, 1144.7720341933405`},
{"TBUR", 66.0878429661093`, 229.72391145526393`, 482.4164248767653`}, {"CURE", 365, 2.4588563650788946`, 89.32229032996814`},
{"TBUR", 95.02166285323783`, 229.73439488471655`, 1061.2373259193018`},
{"CURE", 365, 0.3221869144817853`, 6.877274339834974`}, {"CURE", 365, 2.927355512562018`, 162.8931789074825`},
{"CURE", 365, 0.08565132785496955`, 11.102682605825398`}, {"TBUR", 96.47002992247288`, 229.69996859826867`, 872.136069473467`},
{"TBUR", 36.21221012978158`, 229.76984531256926`, 853.6694734974914`},
{"TBUR", 16.376135513264767`, 229.16201737129157`, 1193.4809629540698`},
{"TBUR", 15.409648258704054`, 228.77204791032815`, 1400.4316696367769`},
{"TBUR", 47.02705398523487`, 229.4885769060168`, 1263.6865505419278`}, {"CURE", 365, 25.84460121793994`, 242.96942284680676`},
{"CURE", 365, 0.4163109559663061 3`, 40.82185611985094`}, {"TBUR", 37.995682188459135`, 229.65705565869135`, 827.1650802461725`},
{"CURE", 365, 1.735231951290143`, 49.97505256795043`}, {"CURE", 365, 0.039408030181972475`, 4.559445327719285`},
{"CURE", 365, 4.924457749120802`, 197.99350693793212`}, {"CURE", 365, 5.676657697655015`, 46.8986680378026`},
{"CURE", 365, 7.752544249995413`, 138.95588683659957`}, {"CURE", 365, 1.6063455017141957`, 89.02187891718323`},
{"TBUR", 116.32561728131724`, 229.7762969404745`, 673.6101910942479`}, {"CURE", 365, 0.6491390050550443`, 100.37525602220252`},
{"CURE", 365, 1.3315949319788896`, 249.722499108796`}, {"TBUR", 21.481545436708682`, 228.3555296513211`, 1167.4512634779278`},
{"CURE", 365, 0.7046956223775455`, 33.49585608431488`}, {"TBUR", 71.93957483223865`, 229.741908307037`, 456.0465939421404`},
{"CURE", 365, 1.1502295464259271`, 22.86869612950168`}, {"TBUR", 38.33089412041798`, 229.60712640127988`, 763.1221837746468`},
{"CURE", 365, 1.5481291843060303`, 101.84697585982212`}, {"CURE", 365, 0.034335755922439135`, 8.18869429430971`},
{"CURE", 365, 1.4095966293694007`, 79.10982500237624`}, {"CURE", 365, 0.45477508898619834`, 36.87908382502167`},
{"CURE", 365, 0.08237997319160201`, 25.27172637607554`}, {"TBUR", 15.801127337111659`, 229.31908005095752`, 1310.3266384727078`},
{"CURE", 365, 0.03420183188744115`, 5.730846365570302`}, {"TBUR", 46.737093090067`, 229.24163399313343`, 981.0811399158677`},
{"TBUR", 19.393023534904145`, 229.75534296145563`, 1159.2732058073345`},

{"CURE", 365, 9.742539769945935`, 660.20422207217`}, {"CURE", 365, 0.17142455097750575`, 73.69166214221723`},
{"CURE", 365, 3.8169324922100887`, 237.60102584632173`}, {"CURE", 365, 0.8462877800316432`, 464.35735177057927`},
{"TBUR", 19.46694616689004`, 229.38396151314623`, 985.3152505975021`}, {"CURE", 365, 7.576901232608726`, 397.29960722438824`},
{"CURE", 365, 0.3885598319720078`, 21.797046725780056`}, {"CURE", 365, 0.29331697875196655`, 39.18453267814502`},
{"CURE", 365, 2.9990519714124595`, 284.54681503756393`}, {"TBUR", 22.56278364231355`, 229.61158132049695`, 1155.2591286207503`},
{"TBUR", 13.931301602506629`, 227.56897240346618`, 1256.2010285728857`},
{"CURE", 365, 0.386198849915191`, 24.63679993154487`}, {"TBUR", 63.99734662698252`, 229.7418867916918`, 862.2854065895082`},
{"CURE", 365, 27.535014317396982`, 550.0753814673544`}, {"TBUR", 98.63876873687674`, 229.33974536500395`, 1312.548891099628`},
{"CURE", 365, 14.361130587212566`, 722.8671535094875`}, {"CURE", 365, 0.49564431235663`, 26.294516119027758`},
{"CURE", 365, 0.9419520299648013`, 167.09333309914228`}, {"CURE", 365, 0.028703811395395684`, 4.639785500009402`},
{"TBUR", 175.0697621483362`, 229.77449964361514`, 935.1782956876252`}, {"CURE", 365, 0.07031689607546604`, 23.07307758889528`},
{"CURE", 365, 1.733357105096643`, 74.44268951948493`}, {"CURE", 365, 0.31704832621076956`, 50.111292162547024`},
{"CURE", 365, 0.1355210614763555`, 16.82613885919262`}, {"TBUR", 44.45389860246566`, 229.48003438157747`, 1170.7473827663223`},
{"CURE", 365, 1.2206269530014073`, 96.86205052789629`}, {"TBUR", 36.40156692235411`, 229.65086856738614`, 1446.4519819460418`},
{"TBUR", 68.50294551457343`, 229.66700239980898`, 733.7433290481569`}, {"TBUR", 75.7342188946373`,
229.7654079851591`, 693.9773154258728`}, {"TBUR", 68.21508526091294`, 229.6934630383136`, 1297.9758340434025`},
{"CURE", 365, 0.3229082930956889 7`, 16.292588346358702`}, {"TBUR", 17.38682280942599`, 228.7400376970469`, 1596.222031994870 1`},
{"TBUR", 34.21834035728973`, 229.6256258726516`, 1136.7824514288652`}, {"CURE", 365, 0.44737074829042406`, 21.010812688456628`},
{"CURE", 365, 0.041658332338816786`, 16.19112809876707`}, {"TBUR", 31.192071200356136`, 229.37188765451216`, 1139.634997217279`},
{"CURE", 365, 0.580463268825095`, 117.58071704160419`}, {"TBUR", 77.41494605028566`, 229.66438328543083`, 626.4156362884922`},
{"TBUR", 52.72650848845317`, 229.5805203605224`, 783.7790334852119`}, {"CURE", 365, 3.90670365585123`, 284.44631814676586`},
{"TBUR", 80.917860850209`, 229.77788707471527`, 784.3142145558409`}, {"CURE", 365, 1.429362759329512`, 99.2982181027259 1`},
{"CURE", 365, 0.4200965540117255`, 34.02757489486844`}, {"CURE", 365, 1.0477948429761017`, 52.06580277125197`},
{"CURE", 365, 2.862600649705849`, 168.47080698039417`}, {"TBUR", 60.04810615543954`, 229.50889788242034`, 1210.4261719553094`},
{"TBUR", 145.64508321771854`, 229.60094578237664`, 941.168291192306`},
{"TBUR", 57.81834167033883`, 229.77591714933774`, 546.5084124866283`}, {"CURE", 365, 8.145327090111017`, 171.94822802196506`},
{"TBUR", 113.05752944028667`, 229.69609719007676`, 559.8292052118402`}, {"CURE", 365, 0.11824279124865042`, 17.756579497932545`},
{"CURE", 365, 0.6776784502765811`, 40.98514001459202`}, {"TBUR", 17.54051866596359`, 229.1831060474765`, 1355.394933349208`},
{"TBUR", 42.73078178866196`, 229.62723103362518`, 1101.3930004521424`}, {"CURE", 365, 6.62713669505142`, 129.3043269399074`},
{"CURE", 365, 0.3983871803240116`, 24.409557832031123`}, {"TBUR", 88.99815837102159`, 229.7668298615634`, 1072.9061141767`},
{"CURE", 365, 0.06258448782478906`, 22.104139453858703`}, {"CURE", 365, 0.4548092928707336`, 70.65448551705983`},

{"CURE", 365, 0.02683664758528678`, 13.506874227445119`}, {"CURE", 365, 0.24854907834608128`, 11.217673047911605`},
{"CURE", 365, 0.5523667090852342`, 72.96978828055805`}, {"TBUR", 66.39062962152059`, 229.79216443312714`, 1065.7861458627801`},
{"TBUR", 47.11723296659388`, 229.7431179006252`, 899.7818316911397`}, {"CURE", 365, 2.51399893408352`, 103.59504073272508`},
{"CURE", 365, 0.8805119898716877`, 146.91323493139075`}, {"TBUR", 85.97877305881495`, 229.7591168559533`, 863.7432380726461`},
{"CURE", 365, 18.596050574581135`, 186.54891924318528`}, {"CURE", 365, 3.912105111273413`, 108.94503592326468`},
{"TBUR", 79.30343919572088`, 229.7721054945368`, 898.4213058572064`}, {"CURE", 365, 1.1235789954057875`, 65.1451811920862`},
{"CURE", 365, 2.7886677556085115`, 67.69773094553045`}, {"CURE", 365, 3.236289995625369`, 79.24577367869783`},
{"CURE", 365, 0.12026061845990453`, 24.252194447302468`}, {"CURE", 365, 0.5473351525333022`, 151.3791401577295`},
{"TBUR", 67.58229900805385`, 229.73712451312068`, 952.3251290070383`}, {"CURE", 365, 0.8743651486988795`, 37.13125545310046`},
{"CURE", 365, 0.508619096232105`, 17.231080257618668`}, {"TBUR", 33.91033364916279`, 229.77087129873703`, 847.9030179977417`},
{"CURE", 365, 13.060666627787144`, 621.88083935863`}, {"CURE", 365, 0.1443514876963271`, 32.716401299450474`},
{"TBUR", 103.67526834698734`, 229.54409267064293`, 800.7224892315113`},
{"TBUR", 40.795817164015325`, 229.78083702420443`, 993.7966213728253`},
{"TBUR", 128.54397154564964`, 229.59861540331363`, 1134.3033489428071`},
{"CURE", 365, 0.3370313680276698`, 102.70304143498653`}, {"CURE", 365, 0.06123026928564246`, 13.988777939359332`},
{"CURE", 365, 0.13734435430602923`, 59.02820916909749`}, {"CURE", 365, 0.12232221475356893`, 7.056487506238071`},
{"CURE", 365, 0.1214528993590194`, 5.1069245711274736`}, {"CURE", 365, 6.597781603056228`, 336.8508593369566`},
{"CURE", 365, 0.5534095849199929`, 28.88081328142719`}, {"TBUR", 125.36889566944771`, 229.70163751527411`, 786.1411062039828`},
{"TBUR", 92.18394466936235`, 229.58191809403672`, 670.0458237999364`},
{"TBUR", 45.62633707954276`, 229.74264785014753`, 1200.5570998944734`}, {"CURE", 365, 0.4349117277031735`, 16.553610682996744`},
{"CURE", 365, 1.7549000405365631`, 151.45658100170104`}, {"TBUR", 24.029859659712557`, 229.64839429631385`, 585.8865184532968`},
{"TBUR", 82.20684281328796`, 229.64708565742444`, 1021.1351256621515`}, {"CURE", 365, 0.2207837360500139`, 24.264774654909758`},
{"TBUR", 109.85067249698618`, 229.78809049967742`, 1087.3310192007768`},
{"CURE", 365, 1.3632562207586216`, 102.68926358597489`}, {"CURE", 365, 0.7443583678006032`, 81.34687068013262`},
{"TBUR", 58.082585463801884`, 229.72422276427673`, 1279.3878251627868`}, {"CURE", 365, 2.5550804213748193`, 131.62383696088952`},
{"CURE", 365, 3.0645350381006145`, 207.4281713709939`}, {"CURE", 365, 3.1480238039537642`, 81.67174553076097`},
{"TBUR", 34.62341112463206`, 229.453893520153`, 1796.4954672361669`}, {"CURE", 365, 0.3155468245196187`, 66.47130802185936`},
{"CURE", 365, 1.7911725562222576`, 36.7788912924726`}, {"CURE", 365, 4.264616557083957`, 506.74681635231383`},
{"CURE", 365, 0.61267624744419`, 33.77988600419588`}, {"CURE", 365, 1.997273787987452`, 197.6016049027727`},
{"CURE", 365, 0.23411985417886627`, 41.105600547839465`}, {"CURE", 365, 0.7450058474151328`, 36.03850339774369`},
{"CURE", 365, 1.2814728273097193`, 60.39700495122651`}, {"TBUR", 21.731039101575412`, 229.30445792885558`, 900.773297360069`},

```
{"CURE", 365, 11.371517127308003`, 376.1272788062472`}, {"CURE", 365, 3.324790230743838`, 493.6114389847917`},
{"CURE", 365, 0.9658472125461566`, 19.611928152410538`}, {"TBUR", 74.91413648692027`, 229.60703278830374`, 642.1532909493698`},
{"TBUR", 172.06054532777702`, 229.32820368979748`, 756.4076556657492`},
{"CURE", 365, 0.1600977996822610 7`, 103.40142958900786`}, {"TBUR", 143.80820041511646`, 229.5227799360978`, 1148.3600704293503`},
{"TBUR", 32.28862163575961`, 229.6768291236565`, 1092.3146463695325`},
{"CURE", 365, 0.8001233057961691`, 149.1210026138588`}, {"CURE", 365, 2.121970972851333`, 159.91510996757873`},
{"CURE", 365, 1.6365323483382825`, 53.31904298717748`}, {"TBUR", 75.43833430505356`, 229.7256003846029`, 492.01023591192137`},
{"TBUR", 90.84541267233584`, 229.7637139868363`, 875.6044460597791`}, {"CURE", 365, 0.09343794756047939`, 30.007807570324818`},
{"TBUR", 75.7881820742075`, 229.67838854379022`, 807.8670031145999`}, {"CURE", 365, 0.6377511631265117`, 137.60939289048852`},
{"CURE", 365, 0.1597923945617538 1`, 14.982976937936655`}, {"TBUR", 91.89329043488713`, 229.78209831937846`, 657.2461861058287`},
{"TBUR", 39.43386732918001`, 229.75189757163943`, 1144.1873236706383`},
{"TBUR", 83.68959051614195`, 229.3286328715545`, 851.5857300005463`},
{"TBUR", 59.670018515839786`, 229.64430159712032`, 940.6011132190104`}, {"CURE", 365, 3.9317068233575583`, 244.03901924593742`},
{"CURE", 365, 0.4040204934843877`, 196.53807657965595`}, {"CURE", 365, 2.6799965146356888`, 392.32134105764766`},
{"CURE", 365, 1.164799692779469`, 36.037041798288875`}, {"TBUR", 128.36828923234611`, 229.68189243396657`, 1127.9697154697617`},
{"TBUR", 28.31286375910967`, 229.68939638734903`, 1339.8575880652977`}, {"CURE", 365, 2.8635722484419666`, 96.58843902477659`},
{"TBUR", 83.39015138723369`, 229.61260955988317`, 1435.857062339783`}, {"CURE", 365, 1.4879127721543597`, 60.70890244368361`},
{"CURE", 365, 11.223610778807043`, 238.37944125352558`}, {"TBUR", 65.66170669202165`, 229.7329087883162`, 1260.0888714037442`},
{"TBUR", 51.609710677480855`, 229.687534501833`, 660.997713365053`}, {"CURE", 365, 0.3693908761346261`, 14.944313629187178`},
{"CURE", 365, 2.1495403797579704`, 69.37832267467483`}, {"CURE", 365, 2.017848545345598`, 86.8371341618166`},
{"TBUR", 79.70638868850733`, 229.69161508359213`, 480.9656064133897`},
{"TBUR", 158.16224968257143`, 229.5140590061437`, 761.0077583162407`},
{"TBUR", 58.454932370440964`, 229.6851031896138`, 1062.1586613906054`},
{"CURE", 365, 0.17271712852422705`, 3.641128426600727`}, {"CURE", 365, 1.21395745964213`, 327.54334479981145`},
{"CURE", 365, 4.8233410466099675`, 115.8197443618551`}, {"CURE", 365, 0.29839650991244904`, 8.14021195882255`},
{"TBUR", 128.6297580592326`, 229.76984639556096`, 1209.8116560986166`}, {"CURE", 365, 9.009377589144927`, 198.619128451424`},
{"CURE", 365, 4.2704934922763895`, 81.27462692968034`}, {"TBUR", 169.38564326048`, 229.70474380312456`, 841.0521743172093`},
{"CURE", 365, 0.5308403872916156`, 51.19495465760549`}, {"CURE", 365, 0.23578965416118652`, 19.56868967462101`},
{"CURE", 365, 2.200016384093682`, 126.03467084829715`}, {"TBUR", 89.72758008360655`, 229.6664167095082`, 900.404237195065`},
{"CURE", 365, 0.5779651322100932`, 144.1729382896517`}, {"TBUR", 82.85030307316461`, 229.67483587795465`, 699.8025049661337`},
{"TBUR", 48.16367472551972`, 229.7172993737496`, 441.07423506285016`},
```

{"TBUR", 43.010850867102285`, 229.6830999259746`, 761.2703082436011`}, {"CURE", 365, 1.2027754520821208`, 48.842213612879426`},
{"CURE", 365, 0.8313598461322332`, 201.65962155574186`}, {"TBUR", 123.79526416296811`, 229.78413326265252`, 1054.5293725164315`},
{"TBUR", 57.85928138023631`, 229.68883185721816`, 556.8644511072259`}, {"CURE", 365, 0.6244069246009805`, 61.81777840309019`},
{"CURE", 365, 0.2815608176117965`, 11.995538573222055`}, {"TBUR", 83.1153292328735`, 229.5646017955762`, 1271.505190949691`},
{"CURE", 365, 0.15488766686246444`, 60.19888359725028`}, {"CURE", 365, 5.225641236481655`, 174.71036718544693`},
{"TBUR", 49.902118002009665`, 229.7130315428575`, 1210.6242897636012`},
{"CURE", 365, 2.4453980255956234`, 358.97648271231253`}, {"TBUR", 36.61986526767716`, 229.71845970242828`, 1006.2815498050892`},
{"CURE", 365, 14.585886468677824`, 218.1717762835453`}, {"TBUR", 81.7012546042678`, 229.74145843062328`, 958.6780482844306`},
{"TBUR", 38.930287381769574`, 229.61093363121935`, 980.8435738714118`},
{"CURE", 365, 2.4685595061756307`, 294.70278188224967`}, {"CURE", 365, 0.14088363878115598`, 17.24724433012472`},
{"CURE", 365, 4.588833721600873`, 79.05455665242881`}, {"CURE", 365, 1.0299139346496964`, 225.98409104545888`},
{"CURE", 365, 0.36011201481194455`, 31.564269966621243`}, {"CURE", 365, 9.862059608131124`, 507.8244781944375`},
{"CURE", 365, 11.085710987337782`, 637.4852179683168`}, {"CURE", 365, 3.403928761601249`, 227.05266603715978`},
{"CURE", 365, 0.5641453948922631`, 9.370706108102922`}, {"TBUR", 87.11304704921031`, 229.6630336467538`, 1310.3608269440501`},
{"CURE", 365, 2.1427467625291854`, 270.01352432118415`}, {"TBUR", 18.558823924276968`, 229.49627945461168`, 914.8799866124206`},
{"CURE", 365, 0.3400836100363643`, 46.476720704224476`}, {"TBUR", 135.1784480064543`, 229.69364494027997`, 1181.7499341462787`},
{"TBUR", 61.81243472750837`, 229.74710364390936`, 1136.6982951917148`}, {"CURE", 365, 9.251841651407819`, 375.7981046485485`},
{"TBUR", 76.61497831292819`, 229.77516691944427`, 1154.6419828816463`}, {"CURE", 365, 1.949587379671923`, 124.2651210363698`},
{"CURE", 365, 1.3884107379079393`, 40.758692293722326`}, {"TBUR", 73.27987007934684`, 229.6761456526711`, 1076.810612929495`},
{"TBUR", 76.32983503766393`, 229.5524974031707`, 1037.0967586416948`}, {"CURE", 365, 1.2917625829047312`, 33.449086782799455`},
{"TBUR", 15.17401258757658`, 229.42446778587652`, 1372.8056315371864`}, {"CURE", 365, 0.4946521183086901`, 37.80808821294862`},
{"CURE", 365, 0.2884839175470515`, 43.0708614739687`}, {"CURE", 365, 0.39624226246046035`, 26.288750744571665`},
{"CURE", 365, 0.4653353481834573`, 24.99520836716303`}, {"CURE", 365, 6.868270555180746`, 159.03790616276027`},
{"CURE", 365, 0.4801340095077254`, 69.95967787409019`}, {"TBUR", 69.53749336575329`, 229.2105722621521`, 995.8365334962544`},
{"TBUR", 44.72740212623164`, 229.71785115605925`, 938.9688328692789`},
{"TBUR", 80.37258606064438`, 229.41233667363872`, 996.6456606513575`}, {"CURE", 365, 7.41235631030548`, 116.76884087644329`},
{"TBUR", 27.4954970315109`, 229.68429748918425`, 936.3152815166272`}, {"CURE", 365, 0.7749750178032045`, 292.6920810004033`},
{"CURE", 365, 3.8405374438855304`, 179.78435657415508`}, {"TBUR", 27.804681938286368`, 229.356222374851`, 1256.712102388081`},
{"CURE", 365, 0.6431695112266637`, 11.071381215391131`}, {"TBUR", 18.343531850165963`, 229.18612599430782`, 1557.062206268311`},
{"CURE", 365, 0.08777743407834658`, 41.78742098852362`}, {"CURE", 365, 49.6455963170378`, 691.6064604347641`},
{"TBUR", 116.4544167913188`, 229.58622624604945`, 1189.34328129417`}, {"CURE", 365, 0.34520861490794236`, 65.4654867456162`},

{"CURE", 365, 0.598803927682531`, 59.25823406803572`}, {"CURE", 365, 0.1308623809664497`, 33.88373583404124`},
{"TBUR", 33.04710944532894`, 229.2709228515011`, 829.2597815864966`}, {"CURE", 365, 0.14299402373836562`, 35.43727484412724`},
{"CURE", 365, 1.3238988359960242`, 90.02444079240833`}, {"CURE", 365, 2.1667866831951943`, 70.282730149504`},
{"CURE", 365, 0.33725532253796214`, 25.069935686766698`}, {"CURE", 365, 1.5952927669858625`, 326.1909859584753`},
{"CURE", 365, 2.4235344156796645`, 58.395063631040095`}, {"TBUR", 42.16367275762186`, 229.7873314178439`, 1019.9674578716881`},
{"CURE", 365, 0.3770524722194498`, 157.4217894920961`}, {"TBUR", 31.083451170874735`, 229.61620509197388`, 1134.4401028281768`},
{"CURE", 365, 0.7905374902229305`, 78.18535393056641`}, {"CURE", 365, 0.057842744805674064`, 29.229050020241285`},
{"TBUR", 43.812165969745266`, 229.72885805642323`, 932.8578627736947`}, {"CURE", 365, 0.4828786777765942`, 48.44583474340646`},
{"CURE", 365, 1.7209625882604997`, 224.83790616324717`}, {"CURE", 365, 0.9741195924376918`, 66.0175022511591`},
{"CURE", 365, 1.6416619858095505`, 147.87671332451058`}, {"TBUR", 42.84587839476453`, 229.78315424194074`, 1192.6727124264364`},
{"CURE", 365, 5.128439696168752`, 138.7602578205695`}, {"TBUR", 111.28094382463499`, 229.7577255001127`, 1197.3039353521247`},
{"CURE", 365, 0.4131613030495764`, 25.61786545619475`}, {"CURE", 365, 0.20808752169641345`, 25.119228330369506`},
{"TBUR", 19.201448694094516`, 229.208485591171`, 962.970893006576`}, {"CURE", 365, 0.11359864470615688`, 44.51282303460948`},
{"CURE", 365, 0.1096143282649717`, 15.138393522328357`}, {"CURE", 365, 0.7422100695284347`, 24.486833172856983`},
{"CURE", 365, 0.8411027312714454`, 87.98053434763345`}, {"TBUR", 50.19180446996147`, 229.6760407104994`, 1092.586400885331`},
{"TBUR", 51.620682824527705`, 229.71451823213815`, 898.8210479836715`},
{"TBUR", 87.49675790205673`, 229.60649049285027`, 533.0986420731795`},
{"TBUR", 89.30843878122542`, 229.71535353253046`, 761.8295965696637`}, {"CURE", 365, 0.23278498388483754`, 73.49325183927627`},
{"CURE", 365, 9.435085842310334`, 461.96315618615137`}, {"CURE", 365, 0.1036737960090308`, 11.207614871250694`},
{"CURE", 365, 0.041238644498106734`, 7.292288879018173`}, {"TBUR", 33.82952945365581`, 229.49094322462332`, 838.3635578657452`},
{"CURE", 365, 0.5778094726490485`, 30.18516950044326`}, {"TBUR", 63.78886686883801`, 229.79640816060007`, 1058.5171508789067`},
{"CURE", 365, 0.06672744568252947`, 38.11098485992051`}, {"CURE", 365, 23.795316424299955`, 516.9794415251143`},
{"CURE", 365, 8.944147525262363`, 72.34955936145298`}, {"CURE", 365, 48.94375602323811`, 253.1619732926393`},
{"CURE", 365, 17.50950234146292`, 293.3812815928982`}, {"CURE", 365, 0.10470706456402613`, 11.51505547225931`},
{"TBUR", 64.77434467727512`, 229.60950035068828`, 844.7440909084521`}, {"CURE", 365, 2.0838349349547136`, 201.6459744260545`},
{"TBUR", 26.486846762156336`, 229.33981107820117`, 1072.8394904889558`}, {"CURE", 365, 2.0895419841031764`, 25.03885035542058`},
{"CURE", 365, 0.5505069537262681`, 47.720993033322024`}, {"TBUR", 22.430124479376925`, 229.6022438784811`, 1205.0366662677968`},
{"CURE", 365, 0.5716161170244909`, 33.12838516374073`}, {"TBUR", 59.93621421820717`, 229.76334986030508`, 890.9822451440907`},
{"CURE", 365, 0.2811254736503091`, 138.6083430428025`}, {"TBUR", 52.71841931477781`, 229.59129232336008`, 1138.5751546056651`},
{"TBUR", 147.46413910718366`, 229.71623654387972`, 832.3424420858684`},
{"CURE", 365, 0.8697059280482096`, 25.34071530711742`}, {"CURE", 365, 1.7681410731406455`, 93.83471840150673`},

```
{"CURE", 365, 1.8500495995923634`, 190.91114104013906`}, {"CURE", 365, 1.3653241470378565`, 38.32983261948075`},
{"TBUR", 75.18637536632997`, 229.6918915320002`, 1210.1833461460312`}, {"CURE", 365, 0.979101944321805`, 52.863174100132255`},
{"CURE", 365, 1.744404235669263 3`, 133.6262657349429`}, {"CURE", 365, 0.038464889351694224`, 10.717548953461424`},
{"TBUR", 32.419579363266784`, 229.62139111111333`, 1201.0041799043356`},
{"CURE", 365, 1.833239922925236`, 60.10643459774058`}, {"CURE", 365, 1.7390699789140376`, 178.2073804979087`},
{"CURE", 365, 0.49229221845817517`, 66.56863813908484`}, {"TBUR", 163.56185599428647`, 229.69518941221583`, 766.8623148767572`},
{"TBUR", 16.773631343645906`, 229.52910214855243`, 764.6944151426616`},
{"TBUR", 85.31609649284722`, 229.47187557745562`, 1055.8758723108392`}, {"CURE", 365, 2.0242393667067`, 53.79068753290875`},
{"CURE", 365, 0.05145159017365117`, 8.516878718496518`}, {"CURE", 365, 18.755952810665057`, 777.0331026765227`},
{"CURE", 365, 7.742923736963619`, 217.46112908457255`}, {"CURE", 365, 0.39399519364282437`, 101.71116940320925`},
{"TBUR", 74.72142440600236`, 229.31553013736317`, 1141.781857892087`},
{"CURE", 365, 1.0590540745639874`, 239.13745261678594`}, {"TBUR", 67.80363608414669`, 229.7630171590422`, 564.0115948727257`},
{"TBUR", 115.29572400141748`, 229.71194697721708`, 854.9511028590957`}, {"CURE", 365, 1.1429963906654859`, 18.000454676825353`},
{"CURE", 365, 5.633358577101365`, 339.6728090736476`}, {"CURE", 365, 5.890856390571676`, 741.7152696663883`},
{"CURE", 365, 7.7976354129752625`, 438.9687728243383`}, {"TBUR", 15.737630896131675`, 229.46799208270176`, 1542.47161865234 4`},
{"TBUR", 86.79742352905289`, 229.70548177091916`, 966.7228585795352`}, {"CURE", 365, 2.08280755726948`, 127.59124865674157`},
{"CURE", 365, 0.23656615269131565`, 36.71769849186109`}, {"CURE", 365, 0.9379457268258242`, 61.45764954904204`},
{"TBUR", 55.935167558397374`, 229.50968625408566`, 1318.3442733162328`},
{"CURE", 365, 1.0852529292438395`, 55.57617741052282`}, {"CURE", 365, 1.5267759135817613`, 193.59552721167307`},
{"CURE", 365, 3.085462221307893`, 465.93536139725467`}, {"TBUR", 79.74102176400397`, 229.7169346328774`, 1251.2577262677642`},
{"CURE", 365, 0.07768305146986983`, 35.24970758423872`}, {"CURE", 365, 0.14242967955001945`, 20.1968652202858`},
{"CURE", 365, 1.572539760770656`, 213.39984302706162`}, {"TBUR", 29.7646787330415`, 229.066346207699`, 2168.665906504581`},
{"CURE", 365, 185.6226341370326`, 771.9582793346566`}, {"CURE", 365, 0.3210287007983127`, 12.749188524618994`},
{"TBUR", 54.86583320025985`, 229.75395080659686`, 447.0225907626906`}, {"CURE", 365, 1.2922507428588885`, 102.66223512007062`},
{"TBUR", 43.70138516655898`, 229.76179842388422`, 1247.260305003116`}, {"CURE", 365, 3.0620996334285184`, 145.00973369983282`}};
```

In[∘]:=

```
TestConstantDose =
  {{{"400 Res", "400 OS", "400 Tox"}, {"TBUR", 21.37411969502965`, 31.735387280178852`}, {"CURE", 365, 125.39080767744953`},
    {"TBUR", 26.67165311380404`, 97.12490277892121`}, {"TBUR", 27.652015703227644`, 2.7705770828803487`},
    {"TBUR", 17.716694445993667`, 12.097388994601605`}, {"CURE", 365, 19.95734876901589`}, {"CURE", 365, 55.45708946919421`},
    {"CURE", 365, 51.41875583721702`}, {"CURE", 365, 125.02847981699978`}, {"TBUR", 70.58541368033269`, 171.77670607138083`},
```

{"TBUR", 54.24018757420318`, 88.8615860383954`}, {"CURE", 365, 76.04700655909836`},
{"TOX", 32.505543968871585`, 235.41012080249052`}, {"TBUR", 42.55198810419821`, 53.27662094292225`},
{"TBUR", 57.0461514031456`, 5.718021152803736`}, {"TBUR", 84.19110843030596`, 19.14654295555638`},
{"CURE", 365, 90.03492216467376`}, {"TBUR", 43.83308101547417`, 97.46763110562352`},
{"TBUR", 42.655962300549675`, 5.558716651324665`}, {"CURE", 365, 70.03600995066394`}, {"CURE", 365, 119.92624496901955`},
{"CURE", 365, 35.10002830841246`}, {"CURE", 365, 20.801549671298996`}, {"CURE", 365, 52.22856668520064`},
{"CURE", 365, 43.04777832399807`}, {"CURE", 365, 137.22178016510927`}, {"CURE", 365, 73.9869585203946`},
{"CURE", 365, 39.74716059459438`}, {"CURE", 365, 24.85491301667912`}, {"TBUR", 86.32311725679925`, 4.7102115471832615`},
{"CURE", 365, 112.65259367521323`}, {"TBUR", 13.345955708483537`, 29.81288162497248`},
{"CURE", 365, 57.10743391579657`}, {"TBUR", 46.47908847279728`, 32.12301209631957`}, {"CURE", 365, 158.00631684203674`},
{"CURE", 365, 186.77533109899534`}, {"CURE", 365, 33.496473645168116`}, {"TBUR", 20.92691752881735`, 9.484760143021099`},
{"TBUR", 71.3868874533562`, 39.02485360219223`}, {"CURE", 365, 84.99242260711628`},
{"TBUR", 17.925303141805266`, 51.40931961472137`}, {"CURE", 365, 80.34319051257705`}, {"CURE", 365, 61.14444480069253`},
{"TBUR", 45.8732857680824`, 68.64492360038872`}, {"CURE", 365, 84.81770679783081`}, {"CURE", 365, 4.428275756998943`},
{"CURE", 365, 94.08205939162666`}, {"CURE", 365, 58.02760303235007`}, {"TBUR", 112.03562801658646`, 116.93442171746109`},
{"CURE", 365, 197.9768785059515`}, {"TBUR", 29.587104219217256`, 88.1844580352803`},
{"TBUR", 41.55039732466129`, 76.17138589561038`}, {"CURE", 365, 5.775763119408803`}, {"CURE", 365, 122.91831664892005`},
{"CURE", 365, 33.74617556276426`}, {"CURE", 365, 86.35021941868055`}, {"CURE", 365, 55.54237776556302`},
{"TBUR", 102.98314130533471`, 57.010974291810946`}, {"TBUR", 37.069400798342784`, 147.92539282658416`},
{"TBUR", 51.985627261507915`, 88.91126621767522`}, {"CURE", 365, 82.24915445858187`},
{"CURE", 365, 4.086845236426597`}, {"TBUR", 33.54199753844605`, 100.37674586544392`}, {"CURE", 365, 64.54401736354724`},
{"TBUR", 64.56238050553391`, 6.198748761425599`}, {"CURE", 365, 77.66986041909328`}, {"CURE", 365, 67.40676383314478`},
{"TBUR", 57.18584177562153`, 90.86117801500608`}, {"CURE", 365, 32.91288459227222`}, {"CURE", 365, 27.356918136038534`},
{"CURE", 365, 53.34128764409417`}, {"CURE", 365, 37.95534695098648`}, {"CURE", 365, 121.90198241231002`},
{"TBUR", 13.175573357951121`, 1.8268685270561422`}, {"TBUR", 79.20633300180975`, 76.93823036878729`},
{"CURE", 365, 97.18005218980025`}, {"TBUR", 96.89160708641116`, 190.52508706612613`}, {"CURE", 365, 61.98240977528097`},
{"TBUR", 75.65259557154286`, 124.32758234173498`}, {"CURE", 365, 43.887910694351305`}, {"CURE", 365, 99.19685861382045`},
{"CURE", 365, 3.2186842079482565`}, {"CURE", 365, 6.394900319287186`}, {"CURE", 365, 4.507321953540303`},
{"CURE", 365, 115.14854039467555`}, {"TBUR", 99.20086286717886`, 88.07758770333842`}, {"CURE", 365, 61.02187396059579`},
{"CURE", 365, 33.861405921973954`}, {"CURE", 365, 6.9315589000199545`}, {"TBUR", 71.32375080480583`, 125.17136528511665`},
{"TBUR", 31.637974155581343`, 8.778407416657979`}, {"TBUR", 89.0508136460763`, 94.65676453389614`},

{"TBUR", 62.438693330751995`, 2.6501147117325186`}, {"CURE", 365, 6.143523420985161`},
{"TBUR", 18.485519313927945`, 1.7992172433829516`}, {"TBUR", 157.6441367736462`, 48.8753878402692`},
{"CURE", 365, 33.04259362537681`}, {"CURE", 365, 173.25980840846086`}, {"TBUR", 80.10938279963479`, 9.365859864942678`},
{"TBUR", 43.5238105324415`, 129.54788061186832`}, {"TBUR", 19.686234736804312`, 9.159366894169006`},
{"TBUR", 127.23925955241175`, 117.82266354466633`}, {"CURE", 365, 65.14850556981442`},
{"TBUR", 90.29598812907517`, 87.59108819713383`}, {"CURE", 365, 58.55112489252078`}, {"CURE", 365, 93.45581555331778`},
{"CURE", 365, 37.03702978445487`}, {"TBUR", 137.70677565915292`, 83.42011728962993`}, {"CURE", 365, 32.63459581847438`},
{"CURE", 365, 96.0869559019103`}, {"CURE", 365, 133.2879049683211`}, {"CURE", 365, 72.84374568273091`},
{"CURE", 365, 16.008776694894003`}, {"CURE", 365, 43.5691065747571`}, {"CURE", 365, 66.0021721294166`},
{"CURE", 365, 53.48812425075963`}, {"CURE", 365, 2.162622625031719`}, {"CURE", 365, 70.11684858607055`},
{"CURE", 365, 63.04497832566579`}, {"CURE", 365, 16.065254289634222`}, {"CURE", 365, 77.60987150062998`},
{"CURE", 365, 156.0761300881037`}, {"CURE", 365, 96.35210188361314`}, {"TBUR", 19.356420261274412`, 5.06914375869628`},
{"CURE", 365, 69.53445731731222`}, {"TBUR", 38.251905174385115`, 55.248166664178534`}, {"CURE", 365, 42.17666009725213`},
{"CURE", 365, 29.888153467727992`}, {"CURE", 365, 110.61287674709054`}, {"CURE", 365, 107.01672528090494`},
{"CURE", 365, 96.63232539241619`}, {"CURE", 365, 105.95522905787348`}, {"TBUR", 71.3039091031672`, 28.73286838030849`},
{"TBUR", 43.930967114570024`, 29.016312611660158`}, {"CURE", 365, 155.2646068507269`},
{"TBUR", 12.756166831645483`, 65.83561269041334`}, {"TBUR", 84.37633799215429`, 21.762269956204392`},
{"TBUR", 61.858494381447734`, 48.09602972569843`}, {"TBUR", 93.46242168609562`, 17.820674373909778`},
{"CURE", 365, 105.02079086636014`}, {"TBUR", 52.80730299996674`, 159.08328878488769`}, {"CURE", 365, 105.60929788330169`},
{"CURE", 365, 135.29645204184754`}, {"CURE", 365, 7.888881779340033`}, {"CURE", 365, 1.1159354503394692`},
{"CURE", 365, 61.3175750702126`}, {"CURE", 365, 153.3390733267975`}, {"TBUR", 35.651302199434554`, 35.69558429697691`},
{"CURE", 365, 69.66336479604867`}, {"CURE", 365, 10.043166097331513`}, {"CURE", 365, 6.702429203124482`},
{"CURE", 365, 53.40507922255386`}, {"CURE", 365, 1.1166922130800143`}, {"TBUR", 88.15706726153778`, 155.945172355656`},
{"TBUR", 49.67318297684931`, 57.493081093261324`}, {"TBUR", 88.24387430288434`, 136.56680459247056`},
{"CURE", 365, 64.98095483309498`}, {"CURE", 365, 79.65521451946897`}, {"CURE", 365, 64.6369684419257`},
{"CURE", 365, 79.78085625884555`}, {"CURE", 365, 14.545810872681523`}, {"TBUR", 88.80250565227777`, 21.177055396468845`},
{"CURE", 365, 1.6210621927484299`}, {"TBUR", 56.91102145511907`, 6.468671951156726`},
{"TBUR", 78.52659987653968`, 66.95903187571147`}, {"CURE", 365, 107.26380077172304`}, {"CURE", 365, 106.665082806054`},
{"CURE", 365, 75.42690784403553`}, {"CURE", 365, 111.29766252406488`}, {"TBUR", 155.69296833140217`, 1.6373568254861859`},
{"CURE", 365, 60.29216036073395`}, {"TBUR", 29.616595224342085`, 118.60958899556482`}, {"CURE", 365, 52.104933302765325`},
{"TBUR", 20.459756124399146`, 6.258966408049343`}, {"TBUR", 50.132517795249235`, 117.32375284330058`},

{"TBUR", 81.53050700808575`, 9.7023872961973`}, {"TBUR", 67.53670647791503`, 137.5468217491539`},
{"TBUR", 21.15004464905791`, 75.1561761013656`}, {"TBUR", 56.86210624605521`, 34.581954921224096`},
{"TBUR", 90.89657734632628`, 94.2551043133807`}, {"TBUR", 79.95082003363054`, 85.18263522078712`},
{"CURE", 365, 77.42958428670464`}, {"CURE", 365, 132.77329250746737`}, {"TBUR", 18.451418084794355`, 6.077164510736032`},
{"CURE", 365, 120.58573534356184`}, {"TBUR", 27.829237692702826`, 38.183424630584796`},
{"TBUR", 53.504103067112446`, 132.49581458584592`}, {"CURE", 365, 59.06839498350843`},
{"CURE", 365, 3.5167880604319417`}, {"TBUR", 24.055182926708216`, 86.45758972445269`}, {"CURE", 365, 82.17112810304278`},
{"CURE", 365, 133.7054217721941`}, {"CURE", 365, 152.85691494838522`}, {"CURE", 365, 100.58813392523263`},
{"CURE", 365, 58.04685420812056`}, {"CURE", 365, 44.085455823674295`}, {"CURE", 365, 159.3914229415243`},
{"TBUR", 13.483097229141942`, 11.55561461696492`}, {"CURE", 365, 89.98580768962381`}, {"CURE", 365, 78.58428736093329`},
{"TBUR", 24.74984794501355`, 21.77773065887662`}, {"TBUR", 56.98696254612827`, 92.87654449993613`},
{"CURE", 365, 83.9692439181273`}, {"TBUR", 65.20168116547751`, 72.50087626162674`}, {"CURE", 365, 1.6448114742165498`},
{"TBUR", 121.29618241527508`, 10.640003627393416`}, {"TBUR", 15.808218214914586`, 77.61432269063434`},
{"CURE", 365, 154.94386637393632`}, {"CURE", 365, 3.9730114683515265`}, {"CURE", 365, 118.6061456693172`},
{"CURE", 365, 2.702867357244278`}, {"CURE", 365, 84.32841995461393`}, {"CURE", 365, 11.397881504872142`},
{"TBUR", 74.83073245038976`, 103.20566696236413`}, {"CURE", 365, 18.77113129710717`},
{"TBUR", 39.28254247154868`, 12.481998478469592`}, {"TBUR", 79.85622010507723`, 144.67983455642624`},
{"CURE", 365, 69.46099225059899`}, {"TBUR", 49.99512666164919`, 189.50815185720944`}, {"CURE", 365, 65.29668238143016`},
{"CURE", 365, 92.40727368113174`}, {"CURE", 365, 11.622798200215282`}, {"CURE", 365, 62.088024348051604`},
{"CURE", 365, 122.9946081909826`}, {"CURE", 365, 129.59881245450325`}, {"CURE", 365, 62.0458117071941`},
{"CURE", 365, 114.52731985151365`}, {"CURE", 365, 72.53309019968923`}, {"CURE", 365, 7.0397756653546555`},
{"CURE", 365, 44.32869581827059`}, {"CURE", 365, 33.69647994581631`}, {"CURE", 365, 63.96648176197191`},
{"CURE", 365, 94.6934794042434`}, {"CURE", 365, 78.96947476042479`}, {"CURE", 365, 71.22401408965041`},
{"TBUR", 22.41774572051581`, 90.22689852908108`}, {"TBUR", 27.899984052443486`, 61.49802175801233`},
{"TBUR", 156.39940410589068`, 58.34597105089197`}, {"TBUR", 19.557053185385232`, 38.985691140253266`},
{"CURE", 365, 53.050198389041306`}, {"CURE", 365, 60.94902218480326`}, {"CURE", 365, 113.3558485252997`},
{"TBUR", 26.292054723526913`, 5.138297877975904`}, {"TBUR", 30.874205414792954`, 18.006445612157933`},
{"CURE", 365, 1.5168304357344466`}, {"CURE", 365, 2.2722329703514372`}, {"TBUR", 18.594286493067674`, 20.338138368504076`},
{"CURE", 365, 182.2896646044458`}, {"CURE", 365, 40.83126378902958`}, {"CURE", 365, 177.84390770877513`},
{"TBUR", 38.214092670905394`, 79.49910901151691`}, {"CURE", 365, 46.87501193263527`},
{"TBUR", 15.12817869093827`, 105.15120183025655`}, {"CURE", 365, 43.40445996978168`},

{"CURE", 365, 90.90565187256568`}, {"CURE", 365, 1.9592357136793734`}, {"CURE", 365, 66.49365247935938`},
{"CURE", 365, 17.44225551203387`}, {"CURE", 365, 8.410684079367421`}, {"CURE", 365, 102.04362755131002`},
{"TBUR", 21.422165901557417`, 109.91966956624107`}, {"TBUR", 13.600484548297835`, 79.17488767011125`},
{"CURE", 365, 51.47191458468321`}, {"TBUR", 27.976413029249326`, 74.67928340796867`},
{"CURE", 365, 4.92454149069859`}, {"CURE", 365, 49.413416330419665`}, {"CURE", 365, 33.3965725712805`},
{"CURE", 365, 185.3133487117607`}, {"CURE", 365, 69.05439104747389`}, {"CURE", 365, 38.63741124703214`},
{"CURE", 365, 1.457869259516106`}, {"TBUR", 69.64915480595255`, 74.1856481699111`}, {"CURE", 365, 64.03253066602821`},
{"TBUR", 151.09877067563335`, 68.35452056883787`}, {"TBUR", 49.32815312624516`, 6.774234258603108`},
{"CURE", 365, 66.83446546803968`}, {"CURE", 365, 47.80104513563248`}, {"TBUR", 20.438169427400062`, 64.49272103188693`},
{"CURE", 365, 31.02347970599286`}, {"CURE", 365, 85.65057517074418`}, {"TBUR", 89.8024143408433`, 5.209970526761389`},
{"TBUR", 97.73559094657497`, 88.77033451247276`}, {"TBUR", 16.500703521947244`, 33.48661421164864`},
{"CURE", 365, 71.7921534474982`}, {"TBUR", 26.682500362305124`, 79.79590657988106`}, {"CURE", 365, 8.121476567014941`},
{"CURE", 365, 102.64689711426941`}, {"CURE", 365, 60.694009627568946`}, {"CURE", 365, 168.95697244833303`},
{"CURE", 365, 121.2848335977957`}, {"CURE", 365, 84.38941817254678`}, {"CURE", 365, 49.461114815916204`},
{"CURE", 365, 178.67363015478043`}, {"TBUR", 29.228015201097673`, 24.818876858622613`}, {"CURE", 365, 16.201427735492903`},
{"CURE", 365, 13.623571241220988`}, {"CURE", 365, 65.99062538733665`}, {"CURE", 365, 59.94498199704398`},
{"CURE", 365, 100.5815609890632`}, {"CURE", 365, 23.058352273756483`}, {"TBUR", 23.136936200461182`, 128.13744199503773`},
{"TBUR", 87.7385433519351`, 1.9479251158642152`}, {"CURE", 365, 101.61625078445964`},
{"TBUR", 38.03751078910932`, 123.6970812812927`}, {"CURE", 365, 33.12458935393802`}, {"CURE", 365, 106.46980433079797`},
{"CURE", 365, 3.5578973447029876`}, {"TBUR", 21.75207213984297`, 36.7486515835763`}, {"CURE", 365, 53.30775347336408`},
{"CURE", 365, 52.61138296559014`}, {"TBUR", 28.392215764787043`, 25.528943464371586`}, {"CURE", 365, 55.0841657155933`},
{"TBUR", 35.32610044497362`, 25.69483372385784`}, {"CURE", 365, 95.7648108169653`}, {"CURE", 365, 72.26351326594084`},
{"CURE", 365, 82.57716020102552`}, {"CURE", 365, 54.801715337020376`}, {"CURE", 365, 62.45748329749818`},
{"CURE", 365, 90.14191194244503`}, {"CURE", 365, 113.38083820078562`}, {"TBUR", 48.948045140652944`, 85.5454317225582`},
{"CURE", 365, 113.796372241002`}, {"CURE", 365, 69.86441630379011`}, {"TBUR", 52.60012599897225`, 18.13130178207005`},
{"TBUR", 16.6233472322856`, 12.256598595666619`}, {"CURE", 365, 0.6720342741440326`}, {"CURE", 365, 100.18683020170542`},
{"CURE", 365, 98.50876491800541`}, {"CURE", 365, 78.99122127819926`}, {"TBUR", 137.48992738931514`, 184.67187710249218`},
{"CURE", 365, 141.5361040225076`}, {"CURE", 365, 29.088111302573072`}, {"TBUR", 20.36259619285672`, 53.94880305164814`},
{"TBUR", 29.06658961646769`, 6.213294671817894`}, {"CURE", 365, 175.773509607578`},
{"TBUR", 38.6568062834415`, 169.34901279731747`}, {"TBUR", 22.392302091734177`, 19.876483107554233`},
{"CURE", 365, 166.64443964695704`}, {"CURE", 365, 25.447816017948252`}, {"CURE", 365, 54.69887097613882`},

{"CURE", 365, 114.5884312625902`}, {"CURE", 365, 1.9706784059214104`}, {"TBUR", 55.536523008728715`, 32.423599845385326`},
{"CURE", 365, 107.09900342572634`}, {"TBUR", 45.931086510760295`, 25.887842198714477`},
{"TBUR", 38.20557424732755`, 169.82569381115326`}, {"CURE", 365, 2.914479592839356`}, {"CURE", 365, 143.94342220566065`},
{"CURE", 365, 84.36175745719622`}, {"CURE", 365, 78.7784923677926`}, {"CURE", 365, 101.49997891972455`},
{"CURE", 365, 41.154847826469926`}, {"TBUR", 35.38673775853869`, 128.47761037824273`}, {"CURE", 365, 76.93119112908461`},
{"CURE", 365, 136.79322583838083`}, {"CURE", 365, 87.88727136653874`}, {"TBUR", 54.20454474438432`, 93.60296877646984`},
{"CURE", 365, 111.31604146842474`}, {"CURE", 365, 78.44755022869238`}, {"CURE", 365, 35.307632821141866`},
{"CURE", 365, 123.37539197723312`}, {"CURE", 365, 147.54597998037934`}, {"CURE", 365, 37.308576635153784`},
{"TBUR", 63.744849268016345`, 96.00967927258498`}, {"TBUR", 45.74083355587658`, 124.02208326059285`},
{"CURE", 365, 7.264692212323639`}, {"CURE", 365, 88.98175994315513`}, {"CURE", 365, 42.97458901099365`},
{"TBUR", 14.265036714268824`, 6.706874887911726`}, {"TBUR", 13.241389688041155`, 25.3774011116367`},
{"TBUR", 30.242407454574543`, 43.32732139286461`}, {"CURE", 365, 85.90029153003536`}, {"CURE", 365, 14.969767127668554`},
{"CURE", 365, 74.45048837347714`}, {"TBUR", 77.79124923275677`, 139.93270152667918`}, {"CURE", 365, 77.98834677330942`},
{"CURE", 365, 69.83284994448697`}, {"CURE", 365, 61.82026951480552`}, {"TBUR", 16.87735984677906`, 28.72843903972451`},
{"TBUR", 13.522658451039321`, 70.94023378695579`}, {"TBUR", 70.64866212543237`, 32.32388885904532`},
{"CURE", 365, 126.91948420926363`}, {"TBUR", 49.24966445474286`, 74.84447654020245`},
{"CURE", 365, 99.23982447937126`}, {"CURE", 365, 75.74479586781558`}, {"CURE", 365, 64.26025360683963`},
{"TBUR", 100.24236378713161`, 26.545570960555747`}, {"CURE", 365, 45.0719098254561`}, {"CURE", 365, 177.93778463740153`},
{"CURE", 365, 91.0188960472722`}, {"CURE", 365, 75.51745685231239`}, {"TBUR", 32.90204728960697`, 65.71792817793543`},
{"TBUR", 76.61072500363913`, 62.381436398846624`}, {"CURE", 365, 47.71942184771217`},
{"TBUR", 15.876165574126404`, 54.05149188253251`}, {"TBUR", 17.065060533123106`, 50.95041808460513`},
{"CURE", 365, 38.055235585928564`}, {"CURE", 365, 71.64317991052182`}, {"TBUR", 97.29235254102485`, 6.012138243001198`},
{"TBUR", 12.943947170430038`, 50.89015996218987`}, {"TBUR", 20.480345142850375`, 12.132683683630885`},
{"CURE", 365, 111.659087388366`}, {"CURE", 365, 22.070185491506518`}, {"CURE", 365, 84.17967176777395`},
{"CURE", 365, 4.599703131873197`}, {"CURE", 365, 67.79752656636359`}, {"CURE", 365, 2.5866349976508314`},
{"CURE", 365, 79.19742211576295`}, {"CURE", 365, 68.73402381973477`}, {"TBUR", 114.72228163010769`, 84.45299244744268`},
{"CURE", 365, 6.4641335416418295`}, {"CURE", 365, 42.658667769393304`}, {"CURE", 365, 171.03763704020182`},
{"TBUR", 24.74503428089822`, 38.21768658706064`}, {"CURE", 365, 1.5866718860520959`}, {"CURE", 365, 15.187274838835664`},
{"CURE", 365, 120.59557864000396`}, {"CURE", 365, 3.8824607164948715`}, {"CURE", 365, 85.15814931353366`},
{"CURE", 365, 139.11005886212868`}, {"CURE", 365, 54.91747163074165`}, {"CURE", 365, 6.984633646220069`},
{"TBUR", 148.4763101502527`, 1.6252033319703796`}, {"CURE", 365, 104.96917192807862`},

{"TBUR", 13.328711927541526`, 13.049949949051173`}, {"TBUR", 50.57614787260857`, 199.62277540094607`},
{"CURE", 365, 72.96572252598811`}, {"CURE", 365, 81.02188438929194`}, {"TBUR", 84.82297076212076`, 72.04060255709813`},
{"CURE", 365, 45.47261653253527`}, {"CURE", 365, 204.73636325819203`}, {"CURE", 365, 59.70800519416272`},
{"TBUR", 102.17744446287837`, 3.456169281764965`}, {"CURE", 365, 59.757983774346`}, {"CURE", 365, 15.149360778144606`},
{"CURE", 365, 157.95342810610936`}, {"CURE", 365, 58.99697191305219`}, {"CURE", 365, 3.9452949908066777`},
{"CURE", 365, 63.60863976466542`}, {"TBUR", 21.92116679480445`, 76.05227961855977`}, {"CURE", 365, 75.94945931694663`},
{"CURE", 365, 63.95488583395388`}, {"CURE", 365, 47.67124549690993`}, {"CURE", 365, 109.69625022844791`},
{"CURE", 365, 9.127661248979994`}, {"TBUR", 77.82297973077193`, 148.39938769495237`}, {"CURE", 365, 60.87933529501473`},
{"CURE", 365, 6.035751856751758`}, {"CURE", 365, 21.143249580456942`}, {"CURE", 365, 180.74337810910578`},
{"CURE", 365, 69.87551180596513`}, {"CURE", 365, 128.02004413539473`}, {"CURE", 365, 62.82663949283482`},
{"CURE", 365, 146.986008475049`}, {"CURE", 365, 136.3638477702121`}, {"TBUR", 70.43712032213358`, 17.407892255329706`},
{"CURE", 365, 57.124410204262986`}, {"TBUR", 131.17586904647402`, 58.48324537651859`},
{"TBUR", 17.069404624845685`, 11.449336806888594`}, {"TBUR", 21.386511436170466`, 7.64898163902746`},
{"CURE", 365, 50.2226437187267`}, {"CURE", 365, 83.23497367415845`}, {"TBUR", 49.581320162950654`, 31.967269913301116`},
{"CURE", 365, 131.39716455726062`}, {"CURE", 365, 124.53166514402328`}, {"TBUR", 18.52764254660557`, 13.499955388987733`},
{"TBUR", 24.517800108169418`, 29.777713403842125`}, {"TBUR", 33.58421362342561`, 10.532057786892244`},
{"TBUR", 25.92869437016407`, 92.91995796326212`}, {"TBUR", 43.775531948909034`, 79.92633592669213`},
{"CURE", 365, 102.13081905015211`}, {"TBUR", 76.85279996119341`, 131.11564444599432`},
{"CURE", 365, 29.94431885829159`}, {"CURE", 365, 99.97770736392638`}, {"TBUR", 27.52434588652844`, 85.03075464524252`},
{"TBUR", 25.824582293353625`, 5.594705915067436`}, {"CURE", 365, 139.428022189117`}, {"CURE", 365, 57.915297118404986`},
{"TBUR", 34.6450850715742`, 3.0978109597568273`}, {"TBUR", 19.321894449183365`, 2.184378860428992`},
{"CURE", 365, 46.10397298199079`}, {"CURE", 365, 67.10843402865731`}, {"CURE", 365, 76.0818011911951`},
{"TBUR", 38.362261612085554`, 50.258873426396065`}, {"CURE", 365, 81.29653298786141`},
{"CURE", 365, 115.34352713181377`}, {"CURE", 365, 53.05664265392879`}, {"TBUR", 88.21937275903525`, 18.492202505862448`},
{"CURE", 365, 100.01346300080158`}, {"TBUR", 97.11780006011699`, 2.0375386875922072`},
{"CURE", 365, 26.910448456851945`}, {"TBUR", 77.21030665289423`, 65.4326346332567`},
{"CURE", 365, 6.150181967611863`}, {"TBUR", 42.82662370312319`, 37.167913148103935`},
{"CURE", 365, 65.69272115775715`}, {"TBUR", 34.0458713366849`, 74.05338908167738`}, {"CURE", 365, 24.58165691215235`},
{"TBUR", 20.57703933925313`, 50.829298117528396`}, {"CURE", 365, 68.25097685821949`},
{"TBUR", 16.323092391002486`, 82.50779605246201`}, {"CURE", 365, 73.36962292551547`},
{"TBUR", 16.09648514175443`, 18.43426741577079`}, {"TBUR", 63.977272649926185`, 55.29849800887826`},

```
{"CURE", 365, 133.68548720580435`}, {"CURE", 365, 5.860048491335502`}, {"CURE", 365, 57.24402639236171`},
{"CURE", 365, 23.20149647525105`}, {"CURE", 365, 84.49494878529346`}, {"CURE", 365, 31.100619814605015`},
{"TBUR", 49.45750498335458`, 104.44742591522702`}, {"TBUR", 77.70051188611988`, 65.24945854143103`},
{"CURE", 365, 124.08487624536902`}, {"CURE", 365, 32.83308501322071`}, {"CURE", 365, 168.12978644926918`},
{"CURE", 365, 110.62818719820496`}, {"CURE", 365, 84.17406171885916`}, {"CURE", 365, 107.7495536544788`},
{"TBUR", 134.19334483038605`, 172.20805140007587`}, {"TBUR", 39.02690538810104`, 183.9137683007787`},
{"CURE", 365, 50.919651497759716`}, {"CURE", 365, 86.74631822008924`}, {"CURE", 365, 94.42528536900087`},
{"TBUR", 39.66250537117584`, 31.763136807772177`}, {"TBUR", 16.179411743316162`, 6.071595828021324`},
{"CURE", 365, 0.8535932541074861`}, {"CURE", 365, 64.14621905081533`}, {"CURE", 365, 114.32195660754405`},
{"TBUR", 20.14880034720517`, 90.4960822878003`}, {"CURE", 365, 127.94258650991308`}, {"CURE", 365, 169.96849528127805`},
{"CURE", 365, 36.842873681667825`}, {"CURE", 365, 81.24343517157685`}, {"TBUR", 34.70365284665551`, 6.438885707869024`},
{"CURE", 365, 191.14840493063224`}, {"TBUR", 36.648891565106005`, 60.189579706189726`},
{"TBUR", 57.46959851567043`, 39.76665708182943`}, {"TBUR", 64.3245965241776`, 49.37454043345017`},
{"CURE", 365, 176.90227151218215`}, {"CURE", 365, 68.89171785040783`}, {"TBUR", 91.95626995407923`, 153.4506230173282`},
{"CURE", 365, 119.38689425868635`}, {"CURE", 365, 104.19793151052896`}, {"TBUR", 54.1119717332647`, 99.85742921167508`},
{"TBUR", 87.57278357584075`, 96.58840696470678`}, {"CURE", 365, 46.05943373850624`}, {"CURE", 365, 37.9313564573026`},
{"CURE", 365, 8.404425184387794`}, {"CURE", 365, 72.44116909401721`}, {"CURE", 365, 85.8496667806691`},
{"TBUR", 33.283141533403374`, 44.179948621366904`}, {"TBUR", 43.3068646909743`, 57.167083284521475`},
{"CURE", 365, 113.94187950846488`}, {"TBUR", 41.172831492491895`, 52.2196262450745`},
{"CURE", 365, 61.99341160064836`}, {"CURE", 365, 23.60343032030991`}, {"TBUR", 20.092630719805136`, 8.25971548515332`},
{"CURE", 365, 41.74107392848736`}, {"CURE", 365, 135.59909918378122`}, {"CURE", 365, 53.10769384061057`},
{"TBUR", 42.55218343891897`, 52.01579118158804`}, {"CURE", 365, 8.43484422568755`},
{"TBUR", 25.658531415158098`, 121.66991634929244`}, {"CURE", 365, 130.8259796597159`}, {"CURE", 365, 82.24077802463302`},
{"TBUR", 99.634896190584`, 25.461809377061602`}, {"CURE", 365, 127.8153450068926`}, {"CURE", 365, 15.132120267571393`},
{"TBUR", 84.17248675161044`, 189.30974043200953`}, {"TBUR", 28.380972188055473`, 66.25422811438263`},
{"CURE", 365, 138.56530638444738`}, {"TBUR", 42.81753873089092`, 69.51604521617344`}, {"CURE", 365, 37.38870815462628`},
{"TBUR", 41.27963596496956`, 48.71369829945975`}, {"TBUR", 23.91927341999707`, 16.40756391558597`},
{"CURE", 365, 108.77169375606124`}, {"TBUR", 41.33082623862365`, 33.33007945791013`},
{"CURE", 365, 46.153778427310044`}, {"TBUR", 23.458578941694444`, 41.27865854446501`}, {"CURE", 365, 70.19268857540301`},
{"TBUR", 23.07738397605274`, 39.814552074451235`}, {"TBUR", 106.08334999960725`, 142.6395554370277`},
{"CURE", 365, 82.43703469560914`}, {"TBUR", 32.99792025373741`, 60.430051992493354`},
```

{"TBUR", 66.02009482823483`, 62.09642504017367`}, {"TBUR", 13.771781832924777`, 57.84849757911429`},
{"CURE", 365, 2.069368624019367`}, {"TBUR", 108.12901925673856`, 111.30546593171762`},
{"TBUR", 101.70247653577974`, 97.2431534262058`}, {"CURE", 365, 81.03132807497676`},
{"TBUR", 38.408228472077795`, 8.398536855651596`}, {"CURE", 365, 54.58539259464988`}, {"CURE", 365, 156.9358037079806`},
{"CURE", 365, 76.44268748918019`}, {"CURE", 365, 90.90139571144142`}, {"TBUR", 27.748110686821803`, 29.062767246447887`},
{"TBUR", 85.02614974735303`, 74.86344181206691`}, {"TBUR", 13.042043461819132`, 2.183919068511966`},
{"TBUR", 14.987710459316895`, 11.047396771038427`}, {"TBUR", 52.50108241491306`, 109.98917818006366`},
{"CURE", 365, 66.86917846355936`}, {"TBUR", 51.062706852384245`, 60.803237479895934`}, {"CURE", 365, 10.755016659861091`},
{"TBUR", 217.69563081136482`, 132.66606578519222`}, {"CURE", 365, 69.4423768739203`}, {"CURE", 365, 130.3415459731218`},
{"TBUR", 48.9721721721283`, 21.47149998141249`}, {"TBUR", 29.884399494170037`, 72.56994876721602`},
{"CURE", 365, 60.55800360157881`}, {"CURE", 365, 75.1594737627776`}, {"CURE", 365, 11.4907481223701`},
{"CURE", 365, 52.92607760472212`}, {"CURE", 365, 116.80281579959414`}, {"TBUR", 20.592339971896862`, 75.7314316717856 4`},
{"CURE", 365, 35.615281256363396`}, {"TBUR", 38.208061060451286`, 2.473390358628564`}, {"CURE", 365, 38.653306701943414`},
{"CURE", 365, 118.9698899175482`}, {"CURE", 365, 45.802007630752605`}, {"CURE", 365, 69.4467570811751`},
{"TBUR", 15.849313527483899`, 90.46963070981386`}, {"TBUR", 87.77317879634674`, 71.2737092797677`},
{"TBUR", 85.96736850075924`, 132.5326252093385`}, {"CURE", 365, 121.76018115816632`},
{"CURE", 365, 81.57959518536526`}, {"CURE", 365, 89.83302895492946`}, {"CURE", 365, 124.37512911683652`},
{"TBUR", 18.866761175029232`, 3.085679167387034`}, {"TBUR", 79.08038622729126`, 130.7832094537024`},
{"TBUR", 147.63066486757847`, 39.397205975948864`}, {"TBUR", 65.98429700151051`, 183.73946434952293`},
{"CURE", 365, 11.146735422680722`}, {"TBUR", 90.71287488363437`, 65.40597699004836`}, {"CURE", 365, 154.65373931586288`},
{"CURE", 365, 7.2320261124810825`}, {"CURE", 365, 92.71211561197671`}, {"TBUR", 94.69640156001263`, 77.51152966311832`},
{"TBUR", 33.00922426698364`, 101.00001126578722`}, {"TBUR", 15.37368333824546`, 49.43918221443864`},
{"TBUR", 13.762030320250526`, 24.705370057404`}, {"TBUR", 16.91915490514086`, 6.484270399123638`},
{"CURE", 365, 69.71610100904255`}, {"CURE", 365, 67.61840398393679`}, {"TBUR", 37.01237989888682`, 83.22188472821493`},
{"CURE", 365, 14.058557242225733`}, {"CURE", 365, 191.2625465953045`}, {"CURE", 365, 34.70564681374046`},
{"CURE", 365, 77.06473243517827`}, {"CURE", 365, 41.69559353204865`}, {"CURE", 365, 7.3517204566407965`},
{"TBUR", 115.07647402100237`, 131.0679884639467`}, {"CURE", 365, 2.726396587631069`}, {"CURE", 365, 48.52155427373457`},
{"TBUR", 18.893891242071643`, 23.382740016588883`}, {"CURE", 365, 54.92022151148585`},
{"TBUR", 71.35347187812398`, 197.78879649049298`}, {"CURE", 365, 136.86288081372194`},
{"TBUR", 36.41395832397003`, 75.10956828258296`}, {"CURE", 365, 71.63548901467307`}, {"CURE", 365, 119.6146564390445`},
{"CURE", 365, 145.76398951051772`}, {"CURE", 365, 112.4282118274608`}, {"CURE", 365, 146.42994509123767`},

```
{"TBUR", 14.260553040099886`, 48.18732489227119`}, {"CURE", 365, 56.964780524936444`},
{"TBUR", 23.2140894902205`, 5.994920555666963`}, {"TBUR", 18.762044215479474`, 71.04343278346967`},
{"TBUR", 58.43325470759082`, 3.93133680690393`}, {"CURE", 365, 0.9778218200401866`}, {"CURE", 365, 6.493741637200496`},
{"TBUR", 136.00431977192588`, 0.7216045082282081`}, {"TBUR", 18.683574016404712`, 66.15858317128637`},
{"CURE", 365, 8.553232865532062`}, {"CURE", 365, 54.6696750889689`}, {"CURE", 365, 143.16482072051025`},
{"CURE", 365, 22.69770338580125`}, {"TBUR", 20.322511572324096`, 67.5651972288776`},
{"TBUR", 13.16570187272746`, 46.24836949221434`}, {"CURE", 365, 8.777524814848737`},
{"TBUR", 62.24065625395157`, 91.4634948793198`}, {"TBUR", 31.381494162127535`, 19.45066019530328`},
{"TBUR", 57.24448368826522`, 1.7910786517753567`}, {"TBUR", 41.06029805331965`, 7.1440609415841525`},
{"CURE", 365, 140.4689922163336`}, {"CURE", 365, 22.039542993297488`}, {"CURE", 365, 141.60908469639975`},
{"TBUR", 171.907220869053`, 90.16274504985937`}, {"CURE", 365, 126.70863534199708`}, {"CURE", 365, 40.309968508838274`},
{"CURE", 365, 169.5595273000696`}, {"CURE", 365, 73.22308214569226`}, {"TBUR", 22.59425164073345`, 3.7186371691247846`},
{"CURE", 365, 125.4603634646234`}, {"TBUR", 26.05565942821974`, 22.86118380208824`},
{"TBUR", 67.3006256033014`, 94.42477996849908`}, {"TBUR", 75.0350814771907`, 124.54343426531841`},
{"TBUR", 63.32257834882314`, 32.927336854924725`}, {"CURE", 365, 89.18906274213784`},
{"TBUR", 15.419845886308089`, 27.30539787950235`}, {"TBUR", 32.07852014878328`, 58.647986459313934`},
{"CURE", 365, 107.3060290319928`}, {"CURE", 365, 36.70227424037692`}, {"TBUR", 30.123589865876294`, 64.7496431648311`},
{"CURE", 365, 30.71861252722088`}, {"TBUR", 77.11499840133769`, 121.77755549887743`},
{"TBUR", 52.052697465858444`, 71.57299668551124`}, {"CURE", 365, 65.69681104740309`},
{"TBUR", 77.1868423890277`, 111.11935538809065`}, {"CURE", 365, 45.50714846709989`}, {"CURE", 365, 64.63228995425757`},
{"CURE", 365, 103.83083889576369`}, {"CURE", 365, 6.873696080893667`}, {"TBUR", 17.33441356590885`, 9.545559937661494`},
{"TBUR", 145.04787275615615`, 39.244788584069724`}, {"TBUR", 56.87434119717797`, 165.66982051194407`},
{"CURE", 365, 19.186238755950995`}, {"TBUR", 112.86210897653183`, 148.87468386293028`},
{"CURE", 365, 65.1295492848478`}, {"CURE", 365, 156.46655454486088`}, {"TBUR", 15.563597410210194`, 37.81587315464453`},
{"TBUR", 39.36335038015242`, 50.11021076458499`}, {"CURE", 365, 74.64812113205619`}, {"CURE", 365, 165.7015498500929`},
{"TBUR", 84.59773617886849`, 74.310649011671`}, {"CURE", 365, 185.9381057849311`}, {"CURE", 365, 35.95487784291274`},
{"CURE", 365, 96.08836055287603`}, {"CURE", 365, 75.25006477616363`}, {"CURE", 365, 84.906942781086`},
{"TBUR", 63.45651519009266`, 83.03807108071551`}, {"TBUR", 44.56281782869434`, 85.41722717926405`},
{"CURE", 365, 9.876255213017767`}, {"CURE", 365, 26.98647727701158`}, {"TBUR", 81.9264961918049`, 94.56199680044864`},
{"CURE", 365, 71.68085120046322`}, {"CURE", 365, 14.602173238322603`}, {"TBUR", 76.46643568699658`, 93.73862576158638`},
{"CURE", 365, 23.6052385246055`}, {"CURE", 365, 16.610386824202227`}, {"CURE", 365, 16.60280256347393`},
```

{"CURE", 365, 19.213683715873962`}, {"CURE", 365, 29.25445035803779`}, {"TBUR", 67.29881842086809`, 78.3830144031036`},
{"CURE", 365, 116.40368889571744`}, {"CURE", 365, 143.21303103778092`}, {"TBUR", 31.46969505835453`, 103.07209914544399`},
{"TBUR", 63.844620143681325`, 7.393105368347291`}, {"CURE", 365, 173.6529801139538`},
{"TBUR", 102.13809617192516`, 49.80897623659201`}, {"TBUR", 39.14251326063107`, 85.99809214405695`},
{"TBUR", 118.40067387832335`, 14.802095102606145`}, {"CURE", 365, 70.22809869641146`},
{"CURE", 365, 1.872779975795866`}, {"CURE", 365, 119.06133918459537`}, {"CURE", 365, 137.8287513348273`},
{"CURE", 365, 71.99279842263236`}, {"CURE", 365, 25.686804452491074`}, {"CURE", 365, 59.476470863525044`},
{"TBUR", 122.76098488090669`, 91.90262124388371`}, {"TBUR", 90.64105211256373`, 92.58777459839021`},
{"TBUR", 40.367302254432474`, 56.32304805126638`}, {"CURE", 365, 82.37228198641401`}, {"CURE", 365, 55.34296504916372`},
{"TBUR", 22.52123239920429`, 143.098983134875`}, {"TBUR", 80.39488255686756`, 42.594627071784124`},
{"CURE", 365, 49.00975196700369`}, {"TBUR", 109.11784304922816`, 79.93365753075912`}, {"CURE", 365, 102.93666417895646`},
{"CURE", 365, 3.7257680068485346`}, {"TBUR", 54.96746097594563`, 45.95275928463627`}, {"CURE", 365, 73.96763548718879`},
{"CURE", 365, 11.816774746772824`}, {"CURE", 365, 144.0738871681256`}, {"TBUR", 15.173552032916275`, 6.623571935427017`},
{"CURE", 365, 139.1962316431748`}, {"CURE", 365, 131.75017878925092`}, {"TBUR", 100.61077901968808`, 3.3420350997069734`},
{"CURE", 365, 110.99565498031956`}, {"CURE", 365, 39.361770033820626`}, {"CURE", 365, 42.584979693430704`},
{"CURE", 365, 120.49539930753187`}, {"CURE", 365, 8.659102867137241`}, {"TBUR", 20.904600093790446`, 68.07236684677042`},
{"CURE", 365, 18.398055302144822`}, {"TBUR", 78.65180876151821`, 2.4104732563743867`}, {"CURE", 365, 68.70738462123502`},
{"TBUR", 74.51769153154721`, 106.51851230583617`}, {"TBUR", 149.69717475974568`, 16.378881965585343`},
{"CURE", 365, 0.6416497706644069`}, {"TBUR", 81.72301936841525`, 7.822366814241709`},
{"TBUR", 30.374014310186848`, 54.66747773240856`}, {"CURE", 365, 2.1987958957535954`},
{"CURE", 365, 5.429348130732761`}, {"CURE", 365, 106.52348779621303`}, {"TBUR", 75.1363534253982`, 179.5810534433977`},
{"TBUR", 89.39648507501671`, 94.34745120213469`}, {"CURE", 365, 174.28640720921072`},
{"TBUR", 73.7915170572194`, 82.41705222595441`}, {"CURE", 365, 1.9928056546858421`}, {"CURE", 365, 92.80438335890278`},
{"TBUR", 89.34812573198708`, 135.79136494476728`}, {"TBUR", 38.591131813820205`, 69.97814028860064`},
{"TBUR", 63.13372493862804`, 6.876332213615438`}, {"TBUR", 57.553588362286014`, 53.33716163923441`},
{"CURE", 365, 36.01733475656048`}, {"CURE", 365, 17.220221896117014`}, {"CURE", 365, 5.822333991397865`},
{"CURE", 365, 107.57205680368827`}, {"TBUR", 127.35161576264922`, 42.28880480668772`},
{"TBUR", 24.926086761908902`, 40.706366676578156`}, {"CURE", 365, 60.3514026022623`},
{"TBUR", 65.51123605691829`, 11.97273911442326`}, {"CURE", 365, 86.85355707118326`}, {"CURE", 365, 44.71573509164127`},
{"TBUR", 63.42171837600915`, 49.92633819388099`}, {"TBUR", 50.70243919425854`, 116.28675432396041`},
{"CURE", 365, 158.90471651496617`}, {"CURE", 365, 43.059439006267134`}, {"CURE", 365, 9.432945368147099`},

{"TBUR", 79.38853353753716`, 181.7507434055894`}, {"TBUR", 155.08795127359014`, 52.07668834590822`},
{"TBUR", 57.0748449560238`, 50.63027873788688`}, {"CURE", 365, 75.16667307338422`}, {"CURE", 365, 9.117911042288108`},
{"CURE", 365, 67.81718693269012`}, {"CURE", 365, 63.77266512877044`}, {"TBUR", 118.26280207494356`, 64.84290355552835`},
{"CURE", 365, 109.52825913188146`}, {"CURE", 365, 43.27954495959817`}, {"TBUR", 165.12673397087886`, 83.54441579446765`},
{"CURE", 365, 34.39214157935073`}, {"CURE", 365, 114.0332204257644`}, {"CURE", 365, 48.571062802007106`},
{"TBUR", 84.95457630532964`, 63.96213623951197`}, {"CURE", 365, 28.410634186582527`},
{"TBUR", 82.28060510026513`, 103.85972110091552`}, {"TBUR", 47.71280274718112`, 204.48464618824835`},
{"TBUR", 41.79236831363921`, 97.75251550554465`}, {"CURE", 365, 97.03279517356218`}, {"CURE", 365, 33.24431771229411`},
{"TBUR", 120.99065651052842`, 81.23605800481984`}, {"TBUR", 57.39109151778137`, 149.19133419713455`},
{"CURE", 365, 4.149760845970239`}, {"CURE", 365, 98.93159557654367`}, {"TBUR", 63.59502329964479`, 7.949161690298208`},
{"CURE", 365, 5.978989225546947`}, {"CURE", 365, 12.047936752148852`}, {"TBUR", 48.33663839757657`, 49.2736450919936`},
{"CURE", 365, 2.7504971480706457`}, {"TBUR", 32.02053193158334`, 67.41456965781069`},
{"CURE", 365, 61.86828874436886`}, {"TBUR", 80.28897825146097`, 77.99442404480602`},
{"TBUR", 32.41327897600387`, 41.18525872970006`}, {"CURE", 365, 68.06903938138392`}, {"CURE", 365, 145.92854562233998`},
{"CURE", 365, 70.99415181458045`}, {"CURE", 365, 13.477281280146128`}, {"CURE", 365, 72.10578526559256`},
{"TBUR", 107.97787924143276`, 6.9717996846104535`}, {"TBUR", 59.33867970920854`, 6.84600257496782`},
{"CURE", 365, 111.8625801838132`}, {"CURE", 365, 116.18786755039271`}, {"TBUR", 80.95365956865403`, 21.49383137211288`},
{"CURE", 365, 33.795054682037176`}, {"TBUR", 18.241609886546314`, 79.2113564078525`},
{"CURE", 365, 139.32490816949073`}, {"TBUR", 133.04056475325459`, 41.618569946614166`},
{"TBUR", 57.896264964248346`, 62.71911787587473`}, {"CURE", 365, 9.874784436493718`},
{"TBUR", 75.67448518917055`, 71.0094981743109`}, {"CURE", 365, 58.03497113363051`}, {"CURE", 365, 73.93910281271266`},
{"TBUR", 69.28396633705073`, 45.83068927034733`}, {"TBUR", 70.77914724105753`, 11.921641574590732`},
{"CURE", 365, 86.20362028128187`}, {"TBUR", 14.621795089339845`, 46.83752724641583`}, {"CURE", 365, 18.141931496644606`},
{"CURE", 365, 93.11958597832381`}, {"CURE", 365, 166.256717667388`}, {"CURE", 365, 131.43073724424548`},
{"CURE", 365, 68.56853177799508`}, {"CURE", 365, 94.80496947918436`}, {"TBUR", 36.678342917806`, 9.586221332933697`},
{"TBUR", 42.669591847422026`, 80.96421098953371`}, {"TBUR", 60.501892152309665`, 15.504815095439112`},
{"CURE", 365, 45.12863435098112`}, {"TBUR", 25.71628368787192`, 83.86040688794772`}, {"CURE", 365, 16.502534757571404`},
{"CURE", 365, 59.82427500696879`}, {"TBUR", 19.945325397831635`, 21.996810734427896`},
{"CURE", 365, 199.91606802632504`}, {"TBUR", 14.327744528258476`, 11.530962146438977`}, {"CURE", 365, 89.01177657860671`},
{"TBUR", 39.77492413673117`, 26.712929555812128`}, {"TBUR", 100.26159325706733`, 9.582191016299753`},
{"CURE", 365, 2.199652635687504`}, {"CURE", 365, 4.14470437639796`}, {"CURE", 365, 72.16357625168945`},

{"TBUR", 26.382493183914583`, 37.43050818684694`}, {"CURE", 365, 2.277214105754749`}, {"CURE", 365, 62.95778900937692`},
{"CURE", 365, 93.25510679343304`}, {"CURE", 365, 179.79268770668784`}, {"CURE", 365, 40.35777667470332`},
{"CURE", 365, 75.42439722639605`}, {"TBUR", 40.80865259216797`, 86.11687789037697`}, {"CURE", 365, 6.774242113762082`},
{"TBUR", 24.085958729823876`, 35.060490662883666`}, {"CURE", 365, 25.402734860475135`}, {"CURE", 365, 75.44253794326569`},
{"TBUR", 42.19793372196124`, 77.49071669077189`}, {"CURE", 365, 50.82619874571982`}, {"CURE", 365, 3.146723696692009`},
{"CURE", 365, 11.521396606927029`}, {"CURE", 365, 59.8929277910728`}, {"TBUR", 41.82191482239538`, 71.71457731424788`},
{"CURE", 365, 95.03375131712124`}, {"TBUR", 106.96205580274983`, 61.765974823794785`}, {"CURE", 365, 181.7245568034679`},
{"CURE", 365, 81.96255697575717`}, {"TBUR", 18.083685460539694`, 64.40589046713995`}, {"CURE", 365, 1.052960949222095`},
{"CURE", 365, 33.302167159636156`}, {"CURE", 365, 137.759034592831`}, {"CURE", 365, 3.9725937168566436`},
{"TBUR", 46.60759217745594`, 50.48708340235762`}, {"TBUR", 49.9115418174276`, 81.67044116064338`},
{"TBUR", 87.35592133525536`, 147.81048381528495`}, {"TBUR", 88.31767227301059`, 99.8661801294719`},
{"CURE", 365, 9.41453865658717`}, {"TBUR", 71.31534354241842`, 8.143996770765613`}, {"CURE", 365, 56.870479859822225`},
{"CURE", 365, 143.37334975995063`}, {"TBUR", 32.49240305852385`, 80.76451487265572`},
{"CURE", 365, 7.782704530077411`}, {"TBUR", 53.87578565821431`, 84.94357669588585`}, {"CURE", 365, 68.44379767752767`},
{"TBUR", 63.329227229122274`, 3.6109606034221304`}, {"CURE", 365, 58.95247698733908`}, {"CURE", 365, 79.909469868638`},
{"CURE", 365, 43.141321824108736`}, {"CURE", 365, 226.55301341835286`}, {"TBUR", 62.45333252717541`, 57.693286422434774`},
{"CURE", 365, 77.7897312605486`}, {"TBUR", 24.440604540186442`, 63.39942856154084`}, {"CURE", 365, 185.2113832158945`},
{"CURE", 365, 63.15194142283356`}, {"TBUR", 19.493421101467938`, 63.60114159557648`},
{"CURE", 365, 35.413140117580554`}, {"TBUR", 58.75774416605372`, 93.56501322235285`}, {"CURE", 365, 0.8332662901785752`},
{"TBUR", 26.451387063183237`, 14.421523651873677`}, {"TBUR", 145.79022539224664`, 87.83947575914877`},
{"CURE", 365, 77.03548778120084`}, {"CURE", 365, 7.8101402568539715`}, {"CURE", 365, 56.44176959417406`},
{"CURE", 365, 36.690010867977016`}, {"TBUR", 70.82496612395282`, 38.78170381310555`}, {"CURE", 365, 7.5554949634532385`},
{"CURE", 365, 50.487540670124034`}, {"CURE", 365, 40.36223099450429`}, {"TBUR", 26.0128572406186`, 25.348394567849088`},
{"CURE", 365, 137.910487371618`}, {"CURE", 365, 3.9486749112741855`}, {"CURE", 365, 58.76395691847418`},
{"TBUR", 162.21068941782565`, 93.971591680958`}, {"TBUR", 16.5244355118097`, 102.36205188543613`},
{"TBUR", 40.537541866372514`, 5.797571662650763`}, {"CURE", 365, 72.62841559918789`},
{"CURE", 365, 94.76102158956577`}, {"TBUR", 24.625976624898183`, 3.338830247714666`}, {"CURE", 365, 44.98313821167756`},
{"CURE", 365, 77.56388866213545`}, {"TBUR", 20.342737884597714`, 20.012490482443305`},
{"CURE", 365, 1.834490670745225`}, {"TBUR", 66.08191289725619`, 157.19995412911882`},
{"TBUR", 113.2404664569553`, 83.30524792793778`}, {"CURE", 365, 84.71821841427223`}, {"CURE", 365, 19.931658111675958`},
{"TBUR", 74.1779698796035`, 2.7116167629205927`}, {"TBUR", 27.71775968166377`, 1.6572597394210198`},

{"TBUR", 13.087771424249805`, 14.449937263233453`}, {"TBUR", 83.92927738515213`, 66.64124572129045`},
{"CURE", 365, 110.43522625119702`}, {"CURE", 365, 94.58399883287456`}, {"CURE", 365, 43.88447838068325`},
{"TBUR", 20.27403561867634`, 1.1265220184637057`}, {"CURE", 365, 74.78241790456579`}, {"CURE", 365, 75.47369708406484`},
{"TBUR", 63.1988192013355`, 0.9061812847467914`}, {"TBUR", 76.20906858215152`, 44.20029361017386`},
{"CURE", 365, 0.9245982083718938`}, {"CURE", 365, 126.87882824374353`}, {"CURE", 365, 52.20645918543176`},
{"TBUR", 14.326213817017177`, 0.638368089605249`}, {"TBUR", 162.90978889135604`, 76.0452726310438`},
{"CURE", 365, 24.893811055755695`}, {"TBUR", 54.57152594249798`, 203.1592744208608`}, {"CURE", 365, 107.34738618833639`},
{"TBUR", 41.615276159374176`, 62.23575026670608`}, {"CURE", 365, 158.1243420930033`}},
{{"410 Res", "410 OS", "410 Tox"}, {"TBUR", 21.438978435630705`, 33.38493637467016`}, {"CURE", 365, 130.33767186106158`},
{"TBUR", 26.741923743343875`, 100.81605036771252`}, {"TBUR", 27.99635204112119`, 3.261534103402723`},
{"TBUR", 17.802094079524892`, 13.142245706284626`}, {"CURE", 365, 22.2615371883325`}, {"CURE", 365, 57.53336187780042`},
{"CURE", 365, 53.73989225794082`}, {"CURE", 365, 129.4809250978729`}, {"TBUR", 70.6147725606471`, 176.8555835905582`},
{"TBUR", 54.310834226236985`, 91.36195831418713`}, {"CURE", 365, 79.19607979269081`},
{"TOX", 26.205521772656176`, 241.39658257754206`}, {"TBUR", 42.68083722903007`, 55.40215403642059`},
{"TBUR", 58.843924809643774`, 5.866249179046376`}, {"TBUR", 85.35726008515687`, 21.51165613824775`},
{"CURE", 365, 92.54748840209658`}, {"TBUR", 43.99640243698688`, 101.33572874885701`},
{"TBUR", 43.079004598399294`, 5.803510631097066`}, {"CURE", 365, 73.53097142181817`}, {"CURE", 365, 123.4542652822955`},
{"CURE", 365, 38.417859255294154`}, {"CURE", 365, 22.953894280441713`}, {"CURE", 365, 55.13039670488793`},
{"CURE", 365, 46.4520035392985`}, {"CURE", 365, 142.47762119123036`}, {"CURE", 365, 79.76298280897727`},
{"CURE", 365, 42.2542961906393`}, {"CURE", 365, 27.03822399692133`}, {"CURE", 365, 4.875467682260612`},
{"CURE", 365, 117.77076998930495`}, {"TBUR", 13.362928291033159`, 31.064396543333444`},
{"CURE", 365, 59.69594326731961`}, {"TBUR", 46.933341517965054`, 34.10428536536998`}, {"CURE", 365, 163.5283336098805`},
{"CURE", 365, 192.6153187900356`}, {"CURE", 365, 35.52327368687306`}, {"TBUR", 21.052704186821934`, 9.811562277138918`},
{"TBUR", 71.53116262915137`, 41.4584971470404`}, {"CURE", 365, 87.24334658202066`},
{"TBUR", 17.949777983885554`, 53.5488354279586`}, {"CURE", 365, 84.28379418503219`}, {"CURE", 365, 63.60199822044368`},
{"TBUR", 45.89136760208776`, 70.7359005611918`}, {"CURE", 365, 87.29534765465837`}, {"CURE", 365, 4.540621318595501`},
{"CURE", 365, 96.9619775688007`}, {"CURE", 365, 60.17911171176247`}, {"TBUR", 112.14337548449782`, 119.89555074409648`},
{"CURE", 365, 203.63190685183784`}, {"TBUR", 29.6092185732819`, 90.62014868369057`},
{"TBUR", 41.58514515335005`, 78.18803438007856`}, {"CURE", 365, 5.92401439058454`}, {"CURE", 365, 126.39758969450689`},
{"CURE", 365, 36.8072339330575`}, {"CURE", 365, 88.8007132409021`}, {"CURE", 365, 58.76140312370357`},
{"TBUR", 103.06535630722317`, 62.58919822518805`}, {"TBUR", 37.14670924548391`, 152.47250711101356`},

{"TBUR", 52.08488534688098`, 91.34351350079372`}, {"CURE", 365, 84.46723639579871`}, {"CURE", 365, 4.191965305945565`},
{"TBUR", 33.554407185122564`, 103.25571039300112`}, {"CURE", 365, 68.51676320966419`},
{"TBUR", 66.30040088529817`, 6.364854321574972`}, {"CURE", 365, 80.076750997337`}, {"CURE", 365, 70.2577205591536`},
{"TBUR", 57.23362357252988`, 97.04218132691075`}, {"CURE", 365, 35.185292382311644`}, {"CURE", 365, 29.42150901239518`},
{"CURE", 365, 56.607815656340954`}, {"CURE", 365, 40.634415155754176`}, {"CURE", 365, 126.06177159329489`},
{"TBUR", 13.230234454975216`, 1.9064944817740468`}, {"TBUR", 79.27858638550242`, 80.0749547042862`},
{"CURE", 365, 101.87433816297977`}, {"TBUR", 96.9119449934974`, 196.09631856293365`}, {"CURE", 365, 64.05884220591993`},
{"TBUR", 75.68590851586002`, 127.75740468303691`}, {"CURE", 365, 46.892864392639794`}, {"CURE", 365, 102.30665477868915`},
{"CURE", 365, 3.3071719611615817`}, {"CURE", 365, 6.55723871272345`}, {"CURE", 365, 4.626176120921831`},
{"CURE", 365, 118.64673367574788`}, {"TBUR", 99.27394796315706`, 90.75328073752503`}, {"CURE", 365, 67.37323203025406`},
{"CURE", 365, 36.5321809014034`}, {"CURE", 365, 7.108851852663127`}, {"TBUR", 71.34249337108203`, 130.93022244012877`},
{"TBUR", 31.917436988534405`, 9.730232658212175`}, {"TBUR", 89.10034000480873`, 97.72868928899199`},
{"TBUR", 63.84462418482995`, 2.7198199894117785`}, {"CURE", 365, 6.30120108007141`},
{"TBUR", 18.658496674258732`, 1.8661513027489962`}, {"TBUR", 157.85139186602797`, 51.04671514962544`},
{"CURE", 365, 35.375758815440186`}, {"CURE", 365, 178.29858305089147`}, {"TBUR", 82.56481820031868`, 9.618072108081742`},
{"TBUR", 43.577318504001035`, 135.21365402708395`}, {"TBUR", 19.87351113304814`, 9.625237158893036`},
{"TBUR", 127.2649201008624 7`, 122.49820575301683`}, {"CURE", 365, 67.67101584235363`},
{"TBUR", 90.43702399228849`, 89.88271014201106`}, {"CURE", 365, 61.008453956967585`}, {"CURE", 365, 97.62852947279353`},
{"CURE", 365, 39.08035383047675`}, {"TBUR", 137.978415435085`, 85.61718291180951`}, {"CURE", 365, 34.77961251139477`},
{"CURE", 365, 99.71845829794013`}, {"CURE", 365, 137.85931379907024`}, {"CURE", 365, 75.26193252026151`},
{"CURE", 365, 18.110378457522653`}, {"CURE", 365, 46.112920806955294`}, {"CURE", 365, 68.04715665335549`},
{"CURE", 365, 55.709828698874716`}, {"CURE", 365, 2.3309458784335972`}, {"CURE", 365, 73.2389822575199`},
{"CURE", 365, 68.36317963092148`}, {"CURE", 365, 18.30351806422875`}, {"CURE", 365, 79.9728662534141`},
{"CURE", 365, 160.8119507349836`}, {"CURE", 365, 100.06566383005287`}, {"TBUR", 19.505368247746677`, 5.24122526746205`},
{"CURE", 365, 71.6739267604126`}, {"TBUR", 38.41849629479499`, 58.75326936665495`}, {"CURE", 365, 46.864288526669355`},
{"CURE", 365, 32.360285886485876`}, {"CURE", 365, 114.66127066336152`}, {"CURE", 365, 110.76970621080612`},
{"CURE", 365, 99.51783864107614`}, {"CURE", 365, 109.0242150334739`}, {"TBUR", 71.43474836408872`, 31.480589092904115`},
{"TBUR", 44.554425898809`, 32.31161484000833`}, {"CURE", 365, 159.66820769845344`},
{"TBUR", 12.75991528882548`, 68.01864990208105`}, {"TBUR", 86.97377684757836`, 22.36962688440258`},
{"TBUR", 62.005139096256094`, 50.758429973962436`}, {"TBUR", 95.91968014232238`, 18.273082747896797`},
{"CURE", 365, 107.913417517256`}, {"TBUR", 52.856317546280444`, 163.17251918475688`}, {"CURE", 365, 109.29159337043389`},

{"CURE", 365, 141.21755017998785`}, {"CURE", 365, 12.087863681472344`}, {"CURE", 365, 1.169583519341947`},
{"CURE", 365, 64.20909447373249`}, {"CURE", 365, 158.140117118979`}, {"TBUR", 36.06405871055059`, 37.72666914204654`},
{"CURE", 365, 72.17981207306563`}, {"CURE", 365, 12.146530494167068`}, {"CURE", 365, 6.8790056041535115`},
{"CURE", 365, 56.89561177091404`}, {"CURE", 365, 1.149284047828874`}, {"TBUR", 88.26799337669777`, 160.6298857816902`},
{"TBUR", 49.794903139881725`, 61.25565904990612`}, {"TBUR", 88.27476693933812`, 141.45384797304823`},
{"CURE", 365, 68.30979460944516`}, {"CURE", 365, 82.81041162638142`}, {"CURE", 365, 66.77374493820574`},
{"CURE", 365, 82.86833100899722`}, {"CURE", 365, 17.631600312358113`}, {"TBUR", 91.37094133284361`, 21.72460821956793`},
{"CURE", 365, 1.6624664575713088`}, {"TBUR", 58.65958676947526`, 7.145130051924404`},
{"TBUR", 78.60513155474557`, 70.39995550824275`}, {"CURE", 365, 110.10645666142062`}, {"CURE", 365, 109.77728084832171`},
{"CURE", 365, 78.23768778916623`}, {"CURE", 365, 114.18449472277781`}, {"CURE", 365, 1.6843111646594198`},
{"CURE", 365, 62.578136258501964`}, {"TBUR", 29.65716247027094`, 122.2887957608241`}, {"CURE", 365, 54.336624503535674`},
{"TBUR", 20.621793323967708`, 6.480620600837197`}, {"TBUR", 50.16705445901241`, 120.95709463065864`},
{"TBUR", 83.35831035341404`, 9.973601882263457`}, {"TBUR", 67.61969383049653`, 143.139816162965`},
{"TBUR", 21.16576325554795`, 77.19972661269658`}, {"TBUR", 57.56417119658627`, 37.327186825392744`},
{"TBUR", 90.95295276290905`, 96.67501218705732`}, {"TBUR", 80.05580566689365`, 91.23285406961817`},
{"CURE", 365, 79.66477483209906`}, {"CURE", 365, 136.94031573962258`}, {"TBUR", 18.824254702013437`, 6.899835928927899`},
{"CURE", 365, 124.00511335738686`}, {"TBUR", 27.87601604146823`, 39.88625164249834`},
{"TBUR", 53.527609916782474`, 136.18929278033883`}, {"CURE", 365, 62.486054771606376`},
{"CURE", 365, 3.6082344745837562`}, {"TBUR", 24.110904499413596`, 89.11451091155335`}, {"CURE", 365, 85.01096749470257`},
{"CURE", 365, 137.0916402105861`}, {"CURE", 365, 157.80688498058447`}, {"CURE", 365, 103.94158009758912`},
{"CURE", 365, 60.513284681178504`}, {"CURE", 365, 46.10688634888426`}, {"CURE", 365, 164.487275289505`},
{"TBUR", 13.540542441689716`, 12.325440137307474`}, {"CURE", 365, 92.69706519050592`}, {"CURE", 365, 82.01469004742106`},
{"TBUR", 25.3171508784503`, 22.355915929953824`}, {"TBUR", 57.025555330240415`, 95.44977993466316`},
{"CURE", 365, 88.96753590683957`}, {"TBUR", 65.21235902190573`, 74.55257517755106`}, {"CURE", 365, 1.6880786173668518`},
{"TBUR", 124.62120634411606`, 11.561110714244231`}, {"TBUR", 15.827408521081255`, 80.34242434788291`},
{"CURE", 365, 160.76707656761954`}, {"CURE", 365, 4.3279187640106995`}, {"CURE", 365, 122.0725175487397`},
{"CURE", 365, 2.772178610366293`}, {"CURE", 365, 86.496090714862`}, {"CURE", 365, 13.597044083941007`},
{"TBUR", 75.08261101610965`, 105.84480580381336`}, {"CURE", 365, 21.00601388762146`},
{"TBUR", 40.996771693368515`, 12.835544824925531`}, {"TBUR", 79.8916101425588`, 148.68909761727454`},
{"CURE", 365, 71.4677548882231`}, {"TBUR", 50.02070191384268`, 194.47745082787722`}, {"CURE", 365, 67.35073599161687`},
{"CURE", 365, 95.5222593370533`}, {"CURE", 365, 11.92498074409974`}, {"CURE", 365, 64.52099111559636`},

{"CURE", 365, 126.93388597647193`}, {"CURE", 365, 133.20178526011978`}, {"CURE", 365, 64.07862009861904`},
{"CURE", 365, 118.02593045078855`}, {"CURE", 365, 75.07883050862478`}, {"CURE", 365, 7.2292527092876`},
{"CURE", 365, 46.8064560542598`}, {"CURE", 365, 36.88770577323089`}, {"CURE", 365, 66.01730151815276`},
{"CURE", 365, 97.53047264767014`}, {"CURE", 365, 81.25058880641338`}, {"CURE", 365, 73.7083259276776`},
{"TBUR", 22.495697581340984`, 93.50448555177557`}, {"TBUR", 27.944322857417244`, 63.52693400436102`},
{"TBUR", 156.45939035269473`, 61.367855930266096`}, {"TBUR", 19.88692067685682`, 41.05244886102208`},
{"CURE", 365, 55.10832885350133`}, {"CURE", 365, 63.55196919901302`}, {"CURE", 365, 116.88849488698476`},
{"TBUR", 26.491231711366684`, 5.317749601487443`}, {"TBUR", 31.09105568725494`, 19.663432532193983`},
{"CURE", 365, 1.5573043189918954`}, {"CURE", 365, 2.3301416494842466`}, {"TBUR", 18.992848180085634`, 20.92722860579165`},
{"CURE", 365, 187.44986131964512`}, {"CURE", 365, 44.323474392603984`}, {"CURE", 365, 183.12116073477944`},
{"TBUR", 38.33651236001247`, 83.27520330353191`}, {"CURE", 365, 51.674330067649734`},
{"TBUR", 15.157870241242719`, 108.63322896909875`}, {"CURE", 365, 45.911715377521574`},
{"CURE", 365, 93.64531057402168`}, {"CURE", 365, 2.0588956772090232`}, {"CURE", 365, 68.82685112509047`},
{"CURE", 365, 19.932304103260506`}, {"CURE", 365, 8.624641793427166`}, {"CURE", 365, 106.37695328417752`},
{"TBUR", 21.470626585892166`, 113.14340583350962`}, {"TBUR", 13.62512994389988`, 81.55192524032128`},
{"CURE", 365, 53.71064947958336`}, {"TBUR", 27.98852996056516`, 76.73302211964095`}, {"CURE", 365, 5.065627592965355`},
{"CURE", 365, 52.301939030079396`}, {"CURE", 365, 37.79696476740912`}, {"CURE", 365, 191.14977250938742`},
{"CURE", 365, 74.13244257326771`}, {"CURE", 365, 40.688621072714255`}, {"CURE", 365, 1.4957075170278182`},
{"TBUR", 69.73452651490288`, 77.66413704305997`}, {"CURE", 365, 66.65458657470873`},
{"TBUR", 151.16393729633162`, 70.90216833968567`}, {"TBUR", 50.83716717025143`, 7.714882942949822`},
{"CURE", 365, 70.66612439592154`}, {"CURE", 365, 50.078187849605996`}, {"TBUR", 20.480924572160625`, 66.67048868583902`},
{"CURE", 365, 33.938408031794076`}, {"CURE", 365, 89.65537804658382`}, {"TBUR", 113.8236012897201`, 6.870221896129426`},
{"TBUR", 97.7632906020069`, 91.30590030253637`}, {"TBUR", 16.520417807801945`, 34.87564844471762`},
{"CURE", 365, 73.87427312104772`}, {"TBUR", 26.825076296744598`, 83.63615226428374`}, {"CURE", 365, 8.328748453510446`},
{"CURE", 365, 106.14037954727678`}, {"CURE", 365, 62.87674902440345`}, {"CURE", 365, 175.10249220249258`},
{"CURE", 365, 124.52440781586027`}, {"CURE", 365, 86.94143184217731`}, {"CURE", 365, 51.951917896246805`},
{"CURE", 365, 183.48817083348283`}, {"TBUR", 29.58585311181158`, 26.47846468266182`}, {"CURE", 365, 16.617729265159713`},
{"CURE", 365, 17.824959811695653`}, {"CURE", 365, 71.42381305552708`}, {"CURE", 365, 65.56962687149594`},
{"CURE", 365, 103.37918975500504`}, {"CURE", 365, 26.467594265836794`}, {"TBUR", 23.16035862468263`, 131.8757756211035`},
{"CURE", 365, 2.015573978597178`}, {"CURE", 365, 106.23481814308963`}, {"TBUR", 38.14907810925798`, 128.27260384960152`},
{"CURE", 365, 39.02412756930968`}, {"CURE", 365, 109.5815909496696`}, {"CURE", 365, 3.6485876634570884`},

{"TBUR", 21.900922246239766`, 38.989605175712484`}, {"CURE", 365, 57.01468071073418`},
{"CURE", 365, 56.67746827394718`}, {"TBUR", 28.56631586236138`, 27.06325849018784`}, {"CURE", 365, 58.110416267458895`},
{"TBUR", 35.694417471156775`, 27.4528020922978`}, {"CURE", 365, 98.28108694952338`}, {"CURE", 365, 75.3664411209966`},
{"CURE", 365, 84.83472034473716`}, {"CURE", 365, 57.87757918065945`}, {"CURE", 365, 64.78131018585144`},
{"CURE", 365, 92.94684057025414`}, {"CURE", 365, 118.7725551531852`}, {"TBUR", 48.98498724282284`, 88.15004890235872`},
{"CURE", 365, 118.1559751696697`}, {"CURE", 365, 72.38496258125598`}, {"TBUR", 54.16664827421289`, 18.60087145332475`},
{"TBUR", 16.738388362432456`, 13.196661041998288`}, {"CURE", 365, 0.6980636004227804`}, {"CURE", 365, 103.81006751403484`},
{"CURE", 365, 103.32626590823506`}, {"CURE", 365, 81.45429095993065`}, {"TBUR", 137.7915755424083`, 189.37402450350135`},
{"CURE", 365, 145.86247629636895`}, {"CURE", 365, 32.734667439074826`}, {"TBUR", 20.397950415514465`, 56.06647339561241`},
{"TBUR", 29.925987152644268`, 6.475003365335999`}, {"CURE", 365, 180.50532764580683`},
{"TBUR", 38.75199253054658`, 174.90835952035502`}, {"TBUR", 22.710875404652775`, 21.720865258845336`},
{"CURE", 365, 172.19487659531893`}, {"CURE", 365, 27.706369540778997`}, {"CURE", 365, 58.65787777364946`},
{"CURE", 365, 119.63983201438039`}, {"CURE", 365, 2.0216520992377296`}, {"TBUR", 55.54639787971482`, 34.533183673127525`},
{"CURE", 365, 110.44595328376597`}, {"TBUR", 49.001639395804865`, 26.556655257868886`},
{"TBUR", 38.29730878823628`, 175.43199839262363`}, {"CURE", 365, 2.996382526188085`}, {"CURE", 365, 148.2407560837341`},
{"CURE", 365, 86.9918413799278`}, {"CURE", 365, 81.2673029468899`}, {"CURE", 365, 104.36285580430304`},
{"CURE", 365, 43.20935688822451`}, {"TBUR", 35.43353284838796`, 132.1857035072715`}, {"CURE", 365, 80.05821329716984`},
{"CURE", 365, 141.05348922411224`}, {"CURE", 365, 91.01017548650081`}, {"TBUR", 54.2478981334118`, 96.33081452751767`},
{"CURE", 365, 114.20466986879273`}, {"CURE", 365, 81.72647013128372`}, {"CURE", 365, 37.814694990343604`},
{"CURE", 365, 126.7947617088958`}, {"CURE", 365, 152.71063580998887`}, {"CURE", 365, 39.5385156466153`},
{"TBUR", 63.82489330281331`, 99.46464299183194`}, {"TBUR", 45.78027571174047`, 127.6458429086473`},
{"CURE", 365, 9.47472121735395`}, {"CURE", 365, 91.73334127425187`}, {"CURE", 365, 45.218229503757726`},
{"TBUR", 14.344260847642863`, 7.205457451696313`}, {"TBUR", 13.285059492120471`, 26.56978007936807`},
{"TBUR", 30.750046875585323`, 44.583669210992475`}, {"CURE", 365, 88.56376695046751`}, {"CURE", 365, 17.76438253850004`},
{"CURE", 365, 77.63404986838725`}, {"TBUR", 77.8719554586635`, 144.9569064993344`}, {"CURE", 365, 80.85317284999533`},
{"CURE", 365, 71.95880389660681`}, {"CURE", 365, 66.4005693062754`}, {"TBUR", 16.9525921946333`, 30.409757791932584`},
{"TBUR", 13.54566527651752`, 73.50434529326488`}, {"TBUR", 72.4437065536523`, 33.1559936551317`},
{"CURE", 365, 130.50868478045578`}, {"TBUR", 49.38487364761175`, 77.56971193627597`},
{"CURE", 365, 103.29089380795679`}, {"CURE", 365, 78.00566018594795`}, {"CURE", 365, 66.9051474177668`},
{"TBUR", 100.61367353632915`, 29.090330949565324`}, {"CURE", 365, 47.09787960670651`}, {"CURE", 365, 183.48090464648783`},
{"CURE", 365, 94.1107231287039`}, {"CURE", 365, 77.58944720343156`}, {"TBUR", 32.94458052535498`, 67.99613775864943`},

{"TBUR", 76.67963593321164`, 64.7597124790046`}, {"CURE", 365, 49.79824807130322`},
{"TBUR", 15.892173559226336`, 56.00129737943027`}, {"TBUR", 17.071059719325586`, 52.65987731026324`},
{"CURE", 365, 41.67536014610743`}, {"CURE", 365, 74.2350696087433`}, {"TBUR", 99.25662245834923`, 6.169149286693382`},
{"TBUR", 12.947097483804761`, 52.485538612600735`}, {"TBUR", 20.601185561685664`, 13.149200655287467`},
{"CURE", 365, 116.3152830429505`}, {"CURE", 365, 24.90053616030661`}, {"CURE", 365, 87.32223868410348`},
{"CURE", 365, 4.7162235710736935`}, {"CURE", 365, 71.2799815799656`}, {"CURE", 365, 2.6534575678040273`},
{"CURE", 365, 82.9075631307448`}, {"CURE", 365, 73.03609892187008`}, {"TBUR", 114.74896195005891`, 88.462635427739`},
{"CURE", 365, 6.629234476421067`}, {"CURE", 365, 43.72709677888637`}, {"CURE", 365, 176.10387176215042`},
{"TBUR", 24.822162516710534`, 39.93496851560027`}, {"CURE", 365, 1.6318066936842437`}, {"CURE", 365, 17.92897441436969`},
{"CURE", 365, 123.9397181623505`}, {"CURE", 365, 7.010177235323111`}, {"CURE", 365, 89.56758953316047`},
{"CURE", 365, 144.6273530915543`}, {"CURE", 365, 57.157591063826`}, {"CURE", 365, 7.164125769488966`},
{"TBUR", 152.28250240351767`, 1.8587395550213355`}, {"CURE", 365, 109.02153028940911`},
{"TBUR", 13.360562132644205`, 13.910339513922382`}, {"TBUR", 50.79796985139788`, 205.21194889885348`},
{"CURE", 365, 75.07660142860387`}, {"CURE", 365, 85.3376698757945`}, {"TBUR", 84.83498764625692`, 74.17404696884196`},
{"CURE", 365, 47.676693130007294`}, {"CURE", 365, 210.12107813625863`}, {"CURE", 365, 63.173271885376224`},
{"TBUR", 104.43271356859785`, 3.5471454336548365`}, {"CURE", 365, 63.539912500901764`}, {"CURE", 365, 17.53591074932322`},
{"CURE", 365, 162.11660621567717`}, {"CURE", 365, 61.06409703206565`}, {"CURE", 365, 4.0463016505750184`},
{"CURE", 365, 68.01959197070967`}, {"TBUR", 21.945929337912734`, 78.31181618883777`}, {"CURE", 365, 78.7984148578762`},
{"CURE", 365, 67.08908872320511`}, {"CURE", 365, 49.87377083719317`}, {"CURE", 365, 112.85981525952832`},
{"CURE", 365, 9.369514267683524`}, {"TBUR", 77.87923220087518`, 153.09892107722393`}, {"CURE", 365, 62.90950760529918`},
{"CURE", 365, 6.189149044982684`}, {"CURE", 365, 23.491741809787793`}, {"CURE", 365, 186.1906925499086`},
{"CURE", 365, 72.34122786357747`}, {"CURE", 365, 132.01469395643502`}, {"CURE", 365, 67.41312935783164`},
{"CURE", 365, 151.77432544923525`}, {"CURE", 365, 140.83252704066868`}, {"TBUR", 71.80824294091889`, 19.777157583781978`},
{"CURE", 365, 59.73557921582043`}, {"TBUR", 131.20968888150915`, 60.61899439102391`},
{"TBUR", 17.23896971429079`, 12.470028938987111`}, {"TBUR", 21.951068203058103`, 7.9635169260569665`},
{"CURE", 365, 53.63186907771703`}, {"CURE", 365, 87.07223946277173`}, {"TBUR", 50.52142694371837`, 35.961756039830945`},
{"CURE", 365, 136.54393319109033`}, {"CURE", 365, 128.91382229742044`}, {"TBUR", 18.609265459385778`, 14.510427177770383`},
{"TBUR", 24.68708157545162`, 31.43147796153408`}, {"TBUR", 37.75881273370592`, 11.311903967676392`},
{"TBUR", 26.005875214332338`, 97.10466608510922`}, {"TBUR", 43.83178765284537`, 83.78975297663673`},
{"CURE", 365, 105.87563724813629`}, {"TBUR", 76.87114121237636`, 136.47536779763004`},
{"CURE", 365, 32.19255271996882`}, {"CURE", 365, 102.9341626650447`}, {"TBUR", 27.541897580538325`, 87.41080095158772`},

{"TBUR", 26.102035263709222`, 5.751590489022368`}, {"CURE", 365, 143.51284787061476`}, {"CURE", 365, 60.39629904027972`},
{"TBUR", 35.452624755542075`, 3.1992439840823548`}, {"TBUR", 19.805839293431283`, 2.269822989334806`},
{"CURE", 365, 48.597678931662486`}, {"CURE", 365, 70.4360762130968`}, {"CURE", 365, 80.45133908561651`},
{"TBUR", 38.58709130833812`, 52.84097724278256`}, {"CURE", 365, 83.5631909316737`}, {"CURE", 365, 118.59154009840036`},
{"CURE", 365, 56.426484155136386`}, {"TBUR", 91.34811191161313`, 21.354936646476567`},
{"CURE", 365, 102.99275280696259`}, {"TBUR", 99.2839781572611`, 2.1640324813569625`},
{"CURE", 365, 29.284358383012155`}, {"TBUR", 77.33696030013839`, 68.24842799177209`},
{"CURE", 365, 6.308137561313698`}, {"TBUR", 43.298135710357265`, 40.53822662272232`},
{"CURE", 365, 67.70534071430146`}, {"TBUR", 34.14120265186922`, 76.71510381001063`}, {"CURE", 365, 30.422344198088485`},
{"TBUR", 20.684392646344048`, 53.17917138579724`}, {"CURE", 365, 70.58964871189683`},
{"TBUR", 16.334446725602714`, 85.48027144248302`}, {"CURE", 365, 75.75378001334562`},
{"TBUR", 16.145129670624552`, 19.588175224282075`}, {"TBUR", 64.14047051222649`, 59.59716257557963`},
{"CURE", 365, 139.44025485555682`}, {"CURE", 365, 6.008657796215113`}, {"CURE", 365, 59.478050369840474`},
{"CURE", 365, 25.875071396863355`}, {"CURE", 365, 87.19067316812061`}, {"CURE", 365, 33.5819715315326`},
{"TBUR", 49.59155559734444`, 107.18411012750498`}, {"TBUR", 77.7663632364552`, 67.72396523179339`},
{"CURE", 365, 129.3647766904336`}, {"CURE", 365, 36.465163638880846`}, {"CURE", 365, 173.06953440525245`},
{"CURE", 365, 114.24164511828073`}, {"CURE", 365, 88.8683880170132`}, {"CURE", 365, 111.10207462090418`},
{"TBUR", 134.31133083780364`, 177.22222294249775`}, {"TBUR", 39.211858915032856`, 189.84275673400992`},
{"CURE", 365, 53.296026693023116`}, {"CURE", 365, 90.07220579396021`}, {"CURE", 365, 98.10244265880215`},
{"TBUR", 39.7545922734957`, 33.502148656769585`}, {"TBUR", 16.286391644873095`, 6.567257990262936`},
{"CURE", 365, 0.8851908379242198`}, {"CURE", 365, 67.48808281316806`}, {"CURE", 365, 117.53295642122879`},
{"TBUR", 20.194756591889078`, 93.677115352567`}, {"CURE", 365, 131.82607674106805`}, {"CURE", 365, 175.3916411253408 7`},
{"CURE", 365, 39.731630486328484`}, {"CURE", 365, 87.01608481560845`}, {"TBUR", 36.211531702984715`, 6.708243296982336`},
{"CURE", 365, 196.2994828161312`}, {"TBUR", 36.684352381933614`, 62.31428028862855`},
{"TBUR", 57.64281877929005`, 41.88857373473849`}, {"TBUR", 64.52353290538008`, 51.50701407505054`},
{"CURE", 365, 182.14180022486127`}, {"CURE", 365, 71.48369896306525`}, {"TBUR", 91.99619314326914`, 159.8078048364484`},
{"CURE", 365, 122.55689925278699`}, {"CURE", 365, 107.8512213458778`}, {"TBUR", 54.230419790038596`, 103.92561424255928`},
{"TBUR", 87.6223684742105`, 101.85744187031943`}, {"CURE", 365, 48.181067787075015`}, {"CURE", 365, 40.05950291478733`},
{"CURE", 365, 8.662211676522325`}, {"CURE", 365, 75.00701435743292`}, {"CURE", 365, 88.74136994761145`},
{"TBUR", 33.49517511647438`, 46.41216096488814`}, {"TBUR", 43.53802252522176`, 59.9274842273337`},
{"CURE", 365, 118.46704392744051`}, {"TBUR", 41.35049321023037`, 54.768305445188744`},

{"CURE", 365, 64.38312102794514`}, {"CURE", 365, 27.062450054563836`}, {"TBUR", 20.279460841926976`, 9.067748568971169`},
{"CURE", 365, 43.90741301773964`}, {"CURE", 365, 139.37073945051773`}, {"CURE", 365, 56.15528059204203`},
{"TBUR", 42.636229850200955`, 54.004246743051446`}, {"CURE", 365, 10.669221789011582`},
{"TBUR", 25.70807203894738`, 125.89090636521271`}, {"CURE", 365, 135.00344972968912`}, {"CURE", 365, 84.64716540669818`},
{"TBUR", 99.97235997611587`, 28.83796236719511`}, {"CURE", 365, 131.08447908942443`}, {"CURE", 365, 15.513520672173065`},
{"TBUR", 84.19919019716309`, 194.88325054593196`}, {"TBUR", 28.42762763853941`, 68.2233689661592`},
{"CURE", 365, 144.48661269839747`}, {"TBUR", 42.88359525099704`, 71.65625502856457`}, {"CURE", 365, 39.41378603790983`},
{"TBUR", 41.385556865119696`, 50.83406777402528`}, {"TBUR", 24.12358172573506`, 17.1194634990935`},
{"CURE", 365, 113.26420801470906`}, {"TBUR", 41.67427560136814`, 35.42690932930922`},
{"CURE", 365, 50.8551904608068`}, {"TBUR", 23.89664976249805`, 42.92916795148364`}, {"CURE", 365, 72.2520641679574 7`},
{"TBUR", 23.123417194317962`, 41.554866675192905`}, {"TBUR", 106.12465501602722`, 146.97665084522654`},
{"CURE", 365, 88.66814329596274`}, {"TBUR", 33.10803315702048`, 62.50501582310795`},
{"TBUR", 66.11943272003589`, 64.09812760053772`}, {"TBUR", 13.786327108674799`, 60.05652204901539`},
{"CURE", 365, 2.436750589202353`}, {"TBUR", 108.17967821882044`, 114.48740521687371`},
{"TBUR", 101.74754487261465`, 100.507420213544`}, {"CURE", 365, 83.91371469617083`},
{"TBUR", 39.846540285366096`, 8.62038113800503`}, {"CURE", 365, 59.65352032614297`}, {"CURE", 365, 162.9867795611371`},
{"CURE", 365, 78.9346454746704`}, {"CURE", 365, 94.2499028803948`}, {"TBUR", 27.921402991244943`, 30.903066609319104`},
{"TBUR", 85.06260018157204`, 77.4719856516349`}, {"TBUR", 13.106817678948579`, 2.2790467698108774`},
{"TBUR", 15.086536761279767`, 11.765470904748002`}, {"TBUR", 52.578854228275596`, 114.22878915857873`},
{"CURE", 365, 70.12784057976198`}, {"TBUR", 51.151956637843256`, 63.07374886532701`}, {"CURE", 365, 14.623810970717244`},
{"TBUR", 217.7916889168146`, 136.74557815107775`}, {"CURE", 365, 72.10844640964302`}, {"CURE", 365, 135.58428849739067`},
{"TBUR", 50.28277785894905`, 22.038819690188877`}, {"TBUR", 30.00488331667513`, 75.93877288687469`},
{"CURE", 365, 62.92390480375961`}, {"CURE", 365, 77.61853955659208`}, {"CURE", 365, 13.67589347403632`},
{"CURE", 365, 55.001549128156555`}, {"CURE", 365, 121.74336442907027`}, {"TBUR", 20.63065218741124`, 78.31335131102512`},
{"CURE", 365, 38.028093168827155`}, {"TBUR", 39.35664484633947`, 2.587279164741017`}, {"CURE", 365, 41.07986556567553`},
{"CURE", 365, 123.49767012614224`}, {"CURE", 365, 48.22659765324519`}, {"CURE", 365, 72.62418916467644`},
{"TBUR", 15.860962532500524`, 93.06854414388948`}, {"TBUR", 87.90417146883945`, 74.1461995402566`},
{"TBUR", 86.04420195310504`, 137.14485347157654`}, {"CURE", 365, 124.9042751771436`},
{"CURE", 365, 84.97328228487653`}, {"CURE", 365, 92.88693792262636`}, {"CURE", 365, 128.72946698644182`},
{"TBUR", 19.0595810483346`, 3.583889077163942`}, {"TBUR", 79.15674846634916`, 134.4912867201009`},
{"TBUR", 147.8069698621884`, 41.953326443237366`}, {"TBUR", 65.99812880910241`, 189.31891590607484`},

{"CURE", 365, 11.432754449934446`}, {"TBUR", 90.90033010806651`, 67.88651666312505`}, {"CURE", 365, 159.2240271911829`},
{"CURE", 365, 7.414733841561085`}, {"CURE", 365, 96.77621560208866`}, {"TBUR", 94.75978795638753`, 80.7362597943044`},
{"TBUR", 33.08636285194738`, 103.80033825213499`}, {"TBUR", 15.396117216959242`, 51.380411589353585`},
{"TBUR", 13.788362414732745`, 26.092069948709053`}, {"TBUR", 17.19311818892311`, 6.852807383420654`},
{"CURE", 365, 72.50315102710377`}, {"CURE", 365, 70.62926575899733`}, {"TBUR", 37.057081597556916`, 86.60310747817593`},
{"CURE", 365, 14.41129749624207`}, {"CURE", 365, 197.26710168803427`}, {"CURE", 365, 36.7791179615678`},
{"CURE", 365, 79.09272790944131`}, {"CURE", 365, 44.21539620645323`}, {"CURE", 365, 7.54469762101277`},
{"TBUR", 115.1348104719985`, 134.6757299306336`}, {"CURE", 365, 2.83944010591272`}, {"CURE", 365, 51.66082120646827`},
{"TBUR", 18.957443450662637`, 25.030600223846555`}, {"CURE", 365, 57.46828944328648`},
{"TBUR", 71.45866226905905`, 203.49016930523862`}, {"CURE", 365, 140.45732345952734`},
{"TBUR", 36.54862976195449`, 79.31131735054416`}, {"CURE", 365, 74.69340625369404`},
{"CURE", 365, 123.4899111312023`}, {"CURE", 365, 150.26299681989286`}, {"CURE", 365, 116.3910905302553`},
{"CURE", 365, 151.41065038618487`}, {"TBUR", 14.28528363216293`, 49.9275369187313`}, {"CURE", 365, 59.28933822746482`},
{"TBUR", 23.538598411911476`, 6.694403328682534`}, {"TBUR", 18.771323364573576`, 73.05654105986937`},
{"TBUR", 60.1013509661591 5`, 4.038819504400528`}, {"CURE", 365, 1.0055798161991463`}, {"CURE", 365, 6.662895471993588`},
{"TBUR", 139.19308106417284`, 0.7409071657154488`}, {"TBUR", 18.705278291314954`, 68.65454812946464`},
{"CURE", 365, 12.512360358095654`}, {"CURE", 365, 56.84729888276139`}, {"CURE", 365, 147.13439411236493`},
{"CURE", 365, 26.681879924541096`}, {"TBUR", 20.359143440564264`, 69.60754301707148`},
{"TBUR", 13.182765100881685`, 48.02892319194186`}, {"CURE", 365, 12.77395869007446`},
{"TBUR", 62.3100501848158`, 94.43102455917375`}, {"TBUR", 32.01568905500208`, 19.951235922344008`},
{"TBUR", 58.57960431015752`, 1.8468472929074593`}, {"TBUR", 41.98557397621385`, 7.329298415257727`},
{"CURE", 365, 144.6469797899112`}, {"CURE", 365, 24.211811252911573`}, {"CURE", 365, 145.63922663403153`},
{"TBUR", 171.9921653734002`, 92.77152967297607`}, {"CURE", 365, 130.32106307177418`}, {"CURE", 365, 42.640956633367345`},
{"CURE", 365, 175.16975490589456`}, {"CURE", 365, 75.82123244524482`}, {"TBUR", 23.330045764398044`, 4.34247327753842`},
{"CURE", 365, 129.55785565813403`}, {"TBUR", 26.284486817470462`, 24.24960578253411`},
{"TBUR", 67.34797470416764`, 98.47891927640185`}, {"TBUR", 75.06769984920155`, 128.12277714632776`},
{"TBUR", 63.55456165693759`, 35.122528238470025`}, {"CURE", 365, 91.50286211115095`},
{"TBUR", 15.454052052820497`, 28.611826877841178`}, {"TBUR", 32.156193449890324`, 60.89448693384079`},
{"CURE", 365, 110.33364103260085`}, {"CURE", 365, 39.82493157106758`}, {"TBUR", 30.147534452879587`, 66.86248898179068`},
{"CURE", 365, 33.65784589712671`}, {"TBUR", 77.13343539784537`, 126.54104588871392`},
{"TBUR", 52.08983678076866`, 75.68243209815778`}, {"CURE", 365, 71.14792414688169`},

{"TBUR", 77.29267575526228`, 114.20852761146156`}, {"CURE", 365, 47.95624180320876`}, {"CURE", 365, 68.39031638495707`},
{"CURE", 365, 109.88158034142819`}, {"CURE", 365, 7.0512115962896225`}, {"TBUR", 17.556678213458923`, 9.909819331269055`},
{"TBUR", 145.064064758703`, 42.76418490156939`}, {"TBUR", 56.942673749736905`, 170.03892543313344`},
{"CURE", 365, 19.677677036265262`}, {"TBUR", 112.87512368364025`, 153.9317221472241`},
{"CURE", 365, 67.52625513012367`}, {"CURE", 365, 162.07193232377563`}, {"TBUR", 15.597251976758002`, 39.44439902145213`},
{"TBUR", 39.45582739921073`, 52.40614289494851`}, {"CURE", 365, 77.80870827138679`}, {"CURE", 365, 170.5362689101146`},
{"TBUR", 84.69710530117337`, 76.6221810404563`}, {"CURE", 365, 191.57494356836725`}, {"CURE", 365, 38.607820703250766`},
{"CURE", 365, 99.37701002786214`}, {"CURE", 365, 77.40074828860962`}, {"CURE", 365, 87.48222487808906`},
{"TBUR", 63.517558276871966`, 85.23821708606287`}, {"TBUR", 44.63936314163622`, 88.30697989265003`},
{"CURE", 365, 10.127449342347619`}, {"CURE", 365, 29.11400922128254`}, {"TBUR", 82.0197766949574`, 97.4805479161773`},
{"CURE", 365, 76.35712333284565`}, {"CURE", 365, 14.972752120714002`}, {"TBUR", 76.5423246468129`, 96.46877585953887`},
{"CURE", 365, 29.15450798448453`}, {"CURE", 365, 17.027897167777493`}, {"CURE", 365, 17.02006487778151`},
{"CURE", 365, 21.36805472310838`}, {"CURE", 365, 31.35654390923404`}, {"TBUR", 67.30729846176654`, 81.12361579756191`},
{"CURE", 365, 120.98703357397625`}, {"CURE", 365, 147.33422028976887`}, {"TBUR", 31.53888550400797`, 105.89351928289032`},
{"TBUR", 65.68511459706788`, 7.585574012410525`}, {"CURE", 365, 178.51456415813848`},
{"TBUR", 102.2451165694527`, 54.296459612448075`}, {"TBUR", 39.18903082510686`, 88.41849982127616`},
{"TBUR", 120.7145476990507`, 17.733938801191925`}, {"CURE", 365, 72.84462057732331`},
{"CURE", 365, 1.9477480111295447`}, {"CURE", 365, 123.20370547340997`}, {"CURE", 365, 141.73609299059453`},
{"CURE", 365, 74.69375791264996`}, {"CURE", 365, 28.780565462612`}, {"CURE", 365, 61.91758927185351`},
{"TBUR", 122.83565072154937`, 95.47243780306954`}, {"TBUR", 90.70583650980944`, 97.65658553051908`},
{"TBUR", 40.52554308739622`, 58.4856090338353`}, {"CURE", 365, 84.75414463959625`}, {"CURE", 365, 57.495013868637486`},
{"TBUR", 22.602503554399245`, 147.58648648100723`}, {"TBUR", 80.4769684115683`, 45.60948092014601`},
{"CURE", 365, 51.27836093143722`}, {"TBUR", 109.13622197918212`, 82.11394447705771`}, {"CURE", 365, 108.87693824237198`},
{"CURE", 365, 3.8211490156919963`}, {"TBUR", 55.06936790429714`, 48.01947301180935`}, {"CURE", 365, 77.50054909764836`},
{"CURE", 365, 13.964509765336892`}, {"CURE", 365, 148.48725347218772`}, {"TBUR", 15.337135026105665`, 6.888008252851118`},
{"CURE", 365, 143.9891565433581`}, {"CURE", 365, 135.32724816343128`}, {"TBUR", 102.95409418642645`, 3.4281585845384916`},
{"CURE", 365, 114.24252718908244`}, {"CURE", 365, 43.00446144228104`}, {"CURE", 365, 47.45832629706181`},
{"CURE", 365, 124.5441854536429`}, {"CURE", 365, 8.895405448150932`}, {"TBUR", 20.927666160892663`, 70.87886345295001`},
{"CURE", 365, 21.348460235152807`}, {"TBUR", 80.47032171534966`, 2.477734519676743`}, {"CURE", 365, 72.05935358868146`},
{"TBUR", 74.54023466802276`, 111.60278370023599`}, {"TBUR", 153.9356258799236`, 17.27911243716353`},
{"CURE", 365, 0.6586541751295105`}, {"TBUR", 84.31999155303856`, 8.075830706272207`},

{"TBUR", 30.41944094938212`, 56.93038473081048`}, {"CURE", 365, 2.258291840241647`}, {"CURE", 365, 5.568191050463289`},
{"CURE", 365, 109.5449121639894 2`}, {"TBUR", 75.17099126945152`, 185.03118063588528`},
{"TBUR", 89.43007807126143`, 97.19528060661825`}, {"CURE", 365, 179.7989188345317`},
{"TBUR", 73.89205772125064`, 86.02433641058056`}, {"CURE", 365, 2.063591681608951`}, {"CURE", 365, 95.37271110756716`},
{"TBUR", 89.45327182218287`, 139.44594004689864`}, {"TBUR", 38.60897297146759`, 72.0718059116611`},
{"TBUR", 64.68415305220134`, 7.875336455497699`}, {"TBUR", 57.63304078853209`, 56.60487562048757`},
{"CURE", 365, 38.07235138872248`}, {"CURE", 365, 19.313286632702916`}, {"CURE", 365, 9.913456603439668`},
{"CURE", 365, 110.48964406905928`}, {"TBUR", 127.39496630463776`, 44.86357261253387`},
{"TBUR", 25.01568297512417`, 42.492572343955864`}, {"CURE", 365, 62.4941285487585`},
{"TBUR", 67.37880676509343`, 12.349337080515976`}, {"CURE", 365, 90.71424041256707`}, {"CURE", 365, 47.07362648736755`},
{"TBUR", 63.50362787869176`, 52.017580291219154`}, {"TBUR", 50.74821304100155`, 120.63049943748472`},
{"CURE", 365, 163.02363151628356`}, {"CURE", 365, 46.832640309068964`}, {"CURE", 365, 9.67303615761506`},
{"TBUR", 79.43135979441138`, 187.66767036805265`}, {"TBUR", 155.2807157712261`, 56.99507220855348`},
{"TBUR", 57.1078875109415`, 53.337799933894914`}, {"CURE", 365, 77.37653391140026`},
{"CURE", 365, 12.444690043130176`}, {"CURE", 365, 70.09180166235568`}, {"CURE", 365, 65.8511755076946`},
{"TBUR", 118.4541198962538 3`, 66.88108856343885`}, {"CURE", 365, 114.53026080899633`}, {"CURE", 365, 45.99544282674255`},
{"TBUR", 165.26351843452736`, 86.85896842635216`}, {"CURE", 365, 37.00239000728439`}, {"CURE", 365, 117.40996690481381`},
{"CURE", 365, 51.03956818097078`}, {"TBUR", 85.1224587201681`, 67.27838613305777`}, {"CURE", 365, 30.92063102736454`},
{"TBUR", 82.30260747092368`, 108.05731396680822`}, {"TBUR", 47.82412616165822`, 210.6235914785248`},
{"TBUR", 41.84176110103977`, 101.36940781748606`}, {"CURE", 365, 100.08694090335025`}, {"CURE", 365, 35.42552840138666`},
{"TBUR", 121.04169087813248`, 83.5058443710187`}, {"TBUR", 57.42565634777793`, 154.32119689169824`},
{"CURE", 365, 4.256207209322158`}, {"CURE", 365, 101.47157931545162`}, {"TBUR", 65.6632390354735`, 8.44702544402191`},
{"CURE", 365, 11.170904963568539`}, {"CURE", 365, 12.351483053901715`}, {"TBUR", 48.3946614900177`, 51.489237765346736`},
{"CURE", 365, 2.8289222091859623`}, {"TBUR", 32.168561735615235`, 70.023212846802`}, {"CURE", 365, 64.46655017682417`},
{"TBUR", 80.33136440374733`, 80.71142020634726`}, {"TBUR", 32.88870462268038`, 44.048074348122135`},
{"CURE", 365, 74.26888351033197`}, {"CURE", 365, 150.09545769455838`}, {"CURE", 365, 73.28954162107628`},
{"CURE", 365, 16.10607588993565`}, {"CURE", 365, 75.23296485523`}, {"TBUR", 110.04553116578114`, 7.1553301170823245`},
{"TBUR", 61.32800602476362`, 7.0222542815312465`}, {"CURE", 365, 118.1071615248378`},
{"CURE", 365, 119.67082207650155`}, {"TBUR", 81.37562401740162`, 23.786582032023276`},
{"CURE", 365, 36.214307064349015`}, {"TBUR", 18.250226552596608`, 81.900663522619`}, {"CURE", 365, 143.8833139417625`},
{"TBUR", 133.13005066148224`, 44.02510464138093`}, {"TBUR", 57.99744384984438`, 64.98886751277104`},

{"CURE", 365, 10.131397309572113`}, {"TBUR", 75.69291128264969`, 73.11369321463461`},
{"CURE", 365, 60.46463619533152`}, {"CURE", 365, 76.3500023668754`}, {"TBUR", 69.4567015948261`, 48.551120096231976`},
{"TBUR", 72.00266667468995`, 15.368536401683922`}, {"CURE", 365, 88.56180993276776`},
{"TBUR", 14.632115848025759`, 48.496967610254394`}, {"CURE", 365, 20.14932045359305`},
{"CURE", 365, 96.1059707985946`}, {"CURE", 365, 171.4917111430506`}, {"CURE", 365, 136.0414745589528`},
{"CURE", 365, 72.58549185803271`}, {"CURE", 365, 97.89378671618107`}, {"TBUR", 38.57782158685086`, 9.895733379262236`},
{"TBUR", 42.73442354642043`, 83.65018041477657`}, {"TBUR", 62.50895881162036`, 15.938311050574027`},
{"CURE", 365, 49.44421159596402`}, {"TBUR", 25.763518792597896`, 86.47950305098921`}, {"CURE", 365, 18.58556968158674`},
{"CURE", 365, 62.36022894953846`}, {"TBUR", 20.19330268918182`, 23.656280014689223`}, {"CURE", 365, 206.210359795377`},
{"TBUR", 14.397390024364647`, 12.505407690830841`}, {"CURE", 365, 92.12378549127656`},
{"TBUR", 40.76976202191309`, 27.416511267679695`}, {"TBUR", 101.93344700972575`, 10.918801599094579`},
{"CURE", 365, 2.264184339634196`}, {"CURE", 365, 4.25243163669747`}, {"CURE", 365, 74.34900438367691`},
{"TBUR", 26.679713674966596`, 40.50895535836756`}, {"CURE", 365, 5.676428042714131`}, {"CURE", 365, 65.97145155084702`},
{"CURE", 365, 96.96280857316663`}, {"CURE", 365, 184.67804777591743`}, {"CURE", 365, 46.603954084163824`},
{"CURE", 365, 81.69942584758007`}, {"TBUR", 40.83978383478393`, 88.41660330541798`}, {"CURE", 365, 9.71243127315047`},
{"TBUR", 24.336989843865695`, 37.20283867323996`}, {"CURE", 365, 27.59095575568984`}, {"CURE", 365, 81.09080438101897`},
{"TBUR", 42.26325258456405`, 80.34415078960245`}, {"CURE", 365, 54.14031946853859`}, {"CURE", 365, 3.2405062918006906`},
{"CURE", 365, 14.495535272825073`}, {"CURE", 365, 62.198132303418554`}, {"TBUR", 41.837768051519475`, 73.68195277122136`},
{"CURE", 365, 98.46635202068406`}, {"TBUR", 107.0937474348071`, 63.87323011356896`}, {"CURE", 365, 186.75426781745196`},
{"CURE", 365, 84.25421556382486`}, {"TBUR", 18.115999132929282`, 66.96709189203084`}, {"CURE", 365, 1.0800074728333624`},
{"CURE", 365, 35.44080380387655`}, {"CURE", 365, 141.4868942349965`}, {"CURE", 365, 4.086596732379633`},
{"TBUR", 46.762303904216544`, 53.066613357152704`}, {"TBUR", 49.975359036531415`, 84.63789737147546`},
{"TBUR", 87.36759877725345`, 153.95696852139343`}, {"TBUR", 88.34722001453217`, 103.4556510093653`},
{"CURE", 365, 13.445916465022787`}, {"TBUR", 73.35826264608835`, 8.352070875052092`},
{"CURE", 365, 58.98593068977562`}, {"CURE", 365, 147.22475312565615`}, {"TBUR", 32.55067288235379`, 84.03306912183454`},
{"CURE", 365, 7.978214958465271`}, {"TBUR", 54.042096114748944`, 87.14662438814254`},
{"CURE", 365, 72.494433665254`}, {"TBUR", 64.98855465141227`, 3.936319527126211`},
{"CURE", 365, 61.54161700324786`}, {"CURE", 365, 82.0176234274487`}, {"CURE", 365, 45.5436917268669`},
{"TOX", 40.23840382358852`, 232.78263773137405`}, {"TBUR", 62.54518950658499`, 61.56626270755825`},
{"CURE", 365, 81.58386984611784`}, {"TBUR", 24.491414207681775`, 65.68540097861516`},
{"CURE", 365, 190.08778279501647`}, {"CURE", 365, 67.4148140918558`}, {"TBUR", 19.545276460288722`, 65.57863277712053`},

{"CURE", 365, 38.88521284081879`}, {"TBUR", 58.79604593465504`, 96.31566180192473`}, {"CURE", 365, 0.854939369769405`},
{"TBUR", 28.38195402988968`, 16.071592012671168`}, {"TBUR", 145.8594111879919`, 91.12135518148587`},
{"CURE", 365, 79.06610988005062`}, {"CURE", 365, 8.858172647944189`}, {"CURE", 365, 59.047044190054606`},
{"CURE", 365, 39.06189801069651`}, {"TBUR", 71.01863145786642`, 41.14053781019635`}, {"CURE", 365, 7.765449251165912`},
{"CURE", 365, 52.689612166301174`}, {"CURE", 365, 42.72959789794297`}, {"TBUR", 26.34000879509858`, 27.24192737530458`},
{"CURE", 365, 141.9453417176988`}, {"CURE", 365, 4.049125464897056`}, {"CURE", 365, 61.50677237591772`},
{"TBUR", 162.2629976104537`, 97.67161595741467`}, {"TBUR", 16.53248556258866`, 105.64072478867621`},
{"TBUR", 41.74314575239947`, 6.215550781438026`}, {"CURE", 365, 77.42790013274319`}, {"CURE", 365, 97.91904297303692`},
{"TBUR", 25.533194761484253`, 3.4538415698473046`}, {"CURE", 365, 50.04908430114718`},
{"CURE", 365, 81.07780493632329`}, {"TBUR", 20.848006093859976`, 20.559085106656244`},
{"CURE", 365, 1.8899668164999701`}, {"TBUR", 66.1910476603848`, 161.62541356221564`},
{"TBUR", 113.31417472223336`, 86.52388117742845`}, {"CURE", 365, 86.90501796346517`}, {"CURE", 365, 22.29737948207387`},
{"TBUR", 76.28246739469103`, 2.7844536260593413`}, {"TBUR", 29.542435013230367`, 2.096454993164994`},
{"TBUR", 13.127813418984662`, 15.430188872536124`}, {"TBUR", 84.06098299433069`, 69.51155991119052`},
{"CURE", 365, 114.82377652505218`}, {"CURE", 365, 97.70903779060376`}, {"CURE", 365, 46.04321324018514`},
{"TBUR", 20.722707755115646`, 1.3257954069617697`}, {"CURE", 365, 76.91807291064347`}, {"CURE", 365, 79.04650578457101`},
{"TBUR", 64.61465388081112`, 0.9606086416510572`}, {"TBUR", 76.32087176893637`, 46.382296348748355`},
{"CURE", 365, 0.9503655552134854`}, {"CURE", 365, 131.19123347494877`}, {"CURE", 365, 55.06502360515707`},
{"TBUR", 14.398666879754419`, 0.665403542700765`}, {"TBUR", 162.99779686081686`, 78.99153080674255`},
{"CURE", 365, 27.01144394955724`}, {"TBUR", 54.635151459023`, 208.81326521166758`}, {"CURE", 365, 112.47802489766927`},
{"TBUR", 41.64782427593019`, 64.15576278605508`}, {"CURE", 365, 164.19339574540246`}},
{{"420 Res", "420 OS", "420 Tox"}, {"TBUR", 21.50207134146656 8`, 35.05476849649342`}, {"CURE", 365, 135.28451671263772`},
{"TBUR", 26.81051095605258`, 104.52879971269478`}, {"TBUR", 28.370844341064355`, 3.8365759719204915`},
{"TBUR", 17.886637309787528`, 14.229362204531265`}, {"CURE", 365, 24.566806085564544`}, {"CURE", 365, 59.60960611248154`},
{"CURE", 365, 56.060912811873706`}, {"CURE", 365, 133.93329494945417`}, {"TBUR", 70.6435053144324`, 181.93444238610962`},
{"TBUR", 54.379348538422455`, 93.86241563462464`}, {"CURE", 365, 82.3450602619701`},
{"TOX", 22.76319006389425`, 247.38281978308805`}, {"TBUR", 42.80523995096885`, 57.54146518672545`},
{"TBUR", 60.60850752629099`, 6.014639032068125`}, {"TBUR", 86.22922468627696`, 23.874695942296846`},
{"CURE", 365, 95.060049083349`}, {"TBUR", 44.15382871388872`, 105.20637403565942`},
{"TBUR", 43.52441182088585`, 6.107086424871223`}, {"CURE", 365, 77.0258544298675`}, {"CURE", 365, 126.98224233413534`},
{"CURE", 365, 41.735264805243865`}, {"CURE", 365, 25.105873556501695`}, {"CURE", 365, 58.03214619039768`},

{"CURE", 365, 49.856256610173`}, {"CURE", 365, 147.73344445637085`}, {"CURE", 365, 85.53844008931625`},
{"CURE", 365, 44.761375679834664`}, {"CURE", 365, 29.2212314971878`}, {"CURE", 365, 5.072591098100711`},
{"CURE", 365, 122.88889404305317`}, {"TBUR", 13.37943737969128`, 32.326817686662096`}, {"CURE", 365, 62.28428687578552`},
{"TBUR", 47.35967760077464`, 36.098165846481066`}, {"CURE", 365, 169.0503095166062`}, {"CURE", 365, 198.45519930295393`},
{"CURE", 365, 37.550048467455056`}, {"TBUR", 21.178510170104218`, 10.140720619663549`},
{"TBUR", 71.66163421128685`, 43.89187985439789`}, {"CURE", 365, 89.4942378770868 3`},
{"TBUR", 17.97365412769262`, 55.706832988486994`}, {"CURE", 365, 88.22435388350563`}, {"CURE", 365, 66.05951875235147`},
{"TBUR", 45.90905595364855`, 72.82683785348958`}, {"CURE", 365, 89.77295690855408`}, {"CURE", 365, 4.6531406720323165`},
{"CURE", 365, 99.84188632524332`}, {"CURE", 365, 62.3306051330459`}, {"TBUR", 112.25004445456405`, 122.85667526851053`},
{"CURE", 365, 209.28683697640196`}, {"TBUR", 29.630943372964154`, 93.05837318533355`},
{"TBUR", 41.61928157424184`, 80.20530748650404`}, {"CURE", 365, 6.072463580590474`}, {"CURE", 365, 129.8768543009997`},
{"CURE", 365, 39.86742485684651`}, {"CURE", 365, 91.25119264811134`}, {"CURE", 365, 61.98039371267184`},
{"TBUR", 103.1392236472482`, 68.16667227567049`}, {"TBUR", 37.22231336535262`, 157.02550946889278`},
{"TBUR", 52.18286559450289`, 93.7761779455442`}, {"CURE", 365, 86.68530700613162`}, {"CURE", 365, 4.297574414169506`},
{"TBUR", 33.56656679810426`, 106.1389829088571`}, {"CURE", 365, 72.48925359216827`},
{"TBUR", 68.0178061902539`, 6.533010458031833`}, {"CURE", 365, 82.48362571347049`}, {"CURE", 365, 73.10857466036599`},
{"TBUR", 57.277846681644704`, 103.22892366056607`}, {"CURE", 365, 37.45755189688994`}, {"CURE", 365, 31.485902329557923`},
{"CURE", 365, 59.87423858344638`}, {"CURE", 365, 43.31324847967281`}, {"CURE", 365, 130.22148394035855`},
{"TBUR", 13.284944440291625`, 1.9883855550208522`}, {"TBUR", 79.34801229348172`, 83.21155988856246`},
{"CURE", 365, 106.5684934869211`}, {"TBUR", 96.93191039120796`, 201.66759903918762`}, {"CURE", 365, 66.13526065306772`},
{"TBUR", 75.71810013046385`, 131.18738294402164`}, {"CURE", 365, 49.89758602799733`}, {"CURE", 365, 105.41643983650214`},
{"CURE", 365, 3.3978789629751005`}, {"CURE", 365, 6.719912638227009`}, {"CURE", 365, 4.74647575445704`},
{"CURE", 365, 122.1448816375103`}, {"TBUR", 99.34537619147765`, 93.42894709587657`}, {"CURE", 365, 73.71941043239093`},
{"CURE", 365, 39.20264153043345`}, {"CURE", 365, 7.286802472050906`}, {"TBUR", 71.360201393378`, 136.68907353643223`},
{"TBUR", 32.194705138818804`, 10.751644211581969`}, {"TBUR", 89.14815523306541`, 100.80057167653646`},
{"TBUR", 65.24476608671448`, 2.7894966596745463`}, {"CURE", 365, 6.459124121851256`},
{"TBUR", 18.832392866111064`, 1.9355331666133733`}, {"TBUR", 158.04800588518242`, 53.218009770625315`},
{"CURE", 365, 37.708603133257306`}, {"CURE", 365, 183.33734793466647`}, {"CURE", 365, 9.877253783600093`},
{"TBUR", 43.62768039835902`, 140.88598150349972`}, {"TBUR", 20.067429604665293`, 10.131043645435389`},
{"TBUR", 127.29019180856697`, 127.17370128066345`}, {"CURE", 365, 70.19343630882899`},
{"TBUR", 90.57604981588358`, 92.1743071521968`}, {"CURE", 365, 63.46575436517634`}, {"CURE", 365, 101.8012223186999`},

{"CURE", 365, 41.12351398336121`}, {"TBUR", 138.24912593922227`, 87.81418780703785`}, {"CURE", 365, 36.92459338713945`},
{"CURE", 365, 103.34989606167343`}, {"CURE", 365, 142.43070077578574`}, {"CURE", 365, 77.68010572853242`},
{"CURE", 365, 20.211486098978476`}, {"CURE", 365, 48.65659348077702`}, {"CURE", 365, 70.09213015546862`},
{"CURE", 365, 57.93149532942539`}, {"CURE", 365, 5.2705240636189865`}, {"CURE", 365, 76.36106350381594`},
{"CURE", 365, 73.68134245297925`}, {"CURE", 365, 20.542491697953505`}, {"CURE", 365, 82.33581377689181`},
{"CURE", 365, 165.54769451068933`}, {"CURE", 365, 103.77914825161135`}, {"TBUR", 19.655608307708164`, 5.415753729987153`},
{"CURE", 365, 73.81338698398658`}, {"TBUR", 38.56568054576087`, 62.259259995935714`}, {"CURE", 365, 51.551321923184815`},
{"CURE", 365, 34.83213480164497`}, {"CURE", 365, 118.7096165629850`}, {"CURE", 365, 114.52263827340123`},
{"CURE", 365, 102.40334457860295`}, {"CURE", 365, 112.09318623518455`}, {"TBUR", 71.54804221304326`, 34.22765498634904`},
{"TBUR", 45.00804564445564`, 35.60534999373537`}, {"CURE", 365, 164.07177931890558`},
{"TBUR", 12.763574104693761`, 70.21201240545842`}, {"TBUR", 89.53429389414302`, 23.00541343035582`},
{"TBUR", 62.13875483862895`, 53.43081086435368`}, {"TBUR", 98.35976019765044`, 18.7255695091391`},
{"CURE", 365, 110.80600457603872`}, {"TBUR", 52.90487294781762`, 167.26181736207826`}, {"CURE", 365, 112.97384965672312`},
{"CURE", 365, 147.1385239064372`}, {"CURE", 365, 17.7975295247943`}, {"CURE", 365, 1.247773462034619`},
{"CURE", 365, 67.10054952202128`}, {"CURE", 365, 162.94110447345366`}, {"TBUR", 36.45558008120825`, 39.778003315813976`},
{"CURE", 365, 74.69623866558321`}, {"CURE", 365, 14.250862390980572`}, {"CURE", 365, 7.058385357714478`},
{"CURE", 365, 60.38586237136482`}, {"CURE", 365, 1.18278643625943`}, {"TBUR", 88.3775116679709 4`, 165.3149165561468`},
{"TBUR", 49.90574123275177`, 65.01777963296603`}, {"TBUR", 88.30540488106097`, 146.34107630925902`},
{"CURE", 365, 71.63838377622979`}, {"CURE", 365, 85.9655656561745`}, {"CURE", 365, 68.9104730668499`},
{"CURE", 365, 85.95569275467727`}, {"CURE", 365, 20.716699932033375`}, {"TBUR", 93.89947947113899`, 22.27221386090412`},
{"CURE", 365, 1.703935003300205`}, {"TBUR", 60.458301579573735`, 7.995434941781651`},
{"TBUR", 78.67858072693902`, 73.84061365742546`}, {"CURE", 365, 112.94910207331111`}, {"CURE", 365, 112.8894655720218`},
{"CURE", 365, 81.0484200923112`}, {"CURE", 365, 117.07131730192913`}, {"CURE", 365, 1.73432494626005`},
{"CURE", 365, 64.86407658418545`}, {"TBUR", 29.696897682318642`, 125.97708634811119`}, {"CURE", 365, 56.56823691313473`},
{"TBUR", 20.785957018722602`, 6.7080771888895105`}, {"TBUR", 50.20047519352523`, 124.59577850651552`},
{"TBUR", 85.24287202791746`, 10.244834893023736`}, {"TBUR", 67.70142612388062`, 148.73270350620479`},
{"TBUR", 21.181192046518415`, 79.24531809522323`}, {"TBUR", 58.21124980125718`, 40.119486140072986`},
{"TBUR", 91.00894382844199`, 99.09491158384745`}, {"TBUR", 80.15057086393945`, 97.29309262528994`},
{"CURE", 365, 81.89995578036536`}, {"CURE", 365, 141.10730335220208`}, {"TBUR", 19.219939573838726`, 7.807163576700562`},
{"CURE", 365, 127.42447784839725`}, {"TBUR", 27.921369353880504`, 41.601141886115236`},
{"TBUR", 53.550336630591254`, 139.88279682485216`}, {"CURE", 365, 65.90360941117211`},

{"CURE", 365, 3.700489533196376`}, {"TBUR", 24.165616960775743`, 91.7774770807329`}, {"CURE", 365, 87.85074610320395`},
{"CURE", 365, 140.47785784784588`}, {"CURE", 365, 162.75679528355883`}, {"CURE", 365, 107.29500438111027`},
{"CURE", 365, 62.97966764224842`}, {"CURE", 365, 48.128278967230216`}, {"CURE", 365, 169.5830768390959`},
{"TBUR", 13.596841230220942`, 13.124517220827322`}, {"CURE", 365, 95.40830536188099`}, {"CURE", 365, 85.44492096159345`},
{"TBUR", 25.90654933152393`, 22.932878848589706`}, {"TBUR", 57.06322758317605`, 98.02416622476437`},
{"CURE", 365, 93.96580807801332`}, {"TBUR", 65.22296275646033`, 76.6042572147724`}, {"CURE", 365, 1.73159704113818`},
{"TBUR", 141.66019679710777`, 15.307424564161085`}, {"TBUR", 15.84647088887456`, 83.08822084509073`},
{"CURE", 365, 166.5901905026977`}, {"CURE", 365, 6.803396107327299`}, {"CURE", 365, 125.53886757459334`},
{"CURE", 365, 2.8416914422001787`}, {"CURE", 365, 88.66373968220373`}, {"CURE", 365, 15.79460662486257`},
{"TBUR", 75.33310242504322`, 108.48386850115931`}, {"CURE", 365, 23.2393689674806`},
{"TBUR", 42.678850910063744`, 13.208278856870393`}, {"TBUR", 79.92644748447411`, 152.69839964884252`},
{"CURE", 365, 73.47450053436474`}, {"TBUR", 50.04623733921879`, 199.4496717542974`}, {"CURE", 365, 69.4047721913414`},
{"CURE", 365, 98.63722258250185`}, {"CURE", 365, 12.233089133644237`}, {"CURE", 365, 66.95392588518708`},
{"CURE", 365, 130.8730920967796`}, {"CURE", 365, 136.80474129476434`}, {"CURE", 365, 66.11138796509373`},
{"CURE", 365, 121.52449869869898`}, {"CURE", 365, 77.62454024784171`}, {"CURE", 365, 7.4231467143662`},
{"CURE", 365, 49.28412587920846`}, {"CURE", 365, 40.07860458103309`}, {"CURE", 365, 68.06810853337545`},
{"CURE", 365, 100.36743984761944`}, {"CURE", 365, 83.53169584256726`}, {"CURE", 365, 76.19260308371507`},
{"TBUR", 22.57073657884287`, 96.79470585812874`}, {"TBUR", 27.987418179381716`, 65.56105868445613`},
{"TBUR", 156.517995849238`, 64.3896871967467`}, {"TBUR", 20.22241609056634`, 43.17793083229169`},
{"CURE", 365, 57.16641639975732`}, {"CURE", 365, 66.15475177853124`}, {"CURE", 365, 120.42113292700812`},
{"TBUR", 26.69345541627385`, 5.4974785131413055`}, {"TBUR", 31.301250884348587`, 21.381739579983098`},
{"CURE", 365, 1.5982647737099585`}, {"CURE", 365, 2.3881153091950886`}, {"TBUR", 19.41211690811581`, 21.513721093366627`},
{"CURE", 365, 192.61003640086037`}, {"CURE", 365, 47.815308318540666`}, {"CURE", 365, 188.39835329041205`},
{"TBUR", 38.45385768726937`, 87.09042767514865`}, {"CURE", 365, 56.47385020857445`},
{"TBUR", 15.187404109325364`, 112.13640472736294`}, {"CURE", 365, 48.4185896600253`},
{"CURE", 365, 96.38494608132417`}, {"CURE", 365, 3.456514589953876`}, {"CURE", 365, 71.16000456687213`},
{"CURE", 365, 22.421977454931756`}, {"CURE", 365, 8.83946558546688`}, {"CURE", 365, 110.71013348318544`},
{"TBUR", 21.51876720168190 7`, 116.37568162330095`}, {"TBUR", 13.649650362185918`, 83.93736827323879`},
{"CURE", 365, 55.94935975533372`}, {"TBUR", 28.000416485991366`, 78.78879929524132`},
{"CURE", 365, 5.215806197209865`}, {"CURE", 365, 55.19021286032905`}, {"CURE", 365, 42.19701662060126`},
{"CURE", 365, 196.98615162827815`}, {"CURE", 365, 79.21028747907982`}, {"CURE", 365, 42.73979370932474`},

{"CURE", 365, 1.533703227993202`}, {"TBUR", 69.814861993729`, 81.1423904732872`}, {"CURE", 365, 69.27661328206267`},
{"TBUR", 151.22643952790034`, 73.4497944403853`}, {"TBUR", 52.281914171350095`, 8.835696871637953`},
{"CURE", 365, 74.49757403132628`}, {"CURE", 365, 52.35529965299914`}, {"TBUR", 20.523194920581474`, 68.85771167403946`},
{"CURE", 365, 36.85216169652803 6`}, {"CURE", 365, 93.6600397455014`}, {"CURE", 365, 10.384181974351614`},
{"TBUR", 97.79035597150674`, 93.84145202129699`}, {"TBUR", 16.539573006019282`, 36.27480113294189`},
{"CURE", 365, 75.95638178881836`}, {"TBUR", 26.960350349231614`, 87.50559343269889`}, {"CURE", 365, 8.536731638943284`},
{"CURE", 365, 109.6337550654497`}, {"CURE", 365, 65.05948289355567`}, {"CURE", 365, 181.2479459115435`},
{"CURE", 365, 127.7639254709263 1`}, {"CURE", 365, 89.49342145098203`}, {"CURE", 365, 54.44263586929524`},
{"CURE", 365, 188.30270374404674`}, {"TBUR", 29.931863673028317`, 28.173363262891503`},
{"CURE", 365, 17.035845363373927`}, {"CURE", 365, 22.023659073366964`}, {"CURE", 365, 76.85601913619064`},
{"CURE", 365, 71.19417038221073`}, {"CURE", 365, 106.17678885814522`}, {"CURE", 365, 29.875997083844776`},
{"TBUR", 23.183775334670457`, 135.62423250857358`}, {"CURE", 365, 2.0946659615121797`}, {"CURE", 365, 110.85305679668024`},
{"TBUR", 38.25645368784409`, 132.86448383113566`}, {"CURE", 365, 44.91814268946924`}, {"CURE", 365, 112.69336903567637`},
{"CURE", 365, 3.73942530935267`}, {"TBUR", 22.044515632576903`, 41.27167915522003`}, {"CURE", 365, 60.721102537295266`},
{"CURE", 365, 60.74333123368702`}, {"TBUR", 28.734593993932684`, 28.62276471761958`}, {"CURE", 365, 61.13661389849591`},
{"TBUR", 36.05364584978975`, 29.243764001802138`}, {"CURE", 365, 100.79736124001626`}, {"CURE", 365, 78.4692812388662`},
{"CURE", 365, 87.09226965249951`}, {"CURE", 365, 60.95306461115204`}, {"CURE", 365, 67.10510583937761`},
{"CURE", 365, 95.75176290785065`}, {"CURE", 365, 124.16395846894225`}, {"TBUR", 49.0206474568324`, 90.75468405166701`},
{"CURE", 365, 122.51554393849192`}, {"CURE", 365, 74.90546787721334`}, {"TBUR", 55.71171997944698`, 19.07085241592466`},
{"TBUR", 16.852528550157018`, 14.175261831012213`}, {"CURE", 365, 0.72738344229452`}, {"CURE", 365, 107.43324985176277`},
{"CURE", 365, 108.14354347120977`}, {"CURE", 365, 83.91734147430681`}, {"TBUR", 138.09290720125412`, 194.07613810533178`},
{"CURE", 365, 150.188830434699`}, {"CURE", 365, 36.380200382678`}, {"TBUR", 20.43244273446799`, 58.19794384001083`},
{"TBUR", 31.09102129370592`, 6.8011321433847955`}, {"CURE", 365, 185.23712097598454`},
{"TBUR", 38.8454442899034`, 180.4829267656431 3`}, {"TBUR", 23.040205464538943`, 23.684746237237015`},
{"CURE", 365, 177.74525085958234`}, {"CURE", 365, 29.964618724382426`}, {"CURE", 365, 62.61668777245723`},
{"CURE", 365, 124.691187800832`}, {"CURE", 365, 2.0728174365092187`}, {"TBUR", 55.55593057727824`, 36.64247384033329`},
{"CURE", 365, 113.79287006582697`}, {"TBUR", 52.03062479752684`, 27.22534075147206`},
{"TBUR", 38.38798299690996`, 181.06031068025928`}, {"CURE", 365, 3.081205267250827`}, {"CURE", 365, 152.53805101862446`},
{"CURE", 365, 89.62189273947571`}, {"CURE", 365, 83.75609804775988`}, {"CURE", 365, 107.22572938413612`},
{"CURE", 365, 45.26377198442498`}, {"TBUR", 35.4792602100867`, 135.8946639714503`}, {"CURE", 365, 83.18517571373009`},
{"CURE", 365, 145.3137009799952`}, {"CURE", 365, 94.13305797841686`}, {"TBUR", 54.2899847848999`, 99.06071279906693`},

{"CURE", 365, 117.0932913555538`}, {"CURE", 365, 85.00536123721226`}, {"CURE", 365, 40.321676863939686`},
{"CURE", 365, 130.21404042568048`}, {"CURE", 365, 157.8752189541416`}, {"CURE", 365, 41.768309984777055`},
{"TBUR", 63.902460975249305`, 102.91949898335349`}, {"TBUR", 45.81910801702579`, 131.2698623592919 8`},
{"CURE", 365, 11.682478327686663`}, {"CURE", 365, 94.48491264718686`}, {"CURE", 365, 47.461900202126614`},
{"TBUR", 14.42315958926159`, 7.735301045857566`}, {"TBUR", 13.327835026132338`, 27.780888594385235`},
{"TBUR", 31.280959192144866`, 45.833235859077256`}, {"CURE", 365, 91.22722177943686`}, {"CURE", 365, 20.55729641441209`},
{"CURE", 365, 80.81751640332232`}, {"TBUR", 77.95246778117568`, 149.9808859874078`}, {"CURE", 365, 83.71793563503223`},
{"CURE", 365, 74.08472609426407`}, {"CURE", 365, 70.9804753208289`}, {"TBUR", 17.026247435057083`, 32.12619928241202`},
{"TBUR", 13.568378595833925`, 76.08694813690396`}, {"TBUR", 74.23539812202897`, 33.99059586675044`},
{"CURE", 365, 134.0978723352317`}, {"TBUR", 49.51601491075824`, 80.29824392480117`},
{"CURE", 365, 107.34173471115297`}, {"CURE", 365, 80.26651163317246`}, {"CURE", 365, 69.55002771602786`},
{"TBUR", 100.89886817337677`, 31.63534384283012`}, {"CURE", 365, 49.1237885480155`}, {"CURE", 365, 189.02401020574231`},
{"CURE", 365, 97.20250020066302`}, {"CURE", 365, 79.6614353955787`}, {"TBUR", 32.9857299514485`, 70.28088464726243`},
{"TBUR", 76.74599432830998`, 67.1405214247916`}, {"CURE", 365, 51.87706298240694`},
{"TBUR", 15.907764605741031`, 57.96217642632659`}, {"TBUR", 17.07692005360403`, 54.3758950255161`},
{"CURE", 365, 45.29542967024834`}, {"CURE", 365, 76.82688918360579`}, {"TBUR", 101.21369748875952`, 6.32693453949383`},
{"TBUR", 12.950166982280304`, 54.08609388351262`}, {"TBUR", 20.718798042797967`, 14.199258644449685`},
{"CURE", 365, 120.97131732244917`}, {"CURE", 365, 27.73006436598029`}, {"CURE", 365, 90.4648000202498`},
{"CURE", 365, 4.832923335065546`}, {"CURE", 365, 74.76237906726834`}, {"CURE", 365, 2.7204617473473527`},
{"CURE", 365, 86.61752405380965`}, {"CURE", 365, 77.33804124320808`}, {"TBUR", 114.77457476361232`, 92.47232089025259`},
{"CURE", 365, 6.794752144143361`}, {"CURE", 365, 44.795595083137286`}, {"CURE", 365, 181.1700930304083`},
{"TBUR", 24.896537290972482`, 41.66453280140113`}, {"CURE", 365, 1.678080075704538`}, {"CURE", 365, 20.668525619182414`},
{"CURE", 365, 127.28381133194557`}, {"CURE", 365, 10.136414358747622`}, {"CURE", 365, 93.97669677330398`},
{"CURE", 365, 150.14452570226092`}, {"CURE", 365, 59.397684662805425`}, {"CURE", 365, 7.344563694243789`},
{"TBUR", 162.85905485446693`, 2.4004410733169244`}, {"CURE", 365, 113.07379664138963`},
{"TBUR", 13.391641714387424`, 14.790018664466162`}, {"TBUR", 51.01854739984282`, 210.8015154335689`},
{"CURE", 365, 77.18746375858503`}, {"CURE", 365, 89.65332202802584`}, {"TBUR", 84.84662425879851`, 76.30747117714996`},
{"CURE", 365, 49.880694835534094`}, {"CURE", 365, 215.5057768198169`}, {"CURE", 365, 66.63835295355929`},
{"TBUR", 106.67243710012933`, 3.6381872385430647`}, {"CURE", 365, 67.32176812206421`},
{"CURE", 365, 19.921891062066255`}, {"CURE", 365, 166.27977144867134`}, {"CURE", 365, 63.13121605980081`},
{"CURE", 365, 4.147712249710851`}, {"CURE", 365, 72.42973249586483`}, {"TBUR", 21.97037233341102`, 80.57630648568623`},

{"CURE", 365, 81.6473443783709`}, {"CURE", 365, 70.22312673452713`}, {"CURE", 365, 52.07620625991026`},
{"CURE", 365, 116.02334714459357`}, {"CURE", 365, 9.616210898421548`}, {"TBUR", 77.93439441448048`, 157.79855654671078`},
{"CURE", 365, 64.93965553894321`}, {"CURE", 365, 6.342710845017859`}, {"CURE", 365, 25.839879840996396`},
{"CURE", 365, 191.6379429578545`}, {"CURE", 365, 74.80692239801425`}, {"CURE", 365, 136.00930988775457`},
{"CURE", 365, 71.99901691961864`}, {"CURE", 365, 156.562625835077`}, {"CURE", 365, 145.3011625462381`},
{"TBUR", 72.92827362047917`, 22.5715401736381`}, {"CURE", 365, 62.34670260165861`},
{"TBUR", 131.24279146636`, 62.754718627457216`}, {"TBUR", 17.407096967564335`, 13.53436941512073`},
{"TBUR", 22.5568902979218`, 8.284223436651796`}, {"CURE", 365, 57.040674142469584`}, {"CURE", 365, 90.90940756146844`},
{"TBUR", 51.372691944930985`, 40.261185663025515`}, {"CURE", 365, 141.69060904613696`}, {"CURE", 365, 133.29595704642256`},
{"TBUR", 18.688919116027243`, 15.551070098208685`}, {"TBUR", 24.84970268011269`, 33.11007548867962`},
{"TBUR", 66.44358413012078`, 13.382113765029546`}, {"TBUR", 26.079987478966125`, 101.3278734512427`},
{"TBUR", 43.88404880364781`, 87.65642325989903`}, {"CURE", 365, 109.6203617035677`},
{"TBUR", 76.8888988539594`, 141.83518373704362`}, {"CURE", 365, 34.44224080809236`}, {"CURE", 365, 105.89058842630638`},
{"TBUR", 27.559090814626277`, 89.79378748023228`}, {"TBUR", 26.381621809514904`, 5.908213823450051`},
{"CURE", 365, 147.59764606768803`}, {"CURE", 365, 62.87725188522643`}, {"TBUR", 36.29855299496048`, 3.302343742958491`},
{"TBUR", 20.3233461043475`, 2.357445483078195`}, {"CURE", 365, 51.09133829816537`}, {"CURE", 365, 73.7635918665867`},
{"CURE", 365, 84.8207539150113`}, {"TBUR", 38.79315547754634`, 55.43705645696084`}, {"CURE", 365, 85.82983978013665`},
{"CURE", 365, 121.83952982956639`}, {"CURE", 365, 59.79606781252772`}, {"TBUR", 92.97033408655079`, 24.213670083169244`},
{"CURE", 365, 105.9720301619605`}, {"TBUR", 101.65643392792946`, 2.365859770960882`},
{"CURE", 365, 31.657820167396224`}, {"TBUR", 77.4573389732691`, 71.06405265938938`},
{"CURE", 365, 6.466883870519624`}, {"TBUR", 43.75340470525933`, 44.03459436396953`},
{"CURE", 365, 69.71795258639192`}, {"TBUR", 34.233327654309505`, 79.38254272105739`},
{"CURE", 365, 36.262143724814344`}, {"TBUR", 20.7883224044095`, 55.55073911406397`}, {"CURE", 365, 72.92823438632087`},
{"TBUR", 16.345529531739302`, 88.47201145212813`}, {"CURE", 365, 78.13788365684209`},
{"TBUR", 16.19277587300641`, 20.767687437678145`}, {"TBUR", 64.28125635382932`, 63.89405630448909`},
{"CURE", 365, 145.1947821750723`}, {"CURE", 365, 6.1574998026305865`}, {"CURE", 365, 61.712062066931985`},
{"CURE", 365, 28.5479298898516`}, {"CURE", 365, 89.88638135540863`}, {"CURE", 365, 36.063190783735365`},
{"TBUR", 49.724126389259894`, 109.92054700118403`}, {"TBUR", 77.82882680985846`, 70.19841210457089`},
{"CURE", 365, 134.644553585868`}, {"CURE", 365, 40.09697333847324`}, {"CURE", 365, 178.0092499720389`},
{"CURE", 365, 117.85507386638228`}, {"CURE", 365, 93.5625086875174`}, {"CURE", 365, 114.45457065196568`},
{"TBUR", 134.4300341968592`, 182.2363370627167`}, {"TBUR", 39.39528761187041`, 195.77753059116995`},

{"CURE", 365, 55.67228213352597`}, {"CURE", 365, 93.39804185805119`}, {"CURE", 365, 101.77954562921347`},
{"TBUR", 39.84299613128159`, 35.25856389440329`}, {"TBUR", 16.392340276527268`, 7.093593845061254`},
{"CURE", 365, 0.920072408902781`}, {"CURE", 365, 70.82971071519403`}, {"CURE", 365, 120.74394749871018`},
{"TBUR", 20.239873978211367`, 96.87675015609177`}, {"CURE", 365, 135.70952855473408`},
{"CURE", 365, 180.81475253488065`}, {"CURE", 365, 42.61684097868601`}, {"CURE", 365, 92.78803107140088`},
{"TBUR", 37.74487763689221`, 7.017311391244689`}, {"CURE", 365, 201.45054977356227`},
{"TBUR", 36.7188781605375`, 64.4392269792693`}, {"TBUR", 57.79965090834926`, 44.01033111686088`},
{"TBUR", 64.70995702210922`, 53.640106622616095`}, {"CURE", 365, 187.38128688280764`}, {"CURE", 365, 74.07566576588829`},
{"TBUR", 92.03551190347734`, 166.16501519771813`}, {"CURE", 365, 125.72688855345724`}, {"CURE", 365, 111.50444067297063`},
{"TBUR", 54.34363398049555`, 107.9958765230143`}, {"TBUR", 87.66837666803355`, 107.1267096417147`},
{"CURE", 365, 50.30247276807131`}, {"CURE", 365, 42.18764144218531`}, {"CURE", 365, 10.357753379267049`},
{"CURE", 365, 77.57285184956761`}, {"CURE", 365, 91.63296367620478`}, {"TBUR", 33.69231731619724`, 48.65242961046364`},
{"TBUR", 43.74854682041269`, 62.69211343647692`}, {"CURE", 365, 122.99215493324851`},
{"TBUR", 41.51185751441066`, 57.317295702928455`}, {"CURE", 365, 66.77279977502188`}, {"CURE", 365, 30.52180647773689`},
{"TBUR", 20.463219281334624`, 9.917607782400257`}, {"CURE", 365, 46.07372116283931`}, {"CURE", 365, 143.14235958534428`},
{"CURE", 365, 59.20261291909027`}, {"TBUR", 42.71767141361701`, 55.99885660670473`}, {"CURE", 365, 12.901828131690758`},
{"TBUR", 25.75640730596536`, 130.1315480950726`}, {"CURE", 365, 139.18087759529485`}, {"CURE", 365, 87.05354177102186`},
{"TBUR", 100.24687354674097`, 32.212890068091156`}, {"CURE", 365, 134.35360719570176`}, {"CURE", 365, 15.895194706027533`},
{"TBUR", 84.2250371390577`, 200.45866059602824`}, {"TBUR", 28.473409887675967`, 70.1953707273416`},
{"CURE", 365, 150.40784493710643`}, {"TBUR", 42.94705652025979`, 73.79705831564796`}, {"CURE", 365, 41.43874351965354`},
{"TBUR", 41.48669113571581`, 52.96361619986895`}, {"TBUR", 24.332864536422928`, 17.87881046194544`},
{"CURE", 365, 117.75650710705182`}, {"TBUR", 41.97128483597807`, 37.5225384781809`}, {"CURE", 365, 55.55659255512763`},
{"TBUR", 24.338459226215086`, 44.631419889711374`}, {"CURE", 365, 74.31142605954422`},
{"TBUR", 23.168006958067043`, 43.30788244059795`}, {"TBUR", 106.16538793420496`, 151.31382373406984`},
{"CURE", 365, 94.8992167231969`}, {"TBUR", 33.21339510399529`, 64.58150091419185`},
{"TBUR", 66.21447027512504`, 66.10021987888271`}, {"TBUR", 13.80051946540677`, 62.28146829733786`},
{"CURE", 365, 4.559787040397829`}, {"TBUR", 108.22909603438106`, 117.66932565075771`},
{"TBUR", 101.79141459662604`, 103.77164342143534`}, {"CURE", 365, 86.79607422027846`},
{"TBUR", 41.37705565358592`, 8.842116218980214`}, {"CURE", 365, 64.72121705469883`}, {"CURE", 365, 169.0374602161964`},
{"CURE", 365, 81.42656364791016`}, {"CURE", 365, 97.59836514553017`}, {"TBUR", 28.09021874569974`, 32.7738843925484`},
{"TBUR", 85.0978995073666`, 80.08050269301052`}, {"TBUR", 13.1721559765612`, 2.375622918809288`},

{"TBUR", 15.184757874387143`, 12.513428613620063`}, {"TBUR", 52.6545357368715`, 118.48642610665672`},
{"CURE", 365, 73.38645321470408`}, {"TBUR", 51.23658944021386`, 65.34560013320238`}, {"CURE", 365, 18.499915168404787`},
{"TBUR", 217.88620697794263`, 140.82507502723467`}, {"CURE", 365, 74.77444981517279`}, {"CURE", 365, 140.8269434378147`},
{"TBUR", 51.62791966284921`, 22.614947837430606`}, {"TBUR", 30.117382985728575`, 79.31637531378458`},
{"CURE", 365, 65.28975898204293`}, {"CURE", 365, 80.07759200543465`}, {"CURE", 365, 15.859334458286291`},
{"CURE", 365, 57.07699269897471`}, {"CURE", 365, 126.68369807323643`}, {"TBUR", 20.6683902150265`, 80.90723886956792`},
{"CURE", 365, 40.44072780577174`}, {"TBUR", 40.53427562605297`, 2.7257948503860088`}, {"CURE", 365, 43.50635720237153`},
{"CURE", 365, 128.0253656493398`}, {"CURE", 365, 50.65099810282245`}, {"CURE", 365, 75.8015227438415`},
{"TBUR", 15.872568986580887`, 95.6739382741384`}, {"TBUR", 88.02817127798703`, 77.01937994800346`},
{"TBUR", 86.12036306677008`, 141.7568981784514`}, {"CURE", 365, 128.04835770444453`},
{"CURE", 365, 88.36684642303432`}, {"CURE", 365, 95.9407972058439`}, {"CURE", 365, 133.0837182531863`},
{"TBUR", 19.252529915699967`, 4.1274067834371895`}, {"TBUR", 79.23190184053995`, 138.19929870826513`},
{"TBUR", 147.969955534052`, 44.509344404458496`}, {"TBUR", 66.01155736549015`, 194.89838241863774`},
{"CURE", 365, 11.720691873347015`}, {"TBUR", 91.0755601722872`, 70.3675449556583`}, {"CURE", 365, 163.79428278590004`},
{"CURE", 365, 7.597646879300503`}, {"CURE", 365, 100.84020039455703`}, {"TBUR", 94.82049240771899`, 83.96086488205339`},
{"TBUR", 33.163009940049086`, 106.6036042883016`}, {"TBUR", 15.417979322648764`, 53.33607948835076`},
{"TBUR", 13.814184458167015`, 27.502085459861366`}, {"TBUR", 17.479996355719315`, 7.2581318784344475`},
{"CURE", 365, 75.29002694049643`}, {"CURE", 365, 73.63995434920187`}, {"TBUR", 37.099386884860216`, 89.98878777888612`},
{"CURE", 365, 14.764122170594502`}, {"CURE", 365, 203.27161202398932`}, {"CURE", 365, 38.852511458767054`},
{"CURE", 365, 81.12071949257977`}, {"CURE", 365, 46.734891066786105`}, {"CURE", 365, 7.7405370965937506`},
{"TBUR", 115.19164720887693`, 138.28345818695541`}, {"CURE", 365, 4.516210582515574`}, {"CURE", 365, 54.8000219070446`},
{"TBUR", 19.019382919405505`, 26.721983530926163`}, {"CURE", 365, 60.016289135593254`},
{"TBUR", 71.56356814638043`, 209.19145266907097`}, {"CURE", 365, 144.05175793262632`},
{"TBUR", 36.67050603566615`, 83.51960980466158`}, {"CURE", 365, 77.75128805623018`}, {"CURE", 365, 127.36514889595283`},
{"CURE", 365, 154.76198800248127`}, {"CURE", 365, 120.35392454656481`}, {"CURE", 365, 156.3912583815225`},
{"TBUR", 14.309582650986759`, 51.67767097005738`}, {"CURE", 365, 61.61385931507265`},
{"TBUR", 23.880580578329052`, 7.4694783279781`}, {"TBUR", 18.780543133213452`, 75.0731042587504`},
{"TBUR", 61.74163606090838`, 4.146114582598148`}, {"CURE", 365, 1.0339444139622729`}, {"CURE", 365, 6.833488832119688`},
{"TBUR", 142.3778785249349`, 0.7602700917435417`}, {"TBUR", 18.726409915407753`, 71.1665422053417`},
{"CURE", 365, 16.47074151142777`}, {"CURE", 365, 59.0248584391548`}, {"CURE", 365, 151.10393393928783`},
{"CURE", 365, 30.6657187071795`}, {"TBUR", 20.39535969299612`, 71.65480136160446`},

{"TBUR", 13.19938329646217`, 49.823544125326805`}, {"CURE", 365, 16.83163650596403`},
{"TBUR", 62.37660618939923`, 97.4005591068384`}, {"TBUR", 32.65245997926843`, 20.451740790352225`},
{"TBUR", 59.95143041129261`, 1.902830307232209`}, {"TBUR", 42.928168842498124`, 7.51454822910164`},
{"CURE", 365, 148.82493746913477`}, {"CURE", 365, 26.383976729900063`}, {"CURE", 365, 149.66934920171866`},
{"TBUR", 172.07512654250544`, 95.38030853780812`}, {"CURE", 365, 133.93347261400024`}, {"CURE", 365, 44.97177493620322`},
{"CURE", 365, 180.77995211613455`}, {"CURE", 365, 78.41932870916901`}, {"TBUR", 24.0993347599451`, 5.040205624320784`},
{"CURE", 365, 133.65532800814952`}, {"TBUR", 26.511368252037183`, 25.67473084380441`},
{"TBUR", 67.3938841031718`, 102.53286083988978`}, {"TBUR", 75.0993083792457`, 131.70210653999965`},
{"TBUR", 63.76451512275962`, 37.317269042495475`}, {"CURE", 365, 93.81664343297513`},
{"TBUR", 15.48740983565209`, 29.932783619946665`}, {"TBUR", 32.22992533636844`, 63.1466760161308`},
{"CURE", 365, 113.36122811796572`}, {"CURE", 365, 42.94747471064583`}, {"TBUR", 30.170891360740924`, 68.98119420928315`},
{"CURE", 365, 36.596946236571604`}, {"TBUR", 77.15109677553822`, 131.3047137196243`},
{"TBUR", 52.12410604731845`, 79.79265933329573`}, {"CURE", 365, 76.59782995383178`},
{"TBUR", 77.39764892404068`, 117.2975900259934`}, {"CURE", 365, 50.40520522781943`}, {"CURE", 365, 72.14830001088205`},
{"CURE", 365, 115.93215332623042`}, {"CURE", 365, 7.2301561782034005`}, {"TBUR", 17.796737922396165`, 10.305903791005123`},
{"TBUR", 145.07942723741624`, 46.28334644907494`}, {"TBUR", 57.01029886302818`, 174.40915899250626`},
{"CURE", 365, 20.17222469084163`}, {"TBUR", 112.88799546161877`, 158.98870464660877`}, {"CURE", 365, 69.92290974052507`},
{"CURE", 365, 167.67714684899883`}, {"TBUR", 15.630204617394204`, 41.087843998869936`},
{"TBUR", 39.5453038849339`, 54.71888622012097`}, {"CURE", 365, 80.96923686147635`}, {"CURE", 365, 175.3709798670709`},
{"TBUR", 84.79394142643929`, 78.93366326393331`}, {"CURE", 365, 197.21176627354308`}, {"CURE", 365, 41.26053619558263`},
{"CURE", 365, 102.66563236181119`}, {"CURE", 365, 79.55142822143517`}, {"CURE", 365, 90.05748159198748`},
{"TBUR", 63.577486638726995`, 87.43862957531567`}, {"TBUR", 44.71358600633425`, 91.19669425967172`},
{"CURE", 365, 10.379266927043203`}, {"CURE", 365, 31.241447715599335`}, {"TBUR", 82.11259368941353`, 100.39892699018999`},
{"CURE", 365, 81.03262599953032`}, {"CURE", 365, 15.344114251503457`}, {"TBUR", 76.61662894628229`, 99.19888265191214`},
{"CURE", 365, 34.70533792557756`}, {"CURE", 365, 17.445682108098918`}, {"CURE", 365, 17.43740603117968`},
{"CURE", 365, 23.522360088184712`}, {"CURE", 365, 33.45848284508753`}, {"TBUR", 67.31548329955082`, 83.86418716665007`},
{"CURE", 365, 125.5702044498093`}, {"CURE", 365, 151.45536701501086`}, {"TBUR", 31.60686429534309`, 108.71561176116639`},
{"TBUR", 67.53696836895223`, 7.778195573795725`}, {"CURE", 365, 183.37613686150934`},
{"TBUR", 102.33847750472343`, 58.783577392945766`}, {"TBUR", 39.23415011278942`, 90.83897313468567`},
{"TBUR", 122.00790409719536`, 20.664559164327578`}, {"CURE", 365, 75.46111085640518`},
{"CURE", 365, 2.3415653135988577`}, {"CURE", 365, 127.34600989919863`}, {"CURE", 365, 145.6434242459714`},

{"CURE", 365, 77.39471174963951`}, {"CURE", 365, 31.873785498255344`}, {"CURE", 365, 64.35863422158666`},
{"TBUR", 122.90951527849381`, 99.04216969060397`}, {"TBUR", 90.7695186241429`, 102.72609282087578`},
{"TBUR", 40.67534171505103`, 60.64893898858884`}, {"CURE", 365, 87.13598534237508`}, {"CURE", 365, 59.64700738672475`},
{"TBUR", 22.683728849376756`, 152.09576693418038`}, {"TBUR", 80.55191628004799`, 48.62396187112601`},
{"CURE", 365, 53.546925341187006`}, {"TBUR", 109.1540781338129`, 84.2942275160764`}, {"CURE", 365, 114.81694549511505`},
{"CURE", 365, 3.9168113039316044`}, {"TBUR", 55.16462934185558`, 50.08882167964998`}, {"CURE", 365, 81.03325521342877`},
{"CURE", 365, 16.10971508182223`}, {"CURE", 365, 152.90054216526727`}, {"TBUR", 15.504090805177068`, 7.168267383898794`},
{"CURE", 365, 148.7820523133931`}, {"CURE", 365, 138.90430359342028`}, {"TBUR", 105.28930138126371`, 3.514329610996405`},
{"CURE", 365, 117.48937529709461`}, {"CURE", 365, 46.64679973099077`}, {"CURE", 365, 52.331631414340244`},
{"CURE", 365, 128.5929582665286`}, {"CURE", 365, 9.147824713359437`}, {"TBUR", 20.950237373581793`, 73.70968778681289`},
{"CURE", 365, 24.294940551738943`}, {"TBUR", 82.28092502039372`, 2.5464384156169073`}, {"CURE", 365, 75.41127507739613`},
{"TBUR", 74.56178302794169`, 116.68685928089118`}, {"TBUR", 169.01692067418756`, 20.22583331088524`},
{"CURE", 365, 0.6757514146042323`}, {"TBUR", 87.00176566171511`, 8.369070355170901`},
{"TBUR", 30.46372278013861`, 59.20795603144872`}, {"CURE", 365, 2.3186418210617825`}, {"CURE", 365, 5.7072032782350215`},
{"CURE", 365, 112.56632055300155`}, {"TBUR", 75.20509397083956`, 190.48123704212384`},
{"TBUR", 89.4634289941553`, 100.04306997126496`}, {"CURE", 365, 185.31140550653652`},
{"TBUR", 73.98608883971495`, 89.63196318634105`}, {"CURE", 365, 2.1467111405083075`}, {"CURE", 365, 97.94102499865774`},
{"TBUR", 89.55768448800407`, 143.10044161403135`}, {"TBUR", 38.62641535902931`, 74.1683136379846`},
{"TBUR", 66.24148156114646`, 9.064973458298272`}, {"TBUR", 57.70718898565587`, 59.87201290747512`},
{"CURE", 365, 40.127304066905396`}, {"CURE", 365, 21.40601923979195`}, {"CURE", 365, 13.993860961568798`},
{"CURE", 365, 113.40720258071508`}, {"TBUR", 127.43498193106906`, 47.43828222129214`},
{"TBUR", 25.10211435384886`, 44.29204262680461`}, {"CURE", 365, 64.6368082679504`},
{"TBUR", 69.16936323246978`, 12.755495866163107`}, {"CURE", 365, 94.57489193719182`}, {"CURE", 365, 49.43138222457806`},
{"TBUR", 63.58059461976206`, 54.108759621870796`}, {"TBUR", 50.79249963076034`, 124.97434007322518`},
{"CURE", 365, 167.14253988909152`}, {"CURE", 365, 50.60543008866301`}, {"CURE", 365, 9.913981752027095`},
{"TBUR", 79.47302050397934`, 193.5859056243328`}, {"TBUR", 155.44601491568878`, 61.91288907348309`},
{"TBUR", 57.139602275614`, 56.04509059217703`}, {"CURE", 365, 79.58638121792654`}, {"CURE", 365, 15.764712533206037`},
{"CURE", 365, 72.36636660342555`}, {"CURE", 365, 67.92966333160787`}, {"TBUR", 118.64119204935803`, 68.91921847389816`},
{"CURE", 365, 119.53220865511317`}, {"CURE", 365, 48.71126246370613`}, {"TBUR", 165.3960062539895`, 90.17347422496223`},
{"CURE", 365, 39.612545661052934`}, {"CURE", 365, 120.78670211482917`}, {"CURE", 365, 53.50800928208997`},
{"TBUR", 85.28171068164892`, 70.59429577141974`}, {"CURE", 365, 33.43042644756054`},

{"TBUR", 82.3242549823264`, 112.2547850125817`}, {"TBUR", 47.9344064457124`, 216.76537327351625`},
{"TBUR", 41.88946045871725`, 104.9897517253997`}, {"CURE", 365, 103.14106487310906`}, {"CURE", 365, 37.60653096483536`},
{"TBUR", 121.09215048511308`, 85.77561951246045`}, {"TBUR", 57.45935011091166`, 159.4513338239628`},
{"CURE", 365, 4.362980406408707`}, {"CURE", 365, 104.01152929594619`}, {"TBUR", 67.62833445477187`, 9.025675769069993`},
{"CURE", 365, 16.36940993103046`}, {"CURE", 365, 12.655139173032124`}, {"TBUR", 48.44852000226736`, 53.70593371682508`},
{"CURE", 365, 2.9117463186984787`}, {"TBUR", 32.31050310248697`, 72.63785803788552`}, {"CURE", 365, 67.06473943704087`},
{"TBUR", 80.37226569593274`, 83.42834873636063`}, {"TBUR", 33.32628937949292`, 46.97711695557644`},
{"CURE", 365, 80.46758084309405`}, {"CURE", 365, 154.26235340782196`}, {"CURE", 365, 75.5849145438912`},
{"CURE", 365, 18.732561858072263`}, {"CURE", 365, 78.36004020476305`}, {"TBUR", 112.10678368057863`, 7.340010524062542`},
{"TBUR", 63.27985799667683`, 7.198546198464817`}, {"CURE", 365, 124.35110134919623`},
{"CURE", 365, 123.15377313007595`}, {"TBUR", 81.73201106723029`, 26.078428181730438`},
{"CURE", 365, 38.633094035247545`}, {"TBUR", 18.258663779774047`, 84.60321650671594`},
{"CURE", 365, 148.44162800152606`}, {"TBUR", 133.21307783791448`, 46.431568123778256`},
{"TBUR", 58.094877109042876`, 67.25848546723067`}, {"CURE", 365, 10.391356003597188`},
{"TBUR", 75.71091173741281`, 75.21786545390442`}, {"CURE", 365, 62.89427113815111`}, {"CURE", 365, 78.76088350508094`},
{"TBUR", 69.61525345722218`, 51.27117365275776`}, {"TBUR", 72.72678443686793`, 18.8058859931995`},
{"CURE", 365, 90.9199521549533`}, {"TBUR", 14.64219200004885`, 50.165046829078506`}, {"CURE", 365, 22.156567784570704`},
{"CURE", 365, 99.0923377582683`}, {"CURE", 365, 176.72668105527524`}, {"CURE", 365, 140.65213394420402`},
{"CURE", 365, 76.60238012616404`}, {"CURE", 365, 100.98255193973036`}, {"TBUR", 40.480298933255284`, 10.20194150380782`},
{"TBUR", 42.797206530513435`, 86.34134204084428`}, {"TBUR", 64.45018580970732`, 16.389977183644824`},
{"CURE", 365, 53.759702001879404`}, {"TBUR", 25.80972134480884`, 89.10479494857795`}, {"CURE", 365, 20.668291566752583`},
{"CURE", 365, 64.89607000603688`}, {"TBUR", 20.437140942210114`, 25.367015657877978`},
{"CURE", 365, 212.50464028699133`}, {"TBUR", 14.46597968390889`, 13.5124466033622`}, {"CURE", 365, 95.23576430928087`},
{"TBUR", 41.81115273170377`, 28.119249653827374`}, {"TBUR", 103.61920279030333`, 12.513121793597724`},
{"CURE", 365, 2.3336926941425706`}, {"CURE", 365, 4.361000263861266`}, {"CURE", 365, 76.53440888165822`},
{"TBUR", 26.971271644987986`, 43.69987154444396`}, {"CURE", 365, 9.12135306828542`}, {"CURE", 365, 68.98493008625745`},
{"CURE", 365, 100.67045182061743`}, {"CURE", 365, 189.56340160982342`}, {"CURE", 365, 52.84752682387651`},
{"CURE", 365, 87.97402989376852`}, {"TBUR", 40.87029846598101`, 90.71735338505773`}, {"CURE", 365, 12.646024497097168`},
{"TBUR", 24.58148515187632`, 39.386511175007975`}, {"CURE", 365, 29.77903326054853`}, {"CURE", 365, 86.738917105463`},
{"TBUR", 42.32519452617096`, 83.19785053028342`}, {"CURE", 365, 57.45405267544114`}, {"CURE", 365, 3.3419137575470277`},
{"CURE", 365, 17.47418326195087`}, {"CURE", 365, 64.50326251874085`}, {"TBUR", 41.85345049684838`, 75.65066407792374`},

{"CURE", 365, 101.89877141491165`}, {"TBUR", 107.21971269053361`, 65.98046433624984`}, {"CURE", 365, 191.78395877345127`},
{"CURE", 365, 86.54586460079915`}, {"TBUR", 18.147433088412658`, 69.54859318556026`}, {"CURE", 365, 1.1071165580827538`},
{"CURE", 365, 37.57942590366153`}, {"CURE", 365, 145.21474657077476`}, {"CURE", 365, 4.207018390816386`},
{"TBUR", 46.90392648079693`, 55.64737597344622`}, {"TBUR", 50.03625354338124`, 87.60537547173277`},
{"TBUR", 87.3789016058532`, 160.10323810842243`}, {"TBUR", 88.37656447519775`, 107.04504874010004`},
{"CURE", 365, 17.477487626207896`}, {"TBUR", 75.38135928710955`, 8.560239071840654`},
{"CURE", 365, 61.10135655668561`}, {"CURE", 365, 151.07614567030532`}, {"TBUR", 32.60611397920839`, 87.31271605497835`},
{"CURE", 365, 8.173798858054802`}, {"TBUR", 54.207453777540806`, 89.34949659858503`},
{"CURE", 365, 76.5447738478457`}, {"TBUR", 66.61555367547147`, 4.3446147298445785`},
{"CURE", 365, 64.13079298826038`}, {"CURE", 365, 84.12570382426713`}, {"CURE", 365, 47.94593264369842`},
{"TOX", 29.418290439296733`, 239.01224203365734`}, {"TBUR", 62.63065462007421`, 65.43851325458257`},
{"CURE", 365, 85.37765856425747`}, {"TBUR", 24.540902609053532`, 67.98135878736461`}, {"CURE", 365, 194.9640434458893`},
{"CURE", 365, 71.67741447293055`}, {"TBUR", 19.596221381244714`, 67.56059563649713`}, {"CURE", 365, 42.3569916627353`},
{"TBUR", 58.833195972567495`, 99.06804263851893`}, {"CURE", 365, 0.8766830410191684`},
{"TBUR", 31.653768765253034`, 18.083979019147428`}, {"TBUR", 145.92550203743443`, 94.40320445653603`},
{"CURE", 365, 81.09671807664665`}, {"CURE", 365, 12.875287888515926`}, {"CURE", 365, 61.65215773459341`},
{"CURE", 365, 41.43374903583754`}, {"TBUR", 71.19584923518235`, 43.49911647407719`}, {"CURE", 365, 8.018157940007326`},
{"CURE", 365, 54.89163249023105`}, {"CURE", 365, 45.09693057151524`}, {"TBUR", 26.651232037282696`, 29.19992375507601`},
{"CURE", 365, 145.98017735921616`}, {"CURE", 365, 4.149702994383933`}, {"CURE", 365, 64.24955603761066`},
{"TBUR", 162.31348164160423`, 101.37159187739883`}, {"TBUR", 16.540424352785276`, 108.93436011387537`},
{"TBUR", 43.065503982888316`, 6.71708890181488`}, {"CURE", 365, 82.22747516180378`}, {"CURE", 365, 101.0770373711256`},
{"TBUR", 26.50496730552425`, 3.5690293426788515`}, {"CURE", 365, 55.113046883263486`},
{"CURE", 365, 84.59166512146192`}, {"TBUR", 21.39022120674951`, 21.102882117985107`},
{"CURE", 365, 1.948225088106185`}, {"TBUR", 66.29948276011739`, 166.05070251882032`},
{"TBUR", 113.38494012267704`, 89.74246588351984`}, {"CURE", 365, 89.09180774451609`}, {"CURE", 365, 24.662158840126317`},
{"TBUR", 78.35207638357153`, 2.8572492801771308`}, {"TBUR", 37.304267306206924`, 2.911596577972113`},
{"TBUR", 13.167141478931974`, 16.43847585544677`}, {"TBUR", 84.18358170578449`, 72.38264865309735`},
{"CURE", 365, 119.21222674494624`}, {"CURE", 365, 100.8340451968049`}, {"CURE", 365, 48.20185761164502`},
{"TBUR", 21.198849093690704`, 1.5666698613651568`}, {"CURE", 365, 79.0536906160431`}, {"CURE", 365, 82.61915668926362`},
{"TBUR", 66.05150027552308`, 1.036564630506876`}, {"TBUR", 76.42624003377556`, 48.56407236142446`},
{"CURE", 365, 0.9766509321976996`}, {"CURE", 365, 135.50358602082375`}, {"CURE", 365, 57.923318029431265`},

{"TBUR", 14.47166817330794`, 0.6929423433470233`}, {"TBUR", 163.0827471105636`, 81.93775929364142`},
{"CURE", 365, 29.12915043198377`}, {"TBUR", 54.698187040090254`, 214.46822337766824`}, {"CURE", 365, 117.60859000790909`},
{"TBUR", 41.67991384766716`, 66.07867637949435`}, {"CURE", 365, 170.26231771148744`}},
{{"430 Res", "430 OS", "430 Tox"}, {"TBUR", 21.563485096940045`, 36.744014934096946`}, {"CURE", 365, 140.2313435956807`},
{"TBUR", 26.87751632032816`, 108.26231041614348`}, {"TBUR", 28.78108432105769`, 4.502736764803938`},
{"TBUR", 17.97032948282975`, 15.357930686580564`}, {"CURE", 365, 26.872642700487834`}, {"CURE", 365, 61.685824168161666`},
{"CURE", 365, 58.38182832815452`}, {"CURE", 365, 138.3855952537237`}, {"TBUR", 70.67165770077295`, 187.01328591001095`},
{"TBUR", 54.44587667896684`, 96.36295201727195`}, {"CURE", 365, 85.49395599987851`},
{"TOX", 20.417984212596295`, 253.3688371717045`}, {"TBUR", 42.92543277701032`, 59.69370813858525`},
{"TBUR", 62.34379436439707`, 6.163224497060282`}, {"TBUR", 86.91818025487179`, 26.236272023294298`},
{"CURE", 365, 97.57260458202799`}, {"TBUR", 44.30593648343298`, 109.07924332885624`},
{"TBUR", 43.99507014235725`, 6.485395208123885`}, {"CURE", 365, 80.52066668573565`}, {"CURE", 365, 130.5101792770238`},
{"CURE", 365, 45.052294577168574`}, {"CURE", 365, 27.257548401567607`}, {"CURE", 365, 60.933823637386766`},
{"CURE", 365, 53.260515640396584`}, {"CURE", 365, 152.98925126202337`}, {"CURE", 365, 91.31339093190044`},
{"CURE", 365, 47.268405148910226`}, {"CURE", 365, 31.403983230857165`}, {"CURE", 365, 5.332640232372611`},
{"CURE", 365, 128.0069705180266`}, {"TBUR", 13.39550669346527`, 33.599753075717864`}, {"CURE", 365, 64.87248001154771`},
{"TBUR", 47.759437598636154`, 38.102748132367644`}, {"CURE", 365, 174.57224766022077`},
{"CURE", 365, 204.29498069902422`}, {"CURE", 365, 39.576797551329896`}, {"TBUR", 21.304325744996945`, 10.473413298983793`},
{"TBUR", 71.78046817160529`, 46.32503687394954`}, {"CURE", 365, 91.74509718581677`},
{"TBUR", 17.996962330019805`, 57.88268914165985`}, {"CURE", 365, 92.16487227611222`}, {"CURE", 365, 68.51700926812495`},
{"TBUR", 45.9263773392024`, 74.91773811092912`}, {"CURE", 365, 92.25053614288427`}, {"CURE", 365, 4.765856319896335`},
{"CURE", 365, 102.72178639289194`}, {"CURE", 365, 64.48208462088112`}, {"TBUR", 112.35569131283097`, 125.81779540581782`},
{"CURE", 365, 214.94167177812903`}, {"TBUR", 29.652299044670595`, 95.49903906747167`},
{"TBUR", 41.65283891634102`, 82.22317797692376`}, {"CURE", 365, 6.221134469781657`}, {"CURE", 365, 133.3561108884225`},
{"CURE", 365, 42.9268754495789`}, {"CURE", 365, 93.70165862968017`}, {"CURE", 365, 65.19935249198451`},
{"TBUR", 103.20661902557536`, 73.74352405241507`}, {"TBUR", 37.296325985308044`, 161.58413604758522`},
{"TBUR", 52.27963910501162`, 96.20922606680182`}, {"CURE", 365, 88.90336704873263`}, {"CURE", 365, 4.403794487914908`},
{"TBUR", 33.578489551795045`, 109.02640375381038`}, {"CURE", 365, 76.46151236694854`},
{"TBUR", 69.71675461326839`, 6.704038257028256`}, {"CURE", 365, 84.89048569433427`}, {"CURE", 365, 75.95933496729423`},
{"TBUR", 57.31906091195813`, 109.42081353166917`}, {"CURE", 365, 39.729679746332046`}, {"CURE", 365, 33.550126009255614`},
{"CURE", 365, 63.14056811769711`}, {"CURE", 365, 45.99187779268498`}, {"CURE", 365, 134.38112554501103`},

{"TBUR", 13.339686442177621`, 2.0730259587209803`}, {"TBUR", 79.4148804316784`, 86.34805376789751`},
{"CURE", 365, 111.26252905345557`}, {"TBUR", 96.95152858956703`, 207.23892701352958`}, {"CURE", 365, 68.2116616560004`},
{"TBUR", 75.7492418199354 9`, 134.6175092531192`}, {"CURE", 365, 52.902107451723744`}, {"CURE", 365, 108.52621459737085`},
{"CURE", 365, 3.4918944921154944`}, {"CURE", 365, 6.882978874853419`}, {"CURE", 365, 4.868776058366759`},
{"CURE", 365, 125.64298771315478`}, {"TBUR", 99.41527142486244`, 96.10458784560946`}, {"CURE", 365, 80.06151256128237`},
{"CURE", 365, 41.87282987961043`}, {"CURE", 365, 7.4655783696501645`}, {"TBUR", 71.37698153331216`, 142.44792706791776`},
{"TBUR", 32.46967699275748`, 11.842192256412993`}, {"TBUR", 89.19438538674386`, 103.8724147481944`},
{"TBUR", 66.63949023392712`, 2.8591628991696876`}, {"CURE", 365, 6.617327554470817`},
{"TBUR", 19.007342390128816`, 2.008235171226393`}, {"TBUR", 158.23499575914715`, 55.389274672460374`},
{"CURE", 365, 40.04116591684299`}, {"CURE", 365, 188.3761036386259`}, {"CURE", 365, 10.148611689331101`},
{"TBUR", 43.675229862690294`, 146.56441728799945`}, {"TBUR", 20.26849950728426`, 10.677889757175118`},
{"TBUR", 127.3151090099417`, 131.8491538937725`}, {"CURE", 365, 72.71577469152979`},
{"TBUR", 90.71315350614674`, 94.46587975434721`}, {"CURE", 365, 65.92302855860211`}, {"CURE", 365, 105.97389589358086`},
{"CURE", 365, 43.16652984184306`}, {"TBUR", 138.51895874708808`, 90.01113329410337`}, {"CURE", 365, 39.069539786749345`},
{"CURE", 365, 106.98127403952533`}, {"CURE", 365, 147.00206755647528`}, {"CURE", 365, 80.09826619631136`},
{"CURE", 365, 22.312172133366207`}, {"CURE", 365, 51.20014100662597`}, {"CURE", 365, 72.13709346901557`},
{"CURE", 365, 60.153126281801335`}, {"CURE", 365, 9.340032897016858`}, {"CURE", 365, 79.48309708876567`},
{"CURE", 365, 78.99943247727235`}, {"CURE", 365, 22.781754179222244`}, {"CURE", 365, 84.69871631737531`},
{"CURE", 365, 170.28336288806054`}, {"CURE", 365, 107.49256183038`}, {"TBUR", 19.807230393006957`, 5.593787394118368`},
{"CURE", 365, 75.95283866922033`}, {"TBUR", 38.69717046338108`, 65.76600244446519`}, {"CURE", 365, 56.23786191345349`},
{"CURE", 365, 37.30373950383636`}, {"CURE", 365, 122.75791698892806`}, {"CURE", 365, 118.2755253802562`},
{"CURE", 365, 105.28884374468747`}, {"CURE", 365, 115.16214373478955`}, {"TBUR", 71.64759947522936`, 36.97417311759543`},
{"TBUR", 45.35833485512875`, 38.89771646024535`}, {"CURE", 365, 168.47532377580717`},
{"TBUR", 12.767147843815156`, 72.41538833337714`}, {"TBUR", 92.0657875747918`, 23.68275199545457`},
{"TBUR", 62.26110531167903`, 56.111635843612966`}, {"TBUR", 100.78397425935633`, 19.178144909622155`},
{"CURE", 365, 113.69855279464603`}, {"TBUR", 52.95299507096851`, 171.3511810491505`}, {"CURE", 365, 116.65606989047434`},
{"CURE", 365, 153.05938412517438`}, {"CURE", 365, 23.541883658848086`}, {"CURE", 365, 2.304297961580209`},
{"CURE", 365, 69.99194653181812`}, {"CURE", 365, 167.74203964410506`}, {"TBUR", 36.825917969940164`, 41.84694799603487`},
{"CURE", 365, 77.21264606509285`}, {"CURE", 365, 16.355217437625736`}, {"CURE", 365, 7.242252358302582`},
{"CURE", 365, 63.87586451585434`}, {"CURE", 365, 1.2174542978937937`}, {"TBUR", 88.48572190042158`, 170.0002324987428`},
{"TBUR", 50.00752843715839`, 68.77951563422604`}, {"TBUR", 88.33580502147149`, 151.2284786256906`},

{"CURE", 365, 74.96674816415045`}, {"CURE", 365, 89.12068008688664`}, {"CURE", 365, 71.04715652346975`},
{"CURE", 365, 89.04295004465139`}, {"CURE", 365, 23.801176071176144`}, {"TBUR", 96.39290710741801`, 22.819902643314272`},
{"CURE", 365, 1.7454732699409778`}, {"TBUR", 63.17762993471151`, 9.210265861669159`},
{"TBUR", 78.74755522968103`, 77.28103361509454`}, {"CURE", 365, 115.79173748204215`}, {"CURE", 365, 116.00163793758168`},
{"CURE", 365, 83.85910823763041`}, {"CURE", 365, 119.9581307859073`}, {"CURE", 365, 1.787557544722784`},
{"CURE", 365, 67.14998421002946`}, {"TBUR", 29.735848060375755`, 129.67412492353782`}, {"CURE", 365, 58.79977580242151`},
{"TBUR", 20.95237621227256`, 6.944601149479452`}, {"TBUR", 50.23284769510168`, 128.23954980526852`},
{"TBUR", 87.18193354750385`, 10.516253915637876`}, {"TBUR", 67.78201953745088`, 154.3254871009476`},
{"TBUR", 21.196345805724988`, 81.29287999982299`}, {"TBUR", 58.803391199734655`, 42.95003855065119`},
{"TBUR", 91.06457189577631`, 101.51480264151759`}, {"TBUR", 80.23678623558834`, 103.36190408764843`},
{"CURE", 365, 84.13512742673704`}, {"CURE", 365, 145.27425707838955`}, {"TBUR", 19.6416999512436`, 8.806408597227938`},
{"CURE", 365, 130.8438297824165`}, {"TBUR", 27.96538096722387`, 43.3275215610135`},
{"TBUR", 53.572333549278575`, 143.57632731354704`}, {"CURE", 365, 69.3210704343655`},
{"CURE", 365, 3.7938551457882297`}, {"TBUR", 24.21937533316375`, 94.44625464795753`}, {"CURE", 365, 90.69046859551317`},
{"CURE", 365, 143.86407471270104`}, {"CURE", 365, 167.7066502945661`}, {"CURE", 365, 110.64840801860724`},
{"CURE", 365, 65.44600669164315`}, {"CURE", 365, 50.14963700067169`}, {"CURE", 365, 174.678830649961`},
{"TBUR", 13.652030270513555`, 13.95237026813305`}, {"CURE", 365, 98.11952948086709`}, {"CURE", 365, 88.87499549631465`},
{"TBUR", 26.518405754786162`, 23.508825618494825`}, {"TBUR", 57.10003148374778`, 100.5996484334477`},
{"CURE", 365, 98.96405974015768`}, {"TBUR", 65.23349694932791`, 78.65592337846127`},
{"CURE", 365, 1.7754036753032227`}, {"TBUR", 142.84312517759182`, 20.97221611742465`},
{"TBUR", 15.865413744785586`, 85.85128123991545`}, {"CURE", 365, 172.41321581003498`}, {"CURE", 365, 9.795666951369789`},
{"CURE", 365, 129.00519732262205`}, {"CURE", 365, 2.9114367053960803`}, {"CURE", 365, 90.8313674189226`},
{"CURE", 365, 17.990991334124093`}, {"CURE", 365, 111.12288243588777`}, {"CURE", 365, 25.471512815193837`},
{"TBUR", 44.332351188030856`, 13.608922086133356`}, {"TBUR", 79.96076591385889`, 156.7077389500386`},
{"CURE", 365, 75.48123042819627`}, {"TBUR", 50.07173532763343`, 204.42471667659566`}, {"CURE", 365, 71.4587920626059`},
{"CURE", 365, 101.75216488006278`}, {"CURE", 365, 12.556067898198691`}, {"CURE", 365, 69.38683129816008`},
{"CURE", 365, 134.81223213370848`}, {"CURE", 365, 140.40768176333543`}, {"CURE", 365, 68.14411884237018`},
{"CURE", 365, 125.02302722310604`}, {"CURE", 365, 80.17022194815242`}, {"CURE", 365, 7.624858082903105`},
{"CURE", 365, 51.76171496189119`}, {"CURE", 365, 43.26922130852993`}, {"CURE", 365, 70.11890374071143`},
{"CURE", 365, 103.2043827873808`}, {"CURE", 365, 85.81279634997901`}, {"CURE", 365, 78.67684786644107`},
{"TBUR", 22.643053647428626`, 100.09692820515288`}, {"TBUR", 28.02933756774232`, 67.60015681430276`},

{"TBUR", 156.57538341644562`, 67.41146847694178`}, {"TBUR", 20.56230769453341`, 45.36052302478972`},
{"CURE", 365, 59.22446433333083`}, {"CURE", 365, 68.75738508485223`}, {"CURE", 365, 123.95376305422657`},
{"TBUR", 26.898833729436017`, 5.677424796929406`}, {"TBUR", 31.50494434594707`, 23.15951593856305`},
{"CURE", 365, 1.639844484117842`}, {"CURE", 365, 2.4461594683028656`}, {"TBUR", 19.853527716650863`, 22.097748925784867`},
{"CURE", 365, 197.77019131863833`}, {"CURE", 365, 51.306801474572225`}, {"CURE", 365, 193.67548984512754`},
{"TBUR", 38.566424645724155`, 90.94268350364226`}, {"CURE", 365, 61.27351061346631`},
{"TBUR", 15.216791830792658`, 115.66031844663864`}, {"CURE", 365, 50.92512589973363`},
{"CURE", 365, 99.12456013212753`}, {"CURE", 365, 6.048263036176831`}, {"CURE", 365, 73.49311576578232`},
{"CURE", 365, 24.911351660998196`}, {"CURE", 365, 9.055505301347987`}, {"CURE", 365, 115.04317656269039`},
{"TBUR", 21.56660562005007`, 119.61625005497393`}, {"TBUR", 13.674053593915813`, 86.3310171732924`},
{"CURE", 365, 58.18804744773575`}, {"TBUR", 28.012084473473976`, 80.84653928224226`},
{"CURE", 365, 5.386474259657674`}, {"CURE", 365, 58.078263913517695`}, {"CURE", 365, 46.59678995604556`},
{"CURE", 365, 202.82248953088163`}, {"CURE", 365, 84.28795262450662`}, {"CURE", 365, 44.79093253465958`},
{"CURE", 365, 1.5718766455821638`}, {"TBUR", 69.89074133934453`, 84.62043162644623`}, {"CURE", 365, 71.89861320405785`},
{"TBUR", 151.28657718481207`, 75.99740066519246`}, {"TBUR", 53.68771051173976`, 10.201554077253698`},
{"CURE", 365, 78.32883726912826`}, {"CURE", 365, 54.63238331676338`}, {"TBUR", 20.565006656751503`, 71.05407741347092`},
{"CURE", 365, 39.76494773599276`}, {"CURE", 365, 97.66457340248813`}, {"CURE", 365, 13.886406853865898`},
{"TBUR", 97.81679944995537`, 96.3769906133794`}, {"TBUR", 16.558197907942514`, 37.683676158718576`},
{"CURE", 365, 78.03848027244925`}, {"TBUR", 27.08891903040844`, 91.40226337753984`}, {"CURE", 365, 8.745613722530642`},
{"CURE", 365, 113.12703240034384`}, {"CURE", 365, 67.24221146616898`}, {"CURE", 365, 187.39333895612555`},
{"CURE", 365, 131.00338787615232`}, {"CURE", 365, 92.0453886714441`}, {"CURE", 365, 56.93327819414012`},
{"CURE", 365, 193.11722941155446`}, {"TBUR", 30.265540067597094`, 29.90053399767146`},
{"CURE", 365, 17.456458072455757`}, {"CURE", 365, 26.22037884656345`}, {"CURE", 365, 82.28736240504051`},
{"CURE", 365, 76.81862312298854`}, {"CURE", 365, 108.97435993898782`}, {"CURE", 365, 33.28366882908224`},
{"TBUR", 23.207188010365403`, 139.38254702200604`}, {"CURE", 365, 2.198200534405933`}, {"CURE", 365, 115.47099811917872`},
{"TBUR", 38.359959474303984`, 137.47181994563968`}, {"CURE", 365, 50.807951355585246`}, {"CURE", 365, 115.80513921740652`},
{"CURE", 365, 3.8304263160921233`}, {"TBUR", 22.183008456243737`, 43.59284963127397`}, {"CURE", 365, 64.42707632120616`},
{"CURE", 365, 64.80899871002521`}, {"TBUR", 28.897258417504407`, 30.20591147300008`}, {"CURE", 365, 64.16275966395652`},
{"TBUR", 36.40319349251432`, 31.064871672374696`}, {"CURE", 365, 103.31363381093334`}, {"CURE", 365, 81.57204139336893`},
{"CURE", 365, 89.34980889026056`}, {"CURE", 365, 64.02821216032329`}, {"CURE", 365, 69.42887267335088`},
{"CURE", 365, 98.55667942347031`}, {"CURE", 365, 129.55507851570943`}, {"TBUR", 49.05511236445053`, 93.35933706755017`},

```
{"CURE", 365, 126.87508117024525`}, {"CURE", 365, 77.42593568303352`}, {"TBUR", 57.23734457753941`, 19.54177121164215`},
{"TBUR", 16.965751154144836`, 15.191862949166813`}, {"CURE", 365, 0.7621812273888818`}, {"CURE", 365, 111.05638173408092`},
{"CURE", 365, 112.96061629165003`}, {"CURE", 365, 86.3803742553707`}, {"TBUR", 138.39395302387723`, 198.77821855420734`},
{"CURE", 365, 154.5151676799985`}, {"CURE", 365, 40.0248264671581`}, {"TBUR", 20.466118660852086`, 60.34269757962447`},
{"TBUR", 33.08039724946631`, 7.235552164591352`}, {"CURE", 365, 189.96889105756554`},
{"TBUR", 38.937275860203236`, 186.072090289363`}, {"TBUR", 23.380428634677216`, 25.771939584114424`},
{"CURE", 365, 183.29556681198684`}, {"CURE", 365, 32.222593086966825`}, {"CURE", 365, 66.5753211289246`},
{"CURE", 365, 129.74250261333552`}, {"CURE", 365, 2.1241996117317252`}, {"TBUR", 55.56517284625231`, 38.7515002991585`},
{"CURE", 365, 117.13975627026367`}, {"TBUR", 54.96807220903299`, 27.89393012134679`},
{"TBUR", 38.477662294085036`, 186.70988231703515`}, {"CURE", 365, 3.1707730485409984`}, {"CURE", 365, 156.8353097015955`},
{"CURE", 365, 92.25191394977196`}, {"CURE", 365, 86.24487864192065`}, {"CURE", 365, 110.08859988805318`},
{"CURE", 365, 47.3181016605225`}, {"TBUR", 35.52398969980615`, 139.60445741708466`}, {"CURE", 365, 86.31208371456279`},
{"CURE", 365, 149.57386495775754`}, {"CURE", 365, 97.2559205876141`}, {"TBUR", 54.33087857882224`, 101.7925615165692`},
{"CURE", 365, 119.98190609984968`}, {"CURE", 365, 88.28422590847157`}, {"CURE", 365, 42.82858198576801`},
{"CURE", 365, 133.63323041030665`}, {"CURE", 365, 163.03973514114014`}, {"CURE", 365, 43.99797536347094`},
{"TBUR", 63.97776835956548`, 106.37425003634259`}, {"TBUR", 45.85736850958374`, 134.89412906523972`},
{"CURE", 365, 13.88594425996874`}, {"CURE", 365, 97.23647474096067`}, {"CURE", 365, 49.70558437014432`},
{"TBUR", 14.501736486699931`, 8.296173490125119`}, {"TBUR", 13.36975617690309`, 29.010191013353086`},
{"TBUR", 31.83701604292971`, 47.07629219412444`}, {"CURE", 365, 93.89065756338117`}, {"CURE", 365, 23.34895331033218`},
{"CURE", 365, 84.00089634088003`}, {"TBUR", 78.03279995565367`, 155.0046443024778`}, {"CURE", 365, 86.5826405059808`},
{"CURE", 365, 76.21061792648474`}, {"CURE", 365, 75.56003366838097`}, {"TBUR", 17.098361379434014`, 33.876713035509695`},
{"TBUR", 13.590815018647968`, 78.68759990171178`}, {"TBUR", 76.02759984101937`, 34.829183966731705`},
{"CURE", 365, 137.68704763643862`}, {"TBUR", 49.643389523028354`, 83.02979252273077`},
{"CURE", 365, 111.39236745698936`}, {"CURE", 365, 82.52735069664166`}, {"CURE", 365, 72.19489563527803`},
{"TBUR", 101.13044156782571`, 34.18038885047495`}, {"CURE", 365, 51.149640602545645`}, {"CURE", 365, 194.56710215088256`},
{"CURE", 365, 100.29423086474168`}, {"CURE", 365, 81.73342157448913`}, {"TBUR", 33.02557892946077`, 72.57184843949129`},
{"TBUR", 76.81002449931347`, 69.52364778269998`}, {"CURE", 365, 53.955867346671724`},
{"TBUR", 15.92296028461464`, 59.9337524655822`}, {"TBUR", 17.082648723703397`, 56.09823371634407`},
{"CURE", 365, 48.9154136934804`}, {"CURE", 365, 79.41864427187443`}, {"TBUR", 103.16391871729125`, 6.485780276378314`},
{"TBUR", 12.95315971967696`, 55.691651806504744`}, {"TBUR", 20.83328521668728`, 15.281759215050169`},
{"CURE", 365, 125.62720331087777`}, {"CURE", 365, 30.558926427938363`}, {"CURE", 365, 93.6073560672341`},
```

{"CURE", 365, 4.949829176742791`}, {"CURE", 365, 78.24472189554864`}, {"CURE", 365, 2.787673720078368`},
{"CURE", 365, 90.32732202229641`}, {"CURE", 365, 81.63986230340288`}, {"TBUR", 114.79922633783018`, 96.48204465834412`},
{"CURE", 365, 6.960782134710097`}, {"CURE", 365, 45.86416892021887`}, {"CURE", 365, 186.2363018690109`},
{"TBUR", 24.968317952692498`, 43.405713980811036`}, {"CURE", 365, 1.7258688953577004`}, {"CURE", 365, 23.406360640160354`},
{"CURE", 365, 130.62785989692804`}, {"CURE", 365, 13.260460003134929`}, {"CURE", 365, 98.38550562371039`},
{"CURE", 365, 155.66158545034776`}, {"CURE", 365, 61.63775400250343`}, {"CURE", 365, 7.526221664386002`},
{"CURE", 365, 4.346581282093501`}, {"CURE", 365, 117.12597892504162`}, {"TBUR", 13.421985390832946`, 15.688329383677775`},
{"TBUR", 51.237940484962465`, 216.39125966321973`}, {"CURE", 365, 79.29831075373431`}, {"CURE", 365, 93.9688525003332`},
{"TBUR", 84.857904030591`, 78.44087671605222`}, {"CURE", 365, 52.084628359378755`}, {"CURE", 365, 220.89045997765902`},
{"CURE", 365, 70.10326172416168`}, {"TBUR", 108.89834034264294`, 3.7293037233518667`},
{"CURE", 365, 71.10355858104842`}, {"CURE", 365, 22.307373056294043`}, {"CURE", 365, 170.4429246883126`},
{"CURE", 365, 65.19832922320252`}, {"CURE", 365, 4.249630302147072`}, {"CURE", 365, 76.83918709553767`},
{"TBUR", 21.994512641583842`, 82.8455828809762`}, {"CURE", 365, 84.4962499725343`}, {"CURE", 365, 73.35701670786338`},
{"CURE", 365, 54.27855856092467`}, {"CURE", 365, 119.18684810237863`}, {"CURE", 365, 9.87126357188821`},
{"TBUR", 77.98854892113127`, 162.49828833078433`}, {"CURE", 365, 66.9697809747177`}, {"CURE", 365, 6.496461166345577`},
{"CURE", 365, 28.187646586344083`}, {"CURE", 365, 197.08513406095148`}, {"CURE", 365, 77.2725968440559`},
{"CURE", 365, 140.00389436953347`}, {"CURE", 365, 76.58437396708631`}, {"CURE", 365, 161.3509108622916`},
{"CURE", 365, 149.76975762284712`}, {"TBUR", 73.44299635906208`, 25.5038645437036`}, {"CURE", 365, 64.95778081957332`},
{"TBUR", 131.27523111381194`, 64.8904198016312`}, {"TBUR", 17.573730162494574`, 14.641764936074154`},
{"TBUR", 23.20739362011253`, 8.611447928979066`}, {"CURE", 365, 60.44911042742992`}, {"CURE", 365, 94.74648742674054`},
{"TBUR", 52.11201254534474`, 44.81306508022801`}, {"CURE", 365, 146.83719973153404`}, {"CURE", 365, 137.67807119669578`},
{"TBUR", 18.766673118188177`, 16.620856805387326`}, {"TBUR", 25.00591934700578`, 34.81203223977689`},
{"CURE", 365, 16.13941617696123`}, {"TBUR", 26.151227272960654`, 105.58780814392543`},
{"TBUR", 43.93281805046091`, 91.52603823801671`}, {"CURE", 365, 113.36499757387763`},
{"TBUR", 76.9061254649613`, 147.19509187094076`}, {"CURE", 365, 36.69281215926684`}, {"CURE", 365, 108.84698617416076`},
{"TBUR", 27.57594427443301`, 92.1796072068621`}, {"TBUR", 26.663366949391637`, 6.06462266668262`},
{"CURE", 365, 151.68241741086723`}, {"CURE", 365, 65.35816022169683`}, {"TBUR", 37.183272459267435`, 3.407936038983723`},
{"TBUR", 20.8772485234654`, 2.4478155046222856`}, {"CURE", 365, 53.58495585510227`}, {"CURE", 365, 77.09099094216309`},
{"CURE", 365, 89.19005725107546`}, {"TBUR", 38.98249739267547`, 58.04514217951379`}, {"CURE", 365, 88.09648020563306`},
{"CURE", 365, 125.08749795490671`}, {"CURE", 365, 63.165420905472146`}, {"TBUR", 94.01808109000714`, 27.07013204030245`},
{"CURE", 365, 108.95129590298757`}, {"TBUR", 104.60329901756486`, 2.702881131562663`},

{"CURE", 365, 34.03088913409611`}, {"TBUR", 77.57204811264411`, 73.87952275395998`},
{"CURE", 365, 6.626672156277706`}, {"TBUR", 44.19070128795293`, 47.64947762256621`},
{"CURE", 365, 71.73055728088971`}, {"TBUR", 34.3224689015061`, 82.05534280211724`}, {"CURE", 365, 42.10087903489909`},
{"TBUR", 20.88898633274891`, 57.94289761502795`}, {"CURE", 365, 75.26673841766448`},
{"TBUR", 16.356354883249498`, 91.48245189082445`}, {"CURE", 365, 80.52193807725064`},
{"TBUR", 16.239460646634043`, 21.971969690237856`}, {"TBUR", 64.40459803494643`, 68.18949385940155`},
{"CURE", 365, 150.9490899668249`}, {"CURE", 365, 6.306610370836538`}, {"CURE", 365, 63.94606244690407`},
{"CURE", 365, 31.220188645320714`}, {"CURE", 365, 92.5820744670509`}, {"CURE", 365, 38.54429563403897`},
{"TBUR", 49.85530129221083`, 112.6567408420456`}, {"TBUR", 77.8881971960871`, 72.6728045533433`},
{"CURE", 365, 139.92421762026612`}, {"CURE", 365, 43.72855321205826`}, {"CURE", 365, 182.94893531690718`},
{"CURE", 365, 121.46847557785848`}, {"CURE", 365, 98.25644540240488`}, {"CURE", 365, 117.80704300452338`},
{"TBUR", 134.54828865433063`, 187.25039572811036`}, {"TBUR", 39.57727012174162`, 201.71742305157085`},
{"CURE", 365, 58.04842943809287`}, {"CURE", 365, 96.72383057408445`}, {"CURE", 365, 105.45659862993544`},
{"TBUR", 39.92795787969313`, 37.0313394254894`}, {"TBUR", 16.497260186071024`, 7.650117220274108`},
{"CURE", 365, 0.9602148928270139`}, {"CURE", 365, 74.17112816555826`}, {"CURE", 365, 123.9549304438517`},
{"TBUR", 20.284198316614106`, 100.09444360119693`}, {"CURE", 365, 139.59294484018068`}, {"CURE", 365, 186.23783208053348`},
{"CURE", 365, 45.49941004719639`}, {"CURE", 365, 98.55936284964172`}, {"TBUR", 39.29445809174789`, 7.373566522287068`},
{"CURE", 365, 206.60160657927614`}, {"TBUR", 36.75255095224121`, 66.5643910749984`},
{"TBUR", 57.94266611935381`, 46.1319430864975`}, {"TBUR", 64.88524497221894`, 55.77373192788346`},
{"CURE", 365, 192.6207345170557`}, {"CURE", 365, 76.6676193498152`}, {"TBUR", 92.07428134425956`, 172.52225661258987`},
{"CURE", 365, 128.8968629238049`}, {"CURE", 365, 115.1575952669505`}, {"TBUR", 54.45215934351936`, 112.06802885412861`},
{"TBUR", 87.71130851673728`, 112.39619266057984`}, {"CURE", 365, 52.42367469087667`}, {"CURE", 365, 44.315771872576356`},
{"CURE", 365, 13.132233005947421`}, {"CURE", 365, 80.13868206650295`}, {"CURE", 365, 94.52445497000907`},
{"TBUR", 33.87594145340069`, 50.899745905318014`}, {"TBUR", 43.94119650305586`, 65.4603460848289`},
{"CURE", 365, 127.51721604572296`}, {"TBUR", 41.659315812559896`, 59.86654579590285`},
{"CURE", 365, 69.16245023356848`}, {"CURE", 365, 33.98114252533782`}, {"TBUR", 20.64386296569344`, 10.80878589941575`},
{"CURE", 365, 48.24000121290429`}, {"CURE", 365, 146.91396100040748`}, {"CURE", 365, 62.24971886947606`},
{"TBUR", 42.796661244350275`, 57.999258050106704`}, {"CURE", 365, 15.132787013245101`},
{"TBUR", 25.80360858148479`, 134.39116317446894`}, {"CURE", 365, 143.3582664508688`}, {"CURE", 365, 89.45990787984788`},
{"TBUR", 100.47623323659062`, 35.58686036325051`}, {"CURE", 365, 137.62272943649617`}, {"CURE", 365, 16.277188433012153`},
{"TBUR", 84.25008517013192`, 206.03587424646352`}, {"TBUR", 28.518365461736654`, 72.17010942876635`},

{"CURE", 365, 156.32900979799243`}, {"TBUR", 43.00810689747832`, 75.93841575782109`}, {"CURE", 365, 43.46359185291327`},
{"TBUR", 41.58339076898395`, 55.10166440975747`}, {"TBUR", 24.547403871495934`, 18.68717981686464`},
{"CURE", 365, 122.2486095142831 9`}, {"TBUR", 42.23179253939675`, 39.617160984123636`},
{"CURE", 365, 60.257969646841644`}, {"TBUR", 24.783247544587216`, 46.3877209446141`}, {"CURE", 365, 76.3707752724421`},
{"TBUR", 23.211232106171995`, 45.07301292331515`}, {"TBUR", 106.20558662016235`, 155.65106929422703`},
{"CURE", 365, 101.13025258193152`}, {"TBUR", 33.31436534600439`, 66.65938705957096`},
{"TBUR", 66.30552947385411`, 68.10266866079233`}, {"TBUR", 13.81437679461041`, 64.52283381987934`},
{"CURE", 365, 6.71364691593278`}, {"TBUR", 108.27731266086774`, 120.85122846353978`},
{"TBUR", 101.8341768452756`, 107.03582592731226`}, {"CURE", 365, 89.67840876871647`},
{"TBUR", 42.99766572456465`, 9.063800099937446`}, {"CURE", 365, 69.78847134369491`}, {"CURE", 365, 175.0878703446108`},
{"CURE", 365, 83.91844531595875`}, {"CURE", 365, 100.94678623047807`}, {"TBUR", 28.254623640106335`, 34.67346483027211`},
{"TBUR", 85.13213676111508`, 82.68899491090643`}, {"TBUR", 13.238069681312089`, 2.473625933707346`},
{"TBUR", 15.282370275181007`, 13.2908800980191`}, {"TBUR", 52.72826059706042`, 122.76120677518519`},
{"CURE", 365, 76.64502079535046`}, {"TBUR", 51.31702100698456`, 67.6186662111223`}, {"CURE", 365, 22.378182646301962`},
{"TBUR", 217.97929736888364`, 144.90455726661958`}, {"CURE", 365, 77.44039272204405`}, {"CURE", 365, 146.0695171367572`},
{"TBUR", 53.04244079708365`, 23.20933523186115`}, {"TBUR", 30.222805025598788`, 82.70192487929071`},
{"CURE", 365, 67.65557015193983`}, {"CURE", 365, 82.5366320895239`}, {"CURE", 365, 18.041490375294547`},
{"CURE", 365, 59.15241050860012`}, {"CURE", 365, 131.62383661173345`}, {"TBUR", 20.70558512374616`, 83.51270739168726`},
{"CURE", 365, 42.85320905039495`}, {"TBUR", 41.7501756333242`, 2.9016040698165066`}, {"CURE", 365, 45.93278804372792`},
{"CURE", 365, 132.55298305654065`}, {"CURE", 365, 53.075229205397264`}, {"CURE", 365, 78.97876718385866`},
{"TBUR", 15.884135543014308`, 98.28564429028403`}, {"TBUR", 88.14587589844338`, 79.89317575690382`},
{"TBUR", 86.19581951303101`, 146.36876780474316`}, {"CURE", 365, 131.19242927570633`},
{"CURE", 365, 91.76030037881858`}, {"CURE", 365, 98.99461054082313`}, {"CURE", 365, 137.43788977417745`},
{"TBUR", 19.445562705011582`, 4.716396417972461`}, {"TBUR", 79.30594043000657`, 141.90724790285316`},
{"TBUR", 148.1216884774475`, 47.06527139252819`}, {"TBUR", 66.02461162621599`, 200.47786688562414`},
{"CURE", 365, 12.011491862617865`}, {"TBUR", 91.23984713370754`, 72.84900493996386`}, {"CURE", 365, 168.36450817857448`},
{"CURE", 365, 7.780797791314916`}, {"CURE", 365, 104.90407854864688`}, {"TBUR", 94.8787153943805`, 87.18535543687658`},
{"TBUR", 33.23919008405121`, 109.4096778091728`}, {"TBUR", 15.439299290014317`, 55.305702278647175`},
{"TBUR", 13.839521124486794`, 28.93473649233467`}, {"TBUR", 17.78109176651134`, 7.702228312806451`},
{"CURE", 365, 78.07674832783128`}, {"CURE", 365, 76.65048535532439`}, {"TBUR", 37.13952948039495`, 93.37857294320516`},
{"CURE", 365, 15.117040212069199`}, {"CURE", 365, 209.2760808001242`}, {"CURE", 365, 40.925836471704436`},

{"CURE", 365, 83.14870733094588`}, {"CURE", 365, 49.2541154823884`}, {"CURE", 365, 7.940991030910171`},
{"TBUR", 115.24706326921788`, 141.89117406125374`}, {"CURE", 365, 10.219557871228512`},
{"CURE", 365, 57.939163485247974`}, {"TBUR", 19.079775470813757`, 28.455783844821177`},
{"CURE", 365, 62.564225756305134`}, {"TBUR", 71.66820418762673`, 214.89264754861517`},
{"CURE", 365, 147.64618477340136`}, {"TBUR", 36.78151757198519`, 87.7336825579896`}, {"CURE", 365, 80.80913760225441`},
{"CURE", 365, 131.24037098482026`}, {"CURE", 365, 159.26096425287193`}, {"CURE", 365, 124.31671728653836`},
{"CURE", 365, 161.3717766250035`}, {"TBUR", 14.333472554912444`, 53.43740723915235`}, {"CURE", 365, 63.93834627500273`},
{"TBUR", 24.24218780683989 7`, 8.324952414522519`}, {"TBUR", 18.78970669420284`, 77.09301151879437`},
{"TBUR", 63.35550002217274`, 4.253303390416354`}, {"CURE", 365, 1.0630689619387907`}, {"CURE", 365, 7.006084668852128`},
{"TBUR", 145.55874143872867`, 0.7796980924185976`}, {"TBUR", 18.746999470959892`, 73.69401787420219`},
{"CURE", 365, 20.42344918367849`}, {"CURE", 365, 61.2023588071755`}, {"CURE", 365, 155.07344171168143`},
{"CURE", 365, 34.64927747244514`}, {"TBUR", 20.431182171655955`, 73.70680217346772`},
{"TBUR", 13.215578513433023`, 51.63178565292528`}, {"CURE", 365, 20.90750945639127`},
{"TBUR", 62.440525676505445`, 100.37196890414695`}, {"TBUR", 33.29151672721448`, 20.952294900995902`},
{"TBUR", 61.35664922574145`, 1.9591135650954976`}, {"TBUR", 43.886949494581`, 7.699830388350814`},
{"CURE", 365, 153.0028674471469`}, {"CURE", 365, 28.556053813311493`}, {"CURE", 365, 153.69945375920204`},
{"TBUR", 172.15622009019492`, 97.98908194857617`}, {"CURE", 365, 137.54586525775287`}, {"CURE", 365, 47.30244143810776`},
{"CURE", 365, 186.39012128534426`}, {"CURE", 365, 81.01737512746102`}, {"TBUR", 24.90288514524988`, 5.818853981577398`},
{"CURE", 365, 137.75278200883795`}, {"TBUR", 26.736108131876318`, 27.13501076367827`},
{"TBUR", 67.43850056110001`, 106.58662055692847`}, {"TBUR", 75.12997588107275`, 135.28142387713714`},
{"TBUR", 63.955976368172244`, 39.51162366002032`}, {"CURE", 365, 96.13040723173762`},
{"TBUR", 15.51996071522735`, 31.267718728771495`}, {"TBUR", 32.30002985545188`, 65.40411454136033`},
{"CURE", 365, 116.38879199969061`}, {"CURE", 365, 46.0699130075031`}, {"TBUR", 30.19369264544443`, 71.10551575874359`},
{"CURE", 365, 39.535932700348496`}, {"TBUR", 77.16805237482893`, 136.0685513427075`},
{"TBUR", 52.15592311461981`, 83.90360585645388`}, {"CURE", 365, 82.04670003127805`},
{"TBUR", 77.5018918627161 4`, 120.38654545773494`}, {"CURE", 365, 52.854052392733834`}, {"CURE", 365, 75.9062442378876`},
{"CURE", 365, 121.98257121906865`}, {"CURE", 365, 7.41123392115017`}, {"TBUR", 18.056909685152075`, 10.737131170746427`},
{"TBUR", 145.0941249352877`, 49.80230431503794`}, {"TBUR", 57.07725640266579`, 178.78047142148884`},
{"CURE", 365, 20.671965313313233`}, {"TBUR", 112.90073485535656`, 164.04563584244184`},
{"CURE", 365, 72.31951703646735`}, {"CURE", 365, 173.28221075512513`}, {"TBUR", 15.66249169340815`, 42.745703576996924`},
{"TBUR", 39.63195471795792`, 57.04751425886573`}, {"CURE", 365, 84.12971233233803`}, {"CURE", 365, 180.20568312214036`},

{"TBUR", 84.88842224818916`, 81.24509667012215`}, {"CURE", 365, 202.84857504784026`}, {"CURE", 365, 43.91305206433408`},
{"CURE", 365, 105.95422945411902`}, {"CURE", 365, 81.70210481289104`}, {"CURE", 365, 92.6327146594251`},
{"TBUR", 63.63636229767466`, 89.63929444420681`}, {"TBUR", 44.78567178196607`, 94.0863648174088`},
{"CURE", 365, 10.631876678631825`}, {"CURE", 365, 33.368805166112516`}, {"TBUR", 82.20497408413313`, 103.31713701957966`},
{"CURE", 365, 85.7074811142853`}, {"CURE", 365, 15.716501468672893`}, {"TBUR", 76.68945444034749`, 101.9289470547651`},
{"CURE", 365, 40.256408991868696`}, {"CURE", 365, 17.863797638415267`}, {"CURE", 365, 17.854834622557902`},
{"CURE", 365, 25.67660011619255`}, {"CURE", 365, 35.5602858228631`}, {"TBUR", 67.32339671298027`, 86.60473121523461`},
{"CURE", 365, 130.15321601012297`}, {"CURE", 365, 155.5764741127295`}, {"TBUR", 31.67370239470471`, 111.53833644116534`},
{"TBUR", 69.39472636582863`, 7.971002653533851`}, {"CURE", 365, 188.2376990359797`},
{"TBUR", 102.42117896502037`, 63.27039309387638`}, {"TBUR", 39.277960498500775`, 93.25950904557932`},
{"TBUR", 122.86622231595977`, 23.59401354252617`}, {"CURE", 365, 78.07757204671992`},
{"CURE", 365, 4.496242618907362`}, {"CURE", 365, 131.48825737369967`}, {"CURE", 365, 149.55074564825736`},
{"CURE", 365, 80.09565960310033`}, {"CURE", 365, 34.966563528385805`}, {"CURE", 365, 66.79961176692156`},
{"TBUR", 122.98265421882584`, 102.61182018179471`}, {"TBUR", 90.83221961394513`, 107.79621086888281`},
{"TBUR", 40.81755021414675`, 62.81293365910932`}, {"CURE", 365, 89.5178054225204`}, {"CURE", 365, 61.798949052789744`},
{"TBUR", 22.764917141850525`, 156.62629247865306`}, {"TBUR", 80.62095009108735`, 51.6381162082482`},
{"CURE", 365, 55.81544905280303`}, {"TBUR", 109.17144331890215`, 86.47450693760649`}, {"CURE", 365, 120.75671457143504`},
{"CURE", 365, 4.012799921438469`}, {"TBUR", 55.25399772836695`, 52.16046311033644`}, {"CURE", 365, 84.56577411894011`},
{"CURE", 365, 18.253120819489613`}, {"CURE", 365, 157.31375845390008`}, {"TBUR", 15.674106449621299`, 7.4685487778513515`},
{"CURE", 365, 153.57492125367628`}, {"CURE", 365, 142.4813454856393`}, {"TBUR", 107.6173172639058`, 3.6005534007279305`},
{"CURE", 365, 120.73620097419939`}, {"CURE", 365, 50.28883754765432`}, {"CURE", 365, 57.20488305667816`},
{"CURE", 365, 132.6417186746645`}, {"CURE", 365, 9.551479317410578`}, {"TBUR", 20.972340301727197`, 76.56407872895005`},
{"CURE", 365, 27.238807215787986`}, {"TBUR", 84.08450747675211`, 2.6171329769931395`}, {"CURE", 365, 78.76314828795596`},
{"TBUR", 74.58245704849783`, 121.77076128644634`}, {"TBUR", 170.86790106403922`, 26.407491084600483`},
{"CURE", 365, 0.6929497492618913`}, {"TBUR", 89.82780335511154`, 8.732675479331082`},
{"TBUR", 30.506922589793113`, 61.499552676407944`}, {"CURE", 365, 2.3800772008758675`},
{"CURE", 365, 5.846404795031382`}, {"CURE", 365, 115.58771392498045`}, {"TBUR", 75.238744517177`, 195.93122921065216`},
{"TBUR", 89.49651396472828`, 102.89082133694266`}, {"CURE", 365, 190.82386889350593`},
{"TBUR", 74.07437089060055`, 93.23990076008329`}, {"CURE", 365, 2.261339539907939`}, {"CURE", 365, 100.50932574301292`},
{"TBUR", 89.66140714871469`, 146.75487033931097`}, {"TBUR", 38.643480457571876`, 76.26753958809284`},
{"TBUR", 67.82653476964573`, 10.48628015173296`}, {"TBUR", 57.776794002934615`, 63.138632339159926`},

{"CURE", 365, 42.182199826363835`}, {"CURE", 365, 23.498478871233065`}, {"CURE", 365, 18.069036664772767`},
{"CURE", 365, 116.3247333964128`}, {"TBUR", 127.47210829857407`, 50.01294001547557`},
{"TBUR", 25.18555490803365`, 46.10402047713491`}, {"CURE", 365, 66.77944553219405`},
{"TBUR", 70.87873025391019`, 13.198501767044068`}, {"CURE", 365, 98.43551447178832`}, {"CURE", 365, 51.789019680247655`},
{"TBUR", 63.653125177768906`, 56.199882388939756`}, {"TBUR", 50.83542826673326`, 129.31827740959076`},
{"CURE", 365, 171.26144202650295`}, {"CURE", 365, 54.377865214599645`}, {"CURE", 365, 10.156094898026025`},
{"TBUR", 79.51360516778405`, 199.50538351012239`}, {"TBUR", 155.59156573879832`, 66.83025099437253`},
{"TBUR", 57.170150694741565`, 58.75216907341732`}, {"CURE", 365, 81.79621590265235`},
{"CURE", 365, 19.080169284826404`}, {"CURE", 365, 74.64088579264036`}, {"CURE", 365, 70.00813024655619`},
{"TBUR", 118.82430576481191`, 70.95729452498183`}, {"CURE", 365, 124.53410723865767`}, {"CURE", 365, 51.42700981620639`},
{"TBUR", 165.5246027143204`, 93.48793577047898`}, {"CURE", 365, 42.22261524103716`}, {"CURE", 365, 124.1634268676464`},
{"CURE", 365, 55.97639243843291`}, {"TBUR", 85.43335973742218`, 73.90988841940379`}, {"CURE", 365, 35.94004791543402`},
{"TBUR", 82.34557573076447`, 116.45214453781347`}, {"TBUR", 48.04370691658002`, 222.9098333860876`},
{"TBUR", 41.93560376085534`, 108.6133093628485`}, {"CURE", 365, 106.19516866766882`}, {"CURE", 365, 39.78734998769628`},
{"TBUR", 121.14208038687138`, 88.04538382832615`}, {"TBUR", 57.49224033438311`, 164.5817377829006`},
{"CURE", 365, 4.470130869123641`}, {"CURE", 365, 106.55144654782819`}, {"TBUR", 69.485909769142`, 9.69309038279598`},
{"CURE", 365, 21.56897688420278`}, {"CURE", 365, 12.958915495354004`}, {"TBUR", 48.49873701635619`, 55.92358997122445`},
{"CURE", 365, 3.0033076897725435`}, {"TBUR", 32.44681381131348`, 75.25802915554085`},
{"CURE", 365, 69.66286311985644`}, {"TBUR", 80.41180735003793`, 86.14521480092533`},
{"TBUR", 33.72604392867868`, 49.96137940852525`}, {"CURE", 365, 86.66529947018924`}, {"CURE", 365, 158.42923389585246`},
{"CURE", 365, 77.88027184218971`}, {"CURE", 365, 21.357271320458146`}, {"CURE", 365, 81.48702060648222`},
{"TBUR", 114.16186395138484`, 7.526255372578434`}, {"TBUR", 65.19671807354575`, 7.374887261947927`},
{"CURE", 365, 130.5944678969297`}, {"CURE", 365, 126.63672094290838`}, {"TBUR", 82.0392046182986`, 28.369553698780948`},
{"CURE", 365, 41.05147753726589`}, {"TBUR", 18.26693098130351`, 87.31860375989825`}, {"CURE", 365, 152.99985754457134`},
{"TBUR", 133.2903806955726`, 48.837968352313105`}, {"TBUR", 58.18886445811377`, 69.52797938114131`},
{"CURE", 365, 10.657204113498763`}, {"TBUR", 75.72852059365057`, 77.32201646227584`},
{"CURE", 365, 65.32387864034145`}, {"CURE", 365, 81.17174763582243`}, {"TBUR", 69.76170087852384`, 53.99089441967323`},
{"TBUR", 73.22022807903384`, 22.23775837827653`}, {"CURE", 365, 93.27804809054822`},
{"TBUR", 14.652036073428242`, 51.84147169693585`}, {"CURE", 365, 24.163683293531747`},
{"CURE", 365, 102.07868763259468`}, {"CURE", 365, 181.96162892721657`}, {"CURE", 365, 145.26272158860152`},
{"CURE", 365, 80.61920502846947`}, {"CURE", 365, 104.07126893264441`}, {"TBUR", 42.35756190743097`, 10.507003108042165`},

{"TBUR", 42.858064370692055`, 89.03742349865952`}, {"TBUR", 66.32114639696988`, 16.868312485074984`},
{"CURE", 365, 58.07503192445746`}, {"TBUR", 25.85494878924351`, 91.73603465514839`}, {"CURE", 365, 22.7507567468383`},
{"CURE", 365, 67.43180873459487`}, {"TBUR", 20.676496635641254`, 27.127090131564152`},
{"CURE", 365, 218.7989102624928`}, {"TBUR", 14.53352701527474`, 14.551236278540268`}, {"CURE", 365, 98.34771523657001`},
{"TBUR", 42.900427760858506`, 28.821313778891408`}, {"TBUR", 105.44891821289467`, 14.41945779326809`},
{"CURE", 365, 2.4149006077356114`}, {"CURE", 365, 4.47068443185853`}, {"CURE", 365, 78.7197915248939`},
{"TBUR", 27.25637394293075`, 46.99962223892936`}, {"CURE", 365, 12.57033208599834`}, {"CURE", 365, 71.99824287586571`},
{"CURE", 365, 104.37804155816914`}, {"CURE", 365, 194.4487495688116`}, {"CURE", 365, 59.08900514540208`},
{"CURE", 365, 94.24826211369933`}, {"TBUR", 40.90022934032731`, 93.01908529324785`}, {"CURE", 365, 15.576365656279103`},
{"TBUR", 24.819154955948417`, 41.608886204969714`}, {"CURE", 365, 31.966986996430393`}, {"CURE", 365, 92.38689293701214`},
{"TBUR", 42.38406570893857`, 86.05179848172855`}, {"CURE", 365, 60.76743947594726`}, {"CURE", 365, 3.457651988384073`},
{"CURE", 365, 20.45417750321073`}, {"CURE", 365, 66.80832441045288`}, {"TBUR", 41.86897107785371`, 77.6206562907009`},
{"CURE", 365, 105.33101658516306`}, {"TBUR", 107.3403917979419`, 68.08767929039652`}, {"CURE", 365, 196.8136310233993 2`},
{"CURE", 365, 88.8375045966627`}, {"TBUR", 18.178034413047115`, 72.14972480018876`}, {"CURE", 365, 1.1342927135975982`},
{"CURE", 365, 39.71803269362239`}, {"CURE", 365, 148.94259201558205`}, {"CURE", 365, 4.340350230122707`},
{"TBUR", 47.03438273734227`, 58.22916665964089`}, {"TBUR", 50.09448885434925`, 90.57287599889767`},
{"TBUR", 87.38985431866071`, 166.24931223323543`}, {"TBUR", 88.40571831882366`, 110.63437805951021`},
{"CURE", 365, 21.508692500775407`}, {"TBUR", 77.38652176626306`, 8.768512820229608`},
{"CURE", 365, 63.21675933641626`}, {"CURE", 365, 154.9275281153263`}, {"TBUR", 32.658964818874765`, 90.60269275900073`},
{"CURE", 365, 8.36946258314576`}, {"TBUR", 54.37190556734955`, 91.55219889780949`},
{"CURE", 365, 80.59484729778116`}, {"TBUR", 68.26095539081103`, 4.871500347483052`},
{"CURE", 365, 66.71994096911946`}, {"CURE", 365, 86.2337129185453`}, {"CURE", 365, 50.34806001169624`},
{"TOX", 24.86708990034711`, 245.2418275108366`}, {"TBUR", 62.710854438275206`, 69.31011627839482`},
{"CURE", 365, 89.17113265662778`}, {"TBUR", 24.58914088458058`, 70.2868792646412`}, {"CURE", 365, 199.84016920537917`},
{"CURE", 365, 75.9397684056606`}, {"TBUR", 19.64630608000293`, 69.54684050379834`}, {"CURE", 365, 45.82847090838565`},
{"TBUR", 58.86926442589719`, 101.82206457363036`}, {"CURE", 365, 0.898503361842926`},
{"TBUR", 37.588645152491246`, 20.646435960294873`}, {"TBUR", 145.98871904348366`, 97.68502612622241`},
{"CURE", 365, 83.12731273815277`}, {"CURE", 365, 16.97611296944015`}, {"CURE", 365, 64.25712583389276`},
{"CURE", 365, 43.805561932686686`}, {"TBUR", 71.35900922980615`, 45.857473234261306`},
{"CURE", 365, 10.088094669985868`}, {"CURE", 365, 57.093606438811285`}, {"CURE", 365, 47.46423174637087`},
{"TBUR", 26.946383496480173`, 31.218907359226026`}, {"CURE", 365, 150.01499516835437`},

{"CURE", 365, 4.2504207218997`}, {"CURE", 365, 66.99231076502551`}, {"TBUR", 162.36235838611583`, 105.071523461221`},
{"TBUR", 16.548257858295006`, 112.2425604580146`}, {"TBUR", 44.534147260167764`, 7.3163408043217135`},
{"CURE", 365, 87.02711560327077`}, {"CURE", 365, 104.2350067712493`}, {"TBUR", 27.542142979264355`, 3.684155378085776`},
{"CURE", 365, 60.17536925629358`}, {"CURE", 365, 88.10547448100598`}, {"TBUR", 21.97355868403262`, 21.64430935555572`},
{"CURE", 365, 2.010660042292048`}, {"TBUR", 66.40725713486786`, 170.47582485027894`},
{"TBUR", 113.45303100228945`, 92.96100569912743`}, {"CURE", 365, 91.27858803984786`}, {"CURE", 365, 27.026190599848224`},
{"TBUR", 80.39096558633605`, 2.9300256377650227`}, {"TBUR", 68.78686976009813`, 5.482176853042062`},
{"TBUR", 13.205783211231811`, 17.474135393952395`}, {"TBUR", 84.29813367110602`, 75.25442072506125`},
{"CURE", 365, 123.6005849217974`}, {"CURE", 365, 103.95902347247059`}, {"CURE", 365, 50.36042046350282`},
{"TBUR", 21.705429879423797`, 1.8523136247150633`}, {"CURE", 365, 81.18927262261565`}, {"CURE", 365, 86.1916645294203`},
{"TBUR", 67.56752469783407`, 1.1529587438851001`}, {"TBUR", 76.52603607468097`, 50.74564421172362`},
{"CURE", 365, 1.003599322639166`}, {"CURE", 365, 139.81588999071084`}, {"CURE", 365, 60.78137050083603`},
{"TBUR", 14.545225497850014`, 0.7210146819289527`}, {"TBUR", 163.1647692013061`, 84.88396033229454`},
{"CURE", 365, 31.246906302777514`}, {"TBUR", 54.76066686067967`, 220.12411318477226`}, {"CURE", 365, 122.7390887366043`},
{"TBUR", 41.71156975705225`, 68.00436149500023`}, {"CURE", 365, 176.33111842808955`}},
{{"440 Res", "440 OS", "440 Tox"}, {"TBUR", 21.62330071600892`, 38.45186063565146`}, {"CURE", 365, 145.17815376541225`},
{"TBUR", 26.94303309183048`, 112.0157957610388`}, {"TBUR", 29.23432778028681`, 5.268330700031764`},
{"TBUR", 18.053176188367775`, 16.52715848943704`}, {"CURE", 365, 29.17879233350198`}, {"CURE", 365, 63.762017863067825`},
{"CURE", 365, 60.70264830773269`}, {"CURE", 365, 142.837831269707`}, {"TBUR", 70.69927116517698`, 192.09211721465306`},
{"TBUR", 54.510551075351124`, 98.86356198527608`}, {"CURE", 365, 88.64277413561112`},
{"TOX", 18.656851991041332`, 259.3546391921529`}, {"TBUR", 43.04163666105865`, 61.85810413566184`},
{"TBUR", 64.05319645152201`, 6.312045021813916`}, {"TBUR", 87.48337478105753`, 28.59675942469984`},
{"CURE", 365, 100.08515523747445`}, {"TBUR", 44.4532258677573`, 112.95405116069074`},
{"TBUR", 44.49244130466716`, 6.945360352412817`}, {"CURE", 365, 84.0154148909686`}, {"CURE", 365, 134.0380789591503`},
{"CURE", 365, 48.368991425363234`}, {"CURE", 365, 29.408966081359928`}, {"CURE", 365, 63.835436353982686`},
{"CURE", 365, 56.6647662038807`}, {"CURE", 365, 158.2450427892966`}, {"CURE", 365, 97.08788768468305`},
{"CURE", 365, 49.77538980774391`}, {"CURE", 365, 33.58651686785801`}, {"CURE", 365, 5.786032347766682`},
{"CURE", 365, 133.12500356666968`}, {"TBUR", 13.411158198740752`, 34.882833343512374`},
{"CURE", 365, 67.4605360589061`}, {"TBUR", 48.13413444759603`, 40.116441089924294`}, {"CURE", 365, 180.09415079239142`},
{"CURE", 365, 210.13467023366925`}, {"CURE", 365, 41.60352106841676`}, {"TBUR", 21.43029263985034`, 10.812073586864777`},
{"TBUR", 71.88937607208254`, 48.75799701578932`}, {"CURE", 365, 93.99592517458`},

{"TBUR", 18.019731173354536`, 60.0758159279638`}, {"CURE", 365, 96.10535191308105`}, {"CURE", 365, 70.97447230401876`},
{"TBUR", 45.94335575284304`, 77.00860369721575`}, {"CURE", 365, 94.72808679091318`}, {"CURE", 365, 4.878795402751025`},
{"CURE", 365, 105.60167843263028`}, {"CURE", 365, 66.63355134490344`}, {"TBUR", 112.46036259554673`, 128.7789126248022`},
{"CURE", 365, 220.5964139506503`}, {"TBUR", 29.673304474461037`, 97.94205941584713`},
{"TBUR", 41.68584692941511`, 84.24162040580389`}, {"CURE", 365, 6.370055221556606`}, {"CURE", 365, 136.83536061809292`},
{"CURE", 365, 45.98568743091318`}, {"CURE", 365, 96.15211208096079`}, {"CURE", 365, 68.4182821171264`},
{"TBUR", 103.2688833609064`, 79.3198515614467`}, {"TBUR", 37.368849453966895`, 166.14814114125915`},
{"TBUR", 52.3752711427459`, 98.64262687197423`}, {"CURE", 365, 91.12141721200926`}, {"CURE", 365, 4.510798926865639`},
{"TBUR", 33.590187682381085`, 111.9178227671947`}, {"CURE", 365, 80.4335607489533`},
{"TBUR", 71.39978549667124`, 6.879268066323761`}, {"CURE", 365, 87.29733194825042`}, {"CURE", 365, 78.81000930644515`},
{"TBUR", 57.35769931921364`, 115.61735984414544`}, {"CURE", 365, 42.00169009937475`}, {"CURE", 365, 35.614202735058235`},
{"CURE", 365, 66.40681421636862`}, {"CURE", 365, 48.67032837055224`}, {"CURE", 365, 138.54070184863878`},
{"TBUR", 13.394442810090679`, 2.161161917416201`}, {"TBUR", 79.47942552343747`, 89.48444324723418`},
{"CURE", 365, 115.95645463268535`}, {"TBUR", 96.97082252705079`, 212.810301088048`}, {"CURE", 365, 70.28805968155827`},
{"TBUR", 75.77939855694116`, 138.04777638145796`}, {"CURE", 365, 55.906454758277896`}, {"CURE", 365, 111.6359797953976`},
{"CURE", 365, 3.59138028254596`}, {"CURE", 365, 7.046509934301143`}, {"CURE", 365, 4.994030365181804`},
{"CURE", 365, 129.1410550126184`}, {"TBUR", 99.48372884075151`, 98.78020395534786`}, {"CURE", 365, 86.40032744515129`},
{"CURE", 365, 44.54278048689984`}, {"CURE", 365, 7.645419788946878`}, {"TBUR", 71.3929254242773`, 148.20679020425922`},
{"TBUR", 32.74224726931143`, 13.00135766967312`}, {"TBUR", 89.2391439235479`, 106.94422125701388`},
{"TBUR", 68.02914411430963`, 2.9288369260491307`}, {"CURE", 365, 6.775853742936077`},
{"TBUR", 19.18368023646754`, 2.0856145213186186`}, {"TBUR", 158.41323823585716`, 57.56051243898217`},
{"CURE", 365, 42.37348010323288`}, {"CURE", 365, 193.41485068872836`}, {"CURE", 365, 10.440667601137017`},
{"TBUR", 43.720252394207314`, 152.248565363038`}, {"TBUR", 20.477294537092916`, 11.266939780472953`},
{"TBUR", 127.33971333584873`, 136.5245668620534`}, {"CURE", 365, 75.23803781659059`},
{"TBUR", 90.84849793972401`, 96.75742844668567`}, {"CURE", 365, 68.38027869550245`}, {"CURE", 365, 110.14655180721591`},
{"CURE", 365, 45.20941781822499`}, {"TBUR", 138.78796028124466`, 92.20802065204518`}, {"CURE", 365, 41.21445330466278`},
{"CURE", 365, 110.6125966244071`}, {"CURE", 365, 151.57341566716784`}, {"CURE", 365, 82.51641472571897`},
{"CURE", 365, 24.412498537544096`}, {"CURE", 365, 53.74357722724271`}, {"CURE", 365, 74.18204734339356`},
{"CURE", 365, 62.37472360005112`}, {"CURE", 365, 13.408587733701763`}, {"CURE", 365, 82.60508720442384`},
{"CURE", 365, 84.31742846573704`}, {"CURE", 365, 25.02111698977729`}, {"CURE", 365, 87.06157590184532`},
{"CURE", 365, 175.01895726855324`}, {"CURE", 365, 111.20591047739975`}, {"TBUR", 19.96045093280183`, 5.777033048154818`},

{"CURE", 365, 78.09228243197036`}, {"TBUR", 38.815752405539605`, 69.2733963688277`}, {"CURE", 365, 60.923986618883056`},
{"CURE", 365, 39.77513169740786`}, {"CURE", 365, 126.80617422180488`}, {"CURE", 365, 122.02837103627019`},
{"CURE", 365, 108.17433662658676`}, {"CURE", 365, 118.23108850874652`}, {"TBUR", 71.73614555308843`, 39.72022637561652`},
{"TBUR", 45.64020417111307`, 42.18896360865641`}, {"CURE", 365, 172.8788429288243`},
{"TBUR", 12.770640707906743`, 74.62848202355744`}, {"TBUR", 94.58317743604547`, 24.41909198222989`},
{"TBUR", 62.37367534176815`, 58.79965480609796`}, {"CURE", 365, 19.63082014936204`}, {"CURE", 365, 116.59106289526937`},
{"TBUR", 53.000707661873804`, 175.44060808426534`}, {"CURE", 365, 120.33825688207905`},
{"CURE", 365, 158.98014045797612`}, {"CURE", 365, 29.2956478719255`}, {"CURE", 365, 7.249129324396991`},
{"CURE", 365, 72.88329099759538`}, {"CURE", 365, 172.54292646606797`}, {"TBUR", 37.175461928232444`, 43.93120166176402`},
{"CURE", 365, 79.72903562452369`}, {"CURE", 365, 18.459411694647113`}, {"CURE", 365, 7.434502707407295`},
{"CURE", 365, 67.36564637855949`}, {"CURE", 365, 1.2536670029151502`}, {"TBUR", 88.59271395794917`, 174.68580413801996`},
{"TBUR", 50.10169036296888`, 72.54092456837365`}, {"TBUR", 88.36598275114673`, 156.11604485016676`},
{"CURE", 365, 78.29491007223854`}, {"CURE", 365, 92.27575803424351`}, {"CURE", 365, 73.18379863043627`},
{"CURE", 365, 92.13011045706021`}, {"CURE", 365, 26.88511360221486`}, {"TBUR", 98.85528576182497`, 23.36770909541499`},
{"CURE", 365, 1.7870873959243443`}, {"TBUR", 68.90758700880474`, 11.568103251641835`},
{"TBUR", 78.81256501787412`, 80.72123880710446`}, {"CURE", 365, 118.63436332033318`}, {"CURE", 365, 119.11379881695753`},
{"CURE", 365, 86.66975531629527`}, {"CURE", 365, 122.84493565091383`}, {"CURE", 365, 1.8463824988297368`},
{"CURE", 365, 69.43586168297591`}, {"TBUR", 29.77405706928472`, 133.37959663263024`}, {"CURE", 365, 61.03124612388645`},
{"TBUR", 21.121401193394608`, 7.195240024139629`}, {"TBUR", 50.264233838910044`, 131.88817210577372`},
{"TBUR", 89.17241301565925`, 10.788064601384448`}, {"TBUR", 67.8615757649861`, 159.91816930888436`},
{"TBUR", 21.211238290147147`, 83.34234594376609`}, {"TBUR", 59.34263393806604`, 45.81154935501196`},
{"TBUR", 91.11985647752972`, 103.93468548825106`}, {"TBUR", 80.31576175803553`, 109.43814567068605`},
{"CURE", 365, 86.37029004245234`}, {"CURE", 365, 149.44117849019727`}, {"TBUR", 20.093545324162577`, 9.906096251394269`},
{"CURE", 365, 134.26317003482376`}, {"TBUR", 28.0081275836014`, 45.06485738620714`},
{"TBUR", 53.5936464856202`, 147.26988469805912`}, {"CURE", 365, 72.73844770493571`}, {"CURE", 365, 3.8888404859038235`},
{"TBUR", 24.272230389367273`, 97.12062418662266`}, {"CURE", 365, 93.53013917370775`},
{"CURE", 365, 147.2502908304549`}, {"CURE", 365, 172.65645403914`}, {"CURE", 365, 114.00179214503619`},
{"CURE", 365, 67.91230498061557`}, {"CURE", 365, 52.17096338873946`}, {"CURE", 365, 179.77453947926938`},
{"TBUR", 13.706144602469468`, 14.80853106695534`}, {"CURE", 365, 100.83073870370892`}, {"CURE", 365, 92.30492720762459`},
{"TBUR", 27.152926040270145`, 24.083956622893062`}, {"TBUR", 57.136014877949336`, 103.17617558066443`},
{"CURE", 365, 103.9622907500019`}, {"TBUR", 65.24396589159136`, 80.7075745823891`}, {"CURE", 365, 1.8195441004804085`},

{"TBUR", 143.1793549876576`, 26.63649484030657`}, {"TBUR", 15.884244866343932`, 88.63119767353732`},
{"CURE", 365, 178.23615935617158`}, {"CURE", 365, 12.784964038421707`}, {"CURE", 365, 132.47150821699174`},
{"CURE", 365, 2.981452807771265`}, {"CURE", 365, 92.99897445371704`}, {"CURE", 365, 20.186474394968172`},
{"CURE", 365, 113.76179859391041`}, {"CURE", 365, 27.702703402006424`}, {"TBUR", 45.96036316599377`, 14.045141313009278`},
{"TBUR", 79.994596241077`, 160.71711390567341`}, {"CURE", 365, 77.4879456902568`},
{"TBUR", 50.097198112853874`, 209.402493069299`}, {"CURE", 365, 73.51279657599403`}, {"CURE", 365, 104.86708753978802`},
{"CURE", 365, 13.230819577157273`}, {"CURE", 365, 71.81970971147872`}, {"CURE", 365, 138.7513110964102`},
{"CURE", 365, 144.01060774683796`}, {"CURE", 365, 70.17681587387571`}, {"CURE", 365, 128.52151839160354`},
{"CURE", 365, 82.71587785869235`}, {"CURE", 365, 7.843204896306892`}, {"CURE", 365, 54.239231598227576`},
{"CURE", 365, 46.459592714865586`}, {"CURE", 365, 72.16968796478719`}, {"CURE", 365, 106.04130307662807`},
{"CURE", 365, 88.09389076251102`}, {"CURE", 365, 81.16106233597459`}, {"TBUR", 22.71282340897655`, 103.41057010388676`},
{"TBUR", 28.070143437718034`, 69.6440060913556`}, {"TBUR", 156.63168710913786`, 70.43320291484396`},
{"TBUR", 20.905178925984565`, 47.59833311063474`}, {"CURE", 365, 61.282475632991364`},
{"CURE", 365, 71.35988243240433`}, {"CURE", 365, 127.48638563651555`}, {"TBUR", 27.107478752588765`, 5.857536888194517`},
{"TBUR", 31.702294860555586`, 24.994916160208685`}, {"CURE", 365, 1.6822388140298643`},
{"CURE", 365, 2.5042803383904717`}, {"TBUR", 20.31856738916284`, 22.679456672574354`}, {"CURE", 365, 202.9303274106147`},
{"CURE", 365, 54.79798655212166`}, {"CURE", 365, 198.95257444508186`}, {"TBUR", 38.67449045717641`, 94.83001810725538`},
{"CURE", 365, 66.07326906945985`}, {"TBUR", 15.246044009160215`, 119.20458048754426`}, {"CURE", 365, 53.43136069873262`},
{"CURE", 365, 101.86415429366677`}, {"CURE", 365, 8.64558522927808`}, {"CURE", 365, 75.82618737770639`},
{"CURE", 365, 27.400482912118107`}, {"CURE", 365, 9.273377518607756`}, {"CURE", 365, 119.37608992973017`},
{"TBUR", 21.61415833873281`, 122.86487617989039`}, {"TBUR", 13.69834682173127`, 88.73268198506847`},
{"CURE", 365, 60.42671437579927`}, {"TBUR", 28.023544902428355`, 82.90617098778782`},
{"CURE", 365, 5.682487565064713`}, {"CURE", 365, 60.96611469411468`}, {"CURE", 365, 50.99633170579027`},
{"CURE", 365, 208.65878932051447`}, {"CURE", 365, 89.36546019956748`}, {"CURE", 365, 46.842040566118406`},
{"CURE", 365, 1.6102521718699685`}, {"TBUR", 69.96265519092779`, 88.09828037692554`}, {"CURE", 365, 74.52058849485775`},
{"TBUR", 151.34453169319434`, 78.54498860780663`}, {"TBUR", 55.1402545571145`, 11.952685133972354`},
{"CURE", 365, 82.159933670409`}, {"CURE", 365, 56.90944125341956`}, {"TBUR", 20.60638401835439`, 73.25929080359688`},
{"CURE", 365, 42.676924147383126`}, {"CURE", 365, 101.66899048947747`}, {"CURE", 365, 17.381522983591697`},
{"TBUR", 97.84267975650728`, 98.91251692947716`}, {"TBUR", 16.576319193280774`, 39.101901745042134`},
{"CURE", 365, 80.12056931385334`}, {"TBUR", 27.211323535808084`, 95.32438467060543`}, {"CURE", 365, 8.955667428551287`},
{"CURE", 365, 116.6202193476328`}, {"CURE", 365, 69.42493498353754`}, {"CURE", 365, 193.5386761562453`},

{"CURE", 365, 134.24279628835095`}, {"CURE", 365, 94.59733501551908`}, {"CURE", 365, 59.423852930069216`},
{"CURE", 365, 197.9317483108331`}, {"TBUR", 30.5865053756984`, 31.65705616026168`}, {"CURE", 365, 17.880659015068545`},
{"CURE", 365, 30.415601028823886`}, {"CURE", 365, 87.71794357191261`}, {"CURE", 365, 82.44299452983974`},
{"CURE", 365, 111.77190448403283`}, {"CURE", 365, 36.69070381498997`}, {"TBUR", 23.23059820076578`, 143.15046790856547`},
{"CURE", 365, 2.3610417183594943`}, {"CURE", 365, 120.08866948145162`}, {"TBUR", 38.459885544869714`, 142.0937870171874`},
{"CURE", 365, 56.694451033298904`}, {"CURE", 365, 118.91690206786706`}, {"CURE", 365, 3.921609434211421`},
{"TBUR", 22.31656247021499`, 45.95118247385465`}, {"CURE", 365, 68.13265083064213`}, {"CURE", 365, 68.87449331530051`},
{"TBUR", 29.054514317163765`, 31.81124106610575`}, {"CURE", 365, 67.18885513776759`},
{"TBUR", 36.742585243015675`, 32.91346349584081`}, {"CURE", 365, 105.82990477271788`}, {"CURE", 365, 84.67472841851686`},
{"CURE", 365, 91.60733875360376`}, {"CURE", 365, 67.10305658067985`}, {"CURE", 365, 71.75261285753747`},
{"CURE", 365, 101.3615905422281`}, {"CURE", 365, 134.94594178237517`}, {"TBUR", 49.088460356696245`, 95.96400780189654`},
{"CURE", 365, 131.23458918910634`}, {"CURE", 365, 79.94636910396392`}, {"TBUR", 58.74522078425124`, 20.01437888122003`},
{"TBUR", 17.078039030539212`, 16.24591375188324`}, {"CURE", 365, 0.8076965389625859`},
{"CURE", 365, 114.67946717765827`}, {"CURE", 365, 117.77750113776662`}, {"CURE", 365, 88.84339059542206`},
{"TBUR", 138.69471161953936`, 203.48026647405277`}, {"CURE", 365, 158.84148916273696`}, {"CURE", 365, 43.66865082324857`},
{"TBUR", 20.49902039783375`, 62.5002492673914`}, {"CURE", 365, 8.884257456836544`}, {"CURE", 365, 194.70063921655438`},
{"TBUR", 39.02759117753881`, 191.67526551280284`}, {"TBUR", 23.731544683282635`, 27.986168201793145`},
{"CURE", 365, 188.84582839723788`}, {"CURE", 365, 34.48031978014506`}, {"CURE", 365, 70.53379544133229`},
{"CURE", 365, 134.793779985678`}, {"CURE", 365, 2.1758290930671227`}, {"TBUR", 55.574167360301786`, 40.86028888789809`},
{"CURE", 365, 120.48661416348426`}, {"TBUR", 57.791131299423824`, 28.562452659627798`},
{"TBUR", 38.566406633646515`, 192.38000566597822`}, {"CURE", 365, 3.2694092383008098`}, {"CURE", 365, 161.13253456412204`},
{"CURE", 365, 94.88190719396016`}, {"CURE", 365, 88.73364560621121`}, {"CURE", 365, 112.9514675225396`},
{"CURE", 365, 49.37235346832318`}, {"TBUR", 35.56778469062446`, 143.31505167094284`}, {"CURE", 365, 89.43894201647117`},
{"CURE", 365, 153.83398466550753`}, {"CURE", 365, 100.37876487640041`}, {"TBUR", 54.37064748371499`, 104.52626623094078`},
{"CURE", 365, 122.87051426205329`}, {"CURE", 365, 91.56306625672924`}, {"CURE", 365, 45.33541423260964`},
{"CURE", 365, 137.0523338015904`}, {"CURE", 365, 168.20418948525543`}, {"CURE", 365, 46.22752525449263`},
{"TBUR", 64.05100550150381`, 109.82889858267357`}, {"TBUR", 45.89509191549454`, 138.51863134210132`},
{"CURE", 365, 16.086487912289194`}, {"CURE", 365, 99.98802816827906`}, {"CURE", 365, 51.949270953389465`},
{"TBUR", 14.579994409151748`, 8.887838644317094`}, {"TBUR", 13.410860371092502`, 30.257176500636543`},
{"TBUR", 32.42025153089274`, 48.31317838435022`}, {"CURE", 365, 96.55407568920448`}, {"CURE", 365, 26.139644705451307`},
{"CURE", 365, 87.18419705905234`}, {"TBUR", 78.11296314394333`, 160.02818533631816`}, {"CURE", 365, 89.44729224299658`},

{"CURE", 365, 78.33648065567353`}, {"CURE", 365, 80.13928242756128`}, {"TBUR", 17.16896968946457`, 35.66028469324424`},
{"TBUR", 13.612989898268372`, 81.30588163199282`}, {"TBUR", 77.83408711135317`, 35.67464870490318`},
{"CURE", 365, 141.27621137645446`}, {"TBUR", 49.76726882058416`, 85.7641068615973`}, {"CURE", 365, 115.44280993611409`},
{"CURE", 365, 84.78817781905373`}, {"CURE", 365, 74.83975218191918`}, {"TBUR", 101.32548776163813`, 36.72538487996206`},
{"CURE", 365, 53.17543953183737`}, {"CURE", 365, 200.11018126541907`}, {"CURE", 365, 103.38591834327686`},
{"CURE", 365, 83.80540587265239`}, {"TBUR", 33.06420399554949`, 74.86873177210886`},
{"TBUR", 76.87192484499592`, 71.90890048331603`}, {"CURE", 365, 56.03466187631331`},
{"TBUR", 15.937780580019712`, 61.9156701929829 7`}, {"TBUR", 17.088252307240232`, 57.8266700489489`},
{"CURE", 365, 52.535299825563186`}, {"CURE", 365, 82.01033991493156`}, {"TBUR", 105.10775252183426`, 6.646152562999573`},
{"TBUR", 12.956079458299843`, 57.30204822042928`}, {"TBUR", 20.944746892199927`, 16.39564017743415`},
{"CURE", 365, 130.2829527029254`}, {"CURE", 365, 33.38723986900175`}, {"CURE", 365, 96.74990710728869`},
{"CURE", 365, 5.066974287954027`}, {"CURE", 365, 81.72701294525736`}, {"CURE", 365, 2.855125520442264`},
{"CURE", 365, 94.03697203462812`}, {"CURE", 365, 85.94157252637537`}, {"TBUR", 114.82300801803659`, 100.4918032592237`},
{"CURE", 365, 7.127456622323215`}, {"CURE", 365, 46.932825397337275`}, {"CURE", 365, 191.3024991632211`},
{"TBUR", 25.037652007316314`, 45.15789548249104`}, {"CURE", 365, 1.7757685958704807`}, {"CURE", 365, 26.14279841256926`},
{"CURE", 365, 133.97186545458834`}, {"CURE", 365, 16.38264571014605`}, {"CURE", 365, 102.79404572756266`},
{"CURE", 365, 161.17854030785477`}, {"CURE", 365, 63.877800564221275`}, {"CURE", 365, 7.709513807630091`},
{"CURE", 365, 6.400752955943211`}, {"CURE", 365, 121.17808418907518`}, {"TBUR", 13.451625713622555`, 16.604648187555682`},
{"TBUR", 51.45620448762366`, 221.98098573193093`}, {"CURE", 365, 81.40914353294535`}, {"CURE", 365, 98.28427168469254`},
{"TBUR", 84.8688498016617`, 80.57426496678377`}, {"CURE", 365, 54.28849963020253`}, {"CURE", 365, 226.27512821460104`},
{"CURE", 365, 73.56801061121291`}, {"TBUR", 111.11196184516866`, 3.8205046795532507`},
{"CURE", 365, 74.88529075494525`}, {"CURE", 365, 24.69242100771227`}, {"CURE", 365, 174.60606673657156`},
{"CURE", 365, 67.26543675833655`}, {"CURE", 365, 4.352204535625844`}, {"CURE", 365, 81.24805643074782`},
{"TBUR", 22.01836588601527`, 85.1194873301251`}, {"CURE", 365, 87.34513350711022`}, {"CURE", 365, 76.49077320123001`},
{"CURE", 365, 56.480834316135386`}, {"CURE", 365, 122.3503201362841`}, {"CURE", 365, 10.143431780390928`},
{"TBUR", 78.04176963519363`, 167.1981110226657`}, {"CURE", 365, 68.99988559812161`}, {"CURE", 365, 6.65042937660256`},
{"CURE", 365, 30.53505224715221`}, {"CURE", 365, 202.53227012532432`}, {"CURE", 365, 79.73825252305981`},
{"CURE", 365, 143.99844959251243`}, {"CURE", 365, 81.1692609038099`}, {"CURE", 365, 166.13918161903925`},
{"CURE", 365, 154.23831527035395`}, {"TBUR", 73.70683432891585`, 28.455408318544702`}, {"CURE", 365, 67.56881478683862`},
{"TBUR", 131.30705903025168`, 67.02609944044616`}, {"TBUR", 17.73881345764681`, 15.79159942834036`},
{"TBUR", 23.9058870280 6632`, 8.94561690680303`}, {"CURE", 365, 63.85722096449987`}, {"CURE", 365, 98.58348730144344`},

{"TBUR", 52.73572811993428`, 49.56017761838668`}, {"CURE", 365, 151.9837120521245`}, {"CURE", 365, 142.06016633984336`},
{"TBUR", 18.84259389506382`, 17.71880279142498`}, {"TBUR", 25.155986165595376`, 36.53595573354499`},
{"CURE", 365, 18.882663869723057`}, {"TBUR", 26.219774950196367`, 109.88281716962094`},
{"TBUR", 43.97851307909238`, 95.39833416004421`}, {"CURE", 365, 117.10954946257628`},
{"TBUR", 76.92286702036557`, 152.5550915508496`}, {"CURE", 365, 38.943940315576235`}, {"CURE", 365, 111.80335728510104`},
{"TBUR", 27.59247531530016`, 94.56815953560624`}, {"TBUR", 26.9472908454588`, 6.2208706878631475`},
{"CURE", 365, 155.7671624790015`}, {"CURE", 365, 67.83902805385128`}, {"TBUR", 38.106537934838315`, 3.5174016537247317`},
{"TBUR", 21.47035542945026`, 2.541814808457038`}, {"CURE", 365, 56.07853573328178`}, {"CURE", 365, 80.41828243127458`},
{"CURE", 365, 93.55925928163099`}, {"TBUR", 39.156950954195146`, 60.66359724184995`}, {"CURE", 365, 90.36311274183679`},
{"CURE", 365, 128.33544595454998`}, {"CURE", 365, 66.53456695087931`}, {"TBUR", 94.77367578419182`, 29.92516945330489`},
{"CURE", 365, 111.93055079259334`}, {"TBUR", 128.301538556931`, 3.5185016353726346`},
{"CURE", 365, 36.40361182967301`}, {"TBUR", 77.68161708692749`, 76.69485061475065`},
{"CURE", 365, 6.787891250221957`}, {"TBUR", 44.60870078169125`, 51.37513783300899`}, {"CURE", 365, 73.7431552619527`},
{"TBUR", 34.408828510558195`, 84.73317301704782`}, {"CURE", 365, 47.93853836126625`},
{"TBUR", 20.98653516055197`, 60.35461767586018`}, {"CURE", 365, 77.60516487921149`},
{"TBUR", 16.366935818392946`, 94.51105864362984`}, {"CURE", 365, 82.90594705746288`},
{"TBUR", 16.285219065231427`, 23.20022782774634`}, {"TBUR", 64.51408367702395`, 72.48371488265401`},
{"CURE", 365, 156.70319653256024`}, {"CURE", 365, 6.456034202602622`}, {"CURE", 365, 66.18005236683692`},
{"CURE", 365, 33.89193867859011`}, {"CURE", 365, 95.27775351616597`}, {"CURE", 365, 41.02530096140699`},
{"TBUR", 49.98515725401287`, 115.39269590621886`}, {"TBUR", 77.94473386302924`, 75.14714734226939`},
{"CURE", 365, 145.2037782755819`}, {"CURE", 365, 47.3599350658538`}, {"CURE", 365, 187.88859242022883`},
{"CURE", 365, 125.08185216835255`}, {"CURE", 365, 102.95021682524265`}, {"CURE", 365, 121.15949281229844`},
{"TBUR", 134.66616477796475`, 192.26440078259574`}, {"TBUR", 39.75787824873831`, 207.6618195035144`},
{"CURE", 365, 60.424478743434726`}, {"CURE", 365, 100.0495756647766`}, {"CURE", 365, 109.13360553991615`},
{"TBUR", 40.00969775337775`, 38.81951624784094`}, {"TBUR", 16.60115385580605`, 8.236341372726555`},
{"CURE", 365, 1.0101942548944673`}, {"CURE", 365, 77.51235690726995`}, {"CURE", 365, 127.16590580227873`},
{"TBUR", 20.32777201021703`, 103.32968251953002`}, {"CURE", 365, 143.4763282010216`},
{"CURE", 365, 191.66088212206432`}, {"CURE", 365, 48.38014001596851`}, {"CURE", 365, 104.33015397761784`},
{"TBUR", 40.860446439142436`, 7.786405088141161`}, {"CURE", 365, 211.75265394001`},
{"TBUR", 36.78544280892825`, 68.68974728106505`}, {"TBUR", 58.07390151474909`, 48.25342264700932`},
{"TBUR", 65.05057674579417`, 57.90781842052806`}, {"CURE", 365, 197.8601458675351`}, {"CURE", 365, 79.25956069687396`},

{"TBUR", 92.1125507362695`, 178.87953106137434`}, {"CURE", 365, 132.0668230587708`}, {"CURE", 365, 118.81069029394135`},
{"TBUR", 54.55646480561314`, 116.14190667966852`}, {"TBUR", 87.75157058928687`, 117.66587568529287`},
{"CURE", 365, 54.5446956684337`}, {"CURE", 365, 46.44389422906366`}, {"CURE", 365, 15.912662132723785`},
{"CURE", 365, 82.70450545654698`}, {"CURE", 365, 97.41585010047218`}, {"TBUR", 34.04729455578508`, 53.15325725854667`},
{"TBUR", 44.1182773486153`, 68.23167921568655`}, {"CURE", 365, 132.04223050826442`},
{"TBUR", 41.79479907542043 5`, 62.41601671531882`}, {"CURE", 365, 71.55207458833563`}, {"CURE", 365, 37.44028542954089`},
{"TBUR", 20.821354950014694`, 11.740760544271483`}, {"CURE", 365, 50.40625569123428`}, {"CURE", 365, 150.68554497805815`},
{"CURE", 365, 65.29662236274108`}, {"TBUR", 42.87334013281769`, 60.0051183658055 1`}, {"CURE", 365, 17.36233802492764`},
{"TBUR", 25.849741527180658`, 138.6691150712079`}, {"CURE", 365, 147.53561917602582`}, {"CURE", 365, 91.86626442809629`},
{"TBUR", 100.67186882638981`, 38.96006191935832`}, {"CURE", 365, 140.89184591726053`}, {"CURE", 365, 16.659560354517765`},
{"TBUR", 84.27438667825301`, 211.6148026476915`}, {"TBUR", 28.562537432674112`, 74.14746932047004`},
{"CURE", 365, 162.25011317392367`}, {"TBUR", 43.06691285865751`, 78.08029183065507`}, {"CURE", 365, 45.48834092020366`},
{"TBUR", 41.675975089146775`, 57.24759837755166`}, {"TBUR", 24.767522176051195`, 19.54622264989596`},
{"CURE", 365, 126.7405316531945`}, {"TBUR", 42.462975908911716`, 41.710931775319104`},
{"CURE", 365, 64.9593128060602`}, {"TBUR", 25.230170040749083`, 48.200401316824`}, {"CURE", 365, 78.4301127270864`},
{"TBUR", 23.253165589289228`, 46.849711414464274`}, {"TBUR", 106.24528519321754`, 159.98838318048766`},
{"CURE", 365, 107.36124996506305`}, {"TBUR", 33.411267486119364`, 68.73856721149615`},
{"TBUR", 66.39289960297995`, 70.10544456640422`}, {"TBUR", 13.827915662059917`, 66.78014279327687`},
{"CURE", 365, 8.870859273191593`}, {"TBUR", 108.3243922025488`, 124.0331147676527`},
{"TBUR", 101.87591643887352`, 110.29997031506629`}, {"CURE", 365, 92.56072024682261`},
{"TBUR", 44.70318972608537`, 9.28548066397762`}, {"CURE", 365, 74.85529211879833`}, {"CURE", 365, 181.13803183573378`},
{"CURE", 365, 86.41029342233803`}, {"CURE", 365, 104.29516945320486`}, {"TBUR", 28.414692819582662`, 36.600159591343434`},
{"TBUR", 85.16539116914774`, 85.29746406367038`}, {"TBUR", 13.304570289578662`, 2.573036351630501`},
{"TBUR", 15.379370051895433`, 14.097432077328287`}, {"TBUR", 52.80015134311301`, 127.05231387263191`},
{"CURE", 365, 79.90354718120676`}, {"TBUR", 51.39361793718858`, 69.89283788508016`}, {"CURE", 365, 26.25706992343485`},
{"TBUR", 218.07107087281864`, 148.9840256387138`}, {"CURE", 365, 80.10628012498938`}, {"CURE", 365, 151.3120153578012`},
{"TBUR", 54.609240631861766`, 23.839566184690693`}, {"TBUR", 30.32192438656244`, 86.09470553625621`},
{"CURE", 365, 70.02134187617136`}, {"CURE", 365, 84.99566068789653`}, {"CURE", 365, 20.22264368939889`},
{"CURE", 365, 61.227804526357254`}, {"CURE", 365, 136.56379746899484`}, {"TBUR", 20.742265657294762`, 86.12939199964396`},
{"CURE", 365, 45.265556481322974`}, {"TBUR", 43.00832842425244`, 3.126495659004325`}, {"CURE", 365, 48.35916375830092`},
{"CURE", 365, 137.08052826254476`}, {"CURE", 365, 55.49930828501181`}, {"CURE", 365, 82.1559305680778 8`},

{"TBUR", 15.895664679870418`, 100.90350220682193`}, {"TBUR", 88.25788982941741`, 82.76752308445802`},
{"TBUR", 86.27067534787737`, 150.9804701488554`}, {"CURE", 365, 134.33649037892565`},
{"CURE", 365, 95.1536551916874`}, {"CURE", 365, 102.04838130406341`}, {"CURE", 365, 141.7919876792644`},
{"TBUR", 19.638632086152164`, 5.351012020424436`}, {"TBUR", 79.37892757765526`, 145.61513654742683`},
{"TBUR", 148.2636535193825`, 49.62111697211552`}, {"TBUR", 66.03731766711034`, 206.057371890924`},
{"CURE", 365, 12.3070288806778`}, {"TBUR", 91.39429676661848`, 75.33084855287096`}, {"CURE", 365, 172.9347052771825`},
{"CURE", 365, 7.964227445433002`}, {"CURE", 365, 108.967857826693`}, {"TBUR", 94.93472429160232`, 90.40974073078941`},
{"TBUR", 33.31492586839451`, 112.2184344068962`}, {"TBUR", 15.460104588800741`, 57.288823682457725`},
{"TBUR", 13.864395434564795`, 30.389378842239893`}, {"TBUR", 18.097924676086706`, 8.187304891301341`},
{"CURE", 365, 80.86333176672048`}, {"CURE", 365, 79.66087254199334`}, {"TBUR", 37.17771213327045`, 96.7721521672904`},
{"CURE", 365, 15.470062051193244`}, {"CURE", 365, 215.28051087274426`}, {"CURE", 365, 42.999100753136375`},
{"CURE", 365, 85.17669155689862`}, {"CURE", 365, 51.77310129181351`}, {"CURE", 365, 8.150141125521282`},
{"TBUR", 115.30116282422031`, 145.49887830322203`}, {"CURE", 365, 15.960980878524927`},
{"CURE", 365, 61.07825192774318`}, {"TBUR", 19.138683480337118`, 30.230933711468108`},
{"CURE", 365, 65.11210399174692`}, {"TBUR", 71.77258407228345`, 220.59375473823232`},
{"CURE", 365, 151.24060447191295`}, {"TBUR", 36.883215084355186`, 91.95290888051545`}, {"CURE", 365, 83.86695768342098`},
{"CURE", 365, 135.11557851108503`}, {"CURE", 365, 163.75992664831205`}, {"CURE", 365, 128.2794718031026`},
{"CURE", 365, 166.35221181123694`}, {"TBUR", 14.35697419271976`, 55.20644454047053`}, {"CURE", 365, 66.26280138394287`},
{"TBUR", 24.62598948957463`, 9.266243443399919`}, {"TBUR", 18.798816984053282`, 79.11615824399804`},
{"TBUR", 64.94476375472547`, 4.360464832550073`}, {"CURE", 365, 1.0931722435474378`}, {"CURE", 365, 7.18163808963157`},
{"TBUR", 148.73633834778167`, 0.7991964177961337`}, {"TBUR", 18.767075265228186`, 76.23645887498571`},
{"CURE", 365, 24.37253822647612`}, {"CURE", 365, 63.37980453110753`}, {"CURE", 365, 159.04291880302313`},
{"CURE", 365, 38.63260372340441`}, {"TBUR", 20.466631153177428`, 75.76338506082809`},
{"TBUR", 13.231371252328346`, 53.45322504183886`}, {"CURE", 365, 24.99163072614923`},
{"TBUR", 62.50199072246883`, 103.34513622058056`}, {"TBUR", 33.932619605316134`, 21.453046820588384`},
{"TBUR", 62.791401709856025`, 2.015807123583095`}, {"TBUR", 44.86070156214976`, 7.885163790297059`},
{"CURE", 365, 157.18077171879537`}, {"CURE", 365, 30.728054303806925`}, {"CURE", 365, 157.72954153916214`},
{"TBUR", 172.23556604829864`, 100.59785019282859`}, {"CURE", 365, 141.15824217087481`}, {"CURE", 365, 49.63297167213179`},
{"CURE", 365, 192.0002645560187`}, {"CURE", 365, 83.61537546890342`}, {"TBUR", 25.7419994298987 6`, 6.686951495473885`},
{"CURE", 365, 141.85021899851708`}, {"TBUR", 26.95851468280593`, 28.628912909354703`},
{"TBUR", 67.4819505622340 5`, 110.64021229611951`}, {"TBUR", 75.15976480776818`, 138.86073042484364`},

```
{"TBUR", 64.13170912188117`, 41.705644004565585`}, {"CURE", 365, 98.4441539988521`},
{"TBUR", 15.551743396556107`, 32.616115346055196`}, {"TBUR", 32.36678916550318`, 67.66640884058657`},
{"CURE", 365, 119.41633422803804`}, {"CURE", 365, 49.19225517679273`}, {"TBUR", 30.215967976312815`, 73.23522661433311`},
{"CURE", 365, 42.47482079520854`}, {"TBUR", 77.18436327792075`, 140.832551687281`},
{"TBUR", 52.18562355856634`, 88.01521064460886`}, {"CURE", 365, 87.49467332346028`},
{"TBUR", 77.60542943079149`, 123.47539653221664`}, {"CURE", 365, 55.302795062824906`}, {"CURE", 365, 79.66415218970256`},
{"CURE", 365, 128.03284624698355`}, {"CURE", 365, 7.595808553428923`}, {"TBUR", 18.34070000602852`, 11.206779071423863`},
{"TBUR", 145.10826776704215`, 53.32108412848601`}, {"TBUR", 57.143583222361244`, 183.1528160319903`},
{"CURE", 365, 21.18162891325208`}, {"TBUR", 112.91334716983347`, 169.10251974256718`},
{"CURE", 365, 74.71608054225256`}, {"CURE", 365, 178.88713536425587`}, {"TBUR", 15.69414698354964`, 44.41750235689724`},
{"TBUR", 39.71594126693705`, 59.391171419670286`}, {"CURE", 365, 87.2901394358496`}, {"CURE", 365, 185.0403790445819`},
{"TBUR", 84.9807079625145`, 83.55648217736821`}, {"CURE", 365, 208.4853709063455`}, {"CURE", 365, 46.5653915662639`},
{"CURE", 365, 109.24280302437441`}, {"CURE", 365, 83.85277828201454`}, {"CURE", 365, 95.20792564384764`},
{"TBUR", 63.694242459356836`, 91.84019864808212`}, {"TBUR", 44.85578516662774`, 96.9759865755067`},
{"CURE", 365, 10.885522883721338`}, {"CURE", 365, 35.4960917082613`}, {"TBUR", 82.2969424304777`, 106.23518084975628`},
{"CURE", 365, 90.3817848610277`}, {"CURE", 365, 16.090284623081555`}, {"TBUR", 76.76089741493439`, 104.65896994036541`},
{"CURE", 365, 45.80719516737114`}, {"CURE", 365, 18.282318757575382`}, {"CURE", 365, 18.272360682213648`},
{"CURE", 365, 27.830777373273705`}, {"CURE", 365, 37.66196852391155`}, {"TBUR", 67.33105992341264`, 89.34525037699667`},
{"CURE", 365, 134.7360811508258`}, {"CURE", 365, 159.6975442099691`}, {"TBUR", 31.739464766265`, 114.3616557532066`},
{"TBUR", 71.25360096576837`, 8.164033200447616`}, {"CURE", 365, 193.0992514235539`},
{"TBUR", 102.4953784203973`, 67.7569557306676`}, {"TBUR", 39.32054339889921`, 95.6801047019244`},
{"TBUR", 123.49186765509941`, 26.522602722461226`}, {"CURE", 365, 80.69400638145264`},
{"CURE", 365, 6.667234377341868`}, {"CURE", 365, 135.63045228204018`}, {"CURE", 365, 153.45805769635794`},
{"CURE", 365, 82.79660127276934`}, {"CURE", 365, 38.05897433788823`}, {"CURE", 365, 69.24052731675079`},
{"TBUR", 123.05512841613555`, 106.18139220072446`}, {"TBUR", 90.89404151561628`, 112.86686716271257`},
{"TBUR", 40.95290776117067`, 64.97750468645202`}, {"CURE", 365, 91.89960608400636`}, {"CURE", 365, 63.95084192771644`},
{"TBUR", 22.846076371059418`, 161.1775495431219`}, {"TBUR", 80.6848553861814`, 54.65198229070198`},
{"CURE", 365, 58.083935488491775`}, {"TBUR", 109.18834660415882`, 88.65478299609359`}, {"CURE", 365, 126.69627001924432`},
{"CURE", 365, 4.1091716167436525`}, {"TBUR", 55.33811901858552`, 54.23411091131206`}, {"CURE", 365, 88.09812348761918`},
{"CURE", 365, 20.395187070257542`}, {"CURE", 365, 161.72690703808945`}, {"TBUR", 15.84674664990138`, 7.79125274025434`},
{"CURE", 365, 158.36776544846174`}, {"CURE", 365, 146.0583742147614`}, {"TBUR", 109.93876817733107`, 3.6868354877643803`},
```

{"CURE", 365, 123.98300572864805`}, {"CURE", 365, 53.93061701261477`}, {"CURE", 365, 62.07807634827993`},
{"CURE", 365, 136.6904675143727`}, {"CURE", 365, 12.13798577917324`}, {"TBUR", 20.993999468351177`, 79.44131751607695`},
{"CURE", 365, 30.18081313637761`}, {"TBUR", 85.8824180620177`, 2.6906958333830078`}, {"CURE", 365, 82.11497332489698`},
{"TBUR", 74.60234989472667`, 126.85450873217796`}, {"TBUR", 171.2610902531934`, 32.62889810059776`},
{"CURE", 365, 0.7102585850846388`}, {"TBUR", 92.84465497949839`, 9.197642152321775`},
{"TBUR", 30.549098383808225`, 63.80457878536642`}, {"CURE", 365, 2.4429365259067706`},
{"CURE", 365, 5.985819250416224`}, {"CURE", 365, 118.60909314856984`}, {"TBUR", 75.27191953753493`, 201.38116171120038`},
{"TBUR", 89.52935717649251`, 105.73853654865616`}, {"CURE", 365, 196.33631050201473`},
{"TBUR", 74.15754528995713`, 96.84812178149998`}, {"CURE", 365, 2.704489103986928`}, {"CURE", 365, 103.0776139876183`},
{"TBUR", 89.76447948262695`, 150.40922689450213`}, {"TBUR", 38.66018815573057`, 78.36936808356809`},
{"TBUR", 69.48655159024455`, 12.202372372910103`}, {"TBUR", 57.8424705753005`, 66.40478336877358`},
{"CURE", 365, 44.2370446174899`}, {"CURE", 365, 25.590710560991702`}, {"CURE", 365, 22.14107064071075`},
{"CURE", 365, 119.24223748391604`}, {"TBUR", 127.50671102255562`, 52.58755145349185`},
{"TBUR", 25.266166788857888`, 47.927804703260904`}, {"CURE", 365, 68.92204368966077`},
{"TBUR", 72.50630392650667`, 13.68483951130741`}, {"CURE", 365, 102.29611049398817`}, {"CURE", 365, 54.14655326611655`},
{"TBUR", 63.72165500960094`, 58.29095400117362`}, {"TBUR", 50.87711279148846`, 133.6623120966936`},
{"CURE", 365, 175.38033828799536`}, {"CURE", 365, 58.14999245665073`}, {"CURE", 365, 10.399890094413005`},
{"TBUR", 79.55319389437524`, 205.4260435935895`}, {"TBUR", 155.7224572391856`, 71.74723875593074`},
{"TBUR", 57.199667896426114`, 61.45905127575457`}, {"CURE", 365, 84.00603879796861`}, {"CURE", 365, 22.39232054784003`},
{"CURE", 365, 76.91536283659688`}, {"CURE", 365, 72.08657775442295`}, {"TBUR", 119.00371733496938`, 72.99531786293548`},
{"CURE", 365, 129.5359605548035`}, {"CURE", 365, 54.142690885684495`}, {"TBUR", 165.6496539605562`, 96.80235535681578`},
{"CURE", 365, 44.8326053012101`}, {"CURE", 365, 127.5401418911823`}, {"CURE", 365, 58.444723122289766`},
{"TBUR", 85.57828267436506`, 77.22518403187426`}, {"CURE", 365, 38.449517913231155`},
{"TBUR", 82.36659529237353`, 120.64940165111473`}, {"TBUR", 48.15208502962542`, 229.05682354698286`},
{"TBUR", 41.98031268273792`, 112.2398676954244`}, {"CURE", 365, 109.24925371390496`}, {"CURE", 365, 41.968006146063914`},
{"TBUR", 121.19150124812774`, 90.31513767796687`}, {"TBUR", 57.52438739314779`, 169.71240182403523`},
{"CURE", 365, 4.577721565146532`}, {"CURE", 365, 109.09133205583925`}, {"TBUR", 71.234797293649`, 10.459080256519986`},
{"CURE", 365, 26.768391161331685`}, {"CURE", 365, 13.262823854777876`}, {"TBUR", 48.54574987740565`, 58.14208856594091`},
{"CURE", 365, 3.1181154529874333`}, {"TBUR", 32.577908685717475`, 77.88329976146409`}, {"CURE", 365, 72.26092699987989`},
{"TBUR", 80.45009718011134`, 88.86202301297001`}, {"TBUR", 34.08949445866009`, 52.99129953771477`},
{"CURE", 365, 92.86217282268646`}, {"CURE", 365, 162.5961001853289`}, {"CURE", 365, 80.17561463792025`},

```
{"CURE", 365, 23.98057292197991`}, {"CURE", 365, 84.61391426775731`}, {"TBUR", 116.2112222541051`, 7.7147295502640425`},
{"TBUR", 67.08149032777199`, 7.551286800510426`}, {"CURE", 365, 136.83731945927985`},
{"CURE", 365, 130.11966572474694`}, {"TBUR", 82.30835671661353`, 30.660091721110117`},
{"CURE", 365, 43.46950849372692`}, {"TBUR", 18.275036882715973`, 90.04643623786247`},
{"CURE", 365, 157.55800903039386`}, {"TBUR", 133.36265199851013`, 51.24431210096239`},
{"TBUR", 58.27967234498509`, 71.79735605247788`}, {"CURE", 365, 10.935670825163802`},
{"TBUR", 75.7458160758054`, 79.42614765921394`}, {"CURE", 365, 67.75346106287742`}, {"CURE", 365, 83.5825960206757`},
{"TBUR", 69.89771595266897`, 56.71031921614555`}, {"TBUR", 73.58447809523136`, 25.66603351853326`},
{"CURE", 365, 95.6360988227035`}, {"TBUR", 14.66165966961657`, 53.525965860106886`}, {"CURE", 365, 26.170678172168714`},
{"CURE", 365, 105.0650211229893`}, {"CURE", 365, 187.19655611978519`}, {"CURE", 365, 149.87324303239367`},
{"CURE", 365, 84.63597368914274`}, {"CURE", 365, 107.15994108306812`}, {"TBUR", 44.188081455628954`, 10.814367723163297`},
{"TBUR", 42.917110522935104`, 91.73817346344218`}, {"TBUR", 68.11987300913587`, 17.3809391548065`},
{"CURE", 365, 62.39017856147486`}, {"TBUR", 25.899254208748392`, 94.37298997741814`}, {"CURE", 365, 24.83300798296857`},
{"CURE", 365, 69.9674543892695`}, {"TBUR", 20.91104827950352`, 28.934516681019385`}, {"CURE", 365, 225.09317043769653`},
{"TBUR", 14.600045719297393`, 15.620956884179614`}, {"CURE", 365, 101.45964025379357`},
{"TBUR", 44.038504904694925`, 29.52285173410661`}, {"TBUR", 107.71079754603839`, 16.703521007870137`},
{"CURE", 365, 2.575776919904139`}, {"CURE", 365, 4.581915863098254`}, {"CURE", 365, 80.90515391648334`},
{"TBUR", 27.534328128621212`, 50.40435584380591`}, {"CURE", 365, 16.01992066223462`}, {"CURE", 365, 75.01140578957533`},
{"CURE", 365, 108.08558222875739`}, {"CURE", 365, 199.33409198298276`}, {"CURE", 365, 65.32876314867175`},
{"CURE", 365, 100.52216729314536`}, {"TBUR", 40.929606853247286`, 95.32175897751775`}, {"CURE", 365, 18.504320503921512`},
{"TBUR", 25.049781863777778`, 43.8674485865615`}, {"CURE", 365, 34.154833074276326`}, {"CURE", 365, 98.03474633705159`},
{"TBUR", 42.4401343637111`, 88.90597917483903`}, {"CURE", 365, 64.08051529627257`}, {"CURE", 365, 3.6022659685530525`},
{"CURE", 365, 23.43448994217387`}, {"CURE", 365, 69.11332327104029`}, {"TBUR", 41.88433799602727`, 79.59187807339718`},
{"CURE", 365, 108.76309391856992`}, {"TBUR", 107.45617866122652`, 70.19487656140386`}, {"CURE", 365, 201.84328578963706`},
{"CURE", 365, 91.12913601413638`}, {"TBUR", 18.20784679224633`, 74.76985516098351`}, {"CURE", 365, 1.1615409511623986`},
{"CURE", 365, 41.856623928850595`}, {"CURE", 365, 152.67043095098666`}, {"CURE", 365, 4.5144408989350735`},
{"TBUR", 47.15523153410218`, 60.811823831796964`}, {"TBUR", 50.150296145843974`, 93.54039933242113`},
{"TBUR", 87.40006391867828`, 172.3952049375295`}, {"TBUR", 88.434700777491`, 114.22364320472911`},
{"CURE", 365, 25.539441154322066`}, {"TBUR", 79.37537676309294`, 8.976904755253686`},
{"CURE", 365, 65.33214071962166`}, {"CURE", 365, 158.77890111190862`}, {"TBUR", 32.7094372558674`, 93.9023111782468`},
{"CURE", 365, 8.565213346937776`}, {"TBUR", 54.535495152613386`, 93.75473662485598`},
```

{"CURE", 365, 84.64467937577587`}, {"TBUR", 70.23229500029692`, 5.617685397639729`},
{"CURE", 365, 69.30902937600331`}, {"CURE", 365, 88.34165248084383`}, {"CURE", 365, 52.750086795765995`},
{"TOX", 21.997762061905924`, 251.47139524673`}, {"TBUR", 62.78665139507932`, 73.1811356069713 1`},
{"CURE", 365, 92.96432266941544`}, {"TBUR", 24.636194892447204`, 72.6015675555872`}, {"CURE", 365, 204.71616392820462`},
{"CURE", 365, 80.20189868802206`}, {"TBUR", 19.695576839331313`, 71.5371893336805`}, {"CURE", 365, 49.29965738071418`},
{"TBUR", 58.90431553901021`, 104.57764343984164`}, {"CURE", 365, 0.920407197717919`},
{"TBUR", 42.632512794610854`, 23.60179437020251`}, {"TBUR", 146.04931854921097`, 100.96682239823866`},
{"CURE", 365, 85.1578942057839`}, {"CURE", 365, 21.085534064549208`}, {"CURE", 365, 66.86196209707947`},
{"CURE", 365, 46.1773366114401`}, {"TBUR", 71.51003322492109`, 48.21563549659347`}, {"CURE", 365, 12.664549095975321`},
{"CURE", 365, 59.2955382072514`}, {"CURE", 365, 49.83150390561452`}, {"TBUR", 27.225507020189582`, 33.29512114649626`},
{"CURE", 365, 154.04979593142318`}, {"CURE", 365, 4.351294002597327`}, {"CURE", 365, 69.735039082626`},
{"TBUR", 162.40977663343207`, 108.77141427246067`}, {"TBUR", 16.555991666753872`, 115.56494900466733`},
{"TBUR", 46.198027584569644`, 8.03340531031299`}, {"CURE", 365, 91.8268032463793`}, {"CURE", 365, 107.39295292455269`},
{"TBUR", 28.64375341046014`, 3.7989632845911103`}, {"CURE", 365, 65.23631849235157`},
{"CURE", 365, 91.61923762149054`}, {"TBUR", 22.602686065523468`, 22.183815716845128`},
{"CURE", 365, 2.08001095562787`}, {"TBUR", 66.51438914831147`, 174.9007839972679`},
{"TBUR", 113.51867948128191`, 96.17950386652616`}, {"CURE", 365, 93.46535911197778`},
{"CURE", 365, 29.389615553450863`}, {"TBUR", 82.40291919870315`, 3.0028017657043633`}, {"CURE", 365, 8.06191170393786`},
{"TBUR", 13.243764662970115`, 18.536530285419794`}, {"TBUR", 84.40553645216042`, 78.12679953114542`},
{"CURE", 365, 127.98885813396241`}, {"CURE", 365, 107.08397479547261`}, {"CURE", 365, 52.51890959828215`},
{"TBUR", 22.246174144533388`, 2.1864100156715356`}, {"CURE", 365, 83.32482038541656`}, {"CURE", 365, 89.76404211925983`},
{"TBUR", 69.25850064457582`, 1.3369825593794042`}, {"TBUR", 76.62045272343127`, 52.92703136772024`},
{"CURE", 365, 1.0314262088410786`}, {"CURE", 365, 144.12814907703805`}, {"CURE", 365, 63.639205204541575`},
{"TBUR", 14.619345989898447`, 0.7496613236291427`}, {"TBUR", 163.24420505550125`, 87.83013590585666`},
{"CURE", 365, 33.364695500076934`}, {"TBUR", 54.82262231736413`, 225.780900880063`}, {"CURE", 365, 127.86952732963464`},
{"TBUR", 41.742815007736105`, 69.93269744155519`}, {"CURE", 365, 182.39980714691234`}},
{{"450 Res", "450 OS", "450 Tox"}, {"TBUR", 21.681593913633254`, 40.177539942251535`}, {"CURE", 365, 150.12494835334138`},
{"TBUR", 27.007147121060882`, 115.78851782124755`}, {"TBUR", 29.740207880882615`, 6.143331826056098`},
{"TBUR", 18.13518315718996`, 17.736268180123503`}, {"CURE", 365, 31.485117973358104`}, {"CURE", 365, 65.83818886313749`},
{"CURE", 365, 63.02338112075268`}, {"CURE", 365, 147.29000773974474`}, {"TBUR", 70.72638303284765`, 197.170938999826`},
{"TBUR", 54.57349183534134`, 101.36424050668015`}, {"CURE", 365, 91.79152101048093`},

{"TOX", 17.258394612525016`, 265.3402290799942`}, {"TBUR", 43.15405818091577`, 64.03393554249858`},
{"TBUR", 65.73963567825743`, 6.461148185445151`}, {"TBUR", 87.95986134749559`, 30.95640544179281`},
{"CURE", 365, 102.59770135681026`}, {"TBUR", 44.59613303166754`, 116.83054454683376`},
{"TBUR", 45.02002146076806`, 7.492568547818287`}, {"CURE", 365, 87.5101049112166`}, {"CURE", 365, 137.56594395932234`},
{"CURE", 365, 51.68539225098627`}, {"CURE", 365, 31.560163986157587`}, {"CURE", 365, 66.73699066312464`},
{"CURE", 365, 60.0689988608787`}, {"CURE", 365, 163.5008201191205`}, {"CURE", 365, 102.8619757876167`},
{"CURE", 365, 52.28233413992836`}, {"CURE", 365, 35.76886264423089`}, {"CURE", 365, 8.378473324828134`},
{"CURE", 365, 138.2429968497628`}, {"TBUR", 13.42641233060402`, 36.17571019228005`}, {"CURE", 365, 70.04846680094802`},
{"TBUR", 48.48536636035118`, 42.13791691620267`}, {"CURE", 365, 185.616021375836`}, {"CURE", 365, 215.9742744525341`},
{"CURE", 365, 43.63021951057949`}, {"TBUR", 21.557062549863595`, 11.16159376156995`},
{"TBUR", 71.98973179575574`, 51.190784139205235`}, {"CURE", 365, 96.24672248490603`},
{"TBUR", 18.041987240344714`, 62.28565820054761`}, {"CURE", 365, 100.04579519134008`}, {"CURE", 365, 73.43191010888498`},
{"TBUR", 45.96001294001791`, 79.09943673375881`}, {"CURE", 365, 97.20561015496932`}, {"CURE", 365, 4.991991062030502`},
{"CURE", 365, 108.48156303888737`}, {"CURE", 365, 68.78500635062807`}, {"TBUR", 112.56411471509611`, 131.74002293734625`},
{"CURE", 365, 226.25106596674723`}, {"TBUR", 29.693977230248265`, 100.3873523794494`},
{"TBUR", 41.718333359950634`, 86.2606109841368`}, {"CURE", 365, 6.51925954375214`}, {"CURE", 365, 140.3146033959164`},
{"CURE", 365, 49.04394341003214`}, {"CURE", 365, 98.6025538119511`}, {"CURE", 365, 71.63718499143668`},
{"TBUR", 103.327003079774`, 84.8957316358101`}, {"TBUR", 37.43997706177126`, 170.71729546075383`},
{"TBUR", 52.469821990428436`, 101.07635168512851`}, {"CURE", 365, 93.33945812054125`},
{"CURE", 365, 4.618846040383499`}, {"TBUR", 33.601672575545116`, 114.81309878394596`}, {"CURE", 365, 84.40541759446432`},
{"TBUR", 73.07100288779819`, 7.060964513083741`}, {"CURE", 365, 89.70416539477026`}, {"CURE", 365, 81.66060464157884`},
{"TBUR", 57.394108913172616`, 121.81814937009007`}, {"CURE", 365, 44.27359512350647`}, {"CURE", 365, 37.67815114901568`},
{"CURE", 365, 69.6729854180864`}, {"CURE", 365, 51.34862114758355`}, {"CURE", 365, 142.70021772265378`},
{"TBUR", 13.449202445504826`, 2.253959890738593`}, {"TBUR", 79.54185280483898`, 92.62073441470487`},
{"CURE", 365, 120.650279007722`}, {"TBUR", 96.98981321553023`, 218.3817198763771`}, {"CURE", 365, 72.36444204493758`},
{"TBUR", 75.80862964897912`, 141.47817763122575`}, {"CURE", 365, 58.910649543857446`}, {"CURE", 365, 114.74573608816632`},
{"CURE", 365, 3.701714353269107`}, {"CURE", 365, 7.210600470808908`}, {"CURE", 365, 5.124096143544366`},
{"CURE", 365, 132.63908632626152`}, {"TBUR", 99.55084414456853`, 101.45579630068274`}, {"CURE", 365, 92.73643895546732`},
{"CURE", 365, 47.21252201326419`}, {"CURE", 365, 7.8266884309209335`}, {"TBUR", 71.40811224827466`, 153.96566902388358`},
{"TBUR", 33.01230768994635`, 14.228550950085252`}, {"TBUR", 89.28253540969945`, 110.01599366360655`},
{"TBUR", 69.41402389705011`, 2.9985372760392575`}, {"CURE", 365, 6.934754657013155`},

```
{"TBUR", 19.36218258954332`, 2.1698153639091844`}, {"TBUR", 158.58351530325427`, 59.731725335864844`},
{"CURE", 365, 44.70557349529483`}, {"CURE", 365, 198.45358956167252`}, {"CURE", 365, 10.76441760461726`},
{"TBUR", 43.762993852389314`, 157.93807242782984`}, {"TBUR", 20.694455597684843`, 11.899424325119293`},
{"TBUR", 127.36403039600869`, 141.1999431709942`}, {"CURE", 365, 77.7602317550847`},
{"TBUR", 90.98206790608134`, 99.04895369723224`}, {"CURE", 365, 70.83750669899794`}, {"CURE", 365, 114.31919147658668`},
{"CURE", 365, 47.25219179390422`}, {"TBUR", 139.05617475157604`, 94.4048511211741`}, {"CURE", 365, 43.359335652616295`},
{"CURE", 365, 114.24386781007287`}, {"CURE", 365, 156.14474645997606`}, {"CURE", 365, 84.93455203728423`},
{"CURE", 365, 26.512516780396183`}, {"CURE", 365, 56.2869139190525`}, {"CURE", 365, 76.22699245501974`},
{"CURE", 365, 64.59628922483893`}, {"CURE", 365, 17.474113052705576`}, {"CURE", 365, 85.727037553286`},
{"CURE", 365, 89.63531787970344`}, {"CURE", 365, 27.26048987211973`}, {"CURE", 365, 89.42439437094612`},
{"CURE", 365, 179.7544789871229`}, {"CURE", 365, 114.91919945434948`}, {"TBUR", 20.11577413718333`, 5.968245195627265`},
{"CURE", 365, 80.23171882919411`}, {"TBUR", 38.923563131929136`, 72.78136517383903`},
{"CURE", 365, 65.6097573424163`}, {"CURE", 365, 42.24633731499965`}, {"CURE", 365, 130.85439031686695`},
{"CURE", 365, 125.78117838464141`}, {"CURE", 365, 111.05982366399857`}, {"CURE", 365, 121.30002143061878`},
{"TBUR", 71.81570987960126`, 42.46588036358657`}, {"TBUR", 45.87395879210216`, 45.47930238260016`},
{"CURE", 365, 177.2823384979206`}, {"TBUR", 12.774056620781595`, 76.85101345570675`}, {"CURE", 365, 25.24614103593384`},
{"TBUR", 62.477717431962176`, 61.49384488572377`}, {"CURE", 365, 20.08360762249232`}, {"CURE", 365, 119.48353557237998`},
{"TBUR", 53.04803260062576`, 179.53009638829133`}, {"CURE", 365, 124.02041315442963`},
{"CURE", 365, 164.9008014856199`}, {"CURE", 365, 35.05292703923925`}, {"CURE", 365, 12.237224149015596`},
{"CURE", 365, 75.77458772912175`}, {"CURE", 365, 177.34376840609332`}, {"TBUR", 37.50486227925405`, 46.028769484001316`},
{"CURE", 365, 82.24540857335967`}, {"CURE", 365, 20.563417448043364`}, {"CURE", 365, 7.648505064767802`},
{"CURE", 365, 70.8552318595884`}, {"CURE", 365, 1.292025390622302`}, {"TBUR", 88.69856922124018`, 179.3716044379514`},
{"TBUR", 50.18935564811535`, 76.30205247854968`}, {"TBUR", 88.39595186637534`, 161.00376587511013`},
{"CURE", 365, 81.6228888632086`}, {"CURE", 365, 95.43080229359468`}, {"CURE", 365, 75.32040238181878`},
{"CURE", 365, 95.21718073588264`}, {"CURE", 365, 29.968590723996506`}, {"TBUR", 101.2900342512816`, 23.915673921546745`},
{"CURE", 365, 1.8287843478923342`}, {"TBUR", 70.0918087791948`, 14.038510930137996`},
{"TBUR", 78.87404466447808`, 84.1612494787993`}, {"CURE", 365, 121.47697998247314`}, {"CURE", 365, 122.2259489954168`},
{"CURE", 365, 89.48036409930468`}, {"CURE", 365, 125.73173233109776`}, {"CURE", 365, 1.9163993255378946`},
{"CURE", 365, 71.72171126785823`}, {"TBUR", 29.81156484118249`, 137.09320559954548`}, {"CURE", 365, 63.262652493439866`},
{"TBUR", 21.293549082963064`, 7.466550080526059`}, {"TBUR", 50.2946905182695`, 135.54142572686916`},
{"TBUR", 91.21039840587224`, 11.060537149749265`}, {"TBUR", 67.94018332382898`, 165.51075167698133`},
```

{"TBUR", 21.22588224705616`, 85.39365333553228`}, {"TBUR", 59.83234315354901`, 48.69810773459688`},
{"TBUR", 91.17481567567938`, 106.35456024310896`}, {"TBUR", 80.3885409183217`, 115.52089795848123`},
{"CURE", 365, 88.60544387681708`}, {"CURE", 365, 153.60806900222758`}, {"TBUR", 20.58054420313793`, 11.11635892963677`},
{"CURE", 365, 137.68249939932397`}, {"TBUR", 28.04967982617569`, 46.812652955664596`},
{"TBUR", 53.614317272253494`, 150.9634693224472`}, {"CURE", 365, 76.15574972346654`},
{"CURE", 365, 3.986409614145104`}, {"TBUR", 24.324229126283033`, 99.80037928287891`}, {"CURE", 365, 96.36976163051305`},
{"CURE", 365, 150.63650622580286`}, {"CURE", 365, 177.60621015258636`}, {"CURE", 365, 117.35515778361142`},
{"CURE", 365, 70.37856526723728`}, {"CURE", 365, 54.19226073508568`}, {"CURE", 365, 184.870205808752`},
{"TBUR", 13.759217771798962`, 15.692538920560608`}, {"CURE", 365, 103.5419340771995`}, {"CURE", 365, 95.73472808640054`},
{"TBUR", 27.810135489199084`, 24.6584697567284`}, {"TBUR", 57.17122207481299`, 105.75370026797204`},
{"CURE", 365, 108.96050134572089`}, {"TBUR", 65.25437339483707`, 82.75921164928658`},
{"CURE", 365, 1.8640754750468755`}, {"TBUR", 143.35409551529077`, 32.2983337289578`},
{"TBUR", 15.902971439279971`, 91.42758305253464`}, {"CURE", 365, 184.0590272984331`}, {"CURE", 365, 15.771089757338096`},
{"CURE", 365, 135.9378015471369`}, {"CURE", 365, 3.051788375645261`}, {"CURE", 365, 95.16656128672092`},
{"CURE", 365, 22.381246198301074`}, {"CURE", 365, 116.400645244032`}, {"CURE", 365, 29.93313350012947`},
{"TBUR", 47.573617386282166`, 14.524757156872766`}, {"TBUR", 80.0279667267855`, 164.726523006219`},
{"CURE", 365, 79.49464733684133`}, {"TBUR", 50.12262775806517`, 214.38291357337374`}, {"CURE", 365, 75.56678661165623`},
{"CURE", 365, 107.98199174142934`}, {"CURE", 365, 15.622870266940906`}, {"CURE", 365, 74.25256323381713`},
{"CURE", 365, 142.69033349750208`}, {"CURE", 365, 147.61352023582967`}, {"CURE", 365, 72.20948182074864`},
{"CURE", 365, 132.0199743484178`}, {"CURE", 365, 85.26150999131019`}, {"CURE", 365, 8.111693831049772`},
{"CURE", 365, 56.71668294833779`}, {"CURE", 365, 49.64974920108383`}, {"CURE", 365, 74.22046199405574`},
{"CURE", 365, 108.87820217356467`}, {"CURE", 365, 90.3749794735314`}, {"CURE", 365, 83.64524833680008`},
{"TBUR", 22.780205963965916`, 106.73509276319547`}, {"TBUR", 28.109893638887616`, 71.69239921271591`},
{"TBUR", 156.68702483094657`, 73.45489325610838`}, {"TBUR", 21.249458109624953`, 49.889208989308635`},
{"CURE", 365, 63.3404529900671`}, {"CURE", 365, 73.96225556194517`}, {"CURE", 365, 131.01900100876048`},
{"TBUR", 27.31950675896698`, 6.0377718769835615`}, {"TBUR", 31.89346569151982`, 26.88610517666955`},
{"CURE", 365, 1.725752754419538`}, {"CURE", 365, 2.5624849490110573`}, {"TBUR", 20.808756633379637`, 23.25899905919438`},
{"CURE", 365, 208.09044589936514`}, {"CURE", 365, 58.28889289216423`}, {"CURE", 365, 204.22961073803128`},
{"TBUR", 38.77831446351168`, 98.75061319456125`}, {"CURE", 365, 70.87309609855764`},
{"TBUR", 15.275170449885223`, 122.7688201590046`}, {"CURE", 365, 55.93732538268919`},
{"CURE", 365, 104.60372998451835`}, {"CURE", 365, 11.24314155068969`}, {"CURE", 365, 78.15922178582869`},

{"CURE", 365, 29.889413594969668`}, {"CURE", 365, 9.494332742288375`}, {"CURE", 365, 123.70888014872426`},
{"TBUR", 21.661440590703084`, 126.121336233228`}, {"TBUR", 13.722536693950405`, 91.14218189121235`},
{"CURE", 365, 62.66536216635174`}, {"TBUR", 28.03480794316479`, 84.96762749625641`}, {"CURE", 365, 8.340356267762834`},
{"CURE", 365, 63.853784718991676`}, {"CURE", 365, 55.3956782178886`}, {"CURE", 365, 214.49505380472633`},
{"CURE", 365, 94.44282871954103`}, {"CURE", 365, 48.89312049718113`}, {"CURE", 365, 1.6488595692153816`},
{"TBUR", 70.03102198925131`, 91.57595387429681`}, {"CURE", 365, 77.14254108519113`},
{"TBUR", 151.4004198974849`, 81.09255967507782`}, {"TBUR", 57.794124375967876`, 14.719699062938412`},
{"CURE", 365, 85.9908800390565`}, {"CURE", 365, 59.18647557418903`}, {"TBUR", 20.647349422886585`, 75.47307310333535`},
{"CURE", 365, 45.588214203596095`}, {"CURE", 365, 105.67330103813318`}, {"CURE", 365, 20.872460445767278`},
{"TBUR", 97.86802543185588`, 101.44803174880418`}, {"TBUR", 16.593961653658646`, 40.52912838444825`},
{"CURE", 365, 82.20264958471591`}, {"TBUR", 27.328054404497255`, 99.27034754310166`}, {"CURE", 365, 9.167311339464852`},
{"CURE", 365, 120.11332289192359`}, {"CURE", 365, 71.60765367736447`}, {"CURE", 365, 199.68396182641752`},
{"CURE", 365, 137.4821519121524`}, {"CURE", 365, 97.14926185446387`}, {"CURE", 365, 61.91436697583385`},
{"CURE", 365, 202.74626087153518`}, {"TBUR", 30.894518848092787`, 33.440155903098045`},
{"CURE", 365, 18.31030410023945`}, {"CURE", 365, 34.60965770296192`}, {"CURE", 365, 93.14784832402293`},
{"CURE", 365, 88.06729291651355`}, {"CURE", 365, 114.56942384263363`}, {"CURE", 365, 40.097182573978706`},
{"TBUR", 23.254007249050698`, 146.92775594219614`}, {"CURE", 365, 3.775734575083159`}, {"CURE", 365, 124.7060948832854`},
{"TBUR", 38.556493653483855`, 146.72962793340582`}, {"CURE", 365, 62.57828176611282`}, {"CURE", 365, 122.02865810080328`},
{"CURE", 365, 4.012996782195931`}, {"TBUR", 22.445343365960518`, 48.34483323601411`}, {"CURE", 365, 71.83786782493685`},
{"CURE", 365, 72.93983426586293`}, {"TBUR", 29.206563413628558`, 33.43738518046275`}, {"CURE", 365, 70.21490219537597`},
{"TBUR", 37.071463438244336`, 34.78706741074242`}, {"CURE", 365, 108.34617422658373`}, {"CURE", 365, 87.77734833422112`},
{"CURE", 365, 93.86485987149479`}, {"CURE", 365, 70.1776278597587`}, {"CURE", 365, 74.0763283455592`},
{"CURE", 365, 104.16649664746399`}, {"CURE", 365, 140.3365714665372`}, {"TBUR", 49.12076257620074`, 98.56869608531063`},
{"CURE", 365, 135.59407006239763`}, {"CURE", 365, 82.46677090613787`}, {"TBUR", 60.237131945508054`, 20.48986212357248`},
{"TBUR", 17.189374767774257`, 17.336851917181868`}, {"CURE", 365, 0.8837621505209333`}, {"CURE", 365, 118.3025097737938`},
{"CURE", 365, 122.59421306593984`}, {"CURE", 365, 91.3063916590417`}, {"TBUR", 138.99520730800987`, 208.182282471549`},
{"CURE", 365, 163.16779587772893`}, {"CURE", 365, 47.31176574216525`}, {"TBUR", 20.53118707226683`, 64.67014229312618`},
{"CURE", 365, 14.24111167567059`}, {"CURE", 365, 199.43236665806373`}, {"TBUR", 39.116485039656965`, 197.29190377991586`},
{"TBUR", 24.0933803496922`, 30.330988838363567`}, {"CURE", 365, 194.39603918074613`}, {"CURE", 365, 36.737823254796425`},
{"CURE", 365, 74.49212611572794`}, {"CURE", 365, 139.8450230427479`}, {"CURE", 365, 2.2277432045819245`},
{"TBUR", 55.5829456629309`, 42.968861959015044`}, {"CURE", 365, 123.83344578256143`},

{"TBUR", 60.49703236561716`, 29.23093662358552`}, {"TBUR", 38.654271026440036`, 198.07001080063776`},
{"CURE", 365, 3.392865269494908 6`}, {"CURE", 365, 165.4297278081638`}, {"CURE", 365, 97.51187444993239`},
{"CURE", 365, 91.2223997391357`}, {"CURE", 365, 115.81433247552397`}, {"CURE", 365, 51.426534043402256`},
{"TBUR", 35.610702884912584`, 147.02641649647111`}, {"CURE", 365, 92.56575480313344`},
{"CURE", 365, 158.09406325407966`}, {"CURE", 365, 103.50159224751775`}, {"TBUR", 54.40935417226095`, 107.26173915861499`},
{"CURE", 365, 125.7591159923357`}, {"CURE", 365, 94.84188417664174`}, {"CURE", 365, 47.842177588696494`},
{"CURE", 365, 140.47135261086999`}, {"CURE", 365, 173.36858655528005`}, {"CURE", 365, 48.456971275279386`},
{"TBUR", 64.1223404223068`, 113.28344674425065`}, {"TBUR", 45.93230972746142`, 142.14335816555487`},
{"CURE", 365, 18.284989654522583`}, {"CURE", 365, 102.73957348182873`}, {"CURE", 365, 54.192952659661884`},
{"TBUR", 14.657935603440771`, 9.510056483883474`}, {"TBUR", 13.451182761435824`, 31.52135757173175`},
{"TBUR", 33.032870731341795`, 49.54430291652335`}, {"CURE", 365, 99.21747741021657`}, {"CURE", 365, 28.929572068180413`},
{"CURE", 365, 90.36742509963038`}, {"TBUR", 78.19296573284649`, 165.05151255961943`}, {"CURE", 365, 92.3118951162459`},
{"CURE", 365, 80.46231543506993`}, {"CURE", 365, 84.71825339065128`}, {"TBUR", 17.238107831478434`, 37.47593510112497`},
{"TBUR", 13.634917490738811`, 83.94139574009003`}, {"TBUR", 79.70012767524892`, 36.53306857471113`},
{"CURE", 365, 144.86536418729182`}, {"TBUR", 49.88789770961453`, 88.50096114939878`}, {"CURE", 365, 119.49307802025524`},
{"CURE", 365, 87.04899340247185`}, {"CURE", 365, 77.48459824673269`}, {"TBUR", 101.4940750963087`, 39.27030167576299`},
{"CURE", 365, 55.20118887771086`}, {"CURE", 365, 205.65324825851982`}, {"CURE", 365, 106.47756552600428`},
{"CURE", 365, 85.87738841107718`}, {"TBUR", 33.101675402308025`, 77.17125865082794`},
{"TBUR", 76.9318711152509`, 74.29610920546365`}, {"CURE", 365, 58.113447228310854`},
{"TBUR", 15.952244017126644`, 63.90759419608308`}, {"TBUR", 17.093737025191402`, 59.56099356993961`},
{"CURE", 365, 56.155085038182456`}, {"CURE", 365, 84.60198063552075`}, {"TBUR", 107.04624350303816`, 6.808876365236141`},
{"TBUR", 12.958929705113176`, 58.91712787799842`}, {"TBUR", 21.053280135259108`, 17.53987481447614`},
{"CURE", 365, 134.93857599736282`}, {"CURE", 365, 36.215094689621104`}, {"CURE", 365, 99.8924534092044`},
{"CURE", 365, 5.184400540728654`}, {"CURE", 365, 85.20925504388703`}, {"CURE", 365, 2.922856899882258`},
{"CURE", 365, 97.74648726835078`}, {"CURE", 365, 90.24318130054844`}, {"TBUR", 114.84599855922995`, 104.50159370907748`},
{"CURE", 365, 7.294965509725478`}, {"CURE", 365, 48.001572662554686`}, {"CURE", 365, 196.36868576101946`},
{"TBUR", 25.10467605235821`, 46.92050516756156`}, {"CURE", 365, 1.8287973664948929`},
{"CURE", 365, 28.87807968097449`}, {"CURE", 365, 137.31582946410902`}, {"CURE", 365, 19.503335034653592`},
{"CURE", 365, 107.2023426533133`}, {"CURE", 365, 166.69539754994`}, {"CURE", 365, 66.11782573529113`},
{"CURE", 365, 7.895109283512181`}, {"CURE", 365, 8.452578431845888`}, {"CURE", 365, 125.23011870484326`},
{"TBUR", 13.480593204353381`, 17.538383717961167`}, {"TBUR", 51.67339061635388`, 227.5705156541556`},

{"CURE", 365, 83.5199631045017`}, {"CURE", 365, 102.59958881478076`}, {"TBUR", 84.87948415540177`, 82.70763717437539`},
{"CURE", 365, 56.49231392078648`}, {"TOX", 39.316909692217905`, 231.65978210472676`}, {"CURE", 365, 77.03261109992918`},
{"TBUR", 113.31462785804352`, 3.911800741122546`}, {"CURE", 365, 78.6669706080475`}, {"CURE", 365, 27.07709044356122`},
{"CURE", 365, 178.769198325109`}, {"CURE", 365, 69.33253890182415`}, {"CURE", 365, 4.455659518631307`},
{"CURE", 365, 85.65642187537668`}, {"TBUR", 22.041946524611713`, 87.39787096116856`}, {"CURE", 365, 90.19399665994848`},
{"CURE", 365, 79.62440888341503`}, {"CURE", 365, 58.683039712278564`}, {"CURE", 365, 125.5137650627667`},
{"CURE", 365, 10.470369021888393`}, {"TBUR", 78.09412295150155`, 171.89801952530988`}, {"CURE", 365, 71.02997091953755`},
{"CURE", 365, 6.80465210033289`}, {"CURE", 365, 32.882118411987484`}, {"CURE", 365, 207.9793550172507`},
{"CURE", 365, 82.2038906448821`}, {"CURE", 365, 147.99297754442983`}, {"CURE", 365, 85.75372894325086`},
{"CURE", 365, 170.92743913450394`}, {"CURE", 365, 158.70683819655838`}, {"TBUR", 73.87698371800374`, 31.41170973756782`},
{"CURE", 365, 70.17980572198843`}, {"TBUR", 131.3383211209146`, 69.16175890779355`},
{"TBUR", 17.902291656466623`, 16.98323467684268`}, {"TBUR", 24.655297528530284`, 9.28732262863866`},
{"CURE", 365, 67.26504202088609`}, {"CURE", 365, 102.42041441453917`}, {"TBUR", 53.25538078884255`, 54.452646429970024`},
{"CURE", 365, 157.1301520840708`}, {"CURE", 365, 146.44224393061492`}, {"TBUR", 18.91674505475628`, 18.843964408649512`},
{"TBUR", 25.30015504209476`, 38.2805328620718`}, {"CURE", 365, 21.618878499723316`},
{"TBUR", 26.28579656238581`, 114.21135698488523`}, {"TBUR", 44.021484512672764`, 99.27308390141067`},
{"CURE", 365, 120.8540214948818`}, {"TBUR", 76.93916395760834`, 157.91518192317986`},
{"CURE", 365, 41.19542661340587`}, {"CURE", 365, 114.7597030034508`}, {"TBUR", 27.608700015318465`, 96.95934970993164`},
{"TBUR", 27.2334086599007`, 6.377020420746193`}, {"CURE", 365, 159.85188179826025`}, {"CURE", 365, 70.31985890704043`},
{"TBUR", 39.067277477143286`, 3.6331613233366786`}, {"TBUR", 22.105248609061768`, 2.6408597088421732`},
{"CURE", 365, 58.572081525261225`}, {"CURE", 365, 83.74547447283422`}, {"CURE", 365, 97.92836899618452`},
{"TBUR", 39.318146361729795`, 63.29105658428266`}, {"CURE", 365, 92.62973792801208`}, {"CURE", 365, 131.5833751757947`},
{"CURE", 365, 69.90352633003887`}, {"TBUR", 95.35610650609961`, 32.77922991477194`}, {"CURE", 365, 114.909795523058`},
{"CURE", 365, 7.784454639436882`}, {"CURE", 365, 38.77602765252614`}, {"TBUR", 77.78649866006536`, 79.51004707345739`},
{"CURE", 365, 6.9511871668837735`}, {"TBUR", 45.006504859040085`, 55.20380218762177`},
{"CURE", 365, 75.75574695376378`}, {"TBUR", 34.49259039631198`, 87.41573086849964`}, {"CURE", 365, 53.77517387220787`},
{"TBUR", 21.081112724177366`, 62.78493912891982`}, {"CURE", 365, 79.94351743511892`},
{"TBUR", 16.377284463290785`, 97.55732476970414`}, {"CURE", 365, 85.28991399985792`},
{"TBUR", 16.330084503340288`, 24.451705439166048`}, {"TBUR", 64.61234896248514`, 76.77690559657637`},
{"CURE", 365, 162.45711804661764`}, {"CURE", 365, 6.605827974509758`}, {"CURE", 365, 68.41403259348527`},
{"CURE", 365, 36.5632523084522`}, {"CURE", 365, 97.97341944038307`}, {"CURE", 365, 43.50621912278467`},

{"TBUR", 50.113765018503365`, 118.12841638990614`}, {"TBUR", 77.99866631520601`, 77.62144468853002`},
{"CURE", 365, 150.48324397648037`}, {"CURE", 365, 50.99114496810487`}, {"CURE", 365, 192.82822309223266`},
{"CURE", 365, 128.69520537939246`}, {"CURE", 365, 107.64383912537653`}, {"CURE", 365, 124.51192110004591`},
{"TBUR", 134.782281044951`, 197.27835384412603`}, {"TBUR", 39.937177904959334`, 213.61015346649194`},
{"CURE", 365, 62.80043893321672`}, {"CURE", 365, 103.3752804726555`}, {"CURE", 365, 112.81056982892207`},
{"TBUR", 40.088417551207584`, 40.62221089243157`}, {"TBUR", 16.704023675223286`, 8.851779314755959`},
{"CURE", 365, 1.0859574429144798`}, {"CURE", 365, 80.85341567127682`}, {"CURE", 365, 130.3768740731722`},
{"TBUR", 20.370634355260663`, 106.5819816721453`}, {"CURE", 365, 147.35968099426913`},
{"CURE", 365, 197.0839047880504`}, {"CURE", 365, 51.25953537090469`}, {"CURE", 365, 110.10046637957073`},
{"TBUR", 42.45597845559633`, 8.269365931638331`}, {"CURE", 365, 216.9036924967579`},
{"TBUR", 36.817617052158674`, 70.81527321764366`}, {"TBUR", 58.19499452910366`, 50.37478169008764`},
{"TBUR", 65.20696951358455`, 60.04230618958128`}, {"CURE", 365, 203.09952340983264`}, {"CURE", 365, 81.85149069643404`},
{"TBUR", 92.15036265321025`, 185.23684006331644`}, {"CURE", 365, 135.23676959152695`}, {"CURE", 365, 122.46373035875465`},
{"TBUR", 54.65695592264323`, 120.21736411548423`}, {"TBUR", 87.7894972898788`, 122.93574543089323`},
{"CURE", 365, 56.66555461354573`}, {"CURE", 365, 48.572008658049555`}, {"CURE", 365, 18.69421759849265`},
{"CURE", 365, 85.2703224251024`}, {"CURE", 365, 100.3071547023844`}, {"TBUR", 34.20750278744805`, 55.41224006968941`},
{"TBUR", 44.28172494322984`, 71.00570355918886`}, {"CURE", 365, 136.56720132499407`},
{"TBUR", 41.9198847320278`, 64.96567821542368`}, {"CURE", 365, 73.94167482716945`}, {"CURE", 365, 40.89915419722368`},
{"TBUR", 20.995664400879647`, 12.712995020919534`}, {"CURE", 365, 52.572486837893486`}, {"CURE", 365, 154.4571126928O338`},
{"CURE", 365, 68.34334393958413`}, {"TBUR", 42.94783809686367`, 62.01613159579659`}, {"CURE", 365, 19.590705846112883`},
{"TBUR", 25.894866579018267`, 142.96480516662496`}, {"CURE", 365, 151.71293837074117`}, {"CURE", 365, 94.27261204774724`},
{"TBUR", 100.84153317565867`, 42.33263293320088`}, {"CURE", 365, 144.16095673692072`}, {"CURE", 365, 17.04238628998736`},
{"TBUR", 84.29798913174486`, 217.19536370256557`}, {"TBUR", 28.605965616619933`, 76.12734212449239`},
{"CURE", 365, 168.17116031832887`}, {"TBUR", 43.123625266414386`, 80.22265423569104`}, {"CURE", 365, 47.51299944916101`},
{"TBUR", 41.764734226665816`, 59.40086200576351`}, {"TBUR", 24.9935714854763`, 20.457680467139088`},
{"CURE", 365, 131.23228814505416`}, {"TBUR", 42.6701647219933`, 43.80397537039755`}, {"CURE", 365, 69.66061699464808`},
{"TBUR", 25.678296536420916`, 50.07180919867335`}, {"CURE", 365, 80.48943925487045`},
{"TBUR", 23.293875021499254`, 48.63746730816087`}, {"TBUR", 106.28451493398074`, 164.32576117526628`},
{"CURE", 365, 113.5922089933392`}, {"TBUR", 33.50439360811532`, 70.8189456099769`},
{"TBUR", 66.47684124329861`, 72.1085215279164`}, {"TBUR", 13.841151528894057`, 69.05294405172901`},
{"CURE", 365, 11.0292900067682`}, {"TBUR", 108.37035757625932`, 127.21498556979779`},

{"TBUR", 101.9167031495993`, 113.56407890595058`}, {"CURE", 365, 95.44301036991781`},
{"TBUR", 46.485162880720424`, 9.507198783051033`}, {"CURE", 365, 79.9216985303033`}, {"CURE", 365, 187.1879641730379`},
{"CURE", 365, 88.90211060150706`}, {"CURE", 365, 107.64351777325723`}, {"TBUR", 28.570509349182082`, 38.5524223162778`},
{"TBUR", 85.1977335919929`, 87.90591172721702`}, {"TBUR", 13.371669460544611`, 2.6738370619940617`},
{"TBUR", 15.47575286926437`, 14.932688137840254`}, {"TBUR", 52.870319990728554`, 131.35898876288687`},
{"CURE", 365, 83.16203573279661`}, {"TBUR", 51.4667048398256`, 72.16801936186658`}, {"CURE", 365, 30.136011490030345`},
{"TBUR", 218.16161217909232`, 153.06348083878567`}, {"CURE", 365, 82.77211647459056`}, {"CURE", 365, 156.5544433459689`},
{"TBUR", 56.43394545645672`, 24.52788169636867`}, {"TBUR", 30.415406883431654`, 89.49409659934047`},
{"CURE", 365, 72.38707732558785`}, {"CURE", 365, 87.45467859166205`}, {"CURE", 365, 22.40299377655504`},
{"CURE", 365, 63.30317652849553`}, {"CURE", 365, 141.50359599105573`}, {"TBUR", 20.77845847044545`, 88.75694829032557`},
{"CURE", 365, 47.677786323601744`}, {"TBUR", 44.314165115880854`, 3.41086664956513`}, {"CURE", 365, 50.78548935142725`},
{"CURE", 365, 141.60800658795324`}, {"CURE", 365, 57.92325027936823`}, {"CURE", 365, 85.33301987198949`},
{"TBUR", 15.90715871343625`, 103.5273601565576`}, {"TBUR", 88.3647395477889`, 85.64236691519268`},
{"TBUR", 86.34498073196903`, 155.59201216624663`}, {"CURE", 365, 137.48054146062609`},
{"CURE", 365, 98.54692044788386`}, {"CURE", 365, 105.1021125391888`}, {"CURE", 365, 146.14601747474973`},
{"TBUR", 19.831688519444203`, 6.031396551422335`}, {"TBUR", 79.45093897668472`, 149.3229667005811`},
{"TBUR", 148.39713650179476`, 52.176889158169885`}, {"TBUR", 66.04969871620452`, 211.63689963710533`},
{"CURE", 365, 12.611973036899307`}, {"TBUR", 91.53986787976514`, 77.81303490026494`}, {"CURE", 365, 177.50487583441682`},
{"CURE", 365, 8.147988076665607`}, {"CURE", 365, 113.03154529239016`}, {"TBUR", 94.98871677199544`, 93.63402895447295`},
{"TBUR", 33.39023826833429`, 115.02975623801115`}, {"TBUR", 15.480420770032481`, 59.28501267035198`},
{"TBUR", 13.888828942040481`, 31.865401359873115`}, {"TBUR", 18.432284684673295`, 8.715835034702582`},
{"CURE", 365, 83.64979140100178`}, {"CURE", 365, 82.67112810212062`}, {"TBUR", 37.21411175452077`, 100.16925036967837`},
{"CURE", 365, 15.823199949768163`}, {"CURE", 365, 221.28490488982385`}, {"CURE", 365, 45.07231089324706`},
{"CURE", 365, 87.20467229322766`}, {"CURE", 365, 54.29187564692055`}, {"CURE", 365, 8.382475175036154`},
{"TBUR", 115.35408217184775`, 149.10657159419864`}, {"CURE", 365, 21.70728408145223`},
{"CURE", 365, 64.21729232192799`}, {"TBUR", 19.19616610450291`, 32.046402580885555`}, {"CURE", 365, 67.65992809134319`},
{"TBUR", 71.87672176566474`, 226.29477493460274`}, {"CURE", 365, 154.83501747338943`},
{"TBUR", 36.97685907567977`, 96.17676700174583`}, {"CURE", 365, 86.92475076039688`}, {"CURE", 365, 138.99077251621344`},
{"CURE", 365, 168.25887615975472`}, {"CURE", 365, 132.24219086115485`}, {"CURE", 365, 171.33257006701166`},
{"TBUR", 14.380106892518919`, 56.98449832472873`}, {"CURE", 365, 68.58722673021741`},
{"TBUR", 25.03508385917365`, 10.299499288809832`}, {"TBUR", 18.80787673873666`, 81.14244559056925`},

{"TBUR", 66.51142689764067`, 4.467677874043376`}, {"CURE", 365, 1.1245814659599807`}, {"CURE", 365, 7.361951317073146`},
{"TBUR", 151.91122027818096`, 0.8187708500324089`}, {"TBUR", 18.786663516733544`, 78.79337780915662`},
{"CURE", 365, 28.319160857305672`}, {"CURE", 365, 65.5571997079320 9`}, {"CURE", 365, 163.01236646816918`},
{"CURE", 365, 42.61573534217873`}, {"TBUR", 20.501725384749744`, 77.82439854669336`},
{"TBUR", 13.246780593431472`, 55.28746155589619`}, {"CURE", 365, 29.08018325134224`},
{"TBUR", 62.56116648409728`, 106.3199539458487`}, {"TBUR", 34.575614493599836`, 21.954189662941268`},
{"TBUR", 64.25137066388106`, 2.073055390589427`}, {"TBUR", 45.84815081123349`, 8.07056671242937`},
{"CURE", 365, 161.35865207507254`}, {"CURE", 365, 32.899987958388564`}, {"CURE", 365, 161.7596136601562`},
{"TBUR", 172.31326433499623`, 103.20661352189283`}, {"CURE", 365, 144.7706044078737`},
{"CURE", 365, 51.96337909260407`}, {"CURE", 365, 197.6103838086829`}, {"CURE", 365, 86.21333312961245`},
{"TBUR", 26.61918240798909`, 7.655080118190356`}, {"CURE", 365, 145.94764018256626`},
{"TBUR", 27.178401872588562`, 30.154925452119095`}, {"TBUR", 67.52434365656646`, 114.69364821520641`},
{"TBUR", 75.18873196956768`, 142.44002731559644`}, {"TBUR", 64.29392025273725`, 43.89937251697265`},
{"CURE", 365, 100.75788419593599`}, {"TBUR", 15.58279392820622`, 33.97748671779751`},
{"TBUR", 32.43045726967164`, 69.93320522864732`}, {"CURE", 365, 122.44385621142547`},
{"CURE", 365, 52.3145092147567`}, {"TBUR", 30.237744698600853`, 75.37011404290038`}, {"CURE", 365, 45.41362321443087`},
{"TBUR", 77.20008329884453`, 145.59670817958772`}, {"TBUR", 52.21348068352795`, 92.12742179631867`},
{"CURE", 365, 92.9418638198554`}, {"TBUR", 77.70830705755009`, 126.56414571089283`}, {"CURE", 365, 57.75144343320001`},
{"CURE", 365, 83.42202671799011`}, {"CURE", 365, 134.08298952441424`}, {"CURE", 365, 7.7870624874990595`},
{"TBUR", 18.653009593228493`, 11.718672278180527`}, {"TBUR", 145.12195045405434`, 56.83970720952676`},
{"TBUR", 57.209313082068576`, 187.52614886523872`}, {"CURE", 365, 21.715315938115527`},
{"TBUR", 112.92585080504327`, 174.15935995809633`}, {"CURE", 365, 77.1126034382561`}, {"CURE", 365, 184.4919308536166`},
{"TBUR", 15.725201823973837`, 46.102791787796754`}, {"TBUR", 39.797412871273195`, 61.74906637145882`},
{"CURE", 365, 90.45052233290619`}, {"CURE", 365, 189.87506796301955`}, {"TBUR", 85.07094358306321`, 85.86782062310618`},
{"CURE", 365, 214.12215480333055`}, {"CURE", 365, 49.21757435836811`}, {"CURE", 365, 112.53135465268458`},
{"CURE", 365, 86.00344882813415`}, {"CURE", 365, 97.78311595884739`}, {"TBUR", 63.75117988658032`, 94.04133008082319`},
{"TBUR", 44.92407318365596`, 99.86555495456739`}, {"CURE", 365, 11.140576380565607`}, {"CURE", 365, 37.62331571218177`},
{"TBUR", 82.38852110460391`, 109.15306117864533`}, {"CURE", 365, 95.05561433084829`}, {"CURE", 365, 16.4660728473915`},
{"TBUR", 76.83104521315902`, 107.38895208884651`}, {"CURE", 365, 51.35748710213723`}, {"CURE", 365, 18.701349165543643`},
{"CURE", 365, 18.6899960077825`}, {"CURE", 365, 29.984895455196934`}, {"CURE", 365, 39.76354424298787`},
{"TBUR", 67.3384916561459`, 92.08574677602617`}, {"CURE", 365, 139.3188113894687`}, {"CURE", 365, 163.81857969137675`},

{"TBUR", 31.804211263859123`, 117.18553477196244`}, {"TBUR", 73.10953706899319`, 8.357332572431785`},
{"CURE", 365, 197.96079469057233`}, {"TBUR", 102.56267001148215`, 72.24330379562231`},
{"TBUR", 39.36197314138915`, 98.10075745106256`}, {"TBUR", 123.9756218657646`, 29.450544963614952`},
{"CURE", 365, 83.31041585216806`}, {"CURE", 365, 8.840584525246095`}, {"CURE", 365, 139.77259858340963`},
{"CURE", 365, 157.3653608437048`}, {"CURE", 365, 85.49753665554431`}, {"CURE", 365, 41.1510756240354`},
{"CURE", 365, 71.68138571983167`}, {"TBUR", 123.12699714961886`, 109.75088837308196`},
{"TBUR", 90.95506975914397`, 117.93799950303301`}, {"TBUR", 41.08205852365916`, 67.14257665814692`},
{"CURE", 365, 94.28138842120667`}, {"CURE", 365, 66.10268874068137`}, {"TBUR", 22.92721350715727`, 165.7490413900579`},
{"TBUR", 80.74440073176544`, 57.66559229040317`}, {"CURE", 365, 60.35238769226915`},
{"TBUR", 109.20481464477454`, 90.83505592512849`}, {"CURE", 365, 132.63563300235862`}, {"CURE", 365, 4.205999257378055`},
{"TBUR", 55.41754918313941`, 56.309523781413915`}, {"CURE", 365, 91.63031879149545`}, {"CURE", 365, 22.536220236489346`},
{"CURE", 365, 166.13999215017378`}, {"TBUR", 16.02175117656528`, 8.137307107576179`}, {"CURE", 365, 163.16058674240162`},
{"CURE", 365, 149.63539012619879`}, {"TBUR", 112.25449012941453`, 3.7731818191680646`},
{"CURE", 365, 127.22979092735828`}, {"CURE", 365, 57.572172316782265`}, {"CURE", 365, 66.95121035439992`},
{"CURE", 365, 140.7392055447695`}, {"CURE", 365, 14.912804524745058`}, {"TBUR", 21.015237689638607`, 82.34072450500564`},
{"CURE", 365, 33.12142864408459`}, {"TBUR", 87.67770911284435`, 2.7686236446043924`}, {"CURE", 365, 85.46675090108755`},
{"TBUR", 74.62154260133295`, 131.93811816045576`}, {"TBUR", 171.4433325379737`, 38.8525254790387`},
{"CURE", 365, 0.7276886958990086`}, {"TBUR", 96.14524371599052`, 9.796804268029234`},
{"TBUR", 30.590303884885547`, 66.12247744736845`}, {"CURE", 365, 2.5077434480483456`},
{"CURE", 365, 6.125474946875358`}, {"CURE", 365, 121.63045901439574`}, {"TBUR", 75.30465853732409`, 206.83103943613435`},
{"TBUR", 89.56197859304015`, 108.58621728103236`}, {"CURE", 365, 201.8487316917484`},
{"TBUR", 74.23615746642272`, 100.45660258962388`}, {"CURE", 365, 5.507062238819746`}, {"CURE", 365, 105.64589032228592`},
{"TBUR", 89.86693799412025`, 154.0635119373383`}, {"TBUR", 38.67655680596958`, 80.47369110319504`},
{"TBUR", 71.34218237519057`, 14.322398310932414`}, {"TBUR", 57.90472414900863`, 69.67050794074858`},
{"CURE", 365, 46.291843504736015`}, {"CURE", 365, 27.682749348977243`}, {"CURE", 365, 26.21099351790934`},
{"CURE", 365, 122.15971573228185`}, {"TBUR", 127.53909303494643`, 55.162121229024635`},
{"TBUR", 25.344101126162084`, 49.762745143980254`}, {"CURE", 365, 71.06460572927949`},
{"TBUR", 74.05249736215876`, 14.221914657314924`}, {"CURE", 365, 106.1566821918568`}, {"CURE", 365, 56.503995048255945`},
{"TBUR", 63.78656107701856`, 60.38197922049323`}, {"TBUR", 50.91765379865005`, 138.00644430551972`},
{"CURE", 365, 179.4992289975075`}, {"CURE", 365, 61.92185051509873`}, {"CURE", 365, 10.646298798608878`},
{"TBUR", 79.59185859932852`, 211.34783032227153`}, {"TBUR", 155.84211861617845`, 76.66391249318802`},

{"TBUR", 57.228268031244326`, 64.16575104828713`}, {"CURE", 365, 86.21585066381947`},
{"CURE", 365, 25.70196230612769`}, {"CURE", 365, 79.18980096971991`}, {"CURE", 365, 74.16500718979148`},
{"TBUR", 119.17965985885313`, 75.03328955284546`}, {"CURE", 365, 134.5377721113537`}, {"CURE", 365, 56.858311390884644`},
{"TBUR", 165.7714753156173`, 100.11673502534`}, {"CURE", 365, 47.44252204370091`}, {"CURE", 365, 130.91684785233008`},
{"CURE", 365, 60.91300609070352`}, {"TBUR", 85.71721286045008`, 80.54019987532239`}, {"CURE", 365, 40.9588550455884`},
{"TBUR", 82.3873352945812`, 124.8465644346635`}, {"TOX", 25.253020921279425`, 235.20620505058852`},
{"TBUR", 42.023695517930015`, 115.86923529647662`}, {"CURE", 365, 112.30332129770078`}, {"CURE", 365, 44.14851697385713`},
{"TBUR", 121.24044932290181`, 92.58488139195758`}, {"TBUR", 57.5558452826561`, 174.8433192443987`},
{"CURE", 365, 4.685832527144529`}, {"CURE", 365, 111.63118676277412`}, {"TBUR", 72.87571612741998`, 11.336048374732663`},
{"CURE", 365, 31.967327327289393`}, {"CURE", 365, 13.566877826932831`}, {"TBUR", 48.58992709176588`, 60.36133123827985`},
{"CURE", 365, 3.827798264950585`}, {"TBUR", 32.70416405548812`, 80.51328664023653`}, {"CURE", 365, 74.85893615974211`},
{"TBUR", 80.48722126242468`, 91.57877749859766`}, {"TBUR", 34.41915184626374`, 56.05885921934018`},
{"CURE", 365, 99.05830856482788`}, {"CURE", 365, 166.7629532023069`}, {"CURE", 365, 82.47094394358865`},
{"CURE", 365, 26.60273255105521`}, {"CURE", 365, 87.74072846737752`}, {"TBUR", 118.25619102098453`, 7.906582364189719`},
{"TBUR", 68.93714219671762`, 7.727754743248773`}, {"CURE", 365, 143.0797061853075`},
{"CURE", 365, 133.60260766950842`}, {"TBUR", 82.54718424914033`, 32.95014183775475`},
{"CURE", 365, 45.88722922843023`}, {"TBUR", 18.28298956991749`, 92.78634582700967`},
{"CURE", 365, 162.11608825358132`}, {"TBUR", 133.43048492143546`, 53.650605181307625`},
{"TBUR", 58.36753790327382`, 74.06662155587568`}, {"CURE", 365, 11.260522659049139`},
{"TBUR", 75.76274633398482`, 81.53026031157398`}, {"CURE", 365, 70.18302049499023`}, {"CURE", 365, 85.99342979440259`},
{"TBUR", 70.02466056321981`, 59.42947878846585`}, {"TBUR", 73.86773636841073`, 29.09174973313069`},
{"CURE", 365, 97.9941053797608`}, {"TBUR", 14.67107355760249`, 55.21826828070117`},
{"CURE", 365, 28.177563561887514`}, {"CURE", 365, 108.05133886923434`}, {"CURE", 365, 192.43146385175558`},
{"CURE", 365, 154.48370326737742`}, {"CURE", 365, 88.6526921453624`}, {"CURE", 365, 110.24857144683784`},
{"TBUR", 45.95703484497892`, 11.129178581972738`}, {"TBUR", 42.974449238608166`, 94.44335950274176`},
{"TBUR", 69.84790546740776`, 17.934190313151113`}, {"CURE", 365, 66.70514103765865`},
{"TBUR", 25.942686539473147`, 97.01544343079291`}, {"CURE", 365, 26.915078479893285`}, {"CURE", 365, 72.50301511615768`},
{"TBUR", 21.14050127246823`, 30.787265024481865`}, {"TOX", 47.62547933360227`, 231.38742143554012`},
{"TBUR", 14.665549724464121`, 16.720811001409498`}, {"CURE", 365, 104.5715411446652`},
{"TBUR", 45.2257971843532`, 30.223993138062983`}, {"TBUR", 110.34995206251878`, 19.35510435339236`},
{"CURE", 365, 4.50094464552359`}, {"CURE", 365, 4.695432088006058`}, {"CURE", 365, 83.09049750492615`},

{"TBUR", 27.804552535226275`, 53.910057665251934`}, {"CURE", 365, 19.46918942831307`}, {"CURE", 365, 78.02443269467689`},
{"CURE", 365, 111.7930777705215`}, {"CURE", 365, 204.21942914767743`}, {"CURE", 365, 71.56708352311703`},
{"CURE", 365, 106.79578348619006`}, {"TBUR", 40.958459196693575`, 97.62533690048785`}, {"CURE", 365, 21.430459077263063`},
{"TBUR", 25.2732180500390 8`, 46.15979989920306`}, {"CURE", 365, 36.342584851405064`}, {"CURE", 365, 103.68248978725254`},
{"TBUR", 42.493636655701906`, 91.76037881181253`}, {"CURE", 365, 67.39331082947625`}, {"CURE", 365, 3.811796751844552`},
{"CURE", 365, 26.41473774345793`}, {"CURE", 365, 71.4182638045728`}, {"TBUR", 41.8995589344309`, 81.5642812562694 1`},
{"CURE", 365, 112.19500919469476`}, {"TBUR", 107.56742388499633`, 72.30205756903425`}, {"CURE", 365, 206.87292418530814`},
{"CURE", 365, 93.4207592737272`}, {"TBUR", 18.23691067980992`, 77.4083865514879`}, {"CURE", 365, 1.188866862454907`},
{"CURE", 365, 43.9951996907111`}, {"CURE", 365, 156.39826372029873`}, {"CURE", 365, 6.6643379760427415`},
{"TBUR", 47.26774955847277`, 63.3952180267111`}, {"TBUR", 50.203879101035184`, 96.50794564496292`},
{"TBUR", 87.41022675849055`, 178.540937152053`}, {"TBUR", 88.46351419883598`, 117.81284796723354`},
{"CURE", 365, 29.569755656530106`}, {"TBUR", 81.34933392893072`, 9.185428957777809`},
{"CURE", 365, 67.44750223824597`}, {"CURE", 365, 162.63026525464437`}, {"TBUR", 32.7577202312344`, 97.21094919842982`},
{"CURE", 365, 8.76105938451963`}, {"TBUR", 54.69826229947602`, 95.95711489989866`},
{"CURE", 365, 88.6942922965054`}, {"TBUR", 78.20213728690189`, 7.510163500867667`},
{"CURE", 365, 71.89804357241758`}, {"CURE", 365, 90.44952420224237`}, {"CURE", 365, 55.152023982624904`},
{"TOX", 19.92508935753809`, 257.7009462056286`}, {"TBUR", 62.85871894217623`, 77.05162398486014`},
{"CURE", 365, 96.7572552324731`}, {"TBUR", 24.682125721211605`, 74.92505416663994`}, {"CURE", 365, 209.59203129859063`},
{"CURE", 365, 84.46382549447628`}, {"TBUR", 19.744076397209856`, 73.53147487777295`}, {"CURE", 365, 52.770564165594884`},
{"TBUR", 58.93840820450825`, 107.33470144019901`}, {"CURE", 365, 0.9424023720928213`},
{"TBUR", 45.051641541669255`, 26.597300646013366`}, {"TBUR", 146.10749629543488`, 104.24859532804412`},
{"CURE", 365, 87.1884627970988`}, {"CURE", 365, 25.19615497832362`}, {"CURE", 365, 69.4666784515227`},
{"CURE", 365, 48.54907404881904`}, {"TBUR", 71.65048730729733`, 50.57362600140968`}, {"CURE", 365, 15.245613940368688`},
{"CURE", 365, 61.49743149849963`}, {"CURE", 365, 52.198749304523034`}, {"TBUR", 27.488822370373118`, 35.424609310160406`},
{"CURE", 365, 158.08458036674202`}, {"CURE", 365, 4.452340806882482`}, {"CURE", 365, 72.47774322088368`},
{"TBUR", 162.45577275043803`, 112.471267470159`}, {"TBUR", 16.5636309532337`, 118.90116787715942`},
{"TBUR", 48.150102299646`, 8.89923838117529`}, {"CURE", 365, 96.6265247064224`}, {"CURE", 365, 110.5508774395454`},
{"TBUR", 29.806616703912738`, 3.913196880049991`}, {"CURE", 365, 70.29610499619476`},
{"CURE", 365, 95.13295860390147`}, {"TBUR", 23.282785085031787`, 22.721866937476378`},
{"CURE", 365, 2.162947258072236`}, {"TBUR", 66.62096424725432`, 179.3255837269688`},
{"TBUR", 113.58208080881195`, 99.39796324951`}, {"CURE", 365, 95.65212120510144`}, {"CURE", 365, 31.752538865394214`},

```
{"TBUR", 84.39127838818516`, 3.0755948379244646`}, {"CURE", 365, 10.614914998432253`},
{"TBUR", 13.281110495605732`, 19.625047727878513`}, {"TBUR", 84.50655512146528`, 80.999720216658`},
{"CURE", 365, 132.3770526902393`}, {"CURE", 365, 110.20890112718`}, {"CURE", 365, 54.67733184156940 5`},
{"TBUR", 22.825752527171478`, 2.573333916696971`}, {"CURE", 365, 85.46033523376916`}, {"CURE", 365, 93.33630065906333`},
{"TBUR", 71.33263624638562`, 1.6260507210388262`}, {"TBUR", 76.71046291901257`, 55.108250765831784`},
{"CURE", 365, 1.0604722426548538`}, {"CURE", 365, 148.4403666025348`}, {"CURE", 365, 66.49684311242487`},
{"TBUR", 14.6940357064607`, 0.7789373015899729`}, {"TBUR", 163.32114337910238`, 90.77628777699512`},
{"CURE", 365, 35.48250711271569`}, {"TOX", 31.963297990721003`, 231.43855452219623`}, {"CURE", 365, 132.9991124540927`},
{"TBUR", 41.77367087090102`, 71.8635715661327`}, {"CURE", 365, 188.46839209239508`}},
{{"460 Res", "460 OS", "460 Tox"}, {"TBUR", 21.73843568780277`, 41.92033272923493`}, {"CURE", 365, 155.0717283902031`},
{"TBUR", 27.069937648655596`, 119.57978315679883`}, {"TBUR", 30.311867447069318`, 7.139916868922963`},
{"TBUR", 18.21635633275636`, 18.984497104329336`}, {"CURE", 365, 33.79154169193708`}, {"CURE", 365, 67.91433869207042`},
{"CURE", 365, 65.3440341745725`}, {"CURE", 365, 151.7421289766571`}, {"TBUR", 70.75302723191012`, 202.24975364347978`},
{"TBUR", 54.634808168027774`, 103.86498293521159`}, {"CURE", 365, 94.94020228832731`},
{"TOX", 16.106708380495853`, 271.3256128702558`}, {"TBUR", 43.26289069364511`, 66.22054017620279`},
{"TBUR", 67.40558325830052`, 6.610592958905187`}, {"TBUR", 88.37000177046173`, 33.31538187800076`},
{"CURE", 365, 105.11024321936479`}, {"TBUR", 44.73504017422189`, 120.70849833971398`},
{"TBUR", 45.582383087098556`, 8.133286713016869`}, {"CURE", 365, 91.00474189106355`}, {"CURE", 365, 141.09377661810726`},
{"CURE", 365, 55.00152886614763`}, {"CURE", 365, 33.711172165524914`}, {"CURE", 365, 69.63849206772791`},
{"CURE", 365, 63.47320754122287`}, {"CURE", 365, 168.75658423393637`}, {"CURE", 365, 108.63569482414135`},
{"CURE", 365, 54.7892420287165`}, {"CURE", 365, 37.95104517637579`}, {"CURE", 365, 11.16386031979073`},
{"CURE", 365, 143.36095362663963`}, {"TBUR", 13.441288062515598`, 37.478054508000405`}, {"CURE", 365, 72.63628266450763`},
{"TBUR", 48.81475108417904`, 44.16606701288138`}, {"CURE", 365, 191.13786162616609`}, {"CURE", 365, 221.81379925998635`},
{"CURE", 365, 45.656893603355904`}, {"TBUR", 21.686073107084383`, 11.530035864826099`},
{"TBUR", 72.08265306551961`, 53.62341822202024`}, {"CURE", 365, 98.49748973484786`},
{"TBUR", 18.063755216931373`, 64.51169088691904`}, {"CURE", 365, 103.98620437309673`}, {"CURE", 365, 75.88932468590976`},
{"TBUR", 45.97636871744456`, 81.19023914506366`}, {"CURE", 365, 99.68310742093342`}, {"CURE", 365, 5.105484342807204`},
{"CURE", 365, 111.36144074902181`}, {"CURE", 365, 70.9364505652878`}, {"TBUR", 112.66698777470656`, 134.70113052248013`},
{"TOX", 39.93887227394403`, 231.9056301381912`}, {"TBUR", 29.71433355010109`, 102.83484090908546`},
{"TBUR", 41.75032389653955`, 88.280127332977`}, {"CURE", 365, 6.668788252872129`}, {"CURE", 365, 143.79383988143664`},
{"CURE", 365, 52.101711358875534`}, {"CURE", 365, 101.05298455961804`}, {"CURE", 365, 74.8560632754609`},
```

{"TBUR", 103.381720991187`, 90.4712255815032`}, {"TBUR", 37.50979391849824`, 175.29138446008074`},
{"TBUR", 52.56334722481858`, 103.51037388762074`}, {"CURE", 365, 95.55749034401957`}, {"CURE", 365, 4.728344699822509`},
{"TBUR", 33.612954685458924`, 117.71209858397732`}, {"CURE", 365, 88.37709967166943`},
{"TBUR", 74.73906150881689`, 7.253145435142288`}, {"CURE", 365, 92.11098687515374`}, {"CURE", 365, 84.51112718466432`},
{"TBUR", 57.42857150977144`, 128.02283008388946`}, {"CURE", 365, 46.54540532755852`}, {"CURE", 365, 39.74198674049289`},
{"CURE", 365, 72.9390890889253`}, {"CURE", 365, 54.026773650961665`}, {"CURE", 365, 146.85967755121897`},
{"TBUR", 13.503977900330216`, 2.3532404425897497`}, {"TBUR", 79.60234331803849`, 95.75693267834636`},
{"CURE", 365, 125.34401007450627`}, {"TBUR", 97.0085195145681`, 223.9531820986298`}, {"CURE", 365, 74.44081401401507`},
{"TBUR", 75.83698957281042`, 144.90870678106634`}, {"CURE", 365, 61.914709862299304`}, {"CURE", 365, 117.8554840701021`},
{"CURE", 365, 3.8451481799037097`}, {"CURE", 365, 7.375377336855351`}, {"CURE", 365, 5.263444391300268`},
{"CURE", 365, 136.13708418790927`}, {"TBUR", 99.61668830451254`, 104.13136568254833`}, {"CURE", 365, 99.07029120068835`},
{"CURE", 365, 49.88207848608857`}, {"CURE", 365, 8.00996565075459`}, {"TBUR", 71.42261091991946`, 159.7245686578315`},
{"TBUR", 33.27974701751269`, 15.523111422745165`}, {"TBUR", 89.32465189580725`, 113.08773418958846`},
{"TBUR", 70.79443868258934`, 3.0682830738981153`}, {"CURE", 365, 7.094095025617768`},
{"TBUR", 19.544490116273717`, 2.2641871107645932`}, {"TBUR", 158.7464959635379`, 61.902915351112725`},
{"CURE", 365, 47.03746973586045`}, {"CURE", 365, 203.49232069422274`}, {"CURE", 365, 11.131919449957877`},
{"TBUR", 43.8036672075135`, 163.6326209426125`}, {"TBUR", 20.920699465284123`, 12.576645619400546`},
{"TBUR", 127.3880848667527`, 145.87528545392695`}, {"CURE", 365, 80.28236191444365`},
{"TBUR", 91.1140457876344`, 101.34045594895612`}, {"CURE", 365, 73.2947142864054`}, {"CURE", 365, 118.49181618174106`},
{"CURE", 365, 49.29486359690241`}, {"TBUR", 139.32364314736662`, 96.60162590571522`}, {"CURE", 365, 45.50418857985468`},
{"CURE", 365, 117.87509122446127`}, {"CURE", 365, 160.71606115458027`}, {"CURE", 365, 87.35267878499272`},
{"CURE", 365, 28.612269312072623`}, {"CURE", 365, 58.83016116616137`}, {"CURE", 365, 78.2719294158119`},
{"CURE", 365, 66.81782498422572`}, {"CURE", 365, 21.537126926963733`}, {"CURE", 365, 88.84895142253788`},
{"CURE", 365, 94.95309403038206`}, {"CURE", 365, 29.49982884853668`}, {"CURE", 365, 91.78717339720586`},
{"CURE", 365, 184.48992931822562`}, {"CURE", 365, 118.63243345413034`}, {"TBUR", 20.274239425361348`, 6.171701109864585`},
{"CURE", 365, 82.37114836758519`}, {"TBUR", 39.02227251530124`, 76.28984891639907`},
{"CURE", 365, 70.29522305521719`}, {"CURE", 365, 44.717377822482774`}, {"CURE", 365, 134.90256713047216`},
{"CURE", 365, 129.53395025634177`}, {"CURE", 365, 113.94530525868025`}, {"CURE", 365, 124.36894330354147`},
{"TBUR", 71.88781661223038`, 45.21118789059116`}, {"TBUR", 46.07232440797163`, 48.768899351260906`},
{"CURE", 365, 181.6858120311094`}, {"TBUR", 12.7773992347504`, 79.08271687581733`}, {"CURE", 365, 26.28172167717209`},
{"TBUR", 62.57429062330509`, 64.19336387282725`}, {"CURE", 365, 20.536521181612148`}, {"CURE", 365, 122.37597149505858`},

{"TBUR", 53.09499003539689`, 183.61964398371651`}, {"CURE", 365, 127.70254096697828`},
{"CURE", 365, 170.8213748336953`}, {"CURE", 365, 40.8116607744828`}, {"CURE", 365, 17.231733198722367`},
{"CURE", 365, 78.66584095210126`}, {"CURE", 365, 182.14456861253328`}, {"TBUR", 37.81495773244886`, 48.13793032950125`},
{"CURE", 365, 84.76176603191341`}, {"CURE", 365, 22.667245960521697`}, {"CURE", 365, 8.563956356263688`},
{"CURE", 365, 74.34464138848071`}, {"CURE", 365, 1.3335668422951261`}, {"TBUR", 88.80336130952969`, 184.0576084170542`},
{"TBUR", 50.271431035995626`, 80.06293655088876`}, {"TBUR", 88.4257249164046`, 165.89163320036317`},
{"CURE", 365, 84.95070143577728`}, {"CURE", 365, 98.58581538687702`}, {"CURE", 365, 77.45697048335478`},
{"CURE", 365, 98.30416690607798`}, {"CURE", 365, 33.051673941877574`}, {"TBUR", 103.70002383730225`, 24.463846673423777`},
{"CURE", 365, 1.8705720753967463`}, {"TBUR", 70.68344123130497`, 16.505305239573904`},
{"TBUR", 78.93236405937864`, 87.60108323807836`}, {"CURE", 365, 124.31958782980502`}, {"CURE", 365, 125.33808918803936`},
{"CURE", 365, 92.29093704836738`}, {"CURE", 365, 128.61852122275332`}, {"CURE", 365, 2.0130663591635667`},
{"CURE", 365, 74.00753498878855`}, {"TBUR", 29.848408476265394`, 140.81467334798958`}, {"CURE", 365, 65.49399919715357`},
{"TBUR", 21.469069836326312`, 7.76478744561081`}, {"TBUR", 50.3242700466488`, 139.19910606860742`},
{"TBUR", 93.2911751502414`, 11.33405621054103`}, {"TBUR", 68.01792027545082`, 171.10323516748664`},
{"TBUR", 21.240289550171376`, 87.44674303809303`}, {"TBUR", 60.276608746438335`, 51.60498917572625`},
{"TBUR", 91.2294661508954`, 108.77442701996446`}, {"TBUR", 80.45596689191372`, 121.60941002449393`},
{"CURE", 365, 90.84058916029971`}, {"CURE", 365, 157.7749299068957`}, {"TBUR", 21.109229068991752`, 12.449407720217195`},
{"CURE", 365, 141.1018186006203`}, {"TBUR", 28.090102851792338`, 48.570445070998964`},
{"TBUR", 53.6343841916008`, 154.65708142954205`}, {"CURE", 365, 79.57298385199573`}, {"CURE", 365, 4.0887661829543`},
{"TBUR", 24.375415070781536`, 102.48532550546476`}, {"CURE", 365, 99.20933939887324`},
{"CURE", 365, 154.0227209201493`}, {"CURE", 365, 182.55592191396218`}, {"CURE", 365, 120.70850587199689`},
{"CURE", 365, 72.84478999670999`}, {"CURE", 365, 56.21353135891648`}, {"CURE", 365, 189.96583188415286`},
{"TBUR", 13.811281938172938`, 16.603940737315593`}, {"CURE", 365, 106.25311656211653`}, {"CURE", 365, 99.16440877836803`},
{"TBUR", 28.489857856297824`, 25.23256680715187`}, {"TBUR", 57.20569396627706`, 108.33217831048162`},
{"CURE", 365, 113.95869201244463`}, {"TBUR", 65.26472296255524`, 84.81083532931746`},
{"CURE", 365, 1.9090707487458696`}, {"TBUR", 143.46940459102342`, 37.95826627507041`},
{"TBUR", 15.921600137780612`, 94.24006983941456`}, {"CURE", 365, 189.88182520785978`}, {"CURE", 365, 18.754782999356518`},
{"CURE", 365, 139.40407848562543`}, {"CURE", 365, 3.1225061860466785`}, {"CURE", 365, 97.33412839188962`},
{"CURE", 365, 24.57544346675131`}, {"CURE", 365, 119.03942440828568`}, {"CURE", 365, 32.162946637573796`},
{"TBUR", 49.195009422809505`, 15.058626422177127`}, {"TBUR", 80.06090341069434`, 168.73596478997658`},
{"CURE", 365, 81.50133629180839`}, {"TBUR", 50.14802610334132`, 219.3658956270596`}, {"CURE", 365, 77.62076296694075`},

{"CURE", 365, 111.09687854674812`}, {"CURE", 365, 18.091925657493707`}, {"CURE", 365, 76.68539375769141`},
{"CURE", 365, 146.62930340696175`}, {"CURE", 365, 151.21642012511458`}, {"CURE", 365, 74.2421191653727`},
{"CURE", 365, 135.51839703582957`}, {"CURE", 365, 87.80712014777346`}, {"CURE", 365, 9.228462558219508`},
{"CURE", 365, 59.19407523156449`}, {"CURE", 365, 52.83971613569912`}, {"CURE", 365, 76.27122650364623`},
{"CURE", 365, 111.71508140153296`}, {"CURE", 365, 92.656062839772`}, {"CURE", 365, 86.12940752426191`},
{"TBUR", 22.84534816973437`, 110.06999712715822`}, {"TBUR", 28.14864181900384`, 73.74514270316965`},
{"TBUR", 156.74149520167686`, 76.47654191375949`}, {"TBUR", 21.593461522617012`, 52.2307676695029`},
{"CURE", 365, 65.39839883874433`}, {"CURE", 365, 76.56451487434644`}, {"CURE", 365, 134.55160947479567`},
{"TBUR", 27.535038298203006`, 6.218096150844694`}, {"TBUR", 32.078623654762005`, 28.831263098770066`},
{"CURE", 365, 1.7709022584944538`}, {"CURE", 365, 2.6207812972808133`}, {"TBUR", 21.325625370124722`, 23.836542412149704`},
{"CURE", 365, 213.25054790445202`}, {"CURE", 365, 61.77954665513329`}, {"CURE", 365, 209.50660204820682`},
{"TBUR", 38.87813918386166`, 102.70277485157756`}, {"CURE", 365, 75.67297076831605`},
{"TBUR", 15.304180237852856`, 126.35268401346026`}, {"CURE", 365, 58.44304694440637`},
{"CURE", 365, 107.3432884853412`}, {"CURE", 365, 13.84009951956083`}, {"CURE", 365, 80.4922211348962`},
{"CURE", 365, 32.37817633554512`}, {"CURE", 365, 9.72160757831791`}, {"CURE", 365, 128.04155306581572`},
{"TBUR", 21.70846647020678`, 129.38541670856995`}, {"TBUR", 13.746629340916744`, 93.55934433072223`},
{"CURE", 365, 64.90399228454123`}, {"TBUR", 28.04588304263847`, 87.03084580360442`},
{"CURE", 365, 11.236358272761441`}, {"CURE", 365, 66.74129100160292`}, {"CURE", 365, 59.794858159151474`},
{"CURE", 365, 220.33128550842684`}, {"CURE", 365, 99.52007378887173`}, {"CURE", 365, 50.94417472931513`},
{"CURE", 365, 1.687735649192973`}, {"TBUR", 70.09620096433413`, 95.05346700643938`}, {"CURE", 365, 79.7644727099079`},
{"TBUR", 151.45443457766507`, 83.64011515707254`}, {"TBUR", 59.83367421703959`, 19.775495679119032`},
{"CURE", 365, 89.8216909147257`}, {"CURE", 365, 61.463488134510555`}, {"TBUR", 20.687923692057293`, 77.69516024172336`},
{"CURE", 365, 48.49891587377812`}, {"CURE", 365, 109.67751390178162`}, {"CURE", 365, 24.360632521557886`},
{"TBUR", 97.89286253266609`, 103.98353577013731`}, {"TBUR", 16.611148331444017`, 41.96502718308448`},
{"CURE", 365, 84.28472169413685`}, {"TBUR", 27.439556158334998`, 103.23869066971965`},
{"CURE", 365, 9.381236687702643`}, {"CURE", 365, 123.60634931032513`}, {"CURE", 365, 73.79036777388349`},
{"CURE", 365, 205.82919984865126`}, {"CURE", 365, 140.7214559043479`}, {"CURE", 365, 99.70117043486565`},
{"CURE", 365, 64.4048262764909`}, {"CURE", 365, 207.56076748744056`}, {"TBUR", 31.189476243450482`, 35.24722851026625`},
{"CURE", 365, 18.748785702121367`}, {"CURE", 365, 38.80278489837227`}, {"CURE", 365, 98.5771499238061`},
{"CURE", 365, 93.69152561104907`}, {"CURE", 365, 117.36691924366538`}, {"CURE", 365, 43.50317337956274`},
{"TBUR", 23.27741644049348`, 150.7141838764897`}, {"CURE", 365, 7.89598348037146`}, {"CURE", 365, 129.32329545783503`},

{"TBUR", 38.65002056591809`, 151.37864596408272`}, {"CURE", 365, 68.45991638122861`}, {"CURE", 365, 125.1404077861486`},
{"CURE", 365, 4.104614701349822`}, {"TBUR", 22.56951911919373`, 50.77204687139605`}, {"CURE", 365, 75.54276327792213`},
{"CURE", 365, 77.00503795309201`}, {"TBUR", 29.35360326975487`, 35.08306094331483`}, {"CURE", 365, 73.2409028811635`},
{"TBUR", 37.38958454008781`, 36.68340051689435`}, {"CURE", 365, 110.86244226471283`}, {"CURE", 365, 90.87990647591015`},
{"CURE", 365, 96.12237281768049`}, {"CURE", 365, 73.25195202753292`}, {"CURE", 365, 76.40002090178882`},
{"CURE", 365, 106.971398086179`}, {"CURE", 365, 145.72698800735114`}, {"TBUR", 49.1520837137262`, 101.17340169728868`},
{"CURE", 365, 139.9535256358583`}, {"CURE", 365, 84.98714356891429`}, {"TBUR", 61.71589295588162`, 20.970243339375596`},
{"TBUR", 17.29974075029923`, 18.464104460416987`}, {"CURE", 365, 2.2469809580101727`}, {"CURE", 365, 121.9255127444682`},
{"CURE", 365, 127.41076561934946`}, {"CURE", 365, 93.76937849877608`}, {"TBUR", 139.29545813624821`, 212.88426713567216`},
{"CURE", 365, 167.49408875852455`}, {"CURE", 365, 50.95425125415274`}, {"TBUR", 20.562655102744603`, 66.85194701036622`},
{"CURE", 365, 19.52981440617231`}, {"CURE", 365, 204.16407447911237`}, {"TBUR", 39.204044122176`, 202.9214895622962`},
{"TBUR", 24.465549860436326`, 32.80970209513323`}, {"CURE", 365, 199.9462023928606`}, {"CURE", 365, 38.99512527695054`},
{"CURE", 365, 78.45032667353408`}, {"CURE", 365, 144.89623453898147`}, {"CURE", 365, 2.2799883414101507`},
{"TBUR", 55.591535181964375`, 45.07723898915319`}, {"CURE", 365, 127.18025297557699`},
{"TBUR", 63.09424549729893`, 29.899410328039952`}, {"TBUR", 38.741306016726675`, 203.77926228075037`},
{"CURE", 365, 4.162522336360827`}, {"CURE", 365, 169.72689143598845`}, {"CURE", 365, 100.14181751217738`},
{"CURE", 365, 93.71114176197727`}, {"CURE", 365, 118.67719491765503`}, {"CURE", 365, 53.48064930405309`},
{"TBUR", 35.65279686011341`, 150.7385234343006`}, {"CURE", 365, 95.69252579191595`}, {"CURE", 365, 162.3541035769764`},
{"CURE", 365, 106.62440396309775`}, {"TBUR", 54.447056309948614`, 109.99889893935567`},
{"CURE", 365, 128.6477114319323`}, {"CURE", 365, 98.1206813684068`}, {"CURE", 365, 50.34887600469683`},
{"CURE", 365, 143.8902887344859`}, {"CURE", 365, 178.5329304622954`}, {"CURE", 365, 50.686323506384134`},
{"TBUR", 64.19192233766735`, 116.73789637801572`}, {"TBUR", 45.969050909738996`, 145.76829923711688`},
{"CURE", 365, 20.481997631270946`}, {"CURE", 365, 105.49111118379678`}, {"CURE", 365, 56.43662470563163`},
{"TBUR", 14.735561723022702`, 10.162583384667446`}, {"TBUR", 13.490756406256509`, 32.802268568717835`},
{"TBUR", 33.67725348266924`, 50.77014313617045`}, {"CURE", 365, 101.88086385830323`}, {"CURE", 365, 31.718880430979283`},
{"CURE", 365, 93.55058627675376`}, {"TBUR", 78.27281791410606`, 170.07462924458312`}, {"CURE", 365, 95.17645294551102`},
{"CURE", 365, 82.58812331766337`}, {"CURE", 365, 89.29697335447618`}, {"TBUR", 17.305810883889`, 39.32271911211715`},
{"TBUR", 13.65661104809947`, 86.59376504494062`}, {"TBUR", 81.76541349315974`, 37.417637276672885`},
{"CURE", 365, 148.45450664648152`}, {"TBUR", 50.005497929600004`, 91.24015181898802`},
{"CURE", 365, 123.54318583471826`}, {"CURE", 365, 89.30979781609895`}, {"CURE", 365, 80.12943462784806`},
{"TBUR", 101.6426224184019`, 41.81512975401604`}, {"CURE", 365, 57.22689194378965`}, {"CURE", 365, 211.19630379607057`},

{"CURE", 365, 109.5691750157686`}, {"CURE", 365, 87.9493693026245`}, {"TBUR", 33.13805792710815`, 79.47917198084872`},
{"TBUR", 76.99001988974656`, 76.68512170943144`}, {"CURE", 365, 60.19222401020022`},
{"TBUR", 15.966367846856393`, 65.90920745864472`}, {"TBUR", 17.099108568070758`, 61.30100582435225`},
{"CURE", 365, 59.77477116083596`}, {"CURE", 365, 87.19357050906527`}, {"TBUR", 108.98273985045775`, 6.975581009805179`},
{"TBUR", 12.961713707708691`, 60.53674406438919`}, {"TBUR", 21.158979266482877`, 18.713471084418174`},
{"CURE", 365, 139.59408267281444`}, {"CURE", 365, 39.04256107769541`}, {"CURE", 365, 103.03499522862016`},
{"CURE", 365, 5.302161793719097`}, {"CURE", 365, 88.69145091998892`}, {"CURE", 365, 2.9909179863609867`},
{"CURE", 365, 101.45587935960268`}, {"CURE", 365, 94.54469707749867`}, {"TBUR", 114.8682666357703`, 108.51141343021435`},
{"CURE", 365, 7.463595166411529`}, {"CURE", 365, 49.07042011802559`}, {"CURE", 365, 201.4348623868977`},
{"TBUR", 25.169516823262718`, 48.69301153378479`}, {"CURE", 365, 1.886902401341974`},
{"CURE", 365, 31.612390034576404`}, {"CURE", 365, 140.6597532687234`}, {"CURE", 365, 22.62281958580658`},
{"CURE", 365, 111.61041858173179`}, {"CURE", 365, 172.21216382447156`}, {"CURE", 365, 68.3578308081431`},
{"CURE", 365, 8.084197507390677`}, {"CURE", 365, 10.502966117410379`}, {"CURE", 365, 129.28208808001114`},
{"TBUR", 13.508916511392078`, 18.48897466079086`}, {"TOX", 25.84878063397265`, 233.15968787157576`},
{"CURE", 365, 85.63077038605162`}, {"CURE", 365, 106.91481215826376`}, {"TBUR", 84.88982440505065`, 84.84099446837858`},
{"CURE", 365, 58.69607592565429`}, {"TOX", 27.339530663105542`, 237.0444221451317`}, {"CURE", 365, 80.4970737775615`},
{"TBUR", 115.50747485530894`, 4.003203595347902`}, {"CURE", 365, 82.4486033183301`}, {"CURE", 365, 29.461428619633203`},
{"CURE", 365, 182.9323201169916`}, {"CURE", 365, 71.39963588929133`}, {"CURE", 365, 4.560357417578203`},
{"CURE", 365, 90.06434989220463`}, {"TBUR", 22.06526806258146`, 89.68059290060913`}, {"CURE", 365, 93.04284094152638`},
{"CURE", 365, 82.75793482537924`}, {"CURE", 365, 60.88518046128533`}, {"CURE", 365, 128.6771845265268`},
{"CURE", 365, 13.083208638079117`}, {"TBUR", 78.1456686415018`, 176.59800904519977`}, {"CURE", 365, 73.0600383020177`},
{"CURE", 365, 6.959175820933164`}, {"CURE", 365, 35.22887065904443`}, {"CURE", 365, 213.4263922426518`},
{"CURE", 365, 84.6695123275385`}, {"CURE", 365, 151.98748001714605`}, {"CURE", 365, 90.33782182365724`},
{"CURE", 365, 175.71568429518496`}, {"CURE", 365, 163.17532885172562`}, {"TBUR", 74.00150965642753`, 34.3698409802683`},
{"CURE", 365, 72.79075504238476`}, {"TBUR", 131.36905835794192`, 71.29739942714738`},
{"TBUR", 18.06411047816143`, 18.216011097282475`}, {"TBUR", 25.45774825620703`, 9.63748915320388`},
{"CURE", 365, 70.6726044254533`}, {"CURE", 365, 106.25727512870162`}, {"TBUR", 53.68852919569536`, 59.452362948249636`},
{"CURE", 365, 162.27652529739498`}, {"CURE", 365, 150.82430524199222`}, {"TBUR", 18.989187410522224`, 19.995437049469324`},
{"TBUR", 25.438673835786478`, 40.04452740640189`}, {"CURE", 365, 24.35097195097887`},
{"TBUR", 26.3494451408607`, 118.5719846545565`}, {"TBUR", 44.0620290061974`, 103.15009041429612`},
{"CURE", 365, 124.5984173800417`}, {"TBUR", 76.95505175697389`, 163.27536194723328`}, {"CURE", 365, 43.44714464291937`},

{"CURE", 365, 117.71602445804798`}, {"TBUR", 27.624633283223044`, 99.35308850451204`},
{"TBUR", 27.521729757034375`, 6.533146172349656`}, {"CURE", 365, 163.93657588698753`}, {"CURE", 365, 72.80065589567486`},
{"TBUR", 40.06351172727508`, 3.759558241602726`}, {"TBUR", 22.783978029005734`, 2.747254728443676`},
{"CURE", 365, 61.06559637261357`}, {"CURE", 365, 87.07257442413031`}, {"CURE", 365, 102.29739436900648`},
{"TBUR", 39.46752351230035`, 65.9263778520934`}, {"CURE", 365, 94.8963562427958`}, {"CURE", 365, 134.83128684965843`},
{"CURE", 365, 73.27231679106086`}, {"TBUR", 95.8255965758851`, 35.632575991153686`}, {"CURE", 365, 117.88903072639819`},
{"CURE", 365, 12.057481254225324`}, {"CURE", 365, 41.14817016679874`}, {"TBUR", 77.88709992799005`, 82.32512168735093`},
{"CURE", 365, 7.117745991416628`}, {"TBUR", 45.38363369010701`, 59.1278118524851`},
{"CURE", 365, 77.76833274776341`}, {"TBUR", 34.57392243430188`, 90.10273903965347`},
{"CURE", 365, 59.61086067815402`}, {"TBUR", 21.17285611089091`, 65.23296527338005`},
{"CURE", 365, 82.28179939780082`}, {"TBUR", 16.38741208744`, 100.6207692745505`}, {"CURE", 365, 87.67384196937945`},
{"TBUR", 16.37408874102941`, 25.725681599175406`}, {"TBUR", 64.70138346370753`, 81.06921360759557`},
{"CURE", 365, 168.2108688501756`}, {"CURE", 365, 6.756064981503164`}, {"CURE", 365, 70.64800381442905`},
{"CURE", 365, 39.23418791617683`}, {"CURE", 365, 100.66907309121584`}, {"CURE", 365, 45.98706045117322`},
{"TBUR", 50.24118977168637`, 120.86390643213808`}, {"TBUR", 78.05019841533876`, 80.0957003484362`},
{"CURE", 365, 155.7626222719634`}, {"CURE", 365, 54.62220445934123`}, {"CURE", 365, 197.767828998976`},
{"CURE", 365, 132.3085367799126`}, {"CURE", 365, 112.33732638829706`}, {"CURE", 365, 127.86432879811255`},
{"TBUR", 134.90012822825943`, 202.2922566230594`}, {"TBUR", 40.11522968747093`, 219.56190240018694`},
{"CURE", 365, 65.1763178304038`}, {"CURE", 365, 106.70094800646662`}, {"CURE", 365, 116.48749460933183`},
{"TBUR", 40.164302371955955`, 42.43860845055252`}, {"TBUR", 16.805872006751887`, 9.495944271731323`},
{"CURE", 365, 2.424386903122696`}, {"CURE", 365, 84.19432069033566`}, {"CURE", 365, 133.58783571107892`},
{"TBUR", 20.412821819964346`, 109.85088131057641`}, {"CURE", 365, 151.24300535373874`},
{"CURE", 365, 202.50690199782198`}, {"CURE", 365, 54.13792243752786`}, {"CURE", 365, 115.87035243521666`},
{"TBUR", 44.11931118861722`, 8.843995018200259`}, {"CURE", 365, 222.0547228355211`},
{"TBUR", 36.84912999478473`, 72.94094895204738`}, {"TBUR", 58.30727844742349`, 52.496030987472`},
{"TBUR", 65.35530428337236`, 62.17714465018735`}, {"CURE", 365, 208.3388693910991`}, {"CURE", 365, 84.44341015443342`},
{"TBUR", 92.18775427334353`, 191.59418473379012`}, {"CURE", 365, 138.40670310074037`}, {"CURE", 365, 126.11671960957662`},
{"TBUR", 54.753985580903716`, 124.29427138436172`}, {"TBUR", 87.82536658014229`, 128.20579016459905`},
{"CURE", 365, 58.78626782385953`}, {"CURE", 365, 50.70011538568112`}, {"CURE", 365, 21.475908801159516`},
{"CURE", 365, 87.83613334095867`}, {"CURE", 365, 103.19837385348339`}, {"TBUR", 34.35757956004133`, 57.67607766693298`},
{"TBUR", 44.43317201288527`, 73.78208269071145`}, {"CURE", 365, 141.09213126505125`},

{"TBUR", 42.0358749508949`, 67.51550645569519`}, {"CURE", 365, 76.33125275742383`}, {"CURE", 365, 44.35771552479139`},
{"TBUR", 21.166766523810264`, 13.724938996522441`}, {"CURE", 365, 54.73869664243227`}, {"CURE", 365, 158.2286652064022`},
{"CURE", 365, 71.38990135236567`}, {"TBUR", 43.020275068055476`, 64.03201612882364`}, {"CURE", 365, 21.818078253662154`},
{"TBUR", 25.939039479141826`, 147.27766920024192`}, {"CURE", 365, 155.89022638962052`}, {"CURE", 365, 96.67895131571389`},
{"TBUR", 100.99068664148949`, 45.704677740533626`}, {"CURE", 365, 147.43006199330026`}, {"CURE", 365, 17.425766891083885`},
{"TBUR", 84.32093602541885`, 222.777481362818`}, {"TBUR", 28.648686989049143`, 78.10962635215043`},
{"CURE", 365, 174.09215580264393`}, {"TBUR", 43.17838114845258`, 82.365473665841`}, {"CURE", 365, 49.53757516502009`},
{"TBUR", 41.84993226693477`, 61.56095053164008`}, {"TBUR", 25.22593505777123`, 21.42339360659355`},
{"CURE", 365, 135.72389206049436`}, {"TBUR", 42.85740979318713`, 45.89639272288905`}, {"CURE", 365, 74.36187967794272`},
{"TBUR", 26.126611739082716`, 52.004298888087185`}, {"CURE", 365, 82.54875560823649`},
{"TBUR", 23.33342312593196`, 50.43580326325203`}, {"TBUR", 106.32330404994252`, 168.6631995712509`},
{"CURE", 365, 119.8231303799543`}, {"TBUR", 33.59400808215811`, 72.90043629909769`},
{"TBUR", 66.5575901241072`, 74.11187631141956`}, {"TBUR", 13.85409874978721`, 71.34080923108772`},
{"CURE", 365, 13.188260071111232`}, {"TBUR", 108.4153207583991`, 130.3968417891419`},
{"TBUR", 101.9566104806899`, 116.82815377411517`}, {"CURE", 365, 98.32528068688629`},
{"TBUR", 48.33199513475779`, 9.728991404222107`}, {"CURE", 365, 84.98771441174497`}, {"CURE", 365, 193.23768475025136`},
{"CURE", 365, 91.39389921617139`}, {"CURE", 365, 110.99183384472292`}, {"TBUR", 28.72216293430766`, 40.52880323212201`},
{"TBUR", 85.2292276423934`, 90.5143393163154`}, {"TBUR", 13.43937906208463`, 2.776013628030693`},
{"TBUR", 15.57151404339207`, 15.796249224924557`}, {"TBUR", 52.93886964367178`, 135.68052599874432`},
{"CURE", 365, 86.42048940708776`}, {"TBUR", 51.536569762483836`, 74.44412614741945`}, {"CURE", 365, 34.014784452147474`},
{"TBUR", 218.25101721854745`, 157.14292349762164`}, {"CURE", 365, 85.43790574878787`}, {"CURE", 365, 161.7968058618609`},
{"TBUR", 58.69599360785595`, 25.300229319382453`}, {"TBUR", 30.503827153636873`, 92.89955658076515`},
{"CURE", 365, 74.75277933200012`}, {"CURE", 365, 89.91368651470222`}, {"CURE", 365, 24.58268662006628`},
{"CURE", 365, 65.37852811713184`}, {"CURE", 365, 146.4432457504141`}, {"TBUR", 20.814188256032033`, 91.3950505669661`},
{"CURE", 365, 50.089912164147634`}, {"TBUR", 45.685322751215764`, 3.767185471802455`}, {"CURE", 365, 53.21176925473099`},
{"CURE", 365, 146.1354228485497`}, {"CURE", 365, 60.347068144744874`}, {"CURE", 365, 88.51004120591794`},
{"TBUR", 15.918619752378964`, 106.15707381643873`}, {"TBUR", 88.46688598945312`, 88.5176596131213`},
{"TBUR", 86.4188376399758`, 160.2034002382332`}, {"CURE", 365, 140.62458292888667`},
{"CURE", 365, 101.94010451679539`}, {"CURE", 365, 108.15580701700632`}, {"CURE", 365, 150.49998411657455`},
{"TBUR", 20.024680236051452`, 6.757680845368987`}, {"TBUR", 79.52203227126236`, 153.03074024517264`},
{"TBUR", 148.52327966761786`, 54.7325947252298`}, {"TBUR", 66.0617761607777`, 217.21645204382025`},

{"CURE", 365, 12.945900473314774`}, {"TBUR", 91.67739579890898`, 80.2955289800574`}, {"CURE", 365, 182.07502146194906`},
{"CURE", 365, 8.332147899834604`}, {"CURE", 365, 117.09514733467684`}, {"TBUR", 95.0408495914071`, 96.85822738074252`},
{"TBUR", 33.46514670582551`, 117.84353162075878`}, {"TBUR", 15.500271517700885`, 61.29386135520477`},
{"TBUR", 13.912841794587552`, 33.36222369182695`}, {"TBUR", 18.7863008335704`, 9.290609766030672`},
{"CURE", 365, 86.43613938521958`}, {"CURE", 365, 85.68126287717234`}, {"TBUR", 37.24888345026232`, 103.56962272406524`},
{"CURE", 365, 16.17646845691577`}, {"CURE", 365, 227.28926523898747`}, {"CURE", 365, 47.14547254712428`},
{"CURE", 365, 89.23264965142812`}, {"CURE", 365, 56.81046177833463`}, {"CURE", 365, 9.1083309115633`},
{"TBUR", 115.40579157647444`, 152.71425455248445`}, {"CURE", 365, 27.45418047737146`},
{"CURE", 365, 67.35628901419265`}, {"TBUR", 19.252279533642593`, 33.901195358810824`},
{"CURE", 365, 70.2077019100468`}, {"TOX", 38.487832457187146`, 231.9957086267093`}, {"CURE", 365, 158.42942418472657`},
{"TBUR", 37.06348537790819`, 100.40481773794636`}, {"CURE", 365, 89.98251901337377`},
{"CURE", 365, 142.86595390563696`}, {"CURE", 365, 172.75781366524723`}, {"CURE", 365, 136.20487695923765`},
{"CURE", 365, 176.3128568562333`}, {"TBUR", 14.40288867904919`, 58.77129983521731`}, {"CURE", 365, 70.91162423905674`},
{"TBUR", 25.473247233385322`, 11.431747601125666`}, {"TBUR", 18.81688849876454`, 83.17178001047643`},
{"TBUR", 68.0574968673655`, 4.575024759351925`}, {"CURE", 365, 1.1578174113031294`}, {"CURE", 365, 7.551029394012972`},
{"TBUR", 155.08347533132581`, 0.838427802919249`}, {"TBUR", 18.805788612657675`, 81.3643145168212`},
{"CURE", 365, 32.263996143483396`}, {"CURE", 365, 67.73454804805002`}, {"CURE", 365, 166.981785847873`},
{"CURE", 365, 46.59870245693602`}, {"TBUR", 20.536482234009686`, 79.88969933997116`},
{"TBUR", 13.2618243466071`, 57.134115297209505`}, {"CURE", 365, 33.171347344479344`},
{"TBUR", 62.618203233779795`, 109.2963242478686`}, {"TBUR", 35.22050988244697`, 22.45598818251369`},
{"TBUR", 65.73189425215736`, 2.1310535127318864`}, {"TBUR", 46.847988443067344`, 8.256057439506689`},
{"CURE", 365, 165.53651016186046`}, {"CURE", 365, 35.07186290470656`}, {"CURE", 365, 165.78967115081588`},
{"TBUR", 172.3894030523308`, 105.81537216784797`}, {"CURE", 365, 148.3829529402061`}, {"CURE", 365, 54.293675407551625`},
{"CURE", 365, 203.22048076429112`}, {"CURE", 365, 88.81125118267406`}, {"TBUR", 27.53933336291121`, 8.736683923364147`},
{"CURE", 365, 150.04504664177713`}, {"TBUR", 27.395591173743206`, 31.71156226768339`},
{"TBUR", 67.56577517572407`, 118.74693900531254`}, {"TBUR", 75.21692912729627`, 146.01931555963162`},
{"TBUR", 64.44439728054876`, 46.092844328082414`}, {"CURE", 365, 103.07159825760458`},
{"TBUR", 15.613146032445494`, 35.35137348103256`}, {"TBUR", 32.49126332831765`, 72.20418495287974`},
{"CURE", 365, 125.47135923111796`}, {"CURE", 365, 55.43668240335896`}, {"TBUR", 30.259048177886932`, 77.50997859803027`},
{"CURE", 365, 48.35235046745698`}, {"TBUR", 77.21525980276517`, 150.3610146821099`},
{"TBUR", 52.23971964451543`, 96.24019453047529`}, {"CURE", 365, 98.388366114034`},

{"TBUR", 77.81051560271455`, 129.65279529967057`}, {"CURE", 365, 60.20000638972917`}, {"CURE", 365, 87.1798704195879`},
{"CURE", 365, 140.13301115774254`}, {"CURE", 365, 7.995802003572845`}, {"TBUR", 19.00055053628276`, 12.2774797934029`},
{"TBUR", 145.13521230561676`, 60.35819146528604`}, {"TBUR", 57.27447730585588`, 191.90042851961525`},
{"CURE", 365, 22.535881114299453`}, {"TBUR", 112.93824078318875`, 179.2161597055292`},
{"CURE", 365, 79.5090886012805`}, {"CURE", 365, 190.09660639372723`}, {"TBUR", 15.755685458578899`, 47.80114803764219`},
{"TBUR", 39.876507772552664`, 64.12046605246137`}, {"CURE", 365, 93.6108647023708`}, {"CURE", 365, 194.7097501894441`},
{"TBUR", 85.15926076127921`, 88.17911274882658`}, {"CURE", 365, 219.75892754136885`}, {"CURE", 365, 51.86961718398602`},
{"CURE", 365, 115.81988578103042`}, {"CURE", 365, 88.15411663451151`}, {"CURE", 365, 100.35828688493781`},
{"TBUR", 63.80722333917153`, 96.24267747926746`}, {"TBUR", 44.990667490627594`, 102.75506575948958`},
{"CURE", 365, 11.397635343303433`}, {"CURE", 365, 39.750484172472056`}, {"TBUR", 82.47973054245215`, 112.07078057828282`},
{"CURE", 365, 99.72903215582197`}, {"CURE", 365, 16.844966935989284`}, {"TBUR", 76.89997751861722`, 110.11889422489112`},
{"CURE", 365, 56.90721362934084`}, {"CURE", 365, 19.121038053178466`}, {"CURE", 365, 19.10775461490201`},
{"CURE", 365, 32.13895833457558`}, {"CURE", 365, 41.86502432349794`}, {"TBUR", 67.34570887757395`, 94.82622236214398`},
{"CURE", 365, 143.90141704309235`}, {"CURE", 365, 167.939582727973`}, {"TBUR", 31.86799672547541`, 120.00994057628209`},
{"TBUR", 74.95921926077061`, 8.550956292306653`}, {"CURE", 365, 202.82232946491158`},
{"TBUR", 102.62426040272707`, 76.72946806841314`}, {"TBUR", 39.40231778360715`, 100.52146478605724`},
{"TBUR", 124.36513964411296`, 32.37798948841091`}, {"CURE", 365, 85.92680224048748`},
{"CURE", 365, 11.014849293456724`}, {"CURE", 365, 143.91469985015584`}, {"CURE", 365, 161.27265550817896`},
{"CURE", 365, 88.19846572292374`}, {"CURE", 365, 44.24291274244211`}, {"CURE", 365, 74.122191330776`},
{"TBUR", 123.19830780899534`, 113.3203110663236`}, {"TBUR", 91.0153785834677`, 123.0095543766531`},
{"TBUR", 41.205566365507636`, 69.30808479109047`}, {"CURE", 365, 96.66315343096984`}, {"CURE", 365, 68.25449192908708`},
{"TBUR", 23.008334854693`, 170.34028741512913`}, {"TBUR", 80.80005591170686`, 60.67897339512163`},
{"CURE", 365, 62.62080838372254`}, {"TBUR", 109.22087188640485`, 93.01532594133462`}, {"CURE", 365, 138.57482189524802`},
{"CURE", 365, 4.303378547376758`}, {"TBUR", 55.49276917915016`, 58.38649720060914`}, {"CURE", 365, 95.1623736390655`},
{"CURE", 365, 24.676434053939616`}, {"CURE", 365, 170.553017622838`}, {"TBUR", 16.199060509122226`, 8.507113032621678`},
{"CURE", 365, 167.95338681947885`}, {"CURE", 365, 153.21239354064048`}, {"TBUR", 114.56475937896504`, 3.85959887453791`},
{"CURE", 365, 130.47655780877994`}, {"CURE", 365, 61.21353155424244`}, {"CURE", 365, 71.82428628473433`},
{"CURE", 365, 144.7879334539852`}, {"CURE", 365, 17.702682769142427`}, {"TBUR", 21.03607613005762`, 85.261655985756`},
{"CURE", 365, 36.06096695740735`}, {"TBUR", 89.47841067106135`, 2.8536546088356727`},
{"CURE", 365, 88.81848214150654`}, {"TBUR", 74.64011127879375`, 137.02160412503613`},
{"TBUR", 171.55159574409703`, 45.07551145023169`}, {"CURE", 365, 0.745252500706311 5`},

{"TBUR", 100.03225362460596`, 10.587364720706518`}, {"TBUR", 30.630588844680045`, 68.4527275687499`},
{"CURE", 365, 2.57537153645601`}, {"CURE", 365, 6.265406133092203`}, {"CURE", 365, 124.65181223776683`},
{"TBUR", 75.3370347362792`, 212.28086733032808`}, {"TBUR", 89.59439561574727`, 111.43386504823827`},
{"CURE", 365, 207.361133686166`}, {"TBUR", 74.31067457989664`, 104.06532252627218`},
{"CURE", 365, 8.355576375645448`}, {"CURE", 365, 108.21415528606259`}, {"TBUR", 89.96881624149437`, 157.717726111141`},
{"TBUR", 38.69260345044224`, 82.58040741554305`}, {"TBUR", 73.80803784587849`, 17.07732264725019`},
{"TBUR", 57.96397616735996`, 72.93584200026153`}, {"CURE", 365, 48.3466008280856`}, {"CURE", 365, 29.774623012138424`},
{"CURE", 365, 30.27939553943683`}, {"CURE", 365, 125.07716896033682`}, {"TBUR", 127.56950761417148`, 57.7366533950291`},
{"TBUR", 25.419498955662355`, 51.60823834819282`}, {"CURE", 365, 73.20713432461837`},
{"TBUR", 75.51700394711813`, 14.819827378963007`}, {"CURE", 365, 110.01723150230886`}, {"CURE", 365, 58.86135522016863`},
{"TBUR", 63.84817131249365`, 62.47296221893218`}, {"TBUR", 50.95714062807861`, 142.35067384138`},
{"CURE", 365, 183.6181144506114`}, {"CURE", 365, 65.69347164573497`}, {"CURE", 365, 10.897273553612651`},
{"TBUR", 79.62966413773985`, 217.27069234675292`}, {"TBUR", 155.9529904093303`, 81.58031816872894`},
{"TBUR", 57.25604759462311`, 66.87228050318443`}, {"CURE", 365, 88.42565219745963`}, {"CURE", 365, 29.009632118882845`},
{"CURE", 365, 81.46420309811782`}, {"CURE", 365, 76.24341978393143`}, {"TBUR", 119.35234999702307`, 77.07121058813463`},
{"CURE", 365, 139.53954500331506`}, {"CURE", 365, 59.573876629509506`}, {"TBUR", 165.89032072211262`, 103.43107655748764`},
{"CURE", 365, 50.052371246197765`}, {"CURE", 365, 134.29354534612872`}, {"CURE", 365, 63.38124549745927`},
{"TBUR", 85.85078314050733`, 83.85495098275125`}, {"CURE", 365, 43.46807485566375`},
{"TBUR", 82.40781372852348`, 129.04364008691473`}, {"TOX", 21.936029839622023`, 241.35784763354897`},
{"TBUR", 42.065849040677115`, 119.5012393604814`}, {"CURE", 365, 115.35737257796876`}, {"CURE", 365, 46.328897456748564`},
{"TBUR", 121.28894826316333`, 94.85461527191832`}, {"TBUR", 57.58666252562055`, 179.97448354132555`},
{"CURE", 365, 4.794567562365661`}, {"CURE", 365, 114.17101157180984`}, {"TBUR", 74.41015965330996`, 12.340235083658461`},
{"CURE", 365, 37.16571732731463`}, {"CURE", 365, 13.871093095272004`}, {"TBUR", 48.63158124421695`, 62.58123534729959`},
{"CURE", 365, 6.355127266467752`}, {"TBUR", 32.825921938944425`, 83.14764436317937`}, {"CURE", 365, 77.45689509157594`},
{"TBUR", 80.52326334870642`, 94.29548197016861`}, {"TBUR", 34.718027854000375`, 59.15749026141689`},
{"CURE", 365, 105.25379474370882`}, {"CURE", 365, 170.92979379788275`}, {"CURE", 365, 84.76626065733352`},
{"CURE", 365, 29.22394829225287`}, {"CURE", 365, 90.8674696791797`}, {"TBUR", 120.30151449186954`, 8.103996782177294`},
{"TBUR", 70.76650207920797`, 7.904301921041477`}, {"CURE", 365, 149.3216714740572`},
{"CURE", 365, 137.0855469542075`}, {"TBUR", 82.76141235290108`, 35.23978056489883`},
{"CURE", 365, 48.30467527433827`}, {"TBUR", 18.290796574571814`, 95.53798353332672`},
{"CURE", 365, 166.67410046803764`}, {"TBUR", 133.49433115236096`, 56.05685260624146`},

{"TBUR", 58.45267449077485`, 76.3357813367823`}, {"CURE", 365, 14.109312066405888`},
{"TBUR", 75.77938385163749`, 83.63435558585552`}, {"CURE", 365, 72.61255878846747`}, {"CURE", 365, 88.40424997864352`},
{"TBUR", 70.14365772565873`, 62.14839908351223`}, {"TBUR", 74.09622754772828`, 32.51554635852737`},
{"CURE", 365, 100.35206873931968`}, {"TBUR", 14.680287764454004`, 56.9181324835933`},
{"CURE", 365, 30.184349877144715`}, {"CURE", 365, 111.03764145342382`}, {"CURE", 365, 197.66635324719806`},
{"CURE", 365, 159.09410678502667`}, {"CURE", 365, 92.66936556338452`}, {"CURE", 365, 113.33716277497969`},
{"TBUR", 47.655119279832284`, 11.458408307818264`}, {"TBUR", 43.03017672671192`, 97.15276627153`},
{"TBUR", 71.50804447627841`, 18.5349318892205`}, {"CURE", 365, 71.01992788920724`},
{"TBUR", 25.98529112328901`, 99.66319068147655`}, {"CURE", 365, 28.996994507679872`}, {"CURE", 365, 75.03849811571239`},
{"TBUR", 21.364592230569162`, 32.68327787288995`}, {"TOX", 30.98809003036838`, 237.6816638665374`},
{"TBUR", 14.730053147340618`, 17.850023252573827`}, {"CURE", 365, 107.68341951869542`},
{"TBUR", 46.462123216204624`, 30.924851390130158`}, {"TBUR", 112.01107424530092`, 22.164592008900375`},
{"CURE", 365, 6.556302816265598`}, {"CURE", 365, 4.812653796804807`}, {"CURE", 365, 85.2758236063533`},
{"TBUR", 28.06658121414043`, 57.512603283759056`}, {"CURE", 365, 22.917841449737633`}, {"CURE", 365, 81.03733576332124`},
{"CURE", 365, 115.5005316952275`}, {"CURE", 365, 209.10476133625411`}, {"CURE", 365, 77.80418519717958`},
{"CURE", 365, 113.06914311881845`}, {"TBUR", 40.98681244633936`, 99.92978389874824`}, {"CURE", 365, 24.35517286574881`},
{"TBUR", 25.48938085910039`, 48.48366523293757`}, {"CURE", 365, 38.53025350029982`}, {"CURE", 365, 109.33013411979182`},
{"TBUR", 42.54478148192615`, 94.61498501190316`}, {"CURE", 365, 70.7058528032577`}, {"CURE", 365, 5.677126024038959`},
{"CURE", 365, 29.394773929371137`}, {"CURE", 365, 73.72315020938657`}, {"TBUR", 41.91464090761807`, 83.53782064866697`},
{"CURE", 365, 115.62676767033955`}, {"TBUR", 107.6744451414995`, 74.40922358121134`}, {"CURE", 365, 211.90254722292812`},
{"CURE", 365, 95.71237475901087`}, {"TBUR", 18.265263691059285`, 80.06475354991585`}, {"CURE", 365, 1.216276711828599`},
{"CURE", 365, 46.13376028151415`}, {"CURE", 365, 160.126090638196`}, {"CURE", 365, 10.759083027453228`},
{"TBUR", 47.37299203698744`, 65.97924408798463`}, {"TBUR", 50.255418047210185`, 99.4755149242393`},
{"TBUR", 87.42209094571582`, 184.68653833550567`}, {"TBUR", 88.49217000443679`, 121.40199574535364`},
{"CURE", 365, 33.59968379882366`}, {"TBUR", 83.3096542079834`, 9.394101297444548`}, {"CURE", 365, 69.56284528036254`},
{"CURE", 365, 166.48162108547396`}, {"TBUR", 32.80398267034364`, 100.52804273787973`},
{"CURE", 365, 8.957010154325538`}, {"TBUR", 54.86024483920527`, 98.15933863663939`},
{"CURE", 365, 92.74370561173448`}, {"TBUR", 80.60855913690018`, 9.95594274744626`},
{"CURE", 365, 74.48697803708912`}, {"CURE", 365, 92.5573296977732`}, {"CURE", 365, 57.55388095168141`},
{"TOX", 18.317786096307753`, 263.9304812788727`}, {"TBUR", 62.92759340784274`, 80.92162555112228`},
{"CURE", 365, 100.54995367599619`}, {"TBUR", 24.726990112577944`, 77.25699302525163`}, {"CURE", 365, 214.46777484471235`},

{"CURE", 365, 88.72556669971306`}, {"TBUR", 19.791844231188417`, 75.52953968828287`}, {"CURE", 365, 56.24120724476305`},
{"TBUR", 58.971596667153676`, 110.09316651097147`}, {"CURE", 365, 0.9644978517518376`},
{"TBUR", 46.4041529976072`, 29.581930282186093`}, {"TBUR", 146.1634720918273`, 107.53034666847877`},
{"CURE", 365, 89.21901880954177`}, {"CURE", 365, 29.306372065828953`}, {"CURE", 365, 72.07128539781671`},
{"CURE", 365, 50.9207758038263`}, {"TBUR", 71.78166371001241`, 52.93146380764639`}, {"CURE", 365, 17.827694204800736`},
{"CURE", 365, 63.699289585638894`}, {"CURE", 365, 54.56596998685729`}, {"TBUR", 27.7367077011145`, 37.603307736144494`},
{"CURE", 365, 162.11934912866852`}, {"CURE", 365, 4.553582351432952`}, {"CURE", 365, 75.22042516137432`},
{"TBUR", 162.50059684399787`, 116.17108587547044`}, {"TBUR", 16.57118049034488`, 122.25087727912424`},
{"TBUR", 50.60966524849478`, 9.968266369588909`}, {"CURE", 365, 101.42627004896181`},
{"CURE", 365, 113.7087817985229`}, {"TBUR", 31.025099592994493`, 4.026622074722913`}, {"CURE", 365, 75.35489674653567`},
{"CURE", 365, 98.64664101291397`}, {"TBUR", 24.019551655227254`, 23.258946884223278`},
{"CURE", 365, 2.286560460636551`}, {"TBUR", 66.7269641108241`, 183.7502267934328`},
{"TBUR", 113.64342804700102`, 102.61638640846574`}, {"CURE", 365, 97.83887454704501`},
{"CURE", 365, 34.11504110861314`}, {"TBUR", 86.35894167726678`, 3.148420687809349`}, {"CURE", 365, 13.156390573752219`},
{"TBUR", 13.317844047426641`, 20.73909808077111`}, {"TBUR", 84.60184587519898`, 83.8731274451308`},
{"CURE", 365, 136.76517420716522`}, {"CURE", 365, 113.33380424035043`}, {"CURE", 365, 56.83569318606563`},
{"TBUR", 23.450190679322876`, 3.0183743027580645`}, {"CURE", 365, 87.59581838317106`}, {"CURE", 365, 96.90844999035082`},
{"TBUR", 74.96425508982705`, 2.114303356946238`}, {"TBUR", 76.79604658531306`, 57.28931718376526`},
{"CURE", 365, 1.091324127035208`}, {"CURE", 365, 152.75254556882004`}, {"CURE", 365, 69.3543025205347`},
{"TBUR", 14.76929927626078`, 0.8089174137834924`}, {"TBUR", 163.3958863748853`, 93.72241752014749`},
{"CURE", 365, 37.600333585439564`}, {"TOX", 25.21104512970722`, 237.09704380880726`}, {"CURE", 365, 138.13024534555475`},
{"TBUR", 41.80415703440645`, 73.7968785263303`}, {"CURE", 365, 194.53688060650984`}},
{{"470 Res", "470 OS", "470 Tox"}, {"TBUR", 21.793892559450377`, 43.67956113943894`}, {"CURE", 365, 160.01849481845738`},
{"TBUR", 27.131477732742088`, 123.38893961374842`}, {"TBUR", 30.967835794496704`, 8.273267898019798`},
{"TBUR", 18.296701809914023`, 20.27109760692099`}, {"CURE", 365, 36.09801737713`}, {"CURE", 365, 69.99046874119807`},
{"CURE", 365, 67.66461405175747`}, {"CURE", 365, 156.19419884724513`}, {"TBUR", 70.77923478216337`, 207.3285632900712`},
{"TBUR", 54.69459982792819`, 106.36578498499918`}, {"CURE", 365, 98.08882304471199`},
{"TOX", 15.133496540567334`, 277.31079486734365`}, {"TBUR", 43.36831509117624`, 68.4173062705441`},
{"TBUR", 69.05311686392922`, 6.760454463957782`}, {"TBUR", 88.72884842127498`, 35.673813022960225`},
{"CURE", 365, 107.622781079918`}, {"TBUR", 44.87028374432255`, 124.58771145513484`},
{"TBUR", 46.18525561563561`, 8.87473139347897`}, {"CURE", 365, 94.49933037254475`}, {"CURE", 365, 144.6215790658597 8`},

{"CURE", 365, 58.317428757807726`}, {"CURE", 365, 35.86201511772772`}, {"CURE", 365, 72.5399453809182`},
{"CURE", 365, 66.87738848327801`}, {"CURE", 365, 174.01233603028604`}, {"CURE", 365, 114.40907941454914`},
{"CURE", 365, 57.29611685485341`}, {"CURE", 365, 40.13308474966378`}, {"CURE", 365, 13.938564988398044`},
{"CURE", 365, 148.4788768230163`}, {"TBUR", 13.455803122064996`, 38.78955520684515`}, {"CURE", 365, 75.22399290640135`},
{"TBUR", 49.123878396773435`, 46.199964366641886`}, {"CURE", 365, 196.65967352918193`},
{"CURE", 365, 227.65325004680548`}, {"CURE", 365, 47.68354421422423`}, {"TBUR", 21.818371870538627`, 11.92551226715759`},
{"TBUR", 72.16906034151711`, 56.0559161672895`}, {"CURE", 365, 100.74822752098913`},
{"TBUR", 18.085058177097`, 66.75341681259316`}, {"CURE", 365, 107.92658156491319`}, {"CURE", 365, 78.3467178248652`},
{"TBUR", 45.992441135949235`, 83.28101265968077`}, {"CURE", 365, 102.1605796725728`}, {"CURE", 365, 5.219326909600201`},
{"CURE", 365, 114.24131204609517`}, {"CURE", 365, 73.0878848238926`}, {"TBUR", 112.76902954663584`, 137.66223410385928`},
{"TOX", 28.098804115577835`, 237.5601086197662`}, {"TBUR", 29.73438863098668`, 105.2844522264449`},
{"TBUR", 41.78184237303424`, 90.3001486091342`}, {"CURE", 365, 6.818691418955254`}, {"CURE", 365, 147.27307049113747`},
{"CURE", 365, 55.159047832122475`}, {"CURE", 365, 103.50340499507206`}, {"CURE", 365, 78.0749189153484`},
{"TBUR", 103.43360807499825`, 96.04638300067599`}, {"TBUR", 37.578377915933025`, 179.8702072767869`},
{"TBUR", 52.65589833957032`, 105.9446687429816`}, {"CURE", 365, 97.77551440201026`}, {"CURE", 365, 4.839998430826178`},
{"TBUR", 33.624043829515934`, 120.61469673502667`}, {"CURE", 365, 92.34862192005961`},
{"TBUR", 76.42370063761436`, 7.463037044279552`}, {"CURE", 365, 94.51779715919939`}, {"CURE", 365, 87.36158250331749`},
{"TBUR", 57.46131930493868`, 134.23109943953773`}, {"CURE", 365, 48.81712983882141`}, {"CURE", 365, 41.80572251579488`},
{"CURE", 365, 76.20513162838567`}, {"CURE", 365, 56.70480067953188`}, {"CURE", 365, 151.01908527909745`},
{"TBUR", 13.558833504950423`, 2.4617405937323134`}, {"TBUR", 79.66105685830794`, 98.89304285407351`},
{"CURE", 365, 130.0376549541871`}, {"TBUR", 97.02695876306389`, 229.52468649195464`}, {"CURE", 365, 76.51717627508225`},
{"TBUR", 75.86452834633404`, 148.33935811043955`}, {"CURE", 365, 64.9186509311076`}, {"CURE", 365, 120.96522428217638`},
{"CURE", 365, 5.187385055185166`}, {"CURE", 365, 7.541016089147665`}, {"CURE", 365, 5.429062298025384`},
{"CURE", 365, 139.6350509135281`}, {"TBUR", 99.68134958573901`, 106.80691282851008`}, {"CURE", 365, 105.40222976840292`},
{"CURE", 365, 52.55147021896581`}, {"CURE", 365, 8.196275757278073`}, {"TBUR", 71.4364816732866`, 165.48349345787724`},
{"TBUR", 33.5444517679305`, 16.88430678624263`}, {"TBUR", 89.3655781027991`, 116.15944484816481`},
{"TBUR", 72.17065834250637`, 3.1380943822901997`}, {"CURE", 365, 7.253956827218402`},
{"TBUR", 19.73356194894`, 2.3736420756631764`}, {"TBUR", 158.90277899689372`, 64.0740842399547`},
{"CURE", 365, 49.36918907121949`}, {"CURE", 365, 208.53104448419535`}, {"CURE", 365, 11.559998720799447`},
{"TBUR", 43.842458347607135`, 169.33192540560876`}, {"TBUR", 21.15683011649847`, 13.299983308753916`},
{"TBUR", 127.41190036930679`, 150.5505960027829`}, {"CURE", 365, 82.80443313343443`},

{"TBUR", 91.24449047206414`, 103.6319356217739`}, {"CURE", 365, 75.7519029954397`}, {"CURE", 365, 122.66442705872075`},
{"CURE", 365, 51.3374433755955`}, {"TBUR", 139.59040172381418`, 98.79834617465897`}, {"CURE", 365, 47.64901379550728`},
{"CURE", 365, 121.50627017310703`}, {"CURE", 365, 165.28736085740027`}, {"CURE", 365, 89.7707955596049`},
{"CURE", 365, 30.71179120229616`}, {"CURE", 365, 61.37332767327205`}, {"CURE", 365, 80.31685878108549`},
{"CURE", 365, 69.03933260108305`}, {"CURE", 365, 25.59818069572806`}, {"CURE", 365, 91.97083173695107`},
{"CURE", 365, 100.27075413998112`}, {"CURE", 365, 31.73911281908348`}, {"CURE", 365, 94.1499145075375`},
{"CURE", 365, 189.2253094878968`}, {"CURE", 365, 122.3456166809528`}, {"TBUR", 20.43752866786403`, 6.393338605465769`},
{"CURE", 365, 84.51057150652176`}, {"TBUR", 39.113207002628`, 79.79879955654552`},
{"CURE", 365, 74.98042345427903`}, {"CURE", 365, 47.188271180805536`}, {"CURE", 365, 138.95070634729421`},
{"CURE", 365, 133.2866892058122`}, {"CURE", 365, 116.8307817755581`}, {"CURE", 365, 127.43785485379335`},
{"TBUR", 71.95365311040491`, 47.95619215496452`}, {"TBUR", 46.24373993913676`, 52.05788441780703`},
{"CURE", 365, 186.0892649462568`}, {"TBUR", 12.780671952625495`, 81.32333963610067`}, {"CURE", 365, 30.016715590480512`},
{"TBUR", 62.664293391212354`, 66.8975138820193`}, {"CURE", 365, 20.989576481632618`}, {"CURE", 365, 125.2683713090551 3`},
{"TBUR", 53.1415987066066`, 187.70924897895995`}, {"CURE", 365, 131.3846423656674`}, {"CURE", 365, 176.7418673319534`},
{"CURE", 365, 46.57098555315067`}, {"CURE", 365, 22.22806749203023`}, {"CURE", 365, 81.55705439763196`},
{"CURE", 365, 186.94532993807425`}, {"TBUR", 38.1067117126491`, 50.257203819671055`}, {"CURE", 365, 87.27810901969981`},
{"CURE", 365, 24.77091710498010 4`}, {"CURE", 365, 13.121970400915746`}, {"CURE", 365, 77.83389254635608`},
{"CURE", 365, 1.3803282507860661`}, {"TBUR", 88.90715731058086`, 188.74379304987198`},
{"TBUR", 50.348654439786586`, 83.8236070995326`}, {"TBUR", 88.455313292213`, 170.77963888343783`},
{"CURE", 365, 88.27836261816597`}, {"CURE", 365, 101.74079958843689`}, {"CURE", 365, 79.59350538772537`},
{"CURE", 365, 101.39107436375531`}, {"CURE", 365, 36.134418486990796`}, {"TBUR", 106.08764799463674`, 25.012289741304894`},
{"CURE", 365, 1.9124597056187638`}, {"TBUR", 71.06559712711542`, 18.96966460281164`},
{"TBUR", 78.98784042135453`, 91.04075545341338`}, {"CURE", 365, 127.16218719316004`}, {"CURE", 365, 128.4502200463252 8`},
{"CURE", 365, 95.10147638544706`}, {"CURE", 365, 131.50530268881`}, {"CURE", 365, 2.1830608377578486`},
{"CURE", 365, 76.29333465772937`}, {"TBUR", 29.884622285301436`, 144.54373735533622`}, {"CURE", 365, 67.72529020261146`},
{"TBUR", 21.64773580216636`, 8.093893148221728`}, {"TBUR", 50.35302069324431`, 142.86102220799725`},
{"TBUR", 95.40927091586374`, 11.609220188812667`}, {"TBUR", 68.09485562545788`, 176.69562017155346`},
{"TBUR", 21.254471232507193`, 89.50155915342116`}, {"TBUR", 60.67977961079943`, 54.52844531136138`},
{"TBUR", 91.28382354000267`, 111.19428592542832`}, {"TBUR", 80.51872999920374`, 127.70306032869154`},
{"CURE", 365, 93.07572610564642`}, {"CURE", 365, 161.9417623842768`}, {"TBUR", 21.688202493276894`, 13.920194130739683`},
{"CURE", 365, 144.52112829887042`}, {"TBUR", 28.129456959537485`, 50.33780121426202`},

{"TBUR", 53.6538823916201`, 158.35072117136414`}, {"CURE", 365, 82.99015649613878`}, {"CURE", 365, 4.203269712077439`},
{"TBUR", 24.42582860961019`, 105.17527944588345`}, {"CURE", 365, 102.04887559171047`},
{"CURE", 365, 157.4089349338995`}, {"CURE", 365, 187.5055923659009`}, {"CURE", 365, 124.06183725082693`},
{"CURE", 365, 75.3109813263343`}, {"CURE", 365, 58.234777329185135`}, {"CURE", 365, 195.06141974390925`},
{"TBUR", 13.862367873250685`, 17.542291055725492`}, {"CURE", 365, 108.96428701324362`}, {"CURE", 365, 102.5939787697962`},
{"TBUR", 29.19169847151401`, 25.80646194958711`}, {"TBUR", 57.2394684662013`, 110.91156835720693`},
{"CURE", 365, 118.95686335444925`}, {"TBUR", 65.27501792372969`, 86.86244630873`}, {"CURE", 365, 1.9546249917568832`},
{"TBUR", 143.55512449413524`, 43.616746521389196`}, {"TBUR", 15.940137134592455`, 97.06830873199777`},
{"CURE", 365, 195.70455810045414`}, {"CURE", 365, 21.736613629371316`}, {"CURE", 365, 142.870340097193`},
{"CURE", 365, 3.193689166078811`}, {"CURE", 365, 99.5016762186022`}, {"CURE", 365, 26.76916765410677`},
{"CURE", 365, 121.67813802791945`}, {"CURE", 365, 34.392251202571906`}, {"TBUR", 50.871335903739215`, 15.664600330901052`},
{"TBUR", 80.09342994681839`, 172.74543790079812`}, {"CURE", 365, 83.50801339469119`},
{"TBUR", 50.17339494513037`, 224.35136094965563`}, {"CURE", 365, 79.67472636900648`}, {"CURE", 365, 114.21174891963797`},
{"CURE", 365, 20.570723310410774`}, {"CURE", 365, 79.11820298611305`}, {"CURE", 365, 150.5682245223795`},
{"CURE", 365, 154.8193082321124`}, {"CURE", 365, 76.27473010350688`}, {"CURE", 365, 139.01678821924924`},
{"CURE", 365, 90.35270994740269`}, {"CURE", 365, 11.616078906385585`}, {"CURE", 365, 61.67141387450317`},
{"CURE", 365, 56.02951488708546`}, {"CURE", 365, 78.32198213173469`}, {"CURE", 365, 114.5519419642073`},
{"CURE", 365, 94.93714118608008`}, {"CURE", 365, 88.61354138988519`}, {"TBUR", 22.908385115916527`, 113.41481973637592`},
{"TBUR", 28.186437821823564`, 75.80205563753626`}, {"TBUR", 156.79518322753015`, 79.49815100495678`},
{"TBUR", 21.935447931869472`, 54.62043368279189`}, {"CURE", 365, 67.45631538834701`}, {"CURE", 365, 79.16666961524191`},
{"CURE", 365, 138.08421131207857`}, {"TBUR", 27.754198287617807`, 6.398486283025107`},
{"TBUR", 32.25793808884231`, 30.82858958968802`}, {"CURE", 365, 1.8186694055502977`}, {"CURE", 365, 2.6791785418913387`},
{"TBUR", 21.870679363151233`, 24.412269364169145`}, {"CURE", 365, 218.4106344496307`}, {"CURE", 365, 65.2699710978443`},
{"CURE", 365, 214.78355138299364`}, {"TBUR", 38.974191325989445`, 106.684924057209`}, {"CURE", 365, 80.47287803075908`},
{"TBUR", 15.333081800865191`, 129.95583617784294`}, {"CURE", 365, 60.948548784081424`},
{"CURE", 365, 110.08283096370502`}, {"CURE", 365, 16.43631046491999`}, {"CURE", 365, 82.82518736278259`},
{"CURE", 365, 34.86679671309992`}, {"CURE", 365, 9.970266921369308`}, {"CURE", 365, 132.37411389402345`},
{"TBUR", 21.75524907495498`, 132.6569137740707`}, {"TBUR", 13.770630497255775`, 95.984004630217`},
{"CURE", 365, 67.14260604426346`}, {"TBUR", 28.056779095999232`, 89.09576642186849`},
{"CURE", 365, 14.134360008703332`}, {"CURE", 365, 69.62864844408392`}, {"CURE", 365, 64.19389449359116`},
{"CURE", 365, 226.16748671556178`}, {"CURE", 365, 104.59720864856595`}, {"CURE", 365, 52.995205409026454`},

{"CURE", 365, 1.7269266709152833`}, {"TBUR", 70.15850271342494`, 98.53083276592807`}, {"CURE", 365, 82.38638492965588`},
{"TBUR", 151.5067085097425`, 86.18765616717117`}, {"TBUR", 60.14031698280403`, 24.937897790033972`},
{"CURE", 365, 93.65237894478764`}, {"CURE", 365, 63.74048057247315`}, {"TBUR", 20.72812615627609`, 79.92530211068352`},
{"CURE", 365, 51.40910826874423`}, {"CURE", 365, 113.6816368803959`}, {"CURE", 365, 27.846831019561005`},
{"TBUR", 97.91722684703772`, 106.51902963177908`}, {"TBUR", 16.627900681021213`, 43.4092880542886`},
{"CURE", 365, 86.36678619656216`}, {"TBUR", 27.546231556063432`, 107.2280847299462`}, {"CURE", 365, 9.59871160249295`},
{"CURE", 365, 127.0993042655501`}, {"CURE", 365, 75.9730774952557`}, {"CURE", 365, 211.9743937203705`},
{"CURE", 365, 143.96070937772407`}, {"CURE", 365, 102.25306189264717`}, {"CURE", 365, 66.89523599480079`},
{"CURE", 365, 212.3752685182097`}, {"TBUR", 31.47140428639931`, 37.075853495296236`},
{"CURE", 365, 19.20289747509538`}, {"CURE", 365, 42.995155512170776`}, {"CURE", 365, 104.00591131550117`},
{"CURE", 365, 99.3156990285199`}, {"CURE", 365, 120.16439180994865`}, {"CURE", 365, 46.908734070627524`},
{"TBUR", 23.300826907109464`, 154.50953531647778`}, {"CURE", 365, 11.997513838641447`}, {"CURE", 365, 133.94028990203188`},
{"TBUR", 38.74068093390788`, 156.0401986771804`}, {"CURE", 365, 74.33971420668801`}, {"CURE", 365, 128.2521515502466 8`},
{"CURE", 365, 4.196494897022179`}, {"TBUR", 22.689258531102602`, 53.23115589030034`}, {"CURE", 365, 79.24736837390212`},
{"CURE", 365, 81.07011850283925`}, {"TBUR", 29.495826926586354`, 36.74706696787679`}, {"CURE", 365, 76.2668593174393`},
{"TBUR", 37.69681262647115`, 38.60036537412746`}, {"CURE", 365, 113.37870897093202`}, {"CURE", 365, 93.98240758560995`},
{"CURE", 365, 98.37987811366348`}, {"CURE", 365, 76.32605181065156`}, {"CURE", 365, 78.72369212120496`},
{"CURE", 365, 109.77629517585783`}, {"CURE", 365, 151.11720945958228`}, {"TBUR", 49.18248289426289`, 103.77812442788482`},
{"CURE", 365, 144.31295756329857`}, {"CURE", 365, 87.50748931230116`}, {"TBUR", 63.18754311979447`, 21.45908266462014`},
{"TBUR", 17.409119349890677`, 19.62708863653837`}, {"CURE", 365, 5.381690106414668`}, {"CURE", 365, 125.54847898436097`},
{"CURE", 365, 132.22717100023056`}, {"CURE", 365, 96.23235207212095`}, {"TBUR", 139.59547235920007`, 217.58622103938157`},
{"CURE", 365, 171.8203686308701`}, {"CURE", 365, 54.59617649327205`}, {"TBUR", 20.593458359394006`, 69.04525805478475`},
{"CURE", 365, 24.794490710815154`}, {"CURE", 365, 208.8957636859165`}, {"TBUR", 39.290347717890526`, 208.56353703515353`},
{"TBUR", 24.84741538694145`, 35.425249111964874`}, {"CURE", 365, 205.49632096883417`},
{"CURE", 365, 41.252245132685054`}, {"CURE", 365, 82.4084090048012`}, {"CURE", 365, 149.9474169411449`},
{"CURE", 365, 2.3326231474485137`}, {"TBUR", 55.59996122927571`, 47.18543704179725`}, {"CURE", 365, 130.5270374122785`},
{"TBUR", 65.59569673487168`, 30.567902696703268`}, {"TBUR", 38.827558222901516`, 209.50715665104804`},
{"CURE", 365, 6.547011771702155`}, {"CURE", 365, 174.02402726768995`}, {"CURE", 365, 102.77173801542946`},
{"CURE", 365, 96.19987233274945`}, {"CURE", 365, 121.5400550048038`}, {"CURE", 365, 55.53470450736930 5`},
{"TBUR", 35.69411478362804`, 154.45134572233624`}, {"CURE", 365, 98.81925829717675`},
{"CURE", 365, 166.61410824874457`}, {"CURE", 365, 109.74720116184773`}, {"TBUR", 54.48380728828143`, 112.7376696780415`},

{"CURE", 365, 131.5363007144218`}, {"CURE", 365, 101.39945937025553`}, {"CURE", 365, 52.8555133178703`},
{"CURE", 365, 147.30914396544267`}, {"CURE", 365, 183.6972249071862`}, {"CURE", 365, 52.91559073557086`},
{"TBUR", 64.2598842648208`, 120.19224912004915`}, {"TBUR", 46.005341923466624`, 149.3934448716632`},
{"CURE", 365, 22.677869851372236`}, {"CURE", 365, 108.24264172753648`}, {"CURE", 365, 58.68028401111903`},
{"TBUR", 14.812873841924674`, 10.845172195019227`}, {"TBUR", 13.52961244325632`, 34.09946463558873`},
{"TBUR", 34.35595284529478`, 51.991252622803145`}, {"CURE", 365, 104.54423605980581`}, {"CURE", 365, 34.5076776402532`},
{"CURE", 365, 96.73368578582108`}, {"TBUR", 78.35252859651459`, 175.09753840414012`}, {"CURE", 365, 98.04096916440197`},
{"CURE", 365, 84.71390527207568`}, {"CURE", 365, 93.87546509529022`}, {"TBUR", 17.372113611837698`, 41.1997247862900 2`},
{"TBUR", 13.678082864755734`, 89.26263060884716`}, {"TBUR", 84.42630059647163`, 38.35579366189842`},
{"CURE", 365, 152.0436392829063`}, {"TBUR", 50.12027061013598`, 93.98149465368195`}, {"CURE", 365, 127.5931459999546 7`},
{"CURE", 365, 91.57059139627745`}, {"CURE", 365, 82.77426203811719`}, {"TBUR", 101.77546959063491`, 44.35986868685715`},
{"CURE", 365, 59.25255180435936`}, {"CURE", 365, 216.73934848471805`}, {"CURE", 365, 112.66074915988166`},
{"CURE", 365, 90.0213486474586`}, {"TBUR", 33.173411267468495`, 81.79223228872276`},
{"TBUR", 77.04651079849658`, 79.07580154972092`}, {"CURE", 365, 62.27099278254997`},
{"TBUR", 15.980168125501521`, 67.92020961938155`}, {"TBUR", 17.104372281936143`, 63.046519399032405`},
{"CURE", 365, 63.39436247812967`}, {"CURE", 365, 89.78511319683136`}, {"TBUR", 110.93098593207944`, 7.149969510468919`},
{"TBUR", 12.964434495050009`, 62.16075765630521`}, {"TBUR", 21.261935801260414`, 19.91547075969611 7`},
{"CURE", 365, 144.24948124842084`}, {"CURE", 365, 41.869694693508585`}, {"CURE", 365, 106.17753280756521`},
{"CURE", 365, 5.420328938473176`}, {"CURE", 365, 92.17360318509292`}, {"CURE", 365, 3.0593731617199063`},
{"CURE", 365, 105.16515861276301`}, {"CURE", 365, 98.84612745545098`}, {"TBUR", 114.8898723706838`, 112.5212601339314`},
{"CURE", 365, 7.633805542285511`}, {"CURE", 365, 50.139378699056195`}, {"CURE", 365, 206.5010297311662`},
{"TBUR", 25.232291964678762`, 50.4749201784563`}, {"CURE", 365, 1.9545119746565691`},
{"CURE", 365, 34.34587525417114`}, {"CURE", 365, 144.00363810177058`}, {"CURE", 365, 25.74132265637524`},
{"CURE", 365, 116.01829287394843`}, {"CURE", 365, 177.72884520709812`}, {"CURE", 365, 70.59781699189689`},
{"CURE", 365, 8.279224251408097`}, {"CURE", 365, 12.552418042007371`}, {"CURE", 365, 133.3339973380003`},
{"TBUR", 13.536622616819834`, 19.455887560087305`}, {"TOX", 21.834938388122623`, 238.7483558106081`},
{"CURE", 365, 87.7415662110419`}, {"CURE", 365, 111.2299491270882`}, {"TBUR", 84.89989000620514`, 86.9743378734302`},
{"CURE", 365, 60.89978984055777`}, {"TOX", 23.01564232196013`, 242.42904881729103`}, {"CURE", 365, 83.96140836926014`},
{"TBUR", 117.69153472207424`, 4.094726453087792`}, {"CURE", 365, 86.23019341491822`}, {"CURE", 365, 31.845475580116062`},
{"CURE", 365, 187.09543272142207`}, {"CURE", 365, 73.4667279510343`}, {"CURE", 365, 4.666938638729245`},
{"CURE", 365, 94.47189531503783`}, {"TBUR", 22.08834294176088`, 91.96752002908738`}, {"CURE", 365, 95.89166771493863`},

{"CURE", 365, 85.89136076024496`}, {"CURE", 365, 63.08726179006272`}, {"CURE", 365, 131.84058002681883`},
{"CURE", 365, 17.16841425028772`}, {"TBUR", 78.1964607988404`, 181.2980750592895`}, {"CURE", 365, 75.09008897353091`},
{"CURE", 365, 7.1140607436330034`}, {"CURE", 365, 37.575335035006226`}, {"CURE", 365, 218.87338499299292`},
[truncated: 1,642,292 more chars]
