# Supplementary material for: Mathematical Modeling Unveils Optimization Strategies for Targeted Radionuclide Therapy of Blood Cancers
Source: Cancer Res Commun. 2024 Nov 14;4(11):2955–67. doi: 10.1158/2767-9764.CRC-24-0306 (PMC11562018; doi:10.1158/2767-9764.CRC-24-0306)
Supplement: Computational codes — Designed in Wolfram Mathematica, version 13.3.1.0. [file crc-24-0306_computational_codes_suppscc.zip › CodesPDF/04-Impure-drug-Hetero.pdf]

```
(* This file contains simulations for the paper
  "Mathematical Modeling Unveils Optimization Strategies
for Targeted Radionuclide Therapy of Blood Cancers"
  by Maxim Kuznetsov, Vikram Adhikarla, Enrico Caserta,
 Flavia Pichiorri, John E.Shively, Xiuli Wang and Russell C.Rockne *)

(****************************************************************************************************)

(* Supplementary S.4.1 Augmentation of the model, accounting for heterogeneity of cancer cells *)

In[•]:=
(* Experimental data and the necessary scripts are hidden in this cell, which has to be initialized first *)

In[•]:=
(* 1 -- Vehicle control (no treatment) *)
ControlData = (* {days, BLI signal} *)
    {{{0, 6210000}, {7, 64900000}, {22, 7360000000}},
      {{0, 5140000}, {7, 62500000}, {22, 7870000000}},
      {{0, 8320000}, {7, 89100000}, {22, 14800000000}},
      {{0, 4800000}, {7, 34000000}, {22, 5300000000}},
      {{0, 3460000}, {7, 23400000}, {22, 5990000000}},
      {{0, 3030000}, {7, 45500000}, {22, 13100000000}},
      {{0, 7420000}, {7, 97600000}, {22, 7190000000}},
      {{0, 3840000}, {7, 56100000}, {22, 5550000000}}};
(* Day 0 here corresponds to day 8 after injection of cancer cells, when the first BLI measurement was performed
   note that injection of antibodies were performed on day 1 in current notation (day 9 of experiment) *)

(* Logarithms of BLI will be used for statistical processing --
   to avoid giving much weight to greater values due to the exponential nature of tumor cell proliferation --
   e.g., the average of 10^7 and 10^9 is 5.05·10^8, but for fitting purposes let's better assume that on average tumors grew to 10^8 cells *)
ControlData[[All, All, {2}]] = Log10[ControlData[[All, All, {2}]]];

(* Find means and errors *)
daysControl = ControlData[[1, All, 1]];
```

```
meansControl = Mean /@ Table[ControlData[[All, k, 2]], {k, 1, Length[daysControl]}];
errorsControl = N[StandardDeviation /@ Table[ControlData[[All, k, 2]], {k, 1, Length[daysControl]}]];
ControlErr = Table[{daysControl[[k]], Around[meansControl[[k]], errorsControl[[k]]]}, {k, 1, Length[daysControl]}];
ControlMean = Table[{daysControl[[k]], meansControl[[k]]}, {k, 1, Length[daysControl]}];

(* 2 -- Treatment by 600 nCi of of 225Ac-DOTA-trastumumab, which does not bind to multiple myeloma cells *)
TrasData =
  {{{0, 4620000}, {7, 20200000}, {22, 488000000}, {28, 2440000000}},
   {{0, 5540000}, {7, 27500000}, {22, 651000000}, {28, 2920000000}},
   {{0, 5050000}, {7, 25700000}, {22, 1240000000}, {28, 6180000000}},
   {{0, 5980000}, {7, 29300000}, {22, 1320000000}, {28, 4550000000}
     (*,{35,9590000000},{42,23500000000} -- this one mouse survived for a longer time, let's neglect these data points *) }};

TrasData[[All, All, {2}]] = Log10[TrasData[[All, All, {2}]]];
daysTras = TrasData[[1, All, 1]];
meansTras = Mean /@ Table[TrasData[[All, k, 2]], {k, 1, Length[daysTras]}];
errorsTras = N[StandardDeviation /@ Table[TrasData[[All, k, 2]], {k, 1, Length[daysTras]}]];
TrasErr = Table[{daysTras[[k]], Around[meansTras[[k]], errorsTras[[k]]]}, {k, 1, Length[daysTras]}];
TrasMean = Table[{daysTras[[k]], meansTras[[k]]}, {k, 1, Length[daysTras]}];

(* 3 -- Treatment by 25 nCi of 225Ac-DOTA-daratumumab *)
Dara25Data =
  {{{0, 6280000}, {8, 11235000}, {14, 54750000}, {21, 481000000}, {31, 6695000000}, {37, 37000000000}},
   {{0, 5705000}, {8, 11805000}, {14, 73850000}, {21, 608000000}, {31, 5400000000}, {37, 22650000000}},
   {{0, 5515000}, {8, 8595000}, {14, 46550000}, {21, 408000000}, {31, 217500000}, {37, 24050000000}},
   {{0, 8370000}, {8, 17750000}, {14, 11630000}, {21, 994000000}, {31, 1465000000}, {37, 32150000000}},
   {{0, 4665000}, {8, 9320000}, {14, 65100000}, {21, 559000000}, {31, 3985000000}, {37, 25200000000}},
   {{0, 4875000}, {8, 8445000}, {14, 63500000}, {21, 659500000}, {31, 4735000000}, {37, ""}}};

Dara25Data[[All, All, {2}]] = Log10[Dara25Data[[All, All, {2}]]];
daysDara25 = Dara25Data[[1, All, 1]];
```

```
meansDara25 = Join[N[Mean /@ Table[Dara25Data[[All, k, 2]], {k, 1, 5}]], N[Mean /@ Table[Dara25Data[[1 ;; 5, k, 2]], {k, {6}}]]];
errorsDara25 = Join[N[StandardDeviation /@ Table[Dara25Data[[All, k, 2]], {k, 1, 5}]],
   N[StandardDeviation /@ Table[Dara25Data[[1 ;; 5, k, 2]], {k, {6}}]]];
Dara25Err = Table[{daysDara25[[k]], Around[meansDara25[[k]], errorsDara25[[k]]]}, {k, 1, Length[daysDara25]}];
Dara25Mean = Table[{daysDara25[[k]], meansDara25[[k]]}, {k, 1, Length[daysDara25]}];

(* 4 -- Treatment by 50 nCi of 225Ac-DOTA-daratumumab *)
Dara50Data =
  {{{0, 9490000}, {8, 14970000}, {14, 89000000}, {21, 787000000}, {31, 6710000000}, {37, 31050000000}},
   {{0, 5590000}, {8, 9435000}, {14, 46550000}, {21, 406000000}, {31, 4265000000}, {37, 12670000000}},
   {{0, 6155000}, {8, 13645000}, {14, 51700000}, {21, 316500000}, {31, 3875000000}, {37, 27900000000}},
   {{0, 6650000}, {8, 15530000}, {14, 73350000}, {21, 527000000}, {31, 3120000000}, {37, 18050000000}},
   {{0, 6010000}, {8, 15030000}, {14, 55800000}, {21, 554000000}, {31, 5510000000}, {37, 20800000000}},
   {{0, 4670000}, {8, 12650000}, {14, 49000000}, {21, 519500000}, {31, 9415000000}, {37, 34350000000}}};

Dara50Data[[All, All, {2}]] = Log10[Dara50Data[[All, All, {2}]]];
daysDara50 = Dara50Data[[1, All, 1]];
meansDara50 = Mean /@ Table[Dara50Data[[All, k, 2]], {k, 1, Length[daysDara50]}];
errorsDara50 = N[StandardDeviation /@ Table[Dara50Data[[All, k, 2]], {k, 1, Length[daysDara50]}]];
Dara50Err = Table[{daysDara50[[k]], Around[meansDara50[[k]], errorsDara50[[k]]]}, {k, 1, Length[daysDara50]}];
Dara50Mean = Table[{daysDara50[[k]], meansDara50[[k]]}, {k, 1, Length[daysDara50]}];

(* 5 -- Treatment by 100 nCi of 225Ac-DOTA-daratumumab *)
Dara100Data =
  {{{0, 4420000}, {8, 5405000}, {14, 4515000}, {21, 8010000}, {31, 36790000},
     {37, 108000000}, {44, 766000000}, {51, 3450000000}, {58, 14930000000} (*,{65,128250000000}*)},
   {{0, 4545000}, {8, 3295000}, {14, 3910000}, {21, 5695000}, {31, 22150000},
     {37, 169850000}, {44, 1431000000}, {51, 9245000000}, {58, 297000000} (*,{65,117450000000}*)},
   {{0, 3960000}, {8, 4275000}, {14, 3535000}, {21, 8120000},
     {31, 72850000}, {37, 84200000}, {44, 1252000000}, {51, 354500000}, {58, 20300000000}},
   {{0, 6855000}, {8, 4545000}, {14, 3235000}, {21, 4925000},
```

```
    {31, 16 815 000}, {37, 11 445 000}, {44, 62 950 000}, {51, 884 000 000}, {58, 7 670 000 000}},
  {{0, 5 425 000}, {8, 4 215 000}, {14, 3 595 000},
    {21, 5 430 000}, {31, 27 350 000}, {37, 126 500 000}, {44, 588 500 000}, {51, 4 660 000 000}, {58, ""}},
  {{0, 4 920 000}, {8, 3 370 000}, {14, 4 420 000},
    {21, 18 880 000}, {31, 297 300 000}, {37, 612 000 000}, {44, 7 435 000 000}, {51, ""}, {58, ""}}};

Dara100Data⟦All, All, {2}⟧ = Log10[Dara100Data⟦All, All, {2}⟧];
daysDara100 = Dara100Data⟦1, All, 1⟧;
meansDara100 = Join[N[Mean /@ Table[Dara100Data⟦All, k, 2⟧, {k, 1, 7}]],
    N[Mean /@ Table[Dara100Data⟦1 ;; 5, k, 2⟧, {k, {8}}]], N[Mean /@ Table[Dara100Data⟦1 ;; 4, k, 2⟧, {k, {9}}]]];
errorsDara100 = Join[N[StandardDeviation /@ Table[Dara100Data⟦All ;;, k, 2⟧, {k, 1, 7}]],
    N[StandardDeviation /@ Table[Dara100Data⟦1 ;; 5, k, 2⟧, {k, {8}}]],
    N[StandardDeviation /@ Table[Dara100Data⟦1 ;; 4, k, 2⟧, {k, {9}}]]];
Dara100Err = Table[{daysDara100⟦k⟧, Around[meansDara100⟦k⟧, errorsDara100⟦k⟧]}, {k, 1, Length[daysDara100]}];
Dara100Mean = Table[{daysDara100⟦k⟧, meansDara100⟦k⟧}, {k, 1, Length[daysDara100]}];

(* 6 -- Treatment by 300 nCi of 225Ac-DOTA-daratumumab *)
Dara300Data =
  {{{0, 3 540 000}, {7, 3 780 000}, {22, 5 850 000}, {28, 13 500 000}, {35, 69 400 000}, {42, 455 000 000}, {56, 18 400 000 000}},
   {{0, 4 470 000}, {7, 4 010 000}, {22, 7 340 000}, {28, 4 010 000}, {35, 6 690 000}, {42, 53 900 000}, {56, 6 890 000 000}},
   {{0, 7 660 000}, {7, 4 890 000}, {22, 19 300 000}, {28, 4 610 000}, {35, 129 000 000}, {42, 1 570 000 000}, {56, 36 700 000 000}},
   {{0, 6 340 000}, {7, 4 570 000}, {22, 5 840 000}, {28, 13 800 000}, {35, 29 000 000}, {42, 58 300 000}, {56, 9 860 000 000}}};

Dara300Data⟦All, All, {2}⟧ = Log10[Dara300Data⟦All, All, {2}⟧];
daysDara300 = Dara300Data⟦1, All, 1⟧;
meansDara300 = Mean /@ Table[Dara300Data⟦All, k, 2⟧, {k, 1, Length[daysDara300]}];
errorsDara300 = N[StandardDeviation /@ Table[Dara300Data⟦All, k, 2⟧, {k, 1, Length[daysDara300]}]];
Dara300Err = Table[{daysDara300⟦k⟧, Around[meansDara300⟦k⟧, errorsDara300⟦k⟧]}, {k, 1, Length[daysDara300]}];
Dara300Mean = Table[{daysDara300⟦k⟧, meansDara300⟦k⟧}, {k, 1, Length[daysDara300]}];

(* 7 -- Treatment by 600 nCi of 225Ac-DOTA-daratumumab *)
```

```
Dara600Data =
  {{{0, 7080000}, {7, 2490000}, {22, 3220000}, {28, 2720000}, {35, 2410000}, {42, ""}, {56, ""}}, (* this mouse died due to toxicity *)
   {{0, 4670000}, {7, 2280000}, {22, 2000000}, {28, 2580000}, {35, 3520000}, {42, 14000000}, {56, 102000000}},
   {{0, 3860000}, {7, 2820000}, {22, 2960000}, {28, 2960000}, {35, 2980000}, {42, 17100000}, {56, 119000000}},
   {{0, 6170000}, {7, 3410000}, {22, 3650000}, {28, 3400000}, {35, 4480000}, {42, 179000000}, {56, 257000000}}};

Dara600Data[[All, All, {2}]] = Log10[Dara600Data[[All, All, {2}]]];
daysDara600 = Dara600Data[[1, All, 1]];
meansDara600 = Join[N[Mean /@ Table[Dara600Data[[All, k, 2]], {k, 1, 5}]], N[Mean /@ Table[Dara600Data[[2 ;;, k, 2]], {k, 6, 7}]]];
errorsDara600 = Join[N[StandardDeviation /@ Table[Dara600Data[[All, k, 2]], {k, 1, 5}]],
   N[StandardDeviation /@ Table[Dara600Data[[2 ;;, k, 2]], {k, 6, 7}]]];
Dara600Err = Table[{daysDara600[[k]], Around[meansDara600[[k]], errorsDara600[[k]]]}, {k, 1, Length[daysDara600]}];
Dara600Mean = Table[{daysDara600[[k]], meansDara600[[k]]}, {k, 1, Length[daysDara600]}];

(* Setting colorcode corresponding to the figures in experimental paper *)
ControlColor = RGBColor[51 / 255, 75 / 255, 160 / 255];
TrasColor = RGBColor[13 / 255, 156 / 255, 72 / 255];
Dara25Color = RGBColor[98 / 255, 186 / 255, 70 / 255];
Dara50Color = RGBColor[170 / 255, 121 / 255, 181 / 255];
Dara100Color = RGBColor[235 / 255, 11 / 255, 124 / 255];
Dara300Color = RGBColor[127 / 255, 70 / 255, 154 / 255];
Dara600Color = RGBColor[238 / 255, 43 / 255, 36 / 255];

Dara600ErrRegr = Table[Dara600Err[[Length[Dara600Err] - ii]], {ii, 2, 0, -1}];
Dara600MeanRegr = Table[Dara600Mean[[Length[Dara600Mean] - ii]], {ii, 2, 0, -1}];
Dara300ErrRegr = Table[Dara300Err[[Length[Dara300Err] - ii]], {ii, 2, 0, -1}];
Dara300MeanRegr = Table[Dara300Mean[[Length[Dara300Mean] - ii]], {ii, 2, 0, -1}];
```

In[ ]:=

```
(* Script for fitting cancer cell proliferation rate during regrowth *)
FitRhoRegrowth[] := (DaraErrRegr = Table[DaraErr[[Length[DaraErr] - ii]], {ii, 2, 0, -1}];
```

```
DaraMeanRegr = Table[DaraMean[[Length[DaraMean] - ii]], {ii, 2, 0, -1}];

(* Setting boundaries and step of variation*)
rhomin = 0.1; rhomax = 0.4; rhostep = 0.01;

(* Table which will contain the deviations of simulations from the experimental data *)
result = Array[f, {Round[(rhomax - rhomin) / rhostep, 1] + 2, 2}];
result[[1, 1]] = "rho";
result[[1, 2]] = "SSD"; (* sum of squared deviations *)

N0 = 10^DaraMeanRegr[[1, 2]]; (* Initial "number of tumor cells"
  (coefficient of proportionality between them and radiance can be set to 1, since it does not change in dynamics) *)

ii = 2;
For[rho = rhomin, rho ≤ rhomax, rho = rho + rhostep,

  NotebookDelete[pr]; (* To see the code running *)
  pr = PrintTemporary["Current rho is " <> ToString[rho] <> "; max value is " <> ToString[rhomax]];

  (* The day of the last experimental value *)
  tBegin = DaraMeanRegr[[1, 1]];
  tEnd = DaraMeanRegr[[Length[DaraMeanRegr], 1]];
  Clear[NN];

  (* Straightforward solution of highly reduced model, which is now boils down to literally one exponential equation *)
  sol = NDSolve[{
        (* Initial conditions *)
        NN[tBegin] == N0,
        (* We need only the equation for the number of viable tumor cells, there are no damaged cells now *)
        NN'[t] == rho * NN[t]}
      , {NN}, {t, tBegin, tEnd}];
```

```
    NN = First[NN /. sol]; (* Save solution *)

    (* Save sum of squared deviations *)
    result〚ii, 1〛 = rho;
    result〚ii, 2〛 = Sum[(Log10[NN[DaraMeanRegr〚kk, 1〛]] - DaraMeanRegr〚kk, 2〛) ^2, {kk, 1, Length[DaraMeanRegr]}];

    ii++;
  ];

  (* Find optimal rho *)
  DevMin = Min[result〚All, 2〛〚2 ;;〛];
  PosOpt = Position[result〚All, 2〛, DevMin]〚1, 1〛;
  rhoOpt = result〚PosOpt, 1〛;

  IS = 300;
  (* Visualize results *)
  PicVar = ListLogPlot[result, AxesLabel → {"ρ", "SSD"}, PlotLabel → "Optimal ρ is " <> ToString[rhoOpt] <>
        "; " <> ToString[PercentForm[Round[rhoOpt / 0.34, 0.01]]] <> "of basic value", ImageSize → IS];

  (* Run simulation with it *)
  Clear[NN];
  sol = NDSolve[{
        NN[tBegin] == N0,
        NN'[t] == rhoOpt * NN[t]}
      , {NN}, {t, tBegin, tEnd}];
  NN = First[NN /. sol];

  (* Plot simulation with optimal rho along with experimental data *)
  PicOptSim =
    Show[Plot[{{Log10[NN[t]]}}, {t, tBegin, 1.01 * tEnd}, Filling → {1 → Axis}, PlotStyle → {Directive[Lighter[Gray], Thickness[0.002]]},
        PlotRange → {0.95 * Log10[N0], 1.02 * NMaximize[{Log10[NN[t]], t > tBegin, t < tEnd}, t]〚1〛},
```

```
        AxesLabel → {"days", "Log (Radiance) "},
        (*PlotLegends→Placed[{"N"},Above],*) ImageSize → 280], ListPlot[DaraErrRegr], ImageSize → IS];

    PicRho = GraphicsGrid[{{PicVar, PicOptSim}}, ImageSize → {2.2 * IS, IS}])
```

In[ ]:=

```
(* Script for the solution of full system with variable ρ and α *)

FullSystemSolutionVarRAO[] := (

    (* Radiation damage function *)
    RD[NN_, DD_, fAN_, fAD_, a_, p_, rho_] :=
     ((rho - rhomin)/(rhomax - rhomin) * (alphamax - alphamin) + alphamin) * (ks * (lambda * gamma * fAN)/nu (*self-dose*) +
        (1 - ks) * (lambda * gamma * (fAN * NN + fAD * DD))/(nu * (NN + DD)) (*cross-fire*) + kf * lambda * (a + p)) (*dose from unanchored nuclides*);

    Clear[a, b, NN, DD, p, fFN, fAN, fFD, fAD, rho];
    sol = NDSolve[{
        (* Initial conditions *)
        a[0] == 0, b[0] == 0, NN[0] == N0, DD[0] == 0.0000000000000001
        (*to avoid division by zero*), p[0] == 0, fFN[0] == 1, fAN[0] == 0, fFD[0] == 1, fAD[0] == 0, rho[0] == rhomax,
        (* Equations *)
        (* Active antibodies *)
        a'[t] == AInjFunc[t] (*injection*) - lambda * a[t] (*decay*) -
          kon * gamma/V * (fFN[t] * NN[t] + fFD[t] * DD[t]) * a[t] (*binding*) - kappac * a[t] (*clearance*),
        (* Inert antibodies *)
        b'[t] == eta * AInjFunc[t] (*injection*) + lambda * a[t] (*decay of a*) -
          kon * gamma/V * (fFN[t] * NN[t] + fFD[t] * DD[t]) * b[t] (*binding*) - kappac * b[t] (*clearance*),
        (* Viable cells *)
```

```
NN'[t] == rho[t] * NN[t] (*proliferation*) - RD[NN[t], DD[t], fAN[t], fAD[t], a[t], p[t], rho[t]] * NN[t] (*damage*),
(* Damaged cells *)
DD'[t] == RD[NN[t], DD[t], fAN[t], fAD[t], a[t], p[t], rho[t]] * NN[t] (*damage*) - (* rho[t]/rhomax **) omega * DD[t] (*death*),
(* Active fragments *)
p'[t] == (* rho[t]/rhomax **) omega * DD[t] * (gamma * fAD[t])/V (*release*) - lambda * p[t] (*decay*) - kappap * p[t] (*clearance*),
(* Free receptors on viable cells *) fFN'[t] == (1 - fFN[t]) * rho[t] - kon * (a[t] + b[t]) * fFN[t],
(* Active receptors on viable cells *) fAN'[t] == kon * a[t] * fFN[t] - (lambda + rho[t]) * fAN[t],
(* Free receptors on damaged cells *)
fFD'[t] == (fFN[t] - fFD[t]) * RD[NN[t], DD[t], fAN[t], fAD[t], a[t], p[t], rho[t]] * NN[t]/DD[t] - kon * (a[t] + b[t]) * fFD[t],
(* Active receptors on damaged cells *) fAD'[t] ==
  (fAN[t] - fAD[t]) * RD[NN[t], DD[t], fAN[t], fAD[t], a[t], p[t], rho[t]] * NN[t]/DD[t] + kon * a[t] * fFD[t] - lambda * fAD[t],
(* Proliferation rate *) rho'[t] == -kD * rho[t] * Log[rho[t] / rhomin] * RD[NN[t], DD[t], fAN[t], fAD[t], a[t], p[t], rho[t]]}
, {a, b, NN, DD, p, fFN, fAN, fFD, fAD, rho}, {t, 0, tEnd}];
a = First[a /. sol]; b = First[b /. sol]; NN = First[NN /. sol]; DD = First[DD /. sol];
p = First[p /. sol]; fFN = First[fFN /. sol]; fAN = First[fAN /. sol];
fFD = First[fFD /. sol]; fAD = First[fAD /. sol]; rho = First[rho /. sol]; (* Save solution *))
```

In[ ]:=

```
SetBasicParameterValuesReduced[] := (
  lambda = 0.07; (* -225Ac decay rate *)
  kon = 11.15; (* daratumumab-CD38 association rate *)
  kappac = 0.1; (* antibody clearance rate (0.04-0.28) *)
  kappap = 1; (* antibody fragments clearance rate (0.4-4.) *)
  gamma = 2.1; (* number of receptors on 10^7 cancer cells (0.13-10) *)
  V = 1.; (* volume of drug distribution (0.75-1.5) *)
  nu = 0.015; (* volume of lesion with 10^7 cancer cells *)
  ks = 0.3; (* relative significance of self-dose (0.1-0.5) *)
  rhomax = 0.34; (* maximum cancer cells proliferation rate -- already estimated *)
  omega = 0.05; (* damaged cells death rate -- already estimated *)
  kf = 0.05; (* significance of unanchored nuclides decays (0.01-0.25) *)
  N0 = 3.; (* initial number of cancer cells, x10^7 (1-10) *)
  nCpm = 0.000076; (* factor of conversion from nCi to pmol *))
```

In[ ]:=

```
(* Script for the solution of full system *)

FullSystemSolutionHetMD[] :=(

  (* maximum time of simulation-- where little radioactivity of the last dose remains, namely 0.17% of the last dose *)
  tEnd = Ainj[[Length[Ainj], 1]] + (-Log[0.0017] / lambda);
  lAinj = Length[Ainj]; (* number of injections*)
  If[Ainj[[1, 1]] == 0, Npw = lAinj, Npw = lAinj + 1];
  (* the injections are treated as new initial conditions for a new system, which as well takes the actual vaules of other parameters *)
  (* therefore the number of injections has to me remembered and logic differs whether the first injection is made at t=0 or t>0 *)
  apw = Array[ff, Npw]; bpw = Array[ff, Npw]; NNpw = Array[ff, Npw]; DDpw = Array[ff, Npw];
  papw = Array[ff, Npw]; pbpw = Array[ff, Npw]; fFNpw = Array[ff, Npw]; fANpw = Array[ff, Npw];
  dFpw = Array[ff, Npw];
  dApw = Array[ff, Npw];
  rhopw = Array[ff, Npw]; (* cancer cell proliferation rate -- is now an explicit variable,
  while variables alpha and omega can be expressed through it *)
```

```
(* inert antibody fragments are as well accounted for as pb *)

(* times of beginning and end for solution of separate systems *)
tB = Array[ff, Npw]; tE = Array[ff, Npw];

(* for monitoring the paths of activity *)
ActBloodpw = Array[ff, Npw]; ActBloodFragpw = Array[ff, Npw];
ActOutpw = Array[ff, Npw]; ActOutFragpw = Array[ff, Npw]; ActTumorpw = Array[ff, Npw];

(* for monitoring influence of self-dose, croos-fire and decays in blood *)
SDpw = Array[ff, Npw]; CFNpw = Array[ff, Npw]; CFDpw = Array[ff, Npw]; UNpw = Array[ff, Npw];

(* for monitoring the number of new cancer cells appearing during treatment *)
NewCellspw = Array[ff, Npw];

Clear[a, b, NN, DD, pa, pb, fFN, fAN, dF, dA, rho, ActBlood, ActBloodFrag, ActOut, ActOutFrag, ActTumor, SD, CFN, CFD, UN, NewCells];

(* EQUATIONS *)

(* Radiation damage function *)
RD[NN_, DD_, fAN_, dA_, a_, pa_, rho_] :=
```

$$\left(\frac{rho - rhomin}{rhomax - rhomin} * (alphamax - alphamin) + alphamin\right) (*alpha*) * \left(ks * \frac{lambda * gamma * fAN}{nu} (*self-dose*) + (1 - ks) * \frac{lambda * gamma * (fAN * NN + dA)}{nu * (NN + DD)} (*cross-fire*) + kf * lambda * (a + pa) (*dose from unanchored nuclides*)\right);$$

```
(* Active antibodies *)
Fa[t_] := (*injections are considered as initial conditions*)
```

$$- lambda * a[t] (*decay*) - kon * \frac{gamma}{V} * (fFN[t] * NN[t] + dF[t]) * a[t] (*binding*) - kappac * a[t] (*clearance*);$$

```
(* Inert antibodies *)
Fb[t_] := (*injections are considered as initial conditions*)
  + lambda * a[t] (*decay of a*) - kon * gamma/V * (fFN[t] * NN[t] + dF[t]) * b[t] (*binding*) - kappac * b[t] (*clearance*);

(* Viable cells *)
FNN[t_] := rho[t] * NN[t] (*proliferation*) - RD[NN[t], DD[t], fAN[t], dA[t], a[t], pa[t], rho[t]] * NN[t] (*damage*);

(* Damaged cells *)
FDD[t_] := RD[NN[t], DD[t], fAN[t], dA[t], a[t], pa[t], rho[t]] * NN[t] (*damage*) - omega * DD[t] (*death*);

(* Active fragments *) Fpa[t_] := omega * (gamma * dA[t])/V (*release*) - lambda * pa[t] (*decay*) - kappap * pa[t] (*clearance*);

(* Inert fragments *)
Fpb[t_] := omega * (gamma * (DD[t] - dF[t] - dA[t]))/V (*release*) + lambda * pa[t] (*decay*) - kappap * pb[t] (*clearance*);

(* Free receptors of viable cells *) FfFN[t_] := (1 - fFN[t]) * rho[t] - kon * (a[t] + b[t]) * fFN[t];

(* Active receptors of viable cells *) FfAN[t_] := kon * a[t] * fFN[t] - (lambda + rho[t]) * fAN[t];

(* Free receptors of damaged cells *)
FdF[t_] :=
  RD[NN[t], DD[t], fAN[t], dA[t], a[t], pa[t], rho[t]] * fFN[t] * NN[t] - kon * (a[t] + b[t]) * dF[t] - omega * dF[t];

(* Active receptors of damaged cells *)
FdA[t_] :=
  RD[NN[t], DD[t], fAN[t], dA[t], a[t], pa[t], rho[t]] * fAN[t] * NN[t] + kon * a[t] * dF[t] - lambda * dA[t] - omega * dA[t];
```

```
(* Cancer cell proliferation rate *)
Frho[t_] := -kD * rho[t] * Log[rho[t] / rhomin] * RD[NN[t], DD[t], fAN[t], dA[t], a[t], pa[t], rho[t]];

(* Initial conditions *)

If[Ainj⟦1, 1⟧ == 0
  , a0 = Ainj⟦1, 2⟧ / V; b0 = Ainj⟦1, 3⟧ / V
  , a0 = 0; b0 = 0]; (*complexes in blood*)
NN0 = N0;
DD0 = 0;
pa0 = 0; pb0 = 0; fFN0 = 1; fAN0 = 0;
dF0 = 0; dA0 = 0; rho0 = rhomax;
ActBlood0 = 0; ActBloodFrag0 = 0; ActOut0 = 0; ActOutFrag0 = 0; ActTumor0 = 0; NewCells0 = 0;
SD0 = 0; CFN0 = 0; CFD0 = 0; UN0 = 0;

(* SOLVER *)

tB⟦1⟧ = 0; If[Ainj⟦1, 1⟧ == 0, If[lAinj > 1, tE⟦1⟧ = Ainj⟦2, 1⟧, tE⟦1⟧ = tEnd], tE⟦1⟧ = Ainj⟦1, 1⟧];

For[npw = 1, npw ≤ Npw, npw++,

  Clear[a, b, NN, DD, pa, pb, fFN, fAN, dF, dA, rho, ActBlood, ActBloodFrag, ActOut, ActOutFrag, ActTumor, SD, CFN, CFD, UN, NewCells];
   (*fFD,fAD,*)

  sol = NDSolve[{
        (*INITIAL CONDITIONS*)
        a[tB⟦npw⟧] == a0, b[tB⟦npw⟧] == b0, NN[tB⟦npw⟧] == NN0, DD[tB⟦npw⟧] == DD0, pa[tB⟦npw⟧] == pa0, pb[tB⟦npw⟧] == pb0,
        fFN[tB⟦npw⟧] == fFN0, fAN[tB⟦npw⟧] == fAN0, dF[tB⟦npw⟧] == dF0, dA[tB⟦npw⟧] == dA0, rho[tB⟦npw⟧] == rho0,
        ActBlood[tB⟦npw⟧] == ActBlood0, ActBloodFrag[tB⟦npw⟧] == ActBloodFrag0, ActOut[tB⟦npw⟧] == ActOut0,
        ActOutFrag[tB⟦npw⟧] == ActOutFrag0, ActTumor[tB⟦npw⟧] == ActTumor0, SD[tB⟦npw⟧] == SD0,
```

```
        CFN[tB⟦npw⟧] == CFN0, CFD[tB⟦npw⟧] == CFD0, UN[tB⟦npw⟧] == UN0, NewCells[tB⟦npw⟧] == NewCells0,

        a'[t] == Fa[t], b'[t] == Fb[t], NN'[t] == FNN[t], DD'[t] == FDD[t], pa'[t] == Fpa[t],
        pb'[t] == Fpb[t], fFN'[t] == FfFN[t], fAN'[t] == FfAN[t], dF'[t] == FdF[t], dA'[t] == FdA[t], rho'[t] == Frho[t],

        ActBlood'[t] == V * lambda * (a[t] + pa[t]),
        ActBloodFrag'[t] == V * lambda * pa[t],
        ActOut'[t] == V * (kappac * a[t] + kappap * pa[t]),
        ActOutFrag'[t] == V * kappap * pa[t],
        ActTumor'[t] == (lambda * gamma) * (fAN[t] * NN[t] + dA[t]),

        SD'[t] == ks * (lambda * gamma) * (fAN[t] * NN[t]),
        CFN'[t] == (1 - ks) * (lambda * gamma) * (fAN[t] * NN[t]) * NN[t] / (NN[t] + DD[t]),
        CFD'[t] == (1 - ks) * (lambda * gamma) * dA[t] * NN[t] / (NN[t] + DD[t]),
        UN'[t] == kf * lambda * (a[t] + pa[t]) * nu * NN[t],
        NewCells'[t] == If[t > Ainj⟦1, 1⟧, rho[t] * NN[t], 0], (* start counting new cells from the moment of the first injection *)
        WhenEvent[NN[t] > Cd / Nnor, NN[t] → 0.99 * Cd / Nnor]
      }
    , {a, b, NN, DD, pa, pb, fFN, fAN, dF, dA, rho, ActBlood,
        ActBloodFrag, ActOut, ActOutFrag, ActTumor, SD, CFN, CFD, UN, NewCells}, {t, tB⟦npw⟧, tE⟦npw⟧}
    , AccuracyGoal → 10, PrecisionGoal → 10];

  apw⟦npw⟧ = First[a /. sol]; bpw⟦npw⟧ = First[b /. sol]; NNpw⟦npw⟧ = First[NN /. sol]; DDpw⟦npw⟧ = First[DD /. sol];
  papw⟦npw⟧ = First[pa /. sol]; pbpw⟦npw⟧ = First[pb /. sol]; fFNpw⟦npw⟧ = First[fFN /. sol]; fANpw⟦npw⟧ = First[fAN /. sol];
  dFpw⟦npw⟧ = First[dF /. sol]; dApw⟦npw⟧ = First[dA /. sol]; rhopw⟦npw⟧ = First[rho /. sol];
  ActBloodpw⟦npw⟧ = First[ActBlood /. sol];
  ActBloodFragpw⟦npw⟧ = First[ActBloodFrag /. sol];
  ActOutpw⟦npw⟧ = First[ActOut /. sol];
```

```
ActOutFragpw⟦npw⟧ = First[ActOutFrag /. sol];
ActTumorpw⟦npw⟧ = First[ActTumor /. sol];
SDpw⟦npw⟧ = First[SD /. sol];
CFNpw⟦npw⟧ = First[CFN /. sol];
CFDpw⟦npw⟧ = First[CFD /. sol];
UNpw⟦npw⟧ = First[UN /. sol];
NewCellspw⟦npw⟧ = First[NewCells /. sol];

If[npw < Npw,
  (*renew initial conditions*)
  If[Ainj⟦1, 1⟧ == 0
    , a0 = apw⟦npw⟧[tE⟦npw⟧] + Ainj⟦npw + 1, 2⟧/V; b0 = bpw⟦npw⟧[tE⟦npw⟧] + Ainj⟦npw + 1, 3⟧/V
    , a0 = apw⟦npw⟧[tE⟦npw⟧] + Ainj⟦npw, 2⟧/V; b0 = bpw⟦npw⟧[tE⟦npw⟧] + Ainj⟦npw, 3⟧/V];

  NN0 = NNpw⟦npw⟧[tE⟦npw⟧];
  DD0 = DDpw⟦npw⟧[tE⟦npw⟧];
  pa0 = papw⟦npw⟧[tE⟦npw⟧];
  pb0 = pbpw⟦npw⟧[tE⟦npw⟧];
  fFN0 = fFNpw⟦npw⟧[tE⟦npw⟧]; fAN0 = fANpw⟦npw⟧[tE⟦npw⟧];
  dF0 = dFpw⟦npw⟧[tE⟦npw⟧];
  dA0 = dApw⟦npw⟧[tE⟦npw⟧];
  rho0 = rhopw⟦npw⟧[tE⟦npw⟧];
  ActBlood0 = ActBloodpw⟦npw⟧[tE⟦npw⟧];
  ActBloodFrag0 = ActBloodFragpw⟦npw⟧[tE⟦npw⟧];
  ActOut0 = ActOutpw⟦npw⟧[tE⟦npw⟧];
  ActOutFrag0 = ActOutFragpw⟦npw⟧[tE⟦npw⟧];
  ActTumor0 = ActTumorpw⟦npw⟧[tE⟦npw⟧];
  SD0 = SDpw⟦npw⟧[tE⟦npw⟧];
  CFN0 = CFNpw⟦npw⟧[tE⟦npw⟧];
  CFD0 = CFDpw⟦npw⟧[tE⟦npw⟧];
  UN0 = UNpw⟦npw⟧[tE⟦npw⟧];
```

```
          NewCells0 = NewCellspw[[npw]][tE[[npw]]];

          (*renew time frame*)
          tB[[npw + 1]] = tE[[npw]];

          If[Ainj[[1, 1]] == 0, If[Npw > npw + 1, tE[[npw + 1]] = Ainj[[npw + 2, 1]], tE[[npw + 1]] = tEnd],
            If[Npw > npw + 1, tE[[npw + 1]] = Ainj[[npw + 1, 1]], tE[[npw + 1]] = tEnd]];]
       ];
       npw--;
       (* It will be convenient to have estimation of minimal viable cell number here *)
       TableNN = If[Ainj[[1, 1]] == 0, Table[NMinimize[{Nnor* (NNpw[[nn]][t]), t > tB[[nn]], t < tE[[nn]]}, t], {nn, 1, npw}],
          Table[NMinimize[{Nnor* (NNpw[[nn]][t]), t > tB[[nn]], t < tE[[nn]]}, t][[1]], {nn, 2, npw}]];

       Nn = Min[TableNN[[All, 1]]];

       tminNn = t /. TableNN[[Position[TableNN[[All, 1]], Nn][[1, 1]]]][[2]];

       Return[Nn])
```

In[∘]:=

```
   SetBasicParameterValuesHet[] := (
       Nnor = 10^5;  (* due to numerical pecularities, it is better to use this normalization number of cells *)

       lambda = 0.07; (* radionuclide decay rate *)
       kon = 11.15;  (* antibody−receptor association rate *)
       kappac = 0.1; (* antibody clearance rate (0.04−0.28) *)
       kappap = 1;  (* antibody fragments clearance rate (0.4−4.) *)
       gamma = 2.1*Nnor / 10^7;  (* number of receptors of Nnor cancer cells (0.13−10)*Nnor/10^7 *)
       V = 1.;  (* volume of drug distribution (0.75−1.5) *)
       nu = 0.015*Nnor / 10^7;  (* volume of lesion with Nnor cancer cells *)
       ks = 0.3;  (* relative significance of self−dose (0.1−0.5) *)
```

```
    rhomax = 0.34; (* initial (and maximum) cancer cells proliferation rate (0.15-0.7) *)
    rhomin = 0.2; (* minimum cancer cells proliferation rate 0.05-min (0.25,ρ0) *)
    omega = 0.05; (* damaged cells death rate (0.005-0.5) *)
    kf = 0.05; (* significance of unanchored nuclides decays (0.01-0.25) *)
    N0 = 3. * 10^7 / Nnor; (* initial number of viable cancer cells, xNnor (1-10) *Nnor / 10^7 *)
    eta = 1780; (* coefficient of drug impurity (0-10^5) *)
    kD = 0.6; (* sensitivity of population-based parameters to radiation (0.1-1.5) *)
    alphamax = 2400.; (* initial (and maximum) cancer cell radiosensitivity (2000-4000) *)
    alphamin = 90; (* minimum cancer cell radiosensitivity (20-400) *)

    nCpm = 0.000076; (* convenient parameter of conversion from nCi to pmol *)
    DA1 = 50 * nCpm; (* injected amount of radionuclides *)
    t1 = 0; (* moment of their injection *)
    Ncur = 0.01; (* number of viable cancer cells (in cells), corresponding to cancer cure *)
    Abld = 0.0175; (* critical amount of decays in blood, leading to death of toxicity (230 nCi) *)
    Cd = 10^11; (* critical number of cacner cells, leading to death of tumor burden *)
    Ainj = {{t1, DA1, eta * DA1}}; (* Schedule of injections *)

    kGy = 2500; (* coefficient of conversion of nM of 225-Ac decay in bone marrow into Gy *)
  )
SetBasicParameterValuesHet[];
```

In[ ]:=

```
(* Script for finding curative dose -- initialize it *)
(*FindCurDose[np_]*)
FindCurDoseHet[] := (Quiet[DA1 = (nu * N0 * rhomax)/(alphamax * lambda) / 2; (* at this dose N'(0)=0 for approximated system *)
    Ainj = {{t1, DA1, eta * DA1}};

    While[FullSystemSolutionHetMD[] > Ncur, DA1 = 2 * DA1; Ainj = {{t1, DA1, eta * DA1}}; ];
    Dmax = DA1; Dmin = DA1 / 2;

    While[Abs[Dmin - Dmax] / Dmax > 0.000001,
      DA1 = (Dmin + Dmax) / 2; Ainj = {{t1, DA1, eta * DA1}};
      If[FullSystemSolutionHetMD[] > Ncur, Dmin = DA1, Dmax = DA1]];
    Dcur = Dmax;
    Return[Dcur / nCpm]])
```

In[ ]:=

```
(* This is the script for finding minimal lethally toxic dose -- initialize it *)
FindToxDoseHet[] := (DA1 = Abld;
   Ainj = {{t1, DA1, eta * DA1}};

   While[FullSystemSolutionToxicityHet[] < Abld, DA1 = 2 * DA1; Ainj = {{t1, DA1, eta * DA1}}; ];
   Dmax = DA1; Dmin = DA1 / 2;

   While[Abs[Dmin - Dmax] / Dmax > 0.000001,
     DA1 = (Dmin + Dmax) / 2; Ainj = {{t1, DA1, eta * DA1}};
     If[FullSystemSolutionToxicityHet[] < Abld, Dmin = DA1, Dmax = DA1]];
   Dtox = Dmax;
   Return[Dtox / nCpm])
```

```
FullSystemSolutionToxicityHet[] := (

  (* maximum time of simulation-- where little radioactivity of the last dose remains, namely 0.17% of the last dose *)
  tEnd = Ainj[[Length[Ainj], 1]] + (-Log[0.0017] / lambda);
  lAinj = Length[Ainj]; (* number of injections*)
  If[Ainj[[1, 1]] == 0, Npw = lAinj, Npw = lAinj + 1];
  (* the injections are treated as new initial conditions for a new system, which as well takes the actual vaules of other parameters *)
  (* therefore the number of injections has to me remembered and logic differs whether the first injection is made at t=0 or t>0 *)
  apw = Array[ff, Npw]; bpw = Array[ff, Npw]; NNpw = Array[ff, Npw]; DDpw = Array[ff, Npw];
  papw = Array[ff, Npw]; pbpw = Array[ff, Npw]; fFNpw = Array[ff, Npw]; fANpw = Array[ff, Npw];
  dFpw = Array[ff, Npw];
  dApw = Array[ff, Npw];
  rhopw = Array[ff, Npw]; (* cancer cell proliferation rate -- is now an explicit variable,
  while variables alpha and omega can be expressed through it *)
  (* inert antibody fragments are as well accounted for as pb *)

  (* times of beginning and end for solution of separate systems *)
  tB = Array[ff, Npw]; tE = Array[ff, Npw];

  (* for monitoring the paths of activity *)
  ActBloodpw = Array[ff, Npw]; ActBloodFragpw = Array[ff, Npw];
  ActOutpw = Array[ff, Npw]; ActOutFragpw = Array[ff, Npw]; ActTumorpw = Array[ff, Npw];

  (* for monitoring influence of self-dose, croos-fire and decays in blood *)
  SDpw = Array[ff, Npw]; CFNpw = Array[ff, Npw]; CFDpw = Array[ff, Npw]; UNpw = Array[ff, Npw];

  (* for monitoring the number of new cancer cells appearing during treatment *)
  NewCellspw = Array[ff, Npw];

  Clear[a, b, NN, DD, pa, pb, fFN, fAN, dF, dA, rho, ActBlood, ActBloodFrag, ActOut, ActOutFrag, ActTumor, SD, CFN, CFD, UN, NewCells];
```

```
(* EQUATIONS *)

(* Radiation damage function *)
RD[NN_, DD_, fAN_, dA_, a_, pa_, rho_] :=
 ((rho - rhomin)/(rhomax - rhomin) * (alphamax - alphamin) + alphamin) (*alpha*) * (ks * (lambda * gamma * fAN)/nu (*self-dose*) +
    (1 - ks) * (lambda * gamma * (fAN * NN + dA))/(nu * (NN + DD)) (*cross-fire*) + kf * lambda * (a + pa) (*dose from unanchored nuclides*));



(* Active antibodies *)
Fa[t_] := (*injections are considered as initial conditions*)
  - lambda * a[t] (*decay*) - kon * gamma/V * (fFN[t] * NN[t] + dF[t]) * a[t] (*binding*) - kappac * a[t] (*clearance*);

(* Inert antibodies *)
Fb[t_] := (*injections are considered as initial conditions*)
  + lambda * a[t] (*decay of a*) - kon * gamma/V * (fFN[t] * NN[t] + dF[t]) * b[t] (*binding*) - kappac * b[t] (*clearance*);

(* Viable cells *)
FNN[t_] := rho[t] * NN[t] (*proliferation*) - RD[NN[t], DD[t], fAN[t], dA[t], a[t], pa[t], rho[t]] * NN[t] (*damage*);

(* Damaged cells *)
FDD[t_] := RD[NN[t], DD[t], fAN[t], dA[t], a[t], pa[t], rho[t]] * NN[t] (*damage*) - omega * DD[t] (*death*);

(* Active fragments *) Fpa[t_] := omega * (gamma * dA[t])/V (*release*) - lambda * pa[t] (*decay*) - kappap * pa[t] (*clearance*);

(* Inert fragments *)
```

```
Fpb[t_] := omega * (gamma * (DD[t] - dF[t] - dA[t]))/V (*release*) + lambda * pa[t] (*decay*) - kappap * pb[t] (*clearance*);

(* Free receptors of viable cells *) FfFN[t_] := (1 - fFN[t]) * rho[t] - kon * (a[t] + b[t]) * fFN[t];

(* Active receptors of viable cells *) FfAN[t_] := kon * a[t] * fFN[t] - (lambda + rho[t]) * fAN[t];

(* Free receptors of damaged cells *)
FdF[t_] :=
  RD[NN[t], DD[t], fAN[t], dA[t], a[t], pa[t], rho[t]] * fFN[t] * NN[t] - kon * (a[t] + b[t]) * dF[t] - omega * dF[t];

(* Active receptors of damaged cells *)
FdA[t_] :=
  RD[NN[t], DD[t], fAN[t], dA[t], a[t], pa[t], rho[t]] * fAN[t] * NN[t] + kon * a[t] * dF[t] - lambda * dA[t] - omega * dA[t];

(* Cancer cell proliferation rate *)
Frho[t_] := -kD * rho[t] * Log[rho[t] / rhomin] * RD[NN[t], DD[t], fAN[t], dA[t], a[t], pa[t], rho[t]];

(* Initial conditions *)

If[Ainj[[1, 1]] == 0
  , a0 = Ainj[[1, 2]] / V; b0 = Ainj[[1, 3]] / V
  , a0 = 0; b0 = 0]; (*complexes in blood*)
NN0 = N0;
DD0 = 0;
pa0 = 0; pb0 = 0; fFN0 = 1; fAN0 = 0;
dF0 = 0; dA0 = 0; rho0 = rhomax;
ActBlood0 = 0; ActBloodFrag0 = 0; ActOut0 = 0; ActOutFrag0 = 0; ActTumor0 = 0; NewCells0 = 0;
SD0 = 0; CFN0 = 0; CFD0 = 0; UN0 = 0;

(* SOLVER *)
```

```
tB[[1]] = 0; If[Ainj[[1, 1]] == 0, If[lAinj > 1, tE[[1]] = Ainj[[2, 1]], tE[[1]] = tEnd], tE[[1]] = Ainj[[1, 1]]];

For[npw = 1, npw ≤ Npw, npw++,

  Clear[a, b, NN, DD, pa, pb, fFN, fAN, dF, dA, rho, ActBlood, ActBloodFrag, ActOut, ActOutFrag, ActTumor, SD, CFN, CFD, UN, NewCells];
  (*fFD,fAD,*)

  sol = NDSolve[{
      (*INITIAL CONDITIONS*)
      a[tB[[npw]]] == a0, b[tB[[npw]]] == b0, NN[tB[[npw]]] == NN0, DD[tB[[npw]]] == DD0, pa[tB[[npw]]] == pa0, pb[tB[[npw]]] == pb0,
      fFN[tB[[npw]]] == fFN0, fAN[tB[[npw]]] == fAN0, dF[tB[[npw]]] == dF0, dA[tB[[npw]]] == dA0, rho[tB[[npw]]] == rho0,
      ActBlood[tB[[npw]]] == ActBlood0, ActBloodFrag[tB[[npw]]] == ActBloodFrag0, ActOut[tB[[npw]]] == ActOut0,
      ActOutFrag[tB[[npw]]] == ActOutFrag0, ActTumor[tB[[npw]]] == ActTumor0, SD[tB[[npw]]] == SD0,
      CFN[tB[[npw]]] == CFN0, CFD[tB[[npw]]] == CFD0, UN[tB[[npw]]] == UN0, NewCells[tB[[npw]]] == NewCells0,

      a'[t] == Fa[t], b'[t] == Fb[t], NN'[t] == FNN[t], DD'[t] == FDD[t], pa'[t] == Fpa[t],
      pb'[t] == Fpb[t], fFN'[t] == FfFN[t], fAN'[t] == FfAN[t], dF'[t] == FdF[t], dA'[t] == FdA[t], rho'[t] == Frho[t],

      ActBlood'[t] == V*lambda*(a[t] + pa[t]),
      ActBloodFrag'[t] == V*lambda*pa[t],
      ActOut'[t] == V*(kappac*a[t] + kappap*pa[t]),
      ActOutFrag'[t] == V*kappap*pa[t],
      ActTumor'[t] == (lambda*gamma)*(fAN[t]*NN[t] + dA[t]),

      SD'[t] == ks*(lambda*gamma)*(fAN[t]*NN[t]),
      CFN'[t] == (1 - ks)*(lambda*gamma)*(fAN[t]*NN[t])*NN[t]/(NN[t] + DD[t]),
      CFD'[t] == (1 - ks)*(lambda*gamma)*dA[t]*NN[t]/(NN[t] + DD[t]),
```

```
      UN'[t] == kf * lambda * (a[t] + pa[t]) * nu * NN[t],
      NewCells'[t] == If[t > Ainj[[1, 1]], rho[t] * NN[t], 0], (* start counting new cells from the moment of the first injection *)
      WhenEvent[NN[t] < Ncur / Nnor, NN[t] → 0] (*treatment wins*)
    }
    , {a, b, NN, DD, pa, pb, fFN, fAN, dF, dA, rho, ActBlood,
      ActBloodFrag, ActOut, ActOutFrag, ActTumor, SD, CFN, CFD, UN, NewCells}, {t, tB[[npw]], tE[[npw]]}
    , AccuracyGoal → 10, PrecisionGoal → 10];

apw[[npw]] = First[a /. sol]; bpw[[npw]] = First[b /. sol]; NNpw[[npw]] = First[NN /. sol]; DDpw[[npw]] = First[DD /. sol];
papw[[npw]] = First[pa /. sol]; pbpw[[npw]] = First[pb /. sol]; fFNpw[[npw]] = First[fFN /. sol]; fANpw[[npw]] = First[fAN /. sol];
dFpw[[npw]] = First[dF /. sol];  dApw[[npw]] = First[dA /. sol]; rhopw[[npw]] = First[rho /. sol];
ActBloodpw[[npw]] = First[ActBlood /. sol];
ActBloodFragpw[[npw]] = First[ActBloodFrag /. sol];
ActOutpw[[npw]] = First[ActOut /. sol];
ActOutFragpw[[npw]] = First[ActOutFrag /. sol];
ActTumorpw[[npw]] = First[ActTumor /. sol];
SDpw[[npw]] = First[SD /. sol];
CFNpw[[npw]] = First[CFN /. sol];
CFDpw[[npw]] = First[CFD /. sol];
UNpw[[npw]] = First[UN /. sol];
NewCellspw[[npw]] = First[NewCells /. sol];

If[npw < Npw,
  (*renew initial conditions*)
  If[Ainj[[1, 1]] == 0
    , a0 = apw[[npw]][tE[[npw]]] + Ainj[[npw + 1, 2]]/V; b0 = bpw[[npw]][tE[[npw]]] + Ainj[[npw + 1, 3]]/V
    , a0 = apw[[npw]][tE[[npw]]] + Ainj[[npw, 2]]/V; b0 = bpw[[npw]][tE[[npw]]] + Ainj[[npw, 3]]/V];

  NN0 = NNpw[[npw]][tE[[npw]]];
  DD0 = DDpw[[npw]][tE[[npw]]];
```

```
      pa0 = papw[[npw]][tE[[npw]]];
      pb0 = pbpw[[npw]][tE[[npw]]];
      fFN0 = fFNpw[[npw]][tE[[npw]]]; fAN0 = fANpw[[npw]][tE[[npw]]];
      dF0 = dFpw[[npw]][tE[[npw]]];
      dA0 = dApw[[npw]][tE[[npw]]];
      rho0 = rhopw[[npw]][tE[[npw]]];
      ActBlood0 = ActBloodpw[[npw]][tE[[npw]]];
      ActBloodFrag0 = ActBloodFragpw[[npw]][tE[[npw]]];
      ActOut0 = ActOutpw[[npw]][tE[[npw]]];
      ActOutFrag0 = ActOutFragpw[[npw]][tE[[npw]]];
      ActTumor0 = ActTumorpw[[npw]][tE[[npw]]];
      SD0 = SDpw[[npw]][tE[[npw]]];
      CFN0 = CFNpw[[npw]][tE[[npw]]];
      CFD0 = CFDpw[[npw]][tE[[npw]]];
      UN0 = UNpw[[npw]][tE[[npw]]];
      NewCells0 = NewCellspw[[npw]][tE[[npw]]];

      (*renew time frame*)
      tB[[npw + 1]] = tE[[npw]];

      If[Ainj[[1, 1]] == 0, If[Npw > npw + 1, tE[[npw + 1]] = Ainj[[npw + 2, 1]], tE[[npw + 1]] = tEnd],
        If[Npw > npw + 1, tE[[npw + 1]] = Ainj[[npw + 1, 1]], tE[[npw + 1]] = tEnd]];]
    ];
    npw--;
    Return[ActBloodpw[[npw]][tEnd]]
  )
```

In[ ]:=

```
(* "for 300 nCi and 600 nCi of injected 225Ac-DOTA-daratumumab,the parts of the experimental data starting from day 35,
by which less than 9% of activity should remain... *)
SetBasicParameterValuesReduced[];
PercentForm[Exp[-lambda*35]]
```

Out[ ]//PercentForm=

8.629%

```
(* ...are better fitted by exponential growth with ρ=0.28 and 0.2, respectively (...)
This is about 82% and 59% of the estimated value of proliferation rate of untreated cancer cells" *)

Print["300 nCi"];
DaraErrRegr = Dara300ErrRegr;
DaraMeanRegr = Dara300MeanRegr;
DaraErr = Dara300Err;
DaraMean = Dara300Mean;
FitRhoRegrowth[]

Print["600 nCi"];
DaraErrRegr = Dara600ErrRegr;
DaraMeanRegr = Dara600MeanRegr;
DaraErr = Dara600Err;
DaraMean = Dara600Mean;
FitRhoRegrowth[]
```

300 nCi

Out[ ]=

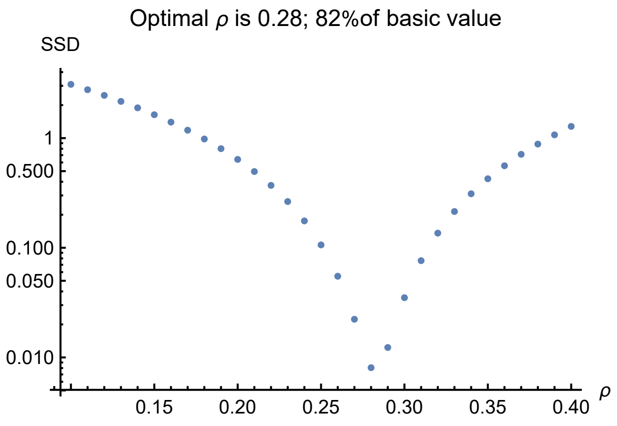

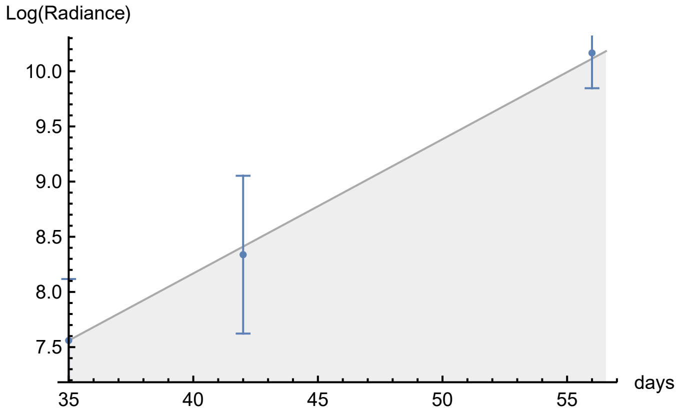

600 nCi

Out[ ]=

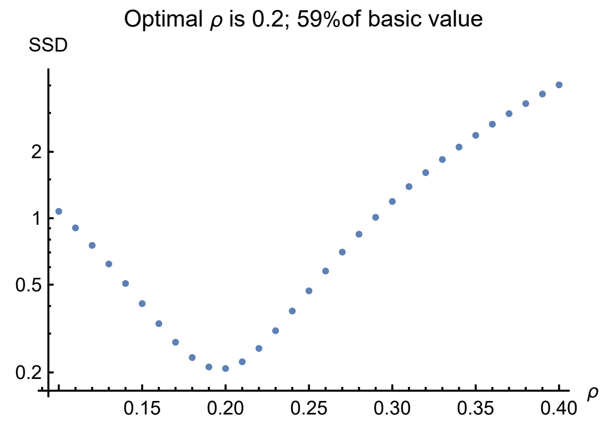

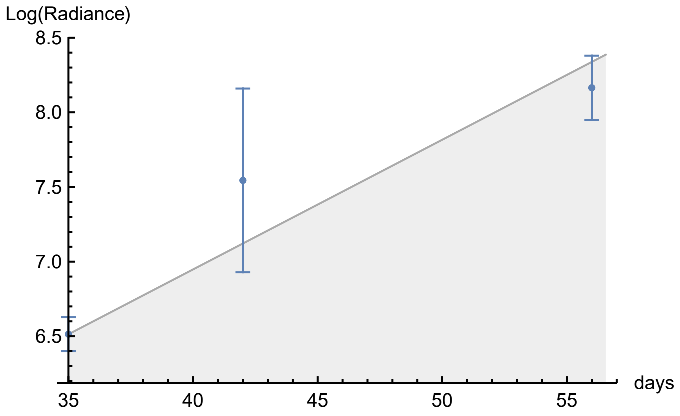

(* Figure S.19 *)

(* "Four values of parameters were optimized by simultaneous fitting to experimental data of all

```
five mice groups, treated with different doses of 225 Ac -DOTA -trastuzumab." *)

SetBasicParameterValuesReduced[];

(* Setting boundaries and steps of variation *)
nnn = 6;
rhominmin = 0.1;
rhominmax = 0.2;
rhominstep = N[(rhominmax - rhominmin) / nnn]; (* minimum cancer cells proliferation rate *)
alphamaxmin = 1000;
alphamaxmax = 4000;
alphamaxstep = N[(alphamaxmax - alphamaxmin) / nnn]; (* maximum cancer cell radiosensitivity *)
alphaminmin = 50;
alphaminmax = 400;
alphaminstep = N[(alphaminmax - alphaminmin) / nnn]; (* minimum cancer cell radiosensitivity *)
kDmin = 0.1; kDmax = 2; kDstep = N[(kDmax - kDmin) / (2 * nnn)]; (* sensitivity of proliferation rate to radiation *)

(* Table which will contain the deviations of simulations from the experimental data *)
result = Array[f, {Round[((rhominmax - rhominmin) / rhominstep + 1) * ((alphamaxmax - alphamaxmin) / alphamaxstep + 1) *
          ((alphaminmax - alphaminmin) / alphaminstep + 1) * ((kDmax - kDmin) / kDstep + 1) + 1], 5}];
result[[1, 1]] = "rhomin";
result[[1, 2]] = "alphamax";
result[[1, 3]] = "alphamin";
result[[1, 4]] = "kD";
result[[1, 5]] = "SSD"; (* sum of squared deviations *)

ii = 2;
For[rhomin = rhominmin, rhomin ≤ rhominmax, rhomin = rhomin + rhominstep,
  For[alphamax = alphamaxmin, alphamax ≤ alphamaxmax, alphamax = alphamax + alphamaxstep,
```

```
NotebookDelete[pr]; (* To see the code running *)
pr = PrintTemporary["Current rhomin is " <> ToString[rhomin] <> "; max value is " <> ToString[rhominmax] <> "
" <> "Current alphamax is " <> ToString[alphamax] <> "; max value is " <> ToString[alphamaxmax]];

For[alphamin = alphaminmin, alphamin ≤ alphaminmax, alphamin = alphamin + alphaminstep,
  For[kD = kDmin, kD ≤ kDmax, kD = kD + kDstep,

    SSD = 0;

    Do[If[DnCi == 25, DaraMean = Dara25Mean; DaraData = Dara25Data; daysDara = daysDara25; DaraErr = Dara25Err];
      If[DnCi == 50, DaraMean = Dara50Mean; DaraData = Dara50Data; daysDara = daysDara50; DaraErr = Dara50Err];
      If[DnCi == 100, DaraMean = Dara100Mean; DaraData = Dara100Data; daysDara = daysDara100; DaraErr = Dara100Err];
      If[DnCi == 300, DaraMean = Dara300Mean; DaraData = Dara300Data; daysDara = daysDara300; DaraErr = Dara300Err];
      If[DnCi == 600, DaraMean = Dara600Mean; DaraData = Dara600Data; daysDara = daysDara600; DaraErr = Dara600Err];

      (* The day of the last experimental value *)
      tEnd = DaraData[[1, All, 1]][[Length[daysDara]]];
      DA1 = nCpm * DnCi; (* injected amount of radionuclides *)
      t1 = 1; (* moment of their injection *)
      eta = Round[202.7 / DA1 - 1]; (* coefficient of drug impurity *)

      (* the coeffient of transition of cell number to radiance *)
      kNR = 10^DaraMean[[1, 2]] / N0;

      (* Close-to-delta function of drug injection (it is smooth function, that will allow to avoid numerical difficulties) *)
      kk = 250; (* Technical parameter, which largeness yields sharp function *)
      AInjFunc[t_] := DA1/2 * kk * Sech[kk * (t - t1)]^2 / V;

      FullSystemSolutionVarRAO[];
```

```
      Quiet[SSD = SSD + Log10[Sum[
            (Log10[kNR * (NN[DaraMean[[kk, 1]]] + DD[DaraMean[[kk, 1]]])] - DaraMean[[kk, 2]])^2, {kk, 1, Length[daysDara]}]]],

      {DnCi, {25, 50, 100, 300, 600}}];

     (* Save sum of squared deviations *)
     result[[ii, 1]] = rhomin;
     result[[ii, 2]] = alphamax;
     result[[ii, 3]] = alphamin;
     result[[ii, 4]] = kD;
     result[[ii, 5]] = SSD;

     ii++;]]]];

(* Find optimal parameters *)

FitInt = Interpolation[Thread[{result[[2 ;;, {1, 2, 3, 4}]], result[[2 ;;, 5]]}]];
OptVal = NMinimize[{FitInt[rm, aM, am, kd], rhominmin ≤ rm ≤ rhominmax,
     alphamaxmin ≤ aM ≤ alphamaxmax, alphaminmin ≤ am ≤ alphaminmax, kDmin ≤ kd ≤ kDmax}, {rm, aM, am, kd}];

rhomin = rm /. OptVal[[2]];
Print["Optimal rhomin is " <> ToString[rhomin]];
alphamax = aM /. OptVal[[2]];
Print["Optimal alphamax is " <> ToString[alphamax]];
alphamin = am /. OptVal[[2]];
Print["Optimal alphamin is " <> ToString[alphamin]];
kD = kd /. OptVal[[2]];
Print["Optimal kD is " <> ToString[kD]];
```

```
Do[If[DnCi == 25, DaraMean = Dara25Mean; DaraData = Dara25Data; daysDara = daysDara25; DaraErr = Dara25Err];
    If[DnCi == 50, DaraMean = Dara50Mean; DaraData = Dara50Data; daysDara = daysDara50; DaraErr = Dara50Err];
    If[DnCi == 100, DaraMean = Dara100Mean; DaraData = Dara100Data; daysDara = daysDara100; DaraErr = Dara100Err];
    If[DnCi == 300, DaraMean = Dara300Mean; DaraData = Dara300Data; daysDara = daysDara300; DaraErr = Dara300Err];
    If[DnCi == 600, DaraMean = Dara600Mean; DaraData = Dara600Data; daysDara = daysDara600; DaraErr = Dara600Err];

    (* The day of the last experimental value *)
    tEnd = DaraData[[1, All, 1]][[Length[daysDara]]];

    DA1 = nCpm * DnCi; (* injected amount of radionuclides *)
    t1 = 1; (* moment of their injection *)
    eta = Round[202.7 / DA1 - 1]; (* coefficient of drug impurity *)

    (* the coeffient of transition of cell number to radiance *)
    kNR = 10^DaraMean[[1, 2]] / N0;

    (* Close-to-delta function of drug injection (it is smooth function, that will allow to avoid numerical difficulties) *)
    kk = 250; (* Technical parameter, which largeness yields sharp function *)
    AInjFunc[t_] := DA1/2 * kk * Sech[kk * (t - t1)]^2 / V;

    FullSystemSolutionVarRAO[];

    IS = 300;
    (* Plot simulation with optimal rho along with experimental data *)
    PicOptSim = Show[Plot[{{Log10[kNR * NN[t]], Log10[kNR * (NN[t] + DD[t])]}}, {t, 0, 1.01 * tEnd}, Filling → {1 → Axis, 2 → Axis},
        PlotStyle → {Directive[Lighter[Gray], Thickness[0.002]], Directive[Darker[Gray], Thickness[0.002]]},
        PlotRange → {Automatic, {5, 11}}, AxesLabel → {"days", "Log (Radiance) "},
        PlotLabel → "Dara " <> ToString[DnCi] <> " nCi", ImageSize → 280], ListPlot[DaraErr], ImageSize → IS];

    Print[PicOptSim],
```

```
{DnCi, {25, 50, 100, 300, 600}}];
```

Optimal rhomin is 0.2

Optimal alphamax is 2354.51

Optimal alphamin is 87.9684

Optimal kD is 0.575067

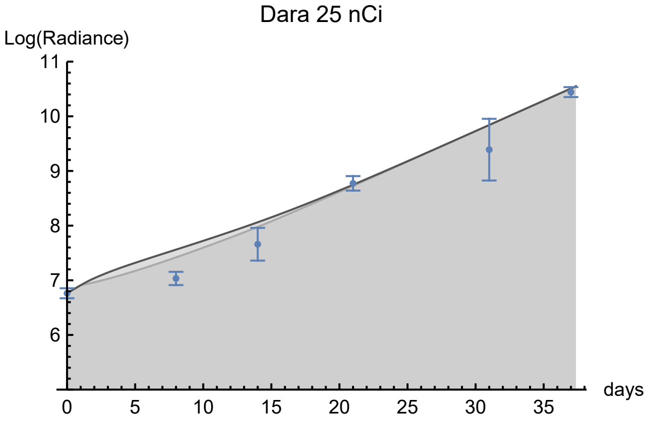

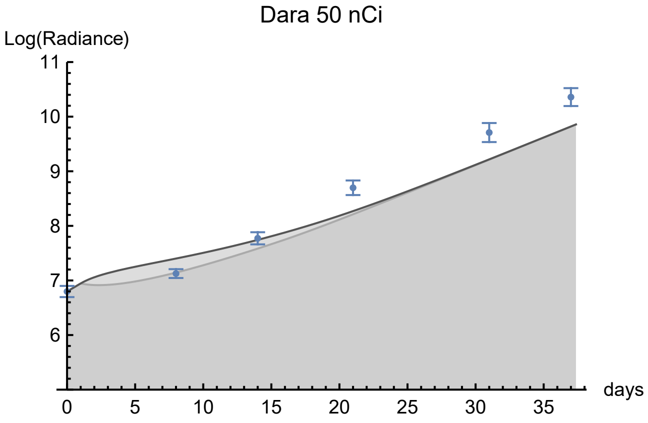

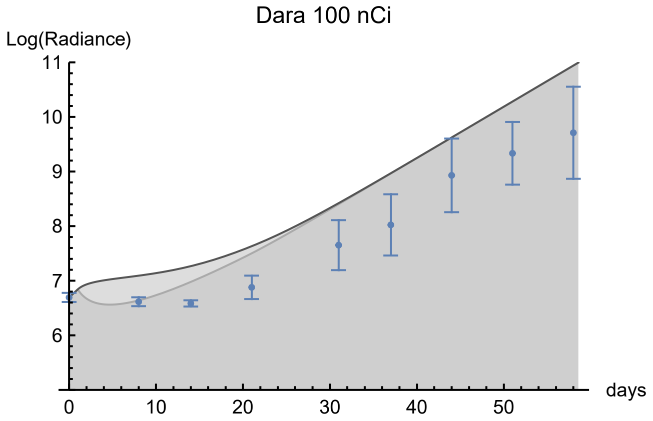
Dara 100 nCi
Log(Radiance)
11
10
9
8
7
6
days
0
10
20
30
40
50

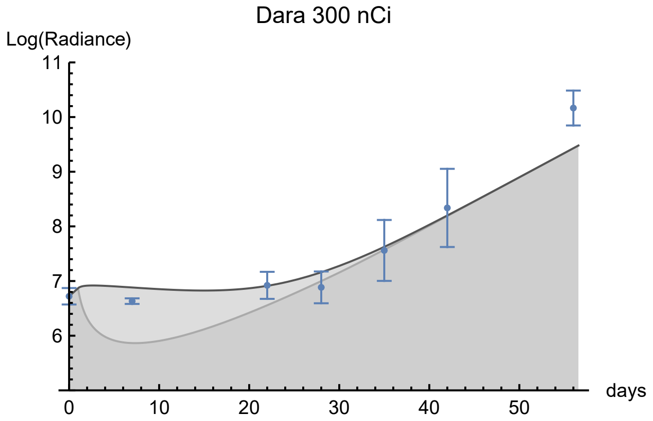
Dara 300 nCi
Log(Radiance)
11
10
9
8
7
6
days
0
10
20
30
40
50

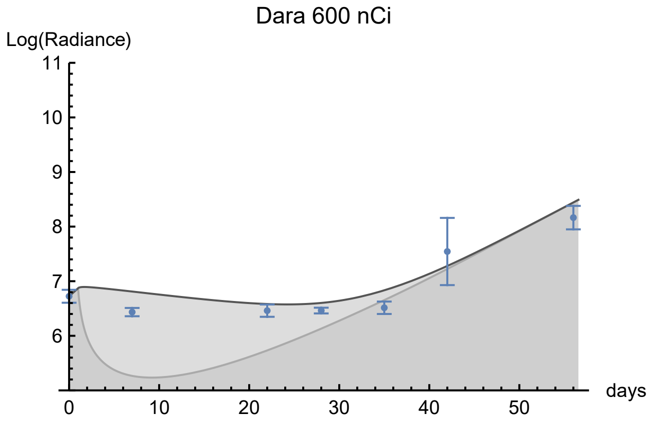
Dara 600 nCi
Log(Radiance)
11
10
9
8
7
6
days
0
10
20
30
40
50

In[∘]:=

```
(* "The simulations of 600 nCi setting suggest that in it about 39 % of injected activity
is spent in blood in form of decays" *)
(* it was the last setting in the previous script, so it has to be run in advance *)
ToxDecays = NIntegrate[V * lambda * (a[t] + p[t]), {t, 0, tEnd}] / nCpm (* in nCi *)
PercentForm[ToxDecays / 600]
```

Out[∘]=

234.176

Out[∘]//PercentForm=

39.03%

In[∘]:=

```
(* "The basic set of parameters (...) corresponds to minimal single curative dose
of 220 nCi for the case of pure radioconjugates.
While the corresponding amount of them still can occupy less than one percent of cancer receptors..." *)
SetBasicParameterValuesHet[];
eta = 0;
Acur0 = FindCurDoseHet[]
PercentForm[Acur0 * nCpm / (gamma * N0)]
```

Out[∘]=

219.867

Out[∘]//PercentForm=

0.2652%

In[∘]:=

```
(* "...the use of labeling ratio of 1.85 kBq / μg prevents their fitting onto
the cancer cells and escalates the formal minimal curative dose by a factor of ≈500, rendering such treatment
lethally toxic." *)
SetBasicParameterValuesHet[];
eta = 1780;
Acur = FindCurDoseHet[]
Acur / Acur0
ActBloodpw[[npw]][tEnd] / nCpm (* toxic decays in blood *)
```

Out[∘]=

113 331.

Out[∘]=

515.453

Out[∘]=

46 647.7

(* S.4.2 Global parameter sweep with pure radioconjugates *)

In[∘]:=

```
(* The outcome of the global parameter sweep, that was generated in this study
  is saved here in a closed cell -- open this group of cells to see it, and run it to upload it in the array "result" *)
```

In[∘]:=

```
Npar = 1000;
result = {{"κc", "κp", "γ", "V", "ks", "ρ0", "ρm", "ω", "α0", "αm", "kf", "N0", "kD", "A^sim_cur", "A^est_min", "A^est_max", "Mm", "Canc", "Bl^sim_Frg", "Bl^est_Frg", "Err",
    "Bl^sim_Ab", "Bl^est_Ab", "Err", "Cld^sim_Frg", "Cld^sim_Ab", "ToVbl", "fD^sim_Max", "fD^est_Max", "Err", "Nnew"}, {0.08287723093332094`, 3.0858822376571258`,
    1.0544020640762781`, 0.8913551057392846`, 0.07800980208832753`, 0.18650785858589003`, 0.06335580187071355`,
    0.005963202911440555`, 1833.7547379259959`, 302.5435916235979`, 0.054836866330129685`, 9.998403612912536`, 1.2206910575241197`,
    178.8241471781496`, 39.93994150277729`, 188.67001136781187`, 0, 164.67235963287357`, 0.30538026965918763`, 0.31137212667192893`,
    0.019620969682908385`, 0.09474929512729245`, 0.09481128010528182`, 0.0006541998851399278`, 13.46239356960328`,
    0.11217941732905562`, 3.121385390637066`, 0.0012818419195219644`, 0.0012891481899085292`, 0.005699821698208707`,
    0.24939863756652184`}, {0.16008991075718454`, 0.9889641902273629`, 4.070194520482062`, 1.048412343563395`, 0.9623075048370111`,
    0.2561461923913073`, 0.06710665317178915`, 0.4051510354125659`, 2424.3911699885257`, 364.2049627898059`, 0.11376488110702476`,
    6.677727754478127`, 0.46263232179329394`, 156.2065584325259`, 61.32021353754079`, 172.62016798875143`, 0, 23.86824003266899`,
```

8.739663271315978`, 8.804431175340582`, 0.007410800852840182`, 0.03777585942370417`, 0.03779767865425641`,
0.0005775971979222749`, 123.4744858568119`, 0.08639334234156137`, 1.0262897879616137`, 0.00043495478850097413`,
0.00043678627358973473`, 0.004210748190800695`, 0.10419806999296415`}, {0.23888202110078255`, 0.8810916509901707`,
5.093732295047118`, 1.4600046563601987`, 0.26112133308478946`, 0.18414540656565503`, 0.10719043869683506`, 0.01905226907504459`,
1361.794327483607`, 157.34664623392825`, 0.2248991829845473`, 7.770022547327725`, 1.3856793740595026`, 320.2706074734752`,
47.02938615353104`, 344.5333262086874`, 0, 252.22788669907231`, 4.976623600096425`, 5.043063896579252`, 0.0133504765121355`,
0.07407565449233257`, 0.07409561672724449`, 0.0002694844216866165`, 62.640878630937266`, 0.25279060084989424`,
3.874819088955209`, 0.0006129422096077253`, 0.0006149959845967612`, 0.003350682914055491`, 0.17687305969303108`},
{0.20921512222542155`, 0.8329034625019238`, 5.512850623987205`, 1.0082829806829832`, 0.6432484314226474`, 0.16609066867476052`,
0.06366860478505312`, 0.09015760505638287`, 3121.845933283612`, 31.743667621759982`, 0.23453419786488217`, 5.669698519018798`,
0.216211427919029`, 367.5462113807418`, 19.84468464221906`, 1326.0340009206461`, 0, 160.8024581240046`, 16.005345458186557`,
16.040705768234723`, 0.0022092812767173786`, 0.0743784325999251`, 0.07437563414831408`, -0.000037624503679389854`,
190.44153786661457`, 0.2223013266756827`, 0.515170955662475`, 0.000891550691288101`, 0.0008936950478562457`,
0.002405198705018652`, 0.01779144780608476`}, {0.15821344070119114`, 2.676629891143981`, 7.114918370652138`,
1.231756200149393`, 0.7440882968059128`, 0.609542391688829`, 0.20113875027795403`, 0.00829343649930974`, 3321.626865604231`,
300.9780595191306`, 0.1294374982810616`, 4.573476150462291`, 0.6252568014280515`, 184.40001884554837`, 36.20278448216762`,
203.17221829309`, 0, 164.67769092367712`, 0.49524214427529506`, 0.4978150555569566`, 0.005195259150302167`, 0.04371660347612581`,
0.04378811002738489`, 0.001635684055329989`, 18.936856096021383`, 0.09880791788179769`, 0.6635964048305241`,
0.00042751530539764104`, 0.000430683479331421`, 0.007410667860962672`, 0.09284546767380224`}, {0.04416264589453098`,
1.237497547847834`, 1.3522815348119845`, 1.466732288470342`, 0.6840124021467311`, 0.46728396632651026`, 0.0737811771799529`,
0.04606361653599369`, 2651.0081518614816`, 102.21078555052515`, 0.21006656956644504`, 2.3728441322292344`, 0.992859548694442`,
165.33622945678897`, 17.61522521060715`, 180.6367766896037`, 0, 99.63963350891684`, 3.4760122612726088`, 3.5130725551174824`,
0.010661727019140477`, 0.4692903462088751`, 0.4722564361755873`, 0.006320372858025891`, 61.45080928019789`, 0.2960729054478448`,
0.9824378010387709`, 0.0038464643224145334`, 0.003916023528244958`, 0.01808393371155992`, 0.04898016066966178`},
{0.2369260528945452`, 2.0701500057376476`, 2.467879519617215`, 1.3729060512118858`, 0.7839988344536148`, 0.6604044950304033`,
0.16113998364121102`, 0.05466214114010759`, 886.5316249876132`, 108.21460952627575`, 0.04090907280605699`, 1.8765499412863633`,
0.31190921443437514`, 183.14070081531148`, 62.416964263047916`, 210.07751445133314`, 0, 102.32175973991025`, 2.5953783564152144`,
2.6265807343621415`, 0.01202228487025847`, 0.33452109436815614`, 0.338092171149424`, 0.010675191613888968`,
76.75460742034822`, 1.1322394642664984`, 0.7105398160665205`, 0.0028939955808313655`, 0.003005483743302803`,
0.038523957399897`, 0.07161900505670984`}, {0.10845014170633277`, 2.595808559087966`, 3.825660598104694`, 0.9092167031122353`,
0.4998129490409222`, 0.3952109813060457`, 0.14370479224814003`, 0.0648286156513408`, 2096.8410035229635`, 61.50816633658093`,

0.11793078229478832`, 9.815002337220978`, 0.4969144428042134`, 1119.8167592228438`, 67.7432671674452`, 1464.6527401349804`, 0, 582.1928030822862`, 14.10563652706238`, 14.138414740236005`, 0.002323767035308011`, 0.17022984450449774`, 0.17016585621649827`, -0.0003758934761747268`, 523.0790289761767`, 0.263735010844932`, 2.1742617295686992`, 0.002259219982126859`, 0.002266541679875057`, 0.0032408078036318955`, 0.07555369445763135`}, {0.1958274434061072`, 1.1625250352222158`, 4.38654338158619`, 1.0335502224347561`, 0.757936701785614`, 0.28764737226179815`, 0.053841290852541135`, 0.15446581430564627`, 1541.8645934819597`, 81.13506069010731`, 0.032171888494962775`, 6.764038142553707`, 0.3026234451737826`, 458.0778203092906`, 74.25362832784279`, 627.3130938716943`, 0, 143.8287014718003`, 17.82587449033454`, 17.902913970578116`, 0.004321778451056968`, 0.10007108726451526`, 0.10009330340551446`, 0.00022200359371016276`, 296.04321956775806`, 0.2799523596838094`, 1.5952591756938463`, 0.0011685544066463166`, 0.0011733413601060752`, 0.004096474612164114`, 0.06577324481722528`},
{0.11706495920321314`, 3.996131916383794`, 3.4445284668439964`, 1.0745666843500223`, 0.4349463063881305`, 0.49843595508242333`, 0.15623150182584739`, 0.02045656151514831`, 3837.5091768132115`, 201.1196769174046`, 0.10740962694701128`, 6.186844269751422`, 0.8954099169722194`, 240.62613668017184`, 24.20528030320615`, 271.99935755214216`, 0, 186.27335796004928`, 0.9311082930869078`, 0.9368109002013835`, 0.00612453691672088`, 0.07599676350764463`, 0.07610849562880054`, 0.0014702220989275538`, 53.15473668020336`, 0.12709368599419765`, 1.220235327264689`, 0.0008522732589788529`, 0.0008581385812539323`, 0.006881973842646438`, 0.11983511922235734`}, {0.15007898341668874`, 1.7157935178042445`, 2.2029422939279666`, 1.1981889493296447`, 0.6780839054962491`, 0.6847631092713093`, 0.13190149021010728`, 0.01567939294607179`, 3588.049208356785`, 265.194158460061`, 0.020899950425706137`, 5.763952406784453`, 0.2759848394189104`, 173.49498502368857`, 40.89722881618427`, 221.38188119231015`, 0, 141.5251643994773`, 1.2377772446463615`, 1.2445334877347876`, 0.005458367503238692 5`, 0.10214853528838774`, 0.10258970071174117`, 0.004318861960261433`, 30.33957389785466`, 0.21900497619405748`, 0.550241273058237`, 0.0010210897719211687`, 0.0010384294850109119`, 0.016981575534851023`, 0.14649854758573694`},
{0.05919792771600385`, 1.1566416574477563`, 7.068615334313819`, 0.9063681130718225`, 0.7973057249073783`, 0.32056833480557556`, 0.14802374216985142`, 0.006390669590825526`, 3472.301841329897`, 218.24620282866817`, 0.09503155541006275`, 6.650147058357586`, 0.752916478623388`, 332.08604644455625`, 36.80586922834271`, 372.5485566129848`, 0, 304.03578927645555`, 1.578392573550217`, 1.5853932540135456`, 0.004435322733166602`, 0.040179874219804776`, 0.04018979604684297`, 0.00024693524384655774`, 26.080494319633612`, 0.033979504138628915`, 1.0600440985654158`, 0.0005356580374147324`, 0.0005369064513849291`, 0.002330617451801853`, 0.06636089571723593`}, {0.12780221907252537`, 3.8001966548501374`, 9.893992295428603`, 0.9054601762352339`, 0.3495759940365448`, 0.1520403910810577`, 0.10896185913129744`, 0.3816734295429438`, 1447.6496724709095`, 224.52426056149727`, 0.14587747065522566`, 4.530340760708304`, 0.2310603840049561`, 103.066109251912`, 25.340311886488394`, 146.6646402741003`, 0, 16.634903549994725`, 1.562608025403928`, 1.5752455905749423`, 0.008087482571163207`, 0.013063911694492374`, 0.013066231953089664`, 0.00017760825789014945`, 84.83168272831419`, 0.023851384347504927`, 0.7966324258259161`, 0.00017446100006734966`, 0.00017475396425496148`, 0.001679253171188444`, 0.0734609820271068`},

{0.052097538451470415`, 0.8314166561245999`, 5.481254262333325`, 1.3932692734662013`, 0.3669026419040864`,
0.3620022667552303`, 0.11591227570051149`, 0.01952536971625568`, 3813.0335975189155`, 109.62712481259092`, 0.17741871372248502`,
1.8758946193559272`, 0.37818057550442363`, 83.73535610204333`, 5.641914881430802`, 124.3157171632235`, 0, 65.3988061075936`,
1.4124164347464734`, 1.4181901866089166`, 0.004087853780517348`, 0.07092258092990109`, 0.07112704793445672`,
0.002882960572990534`, 16.775807846176306`, 0.05278416981534219`, 0.19211189607525558`, 0.0006130794165877251`,
0.0006189193741823612`, 0.00952561354471837`, 0.020938279521232093`}, {0.16758241286506004`, 2.4053214221448718`,
3.775183470319785`, 1.3879685907097887`, 0.849157385981911`, 0.1600626918561956`, 0.12028733740307626`, 0.23825099986429926`,
3824.577207611347`, 166.9670016449728`, 0.011876010376545704`, 5.836418598900732`, 0.8350403039197589`, 349.4316392822970`,
20.654011824345407`, 402.7081935084246`, 0, 79.93457361339934`, 7.607903002515856`, 7.63763506075885`, 0.003908049068601649`,
0.138012404324384`, 0.1380060095955405`, -0.00004633444997070857`, 261.42074385073823`, 0.33040645317220063`,
0.8937285125333342`, 0.0012012371570988556`, 0.0012052893589790442`, 0.0033733570895986276`, 0.03330587904736248`},
{0.2134810376991253`, 1.4110142375655572`, 5.989091842275013`, 1.4582774889459504`, 0.06026935559352742`, 0.21384589376924967`,
0.11902027597851936`, 0.012348918184910978`, 2514.3629410926587`, 346.5120508621949`, 0.0374109599718197`, 6.153474798173264`,
0.7147791109959445`, 98.63742784465616`, 18.225307820177722`, 112.03110267851086`, 0, 83.86842263260644`, 0.6907743716134402`,
0.6991197325639826`, 0.01208116759029454`, 0.024463218138573356`, 0.024478623533215026`, 0.0006297370425429882`,
13.9241781898852`, 0.0746061884811799`, 1.0501567046610194`, 0.00020257349248820056`, 0.00020341076433052526`,
0.004133175728179061`, 0.15524870472035768`}, {0.183290589228506`, 0.5116934633594044`, 9.423512566077996`, 1.207136012185702`,
0.9255825329486453`, 0.5109486403531394`, 0.061891644358164966`, 0.06517375803184387`, 1490.0042360738662`, 338.5269107534542`,
0.2295202271938529`, 6.23442268470835`, 0.9091680440302747`, 162.23153178875305`, 145.76147563703694`, 161.90980293203245`, 0,
84.96140722132593`, 9.289352768715908`, 9.412814524222783`, 0.013290673589516633`, 0.02090038374785787`, 0.020917170061770275`,
0.0008031581675684851`, 67.90430129416472`, 0.054726337889122664`, 2.055520031567225`, 0.00020895304133372594`,
0.00020986489753526237`, 0.00436392883164638`, 0.20639302609834936`}, {0.07608249414500318`, 1.6334157835018592`,
2.1569194913061693`, 1.197668491595115`, 0.09742648797035525`, 0.6759576090965576`, 0.07148505885394518`, 0.13535119072972454`,
1569.9807697844644`, 192.45980341337224`, 0.19113163797167387`, 2.99326845694066`, 0.7712705061277614`, 83.6156700674129`,
23.912317131603086`, 87.54025878604611`, 0, 29.215980940861993`, 2.2272330185041342`, 2.2648002417049087`, 0.0168672172550699`,
0.09634501955305189`, 0.09714347438372989`, 0.008287453097026276`, 51.971393799444904`, 0.1047167055149684`, 1.2312764575791522`,
0.0009587952381323595`, 0.0009842868036412074`, 0.026587079800795665`, 0.16546828694107477`}, {0.2761380591152234`,
0.5948415416580923`, 4.492630890025399`, 1.4137462343952987`, 0.19259719440314438`, 0.2905045944613839`, 0.092594223894082`,
0.006415584066097922`, 2524.964006213205`, 55.97986174003478`, 0.1939779490397887`, 7.138405092365562`, 0.3980368971508512`,
550.3576994816564`, 26.12363138165174`, 821.2272516238796`, 0, 503.0816668497942`, 4.842987014999658`, 4.864959194799504`,
0.00453690660986572`, 0.15205459528199367`, 0.1521051179216729`, 0.00033226644407258`, 41.15442660332174`, 0.599829440296061`,

1.1554295597155706`, 0.00129816880931799`, 0.001304237581574978`, 0.00467487141381584`, 0.047274860934918776`},
{0.2691641473228049`, 2.5948088236594433`, 9.806966931633422`, 1.0098220223982928`, 0.036933113005692464`, 0.496552488389817`,
0.11856800340468354`, 0.008793318254398408`, 2701.46341207771`, 37.07745796913986`, 0.05351989235558863`, 6.680707066067022`,
0.2241309195665182`, 452.10125746109827`, 25.511480940315543`, 1123.3353634082928`, 0, 401.2375965581072`, 1.3214476271390425`,
1.325351575076996`, 0.0029542963775306408`, 0.04370940994845909`, 0.0437263290875584`, 0.00038708230377082486`,
48.984342327203215`, 0.16807151512511384`, 0.7712164850838945`, 0.0005227038909747517`, 0.0005244356024209601`,
0.0033129874793529357`, 0.06308825951605102`}, {0.18781219936172783`, 1.4023238769545223`, 1.2203108039534882`,
1.4529300029349537`, 0.3944110423683815 4`, 0.21312489114159694`, 0.11407119247069003`, 0.05613185190396572 5`,
1464.0480188461024`, 295.31541235105215`, 0.1586938998074992`, 9.00673891342328`, 0.8723355545572158`, 222.72412748531806`,
60.12608734247`, 238.18407130974305`, 0, 124.36161833044693`, 4.6442019289126035`, 4.71244776820585`, 0.014694847540624778`,
0.18400561723912115`, 0.1842772282557668`, 0.001476101766462401`, 93.03821791874867`, 0.49369285240819766`,
2.5902946717097928`, 0.0015236231042210502`, 0.001540078395816354`, 0.010800106371264606`, 0.2531973044258862`},
{0.2094783821158741`, 2.6664932858575137`, 7.297360825644024`, 0.9801370202263215`, 0.5993287287749127`, 0.5949313991226747`,
0.07854776414147877`, 0.007471189820589976`, 3737.8249085720936`, 225.90376084120953`, 0.12230719137003099`,
4.645596524735494`, 0.773898586938345`, 145.12317994090355`, 25.37143643987266`, 158.4940749917159`, 0, 131.0125474895354`,
0.35506274802031246`, 0.3580057350452267`, 0.008288639237214124`, 0.026290313485557066`, 0.026322360173716867`,
0.0012189542044602764`, 13.525320480775447`, 0.0786750333467609`, 0.9694382648217789`, 0.0003235210691387991`,
0.000325344200334294`, 0.005635278099036967`, 0.0918318164073313`}, {0.04064294172797539`, 1.2711785498005437`,
6.4142221576096325`, 1.3607991978128624`, 0.4152034158458744`, 0.43390213144020495`, 0.1732390453420793`, 0.044490756173200585`,
3317.76480386853`, 267.9877730423784`, 0.047319616563592015`, 9.540745693864437`, 1.14427781856239`, 298.62490315049274`,
39.65179956617213`, 326.469834033415`, 0, 183.3595954953579`, 6.012115514858708`, 6.056710650265862`, 0.00741754467241007`,
0.041653398561443566`, 0.04167936580758059`, 0.0006234124233277516`, 109.17817544873458`, 0.024184523578698318`,
2.172867221054613`, 0.0003695784956447845`, 0.00037086282014271065`, 0.003475106135938599 3`, 0.24157074631673656`},
{0.09357862879768009`, 0.6953322280042711`, 5.170280682368036`, 1.0934248327219471`, 0.7339854764578926`, 0.4916807813635117`,
0.08077481700228195`, 0.04710573802150511`, 2462.550423949988`, 223.30073476186828`, 0.13718177246897728`, 6.470496132991109`,
0.9927113806256207`, 238.64487178086233`, 59.45480656036568`, 250.6420846810697`, 0, 143.377107989017`, 8.702529248386716`,
8.780025586417796`, 0.008905036204899464`, 0.04889458900337652`, 0.04894454717192453`, 0.0010217524999456273`,
86.444986450758`, 0.06536412277949048`, 1.9986077480136883`, 0.0005393953982598676`, 0.0005421431764639854`,
0.005094181769036865`, 0.13600478148250086`}, {0.18983638851163714`, 1.3904426162746537`, 6.462224486154522`,
1.3121224928907784`, 0.33702569938499116`, 0.4827790093546729`, 0.17642716165084854`, 0.14070582325373504`,
1445.2238937860711`, 169.08245719423826`, 0.08107423780437323`, 3.772942227886274`, 0.5974646397153294`, 164.37044701490487`,

34.2523236613152`, 187.6466325996898`, 0, 55.287065400199126`, 5.2185904391861415`, 5.261054637887615`, 0.008137101233814503`,
0.05536091134240438`, 0.055464458788168594`, 0.001870407174545452`, 103.65929347896954`, 0.15013593534220343`,
1.149629896338596`, 0.0005076310303241405`, 0.0005123599006018277`, 0.009315565824783656`, 0.12430499405234274`},
{0.14369943000217644`, 1.7114006053749717`, 7.290050955062047`, 0.8291375841791329`, 0.041053311258442804`,
0.588971030507233`, 0.22011431905536544`, 0.14621434360249197`, 2238.8977574406254`, 92.23431071779362`, 0.19794610644205557`,
8.103641190648325`, 1.3952824759429032`, 576.513560284603`, 40.98331791405394`, 656.8967743985768`, 0, 189.49210484352633`,
15.201888016128526`, 15.31974190461617`, 0.007752582334681346`, 0.05076861854250465`, 0.05078456382663051`,
0.0003140775657017869`, 371.66457648064073`, 0.10422030780803773`, 4.560470372140489`, 0.0007394358257580835`,
0.0007416727998546376`, 0.0030252444074649887`, 0.2593768862591868`}, {0.07643772187175424`, 1.9519546589360584`,
6.900928196416569`, 0.876872363876414`, 0.7662923029927724`, 0.15407458175108746`, 0.15169600745126946`, 0.11514584917672349`,
3018.1218065404273`, 170.6089829682045`, 0.07107865878327907`, 5.366481527317603`, 0.4272921221427337`, 279.260202913359`,
21.281102016818423`, 373.4228533693074`, 0, 105.90365998650417`, 5.998591534274334`, 6.01270620462232`, 0.0023529974106983875`,
0.04149844398822018`, 0.04149888479858399`, 0.000010622334753795926`, 167.27112417687374`, 0.04531495028111665`,
0.5698856456897808`, 0.0005721514170435871`, 0.0005730935150466153`, 0.0016465886039331856`, 0.025896387008619782`},
{0.2710591123734774`, 3.4140873238389613`, 0.9957406410201042`, 1.2040917290884572`, 0.20012464577442923`,
0.17413508978502545`, 0.1542723564673828`, 0.3932031693142617`, 919.8622664894237`, 351.88082244581324`, 0.07678285799586054`,
9.51148011674945`, 0.10358667667962984`, 142.41548321391605`, 80.73833692065708`, 203.21266781776094`, 0, 23.339254045738222`,
2.381331133354817`, 2.4289115707696514`, 0.019980605279284802`, 0.11309017665230736`, 0.11322960439724493`,
0.0012328899738678345`, 116.143891946425`, 0.4379160414504264`, 2.282573187250066`, 0.0011298906578447454`,
0.001142816395801653`, 0.011439813106839392`, 0.41160557036020456`}, {0.10385665451428133`, 0.45328132552019573`,
1.5503843984121968`, 1.4694004919600818`, 0.4251249704277895`, 0.18123604867037413`, 0.08236798590439715`,
0.07631033842692926`, 1502.3783558556506`, 20.10790624452443`, 0.14290440434604346`, 4.641987001123601`, 1.0114789952636625`,
1353.6696877052605`, 28.470075211204115`, 1625.0720112434083`, 0, 647.3792884184513`, 93.90295064151277`, 94.44606594026541`,
0.005783793747078825`, 1.7398339476319358`, 1.7296265409526024`, -0.005866885568721503`, 608.0636276720353`, 2.581333331591716`,
3.5035073571877646`, 0.01422073642590116`, 0.014294961397219336`, 0.005219488576061693`, 0.02551800460122676`},
{0.08203524638521087`, 2.7438300558988873`, 0.5228963098315518`, 1.1830383771323514`, 0.45293779314745897`,
0.15188403574429454`, 0.10479303433979437`, 0.2038448791081171`, 983.5363393912057`, 168.65049566992798`,
0.019976684129921263`, 1.733382066252318`, 1.4812211807580051`, 74.07114437053829`, 15.213379211165089`, 77.61880187012376`, 0,
19.33925294154096`, 1.329397095484475`, 1.3716534870592167`, 0.031786132012980195`, 0.5954862326872417`, 0.596348066388643`,
0.0014472772905465003`, 52.109138668785626`, 0.6978694259642694`, 1.003934698880138`, 0.006018006164784873`,
0.006210874742467028`, 0.03204858426545831`, 0.06132501293089897`}, {0.04913245961465748`, 2.276131341102663`,

```
7.044266329747086`, 0.9173667594455086`, 0.41644326647059726`, 0.25990231144478826`, 0.18555590900866242`, 0.210063875037094`,
1773.4485121819462`, 75.91056730044647`, 0.184796166571025`, 2.2167031467842424`, 0.9244440644631444`, 222.106449408703`,
12.939747801168876`, 262.23533381564926`, 0, 56.09827107996286`, 4.948923427750774`, 4.97051246813473`, 0.004362371069008164`,
0.08182577615924427`, 0.08186749719643348`, 0.000509876461275649`, 160.91999598029642`, 0.05743288060781126`,
0.8413488596125969`, 0.0010763430035823918`, 0.0010810143438943882`, 0.004340010848260078 5`, 0.028027314155551597`},
{0.15077968496954103`, 0.971820860662203`, 0.23396787648709783`, 0.9304382139491995`, 0.8434070020798108`,
0.5328351579449485`, 0.0913866934528598`, 0.005065303834986521`, 1783.900832424436`, 398.4573153532763`,
0.17963340543276235`, 6.010950437900332`, 0.19621378169468162`, 147.1808805737204`, 100.62198670607387`,
151.85881329144362`, 0, 135.42327248521173`, 0.6537859559123055`, 0.6673023311331332`, 0.020674006681539625`,
0.5928742766982545`, 0.6034070755760077`, 0.017765653346289323`, 9.076611862336549`, 1.2770485238158167`,
0.9548604175392305`, 0.007475793212911697`, 0.007953634118045068`, 0.06391842196866504`, 0.3612128782431417`},
{0.15622813333517427`, 1.117114824910261`, 4.688994947991285`, 1.188191128841129`, 0.5951306703894546`, 0.30511551468851006`,
0.2217863487164401`, 0.33567754538344835`, 2568.8944572362634`, 80.78669839983178`, 0.03729030447311804`, 7.7879419731274115`,
0.9647929000978697`, 998.6374174371173`, 42.74315236446`, 1159.6047262219345`, 0, 174.0751796408241`, 48.58268055220434`,
48.72529383977`, 0.0029354758927397917`, 0.20393717622223242`, 0.2038582197573035`, -0.00038716072464828333`,
775.3204668392423`, 0.4551532051263908`, 2.2900697030075277`, 0.0020718721545402996`, 0.0020783512875781397`,
0.00312718766147890 3`, 0.06947709987357452`}, {0.11331162134690587`, 1.2483595201539153`, 4.922077543835222`,
1.0559046192245036`, 0.27438155190999924`, 0.25039971454185184`, 0.17415638432949032`, 0.1908750894543732`,
1293.217041147549`, 78.02731777513316`, 0.14932153866568026`, 5.182262423333571`, 0.7834976909752784`, 449.2658318454003`,
36.94105042569882`, 535.1356450492154`, 0, 122.00120975154046`, 17.36031630916318`, 17.453567633841324`, 0.005371522212928914`,
0.11666069584108792`, 0.11667771980621754`, 0.00014592716944550332`, 309.5988019632467`, 0.18884303704520491`,
2.1419120325536336`, 0.0013333433653327509`, 0.001338594788455745`, 0.003938537708689571`, 0.07691456565527477`},
{0.13914220002486866`, 3.6703502896291074`, 6.379985611157895`, 1.2081851433506285`, 0.7677675288971362`,
0.4439146253901465`, 0.18717370496505287`, 0.22151595381283107`, 1066.171533351664`, 40.74945145753361`,
0.011595132472834446`, 8.264527016617006`, 0.7438205368393507`, 2387.4387495258375`, 175.5291210649724`,
2741.3317845490583`, 0, 576.7076682399521`, 33.86830105337784`, 33.9516343193639`, 0.0024605091898386444`,
0.3436227116704194`, 0.34329271006328926`, -0.0009603602902903408`, 1775.83612257873`, 0.6830345725767526`,
4.85443940227977 1`, 0.0034301627298499726`, 0.0034411858446544564`, 0.0032135836322162437`, 0.07080622029138861`},
{0.22919129490265872`, 2.16424636204603`, 7.737422782052235`, 1.4860287946100363`, 0.8497692228491351`,
0.6288301911078309`, 0.10881904848202179`, 0.012090233236638177`, 2725.277273810464`, 77.99221256357112`,
0.11691550532917167`, 1.34040213575469`, 0.8482131070094612`, 157.40188709977625`, 15.88636674737511`,
```

177.47918945964864`, 0, 133.70412843006045`, 0.7208534577713737`, 0.7263067910002478`, 0.007565106569279667`,
0.14048479162379657`, 0.1410465066931605`, 0.003998404829955726`, 22.287206764999983`, 0.45996987580551`,
0.4909142862080487`, 0.0011387793830566206`, 0.001153432244561043`, 0.01286716437128721`, 0.018572587636337276`},
{0.14382797385454654`, 3.902431027294017`, 8.163450688536429`, 0.8614076883883874`, 0.9648536276916164`,
0.1955756537598935`, 0.13123090751766925`, 0.17894545684886398`, 1385.50217697701`, 120.46271140783404`,
0.09423544317816807`, 8.260526259890764`, 0.9048289425944`, 874.7343828386483`, 110.04949912247456`,
962.3685316187962`, 0, 248.20644876040487`, 11.03655532029534`, 11.079861813455814`, 0.003923913929995582`,
0.07015247065658954`, 0.0701326496143413`, -0.0002825423262035054`, 615.2770845195307`, 0.14414125307875428`,
3.2076714336673833`, 0.0009846261670662937`, 0.0009858442322397034`, 0.0012370838945292562`, 0.06883884557405148`},
{0.26055110138040244`, 2.367351112459766`, 4.465438967228517`, 1.1966516268556373`, 0.9350425579579571`,
0.17322311915607413`, 0.07783685162029783`, 0.005169562389067373`, 1562.7816595558625`, 214.0110363941617`,
0.12855831417818353`, 8.432278535705368`, 1.4776941418404932`, 380.6833055363129`, 87.43930157160808`,
395.55075637162946`, 0, 354.03726165748316`, 0.7433457841185143`, 0.7518921225487617`, 0.011497123697798317`,
0.07587571939477733`, 0.07588163119550513`, 0.00007791426262526713`, 25.1394352710749`, 0.28242146080484437`,
3.556452820094166`, 0.0007662634658406331`, 0.0007683663240321434`, 0.0027443017777226686`, 0.11609597929217713`},
{0.14680250287088714`, 2.9795896823389043`, 0.5962523026989057`, 1.2784383052923718`, 0.4427855138400896`,
0.39715884806633817`, 0.14338219057113172`, 0.008545850364486081`, 3591.485740200138`, 175.10126732731499`,
0.10771590512275864`, 1.6757545718316624`, 0.6195088999107756`, 67.56947904186589`, 6.18761498808319`,
80.63335594790892`, 0, 58.758790804192245`, 0.16395786622413897`, 0.16874823505825945`, 0.02921707231522408`,
0.5216603323683195`, 0.5295909357816782`, 0.015202619254858663`, 6.97895952199638`, 1.094014892001825`,
0.25333270867914953`, 0.004905610535110849`, 0.0051395332567881315`, 0.047684731595186935`, 0.053181955213308096`},
{0.1718184288134474`, 1.149839439352271`, 8.637641060035964`, 0.8458363550999337`, 0.6513629677556381`,
0.2037277594433513`, 0.17428171056999092`, 0.14466402071943818`, 1236.767232462591`, 142.28311915163187`,
0.08823953882269175`, 7.82350558871963`, 1.0042870130174983`, 576.5365492555388`, 77.70646496275542`,
626.4072268007968`, 0, 190.1173026687046`, 22.16553476295557`, 22.29582185376466`, 0.00587791326500442`,
0.04529667045710172`, 0.04529204577893711`, -0.00010209752986134468`, 364.0972294968591`, 0.11118289640517365`,
3.2418163891778127`, 0.0006475197318147607`, 0.0006484013146132436`, 0.0013614763460754187`, 0.121265464737932`},
{0.06783774432411926`, 1.4183998935859234`, 4.514906377341504`, 1.3074717506903661`, 0.44121610557851443`,
0.3859898733629271`, 0.18885629375508112`, 0.08215446401079224`, 1996.0572117605661`, 32.84560309478019`,
0.14544575809597998`, 5.16703571475392`, 0.8091181446129996`, 1200.25063149021`, 34.037865761911405`,
1495.8512448798494`, 0, 552.877137633676`, 30.407051125191`, 30.47872678881445`, 0.0023572053510991786`,

0.4222905017048736`, 0.4220232944907544`, -0.0006327568653342608`, 616.1336868604669`, 0.40924621550166895`,
2.0196617181687526`, 0.003893815459759198`, 0.003910167151159315`, 0.00419940070840652`, 0.037235514623978574`},
{0.09592803445036208`, 0.8366316591486305`, 5.0931225791851045`, 1.1660530096752404`, 0.2059334355486433`,
0.4873422492046261`, 0.1598589026416986`, 0.029544379859417352`, 2582.9507509173`, 296.85131956979524`,
0.07927444657096727`, 2.8630420963700924`, 1.2775475419622428`, 65.59694692094624`, 12.80376281727538`,
70.53139726730839`, 0, 46.29989706905996`, 1.4832929363366867`, 1.503172837025065`, 0.013402545243339503`,
0.032798130931107`, 0.032892487823146035`, 0.0028768984500133765`, 17.728140433297234`, 0.04494657476952987`,
0.812888892651307`, 0.0003381506886520613`, 0.00034188917981980353`, 0.011055695857502323`, 0.1270373434769954`},
{0.06735695081703474`, 1.6218831494512376`, 8.01632413374589`, 1.2380602139197665`, 0.3870710635961401`,
0.2680531526020482`, 0.07829277488965869`, 0.011053339337592866`, 594.5995475908121`, 283.2125249310004`,
0.06462100682727939`, 4.113525656200814`, 1.255132039019542`, 98.12700137195351`, 71.97731559479051`,
97.85662102850388`, 0, 84.96074996467637`, 0.5403174486217913`, 0.5536544481928233`, 0.024683636638148876`,
0.023100907609128068`, 0.023118687029651037`, 0.0007696416445535093`, 12.519025218202394`, 0.022228667109390154`,
2.2916762904400887`, 0.00022518675664140453`, 0.00022615835692884345`, 0.004314642219329645`, 0.19442317486074473`},
{0.25318695512908856`, 3.592770590245955`, 0.3860629768890025`, 0.8375102060070931`, 0.25324293087688066`,
0.22639700269651497`, 0.18616406291052912`, 0.3042902902243334`, 535.9279722175538`, 147.98665712717872`,
0.04054733608151545`, 8.866857733517588`, 0.9845499556289559`, 466.8751600617524`, 147.48199530002978`,
497.8565200699209`, 0, 91.65137585685935`, 7.10807660068774`, 7.253850876961717`, 0.020508259049976107`,
0.7129339956644228`, 0.7120610022644251`, -0.0012245080264184915`, 364.82412234523406`, 2.5786512510041213`,
6.278145615381368`, 0.010150308419213827`, 0.01036540951496305`, 0.021191582252028107`, 0.4039178899063425`},
{0.10623464305011832`, 2.9795950019626645`, 8.394466481175808`, 1.3150375502652678`, 0.3977517440977254`,
0.1685394428801441`, 0.14812837191515202`, 0.06701796399112063`, 3705.0856036562427`, 41.40776885031511`,
0.10405528061386266`, 6.850682270825037`, 0.7771134705080516`, 1066.5853607369756`, 16.36227414652897`,
1397.0581359755809`, 0, 545.2809733179336`, 11.957007537987652`, 11.974715699917342`, 0.0014809860973516997`,
0.15312978595395899`, 0.15306400765701209`, -0.000429559125529555`, 508.95771283739793`, 0.2323955450177473`,
1.1991945179695014`, 0.001407710011086638`, 0.0014095558415598885`, 0.001311229201123254`, 0.01922369508140909`},
{0.16614727413225283`, 2.422977153037391`, 4.380699036993274`, 1.2650547043993527`, 0.7289628812438445`,
0.5253372649543967`, 0.1282487061525488`, 0.005846875030812566 5`, 1525.6199865357175`, 306.5080581733838`,
0.2463161390095024`, 8.0298979658821`, 0.3034478973618291`, 258.04791656645295`, 124.01665930686386`,
279.5074579430823`, 0, 237.86071107924104`, 0.5540783414537096`, 0.5585553525634167`, 0.008080104878239736`,
0.0581384727094868 8`, 0.05821685370693774`, 0.0013481777865498312`, 19.17884517621698`, 0.13799355375563577`,

1.6198764545697169`, 0.0005533587655377481`, 0.0005575200688843759`, 0.007520082098245728`, 0.28108968758933217`},
{0.09439363668230882`, 0.5823127951342895`, 9.668824509749783`, 1.1792283442388243`, 0.7110022446147217`,
0.31000063243129616`, 0.126665851525797`, 0.01012852201633408`, 3883.168665953424 4`, 167.0440580830566`,
0.14786211145387196`, 1.794206476801321`, 0.758471056166536`, 91.06996158696003`, 7.302627022975878`,
105.84183178597655`, 0, 79.46156047790687`, 1.229211773854964`, 1.235310805737734`, 0.004961742160703997`,
0.03877464225002582`, 0.038825324842848806`, 0.0013071066522336583`, 10.225510626363748`, 0.05228684990049916`,
0.2900979068347752`, 0.00039684314855126246`, 0.00039897221126975217`, 0.005364998050897851`, 0.017717374472289535`},
{0.10782261295184936`, 0.4917827154911696`, 7.249268882781941`, 1.3611009578260718`, 0.7853661815311994`,
0.41488889777264726`, 0.055591995773018654`, 0.052207153417698195`, 693.7280684674711`, 321.57606910090226`,
0.24632437610376973`, 5.428213742379803`, 0.6782641447302435`, 134.8158698606405`, 172.7256940261541`,
129.31790814396965`, 0, 78.22907718244986`, 7.041373413257367`, 7.176357591904281`, 0.019170149163339145`,
0.02922640606253776`, 0.029259296243231876`, 0.0011253583702264258`, 49.468939113700436`, 0.045018106697919655`,
2.4742758707583894`, 0.000258834103756711`, 0.0002603775053987467`, 0.005962899091096485`, 0.28440418499972675`},
{0.05850570936819943`, 2.3562511218485964`, 4.849470518860397`, 1.0037692866900862`, 0.20135980488529004`,
0.6258574217265285`, 0.20434104039710765`, 0.046458478403025076`, 1143.066165281296`, 382.63411260362705`,
0.03938722953573187`, 7.245742033238452`, 0.7325066156705251`, 149.77161720413048`, 84.97208117850828`,
154.2560564211639`, 0, 90.87279249968644`, 1.697765488414136`, 1.7237952526914133`, 0.01533177841987543`,
0.026813635119327908`, 0.026851369601958577`, 0.001407287093403653`, 57.14802623873774`, 0.022410724905718906`,
2.3216672852342373`, 0.00032166312462633684`, 0.00032394100113688763`, 0.007081559358713907`, 0.6371278057905626`},
{0.26935136701037576`, 2.6617924377127427`, 1.8653214881187097`, 1.3783772798670109`, 0.352129512501022`,
0.17316862578533354`, 0.07359966841424637`, 0.03044640192221252`, 2578.790502033272`, 266.15340429326216`,
0.24670889410575075`, 9.827796845989266`, 0.8840863683155358`, 225.67921943029836`, 33.28593266193509`,
248.53333427636892`, 0, 157.52683084998068`, 1.732532425264037`, 1.7528449599685971`, 0.011724187327382563`,
0.1062260587167546`, 0.10628703191089653`, 0.0005739946946965446`, 65.88059582371334`, 0.4087447732496501`,
2.104987967125095`, 0.0009301186085978186`, 0.0009356109578918724`, 0.005904998828411445`, 0.14865546649481137`},
{0.2583950802004026`, 3.7896744268241553`, 0.3341722318602649`, 0.884753511926686`, 0.6013312124575798`,
0.2239296898773392`, 0.10126995172624759`, 0.005456842095783797 5`, 755.0573605362365`, 345.1189599908564`,
0.23686856237319676`, 4.65826036739878`, 1.1534719871772796`, 116.6590416243335`, 74.98245150156563`,
116.19979774444907`, 0, 106.48423268008636`, 0.14774656215202617`, 0.15300602511341047`, 0.03559787033130757`,
0.40689390600167286`, 0.4094114030765323`, 0.006187109312099315`, 7.9987338319817`, 1.501991192490817`,
2.006057989264579`, 0.005471630116634984 6`, 0.00569557897469218`, 0.0409290930277506 1`, 0.2162317713600932 8`},

```
{0.11828518723170395`, 1.6435868292536613`, 7.819044307747686`, 1.2773743957591923`, 0.9657225148907191`,
  0.25540329193006406`, 0.2479438125086047`, 0.3343373077742329`, 1140.611649524565`, 164.9221880908642`,
  0.023651248717273365`, 8.003232493785013`, 0.21949174382120273`, 721.4250019706934`, 157.9519247512417`,
  1068.0778568075198`, 0, 126.266086007514`, 24.302079171850536`, 24.36823867893834`, 0.0027223805263723033`,
  0.0924199337268112`, 0.09241953647495461`, -4.298335224639516`*^-6, 570.6082464333335`, 0.1561701309256717`,
  1.710699044630412`, 0.0008740305557435324`, 0.0008761645433126499`, 0.0024415480157922165`, 0.07344516281057677`},
{0.26141584699667636`, 0.7473599798954296`, 9.058762118324648`, 0.765952368266298`, 0.21904751242478016`,
  0.26721271327445206`, 0.2224524942406871 4`, 0.03093590173170892`, 2551.7003206225927`, 385.7759782876328`,
  0.1131827887828083`, 7.852734975537449`, 1.3811110977409826`, 158.66031481034454`, 28.147162774574614`,
  173.6595211385866`, 0, 110.5118169318338`, 4.117721313767918`, 4.164569309377514`, 0.011377165194000893`,
  0.010719896932703117`, 0.010721720471103209`, 0.00017010782953774672`, 43.96314454675107`, 0.04003358480540895`,
  1.78738744232212`, 0.00016923805858026775`, 0.00016950874484732988`, 0.0015994408665103155`, 0.3126621660872811`},
{0.2597492463712146`, 3.976490463784595`, 9.94323613361071`, 0.955277937303862`, 0.36781597694318213`,
  0.3161337854088553`, 0.14241883254664206`, 0.0371161903088566`, 1242.5354975091168`, 278.37240293523723`,
  0.07851375810023631`, 8.744407246757977`, 1.3989634873529022`, 248.0058573920434`, 79.10493400656651`,
  257.8917711204431`, 0, 163.2216987433427`, 1.4650306848906403`, 1.4865849493017433`, 0.014712500313747379`,
  0.017097373504041575`, 0.017100768988154515`, 0.00019859682612288765`, 83.2240078231349`, 0.06344328403714314`,
  3.583156702957147`, 0.00021638028696158162`, 0.00021677909638551335`, 0.0018430949950747344`, 0.3494610452006492`},
{0.05750955566796961 4`, 0.5897063506624876`, 7.194630155059524`, 1.2755298225691563`, 0.689564930155854`,
  0.36119278217271744`, 0.2498841160696511`, 0.4009780889195491`, 1320.9907894027438`, 256.63876030059`,
  0.13571670185918522`, 7.16857463714371`, 1.3676255250557143`, 384.6911637710406`, 94.98543044537928`,
  397.8797792559164`, 0, 59.500177630424396`, 34.49376937607734`, 34.75197766968603`, 0.007485650257398779`,
  0.059684293226773454`, 0.05971203932724461`, 0.00046488110977094266`, 290.58849799085806`, 0.04903453119780365`,
  2.780953144585598`, 0.0005648577312180603`, 0.0005668713631281093`, 0.003564847923222869`, 0.22163064220370668`},
{0.1397208239381057`, 1.3512320351631422`, 4.315748656362622`, 1.0234149323507424`, 0.4387218679676288`,
  0.5908017035553538`, 0.24436343171483993`, 0.05514077662518975`, 1030.1549542852385`, 113.10980109596312`,
  0.2358198015899574`, 7.7106342544090705`, 0.46099446073278916`, 613.4272706931692`, 124.79065016189236`,
  729.551617580889`, 0, 344.1447447412435`, 13.245177405594161`, 13.312823180951462`, 0.005107200401009981`,
  0.11827041065071452`, 0.11836971587157198`, 0.0008396455234329636`, 255.67582888368403`, 0.2360691317662791`,
  2.8100287777255737`, 0.001391428423118124`, 0.0014009751247043307`, 0.006861079900044586 6`, 0.22672848084962458`},
{0.04583854888632438`, 2.3264689225741337`, 6.981580565170444`, 1.2372980328075218`, 0.8253651804903572`,
```

0.45495452249062196`, 0.0835295837141499`, 0.03439901257655977`, 1013.5313447757526`, 64.02695030885803`,
0.1777154613277634`, 6.347498471152688`, 0.28284114950471473`, 706.2082515692939`, 159.36596652342857`,
931.7939567542318`, 0, 474.0610262882956`, 6.773405864128696`, 6.7968644920905215`, 0.00346334302600404`,
0.12369432701423846`, 0.12375078662068126`, 0.00045644459051308317`, 225.1159748982405 5`, 0.08099954936851167`,
2.1846818977820055`, 0.001205574089574557`, 0.0012111279340655335`, 0.004606804790352026`, 0.08836351591558417`},
{0.08906331353528191`, 1.5482338770119695`, 5.590038221637837`, 1.3588168835620769`, 0.3085034668822382`,
0.2676722221657748`, 0.07831428730380899`, 0.019104385555027914`, 2856.0098758390477`, 29.221123010272038`,
0.07226770477125283`, 4.247245472589608`, 0.2276638271182967`, 308.2456179562463`, 14.420058404634487`,
939.0024545235459`, 0, 241.98291779110258`, 2.8514306230150432`, 2.85882556535675`, 0.002593414786955961`,
0.1105926423843846`, 0.11066350984536738`, 0.0006407972488484681`, 63.06687840716053`, 0.1407106740482688`,
0.4503932653413911`, 0.0009823613863110259`, 0.0009867073520556046`, 0.004423998953072328`, 0.022864357032671423`},
{0.14523765830527252`, 1.8881182449998724`, 9.138446692610067`, 0.8999286193853917`, 0.5310683312029048`,
0.6925914130374311`, 0.2344374703010791`, 0.005837765173497995 5`, 3489.658152730246`, 207.59827142911195`,
0.0635989978100086`, 4.4743246714218365`, 0.16854984601616874`, 129.63290438670268`, 26.094520011893618`,
244.02115587761648`, 0, 119.50988057201783`, 0.3552755176663163`, 0.35672658164208165`, 0.004084334280326729`,
0.017883193707099402`, 0.017904497314145885`, 0.0011912641218008169`, 9.582888384393529`, 0.0371044739576916`,
0.34052009832102387`, 0.00023949357151120676`, 0.00024095112931643003`, 0.006085999703566491 4`, 0.1048175244051432`},
{0.23258902546586485`, 2.08539598507119`, 3.699686103649381`, 1.0931833498928993`, 0.05064531761576041`,
0.520823194388518`, 0.08776225589270567`, 0.3513344393005501`, 3745.4841551781074`, 189.61513791631432`,
0.22360193983114673`, 3.043950615318625`, 0.6185359661549543`, 77.92662898485942`, 8.54005423686738`,
92.01703580652752`, 0, 13.338230642646732`, 2.0909847771008003`, 2.110331221031082`, 0.0092523121842647`,
0.047218115210558166`, 0.04736494342541607`, 0.003109573819352285`, 62.29330370015686`, 0.1568916485880485`,
0.5256976029757305`, 0.0005191173315149955`, 0.0005258925292197863`, 0.013051380282407488`, 0.07366817383415888`},
{0.1820955139503701`, 2.1301463589452796`, 1.0837335286589127`, 0.8319946959595101`, 0.024409178193170122`,
0.6747387048956719`, 0.18033910089153304`, 0.00804054909792445`, 2554.5651932014816`, 147.37665368444442`,
0.18624166889237875`, 4.7992891492518055`, 0.5892958331460565`, 184.34973804480404`, 22.402215948165598`,
223.37334143823193`, 0, 164.73083165993174`, 0.5983844537173745`, 0.6043024910695086`, 0.009890025242750289`,
0.1833599738072683`, 0.1844671141962574`, 0.006038070174207322`, 18.209235219078966`, 0.4769861238336974`,
1.0346477696473968`, 0.0026322090774766327`, 0.0026937463003469777`, 0.023378546710786985`, 0.17262446978824741`},
{0.11714865623011333`, 0.7803444845933583`, 3.7286455817586397`, 0.8712432490902873`, 0.9005891902672722`,
0.6478151883640173`, 0.15581100010071458`, 0.01574292115738763`, 1037.936039940223`, 180.67525214379884`,

0.029753801751762954`, 7.189499669067537`, 0.709240932965888`, 558.3547603465491`, 273.44599654046567`,
570.1532907970316`, 0, 455.97845210744345`, 8.38351623592943`, 8.439183090056304`, 0.006640036538403837`,
0.1137542902619997`, 0.11386393869609103`, 0.0009639059224824109`, 93.4575808029486`, 0.19037374635150872`,
3.1354881550167595`, 0.0015715666517484017`, 0.0015829749521372271`, 0.007259189660288046`, 0.20363266380306302`},
{0.25987401963349727`, 2.104611334162005`, 6.476348965574324`, 1.4421427522116774`, 0.618645235230914`,
0.3805640132205469`, 0.11350606280121234`, 0.0053044995482471255`, 2985.7971643892297`, 372.29941068623043`,
0.13537534314965316`, 9.556057499862177`, 1.2376769397336673`, 227.193696185474`, 52.696199698126534`,
236.8176069143273`, 0, 210.97444564963808`, 0.5093678551641657`, 0.515152912974450З`, 0.011357328012817192`,
0.033193601587804995`, 0.033213864147198985`, 0.0006104356991931681`, 15.314590874804177`, 0.1232307810105349`,
2.171061223316668`, 0.00027795901801674905`, 0.00027899786072906984`, 0.0037373952452881554`, 0.23304574291902133`},
{0.14562691763665958`, 0.7916589364478996`, 6.589457190185791`, 1.2275476972045025`, 0.23387470631242246`,
0.5473498244385164`, 0.05668225254693815`, 0.02480814693768771`, 3264.7917840803066`, 42.76592076077594`,
0.07262816395108834`, 7.451596380699327`, 1.1772112101747596`, 910.7979094237692`, 29.37108723470624`,
1031.8815101123764`, 0, 673.3648786295357`, 19.239414594633367`, 19.36124746543737`, 0.006332462466814626`,
0.14285142959924388`, 0.14288106973869347`, 0.00020748927422520325`, 217.5864927981091`, 0.2971859053225032`,
5.194076467268623`, 0.0014056718760696585`, 0.0014097323096266918`, 0.0028886069545521753`, 0.06510727274895176`},
{0.12870736792434306`, 1.8011116706209283`, 5.964705884730831`, 1.0934961970990227`, 0.1765898735142728`,
0.17954421546189758`, 0.07865767771997476`, 0.015380472377885535`, 1239.7199915121791`, 204.53201197549618`,
0.09598785640324625`, 4.188391285134756`, 0.8112464540523976`, 113.04713078030159`, 25.440187587848772`,
123.49287556342261`, 0, 92.83879780438248`, 0.7509268815885457`, 0.7618491675675292`, 0.014545072558699745`,
0.031024549405094838`, 0.031040240331272697`, 0.0005057583906531438`, 19.321473860173036`, 0.05704411564239587`,
1.5133424452764253`, 0.0003426638237123969`, 0.0003439037055223431З`, 0.0036183621501488794`, 0.09683869455895813`},
{0.14650986127607446`, 2.5172437701709836`, 0.1805184658738579`, 0.7600994183135076`, 0.9075051465461681`,
0.2801821328714206`, 0.17102217375046636`, 0.35873020461861416`, 1565.8031681921566`, 125.39398648792553`,
0.18475404806650503`, 5.639231846073234`, 1.0926853341214517`, 647.2938657731713`, 77.53873720322431`,
693.1140230632276`, 0, 106.09920077646605`, 14.38567317608773`, 14.65365780220056`, 0.01862857739311652`,
3.0686049989996365`, 2.990900383633355`, -0.025322456097025547`, 517.3178026031812`, 6.422584181634889`,
2.387303493248516`, 0.04745580945588712`, 0.048325182126730135`, 0.018319625791045624`, 0.11228728191403795`},
{0.21477552815648338`, 3.895561319530766`, 6.525902778767705`, 1.1779397940067482`, 0.3665320870360802`,
0.26560577062434`, 0.16739034513753454`, 0.18566064627492787`, 2084.753342798819`, 127.5970184006917`,
0.1877200023200773`, 6.725934789039004`, 0.6926175903352805`, 379.09576255621755`, 33.30064650793632`,

453.6067293365148`, 0, 104.85385781283706`, 4.836329594936657`, 4.859574849361584`, 0.004806383429545979 5`,
0.06381075812624785`, 0.06382699112058844`, 0.000254392751587007`, 269.1459785505307`, 0.1957855611232157`,
1.579177865766423`, 0.000654325302541392`, 0.0006564010551217228`, 0.003172355665092885 2`, 0.08762243769813427`},
{0.16557107707187058`, 3.9808920811325805`, 5.098629354330779`, 1.349285917396553`, 0.636892795916012`,
0.22358004297978562`, 0.184200177687603`, 0.010072170136625726`, 2379.9993388287503`, 250.88159080337186`,
0.22088678734814615`, 3.6464862549045485`, 1.4194198069422335`, 153.3761032599579`, 18.917322392712567`,
163.21864062304766`, 0, 133.91682243611498`, 0.33040119451567573`, 0.33338535675230857`, 0.009031935374831823`,
0.06970503946467015`, 0.06977365115285558`, 0.0009843146021057958`, 18.78987855491712`, 0.164873406592906 3`,
1.044759629607236`, 0.0006232349486274824`, 0.0006269649593787027`, 0.005984919105442854`, 0.07636993348747334`},
{0.2183191832780194`, 0.7656891998585413`, 0.2734070058927056`, 0.9147050043440094`, 0.3441094983626165`,
0.18539360977555885`, 0.1738037138598677`, 0.02857315375757566`, 2216.527228285475`, 152.12573952853177`,
0.143511762959395`, 2.048281105987993`, 0.3150624921161962`, 76.42222408559353`, 8.116651528401766`,
115.44998058554846`, 0, 52.152276990895196`, 1.7742419483216239`, 1.8555490953839262`, 0.04582641456494496`,
0.747434765727396`, 0.7518976434401156`, 0.00597092604914673`, 19.407398539512027`, 2.3311335372457505`,
0.26339404431033203`, 0.009848031047324679`, 0.010371320781604977`, 0.05313648299498963`, 0.04652647551924745`},
{0.07967342618536455`, 2.504619914171559`, 1.5706511952625795`, 1.0567028274669426`, 0.8710362851311728`,
0.17476539269891578`, 0.1454017836571002`, 0.013152148214347218`, 996.5768638290242`, 179.4306604453801`,
0.15492643832143987`, 3.5116876584841332`, 0.7677856536659751`, 235.45523845012718`, 50.80226292323742`,
252.53938121253887`, 0, 197.81518803190505`, 1.0036681821280828`, 1.0125500369454086`, 0.008849393629768665`,
0.2822194064308883`, 0.2824706374460388`, 0.0008901975180506394`, 35.91153308826236`, 0.3212198149478178`,
1.341847723811846`, 0.003211981173717904`, 0.003244339685481265`, 0.010074315512225152`, 0.05559414214502557`},
{0.07456457809354955`, 0.9644871227153224`, 4.441895089579111`, 1.07362216198562 7`, 0.23632853710586565`,
0.6486715280931723`, 0.07294638203638015`, 0.02103243083607793`, 1904.5570203562393`, 282.8945937517137`,
0.14375820330010797`, 5.555062182602162`, 0.9932669340579485`, 118.06130187224556`, 41.00154899601797`,
121.31585404075494`, 0, 91.14188486979282`, 1.8149919487070103`, 1.8457541288435648`, 0.01694893476440451`,
0.03216199790769228`, 0.03223146575618386 6`, 0.002159935731945728`, 25.0076623194271`, 0.03425922578048288`,
1.9217925840908565`, 0.00036035899023845186`, 0.00036363354385364314`, 0.009086920831431033`, 0.262555712239004`},
{0.2103616483258971`, 1.9343221969844642`, 6.714766349656301`, 1.0369410555646754`, 0.10623162247280038`,
0.4483194849728549`, 0.05288545252588117`, 0.03973487414634300 4`, 3533.119265437819`, 182.57420336612938`,
0.1697677503867407`, 5.3789812588786425`, 1.1987739832103812`, 149.57957581226793`, 15.730233982309967`,
161.4026400574431`, 0, 96.00242430602643`, 1.8671468836944227`, 1.8916029084089154`, 0.013098072212777812`,

0.026917281328740456`, 0.02694047238314265`, 0.0008615674859195899`, 51.59519517372124`, 0.08089090955378238`,
1.8595588598199577`, 0.00031341592015277087`, 0.00031474218328743576`, 0.004231639330951831`, 0.10405743421598102`},
{0.20281939839069102`, 1.9616090683543987`, 9.233817142633974`, 0.9596707111380289`, 0.0428378421346598`,
0.4996155069836299`, 0.1873396325631559`, 0.010300259610186683`, 2982.0763963636246`, 256.635560614439`,
0.10323853661350679`, 1.5583642397770507`, 1.1284223856531295`, 36.58679555143331`, 5.2559077936160845`,
41.03846627420804`, 0, 31.86886154891402`, 0.15966671245467826`, 0.1617012929175371`, 0.012742671478479739`,
0.015254745712010692`, 0.015293659961595953`, 0.002550960227060583 6`, 4.47433815807759`, 0.04419940497018293`,
0.3977155256141884`, 0.00019122283557171382`, 0.0001932358590017781`, 0.010527107936904034`, 0.0676992296160776`},
{0.09628613710357908`, 1.5426640827734222`, 0.6350126000666823`, 1.1824558202170816`, 0.4624355823492319`,
0.315548754668174`, 0.24429500934345866`, 0.021157750682009126`, 1240.1379477304627`, 114.8299639556875`,
0.022101718613827426`, 8.73583411100617`, 1.006336055790952`, 749.2384161897579`, 87.32438209167819`,
838.5176672590374`, 0, 574.2928418823395`, 7.482498769218552`, 7.548315747134032`, 0.008796122785377225`,
0.9999428362480052`, 0.9994544705483395`, -0.0004883936180773985`, 164.89974429528294`, 1.3754376146671883`,
4.047091551488218`, 0.010106354502865678`, 0.010264715297334684`, 0.01566942802413096`, 0.2282759390623346`},
{0.2497495903654815`, 1.9922654552033938`, 4.7803148098485195`, 1.4608470462421037`, 0.7453342000999874`,
0.579116855343716`, 0.20607471500549013`, 0.11029005763163223`, 2995.828190949672`, 372.8602258250605`,
0.24417612053219007`, 5.214690665497957`, 0.479039467804907`, 171.88275466740424`, 44.52491978653581`,
190.84039946351044`, 0, 67.02603386540717`, 3.5494129446265084`, 3.5690313631022277`, 0.005527229088804653`,
0.06297993903580167`, 0.06313147386927545`, 0.002406080980606351`, 101.0196113690166`, 0.22470305679184396`,
0.6620154927125093`, 0.0005182438370429088`, 0.0005240356682714178`, 0.011175880569187502`, 0.1265661466195685`},
{0.1826369436867583`, 3.4688854117825922`, 2.4438258950850287`, 1.4614655744939826`, 0.6869584196330984`,
0.32705660778535306`, 0.23164888048001758`, 0.012304471968752876`, 769.9733124793984`, 249.22075665117416`,
0.24824092697008104`, 9.063288482525959`, 1.0083702284112093`, 488.6415540010345`, 195.53159835634162`,
500.4418270687013`, 0, 415.3623300748584`, 1.429688260281041`, 1.4449788294986012`, 0.010695037262566931`,
0.2019142309024812`, 0.20211441439829456`, 0.0009914283649974376`, 70.8489249926534`, 0.5268142574127256`,
4.210031442030268`, 0.0016623390675764016`, 0.0016766712702922105`, 0.008621708407962947`, 0.329462593821324`},
{0.07327234901270369`, 0.8531314502177398`, 8.241277338171692`, 1.2515317235008665`, 0.44747974375408006`,
0.36044147530449333`, 0.1678069040859092`, 0.08333627347055737`, 3911.155878788726`, 119.81486891303797`,
0.14940939146949633`, 5.454641004053358`, 0.6006995465954545`, 327.30900649573846`, 17.859557764172177`,
416.00235709719266`, 0, 149.75372866213542`, 13.454918324345222`, 13.48905795414784`, 0.002537334599857610 2`,
0.05715932026276185`, 0.05718826902807442 5`, 0.0005064574802411403`, 163.98305689442694`, 0.05983139519456727`,

0.718665384225879`, 0.000551567855172852`, 0.000553639284944788`, 0.003256305284621508`, 0.045818718889910966`},
{0.1930703354969151`, 1.921406348468734`, 8.333479032313342`, 1.3125156750016385`, 0.34274795388496426`,
0.18757059400837783`, 0.06254344682622745`, 0.48499736430114065`, 1025.3342142153256`, 313.5826206578507`,
0.06795986160544415`, 8.852485488110243`, 1.3326254900136894`, 178.11514443403297`, 76.64497862073068`,
180.7240089584147`, 0, 25.617403349900883`, 5.357827364667006`, 5.471261316733589`, 0.021171632519300543`,
0.019882379639230056`, 0.019886331894410123`, 0.00019878179834509346`, 147.06519303529572`, 0.05483853867747364`,
3.6672300900574184`, 0.0001831593543419885`, 0.00018349442299893878`, 0.0018293832611171457`, 0.2571428362093912`},
{0.075070301444424`, 1.1158533918226947`, 4.504617389718993`, 1.302858138181639`, 0.7006793741520845`,
0.20393211135209022`, 0.13347728349830462`, 0.12945769800166135`, 2084.9216842923897`, 207.91665221421215`,
0.1979670492115873`, 1.2145592445361792`, 1.330809319256201`, 54.29152172033512`, 7.113535345271648`,
57.669930190172806`, 0, 19.25446862637522`, 2.0583351893160193`, 2.080061291741055`, 0.010555181944032821`,
0.08071876412920682`, 0.08091559047597745`, 0.0024384212133818917`, 32.81143289294691`, 0.08656545650573656`,
0.4031633137634961`, 0.0007460626743833743`, 0.0007541695504463955`, 0.010866213176689898`, 0.024067734918643047`},
{0.04793776202493774`, 3.0088158689008297`, 5.170365665010708`, 1.3304854892588214`, 0.4098476660063537`,
0.28021047255972975`, 0.1484797686973009`, 0.06051575982799722`, 541.2425249553703`, 199.10653072626633`,
0.20919118463500602`, 1.1760815484955796`, 1.3182149861572752`, 46.496614771044015`, 22.707270233192244`,
47.436577501373215`, 0, 25.253214319565682`, 0.48055284252422237`, 0.4901637535329115`, 0.0199996965124698`,
0.06343328144393083`, 0.06372238253554167`, 0.004557561662112297`, 20.655643120461146`, 0.04344070786171231`,
0.8229813659603871`, 0.0005708239593449349`, 0.0005811339286496829`, 0.018061556695306846`, 0.06508634175126277`},
{0.27316321779569186`, 1.2038217889581082`, 3.7625718380424993`, 0.7524163201956243`, 0.8286278988606164`,
0.5838413652579414`, 0.1289047793447199`, 0.04536097191427075`, 2261.212930013986`, 291.3114882787695`,
0.1550429747126918`, 5.46528595832512`, 1.2275549298228752`, 214.83100994772144`, 73.52502687873006`,
218.95701158619445`, 0, 130.86635673896546`, 4.600489581167228`, 4.642049656056249`, 0.009033837411382084`,
0.04922949821125329`, 0.04929375070553889 5`, 0.0013051624863182898`, 79.11670853834092`, 0.19210983059795708`,
1.6840502441363894`, 0.000788433126853727`, 0.0007939860488607035`, 0.007042984138852182`, 0.14522422700416132`},
{0.2494292406608673`, 1.335820360094238`, 3.8994390375054255`, 0.9165975453056769`, 0.13563044706422311`,
0.5269987018012078`, 0.205844612334116`, 0.012716247145242394`, 1091.290454779412`, 128.1406782295989`,
0.24389684971209813`, 1.43576099827777`, 0.4903794001833075`, 69.43284851349263`, 14.684534304019103`,
83.22090467035795`, 0, 58.53185310496875`, 0.5248922896756743`, 0.5314976294117245`, 0.012584181299617914`,
0.0706003854284146`, 0.07103140073308313`, 0.006104999314848758`, 10.016597248646415`, 0.2515685789681933`,
0.5217401313050798`, 0.0009192830812310149`, 0.0009425280593290727`, 0.02528598488610312`, 0.06641505493107128`},

{0.12542240143398464`, 0.5428740326561194`, 7.689007709078183`, 0.7695505557683522`, 0.47414990819814995`,
0.5751602267111138`, 0.1894118267745437`, 0.4188796421855669`, 2595.8116503362608`, 121.7056153778716`,
0.02093687945319478`, 2.468481199752018`, 0.4696061183497453`, 151.86218896220618`, 15.842105119541863`,
193.96116487322854`, 0, 22.082778523992854`, 14.810581275998494`, 14.861538669330226`, 0.003440607251135485 7`,
0.0385731093831492`, 0.03862792290944372`, 0.0014210294988161731`, 114.8611426183213`, 0.06911331442333353`,
0.41792281966588124`, 0.0006037914492955654`, 0.000608083371670794`, 0.007108286114743567`, 0.03978475514287984 5`},
{0.09391968152154795`, 3.06447396666384`, 0.887078247635884`, 1.3969498711006763`, 0.9012814324673109`,
0.6019227650187913`, 0.056809355713011156`, 0.11429079373151174`, 3926.488447769728`, 52.970576122622276`,
0.24789590005226048`, 5.914871844531001`, 0.869797459980445`, 808.4123391506231`, 56.07692286408627`,
930.9671385405933`, 0, 307.2990311910298`, 11.12050874081094`, 11.196284227384206`, 0.00681403057534391`,
1.3481200408360194`, 1.3459629749927011`, −0.0016000547265663245`, 486.83585046103974`, 1.808785784116393`,
2.3305828917189935`, 0.01159239697685066`, 0.011709524422972501`, 0.010103816005933686`, 0.06114428315390263`},
{0.15904117838601922`, 2.05514897407154`, 8.990862054448137`, 1.399223458930086`, 0.16448814747402052`,
0.679414685909332`, 0.20987970122910526`, 0.011549865816715998`, 3644.479968128845`, 344.7039952488134`,
0.14067273235487315`, 5.192736401046902`, 1.265673086788253`, 109.18052993011909`, 19.195313903582942`,
119.32319285898598`, 0, 93.76148846753595`, 0.5035270174393421`, 0.5093397210972638`, 0.011543975708556475`,
0.020497247893962994`, 0.020530075629804842`, 0.0016015679769143532`, 14.783186190110946`, 0.04657009226750388`,
1.170610629351623`, 0.0001765035498156875`, 0.00017773017918574677`, 0.006949601701156549`, 0.25296477178796584`},
{0.10596194132792924`, 2.903583061514107`, 0.8170701309155923`, 1.3631505832711714`, 0.6107209987788129`,
0.24870490908317233`, 0.18241426717129616`, 0.018338650769170847`, 1588.8736918382`, 58.688447621427315`,
0.2068253327055226`, 8.58249867744179`, 0.3849797974029039`, 1144.437855313107`, 70.3179188627623`,
1641.2852717316282`, 0, 904.1485368729643`, 5.565230415307216`, 5.592767931534231`, 0.004948135867164360 5`,
1.3970303770190577`, 1.3923739525926913`, −0.003333087456768191`, 230.84441096155948`, 2.1147435834714496`,
1.7704840963492006`, 0.012292023099648941`, 0.012403170887781537`, 0.009042269708700035`, 0.06744116057649334`},
{0.18968282299824824`, 0.519816328948473`, 1.9818995109191384`, 0.8631954007357836`, 0.033894385168637076`,
0.36152588787936835`, 0.13701549203954322`, 0.2296501432463657`, 2230.7628620359865`, 54.21442465485268`,
0.025809495734432863`, 5.0872858624540225`, 0.22420423998520422`, 269.29952297655626`, 20.211725259530517`,
600.3364448257337`, 0, 63.285368386372376`, 24.386441439202162`, 24.494527335411647`, 0.00443221273095351 1`,
0.14428930881824056`, 0.14445580930907423`, 0.0011539350503328993`, 181.09243521489975`, 0.3909886203587892`,
0.6594513233178166`, 0.0020104723450591333`, 0.002029931598234824`, 0.009678945956910656`, 0.06444412354966932`},
{0.1333252885737945`, 0.6199239307530391`, 1.8571978251971686`, 0.9095514210206579`, 0.1119169900929906 1`,

0.2478793840048047`, 0.08625348528814925`, 0.24475415564717562`, 2619.34383923971`, 229.53625600516762`,
0.07038297161992796`, 1.9415562105060538`, 1.123956994156356`, 44.90308442957719`, 5.842898243026262`,
49.090432738657384`, 0, 10.35359253116544`, 3.484609871437473`, 3.542677397729449`, 0.016663996382476398`,
0.07057015429347958`, 0.07078241828935034`, 0.003007843726513748`, 30.85990069489089`, 0.13441123122675272`,
0.5457763452539046`, 0.000933013214413303`, 0.0009464150360715737`, 0.014364021271336513`, 0.05035336290990877`},
{0.15956630372935293`, 0.48645969647201426`, 2.666967707997488`, 1.3223736190803834`, 0.3256325807266449`,
0.3482049071188411`, 0.2077761075845453`, 0.13946170160498525`, 1352.5873448025677`, 303.93582335946746`,
0.20467290406100863`, 9.626982891792906`, 0.24128691898319654`, 229.7643019028084`, 80.5681602905176 5`,
289.0010076759628`, 0, 77.91546826664622`, 19.07128497450129`, 19.244082586132915`, 0.009060617145759231`,
0.0741258595738148`, 0.07421499350061755`, 0.0012024673617982984`, 132.53445000038838`, 0.1689712774708895`,
1.8567691869423202`, 0.0006749854450628368`, 0.0006801241388269574`, 0.007613043809622022`, 0.37209557580240604`},
{0.1706421742509121`, 2.0231510661206737`, 3.9336130035151675`, 1.4585682836966742`, 0.11643260032474756`,
0.3849941275209635`, 0.09390422638545698`, 0.012272074152593612`, 1400.0032271122882`, 356.7106633003026`,
0.22727785977132925`, 4.752121167794142`, 0.5739518122089307`, 77.10424967180968`, 32.89247422096266`,
81.39289412743169`, 0, 65.63865041794183`, 0.37766815061117365`, 0.38462836292774844`, 0.018429439457129604`,
0.0376164033710938`, 0.037706687019444525`, 0.002400113787063951`, 10.91542459355456`, 0.0916992122677512`,
1.2658960673045685`, 0.0003099238597302767`, 0.0003134821170125058`, 0.011481069206242589`, 0.2611493584598003`},
{0.15918053794813153`, 2.206413285621343`, 5.933314299085708`, 1.101458457677796`, 0.578828119858223`,
0.5102982514637783`, 0.14381010711428127`, 0.005244970313706263`, 866.7690619050336`, 266.3421407794259`,
0.02684631402674248`, 2.069701061730642`, 0.9768437925368372`, 72.09055788478798`, 42.263211524349494`,
72.32687352945604`, 0, 66.9380756790139`, 0.15203248138783373`, 0.15452219691792943`, 0.016376207948250565`,
0.04037644083055421`, 0.04051956528476207 5`, 0.003544751624059872`, 4.7920926682870855`, 0.09181633674054211`,
0.9662614811264116`, 0.0004395303924886 74`, 0.0004461562712275264`, 0.015074904607474071`, 0.11456782302937603`},
{0.05059760434947658`, 2.210267681954983`, 2.4323928454491863`, 1.1361578898617037`, 0.6967300845119604`,
0.5269320202068383`, 0.09927434886429598`, 0.24328677204756152`, 3807.4700659446307`, 207.19795070674797`,
0.16480436384127967`, 4.45009968475919`, 0.7729577502629965`, 175.81861191981164`, 25.695461084329075`,
194.16942285048262`, 0, 39.903940137993196`, 4.166225148047493`, 4.191345800645228`, 0.006029595546344302`,
0.11541245591403411`, 0.11572587942696042`, 0.0027156818598486065`, 131.5496114353924`, 0.08342276830478496`,
0.8619817184915652`, 0.0012220721413219726`, 0.0012344542022546645`, 0.010132021272735825`, 0.07971436943940255`},
{0.04928925392103686`, 1.1598100203546071`, 3.5959706980197943`, 1.0567798767912857`, 0.5147503710857468`,
0.3483622087425814`, 0.2380152845766259`, 0.2996884511067484`, 3369.979145995473`, 363.0241772367476`,

0.014634320972586101`, 4.5222312521830705`, 0.7343151280843321`, 122.63988659232628`, 18.114361432229266`,
139.74207876832804`, 0, 23.685773203013333`, 5.6275618540160135`, 5.658819956824939`, 0.005554466324811491`,
0.049928839779534176`, 0.049999849540892455`, 0.0014222193359956226`, 93.24146612075891`, 0.0351565037411137`,
0.6015640763087992`, 0.0005690547494725529`, 0.0005731607223482895`, 0.007215426774914713`, 0.10053672037748909`},
{0.21304011543754336`, 1.277940262843642`, 8.381125715629981`, 0.9676658592923792`, 0.3190964277032775`,
0.4221745616471033`, 0.15115883673524522`, 0.016195119502937567`, 3029.1645990840216`, 191.62516925020475`,
0.15425858593294461`, 3.4086567800829783`, 0.2418820655638112`, 88.24549669013408`, 13.497231012496432`,
139.22361028923953`, 0, 71.63737548723175`, 0.8567401451273298`, 0.8610362323676963`, 0.005014457726535193`,
0.018728590647263964`, 0.01874917898211898`, 0.0010992997413836747`, 15.640896089324563`, 0.056999158763938115`,
0.3318198594335032`, 0.00023336676361795927`, 0.00023475806170294427`, 0.005961851908194982`, 0.06580805980690421`},
{0.24406698410388555`, 1.4917825523241879`, 1.8330046648415304`, 0.8025451323745518`, 0.3882804043356616`,
0.6182505929388649`, 0.23260014303933024`, 0.008919285535719272`, 1172.1683108026946`, 354.24325796754124`,
0.11125790867650998`, 4.298846537988302`, 1.0733397741970694`, 116.21375995076808`, 58.542816952529826`,
119.4055404501654`, 0, 102.87626099035326`, 0.5790112780342527`, 0.5886836150896041`, 0.016704919959744213`,
0.07375743795856494`, 0.07409528190091298`, 0.004580472853976225`, 12.339413173863216`, 0.2571679348254047`,
1.5385544072839465`, 0.0010984008624363994`, 0.0011208709996147952`, 0.02045713723180631`, 0.3243338232856431`},
{0.18459742862139494`, 2.6783696392861316`, 5.280110904616961`, 0.799376053447836`, 0.38050898992691184`,
0.5204372051986464`, 0.12691110515834225`, 0.11946707964132822`, 2456.6727054183984`, 281.1798234579603`,
0.015729828687073455`, 5.211513473652531`, 0.2697983327311402`, 114.69026471684204`, 30.5561510463887`,
145.19267698649068`, 0, 42.76218524023085`, 1.830048434753909`, 1.8418910568653397`, 0.00647120692902492`,
0.020878980229017293`, 0.02090287249681153`, 0.0011443215871735912`, 70.02208808668533`, 0.055060086607352`,
0.6730603030125126`, 0.00031478704469523056`, 0.00031676211777009777`, 0.006274314995330821`, 0.16985760451050605`},
{0.10846037232055339`, 2.4223111603013434`, 3.931890423132195`, 1.3804697934424115`, 0.1958381176894204`,
0.37535535595877956`, 0.06969589452072714`, 0.03328599418804682`, 502.38138813825617`, 240.4375056150992`,
0.057245528883930086`, 5.6090889313070385`, 0.20778481434400486`, 139.9397725748632`, 114.66175870420382`,
140.01325277097266`, 0, 95.63571385539821`, 1.2400845003117378`, 1.266651204333121`, 0.021423301407851536`,
0.054836723466464204`, 0.05493670847671959`, 0.0018233221085233176`, 42.912436068883444`, 0.08496587777165404`,
2.8980543655064634`, 0.000477651233330989`, 0.0004822375500192389`, 0.009601810627110519`, 0.5016611445590666`},
{0.06691193258644623`, 0.6501044545968662`, 4.921653023328441`, 1.039296561059753`, 0.3007307833256643`,
0.63941363731021`, 0.1869993853971682`, 0.22083355826369067`, 1064.9317102210284`, 220.88788456016823`,
0.19206991947921326`, 8.047969449780908`, 0.8982344488011158`, 296.68756185510983`, 113.25296718141817`,

311.4368170836702`, 0, 74.02410113740495`, 21.635418061364565`, 21.89890681529264`, 0.012178583893352135`,
0.04880007035867087`, 0.04885677419772162`, 0.0011619622396850104`, 200.93259512512276`, 0.046647243115012026`,
3.607591316377752`, 0.000565645768107137`, 0.0005692664904801478`, 0.006401042095881326`, 0.48297562305491853`},
{0.05561357895854885`, 2.541859626964298`, 6.751848587656245`, 1.0550493742938256`, 0.47524313475951674`,
0.6531938104590875`, 0.23105177416584038`, 0.08329104995794483`, 3011.985435651468`, 344.16286415130503`,
0.16793335434061596`, 8.98503885095289`, 0.954537509095668`, 261.4075389726544`, 55.33150625205841`,
283.7642005888341`, 0, 120.29563932998424`, 3.780538178113984`, 3.8066930048223426`, 0.006918281333534004`,
0.028512533580793514`, 0.028535555429161644`, 0.0008074290663402373`, 137.27996233064388`, 0.022652629108688038`,
1.7879977950579446`, 0.0003260806074651956`, 0.00032748321615463114`, 0.004301417064752178`, 0.3203568187185204`},
{0.2794959518803075`, 2.632356308938026`, 9.83032757153688`, 1.3936963350666467`, 0.29701793223992223`,
0.20626280407223174`, 0.16564637606336619`, 0.029273176379366037`, 3072.6434786295413`, 349.76316755112407`,
0.019513472485920585`, 8.521067400493664`, 0.14808884776404962`, 100.73624981980332`, 24.542818523460927`,
199.47662631792193`, 0, 71.17649613587852`, 0.7640235837504594`, 0.7694477603776957`, 0.007099488474701321`,
0.010514205658474983`, 0.010516922746221468`, 0.0002584206391562649`, 28.73117572661377`, 0.041981113125433234`,
0.6511826303415359`, 0.0000912124697493022`, 0.00009139814488415659`, 0.0020356332348496764`, 0.16315849558774342`},
{0.08563187236977793`, 0.7699393339104481`, 2.55010147552081`, 0.886086358774756`, 0.5489407388189`,
0.6967816713320905`, 0.17330539558613361`, 0.06157221588457279`, 1722.9132401400711`, 240.8998329943014`,
0.21060998547292448`, 8.988787778446675`, 1.3846037250141348`, 372.9635823415463`, 111.07578324163194`,
384.8541065537955`, 0, 200.02664847554354`, 14.395503856669833`, 14.545784282649382`, 0.010439400209664829`,
0.09032309110859371`, 0.09046337698641745`, 0.0015531562981505598`, 158.33806643870972`, 0.11049336299757687`,
3.720710332830861`, 0.0012265928081278688`, 0.0012365777569578542`, 0.008140394076845636`, 0.3745754139549641`},
{0.23109541414596008`, 0.5235141892941444`, 4.716797836648434`, 1.149537093670705`, 0.6317810219681328`,
0.3294586442905981`, 0.09670796472340015`, 0.006726344420984968`, 700.9172696415767`, 217.90397850913655`,
0.1081952457359675`, 4.152209762717746`, 1.275784993485201`, 160.9186258023874`, 89.46846642803209`,
161.70408201586758`, 0, 146.6624335762295`, 1.6336876861383187`, 1.663825758267212`, 0.01844787861511832`,
0.05912415718901349`, 0.05920223786687377`, 0.0013206222561561454`, 12.217981208121833`, 0.19519030845180046`,
2.5146966990870516`, 0.0006195891325231351`, 0.0006244438023597375`, 0.007835305013877303`, 0.15745533244458096`},
{0.23775769207863484`, 1.2489503941392437`, 0.7600016230767555`, 1.095753134818721`, 0.8325305358876416`,
0.5578220865599602`, 0.11931867110919586`, 0.014230310160314848`, 1755.4498344511894`, 146.4106779572221`,
0.028427482579488184`, 4.979696715264749`, 1.3071286627299754`, 373.578771224552`, 84.56002073439514`,
383.6884921270349`, 0, 308.30684669146507`, 3.298394753428062`, 3.3496369094807714`, 0.015535483131439154`,

0.6705576469214508`, 0.6736647024405723`, 0.004633539761101923 6`, 58.850448961725746`, 2.2775748362531583`,
2.5938617131020725`, 0.007319381987125562`, 0.007502022608245693`, 0.024953011257150992`, 0.13861788334799494`},
{0.2685717150273603`, 3.803415502373147`, 9.096737964614093`, 1.4671460946906174`, 0.7887407820119334`,
0.32631565166991583`, 0.10844882100921999`, 0.25610077987407076`, 3027.035564030042`, 145.16929800361856`,
0.22414767286679493`, 5.11102243526882`, 0.8167747408960464`, 320.35353365649087`, 32.09293604415167`,
360.4669497662974`, 0, 69.61023798749238`, 4.525869852400767`, 4.546660599013952`, 0.004593757065761927 5`,
0.06337281827848744`, 0.06340399746600538`, 0.0004919962274823053`, 245.91090797634857`, 0.2431449498739324`,
1.1635756100292096`, 0.0005217339144807376`, 0.0005236602864147933`, 0.0036922497859332992`, 0.04854698399958145`},
{0.26187896590333215`, 2.5078884075536303`, 9.466913557279852`, 1.4296579631390225`, 0.32008183029716575`,
0.24032929426056993`, 0.0616102099815665 2`, 0.06072142573048648`, 3034.015450440843`, 187.9292507538172`,
0.24599280561665748`, 9.218911969505061`, 0.5310393366539319`, 258.38734784057334`, 29.142678470123563`,
310.04256483760076`, 0, 138.9714163717222`, 3.2391538644281455`, 3.2591200270610607`, 0.006164005622635082 6`,
0.026553895505741915`, 0.02655992117331861`, 0.0002269221694946033`, 116.04909181259859`, 0.09934152422506545`,
1.4709164345900574`, 0.0002245512334230204`, 0.0002250073770190434`, 0.002031356448457755`, 0.10106694895725504`},
{0.2642492231861682`, 0.678656463776123`, 5.229669084224717`, 1.4960336643896621`, 0.09351401012737992`,
0.6251695134859938`, 0.20442619944666085`, 0.006818687130271267`, 2270.7823160691296`, 72.58428376873474`,
0.07107729542645586`, 9.843971184435041`, 0.8047286671187344`, 849.4178770724275`, 53.14686686695808 5`,
1032.7031604416616`, 0, 772.9295408275059`, 7.00939138539277`, 7.049701713766057`, 0.005750902775566447`,
0.1547083745344915`, 0.15483289094123062`, 0.0008048459374858652`, 67.95669672619258`, 0.5840223970161987`,
3.3844669214335625`, 0.0012466869533516167`, 0.001253979528233296`, 0.005849563807557079`, 0.18788606203958264`},
{0.08663733907452292`, 1.1055276206514`, 6.650123879097649`, 1.442255346530818`, 0.5561115773214398`,
0.5670470763741954`, 0.06364195502160275`, 0.013792669120976587`, 1277.4869438309242`, 50.05278631474647`,
0.06972164372518636`, 6.513399093256677`, 1.399747572725158`, 870.2457894840405`, 95.92171318057683`,
924.211497464172`, 0, 727.6457053101311`, 8.441862082310866`, 8.530007755406357`, 0.010441496465595224`,
0.18172132647267167`, 0.18183050171168158`, 0.0006007838547577027`, 133.32445288177684`, 0.2249121739811891`,
8.214535501883985`, 0.0015202288368650896`, 0.0015269263796650653`, 0.004405614890049758`, 0.10729762936729531`},
{0.19280830132567833`, 3.755171648384974`, 8.021128419894612`, 1.335462575824002`, 0.48264871431934253`,
0.18420836982360544`, 0.06051840370015368`, 0.009206053536078937`, 2566.2923313365445`, 129.64164707934253`,
0.24657184473865845`, 2.9358400550970494`, 1.228808415909655`, 134.82454164139838`, 10.96905199579468`,
147.83310317535918`, 0, 119.03810434721481`, 0.2837795475002069`, 0.28676832884969367`, 0.010532053404886721`,
0.04791013273076969`, 0.04793755287349145`, 0.0005723244992001941`, 15.223441588061316`, 0.13196387583008526`,

```
  1.1355694965636776`, 0.0004335099141802301`, 0.00043512564871913523`, 0.0037270993950864195`, 0.03159194099813638`},
 {0.16230945707171968`, 3.49945541680664`, 3.914597683293998`, 1.1051912745206218`, 0.5225762284594742`,
  0.37657675843017935`, 0.10709361805155937`, 0.30774950760646624`, 1978.9594818216538`, 231.32180409655837`,
  0.1878700890542328`, 3.1200520265606095`, 0.5684357627808438`, 97.2029329409237`, 22.152575304207726`,
  108.5364970401756`, 0, 18.538225487323952`, 1.5391014738877618`, 1.5529908866816104`, 0.009024364559124365`,
  0.05499312189322748`, 0.055115406408776234`, 0.0022236329078784145`, 76.94309985445139`, 0.12751291081680588`,
  0.6957701265625016`, 0.0005983724370750565`, 0.0006048448551946938`, 0.01081670497938636`, 0.07817978255394147`},
 {0.1178483592983135`, 1.9540292922377533`, 2.158901124676431`, 0.8036721080520579`, 0.022324771726093706`,
  0.23728547762141228`, 0.05703389546493082`, 0.19717089259438486`, 1678.2311544802042`, 81.55285235418881`,
  0.08629691407172851`, 9.548029647858971`, 0.23903934985577635`, 387.9516199663889 3`, 43.28944592467017`,
  641.0347605350382`, 0, 102.87224898916007`, 9.850514901502457`, 9.901761863411323`, 0.005202465294585679`,
  0.09489169056050287`, 0.094896010588795`, 0.000004552588605610808`, 274.9742094451481`, 0.1597547149085197`,
  1.773059646491535`, 0.001424791445627016`, 0.001430357674559 0485`, 0.00390669732690796`, 0.11425273357756482`},
 {0.07697798125264199`, 2.659703661710245`, 6.212164355972867`, 1.1315710739609641`, 0.8617717680117074`,
  0.1678172363037963`, 0.11812638083909416`, 0.3038591454145657`, 1784.188819853568`, 30.892067935544162`,
  0.199805435190976`, 6.1761313696591404`, 0.7335007728106389`, 1846.7946573381373`, 49.026769060082486`,
  2313.7496275135936`, 0, 347.34552924812533`, 38.43315318723845`, 38.49155447436544`, 0.001519554922867261 1`,
  0.3423641524964752`, 0.34181832856426086`, -0.0015942788642860917`, 1460.2971180452696`, 0.3764928758925429`,
  2.090945796713329`, 0.003653355878107112`, 0.003658244592258882`, 0.0013381434261758862`, 0.01575006506422772`},
 {0.2783243544518275`, 1.6065452524928183`, 9.36513522454521`, 1.0091499802528594`, 0.9718534638380145`,
  0.5642633711867375`, 0.20737635968923002`, 0.08580910729338392`, 3111.9739304829964`, 94.14000266759376`,
  0.09401444314711727`, 7.942384951366636`, 0.4018498645586557`, 1190.5013021506002`, 111.1508544213112`,
  1642.9620318556488`, 0, 535.1743221465656`, 27.3404624600914`, 27.374940200889576`, 0.0012610518512079505`,
  0.1013587994363121`, 0.10135840010618859`, -3.939767693861285`*^-6, 627.4812880888242`, 0.40300889173043364`,
  0.9953798255023957`, 0.0012130391900948556`, 0.0012164057988160253`, 0.0027753503338225904`, 0.04153851140969137`},
 {0.13125352185182698`, 1.2184353998248145`, 9.320259419097251`, 0.7781699346988293`, 0.39678231153919463`,
  0.199523418988783`, 0.19269759399037872`, 0.23378140134846623`, 1538.2313512668088`, 153.62701919814924`,
  0.16196850129753104`, 1.3309378497731819`, 0.3512246763428848`, 55.22436842464985`, 7.734121306646269`,
  76.35953020756449`, 0, 12.90034950442511`, 2.296050492854035`, 2.308952111517015`, 0.005619048319335018`,
  0.0217069007338258`, 0.02172456717424802`, 0.0008138628650331547`, 39.96556000397954`, 0.040701530997175495`,
  0.24836033728468387`, 0.0003365556527452629`, 0.00033834407335472293`, 0.005313892650062657`, 0.018632692128691532`},
```

{0.19506025087199597`, 3.126229772881034`, 2.308505447241318`, 0.9330443187011872`, 0.44656066575469633`,
0.6840356045619456`, 0.09118120944389022`, 0.034391869601605236`, 2773.7056708459277`, 224.4415538260822`,
0.0593475599896775`, 3.3310563178619557`, 0.5039179573441945`, 96.03473083671591`, 21.659625548704046`,
107.77511323873885`, 0, 64.41212291553637`, 0.6863867273464765`, 0.692911039655 8165`, 0.009505300801142313`,
0.07254394399051398`, 0.07294407200914123`, 0.0055156639771276605`, 30.65432318201322`, 0.20214914162914738`,
0.6336705335270624`, 0.0009303852198747631`, 0.0009491372907488309`, 0.020155168497401732`, 0.11430481336970229`},
{0.06456964840349072`, 3.9537241675412282`, 3.641109216568884`, 1.3108140470595528`, 0.011724209788321671`,
0.438559457390715`, 0.1826458897676254`, 0.0058767891936883`, 1354.5450008176776`, 86.5151446772075`,
0.010849475740387471`, 9.258377423570199`, 0.4300240953856598`, 567.8660734340664`, 65.72923258004367`,
743.7645589145811`, 0, 523.3148859603242`, 0.7605700383975144`, 0.765150610187679`, 0.006022550927480275`,
0.13844991508742382`, 0.1385599862078613`, 0.0007950248316728192`, 42.95834488457154`, 0.127709461981313`,
2.6272408258386317`, 0.0012723708491446661`, 0.0012802386321123882`, 0.006183561163014106`, 0.20603729777957752`},
{0.1755298706210477`, 1.1941209557460892`, 3.229216030497655`, 1.0124753772100286`, 0.8865998145070868`,
0.346236517225073`, 0.10475625859225324`, 0.48133977507102055`, 1150.8999752212085`, 275.6916176605181`,
0.02747195653647755`, 4.902957385276519`, 0.7900769747759009`, 191.95618781458083`, 100.51449898735049`,
196.07258156144735`, 0, 25.83695474567208`, 9.183832560066703`, 9.279914001176245`, 0.010462020129518246`,
0.07685874261730717`, 0.07695616295936397`, 0.0012675245357820497`, 156.66581305769915`, 0.19272864496717543`,
1.7222855329614724`, 0.0009140044151789706`, 0.0009214260589232174`, 0.008119921108689088`, 0.13179369991573187`},
{0.23831756341954657`, 2.9118296516681834`, 0.7702789156400254`, 0.809756039261601`, 0.15598009967599835`,
0.15072565384210967`, 0.12556989454649928`, 0.015474985134933292`, 1269.344776285695`, 338.73985440309866`,
0.16681668263344573`, 4.906919687762464`, 0.7266912138912884`, 89.33988472539919`, 27.46690718452094`,
97.75815788806175`, 0, 72.9350379434996`, 0.37190798651583346`, 0.37970979928491755`, 0.020977803790056315`,
0.1192460985692328`, 0.11945397141126049`, 0.0017432255186697887`, 15.470467183271667`, 0.40597770940435696`,
1.3056634514636478`, 0.0017698921346539498`, 0.0017963957537134687`, 0.014974708650649315`, 0.16061209097629545`},
{0.1708861337350846`, 3.1382784382473305`, 0.22397208377012315`, 1.1376251902182828`, 0.5276068694742282`,
0.6173335030361042`, 0.09813160076689689`, 0.04549667808286232`, 3051.102513690643`, 71.6500993647141`,
0.0653128233426199`, 7.970153906640311`, 0.19805272219502368`, 449.0159686097235`, 50.67153006194715`,
912.6395188732379`, 0, 268.78166261053644`, 3.7999619595839325`, 3.8592060645789488`, 0.015590710018976939`,
1.7610448384645117`, 1.7720865061269475`, 0.006269952599312178 5`, 170.36198119889067`, 4.299116339704506`,
0.8319650730257255`, 0.01842919302378687 2`, 0.019116781670927542`, 0.03730975340337417`, 0.2276219805234334`},
{0.08731955025115129`, 0.716566084545263`, 3.9517372388296312`, 0.9979934558679373`, 0.7980323468748562`,

0.2894395727404939`, 0.23900619972839288`, 0.013550707634954815`, 3303.670456871152`, 218.2198703581039`,
0.21020148991020782`, 6.102509356835768`, 0.9882047778905934`, 411.3514364545894`, 33.25700226265034`,
448.06464645390605`, 0, 344.44555472446586`, 5.91443134654498`, 5.937282104510676`, 0.0038635595929346067`,
0.10677944330980611`, 0.10681047833470027`, 0.00029064606381301594`, 60.54401303293575`, 0.1331990423697229`,
1.1225236387334798`, 0.00129137594536477`, 0.0012963733574253896`, 0.003869835177399139`, 0.06781810363047557`},
{0.22902394998753428`, 3.040671713374585`, 4.278745423137005`, 1.1872158647718087`, 0.928367976025805`,
0.6049768179185504`, 0.23137362815653145`, 0.010091922508071186`, 2258.092758703583`, 198.0963189618003`,
0.016671766650619035`, 7.17480421825168`, 0.31956308961413415`, 565.3943322036649`, 128.3481770366565`,
704.3872595592933`, 0, 493.4333403825793`, 1.5975350126455696`, 1.6031734029643163`, 0.0035294314516520764`,
0.13696920016098682`, 0.13712846819570745`, 0.0011628018162728182`, 69.39399320109841`, 0.44813181782141603`,
1.0112644020557273`, 0.0013888628038897233`, 0.001399711531280847`, 0.007811230425885496`, 0.09803832806862381`},
{0.06329193275668865`, 1.9001497619691943`, 5.252505884188258`, 1.1790665711161483`, 0.6185584326542477`,
0.29903601211054487`, 0.18805549054580545`, 0.08613649661622771`, 1994.6987223768147`, 102.68040788991027`,
0.2346504641795843`, 5.384687813670801`, 1.1000521056786416`, 531.21873150609`, 38.32032762664975`,
590.7565244912199`, 0, 239.18276865195665`, 10.366711064326598`, 10.4124985378026`, 0.004416779168618268`,
0.13892870218499614`, 0.13896051752685157`, 0.00022900481581600296`, 281.4043365897667`, 0.12561522966681588`,
2.0970132370311316`, 0.0014221070143938253`, 0.0014274466828896734`, 0.0037547585672546013`, 0.0719270551670227`},
{0.1794793632102442`, 3.220429652904432`, 3.9791446112229796`, 1.2467997677613296`, 0.11361466513971896`,
0.5165426921925539`, 0.22271304495125288`, 0.2987687877435581`, 2176.453438414389`, 103.4889254155421`,
0.20600780354143933`, 7.995100635365489`, 1.3485017278608633`, 538.6703362238892`, 41.31082902602779`,
610.1712806179445`, 0, 105.26661739432683`, 9.210120401015258`, 9.284308423749819`, 0.008055054603453682`,
0.1323036599654237`, 0.13241848922570434`, 0.0008679220235530138`, 423.72206923213747`, 0.33922538058578144`,
4.049480445192826`, 0.0012790363758261591`, 0.0012868353871256155`, 0.006097568018281541`, 0.2500138557649401`},
{0.25718420980427975`, 3.2208936021473447`, 9.13093887998258`, 0.7880508822382197`, 0.19579212797696832`,
0.6844641096337076`, 0.10728371238227297`, 0.15509978165260505`, 1971.6454958829345`, 171.21565981007905`,
0.07048499237249867`, 2.8101083115304704`, 0.7477956360854239`, 96.56247150680268`, 19.59495107185513`,
106.14316431166327`, 0, 30.630556737166728`, 1.4005793230304038`, 1.415235403389692`, 0.010464298678618977`,
0.018572086156479987`, 0.018601847906818774`, 0.0016024990455045796`, 64.44452829783538`, 0.06823496146530392`,
0.9448456169456666`, 0.0002838538724619921`, 0.0002860114831084539`, 0.007601131623633961`, 0.10606206568673397`},
{0.0634556155295738`, 3.680544991233944`, 1.898464422832438`, 1.0496696027333048`, 0.16836359340314266`,
0.3732784807189835`, 0.2175697213065485`, 0.48318139091353535`, 714.7371038686642`, 368.95513713314926`,

0.1959410717243747`, 1.9243643979934077`, 1.1366603632987364`, 40.876859208691755`, 26.252088615541982`,
40.78595823735657`, 0, 5.86577110626676`, 0.6508503488827988`, 0.6663828588437607`, 0.023864948351988824`,
0.07292797749561192`, 0.07348058154908911`, 0.007577394471284249`, 34.221199880335035`, 0.06610985287591126`,
0.8517788806617157`, 0.0008247583673449688`, 0.0008503570860023306`, 0.031037840500810265`, 0.1868398997179531`},
{0.22344776749508172`, 2.0217933679260236`, 8.515379660775892`, 1.0649182548208418`, 0.4042870031976431`,
0.16656241643092373`, 0.13003071453691661`, 0.03138725525414384`, 1808.0816946786463`, 137.22272185069306`,
0.15677750076118624`, 3.7329055172229837`, 0.8930251877812045`, 188.69706518623647`, 17.924159855255905`,
216.0620448833551`, 0, 130.49593010659396`, 1.9414616528439999`, 1.9548542244580926`, 0.006898190131375603`,
0.0396428304556062`, 0.03965252315074782`, 0.0002445005825824076`, 56.07477562575271`, 0.12654431374985267`,
1.1239142335717447`, 0.0004498834508891436`, 0.00045115702510118387`, 0.002830898112662572`, 0.04818992278060726`},
{0.21641932432281485`, 3.6568426450830875`, 1.306554690894977`, 1.4626425107711718`, 0.8835071341302185`,
0.5676582474171099`, 0.11158412214739238`, 0.023181801672309155`, 527.0796987655522`, 75.44193071736254`,
0.13812060798969733`, 3.0114637760169054`, 0.9949014306145822`, 443.890625213387 3`, 191.0964085425386`,
449.135852964148`, 0, 331.12125595666424`, 2.038115585257326`, 2.0741924034890786`, 0.01770106587315934`,
1.0184748629844675`, 1.0261354078916378`, 0.007521584661129843`, 106.47239982539223`, 3.148823452669642`,
3.4737498375250806`, 0.008284889545931429`, 0.008574016762257696`, 0.03489813771485384`, 0.11253111069472897`},
{0.27575001701682567`, 3.4736287001446966`, 8.538624919070354`, 1.4033945242686854`, 0.5325831427167897`,
0.4946990317811615`, 0.09780340245600855`, 0.03288516063918166`, 2535.414355248974`, 43.33184327775365`,
0.19548398944691353`, 1.2011151230670372`, 1.3252105639060514`, 187.6211292081231`, 7.650076869827188`,
210.032410009639`, 0, 127.3979230483034`, 1.173695128751497`, 1.1846192966501579`, 0.00930750041561601`,
0.16011134804939808`, 0.16049932860938262`, 0.002423192139165753`, 58.242586920731775`, 0.6307243849886496`,
0.9356643894724548`, 0.001376415454786839`, 0.0013903455977705803`, 0.010120594719636111`, 0.015614479309006352`},
{0.232764074403509`, 1.2733235890477337`, 2.798704761143199`, 1.2089738970000061`, 0.2230756746500473`,
0.581248946739561`, 0.23598452021555733`, 0.3774029175060033`, 1879.298545402161`, 390.18642709154335`,
0.06147319592641398`, 6.399283876049893`, 0.6943722912689665`, 131.64646763203405`, 44.69168804341732`,
142.79382821559153`, 0, 21.688120182971545`, 5.717366450903551`, 5.786727753999687`, 0.012131687498388999`,
0.055525389414245306`, 0.05568849994526809`, 0.0029375846390899962`, 104.00082241665167`, 0.18463308389862526`,
1.397902454843019`, 0.0005508899077104612`, 0.000558642972663201`, 0.014073710271734985`, 0.4000336376043883`},
{0.1797514088363001`, 3.6024179997471064`, 3.854320723033281`, 1.3651410466183755`, 0.3139114488968535`,
0.24530825544465074`, 0.2138602169809023`, 0.009505828045846924`, 3546.9761969830042`, 314.2947763723504`,
0.014606932674341683`, 5.351501403328509`, 1.3913390486692032`, 137.5685773718145`, 14.289237738093975`,

152.7771673876927`, 0, 120.98326469679284`, 0.3103752311661663`, 0.31351344314619634`, 0.010111025832309162`,
0.057014772818489184`, 0.057075943140546176`, 0.0010728854827104506`, 15.972875991838105`, 0.14640693912293165`,
1.109190419197373`, 0.0005037448728711924`, 0.0005068848893734577`, 0.006233346821711949`, 0.1500181169691899`},
{0.08593348279794505`, 1.5299819772683403`, 7.853142207396741`, 0.8569688383589438`, 0.17350357661082394`,
0.3599516000206078`, 0.22245867865147873`, 0.35987771942812175`, 2188.9020302076506`, 267.66973385037716`,
0.21174504666607785`, 1.9987769240506328`, 0.6930609981274307`, 50.5063046896732`, 8.780655444651597`,
59.00533138529461`, 0, 8.538616415741519`, 1.8344236571835955`, 1.8498587458726996`, 0.008414135212801321`,
0.01727264311903801`, 0.017297946173923908`, 0.0014649208410963954`, 40.09478763093676`, 0.02120426257635101`,
0.3972639131034846`, 0.00024279029052376444`, 0.00024454083901311263`, 0.007210125600870532`, 0.0680106630676453`},
{0.06256964883486332`, 3.548483703832706`, 5.540385857803061`, 1.459008903547168`, 0.4922186967390012`,
0.21082467659119386`, 0.05317469777445105`, 0.051461677692841074`, 2370.1608114198016`, 100.46792579677714`,
0.24298196986117648`, 3.038179651692209`, 1.2632718479945817`, 175.31541382437862`, 13.14811222865647`,
191.76574387104856`, 0, 101.55896177161617`, 1.42328449505042`, 1.4369318959491877`, 0.009588666880182783`,
0.09522424062074947`, 0.09530164931606946`, 0.0008129095576439038`, 72.15002623720231`, 0.08511638994595831`,
1.4961018403878594`, 0.0007882985137966925`, 0.0007915534219198065`, 0.004129029886707824б`, 0.03224166660705731б`},
{0.23779270937803498`, 3.159033522410793`, 8.923446211238858`, 1.0790485164876151`, 0.3460136054742138`,
0.6482331083335031`, 0.1636040824670958`, 0.011344127288819656`, 1091.512574756292`, 48.569342600520315`,
0.22296455933807102`, 5.246566496412501`, 0.7138062053370233`, 764.9319663223441`, 75.45094268048634`,
899.708806274822`, 0, 657.8853026823189`, 2.2999861796687795`, 2.3125609267978504`, 0.005467314212680208`,
0.11054621053638554`, 0.11061217659542112`, 0.000596728361067278`, 103.79619203793153`, 0.37552975592748034`,
3.273139469751476`, 0.001235322115619164`, 0.0012417341091282407`, 0.0051905437683053`, 0.10222546661198603`},
{0.22757425351270916`, 2.581225270499978`, 0.37748773050037343`, 0.887385361142591`, 0.4937063046079888`,
0.23328592019488925`, 0.08261142866202484`, 0.0067329461689494274`, 1345.0595989079266`, 159.82616980913542`,
0.14361492663679165`, 1.2675214995529185`, 1.4755532059142284`, 55.97703188439445`, 10.311646408489446`,
57.92564881632609`, 0, 48.70326487947704`, 0.1218850461880316`, 0.1296834468543122`, 0.06398160324154989`,
0.6133740639947546`, 0.6210207712910166`, 0.01246662965574541`, 4.494468018808634`, 1.9941163533951802`,
0.7182722625117419`, 0.008277256386990506`, 0.008891300415446904`, 0.07418448816222489`, 0.050490113916438116`},
{0.07923961132457574`, 1.4534207730102917`, 0.3049651947786387`, 0.809144094297255`, 0.9651646059166545`,
0.27855980457998597`, 0.18910876827885104`, 0.18936230186753644`, 1265.284678743018`, 224.56622155669697`,
0.05882269816842833`, 4.935952740691619`, 1.3773991041706521`, 381.0312795328494`, 92.67007476068274`,
391.06505830249023`, 0, 103.61541954927561`, 12.62152929231382`, 12.782784523551136`, 0.012776203857920443`,

1.2812300770278597`, 1.2766591456865357`, -0.003567611643903623`, 262.06275515152566`, 1.4503453331577825`,
2.0817114387873685`, 0.018804486597388093`, 0.019237691310782543`, 0.023037306078572906`, 0.12788626544887932`},
{0.18119347985627088`, 3.338563344897951`, 0.31616309891789207`, 0.8233375529098934`, 0.07175467184830686`,
0.559491101800496`, 0.06295447855153882`, 0.018102651459346017`, 3519.8202135278207`, 127.22666926323683`,
0.07035715001733955`, 6.64501017919611`, 1.131299832263819`, 270.3485164936642`, 21.432865128874447`,
294.78901519766237`, 0, 213.39020328595524`, 1.1195472133638065`, 1.1407851848791504`, 0.01897014369901573`,
0.656212605286027`, 0.6593268468905974`, 0.004745781442605734`, 53.39541842027225`, 1.6985920782475024`,
2.6598324737586028`, 0.009537315170971561`, 0.009779817550179825`, 0.02542669240357709`, 0.1803896541062044`},
{0.16049167400816333`, 2.9496377174467003`, 2.796051273230102`, 0.7713172859306066`, 0.5895053698909607`,
0.5330035027095973`, 0.21946432427500168`, 0.006395869608132028`, 3721.6840074196953`, 354.1366035930697`,
0.05846005834212181`, 3.723454931340358`, 1.352543221697399`, 115.09163522540933`, 18.737988417976716`,
122.14447776980205`, 0, 105.26026564860538`, 0.22129994587024848`, 0.2233658018086767`, 0.009335094639559616`,
0.053315386983789126`, 0.05344956115669023`, 0.0025166125670605943`, 9.325066674397117`, 0.12223822439173984`,
0.7904804173875817`, 0.0008315132753468069`, 0.000840167968944759`, 0.01040836491076158`, 0.11705107717762926`},
{0.2459634383839392`, 2.317311476187519`, 5.114630918885933`, 1.230187162908409`, 0.6324239129659683`,
0.3459102702870417`, 0.21279405046832994`, 0.012571031115264336`, 3926.1200815627817`, 399.1203840176114`,
0.14512335124342485`, 1.859263107025912`, 0.81357096327371`, 49.198075000343046`, 7.185337238043029`,
54.658055821343126`, 0, 41.56885000237225`, 0.21766585004331343`, 0.21962423303423703`, 0.008997199103735865`,
0.03968878113644597`, 0.039810548634676535`, 0.003068058396954454`, 7.205708175421159`, 0.13945698676554652`,
0.23468676637126754`, 0.00038798822985330883`, 0.0003931937003377155`, 0.013416568039640753`, 0.04021272991286724`},
{0.07535246017332142`, 2.911051338058316`, 5.442775177543007`, 0.8607203087442552`, 0.8022890490197512`,
0.40209854436599723`, 0.144612694452116`, 0.016940102043156918`, 3925.350442074646`, 91.64171206007711`,
0.21807597998523465`, 2.8164983201304956`, 1.3696264396607019`, 331.62314740580933`, 15.660272367786153`,
361.39171009378026`, 0, 266.9263822056112`, 1.5106475328992783`, 1.5172924544376958`, 0.004398723986702713`,
0.11674699314667422`, 0.11681064000960086`, 0.0005451691834723604`, 62.82246459972761`, 0.12567390216343308`,
1.0922595841040115`, 0.0016375109401589594`, 0.0016441011651168527`, 0.004024537971791142`, 0.027594999521892625`},
{0.2204293092455124`, 2.4745010079982768`, 1.6220990910883482`, 1.0541684584544448`, 0.6868676732591923`,
0.6458136221345077`, 0.24561205720827334`, 0.010048142648758356`, 2710.1004320631428`, 99.89140315660762`,
0.1947182155448685`, 4.969891992321378`, 0.16288064769797317`, 290.22395659939156`, 45.484130262675244`,
693.4097194146273`, 0, 252.82265876055155`, 0.9964942529908587`, 1.0022203197860384`, 0.005746211559167103`,
0.23640058550070503`, 0.23744629704331047`, 0.004423472726984068`, 35.22608619271959`, 0.7444231109594055`,

0.47803303263546565`, 0.0026838015313725982`, 0.0027360404846974574`, 0.019464536670914745`, 0.0945352806007523`},
{0.25338042742565964`, 2.965005843458032`, 9.015448508781162`, 0.9648345670574521`, 0.4738085996714536`,
0.5217539624890015`, 0.09898738245676192`, 0.10703385809743371`, 584.7617542091971`, 399.01325318858073`,
0.22656752967920823`, 7.539332683081733`, 0.5787011055505342`, 158.4076643656389`, 208.7611437203211`,
147.51369292402487`, 0, 64.60548424212719`, 2.16196974982267`, 2.208917750251445`, 0.021715382665564942`,
0.014102789429844437`, 0.014110851274643735`, 0.0005716489521030432`, 91.5750420229105`, 0.05104815448041301`,
3.3828439981568152`, 0.00017648830871719312`, 0.00017712078895473806`, 0.0035836948188925355`, 0.7189667888043897`},
{0.13715202199195703`, 2.280611332139774`, 9.546074571903652`, 1.3294680551764548`, 0.96176455508361`,
0.2027171739690981 6`, 0.1365163915603838`, 0.01317998381974412`, 622.5257559162319`, 109.88086672718458`,
0.09246427533408269`, 2.547747841165865`, 0.5344581094207239`, 283.0434601311706`, 73.69201699601584`,
317.53570044665986`, 0, 238.04696544348386`, 1.3270860440438976`, 1.3355691682732267`, 0.006392294054633485`,
0.09698874642590485`, 0.09703582557128076`, 0.00048540832942789436`, 43.23667815387226`, 0.19003146689677522`,
1.3615316456796565`, 0.0008803475400757099`, 0.0008844747763025535`, 0.004688189651201435`, 0.033039803612461206`},
{0.26021306080280165`, 2.6412569822545393`, 9.393216711347169`, 0.7980583185168122`, 0.12902542313562004`,
0.41288706652580376`, 0.11050318145828913`, 0.08290531025525745`, 2885.381565930523`, 235.35959645539515`,
0.24979107480174895`, 7.498842240586485`, 1.2653883281677927`, 190.84973939457367`, 26.53449404430071`,
206.6557294871179`, 0, 88.53530931232577`, 2.6399260205999484`, 2.671644744616608`, 0.012015004878603186`,
0.013566380121562548`, 0.01357047703455266`, 0.00030199013689746756`, 99.61032906492932`, 0.05043070422068932`,
2.277384097997084`, 0.00020547190557085315`, 0.0002059190871792106`, 0.002176363756957622`, 0.21554181366710892`},
{0.21577611209111464`, 2.7972286676872242`, 9.151464868370521`, 1.0125832913995234`, 0.03171655307538246`,
0.37863990617192567`, 0.08961735731262133`, 0.24231024163547266`, 1129.2395603552968`, 145.3261697746342`,
0.1783001304868151`, 2.7363213922238323`, 0.4255887662905571`, 91.07585603526746`, 21.501416629529267`,
106.76524185613711`, 0, 21.09743899213494`, 1.7061409971053771`, 1.7251406791226966`, 0.011136056193218558`,
0.02306986230115528`, 0.02309668240514376`, 0.001162560211169339`, 68.17809297456614`, 0.07111321705456888`,
0.938092465297313`, 0.0002746224797274266`, 0.00027641342460980677`, 0.0065214795385935`, 0.09592854454607451`},
{0.18983832850361748`, 0.47273123425625707`, 0.543837247642804`, 1.0885560496178721`, 0.427736490811788`,
0.6656708549524757`, 0.12908120643315885`, 0.022867246439630798`, 898.0605548746116`, 139.83397705936972`,
0.09941082510128124`, 1.7064291808810135`, 1.0668304792785985`, 97.9622028968095`, 33.23547982679414`,
98.12660428056266`, 0, 72.21080086189338`, 2.992360058009184`, 3.1111651839870706`, 0.03970281773408235`,
0.6813648219441194`, 0.7022037308768678`, 0.030584069299746552`, 20.208315193740063`, 1.847845127129108`,
1.2186266163330686`, 0.007344773355912104`, 0.008022595885440945`, 0.09228637790208105`, 0.14365952111587588`},

```
{0.17497599862759233`, 3.3657454284285517`, 2.034553169278075`, 1.2437246851025225`, 0.5208306933739273`,
  0.3059877216844632`, 0.14568158867858877`, 0.07100111598759662`, 1376.7566751341965`, 41.042975981802954`,
  0.07580837625359882`, 2.9604840388353644`, 0.18481744658999433`, 243.56499977248575`, 27.125525808593707`,
  652.123727032864`, 0, 120.65566405989799`, 2.481789500916075`, 2.49881766879133`, 0.006861245834495655`,
  0.31354336907481495`, 0.3143147244297306`, 0.0024601233226257957`, 119.32959524328928`, 0.7837509159556926`,
  0.5645111396824851`, 0.0030273513339004188`, 0.0030732416119068815`, 0.01515855708340852`, 0.039312870649780694`},
{0.18088366879997198`, 3.821865573482567`, 4.692160635426452`, 1.0482928849004183`, 0.06532272769142566`,
  0.687087343414349`, 0.24770159954715665`, 0.017305980256248125`, 3393.812324444456`, 259.70338252927684`,
  0.20399575556521798`, 4.91689758771607`, 1.287491266139321`, 132.0559861213635`, 18.00654691721507`,
  147.83246800899246`, 0, 105.97707942292953`, 0.4658011582013101`, 0.4708152402933033`, 0.01076442598673455`,
  0.037546605948320134`, 0.03763189410877177`, 0.0022715278331422173`, 25.43184872311278`, 0.0970223976417134`,
  1.3104716610136635`, 0.0004309258014815187`, 0.0004350184239054764`, 0.00949727867277228`, 0.24709714871088131`},
{0.05949240242631926`, 3.2421313428658216`, 6.819444043498855`, 1.0897919998234773`, 0.4914757475836029`,
  0.6217813654383784`, 0.14811683608778792`, 0.1990515558123701`, 2177.5035606095144`, 392.178441681785`,
  0.16794023089571636`, 3.103361276547407`, 0.452347159579072`, 64.90758204272892`, 25.551721493830943`,
  70.0998305775464`, 0, 17.264064121748984`, 1.0061006807994015`, 1.0148830266475821`, 0.00872909244152642`,
  0.020917515190776587`, 0.020971102520740442`, 0.0025618401361306997`, 46.59872216140525 6`, 0.01777761759275862`,
  0.534005789865411`, 0.00023056508950913823`, 0.00023309218681777646`, 0.010960450751752226`, 0.14716565824360694`},
{0.22260435016590752`, 0.807488655841027`, 7.62907904124237`, 1.0059991047708425`, 0.2817863964260634`,
  0.32047133388609383`, 0.11883206718041817`, 0.029206686321015127`, 2029.3046388765315`, 71.8362522665995`,
  0.05374037941301346`, 8.93317694888524`, 1.0534684445303992`, 794.4066877992785`, 45.982868118617176`,
  908.8209169912506`, 0, 561.4665363056466`, 18.549992480459824`, 18.65695498824786`, 0.00576617526399037`,
  0.07359340017498282`, 0.0735897635697022`, -0.00004941482893805471`, 213.98440705582394`, 0.23403158603440744`,
  4.231099128449366`, 0.0008841233450185282`, 0.0008858872126450546`, 0.0019950469993408326`, 0.11191550133319558`},
{0.06601763360963414`, 2.324714740822664`, 5.47799549721827`, 1.4572263706661284`, 0.6058468419307841`,
  0.40657776744209273`, 0.16352063484885299`, 0.154889405316064`, 3091.1261645885334`, 170.4797745198008`,
  0.16879539576852792`, 3.0092390791565844`, 1.1781015424249213`, 165.44076909106352`, 15.812749492900197`,
  180.47524468707854`, 0, 52.033490275726365`, 3.309813248676932`, 3.330730616294859`, 0.006319802975677913`,
  0.09153920490046213`, 0.09171625047144885`, 0.0019340955733582188`, 109.9195949795629`, 0.0863314527147772`,
  0.8726602722851782`, 0.000756948841102334`, 0.0007627421885001205`, 0.00765355210710128`, 0.05633344107234941`},
{0.09129438454580396`, 0.5458728552745402`, 8.578700312104903`, 0.7708504139325052`, 0.9169389731376072`,
```

```
  0.22022269715135345`, 0.2062604041361082`, 0.19807010574894265`, 1283.9010613736064`, 230.53840695892347`,
  0.015448919626635749`, 5.258490435173719`, 1.2826107951025567`, 385.51784646817174`, 74.52593556152239`,
  396.8693716628985`, 0, 102.11326381371825`, 32.200886110723026`, 32.37591209414873`, 0.005435439969691425`,
  0.04134748718588183`, 0.04134736566403593`, -2.9390382385585667`*^-6, 251.108423480438`, 0.05392561993107284`,
  1.9923848513725253`, 0.0006483470248567214`, 0.0006494945254837477`, 0.0017698864697959227`, 0.08639375225403821`},
{0.14843032745543416`, 2.891906313088681`, 7.801883637284866`, 1.3483733856378388`, 0.31815406883941244`,
  0.21186149946857102`, 0.16733027048593607`, 0.016108958562959286`, 2806.829919143951`, 114.3420493392897`,
  0.021367639016282636`, 6.332693333253493`, 0.6091184877547111`, 358.0407415965272`, 20.330617635293017`,
  457.9078397090219`, 0, 290.97468711269636`, 1.5771449443269308`, 1.5829904625374782`, 0.0037063925110840668`,
  0.06129937655299866`, 0.0613120359610211`, 0.00020651772879753 95`, 65.15650601649916`, 0.129981236208314`,
  0.9981011224395389`, 0.0005493690869498025`, 0.0005507545759931752`, 0.002521963969733365`, 0.05089753777990752`},
{0.19262113260207286`, 0.4100732369307054`, 0.6039207257051533`, 0.8273370736078748`, 0.5063544182124549`,
  0.2807655019410843`, 0.08795158701010541`, 0.1942202595284532`, 2504.9035989294416`, 81.69837632668111`,
  0.149947676078880 5`, 6.632628072950256`, 0.568480342643902`, 497.0402308395948`, 32.32777213205835`,
  622.8979964443054`, 0, 132.07549447820273`, 52.86422058582274`, 53.27353902188130 5`, 0.0077428255164389`,
  0.6428806867775789`, 0.6413926161162834`, -0.0023146918112511017`, 309.6886007635314`, 1.7690343716437926`,
  1.4250532963739064`, 0.009331646002646132`, 0.00943060501602655`, 0.010604668603197753`, 0.0648351252940906`},
{0.17039397430438102`, 1.0518028751020552`, 7.3508889662987045`, 1.2231850486496643`, 0.08959309467655463`,
  0.438007460954018`, 0.13336650260220756`, 0.015835766380232735`, 635.4248848043253`, 46.431191145002344`,
  0.019547600805230075`, 2.463817783746629`, 1.1305047170660831`, 306.12979137438873`, 38.372112665979465`,
  339.72353889719665`, 0, 249.76234458899162`, 3.4817728888248025`, 3.5241783904032813`, 0.012179284213104324`,
  0.12939628088178112`, 0.12961061327847545`, 0.0016564030684169673`, 52.31626764169022`, 0.31497637942363166`,
  3.2365294086014518`, 0.001273313932677711`, 0.0012846083461541926`, 0.008870093373383625`, 0.07926426959271456`},
{0.2503454420826467`, 0.6816060830582531`, 6.557163545095662`, 1.251247380535385`, 0.5924064529900261`,
  0.3249563797350232`, 0.13275565091305608`, 0.008288831069377075`, 3729.443509243899`, 228.98969063119478`,
  0.16939843347227213`, 6.2896214012738305`, 1.016677469632238`, 236.3717158596898`, 24.33771536119123`,
  260.3167208569051`, 0, 211.1249163166133`, 2.3143679426198966`, 2.330755627031262`, 0.007080846614568204`,
  0.04495639088707173`, 0.044982433877864776`, 0.0005792945180691511`, 22.535532401781946`, 0.16078039358664065`,
  1.2593666289037504`, 0.0004339313891454921`, 0.0004355806515053985 4`, 0.003800744544325773 7`, 0.09162061286753594`},
{0.21895003474025426`, 1.413342329996616`, 6.660357061219696`, 1.3885224416718682`, 0.6085057775930085`,
  0.3063420766693563`, 0.18696649922653613`, 0.1721695479130944`, 1066.3987466436151`, 245.09142596090578`,
```

0.1673351585832708`, 3.1429897838085274`, 0.7494357676944992`, 131.5011099606298`, 41.193545032101454`,
140.09624161855953`, 0, 38.71501418660904`, 4.368009585216303`, 4.411871719784411`, 0.010041675438754005`,
0.054587948449984304`, 0.05466623051324085`, 0.001434053952920955`, 88.19275492310211`, 0.17074333156460716`,
1.095251475192973`, 0.0004734735791804834`, 0.0004774221115157995`, 0.008339498778686671`, 0.09788002265820878`},
{0.1254259042141826`, 0.6960838019109872`, 5.192144520750629`, 0.8756261738238285`, 0.8328612976967942`,
0.22677152638905884`, 0.14617978450286806`, 0.02130952691785612`, 2632.7908352385166`, 212.41036911499543`,
0.027687267864634157`, 4.124602621711544`, 1.0784411017781297`, 224.33911343100576`, 25.235442488567468`,
241.2943899561976`, 0, 172.05191556202874`, 4.757842085583195`, 4.783926242195498`, 0.005482350221614363`,
0.057525048201797005`, 0.057544908304472835`, 0.0003452426950805165`, 47.312240111783574`, 0.10307330265252615`,
1.0039285303963177`, 0.0007934392419426395`, 0.0007961404788900595`, 0.00340446603171074`, 0.04842878200111491`},
{0.21183190072031832`, 2.1405207408150053`, 6.713672348966824`, 1.4852810672850318`, 0.9707621809425402`,
0.24869479497663283`, 0.14712840909677755`, 0.010718332694701214`, 2858.107536317915`, 341.2444641156959`,
0.09413983850760904`, 3.9282483342993455`, 1.4253439441659714`, 163.2809620248606`, 29.82395573893237`,
170.7817803166572`, 0, 141.42948833063116`, 0.6812945427496191`, 0.6865845827084635`, 0.007764688584608992`,
0.057599156904847446`, 0.05764883057217349`, 0.0008624026807908525`, 20.83321570513767`, 0.17430484124344595`,
0.9183847097640454`, 0.00046814216637791617`, 0.0004705329763174482`, 0.005107016866329417`, 0.05932369886090558`},
{0.0808570843788532`, 1.9944028866868502`, 1.847446007042576`, 1.1962230350350178`, 0.9881944572508863`,
0.5065109438103407`, 0.13986962480807952`, 0.21324716376368485`, 3595.9260786344357`, 95.54926882555071`,
0.18522194155873545`, 5.182795178847357`, 1.4775938218806233`, 745.6345907215933`, 59.29193854357404`,
805.4232377741208`, 0, 185.764991845659`, 18.941427683457732`, 19.0347564033793`, 0.0049272273178793125`,
0.5842216030990117`, 0.5838376610116375`, -0.000657185707165886`, 539.6691149979749`, 0.6748350779677916`,
2.4030619707257594`, 0.00587907695588874`, 0.005918393522613153`, 0.006687540751619592`, 0.05839575901393171 5`},
{0.18422677214256195`, 0.6743311410267894`, 3.1036416049630393`, 1.492376851920162`, 0.6594088757028673`,
0.5950695833857709`, 0.1860537678137455`, 0.02943690821344356`, 3269.4748824588514`, 385.0414868746369`,
0.02235734672967704`, 2.542522241338192`, 0.26953310124704477`, 60.74720529080582`, 17.18063283634961`,
75.55595352862153`, 0, 42.64641183839458`, 1.6772382503315473`, 1.691230238045565`, 0.008342277974671797`,
0.07126714556654831`, 0.07181628896536273`, 0.007705421543811264`, 16.157342615997823`, 0.1875616598219786`,
0.23751084354782695`, 0.0005693464660316838`, 0.0005850642603460808`, 0.0276067302638221`, 0.08591783202631771`},
{0.12205814528256814`, 3.0220310797867835`, 4.8913469029191035`, 0.8430601347747492`, 0.11080265100724662`,
0.5338876009745465`, 0.22020963395963772`, 0.11433363332445597`, 1824.128984278892`, 211.39670746978072`,
0.11084187323527839`, 8.781693512702038`, 0.22047341418167488`, 227.06625042816896`, 55.003557128494066`,

```
326.9141293065958`, 0, 87.00833946517977`, 3.169012476882297`, 3.1884249530996818`, 0.006125717825031351 5`,
 0.02794913215056802 5`, 0.02796924710127864`, 0.0007196985796285027`, 136.81220281957692`, 0.048734560465248246`,
 1.3427756821547663`, 0.0003999300043947551`, 0.0004017532882580799`, 0.004559007434523865`, 0.3443568492267541`},
{0.1379604951115434`, 1.4163155012457889`, 4.400902234814488`, 0.7945346450123854`, 0.8193909852858012`,
 0.6724735717160395`, 0.06698309689845514`, 0.09275337016012092`, 3209.7522511906463`, 80.79361285288883`,
 0.15292409302459276`, 2.596140589311666`, 1.3399665214161485`, 245.4352171419699`, 26.118619534341654`,
 263.06965780507204`, 0, 106.3397939920806`, 6.535931652415579`, 6.587545382107669`, 0.007896920047046896`,
 0.10682972168661924`, 0.10701386166985406`, 0.0017236774591158355`, 132.24201877713122`, 0.21054687566457597`,
 1.6122633189815652`, 0.0016215385998321619`, 0.001632602823763869`, 0.006823287421497071`, 0.0354081707454484`},
{0.2598992397477407`, 1.4339452918245774`, 1.0784655091863726`, 1.4648755135291642`, 0.5315691563517717`,
 0.48571691620677493`, 0.23359094834009136`, 0.11846678481017199`, 3222.0061450547964`, 66.19766765214632`,
 0.2128172489806378`, 7.890656547448669`, 1.1120638442139867`, 1191.2800602383477`, 41.165954415427784`,
 1379.3255192466313`, 0, 441.9508067577132`, 34.59546658616566`, 34.853119417613755`, 0.007447589435059854`,
 1.2832870083457042`, 1.2808906166618101`, -0.0018673856029940428`, 708.6858061386675`, 4.764647397815393`,
 2.8219497574388765`, 0.010509922855096776`, 0.010639177732765077`, 0.012298365977597925`, 0.10742190620815543`},
{0.15978649721954924`, 2.191594266371557`, 3.0757440378000283`, 1.4265108854971194`, 0.1706212563060796`,
 0.5071304116694296`, 0.24651071826762905`, 0.09495214929797174`, 590.7999831566981`, 288.3901791663929 5`,
 0.04539479043869582`, 1.2808934229971172`, 0.510577419948266`, 35.425429421659686`, 24.443146685348577`,
 36.13353131634994`, 0, 15.268046151375543`, 0.615893630380465`, 0.6311684618378122`, 0.02480108691481564`,
 0.07883204833226364`, 0.07993214706836331`, 0.013954968307596793`, 19.28269927195125`, 0.17994709816648807`,
 0.6339738994822871`, 0.00064914144298122 7`, 0.0006833851371904014`, 0.05275228469762738`, 0.1583235162968756`},
{0.07381047173055871`, 2.1206143008004066`, 2.7168272133152236`, 1.2500057406362022`, 0.5621616976632486`,
 0.5554267209678074`, 0.23180667473506628`, 0.07059925918611996`, 921.2671327671924`, 62.71094421988312`,
 0.17012421692254281`, 1.3889827292164867`, 1.4926610431972103`, 232.70499621672502`, 27.222293811788273`,
 248.75810765797604`, 0, 116.1173353045808`, 3.694064180412223`, 3.733833439049779`, 0.010765719461083734`,
 0.4785467231455109`, 0.48187068830323965`, 0.006945957410135817`, 111.90979041509526`, 0.5045965625785686`,
 1.5271922754979956`, 0.004574929129916638`, 0.004686627490105095`, 0.024415320328796453`, 0.05233234540109977`},
{0.06137349343986376`, 1.2320478370611792`, 8.061209423245938`, 0.9154821233959622`, 0.94847137331962`,
 0.5807992742846495`, 0.08922764994064156`, 0.006685723224911482`, 1682.5088546386705`, 136.82405088198965`,
 0.1751207515405141`, 5.423583339438931`, 0.7354883118939113`, 397.4524698002729`, 132.08422627178751`,
 425.54412580046545`, 0, 362.5586455659769`, 1.850804888413934`, 1.8629027148037445`, 0.006536521740104995`,
```

0.0522009803239463\`, 0.0522353591610029\`, 0.0006585860427001933\`, 32.57543085132346\`, 0.04576795033520573\`,
2.095498440803108\`, 0.0006880713950414563\`, 0.0006908953512231245\`, 0.004104161576863774\`, 0.09396438929230894\`},
{0.1743385118675298\`, 3.075048744775078\`, 5.850960000178599\`, 1.4590538879216268\`, 0.970972084241031\`,
0.3933384319595665\`, 0.1949697002211620\`, 0.08582933681764099\`, 1421.4342985094609\`, 163.5857848008759\`,
0.2259813185949704\`, 6.6523561809248974\`, 0.7554779380569299\`, 691.4228861157213\`, 149.200744185414\`,
750.8592212370442\`, 0, 311.5395717975885\`, 8.442505631344625\`, 8.476195093650595\`, 0.003990457783159984\`,
0.1625045495536969\`, 0.1625844565121205\`, 0.00049172136191266\`, 370.8730906346119\`, 0.4047257334415824\`,
2.183967661860681\`, 0.001343169806090949\`, 0.001350065127873579\`, 0.005133618810787555\`, 0.0895729757218519\`},
{0.0799812184424410\`, 2.715580846021229\`, 9.746524688173917\`, 0.8568934646193223\`, 0.7996741613090028\`,
0.4248586468427789\`, 0.06068894221141682\`, 0.01269146766349089\`, 3356.3929343624186\`, 81.65136803572034\`,
0.1060762194686020\`, 3.6515832472165357\`, 0.6293869420871867\`, 292.0043826610001\`, 24.714589820170275\`,
351.67214369029176\`, 0, 247.1057078209606\`, 1.121957195420502\`, 1.126218843914296\`, 0.0037984055997757338\`,
0.0441026761164360\`, 0.0441232761999213\`, 0.000467093729888068\`, 43.525220999137304\`, 0.05039122531955838\`,
0.8518602552766937\`, 0.000621755841741622\`, 0.000623550983995115\`, 0.0028872141329054912\`, 0.025457044088201798\`},
{0.2430469449816875\`, 1.1442280451992772\`, 8.167052671305406\`, 1.1537428575449382\`, 0.8133574376235939\`,
0.588378131385339\`, 0.08683207301011828\`, 0.2314931244773344\`, 1071.7662116086185\`, 394.7462751939248\`,
0.2316660985974891\`, 7.333555001844963\`, 0.7455281507224547\`, 183.53998603550278\`, 204.2226332707931\`,
177.135807039989\`, 0, 44.47222697419063\`, 8.01151354843255\`, 8.124359992281914\`, 0.014085533671904349\`,
0.02216501790474671\`, 0.0221844243938054\`, 0.000875545832724755\`, 130.95712123729305\`, 0.07695914124592265\`,
2.4661665459315785\`, 0.000231742312662319\`, 0.000232897258621338\`, 0.004983750898789996\`, 0.38015855031829326\`},
{0.1801027988172071\`, 1.3432512708969133\`, 4.741465237867114\`, 1.304640592667152\`, 0.29820882042399743\`,
0.1829691960165180\`, 0.12795168258022568\`, 0.15240389940354152\`, 1270.873559520909\`, 206.3480725013959\`,
0.0878305712829499\`, 2.6716194652601057\`, 1.4440291039953363\`, 88.20418646632857\`, 17.196792001904782\`,
93.91378197989496\`, 0, 28.507890894118727\`, 2.9467703683949846\`, 2.9937913936287757\`, 0.0159567999387078\`,
0.0568314923984414\`, 0.0569002197315599\`, 0.00120931776059274\`, 56.54647203411333\`, 0.1462215834560956\`,
1.211698359301423\`, 0.000525182273674373\`, 0.000529194864588025\`, 0.007640377664650533\`, 0.08177070755902469\`},
{0.2317786010421932\`, 0.964191776963677\`, 8.537582892707828\`, 1.3413871161177928\`, 0.724114448343743\`,
0.6184241033984961\`, 0.2192767238378909\`, 0.15745254433360553\`, 952.8150091975604\`, 129.0382057619487\`,
0.1301097597429999\`, 9.838389368601312\`, 0.4512990117725310\`, 963.2921567041767\`, 269.83761587553977\`,
1069.4951078087568\`, 0, 298.91250469521793\`, 44.94084775850364\`, 45.1350437288807\`, 0.004321146130144449\`,
0.096495759719043\`, 0.0965358702179198\`, 0.000415671102990122\`, 619.0227979789368\`, 0.3195093170613829\`,

3.756726519274266`, 0.0008679124480794886`, 0.0008715908788607404`, 0.004238250977263158`, 0.2421101678601708`},
{0.18229092854924844`, 2.666175072988888`, 1.1843338363668607`, 1.0835916247483197`, 0.8465995494931746`,
0.28296103301830944`, 0.11340515234724796`, 0.021054051155640643`, 2912.6266504267696`, 38.65103872844048`,
0.22568389644671255`, 7.751508465891021`, 0.13543768169398884`, 334.9850493459807`, 51.692995798805974`,
2260.2435053452696`, 0, 256.92913326106793`, 1.971958185588924`, 1.981599952713885`, 0.004889437917813488`,
0.24731640409246`, 0.24756858318197908`, 0.0010196617989999268`, 75.10836799133705`, 0.6440505278211961`,
0.6576514873309645`, 0.002745908202657965`, 0.0027731837960549 23`, 0.009933177434903406`, 0.07355207875317156`},
{0.20049846933924464`, 3.760252667630848`, 8.551337156929279`, 1.0134432778283617`, 0.24009952242243937`,
0.3670355852238165`, 0.13721671891900983`, 0.05322626702251803`, 601.1668980568775`, 144.18193811625173`,
0.13298602138197724`, 6.629891741121696`, 0.6106305978341504`, 313.4841858083295`, 116.96830133524378`,
337.5870067068193`, 0, 179.69222277923748`, 2.4425619682900885`, 2.47462256894183`, 0.013125808502695069`,
0.035151967574541726`, 0.03516490869727406`, 0.00036814789114969493`, 131.20928795880656`, 0.10068450989923725`,
4.04081537283614`, 0.0004189005132509971`, 0.00042023165398181984`, 0.0031777013603828674`, 0.3043638010946555`},
{0.062486454467917896`, 1.2059427569006989`, 6.5765672576808525`, 1.393503279917707`, 0.48537073645078954`,
0.6632121477086064`, 0.05231057461071709`, 0.07259823309572629`, 1438.7234727065425`, 224.83427991463657`,
0.2172117922601684`, 1.0640862319201538`, 0.7673574870722413`, 28.363552578440938`, 13.354480982439583`,
28.554897519071453`, 0, 14.112829197076403`, 0.7781710195693866`, 0.7922082881107692`, 0.018038796342159324`,
0.03505307608573341`, 0.03537442134274405`, 0.00916739107930753`, 13.406138638281922`, 0.03129060632559638`,
0.44395911311979736`, 0.00029976179484314525`, 0.0003080336261445463`, 0.027594681656244457`, 0.05788926761748161`},
{0.19627224659336223`, 0.5402822985479045`, 2.0302490383203153`, 0.7794095357324428`, 0.7903414997496967`,
0.18384078811354698`, 0.12435150563892122`, 0.011407347781693793`, 1336.2245441274245`, 314.05659164167207`,
0.0727358338086812`, 6.3699550531919975`, 1.0324129890894564`, 216.73656002916147`, 65.07976447212509`,
224.63957372493866`, 0, 186.25769434888707`, 3.4449608389512516`, 3.483537734857062`, 0.011198065147687952`,
0.08186150986873482`, 0.08188621186888839`, 0.00030175353707972974`, 26.58930514965856`, 0.22953060644945378`,
1.9471889213942257`, 0.0012671881173126343`, 0.00127367928928768`, 0.005122500666129737`, 0.12880240566466555`},
{0.12533935047015737`, 1.521368835274294`, 3.474516335185184`, 0.949787360467496`, 0.3211564169863861`,
0.652599038806982`, 0.05499170112558746`, 0.016940628240370355`, 2778.039504369459`, 65.83823943133149`,
0.237336473550759`, 8.791957984840526`, 0.6376701649045213`, 693.9804531720469`, 49.1195071146809`,
822.3203088580074`, 0, 558.828448694285`, 5.917140270305376`, 5.948139801281646`, 0.005238937993719439`,
0.13530199770127257`, 0.13538805116087768`, 0.0006360102664195733`, 128.60218287413022`, 0.24226663584560837`,
3.016059071890182`, 0.0017177145186821896`, 0.0017265567934649827`, 0.005147697528677053`, 0.10656922636055889`},

```
{0.05859295952350296`, 0.6393158910447498`, 7.38865195012022`, 1.4291671399819033`, 0.009131117329247873`,
  0.22529958337877898`, 0.1303909880662386`, 0.09497257153796428`, 2573.212680919086`, 234.8157611604919`,
  0.05429505063598533`, 9.846003813495852`, 0.7602792516330825`, 229.28322313206965`, 28.375229461922107`,
  266.56322215699623`, 0, 98.31974417880109`, 12.919213303506673`, 13.026180203092666`, 0.008279675942571396`,
  0.028264402379797608`, 0.028271892296966354`, 0.0002649947120090257`, 117.99226235326513`, 0.023658499779937513`,
  1.887619594385745`, 0.00023904954333076045`, 0.00023953041207918648`, 0.002011586141206889`, 0.1984355695719302`},
{0.22902870315185453`, 2.2080085479773457`, 7.247023111118531`, 1.0318589243161904`, 0.282713068412896`,
  0.6376211846150526`, 0.2313240848611663`, 0.02849242642499769`, 1791.1158452651816`, 374.3905300694138`,
  0.0826563501876693`, 3.8730202440010704`, 1.4480774422437706`, 88.1957780220842`, 31.27481775796266`,
  92.26745829091008`, 0, 62.954733141929`, 0.7726075241623229`, 0.7840018457447379`, 0.014747878096021516`,
  0.020297497552600324`, 0.020335455458340607`, 0.0018700781040577041`, 24.370343108314586`, 0.06641013630996581`,
  1.2362806591384363`, 0.00023674568600462464`, 0.00023880970640172423`, 0.008718302039342207`, 0.2680655378077335`},
{0.11087514755950806`, 2.820953169055314`, 7.453143180044123`, 0.7876974831919437`, 0.32208463912551366`,
  0.485410651626032 7`, 0.2381966627750387`, 0.019508031907783826`, 3675.7660426974826`, 393.486383654267`,
  0.19475280794369443`, 3.2333842106996737`, 1.137763300389826`, 68.77202826254066`, 11.34823846401575`,
  76.17165625588599`, 0, 53.86068931205953`, 0.3596907674702874`, 0.36292782473674756`, 0.008999556172170964`,
  0.014087579086729807`, 0.014104817445899838`, 0.001223656603019041`, 14.495297291074902`, 0.022313748714273194`,
  0.577692919110931 1`, 0.0002156464318631146`, 0.0002168845659470468`, 0.005741500442344893`, 0.12478390501535985`},
{0.14807057579619815`, 1.4220065645386768`, 3.077692541817422`, 1.3463149673717403`, 0.07642859383354339`,
  0.6424799174126323`, 0.13735279371122205`, 0.011944441122857653`, 3609.93932375683`, 78.77118206979054`,
  0.05838602009446581`, 3.355260507755876`, 1.0164328161645106`, 234.69921810645087`, 11.162545546163662`,
  275.91795356150277`, 0, 200.0341923715157`, 1.5921141299302843`, 1.6050378753691414`, 0.008117348622126208`,
  0.19088626429739197`, 0.1916125845086976`, 0.003804989395014946`, 32.34281063222353`, 0.4037805580871439`,
  1.1988970781484103`, 0.001705816139000449`, 0.0017273238031755018`, 0.012608430465229192`, 0.0680770879525394`},
{0.1489453640293268`, 3.0321837029004524`, 5.620984907920828`, 0.8681722630051818`, 0.8115372327820141`,
  0.35816484450213293`, 0.1426190893322286`, 0.05255336055473145`, 2844.388518993981`, 198.5585650329739`,
  0.018535462639950012`, 9.846692622248703`, 0.39135970086161165`, 492.31353233029904`, 74.15763632278423`,
  612.7724915283267`, 0, 281.6354876316548`, 4.750316914053079`, 4.763734726793104`, 0.002824614227385558`,
  0.048458818331935126`, 0.048465679521003245`, 0.00014158803917019291`, 205.76905043434402`, 0.10311023338457319`,
  1.2306407422702756`, 0.0006743440744438889`, 0.0006760091362870854`, 0.0024691576693540718`, 0.09632993480515328`},
{0.24690212899704622`, 2.8546371760166815`, 5.1085465841448325`, 0.8777130205154242`, 0.2272138406192541`,
```

0.3209103618486969`, 0.13407179467911823`, 0.026501037209412173`, 2259.637457560987`, 234.32947168493797`,
0.12630638676582567`, 7.901213702591074`, 0.632382460130227`, 212.4940061136823`, 34.7337884999784`,
245.25080758526136`, 0, 154.44379580013992`, 1.3854857396250913`, 1.396701561805872`, 0.008095227442626474`,
0.028979241356991637`, 0.028990980661568223`, 0.0004050935782606846`, 56.500844273925146`, 0.1022148055393637`,
1.5780679187149593`, 0.00039877388832953997`, 0.0004001005149283976`, 0.003326763957426815`, 0.1824369421333507`},
{0.20217859061685106`, 2.487845857605743`, 0.8626257496221044`, 1.199588786641617`, 0.6707739359709337`,
0.41572085938323444`, 0.1621256536590063`, 0.024225003575992 82`, 546.6195403678685`, 211.4653119641191`,
0.06380462964198375`, 1.3445609170340642`, 0.9773390980954635`, 70.35795412952083`, 44.30089752297096`,
68.64688229568878`, 0, 51.222997297131904`, 0.4769168130648208`, 0.49503346691447186`, 0.03798703118312741`,
0.43591823263608465`, 0.4455920088586871`, 0.022191721975250323`, 16.94993596865492`, 1.2590476271221949`,
0.8768638625678784`, 0.004263889591663239`, 0.0046102438135936265`, 0.08122964126641086`, 0.10038652877469689`},
{0.08931462102812832`, 1.1252555996419966`, 6.092718311835377`, 1.0218956744375698`, 0.19346465860989204`,
0.176574671865492 8`, 0.12857456324762098`, 0.36563376111510176`, 3469.1850778098988`, 184.0525308849526`,
0.10979923715549866`, 4.959384809772031`, 1.0536081443626233`, 161.93555055381262`, 10.890990972086833`,
186.42765114353375`, 0, 26.845407544452073`, 7.906958483924072`, 7.959837749664397`, 0.006687687288081223`,
0.034357308350532086`, 0.03436554357630391`, 0.00023969356644037987`, 127.10499014531918`, 0.04383728535545835`,
1.0232501457455756`, 0.00040633077895080305`, 0.0004073023232885591`, 0.002391018323210980 5`, 0.0672067406790945`},
{0.06880098426216452`, 3.7509374271202`, 6.144272737202943`, 0.9237943864650779`, 0.699854200915176`,
0.3107815967047757`, 0.08743219256910584`, 0.0070907725066866245`, 1964.75988825503`, 142.8027500827095`,
0.1044689728863506`, 3.2094136485070344`, 0.6936760287011401`, 169.56313763882827`, 25.939423275174565`,
190.58717523368878`, 0, 153.8237680517652`, 0.2837639524129756`, 0.2857266261956376`, 0.006916571911169456`,
0.04979863730320142`, 0.0498402900023169`, 0.0008364224679859866`, 15.205440422476931`, 0.04894564659108041`,
0.9170814856730002`, 0.0006504697513520785`, 0.0006535049117292117`, 0.004666105335143111`, 0.04336116306272698`},
{0.2326571783338366`, 3.2198726087577585`, 0.7879059046396755`, 0.9520817800309282`, 0.3381503807716493`,
0.6953568861265582`, 0.09096644213287763`, 0.03053119099726608`, 3051.5436637428375`, 193.8478952968402`,
0.1252944454366426`, 7.5675708168133315`, 0.1396895662891593`, 155.42872722559378`, 40.68201955015994`,
273.56508948179095`, 0, 107.95957079858485`, 0.9955020060062407`, 1.00436869473287`, 0.008906751240211674`,
0.1540040840943055`, 0.15513849400369056`, 0.007366102763160631`, 45.791280587184275`, 0.5118593665323736`,
0.7040286199270075`, 0.0019222720550353678`, 0.0019811348335063293`, 0.030621460847215287`, 0.32731859007654407`},
{0.20686587279815516`, 2.3834956380504195`, 3.228648073866996`, 1.4616470372504198`, 0.35618390382684884`,
0.6609631833857008`, 0.09990239078555083`, 0.2797108933201619`, 2467.4094286971804`, 85.00316013652997`,

0.14363808446123288`, 3.440698993285203`, 1.2812874699661427`, 262.6740519396737`, 22.229926604572228`,
285.828327556423`, 0, 54.05204981912301`, 5.9278148916758315`, 5.994185005427342`, 0.011196387701767119`,
0.21552735428676517`, 0.2162712911043928`, 0.003451704866370697`, 201.84172767828042`, 0.6369322036633509`,
2.1021281268496157`, 0.00177351764835465`, 0.0017970633053245113`, 0.013276246217062138`, 0.08156510417038738`},
{0.14277465652344312`, 2.2128169765977077`, 1.5395722189285141`, 1.2588391498483595`, 0.06601455592248096`,
0.5924077255302849`, 0.2026072042186735`, 0.006704812631657588`, 2107.7558344556182`, 393.2226011972627 5`,
0.1318126775401366`, 3.5561906346112124`, 0.787606393777565`, 60.72293423646912`, 19.288390017033393`,
65.53079093317909`, 0, 55.18968565991112`, 0.1598944430323653 6`, 0.16275851851394993`, 0.017912289053127584`,
0.08642386026020027`, 0.08726894124686647`, 0.009778329550680587`, 5.054530542937905`, 0.17627338520115096`,
0.7937340796041448`, 0.0008149715254075485`, 0.0008429100692055134`, 0.03428161957436915`, 0.2704527083977666`},
{0.16055207404865252`, 0.7029113592886698`, 3.6403774574439822`, 1.1854100604530788`, 0.5916505631446303`,
0.6508026186847373`, 0.196607205292044`, 0.08269873784662057`, 2208.609406288604`, 140.20841727601464`,
0.08922686090021398`, 7.843962027709463`, 0.25680184922543736`, 445.9265303236443`, 76.82640026211152`,
633.8965705578669`, 0, 204.83677010661376`, 21.800070530372448`, 21.872332299326047`, 0.0033147493194078947`,
0.11593399598568319`, 0.11611827161590081`, 0.0015894874376656265`, 218.9073887013284`, 0.2659063358319755`,
1.0994517167547413`, 0.0011761488533380549`, 0.001186847733277185`, 0.009096535620270796`, 0.1529969525163264`},
{0.10433679528385242`, 1.2124558701074761`, 1.611384270831014`, 0.8947495690924911`, 0.7004462194859642`,
0.22790601678310374`, 0.1107678931866184`, 0.03745668981933727`, 3402.687823353078`, 290.01464659756004`,
0.06742303128382326`, 2.428172996695036`, 0.7057307677392819`, 69.03666806439622`, 9.48219543228334`,
77.97613441946815`, 0, 44.93013868222139`, 1.302193529531467`, 1.313503908687997`, 0.008685636120922435`,
0.09849615311426375`, 0.09875877562978826`, 0.00266632256408883`, 22.555031269948532`, 0.14681104233903042`,
0.34700593983576744`, 0.0013251764088615392`, 0.0013409564385672883`, 0.011907870982479807`, 0.035945993770156795`},
{0.05355871135675744`, 3.309279473250874`, 4.916547265237652`, 0.9710244117509734`, 0.2158289016549566`,
0.18208484161878558`, 0.142360576114893`, 0.05203968288186598`, 866.5149045887983`, 189.9142460402237`,
0.14005711660330905`, 2.6475969017133205`, 0.76310092982505`, 89.2149437878378`, 23.418823952980325`,
98.6917485648539`, 0, 51.59819265035633`, 0.7776580948848769`, 0.7880342742877541`, 0.01334285526136414`,
0.04170898335817777`, 0.041746492013814494`, 0.0008992944113410672`, 36.76411386585567`, 0.03191256286663308`,
1.128108876348878`, 0.0005180245436751107`, 0.0005208817190849378`, 0.005515521310162086`, 0.0826874197966507`},
{0.1293133825399687`, 3.5858476083752784`, 9.218617588443664`, 0.956805767292237`, 0.24196147957252068`,
0.1873204590192723`, 0.13102150029404855`, 0.057479405562115365`, 1692.2136201181738`, 281.3785219692511`,
0.06648388834980773`, 9.468482191548883`, 1.1484858817527992`, 228.55708150810247`, 45.97605224392732`,

246.90978239354112`, 0, 126.71598532371068`, 1.9490949559080089`, 1.9732262128318185`, 0.012380749768329258`,
0.0157244105589515`, 0.015725714326103354`, 0.00008291357866574423`, 99.84510694484355`, 0.029048238826225144`,
2.8182846454958193`, 0.00019878097541914386`, 0.0001990041383085191`, 0.001122657180369968`, 0.2576721939725537`},
{0.19221768216079177`, 1.1625228381265575`, 8.172090892620595`, 0.9088617496226665`, 0.5295960957742345`,
0.666157439310989`, 0.15242811761548303`, 0.007090627883866567`, 1515.1410756583437`, 333.01297852270045`,
0.2052004181173977`, 5.919585120700083`, 0.3172585313240366`, 156.411579236585`, 77.98250799012574`,
168.32715287858227`, 0, 141.94759483599174`, 0.8095813085638489`, 0.8170621208028049`, 0.009240347028548035`,
0.018422510790656235`, 0.018440538415820253`, 0.0009785650484277486`, 13.445096578940841`, 0.05058760462516767`,
1.2388665843715143`, 0.0002444110824000312`, 0.0002457299176030838`, 0.005395971369637431`, 0.31599374894866783`},
{0.16098880364150353`, 3.127450251430191`, 2.20024435719991`, 0.7637976091034757`, 0.9064557867648941`,
0.5784333391778734`, 0.10152394954071559`, 0.028389209793756094`, 1613.7605012073036`, 376.897672127746`,
0.043498602143453025`, 2.961300492740582`, 0.7808989235332793`, 85.64758062879582`, 65.46469120456905`,
85.16464550601404`, 0, 61.013274875072945`, 0.5345410213397682`, 0.541022310910516`, 0.01212496199918034`,
0.06258591984012998`, 0.06287959843170164`, 0.004692406731767074`, 23.882149308411513`, 0.14393760514095705`,
0.8103611830233739`, 0.0009808253325354066`, 0.0009990225633413757`, 0.018552977989393593`, 0.118579581428003`},
{0.23820794255869648`, 2.736768610725435`, 5.161003241600229`, 1.3463074049058292`, 0.830070705025693`,
0.6819229399748419`, 0.14093440819681458`, 0.012428436330344567`, 3231.77982743126`, 163.97686949725403`,
0.16644222788576973`, 5.029839035337567`, 1.3104845535759155`, 358.60644163507345`, 53.06421073453003`,
375.3895149335204`, 0, 304.2083946070913`, 1.3389700638324367`, 1.3484937750947306`, 0.007112714107315288`,
0.11637942172076945`, 0.11659370963916309`, 0.0018412870181445662`, 52.34930344853771`, 0.3960357514896974`,
1.8269403133714643`, 0.0010418601018071794`, 0.001049889179921447`, 0.007706483913090256`, 0.0914718943127048`},
{0.2556095337978518`, 3.423935597729149`, 0.44377078820861904`, 1.3028637036663855`, 0.63065566823863`,
0.2812042846574222`, 0.0670032727620068`, 0.017466077681157405`, 3127.9987163099076`, 356.3279534853283`,
0.04932332227468639`, 8.912956605460284`, 0.5977251839966713`, 175.71294543410997`, 40.76321758996798`,
192.56233649509107`, 0, 139.5072932085658`, 0.6907508090068148`, 0.7029794653957101`, 0.017703426806662703`,
0.3583971398649633`, 0.3599049301855349`, 0.004207037816037484`, 33.78694691598039`, 1.3087103690768047`,
1.3379349798655105`, 0.0032985833442329415`, 0.0033762674992030436`, 0.023550763119543516`, 0.19818256104065252`},
{0.17842055683347624`, 1.0601527835649565`, 1.9863486015726688`, 1.4771465685197964`, 0.5130989252089981`,
0.50009800158305`, 0.10443547318598384`, 0.0065079948116299165`, 919.6368613396894`, 101.38339784479837`,
0.013661722290118639`, 4.3887706359180285`, 0.5048436819791595`, 312.6333112968678`, 78.34389067269089`,
347.94169383335793`, 0, 284.879608709311`, 1.6282694244285103`, 1.6471627404773796`, 0.011603310708546033`,

0.3297477230565703`, 0.33131965913422184`, 0.004767086981164459`, 24.660205181451307`, 0.8404824623189382`,
2.130861904123557`, 0.002668896413071775`, 0.002725527004215816`, 0.021218729534302705`, 0.14200414355669147`},
{0.09313560387857911`, 0.7038695534977393`, 3.6724738159211565`, 0.9475746331523089`, 0.19159788938387368`,
0.6119542230262267`, 0.1716716908175685`, 0.15713357297550534`, 2263.7776933944124`, 51.55094049564468`,
0.21456002491809933`, 4.890757314915582`, 1.3381695004184304`, 624.4770405882699`, 28.119070532181137`,
715.1426668842546`, 0, 194.66847268848264`, 38.83459148846706`, 39.07818714940892`, 0.006272646411491056`,
0.20652586595330674`, 0.20667272097552236`, 0.0007110732669624031`, 390.4926653036339`, 0.2747844463158247`,
3.5574025889564447`, 0.0026260340216985867`, 0.0026423798378694442`, 0.006224525667144487`, 0.09979066043181249`},
{0.14593842634629578`, 1.5677173946402423`, 2.2260911107394623`, 1.0410408322166074`, 0.5774262288207519`,
0.5605768734730473`, 0.06528103792572038`, 0.18580384429276964`, 1821.6991564326672`, 281.5049269480922 5`,
0.09810886942125424`, 9.103899808381804`, 0.814497273403965`, 232.2063465654892 7`, 97.04961673419538`,
237.46222150134534`, 0, 65.5371136033605`, 7.114000558483269`, 7.209095722963875`, 0.013367325979080302`,
0.07466870025546847`, 0.07481053821485417`, 0.0018995637917953445`, 159.32489172878`, 0.15567189446586116`,
2.8682120980384376`, 0.0008626957819135583`, 0.0008707976991574972`, 0.009391395453409723`, 0.32829690993209676`},
{0.10172713838830055`, 2.2418772278235775`, 3.04935119913241`, 1.1880003837277453`, 0.642679290083189`,
0.5307574217574084`, 0.20278574850964748`, 0.28989270792503147`, 1645.7112772203664`, 50.385249878477794`,
0.23495391386029668`, 2.9089393035407407`, 0.6416895284655368`, 557.0267009138922`, 35.45266700348367`,
678.4944743025469`, 0, 108.94067206643078`, 13.532646517640677`, 13.585434415740357`, 0.0039007815678084867`,
0.4670474336201061`, 0.4673892937943818`, 0.0007319602885427212`, 433.40760085835177`, 0.6787342701968468`,
1.029035107836426`, 0.00472543818972504`, 0.004772517180282261`, 0.009962883581799753`, 0.03602763352512917`},
{0.10397296284216978`, 2.835686168016201`, 8.434918863116089`, 0.8374208138967196`, 0.6614675061514772`,
0.2543464777963602`, 0.10409619202490922`, 0.05685431008257157`, 2232.998807497718`, 265.245542499567`,
0.1684824275948374`, 9.30545987088001`, 0.3476653622341219`, 264.4920671451387`, 58.317018285584176`,
323.0486942181233`, 0, 146.514333303095`, 2.841042736682317`, 2.855749773335161`, 0.0051766333758211935`,
0.017710855282285932`, 0.01771285364279469`, 0.00011283252428562207`, 115.09007987361358`, 0.026306429973898836`,
1.3222555213226312`, 0.0002557467148005532`, 0.00025609877030743455`, 0.0013765788043687`, 0.1320665828490178`},
{0.19614726189645104`, 0.5421409973222318`, 7.311205144883296`, 1.4430957843086838`, 0.5642408555529124`,
0.4388312833001765`, 0.24379751153424895`, 0.027088524601856054`, 3097.2470344285757`, 24.388568117880652`,
0.033832395593974274`, 8.563316908406161`, 1.3116329767787245`, 3652.905000791802`, 45.69141715558451`,
4284.162323991907`, 0, 2633.3573443814635`, 116.29462762900754`, 116.54734946931457`, 0.002173117068771546`,
0.5291460291876721`, 0.5283075826312281`, -0.0015845277299559868`, 900.6869343715397`, 1.4827220681215048`,

```
5.37616120686446`, 0.004424457959070427`, 0.004434258260034274`, 0.0022150286101725314`, 0.05445967588600048`},
{0.14550969692078547`, 0.6504460416509024`, 3.203391452599939`, 0.9809775787785509`, 0.8932996308568291`,
 0.28864614416923273`, 0.12555971991665899`, 0.04147738167353124`, 2412.5327079966373`, 332.6211608259723`,
 0.10031126168945415`, 7.026514484853989`, 1.271775072831928`, 254.4155746463552`, 61.9975173089058`,
 263.29723272467515`, 0, 160.27571545093417`, 9.124996909244226`, 9.197395200750735`, 0.007934062030548805`,
 0.06951145922579813`, 0.06955210982357637`, 0.0005848042643761264`, 84.79025885278034`, 0.14449416234949133`,
 1.7815951417516027`, 0.000855159462303634`, 0.0008590280162694221`, 0.0045237808108524735`, 0.12491870792943947`},
{0.26324845548994974`, 0.6443701830080291`, 0.83901460272339`, 1.148634249041292`, 0.7108758830917159`,
 0.6261909633849789`, 0.1815221463640686`, 0.007774421269715129`, 2091.205586215532`, 76.86431957676541`,
 0.07691551140505637`, 2.5668632188750475`, 1.1962425546812079`, 376.2061522575128`, 30.914547892705485`,
 404.22515195534345`, 0, 333.1779283696722`, 3.6100871836071935`, 3.6849525157950325`, 0.02073781833518873`,
 1.2332473041732466`, 1.2399103753908804`, 0.00540286704466042`, 33.23189341679868`, 4.6378635437250235`,
 1.4380724799064597`, 0.01282639640079064`, 0.013275999861632812`, 0.035052983456402176`, 0.06766684826658007`},
{0.16309305365471205`, 3.729196744300981`, 9.307515882091003`, 0.8570302037138485`, 0.8937228771832244`,
 0.5152206015210095`, 0.1870688649861051`, 0.010071822941243438`, 3256.3985186156888`, 304.0187763152717`,
 0.14293680908473783`, 5.902796930663761`, 1.3773483940741578`, 298.72801881707755`, 58.52153731949297`,
 306.9813985026297`, 0, 261.0732155785285`, 0.6882442627966896`, 0.6923259029075373`, 0.005930510912305786`,
 0.02923230343309595`, 0.02924570043797866`, 0.0004582945341058408 7`, 36.66568948721782`, 0.06810836617520057`,
 1.4607675967909255`, 0.0004120242585179401`, 0.0004132357841041897`, 0.002940422951326793`, 0.10801722357652022`},
{0.16894031536983917`, 1.5924806965086606`, 5.6931408636729035`, 0.9867223743305529`, 0.8224539897688026`,
 0.597939456714043`, 0.09503923786081717`, 0.017883705208976936`, 748.6938025465079`, 285.65032417980353`,
 0.22927316540826853`, 3.929313941874591 7`, 0.9067902983749052`, 139.62278470678754`, 158.60533827642251`,
 134.6873368766289`, 0, 111.46062194642361`, 1.1782605693760235`, 1.1963186357689608`, 0.01532603811269051`,
 0.03854972837445115`, 0.03862739921538592 4`, 0.0020148220029030384`, 26.80510303126602`, 0.093037189557187`,
 1.937513219397711`, 0.0004697687706984865 5`, 0.00047435253852436176`, 0.009757497968755358`, 0.20806721165708883`},
{0.1623342771322262`, 3.665527787592313`, 5.80147646237554`, 0.8352024744734798`, 0.7018868526857065`,
 0.24394785423193066`, 0.08747688957871178`, 0.16089532027636136`, 3759.8814706106723`, 381.8961690598002`,
 0.2313099688547675`, 3.097651818220701`, 0.4382846191648839`, 57.89139696107907`, 11.446324030649732`,
 68.83276048571678`, 0, 17.74494255307947`, 0.7512551213376619`, 0.7559418339679335`, 0.006238510057577473`,
 0.016861335694358243`, 0.01687475467857249`, 0.0007958434881725207`, 39.33923604048903`, 0.0391024677346731`,
 0.30450371261595327`, 0.00024368407319041996`, 0.00024482540780034466`, 0.004683665185754027`, 0.04197781806715173`},
```

{0.22071774239833053`, 2.103280372140615`, 9.912636083572693`, 1.1033554615041679`, 0.6309736957880776`,
0.6000918403254052`, 0.08729060724669563`, 0.08702471997827647`, 909.0462708578816`, 192.71874762961875`,
0.022259580341630003`, 7.880477191609103`, 0.5653775848064009`, 336.5993707506328`, 189.06302125231198`,
341.25851330016167`, 0, 151.97524854310424`, 5.9426314906520865`, 6.008557048756218`, 0.011093664180226392`,
0.02981926000193892`, 0.029836668759555283`, 0.0005838091761911635`, 178.5574310450463`, 0.0940234249661048`,
3.6146401578161225`, 0.0003262357528190929`, 0.00032748032532208487`, 0.003814948215323577`, 0.3486809123637021`},
{0.06066901211156295`, 0.5118142470076563`, 2.3575039706529495`, 1.153198866823781`, 0.34052857777406675`,
0.48310824315452683`, 0.1564442910712987`, 0.01838618195497249`, 3533.672368817396`, 112.99427366098905`,
0.18169108406320317`, 9.689478468414666`, 0.6768155401525213`, 585.6953485405761`, 36.95468260101012`,
714.706442214136`, 0, 463.7950459086865`, 14.601309310296308`, 14.658605027444583`, 0.003924012287574108`,
0.1853701698378216`, 0.18551945280775756`, 0.0008053235861336105`, 106.75940185678843`, 0.16066035827159109`,
1.7559499036517698`, 0.0019369788323263526`, 0.0019486441246681226`, 0.006022416015646348`, 0.12336873633009054`},
{0.17215213474108`, 3.1043854956757873`, 4.4538232156171205`, 1.2105616525577976`, 0.07070121186202716`,
0.43467897429752544`, 0.1858388910260701`, 0.10113399844294489`, 1450.8531111854936`, 101.3426083924827`,
0.1798803264840549`, 9.89915477207009`, 1.3540588713189834`, 639.3198239883807`, 68.52461493062177`,
710.6453699988288`, 0, 264.8917831304964`, 8.248303908229252`, 8.331389248795094`, 0.010073021252641867`,
0.11009038899907285`, 0.11013827542551737`, 0.00043497372368195286`, 365.79878595189757`, 0.27074707829532846`,
6.311746680448452`, 0.0010971990274031285`, 0.0011020484151509791`, 0.004419788595081187`, 0.33935222962016076`},
{0.24067020849458426`, 3.9616105393925194`, 6.770909075338004`, 0.8588077802255987`, 0.4748383175874904`,
0.6078443186142359`, 0.2469104749167066`, 0.3360706312022538`, 1507.4555524848456`, 50.50450753574768`,
0.19906071527290714`, 6.968998972407538`, 1.347049135136762`, 1366.3709952458225`, 81.66631214221587`,
1532.1385656383911`, 0, 240.17122397515206`, 19.5419402875168`, 19.634375103610186`, 0.004730073612620567`,
0.15608684373615792`, 0.1560451284779942`, -0.00026725672174021664`, 1105.9650943315091`, 0.5366493317888071`,
5.895399848167941`, 0.0021930342897086286`, 0.0022007202741123936`, 0.0035047260500364885`, 0.13753270159297015`},
{0.21944864620047738`, 1.5851568289063627`, 2.8253271766672086`, 1.4978148427385942`, 0.6625588078225084`,
0.3414024356763743`, 0.10952337049286448`, 0.007987153739989428`, 796.8444419321349`, 241.79415763040436`,
0.07683828049172353`, 5.204625250868856`, 0.2239996119446115`, 182.17130817846783`, 105.7182289037189`,
198.77450671453042`, 0, 163.1227102501771`, 0.7789828936245222`, 0.7890560252037957`, 0.012931133227334746`,
0.11590040140367264`, 0.11618664637138337`, 0.002469749580191305`, 17.640143620430752`, 0.36334551688749983`,
1.7050261463931966`, 0.0009286490010984583`, 0.0009415325916102732`, 0.013873476950468255`, 0.21274105151658376`},
{0.22738531013884145`, 1.6748591792805003`, 7.320466644196401`, 1.2670610367241493`, 0.9156886696389757`,

0.27273514068006444`, 0.2379234232685784`, 0.022570855753759948`, 3790.0561229040195`, 356.22460568492386`,
0.13302881383052462`, 2.695355303099337`, 1.0187081757900263`, 132.57432155280665`, 14.650032216505942`,
141.50727658813713`, 0, 100.15172971164807`, 1.2904684778312334`, 1.2967934778285275`, 0.0049013208039951905`,
0.053298506656741164`, 0.053355862347198316`, 0.0010761219038750713`, 30.87647108096822`, 0.17313282094397822`,
0.39060745896725707`, 0.0005075369537608232`, 0.0005106439810593284`, 0.006121775518969086`, 0.032742271841650766`},
{0.12775499399576257`, 3.9034479686029915`, 9.751449972499827`, 0.7624853991106708`, 0.7599399040180521`,
0.19562916289510757`, 0.12110783011333992`, 0.024828285786588924`, 1328.3636666047569`, 234.8344441201524`,
0.018565368539142812`, 9.598538649304729`, 1.107753026473853`, 421.0941753922051`, 99.57791059703924`,
439.75295320630414`, 0, 311.6479583917216`, 1.92571015010006`, 1.9423101500861046`, 0.008620196546807568`,
0.02153326027015132`, 0.0215326202392051`, −0.000029722900209194 77`, 107.38441962180366`, 0.03929973623589201`,
3.478747973889404`, 0.0003416153293095414`, 0.0003419152815240996 4`, 0.0008780408512827353`, 0.16921301653374232`},
{0.09993396962410062`, 1.749743490015244`, 1.6240016651924947`, 1.3301699142654`, 0.8818156710905265`,
0.1568106112519350 7`, 0.135253256362938 5`, 0.07239358889803704`, 2734.4752174906116`, 304.1441804766208`,
0.060678774409857306`, 8.403610161636486`, 0.537751994770234`, 307.15971054619445`, 43.43157566377896`,
357.57229142370886`, 0, 151.31516143482577`, 5.977316713789077`, 6.007057462395928`, 0.004975601934935625`,
0.18761970010395612`, 0.18767115558614758`, 0.0002742541543503396`, 149.41101439588203`, 0.2678511630153878`,
1.0667501710563017`, 0.0017013705164397042`, 0.001710509435494037`, 0.005371504305515362`, 0.07242687772160757`},
{0.14782514342208763`, 2.652895873833227`, 4.391872450348963`, 1.4876628412878459`, 0.36986838675832656`,
0.39309495169020825`, 0.13298297698236788`, 0.014228765232101433`, 3735.496122883863`, 215.5408051024042`,
0.11408789381411022`, 7.212929997484123`, 0.10464527448884264`, 88.04668646202823`, 23.991542311378637`,
267.29078771475565`, 0, 73.14949350987193`, 0.3798454258931243`, 0.3823726336138323`, 0.006653253003549464`,
0.02589623717975552`, 0.02593468713493306`, 0.0014847699652518198`, 14.395576614947041`, 0.05468735678842028`,
0.46458437729122676`, 0.00020964001061418713`, 0.000211234656407761`, 0.007606590883591169`, 0.20700849365713214`},
{0.2706278159985153`, 3.6076079533892003`, 4.017828860181465`, 0.931912448165567`, 0.07202782033095079`,
0.3509804409724484`, 0.12413238666840237`, 0.15319152742338107`, 1515.3910453914696`, 86.92889855061799`,
0.05735931907629604 5`, 7.405405977315304`, 0.4162338517487447`, 417.6641392610411`, 44.41195635721702`,
550.0970589795371`, 0, 132.15006393484816`, 5.4269101697226505`, 5.4565336821429895`, 0.005458633272688251`,
0.08201695817498234`, 0.08204834308483461`, 0.00038266366554728926`, 279.6880612945742`, 0.3170867180825485`,
1.9045206970547375`, 0.001061746163022792`, 0.0010668429073074088`, 0.004800341609058734`, 0.1218954905055062`},
{0.10989408866872064`, 2.7814923653363133`, 7.842262663464009`, 1.0963577256835755`, 0.7584107639763666`,
0.4703739490649106`, 0.21862941653484513`, 0.05112895789567924`, 3757.6555119131663`, 58.131608822022315`,

0.16080796994998975`, 8.15898170434339`, 1.4928178868639161`, 1869.806337113886`, 50.17389080399913`,
2041.4834122578436`, 0, 1081.9736411749425`, 19.32670133429065`, 19.37498676103892`, 0.002498379103246018`,
0.2011952056469804`, 0.20108269333197148`, -0.0005592196625517909`, 767.9581744066355`, 0.31585948241570333`,
4.146207450381202`, 0.002216671026461503`, 0.002220919801192803`, 0.0019167367104004907`, 0.06919592034012671`},
{0.09639258202167578`, 1.466780969423465`, 4.239504689160903`, 1.0209716259894424`, 0.025576954982881928`,
0.3026304446672493`, 0.21752127412681987`, 0.031317816620157334`, 525.4375097331736`, 358.0480696321463`,
0.16662257108917217`, 7.934540763419077`, 1.3681027859387838`, 161.2958041195384`, 125.070531133007`,
163.25709788637516`, 0, 112.58991789489511`, 2.2144730332228284`, 2.270987745330854`, 0.0255206142771478`,
0.030700895222470426`, 0.03072036257171775`, 0.0006340971201737045`, 46.40209860618146`, 0.0422762651553229`,
4.144706487744108`, 0.0003628925544640893`, 0.0003644179382032075`, 0.0042034032397573196`, 0.7834359334910719`},
{0.07632945356776594`, 2.5680095036868336`, 1.636586934722592`, 1.0974626755451315`, 0.20070740407760934`,
0.2792825326359145`, 0.08134798544013919`, 0.2967486253326174`, 1516.758023227967`, 278.84396411497187`,
0.08326880329861536`, 5.936954264251678`, 0.30521315193193943`, 112.29312565751323`, 35.64955292296975`,
132.78275074179373`, 0, 22.35013713966202`, 2.382250188166138`, 2.4109881500677837`, 0.012063368509488193`,
0.07934542099694626`, 0.07950968436553876`, 0.002070231231098063`, 87.39487319100547`, 0.08651989468332386`,
1.1970779286296838`, 0.0008686425209796234`, 0.0008783426666536055`, 0.01116701685641952`, 0.20168343541093628`},
{0.16556454843244983`, 1.858436546204688`, 5.85841964381955`, 1.4381472842647658`, 0.6188369558385702`,
0.43793021380482466`, 0.22826919863757877`, 0.3543109226514421`, 3912.388498432202`, 125.48108540961005`,
0.039979729590999546`, 4.62864467759934`, 0.44406393527696175`, 334.6061886902452`, 20.67369175638443`,
454.2957251437215`, 0, 55.52083009096833`, 10.116892489951589`, 10.142078910230762`, 0.002489541161398101`,
0.11117923612221937`, 0.11128582278104038`, 0.0009586921311803831`, 268.59432481928235`, 0.2629620003374453`,
0.4590059768098758 7`, 0.0009321762490287488`, 0.0009378065054586319`, 0.006039905474688023`, 0.03840420415892291`},
{0.17960696499390383`, 2.6986368926848217`, 0.39096283943110777`, 1.4614976941662214`, 0.0584358271913763`,
0.28608789101372956`, 0.23241827692103179`, 0.025712440835301015`, 1101.1873067230754`, 291.87610702538757`,
0.09533779650969992`, 6.256274031322354`, 0.1260305021418897`, 108.82142418540526`, 47.45913141986832`,
166.067583208332`, 0, 78.88166089350811`, 0.7206003253097646`, 0.7391308978833692`, 0.02571546517917378`,
0.39843043112998927`, 0.40282259438755724`, 0.01102366414410505`, 27.78055175373738`, 1.0222982928066877`,
1.3305706336112233`, 0.0032132465344509864`, 0.003381245889363872`, 0.05228336920671106`, 0.4069357513127763`},
{0.06965658134691888`, 0.9261903057977481`, 7.316991447780513`, 1.3759095728452715`, 0.2908565323439223`,
0.2820661078343176`, 0.18803658000898693`, 0.00943996945527825`, 744.7814906580452`, 341.56345427898043`,
0.1305415452351143`, 4.644709496928025`, 0.9315609895678807`, 109.54015529150739`, 61.01688812496366`,

113.55802777991248`, 0, 96.62709571596939`, 0.8978670637375975`, 0.9146619266136777`, 0.018705288961338384`,
0.027802974326853032`, 0.02782756095769917`, 0.0008843165683318777`, 11.879939576126453`, 0.027666573469777807`,
1.929339495426521`, 0.00024375428656098208`, 0.00024496037651166116`, 0.004947974321581228`, 0.2863345682271773`},
{0.14063443384893737`, 0.6174318191344224`, 9.816767595769278`, 1.0438016034352748`, 0.579595650707708`,
0.4622649825890621`, 0.24429017498983885`, 0.01619284881329759`, 2900.32749785945`, 335.82024218042955`,
0.09977744332778332`, 6.127347054973281`, 1.0166995269478085`, 206.7059542113607`, 36.349998446262035`,
224.18180366480294`, 0, 167.97515807982452`, 3.9285815270305555`, 3.9543346682606755`, 0.006555328189812526`,
0.022499245304950132`, 0.022511829151134827`, 0.0005593008127222632`, 34.65187484074799`, 0.04520240893560193`,
1.206226627473104`, 0.0002603046934608333`, 0.00026117138364721 6`, 0.0033295219339295468`, 0.1661255821512478`},
{0.18537094617034594`, 1.96648991280105`, 7.838266997084801`, 1.0125623241231316`, 0.014033931484803919`,
0.3423492209947736`, 0.1167129177170042`, 0.32838459574903894`, 3257.498914359846`, 126.9547671220904`,
0.1116209985614412`, 2.857468681315895`, 0.2336256655095057`, 71.24702100209345`, 7.36737182106995`,
134.0880731104634`, 0, 12.717463106624459`, 2.009298536353016`, 2.018657902270879`, 0.004658026544353655`,
0.020178086918281487`, 0.020200432591143635`, 0.0011074227677105863`, 56.44664719348723`, 0.05343472948499932`,
0.26017485994063905`, 0.00024027410440563557`, 0.0002417568400831566`, 0.006171017393609146`, 0.043938387435693324`},
{0.24032952463671997`, 3.1818327111380986`, 8.060728243579248`, 1.022907398702028`, 0.8948393046349232`,
0.3515250656278254`, 0.11210328340390646`, 0.4812285429766681`, 3331.6694795416133`, 199.3192679768665`,
0.1656921504225664`, 6.971086437067735`, 1.1039536289023264`, 381.0362910539855`, 51.34240761098081`,
408.9695366708799`, 0, 49.90651675400414`, 7.123853680901351`, 7.160706391467594`, 0.005173142545732423`,
0.04351424724596699`, 0.04352423091046273`, 0.00022943438362399426`, 323.8130095893353`, 0.14939654793506876`,
1.7734064832207834`, 0.0005140885363050796`, 0.0005153534478661421`, 0.0024604936148817647`, 0.0783900297039514`},
{0.06861147798506062`, 2.729021236857605`, 1.928866504736478`, 1.4151352288745787`, 0.45478865294711146`,
0.6487874608126984`, 0.19220035768006005`, 0.027739198253996934`, 956.7552237848031`, 40.992278320550554`,
0.10039930252063617`, 4.815840989066018`, 1.4694203679389397`, 1045.772771920581`, 89.49831961534596`,
1137.4777113456091`, 0, 749.3232439689369`, 7.3608604705068155`, 7.422570118575989`, 0.008383482925186536`,
0.9970158857624476`, 0.9983031002603309`, 0.0012910671898662684`, 286.9706363651254`, 0.9772390499534934`,
6.744847408526777`, 0.008435714207573608`, 0.008556114980322478`, 0.014272742033007102`, 0.14108189457619433`},
{0.16505190858637997`, 0.8247371132573029`, 2.6996089778841377`, 0.7893797405853178`, 0.33037619005051444`,
0.49339259475674924`, 0.09174509005830872`, 0.3021633567730715`, 3738.8764636919877`, 330.53731575160793`,
0.1571748109049641`, 2.9654306879318377`, 0.993830283737029`, 56.393436658153554`, 10.400678454581726`,
60.4429576935098`, 0, 11.066031100281041`, 3.537078236238278`, 3.582113445582651`, 0.012732319258017988`,

0.03472940671580638`, 0.03483752148504822`, 0.003113061220036295`, 41.67370991314843`, 0.08188792660736303`,
0.6060260061754841`, 0.0005288400515247593`, 0.0005353693604129057`, 0.01234647199908756`, 0.09132491182376044`},
{0.07254416598733587`, 2.8849957402027044`, 0.27388492744414883`, 1.0617374480096804`, 0.016195895901701673`,
0.5364706306281042`, 0.056724271897686834`, 0.010490294664589572`, 1785.3192582331621`, 96.17787055857661`,
0.24599567314403442`, 2.275980053816065`, 0.5487867290449304`, 109.30580642145082`, 13.398407503855692`,
124.76688020834096`, 0, 93.12885536787711`, 0.32767470625045986`, 0.3374648809001152`, 0.029877724654682858`,
1.1163494406608614`, 1.143909715401537`, 0.024687856451435453`, 13.504859024353511`, 1.1569234160453186`,
0.8547378482446581`, 0.01252553607161011`, 0.013326626946535872`, 0.06395661394017971`, 0.1245318077188732`},
{0.24080755705319862`, 2.3759884700193172`, 5.524917589968679`, 1.2325829370203099`, 0.2181690975782502`,
0.6636754905333937`, 0.21732363084691986`, 0.11964660140986366`, 3828.961095769544`, 112.06990257277658`,
0.03236755171123323`, 3.877460599831684`, 0.7029311637647315`, 229.47558695358063`, 14.076900575755303`,
286.5219239369245`, 0, 84.99075679655243`, 4.124415772993861`, 4.14319550165722`, 0.004553306382524935`,
0.08257246166110682`, 0.08275736166945355`, 0.002239245441241655`, 139.99377603141707`, 0.2840581824639387`,
0.6933357463632828`, 0.0008064558972991076`, 0.0008140983672279098`, 0.009476612365781456`, 0.06877026280999016`},
{0.15494186822563605`, 2.0029420912170632`, 3.8996790997983233`, 1.319513511447182`, 0.5220998731870559`,
0.2887737008742528`, 0.22727260445012404`, 0.040774601815574976`, 1826.996357882992`, 128.81708545308533`,
0.21865899508635123`, 9.155723124997508`, 0.5134933136755975`, 646.735726977204`, 63.45064537455784`,
804.6941257414771`, 0, 409.0322404728458`, 8.009681569460609`, 8.038726517226676`, 0.003626230021029686`,
0.14991182782697177`, 0.14994040156865848`, 0.00019060365083189978`, 229.18469075311774`, 0.331823123894179`,
1.8450934175621279`, 0.0013707678143255464`, 0.0013766353691040804`, 0.004280487707118308`, 0.10646679447210074`},
{0.22396249520436795`, 1.9570085864611029`, 1.4759242627722013`, 1.3116517086556398`, 0.6599681888830524`,
0.6953504264310013`, 0.09804476293632919`, 0.007430663549575122`, 1204.900095750464`, 62.93764873891854`,
0.10119557783088817`, 5.061320104116531`, 1.1487439857805883`, 671.0283261791079`, 104.73347623191799`,
708.5577982223468`, 0, 603.7781988308169`, 2.1959216954827867`, 2.2238112429934525`, 0.012700611122899774`,
0.7338712996665199`, 0.7362899505382101`, 0.003295742554299652`, 61.391965903657685`, 2.347994963317162`,
4.78083000194279`, 0.006701847314186438`, 0.006826947582692163 5`, 0.01866653515679384`, 0.12926456247279647`},
{0.10601932444652468`, 3.342699233573309`, 6.832537694215926`, 1.4208647912460013`, 0.5802751516427387`,
0.2570998642455964`, 0.07303385433227993`, 0.04213475658223818`, 3584.7181661863006`, 57.24897785837004`,
0.15446538158110595`, 5.395898684860962`, 0.7872614775312905`, 576.4662755758716`, 18.904561867908015`,
704.1837944474191`, 0, 360.1254572694688`, 4.429873516343443`, 4.442978125248937`, 0.0029582354568693514`,
0.13938297549769266`, 0.13939264573131666`, 0.00006937887205715754`, 211.5390686843992`, 0.21110412716577823`,

1.336233396962081`, 0.001185464276467818`, 0.0011883426233246007`, 0.0024280333991666403`, 0.02397855645151783`},
{0.041347104778859584`, 0.6250053944652225`, 5.297803080507409`, 1.3565417270883027`, 0.7676292422012339`,
0.6702284155028233`, 0.05669620363458108`, 0.027633397705997945`, 3343.0533516535834`, 245.48920969891526`,
0.16311205424117575`, 9.389474878516673`, 1.0487847358699702`, 287.41967522136844`, 84.11818618220181`,
298.576727580039`, 0, 206.77173831510447`, 8.110709380767126`, 8.193363881368485`, 0.010190785629350385`,
0.049134191002104206`, 0.049194602037428495`, 0.001229511142692985`, 72.41767308455925`, 0.029022236337000847`,
2.7903656923245914`, 0.00043684699926316295`, 0.000439129861438767`, 0.005225770531684049`, 0.20496176667854535`},
{0.04498655055054335`, 1.4491138993771555`, 1.16394731374956`, 1.169713814519905`, 0.8457893355464972`,
0.3136806500838595`, 0.06778013214591205`, 0.09196917164932833`, 3408.380878728516`, 174.30204224938495`,
0.10551969253057247`, 1.6463871196433963`, 0.4159425601435516`, 61.77719913290472`, 9.526496863056764`,
78.02172832069927`, 0, 26.652342108223127`, 1.600831408623305`, 1.6163887930374434`, 0.009718315326857185`,
0.23387888373451057`, 0.23525586898390374`, 0.005887599715741265`, 33.139814925650526`, 0.15030577465479045`,
0.21110247905929638`, 0.0024072040437472797`, 0.0024500588966500773`, 0.017802750462351913`, 0.029233682189335166`},
{0.1215137090416612`, 1.1969038422236755`, 9.326465300388158`, 1.005636980391936`, 0.6897518274648109`,
0.6108427950530713`, 0.10731717880916097`, 0.23857365224573474`, 2004.0983212715491`, 148.7170844059682`,
0.23834887248578368`, 9.94205781935025`, 0.30611051261382083`, 516.3486062097153`, 121.06016239303418`,
639.8130646133907`, 0, 118.49888344607123`, 21.97700432621446`, 22.057741056936596`, 0.0036736913513655267`,
0.03513789918336072`, 0.03515063375957189`, 0.0003624171196097148`, 375.77658455160594`, 0.060996235108945036`,
1.8068332561864022`, 0.0004220035082178608`, 0.0004232171303254443`, 0.0028758578636198795`, 0.19223540949141793`},
{0.279562619019907`, 2.2124846272464014`, 7.584786512534812`, 1.3700002017675512`, 0.23921169075395055`,
0.3169220525365052`, 0.13344245040196623`, 0.14640355547821746`, 3971.2444101007122`, 129.66148944966505`,
0.11735939166830095`, 3.485518254759493`, 0.4332823244894972`, 136.0017854550851`, 8.570801889756154`,
191.52444906008506`, 0, 44.1563962158364`, 2.8099865962255595`, 2.821772656270117`, 0.0041943474251404655`,
0.04412954778742357`, 0.04417460617758276`, 0.0010210480827095303`, 88.81503067024973`, 0.17624245650880377`,
0.35145698899014316`, 0.00038864923087233993`, 0.00039097364147734165`, 0.005980741553982982`, 0.03339010709446208`},
{0.21456925154594686`, 2.7215826602358453`, 4.860741945426051`, 1.4762193210184287`, 0.8476298137931575`,
0.5583227752271869`, 0.16730998643348832`, 0.023150536959116674`, 1148.3854317955947`, 98.18795037270098`,
0.13536301841717513`, 8.630410561575445`, 0.520586144335875`, 1103.648956405251`, 234.13344415928538`,
1235.9160861189268`, 0, 829.2607453673285`, 6.849810525738683`, 6.877873926581179`, 0.004096960162189189`,
0.24346176186062632`, 0.24360215378152622`, 0.0005766487510274754`, 266.31893646787626`, 0.7462772574640429`,
3.2898190474507754`, 0.001986280407237362`, 0.0019994499058465995`, 0.006630231341583137`, 0.136500827101347`},

```
{0.2192605474178046`, 3.4277372937593142`, 0.317243264743297`, 1.207905605629452`, 0.354638331302892`,
  0.2232326751046343`, 0.19168786121654457`, 0.1758842660002451`, 1819.052685961844`, 205.84067214106688`,
  0.2440876719932876 2`, 9.526826970263976`, 0.6180461674628193`, 359.922253817581`, 50.80747588949222`,
  423.4067233042655`, 0, 102.81013744063728`, 5.071655128300662`, 5.152473934788883`, 0.015935390803140104`,
  0.8937646828789466`, 0.8938084960003275`, 0.00004902086893809887`, 248.3471632051694`, 2.799533337581987`,
  2.004847434632448`, 0.008841140415975723`, 0.009050688209130313`, 0.023701443851739112`, 0.2187841230238138`},
{0.2002208994690245`, 3.902908071048812`, 5.625275625663315`, 1.4917900472855687`, 0.70165150687693`,
  0.17917645896398615`, 0.1075286064543329`, 0.022727063680328688`, 1296.438202913817`, 261.4435185272306`,
  0.04322514446110226`, 5.849567498164653`, 1.0949838652058799`, 203.45485632275992`, 54.504889961214644`,
  212.42252546804835`, 0, 153.88711983417582`, 0.8686429575336146`, 0.8786064878638501`, 0.011470225187256933`,
  0.057817028052300136`, 0.05784348785216213`, 0.00045764718030905094`, 48.4319087116808`, 0.1653739623037478`,
  2.052270444515635`, 0.0004680719433662883`, 0.0004699093532731679`, 0.003925486098707909`, 0.1173878937132119`},
{0.09281080427028687`, 3.9541950243176203`, 2.7903763295535473`, 1.1746304733040271`, 0.7021084302247975`,
  0.18301687365086206`, 0.15380914186275987`, 0.31491382108322413`, 1955.6345018254533`, 45.589098202841626`,
  0.16844466131119568`, 4.505740157817099`, 0.739135067608975`, 888.1638457884001`, 27.893957831867557`,
  1097.3935714852926`, 0, 162.34939535952248`, 12.604289609566463`, 12.639803627440234`, 0.002817613604087388`,
  0.5214150564157908`, 0.5202313744177923`, -0.002270133904715199`, 711.997417988659`, 0.69132786777892`,
  1.2915030404394572`, 0.005351646868416271`, 0.00536880661451538`, 0.003206442151551636`, 0.02053092334453282`},
{0.07553841622273444`, 3.998538946411479`, 5.0568584828370255`, 0.9418368972909419`, 0.5147743656673969`,
  0.5745728154097487`, 0.1277108500152948`, 0.02699422488607613`, 3283.474706944715`, 85.73668262235145`,
  0.1900835508559061`, 1.7563639079857047`, 1.1017643436886124`, 151.93718403556502`, 9.294094911558208`,
  171.14467005646603`, 0, 109.67501011532059`, 0.7231424999467624`, 0.7275261702995987`, 0.006061973059471759`,
  0.10073126794544322`, 0.101010594885273`, 0.0027729914010519163`, 41.30733499774991`, 0.10870114921007212`,
  0.6945243475687666`, 0.001288617760920041`, 0.0013001167730775634`, 0.008923524497529867`, 0.02826959122265116`},
{0.17556485477494027`, 3.9790149464214117`, 7.860200999240184`, 1.4870239255992979`, 0.43953202100784705`,
  0.2354118710730827`, 0.08492480553651033`, 0.015229986606251729`, 2122.168041719343`, 48.98316603866812`,
  0.24455585463944812`, 1.202710250977347`, 0.4184441960486769`, 111.23015755050835`, 5.641063928758047`,
  166.97091236196292`, 0, 91.04199430788717`, 0.34157138409652965`, 0.34361990520117114`, 0.005997344039987196`,
  0.10926568077219666`, 0.10946293621796765`, 0.001805282723513546`, 19.415966322713412`, 0.2740459053808052`,
  0.23294896709123838`, 0.0008867734276946093`, 0.000894214031351032`, 0.008390647964910736`, 0.008461701966301513`},
{0.13038773573510337`, 1.934638714312162`, 3.5362126783730226`, 1.036300296330771`, 0.1950167370197864`,
```

```
  0.2191580432274216`, 0.06372829025507237`, 0.4487751055567468`, 2715.7233289184524`, 201.32035054817607`,
  0.24618537045557576`, 1.5657139002447844`, 1.056666818669949`, 39.86386646384336`, 4.580178079869235`,
  43.69270592352921`, 0, 5.7390139123148005`, 1.186946934401836`, 1.2041787980546066`, 0.01451780459035823`,
  0.04660925727483693`, 0.04668517583255037`, 0.001628830025455441`, 32.80447844468475`, 0.08681822171943843`,
  0.445543169718303`, 0.0005419955561342116`, 0.0005471951350428441`, 0.00959339767602252`, 0.029073292270528475`},
 {0.24054054866212005`, 3.45017251549384`, 2.434688830833908`, 1.0572260280057717`, 0.42678683298038966`,
  0.6433017075143015`, 0.14198529935191645`, 0.0642120318165855`, 1461.85471205871`, 46.12110813109672`,
  0.14351244735387614`, 4.149492178713276`, 0.5419589373402318`, 603.5712020213003`, 48.43665209572478`,
  754.0902522056126`, 0, 314.6578602591031`, 5.710282278959699`, 5.742322287362748`, 0.005610932496473042`,
  0.3946160930246094`, 0.3953822602027625`, 0.0019415507671789456`, 281.4494139225474`, 1.3560167361000877`,
  1.63787331700127`, 0.004479953600526776`, 0.004540500091746163`, 0.013514981765049505`, 0.06817445332706491`},
 {0.2576647285795562`, 1.7400215170432691`, 5.671933058359681`, 1.159126105619701`, 0.5426396846396173`,
  0.16289934098722736`, 0.12398680737225279`, 0.2145763112631837`, 795.8175406593573`, 85.6025153828084`,
  0.04739051555756513`, 6.248380217555781`, 0.8502018161731435`, 579.5009132683543`, 77.49333344678588`,
  645.7112541921704`, 0, 145.20197423766464`, 16.774368128886163`, 16.898628424084087`, 0.007407748193146046 5`,
  0.11889742305293427`, 0.11887546300549692`, -0.00018469742130211575`, 416.96802112952633`, 0.43765246056790874`,
  3.741192270520479`, 0.001239137888163766`, 0.0012427082219871328`, 0.002881304701817688 5`, 0.08476193838313434`},
 {0.25257809880572757`, 1.2556801180516963`, 6.596575825508853`, 1.3398305186381605`, 0.284033409354278`,
  0.1515369008103068`, 0.1400005745311825`, 0.02101007059419062`, 611.632102430428`, 356.19419891984376`,
  0.010524324923865458`, 2.8673905561599544`, 0.6614014808678237`, 57.282326061329584`, 35.629591470825126`,
  59.62673829325813`, 0, 44.227474471205724`, 0.6823807559894701`, 0.6982613479747903`, 0.023272332705650545`,
  0.025400343298756414`, 0.02542138379499571`, 0.0008283547978789052`, 12.240742117672351`, 0.0916510059916164`,
  1.1965365041021208`, 0.00022876583685671 47`, 0.00023015945891619532`, 0.006091915115601365`, 0.1356926114847002`},
 {0.2042789431730157`, 3.7869781485826124`, 7.097464150909694`, 1.0298616729281707`, 0.4360801596581312`,
  0.5374359749706832`, 0.19209770462574638`, 0.13005052253847288`, 2094.968893303633`, 292.0969799908029`,
  0.013655036785018004`, 7.349385967933083`, 0.6499659559020465`, 219.52850965588573`, 55.19621616000367`,
  242.54794142945454`, 0, 77.7671253737187`, 2.5708838105734113`, 2.590086105164405`, 0.007469141355987974`,
  0.027176839079571483`, 0.027197429586093343`, 0.0007576490577720918`, 139.08401161694727`, 0.07930937094222412`,
  1.5547662308208585`, 0.0003184099430576514 7`, 0.00031985287439131947`, 0.004531677999159545`, 0.25549715458986144`},
 {0.06006756967417276`, 3.885393698216971`, 2.4182637836940106`, 1.1497042341120063`, 0.852306387023593`,
  0.3634054456246256`, 0.23851842406334756`, 0.1905044887986902`, 1969.8862529979606`, 336.32300959810993`,
```

0.04225162262081883`, 6.207670246795658`, 1.4904811957625173`, 318.4612407408287`, 72.44301544227577`,
320.95851812178336`, 0, 86.80239379853938`, 4.0947241284302835`, 4.121496237509937`, 0.0065381960395745065`,
0.15279189075179506`, 0.15298390701221498`, 0.0012567176142341818`, 227.28021892200024`, 0.13111196490534596`,
1.7922676675580886`, 0.0015995808736413153`, 0.0016122703385748722`, 0.00793299366269018`, 0.15239980894255178`},
{0.2595298239660976`, 3.319555998413656`, 5.7932515179865565`, 0.881744759172532`, 0.6648616448212201`,
0.6517725519271407`, 0.19583264477028905`, 0.005149860640734175`, 3979.7041048852343`, 156.14061322234556`,
0.24304909070853137`, 9.19022988333834`, 0.6081568865700206`, 612.3541994257707`, 56.26392813139535`,
724.0254576365425`, 0, 569.5943555008841`, 0.8634125011205425`, 0.8666130499262983`, 0.003706859469375434`,
0.06360886673965056`, 0.06363667575715302`, 0.00043718775271184285`, 40.94494496000041`, 0.2358342569661145`,
1.3077837478976937`, 0.0008707788579478404`, 0.0008741129085612037`, 0.0038288143802902574`, 0.10635481837967951`},
{0.12106147370268983`, 1.1502892713218662`, 9.893056355486525`, 1.0779684733916626`, 0.2654952298373383`,
0.5544611241456747`, 0.21486298948769583`, 0.008770181568324542`, 1487.6775848537964`, 210.36931291761118`,
0.08815050293390886`, 9.314054867228961`, 1.3778011559930095`, 363.4293393002092`, 84.02347271818466`,
388.79906044207445`, 0, 323.10531679624245`, 2.2928638289124543`, 2.321143387704253`, 0.012333727993437904`,
0.02667542879925023`, 0.026686578136772336`, 0.0004179628228664356`, 37.677952328570946`, 0.046133810315560735`,
4.227532790614067`, 0.0002989122315912862`, 0.00029975352700889116`, 0.002814523223510168`, 0.46382378886214093`},
{0.08502696111412816`, 3.6275157197896295`, 7.363159164909369`, 1.488436793334436`, 0.26568493648971336`,
0.6629645451225747`, 0.17264441279507348`, 0.30453583199957635`, 1224.7690817107496`, 42.94433873275722`,
0.17512531432720302`, 7.6510105838564435`, 0.2548403509235575`, 870.9536131290735`, 92.11916700592842`,
1439.7690230948174`, 0, 164.34293481656738`, 13.371242220449892`, 13.406890504764817`, 0.002666041324149049`,
0.14429194102772086`, 0.14441293512747225`, 0.0008385367809844446`, 692.9198763970975`, 0.17526721798322298`,
2.022454685879851`, 0.0011677310415728615`, 0.0011749668837115763`, 0.00619649720792603`, 0.13219821451961963`},
{0.04600515107837966`, 1.2443774572294632`, 7.3914675121007605`, 0.8524218043851708`, 0.25925086237615913`,
0.25962408201227716`, 0.17007776716368422`, 0.3647699508957847`, 2650.181739077888`, 363.5928673248461`,
0.07845934701674573`, 6.64885092299725`, 0.9963780523907508`, 132.13771103759032`, 23.331495669396794`,
145.64209385323306`, 0, 22.315897500905823`, 5.847527655506493`, 5.904243389548382`, 0.009699096333213753`,
0.014382182634035777`, 0.014386291928907816`, 0.00028572122720182946`, 103.95045135768744`, 0.009452206927364752`,
1.2818697962050276`, 0.00020392937654578613`, 0.00020434457094801277`, 0.0020359715174895765`, 0.1920670361181569`},
{0.1217700466706656`, 2.558519459791139`, 5.216939277492001`, 1.0963651311366025`, 0.09816261776323953`,
0.6044055975062239`, 0.09115244000304745`, 0.06193230544868139`, 3735.1363049157717`, 184.7914603929239`,
0.05165113964049522`, 2.282481339681045`, 0.8427745163468003`, 63.964223741287974`, 7.1619144501206105`,

72.2611466413348`, 0, 34.1298750610493`, 0.7918234360552839`, 0.7996318288873491`, 0.00986128027602362`,
0.036779711520228645`, 0.03691520721653861`, 0.0036839793111331787`, 28.941366712373277`, 0.06398095983345882`,
0.540783823358831`, 0.00040325876190228094`, 0.00040825142429111726`, 0.012380790848249834`, 0.06266094945888194`},
{0.07220853239716318`, 1.7719009634055194`, 4.958541757826833`, 0.8715174582622381`, 0.41441036947741816`,
0.32423173571278785`, 0.1998499231948091`, 0.007134676741262494 5`, 2969.140628850977`, 169.59950979095402`,
0.08473674334867182`, 5.398547675716223`, 1.090541691203971`, 266.91946717019687`, 21.359806686686667`,
302.757900600032`, 0, 242.03888272520814`, 0.9323164579805832`, 0.93828905684363`, 0.006406192674087929`,
0.05450775860273543`, 0.05453411875016842`, 0.0004836035843100994`, 23.59960614420881`, 0.056227503613761186`,
1.3722734240791299`, 0.0007550614592142235`, 0.0007578145256437438`, 0.003646148794808 5125`, 0.0983807969393714`},
{0.16122326429150147`, 3.7922162994918844`, 7.367254304672895`, 0.921371724322694`, 0.6381223018466633`,
0.5967903533432476`, 0.1832909738697283`, 0.43512505909917726`, 2133.3461489698684`, 214.82179755577738`,
0.22692868851616532`, 5.899255886059763`, 0.6939454361655824`, 286.3063045481693`, 60.31434444243885`,
312.94324708177544`, 0, 40.86576113511818`, 4.446161667138933 5`, 4.470000174677839`, 0.005361592610339194`,
0.038058391321469515`, 0.03808861501125126`, 0.0007941399710371666`, 240.8686677757176`, 0.08765568689416589`,
1.4184245750913962`, 0.000498268672022073`, 0.0005006585162690918`, 0.004796296418396739`, 0.14367372825701383`},
{0.14827779601188723`, 0.9768409840676426`, 0.6891894673357726`, 0.9389444267785162`, 0.30176378998265774`,
0.1751647596247058`, 0.07879547499477868`, 0.023619248417657335`, 3252.235532426287`, 167.1595777298993`,
0.05956658968902967`, 2.6817550689507357`, 0.5206583529379527`, 80.68187912880265`, 6.710526464600179`,
102.5460728575649`, 0, 59.81154211811191`, 1.341497471247479`, 1.3611132172696485`, 0.014622275809382357`,
0.2540326703409185`, 0.2547906471028934`, 0.0029837766967442114`, 18.720424427680584`, 0.5381057781880806`,
0.35685739215510737`, 0.003259297765562552`, 0.003317659835247453`, 0.017906332554683768`, 0.034398208147378144`},
{0.2570676551725277`, 1.8968814761831432`, 5.846591463128114`, 1.1172410815007583`, 0.43583764365067834`,
0.15357951833619232`, 0.128960566744062`, 0.20068004601330075`, 2555.9384884114397`, 300.14317051223054`,
0.1562959949020017`, 5.731951708372693`, 0.952499750656465`, 141.836555291966`, 19.66818504152704`,
157.24350781767902`, 0, 37.45305201909437`, 3.7100089651866828`, 3.7424497290028578`, 0.008744120060244276`,
0.029649283739575646`, 0.029657085877813658`, 0.0002631476128240884`, 100.5349611790859`, 0.1088838835496507`,
1.1070562018136882`, 0.0003207423813622601`, 0.0003216595844949982`, 0.002859625624909823`, 0.0933451452761865`},
{0.055135962685152806`, 2.9220194449851506`, 9.20311067851182`, 0.9951015075863325`, 0.9088587426511963`,
0.6564149766220422`, 0.21100197083140715`, 0.41165435784921034`, 2658.942850929282`, 386.04298432332337`,
0.012398563555194947`, 5.099390369220371`, 1.471358614723604`, 229.39000091635114`, 77.34660139739466`,
230.11213016333127`, 0, 34.439648601491506`, 4.559698968044853`, 4.586752800394118`, 0.005933249659431983`,

0.030500259035714412`, 0.03052873428579661`, 0.0009336068276946108`, 190.33612925579672`, 0.024023730630343534`,
1.3016184114172804`, 0.0003698034556638419`, 0.00037147977260421023`, 0.004532994255987077`, 0.13094514544175315`},
{0.09951701144549052`, 3.3599674683318668`, 5.87127293585465`, 1.0675363096846835`, 0.6922846316607385`,
0.4471508361318377`, 0.09256546388382819`, 0.022405598476994366`, 2159.6702917097355`, 22.498867590732686`,
0.07537929727214032`, 4.117708447722572`, 0.7807622026379022`, 1293.6350612944814`, 37.45425903741472`,
1595.5772817996572`, 0, 979.581292668245`, 6.38572449692411`, 6.401445928607744`, 0.002461965230602603`,
0.3586958987920451`, 0.35837547986142193`, −0.0008932885257462164`, 306.5118081627836`, 0.5099477695079402`,
2.02471401103693`, 0.004053561815375417`, 0.004066656655984432`, 0.0032304529215132494`, 0.019318149692621772`},
{0.13155432244432247`, 2.613811333361477`, 8.357167885800742`, 0.8056721180002253`, 0.6603026707260147`,
0.5801438627936167`, 0.11636775006500844`, 0.26451755332760135`, 2521.6309343959783`, 242.4992509443806`,
0.160383814941372`, 4.821776617001756`, 1.0110903713708876`, 178.10498009242363`, 42.189945840760885`,
187.0892916529765`, 0, 38.345095735308036`, 3.6435836821552736`, 3.673311056381809`, 0.008158828455656852`,
0.02233010052880394`, 0.02234778185657041`, 0.0007918158605537684`, 136.05200460669084`, 0.04196601778836636`,
1.3916678978718862`, 0.00033450570472814967`, 0.00033591039390204854`, 0.0041992981107465965`, 0.1217724764818643`},
{0.10755292243344333`, 3.7450945369693454`, 9.057610815009983`, 1.4262360104426115`, 0.8847828962958162`,
0.32323944244008773`, 0.10925879484006157`, 0.04648694210521617`, 2597.7878756158707`, 114.3810122188126`,
0.2151089301912223`, 9.739939811090224`, 0.6433246757255959`, 841.7590253465713`, 85.91934839370788`,
983.8505522935309`, 0, 506.5274083845961`, 6.146526078230242`, 6.163597241244235`, 0.002777367702783362`,
0.08540676176821121`, 0.085412342443006`, 0.000006534230638455263`, 328.84744624170963`, 0.13122495462421552`,
2.0982852074539067`, 0.0007237157818643647`, 0.0007251559999049307`, 0.0019900326573725202`, 0.06722283470679771`},
{0.11067094408083894`, 3.800544284934695`, 7.350733993347461`, 1.2140806206228738`, 0.3865719277011892`,
0.3640208182074153`, 0.2468843803041872`, 0.02524798025663777`, 519.3604951232796`, 258.54462558963803`,
0.248859794496116`, 2.6642578190064192`, 1.225620094151966`, 99.49008897831348`, 60.737298076178185`,
102.07110911530218`, 0, 73.44946504542625`, 0.4688400229332147`, 0.4769542084858173`, 0.017306938733254285`,
0.038613569115547676`, 0.0386818760482319`, 0.001768987800113031`, 25.45496099582115`, 0.061048573547840726`,
1.6254475405792645`, 0.00038264119290087883`, 0.0003860881352445108`, 0.009008288724744906`, 0.1975799000097067`},
{0.17620161354602198`, 3.207637663312279`, 8.038302489204167`, 0.9527508465697585`, 0.2777263075882186`,
0.3259017504461914`, 0.20696733159344116`, 0.041067631602668214`, 3666.144435165166`, 184.14344105031228`,
0.011349261156917284`, 2.0150600100651097`, 0.6578483910293456`, 72.93639717195593`, 5.530031871790882`,
91.24818910902312`, 0, 46.003735109607575`, 0.573115133383746`, 0.5759616294741163`, 0.0049667089988783`,
0.026863057257787887`, 0.026898669362823254`, 0.0013256906946077507`, 26.262081246513517`, 0.06761877190863078`,

```
0.268478666973489`, 0.00033999297847964183`, 0.0003422201439361728`, 0.006550621917223998`, 0.030479013488267762`},
{0.098793524136433`, 2.9572290906746908`, 8.636235866475928`, 1.1358825426984174`, 0.44571351797486836`,
 0.28917170815364945`, 0.20954073160539777`, 0.05617013174448901`, 938.6734643463747`, 177.03868080567804`,
 0.11564637982987985`, 4.570821554383135`, 0.26856807485341383`, 199.03080641776728`, 55.6943098678135`,
 256.13724809942624`, 0, 110.93849198647166`, 2.0349479203664855`, 2.0489055575396677`, 0.006858965300039932`,
 0.03591996948009746`, 0.03593924900484882`, 0.0005367355549132924`, 85.96867411593904`, 0.05069514816874253`,
 1.282020680587036`, 0.00038175096043024936`, 0.0003831908526724747`, 0.0037718103985973084`, 0.11889649138420616`},
{0.1873960215776312`, 2.8261178368849595`, 9.525044121358242`, 1.3550980694568904`, 0.08983672188035041`,
 0.6607774222729332`, 0.06657454788578898`, 0.12572829008110223`, 835.4208642240601`, 103.25606657618908`,
 0.08845188667521903`, 5.652525212839048`, 0.4964020494140271`, 285.0697458735694`, 84.2107841881027`,
 309.9607841924893`, 0, 103.98992249892946`, 4.372755138094899`, 4.426009556285129`, 0.012178687465548732`,
 0.04496221800014294`, 0.045017696063736484`, 0.0012338818248105454`, 176.5417327442901`, 0.12036772535043876`,
 3.390815069760272`, 0.00039975508245904123`, 0.0004023974469377683`, 0.0066099584337313555`, 0.27031188764274505`},
{0.261714977559417`, 0.5482487201542185`, 3.431220669456936`, 0.9478503018778801`, 0.34449029105022966`,
 0.1671252432139082`, 0.1374901654337824`, 0.05820678844321773`, 3721.854350957653`, 371.28139834560955`,
 0.08783171860573763`, 6.880528123303602`, 0.4952942413378081`, 118.86605595677261`, 15.64558978999501`,
 146.72534714244145`, 0, 65.10670153710997`, 6.070619839853191`, 6.110197993759519`, 0.006519623193417701`,
 0.029913581357126403`, 0.02992484647719799`, 0.0003765888121887162`, 47.54585082489015`, 0.11184046105154186`,
 0.6338673921336118`, 0.0003812617880702973`, 0.00038264938650643985`, 0.00363948992414298`, 0.08619424647147339`},
{0.19384041765838167`, 0.8310343395086068`, 9.704183649775683`, 1.2913450048559905`, 0.5670495339238457`,
 0.6409270851330959`, 0.14192751733077513`, 0.022909401085561467`, 1113.6891447077023`, 383.5158989221919`,
 0.1816390006173258`, 4.168222848202584`, 1.1878448557299315`, 103.38007435062605`, 76.18473439589869`,
 102.03907048472615`, 0, 78.18804002696284`, 1.9493291148807208`, 1.9803758087329977`, 0.01592686099811136`,
 0.020670020307106827`, 0.020704483662287592`, 0.0016673111428400222`, 23.142277620997117`, 0.05723836241911405`,
 1.5445504343109258`, 0.00019271039907509113`, 0.00019424081683414653`, 0.007941542160675441`, 0.25917449726119784`},
{0.24542600753982147`, 1.0072788201913916`, 4.873030125775312`, 1.3215761420654866`, 0.934997683525973`,
 0.5336155406906149`, 0.1530570292862033`, 0.37775895009481864`, 1381.366911360472`, 23.478593484050236`,
 0.23171577878109345`, 1.0189498874051495`, 0.9824058311213721`, 518.6457721506471`, 25.715336220067883`,
 599.3514602428256`, 0, 81.20947922329417`, 28.171344384397827`, 28.432251211260134`, 0.009261426196145894`,
 0.8627945656946008`, 0.8601546388688478`, -0.0030597397465382814`, 405.3771219245949`, 3.0250317940781737`,
 0.8739093970918868`, 0.00785453358343835`, 0.007938391482429173`, 0.01067636901669644 1`, 0.010448498433364858`},
```

```
{0.21619628547349445`, 3.9792600826840445`, 2.630502577782165`, 1.4259463002457018`, 0.22306595661200035`,
  0.38072127406591705`, 0.0850183629201968`, 0.035809155258912144`, 1510.787355159533`, 321.0088965168211`,
  0.03982099495880792`, 2.097097341241465`, 0.5759181842297432`, 37.963910496149374`, 14.388710009634922`,
  40.25873605811438`, 0, 25.132378001521865`, 0.2174817892077439`, 0.22210796729311982`, 0.02127156532152852`,
  0.060768603711668215`, 0.061202332728842054`, 0.007137386589163297`, 12.363094321501194`, 0.18768494851248033`,
  0.5552882352589724`, 0.0005083201003212023`, 0.0005230307368881843`, 0.028939710543979036`, 0.10862182658854785`},
{0.12757698942849355`, 3.9475071221290925`, 7.046874473492651`, 0.9016631589117734`, 0.33636145019883923`,
  0.46329966420746505`, 0.22687767004632875`, 0.06279665921261046`, 3797.4437504167317`, 319.7090357145597`,
  0.1993551083304292`, 8.931980068156673`, 0.8508236103120903`, 232.06421989796212`, 30.818930146488885`,
  265.28136677983474`, 0, 122.91407889213623`, 1.9007550036873582`, 1.912048746896106`, 0.005941714311859414`,
  0.020856077948065547`, 0.020865133831463184`, 0.0004342083597974611`, 107.18919877826184`, 0.03801079479850521`,
  1.3489111369268303`, 0.00027941371917550306`, 0.0002802060427900148`, 0.0028356646797793594`, 0.23316066580714506`},
{0.07009559015680356`, 2.7849471367228507`, 3.0258579570105155`, 1.1102521989309375`, 0.4599988219148854`,
  0.6809018065475554`, 0.21885987895906978`, 0.1581032784122877`, 1010.998941039169`, 191.1453687810041`,
  0.0813026067841443`, 7.337454751758624`, 0.7004457216179918`, 375.0371573024324`, 134.83398892511696`,
  399.239149732994`, 0, 117.14056232633567`, 6.317564649945027`, 6.373582553688027`, 0.008867009179476693`,
  0.11740915985415522`, 0.1176663689156482`, 0.0021907069415409985`, 251.344051184656`, 0.11756949071180574`,
  3.14213807791533`, 0.0012694462523269445`, 0.0012837897896762437`, 0.011299050529320898`, 0.3768629499764321`},
{0.16572747960623446`, 1.3999561572838486`, 8.592203336105698`, 1.0149199248092395`, 0.9204720561931288`,
  0.6093468506279616`, 0.11483304130851824`, 0.012170616273068445`, 1337.06098163951`, 286.8021266598324`,
  0.027824790783174902`, 6.4485130480990716`, 1.1250259465267032`, 273.3679575002954`, 190.82275846513033`,
  273.1160717515755`, 0, 233.02939591271146`, 1.9085291800936157`, 1.9281343504055595`, 0.010272397465247218`,
  0.03140044676987691`, 0.03142463757594572`, 0.000770396875117596`, 38.16938824325645`, 0.07434167002402006`,
  2.5161675006294684`, 0.00037332176153737073`, 0.00037497047342959525`, 0.004416329456485402`, 0.20524226328818107`},
{0.15460498360139963`, 1.7922898385073154`, 5.637575606203491`, 1.3053191224828369`, 0.530866536081585`,
  0.39279997373545783`, 0.23407598158461806`, 0.3220260634134779`, 3258.312065268822`, 390.4363804447645`,
  0.21449854536638602`, 8.936127787959748`, 0.6328205146062444`, 232.23497643543195`, 40.960793950788606`,
  264.74807571195686`, 0, 42.35299125405255`, 7.132758801077703`, 7.170583153837914`, 0.005302906465096795`,
  0.03773172265329381`, 0.037757143943510686`, 0.000673737863772228`, 182.6281588527877`, 0.08333589088692471`,
  1.1206062589847043`, 0.0003488693316108238`, 0.0003503478614618597 3`, 0.004238061982144492`, 0.19971295513794995`},
{0.08186552922698848`, 3.5534207813275085`, 2.793085272759267`, 1.44138898302728`, 0.2645186976244649`,
```

0.667000460134499`, 0.1031352647835832`, 0.11738652278758988`, 1616.7923697759452`, 22.69493932260025`,
0.19900500778151703`, 7.38154340093187`, 0.5255971444464016`, 1676.961244081816`, 67.46671729665387`,
2246.122729043935`, 0, 627.7666314561877`, 20.238307286182117`, 20.29467873753158`, 0.002785383705877109`,
0.7356176121842228`, 0.7353306695278605`, -0.00039007040017746686`, 1027.3603098515914`, 0.8603103590016619`,
3.1077292200777893`, 0.006135796021527917`, 0.006181661865206362`, 0.007475125235180569`, 0.0727250760109647`},
{0.08442779942280981`, 0.8533376706252112`, 5.086343297092757`, 1.4765588559370955`, 0.4420393122680226`,
0.47437344679325344`, 0.24643808934122824`, 0.005941536592530679`, 2172.43229526953`, 242.96995635142855`,
0.157510417696612`, 9.228156626391328`, 0.9577175035728991`, 371.76063514543347`, 63.21182816251446`,
411.49537140082504`, 0, 342.3687568048335`, 2.187902522220737`, 2.2050593486256087`, 0.007841677693875226`,
0.07331837854284985`, 0.07338841915789605`, 0.0009552941082195954`, 26.67170916952662`, 0.0884301336803059`,
2.3890678629155624`, 0.0005987045803427238`, 0.0006019443849627215`, 0.005411357665149552`, 0.2867529650909741`},
{0.16851133973676596`, 2.4752237313475174`, 2.42575793497873`, 1.0578005973522087`, 0.5315655422073631`,
0.41472938652046143`, 0.24177717448224295`, 0.0988063583504681`, 1028.5760265584995`, 54.93252411146938`,
0.04650878688675153`, 2.5043219440736237`, 0.71806339741822`, 433.9585800790301`, 36.26591306309482`,
515.7348805026295`, 0, 180.03269179931442`, 6.939371079061469`, 6.985804823522958`, 0.006691347664285585`,
0.471900402401727`, 0.4726318613549203`, 0.001550028246364299`, 245.37851393599567`, 1.1360081290158182`,
1.2943832417676269`, 0.005350784658687102`, 0.005429055133788689`, 0.01462784994991595`, 0.04484327625695699`},
{0.2040419029224893`, 1.8168800092917534`, 6.174811187872505`, 0.9288754688257587`, 0.6581669676988375`,
0.5754364926136093`, 0.20349981430348302`, 0.3349223421963805`, 3304.529174698706`, 164.1808894290948`,
0.03840187502351833`, 7.840532439325425`, 0.2974189092407089`, 416.37083216341335`, 52.592520271469056`,
590.9904857428097`, 0, 72.57785901181529`, 12.746851151091406`, 12.776339233815303`, 0.002313362129546137`,
0.05009995323895911`, 0.05012876228682785`, 0.0005750314322916505`, 330.84998625479324`, 0.1460355685028491`,
0.7684050617517807`, 0.000650789812854935`, 0.0006536189762247985`, 0.004347276669025124`, 0.09658793168721103`},
{0.17393597413635692`, 1.5761991979266723`, 3.500768537824096`, 1.241966650800916`, 0.7553239598200872`,
0.5647220891577981`, 0.0698456460365596`, 0.07461556744160115`, 1637.6446967775864`, 102.27592905310792`,
0.14712502478326317`, 2.669212259273973`, 0.5885270492629138`, 187.9445166516793`, 40.995334558812644`,
208.92650871098598`, 0, 91.236445745871`, 4.089146593321728`, 4.12344032605316`, 0.008386525635309727`,
0.15573268646898153`, 0.15637065301273748`, 0.004096548760706087`, 92.07585115140412`, 0.3869645217978715`,
1.0260734157166018`, 0.0015054093652142608`, 0.0015286101667969338`, 0.015411622990249452`, 0.057558087640503824`},
{0.06303562103190802`, 2.7937374046310888`, 1.991826202033483`, 1.3853239568680058`, 0.8642226718049046`,
0.42627609641461395`, 0.1739341576099066`, 0.013248801141587125`, 3504.113575209887`, 145.60993330770657`,

0.09071202532973738`, 5.892751585058276`, 1.3807549697256851`, 555.1797751967358`, 44.22095650012901`,
587.1866182549933`, 0, 466.24024813853106`, 2.1480028560765017`, 2.15971815086658`, 0.005454040601918564`,
0.41036760604216255`, 0.410797149586286`, 0.0010467286837434475`, 85.72794177536211`, 0.369539669974822`,
1.9491640596499893`, 0.0035671834426065363`, 0.0035948241821720907`, 0.00774861736444854`, 0.08078615140337869`},
{0.2432575073739432`, 0.6270765712575339`, 1.9550603903514805`, 1.0314330185313336`, 0.13493745232200305`,
0.4469694845616632`, 0.19582833943170452`, 0.02468113928377954`, 1814.663806970997`, 145.9448155579372`,
0.17557852480928543`, 9.315910864758589`, 1.2079900473113105`, 442.1617660975512`, 55.01730630813117`,
492.8974447463634`, 0, 327.33210036296356`, 11.45235685036908`, 11.574452786502457`, 0.010661206049429106`,
0.15678232334553904`, 0.156952939922248`, 0.0010882386039765546`, 102.59292380781663`, 0.5448353882475843`,
4.057862290534077`, 0.001829595966986397`, 0.0018450548312817134`, 0.008449332297544965`, 0.3308863356889017`},
{0.11357589384964273`, 2.240672872189519`, 7.052253737321249`, 0.7646938475078471`, 0.009142046869567766`,
0.5509926958854778`, 0.24090899686155176`, 0.02638287046938754`, 2863.3930915889323`, 84.10264867474814`,
0.16678159718247448`, 7.7951222229657064`, 0.5053738528157279`, 506.79274782785944`, 29.02335448601901`,
698.1094839727773`, 0, 368.25926987931575`, 4.1908580192444775`, 4.202542893884992`, 0.002788181939559209`,
0.04423445018250196`, 0.04424771516260453`, 0.0002998789415904479`, 134.14774107027142`, 0.07177096026332286`,
1.2443232156820176`, 0.0006985064619269288`, 0.00070063709165407`, 0.003050264876954145`, 0.10786033058886779`},
{0.1116182977145373`, 2.360842887008661`, 0.9627021602560966`, 1.1405981578601385`, 0.14103896539205096`,
0.5791003705327337`, 0.05180942465934579`, 0.03555380982336255`, 762.58124752924`, 235.4407827441829`,
0.05499220801683008`, 8.486879923307693`, 1.2577154495282135`, 207.68749054764754`, 136.2438341030107`,
203.4558316559039`, 0, 139.16870157365454`, 1.9592018714029928`, 2.0144835288280065`, 0.02821641722168544`,
0.18063857258418878`, 0.18160999168890726`, 0.005377694757113582 5`, 66.07668289022584`, 0.28803671390627017`,
5.352273729180785`, 0.0018904553962804105`, 0.0019318968180786396`, 0.021921396230647927`, 0.548802625120759`},
{0.1166411660555075`, 0.7450165479373432`, 8.310812790636028`, 1.063932072189387`, 0.9753313471385676`,
0.15156075882067166`, 0.057187743785721765`, 0.12429446652524245`, 3685.6740694089303`, 232.00827662364395`,
0.20692272239979642`, 9.093961104192626`, 0.5500507674166981`, 290.6098955942164`, 38.8209954632563`,
348.4293127965795`, 0, 105.38427728793954`, 15.90274517722124`, 15.96736946088224`, 0.0040637187442058575`,
0.025676378949796196`, 0.025677252676437078`, 0.000034028421320275726`, 169.25440449515176`, 0.042784611154155994`,
1.108914879187778`, 0.0002919178616834772`, 0.0002922317500372150 6`, 0.0010752625821786044`, 0.04961690591048378`},
{0.12701256602307204`, 3.4510594830271417`, 0.17236039015202387`, 1.4218046895736245`, 0.5977738378490018`,
0.6174455495269762`, 0.06547152715546964`, 0.021505928148282425`, 2080.7788466921183`, 285.32441857492176`,
0.13527159522812743`, 2.0719517072723157`, 0.8493272554882347`, 56.4908988438072`, 21.73768657863022`,

54.445817151018794`, 0, 40.67351392325777`, 0.2443570030634067`, 0.26394420002498237`, 0.08015811585515742`,
1.2481278474166304`, 1.319168122802724`, 0.05691746685496413`, 12.047007895229468`, 2.2646845803605524`,
0.6773555230267962`, 0.010444801884769928`, 0.012021951735114224`, 0.15099854145094094`, 0.22229247754610534`},
{0.16525167732730567`, 1.567211428071798`, 0.46961269510991777`, 1.0926048049265726`, 0.5982927168612024`,
0.4872752592438997`, 0.13461015322956665`, 0.008651638441061493`, 1318.8282746769155`, 92.92105735217046`,
0.1547378735694622 8`, 9.951552143172052`, 0.8370529835607092`, 959.7549504226304`, 139.79241656466428`,
1047.3131484657997`, 0, 849.8735538222058`, 4.4646047117585015`, 4.513819838680406`, 0.011023400748622958`,
1.4032473071185014`, 1.4017771687594143`, -0.001047668754915354`, 99.95685037273005`, 3.312699588662167`,
5.049682048310935`, 0.015303585538575915`, 0.015607857867099034`, 0.019882420871640605`, 0.239130816335155`},
{0.26512296149188996`, 3.233300402128563`, 3.292718317720548`, 1.2400916214421978`, 0.5963811820900797`,
0.3451658167692204`, 0.10292393665820598`, 0.09694787300120739`, 3191.5882949078978`, 195.13476182743136`,
0.1886240896532882`, 4.4133830990517655`, 1.1858146199794088`, 178.1532549997033`, 20.52454800587001`,
192.079957884837`, 0, 75.23380823465081`, 2.1713143106444757`, 2.192305767590827`, 0.009667627042038385`,
0.09505358887424069`, 0.09519892387106935`, 0.001528979584568102`, 100.29302048220397`, 0.36001269975392514`,
1.2647097299706511`, 0.0009241317466391896`, 0.00093171083649296`, 0.008201308829972831`, 0.07596564959062667`},
{0.15032567895129462`, 2.925200495412599`, 9.469770009612255`, 1.2491630117650723`, 0.5515247294638286`,
0.2864898115312603`, 0.05595545885252959`, 0.4795641847786323`, 1954.175399450165`, 168.25938072536803`,
0.17272189335079902`, 2.4562744379868438`, 1.1923215617571556`, 90.61032933482842`, 15.731113824485663`,
95.80457625179253`, 0, 12.451291339038939`, 1.8243905016474309`, 1.8478987403117948`, 0.012885530067787476`,
0.030488155380064427`, 0.030517085398993078`, 0.0009488937119352947`, 76.23868570350123`, 0.0654736093926598`,
1.0664180843428048`, 0.0002946091862596756`, 0.0002960567593688337 6`, 0.004913536904725957`, 0.04316331625107979`},
{0.051008388371518276`, 3.802328793778008`, 0.48994620522738513`, 1.2897891346361594`, 0.07123389103998612`,
0.5215810111010959`, 0.05373739522257526`, 0.03556482649077374`, 3356.8546057655267`, 95.53240615712758`,
0.0371102186886971 8`, 1.106351331760008`, 0.48227443908576983`, 48.03128919943497`, 3.520122038908093 4`,
60.349535193088734`, 0, 31.138082462343583`, 0.28398498801099586`, 0.29251727172531256`, 0.030044840658923544`,
0.6827249874674151`, 0.6994424003213406`, 0.024486305849064083`, 15.425775670213058`, 0.497495733023963`,
0.22243577431698774`, 0.006397809065429416`, 0.006734360710349156`, 0.0526042027009368`, 0.05084517403994347`},
{0.10228292810378975`, 0.7288905389375508`, 3.433004371974512`, 1.274497031997786`, 0.6101427090150058`,
0.660255061516275`, 0.2170030685035811`, 0.012721245103120268`, 2195.647120536849`, 85.90708396049939`,
0.06296262837152478`, 5.578028384550223`, 0.44836886971981804`, 606.5689073533044`, 56.540967488718465`,
777.377736224291`, 0, 512.6550095843131`, 8.145528013165963`, 8.173413378903296`, 0.003423395719990285 4`,

0.2527104263520099`, 0.2531865795185806`, 0.0018841848887842794`, 84.81711862067796`, 0.36925660528049076`,
1.0948705748152512`, 0.0023837293406056004`, 0.0024073468095448212`, 0.00990778128074754`, 0.07785714909015277`},
{0.19207820059415281`, 3.655701792168804`, 2.510935445862126`, 1.2073260918388902`, 0.9635406057316576`,
0.42073159663388215`, 0.061393125488842015`, 0.03366936773322557`, 1456.9751171045727`, 203.989380011923`,
0.13700463901391435`, 8.26369238323722`, 0.48805011827851597`, 347.5122423568788`, 188.99510690017965`,
368.6394816554474`, 0, 235.10523890189492`, 2.1025203400109813`, 2.1205307237928532`, 0.008566092531488989`,
0.12659813578507806`, 0.1267694559810951`, 0.0013532600219949842`, 109.80267678642146`, 0.34738203028809483`,
2.4527080733531212`, 0.0012620512727590238`, 0.0012728405758967464`, 0.008549021240742194`, 0.18649940821689043`},
{0.2627532955765636`, 1.639112549779572`, 2.4635443376968276`, 1.095970586814591`, 0.7703245554604254`,
0.3899598370308347`, 0.22903116483622232`, 0.3600410344547181`, 1714.6905884118041`, 141.27158792075448`,
0.22143418867904247`, 4.170924599810817`, 1.4302741156765273`, 420.3990516390695`, 49.96298954312423`,
438.9119617078995`, 0, 69.85984908825992`, 14.302417755837073`, 14.415549690651286`, 0.00790998674109078`,
0.28039538710052786`, 0.28061498528119205`, 0.0007831733001564256`, 334.9038919397534`, 1.0524973146440684`,
1.9842794298917932`, 0.003076971027407893`, 0.0031094429859598446`, 0.010553222068946821`, 0.08783139825078556`},
{0.23274157877277596`, 1.9065983093139076`, 3.8223892292706183`, 1.0506044130310364`, 0.03733185970169073`,
0.2700778128853568`, 0.09296420307934006`, 0.03280802595849235`, 1773.9605198177533`, 179.0899731082768`,
0.06001861030669786`, 8.285893379373807`, 1.1764377117465887`, 254.09521154462635`, 37.44380118041848`,
275.87057677138426`, 0, 173.87062475311444`, 2.8322220341771382`, 2.8716346228991894`, 0.013915783524896419`,
0.052844230003717615`, 0.052868093338462784`, 0.00045157881463109284`, 77.14156774233882`, 0.17570070743003527`,
3.3397443548766836`, 0.0006073386738238984`, 0.0006097275572038734`, 0.003933362854919631`, 0.21446589563012822`},
{0.26351647839883513`, 0.8673354775463569`, 5.854038424853368`, 0.9424071816766871`, 0.9232227163729747`,
0.413019745414411`, 0.17796832053957906`, 0.014103729537635504`, 3690.4717118898934`, 307.30915173008475`,
0.17184547983019266`, 6.1008526886318215`, 1.0313875805176784`, 299.60544728205286`, 48.486252334511576`,
317.86242707339807`, 0, 249.2329594398464`, 3.7334699130932507`, 3.7520740973641264`, 0.004983081343613138`,
0.049565780892693444`, 0.04959326597278503`, 0.0005545172414631594`, 46.259584428252644`, 0.18659142899897233`,
1.0971004148755992`, 0.0006350258195013669`, 0.0006375543160629595`, 0.003981722449613034`, 0.08222460329297347`},
{0.22251678082176873`, 2.4705119403336626`, 3.219964315804703`, 1.347383920776866`, 0.9659234897125795`,
0.3313519776076561`, 0.0865111078215019`, 0.112897975300224`, 1782.1419072767694`, 367.3360449099217`,
0.11075042639430366`, 6.566322730486396`, 1.1397550110996595`, 189.47732511989395`, 101.01348124535997`,
193.415994746943`, 0, 73.55474790160682`, 3.1853705210620125`, 3.222636341315235`, 0.011699053534531378`,
0.07557770288981358`, 0.07567858994320391`, 0.0013348785360334237`, 112.42137009529391`, 0.24024724498448125`,

1.8771682076029639`, 0.0006759712749655478`, 0.0006810791014209626`, 0.0075562773812765105`, 0.15122967869762047`},
{0.1726869732489258`, 1.0689003029764699`, 9.291822504855592`, 1.1990435589159916`, 0.8172577994876133`,
0.22377486544985759`, 0.217477012217474`, 0.16181368479198038`, 1626.0973452248018`, 102.05318105921833`,
0.2209045520339541`, 2.2233716047935985`, 1.1920782532980323`, 303.1518299228296`, 20.88518028781105`,
327.06325675172684`, 0, 92.09786177331591`, 12.948467386527351`, 13.006148339711835`, 0.004454654860891 0275`,
0.11028330315549476`, 0.11032071946319963`, 0.00033927445618964214`, 197.72315303628568`, 0.2720641403116801`,
0.9671468743170201`, 0.0011102777405285202`, 0.0011152207113347661`, 0.004452012884535339`, 0.0252490848931536`},
{0.25925369048850816`, 0.417309499595206`, 6.855645959180599`, 1.1773438518732138`, 0.2888562944151887`,
0.22102634108263253`, 0.2071754486228008`, 0.04320874178397733`, 1948.065661484895`, 384.2385254384834`,
0.1833279639586075`, 5.15481721625838`, 0.9762665296404283`, 105.39726676332255`, 23.515347601945177`,
116.34213454686093`, 0, 65.51654904921392`, 5.713265174325113`, 5.778487357845341`, 0.01141592093665289`,
0.022010879159502908`, 0.022022526938959633`, 0.0005291828360112216`, 34.059997585033294`, 0.0815200236143306`,
1.1139554472383872`, 0.00022579146875667533`, 0.00022666334258796895`, 0.003861411753484756`, 0.17678063001145464`},
{0.16707182228455675`, 3.9273258352780225`, 7.016188766085528`, 0.799128142527102`, 0.012996916454743213`,
0.30869623835953575`, 0.08235696915123317`, 0.07054168097616643`, 791.6946431232782`, 51.289432144121236`,
0.13635302362949547`, 6.005214476564535`, 0.8946308584625005`, 589.736261527825`, 62.262224502649694`,
665.8611002894562`, 0, 296.31024938667184`, 5.134198145749433`, 5.1835463076570925`, 0.00961165901797445`,
0.07019508153900988`, 0.07019279591535509`, −0.000032561022862087 62`, 288.05241458911786`, 0.16753743125933063`,
5.530817332320156`, 0.0010610504550273 57`, 0.0010637550549300154`, 0.0025489833116265626`, 0.11336365279597051`},
{0.22880237101805018`, 3.9696692691682696`, 1.9380469146031594`, 1.4177879339964377`, 0.6814855489346618`,
0.5439318492089463`, 0.21630816993687996`, 0.03709761261377448`, 3976.9551811029105`, 217.4437376330418`,
0.19674001267675278`, 8.838870490399184`, 0.20990582924569878`, 320.6279369375228`, 49.61778440153105`,
536.160421070648`, 0, 209.30187531982284`, 1.9164868422988657`, 1.9245081833482212`, 0.004185440187908673`,
0.16579542139076273`, 0.1662312073020424`, 0.0026284556450602015`, 108.68312746627365`, 0.5419197931162707`,
0.6143807607143593`, 0.0014041737238090768`, 0.0014225053107489105`, 0.01305507048665322`, 0.14012518007368835`},
{0.22057802576642993`, 2.653890334277995`, 3.4863828466021776`, 1.1742912829666252`, 0.8455831985967934`,
0.6315585043048466`, 0.21227533056536496`, 0.0900482536994353`, 1462.898116977769`, 366.27377580582504`,
0.07912574234824876`, 7.4931206124945415`, 1.2283305207046857`, 333.6570096595093`, 173.0944273987836`,
329.5559328026428`, 0, 147.10072793285937`, 4.7842049790355015`, 4.824285815369922`, 0.008377742281122202`,
0.09387725558101742`, 0.09404874495019291`, 0.001826740333578325`, 181.3822193009567`, 0.29581799572034695`,
2.39363378234643`, 0.000961496446750254`, 0.0009706799209555603`, 0.00955123051816309`, 0.2724816982972714`},

```
{0.18767448152183636`, 2.9367397535675925`, 4.254981609198115`, 0.9658072683472699`, 0.6065533427003664`,
  0.6047075695535695`, 0.22956691915882899`, 0.016808738900599973`, 3085.080662132773`, 300.13204027351287`,
  0.16815220633174727`, 7.212084588140897`, 0.5334832326025793`, 259.19823914173617`, 49.27597636912892`,
  296.91144923278574`, 0, 208.96875217960965`, 1.1627817941198053`, 1.1684386954869763`, 0.004864972427138126`,
  0.05111220544150028`, 0.051176600227224804`, 0.0012598710067055485`, 48.782678850232585`, 0.13703509508104117`,
  0.9804200053002982`, 0.0006375416292260239`, 0.0006419292129293136`, 0.006882034838440632`, 0.16778257818143635`},
{0.16674052895366065`, 1.1207156804364002`, 9.241855378678228`, 1.116892231935736`, 0.24719875970191896`,
  0.3952404123967743`, 0.24464165205494265`, 0.025889397470345094`, 2185.868573954095`, 178.3786216911565 8`,
  0.08745488697820436`, 7.480105474877458`, 0.1628128610930062`, 191.66228719031074`, 37.91332098269115`,
  378.5946962347414`, 0, 140.0697746210134`, 3.0273108924426713`, 3.04213152795944`, 0.004895643706025243`,
  0.01942480025681576`, 0.01943368977202682`, 0.000457637406487299`, 48.4679255245202`, 0.04627002099488381`,
  0.8455875025908096`, 0.00021006966475423106`, 0.00021070913721238008`, 0.0030440971041543197`, 0.1802276754928562`},
{0.1343775710472654`, 3.3862425535357383`, 3.6167615926265064`, 1.4522710413875881`, 0.41930411990329275`,
  0.6633621503450218`, 0.2054877494749729`, 0.014727056691440865`, 2132.348768069086`, 153.41895938212008`,
  0.07798120175302098`, 2.3819365486087474`, 1.4841799853609898`, 136.3634991387807`, 18.99885930171142`,
  146.38684531062754`, 0, 112.4622486418938`, 0.4743902149921034`, 0.48004925794389985`, 0.011929088697351542`,
  0.14296769268357787`, 0.1438729085330747`, 0.006331611236814805`, 22.94857618553184`, 0.27445216115781684`,
  1.1627806658890922`, 0.0011796956966975136`, 0.0012029890067832753`, 0.0197451852634285`, 0.09912819297012021`},
{0.18395565451029489`, 3.44119115260109`, 6.476610943613181`, 1.4097688201668712`, 0.3016652649845466`,
  0.23377636743924812`, 0.22702955380114842`, 0.044315995698336386`, 2035.6758637667072`, 215.7759126319412`,
  0.23319048350186838`, 1.2339772874754598`, 0.4032881579686409`, 37.414793992872326`, 5.333788525162513`,
  49.72903660911365 6`, 0, 22.888878587381964`, 0.2865935349457901`, 0.2891614760269959`, 0.008960219851754436`,
  0.04114786603399779`, 0.041268443925921036`, 0.002930355898010051`, 14.08890195497318`, 0.1081340375426553 1`,
  0.18447977125686824`, 0.0003507680676443359`, 0.0003557967486589161 3`, 0.01433619955303089 3`, 0.02466355946925294 4`},
{0.1877085328273454`, 2.4698348389855864`, 6.723740484115233`, 0.7818601707856943`, 0.1041858328478078`,
  0.45810434108336306`, 0.1358172536153655`, 0.022077708334214 59`, 1185.161236523103`, 108.74647947804544`,
  0.020759548694208763`, 9.734619845644367`, 1.2692223563341272`, 561.0925084420858`, 86.79021162533793`,
  608.1467023989247`, 0, 427.89722922681096`, 3.6631447203748606`, 3.7078872861412226`, 0.012214250099784163`,
  0.042060242456801476`, 0.04206655094552089`, 0.0001499869794114872`, 129.2480350089702`, 0.11278666288478412`,
  6.748508244675498`, 0.0006499201800066468`, 0.00065150555582003`, 0.002439339263733853`, 0.3386216775621174`},
{0.22900819014324658`, 0.8599246184787699`, 4.575509667907129`, 1.1011546323816885`, 0.08315194396691528`,
```

0.18390636972505037`, 0.1408377449166481`, 0.3122650757195923`, 2947.0026584149073`, 167.82607804795214`,
0.10792455151802871`, 5.931199053322571`, 0.9798220386267653`, 201.8419073321254`, 14.489874346255709`,
235.58660844462923`, 0, 37.955914241861336`, 12.31999496978009`, 12.411390180211317`, 0.00741844543406156`,
0.05134603978199799`, 0.05136041714259 7925`, 0.00028000914308057 29`, 151.34667105783572`, 0.1679809091645654`,
1.3067921207954336`, 0.0005633051613413986`, 0.0005652531054870248`, 0.0034580619516915156`, 0.08983453538698141`},
{0.1416858046718097`, 2.2139997848909125`, 1.7657175272923222`, 0.989271832961955`, 0.13788431085704778`,
0.5209143755701833`, 0.21392682943087216`, 0.02217837535493898`, 1932.3838117923324`, 368.90142938726285`,
0.22169599764103476`, 7.130639713263575`, 1.0904656940263826`, 142.67632660031822`, 42.45702037360813`,
152.78613970816866`, 0, 108.60131558057734`, 1.03683758805616`, 1.0520940889657393`, 0.014714455846630559`,
0.07006791013448956`, 0.07027383551684949`, 0.0029389399792953963`, 32.79368852747358`, 0.14182326041544854`,
1.9217992479470256`, 0.0008498256310464081`, 0.0008612233376808068`, 0.013411817928301817`, 0.45261652192267415`},
{0.10996511298893497`, 0.7862218358455948`, 0.8377076631006055`, 1.462919598856414`, 0.4311311224143921`,
0.3856327187176355`, 0.0881986225258021`, 0.45328063368772087`, 3501.1649053241954`, 360.18740869055375`,
0.12284319478126604`, 4.367675405932559`, 1.4276764859907565`, 84.2652922031809`, 16.23481089907895`,
87.51817428768446`, 0, 12.115115963998765`, 5.85464541908372`, 5.967507508671917`, 0.019277356954925606`,
0.20913546369863903`, 0.21016279285262135`, 0.004912266603729565`, 65.75785813738578`, 0.328537212794426`,
1.0742615220345042`, 0.0017127869654032235`, 0.0017503281595890499`, 0.021918192363747036`, 0.14939649765081453`},
{0.2044811200034678`, 2.892822767813837`, 7.595852124568353`, 1.216932964982977`, 0.7343890780610787`,
0.5330606684550935`, 0.058989793379298994`, 0.012080434529966445`, 2521.088434314187`, 248.82760985623736`,
0.04480645758683227`, 4.109426945013643`, 0.10374011787051507`, 81.00936821632949`, 38.40149456254 5025`,
124.24726490709979`, 0, 69.04312993555638`, 0.2797965066457519`, 0.28168941051857543`, 0.006765287728270675`,
0.019774982840899086`, 0.019808459635581095`, 0.0016928861557730102`, 11.562881496851542`, 0.05776586627650778`,
0.42235488099669577`, 0.0001955850565776851`, 0.0001972382187998938`, 0.008452395347249286`, 0.14122502711414459`},
{0.04803156621959867`, 0.5827754735791681`, 9.214605590518953`, 1.1720588669143222`, 0.8133320484998057`,
0.6073432395586391`, 0.13364149823347654`, 0.026188730330046147`, 821.8332545613489`, 133.84484048922508`,
0.12502450253620295`, 2.6224761178996694`, 0.4958664794043681`, 212.0691133526271`, 94.41347387662846`,
222.81237544200005`, 0, 154.57003582982836`, 6.150502094363651`, 6.191585629960623`, 0.006679704350417426`,
0.06440600478565724`, 0.06454146230200052`, 0.00210318147809474`, 51.20516815417773`, 0.04419316119714904`,
1.2664482276757996`, 0.0006606375070140613`, 0.0006669645020504839`, 0.009577105400841113`, 0.08356100872086808`},
{0.1676455194581572`, 2.242553618614152`, 0.4702874552580045`, 1.338910822252204`, 0.5222950299457514`,
0.5002656056336167`, 0.1815577312255944 3`, 0.023205215351886358`, 1812.861489403048`, 208.09707961592778`,

0.04482084976511336`, 4.8128979171410045`, 1.2921971759159723`, 225.34526715302866`, 44.59617336687893`,
235.1163613652542`, 0, 167.59321149254157`, 1.6627440855872353`, 1.6982438835039466`, 0.021350127313292333`,
0.8171936462921378`, 0.826441256680953 7`, 0.011316302360860453`, 53.26846808518481`, 1.9571264761507607`,
1.9268059788870087`, 0.007250533680469684`, 0.007566446905256915`, 0.04357103059022904`, 0.2284169256894474`},
{0.18135996247125585`, 0.9876459274110312`, 2.54354039452296`, 1.3578342543318755`, 0.8930073147262432`,
0.19298717253815956`, 0.057956724513287594`, 0.0261548554577047`, 3458.0576394641494`, 159.9558341984582`,
0.04888106360623701`, 2.7801567336765522`, 1.2025006457139424`, 129.5226479400083`, 12.638791350813488`,
141.01573886320986`, 0, 94.09503255561964`, 2.306488086140044`, 2.33176381218727`, 0.010958533104554302`,
0.15525298698536172`, 0.15546471806001771`, 0.0013637810052309352`, 32.542765212832606`, 0.40223822704591866`,
0.8256345166824473`, 0.001380425184911327`, 0.0013920389501642194`, 0.008413179779560798`, 0.02667109925368954`},
{0.06724626093304326`, 3.981403022919509`, 8.288988323065759`, 1.0703538801483565`, 0.1653330791428167`,
0.4148194823615189`, 0.14440389123820263`, 0.19094336267448156`, 2003.8545802245517`, 246.03939081881197`,
0.139046230518015`, 4.691983788055152`, 0.20050055393273314`, 91.70057564591112`, 24.405379532719014`,
133.67770331463902`, 0, 25.049972358938266`, 1.1510507332878284`, 1.1593724232840832`, 0.0072296465790739894`,
0.01582431058078089`, 0.015838625639857787`, 0.0009046245018904386`, 65.46852670065525`, 0.015201795977168728`,
0.641575310587423`, 0.00017833518212784227`, 0.00017919571628293525`, 0.004825375143734156`, 0.1673106314178123`},
{0.21881875684613517`, 0.5898239021275495`, 9.93413148158805`, 0.7544358459883748`, 0.3693391180254211`,
0.6579008857369009`, 0.12709107324060326`, 0.48252891951701626`, 2900.587783201154`, 140.00284155203332`,
0.2319315634448451`, 3.003524790772248`, 0.5189129901116001`, 134.59414529048343`, 16.60686013653917`,
163.5452247985029`, 0, 17.52128752507207`, 12.410796920648805`, 12.469943858051025`, 0.004765764662848726`,
0.02132684676009731`, 0.021351391390511832`, 0.0011508794849337534`, 104.57406668928073`, 0.06666734422132228`,
0.5608688403729135`, 0.00034083184649258413`, 0.0003428298562260011`, 0.005862156820079845`, 0.06037816465210098`},
{0.18529128706683434`, 3.034817908847505`, 4.618773666141445`, 1.078127573609324`, 0.45431233294938433`,
0.23204854612180326`, 0.21276533477653692`, 0.005983047100273517`, 2838.235019182518`, 133.97625538243244`,
0.13415379883362671`, 8.373997227624333`, 0.3370984440619733`, 423.99847746124726`, 31.426267449939147`,
641.6290457133032`, 0, 390.0457141734218`, 0.7499510057054601`, 0.7527173356052438`, 0.0036886808321319187`,
0.07414214027024751`, 0.07415168794321804`, 0.00012877525433885317`, 32.5137820410446`, 0.1962556085223143`,
0.8644348771852138`, 0.0008306696964877069`, 0.0008331407858097469`, 0.002974815781156437`, 0.06014840021717278`},
{0.11830138320073424`, 2.557281635675193`, 6.588149241112692`, 0.9721130031371765`, 0.10225884860358048`,
0.23082972097158927`, 0.158140431907085`, 0.0665459599837257`, 1023.3689587458948`, 34.93006289140902`,
0.0483952400928380 8`, 8.085053919685542`, 0.4877285564517453`, 1176.7526427865603`, 62.64875350408371`,

```
1627.9996375070914`, 0, 604.5527102738505`, 15.235620907792153`, 15.279861988215067`, 0.002903792414544082`,
0.13485112252845413`, 0.1347860431352028 4`, -0.0004826017910051217`, 556.5967650800836`, 0.22790106173311608`,
3.078247312917056`, 0.0016757952640189533`, 0.001679006685250063`, 0.0019163565502673396`, 0.062204114054729136`},
{0.09989873104684788`, 3.3669591438562216`, 5.451558876338645`, 1.2627430306397467`, 0.36748912342215223`,
0.31108122243153336`, 0.12542210883620514`, 0.007371687908480407`, 3955.966637225736`, 182.23968139905298`,
0.15451142826141562`, 8.671574224679677`, 1.2389048546471688`, 337.3206557082074`, 24.448858550506912`,
374.42059874309433`, 0, 304.9989732842535`, 0.6495136531014087`, 0.6545630342973769`, 0.007774095543423121`,
0.05652138926120213 5`, 0.05654389316197301`, 0.00039814840125185214`, 31.24122761956063 2`, 0.08066307234572659`,
2.2106354456163886`, 0.0005407635213102724`, 0.0005422978411643778`, 0.0028373212941357195`, 0.13626464886854187`},
{0.22609069450991742`, 1.379608652743129`, 6.641039030693791`, 1.0528553454528264`, 0.9110900221670273`,
0.41585330034333745`, 0.15736472782730937`, 0.24498648200968076`, 2031.8230399130634`, 206.889154659356`,
0.10264734406954457`, 2.177599971086371`, 1.0407277908428236`, 139.8185881526575`, 29.629441039968555`,
147.06403508747815`, 0, 31.54099916356741`, 5.2155993399658085`, 5.251246258746511`, 0.006834673535512925`,
0.06367415873371893`, 0.06378283749602509`, 0.0017067954169704969`, 102.79265683797405`, 0.2056590681490294`,
0.6813754389219765`, 0.0007285795802954853`, 0.0007347919164815038`, 0.008526640540075237`, 0.039294559094035966`},
{0.14149819408460695`, 3.9050575954333624`, 9.383356210665369`, 0.960335311543541`, 0.3424013679631459`,
0.21333414365316794`, 0.05126386183615583`, 0.4919218322625912`, 3186.224620232545`, 108.82685651649962`,
0.06233434212075295`, 5.950211096913752`, 0.24827529292780603`, 186.56826801389326`, 17.18122258610968`,
329.72833275629074`, 0, 23.753424770745088`, 2.8660667160547506`, 2.876156978842834`, 0.003520595920381453`,
0.020136184747616238`, 0.020139635393599992`, 0.00017136543128715154`, 159.88793712211944`, 0.040703339679342886`,
0.5965833069911368`, 0.0002535064664486164`, 0.0002539573550262649`, 0.0017786077963415359`, 0.04303613232027338`},
{0.1828220078605639`, 2.600312322938115`, 2.7331064358556674`, 1.452306554796921`, 0.9684456895629803`,
0.28153635898957774`, 0.13708089241679355`, 0.10257553866825626`, 1556.8951227385596`, 164.52245666603233`,
0.13014520838075316`, 8.353642416878472`, 1.085154972102886`, 694.4508168663989`, 131.29307570821146`,
737.9052681713084`, 0, 283.22151785027916`, 10.75381380729924`, 10.820368261333595`, 0.006188916344188611`,
0.27688187924826235`, 0.27692654724103943`, 0.0001613250852614545`, 399.4753508814608`, 0.7231443014911118`,
3.276260622917067`, 0.0022984245612943655`, 0.0023116530871058116`, 0.005755475308703017 5`, 0.1118238522021153`},
{0.21889388250937136`, 0.5333175361369764`, 2.4121127215145144`, 1.3818810352832893`, 0.021571596232064838`,
0.5244501966414251`, 0.09076416020812753`, 0.010775282992960485`, 1502.4651132234349`, 214.3639418846526`,
0.02290218457939025`, 5.386530044496094`, 1.4845771171901991`, 141.1929673085608`, 37.43859453682166`,
145.93931853746557`, 0, 122.30026243641508`, 2.136519703576679`, 2.1853215747936217`, 0.02284176042713071`,
```

0.09365865887772634`, 0.09401671704285965`, 0.0038230118755038234`, 16.27776320313734`, 0.2928758210338134`,
2.7661861980134805`, 0.0008133196497899231`, 0.0008258857485683249`, 0.015450381386515888`, 0.2684151709797969`},
{0.08341672306903047`, 2.823299860683674`, 4.834884605952357`, 1.4354641770214007`, 0.9014467152160488`,
0.6047529781600969`, 0.2470533033127953`, 0.006202153420058688`, 2417.2420994663553`, 158.0041607308176`,
0.0735259731187195`, 6.516775962062823`, 0.4133308952289383`, 658.3241821206649`, 100.76361797765395`,
797.6522933458414`, 0, 603.8607308626113`, 1.2922941774298211`, 1.2963422115244796`, 0.0031324400940266894`,
0.18793629327688022`, 0.18817601995963648`, 0.001275574177697969`, 52.12191387285622`, 0.22395756758428928`,
0.9927073590645151`, 0.001576360125449816`, 0.0015879414911530644`, 0.007346903487515988`, 0.07483628046444818`},
{0.16837082644708545`, 0.688574652311627`, 5.390405754713122`, 1.3217406774272815`, 0.6156990878136903`,
0.18352461332079384`, 0.0882858818891156`, 0.4733971203783309`, 2234.460265184529`, 202.49554378641346`,
0.1503105450782266`, 3.806358767200937`, 0.9148594831472519`, 137.9681654924613`, 18.70524091765933`,
151.72245582920888`, 0, 18.59633343620029`, 10.997932465140126`, 11.09141313314464`, 0.00849984015639449`,
0.055692363642535325`, 0.05572106993250389`, 0.000515443915306113`, 108.18425033329424`, 0.13395670418985056`,
0.9809756303173014`, 0.000508765981778736`, 0.0005110475428513213`, 0.004484500053656415`, 0.04956598227658542`},
{0.10718623369591462`, 2.960462485410858`, 9.423477882136847`, 1.1211033662754974`, 0.7860054376705241`,
0.5141091538304853`, 0.05867214344346472`, 0.09405923440011967`, 1537.0957508113852`, 239.00629492907444`,
0.09357298190053748`, 9.410898004520863`, 0.8654487723896875`, 309.166899851744`, 158.6070244887968`,
314.74944093462796`, 0, 133.93102800187665`, 4.046299502593468`, 4.094330323699657`, 0.011870307938254054`,
0.02452132526006269`, 0.024531963031859182`, 0.0004338171645974498`, 171.12739831663694`, 0.0375478357124161`,
3.5829193271865316`, 0.00026421657826858347`, 0.0002649502302695105`, 0.00277670691875076`, 0.2665574626176616`},
{0.08165938583300264`, 3.5663918013786944`, 1.9719703539316173`, 1.0863058189118182`, 0.9278717656325091`,
0.29627245453078765`, 0.07723831560352282`, 0.005496209135881868`, 2965.7691899558304`, 36.38856085381832`,
0.19440710566976382`, 4.620185477920922`, 1.4555441950836703`, 1111.2832310595259`, 35.61209605242687`,
1232.8203035933977`, 0, 1027.9117640676527`, 1.5480798662541195`, 1.5573643741676166`, 0.005997434703393312`,
0.8330940791938833`, 0.8304939940385176`, −0.0031209982405366876`, 78.8722763269731`, 0.9718564406869767`,
3.663071647206999`, 0.009232579606140212`, 0.009269974959305852`, 0.004050368885069711`, 0.027912882830669856`},
{0.18162340679997874`, 2.5029500310491875`, 3.7850875297449385`, 0.8714265661124054`, 0.04795600587846183`,
0.17826165391264504`, 0.1376006876951453`, 0.011710367976498486`, 1897.0378575605146`, 378.5017307178937`,
0.04683765268043216`, 7.517716852692461`, 0.3647662687926059`, 106.09874031294105`, 27.81384021112243`,
129.79266940534777`, 0, 90.93472802479782`, 0.40886250557514664`, 0.41368549315700953`, 0.011796111201437753`,
0.020377672034680987`, 0.020384603309140065`, 0.0003401406425267428`, 14.619463157488036`, 0.052872317394122285`,

1.1235628036035368`, 0.00028254735080701643`, 0.0002833752580045284`, 0.00293015381367856`, 0.19546087689614794`},
{0.10569564926832292`, 3.94925758524324`, 2.123437162636053`, 0.9889632230650253`, 0.6091744144977718`,
0.44858710373952704`, 0.06967757399007424`, 0.06579434912735219`, 2048.3556961935665`, 192.929988723693`,
0.1607054226404262`, 3.065879080542288`, 0.48218127318000126`, 103.96239734093291`, 25.894801241559875`,
116.51824556212729`, 0, 53.80269813708268`, 0.8692754877578892`, 0.877274251577394`, 0.009201644279808185`,
0.09849581045330395`, 0.09891150967263392`, 0.004220476154435326`, 49.0427544813409`, 0.14872255194392892`,
0.7072555047741426`, 0.0011942591915860001`, 0.0012136555383039768`, 0.016241320857843355`, 0.07964236426451289`},
{0.1295394448875809`, 2.022900103755206`, 6.745994489858977`, 0.9580215588674443`, 0.9679692042597987`,
0.6904939360195417`, 0.05882320965699680 5`, 0.04912899750957061`, 1189.5399921293747`, 163.6733664974323`,
0.16033185329150101`, 5.9616048020309655`, 1.2439833727010554`, 319.2702189795281`, 253.85476295305983`,
321.076022484709`, 0, 189.11814104519613`, 4.348360937968629`, 4.40380789603914`, 0.012751231754099202`,
0.0476765400646775`, 0.047726830883194085`, 0.0010548336445630468`, 125.66142560831177`, 0.08822846477359128`,
3.880937906773144`, 0.0006000575853438717`, 0.0006033409978828666`, 0.005471829069727319`, 0.16908200433958157`},
{0.24619035501818215`, 2.0773850626788413`, 3.018461478313549`, 0.8871662168885301`, 0.552617674236112`,
0.602322011487733`, 0.20434748314233808`, 0.03902325854898381 5`, 3150.0536016394335`, 203.54823250165975`,
0.24910387895313074`, 1.7034902327529498`, 0.8084130559906435`, 78.52578760000311`, 10.190724289882855`,
88.38879165559429`, 0, 50.29461075295866`, 0.9077559542575393`, 0.9162305875527479`, 0.009335805791701013`,
0.08415443912116369`, 0.08464386164077443`, 0.0058157659265731 8`, 26.939409427605657`, 0.29597158919412114`,
0.3482776495246285`, 0.0011372282495643038`, 0.0011606482147100067`, 0.020593900261161924`, 0.04617738191435557`},
{0.18194781074756367`, 2.220335509721994`, 7.082070220373439`, 1.2346660258162963`, 0.911357302435295`,
0.2993417896543701`, 0.10379984911373669`, 0.06373968962233406`, 1006.0135021400088`, 377.69931275745535`,
0.06750680684166793`, 5.51898040870112`, 1.3240328384144195`, 165.34286441710015`, 122.95833896916115`,
164.9350169637921`, 0, 87.45735278765568`, 2.3768020743602056`, 2.4084299765383994`, 0.013306914580469398`,
0.032744967917252205`, 0.032766432939532256`, 0.0006555212493808416`, 75.3899720754693`, 0.08511250322219613`,
2.0563474398678823`, 0.00032015135555096 83`, 0.00032149925317465766`, 0.004210188713303031`, 0.16409350165932868`},
{0.1644023253774144`, 3.882290753800083`, 5.67814834147805`, 1.1217171634204655`, 0.4202214546941414`,
0.41106474358929035`, 0.06359886932295966`, 0.24993914606767362`, 1680.5799994030467`, 230.96461331819444`,
0.016083788677699107`, 8.870452591991157`, 0.6584854072019537`, 241.24664805751925`, 71.06269132026577`,
256.6988536365437`, 0, 54.83191549512951`, 3.2996391710434407`, 3.337930899891263`, 0.011604823092130179`,
0.03369379739220248`, 0.033714135610466954`, 0.0006036190586573564`, 183.00226635169258`, 0.07913340917253509`,
2.6953034912395206`, 0.00036260003106103333`, 0.0003640175871929822`, 0.003909420878428493`, 0.2536929837683777`},

```
{0.11290442193886385`, 3.342282901193297`, 2.898098788178677`, 1.2208920487711645`, 0.15571856198486667`,
  0.6450091875601396`, 0.07200970199664747`, 0.18140557939535418`, 3133.8759234972686`, 359.44606615721364`,
  0.05885138914392884 5`, 2.4968280987996607`, 0.44903712610868673`, 36.16688804351768`, 10.204365575213874`,
  39.98118346449747`, 0, 10.307185412951616`, 0.5284570991906103`, 0.535352555972579`, 0.013048281104615311`,
  0.037910828026159527`, 0.038204049482729714`, 0.00773450414662169`, 25.232187523414016`, 0.06114714462166147`,
  0.3797326667339076`, 0.0003700017415002588`, 0.0003798594242325433`, 0.02664226036427353`, 0.13028924514782578`},
{0.05717970106523162 5`, 2.855281586486381`, 5.482300889649739`, 1.2083595069262243`, 0.795518738527804`,
  0.6366469580580449`, 0.11918231638825821`, 0.16920403900000133`, 799.767703151836`, 247.48605975500072`,
  0.10228761215396959`, 9.288149547399101`, 1.2673098981919697`, 423.0008229944361`, 355.2405520709499`,
  414.2865797155583`, 0, 127.38159740997992`, 7.071230762807031`, 7.160012690954144`, 0.01255537135261986`,
  0.0629397567955939`, 0.06300150801245887`, 0.0009811162293733577`, 288.4336427262718`, 0.05141252112416643`,
  5.161360226146286`, 0.00062779036772731`, 0.0006313391739802825`, 0.0056528523459506985`, 0.41059881396106285`},
{0.0875578111208043`, 1.1766586937430805`, 5.077956330142905`, 0.8750465005858246`, 0.8596423212250299`,
  0.6288939270221594`, 0.1488852290513452`, 0.012846012396330703`, 3117.450697007702`, 283.31782716202474`,
  0.08644475242948957`, 6.632814251236063`, 1.2827414734862095`, 300.5651896738278`, 73.76769902650184`,
  308.89860900056226`, 0, 254.0096529883742`, 2.5989013640383365`, 2.616892473576971`, 0.0069225826680390234`,
  0.04895892653597319`, 0.049005720578858325`, 0.0009557816356686732`, 43.68599834537795`, 0.0612390920330718`,
  1.79313660087819`, 0.0006749350891978034`, 0.0006782120100408832`, 0.004855164438071657`, 0.14176023297684817`},
{0.1753150595055662`, 3.713574756709245`, 6.279606102538107`, 0.9857257532666506`, 0.18592289869330236`,
  0.21496347741152755`, 0.07049642727707597`, 0.017218370675811997`, 2472.055030254328`, 256.95308160663865`,
  0.22538544536854038`, 4.255341860945123`, 0.22574651763666997`, 66.63235108716884`, 13.886160565210373`,
  98.09897479624235`, 0, 53.510598676782145`, 0.24132753385840994`, 0.24336882303434307`, 0.008458583831262079`,
  0.015409447273636625`, 0.015418610125190713`, 0.0005946255820457047`, 12.802683397649806`, 0.038592973796096636`,
  0.48678087836739914`, 0.0001887806121685376`, 0.0001895098863397403`, 0.0038630776901582564`, 0.07927150472991518`},
{0.16802857593346404`, 0.7065678176512091`, 9.645249981168057`, 1.3116063027060454`, 0.32088080529355767`,
  0.3691390904550721`, 0.21874840715793664`, 0.07094779858961066`, 1630.2542224830704`, 63.80576916127296`,
  0.10975436657116389`, 7.114567585043865`, 0.902530053910509`, 858.0859196573565`, 50.083931868098055`,
  1023.1059629001267`, 0, 427.4987660754567`, 38.781451040301874`, 38.934092319250105`, 0.003935935217834086`,
  0.1029133884800018`, 0.1029243837643627`, 0.00010684017427964321`, 391.45321752704814`, 0.24703414443965446`,
  3.089471085540289`, 0.0009477106793006618`, 0.0009503473557605711`, 0.0027821533696918976`, 0.10102507522659851`},
{0.22967780600547444`, 3.4707114805627004`, 7.357111611957344`, 0.9509066717740751`, 0.7301577279137197`,
```

0.34891003518552344`, 0.14994464399131946`, 0.18785696851097627`, 1448.9102951912027`, 296.3879865990905`,
0.13500832540748392`, 3.275227564043599`, 0.3389151549206586`, 109.09400111272782`, 40.21515534455434`,
123.25511528540363`, 0, 30.06622113247406`, 1.5600993160142742`, 1.571291670093385`, 0.007174129213584290 5`,
0.026972443790005612`, 0.02699931374085162`, 0.0009962000868444232`, 77.35220867012525`, 0.08849959588985903`,
0.6655697489791106`, 0.00034200404308071253`, 0.0003440849184371368`, 0.006084358938216461`, 0.08379816940238215`},
{0.10636965549911737`, 2.776846234427638`, 9.809572024338209`, 1.0633435610610082`, 0.685773597384219`,
0.389828683015383`, 0.18173035709309476`, 0.0701248233933049`, 611.7373128577356`, 348.65549321322396`,
0.0858335193643106`, 8.943700469577841`, 1.0373885202439106`, 316.1134879560495`, 267.8493775251145`,
310.9121265675433`, 0, 159.9477248500539`, 3.838386952794567`, 3.889858098660768 7`, 0.013409577121641592`,
0.02404176072198478`, 0.024048569396908755`, 0.00028320200848463983`, 152.2658622306252`, 0.03653305436560664`,
4.162761085958271`, 0.0002732137958968428`, 0.0002738352247667586`, 0.002274514974164754`, 0.4643981079947567`},
{0.12945851018082827`, 1.6530391126563773`, 0.25296383859783056`, 1.0004082847943978`, 0.6153536990324371`,
0.46726548359691056`, 0.22184757314007908`, 0.029263173246622916`, 2804.4069174712213`, 201.7772707782526`,
0.15804840166726702`, 6.079299069162072`, 0.5497928673982018`, 317.77217472281234`, 39.86738197690221`,
371.66524802557564`, 0, 221.77314641812643`, 3.7508252856904276`, 3.80585321274372`, 0.014670885168452497`,
1.2756971352652462`, 1.282861107146365`, 0.005615730946694652 6`, 88.57515574266864`, 2.359283579619902`,
0.9392146288443952`, 0.01518339529485202`, 0.01570426107990801`, 0.03430496110659731`, 0.17280569962118938`},
{0.17704121544802504`, 3.3101767206813077`, 0.17894892947703944`, 0.9519221706044084`, 0.3716159634368834`,
0.17251563798408087`, 0.06428454684265117`, 0.050202794311224846`, 1196.331297175113`, 92.7477589491034`,
0.08675697489654427`, 7.03036379115748`, 0.9556798715945236`, 453.9075085423879`, 52.2384533451025`,
499.20249998551753`, 0, 261.5446226554828`, 3.8272801104909746`, 3.925900854733879`, 0.025767840710841927`,
2.1371092276020645`, 2.120629158756165`, -0.007711383504899527`, 180.98533607533994`, 5.405091645712257`,
3.9121472924877065`, 0.026719833098640122`, 0.02742041705980619`, 0.026219623400332015`, 0.12935097912916665`},
{0.09051269700635717`, 3.510668586088882`, 4.372912512921815`, 1.3267853376570602`, 0.8120222411850102`,
0.239383728318748`, 0.21506169431109157`, 0.20950664990140405`, 1339.6563194330593`, 329.7735045463686`,
0.2280878170659803`, 2.1716129406234614`, 0.869217412898017`, 93.78990893462105`, 25.603875456937278`,
97.4642287903841`, 0, 23.881472563029966`, 1.3629965760129106`, 1.3743447745682773`, 0.008325918608367289`,
0.08193446215729965`, 0.08210207079750519`, 0.002045642770971634`, 68.3575608907892`, 0.10594441639440488`,
0.5895476396035275`, 0.0007425764691519898`, 0.000750613717384544`, 0.010823462049279087`, 0.05161170858640124`},
{0.10777187590532172`, 0.739057715374912`, 1.4330240793355227`, 0.7755224108621632`, 0.12555368221137964`,
0.545351416919415`, 0.13748218086765884`, 0.05919866095169625`, 3976.4209480252466`, 374.54745407708265`,

0.06213693455604074`, 7.297702295605297`, 0.10338190129278124`, 62.68878372406737`, 21.39231271323534`,
133.4075198281132`, 0, 34.128545903469146`, 2.464612704519918`, 2.4852051836281954`, 0.00835525966027517 2`,
0.02905764503556778 5`, 0.02915107382044864 6`, 0.00321529101090267 67`, 26.021300495521103`, 0.04473709878393864`,
0.45867514763041334`, 0.00044901419981857593`, 0.0004555791137906842`, 0.014620726860666844`, 0.398718169287977`},
{0.05012776858713164`, 2.0872339482718107`, 6.705710827191493`, 1.4141058091423697`, 0.06890952245028781`,
0.47612787079844376`, 0.0847118039582583 4`, 0.016954072038827265`, 2788.124252935484`, 108.68029812587577`,
0.08106980932549152`, 8.734393861782642`, 0.5389481104734093`, 387.61488273547025`, 32.67941178779199`,
478.6302161475129`, 0, 312.19591011714755`, 2.4393932728891357`, 2.4523662861842115`, 0.005318131126807346`,
0.05869632946326963`, 0.05873743479832662`, 0.0007003050349632112`, 72.73692074800122`, 0.04203308600352008`,
1.842392729083462`, 0.0005009699784018773`, 0.0005029634693042126`, 0.003979262207876477`, 0.1335620510184652`},
{0.27321591675550233`, 0.6886264672292262`, 5.359177927011217`, 1.2203089609351563`, 0.741856408018519`,
0.5849406177035197`, 0.07433546983180528`, 0.21690743690508418`, 1384.5673397625906`, 70.87997184926286`,
0.03337979870016538 6`, 1.435341337958821`, 1.463005840477979`, 154.07824453878644`, 25.627025875066813`,
160.91520790028764`, 0, 38.51220874569146`, 10.594689936115735`, 10.748404649780607`, 0.01450865618453645`,
0.15212089061558423`, 0.152709241009631 5`, 0.003867650206795581`, 104.22548431566275`, 0.593740694102756`,
1.4665908020049807`, 0.0014997139116090796`, 0.0015223045492978838`, 0.015063298082342857`, 0.032592848912548836`},
{0.08919135792007882`, 1.9079775156637941`, 0.7718276581060107`, 1.2887666627276828`, 0.6966500412335945`,
0.2943209647073368`, 0.16567285203320248`, 0.2074475031655441`, 1406.902570296439`, 161.54627762916846`,
0.02754527329468942`, 1.9201358824343482`, 0.9977637383308959`, 125.07449864811512`, 20.804019896980947`,
133.335970166387`, 0, 31.736868253850393`, 3.2491223812955305`, 3.309579756200631`, 0.01860729385053017`,
0.6717896756914198`, 0.6744583270888833`, 0.00397245074467234`, 88.56074927359835`, 0.8559690487373051`,
0.7637572734105867`, 0.006228160174682751`, 0.006414016343068348`, 0.02984126341854476`, 0.06069773982781825`},
{0.1308447304032681`, 2.6119158907935276`, 7.388927202269429`, 1.1426009806290098`, 0.6170224096703181`,
0.5032614025378099`, 0.21866608861569725`, 0.005956991186122966`, 1137.1164610175856`, 368.0725112372012`,
0.24678418452608802`, 7.5428083328821245`, 0.14982171102850428`, 214.5317194830491`, 127.09826951151855`,
250.48194579808265`, 0, 197.56190659458443`, 0.43531209561982176`, 0.43914061378164204`, 0.008794881190629322`,
0.027580460427605192`, 0.027601613386443173`, 0.0007669545217892093`, 16.242836857200615`, 0.05155368441499549`,
1.5934222847776116`, 0.00029120630664947 19`, 0.0002925436299882714`, 0.004592357061858676`, 0.38211998356018373`},
{0.06751611881159836`, 2.2474089970429993`, 6.402097457423925`, 0.9363574880786951`, 0.3836841636235191`,
0.3647284912476121`, 0.21335232520714842`, 0.22248453220280073`, 2502.3638711494295`, 397.5911497982886`,
0.16288128573213767`, 9.005290951575024`, 1.0427120513238162`, 201.58055504590823`, 43.958913410096116`,

218.7487432099531`, 0, 49.58596485118722`, 4.589952179845923`, 4.631705174805864`, 0.00909660783466859`,
0.020541897319034226`, 0.020549747164029474`, 0.00038213826470534507`, 147.3642832140411`, 0.01981298828581255`,
1.825970426215847`, 0.00026504068942045933`, 0.00026573098599094725`, 0.002604492812018133`, 0.3190709513587092`},
{0.09177879720493187`, 2.6455642646824806`, 3.5115323646466643`, 1.0647038743499257`, 0.023437829577520386`,
0.20767277287096642`, 0.13833050574986264`, 0.16097998475232683`, 3330.8307600954595`, 36.94740532139957`,
0.0838397051953012`, 4.654426462364112`, 1.1418435912898404`, 649.4449938066768`, 9.96963982821956`,
799.7146878547653`, 0, 197.8598223274629`, 11.624839271368712`, 11.667501410333568`, 0.0036699121569732096`,
0.26557155735581717`, 0.2653513130540573`, -0.0008293218744986897`, 439.3465622715814 3`, 0.3481976872279511`,
1.8248207391193503`, 0.0030111951532916237`, 0.0030199038593956905`, 0.0028921094982989892`, 0.03248547603610966`},
{0.04551056845008081`, 1.6215675270048893`, 9.700566391026626`, 1.40945752023491`, 0.4723808944761221`,
0.3499440053674635`, 0.2064578553856145`, 0.09474747167065985`, 3852.6928203016614`, 220.886718735037`,
0.22402828336678815`, 8.267759522981173`, 1.1674803222752286`, 344.75833929594864`, 28.152299787298514`,
385.5559053695072`, 0, 147.43649267735447`, 8.16291981634292`, 8.204882235119744`, 0.005140613863780841`,
0.03801674869940604`, 0.038029967560275775`, 0.0003477115040597667`, 189.09608142466274`, 0.024716626342139073`,
1.736445574944273`, 0.0003259386031015943`, 0.00032669571918095703`, 0.0023228794385141693`, 0.1499605721129162`},
{0.04140464424456583`, 0.7619598093834323`, 9.994885078827323`, 1.3154910694140878`, 0.5846504333106828`,
0.5277457306392407`, 0.1974778280215821 5`, 0.20923520620939917`, 3747.8984478066395`, 346.71554418059986`,
0.04757978117327022`, 1.731006219467508`, 0.3938580217562748`, 43.560760926098524`, 8.35759113599478`,
53.416498234137656`, 0, 11.034036431590819`, 2.7339815412196815`, 2.746349081417241`, 0.0045236370513468405`,
0.020716813283129538`, 0.020777835059126986`, 0.0029455194273118046`, 29.759772200079684`, 0.012253889769514635`,
0.1648267316007092`, 0.00018918551848268184`, 0.00019135182847043107`, 0.011450717819860845`, 0.04113736682310923`},
{0.2669155508949231`, 0.8202069836030885`, 1.7120733781356066`, 0.7716732548944238`, 0.42105306969825285`,
0.4443694926042744`, 0.19913166298690133`, 0.07350737572131678`, 3667.631913873951`, 382.69157822195143`,
0.19936982959712085`, 3.809394225029287`, 0.38364950845782375`, 75.68388603116374`, 14.292487305830784`,
94.30825711619718`, 0, 36.96290464600693`, 3.023622226396916`, 3.0483663697002474`, 0.008183609409703863`,
0.05582058437447417`, 0.05601854527615719`, 0.0035463781667885996`, 35.42851522668959`, 0.21284831470836968`,
0.35133972673818986`, 0.0008673226741251483`, 0.0008819393536422758`, 0.016852643143304258`, 0.10277539461924784`},
{0.11628555922147576`, 0.9133810470221562`, 6.60919827925733`, 0.784993620588668`, 0.15558932911031897`,
0.27453121261635205`, 0.2030077151739278`, 0.01885747776005145`, 1720.9600172356268`, 220.92402081631303`,
0.1375226538362544`, 8.707812842669803`, 0.5031177384459613`, 255.97005462107475`, 44.59525706427541`,
311.5053886097685`, 0, 201.90196380345978`, 3.838892741065759`, 3.8668292050982935`, 0.007277219218367437`,

0.021910453630447038`, 0.021913998956916154`, 0.00016180981594060562`, 50.091026732005744`, 0.03639813361725627`,
1.7481433038398986`, 0.00033739233575080796`, 0.00033802193701244976`, 0.0018660805090333632`, 0.2130512511338496`},
{0.16627919709086636`, 3.258899380742358`, 5.159743357401544`, 1.0084152828841422`, 0.293274671001003`,
0.35878951423125527`, 0.20447475990027797`, 0.06840907951998164`, 1127.403994426183`, 328.5526444815032`,
0.06047838915389364`, 6.7509258891150505`, 0.42840334463359087`, 161.02148417644224`, 65.99792730236479`,
179.41297405174174`, 0, 82.23786200658316`, 1.6545736553997419`, 1.6735179054459968`, 0.01144962630368851`,
0.029225905904092173`, 0.029247491789491646`, 0.0007385873844358848`, 77.02984372821201`, 0.06942371668544402`,
1.7724493664595518`, 0.00034965802328335016`, 0.00035132250978798526`, 0.004760326930311143`, 0.3333251224031848`},
{0.27348098404578464`, 2.2019037456007027`, 4.59402198031265`, 0.8385696628288007`, 0.6096935872297715`,
0.3604633388375672`, 0.23592549449411898`, 0.025613961789364897`, 724.0273482822054`, 51.59152478645882`,
0.07558496433041467`, 2.104114801800101`, 0.41664957185729956`, 380.6349353194499`, 43.92741337943161`,
492.8138876748365`, 0, 278.1403162613364`, 3.1247936938202034`, 3.141754184355572`, 0.005427715298104596`,
0.20657244699262442`, 0.20675184635644153`, 0.0008684573689710096`, 98.29278483788818`, 0.8070519440041608`,
0.982197133177361`, 0.0029617684515590437`, 0.0029926762569110195`, 0.01043559138990302`, 0.029292918340206486`},
{0.11413129353935564`, 2.5718486613546734`, 2.678541647274315`, 1.0538897286852023`, 0.5347432665041771`,
0.651301492741162`, 0.13507333994249482`, 0.03374013401687532`, 1792.985239436316`, 324.1256033282515`,
0.22051507650373564`, 9.850878408710493`, 0.1276954587958865`, 218.32727876010526`, 110.27084142159853`,
280.5361541451156`, 0, 147.6612626145724`, 1.8681410501880262`, 1.8814734964364563`, 0.007136744972810360 5`,
0.05460397249495065`, 0.05470998832922823`, 0.0019415406871245988`, 68.63680084496839`, 0.08902888590331255`,
1.410543820608825`, 0.0006226281021722846`, 0.0006288516895968042`, 0.00999567382648836`, 0.5295672276922558`},
{0.1382612217370991`, 3.544859312958268`, 4.625085964177936`, 0.9631685672533119`, 0.6192785632371416`,
0.30258401315557804`, 0.17357832021845082`, 0.08489498345439296`, 3734.2868382787856`, 118.6130866776067`,
0.14202527245998875`, 6.035316827283889`, 1.23738828709505`, 497.5592816570811`, 23.19290565637329`,
554.9550148081265`, 0, 225.64585370232123`, 5.2592593030412145`, 5.280756569226904`, 0.004087508325223421`,
0.10770526805089278`, 0.10771361831607099`, 0.000077528846353702 72`, 266.3333474235426`, 0.21273517069057177`,
1.7258157047108151`, 0.0013504999851164934`, 0.0013546856852174204`, 0.0030993707123705416`, 0.06486848683084537`},
{0.20062439708640356`, 3.062479927466259`, 1.8588498709761279`, 1.1971327198257382`, 0.2717889530441755`,
0.5772443937125967`, 0.13761254703825482`, 0.02383075282424653`, 748.4785019436172`, 123.78948146072491`,
0.21845089918107502`, 7.7975595199904255`, 0.4961018196149929`, 434.4765082582245`, 143.42946385779817`,
476.3124053698039`, 0, 324.44712241708964`, 2.437501851369577`, 2.4658610694664964`, 0.01163454217726434`,
0.2242087400569935`, 0.22483247251156765`, 0.0027819274771161417`, 106.64000704258814`, 0.6425963327918401`,

```
  4.177091844845888`, 0.0022424051510944176`, 0.002278121473391917`, 0.015927684736216197`, 0.39269541437671224`},
 {0.2202391290111929`, 2.272383906087442`, 9.526635756893253`, 1.2801331448227915`, 0.9676407538336758`,
  0.2750641951622783`, 0.1295591726514962`, 0.0055115733663000654`, 1435.4738327945734`, 244.53753727132892`,
  0.22242949998676909`, 3.5195011696028082`, 0.5942454573278351`, 178.31324330113233`, 56.86542249119429`,
  194.43985529303131`, 0, 165.03062746363804`, 0.3861573859674271`, 0.38894271134918496`, 0.007212927896691257`,
  0.04266899381634313`, 0.042698226418398245`, 0.0006851017434565954`, 12.535683272702544`, 0.13424831476992624`,
  0.9113539937110909`, 0.00040232727278177727`, 0.0004041815798738923`, 0.0046089520088807845`, 0.05445257064686142`},
 {0.08672713507238722`, 2.273561509630496`, 5.582415438341933`, 0.8483312100313163`, 0.3485115156653926`,
  0.5097094533881305`, 0.23367094415518952`, 0.14443826868757056`, 1562.9150740851455`, 249.7667060966911`,
  0.16191416277041454`, 3.437993337535003`, 0.32781925870094497`, 105.7373023898941`, 29.971381003203433`,
  129.78462017824003`, 0, 34.936688925153106`, 2.112790690549985`, 2.127305640667128`, 0.006870036952578706`,
  0.029273709776671902`, 0.029323777311919992`, 0.0017103242339304714`, 68.62227988485839`, 0.036268928312283975`,
  0.6711769819629165`, 0.000415170061029535`, 0.00041871125479101603`, 0.008529501748511414`, 0.136975138805642`},
 {0.18381668769060344`, 3.1958936164482523`, 3.6493637720914887`, 1.44172169143792`, 0.026838260836304473`,
  0.6302019111021357`, 0.18006792550977113`, 0.04278842418986425`, 2871.506610396247`, 235.17217671272113`,
  0.15357027364724346`, 9.443732417373614`, 1.0754484669415296`, 253.2024975122598`, 38.29569911383381`,
  280.93877323375017`, 0, 157.92249736294963`, 2.0368532612754926`, 2.0588549688256985`, 0.010801812761135343`,
  0.06631198395511716`, 0.06643522138713315`, 0.0018584488755366557`, 92.99380479074497`, 0.17413213206899297`,
  2.597699365266436`, 0.0005536370069975094`, 0.0005583682917413067`, 0.008545824581807082`, 0.40676057202499427`},
 {0.18865219826812762`, 2.4805712334398518`, 8.790023396255851`, 1.3488199759969297`, 0.9395739613202174`,
  0.6166147449809938`, 0.13773668752267337`, 0.19466267340323914`, 3969.2421406756894`, 297.90931549743027`,
  0.10386024186718207`, 2.09437691916957 1`, 0.2651891384942813`, 70.86538046336966`, 21.41782075829836`,
  93.44813854126625`, 0, 18.837079680260185`, 1.424618341088201`, 1.4304895657654777`, 0.004121261469083759`,
  0.03243929034395707`, 0.0325408508395783`, 0.003130786603047664`, 50.48381822191702`, 0.08742490619484775`,
  0.17121399897047443`, 0.0002889074469607511`, 0.0002925518449040847`, 0.01261441330665547`, 0.03824680684086624`},
 {0.1825851308210345`, 2.4396329783561983`, 0.6407630961927655`, 1.1714042800046478`, 0.8170667200239692`,
  0.31301546820459225`, 0.1111924655180566`, 0.011382861853813236`, 3697.837667320504`, 70.61298960423318`,
  0.08120950744691857`, 1.4309282478289982`, 0.7664004986892703`, 174.47358186209476`, 7.381743884401226`,
  210.74438890602477`, 0, 145.80964490975816`, 0.6599618623435516`, 0.680669062429995`, 0.03137635864731836`,
  1.3577930854058118`, 1.3600457889637374`, 0.0016590919353902667`, 23.00092462615287`, 3.541611830381634`,
  0.3033655470038653`, 0.013986644131876114`, 0.014461985472235452`, 0.03398537461005513`, 0.01968711630798534 7`},
```

```
{0.13375040892255174`, 3.276165649558605`, 4.292971358510812`, 1.2543962009506533`, 0.622136912433882`,
  0.38876133504362986`, 0.059362281046229176`, 0.37593623792715036`, 1950.8385805745784`, 382.0130407968936`,
  0.20132818276194275`, 9.006278307672524`, 1.186932922075334`, 169.9817121860911`, 77.14760905018653`,
  172.09010685842634`, 0, 28.877891165375583`, 2.949711578741054`, 2.9977418314845035`, 0.016283033598813335`,
  0.03457132274555829`, 0.0346018522000883 1`, 0.000883086098692587`, 138.05348214823834`, 0.06605612220303407`,
  2.6466312392455276`, 0.0003325238417543064`, 0.00033412766642573007`, 0.004823187002057860 4`, 0.26855065365278813`},
{0.11761221086988144`, 1.0914541886641524`, 0.5591025160362655`, 1.4775466222869675`, 0.9603313566507792`,
  0.2704038317127577`, 0.19659054677243826`, 0.04563469543817772`, 3422.6083976447926`, 269.42977341830294`,
  0.0784511002461219`, 5.408577374365102`, 0.46841691657751894`, 298.69798716690366`, 36.489802050724826`,
  365.9828363857907`, 0, 179.55817265207077`, 7.033452512582327`, 7.104528275474903`, 0.010105387470154437`,
  0.9070559643904534`, 0.908795564520168`, 0.0019178531402783694`, 109.66701722326283`, 1.5240122479238758`,
  0.5508602581297302`, 0.00738127651040299`, 0.007507086060894548`, 0.01704441641147625`, 0.06966838420208907`},
{0.2429320475534284`, 3.9794232967059395`, 1.0019515993916865`, 1.0030250425538783`, 0.21362795630084253`,
  0.32541860695965363`, 0.056903912665728335`, 0.007999914248720421`, 1861.2936578099834`, 133.12444614515988`,
  0.12956497529754557`, 5.59967766310799`, 0.3532739838839296`, 193.69250744221813`, 29.566894849453487`,
  245.4947687362384`, 0, 172.939960827348`, 0.3393263846524392`, 0.34340680017621644`, 0.012025046410572227`,
  0.2156779670718585 7`, 0.21630393788040944`, 0.0029023400816008404`, 19.290333146756005`, 0.7485012878989342`,
  1.194551655372423`, 0.0025797255411852404`, 0.0026237149053409806`, 0.017051955122144413`, 0.11626764038379593`},
{0.08974721760036025`, 3.2713030704465336`, 9.414991051375608`, 1.4492271123735618`, 0.9934682041726086`,
  0.2039523558527424`, 0.14566778076348777`, 0.062238114816719925`, 3000.2912643397094`, 293.40777590216123`,
  0.11936140149668095`, 5.192024217841877`, 0.8616976591715506`, 245.3234139062623`, 34.029098707510464`,
  271.93684774222504`, 0, 130.2091625558204`, 2.409421010575585`, 2.4189169071259915`, 0.003941152878109255`,
  0.04562738038737372`, 0.045641254398160136`, 0.00030407204333515736`, 112.59923356991833`, 0.0584990062311759`,
  0.8578096596851217`, 0.00038049143086171 8`, 0.000381413421102474`, 0.002423156386644365`, 0.0465813237147573`},
{0.26359879599023367`, 1.2163041539701664`, 3.7777909348817094`, 1.254691553657424`, 0.9061229415337297`,
  0.533372724047131`, 0.10691633026129371`, 0.27923770769686623`, 2206.202484705579`, 127.40873012168657`,
  0.13182990312478088`, 9.420656570983855`, 1.2733451287842463`, 820.0420693902848`, 148.417325880361`,
  862.2561807251366`, 0, 167.85533174505696`, 35.444660181014996`, 35.68152734003727`, 0.006682731836406441`,
  0.18122965494303986`, 0.18130898985230756`, 0.000437758982064107`, 615.8783916318492`, 0.6824559834388033`,
  4.593697942065723`, 0.001742242123771387`, 0.0017511793732245616`, 0.005129740195827903`, 0.14131248325185522`},
{0.19365605952074066`, 1.0097214328963666`, 6.254195026060383`, 0.7786863431193308`, 0.8378299450565547`,
```

```
0.5381078852313175`, 0.13430741341317382`, 0.39183098626736546`, 3848.3805063541085`, 285.46142645085683`,
0.1476809547681347`, 3.2123723043853616`, 0.9596643052107194`, 124.9114962302079`, 24.01829847933813`,
132.87636043492782`, 0, 19.437276969619727`, 6.830650049745718`, 6.870754598392278`, 0.005871263840848018`,
0.03032770294358869`, 0.030366378687408282`, 0.001275261232000835`, 98.5293393691839`, 0.08390204923387402`,
0.6177555141874026`, 0.00046966080514376785`, 0.0004725183839812531`, 0.00608434599223262`, 0.05678869800970615`},
{0.11992752605735896`, 1.1695863474317374`, 5.859787436657632`, 1.088943542117163`, 0.7084196598983477`,
0.2068540131141825`, 0.10134897101245235`, 0.042086980108994226`, 642.019354817593`, 42.643211093372884`,
0.14904119135720395`, 5.991168965595664`, 0.5811778237079277`, 1103.0475445892484`, 122.36999039746348`,
1307.7319686656347`, 0, 690.0172034985474`, 23.288714627145673`, 23.388807752967622`, 0.00429792401274387`,
0.21482658313127423`, 0.2146841281359542`, -0.0006631162365646759`, 389.1166096734762`, 0.3680517235187395`,
4.110991332891398`, 0.0023815400650538665`, 0.0023878904258601208`, 0.0026664933752063025`, 0.05030280776343776`},
{0.08593805782066072`, 2.847179937660589`, 9.336517990063154`, 1.1546615909588782`, 0.6233557238718435`,
0.5408319922909341`, 0.12713218439588464`, 0.009396030605633099`, 2805.654341504175`, 67.07123229055907`,
0.22248402654525073`, 4.783547942175421`, 0.25670135456607057`, 378.65983131581737`, 34.01558155063026`,
674.7817761934123`, 0, 333.5524855060317`, 1.0725197541981455`, 1.075300112497549`, 0.0025923609225102506`,
0.06138837333731988`, 0.061437715545195115`, 0.0008037712223469828`, 43.62366752710896`, 0.0753656796768087`,
0.5638120505777736`, 0.0006413812222106907`, 0.0006443587940715875`, 0.004642436912377645 4`, 0.04361843606472140 5`},
{0.13317608837170147`, 1.503932075956504`, 4.603993052764295`, 1.367556919883856`, 0.822788205819833`,
0.29931793037743193`, 0.1910982001847455`, 0.009186595448818861`, 2039.1477227581072`, 222.71270432048289`,
0.13206186826480382`, 1.3638292654387778`, 0.18903235795918816`, 51.169640215443174`, 12.185721210091218`,
84.35694952497907`, 0, 45.04144089469899`, 0.2619099108047367`, 0.26401423137604707`, 0.00803452059085763`,
0.06938638985077565`, 0.06968930095943039`, 0.004365569520279111`, 5.627067369573578`, 0.13200868552228406`,
0.1672813240507028`, 0.0006086579832240702`, 0.0006193430815235396`, 0.017555176460301025`, 0.024457389671539122`},
{0.09391708894580503`, 3.041674977657679`, 1.8104005667515946`, 0.8959187121202952`, 0.2181847991244159`,
0.2824961199777095`, 0.12004816256362977`, 0.10420303973300599`, 1895.0150961574109`, 50.54358082712821`,
0.23566298754715342`, 8.257703012553833`, 1.009710413761872`, 977.4311705757339`, 40.80352894460885`,
1146.9781197940922`, 0, 394.8683003169929`, 13.085905198866055`, 13.152692184590242`, 0.005103734492129197`,
0.36791775943699845`, 0.3674189706679625`, -0.0013557072368544087`, 568.61529147702`, 0.4936252133968467`,
4.2067903500870685`, 0.004948199305848444`, 0.004968963889056892`, 0.004196391843777514`, 0.08789055592718428`},
{0.27769905250843735`, 3.334803063409833`, 9.639698328103112`, 0.972586344049939`, 0.15282290468559845`,
0.3020316347056351`, 0.21057051005264293`, 0.06155018310530056`, 2190.880276553241`, 205.62596489641203`,
```

0.17875345007490812`, 2.046885089160499`, 1.0703002508596509`, 66.58285344494571`, 8.200202080436071`,
76.07670537265776`, 0, 35.63411630509681`, 0.6341737103152915`, 0.6404811292329132`, 0.009945885196795512`,
0.02054990641762885`, 0.020572629261776564`, 0.001105739543817208`, 30.212063312763085`, 0.0815241363044681l`,
0.5780219137465717`, 0.0002548463267048051`, 0.0002564596765067610 4`, 0.006330677090059522`, 0.06479258611709689`},
{0.16775949346003155`, 3.734350305060132`, 8.90335371268981`, 0.9286973988343983`, 0.35313792052011217`,
0.3940821550050888`, 0.14910438190884368`, 0.1880509002120729`, 2527.5769345457447`, 29.455979238374994`,
0.037167259903154726`, 1.2969361348709503`, 1.0523609025190432`, 279.4437373663062`, 5.992657025819448`,
340.20014820898024`, 0, 76.16559565830542`, 3.7315168363988476`, 3.746985041959879`, 0.004145286284158667`,
0.1407842659342219`, 0.14085601135378167`, 0.0005096124846315497`, 199.06844337632904`, 0.33739853057537905`,
0.6900121331164303`, 0.0018297908479817382`, 0.0018392287852791896`, 0.005157932289289624`, 0.01092588465538831`},
{0.2349663788193207`, 3.197269954964022`, 9.218845167290574`, 1.0446779264109838`, 0.588347422519365`,
0.31448476179540896`, 0.13233876836756175`, 0.2796850376475317`, 2984.1524918506084`, 161.29090180593857`,
0.1933086585832205`, 6.384613193914876`, 0.9299276109407706`, 333.4573757378706`, 30.200119234694387`,
373.41981916246544`, 0, 67.92635600691857`, 5.685434108805596`, 5.714069683243543`, 0.005036655757490127`,
0.03712974654145776`, 0.03713847170382846`, 0.00023499116432046385`, 259.6838236715963`, 0.12463202987627275`,
1.5256856814454924`, 0.00042954354704727304`, 0.0004305690494681106`, 0.0023874236451391084`, 0.07850925998578501`},
{0.23753660977363267`, 1.5829476505005262`, 6.77431038963988`, 1.365304095754423`, 0.13883005311148944`,
0.18235912783176345`, 0.11037102150981828`, 0.005907370140418340 5`, 3040.16542258953`, 172.94160910300627`,
0.10069032496396058`, 7.204527027300948`, 0.264985624786922`, 164.45677175727243`, 17.77472723190549`,
271.4014425820212`, 0, 151.44231136790498`, 0.5388397437568029`, 0.5420020630497406`, 0.005868756582226187`,
0.028851117157544407`, 0.028860164125028356`, 0.0003135742520661289`, 12.185072948230523`, 0.09790280796836709`,
0.6671103154161598`, 0.00025538546334480383`, 0.0002560912775805062`, 0.002763721264547714`, 0.07083940281008586`},
{0.23896625246574088`, 1.021768300475304`, 5.961217623879941`, 0.8744606875234477`, 0.07851707385676243`,
0.3191621351693026`, 0.12686157250224162`, 0.07080906253325867`, 754.026010583138`, 128.7873645709799`,
0.07054179811818284`, 9.522685613917606`, 0.14468703962562834`, 315.55071958970166`, 111.61348526408477`,
485.7348933477372`, 0, 158.33861559919137`, 10.071148291715533`, 10.174075873107604`, 0.010220044270099704`,
0.030496839823944112`, 0.030503724668218487`, 0.00022575599026386506`, 147.00542962658457`, 0.10411022178301511`,
3.135471001503577`, 0.0004213733669690134`, 0.0004224626742406963 4`, 0.0025851355521551156`, 0.39467653751694526`},
{0.0664141372741357`, 2.373389311008811`, 6.163630213717491`, 0.7749555716943826`, 0.011151563708291068`,
0.26661102099162604`, 0.06978956594786417`, 0.06307180805406282`, 3305.773752462622`, 398.92218816248055`,
0.15362130283700076`, 6.399110469992867`, 0.5018401596166369`, 76.69112332047582`, 15.043431032741319`,

88.7735203794044`, 0, 40.70258762402681`, 1.0304856408000447`, 1.0413571245467228`, 0.010549864370975381`,
0.009454012071056646`, 0.009457677678056754`, 0.0003877303067265636`, 34.93919435746988`, 0.008969715078219025`,
0.8010734601008715`, 0.00014742415661306296`, 0.00014777552412611916`, 0.0023833781459468018`, 0.15194742209394632`},
{0.07724629482189549`, 2.6662607156107567`, 3.4543142766524646`, 1.1527116922070777`, 0.6194059290185789`,
0.17356203127759262`, 0.12523844349086252`, 0.006404139963734165`, 681.0439615527839`, 333.4912680980369`,
0.21335410013399275`, 5.078097228348419`, 1.1402121008441597`, 141.41134414349153`, 80.8589206376935`,
143.05860274056351`, 0, 129.509259170664`, 0.29790535768737103`, 0.30322760691722195`, 0.0178655707005988`,
0.058249176250832535`, 0.058289259467899625`, 0.0006881336294695117`, 11.347047888168664`, 0.064279043454365`,
2.242941504293047`, 0.0006096310609300382`, 0.0006126818050860694`, 0.005004246587070549`, 0.1785066954931253`},
{0.11115827720243887`, 2.3474129584418977`, 0.7793271183465329`, 1.2896581551764172`, 0.2400074156115546`,
0.5586724719427854`, 0.16881638153305045`, 0.3542961312162605`, 3281.9581897651888`, 363.45984424300025`,
0.19399862158515324`, 1.0793406751665469`, 0.15013258901534354`, 13.649277689245045`, 4.212204091898173`,
22.41821261059365`, 0, 2.271912737807703`, 0.320096231349179`, 0.33003059457453643`, 0.031035551975994613`,
0.12481224163249267`, 0.12818678201454284`, 0.027036934341636565`, 10.73425773453539`, 0.19819876790921048`,
0.09055310057389586`, 0.0011271604457006035`, 0.0012332324084376868`, 0.09410546931599661`, 0.10167167770357054`},
{0.2790792381180839`, 1.4178392715488872`, 7.586791613535141`, 1.4555087381759333`, 0.0017991755342297022`,
0.24850929517479325`, 0.05953650221890522`, 0.3815756253123188`, 2337.3041910345446`, 253.88772622846432`,
0.0832002701070979`, 3.446598610267717`, 0.1790778319506987`, 45.256577756721605`, 10.923741376838823`,
71.41874840985471`, 0, 7.3389583274373775`, 1.7802515793010922`, 1.7991768938197774`, 0.010630696660364869`,
0.015770480666193958`, 0.015787547221966923`, 0.0010821836147043573`, 36.05872289100022`, 0.0628744818439602`,
0.40503408940559726`, 0.0001306558213600706`, 0.00013153654743756275`, 0.006740810078909476`, 0.09131985452497883`},
{0.05958430298107065`, 0.7208194464934747`, 0.8555730299303229`, 1.0890886690114123`, 0.12206612248038917`,
0.22763156357860503`, 0.13379446389365296`, 0.0940380442991097`, 890.0674874512752`, 363.7513660796561`,
0.17126775622478085`, 2.343574670731165`, 0.6210533219689349`, 40.28014542094611`, 20.41851177540731`,
42.236993334944174`, 0, 17.518295376077344`, 1.9926008607722263`, 2.0439522006348527`, 0.02577101158268369`,
0.1353629594314508`, 0.136492463202899`, 0.008344260321969443`, 20.518649279203718`, 0.11522153695965438`,
0.7883455576798303`, 0.0014763603579294182`, 0.0015267528543937 22`, 0.03413292438641391`, 0.14907508884392134`},
{0.17145251727538108`, 2.2285999256796796`, 4.099234007029899`, 0.9794004497404906`, 0.5456157460051374`,
0.6134582400670898`, 0.07617057839260499`, 0.06280998418842913`, 3845.79457797157`, 146.34600196120715`,
0.1780830585354753`, 4.861786782312107`, 1.4658755518344249`, 231.17561494030969`, 24.417069908294312`,
245.48379118610544`, 0, 122.70309235044881`, 3.2958355183759442`, 3.329466368140909`, 0.010204043732600088`,

0.07113587513383128`, 0.07124673195512557`, 0.0015583813523869505`, 104.92998273292973`, 0.17423464086128043`,
2.113830479797152`, 0.0008760600547744746`, 0.0008815703890368646`, 0.006289904707284633`, 0.09018448748385677`},
{0.12996780445602385`, 1.481230978947499`, 0.6236753706365583`, 1.469443592582019`, 0.8723955727041814`,
0.3094468996181755`, 0.06710955059959622`, 0.0369322761528374`, 2374.524656049569`, 217.63840782204932`,
0.06713489355669272`, 5.581729605257106`, 1.404891625361591`, 218.62862394103357`, 50.42063160774444`,
226.20703563719226`, 0, 142.76711857586037`, 3.348861339498343`, 3.40742146595951 1`, 0.01748657842905499`,
0.5727109102404103`, 0.5750166043550505`, 0.0040259301392955305`, 70.86338800377943`, 1.0633425655994484`,
2.1140202647447093`, 0.004681028857186464 4`, 0.004773019685927244 6`, 0.01965183970176842 5`, 0.12029334848998578`},
{0.05377365231121939`, 0.5209314028947571`, 8.011482854018304`, 0.8336514898133974`, 0.5477465381895592`,
0.6097956289357849`, 0.1415470974234781`, 0.1739421756208861`, 1218.4890478272073`, 28.334007654588277`,
0.1640483947776385`, 1.719700868912403`, 0.7776819970376057`, 454.3481120821877`, 26.517576531164316`,
541.8680385203054`, 0, 131.1602571103345`, 38.24779349888239`, 38.376706722659456`, 0.0033704747904172994`,
0.1723110279669711 8`, 0.1724801008119217`, 0.0009812073373673957`, 284.63538178573924`, 0.1323684758184351`,
1.2365383370373326`, 0.0024899526367260227`, 0.0025063198967491098`, 0.006573321830172674`, 0.02051860029639544`},
{0.11080721322368536`, 2.0431071845443514`, 5.511023662772233`, 1.3452026921196156`, 0.11486345745135074`,
0.24454012333479547`, 0.160320934401384`, 0.03396351864461054`, 2929.9466481691034`, 234.80413085200098`,
0.15939227470850165`, 4.957656716995926`, 0.9862870867437366`, 129.1039258977286`, 13.629380746229321`,
148.01402385622634`, 0, 87.1779961600376`, 1.3851171150061266`, 1.3971649095414536`, 0.008698033115614123`,
0.03984192074121591`, 0.03987447885401044`, 0.0008171823092064656`, 40.42775327291942`, 0.06306817438303301`,
1.0357405569179332`, 0.00035747065202762496`, 0.00035912390412402764`, 0.004624861053698304`, 0.1128451695639836`},
{0.0760667810432224`, 2.1066017580574803`, 2.855976527079802`, 0.8406874141346559`, 0.5880407566565378`,
0.5900461865444994`, 0.0836613609935555`, 0.006020335814040566`, 1435.8256232073982`, 78.12343431322768`,
0.05191171052641791`, 2.750551419426905`, 0.6067313927910662`, 237.97860813371227`, 37.78357506381824`,
270.00136515662604`, 0, 218.70844783122487`, 0.6015137480344367`, 0.606105071125796`, 0.007632947885833685`,
0.15915978489891616`, 0.15966612962340748`, 0.003181360950021972`, 18.102141701501274`, 0.1729538929827122`,
1.2411004077365366`, 0.0022730889483500993`, 0.002302380564664735`, 0.012886260494071822`, 0.05861114673407724`},
{0.1959001110368495`, 2.209062410088075`, 0.3560695839737882`, 0.9080944676346985`, 0.9265730083719161`,
0.5325736257413008`, 0.19563779045692792`, 0.10606554062225873`, 521.6446274837854`, 359.26470542430604`,
0.157717920696867 9`, 8.108669567071114`, 0.6211999644108912`, 421.2018346195486`, 570.1863467581098`,
393.9201845319426`, 0, 168.54271749019003`, 7.664606169457787`, 7.793490698468645`, 0.016815544877496924`,
0.819928206166447`, 0.8254954925537915`, 0.006789968128275703`, 241.87990538683343`, 2.294628951861031`,

```
3.875132027038687`, 0.010611286680798138`, 0.011087135781887808`, 0.044843675927703774`, 0.5141262437337657`},
{0.2577200305567735`, 3.2042225311100276`, 2.603156269860186`, 0.8353734407000886`, 0.2430563561244512`,
 0.34928665438367135`, 0.16249163015987889`, 0.02250334329978988`, 1913.3488368943863`, 296.02734369523876`,
 0.11827389650606279`, 8.829720137683108`, 1.3154105657657276`, 218.83051621348167`, 48.39172396556585`,
 233.64767042499207`, 0, 166.03739518827692`, 1.1226474405381002`, 1.1381181473751825`, 0.013780556814583678`,
 0.04984122620688892`, 0.04987706239383446`, 0.0007190069280555988`, 51.38874604950256`, 0.18350117630037555`,
 2.784304968769627`, 0.0007196550585142347`, 0.0007235595808617574`, 0.0054255470052329`, 0.35304471381541286`},
{0.2269507001135535`, 1.5304419335739787`, 3.489177129657852`, 0.8382349859440477`, 0.3185553593952979`,
 0.48806107220778994`, 0.1765391455298561`, 0.025680745838524602`, 3051.647362946771`, 356.25991793699154`,
 0.11555959688444262`, 5.191309509924922`, 1.4793748585613802`, 114.67259100458743`, 22.208556334987506`,
 122.09184633448908`, 0, 84.12093638978418`, 1.32926318924431`, 1.3461728402035316`, 0.012721070662337741`,
 0.0332218006806466`, 0.033274666965921004`, 0.0015913130592346558`, 29.062287508225342`, 0.10771015605007503`,
 1.3224481324455306`, 0.0004774585976921042`, 0.00048114200972518447`, 0.007714620808767059`, 0.2159008694872049`},
{0.08130128725263741`, 1.139181654994668`, 8.356523528104287`, 1.0901388323360164`, 0.43395806919787105`,
 0.6945552692578585`, 0.15512850330366812`, 0.013156059374009372`, 1072.341203797791`, 380.15089775887463`,
 0.2428753710582479`, 6.517091771782727`, 0.9223679840610632`, 152.48348074248315`, 110.34379821834334`,
 151.24319358267647`, 0, 128.6196043747004`, 1.3745026863727583`, 1.396565185710366`, 0.016051259525603045`,
 0.01913577785164338`, 0.01915712590607201`, 0.001115609440814902`, 22.368689215096197`, 0.022225191027407647`,
 2.3478921887582573`, 0.00021159770681244972`, 0.0002127927917675215`, 0.005647910712619719`, 0.49510646395108593`},
{0.2434557780636919`, 0.7224758340812225`, 2.1790233830774692`, 1.461101906273748`, 0.9519880988578835`,
 0.24073725238447052`, 0.12794532446798884`, 0.029247247168962882`, 1555.7186853348103`, 187.90922583014026`,
 0.22271376685703087`, 9.305754826562229`, 1.0292202961269128`, 637.47978230859`, 126.33775481082526`,
 677.1981609620393`, 0, 449.626196092361`, 16.472481478344775`, 16.593765707899724`, 0.007362839030318202`,
 0.28775235368326463`, 0.28779136020144797`, 0.0001355558614344332`, 170.01385422078076`, 1.0007853307944057`,
 3.2929530703158276`, 0.0023741541022636037`, 0.0023892770471722643`, 0.006369824475269681`, 0.12508066236861404`},
{0.11639393264339765`, 3.920728456674687`, 7.161326334970468`, 1.2230707245468682`, 0.4475871708897683`,
 0.6126582241159237`, 0.06411943719695506`, 0.012576022333314589`, 2883.638838241297`, 313.1192616144217`,
 0.21454888313615755`, 9.840193495130112`, 0.5865096618206191`, 200.55549762612114`, 59.03884613896867`,
 214.31641782880007`, 0, 170.15617621255333`, 0.5302936334324081`, 0.535759368815968`, 0.010306997932790551`,
 0.021829020263655012`, 0.021846719018416137`, 0.0008107901567435682`, 29.701961985597936`, 0.03629665020343289`,
 1.9096166714259937`, 0.00021538056069092537`, 0.00021629728129296628`, 0.004256282921263435`, 0.31520352281584296`},
```

```
{0.15100737716165968`, 3.554370686110622`, 4.587082662015709`, 1.0501596960456492`, 0.32405591591236504`,
 0.16453196956775862`, 0.1064831105262615`, 0.33127320693075046`, 902.0301791506135`, 373.05242738162826`,
 0.0458010200387457`, 5.548602584093444`, 0.22333879786319444`, 93.0643425049627`, 50.57988521650585`,
 106.44337586366683`, 0, 17.411333094664073`, 1.4596712274352346`, 1.4838667159074166`, 0.016575985069388288`,
 0.02407636737830708`, 0.024087216110718006`, 0.00045059672999925304`, 74.11732317364158`, 0.05193870127698933`,
 1.4634541680002606`, 0.0002769103318903676`, 0.0002778924319334691`, 0.0035466356072633154`, 0.19947576173189865`},
{0.25418798006115284`, 2.1642591478158595`, 6.996499815725443`, 1.0633791975130336`, 0.9920570228042984`,
 0.41215457570127223`, 0.23951743090069744`, 0.250242517415804`, 977.2894626743205`, 66.34773026593149`,
 0.24515846775975497`, 7.886389497471068`, 0.4453335711584776`, 2129.653973777248 7`, 285.166975252064`,
 2684.564163574747`, 0, 467.48711007448173`, 52.038781632056455`, 52.13815388277183`, 0.0019095806550197825`,
 0.2577413509990364`, 0.2575241984640259`, -0.0008425211327898197`, 1608.934416976713`, 0.935925048408093`,
 2.8725941142378337`, 0.0029240525441391885`, 0.002933347992562231`, 0.0031789608027645855`, 0.05591931318337934 5`},
{0.17142178078611126`, 3.468749463314624`, 4.1220489747329765`, 1.0943061437323611`, 0.026879199595517766`,
 0.33123915944031324`, 0.10298062238993438`, 0.021389796005587176`, 1913.2712703835568`, 144.71515384537201`,
 0.0624079843624904`, 5.28189397954334`, 0.583459387488 6529`, 182.7108303901955`, 23.474500611638057`,
 217.62325145173745`, 0, 140.06739820515782`, 0.8387227423260686`, 0.8459047228538464`, 0.00856299724013665`,
 0.05752962124962364`, 0.05759053338217092`, 0.0010587959945536074`, 41.56170089019026`, 0.1408832874651699`,
 1.3433293527269892`, 0.0006337871775149 44`, 0.0006377861099077738`, 0.006309582356193388`, 0.1134595987678805`},
{0.20869037066000085`, 0.577011692748 3819`, 5.389618587282298`, 1.0412482369515845`, 0.4332354730171122`,
 0.23498113287901135`, 0.16276058881271271`, 0.005129384861959153`, 3721.998380233862`, 47.9738718293973`,
 0.14995337445020457`, 8.81275872972273`, 0.9157809024114361`, 1346.8469909061616`, 24.88819775685616`,
 1666.4604159929313`, 0, 1253.0065860026732`, 9.916121715957589`, 9.94854626288 0775`, 0.0032698819006031776`,
 0.18539071153352032`, 0.18526266120192394`, -0.0006907052167671734`, 81.73883109748107`, 0.5527036615264054`,
 2.0575658508112937`, 0.0021511789802778347`, 0.002155072772801347`, 0.0018100737126993671`, 0.03843086268231107`},
{0.06860802282414658`, 3.9707655631721437`, 5.6945082231101`, 1.3132775124268627`, 0.833710937956144`,
 0.4055938689007862`, 0.09163259509867216`, 0.3768554443970733`, 2744.3744850106896`, 240.0358375972813 3`,
 0.035389680502848464`, 1.2976415449147964`, 1.3504035222444002`, 48.35739573916334`, 10.732593165883886`,
 50.24327609539747`, 0, 7.91464563236649`, 0.6987674571422402`, 0.7064883911855434`, 0.011049361220798248`,
 0.053655157438300495`, 0.053836049124591605`, 0.0033713755569373394`, 39.63773936408252`, 0.052588203802279895`,
 0.42384954459630214`, 0.0004915540035815846`, 0.0004973539032481538`, 0.011799109811555963`, 0.029409230629700706`},
{0.05446295287328684`, 1.6696297250378036`, 9.103903568630106`, 0.7607067601908715`, 0.16998909961411246`,
```

0.4015279687740475`, 0.15306183541311225`, 0.23729003810245272`, 1974.2834194249153`, 302.21506503791556`,
0.16971647699912568`, 4.841445731177878`, 1.0533590220242193`, 106.59925906600706`, 25.291045063795572`,
114.70353741515794`, 0, 25.294499955830823`, 3.2707515478865954`, 3.3122753381312298`, 0.012695488983706316`,
0.011542143308666637`, 0.011548039389886338`, 0.0005108307063970141`, 78.01348582235524`, 0.008980274386849122`,
1.3670416209187353`, 0.00018326667005530428`, 0.00018380827585999648`, 0.002955288075724649`, 0.20408131235438107`},
{0.07612701991269089`, 1.8994519379164023`, 6.640792134140813`, 1.254560879057461`, 0.12469411598778724`,
0.4203442966564226`, 0.10535108099169138`, 0.03365653592102131`, 2960.4487643491357`, 231.61182906412773`,
0.044585724001347915`, 3.1581032850809603`, 1.1020659040461283`, 77.28411138151581`, 10.711951273978247`,
84.60541174545325`, 0, 52.42025107877681`, 0.8813627190034512`, 0.8918988299918115`, 0.011954341568104043`,
0.028948283224740603`, 0.029001373968793473`, 0.0018339859272724635`, 23.915801780262488`, 0.0314820933355385`,
0.8747190946354377`, 0.00027802777338026274`, 0.0002800641978844989`, 0.007324536248581426`, 0.09300915216222543`},
{0.08639685731709784`, 1.2263080297925724`, 1.8023918869870366`, 1.4078333023480911`, 0.09921808861838555`,
0.1733433235749129`, 0.14800205989677365`, 0.20963109628072585`, 1801.4315718325315`, 163.03870519568818`,
0.04013342775966272`, 4.222103734481822`, 0.21608307503029467`, 100.91797991702559`, 16.59083027795759`,
175.01026369220878`, 0, 25.636853404885898`, 4.051063599545638`, 4.085343869393269`, 0.008462041882402405`,
0.11670681276736503`, 0.1169066797857983`, 0.0017125565654 13914`, 70.96931173318872`, 0.14404431215140648`,
0.5923493575331628`, 0.0009969514281955583`, 0.001007868838041268`, 0.010950794127924324`, 0.07142664747105847`},
{0.05971802623282291`, 2.1738091632492136`, 8.683813711430187`, 0.7864060587963683`, 0.5208725203898663`,
0.5102800946784368`, 0.10321808603446814`, 0.07803262912760096`, 1116.648121119908`, 221.4014904292125 7`,
0.07733870760092837`, 7.219382522940123`, 0.8997811624944188`, 259.6829535037563`, 109.44321601755571`,
266.3850486164721`, 0, 124.44565607156369`, 4.217797585693835`, 4.270456987257826`, 0.012485047111460368`,
0.020439176113614763`, 0.020447468082484446`, 0.00040568997613155133`, 130.98124343588088`, 0.01743696079122022`,
3.2150515480024326`, 0.0003139357185789615`, 0.00031480852335971686`, 0.002780202216893679`, 0.28715835632083486`},
{0.25957147156308197`, 2.9787560040757644`, 6.8514986634875745`, 1.2875526348358215`, 0.5271393050674782`,
0.38366555469751695`, 0.09532745198151626`, 0.00993086596705298`, 3516.3450563644556`, 352.0852345112979`,
0.03547806916020174`, 5.814047961801249`, 0.6954919748641395`, 119.05062073083778`, 23.99619164739186`,
130.84769376602964`, 0, 104.19642451765485`, 0.3365612403774093`, 0.3396093494615487`, 0.009056625417476205`,
0.02411228166432958`, 0.02413464547698397`, 0.0009274863725350357`, 14.321911650191337`, 0.08941229191934115`,
0.8812865592472833`, 0.00022598395348705225`, 0.00022713343506174699`, 0.005086562815446172`, 0.12307341263860665`},
{0.07058107847879669`, 1.480448796537483`, 8.054675740834305`, 1.3102914246603354`, 0.04592614768203762`,
0.23013975911080142`, 0.1465551863327878`, 0.10936312847944579`, 1149.9199910201796`, 342.3645344528402`,

0.16288013724428108`, 8.990901684554295`, 0.299529341502206`, 149.00856456958402`, 59.711986962178166`,
175.18511488405403`, 0, 59.36434581386936`, 4.045742224350633`, 4.101942779177112`, 0.013891284147620198`,
0.01691707300292171`, 0.01692200936348`, 0.00029179755608077507`, 85.56448867343956`, 0.017057503674906934`,
2.0674277865036252`, 0.0001560565975684547`, 0.00015637722896567313`, 0.002054584056132569`, 0.3809832827734331`},
{0.2559888963315523`, 2.7036918392629854`, 1.376199541747745`, 1.1184454818096095`, 0.29882869252546684`,
0.4809279853463262`, 0.1762427890412958`, 0.033675551797332184`, 2802.532681882846`, 74.90505814957066`,
0.08607671060416239`, 7.246865433053017`, 1.1485805723085236`, 701.2005737326672`, 33.191226452953124`,
808.2980319808651`, 0, 472.8249405431671`, 5.704323505717835`, 5.748048967241503`, 0.007665319380963931`,
0.4918224192011297`, 0.4920701559631056`, 0.0005037118120363449`, 220.32475587036194`, 1.7985868326058958`,
3.0781898350895416`, 0.005286896491525583`, 0.005343480749905878`, 0.010702736183882955`, 0.1275641014224355`},
{0.12936768312046054`, 1.4931741189869152`, 8.533444012750714`, 1.3526677837265888`, 0.34592179565846926`,
0.5020921142394136`, 0.1702518668235985`, 0.4026569331476235`, 2469.5593778381244`, 155.39036984038785`,
0.09801542749760322`, 2.524614796651056`, 1.2701205529096362`, 122.20503855022004`, 13.679507756560152`,
133.95305015469165`, 0, 18.884890169508537`, 4.620621711012872`, 4.661964317439836`, 0.008947412061114601`,
0.048022635101810374`, 0.048116792539265096`, 0.0019606886888881014`, 98.56275360733507`, 0.08875110057799425`,
0.9754913871520465`, 0.00042761807074509495`, 0.00043110517673764203`, 0.008154720838787544`, 0.07334458599631302`},
{0.10764603393952993`, 1.2107066619029974`, 4.216520809210639`, 1.0534038454487438`, 0.2705194689594228`,
0.49749815429385036`, 0.21553478853143437`, 0.04863912466742637`, 3262.748108283069`, 369.9540993093576`,
0.034536512984739975`, 2.033964341592309`, 0.4918523584430632`, 38.63664655918477`, 7.667257911778504`,
46.33530198371953`, 0, 22.848092441152357`, 0.8588096249607332`, 0.8657761408275979`, 0.008111827888728218`,
0.02960025797889475`, 0.029735199437061138`, 0.004558793313984122`, 14.853807632091083`, 0.04551929107164436`,
0.2416153121357458`, 0.00033649452938966373`, 0.0003423855555859208`, 0.01750704894650834`, 0.07548185199417355`},
{0.16173577525354788`, 1.3192464264843462`, 3.8155846300226686`, 1.0837420201848613`, 0.1916516348586692`,
0.5385828520739745`, 0.0857724509699242`, 0.02449629028204095`, 2335.082397263821`, 344.7269386138737`,
0.14212635984223515`, 7.933933586100813`, 1.1885980026661422`, 140.96811688534518`, 41.76806750456974`,
146.00258320237302`, 0, 104.9123592421127`, 1.8101412587782684`, 1.841302786375817`, 0.017214970072877422`,
0.03161352108504423`, 0.03165900935688628`, 0.0014388865991763478`, 34.11460552964436`, 0.07304339058836526`,
2.3199891470249794`, 0.0003514837920148839`, 0.000353903484876085`, 0.006884223159566494`, 0.3395682690632737`},
{0.2639188435968321`, 3.196402084598291`, 5.8733763940010615`, 1.001723636198023`, 0.8720114445540239`,
0.5139297111136746`, 0.13829965727943472`, 0.009850038986012472`, 3721.325240000034`, 357.3898095475904`,
0.1088650950486454 4`, 4.342058559354168`, 0.9364053798077125`, 145.97914527198137`, 35.38474830899742`,

153.46771876073038`, 0, 127.8256785052566`, 0.3831893670721177`, 0.3859064428227561`, 0.007090686705111704`,
0.035906560593900384`, 0.03595570642547322`, 0.001368714540183058`, 17.49753273864595`, 0.1353773992783181`,
0.7553229499807441`, 0.00043218581312620685`, 0.00043503169466444636`, 0.006584856447864107`, 0.08237598256823697`},
{0.19137997577853977`, 1.0693425445674762`, 5.859392287604482`, 1.4113333162411321`, 0.03799318259045559`,
0.31471592998942965`, 0.1330917250869928`, 0.057621317595080414`, 1667.5088619944472`, 46.210982838293944`,
0.016645464987740083`, 9.646205098992475`, 0.9349271114716617`, 1132.6531070616832`, 49.35384512149585`,
1355.759161248836`, 0, 623.1094584298833`, 31.26444783549952`, 31.419596733257322`, 0.00496247042564546`,
0.1774783310068358`, 0.1774542801672680 2`, −0.0001355142311252333`, 477.6057743272886`, 0.485225695561468`,
5.0752322322728824`, 0.0015185965255701817`, 0.0015230054103816304`, 0.0029032628069480815`, 0.11382269889748621`},
{0.19721869082033683`, 3.2840461091147555`, 6.111568583801249`, 1.2835967155205092`, 0.47111924671273675`,
0.3724666179342665`, 0.13247758167737783`, 0.03750893971444233`, 2173.6667113114927`, 83.61542153296074`,
0.1890720712372439`, 6.476185684583786`, 0.7126657781022314`, 558.7914862742397`, 40.019446070732215`,
664.313815886468`, 0, 364.17858172863237`, 4.051920458680527`, 4.0688249044612395`, 0.004171958939741094`,
0.11363570758991125`, 0.11368211726159441`, 0.00040840746863346 49`, 190.0956230967465`, 0.32015836401910086`,
1.8786417766921994`, 0.0010685849105768197`, 0.001072979416622579`, 0.004112453771583979`, 0.07030286050790366`},
{0.21291226343526198`, 2.8179245900150987`, 9.921791649942335`, 1.3352776575768062`, 0.9588380301695891`,
0.6936227079381379`, 0.0795524915712694`, 0.4777153216598742`, 2688.6717707047637`, 396.8627131234574`,
0.23818107150054235`, 1.6319345031256631`, 1.1969051802794537`, 39.66297147938311`, 28.59350696174632`,
39.86075856634459`, 0, 5.431626847663481`, 0.8277291047705108`, 0.8385167059845072`, 0.013032767788185184`,
0.02041228995383125`, 0.020491765804121143`, 0.003893529362440473`, 33.32111711720006`, 0.06208609794233825`,
0.42834295324800276`, 0.00018358760553838227`, 0.00018616841977989139`, 0.014057671453041154`, 0.05458875384398391`},
{0.08637419772835031`, 2.5843437924117936`, 9.303756737243045`, 0.9248602316926738`, 0.9539236366945725`,
0.30709475006465425`, 0.15355620403975512`, 0.05658228330400466`, 3850.542613273592`, 202.84343087793332`,
0.1503135323154285`, 6.227303197646734`, 1.09139924606 9971`, 427.1471951976873`, 40.51484061703827`,
465.797970841371`, 0, 236.73056987025464`, 5.019007496613277`, 5.035307184378336`, 0.003247591834851349`,
0.042792817484618655`, 0.04279778197066461`, 0.00011601213329170967`, 185.2977266848684`, 0.05280278969671792`,
1.2844618316101424`, 0.0005593213654938589`, 0.0005603157024197024`, 0.0017777560221852173`, 0.05348706927219469 6`},
{0.09166372218259045`, 1.1041359694939814`, 7.465464940650059`, 0.8285398481134653`, 0.01938900145906275`,
0.6417891529291755`, 0.14382119055783732`, 0.023209894299314995`, 3535.4335241265126`, 296.7460220437812`,
0.08660924945570686`, 6.289282036011649`, 0.7877277513120826`, 121.55485792944587`, 20.604575529871035`,
136.84864840017167`, 0, 91.51901486652008`, 1.7873106571740132`, 1.8045258777330473`, 0.009631912890987637`,

0.013451128848379745`, 0.013462938446393128`, 0.0008779633402149489`, 28.19191407493934`, 0.017614007682856085`,
1.1560308354750441`, 0.00019588987652385814`, 0.00019675599844672825`, 0.00442147362712042`, 0.24491916639807387`},
{0.06057989660476104`, 0.9664690813204819`, 5.824893628794225`, 1.1149397320321297`, 0.7552676734434385`,
0.19045278243945962`, 0.155017124958911 8`, 0.20977491640661988`, 1881.802129389779`, 160.06890201789201`,
0.2135476446394321`, 7.516581972853506`, 0.39758727016160034`, 440.1717460263161 6`, 53.48002631771335`,
563.8774975526519`, 0, 111.02578068947356`, 22.220664295036595`, 22.28992633365782`, 0.0031170102613311546`,
0.07034818097007334`, 0.07034914637681343`, 0.000013723265147369546`, 306.79407153649777`, 0.06088122185015979`,
1.2409149591040247`, 0.0007624469981313275`, 0.0007640598127452222`, 0.002115313743574987`, 0.05730612800402354`},
{0.22678054201622128`, 1.767805615425421`, 3.537786208995465`, 1.13577377357013`, 0.9133787654245775`,
0.32400922076880223`, 0.1547055518280115`, 0.1014618010767026`, 3393.1210765994138`, 223.6969184131283`,
0.017411417358218523`, 7.612407230565935`, 0.356714236328328`, 377.2217642234362`, 54.123458758898`,
495.09349774879837`, 0, 154.2652976462002`, 8.476062760597655`, 8.502181928427932`, 0.003081521287418676`,
0.09971105196763784`, 0.099763325339877`, 0.0005242485281984344`, 214.05759064118308`, 0.3230360915750767`,
0.7367302119409969`, 0.0010591479037571627`, 0.001064527135663898`, 0.005078829772172`, 0.06273408710712171`},
{0.21988733193649235`, 2.7975541724377093`, 2.3611483960904547`, 1.1600939571587892`, 0.24697322096821828`,
0.5139658559575447`, 0.06350156651392477`, 0.17257512128303623`, 3203.836504260581`, 102.69376441927619`,
0.05643237763666792`, 2.2035776970401866`, 1.4380067287288245`, 116.17691929270121`, 8.465237050933617`,
125.39648259323393`, 0, 34.202219593873465`, 1.9848093088228007`, 2.017614521010785`, 0.016528143052413125`,
0.16103081237496603`, 0.16168668200765735`, 0.004072944941518886`, 79.3230223341489`, 0.5058376527528673`,
1.2470401856157083`, 0.0016710270953006479`, 0.0016969998234729286`, 0.015542972489986928`, 0.04784396720215145`},
{0.17952419657415108`, 2.390230853640494`, 9.385595862371645`, 1.2510854246897463`, 0.45025706246945174`,
0.39156066611114926`, 0.09061933973189976`, 0.007387745401806454`, 1060.2070144427744`, 157.30384712261866`,
0.017455537733710474`, 7.390923217745433`, 0.4592263583377443`, 315.88407367605106`, 94.15516293502421`,
350.8345926617623`, 0, 285.6192944014491`, 0.8496637743695546`, 0.8580046380868107`, 0.009816663919142599`,
0.03573618361153532`, 0.03575214275138156`, 0.0004465820978456492`, 29.012750981696453`, 0.09165013787836652`,
2.7003096949756196`, 0.00034495637945108903`, 0.0003460834105725893 4`, 0.0032671699630362205`, 0.21302916485464385`},
{0.17085484316695765`, 3.896251009939033`, 5.375947987134143`, 1.4639220165256899`, 0.9541878933608088`,
0.33462154790480914`, 0.21681254773335146`, 0.0069834922249469925`, 3376.9159214269603`, 211.30439423571625`,
0.23015330461401873`, 6.0128254119306135`, 1.4535070849014953`, 521.5989981590093`, 47.682478564226436`,
549.1974995348337`, 0, 473.5395481425547`, 0.8309091228938612`, 0.8350829920933582`, 0.005023255954827332 6`,
0.14808244105802282`, 0.14815610636267304`, 0.0004974614419095413`, 46.24900727489659`, 0.3614371748964077`,

```
  1.6225288835867482`, 0.0012210062564865787`, 0.001226356362933531`, 0.004381719109570303`, 0.07203133680804116`},
 {0.13727970757875596`, 2.8452993900541435`, 0.2811962573637228`, 1.4853711561890859`, 0.35668909434089957`,
  0.6882050716830825`, 0.1434517751263107`, 0.09344751133387784`, 2265.9177865516995`, 139.6613455165895`,
  0.057526142554834014`, 2.484713339974709`, 0.10336153105397905`, 60.79840359873283`, 18.42509700707225`,
  144.44468515618217`, 0, 25.2614309577864`, 0.8004909583856997`, 0.8346347868865035`, 0.04265360919211325`,
  0.7425511114139991`, 0.7806046250616215`, 0.05124699574573288`, 32.53766336626693`, 1.4562457062456085`,
  0.29594390993440506`, 0.005851113827814669`, 0.006613326138342605`, 0.13026789991754684`, 0.2747456861037246`},
 {0.10845833265331112`, 2.205454065801659`, 8.04431016182664`, 0.9250832587897069`, 0.5103893648573781`,
  0.6279318303072325`, 0.0552625938696 4427`, 0.03668517084277843`, 3275.5705391236816`, 196.31559723816565`,
  0.1402871557364922`, 7.884261458348763`, 1.2767651696233133`, 257.17385453330564`, 45.48048644104072`,
  269.39280447798075`, 0, 169.72118957662352`, 2.6879821941811035`, 2.720465797238481`, 0.012084753808152993`,
  0.023529822930149536`, 0.023543965312143592`, 0.0006010407318424171`, 84.68887512798808`, 0.03645721946614004`,
  3.0288713886751766`, 0.00030721380698806033`, 0.00030817015817563505`, 0.003112982443565393`, 0.16527921402309345`},
 {0.13079509007818008`, 3.451126694395393`, 8.265071576900024`, 0.9978113975551193`, 0.28001559296341805`,
  0.46031518977081587`, 0.08917008131877008`, 0.017138702372490412`, 1442.9762283162472`, 33.814218463353484`,
  0.17998121143599194`, 7.358113266662068`, 1.1154000745850698`, 1286.2434303655218`, 62.69191620152687`,
  1458.6645642172543`, 0, 1033.9773688575722`, 4.998315894091522`, 5.029286846553486`, 0.006196277529912475`,
  0.1324786099634216`, 0.13245044222822452`, -0.00021262100504249037`, 246.42602013028596`, 0.2475364531942781`,
  7.2142204221173465`, 0.0016036293181764805`, 0.0016073989268014497`, 0.0023506733022664594`, 0.08543178818297023`},
 {0.15200365596867`, 1.2321714037993603`, 1.2826182456063666`, 1.2525135629617612`, 0.10189417734334105`,
  0.42182447183087945`, 0.16343163967002294`, 0.04597060352203497`, 2744.3901597051326`, 303.2449917542002`,
  0.2131773205429714`, 4.894467965266408`, 0.9895276491017069`, 102.5510604483128`, 17.861027123778456`,
  113.14450046515275`, 0, 62.07231439800787`, 2.154144389273946`, 2.185256043192186`, 0.014442696633128804`,
  0.1272664865812245`, 0.12794432901376762`, 0.0053261659903725`, 37.91821594454562`, 0.2763567320376331`,
  1.1016134415940633`, 0.0012163562176128462`, 0.0012415117779544752`, 0.0206810800794836 6`, 0.20961855564590487`},
 {0.1701034994591067`, 3.2342889044936154`, 4.148994458467392`, 1.1940097836786847`, 0.6849042746613065`,
  0.28365434424986613`, 0.24647764422374546`, 0.16243634058501621`, 3080.6851495717774`, 370.74406137436483`,
  0.0951116860303981`, 9.02678426482985`, 1.2346863975759255`, 324.5846190696541`, 44.79014567611737`,
  344.99778653474607`, 0, 98.86040917795341`, 4.777158968044568`, 4.8053733094227065`, 0.005906092212310865`,
  0.06489409571300801`, 0.06492097374621572`, 0.00041418303024931546`, 220.72446064783898`, 0.15769589678531606`,
  1.7010999963320803`, 0.0006561881067512765`, 0.0006586665530297424`, 0.0037770362689693027`, 0.18765949884214134`},
```

{0.06598975858427436`, 1.9097424631845499`, 9.748507937422985`, 0.8697722369253212`, 0.5875961327091277`,
0.6558947995694533`, 0.20328697625166237`, 0.09192814399763598`, 849.932255943002`, 347.93153422311434`,
0.24798460225057473`, 8.434300711569456`, 1.1653187620032228`, 280.9780000789272`, 214.65463278515017`,
278.2337311050637`, 0, 123.46067910262322`, 5.5682334264784`, 5.640113095842556`, 0.012908882199935912`,
0.01864866508655414`, 0.01865768565271862`, 0.0004837110925963728`, 151.9127402781346`, 0.01758029867150515`,
3.608898487510784`, 0.0002589226028956171`, 0.00025971598392153945`, 0.0030641628697136714`, 0.5533776524686335`},
{0.24510065816515997`, 2.793418227932695`, 5.458971845554931`, 1.0315954512213719`, 0.7555698096939896`,
0.6374757156959183`, 0.22964026454409542`, 0.029915287766295595`, 3671.7453130653475`, 246.1074690301475`,
0.07031820961864549`, 2.73413282232541`, 0.7379537484131258`, 144.38887938074865`, 20.352772396357178`,
159.53065523669855`, 0, 101.07735078676961`, 1.0515473043542378`, 1.0568375652546917`, 0.0050309300195512385`,
0.062358790963852995`, 0.06252999936814047`, 0.0027455375840548246`, 41.963020107380196`, 0.21834543868042156`,
0.4283446303279133`, 0.0007272000443114823`, 0.0007352192595390338`, 0.011027523018297014`, 0.05088407194048422`},
{0.22754574199703576`, 2.0500842079624535`, 1.4317719989732147`, 1.4394511204103708`, 0.9096762268457756`,
0.3629999302632213`, 0.106795983345218`, 0.12347919331546342`, 3709.9742200770725`, 60.75950393256113`,
0.07245771005729762`, 9.46479696180289`, 0.2919149083841388`, 995.5239820150618`, 67.00558249547518`,
1825.6189383440078`, 0, 359.71067933310144`, 20.900009059446734`, 20.97761792329905`, 0.003713341158444905`,
0.662588531059913`, 0.6619990226113093`, −0.0008897051804696021`, 612.0968359863435`, 2.1538456991225603`,
0.8702441120800298`, 0.005544330261078034`, 0.00558316100703427`, 0.007003685590094344`, 0.05140199048224216`},
{0.20503801915719277`, 1.0262506067723134`, 1.0800304574674584`, 1.0922656655058383`, 0.19306135916753409`,
0.5113898504084636`, 0.20945291149465461`, 0.029409960652989255`, 1028.4817812589972`, 23.515618103058102`,
0.10367816050153927`, 1.7590551256620532`, 0.7974605289147969`, 473.7450085705832`, 20.040704362380957`,
592.1643446259717`, 0, 329.2771323636849`, 8.79889042666164`, 8.949471349749285`, 0.017113626353542077`,
1.683444512731969`, 1.6860282454951738`, 0.001534789381927304`, 128.9980948469231`, 4.93100183216582`,
1.3395830676523184`, 0.01837983940183152`, 0.018951464958919053`, 0.03110068290534529`, 0.04136650275041036`},
{0.20946691214847096`, 2.8105866650441875`, 8.539169469770648`, 1.3728902130481506`, 0.8085940550353596`,
0.2919084226376192`, 0.2093509791170406`, 0.014346546703503377`, 2081.7407715487734`, 169.23962157045707`,
0.03811656206153746`, 8.571585131217248`, 0.31122812087193474`, 568.3333842565514`, 76.67249749719875`,
770.6220085188266`, 0, 471.4270930229818`, 2.3420056133973275`, 2.3490936537677656`, 0.003026483083512499`,
0.0668814122164404`, 0.06689308854651897`, 0.00017458258866898113`, 94.0344249496167`, 0.20013489853009683`,
1.1805449400484411`, 0.0005885842947316311`, 0.0005901195251252336`, 0.0026083441358260018`, 0.07222307945758245`},
{0.07543371305305874`, 3.820737964468253`, 7.006759966869079`, 1.4837946743911272`, 0.9807341557825149`,

```
  0.31098343284147656`, 0.07258154131712924`, 0.2086007075373027`, 2270.591159595845`, 320.3654657559956`,
  0.09813455813826927`, 7.706001493171778`, 0.25408983910049043`, 200.6754926158249`, 91.99608601819017`,
  240.56796554553745`, 0, 51.16078919979314`, 2.6886927351988046`, 2.7032982122013056`, 0.005432185244261811`,
  0.0345887322194659`, 0.034609023718478904`, 0.0005866505567262426`, 146.75414868805814`, 0.03727366430155834`,
  1.0129552803613673`, 0.00028144411338992814`, 0.0002824630220196377`, 0.003620287585471393 4`, 0.1309380277934832`},
 {0.21236403141675547`, 1.9573734849300308`, 2.6535583097467583`, 1.4800706545848268`, 0.3008051623864796`,
  0.20266843878015717`, 0.18796743731817545`, 0.01387997496279432`, 631.4288286008941`, 122.32978434555201`,
  0.20929941217323872`, 7.603405617879708`, 1.123897133765297`, 489.36597008121015`, 105.9624204478217`,
  532.1507481759143`, 0, 408.3137418664404`, 2.7590288068544475`, 2.795943777222838`, 0.01337969733287303`,
  0.2248774563730758`, 0.22495546214813703`, 0.00034688125843906903`, 77.14928329564333`, 0.682226903001789`,
  5.461333511099616`, 0.0018308473391921432`, 0.0018433617150500653`, 0.006835291829106804`, 0.2553516636192586`},
 {0.19210055303112722`, 1.6234167219204663`, 6.9088164888327235`, 1.007010553449088`, 0.1818811973938239`,
  0.28265941449906173`, 0.05224493104477926`, 0.18359878228489843`, 3879.878639699238`, 252.77022062868934`,
  0.19702879614057178`, 8.129123272011793`, 1.4896800872011862`, 173.2423015537016`, 19.03652197082293`,
  184.16066934427622`, 0, 49.52422880704717`, 5.111061769267496`, 5.18454649279416`, 0.014377584706278368`,
  0.01948724595449209`, 0.019493093406395635`, 0.0003000655873695024`, 118.53404489996089`, 0.05347872464149838`,
  2.4260476991006468`, 0.0002339433906491939`, 0.00023443376941381918`, 0.00209614284577353`, 0.13616633794045427`},
 {0.25397209407460797`, 0.7718183137480485`, 8.774125702249133`, 0.8493367748387377`, 0.629933366847081`,
  0.44793398136834006`, 0.17220103999190545`, 0.007968172925746744`, 1049.3506020599693`, 93.83027794564788`,
  0.034976329114162485`, 3.5404695212710173`, 1.4516591802815526`, 386.457007202826`, 60.19799149869615`,
  406.8031280893948`, 0, 346.6927152620287`, 3.2543214993021468`, 3.2841444436511895`, 0.009164105130804678`,
  0.0662468664198843`, 0.06628186228824498`, 0.0005282645089788041`, 35.88207045693435`, 0.2403550770077340 4`,
  2.8898094050684846`, 0.000941241993101416`, 0.0009454750363101684`, 0.004497295318077077`, 0.08935973085042757`},
 {0.04503017472676637 5`, 2.4891362382491593`, 7.362757592309742`, 1.3044006364876828`, 0.9149255196562065`,
  0.40547780792622723`, 0.18724836348467627`, 0.040102604909543316`, 2100.7064218407268`, 212.58570231059025`,
  0.17762315543370177`, 6.925880601095635`, 0.30534617107023765`, 420.59850129070384`, 93.88691440261263`,
  535.1471259175289`, 0, 267.6806644029867`, 4.1792092826574745`, 4.190321486361037`, 0.002658924919044958`,
  0.06748839450569234`, 0.06752612054381013`, 0.0005590003791628995`, 148.60887532414316`, 0.04341448852305463 5`,
  0.9869303684495482`, 0.0006243820509050924`, 0.0006268531472229418`, 0.003957667127470144`, 0.08270351943745056`},
 {0.1552219340650659`, 0.8853243438823553`, 3.704192180484896`, 1.4225984400522793`, 0.30727783518964036`,
  0.6899910723821459`, 0.23852460823207766`, 0.04807056691157154`, 2363.417831098286`, 24.86201270145591`,
```

0.05644269407234525 6`, 9.159332366722428`, 1.3338948458352062`, 2945.0992111105083`, 61.278809677638115`,
3485.0411680997167`, 0, 1747.7396799656956`, 87.54699143806694`, 87.85868931267154`, 0.0035603493562095245`,
0.7758721298580715`, 0.7746052359618307`, -0.0016328642923062464`, 1107.2497536254375`, 1.7204624654824041`,
8.18587737071089`, 0.006565439822976082`, 0.006597147472609387`, 0.004829478372849083`, 0.12009988394243662`},
{0.20103480965042153`, 1.3452512500000138`, 4.3677490431720685`, 0.877487125235695`, 0.3607930851168655`,
0.3984625684159562`, 0.053314676617049916`, 0.01650783971749592`, 3182.8108727507833`, 127.76026016049212`,
0.014515185076648118`, 8.964504972667974`, 1.3475969548800526`, 406.6944716063615`, 35.09570736490002`,
437.22704128392047`, 0, 329.56604749930847`, 3.7962029622844145`, 3.8385529724019265`, 0.011155886694748096`,
0.057171508013307934`, 0.05718889195187591`, 0.00030406646898173406`, 72.95495400381235`, 0.16419233186970894`,
4.156200009991825`, 0.0007871177109817218`, 0.0007894013621237313`, 0.002901282883294831`, 0.12463208728739014`},
{0.2769245298620152`, 1.0085946471087137`, 4.542179183090196`, 1.0124324963510387`, 0.5561370973292907`,
0.2204460879068223`, 0.09699841861743363`, 0.4013041825941674`, 2543.8306625681216`, 129.04963561861496`,
0.06920814067274877`, 7.689149017779954`, 0.320100144071481 9`, 336.2729713632035`, 34.76856413855763`,
489.8447798532344`, 0, 50.721759584703214`, 18.512399929759635`, 18.582499639687462`, 0.00378663545481972`,
0.06113532500722681`, 0.06114305490027172`, 0.00012643906029774 5`, 266.73582106130425`, 0.2418553019362405`,
0.9696085390679906`, 0.0007294633263760231`, 0.0007317504755967829`, 0.003135386164130116`, 0.05897513323050532`},
{0.19504311625596543`, 2.4976887333066733`, 0.9780959245602254`, 1.4635414205075887`, 0.2583014895103719`,
0.4579159763055559`, 0.20087332417307124`, 0.20355826875644964`, 1388.181044794952`, 55.982899470557015`,
0.20280310820910474`, 7.420374461676447`, 0.562614521298161`, 885.2740523679362`, 64.51226068211095`,
1126.6181972827596`, 0, 227.33029824876783`, 17.82166153357651`, 17.95859273577116`, 0.007683413913829984`,
1.1152270955710613`, 1.1153775556987566`, 0.00013491434013124248`, 635.8994745888433`, 3.10739097218993`,
2.58863247970498`, 0.00911515711912636`, 0.009270092250276198`, 0.01699752720934855`, 0.13743758136007642`},
{0.13592819841871134`, 2.9064950846732005`, 2.3581026922159136`, 0.7780848542747187`, 0.30526997799339717`,
0.3770830944311493`, 0.13314104598243304`, 0.28747750393724975`, 817.0224638364293`, 99.04079463539466`,
0.1276940051281772`, 7.752046366250018`, 0.9291873586736861`, 551.48867313338`, 108.65567371473395`,
598.7232978362119`, 0, 112.14365861117372`, 10.322151171592676`, 10.430007383530409`, 0.010449005264964617`,
0.14722983865951694`, 0.14725375633559407`, 0.000162451282259779`, 428.58973776411`, 0.2858955246066777`,
5.406946172063374`, 0.0022793289477537337`, 0.002292826082838734`, 0.005921538924121394 5`, 0.23470281791504416`},
{0.09132185718513763`, 1.6890298787004205`, 2.765293267846525`, 1.131503269745704`, 0.5764776999808194`,
0.5819767311690106`, 0.1370413367124192`, 0.18256196348098624`, 2411.1512688147986`, 324.527748891113`,
0.027976143287822552`, 9.606504565586533`, 1.4766385859888795`, 277.66926127476574`, 79.10351757707278`,

284.5148467634269`, 0, 79.04473877965947`, 7.89740103781521`, 7.987207759768189`, 0.011371680572248577`,
0.0740890102221041`, 0.0742049100271347`, 0.0015643319391520638`, 190.55637595642298`, 0.09665637157848488`,
2.993937084740609`, 0.0007885541937499019`, 0.0007943919001142263`, 0.007403050304714886 6`, 0.34139043273963476`},
{0.2668975721045642`, 1.1689144747314444`, 8.555782168057934`, 1.3239106462159618`, 0.5762520317144466`,
0.46812793372285433`, 0.21778203282682917`, 0.19497129015379866`, 2321.6608050102914`, 177.91119737865392`,
0.06980210378448115`, 6.741021070773617`, 0.3841763241252334`, 346.68715440615557`, 50.24404376011543`,
440.6752532245899`, 0, 92.25031148105552`, 14.362387729914369`, 14.413395656672277`, 0.0035514935063107878`,
0.04989331930482043`, 0.0499267251971787`, 0.0006695464006749585`, 239.83432727431614`, 0.1902343683819423`,
0.994622042853188`, 0.0004547617739653065`, 0.0004568419105550096`, 0.004574123659438989`, 0.10653370488857825`},
{0.24059659553061208`, 3.474305761400969`, 0.3551751508042571`, 0.7879508982528726`, 0.7115917578613755`,
0.16553560182957539`, 0.059563521780316875`, 0.007818446748999992`, 3031.006140812062`, 311.2165043893773`,
0.22179274663929038`, 7.521418478036052`, 0.4828099783575506`, 159.22861714023023`, 29.597814392756842`,
185.8140239347897`, 0, 142.04161692733817`, 0.31122696061610294`, 0.31595549086028885`, 0.01519318967362393`,
0.2921524861927906`, 0.29244714839898345`, 0.001008590445464863`, 15.447108891026271`, 1.0041556221970025`,
0.9624184160591398`, 0.004458941836321562`, 0.004529939371029623`, 0.015922507472452496`, 0.09659736359381448`},
{0.2655286560784189`, 1.7978740647108253`, 3.744260697890588`, 1.4979569598721256`, 0.2121339839068337`,
0.5203547954080169`, 0.20358958391513882`, 0.3001328269718666`, 1853.7326098612984`, 25.1210343423665`,
0.20564102192348216`, 6.431952930060138`, 0.9412816530341135`, 1688.47436162765`, 42.530931590121575`,
2095.32254444766`, 0, 321.70091134174726`, 51.102533522630054`, 51.30985805445908`, 0.004057030396295458`,
0.6587946627383139`, 0.6581060990112032`, -0.0010451871668913926`, 1312.5131380193136`, 2.49898373469083`,
3.78452720639372`, 0.005295196034692307`, 0.005328428297116239`, 0.006275926746848848`, 0.06706377156263715`},
{0.11579761780826803`, 3.5947224028634324`, 1.7024337214669405`, 0.9403811808729834`, 0.8023022872362131`,
0.2690829028689966`, 0.10714511128523063`, 0.11270721526081176`, 1656.7229648447174`, 66.45258485414047`,
0.04987530781728505`, 9.865292743118054`, 1.1705893328455934`, 1439.158027218313`, 104.92540266691614`,
1568.8768096315787`, 0, 554.502546362605`, 16.872153502832287`, 16.9574858162848`, 0.005057582805786387`,
0.506564781412956`, 0.5054176236668605`, -0.002264582513801683`, 866.4386883025995`, 0.8379856421881756`,
5.9236571077899205`, 0.006487777447666931`, 0.006512412279491312`, 0.0037971141925094987`, 0.09059124278163248`},
{0.23978880653880486`, 1.194922909654343`, 4.719390315894781`, 1.228676410453662`, 0.2690436202211013`,
0.15286621224443908`, 0.10420745578996382`, 0.13476606932746013`, 2057.7110605479693`, 394.7187402510973`,
0.20560140619917516`, 7.632899208080902`, 0.8870899800709835`, 123.71575783755716`, 28.8845783438613`,
134.44511290355854`, 0, 43.24886022464682`, 4.446505205338859`, 4.5059002102426895`, 0.013357682530657167`,

```
0.026458219978346152`, 0.026466646639542 14`, 0.00031848934670919427`, 75.9032991108097`, 0.09063407131070668`,
 1.5430849253821608`, 0.0002602425016463572`, 0.00026101363053693233`, 0.0029631166534935804`, 0.17581299992546215`},
{0.14115594725086872`, 1.0548987126390301`, 4.221995326207644`, 1.1742416630307742`, 0.9684670314689854`,
 0.2982166752619343`, 0.232586446320313`, 0.2439177749163077`, 3034.8452920870423`, 398.0846179891778`,
 0.1346647166413732`, 6.263806688157171`, 1.2365678782206118`, 313.5428123331356`, 52.22570303168065`,
 328.815773791622`, 0, 70.75726241212494`, 15.091632932088325`, 15.160342465762993`, 0.004552823010197438`,
 0.08728162101791573`, 0.08732857640831425`, 0.0005379756912269329`, 227.43063073829674`, 0.17600456989155508`,
 1.1595623598078595`, 0.0008969272929229`, 0.0009010613205708051`, 0.004609100069230099`, 0.09207204220949683`},
{0.19091547503659084`, 3.8724415822010574`, 1.7819835666756205`, 0.970240031108671`, 0.6126075063757175`,
 0.3126984772797464`, 0.17470342972606634`, 0.006235187142203773`, 3131.6216914066945`, 328.479620734891`,
 0.10630619937563857`, 8.167239786634365`, 0.8931338365031225`, 250.24110149727417`, 38.15362109321355`,
 273.2488799348025`, 0, 229.3072367854833`, 0.3604932045846788`, 0.36340043126344973`, 0.008064581084462619`,
 0.10446478542138679`, 0.10456949997733157`, 0.0010023909542569154`, 19.942698221923166`, 0.2849134876188675`,
 1.3828526830518535`, 0.0012973398597325714`, 0.0013067521826212777`, 0.007255094197635037`, 0.15863437422684998`},
{0.1729303989394022`, 1.7633209846264775`, 4.719997292494861`, 1.358984906012456`, 0.8429652151291751`,
 0.587790420447317`, 0.06264894796386378`, 0.017353544405872348`, 3524.820728982824`, 361.1436357327882`,
 0.017184717238444003`, 5.167166487729965`, 1.3598724575284078`, 116.70114144836133`, 46.1853408772872`,
 118.91741297896097`, 0, 93.64228072140646`, 0.8734665374056514`, 0.8852018124619799`, 0.013435288650191834`,
 0.04068298031899392`, 0.040774794500370304`, 0.00225682043587927`, 22.002883925377326`, 0.10050462880867422`,
 1.3674596782217758`, 0.00036060442846852503`, 0.00036365912695382393`, 0.00847105094707823`, 0.1290674258087965`},
{0.1806934944296955`, 0.9977416627132909`, 2.873305835487411`, 0.9197375741494748`, 0.03558129425516987`,
 0.4888154998532678`, 0.06242566822825624`, 0.4327688666695781`, 577.8384758076331`, 373.1652197475112`,
 0.03824901809490494`, 6.041786978778951`, 0.10163020529956279`, 100.72210668186901`, 106.76029225746541`,
 88.85192519193521`, 0, 16.085094663288412`, 5.540871571044408`, 5.683871949515295`, 0.025808282440289876`,
 0.03339305395234518`, 0.03346167023391938`, 0.0020548070168162624`, 78.97654877392351`, 0.0861986801188624`,
 2.6074376627794025`, 0.00043622982337276905`, 0.00044095180972529807`, 0.010824538120801419`, 1.0037910380346633`},
{0.05413140542514305`, 2.0680722669723384`, 4.8344183696420355`, 0.8194669559280681`, 0.2769337929060782`,
 0.48015527013462056`, 0.18187780015894756`, 0.008280997540164719`, 3109.753891098221`, 77.01250512455277`,
 0.1133305062638244`, 4.98724945291187`, 0.14526723437594202`, 145.13437618558052`, 19.892758804441684`,
 529.8978181816296`, 0, 129.6711829449446`, 0.5006513301432444`, 0.5026576015703172`, 0.0040073226740431345`,
 0.03092094640606034`, 0.030956744813853375`, 0.001157739718675721`, 14.791187589886494`, 0.02391134694338584`,
```

0.40803972668173166`, 0.00045467960901546256`, 0.00045748679187760744`, 0.006173980109254185`, 0.0915394264539363 8`},
{0.100462842594787`, 2.3014533321484603`, 1.3291106382168039`, 0.7767037741986953`, 0.6186202165209542`,
0.5591414823289833`, 0.13230502890488538`, 0.011503939527627875`, 2830.1764339684587`, 75.11206729916319`,
0.04992405177453235`, 4.899377436291939`, 1.144111391119885`, 565.9488757384081`, 35.07755047925669`,
626.5353158561122`, 0, 485.2118977137579`, 2.3427711843236705`, 2.3579177296132534`, 0.006465226049788386`,
0.42290707851088744`, 0.4230215950590996`, 0.00027078418411763394`, 77.02540783747291`, 0.6069492465808966`,
2.2825898224263987`, 0.006544699011659549`, 0.006605242429003854`, 0.009250756564426421`, 0.07232165153721665`},
{0.04855602650750329`, 0.6081046972703708`, 9.327171063477682`, 0.9416092665673466`, 0.16765715967906902`,
0.2590243352245839`, 0.2187512491754251`, 0.05460948171436536`, 1404.1776553137433`, 151.91892927077328`,
0.22495478161340088`, 3.6643606235348707`, 1.0355618722545916`, 168.12337414310247`, 22.136995559785266`,
192.15770338954843`, 0, 95.02186951778917`, 7.540882054493272`, 7.60582537067135`, 0.008612164427022284`,
0.02905763244711748`, 0.029070107842423167`, 0.00042933282084800517`, 65.50922569856`, 0.020156045305042312`,
1.4415794767612857`, 0.0003727233638837202`, 0.0003738466903023756`, 0.003013834193141385`, 0.11027134238006`},
{0.07918907579486661`, 1.9928853739387806`, 6.231134938054945`, 1.0170500506380848`, 0.5386394257050193`,
0.6572554793818131`, 0.08180903968242625`, 0.09102806961295826`, 3292.3299295864044`, 25.797137029704572`,
0.14701504075704036`, 8.245305783801324`, 0.46404074549033125`, 1686.8261826835494`, 50.58048608091667`,
2454.0724251416354`, 0, 734.0052525356871`, 32.31693577713266`, 32.35690866799728`, 0.0012369022589358014`,
0.20965509297583734`, 0.2095785799899108`, −0.000364946946150968`, 920.0564091538096`, 0.23717704355193905`,
1.6458431303611338`, 0.0024881595001888 8`, 0.0024952266665776586`, 0.002840318873545744`, 0.036518173166096846`},
{0.08056415815759815`, 3.9535505406039233`, 0.5752240632800181`, 1.4853820342325343`, 0.15510043342374935`,
0.6600536355917208`, 0.1525299204594313`, 0.16813894995214293`, 3147.186246353149`, 370.3183465224665`,
0.19963532109395127`, 2.5689014931104612`, 0.8619285687304634`, 46.05322505876729`, 10.705668199061575`,
48.732247950366286`, 0, 13.7084828074934`, 0.5522715320405546`, 0.5657004502330724`, 0.024315789269274912`,
0.27921342751660416`, 0.286735542520165`, 0.026940377010032934`, 31.191905915129894`, 0.32135135334525955`,
0.5165359478335356`, 0.002210381875978662`, 0.002368585925756124`, 0.07157317543033859`, 0.20935956652429544`},
{0.2470446862474004`, 0.7616885134704638`, 0.987661776479948`, 1.418205334084091`, 0.19365902643767297`,
0.4570709516573175`, 0.06398975270684754`, 0.008457878153676336`, 2687.6783620781935`, 271.5689565049082`,
0.13340639067828225`, 6.091940716408539`, 0.7457858481599575`, 123.84567514766934`, 25.69161512098768`,
133.3739626729705`, 0, 109.82832042648806`, 1.1025702654912655`, 1.123681024959166`, 0.019146860865592474`,
0.1808823475764464`, 0.18204475756290517`, 0.006426331823051523`, 11.99735866455397`, 0.6383717543530965`,
1.435312548050202`, 0.0015263082085492652`, 0.0015643377387240516`, 0.024916022833247453`, 0.21369667249605684`},

```
{0.21332713902898792`, 1.1214424929874225`, 9.162265965223703`, 1.195968506251218`, 0.6194628623567042`,
  0.6911884704772038`, 0.19705041057272926`, 0.35280397622564785`, 1556.9191271276404`, 81.28832183992904`,
  0.22397741498275942`, 6.620834183658268`, 1.366777947793718`, 883.8428016330948`, 99.23211542105192`,
  945.5505350583958`, 0, 150.22016316226242`, 43.07603661780301`, 43.33056901396351`, 0.005908909364593207`,
  0.10928790966134244`, 0.10934144842720792`, 0.00048988736294219 85`, 690.1042556098075`, 0.3330582442636007`,
  4.824637617416535`, 0.001102678527426404`, 0.0011073196728694915`, 0.00420897417302557`, 0.14838902302144205`},
{0.25495798287734545`, 3.119749211986244`, 7.11103782773306`, 1.024996658386143`, 0.715060033499787`,
  0.6413092223890626`, 0.1635833958172549`, 0.00714360523367534`, 2176.4539355050265`, 202.8994026859108`,
  0.2178929688734338`, 6.062772582072755`, 0.23204754406386696`, 265.7896598899209`, 72.43412814793733`,
  350.26551456861483`, 0, 240.87307489136393`, 0.537584252983774`, 0.540128505625492`, 0.004732751429374016`,
  0.03960171347874999`, 0.03964410505726382`, 0.001070448088984266`, 23.958972137462002`, 0.14423961410042732`,
  0.8319459604117541`, 0.00046564360311307507`, 0.0004685407915159808`, 0.006221901006556285`, 0.13870982547312594`},
{0.13839247434786828`, 2.7262203960757274`, 9.158826513860994`, 1.4613001862440893`, 0.08417876673230018`,
  0.5490480248818964`, 0.16559733844524815`, 0.07310698628337725`, 1278.2933939526329`, 222.03326298760976`,
  0.23267447938843522`, 2.718547094516405`, 0.8865837550934321`, 77.55346272805954`, 23.493469554418432`,
  83.30691675757029`, 0, 38.422049683962115`, 0.9774814690784673`, 0.9918040553198234`, 0.014652539914499885`,
  0.02847031728198757`, 0.028543747614337634`, 0.002579189112041247`, 38.06899882553982`, 0.05628682363034778`,
  1.090014056840952`, 0.00023410361074793773`, 0.0002367217116262833`, 0.011183513445097937`, 0.16564279969002524`},
{0.21091599252169352`, 2.726329060939385`, 6.86461568422051`, 1.1885210188613522`, 0.09348887154977148`,
  0.3177710803820454`, 0.16213356559574865`, 0.08883572559161597`, 2040.1598914696724`, 141.3083228416025`,
  0.09021913069336979`, 2.444490167264778`, 1.4613873909313537`, 105.74031319121526`, 10.313598055484002`,
  117.32788910338819`, 0, 47.1249366493537`, 1.4625986754074487`, 1.48043661883123`, 0.012196061519618162`,
  0.04686504978474022`, 0.04693445976205736`, 0.001481060569357151`, 56.96464676078245`, 0.14120840699912376`,
  1.1376784168826763`, 0.00047535834109269093`, 0.000478905320251714`, 0.007461695425118098`, 0.07777606857312963`},
{0.0736068126313521`, 3.668322450041937`, 9.040034492942976`, 0.8406840844419607`, 0.9489073810268334`,
  0.18492930812213693`, 0.07953103883609952`, 0.21231255359096723`, 2489.458988929943`, 66.10862660280799`,
  0.025206551238390773`, 7.73776692806604`, 0.6102159281560899`, 949.9175762082127`, 53.58273493760965`,
  1197.2554242779117`, 0, 236.75154479911444`, 13.351263345097056`, 13.376813990893394`, 0.001913724951408513`,
  0.07168653801774429`, 0.07166200750556269`, -0.00034219133549906466`, 699.667700932023`, 0.07538025102982474`,
  1.668250276917393`, 0.001031134954112444 8`, 0.0010320810026016036`, 0.0009174827071720681`, 0.024329346203729726`},
{0.24859051354510608`, 1.2059184673508376`, 9.814154373421665`, 0.999806271433672`, 0.5018863929556321`,
```

0.5028910758235472`, 0.0547445799608962 7`, 0.010244417191277673`, 1992.5389939670486`, 81.33759966126644`,
0.05634540009721645`, 5.237660071402415`, 0.5456697726165687`, 358.6121603004655`, 42.75039861002982`,
422.3546679402722`, 0, 312.6694498150313`, 2.4963371100315936`, 2.511727715913219`, 0.006165275442879148`,
0.043741749427537654`, 0.04376556636438089`, 0.0005444898101913953`, 43.0054145960045 6`, 0.1553397707650267 8`,
1.7586806561326278`, 0.000528239660211649`, 0.0005302106055435445`, 0.0037311574278722226`, 0.06983321937295067`},
{0.15548452904006277`, 0.7941888445067193`, 0.96068281269703`, 0.8969171753329834`, 0.8390319554716188`,
0.17764885192591462`, 0.10783704631792568`, 0.4257693977926573`, 1410.5075470475076`, 21.97142523719117`,
0.06053827716094551`, 9.545575914809223`, 1.320581285010927`, 4244.599933076013`, 97.9433537905959`,
4768.146036554343`, 0, 606.6192917574284`, 293.98838637337593`, 295.27105420520644`, 0.004362988101854759`,
2.64834556117061`, 2.6011893144822302`, -0.01780592660556579`, 3335.461383889515`, 5.882525175914634`,
9.959524063673463`, 0.03508320191124792`, 0.035177761356340674`, 0.002695291191834981`, 0.04751012379079507`},
{0.23828594722598612`, 2.796383207017321`, 7.808028439446116`, 0.842565467286883`, 0.02113404997076329`,
0.6079237099359636`, 0.11031479903153951`, 0.30382867676630676`, 3734.3994148280126`, 266.6055228534507`,
0.0826889096493309`, 9.854969604997791`, 0.9421568133509814`, 207.0820627147839`, 30.022351031643403`,
227.73024064811642`, 0, 40.44348861329633`, 4.067953983331795`, 4.110194939441862`, 0.01038383331845627`,
0.014224489772369543`, 0.014231188510744546`, 0.00047092995827613393`, 162.50797437011835`, 0.04842137170324865`,
2.1520838328696805`, 0.00020394211720853317`, 0.00020453112111517942`, 0.002888093517456003`, 0.3172233084201244`},
{0.25363168051984797`, 1.914628055312483`, 6.622889612666614`, 0.7775608926930352`, 0.30452546646747747`,
0.5186902253252116`, 0.23177406235661663`, 0.009523543254042525`, 2339.914222113791`, 312.39264418675145`,
0.06116037168819993`, 6.862836561311433`, 1.3371389516738463`, 188.14506373767708`, 39.21863083041139`,
203.14371801286788`, 0, 165.63857570328622`, 0.7857707517626323`, 0.794720797444234`, 0.011390148668075195`,
0.020184506920354884`, 0.020196850203104404`, 0.0006115226296199427`, 21.492267519553092`, 0.07313472015250803`,
1.9606015024002272`, 0.00031338970146554956`, 0.00031459748613794474`, 0.0038539386161926537`, 0.31840046473888894`},
{0.11287267839860893`, 0.6274623554262155`, 1.4782831154053753`, 1.2121235395747008`, 0.42002460109961204`,
0.37694632007071904`, 0.10874502522899515`, 0.13201309589288884`, 1491.5919687682626`, 61.86932965722724`,
0.04486960703076642`, 6.490904378609384`, 0.9946688583085637`, 721.7261532509043`, 55.69688247084482`,
810.908722937453`, 0, 252.13189475170122`, 46.98050805531155`, 47.335525482253885`, 0.007556696205250901`,
0.5712259646052915`, 0.5711902843467451`, -0.0000624625993165262`, 421.1214320500872`, 0.9210829227969543`,
3.9848886985665506`, 0.005664769244066892`, 0.005716407858365161`, 0.009115748951707747`, 0.1035432461805923`},
{0.2430762813238787`, 2.3310866531539265`, 1.0183842540902877`, 1.0634798069102733`, 0.11637048805058381`,
0.6527039790628004`, 0.1653633422639308`, 0.011869416806071493`, 3558.3378742172754`, 124.12326765089796`,

0.15620828250230562`, 4.139014546068248`, 0.5423323870297714`, 185.67878049650463`, 14.708721268848858`,
236.24338320692348`, 0, 157.650733441625`, 0.7761969952834772`, 0.7848021163362862`, 0.011086259165002721`,
0.28986389998814494`, 0.2920385940392261`, 0.007502465988935114`, 25.848320798907057`, 1.0065576985593503`,
0.6485675707101902`, 0.003260829565593814 6`, 0.003347859617879819 7`, 0.02668954342302654`, 0.1089047125200582 7`},
{0.12740998379951485`, 2.0965772202079593`, 9.26399010812127`, 1.4391840070681714`, 0.39059961846309776`,
0.5024732906844885`, 0.2058431543840883`, 0.008446302531916432`, 3264.970078645587`, 50.15257581792508`,
0.04320568862225377`, 1.5793394493087352`, 0.8400374654569864`, 227.3488628433876`, 6.710613420553291`,
285.39755660563185`, 0, 202.40394108506925`, 0.787400089246165`, 0.7908803918307415`, 0.004419992621423896`,
0.13985007772715538`, 0.140153203040363`, 0.002167501928737048`, 23.583501290046105`, 0.25454708767967976`,
0.4285637929918271`, 0.0011719203415987423`, 0.001180953540302612`, 0.007708031325360132`, 0.016143603347283743`},
{0.2006523908963831`, 1.4235542095429166`, 8.760081949765812`, 0.898891491102243`, 0.20916515630212307`,
0.606680748263352`, 0.1734284929050921`, 0.008390530446632872`, 1251.1518948117655`, 294.61074552228456`,
0.1942512237263747`, 3.244161217546491`, 0.31687231038469954`, 74.10551919459263`, 33.82710429223264`,
83.1881955781802`, 0, 66.15353197749045`, 0.3672663620778794`, 0.3717520898028097`, 0.012213826770171687`,
0.014680805319483832`, 0.014704053931569042`, 0.0015836060474390123`, 7.4689082251354035`, 0.042081981251978706`,
0.809542089792373`, 0.0001966026603412723`, 0.00019817713675080072`, 0.007989932103185549`, 0.23259485320477705`},
{0.2188265776716416`, 1.1127955725409375`, 6.7824679934503616`, 1.4242069861528708`, 0.19844568020247255`,
0.5897838311529561`, 0.14866055702548336`, 0.11878670475760818`, 3389.027367705158`, 288.45426002597424`,
0.06947315366014212`, 7.544154049880284`, 0.26623655067609353`, 141.29466362310515`, 28.85256874055941`,
192.12493142008188`, 0, 52.79441628282018`, 5.231588895777681`, 5.261511675822909`, 0.005719635208603302`,
0.024640228241835028`, 0.024672361995584577`, 0.0013041175363379676`, 83.16698515108182`, 0.07702766884590974`,
0.7183622803099865`, 0.00020846243140459908`, 0.0002098655003736052`, 0.006730560319921253`, 0.2426868350537366`},
{0.09304029298502164`, 1.718583593389087`, 3.3604210127127847`, 1.2870363565175618`, 0.488105058647589`,
0.628165881843018`, 0.2385374614927307`, 0.005700260097503202`, 2964.7790422043363`, 284.02452311663967`,
0.049929771885406815`, 9.399697265119372`, 0.1891315432188958`, 243.0297147223457`, 58.48492277542652`,
370.1740051103994`, 0, 224.43197109725034`, 0.7125575216316288`, 0.7162184021023346`, 0.005137663079217702 5`,
0.06201444120883341`, 0.06213082627721966`, 0.0018767413866445093`, 17.49413808603011`, 0.08242631113390804`,
0.8507851885297776`, 0.000579348259379775`, 0.0005847435110930459`, 0.00931262263400412`, 0.27349619711317613`},
{0.1372470868668202`, 0.5235381747902137`, 5.631494663469729`, 0.937247593354749`, 0.03802747675264895`,
0.555726088455389`, 0.17904217377421067`, 0.005332568756650943`, 2773.350295110703`, 213.09690601297643`,
0.06159806784551985 5`, 6.563446448883308`, 1.299385122835837`, 193.8911356188973`, 25.83224751462325`,

214.1095017988333`, 0, 180.03720012370468`, 1.598953096052022`, 1.6186800892365067`, 0.012337443314124119`,
0.03082122403892334`, 0.03085142269130597`, 0.0009798005538161902`, 11.958756935460517`, 0.060430331614468515`,
2.1562583709960896`, 0.0003966740069386576`, 0.0003986721731729854`, 0.0050373006533719344`, 0.26902915761355195`},
{0.1326843363181519`, 3.8211461387770695`, 8.543417929499473`, 1.102470057077619`, 0.6181175624574178`,
0.32965325202173645`, 0.07539452320036058`, 0.007386442292018944`, 562.3263637037148`, 31.555160873736668`,
0.05487832008430005`, 6.002761669320297`, 1.041478797158169`, 1357.4561615078567`, 160.06268848705972`,
1476.9335098992926`, 0, 1227.2473649924057`, 2.31162252664017`, 2.3308629537094574`, 0.008323342953943325`,
0.1832092382233299`, 0.18313162828199778`, -0.00042361368937926525`, 126.18639274258871`, 0.3472713740144541`,
9.548868040134987`, 0.002006449458411219`, 0.0020116695995584786`, 0.0026016808573852135`, 0.07835323005408247`},
{0.22025109593540232`, 0.8195100379779809`, 7.427283096510775`, 1.0066916952384835`, 0.042788382562981786`,
0.22140131473144997`, 0.09976052534424382`, 0.3169961605914291`, 1982.7909761773044`, 250.35193827375224`,
0.1491805579320653`, 4.623227908408321`, 0.5222292355158549`, 93.14983110161575`, 17.203532073122155`,
109.72938519976313`, 0, 17.546140768024003`, 5.944045481590515`, 6.004496612606125`, 0.010170031706997484`,
0.017123804016650594`, 0.017131489965256406`, 0.0004488458638243209`, 69.588641976587`, 0.05387909430356104`,
0.8877776663981978`, 0.00020549092320965023`, 0.00020616758477175955`, 0.0032929024384154015`, 0.10350931596742509`},
{0.1573792753543678`, 0.7169949173367489`, 0.7718194209824034`, 0.9372436231934692`, 0.09272278999885297`,
0.4028207043118923`, 0.14589558430857152`, 0.11971411886775803`, 3682.2389249814178`, 277.8795147586992`,
0.21886799519974814`, 6.424836122129667`, 1.423509413643259`, 144.4896707104504`, 17.098920185857086`,
157.3858728063569`, 0, 54.11030571926659`, 7.989712778998303`, 8.109771804253475`, 0.015026701031200851`,
0.17016419864238483`, 0.17079061701695614`, 0.0036812583350025463`, 81.83690647888919`, 0.38257597533730336`,
1.659950007772212`, 0.0021788271378945234`, 0.002214484458114401`, 0.016365373645168768`, 0.2367726541447332`},
{0.18899782585068592`, 2.4715170036459453`, 7.1946744158464355`, 0.9247708579288002`, 0.021153717979273567`,
0.5975157085148236`, 0.06082076754336166`, 0.3491165783662039`, 1598.0596114559885`, 238.85974939012692`,
0.11421989314530723`, 3.1171043316464786`, 1.3648370017589126`, 68.16378433364996`, 21.471628713374148`,
69.10138471175253`, 0, 12.583569224555939`, 1.5290308065262976`, 1.5638473072755275`, 0.022770306916397143`,
0.017595817227776495`, 0.017629205176401094`, 0.0018974934890716444`, 53.986080534689044`, 0.04750816000165467`,
1.4980774113702897`, 0.0002290910150282155`, 0.00023099618210099834`, 0.00831620163081559`, 0.1460902304881338`},
{0.26630725498764907`, 1.3537243132764054`, 3.4681976762260653`, 0.8182294329126494`, 0.5922927850334725`,
0.35114377894258253`, 0.24401965334161962`, 0.12295914082921328`, 3688.719544583101`, 187.1594487192641`,
0.18710725308822346`, 1.0160874849867394`, 1.302279127254418`, 57.779397972490315`, 3.9398611266152144`,
64.08906496602934`, 0, 20.97322011014029`, 1.7899552723542813`, 1.8102585775274513`, 0.011342912019508544`,

0.08334493212677702`, 0.0836383491618596`, 0.0035205144163565905`, 34.61579959804687`, 0.31707657274016865`,
0.25216197795962086`, 0.0012248544876398704`, 0.0012460964522791444`, 0.01734243932942947`, 0.02256615089001676`},
{0.10674642605203749`, 3.74860921502051`, 5.369830496673435`, 1.1859139787437987`, 0.18962703041335294`,
0.5643238513377844`, 0.22288847642351867`, 0.016526944643235637`, 1801.300442728153`, 341.7838883552571`,
0.21833110166851838`, 9.013661833764864`, 0.39778302570712243`, 190.9485885670551`, 63.074586118666126`,
221.05894457029083`, 0, 154.66326320806027`, 0.6624704507691235`, 0.6685755344108026`, 0.00921563163246164`,
0.02932924494552042`, 0.029360424693955514`, 0.0010630941400984195`, 35.47632623474176`, 0.044725601096268275`,
1.659090067154246`, 0.0002981128483561246`, 0.0002998252518899455`, 0.005744145357248431`, 0.4876097869426688`},
{0.08668509537236802`, 3.2156402873225405`, 9.589080153119365`, 1.1094271452742452`, 0.38296738962607746`,
0.3187881217442111`, 0.09471255988041424`, 0.04715614001519508`, 3816.5009707519202`, 190.70162191213433`,
0.12608220202713782`, 5.338078693011617`, 0.15622194317981242`, 88.51680762771142`, 15.791280807106174`,
200.53660296570206`, 0, 53.01743437668821`, 0.7556897002311279`, 0.7590614954802631`, 0.004461878001121411`,
0.012034197546722516`, 0.012040749710472896`, 0.0005444620403596279`, 34.7146606396844`, 0.014902650886731051`,
0.36700350271759175`, 0.00013101958394690083`, 0.00013142482709059090`, 0.003092996722186880 4`, 0.08787738291205155`},
{0.2778234194471715`, 3.6898619438208646`, 9.09826349254168`, 0.7771496857479276`, 0.47494449837227326`,
0.40530279224610744`, 0.24790715727254958`, 0.03660245395755471`, 1572.9621965794513`, 202.7146387762226`,
0.11027468006890989`, 9.901732606435996`, 0.25331571472189185`, 403.18269062913623`, 89.6857629711084`,
549.22095075161`, 0, 265.2392611515495`, 2.565710749574716`, 2.5773358876076182`, 0.004530962048169052`,
0.021826409803437377`, 0.021829468840602415`, 0.0001401530161206921`, 135.24454933868344`, 0.0866269686549177`,
1.7024265950017128`, 0.0003395012715743384`, 0.00034013064820831305`, 0.001853827030031674`, 0.22313914974447907`},
{0.055450231563250685`, 1.3594142714077613`, 6.005951115471895`, 1.0656701550312766`, 0.9603365707697988`,
0.4169951955673704`, 0.09937287247557686`, 0.03650907654126198`, 3150.102571227402`, 288.6133211175843`,
0.0673017768034384`, 1.580996224465487`, 0.5598870619846228`, 53.79709237829971`, 15.45716320590283`,
60.2103697232662`, 0, 35.391418756528644`, 0.8978682948352967`, 0.9030524153963418`, 0.005773809578604405`,
0.03773992809528381 5`, 0.03785681803280137`, 0.003097248548604492`, 17.436785340623615`, 0.029895539315210516`,
0.2378515086920717`, 0.00042600788892921404`, 0.0004305858660656748`, 0.010746226197753339`, 0.027453170137897262`},
{0.18890773944419526`, 3.611362633522033`, 5.813075251756651`, 1.273103869446512`, 0.8898144869747651`,
0.6114578055859192`, 0.07139582948563947`, 0.027880925348167697`, 2889.9541028194217`, 350.19982759883226`,
0.08313976867192174`, 6.993039414663205`, 0.8874083489329938`, 179.50636637955034`, 88.70840983566373`,
183.95270603648578`, 0, 128.7208007036521`, 0.9627326540252369`, 0.9722510329354165`, 0.009886835011134965`,
0.03521856128258805`, 0.03526787033585875`, 0.0014000870982504576`, 49.66823904026049`, 0.09504369711963392`,

1.606639616381086`, 0.00033344249476452426`, 0.0003355996715213487`, 0.006469411639772549`, 0.18165101476390014`},
{0.09328585740614403`, 2.929301480876771`, 5.799770741738055`, 0.9079806695024137`, 0.4314055581005425`,
0.32164171745260006`, 0.17247524633063527`, 0.16386897475111406`, 712.2588807999496`, 70.83959017853539`,
0.20999817663531567`, 4.513003403554821`, 0.1126618170824738`, 244.0751327648031`, 75.06617621545819`,
576.7772120126691`, 0, 74.04576983769456`, 3.9653851708496424`, 3.9914029784519247`, 0.006561230871982904`,
0.053095796743406226`, 0.05312834054850511`, 0.0006129262031071558`, 165.94012361738112`, 0.07075838462693126`,
1.482871281415991`, 0.000705270261266544`, 0.0007086970723922545`, 0.004858862359454319`, 0.12506675940280046`},
{0.06022915767996573`, 2.387138635828979`, 4.788528842745`, 0.9281538320395417`, 0.2752953410792893`,
0.5017610843522332`, 0.23773129300939827`, 0.14096915249034483`, 1499.4959807822306`, 322.9162482919975`,
0.15516909665873135`, 4.726500227282653`, 1.1616754765054393`, 123.99175603804568`, 39.88555723612112`,
131.89307130613918`, 0, 42.118166382807196`, 2.330760804021554`, 2.3602951855311223`, 0.012671562632514233`,
0.031857824045600024`, 0.031907015912604295`, 0.0015441063060006588`, 79.48355951650915`, 0.027410998682749717`,
1.5563552115083579`, 0.00041325355725030377`, 0.0004163557936203245`, 0.007506859446443137`, 0.2860818493653741`},
{0.24946839754186306`, 0.6156533776470687`, 5.962248269725286`, 1.0862299419007784`, 0.6836705094026099`,
0.5738298940987708`, 0.24726310480002844`, 0.11371634775304798`, 3850.890468269413`, 155.80403877473225`,
0.1865059666282849`, 5.974825484126367`, 0.19524283912722917`, 269.41889359999055`, 35.53337775396924`,
536.1702872271123`, 0, 102.82163611165112`, 16.984329904355032`, 17.02537037160552`, 0.002416372472838324`,
0.05147180856272032`, 0.05152979947750416`, 0.0011266539180019297`, 149.37800103840394`, 0.18343699429619462`,
0.41648975846463815`, 0.0005710579757149947`, 0.0005747862357863887`, 0.006528689257383924`, 0.07364388241019157`},
{0.16738121319303478`, 0.840466372179935`, 6.567452802957117`, 1.3673648738160649`, 0.9708045560189591`,
0.43291645558985303`, 0.10721701325461769`, 0.4069236600033958`, 2609.682510998089`, 166.81040184723918`,
0.052553234892402045`, 3.550175474274951`, 0.3845611655536718`, 205.33957324598805`, 45.83932681573903`,
256.10246658942793`, 0, 30.55365213164506`, 13.418524191341344`, 13.47010121688643`, 0.0038437181920771835`,
0.07540375959261408`, 0.07550775585439655`, 0.0013791919971142264`, 161.1116906729333`, 0.18030246799938965`,
0.5308863022598581`, 0.0006644418840561217`, 0.0006693287310103126`, 0.0073548147271493836`, 0.04202264648688574`},
{0.27699847669745875`, 2.1853512734955096`, 5.84751172204828`, 0.9661484791489172`, 0.9868204791467026`,
0.40527218684546984`, 0.18624243293429998`, 0.043237932216039034`, 919.7606571301644`, 386.4267934374768`,
0.13084528987555377`, 5.85403217789433`, 0.6639489535590795`, 273.61654097963174`, 216.0479886585115`,
276.46606083134856`, 0, 169.70359541572523`, 3.217441265950361`, 3.242643324968234`, 0.007832950762639346`,
0.04840632621170831`, 0.04843974983551454 4`, 0.0006904804892660099`, 100.44627668488037`, 0.19154969461669366`,
1.8781897931639773`, 0.0006043093451915116`, 0.0006074768526855123`, 0.005241533196870929`, 0.17774538833171785`},

{0.19754063204381567`, 1.2678132216826858`, 8.502246050318615`, 1.3049855200762384`, 0.9149570338409301`,
0.5843100223668893`, 0.12148336049510372`, 0.006360783777788973`, 2748.553955173861`, 51.9861836865702`,
0.0949023054382408`, 9.034485905171376`, 0.4708246470769286`, 1658.8731065048207`, 125.31180922852893`,
2197.5552969365185`, 0, 1518.7684250863122`, 7.212431674967453`, 7.230294351028798`, 0.0024766509918341395`,
0.1768716504953709`, 0.1768593056932935`, -0.000069795255728255 51`, 130.62880340009642`, 0.49913339470689794`,
1.7500579052570886`, 0.0016365092617728338`, 0.0016413063321645855`, 0.0029312821526930044`, 0.046375411708622284`},
{0.04026554170616964`, 0.5302615276235345`, 1.524346923969 6097`, 1.4195352782408457`, 0.35050648466256606`,
0.19736961374591044`, 0.19256070262690145`, 0.16778953261543247`, 3042.5326446416893`, 157.0549085671795`,
0.10950358517591147`, 5.218120753168542`, 0.25613055846451904`, 165.34145096583853`, 15.374385954000783`,
295.06223895153494`, 0, 48.903391052952266`, 13.544596556753897`, 13.605402875897425`, 0.004489341479367193`,
0.18468114449847875`, 0.18492148184987914`, 0.00130136377513268`, 102.60254944612555`, 0.1062326618021305`,
0.4511159153411628`, 0.0015662000605372 04`, 0.0015797828436684817`, 0.00867244451939242`, 0.0538496573285559`},
{0.11555514134387701`, 3.2236896162066904`, 2.732079118025373`, 1.164987224676954`, 0.23114456633378633`,
0.6224082143049414`, 0.17910660716870297`, 0.49555341188081165`, 1154.204177573869`, 364.61723023266256`,
0.21380198259541727`, 1.1657297203283505`, 0.5170897309434226`, 23.79286822432608`, 13.40141156634526`,
24.091456178239607`, 0, 3.221027357393116`, 0.434194856566482`, 0.4430768020760923`, 0.020456127877346963`,
0.053492303267762095`, 0.05430806214068563`, 0.015250023332144869`, 19.995849293195985`, 0.08830443807022406`,
0.35146980476837775`, 0.0005383872848223614`, 0.0005677658286857752`, 0.05456767775098381`, 0.12263400565395209`},
{0.27279971271031717`, 1.1118456280045157`, 7.947060215794888`, 0.8164028833969883`, 0.5079045986266995`,
0.311953435377873`, 0.24456124910810323`, 0.015036338564254712`, 1857.2767207919096`, 90.80006961498503`,
0.07729824929242363`, 4.7437001414515265`, 0.238261523479399`, 324.7897639921041`, 32.40588565084819`,
590.1457681616845`, 0, 267.1850019889465`, 3.3906737982793778`, 3.4015523898558935`, 0.003208386363216631`,
0.044117220718472905`, 0.04412830488356261`, 0.0002512435033121818`, 53.85579769437701`, 0.1719309305367926`,
0.6744299831137471`, 0.000652638636820857`, 0.0006547751573164125`, 0.003273665356318789`, 0.044696740599083615`},
{0.13808283652405223`, 3.3382876893654982`, 1.764700397616487`, 1.2575917791512339`, 0.24597284032716238`,
0.16023118424518423`, 0.11783424678280677`, 0.22421613327193346`, 2741.9690444157613`, 110.60741736270495`,
0.06748966623215114`, 5.495223824255797`, 0.7719457903315097`, 286.52282982236284`, 15.4384720258035`,
348.6941119982002`, 0, 68.84621714736184`, 4.456471171058436`, 4.48457613094757`, 0.0063065503647044`,
0.23272969202667212`, 0.23271047387369417`, -0.00008257714265247351`, 212.52832640509513`, 0.45908537169170094`,
1.1213466714698013`, 0.0022322442538695864`, 0.0022455175531960153`, 0.005946167989197226`, 0.04963813590966182 5`},
{0.2720572435314704`, 2.092047115388646`, 0.9775412869939437`, 1.361730496090756`, 0.8643513713503745`,

0.6446371862205038`, 0.07237861696827474`, 0.06459834622446187`, 2040.0072337232305`, 288.27046449548277`,
0.2127097516522497`, 3.2460958268505635`, 0.7031503645327328`, 99.89302282307683`, 56.29231602127329`,
100.56135931668807`, 0, 51.71195628162505`, 1.5184818722882532`, 1.5522072851619062`, 0.022209954224103434`,
0.2619771000053276`, 0.26562741533780404`, 0.013937191395820 8`, 45.38193743843706`, 1.0181823956545062`,
0.8946885613797184`, 0.0022868899807811927`, 0.002392501959623059`, 0.0461814865294873 9`, 0.1497375076090235`},
{0.13280526554371425`, 1.6805260743084913`, 5.020140137415806`, 1.1878313111770886`, 0.7515564764604186`,
0.6825215664361661`, 0.05451094190532624`, 0.03202911588893443`, 2354.572550424513`, 215.4436338815351`,
0.062416682151146596`, 4.270435440493394`, 0.24902957081957822`, 127.10598061832509`, 52.18093400133016`,
146.56636672644274`, 0, 87.31899640172028`, 1.5854215640537828`, 1.5955714950073658`, 0.006402039169715046`,
0.04404276335153856`, 0.04416909161150287`, 0.0028683091239301017`, 38.062032530904716`, 0.08355872688828916`,
0.6731766829159878`, 0.000445147679972501 8`, 0.00045060040370949325`, 0.012249246670966052`, 0.13725725319260423`},
{0.04136924877145362`, 3.2505996769323806`, 7.3279282523448686`, 0.890763406913394`, 0.44633950095226127`,
0.48929728316730614`, 0.1843515006058281`, 0.4646336778944382`, 1146.503881369189`, 33.19464868615876`,
0.20060766821265197`, 6.423062520789202`, 1.3180525740121252`, 1643.0417391513147`, 84.3880228835433`,
1842.7995762397838`, 0, 221.44123712306163`, 29.961541230948093`, 30.101247025063024`, 0.0046628373700157155`,
0.1953052634785692`, 0.19517702409522922`, -0.0006566099707498996`, 1391.3282320816556`, 0.11542331473186238`,
7.492034009222409`, 0.0026460499773913737`, 0.002653009655721251`, 0.0026302142398453 3`, 0.10118862528070802`},
{0.12443960147958688`, 1.4681483834231432`, 1.2961197051090405`, 1.2383341395061327`, 0.20461077825222174`,
0.2180798495866123`, 0.13955102593271534`, 0.036090000549476184`, 981.8387463156896`, 155.4019202626156`,
0.2259516918970782`, 6.485966100174822`, 0.46818640769970843`, 254.31526494034034`, 55.13605283656998`,
301.16549569906886`, 0, 168.08623419166756`, 3.893852420517424`, 3.937174580904122`, 0.011125783853138893`,
0.23424885933488862`, 0.2345845906052708`, 0.0014332247821202682`, 81.66790194958489`, 0.41642621003837577`,
2.175808528521803`, 0.0022727409764597972`, 0.0022991443207308216`, 0.011617401430475516`, 0.16605113620703235`},
{0.09633016509755776`, 2.8964637462473926`, 5.167447635911598`, 1.1556815734309027`, 0.8460405566030094`,
0.6768234428979001`, 0.20660641079063646`, 0.37899350311781294`, 2084.615204649709`, 251.12222995868854`,
0.0719093058983113`, 5.055969069135582`, 0.9365067275794634`, 305.5683522870098`, 85.30127424428147`,
314.46111567351437`, 0, 48.794987418276484`, 6.054365505014646`, 6.0863801645029705`, 0.005287863684775518`,
0.08464943555719324`, 0.08480134892139973`, 0.0017946175684047105`, 250.51785988293824`, 0.11648991575226966`,
1.4126849030631943`, 0.0008814932682106313`, 0.0008888765967759747`, 0.008375933012320091`, 0.12548958687325867`},
{0.0668112344511611`, 2.714918834733731`, 2.085154017953151`, 1.4476779676976927`, 0.2539160749138807`,
0.4870680566357908`, 0.17330271142201725`, 0.24053269905137525`, 735.8247906168481`, 182.42768732112722`,

```
0.2338897837659121 3`, 1.775892168476009`, 0.1844693395246435`, 60.95819265822625`, 28.88262885527235`,
  74.7103032536908`, 0, 14.157331803556794`, 1.1691364421595567`, 1.1868186609819766`, 0.015124170443065132`,
  0.1469920499753866 2`, 0.1488998243283233`, 0.012978758737334006`, 45.34443638846528`, 0.14029600447664237`,
  0.6423829886160145`, 0.00119354274615735`, 0.0012510968861209957`, 0.048221264088733395`, 0.1419424545208815 8`},
 {0.18113629662628528`, 0.6738122810255058`, 2.859320071162905`, 1.31555093037265`, 0.36681457284995544`,
  0.471103526437621`, 0.08814722965023356`, 0.04506210537052006`, 2186.7720797416923`, 139.59007135140308`,
  0.07150479816488481`, 8.93352349425783`, 1.2069106988965403`, 422.93598710377444`, 55.76842642476375`,
  453.59628290360695`, 0, 258.5818803066168`, 15.420062055038056`, 15.587931837424103`, 0.010886453101607296`,
  0.13642629565706468`, 0.13658922287199132`, 0.0011942508161051357`, 148.4318169551436`, 0.3530250565394172`,
  4.128211307729085`, 0.00124984863167521`, 0.0012583535418722652`, 0.006804752176794215`, 0.20214685026275547`},
 {0.06896509664813616`, 2.160626722426797`, 4.988265718811416`, 1.2639821322192157`, 0.4392795364272064`,
  0.4617851413606676`, 0.11584839231589822`, 0.2654092351920312`, 1506.9815785286419`, 346.2179045294025`,
  0.0968394930023978`, 3.8794768609952577`, 0.5989898263674145`, 84.16203345723434`, 36.8147176043394`,
  87.87625622508894`, 0, 18.324472859335298`, 2.063926006657155`, 2.089913799383404`, 0.012591436244528964`,
  0.03440246003213142`, 0.034482932324689325`, 0.0023391435520234705`, 63.70533832992963`, 0.03389384258655848`,
  0.998952867175967`, 0.00032710363501331496`, 0.0003305270950300647`, 0.010465979739449738`, 0.1840282800165636`},
 {0.1192842170105507`, 2.0587406052635338`, 2.982429260158481`, 0.9607554862496793`, 0.6846341105753171`,
  0.4253089041287377`, 0.144815555900927 7`, 0.03303667191414802`, 3625.499229568628`, 138.38440688618937`,
  0.05121864534109544`, 6.262852463653789`, 0.9148148813580201`, 432.1762671202995`, 32.94427350758532`,
  485.26493902990484`, 0, 293.7785149394755`, 4.537371352948909`, 4.556597605295066`, 0.004237310735798916`,
  0.13934521710489112`, 0.13943621007564105`, 0.0006530039038328272`, 133.4467235067922`, 0.23745264452159365`,
  1.4196426425283544`, 0.0017491990693774273`, 0.0017584587043714844`, 0.005293642762657535 5`, 0.07149865189614732`},
 {0.19942002949430754`, 3.5293725272813257`, 4.475631886088314`, 1.3690551967616487`, 0.1535320198116885`,
  0.586863827463982`, 0.20957264255775987`, 0.0070808196203240945`, 2259.133238896783`, 93.9809316080586`,
  0.18155601470027005`, 6.2714522403203645`, 0.9597826662416344`, 449.53224433135`, 34.33529199664976`,
  527.4742004697223`, 0, 407.60317259501363`, 0.7973439963012198`, 0.8030993939815951`, 0.007218211596342172`,
  0.13727632530720782`, 0.13748995122337326`, 0.00155617449467238`, 40.20177136197471`, 0.3910806977376453`,
  2.3843122148866662`, 0.0012074757936134661`, 0.0012171720253176636`, 0.008030166530445193`, 0.1541079489485389`},
 {0.19290552010423068`, 2.5311022785006196`, 4.590275609347875`, 1.0183350360370953`, 0.6768490777669534`,
  0.27037924456465456`, 0.13002648835247615`, 0.039251036869391874`, 2833.896145381659`, 40.558338114360765`,
  0.02944272635318218`, 3.3740003853748863`, 0.42763776068514936`, 530.7648748580722`, 17.000659064536165`,
```

824.0355645995788`, 0, 339.7014791177067`, 5.1190291090206745`, 5.131783795286887`, 0.0024916221405610894`,
0.21880216706641495`, 0.21875568732008646`, −0.0002124281809072759`, 185.09694630790355`, 0.6029735119696797`,
0.47206586699293857`, 0.0025936750976227607`, 0.002604545066522133`, 0.004190952409318882`, 0.01246066471635926`},
{0.16407676745668215`, 2.743210330542553`, 9.218463902446366`, 1.4442040388312085`, 0.8033045111109769`,
0.5898809766156388`, 0.14591263450208874`, 0.06903241777052908`, 3856.2015344843503`, 358.8528117340675`,
0.03175828336990505`, 3.8731976390854896`, 1.2881241509575583`, 125.39385991556173`, 28.854938592209756`,
129.25873848443808`, 0, 63.49193014868158`, 1.5376283190228484`, 1.5492058087705545`, 0.007529446228633052`,
0.03176139653053692`, 0.031814943550524555`, 0.00016859151623309643`, 60.25768413254646`, 0.0744472467520042`,
0.8350503688091695`, 0.0002651186994711008`, 0.0002669080335263574`, 0.006749180871912452`, 0.09123282913338802`},
{0.19253541459312512`, 0.9166337410286696`, 9.734161690686161`, 0.9309028594902833`, 0.7413366163896413`,
0.3490367975563471`, 0.060904155020362505`, 0.013080541604234533`, 2740.950745040873`, 120.34372569041233`,
0.17782424959038423`, 7.718689374811543`, 1.0556383976941355`, 461.78859214699577`, 52.367200883818825`,
502.4152973835503`, 0, 389.236347091746`, 5.120726686817378`, 5.158360427031423`, 0.007349296792372728`,
0.03590439620552893`, 0.03590888670405347`, 0.0001250682088853594`, 67.05472656746798`, 0.09875525441631237`,
2.9879332349315777`, 0.0004662963611242743`, 0.0004671051683325535`, 0.001734534677322097`, 0.07913011353297071`},
{0.2344407889708734`, 1.4871626351148812`, 6.830413107063742`, 1.1276656092803836`, 0.7850884426482851`,
0.26642284093684543`, 0.1715246889391343`, 0.04980986785514341`, 2193.834713356332`, 311.9394292836438`,
0.1837203477628585`, 1.994457399163636`, 0.8876922575805768`, 75.01227396477513`, 14.689918568571782`,
79.97962067663646`, 0, 43.901418177956806`, 1.3908873454343116`, 1.4019069780329885`, 0.00792273553631051`,
0.03883525927385314`, 0.0388940925087177`, 0.00015149437898607232`, 29.54965271120047`, 0.130065268915014`,
0.42151848851778967`, 0.0004150583268051067`, 0.00041847950004416225`, 0.008242632464188437`, 0.037402865889426685`},
{0.1574181424723891`, 2.4334976996958355`, 7.454513230702137`, 1.3609349886556783`, 0.33361805963053826`,
0.5460996944909747`, 0.19878306773728516`, 0.24115086657234458`, 1578.7144043122416`, 80.91704790184991`,
0.12351463962730552`, 9.357025478355425`, 1.447433410261179`, 947.9587447886703`, 84.53313521714132`,
1042.9278998396571`, 0, 218.32749756165194`, 20.390591824336802`, 20.54272690093053`, 0.0074610427154031544`,
0.11603105115757659`, 0.11607002918851744`, 0.00033592758621070473`, 708.8636899994299`, 0.26093417917691375`,
6.869915395768494`, 0.0010291424338215727`, 0.0010328706283270622`, 0.003622622469900083`, 0.25550431749877034`},
{0.1378028697180832`, 3.3038928286894196`, 8.205199135932347`, 1.1443333838214729`, 0.43666320558690463`,
0.2863993034286605`, 0.21178222433889116`, 0.37748074045027574`, 3064.1095742596744`, 356.28526904181854`,
0.15861739359359306`, 8.78386257360005`, 0.18434483317647987`, 150.7395581946359`, 32.97994821726236`,
248.7933028754912`, 0, 24.16242485303062`, 2.625240147674427`, 2.638240877680395`, 0.004952205998176806`,

0.015016306765901672`, 0.015021033990957064`, 0.00031480610572809375`, 123.90731567841549`, 0.029561288069893363`,
0.704116719839565`, 0.00015859428605335957`, 0.0001589520642254095`, 0.0022559335582212903`, 0.16575216231790066`},
{0.24756545924701062`, 1.5758450559815111`, 8.280763168619`, 0.7891779726588277`, 0.6872262965666405`,
0.4388878534659798`, 0.12835670237954216`, 0.07541218293771562`, 709.0120611967568`, 337.23881046719544`,
0.12174060791630043`, 8.605194748975368`, 1.4619068526165075`, 267.2626168506013`, 238.9199317898913`,
262.329400651287`, 0, 130.74210317984375`, 5.802795974765914`, 5.895057254341085`, 0.01589945260463721`,
0.01857133662548762`, 0.018576688926117916`, 0.0002882022300403353`, 130.6329621100611`, 0.06568030686450381`,
4.135037965937642`, 0.00028436060358227877`, 0.000285049800398327`, 0.0024236719410704044`, 0.41726064631832743`},
{0.13084693466492658`, 3.6481233497064993`, 4.2504273066825995`, 1.0067280367884681`, 0.5994607748865513`,
0.5813901581577328`, 0.20650373563828406`, 0.1827740239544238`, 3288.755010061308`, 116.07278762719005`,
0.07676913646394057`, 7.439949797465348`, 1.2495449290646898`, 679.2697279555829`, 46.09091343695347`,
747.6671703276448`, 0, 189.893699578175`, 9.206010771498976`, 9.246950615132135`, 0.004470775289451 31`,
0.13561751383361142`, 0.13568318242453772`, 0.0004842191031968479`, 479.7808979022129`, 0.2535019424573938`,
2.658691833012579`, 0.0016249778348662591`, 0.0016325002431650387`, 0.004629237480890724`, 0.12204858594187727`},
{0.15289027955876405`, 3.4056340118853177`, 8.85602064850179`, 1.1900353929127663`, 0.4946084696569088 6`,
0.2120137239983716`, 0.13358418114947193`, 0.3517341695519152`, 1451.4695761117755`, 341.1785262828431`,
0.2218032559662697`, 9.743566224950836`, 0.41975629001252956`, 224.6083879824341`, 71.82304687038265`,
253.81374877740114`, 0, 38.89990616734354`, 3.7389612996438832`, 3.7728170243064816`, 0.009054847576470904`,
0.019438567989355268`, 0.019441633237482943`, 0.00015768898868229186`, 181.90762530271593`, 0.04245668705890859`,
1.9756566972842824`, 0.00019751382495714065`, 0.00019782583223049402`, 0.001579673085775557`, 0.22993232153682328`},
{0.10700117050359564`, 1.3986545196260032`, 5.134806342648034`, 1.3393940232844244`, 0.8110706849311933`,
0.31760122726745454`, 0.23195078300551503`, 0.016714409489937344`, 2707.277188066014`, 260.0392856094112`,
0.179990816902801`, 9.35690893256919`, 0.5478079740525017`, 511.50412499003`, 68.36169153862481`,
585.7646688036432`, 0, 412.8207418535374`, 4.683580355243104`, 4.699236486018964`, 0.0033427697591079664`,
0.08945133563418695`, 0.08948125109367523`, 0.00033443278712597113`, 93.58158331274751`, 0.1367342516567072`,
1.2814519084377247`, 0.000806294012399289`, 0.000809107563641288`, 0.003489485471468212`, 0.1005038255533244`},
{0.27215562413942285`, 2.0660135805917337`, 5.081795444601147`, 0.8142657507592871`, 0.26635598111633185`,
0.328163277836687`, 0.07966098389627496`, 0.17309045811215876`, 992.4120776836976`, 330.82978884166914`,
0.11630419854588847`, 1.9092525381665606`, 1.4920575883264906`, 35.42570715745277`, 19.217168028012573`,
35.55441086117641`, 0, 10.75975267416344`, 0.805360266346037`, 0.8266425840961118`, 0.026425835293109046`,
0.01857738684734011`, 0.018617025556676547`, 0.00213370748330588`, 23.769789250569847`, 0.07222771874739412`,

```
  0.8394807699890066`, 0.00027449099139165156`, 0.0002774927010869237`, 0.010935549032242031`, 0.0950341996449077`},
 {0.16724732499606915`, 3.8236591860031757`, 7.535496783883705`, 0.9286997421550199`, 0.15670002022620788`,
  0.2590202034037111`, 0.17595862302525594`, 0.2753459074292645`, 1491.5245062190434`, 377.6558909388741`,
  0.10962242795283667`, 4.774971062285175`, 0.9122667174222503`, 86.90823989257579`, 27.14813487724858`,
  93.66355338809932`, 0, 18.56788883137034`, 1.2277620673288017`, 1.245734154501288`, 0.014638086361136393`,
  0.014067761658890317`, 0.014074675037979113`, 0.0004914341923349941`, 67.06491009954325`, 0.033611364373326194`,
  1.250843386314504`, 0.00018296770161385645`, 0.00018356586028172908`, 0.003269203594932879`, 0.2163144249094715`},
 {0.1513970595453768`, 3.565269862926928`, 2.882233750328341`, 1.46147567899756`, 0.43028383944211446`,
  0.5449527314944854`, 0.05024950045158358`, 0.013582696865047886`, 1199.944383613747`, 41.76183563022494`,
  0.15753768337487067`, 1.280336274118845`, 0.8861263254663858`, 163.44008653992236`, 16.20147530529498`,
  181.01202420039922`, 0, 135.95540689851168`, 0.5033190778419326`, 0.5114339994150535`, 0.01612281737444765`,
  0.40010056479519973`, 0.4031978710678742`, 0.007741319421180615`, 25.635261995228774`, 0.8653435576062363`,
  1.262068173724518`, 0.0032881263679290784`, 0.0033660373179691516`, 0.02369463375859948`, 0.029953629933454233`},
 {0.054023975296500326`, 3.9030377038282893`, 2.6328389166852855`, 1.06539261997144`, 0.07270428031369969`,
  0.6221832003428629`, 0.16473607550076202`, 0.3329139837617463`, 3016.8950283011136`, 162.89707817078545`,
  0.17967527091419977`, 4.813593384225332`, 0.9924030037454137`, 179.19394301191377`, 18.88686359913677`,
  203.02853625653327`, 0, 32.207183464394326`, 2.5867839066659637`, 2.6086653915024836`, 0.008458953521449208`,
  0.09420094108777251`, 0.0944836329388146`, 0.0030009450837511498`, 144.2330731339071`, 0.07270156163176607`,
  1.400938861287397`, 0.0010622929524742863`, 0.0010745910714587907`, 0.011576956202015376`, 0.15819712058671795`},
 {0.25659351647641615`, 2.0548774277019515`, 7.952450585873037`, 1.1700596392648208`, 0.24509849180489507`,
  0.5476063706170384`, 0.05117766409060387`, 0.047107635368495424`, 1627.113553842868`, 255.20388289511038`,
  0.18601650100638534`, 1.4517697640535214`, 0.19929851500937135`, 26.344225740700082`, 11.19469090492552`,
  31.17464834465072`, 0, 15.811116669040933`, 0.34441518557422374`, 0.34910469459416166`, 0.013615860206974917`,
  0.016636724634682108`, 0.016712082705667056`, 0.00452962182398875`, 10.110442722774993`, 0.060983938238038174`,
  0.28470582250252274`, 0.00017005309460438323`, 0.00017342044850710093`, 0.019801779618016546`, 0.088257470267304`},
 {0.1846136822622525`, 2.83222539763279`, 6.185815327147518`, 0.9985231745146829`, 0.7838598641352239`,
  0.6970792682180826`, 0.05665196048496565`, 0.0105368043370621`, 1139.0438628747534`, 42.309712199780904`,
  0.09405173581505571`, 1.6109366539180776`, 0.8793784843352515`, 256.89415280375994`, 42.8916447242039`,
  279.59973660661177`, 0, 222.84735280131093`, 0.8030429318335214`, 0.8106542119483162`, 0.00947804882289005`,
  0.16071862630985695`, 0.16123804354525187`, 0.003231842178600397`, 32.491408384691375`, 0.42386939158842957`,
  1.663375379722879`, 0.0019363236254845706`, 0.0019592614708601277`, 0.011846080414278148`, 0.029199491571709892`},
```

```
{0.10141299379366919`, 1.6007716008532835`, 0.6398899685364796`, 1.0164701283406912`, 0.8225647972224088`,
  0.6292458866768325`, 0.13571266692666845`, 0.07872745868282192`, 1791.1473855018166`, 192.97393158172054`,
  0.14848279636744627`, 1.8821154571770222`, 1.1130294782612764`, 111.63094556424964`, 32.60194520887422`,
  112.87792219557637`, 0, 52.28721966887102`, 2.4273676089540532`, 2.475714921607186`, 0.01991758993355175`,
  0.57447894999371`, 0.5839182477603254`, 0.016431059426492256`, 55.50944476063986`, 0.8322804312900641`,
  0.7786894459162983`, 0.006720784854097439`, 0.007044442562663666`, 0.0481577249670333`, 0.08794450296242397`},
{0.11981560320968349`, 3.640754447753876`, 5.349606530517274`, 1.2002498449288461`, 0.9084970459884618`,
  0.2543242557071712`, 0.166873467304054`, 0.28421934758961104`, 1487.006076738341`, 292.7577364926251`,
  0.15406116741524772`, 6.480379092988072`, 1.4335012083087681`, 327.1770421109653`, 87.05103796549606`,
  333.4497907086179`, 0, 66.41114264560177`, 4.9154767310529905`, 4.9522208524105285`, 0.007475189766520618`,
  0.07104692831877508`, 0.07107215839774791`, 0.00035511850504810205`, 255.65776816302633`, 0.1216075796100637`,
  2.2405723432949443`, 0.0007147132955077629`, 0.0007172559530787197`, 0.003557590976603242`, 0.1287260251476444`},
{0.1768569128079565`, 2.3909839842824203`, 9.326880330280886`, 1.4539501621465738`, 0.2185806450894794`,
  0.20927947571084793`, 0.1254436846064726`, 0.4971843180024683`, 563.0454599592058`, 164.61314507136376`,
  0.20371869506435597`, 2.3340090949236654`, 0.42101883446758315`, 86.37771184855002`, 33.32315500921454`,
  96.479621640613`, 0, 11.669751101999056`, 2.1213622513590247`, 2.1536947790238`, 0.015241398607928502`,
  0.036129129764423604`, 0.03616543734929301`, 0.0010049393690394481`, 72.45918811229623`, 0.09128123360818788`,
  1.1956977810786562`, 0.0002996052493405843`, 0.0003015618084577566`, 0.006530456730910572`, 0.08720610889721567`},
{0.2133813191703517`, 1.451835237764156`, 4.809264415807901`, 1.4381107471220305`, 0.9545564566791402`,
  0.6082425656359232`, 0.08609247081289534`, 0.07332616905477864`, 1657.6679333430675`, 311.00628516031566`,
  0.15574013896641808`, 2.719528414156583`, 0.38330836635142496`, 88.49793890209897`, 69.55754457429003`,
  90.18234927166462`, 0, 43.409159674888386`, 2.0626551846719785`, 2.0825577974052494`, 0.009649025625403373`,
  0.06062217942907313`, 0.06092063573691975`, 0.0049232196971045195`, 42.78050686376737`, 0.18479486596505`,
  0.6011454678802454`, 0.0005042835177698768`, 0.0005142502272006295`, 0.01976409912191679`, 0.10404893015072514`},
{0.07679027731361865`, 1.6374208448422314`, 5.731071459295233`, 1.355982875935464`, 0.05181959233087374`,
  0.15382155253913876`, 0.1281658546365993`, 0.05032482400738273`, 1230.193557333262`, 218.81347681784587`,
  0.015802664299199992`, 1.3298614212710635`, 0.20143294852402427`, 24.215881769931606`, 6.892701196660307`,
  36.874501899895776`, 0, 14.163670376753325`, 0.4097827141845563`, 0.4152262279324706`, 0.013283902808703285`,
  0.026927359377385014`, 0.02698481311247599`, 0.0021336564898832844`, 9.585525115168847`, 0.029539419913042618`,
  0.2648446273536471`, 0.0002389180559078108`, 0.0002414747186690985 3`, 0.01070100269974672`, 0.03487704187494566`},
{0.2370685516334804`, 3.237122537432753`, 3.9976792452307883`, 1.4537281576817178`, 0.8596265155383829`,
```

0.4350695278152106`, 0.13391759982092372`, 0.21967358388020922`, 1928.121818584993`, 246.10473403948492`,
0.1821805288264413`, 9.36511897288407`, 0.6656126516266203`, 449.26108796991053`, 130.27978586235983`,
479.00821494588286`, 0, 110.1391436405093`, 7.167854344495377`, 7.211332167941226`, 0.006065667821394616`,
0.1093028078273809`, 0.1094006161104541`, 0.0008948377906967675`, 331.47461205144816`, 0.3701751191586599`,
2.237956707882946`, 0.0009063292987832039`, 0.0009119922596873824`, 0.006248237712034088`, 0.17801976584667314`},
{0.21885531656429402`, 2.687374858822965`, 1.1296170207345284`, 1.325784614121181`, 0.40511738093112615`,
0.5937209983207841`, 0.0879699618250398`, 0.34321707574230476`, 3195.5291529724673`, 92.2297421582175`,
0.21288285544599406`, 8.464472481658461`, 0.25864431062726`, 416.05286248662367`, 42.812165412336874`,
665.6821960169743`, 0, 71.01795490444023`, 8.721521117609548`, 8.772863451594056`, 0.005886855434064531`,
0.3598357515885593`, 0.36087459249552173`, 0.0028869863607947632`, 334.82852260225013`, 1.1250281046435961`,
0.963812349019673`, 0.0032534252622935522`, 0.0033069755320727094`, 0.016459658809376165`, 0.147861831266885`},
{0.14216552227340323`, 2.5649444615040133`, 6.744792074075217`, 0.7596927543658916`, 0.15436598317484496`,
0.17272955179378358`, 0.11731963299504705`, 0.1434376311009391`, 3629.5744652923077`, 39.43043872717277`,
0.05961813000749189`, 7.187412396441442`, 0.1404254814851862`, 130.95887742340355`, 14.750346116264245`,
1216.3569170783123`, 0, 43.25244959625578`, 2.328974159930835`, 2.338049114515421`, 0.0038965458443969148`,
0.012878576795855984`, 0.012880285401214867`, 0.00013267035527020354`, 85.33841960715087`, 0.026155565661764982`,
0.4718056848222064`, 0.00020500635566500502`, 0.0002053087248494483`, 0.0014749259039430473`, 0.06279084920097142`},
{0.20003331751651454`, 0.9418735138273977`, 0.3780377423788224`, 1.1513921169618064`, 0.6645698632364119`,
0.4730945551961576`, 0.051565459379669804`, 0.1952333288925963`, 2322.1212203613577`, 230.2978627277754`,
0.10438353064420558`, 8.70346208169968`, 0.8526709835745097`, 260.8801471205806`, 75.18215516661203`,
271.5702245819633`, 0, 70.24964254738782`, 13.036696553860256`, 13.284300951967232`, 0.018992878838900218`,
0.5652908499742902`, 0.5683228607568199`, 0.005363629683140303`, 175.4131313126562`, 1.6153857726009024`,
2.707575130615687`, 0.005866471039066567 5`, 0.006025976317095956`, 0.02718930630820382`, 0.2668477494873089`},
{0.045463415265626916`, 1.0604864030915095`, 1.5457624129661234`, 0.9218745066798075`, 0.3077029567700027`,
0.3372897124352183`, 0.13368077049637672`, 0.019951884664535027`, 2805.850760096928`, 274.4324431552926`,
0.0964209850306606`, 7.937294323580787`, 0.5250555034364572`, 188.28494184148033`, 30.861418264857722`,
222.07278286379346`, 0, 146.62083098487298`, 2.567515275612516`, 2.5859608395211002`, 0.007184208048843565 6`,
0.0886178679585739`, 0.08874779123654092`, 0.0014661070161128276`, 38.89735770738315`, 0.057555299013655824`,
1.16168710835715`, 0.0011569457291117358`, 0.0011663098767079846`, 0.008093852080199415`, 0.1763029956551092`},
{0.09737410253356232`, 2.2163561335231643`, 6.975749882171211`, 0.9583917748679426`, 0.07040378529617852`,
0.6807220990959502`, 0.09176157414171682`, 0.0913364004962977 1`, 816.8955105959267`, 131.23291821104817`,

0.07570904922141436`, 6.1606408812314815`, 0.7251058774037329`, 268.1028719039788`, 94.01037177946847`,
280.81389648525436`, 0, 118.48215146399255`, 4.578100627931478`, 4.646940974430912`, 0.015036879285577731`,
0.03748318595959976`, 0.03752369183211862`, 0.0010806411323336373`, 144.95287723717388`, 0.05214130846992511`,
4.057458565942674`, 0.0004713273946767016`, 0.0004741309049376177`, 0.005948116516416579 5`, 0.3591510371251133`},
{0.12981111976782828`, 1.1084267930609286`, 4.931023641639941`, 1.2438579943673111`, 0.8299266795906743`,
0.2963366491664211`, 0.21441842262722777`, 0.013448184708505154`, 893.4222083111035`, 95.2241911833517`,
0.18109496842042927`, 2.912115357106975`, 1.136648183382977`, 445.65942269769283`, 61.212782075464865`,
468.2445872206725`, 0, 373.3818070611253`, 4.239001105124271`, 4.266240133792338`, 0.006425813061275098 5`,
0.2417969178929594`, 0.24197905962334934`, 0.0007532839209742903`, 67.12317715335169`, 0.44839898097287245`,
1.9252805732750118`, 0.0023409014607841394`, 0.00235869087162167`, 0.007599384739403581`, 0.04971214425340676`},
{0.04881288196111078`, 1.5001688796472123`, 0.7565632194730121`, 1.206042774752989`, 0.6805558920404622`,
0.3640356697656889`, 0.08453170981106164`, 0.19820032009109562`, 3391.2112266676813`, 283.705794296156`,
0.20144392981632914`, 6.84055461750941`, 1.1851088923020137`, 195.11543023790574`, 35.03168890481296`,
205.23537827167183`, 0, 52.031150126313015`, 6.357384430223181`, 6.428184749094661`, 0.011136705613536968`,
0.2839120751186465`, 0.28475007671267955`, 0.0029516236450415345`, 136.24500397392188`, 0.19797952300122748`,
1.6838914412306796`, 0.00282843467264815`, 0.0028652905618359974`, 0.01303048981270627`, 0.1508188264714158`},
{0.16605942412072794`, 0.6402554572286978`, 3.356342996009028`, 1.3219512159205855`, 0.7993093802210203`,
0.15372675446550776`, 0.10521895558502489`, 0.005367878364442133`, 806.3427040773236`, 321.0178159818362`,
0.15297743964506222`, 6.095134085467343`, 0.509077325139712`, 186.0384756773356`, 94.34390622238328`,
194.8053922616763`, 0, 172.51828659142151`, 1.2881856698053238`, 1.3058780121520344`, 0.013734310791847504`,
0.0753309207376293`, 0.07536991553943795`, 0.0005176466904535104`, 11.782398643095194`, 0.17870584737392872`,
2.0570940249011174`, 0.0006877149270471117`, 0.000691141170636126`, 0.004982069538211009`, 0.13817907617762956`},
{0.27402590763234025`, 2.0480444129965374`, 8.367988840463223`, 1.356112416883951`, 0.549124019732967`,
0.6110139101654402`, 0.05091107534805153`, 0.12010047430936349`, 1627.3034033398817`, 297.4011443552333`,
0.1966061654681946`, 8.76675255073281`, 1.4917606486762143`, 198.14981293510252`, 105.78125649462716`,
197.92476520968904`, 0, 75.28799022107629`, 4.056773866463875`, 4.137310984684612`, 0.01985250370658176`,
0.022963165033888656`, 0.022982912081308104`, 0.000859944497646925`, 118.69218645716713`, 0.08989288772169644`,
3.7836390620070888`, 0.00020435073963231964`, 0.0002052803683021701`, 0.004549181821035253`, 0.3205180593775793`},
{0.10823031336368771`, 0.8296257610813722`, 5.332629740125888`, 0.8124935731916766`, 0.40472412917038403`,
0.20010405592172198`, 0.09023994109265432`, 0.01008956636997111`, 1875.5688641041388`, 260.72528052100415`,
0.14449594942054067`, 1.5456312543093684`, 1.2237716091504276`, 38.03734873811938`, 7.466981050506643`,

40.52698038165148`, 0, 33.2318692470989`, 0.36723158087824065`, 0.37285756108979157`, 0.015319979284178942`,
0.02347000950958927`, 0.023502942363317774`, 0.0014031887679912813`, 4.35235399684608`, 0.036288092626737864`,
0.4938380505384807`, 0.0003483559860759655`, 0.0003507329023993867`, 0.006823239497606437`, 0.03709065853839157`},
{0.20273952773619414`, 0.8775156867465963`, 8.853127710010899`, 1.0632724405144907`, 0.5470610681912369`,
0.35564991449731365`, 0.24613003734690775`, 0.2102067712155018`, 2109.2588977466867`, 152.77073389943882`,
0.07849302645811645`, 3.0982028200464633`, 0.7906746942794358`, 204.03613183100776`, 20.591603427835743`,
236.19700467672692`, 0, 51.4850296146401`, 11.255811225922292`, 11.308025740552004`, 0.004638893952793266`,
0.04956912577764218`, 0.04960856033819797`, 0.0007955468234943641`, 141.1021559686463 7`, 0.14356601643505118`,
0.7597577674183303`, 0.0005624399735700836`, 0.0005653457083480266`, 0.005166302031306413`, 0.05182377227520159`},
{0.18840966187391878`, 3.686049426661911`, 6.507340210098313`, 1.3405973621357103`, 0.6314264717273861`,
0.6528641596796794`, 0.05949520733205324`, 0.005267163480527638`, 1036.4467237949607`, 79.36725884036872`,
0.16755847890105047`, 9.771579716601678`, 1.20892036427258`, 875.5585721805138`, 218.21479101333458`,
906.7140074666745`, 0, 813.7230867361372`, 1.127238079432022`, 1.1418871725476611`, 0.012995562679199324`,
0.11576231085069763`, 0.11583139744058717`, 0.0005967969141411622`, 59.35793252002698`, 0.3115819692159715`,
9.95112909883333`, 0.0010417389207514338`, 0.0010464789686080696`, 0.004550130327487967`, 0.2295380562315712`},
{0.1000404296450419`, 3.096045401845493`, 7.6799040120684445`, 0.803333640129631`, 0.6198933615423707`,
0.29060269426384966`, 0.18836459448376747`, 0.04739137692785461`, 3452.9247864535073`, 66.50695651270411`,
0.1918335051763443`, 8.136439035690735`, 0.9349024413134766`, 1181.5231919028201`, 33.4257764507725`,
1400.410956741281`, 0, 705.1730133770193`, 10.526187520778413`, 10.545963956539078`, 0.0018787842912380004`,
0.09538274895388107`, 0.09534239826654377`, -0.00042303967729862`, 465.5650638952777`, 0.13631615980072928`,
1.9756559700030274`, 0.001435106325913038`, 0.0014370294757522653`, 0.0013400748115326433`, 0.04437517827134488`},
{0.16207753282118065`, 2.4074661619125743`, 6.439531987652202`, 1.216188209247578`, 0.5635250461042756`,
0.41870015151777173`, 0.15568766166521003`, 0.14566116758733688`, 2407.082097704033`, 343.2271284427226`,
0.14229137239354006`, 4.642019464618446`, 0.8431314645967629`, 123.40858578247371`, 30.50221978063873`,
132.0890131585702`, 0, 40.66404201329845`, 2.334973532862704`, 2.3550900561276507`, 0.008615311043925988`,
0.031455537078152275`, 0.03149491608218194`, 0.0012518941873993494`, 80.30528241897716`, 0.07283194061686646`,
0.9636727704069243`, 0.0003117770932691277`, 0.0003137600934957939`, 0.006360314049610016`, 0.13429966234439497`},
{0.22160418402095905`, 3.3706460558066524`, 3.148818263883834`, 0.8683984407887995`, 0.063094397851442`,
0.5589539148331857`, 0.058740794885626`, 0.0138702175197992`, 2238.370585799582`, 370.4864263128153`,
0.21378440885525551`, 5.817374569517135`, 0.5948516756530484`, 82.02605955000108`, 28.977246489080848`,
86.22568333342932`, 0, 68.54849612337034`, 0.2713320986394851 5`, 0.2759849457853778`, 0.017148163336453637`,

0.024332080807456826`, 0.024382672956256894`, 0.002079236428664366`, 13.06520668704175`, 0.07702987018382869`,
1.2880076995437115`, 0.00033697928857112647`, 0.0003403226210878476`, 0.009921477758759734`, 0.299863851912606`},
{0.09473876647477247`, 3.4861652630821096`, 6.982828746666236`, 1.2832000030310025`, 0.6886348717794659`,
0.4656186082808249`, 0.150625912685666`, 0.03239358546984584`, 1673.775609656389`, 245.14838945542726`,
0.016860445211711483`, 6.847475541460803`, 0.5191626673367391`, 281.48753638642506`, 83.12528891828873`,
307.17222486918655`, 0, 192.84140756730284`, 1.7422288611343124`, 1.7529177724391924`, 0.006135193569185127`,
0.04736689959785339`, 0.04740701887544594`, 0.0008469897319258735`, 86.76711051465055`, 0.0641068805661927`,
1.5917848196873836`, 0.000445223153706542l`, 0.0004474153517286561`, 0.004923818547763359`, 0.1723097655255914`},
{0.1760690989521051`, 0.7026153943847899`, 3.261997951042007`, 0.993553588623698`, 0.646826501995915`,
0.3736019899194698`, 0.21533349413201053`, 0.25320537109656943`, 1549.1302534082051`, 389.4106290690562`,
0.0763719271638561`, 2.133344446029941`, 1.4125782678502792`, 64.67922535603616`, 22.343288032123997`,
65.99923370820483`, 0, 14.463140815400271`, 4.531304327751097`, 4.590857760645791`, 0.013142668994878814`,
0.057587256512493946`, 0.05779208517669389`, 0.003556840117144633`, 45.48234539029069`, 0.1448476623612148`,
0.6450201717303224`, 0.0006951207319653152`, 0.0007063724242667415`, 0.016186673456874523`, 0.09132863435816824`},
{0.06662682417897325`, 3.243608945481448`, 8.237889478852154`, 1.2035188774949521`, 0.1279184849350048`,
0.6784394756208973`, 0.18789313451307382`, 0.086116484822983`, 685.6358133562785`, 181.08244593249674`,
0.08115584053395464`, 7.371846442202106`, 0.7077387695529302`, 293.3868432318669`, 141.04942792618095`,
306.0132888741719`, 0, 133.79299607189722`, 3.3699130309919174`, 3.418809880432286`, 0.014509825325069725`,
0.036457279186523604`, 0.03649379437540395`, 0.0010015884261005414`, 156.15257218314116`, 0.03470046757726098`,
4.361004663088988`, 0.0003651510726413454`, 0.000367165600181317`, 0.00551697007323404`, 0.6147443848240668`},
{0.13921193952574745`, 0.46815880576040936`, 4.3864048931388595`, 0.7514079591567522`, 0.40824219063798206`,
0.5617438808850261`, 0.24339108461093562`, 0.020519348847045586`, 1920.4537040361229`, 81.58086121632874`,
0.1022235991904683`, 4.414511511279496`, 0.3646779989849891`, 388.340488413041`, 35.47514000914551`,
552.0562025298952`, 0, 300.19430049648554`, 11.41488123560982`, 11.450446325581492`, 0.0031156776174527945`,
0.09444501581401746`, 0.09453751160404288`, 0.0009793612635 69123`, 76.34253095942859`, 0.1878267690000328`,
0.8103126384337929`, 0.0015134141783442745`, 0.0015241752101851178`, 0.007110434139460997`, 0.06362908544823094`},
{0.22961340343556502`, 1.6030194574854644`, 2.9811826554982517`, 1.0274834008587208`, 0.6451263943126482`,
0.5097595717714782`, 0.19295611967128257`, 0.020325995630318172`, 1782.352038334574`, 109.59991443386025`,
0.16872014474364577`, 5.851396867706905`, 0.6578614287996216`, 547.9594891853092`, 65.70494295695009`,
628.8275007812234`, 0, 424.2549690488905`, 5.133473529590255`, 5.159226216536643`, 0.005016620188639465`,
0.20209548127488122`, 0.2023072567776024`, 0.0010478982577206697`, 117.55797074885365`, 0.6629118753497214`,

1.7331537906160277`, 0.0023668495976801873`, 0.002387337960404571`, 0.00865638557873094`, 0.09473934641356004`},
{0.26748619026781384`, 2.88696602814122`, 2.7118514018243634`, 0.9885856963040458`, 0.34646533154127157`,
0.2537466476440833`, 0.09945272546945017`, 0.07388745663205695`, 3603.205135648420 8`, 34.51441620104788`,
0.19141821235831952`, 6.868891191349718`, 0.1593219725686645 4`, 223.00560217901332`, 18.98235833752477`,
1428.428000680557`, 0, 108.56692784523321`, 2.700624249606343`, 2.7109109986760385`, 0.003809026402393778`,
0.07413785344981923`, 0.07418287650079015`, 0.0006072882997805173`, 111.38014947697003`, 0.28329788534159844`,
0.4914247361460958`, 0.0009046952770004335`, 0.0009098647508281783`, 0.005714049757045814`, 0.0550181121026241 5`},
{0.10551271069457996`, 0.6900129952362093`, 8.232394419645587`, 1.130400293700848`, 0.7046770328984258`,
0.2711667378009396`, 0.05003959656184298`, 0.014784496484154912`, 540.5369479440305`, 45.69515764102175`,
0.16942738870919372`, 8.057748743914669`, 1.27779344817754`, 1204.8433151161437`, 227.35086437469698`,
1266.4470610797055`, 0, 996.3679479607634`, 19.121336423217322`, 19.35074487766426`, 0.011997511542571226`,
0.12890208152723326`, 0.12886341481825342`, −0.0002999696243980443`, 188.48529454719167`, 0.19429725765859956`,
13.427935536046075`, 0.0013778173746760158`, 0.001380397849181102`, 0.0018728712182867202`, 0.11338973407641413`},
{0.044118696420245185`, 1.9220692229229748`, 8.936584527376546`, 1.198231045412435`, 0.8133241613996403`,
0.3641703381601298`, 0.06715507154740682`, 0.009124556845180784`, 2953.448531772523`, 220.86580819426717`,
0.07551922906053443`, 5.11062195030733`, 1.275640621281854`, 184.7883672091885`, 36.78842616997562`,
193.96515801964333`, 0, 163.504916571609`, 0.741308705092144`, 0.7488049950441358`, 0.010112237857857087`,
0.030409502863317384`, 0.030428212718289174`, 0.0006152634278793201`, 20.354952096321348`, 0.01916610893025269`,
1.6637368174818745`, 0.0003065743378449781`, 0.0003074984258485518 6`, 0.0030142379498214744`, 0.07867968467878897`},
{0.08620550491180146`, 0.4949942611001066`, 5.984234002717921`, 0.7531376788437371`, 0.21717148283204812`,
0.22262766010657997`, 0.11267502779798616`, 0.05995928028643471`, 1301.4568875111263`, 314.2464318877011`,
0.04884398840792192`, 1.2952896203900384`, 0.6666662921648308`, 24.40470292030185`, 8.022665462390247`,
26.599289436807602`, 0, 13.288318060351628`, 1.3731369266047542`, 1.39500782108783`, 0.015927686496025162`,
0.014841103934385732`, 0.01486636745201342`, 0.0017022667410309111`, 9.709927119628453`, 0.018276926544460843`,
0.35822904330679356`, 0.00023729752556322925`, 0.00023928290067771994`, 0.008366606898990492`, 0.04793941349055572`},
{0.18748668988618272`, 2.2339195778761853`, 8.488097011441944`, 1.47468717511323`, 0.23541263239484844`,
0.20987853326618222`, 0.07676134969948037`, 0.061849152465116755`, 2541.5254126664167`, 387.5680307693225`,
0.09509361772923897`, 9.161664648477608`, 0.10551879857006874`, 80.2267998437878`, 30.64547987483623`,
150.40365433495853`, 0, 42.96604269725655`, 1.1310117733515648`, 1.1434221582128652`, 0.010972816688304077`,
0.009543949657726432`, 0.009547016536767029`, 0.000321342751228082`, 36.09413347569313`, 0.025562336139590247`,
0.8472331317860291`, 0.00007823532936968025`, 0.00007840582625943619`, 0.002179282571308727`, 0.27205918686092634`},

{0.08696471520703919`, 3.613913666113315`, 7.0626231358127285`, 1.3208938988261034`, 0.28024078243683714`,
0.37947312403263656`, 0.23429714063319607`, 0.06281923284932987`, 2203.521948663617`, 29.0776175939651`,
0.14152777414422596`, 8.483647124571803`, 0.7235011997593546`, 2015.4914979815048`, 43.27854454633598 6`,
2671.7069886206446`, 0, 1063.05240414949`, 18.085694505687965`, 18.113452131716087`, 0.0015347835284618139`,
0.27904604096171515`, 0.2788615827416359`, -0.0006610314894398561`, 933.7162647893739`, 0.3466737068835274`,
2.4951204844019523`, 0.002550333883753697`, 0.0025565008071614546`, 0.0024180847249226645`, 0.04756056197605703`},
{0.09172278717373272`, 1.2157183406525425`, 2.2812242417200608`, 0.9103021242940673`, 0.7524885408365471`,
0.5328408138361465`, 0.11958813531559015`, 0.005068237864616913`, 874.3804057883599`, 319.73415041813746`,
0.21547584642190498`, 9.166843855230969`, 0.9284914101950568`, 310.4988165553299`, 261.01971351477516`,
304.0406303357188`, 0, 289.30116140238476`, 1.1252423039930006`, 1.141334428078115`, 0.01430103012303241`,
0.08468189214374207`, 0.08480212710397139`, 0.0014198426273379638`, 19.54253866632021`, 0.11096084529381728`,
3.860062773926824`, 0.0011189928674473215`, 0.0011284588573504952`, 0.00845938359264764`, 0.4415116149971411`},
{0.13868488683280167`, 1.226702016867053`, 6.040570905634304`, 0.759875582646631`, 0.27881848833013434`,
0.595446367026503`, 0.23094105751878413`, 0.2051375441364418`, 1528.2920750506055`, 270.40251576187586`,
0.23385239775529226`, 1.5893916227857634`, 0.2397907655262943`, 39.81711579710742`, 14.129222527331965`,
50.79638203711839`, 0, 10.328925983711262`, 1.588696617525207`, 1.60259191456256`, 0.008746350237088363`,
0.0196812553798651`, 0.019755303207486 64`, 0.003762352867860974`, 27.840819212971933`, 0.03899275250119557`,
0.29553892529380593`, 0.000310088525798502`, 0.0003151914907204521`, 0.016456477739089426`, 0.09091357223227563`},
{0.1306914623455918`, 0.7537584653459817`, 0.21965733160577017`, 1.2102680433379889`, 0.2532343966945656`,
0.6654185660627909`, 0.08400179279005554`, 0.3103404088337731`, 3637.61631387517`, 133.45404753770885`,
0.03716426964372 4435`, 2.2620412269050973`, 0.956581036605332`, 98.88948248672286`, 9.223181246756917`,
110.0965641957919`, 0, 18.029647661721256`, 6.516778975087656`, 6.856683646039431`, 0.05215838564590913`,
1.4546537603867749`, 1.4486847802401688`, -0.0041033683128960385`, 70.17253313230046`, 2.715868959306548`,
0.7516827599678038`, 0.014375901426500137`, 0.015125769467286325`, 0.052161462334730624`, 0.13328074084796407`},
{0.26277447700446094`, 1.2458735546481199`, 6.447910682321009`, 1.49065802757454`, 0.9176757405073024`,
0.4907046225412167`, 0.15937178447602146`, 0.008327156523724668`, 782.8427424300617`, 244.66740068612717`,
0.09440964708909416`, 9.330818440145894`, 0.2131775359264001`, 548.7600490177299`, 399.77562265257495`,
572.9289522865931`, 0, 489.98996738235167`, 3.0814140858725065`, 3.103492745629528`, 0.007165106390032 6495`,
0.08523382866930901`, 0.08529506523093855`, 0.0007184537241327 06`, 54.84360457869663`, 0.3199610678809295`,
3.0405328223863473`, 0.0006894016623776311`, 0.0006931980408529947`, 0.005506773021507705`, 0.3127166127371895`},
{0.13025097964415272`, 1.0189597502038827`, 8.886210776319402`, 0.9111028640704337`, 0.2284753731773581`,

0.5590994693246798`, 0.2266682667598287`, 0.0061069720799473886`, 2065.306144054668`, 113.92755603976332`,
0.14827318077057916`, 4.714739506212668`, 0.4593044246185465`, 270.2263916058815`, 29.245672208907873`,
352.5979186604657`, 0, 248.27697540343522`, 1.3871332865303618`, 1.3938481560653218`, 0.0048408250311373635`,
0.036849556369836556`, 0.0368786017769473`, 0.0007882159236662911`, 20.19189981632094`, 0.0685670116660472`,
0.9338188180900945`, 0.00048788745238437237`, 0.0004901929281003966`, 0.004725425310196263`, 0.095338568248805`},
{0.09142437343902587`, 2.458407615666487`, 5.031014986619411`, 1.0519598677125233`, 0.487417438430646`,
0.4280564058665117`, 0.2026543236563062`, 0.1589331435911828`, 3989.0362964026026`, 249.7709598067779`,
0.24152899778887077`, 6.187241064247978`, 0.924037240334326`, 224.88009757599536`, 22.787606434365237`,
254.4459719339861`, 0, 69.43313828642292`, 4.3005707226934184`, 4.322229000804096`, 0.005036140435126679`,
0.047646791280754795`, 0.0476879096330634`, 0.0008629826102311`, 151.03651166259752`, 0.062229686274506237`,
1.036679840333957`, 0.0005464738427158888`, 0.0005490500876101416`, 0.004714305961012188 5`, 0.1168225773470653`},
{0.05362058570834477`, 1.4723855364180096`, 9.67162648709779`, 1.3845527897944452`, 0.7224375135481673`,
0.2670471970549346`, 0.10991701130195836`, 0.006877132758778975`, 886.1772845072878`, 273.92553716604675`,
0.12489313995376372`, 6.85687873202156`, 0.7259468075744833`, 239.29544289380024`, 119.2317842129614`,
245.57929422599588`, 0, 217.8765852103865`, 0.9596426409249246`, 0.9715155469697727`, 0.012372216008872616`,
0.031346333586765765`, 0.03135744140220071`, 0.00035435772429970314`, 20.18519920896915`, 0.024011553810449844`,
2.657404449619667`, 0.0002735411906980012`, 0.00027423446552311554`, 0.002534443983903456`, 0.20065298680516974`},
{0.15232520315695658`, 1.456608465380997`, 9.96731462021549`, 1.4944244110861535`, 0.5157767046786654`,
0.3722073718864378`, 0.22976562023274222`, 0.07428975878835796`, 1050.2078985192002`, 33.60662262776219`,
0.220518601124694`, 7.093566043209907`, 0.6560171292141301`, 1894.598853313323`, 95.67486192016392`,
2365.929648836791`, 0, 920.5305277824572`, 44.6274332645572`, 44.72816534177121`, 0.002257178373151314`,
0.251395908521653`, 0.2512977602283452`, -0.0003904132485088585`, 928.6385297339943`, 0.5470561834066587`,
3.5022007209857104`, 0.0020306793775768384`, 0.0020365171832690465`, 0.002874804243678364`, 0.05822873311194684 5`},
{0.0694009118290565`, 3.678178592599034`, 1.755843032698575`, 1.2142042066341803`, 0.3177757036060529`,
0.6847455380724545`, 0.08295711309717257`, 0.10240501118816868`, 536.5981094108779`, 211.10249067816517`,
0.18136782974370297`, 8.788955479280464`, 0.4777460308714221`, 295.9825552568471`, 260.51647263195275`,
281.93472724533507`, 0, 123.20509712417507`, 3.221334318226977`, 3.283335055825396`, 0.019246911830171243`,
0.1454971162099656`, 0.14605992234096357`, 0.00386816004095758`, 169.26632755581556`, 0.14425189333505398`,
5.505991667682314`, 0.0014317814455506728`, 0.0014576617383433262`, 0.018075588891780514`, 0.8809177318567568`},
{0.16483058262413092`, 2.446957095238485`, 8.164140625789859`, 1.1581574304294189`, 0.8637023907646861`,
0.5910929885256712`, 0.17429603155004098`, 0.036201628416469234`, 3040.5552834584214`, 70.89960300369222`,

0.19751315642431794`, 1.918185462218652`, 0.5717895645919415`, 300.9660508816507`, 20.281203440921`,
376.273306509112`, 0, 198.17638061846858`, 2.8451931244274067`, 2.853229250501102`, 0.0028244571536113483`,
0.1392696091412656`, 0.13951833745279685`, 0.001785948227074785`, 99.45807861630553`, 0.32794129737999544`,
0.361867000893801`, 0.0014499886807350926`, 0.001460595741473023`, 0.007315271407879686 5`, 0.01539218084059605 5` },
{0.26384722865028404`, 3.0959478641925884`, 9.455492188682882`, 1.339136584268803`, 0.7399277527624639`,
0.3172867286222486`, 0.06239618913281497`, 0.23953121953891282`, 1016.1140721617635`, 88.38662693497645`,
0.16528145715165937`, 7.22137987568804`, 1.361790640282516`, 616.5256794877836`, 124.32642543986617`,
643.7463295665209`, 0, 144.36303202173164`, 10.431650706338202`, 10.548801462005136`, 0.011230318092970037`,
0.07585215722395511`, 0.07586473396158724`, 0.00016580593212389694`, 461.3692389184421`, 0.28590544958108`,
6.519254302630731`, 0.0006844224096617113`, 0.0006862153541971568`, 0.002619646157307587`, 0.11558420953979827` },
{0.17041949742674112`, 0.566658425910223`, 8.176154533792225`, 1.187496781729836`, 0.9612002380635534`,
0.5389795298813838`, 0.1929826430211613`, 0.013244827072013646`, 716.151240381786`, 217.26337969751137`,
0.13800797665502906`, 7.448468008162791`, 0.8606351443610007`, 618.281812659997`, 420.70482286433923`,
620.5694325034558`, 0, 520.0386211658091`, 10.73775471557181`, 10.816009431568094`, 0.0072878099816156006`,
0.07561627515841023`, 0.07565601776053196`, 0.0005255826478951509`, 86.92341692765709`, 0.18409268013971694`,
3.855985975856596`, 0.0007682015276779808`, 0.0007715854256699794`, 0.004404961289555143`, 0.22382122985831218` },
{0.04627776053028976`, 2.3407537273567067`, 0.9761597154152462`, 1.37527944112939`, 0.3584342070422313`,
0.22640865017403333`, 0.13240363624591456`, 0.038325642841679504`, 1249.254134864137`, 143.93359215534173`,
0.13242235789061851`, 6.269547025830331`, 1.0591127938814697`, 314.5908363883492`, 48.10440696104188`,
343.36639116159756`, 0, 203.80097378447613`, 3.195151823461897`, 3.2318368925141256`, 0.011481479153150564`,
0.4420574879639394`, 0.4427785660781542`, 0.001631186291032094`, 106.84376486055746`, 0.29224900812313565`,
2.880410871171927`, 0.003859236099935659`, 0.003906633006845621`, 0.01228142194014814`, 0.1580824112429125` },
{0.1264814146333596`, 1.0630284736090054`, 7.0368315732194056`, 0.7618799049872292`, 0.05995838274087717`,
0.17364177343291576`, 0.061949148356618716`, 0.006022075969931666`, 3448.0521144594068`, 47.239369625587926`,
0.19227473651596427`, 8.592453879074284`, 1.3325684049534696`, 884.5795164563353`, 17.556174353386353`,
1019.8552375987382`, 0, 813.8786160336252`, 4.301860058908045`, 4.3291297210752475`, 0.006339039809241109`,
0.06998775334001091`, 0.06996096357475902`, -0.00038277790003837797`, 65.32856760143659`, 0.12645928642073098`,
4.537159995330293`, 0.0011109474487365212`, 0.0011118753568355637`, 0.0008352403168105926`, 0.0485263699002406` },
{0.04081744499533296`, 2.3413945804849527`, 2.3401462602509717`, 1.006052431149826`, 0.9376889728762128`,
0.63499912288976`, 0.07105146864527681`, 0.02628017367597553`, 775.1156602545625`, 104.41750831638893`,
0.12180266898464448`, 8.067006250284603`, 0.5522058225859034`, 718.1308154016845`, 450.9545649826378`,

740.2049753307122`, 0, 523.1547517315789`, 5.645629166839046`, 5.6901627521722755`, 0.007888152766888812`,
0.23983130734398794`, 0.24013860694748487`, 0.001281315633476554`, 188.8377933523531`, 0.1398471599381117`,
5.135760293890763`, 0.002862507354170152`, 0.002891090987930289`, 0.009985523257606932`, 0.2366275470894232`},
{0.22961450923300392`, 3.8785966005678736`, 7.959504018056748`, 1.2921046945277759`, 0.1917613861571974`,
0.3981097458569077`, 0.1102143743054892`, 0.20929581166220373`, 1753.2557317520059`, 231.02169920905055`,
0.10007865476065686`, 4.452819250691443`, 1.402721807760341`, 121.16783831937573`, 26.542136530168364`,
127.34203624739558`, 0, 31.68214486218406`, 1.584286054473345`, 1.6096770315999687`, 0.016026763004654443`,
0.027675613079569415`, 0.02770524034136362`, 0.0010705187165691576`, 87.78295007439202`, 0.09078174735671464`,
1.848634675238061`, 0.0002583688039843324`, 0.00025982436788499235`, 0.005633667370880557`, 0.16816572904605018`},
{0.12232907903298573`, 1.3289405346143397`, 5.1200974038340075`, 1.1450551183026931`, 0.6675798963164832`,
0.5719242545987869`, 0.08761535581144905`, 0.11107132883791111`, 3964.0786866225717`, 279.32313661047124`,
0.2162046831451349`, 8.020270743802456`, 1.0641453807394075`, 233.2830474057661`, 45.381459198924055`,
246.55534213250874`, 0, 91.25964841135544`, 7.100941061587705`, 7.160349892292607`, 0.0083663320381957`,
0.04077592550961289`, 0.040818463692399755`, 0.0010432180816308811`, 134.81040586644693`, 0.07125830591824672`,
1.8403331969814034`, 0.00042964690146685136`, 0.00043174719032977436`, 0.004888406865620087`, 0.16537795273039538`},
{0.11461934044260397`, 2.0495668840197947`, 1.6301061314690006`, 1.1441247153256733`, 0.9293434036316242`,
0.32161099339559507`, 0.24691379277403247`, 0.09383957046935675`, 3183.5471916851793`, 210.97634406977602`,
0.19279849145037242`, 7.844330233274125`, 0.7023420045147568`, 673.3281585903151`, 61.22330231347448`,
761.4755549926938`, 0, 287.89904739359685`, 12.696123841073844`, 12.736347563148724`, 0.003168189171623536 6`,
0.37773849625032735`, 0.37766664743498657`, -0.00019020781851464275`, 371.73649971541624`, 0.6185162471426863`,
1.1370769985653555`, 0.003975452829198867`, 0.004001922001737291`, 0.006658152838341591`, 0.0685702693235186`},
{0.10880423027814301`, 3.010977164978767`, 0.4477158763471074`, 1.3560425240667602`, 0.3025579540978818`,
0.35180253133086303`, 0.2109756008586861`, 0.03552671363577697`, 2668.2716874123935`, 170.59395733846873`,
0.23332643955483107`, 9.954767028855915`, 1.4592401570775992`, 478.43277465886`, 41.71549848366798`,
527.9691829039227`, 0, 316.95895422088455`, 3.6152935954152103`, 3.6595114095082275`, 0.012230767135784637`,
0.9074855151219051`, 0.9094314749355292`, 0.002144342561062995`, 155.508092292703`, 1.4105466137340854`,
3.5710513208605863`, 0.008003938980239877`, 0.008158323447667561`, 0.01928856127074785`, 0.32474913187459303`},
{0.22749339290790638`, 1.7311095241107948`, 1.2544972871049804`, 1.3263167066853254`, 0.7333948539753357`,
0.4493142943490447`, 0.2441212061859469`, 0.014870392763137733`, 1580.7600138635335`, 264.3754143437418`,
0.09646148923631659`, 4.446033883263464`, 0.6510175252381905`, 232.05413366704894`, 59.702146284180074`,
247.95027289771753`, 0, 190.3023614687392`, 1.56216525590488 5`, 1.5802050805835786`, 0.01154796178605566`,

```
0.3422984460341763`, 0.3442472567220693`, 0.005693308603856195`, 38.63255932474178`, 1.1124376410774077`,
  1.0972988310216099`, 0.003081873511346589`, 0.0031619893784351656`, 0.025995832338223`, 0.13712667826784378`},
 {0.10680853367942073`, 2.671856809998241`, 2.225956166276376`, 0.9564945044055834`, 0.04023151222298815`,
  0.66439395337897`, 0.05090336466790368`, 0.0058123339013462704`, 3923.759723431366`, 155.48006966286152`,
  0.1590662885693253`, 5.07114362920246`, 0.8193607123659179`, 155.13445264577044`, 15.45997296157163`,
  172.87264272760237`, 0, 143.01197818251606`, 0.3002177745655264`, 0.30364925207425747`, 0.011429961179670656`,
  0.08214443938901227`, 0.08241537505989717`, 0.0032982837657680175`, 11.459127220791704`, 0.12533895887224583`,
  1.4232198315537783`, 0.001032871837447491`, 0.0010444780820361727`, 0.011236868087490803`, 0.11675418500730272`},
 {0.1306588579678935`, 3.206018430436262`, 4.178329357094787`, 1.096081964467313`, 0.37321063526655895`,
  0.6088846209535219`, 0.1822932929157695 7`, 0.013520486536436158`, 782.6656351062597`, 123.9415631086822`,
  0.023548806194033767`, 9.649188633359959`, 0.8429625600097688`, 657.6525956747946`, 194.9983578834161`,
  703.1453759745679`, 0, 551.6336282431744`, 2.251472789934295`, 2.2748226649251637`, 0.010370933681836725`,
  0.11210079129533658`, 0.11219069756611874`, 0.0008020128113572778`, 103.11804657364411`, 0.2092423052563469`,
  6.273713664551445`, 0.0012319051693533423`, 0.0012397001792143801`, 0.006327605448014806`, 0.43835578521403534`},
 {0.06490925302735506`, 3.8934906583752236`, 0.9790395136780409`, 1.48284195083516`, 0.9349692905507263`,
  0.3703362249698605`, 0.1491469107421124`, 0.13667677484236057`, 1004.3258994396238`, 82.66219089427364`,
  0.140719161966 7938`, 7.134069953930672`, 0.6810640411318756`, 1138.1787159505802`, 198.41838319980116`,
  1261.245596442762`, 0, 386.826329062086`, 13.21817362121154`, 13.293327595476615`, 0.005685654948916374`,
  1.5162701184647511`, 1.5131317479392272`, -0.0020697964612673703`, 735.2119359281272`, 1.4059994396745794`,
  3.527902418150927`, 0.012232010925906978`, 0.012384727879966142`, 0.012485024333628969`, 0.10719652796441503`},
 {0.09335758271301797`, 3.886141513910534`, 2.1262193060875347`, 1.2374865217449704`, 0.20774186356921476`,
  0.6885354117013798`, 0.06828062526335338`, 0.04634612342186317`, 2337.7355280609045`, 265.47200855531526`,
  0.06367324855715212`, 6.746431099417454`, 1.2698799762745048`, 149.86756919163565`, 41.026364717684615`,
  153.86105811206693`, 0, 91.03538557977181`, 1.0375754585786836`, 1.0563197051535953`, 0.018065429766995766`,
  0.0807421651814422`, 0.08106634560732244`, 0.004015007835766582`, 57.602358048534036`, 0.1076841909193933`,
  2.5321298939773675`, 0.0007829825043355054`, 0.0007940340181898691`, 0.014114637036165778`, 0.2907013742012097`},
 {0.08231211479941364`, 2.387884906790136`, 8.578001165552415`, 0.9642461558703308`, 0.8238919598475782`,
  0.2020584877079309`, 0.19955393113038733`, 0.1095787053029616`, 2239.622315346988`, 371.59873105616873`,
  0.17019241214364111`, 3.55759780947197`, 0.4666380334441058`, 121.66650867018168`, 23.41272235620549`,
  140.05970297704337`, 0, 47.697226394373985`, 2.105133590002235`, 2.1143596965839566`, 0.004382670356664509`,
  0.02411475463411544`, 0.02412413630129606`, 0.000389042614074242`, 71.8116675191897`, 0.028356235025848685`,
```

```
  0.47974923159302124`, 0.00030213419256552854`, 0.000302999343895247`, 0.002863467131515751`, 0.04320680478618635`},
 {0.17519323235207396`, 1.2618694118815563`, 6.2707952492510515`, 1.261446257781183`, 0.5333912181747638`,
  0.18672075814011158`, 0.09419037155450471`, 0.010359125778073429`, 2462.605666629158`, 256.6607054858181`,
  0.10840920502450685`, 9.870044854094623`, 1.435459480935227`, 293.2600626005404`, 42.17826574140466`,
  310.6536706473716`, 0, 255.5503860676756`, 1.964736097886957`, 1.9869055406099159`, 0.011283674559042112`,
  0.03750116732567713`, 0.037506669292916094`, 0.0001467145593410102`, 35.417719776329676`, 0.09385643886804715`,
  2.9671734968815664`, 0.00035943416360872 95`, 0.00036010136554083663`, 0.0018562563040986912`, 0.16855305205378668`},
 {0.23749378089306467`, 0.4157158512844461`, 5.854529102702003`, 1.3110653325714712`, 0.1450893072377843 5`,
  0.1573757215384779`, 0.06204301966356823`, 0.16029852320715812`, 2190.57271228382`, 132.40666721155492`,
  0.12799517810137861`, 9.84238745503276`, 1.14600445718771`, 392.719291091847`, 32.67290272445369`,
  435.0538754959596`, 0, 121.85406171869109`, 39.00084383251472`, 39.39457019342676`, 0.010095329285767729`,
  0.0560595520881073`, 0.05606152319538198`, 0.000035160952973622 98`, 231.61812849493705`, 0.1901970711511688`,
  3.74565750041082`, 0.0005170088731456524`, 0.0005179685615559545`, 0.0018562319916541892`, 0.11381021928188183`},
 {0.06958654560186628`, 1.2810573894719157`, 1.5604323721050744`, 1.2865370396261684`, 0.41858142719439173`,
  0.3987961369914276`, 0.21356300651181537`, 0.006245930962717586 4`, 2941.8823280388497`, 383.1127710935082`,
  0.10442332143534439`, 6.649327249023878`, 0.3474394649692636`, 137.62118962267735`, 29.71517653886926`,
  171.56470829026205`, 0, 126.08695460519328`, 0.5796961724618128`, 0.5841038362576082`, 0.007603403308800827`,
  0.10659965818629895`, 0.1069632263845569`, 0.0034105944094358076`, 10.608915219725572`, 0.1059700282217668`,
  0.6960997230644941`, 0.0009933029306581354`, 0.0010080364612232299`, 0.01483286730598143 4`, 0.18788146589197877`},
 {0.12162705978453553`, 2.1459364566550487`, 0.8994451186914905`, 1.339976475017456`, 0.23917524394620604`,
  0.5256296133560587`, 0.21370599200931129`, 0.027381167717735945`, 996.433258066003`, 270.7208781713538`,
  0.090253513817738`, 8.675427486273929`, 0.7193288794908814`, 260.34637290978816`, 110.90363259732038`,
  275.2406666143308`, 0, 187.46281702066852`, 2.2771134332577994`, 2.312433522097973`, 0.015510904430282135`,
  0.2782018897561707`, 0.2798507521117908`, 0.005926855338995729`, 69.80772474809801`, 0.48338396967919534`,
  3.3146501392819303`, 0.002470605816370708`, 0.002535709925208346`, 0.02635147557989459`, 0.6287935870360404`},
 {0.2211475447831816`, 0.6764621696057107`, 7.070732773871981`, 1.484318092913942`, 0.39213098277018266`,
  0.49361758237854214`, 0.08219155305388848`, 0.007529395154295265`, 1570.1936066911949`, 345.9142373695621`,
  0.2485313792002623`, 1.2269777403584339`, 0.7159550405850554`, 22.7565089668544`, 10.710529885384396`,
  23.266585672282698`, 0, 20.47148062864608`, 0.20300480514608202`, 0.20724762871699512`, 0.020900114004001047`,
  0.02417864951285219 5`, 0.024334296576484216`, 0.00643737622935836`, 1.9617867275643301`, 0.07638641394200249`,
  0.3517388701415878`, 0.00019454317645473118`, 0.0001993508642353017`, 0.02471270320647423`, 0.06850441060309118`},
```

```
{0.1091983534434775`, 0.4123168888384443`, 1.8612158499296871`, 0.8333258155688079`, 0.39517409251864644`,
  0.5848465884265365`, 0.12470223152921811`, 0.040839984644619944`, 3265.644855437202`, 125.19293803224065`,
  0.0879734848590002`, 8.526937482297015`, 1.0338521843728818`, 488.6789050142701`, 41.26918913707241`,
  545.5597187964001`, 0, 309.35163756354785`, 25.963275493646478`, 26.13233854230154`, 0.006511622491408353`,
  0.1607963517354941`, 0.16095535816108222`, 0.000988868365929596`, 152.92995679422484`, 0.2508385264179364`,
  2.7809645053497203`, 0.002323947789736436`, 0.002340169761090931 6`, 0.0069803510329014`, 0.1592840151397116 3`},
{0.1605397117399036`, 2.9861451600751723`, 5.985367280571584`, 0.9013600575793626`, 0.4265711037361093`,
  0.6369377322655665`, 0.2099395794597424`, 0.0440322954061731`, 1331.4501176292097`, 178.64680303201567`,
  0.0465601985112431`, 1.120746116836358`, 1.298216136515848`, 55.3760038051425`, 13.833301202449599`,
  58.53759636474258`, 0, 34.115843770088205`, 0.48342352704903624`, 0.4897665638730066`, 0.013121075969740659`,
  0.04631303635753214`, 0.04658436075323415`, 0.005858488603671219`, 20.622468936627847`, 0.10621545009481927`,
  0.5804205101632518`, 0.0006148385292085168`, 0.0006273893699937702`, 0.020413230773631108`, 0.05940846848601740 6`},
{0.2316510553565545`, 1.3345287101431156`, 2.181207240226371`, 1.1295787756210554`, 0.4108038804094787`,
  0.55044788544399`, 0.15705941808893342`, 0.0693266889389808`, 2764.047895868124`, 88.55030846959835`,
  0.19804190063549815`, 3.6671215326087623`, 1.3965489185868663`, 320.7069665965303`, 20.27069475210782`,
  354.42235895785774`, 0, 161.45954147403657`, 7.875971163568017`, 7.953200942306792`, 0.009805746762509449`,
  0.2825088270376864`, 0.28324990986709264`, 0.0026232200854643395`, 150.15299482915432`, 0.9349066847261286`,
  1.9450752483320237`, 0.003008066994996228`, 0.0030471915301796635`, 0.013006537171052823`, 0.08238109984690511`},
{0.23613672937320296`, 2.4419271800403806`, 3.4588792688353487`, 1.4108864845746094`, 0.7134807082114705`,
  0.592154370313409`, 0.22091066377388413`, 0.019900714899270838`, 1522.4223604141134`, 78.60644958620696`,
  0.18909869538832608`, 2.793726035246588`, 0.7950281959201368`, 423.7806662181885`, 44.31081903235205`,
  474.49664155371016`, 0, 328.8289823803792`, 2.5957357502126888`, 2.614193117204647`, 0.007110649452836570 5`,
  0.385577024750259`, 0.38690082098240813`, 0.0034332860807941845`, 90.55139543781237`, 1.3006985363711363`,
  1.1951350530953093`, 0.0032810528885194312`, 0.0033330015315075766`, 0.015832918503056348`, 0.05191816448201239`},
{0.0484675208754865`, 0.6825441464113089`, 2.084792805230027`, 1.4119489784121344`, 0.5877996575498201`,
  0.6759892639478722`, 0.22086781309234377`, 0.0387732952545585`, 2548.5220980600216`, 370.2048073952212`,
  0.03857632786812504`, 3.327588495854709`, 1.4128853163273263`, 101.32068293344796`, 28.197724593713893`,
  104.21691878289695`, 0, 65.37468514253099`, 3.3229516943238875`, 3.3594995957767058`, 0.01099862556390696`,
  0.12810658414119785`, 0.12918426963084406`, 0.008412412967458316`, 32.400874682404464`, 0.0887001220163958`,
  0.8959686189757322`, 0.0010830134135463299`, 0.001109990308519423`, 0.024909105128031017`, 0.16670803719338864`},
{0.15820801401395784`, 2.3900304014986444`, 6.76991723319799`, 1.147995471223545`, 0.5011235356714243`,
```

0.39186104654424503`, 0.19387948138295608`, 0.05551860155117706`, 723.5959050648971`, 29.172220754504224`,
0.17373406698962968`, 6.019570931242669`, 1.3786373610317848`, 1900.8111528638758`, 118.44717481179086`,
2105.3295824705615`, 0, 1063.5476105484422`, 23.792481500076605`, 23.923623746754668`, 0.00551191966578357`,
0.336280808727089`, 0.33597200966619123`, -0.0009182773827226542`, 812.353630175388`, 0.7600331271391499`,
9.495104444562687`, 0.0035324956463018697`, 0.0035448973868644393`, 0.0035107589093712477`, 0.1045617896669757`},
{0.07579699484940727`, 2.2173315933991757`, 7.471866642915501`, 0.8771923938554881`, 0.8171799463467477`,
0.18907166793764196`, 0.16741258135296538`, 0.034704832899603626`, 2559.9885855263465`, 365.24466351817625`,
0.21912261270377126`, 5.637070181749923`, 1.0559796296250288`, 194.44266116256762`, 31.447687415677308`,
205.50810070683278`, 0, 130.3011340561626`, 1.9608891179766637`, 1.972348695740237`, 0.005844072292775948`,
0.025411893104448375`, 0.025416092330082747`, 0.00016524647011206817`, 62.11344846346148`, 0.02751635901075935`,
1.0667081598309536`, 0.0003502134111666999`, 0.0003508508997751341`, 0.0018202861115754754`, 0.08161527894768979`},
{0.06741497368958249`, 1.0249716767004005`, 9.449217531688483`, 1.0872407203117702`, 0.5615736740328037`,
0.5521415654810891`, 0.18288037923286848`, 0.005326201838481523`, 3978.4896243421163`, 351.6470055302269`,
0.09021448343705185`, 1.426476556917251`, 0.13651689047774385`, 21.293939312209314`, 6.4290213802314256`,
40.67797300418529`, 0, 19.754340317497643`, 0.09564554652195989`, 0.09625487115316558`, 0.006370653452911101`,
0.010733585333374881`, 0.010772410545115052`, 0.003617170792332582`, 1.4004853741077052`, 0.010337205326709682`,
0.09067503537915761`, 0.00011833025463514613`, 0.00012006297999929966`, 0.014643130529010673`, 0.0477943944144375`},
{0.05715322142426921`, 2.4704612263538817`, 0.36234457179092416`, 0.9684061618557824`, 0.863166347709152`,
0.23804186668051197`, 0.13749867219091239`, 0.1430597139428923`, 3758.60599725621`, 74.20395287068635`,
0.1109041971133704`, 1.6071731513826233`, 0.773390027344468`, 214.07104025937485`, 7.314456895401228`,
264.0834583023267`, 0, 69.19064043451664`, 3.880049823828504`, 3.960585574386611`, 0.02075637020522625`,
2.2375508153239956`, 2.1932869161021133`, -0.019782298984558633`, 136.93589494413607`, 1.8269033885180515`,
0.2909410056536169`, 0.027786664345384016`, 0.027937470599552608`, 0.00542728887116839`, 0.019665243741709533`},
{0.060904719608809554`, 1.0227281808325657`, 0.3338146022683226`, 1.4161209446138403`, 0.8561719858721175`,
0.4775008498746448`, 0.07550639206271559`, 0.17875509724795088`, 3800.334358301937`, 331.7811845820245`,
0.1313746491970903`, 6.20009884490432`, 1.2664955877170798`, 168.27439436272707`, 46.11422660391681`,
172.77401651030726`, 0, 48.055922444236046`, 7.615913029393943`, 7.746229837931914`, 0.017111120890562548`,
0.7117408948978249`, 0.7170721719273534`, 0.007490474507993339`, 111.27155539901588`, 0.6192625662554189`,
1.509096359324252`, 0.006013366818074517`, 0.006179135015855581`, 0.027566619964511485`, 0.19721970542296474`},
{0.09804650260585268`, 0.6377788755200506`, 9.103001426868161`, 0.9608072109179064`, 0.12522756138173952`,
0.16039700353140673`, 0.1476329494772725`, 0.03181180155196076`, 3994.864550131647`, 201.1651941507272`,

0.22810874694301092`, 5.8973245769190275`, 0.5910947751289481`, 157.47338641524934`, 10.47465857019683`,
203.0651991459402`, 0, 108.4154951390211`, 4.846133793506075`, 4.86628738015045`, 0.004158693817199355`,
0.017686831363706045`, 0.017689266262723973`, 0.00013766733949438503`, 44.15373944917174`, 0.024773313677006838`,
0.6019527475392836`, 0.00022261763223729858`, 0.00022293646851868654`, 0.0014322148617953445`, 0.051733882922860255`},
{0.10576408085084482`, 2.4399851863533764`, 2.261754328254943`, 0.8217420902241768`, 0.7416397192385102`,
0.2463678861595856`, 0.2027535408226292`, 0.2140679841559756`, 1564.164909651323`, 70.02516408668095`,
0.19509596638498505`, 2.4981390117767504`, 1.4295314398093057`, 433.51226227580935`, 23.36278144356929`,
468.0438673247004`, 0, 107.77849319834256`, 9.056572494256432`, 9.110824858735878`, 0.005990385933955844`,
0.3954221936740593`, 0.394914913627529 7`, -0.0012828820805839136`, 315.6843246445885`, 0.5974494980283243`,
1.6040672334008987`, 0.005789329897054607`, 0.005831132998361656`, 0.007220715013721568`, 0.03466771855415396`},
{0.18148677226867904`, 2.435655182727153`, 9.932013575043552`, 1.3523612276244852`, 0.270501525518553`,
0.3129668058670274`, 0.09479199345181186`, 0.24780135341453666`, 2571.145269598781`, 147.08034915528242`,
0.05706779714237542`, 9.481295153852962`, 0.5023669027433282`, 357.514348912612`, 37.80535478215227`,
440.5809147086804`, 0, 80.01384877609603`, 7.7492439871944745`, 7.787860140331559`, 0.004983215549916542`,
0.03221514019173617`, 0.0322228343699785`, 0.00023883733538121454`, 269.6355182803919`, 0.08352316873722467`,
1.6794761275480756`, 0.0002879282431096364`, 0.0002885373895708553`, 0.002115618998122848`, 0.12234886979742188`},
{0.045935603836807504`, 2.302483508903091`, 2.46282437804547`, 1.0004914316838749`, 0.17651744242150835`,
0.27731852138484747`, 0.16499475989489315`, 0.14699844290776085`, 3995.605017809292`, 299.6167282278376`,
0.2482978016224947`, 6.149511597608322`, 0.9467803390801968`, 132.7731513227521`, 13.7131176842289`,
152.31321975922168`, 0, 43.42318846390166`, 2.633579789652454`, 2.6537573976107556`, 0.007661665705964493`,
0.05496749585324919`, 0.055026835089255086`, 0.0010795331874735558`, 86.62534335793212`, 0.03607093019167796`,
0.9492233279913205`, 0.0006624387013365318`, 0.0006662692758764001`, 0.005782534341879142`, 0.13614021269839724`},
{0.24610403760574778`, 2.8350518587284927`, 2.483700295848834`, 1.2187293428205228`, 0.8051620910181194`,
0.2877775756384263`, 0.0943064533423209`, 0.11494360430719369`, 621.5470695356762`, 130.28571465739265`,
0.12477806190504986`, 6.74453372259727`, 1.1595275933030642`, 498.36559767337775`, 197.86058481840206`,
508.77386707438825`, 0, 191.57051080640292`, 7.367815717792286`, 7.463416868822375`, 0.012975507897031946`,
0.22703772876087983`, 0.22716035056381928`, 0.0005400943869933172`, 298.40199493565797`, 0.7982128819555296`,
5.383118443280992`, 0.002243162204526139`, 0.0022610518830565216`, 0.007975205045041234`, 0.16621461868687146`},
{0.2282743224553333`, 3.8004017582328853`, 8.466984049452861`, 1.390127484711968`, 0.35687133565138884`,
0.6519422926243725`, 0.17290926755870883`, 0.3014446142578311`, 2372.838415127524`, 111.84440462244766`,
0.08570923572829736`, 3.872670068176556`, 0.42950313779436455`, 227.19308607115659`, 25.843459315814194`,

293.84094746900337`, 0, 43.33381982682349`, 3.320628397484751`, 3.334652365410713`, 0.004223287356268202`,
0.06028903963828412`, 0.06040062227599712`, 0.0018507947444919015`, 180.28174286055878`, 0.19660628107026293`,
0.7100216296301142`, 0.0005219841160816197`, 0.0005265861866911047`, 0.008816495498045862`, 0.07602522499079656`},
{0.11402356544669451`, 0.8158491547123496`, 4.200202309736193`, 0.971641321517407`, 0.6807220168616288`,
0.6359800608186865`, 0.13923173061454314`, 0.037851089653330894`, 2281.1440386375243`, 126.7093813407077`,
0.1191355231677683`, 8.934189056957887`, 0.5820810660938738`, 654.2351214858214`, 96.36813232310024`,
748.75270015922`, 0, 425.180884017484`, 18.074913204695388`, 18.143704699533227`, 0.003805910106383105`,
0.10623428751662466`, 0.10630461071350608`, 0.0006619632749966797`, 210.66289513642582`, 0.1730458890764064`,
2.1782411449848706`, 0.0013180444816310866`, 0.0013250189799076555`, 0.005291549999843603`, 0.14209803397018728`},
{0.15189623977997108`, 2.7915541399359975`, 9.913479700081336`, 0.7741019472122475`, 0.15478570831213023`,
0.4331484807185151`, 0.23125927282289244`, 0.006529023247839043`, 823.7432504407011`, 34.92144293228938`,
0.20656993466421963`, 9.240164591756866`, 0.7039592626126989`, 1760.1180042585152`, 120.2541926278601`,
2190.2824177392404`, 0, 1608.6933975103154`, 3.6564574973474984`, 3.6733296380208063`, 0.004614340706967646`,
0.09340344020298011`, 0.09336503335859535`, -0.00041119303851433475`, 145.81712948886334`, 0.20268044784759787`,
6.280870505933138`, 0.0014578532521160037`, 0.0014603250962788876`, 0.0016955370228768363`, 0.1463497847599679`},
{0.19262437700106766`, 3.938833744371137`, 3.6810076480536082`, 1.109072783031779`, 0.7645216483353034`,
0.616702884118477`, 0.13777110888715882`, 0.010064854238404494`, 3438.969205276325`, 98.49201035779356`,
0.20668453166808726`, 2.7590741497101465`, 0.5477570556056697`, 251.4576129343318`, 21.822183991967442`,
308.72805915436726`, 0, 219.1941169248209`, 0.5491485373396952`, 0.5519634944630999`, 0.005126039553964068`,
0.17144336004500974`, 0.1719500459794046`, 0.002955412996233031`, 30.900068422080576`, 0.47177386313771097`,
0.46781498467019994`, 0.0018608420530803471`, 0.0018816909179536398`, 0.011203994900470304`, 0.032667829480698296`},
{0.15161686088493992`, 2.6676538201668016`, 4.769091008698259`, 0.9792911695535791`, 0.8799626502245399`,
0.5483041230008869`, 0.08973546330141513`, 0.2940210093980764`, 2792.4321626291066`, 123.07394825008441`,
0.21143511654972236`, 3.6673259692190108`, 1.1506144153300002`, 278.0492788949217`, 42.3458931756786`,
297.23837038308534`, 0, 54.646753004966094`, 5.704361779979218`, 5.742396781361008`, 0.006667704968377 43`,
0.09750605212640658`, 0.09763119480982571`, 0.0012834350349544277`, 217.38946419964338`, 0.21119373629560767`,
1.5383165882581802`, 0.001200678390947174`, 0.0012082317428459021`, 0.006290903505616896`, 0.05248692257542572 6`},
{0.15928964461807182`, 2.676103796250932`, 3.0276164230907305`, 1.1887325681254923`, 0.14578762490346686`,
0.4353007161971356`, 0.22317821296952212`, 0.2148623762619366`, 3792.9286804177964`, 76.64814683698182`,
0.1595894948400946`, 1.5825099373005287`, 0.48932638994153477`, 108.6066456177687`, 4.277397562256068`,
159.54633100527897`, 0, 26.68842984753557`, 2.0741407795393663`, 2.088155198318619`, 0.006756734604275394`,

0.1677757159099908`, 0.16830858496745726`, 0.003176079771594286`, 79.2945144869168`, 0.3817847737547313`,
0.1823827003647925`, 0.0016985716309687593`, 0.0017227517293014155`, 0.014235548205209003`, 0.02136731234268877`},
{0.16818423551071837`, 1.2425750232039041`, 0.8451308809778482`, 1.0511110147264935`, 0.32337126733921173`,
0.6302171747780823`, 0.06186616786607688`, 0.012735137580066362`, 2086.5412121340623`, 307.75958728157127`,
0.20485938116823293`, 3.379450004526051`, 0.9974668662640704`, 66.75586591839279`, 24.35226926950955`,
67.42309042225656`, 0, 56.181358862973845`, 0.5346231159545513`, 0.547995491564944`, 0.02501271495998969`,
0.1510256709308148`, 0.15303481844129888`, 0.013303351000536034`, 9.490133295893887`, 0.36285910011414635`,
1.1028102982074206`, 0.0017076662765184158`, 0.0017763683597601134`, 0.04023156291507202`, 0.19066790808284595`},
{0.049399735586138194`, 2.5477631442089104`, 4.041803486797548`, 1.3304361527101167`, 0.993281383326639`,
0.3001497243237138`, 0.21152425523664786`, 0.4874448677287525`, 780.8123462023354`, 361.74617623884797`,
0.046928831923920444`, 5.346338091727542`, 1.4411222226599376`, 296.28674959924956`, 183.14775021727928`,
299.16765633168785`, 0, 39.10808263104243`, 6.8718418811596695`, 6.927930167572917`, 0.008162045545172303`,
0.11434085317802099`, 0.11444889009917611`, 0.0009448671944656262`, 250.1117925378543`, 0.08069154162401945`,
2.2044662191708966`, 0.0010351332582424488`, 0.0010420635580060814`, 0.00669507979619887`, 0.14997193998419875`},
{0.20618269294314817`, 1.8587454568359414`, 4.34452965291473`, 0.8960498853155586`, 0.6640333303615127`,
0.5390503581736582`, 0.2329294063957692`, 0.4180316503514536`, 633.3353428142332`, 244.55036900145114`,
0.12942298196931046`, 3.095308470158429`, 1.4194968692598873`, 165.2052713657773`, 102.41979900215996`,
163.8287082111389`, 0, 25.23075939346532`, 5.070242605724032`, 5.1358011596551`, 0.012930062529366015`,
0.06882567217882068`, 0.06899480026487392`, 0.002457340127588248`, 134.63272012065087`, 0.20272374904972287`,
1.8617287920840155`, 0.0009220309994020237`, 0.0009336643812157615`, 0.0126171265622117`, 0.17580054292681443`},
{0.09869674443849347`, 1.9183657949501018`, 3.7344926730748456`, 1.0943839787542378`, 0.42780632576683675`,
0.5861386946846783`, 0.14629063580280066`, 0.03398060371034166`, 2435.986502306805`, 302.9804614286604`,
0.13730947981168928`, 9.171543304681595`, 0.26064124405682665`, 211.66915555435654`, 61.93859448191102`,
263.19522317547944`, 0, 142.79180715921694`, 2.4206319927605007`, 2.4352193697330247`, 0.006026267940005381`,
0.042380716716437845`, 0.042439137239746905`, 0.0013784694510936557`, 66.3379659581949`, 0.0597548395269118450`,
1.168938833569676`, 0.0004662968520746657 6`, 0.0004696746677226219`, 0.007243916901706227`, 0.32482522832182126`},
{0.1902308570197676`, 3.972029979384046`, 4.634110792119705`, 1.2197488970650956`, 0.8492525180509605`,
0.5006542712905349`, 0.18652832755791343`, 0.005076576077217329`, 1943.0673920285199`, 83.26055229551639`,
0.0580258116705662 5`, 7.38223678334383`, 0.9931576433164864`, 1220.1062802241013`, 109.2704789944178`,
1325.4261871592746`, 0, 1135.6560780329178`, 1.4223018178396893`, 1.4287709551626404`, 0.004548357628324506`,
0.2728999083973212`, 0.27288247106478947`, -0.000063896439665867`, 80.70607800273832`, 0.7416283350719045`,

3.191357686765922`, 0.0026978276504009413`, 0.002710546556867927`, 0.004714499262062022`, 0.0805616263038687`},
{0.06272312309945755`, 3.461529579186063`, 7.564878788392824`, 1.3521314303721368`, 0.32784603357531017`,
0.6503539369626794`, 0.11129074624350982`, 0.1957733709006163`, 2103.0882520472996`, 262.6269091211209`,
0.16330910334057364`, 4.05183701830078`, 1.3383335426462928`, 107.14298735101906`, 29.54162667278792`,
110.83493598966515`, 0, 29.297062737608687`, 1.5419061226645243`, 1.564375828726935`, 0.014572681003160781`,
0.029592792574347913`, 0.02965677381033182`, 0.002162054690281856`, 76.24790931330482`, 0.02651646244997059`,
1.5228180829682039`, 0.0002634185421891422`, 0.0002656583077138015`, 0.008502687419214006`, 0.17789485459036736`},
{0.1334851760745519`, 3.2697077297961004`, 8.047111995239536`, 0.842079209613761`, 0.8088703615622166`,
0.5692447244516433`, 0.16235903984142924`, 0.09701377075706732`, 2643.3784916344785`, 292.46187756743905`,
0.08266433823104813`, 7.391813212062153`, 0.547699130297068`, 303.50660292440494`, 80.76909107738538`,
330.7440724671764`, 0, 127.87704516690566`, 3.6795372067527103`, 3.6952074582829164`, 0.004258756101568395`,
0.026954050936865665`, 0.026967522110524996`, 0.0004997828968595908`, 171.87158924273848`, 0.0513995176459152`,
1.2142186196998683`, 0.0003864947137758312`, 0.0003877847325658462`, 0.003337739803507933`, 0.1457832627405855`},
{0.09401709418411008`, 0.8110971536952984`, 8.245292807864445`, 1.0264842776394885`, 0.12930370633004684`,
0.1740236808265636`, 0.07252752048975858`, 0.09884601796368213`, 3866.984141926244`, 274.6428268585172`,
0.20040469873166988`, 4.952717873499136`, 0.15734837909658306`, 48.380924078320284`, 9.288938552801362`,
105.10674777868473`, 0, 20.237329052948947`, 2.234486264437976`, 2.250177389540437`, 0.007022251759693843`,
0.0076294147869413066`, 0.00763201837196782`, 0.00034125619057556733`, 25.891220700812546`, 0.010247077265591546`,
0.3226373241653887`, 0.00008984432708081247`, 0.00009004053750870619`, 0.002183893343841614`, 0.07465978190489962`},
{0.16503822022515502`, 3.799827999093722`, 2.226332530621681`, 1.4474524276394465`, 0.4523982192034186`,
0.1981242269935809`, 0.06722881851519197`, 0.02132348079412978`, 3790.4878202500677`, 323.4156208714485`,
0.10032103134111592`, 5.6566553483532305`, 0.498005589518143`, 99.7417800416859`, 14.684418035595485`,
118.84101314717677`, 0, 76.39232598452736`, 0.41756367497588487`, 0.4212686443633997`, 0.0088728249355714`,
0.07169311410805035`, 0.07179660324116546`, 0.0014435017142531148`, 22.66671633682628`, 0.16903005649700945`,
0.6167641311056427`, 0.0005971561550339732`, 0.0006019230244950331`, 0.007982617981704854`, 0.07353691952525424`},
{0.2175565696926961`, 2.150769749820693`, 7.849181160138611`, 1.1076816333069537`, 0.23775832216164305`,
0.4289452843039179`, 0.23138213560150006`, 0.007541689166030151`, 1830.5976676958226`, 333.35844770130507`,
0.13740519457686395`, 6.97796965521683`, 1.0770120195809052`, 168.60578083422047`, 43.46369065605549`,
183.26174235283813`, 0, 152.17616759155402`, 0.5105379336451402`, 0.5168926290393454`, 0.012447058240773412`,
0.02138216942582173`, 0.02139590713501003`, 0.0006424843482770726`, 15.686422054570501`, 0.06645473475528249`,
1.9141117324861345`, 0.00023303757197534747`, 0.000233955157616751`, 0.003937500865742738`, 0.34847027627770094`},

{0.04261251981259187`, 1.6956083841270555`, 8.388949518118906`, 0.8816084343593602`, 0.6157741880137009`,
0.15816307961776666`, 0.1228282175848856`, 0.05282284640298743`, 1555.166107990478`, 26.393755355375106`,
0.19116960681111228`, 7.474128794459224`, 0.8297590847908434`, 2164.3617752231917`, 50.43677700203865`,
2666.369409635601`, 0, 1234.7534730144812`, 36.84219066637532`, 36.90423055151734`, 0.0016839358360585255`,
0.19124616903192776`, 0.1910288389692449`, -0.001136389104069102`, 892.4275340501933`, 0.11642115952969752`,
3.320713724188868`, 0.0026212631629801697`, 0.002623465088335324`, 0.0008400245294910569`, 0.02341750058207573`},
{0.12052689532243871`, 2.1969784256629277`, 8.110278600920065`, 0.9177559121464718`, 0.6615840119954921`,
0.34704707074624996`, 0.07379725368855697`, 0.00573204546098014`, 2320.908483262354`, 384.0513502933301`,
0.016004877265817924`, 9.641293645315589`, 0.17473869996337155`, 161.022955842351`, 68.89582811937123`,
202.53590118320292`, 0, 148.73923221237936`, 0.3732718440370699`, 0.3763296068285262`, 0.00819178526401942 4`,
0.011859237435454693`, 0.011862602623242693`, 0.0002837608915680079`, 11.715288403669412`, 0.020419386699811224`,
1.1156073238902475`, 0.00015619116227039154`, 0.00015650575187279146`, 0.002014131899827465`, 0.2662636731372991`},
{0.2044156153713736`, 2.9629304518198207`, 4.386784427445061`, 1.111833791211073`, 0.9591350877217695`,
0.43840802875011997`, 0.2469979148221132`, 0.01642865649440784`, 2743.650369521729`, 173.03541841863364`,
0.08406686391515611`, 5.337297453020938`, 0.28953137005492313`, 450.8167145865481`, 65.17903092102092`,
658.5210691071982`, 0, 364.6669929267498`, 1.9722189099103038`, 1.9777886718992785`, 0.002824109413507303`,
0.13412752000825254`, 0.13424189803333453`, 0.0008527558332172003`, 83.47924951183035`, 0.3916822791533013`,
0.568114539424493`, 0.00145378171291477`, 0.0014633422158221304`, 0.006576298781604484`, 0.04481657130607202`},
{0.12469439922923026`, 3.2167561610705597`, 1.2966549629505941`, 1.3380513288544775`, 0.9647032506590352`,
0.3520228082837078`, 0.24731936603055854`, 0.03725013837412559`, 3227.904692724528`, 130.80077454497865`,
0.06370555142915824`, 7.153312146366998`, 1.3824196402345823`, 1117.5038694620935`, 63.83687711755748`,
1196.4455981142053`, 0, 728.2908574694399`, 8.228009428363302`, 8.266270620922949`, 0.004650115303435953`,
1.0114885924978283`, 1.0095336221585398`, -0.0019327655831102852`, 378.1071431719186`, 1.801813748125047`,
2.2984718182310555`, 0.009080891966867455`, 0.009156532150128226`, 0.008329598406935368`, 0.07730559215051233`},
{0.0769260990011068`, 2.196578391656532`, 0.8056525760561577`, 0.8210657722273409`, 0.8826879394098923`,
0.3537183279427859`, 0.2409594091636174`, 0.049834595534127876`, 2188.2120164663`, 216.0992703121101`,
0.032694389865458584`, 6.1007061352710465`, 0.6801916687054326`, 474.15843323976617`, 66.82905997512145`,
519.4865002437508`, 0, 276.8563422437266`, 6.06095621694492`, 6.08975048267633`, 0.004750779365623625`,
0.49614671296845325`, 0.49618284316465017`, 0.0000728215974277191`, 190.19093512739337`, 0.5452375880124766`,
1.195251037658997`, 0.007245850440952384`, 0.007331775619557702`, 0.011858536041495338`, 0.08866178039636952`},
{0.09180583003794474`, 1.0245902356282572`, 8.242121052091868`, 1.338438298182017`, 0.7912235553845766`,

```
  0.4563538789330911`, 0.0736594669866128`, 0.007188071678387363 5`, 2109.8793350532806`, 273.6588416089354`,
  0.0378211628956065`, 4.589600480914077`, 0.9767739503530284`, 138.92045192799654`, 51.411315078958566`,
  143.43364308394416`, 0, 125.93702365658818`, 0.817839760580945`, 0.8273225569319141`, 0.011594931926803165`,
  0.030805869805655348`, 0.030842594914487802`, 0.001192146466376220 5`, 11.970723328568381`, 0.040402263536415045`,
  1.3956500113732793`, 0.00027759027038576356`, 0.000279103902304899`, 0.005452755664065334`, 0.11208430149112797`},
 {0.25211089910042056`, 2.9465650077307215`, 6.640985006189094`, 1.09304531251437`, 0.4582346106456783`,
  0.5101745978688277`, 0.23290591035097613`, 0.10611238144646964`, 2453.147250432534`, 22.888736398204685`,
  0.22056464944244836`, 3.285214614677961 7`, 1.410915433494099`, 1272.7074255458847`, 20.59870023849463`,
  1493.0661847068623`, 0, 506.8732987548052`, 17.728609984518176`, 17.794676646385298`, 0.0037265562232355887`,
  0.400093322409015`, 0.39972890213927365`, -0.0009108381703226698`, 746.2643116583837`, 1.4409698176660806`,
  2.964707414332088`, 0.004413468937865428`, 0.00443349195152883`, 0.004536797232583822`, 0.03377465390145257`},
 {0.10187608814553512`, 3.02527275016552`, 8.112504151515616`, 1.0430646419869034`, 0.02000177204588316`,
  0.406606876656344`, 0.10224573377309781`, 0.465955444901958`, 2596.0557042261407`, 108.81287726186605`,
  0.13773652673586545`, 3.785014828823544`, 0.37908929500916355`, 146.54936493205884`, 13.339723230901736`,
  203.7839107142125`, 0, 19.63481839547059`, 2.868456168477951`, 2.8813682085949934`, 0.004501390071403488`,
  0.031208784234473846`, 0.031236938841904097`, 0.0009021372706712327`, 123.96946116486465`, 0.04542041219403088`,
  0.5911026972740173`, 0.00036091688764261 5`, 0.00036272293397307894`, 0.005004050495559742`, 0.05879398843936458`},
 {0.27609243280065304`, 2.4334000704041427`, 3.768968853050694`, 0.9897634681270504`, 0.04043037685648332`,
  0.4200152717945378`, 0.22935860430417082`, 0.1525987200218085`, 3069.7266486703747`, 262.01073398400206`,
  0.24155932723410417`, 8.399632627499091`, 0.7132175733358337`, 204.00395607435718`, 26.327634938971173`,
  243.32131196839003`, 0, 64.97835231430733`, 3.8819043103556523`, 3.9105207744594708`, 0.00737175927481748`,
  0.03996831110213776`, 0.04000268038458571`, 0.0008599133038200701`, 134.94608888745086`, 0.15764211781607676`,
  1.355252767604441`, 0.0004870284965574623`, 0.000489744245128164`, 0.005576159485323417 5`, 0.2536412285286762`},
 {0.0978918800897613`, 2.777864894021529`, 4.606090716083992`, 1.4880029918673265`, 0.6064208129951822`,
  0.3601049854666406`, 0.18842765361648817`, 0.044059072442558106`, 799.5331038282079`, 182.92623376411473`,
  0.07894991488107306`, 2.020622256243092`, 0.5601073680228721`, 110.23271975164187`, 37.96036380247329`,
  119.0620576740567`, 0, 67.81589415830172`, 1.0360321261863785`, 1.0466336541546308`, 0.010232817786526072`,
  0.1099307470147125`, 0.11037624973657673`, 0.004052576135088026`, 41.11367532016605`, 0.15373325007060845`,
  0.859747935080134`, 0.000884773044382503`, 0.0009001326103990511`, 0.017359893719713915`, 0.0733330922037534`},
 {0.08900054818761971`, 2.823318859242785`, 7.144670702377984`, 0.8315383304970898`, 0.2795052665240947`,
  0.4576393886321606`, 0.05117478065859138`, 0.06552086595628574`, 2102.911960214631`, 101.67438536233578`,
```

0.08762396482794987`, 9.096737685205667`, 0.25171195782463274`, 370.6443630841629`, 53.27522599811497`,
533.7273623082874`, 0, 192.17055432040902`, 4.316260998180053`, 4.335434230833379`, 0.004442092973851786`,
0.02975694203787886`, 0.02976567449407895`, 0.0002934594619625752`, 174.08830110822632`, 0.03783405933938403`,
1.5893621030000493`, 0.00043230045033870734`, 0.0004334141673776541`, 0.002576256948319644`, 0.15405289955818888`},
{0.1517146567463153`, 0.8404448307598287`, 8.427449210104406`, 1.2489405079089722`, 0.7937040652491076`,
0.6477616643753492`, 0.08512282603978563`, 0.016388604299130645`, 3405.4110812811105`, 399.1826202269507`,
0.20314112870963702`, 6.737758928823055`, 0.8484255316811282`, 151.76174525803793`, 60.481655147020746`,
156.46155342873104`, 0, 123.1115648648609`, 2.19313416704271`, 2.2135647469774815`, 0.009315699988531`,
0.020924312956928124`, 0.020947187414603972`, 0.0010931999403245296`, 26.33154676934007`, 0.045350356541631026`,
1.2775384279412674`, 0.00020208393872667152`, 0.00020312542757228085`, 0.005153743796620924`, 0.18477479328848262`},
{0.07742729394690523`, 3.317040912283317`, 9.880999371532482`, 0.8010465312053925`, 0.28743469962710155`,
0.6365305604148241`, 0.19411226430840994`, 0.47831260845896817`, 3458.518042369643`, 116.53097484036505`,
0.09815493609620851`, 4.832652127106721`, 0.5129478012500222`, 268.7520007497255`, 20.37163699828287`,
349.73691122893763`, 0, 34.90031920441917`, 4.831783551583209`, 4.845213293446963`, 0.0027794585002371974`,
0.028279382198461916`, 0.028297619226262838`, 0.0006448877727576185`, 228.96033885570117`, 0.03127994340191024`,
0.6980906592837757`, 0.00042611469472331187`, 0.0004277390421960821`, 0.003811995908343574`, 0.07040357675318752`},
{0.2511800957944456`, 0.4969745065700919`, 2.29184449492171`, 0.9917654079551637`, 0.683514928780494`,
0.655379840437561`, 0.10467228571183546`, 0.22778373737657998`, 3254.986072380726`, 170.41822686556145`,
0.16061682605470035`, 9.463970581786636`, 1.0777227394408708`, 489.0360396593355`, 73.48055914776239`,
522.7756652820509`, 0, 117.09636838170296`, 45.84121960400796`, 46.18459208383119`, 0.0074904743545090025`,
0.14002772033052716`, 0.1401983254215754`, 0.0012183665537477406`, 325.45596418961486`, 0.5024596600931067`,
3.02372898191413`, 0.0017008693538074215`, 0.001713546639653961`, 0.007453415406751374`, 0.18128392246771188`},
{0.2292340810710673`, 2.064324787257691`, 0.2897872149067009`, 0.9514880626424775`, 0.10199246953503383`,
0.4890381533823508`, 0.20734257158694452`, 0.0515148990758617`, 1032.7042553842448`, 333.02101571525463`,
0.23061763375632705`, 3.9940977361306764`, 0.21053286147363237`, 80.41894205010918`, 42.118508286250616`,
91.6689182702137`, 0, 45.76969575905685`, 1.0807865925677314`, 1.1181477909389437`, 0.03456852502439878`,
0.39637205774899936`, 0.4060783089629023`, 0.024487728194123592`, 31.87277932533364`, 1.2980283488619522`,
1.0469476770118413`, 0.004808614644577047`, 0.00528048774272739`, 0.09813077841088846`, 0.45182768402183443`},
{0.25996807730167304`, 3.8602372982487374`, 0.7583354986548105`, 0.984076713917565`, 0.09643958841060729`,
0.4475162082476123`, 0.14281046726777719`, 0.2769302537407711`, 2666.244743594094`, 250.78576435414914`,
0.15930752740741816`, 6.009785296615535`, 0.38944531154385054`, 130.5797260693887`, 23.27804058076261`,

162.5954124133564`, 0, 26.826031879157732`, 1.8332281210721992`, 1.8564499227675462`, 0.012667164238002737`,
0.17497696953641784`, 0.1758900138508659`, 0.005218082796079271`, 101.09565098801974`, 0.6498346620351982`,
0.8333487358180321`, 0.002122053225132703`, 0.0021775542865115514`, 0.02615441531886087`, 0.21080211491390202`},
{0.1007555201432343`, 3.8180664304949197`, 4.270424159744305`, 1.4627535709622805`, 0.3807188794187142`,
0.17571212182398588`, 0.053627366653205444`, 0.4983255094785715`, 1310.607317823291`, 380.8287865986265`,
0.23700173797672958`, 3.1928180922211973`, 0.4025308666594032`, 47.185464670674044`, 21.073203453941264`,
50.73444786296926`, 0, 6.4381717372634855`, 0.732214847733402`, 0.7448838686227955`, 0.017302327218044145`,
0.031685880434621574`, 0.03172812775559575`, 0.0013333169346940732`, 39.937784714869366`, 0.04560753377692503`,
0.7345559995503558`, 0.00026100152079600036`, 0.0002630126513758008`, 0.0077054362505892815`, 0.09342143188265147`},
{0.11012573052984026`, 2.7973646534087315`, 6.317504258164328`, 1.4230817420807518`, 0.4665277158140053`,
0.4964083914255707`, 0.11975389672549558`, 0.008384974576684757`, 3433.4182170381464`, 104.6436908016745`,
0.1542234498742236`, 1.795119919919351`, 0.21223525603288929`, 66.68439341899203`, 7.874854500227617`,
135.07055842840575`, 0, 59.40074413054946`, 0.1732415116616431`, 0.1741438918733993`, 0.005208798994542629`,
0.05220866863674009`, 0.05242744752053352`, 0.00419047046220733`, 6.923138303219667`, 0.08213596819444724`,
0.15808324044685532`, 0.00044040933810785`, 0.00044688809760098534`, 0.014710767762033283`, 0.03063193360122708`},
{0.08998066206695893`, 2.280866979574526`, 7.329281823836337`, 0.8411001090310725`, 0.6029746925458184`,
0.27522739162036525`, 0.10930338132980638`, 0.008444888139321425`, 599.859329243076`, 286.89260187390653`,
0.08925071767070175`, 9.77708834471905`, 0.3030303902445597`, 299.6595456833217`, 221.49784791716863`,
305.9587729204112`, 0, 267.58021306267176`, 0.9466347794122221`, 0.9605663179237213`, 0.014716909640853704`,
0.022074895738669574`, 0.02207775370737228`, 0.0001294669173770302`, 30.844971572545063`, 0.02837591048033096`,
4.097346875727265`, 0.0003172910128347306`, 0.00031781233430638764`, 0.0016430388840813492`, 0.44085035701359654`},
{0.21313668431881322`, 1.7635041678835899`, 2.1912925923575113`, 0.8252025033116523`, 0.016757328021256512`,
0.6998740735780284`, 0.1801084581445288`, 0.26779805577049626`, 1220.0176309294266`, 152.79020888909633`,
0.1629579286674429`, 2.280125156684093`, 0.10691523188617968`, 54.78014200131796`, 22.278899189834885`,
99.47451691587531`, 0, 11.668921998159611`, 1.6372529522808352`, 1.6580192413965387`, 0.012683616839275588`,
0.056069348815492547`, 0.056562709852191785`, 0.00879912192885901`, 41.24717721752802`, 0.17072050140645872`,
0.48354721806828543`, 0.0008039479890079582`, 0.0008332545411113429`, 0.03645329362605643`, 0.2052529555039463`},
{0.09290951514101703`, 0.41749240574692914`, 4.773370270407071`, 0.8850952467526404`, 0.9766517437912483`,
0.4208978561725222`, 0.06525352787617444`, 0.011206616294756987`, 2604.3347674109446`, 377.7615808940852`,
0.1923606467616938`, 6.325030382869231`, 0.9625675284230475`, 152.13387396729792`, 82.96563031497529`,
156.66907343420513`, 0, 131.20493434291316`, 2.982116320667766`, 3.014665961578155`, 0.010914946772800693`,

```
0.02796414349030593`, 0.027987582448987143`, 0.0008381790305622694`, 17.78587024189669`, 0.03711621447167167`,
 1.4668411275329862`, 0.0003812768709606784`, 0.00038295855583557405`, 0.004410665851978912`, 0.13586537921014122`},
{0.1404288473454146`, 2.9064356368530344`, 5.23236147204817`, 1.4484340156797024`, 0.10732611967199035`,
 0.27136542485299375`, 0.18681354316538268`, 0.3953864116976934`, 677.7158408842279`, 88.88656461439052`,
 0.043948668205834374`, 4.975328880565433`, 0.3586361093450223`, 316.78256303862986`, 61.1517628473689`,
 409.309836705922`, 0, 49.432366955564135`, 6.279749912169693`, 6.329521125722416`, 0.007925668099659333`,
 0.11045226410534092`, 0.11053704035400536`, 0.0007675374457112305`, 260.7384133607818`, 0.2215812019285914 6`,
 2.206231353812065`, 0.0009189634589740203`, 0.0009248159847010772`, 0.006368616368697566`, 0.1283487215206620 7`},
{0.12849833171545855`, 1.198455475456262`, 3.2539226138784088`, 1.3648636137864978`, 0.5280448025145577`,
 0.6072554649082684`, 0.24978634440506725`, 0.03994982606054573`, 2204.2740688947933`, 354.6777076623247`,
 0.08439759959738258`, 4.855199768823253`, 0.40974365814152147`, 139.32163146354804`, 41.31358370492168`,
 159.73519130350044`, 0, 88.82497015450855`, 2.774552552960507`, 2.793583841851358`, 0.006859228119699523`,
 0.07515618053358349`, 0.07544825911556756`, 0.0038862882588022174`, 47.502538557666774`, 0.13796348309523576`,
 0.7415684451448805`, 0.0006594315253982685`, 0.0006702206733124313`, 0.016361286196692637`, 0.19060867641649445`},
{0.17543069017848928`, 1.9027743233680443`, 8.420650851113926`, 1.2989717187578058`, 0.4754143700193414`,
 0.44151569893640996`, 0.17410928694054217`, 0.39942064454570464`, 1461.6707316679758`, 62.00370069117048`,
 0.1325925609245337`, 5.29940719568374 7`, 0.7461186052859023`, 687.384493794975`, 53.18491151759484`,
 811.2975080173674`, 0, 104.35793613247976`, 20.67193359099289`, 20.75337277478891`, 0.00393960165543028`,
 0.12549053675249888`, 0.1255369195901833`, 0.00036961223439413 44`, 561.9146350187224`, 0.31449844961964346`,
 2.2910648556287883`, 0.001165692145032926`, 0.0011706858571753813`, 0.004283903055994465`, 0.07030428621136905`},
{0.27774537273159505`, 2.913102479760642`, 7.942633412240518`, 1.0326494422606074`, 0.869193199676557`,
 0.6849829313653006`, 0.16756021109529395`, 0.031844382721063484`, 1157.358808485019`, 382.8520115993825 4`,
 0.04020804442348902`, 7.095647344980355`, 1.3824063653570637`, 273.1922978115347`, 232.6841345732932`,
 266.25937956234054`, 0, 188.46036689502267`, 1.9840113354422673`, 2.0044446692101543`, 0.010299000516210244`,
 0.031379718234021865`, 0.031407971477112016`, 0.0009003663729370004`, 82.56611915928701`, 0.12450816481598857`,
 2.611978603305158`, 0.0003665003597869143`, 0.0003684049883098843`, 0.005196798508130707`, 0.2972113473775722`},
{0.11633291490443043`, 1.4450658892853943`, 9.421948807050416`, 1.0073582836381636`, 0.9376244508422855`,
 0.2923945304421177`, 0.08314225478001569`, 0.015377391816799622`, 1342.5955234153207`, 117.35920160680735`,
 0.24930648416353973`, 5.058158894639092`, 1.4288639894036779`, 420.2758683582103`, 87.17869086014393`,
 439.63576507222444`, 0, 344.91204251674236`, 3.466916648737199`, 3.4973634787730337`, 0.008782106153871538`,
 0.05574163973055671 5`, 0.05575130006040287`, 0.0001733054480070883 6`, 71.5703284297964`, 0.09263696330594097`,
```

```
  3.3284209840858723`, 0.0006686795351256158`, 0.0006702160721732022`, 0.002297867613516358`, 0.07062532886261152`},
 {0.09982764185325876`, 2.300171702541898`, 0.4858627002636364`, 0.9919442401423758`, 0.5310842871377108`,
  0.5692781547371908`, 0.20674425403353813`, 0.29388576255746934`, 1499.3990415110002`, 267.8323361172651`,
  0.11758456647217047`, 5.8552111813994445`, 0.6586971862135473`, 222.94062513178866`, 71.36064543749086`,
  237.01910015175844`, 0, 43.95874881912432`, 5.251515110718016`, 5.317664446036928`, 0.012596238213978594`,
  0.4813043644303288`, 0.4854484862055924`, 0.008610189479932417`, 172.56266361635366`, 0.6863925673542339`,
  1.6401593541034736`, 0.005741483897784616`, 0.0059558902156166245`, 0.037343363083320424`, 0.3032822176693308`},
 {0.16308341937411774`, 2.9768832237222593`, 6.137404711598409`, 1.4978818887932133`, 0.6270064524377599`,
  0.3856550031514199`, 0.07688210862674477`, 0.2679284542888144`, 1679.641743573421`, 154.41066935622916`,
  0.011847012076814933`, 5.409061271016132`, 0.714419786721034`, 252.16756413883186`, 53.14749547783482`,
  274.47787357953734`, 0, 53.65634476971938`, 4.555202348126738`, 4.593307607823167`, 0.008365217784913614`,
  0.0712864946387075`, 0.07136312594764588`, 0.0010749765341495632`, 193.71864929712487`, 0.16608064715548354`,
  1.8697768840376536`, 0.0005738577793223953`, 0.0005772928149641779`, 0.005985865776427568`, 0.10417643749616823`},
 {0.10408875617257995`, 0.7208211774242166`, 9.395809932215364`, 0.8438330742384919`, 0.9431300864694563`,
  0.42081055780340804`, 0.06365903598523207`, 0.007548231362222756`, 2388.75487627468`, 266.1557043275724`,
  0.07149291165582927`, 4.419002029188226`, 0.6654732054016175`, 138.77864143358954`, 57.0921529838827`,
  147.6164469162356`, 0, 125.22212152488629`, 1.1854521824028539`, 1.1956819877603115`, 0.008629454236376066`,
  0.017690684702766832`, 0.01770133970824891`, 0.000602294691308014`, 12.207129112853295`, 0.026305733807900723`,
  1.0129462459075746`, 0.0002531685199863576`, 0.0002540257838291448`, 0.003386139172569136`, 0.08214271786537033`},
 {0.1859503570691094`, 3.4941122484776246`, 1.6297837870350822`, 1.1031963489157173`, 0.4293650493894907`,
  0.20959825245659025`, 0.17774273230533655`, 0.18327500412709075`, 1189.2239199460373`, 282.6299270365462`,
  0.11232193397712892`, 9.90920375985121`, 0.8278911236090769`, 304.5245875813241`, 83.59779471259966`,
  329.46031638712145`, 0, 86.20642244318577`, 4.278460536735511`, 4.327927104127533`, 0.011561767829174618`,
  0.13032975580275089`, 0.13039145462779547`, 0.0004734055140713789`, 213.5631623719388`, 0.34621235183255394`,
  3.0596462690019544`, 0.0014238451690773424`, 0.001433069338439032`, 0.0064783514120898555`, 0.2963741254932882`},
 {0.2642909093671957`, 3.7936229586154386`, 9.112438874043047`, 1.343319643044862`, 0.7298819945671444`,
  0.2416317029456937`, 0.21308855511768615`, 0.0296357748833522`, 1462.8216839780898`, 69.98981659671449`,
  0.07124306277278569`, 2.5551101499765654`, 0.4563514754130473`, 366.33638321690165`, 24.884564240710755`,
  485.14255446532815`, 0, 257.07757101794385`, 1.9672998057557387`, 1.9741690534834817`, 0.003491713722354683 5`,
  0.13240103703011696`, 0.1324609183450315`, 0.00045227224995891824`, 106.61705299420964`, 0.49989129254054837`,
  0.6011572854462511`, 0.001189481900971456 3`, 0.0011957747824243852`, 0.005290439011967685`, 0.01665838590608277 3`},
```

```
{0.24431991434407568`, 1.8546418038040455`, 9.014242525232781`, 1.0012178057162573`, 0.9513564875697376`,
 0.5540264355265693`, 0.16546833659186994`, 0.010160023644511053`, 3069.219263999773`, 161.6333371189296`,
 0.2033203481299034`, 5.464378114605116`, 1.4999257720510482`, 510.55691298037294`, 70.7912894081629`,
 534.9136477391231`, 0, 445.55726160699146`, 2.3408530824446534`, 2.353581102913419`, 0.0054373427210019365`,
 0.0650845700372762`, 0.06511441398351231`, 0.00045854103697706705`, 62.02062833236305`, 0.22716366538042673`,
 2.1132250899534952`, 0.000785116936737329`, 0.00078774883852781`, 0.0033522417710389885`, 0.07580657003993398`},
{0.2550662278820944`, 2.7952392159553643`, 1.7242958226447735`, 0.9892662747315195`, 0.6466530652803972`,
 0.41633012667926605`, 0.0937340972126295 1`, 0.04014553436100187`, 3927.541477581917`, 214.6071511864991`,
 0.12072625793793595`, 4.810615700541705`, 1.0908243473679682`, 177.48815555169776`, 21.691540653228298`,
 191.44224963295213`, 0, 112.82341487722123`, 1.564626263310993`, 1.5804358716826596`, 0.010104399205348225`,
 0.13212558953805553`, 0.1324298580278861`, 0.0023028732805987495`, 62.47863842172269`, 0.48143965328826044`,
 1.164647274145918`, 0.0016083106959164928`, 0.0016261871617484038`, 0.011115057480684198`, 0.0840810429706326 7`},
{0.07397988502541059`, 0.707910258007638`, 2.664085400294777`, 0.9518019482057067`, 0.7875244424943906`,
 0.2321255676355145`, 0.14195245680232`, 0.005701405567921862`, 2118.0076336028906`, 265.7950362273598`,
 0.09877063847703516`, 6.01326524592864`, 1.001781869102726`, 251.26781810990522`, 44.02544745333544`,
 266.7567169501412`, 0, 232.04208408593286`, 1.6896430115475018`, 1.7028774813636862`, 0.007832701775307704`,
 0.09360266071513354`, 0.09365156928767339`, 0.00052251263122427`, 17.087366003505636`, 0.09892448682538883`,
 1.486230253795584`, 0.0011863916352758963`, 0.0011920430973900635`, 0.004763572117442383`, 0.09534782119816398`},
{0.2536234425791481`, 3.084120548082856`, 3.3542268996076317`, 1.2342076675511184`, 0.07971619232511307`,
 0.37753168173048746`, 0.24305535176309745`, 0.09244094904749854`, 1429.525330090045`, 199.04655556460716`,
 0.17472732471161218`, 3.7784584150910736`, 1.055845732825249`, 131.93119479334297`, 24.546142447676267`,
 147.8414824627025`, 0, 57.4676401372047`, 1.644351217378095`, 1.6662345104534502`, 0.013308162419369163`,
 0.08022685483537244`, 0.08043147439450005`, 0.0025505120392355085`, 72.44824825401219`, 0.29067730158062705`,
 1.4406879049220709`, 0.000780812453391011`, 0.0007911412031957908`, 0.013228208335974712`, 0.1858796166431398`},
{0.23487265302124444`, 2.745668437703702`, 1.8560463908813354`, 1.0225932800667423`, 0.519798686110498`,
 0.6167124420851566`, 0.13872112580125617`, 0.02304360370209753`, 1950.6949460047217`, 63.792561577309925`,
 0.17121277633362675`, 1.4754433886909606`, 1.2090151658939319`, 182.77342982799544`, 13.86652468848771`,
 200.83120838433078`, 0, 136.34219520062467`, 1.1077595807851237`, 1.125365155203139`, 0.015892956128203517`,
 0.421253942186589`, 0.4241466416205445`, 0.006866878014103461`, 43.450578821794274`, 1.4134432999574538`,
 1.0038255511629224`, 0.004940879586846969`, 0.005072420817527299`, 0.026623039150863725`, 0.03849931476655650 6`},
{0.19404987421370834`, 1.8913121151690095`, 6.875162123920855`, 0.9378067865957597`, 0.9620319910139619`,
```

0.17047428599364534`, 0.1676311609323027`, 0.36688161374531547`, 2436.6727196810125`, 312.9444240665532`,
0.08367109576690585`, 6.310397829770168`, 0.3372839322361041`, 252.49049863331226`, 42.7364504135267`,
328.89559059609036`, 0, 41.03096463484105`, 7.54246358986224`, 7.567606628642029`, 0.003335313429398994`,
0.03424415468595689`, 0.03424677198711993`, 0.000007643059631767812`, 203.78789665325118`, 0.09492962727680121`,
0.7102536573297733`, 0.00044142927875445714`, 0.00044230204663112577`, 0.0019771408890938424`, 0.04565425019211554`},
{0.14433043129139256`, 1.3202167920086731`, 8.463992130027801`, 1.1010419605014736`, 0.14350706526043644`,
0.4137152347701779`, 0.23975541502065495`, 0.00799616367743015`, 1911.0537077498657`, 153.77127625620255`,
0.2417426282164734`, 8.21507395149112`, 1.2501529132389022`, 391.7473103724207`, 44.56042824385376`,
442.7597310385029`, 0, 351.5131765758041`, 2.003567863137137`, 2.0222272896944093`, 0.009313099346710318`,
0.03891895526714055`, 0.03893266784328891 5`, 0.00035233669697043446`, 37.78777052632267`, 0.0802455657016692`,
3.3221554111575644`, 0.00042698688653153294`, 0.00042818641810334074`, 0.0028092937034946974`, 0.29051865880254985`},
{0.047715308213800045`, 1.0917315932828888`, 7.945801231718313`, 1.3183453869548736`, 0.37495408831467225`,
0.3679377030871549`, 0.15114689724124963`, 0.44212694961805754`, 1904.8096035348635`, 21.765700565880422`,
0.02920214850379993`, 1.0562137220921812`, 0.16322301362739666`, 63.526281997992555`, 6.355994129288801`,
380.1407596207104`, 0, 8.79255617035026`, 3.291653476418555`, 3.3045706067892766`, 0.003924207229971222`,
0.062378989529825`, 0.06254558529772208`, 0.0026707032151815913`, 51.33717277636546`, 0.04252046730687875`,
0.14418907533142072`, 0.0005683380068483013`, 0.0005752776851637434`, 0.012210477272012632`, 0.015393509819813562`},
{0.20298433403939725`, 3.2126236854576584`, 5.763393946680621`, 1.2592915169856764`, 0.03637647134670963`,
0.17490854387375587`, 0.13159008302912958`, 0.006425245135071102`, 1770.122374418922`, 374.53287469529414`,
0.12888233713533293`, 8.436918800144038`, 0.31480413938383744`, 116.62635266246883`, 33.13876630619161`,
145.11817853469074`, 0, 106.75861171177004`, 0.20648773091620742`, 0.2090867423169494`, 0.012586759461251651`,
0.01894410312117547`, 0.01894997710446039`, 0.00031006922034526596`, 9.47667678711164`, 0.05493365937180517`,
1.2894081841011185`, 0.000181821733373555`, 0.00018228381560994722`, 0.0025414026575296678`, 0.22447176448115347`},
{0.05881255333180735`, 2.3801271276267855`, 7.362434757054917`, 1.0646703054096496`, 0.806093766428617`,
0.17381221060809304`, 0.15459898392254903`, 0.11200780858178885`, 3179.4524228830724`, 78.98698912374886`,
0.05455878974952594`, 9.203168125761906`, 0.21263297657280256`, 555.2328198856336`, 39.529158263495155`,
1490.9932593871174`, 0, 213.95262858294015`, 9.74747635578931`, 9.762090518857054`, 0.0014992765854788814`,
0.0547661890479952 4`, 0.05476155849667482`, −0.00008455127882578406`, 331.4318985759242`, 0.04601342020284263`,
0.7311537931592459`, 0.0006219879256402772`, 0.0006227731876102576`, 0.0012625035593287 76`, 0.03166767543893665`},
{0.11153574406001432`, 1.6124096945608146`, 2.7742957521629403`, 1.2244678342329687`, 0.3686430365196074`,
0.6752573668077164`, 0.2389841389881603`, 0.023498980178372084`, 1283.4055152922938`, 282.1703009016943`,

```
0.01923556355775 6297`, 3.0024732562806733`, 0.7791774112114904`, 97.08939942143643`, 38.138294743687645`,
 102.81717472719016`, 0, 72.7465024399127`, 1.0024382238295868`, 1.0152669140184731`, 0.0127974870509997`,
 0.08873994037527203`, 0.08938657463343455`, 0.0072868457588317614`, 23.090587290016366`, 0.1413953611085438`,
 1.0123843950041287`, 0.0008627955939581877`, 0.0008858363598248716`, 0.026704779240910748`, 0.2098206673647145`},
{0.06579519568062725`, 3.2190545867000324`, 6.479421382941614`, 1.2315548176863993`, 0.37078258595806113`,
 0.3204788641456453`, 0.18121938321856745`, 0.0745089189861041`, 2845.013880184243`, 202.75756338852523`,
 0.11614494172591355`, 2.5591762204175446`, 0.6923322117865267`, 91.5094163206328`, 9.852967488198749`,
 108.67359858955102`, 0, 44.5098907056469`, 0.9985162974752948`, 1.0041679460927289`, 0.005660046442631073`,
 0.04256079060391405`, 0.04262983292481763`, 0.0016222048492029462`, 45.918263818322544`, 0.04000422208727438`,
 0.4404857168840786`, 0.0004164097144702117`, 0.0004194140367076811`, 0.007214822644788121`, 0.046916235333918865`},
{0.11289150267992315`, 0.6299562074506553`, 2.6857393547101953`, 0.7515616774015286`, 0.8067525150133392`,
 0.6856411274749656`, 0.203218351458197`, 0.13643075142532088`, 665.9502014092905`, 389.69992150044357`,
 0.0982534706706813`, 2.604584539279596`, 0.7279001379553152`, 101.996453613077`, 124.6834537061381`,
 95.58001436431347`, 0, 35.24139556329761`, 6.658077068639076`, 6.741400362677606`, 0.012514618437055924`,
 0.06830066180776284`, 0.0686761379887818`, 0.005497401797888379`, 59.91852827248624`, 0.11015091922160719`,
 1.0904763102452486`, 0.0010831794891142943`, 0.0011081443658679478`, 0.02304777463434715`, 0.18897367711186022`},
{0.07532861497660431`, 3.2456716657650855`, 3.170566131100358`, 0.7667747544642987`, 0.6612666356256824`,
 0.6262282997594197`, 0.0817137209363257`, 0.09276751126759113`, 725.6513719895092`, 116.55380786905249`,
 0.24676733030978965`, 3.599391507892623`, 0.6435082289815082`, 243.16337534880617`, 114.27792882978159`,
 249.21847018251768`, 0, 105.87123703341946`, 2.89401033905504`, 2.9258554597472735`, 0.011003803359815212`,
 0.10221762514773819`, 0.10248093343088188`, 0.002575957744695323`, 134.18581939860232`, 0.1099987446935459`,
 2.463119458020521`, 0.0015992786369192213`, 0.0016193691138287037`, 0.012562211765789533`, 0.145940155158870 5`},
{0.1447096604875236`, 0.781557395333436`, 5.079515305869995`, 0.8829448417099249`, 0.5606054450578024`,
 0.17333438670886359`, 0.13484876640308333`, 0.3428154279908 5973`, 2132.4017848523063`, 228.9439898899625`,
 0.07892443810945204`, 8.583015855961246`, 1.0651655314559392`, 322.7535693266664`, 41.90868935532836`,
 351.6464000759475`, 0, 56.54242649726797`, 21.872833040491187`, 22.032288402378892`, 0.007290110137654349`,
 0.04101968286164954`, 0.04102005861582557`, 9.160338398928047`*^-6, 244.21249170984808`, 0.08479920543157068`,
 2.235436455655037`, 0.0005616418476132035`, 0.0005626294461812417`, 0.0017584134306145582`, 0.13093598878248397`},
{0.08073679542779827`, 3.436765213609185`, 9.447896083410843`, 1.2321734810402918`, 0.28274585136082786`,
 0.4537000076509107`, 0.2378954237232333`, 0.016202557156482104`, 1882.6285138697513`, 143.19709501896392`,
 0.04435960951477524 4`, 6.7684345441443945`, 0.3648612319782283`, 322.466632771675`, 43.902979978027176`,
```

```
433.30454403401126`, 0, 261.90532220272286`, 1.20469970459108`, 1.2098723340813509`, 0.004293708606848856`,
0.03897601489532516`, 0.03899809174442398`, 0.0005664214045000282`, 59.14671482262689`, 0.04495426487422782`,
1.1854697874223277`, 0.00038184083567305294`, 0.0003832437082989862`, 0.003673972228403599`, 0.13688921504384333`},
{0.17570347686215215`, 3.233960190352999`, 8.02035493411961`, 1.3345169115484334`, 0.9514022176165366`,
0.3640798512190224`, 0.2136248279814617`, 0.18054342141341007`, 1968.2648913603198`, 103.08834326532372`,
0.05518740857004362`, 6.915277843565254`, 1.0364954394923056`, 1175.0405498037644`, 100.3488229877761`,
1280.916518803755`, 0, 329.9739493885558`, 17.890974060621897`, 17.939682508577985`, 0.0027225151515530843`,
0.17743315896667938`, 0.17740533467093889`, -0.00015681564766445444`, 826.5528268384192`, 0.4453660420163754`,
2.5706942331925413`, 0.0016055076132626134`, 0.0016101384620778232`, 0.0028843518255259326`, 0.05987162119451245`},
{0.11925732953940865`, 3.6343980885034854`, 9.962959338328435`, 1.011868751364569`, 0.06493978743288742`,
0.17742779913292284`, 0.1769046107233871`, 0.005362579496616395`, 3376.132332358329`, 358.9394383628304`,
0.05158147181807593`, 6.474636554195383`, 0.334042905285048`, 91.21649361568437`, 13.520518367865936`,
127.05968122879956`, 0, 84.65253168851524`, 0.12177594475699126`, 0.12265118564275718`, 0.007187305239244823`,
0.008978786732358227`, 0.008980517430802826`, 0.00019275415445174637`, 6.322603726435936`, 0.015296944688635373`,
0.5372389724455356`, 0.00010730857649809966`, 0.00010746900139776057`, 0.0014949867465974265`, 0.0999484504192084`},
{0.1849591682194595`, 2.435795929316444`, 3.5195053556656077`, 1.1867529613387038`, 0.18511861536763652`,
0.2725329941056882`, 0.1913335103508631`, 0.007193119734412014`, 888.0411283184822`, 208.53516984841042`,
0.12216322134268065`, 5.549467591217473`, 0.7979574318926956`, 189.221856545856`, 55.55052655732122`,
207.9066760861426`, 0, 171.41318830320816`, 0.48548148262196533`, 0.4925636243614704`, 0.014587872026047721`,
0.07200425604347542`, 0.07208067781441548`, 0.0010613507470158545`, 16.893340273272784`, 0.1902549615152036`,
2.3680742875327394`, 0.0007310118138914579`, 0.0007362951531662044`, 0.007227433502916103`, 0.24168943586231206`},
{0.04791496352556143`, 3.7363958898231298`, 7.859362293932024`, 1.2955394392367978`, 0.21077880799821336`,
0.6986917555356797`, 0.24146054265338857`, 0.25080131253737453`, 3174.4582657930478`, 214.6472473246855`,
0.12886781736255348`, 2.1496769931608686`, 0.9494229236050717`, 73.48476444276658`, 9.49951295090009`,
84.16433298335035`, 0, 16.396382258122987`, 1.0487700815862273`, 1.056513520313702`, 0.007383352045820191`,
0.03521710992377133`, 0.0353474883230839`, 0.0037021322758961084`, 55.98028888868939`, 0.024106093392250692`,
0.5024760816633072`, 0.00032634077580318`, 0.00033056006195290703`, 0.012929080466094423`, 0.08429772420864426`},
{0.2320051986600553`, 0.8210990691375057`, 3.0745548516676084`, 1.0653538281011379`, 0.9016142110480403`,
0.45929895353677397`, 0.05711960119403317`, 0.10928247272113006`, 3933.7984981846193`, 65.47103817619023`,
0.11710947379300074`, 4.724190385949658`, 0.932323733177236`, 521.7548015741854`, 36.00358356838842`,
596.480806225369`, 0, 204.36613278916832`, 24.851121967935672`, 24.98337945165485`, 0.005321992459327518`,
```

0.23970368003515655`, 0.23977942855516132`, 0.0003160089990845716`, 291.5033302127809`, 0.794464284372192`,
1.731881780479255`, 0.0027150346881319454`, 0.0027300487974899258`, 0.005529988041629963`, 0.03301240977656442`},
{0.2740885790936093`, 3.0208727150533035`, 3.9044562082788996`, 0.9239050692909432`, 0.13393679798267133`,
0.2935571665601744`, 0.17832264013603433`, 0.12993009746044476`, 1616.3784196961215`, 45.86996911978616`,
0.06338312332920809`, 5.191606790783375`, 1.0490428175043491`, 695.2926283326276`, 28.195678742226075`,
831.8870980671572`, 0, 244.9341602061503`, 10.177289738642374`, 10.233310003406439`, 0.005504438431320313`,
0.19874243265394695`, 0.19867598024908945`, -0.00033436445337875487`, 439.2042412093821`, 0.7781861567381233`,
2.932246578892735`, 0.002594754347874262`, 0.002606868669683055336`, 0.004668118367811891`, 0.07008541625821241`},
{0.07782021540679757`, 2.7209102520090163`, 8.736874384750735`, 1.114706684991148`, 0.7103121173081006`,
0.20678262886709498`, 0.11036600245925934`, 0.19154162173345113`, 2448.698607159915`, 243.62539193659143`,
0.13554193141415577`, 3.0074065943997237`, 0.3291541909818123`, 88.68947831348211`, 15.752924500560878`,
116.37432659120621`, 0, 23.994841989022547`, 1.6213838488392434`, 1.6290957742709353`, 0.00475638476182727`,
0.023594462851740484`, 0.023608231005823037`, 0.0005835332708807872`, 63.02342766783598`, 0.026230374021701114`,
0.371296791298601`, 0.00025560406222080445`, 0.0002565295818654693`, 0.003620911329121679`, 0.03344946903586935`},
{0.20127067113693758`, 0.7134036992984543`, 1.5848778254201719`, 0.9599985271802891`, 0.5544064241430855`,
0.6980173556057399`, 0.23861657897987476`, 0.010226664436870918`, 3481.525245131613`, 209.51927751371306`,
0.18791735173124585`, 4.5381928864523005`, 1.4066869656787473`, 236.45247696133137`, 27.62201722952395`,
254.26659674120432`, 0, 205.68101309459234`, 2.667125459617437`, 2.6932183181189204`, 0.009783138774891453`,
0.19662242507621622`, 0.19749262223285724`, 0.004425726904262017`, 27.181959562631082`, 0.5653475350808429`,
1.3835447240235026`, 0.0024570748367245265`, 0.0024984963556285334`, 0.016858061579933414`, 0.14716377191548388`},
{0.17004475261494956`, 0.4834545237544501`, 3.4349181823258377`, 0.9765473132257132`, 0.7016074781874622`,
0.38572946598569846`, 0.23641719832548008`, 0.044465090938447804`, 728.0925200892948`, 360.1524759911083`,
0.2343727375251467`, 4.114001391225679`, 0.6989801734218899`, 156.86252261006373`, 103.0243159057653`,
157.58268916577984`, 0, 96.391883893052`, 7.618156835062645`, 7.706930108915782`, 0.01165285459135701`,
0.06782673595820776`, 0.06795308603873845`, 0.0018628359266550376`, 52.61474835117028`, 0.16476543623840395`,
1.585062002173324`, 0.0008347798772938564`, 0.0008436298853086637`, 0.010601606789440998`, 0.20348971807208166`},
{0.14843112442757472`, 3.2599821678006826`, 5.742419985764322`, 1.482241642317249`, 0.2702489297613082`,
0.3314664434605483`, 0.1749862727560041`, 0.21934886574956672`, 1289.3717845828933`, 179.7867019868338`,
0.03877340906995197`, 2.6701245702655445`, 1.4405073568802078`, 109.42261613524056`, 21.038401000272273`,
117.02397787317423`, 0, 27.421154766444683`, 1.7194252434802961`, 1.7437203453419239`, 0.014129780840283601`,
0.06613939096113622`, 0.0662828411420458`, 0.0021689068923218535`, 80.07565189445862`, 0.1402449167047343`,

```
  1.3642143251285925`, 0.0005368557336459245`, 0.0005423681905176559`, 0.010268041349386081`, 0.11076109007643024`},
 {0.0976585079740021`, 1.3728751665341834`, 5.874627728896462`, 1.1995783460582101`, 0.28537590201313856`,
  0.6491235286522472`, 0.13987394486973953`, 0.022879541288345737`, 1880.8681942009343`, 204.3983134067610 8`,
  0.24472299006607134`, 8.011571455649513`, 0.7571200668542781`, 275.2489849708273`, 63.37326330201056`,
  300.5018414687285 7`, 0, 207.91132143316338`, 3.2588468092288787`, 3.2894421914539023`, 0.009388407622714645`,
  0.043974316703556074`, 0.04402638669320281`, 0.001184100028154056`, 63.9141407989927`, 0.061349516549208206`,
  2.4686680268211316`, 0.0004417970226409107`, 0.0004444687758502025`, 0.006047467665854844`, 0.310202563342363`},
 {0.1434955367081326`, 3.5028874653292856`, 9.961555440173449`, 1.4583790499213787`, 0.7878184950293752`,
  0.3804561019338192`, 0.248272531578832`, 0.013284854321714896`, 2386.8492620887737`, 259.97790040425207`,
  0.11312758713523102`, 1.6001299135943265`, 0.42901490870982695`, 79.55824738663449`, 13.5082539736143`,
  94.86572469232024`, 0, 66.75232570532546`, 0.2473818560606121`, 0.2486306559980066`, 0.005048066003225937`,
  0.04550594946003592`, 0.045617999322596865`, 0.002462312376524389 3`, 12.379297182065878`, 0.09328429487401271`,
  0.22035618422653483`, 0.00037539377916062655`, 0.00037932930830337087`, 0.01048373564299343`, 0.0252322902195946 2`},
 {0.14734788675597893`, 0.461750217153563`, 2.7608760404738693`, 0.8246000601521355`, 0.6486099382776269`,
  0.46153306705501707`, 0.07562875863771559`, 0.011120543224542678`, 2174.875101604558`, 76.28988785327772`,
  0.1816491692635701`, 7.465741450424737`, 1.3681893457620165`, 729.7392942334805`, 65.43921440404253`,
  782.626567183404`, 0, 629.535620306918`, 13.055227035774356`, 13.16901367491195`, 0.008715791676835005`,
  0.18314890619154753`, 0.18313690338847843`, -0.000065535761685331 37`, 86.11791312511251`, 0.3855229184141351`,
  5.249555270667345`, 0.0026791967739534517`, 0.0026906764099358126`, 0.004284730443826712`, 0.09969208450461689`},
 {0.2174892525974506`, 1.1555616271784261`, 6.330891972593395`, 0.8691848701006539`, 0.5548185878553895`,
  0.5753044260463678`, 0.11961066111713925`, 0.21454325341169517`, 3637.70292743186`, 299.3308064827128`,
  0.20558605918965206`, 2.653266548572333`, 0.34524692314089034`, 61.611733552946724`, 13.512556803689833`,
  75.68902085031952`, 0, 15.341025138323111`, 2.638150475116581`, 2.653339872462969`, 0.005757593241801917`,
  0.019943713989563395`, 0.01998821146815556`, 0.0022311530648431166`, 43.55064936810364`, 0.06196490642293608`,
  0.27394536280582793`, 0.00027595274509306655`, 0.0002787605009226317`, 0.010174770425342983`, 0.06645855929448871`},
 {0.06749293860797512`, 3.7228921901379612`, 3.4260664843308817`, 1.4192699415393606`, 0.03614877922192594`,
  0.4559586129932802`, 0.13413544415463274`, 0.029486897805871767`, 2795.273468589703`, 381.3448566753295 4`,
  0.1616868776536713`, 3.6141605923908813`, 1.2368738510133124`, 55.97819126123808`, 12.62301179076042`,
  59.653409912887625`, 0, 39.57348543940409`, 0.3011843746958609`, 0.3062031111236101`, 0.016663335980882543`,
  0.04006937341173450 6`, 0.0402244769146452`, 0.003870874179062511`, 16.018242233525797`, 0.03863428228194692`,
  0.8723951384691414`, 0.00033882722939382326`, 0.0003435810648603203`, 0.0140302639637373`, 0.18235681099218234`},
```

{0.17265939500359245`, 0.9179315961122354`, 9.1852760379758`, 1.297645317836566`, 0.4142675534047153`,
0.41824524574451727`, 0.08521501500767603`, 0.21771628238910337`, 2996.1049031716466`, 204.18324706241947`,
0.025729542442735343`, 1.6360785031153569`, 0.4558745456460018`, 44.83147152065541`, 7.048370914338896`,
54.29485910219759`, 0, 11.073057177299551`, 2.3860128751122796`, 2.4037020211917097`, 0.007413684252897346`,
0.02419645144525325`, 0.024257745932394218`, 0.002533201501866863`, 31.288522954230757`, 0.05968206668254919`,
0.24928575128879749`, 0.00022431058041383345`, 0.00022672543075120992`, 0.010765655070399616`, 0.033757821342509355`},
{0.11391904455060164`, 3.7004347183812403`, 4.98869805344277`, 0.9900086895027544`, 0.7790608041506635`,
0.5499151960612685`, 0.1741544112916672`, 0.1512134845165499 2`, 2279.078015486494`, 277.9770114196665`,
0.21022773210729895`, 8.293592982203787`, 1.3768230125107617`, 384.78665843304`, 96.99535297609322`,
393.1460773158572`, 0, 123.49971132891785`, 4.84810593645516`, 4.883211607260885`, 0.007241110500855408`,
0.0577389420998973`, 0.057780443135604355`, 0.0007187702821997988`, 256.28713608069796`, 0.09396521596256101`,
2.633603610923708`, 0.0007036685495976913`, 0.0007068115881917216`, 0.004466646400250962`, 0.21300076755922526`},
{0.1144334519289396`, 3.850377768547795`, 8.00437153143687`, 1.3020216404842988`, 0.3359559076583225`,
0.49678442177055226`, 0.2301468632075337`, 0.14684067776603849`, 2776.269241624055`, 290.27051916223877`,
0.07166092983110212`, 1.5123971688855153`, 0.6362818855430454`, 40.31270152272498`, 7.085652134541958 5`,
47.09403236189929`, 0, 13.152703587564048`, 0.4836798871419129`, 0.4874360543046031`, 0.007765812188068155`,
0.027066523705596514`, 0.0271717891287793 1`, 0.003889137161749101`, 26.605004064927495`, 0.04424736770492106`,
0.24612603007818756`, 0.0002493272532885227`, 0.00025308266611108915`, 0.015062183427740461`, 0.052220510096624685`},
{0.07628805291838442`, 0.7253635030519439`, 0.2787614932535387`, 1.2938825783187968`, 0.6322486251684503`,
0.6415725594877175`, 0.11902418817624266`, 0.020293492548644005`, 3744.3615927821575`, 331.54916368743454`,
0.0324650937333269`, 2.843278699811469`, 1.2796927021756468`, 78.98189068203662`, 17.213372470168522`,
79.622488059236`, 0, 60.1240446951027`, 1.5161028315057743`, 1.562284959429072`, 0.030461078868529112`,
0.7702753165528506`, 0.7924828828009892`, 0.02883068660115229`, 15.710366583542852`, 0.8394686301558427`,
0.7106214870948876`, 0.007081043582750857`, 0.007573365278047181`, 0.06952671446559111`, 0.17692438629930052`},
{0.26704012645507424`, 1.8838541538663058`, 7.9577608030460505`, 1.250964933852727`, 0.37177913789440953`,
0.5400109924589773`, 0.2090957264740766`, 0.02799412229122407`, 2680.237536243104`, 60.33735070739806`,
0.08963423619120237`, 8.220004781923546`, 0.7126427956038843`, 1030.2507602618189`, 45.14346784558157`,
1288.6182288209254`, 0, 736.0024405764824`, 10.515630042286093`, 10.544249390995382`, 0.002721600949653 5687`,
0.12359620734659846`, 0.12362244145019825`, 0.00021225654219492185`, 282.9987619383138`, 0.47150209770274804`,
2.0930333938286014`, 0.001192816602180624 1`, 0.0011969984414392973`, 0.0035058526608602314`, 0.07973906133085493`},
{0.14352951682222453`, 2.7122840992738713`, 1.912849383145993`, 1.4933758944571824`, 0.4317056886375634`,

0.3933320053063153`, 0.10505217994551203`, 0.4954512847212043`, 1346.891754031455`, 320.6507144731578`,
0.20904379157314928`, 1.5308675559916907`, 0.6151100951642487`, 33.94656395012595`, 14.562202589052562`,
35.11817959993979`, 0, 4.5404079281222804`, 0.7316558804173963`, 0.7483386081188741`, 0.022801330718425383`,
0.10657261893248067`, 0.10763383846474206`, 0.009957712805516605`, 28.34940872280469`, 0.2185188071693601`,
0.4405822631192905`, 0.0008453807798997071`, 0.0008810306552016642`, 0.04217019850650794`, 0.08747122542815122`},
{0.1069158661028563`, 2.292840293600788`, 3.2223682955272235`, 0.954682665552236`, 0.20354287774001567`,
0.3896645344037619`, 0.21864993682483302`, 0.034752231866091665`, 2419.249949369646`, 316.3106467196254`,
0.1946047812364281`, 6.8989000582764195`, 0.6984200141969727`, 158.31490059730984`, 30.14196739992648`,
182.064879613249`, 0, 106.11196917956435`, 1.5429981568653202`, 1.5559831176874612`, 0.008415409159347575`,
0.042604282736868775`, 0.04265335030712268`, 0.0011517051127689637`, 50.54069067160797`, 0.06507248269290117`,
1.2446124157343648`, 0.0005377664455626929`, 0.0005412281233930543`, 0.006437139875358433`, 0.23944571295165049`},
{0.2686042849529644`, 0.7627258559442236`, 7.922182847008894`, 1.344754730879767`, 0.5223454903809039`,
0.3059455193661864`, 0.17153078163225305`, 0.02722979062605091`, 1945.288646450902`, 327.63559196356823`,
0.15374403181997548`, 2.2340529482759806`, 0.8539303526410174`, 60.23704453128206`, 14.352050194724317`,
65.21428095173985`, 0, 43.402136849782515`, 1.4027794013710424`, 1.4180926215447835`, 0.010916342340623553`,
0.02861665160764164`, 0.02866755468856784`, 0.0017787923487389001`, 15.284801708737906`, 0.10980793204026462`,
0.510428492012637`, 0.00025627606128209024`, 0.0002586657479284635`, 0.00932465808323335`, 0.06610235661839925`},
{0.25819223047450574`, 1.7095391280955443`, 6.520962454670094`, 1.1430189535372957`, 0.9365205495994724`,
0.41508933167510853`, 0.1765660191827808`, 0.05380792374101585`, 3754.7043853290033`, 182.2517379088373`,
0.07048991272145094`, 9.71641095911452`, 1.30627739480544`, 829.2635753252227`, 79.9852403327084`,
881.4462486852605`, 0, 469.73853466664525`, 14.124550107946444`, 14.176887143507374`, 0.003705394873531942`,
0.09386094099426376`, 0.09386875831302567`, 0.00008328617504882452`, 344.949586804008`, 0.34620236728190423`,
2.6144527226709`, 0.0009921266764760794`, 0.000994691959116464`, 0.0025856402223718433`, 0.10217883479204046`},
{0.11085289703164392`, 3.6133570849476584`, 4.542500201931212`, 1.3471228260354866`, 0.6367711489528869`,
0.3976210616429948`, 0.050489910733311105`, 0.00824863073511752`, 1898.7553106453079`, 276.59295465999946`,
0.21653605876058957`, 5.75252417538405`, 0.6232368998851006`, 136.16564018302634`, 51.6560481785979`,
142.95585788913746`, 0, 121.77059153710346`, 0.26932899239541236`, 0.2727890063726776`, 0.012846793605441054`,
0.04396709330854379`, 0.04403332610672099`, 0.0015064174861958435`, 13.902597469339698`, 0.06962685239019868`,
1.5150912863922619`, 0.0003932002953327407`, 0.00039602966347192047`, 0.007195742660329074`, 0.1574266163333131`},
{0.0604034128862494`, 3.158158875209411`, 7.876995843447027`, 1.2883875500461601`, 0.12376268888818709`,
0.6326665048379214`, 0.11422725471154377`, 0.15234673981455935`, 2211.579578858029`, 40.246357658710394`,

0.057745413831265746`, 5.692997033958765`, 0.2028088406309343`, 386.96829724253854`, 32.20526057385506`,
913.346950044915`, 0, 122.33089557289155`, 5.735631485232575`, 5.7493788211643935`, 0.0023968304043266997`,
0.06969442313363426`, 0.06977463923521823`, 0.0011509687285904402`, 258.77193542882617`, 0.060139728805152545`,
0.8045851282356685`, 0.0006517759562602032`, 0.0006558241498011647`, 0.006211020063074057`, 0.0843359264109651`},
{0.17311414162710254`, 0.8702497549340356`, 0.34304718385733324`, 1.0485386108632935`, 0.3716017788116761`,
0.6371600148503609`, 0.055104118812441344`, 0.2168377629398712`, 703.8776101728622`, 164.53996042397466`,
0.027022709136328538`, 3.1628771308577264`, 0.32716182080568834`, 121.71089180054156`, 72.43468374711277`,
118.78509469145317`, 0, 30.338140287044155`, 6.620847139717974`, 6.849878166155815`, 0.034592405111409485`,
0.7027263168051281`, 0.7231775825187836`, 0.02910274629621834`, 82.31129429707543`, 1.7378837590355924`,
1.6803181423561275`, 0.007729308644820798`, 0.008525244777191309`, 0.10297636812625499`, 0.34399011349854797`},
{0.20458649602921813`, 3.3198040898334424`, 1.372039361136828`, 1.1755123934413545`, 0.5154858348933944`,
0.2152893723924586`, 0.1607481890336261`, 0.022174783215048698`, 2483.3920145693382`, 114.03490520349692`,
0.026176842372398812`, 2.8250322344315557`, 1.2559643496212312`, 208.03273853739296`, 12.094894959770683`,
232.30130984025223`, 0, 157.0216902736322`, 1.0206194461018656`, 1.0334807334273617`, 0.012601452357799081`,
0.39251374735252686`, 0.39315206054955665`, 0.0016262187027464847`, 48.40366587617882`, 1.1471858887736999`,
1.0174405373408943`, 0.004017895835344398`, 0.004079014979665243`, 0.01521172942892024`, 0.0462916687959172`},
{0.1578531250336248`, 0.5854218989222861`, 2.149284744894146`, 1.0306646027288366`, 0.9441735295761224`,
0.33751540864617946`, 0.1297018205371923`, 0.005489306207108587 4`, 3639.0053252131047`, 207.4000118567045`,
0.1305358640736498`, 4.5222314256344625`, 0.9549981466481912`, 264.31355019660356`, 32.440392711275166`,
289.57978155913287`, 0, 244.37764920993104`, 2.03887563339627`, 2.052714469732732`, 0.006787484292708035`,
0.17535672238896172`, 0.1755792305538403`, 0.0012688887078138311`, 17.05146349956033`, 0.3954372374964426`,
0.9056300691440374`, 0.0020506549963837 94`, 0.002066742072856576`, 0.007844847866243176`, 0.050840097276109`},
{0.21896388001044664`, 3.129900208151482`, 6.123839398439741`, 0.8969815671266188`, 0.011595810869147183`,
0.37111266340117355`, 0.1718245052376185`, 0.005208778420460518`, 3863.605974823663`, 250.7012639800747`,
0.15570381237665487`, 7.7211234554844195`, 1.4785494708568354`, 188.01829024482603`, 17.778573181053858`,
209.11795689481616`, 0, 174.858060397256`, 0.28150932971519754`, 0.28485841458310757`, 0.011896887649508026`,
0.022371489081687408`, 0.02238149212483861`, 0.00044713354192360555`, 12.587087281031156`, 0.06997925787055127`,
2.0212080130684824`, 0.0003013130613209736`, 0.0003022103976128292`, 0.0029780862731991764`, 0.2530940313287897`},
{0.17565226905516895`, 1.7038906647375853`, 4.512441985203527`, 1.1172179676607337`, 0.956149515870736`,
0.40808719408253036`, 0.2318723848780873`, 0.25398457087869114`, 3825.126366136904`, 43.499586017725164`,
0.07242686582534269`, 9.792971274976665`, 0.9137108964160303`, 3914.9130367576345`, 82.0685618700799`,

4663.753075372082`, 0, 847.2546042413001`, 120.96748377722398`, 121.10897197812774`, 0.0011696382902726121`,
0.6228320278744022`, 0.6210338258533029`, -0.0028871380093219434`, 2944.505233497247`, 1.5628836990904202`,
2.394052580213492`, 0.006720003295753396`, 0.006733015273557265`, 0.0019363052711731665`, 0.029377486531541732`},
{0.06615318131527015`, 2.492376536317514`, 9.394184914213039`, 1.4348602118065803`, 0.7341697528786502`,
0.24813924799413356`, 0.2232148533154487`, 0.026418827644963606`, 737.3840226181687`, 59.19942623358838`,
0.013447593432626304`, 4.186359481235684`, 0.5021329151763947`, 810.1344217866688`, 83.89598550269623`,
985.1190811110479`, 0, 588.3436972441264`, 6.045680309337464`, 6.064065330037634`, 0.003041017678651503`,
0.1854881441759038`, 0.18548157897930623`, -0.000035394157544388 35`, 215.2587392724212`, 0.1752947261928403`,
1.973137661558671`, 0.00156017470607983 1`, 0.0015655792431956558`, 0.0034640589254293364`, 0.03828568446159219 5`},
{0.1576166709633643`, 1.3075085634970804`, 7.656001845154428`, 0.9231504863095856`, 0.8454152618498512`,
0.25647657664732126`, 0.07864463104098307`, 0.005296200134401313`, 3012.7436478007676`, 197.2045635665247`,
0.12709746544272688`, 9.860728262025162`, 1.31076068876941`, 445.03861442911744`, 59.90045236771202`,
472.23721067268116`, 0, 413.4146046122799`, 1.5775382313241362`, 1.590716389008151`, 0.00835362175213561`,
0.034155139611423566`, 0.034156546381299155`, 0.0000041187648230911 48`, 29.466353524290607`, 0.07690599145490215`,
3.0907654320453593`, 0.00044740746763549666`, 0.0004480229556510267`, 0.0013756766707155865`, 0.10996959649049787`},
{0.17154635825703612`, 1.53373134752687`, 8.885182514407113`, 0.952500439197914`, 0.18392969628669165`,
0.39214328491936956`, 0.11223174543931136`, 0.01936642254266069`, 1934.647469413505`, 216.7923390085249`,
0.0451455537258712`, 2.0009219573176864`, 0.3777871026684809`, 48.252108879197024`, 10.440261637376441`,
59.254601265875394`, 0, 37.81949271146656`, 0.4523054446288851`, 0.45641273226374907`, 0.009080783093897926`,
0.016183690275560544`, 0.0162107978282374`, 0.0016749920577627808`, 9.910214844062853`, 0.03966075899904738`,
0.3722994901913887`, 0.0002045901456313448`, 0.0002062687269038987`, 0.008204604710426944`, 0.06159920194369284`},
{0.0855854522942322`, 1.8098534994044577`, 9.52981117766399`, 1.2278891902893156`, 0.1610609466726174`,
0.1996687073144625`, 0.062022895674954254`, 0.00706028348998438`, 1475.3823287257756`, 92.05986706201287`,
0.09971055550215713`, 4.508838235939827`, 0.938398866245965`, 249.20061173870013`, 23.62637883107007`,
280.18090191570246`, 0, 226.2780263857607`, 0.8415393024709317`, 0.8501874230532462`, 0.01027654983780546`,
0.04467837542193818`, 0.04468989774045833`, 0.00025789475134985196`, 21.758040735191447`, 0.05462598526082583`,
2.2419525696804814`, 0.00043976429987202525`, 0.0004407717443855432`, 0.002290873801741311`, 0.05747715515118872`},
{0.1989224969181887`, 2.0656981604697133`, 7.019145766333462`, 1.189741191045774`, 0.9813366885674477`,
0.4707046727829801`, 0.138351198132442`, 0.006338022742977132`, 1353.111767359248`, 264.1038821274834`,
0.2333484554805464`, 8.079661388491221`, 1.0209030718005039`, 442.9019788984805`, 229.38175261399897`,
454.1131455253719`, 0, 405.78074746205465`, 1.19545505429247`, 1.2052542552162124`, 0.0081970467133472`,

```
0.0582706413152922`, 0.0583023665879483`, 0.0005444469451507672`, 35.2778472368025`, 0.16559059239232407`,
2.9412131946234736`, 0.0005911607961599508`, 0.0005935315251761041`, 0.004010294714319684`, 0.1868840255151206`},
{0.252908269598038`, 1.3368065237315108`, 7.681913157962232`, 0.9670562213906954`, 0.8885087458364935`,
0.6712630307685601`, 0.19827927021086678`, 0.0287236993413668`, 1511.0621870028344`, 393.3001097237484`,
0.20663414848444228`, 4.051732672816565`, 0.5729449981349937`, 165.33725349360975`, 103.48338388804642`,
165.53841760173464`, 0, 117.3870532834795`, 2.3774801944372848`, 2.393609704421761`, 0.006784287844843195`,
0.032170761887474614`, 0.03222143186144471`, 0.0015750318300613575`, 45.403300485231775`, 0.11623216743729951`,
0.9470514525843948`, 0.0004004722805642791`, 0.0004037143015379072`, 0.008095494072798193`, 0.1463064926795078`},
{0.19935758638978301`, 1.4210929413876547`, 6.276637411024748`, 1.1091985064517424`, 0.2725861619363459`,
0.30993881233332166`, 0.061372778664464`, 0.007027369768062639`, 3344.583850960579`, 315.8180746154343`,
0.0888647235789749`, 8.724591660226011`, 0.9460844323713795`, 157.78826677186072`, 26.623284546148927`,
170.21168812437378`, 0, 143.35739889979112`, 0.6671649045700555`, 0.6757962366307391`, 0.012937329289294475`,
0.020046405335099486`, 0.020054994510473337`, 0.0004284646164871564`, 13.54433338037248`, 0.05709147119138564`,
1.8197322158709766`, 0.0002183778460820296`, 0.00021898589733847365`, 0.0027843999167187494`, 0.174736124859483`},
{0.15752424015374422`, 1.1744034635310063`, 9.19368700774531`, 1.4814874364707953`, 0.3259429279011785`,
0.1698219186283283`, 0.0532987651599028`, 0.11291488360711258`, 1392.2159030202329`, 251.1254711586031`,
0.20643313217875114`, 1.2559120984693628`, 1.2401551704457958`, 27.169831914415816`, 7.1522186373535686`,
28.417507190513163`, 0, 10.675254257140125`, 0.9238656810857753`, 0.9434652889111406`, 0.02121478070527627`,
0.02179396016259331`, 0.02182848312515663`, 0.0015840610107460407`, 15.499872224351037`, 0.04904395735076136`,
0.500533724923382`, 0.00017739916386860788`, 0.00017883461554706127`, 0.008091648501322934`, 0.02963610730113978`},
{0.12243829123563332`, 2.3703577767539246`, 4.839202410108355`, 1.1566976539300236`, 0.5361220364781598`,
0.5850654781694432`, 0.18441510087344481`, 0.008912199456594784`, 1964.6638548143164`, 274.09302664051813`,
0.22153291468521247`, 9.911211328491756`, 1.3136185654470256`, 364.1398331139607`, 95.10317909855516`,
379.54539438870944`, 0, 323.0102157915799`, 1.1675119507296152`, 1.1796508253396905`, 0.010397216578801993`,
0.05506035390197325`, 0.05510994304714763`, 0.0009006325179576891`, 39.534586169501395`, 0.09630708066542862`,
3.375641091066889`, 0.0005740708694405017`, 0.0005770072326741508`, 0.005114983863422573`, 0.37282057769525284`},
{0.06243550103024703`, 1.4478067318733245`, 4.568123699237523`, 1.4686635133498154`, 0.7846213517525722`,
0.6840252274506751`, 0.19851402241018223`, 0.005300678072293084`, 2514.1415778711344`, 343.58199097307386`,
0.07822848450027337`, 7.993810681409931`, 0.8901709005463876`, 322.0838996604266`, 99.5953229843944`,
331.6006601955318`, 0, 299.07557922755814`, 1.0384047244980403`, 1.0456422502752596`, 0.006969850585683668`,
0.08113347309054174`, 0.08128592733772402`, 0.001879054863239471`, 21.477276436247507`, 0.07236584346760022`,
```

1.732066970215154`, 0.0006648394502855037`, 0.00067033345386638`, 0.00826365459889144 6`, 0.22934204939780586`},
{0.14543352811569443`, 1.222289804727784`, 2.326184459246356`, 1.2918343464415338`, 0.9224834580379948`,
0.39580748300164703`, 0.184644232925161 3`, 0.21500168948406848`, 3817.4733751688063`, 116.03894445631153`,
0.16529510077911613`, 3.062979779789736`, 1.0702400368818767`, 381.2650436241427`, 22.23810321410547`,
422.38528611540903`, 0, 93.89546919899709`, 15.493986411768976`, 15.579716432227091`, 0.005533115763739049`,
0.4323817679233751`, 0.43246401371401877`, 0.0001902156768511798`, 270.5448803670871`, 0.8983258000283563`,
0.7701740118643416`, 0.004031876380900967`, 0.004066795189678971`, 0.008660684375992878`, 0.030912332185168583`},
{0.1313087568846084`, 3.644656164823556`, 9.460965509789986`, 0.8885448984294761`, 0.9355716622019723`,
0.25119735021109135`, 0.1694476044963361`, 0.3877738779001962`, 921.248806442728`, 163.92044778354148`,
0.11679660196129243`, 3.129177349018949`, 1.1599711195943847`, 282.7163846906046`, 68.74129136901948`,
293.40060020833926`, 0, 44.60157682376953`, 4.484216137311866`, 4.5129231778261225`, 0.006401796799086812`,
0.0532288491921057`, 0.0532417866581154`, 0.0002430536486521806`, 233.47751413221397`, 0.09984877168262164`,
1.6479735373833833`, 0.0007234722656731529`, 0.0007257698682991272`, 0.003175799177092875`, 0.05411281851404184`},
{0.08032938050984856`, 3.6316655074823947`, 4.889233151652448`, 1.047924531280927`, 0.6480389250926271`,
0.41632199836938133`, 0.08565204097388288`, 0.014661199276299969`, 1480.2883480287883`, 317.22766308099494`,
0.1835562909337356`, 7.402391506079066`, 0.616770981275718`, 189.06347338362602`, 89.58540054862539`,
195.24220156375026`, 0, 156.53928086312874`, 0.6120434514382307`, 0.6191467258793673`, 0.011605833580025537`,
0.03432505427613655`, 0.034354094583357205`, 0.0008460382025046176`, 31.75338702383854`, 0.03939014779959578`,
2.0289587854658295`, 0.0003950983822591514`, 0.00039701638067826277`, 0.004854483098979845`, 0.25235577719296076`},
{0.13052094951744303`, 1.9253311736310001`, 1.1697698857489094`, 1.0141116955010334`, 0.850854427903335`,
0.33601527543931153`, 0.2494690024453417`, 0.2360086443642378`, 1519.2042074690844`, 278.4809507811633`,
0.016312333738175505`, 8.02616595157577`, 1.4482171660091545`, 516.233886441131`, 115.95609000352269`,
519.8852590486078`, 0, 120.02024377915045`, 13.864829867774242`, 13.967663914062884`, 0.007416899252955078`,
0.34922900368865906`, 0.3493848749381423`, 0.0004463296227887880 7`, 381.34841659308404`, 0.6511671594354579`,
2.8118151978898447`, 0.004135466584948677`, 0.004178799454286362`, 0.01047835073686687`, 0.19896767520408382`},
{0.1763567523046698`, 0.4764578027902213`, 1.019702924150577`, 1.411009603642741`, 0.10601942889051807`,
0.5663043206905454`, 0.11523580705445918`, 0.20578529268229545`, 886.8032845661132`, 265.22978093426616`,
0.0734753688299642`, 5.606338149623012`, 0.8756860344077033`, 136.18052142036134`, 73.78388318178362`,
135.99743517560543`, 0, 36.29155432174177`, 12.701824406261096`, 13.016662125323876`, 0.024786810854320107`,
0.20789622846597774`, 0.20987138970648964`, 0.009500707420650123`, 86.45547640049085`, 0.5237700524092996`,
2.678697686290554`, 0.0017458443117359668`, 0.0018104045454158232`, 0.036979376251288665`, 0.4817946716744358`},

{0.21878401484644522`, 2.7819817431568543`, 8.543045035400766`, 0.9200940784159304`, 0.4855794665787383`,
0.5527693274070447`, 0.20427128258242377`, 0.013404369082852467`, 3643.8303336510735`, 176.1607363330378`,
0.03966906929471847`, 8.016907827776176`, 0.33840217640400705`, 339.3201022603629`, 37.39849669388815`,
472.13451294774006`, 0, 284.6653152477236`, 1.3343739101626069`, 1.3385002730257558`, 0.003092358769699155`,
0.02859557305039151`, 0.028608449893682292`, 0.00045030897852949003`, 53.03148366596005`, 0.08937506112557225`,
0.759092985901998`, 0.0003753240978385808`, 0.00037653347749122746`, 0.0032222275617559415`, 0.10897833228066924`},
{0.155933613154269`, 0.7900752087685747`, 5.426020030668559`, 1.1675266200556353`, 0.3526751580238494`,
0.32689871232148693`, 0.08872571343828128`, 0.47047660149359233`, 1973.8960115130649`, 259.1539886898336`,
0.014745308659178585`, 4.1998835891931225`, 0.8551070030738948`, 103.50100321530753`, 23.45473384291949`,
110.94665355903537`, 0, 14.384409061582085`, 7.244316069746324`, 7.332757149704422`, 0.012208340871189494`,
0.033217451104523475`, 0.033255635777732544`, 0.0011495365218996412`, 81.76506473129122`, 0.07399595957855198`,
1.1619359309337898`, 0.0003430366515505545`, 0.00034517513708941325`, 0.006233985579070289`, 0.11400689569970718`},
{0.2479337375923964`, 3.025341092699729`, 2.1453632749289397`, 0.9396124783291141`, 0.0672772492322784`,
0.28991645499385843`, 0.23601049551242942`, 0.03982040313739142`, 3865.9337586257416`, 194.53557465876634`,
0.20896839632361264`, 7.896823118164437`, 0.4853457216015631`, 233.03478578169694`, 17.171837910204506`,
316.6001004307092`, 0, 148.56237111402052`, 1.9017563984006522`, 1.9108777334524014`, 0.004796268890916`,
0.08095038209676443`, 0.08101271275289798`, 0.0007699859410057464`, 82.19231114837312`, 0.2867190113254599`,
0.7501831584519132`, 0.0010386729044675747`, 0.001045396626495023`, 0.00647337770969858`, 0.10767090292939197`},
{0.1460076457981399`, 2.5733113885431225`, 9.325775684390766`, 1.4910714614077438`, 0.8342963441408033`,
0.1882740945287651`, 0.13611188525292933`, 0.04396527811717692`, 667.8083571719217`, 108.22134685664759`,
0.1548306029558182`, 2.291019500853446`, 1.424632992657926`, 239.51801791359784`, 48.39517829622044`,
247.6425401696026`, 0, 147.65202744926887`, 2.424029768327623`, 2.4469504330916023`, 0.00945560366603626`,
0.10473302719243886`, 0.1047995206565421`, 0.0006348853450122149`, 89.11119155721454`, 0.21845461053839027`,
1.9200134910657207`, 0.0008476185011575543`, 0.0008519969311804108`, 0.005165566840361624`, 0.043092331035191216`},
{0.26317109341632255`, 3.059519748021583`, 7.63492936446163`, 1.3537697529302852`, 0.126934299410153`,
0.5548921430733772`, 0.20372329915496434`, 0.22074667207931783`, 2265.8574749330373`, 181.20735656268073`,
0.09466585200925526`, 2.880276570782346`, 0.773128569372235`, 102.72575093405672`, 14.66547510939779`,
119.22785218547254`, 0, 25.22712414908217`, 1.7292536407744634`, 1.744532565177476`, 0.008835560060564296`,
0.03953060318069686`, 0.03962871051626843`, 0.002481807199427033`, 75.58122376125239`, 0.14861874374976905`,
0.7435836752433965`, 0.00035108293894126064`, 0.00035502072108509197`, 0.011216102256937699`, 0.1029110615480467`},
{0.1645690290583518`, 3.8533306281399318`, 1.0369585072362497`, 1.3664278344740945`, 0.6261111939893407`,

```
0.20406169893014847`, 0.18309667446609262`, 0.4646249368336043`, 2770.959571622493`, 166.0796256998841`,
0.048578133356614095`, 3.3069773434670147`, 1.4087558492193972`, 203.27703142561643`, 13.965384930241715`,
221.21162190770156`, 0, 27.278171605633606`, 3.1099358002501027`, 3.151989370781619`, 0.013522327543910917`,
0.5056669569581327`, 0.5042897584235958`, -0.002723528827791033`, 171.19444100932327`, 1.1888160019070695`,
1.0306501183728847`, 0.004446176962810022`, 0.004505150499883676`, 0.013263875362347743`, 0.06297573378825662`},
{0.22921820594790826`, 0.8201682437489284`, 5.405881273282434`, 1.1219214796772738`, 0.17914795775514958`,
0.5820849501977261`, 0.05414216426604684 4`, 0.17169981881854485`, 1492.1967102291455`, 65.66187445337869`,
0.1524226111094261 2`, 1.916490304748569`, 0.9747174266139518`, 145.2355208640006 8`, 15.78067415525512`,
159.07458443555174`, 0, 42.98996924743462`, 8.007283515087245`, 8.113202234974668`, 0.013227796878660714`,
0.0981194409640175`, 0.09845212689803608`, 0.003390621988363929`, 93.8188522538406`, 0.32129660323414216`,
1.5097705002951676`, 0.001051956417945532`, 0.0010654015125982508`, 0.012781037715399846`, 0.04461737417606176`},
{0.22532476518940014`, 1.9533397508457995`, 5.9287214686276055`, 1.441991810272455`, 0.5762935247594674`,
0.4183646366318182`, 0.09350661705723778`, 0.3294355875363928`, 558.5643880556918`, 49.17304258267865`,
0.1200905527225728`, 2.331392728849849`, 0.39194349265472495`, 311.65021677736536`, 65.889040980094`,
373.5453079661959`, 0, 55.80308642511139`, 8.821710679314007`, 8.892429204974311`, 0.008016418609843035`,
0.20310263717986168`, 0.2035534790984459`, 0.002219773828859628`, 246.1685448623559`, 0.6537722004553904`,
1.6365655431632717`, 0.001691084800313214`, 0.0017135806139767795`, 0.013302593494660231`, 0.05206373630602152`},
{0.22156365739259543`, 2.454378375997047`, 0.8895152478568865`, 1.026128317479294`, 0.516725505225655`,
0.686434221522195`, 0.23046056758531142`, 0.005492504017628765`, 1555.2392746607857`, 206.29023226314905`,
0.041365082475744275`, 1.4870135325767126`, 0.5063457915321476`, 70.99310165865442`, 18.850127837391437`,
78.44682487289265`, 0, 64.51247992722057`, 0.13952431285164993`, 0.1432274605700345`, 0.026541236023301273`,
0.33052695139793536`, 0.33888382558599406`, 0.025283487935594984`, 4.892077948413372`, 1.046182288836439`,
0.39252612986240565`, 0.003793131168162045`, 0.004079072506891202`, 0.07538398385197675`, 0.09683160932153188`},
{0.11472120130152375`, 1.4874886419048003`, 7.95738176456699`, 0.7931641460551735`, 0.8816884572777486`,
0.3579341724415851`, 0.08277045109409453`, 0.048113870328459145`, 3701.2844061656924`, 204.25872179904388`,
0.20301688272576546`, 3.1735817377996653`, 1.31887862582745`, 139.3355290928204`, 20.178140313014353`,
148.02094183092322`, 0, 82.91366241937853`, 2.5324400059448777`, 2.5509645045356675`, 0.007314881516365057`,
0.027442507184277137`, 0.027460112640607253`, 0.0006415396454813038`, 53.81393921640465`, 0.04497481987007285`,
0.9387253619972221`, 0.0004179616630530791 4`, 0.00041932965872155617`, 0.003273017095597241 5`, 0.04053708971262017`},
{0.06425688602740343`, 2.6618634637938463`, 2.643754911431923`, 0.8785376073839924`, 0.5804636394156946`,
0.5001986382462079`, 0.14138114151328934`, 0.3139131008244074`, 2397.9696216564225`, 78.15653575226656`,
```

0.17684868004389004`, 9.538390690744457`, 0.6033965244651656`, 982.6219573903238`, 72.58026836259924`,
1191.7792037765735`, 0, 180.97959392033013`, 20.530350943293204`, 20.587424671510124`, 0.0027799684659344237`,
0.2148850507709695`, 0.2148283287102489`, -0.00026396466304628`, 780.6998724973956`, 0.19725491737679857`,
2.3364924412302917`, 0.0029479209382676785`, 0.0029614455923347633`, 0.004587861869535859`, 0.09637129779122276`},
{0.1767902158998531`, 3.597571059730683`, 4.894388517781371`, 1.2037647529410567`, 0.8088893202611276`,
0.4862113706827499`, 0.053623813047437074`, 0.369409668420823`, 1883.086591548903`, 266.54822065935684`,
0.18383750630418133`, 6.949409820987145`, 0.17339220023403645`, 174.08560156733955`, 95.44065950271633`,
201.72167143826883`, 0, 28.611035731054383`, 2.7739594463038375`, 2.793322018897763`, 0.006980121003472339`,
0.03860051696825928`, 0.03864926310287464`, 0.0012628363152609179`, 142.56451749841784`, 0.09748848183808763`,
1.0763966648885726`, 0.0003862681769788434`, 0.00038898251538400615`, 0.007027082651210437`, 0.2228737065473959`},
{0.07711195041153773`, 1.310646675677738`, 7.287601935184053`, 0.9421992854061032`, 0.6629854601936369`,
0.19600775055309494`, 0.11597851857342667`, 0.34647099332821635`, 1165.7488565303056`, 392.06574230549916`,
0.08156422577601757`, 9.98958158861084`, 1.355669623216047`, 242.76944364327497`, 105.50026085936771`,
246.45357240681707`, 0, 43.521823575056835`, 10.09992822262085`, 10.239803765811484`, 0.013849162103682477`,
0.01972023517822776`, 0.019722132490591387`, 0.0000962114470988151З`, 189.10624785088234`, 0.02172379710235962`,
3.3017255322870436`, 0.0002531120969776479`, 0.0002534403003281438`, 0.0012966719268454607`, 0.2843158483044925`},
{0.07030228204141997`, 2.841321925057506`, 9.523175071752728`, 0.957721444616676`, 0.5285225389857424`,
0.687368450677392`, 0.09615328133104123`, 0.49849774355724874`, 811.2137274256684`, 346.8639129623606`,
0.02062867223765208`, 4.642931209436085`, 0.8694461988489701`, 111.01395861630762`, 115.61779961677617`,
105.51617993008246`, 0, 15.41755582956246`, 2.297799241238104`, 2.340560383484917`, 0.01860960761035524`,
0.015073810060062654`, 0.015092028030449007`, 0.0012085843137044083`, 93.26839090728996`, 0.015138903518326925`,
2.0351228372786023`, 0.00018967585318041547`, 0.00019081704413987893`, 0.00601653262831503`, 0.359400312702425`},
{0.15160173019169382`, 2.757072174383392`, 6.0862251898613255`, 0.9273854095685228`, 0.9702354173159373`,
0.3952766424689479`, 0.139970431273921`, 0.019384819518267777`, 1328.4246699458445`, 112.51314475175559`,
0.06030008858760033`, 1.0538552754157138`, 0.12506996939343384`, 59.336326250311004`, 23.629902825576043`,
127.32360292799598`, 0, 46.36388466447133`, 0.3165824564857915`, 0.3186249586884938`, 0.006451722642419888`,
0.053493707867677265`, 0.05370675171091477`, 0.003982596303933272`, 12.469152595355943`, 0.11585340953010018`,
0.19333600484827032`, 0.0006913042046471363`, 0.0007030808273803934`, 0.017035369746186246`, 0.022129685518206803`},
{0.07720185370449029`, 1.8816151498237366`, 1.2505047699374146`, 1.3773873945814676`, 0.6708624603801276`,
0.176415527179277`, 0.09395893068196878`, 0.03821422736625399`, 2260.6042381201514`, 369.60131923785855`,
0.06295297192499111`, 8.500378555792118`, 1.404314834211414`, 196.73982486247195`, 44.18023598041904`,

204.24711197438123`, 0, 127.79996343942179`, 2.4602987882361314`, 2.4919358607448174`, 0.012859036739748086`,
0.15960744516508749`, 0.15977382405408333`, 0.001042425613816178`, 66.1333639005441`, 0.1760284375969617`,
2.167028988419588`, 0.0013966808748323567`, 0.0014066377002915698`, 0.007128919453707194`, 0.17179839784736772`},
{0.22937140077917728`, 2.9197558499797838`, 5.991654392537161`, 0.7609979028883141`, 0.7071044023774187`,
0.2974885161887877`, 0.18825283303648666`, 0.032338266898960494`, 2797.371157028768`, 202.1761455304635`,
0.23680881860212954`, 3.62202546942657`, 0.8089322407730246`, 195.49746089987985`, 20.345236163329673`,
219.4919768775447`, 0, 133.7995721752817`, 1.439832993886576`, 1.4463790413646316`, 0.004546393578873209`,
0.04297571346199432`, 0.04299726217979726`, 0.0005014161736254241`, 60.05658295563198`, 0.1408199942322048`,
0.6881005436686636`, 0.0006818069087948242`, 0.0006846308406230721`, 0.004141835161570073`, 0.04506664758121585`},
{0.2310975321060973`, 1.6649278018384956`, 6.117367353219549`, 1.3711053926671974`, 0.5776666118144214`,
0.34300872993879494`, 0.05030597587094107`, 0.013880459047725028`, 1272.690119313539`, 46.12165613423167`,
0.07701873188143649`, 9.481774546264408`, 1.4810089454694788`, 1310.1278762283143`, 109.92829736137963`,
1398.987377310036`, 0, 1094.1228670684122`, 8.655908016732804`, 8.747286505557247`, 0.010556776787345568`,
0.1943487741465267`, 0.194301599711117`, -0.00024273081019876575`, 205.8780272459304`, 0.6416217439016669`,
12.280398192475358`, 0.001712189592357416`, 0.0017166157546311487`, 0.0025850888788774284`, 0.10552566314405562`},
{0.21166318477480112`, 1.2271964963163153`, 9.968674707787947`, 1.4226030031673544`, 0.5017683729752407`,
0.4896306570856972`, 0.14845091745225086`, 0.13950989408153797`, 3248.3492688454417`, 221.1597513183293`,
0.152837589460797`, 8.879252520814859`, 1.0157863292829834`, 340.4756409843888`, 44.38631153012762`,
372.4939719883037`, 0, 115.09764405014202`, 12.154513435502016`, 12.234290567373678`, 0.006563580870184493`,
0.03432263344164034`, 0.0343402000431917`, 0.0005118080925003987`, 213.08537574968045`, 0.10378339863056954`,
2.1176677537133135`, 0.00029142531934711613`, 0.0002923383602363549`, 0.0031330184051416854`, 0.184638009914567`},
{0.20922455243011878`, 3.6942310466303763`, 5.573446425852463`, 1.1411804023081409`, 0.9621527847139406`,
0.28451282132289957`, 0.08975759414482132`, 0.051227081701645576`, 3447.2439359977625`, 280.22849496162496`,
0.2438675699856881`, 2.418161847018231`, 0.8016087345021319`, 82.89712247468385`, 16.28601560573895`,
91.24242767134707`, 0, 47.939585023090864`, 0.6467916544041324`, 0.6514201503738974`, 0.007156084866353218`,
0.04390770134658125`, 0.04397317473596096`, 0.0014911595772892916`, 34.134254434302406`, 0.13123670232111204`,
0.4130499575096553`, 0.0004640896985590448`, 0.0004674593515158824 7`, 0.00726077947280479`, 0.029840506768280692`},
{0.2765607651857069`, 1.2704530487690837`, 6.006554660027694`, 1.1061086441305394`, 0.12440018829651067`,
0.36025788190000807`, 0.19633101014307436`, 0.019373612586789046`, 1432.0769734472487`, 313.8129593237045`,
0.16870756948717214`, 9.609773835891207`, 1.2876583132286008`, 220.36040336766962`, 64.66489123145168`,
234.9067683338674`, 0, 173.13242078968918`, 2.45603939935264`, 2.4944872576027173`, 0.015654414281876416`,

0.026482353194777482`, 0.02649459731463701`, 0.0004623501457543533`, 44.57546775435077`, 0.1046282837638077`,
3.349940894221222`, 0.00028915818469810173`, 0.00029014063062858785`, 0.003397607200750219`, 0.5311215055764936`},
{0.21142405672597675`, 2.9545989174954075`, 0.2974772339667986`, 0.9658276187146195`, 0.64106654096473`,
0.6263249493059542`, 0.09733527159806632`, 0.007468085156923343`, 3517.602581345549`, 75.36708608662542`,
0.23543132772166975`, 9.603866416931954`, 0.19450738989435057`, 537.3869310755415`, 62.967132080359875`,
1134.6761617630461`, 0, 481.1513793511758`, 1.1859397532106117`, 1.1989574986926905`, 0.010976734228561602`,
1.127202233893463`, 1.1308892269950745`, 0.003270924232359196`, 50.056804443583346`, 3.404538129147651`,
0.8586249002105343`, 0.013926684262581923`, 0.01429554929398762`, 0.026486206224747955`, 0.19446965075711894`},
{0.08169573554069165`, 2.8299360298510665`, 4.320933911813235`, 1.1825738775709533`, 0.3027516653278022`,
0.5305834377007563`, 0.1475083038117065`, 0.007523165551877513`, 585.1893929943135`, 275.5228279325412`,
0.17684956635249888`, 4.120555621767931`, 1.4111853521392486`, 116.83566643472679`, 94.00709066893859`,
115.48776055006726`, 0, 105.54667052827755`, 0.2675330201797033`, 0.273687008702602`, 0.02300272511694068`,
0.04854039347673793`, 0.04867446902598498`, 0.00276214384853124`, 10.815733328305948`, 0.056650616407366385`,
2.4970974196516162`, 0.0004926415852664068`, 0.0004987186710404344`, 0.012335714149549926`, 0.34076681174788803`},
{0.1982041419042297`, 2.2268219215065645`, 0.4137546724148198`, 1.378661149527917`, 0.7206026710685052`,
0.68793041191814`, 0.15316897603405166`, 0.07890511093433691`, 2517.4293419356973`, 86.78917960165899`,
0.20777476794521893`, 3.6952651421831053`, 0.2532217765575610 6`, 310.53218141153`, 40.7241872121191`,
487.16044496735526`, 0, 143.1719191325059`, 4.901832556240777`, 5.015022200236377`, 0.023091291409269354`,
1.7022599691729328`, 1.72059898868866`, 0.01077333653368906`, 155.9358313113074`, 4.8199282355407425`,
0.49476294005684995`, 0.014757624354193588`, 0.015435890530346396`, 0.045960390363240844`, 0.12218956492413757`},
{0.22157133430375964`, 2.1006687367628096`, 5.865585434381025`, 0.862163364473078`, 0.7961242271327948`,
0.1679554170546661`, 0.16778560934705797`, 0.21616705792607036`, 1509.8006246813002`, 104.18673597092118`,
0.04226318913943389`, 2.3850476310310498`, 1.1811985793876256`, 265.89883348525245`, 19.91300861866803`,
288.4026589403262`, 0, 65.73613352027319`, 6.441074987967013`, 6.4772520024800855`, 0.005616611292471596`,
0.10271540001246286`, 0.10271205352539227`, -0.000032580188269570876`, 193.29378369096008`, 0.3251255462047928`,
1.062322965571054`, 0.0014384024178162313`, 0.0014445134930227467`, 0.004248515666285479 5`, 0.025661986750716518`},
{0.18526653849571717`, 2.309967457429205`, 7.235178488173702`, 0.8111816500462055`, 0.21148492456283652`,
0.18475826854386967`, 0.15626027102897042`, 0.057689140810915564`, 766.0020232731708`, 185.02183046070866`,
0.06106549734909339`, 7.0384168617832215`, 0.11685665088837416`, 170.59564493389058`, 72.79558537680839`,
285.5129488381206`, 0, 94.40720945612298`, 2.238991980591547`, 2.2669141193777698`, 0.012470852521251796`,
0.0170517582683261`, 0.017054015211608313`, 0.000013235839065361965`, 73.8856944658777`, 0.04513028899482026`,

2.070050557830143`, 0.0002541698516302926`, 0.0002545993542803952`, 0.0016898253169985722`, 0.23072015236529084`},
{0.10801077582204055`, 0.4767848668332846`, 7.25138663495552`, 1.4325801889738408`, 0.33847640924280453`,
0.5718972515878133`, 0.105744974897243`, 0.11534684356295588`, 2594.1807156101713`, 318.6618729662216`,
0.06179469905331658`, 5.34696732616077`, 1.3364569926001173`, 116.2079322334835`, 29.910787048045535`,
120.76190421474388`, 0, 44.842988440117786`, 9.12746130663036`, 9.258440663150852`, 0.014350031418412623`,
0.026896389647797107`, 0.026939726988973404`, 0.001611269830032569`, 62.16907748011004`, 0.0415014273240051 1`,
1.6143845167616397`, 0.00022623401597865467`, 0.00022778271955477606`, 0.006845582303005582`, 0.20904683763911774`},
{0.23792597358173623`, 2.688465494136551`, 6.845829046034535`, 1.1002091615985592`, 0.37282035219472753`,
0.3011297207106588`, 0.2473335682094463`, 0.01991156261653569`, 2038.6789052153845`, 210.83183249328954`,
0.08037072870463724`, 9.093415216874622`, 0.3344692931868205`, 324.79054026040365`, 49.352384940568335`,
438.37338121688845`, 0, 252.95166669870554`, 1.8166914553101072`, 1.8252554516702943`, 0.004714062112834316`,
0.036009773395730874`, 0.036019427816549925`, 0.0002681055699227386`, 69.77303272991331`, 0.12239514848059753`,
1.2864149708649992`, 0.0003954478795006322`, 0.0003965188995336290 6`, 0.0027083721737217736`, 0.15286847361955413`},
{0.22993078275515533`, 2.525474685703524`, 6.125260191496192`, 1.4929402058003913`, 0.486733717931074`,
0.6225356403190192`, 0.23641243849582955`, 0.0803729054195917`, 1157.473735647006`, 169.73954640708985`,
0.04795903068483898`, 8.28333454337778`, 1.445122239815786`, 515.2069865708329`, 130.71558497289834`,
540.8860515102126`, 0, 242.1601165929171`, 7.353085841222331`, 7.426822822475091`, 0.010028032154796973`,
0.09499005095400345`, 0.09509856696289586`, 0.0011423934170216032`, 265.2861736258847`, 0.3120162395691106`,
4.827664105065399`, 0.0007666238282054705`, 0.0007717303721494873`, 0.006661081688486448`, 0.37496315179532325`},
{0.1869769669715985`, 1.485119860176268`, 7.03335904463459`, 0.8235833311873434`, 0.33587109510820534`,
0.6579510066594008`, 0.17782007925307802`, 0.007365768570817107`, 1161.9126891061214`, 206.93000870629214`,
0.23913461974132133`, 1.154555664614577`, 0.17860914505549097`, 34.820325481902515`, 15.060243508639793`,
45.194006162277844`, 0, 31.430008243517886`, 0.1476038209404259`, 0.1492231569941317`, 0.010970827471731726`,
0.022000349752912925`, 0.02211932702073137`, 0.005407971652936716`, 3.131562370236116`, 0.05876512384448559`,
0.27354282221278786`, 0.0003186981517857568`, 0.0003258882889478826`, 0.022560962847878008`, 0.07798683760705004`},
{0.2594386598277771`, 0.4907291838119914`, 8.253740230346917`, 1.2562319857439164`, 0.630751008353968`,
0.6003942152343791`, 0.16893163054122257`, 0.12552255641031881`, 2555.8077872611675`, 287.20210893930584`,
0.11105710767727667`, 7.093346066656089`, 1.3316012208831718`, 260.43462940969096`, 60.378428952789`,
270.65425530429303`, 0, 94.5198664675246`, 20.69179471061431`, 20.872214031667095`, 0.008719365505797994`,
0.03502870390817436`, 0.03506021412320517`, 0.000899554123195756`, 145.05810757064302`, 0.12982571424931055`,
2.114430958339101`, 0.0003364588581080685`, 0.00033807291512671147`, 0.004797189848764649`, 0.21774052142086286`},

```
{0.24168594197714738`, 2.487527173714957`, 2.5005074702928987`, 1.3162259499649314`, 0.4221537034288636`,
  0.3099363412058589`, 0.20921038714233392`, 0.06741194388477754`, 2733.252958969397`, 173.34117039752516`,
  0.11909087418011138`, 8.486444686364809`, 0.9620364764733642`, 424.2865911759828`, 36.51773448477484`,
  485.2980805613829`, 0, 216.71456143194217`, 5.661155587556455`, 5.697042474152249`, 0.006339145081028175`,
  0.16481574871012136`, 0.16493264547264697`, 0.0007092572368869732`, 201.1754051239278`, 0.5690521354238051`,
  1.9790097470786243`, 0.0015093780099026954`, 0.0015195638663247297`, 0.006748380031514234`, 0.15136954678977227`},
 {0.12452706981171563`, 3.242843098104845`, 5.361835103883211`, 1.0740271680209217`, 0.6460165689100792`,
  0.2944176768104616`, 0.22857190710285924`, 0.07930287454728857`, 1257.5807309824504`, 295.430402570252`,
  0.10895057078510323`, 6.841864249157172`, 1.163933956705701`, 286.0293022970964`, 79.90920171764309`,
  298.1664018061183`, 0, 135.26182141428902`, 3.182608013530935`, 3.2101731632797685`, 0.008661182788341826`,
  0.05252235475555167`, 0.05254595483972141`, 0.00044933408411673703`, 147.4385490093136`, 0.09343507053320034`,
  2.337638841490439`, 0.0005903314885579158`, 0.0005925652948890161`, 0.0037839864116973043`, 0.21419411450761516`},
 {0.2557093973805171`, 0.4051303627282947`, 4.693462565645005`, 0.8860437827795489`, 0.9194156940466172`,
  0.4581557032512626 5`, 0.1510910941798535`, 0.07870004480420398`, 3132.5620218229606`, 302.90817331129847`,
  0.08637897198804784`, 9.737653549202872`, 0.46859993602327443`, 410.4250349865878`, 99.43219117652139`,
  468.5991510399556`, 0, 193.74115296768792`, 31.889291170553825`, 32.002431721192764`, 0.003547916760953562 2`,
  0.04990479375606386`, 0.049925065249836725`, 0.0004062033373377627`, 184.5617157010668`, 0.1823017819680199 7`,
  1.2202779891463555`, 0.0006799459623447746`, 0.0006824954475702304`, 0.0037495409439067817`, 0.13038527665289476`},
 {0.1829582147550864`, 1.5256866045998043`, 2.03189641777327`, 1.1222778010348193`, 0.03811462288929479`,
  0.5618740829076075`, 0.11263433834449038`, 0.2791855460572071`, 3277.6533486117487`, 172.65623022385375`,
  0.030075112520539238`, 2.59232430828043`, 0.783030452394347`, 78.3465733094254`, 8.542082506374587`,
  89.92128205389362`, 0, 16.095059691398735`, 2.714431622198104`, 2.74793919581682`, 0.012344231972799324`,
  0.10366842661100456`, 0.104293788760255`, 0.006032329897288635`, 59.162456644139475`, 0.27095700370312603`,
  0.6027936164894637`, 0.00110641057453853`, 0.0011304274674380252`, 0.021707034849619333`, 0.08305906765630879`},
 {0.06140104313465666`, 2.460539185106426`, 5.961918171032632`, 1.1102491165917632`, 0.7798976124509112`,
  0.16029666400283926`, 0.13508017803518807`, 0.4919794640607069`, 2590.2148343601484`, 305.84622942181363`,
  0.11973020837325932`, 3.9806430906660957`, 1.1111030420450256`, 136.80693943136393`, 18.86879184828117`,
  146.02212050237392`, 0, 17.755716192599277`, 3.2911208863415884`, 3.3129862222920154`, 0.006643735282155561`,
  0.04014620742588554`, 0.040158096151200647`, 0.00029613570191044936`, 115.68474148236679`, 0.03521455734039073`,
  0.8316879393388863`, 0.0004368557810525697`, 0.0004381092962818135 4`, 0.002869402863854953`, 0.05346071483712627`},
 {0.1711775238741562`, 0.5019625567967068`, 9.586274753416383`, 0.9484474385429034`, 0.980521223518446`,
```

0.4330626846988278`, 0.21438996930373572`, 0.2245552941607962`, 3058.4173866521887`, 152.6790489040933`,
0.0944629158119748`, 2.577057545046525`, 1.1465158474128474`, 299.7521916870493`, 28.793793766711087`,
327.4920417381706`, 0, 71.7238003654546`, 27.87697915191431`, 27.96721840498601`, 0.003237052787532946`,
0.07214240439337409`, 0.07218788523843973`, 0.0006304315117866555`, 199.90285329804902`, 0.17641654500520923`,
0.719203556582276`, 0.0009182117591037819`, 0.0009221508500513421`, 0.00428995916084185`, 0.0264281523116543`},
{0.26691735492143615`, 2.5320514430618486`, 8.550940885105003`, 1.4373682402592758`, 0.4771646947972308`,
0.18844330768909645`, 0.11201955955138962`, 0.11789932012031278`, 2886.771279587132`, 93.01623349049612`,
0.010902609553686937`, 2.9431133951513715`, 0.705430973142406`, 201.83659977089596`, 9.824373947360769`,
249.7259779404756`, 0, 75.41710066529588`, 3.3915676907498473`, 3.4069689445845324`, 0.004541042738639645`,
0.0722158653336108`, 0.07224725828609524`, 0.000434709912280562`, 122.68034093721583`, 0.2753666822600639`,
0.5693709011197111`, 0.0006069850689500722`, 0.0006095265311907134`, 0.004187025959365487`, 0.01905138970818696`},
{0.08703489710225831`, 1.1696029176126457`, 2.8929571896991018`, 0.8551088804080895`, 0.7972556998184932`,
0.6510731694726453`, 0.11258011739373719`, 0.045162705810826695`, 3535.7555380745443`, 62.84409202124414`,
0.17813120427323514`, 8.03275953302387`, 1.2552854307001606`, 1248.570755956766`, 70.58373065339602`,
1366.4766221621733`, 0, 760.1664190979801`, 27.54145963930682`, 27.65009974472828`, 0.003944602313902479`,
0.28847054428926044`, 0.2882875993726777`, -0.0006341892446366515`, 460.1795935634878`, 0.3586714877037185`,
4.246428701535613`, 0.004067682386168725`, 0.004083381696711756`, 0.0038595222174702126`, 0.07601746782081273`},
{0.13518231998646707`, 3.6948829381267645`, 2.131072716483498`, 1.4937759276297344`, 0.7767923162254364`,
0.5165458326040475`, 0.16227719412689456`, 0.04519981251511891`, 3983.684815901678`, 191.70315936858992`,
0.13930377370940616`, 6.306826705642012`, 1.032935178026166`, 382.3060941276819`, 40.01639022689893`,
412.7199304568539`, 0, 232.3676745356371`, 2.7731123473947537`, 2.7889623074805843`, 0.005715585270362755`,
0.26559324327097394`, 0.266209383525165`, 0.0023198641900781247`, 146.37607854139353`, 0.5129072971155486`,
1.3929483296304344`, 0.002140100761393482`, 0.0021618006659940587`, 0.010139664912995672`, 0.09973991504900215`},
{0.10774309849896646`, 3.736554195863712`, 2.2341742787936347`, 1.2045029497948032`, 0.9303574820449074`,
0.4582352004403937`, 0.19359404123783952`, 0.039983935436586286`, 2352.3551825652967`, 320.72717703493936`,
0.06514512285730684`, 6.779792172665335`, 0.8797330310810398`, 348.60783894281593`, 93.64613598950203`,
363.75742889587553`, 0, 222.1078197533552`, 2.3178609380770756`, 2.3305555186175986`, 0.005476851666111671`,
0.17349751883052203`, 0.17381393208119453`, 0.0018237335773174657`, 123.72590019429266`, 0.26704514658153283`,
1.445307357249098`, 0.0017323918083049783`, 0.001749110762337256`, 0.009650792593296709`, 0.13257580083724357`},
{0.20770433175112651`, 3.109204400403325`, 0.20099665267948374`, 0.8742255636364782`, 0.024813473089767113`,
0.240282569360604`, 0.18576559892192723`, 0.1317848182037604`, 1411.5851094618392`, 158.08107833990675`,

```
0.17690068584227342`, 3.904436541828723`, 1.357207699254508`, 158.37355827596463`, 21.182120918846262`,
 173.94322762124457`, 0, 54.624613101813225`, 2.1905537649213698`, 2.2773991669468683`, 0.039645409948938504`,
 1.0738318882665576`, 1.0776973120820106`, 0.0035996545247811707`, 97.29827721733696`, 3.186279068077828`,
 1.8846656261603514`, 0.014539457133268874`, 0.01533730566926631`, 0.05487471290601475 5`, 0.20123362918459525`},
{0.09448668201105037`, 2.410919641885151`, 1.350132874851214`, 1.3762843812070134`, 0.004651491449426448`,
 0.6365872610465713`, 0.23322630347732265`, 0.08615671221466674`, 2950.762127886288`, 316.0892657764441`,
 0.16180448088816285`, 6.47689574973119`, 0.7706785449987734`, 133.17289340670663`, 25.08585044133976`,
 151.68561358138692`, 0, 60.183693184692984`, 2.050767153284565`, 2.0731448120674956`, 0.010911847669829333`,
 0.13039972783132586`, 0.1312794063239999`, 0.006746014790858457`, 70.63192586838069`, 0.17601482311291272`,
 1.2043963474961785`, 0.0011301665777458947`, 0.0011574067448294292`, 0.024102789464774954`, 0.361148377790394`},
{0.08289232854140277`, 1.1468625434643034`, 6.113671903882498`, 1.141476131790856`, 0.8113048716462563`,
 0.5114785013510522`, 0.1437446486191194`, 0.02760266646248284`, 3012.3201335015347`, 281.64171273471936`,
 0.03564353737323955`, 9.384615831703261`, 0.7944935894043699`, 386.88295347951976`, 84.56282467583826`,
 412.6321744785477`, 0, 277.8634150594741`, 6.262139674566524`, 6.293981564782374`, 0.005084825933406645`,
 0.048280780184466535`, 0.04830949653827655`, 0.0005947781643191874`, 102.59733478145831`, 0.05717294704699033`,
 1.8019232251734603`, 0.0005105877400430892`, 0.0005124772131754617`, 0.0037005846090489847`, 0.16668212684268421`},
{0.0849153683065022`, 0.6319892236147719`, 2.114551707925985`, 1.2461670457408607`, 0.7306014894102357`,
 0.32449846710553176`, 0.08929492495989536`, 0.016408600115246787`, 558.733384495335`, 217.00633714400976`,
 0.13070090260521539`, 5.821164045556291`, 0.7435891013457101`, 241.86381482845349`, 181.0120732822622`,
 240.6220503276592`, 0, 196.2332763824145`, 4.506848056858475`, 4.579868993394991`, 0.016202218405254154`,
 0.15320136426954`, 0.15350825692944528`, 0.0020031979569405944`, 40.68970577719622`, 0.1858450038857678`,
 3.4366338276445147`, 0.0014764127745685585`, 0.001493331901944835`, 0.011459618656592063`, 0.23870199836983014`},
{0.09040683538793876`, 3.340658966022863`, 2.160986355031987`, 1.2703609168223777`, 0.7359849646797021`,
 0.4196403007867846`, 0.14182087201107557`, 0.34521905027534144`, 2988.2505759920587`, 354.16133220107486`,
 0.21419902011703607`, 5.4345853130842805`, 0.9728978889757345`, 162.69524181210107`, 37.188623997565934`,
 170.76081902170702`, 0, 28.268263821291686`, 2.7537893974604692`, 2.7762084914878096`, 0.008141179586214964`,
 0.1100448890385067`, 0.11031419567115891`, 0.0024472434386115793`, 131.42101773092867`, 0.1421258595509075`,
 1.0707442307813622`, 0.0010414770261638617`, 0.001052858502263374`, 0.010928206588900258`, 0.13089041205098328`},
{0.20859643515675713`, 1.8877506327350426`, 3.610942473179815`, 0.9188330116771175`, 0.07225013193132535`,
 0.31512009685680187`, 0.2084254211369836`, 0.13036688863188475`, 1423.8303839771024`, 148.24888484193133`,
 0.09090215154618603`, 2.653159557091028`, 1.1769997259400542`, 115.5468418633593`, 15.77644609837419`,
```

129.98806888470622`, 0, 41.01630901757191`, 2.65500491343465`, 2.688067023722103`, 0.012452749190841272`,
0.06927463570725195`, 0.06940558518580144`, 0.0018902947263823577`, 71.59981721786987`, 0.20643488650479103`,
1.2070850858687445`, 0.0009070377326115819`, 0.0009166166332847416`, 0.010560641888160083`, 0.10369302516100053`},
{0.2775711100993279`, 3.8796646995160193`, 2.028692966768544`, 0.959772508161437`, 0.14615379499145553`,
0.5344882702156523`, 0.050540537283472214`, 0.008717405363258768`, 605.4303926477064`, 86.02465982441038`,
0.19867732233067387`, 8.175417405675173`, 0.7557300476034672`, 512.5940717511342`, 159.08659861668394`,
534.3492252089459`, 0, 455.52663393911996`, 0.9880718514435834`, 1.0060692179634865`, 0.018214633372672928`,
0.185634921306256`, 0.18589002403026875`, 0.0013742173197675012`, 54.76267832330149`, 0.7360984454311793`,
8.064544656712581`, 0.0023236032664599726`, 0.00234888044646224`, 0.010878440552709812`, 0.33494148319668243`},
{0.2734564636981751`, 2.078794252055899`, 5.58118723900359`, 1.49140438303907`, 0.8827845137379815`,
0.5395409262732532`, 0.10413442884619478`, 0.18779130214107398`, 2950.6303080099433`, 94.68533320113221`,
0.033296934697099045`, 2.835225178912273`, 0.8590623879542898`, 286.4326220960948`, 30.60313588937996`,
321.2065516029762`, 0, 78.21146447051188`, 6.756139414221511`, 6.797244597946985`, 0.006084123077588988`,
0.1686604237517064`, 0.16899268119652547`, 0.001969978714794429`, 200.63748257674303`, 0.6588754720706241`,
0.9067210017713346`, 0.0013631292347240365`, 0.0013756942476116478`, 0.009217770822848692`, 0.032813660446638304`},
{0.1042141776090886 9`, 3.9806755529248097`, 3.6408344459764717`, 1.322172627166737`, 0.1000592332348238`,
0.48996158640872345`, 0.07063639655536227`, 0.07096628857497574`, 3218.761349505553`, 296.2823833741422`,
0.08840787957878266`, 1.0269425117351112`, 0.10667022544527116`, 9.44522651696954`, 3.305388840340097`,
18.744519868918125`, 0, 4.706023561760364`, 0.08101242642037666`, 0.08217122967675326`, 0.01430401862503805`,
0.020591639885311146`, 0.02085372679889513`, 0.012727831053948258`, 4.60691693335304`, 0.030656297375289538`,
0.08467497174982966`, 0.0001837269033834188`, 0.00019199013152967385`, 0.04497560234284559`, 0.06848150583314123`},
{0.2754934613307095`, 2.4772150697863067`, 4.699383051224052`, 0.8551969359601641`, 0.3181125413370589`,
0.21381447461486702`, 0.12130217582978334`, 0.022512766834479486`, 795.9986377450518`, 357.5238954990325`,
0.24036872560514078`, 2.0345694437060526`, 0.6072647478635216`, 39.13462909452137`, 22.335485375469272`,
40.75897866064123`, 0, 29.696000514602193`, 0.2561766708089365`, 0.2617103210231677`, 0.021600913919122444`,
0.021881887384823436`, 0.02191468218667692`, 0.0014987190673609874`, 9.065781563651194`, 0.08611881280134172`,
0.7269182894256263`, 0.0003081556869041213`, 0.00031107237886475534`, 0.009464994756178235`, 0.09956385076024207`},
{0.06619581350761466`, 2.7881066711576734`, 2.8537509705176394`, 0.9891677990616923`, 0.8373344986239073`,
0.19790897524975626`, 0.09185213371202688`, 0.00857321041500942`, 2055.3736975529255`, 31.88640228370963`,
0.03523831218645268`, 5.696097023837108`, 0.29187148351753467`, 863.8350263186665`, 41.83132180398387`,
1767.7558026716686`, 0, 768.5041748664682`, 2.302567549757074`, 2.3084443029956905`, 0.002552260948538443`,

```
  0.33015713543141173`, 0.32976746808589863`, -0.0011802481415520427`, 91.71148494669818`, 0.3122145737889207`,
  0.8665887484847716`, 0.004026737203530084`, 0.004038787016801549`, 0.0029924508758358837`, 0.017813486639113453`},
 {0.1298132756954506`, 2.8585402154144006`, 6.437713761400808`, 1.4980481550734701`, 0.9611669791610145`,
  0.5357765761577084`, 0.16040271618955604`, 0.016100163586007913`, 522.564427621146`, 43.6121866921153`,
  0.20871550454007498`, 5.59776191182581`, 0.8806650157270011`, 1885.461965128794`, 426.7601518271345`,
  2019.2769730979123`, 0, 1532.4220891951961`, 8.387265303272697`, 8.427348149870655`, 0.004779012604062771`,
  0.4918423539859588`, 0.49169662608588494`, -0.00029628985566798605`, 342.50478809649803`, 0.912109529952613`,
  6.812408638352774`, 0.003951901660159796`, 0.003976356211315848`, 0.006188046479644438`, 0.0853113587000659`},
 {0.20556043625089965`, 1.8192901890738584`, 4.292636640368679`, 1.2838668900928696`, 0.08760791600423734`,
  0.6818222490294337`, 0.142672004987712`, 0.006686505560345256`, 1995.540026133921`, 283.4866335300326`,
  0.2085798635478609`, 3.280310512987043`, 0.6015416808762613`, 69.44050991313887`, 20.58081694953185`,
  76.16856476205429`, 0, 63.25074570129109`, 0.22119411382016105`, 0.22433267432649037`, 0.01418916829261141`,
  0.03946793532785715`, 0.039658792328056094`, 0.004835748275492824`, 5.748804016484364`, 0.11590065719880394`,
  0.7878124151145204`, 0.00036770738454394714`, 0.0003747894949991435`, 0.01926018011299946`, 0.20002313464090485`},
 {0.24058104513431994`, 2.1873687346811277`, 5.659026523633111`, 1.004047709044065`, 0.8328913078837221`,
  0.623891871350166`, 0.08766340991931373`, 0.008430134039147183`, 2601.7165814407754`, 249.12373989835567`,
  0.030474235896864155`, 8.991227710673165`, 1.2833381452421007`, 345.4234496659149`, 112.967580476706`,
  354.04447819534346`, 0, 308.2146398503855`, 1.1394620246603604`, 1.1513274895745875`, 0.0104132166385833`,
  0.04273695462175679`, 0.04276908554350317`, 0.0007518299333855172`, 35.60605152997902`, 0.14688144583942567`,
  3.099920568155126`, 0.000513728721642126`, 0.000515946514840784`, 0.0043170512086008195`, 0.2053753286756565`},
 {0.1843088049905594`, 0.4280411676285585`, 1.9800464046244353`, 1.4120188565898317`, 0.7240044582523009`,
  0.296746547744528`, 0.10234889391033763`, 0.005447859153693297`, 2517.6110832821096`, 241.50069071394864`,
  0.10993322637089764`, 3.2673971617867004`, 1.4127349426750984`, 120.52965810051248`, 20.86684339434545`,
  125.71668359676093`, 0, 111.2352272153975`, 1.2050003473818842`, 1.2232230943278741`, 0.015122607213834183`,
  0.1637802384347403`, 0.16433261590733717`, 0.003372674737049941`, 7.368425081230872`, 0.4312305718139374`,
  1.0614729646992118`, 0.0013955908875861178`, 0.0014158923030225753`, 0.014546824300043726`, 0.07146150509977293`},
 {0.14879926745595878`, 1.0145913123947308`, 5.175666788640903`, 1.4216396007747538`, 0.30528652163260794`,
  0.41852006684217935`, 0.08636270796505932`, 0.024569495844874063`, 1528.3687993879967`, 399.50130012547027`,
  0.11493939543886256`, 2.7975290190897475`, 0.9743823827588485`, 44.87238030803499`, 21.479697981684037`,
  45.63018322256206`, 0, 33.3542971924682`, 0.737290011208397`, 0.752412839033762`, 0.020511369468547702`,
  0.027517102932454345`, 0.02760672718838515`, 0.003257038219132369`, 10.686400572677899`, 0.0584932108408295 5`,
```

0.8271046778835139`, 0.0002323489075293228`, 0.0002355330149694118`, 0.01370399144092005`, 0.1460131203866859`},
{0.1829951377410336`, 1.361564914411157`, 4.694817997056893`, 1.2880900199343872`, 0.9454435675757777`,
0.5028590377428427`, 0.0646889402866752 7`, 0.1666609451608123`, 604.7693853692326`, 275.5852017799763`,
0.22566362443421`, 2.928657758289848`, 1.3281605404183678`, 97.03698505858678`, 171.20003674719035`,
93.9316161690456`, 0, 29.863002785281804`, 3.2746131190562946`, 3.341423590694768`, 0.02040255419783077`,
0.05675013121421175`, 0.05695044356828762`, 0.003529724950234181`, 63.69426187396466`, 0.1483571154052433`,
1.7377975026684627`, 0.0005283468862844476`, 0.0005363687943572561`, 0.01518303274052002`, 0.13854645569550436`},
{0.2341957938761366`, 2.5114017057248956`, 4.373268682378841`, 1.4631865500968817`, 0.20458772158961103`,
0.577553240998902`, 0.11818082574498356`, 0.03355766009617581`, 3153.579024983129`, 307.79686137602005`,
0.09194856938832352`, 3.1157373823819228`, 0.10640897514406333`, 34.56037089036711`, 12.509890621754069`,
67.93587884059397`, 0, 23.370940608146633`, 0.3006245390601783`, 0.3036898546680783`, 0.01019649166858727`,
0.023113787048073507`, 0.02323078072317085`, 0.005061640260594746`, 10.785556859692749`, 0.07733073867438192`,
0.25269001524071066`, 0.00018863729263252083`, 0.00019276357926577953`, 0.02187418285999798`, 0.1814551034236776`},
{0.20513991416113786`, 2.033431069067677`, 9.422225723793833`, 1.0288902090438978`, 0.6375890014689067`,
0.2390905203768514`, 0.1852902306229 6823`, 0.013541725424023602`, 2549.6747377089105`, 150.27313290555605`,
0.19604710287572352`, 2.636313809911791`, 0.4621075910698911`, 150.6016668665475`, 13.066803148124883`,
195.3429260099746`, 0, 126.05706203027415`, 0.8092071366500386`, 0.8124003413700016`, 0.003946090655085 2215`,
0.0391001017830475`, 0.03912259982870464`, 0.0005753960892984633`, 23.50667047107831`, 0.11458559319239267`,
0.36244434837953576`, 0.0004588803633535088`, 0.00046077908935602255`, 0.004137736443193818`, 0.021889446561530287`},
{0.17276893398546517`, 1.4696753313802207`, 3.9973169318549147`, 0.8654667209129807`, 0.10876030003077308`,
0.19704558070570188`, 0.12440236076545855`, 0.1569376119224937`, 3938.384639642497`, 57.46421942479361`,
0.12445676420469665`, 2.5408037017095726`, 0.5238442802739391`, 188.9434459026349`, 4.7063821614034`,
281.529902668244`, 0, 58.367775653799484`, 5.9206036038119425`, 5.9404764620471635`, 0.003356559493769673`,
0.10087626328701368`, 0.1008929111327983`, 0.00016503234003861778`, 124.30521519147476`, 0.24897549246476758`,
0.2850176978701658`, 0.0014076702178674738`, 0.0014138577843286362`, 0.004395607992997208`, 0.011509349975774286`},
{0.11204311855852472`, 1.7013572433137565`, 3.058168037173486`, 1.4867504783146572`, 0.8996771100732928`,
0.6819517798967545`, 0.19493702157072262`, 0.24346814437911918`, 2914.354454850107`, 387.06404549612205`,
0.01985945025481517`, 5.249142162935986`, 1.0430073575453092`, 218.02919760665836`, 73.17307483893188`,
221.467754685356`, 0, 49.455079861848816`, 6.648704189971032`, 6.691989820406943`, 0.006510385963809817`,
0.12611250150946984`, 0.1265814378171108`, 0.0037183966857221584`, 161.59744331796807`, 0.2018576851190365`,
1.0852699352468795`, 0.0010181837477523104`, 0.001032234972178758`, 0.01380028355143792`, 0.14374131721443303`},

{0.16473183239015682`, 1.8712094759941964`, 3.0571210356797724`, 1.4644853700911185`, 0.18431094252367552`,
0.5500426776194254`, 0.09375157274845106`, 0.031874339239305535`, 3596.1155435077626`, 188.36890996702562`,
0.22217643971583478`, 4.716624148360237`, 1.2836160756903663`, 146.6462277238922`, 16.023924324517214`,
158.70940488613206`, 0, 101.01955855973162`, 1.633548834937414`, 1.6545234191388467`, 0.012839888072422534`,
0.09299802065533573`, 0.09330557783835794`, 0.0033071368708164073`, 43.66731513477386`, 0.21885334787439978`,
1.5787800722881022`, 0.0007640956538591537`, 0.0007729307483473031`, 0.011562812121135302`, 0.13060050193287773`},
{0.24923018426453097`, 3.1125598045900347`, 2.019868693874246`, 1.2491757763779443`, 0.3111545672890146`,
0.6578188710626947`, 0.1476722707575428`, 0.3114765600131322`, 2384.9574981509613`, 297.0737195350481`,
0.08236658901774402`, 8.424499891492555`, 1.0114329054147206`, 214.08966366801928`, 54.53213571105593`,
226.10190082683206`, 0, 41.029707835049194`, 3.7965870457854445`, 3.8448081891042514`, 0.01270118207149662`,
0.09814729818717752`, 0.09846079311778383`, 0.0031941269540445294`, 168.81577476198598`, 0.34944670303211167`,
2.3086696399282305`, 0.0009427673454209806`, 0.0009561852170553142`, 0.014232431468383044`, 0.3855711737522299`},
{0.2428184603969214`, 2.139771549805288`, 8.962038430839804`, 1.2513955518769984`, 0.15789830342021016`,
0.6821214869737191`, 0.08731003079523225`, 0.005960116741721571`, 2057.9200252572427`, 141.81337749372574`,
0.11756230704325321`, 2.6769215386493905`, 0.895060254520057`, 104.05986888603967`, 17.232471221922367`,
113.52307941071585`, 0, 95.7451941711425`, 0.2553192021416757`, 0.2586443997746597`, 0.013023688015203927`,
0.033938472941839465`, 0.03402698881141924`, 0.0026081276470943937`, 7.804639498024901`, 0.11772696782800106`,
1.115497846867859`, 0.00032624847958773184`, 0.00032965101907304314`, 0.01042928840499413`, 0.09058921696701758`},
{0.16708285607632617`, 2.514917463798218`, 5.621232009809386`, 1.0053867061169064`, 0.033964338040640696`,
0.565944967748532`, 0.21275159258566828`, 0.33969022847136165`, 3900.0636957226443`, 236.24950114350304`,
0.17313930544506606`, 5.330368544786425`, 0.772540585340217`, 138.08297443762865`, 14.944513644493819`,
164.0165282811315`, 0, 24.25121642797089`, 3.0799208100369997`, 3.10040866055229`, 0.006652070549516553`,
0.029027722578659088`, 0.029066849048243238`, 0.0013479000799365704`, 110.65352331825133`, 0.0692862113404887`,
0.854982143156961`, 0.0003479387962095348 3`, 0.0003502393818845194`, 0.006612041255667167`, 0.16013182514336388`},
{0.26506128276311264`, 1.6499267057635905`, 8.348095275849516`, 0.7847265312910929`, 0.6385591297525388`,
0.153373190400086`, 0.07053246573144985`, 0.21097306215358952`, 2309.981041141632`, 237.73247710249848`,
0.1662652738138321`, 1.110923557283094`, 0.6469777658769382`, 29.354057702741503`, 4.756396602261252`,
33.60779171935273`, 0, 7.452981203437387`, 0.8883333063178241`, 0.8970539554849404`, 0.009816866152709824`,
0.015540973563757887`, 0.015553752992694703`, 0.0008223055579104077`, 20.93835493875784`, 0.05884729125996281`,
0.21218226081427252`, 0.00023910342265953677`, 0.00024055265952407164`, 0.006061129733799131`, 0.013309140004397703`},
{0.13234258341034721`, 3.856159276277494`, 1.0641752125958632`, 1.2393517449503635`, 0.10012444404217513`,

```
  0.25632211545729044`, 0.16888027141449713`, 0.016585410322055873`, 915.4984496215893`, 322.7924808984179`,
  0.249680065848771`, 3.0405535188651083`, 0.4380264905508511`, 59.707720876954404`, 26.70263290391044`,
  65.76611097892155`, 0, 48.09618387089255`, 0.19932550460627435`, 0.2039116885131486`, 0.023008515222039838`,
  0.14158836190493168`, 0.14258510589522513`, 0.007039731068876343`, 10.980441336945386`, 0.26768813707625533`,
  0.9452178355104509`, 0.0013593447641333523`, 0.001402420641187554`, 0.031688706346446605`, 0.18545949930020336`},
 {0.08790421115050512`, 0.939015726321446`, 6.470926650783501`, 0.9066579200686449`, 0.8639128923505324`,
  0.5386650165495622`, 0.10434830834744135`, 0.024067546956899128`, 1142.301427947522`, 369.2550313603065`,
  0.13202765600512467`, 1.8671579613852387`, 0.37302887352364733`, 53.65508150994393`, 48.978559654538984`,
  52.32876035619161`, 0, 40.005245846027094`, 0.9422852528991438`, 0.9523640052225169`, 0.01069607350042201`,
  0.025168983509671294`, 0.02525042366950529`, 0.0032357349593676865`, 12.640295302186825`, 0.0316065662982515`,
  0.502122861304608`, 0.0003328950611292125`, 0.0003375024372696708`, 0.013840325911804285`, 0.09329016702394759`},
 {0.20144727254246125`, 3.9392724652693136`, 7.1335815410177625`, 0.970870453628196`, 0.39841924348884183`,
  0.5432660762964611`, 0.1877336667874709`, 0.10436699241700248`, 3471.7993430199795`, 68.39740948767945`,
  0.03176666869513867`, 6.4702458499294195`, 1.1303015452765406`, 748.9056052929475`, 28.174727851866013`,
  871.0230257606944`, 0, 301.92335040997517`, 7.7974060193317305`, 7.8263351433294694`, 0.0037100958865059486`,
  0.09882481732730512`, 0.09884638387313432`, 0.00021823006014543012`, 438.8015261782665`, 0.28439985585837707`,
  2.442376489833197`, 0.0012291856513889643`, 0.0012331396458876217`, 0.00321675940016819`, 0.07898389433238642`},
 {0.05157825200138594`, 0.5074659893035784`, 0.4819934303065594`, 1.4763707530169474`, 0.8893682022937035`,
  0.32243152253555507`, 0.2150347661898181`, 0.2715592826957914`, 590.6719840967398`, 259.49776063587854`,
  0.04318917041730147`, 4.420302579747371`, 0.3690391195033085`, 286.4417946205615`, 169.1912977940964`,
  291.8862639545607`, 0, 59.78194041617925`, 27.216659002697657`, 27.606196511223725`, 0.014312466070411478`,
  1.2296185806546671`, 1.2367808304654582`, 0.005824773570823671`, 197.30755409060149`, 0.90602252883672`,
  1.91242512379325`, 0.009824515476058338`, 0.010217787367305737`, 0.040029647488039055`, 0.19308169402125722`},
 {0.2034753024853403`, 2.7863395292250592`, 3.7168431579559567`, 1.0150680060811876`, 0.5649574956583596`,
  0.4443788859910486`, 0.06710359282875367`, 0.04727087216660339`, 1537.3049723874492`, 264.87714036516047`,
  0.039914831594556555`, 2.4435728162566726`, 1.470321086577068`, 63.8035123300818`, 25.621783341090772`,
  64.53362921247144`, 0, 38.411457112787865`, 0.6179836156831428`, 0.6302839628809814`, 0.019904002121870823`,
  0.04449265599998944`, 0.04464517445704493`, 0.003427946784195779`, 24.5987453827338`, 0.1293308091138885`,
  1.0814293696941368`, 0.0005267269362211335`, 0.000533898448482878`, 0.013615237362255783`, 0.08708028712263004`},
 {0.06436950144190184`, 1.0629347664880244`, 4.764095504810017`, 0.9320606212428386`, 0.7789254637724665`,
  0.15639368410950716`, 0.08900209182216046`, 0.013774341985629202`, 2340.6363425632753`, 63.84867242722913`,
```

0.2371034531227812`, 2.23106662065706`, 0.9122412114799938`, 266.56397909713576`, 11.291992844613786`,
312.8291448967664`, 0, 222.51224588683934`, 2.6963229510624567`, 2.7080360060971014`, 0.004344084609756882`,
0.14660331700342102`, 0.1465943033211909`, -0.000061483480826907 13`, 40.94307723376964`, 0.13481117750344462`,
0.7352949544874028`, 0.0018999737399216787`, 0.001905996317030538`, 0.0031698212361122025`, 0.011468320253314766`},
{0.24480551289378455`, 2.7380941448464746`, 6.271736317361168`, 0.9720924783254401`, 0.03239591203313119`,
0.6916885160816673`, 0.2288933066843994`, 0.006623283304233729`, 2475.282531422873`, 397.0207555711145`,
0.19280932100568626`, 5.189856377538095`, 0.933411035896893`, 89.19156172698784`, 25.48061376875532`,
97.19323178901861`, 0, 81.42993686911326`, 0.18953783502742186`, 0.1921864297063887 5`, 0.013973962921881578`,
0.016680873721598252`, 0.016708803412607446`, 0.0016743542020243307`, 7.413891947363724`, 0.05833671209903526`,
1.1445834218264574`, 0.00020658557864261518`, 0.00020825442121035886`, 0.00807821426214228`, 0.3652106251275175`},
{0.14578019322843683`, 1.8234631063666207`, 5.479118994270218`, 1.0733190277156792`, 0.6408034729339063`,
0.35566594296423915`, 0.17953675903981858`, 0.2394318445417401`, 2582.269870386629`, 303.8771057624848`,
0.24431388570309442`, 8.496328107267644`, 1.3584441574118422`, 305.0067820136813`, 53.567936901086874`,
320.43076936459573`, 0, 70.67986657375162`, 8.657874512524085`, 8.725043360515059`, 0.007758122145777202`,
0.04411020359343769`, 0.04412916940543402`, 0.00042996428153307953`, 225.53306790199318`, 0.09186277147427292`,
2.240611956361848`, 0.0004963218561782057`, 0.0004979446615217199`, 0.003269663270544232`, 0.20625520988041743`},
{0.18481151841666188`, 0.509983650803119`, 2.442367304149805`, 1.4454403198705184`, 0.8862858075507192`,
0.20520962400760967`, 0.08831882715621944`, 0.028416610934367554`, 2887.1745513215255`, 96.99849051015605`,
0.012592247838918769`, 7.919739870540438`, 0.6672717811625197`, 695.816730024459`, 46.09727170063247`,
838.0726467850207`, 0, 494.4502951030173`, 24.14895464490511`, 24.24828516471352`, 0.004113243047949577`,
0.32599054689616735`, 0.32587817759477433`, -0.0003447011039520165`, 175.93674361268108`, 0.8606686851623686`,
1.5586922398375018`, 0.0027219967157514624`, 0.002733924798252666 6`, 0.004382107602180385`, 0.04094604292628066 5`},
{0.09189072955199334`, 0.5859834843121772`, 9.928586025072377`, 1.1393198615050828`, 0.6662151934740392`,
0.2733768925781913`, 0.07354107739402921`, 0.00787232194478042`, 1945.927425117371`, 381.272657803446`,
0.10334632248372438`, 1.3094920948276538`, 0.643540323804628`, 24.236039348160233`, 9.2325707114852`,
25.661843313661624`, 0, 21.7677212327486`, 0.2579373457854825`, 0.26144871151304794`, 0.01361325060111973`,
0.013291097365224729`, 0.013316435435178561`, 0.0019063941266526108`, 2.159243208823024`, 0.01744755190624741`,
0.27402846859625957`, 0.0001404902382274864`, 0.00014167230409601518`, 0.00841386478834738`, 0.03527358036214791`},
{0.1929089042890706`, 2.044772709758891`, 3.6659878682330156`, 0.820711070285316`, 0.988447409153429`,
0.31657524049709074`, 0.23150398070670547`, 0.04424265567984524`, 3720.3695113921785`, 173.54302693598845`,
0.04329067746612414`, 6.852489239335057`, 0.8460112403464835`, 747.2409726127319`, 51.38438838621834`,

857.5771963604986`, 0, 457.9106622291771`, 9.557163496049307`, 9.578729680413227`, 0.002256546555139849`,
0.1532008256298812`, 0.15314412145957`, -0.00037012966528127134`, 279.17467284893576`, 0.4221971915487851`,
1.0840277982584747`, 0.0022532652176217027`, 0.002260658093181315`, 0.003280961114473513`, 0.04264449171132139`},
{0.10029172339419551`, 3.9666265224186326`, 5.94698722721138`, 1.3749912659713441`, 0.4161641564514196`,
0.2013069921355808`, 0.0801151159349077`, 0.014791692046983768`, 2279.1361385342816`, 360.73732873948904`,
0.013735250572300073`, 8.514487693936715`, 0.49310119875702574`, 147.57752185455973`, 36.2220588156515`,
167.27696015867048`, 0, 121.95920702875009`, 0.4420553088955077`, 0.446441171035797`, 0.00992152351081943`,
0.02513901614237118`, 0.025148247216557073`, 0.0003672010922628477`, 25.049547323441203`, 0.0360176464764415l`,
1.3364348962448545`, 0.0002209307317978526`, 0.0002215023191797975`, 0.002587179145669305`, 0.1607264754678864`},
{0.2302575021554365`, 0.7722117915953741`, 6.903211159271258`, 0.7585731891993218`, 0.822213946918968`,
0.5878727883839281`, 0.2427267432025807`, 0.09976117360743081`, 2459.4926457112624`, 40.66955655789843`,
0.10301492463748846`, 3.6967733258702205`, 1.4430046145797544`, 1383.068436532143`, 44.75885676762198 5`,
1497.1769569560681`, 0, 571.4466410786403`, 67.36546580344341`, 67.55283398875264`, 0.002781368510921034`,
0.25816050500753895`, 0.25789495915127697`, -0.0010286075953184204`, 743.148671996199`, 0.849191329117432`,
2.745145910285414`, 0.004104650763908646`, 0.004118919295479808 6`, 0.003476186499622002 7`, 0.0338834029246803 8`},
{0.22600110400418738`, 1.9843308027955775`, 5.115418048953915`, 1.1683391486965746`, 0.6057502933993939`,
0.6602202205935461`, 0.11123798530174628`, 0.4580159171910898`, 500.4249678579999 5`, 188.2858250407997 5`,
0.18938415994147217`, 1.0587108294757588`, 0.29531889665054534`, 50.087172418575804`, 45.024438779943296`,
47.07704529271664`, 0, 7.1340630112853045`, 1.4539813003395283`, 1.4804295099129285`, 0.018190199259938256`,
0.06675343130994808`, 0.06744969819300299`, 0.010430428359884836`, 41.21685544217847`, 0.21551927388735706`,
0.62550371308623`, 0.0006745825686657048`, 0.0007028807031653339`, 0.04194910425213272`, 0.09942703573935491`},
{0.0717975612264255`, 0.4997816218149955`, 9.589567616976403`, 0.9450897781833669`, 0.6186434889471488`,
0.2554640898762033`, 0.06713444953336731`, 0.20940488388432735`, 1744.4191807628713`, 383.9973692232637`,
0.15609378466736462`, 9.862078934227451`, 0.38202361199438517`, 178.81819623110457`, 75.4036804807058`,
195.34524036938737`, 0, 46.13993005924407`, 16.29727592392527`, 16.464711526494234`, 0.010273839833757847`,
0.011215508597043889`, 0.011217220550161723`, 0.0001526415947186166`, 116.35827132036908`, 0.011503516645440854`,
1.8109525878967734`, 0.0001435143851219456`, 0.00014370034211156097`, 0.0012957376325541947`, 0.2474866861050225`},
{0.26375231265942045`, 3.5837756528213704`, 7.490302795205832`, 1.1022035672057404`, 0.22095637191261486`,
0.15013919278259136`, 0.12602227786755316`, 0.062932127995949`, 2037.490516700962`, 396.7515615806642`,
0.09025977921836642`, 2.8179238772656654`, 0.8710125696526174`, 44.25672041398656`, 10.016708948393898`,
48.79866951717266`, 0, 23.524899739643846`, 0.3958584489034092`, 0.4014004459969777`, 0.013999446468038482`,

0.014477664839408568`, 0.014486293241809148`, 0.0005959802562285343`, 20.26668387348099`, 0.05455025119001284`,
0.5544708164179857`, 0.00015865103257073354`, 0.0001593544474764907`, 0.004433724094695446`, 0.07318065725908332`},
{0.048513291302257844`, 0.4457687353343176`, 2.3881995464771766`, 1.1956444252802232`, 0.6250208921524281`,
0.23033921635096732`, 0.16695818246187422`, 0.0058846456882393735`, 1714.5960674937287`, 267.0960709959734`,
0.21708290835986865`, 1.1611050275443535`, 0.4203748665144109`, 37.261193146812424`, 8.072532492886786`,
44.30128803849542`, 0, 34.19780755781158`, 0.3877420815443633`, 0.3921626180993675`, 0.0114007139421064`,
0.09978653155541332`, 0.10040469503071148`, 0.006194858821752636`, 2.4691899617989503`, 0.06915675819128295`,
0.20933876412967428`, 0.0009999437211769546`, 0.0010212409774209216`, 0.021298454895941177`, 0.027655321990792715`},
{0.14659244900446955`, 3.2113358110509322`, 7.247014044575871`, 1.1315336622196723`, 0.22569695706644644`,
0.6068766796842944`, 0.10140090760989762`, 0.07092703696530009`, 1627.6855361153575`, 309.5759062179927`,
0.23915839319989673`, 9.196051471747733`, 0.7212967644528518`, 193.18999166890407`, 76.59283288843133`,
200.97527593291045`, 0, 97.24979051932904`, 2.0453016684228835`, 2.074194517020885`, 0.014126448457004726`,
0.020569691901777105`, 0.020585980806273607`, 0.0007918885987345803`, 93.8307213172659`, 0.04307659301667212`,
2.7184043191744696`, 0.00021935036970943678`, 0.00022031178014688524`, 0.0043829898199942985`, 0.4978915392990795`},
{0.12214842072252496`, 1.1315944174857293`, 0.8186389490806132`, 1.4945075781385797`, 0.27658322947733693`,
0.6814994349474557`, 0.172591676587236`, 0.06293351186433137`, 2301.8386453341027`, 260.5275220937808`,
0.06414760022752092`, 1.8028968417694637`, 0.30689034634076684`, 45.074973104936646`, 11.691694249854823`,
54.635069429335495`, 0, 23.467769674142154`, 1.2151266128309632`, 1.2431487665107592`, 0.023061097818038112`,
0.2726876830814635`, 0.2816309560198894`, 0.03279676161887424`, 19.643292737398017`, 0.47583385484120777`,
0.27566463992402607`, 0.0021368965816732644`, 0.002321056898981545`, 0.08618120263175166`, 0.14412487817350572`},
{0.09472256798275819`, 0.9080295101270028`, 6.479473262813432`, 1.475083725476`, 0.4311907138108162`,
0.6630526571829747`, 0.09230911068175318`, 0.22906599144409456`, 1061.3916072436969`, 256.8481876635019`,
0.14505807910429386`, 3.6658722632692093`, 0.8887817463762948`, 103.626737530371`, 60.027417173693095`,
103.19955541675678`, 0, 25.4754101872236`, 5.586708159019208`, 5.68082607211192`, 0.016846756697102006`,
0.04023414426701258`, 0.04036420311479637`, 0.0032325491234672654`, 72.46994104081082`, 0.05444402093644664`,
1.6595829048305464`, 0.0003272343883202744`, 0.00033156477672580847`, 0.013233292588112011`, 0.22411893134343952`},
{0.08360100641365104`, 1.0333139170716557`, 1.9968821416355915`, 0.9506164337764446`, 0.3637549658684145`,
0.6013653723888159`, 0.09205612227237822`, 0.02498916600861499`, 2612.089754066442`, 275.94814368199616`,
0.06598092927631555`, 5.08476480449065`, 0.8697872625899152`, 121.95963648651829`, 29.837626959237948`,
129.27175820375865`, 0, 90.08338730598133`, 2.0110783056185104`, 2.0356014540525416`, 0.01219402962357008`,
0.07132145256238324`, 0.07159151359570015`, 0.00378653299413223`, 29.686788593092714`, 0.08517921733000995`,

1.3189133933254482`, 0.0009001870435694226`, 0.0009128646948457995`, 0.01408335230654667`, 0.18881546320587142`},
{0.14651792469342528`, 3.8182021256883862`, 6.121567760696932`, 0.9268713362782599`, 0.31845465027744546`,
0.3641583988116113`, 0.18264126532755154`, 0.03701030981443568`, 3175.4599520184383`, 92.90395359335434`,
0.14653570379145064`, 5.086657320457199`, 0.6916416664989691`, 368.94735387099666`, 18.06163513056803`,
463.73541529851053`, 0, 241.51064244719666`, 2.2900385093152513`, 2.297262935908014`, 0.0031547183872131512`,
0.06887904018794071`, 0.06890661620161241`, 0.00040035420929873666`, 124.9118557739393`, 0.14417162890296037`,
0.9412856330861468`, 0.0008972223219985143`, 0.0009004982021861946`, 0.003651135406867123`, 0.04983298054670219`},
{0.19531388174641623`, 1.6812423772528433`, 1.9285683163805027`, 0.9563894243653364`, 0.3037850003884799`,
0.25494604947946875`, 0.19624000618521847`, 0.006277091862366441`, 3495.8037135332197`, 160.73634765492886`,
0.21386501651912365`, 6.171699440629407`, 0.5167732609586244`, 251.99981869317352`, 16.916919541272488`,
332.49263564161004`, 0, 230.6548148974947`, 0.824295415658893`, 0.8289271569228446`, 0.00561903072122405`,
0.12678258656028576`, 0.12687872539829287`, 0.0007582968656456579`, 19.797719774013913`, 0.3537485588420251`,
0.6851666809311641`, 0.0015978597152876972`, 0.001609066618162689`, 0.007013696363809885`, 0.06446267094806707`},
{0.0992057581918559`, 1.9152747962131755`, 1.1385439717492112`, 0.8734081572386667`, 0.11569399031757222`,
0.6355935198684373`, 0.242859485933678`, 0.028089883137919644`, 3591.0701912926534`, 270.70715346890586`,
0.15233109233560077`, 2.1031889712747507`, 0.926279804309432`, 53.46067804880361`, 7.177219751352795`,
61.15158851035912`, 0, 38.04919419876631`, 0.532904514961366`, 0.539805852589501`, 0.012950420637054094`,
0.1202206534992196`, 0.12174454709392997`, 0.01267580528266099`, 14.580836947053022`, 0.17037972972443022`,
0.40819172790941655`, 0.0016371867729692013`, 0.0016967581778183478`, 0.03638644401036051`, 0.11114202715272714`},
{0.04636398495503513`, 3.7018219205829457`, 3.897884943302158`, 1.3406897199868504`, 0.4374398316223367`,
0.6128218100825888`, 0.1104350576389786`, 0.15302416148791886`, 2822.82344897813`, 307.2546204697094`,
0.07105282376647054`, 2.3240488998943984`, 1.1737510413014665`, 54.94390397151344`, 13.615443334741812`,
57.23515298949648`, 0, 17.651649246691175`, 0.6905312868993652`, 0.6996397364213929`, 0.013190495050451112`,
0.050675324866176666`, 0.05097138373012584`, 0.005842268692524577`, 36.51748363846318`, 0.033564428566916285`,
0.6443902067895659`, 0.00045288986029889955`, 0.0004609553176313723`, 0.017808871514919167`, 0.09536235397659545`},
{0.24799856399906778`, 3.665797094880114`, 6.286133411580671`, 1.3307944188010161`, 0.12338821047601245`,
0.6316889254705509`, 0.13912514557691014`, 0.04125136326689172`, 2913.271077872927`, 274.142281421658`,
0.16386049841393313`, 5.467517906165242`, 0.14206080539009291`, 79.08163567957024`, 23.45037664187552`,
135.48337504804874`, 0, 49.88367090829297`, 0.5454099342610883`, 0.5494439143682126`, 0.007396235113666272`,
0.019163559453558996`, 0.019202450389801062`, 0.002029421326257941`, 28.562316464757902`, 0.06789336036559766`,
0.5057338625298468`, 0.00017315238017245882`, 0.00017487004741967256`, 0.009199747962051`, 0.2738794821018715`},

{0.06486156480593103`, 2.9105746808401234`, 9.680954493261392`, 1.2128101831918736`, 0.9623327252795202`,
0.40985068402002933`, 0.2170918483208174`, 0.22870254244919774`, 3443.315648780802`, 310.5579520138933`,
0.21496605031805494`, 3.304326571943408`, 1.3905287372116648`, 194.81879614282727`, 30.116577678231423`,
203.61073813123878`, 0, 46.19922673040528`, 3.4882953058504085`, 3.5031679659721195`, 0.004263589753071617`,
0.0463080977512235`, 0.04634966641683646`, 0.0008976543548879778`, 145.04205709288075`, 0.04290879547633825`,
0.7389818353169553`, 0.00046084876978169653`, 0.00046285323787928837`, 0.004349513829756679`, 0.050875337026927814`},
{0.271295688290387`, 3.0581992057736915`, 1.212750414175316`, 1.3253429727133483`, 0.8276664257363009`,
0.6720258079110331`, 0.1884616475047557`, 0.2223467966408117`, 2524.6082917357207`, 148.45022765754868`,
0.13442291461399386`, 4.368234255778699`, 0.5211143399174365`, 369.6906579376342`, 58.15760257655526`,
424.4647845488239`, 0, 88.47520622348394`, 6.230207459066354`, 6.29179627813131`, 0.009885516569007713`,
0.5735542631684568`, 0.5762348016917629`, 0.004673556968259884`, 272.18879290174397`, 2.2228971228306618`,
0.8277319867391356`, 0.0051805540798235405`, 0.005303648293274622`, 0.023760820088818413`, 0.0928642396710543`},
{0.19444530138658833`, 2.174470015755692`, 3.8344558373626527`, 1.2294570245280045`, 0.29239376697066977`,
0.28694291912822245`, 0.19808018049330495`, 0.005960265699843253`, 3278.3081237334245`, 293.3280672381975`,
0.14107283306589785`, 3.3795768777624478`, 1.417577504179854`, 88.25008961019421`, 10.09618028321883`,
97.33372987102447`, 0, 81.12350450764909`, 0.213370847440684`, 0.21596259251283864`, 0.012146669065815852`,
0.0523480932260287`, 0.05244565180116919`, 0.0018636509780642907`, 6.62812157137358`, 0.145412010919246`,
0.7987444098279379`, 0.0005129015878310161`, 0.0005175623510350764`, 0.009087051618946962`, 0.1062386776293008`},
{0.2440718787450452`, 1.76561180349694`, 4.95225717475204`, 1.4224475381097175`, 0.8297144603659123`,
0.6870441812099664`, 0.08604894661393966`, 0.28377285612513553`, 3055.531967355695`, 255.76266261457465`,
0.07376742267862452`, 5.8076740025606295`, 0.30546127533463907`, 186.0388027623724`, 65.38601662135984`,
216.66786803934662`, 0, 37.3420138531296`, 5.660620602110749`, 5.69071816881355`, 0.00531700829615378`,
0.057544887033955695`, 0.05768367852425067`, 0.0024118822270531304`, 142.77797928863845`, 0.20064412415090027`,
0.7287512538372246`, 0.00048621819798277954`, 0.0004915998117638079`, 0.01106831007838771`, 0.14206804544670973`},
{0.0730994714406894`, 2.8447930433832704`, 4.637114941779341`, 0.8747237021104985`, 0.8742653369550875`,
0.4602914635394686`, 0.05825966522387901 5`, 0.01073521329241528`, 2525.027865177576`, 85.21306353882699`,
0.10502971061086047`, 4.901287944789633`, 0.3817190308545939`, 362.2652554142097`, 54.74396740961853`,
471.44486052946394`, 0, 313.8641521367833`, 1.1524971927729037`, 1.1568170462039984`, 0.00374825505709131`,
0.08742197368813368`, 0.08748794358771113`, 0.000754614621408578`, 46.8373713788443`, 0.09129285812731773`,
0.9378561846644692`, 0.0012053388254725794`, 0.0012113850144449174`, 0.005016173746803076 5`, 0.04631543022316267`},
{0.21011190738341773`, 1.8519766611225768`, 0.8172862439758575`, 1.0025174016275906`, 0.4801024655035677`,

0.5883298648617231`, 0.199167083104676`, 0.2995026554507827`, 1201.2334950278255`, 265.10269406466296`,
0.16527637611813623`, 2.4641537957227406`, 1.185487228781699`, 90.95112028255596`, 35.29657767772614`,
92.36517754580426`, 0, 17.854220261169864`, 2.621906076218908`, 2.684980257078891`, 0.0240566134050626`,
0.2768205194962166`, 0.28072667788608713`, 0.01411079784468039`, 69.3672694401841`, 0.830904105060327`,
1.0558284733943601`, 0.0032574762158241732`, 0.0034322557861044796`, 0.05365490296790565`, 0.17593560883044532`},
{0.13811436070524757`, 1.6573442876198152`, 8.270384729474788`, 0.8471995866439316`, 0.9496826491555777`,
0.3970545948223342`, 0.06715784938015576`, 0.0832034730885496`, 1059.2542132237509`, 302.271366930348`,
0.038752991973303785`, 7.756453819144459`, 1.2408044408349754`, 241.27260390514454`, 223.92718215625504`,
240.74613934789653`, 0, 112.03980918107318`, 5.234694156258995`, 5.310066836704672`, 0.014398678928654318`,
0.01999325196769876`, 0.019999583572236235`, 0.0003166870776050157`, 123.93843510446689`, 0.03944793162779667 4`,
3.3684933375587924`, 0.0002851620292083634`, 0.0002858463687622132`, 0.0023998270588463377`, 0.2274928003116164`},
{0.13575534771417241`, 3.107967888976927`, 0.2262387959194534`, 1.3197095272988466`, 0.6342015998603714`,
0.3957050275427402`, 0.21335490357997616`, 0.008750656688013836`, 1614.3899114822752`, 338.63558488350077`,
0.10329242712159703`, 6.6095853898508246`, 0.24958476272321328`, 204.5497642027175`, 70.7247047016965`,
243.04799088873335`, 0, 178.93247724109062`, 0.4896931123599614`, 0.5006497229665389`, 0.022374442952188378`,
1.0987933235593623`, 1.1151756662851828`, 0.014909394127689657`, 21.74214955239897`, 2.1309581386544694`,
1.102816920755234`, 0.009825076040643088`, 0.010396119839029045`, 0.05812105636879927`, 0.32124116547915516`},
{0.04766948135443208`, 3.0149795060421347`, 8.932446832206747`, 1.1136088500069867`, 0.021594711979614933`,
0.15138413756352542`, 0.08685938210991187`, 0.005007564365564 6575`, 1362.2649899849648`, 276.8149684326663`,
0.20202028836193903`, 3.9701409072362193`, 0.8085370569094716`, 74.44291054040077`, 18.811622823186667`,
81.47755159143661`, 0, 69.45118327483569`, 0.11088127419543288`, 0.1127692204657247`, 0.01702673678663036`,
0.014666007516982008`, 0.014670973453146658`, 0.0003386017741295877`, 4.775782418615267`, 0.009987442455359664`,
1.169750436548314`, 0.0001591822666687781`, 0.00015953668960471364`, 0.002226522736185288`, 0.10465194871589364`},
{0.18773746739616126`, 0.4299564973161223`, 3.9318971489849446`, 1.0624860251059998`, 0.544093066833734`,
0.43356539469874333`, 0.16330330819292005`, 0.040590232235511496`, 2399.820966791748`, 233.03213128875973`,
0.16697913284338012`, 7.094059719533927`, 0.9913896416500063`, 279.33512230773266`, 47.057304068541846`,
302.47168984720423`, 0, 177.3261312326786`, 14.246302841877029`, 14.354766571225667`, 0.007613465090031024`,
0.06667678168595527`, 0.0667410027821284`, 0.0009631703053036844`, 87.50414956568821`, 0.1788247161119522`,
1.8795604497768539`, 0.0007566841352966147`, 0.0007611007474802132`, 0.0058367976511986`, 0.1722227957084854`},
{0.11479403264996152`, 3.823640197501849`, 7.042467218439598`, 0.8671713775083026`, 0.2915521094222331`,
0.3428757834262214`, 0.1114957061871538 8`, 0.0062188234292322`, 2055.9308370344506`, 383.5883910698325`,

0.2126209830320115`, 4.650760077469522`, 0.9718780818420139`, 80.89022177474669`, 24.127923255354673`,
85.5487589927382`, 0, 74.27518336339435`, 0.11688137477031861`, 0.11865450583747973`, 0.015170347462506006`,
0.013430215660637509`, 0.013439553358205337`, 0.000695275325711675`, 6.384461755872414`, 0.022024408786325434`,
1.1215722882229573`, 0.00018697456618987918`, 0.00018769852012315822`, 0.0038719380289609084`, 0.17311513584156038`},
{0.1495588092693269`, 2.9562247150199887`, 9.2229842597602`, 1.0688736942010013`, 0.2958780650578341`,
0.6794653079911299`, 0.137246604211619`, 0.025056259065215164`, 2160.2080931306828`, 208.1776104802966`,
0.2306160093069518`, 8.141052229334282`, 1.0329085826709994`, 279.99167955853324`, 58.18726173571833`,
300.3704731060498`, 0, 206.81828461272863`, 1.6896329687364922`, 1.7071728839865916`, 0.010380902583367435`,
0.024998939155944633`, 0.025016177828755166`, 0.0006895761737326644`, 71.35621059273456`, 0.05341159390221999`,
2.834939479684725`, 0.0002823223511086992`, 0.0002834044789198234`, 0.0038329512589938552`, 0.30490058138382287`},
{0.15518171148576348`, 2.3499047639493327`, 4.360850342323632`, 0.8133940186135149`, 0.676314303847662`,
0.37760044288930084`, 0.07941455326257985`, 0.04333405133254413`, 3294.797232932802`, 200.86295390407247`,
0.24389739709186076`, 4.759995129088669`, 0.4543298663517916`, 160.50239867324962`, 25.148473298680738`,
193.2241324224549`, 0, 99.30136112945125`, 1.7665241081297627`, 1.7752117854025913`, 0.0049179500199554305`,
0.03941801971615287`, 0.03945339950194759`, 0.0008975536074486978`, 59.302334533221114`, 0.08738508232747373`,
0.6572969392588821`, 0.0005846040657913054`, 0.0005876482248535631`, 0.005207215003093069`, 0.06232027308182183 6`},
{0.11690059278696574`, 0.8783363086389482`, 5.7539466958675`, 1.1072974777335425`, 0.9679537377157941`,
0.4053342552984798`, 0.06890031093641119`, 0.23033316548333493`, 3511.029547228316`, 369.67514830597565`,
0.18966854703112757`, 4.698501468560957`, 0.7333804491317486`, 112.57135671494616`, 42.811552329780966`,
120.05070713884942`, 0, 26.81345587029441`, 6.324394475761433`, 6.372601248894629`, 0.007622353937274395`,
0.02889313231281016`, 0.028926248834625256`, 0.0011461727810111455`, 79.35636140309792`, 0.048251775640543455`,
0.7754693759440615`, 0.00031473958917827005`, 0.0003164581546737781`, 0.005460277494785171`, 0.08221663767049586`},
{0.104439705960304`, 1.3659305120838559`, 9.102673754552008`, 1.4903475140374016`, 0.6812753308651178`,
0.23307188642089138`, 0.0933185162696803`, 0.15281827719772503`, 2644.042120762095`, 355.84712203695506`,
0.219313267777153`, 8.034413215209995`, 1.3985960311786072`, 192.9803646189174`, 41.440463336438`,
200.84930868390364`, 0, 61.97659289550224`, 6.383289366320609`, 6.452116880484416`, 0.01078245246517473`,
0.02467350926715593`, 0.024680972311029607`, 0.0003024719261810471`, 124.55899589882492`, 0.036812772183844746`,
2.0122015216202573`, 0.0002001268649590715`, 0.00020054118333426508`, 0.0020702786468889123`, 0.15327390704151653`},
{0.06252530163633607`, 1.277236277016332`, 7.142814422389127`, 1.3604161019492071`, 0.8603611689699608`,
0.477237284782289`, 0.23913503177709117`, 0.03184426252613322`, 3236.2308449191105`, 49.142351256039206`,
0.06457622252014433`, 7.960619025437833`, 0.9912633563842763`, 2534.2122488904156`, 70.16910649998678`,

```
2881.8361501779445`, 0, 1741.959226703144`, 41.11396559319216`, 41.17104449778879`, 0.0013883093925164403`,
 0.3809029592942069`, 0.3805381770207399`, -0.0009576777091544475`, 750.1749763946615`, 0.34022960605820546`,
 2.544644618109399`, 0.0033794616058058224`, 0.0033871963362601797`, 0.002288746361571059`, 0.03884485120201967` },
{0.05927125459941385`, 2.080731116332318`, 1.403515084093046`, 1.4338880552058586`, 0.836296749585115`,
 0.24670417168146108`, 0.17625476342874957`, 0.3553142034940628`, 3624.424617443623`, 371.6887670168725`,
 0.06984462665929475`, 7.678587831183411`, 0.5475538898722119`, 256.3552528487764`, 37.00329580109272`,
 294.0566953529251`, 0, 42.776682146957874`, 6.938516540357911`, 6.970385932416283`, 0.004593113221392997`,
 0.21363237879945188`, 0.2138025041636399`, 0.0007963463457369979`, 206.24553238155997`, 0.18088941592187793`,
 0.7803168869845066`, 0.0017937656142992253`, 0.0018078290977108522`, 0.007840201250106649`, 0.09035018027648788` },
{0.272219924072492`, 3.699843372922853`, 0.8152026115407764`, 0.8049313533010627`, 0.8541230874020713`,
 0.3759570960402523`, 0.18901354163393813`, 0.05512718200659539`, 2420.333744013932`, 22.324560553991944`,
 0.136832191006448`, 5.284550340747209`, 0.8839580209731261`, 2954.1077207898497`, 51.52886492451052`,
 3564.5778139342324`, 0, 1643.8615989608522`, 24.008080344350248`, 24.16657974407832`, 0.006601918914578064`,
 3.530424578081461`, 3.4454922713279026`, -0.024057250020538046`, 1268.9448136949115`, 13.729313008412289`,
 2.107410213822374`, 0.05167975882710418`, 0.052115429147984414`, 0.008430192608633869`, 0.025159079351688155` },
{0.2192562547626593`, 1.328996578641208`, 4.408276687847465`, 1.066557441081671`, 0.8788419772651497`,
 0.420358813032901`, 0.18871280922209183`, 0.12866248281712975`, 2376.751601415135`, 21.932515037813403`,
 0.11160054269697284`, 8.38604448753847`, 0.8324170692270734`, 4953.548857225716`, 95.843589810574`,
 6030.021497104598`, 0, 1746.170980441006`, 160.29771137300943`, 160.5217580041764`, 0.0013976907670605332`,
 0.9005728404283916`, 0.896546743537153`, -0.004470595503772157`, 3043.3587139820793`, 2.8208032590490575`,
 3.2063538652238455`, 0.010160387250536251`, 0.010183663388907024`, 0.0022908711840232687`, 0.024575018964286675` },
{0.054226264658999324`, 1.41058877238651`, 8.629501826749408`, 1.1478067099640077`, 0.4503418362410423`,
 0.2944196840300318`, 0.21119418614347968`, 0.01982525406608443`, 1227.9750083569616`, 89.64728336361748`,
 0.015407425530496982`, 1.3479803050296402`, 1.4374405820845841`, 130.9698276070016`, 12.258825763192185`,
 144.39059776147369`, 0, 102.12402453056289`, 1.3552409681492303`, 1.3666432212449418`, 0.008413450717389859`,
 0.08090174733888543`, 0.0810432286125256`, 0.00174880368216912`, 27.309824193564687`, 0.06267142232247151`,
 0.9337920430037282`, 0.0008491061345554662`, 0.0008556882653333572`, 0.00775183514760136`, 0.03569849143398065` },
{0.12963957180850916`, 3.3365224852422513`, 8.071825011712662`, 0.9114075438466118`, 0.39364722797662766`,
 0.55978745554174`, 0.07369835156013937`, 0.12865812035500993`, 1440.5511589977796`, 134.4701033570321`,
 0.035366553272213386`, 5.503562356456834`, 0.5009896530595863`, 246.36788041780926`, 58.179829850017796`,
 275.2736611973459`, 0, 88.0060140913561`, 3.2522910335467565`, 3.278700504497161`, 0.00812026681437672`,
```

```
0.03169630450731551`, 0.03172084474613374`, 0.0007742302832991488`, 155.01917374257857`, 0.05870136206050019`,
1.9284747422225206`, 0.0004195610124623217`, 0.00042148492845319054`, 0.004585545209689013`, 0.1572821137215756`},
{0.16726133018607092`, 1.930958758925592`, 3.0083121233683894`, 1.210686290991208`, 0.7910212105934074`,
0.3434585725088355`, 0.1642660591272488`, 0.14345094159717983`, 3630.780310378209`, 105.55549533534378`,
0.20790081387395154`, 3.589057052449082`, 0.2001887825986941`, 176.8164574292167`, 19.279101869018422`,
427.70039949201714`, 0, 58.02499695407872`, 4.141010883045535`, 4.157075534810627`, 0.0038794034159304047`,
0.12395693919468603`, 0.12417644154264759`, 0.0017707951598968652`, 114.2303033631854`, 0.29618860764987875`,
0.2643790405699665`, 0.0012329328198724676`, 0.0012446092416786491`, 0.009470444470274675`, 0.03257888512720775`},
{0.08241763840187705`, 2.142561606833244`, 5.369049459923637`, 0.9852636250226738`, 0.24182715902115692`,
0.28976691554543155`, 0.24065607285215418`, 0.27404651530612695`, 2847.588493754236`, 186.1353278207099`,
0.17363556899862892`, 9.029927532666129`, 0.11169436731877491`, 128.62837239441455`, 30.659763015580115`,
435.7632168841241`, 0, 26.733358843410333`, 3.2225775716648237`, 3.2415040077005677`, 0.005873073840691356`,
0.016399500137949188`, 0.016406279145792565`, 0.00041336673595848694`, 98.63672828701381`, 0.019308686747703613`,
0.7240969065090918`, 0.0002010803187146859`, 0.00020163628944344444`, 0.002764918676836814`, 0.21525337062667502`},
{0.13700552002384053`, 3.065949790291736`, 4.950454801095971`, 1.0391958372853642`, 0.9776337698753284`,
0.6909746470667117`, 0.057475002733817115`, 0.00904662991314958`, 1580.3042677972962`, 74.3483640784055`,
0.03877364360478597`, 4.297289530434117`, 1.330917817336955`, 479.13410638403354`, 140.80814486393734`,
500.5508414554062`, 0, 424.06128465659816`, 1.211643955821506`, 1.224022754170985`, 0.010216531259041428`,
0.146600881993935`, 0.146806151741839`, 0.0014001944948223155`, 53.0691361751313`, 0.28693042962190096`,
4.047330549284514`, 0.001700338112894273`, 0.0017117130583090428`, 0.0066898138249738`, 0.07173452665643489`},
{0.05241322492447886`, 1.041476241641588`, 8.16095761491868`, 1.1603610389656813`, 0.26866448983520885`,
0.3320545378754204`, 0.11976413567707805`, 0.00713319584941315`, 967.2404027877401`, 264.4900675349543`,
0.2287367563952014`, 6.379237490331231`, 0.83403545199883`, 165.50614020506922`, 68.50872159503166`,
173.09482512755613`, 0, 150.26280733929295`, 0.9481359884915196`, 0.9639506246144862`, 0.01667971294721937`,
0.023141067923152684`, 0.023153373033836185`, 0.0005317434236122853`, 14.106587226561118`, 0.01732711425783239`,
2.5866339743916837`, 0.00024083004603714375`, 0.0002416116158852633`, 0.0032453170232713546`, 0.29388507796853325`},
{0.18364277582298344`, 0.43366671305956395`, 6.703835279617622`, 1.4870472910528665`, 0.4047782007506182`,
0.5684293651673247`, 0.22561255273502645`, 0.1213725120163492`, 2694.706223544171`, 135.0368961894007`,
0.022200225329951195`, 8.985808481864087`, 0.4546405408559282`, 520.021041298101`, 52.48460100928635`,
670.1012123653221`, 0, 190.95933654026285`, 45.692725438237204`, 45.837032115368665`, 0.003158198066484763`,
0.08047976228567001`, 0.08054610892441902`, 0.0008243890993802072`, 283.07734359333546`, 0.2111360991963842`,
```

1.3636973898444713`, 0.0006526854322680409`, 0.00065607579615473`, 0.0051944837728457O4`, 0.14255713511605897`},
{0.17373830102452775`, 0.5396236269330235`, 8.716548818231772`, 1.2781997926114008`, 0.2994029165619403`,
0.6311279153766021`, 0.2004572873058531`, 0.029829077121486105`, 1206.6484556475298`, 212.29803760588413`,
0.13764513912866333`, 6.42052885239311`, 0.6565129788897008`, 241.3341426867935`, 78.66650036568984`,
262.98366239761754`, 0, 169.783412082221`, 8.198690060425296`, 8.280141876004219`, 0.009934735302665754`,
0.0345489665438071`, 0.03458665326436163`, 0.0010908204882698058`, 63.20295523580615`, 0.08574969642104273`,
2.3030505222693574`, 0.00032580328101472 49`, 0.00032773059879133334`, 0.005915587377161247`, 0.3388667553453987`},
{0.06373356464631308`, 2.4033771734928857`, 7.841805250818684`, 1.218475494212359`, 0.1766490969017389`,
0.5314535158814123`, 0.18470611278961951`, 0.013035445084968872`, 3902.45780595225`, 188.60058905321694`,
0.11342781551209663`, 2.042548476018199`, 0.8129410978823182`, 67.71824218904842`, 6.108572743647684`,
80.41544939925379`, 0, 57.06147397362041`, 0.29885945491330457`, 0.3008678027558085`, 0.006720041174827607`,
0.03222074536657963`, 0.032311799519398804`, 0.0028259480587193853`, 10.261028457445192`, 0.02933632796817983`,
0.36717886161619`, 0.0003181552158559864`, 0.0003213148840757597`, 0.009931216155838696`, 0.05064247028289126`},
{0.0964110511384016`, 2.2365286327741067`, 5.8526191264605`, 1.4618699408667182`, 0.8182028658978528`,
0.387343137712196`, 0.24955267111553703`, 0.1017336334795354`, 1300.5591312646511`, 204.02951635694023`,
0.010778216795093748`, 2.376136268071548`, 0.7014041691125024`, 179.17668570936544`, 39.839762606355215`,
191.03004065471043`, 0, 73.34740428330163`, 3.203276989472508`, 3.2212904572148853`, 0.005623449923805479`,
0.11776550045437978`, 0.11806222754336448`, 0.0025196435954488727`, 102.34601008088187`, 0.16219850980910802`,
0.7250265168040482`, 0.0009680954322925928`, 0.000979204692165645`, 0.011475376809436888`, 0.051176411665160196`},
{0.22055192958492525`, 2.063754319806727`, 9.746892660619345`, 1.1263743900346668`, 0.3824619170868737`,
0.3377396468040734`, 0.07248726297246674`, 0.022971440771960824`, 3783.69159517103`, 308.71982775738024`,
0.16114587515785966`, 9.879452049970482`, 0.6820463020022816`, 194.62257129955546`, 30.79907367502361`,
217.7232135124382`, 0, 146.82440213453074`, 1.5647658374524147`, 1.577558621760038`, 0.008175526332074856`,
0.014284058935901121`, 0.014287878450739814`, 0.0002673970232014433`, 46.13274652183443`, 0.04500538229455161`,
1.4968470738473105`, 0.0001533106325382061`, 0.00015360585048135707`, 0.001925619497247899`, 0.17051908620789302`},
{0.22700143296888314`, 1.2844277954779395`, 7.271913694665059`, 1.375337551210531`, 0.5655186709000168`,
0.37705025836034534`, 0.20454803556589107`, 0.08200704828690611`, 929.9638116909423`, 312.52970459059486`,
0.17194018780473708`, 2.7869672276628847`, 0.6271449892742724`, 92.49644187668702`, 44.238962657447324`,
96.80445116019284`, 0, 43.02329209187011`, 2.5482747354946174`, 2.579019724205654`, 0.012065021201518533`,
0.03925762554044924`, 0.03933621620088O555`, 0.0020019208841435265`, 46.75821286833552`, 0.1273076750374853`,
0.9511720729434662`, 0.0003432731152935853`, 0.0003468630522013 6464`, 0.010457961162233964`, 0.1300400698257603`},

```
{0.18044570897271706`, 0.9524926761671173`, 6.511131206394843`, 1.1225857366708927`, 0.011276794855722416`,
 0.6477197715834044`, 0.16181965045393948`, 0.013358577208209321`, 2629.2386884676316`, 244.22468112889408`,
 0.24315714767663638`, 3.8935447375778236`, 0.17056295407521116`, 64.20777104657775`, 16.970156348894278`,
 104.15072584279031`, 0, 53.901703693004805`, 0.6989520310361109`, 0.7044259386881585`, 0.007831592740253024`,
 0.017789631840592138`, 0.01783187156250249`, 0.0023744011280757427`, 9.510667007914675`, 0.04585803899770852`,
 0.4151832562772966`, 0.0001903867384495106`, 0.00019248618716274468`, 0.011027284412411076`, 0.19888262876501572`},
{0.1031747291007129`, 1.0753817850602383`, 2.3901827825214443`, 0.9494324054212877`, 0.8655243169054956`,
 0.5875365461047798`, 0.09875542522403158`, 0.020636236338079122`, 3668.9306940550296`, 32.944367358953286`,
 0.21955795791262667`, 5.455907508975919`, 0.3138297355806803`, 931.5929302338438`, 49.42988267080978`,
 1740.933134942887`, 0, 718.6240777176369`, 12.936203838258251`, 12.962912263888613`, 0.0020646262198940057`,
 0.4255028019687354`, 0.42532849871012296`, -0.00040964068345961113`, 198.73368536413224`, 0.6271590903532647`,
 0.6103969693299662`, 0.005398245052307349`, 0.005429272793621827`, 0.005747745982968233`, 0.029673175557081884`},
{0.2492174803391337`, 3.8186304877300854`, 0.6593165104089422`, 1.3669144880495308`, 0.40531282588620576`,
 0.25181467777873967`, 0.15220457771402102`, 0.006740465790302422`, 796.5961892440373`, 51.67728195440782`,
 0.07309861388864569`, 3.0773122735176646`, 1.432188704910093`, 465.1364883798607`, 40.16771912361044`,
 506.21173396746093`, 0, 416.17402843033307`, 0.7153337190479022`, 0.7354400065053147`, 0.02810756283678839`,
 1.9300595234520717`, 1.9301119043431862`, 0.000027139521076O2502`, 39.022787835109526`, 6.871493876275327`,
 3.567936576694838`, 0.016828377215221124`, 0.017423222459633365`, 0.035347748437336346`, 0.08786399879814265`},
{0.27928795263916784`, 0.5798885963915357`, 3.438729573463075`, 0.7859704525714069`, 0.13732274355578267`,
 0.6009735798147406`, 0.18539967791007028`, 0.1369068620522905`, 2588.2655297569117`, 78.78627673888747`,
 0.13662571551540947`, 7.147737978252394`, 0.16582779201112174`, 266.01875824350225`, 34.19428690603585`,
 683.343810573242`, 0, 90.39445384471452`, 18.887989100385322`, 18.95927419778893`, 0.003774096703716978`,
 0.05328676687915299`, 0.053350707251940126`, 0.001199929673574296`, 156.47042125830103`, 0.21260502892054126`,
 0.7455777899385185`, 0.0008162672904363788`, 0.0008225441287589338`, 0.007689684979535816`, 0.15486252974704307`},
{0.158309183474712`, 2.284367344540179`, 3.1130137774580398`, 0.8245076604975312`, 0.2939127991946775`,
 0.5448027938340341`, 0.15596740404331277`, 0.060580071785009876`, 1917.5874264102922`, 102.41199828758255`,
 0.15653233667838135`, 8.020771066756815`, 0.16368718495509893`, 312.44245453281894`, 57.02310506220705`,
 625.8741338703402`, 0, 167.89187254906838`, 4.291439326571326`, 4.309696986916792`, 0.004254437487306362`,
 0.0646603149913195`, 0.06472874610933113`, 0.001058317114923213`, 140.04605512421463`, 0.14623316670714115`,
 1.1264629805642916`, 0.0009442995541204535`, 0.0009510130354370054`, 0.007109482671317169`, 0.19896272534323953`},
{0.09855067343586066`, 0.42248858314270876`, 5.888441909786696`, 1.374275487614058`, 0.713481343784103`,
```

0.39177356523031326`, 0.12647140495570885`, 0.021778948867459624`, 3561.4755687032084`, 75.36389346411136`,
0.14783328914983274`, 7.192274801142693`, 1.4077827751177328`, 906.9281875814153`, 38.4709948604879`,
992.3772176577374`, 0, 692.0175303425053`, 30.451385206517568`, 30.589234495115377`, 0.004526864300685496`,
0.18466107605051596`, 0.18466739572027108`, 0.000034223074457662`, 183.79089415192058`, 0.2599781914598141`,
3.408463089477476`, 0.0016229747672380501`, 0.0016274956065314079`, 0.0027855265433678333`, 0.06710205826548107`},
{0.09888970514692547`, 1.0773269521808775`, 4.509787207697677`, 1.034834313165084`, 0.46524158243265124`,
0.6370595817977545`, 0.17795346839566295`, 0.052840345624928245`, 1394.9195521303727`, 145.8914281907807`,
0.14189384893594092`, 6.214985461828465`, 0.5648213653786103`, 360.1710459254832`, 79.74835700269983`,
406.43944052091473`, 0, 205.98684302792563`, 9.394406415414613`, 9.452447222149084`, 0.006178230339198176`,
0.0833157836240662`, 0.0834379861192589`, 0.0014667388323932329`, 144.58353187238694`, 0.11770104680950988`,
2.0450671506722338`, 0.0009688308302697735`, 0.0009766213672813824`, 0.008041173720121586`, 0.19598420074976286`},
{0.2574834517331688`, 1.8047481810399715`, 6.678550158926733`, 0.8506612742210977`, 0.9197441161866675`,
0.28136838946825016`, 0.14128624992883748`, 0.0064270584722317895`, 1966.6222967379854`, 284.61250325943354`,
0.23333938304045732`, 6.6167222434451745`, 0.3934211369713194`, 275.36217612319285`, 73.7884663423755`,
320.2566654713368`, 0, 251.91376949314426`, 0.8599498623410293`, 0.864618433829832`, 0.005428888000625465`,
0.033251550312576254`, 0.033259334006919244`, 0.0002340851560249657`, 22.171327854936365`, 0.12231034214229332`,
1.0615279201744123`, 0.00047228939353782806`, 0.0004735793761734027`, 0.002731339414403511`, 0.08547173132075268`},
{0.264103406072841l`, 0.6388918081456714`, 7.451950258224173`, 1.0512911558430225`, 0.698530834423349`,
0.4711872720679928`, 0.06135082806583303`, 0.2926679041436665`, 1310.8742778358974`, 123.75694501020632`,
0.09712150854448914`, 7.74080995928431`, 0.8193975183528215`, 452.9236254833473`, 123.24680456607639`,
479.1721433559594`, 0, 90.6272749825212`, 35.75079732211533`, 36.09184073673738`, 0.009539463177540952`,
0.05177194497226106`, 0.05179379123932799`, 0.0004219711482469357`, 326.2984506253675`, 0.19533067151719796`,
4.0616029140180965`, 0.0005946128731354472`, 0.0005967359666218784`, 0.0035705474643290547`, 0.1601467535614001`},
{0.2613599171041266`, 2.58453322148865`, 6.473133758347821`, 1.1092209897073517`, 0.7935873547661896`,
0.22081691696159222`, 0.21037162231016598`, 0.22559388527799015`, 2971.40901940996`, 103.45193855805343`,
0.031105461375102783`, 7.240063151509036`, 0.6982732456981431`, 868.2173335715784`, 37.246972244069156`,
1040.0905618445931`, 0, 206.35724707124672`, 17.437142564234048`, 17.473109768134062`, 0.0020626776300944893`,
0.1289589435734121`, 0.12891617289833152`, -0.00033166117754535O4`, 643.8124892156667`, 0.4814956971741896`,
1.180107794838294`, 0.0014047867531935543`, 0.0014079431163307912`, 0.0022468628281562264`, 0.0337132645788582`},
{0.05972716054183652`, 2.4084080668442445`, 4.924113152287115`, 1.0973018723863064`, 0.45846865431577455`,
0.17436075418224495`, 0.13306789950554873`, 0.08269850672896895`, 1061.1497496191419`, 231.6428562776456`,

0.027554419387727458`, 5.529015160121926`, 1.014914954734902`, 189.31527575875504`, 48.819523981047865`,
201.84576364974575`, 0, 87.90186896244933`, 2.861803387888808`, 2.8958346614348813`, 0.011891548416671283`,
0.04786379208734585`, 0.04788015429015719`, 0.000341849278918005`, 98.46271950162273`, 0.04083954848787512`,
2.168344781510942`, 0.0005268368254172096`, 0.0005284737811728395`, 0.0031071399656493`, 0.14262689452213825`},
{0.20906782584649525`, 2.253200985640894`, 8.714537859819917`, 1.139406043052919`, 0.18448556976959418`,
0.3412765002806809`, 0.08047235865027924`, 0.007526938093912886`, 3981.3819787959537`, 150.99126827565385`,
0.12762508996370686`, 7.834813706411341`, 1.133986173662005`, 288.92486991339257`, 19.351839055353157`,
323.0572344779211`, 0, 260.7529505833792`, 0.8377262637890686`, 0.8452046843023903`, 0.008927045547666657`,
0.030248110262002973`, 0.030257443098132282`, 0.0003085427832836274`, 26.965223475239892`, 0.0903415235491919`,
2.2287002150069166`, 0.00032088700223864564`, 0.00032160699286363625`, 0.0022437512892938827`, 0.10864121264455026`},
{0.14168212332279645`, 3.629529656644613`, 0.31240616809545685`, 0.7793905839088386`, 0.6288942527555839`,
0.6686461551742169`, 0.051179205682700984`, 0.015333271439627172`, 3119.547847449303`, 332.77374740974926`,
0.18323666728985671`, 8.115016271373861`, 1.3496472408131814`, 169.76010622310696`, 60.93362138325542`,
170.8156633229224`, 0, 138.84331737594476`, 0.5651490158439009`, 0.5771697928949935`, 0.0212701017149306`,
0.3229934721786321`, 0.32574485279615406`, 0.008518378402397886`, 29.30321590612787`, 0.6537485851095833`,
2.5145186840431615`, 0.004948934271257865 4`, 0.005089091222226944`, 0.02832063294577858`, 0.312272641329606`},
{0.11363747349097014`, 3.3902353384962547`, 6.9199640132703095`, 0.8644073024825804`, 0.6214378859880609`,
0.26046821126038044`, 0.13701105918453266`, 0.15359214599837653`, 2561.5661019345407`, 185.76122258296698`,
0.15708434183781178`, 8.31641484873343`, 0.7597169041059111`, 393.0429316924564`, 43.64224433482965`,
445.3471903660348`, 0, 124.17992147783511`, 5.437088762720114`, 5.461913997954963`, 0.00456590582170846`,
0.03705126276699889`, 0.03705392231100423`, 0.00007178011778075089`, 263.32872088449466`, 0.060148741292592005`,
1.7034301532694813`, 0.0005181401221951987`, 0.0005190552662636434`, 0.0017662096202231137`, 0.0968107591900394`},
{0.22123407652153293`, 2.082927160793078`, 3.494558969518348`, 1.0455662143345417`, 0.1785350961948371`,
0.6180201085084245`, 0.0648475297068132`, 0.006469955463562222`, 2336.7510217065665`, 120.4669332891844`,
0.020699881273580856`, 5.403351657201977`, 1.2197076183004314`, 245.52375345705082`, 29.985541609946882`,
262.92468541275355`, 0, 224.45967380082973`, 0.665817480995908`, 0.6754180645368968`, 0.0144192422323135`,
0.08505942400736642`, 0.08522852040476844`, 0.001987979572814602`, 19.812133075674367`, 0.26882918742461714`,
2.966560000007051`, 0.0009798621407347552`, 0.0009882151342219716`, 0.00852466192943524`, 0.13601379266281904`},
{0.26969936534459843`, 3.0645680107332938`, 7.896848185396699`, 1.1676096938151472`, 0.03246672129411676`,
0.3430750433397468`, 0.18007472085753745`, 0.00937848460863108`, 691.2033005272847`, 393.60922326457455`,
0.10952344673101261`, 8.268833108688174`, 1.2664329385455138`, 145.35967746927395`, 104.79542010740711`,

```
146.8347030614722`, 0, 128.43037455677552`, 0.3739299454681952`, 0.3835254250016647 5`, 0.02566116902313098`,
0.01630158869400683 3`, 0.0163091103082055`, 0.00046140375271730427`, 16.370482130529656`, 0.06280754464117495`,
3.629845702496808`, 0.00016864844029562676`, 0.00016918409300350113`, 0.003176149787898596 7`, 0.7432799838881989`},
{0.09707681532069068`, 2.1085331709443667`, 7.06540706713491`, 0.9411343900767416`, 0.9036401467365582`,
0.2916719417534003`, 0.09652690802667035`, 0.03828184000699648`, 3174.8977687655497`, 60.92568989682479`,
0.05469741369744979`, 2.8213654015691247`, 1.0633604979013946`, 424.9001152751589`, 18.78814805422754`,
485.10397182717634`, 0, 274.8507083840537`, 4.810969231322616`, 4.826784270182999`, 0.003287287467443445`,
0.1258466347958526`, 0.12585138712593774`, 0.000037762869804724986`, 144.91554583766367`, 0.1745255789259245`,
1.0704752319046509`, 0.0016154395709825353`, 0.0016199586028070717`, 0.002797400723437704`, 0.015533094237148215`},
{0.14835712091109915`, 0.7730242131483069`, 3.477772408678769`, 0.9950646362476758`, 0.43363684394770785`,
0.4723829080128319`, 0.247934187727751`, 0.09606686055299227`, 3641.8956482526974`, 352.02514132734564`,
0.05990938105658999 4`, 4.715269674310706`, 0.9126567361088749`, 125.92286211221575`, 18.727354609425085`,
141.4595460740006`, 0, 53.43305548626154`, 6.0067574770044105`, 6.048576815999748`, 0.006962048851719915`,
0.04781952934305495`, 0.047913244418879467`, 0.0019597657507710586`, 66.33384246048624`, 0.10134810995222916`,
0.739828034662076`, 0.000578351722593573`, 0.0005835935523366616`, 0.009063394364214883`, 0.1400816548853104`},
{0.07690717877213288`, 1.853650900512208`, 4.705122089204835`, 1.177168847413763`, 0.20514199762323693`,
0.5853606940036812`, 0.13349096224239904`, 0.025824642502794638`, 3833.38590479347`, 77.1112215680152`,
0.1481478527297927`, 3.868716389694623`, 0.3394641684298805`, 220.15867947490995`, 12.915359291618138`,
350.5968990238639`, 0, 160.77078885757732`, 2.1529547965815823`, 2.159059952829594`, 0.002835710372417033`,
0.08910957954472547`, 0.08930793914716625`, 0.0022260188349472187`, 57.01180853493606`, 0.09790237663360318`,
0.40277556221973526`, 0.0009112184189746797`, 0.0009192033818887857`, 0.008762951612732772`, 0.046766957961402435`},
{0.2792305556244907`, 3.773985442432103`, 1.91428975676104`, 1.3562902020721337`, 0.4175948252900008`,
0.22518067870989456`, 0.1671047483185617`, 0.02746479862850403`, 1101.9393721601946`, 344.3881723084337`,
0.08960284517652922`, 9.994170351350675`, 1.3158131558054151`, 253.95500681529953`, 92.79862179898906`,
263.9872605170865`, 0, 182.955135090735`, 1.2820383268135818`, 1.3031719232298176`, 0.016484371780648654`,
0.11267836433048971`, 0.11277553738283329`, 0.0008623931747764324`, 69.11991402906392`, 0.44947488969811733`,
3.5939875356554962`, 0.0010011532870245476`, 0.0010088252613357433`, 0.007663136515285274`, 0.4082682011318163`},
{0.22478599831633878`, 3.8226352096828684`, 0.3445246491273739`, 1.3626729827827502`, 0.6328825985865549`,
0.4911616627377584`, 0.2351834938983145`, 0.020644881197453675`, 1711.2523897617402`, 90.3557844360617`,
0.22332245485704472`, 4.143025581982037`, 1.4109410839085532`, 558.4417294002575`, 47.16199653584685`,
594.3196999564491`, 0, 421.1179921785847`, 2.2200567226101926`, 2.2871850345775777`, 0.03023720578114797`,
```

3.2591918175562817`, 3.2645950282349854`, 0.0016578375809603862`, 121.23524279061223`, 10.466009805912023`,
2.575884902877554`, 0.028269787726269757`, 0.02973401400149468`, 0.0517947389418949`, 0.1509918448208039`},
{0.20246997439609643`, 2.796989949233529`, 8.601219291155875`, 1.0746358846435033`, 0.23672394588378642`,
0.5308972062471274`, 0.07979598008259464`, 0.06626312934080632`, 1425.6928357201414`, 284.5057677770119`,
0.012297934961132934`, 4.043474061276271`, 1.0687107503597373`, 86.81974170143934`, 35.50918749158596`,
88.58521930822452`, 0, 45.31726033697542`, 1.0117121512801992`, 1.0308226260381503`, 0.018889241108519972`,
0.016806970951902523`, 0.0168290855230536`, 0.0013157975470037275`, 40.424981694973575`, 0.048612956833016606`,
1.5868931100165833`, 0.00018850049304786065`, 0.00018972187144029328`, 0.006479444019928948`, 0.2008948912610926 7`},
{0.05455278033197386`, 2.653355923086507`, 2.8901946723921075`, 1.0586559640275577`, 0.4813227217769469`,
0.39307761087509174`, 0.2243292319383302`, 0.14625278281931525`, 3083.4776957133126`, 151.39135551576533`,
0.22240872343945634`, 6.123940268706669`, 0.19893741817415211`, 217.94960471297856`, 27.697283904559793`,
431.53545971963234`, 0, 70.84999475244284`, 3.777251304880161`, 3.7887168983587167`, 0.0030354330578277 19`,
0.08167567773523289`, 0.08178732173697134`, 0.0013669185837719144`, 143.1770303255715`, 0.06365193294239394`,
0.5184246335228679`, 0.0009286986093495209`, 0.0009358616171828329`, 0.007712952039768073`, 0.08577794681675821`},
{0.1261136314023228`, 2.7924651112189345`, 3.1510744750802027`, 1.10573405830265`, 0.8251438792845518`,
0.38426336320599863`, 0.09970139546376985`, 0.07482975828652592`, 1939.6449909308958`, 136.19904432267293`,
0.18392510915657945`, 2.6983520820510374`, 0.2768728179957576`, 143.86646508010193`, 30.715871758503077`,
196.38892970848963`, 0, 69.60781702704502`, 1.807941484400879`, 1.817749137474491`, 0.005424762448471565`,
0.11685380595162652`, 0.11718820812301822`, 0.0028617139909858924`, 72.12305026164043`, 0.21052654016784603`,
0.45021226347435844`, 0.0012696503892899447`, 0.0012859257156205988`, 0.012818746379273938`, 0.0428062530011539`},
{0.19898919294397377`, 1.094863215028309`, 5.141348317963875`, 1.122536541475456`, 0.25105837287153987`,
0.22994429074756872`, 0.06388318517905306`, 0.28661130713234945`, 1651.4164658316613`, 309.756100189421`,
0.22912307516081604`, 6.461389927282495`, 0.7712732446594246`, 119.12997644121873`, 34.468681908754604`,
126.89223345221336`, 0, 24.745039945386385`, 5.666035837227724`, 5.7536358986488905`, 0.01546055548141867`,
0.025238295912612528`, 0.025251538920225112`, 0.0005247187709676027`, 88.6219173316109`, 0.07174497335618371`,
1.748896118575913`, 0.00027153252445022424`, 0.0002725408902828 6505`, 0.003713609758839942`, 0.1702908740360037`},
{0.08906555841605851`, 3.099177869846099`, 0.23414284508699804`, 0.9801911457566903`, 0.5103708245250258`,
0.6482190821775309`, 0.09673419046052228`, 0.05503363591579261`, 1161.2636269890509`, 33.10846443598513`,
0.20242548439112923`, 4.392158138354276`, 0.5844178562263633`, 877.7484304100631`, 72.79268954561743`,
1046.442031288759`, 0, 485.87420489437073`, 8.39129863359881`, 8.533414331070409`, 0.016936079107298996`,
5.261801677103096`, 5.181788677391094`, -0.015206388347964728`, 371.5161003502747`, 6.694932923510491`,

2.715214880841546`, 0.06266692441931954`, 0.06486714657109487`, 0.03510978354471517`, 0.1325145210095951`},
{0.12731801883579802`, 0.6615371738972167`, 6.357864878818987`, 1.255082973105248`, 0.4534404170796209`,
0.35401897300932406`, 0.1793246308628349`, 0.24542217350407658`, 3327.8256670682013`, 361.04151598602084`,
0.1826398340506512`, 5.639198085050134`, 1.1435146635553615`, 135.8970712040658`, 21.672434301600198`,
147.46225323590784`, 0, 30.963242521989297`, 10.032960594513616`, 10.117968310589546`, 0.008472844608042651`,
0.0298217387730414`, 0.029847509908902243`, 0.0008641728122218417`, 94.81680567880971`, 0.05424063855460429`,
1.0807263747128595`, 0.0002867378620305949`, 0.0002880680186417943`, 0.004638929096351774`, 0.15340178447204517`},
{0.19766525289867687`, 1.5539440876458963`, 7.208959079796127`, 1.2770132142294717`, 0.17265151827851555`,
0.3201118036294218`, 0.21136859719947498`, 0.44059120086782566`, 1120.1312471078827`, 306.58844853012556`,
0.02781674233524073`, 4.752053599009571`, 1.1633837773022284`, 117.11943341002006`, 39.67529696660062`,
123.50824335818906`, 0, 17.520278744060054`, 4.288705210670776`, 4.356306454483267`, 0.01576262309759402`,
0.027360139531086385`, 0.027384522567802428`, 0.0008911883175279911`, 95.20583008254306`, 0.07725926999632257`,
1.7865003888525404`, 0.00025843644752499007`, 0.00025982962361035085`, 0.005390787943043707`, 0.27443676050774396`},
{0.16821931653812278`, 3.9393436886276305`, 3.179019625415814`, 1.1903697350607159`, 0.6888287521080438`,
0.2381411552279049`, 0.2058483354518103`, 0.007287906278596642`, 1880.9980419826588`, 370.4098828230061`,
0.17633160606015752`, 7.349472380855945`, 1.3310916733121423`, 245.98674283744526`, 54.282064515870346`,
255.47032651404265`, 0, 222.5313986854835`, 0.4010790996299425`, 0.404974513287245`, 0.009712332706681925`,
0.07854561001806068`, 0.07859499682137229`, 0.0006287659272141699`, 22.571263139538082`, 0.18875555477582368`,
1.898042803652368`, 0.0007960483086906223`, 0.0008001583157412743`, 0.005163012100876774`, 0.18989033972328087`},
{0.27267841809763693`, 2.4698162119019047`, 8.674593338753226`, 0.9507011512806018`, 0.410886345842024`,
0.5761365147011471`, 0.24420414171291205`, 0.043929586623096185`, 2067.2152285645698`, 299.4614654667555`,
0.08071123306974876`, 3.4242661195281348`, 0.9268436590103244`, 106.47408784026898`, 25.857609975754013`,
116.2091782896755`, 0, 65.67857330085525`, 1.1213943413253702`, 1.1315148868415492`, 0.009024965744180324`,
0.021342636686979312`, 0.021373099367920565`, 0.0014273157242956636`, 39.566256059149325`, 0.08313823442630436`,
0.8702695675402575`, 0.0002704643697213349`, 0.00027242119094315887`, 0.007235042544939008`, 0.14875410697363792`},
{0.09734540105037814`, 1.0928119036432262`, 3.3746924724651315`, 0.9148152167379004`, 0.5520222973975617`,
0.5701876550674219`, 0.07525801655581627`, 0.11060319201619584`, 3458.3887900851914`, 360.58656442125186`,
0.07476322979210304`, 5.145406927076468`, 0.3705928304372572`, 93.24249077258001`, 27.768641261774626`,
105.64673858204908`, 0, 36.48174105032574`, 3.4125095093561653`, 3.4375155890606517`, 0.007327768504652221`,
0.030746463584550254`, 0.030815784117670497`, 0.0022545855698044015`, 53.27472875885886`, 0.04275752612177114`,
0.612579307871695`, 0.0004040937944265188`, 0.0004081065159528 4814`, 0.009930173592554326`, 0.15780503478637484`},

{0.0485781063525863`, 1.7849942773456657`, 1.173858124805676`, 1.3582711053956302`, 0.42211815922461504`,
0.5511485692462849`, 0.057310028881033426`, 0.193275446076273`, 3997.3620434488857`, 116.86597951838672`,
0.12079571732839778`, 1.998996511553914`, 0.9355040158846233`, 94.89982092806228`, 7.645720031419541`,
105.67802930872963`, 0, 25.546748549927898`, 2.5953409600563178`, 2.6289794163355973`, 0.012961093280996039`,
0.3404712800957438`, 0.342753676464765`, 0.0067036384636594`, 66.18098230659042`, 0.23627785792124284`,
0.6464801519325886`, 0.003013981952150857`, 0.0030736282468305618`, 0.019789864580024963`, 0.0483037632007183 9`},
{0.2386999966275894`, 2.722739872786626`, 1.3062557112348774`, 1.1073558597851454`, 0.32667496326027057`,
0.18792754690523328`, 0.1188035955613946 1`, 0.03176567183554873`, 2751.8624934955715`, 349.72147263095553`,
0.1223537595062607`, 3.6928606229815575`, 0.1168338292618869`, 35.40231725629162`, 11.505291038426357`,
77.50931701896425`, 0, 24.29191114212553`, 0.2728113264346988`, 0.2769848305455435`, 0.015298133568672379`,
0.050539151625127315`, 0.05069900962034537`, 0.003163052605310801`, 10.611346803308043`, 0.17233850460684813`,
0.31049424247485796`, 0.0005476665332837527`, 0.0005577687300823994`, 0.018445890308606216`, 0.09831713695693572`},
{0.11566857496325095`, 2.3986647351668298`, 8.513377414925483`, 0.9662333740036599`, 0.8541931022288194`,
0.36101996809007564`, 0.11530341629473645`, 0.14705784731340077`, 1645.8753074989063`, 310.3763160081718`,
0.036104725588746445`, 9.667079447216771`, 1.4942192927137747`, 354.7566872039095`, 136.84383106361074`,
359.0173883998134`, 0, 116.70951459327344`, 6.747959273359064`, 6.815209808773096`, 0.009966055319796885`,
0.026137554205811517`, 0.02614289785766447`, 0.00020444345369385886`, 231.22988490497693`, 0.04318990925696078 5`,
3.442525023732803`, 0.0003269793472229221`, 0.00032760234985892517`, 0.001905327175230731`, 0.2300908162663768`},
{0.12899904957253838`, 3.8001456108425966`, 4.68277328679647`, 0.7500997606167263`, 0.7705050389454449`,
0.5067773892687756`, 0.08071179965828676`, 0.006247983675383929`, 2714.029884924169`, 310.5929142878384`,
0.10714318122808536`, 8.497633727984809`, 1.3195673297775237`, 239.73670657912515`, 77.77453193949998`,
245.5519101520532 7`, 0, 220.01250666911352`, 0.3511154585832344`, 0.355317659057069`, 0.011968144298717842`,
0.02834532043719586`, 0.02836152044311659`, 0.0005715231181324665`, 19.061283840486606`, 0.0522359913746737`,
2.5328877458324857`, 0.00045627667371916925`, 0.00045787505839512663`, 0.003503104077025698 3`, 0.20068120355031951`},
{0.042020095209803626`, 0.8125545855681908`, 0.8820021130226205`, 0.8679479110418857`, 0.336031336984445`,
0.27953459644666345`, 0.05504645570926081`, 0.01654003058638047`, 2110.5518526782043`, 28.793440121823267`,
0.16487272926817176`, 7.902234236019242`, 1.1698954211202324`, 1480.0196665889825`, 38.61823651310428`,
1695.9581792736178`, 0, 1196.4680423454531`, 22.29714774864691`, 22.43588547970414`, 0.006222218761843479`,
1.1630919394601884`, 1.1556379273143074`, -0.006408790133427211`, 258.8235664036349`, 0.6981890576264617`,
6.770050771448759`, 0.01607179619745913`, 0.016138440087164023`, 0.0041466360627089305`, 0.05400920404490355`},
{0.19490440880029264`, 2.2807650240422737`, 4.164948405329779`, 0.8920125211738439`, 0.9916182528214048`,

0.24033665404408822`, 0.09749261388251534`, 0.009353219207928497`, 558.6138953254322`, 192.81883910349768`,
0.12112913056266378`, 4.477996891709701`, 1.3978148126017071`, 268.86270674045124`, 177.47719450091026`,
272.04103383413553`, 0, 237.10156116786362`, 0.9308521044295804`, 0.9436608746535763`, 0.013760263486587876`,
0.08060301586835854`, 0.08063768825746316`, 0.0004301624291733752`, 30.32935603341614`, 0.22442690221919032`,
3.143654409529704`, 0.0010901170639644464`, 0.0010955967750259203`, 0.005026717994437879`, 0.10921871408971505`},
{0.16009076205688888`, 0.5349813484836639`, 5.374724113132956`, 1.4400444163269621`, 0.6922562033282762`,
0.5453216758418981`, 0.13981434114543434`, 0.007280151675390544`, 3925.9979560926067`, 341.46046547891854`,
0.19348925902086372`, 8.443436699305352`, 1.4876822395937013`, 256.4946530860169`, 48.8137716507635`,
265.58049659367396`, 0, 232.16530230576672`, 2.7692927097822855`, 2.795804711106145`, 0.009573564119895295`,
0.05100594126771989 4`, 0.05106433192862591`, 0.0011447815578882192`, 21.164570688932955`, 0.11665114295684528`,
2.026377985031305`, 0.0004273108879206955`, 0.0004295527265478504`, 0.005246387795227347 4`, 0.2065993599608988`},
{0.1933638890845395`, 3.103067052973441`, 5.111571620050322`, 0.8661544577982099`, 0.8149222692447449`,
0.1595760565881339`, 0.15190282082017698`, 0.005535189167289717`, 2999.703437637353`, 300.1128617408108`,
0.10100183998523121`, 2.2676395814752386`, 1.3326114208284863`, 86.80394003506119`, 9.45283364194059`,
91.91197283685018`, 0, 80.25435833410053`, 0.13914844209709248`, 0.14032704342940758`, 0.008470100811424963`,
0.04062306544582584`, 0.04065028701493796`, 0.0006701013036158887`, 6.168384944915279`, 0.11221477030201114`,
0.4871903485269293`, 0.000566316698372149`, 0.0005691471315181114`, 0.004997968723327917`, 0.031272725622458405`},
{0.06217291008845033`, 1.247503477484723`, 2.5904087629086465`, 1.4683499815329046`, 0.08458788439332876`,
0.3930546684373296`, 0.18769435268404766`, 0.1379057398065497`, 1008.6694100163231`, 278.1009416145228 7`,
0.21639096057090101`, 7.204492217199894`, 0.5358436507882129`, 172.7777273600434`, 68.78525682077775`,
188.9982852424403`, 0, 59.624761752045444`, 6.0033720804976705`, 6.089054904393776`, 0.014272449341337978`,
0.08506982015242676`, 0.0852636602788789`, 0.0022786004026436046`, 106.98896495793603`, 0.07555768970815849`,
2.376459387515013`, 0.0006957680879485784`, 0.0007036062237893809`, 0.011265443150623167`, 0.4493426696001564`},
{0.22725673950089992`, 2.3639145368218726`, 8.344676289930643`, 1.325883986841482`, 0.6505898944316628`,
0.43112151917918207`, 0.20810105395654238`, 0.010151436710313634`, 754.9801668003361`, 190.32738043229529`,
0.07958968362345098`, 7.5054987393450965`, 0.6093046711635028`, 463.3114021427394`, 182.5482850625461`,
488.55501876670763`, 0, 404.55921637036704`, 1.673186731290513`, 1.6876481344761867`, 0.008643030042749578`,
0.06150952272880801`, 0.06154143194933328`, 0.0005187687874925739`, 56.503863384501734`, 0.1996921940513923`,
3.4235152069383465`, 0.00055986230756244 2`, 0.0005622086590885677`, 0.004190943906085209`, 0.2624864194745849`},
{0.2309391357161658`, 2.323989179094352`, 2.3593321069217`, 1.3993677346710833`, 0.7027057115205266`,
0.26166872252110007`, 0.14152733686240188`, 0.2848639929776201`, 2532.106437595179`, 386.9846027360387`,

0.07114341876102281`, 5.5959659257370316`, 0.47197407419590953`, 137.33886305174508`, 32.65105448199968`,
155.81710423095606`, 0, 27.60867397916265`, 3.1970627068701436`, 3.2236280594409967`, 0.008309299818789029`,
0.09098087805185107`, 0.09112629792147493`, 0.001598356410025037`, 106.1419876521758`, 0.300157790628583 7`,
0.7449111276593707`, 0.0007824785455317063`, 0.0007905746798961351`, 0.010346781276830308`, 0.1038307924877516`},
{0.11128373013292153`, 3.3734167013736274`, 6.971024005221597`, 0.8270211193396084`, 0.0351306115056782`,
0.4871945686666914`, 0.14931850539545372`, 0.02878252625021986`, 527.2114941578984`, 74.82432219823664`,
0.24191356032713884`, 9.568158477220692`, 1.3339247715553384`, 802.0022022989866`, 186.73785722440502`,
853.3440946197896`, 0, 571.5774522252808`, 4.678893999831507`, 4.750428657325504`, 0.01528879634729341`,
0.06241244682582216`, 0.062416994612175196`, 0.000072866656962267 13`, 225.48370232840634`, 0.09922128413573014`,
12.411791873235211`, 0.0009114096464324373`, 0.0009138274826018035`, 0.002652853389066623`, 0.47743431269743114`},
{0.18315330325553064`, 2.869805671153377`, 9.468498073936939`, 1.1725531828202505`, 0.3267495326537466`,
0.6971808886096422`, 0.09399727517157247`, 0.4622104919604099`, 2265.8497866660555`, 116.1383358572474`,
0.02715190911437171`, 5.561797604651474`, 0.6165440590050792`, 288.7256990249196`, 39.422503389796134`,
331.2791548278614`, 0, 39.37448909243134`, 5.933855952232634`, 5.97064466809765`, 0.006199799280798 7234`,
0.040296931563426425`, 0.04033902487142212`, 0.0010445784917751944`, 243.2716209074925`, 0.10543594467004416`,
1.666954780998257`, 0.0004144376621015855`, 0.00041668009822918976`, 0.005410792340235 1155`, 0.12507953095761645`},
{0.22883543071148366`, 3.066706533284199`, 1.1439898434307647`, 0.7535281320523861`, 0.1149711112081426 3`,
0.31581178336786275`, 0.15168936854496579`, 0.1415060368713317`, 2986.3979531848126`, 146.71341498099025`,
0.14475182625551103`, 7.74072484577729`, 0.18905729960113726`, 177.63453832670774`, 23.369032049887267`,
369.92168759759517`, 0, 59.10665779910867`, 2.6361075725506034`, 2.6521846880185347`, 0.006098808574938275`,
0.09455281887641813`, 0.09467971092224302`, 0.0013420228749683183`, 115.48811878829802`, 0.3091005004651412`,
0.6825848074561507`, 0.0015090096009932052`, 0.0015245343179922674`, 0.010288017378314862`, 0.13546886445431913`},
{0.1197189938163688`, 2.6914481530927663`, 7.902408409631434`, 0.8148212240990508`, 0.32365266714619123`,
0.3812630996449249`, 0.2044952740314684`, 0.28795554719398225`, 1263.0479831599341`, 345.11453037398417`,
0.1466603335899901`, 5.094081228711747`, 0.9032476272618188`, 123.60099927465367`, 46.700436698795365`,
130.89062274999654`, 0, 25.38918572595484`, 2.4884949622966066`, 2.520458090353494`, 0.012844361166553853`,
0.01569241280685587`, 0.015701191187454583`, 0.0005594028596340728`, 95.68078814648365`, 0.026838283882663235`,
1.5738803628975107`, 0.00023253897536001933`, 0.00023335130682168736`, 0.0034933131549683694`, 0.26572539612615736`},
{0.089875647559861`, 1.0302327249870382`, 2.0596378930749815`, 1.3241139107745634`, 0.7453299280714754`,
0.5095722380183404`, 0.17917298341587806`, 0.222730051899884`, 744.7092202123449`, 118.59827741373601`,
0.0989210230322109`, 4.9882080789644085`, 0.660240574996108`, 504.2320427248888`, 157.23812779156611`,

531.5215606169223`, 0, 122.50895954701404`, 24.227319144663745`, 24.409305941628194`, 0.007511635764476665`,
0.4061059786841575`, 0.4072318146624505`, 0.002772271370987678`, 356.568243164822`, 0.5214148258882697`,
2.8542109951369206`, 0.003669817428654909`, 0.0037299979927037 37`, 0.016398789645207357`, 0.15234753137010143`},
{0.17809194642727977`, 1.7697866199706649`, 7.176315122174 1985`, 0.9277024880907543`, 0.02661598115385022`,
0.6083929126896732`, 0.174056840727485`, 0.0393026670208333`, 1347.608160247999`, 208.03482783091147`,
0.17902076650274934`, 4.82657960035886`, 0.6328054071662224`, 141.5054725662397 2`, 40.34030544616048`,
157.2649600638452`, 0, 91.11927908357184`, 1.913623417876065`, 1.9359542186520953`, 0.011669381011659707`,
0.023749703997820497`, 0.023779647796476348`, 0.0012608072361068867`, 48.38150172313707`, 0.06042330017206142`,
1.6288210113945378`, 0.00030844902460502865`, 0.0003104887395994279`, 0.0066128106484082405`, 0.2816550673022355`},
{0.16322971965863348`, 1.170728402327721`, 4.129867013138943`, 0.9550069661931267`, 0.7632521383792994`,
0.23062906697550656`, 0.15191938086081902`, 0.036668793904547074`, 984.9683279569181`, 143.85195072672764`,
0.06218474573819438`, 6.894986630118397`, 0.7118840534242801`, 526.685696233698`, 105.05574096003002`,
573.0429440807337`, 0, 346.3323896968619`, 10.152574309026878`, 10.2148520515113`, 0.006134182384565312`,
0.11082215091590612`, 0.11081720108090651`, -0.000044664671807082`, 169.79867286172168`, 0.25842098037059436`,
2.8203582760355608`, 0.001400827957392381`, 0.0014057096323361168`, 0.003484849740451379`, 0.10475321695223853`},
{0.0673211374947203`, 3.556048182108581`, 2.056072368577791`, 0.8749285572736749`, 0.3104681364634243`,
0.18652482922479574`, 0.08201020657253827`, 0.044529186328014586`, 1689.51074710815`, 162.90358092109705`,
0.0729615101290263`, 8.407666834293693`, 0.4031543367979451`, 277.0947146525168`, 42.898826054268085`,
346.3601253195717`, 0, 169.9081379536514`, 2.0657197648273273`, 2.079798667484124`, 0.006815494965249247`,
0.08797501221551797`, 0.08799125048370769`, 0.00018457818624617595`, 104.93998592085693`, 0.08460825562076002`,
1.71203914102561`, 0.0012135540438676928`, 0.0012182262234241505`, 0.003849997105664249`, 0.11122892524898297`},
{0.04130707589470009`, 3.0971581646939166`, 5.095324578907563`, 1.2851498582955627`, 0.14534319212364366`,
0.3324535366896153`, 0.10589589728908583`, 0.27538535378439405`, 3967.498675941073`, 98.17019510308785`,
0.19069934275540157`, 9.378576326653985`, 0.907790255687607`, 525.6584329941307`, 22.36790359379976`,
623.8807588652744`, 0, 108.23898458595308`, 9.222617308364915`, 9.263366469731682`, 0.00441839447569925`,
0.08870648404007521`, 0.08872688428090877`, 0.00022997462986285022`, 408.05577852071156`, 0.05234579240838 7506`,
2.2232250706385406`, 0.0008338382576447145`, 0.0008360041951392621`, 0.002597551113408425`, 0.09291875084146171`},
{0.15433792416794267`, 1.8940009891686929`, 3.062826353150152`, 1.3594047846621364`, 0.2865379799536014`,
0.5165321095788333`, 0.13923535270760634`, 0.41888186557593104`, 1933.0228901580695`, 124.09667777854662`,
0.10026068856071241`, 1.5843039926062341`, 0.6139086768148141`, 78.95441521065008`, 10.416962687156445`,
92.91530863535512`, 0, 11.617650397660729`, 2.3843085948278575`, 2.4111286866956094`, 0.011248582472055624`,

```
0.13724433401581415`, 0.1380849774662585`, 0.006125159602927477`, 64.51261195839106`, 0.30260008022596857`,
0.44914497530854885`, 0.0012071865942627769`, 0.001236599656777897`, 0.024364967814344052`, 0.049488487520270213`},
{0.2448264512865554`, 1.018619142628224`, 0.3220150078672237`, 1.1814459661349885`, 0.2096682202943383`,
0.4340268291716256`, 0.23122304821991574`, 0.028723954302408285`, 3147.8296084170843`, 26.23322055856977`,
0.011843614667667979`, 1.6271943864377292`, 0.8914600074049073`, 414.00219708526834`, 5.919891083371849`,
539.6520828774846`, 0, 275.8154138200879`, 7.263925480524431`, 7.745445526278106`, 0.0662892325980895`,
5.596351610514111`, 5.509594382656012`, -0.01550246194236693`, 105.70247921553029`, 19.573355785056457`,
0.5311597727068431`, 0.05619615742077555`, 0.060048296004684205`, 0.06854807803076812`, 0.035399412089886464`},
{0.17570694085504118`, 3.3716783189083603`, 9.273626081950546`, 1.268846725094217`, 0.2584124599965636`,
0.4223453277058432`, 0.22832672513046204`, 0.20979915181246073`, 1007.8354949521076`, 148.0706036186794`,
0.04379179030226882`, 1.2158335564871514`, 0.28231812649411747`, 51.9515783038739`, 13.31706028063578`,
68.96096028586979`, 0, 13.218499850960182`, 0.7851843012216198`, 0.7922893981896093`, 0.009048954439021761`,
0.03648128662544517`, 0.036612696615714105`, 0.00360212049586206`, 37.819841211087756`, 0.09157164673451772`,
0.34564058387439306`, 0.0003444741903466664`, 0.00035017781772033954`, 0.016557488292325173`, 0.04777044247710962`},
{0.2345875520938262`, 0.8974705100407228`, 2.4400268875162734`, 1.1692882451601165`, 0.6149116315758598`,
0.4838775739193003`, 0.16902409799301954`, 0.4305314166593524`, 3073.906515744251`, 376.94525437674156`,
0.0327953725790982`, 6.842916385098416`, 0.9809907681036996`, 185.30262236317418`, 41.49916974103216`,
195.85555523511619`, 0, 26.971635250198744`, 11.43027338048175`, 11.532285242968891`, 0.008924708892906752`,
0.08114801459553457`, 0.08131308705878197`, 0.0020342144422160757`, 146.5476182955125`, 0.2719473443030288`,
1.3026326065626856`, 0.0008347615944199749`, 0.0008434499413681624`, 0.0104081776237257`, 0.19727594741006296`},
{0.26599408746951475`, 1.69869318027059`, 1.7982317117152924`, 1.282444106635544`, 0.20521633822817087`,
0.28537099789283193`, 0.17587118168619353`, 0.016831793401851286`, 735.9167536628461`, 185.12449867405962`,
0.08818428944304713`, 9.590487549937748`, 0.2554550886680036`, 331.1106165531643`, 121.91231011789202`,
406.7282959733328`, 0, 266.87690890046014`, 2.5080667418927978`, 2.5402137683357218`, 0.01281745254460942`,
0.1540624677917368`, 0.15423264104049045`, 0.0011045730423044908`, 60.863369573096584`, 0.5854243647652598`,
3.401421145303967`, 0.001445077762333713`, 0.001459151413558684`, 0.00973902691730788`, 0.3902180306690243`},
{0.26593765918786066`, 1.469820509215408`, 3.318163900631456`, 0.910737040027697`, 0.772245323565576`,
0.6475514852601838`, 0.05903920363599993`, 0.25524741893447345`, 3882.180697561048`, 173.8977279389328`,
0.22560057599213157`, 8.523060233161317`, 0.6568135548380971`, 349.64160717248984`, 64.67015889508639`,
388.29228556013265`, 0, 76.56768952651805`, 12.39850751283555`, 12.473793450924491`, 0.006072177478700702`,
0.07054309564051035`, 0.07061951308132258`, 0.0010832731413101815`, 260.3368660861011`, 0.26800093894943605`,
```

1.7832740930398783`, 0.0009338732968801056`, 0.0009396006494895587`, 0.006132901142571478 6`, 0.129828124791279`},
{0.13345787440765705`, 3.0774883151503953`, 6.064298363382008`, 0.9302596892930706`, 0.6158208110956915`,
0.2585099500502801`, 0.21374523912969606`, 0.4331393141373296`, 3227.8457633864045`, 202.63915585725056`,
0.17639348124623005`, 1.682515063867495`, 1.3735184213990381`, 87.3229491943655`, 6.587603389320726`,
95.34527305684396`, 0, 12.500872462689733`, 1.6608171107031804`, 1.6718667456573117`, 0.006653131692178293 5`,
0.04985294520933762`, 0.04989928589035580 5`, 0.0009295475086497085`, 73.01636073986953`, 0.0950466871514215`,
0.43865928622002187`, 0.000646261561664363`, 0.0006504328476074172`, 0.006454485599161508`, 0.031081583135695107`},
{0.24299864830682322`, 0.6348382188885924`, 8.738994620341877`, 1.2356500801881907`, 0.5770135193458605`,
0.3643052268917296`, 0.09417548384960592`, 0.18321093257407522`, 2194.4027446042355`, 392.84971084704546`,
0.021287183084439776`, 6.026738164007792`, 0.24540222372747866`, 106.06879879190438`, 41.256525579470456`,
125.48040118351318`, 0, 29.87119711112041`, 7.560527378198031`, 7.621933900584196`, 0.008121989289165299`,
0.015602211979300467`, 0.01561264909170407`, 0.0006689508139905431`, 68.56731049476703`, 0.05416166316520224`,
0.7925727796537625`, 0.0001524330574037558`, 0.0001530584980156373 8`, 0.004103051021439263 5`, 0.182311672246072`},
{0.23128596410211183`, 3.0231614800775644`, 9.282448241767675`, 0.9502905953068014`, 0.1244248471921543`,
0.513834937748406`, 0.07158073583305624`, 0.006593831926001459`, 1596.9261281907548`, 180.24662525069675`,
0.024392561873633367`, 8.693689234388824`, 0.44515269072814956`, 252.18442221777084`, 61.705581161343666`,
285.673113386978`, 0, 230.3897663302207`, 0.4860747902888683 5`, 0.4913124602122360 7`, 0.010775440380800472`,
0.018629996874698875`, 0.01863773117646832`, 0.00041515314368889555`, 20.992608320544083`, 0.061555096976926564`,
2.4377692924537837`, 0.00023682219923593095`, 0.00023750086749636608`, 0.002865729068578515 3`, 0.30530796646811525`},
{0.14763336174315628`, 0.9609083924153881`, 5.767031511187355`, 0.7806215764240099`, 0.6823220865642827`,
0.65198969122125`, 0.1566301693296936`, 0.37595000066945006`, 3860.5140609631935`, 333.3928376138639`,
0.19492955999326989`, 4.361121532997624`, 0.15723055392755292`, 90.57029599200469`, 27.803504956912697`,
143.29293291322216`, 0, 14.461241432900222`, 5.164184632612333`, 5.184509480261726`, 0.003935732181425067`,
0.017610077438315667`, 0.017637457245496466`, 0.0015547806235778339`, 70.89011933513963`, 0.0371404990396634 1`,
0.3025553755023733`, 0.00027157696427937417`, 0.0002736836577053784`, 0.007757261119676562`, 0.11885329552026441`},
{0.08020873136305112`, 2.3560872112839712`, 7.656859171402218`, 1.2445222240766278`, 0.028603428928333008`,
0.3751343060067356`, 0.17582324568527535`, 0.011898009060677216`, 1487.4044642092413`, 223.64331235141128`,
0.06070604179895395`, 4.4126063398287325`, 0.25472858441799273`, 99.69307979111217`, 26.469084471206966`,
133.90782254682873`, 0, 85.23636596958859`, 0.4140306366723649`, 0.41788578223558553`, 0.00931125675675859`,
0.02301726269804465`, 0.023042497242707752`, 0.0010963312620682064`, 13.935604116335979`, 0.026374077720863545`,
0.8359308199421417`, 0.00022296249582376415`, 0.00022425015969189602`, 0.005775248717836634`, 0.17524987351178123`},

{0.2593624262161626`, 2.295431745726197`, 3.3398768253362086`, 1.233697621211021`, 0.17962106789139365`,
0.6119977215212877`, 0.1479404509305488`, 0.2392187562484209`, 1384.473686906931`, 390.9701237284811`,
0.07023942191875382`, 2.3245474291144017`, 1.4493220736835069`, 41.52428970186202`, 21.345422172608412`,
41.545785861320454`, 0, 9.980449207453688`, 0.9277736266746448`, 0.9506468868988142`, 0.024653923722915305`,
0.04093862159437126`, 0.041231720796006066`, 0.007159479001000335`, 30.42344336452155`, 0.15168486032371195`,
0.8407010719188366`, 0.0003955699612517849`, 0.0004064872636293486`, 0.027598916618986502`, 0.19291065260436593`},
{0.10144857451882727`, 3.09793869586695`, 1.9042542140912566`, 1.128489317749993`, 0.9516046831815148`,
0.5718665343974156`, 0.24712737605206275`, 0.10884528767737062`, 614.5820043578747`, 372.33327243103304`,
0.18531918510116352`, 6.894885736301427`, 1.4197916184504975`, 407.48214483569024`, 462.4955261893786`,
393.70557255144394`, 0, 161.2165114143374`, 5.429729246034782`, 5.479765637232451`, 0.009215264505906928`,
0.21897198101811724`, 0.21958548006333709`, 0.0028017239574094077`, 240.29954770530892`, 0.31734850476940335`,
3.191897072818294`, 0.0023229641957674962`, 0.0023586862593373945`, 0.01537779343951362`, 0.30705070894812436`},
{0.10212230421521268`, 2.1055462656508395`, 0.1549942156465125`, 1.1640680269346606`, 0.2898972915158997`,
0.471231711737255`, 0.20995913095460078`, 0.12219762483818075`, 1171.3965983974595`, 79.93077709858363`,
0.21373183331424755`, 1.9880929888308272`, 0.4174963770861413`, 173.94811032957583`, 21.82130919756075`,
222.45441652048692`, 0, 60.28644153359416`, 3.3502053978729784`, 3.558477137066031`, 0.06216685679190981`,
3.87973916917079`, 3.8981061543788362`, 0.004734077319938024`, 100.77160663792469`, 5.660112910139008`,
0.6654248822816686`, 0.039682480912374585`, 0.042902354098065094`, 0.08114092444977206`, 0.14252465410918977`},
{0.24870831012490324`, 2.875980304064811`, 9.212538429441743`, 1.2722063248788578`, 0.3282913490792485`,
0.2805639294283834`, 0.1572124446820367`, 0.027187541841276704`, 1491.5273167119358`, 343.00060613142807`,
0.23102970098548903`, 4.50321802361168`, 1.2980803493745015`, 100.60754532951212`, 30.450714028189378`,
105.86655823981083`, 0, 72.78185728962119`, 0.6587328738801108`, 0.6687412290974001`, 0.015193344091569916`,
0.019339420439041532`, 0.01935211686944681`, 0.0006565052166529473`, 27.06432529884585`, 0.06871249394551096`,
1.4411294493125837`, 0.00018354607399562184`, 0.0001843070122371923`, 0.004145761470161657`, 0.18760546412691537`},
{0.044603778808110894`, 0.8401502429199104`, 7.1121926093305605`, 1.2733813165569137`, 0.08797724114115923`,
0.30964677383308725`, 0.08962339338921654`, 0.02592380473764512`, 3666.957163896842`, 393.9775757725391`,
0.24527553411695868`, 5.578947990675762`, 0.6585937869939971`, 74.83370436631942`, 13.184735701294594`,
84.75552676109723`, 0, 54.780033273121845`, 1.5394839305572865`, 1.5554445724340733`, 0.010367527429149037`,
0.015060554124779912`, 0.015072310626965546`, 0.0007806155130964942`, 18.47711140327145`, 0.009596537498727851`,
0.7496301256016816`, 0.00014277810173257688`, 0.0001433359119611393`, 0.003906833203366045`, 0.13989689793085341`},
{0.08578994575352372`, 0.9866526872309773`, 0.8038090794361139`, 0.9523570781926003`, 0.948882623737388`,

```
  0.4631164799606274`, 0.2441006263954396`, 0.07831609826614802`, 1671.5327042927038`, 210.47342650267535`,
  0.12673136991162653`, 7.353061586513307`, 1.0620625305759188`, 708.4005153409929`, 151.87260885622555`,
  734.6970370514715`, 0, 334.87956580242485`, 24.639135654406648`, 24.780347782624535`, 0.005731212742141615`,
  0.7149647356893505`, 0.7149967926485951`, 0.00004483711943303881`, 347.2895629209844`, 0.8762397984353409`,
  2.455796521397034`, 0.008981861980477901`, 0.009109013585481184`, 0.014156486180665784`, 0.14949189945490898`},
{0.07858821042838116`, 1.6977143802882821`, 8.212860235255583`, 1.2964521187700828`, 0.2987911497011755`,
  0.506126935243553`, 0.11440340360512286`, 0.05206277291990468`, 1339.867027105688`, 383.7096297624307`,
  0.2078889107072941`, 8.822910683955193`, 0.960825500308768`, 165.54026384702715`, 86.93178799592657`,
  167.95884002335995`, 0, 96.07828336872889`, 2.748972974100831 7`, 2.7959773692370087`, 0.017098893142647897`,
  0.018576525380796113`, 0.018589745593663727`, 0.0007116623047969117`, 66.67101355935479`, 0.02085565550919544`,
  2.821786308908423`, 0.00017295475247103287`, 0.00017362446494360926`, 0.003872183117307193`, 0.5170554492903957`},
{0.22377870799620725`, 3.1928985468211346`, 8.059299312075755`, 1.3921709162211404`, 0.8873395666266357`,
  0.17674042984905347`, 0.1675175234386073`, 0.03730263939446273`, 3395.3924465278315`, 247.66374801748043`,
  0.05539393372492046`, 2.5087357156089176`, 1.4318151504319032`, 135.41889883737113`, 10.819240367798091`,
  143.41379059769338`, 0, 88.37743705010395`, 1.0037706128547688`, 1.0099570490776635`, 0.006163197192334824`,
  0.05839415326476024 6`, 0.05843265570967286`, 0.0006593544517725647`, 45.7848247303679`, 0.1866766881731562`,
  0.5779835452586732`, 0.0005066014936627727`, 0.0005090268281932539`, 0.004787460283517397`, 0.029298546670823197`},
{0.1736316989567453`, 1.5454802872078899`, 7.071248146205292`, 1.1599254463866382`, 0.1492821504834294`,
  0.5667749351814715`, 0.17572322700248577`, 0.00782459398724836`, 1916.2311636952181`, 145.94896290337653`,
  0.12610631603338446`, 7.247312880006737`, 1.199936026275772`, 335.87982409557736`, 45.87002706553747`,
  370.74963099140444`, 0, 302.0220597023984`, 1.4476943713943595`, 1.4632726054958096`, 0.010760720224701759`,
  0.04766396362685495`, 0.04770340141094018`, 0.000827413019907075`, 31.962615898454338`, 0.11822821405059934`,
  3.2588744632681252`, 0.0004957639474907349`, 0.0004981090362989846`, 0.004730252815113189 5`, 0.26375510185955736`},
{0.09782332022288404`, 3.741906492613495`, 0.4566213801268671`, 0.9419423117532648`, 0.9082792827328243`,
  0.61004571220758`, 0.08136955531902801`, 0.05934353731887512`, 1613.3231664795385`, 279.2732289747655`,
  0.2228587683695682`, 2.342091864078169`, 0.3301362991730612`, 80.11405722431705`, 54.410635937224846`,
  80.97183783170513`, 0, 43.04312803568883`, 0.6619439357609436`, 0.6749836041283007`, 0.019699052537383244`,
  0.42695532288923194`, 0.43719629435770724`, 0.023986049404827847`, 35.3847472995715`, 0.596659818169403`,
  0.5274166780723333`, 0.005337817777885889`, 0.005693274854785428`, 0.06659220896827289`, 0.14750851826260314`},
{0.08130667751836484`, 0.5260337564871658`, 8.449122941731599`, 1.4306920647099228`, 0.5994021973571051`,
  0.5116121891099925`, 0.1394200086694753`, 0.006254931094025137 5`, 1045.8518042423143`, 239.77908991527943`,
```

0.20556349167963178`, 7.684382915701004`, 1.0762288196903413`, 308.8057960226219`, 138.68036069151805`,
313.98894251906984`, 0, 283.40370581961895`, 2.937141571564891`, 2.9748647325023714`, 0.012843494267585243`,
0.04267353299468425`, 0.042707515866418126`, 0.0007963454007453574`, 22.07193734606997`, 0.04956633122527226`,
3.5314316063997424`, 0.00035985851378317335`, 0.0003614751179205398`, 0.0044923326125347884`, 0.31734368646907807`},
{0.1509673631351663`, 1.3366819546761866`, 1.080587080528149`, 1.0668100793723834`, 0.024191324402023318`,
0.40903296900163466`, 0.12044262077141116`, 0.013316823826732663`, 3093.793000213601`, 297.44428012608614`,
0.05031916378077894`, 4.50357864911024`, 1.4241445207811716`, 86.99965123009162`, 13.506398824876664`,
93.15643070635652`, 0, 72.93695993163078`, 0.6789572260812541`, 0.6919705064804276`, 0.019166568819485752`,
0.11858275790078093`, 0.1192141042335844`, 0.005324098916064557`, 12.964998172854447`, 0.2557446610510957`,
1.2692360428020408`, 0.0013326548261342053`, 0.0013586687342847154`, 0.019520364643837995`, 0.19068682287353025`},
{0.09526052730311735`, 2.7416128013280456`, 5.368501678201278`, 1.47877684019711`, 0.27293015978313817`,
0.15337029979855754`, 0.05033287162982811`, 0.0696637301808707`, 1979.7812280561757`, 208.2796492673417`,
0.10966372378753608`, 9.228791958515647`, 0.5867218918142831`, 229.5057632286618`, 36.650196048742046`,
262.9789485717359`, 0, 115.99136180649477`, 2.823595108302154`, 2.850094184221958`, 0.009384871025555297`,
0.04297696091545039`, 0.04298623190169137`, 0.0002157199123320463 5`, 110.58863563840606`, 0.05848582798138331`,
2.053469794171825`, 0.0003512987422760183`, 0.0003520539641894947`, 0.002149799650813966`, 0.11278433481923361`},
{0.2168252075927255`, 1.2147794793098745`, 1.3294046517319291`, 0.8204909797710571`, 0.5261154919084852`,
0.32065750745864985`, 0.23412216559654364`, 0.026797285872998095`, 2049.1492401808873`, 336.0554255621465`,
0.1855137474935905`, 1.9365416597785146`, 1.1594140358846108`, 59.385609628298795`, 12.163160154464155`,
63.58920424311334`, 0, 42.7237262937386`, 0.8811669576187641`, 0.8957324274831745`, 0.016529750393468712`,
0.11714087973592668`, 0.11785503337166453`, 0.006096536387192764`, 15.291764828017014`, 0.3628442223762187`,
0.4864187159635603`, 0.0017067643803637678`, 0.0017531164834048998`, 0.027157880475132012`, 0.07636916652205634`},
{0.2246406125901032`, 2.0919939936953575`, 9.60889385739615`, 1.3369466676292021`, 0.09066206605235938`,
0.26041948811653903`, 0.1688935708724063`, 0.006310725218569426`, 2524.013414165627`, 287.3446683034351`,
0.19227735577509142`, 2.226723678369055`, 0.32009387356875707`, 38.675342069697464`, 6.98390543910818`,
52.939248355129976`, 0, 35.40562505298955`, 0.10260297185268871`, 0.10355508039095723`, 0.00927954152863641 5`,
0.015126661373822063`, 0.0151466012650861`, 0.0013181951239116252`, 3.066354297873119`, 0.04854374967797912`,
0.25868838681958684`, 0.00013637923069154123`, 0.00013737510344714852`, 0.007302231802874148`, 0.05291573388330987`},
{0.06878866730089028`, 3.1245944973527457`, 4.360637333709242`, 0.9232062619650272`, 0.8528374363112068`,
0.38941545287751145`, 0.1888087204525729`, 0.06440167068123348`, 613.447059996161`, 135.80840514252725`,
0.0694695796129513 6`, 9.642980612869511`, 1.3375272307560842`, 1078.9190870839911`, 384.79191652111206`,

1084.539973888157`, 0, 565.4474616698586`, 11.244619976386879`, 11.328271356727035`, 0.00743923587598494`,
0.14870858145917493`, 0.1486725731206111`, -0.00024214028679780952`, 501.9268243291597`, 0.14613521621297998`,
7.696644006216284`, 0.0019434176592684205`, 0.001950029611366854`, 0.00340222909208 4541`, 0.2510296470145902`},
{0.05526353896520764`, 2.7240387277783293`, 3.0534884167300707`, 1.3987200069968335`, 0.586176455097533`,
0.6957346200792549`, 0.06800542073977661`, 0.01575809860169522`, 1863.0130158242628`, 26.33179097220551`,
0.24642894997981157`, 6.726887116499235`, 1.3266658102153124`, 1718.3221820108681`, 80.49953669302113`,
1887.4659982723028`, 0, 1402.6542165284022`, 7.8580659789998`, 7.910408022350876`, 0.006660932027162447`,
0.7346350700098182`, 0.7340337427299344`, -0.0008185387608513617`, 305.7953721747547`, 0.5799790545237231`,
9.43241365292605`, 0.006323692987289187`, 0.006357813322234426`, 0.00539563432535739`, 0.07426358069131979`},
{0.2242164690648932 3`, 0.8944001471401766`, 8.80542685575837`, 1.1224114425628389`, 0.07399275239021863`,
0.6541781208595552`, 0.1361819449849342`, 0.011426236224829198`, 1677.1018012951417`, 95.2905099154865`,
0.2261399510764292`, 3.273626323923104`, 0.6313176099847766`, 186.59008808829986`, 23.58043778884421`,
221.17950456185628`, 0, 160.30799452449938`, 1.8855453129246391`, 1.9005013364690844`, 0.007931935362108788`,
0.045482840313010975`, 0.04556572857427442`, 0.0018224073231356552`, 24.091885790275224`, 0.14568574082895866`,
1.1994758714445402`, 0.00048781551964893755`, 0.0004919519018307888`, 0.008479398492339918`, 0.09542344232297705`},
{0.04830398547205017`, 1.275316218137875`, 4.614953899177735`, 1.2829927355973278`, 0.16612061807564849`,
0.1639575953122483`, 0.12272200896549845`, 0.011345722370418136`, 3563.2586080627198`, 299.98688385512116`,
0.24385149927488797`, 1.0836535164777654`, 1.0916200214657223`, 20.341899769166933`, 2.113535622311134`,
23.01987068048485`, 0, 17.4727573611162`, 0.14577690623249806`, 0.1476259156571053`, 0.012683829506288768`,
0.03258129058459587`, 0.03267386552648351`, 0.002841352820179832`, 2.6558807535467004`, 0.022482945529412802`,
0.19211226942634957`, 0.00030588332766068316`, 0.00030913436820970315`, 0.010628367926696525`, 0.023655361277705098`},
{0.16680821963704395`, 0.5163225257189996`, 0.5139271367473146`, 0.8080955581319136`, 0.922329371415127`,
0.31540072696317234`, 0.14858531583274454`, 0.09051887795629471`, 1828.24438708676`, 386.07575250791444`,
0.1874671503356095`, 9.751925633231235`, 0.9375414879403592`, 354.89733019707455`, 129.98269051268483`,
366.7909469025148`, 0, 155.52385041192784`, 23.65835046809882`, 23.893360702294466`, 0.009933500415108654`,
0.35769981870549256`, 0.35802389091980547`, 0.0009059893166447619`, 174.50484668620078`, 0.8523895703251427`,
2.2953573726306917`, 0.005306318405501775`, 0.005381760831689129`, 0.014217470649543484`, 0.22582304273237883`},
{0.19224907453000317`, 0.9379671824303957`, 0.1768927357330181`, 1.2227110146173203`, 0.42649904659884763`,
0.4545029045501904`, 0.23904198572269159`, 0.01364047530387634`, 3771.0454255220884`, 186.69997665609355`,
0.04831449314300046`, 2.795709036233411`, 0.30562138724668775`, 108.71439694270022`, 10.861373183208922`,
160.69055669877486`, 0, 86.08704415345464`, 1.1617763649736945`, 1.2312655265833945`, 0.0598128553004893`,

```
  1.5604743087044244`, 1.594719247083288`, 0.02194521126547433`, 15.567258623837185`, 4.285710595375328`,
  0.23699165825806295`, 0.015362512369170855`, 0.016707008156596998`, 0.08751796289025271`, 0.128224716473795`},
 {0.09905125996953207`, 1.8406681837351115`, 7.615683537936391`, 1.104455595350767`, 0.8507777546248461`,
  0.6055060761025552`, 0.22355503841638658`, 0.019159603171833028`, 3698.4676016336716`, 125.34182227908332`,
  0.18683452817866786`, 1.0248116987499856`, 1.4496781158120586`, 120.63099369541925`, 8.628227633026386`,
  127.37114122614238`, 0, 94.56001029569377`, 0.9443942427731385`, 0.9497079128148389`, 0.005626537944679821`,
  0.10655286702804934`, 0.10694157387618367`, 0.0036480186688172545`, 24.8330919367875`, 0.15077422474989421`,
  0.382019100659233 4`, 0.0011620334488000594`, 0.0011746798904964622`, 0.010883027256626487`, 0.01738024081025804`},
 {0.2122053342005913`, 3.2723497606887317`, 8.074911535839025`, 0.761570924685792`, 0.7803544809949507`,
  0.2686687403920138`, 0.24235001021375163`, 0.4921495003944174`, 553.2367270947411`, 320.67673970953626`,
  0.04374240634138288`, 8.97316011873557`, 0.13752746723967268`, 402.0725610287867`, 275.04181413570865`,
  446.4349388269953`, 0, 53.115474327614095`, 7.306090888719915`, 7.372177802511445`, 0.009045454648471951`,
  0.026522969087160692`, 0.026524014513684836`, 0.000039415893473560004`, 341.5440681610386`, 0.08040450741612257`,
  3.5136110232841884`, 0.00042106748100823044`, 0.00042173033209135587`, 0.0015742158039331322`, 0.31217979262377465`},
 {0.250195686307457`, 1.9873181983813915`, 2.436986915389067`, 1.2297238031002529`, 0.8787886927027242`,
  0.17801285885202778`, 0.10899785398220965`, 0.042181056020100846`, 2975.5972306347185`, 176.4908556989327`,
  0.20657495695660671`, 8.247111235919043`, 1.3291652831072591`, 497.37469743795197`, 42.153576812326`,
  533.2935270559263`, 0, 310.70385863764363`, 6.3211336282123165`, 6.363238255192219`, 0.006660929740827104`,
  0.1907488088549945`, 0.19072032014912632`, -0.000149351946359233`, 179.45862705352778`, 0.6817789877687077`,
  2.408340194132978`, 0.0018729946700151157`, 0.0018807983302286304`, 0.004166408126218268`, 0.07827252332829557`},
 {0.2026768701716405`, 2.320679359442215`, 5.20658497151164`, 0.8289159455996751`, 0.9558590658000565`,
  0.5980005929117111`, 0.20724756852094034`, 0.0547372318523302`, 1071.5776650859361`, 208.90678381552232`,
  0.10872472503561953`, 4.389182379894066`, 1.1547524475482653`, 384.28186415571395`, 174.59601196081223`,
  388.80896159639997`, 0, 216.37643101384617`, 4.906239590810715`, 4.937570680080701`, 0.006385968049474888`,
  0.08733251209386637`, 0.08743028588973241`, 0.0011195578086766744`, 162.65441358389512`, 0.2528611459342083`,
  2.096539497011282`, 0.0012687525136930722`, 0.0012779883460741876`, 0.007279459375597153`, 0.11610709775444437`},
 {0.2780417648717225`, 2.452933782766964`, 5.240377582277642`, 1.0300585799728414`, 0.04659296483118158`,
  0.5270409461030353`, 0.07214810969171709`, 0.4018520591476553`, 1664.9812973970193`, 368.4059321600538`,
  0.19070038794765692`, 6.66208935797424`, 0.8907914326786526`, 101.20733674795177`, 42.84916086930728`,
  102.48492775337655`, 0, 16.65145860950194`, 2.34346254758509`, 2.3914677464264242`, 0.02048473055001576`,
  0.018706479427135794`, 0.018729417537099148`, 0.0012262120220269956`, 82.11940645172304`, 0.07430260792080155`,
```

2.0105584064072013`, 0.00021888310875761707`, 0.00022031930618050032`, 0.00656148129033407`, 0.3768502670304885`},
{0.07805827059301479`, 2.683721229977123`, 9.002208120629334`, 1.281556673983075`, 0.9431208960986435`,
0.16472833391173414`, 0.07811354421183636`, 0.31224015378681164`, 1280.994389531913`, 98.15018684851424`,
0.09634892070904322`, 1.4706754835946878`, 0.8821078383157976`, 126.79674202901373`, 16.98858084853399`,
141.21989130031184`, 0, 23.742588732902373`, 2.615516218406901`, 2.632925720011482`, 0.006656239208941095`,
0.0769168784640105`, 0.07695650033442594`, 0.0005151258242230838`, 100.27594860982973`, 0.08577140731891345`,
0.6763943575369271`, 0.0007246791228441296`, 0.000727873293970636`, 0.004407704080076513`, 0.013581119420306932`},
{0.04941033680222237`, 1.4372745957329531`, 3.017421402893529`, 1.227554635491979`, 0.595314167669632`,
0.39465993131783816`, 0.0709805192571406`, 0.011053419404823772`, 580.3765952495469`, 188.40590324275774`,
0.07356671197341247`, 3.331653425139695`, 1.2649663092466472`, 130.7725974259874`, 90.71221551354309`,
129.67653118796815`, 0, 113.05677188639841`, 0.8110322455509793`, 0.8282238227245786`, 0.02119715617708895`,
0.09978351000493614`, 0.10011920856037024`, 0.0033642688598296555`, 16.65251489786678`, 0.07043338338074402`,
2.483914515376221`, 0.0009750837354062236`, 0.0009886317595815726`, 0.013894216141042248`, 0.14979244046733403`},
{0.08087154180018502`, 0.9611156520127571`, 3.21224132650018`, 1.479145162275284`, 0.656808362332749`,
0.6575245079334169`, 0.06402086777278035`, 0.03166366563194078`, 3830.357327788698`, 29.178148219068248`,
0.1565085846563346`, 2.9278897142269216`, 0.2225569823133069`, 271.09379792749684`, 17.972474018448448`,
733.6380182026467`, 0, 186.33530799893026`, 5.71332556853335`, 5.731993226845784`, 0.003267389209403282`,
0.26599644953577445`, 0.26709604516692015`, 0.004133873339530414`, 78.44523755660104`, 0.3073077569619223`,
0.28249840978912477`, 0.002165006770937117`, 0.0021906363766957346`, 0.011838118061646474`, 0.0326081082442218`},
{0.05254670070328532`, 1.9573141803351248`, 1.2965014076065788`, 1.332575014963222`, 0.16006264378785184`,
0.5916179072285757`, 0.24512530080047995`, 0.22325909451251788`, 1444.5445370368107`, 345.46000244130437`,
0.09820743687290096`, 1.9309038402941605`, 1.3978069307443537`, 44.311668762861004`, 16.341454128295684`,
45.51763585294383`, 0, 11.028925078562617`, 1.1404635148784132`, 1.1648037424882647`, 0.021342399202000317`,
0.14455062411241332`, 0.14721969621290443`, 0.018464618308499547`, 31.88922014037663`, 0.10850940545293832`,
0.7129356600836217`, 0.001274305073151627`, 0.0013452346931441413`, 0.05566141223709509`, 0.19753451235166306`},
{0.18436703585318248`, 1.635211707023542`, 8.369994731341517`, 1.0460883726353645`, 0.04194749676902032`,
0.5545482251392689`, 0.12087395792220468`, 0.0196688212581193`, 3332.658025818686`, 221.69628027119882`,
0.14765622082279756`, 3.2016279616467553`, 0.8091642391531453`, 77.73522583751895`, 10.345299704612975`,
88.45463648498598`, 0, 60.757113966138924`, 0.6932106642009671`, 0.6999620137425763`, 0.009739246509415933`,
0.019002396101171264`, 0.01903385578585954`, 0.0016555640941688932`, 16.193517050499775`, 0.0500487920468914`,
0.684663201697026`, 0.00021886514484126263`, 0.00022046284524931492`, 0.007299930782542319`, 0.09834265750921191`},

{0.23462399893919866`, 1.1910919878969217`, 8.83980004261467`, 0.9529401154166846`, 0.02195492110865227`,
0.5092860902949421`, 0.15815979544916958`, 0.02056729012396142`, 2175.860445178102`, 128.66287522198218`,
0.01279382216028177`, 3.236783194651748`, 0.9208763725654232`, 141.61846131688316`, 15.108853263592536`,
163.3625199447715`, 0, 109.57572970400118`, 1.7693811579828786`, 1.7851587622591847`, 0.008917018362675888`,
0.029548460079203805`, 0.02958404893894316`, 0.0012044235010542081`, 30.107081725845436`, 0.09903968380402113`,
1.1353322775363748`, 0.0003738828344039824`, 0.00037616415755148186`, 0.006101706036160115`, 0.09481984360161998`},
{0.17738230405709043`, 3.796341718770906`, 2.6191444768927727`, 0.8810359048502269`, 0.8432004845871259`,
0.5364474114112706`, 0.12890044163052178`, 0.09199163668005597`, 2908.5735595435463`, 327.07488963843366`,
0.2488393941661181`, 4.302397057177101`, 1.240771752535033`, 150.7987230047274`, 43.40549404677515`,
154.60788177045433`, 0, 65.66976061914228`, 1.5365400182593114`, 1.5504266352416576`, 0.009037588879772684`,
0.07370912648665975`, 0.07389104264287519`, 0.002468027568449882`, 83.33187105541184`, 0.18678135266058812`,
1.0957952645965794`, 0.0010065712981923713`, 0.0010170476782700158`, 0.010407986097416222`, 0.10712835569804653`},
{0.1997594423184546`, 2.9770319172231083`, 0.9793144795904194`, 1.1541884780655445`, 0.8809204935983468`,
0.3405913744561432`, 0.1996665175564089`, 0.005381059121025975`, 3350.374432264831`, 76.29678229001865`,
0.1902613732440958`, 8.131907933048556`, 0.2784170059108251`, 936.8256464018312`, 57.08824557894052`,
1750.3749809128522`, 0, 866.0519391761051`, 1.5281693993107226`, 1.5363329198622901`, 0.00534202592673938`,
0.8509137231269999`, 0.8494227620747121`, -0.0017521882792166377`, 64.99155823816687`, 2.4282578684709883`,
0.7123410409450054`, 0.008865944850264262`, 0.008940416159561353`, 0.008399703647476553`, 0.04796612592689146`},
{0.20820380555279266`, 1.947139162442057`, 0.8103918093452425`, 0.8157066692238308`, 0.08411468051466398`,
0.597917518430226`, 0.19620399803346056`, 0.2948101237919289`, 3907.9815148798407`, 119.17289873329406`,
0.10826075744997776`, 6.209951432179045`, 1.4468389844056118`, 339.9698896180337`, 18.750382694440546`,
384.8058173967723`, 0, 66.84486454501673`, 9.430726055236944`, 9.534065786517791`, 0.01095777044901669`,
0.34386632608143`, 0.34455736395666364`, 0.0020096119416765923`, 262.32765760592247`, 1.022775395594737`,
2.4530484886928714`, 0.005057157286088687`, 0.005134176564449816`, 0.015229757352612783`, 0.19343231729474983`},
{0.16610260493961904`, 1.5013302413059524`, 6.689821314230944`, 1.0482229687817899`, 0.1182529778116388`,
0.3276703522967399`, 0.18950977903448152`, 0.011602315706762772`, 1976.8505440085055`, 128.55302132767815`,
0.1206362258416388`, 2.990415653764348`, 0.8762156912964318`, 143.5097780671747`, 13.509526837112668`,
169.39498579166272`, 0, 123.02285510493448`, 0.9017715542447361`, 0.9089798570338483`, 0.007993490984697704`,
0.04710238646652662`, 0.0471553174034306`, 0.0011237421471541609`, 19.3408129305299`, 0.11176898701375901`,
0.9241687454008943`, 0.0005418321518462221`, 0.000545191416659233`, 0.0061998255392645 7`, 0.0692836205423352`},
{0.18623015008520377`, 1.9857475843613202`, 5.839525692796151`, 1.2062427816326777`, 0.7227315679666446`,

0.4380351155851495`, 0.06781424675520398`, 0.3047046595907428`, 3879.8004023641743`, 31.750244384294604`,
0.1136575036642325`, 7.396681541568938`, 0.3865663286027079`, 1149.5266271462528`, 39.50880819812861`,
1875.8967884758138`, 0, 215.3848575117069`, 31.78323110236881`, 31.830047650597976`, 0.001472995243258168
3`,
0.20147253547211494`, 0.2014112922190672 6`, -0.0003039781720329815`, 901.6210626389463`, 0.5360037217002082`,
0.9563691078500491`, 0.0020170070061514833`, 0.0020226378654646555`, 0.002791690507766731`, 0.02485183588676648
2`},
{0.11240037278862203`, 0.6491092968888421`, 0.8347611214383086`, 1.4549390021490687`, 0.14670428342309072`,
0.37243292165179553`, 0.23972369902467655`, 0.01185953233820889`, 3372.5164268352446`, 237.4368160205147`,
0.1138398904730133`, 5.916126824166563`, 0.20110182675101163`, 113.41935834463696`, 17.31844760528657`,
205.90239634340364`, 0, 96.53476263237445`, 1.584518270172341`, 1.5995161409210636`, 0.009465255801115902`,
0.2073580753541121`, 0.20876968203987586`, 0.006807579995874891`, 14.693222003701319`, 0.3329589281504587`,
0.4569629200616540 7`, 0.0017006466826164912`, 0.0017454245856226322`, 0.02632992700003322 6`, 0.1653001106266050 7`},
{0.07362676393672318`, 0.7355538194163032`, 3.988559823436466`, 1.1003960033820657`, 0.2330826487371216`,
0.30236238420511485`, 0.203470150172021 7`, 0.007403410519656421`, 3383.0372997062996`, 216.63011163159672`,
0.15749031565529237`, 1.8803990102478743`, 0.9980728680407593`, 59.59167464878472`, 5.198564659371774`,
69.3679549342544`, 0, 53.77805581743797`, 0.4909153732174914`, 0.495291419117269`, 0.008914053497849572`,
0.0546279404283284`, 0.05478625767713577`, 0.0028981002682149093`, 5.158495396861511`, 0.05745826391808171`,
0.37005352145046194`, 0.0005972782486612926`, 0.0006038555444098701`, 0.011012113304510018`, 0.04585100366569754`},
{0.1783157725484701`, 2.833766552573177`, 0.3970480956539326`, 0.7937141247518862`, 0.3426969414627741`,
0.17660776442515302`, 0.06749542877910886`, 0.2962004793965935`, 2934.9500228426696`, 381.96465886346436`,
0.2370505433342167`, 2.5471608872904294`, 0.782904324178523`, 38.38225827943162`, 7.354270449992673`,
42.13239500141911`, 0, 7.547457429693845`, 0.7274555839853761`, 0.7483998877221166`, 0.02879117873011139`,
0.18554949663977657`, 0.1858631284716827`, 0.0016902866220920298`, 29.449132891147748`, 0.4726628834186056`,
0.4232851039920764`, 0.002805433998653961`, 0.0028843278349920754`, 0.028121793767369674`, 0.0642307600824683`},
{0.19113040953496924`, 3.008225434073468`, 6.355992034495486`, 1.3442852038382949`, 0.5652481966732263`,
0.17677420590456394`, 0.15942860908355244`, 0.011393875146253816`, 1546.892426029899`, 241.3481933910041`,
0.08634784635015025`, 8.231809963935362`, 0.7429779031641539`, 309.7403500353932`, 56.06413404006201`,
343.742118871018`, 0, 266.3463434446389`, 0.9783011781088554`, 0.9859959545762771`, 0.007865447409862503`,
0.04992267664253842`, 0.04993125133724675 4`, 0.00017175951461356753`, 42.04214980244416`, 0.1363105947395821`,
2.059346606283618`, 0.000448831883946732`, 0.00044991730427398706`, 0.0024183226862373175`, 0.13822700182916067`},
{0.1657364273353646`, 1.7016560013922657`, 2.864207314541927`, 1.4862862793345373`, 0.7297929998129427`,
0.24571335694321284`, 0.16930689708064994`, 0.03637877544295533`, 2361.9950805518038`, 368.27061311393015`,

0.19309626758691123`, 5.461234510584882`, 0.819553318889934`, 167.2272772084036`, 34.14285930748782`,
179.3464921638975`, 0, 110.14704526668844`, 2.2416541380265054`, 2.25953383130881`, 0.007976115931088934`,
0.09941311938036689`, 0.0995556481186345`, 0.0014337014989167507`, 54.49320310026578`, 0.23537678909110682`,
0.9844087048701796`, 0.0008057513637762526`, 0.0008125038648644373`, 0.008380378106390474`, 0.1044924157494375`},
{0.243670782866162`, 2.468817897966531`, 5.276425534951404`, 1.2860325806374826`, 0.9986905753378303`,
0.15094415892422752`, 0.11186418412899801`, 0.017007046631466297`, 3882.4128529746977`, 364.42553589609224`,
0.22618030191028127`, 8.141676219478274`, 0.7328485154782189`, 259.40362592151627`, 33.91495078064327`,
294.57960962222063`, 0, 208.62921955094575`, 1.3913278392790913`, 1.398031659069876`, 0.004818289120311237`,
0.04870432986945969`, 0.048711550284107535`, 0.00014824995369400185`, 49.07050102216173`, 0.1695403169752026`,
0.9296634989419084`, 0.0004578271952893065`, 0.0004589189528465883`, 0.0023846498602859 7`, 0.0572202243136074 4`},
{0.24765743502672427`, 0.8633830393481601`, 4.686988270445781`, 1.3325105661709873`, 0.9808367367869013`,
0.6497982062118903`, 0.12182607994660094`, 0.10862851464254074`, 1618.8129303586047`, 29.218901620977476`,
0.12364149245529477`, 6.112534098964166`, 0.4592681454217531`, 2087.4495512593935`, 189.83689206883247`,
2822.8893085086606`, 0, 818.1687091635947`, 94.98361028744944`, 95.20224234387926`, 0.0023017871795796285`,
0.6093160541593914`, 0.6087228020220821`, -0.0009736361503354107`, 1171.5319734034183`, 2.155737872765659`,
2.0330399915150115`, 0.005505546571871189`, 0.005537508669466298`, 0.005805435877776155`, 0.038149337431160266`},
{0.05305812976899127`, 2.2171177144674177`, 7.758771838981232`, 0.928469480102368 3`, 0.19067769039866356`,
0.644253374243589`, 0.22681045533276267`, 0.012934901517196042`, 1447.005934926009`, 36.93966570702122`,
0.23490962595246417`, 9.29802294373275`, 0.16252108168662027`, 743.8045317854634`, 87.10371791429012`,
2126.303854529301`, 0, 627.6410468960726`, 3.540106305438367`, 3.550537758904527`, 0.002946649780018129`,
0.0600703925473879`, 0.06009029648595032`, 0.00033134357406972015`, 112.12617715550273`, 0.045531752615357066`,
1.6972069993700278`, 0.000780861742482708`, 0.0007835898791463374`, 0.003493751217668039 6`, 0.17315753931461`},
{0.15871988201336973`, 0.69579697553139`, 0.8043899133503648`, 1.3164539868322986`, 0.019311018286041204`,
0.4418003624772314`, 0.15298309339567961`, 0.16062079917918642`, 1028.6861638399246`, 387.4645099104921`,
0.1109630441095813`, 9.931289248375212`, 0.6360820981063933`, 167.1202507609636`, 94.12234183588787`,
171.24590255232397`, 0, 52.678004407751985`, 10.409741114254071`, 10.639391291366914`, 0.022061084381664653`,
0.17142786529737472`, 0.17231381349055636`, 0.005168052414610491`, 103.47237690518199`, 0.3887001507688598`,
3.216615419855074`, 0.0015522152519045207`, 0.0015899022498191023`, 0.024279492079684717`, 0.8713769735646605`},
{0.047521269126133275`, 3.6122781353018008`, 6.744377644847237`, 0.8245850034533604`, 0.6257570737736955`,
0.5030346499913471`, 0.1805220904232982`, 0.04172896692907123`, 2236.066689248032`, 158.14626584756797`,
0.1995362178990888 7`, 3.2116522076145895`, 1.150911515892405`, 206.08953096046935`, 27.2160032500319`,

222.02535206354074`, 0, 129.49308620617433`, 1.4543756045250327`, 1.463218662287847`, 0.006080312221478756`,
0.04917901641969704`, 0.04923454446006968`, 0.0011291002629811864`, 75.05155995345865`, 0.03338641820910862`,
1.1705279613573238`, 0.000719190322936325`, 0.0007231012859259197`, 0.005438008361440261`, 0.07043083219650269`},
{0.25961180109703935`, 3.304460665024532`, 9.731602575575781`, 0.7572846132780213`, 0.5757688453852501`,
0.6201294851486256`, 0.16223330053532697`, 0.09572577856146514`, 1068.913375906082`, 270.60830273991644`,
0.06817614592711113`, 6.616513224635819`, 1.43430723538359`, 247.5073465464486`, 126.39031410638759`,
248.75199279973899`, 0, 106.3041731942984`, 2.927341437775144`, 2.965654836864226`, 0.013088121048906887`,
0.018259557628419847`, 0.018268625921844774`, 0.0004966327010471616`, 138.1897804889192`, 0.06771995204484599`,
3.162116064693851`, 0.0002911779897416533`, 0.0002921380573532056`, 0.003297184695876698`, 0.3304429442406201`},
{0.2570743120023581`, 3.9645616544323667`, 0.778960090135616`, 1.0710848615453759`, 0.4119983463981953`,
0.5200874369461032`, 0.1117389487686139`, 0.10363949916728431`, 1639.730583125267`, 312.26786162753615`,
0.028234231304594515`, 5.869357124529651`, 1.28422648371814`, 143.72881755284712`, 53.84861863486312`,
145.91846414836684`, 0, 58.79141807444474`, 1.45677005988984`, 1.488409102181344`, 0.021718624759418992`,
0.20847836324565483`, 0.20994757150333515`, 0.007047293708599911`, 82.50649598234844`, 0.765634739982282`,
2.125764221803037`, 0.002322009225275811 8`, 0.0023891955791945176`, 0.028934576653426136`, 0.3034590864169802`},
{0.2562944092973924`, 3.075529570162291`, 2.568785124728583`, 0.8870148603223287`, 0.9606731370217558`,
0.2809776665243148`, 0.1016902992050977`, 0.08251508016369583`, 3903.9002781741165`, 210.26921647621452`,
0.17009591440353533`, 5.238706912116847`, 0.8143837239091778`, 259.3950257300158`, 31.69526518229395`,
291.9966987972128`, 0, 119.2962686000777`, 3.1066174173385654`, 3.123104024677179`, 0.005306931985444 6295`,
0.10707739271277956`, 0.10713390149385649`, 0.0005277377385206616`, 136.49276757437102`, 0.39204767306303484`,
0.8719126947128926`, 0.0014567506026978982`, 0.001464951869770803`, 0.005629836059594773`, 0.04538002892147769`},
{0.19549413117658376`, 0.48089214427271987`, 1.3626169663716503`, 0.9415198179325406`, 0.6690196233409793`,
0.21285229920071647`, 0.06541758209495863`, 0.01678513797168908`, 1726.2120585826779`, 324.36081581294445`,
0.2027319827154383`, 9.808516157446391`, 0.15828659811715773`, 175.03821126130066`, 73.46216859794144`,
235.26617490221673`, 0, 141.16908269709634`, 4.257922057816758`, 4.301737291947117`, 0.010290285621814554`,
0.07722151208875357`, 0.07728238794328446`, 0.0007883276678255502`, 29.251446693280215`, 0.21566217734191467`,
1.4227369298278523`, 0.0009884677669875774`, 0.000995335180955782`, 0.006947534555561186`, 0.22077772184731942`},
{0.05227980057052345`, 2.0688773882101916`, 3.6600804041449706`, 1.1141893430147094`, 0.9026814754625292`,
0.2868484255785748`, 0.07257484506693682`, 0.008111547996607639`, 2542.960019998747`, 267.5073034447911`,
0.05331315230928124`, 5.17983990011182`, 0.732439763493062`, 165.76181751272372`, 43.44671906031177`,
179.33705178573345`, 0, 148.44469524729672`, 0.5588301445364549`, 0.5633588852836234`, 0.008103966458225198`,

0.061057953836179735`, 0.061119513925743295`, 0.0010082239199944443`, 16.516443569738627`, 0.04560139499713933`,
1.0880403120807631`, 0.0006610368142329071`, 0.0006644941005097705`, 0.005230096421899644`, 0.07543209653374446`},
{0.16735387248889688`, 2.2901749191726726`, 8.076219774814714`, 0.8654381827857112`, 0.8324588302247302`,
0.6842201049055578`, 0.20618844268782105`, 0.08330233274804327`, 3386.0149520877076`, 231.00380607589523`,
0.24136440782861118`, 8.110578461374818`, 0.1918239477431709`, 350.90578084926614`, 83.40097481070718`,
546.2496678937277`, 0, 160.54079912917578`, 5.64306654409292`, 5.655262545307958`, 0.002161236469522931 4`,
0.02908155803336398`, 0.029098302836501073`, 0.0005757876905316817`, 184.62299237862905`, 0.06952730506976218`,
0.6602049624737948`, 0.0004055949421557514`, 0.0004071403601295 8545`, 0.003810249619041084`, 0.12698426758116269`},
{0.13514798653424465`, 2.2171967161009567`, 6.79713748604177`, 1.0120008311676625`, 0.6388353844515477`,
0.24746546435416983`, 0.06301863255527632`, 0.010147071600538154`, 1724.1318426660982`, 61.023906450601714`,
0.20149638211275833`, 9.77881328490188`, 0.3825890641127949`, 879.7368889127523`, 76.24671555017035`,
1207.3956085153202`, 0, 767.9136616996283`, 3.396577233759929`, 3.4087911241864512`, 0.003595940732665559`,
0.08408187401115952`, 0.08406650537366266`, -0.00018278181448272424`, 107.58399840965411`, 0.16233565680911927`,
2.299577127437494`, 0.001004197502519677`, 0.0010058985613109854`, 0.0016939484384697057`, 0.05836335219110249 5`},
{0.1787587102327428`, 2.3010027450563504`, 2.4341725113616057`, 1.2732377481963428`, 0.4302268260704325`,
0.6620369180993946`, 0.07196824748401134`, 0.018432445015582038`, 2032.846726882145`, 239.6521240526355`,
0.16901168551833623`, 7.60355154430442`, 1.2541734909464242`, 214.35893876267366`, 66.01102985676056`,
219.28840273533618`, 0, 170.00568833265135`, 1.297759857952506`, 1.319103570454466`, 0.016446580907221398`,
0.09215790205196364`, 0.0924358405560807`, 0.0030158944369236274`, 42.659271365323605`, 0.23534325297947925`,
3.1239701401300684`, 0.0008697478912587098`, 0.0008802121164627931`, 0.012031331503361775`, 0.2860593509051131`},
{0.040040522389580085`, 2.419089216699528`, 0.6029402922994809`, 0.9540354124914854`, 0.5857842300398015`,
0.29335248867278896`, 0.08466193409101153`, 0.02752786168327366`, 1658.0955376199636`, 315.19357733141317`,
0.05596420932223484`, 9.548403397933097`, 0.8186383788896436`, 234.18444921171977`, 79.2437946350975`,
244.40372663390386`, 0, 168.51977741045135`, 1.8350137008254526`, 1.858914455256816`, 0.013024837046509141`,
0.2427153533949096`, 0.24323774493705685`, 0.002152280582338406`, 63.41516937375346`, 0.1388349934557447`,
2.6230241319964684`, 0.003052204451909124`, 0.003091480982659094`, 0.012868250265935854`, 0.27505749732442597`},
{0.2738861805350336`, 1.7653732412133003`, 3.204285049736129`, 0.8904691615720403`, 0.006777008376177118`,
0.6422126566237496`, 0.21196430882173833`, 0.02384817430133364`, 3509.269393393407`, 210.0509220408095`,
0.16655185586266846`, 5.6829810466015855`, 1.030785881009633`, 168.45123424045212`, 18.561193746207547`,
193.45924857329717`, 0, 125.75750822410834`, 1.6173950478701058`, 1.6325900698896416`, 0.009394749934189317`,
0.051527313312036624`, 0.051636215484046455`, 0.0021134843835219908`, 40.79008482829699`, 0.20160884337520213`,

```
  1.3620113383776524`, 0.0006962466681675883`, 0.0007030406528934117`, 0.009758013986198577`, 0.21606637311326796`},
 {0.15392813884203022`, 1.030461564252417`, 8.114572747609092`, 0.8272078545061425`, 0.12582813602684628`,
  0.5315104372547351`, 0.15396277604540914`, 0.15739980098951944`, 2790.789894250489`, 293.11851381168844`,
  0.07517370467239265`, 9.08396266539139`, 0.493350448003097`, 187.43362187823823`, 37.6057384379116`,
  220.4466897835582`, 0, 58.60449221321561`, 8.192093485432625`, 8.252485262735137`, 0.007371959000455952`,
  0.013196558480517196`, 0.013202204398997743`, 0.00042783264203927196`, 120.59482096429946`, 0.02901888122864049`,
  1.375206657687389`, 0.00019272168876915963`, 0.00019325026180089808`, 0.0027426753839396856`, 0.31270112926283905`},
 {0.07916527150572594`, 1.5183903543815438`, 4.168943084064299`, 0.867458547779902`, 0.553589800680331`,
  0.48176316193720536`, 0.2283008873811488`, 0.05557602912612958`, 764.3466604125074`, 98.1402416311065`,
  0.08270787403012858`, 8.344308234825018`, 0.19844893248168471`, 696.4903907836533`, 187.33975350468359`,
  993.3658288119424`, 0, 389.3614521419654`, 13.52452519573088`, 13.584282210864465`, 0.004418418707404781`,
  0.10896890771624596`, 0.10899984287024471`, 0.00028388973191617595`, 293.364408639828`, 0.12323647378553663`,
  2.849648896705384`, 0.001514095284000594`, 0.0015216417542641683`, 0.004984144883956709`, 0.217518467661601`},
 {0.21281579254928695`, 2.151234091423703`, 4.99405590028015`, 1.327714565612129`, 0.5224256111223291`,
  0.6816332648036347`, 0.12550320306228152`, 0.04036971585137323`, 3491.0437940299144`, 389.9199715703103`,
  0.034574092345333096`, 7.184915600941272`, 1.1300147670199618`, 155.61880861644343`, 41.47338230969908`,
  161.9411761428872`, 0, 99.1551463378274`, 1.7745907510517942`, 1.7937898566163524`, 0.01081889193504404`,
  0.036049224620584526`, 0.03611665256141364`, 0.0018704408080558732`, 54.536573171254275`, 0.10959777583453023`,
  1.5393172492612324`, 0.0003269631766306125`, 0.0003296101737574593`, 0.008095704091587441`, 0.2553518268315031`},
 {0.1629622003008503`, 2.9652820153229094`, 3.631698402387231`, 1.3160868122315574`, 0.8459043277254139`,
  0.3376309499507988`, 0.22909924766435485`, 0.29198486983597477`, 2152.5623501482423`, 151.56102061804546`,
  0.24318844331582645`, 4.2450714851638445`, 1.1792634690726134`, 438.4336386342324`, 42.0899158844314`,
  464.03578665340444`, 0, 85.7750631094551`, 8.115058016412018`, 8.155916830914588`, 0.0050349380645138275`,
  0.2344184759627563`, 0.23455401532483727`, 0.0005781940247000517`, 343.7633655624077`, 0.5457335804868848`,
  1.4243089336780226`, 0.002145964078692386`, 0.0021613379401723993`, 0.0071640814646725115`, 0.060285585830694925`},
 {0.1743683640254448`, 2.5950167332919714`, 4.4795399396250115`, 1.4920254306676737`, 0.7406209345210579`,
  0.5005424929143194`, 0.1556663350770055`, 0.009754157595577621`, 589.2836300166855`, 248.33057190508907`,
  0.03897335434322141`, 4.862373453735419`, 0.5620446056385968`, 233.17562550691576`, 189.656960400544`,
  228.48694705090637`, 0, 204.50895408472215`, 0.7395080884392445`, 0.7490618626223383`, 0.012919093560230577`,
  0.09987539722085524`, 0.10012656348465264`, 0.002514796143858966`, 27.41479805577994`, 0.24878728028271982`,
  2.4792683979120747`, 0.0008033539568025327`, 0.0008136076397013454`, 0.012763592949269542`, 0.2623514437402908`},
```

{0.21708195818330828`, 2.190522036446822`, 0.6621285433489934`, 1.031682155458868`, 0.05602003593078142`,
0.3042918074708675`, 0.06460489028881022`, 0.10032641034223391`, 967.4633500940654`, 32.783765325462525`,
0.23824254150550733`, 7.5221087110888885`, 0.8625924583250821`, 1117.2365946884786`, 66.20589057262717`,
1293.2024519811205`, 0, 460.78489345926874`, 20.14337317362714`, 20.378289480556436`, 0.01166221292255365`,
1.452773484143839`, 1.4451810734404682`, -0.005226149008250358`, 630.3500403600295`, 4.5052987533526`,
7.093887800217955`, 0.016838893434309`, 0.017048133163304748`, 0.01242597857228711`, 0.10433098912905506`},
{0.23286963035122604`, 0.6749345188436879`, 0.20207668159083703`, 1.2334071076981241`, 0.15415172519231857`,
0.5502451893184188`, 0.06023408333501554`, 0.2884596823004714`, 1454.378698010155`, 160.3197874285933`,
0.13928726255657442`, 2.5761586107644754`, 0.6603399169512614`, 90.41575517976906`, 22.07171373787987`,
94.87891829453761`, 0, 17.476841582324397`, 6.349369660099105`, 6.837051641579154`, 0.07680793647041129`,
1.2409937528099724`, 1.263564595068327`, 0.018187716261462095`, 61.22012509285281`, 4.1284250926434245`,
1.1168807002819867`, 0.011848144209948841`, 0.0131998472744765`, 0.11408563573969999`, 0.22112541919733147`},
{0.14295588103954704`, 1.412350123248217`, 3.177274858880626`, 1.1913477441560478`, 0.681260929106482`,
0.5789699232051367`, 0.09066128493810405`, 0.04793649880497841`, 2544.3651836876206`, 337.2728729655139`,
0.011905134502266851`, 4.430427622941885`, 0.74003827479563`, 108.79215269650051`, 39.65129838352222`,
113.0342550610454`, 0, 64.85589059613972`, 2.0663944284655`, 2.0881557894716622`, 0.01053107804898712`,
0.05751116257564331`, 0.05771084375858582`, 0.003472042191459357`, 41.692463224609796`, 0.11745084165154815`,
0.9651532044012363`, 0.0005795122394560703`, 0.0005873686056825598`, 0.013556859875579974`, 0.14745194236538706`},
{0.11809903796554244`, 3.7153066081905903`, 9.394589088557098`, 0.7843066157779603`, 0.3510759725555237`,
0.22803969658113876`, 0.10165008188335536`, 0.005399145823047646`, 3902.7306906909416`, 388.8592048663462`,
0.07729944387610005`, 2.9718049006587925`, 0.2891160085661364`, 38.87401728801607`, 7.090405750389786`,
53.624917430212655`, 0, 36.05175808696726`, 0.05110192247506896`, 0.0514772137100652 8`, 0.007343974880385629`,
0.006849283546662468`, 0.006852745786978575`, 0.0005054894125084974`, 2.7122758608980986`, 0.011555625680202939`,
0.22591900238700227`, 0.0001055048456017893`, 0.00010582174823189218`, 0.0030036784405047534`, 0.04762394951427048`},
{0.16202429237235855`, 1.84055090570393`, 9.090225524744397`, 1.4016003618243056`, 0.18111565798783458`,
0.3854966323949548`, 0.18149685472874316`, 0.020436792872453445`, 1570.1908216352922`, 182.31609839273892`,
0.10515949401254626`, 5.892117812074294`, 0.4606489730839265`, 204.00175916387408`, 38.55241027909658`,
247.6603591867031`, 0, 158.07429022210505`, 1.6766555526130689`, 1.6890438685848508`, 0.007388706614471108`,
0.033471950030169936`, 0.03349644951005816`, 0.000731940620912086`, 44.085284227364404`, 0.0774752716851372`,
1.3640465589253183`, 0.00028819440885430314`, 0.00028946857610053753`, 0.004421207376297698`, 0.16965186838111204`},
{0.23592081280505794`, 2.903542817229688`, 5.375766226090379`, 1.0406060233325234`, 0.5645881743757346`,

0.6023633607842607`, 0.10170656063773648`, 0.2230968616100032`, 1009.1076309440446`, 206.38966170547087`,
0.05554284619714861`, 3.437370706600392`, 0.2602834274636374`, 126.22891976540133`, 66.23002868446201`,
136.07468681594563`, 0, 30.893524093009965`, 2.239712729868957`, 2.261856258605623 4`, 0.00988677183522646`,
0.044442908425537705`, 0.044558557353715084`, 0.0026021908168125663`, 92.90145442098427`, 0.14978581541676989`,
1.0592112072345194`, 0.0005125827948763151`, 0.0005191653651935423`, 0.012841965011360923`, 0.18067606018297816`},
{0.16797762422450352`, 3.393739269293537`, 1.1723349699849432`, 0.7876651455122983`, 0.44858157260876874`,
0.38511915317210543`, 0.11126403235021692`, 0.0942998855830809`, 1121.8751632200092`, 393.3700502870249`,
0.18309808816527456`, 7.014749603006731`, 1.3459972330025094`, 141.84181690791576`, 83.6045952576875 9`,
142.06959985579581`, 0, 61.74438302114237`, 1.612883774981315`, 1.6452467332373923`, 0.02006527609619746`,
0.08491637037244128`, 0.08511738702925298`, 0.0023672309111899548`, 78.19581434229258`, 0.20377214504191057`,
2.548628769370118`, 0.0012943541280843185`, 0.0013108529775930937`, 0.012746781696593157`, 0.36056229185290123`},
{0.17119714315024748`, 2.9315531156526395`, 7.9309025517292255`, 1.4075914943739267`, 0.478244345688974`,
0.6490520691087696`, 0.11764936739953108`, 0.10875560486489166`, 3501.9685138343757`, 327.0147307274891`,
0.208688235529143`, 1.76928881617237`, 0.15623787817751156`, 27.789039242566833`, 9.03264550965156`,
42.680712423985135`, 0, 10.943161964618318`, 0.391470923802353`, 0.39429159479739695`, 0.007205314171603971 5`,
0.017373620187285974`, 0.01746266851557419`, 0.005125490676570754`, 16.39454009086005`, 0.04249020203201474`,
0.1411215381445849`, 0.00014747298356876382`, 0.00015051014646680843`, 0.02059470707479405`, 0.07551459435822659`},
{0.217831918966031`, 1.9579162032970752`, 8.666870397376897`, 1.3889143705395077`, 0.46152386514296384`,
0.5340210525452818`, 0.23464141923209442`, 0.08443497471803191`, 2881.1816355963792`, 40.761442588478076`,
0.026544318054481375`, 2.7988265733593654`, 0.6550641924577596`, 539.6315162984331`, 15.32966680593877`,
715.1786796829259`, 0, 244.5661245367648`, 10.15764288338494`, 10.18408597617049`, 0.0026032705706562353`,
0.1935093475590005`, 0.19369401705728834`, 0.0009543182312241605`, 284.1116226954946 5`, 0.6021787502375139`,
0.6116263980507831`, 0.0016806301442706673`, 0.0016907235710337846`, 0.006005739452862979`, 0.020368545125575066`},
{0.05909313438823349`, 3.998253006220927`, 2.7606012432005738`, 1.485525656051693`, 0.006754191626425765`,
0.414858609675688`, 0.1539104163421624`, 0.018873368421200944`, 1163.2065814066136`, 134.3441176716297`,
0.2419809034541145`, 8.466048197729386`, 1.4640823465517014`, 386.24252126707546`, 67.88099528049129`,
415.2622866172925`, 0, 305.0472616470657`, 1.3901571626335931`, 1.4113279624292558`, 0.015229069320159105`,
0.15378278516327437`, 0.15401375635428383`, 0.0015019313817488111`, 79.40285792313331`, 0.12982152557493518`,
5.6758086112666986`, 0.0012459578899502999`, 0.0012559989021899301`, 0.008058869662144641`, 0.3872189655154143`},
{0.05150190326045606`, 3.439128676554682`, 6.3456448543065065`, 1.3220565831174902`, 0.02053589536141165`,
0.5629905593040019`, 0.12931144501098696`, 0.09719268107904352`, 992.6848015373967`, 364.3637294024504`,

0.08014172250870272`, 4.69481466986781`, 1.4428291086778402`, 80.67581803533719`, 50.87593508331862`,
80.27982969382828`, 0, 34.87654542190592`, 0.9128259250637576`, 0.9355315199525687`, 0.02487395927895597`,
0.022419306904710536`, 0.02246530994917391`, 0.00205193874453391`, 44.84751165127603`, 0.016494813933888245`,
2.014993813332703`, 0.0002039566176019747`, 0.00020580823809774416`, 0.009078501681092366`, 0.41014063387709215`},
{0.07096987014316519`, 2.2631739720341697`, 4.701888958284682`, 1.1268609277641355`, 0.5889482309057459`,
0.3638885919169875`, 0.05633232701579177`, 0.3132395508309391`, 2469.4656057395405`, 63.55428754000428`,
0.06344769284580837`, 7.209878698655041`, 1.262286526290143`, 724.9749836912831`, 44.72521338313976`,
793.441409324752`, 0, 136.25860469072185`, 17.65356013527776`, 17.7778897248938`, 0.007042748808926413`,
0.1512344897204392`, 0.15122852209133092`, -0.00003945944552274927`, 570.7582544557238`, 0.1533298870920536`,
4.802553573689234`, 0.0016209277548681067`, 0.0016253102964359895`, 0.002703724181858602`, 0.06680036916263682`},
{0.13424044754942377`, 3.4568739781145084`, 0.7268053299269095`, 0.8378137298915767`, 0.4654803229022981`,
0.5071605573546728`, 0.16764031212635366`, 0.06824087778957048`, 600.6265082502678`, 271.9873255003124`,
0.09465386647177981`, 2.8029066087185885`, 0.5029743243735594`, 95.51693520150762`, 72.29265453924974`,
94.08817970502018`, 0, 48.67431832750189`, 0.9157241937421908`, 0.9358292151225108`, 0.021955324013182453`,
0.24146018614028522`, 0.2447737645491201`, 0.013723083965941019`, 45.22204480681816`, 0.4630531921834027`,
1.3840583621196267`, 0.003382220070984099`, 0.003563421863410124`, 0.05357480844624707`, 0.25898723155062586`},
{0.1814998220796576`, 0.5457041309396096`, 6.628442116729424`, 1.4816442478897651`, 0.04014018359229943`,
0.36141091722571295`, 0.13153411394886588`, 0.008451051131756494`, 2397.6906312548626`, 255.11228411318268`,
0.24580682176341695`, 6.012737749288213`, 0.971372885437352`, 135.83926269384904`, 22.39800044671929`,
150.46216696003302`, 0, 121.16955159308687`, 1.6425292627128831`, 1.6636549276722135`, 0.012861667331538484`,
0.0316451804974538`, 0.03167704614721753`, 0.001006966914481433`, 12.804785769308719`, 0.08205135185668339`,
1.5502770850372716`, 0.00025765435724378616`, 0.00025903304908257864`, 0.005350935468512175`, 0.199384662946653`},
{0.25116849017091947`, 1.0524160237641933`, 0.8392172051883035`, 1.4326662099306988`, 0.4925377955188637`,
0.4563121318749678`, 0.10417530701605077`, 0.012130927300066367`, 2850.5603662771573`, 179.7348471844905`,
0.14797581172978125`, 6.731564570008534`, 0.3667284512812037`, 239.50374638143637`, 36.45967137242655`,
302.17501338385387`, 0, 202.6771052154952`, 2.1805308861830626`, 2.206194343860149`, 0.011769362149237628`,
0.3764334060394044`, 0.37855516129506755`, 0.00563646908489579`, 32.78322349902552`, 1.3506887177830733`,
0.9258284157773601`, 0.003144764927351828 6`, 0.003222073985698079 4`, 0.02458341406502273 5`, 0.15422950184721917`},
{0.10627844972153494`, 1.9122914449389148`, 4.879880104768988`, 1.1152938555639593`, 0.36058806344071925`,
0.2510558996666748 5`, 0.133980532681923`, 0.018281043179813147`, 2529.9927880778405`, 195.84606271036728`,
0.1085822670929286 7`, 2.0748738252120926`, 0.9066780951872615`, 71.11123400558277`, 7.934565025486043`,

```
  80.68989788265371`, 0, 56.369281464075314`, 0.5154043231124962`, 0.5199987102998888`, 0.00891414173565197`,
  0.04900110137273918`, 0.049090132681318475`, 0.0018169246422046204`, 14.080046825322302`, 0.07439658697912946`,
  0.49050646003805504`, 0.0005294402909884477`, 0.0005337660700599999`, 0.008170475774475161`, 0.039765658022270146`},
 {0.2706982412362611`, 0.6287160520084307`, 4.14855201722539`, 0.8172328386793793`, 0.4851216922484758`,
  0.20982300489352756`, 0.08395856094354753`, 0.16391690500747624`, 3554.6452712204655`, 185.42012094476195`,
  0.05154754434614739`, 8.513250180668177`, 0.37417348469464895`, 252.72609766784626`, 25.17917427194304`,
  344.1715338195005`, 0, 76.16653856794959`, 17.670513390755023`, 17.74228483340471`, 0.004061650109568271`,
  0.03668287122273468`, 0.036687674042427725`, 0.00013092812893211736`, 158.71050594282303`, 0.1418569817641186 2`,
  0.8481993478208978`, 0.0005424803673901302`, 0.0005438407517549153`, 0.0025077117008491 8`, 0.06621849374214885`},
 {0.2598662442063618`, 1.900211126429065`, 0.6320497826451614`, 1.3904132145962274`, 0.3261450588717545`,
  0.21444284573744743`, 0.1520608211075556`, 0.47681332398299126`, 3867.6546770468185`, 328.5593273663678`,
  0.12810041704772773`, 2.5338343017479055`, 0.3750039812454511`, 45.5486270760802`, 5.943894363853591`,
  61.66529518665966`, 0, 5.862041121898542`, 1.369351745907161`, 1.411139206025176`, 0.030516235322964436`,
  0.24297415309426856`, 0.2420465534741227`, -0.0038176884591750726`, 37.17224890811217`, 0.9020111514832447`,
  0.20652726238878816`, 0.002097832793698018`, 0.00216152063499993`, 0.03035887392609804`, 0.05859464750441716`},
 {0.15997673085841746`, 3.7261077489541483`, 1.1512593893886542`, 1.376812544747029`, 0.7263504373210632`,
  0.29998090848320735`, 0.07358394021576772`, 0.4410919571058908`, 3522.7326806197016`, 234.3351737610849`,
  0.05138796494631709`, 5.103095752677643`, 0.43578634794289095`, 148.05510534633962`, 23.865050633410085`,
  179.54346039170994`, 0, 20.70649865482566`, 2.3351894271020175`, 2.356204542581781`, 0.008999319385341487`,
  0.21642097295791649`, 0.21678053979687376`, 0.001661423262463435`, 124.30239170858033`, 0.49460456775711076`,
  0.6122205438110843`, 0.0018896748044119338`, 0.0019152703142029837`, 0.013544928329091599`, 0.07635158212601958`},
 {0.25099622201635424`, 3.95450763611092`, 4.239563708075597`, 1.1463399035908803`, 0.8567090221336071`,
  0.6807931178480928`, 0.18419051557334198`, 0.2564924471830997`, 698.4202789859287`, 154.98713283086113`,
  0.08058524622619823`, 2.246868522171841`, 1.4084793726849045`, 211.56087179328927`, 113.98950597305468`,
  208.28418301686932`, 0, 46.56446830569935`, 2.857210283714825`, 2.8908268133865374`, 0.011765507727350633`,
  0.15851786016384067`, 0.15928361200218077`, 0.0048306975475738145`, 161.4122840703562`, 0.5683912003319697`,
  1.7409420249268135`, 0.0016547227968602973`, 0.0016879132192980765`, 0.02005799551487142`, 0.09274710547976824`},
 {0.24225332438192398`, 3.651195248984296`, 0.388765132686828`, 0.8400870426253413`, 0.1821318445823885`,
  0.5573875510223865`, 0.23605316030342982`, 0.08377442811214376`, 1289.2099511812157`, 318.7461100660206`,
  0.19657856943178847`, 4.498486658412734`, 0.6713610627619064`, 111.77828534756699`, 43.281645873070126`,
  119.1462050644325`, 0, 50.91949371421799`, 1.1173388206445594`, 1.1455139353361616`, 0.025216267591372965`,
```

0.3275316733930953`, 0.33261954353634327`, 0.015533979021142175`, 58.28031704918758`, 1.133509095997867`,
1.3583794604767823`, 0.004571449714318332`, 0.004857550133867894`, 0.06258417732419996`, 0.3953570235822832`},
{0.18294446021192495`, 2.0751042203060504`, 7.88640633654303`, 1.253927303712154`, 0.8981192233254209`,
0.26591266597763197`, 0.15456768387072084`, 0.011263961326455277`, 2103.388409363515`, 330.13115223762657`,
0.1896947651115678`, 4.4078344078613245`, 0.7904477019841809`, 174.96837610573732`, 41.78969814719652`,
186.25667694244765`, 0, 150.62433958121798`, 0.7862413834515414`, 0.7914115164140046`, 0.0065757578668357З`,
0.039566528214946384`, 0.039590905047747156`, 0.000616097340366828`, 23.307611613993718`, 0.103406816382052`,
0.8912705679159064`, 0.00038097523389690746`, 0.00038253284719407796`, 0.004088489640751813`, 0.06842085075028168`},
{0.15110272633071126`, 1.2120684691778738`, 9.821466182804322`, 0.999249111664473`, 0.8884716643403618`,
0.5717874967013771`, 0.23862826359871653`, 0.2965485968483864`, 799.8579275077459`, 260.7684224687745`,
0.20976611603443018`, 5.031757425833598`, 0.8119791706180317`, 356.46227328512543`, 215.8984383308125`,
353.034517198484`, 0, 69.8196789138042`, 15.642680968510962`, 15.745799068559744`, 0.006592098902762З556`,
0.045198784826790656`, 0.04523143808435057`, 0.0007224366249014569`, 270.85714821915633`, 0.09756656591687837`,
2.199699487250784`, 0.0005455708242715351`, 0.0005481900428354494`, 0.004800877259907521`, 0.17455857702401845`},
{0.15717196764210273`, 1.1630073112625743`, 2.634220279774457`, 1.0045604176965792`, 0.8286996726157703`,
0.4235785617926241`, 0.10102087300007184`, 0.09118767410472639`, 1523.7756565066265`, 301.9435543713025`,
0.21266930821707325`, 6.460470764249699`, 0.28210830372520523`, 201.9745611450493`, 103.5581903188853`,
221.44889563734165`, 0, 88.32272934964202`, 6.438458676177841`, 6.486842765916815`, 0.007514855988436153`,
0.07463559532315447`, 0.07475799795172318`, 0.0016400033796035718`, 106.97106448081118`, 0.16758033390134167`,
1.2705919937629073`, 0.0008935550057661557`, 0.0009019739837250175`, 0.00942189110299152`, 0.1903709698600057`},
{0.1799797654068389`, 2.619455956239074`, 7.697999886354548`, 1.3015491336074843`, 0.555451962044029`,
0.4433835581064074`, 0.0862654213077988`, 0.03372825256230826`, 2391.7036278961978`, 159.9832142066033`,
0.13737033538391902`, 3.425775682450297`, 0.8987360464358476`, 150.2624868008002`, 22.96004787720416`,
163.78506593163783`, 0, 101.63989863319316`, 1.2608986299412104`, 1.2716891183925758`, 0.008557776331209466`,
0.046438679981453033`, 0.04650688007378581`, 0.001468605316947258`, 47.183834663045644`, 0.11940032469808352`,
1.1095086760043509`, 0.00043020017464001636`, 0.0004330393295629726`, 0.006599613599255205`, 0.0655453889295928`},
{0.16862146125981442`, 2.3151392610028543`, 3.2020316316267774`, 1.3239310858987743`, 0.7072607224053895`,
0.3500350043589193`, 0.17362573378910773`, 0.060358617251082186`, 2001.4962431618678`, 114.46542957104901`,
0.20499895995123107`, 5.257752800260672`, 0.4338865062331345`, 431.95559638393917`, 45.95666008208979`,
547.7912767473758`, 0, 232.00756326321925`, 5.846784396978129`, 5.8697950910481`, 0.003935615289981209`,
0.21268457406806757`, 0.21289800381096072`, 0.0010035036336242253`, 193.37314440089963`, 0.5123311952396632`,

0.9709111355454387`, 0.0019346579487646842`, 0.0019499654485063292`, 0.007912251233567646`, 0.05598838120956324 5`},
{0.12274664777380989`, 1.2488875899911855`, 5.516183439796784`, 1.1021378510059003`, 0.9773171588432767`,
0.5961273492690635`, 0.12759486552792382`, 0.1532767587236556`, 2513.3632749376284`, 182.05354083120335`,
0.05801301004138587`, 1.8616328305112617`, 0.5158476130411485`, 118.08687997516812`, 32.61900877682983`,
134.96483663036182`, 0, 37.19889948594853`, 4.281567767497223`, 4.302537808191299`, 0.004897748169085725`,
0.07912994211241642`, 0.0794187426604682`, 0.0036496999788206974`, 76.38852643619362`, 0.13875621618334746`,
0.3579010596841339`, 0.0008626808195977276`, 0.0008739417982065546`, 0.01305347047599592`, 0.03299736095056796`},
{0.12291711454694165`, 1.1877487218897347`, 9.942019369479574`, 0.7752358604533577`, 0.7160618607453235`,
0.17825216453319837`, 0.07028904237235806`, 0.009021701114390435`, 805.4612461749266`, 63.6125839558365`,
0.06956787912807044`, 9.33154348593904`, 1.458279492320873`, 1137.0782051948272`, 144.08909403760782`,
1202.5515273610133`, 0, 1007.5332966759881`, 7.152962065051409`, 7.224978367064091`, 0.010068039136478246`,
0.05966262797255738`, 0.05964258907564895`, −0.00033587016846881035`, 121.37030786415126`, 0.10476511538088237`,
10.31489092566989`, 0.0009307703376715271`, 0.0009314849415035634`, 0.0007677552701390589`, 0.11051603645943453`},
{0.08216873811896613`, 1.3179095560013607`, 1.6483112503948085`, 1.3628139186636958`, 0.6342779575022122`,
0.2115034893488278`, 0.10646590409334977`, 0.055264018212609206`, 510.50583604116036`, 394.4203282160594`,
0.13986107835869105`, 2.2752923009835655`, 0.9070556531583542`, 51.2254295433115`, 52.683870676059705`,
49.968510117557855`, 0, 28.950092368292854`, 1.110760614741757`, 1.1398261148767344`, 0.02616720448062959`,
0.11563648172076108`, 0.11628414282798312`, 0.005600837188959318`, 20.912600408544435`, 0.13573862547873394`,
1.0645178705645248`, 0.0010127840571094726`, 0.0010380607754231885`, 0.02495765818614548`, 0.1241852785402833`},
{0.0476259458526086`, 3.971655945581807`, 1.7442422392857733`, 1.129286089919653`, 0.5285852533702986`,
0.39600384447582293`, 0.2215904518169189`, 0.024542351730056703`, 3543.890003102313`, 364.0135445894731`,
0.06778311290485467`, 8.573488767539896`, 0.9317909345539408`, 240.34246566174133`, 36.15536298895538`,
264.83914867544394`, 0, 178.1206481099615`, 1.0735886651052535`, 1.080585129174207`, 0.006516894501924941`,
0.11365855089103266`, 0.11385377577903734`, 0.0017176436482269075`, 60.91321149820743`, 0.07732994272035744`,
1.3436490050071141`, 0.00121112179013394`, 0.0012214613292204086`, 0.008537158831338765`, 0.2144819256136845`},
{0.15063755404470214`, 2.71723210471837`, 7.148609543518862`, 1.1908848946871171`, 0.5800141434913164`,
0.29138799856290376`, 0.0950097459950863`, 0.009655692674775408`, 856.5663047299263`, 347.96462878162436`,
0.12103625881441793`, 4.831460173736216`, 0.8148377888737439`, 112.67193881349797`, 75.14701375243729`,
113.23340717699175`, 0, 99.09733376347155`, 0.33698493526852985`, 0.34301039976341`, 0.01788051590519535`,
0.024354755382606206`, 0.02437319869160757`, 0.0007572775300603851`, 13.080946927401305`, 0.05241058257418719`,
1.842036093458216`, 0.0002468026012590885`, 0.0002479301706291073 5`, 0.004568709423103501`, 0.20472686678047317`},

```
{0.25325667629893317`, 2.6359345155712095`, 6.284411921985339`, 1.14262610260638`, 0.592877633639628`,
  0.19560008128978879`, 0.18445820883756597`, 0.009163632115762594`, 1288.7476768868028`, 192.8490635634654`,
  0.16457035981631868`, 2.082802800802952`, 1.1518950127144034`, 105.82046476713528`, 17.504634523333014`,
  113.82922751658909`, 0, 93.35905712923676`, 0.313437205126251 5`, 0.3168781953398241`, 0.010978244309530982`,
  0.057784366664116786`, 0.05784774604009494`, 0.0010968256578212099`, 11.802856392235123`, 0.2090610949056313`,
  0.8124454839679826`, 0.0006097817456727217`, 0.0006144272380444716`, 0.007618287042398863 6`, 0.04796392123189381 6`},
{0.14655389849752304`, 3.5234321911845736`, 9.93741755620114`, 1.0550925690751938`, 0.4448537382022568`,
  0.18176809875202415`, 0.0743479769003609 6`, 0.27672257287602875`, 2966.99983242307`, 232.8915968291967 1`,
  0.13795973482450868`, 7.950903042528848`, 1.2964183380427592`, 223.23877626806674`, 25.526939475484763`,
  240.19311051715945`, 0, 46.784826187598036`, 3.4361926027164245`, 3.470728878554823`, 0.010050739242932982`,
  0.018708480276336287`, 0.01871030008342039`, 0.0000972717750038487 6`, 172.95988045030438`, 0.03916858170665476`,
  2.1902500401443405`, 0.00021447099024785032`, 0.00021473024980407185`, 0.0012088327466661664`, 0.10981447508837844`},
{0.07405512950518717`, 1.967002000369666`, 3.1335932173998042`, 1.0217576314214762`, 0.5481985257231932`,
  0.23482039961813828`, 0.1719074884408075`, 0.010212529243490897`, 3198.221153713561`, 38.498265037806334`,
  0.12854240418059937`, 5.86125181611254`, 1.042171003495977`, 1281.11748367432`, 21.334192120769437`,
  1541.407409887598`, 0, 1116.6977286853087`, 5.588811189331977`, 5.605142332222992`, 0.0029221139054023126`,
  0.4479345443517865`, 0.4471106261480509`, -0.0018393718772635737`, 157.04575413006245`, 0.473883581311679`,
  1.9422649569136325`, 0.00528712391685914 9`, 0.005301143390257788`, 0.002651625651128642`, 0.028643287463176455`},
{0.2776663951441912`, 0.8310897457250155`, 2.0994479814067635`, 1.1003401269753144`, 0.30863525720948326`,
  0.2434882924277243`, 0.08746325015577566`, 0.02243947015640089`, 2140.6294222376637`, 299.3219451566707`,
  0.02431460260750562`, 6.100643757524728`, 0.33277870666165055`, 112.22033787145587`, 26.928712147873664`,
  137.57542848783004`, 0, 84.948540853963`, 2.0933438071368347`, 2.1162000241318037`, 0.01091852036777019`,
  0.06027916416303146`, 0.06036415086435655`, 0.0014098851983952443`, 24.853665319834143`, 0.23910711736363854`,
  0.8970725489516701`, 0.0006597330864116513`, 0.0006658928938692508`, 0.009336817547082932`, 0.1386921768588099`},
{0.11907642171566374`, 1.274849235584174`, 6.899983100552333`, 1.1682344315973607`, 0.7800022001471016`,
  0.41036075345185574`, 0.1345484896240624`, 0.0075107638021500 46`, 2156.4467856375795`, 343.9093505673661`,
  0.08426365817638315`, 2.310160002798696`, 0.6194154558015803`, 69.19931609824549`, 22.639640378052935`,
  73.7100301021381`, 0, 62.405703761731814`, 0.34604258642304164`, 0.3490184053422688`, 0.008599574260461651`,
  0.031728219024315496`, 0.03179988007160023`, 0.002258590286136508`, 6.302173239728358`, 0.05397261126894779`,
  0.4437815843617255`, 0.00032674929951315157`, 0.00032993265902202197`, 0.009742513644600104`, 0.05887486054976496`},
{0.17937371459880497`, 2.8718008248848648`, 2.8198656683422243`, 0.9976096277706823`, 0.8604494338327144`,
```

0.3675397910291335`, 0.21060856912564696`, 0.0072115171001387954`, 3962.8369792288295`, 287.0010951200238`,
0.23748423652768247`, 9.572841373703643`, 0.6693837986608342`, 492.37463988592145`, 57.698976832853496`,
551.2891551393486`, 0, 445.72994269886897`, 1.0898880472596326`, 1.0942712841516478`, 0.0040217313173001035`,
0.11410078986126389`, 0.11414355166348854`, 0.00037477218410719715`, 44.713448473605204`, 0.2923811788010524`,
1.1263481323378746`, 0.0013798750019634598`, 0.0013862449781857653`, 0.004616342939209206`, 0.09651513830273858`},
{0.1446481422691977`, 3.7473728388010814`, 7.773074810909127`, 0.7768035182896058`, 0.045349053147926144`,
0.5946440249560018`, 0.18594588773391896`, 0.007600014271866709`, 2201.8419670169405`, 172.3692269613906`,
0.22887729014375824`, 6.4667868323956865`, 0.5687524909215391`, 220.4546507886182`, 33.37744303851937`,
264.8595589606935`, 0, 198.76896842265126`, 0.39300607269658616`, 0.3959180783521168`, 0.007409569108054903`,
0.021368221510262204`, 0.02138174013574276`, 0.0006326509426188842`, 21.03914689010101`, 0.04415533635794813`,
1.4229983215134472`, 0.0003320276926235266`, 0.00033331239536839075`, 0.003869263839750836`, 0.21242096019996798`},
{0.2743682788249912`, 2.567007165847575`, 8.880180070850319`, 1.3334617648385514`, 0.053478262105743024`,
0.587031003621043`, 0.14269834247006347`, 0.006777940660941963`, 3288.224802818225`, 343.8350462827967`,
0.07407598386826336`, 1.0311140447256584`, 1.1022538897878755`, 17.175437918764523`, 3.4356317123010784`,
18.613815752376674`, 0, 15.594819126852595`, 0.03951067445460211`, 0.04024907020366181`, 0.018688512895625564`,
0.015537281758874618`, 0.01563270358822471`, 0.006141475119712636`, 1.4489169207490629`, 0.0608991036257337`,
0.2343964958462648`, 0.00013947055595253666`, 0.00014255844071507296`, 0.022140047707181587`, 0.05515361564210949`},
{0.08682640281398363`, 0.4802834262657396`, 6.166420960796351`, 1.3554629143634556`, 0.6132401723597833`,
0.3572005740934433`, 0.19494034836925994`, 0.012289451288465592`, 600.315756452052`, 42.934435033309114`,
0.23123185628174636`, 1.349114625805871`, 1.1058635186946257`, 311.58742648162126`, 33.47086316538659`,
341.60733186452734`, 0, 264.5408426417325`, 5.8716899370343105`, 5.919429569788912`, 0.008130475768738155`,
0.3172193393055855`, 0.31799083329192007`, 0.0024320521820120344`, 40.28679087041284`, 0.39347163049909417`,
1.6214093604128592`, 0.0028122175931255544`, 0.0028465020227743765`, 0.012191243569711574`, 0.029525015004041887`},
{0.04351054041483948`, 3.010882988409519`, 9.401958493592993`, 1.3186120593482111`, 0.49329539098927055`,
0.4698069940559316`, 0.20226774754753019`, 0.03712775492888272`, 1947.5927514696796`, 172.9604174112643`,
0.17909729794724766`, 1.3306147942525488`, 0.46866213033580717`, 61.92390655447033`, 10.203035842434474`,
75.41521796098039`, 0, 40.50715347779592`, 0.4850197215696152`, 0.48761591030672446`, 0.005352748809280383`,
0.040786800744278165`, 0.040931921110628135`, 0.0035580227843765577`, 20.8619661245297`, 0.025352224888196155`,
0.2635037899775814`, 0.0003713217254608381`, 0.0003761848155044558`, 0.01309670215924541`, 0.03260790035217089`},
{0.13996819046085934`, 1.009532661617322`, 6.145072622160212`, 1.3450345414096851`, 0.2868040480361447`,
0.18183470956578074`, 0.13804571299234253`, 0.12631256619855588`, 3863.110841624364`, 279.40163171428946`,

0.18548475334584474`, 9.210069253579718`, 1.2809334264760701`, 227.97972396496814`, 20.049948826408706`,
253.14405855977654`, 0, 82.38759886943728`, 9.434000051527955`, 9.511673522183315`, 0.008233354911078239`,
0.03399209936974498`, 0.033999192443522515`, 0.00020866830554888693`, 136.05615973881405`, 0.06796875198182178`,
1.8041031177329259`, 0.0003055226945161138`, 0.00030613987478205645`, 0.002020079938480856 5`, 0.16267004860386908`},
{0.0871063251493665`, 2.6634842127081404`, 9.193238345408428`, 1.4387089146023326`, 0.7548565000722143`,
0.6823132747448253`, 0.19549587941758378`, 0.13611301540454773`, 3578.7577781208274`, 129.49687931265402`,
0.027673492175817715`, 9.652606809319298`, 1.320301893923864`, 957.7027014398086`, 79.78870964558926`,
1020.5935677126732`, 0, 327.4535921141869`, 16.13403590820093`, 16.19594975650198`, 0.003837468111099307`,
0.09741606058596691`, 0.09745732428253358`, 0.0004235820697167103`, 613.8964275537066`, 0.12122221497425674`,
3.468616192223637`, 0.0008177399378777039`, 0.0008202215376489774`, 0.0030347053584223183`, 0.14056025819872445`},
{0.04655003226957066`, 2.717305676329323`, 6.261066689781977`, 1.313518883642772`, 0.22988974709922583`,
0.48948284819450716`, 0.1924496553607991`, 0.40105381179739646`, 1594.1735712767286`, 296.74834364325125`,
0.10951121773307326`, 3.7043174616791976`, 0.46788624174476623`, 87.96151092070284`, 27.65134404225796`,
99.60743994901847`, 0, 13.73864453888857`, 1.8627184082147525`, 1.8807810804058862`, 0.009696941905698475`,
0.0311888926249419`, 0.03125637594370881`, 0.002163697171888135`, 72.30821862921538`, 0.020740627973487625`,
0.8203039216116519`, 0.00028541600545561874`, 0.0002882370102501855`, 0.009883835316325529`, 0.18385637424230059`},
{0.24058904599557956`, 2.305486806402752`, 6.870865029896366`, 1.1663266064905513`, 0.291316248043183`,
0.4756853450103681`, 0.11657670703629042`, 0.014427542030819147`, 3856.0018830548897`, 333.80293042286075`,
0.20384852921840946`, 8.723427410033644`, 0.10055245356998821`, 85.60136511470478`, 28.716893498660014`,
190.98913696359887`, 0, 70.99555802542906`, 0.4278728876364807`, 0.43105661128432615`, 0.007440816513128556`,
0.010443806233458138`, 0.010451777003740512`, 0.0007632054927291243`, 14.09221853233363`, 0.03589521968957719`,
0.5558756255737741`, 0.00010807122276756953`, 0.00010854147371788731`, 0.004351305909891856`, 0.3511826990973395 7`},
{0.09672875865983738`, 3.3181066447219294`, 8.772587658219397`, 1.2949486969635013`, 0.026680322952838287`,
0.3340762083993216`, 0.2420609904328586`, 0.3944381790098702`, 1841.1432974548734`, 89.15662847720563`,
0.1287997244865557`, 3.057938101959733`, 0.8332397440047616`, 206.65529459903553`, 13.957772647286477`,
254.8753393563051`, 0, 31.933555593934486`, 3.606762737729233`, 3.6260896425656712`, 0.005358518494789077`,
0.06252673311591557`, 0.06258244032717103`, 0.0008909343008884907`, 170.96604865706897`, 0.08640190396224919`,
0.9964468501503044`, 0.0005823164563713012`, 0.0005854687539894723`, 0.005413375465661119`, 0.06245143741754205`},
{0.14493124545339742`, 3.446306300213565`, 2.261950562129204`, 1.3227848229460633`, 0.575877479620123 1`,
0.35943404017729164`, 0.23613553647425112`, 0.03288786445085886`, 1106.0434527992074`, 293.5700758832494`,
0.23807677975419816`, 3.6561682593343647`, 0.5615502445855836`, 136.48414230871015`, 48.619730102133026`,

```
147.80475762862085`, 0, 92.89823700935497`, 0.8591234205547481`, 0.868490469204133`, 0.010903030257674029`,
0.13609474115473472`, 0.1366307091779044`, 0.003938197895246409`, 42.29717795598364`, 0.28177686193159107`,
1.0579694030270006`, 0.0012313553009939415`, 0.0012542569163004253`, 0.01859870606639502`, 0.1443305395350015`},
{0.15370210593846179`, 1.1469734999246084`, 8.184859582737346`, 0.8081018637833421`, 0.7671904423744005`,
0.5546779173711273`, 0.13880357299862195`, 0.3920406444124731`, 2732.468857403337`, 28.150193121997006`,
0.06240913374814305`, 6.929933598007185`, 0.8840553716357653`, 2370.992929239997`, 66.1032389446229`,
2821.0945221667107`, 0, 361.6749679207825`, 115.53643350923743`, 115.71724647485988`, 0.0015649865599147716`,
0.2122440836312134`, 0.21200939807515026`, -0.00110573426617111`, 1893.1032501556722`, 0.4660337518154405`,
3.0408454981533275`, 0.003171173595246657`, 0.003176900001508627`, 0.001805768347967307`, 0.030126790265906127`},
{0.06413533732712456`, 2.7505505433928237`, 9.582115463692716`, 1.419324092949555`, 0.0693862133505021`,
0.4085416515074848`, 0.14517410092691557`, 0.08408491973612985`, 1524.8762411053558`, 131.37866475928564`,
0.011227169364072448`, 3.5456702127620083`, 1.1107372198382217`, 159.33481622652934`, 22.104930429794425`,
176.6709270664162`, 0, 73.25494013621206`, 2.134330244599239`, 2.1579074517298786`, 0.011046653717389887`,
0.04171645312887283`, 0.04176740441550112`, 0.0012213714927031827`, 83.86547448660245`, 0.03822141133571386`,
1.7344931771076821`, 0.0003543907678961178`, 0.0003564220283957246`, 0.005731696995566970`, 0.1194662093673024`},
{0.10991493466216079`, 3.7381599633374956`, 1.5402547174363423`, 0.8574896025687118`, 0.9021341382697974`,
0.5521862271024119`, 0.19447159277653675`, 0.017387026381523858`, 877.7862619046055`, 398.7215907021463`,
0.023981889033480308`, 9.30826908769696`, 0.3374448683929794`, 403.9318588429712`, 374.52801484376323`,
390.1502705450047`, 0, 323.6874536574312`, 1.465268059961902`, 1.4773021063154552`, 0.008212863354071898`,
0.1512583937994995`, 0.151523412610824`, 0.0017520932535868283`, 78.24866281866842`, 0.23750794959398716`,
2.765400334233549`, 0.002115710373855606`, 0.002141214734239512`, 0.012054750356698118`, 0.42400360413787347`}};
(*MakeOutputForm[];*)
PatientsTrainingSet = result[[All, 1 ;; 10]];
```

In[•]:=

```
(* The scripts hidden in this group of cells have to be initialized to run the following codes *)
```

In[•]:=

```
CreateResultArray[Njj_] := ( (* argument is how many random sets will be chosen *)
    result = {};
    (* Table that will contatin the results *)
    result = Array[f, {Njj + 1, 31}];
```

```
result⟦1, 1⟧ = "κ c";
result⟦1, 2⟧ = "κ p";
result⟦1, 3⟧ = "γ";
result⟦1, 4⟧ = "V";
result⟦1, 5⟧ = "ks";
result⟦1, 6⟧ = "ρ0";
result⟦1, 7⟧ = "ρm";
result⟦1, 8⟧ = "ω";
result⟦1, 9⟧ = "α0";
result⟦1, 10⟧ = "αm";
result⟦1, 11⟧ = "kf";
result⟦1, 12⟧ = "N0";
result⟦1, 13⟧ = "kD";

(* minimal curative dose *)
result⟦1, 14⟧ = "A^sim_cur"; (* simulation result *)
result⟦1, 15⟧ = "A^est_min"; (* analytical estimation for most radiosensitive population *)
result⟦1, 16⟧ = "A^est_max"; (* analytical estimation for most radioresistant population *)
result⟦1, 17⟧ = "Mm"; (* minimal curative number of radioconjugate molecules of cancer cell *)

(* paths of activity *)
result⟦1, 18⟧ = "Canc"; (* released in cancer *)
result⟦1, 19⟧ = "Bl^sim_Frg"; (* released in blood from fragments, simulation result *)
result⟦1, 20⟧ = "Bl^est_Frg"; (* analytical estimation *)
result⟦1, 21⟧ = "Err"; (* error in estimation *)
result⟦1, 22⟧ = "Bl^sim_Ab"; (* released in blood from antibodies, simulation result *)
result⟦1, 23⟧ = "Bl^est_Ab"; (* analytical estimation *)
result⟦1, 24⟧ = "Err"; (* error in estimation *)
result⟦1, 25⟧ = "Cld^sim_Frg"; (* cleared in form of fragments,
```

```
simulation result *) (* no need to estimate -- error will be the same as for release *)
result[[1, 26]] = "Cld^sim_Ab"; (* cleared in form of fragments, simulation result *)

result[[1, 27]] = "ToVbl"; (* How much activity affects viable cells *)

(* receptors occupancy *)
result[[1, 28]] = "fD^sim_Max"; (* maximum occupancy of receptors on damaged cancer cells, simulation result *)
result[[1, 29]] = "fD^est_Max"; (* analytical estimation *)
result[[1, 30]] = "Err"; (* error in estimation *)
result[[1, 31]] = "Nnew"; (*number of newborn cells until Ncur is achieved*)
)

WriteDownMeasures[] := (

result[[ij, 1]] = kappac;
result[[ij, 2]] = kappap;
result[[ij, 3]] = gamma * 10^7 / Nnor;
result[[ij, 4]] = V;
result[[ij, 5]] = ks;
result[[ij, 6]] = rhomax;
result[[ij, 7]] = rhomin;
result[[ij, 8]] = omega;
result[[ij, 9]] = alphamax;
result[[ij, 10]] = alphamin;
result[[ij, 11]] = kf;
result[[ij, 12]] = N0 * Nnor / 10^7;
result[[ij, 13]] = kD;
```

```
(* minimal curative dose *)
result[[ij, 14]] = DA1 / nCpm; (* simulation result *)
```

$$result[[ij, 15]] = -\frac{kappac + lambda + kon * gamma * N0 / V}{kon * gamma * N0 / V} * N0 * \frac{\frac{rhomax}{lambda} * \frac{nu}{alphamax} * ProductLog\left[-1, -E^{-2} * \left(\frac{Ncur}{N0 * Nnor}\right)^{\frac{lambda}{rhomax}}\right]}{1 - \left(1 - \frac{ProductLog\left[-1, -E^{-2} * \left(\frac{Ncur}{N0 * Nnor}\right)^{\frac{lambda}{rhomax}}\right]}{ProductLog\left[-1, -E^{-1} * \left(\frac{Ncur}{N0 * Nnor}\right)^{\frac{lambda + rhomax}{rhomax}}\right]}\right) * ks} / nCpm;;$$

```
(* analytical estimation for most radiosensitive population *)
```

$$result[[ij, 16]] = -\frac{kappac + lambda + kon * gamma * N0 / V}{kon * gamma * N0 / V} * N0 * \frac{\frac{rhomin}{lambda} * \frac{nu}{alphamin} * ProductLog\left[-1, -E^{-2} * \left(\frac{Ncur}{N0 * Nnor}\right)^{\frac{lambda}{rhomin}}\right]}{1 - \left(1 - \frac{ProductLog\left[-1, -E^{-2} * \left(\frac{Ncur}{N0 * Nnor}\right)^{\frac{lambda}{rhomin}}\right]}{ProductLog\left[-1, -E^{-1} * \left(\frac{Ncur}{N0 * Nnor}\right)^{\frac{lambda + rhomin}{rhomin}}\right]}\right) * ks} / nCpm;;$$

```
(* analytical estimation for most radioresistant population *)
result[[ij, 17]] = 0; (* minimal curative number of radioconjugate molecules of cancer cell *)

(* paths of activity *)
result[[ij, 18]] = ActTumorpw[[Npw]][tEnd] / nCpm; (* released in cancer *)
result[[ij, 19]] = ActBloodFragpw[[Npw]][tEnd] / nCpm; (* released in blood from fragments, simulation result *)
```

$$result[[ij, 20]] = \frac{lambda * omega * DA1}{(lambda + omega) * (lambda + kappap)} / nCpm; \text{ (* analytical estimation *)}$$

```
result[[ij, 21]] = result[[ij, 20]] / result[[ij, 19]] - 1; (* error in estimation *)
result[[ij, 22]] = (ActBloodpw[[Npw]][tEnd] - ActBloodFragpw[[Npw]][tEnd]) / nCpm;
(* released in blood from antibodies, simulation result *)
```

$$result[[ij, 23]] = \frac{lambda * DA1}{lambda + kappac + kon * gamma * N0 / V} / nCpm; \text{ (* analytical estimation *)}$$

```
result[[ij, 24]] = result[[ij, 23]] / result[[ij, 22]] - 1; (* error in estimation *)
result[[ij, 25]] = ActOutFragpw[[Npw]][tEnd] / nCpm; (* cleared in form of fragments,
simulation result *) (* no need to estimate -- error will be the same as for release *)
```

```
result[[ij, 26]] = (ActOutpw[[Npw]][tEnd] - ActOutFragpw[[Npw]][tEnd]) / nCpm; (* cleared in form of fragments, simulation result *)

result[[ij, 27]] = (SDpw[[Npw]][tEnd] + CFNpw[[Npw]][tEnd] + CFDpw[[Npw]][tEnd] + UNpw[[Npw]][tEnd]) / nCpm;
(* How much activity affects viable cells *)

(* receptors occupancy *)
result[[ij, 28]] = 1 - If[Ainj[[1, 1]] == 0
    , Min[Table[NMinimize[{dFpw[[nn]][t] / DDpw[[nn]][t], t > tB[[nn]], t < 10}, t][[1]], {nn, 1, npw}]]
    , Min[Table[NMinimize[{dFpw[[nn]][t] / DDpw[[nn]][t], t > tB[[nn]], t < 10}, t][[1]], {nn, 2, npw}]]];
(* maximum occupancy of receptors on damaged cancer cells, simulation result *)
result[[ij, 29]] = DA1 / (gamma * N0); (* analytical estimation *)
result[[ij, 30]] = result[[ij, 29]] / result[[ij, 28]] - 1; (* error in estimation *)

result[[ij, 31]] = NewCellspw[[npw]][tminNn] * Nnor / 10^7; (*number of newborn cells until Ncur is achieved*)

)
```

In[ ]:=

```
FullSystemSolutionHetMDStopNmin[TMIN_] := (

  (* maximum time of simulation-- where little radioactivity of the last dose remains, namely 0.17% of the last dose *)
  tEnd = Ainj[[Length[Ainj], 1]] + (-Log[0.0017] / lambda);
  lAinj = Length[Ainj]; (* number of injections*)
  If[Ainj[[1, 1]] == 0, Npw = lAinj, Npw = lAinj + 1];
  (* the injections are treated as new initial conditions for a new system, which as well takes the actual vaules of other parameters *)
  (* therefore the number of injections has to me remembered and logic differs whether the first injection is made at t=0 or t>0 *)
```

```
apw = Array[ff, Npw]; bpw = Array[ff, Npw]; NNpw = Array[ff, Npw]; DDpw = Array[ff, Npw];
papw = Array[ff, Npw]; pbpw = Array[ff, Npw]; fFNpw = Array[ff, Npw]; fANpw = Array[ff, Npw];
dFpw = Array[ff, Npw];
dApw = Array[ff, Npw];
rhopw = Array[ff, Npw]; (* cancer cell proliferation rate -- is now an explicit variable,
while variables alpha and omega can be expressed through it *)
(* inert antibody fragments are as well accounted for as pb *)

(* times of beginning and end for solution of separate systems *)
tB = Array[ff, Npw]; tE = Array[ff, Npw];

(* for monitoring the paths of activity *)
ActBloodpw = Array[ff, Npw]; ActBloodFragpw = Array[ff, Npw];
ActOutpw = Array[ff, Npw]; ActOutFragpw = Array[ff, Npw]; ActTumorpw = Array[ff, Npw];

(* for monitoring influence of self-dose, croos-fire and decays in blood *)
SDpw = Array[ff, Npw]; CFNpw = Array[ff, Npw]; CFDpw = Array[ff, Npw]; UNpw = Array[ff, Npw];

(* for monitoring the number of new cancer cells appearing during treatment *)
NewCellspw = Array[ff, Npw];

Clear[a, b, NN, DD, pa, pb, fFN, fAN, dF, dA, rho, ActBlood, ActBloodFrag, ActOut, ActOutFrag, ActTumor, SD, CFN, CFD, UN, NewCells];

(* EQUATIONS *)

(* Radiation damage function *)
RD[NN_, DD_, fAN_, dA_, a_, pa_, rho_] :=
```

$$\left(\frac{\text{rho} - \text{rhomin}}{\text{rhomax} - \text{rhomin}} * (\text{alphamax} - \text{alphamin}) + \text{alphamin}\right) (*\text{alpha}*) * \left(\text{ks} * \frac{\text{lambda} * \text{gamma} * \text{fAN}}{\text{nu}} (*\text{self-dose}*) + \right.$$

```
(1 - ks) * (lambda * gamma * (fAN * NN + dA))/(nu * (NN + DD)) (*cross-fire*) + kf * lambda * (a + pa) (*dose from unanchored nuclides*));


(* Active antibodies *)
Fa[t_] := (*injections are considered as initial conditions*)
  - lambda * a[t] (*decay*) - kon * gamma/V * (fFN[t] * NN[t] + dF[t]) * a[t] (*binding*) - kappac * a[t] (*clearance*);

(* Inert antibodies *)
Fb[t_] := (*injections are considered as initial conditions*)
  + lambda * a[t] (*decay of a*) - kon * gamma/V * (fFN[t] * NN[t] + dF[t]) * b[t] (*binding*) - kappac * b[t] (*clearance*);

(* Viable cells *)
FNN[t_] := rho[t] * NN[t] (*proliferation*) - RD[NN[t], DD[t], fAN[t], dA[t], a[t], pa[t], rho[t]] * NN[t] (*damage*);

(* Damaged cells *)
FDD[t_] := RD[NN[t], DD[t], fAN[t], dA[t], a[t], pa[t], rho[t]] * NN[t] (*damage*) - (* rho[t]/rhomax **) omega * DD[t] (*death*);

(* Active fragments *)
Fpa[t_] := (* rho[t]/rhomax **) omega * (gamma * dA[t])/V (*release*) - lambda * pa[t] (*decay*) - kappap * pa[t] (*clearance*);

(* Inert fragments *)
Fpb[t_] := (* rho[t]/rhomax **)
  omega * (gamma * (DD[t] - dF[t] - dA[t]))/V (*release*) + lambda * pa[t] (*decay*) - kappap * pb[t] (*clearance*);

(* Free receptors of viable cells *) FfFN[t_] := (1 - fFN[t]) * rho[t] - kon * (a[t] + b[t]) * fFN[t];
```

```
(* Active receptors of viable cells *) FfAN[t_] := kon * a[t] * fFN[t] - (lambda + rho[t]) * fAN[t];

(* Free receptors of damaged cells *)
FdF[t_] := RD[NN[t], DD[t], fAN[t], dA[t], a[t], pa[t], rho[t]] * fFN[t] * NN[t] -
    kon * (a[t] + b[t]) * dF[t] - (* rho[t]/rhomax **) omega * dF[t];

(* Active receptors of damaged cells *)
FdA[t_] := RD[NN[t], DD[t], fAN[t], dA[t], a[t], pa[t], rho[t]] * fAN[t] * NN[t] +
    kon * a[t] * dF[t] - lambda * dA[t] - (* rho[t]/rhomax **) omega * dA[t];

(* Cancer cell proliferation rate *)
Frho[t_] := -kD * rho[t] * Log[rho[t] / rhomin] * RD[NN[t], DD[t], fAN[t], dA[t], a[t], pa[t], rho[t]];

(* Initial conditions *)

If[Ainj[[1, 1]] == 0
  , a0 = Ainj[[1, 2]]/V; b0 = Ainj[[1, 3]]/V
  , a0 = 0; b0 = 0]; (*complexes in blood*)
NN0 = N0;
DD0 = 0;
pa0 = 0; pb0 = 0; fFN0 = 1; fAN0 = 0;
dF0 = 0; dA0 = 0; rho0 = rhomax;
ActBlood0 = 0; ActBloodFrag0 = 0; ActOut0 = 0; ActOutFrag0 = 0; ActTumor0 = 0; NewCells0 = 0;
SD0 = 0; CFN0 = 0; CFD0 = 0; UN0 = 0;

(* SOLVER *)

tB[[1]] = 0; If[Ainj[[1, 1]] == 0, If[lAinj > 1, tE[[1]] = Ainj[[2, 1]], tE[[1]] = tEnd], tE[[1]] = Ainj[[1, 1]]];
```

```
For[npw = 1, npw ≤ Npw, npw++,

  Clear[a, b, NN, DD, pa, pb, fFN, fAN, dF, dA, rho, ActBlood, ActBloodFrag, ActOut, ActOutFrag, ActTumor, SD, CFN, CFD, UN, NewCells];
  (*fFD,fAD,*)

  sol = NDSolve[{
      (*INITIAL CONDITIONS*)
      a[tB[[npw]]] == a0, b[tB[[npw]]] == b0, NN[tB[[npw]]] == NN0, DD[tB[[npw]]] == DD0, pa[tB[[npw]]] == pa0, pb[tB[[npw]]] == pb0,
      fFN[tB[[npw]]] == fFN0, fAN[tB[[npw]]] == fAN0, dF[tB[[npw]]] == dF0, dA[tB[[npw]]] == dA0, rho[tB[[npw]]] == rho0,
      ActBlood[tB[[npw]]] == ActBlood0, ActBloodFrag[tB[[npw]]] == ActBloodFrag0, ActOut[tB[[npw]]] == ActOut0,
      ActOutFrag[tB[[npw]]] == ActOutFrag0, ActTumor[tB[[npw]]] == ActTumor0, SD[tB[[npw]]] == SD0,
      CFN[tB[[npw]]] == CFN0, CFD[tB[[npw]]] == CFD0, UN[tB[[npw]]] == UN0, NewCells[tB[[npw]]] == NewCells0,

      a'[t] == Fa[t], b'[t] == Fb[t], NN'[t] == FNN[t], DD'[t] == FDD[t], pa'[t] == Fpa[t],
      pb'[t] == Fpb[t], fFN'[t] == FfFN[t], fAN'[t] == FfAN[t], dF'[t] == FdF[t], dA'[t] == FdA[t], rho'[t] == Frho[t],

      ActBlood'[t] == V*lambda*(a[t] + pa[t]),
      ActBloodFrag'[t] == V*lambda*pa[t],
      ActOut'[t] == V*(kappac*a[t] + kappap*pa[t]),
      ActOutFrag'[t] == V*kappap*pa[t],
      ActTumor'[t] == (lambda*gamma)*(fAN[t]*NN[t] + dA[t]),

      SD'[t] == ks*(lambda*gamma)*(fAN[t]*NN[t]),
      CFN'[t] == (1 - ks)*(lambda*gamma)*(fAN[t]*NN[t])*NN[t]/(NN[t] + DD[t]),
      CFD'[t] == (1 - ks)*(lambda*gamma)*dA[t]*NN[t]/(NN[t] + DD[t]),
      UN'[t] == kf*lambda*(a[t] + pa[t])*nu*NN[t],
      NewCells'[t] == If[t > Ainj[[1, 1]], rho[t]*NN[t], 0] (* start counting new cells from the moment of the first injection *)
```

```
(* The simulations run until the injected activity decays to negligible amounts
  If viable cancer cell number becomes too small (cure) or too great (host death) it just stays there to allow assessment of activity paths
  You can make simulations just stop there by using commented functions *)
 , WhenEvent[t > TMIN, NN[t] → 0]
 , WhenEvent[NN[t] > 10^9 / Nnor, NN[t] → 0.99 * 10^9 / Nnor (*tE⟦npw⟧=t;
  tEnd=t;
  "StopIntegration"*)] (* cancer wins *)
 (*,WhenEvent[NN[t]<0.01/Nnor,NN[t]→0(*tE⟦npw⟧=t;
  tEnd=t;
  "StopIntegration"*)] (* treatment wins *) *)
}
, {a, b, NN, DD, pa, pb, fFN, fAN, dF, dA, rho, ActBlood,
  ActBloodFrag, ActOut, ActOutFrag, ActTumor, SD, CFN, CFD, UN, NewCells}, {t, tB⟦npw⟧, tE⟦npw⟧}
, AccuracyGoal → 10, PrecisionGoal → 10];

apw⟦npw⟧ = First[a /. sol]; bpw⟦npw⟧ = First[b /. sol]; NNpw⟦npw⟧ = First[NN /. sol]; DDpw⟦npw⟧ = First[DD /. sol];
papw⟦npw⟧ = First[pa /. sol]; pbpw⟦npw⟧ = First[pb /. sol]; fFNpw⟦npw⟧ = First[fFN /. sol]; fANpw⟦npw⟧ = First[fAN /. sol];
dFpw⟦npw⟧ = First[dF /. sol]; dApw⟦npw⟧ = First[dA /. sol]; rhopw⟦npw⟧ = First[rho /. sol];
ActBloodpw⟦npw⟧ = First[ActBlood /. sol];
ActBloodFragpw⟦npw⟧ = First[ActBloodFrag /. sol];
ActOutpw⟦npw⟧ = First[ActOut /. sol];
ActOutFragpw⟦npw⟧ = First[ActOutFrag /. sol];
ActTumorpw⟦npw⟧ = First[ActTumor /. sol];
SDpw⟦npw⟧ = First[SD /. sol];
CFNpw⟦npw⟧ = First[CFN /. sol];
CFDpw⟦npw⟧ = First[CFD /. sol];
UNpw⟦npw⟧ = First[UN /. sol];
NewCellspw⟦npw⟧ = First[NewCells /. sol];

If[npw < Npw,
```

```
(*renew initial conditions*)
If[Ainj[[1, 1]] == 0
  , a0 = apw[[npw]][tE[[npw]]] + Ainj[[npw + 1, 2]]/V; b0 = bpw[[npw]][tE[[npw]]] + Ainj[[npw + 1, 3]]/V
  , a0 = apw[[npw]][tE[[npw]]] + Ainj[[npw, 2]]/V; b0 = bpw[[npw]][tE[[npw]]] + Ainj[[npw, 3]]/V];

NN0 = NNpw[[npw]][tE[[npw]]];
DD0 = DDpw[[npw]][tE[[npw]]];
pa0 = papw[[npw]][tE[[npw]]];
pb0 = pbpw[[npw]][tE[[npw]]];
fFN0 = fFNpw[[npw]][tE[[npw]]]; fAN0 = fANpw[[npw]][tE[[npw]]];
dF0 = dFpw[[npw]][tE[[npw]]];
dA0 = dApw[[npw]][tE[[npw]]];
rho0 = rhopw[[npw]][tE[[npw]]];
ActBlood0 = ActBloodpw[[npw]][tE[[npw]]];
ActBloodFrag0 = ActBloodFragpw[[npw]][tE[[npw]]];
ActOut0 = ActOutpw[[npw]][tE[[npw]]];
ActOutFrag0 = ActOutFragpw[[npw]][tE[[npw]]];
ActTumor0 = ActTumorpw[[npw]][tE[[npw]]];
SD0 = SDpw[[npw]][tE[[npw]]];
CFN0 = CFNpw[[npw]][tE[[npw]]];
CFD0 = CFDpw[[npw]][tE[[npw]]];
UN0 = UNpw[[npw]][tE[[npw]]];
NewCells0 = NewCellspw[[npw]][tE[[npw]]];

(*renew time frame*)
tB[[npw + 1]] = tE[[npw]];

If[Ainj[[1, 1]] == 0, If[Npw > npw + 1, tE[[npw + 1]] = Ainj[[npw + 2, 1]], tE[[npw + 1]] = tEnd],
  If[Npw > npw + 1, tE[[npw + 1]] = Ainj[[npw + 1, 1]], tE[[npw + 1]] = tEnd]];]
];
```

```
npw--;
(* It will be convenient to have estimation of minimal viable cell number here *)
TableNN = If[Ainj[[1, 1]] == 0, Table[NMinimize[{Nnor * (NNpw[[nn]][t]), t > tB[[nn]], t < tE[[nn]]}, t], {nn, 1, npw}],
    Table[NMinimize[{Nnor * (NNpw[[nn]][t]), t > tB[[nn]], t < tE[[nn]]}, t][[1]], {nn, 2, npw}]];

Nn = Min[TableNN[[All, 1]]];

tminNn = t /. TableNN[[Position[TableNN[[All, 1]], Nn][[1, 1]]]][[2]];

Return[Nn])
```

In[ ]:=

```
(* The outcome of the global parameter sweep was obtained by the code hidden here.
  It uses randomization and will generate a new array of data.
  It takes a long time, at least half a day. *)
```

In[ ]:=

```
Npar = 1000; (*how many sets will be tested*)
SetBasicParameterValuesHet[];
eta = 0;
CreateResultArray[Npar];
Nnres = Array[f, {Npar + 1, 2}];
(* The minimal viable cell number achieved in simulations can be monitored.
 Numerical acurracy can be adjusted if desired. *)
Nnres[[1, 1]] = "#";
Nnres[[1, 2]] = "Nn";
Quiet[For[ij = 2, ij ≤ Npar + 1, ij++,

   NotebookDelete[pr]; (* To see the code running *)
   pr = PrintTemporary["Set " <> ToString[ij - 1] <> " of " <> ToString[Npar]];

   kappac = RandomReal[{0.04, 0.28}];
```

```
  kappap = RandomReal[{0.4, 4}];
  gamma = RandomReal[{0.13, 10}] * Nnor / 10^7;
  V = RandomReal[{0.75, 1.5}];
  ks = RandomReal[{0, 1}];
  rhomax = RandomReal[{0.15, 0.7}];
  rhomin = RandomReal[{0.05, Min[0.25, rhomax]}];
  omega = 0.05 * 10^RandomReal[{-1, 1}];
  alphamax = RandomReal[{500, 4000}];
  alphamin = RandomReal[{20, 400}];
  kf = RandomReal[{0.01, 0.25}];
  N0 = RandomReal[{1, 10}] * 10^7 / Nnor;
  kD = RandomReal[{0.1, 1.5}];

  ACurSim = FindCurDoseHet[]; (* find curative dose *)
  DA1 = ACurSim * nCpm;  (* set curative dose *)
  Ainj = {{t1, DA1, eta * DA1}};  (* set schedule *)
  FullSystemSolutionHetMD[];  (* find time of achieving of minimal N *)
  FullSystemSolutionHetMDStopNmin[tminNn];  (* solve it with cell proliferation stopping at this moment*)

  Nnres[[ij, 1]] = ij;
  Nnres[[ij, 2]] = Nn;

  WriteDownMeasures[];

  check = 11; OkFlg = 1; (* to avoid possible bugs *)
  While[check < 31, If[ ! NumericQ[result[[ij, check]]], OkFlg = 0]; check++];
  If[OkFlg == 0, ij--];
]];
```

In[ ]:=

```
(* This cell contains some of the results for homogeneous cancer, for comparison *)
```

In[ ]:=

```
(* Fraction of injected activity,released in blood from antibodies *)
```

In[ ]:=

```
FrAcBlAbHomo = {0.01543672406214353`, 0.00039554342945341135`, 0.0006036537894052479`, 0.00018692447958931755`,
    0.0004885262397575715`, 0.0002238593669519052`, 0.00014987601658040408`, 0.00021358778666575194`, 0.0006267068231075658`,
    0.0002459883299254856`, 0.0001911882069246045`, 0.0003432295527617612`, 0.001824416526363635`, 0.000096541038076823`,
    0.00048684395202834417`, 0.0038386273256109547`, 0.0024826371316333706`, 0.0005035058480535904`, 0.0005155702258008334`,
    0.00011885766826965727`, 0.0011904047526438428`, 0.0005531317204380414`, 0.0010285153856310217`, 0.0035095764710330493`,
    0.0002093317163377486`, 0.00011586365230537624`, 0.00014177250576843345`, 0.00019440878020474123`, 0.00008995116918724968`,
    0.0005084227810472644`, 0.001705629402042491`, 0.001696251555979675`, 0.0007620636861465379`, 0.0003539292926691554`,
    0.0004427362146475121`, 0.00009758793318499563`, 0.000543246646163142`, 0.00008549174517773163`, 0.00019981540041703655`,
    0.0005650859222577964`, 0.0046644607506802 9`, 0.0005553988969237436`, 0.0002328935298484398`, 0.0002518025803212933`,
    0.001200232391224219`, 0.0004241492623828353`, 0.0001460153786869606 3`, 0.00006916793825883142`, 0.00007074303209156089`,
    0.00046818341741003536`, 0.004634028250586125`, 0.0003040136963283482`, 0.0001379128158113262 8`, 0.0012391085384642442`,
    0.00047483559784678125`, 0.00013689282489119 21`, 0.0001087281056448624`, 0.001154165459139413`, 0.0001201168546151179 9`,
    0.0046694575577979 4`, 0.00014335482959478718`, 0.0004605170299011154`, 0.0002808187554465782`, 0.00006221762436513 29`,
    0.00014755776094162922`, 0.00039153631913991746`, 0.00023962576963838838`, 0.00013858173224712107`, 0.00026552438660334 78`,
    0.0004673002705341983`, 0.00007221136990533873`, 0.0003068162245489707`, 0.00035947589719100687`, 0.0011443359376586415`,
    0.00041585066240325903`, 0.00010425915991412772`, 0.0001837530946632768`, 0.0003918320049256207`, 0.00032380160773149607`,
    0.0008866395650005743`, 0.00009598642066307851`, 0.0007844127016049466`, 0.0010333458283637094`, 0.0026172799897802236`,
    0.0004336224329251398`, 0.0007826936686103556`, 0.0001998717216787141 7`, 0.0002695168695818763 7`, 0.00031038542441199`,
    0.00027850737102800763`, 0.0018261016116261374`, 0.00023453354857253913`, 0.0000998012065895943`, 0.00012683151327118808`,
    0.003783838261966099`, 0.0005394240959576425`, 0.0002679368706273213 5`, 0.00011757241912307895`, 0.0003659889901435788`,
    0.001733230715675632 4`, 0.000701366803785921`, 0.0009960523918209761`, 0.0000757226681367486`, 0.0002896702940463585 6`,
    0.0003481686240848211`, 0.0032221245682552443`, 0.011466762928392455`, 0.00010660419996054005`, 0.0007581388983077692`,
    0.0003912850618457991`, 0.0004440835906821598`, 0.0010595940406175957`, 0.00015287088476543785`, 0.00023739771502439451`,
    0.00018080260470085773`, 0.0008337988261118932`, 0.0003415727788586573`, 0.0005389515583844079`, 0.00013009247818262428`,
    0.0011303682825404356`, 0.0002718639483348742`, 0.00009223041587335874`, 0.00013956562642671283`, 0.0003397047606263632`,
    0.0007781117639626292`, 0.00026713238511039827`, 0.0006360020954255856`, 0.0005460877386278191`, 0.0011828124331333938`,
```

0.00011725269661701403`, 0.0008947156483737692`, 0.00019855518476084229`, 0.00008271925487897318`, 0.00021104225093106354`,
0.000755409932793092`, 0.0002578151958934732`, 0.0019647316266170373`, 0.00034945338432392653`, 0.0007245729155765386`,
0.00015612610694951248`, 0.0005428349333425184`, 0.00032674369147809413`, 0.0019373124303390056`, 0.00011237185506512016`,
0.00012056315471247676`, 0.0012362219650860632`, 0.0002385391606696813`, 0.00029353878229632216`, 0.00018743546540804106`,
0.001277151976630396`, 0.0004013850826554868`, 0.0005912903015865677`, 0.0018857595509211248`, 0.0012481643324676241`,
0.00694264671239622`, 0.002946110631643848`, 0.00017224869150428616`, 0.00006309809319919224`, 0.00017168670319789322`,
0.0002478388341017889`, 0.00032544340564336777`, 0.00008587398855340524`, 0.0006461446926887216`, 0.0047098898647363035`,
0.0001038162064860029`, 0.0011679839976292314`, 0.002061017630326309`, 0.000170755818867773`, 0.0001532249746111383`,
0.00034452589153835213`, 0.00024178151122646987`, 0.0001352185127734316`, 0.02361892420899003`, 0.00017601166208154278`,
0.0003296292867346608`, 0.0003096556109905996`, 0.00018988937212102214`, 0.00031895691516538354`, 0.00007833063072632451`,
0.005656130119774263`, 0.0007621760150962372`, 0.0008355500911153735`, 0.00015619464855969623`, 0.0002440987422816374`,
0.00021181418132309304`, 0.001298942555636356`, 0.00007712574239899715`, 0.0030620705678861596`, 0.00046191259463829 78`,
0.0012768989230538303`, 0.0001695615400109141 7`, 0.002473205522007472`, 0.0001037014221260692 8`, 0.0005812306146249156`,
0.0029019241112806997`, 0.0006390840349368897`, 0.0001966491045036023`, 0.0007876115100112993`, 0.0009596792039047044`,
0.0002254709541724992`, 0.0025844519202611136`, 0.0003557594533201087`, 0.0002829117097745258 3`, 0.0002817755735185621 3`,
0.00011480002686898493`, 0.0006596143869146829`, 0.0004770289439069439`, 0.00010931240495885489`, 0.00008732916140545393`,
0.0022336620274043787`, 0.0014095174996337646`, 0.0001493685585328822 3`, 0.0001116661992843337 6`, 0.0013021395432259058`,
0.00021939094013396265`, 0.0003616274485718857`, 0.0002053163931857605`, 0.0008701460327521186`, 0.0003330272008008824 4`,
0.0014263929308820308`, 0.0001084356902594565 8`, 0.0009452142952051472`, 0.0029418532815150555`, 0.0068793731566064945`,
0.00047564973365119207`, 0.00013747969401174673`, 0.0011732530223924028`, 0.00029096818302703115`, 0.0002037476819796683`,
0.0001101360628791262`, 0.00011742777230212198`, 0.0001174102262259123`, 0.00014241989922872113`, 0.0013257929420731473`,
0.00016993632344937442`, 0.00011078226722434374`, 0.0006767416317507629`, 0.0024599320970157875`, 0.00006333355848256762`,
0.00022897707953565318`, 0.0010514088097899308`, 0.00044493727181240414`, 0.023326153338741867`, 0.0012345730856697975`,
0.0007097530419761938`, 0.0001934346250846515 3`, 0.00008275936935715258`, 0.00013278629027678817`, 0.00025758118163940523`,
0.00029724720282480507`, 0.00024319155729918274`, 0.0007477260344070045`, 0.0005679876233277326`, 0.00008951167389063115`,
0.00009167000784821662`, 0.0014714983952168635`, 0.005118644901594565`, 0.0011748964672611312`, 0.0007200485371996004`,
0.002321402537624386`, 0.0001670446185108587`, 0.0001268298993825742 6`, 0.00079359880757839`, 0.0014530620125586392`,
0.00021029219030146504`, 0.000646835221131877`, 0.0001692627957323412 3`, 0.00012285487855132196`, 0.0005518277960626007`,
0.00030496964699774985`, 0.0006504200541754929`, 0.000326278283736423`, 0.0001227259795408654 8`, 0.0000888851628984426 3`,
0.00009317130253346817`, 0.00019056053699720524`, 0.0005522691004854435`, 0.0001280774702404661`, 0.00027299695606280793`,
0.0000721113058872252`, 0.00019105765134159113`, 0.00026328447258809834`, 0.000787653374386238`, 0.0006475976319042315`,

0.00043754393048066375`, 0.00023101758039220497`, 0.0005792107181838604`, 0.00032400522359310533`, 0.00068013428645775 41`,
0.0013413416579967762`, 0.0012574166633384078`, 0.0006311856064829459`, 0.00035974742274417023`, 0.00010711515196798345`,
0.001795175630615 4192`, 0.0004042226691904537`, 0.0000865196538765 8482`, 0.00012542685954192195`, 0.0006860721599404783`,
0.0008537352255664516`, 0.0001508341470107854`, 0.0002628611794332127`, 0.00010777135305607738`, 0.00013832765735031298`,
0.00011243973152634887`, 0.00008353462702797918`, 0.0007121831067414404`, 0.0003142309588024 73`, 0.0015362919113228824`,
0.0004391189230178936`, 0.00015273439226940364`, 0.00016817312788135888`, 0.00024770321786138214`, 0.0005439467343109423`,
0.0001359499312625065`, 0.004456949679050421`, 0.003886475714078713`, 0.0002566701726236868`, 0.0012141816808281197`,
0.00022562087133821601`, 0.0005397578133927009`, 0.00018186428139127053`, 0.0015118580973932818`, 0.00045572948435267845`,
0.0003223873565290121`, 0.0001387497709997 6977`, 0.0005406826751101387`, 0.0026412937987246865`, 0.004395403070918802`,
0.0036308457094020976`, 0.0001690357806868 2134`, 0.000394065130006357`, 0.0004156670934315824`, 0.0003314372560958942`,
0.00035580337339958206`, 0.0006416502077722403`, 0.00013278102453169563`, 0.0001970901890575697`, 0.00011499755644205177`,
0.00011297782319293344`, 0.00021905556002388695`, 0.00022933330448151264`, 0.0009810505795330006`, 0.00007821043092985717`,
0.00039760859124604944`, 0.00017864363629167156`, 0.0013213938718386136`, 0.0008354087683118999`, 0.0011632759161880023`,
0.0001734724804710037`, 0.0001008709087969 0061`, 0.00019707036095355246`, 0.0003567979059387555`, 0.00011650389266422477`,
0.00033303899878502996`, 0.00036070690348672627`, 0.003538285726595153`, 0.0002480827892995424`, 0.00023504926066584338`,
0.00019494809356489484`, 0.0004415650886594228`, 0.0007551090208365439`, 0.00008826578913930364`, 0.0006277064367871915`,
0.00040380698766666594`, 0.00014754815916940805`, 0.001470149214272584`, 0.0004499966227000911`, 0.00019026301822736933`,
0.0025010292148430146`, 0.0010773202253228271`, 0.001554029215074857`, 0.0007503443709233191`, 0.0008728075211391894`,
0.00019323626915747466`, 0.00008949297009142316`, 0.00015786882367243833`, 0.00020639110520314947`,
0.0010927857908313186`, 0.0019909624558325733`, 0.0001509735262280 7792`, 0.0017023727545946404`, 0.0006573631980610782`,
0.00010862458497422293`, 0.0020341217130692146`, 0.00012832450107217808`, 0.00047350605630369424`, 0.0002025189296202762`,
0.00014893729377203862`, 0.0013858051717078853`, 0.00007556046885382443`, 0.00018466821834379498`, 0.0002939594832811122`,
0.0020514269570814786`, 0.0000946344632300 4951`, 0.00011153040231582474`, 0.0010435907910203576`, 0.0019699245028595215`,
0.0002870407090393415`, 0.0002986944318109629`, 0.0001267362844670273`, 0.0002017477074103446`, 0.000260491139116795`,
0.0009948607297350493`, 0.0013630134598918676`, 0.0004464417345010608`, 0.004005551388527833`, 0.0002130824483275343`,
0.003849820254869954`, 0.0001260600043893031`, 0.0005355784635430986`, 0.000505190666826 8712`, 0.0001560026911791 3235`,
0.0002703990436489685`, 0.002029772805973578`, 0.000223100893465079`, 0.0000920636535868281`, 0.00027950885012743577`,
0.00008604998781128896`, 0.001379746956891747`, 0.0001290671099952 8297`, 0.00013333959815237413`, 0.00013511743013960994`,
0.0010152627245071332`, 0.0004155792106367389`, 0.0020761823983299382`, 0.0006833431892576626`, 0.00024160998345901106`,
0.00044078927988733447`, 0.0011764732980647356`, 0.0001579716737658179`, 0.00096678380455179 62`, 0.00019244171740365318`,
0.0006424491053389058`, 0.0004953318930548874`, 0.0001579780865034 0272`, 0.00039886490076823037`, 0.00034911150014379984`,

0.0003577289846674186`, 0.00009707210638872356`, 0.0004420582653799137`, 0.0004460394339640207`, 0.00010465825088774183`,
0.0018293004554422322`, 0.00016102590034032895`, 0.000147220737486655`, 0.0002886176766596281`, 0.00015641323954247092`,
0.0000754198708178011 3`, 0.0007149345316728729`, 0.0015594218236801623`, 0.0010093060530602085`, 0.0003447418941342549`,
0.00043063931228915246`, 0.0003627932632631659`, 0.0002980540870082249`, 0.0004633987677815209`, 0.00021299254708965332`,
0.0008042207395720409`, 0.0004952718654436755`, 0.00024031345544482395`, 0.0006750824820557026`, 0.0002907495981512564`,
0.00022413185619229745`, 0.00013219496952594485`, 0.00036043046414505314`, 0.00032413882434295297`,
0.00010365409984014206`, 0.00014264300874687155`, 0.00018049163016560393`, 0.0002150273307703086`, 0.0001656160346597032`,
0.00017161448039158635`, 0.00127062366704402`, 0.0001077998411596515 5`, 0.0002797739070763638 7`, 0.0001669832088197358`,
0.00010357370928866745`, 0.0006858900909238267`, 0.00021057339448680014`, 0.00017057389777394086`, 0.00016327923860705553`,
0.00023659498325219886`, 0.00009179048117477296`, 0.0001764294752479821`, 0.00423355198438948`, 0.00011494460972972084`,
0.00096180129060683 9`, 0.00028832907562017433`, 0.00023055496865996962`, 0.0007083314566229401`, 0.0005456889408017441`,
0.00028733585738116156`, 0.00008908341249223757`, 0.000056062929525953624`, 0.00009332535491878263`,
0.00015741981131718667`, 0.0001632636327738717`, 0.0009537611502943782`, 0.0024421078595439393`, 0.0031907247188897743`,
0.001059644114746208`, 0.00035059401533248737`, 0.00014225451291465313`, 0.0006057963269148241`, 0.00017497960618739416`,
0.00009500021832200752`, 0.00033988385559080244`, 0.0024221328484622905`, 0.0002741679061203291`, 0.00030574603254261 42`,
0.0002907633924927174`, 0.00014856868556826326`, 0.0008057093487823535`, 0.0001397483472066546`, 0.0007970402040480197`,
0.00011682043330853468`, 0.0006120053399802785`, 0.00018900144323422778`, 0.0005065839302014656`, 0.00272933397366679 1`,
0.00014532996230561326`, 0.00040803807938775565`, 0.00015774448594410814`, 0.00011484988463700667`, 0.00016639913494768285`,
0.00020766062349274097`, 0.0022737204035211986`, 0.00021153968261789896`, 0.0019167513386717078`, 0.00011632973348334594`,
0.00022049998535649814`, 0.00012700702720401974`, 0.00013962538139715834`, 0.004758199486606316`, 0.00018403846035827215`,
0.00026753434924483904`, 0.0002403175691436987`, 0.0022301291401739653`, 0.0011962568794136744`, 0.00018473303840786624`,
0.00035885623489524893`, 0.00012534535688789555`, 0.0004418685196683322`, 0.0006784227686722651`, 0.00012988132480999583`,
0.0018536654287020986`, 0.0028174654004540275`, 0.0011815651220731275`, 0.0000721340525233806`, 0.0006841150546169918`,
0.00017618115514313857`, 0.0003231496670604569`, 0.00013181320629293913`, 0.00028534686398970797`, 0.0009265111240593107`,
0.00159202209876594`, 0.0006882111774211641`, 0.0027489629581670567`, 0.00014304427831483303`, 0.0002141459484588977`,
0.00011494082102220105`, 0.0009643730231879651`, 0.0006384477238437637`, 0.0002091853703272209 3`, 0.00008020846340010117`,
0.0005268856935633506`, 0.00007659506924663864`, 0.00033835835758828995`, 0.0003314308161965593`, 0.00009361741365432463`,
0.00013643805125719457`, 0.0002511834592591807`, 0.001279512046469108`, 0.0014056954685281095`, 0.00018763627010293912`,
0.00019974150816423223`, 0.0009132089304004925`, 0.00009020740078051886`, 0.001058389628143899`, 0.00031314323194042633`,
0.0001666173823847117`, 0.0002970041659623531`, 0.00009288371080472903`, 0.00009405384654230093`, 0.00008548562390626377`,
0.0007166877308736378`, 0.0006900379708237419`, 0.0002622310989442223`, 0.00017678119853299824`, 0.00017551997670104687`,

0.0007594625303237479`, 0.00029953148107792225`, 0.0004715941667265448`, 0.00007912550711937125`, 0.00035077919586170246`,
0.0001445789645199381 4`, 0.0001837182764641603`, 0.0003566081914258265`, 0.00011499006829320033`, 0.0004729466932189008`,
0.00140791471609864`, 0.0004876223836450954`, 0.002760102926978511 3`, 0.00008048519103033302`, 0.0016071695280801888`,
0.0022463819691861384`, 0.0005037183962508837`, 0.00117040761860513 9`, 0.0001444871095291018`, 0.00028324415778532554`,
0.00006350523279149036`, 0.0011808842446655757`, 0.000429977152157859`, 0.00011046185191789126`, 0.0003532622734835684`,
0.00029368257496858145`, 0.0001588776413599533`, 0.0016975131096419892`, 0.0002854358353328405`, 0.0010065148445668709`,
0.000541613120257127`, 0.00010883501774623398`, 0.0012114800045978388`, 0.003151878453114363`, 0.0001343692852602444 5`,
0.0001433047649412817`, 0.0005481832333019411`, 0.00012104778157782168`, 0.0003529588602534607`, 0.0002095634666609018`,
0.001603818760073799`, 0.00011373069076726968`, 0.0000762185745420793`, 0.0005486672108692151`, 0.0001936904871714776`,
0.0001526163892649335`, 0.002692868483793015`, 0.002079747921867116`, 0.0004538555936156797`, 0.00009650319402561709`,
0.0001192236245406175`, 0.0034066699789665556`, 0.00028686865599071023`, 0.00021571713034800922`, 0.0025132028892614062`,
0.00009973498634457136`, 0.00007500463836065236`, 0.00010945247837515762`, 0.0010889318661332378`, 0.001224365437122454`,
0.001117369292323215`, 0.0002393217504340524`, 0.000739649309332833`, 0.001642409174891481`, 0.000191709954335375`,
0.0008399672701171991`, 0.0004108991746867504`, 0.00019243052766699376`, 0.00038292321171753735`, 0.0003984319079247037`,
0.0003092471869529614`, 0.0002312524612063394`, 0.0014617799322783908`, 0.0004467469825530268`, 0.00011482136649125194`,
0.0001495739349963596`, 0.0000840065164974523`, 0.00037417567347261625`, 0.00012117969894804662`, 0.0002609507151281714`,
0.0024431842877321993`, 0.000209195013601126`, 0.0006377854514632889`, 0.00044816996133922784`, 0.0001796997197763025`,
0.00019497062559848213`, 0.0014534697230773675`, 0.00026571761561787927`, 0.00009127841975203844`, 0.00023445985655582766`,
0.00658411703778940 7`, 0.00022215978624169196`, 0.0021530918021949097`, 0.001098223029687964`, 0.0003598299140920452`,
0.000433528389144553`, 0.0003231333793876843`, 0.00012066296842981978`, 0.00014600032452167404`, 0.00016111260147187568`,
0.0003855848823668612`, 0.005599799336631668`, 0.00040492784745006224`, 0.00017094028313900116`, 0.0004508966158119563 3`,
0.0002858006934426796`, 0.00012501412307356957`, 0.0002694457244260422`, 0.0005680943761331936`, 0.0006467628997752634`,
0.00046710608310148783`, 0.0001294078565908638`, 0.00012126282413582753`, 0.00017492877427953304`, 0.0001301195500446362`,
0.0006116833373270718`, 0.00015881191632311875`, 0.00017893493964048183`, 0.00913105505382853 3`, 0.0020414534317212013`,
0.0033760563291682652`, 0.0003407430858580394`, 0.0007457222811784376`, 0.00012820200730190233`, 0.00048005760830068526`,
0.0016284057194586269`, 0.00007643603619217344`, 0.0010474319805740947`, 0.0010401109053680305`, 0.0006318754561764218`,
0.0008482732659120423`, 0.0021189682903948867`, 0.00030937496848925636`, 0.00008747892590741467`, 0.00064660866746002 54`,
0.00016067564483165264`, 0.00057609845981201 47`, 0.00024784979175287617`, 0.000212578496456573`, 0.0005127027574288798`,
0.0001591085865847453`, 0.0013195969963364978`, 0.0001814639986888979`, 0.00011327874841719386`, 0.00023409357076317042`,
0.00013359378179709025`, 0.0012585248196326 24`, 0.00028202419479154663`, 0.000498686618499119`, 0.0002407167623403892`,
0.0013367893278319564`, 0.0008383379340881123`, 0.00013064433637436382`, 0.001182303536654536`, 0.0004301995431951706`,

0.00010494976524813404`, 0.000432228121348655`, 0.0011269285564127378`, 0.00040400841086939837`, 0.0011795047405433448`,
0.00011821845176952321`, 0.00036620718434354435`, 0.002831590827716059`, 0.0002172972366318911`, 0.0001098716839355772`,
0.001794072960208972`, 0.00015691470563152684`, 0.0006801699041859351`, 0.0005930503922636616`, 0.00014674300888506558`,
0.001108548274661418 2`, 0.00011799512836191498`, 0.01003564226102409`, 0.0009601182081125974`, 0.00023278942754891146`,
0.0003670902891456828`, 0.00032803016793598813`, 0.0004122798953244 29`, 0.0008233385507731 08`, 0.00014055119192450492`,
0.00015930480642796518`, 0.0007879182596008527`, 0.00024806941858296843`, 0.00036379912457760094`, 0.00034986798996143836`,
0.00020832241631758434`, 0.0001343214594102507`, 0.00017833782936240068`, 0.00017815677520615226`, 0.0002606946546470176`,
0.00019771655035012055`, 0.000053890284702555294`, 0.00035014629169637145`, 0.0002091167498671167`,
0.0006288165037270747`, 0.00032519750545317134`, 0.00020512522985198452`, 0.00010845362795166803`, 0.0012051897720672428`,
0.0025229878392779314`, 0.00018507440931255507`, 0.00017806675383998682`, 0.00046831924907946113`, 0.0005170134032091286`,
0.00014074819468329257`, 0.00015672647102011393`, 0.0003585574179993647`, 0.00011648337343741644`, 0.0019455379709524664`,
0.00039320329970289006`, 0.005229467656464349`, 0.00020297841251315155`, 0.000090859651071405 29`, 0.0006014691709993681`,
0.0005365545224138659`, 0.00011738872339192245`, 0.00011517352073548093`, 0.00010241470482232844`, 0.000096726565174220 35`,
0.00011367734054191528`, 0.00033950680856331304`, 0.00022828387187874148`, 0.0002913261959480938`, 0.00014767389498425331`,
0.0003328755875927468`, 0.0012553785481184965`, 0.00030820443929045196`, 0.0002729844395592091`, 0.00014853712180273087`,
0.000135902448190778`, 0.002562755603547896`, 0.0003292909338500711`, 0.0003967751447644899 3`, 0.0003416183789508357`,
0.0005669023962333343`, 0.00011569838500157838`, 0.00014076020290730562`, 0.000191804799606703`, 0.00052034762069828`,
0.000473291548210088`, 0.00015747109378100167`, 0.0023638099667852375`, 0.0001944707095638616 6`, 0.0002096369349157255`,
0.0018967712028025857`, 0.0002551810371357427 7`, 0.0003763356547725922`, 0.0004982113039211638`, 0.0013004901156807623`,
0.00018557574744464952`, 0.0003358179419751495`, 0.0001460607715591916`, 0.003910788924875035`, 0.0002879575454519753`,
0.005109068064717797`, 0.0001522977875109771`, 0.0005305118711850911`, 0.0004390776663984451`, 0.0002831162661376518`,
0.00008561704353580028`, 0.000329929238259507`, 0.00017077317829502372`, 0.0006708792217262348`, 0.0005404031769741981`,
0.00012762505204449927`, 0.0002402429007817492`, 0.00008248546849609699`, 0.00047995439051687876`, 0.00008825044791301011`,
0.00021987126687266544`, 0.00036082308599065456`, 0.00081132602315915 72`, 0.00009211363832365281`, 0.000535977212980402`,
0.0010075644587314693`, 0.0001252250998010888`, 0.0005160821875544855`, 0.0003227463159623327`, 0.0001545708356214460 7`,
0.005635954336721332`, 0.004297022470772023`, 0.0015996486336850495`, 0.0001319749141716511 5`, 0.0004316615739482178`,
0.000690495088747835`, 0.0002578535338385737`, 0.0004837519982074425`, 0.0009574691758972197`, 0.0007118571954394321`,
0.00021375331917243795`, 0.00014318480044645833`, 0.0005339490150169489`, 0.0002329876086997944`, 0.0018401388229225381`,
0.00018586933939002343`, 0.0002686604457599913`, 0.0001695083053990831`, 0.0007832370967291489`, 0.001117946783381827`,
0.00009679375975993802`, 0.004124424279481709`, 0.0003422720028044274`, 0.001252866776107676`, 0.00186049103524630 7`,
0.0001379631185860429`, 0.0005325028403735068`, 0.0004718925893992536`, 0.000986988486373164`, 0.0004070091426056196`,

```
0.00011369484574482794`, 0.0018223046386904185`, 0.00030631526829701637`, 0.00010627872534821212`, 0.00017394450765058887`,
0.00041868651938857944`, 0.0002958924245815966`, 0.001193034541782523`, 0.00015677447955823263`, 0.0003297843177424646`,
0.0001673370407756246`, 0.0003125276741409684 7`, 0.003693621200266469`, 0.00033869651455746156`, 0.0010889829005837862`,
0.0007427554812295097`, 0.00009698582737337339`, 0.00015884446878165287`, 0.004338967177358835`, 0.0001685029126477362`,
0.000343392065768695`, 0.00021783827247268348`, 0.00011008811376497146`, 0.00018955787878713803`, 0.00065812170578047 49`,
0.00007121178878703911`, 0.0035957685545082506`, 0.00019637774119408614`, 0.00010929160237796845`, 0.0001495676603070089`,
0.0002285027272110463`, 0.000514809090510052`, 0.00016400553635404012`, 0.0003337717793408367`, 0.00019402208090677186`,
0.0003881974373672657`, 0.00018227356162588552`, 0.0023781706357859627`, 0.00024434742159591574`, 0.00006399442968318267`,
0.00008471703010272685`, 0.00023266463642675797`, 0.0001660607119032098`, 0.00020379516964071662`, 0.00025892811116770423`,
0.0004307654528887173`, 0.0015840522856307884`, 0.002072490058863955`, 0.00026184923505230784`, 0.0007201169446361714`,
0.0004027847357356029`, 0.0008095863175998541`, 0.000734925567497685`, 0.00217742402654881 9`, 0.0008182528442684882`,
0.00026975278628301434`, 0.00013093734451519133`, 0.00012122976698434919`, 0.0003966617236520908`, 0.00012040151150138481`,
0.00022818241720100267`, 0.00066986694810963 99`, 0.0004959234810702948`, 0.0010770949621908598`, 0.00023237210853836257`,
0.0002720869091460717`, 0.0008821477778151811`, 0.002368450587538223`, 0.00024591157005306044`, 0.0006940389571608112`,
0.000681252290661724`, 0.0005743804573736939`, 0.0001649274198458373 4`, 0.00009493530003890993`, 0.004845233191503233`,
0.00027844281738156814`, 0.0010020115013588133`, 0.00035935332231417066`, 0.000910503021078936`, 0.0003228266914975667 4`,
0.005385487694980472`, 0.000521669361354376 4`, 0.00013262873262702668`, 0.00009345073529523432`, 0.002820901063608454 7`,
0.00009741729914548526`, 0.0002951625487634981`, 0.00020842750131722166`, 0.000371145898985096`, 0.00034486614011836476`,
0.0016886260520309754`, 0.0010453437018842356`, 0.000293087164255335 5`, 0.00022015900763025677`, 0.0005023821236148325`,
0.0005334912498324465`, 0.0013031778757252497`, 0.00021249898888878567`, 0.0001183911223706039`, 0.00012153090198788665`};
```

In[∘]:=

```
(* Fraction of injected activity,released in blood from antibody fragments *)
```

In[∘]:=

```
FrAcBlFrHomo = {0.0366229723503204`, 0.03709062251508073`, 0.021749470856548204`, 0.009337795299569008`, 0.01527689646166968 5`,
0.006626027149687339`, 0.0538886100246345`, 0.008400326192351416`, 0.003485472494258085 3`, 0.013735867879785826`,
0.01150499603311203`, 0.03373342343755726`, 0.00389368688752063`, 0.0021607380571826785`, 0.010998742296407309`,
0.02420421779856567`, 0.02408199969399425 8`, 0.00912671799268008`, 0.0030054055532700396`, 0.00771239416677519`,
0.016414285140817254`, 0.004073987295682919`, 0.0203023642005985`, 0.04132831022038098`, 0.00143881148341466`,
0.008722619332015817`, 0.02689164452449001`, 0.02240050493559293`, 0.00479150586227936 5`, 0.00912893368922231 3`,
0.0028807204752278686`, 0.007664102862619336`, 0.028202127530272347`, 0.00580890236409799`, 0.02065453375026299`,
0.00248770590985123`, 0.012639924417316203`, 0.008006993291039132`, 0.021942904718693427`, 0.002489688769481608`,
```

0.024639487942128436`, 0.012774953182708503`, 0.016829646015681383`, 0.014811646194487282`, 0.016199220187983948`,
0.004385616556214261`, 0.028859906134011563`, 0.006803769776225122`, 0.005589379745685074`, 0.025085613060646047`,
0.03494366672368754`, 0.013799100812543989`, 0.020466019909732187`, 0.09165388416009997`, 0.016570438902746516`,
0.03852464764028537`, 0.006835936565846545`, 0.004721068147148078`, 0.017576383613244616`, 0.009170605360822731`,
0.004310102765061963`, 0.041706100139082596`, 0.012342235588778061`, 0.02725342787832741`, 0.008076989918773148`,
0.02145180264976223`, 0.008891893318176806`, 0.009508371054076738`, 0.01210595345231386`, 0.020053513161251015`,
0.006544737645895852`, 0.008230347787207064`, 0.023481756169828907`, 0.05997970561364111`, 0.020972706317126232`,
0.02202257829720026`, 0.004911624526496142 5`, 0.005669716566619654`, 0.011642557467337977`, 0.0055659917187288 5`,
0.019153938827525818`, 0.01665715972069408`, 0.025195565073962964`, 0.015861528152264045`, 0.003091883463124525`,
0.007257718712363288`, 0.0023052699542473656`, 0.014492952730464042`, 0.029797718336761602`, 0.016949324368032773`,
0.004689561337843909`, 0.015038212710532104`, 0.006812376464527543`, 0.0038508153820431355`, 0.007567740447076167`,
0.0026559269045346038`, 0.006441479946826106`, 0.0028921330981537935`, 0.013431985238064836`, 0.036433120515277924`,
0.0020338867821347068`, 0.021584670198069117`, 0.005966789887589628`, 0.02330246238446859`, 0.010127126447707387`,
0.04997419964191249`, 0.014746460864688083`, 0.023316417800343603`, 0.006162552474861383`, 0.001448928454428160 2`,
0.01710782625039047`, 0.017585312250214775`, 0.07306699026680372`, 0.009527247541407671`, 0.07560611661065061`,
0.008561281137285407`, 0.003920832274987655`, 0.02012068992975175`, 0.021009756722142062`, 0.014299682838547758`,
0.03286376672652595`, 0.03052676893443556`, 0.02315825564083531`, 0.0055103723501658`, 0.010162042623959434`,
0.0278820002111237`, 0.021642489417838272`, 0.06076323470830515`, 0.004785217642263606 5`, 0.00892888786065164`,
0.011277638244153416`, 0.016324341568062083`, 0.00857187083431878`, 0.01599264981216402`, 0.01124292959094838`,
0.0018453719295530376`, 0.006379088958024406`, 0.022357556808413226`, 0.008439310954684483`, 0.00350609028481274`,
0.017616271946834138`, 0.0692711895374459`, 0.021911635486946964`, 0.02020363274294747`, 0.11493970514038458`,
0.016739632412517393`, 0.014718196222394784`, 0.004220379749610789`, 0.005443885000086763`, 0.026814988550655918`,
0.015095750022714849`, 0.029376980661088924`, 0.009546859016274657`, 0.007445024826526442 5`, 0.015364885545695133`,
0.05697005477305596`, 0.007314175540320485`, 0.022650359201911212`, 0.019271714123242262`, 0.00875024271870945`,
0.015162418103557564`, 0.0017834168117626926`, 0.015273219135505134`, 0.008260992803740328`, 0.019941014796847272`,
0.0025581856452432565`, 0.02472588412661547`, 0.005205946217814115`, 0.018315405981896914`, 0.009075419868217506`,
0.03768536634940422`, 0.009193873967345004`, 0.02030832286874414`, 0.018376095224234102`, 0.08600436130715383`,
0.02311676018318777`, 0.018799294807426425`, 0.003266185675781458`, 0.008101212535981426`, 0.013273479665681768`,
0.004922595981709151`, 0.022205552310182706`, 0.023003760418511352`, 0.009219466206678548`, 0.009020942291627492`,
0.029639154354815393`, 0.006650184976922107`, 0.019087374937369707`, 0.061981148970878634`, 0.01649800654142572`,
0.007073714701604785`, 0.043839233369484386`, 0.01390923980644204`, 0.011677929922251118`, 0.03335763290790723`,

0.003346444558002149`, 0.004409529198389065`, 0.027567965452499368`, 0.015087506450147753`, 0.03460483147324999`,
0.01172814982963523`, 0.01637458985086837`, 0.007616890230336316`, 0.010894125725014773`, 0.08021536368392358`,
0.019512892801072292`, 0.0037979979647693144`, 0.07087894518360807`, 0.012873514292887167`, 0.012071434723432839`,
0.014026502870362783`, 0.015377663078368199`, 0.019173517385422535`, 0.015645003788552804`, 0.016550228556535207`,
0.004514384136124654`, 0.015478578546266641`, 0.00710737415479074`, 0.010524906438921178`, 0.015672433923618473`,
0.014680012661431253`, 0.008384187057348749`, 0.0018753252878042045`, 0.06695119153588784`, 0.014155486418837774`,
0.04754590070059936`, 0.042124483585675165`, 0.019680488340397054`, 0.004071288387176145`, 0.013769602508787827`,
0.015778070090893347`, 0.020751111657654826`, 0.02052012745713859`, 0.06840896637415071`, 0.0350984604096805 4`,
0.011214164702313251`, 0.007529231412158401`, 0.011141178189765155`, 0.001821473478804148`, 0.05142990315950539`,
0.03252704077739953`, 0.004592697028338971`, 0.009297700590100077`, 0.027419079944612807`, 0.013271195952474028`,
0.009721479787055473`, 0.036385282805363156`, 0.021546041912672943`, 0.008557433622245215`, 0.021012728409612158`,
0.006263228238879296`, 0.01731122212457864`, 0.003656484826075241`, 0.02681118063766201`, 0.0020890709828228593`,
0.029956915616335865`, 0.019210099896463335`, 0.017541423544469595`, 0.003764838845973271`, 0.06914994693522825`,
0.025828286566656065`, 0.013708443069757624`, 0.006991698325013878`, 0.057941594235852496`, 0.012031949935720506`,
0.003040200174525548`, 0.0021521435084473042`, 0.006591702531119413`, 0.006567076873872758`, 0.031252738621569055`,
0.008960417424275396`, 0.0704641477360127`, 0.003629465576159642 3`, 0.05135979589007754`, 0.06188953409331782`,
0.002507039828323648`, 0.07040615869253229`, 0.025353111906103645`, 0.005884308308782019`, 0.02727867145420581`,
0.011314778984160577`, 0.011378144107647507`, 0.032617459676913346`, 0.006540324349455542`, 0.017838862104421566`,
0.022430817654859613`, 0.01635814334093508`, 0.015987535406428943`, 0.04282628679563644`, 0.007025681843254215`,
0.0036559696654851173`, 0.0019307926143865212`, 0.002520106081830254`, 0.01902427006391038`, 0.003831397488084415`,
0.005222832882442736`, 0.0645882105224357`, 0.08911106425967662`, 0.006668293710478993`, 0.017544041851153238`,
0.03144237140705304`, 0.03160258051603025`, 0.020605793055526712`, 0.005844434801964621`, 0.002891545336374 6358`,
0.005042332161938327`, 0.003125949981179884`, 0.028156048695257802`, 0.021325109451831484`, 0.030232855306156842`,
0.002085893167990133`, 0.009153367989699472`, 0.015538112327556869`, 0.01053042901513198`, 0.005050444195347881`,
0.0024618896803089763`, 0.012258127207832344`, 0.004800731720677917`, 0.011611634056930895`, 0.008992379803317473`,
0.07069733385594006`, 0.009673155223415721`, 0.008167685778155425`, 0.005606964227800704`, 0.006503453154908149`,
0.0021185973919214795`, 0.035141709147595596`, 0.016765485278952132`, 0.03000358576227669`, 0.002858567375588496`,
0.017218924471390038`, 0.02687278487062522`, 0.006832854237987094`, 0.0038423397055301262`, 0.008831296384053243`,
0.012184109492510234`, 0.01629338447093443`, 0.004857571592105698`, 0.017271685412691994`, 0.01041717946419907`,
0.02005634319135894`, 0.016310858264014856`, 0.0017592999745428947`, 0.0016756615436286301`, 0.014342075171617471`,
0.07776275713494255`, 0.003203302985189169`, 0.0101856596086544`, 0.0064184982094654`, 0.0121987593605519`,

0.022586602208200728`, 0.01891340935081875`, 0.011576682813826731`, 0.017607538009603893`, 0.009832482058378066`,
0.00450466702670292 8`, 0.0018066969692651091`, 0.015470624098735576`, 0.0016169822590192855`, 0.013588819983302167`,
0.02372386751798236`, 0.004764875980284635`, 0.006210688157831853`, 0.01893423470099829`, 0.014485945146674`,
0.00397637517337404`, 0.037184446422319356`, 0.019774869553884907`, 0.02391262834414653`, 0.00575429545656906`,
0.02279446553491415`, 0.007606686206355786`, 0.04272747055187113`, 0.04193730401983988`, 0.0012771361562248105`,
0.07250148491814518`, 0.0588998587861334`, 0.015645401876334057`, 0.009313572745721095`, 0.009137438532101841`,
0.007269421488926675`, 0.03091739475286841`, 0.007689044438373569`, 0.010463793701361752`, 0.00955929153464249`,
0.0020059336229664437`, 0.0025428516042976697`, 0.01768114975158096`, 0.007280164176681196`, 0.004567204057501289`,
0.03292022644224616`, 0.011095947623049348`, 0.0025532777603591472`, 0.0033881762514238354`, 0.02867323105498688`,
0.039020568870996`, 0.015805612168442503`, 0.03508357264289699`, 0.0024286377458940397`, 0.02220182222605148`,
0.009744051714587516`, 0.0206986006748293`, 0.011518941499536464`, 0.011065036260952458`, 0.018707227467181127`,
0.018194036180924994`, 0.05390884223358861`, 0.00313905653459392`, 0.0166964270015836 4`, 0.015894659244559156`,
0.051388671397842665`, 0.002764509225754258`, 0.029949405122890446`, 0.0020243186652087 05`, 0.013464920307091892`,
0.005600723874326528`, 0.009623380612045103`, 0.00282042280659391`, 0.03283382287304601`, 0.002690691068786 4625`,
0.010677697202229072`, 0.01716294363279227`, 0.02847010610150949`, 0.003463328351299777`, 0.02462869504565291 5`,
0.005111456175701691`, 0.010937742017604536`, 0.024807109292977567`, 0.012971868199095979`, 0.0019015863191574618`,
0.01075526293669248`, 0.022271685474294495`, 0.005688387783803515`, 0.0181948894737847`, 0.005264193737175 0835`,
0.016062985522769226`, 0.0064048519419864485`, 0.004831356207273711`, 0.005722133880319589`, 0.01762634945391042`,
0.016569588302658398`, 0.02942883630733336`, 0.06471184693449282`, 0.05812009682458262`, 0.02345268655819936 2`,
0.01542572839948292`, 0.016139432086833907`, 0.010919373296117751`, 0.025655182005248033`, 0.005070564591832387`,
0.015586662222521279`, 0.00969085110519652 9`, 0.023194859822176464`, 0.006641440426353469`, 0.0287649570971850 5`,
0.011230148783695616`, 0.0016751304211800357`, 0.05892676886893634`, 0.0028235207826192766`, 0.01280005611987739`,
0.006299733478776463`, 0.05352920148408077`, 0.00770367029420005`, 0.003072732700792365`, 0.04342290331106383`,
0.014455041442797875`, 0.004883206258824373`, 0.0022795376860203 26`, 0.013196507005118178`, 0.023858365286420755`,
0.01650728635426511`, 0.010357621590115492`, 0.10354317308239522`, 0.00905880251402456`, 0.003821202625713235`,
0.005470068783992226`, 0.013178403620711716`, 0.0023343177912287017`, 0.00477551122790063`, 0.006592536819228083`,
0.004097486097751791`, 0.0075174987314747515`, 0.008978669464773272`, 0.019198289467293444`, 0.031441895167817056`,
0.014092536440907133`, 0.004360411950503434`, 0.003614547299339609`, 0.004037352809206777`, 0.04129974088531989`,
0.0040292273118604`, 0.008070887521401707`, 0.033123430061485305`, 0.007570944546190409`, 0.017131077218108432`,
0.01250006163383095`, 0.003567170246229224`, 0.014430452880115914`, 0.03782774150025853`, 0.030961913110421575`,
0.002848495114858149`, 0.030116847391026873`, 0.006001721509327415`, 0.046094720969925417`, 0.02177569284451187`,

0.07206443167007139`, 0.0028138831358184083`, 0.01796680818207745`, 0.011698939905815537`, 0.0341159896241569`,
0.021430945894505872`, 0.01894964657943155`, 0.0038445273310367964`, 0.020545303848333283`, 0.01036749474768283`,
0.005083614803303968`, 0.004604969599974139`, 0.006356442685496587`, 0.0414086522255671`, 0.0023926992490022245`,
0.013529460379404043`, 0.016292001119427187`, 0.017341338177348797`, 0.003087495776327079`, 0.016705290451788327`,
0.0064369022612601095`, 0.029147130341036418`, 0.005918824619420428`, 0.008185787272360814`, 0.0040425513033716`,
0.010477769508915982`, 0.005989129476075849`, 0.0018501562103934476`, 0.0042329208647916075`, 0.013157671054404619`,
0.02014867376905707`, 0.0234282915609114 7`, 0.015419273812803717`, 0.014699351450145559`, 0.013920538765430906`,
0.02354963624712361`, 0.003432224818505700 5`, 0.00539917890310516`, 0.00133164225228567 1`, 0.011030829818769428`,
0.024278863656171382`, 0.007538490514253709`, 0.012298461025097823`, 0.005120738176009436`, 0.020740155169958113`,
0.004139801972546744`, 0.00591943545016133`, 0.026044271590318052`, 0.004418417613018194`, 0.014450020066493928`,
0.023145327445119802`, 0.010077748540124361`, 0.005453396914950451`, 0.022983841511848326`, 0.008870820993566666`,
0.006484834215115268`, 0.022484276417936072`, 0.004068398677262535`, 0.0390810111507145`, 0.013039562786952322`,
0.006849191742503632`, 0.008270222188847832`, 0.006819926397141209`, 0.002284432367935109`, 0.0014436351634929815`,
0.0035176072016820563`, 0.023926614827560665`, 0.011917275595117773`, 0.0041757156324165 92`, 0.0028606236665698302`,
0.011066938283880539`, 0.006471372774754293`, 0.006712543776760135`, 0.03474295135462072`, 0.04461576487743342`,
0.021619232428761744`, 0.011194350427522816`, 0.04415681228018038`, 0.005442243622846971`, 0.017878514431690473`,
0.0034359005787061117`, 0.011829383001960542`, 0.0541264221464225 8`, 0.1016711128522629`, 0.01279558959843942`,
0.0061558571260859965`, 0.02575278006058593`, 0.019110446499809402`, 0.039981182034238304`, 0.040476768647235 31`,
0.007576085128912824`, 0.03889201128752442`, 0.004985284568969624`, 0.003323904918109649`, 0.02251470780428466`,
0.023702858550572165`, 0.02067732130379885`, 0.01162123089215552`, 0.02863364650920648`, 0.007338849004408912`,
0.007789878868245522`, 0.0022338714968952924`, 0.002968887375262419`, 0.04953881023887457`, 0.03436054806523548`,
0.04584402890750183`, 0.019635956725391444`, 0.013923009444554947`, 0.00896652788369928`, 0.0022086999552044947`,
0.00323994081532182`, 0.04906458712776652`, 0.0047254302551301185`, 0.02256263354930427`, 0.013553750524847481`,
0.010296088936186058`, 0.053008588950108945`, 0.0023511164972934953`, 0.004864069789656331`, 0.042462736572502845`,
0.015342206811847255`, 0.010360362851243669`, 0.010393964100340878`, 0.01523225066405926`, 0.007576595305603701`,
0.007766653711280235`, 0.015774029584761092`, 0.024194593305527284`, 0.03493428163373792`, 0.016664629764721023`,
0.022366846824672843`, 0.003393617549278414`, 0.002557020301332756`, 0.006992134657909388`, 0.011997653148812116`,
0.029382441873315752`, 0.0297900681071305`, 0.01942672767871566`, 0.0016262514575972466`, 0.014961210585985558`,
0.03943544374550235`, 0.03443319182455581`, 0.002691586438609293`, 0.01864160044466897`, 0.005311589654157116`,
0.009833814024922893`, 0.013923149141609357`, 0.0017866504939282153`, 0.023152571195219213`, 0.004188504527197827`,
0.01718044748909303 5`, 0.03867790260377199`, 0.01908113934989309`, 0.0037235516123471364`, 0.002941378724938877 7`,

```
0.006080325224838115`, 0.007145279432507011`, 0.012489962058232117`, 0.003133580658383863`, 0.00840017456581091`,
0.017742668276311213`, 0.003106205213306464`, 0.004875262137582238`, 0.0030492607344220876`, 0.006967794265594454`,
0.01756633046623729`, 0.014016088424147746`, 0.012386567693899031`, 0.08849277502279097`, 0.01444616198353176`,
0.00478803747635736`, 0.0044033231887811355`, 0.006019230301526749`, 0.0023900646424503576`, 0.023844440462237566`,
0.005799881380637639`, 0.02001244378294907`, 0.01575009410234731`, 0.00478945851989805`, 0.07649776896300732`,
0.007996901076317644`, 0.03870513036111835`, 0.07013956829216234`, 0.01245932122196594 1`, 0.003921752766494185 5`,
0.07745493900163167`, 0.02597477957637381`, 0.012805014697653846`, 0.01320550720484085`, 0.01838595840766351`,
0.006887695233178762`, 0.010512549166181892`, 0.002170234357533212`, 0.05575361255058048`, 0.007683381822231704`,
0.007719247655798254`, 0.003314959643086109`, 0.020856578010077707`, 0.02665793156029295`, 0.010533603201077917`,
0.01858079071569968 3`, 0.00880126207326167`, 0.016705399736491613`, 0.007141693386202371`, 0.004373777144442276`,
0.015464338021534844`, 0.015861082062743143`, 0.016724973171813394`, 0.0019519500105567417`, 0.0027179569890323956`,
0.012600221003454235`, 0.010672462553416334`, 0.027047889542997822`, 0.004326758788379342`, 0.010578990957578973`,
0.06494396119755666`, 0.0035013851680536466`, 0.017939308030297207`, 0.002180940952380869`, 0.06008165970875939`,
0.00962101570293695`, 0.10562025388494438`, 0.03793326483774609`, 0.007658639692707008`, 0.005436941943579108`,
0.004294348553106439`, 0.011343443557197254`, 0.0012928308739438804`, 0.028116094918146903`, 0.11132974443308488`,
0.04840350469517551`, 0.03271788667642433 5`, 0.00265696698522095`, 0.01335295755409721`, 0.01721091882737220 8`,
0.024090618939495354`, 0.03423566503133417`, 0.010895022220873029`, 0.00801365946731488`, 0.06145344658247282`,
0.023989108948698015`, 0.028566319595429997`, 0.056412845561212885`, 0.01647975521997475`, 0.06091157546848282`,
0.0034571113056575536`, 0.018799320577385818`, 0.0028298019079688818`, 0.009761684260116149`, 0.019652212396713914`,
0.020883142094222547`, 0.0018220828749790573`, 0.008814509818614412`, 0.006370734894746709`, 0.03126927299164307`,
0.00612500713330123`, 0.00682084995704920 2`, 0.018477324602298707`, 0.0025328749276757037`, 0.005249879908473197`,
0.0025837220391653893`, 0.0065406911337320 93`, 0.002664103436547395`, 0.0038238322312541613`, 0.025748180474475548`,
0.01773347358219058`, 0.03584954117474992`, 0.028104345231385774`, 0.0019265650255412949`, 0.05912124764139856`,
0.01642133394799025`, 0.009762285399674591`, 0.01780363969902837`, 0.00636025418663401`, 0.012471872891318101`,
0.026721810033141554`, 0.034354197943152975`, 0.02710944624719395`, 0.06410523869767226`, 0.002481749026088049`,
0.04052070623179231`, 0.02466432024785179`, 0.009710989382636427`, 0.04497787484875983`, 0.014125254980040488`,
0.003745802278179612`, 0.008350552694126205`, 0.10930114485345371`, 0.006895645302404294`, 0.006886025566850033`,
0.004226307919093058`, 0.033418946728789575`, 0.010740726182802791`, 0.019604930455093962`, 0.001823626355286313 7`,
0.012510849270152728`, 0.008802632530291343`, 0.023775234967621423`, 0.018965554767069643`, 0.007606489015816836`,
0.00431705051597674`, 0.020269756733984217`, 0.001439111319893344`, 0.012180004769820486`, 0.002867922252598243`,
0.026029136857230364`, 0.012535154322276466`, 0.017012880241350416`, 0.011653409960304917`, 0.023992655583960507`,
```

0.0189230568818782`, 0.014692117369578945`, 0.015278769433526398`, 0.024396897257914867`, 0.019600742225651727`,
0.006781126697617449`, 0.0033661675538641748`, 0.011095304668139818`, 0.01030865276329956`, 0.0021810658100684353`,
0.00693601550885395`, 0.008904183021638177`, 0.04639480564472824`, 0.008492201218084618`, 0.016071499210236786`,
0.012232924210468543`, 0.04470426873568682`, 0.01039492513025353`, 0.031376987370307835`, 0.015718431353325023`,
0.014653245856646346`, 0.001888772189918854`, 0.0028439510812399804`, 0.002466297716077215`, 0.014157877576457934`,
0.013897858430353864`, 0.010162477136736797`, 0.005089299693802259`, 0.005961018943993466`, 0.0015683759162724849`,
0.02896553415896919`, 0.01588363206761706`, 0.0027182229923686206`, 0.011272199814607704`, 0.004748485883378061`,
0.011585875157561747`, 0.017889722586123234`, 0.0020548243383509152`, 0.005271471433907414`, 0.010735906677070084`,
0.012531443905159163`, 0.0028225257799763676`, 0.011152081662283595`, 0.006424109030057716`, 0.007276609590069569`,
0.02557430153484373`, 0.015511048563358948`, 0.014166151308917603`, 0.00911256251208158`, 0.00530752049365615 1`,
0.014882366317390105`, 0.007155056333795803`, 0.008459760110904734`, 0.005654191864070151`, 0.014792762697638514`,
0.001583815941545327`, 0.009536269599478593`, 0.002577926629565395`, 0.034706146713515615`, 0.09970833074261565`,
0.008439429875280924`, 0.00451918427148004`, 0.003257826146842275`, 0.012546315632882579`, 0.034521898129325844`,
0.001313231691343699`, 0.015489857098343843`, 0.046884571876957444`, 0.01646054139364104`, 0.011285306338126773`,
0.016738460949828157`, 0.007286255547796387`, 0.017115834389904234`, 0.009190114835190343`, 0.04122200668193855`,
0.012735447094183613`, 0.004666312206917963`, 0.010150408189981796`, 0.008394719640570859`, 0.0018458128605313984`,
0.003060690743694077`, 0.006685498799995869`, 0.013624449914697133`, 0.001638044918313526`, 0.002679831558418047`,
0.005857345806556986`, 0.00348587643685823`, 0.01838460248425688`, 0.002846944148098 3323`, 0.013229405013195698`,
0.03782852911654539`, 0.028598812506923397`, 0.01714746704975004`, 0.06352455968470282`, 0.04736905540738765`,
0.0048241893258912346`, 0.0091724872830487`, 0.010472932788375473`, 0.00829691204210646`, 0.0862173492556045`,
0.006145662755321882`, 0.04154787105999404`, 0.005195480536133877`, 0.003035930082507439`, 0.02313435966138108`,
0.0018800232046998087`, 0.019545436834796426`, 0.013463910789838609`, 0.01190908844756367`, 0.009191057179878805`,
0.00650623895516122 3`, 0.02020928042284122`, 0.028953684916155496`, 0.00829297256351769`, 0.005669088477351275`,
0.0020626973222996796`, 0.013137126727011507`, 0.011187380603660393`, 0.0022590923904784056`, 0.0063043120721 20435`,
0.0024847032652583587`, 0.014199540808757995`, 0.008057085209389914`, 0.020544817704150932`, 0.003992616581492685`,
0.00781897448716185`, 0.003652603205487951`, 0.012944790975131227`, 0.017235019875742212`, 0.015560005320496059`,
0.01888509847894938`, 0.023250793549557044`, 0.002950690590589612`, 0.003297506907270 2185`, 0.019276357928833626`,
0.007891543129551891`, 0.001566914819491564`, 0.05279190622162654`, 0.023091940622560935`, 0.01241054958982339`,
0.01066214536531188`, 0.041029794285506044`, 0.034126175226875465`, 0.0018648516220668987`, 0.0020377704978743 06`,
0.01177842602567197`, 0.00406188849290047`, 0.01311756405885226`, 0.031121583855861955`, 0.010427501457084543`,
0.008358656899210289`, 0.02668538635747561`, 0.027668467922504377`, 0.04043172782967565`, 0.009231918320823835`,

```
0.0440608508283963`, 0.015997613321454565`, 0.022520342684623836`, 0.017686891913841773`, 0.007522109851441363`,
0.019583250627568573`, 0.021200129189095387`, 0.0504915018485523`, 0.016031731748596328`, 0.07102670582267796`,
0.005948001718895253`, 0.00590423229418042`, 0.048438276498657236`, 0.009514026018064257`, 0.0066225637208553 4`,
0.0168318584238817 1`, 0.00816993705428813`, 0.0167810229134448 7`, 0.046943077638172 92`, 0.041479714735796 79`,
0.02078465594205668 5`, 0.01771273462811341`, 0.024184829476077155`, 0.0023332727474290973`, 0.046321560397223675`,
0.03156410629227227`, 0.00912082361665472`, 0.02710390471405605`, 0.016901424686321734`, 0.021126384558791785`};
```

In[ ]:=

```
(* Fraction of activity, spent on viable cells *)
```

In[ ]:=

```
FrAcVCHomo = {0.019729613292576574`, 0.011686212283865544`, 0.013622566770932798`, 0.010128252477523754`, 0.005662254536252713`,
0.006372680998692563`, 0.004123962212583303`, 0.006209678811607699`, 0.02261864298947508`, 0.018088454809526432`,
0.0061040320624602 3`, 0.015669862895034723`, 0.008033172057305 31`, 0.024657397995326496`, 0.008822217295666245`,
0.032298968107307494`, 0.018819424680632343`, 0.015248532014284907`, 0.02247657632982354`, 0.008049099335934116`,
0.005407648310994484`, 0.013722815897071007`, 0.013480932007953517`, 0.007754844967977932 5`, 0.010597271837278734`,
0.02331788008483285`, 0.006422437673791734`, 0.008445377804881169`, 0.008287854827162922`, 0.021020952174863678`,
0.008689620657800821`, 0.014821412675412272`, 0.005498124242526488`, 0.007749431323551077`, 0.012114210597881137`,
0.006730099053828712`, 0.020642770643255 44`, 0.021599663170613 03`, 0.02467790361269966`, 0.008597386944992147`,
0.008754764638471404`, 0.013899481924542198`, 0.020367398322117 43`, 0.009456214426898575`, 0.0239003915764048 1`,
0.0237073186096642 7`, 0.0190840539788826 7`, 0.0229093454574007 5`, 0.020592511563786203`, 0.0136451930852243 1`,
0.017688582616723663`, 0.012873404927680696`, 0.007647973159784627`, 0.009473963003290793`, 0.018412118846862238`,
0.011044120071321693`, 0.022872625422390318`, 0.005792443305160013`, 0.020328316445284753`, 0.009754216542157657`,
0.014335692076023344`, 0.023242815840708055`, 0.013911706199055004`, 0.013151606361479619`, 0.007463375598865606`,
0.013516876873721604`, 0.020156174355969354`, 0.014875889395472974`, 0.033477584965549236`, 0.012083825515097584`,
0.02394246719605545`, 0.018603306983017 72`, 0.013934434440563219`, 0.023093479513484803`, 0.008395914315521823`,
0.006767775685490 12`, 0.019937490959653147`, 0.004652426596656499`, 0.013422246322333567`, 0.014107287876640397`,
0.015206278274607583`, 0.020647564655669 23`, 0.021562118066689562`, 0.006330540758595874 4`, 0.01749552867168612`,
0.02046481140707182`, 0.006497691061569708`, 0.030165885595211207`, 0.010161068940163992`, 0.014380248128893141`,
0.013170306497917936`, 0.008381693733442509`, 0.007534999359318311`, 0.016902815558762715`, 0.008935319672121007`,
0.016701122626010676`, 0.024376463220803984`, 0.012912104961247 02`, 0.023349591054815082`, 0.025379148509823226`,
0.016652117131329743`, 0.015812432220284`, 0.023327940698601165`, 0.00731500390074838`, 0.01819194926428798`,
0.021751551237901807`, 0.00788323085984822`, 0.008444551811786728`, 0.008130723339131157`, 0.011110139403396967`,
```

0.0157481560987815`, 0.010196108361607671`, 0.006384430760341731`, 0.025237636761327713`, 0.0224864475638689 1`,
0.014448981781136816`, 0.024390148693075993`, 0.006975213603281639`, 0.02054097706948647`, 0.025990905940573506`,
0.02048676844196949`, 0.01621090003573445`, 0.010541031471477813`, 0.015499004313937081`, 0.018230068143852377`,
0.0170885675772389`, 0.019185672699328242`, 0.012964740937125154`, 0.023606271971493245`, 0.018651422667705073`,
0.013907485517104502`, 0.01263245867817114`, 0.016926753330146625`, 0.008994518208644699`, 0.01565330583559274`,
0.02002510560743502`, 0.022140809827871432`, 0.021824835185345`, 0.01435303219815639`, 0.015191209189825517`,
0.030587465857506928`, 0.013567775209469455`, 0.019074057536449227`, 0.021420445475982788`, 0.009669811908533336`,
0.008581345934995878`, 0.018400947840337075`, 0.01628537648159036`, 0.021092136518654447`, 0.004160363001329628 5`,
0.00753295771453171`, 0.007815074425526211`, 0.01981304620485982`, 0.009476536156469465`, 0.011185315912045376`,
0.0203128249025834 2`, 0.019145557172622066`, 0.016370604560164603`, 0.018356125739215187`, 0.0224258632842809 3`,
0.006439475824456443`, 0.015911558644329516`, 0.01805051162904142`, 0.008653217256556489`, 0.005876646942571058`,
0.006058228033209463 5`, 0.022113606369325786`, 0.011680923661716754`, 0.018787727146616306`, 0.01742254168008144`,
0.02243601447356116`, 0.011911743564372804`, 0.017211576233112275`, 0.012237484428617046`, 0.01670403407007614`,
0.025468763696528656`, 0.006284468730034182`, 0.01680884105126542`, 0.028015603947197078`, 0.01317168995058917`,
0.005250071908278774`, 0.006384515265046133`, 0.010509025366761238`, 0.023688295658623744`, 0.013489107333332239`,
0.02260058893930791`, 0.01691468037351536`, 0.021999620048553 74`, 0.020098808307364727`, 0.010703648815161336`,
0.01779229670603788`, 0.023760792629676408`, 0.023962412532717865`, 0.014225217444689223`, 0.011870094720629061`,
0.01241438315539363`, 0.014638216541595613`, 0.012922732548551643`, 0.006423120071592281`, 0.012725491922769725`,
0.022397156056577626`, 0.004998451458388777`, 0.00970114960896501`, 0.013681148571329807`, 0.008293905981601521`,
0.01707258264743615`, 0.0151783823681131`, 0.023311534730962882`, 0.01073556407776931`, 0.016578048500034057`,
0.01764880797854413`, 0.01954612502801341`, 0.016050939670114853`, 0.015931317496031133`, 0.006482568396022686`,
0.005615616727823113`, 0.017501282881183265`, 0.021222169617135397`, 0.006629614064332618`, 0.011795207463589918`,
0.011086971089703853`, 0.016956012401446757`, 0.014243657534140627`, 0.013069367925691657`, 0.026395488738713883`,
0.003798306632231503 3`, 0.005572124329119368`, 0.009082917677582358`, 0.017529694384962 39`, 0.015329702510956309`,
0.003989150372075617`, 0.012160245449326732`, 0.022353924595058305`, 0.025298842649845478`, 0.011859683192703325`,
0.02104037415900368`, 0.013381922211507979`, 0.014953791691073037`, 0.014248294436225417`, 0.02658092014549604`,
0.011626939940509355`, 0.02966810592945757`, 0.008615236659339377`, 0.021476447075112374`, 0.025958699286924767`,
0.013966990403989974`, 0.007765259933643629 5`, 0.003986026982121686`, 0.011829032957857 27`, 0.005980595796298315`,
0.01862047965065061`, 0.010681834051522909`, 0.020758826594615455`, 0.015271370623477172`, 0.025482178455796305`,
0.013757486039625701`, 0.016065479880306047`, 0.017176894335530407`, 0.01639536280923141`, 0.018520274366063922`,
0.027373704802330385`, 0.02505986494048353`, 0.023082016025146465`, 0.010060856235984714`, 0.006838216046027544`,

0.018231231790191864`, 0.023046410830277485`, 0.009494515912686961`, 0.007432138282435856`, 0.021648701013404163`,
0.02101044104477543`, 0.018419198738629083`, 0.01658321411961286`, 0.00912288513156415`, 0.028073081600880796`,
0.02178106170225831`, 0.013342823171276396`, 0.011705502774209132`, 0.011762733218612348`, 0.005663376452532499`,
0.009864352846841534`, 0.004141142626161874`, 0.007356033845618222`, 0.016200348324433955`, 0.02902541520129436`,
0.01214295323016106`, 0.022481294361304714`, 0.004828448318705182`, 0.02689248524763411`, 0.02954920891326464`,
0.027109181013989375`, 0.01751368822000887`, 0.018922735531435662`, 0.014653168569429727`, 0.020205521253713377`,
0.0216495363744288`, 0.005324443479242041`, 0.019241778370914387`, 0.009456158505044394`, 0.024887792827798957`,
0.011298514948085748`, 0.013743000745143783`, 0.009749085216205185`, 0.009138541719350063`, 0.006339736182838292`,
0.00819536289300873`, 0.013388397223227868`, 0.00932527205308568 6`, 0.03177961633311359`, 0.026416275092014684`,
0.018025487243907835`, 0.009943805661010538`, 0.017491810031409333`, 0.014939118993760438`, 0.010306875005233458`,
0.02101389422032538`, 0.012341149118762405`, 0.02162951884272814`, 0.019325941232961993`, 0.02277881943194921`,
0.009440278486571963`, 0.01829722723290335`, 0.02164807948762795`, 0.006633094375668834`, 0.009076919738834628`,
0.029027907125238287`, 0.011143170091555991`, 0.024129621664926937`, 0.0165978102962234 2`, 0.022030511051763856`,
0.017481971800408152`, 0.014703133301423728`, 0.025091172953579446`, 0.012609581307992071`, 0.02374182748523026`,
0.00918006280249747`, 0.020798377405008957`, 0.010004513953912735`, 0.004346063670537757`, 0.009149195432343818`,
0.014955175253967152`, 0.013048996490654831`, 0.011369234553824019`, 0.016595411330369157`, 0.006517287142274344`,
0.022633341120721212`, 0.02483987866537639`, 0.018637900061245886`, 0.018301675441164338`, 0.006958434611223216`,
0.02268736555681023`, 0.007078269731233886`, 0.021662719227619733`, 0.0164827103548072`, 0.019815627821149588`,
0.014271554504460065`, 0.011959192408155659`, 0.02114924185568781`, 0.009250755776028552`, 0.009685413683678392`,
0.013962127202036129`, 0.006955352573143057`, 0.01459647495075125`, 0.013018732358864417`, 0.021540327282641374`,
0.016047365251006`, 0.019741806599461634`, 0.018868935892912702`, 0.011306780070292965`, 0.016193495599493308`,
0.004926638944131967`, 0.010068363987406376`, 0.019791725830631043`, 0.023216058540586032`, 0.028729287544305867`,
0.0286862401068308 2`, 0.016547304772585574`, 0.020815993586212604`, 0.012943780375499203`, 0.017618176555412344`,
0.024526199988224726`, 0.02022786582859519`, 0.007587173370294256`, 0.015318864645812424`, 0.013424938231527107`,
0.017702773733615926`, 0.01789066595365935`, 0.02099601794309859`, 0.019614785207563133`, 0.01688381086527732`,
0.013866638093570062`, 0.010516069890388403`, 0.008597656528538552`, 0.011297649908398577`, 0.02114165988365453`,
0.009698746587940083`, 0.009124858588045216`, 0.006904413014015546`, 0.02371311294285827`, 0.01595560389258882`,
0.02132050747256843`, 0.023826033647449688`, 0.011207300789909742`, 0.01570667864468468`, 0.013292466366180975`,
0.020801623763001917`, 0.011826716364476393`, 0.008633690954651762`, 0.008842697571586889`, 0.007685205919714837`,
0.006356323539802408`, 0.014672499731092014`, 0.027402187759511577`, 0.01614859394715186`, 0.009642458245245138`,
0.02609021063803533`, 0.011832862762528486`, 0.02793251091665774`, 0.007567680681062561`, 0.015813129618991437`,

0.021104457147587`, 0.026479427510052056`, 0.00778402011514316`, 0.030260672493628144`, 0.006571363325283904`,
0.021012326276307072`, 0.021274878011555056`, 0.02343971730089932`, 0.014086543154677772`, 0.02777454781025451`,
0.017120631464203475`, 0.030792077993782255`, 0.017713473777052974`, 0.018880720895297527`, 0.012665919396169916`,
0.016017000865684687`, 0.007924899433736731`, 0.006049519136167097`, 0.01897086366159281`, 0.02253403338688544`,
0.01081419398847743`, 0.014579694322513274`, 0.015426001654717917`, 0.004082667083757819`, 0.006749955882603445`,
0.01874575496698126`, 0.01858051303377414`, 0.013709754035408112`, 0.007254421824106248`, 0.010901280008376025`,
0.005454532237619693`, 0.01914703018278172`, 0.010868845665504907`, 0.02849059306065583`, 0.004248882990940011`,
0.021336009337210242`, 0.006812550039585064`, 0.018157554673549944`, 0.019246841268132163`, 0.02992507593464684`,
0.005803479650816696`, 0.017642901130832275`, 0.0191178581365363`, 0.005745855330461821`, 0.02482244764129715`,
0.024869390179141124`, 0.005215459514095981`, 0.010163306541074401`, 0.02409985198877762`, 0.02342230669922805`,
0.009990240278073924`, 0.006834546803746078`, 0.01474322702577512`, 0.006800945804550681`, 0.012196130800212803`,
0.01251337637861137`, 0.007763388962273636`, 0.01527412598680416`, 0.012185493618838182`, 0.006944854808487501`,
0.016707680377566318`, 0.031234674668860946`, 0.01839532711773268`, 0.019301750961928866`, 0.024471078657044343`,
0.00885599283503378`, 0.013570071391596205`, 0.02111405085156677`, 0.004963673728585053 4`, 0.011597177905919425`,
0.0204717617270606 4`, 0.013704470632169478`, 0.006797530042493650 5`, 0.020370539918562493`, 0.015031149783452656`,
0.019806402216334756`, 0.026362188468291723`, 0.008740578635816863`, 0.015681551683132528`, 0.01366521911357686`,
0.01308862210667612`, 0.020194430706267075`, 0.01963767264465497`, 0.011240839739754002`, 0.020941321191476688`,
0.01337958549856568`, 0.012522415641888544`, 0.012557179241850732`, 0.02006837123759859`, 0.014048863383763663`,
0.010088069082204549`, 0.007249288540926206`, 0.021250196501107165`, 0.026490308877175347`, 0.007834731853382921`,
0.01798396574823732`, 0.015789076228946833`, 0.014997918009298991`, 0.016316577770306095`, 0.026527745784780718`,
0.021927035539563027`, 0.02408004958693488`, 0.005194602317762961`, 0.025270908471239698`, 0.019578123235843996`,
0.015543289000915748`, 0.006058554494764867`, 0.016272064156472393`, 0.017751927916824818`, 0.01868393129637044`,
0.02539902583046086`, 0.012352884579314919`, 0.00960185612292197`, 0.012742691993674069`, 0.016301826820255706`,
0.013216511113036124`, 0.020835258093237666`, 0.021909476362462113`, 0.012413156622109855`, 0.009351672926477433`,
0.010843469493707809`, 0.01692644951724066`, 0.008371182431754217`, 0.00993915427352554`, 0.01705160706667045`,
0.006577738796473075`, 0.012871499495137807`, 0.011193827096284222`, 0.02350950575412937`, 0.014011809917744495`,
0.023117620235997893`, 0.004795554528814472`, 0.02024159898334542`, 0.007305537061985445`, 0.024446984132783685`,
0.007288611199622255`, 0.008194639030457649`, 0.01507476317054006`, 0.011735655268782081`, 0.01489520718620381`,
0.009152510072027106`, 0.008707951146134377`, 0.017864068293817727`, 0.018258216975410874`, 0.020852038266638177`,
0.006770859033366603`, 0.011691128583474113`, 0.01942033673898996`, 0.01987937464528032`, 0.007588317214378421`,
0.012757631086722912`, 0.014358965328803432`, 0.024775962176267548`, 0.020949066215824153`, 0.005644366259215017`,

0.006112539645037204`, 0.017694554001765454`, 0.016759120194601206`, 0.013904195550531804`, 0.015282073411056061`,
0.028876115299774015`, 0.027534760667686353`, 0.005226256309122278`, 0.01843790264233679`, 0.021972793734176824`,
0.009607940013153462`, 0.01789557127505043`, 0.019173532372050228`, 0.005150560722038086`, 0.016883204741230264`,
0.02021004922949395`, 0.004394598472255478`, 0.020652626686179034`, 0.005885106983586667`, 0.015023864397321347`,
0.008877484416268693`, 0.013640461238843122`, 0.018879676044101637`, 0.024277799696961002`, 0.01849307755104175`,
0.014369131459369381`, 0.009592411726647548`, 0.006260300065328111`, 0.02352844682525858`, 0.016764367679657298`,
0.019116637825296903`, 0.017448208304402957`, 0.01627690425774426`, 0.012978964010386711`, 0.020599096353023382`,
0.0132342623338286`, 0.02804321112812184`, 0.01785737127684453`, 0.023978445695569242`, 0.0165589536578507`,
0.02625551331319334`, 0.030276791114644253`, 0.01840814447524396`, 0.01439198820414708`, 0.02539477230954668`,
0.02176722181241538`, 0.014593724879925522`, 0.011429407257470634`, 0.02373536570819324`, 0.01808517324109024`,
0.009648744137813834`, 0.030403776719997333`, 0.020930185124944087`, 0.03061706283233077`, 0.015765268711944024`,
0.007562754154351756`, 0.02435756147674705`, 0.012612943387459668`, 0.028088781051909716`, 0.02551244074934867`,
0.0038771448024636536`, 0.01611498244753796`, 0.022926378636193557`, 0.011375090058308045`, 0.019266865467552987`,
0.016807165278558318`, 0.009865367340305336`, 0.012071982985884508`, 0.02131701877295646`, 0.022979822776651903`,
0.022382389888356852`, 0.010410313098876354`, 0.01980496552430988`, 0.020883742571064984`, 0.020312016353486076`,
0.018005936488884718`, 0.01288092708012278`, 0.01745112789826506З`, 0.019027754550173526`, 0.021672233333549732`,
0.020577654166091544`, 0.012862064766624666`, 0.0180215869396594З`, 0.0222623112162579З`, 0.015249324633059712`,
0.029018957541970536`, 0.008721521429119507`, 0.010324534754587465`, 0.008623134937169891`, 0.01182284956368045`,
0.006413843397584813`, 0.025437631741906955`, 0.009552430291594756`, 0.013176183872680628`, 0.01595292898844852`,
0.01960705338592673`, 0.01961668481931008`, 0.009210356830637971`, 0.0135841439210063`, 0.019864062855774835`,
0.0116151432778011`, 0.009203824742537895`, 0.024790927621412266`, 0.0167837630963663`, 0.024731084145100727`,
0.00751041036208697`, 0.0054364265566525 8`, 0.007916312551843543`, 0.018802936544059765`, 0.018982962604053244`,
0.017768113969713298`, 0.014378084740541676`, 0.009777181606668434`, 0.006259045846059393`, 0.024535535776384378`,
0.00783288349474356`, 0.018243384725547426`, 0.017931332731305063`, 0.015662484954920574`, 0.028311229042489842`,
0.014066317738496125`, 0.006337583289726576`, 0.003807170631882022`, 0.028810058712521916`, 0.01787815199145169`,
0.022261252375861816`, 0.023969745389043673`, 0.027608948148791602`, 0.004924299086788126`, 0.014042709925906716`,
0.019831783922915548`, 0.018919345222487834`, 0.020092324652489925`, 0.010673673773231602`, 0.021817288937210 65`,
0.01573084501727905`, 0.016419115735024768`, 0.020497765858943837`, 0.010546233248642125`, 0.01165119313086624`,
0.011435116008531374`, 0.0156600417529598 1`, 0.009793163619935617`, 0.019575788230574605`, 0.01611894975175652`,
0.014010283090217499`, 0.006149743446449732`, 0.01875613512932146`, 0.023584165814830357`, 0.018155867202638568`,
0.006503780186364567`, 0.005697317729942597`, 0.005935793294856544`, 0.012248869269501937`, 0.029714134730466524`,

0.009552527305111363`, 0.018968798737121026`, 0.010840483335909428`, 0.004671596152399404`, 0.01796061583959934`,
0.010140156154842836`, 0.020736443645761236`, 0.0168035569671566`, 0.02131754701114158`, 0.010652285650028178`,
0.006949883277841524 5`, 0.008748131371362915`, 0.01713549219064272`, 0.00928549791769511`, 0.011393852025447848`,
0.008566936783463933`, 0.006402918438997846`, 0.02076635946779505`, 0.014290235538662717`, 0.016092625303911007`,
0.006239117179271001`, 0.021964162926672703`, 0.007033348988462947`, 0.020890356462087492`, 0.011089836078520564`,
0.006107929249409225`, 0.018911581497647417`, 0.010084535780963156`, 0.014558172939437286`, 0.019747146343892737`,
0.015565816179564622`, 0.022191942286892117`, 0.019017171284639738`, 0.008358949995658085`, 0.008214972753790769`,
0.0139322589886550 7`, 0.0231507294019738 7`, 0.02125331812208513`, 0.018628203350737 2`, 0.015234096196542516`,
0.010225512035665187`, 0.0223972934256548`, 0.01909149102047134`, 0.019204683236111315`, 0.008414066632105777`,
0.020002690694791582`, 0.015001286694830002`, 0.010953250465053707`, 0.03135495866021188`, 0.011879203221275716`,
0.02106339414046047`, 0.012261803611369101`, 0.018529723684657353`, 0.021166722977370444`, 0.022189738539555547`,
0.015138920727748346`, 0.015819546689864846`, 0.0209739385732206`, 0.02152821649497876`, 0.023936573453321872`,
0.020197470946630866`, 0.015420321640082592`, 0.006447571849040193`, 0.0235005401425918 8`, 0.006472370330224052`,
0.0077009131274754115`, 0.013904063619523333`, 0.024121709322180687`, 0.007533348404881671`, 0.025454281428532392`,
0.012918633268043786`, 0.007966210803885663`, 0.012429120395619863`, 0.02054423940217502`, 0.026199430535307117`,
0.01276169312853872`, 0.011864079836708157`, 0.024377123908268565`, 0.021708830924882745`, 0.015245121821309021`,
0.024686203327859566`, 0.01643382790272417`, 0.028545587410140457`, 0.008836664557829624`, 0.0207744653055 6229`,
0.005982108050219798`, 0.024255941703278612`, 0.01712336815818876`, 0.016152475951415116`, 0.019821967099982667`,
0.014403072121745393`, 0.013769209652039996`, 0.020400766535100343`, 0.024404077566922998`, 0.02202986070403341`,
0.009869091189211114`, 0.006489203826641583`, 0.01881651162622934`, 0.010594444549914114`, 0.024506391451433327`,
0.021892919074784623`, 0.025121548636777957`, 0.00888595857878521`, 0.02856441556666327`, 0.01035365183030388`,
0.009490276754475169`, 0.025165413361340174`, 0.021428400911324905`, 0.008274770313462403`, 0.006146908756852933`,
0.022234829253668542`, 0.016316304673674314`, 0.006056027518610857`, 0.013475647278933819`, 0.007201167379884805`,
0.02167640244237721`, 0.01701382340774727`, 0.01226433358585837`, 0.01730223212318462`, 0.010550737262092309`,
0.02022538152167052`, 0.01999078291234421`, 0.017520478633206823`, 0.007187646431654622`, 0.015642014289469812`,
0.022515365827020805`, 0.007858167828219161`, 0.02064050745511301`, 0.010960051866610941`, 0.005302438665204744`,
0.01968201911378729`, 0.003964351599844639`, 0.015996106673004985`, 0.010032311967789377`, 0.01922107807879659`,
0.017227270481355687`, 0.01034551989797446`, 0.024844061304524698`, 0.009881995687088908`, 0.010189304883247407`,
0.020831608969720933`, 0.006876789640760524`, 0.02397588658082481`, 0.02111384108777352`, 0.01605240230742333`,
0.008118976227393262`, 0.022424153551302 29`, 0.026337211899112 37`, 0.005453804592255091`, 0.017547121350730 09`,
0.024674626705152847`, 0.01684622550637095`, 0.02038379950638753`, 0.012410677539741607`, 0.01579663941704209`,

```
0.006126036384243225`, 0.013517326125475326`, 0.017796105719767228`, 0.005416580696471378`, 0.010015483485402299`,
0.015560868922885345`, 0.0219395887213298`, 0.007058257403770326`, 0.022813213977568553`, 0.016072582412972946`,
0.02607798712547468`, 0.016949317109725325`, 0.029254236801465813`, 0.01475721732841339`, 0.0187517074017910 4`,
0.012318417450497226`, 0.011240750200288386`, 0.013916551192204205`, 0.01943461990699667`, 0.017838657449113254`,
0.013697374920314902`, 0.020769357906841004`, 0.022354309632200885`, 0.02150933060901049`, 0.022178876922541998`,
0.01823418556613476`, 0.009679835308268884`, 0.017500003200900508`, 0.005731456289380675`, 0.006689866932636172`,
0.009772046535599812`, 0.02139658759865116`, 0.0074620556955403`, 0.008094416511921694`, 0.01855344597147772`,
0.010601390731904865`, 0.006609375571790433`, 0.015475583642371317`, 0.025378724724360903`, 0.013216369322113569`,
0.023973581281067028`, 0.024264983390263457`, 0.014702746167505247`, 0.004499443667470802`, 0.0158772033892111 3`,
0.014708153028995596`, 0.009496256458042139`, 0.012864156841421224`, 0.010494958945158034`, 0.012919407909703801`,
0.025124141954891537`, 0.019309478372063864`, 0.004093221866158013`, 0.015521582550432773`, 0.010513046434765349`,
0.010188465542234114`, 0.01435832421635947`, 0.01501299481184508`, 0.013970888670349051`, 0.02068026182540555`,
0.01259247868097397`, 0.011894888410004278`, 0.006873133855245812`, 0.024479077280415783`, 0.024848528750984977`,
0.01214627687244494`, 0.016201301907761947`, 0.02611432123777365`, 0.018741248090636455`, 0.006966960829282538`,
0.02190451652207865`, 0.006688110804181893`, 0.021180947537045997`, 0.011317868243595867`, 0.012129877776189743`,
0.005939482942997292`, 0.010966212278603102`, 0.022050295749562365`, 0.021202954108668876`, 0.016222098809309245`,
0.014906022081721553`, 0.0216956034228482 8`, 0.006135184061003609`, 0.006493637603915037`, 0.016567326702021944`,
0.024388629270648717`, 0.008028230692871676`, 0.01027374001077061`, 0.009022301435085157`, 0.02349074819531643`,
0.01513006631077087 7`, 0.012964900895781131`, 0.00467896345187829`, 0.019477195334500517`, 0.00621518351986177`,
0.02233262539777237`, 0.01492129348745499`, 0.02468877593604231`, 0.010951766272428422`, 0.011107288088714749`,
0.008371068452417918`, 0.019242594134628154`, 0.026568469325559375`, 0.01138940418199714`, 0.006719903861604886 6`,
0.012516031123995219`, 0.02225438653428039`, 0.011506271718792059`, 0.028057690186243316`, 0.007025187357470622`,
0.008385742582633134`, 0.020446515334504 06`, 0.009912662125763623`, 0.01437375275016062`, 0.018458413893434326`};
```

In[∘]:=

```
(* Fraction of newborn cells *)
```

In[∘]:=

```
NnewFrSimHomo = {0.09082186298063795`, 0.09058808851268381`, 0.059600253846408505`, 0.04385681270821518`, 0.05475508308449677 4`,
0.056369688326373346`, 0.04365659680255914`, 0.039953309298646315`, 0.1945619462325531`, 0.1316164548841018`,
0.04906707268754625`, 0.11621572905001415`, 0.07148909484676537`, 0.1260157978883043`, 0.05209222389998399`,
0.09020330326236656`, 0.17649784003753244`, 0.15499438624030987`, 0.17065493304287407`, 0.05500151855778686 5`,
0.0658017542235812`, 0.152925182668664`, 0.058052305773580476`, 0.10238003040458646`, 0.09216667263064142`, 0.08592657161352826`,
```

0.04796354579149076`, 0.08106461349510208`, 0.06274660699499537`, 0.0760974657684776`, 0.09113476200746128`,
0.03826369580458364`, 0.05601739242529025`, 0.04688792704130226`, 0.036993886337258274`, 0.05130481780216331`,
0.11340300553717825`, 0.10969775108863794`, 0.06419800208338843`, 0.043592065127486826`, 0.09059690640948051`,
0.151341707187915`, 0.22471557966803848`, 0.07423112296435873`, 0.16074806657574262`, 0.0880515005327867`, 0.16158566957607765`,
0.12401739784793721`, 0.2208089292436225`, 0.09268749319220808`, 0.12279308175197534`, 0.069543919603187 3`,
0.07134703674074229`, 0.10309465058197484`, 0.08799864481572427`, 0.06455370304539583`, 0.16284358450778175`,
0.051440609565482104`, 0.22834852497770633`, 0.0721005081169196`, 0.1431392770626708`, 0.12344423541811869`,
0.08900796656809223`, 0.0323140652138635 8`, 0.05513856382223987`, 0.06173906815369283`, 0.14458752238096087`,
0.056569588030841104`, 0.08108041890169773`, 0.03523448145748977`, 0.0808961802033494`, 0.0518809015702901`,
0.05498835518793916`, 0.14092962134637366`, 0.0553179224130684`, 0.040731624510761996`, 0.22039740159623883`,
0.04612063475906944`, 0.12362266028732372`, 0.14035458861456895`, 0.15467968181837985`, 0.09799271725077158`,
0.20598520217830057`, 0.06337274544362345`, 0.10876116165390619`, 0.2148390334400306`, 0.06529823180286522`,
0.08239213911969928`, 0.11421569814746205`, 0.14065795009962284`, 0.09858924983170005`, 0.08609616808821922`,
0.07683434901201444`, 0.16762873355614089`, 0.06779206304720974`, 0.0789260028183197`, 0.18607708570157735`,
0.06286538406804262`, 0.12227723038298006`, 0.085110504135545`, 0.16197720499365378`, 0.049007486398380894`,
0.10878293754052414`, 0.07618836478729663`, 0.10254865107028568`, 0.15919635914877053`, 0.07518236613411931`,
0.06627528226920558`, 0.06919377894066601`, 0.058460295294495694`, 0.05452371643201598`, 0.10623215476522457`,
0.03565373249768028`, 0.07723985535424642`, 0.18454739211930632`, 0.09977145778651587`, 0.18720241028526377`,
0.06674125897463834`, 0.2023246837079289`, 0.14450296531238813`, 0.21800664836900638`, 0.0732422330270785`,
0.09318269976880321`, 0.1334912342802731`, 0.21179202583148346`, 0.16313517443900413`, 0.20650407864050715`,
0.06170884226046519`, 0.07955439782611841`, 0.06874279592128239`, 0.15659238866749323`, 0.08969106038029123`,
0.09359427179143247`, 0.056835153024947196`, 0.05979915929756001`, 0.10309240540687158`, 0.19676359964288292`,
0.0882138710230067`, 0.04338283389490085`, 0.07271891614193611`, 0.09116959749381226`, 0.07299501139212144`,
0.17139829386765507`, 0.18382040192500604`, 0.032342688652774045`, 0.06182022814598657`, 0.19783997430492123`,
0.13067877909999084`, 0.10284664071855493`, 0.0516403927185534`, 0.044732379257912847`, 0.049485733680091216`,
0.07096991730488221`, 0.07832745539776044`, 0.1627315152757322`, 0.2296402106192177`, 0.20146521965697356`,
0.03876805249007937`, 0.049243900497449235`, 0.0904793884324803`, 0.050906065969476076`, 0.17107498081114897`,
0.19836220744445873`, 0.09615586036199984`, 0.058295314516730874`, 0.044388984999805654`, 0.08881411635140073`,
0.08414135984386928`, 0.15844816361765743`, 0.09738949898989036`, 0.2070207757379409`, 0.12210172659272928`,
0.4239898105435885`, 0.07340424172658681`, 0.15264825975263965`, 0.1398942727979383`, 0.05376606577671574`,
0.08978327639526545`, 0.08029972274994951`, 0.19445213607606648`, 0.0452759800129202 1`, 0.04159524311967801`,

0.052278371144425684`, 0.11680059322946879`, 0.04691499850890544`, 0.18539822387526642`, 0.14157595854550073`,
0.13841481656134888`, 0.20299319708585384`, 0.11451723597468853`, 0.07735068890988006`, 0.1490879468026189`,
0.16881281218731548`, 0.14758323763212833`, 0.03784852843405055`, 0.14438220429720308`, 0.14956314011943161`,
0.10494015496110354`, 0.0753648875317784`, 0.10907561375144044`, 0.19939241478112568`, 0.05159703907601098`,
0.08423280431808595`, 0.03769011691272471`, 0.052087159974144065`, 0.14619900280723544`, 0.10999090891443275`,
0.09247988826002798`, 0.07756867815041876`, 0.20473311189531684`, 0.14761304836608627`, 0.21547449873781058`,
0.1517939675900178`, 0.155744092775819`, 0.03938795298706787`, 0.05173673444496452`, 0.1692156849154219`, 0.13390552146141194`,
0.04762998278593535`, 0.0721914044182236`, 0.03467500334312031`, 0.18723773339879368`, 0.1279048806735845`,
0.05306451020139482`, 0.14208077520761753`, 0.03938451279776463`, 0.050502875687912306`, 0.043921155122797616`,
0.1580636124102564`, 0.10505068966999058`, 0.04165327602756901`, 0.03689398473563645`, 0.062359199630344885`,
0.10322355149961505`, 0.06282728313496429`, 0.1149386307406086`, 0.13066743111160184`, 0.04686280527665849`,
0.1183589248982035`, 0.11748881500201094`, 0.09416147540915892`, 0.08876707045120351`, 0.13444078251102984`,
0.17473113071631227`, 0.1003472629277528`, 0.10257938802254954`, 0.07532265006280592`, 0.04021883120333434`,
0.0564991871756731`, 0.04117018697248856`, 0.08505409765099431`, 0.12188816166378597`, 0.21919492670520835`,
0.05881486778266316`, 0.06767993093334922`, 0.10653270055902277`, 0.049262666437161956`, 0.19903046178554976`,
0.13523619557600433`, 0.2384358448883241`, 0.10083192069042411`, 0.147551151282763`, 0.16868866012671574`, 0.05115141191345097`,
0.04571009140644857`, 0.20911715958958366`, 0.11838709545492723`, 0.06885352917908583`, 0.06435346996988754`,
0.11046185420533586`, 0.06691292291644284`, 0.13280137209304554`, 0.1771383599723533`, 0.03202147916440068`,
0.09875723932122407`, 0.2005138546696321`, 0.039012817407301205`, 0.10084326090235754`, 0.03186176799811728`,
0.053910321683661076`, 0.05764128812466547`, 0.03837832325718675`, 0.044141306856367246`, 0.05611879835336235`,
0.11105760535311483`, 0.10142256943300514`, 0.17817586909768`, 0.04991670148433202`, 0.06743121108045065`,
0.12779559151853895`, 0.11696367075035917`, 0.16837069604088964`, 0.13663796047037782`, 0.07337974669274949`,
0.1022015602970574`, 0.15087263782415505`, 0.040007190375930514`, 0.13454046970294523`, 0.0942910964120108`,
0.0971556218967446`, 0.038100227307116125`, 0.10398168465931254`, 0.0674825413929986`, 0.04207360452206374`,
0.06476643777232081`, 0.03874548962225577`, 0.11617265591889815`, 0.04420320470928756`, 0.08331748450610926`,
0.10140839403565606`, 0.18542586622782667`, 0.060390659287636934`, 0.08265217614074345`, 0.15079453844767973`,
0.1092563193054415`, 0.09995060088829245`, 0.04933565923825343`, 0.06304001568121714`, 0.13534521703353133`,
0.08971177072133604`, 0.05578897735643178`, 0.06463782936698584`, 0.19427265609785066`, 0.06564412180958967`,
0.0554075596180195`, 0.0778761810069443 2`, 0.06705232048362372`, 0.1742968149308777`, 0.15774998799221063`,
0.1878292066360815`, 0.14513351556555515`, 0.04560355675235443`, 0.17669266892855823`, 0.10974405924824758`,
0.1028453970103418`, 0.035106153235292585`, 0.14129570731992033`, 0.0642198024566594 1`, 0.037176315280038404`,

0.03449839238745484`, 0.12019460284049621`, 0.03211647586026894`, 0.12347418951438593`, 0.05041927175190864`,
0.03848477658792874`, 0.17129662221664285`, 0.0679304626558291`, 0.19493357921193072`, 0.06018723196148812`,
0.054737596432997505`, 0.12271537126410798`, 0.06548296311776143`, 0.10093690080037418`, 0.1443453288912488`,
0.14168463072989115`, 0.15006192146432568`, 0.04832416910413868 4`, 0.19299100521770807`, 0.08741091328784878`,
0.09659018143416609`, 0.14424185772561543`, 0.07217242465558672`, 0.04418437930999637`, 0.14624960777166532`,
0.10684898900708378`, 0.06249663971973443`, 0.24124483420573883`, 0.047854663103473506`, 0.09755022635324334`,
0.1546241133488237`, 0.045770915538778496`, 0.06800384146766768`, 0.2010974241538806`, 0.1074730053152365`,
0.07305538594073131`, 0.09874594610141249`, 0.09066921222682625`, 0.1651605081641536`, 0.05852179147796508`,
0.1760278947336364`, 0.06321132933916691`, 0.11229620595547`, 0.08621268939836693`, 0.07054035936755669`,
0.13642074323594439`, 0.11676849303623046`, 0.1255695405014301`, 0.1393129360958742`, 0.17412950378240818`,
0.0540600221682515`, 0.05125414576535611`, 0.09382230219523789`, 0.06185526929945112`, 0.1455005601468288 8`,
0.12602055337603008`, 0.10719375969637855`, 0.0941783723351923`, 0.06525788109748948`, 0.11030922354610773`,
0.08213023825282365`, 0.08366192115226709`, 0.07264216765269438`, 0.09554552114459622`, 0.10541207897976478`,
0.16128844828579952`, 0.23897889084745083`, 0.042657092263616005`, 0.08276641976173914`, 0.08796573818299772`,
0.07591799827119611`, 0.06034436597607808`, 0.0882364128601832`, 0.08318188283854809`, 0.08005087209878767`,
0.11892547812388972`, 0.1365621625609065`, 0.07777063901857798`, 0.06702450811533946`, 0.05640503004599928`,
0.08694911942969528`, 0.22939233366552317`, 0.08310869141376667`, 0.0339183058272417`, 0.07351908751674585`,
0.048525251029716755`, 0.21693467079382106`, 0.2270593661305483`, 0.054420988395641044`, 0.15811929255492652`,
0.13816717804879083`, 0.1734449720590866`, 0.10217021272959435`, 0.16754337302820885`, 0.05109522853403724`,
0.1076870403142938`, 0.17496489824193814`, 0.05599183256355217`, 0.06574222485124662`, 0.17300356523169033`,
0.11985961611975016`, 0.03991063030450882`, 0.16373110842724858`, 0.11631098177493318`, 0.05665151853163168`,
0.03858885048181562`, 0.06411399622510121`, 0.14246359713727444`, 0.1465604360867387`, 0.06556571576496814`,
0.06004923978848311`, 0.06975581210669113`, 0.17307249722984114`, 0.06408997920773511`, 0.09759163422753478`,
0.04553825643424476`, 0.22624202075224023`, 0.07613137657712774`, 0.18716987713139208`, 0.1661630656961352`,
0.10345424442256992`, 0.05644010250728336`, 0.15428908260087748`, 0.08634429044832911`, 0.042288375364905394`,
0.12113551457779285`, 0.11548550920470678`, 0.039501768007025255`, 0.07649823057706213`, 0.07634648345735795`,
0.1464070812345906`, 0.10309831552148797`, 0.056332363365897434`, 0.036920646294195274`, 0.05530241499910762`,
0.06028338660908531`, 0.06985707110244485`, 0.06626114813227926`, 0.10167464699297057`, 0.04305843853465381`,
0.046873054755776945`, 0.1867163706646591`, 0.0858422079632724`, 0.0470717916827574`, 0.14816302253354238`,
0.1322209685052277`, 0.05058198321504504`, 0.13525100897628659`, 0.19012926430217247`, 0.04605794248152038 4`,
0.05699471338826693`, 0.22111707310278994`, 0.13284292071078535`, 0.05371765533992249`, 0.17728808174498614`,

0.06135209317010107`, 0.22142025712584523`, 0.13931730182117916`, 0.07405078316569681`, 0.05972299680307780 6`,
0.16401200268992225`, 0.05342273934891044`, 0.10430958865661727`, 0.07414146653180832`, 0.09883795490783948`,
0.05885684020952743`, 0.1365283149708653`, 0.08945827347689349`, 0.034696112295904885`, 0.05482271105482058`,
0.1354309249457413`, 0.05639571888885535`, 0.04319014777290621`, 0.1295870181330665`, 0.08013778754313769`,
0.08618654774026392`, 0.19098182361566496`, 0.1601928290494663`, 0.05113640602601603`, 0.18165834602093828`,
0.1109541605330966`, 0.20127787314014375`, 0.08752874038202306`, 0.04721731049570247`, 0.06269461163397719`,
0.16970441539083073`, 0.15579149905368098`, 0.04818430247047231`, 0.16580597605825143`, 0.08623664150050192`,
0.1877942864194066`, 0.10943103044907866`, 0.07714025352676997`, 0.0800510388390295`, 0.1391658537948138`, 0.138320226278962`,
0.09813422039909797`, 0.1182478615580564`, 0.09926240335881685`, 0.14135538864480188`, 0.10343253220039032`,
0.12141600802855111`, 0.0685141818746929`, 0.03575621723561029`, 0.09710035704684299`, 0.17846517161799233`,
0.057209202595803144`, 0.14317015705754538`, 0.06538217261522733`, 0.16168226988938786`, 0.13891278524697329`,
0.18410213755667104`, 0.04149085660281756`, 0.13677293065967228`, 0.0739920190807543`, 0.1563749970163827`,
0.06680554618049198`, 0.09844448774072466`, 0.14406743043710182`, 0.0436998197126213`, 0.1594722577789818`,
0.10282407110936974`, 0.03545820155237541`, 0.18613508149117156`, 0.11407746688202454`, 0.15631452508698088`,
0.04688667772564429`, 0.0639777449704375`, 0.0460487284662603`, 0.1811585361756576`, 0.043737381154392366`,
0.13545164387041875`, 0.06734126665420566`, 0.07518730224198472`, 0.18560037514750388`, 0.04492301656197116`,
0.059739946069889555`, 0.14250589925696877`, 0.15313012375263213`, 0.1352597616411127`, 0.06090730537680134`,
0.09818331436029121`, 0.08176794298436932`, 0.04403068175085218`, 0.10705114976281781`, 0.18936353160843475`,
0.07083967164638219`, 0.10407747993689041`, 0.1828851725647493`, 0.05053484379475644`, 0.04618976169834973`,
0.19095545106493422`, 0.04653712006639586`, 0.07365092139633152`, 0.04259697804111592`, 0.12308605984634205`,
0.09169242840835037`, 0.0791274639055393`, 0.09485887972629382`, 0.09394340911806003`, 0.08674181745752453`,
0.05828931200737406`, 0.10178457330443565`, 0.04858891863489084`, 0.09670981782028831`, 0.05623332259392176`,
0.2234144085894496`, 0.05242661991254044`, 0.09529219605411095`, 0.04897760250095241`, 0.09352248490221908`,
0.051925567156892054`, 0.0660486615327343`, 0.1965825461251041`, 0.1229461357194228`, 0.1399789650061219 7`,
0.14921818363558909`, 0.08322615670049073`, 0.04970024332225168`, 0.1506427273162331`, 0.07264882818629645`,
0.13500993272413797`, 0.1656486819747672`, 0.10727330579467247`, 0.21811513706773902`, 0.04926883701794021`,
0.08650688704919406`, 0.08414449131300701`, 0.18191968351204493`, 0.07129443136682645`, 0.11021634230233149`,
0.037575783190534735`, 0.0889112046155259`, 0.035031327864553284`, 0.11024330480071193`, 0.09081013358484055`,
0.03935217190452179`, 0.17559124877854354`, 0.06799680313808658`, 0.04825222400196834`, 0.21499273481863757`,
0.07081435657887249`, 0.05125975137239602`, 0.14650068856644538`, 0.15449922064957097`, 0.07213998734431282`,
0.08133592365073081`, 0.046156982397489166`, 0.061927924026804554`, 0.10879875832072601`, 0.24007553350901764`,

0.1881533747319988`, 0.07238393894738462`, 0.04408250995925393`, 0.10915030338249965`, 0.0778441602191434`,
0.21202029500720412`, 0.10528839659953769`, 0.17790651463334867`, 0.12312117430684023`, 0.167133911224666`,
0.07995478574203602`, 0.07531302953867472`, 0.03720591744642256`, 0.04907615954567911 4`, 0.12454441390217923`,
0.04046502521346068`, 0.10425004487055844`, 0.03635447066613622`, 0.07729945039642586`, 0.03848746196573792`,
0.1406496032369393`, 0.14449426269068427`, 0.07142286288208972`, 0.09103477956271722`, 0.09882430896904215`,
0.0483160791781637`, 0.05058919267228543`, 0.179385259165692`, 0.09277270030517638`, 0.10071510544213498`,
0.0475733785296823`, 0.10159122557896864`, 0.07847062669119231`, 0.18415271939351305`, 0.20476687146714453`,
0.10806784761129197`, 0.15395865863985583`, 0.04119766029758628`, 0.05697230882069684`, 0.058505825848761 3`,
0.07006630719467132`, 0.08464472096840313`, 0.205586030261003`, 0.14672484556022708`, 0.09817747618143235`,
0.15768199534765126`, 0.03993420672630654`, 0.03812136767755679`, 0.10122107508035151`, 0.1326810663305442`,
0.1090165114495076`, 0.0729081557698579`, 0.10459706409373849`, 0.04096666765058599`, 0.1551535322930324`,
0.17612243587637896`, 0.1458382201007723`, 0.17038670617814616`, 0.04743679612732299`, 0.2349295821514945`,
0.11118952639835561`, 0.15533609502468915`, 0.05676404463263911`, 0.07125387146621204`, 0.08028784509190746`,
0.11455305116289091`, 0.12893305049133158`, 0.048289931531682345`, 0.0955257982955916`, 0.13031035129930987`,
0.06771089644243955`, 0.04172622655596817`, 0.21721196571436396`, 0.05958087323912556`, 0.15858112197477986`,
0.07249606957133621`, 0.056463586171690325`, 0.05164798825668597`, 0.03744584872356264`, 0.06921937460896652`,
0.05962937459645100 6`, 0.1919941085180392`, 0.08767917104905036`, 0.03829206060594604 6`, 0.1939916558961255`,
0.03558520697732335`, 0.053120821266341114`, 0.14409928981262135`, 0.21964652442369906`, 0.1136868827737919`,
0.04931438329381048`, 0.06421978780061786`, 0.06284486164347311`, 0.03469758662545275`, 0.11323306777568296`,
0.053752714981699326`, 0.042922446179988215`, 0.14670680767569552`, 0.0871386925223451`, 0.14909446472040955`,
0.0514488131855361`, 0.10675009007303714`, 0.07812041237144046`, 0.08351017783495136`, 0.07713972733873965`,
0.04314448288994954`, 0.15101310011757568`, 0.09446442671494915`, 0.16883076378921386`, 0.1093787486833597`,
0.09759905383265223`, 0.10051390126740703`, 0.12349208086450902`, 0.1644758539946782`, 0.09854317363076918`,
0.12208519957848495`, 0.13390839064442903`, 0.1955036486703117`, 0.14898477881918365`, 0.10271280234205724`,
0.06888524025001354`, 0.06115224271826832`, 0.05667235203665494`, 0.10009223983244506`, 0.0728678651416037`,
0.16457626620207905`, 0.14867548437075864`, 0.09025217952573088`, 0.07298687394475283`, 0.0745118747835995`,
0.10589903400556752`, 0.13540583111732496`, 0.14914084005565872`, 0.13835090871851669`, 0.17858621841404687`,
0.1364868493734364`, 0.06601067415574843`, 0.21446657457944018`, 0.20393203480264713`, 0.05940661924151493`,
0.13212898103505866`, 0.12871593215249297`, 0.04334030617989911`, 0.13042991387713687`, 0.0603451420546176`,
0.056190261260302216`, 0.0423452760989978`, 0.16179029869091976`, 0.033692442080141216`, 0.10179514310475973`,
0.12201090805047791`, 0.12206814198764054`, 0.09272223583941705`, 0.131988580979413`, 0.1284814545569135`,

0.03973712364353566`, 0.09730723787028456`, 0.1311442416742865`, 0.16260849863376745`, 0.11113698898442897`,
0.07997844074605052`, 0.08156415562454548`, 0.07633429109633955`, 0.050970002615611736`, 0.15765830866311276`,
0.06285287926266252`, 0.08332107610731172`, 0.13218354541310268`, 0.09661282807264092`, 0.1417940187198018`,
0.09010482485794813`, 0.10973051385348899`, 0.10181580472863302`, 0.10151161777564464`, 0.18814743449210555`,
0.11430456792563179`, 0.03661496422005864`, 0.1619757344219858`, 0.05455633284881463`, 0.1850340558313811`,
0.14061159205806284`, 0.1122647827071516`, 0.05857678627989742`, 0.09456207783487733`, 0.03135826466512013`,
0.09269552806732911`, 0.09774111030942016`, 0.16495417106550056`, 0.05002310423735649`, 0.05205583995619337`,
0.14734051064614997`, 0.09749201240200768`, 0.0414504935099511`, 0.11653394151924305`, 0.03844581118922915`,
0.19138048590837403`, 0.10880348926163382`, 0.13640691608616992`, 0.1486843479590638`, 0.06731151523926421`,
0.12514477224966133`, 0.049790355867708906`, 0.04250710957049147`, 0.05621609422135781`, 0.06561593455959619`,
0.09857330458477308`, 0.043255039375227095`, 0.16006681646449608`, 0.05119625915005018`, 0.04056070330678039`,
0.05797561907330668`, 0.043622679157157465`, 0.06884769003748219`, 0.11169222441499144`, 0.10271758830016404`,
0.1992874659518284`, 0.1020419945376323`, 0.0678168179494302`, 0.034397608884920855`, 0.07207983841495728`,
0.26262403597335165`, 0.05421741255018923`, 0.11751129053385836`, 0.1186859810542836`, 0.08364071008543164`,
0.03999047313692444`, 0.22126071401186428`, 0.12585438904775503`, 0.050757687171580766`, 0.051807486123321915`,
0.1736119697248811`, 0.06363901100051986`, 0.16350517875737144`, 0.07077899982509037`, 0.10910926969495709`,
0.05852870757217523`, 0.10808938891611702`, 0.14552987055150574`, 0.05580023839479874`, 0.11616744470945117`,
0.08489341647666883`, 0.24274934134552917`, 0.05704030297423485`, 0.20168052484617818`, 0.15480715808327275`,
0.1515865741973381`, 0.13159757046283746`, 0.08258552726772093`, 0.12219672423719641`, 0.13196124281940963`,
0.03494661869405111`, 0.14168659687976864`, 0.15852016641425337`, 0.20180690014920125`, 0.12723853257558868`,
0.1432422051228873`, 0.1895920470084083`, 0.08391575630906874`, 0.07677432112699568`, 0.0970535531452139`,
0.08157337459247811`, 0.050548688738124035`, 0.13331314080833861`, 0.05098123807644228`, 0.06518066747627081`,
0.05342304188316237`, 0.08783220047651476`, 0.039100115830983925`, 0.1050661251009382`, 0.0852142143244226`,
0.045384662883106365`, 0.03539913557196775`, 0.0801671333721911`, 0.15630963350211166`, 0.14598613175480374`,
0.06056514541790643`, 0.13240072822568358`, 0.11607461954734026`, 0.037887948448399655`, 0.10597537447484283`,
0.10901505092953315`, 0.07748160613081378`, 0.07321203106713624`, 0.06663480553504905`, 0.08963368325634108`,
0.08578963383575051`, 0.05973030845647149`, 0.055385440715660275`, 0.1284754311695052`, 0.062244430450569654`,
0.04630261116108546`, 0.085908153792262`, 0.15648450178280904`, 0.1516501575126771`, 0.08422111028341642`,
0.14516520263375474`, 0.12652625753726993`, 0.09311399555784912`, 0.11736588926332814`, 0.09095332364607711`,
0.0889601768857599`, 0.06855736417273198`, 0.10600472170162113`, 0.06984713638840497`, 0.07867286751920669`,
0.06547528061891858`, 0.056321362358013445`, 0.07209735402618127`, 0.07841106218013923`, 0.08206518141681361`,

```
0.05283006783149461`, 0.06501480308910579`, 0.1958939956951422`, 0.18321916530841664`, 0.16294087785537117`,
0.10813207124864749`, 0.16376314650073626`, 0.06881285862471115`, 0.05576412141586883`, 0.08009471898386085`,
0.12722739461446728`, 0.06089466354183432`, 0.10508215219858476`, 0.06793674600370206`, 0.1914995523486643`,
0.138975107582395`, 0.13363618635137825`, 0.041892679680502344`, 0.0543230464866706`, 0.05831923891414387 4`,
0.2552486654268499`, 0.13707481730730536`, 0.11175164846994852`, 0.05383279866519428`, 0.0801407437504392`,
0.03545569636730986`, 0.06841228336080879`, 0.11911956472947088`, 0.07855962478580313`, 0.06765537183939101`,
0.07576658777784469`, 0.22034693028245028`, 0.03908064883979266`, 0.07765104188611315`, 0.07537590241529592`,
0.07926680650810936`, 0.1776665457949405`, 0.0799427046646671`, 0.07039146654350134`, 0.1347683990030349`};

(* "Like in the case of homogeneous model ,the majority of cancer cells' receptors remained free during each
treatment, with the maximum receptors occupancy not exceeding 6.3%" *)
(* Max value *)
PercentForm[Max[result[[2 ;;, 28]]]]
(* average value *)
PercentForm[Mean[result[[2 ;;, 28]]]]
```

Out[ ]//PercentForm=

6.267%

Out[ ]//PercentForm=

0.204%

```
(* "Only one case was lethally toxic due to the backflush of radionuclides to the bloodstream." *)
ToxCases = Select[result[[2 ;;]], (#[[19]] + #[[22]]) * nCpm > Abld &];
Length[Select[result[[2 ;;]], (#[[19]] + #[[22]]) * nCpm > Abld &]]
ToxCases[[1, 19]] (* toxic decays due to fragments *)
```

Out[ ]=

1

Out[ ]=

293.988

In[ ]:=

```
(* "Both parameter sweeps for homogeneous and heterogeneous cancer model yielded similar distributions
   for the fractions of activity, released in blood" *)
FrAcBlAb = result⟦2 ;;, 22⟧/result⟦2 ;;, 14⟧;
FrAcBlFr = result⟦2 ;;, 19⟧/result⟦2 ;;, 14⟧;
GraphicsGrid[{{
    Histogram[{Log10[FrAcBlAb], Log10[FrAcBlAbHomo]},
     Ticks → {Table[{Log10[0.01 * 10^i], PercentForm[0.01 * 10^i]}, {i, -2, 2}], Automatic}, LabelStyle → 14,
     ImageSize → 300, PlotRange → {{Log10[0.00003], Log10[0.03]}, Automatic}, PlotLabel → "Antibodies"],
    Histogram[{FrAcBlFr, FrAcBlFrHomo}, Ticks → {Table[{0.02 * i, PercentForm[0.02 * i]}, {i, 1, 16}], Automatic},
     PlotRange → {{Min[FrAcBlFr], Max[FrAcBlFr]}, Automatic}, LabelStyle → 14, ImageSize → 300, PlotLabel → "Fragments"]}}]
```

Out[ ]=

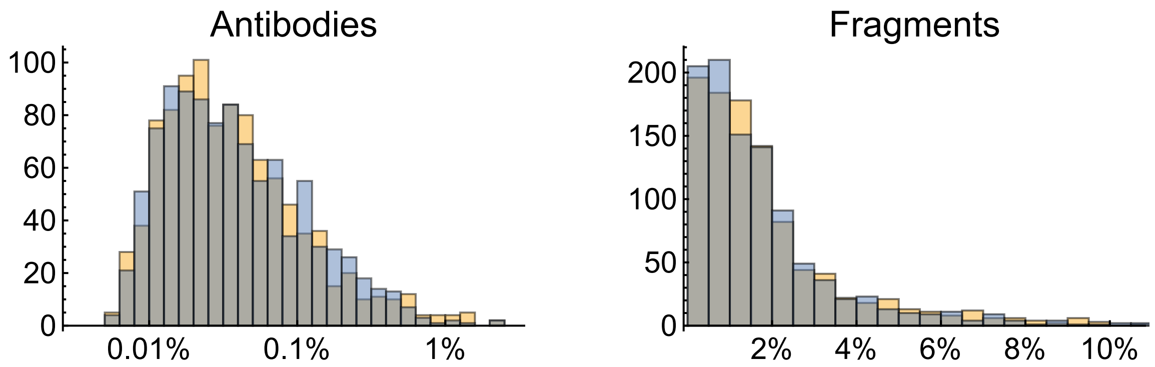

```
(* "with Eqs. (S.8) and (S.9) maintaining good accuracy for the estimation of the level of toxic decays" *)
(*Antibodies, errors*)
PercentForm[Min[result[[2 ;;, 24]]]]
PercentForm[Max[result[[2 ;;, 24]]]]
(*Fragments, errors*)
PercentForm[Min[result[[2 ;;, 21]]]]
PercentForm[Max[result[[2 ;;, 21]]]]
```

Out[•]//PercentForm=

−2.532%

Out[•]//PercentForm=

5.692%

Out[•]//PercentForm=

0.117%

Out[•]//PercentForm=

8.016%

In[•]:=

```
(* Figure S.20 -- estimation of minimal single curative dose *)
```

$$Aest = result[[2;;,15]] + \frac{result[[2;;,13]] - 0.06}{result[[2;;,13]] + 0.03} * (result[[2;;,16]] - result[[2;;,15]]);$$

$$GraphicsGrid\left[\left\{\left\{Show\left[ListPlot\left[Thread\left[\left\{result[[2;;,13]], \frac{result[[2;;,14]] - result[[2;;,15]]}{result[[2;;,16]] - result[[2;;,15]]}\right\}\right], AxesLabel \to \left\{"k_D", "\frac{A_{cur} - A^0_{est}}{A^m_{est} - A^0_{est}}"\right\}\right],\right.\right.\right.$$

$$Plot\left[\frac{x - 0.06}{x + 0.03}, \{x, -1, 1.5\}, PlotStyle \to Darker[Brown]\right]\Big],$$

$$Show\left[ListPlot\left[Thread[\{result[[2;;,14]], Aest\}], AxesLabel \to \left\{"A_{cur}", "A^0_{est} + \frac{k_D - 0.06}{k_D + 0.03} \cdot [A^m_{est} - A^0_{est}]"\right\}\right], Plot[x, \{x, 0, 1200\}]\right]\}\}\Big]$$

Out[•]=

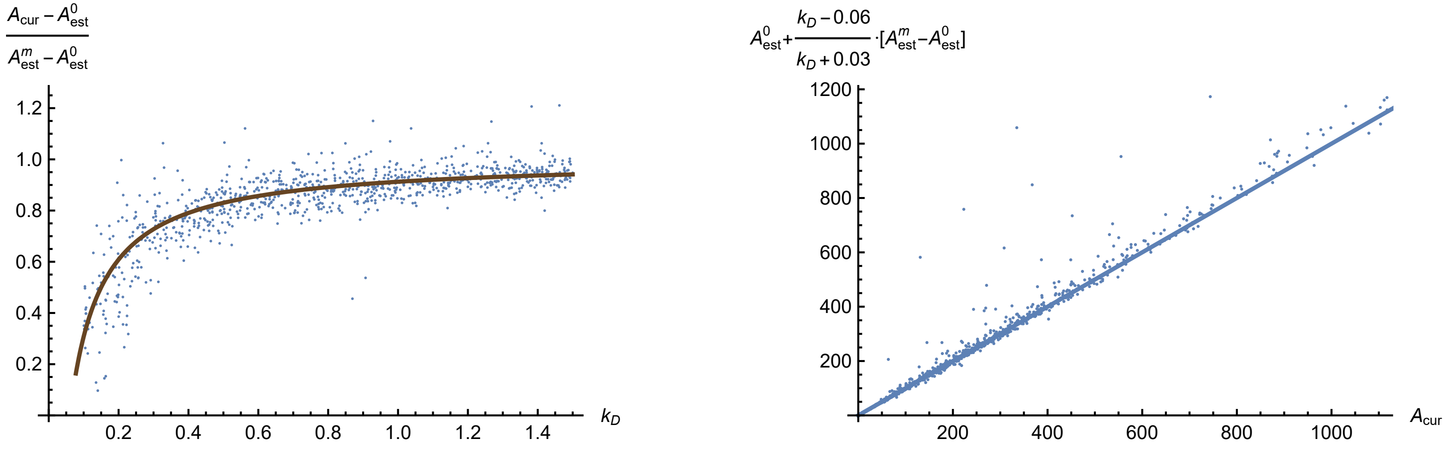

In[ ]:=

```
(* Figure S.21 -- comparison of heterogeneous and homogeneous model *)
(* Fraction of injected activity,spent on viable cancer cells *)
FrAcVC = result[[2 ;;, 27]] / result[[2 ;;, 14]];
(* New cells during treatment *)
NnewFrSim = result[[2 ;;, 31]] / result[[2 ;;, 12]];
GraphicsGrid[{{Histogram[{FrAcVC, FrAcVCHomo}, Ticks → {Table[{0.005 * i, PercentForm[0.005 * i]}, {i, 1, 12}], Automatic}],
    Histogram[{NnewFrSim, NnewFrSimHomo}, Ticks → {Table[{0.05 * i, PercentForm[0.05 * i]}, {i, 1, 12}], Automatic}]}}]
```

Out[ ]=

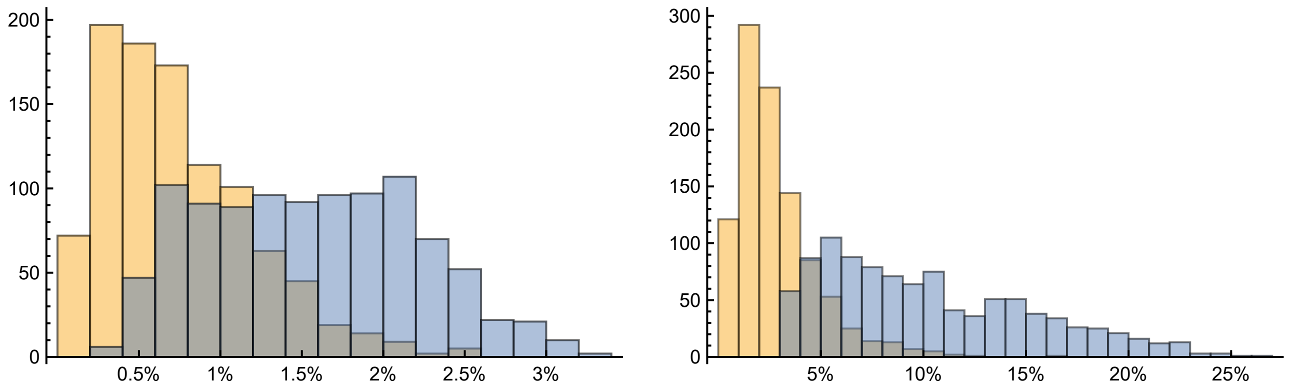


```
(* S.4.3 Parameter sweep for labeling ratio of 1.85 kBq / μg *)
```

In[ ]:=

```
(* The outcome of the global parameter sweep, that was generated in this study
  is saved here in a closed cell -- open this group of cells to see it, and run it to upload it in the array "result" *)
```

In[ ]:=

```
Npar = 1000; SetBasicParameterValuesHet[];
result = {{"κc", "κp", "γ", "V", "ks", "ρ0", "ρm", "ω", "α0", "αm", "kf", "N0", "kD", "A^sim_cur", "A^est_min", "A^est_max",
     "Mm", "Canc", "Bl^sim_Frg", "Bl^est_Frg", "Err", "Bl^sim_Ab", "Bl^est_Ab", "Err", "Cld^sim_Frg", "Cld^sim_Ab", "ToVbl", "fD^sim_Max", "fD^est_Max", "Err", "Nnew"},
   {0.08287723093332094`, 3.0858822376571258`, 1.0544020640762781`, 0.8913551057392846`, 0.07800980208832753`,
     0.18650785858589003`, 0.06335580187071355`, 0.005963202911440555`, 1833.7547379259959`, 302.5435916235979`,
     0.054836866330129685`, 9.998403612912536`, 1.2206910575241197`, 20438.24621443305`, 39.93994150277729`,
     188.67001136781187`, 0, 72.87161446373273`, 0.1359571736926028`, 35.5874768013999`, 260.7550500267288`,
     9322.123317387382`, 10.836211536727538`, -0.9988375811853383`, 5.993540391143838`, 11037.025242343`,
```

3.078668188028717`, 1.000000000000009`, 0.14733987846726693`, -0.8526601215327344`, 0.15574638559244997`},
{0.20921512222542155`, 0.8329034625019238`, 5.512850623987205`, 1.0082829806829832`, 0.6432484314226474`,
0.16609066867476052`, 0.06366860478505312`, 0.09015760505638287`, 3121.845933283612`, 31.743667621759982`,
0.23453419786488217`, 5.669698519018798`, 0.2162114279190297`, 6459.400365862087`, 19.84468464221906`,
1326.0340009206461`, 0, 101.40018968349301`, 10.09155667770734`, 281.9056148580136`, 26.93479974013867`,
1561.334855354292`, 1.307106381600475`, -0.9991628276425663`, 120.07560712709741`, 4666.498037110831`,
0.5150287115461711`, 0.9999999999999999`, 0.015706145078752513`, -0.9842938549212474`, 0.016007005397671203`},
{0.04416264589453098`, 1.237497547847834`, 1.3522815348119845`, 1.466732288470342`, 0.6840124021467311`,
0.46728396632651026`, 0.07378117717995297`, 0.04606361653599369`, 2651.0081518614816`, 102.21078555052515`,
0.21006656956644504`, 2.3728441322292344`, 0.992859548694442`, 25467.42642852931`, 17.61522521060715`,
180.6367766896037`, 0, 14.531225136700968`, 0.5065855173980571`, 541.1331631880652`, 1067.1970656552776`,
15600.427509070021`, 72.74362118463905`, -0.9953370751447455`, 8.95569050793315`, 9842.23079837655`,
0.9736015094514466`, 1.`, 0.6032013759212598`, -0.3967986240787402`, 0.02921205394192425`},
{0.2369260528945452`, 2.0701500057376476`, 2.467879519617215`, 1.3729060512118858`, 0.7839988344536148`,
0.6604044950304033`, 0.16113998364121102`, 0.05466214114010759`, 886.5316249876132`, 108.21460952627575`,
0.04090907280605699`, 1.8765499412863633`, 0.31190921443437514`, 215306.39511096897`, 62.416964263047916`,
210.07751445133314`, 0, 19.656841737283845`, 0.49918212649011523`, 3087.897047820808`, 6184.912683878825`,
49096.527266662204`, 397.4725785221924`, -0.9919042628745743`, 14.76259831453773`, 166174.9488017091`,
0.6963605691241741`, 1.0000000000000082`, 3.5333482260052955`, 2.5333482260052667`, 0.03880933589275128`},
{0.10845014170633277`, 2.595808559087966`, 3.825660598104694`, 0.9092167031122353`, 0.4998129490409222`,
0.3952109813060457`, 0.14370479224814003`, 0.06482861565134085`, 2096.8410035229635`, 61.50816633658093`,
0.11793078229478832`, 9.815002337220978`, 0.4969144428042134`, 56374.410317479706`, 67.7432671674452`,
1464.6527401349804`, 0, 145.05934836264302`, 3.5167380152552545`, 711.7635874264994`, 201.39312235911257`,
22004.348920259865`, 8.566579952805581`, -0.9996106869608435`, 130.4111234295664`, 34091.067979396765`,
2.165967064053776`, 1.0000000000000044`, 0.11410344559552973`, -0.8858965544044708`, 0.05611506430708687`},
{0.1958274434061072`, 1.1625250352222158`, 4.38654338158619`, 1.0335502224347561`, 0.757936701785614`,
0.28764737226179815`, 0.053841290852541135`, 0.15446581430564627`, 1541.8645934819597`, 81.13506069010731`,
0.032171888494962775`, 6.764038142553707`, 0.3026234451737826`, 99885.55689886697`, 74.25362832784279`,
627.3130938716943`, 0, 69.32898960901301`, 8.606001296125655`, 3903.796369918746`, 452.6132677177495`,
26244.57712856938`, 21.825713687156`, -0.9991683724382285`, 142.92417085572686`, 73420.1206051736`,
1.5904366623155475`, 1.000000000000557`, 0.25585140775324355`, -0.7441485922467708`, 0.051234004735160155`},

```
{0.11706495920321314`, 3.996131916383794`, 3.4445284668439964`, 1.0745666843500223`, 0.4349463063881305`,
  0.49843595508242333`, 0.15623150182584739`, 0.02045656151514831`, 3837.5091768132115`, 201.1196769174046`,
  0.10740962694701128`, 6.186844269751422`, 0.8954099169722194`, 2996.958615815443`, 24.20528030320615`,
  271.99935755214216`, 0, 124.7804128907768`, 0.6224248139991994`, 11.667824358083163`, 17.745757070826183`,
  1061.2282058995784`, 0.9479186877136995`, -0.9991067720567132`, 35.53273806816334`, 1774.7519518418965`,
  1.2331083420973075`, 1.0000000000000577`, 0.01068797367623837`, -0.9893120263237623`, 0.1411599994673436`},
{0.15007898341668874`, 1.7157935178042445`, 2.2029422939279666`, 1.1981889493296447`, 0.6780839054962491`,
  0.6847631092713093`, 0.13190149021010728`, 0.01567939294607179`, 3588.049208356785`, 265.194158460061`,
  0.020899950425706137`, 5.763952406784453`, 0.2759848394189104`, 3209.0616694720743`, 40.89722881618427`,
  221.38188119231015`, 0, 79.44701013824357`, 0.6928464313508376`, 23.019597432852596`, 32.224674893643396`,
  989.7943929985461`, 1.8975573051388788`, -0.9980828772939496`, 16.982591653508127`, 2122.104804182289`,
  0.5603709220338514`, 0.9999999999999909`, 0.019207380872381687`, -0.9807926191276182`, 0.20753716154664703`},
{0.16758241286506004`, 2.4053214221448718`, 3.775183470319785`, 1.3879685907097887`, 0.849157385981911`,
  0.1600626918561956`, 0.12028733740307626`, 0.23825099986429926`, 3824.577207611347`, 166.9670016449728`,
  0.011876010376545704`, 5.836418598900732`, 0.8350403039197589`, 47548.01013218152`, 20.654011824345407`,
  402.7081935084246`, 0, 37.86351687953011`, 3.5725315025705826`, 1039.271515311993`, 289.90618642948135`,
  13960.911744222982`, 18.778812233569656`, -0.9986549007272867`, 122.75837934886341`, 33422.90394132911`,
  0.9036459427968329`, 1.0000000000000644`, 0.16400664453469127`, -0.8359933554653193`, 0.05140079901297277`},
{0.07608249414500318`, 1.6334157835018592`, 2.1569194913061693`, 1.197668491595115`, 0.09742648797035525`,
  0.6759576090965576`, 0.07148505885394518`, 0.13535119072972454`, 1569.9807697844644`, 192.45980341337224`,
  0.19113163797167387`, 2.99326845694066`, 0.7712705061277614`, 7619.693959575484`, 23.912317131603086`,
  87.54025878604611`, 0, 17.215410697695667`, 1.3226694340484508`, 206.38577323426242`, 155.03730450060607`,
  3627.536383235064`, 8.852450077564562`, -0.9975596522977752`, 30.863844713288735`, 3942.743080546686`,
  1.2018439078170882`, 1.000000000000827`, 0.08969567792912646`, -0.9103043220709477`, 0.10823322036083843`},
{0.2761380591152234`, 0.5948415416580923`, 4.492630890025399`, 1.4137462343952987`, 0.19259719440314438`,
  0.2905045944613839`, 0.092594223894082`, 0.0064155840660979225`, 2524.964006213205`, 55.97986174003478`,
  0.1939779490397887`, 7.138405092365562`, 0.3980368971508512`, 76737.38078589388`, 26.12363138165174`,
  821.2272516238796`, 0, 217.3413658632372`, 2.0951033904429894`, 678.3301598047477`, 322.76930078917076`,
  15470.6880164779`, 21.20829483158297`, -0.9986291304686001`, 17.803636152917264`, 61029.22517210523`,
  1.145843374679494`, 1.0000000000004179`, 0.1818522317882613`, -0.8181477682118147`, 0.016156499462537017`},
{0.18781219936172783`, 1.4023238769545223`, 1.2203108039534882`, 1.4529300029349537`, 0.39441104236838154`,
```

0.21312489114159694`, 0.11407119247069003`, 0.056131851903965725`, 1464.0480188461024`, 295.31541235105215`,
0.1586938998074992`, 9.00673891342328`, 0.8723355545572158`, 19456.9559473793`, 60.12608734247`,
238.18407130974305`, 0, 45.99990659808499`, 1.7317520676922935`, 411.67470118993424`, 236.72150117223515`,
5260.484819575609`, 16.09827346843673`, −0.9969397738002149`, 34.6925324784324`, 14114.046052478287`,
2.542556229165471`, 1.0000000000002809`, 0.13453970093511616`, −0.8654602990649216`, 0.13726443149749223`},
{0.14369943000217644`, 1.7114006053749717`, 7.290050955062047`, 0.8291375841791329`, 0.041053311258442804`,
0.588971030507233`, 0.22011431905536544`, 0.14621434360249197`, 2238.8977574406254`, 92.23431071779362`,
0.19794610644205557`, 8.103641190648325`, 1.3952824759429032`, 10231.571584150644`, 40.98331791405394`,
656.8967743985768`, 0, 146.5567884665867`, 11.80331699289736`, 271.8843870219003`, 22.034574703492805`,
3205.0838748287465`, 0.9012899885746445`, −0.9997187936341845`, 288.57434067253234`, 6579.553227458001`,
4.476990002623855`, 1.0000000000008993`, 0.013162705730605906`, −0.9868372942694059`, 0.19802454154413382`},
{0.2710591123734774`, 3.4140873238389613`, 0.9957406410201042`, 1.2040917290884572`, 0.20012464577442923`,
0.17413508978502545`, 0.1542723564673828`, 0.3932031693142617`, 919.8622664894237`, 351.88082244581324`,
0.07678285799586054`, 9.51148011674945`, 0.10358667667962984`, 22570.759834061602`, 80.73833692065708`,
203.21266781776094`, 0, 11.28545805322128`, 1.2078469687502464`, 384.9467662140979`, 317.7049155841328`,
4617.835750074684`, 17.945227227275907`, −0.9961139312443095`, 58.90992893067893`, 17881.520850024965`,
2.229795721277233`, 1.0000000000339084`, 0.1811195933332797`, −0.818880406672861 8`, 0.19656170793537192`},
{0.10385665451428133`, 0.45328132552019573`, 1.5503843984121968`, 1.4694004919600818`, 0.4251249704277895`,
0.18123604867037413`, 0.08236798590439715`, 0.07631033842692926`, 1502.3783558556506`, 20.10790624452443`,
0.14290440434604346`, 4.641987001123601`, 1.0114789952636625`, 329299.6616356938`, 28.470075211204115`,
1625.0720112434083`, 0, 25.53607683551696`, 3.712000648572196`, 22975.366767408603`, 6188.483500291405`,
132564.6313501118`, 420.7565847598569`, −0.9968260268182045`, 24.036865347380473`, 196681.70169916673`,
3.4674382724878186`, 1.0000000000000313`, 3.477455389563673`, 2.477455389563564`, 0.010136252518822171`},
{0.08203524638521087`, 2.7438300558988873`, 0.5228963098315518`, 1.1830383771323514`, 0.45293779314745897`,
0.15188403574429454`, 0.10479303433979437`, 0.2038448791081171`, 983.5363393912057`, 168.65049566992798`,
0.019976684129921263`, 1.733382066252318`, 1.4812211807580051`, 205888.2922496714`, 15.213379211165089`,
77.61880187012376`, 0, 1.7921099623986683`, 0.12483011110579617`, 3812.6506132563018`, 30541.715851826808`,
94791.76827829274`, 1657.6102073020948`, −0.9825131418327842`, 4.8930372961897195`, 111089.51522856503`,
0.9806016801261673`, 1.0000000000000029`, 17.263759119290366`, 16.263759119290317`, 0.02995244404895467`},
{0.04913245961465748`, 2.276131341102663`, 7.044266329747086`, 0.9173667594455086`, 0.41644326647059726`,
0.25990231144478826`, 0.18555590900866242`, 0.210063875037094`, 1773.4485121819462`, 75.91056730044647`,

0.184796166571025`, 2.2167031467842424`, 0.9244440644631444`, 9566.36362679048`, 12.939747801168876`,
262.23533381564926`, 0, 29.64844924128039`, 2.605445904498798`, 214.08531723531965`, 81.16839845558128`,
5552.177398162032`, 3.5261211436287323`, -0.9993649120172572`, 84.71910115396135`, 3897.0304541229993`,
0.8472897254148584`, 1.0000000000013187`, 0.04656045030210111`, -0.9534395496979603`, 0.031445894395500676`},
{0.15077968496954103`, 0.971820860662203`, 0.23396787648709783`, 0.9304382139491995`, 0.8434070020798108`, 0.5328351579449485`,
0.0913866934528598`, 0.005065303834986521`, 1783.900832424436`, 398.4573153532763`, 0.17963340543276235`, 6.010950437900332`,
0.19621378169468162`, 9191.98203074127`, 100.62198670607387`, 151.85881329144362`, 0, 9.975362635513184`, 0.04821730972103802`,
41.67546092221666`, 863.3257196083869`, 2910.998918334593`, 37.6849695034855`, -0.9870542825467468`, 0.6694083918845006`,
6270.278569330937`, 0.9253275435825293`, 1.000000000000001`, 0.496733418139468`, -0.5032665818605326`, 0.18291014491538057`},
{0.15622813333517427`, 1.117114824910261`, 4.688994947991285`, 1.188191128841129`, 0.5951306703894546`,
0.30511551468851006`, 0.2217863487164401`, 0.33567754538344835`, 2568.8944572362634`, 80.78669839983178`,
0.03729030447311804`, 7.7879419731274115`, 0.9647929000978697`, 221096.281312041`, 42.74315236446`,
1159.6047262219345`, 0, 47.37584866713935`, 13.23271735381212`, 10787.68037898801`, 814.227899950592`,
68327.99432315155`, 45.13379282232911`, -0.9993394538611967`, 211.17806756844047`, 152496.50010917636`,
2.2820743027812744`, 1.0000000000073668`, 0.4601427233949543`, -0.5398572766084355`, 0.05814312875875228`},
{0.11331162134690587`, 1.2483595201539153`, 4.922077543835222`, 1.0559046192245036`, 0.27438155190999924`,
0.25039971454185184`, 0.17415638432949032`, 0.1908750894543732`, 1293.217041147549`, 78.02731777513316`,
0.14932153866568026`, 5.182262423333571`, 0.7834976909752784`, 36273.717958474626`, 36.94105042569882`,
535.1356450492154`, 0, 51.599684290409`, 7.370719478483488`, 1409.2008446727007`, 190.18904861139026`,
13778.891165165853`, 9.420557719477495`, -0.999316305092583`, 131.44725473355373`, 22304.407118392846`,
2.1210437354650353`, 1.0000000000000064`, 0.10807812741440917`, -0.8919218725855915`, 0.05471515427082215`},
{0.13914220002486866`, 3.6703502896291074`, 6.379985611157895`, 1.2081851433506285`, 0.7677675288971362`,
0.4439146253901465`, 0.18717370496505287`, 0.22151595381283107`, 1066.171533351664`, 40.74945145753361`,
0.011595132472834446`, 8.264527016617006`, 0.7438205368393507`, 1.5695119659789577`*^6, 175.5291210649724`,
2741.3317845490583`, 0, 94.94415955810814`, 5.57266274994294`, 22319.942800361583`, 4004.256338290007`,
525185.0043648202`, 225.6820269775245`, -0.9995702809008219`, 292.19463340369117`, 1.0439342418198775`*^6,
4.855597863052072`, 1.0000000000000784`, 2.2622496017605687`, 1.2622496017603915`, 0.06752550159387713`},
{0.22919129490265872`, 2.16424636204603`, 7.737422782052235`, 1.4860287946100363`, 0.8497692228491351`,
0.6288301911078309`, 0.10881904848202179`, 0.012090233236638177`, 2725.277273810464`, 77.99221256357112`,
0.11691550532917167`, 1.34040213575469`, 0.8482131070094612`, 9360.369937722526`, 15.88636674737511`,
177.47918945964864`, 0, 66.38606693096234`, 0.3569488326180884`, 43.19198694061973`, 120.00330073591329`,

2171.7819705745223`, 8.387748745569615`, -0.996137849535904`, 11.036074463289603`, 7110.764601174554`,
0.4973138673702557`, 1.0000000000011806`, 0.06859226853071415`, -0.9314077314693668`, 0.021302018960837048`},
{0.14680250287088714`, 2.9795896823389043`, 0.5962523026989057`, 1.2784383052923718`, 0.4427855138400896`,
0.39715884806633817`, 0.14338219057113172`, 0.008545850364486081`, 3591.485740200138`, 175.10126732731499`,
0.10771590512275864`, 1.6757545718316624`, 0.6195088999107756`, 47594.045955445996`, 6.18761498808319`,
80.63335594790892`, 0, 6.624970920964577`, 0.01851742341174544`, 118.86152399202253`, 6417.901882247273`,
15364.50242892522`, 373.02900203749766`, -0.9757213743977005`, 0.788204624873392`, 32222.105884743534`,
0.24442823619266507`, 1.0000000000007059`, 3.620143080599406`, 2.6201430805968506`, 0.012708631768262164`},
{0.06783774432411926`, 1.4183998935859234`, 4.514906377341504`, 1.3074717506903661`, 0.44121610557851443`,
0.3859898733629271`, 0.18885629375508112`, 0.08215446401079224`, 1996.0572117605661`, 32.84560309478019`,
0.14544575809597998`, 5.16703571475392`, 0.8091181446129996`, 130634.23739764698`, 34.037865761911405`,
1495.8512448798494`, 0, 79.85485195926299`, 4.392855478588245`, 3317.2781804451693`, 754.1530426198451`,
66253.52400007182`, 45.93264922626452`, -0.9993067138704016`, 89.01179633382705`, 64206.99459555392`,
2.0115863966912517`, 1.000000000000033`, 0.42557920028321466`, -0.5744207997167994`, 0.027452350923563296`},
{0.25318695512908856`, 3.592770590245955`, 0.3860629768890025`, 0.8375102060070931`, 0.25324293087688066`, 0.22639700269651497`,
0.18616406291052912`, 0.3042902902243334`, 535.9279722175538`, 147.98665712717872`, 0.04054733608151545`, 8.866857733517588`,
0.984549955628955 9`, 196216.19686614475`, 147.48199530002978`, 497.8565200699209`, 0, 4.893293326027446`, 0.3964582249062077 5`,
3048.6158902163857`, 7688.627049451713`, 42493.48216756579`, 299.26180219684557`, -0.9929574657822402`, 20.348335010059117`,
153697.07661197416`, 6.011978072585304`, 1.0000000000000269`, 4.3563277894612895`, 3.3563277894611723`, 0.12288680182039509`},
{0.10623464305011832`, 2.9795950019626645`, 8.394466481175808`, 1.3150375502652678`, 0.3977517440977254`, 0.1685394428801441`,
0.14812837191515202`, 0.06701796399112063`, 3705.0856036562427`, 41.40776885031511`, 0.10405528061386266`, 6.850682270825037`,
0.7771134705080516`, 101352.69473873089`, 16.36227414652897`, 1397.0581359755809`, 0, 217.71693116066774`, 4.77553218206657 7`,
1137.9020841596819`, 237.27754494730743`, 40087.94194589014`, 14.544967720941367`, -0.9996371734986901`, 203.27359744853447`,
60838.97433193582`, 1.1959806606149883`, 1.00000000000023`, 0.133943600002255`, -0.8660563999977758`, 0.01467814993231288`},
{0.26935136701037576`, 2.6617924377127427`, 1.8653214881187097`, 1.3783772798670109`, 0.352129512501022`,
0.17316862578533354`, 0.07359966841424637`, 0.03044640192221252`, 2578.790502033272`, 266.15340429326216`,
0.24670889410575075`, 9.827796845989266`, 0.8840863683155358`, 8618.249020319301`, 33.28593266193509`,
248.53333427636892`, 0, 95.44015460782734`, 1.0546811310470496`, 66.93772868036109`, 62.46726675000596`,
1749.556591969121`, 4.058894349914401`, -0.9976800439788311`, 40.10488941170521`, 6732.07799584137`,
2.0816525829480246`, 0.9999999999999936`, 0.035729156816505264`, -0.9642708431834945`, 0.0865802278951815`},
{0.2583950802004026`, 3.7896744268241553`, 0.3341722318602649`, 0.884753511926686`, 0.6013312124575798`,

0.2239296898773392`, 0.10126995172624759`, 0.0054568420957837975`, 755.0573605362365`, 345.1189599908564`,
0.23686856237319676`, 4.65826036739878`, 1.1534719871772796`, 13701.961065331867`, 74.98245150156563`,
116.19979774444907`, 0, 10.824986593040764`, 0.015182471239807635`, 17.971025388810002`, 1182.669318713473`,
2918.1925584031096`, 48.08662086151941`, -0.9835217793551522`, 0.8219517570498869`, 10772.094288125534`,
1.9373046982643918`, 1.0000000000001352`, 0.6689631619558654`, -0.3310368380442251`, 0.07094043592946385`},
{0.1397208239381057`, 1.3512320351631422`, 4.315748656362622`, 1.0234149323507424`, 0.4387218679676288`,
0.5908017035553538`, 0.24436343171483993`, 0.05514077662518975`, 1030.1549542852385`, 113.1098010959631 2`,
0.2358198015899574`, 7.7106342544090705`, 0.46099446073278916`, 15057.356567947305`, 124.7906501618923 6`,
729.551617580889`, 0, 141.56492564206187`, 5.450347583774929`, 326.78026416254136`, 58.95585770260404`,
4941.61225839156`, 2.9055360005609834`, -0.9994120267134219`, 105.20977511386737`, 9863.516233215894`,
2.7968701205328683`, 1.0000000000000109`, 0.03438872545014235`, -0.9656112745498581`, 0.20141566054525656`},
{0.04583854888632438`, 2.3264689225741337`, 6.981580565170444`, 1.2372980328075218`, 0.8253651804903572`,
0.45495452249062196`, 0.0835295837141499`, 0.03439901257655977`, 1013.5313447757526`, 64.02695030885803`,
0.1777154613277634`, 6.347498471152688`, 0.28284114950471473`, 7770.840510438561`, 159.36596652342857`,
931.7939567542318`, 0, 224.67558007348146`, 3.200754394452306`, 74.79004928324025`, 22.366381817008453`,
4493.717208377337`, 1.3617054512372406`, -0.999696975713403`, 106.37793753551261`, 2942.649656250309`,
2.2107604710121675`, 1.0000000000001816`, 0.013326780014884526`, -0.9866732199851179`, 0.13425771723281052`},
{0.08906331353528191`, 1.5482338770119695`, 5.590038221637837`, 1.3588168835620769`, 0.3085034668822382`,
0.2676722221657748`, 0.07831428730380899`, 0.019104385555027914`, 2856.0098758390477`, 29.221123010272038`,
0.07226770477125283`, 4.247245472589608`, 0.2276638271182967`, 50270.60070694661`, 14.420058404634487`,
939.0024545235459`, 0, 138.32393032699315`, 1.6302829619035186`, 466.23494419719384`, 284.98406233284675`,
22045.4082690208`, 18.047689219884937`, -0.9991813402138147`, 36.057990153349266`, 28049.101552529897`,
0.4495267133806677`, 1.000000000000307`, 0.16091833401776587`, -0.8390816659822835`, 0.0159968655078886`},
{0.1820955139503701`, 2.1301463589452796`, 1.0837335286589127`, 0.8319946959595101`, 0.024409178193170122`,
0.6747387048956719`, 0.18033910089153304`, 0.00804054909792445`, 2554.5651932014816`, 147.37665368444442`,
0.18624166889237875`, 4.7992891492518055`, 0.5892958331460565`, 25298.294659879826`, 22.402215948165598`,
223.37334143823193`, 0, 34.832270167587296`, 0.1269762438066296`, 82.92836564302739`, 652.1014239901274`,
7013.852572413777`, 25.314402176478453`, -0.9963907992198121`, 3.8639711916748642`, 18245.58698494013`,
0.9899853196240295`, 1.0000000000000002`, 0.36966251413157214`, -0.6303374858684279`, 0.05384977225495954`},
{0.11714865623011333`, 0.7803444845933583`, 3.7286455817586397`, 0.8712432490902873`, 0.9005891902672722`,
0.6478151883640173`, 0.1558110001007145 8`, 0.01574292115738763`, 1037.936039940223`, 180.67525214379884`,

0.029753801751762954`, 7.189499669067537`, 0.709240932965888`, 19364.920409585935`, 273.44599654046567`,
570.1532907970316`, 0, 170.9774732968534`, 3.1202551484751964`, 292.6886640304325`, 92.80279820176352`,
7164.98113320967`, 3.949041482163129`, -0.9994488413285753`, 34.78391279480925`, 11990.970166708925`,
3.267299160931688`, 0.9999999999999998`, 0.05490090912716432 5`, -0.9450990908728356`, 0.4022327834609539`},
{0.14562691763665958`, 0.7916589364478996`, 6.589457190185791`, 1.2275476972045025`, 0.23387470631242246`,
0.5473498244385164`, 0.05668225254693815`, 0.02480814693768771`, 3264.7917840803066`, 42.76592076077594`,
0.07262816395108834`, 7.451596380699327`, 1.1772112101747596`, 199197.33917080043`, 29.37108723470624`,
1031.8815101123764`, 0, 269.2032249635039`, 7.717871306633284`, 4234.428887284706`, 547.6524352440729`,
64548.13146692452`, 31.249005531679316`, -0.9995158805557727`, 87.28459700358657`, 134284.93463905778`,
5.1275382168830985`, 1.0000000000000004`, 0.3083174896595953`, -0.6916825103404047`, 0.03132097969628807`},
{0.14650986127607446`, 2.5172437701709836`, 0.1805184658738579`, 0.7600994183135076`, 0.9075051465461681`,
0.2801821328714206`, 0.17102217375046636`, 0.35873020461861416`, 1565.8031681921566`, 125.39398648792553`,
0.18475404806650503`, 5.639231846073234`, 1.0926853341214517`, 31008.435037742398`, 77.53873720322431`,
693.1140230632276`, 0, 1.264295259004587`, 0.17141239127866326`, 701.9794563974318`, 4094.266690820685`,
10022.908183722126`, 143.27826224565726`, -0.9857049212045711`, 6.164096772518941`, 20977.926965427934`,
2.358737034689435`, 1.0000000000014606`, 2.3150045904944365`, 1.3150045904910552`, 0.05975223476257216`},
{0.2183191832780194`, 0.7656891998585413`, 0.2734070058927056`, 0.9147050043440094`, 0.3441094983626165`, 0.18539360977555885`,
0.1738037138598677`, 0.02857315375757566`, 2216.527228285475`, 152.12573952853177`, 0.14351176295939 5`, 2.048281105987993`,
0.3150624921161962`, 33404.384285824286`, 8.116651528401766`, 115.44998058554846`, 0, 2.951551387016558`, 0.10069318704858445`,
811.066097395913`, 8053.825963593483`, 8109.124334225635`, 328.6567242134751`, -0.9594707503957812`, 1.1014240831776723`,
25291.105739257873`, 0.2573875481251316`, 1.0000000000012395`, 4.533335545852002`, 3.5333355458463833`, 0.008374029801505447`},
{0.07967342618536455`, 2.504619914171559`, 1.5706511952625795`, 1.0567028274669426`, 0.8710362851311728`,
0.17476539269891578`, 0.1454017836571002`, 0.013152148214347218`, 996.5768638290242`, 179.4306604453801`,
0.15492643832143987`, 3.5116876584841332`, 0.7677856536659751`, 16147.402179480712`, 50.80226292323742`,
252.53938121253887`, 0, 34.979242206767424`, 0.1769602467484448`, 69.44017377157971`, 391.4054981133212`,
7532.473719979031`, 19.371694665869978`, -0.9974282426482964`, 6.33168797175525`, 8573.399841742117`,
1.3509099814273944`, 1.0000000000000115`, 0.2224951886955465`, -0.777504811304456`, 0.06648304038174171`},
{0.09628613710357908`, 1.5426640827734222`, 0.6350126000666823`, 1.1824558202170816`, 0.4624355823492319`, 0.315548754668174`,
0.24429500934345866`, 0.021157750682009126`, 1240.1379477304627`, 114.8299639556875`, 0.022101718613827426`, 8.73583411100617`,
1.006336055790952`, 353699.771890784`, 87.32438209167819`, 838.5176672590374`, 0, 32.036006096145286`, 0.4179017525599568`,
3563.401849438452`, 8525.888981943684`, 148876.27285338394`, 471.8215331855595`, -0.9968307808615667`, 9.20974319717588`,

204781.73170615424`, 3.9868280466719077`, 1.0000000000000007`, 4.845757212576775`, 3.845757212576771`, 0.14873970176018056`},
{0.1826369436867583`, 3.4688854117825922`, 2.4438258950850287`, 1.4614655744939826`, 0.6869584196330984`,
0.32705660778535306`, 0.23164888048001758`, 0.012304471968752876`, 769.9733124793984`, 249.22075665117416`,
0.24824092697008104`, 9.063288482525959`, 1.0083702284112093`, 10053.294287610404`, 195.53159835634162`,
500.4418270687013`, 0, 145.2352817074823`, 0.4986503006838933`, 29.728944035499495`, 58.61882304036833`,
2738.288381469611`, 4.158294911835917`, -0.9984814254992368`, 24.710867908905144`, 7144.466013208112`,
4.248764988012832`, 1.000000000000069`, 0.03449577623067537`, -0.965504223769327`, 0.3883735540180954`},
{0.2494292406608673`, 1.335820360094238`, 3.8994390375054255`, 0.9165975453056769`, 0.13563044706422311`,
0.5269987018012078`, 0.205844612334116`, 0.012716247145242394`, 1091.290454779412`, 128.1406782295989`,
0.24389684971209813`, 1.43576099827777`, 0.4903794001833075`, 12614.675108871405`, 14.684534304019103`,
83.22090467035795`, 0, 35.81419584145573`, 0.32224813016993203`, 96.56337108020774`, 298.65533401012044`,
2755.119229520583`, 12.905102728167199`, -0.9953159548995588`, 6.1495087611898995`, 9817.247104992432`,
0.5042974569073162`, 1.0000000000009355`, 0.1712400614979932`, -0.828759938502167`, 0.03307657489958223`},
{0.09391968152154795`, 3.06447396666384`, 0.887078247635884`, 1.3969498711006763`, 0.9012814324673109`,
0.6019227650187913`, 0.056809355713011156`, 0.11429079373151174`, 3926.488447769728`, 52.970576122622276`,
0.24789590005226048`, 5.914871844531001`, 0.869797459980445`, 63170.98938274777`, 56.07692286408627`,
930.9671385405933`, 0, 14.835552538246157`, 0.5377420644189047`, 874.9004905064097`, 1625.9891243338857`,
26959.811924114463`, 105.17629269757293`, -0.9960987749842759`, 23.54137938845429`, 36172.24213990951`,
2.3066596368997985`, 1.0000000000000007`, 0.9150061264252927`, -0.08499387357470789`, 0.023118657575931102`},
{0.10596194132792924`, 2.903583061514107`, 0.8170701309155923`, 1.3631505832711714`, 0.6107209987788129`,
0.24870490908317233`, 0.18241426717129616`, 0.018338650769170847`, 1588.8736918382`, 58.688447621427315`,
0.2068253327055226`, 8.58249867744179`, 0.3849797974029039`, 64670.31146447088`, 70.3179188627623`,
1641.2852717316282`, 0, 41.22907450182646`, 0.25383505450873395`, 316.03816878450726`, 1244.053286261662`,
25706.00930650732`, 78.68077481983522`, -0.9969392069425604`, 10.529016638429566`, 38912.26642730462`,
1.7624397157827343`, 1.0000000000000153`, 0.7008829013615931`, -0.2991170986384176`, 0.03668340069942483`},
{0.18968282299824824`, 0.519816328948473`, 1.9818995109191384`, 0.8631954007357836`, 0.033894385168637076`,
0.36152588787936835`, 0.13701549203954322`, 0.2296501432463657`, 2230.7628620359865`, 54.21442465485268`,
0.025809495734432863`, 5.0872858624540225`, 0.22420423998520422`, 237940.7261604568`, 20.211725259530517`,
600.3364448257337`, 0, 17.53820543050255`, 6.797887708976921`, 21642.242647612788`, 3182.6716895210284`,
64119.04129256204`, 127.63453787509987`, -0.9980094128779511`, 50.48075762120472`, 173746.86800449164`,
0.6521763930781004`, 1.000000000001645`, 1.7935546012166472`, 0.7935546012163521`, 0.022444432732511108`},

{0.1333252885737945`, 0.6199239307530391`, 1.8571978251971686`, 0.9095514210206579`, 0.1119169009299061`,
0.2478793840048047`, 0.08625348528814925`, 0.24475415564717562`, 2619.34383923971`, 229.53625600516762`,
0.07038297161992796`, 1.9415562105060538`, 1.123956994156356`, 17310.69439995658`, 5.842898243026262`,
49.090432738657384`, 0, 6.181539979841616`, 2.1141148728610024`, 1365.7459519491938`, 645.0131232608702`,
5950.353344248219`, 27.287497673316928`, -0.9954141382713526`, 18.722720029249377`, 11333.322524827703`,
0.535706118408261`, 1.0000000000013336`, 0.3648547014772001`, -0.6351452985232864`, 0.028027487050836235`},
{0.05059760434947658`, 2.210267681954983`, 2.4323928454491863`, 1.1361578898617037`, 0.6967300845119604`,
0.526932020206838З`, 0.09927434886429598`, 0.24328677204756152`, 3807.4700659446307`, 207.19795070674797`,
0.16480436384127967`, 4.45009968475919`, 0.7729577502629965`, 4387.281395542134`, 25.695461084329075`,
194.16942285048262`, 0, 18.67564640217225`, 1.9312859694349418`, 104.588548690404`, 53.15487418520659`,
2499.1661621521494`, 2.8877602447698787`, -0.9988445105057429`, 60.98084232650218`, 1806.4545810882094`,
0.8700123104731403`, 1.000000000000181`, 0.030803894400388117`, -0.9691961055996174`, 0.09122086426591725`},
{0.24406698410388555`, 1.4917825523241879`, 1.8330046648415304`, 0.8025451323745518`, 0.3882804043356616`,
0.6182505929388649`, 0.23260014303933024`, 0.008919285535719272`, 1172.1683108026946`, 354.24325796754124`,
0.11125790867650998`, 4.298846537988302`, 1.0733397741970694`, 8427.312802356477`, 58.542816952529826`,
119.4055404501654`, 0, 54.642646015740496`, 0.3081565329330435`, 42.688757063567714`, 137.5294566279507`,
1864.5779061440476`, 5.373065272324917`, -0.997118347667523`, 6.5671791316318515`, 6501.170088275918`,
1.5080729686202572`, 0.9999999999999978`, 0.08128065496586177`, -0.918719345034138`, 0.25076847590460505`},
{0.08563187236977793`, 0.7699393339104481`, 2.55010147552081`, 0.886086358774756`, 0.5489407388189`,
0.6967816713320905`, 0.17330539558613361`, 0.06157221588457279`, 1722.9132401400711`, 240.8998329943014`,
0.21060998547292448`, 8.988787778446675`, 1.3846037250141348`, 3699.928760748971`, 111.07578324163194`,
384.8541065537955`, 0, 95.73640091391664`, 6.849971809743133`, 144.2992510881127`, 20.065670793399043`,
1584.1205513695372`, 0.8974282373768313`, -0.999433484884341`, 75.3437533214142`, 1937.8744124745463`,
3.780047313978435`, 1.0000000000000069`, 0.01226728244925793`, -0.9877327175507421`, 0.4639434457235676`},
{0.23775769207863484`, 1.2489503941392437`, 0.7600016230767555`, 1.095753134818721`, 0.8325305358876416`,
0.5578220865599602`, 0.11931867110919586`, 0.014230310160314848`, 1755.4498344511894`, 146.4106779572221`,
0.028427482579488184`, 4.979696715264749`, 1.3071286627299754`, 306826.4349381336`, 84.56002073439514`,
383.6884921270349`, 0, 23.502103722304735`, 0.25229613582337895`, 2751.1122966229923`, 10903.298187701617`,
69781.75413164397`, 553.2919826144418`, -0.9920711081356504`, 4.501505118234544`, 237016.41159340498`,
2.532107209416576`, 1.0000000000000073`, 6.161535475284594`, 5.161535475284548`, 0.05539857878373094`},
{0.2642492231861682`, 0.678656463776123`, 5.229669084224717`, 1.4960336643896621`, 0.09351401012737992`, 0.6251695134859938`,

0.20442619944666085`, 0.006818687130271267`, 2270.7823160691296`, 72.58428376873474`, 0.07107729542645586`, 9.843971184435041`,
0.8047286671187344`, 232269.29929449531`, 53.146866866958085`, 1032.7031604416616`, 0, 348.81060957622816`, 3.170839900536306`,
1927.7075765524842`, 606.9485678940894`, 48562.672596143006`, 42.33832140501817`, −0.9991281715123648`, 30.741585629974374`,
183323.55013392883`, 3.2761714556625607`, 1.`, 0.34289476853988377`, −0.6571052314601162`, 0.07127512983015132`},
{0.08663733907452292`, 1.1055276206514`, 6.650123879097649`, 1.442255346530818`, 0.5561115773214398`,
0.5670470763741954`, 0.06364195502160275`, 0.013792669120976587`, 1277.4869438309242`, 50.05278631474647`,
0.06972164372518636`, 6.513399093256677`, 1.399747572725158`, 144858.84784212298`, 95.92171318057683`,
924.211497464172`, 0, 271.051975669259`, 3.149059388035095`, 1419.8828773019866`, 449.89110821372753`,
64591.4084360143`, 30.26703179584003`, −0.9995314077749857`, 49.733887607776985`, 79943.25362817092`,
8.166534496694434`, 1.0000000000000036`, 0.25416818877017017`, −0.7458318112298308`, 0.08436523424550636`},
{0.1178483592983135`, 1.9540292922377533`, 2.158901124676431`, 0.8036721080520579`, 0.022324771726093706`,
0.23728547762141228`, 0.05703389546493082`, 0.19717089259438486`, 1678.2311544802042`, 81.55285235418881`,
0.08629691407172851`, 9.548029647858971`, 0.23903934985577635`, 36771.34885086523`, 43.28944592467017`,
641.0347605350382`, 0, 40.416480325824544`, 3.9005299782209737`, 938.5220243421998`, 239.6139754296308`,
13645.423328860843`, 8.994560482099027`, −0.9993408368311245`, 108.88214046707536`, 22972.725017674024`,
1.7578777785088244`, 1.00000000000018`, 0.13557407245078631`, −0.8644259275492381`, 0.057235371617319`},
{0.07697798125264199`, 2.659703661710245`, 6.212164355972867`, 1.1315710739609641`, 0.8617717680117074`,
0.1678172363037963`, 0.11812638083909416`, 0.3038591454145657`, 1784.188819853568`, 30.892067935544162`,
0.199805435190976`, 6.1761313696591404`, 0.7335007728106389`, 74271.04136986787`, 49.026769060082486`,
2313.7496275135936`, 0, 53.54079232568624`, 5.915429103747229`, 1547.9835960087937`, 260.68576596213404`,
35237.04504290995`, 13.746630206504362`, −0.9996098812999284`, 224.76126354034037`, 38749.665610166034`,
2.0949513616894553`, 1.0000000000012974`, 0.14712065273373154`, −0.8528793472664593`, 0.016757421469314557`},
{0.19506025087199597`, 3.126229772881034`, 2.308505447241318`, 0.9330443187011872`, 0.44656066575469633`,
0.6840356045619456`, 0.09118120944389022`, 0.034391869601605236`, 2773.7056708459277`, 224.4415538260822`,
0.0593475599896775`, 3.3310563178619557`, 0.5039179573441945`, 14153.302672168016`, 21.659625548704046`,
107.77511323873885`, 0, 39.18988829228656`, 0.41785059295621924`, 102.11909362051328`, 243.39140530599397`,
3722.36892738491`, 10.750272534643152`, −0.9971119809066868`, 18.66138520451033`, 10372.660240197525`,
0.6272364336787787`, 1.0000000000036127`, 0.1398809288719731`, −0.8601190711285323`, 0.08640958277760602`},
{0.06456964840349072`, 3.9537241675412282`, 3.641109216568884`, 1.3108140470595528`, 0.011724209788321671`,
0.438559457390715`, 0.1826458897676254`, 0.0058767891936883`, 1354.5450008176776`, 86.5151446772075`,
0.010849475740387471`, 9.258377423570199`, 0.4300240953856598`, 448744.70991910854`, 65.72923258004367`,

743.7645589145811`, 0, 233.05654316879662`, 0.33914008518698474`, 604.6448355977816`, 1781.8763452259288`,
233293.94895263397`, 109.49423416199517`, -0.999530659776417`, 19.155233585510995`, 215195.83226476455`,
2.5958431362985706`, 1.0000000000001046`, 1.0116827549149472`, 0.011682754914841276`, 0.14020937298726627`},
{0.23831756341954657`, 2.9118296516681834`, 0.7702789156400254`, 0.809756039261601`, 0.15598009967599835`,
0.15072565384210967`, 0.12556989454649928`, 0.015474985134933292`, 1269.344776285695`, 338.73985440309866`,
0.16681668263344573`, 4.906919687762464`, 0.7266912138912884`, 13016.761217245079`, 27.46690718452094`,
97.75815788806175`, 0, 23.147441288024655`, 0.11928970361291749`, 55.32346279975283`, 462.77399829302647`,
2948.896022893092`, 17.404363427278316`, -0.9940980070873427`, 4.9621613731257375`, 10039.624499335376`,
1.2677930060330644`, 1.000000000000628`, 0.261733655126526`, -0.7382663448736384`, 0.05613195223424544`},
{0.1708861337350846`, 3.1382784382473305`, 0.22397208377012315`, 1.1376251902182828`, 0.5276068694742282`,
0.6173335030361042`, 0.09813160076689689`, 0.04549667808286232`, 3051.102513690643`, 71.6500993647141`,
0.0653128233426199`, 7.970153906640311`, 0.19805272219502368`, 115093.30256961113`, 50.67153006194715`,
912.6395188732379`, 0, 8.047291194845478`, 0.11392939269081787`, 989.2048441936964`, 8681.613159171358`,
33441.53604687566`, 454.2272495579507`, -0.9864172731503347`, 5.107745093774381`, 81638.49716018626`,
0.8099121282595069`, 1.0000000000002385`, 4.900078595916587`, 3.900078595915418`, 0.04913571002095055`},
{0.06329193275668865`, 1.9001497619691943`, 5.252505884188258`, 1.1790665711161483`, 0.6185584326542477`,
0.29903601211054487`, 0.18805549054580545`, 0.08613649661622771`, 1994.6987223768147`, 102.68040788991027`,
0.2346504641795843`, 5.384687813670801`, 1.1000521056786416`, 9974.89341575625`, 38.32032762664975`,
590.7565244912199`, 0, 96.01331099864426`, 4.144315346104248`, 195.5193914413164`, 46.177730243213546`,
5126.739647141682`, 2.609313770617259`, -0.9994910383693715`, 112.49742597750439`, 4635.446585827731`,
2.1274323785722795`, 1.0000000000000464`, 0.02680370189155514`, -0.9731962981084461`, 0.09706983317672369`},
{0.1794793632102442`, 3.220429652904432`, 3.9791446112229796`, 1.2467997677613296`, 0.11361466513971896`,
0.5165426921925539`, 0.22271304495125288`, 0.2987687877435581`, 2176.453438414389`, 103.4889254155421`,
0.20600780354143933`, 7.995100635365489`, 1.3485017278608633`, 37676.71634301049`, 41.31082902602779`,
610.1712806179445`, 0, 46.084117328025684`, 4.0972268601461534`, 649.3809504617966`, 157.49279833107224`,
10504.526646881475`, 9.26186857086042`, -0.9991182973890965`, 188.49758392987422`, 26933.510762961843`,
3.9016603355875388`, 1.0000000000021747`, 0.09000631480982177`, -0.9099936851903739`, 0.12522365105150793`},
{0.0634556155295738`, 3.680544991233944`, 1.898464422832438`, 1.0496696027333048`, 0.16836359340314266`,
0.3732784807189835`, 0.2175697213065485`, 0.48318139091353535`, 714.7371038686642`, 368.95513713314926`,
0.1959410717243747`, 1.9243643979934077`, 1.1366603632987364`, 1665.111671091218`, 26.252088615541982`,
40.78595823735657`, 0, 4.2959949959167565`, 0.4703148567784196`, 27.144988562133463`, 56.71662997863224`,

857.9073968591547`, 2.993218566800797`, -0.9965110237098326`, 24.72878557741033`, 777.7005990724558`,
0.8511051272110596`, 0.99999999999481`, 0.03463914635096339`, -0.9653608536488568`, 0.18341781798314538`},
{0.21641932432281485`, 3.6568426450830875`, 1.306554690894977`, 1.4626425107711718`, 0.8835071341302185`,
0.5676582474171099`, 0.11158412214739238`, 0.023181801672309155`, 527.0796987655522`, 75.44193071736254`,
0.13812060798969733`, 3.0114637760169054`, 0.9949014306145822`, 150574.9568663771`, 191.0964085425386`,
449.135852964148`, 0, 22.215027130839005`, 0.13724561584204706`, 703.5999724882583`, 5125.575214599321`,
36792.83872378024`, 348.0819057804372`, -0.99053941153077`, 7.1697945837412504`, 113752.58995026907`,
3.380443194490704`, 1.000000000000002`, 2.9084466551369976`, 1.9084466551369919`, 0.04788547107983012`},
{0.27575001701682567`, 3.4736287001446966`, 8.538624919070354`, 1.4033945242686854`, 0.5325831427167897`,
0.4946990317811615`, 0.09780340245600855`, 0.03288516063918166`, 2535.414355248974`, 43.33184327775365`,
0.19548398944691353`, 1.2011151230670372`, 1.3252105639060514`, 99272.24880299871`, 7.650076869827188`,
210.032410009639`, 0, 51.90147998840741`, 0.4795851425058141`, 626.7941252151694`, 1305.950673951645`,
20083.07748635485`, 84.92182809938579`, -0.9957714733632291`, 23.798581645302555`, 79112.98512303672`,
0.9190452483552922`, 1.000000000000007`, 0.7356460047254597`, -0.2643539952745455`, 0.00667077299494982`},
{0.06256964883486332`, 3.548483703832706`, 5.540385857803061`, 1.459008903547168`, 0.4922186967390012`,
0.21082467659119386`, 0.05317469777445105`, 0.051461677692841074`, 2370.1608114198016`, 100.46792579677714`,
0.24298196986117648`, 3.038179651692209`, 1.2632718479945817`, 3518.0298587216407`, 13.14811222865647`,
191.76574387104856`, 0, 72.96107234293052`, 1.0199048075843933`, 28.834711133629877`, 27.271963147152743`,
1791.2306246181522`, 1.9124048511513676`, -0.9989323514097694`, 51.7016512739122`, 1601.0953023515842`,
1.4996813603383854`, 0.9999999999999765`, 0.01588399167158648`, -0.9841160083284132`, 0.03319994420531737`},
{0.23779270937803498`, 3.159033522410793`, 8.923446211238858`, 1.0790485164876151`, 0.3460136054742138`,
0.6482331083335031`, 0.1636040824670958`, 0.011344127288819656`, 1091.512574756292`, 48.569342600520315`,
0.22296455933807102`, 5.246566496412501`, 0.7138062053370233`, 55414.84183885703`, 75.45094268048634`,
899.708806274822`, 0, 300.853152751905`, 1.0537241279513274`, 167.53149775835186`, 157.98990382242658`,
12523.239554788235`, 8.013204495762924`, -0.9993601332577959`, 47.55356919387609`, 42541.92948461818`,
3.2123232017914503`, 1.0000000000000042`, 0.08995636513150841`, -0.9100436348684919`, 0.057798016415542046`},
{0.22757425351270916`, 2.581225270499978`, 0.37748773050037343`, 0.887385361142591`, 0.4937063046079888`,
0.23328592019488925`, 0.08261142866202484`, 0.0067329461689494274`, 1345.0595989079266`, 159.82616980913542`,
0.14361492663679165`, 1.2675214995529185`, 1.4755532059142284`, 42676.797221725064`, 10.311646408489446`,
57.92564881632609`, 0, 3.2496400929380425`, 0.008205190940294007`, 98.87044700486824`, 12048.743598206323`,
10038.255364021443`, 473.4652166195811`, -0.9528339139172979`, 0.3025635172052047`, 32634.978157673286`,

```
0.6875892967795304`, 1.0000000000000095`, 6.778712841565523`, 5.778712841565458`, 0.010404300004332375`},
{0.07923961132457574`, 1.4534207730102917`, 0.3049651947786387`, 0.809144094297255`, 0.9651646059166545`, 0.27855980457998597`,
 0.18910876827885104`, 0.18936230186753644`, 1265.284678743018`, 224.5662215569697`, 0.05882269816842833`, 4.935952740691619`,
 1.3773991041706521`, 41609.60387683968`, 92.67007476068274`, 391.06505830249023`, 0, 3.1570994230296074`, 0.37988519374421625`,
 1395.9132203530937`, 3673.5660092585445`, 19511.367638507316`, 139.41448954765312`, -0.9928547043892245`, 7.887614742098314 5`,
 22086.759829803184`, 2.0966166914874536`, 1.`, 2.1008057814255436`, 1.1008057814255436`, 0.1368064610539071`},
{0.18119347985627088`, 3.338563344897951`, 0.31616309891789207`, 0.8233375529098934`, 0.07175467184830686`,
 0.559491101800496`, 0.06295447855153882`, 0.018102651459346017`, 3519.8202135278207`, 127.22666926323683`,
 0.07035715001733955`, 6.64501017919611`, 1.131299832263819`, 90593.19782550406`, 21.432865128874447`,
 294.7890151976623 7`, 0, 12.408367703283519`, 0.06562444527014523`, 382.2746256223052`, 5824.18639279401`,
 25241.225292048115`, 220.93898737345413`, -0.9912468992761989`, 3.129876678688115`, 65336.363521461615`,
 2.5635011164425836`, 1.0000000000000133`, 3.277195516038798`, 2.2771955160387547`, 0.03775257915843456`},
{0.07535246017332142`, 2.911051338058316`, 5.442775177543007`, 0.8607203087442552`, 0.8022890490197512`,
 0.40209854436599723`, 0.144612694452116`, 0.016940102043156918`, 3925.350442074646`, 91.64171206007711`,
 0.21807597998523465`, 2.8164983201304956`, 1.3696264396607019`, 8816.078128440184`, 15.660272367786153`,
 361.39171009378026`, 0, 92.49278570346353`, 0.5220984971261488`, 40.336656010463905`, 76.25870929047669`,
 4190.438535419954`, 3.105367452826069`, -0.999258939744235`, 21.712221837961554`, 4510.855040699775`,
 1.109827952440159`, 1.0000000000000016`, 0.0437078184563295`, -0.9562921815436706`, 0.03841995995063323 5`},
{0.2204293092455124`, 2.4745010079982768`, 1.6220990910883482`, 1.0541684584544448`, 0.6868676732591923`,
 0.6458136221345077`, 0.24561205720827334`, 0.010048142648758356`, 2710.1004320631428`, 99.89140315660762`,
 0.1947182155448685`, 4.969891992321378`, 0.16288064769797317`, 17680.94147770348`, 45.484130262675244`,
 693.4097194146273`, 0, 52.70576503257963`, 0.20775760102309856`, 61.05698175137952`, 292.8856698898404`,
 4246.971417728326`, 14.46563588792646`, -0.9965938937503696`, 7.344234187870878`, 13373.67108564697`,
 0.4747141974398171`, 1.0000000000000964`, 0.1666842815368903`, -0.8333157184631258`, 0.061881766186274376`},
{0.18983832850361748`, 0.47273123425625707`, 0.543837247642804`, 1.0885560496178721`, 0.427736490811788`,
 0.6656708549524757`, 0.12908120643315885`, 0.022867246439630798`, 898.0605548746116`, 139.83397705936972`,
 0.09941082510128124`, 1.7064291808810135`, 1.0668304792785985`, 78412.05853193086`, 33.23547982679414`,
 98.12660428056266`, 0, 5.286770876779014`, 0.22097801544276097`, 2490.275425576867`, 11268.335642223255`,
 21122.187837552043`, 562.0661685696991`, -0.973389775107935`, 1.4923315711964942`, 57282.86904886157`,
 1.1288232507063234`, 1.0000000000000384`, 6.4215405487549635`, 5.421540548754717`, 0.03647189044784779`},
{0.17497599862759233`, 3.3657454284285517`, 2.034553169278075`, 1.2437246851025225`, 0.5208306933739273`,
```

0.3059877216844632`, 0.14568158867858877`, 0.07100111598759662`, 1376.7566751341965`, 41.042975981802954`,
0.07580837625359882`, 2.9604840388353644`, 0.18481744658999433`, 115704.28796669048`, 27.125525808593707`,
652.123727032864`, 0, 22.259271381287398`, 0.45855086681041546`, 1187.0503536885724`, 2587.6993997971217`,
33048.81531203522`, 149.31357716239802`, -0.9954820293631517`, 22.04807833813006`, 82610.70660974627`,
0.5597731437818949`, 1.0000000000000002`, 1.4599274640750686`, 0.45992746407506835`, 0.01777296328947708`},
{0.22260435016590752`, 0.807488655841027`, 7.62907904124237`, 1.0059991047708425`, 0.2817863964260634`,
0.32047133388609383`, 0.11883206718041817`, 0.029206686321015127`, 2029.3046388765315`, 71.8362522665995`,
0.05374037941301346`, 8.93317694888524`, 1.0534684445303992`, 80648.39507808641`, 45.982868118617176`,
908.8209169912506`, 0, 358.9369153717395`, 11.880127107153516`, 1894.0594281936192`, 158.4309060088362`,
19172.076561069032`, 7.470853930640467`, -0.9996103263041516`, 137.0438266996565`, 60968.394917254154`,
4.189238149033244`, 1.0000000000000504`, 0.08993552423122991`, -0.9100644757687746`, 0.07940419436262978`},
{0.19262113260207286`, 0.4100732369307054`, 0.6039207257051533`, 0.8273370736078748`, 0.5063544182124549`,
0.2807655019410843`, 0.08795158701010541`, 0.19422202595284532`, 2504.9035989294416`, 81.69837632668111`,
0.149947676078805`, 6.632628072950256`, 0.568480342643902`, 59925.54317492346`, 32.32777213205835`,
622.8979964443054`, 0, 7.890149186155234`, 3.176762297950379`, 6422.912200374757`, 2020.8422399808658`,
15964.863876451775`, 77.32935590390363`, -0.9951562784059835`, 18.610074263999145`, 43931.0023102865`,
1.4067058689865377`, 1.0000000000025293`, 1.1369987638604837`, 0.13699876385760779`, 0.018895071362943466`},
{0.17039397430438102`, 1.0518028751020552`, 7.3508889662987045`, 1.2231850486496643`, 0.08959309467655463`, 0.438007460954018`,
0.13336650260220756`, 0.015835766380232735`, 635.4248848043253`, 46.431191145002344`, 0.019547600805230075`,
2.463817783746629`, 1.1305047170660831`, 762900.0119032658`, 38.372112665979465`, 339.72353889719665`, 0, 110.83549658020797`,
1.5541603126618764`, 8782.535420408702`, 5649.984231714475`, 222108.2917815116`, 323.00005160886457`, -0.9985457541948655`,
23.35243264610397`, 540655.922322984`, 3.1326171148810547`, 1.`, 3.201347108924528`, 2.201347108924528`, 0.037085970343700245`},
{0.0808570843788532`, 1.9944028866868502`, 1.847446007042576`, 1.1962230350350178`, 0.9881944572508863`, 0.5065109438103407`,
0.13986962480807952`, 0.21324716376368485`, 3595.9260786344357`, 95.54926882555071`, 0.18522194155873545`, 5.182795178847357`,
1.4775938218806233`, 36112.963865941856`, 59.29193854357404`, 805.4232377741208`, 0, 17.953974518342743`, 1.8189522412585384`,
921.9012620203206`, 505.83093327533135`, 16723.7285598126`, 28.276730476365074`, -0.998309185037581`, 51.82462286730786`,
19317.599018425735`, 2.4224619051206657`, 1.`, 0.28664272565428284`, -0.7133572743457172`, 0.06380461187757126`},
{0.1379604951115434`, 1.4163155012457889`, 4.400902234814488`, 0.7945346450123854`, 0.8193909852858012`,
0.6724735717160395`, 0.06698309689845514`, 0.09275337016012092`, 3209.7522511906463`, 80.79361285288883`,
0.15292409302459276`, 2.596140589311666`, 1.3399665214161485`, 25114.249323823435`, 26.118619534341654`,
263.06965780507204`, 0, 36.948590379951824`, 2.273956064464768`, 674.0730164349465`, 295.4318559046594`,

8424.827136309097`, 10.95023295506636`, -0.9987002424170968`, 46.00913176076191`, 16604.19032791957`,
1.6033083863592825`, 1.0000000000008282`, 0.16705668746415864`, -0.8329433125359798`, 0.025599459146957305`},
{0.2598992397477407`, 1.4339452918245774`, 1.0784655091863726`, 1.4648755135291642`, 0.5315691563517717`, 0.48571691620677493`,
0.23359094834009136`, 0.11846678481017199`, 3222.0061450547964`, 66.19766765214632`, 0.2128172489806378`, 7.890656547448669`,
1.1120638442139867`, 137401.79523465692`, 41.165954415427784`, 1379.3255192466313`, 0, 23.50197698066303`, 1.8455427334855672`,
4019.945718347594`, 2177.1916210389563`, 29141.33703343376`, 147.73744319479889`, -0.9949303134916115`, 37.80581876489551`,
108197.30486031545`, 2.7488125409948005`, 1.`, 1.2271187683692344`, 0.2271187683692344`, 0.03576868239201719`},
{0.07381047173055871`, 2.1206143008004066`, 2.7168272133152236`, 1.2500057406362022`, 0.5621616976632486`,
0.5554267209678074`, 0.23180667473506628`, 0.07059925918611996`, 921.2671327671924`, 62.7109442198831 2`,
0.17012421692254281`, 1.3889827292164867`, 1.4926610431972103`, 64817.70233326014`, 27.222293811788273`,
248.75810765797604`, 0, 14.382719095543104`, 0.45699080541450915`, 1040.0228114952145`, 2274.8068634485367`,
31536.104147495727`, 134.22037062097993`, -0.9957439140233294`, 13.84430338994719`, 33252.78176672416`,
1.5149610910829452`, 1.0000000000004234`, 1.3054142821995545`, 0.3054142821990018`, 0.04072790226018062`},
{0.18229092854924844`, 2.666175072988888`, 1.1843338363668607`, 1.0835916247483197`, 0.8465995494931746`,
0.28296103301830944`, 0.11340515234724796`, 0.021054051155640643`, 2912.6266504267696`, 38.65103872844048`,
0.22568389644671255`, 7.751508465891021`, 0.13543768169398884`, 12611.531668988258`, 51.692995798805974`,
2260.2435053452696`, 0, 52.48533822124235`, 0.4029322259638814`, 74.60336097897155`, 184.1511399975711`,
3480.226386976547`, 9.320472758834617`, -0.9973218774520781`, 15.346969385269002`, 9063.052852050714`,
0.6554218380556868`, 1.0000000000000797`, 0.10440494385094076`, -0.8955950561490675`, 0.04910489684060581`},
{0.19627224659336223`, 0.5402822985479045`, 2.0302490383203153`, 0.7794095357324428`, 0.7903414997496967`,
0.18384078811354698`, 0.12435150563892122`, 0.011407347781693793`, 1336.2245441274245`, 314.05659164167207`,
0.0727358338086812`, 6.3699550531919975`, 1.0324129890894564`, 6619.470835343296`, 65.07976447212509`,
224.63957372493866`, 0, 84.49366371767894`, 1.555821044168725`, 106.39264753764482`, 67.3836023020825`,
1714.3907670100302`, 2.500931966484648`, -0.9985412124151565`, 12.00832242675255`, 4806.96181971394`,
1.9693384847224433`, 1.000000000000118`, 0.03890014175682307`, -0.9610998582431816`, 0.17509116610245565`},
{0.12533935047015737`, 1.521368835274294`, 3.474516335185184`, 0.949787360467496`, 0.3211564169863861`,
0.652599038806982`, 0.05499170112558746`, 0.016940628240370355`, 2778.039504369459`, 65.83823943133149`,
0.237336473550759`, 8.791957984840526`, 0.6376701649045213`, 29444.382669408827`, 49.1195071146809`,
822.3203088580074`, 0, 182.86518804673543`, 1.9405183527024086`, 252.36921829649702`, 129.05247692970391`,
10470.04742162356`, 5.7442793511333905`, -0.9994513607131073`, 42.17491637256078`, 18747.27061740062`,
2.978020990490395`, 1.00000000000017`, 0.07325479946140091`, -0.9267452005386115`, 0.05397816017727885`},

{0.14807057579619815`, 1.4220065645386768`, 3.077692541817422`, 1.3463149673717403`, 0.07642859383354339`, 0.6424799174126323`, 0.13735279371122205`, 0.011944441122857653`, 3609.93932375683`, 78.77118206979054`, 0.0583860200944658`, 3.355260507755876`, 1.0164328161645106`, 179114.13096104184`, 11.162545546163662`, 275.91795356150277`, 0, 65.64988413830865`, 0.5240032206316805`, 1224.9080611589775`, 2336.5964363012185`, 57470.42522934354`, 146.23193819038644`, -0.9974555271236148`, 10.644800279680814`, 121566.84221373254`, 1.1618964095943032`, 1.0000000000000024`, 1.3182323502832238`, 0.3182323502832205`, 0.025350752246408325`},
{0.20217859061685106`, 2.487845857605743`, 0.8626257496221044`, 1.199588786641617`, 0.6707739359709337`, 0.41572085938323444`, 0.1621256536590063`, 0.02422500357599282`, 546.6195403678685`, 211.4653119641191`, 0.06380462964198375`, 1.3445609170340642`, 0.9773390980954635`, 83797.98186702734`, 44.30089752297096`, 68.6468822956878`, 0, 6.581538919421345`, 0.06162095097819873`, 589.5965281722882`, 9567.117966580641`, 21549.234550230332`, 530.7105861789908`, -0.9753721838730779`, 2.1900489661834093`, 62239.91243196451`, 0.83686090629583`, 1.0000000000000062`, 5.490909056038022`, 4.490909056037988`, 0.04212897960252752`},
{0.2326571783338366`, 3.2198726087577585`, 0.7879059046396755`, 0.9520817800309282`, 0.3381503807716493`, 0.6953568861265582`, 0.09096644213287763`, 0.03053119099726608`, 3051.5436637428375`, 193.8478952968402`, 0.1252944454366426`, 7.5675708168133315`, 0.1396895662891593`, 17785.99622119358`, 40.68201955015994`, 273.56508948179095`, 0, 31.20600777124764`, 0.2886927535487017`, 114.93176408294245`, 397.1110113439468 7`, 4103.273630541653`, 17.752784297759746`, -0.9956735070833148`, 13.279341278547435`, 13637.943783049424`, 0.6817675374024624`, 1.0000000000000497`, 0.22670491672543477`, -0.7732950832745765`, 0.1253893444957111`},
{0.20686587279815516`, 2.3834956380504195`, 3.228648073866996`, 1.4616470372504198`, 0.35618390382684884`, 0.6609631833857008`, 0.09990239078555083`, 0.2797108933201619`, 2467.4094286971804`, 85.00316013652997`, 0.14363808446123288`, 3.440698993285203`, 1.2812874699661427`, 87358.09262294849`, 22.229926604572228`, 285.828327556423`, 0, 16.839033164191278`, 1.8822263048779393`, 1993.4994150981884`, 1058.11781698719`, 22065.8099007941`, 71.92582343200469`, -0.9967403950385063`, 64.08968839286185`, 65209.47177322764`, 2.0438852655460185`, 1.0000000000012383`, 0.5976533331579125`, -0.4023466668428276`, 0.031281807193809665`},
{0.14277465652344312`, 2.2128169765977077`, 1.5395722189285141`, 1.2588391498483595`, 0.06601455592248096`, 0.5924077255302849`, 0.2026072042186735`, 0.006704812631657588`, 2107.7558344556182`, 393.22260119726275`, 0.1318126775401366`, 3.5561906346112124`, 0.787606393777565`, 6389.134586047976`, 19.288390017033393`, 65.53079093317909`, 0, 38.703169370014635`, 0.11245060353580956`, 17.125096026516946`, 151.28994321105185`, 2087.9871117054913`, 9.182247495432703`, -0.9956023447443924`, 3.5547514933242352`, 4258.737752844678`, 0.7669295321725982`, 0.9999999999999974`, 0.08868915746259545`, -0.9113108425374044`, 0.17477836164911278`},
{0.16055207404865252`, 0.7029113592886698`, 3.6403774574439822`, 1.1854100604530788`, 0.5916505631446303`,

0.6508026186847373`, 0.196607205292044`, 0.08269873784662057`, 2208.609406288604`, 140.20841727601464`,
0.08922686090021398`, 7.843962027709463`, 0.25680184922543736`, 13648.80609058936`, 76.82640026211152`,
633.8965705578669`, 0, 99.3281008805517`, 10.551727303737648`, 669.4627971243782`, 62.445801607026006`,
4078.50546098499`, 3.554118593727864`, -0.9991285732904549`, 105.95612831305137`, 9354.464439712789`,
1.1065721743270744`, 0.9999999999999999`, 0.03632673426898121`, -0.9636732657310187`, 0.182035351832095`},
{0.10433679528385242`, 1.2124558701074761`, 1.611384270831014`, 0.8947495690924911`, 0.7004462194859642`,
0.22790601678310374`, 0.1107678931866184`, 0.03745668981933727`, 3402.687823353078`, 290.01464659756004`,
0.06742303128382326`, 2.428172996695036`, 0.7057307677392819`, 9694.273143185983`, 9.48219543228334`,
77.97613441946815`, 0, 19.152833040940934`, 0.5540461611167627`, 184.44496269122234`, 331.9054068697922`,
3880.6935516121794`, 13.867913575271992`, -0.996426434246654`, 9.596521719378996`, 5784.273266484656`,
0.3475752362095536`, 0.9999999999999998`, 0.18829990428361035`, -0.8117000957163896`, 0.033881080184610696`},
{0.2556095337978518`, 3.423935597729149`, 0.44377078820861904`, 1.3028637036663855`, 0.63065566823863`,
0.2812042846574222`, 0.0670032727620068`, 0.017466077681157405`, 3127.9987163099076`, 356.3279534853283`,
0.04932332227468639`, 8.912956605460284`, 0.5977251839966713`, 76944.20689219463`, 40.76321758996798`,
192.56233649509107`, 0, 23.549325696948113`, 0.1172548544021334`, 307.8327398857189`, 2624.330451820663`,
16535.25180541936`, 157.6013613641291`, -0.9904687655671215`, 5.735329571343036`, 60379.5429316193`,
1.3079481199572647`, 1.0000000000000844`, 1.4784580859438567`, 0.47845808594373196`, 0.05839756253033285`},
{0.17842055683347624`, 1.0601527835649565`, 1.9863486015726688`, 1.4771465685197964`, 0.5130989252089981`,
0.50009800158305`, 0.10443547318598384`, 0.0065079948116299165`, 919.6368613396894`, 101.38339784479837`,
0.013661722290118639`, 4.3887706359180285`, 0.5048436819791595`, 768397.6091724653`, 78.34389067269089`,
347.94169383335793`, 0, 59.756051375877405`, 0.3426083864781212`, 4048.435870286816`, 11815.511299980531`,
216500.83579768921`, 814.3253605781796`, -0.9962386964577858`, 5.188817637106841`, 551831.4239705233`,
2.0779941069838532`, 1.`, 6.6988652779413735`, 5.6988652779413735`, 0.06465835780006285`},
{0.09313560387857911`, 0.7038695534977393`, 3.6724738159211565`, 0.9475746331523089`, 0.19159788938387368`,
0.6119542230262267`, 0.1716716908175685`, 0.15713357297550534`, 2263.7776933944124`, 51.55094049564468`,
0.21456002491809933`, 4.890757314915582`, 1.3381695004184304`, 49160.289932646774`, 28.119070532181137`,
715.1426668842546`, 0, 41.709529812233235`, 8.36865631159831`, 3076.326086380155`, 366.6009590830735`,
21036.62899489871`, 16.26975889258423`, -0.9992265985725883`, 84.14917687743947`, 27989.416357278977`,
3.474211966019486`, 1.0000000000001223`, 0.2080143071704822`, -0.7919856928295432`, 0.05713210054565385`},
{0.14593842634629578`, 1.5677173946402423`, 2.2260911107394623`, 1.0410408322166074`, 0.5774262288207519`,
0.5605768734730473`, 0.06528103792572038`, 0.18580384429276964`, 1821.6991564326672`, 281.5049269480922 5`,

```
0.09810886942125424`, 9.103899808381804`, 0.814497273403965`, 3265.5551045915163`, 97.04961673419538`,
237.46222150134534`, 0, 44.288110331138725`, 4.763159929433868`, 101.38266970654944`, 20.284750293614312`,
1008.1020908924105`, 1.0520726007626855`, -0.9989563828800004`, 106.67555249752637`, 2101.726182017838`,
2.889846713296147`, 1.0000000000000686`, 0.012246167745239987`, -0.9877538322547609`, 0.3705535899212349`},
{0.10172713838830055`, 2.2418772278235775`, 3.04935119913241`, 1.1880003837277453`, 0.642679290083189`,
0.5307574217574084`, 0.20278574850964748`, 0.28989270792503147`, 1645.7112772203664`, 50.385249878477794`,
0.23495391386029668`, 2.9089393035407407`, 0.6416895284655368`, 56276.68644895397`, 35.45266700348367`,
678.4944743025469`, 0, 12.950592465098174`, 1.6085055318814783`, 1372.5432400872194`, 852.3034005060259`,
22912.757109000053`, 47.2205743338873`, -0.99793911426245`, 51.51531318219065`, 33297.84590406815`,
1.0240344851754117`, 1.000000000000984`, 0.48216979991504805`, -0.5178302000854265`, 0.02412488128056874 3`},
{0.19614726189645104`, 0.5421409973222318`, 7.311205144883296`, 1.4430957843086838`, 0.5642408555529124`,
0.4388312833001765`, 0.24379751153424895`, 0.027088524601856054`, 3097.2470344285757`, 24.388568117880652`,
0.033832395593974274`, 8.563316908406161`, 1.3116329767787245`, 1.6690267268832875`*^6, 45.69141715558451`,
4284.162323991907`, 0, 334.7989778454743`, 14.79501073391258`, 53250.94443176828`, 3598.250138407025`,
438852.43287150736`, 241.38582175980235`, -0.9994499612997919`, 114.58545535252104`, 1.2297100440620286`*^6,
5.296772050669068`, 1.0000000000000002`, 2.0260301180282463`, 1.0260301180282458`, 0.031806588566413926`},
{0.26324845548994974`, 0.6443701830080291`, 0.83901460272339`, 1.148634249041292`, 0.7108758830917159`,
0.6261909633849789`, 0.1815221463640686`, 0.007774421269715129`, 2091.205586215532`, 76.86431957676541`,
0.07691551140505637`, 2.5668632188750475`, 1.1962425546812079`, 250323.7946085231`, 30.914547892705485`,
404.22515195534345`, 0, 14.419565293977403`, 0.15653705151917455`, 2451.930387556854`, 15662.578454820403`,
52578.01855555237`, 825.0239085135145`, -0.9843085774021358`, 1.440968693356399`, 197729.74539244402`,
1.3913684229607575`, 1.0000000000002733`, 8.83371694653031`, 7.833716946527895`, 0.019428735742975715`},
{0.06066901211156295`, 0.5118142470076563`, 2.3575039706529495`, 1.153198866823781`, 0.34052857777406675`,
0.48310824315452683`, 0.15644429107129876`, 0.01838618195497249`, 3533.672368817396`, 112.99427366098905`,
0.18169108406320317`, 9.689478468414666`, 0.6768155401525213`, 20718.771827832228`, 36.95468260101012`,
714.706442214136`, 0, 135.02646281370133`, 4.252135543895795`, 518.543119105714`, 120.94886869260668`,
11007.770927626349`, 6.562686936008557`, -0.9994038132716281`, 31.090050736764468`, 9540.436967563917`,
1.7472539061766823`, 1.000000000000977`, 0.06893261674904298`, -0.9310673832509637`, 0.09616033609241174`},
{0.17215213474108`, 3.1043854956757873`, 4.4538232156171205`, 1.2105616525577976`, 0.07070121186202716`,
0.43467897429752544`, 0.1858388910260701`, 0.10113399844294489`, 1450.8531111854936`, 101.3426083924827`,
0.1798803264840549`, 9.89915477207009`, 1.3540588713189834`, 36612.819331501356`, 68.52461493062177`,
```

710.6453699988288`, 0, 136.6936778931448`, 4.297405227692348`, 477.12527893095876`, 110.02636443414227`,
10487.981751129586`, 6.307442110086814`, -0.9993986028713858`, 190.58289225556337`, 25793.263536892133`,
6.104530463826308`, 1.0000000000001006`, 0.06311254243106895`, -0.9368874575689374`, 0.1798653282166438`},
{0.24067020849458426`, 3.9616105393925194`, 6.770909075338004`, 0.858077802255987`, 0.4748383175874904`,
0.6078443186142359`, 0.2469104749167066`, 0.3360706312022538`, 1507.4555524848456`, 50.50450753574768`,
0.19906071527290714`, 6.968998972407538`, 1.347049135136762`, 79027.35422554782`, 81.66631214221587`,
1532.1385656383911`, 0, 61.50903458096826`, 5.051256030847012`, 1135.601327684157`, 223.81563412134855`,
17726.979657878743`, 9.025245476015801`, -0.9994908751716198`, 285.8729875567641`, 60947.941289156384`,
5.754950693435222`, 1.000000000000712`, 0.12728395235170778`, -0.8727160476483828`, 0.07704060069214108`},
{0.09993396962410062`, 1.749743490015244`, 1.6240016651924947`, 1.3301699142654`, 0.8818156710905265`,
0.15681061125193507`, 0.1352532563629385`, 0.07239358889803704`, 2734.4752174906116`, 304.1441804766208`,
0.060678774409857306`, 8.403610161636486`, 0.537751994770234`, 15539.758600068304`, 43.43157566377896`,
357.57229142370886`, 0, 50.44991302472438`, 1.9841233905194484`, 303.90777063951157`, 152.16979382010499`,
6359.180528484309`, 9.494619098379426`, -0.998506942984894`, 49.59581408497704`, 9078.545053824633`,
1.074497881295947`, 1.0000000000007512`, 0.0865377287387399`, -0.9134622712613251`, 0.10143966052518431`},
{0.2706278159985153`, 3.6076079533892003`, 4.017828860181465`, 0.931912448165567`, 0.07202782033095079`,
0.3509804409724484`, 0.12413238666840237`, 0.15319152742338107`, 1515.3910453914696`, 86.92889855061799`,
0.057359319076296045`, 7.405405977315304`, 0.4162338517487447`, 110215.11203824819`, 44.41195635721702`,
550.0970589795371`, 0, 69.68613090142044`, 2.8849367532918895`, 1439.894916958606`, 498.1079666878651`,
22604.055762637854`, 21.65128981781172`, -0.9990421502209529`, 148.6817253742946`, 87389.80348216198`,
1.8711954072074062`, 1.0000000000000449`, 0.2815233569349063`, -0.7184766430651063`, 0.04849984220506609`},
{0.10989408866872064`, 2.7814923653363133`, 7.842262663464009`, 1.0963577256835755`, 0.7584107639763666`,
0.4703739490649106`, 0.21862941653484513`, 0.05112895789567924`, 3757.6555119131663`, 58.131608822022315`,
0.16080796994998975`, 8.15898170434339`, 1.4928178868639161`, 47175.42782139169`, 50.17389080399913`,
2041.4834122578436`, 0, 276.88573260286574`, 4.9371242586240545`, 488.83313279205123`, 98.01171353712007`,
18170.797037833312`, 5.073339359869587`, -0.9997207970927579`, 196.17962045827875`, 28526.616869385587`,
4.203991350424851`, 1.000000000000042`, 0.056034060693147154`, -0.9439659393068552`, 0.0927620431242018`},
{0.07632945356776594`, 2.5680095036868336`, 1.636586934722592`, 1.0974626755451315`, 0.20070740407760934`,
0.2792825326359145`, 0.08134798544013919`, 0.2967486253326174`, 1516.758023227967`, 278.84396411497187`,
0.08326880329861536`, 5.936954264251678`, 0.30521315193193943`, 7266.820824186787`, 35.64955292296975`,
132.78275074179373`, 0, 14.664772851473728`, 1.5693678512085112`, 156.02218562528648`, 98.41721789772848`,

3440.9347299949795`, 5.1453072188427225`, -0.9985046774720862`, 57.57359366691478`, 3752.0666814694996`,
1.1894193894240483`, 0.9999999999999949`, 0.056840156006318455`, -0.9431598439936812`, 0.16934742707605036`},
{0.17960696499390383`, 2.6986368926848217`, 0.39096283943110777`, 1.4614976941662214`, 0.0584358271913763`,
0.28608789101372956`, 0.23241827692103179`, 0.025712440835301015`, 1101.1873067230754`, 291.87610702538757`,
0.09533779650969992`, 6.256274031322354`, 0.1260305021418897`, 35194.391608171834`, 47.45913141986832`,
166.067583208332`, 0, 13.531945120979794`, 0.12474258560824489`, 239.0454128360485`, 1915.3095880245146`,
9864.76717719874`, 130.27853882284361`, -0.9867935515879211`, 4.809070623018635`, 25311.155615259115`,
1.2725323551100076`, 1.0000000000004061`, 1.0935428647859338`, 0.09354286478548968`, 0.13872295541899854`},
{0.06861147798506062`, 2.729021236857605`, 1.928866504736478`, 1.4151352288745787`, 0.45478865294711146`, 0.6487874608126984`,
0.19220035768006005`, 0.027739198253996934`, 956.7552237848031`, 40.992278320550554`, 0.10039930252063617`, 4.815840989066018`,
1.4694203679389397`, 211083.13674605594`, 89.49831961534596`, 1137.4777113456091`, 0, 50.26346465816486`, 0.49412897400494576`,
1498.202501934665`, 3031.0069875515323`, 106563.10103740345`, 201.50166028826965`, -0.99810908599387`, 19.26412091151657`,
104449.31229782249`, 6.6558509882777415`, 1.`, 1.7270019232663136`, 0.7270019232663136`, 0.09989521661023697`},
{0.07254416598733587`, 2.8849957402027044`, 0.27388492744414883`, 1.0617374480096804`, 0.016195895901701673`,
0.5364706306281042`, 0.056724271897686834`, 0.010490294664589572`, 1785.3192582331621`, 96.17787055857661`,
0.24599567314403442`, 2.275980053816065`, 0.5487867290449304`, 23018.470371378986`, 13.398407503855692`,
124.76688020834096`, 0, 4.052185312348444`, 0.014313572184893816`, 71.06599014904496`, 4963.937419608379`,
11301.501247870732`, 240.89344156135775`, -0.9786848281234546`, 0.5899227825786076`, 11712.256891880219`,
0.8072715388511965`, 1.0000000000000169`, 2.806425180529629`, 1.8064251805295815`, 0.026929122032398705`},
{0.24080755705319862`, 2.3759884700193172`, 5.524917589968679`, 1.2325829370203099`, 0.2181690975782502`,
0.6636754905333937`, 0.21732363084691986`, 0.11964660140986366`, 3828.961095769544`, 112.06990257277658`,
0.03236755171123323`, 3.877460599831684`, 0.7029311637647315`, 79442.67793972418`, 14.076900575755303`,
286.5219239369245`, 0, 59.36885689569964`, 2.884800520759513`, 1434.3423204580354`, 496.2067600987538`,
17855.98619037316`, 28.6499601876063`, -0.9983954983005614`, 97.9178967947189`, 61426.52018970626`,
0.6853862516743361`, 1.000000000000004`, 0.2818345744640136`, -0.7181654255359875`, 0.04694588215595328`},
{0.15494186822563605`, 2.0029420912170632`, 3.8996790997983233`, 1.319513511447182`, 0.5220998731870559`,
0.2887737008742528`, 0.22727260445012404`, 0.040774601815574976`, 1826.996357882992`, 128.81708545308533`,
0.21865899508635123`, 9.155723124997508`, 0.5134933136755975`, 16385.852513974252`, 63.45064537455784`,
804.6941257414771`, 0, 168.78019480806216`, 3.3045987391121945`, 203.67111575404448`, 60.63263132175687`,
5016.158340709074`, 3.798926212834245`, -0.9992426622218036`, 94.55599870215207`, 11103.04206607239`,
1.844067101625537 7`, 0.9999999999999999`, 0.0348787660596568`, -0.9651212339403432`, 0.10029693294306556`},

{0.22396249520436795`, 1.9570085864611029`, 1.4759242627722013`, 1.3116517086556398`, 0.6599681888830524`, 0.6953504264310013`,
0.09804476293632919`, 0.007430663549575122`, 1204.900095750464`, 62.93764873891854`, 0.10119557783088817`, 5.061320104116531`,
1.1487439857805883`, 226615.47185704942`, 104.73347623191799`, 708.5577982223468`, 0, 50.30249800007346`, 0.1836949583153087`,
751.0115661160042`, 4087.3624297783285`, 53949.692160868326`, 248.65521775348117`, −0.9953909798593098`, 5.135608724466758`,
172610.10959793697`, 4.650570595242731`, 1.`, 2.3055538603033345`, 1.3055538603033345`, 0.04439718615502225`},
{0.10601932444652468`, 3.342699233573309`, 6.832537694215926`, 1.4208647912460013`, 0.5802751516427387`, 0.2570998642455964`,
0.07303385433227993`, 0.04213475658223818`, 3584.7181661863006`, 57.24897785837004`, 0.15446538158110595`, 5.395898684860962`,
0.7872614775312905`, 38158.70593173893`, 18.904561867908015`, 704.1837944474191`, 0, 170.85388040774265`, 2.101965314182915`,
294.09924383375164`, 138.91631633944215`, 15066.387048444278`, 9.226980315882003`, −0.9993875784362758`, 100.37482635309867`,
22818.973953227644`, 1.334249205688934`, 1.`, 0.07866135215680364`, −0.9213386478431964`, 0.01928716622690557`},
{0.04498655055054335`, 1.4491138993771555`, 1.16394731374956`, 1.169713814519905`, 0.8457893355464972`,
0.3136806500838595`, 0.06778013214591205`, 0.09196917164932833`, 3408.380878728516`, 174.30204224938495`,
0.10551969253057247`, 1.6463871196433963`, 0.4159425601435516`, 15652.939934801552`, 9.526496863056764`,
78.02172832069927`, 0, 6.218305623436127`, 0.37278076227874485`, 409.55622857341785`, 1097.651727814147`,
9520.011857915368`, 59.60849695681959`, −0.993738611059895`, 7.71716834340773`, 6118.178495541163`,
0.20972467242662088`, 1.0000000000002336`, 0.6207893087461605`, −0.37921069125398454`, 0.01764477841722393`},
{0.2145692515459468 6`, 2.7215826602358453`, 4.860741945426051`, 1.4762193210184287`, 0.8476298137931575`,
0.5583227752271869`, 0.16730998643348832`, 0.023150536959116674`, 1148.3854317955947`, 98.18795037270098`,
0.13536301841717513`, 8.630410561575445`, 0.520586144335875`, 50492.86149808243`, 234.13344415928538`,
1235.9160861189268`, 0, 237.1378168960563`, 1.9570407386399413`, 314.6684763851814`, 159.78790296611933`,
12342.980837980494`, 11.145001986490943`, −0.9990970574990932`, 76.089259137966`, 37834.63086073474`,
3.3026725286922707`, 1.0000000000002116`, 0.09147650308763157`, −0.9085234969123878`, 0.14914406137319133`},
{0.2192605474178046`, 3.4277372937593142`, 0.317243264743297`, 1.207905605629452`, 0.354638331302892`,
0.2232326751046343`, 0.19168786121654457`, 0.1758842660002451`, 1819.052685961844`, 205.84067214106688`,
0.24408767199328762`, 9.526826970263976`, 0.6180461674628193`, 27552.269051174484`, 50.80747588949222`,
423.4067233042655`, 0, 6.440923723966631`, 0.32115951509539864`, 394.42503658697797`, 1227.1281358573985`,
6662.106721790489`, 68.42158799802368`, −0.9897297370253422`, 15.726434959116471`, 20867.673811080243`,
1.954787529093579`, 1.0000000000004263`, 0.6928357276920069`, −0.30716427230828847`, 0.06853988136724469`},
{0.09281080427028687`, 3.9541950243176203`, 2.7903763295535473`, 1.1746304733040271`, 0.7021084302247975`,
0.18301687365086206`, 0.15380914186275987`, 0.31491382108322413`, 1955.6345018254533`, 45.589098202841626`,
0.16844466131119568`, 4.505740157817099`, 0.739135067608975`, 81442.45896055033`, 27.893957831867557`,

1097.3935714852926`, 0, 17.06242243460875`, 1.3245075752048132`, 1159.0391717458729`, 874.0717575674465`,
34975.846022779144`, 47.70394850220675`, −0.9986360887890706`, 74.8194466220837`, 46373.3771343977`,
1.2898261191524343`, 1.0000000000001104`, 0.49230647525588644`, −0.5076935247441678`, 0.01562943327785717`},
{0.07553841622273444`, 3.998538946411479`, 5.0568584828370255`, 0.9418368972909419`, 0.5147743656673969`,
0.5745728154097487`, 0.1277108500152948`, 0.02699422488607613`, 3283.474706944715`, 85.73668262235145`,
0.1900835508559061`, 1.7563639079857047`, 1.1017643436886124`, 13212.325024267619`, 9.294094911558208`,
171.14467005646603`, 0, 48.09498926801176`, 0.3168483469196249`, 63.26504131739701`, 198.66978503266577`,
6322.7615623764095`, 8.783793243176438`, −0.9986107663310532`, 18.099006503774525`, 6823.01992251283`,
0.6945659456997274`, 1.0000000000010583`, 0.11305702079737032`, −0.8869429792027493`, 0.024543781220805246`},
{0.17556485477494027`, 3.9790149464214117`, 7.860200999240184`, 1.4870239255992979`, 0.43953202100784705`,
0.2354118710730827`, 0.08492480553651033`, 0.015229986606251729`, 2122.168041719343`, 48.98316603866812`,
0.24455585463944812`, 1.202710250977347`, 0.4184441960486769`, 16679.90172034924`, 5.641063928758047`,
166.97091236196292`, 0, 57.61196689500861`, 0.21618559119467087`, 51.52870744886456`, 237.35403258889704`,
4734.728231139634`, 16.41489195326788`, −0.9965330867682521`, 12.288652836636333`, 11875.026775697685`,
0.23217788521882649`, 1.0000000000009504`, 0.13409494770444422`, −0.8659050522956833`, 0.005271229581496665`},
{0.24054054866212005`, 3.45017251549384`, 2.434688830833908`, 1.0572260280057717`, 0.42678683298038966`,
0.6433017075143015`, 0.14198529935191645`, 0.0642120318165855`, 1461.85471205871`, 46.12110813109672`,
0.14351244735387614`, 4.149492178713276`, 0.5419589373402318`, 155374.03583381997`, 48.43665209572478`,
754.0902522056126`, 0, 39.16914463589972`, 0.7128981889071542`, 1478.214642875815`, 2072.5284026206627`,
35006.47874885498`, 101.78109435816452`, −0.9970925069302637`, 35.13745339589717`, 120292.53721397763`,
1.602803443084523`, 1.0000000000000273`, 1.1688361233867062`, 0.1688361233866742`, 0.021271272041126027`},
{0.2576647285795562`, 1.7400215170432691`, 5.671933058359681`, 1.159126105619701`, 0.5426396846396173`,
0.16289934098722736`, 0.12398680737225279`, 0.2145763112631837`, 795.8175406593573`, 85.6025153828084`,
0.04739051555756513`, 6.248380217555781`, 0.8502018161731435`, 137087.511874311`, 77.49333344678588`,
645.7112541921704`, 0, 65.97528903239174`, 7.670817486365587`, 3997.5621637604445`, 520.1390012688828`,
29229.948727624153`, 28.12133867817356`, −0.9990379271978812`, 190.67696399411355`, 107593.24007568022`,
3.7130064093803137`, 1.000000000001043`, 0.29397672072187925`, −0.7060232792784273`, 0.06010713835954297`},
{0.06006756967417276`, 3.885393698216971`, 2.4182637836940106`, 1.1497042341120063`, 0.852306387023593`,
0.3634054456246256`, 0.23851842406334756`, 0.1905044887986902`, 1969.8862529979606`, 336.32300959810993`,
0.04225162262081883`, 6.207670246795658`, 1.4904811957625173`, 3923.8618368243033`, 72.44301544227577`,
320.95851812178336`, 0, 32.77731998422521`, 1.4967578543025366`, 50.78226084706333`, 32.928173953513`,

2048.5794203408805`, 1.8849631841447123`, -0.9990798681440278`, 83.07847906948336`, 1757.902672348605`,
1.8961607474468871`, 1.0000000000003517`, 0.019865293614572874`, -0.9801347063854341`, 0.3703420769232921`},
{0.08502696111412816`, 3.6275157197896295`, 7.363159164909369`, 1.488436793334436`, 0.26568493648971336`, 0.6629645451225747`,
0.17264441279507348`, 0.30453583199957635`, 1224.7690817107496`, 42.94433873275722`, 0.17512531432720302`, 7.6510105838564435`,
0.2548403509235575`, 40266.245289300256`, 92.11916700592842`, 1439.7690230948174`, 0, 79.6090359731854`, 6.484266166548099`,
619.8322545470016`, 94.59019303443701`, 17990.979061331258`, 6.676551519086284`, -0.9996288944867132`, 336.0253921493319`,
21853.11824361299`, 2.014371928060684`, 1.`, 0.05432149776192914`, -0.9456785022380708`, 0.10905340541575315`},
{0.14827779601188723`, 0.9768409840676426`, 0.6891894673357726`, 0.9389444267785162`, 0.30176378998265774`,
0.1751647596247058`, 0.07879547499477868`, 0.023619248417657335`, 3252.235532426287`, 167.1595777298993`,
0.05956658968902967`, 2.6817550689507357`, 0.5206583529379527`, 58602.070444886835`, 6.710526464600179`,
102.5460728575649`, 0, 10.253449261493868`, 0.23066700033534746`, 988.6241310092084`, 4284.936564709866`,
18788.83509445322`, 185.06335761448256`, -0.9901503548951198`, 3.2189282799930248`, 39799.529391947544`,
0.3517260443312846`, 1.000000000000001`, 2.409732364648609`, 1.4097323646486064`, 0.010097889642220416`},
{0.09951701144549052`, 3.3599674683318668`, 5.87127293585465`, 1.0675363096846835`, 0.6922846316607385`,
0.4471508361318377`, 0.09256546388382819`, 0.022405598476994366`, 2159.6702917097355`, 22.498867590732686`,
0.07537929727214032`, 4.117708447722572`, 0.7807622026379022`, 317239.24108718213`, 37.45425903741472`,
1595.5772817996572`, 0, 135.72198337048923`, 0.8850505244745601`, 1569.8321025869805`, 1772.7203235023942`,
130926.11359254454`, 87.88472781630321`, -0.9993287456153338`, 42.48201385806534`, 186133.9363557556`,
2.0163998730361796`, 1.0000000000000084`, 0.9972697168672067`, -0.0027302831328017696`, 0.011982520947525936`},
{0.24542600753982147`, 1.0072788201913916`, 4.873030125775312`, 1.3215761420654866`, 0.934997683525973`,
0.5336155406906149`, 0.1530570292862033`, 0.37775895009481864`, 1381.366911360472`, 23.478593484050236`,
0.23171577878109345`, 1.0189498874051495`, 0.9824058311213721`, 243332.09652504788`, 25.715336220067883`,
599.3514602428256`, 0, 5.79667201949011`, 2.0161966067645416`, 13339.507748176922`, 6615.174089085131`,
53992.595970877715`, 403.5571923083322`, -0.9925256938465045`, 29.01245913336675`, 189302.6752263313`,
0.8634023267724725`, 1.0000000000116906`, 3.724440738128676`, 2.7244407380851348`, 0.003950882943234286`},
{0.07009559015680356`, 2.7849471367228507`, 3.0258579570105155`, 1.1102521989309375`, 0.4599988219148854`,
0.6809018065475554`, 0.21885987895906978`, 0.1581032784122877`, 1010.998941039169`, 191.1453687810041`,
0.0813026067841443`, 7.337454751758624`, 0.7004457216179918`, 16343.453968056438`, 134.83398892511696`,
399.239149732994`, 0, 54.57414166908641`, 2.923135204143835`, 277.7494204229141`, 94.0176440792669`,
8079.29034292931`, 5.127691607395754`, -0.9993653289596303`, 116.29681452905862`, 8090.3232090827705`,
3.186338149762233`, 1.0000000000004732`, 0.055945281483975415`, -0.944054718516051`, 0.4430026656564826`},

{0.0818655292269884`, 3.5534207813275085`, 2.793085272759267`, 1.44138898302728`, 0.2645186976244649`, 0.667000460134499`, 0.1031352647835832`, 0.11738652278758988`, 1616.7923697759452`, 22.69493932260025`, 0.19900500778151703`, 7.38154340093187`, 0.5255971444464016`, 159262.02640935045`, 67.46671729665387`, 2246.122729043935`, 0, 57.26981785746889`, 1.849444821270482`, 1927.3979482070522`, 1041.1494742854886`, 73338.70155168147`, 69.83479965517787`, -0.9990477769829894`, 93.8836523117294`, 85770.165933551`, 3.07526878762087`, 1.0000000000001719`, 0.5870761764498703`, -0.4129238235502306`, 0.03323985780060725`},
{0.16851133973676596`, 2.4752237313475174`, 2.42575793497873`, 1.0578005973522087`, 0.5315655422073631`, 0.41472938652046143`, 0.24177717448224295`, 0.0988063583504681`, 1028.5760265584995`, 54.93252411146938`, 0.04650878688675153`, 2.5043219440736237`, 0.71806339741822`, 348998.1530895664`, 36.26591306309482`, 515.7348805026295`, 0, 18.83180545761012`, 0.7274369673946695`, 5618.12369468463`, 7722.17595407071`, 102413.16415973555`, 380.0999779152006`, -0.9962885632815489`, 25.72241778221067`, 246539.70713197833`, 1.2733699898847166`, 1.0000000000009048`, 4.366154517255134`, 3.366154517251183`, 0.02249009710266527`},
{0.17393597413635692`, 1.5761991979266723`, 3.500768537824096`, 1.241966650800916`, 0.7553239598200872`, 0.5647220891577981`, 0.0698456460365596`, 0.07461556744160115`, 1637.6446967775864`, 102.27592905310792`, 0.14712502478326317`, 2.669212259273973`, 0.5885270492629138`, 33076.21500127532`, 40.995334558812644`, 208.92650871098598`, 0, 34.02712103039133`, 1.527959175790097`, 725.6811808041887`, 473.9349277796928`, 9471.492773031534`, 27.519554340203268`, -0.997094486054135`, 34.4052575335007`, 23534.76174289569`, 1.0147585474022478`, 0.9999999999999999`, 0.26901896065324205`, -0.730981039346758`, 0.03601205536062305`},
{0.06303562103190802`, 2.7937374046310888`, 1.991826202033483`, 1.3853239568680058`, 0.8642226718049046`, 0.42627609641461395`, 0.1739341576099066`, 0.013248801141587125`, 3504.113575209887`, 145.60993330770657`, 0.09071202532973738`, 5.892751585058276`, 1.3807549697256851`, 43165.03198862283`, 44.22095650012901`, 587.1866182549933`, 0, 74.24800478270457`, 0.3411042183185655`, 167.9173255825645`, 491.27572268174777`, 22665.797524168163`, 31.93933369860871`, -0.9985908577157034`, 13.61365162277178`, 20410.7517588489`, 1.9797290259776454`, 1.0000000000000002`, 0.2794963140758257`, -0.7205036859241744`, 0.10589773013886339`},
{0.2432575073739432`, 0.6270765712575339`, 1.9550603903514805`, 1.0314330185313336`, 0.13493745232200305`, 0.4469694845616632`, 0.19582833943170452`, 0.02468113928377954`, 1814.663806970997`, 145.9448155579372`, 0.17557852480928543`, 9.315910864758589`, 1.2079900473113105`, 40534.1679147412`, 55.01730630813117`, 492.8974447463634`, 0, 100.85665215483887`, 3.5535845820122605`, 1061.0614683175183`, 297.5890567199274`, 9027.246967366122`, 14.388301543277608`, -0.9984061251901835`, 31.83385193374114`, 31370.651367578233`, 3.8787349137925786`, 1.0000000000000009`, 0.16914117881141613`, -0.830858821188584`, 0.11943075551184866`},
{0.11357589384964273`, 2.240672872189519`, 7.052253737321249`, 0.7646938475078471`, 0.009142046869567766`, 0.5509926958854778`, 0.24090899686155176`, 0.02638287046938754`, 2863.3930915889323`, 84.10264867474814`,

0.16678159718247448`, 7.7951222229657064`, 0.5053738528157279`, 8439.877816814316`, 29.02335448601901`,
698.1094839727773`, 0, 298.32892054674664`, 3.396840359505256`, 69.98708781120563`, 19.603584626920508`,
3061.703369112861`, 0.7368797427472885`, -0.9997593236006528`, 108.7315434957422`, 4967.652812135066`,
1.236064455912526`, 1.0000000000000009`, 0.011668066429192535`, -0.9883319335708075`, 0.0845674690941055`},
{0.1116182977145373`, 2.360842887008661`, 0.9627021602560966`, 1.1405981578601385`, 0.14103896539205096`,
0.5791003705327337`, 0.05180942465934579`, 0.03555380982336255`, 762.58124752924`, 235.4407827441829`,
0.05499220801683008`, 8.486879923307693`, 1.2577154495282135`, 49571.38641645803`, 136.2438341030107`,
203.4558316559039`, 0, 41.564016549856525`, 0.595097774681289`, 480.8221293145871`, 806.9716473013136`,
19082.000543809765`, 43.3471416663642`, -0.9977283754097562`, 20.070462120442922`, 30427.14882411329`,
5.1570824915565`, 1.0000000000000033`, 0.46111011998448315`, -0.5388898800155184`, 0.2658418287590713`},
{0.12701256602307204`, 3.4510594830271417`, 0.17236039015202387`, 1.4218046895736245`, 0.5977738378490018`,
0.6174455495269762`, 0.06547152715546964`, 0.021505928148282425`, 2080.7788466921183`, 285.32441857492176`,
0.13527159522812743`, 2.0719517072723157`, 0.8493272554882347`, 27671.48594250556`, 21.73768657863022`,
54.445817151018794`, 0, 2.062018087135592`, 0.012474632323150228`, 129.29035242989133`, 10363.261573461874`,
9830.924561287926`, 646.1809408780313`, -0.9342705829091037`, 0.6150099739440633`, 17837.87078440606`,
0.6128070212455148`, 1.0000000000000062`, 5.888829444181554`, 4.888829444181518`, 0.04094503193115389`},
{0.16525167732730567`, 1.567211428071798`, 0.46961269510991777`, 1.0926048049265726`, 0.5982927168612024`,
0.4872752592438997`, 0.13461015322956665`, 0.008651638441061493`, 1318.8282746769155`, 92.92105735217046`,
0.15473787356946228`, 9.951552143172052`, 0.8370529835607092`, 74147.72398760846`, 139.79241656466428`,
1047.3131484657997`, 0, 31.02283154984112`, 0.16335724170861105`, 348.7238772572986`, 2133.73166913124`,
22052.551501132708`, 108.29700493395184`, -0.9950891394617811`, 3.6573619437717415`, 52060.301784413896`,
4.936563112065349`, 1.0000000000000033`, 1.2058152309168797`, 0.2058152309168757`, 0.09975127490759018`},
{0.26512296149188996`, 3.233300402128563`, 3.292718317720548`, 1.2400916214421978`, 0.5963811820900797`,
0.3451658167692204`, 0.10292393665820598`, 0.09694787300120739`, 3191.5882949078978`, 195.13476182743136`,
0.1886240896532882`, 4.4133830990517655`, 1.1858146199794088`, 4261.1545362867655`, 20.52454800587001`,
192.079957884837`, 0, 46.35977656740133`, 1.333183760789742`, 52.436615129557126`, 38.33187357344879`,
867.2390678622627`, 2.2770132732264763`, -0.9973744110965396`, 61.57976556961051`, 3284.6427141872696`,
1.2703175088443375`, 0.9999999999993857`, 0.02228510423475633`, -0.97771489576523`, 0.08007597750755265`},
{0.051008388371518276`, 3.802328793778008`, 0.48994620522738513`, 1.2897891346361594`, 0.07123389103998612`,
0.5215810111010959`, 0.05373739522257526`, 0.03556482649077374`, 3356.8546057655267`, 95.53240615712758`,
0.0371102186886971 8`, 1.106351331760008`, 0.48227443908576983`, 134559.70514914623`, 3.5201220389080934`,

60.349535193088734`, 0, 2.6818424265095953`, 0.02451618895515633`, 819.4874318479419`, 33425.37933436161`,
77835.45617003912`, 1959.4885901409007`, -0.974825244348023`, 1.3316944453984747`, 56718.01681993759`,
0.21149220898187826`, 1.0000000000022453`, 18.86631832408191`, 17.866318324039547`, 0.00934978723905567`},
{0.10228292810378975`, 0.7288905389375508`, 3.433004371974512`, 1.274497031997786`, 0.6101427090150058`,
0.660255061516275`, 0.2170030685035811`, 0.012721245103120268`, 2195.647120536849`, 85.90708396049939`,
0.06296262837152478`, 5.578028384550223`, 0.44836886971981804`, 97599.12271081915`, 56.540967488718465`,
777.377736224291`, 0, 121.12179312689635`, 1.924029750253735`, 1315.1316621463288`, 682.5297957180202`,
39597.17057010952`, 40.73863289661397`, -0.9989711731340883`, 20.03438687991895`, 57858.77929337165`,
1.0936562026322298`, 1.0000000000000004`, 0.3873507755243565`, -0.6126492244756436`, 0.0666310930661303`},
{0.19207820059415281`, 3.655701792168804`, 2.510935445862126`, 1.2073260918388902`, 0.9635406057316576`,
0.42073159663388215`, 0.061393125488842015`, 0.03366936773322557`, 1456.9751171045727`, 203.989380011923`,
0.13700463901391435`, 8.26369238323722`, 0.48805011827851597`, 9993.450398116098`, 188.99510690017965`,
368.6394816554474`, 0, 106.42414374234797`, 0.9498337680122754`, 60.980351259517676`, 63.20107740234562`,
2627.2774968819986`, 3.6455241454262954`, -0.9986124327750865`, 49.60441439978466`, 7209.181915180028`,
2.46162872310506`, 1.0000000000000453`, 0.03660322604367633`, -0.9633967739563253`, 0.2010626618963065`},
{0.2627532955765636`, 1.639112549779572`, 2.4635443376968276`, 1.095970586814591`, 0.7703245554604254`,
0.3899598370308347`, 0.22903116483622232`, 0.3600410344547181`, 1714.6905884118041`, 141.27158792075448`,
0.22143418867904247`, 4.170924599810817`, 1.4302741156765273`, 33686.182439865435`, 49.96298954312423`,
438.9119617078995`, 0, 12.797242813310262`, 2.63123361966045`, 1155.104501204102`, 437.99731767381553`,
7070.222656447356`, 22.485416065253315`, -0.9968197018456344`, 61.61268639124796`, 26538.918620594675`,
1.9648197333536572`, 1.0000000000154317`, 0.2491567554755845`, -0.7508432445282605`, 0.0625555531091185`},
{0.23274157877277596`, 1.9065983093139076`, 3.8223892292706183`, 1.0506044130310364`, 0.03733185970169073`,
0.2700778128853568`, 0.09296420307934006`, 0.03280802595849235`, 1773.9605198177533`, 179.0899731082768`,
0.06001861030669786`, 8.285893379373807`, 1.1764377117465887`, 5160.904039924009`, 37.44380118041848`,
275.87057677138426`, 0, 163.9024055687985`, 2.6719297208055615`, 58.32550183222651`, 20.828980522227926`,
1137.9583705102632`, 1.0737988915058028`, -0.9990563812180367`, 72.77566697562095`, 3783.574681860787`,
3.3272729373505507`, 0.9999999999998677`, 0.012384119299602819`, -0.9876158807003955`, 0.19479440805367293`},
{0.16707182228455675`, 3.9273258352780225`, 7.016188766085528`, 0.799128142527102`, 0.012996916454743213`,
0.30869623835953575`, 0.08235696915123317`, 0.07054168097616643`, 791.6946431232782`, 51.289432144121236`,
0.13635302362949547`, 6.005214476564535`, 0.8946308584625005`, 54619.13336701747`, 62.262224502649694`,
665.8611002894562`, 0, 157.22225168556363`, 2.7479809489258322`, 480.0803775547986`, 173.70294972112484`,

16034.588601337233`, 6.500990241929915`, -0.9995945645751467`, 154.17452250811786`, 38270.3991029799`,
5.421759747907101`, 1.0000000000000502`, 0.09852095420508605`, -0.9014790457949189`, 0.05578068505349952`},
{0.22880237101805018`, 3.9696692691682696`, 1.9380469146031594`, 1.4177879339964377`, 0.6814855489346618`,
0.5439318492089463`, 0.21630816993687996`, 0.03709761261377448`, 3976.9551811029105`, 217.4437376330418`,
0.19674001267675278`, 8.838870490399184`, 0.20990582924569878`, 6794.233579799393`, 49.61778440153105`,
536.160421070648`, 0, 84.13891005984597`, 0.7697937971275673`, 40.78109427642014`, 51.976647030142345`,
1561.5551717257683`, 3.5225054355828225`, -0.9977442324809505`, 43.654668286480785`, 5104.107510947934`,
0.6151961841025623`, 0.9999999999999996`, 0.03014345363054404`, -0.969856546369456`, 0.14122766426419953`},
{0.1343775710472654`, 3.3862425535357383`, 3.6167615926265064`, 1.4522710413875881`, 0.41930411990329275`,
0.6633621503450218`, 0.2054877494749729`, 0.014727056691440865`, 2132.348768069086`, 153.41895938212008`,
0.07798120175302098`, 2.3819365486087474`, 1.4841799853609898`, 37811.78878563282`, 18.99885930171142`,
146.38684531062754`, 0, 54.383594947427724`, 0.229385344759802`, 133.11128903784757`, 579.295524882958`,
12928.14792642813`, 39.89404836180367`, -0.9969141714196934`, 11.096491651187192`, 24817.90166418782`,
1.1515179636625714`, 1.0000000000000004`, 0.33357288807640484`, -0.6664271119235954`, 0.07962503457076806`},
{0.1877085328273454`, 2.4698348389855864`, 6.723740484115233`, 0.7818601707856943`, 0.1041858328478078`,
0.45810434108336306`, 0.1358172536153655`, 0.022077708334214 59`, 1185.161236523103`, 108.74647947804544`,
0.020759548694208763`, 9.734619845644367`, 1.2692223563341272`, 20918.045036087802`, 86.79021162533793`,
608.1467023989247`, 0, 381.0595252272154`, 3.261581453545584`, 138.23345005192698`, 41.38233875829066`,
5546.1766364207915`, 1.5682797291921315`, -0.9997172322787389`, 115.0795357736594`, 14872.352560340705`,
6.7368568475874`, 0.9999999999999988`, 0.02428872664107485`, -0.9757112733589252`, 0.3278522496360188`},
{0.1416858046718097`, 2.2139997848909125`, 1.7657175272923222`, 0.989271832961955`, 0.13788431085704778`,
0.5209143755701833`, 0.21392682943087216`, 0.02217837535493898`, 1932.3838117923324`, 368.90142938726285`,
0.22169599764103476`, 7.130639713263575`, 1.0904656940263826`, 3215.489213737869`, 42.45702037360813`,
152.78613970816866`, 0, 74.27232312081146`, 0.7108921167299367`, 23.71099169369251`, 32.353853750357686`,
1031.0561138261303`, 1.5837579050196453`, -0.9984639459639665`, 22.484499907438952`, 2086.943073560635`,
1.883759071183829`, 1.0000000000002012`, 0.019409347149015987`, -0.9805906528509879`, 0.34841739196863414`},
{0.10996511298893497`, 0.7862218358455948`, 0.8377076631006055`, 1.462919598856414`, 0.4311311224143921`,
0.3856327187176355`, 0.0881986225258021`, 0.45328063368772087`, 3501.1649053241954`, 360.18740869055375`,
0.12284319478126604`, 4.367675405932559`, 1.4276764859907565`, 15433.858504073954`, 16.23481089907895`,
87.51817428768446`, 0, 3.9069183865749713`, 1.9307022291201468`, 1092.996465125466`, 565.1134320146109`,
5992.513781869468`, 38.4929871231838`, -0.9935764875101922`, 21.68514644357186`, 9413.820787300358`,

1.0519873176169994`, 1.0000000000019147`, 0.32058652435045865`, -0.6794134756501552`, 0.08056893866488278`},
{0.1676455194581572`, 2.242553618614152`, 0.4702874552580045`, 1.338910822252204`, 0.5222950299457514`,
0.5002656056336167`, 0.18155773122559443`, 0.023205215351886358`, 1812.861489403048`, 208.09707961592778`,
0.04482084976511336`, 4.8128979171410045`, 1.2921971759159723`, 140039.67015250266`, 44.59617336687893`,
235.1163613652542`, 0, 12.811171572150116`, 0.1276558750153165`, 1055.365024031753`, 8266.265599057837`,
41244.55907391927`, 513.5877156338364`, -0.9875477462442167`, 4.089644920756474`, 98778.0790109976`,
1.8514105235823748`, 1.0000000000000007`, 4.70212994586233`, 3.7021299458623265`, 0.08996199595893722`},
{0.18135996247125585`, 0.9876459274110312`, 2.54354039452296`, 1.3578342543318755`, 0.8930073147262432`,
0.19298717253815956`, 0.057956724513287594`, 0.0261548554577047`, 3458.0576394641494`, 159.9558341984582`,
0.04888106360623701`, 2.7801567336765522`, 1.2025006457139424`, 62822.641894580236`, 12.638791350813488`,
141.01573886320986`, 0, 38.354431052009815`, 0.9410137112334104`, 1130.9802979292974`, 1200.8744088722076`,
17480.525636870134`, 75.40537863656147`, -0.995686320869121`, 13.276976564823148`, 45289.535335437`,
0.8219059181623707`, 1.`, 0.6751835749218127`, -0.3248164250781873`, 0.01821913141465735`},
{0.11830138320073424`, 2.557281635675193`, 6.588149241112692`, 0.9721130031371765`, 0.10225884860358048`, 0.23082972097158927`,
0.158140431907085`, 0.0665459599837257`, 1023.3689587458948`, 34.93006289140902`, 0.04839524009283808`, 8.085053919685542`,
0.4877285564517453`, 268244.22690557974`, 62.64875350408371`, 1627.9996375070914`, 0, 202.8842755971497`, 5.123597213499649`,
3483.089493257365`, 678.813292910716`, 99571.39854679561`, 30.72487507047181`, -0.9996914287082547`, 187.17830089541778`,
168277.6310759642`, 3.0543451193371056`, 1.`, 0.3827345135063308`, -0.6172654864936692`, 0.03435196973717193`},
{0.1828220078605639`, 2.600312322938115`, 2.7331064358556674`, 1.452306554796921`, 0.9684456895629803`, 0.28153635898957774`,
0.13708089241679355`, 0.10257553866825626`, 1556.8951227385596`, 164.52245666603233`, 0.13014520838075316`, 8.353642416878472`,
1.085154972102886`, 33606.55407823631`, 131.29307570821146`, 737.9052681713084`, 0, 70.08285861428463`, 2.651952400748875`,
523.6300142345384`, 196.4507589527898`, 9257.388080637018`, 13.401304684973368`, -0.9985523665456995`, 98.51292153589448`,
24177.91823495011`, 3.2934032629475523`, 1.`, 0.11186781352276677`, -0.8881321864772332`, 0.12886777899146162`},
{0.21889388250937136`, 0.5333175361369764`, 2.4121127215145144`, 1.3818810352832893`, 0.021571596232064838`,
0.5244501966414251`, 0.09076416020812753`, 0.010775282992960485`, 1502.4651132234349`, 214.3639418846526`,
0.02290218457939025`, 5.386530044496094`, 1.4845771171901991`, 106475.06269916309`, 37.43859453682166`,
145.93931853746557`, 0, 85.40913203241482`, 1.5053015709337767`, 1647.973380894281`, 1093.779552958283`,
25775.42667260286`, 70.89896921021081`, -0.9972493580761722`, 11.468624642193179`, 80601.18882430949`,
2.678441529840758`, 1.`, 0.6228089014446676`, -0.3771910985553324`, 0.13975930171248327`},
{0.08165938583300264`, 3.5663918013786944`, 1.9719703539316173`, 1.0863058189118182`, 0.9278717656325091`,
0.29627245453078765`, 0.07723831560352282`, 0.005496209135881868`, 2965.7691899558304`, 36.38856085381832`,

```
0.19440710566976382`, 4.620185477920922`, 1.4555441950836703`, 86923.6052242615`, 35.61209605242687`,
 1232.8203035933977`, 0, 62.63069252071696`, 0.09433053929233517`, 121.81568322722563`, 1290.3705798894312`,
 40089.271111946684`, 64.96051597043981`, -0.9983796034657492`, 4.805995170740209`, 46766.64653563282`,
 3.653864600369949`, 1.`, 0.7250893573128431`, -0.2749106426871569`, 0.02020619397460773`},
{0.10569564926832292`, 3.94925758524324`, 2.123437162636053`, 0.9889632230650253`, 0.6091744144977718`,
 0.44858710373952704`, 0.06967757399007424`, 0.06579434912735219`, 2048.3556961935665`, 192.929988723693`,
 0.1607054226404262`, 3.065879080542288`, 0.48218127318000126`, 7295.868281858731`, 25.894801241559875`,
 116.51824556212729`, 0, 25.469639753712737`, 0.41145557432480634`, 61.565311596126676`, 148.6280897327849`,
 2887.2319233247194`, 6.941407322156864`, -0.9975958262077667`, 23.213486398469318`, 4359.5407532004965`,
 0.7034876533184491`, 0.9999999999999993`, 0.0851718618797937`, -0.9148281381202062`, 0.06417062818247701`},
{0.24619035501818215`, 2.0773850626788413`, 3.018461478313549`, 0.8871662168885301`, 0.552617674236112`,
 0.602322011487733`, 0.20434748314233808`, 0.039023258548983815`, 3150.0536016394335`, 203.54823250165975`,
 0.24910387895313074`, 1.7034902327529498`, 0.8084130559906435`, 4637.936547136123`, 10.190724289882855`,
 88.38879165559429`, 0, 24.995970131475435`, 0.45079062414736426`, 54.11495328465479`, 119.04454038281975`,
 1018.1768015042472`, 4.999285857458443`, -0.9950899629120674`, 13.378081557134443`, 3580.9329747658207`,
 0.3471599865404574`, 1.000000000001406`, 0.06855089185213879`, -0.9314491081479576`, 0.03802134441248865`},
{0.16802857593346404`, 0.7065678176512091`, 9.645249981168057`, 1.3116063027060454`, 0.32088080529355767`,
 0.3691390904550721`, 0.21874840715793664`, 0.07094779858961066`, 1630.2542224830704`, 63.80576916127296`,
 0.10975436657116389`, 7.114567585043865`, 0.902530053910509`, 48182.17890510033`, 50.083931868098055`,
 1023.1059629001267`, 0, 255.7163270497367`, 23.207751353763367`, 2186.178981217733`, 93.20037934279327`,
 14018.610442150939`, 5.779282655298622`, -0.9995877421175838`, 234.25500323743594`, 33650.387845151665`,
 3.0752738160552737`, 1.0000000000000107`, 0.053362728915921684`, -0.9466372710840789`, 0.08809293631181297`},
{0.12945851018082827`, 1.6530391126563773`, 0.25296383859783056`, 1.0004082847943978`, 0.6153536990324371`,
 0.46726548359691056`, 0.22184757314007908`, 0.029263173246622916`, 2804.4069174712213`, 201.7772707782526`,
 0.15804840166726702`, 6.079299069162072`, 0.5497928673982018`, 25248.551839666565`, 39.86738197690221`,
 371.66524802557564`, 0, 8.111949485454634`, 0.13729064707110947`, 302.39363223020126`, 2201.5799912908633`,
 8856.950319951087`, 101.9295827116728`, -0.988491571135714`, 3.242097277292097`, 16380.108473806758`,
 0.918746608292876`, 1.`, 1.2477802700176148`, 0.24778027001761482`, 0.07056826770232949`},
{0.17704121544802504`, 3.3101767206813077`, 0.17894892947703944`, 0.9519221706044084`, 0.3716159634368834`,
 0.17251563798408087`, 0.06428454684265117`, 0.050202794311224846`, 1196.331297175113`, 92.7477589491034`,
 0.08675697489654427`, 7.03036379115748`, 0.9556798715945236`, 117449.2971966462`, 52.2384533451025`,
```

499.20249998551753`, 0, 5.449912080112843`, 0.08046922284396561`, 1015.8331544963805`, 12622.87181824955`,
33277.02740516508`, 548.7162023612301`, -0.9835106605022039`, 3.8052478312773035`, 84162.93397582357`,
3.823065877043781`, 1.`, 7.0950769747234075`, 6.0950769747234075`, 0.03139225448527383`},
{0.27321591675550233`, 0.6886264672292262`, 5.359177927011217`, 1.2203089609351563`, 0.741856408018519`,
0.5849406177035197`, 0.07433546983180528`, 0.21690743690508418`, 1384.5673397625906`, 70.87997184926286`,
0.033379798700165386`, 1.435341337958821`, 1.463005840477979`, 308974.4105883591`, 25.627025875066813`,
160.91520790028764`, 0, 14.288661763514668`, 3.983058385773095`, 21553.867006806035`, 5410.386155872912`,
63004.61553698024`, 306.22913620014043`, -0.9951395761470777`, 39.1834203566095З`, 245912.339910916`,
1.4408696491843795`, 1.0000000000029847`, 3.0526902241340657`, 2.0526902241249543`, 0.01595865594165926`},
{0.08919135792007882`, 1.9079775156637941`, 0.7718276581060107`, 1.2887666627276828`, 0.6966500412335945`,
0.2943209647073368`, 0.16567285203320248`, 0.2074475031655441`, 1406.902570296439`, 161.54627762916846`,
0.02754527329468942`, 1.9201358824343482`, 0.9977637383308959`, 173396.53330194767`, 20.804019896980947`,
133.335970166387`, 0, 2.8673513883837463`, 0.2932593601831985`, 4588.222720172724`, 15644.613893812191`,
76241.3902956878`, 935.0326168635487`, -0.9877358923645384`, 7.993318078392785`, 97143.90185981571`,
0.75283073679418`, 1.0000000000001736`, 8.892046024178468`, 7.892046024176924`, 0.03638705485397602`},
{0.09177879720493187`, 2.6455642646824806`, 3.5115323646466643`, 1.0647038743499257`, 0.023437829577520386`,
0.20767277287096642`, 0.13833050574986264`, 0.16097998475232683`, 3330.8307600954595`, 36.94740532139957`,
0.0838397051953012`, 4.654426462364112`, 1.1418435912898404`, 190666.9025923754`, 9.96963982821956`,
799.7146878547653`, 0, 36.84490535294658`, 2.173072125210839`, 3425.396109162528`, 1575.291955256747`,
82447.13059453324`, 77.90299939381364`, -0.9990551156986053`, 82.1285994147913`, 108098.54969948808`,
1.7909114517969034`, 1.000000000000601`, 0.8865965870685214`, -0.1134034129320115`, 0.015469864189319654`},
{0.27348098404578464`, 2.2019037456007027`, 4.59402198031265`, 0.8385696628288007`, 0.6096935872297715`,
0.3604633388375672`, 0.23592549449411898`, 0.025613961789364897`, 724.0273482822054`, 51.59152478645882`,
0.07558496433041467`, 2.104114801800101`, 0.41664957185729956`, 186114.85461953908`, 43.92741337943161`,
492.8138876748365`, 0, 52.67925822038102`, 0.592596529717856`, 1536.1887967034095`, 2591.3013714488197`,
37914.77757942406`, 101.09316370200283`, -0.9973336738296767`, 18.64057883451041`, 148128.152604256`,
0.9720301844948047`, 1.0000000000001759`, 1.4632958112762064`, 0.4632958112759491`, 0.01507487345368329`},
{0.1382612217370991`, 3.544859312958268`, 4.625085964177936`, 0.9631685672533119`, 0.6192785632371416`,
0.30258401315557804`, 0.17357832021845082`, 0.08489498345439296`, 3734.2868382787856`, 118.6130866776067`,
0.14202527245998875`, 6.035316827283889`, 1.23738828709505`, 16188.47887293407`, 23.19290565637329`,
554.9550148081265`, 0, 94.75627825534099`, 2.205013339999241`, 171.813529172501`, 76.91949647459282`,

5371.089930494626`, 3.504546490680833`, −0.9993475166984668`, 111.66374390705073`, 10608.763655000264`,
1.7350293939422723`, 1.000000000000432`, 0.04407575419268901`, −0.95592424580733`, 0.07131541044478593`},
{0.20062439708640356`, 3.062479927466259`, 1.8588498709761279`, 1.1971327198257382`, 0.2717889530441755`,
0.5772443937125967`, 0.13761254703825482`, 0.02383075282424653`, 748.4785019436172`, 123.7894814607249 1`,
0.21845089918107502`, 7.7975595199904255`, 0.4961018196149929`, 37103.96372359961`, 143.42946385779817`,
476.3124053698039`, 0, 81.68285541805356`, 0.6176945378374576`, 210.5826619618841`, 339.91715089327465`,
9569.067957367472`, 19.200522342161634`, −0.9979934804071092`, 27.023958906182195`, 27425.549851795262`,
4.009782077102532`, 1.0000000000002425`, 0.1945498430871421 5`, −0.80545015691290 5`, 0.165577234902949 1`},
{0.1825851308210345`, 2.4396329783561983`, 0.6407630961927655`, 1.1714042800046478`, 0.8170667200239692`,
0.31301546820459225`, 0.11111924655180566`, 0.011382861853813236`, 3697.837667320504`, 70.61298960423318`,
0.08120950744691857`, 1.4309282478289982`, 0.7664004986892703`, 169986.78071593988`, 7.381743884401226`,
210.74438890602477`, 0, 5.8394390928423165`, 0.026449553249303922`, 663.1648265630602`, 25071.817688537434`,
47107.28293028169`, 1325.0705512250584`, −0.9718712167461208`, 0.9218171767112883`, 122872.70594927155`,
0.2974690085023755`, 1.0000000000000022`, 14.090077861352622`, 13.09007786135259`, 0.0038046417763553738`},
{0.11761221086988144`, 1.0914541886641524`, 0.5591025160362655`, 1.4775466222869675`, 0.9603313566507792`,
0.2704038317127577`, 0.19659054677243826`, 0.04563469543817772`, 3422.6083976447926`, 269.42977341830294`,
0.0784511002461219`, 5.408577374365102`, 0.46841691657751894`, 42774.04262795602`, 36.489802050724826`,
365.9828363857907`, 0, 13.669636707222056`, 0.5347383816756186`, 1017.3801242821163`, 1901.5754633398963`,
15951.013340047988`, 130.14101830944713`, −0.9918412068540684`, 8.337749235990637`, 26800.48492197168`,
0.5495253070393858`, 1.0000000000000202`, 1.0750270606979748`, 0.07502706069795306`, 0.04916695718502892`},
{0.2429320475534284`, 3.9794232967059395`, 1.0019515993916865`, 1.0030250425538783`, 0.21362795630084253`,
0.32541860695965363`, 0.056903912665728335`, 0.007999914248720421`, 1861.2936578099834`, 133.12444614515988`,
0.12956497529754557`, 5.59967766310799`, 0.3532739838839296`, 49102.731568707044`, 29.566894849453487`,
245.4947687362384`, 0, 37.378167867822434`, 0.073673120137141`, 87.05660405037436`, 1180.660338103236`,
10974.503995595449`, 54.83492541478143`, −0.9950034256275464`, 4.1882361516393525`, 38086.5532361898`,
1.1674995754574542`, 1.0000000000000264`, 0.6651344980301135`, −0.33486550196990406`, 0.030289455498840188`},
{0.26359879599023367`, 1.2163041539701664`, 3.7777909348817094`, 1.254691553657424`, 0.9061229415337297`,
0.533372724047131`, 0.10691633026129371`, 0.27923770769686623`, 2206.202484705579`, 127.40873012168657`,
0.13182990312478088`, 9.420656570983855`, 1.2733451287842463`, 40367.75916777306`, 148.417325880361`,
862.2561807251366`, 0, 54.54323385858706`, 11.525055363969429`, 1756.4748885040544`, 151.40489811400732`,
8414.59988021626`, 8.925197755710252`, −0.9989393200053761`, 200.25675305617318`, 31686.834245208018`,

```
4.582091546941631`, 1.0000000000023344`, 0.08620434223632084`, -0.9137956577638804`, 0.12287079177463386`},
{0.11992752605735896`, 1.1695863474317374`, 5.859787436657632`, 1.088943542117163`, 0.7084196598983477`,
 0.2068540131141825`, 0.10134897101245235`, 0.042086980108994226`, 642.019354817593`, 42.643211093372884`,
 0.14904119135720395`, 5.991168965595664`, 0.5811778237079277`, 81184.31628991745`, 122.36999039746348`,
 1307.7319686656347`, 0, 163.40020744971417`, 5.518730777253022`, 1721.4166112559285`, 310.9225562426825`,
 29825.17820593624`, 15.800755141071146`, -0.9994702209310546`, 92.2090310318068`, 51097.99766368301`,
 4.100032038675853`, 1.0000000000005982`, 0.17574877216274734`, -0.8242512278373578`, 0.04116357086063133`},
{0.09391708894580503`, 3.041674977657679`, 1.8104005667515946`, 0.895918712120295`, 0.2181847991244159`,
 0.2824961199777095`, 0.12004816256362977`, 0.10420303973300599`, 1895.0150961574109`, 50.54358082712821`,
 0.23566298754715342`, 8.257703012553833`, 1.009710413761872`, 46765.91659178202`, 40.80352894460885`,
 1146.9781197940922`, 0, 44.79623159348607`, 1.490604813414364`, 629.3002762533356`, 421.17781036938084`,
 19923.72546010091`, 17.579432141881938`, -0.9991176634020035`, 64.77050517912252`, 26731.118516687133`,
 4.1437486230578475`, 1.0000000000000384`, 0.23774374889879388`, -0.7622562511012152`, 0.04600634914982359`},
{0.16775949346003155`, 3.734350305060132`, 8.90335371268981`, 0.9286973988343983`, 0.35313792052011217`,
 0.3940821550050888`, 0.14910438190884368`, 0.1880509002120729`, 2527.5769345457447`, 29.455979238374994`,
 0.037167259903154726`, 1.2969361348709503`, 1.0523609025190432`, 487233.49838270264`, 5.992657025819448`,
 340.20014820898024`, 0, 23.32365428958708`, 1.1466267595465531`, 6533.181410999471`, 5696.740225060763`,
 143423.7159220493`, 245.59422167394658`, -0.998287631720493`, 61.17008556146825`, 343724.14190340624`,
 0.6785784698194514`, 1.0000000000000524`, 3.206849020212822`, 2.2068490202126543`, 0.005079785908874456`},
{0.11115827720243887`, 2.3474129584418977`, 0.7793271183465329`, 1.2896581551764172`, 0.2400074156115546`,
 0.5586724719427854`, 0.16881638153305045`, 0.3542961312162605`, 3281.9581897651888`, 363.45984424300025`,
 0.19399862158515324`, 1.0793406751665469`, 0.15013258901534354`, 3519.2226084297645`, 4.212204091898173`,
 22.41821261059365`, 0, 1.0952841439004244`, 0.155218749422731`, 85.09249766494179`, 547.2101742309259`,
 1357.3416128791625`, 33.05067356955419`, -0.9756504381388206`, 5.205178625543788`, 2155.425075183261`,
 0.08559302799756857`, 1.0000000000048905`, 0.3179669629435348`, -0.6820330370580202`, 0.04219397020038265`},
{0.05958430298107065`, 0.7208194464934747`, 0.8555730299303229`, 1.0890886690114123`, 0.12206612248038917`,
 0.22763156357860503`, 0.13379446389365296`, 0.0940380442991097`, 890.0674874512752`, 363.7513660796561`,
 0.17126775622478085`, 2.343574670731165`, 0.6210533219689349`, 6151.694357396041`, 20.41851177540731`,
 42.236993334944174`, 0, 6.717510345198682`, 0.7706294313451196`, 312.15798970016`, 404.0688657910377`,
 3314.7204871150097`, 20.845503583403854`, -0.993711233371129`, 7.935495430768153`, 2821.504425740328`,
 0.7698728091842425`, 1.0000000000019353`, 0.2331698860905342`, -0.766830113909171`, 0.09837772202086133`},
```

{0.17145251727538108`, 2.2285999256796796`, 4.099234007029899`, 0.9794004497404906`, 0.5456157460051374`,
0.6134582400670898`, 0.07617057839260499`, 0.06280998418842913`, 3845.79457797157`, 146.34600196120715`,
0.1780830585354753`, 4.861786782312107`, 1.4658755518344249`, 4722.0268353088795`, 24.417069908294312`,
245.48379118610544`, 0, 79.72388249712499`, 2.1367692977056216`, 68.00816574740892`, 30.82756595222208`,
1325.517628803473`, 1.4552961405857132`, -0.9989020922023502`, 68.02862711516258`, 3246.6190593035685`,
2.1174156875603978`, 0.9999999999999649`, 0.018007085372393677`, -0.9819929146276057`, 0.08927273381393384`},
{0.12996780445602385`, 1.481230978947499`, 0.6236753706365583`, 1.469443592582019`, 0.8723955727041814`, 0.3094468996181755`,
0.06710955059959622`, 0.0369322761528374`, 2374.524656049569`, 217.63840782204932`, 0.0671348935566927`, 5.581729605257106`,
1.404891625361591`, 70921.79558486029`, 50.42063160774444`, 226.20703563719226`, 0, 17.075232022259115`, 0.40212972128500846`,
1105.34679459641`, 2747.731904382164`, 24817.52735742242`, 186.53188835405425`, -0.9924838649045241`, 8.509242867469696`,
46078.279181021506`, 2.0831633407637193`, 1.`, 1.5483385495722926`, 0.5483385495722926`, 0.05910996599603921`},
{0.05377365231121939`, 0.5209314028947571`, 8.011482854018304`, 0.8336514898133974`, 0.5477465381895592`,
0.6097956289357849`, 0.1415470974234781`, 0.1739421756208861`, 1218.4890478272073`, 28.334007654588277`,
0.1640483947776385`, 1.719700868912403`, 0.7776819970376057`, 60513.22576983488`, 26.517576531164316`,
541.8680385203054`, 0, 29.73329417777266`, 8.660693179610472`, 5111.275377745924`, 589.1693169063185`,
34164.56512623389`, 22.972093431627762`, -0.9993276046878762`, 64.45181497279334`, 26245.04923517301`,
1.2368557561420916`, 1.000000000000368`, 0.33380902821045094`, -0.6661909717896719`, 0.01806128823145317`},
{0.0760667810432224`, 2.1066017580574803`, 2.855976527079802`, 0.8406874141346559`, 0.5880407566565378`, 0.5900461865444994`,
0.0836613609935555`, 0.006020335814040566`, 1435.8256232073982`, 78.12343431322768`, 0.05191171052641791`, 2.750551419426905`,
0.6067313927910662`, 78166.44986169627`, 37.78357506381824`, 270.00136515662604`, 0, 54.223573284287426`, 0.14919611399618485`,
199.08126207064285`, 1333.3595670040952`, 37431.63163674291`, 52.44393440946078`, -0.9985989407323088`, 4.489954229138682`,
40675.7675400383`, 1.2313670135692771`, 1.`, 0.756239883835657`, -0.243760116164343`, 0.04223985244174412`},
{0.1959001110368495`, 2.209062410088075`, 0.3560695839737882`, 0.9080944676346985`, 0.9265730083719161`,
0.5325736257413008`, 0.19563779045692792`, 0.10606554062225873`, 521.6446274837854`, 359.26470542430604`,
0.1577179206968679`, 8.108669567071114`, 0.6211999644108912`, 18150.80566284657`, 570.1863467581098`,
393.9201845319426`, 0, 9.02996658137701`, 0.41057503760944575`, 335.8440621011993`, 816.9846102106837`,
4772.425883676374`, 35.57298907407056`, -0.9925461411154137`, 12.956941172907479`, 13355.982293247622`,
3.827178659658837`, 1.0000000000000264`, 0.4777767578253995`, -0.5222232421746131`, 0.4090312777026137`},
{0.2577200305567735`, 3.2042225311100276`, 2.603156269860186`, 0.8353734407000886`, 0.2430563561244512`,
0.34928665438367135`, 0.16249163015987889`, 0.02250334329978988`, 1913.3488368943863`, 296.02734369523876`,
0.1182738965060627 9`, 8.829720137683108`, 1.3154105657657276`, 1792.109846027106`, 48.39172396556585`,

233.64767042499207`, 0, 134.3365746944244`, 0.9067668975807877`, 9.320604699681898`, 9.278942388114125`,
345.02752018176653`, 0.4084675947101395`, -0.9988161303930337`, 41.50689890990143`, 1270.2929006310374`,
2.7948906315676907`, 0.999999999998954`, 0.005925582370717428`, -0.9940744176292764`, 0.3712916542101005`},
{0.2434557780636919`, 0.7224758340812225`, 2.1790233830774692`, 1.461101906273748`, 0.9519880988578835`,
0.24073725238447052`, 0.12794532446798884`, 0.029247247168962882`, 1555.7186853348103`, 187.90922583014026`,
0.22271376685703087`, 9.305754826562229`, 1.0292202961269128`, 23835.487975911023`, 126.33775481082526`,
677.1981609620393`, 0, 107.11385994930272`, 3.9245031360183695`, 620.4439952171938`, 157.0949164042889`,
5289.022944396229`, 10.76057264249376`, -0.9979654895137305`, 40.50512395070343`, 18394.902801781463`,
3.2868206169841163`, 1.0000000000000226`, 0.08933551448918996`, -0.910664485510812`, 0.11002558996978329`},
{0.25418798006115284`, 2.1642591478158595`, 6.996499815725443`, 1.0633791975130336`, 0.9920570228042984`,
0.41215457570127223`, 0.23951743090069744`, 0.250242517415804`, 977.2894626743205`, 66.34773026593149`,
0.24515846775975497`, 7.886389497471068`, 0.4453335711584776`, 35739.29234041563`, 285.166975252064`,
2684.564163574747`, 0, 90.67755204528412`, 10.05881790068062`, 874.9687726973713`, 85.98524830021701`,
7628.071308525095`, 4.321703303430504`, -0.9994334474431826`, 310.9984093965992`, 27699.48625252037`,
2.8904346739167392`, 1.0000000000001321`, 0.04922667378513681`, -0.9507733262148697`, 0.07944968235812694`},
{0.17142178078611126`, 3.468749463314624`, 4.1220489747329765`, 1.0943061437323611`, 0.026879199595517766`,
0.33123915944031324`, 0.10298062238993438`, 0.021389796005587176`, 1913.2712703835568`, 144.71515384537201`,
0.0624079843624904`, 5.28189397954334`, 0.5834593874886529`, 10910.584537806362`, 23.474500611638057`,
217.62325145173745`, 0, 125.52489181954786`, 0.7524796103587106`, 50.513234327250956`, 66.12904061702224`,
3116.0756857074393`, 3.4390210022123187`, -0.9988963615299891`, 37.288046351242436`, 7630.903472975316`,
1.3342218474797114`, 1.0000000000000042`, 0.038085423038835005`, -0.9619145769611651`, 0.0914356895290032`},
{0.20869037066000085`, 0.5770116927483819`, 5.389618587282298`, 1.0412482369515845`, 0.4332354730171122`,
0.23498113287901135`, 0.16276058881271271`, 0.005129384861959153`, 3721.998380233862`, 47.9738718293973`,
0.14995337445020457`, 8.81275872972273`, 0.9157809024114361`, 109136.18374761504`, 24.88819775685616`,
1666.4604159929313`, 0, 327.33093384758905`, 2.5922839190978366`, 806.1393612624939`, 309.976492707267`,
27323.929338825852`, 15.011994659394851`, -0.9994505916600339`, 21.368259032043582`, 81460.58488010339`,
2.034848453827164`, 1.000000000000002`, 0.17462742220160388`, -0.8253725777983965`, 0.019248837970362684`},
{0.08639685731709784`, 1.2263080297925724`, 1.8023918869870366`, 1.4078333023480911`, 0.09921808861838555`,
0.1733433235749129`, 0.14800205989677365`, 0.20963109628072585`, 1801.4315718325315`, 163.03870519568818`,
0.04013342775966272`, 4.222103734481822`, 0.21608307503029467`, 39492.08141237417`, 16.59083027795759`,
175.01026369220878`, 0, 14.425556696165696`, 2.290659164949741`, 1598.7114765899478`, 696.9263877631593`,

17650.384836352037`, 45.74891530277453`, -0.9974080499815193`, 40.129338964226044`, 21784.825432831036`,
0.5884004485975394`, 1.`, 0.3944077976753644`, -0.6055922023246356`, 0.04717496790636307`},
{0.2559888963315523`, 2.7036918392629854`, 1.376199541747745`, 1.1184454818096095`, 0.29882869252546684`,
0.4809279853463262`, 0.1762427890412958`, 0.033675551797332184`, 2802.532681882846`, 74.90505814957066`,
0.08607671060416239`, 7.246865433053017`, 1.1485805723085236`, 215161.55059822186`, 33.191226452953124`,
808.2980319808651`, 0, 50.03782555151892`, 0.6060585299495184`, 1763.7736976206522`, 2909.236570331855`,
46186.00396651317`, 150.9904323046024`, -0.9967308184441747`, 23.408507164860534`, 168901.48830503432`,
2.9735337501738446`, 1.0000000000000016`, 1.6396330048923002`, 0.6396330048922978`, 0.0373373054216828`},
{0.19137997577853977`, 1.0693425445674762`, 5.859392287604482`, 1.4113333162411321`, 0.03799318259045559`,
0.31471592998942965`, 0.1330917250869928`, 0.057621317595080414`, 1667.5088619944472`, 46.210982838293944`,
0.016645464987740083`, 9.646205098992475`, 0.9349271114716617`, 1.313260544539391`*^6, 49.35384512149585`,
1355.759161248836`, 0, 230.45311714032067`, 11.61486123928526`, 36429.61508503458`, 3135.465803122788`,
351591.0985088706`, 205.75028943139668`, -0.9994148023362821`, 177.43236103450673`, 961249.9416653992`,
4.959407102467797`, 1.0000000000001514`, 1.765856555819517`, 0.7658565558192496`, 0.044924328054953486`},
{0.19721869082033683`, 3.2840461091147555`, 6.111568583801249`, 1.2835967155205092`, 0.47111924671273675`,
0.3724666179342665`, 0.13247758167737783`, 0.03750893971444233`, 2173.6667113114927`, 83.61542153296074`,
0.1890720712372439`, 6.476185684583786`, 0.7126657781022314`, 24094.087483592564`, 40.019446070732215`,
664.313815886468`, 0, 192.32898227612824`, 2.141044104562126`, 175.44043818054257`, 80.94153394912088`,
6234.37216373609`, 4.901769167751107`, -0.9992137509537427`, 100.44696515757651`, 17564.781660268276`,
1.8706556795267681`, 1.000000000000047`, 0.04626494956924029`, -0.9537350504307619`, 0.05630186496515804`},
{0.21988733193649235`, 2.7975541724377093`, 2.3611483960904547`, 1.1600939571587892`, 0.24697322096821828`,
0.5139658559575447`, 0.06350156651392477`, 0.17257512128303623`, 3203.836504260581`, 102.69376441927619`,
0.05643237763666792`, 2.2035776970401866`, 1.4380067287288245`, 149918.83848214865`, 8.465237050933617`,
125.39648259323393`, 0, 11.286210535340084`, 0.6688446617393811`, 2603.601707948342`, 3891.6851881832763`,
36192.02895343715`, 208.64625875944859`, -0.9942350217770912`, 26.730416773738572`, 113688.1240562794`,
1.2129207182260862`, 1.0000000000024851`, 2.1898690720012586`, 1.1898690719958163`, 0.014244748785867695`},
{0.13727970757875596`, 2.8452993900541435`, 0.2811962573637228`, 1.4853711561890859`, 0.35668909434089957`,
0.6882050716830825`, 0.1434517751263107`, 0.09344751133387784`, 2265.9177865516995`, 139.6613455165895`,
0.057526142554834014`, 2.484713339974709`, 0.10336153105397905`, 52815.87709287996`, 18.42509700707225`,
144.44468515618217`, 0, 2.2825145545156365`, 0.07260702962175829`, 725.0514110959057`, 9984.967128431159`,
17834.54815251209`, 678.1151394614501`, -0.961977442116136`, 2.9512676728061686`, 34976.02221680142`,

0.2719939309191992`, 1.`, 5.745029471548714`, 4.745029471548714`, 0.06981309894310286`},
{0.13079509007818008`, 3.451126694395393`, 8.265071576900024`, 0.9978113975551193`, 0.28001559296341805`,
0.46031518977081587`, 0.08917008131877008`, 0.017138702372490412`, 1442.9762283162472`, 33.814218463353484`,
0.17998121143599194`, 7.358113266662068`, 1.1154000745850698`, 84654.88603107883`, 62.69191620152687`,
1458.6645642172543`, 0, 363.45136817446866`, 1.7615873613949842`, 331.0055427778546`, 186.90186057859458`,
29354.234832340535`, 8.71730562573989`, -0.9997030307321745`, 86.84944524885387`, 54848.42555817204`,
7.1128867710616435`, 1.0000000000001084`, 0.1057919284502669`, -0.8942080715497446`, 0.048374434139704804`},
{0.15200365596867`, 1.2321714037993603`, 1.2826182456063666`, 1.2525135629617612`, 0.10189417734334105`,
0.42182447183087945`, 0.16343163967002294`, 0.04597060352203497`, 2744.3901597051326`, 303.2449917542002`,
0.2131773205429714`, 4.894467965266408`, 0.9895276491017069`, 10183.102743699374`, 17.861027123778456`,
113.14450046515275`, 0, 28.68258736169041`, 1.0023135059389578`, 216.99128913768436`, 215.49043722543576`,
3195.9119479582523`, 12.704600441236174`, -0.9960247338950147`, 17.643171995140946`, 6939.861432051522`,
1.0631741185196597`, 1.0000000000000508`, 0.1232794857230684`, -0.8767205142769379`, 0.10136370520676609`},
{0.22754574199703576`, 2.0500842079624535`, 1.4317719989732147`, 1.4394511204103708`, 0.9096762268457756`,
0.3629999302632213`, 0.106795983345218`, 0.12347919331546342`, 3709.9742200770725`, 60.75950393256113`,
0.07245771005729762`, 9.46479696180289`, 0.2919149083841388`, 195362.57913588584`, 67.00558249547518`,
1825.6189383440078`, 0, 36.335445682387984`, 2.112881655838392`, 4116.667820826926`, 1947.3664924874515`,
45936.99603750224`, 129.91132185585488`, -0.9971719674105421`, 61.87979022754065`, 149325.25497811948`,
0.8664320189118502`, 1.`, 1.0956448601643254`, 0.09564486016432538`, 0.019982625406693082`},
{0.20503801915719277`, 1.0262506067723134`, 1.0800304574674584`, 1.0922656655058383`, 0.19306135916753409`,
0.5113898504084636`, 0.20945291149465461`, 0.029409960652989255`, 1028.4817812589972`, 23.515618103058102`,
0.10367816050153927`, 1.7590551256620532`, 0.7974605289147969`, 458401.54575658665`, 20.040704362380957`,
592.1643446259717`, 0, 9.931701121848059`, 0.2660201636806102`, 8659.619470836355`, 32551.49282995438`,
116664.31185925372`, 1631.4218407413628`, -0.9860161019703305`, 3.900047919869947`, 341723.1344279774`,
1.284534971235632`, 1.0000000000001652`, 18.337672533442532`, 17.337672533439502`, 0.008007425308883405`},
{0.21236403141675547`, 1.9573734849300308`, 2.6535583097467583`, 1.4800706545848268`, 0.3008051623864796`,
0.20266843878015717`, 0.18796743731817545`, 0.01387997496279432`, 631.4288286008941`, 122.32978434555201`,
0.20929941217323872`, 7.603405617879708`, 1.123897133765297`, 49037.07996358634`, 105.9624204478217`,
532.1507481759143`, 0, 126.46696361593555`, 0.8598781326570235`, 280.16847709009147`, 324.8234701519515`,
12119.087783049612`, 22.541736982187583`, -0.9981399807158988`, 24.04432367334303`, 36766.54769574233`,
5.311080391955371`, 1.0000000000000628`, 0.18471467439332367`, -0.8152853256066879`, 0.1229486195773041`},

{0.1552219340650659`, 0.8853243438823553`, 3.704192180484896`, 1.4225984400522793`, 0.30727783518964036`,
0.6899910723821459`, 0.23852460823207766`, 0.04807056691157154`, 2363.417831098286`, 24.86201270145591`,
0.056442694072345256`, 9.159332366722428`, 1.3338948458352062`, 945149.7614855213`, 61.278809677638115`,
3485.0411680997167`, 0, 149.63778415673437`, 7.510636636760931`, 28195.864823511423`, 3753.1244753482433`,
293678.4703510768`, 248.58855391112633`, -0.9991535349744435`, 94.99070646541651`, 651219.1451594909`,
7.946413967654675`, 1.0000000000000797`, 2.1171756580215275`, 1.1171756580213588`, 0.05479829585638326`},
{0.20103480965042153`, 1.3452512500000138`, 4.3677490431720685`, 0.877487125235695`, 0.3607930851168655`,
0.3984625684159562`, 0.053314676617049916`, 0.01650783971749592`, 3182.8108727507833`, 127.76026016049212`,
0.014515185076648118`, 8.964504972667974`, 1.3475969548800526`, 87657.70313447248`, 35.09570736490002`,
437.22704128392047`, 0, 237.01866131286496`, 2.7349837197681155`, 827.3501618837639`, 301.50643025908414`,
22563.77836842717`, 12.326321755755451`, -0.9994537119823423`, 52.56057525353917`, 64801.49841844371`,
4.134726713002894`, 1.0000000000000109`, 0.17014519519204566`, -0.8298548048079561`, 0.09935681508682173`},
{0.19504311625596543`, 2.4976887333066733`, 0.9780959245602254`, 1.4635414205075887`, 0.2583014895103719`,
0.4579159763055559`, 0.20087332417307124`, 0.20355826875644964`, 1388.181044794952`, 55.982899470557015`,
0.20280310820910474`, 7.420374461676447`, 0.562614521298161`, 126552.42143916685`, 64.51226068211095`,
1126.6181972827596`, 0, 13.84679733809437`, 1.0923228471204065`, 2567.2314581825044`, 2349.2497132146123`,
33409.26414032825`, 159.44636591913886`, -0.9952274804602275`, 38.97546383408716`, 93089.2427106895`,
2.5260625892873847`, 1.0000000000007063`, 1.3251858202541376`, 0.32518582025320164`, 0.0408364819804416`},
{0.13592819841871134`, 2.9064950846732005`, 2.3581026922159136`, 0.7780848542747187`, 0.30526997799339717`,
0.3770830944311493`, 0.13314104598243304`, 0.28747750393724975`, 817.0224638364293`, 99.04079463539466`,
0.1276940051281772`, 7.752046366250018`, 0.9291873586736861`, 40905.16957930275`, 108.65567371473395`,
598.7232978362119`, 0, 27.5295686048479`, 2.58186347353631`, 773.6173769674252`, 298.6352769605675`,
13857.984959794481`, 10.922146124731025`, -0.9992118517839051`, 107.20247850186578`, 26909.870418549395`,
5.286516015210988`, 1.0000000000001728`, 0.1700641269774252`, -0.8299358730226042`, 0.13104858698387706`},
{0.24059659553061208`, 3.474305761400969`, 0.3551751508042571`, 0.7879508982528726`, 0.7115917578613755`,
0.16553560182957539`, 0.059563521780316875`, 0.007818446748999992`, 3031.006140812062`, 311.2165043893773`,
0.22179274663929038`, 7.521418478036052`, 0.4828099783575506`, 11287.113151851538`, 29.597814392756842`,
185.8140239347897`, 0, 17.813603290041684`, 0.039183405187566175`, 22.396887194895086`, 570.591138842679`,
2539.3418415101037`, 20.7304699004489`, -0.9918362823147431`, 1.9447875770638532`, 8727.957170796653`,
0.9498661175436742`, 1.000000000000001`, 0.3211102323824692`, -0.6788897676175312`, 0.02981650608253329`},
{0.2655286560784189`, 1.7978740647108253`, 3.744260697890588`, 1.4979569598721256`, 0.2121339839068337`, 0.5203547954080169`,

0.20358958391513882`, 0.3001328269718666`, 1853.7326098612984`, 25.1210343423665`, 0.20564102192348216`, 6.431952930060138`,
0.9412816530341135`, 353064.38096216327`, 42.530931590121575`, 2095.32254444766`, 0, 33.85556280029627`, 5.417970573275027`,
10729.024782935385`, 1979.2663447191737`, 73621.18329908352`, 137.61169712451596`, -0.9981308138370247`, 139.1546968151088 5`,
279264.7694329798`, 3.672386620579205`, 1.`, 1.11418821687592`, 0.11418821687592007`, 0.01834457412700192`},
{0.11579761780826803`, 3.5947224028634324`, 1.7024337214669405`, 0.9403811808729834`, 0.8023022872362131`,
0.2690829028689966`, 0.10714511128523063`, 0.11270721526081176`, 1656.7229648447174`, 66.45258485414047`,
0.04987530781728505`, 9.865292743118054`, 1.1705893328455934`, 198082.40035152837`, 104.92540266691614`,
1568.8768096315787`, 0, 48.13730618420106`, 1.4667968436748549`, 2333.989340217987`, 1590.21513676734`,
74581.27049945721`, 69.56451910246707`, -0.9990672655663198`, 75.32467820581985`, 123376.19224215978`,
5.896588933957749`, 1.0000000000000082`, 0.8963534455585688`, -0.10364655444143855`, 0.06813412037414344`},
{0.19091547503659084`, 3.8724415822010574`, 1.7819835666756205`, 0.970240031108671`, 0.6126075063757175`,
0.3126984772797464`, 0.17470342972606634`, 0.006235187142203773`, 3131.6216914066945`, 328.479620734891`,
0.10630619937563857`, 8.167239786634365`, 0.8931338365031225`, 9057.514045604581`, 38.15362109321355`,
273.2488799348025`, 0, 100.58009089011921`, 0.1580152291959914`, 13.153332888375703`, 82.2409189626982`,
2400.60503750292`, 3.784908670556047`, -0.9984233521918736`, 8.741496345136968`, 6547.32358728719`,
1.3850212458735598`, 1.0000000000001057`, 0.047298090431181994`, -0.952701909568823`, 0.1586569317804397`},
{0.100462842594787`, 2.3014533321484603`, 1.3291106382168039`, 0.7767037741986953`, 0.6186202165209542`,
0.5591414823289833`, 0.13230502890488538`, 0.011503939527627875`, 2830.1764339684587`, 75.11206729916319`,
0.04992405177453235`, 4.899377436291939`, 1.144111391119885`, 134143.84483382353`, 35.07755047925669`,
626.5353158561122`, 0, 41.68797347409397`, 0.20143815069711293`, 558.884668936707`, 2773.472794763982`,
55065.78095433001`, 100.26655346726636`, -0.9981791495239044`, 6.622864330624225`, 79029.49834819826`,
2.2588845625205565`, 1.0000000000000009`, 1.5656053991273065`, 0.5656053991273051`, 0.04447569830775915`},
{0.07918907579486661`, 1.9928853739387806`, 6.231134938054945`, 1.0170500506380848`, 0.5386394257050193`,
0.6572554793818131`, 0.08180903968242625`, 0.09102806961295826`, 3292.3299295864044`, 25.797137029704572`,
0.14701504075704036`, 8.245305783801324`, 0.46404074549033125`, 91682.41584016682`, 50.58048608091667`,
2454.0724251416354`, 0, 165.6652268041351`, 7.296532481577534`, 1758.663451074826`, 240.02729008815393`,
42839.01210716493`, 11.391019844889001`, -0.9997340970464866`, 207.73075518578722`, 48462.5968104499`,
1.6413883298406597`, 1.0000000000002527`, 0.13562061770745198`, -0.8643793822925823`, 0.024349607926883485`},
{0.08056415815759815`, 3.9535505406039233`, 0.5752240632800181`, 1.4853820342325343`, 0.15510043342374935`,
0.6600536355917208`, 0.1525299204594313`, 0.16813894995214293`, 3147.186246353149`, 370.3183465224665`,
0.19963532109395127`, 2.5689014931104612`, 0.8619285687304634`, 9944.487543646723`, 10.705668199061575`,

48.732247950366286`, 0, 3.3715303677803385`, 0.1372884555391526`, 122.15433498087162`, 888.764070119028`,
4618.130734655911`, 61.91614218708726`, -0.9865928130352722`, 7.753954965935593`, 5315.0830699897615`,
0.48556899842086465`, 1.0000000000000293`, 0.5114597990625327`, -0.4885402009374823`, 0.08224825750476171`},
{0.2470446862474004`, 0.7616885134704638`, 0.987661776479948`, 1.418205334084091`, 0.19365902643767297`,
0.4570709516573175`, 0.06398975270684754`, 0.008457878153676336`, 2687.6783620781935`, 271.5689565049082`,
0.1334063906782825`, 6.091940716408539`, 0.7457858481599575`, 31609.809167016043`, 25.69161512098768`,
133.3739626729705`, 0, 40.05985958353773`, 0.4053189149433345`, 286.80325510926554`, 706.5989906598907`,
6969.184300316828`, 46.46427935055289`, -0.9933328955946192`, 4.410382311494979`, 24595.713555315302`,
1.3838117548198394`, 1.0000000000000044`, 0.3992744787806928`, -0.600725521219309`, 0.0633727651622775`},
{0.21332713902898792`, 1.1214424929874225`, 9.162265965223703`, 1.195968506251218`, 0.6194628623567042`,
0.6911884704772038`, 0.19705041057272926`, 0.35280397622564785`, 1556.9191271276404`, 81.28832183992904`,
0.22397741498275942`, 6.620834183658268`, 1.366777947793718`, 8354.513537188477`, 99.23211542105192`,
945.5505350583958`, 0, 79.6726194014419`, 22.475688708883244`, 409.58168662159375`, 17.22332084799304`,
1949.9027715308089`, 1.0335487366905303`, -0.9994699485780622`, 360.0741768185606`, 5942.388280505138`,
4.933332115467757`, 1.0000000000049216`, 0.010466925996217652`, -0.9895330740038338`, 0.21157230506166982`},
{0.15548452904006277`, 0.7941888445067193`, 0.96068281269703`, 0.8969171753329834`, 0.8390319554716188`,
0.17764885192591462`, 0.10783704631792568`, 0.4257693977926573`, 1410.5075470475076`, 21.97142523719117`,
0.06053827716094551`, 9.545575914809223`, 1.320581285010927`, 621970.2551468952`, 97.9433537905959`,
4768.146036554343`, 0, 9.688654731597536`, 4.71806326209159`, 43266.695523037444`, 9169.435646904967`,
193064.96321467374`, 381.1578021774701`, -0.9980257536332283`, 53.52904586328724`, 428837.35542243725`,
9.887882135891303`, 1.0000000000001632`, 5.154672183779628 5`, 4.154672183778787`, 0.02649916028708113`},
{0.11287267839860893`, 0.6274623554262155`, 1.4782831154053753`, 1.2121235395747008`, 0.42002460109961204`,
0.37694632007071904`, 0.10874502522899515`, 0.13201309589288884`, 1491.5919687682626`, 61.86932965722724`,
0.04486960703076642`, 6.490904378609384`, 0.9946688583085637`, 300649.0387296914`, 55.69688247084482`,
810.908722937453`, 0, 24.915881823934633`, 4.6696705149253255`, 19718.531980449072`, 4221.68164690082`,
115055.07897826826`, 237.94040045114974`, -0.9979319435303148`, 41.85774943370564`, 185522.4989662945`,
3.9194932594930276`, 1.0000000000000093`, 2.3812806559150226`, 1.3812806559150004`, 0.05108011082602363 6`},
{0.2430762813238787`, 2.3310866531539265`, 1.0183842540902877`, 1.0634798069102733`, 0.11637048805058381`,
0.6527039790628004`, 0.1653633422639308`, 0.011869416806071493`, 3558.3378742172754`, 124.12326765089796`,
0.15620828250230562`, 4.139014546068248`, 0.5423323870297714`, 53961.70480932782`, 14.708721268848858`,
236.24338320692348`, 0, 26.76921920160667`, 0.13214092136168704`, 228.07808206318782`, 1725.0215814517305`,

12058.169730296951`, 84.87184352642008`, -0.9929614655105431`, 4.4004562588813165`, 41872.21510875343`,
0.6249371966808006`, 1.`, 0.9729502313620628`, -0.027049768637937177`, 0.023861712934925554`},
{0.12740998379951485`, 2.0965772202079593`, 9.26399010812127`, 1.4391840070681714`, 0.39059961846309776`,
0.5024732906844885`, 0.2058431543840883`, 0.008446302531916432`, 3264.970078645587`, 50.15257581792508`,
0.04320568862225377`, 1.5793394493087352`, 0.8400374654569864`, 186529.3128597386`, 6.710613420553291`,
285.39755660563185`, 0, 97.0605551524385`, 0.377621550525997`, 648.8810816883297`, 1717.3369984697367`,
66103.20833993627`, 114.98927389058059`, -0.9982604585045366`, 11.31018200989162`, 120317.26719553249`,
0.42608586964250444`, 1.000000000000001`, 0.9689182063059909`, -0.031081793694010207`, 0.010930022285577618`},
{0.1326843363181519`, 3.8211461387770695`, 8.543417929499473`, 1.102470057077619`, 0.6181175624574178`,
0.32965325202173645`, 0.07539452320036058`, 0.007386442292018944`, 562.3263637037148`, 31.555160873736668`,
0.05487832008430005`, 6.002761669320297`, 1.041478797158169`, 334807.674758034`, 160.06268848705972`,
1476.9335098992926`, 0, 345.6293180750108`, 0.6523839447568783`, 574.8920870079897`, 880.2174052232889`,
115498.718820679`, 45.16821712433136`, -0.9996089288471289`, 35.61220559297004`, 218926.72646169478`,
9.46890263131316`, 1.0000000000001326`, 0.49616513601548184`, -0.503834863984584`, 0.05349366191020215`},
{0.1573792753543678`, 0.7169949173367489`, 0.7718194209824034`, 0.9372436231934692`, 0.09272278999885297`,
0.4028207043118923`, 0.14589558430857152`, 0.11971411886775803`, 3682.2389249814178`, 277.8795147586992`,
0.21886799519974814`, 6.424836122129667`, 1.423509413643259`, 11944.648458969545`, 17.098920185857086`,
157.3858728063569`, 0, 13.833007644738721`, 2.074351332818922`, 670.4172887097928`, 322.1937030641454`,
3665.7895729449046`, 14.118890785253164`, -0.9961484721083129`, 21.247133748598344`, 8241.70437988095`,
1.5944805431958957`, 1.0000000000001756`, 0.1830666388813015`, -0.8169333611187306`, 0.0920229258811092`},
{0.26630725498764907`, 1.3537243132764054`, 3.4681976762260653`, 0.8182294329126494`, 0.5922927850334725`,
0.35114377894258253`, 0.24401965334161962`, 0.12295914082921328`, 3688.719544583101`, 187.1594487192641`,
0.18710725308822346`, 1.0160874849867394`, 1.302279127254418`, 6748.005730276609`, 3.9398611266152144`,
64.08906496602934`, 0, 9.718136336461628`, 0.8274928932499723`, 211.41852776406014`, 254.49286222110672`,
1399.0242636712019`, 9.76805019124326`, -0.9930179551242301`, 16.00281783794177`, 5322.433018848487`,
0.25261678871368404`, 1.0000000000130953`, 0.145530522911653`, -0.8544694770902528`, 0.020374979274187213`},
{0.19754063204381567`, 1.2678132216826858`, 8.502246050318615`, 1.3049855200762384`, 0.9149570338409301`,
0.5843100223668893`, 0.12148336049510372`, 0.006360783777788973`, 2748.553955173861`, 51.9861836865702`,
0.0949023054382408`, 9.034485905171376`, 0.4708246470769286`, 71014.57429101593`, 125.31180922852893`,
2197.5552969365185`, 0, 522.7785758841642`, 2.4816865374522967`, 309.5211281222584`, 123.72208856804828`,
18431.09730205437`, 7.571156741263013`, -0.9995892183402223`, 44.947357203627774`, 52012.723004412794`,

1.7548101412820736`, 1.0000000000004154`, 0.07026255956695632`, -0.9297374404330728`, 0.052091012474221036`},
{0.04026554170616964`, 0.5302615276235345`, 1.5243469239696097`, 1.4195352782408457`, 0.35050648466256606`,
0.19736961374591044`, 0.19256070262690145`, 0.16778953261543247`, 3042.5326446416893`, 157.0549085671795`,
0.10950358517591147`, 5.218120753168542`, 0.25613055846451904`, 15234.992935629069`, 15.374385954000783`,
295.06223895153494`, 0, 17.56881006994601`, 4.861744312010151`, 1253.6373395169417`, 256.8575217170582`,
9633.610519260914`, 17.03914809729745`, -0.9982312811937715`, 36.82851379716467`, 5541.464944918514`,
0.45035923294230257`, 1.0000000000028952`, 0.1455653154277087`, -0.8544346845727128`, 0.04406546606480105`},
{0.13808283652405223`, 3.3382876893654982`, 1.764700397616487`, 1.2575917791512339`, 0.24597284032716238`,
0.16023118424518423`, 0.11783424678280677`, 0.22421613327193346`, 2741.9690444157613`, 110.60741736270495`,
0.06748966623215114`, 5.495223824255797`, 0.7719457903315097`, 101763.47086926912`, 15.4384720258035`,
348.6941119982002`, 0, 17.2318608324225`, 1.123476819378735`, 1592.773716306091`, 1416.7183621704537`,
34209.48923823643`, 82.65109465692976`, -0.9975839716845422`, 53.57841193456457`, 67482.04728649602`,
1.1075291577107076`, 1.0000000000003502`, 0.7975338658101584`, -0.20246613419012083`, 0.022392660552315317`},
{0.2720572435314704`, 2.092047115388646`, 0.9775412869939437`, 1.361730496090756`, 0.8643513713503745`,
0.6446371862205038`, 0.07237861696827474`, 0.06459834622446187`, 2040.0072337232305`, 288.27046449548277`,
0.2127097516522497`, 3.2460958268505635`, 0.7031503645327328`, 18700.138816851526`, 56.29231602127329`,
100.56135931668807`, 0, 12.460399714088886`, 0.3685228381068807`, 290.57576680270716`, 787.4878133887405`,
3821.993955689645`, 49.72589075791683`, -0.9869895422822708`, 11.01381629166196`, 14854.302005412375`,
0.863054896603257`, 1.0000000000017761`, 0.44788031736491657`, -0.552119682635879`, 0.05563083879247019`},
{0.04136924877145362`, 3.2505996769323806`, 7.3279282523448686`, 0.890763406913394`, 0.44633950095226127`,
0.48929728316730614`, 0.1843515006058281`, 0.4646336778944382`, 1146.503881369189`, 33.19464868615876`,
0.20060766821265197`, 6.423062520789202`, 1.3180525740121252`, 51879.4681366229`, 84.3880228835433`,
1842.7995762397838`, 0, 47.839464278818554`, 6.4421695012758455`, 950.4546650872112`, 146.53642618359817`,
32384.887815067712`, 6.162765048062365`, -0.9998097024425944`, 299.15591570844316`, 19139.121149387964`,
7.522819097687835`, 1.000000000000209`, 0.0837694664843009`, -0.9162305335157166`, 0.10712682229867675`},
{0.12443960147958688`, 1.4681483834231432`, 1.2961197051090405`, 1.2383341395061327`, 0.20461077825222174`,
0.2180798495866123`, 0.13955102593271534`, 0.036090000549476184`, 981.8387463156896`, 155.4019202626156`,
0.2259516918970782`, 6.485966100174822`, 0.46818640769970843`, 21133.103670306344`, 55.13605283656998`,
301.1654956990688б`, 0, 41.61061233396989`, 0.9690901286015559`, 327.1715467251275`, 336.6069336267535`,
7585.458832507248`, 19.49352302497664`, -0.9974301458283001`, 20.325258652824107`, 13484.735345100182`,
2.1367738224250945`, 1.0000000000000013`, 0.1910544193813877`, -0.8089455806186125`, 0.08437986030802387`},

{0.0668112344511611`, 2.714918834733731`, 2.085154017953151`, 1.4476779676976927`, 0.2539160749138807`, 0.4870680566357908`, 0.17330271142201725`, 0.24053269905137525`, 735.8247906168481`, 182.42768732112722`, 0.23388978376591213`, 1.775892168476009`, 0.1844693395246435`, 7430.349197374119`, 28.88262885527235`, 74.7103032536908`, 0, 6.776591268721943`, 0.5599278043549463`, 144.66434617736346`, 257.36249790099487`, 3786.887305063325`, 18.149778429131295`, -0.9952072039733362`, 21.716550601919334`, 3614.3802225530326`, 0.631541334937569`, 0.9999999999999969`, 0.15249938258088927`, -0.8475006174191102`, 0.11136789709470786`},
{0.18113629662628528`, 0.6738122810255058`, 2.859320071162905`, 1.31555093037265`, 0.36681457284995544`, 0.471103526437621`, 0.08814722965023356`, 0.04506210537052006`, 2186.7720797416923`, 139.59007135140308`, 0.0715047981648481`, 8.93352349425783`, 1.2069106988965403`, 66537.81464490388`, 55.76842642476375`, 453.59628290360695`, 0, 116.71990129853418`, 7.0002543347832535`, 2452.34964846212`, 349.322935593461`, 18493.023172360616`, 21.488709097998903`, -0.9988380099404128`, 67.38367630112825`, 47853.68186950706`, 4.056484316159474`, 1.`, 0.1979687169687714`, -0.8020312830312286`, 0.1145264364283316`},
{0.1192842170105507`, 2.0587406052635338`, 2.982429260158481`, 0.9607554862496793`, 0.6846341105753171`, 0.4253089041287377`, 0.1448155559009277`, 0.03303667191414802`, 3625.499229568628`, 138.38440688618937`, 0.05121864534109544`, 6.262852463653789`, 0.9148148813580201`, 50089.85951304339`, 32.94427350758532`, 485.26493902990484`, 0, 94.91928878830153`, 1.4651299490410257`, 528.1163064032104`, 359.4569729455598`, 18472.35888191661`, 16.16086005892573`, -0.9991251328451211`, 43.09032168683489`, 31478.01236524738`, 1.4214047619544834`, 1.0000000000000397`, 0.20380792783546803`, -0.7961920721645401`, 0.06835996914822533`},
{0.19942002949430754`, 3.5293725272813257`, 4.475631886088314`, 1.3690551967616487`, 0.1535320198116885`, 0.586863827463982`, 0.20957264255775987`, 0.0070808196203240945`, 2259.133238896783`, 93.9809316080586`, 0.18155601470027005`, 6.2714522403203645`, 0.9597826662416344`, 48881.532858938284`, 34.33529199664976`, 527.4742004697223`, 0, 190.43575905009249`, 0.37344432312909404`, 87.32795013246847`, 232.84463151226032`, 12645.754315792687`, 14.950472748614006`, -0.9988177476506923`, 18.828916207442784`, 36025.95283761635`, 2.3115942217532726`, 1.`, 0.1323536522703596`, -0.8676463477296403`, 0.07486298470062526`},
{0.19290552010423068`, 2.5311022785006196`, 4.590275609347875`, 1.0183350360370953`, 0.6768490777669534`, 0.27037924456465456`, 0.13002648835247615`, 0.039251036869391874`, 2833.896145381659`, 40.558338114360765`, 0.02944272635318218`, 3.3740003853748863`, 0.42763776068514936`, 432572.0119907971`, 17.000659064536165`, 824.0355645995788`, 0, 73.46697366804463`, 1.107533766874454`, 4182.39063393676`, 3775.3098146793213`, 115144.09032339277`, 178.2853242196616`, -0.9984516328739154`, 40.046874869318664`, 317313.2947251785`, 0.47058834565274554`, 1.000000000000014`, 2.1226975504877816`, 1.1226975504877519`, 0.006076402746822877`},
{0.1574181424723891`, 2.4334976996958355`, 7.454513230702137`, 1.3609349886556783`, 0.33361805963053826`, 0.5460996944909747`, 0.19878306773728516`, 0.24115086657234458`, 1578.7144043122416`, 80.91704790184991`,

0.12351463962730552`, 9.357025478355425`, 1.447433410261179`, 43442.468351443524`, 84.53313521714132`,
1042.9278998396571`, 0, 121.18405068650247`, 11.348487087322523`, 941.4194110788776`, 81.95549916345625`,
13209.495751161201`, 5.319185668462184`, −0.9995973210659465`, 394.5216746003885`, 29705.91834492441`,
6.81047269510482`, 1.00000000000002`, 0.04733375774938077`, −0.9526662422506201`, 0.216333240841746`},
{0.13084693466492658`, 3.6481233497064993`, 4.2504273066825995`, 1.0067280367884681`, 0.5994607748865513`,
0.5813901581577328`, 0.20650373563828406`, 0.1827740239544238`, 3288.755010061308`, 116.07278762719005`,
0.07676913646394057`, 7.439949797465348`, 1.2495449290646898`, 44538.006036441555`, 46.09091343695347`,
747.6671703276448`, 0, 66.5077906778956`, 3.218373537959984`, 606.2992731252142`, 187.38685627198092`,
15439.810109561016`, 8.896404696342973`, −0.9994238008995439`, 167.7289093129905`, 28860.74035206587`,
2.6661484499811454`, 1.`, 0.10703893121133048`, −0.8929610687886695`, 0.12344215612951638`},
{0.1513970595453768`, 3.565269862926928`, 2.882233750328341`, 1.46147567899756`, 0.43028383944211446`,
0.5449527314944854`, 0.05024950045158358`, 0.013582696865047886`, 1199.944383613747`, 41.76183563022494`,
0.15753768337487067`, 1.280336274118845`, 0.8861263254663858`, 148213.52583743827`, 16.20147530529498`,
181.01202420039922`, 0, 22.98469411596362`, 0.0854950930849382`, 463.7872990108331`, 5423.724183293967`,
46852.59205266862`, 365.63476773809384`, −0.9921960610561936`, 4.354472554340888`, 101333.49526933045`,
1.2282747800429856`, 1.0000000000000124`, 3.0524473497188294`, 2.0524473497187916`, 0.008275772990169962`},
{0.05402397529650032 6`, 3.9030377038282893`, 2.6328389166852855`, 1.06539261997144`, 0.07270428031369969`,
0.6221832003428629`, 0.16473607550076202`, 0.3329139837617463`, 3016.8950283011136`, 162.89707817078545`,
0.17967527091419977`, 4.813593384225332`, 0.9924030037454137`, 9497.798842887481`, 18.88686359913677`,
203.02853625653327`, 0, 17.1738036868619`, 1.386639330611196`, 138.26683380277885`, 98.71362469708275`,
5306.445210387531`, 5.0079066541798`, −0.9990562596134269`, 77.3157941283816`, 4095.3609279744`,
1.3802548882482928`, 0.9999999999999959`, 0.05695644430570933`, −0.9430435556942904`, 0.11710805988468657`},
{0.1846136822622525`, 2.83222539763279`, 6.185815327147518`, 0.9985231745146829`, 0.7838598641352239`,
0.6970792682180826`, 0.0566519604849656 5`, 0.0105368043370621`, 1139.0438628747534`, 42.309712199780904`,
0.09405173581505571`, 1.6109366539180776`, 0.8793784843352515`, 142206.3327162521`, 42.8916447242039`,
279.59973660661177`, 0, 64.54611170948901`, 0.23320435209303994`, 448.7457628909728`, 1923.25981274972`,
39075.84526629399`, 89.25493483080182`, −0.9977158540212618`, 9.435532697663014`, 103056.22403029355`,
1.6379825497827136`, 1.0000000000000004`, 1.0845688216816078`, 0.0845688216816074`, 0.01416264904077456 7`},
{0.10141299379366919`, 1.6007716008532835`, 0.6398899685364796`, 1.0164701283406912`, 0.8225647972224088`,
0.6292458866768325`, 0.13571266692666845`, 0.07872745868282192`, 1791.1473855018166`, 192.97393158172054`,
0.1484827963674462 7`, 1.8821154571770222`, 1.1130294782612764`, 22519.796926325074`, 32.60194520887422`,

112.87792219557637`, 0, 4.32830356013052`, 0.2007536052264901`, 499.4367556438731`, 2486.8096464589457`,
9192.693588057085`, 117.79637173788628`, -0.9871858698857401`, 4.590866714506802`, 13317.979682753368`,
0.7606718219254571`, 1.0000000000000602`, 1.4211060845940895`, 0.421106084594004`, 0.04753574334788372`},
{0.21885531656429402`, 2.687374858822965`, 1.1296170207345284`, 1.325784614121181`, 0.40511738093112615`,
0.5937209983207841`, 0.0879699618250398`, 0.34321707574230476`, 3195.5291529724673`, 92.2297421582175`,
0.21288285544599406`, 8.464472481658461`, 0.25864431062726`, 42932.34176828027`, 42.812165412336874`,
665.6821960169743`, 0, 12.088059668824132`, 1.4957688937279598`, 905.2685510663924`, 604.2195328184412`,
10386.837949653975`, 37.23851639406693`, -0.9964148361056016`, 57.42416742305671`, 32474.49582247916`,
0.9473086045347301`, 1.0000000000000508`, 0.3412455881537185`, -0.6587544118462989`, 0.044105305872094`},
{0.20003331751651454`, 0.9418735138273977`, 0.3780377423788224`, 1.1513921169618064`, 0.6645698632364119`,
0.4730945551961576`, 0.051565459379669804`, 0.1952333288925963`, 2322.1212203613577`, 230.2978627277754`,
0.10438353064420558`, 8.70346208169968`, 0.8526709835745097`, 48114.41230062281`, 75.18215516661203`,
271.5702245819633`, 0, 6.553320409757116`, 1.2431253814951593`, 2450.0382270678506`, 1969.869763853656`,
12466.210739684266`, 104.8164099266789`, -0.9915919590872139`, 16.726669588526782`, 35623.67844455777`,
2.6272740304351787`, 1.0000000000000002`, 1.1113774360934137`, 0.11137743609341344`, 0.0774080305644007`},
{0.045463415265626916`, 1.0604864030915095`, 1.5457624129661234`, 0.9218745066798075`, 0.3077029567700027`,
0.3372897124352183`, 0.13368077049637672`, 0.019951884664535027`, 2805.850760096928`, 274.4324431552926`,
0.0964209850306606`, 7.937294323580787`, 0.5250555034364572`, 7195.58167137124`, 30.861418264857722`,
222.07278286379346`, 0, 72.10701028385108`, 1.2621756565994193`, 98.82623771054256`, 77.2983233703004`,
4306.135339439744`, 3.391625340564804`, -0.9992123737241835`, 19.121716030525462`, 2796.737416099152`,
1.1606115033928153`, 1.000000000000001`, 0.04457222064547678`, -0.9554277793545233`, 0.16578468496844626`},
{0.12981111976782828`, 1.1084267930609286`, 4.931023641639941`, 1.2438579943673111`, 0.8299266795906743`,
0.2963366491664211`, 0.21441842262722777`, 0.013448184708505154`, 893.4222083111035`, 95.2241911833517`,
0.18109496842042927`, 2.912115357106975`, 1.136648183382977`, 29472.44740434821`, 61.212782075464865`,
468.2445872206725`, 0, 91.09281371686058`, 1.03129196870907`, 282.13593509680123`, 272.5752276340983`,
10287.093383023508`, 16.002612633056586`, -0.9984443990117301`, 16.330166422652578`, 19076.844445807084`,
1.9529404767205474`, 1.000000000000386`, 0.15598546584336764`, -0.8440145341566926`, 0.06744132497727465`},
{0.04881288196111078`, 1.5001688796472123`, 0.7565632194730121`, 1.206042774752989`, 0.6805558920404622`,
0.3640356697656889`, 0.08453170981106164`, 0.19820032009109562`, 3391.2112266676813`, 283.705794296156`,
0.20144392981632914`, 6.84055461750941`, 1.1851088923020137`, 8020.2621472072115`, 35.03168890481296`,
205.23537827167183`, 0, 10.43636290225484`, 1.2705428013113853`, 264.23193058363603`, 206.9677522952475`,

4702.198136425529`, 11.704713762968082`, -0.9975107995402623`, 27.228982439816154`, 3278.9691798727786`,
1.679783700607946`, 1.0000000000000446`, 0.1177783910058942`, -0.8822216089941111`, 0.12744149072049088`},
{0.18840966187391878`, 3.686049426661911`, 6.507340210098313`, 1.3405973621357103`, 0.6314264717273861`,
0.6528641596796794`, 0.05949520733205324`, 0.005267163480527638`, 1036.4467237949607`, 79.36725884036872`,
0.16755847890105047`, 9.771579716601678`, 1.20892036427258`, 29225.5769552798`, 218.21479101333458`,
906.7140074666745`, 0, 445.1764650025531`, 0.6175325141269045`, 38.11545280451914`, 60.722179695118555`,
7787.143821696469`, 3.866376879055381`, -0.9995034922986419`, 32.517933852036734`, 20959.616205848706`,
9.89303898946089`, 1.0000000000000233`, 0.03493078887112019`, -0.965069211128807`, 0.1903536933537174`},
{0.1000404296450419`, 3.096045401845493`, 7.6799040120684445`, 0.803333640129631`, 0.6198933615423707`,
0.29060269426384966`, 0.18836459448376747`, 0.04739137692785461`, 3452.9247864535073`, 66.50695651270411`,
0.1918335051763443`, 8.136439035690735`, 0.9349024413134766`, 16007.986468021178`, 33.4257764507725`,
1400.410956741281`, 0, 277.49900440197496`, 4.138639845377063`, 142.8830593131531`, 33.52415881820597`,
6398.655010079678`, 1.2917561261083155`, -0.9997981206794125`, 183.04881233106002`, 9144.631376553889`,
1.988834752991986`, 1.0000000000002098`, 0.019469739197368118`, -0.980530260802636`, 0.05431531559569296`},
{0.13921193952574745`, 0.46815880576040936`, 4.3864048931388595`, 0.7514079591567522`, 0.40824219063798206`,
0.5617438808850261`, 0.24339108461093562`, 0.020519348847045586`, 1920.4537040361229`, 81.58086121632874`,
0.1022235991904683`, 4.414511511279496`, 0.3646779989849891`, 33885.690929856646`, 35.47514000914551`,
552.0562025298952`, 0, 111.85617300446243`, 4.2548320935059385`, 999.139406717956`, 233.82463814328224`,
11289.39329825973`, 8.249124145111304`, -0.9992693031479041`, 28.456244451525677`, 22451.6905302816`,
0.8061028644585215`, 1.0000000000001108`, 0.1329959961330362`, -0.8670040038669785`, 0.04792636683285895`},
{0.22961340343556502`, 1.6030194574854644`, 2.9811826554982517`, 1.0274834008587208`, 0.6451263943126482`,
0.5097595717714782`, 0.19295611967128257`, 0.020325995630318172`, 1782.352038334574`, 109.5999144338602 5`,
0.16872014474364577`, 5.851396867706905`, 0.6578614287996216`, 42512.6866861228`, 65.70494295695009`,
628.8275007812234`, 0, 100.92422618161507`, 1.2224500378359848`, 400.2715018450553`, 326.4338332498456`,
9902.012696603784`, 15.695731512018108`, -0.9984148948306841`, 27.99444566364172`, 32480.497658991062`,
1.714431554488437`, 1.0000000000001765`, 0.1852183468443309`, -0.8147816531557018`, 0.05934683250111819`},
{0.26748619026781384`, 2.88696602814122`, 2.7118514018243634`, 0.9885856963040458`, 0.34646533154127157`,
0.2537466476440833`, 0.09945272546945017`, 0.07388745663205695`, 3603.2051356484208`, 34.51441620104788`,
0.19141821235831952`, 6.868891191349718`, 0.1593219725686645 4`, 8475.3966253183`, 18.98235833752477`,
1428.428000680557`, 0, 67.40182456662725`, 1.6786536596933714`, 103.02900781512027`, 60.37597664662996`,
1729.2437760425337`, 2.8193430793119774`, -0.9983696092370712`, 69.23165840785273`, 6607.840424256358`,

0.4896869127799858`, 1.0000000000006477`, 0.03457969021098848`, -0.9654203097890339`, 0.034768482713811104`},
{0.10551271069457996`, 0.6900129952362093`, 8.232394419645587`, 1.130400293700848`, 0.7046770328984258`,
0.2711667378009396`, 0.05003959656184298`, 0.014784496484154912`, 540.5369479440305`, 45.69515764102175`,
0.16942738870919372`, 8.057748743914669`, 1.27779344817754`, 49517.0454696355`, 227.35086437469698`,
1266.4470610797055`, 0, 410.02357796597886`, 7.881318476119783`, 795.2832554714804`, 99.90738724506194`,
19551.208048585846`, 5.296070859066725`, -0.9997291179733802`, 77.68874525882718`, 29470.013693714012`,
13.380187869435757`, 1.0000000000000002`, 0.056732043251199515`, -0.9432679567488005`, 0.09582563960551443`},
{0.08696471520703919`, 3.613913666113315`, 7.0626231358127285`, 1.3208938988261034`, 0.28024078243683714`,
0.37947312403263656`, 0.23429714063319607`, 0.06281923284932987`, 2203.521948663617`, 29.0776175939651`,
0.14152777414422596`, 8.483647124571803`, 0.7235011997593546`, 159796.91308526453`, 43.278544546335986`,
2671.7069886206446`, 0, 234.3756537135418`, 3.989454827931985`, 1436.1130964157985`, 358.97727969268345`,
71064.84886883838`, 22.109356524109444`, -0.9996888847738927`, 205.96493318578746`, 88287.633472997`,
2.4782887460986833`, 1.00000000000001`, 0.20269047906851376`, -0.7973095209314882`, 0.03219740031243343`},
{0.09172278717373272`, 1.2157183406525425`, 2.2812242417200608`, 0.9103021242940673`, 0.7524885408365471`,
0.5328408138361465`, 0.11958813531559015`, 0.005068237864616913`, 874.3804057883599`, 319.73415041813746`,
0.21547584642190498`, 9.166843855230969`, 0.9284914101950568`, 736.3938895797211`, 261.01971351477516`,
304.0406303357188`, 0, 155.90780061502468`, 0.6003421008166319`, 2.706843485356474`, 3.508835015359435`,
246.40628828814974`, 0.20112079303722366`, -0.9991837838456378`, 10.426384323266541`, 322.8724505560479`,
4.022092251037683`, 0.9999999999995267`, 0.002676307164111007`, -0.9973236928358877`, 0.7653702730030812`},
{0.1306914623455918`, 0.7537584653459817`, 0.21965733160577017`, 1.2102680433379889`, 0.2532343966945656`,
0.6654185660627909`, 0.08400179279005554`, 0.3103404088337731`, 3637.61631387517`, 133.45404753770885`,
0.037164269643724435`, 2.2620412269050973`, 0.956581036605332`, 187116.5064286666`, 9.223181246756917`,
110.0965641957919`, 0, 0.692152417301125`, 0.2557972336093466`, 12974.06617236296`, 50719.11917133142`,
65263.843219131115`, 2741.1695175097707`, -0.9579986500594829`, 2.7544190035018254`, 121848.95869431054`,
0.6987225474419219`, 1.000000000000003`, 28.62064871402286`, 27.62064871402277`, 0.01954005276494589`},
{0.15232520315695658`, 1.456608465380997`, 9.96731462021549`, 1.4944244110861535`, 0.5157767046786654`,
0.3722073718864378`, 0.22976562023274222`, 0.07428975878835796`, 1050.2078985192002`, 33.60662262776219`,
0.220518601124694`, 7.093566043209907`, 0.6560171292141301`, 110617.65475706953`, 95.67486192016392`,
2365.929648836791`, 0, 255.4235942441771`, 12.390033755615796`, 2611.4893625320547`, 209.7733856131259`,
34662.92284138692`, 14.67221878317414`, -0.9995767172073079`, 257.82040078266135`, 75429.09662611647`,
3.4858453486455327`, 1.0000000000017073`, 0.11890366886464057`, -0.8810963311355624`, 0.04477171405135425`},

```
{0.0694009118290565`, 3.678178592599034`, 1.755843032698575`, 1.2142042066341803`, 0.3177757036060529`,
  0.6847455380724545`, 0.08295711309717257`, 0.10240501118816868`, 536.5981094108779`, 211.10249067816517`,
  0.18136782974370297`, 8.788955479280464`, 0.4777460308714221`, 10277.415476426948`, 260.5164726319527 5`,
  281.93472724533507`, 0, 51.3260398184252`, 1.3478484666171966`, 114.00738968468126`, 83.58472336346144`,
  5098.762294869514`, 5.071645202367079`, -0.9990053183676615`, 70.82324822826855`, 5055.1250351936615`,
  5.4131399042407224`, 0.9999999999999997`, 0.05061445359860798`, -0.949385546401392`, 0.7412575116757821`},
{0.16483058262413092`, 2.446957095238485`, 8.164140625789859`, 1.1581574304294189`, 0.8637023907646861`,
  0.5910929885256712`, 0.17429603155004098`, 0.036201628416469234`, 3040.5552834584214`, 70.8960300369222`,
  0.19751315642431794`, 1.918185462218652`, 0.5717895645919415`, 7709.679112530568`, 20.281203440921`,
  376.273306509112`, 0, 77.16571430771698`, 1.105876347610658`, 73.08957901201852`, 65.09199949880013`,
  2263.2996335311977`, 3.5739632723485983`, -0.998420905822897`, 38.65759964631884`, 5329.442817827574`,
  0.36629968983567573`, 0.9999999999999962`, 0.03741526476789814`, -0.9625847352321018`, 0.02068643297112587`},
{0.04627776053028976`, 2.3407537273567067`, 0.9761597154152462`, 1.37527944112939`, 0.3584342070422313`,
  0.22640865017403333`, 0.13240363624591456`, 0.038325642841679504`, 1249.254134864137`, 143.93359215534173`,
  0.13242235789061851`, 6.269547025830331`, 1.0591127938814697`, 32679.630898437954`, 48.10440696104188`,
  343.36639116159756`, 0, 29.908693923873845`, 0.46939164224125773`, 335.72254673349335`, 714.2290678429652`,
  19645.127971620303`, 45.99574569715467`, -0.9976586690723726`, 15.696146230947306`, 12987.607540821964`,
  2.8572052040010987`, 1.000000000000006`, 0.4058202272674266 7`, -0.5941797727325757`, 0.12150236482454647`},
{0.1264814146333596`, 1.0630284736090054`, 7.0368315732194056`, 0.7618799049872292`, 0.05995838274087717`,
  0.17364177343291576`, 0.061949148356618716`, 0.006022075969931666`, 3448.0521144594068`, 47.239369625587926`,
  0.19227473651596427`, 8.592453879074284`, 1.3325684049534696`, 33995.40366052841`, 17.556174353386353`,
  1019.8552375987382`, 0, 412.2858175997964`, 2.1840912916961357`, 166.373412032324`, 75.17511807536245`,
  11951.830583632309`, 2.688679935446886`, -0.9997750403240212`, 33.16787474334948`, 21595.491995372442`,
  4.489417181514148`, 1.00000000000022`, 0.042730643059927645`, -0.9572693569400818`, 0.02691206423029527`},
{0.04081744499533296`, 2.3413945804849527`, 2.3401462602509717`, 1.006052431149826`, 0.9376889728762128`,
  0.63499912288976`, 0.07105146864527681`, 0.02628017367597553`, 775.1156602545625`, 104.41750831638893`,
  0.12180266898464448`, 8.067006250284603`, 0.5522058225859034`, 24910.810037179777`, 450.9545649826378`,
  740.2049753307122`, 0, 105.55204705369663`, 1.134456145969615`, 197.3826500130259`, 172.9887881204291`,
  15643.379223042097`, 8.330024407761607`, -0.999467504796183`, 37.94584959958713`, 9121.753871109366`,
  5.205313363311309`, 1.0000000000000004`, 0.10028732489393312`, -0.8997126751060669`, 0.3031043401714111`},
{0.11461934044260397`, 2.0495668840197947`, 1.6301061314690006`, 1.1441247153256733`, 0.9293434036316242`,
```

0.32161099339559507`, 0.24691379277403247`, 0.09383957046935675`, 3183.5471916851793`, 210.97634406977602`,
0.19279849145037242`, 7.844330233274125`, 0.7023420045147568`, 14389.569629675805`, 61.22330231347448`,
761.4755549926938`, 0, 41.01646527061581`, 1.8030842557424418`, 272.1860919516227`, 149.95583641461434`,
5419.675476453273`, 8.071042998483222`, -0.998510788508731`, 52.793453995245684`, 8874.280407486123`,
1.1495084938404105`, 1.0000000000000027`, 0.08552432355880181`, -0.9144756764411984`, 0.09567283473904349`},
{0.10880423027814301`, 3.010977164978767`, 0.4477158763471074`, 1.3560425240667602`, 0.3025579540978818`,
0.35180253133086303`, 0.2109756008586861`, 0.03552671363577697`, 2668.2716874123935`, 170.59395733846873`,
0.23332643955483107`, 9.954767028855915`, 1.4592401570775992`, 27503.333843823817`, 41.71549848366798`,
527.9691829039227`, 0, 22.241127701978495`, 0.2546501037632504`, 210.37180003554994`, 825.1210065366153`,
10754.172847179792`, 52.279857877771235`, -0.9951386444480032`, 10.95350924986602`, 16715.707127364272`,
3.4621601332526546`, 1.0000000000001281`, 0.46899189451869916`, -0.5310081054813609`, 0.1603749274268306`},
{0.22749339290790638`, 1.7311095241107948`, 1.2544972871049804`, 1.3263167066853254`, 0.7333948539753357`,
0.4493142943490447`, 0.2441212061859469`, 0.014870392763137733`, 1580.7600138635335`, 264.3754143437418`,
0.09646148923631659`, 4.446033883263464`, 0.6510175252381905`, 44686.648557326254`, 59.702146284180074`,
247.95027289771753`, 0, 34.69130370917881`, 0.28495351222643966`, 304.29998366612506`, 1066.8934303652718`,
10504.846649797804`, 66.29167054629873`, -0.9936894204402713`, 7.046939127771615`, 34139.760090567914`,
1.08349135943842`, 1.000000000000009`, 0.6089040770929165`, -0.39109592290708906`, 0.0907417402009726`},
{0.10680853367942073`, 2.671856809998241`, 2.225956166276376`, 0.9564945044055834`, 0.04023151222298815`, 0.66439395337897`,
0.05090336466790368`, 0.0058123339013462704`, 3923.759723431366`, 155.48006966286152`, 0.1590662885693253`, 5.07114362920246`,
0.8193607123659179`, 12616.14355140505`, 15.45997296157163`, 172.87264272760237`, 0, 77.8819058477504`, 0.164056065168024`,
24.69395088009842`, 149.52141385207068`, 4961.426123043391`, 6.7023422899664835`, -0.998649109726972`, 6.261918784867122`,
7570.323559443391`, 1.3967984531734452`, 1.`, 0.08494106366787015`, -0.9150589363321299`, 0.06112734204436063`},
{0.1306588579678935`, 3.206018430436262`, 4.178329357094787`, 1.096081964467313`, 0.37321063526655895`,
0.6088846209535219`, 0.18229329291576957`, 0.013520486536436158`, 782.6656351062597`, 123.9415631086822`,
0.023548806194033767`, 9.649188633359959`, 0.8429625600097688`, 139003.54819539117`, 194.9983578834161`,
703.1453759745679`, 0, 259.4115837224227`, 1.059955323803617`, 480.8137670549944`, 452.6170122053909`,
48383.6413320574`, 23.712983327018037`, -0.9995098966784192`, 48.54623290790616`, 90310.73315392566`,
6.209617843408647`, 1.0000000000001155`, 0.2620269801147014`, -0.7379730198853289`, 0.37066123859698225`},
{0.06490925302735506`, 3.8934906583752236`, 0.9790395136780409`, 1.48284195083516`, 0.9349692905507263`,
0.3703362249698605`, 0.1491469107421124`, 0.13667677484236057`, 1004.3258994396238`, 82.66219089427364`,
0.1407191619667938`, 7.134069953930672`, 0.6810640411318756`, 69659.32573795183`, 198.41838319980116`,

1261.245596442762`, 0, 17.90374740202847`, 0.6089960986791493`, 813.5842149721034`, 1334.943229745946`,
36116.59904073074`, 92.60736985940237`, -0.9974358778977234`, 33.87315173134604`, 33490.020937461755`,
3.545157551836183`, 1.0000000000000855`, 0.7579756864860527`, -0.2420243135140121`, 0.10943370041580547`},
{0.09335758271301797`, 3.886141513910534`, 2.1262193060875347`, 1.2374865217449704`, 0.20774186356921476`,
0.6885354117013798`, 0.06828062526335338`, 0.04634612342186317`, 2337.7355280609045`, 265.47200855531526`,
0.06367324855715212`, 6.746431099417454`, 1.2698799762745048`, 10134.581918446996`, 41.026364717684615`,
153.86105811206693`, 0, 66.66871271237488`, 0.7610861987671923`, 71.43212265129856`, 92.85549595696826`,
4295.745018486951`, 5.481996704276955`, -0.9987238542602773`, 42.25269532419352`, 5729.148155391867`,
2.509665134755567`, 0.9999999999999981`, 0.05369542487934066`, -0.9463045751206592`, 0.24010870708181922`},
{0.06958654560186628`, 1.2810573894719157`, 1.5604323721050744`, 1.2865370396261684`, 0.41858142719439173`,
0.3987961369914276`, 0.21356300651181537`, 0.0062459309627175864`, 2941.8823280388497`, 383.1127710935082`,
0.10442332143534439`, 6.649327249023878`, 0.3474394649692636`, 3695.2281744242096`, 29.71517653886926`,
171.56470829026205`, 0, 72.52156828545222`, 0.3328999453164653`, 15.683609177090826`, 46.11208096529271`,
1813.4525828895898`, 2.872039755267451`, -0.9984162586977096`, 6.092341927177932`, 1802.7414408009815`,
0.7002421628317117`, 1.0000000000002418`, 0.02706650583803092`, -0.9729334941619756`, 0.20737418176197944`},
{0.12162705978453553`, 2.1459364566550487`, 0.8994451186914905`, 1.339976475017456`, 0.23917524394620604`,
0.5256296133560587`, 0.21370599200931129`, 0.027381167717735945`, 996.433258066003`, 270.7208781713538`,
0.090253513817738`, 8.675427486273929`, 0.7193288794908814`, 38075.335222439666`, 110.90363259732038`,
275.2406666143308`, 0, 43.220190509014785`, 0.5281353289339901`, 338.1905441947368`, 639.3482699734449`,
13886.74998476111`, 40.92782657125964`, -0.9970527426059969`, 16.190640805813633`, 24128.636722992058`,
3.1841529660838823`, 1.00000000000027`, 0.3708444421564103`, -0.6291555578436898`, 0.3494553590525035`},
{0.1091983534434775`, 0.4123168888384443`, 1.8612158499296871`, 0.8333258155688079`, 0.39517409251864644`,
0.5848465884265365`, 0.12470223152921811`, 0.040839984644619944`, 3265.644855437202`, 125.19293803224065`,
0.0879734848590002`, 8.526937482297015`, 1.0338521843728818`, 42165.438174059665`, 41.26918913707241`,
545.5597187964001`, 0, 75.06531390226804`, 6.315692611591008`, 2254.8170052825085`, 356.01816791151424`,
16424.70066752079`, 13.88796024073632`, -0.9991544466762673`, 37.20095326387371`, 25622.146695646497`,
2.739618317489065`, 1.0000000000000973`, 0.201920488823233`, -0.7980795111767867`, 0.09817039682025894`},
{0.2316510553565545`, 1.3345287101431156`, 2.181207240226371`, 1.1295787756210554`, 0.4108038804094787`,
0.55044788544399`, 0.15705941808893342`, 0.0693266889389808`, 2764.047895868124`, 88.55030846959835`,
0.19804190063549815`, 3.6671215326087623`, 1.3965489185868663`, 64725.020990669975`, 20.27069475210782`,
354.42235895785774`, 0, 30.013568886377957`, 1.4731840558311804`, 1605.1135508429354`, 1088.5539796874325`,

15006.018697040636`, 57.16544469337166`, -0.9961904989026407`, 28.085805969025525`, 49659.429540994795`,
1.8834402160002512`, 1.0000000000001443`, 0.6149836339589022`, -0.3850163660411865`, 0.029928038659350512`},
{0.23613672937320296`, 2.4419271800403806`, 3.4588792688353487`, 1.4108864845746094`, 0.7134807082114705`,
0.592154370313409`, 0.22091066377388413`, 0.019900714899270838`, 1522.4223604141134`, 78.60644958620696`,
0.18909869538832608`, 2.793726035246588`, 0.7950281959201368`, 83765.12215998747`, 44.31081903235205`,
474.49664155371016`, 0, 56.17207243371707`, 0.44376324793984123`, 516.7253328628782`, 1163.416691246428`,
19136.907892099887`, 76.47539663054097`, -0.9960037746399923`, 15.480536237818514`, 64556.097713667164`,
1.178800665654283 8`, 1.000000000000007`, 0.658806082253912`, -0.3411939177460926 5`, 0.0285530248381046 38`},
{0.0484675208754865`, 0.6825441464113089`, 2.084792805230027`, 1.4119489784121344`, 0.5877996575498201`,
0.6759892639478722`, 0.22086781309234377`, 0.0387732952545585`, 2548.5220980600216`, 370.2048073952212`,
0.03857632786812504`, 3.327588495854709`, 1.4128853163273263`, 4829.690534073407`, 28.197724593713893`,
104.21691878289695`, 0, 35.56954884439272`, 1.7900779493741896`, 160.1385119719706`, 88.45896016872055`,
2821.315754599823`, 6.1578744450142855`, -0.9978173749482048`, 17.45438894236156`, 1953.459717605795`,
0.928969737373122`, 0.9999999999999889`, 0.05291031930263210 4`, -0.9470896806973673`, 0.23691217367419143`},
{0.15820801401395784`, 2.3900304014986444`, 6.76991723319799`, 1.147995471223545`, 0.5011235356714243`,
0.39186104654424503`, 0.19387948138295608`, 0.05551860155117706`, 723.5959050648971`, 29.172220754504224`,
0.17373406698962968`, 6.019570931242669`, 1.3786373610317848`, 184078.82397484718`, 118.44717481179086`,
2105.3295824705615`, 0, 170.1576827842618`, 3.81711614146941`, 2316.81749019846`, 605.9549377941103`,
56370.5714975969`, 32.536284488145284`, -0.9994228143582058`, 130.3289089166158`, 127403.94522095122`,
9.335590691543178`, 1.0000000000003115`, 0.3432958298368348`, -0.6567041701632721`, 0.06530909649620746`},
{0.05715322142426921`, 2.4704612263538817`, 0.36234457179092416`, 0.9684061618557824`, 0.863166347709152`,
0.23804186668051197`, 0.13749867219091239`, 0.1430597139428923`, 3758.60599725621`, 74.20395287068635`,
0.1109041971133704`, 1.6071731513826233`, 0.773390027344468`, 55931.17448958841`, 7.314456895401228`,
264.0834583023267`, 0, 1.4249101698568296`, 0.07979761297908763`, 1034.7976193957102`, 12966.776613404427`,
30788.39243717657`, 573.0486153643396`, -0.9813875110065055`, 2.816241554577562`, 25137.94014656069`,
0.28807100787714585`, 1.0000000000000315`, 7.299331759251789`, 6.299331759251559`, 0.006998901513459436`},
{0.060904719608809554`, 1.0227281808325657`, 0.3338146022683226`, 1.4161209446138403`, 0.8561719858721175`,
0.4775008498746448`, 0.07550639206271559`, 0.17875509724795088`, 3800.334358301937`, 331.7811845820245`,
0.1313746491970903`, 6.20009884490432`, 1.2664955877170798`, 16185.70199911145`, 46.11422660391681`,
172.77401651030726`, 0, 4.490131294368294`, 0.7101077444788807`, 745.081675962714`, 1048.2515843627355`,
8646.757641453714`, 68.9725643085872`, -0.9920233031653479`, 10.374960024371447`, 7523.262138258145`,

```
1.4883268744790827`, 1.0000000000018114`, 0.594348524371016`, -0.40565147563006054`, 0.11693568083201208`},
{0.10576408085084482`, 2.4399851863533764`, 2.261754328254943`, 0.8217420902241768`, 0.7416397192385102`,
0.2463678861595856`, 0.2027535408226292`, 0.2140679841559756`, 1564.164909651323`, 70.02516408668095`,
0.19509596638498505`, 2.4981390117767504`, 1.4295314398093057`, 39189.34881326415`, 23.36278144356929`,
468.0438673247004`, 0, 10.547202856309736`, 0.8846205463225567`, 823.6152110004159`, 930.0378494194542`,
15590.758990271273`, 35.700162713881674`, -0.9977101715999741`, 30.8351575510125`, 23556.31849104405`,
1.6049799728991618`, 1.0000000000010054`, 0.5271322749895867`, -0.4728677250109433`, 0.03178007776615023`},
{0.24610403760574778`, 2.8350518587284927`, 2.483700295848834`, 1.2187293428205228`, 0.8051620910181194`,
0.2877775756384263`, 0.0943064533423209`, 0.11494360430719369`, 621.5470695356762`, 130.28571465739265`,
0.12477806190504986`, 6.74453372259727`, 1.1595275933030642`, 61455.28146706734`, 197.86058481840206`,
508.77386707438825`, 0, 48.086426892994474`, 1.867169697370972`, 920.3411843049033`, 491.90709119838965`,
13581.223005181184`, 28.01197222928405`, -0.997937448474368`, 75.62175601561583`, 47748.48310284478`,
5.311686690267407`, 1.000000000000014`, 0.2788185631865174`, -0.7211814368134866`, 0.099951999730397`},
{0.11402356544669451`, 0.8158491547123496`, 4.200202309736193`, 0.971641321517407`, 0.6807220168616288`,
0.6359800608186865`, 0.13923173061454314`, 0.037851089653330894`, 2281.1440386375243`, 126.7093813407077`,
0.11913552316776838`, 8.934189056957887`, 0.5820810660938738`, 13157.37621738727`, 96.36813232310024`,
748.75270015922`, 0, 183.83901004137968`, 7.8014562456423`, 364.8895341582632`, 45.7719772654088 1`,
4897.391903803304`, 2.137900749846566`, -0.9995634613704927`, 90.92587833618074`, 7977.40123230612`,
2.196984126077724`, 0.9999999999999999`, 0.026647565441351117`, -0.9733524345586488`, 0.17367907493078108`},
{0.15189623977997108`, 2.7915541399359975`, 9.913479700081336`, 0.7741019472122475`, 0.15478570831213023`,
0.4331484807185151`, 0.23125927282289244`, 0.006529023247839043`, 823.7432504407011`, 34.92144293228938`,
0.20656993466421963`, 9.240164591756866`, 0.703959262612698 9`, 62753.81165051895`, 120.2541926278601`,
2190.2824177392404`, 0, 624.0781023163338`, 1.4206443541663876`, 130.9658986936707`, 91.18767407168524`,
19581.09409003391`, 3.3287607444240286`, -0.9998300012895541`, 56.65436611785534`, 42489.92232934243`,
6.164943037042884`, 1.`, 0.05206523984113034`, -0.9479347601588697`, 0.08301601074877642`},
{0.19262437700106766`, 3.938833744371137`, 3.6810076480536082`, 1.109072783031779`, 0.7645216483353034`,
0.616702884118477`, 0.13777110888715882`, 0.010064854238404494`, 3438.969205276325`, 98.49201035779356`,
0.20668453166808726`, 2.7590741497101465`, 0.5477570556056697`, 23713.24081852913`, 21.822183991967442`,
308.72805915436726`, 0, 66.07290205997171`, 0.16554037452269232`, 52.05188705365798`, 313.4362044832917`,
6300.384383012286`, 16.215428125183045`, -0.9974262795506724`, 9.31480018894022`, 17337.251666357126`,
0.4654388877646732`, 1.00000000000006`, 0.1774493496648532`, -0.8225506503351574`, 0.02110947602090083 5`},
```

```
{0.15161686088493992`, 2.6676538201668016`, 4.769091008698259`, 0.9792911695535791`, 0.8799626502245399`,
  0.5483041230008869`, 0.08973546330141513`, 0.2940210093980764`, 2792.4321626291066`, 123.07394825008441`,
  0.21143511654972236`, 3.6673259692190108`, 1.1506144153300002`, 4827.886388919361`, 42.3458931756786`,
  297.23837038308534`, 0, 26.191074073000603`, 2.6944754593488094`, 99.70764668296178`, 36.00447385297721`,
  1483.3805024217681`, 1.695211433130247`, -0.9988571971720253`, 102.68468217825017`, 3212.935646787335`,
  1.5564861330779443`, 0.9999999999998137`, 0.020979035116111516`, -0.9790209648838846`, 0.06803086856651631`},
 {0.15928964461807182`, 2.676103796250932`, 3.0276164230907305`, 1.1887325681254923`, 0.14578762490346686`,
  0.4353007161971356`, 0.22317821296952212`, 0.2148623762619366`, 3792.9286804177964`, 76.64814683698182`,
  0.1595894948400946`, 1.5825099373005287`, 0.48932638994153477`, 49695.52676604663`, 4.277397562256068`,
  159.54633100527897`, 0, 8.759957758553645`, 0.6828242968398958`, 955.4845558430966`, 1398.3124735968972`,
  15160.730756202582`, 77.01355420406497`, -0.9949201951117985`, 26.10440989922299`, 34499.24877579697`,
  0.17942823432480723`, 1.0000000000081177`, 0.7882855988026648`, -0.2117144012037343`, 0.006720405090385192`},
 {0.16818423551071837`, 1.2425750232039041`, 0.8451308809778482`, 1.0511110147264935`, 0.32337126733921173`,
  0.6302171747780823`, 0.06186616786607688`, 0.012735137580066362`, 2086.5412121340623`, 307.75958728157127`,
  0.20485938116823293`, 3.379450004526051`, 0.9974668662640704`, 10530.882689975298`, 24.35226926950955`,
  67.42309042225656`, 0, 18.24240178274697`, 0.17520848823603755`, 86.44747778959996`, 492.39777233360724`,
  3088.59245408885`, 24.141574651388893`, -0.9921836321851305`, 3.110138447648797`, 7420.750867072964`,
  1.0552155878872973`, 0.9999999999999999`, 0.28022596296909774`, -0.7197740370309023`, 0.07087144509643623`},
 {0.1902308570197676`, 3.972029979384046`, 4.634110792119705`, 1.2197488970650956`, 0.8492525180509605`,
  0.5006542712905349`, 0.18652832755791343`, 0.005076576077217329`, 1943.0673920285199`, 83.26055229551639`,
  0.05802581167056625`, 7.38223678334383`, 0.9931576433164864`, 157749.43988976517`, 109.2704789944178`,
  1325.4261871592746`, 0, 237.97681376378532`, 0.297923587537409`, 184.728020469064`, 619.0516783380519`,
  42364.61099097346`, 35.28139938620674`, -0.9991671964273737`, 16.90516316091766`, 115129.37508745673`,
  3.1973819282232387`, 1.0000000000000084`, 0.3504507829125431`, -0.6495492170874598`, 0.0798560665113349`},
 {0.04261251981259187`, 1.6956083841270555`, 8.388949518118906`, 0.8816084343593602`, 0.6157741880137009`,
  0.15816307961776666`, 0.1228282175848856`, 0.05282284640298743`, 1555.166107990478`, 26.393755355375106`,
  0.19116960681111228`, 7.474128794459224`, 0.8297590847908434`, 55507.9846456644`, 50.43677700203865`,
  2666.369409635601`, 0, 265.29707445152775`, 7.911329987999166`, 946.4588989991942`, 118.63334868282502`,
  34213.64139669859`, 4.899192908399283`, -0.9998568058613932`, 191.63596367495944`, 20827.56388396758`,
  3.329762807952655`, 1.0000000000002776`, 0.06728230996721282`, -0.9327176900328058`, 0.026522601973569083`},
 {0.12469439922923026`, 3.2167561610705597`, 1.2966549629505941`, 1.3380513288544775`, 0.9647032506590352`,
```

0.3520228082837078`, 0.24731936603055854`, 0.03725013837412559`, 3227.904692724528`, 130.8007745449786 5`,
0.06370555142915824`, 7.153312146366998`, 1.3824196402345823`, 123609.53347814265`, 63.83687711755748`,
1196.4455981142053`, 0, 45.37263141903548`, 0.5115988792283613`, 914.3501718237425`, 1786.2403731666618`,
44417.35016623637`, 111.66670959768837`, -0.9974859664257376`, 23.509840667923267`, 79122.78277618815`,
2.3242498058643006`, 1.0000000000001765`, 1.0128239358130984`, 0.012823935812919629`, 0.0920642331273464`},
{0.0769260990011068`, 2.196578391656532`, 0.8056525760561577`, 0.8210657722273409`, 0.8826879394098923`,
0.3537183279427859`, 0.2409594091636174`, 0.049834595534127876`, 2188.2120164663`, 216.0992703121101`,
0.032694389865458584`, 6.1007061352710465`, 0.6801916687054326`, 65265.73324461061`, 66.82905997512145`,
519.4865002437508`, 0, 21.664449845174403`, 0.4727744411133982`, 838.2262186352673`, 1771.9939390573209`,
31076.889654353225`, 68.29729223894482`, -0.9978023124901312`, 14.835516021102553`, 34151.769859960696`,
1.2087466074637523`, 1.0000000000005478`, 1.009185281227349`, 0.009185281226796072`, 0.112136514930765`},
{0.25211089910042056`, 2.9465650077307215`, 6.640985006189094`, 1.09304531251437`, 0.4582346106456783`,
0.5101745978688277`, 0.23290591035097613`, 0.10611238144646964`, 2453.147250432534`, 22.888736398204685`,
0.22056464944244836`, 3.2852146146779617`, 1.410915433494099`, 246469.73720504314`, 20.59870023849463`,
1493.0661847068623`, 0, 64.44015534702613`, 2.2603728898485036`, 3446.0781705600098`, 1523.5618039557073`,
53526.7582642582`, 77.4106251649077`, -0.9985537957523463`, 95.14765230929706`, 192781.1307418994`,
2.8808854544830615`, 1.000000000000766`, 0.8585803573239141`, -0.14141964267674356`, 0.013655786120648312`},
{0.25118009579444556`, 0.4969745065700919`, 2.29184449492171`, 0.9917654079551637`, 0.683514928780494`,
0.655379840437561`, 0.10467228571183546`, 0.22778373737657998`, 3254.986072380726`, 170.41822686556145`,
0.16061682605470035`, 9.463970581786636`, 1.0777227394408708`, 24775.678306981234`, 73.48055914776239`,
522.7756652820509`, 0, 38.609327563552384`, 15.239006428409267`, 2339.8165031052645`, 152.541276729385`,
5364.450421954279`, 7.1027661115571`, -0.9986759564255663`, 108.19139571967355`, 19249.188155300446`,
2.981165533595215`, 1.0000000000000007`, 0.08681217101637143`, -0.9131878289836286`, 0.10687788982105474`},
{0.2292340810710673`, 2.064324787257691`, 0.2897872149067009`, 0.9514880626424775`, 0.10199246953503383`,
0.4890381533823508`, 0.20734257158694452`, 0.0515148990758617`, 1032.7042553842448`, 333.02101571525463`,
0.23061763375632705`, 3.9940977361306764`, 0.21053286147363237`, 13070.2063972981`, 42.118508286250616`,
91.6689182702137`, 0, 5.086888236134027`, 0.12176316377776376`, 181.72860818224453`, 1491.4760702992803`,
3055.462366948611`, 65.99847220451858`, -0.9783998412422179`, 3.5908388165907974`, 10005.944399067028`,
0.957825524413856`, 1.0000000000000098`, 0.8582190080646062`, -0.14178099193540228`, 0.11673189235711673`},
{0.25996807730167304`, 3.8602372982487374`, 0.7583354986548105`, 0.984076713917565`, 0.09643958841060729`,
0.4475162082476123`, 0.14281046726777719`, 0.2769302537407711`, 2666.244743594094`, 250.78576435414914`,

```
0.1593075274074181`, 6.009785296615535`, 0.38944531154385054`, 23126.708927276522`, 23.27804058076261`,
162.5954124133564`, 0, 6.916075420362135`, 0.48211167691414797`, 328.79206774810973`, 680.9832074025027`,
4898.930525504066`, 31.151522398221534`, -0.993641158567968`, 26.586649673503555`, 18193.793564996617`,
0.8026515436108419`, 1.0000000000000022`, 0.38566220812924207`, -0.6143377918707588`, 0.056380658890275645`},
{0.21313668431881322`, 1.7635041678835899`, 2.1912925923575113`, 0.8252025033116523`, 0.016757328021256512`,
0.6998740735780284`, 0.1801084581445288`, 0.26779805577049626`, 1220.0176309294266`, 152.79020888909633`,
0.1629579286674429`, 2.280125156684093`, 0.10691523188617968`, 5951.6416858170005`, 22.278899189834885`,
99.47451691587531`, 0, 8.230448419025723`, 1.1691545039910982`, 180.1371094062681 7`, 153.07468285101837`,
1461.820950381276`, 6.145310499760613`, -0.9957961264010085`, 29.454412009830932`, 4450.966720457748`,
0.46472334533532184`, 0.9999999999999958`, 0.09052974820063937`, -0.9094702517993603`, 0.12068904681065191`},
{0.1404288473454146`, 2.9064356368530344`, 5.23236147204817`, 1.4484340156797024`, 0.10732611967199035`,
0.27136542485299375`, 0.18681354316538268`, 0.3953864116976934`, 677.7158408842279`, 88.88656461439052`,
0.043948668205834374`, 4.975328880565433`, 0.3586361093450223`, 107907.5693914395`, 61.1517628473689`,
409.309836705922`, 0, 30.251823120559337`, 3.889797333195567`, 2156.0632426766033`, 553.2867810301425`,
35830.80246776677`, 37.65290373911663`, -0.9989491470705132`, 161.506365561935`, 71881.11842871059`,
2.1801707155947647`, 1.0000000000000555`, 0.31502568855494273`, -0.6849743114450748`, 0.08653317241012584`},
{0.17543069017848928`, 1.9027743233680443`, 8.420650851113926`, 1.2989717187578058`, 0.4754143700193414`,
0.44151569893640996`, 0.17410928694054217`, 0.39942064454570464`, 1461.6707316679758`, 62.00370069117048`,
0.1325925609245337`, 5.299407195683747`, 0.7461186052859023`, 49898.59889183843`, 53.18491151759484`,
811.2975080173674`, 0, 50.604670123404595`, 10.034663441774512`, 1506.5283448929065`, 149.13242383607982`,
14136.63248853373`, 9.112973093361513`, -0.9993553646456642`, 272.76714200926517`, 35428.5599180447`,
2.2832765940429103`, 0.999999999999999`, 0.08498240001463565`, -0.9150175999853642`, 0.060360502072617284`},
{0.09982764185325876`, 2.300171702541898`, 0.4858627002636364`, 0.9919442401423758`, 0.5310842871377108`,
0.5692781547371908`, 0.20674425403353813`, 0.29388576255746934`, 1499.3990415110002`, 267.8323361172651`,
0.11758456647217047`, 5.8552111813994445`, 0.6586971862135473`, 21727.21415827408`, 71.36064543749086`,
237.01910015175844`, 0, 4.286508200945169`, 0.5143265758780288`, 518.2457623978821`, 1006.6200350198951`,
8946.633107570158`, 47.310548341576926`, -0.9947119158936399`, 16.900563367141558`, 12758.875509357433`,
1.6039259655359115`, 1.0000000000008145`, 0.5804455878841078`, -0.4195544121163649`, 0.19224626761001426`},
{0.1859503570691094`, 3.4941122484776246`, 1.6297837870350822`, 1.1031963489157173`, 0.4293650493894907`,
0.20959825245659025`, 0.17774273230533655`, 0.18327500412709075`, 1189.2239199460373`, 282.6299270365462`,
0.11232193397712892`, 9.90920375985121`, 0.8278911236090769`, 19362.81196319898`, 83.59779471259966`,
```

329.46031638712145`, 0, 34.30199587957178`, 1.7177319029193414`, 275.18578835698935`, 159.2029908679591 6`,
5262.245084206619`, 8.290776247720082`, -0.9984244792640687`, 85.74211545130318`, 13978.805034191217`,
3.0195304286367706`, 1.0000000000008935`, 0.09111990710113248`, -0.9088800928989489`, 0.2058057375496783`},
{0.2642909093671957`, 3.7936229586154386`, 9.112438874043047`, 1.343319643044862`, 0.7298819945671444`,
0.2416317029456937`, 0.21308855511768615`, 0.0296357748833522`, 1462.8216839780898`, 69.98981659671449`,
0.07124306277278569`, 2.5551101499765654`, 0.4563514754130473`, 10271.106206131331`, 24.884564240710755`,
485.14255446532815`, 0, 122.24856390400268`, 0.9335312753336872`, 55.35049464410144`, 58.291526815013896`,
2114.3614714458295`, 3.713854868949687`, -0.9982435099583942`, 50.59236683844899`, 7982.950228848317`,
0.6068316480123952`, 1.000000000002327`, 0.03352637180354134`, -0.9664736281965367`, 0.02663136409931591`},
{0.2550662278820944`, 2.7952392159553643`, 1.7242958226447735`, 0.9892662747315195`, 0.6466530652803972`,
0.41633012667926605`, 0.09373409721262951`, 0.04014553436100187`, 3927.541477581917`, 214.6071511864991`,
0.12072625793793595`, 4.810615700541705`, 1.0908243473679682`, 25379.642597749746`, 21.691540653228298`,
191.44224963295213`, 0, 39.376412743497674`, 0.5481372676130082`, 225.99196801211733`, 411.29082816472624`,
5451.959457781812`, 18.936601462622722`, -0.996526643015367`, 21.888211230835715`, 19865.867620893117`,
1.1467197918899357`, 1.0000000000010916`, 0.23253410253734963`, -0.7674658974629042`, 0.04221248237529392`},
{0.07397988502541059`, 0.707910258007638`, 2.664085400294777`, 0.9518019482057067`, 0.7875244424943906`,
0.2321255676355145`, 0.14195245680232`, 0.005701405567921862`, 2118.0076336028906`, 265.7950362273598`,
0.09877063847703516`, 6.01326524592864`, 1.001781869102726`, 1472.5752323821066`, 44.02544745333544`,
266.7567169501412`, 0, 112.49748903321668`, 0.8143113160871197`, 9.979850271715057`, 11.255571148967498`,
656.7798929694399`, 0.5488525448429008`, -0.9991643280333059`, 8.235133340996748`, 694.1214424125802`,
1.5233645359329`, 0.999999999999959`, 0.006986064329101048`, -0.9930139356708987`, 0.17252390864913802`},
{0.2536234425791481`, 3.084120548082856`, 3.3542268996076317`, 1.2342076675511184`, 0.07971619232511307`,
0.37753168173048746`, 0.24305535176309745`, 0.09244094904749854`, 1429.525330090045`, 199.04655556460716`,
0.17472732471161218`, 3.7784584150910736`, 1.055845732825249`, 12344.242478982844`, 24.546142447676267`,
147.8414824627025`, 0, 41.957501610846634`, 1.209173297828752`, 155.90249793542017`, 127.93312994536512`,
2649.20878600447`, 7.525632011618124`, -0.9971592907092203`, 53.27480305752525`, 9598.592177391092`,
1.3968399648901344`, 1.0000000000001044`, 0.07402372776704252`, -0.9259762722329652`, 0.11497439305088004`},
{0.23487265302124444`, 2.745668437703702`, 1.8560463908813354`, 1.0225932800667423`, 0.519798686110498`,
0.6167124420851566`, 0.13872112580125617`, 0.02304360370209753`, 1950.6949460047217`, 63.792561577309925`,
0.17121277633362675`, 1.4754433886909606`, 1.2090151658939319`, 97897.91694532946`, 13.86652468848771`,
200.83120838433078`, 0, 15.342710351053507`, 0.12513460873851853`, 602.7730868809783`, 4815.9974154834645`,

22473.080123760563`, 227.18330959311234`, -0.9898908690601377`, 4.908259223967887`, 75404.45643180903`,
0.9688471014789376`, 1.0000000000000016`, 2.71691258610931`, 1.7169125861093057`, 0.010909451305913632`},
{0.11153574406001432`, 1.6124096945608146`, 2.7742957521629403`, 1.2244678342329687`, 0.3686430365196074`,
0.6752573668077164`, 0.2389841389881603`, 0.023498980178372084`, 1283.405515292938`, 282.1703009016943`,
0.019235563557756297`, 3.0024732562806733`, 0.7791774112114904`, 13919.337610428809`, 38.138294743687645`,
102.81717472719016`, 0, 49.98439316690687`, 0.6856531066575344`, 145.5549527047661`, 211.28657945462638`,
5341.648649782389`, 12.815012942472267`, -0.9976009255226859`, 15.793624518290677`, 8511.210809437871`,
1.0317924785456862`, 1.0000000000003477`, 0.1269989868458624`, -0.873001013154181`, 0.24535933705577934`},
{0.07532861497660431`, 3.2456716657650855`, 3.170566131100358`, 0.7667747544642987`, 0.6612666356256824`,
0.6262282997594197`, 0.0817137209363257`, 0.09276751126759113`, 725.6513719895092`, 116.55380786905249`,
0.24676733030978965`, 3.599391507892623`, 0.6435082289815082`, 7750.801067126962`, 114.27792882978159`,
249.21847018251768`, 0, 38.263807918607895`, 1.043466787705577`, 93.26126349059892`, 88.3763602152265`,
3691.06152004447`, 3.2665664681486177`, -0.9991150062250631`, 48.382151243184424`, 3972.036458548429`,
2.4663174569184205`, 1.0000000000003542`, 0.05161718057882511`, -0.9483828194211932`, 0.14586070396642786`},
{0.1849591682194595`, 2.435795929316444`, 3.5195053556656077`, 1.1867529613387038`, 0.18511861536763652`,
0.2725329941056882`, 0.1913335103508631`, 0.007193119734412014`, 888.0411283184822`, 208.53516984841042`,
0.12216322134268065`, 5.549467591217473`, 0.7979574318926956`, 5360.674750729406`, 55.55052655732122`,
207.9066760861426`, 0, 136.1974268200353`, 0.3857167017192691`, 13.954378381243739`, 35.17779141801322`,
1430.5724432052073`, 2.0420530515276805`, -0.9985725622905524`, 13.42181674167375`, 3779.964131041664`,
2.3615974839913845`, 1.0000000000000449`, 0.020859317780269147`, -0.9791406822197318`, 0.22914742514719486`},
{0.2320051986600553`, 0.8210990691375057`, 3.0745548516676084`, 1.0653538281011379`, 0.9016142110480403`,
0.45929895353677397`, 0.05711960119403317`, 0.10928247272113006`, 3933.7984981846193`, 65.47103817619023`,
0.11710947379300074`, 4.724190385949658`, 0.932323733177236`, 111561.96861260048`, 36.00358356838842`,
596.480806225369`, 0, 42.129733394175446`, 5.143024301328874`, 5341.963285844167`, 1037.6813230619755`,
25833.349264429253`, 51.26980145024064`, -0.9980153637484074`, 60.327606662467055`, 85621.01897355`,
1.7149442914613642`, 1.0000000000000058`, 0.5837409015451749`, -0.41625909845482845`, 0.01293720160918428`},
{0.2740885790936093`, 3.0208727150533035`, 3.9044562082788996`, 0.9239050692909432`, 0.13393679798267133`,
0.2935571665601744`, 0.17832264013603433`, 0.12993009746044476`, 1616.3784196961215`, 45.86996911978616`,
0.06338312332920809`, 5.191606790783375`, 1.0490428175043491`, 361949.77351006115`, 28.195678742226075`,
831.8870980671572`, 0, 52.77803056024782`, 2.2098559416932844`, 5327.173174370603`, 2409.6427364168812`,
73603.02212514047`, 103.42512364253813`, -0.9985948250403809`, 95.36705026370952`, 288196.39644678927`,

2.8378248095334264`, 1.0000000000011335`, 1.3570615742205907`, 0.3570615742190524`, 0.020869028139248357`},
{0.20127067113693758`, 0.7134036992984543`, 1.5848778254201719`, 0.9599985271802891`, 0.5544064241430855`,
0.6980173556057399`, 0.23861657897987476`, 0.010226664436870918`, 3481.525245131613`, 209.51927751371306`,
0.18791735173124585`, 4.5381928864523005`, 1.4066869656787473`, 20006.91446823817`, 27.62201722952395`,
254.26659674120432`, 0, 47.23351184809605`, 0.6135299349831148`, 227.8808377368334`, 370.4257850240103`,
5148.711590597228`, 16.710410700273677`, -0.9967544480971141`, 6.252778932104199`, 14804.066247571925`,
1.3519958883036785`, 1.`, 0.2114048604127737`, -0.7885951395872263`, 0.08613495372481568`},
{0.14734788675597893`, 0.461750217153563`, 2.7608760404738693`, 0.8246000601521355`, 0.6486099382776269`,
0.46153306705501707`, 0.07562875863771559`, 0.011120543224542678`, 2174.875101604558`, 76.28988785327772`,
0.1816491692635701`, 7.465741450424737`, 1.3681893457620165`, 39734.88716367293`, 65.43921440404253`,
782.626567183404`, 0, 132.42353818305355`, 2.7540825008127374`, 717.0633081765661`, 259.36377195126096`,
12747.771349367542`, 9.971950598178159`, -0.9992177494932342`, 18.16711704013012`, 26833.673845395835`,
5.18888183628936`, 1.0000000000000682`, 0.14650947864203814`, -0.8534905213579719`, 0.0575413861086939`},
{0.07628805291838442`, 0.7253635030519439`, 0.2787614932535387`, 1.2938825783187968`, 0.6322486251684503`,
0.6415725594877175`, 0.11902418817624266`, 0.020293492548644005`, 3744.3615927821575`, 331.54916368743454`,
0.0324650937333269`, 2.843278699811469`, 1.2796927021756468`, 69655.8275051499`, 17.213372470168522`,
79.622488059236`, 0, 4.659432580962878`, 0.11759408165584159`, 1377.812694886927`, 11715.683998768938`,
33327.94494291052`, 698.9076927443617`, -0.9790293792809138`, 1.218549357257962`, 36321.771820939524`,
0.6798000612514431`, 1.0000000000000002`, 6.679113666256225`, 5.679113666256224`, 0.06960776191721034`},
{0.26704012645507424`, 1.8838541538663058`, 7.9577608030460505`, 1.250964933852727`, 0.37177913789440953`,
0.5400109924589773`, 0.2090957264740766`, 0.02799412229122407`, 2680.237536243104`, 60.33735070739806`,
0.08963423619120237`, 8.220004781923546`, 0.7126427956038843`, 125487.42120735074`, 45.14346784558157`,
1288.6182288209254`, 0, 347.30287138429054`, 4.9668434264725905`, 1284.3190373447821`, 257.5785230312554`,
25961.59484868011`, 15.057558780155356`, -0.9994200064029996`, 133.66869457948556`, 99039.82244809954`,
2.06813612330596`, 1.0000000000000107`, 0.14579775468184353`, -0.854202245318158`, 0.04679672336059954`},
{0.14352951682222453`, 2.7122840992738713`, 1.912849383145993`, 1.4933758944571824`, 0.4317056886375634`,
0.3933320053063153`, 0.10505217994551203`, 0.4954512847212043`, 1346.891754031455`, 320.6507144731578`,
0.20904379157314928`, 1.5308675559916907`, 0.6151100951642487`, 1919.3519600970096`, 14.562202589052562`,
35.11817959993979`, 0, 3.114624707488607`, 0.4930505021957069`, 42.311356649216926`, 84.81546202831419`,
621.7632503812725`, 6.085659188764556`, -0.9902122565381072`, 19.104186246348654`, 1274.876841500569`,
0.4388335317392434`, 1.000000000670488`, 0.04981381672240182`, -0.9501861832809382`, 0.07767576789080405`},

```
{0.0604034128862494`, 3.158158875209411`, 7.876995843447027`, 1.2883875500461601`, 0.12376268888818709`,
  0.6326665048379214`, 0.11422725471154377`, 0.15234673981455935`, 2211.579578858029`, 40.246357658710394`,
  0.057745413831265746`, 5.692997033958765`, 0.2028088406309343`, 9473.65521348625`, 32.20526057385506`,
  913.346950044915`, 0, 106.2539574522403`, 4.980125718999236`, 140.75476733251108`, 27.26329600385991`,
  4905.063490852058`, 1.7082042107070654`, -0.9996517467686417`, 224.6861177016574`, 4232.608218160076`,
  0.804137560868214`, 1.0000000000008202`, 0.016055712884406815`, -0.9839442871156063`, 0.07960254315070053`},
{0.17311414162710254`, 0.8702497549340356`, 0.34304718385733324`, 1.0485386108632935`, 0.3716017788116761`,
  0.6371600148503609`, 0.055104118812441344`, 0.2168377629398712`, 703.8776101728622`, 164.53996042397466`,
  0.027022709136328538`, 3.1628771308577264`, 0.32716182080568834`, 205975.48409347053`, 72.43468374711277`,
  118.78509469145317`, 0, 2.047761697476038`, 0.4610873431534965`, 11592.28192631615`, 25140.18441645679`,
  59304.27126127712`, 1223.8580330917387`, -0.9793630710391874`, 5.73230210546452`, 146662.9716316684`,
  1.5351819598813072`, 1.0000000000000004`, 14.427561855967749`, 13.427561855967742`, 0.08929122775777551`},
{0.20458649602921813`, 3.3198040898334424`, 1.372039361136828`, 1.1755123934413545`, 0.5154858348933944`,
  0.2152893723924586`, 0.1607481890336261`, 0.022174783215048698`, 2483.3920145693382`, 114.03490520349692`,
  0.026176842372398812`, 2.8250322344315557`, 1.2559643496212312`, 370698.79007785855`, 12.094894959770683`,
  232.30130984025223`, 0, 21.90346530059168`, 0.1427788739745504`, 1841.5854165253827`, 12897.16459018745`,
  94494.44159452447`, 700.5675846359219`, -0.9925861503299627`, 6.7713984251789165`, 276175.5242865908`,
  0.9950212053417162`, 1.0000000000001001`, 7.268499796245174`, 6.268499796244446`, 0.01872439674870982`},
{0.1578531250336248`, 0.5854218989222861`, 2.149284744894146`, 1.0306646027288366`, 0.9441735295761224`,
  0.33751540864617946`, 0.1297018205371923`, 0.0054893062071085874`, 3639.0053252131047`, 207.4000118567045`,
  0.1305358640736498`, 4.5222314256344625`, 0.9549981466481912`, 14613.100359300395`, 32.440392711275166`,
  289.57978155913287`, 0, 67.3328034974558`, 0.5610174188672328`, 113.48840244051159`, 201.29033649197464`,
  4467.047481881012`, 9.707246999571272`, -0.9978269210168584`, 4.6918840383105`, 10073.39149555004`,
  0.9101448875266805`, 0.9999999999999999`, 0.11426394638101972`, -0.8857360536189802`, 0.05438954699438275`},
{0.17565226905516895`, 1.7038906647375853`, 4.512441985203527`, 1.1172179676607337`, 0.956149515870736`,
  0.40808719408253036`, 0.2318723848780873`, 0.25398457087869114`, 3825.126366136904`, 43.499586017725164`,
  0.07242686582534269`, 9.792971274976665`, 0.9137108964160303`, 286991.4215434506`, 82.0685618700799`,
  4663.753075372082`, 0, 70.95551800754836`, 10.122188292076178`, 8878.162989401946`, 876.0991739357313`,
  81686.5117501917`, 45.52626810219518`, -0.9994426709241616`, 246.38717339406793`, 204977.4448588981`,
  2.4019229725501474`, 1.0000000000000673`, 0.49357868399353444`, -0.5064213160064988`, 0.029872931673004796`},
{0.06615318131527015`, 2.492376536317514`, 9.394184914213039`, 1.4348602118065803`, 0.7341697528786502`,
```

0.24813924799413356`, 0.2232148533154487`, 0.026418827644963606`, 737.3840226181687`, 59.19942623358838`,
0.013447593432626304`, 4.186359481235684`, 0.5021329151763947`, 263740.0911635962`, 83.89598550269623`,
985.1190811110479`, 0, 214.46862279058618`, 2.197741902148517`, 1974.1626820879355`, 897.2686639218144`,
135443.64583654547`, 60.38371810110758`, -0.9995541782877435`, 78.25143356852543`, 128000.40087180292`,
1.9985841431460034`, 1.0000000000006808`, 0.5096759268833858`, -0.49032407311696113`, 0.06421428200811005`},
{0.14543352811569443`, 1.222289804727784`, 2.326184459246356`, 1.2918343464415338`, 0.9224834580379948`,
0.39580748300164703`, 0.1846442329251613`, 0.21500168948406848`, 3817.4733751688063`, 116.03894445631153`,
0.16529510077911613`, 3.062979779789736`, 1.0702400368818767`, 41420.30677276921`, 22.23810321410547`,
422.38528611540903`, 0, 13.143386663472079`, 2.1634598028513814`, 1692.5670077736345`, 781.3427112178729`,
13441.295032557975`, 46.98251889537623`, -0.9965046136714079`, 37.77678371376608`, 27925.927986126815`,
0.7717313364525813`, 1.`, 0.4418131353908852`, -0.5581868646091148`, 0.026092147998247862`},
{0.13052094951744303`, 1.9253311736310001`, 1.1697698857489094`, 1.0141116955010334`, 0.850854427903335`,
0.33601527543931153`, 0.2494690024453417`, 0.2360086443642378`, 1519.2042074690844`, 278.4809507811633`,
0.016312333738175505`, 8.02616595157577`, 1.4482171660091545`, 149979.76939692209`, 115.95609000352269`,
519.8852590486078`, 0, 16.79366134851516`, 1.9200479123498646`, 4057.9804384533572`, 2112.4787378753317`,
52331.57469582722`, 101.50566313118344`, -0.9980603361599345`, 52.81040143589049`, 97576.66884346229`,
2.846658546842519`, 1.000000000000744`, 1.2140531549188076`, 0.21405315491790433`, 0.25343897611680366`},
{0.1763567523046698`, 0.4764578027902213`, 1.019702924150577`, 1.411009603642741`, 0.10601942889051807`,
0.5663043206905454`, 0.11523580705445918`, 0.20578529268229545`, 886.8032845661132`, 265.22978093426616`,
0.0734753688299642`, 5.606338149623012`, 0.8756860344077033`, 53906.15073906257`, 73.78388318178362`,
135.99743517560543`, 0, 11.370032557603889`, 4.133539109844638`, 5152.558848568503`, 1245.5247604158185`,
15304.536241900614`, 83.0761892474516`, -0.9945717930988326`, 28.135099457486007`, 38557.975816438724`,
2.519256551665463`, 1.000000000000006`, 0.7166365593697729`, -0.28336344063023144`, 0.18792435412111297`},
{0.2479337375923964`, 3.025341092699729`, 2.1453632749289397`, 0.9396124783291141`, 0.0672772492322784`,
0.28991645499385843`, 0.23601049551242942`, 0.03982040313739142`, 3865.9337586257416`, 194.53557465876634`,
0.20896839632361264`, 7.896823118164437`, 0.4853457216015631`, 13580.359879980495`, 17.171837910204506`,
316.6001004307092`, 0, 80.31428431178462`, 1.030711974145597`, 111.35851336475987`, 107.04038000729534`,
2962.291419111802`, 4.721105436458194`, -0.9984062657016123`, 44.54650414457621`, 10492.17119111817`,
0.7386847382317175`, 1.0000000000000107`, 0.06092164462699293`, -0.9390783553730078`, 0.046517379122204085`},
{0.1645690290583518`, 3.8533306281399318`, 1.0369585072362497`, 1.3664278344740945`, 0.6261111939893407`,
0.20406169893014847`, 0.18309667446609262`, 0.4646249368336043`, 2770.959571622493`, 166.0796256998841`, 0.048578133356614095`,

3.3069773434670147`, 1.4087558492193972`, 141215.10839119347`, 13.965384930241715`, 221.21162190770156`, 0, 3.433842899967598`,
0.3953165885247419`, 2189.66460460975`, 5538.015230251852`, 42133.72198025718`, 350.3265095762449`, −0.9916853652345169`,
21.76122169105971`, 99055.7959557925`, 1.013698858075048`, 1.`, 3.129696019751797`, 2.129696019751797`, 0.03374463089578711`},
{0.22921820594790826`, 0.8201682437489284`, 5.405881273282434`, 1.1219214796772738`, 0.17914795775514958`,
0.5820849501977261`, 0.05414216426604684`, 0.17169981881854485`, 1492.1967102291455`, 65.66187445337869`,
0.1524226111094261`, 1.916490304748569`, 0.9747174266139518`, 56641.259265565786`, 15.78067415525512`,
159.07458443555174`, 0, 22.646654704620556`, 4.292609952117885`, 3164.1156965690616`, 736.1076645358735`,
13232.75676071685`, 38.395926917216514`, −0.9970984181443432`, 50.29517665039574`, 43331.2680633797`,
1.4729786462802412`, 1.000000000000166`, 0.41550223346196075`, −0.5844977665381083`, 0.017494210040419556`},
{0.22532476518940014`, 1.9533397508457995`, 5.9287214686276055`, 1.441991810272455`, 0.5762935247594674`,
0.4183646366318182`, 0.09350661705723778`, 0.3294355875363928`, 558.5643880556918`, 49.17304258267865`,
0.1200905527225728`, 2.331392728849849`, 0.39194349265472495`, 137621.18924704683`, 65.889040980094`,
373.5453079661959`, 0, 18.374187786846385`, 2.9374745212887743`, 3926.7955438579925`, 1335.793056552255`,
32595.48361618079`, 89.88689999504781`, −0.9972423510860128`, 81.96979642185876`, 104922.42417215512`,
1.6108481735303486`, 1.0000000000000744`, 0.7566976991215643`, −0.24330230087849203`, 0.026694401800860183`},
{0.22156365739259543`, 2.454378375997047`, 0.8895152478568865`, 1.026128317479294`, 0.516725505225655`,
0.686434221522195`, 0.23046056758531142`, 0.005492504017628765`, 1555.2392746607857`, 206.29023226314905`,
0.041365082475744275`, 1.4870135325767126`, 0.5063457915321476`, 112937.99516086612`, 18.850127837391437`,
78.44682487289265`, 0, 9.2437530907824`, 0.02003952949167644`, 227.85062028332695`, 11369.058382756257`,
27112.299798125947`, 539.1067436121963`, −0.9801157870182057`, 0.7026369692789399`, 85815.71862281903`,
0.37144066834364764`, 1.`, 6.489113171292764`, 5.489113171292764`, 0.03127591448741057`},
{0.06425688602740343`, 2.6618634637938463`, 2.643754911431923`, 0.8785376073839924`, 0.5804636394156946`,
0.5001986382462079`, 0.14138114151328934`, 0.3139131008244074`, 2397.9696216564225`, 78.15653575226656`,
0.17684868004389004`, 9.538390690744457`, 0.6033965244651656`, 25818.46410832859`, 72.58026836259924`,
1191.7792037765735`, 0, 34.67020823750693`, 3.929493281107477`, 540.9360954806776`, 136.66052179842936`,
13363.351687701645`, 5.644630116945925`, −0.9995776037143331`, 149.42535137433356`, 12266.96237629646`,
2.3360034276543633`, 1.0000000000006426`, 0.07781220046977948`, −0.9221877995302705`, 0.08870854768131904`},
{0.07720185370449029`, 1.8816151498237366`, 1.2505047699374146`, 1.3773873945814676`, 0.6708624603801276`,
0.176415527179277`, 0.09395893068196878`, 0.03821422736625399`, 2260.6042381201514`, 369.60131923785855`,
0.06295297192499111`, 8.500378555792118`, 1.404314834211414`, 15213.711116444363`, 44.18023598041904`,
204.2471119743812 3`, 0, 52.23787570929633`, 1.0025365679449`, 192.69912602891318`, 191.21156832605834`,

7196.544364304651`, 12.355164008240909`, -0.9982831810126088`, 26.94839992139176`, 7936.950931298855`,
2.1741442788866814`, 1.0000000000000113`, 0.10877400969882466`, -0.8912259903011766`, 0.18369690892831922`},
{0.2310975321060973`, 1.6649278018384956`, 6.117367353219549`, 1.3711053926671974`, 0.5776666118144214`,
0.34300872993879494`, 0.05030597587094107`, 0.013880459047725028`, 1272.690119313539`, 46.12165613423167`,
0.07701873188143649`, 9.481774546264408`, 1.4810089454694788`, 293840.251723618`, 109.92829736137963`,
1398.987377310036`, 0, 359.5067137792731`, 2.8607015611903877`, 1961.8732761347826`, 684.8014491097118`,
68212.69603437433`, 43.57867045298`, -0.9993611354925626`, 68.04087945698083`, 225196.9387406759`,
12.121470926072252`, 1.000000000000015`, 0.38500883356949667`, -0.6149911664305092`, 0.04669181683188204`},
{0.21142405672597675`, 2.9545989174954075`, 0.2974772339667986`, 0.9658276187146195`, 0.64106654096473`,
0.6263249493059542`, 0.09733527159806632`, 0.007468085156923343`, 3517.602581345549`, 75.36708608662542`,
0.23543132772166975`, 9.603866416931954`, 0.19450738989435057`, 28140.17590789518`, 62.967132080359875`,
1134.6761617630461`, 0, 19.15614274976503`, 0.04725400097786937`, 62.7832069748633`, 1327.632616828928`,
6994.168160548574`, 59.21882342075699`, -0.9915331141514744`, 1.9945231448077076`, 21124.791513240576`,
0.8429042639247889`, 1.0000000000003961`, 0.7485840249737098`, -0.25141597502658675`, 0.04985899063444851`},
{0.1982041419042297`, 2.2268219215065645`, 0.4137546724148198`, 1.378661149527917`, 0.7206026710685052`,
0.68793041191814`, 0.15316897603405166`, 0.07890511093433691`, 2517.4293419356973`, 86.78917960165899`,
0.20777476794521893`, 3.6952651421831053`, 0.25322177655756106`, 51171.58539673176`, 40.7241872121191`,
487.16044496735526`, 0, 5.355848925667566`, 0.18356871031324323`, 826.4091522476031`, 4500.906402444139`,
13352.569491033137`, 283.5318957378211`, -0.9787657427337693`, 5.839640403260255`, 37807.63683124019`,
0.47974249243306166`, 1.`, 2.5436300574639756`, 1.5436300574639756`, 0.027335632483639294`},
{0.22157133430375964`, 2.1006687367628096`, 5.865585434381025`, 0.862163364473078`, 0.7961242271327948`,
0.1679554170546661`, 0.16778560934705797`, 0.21616705792607036`, 1509.8006246813002`, 104.18673597092118`,
0.04226318913943389`, 2.3850476310310498`, 1.1811985793876256`, 45818.235145552484`, 19.91300861866803`,
288.4026589403262`, 0, 26.112428510792448`, 2.538781215123007`, 1116.1246984676723`, 438.6301232335984`,
10974.802254855083`, 17.698780242937808`, -0.9983873258185487`, 76.18769040127985`, 34738.59399040204`,
1.0746156297842537`, 0.9999999999999988`, 0.2489106778947581`, -0.7510893221052416`, 0.03486503888675411`},
{0.24168594197714738`, 2.487527173714957`, 2.5005074702928987`, 1.3162259499649314`, 0.4221537034288636`,
0.3099363412058589`, 0.20921038714233392`, 0.06741194388477754`, 2733.252958969397`, 173.34117039752516`,
0.11909087418011138`, 8.486444686364809`, 0.9620364764733642`, 44956.27188032497`, 36.51773448477484`,
485.2980805613829`, 0, 80.88661222439067`, 2.1198257275420733`, 603.6433762185922`, 283.7608500904994`,
10060.945948248764`, 17.475821781825115`, -0.9982630041079917`, 75.33034429715704`, 34736.988552623785`,

1.9441075148028377`, 1.000000000000074`, 0.16100892117440863`, -0.8389910788256033`, 0.08526796280018055`},
{0.1829582147550864`, 1.5256866045998043`, 2.03189641777327`, 1.1222778010348193`, 0.03811462288929479`,
0.5618740829076075`, 0.11263433834449038`, 0.2791855460572071`, 3277.6533486117487`, 172.65623022385375`,
0.030075112520539238`, 2.59232430828043`, 0.783030452394347`, 109967.28761738329`, 8.542082506374587`,
89.92128205389362`, 0, 8.014206594359395`, 1.375825379443063`, 3857.0087642252424`, 2802.414460770136`,
30419.86107982526`, 146.38681158408528`, -0.9951877882939718`, 29.986833595496027`, 79508.04966089358`,
0.5818670792143109`, 1.0000000000000027`, 1.586668531773457`, 0.5866685317734528`, 0.03100958639736976`},
{0.08703489710225831`, 1.1696029176126457`, 2.8929571896991018`, 0.8551088804080895`, 0.7972556998184932`,
0.6510731694726453`, 0.11258011739373719`, 0.045162705810826695`, 3535.7555380745443`, 62.84409202124414`,
0.17813120427323514`, 8.03275953302387`, 1.2552854307001606`, 40369.49326419106`, 70.58373065339602`,
1366.4766221621733`, 0, 105.47268094017657`, 3.8198574770237417`, 893.9986060651179`, 233.0397806573874`,
17917.955261441475`, 9.321077115975724`, -0.9994797912496168`, 63.82452071416339`, 22278.391320891824`,
4.245379099016576`, 1.0000000000000482`, 0.1320261980461076`, -0.8679738019538987`, 0.06805770251472217`},
{0.13518231998646707`, 3.6948829381267645`, 2.131072716483498`, 1.4937759276297344`, 0.7767923162254364`,
0.5165458326040475`, 0.16227719412689456`, 0.04519981251511891`, 3983.684815901678`, 191.70315936858992`,
0.13930377370940616`, 6.306826705642012`, 1.032935178026166`, 25077.89631777037`, 40.01639022689893`,
412.7199304568539`, 0, 61.38063126879721`, 0.7316193362072125`, 182.9458349094713`, 249.05604124390797`,
8521.208969145184`, 17.46237222321658`, -0.9979507165841788`, 38.617825750795085`, 16455.95425055051`,
1.3954741044681636`, 1.`, 0.1418063007475362`, -0.8581936992524638`, 0.09224432387765986`},
{0.10774309849896646`, 3.736554195863712`, 2.2341742787936347`, 1.2045029497948032`, 0.9303574820449074`,
0.4582352004403937`, 0.19359404123783952`, 0.039983935436586286`, 2352.3551825652967`, 320.72717703493936`,
0.06514512285730684`, 6.779792172665335`, 0.8797330310810398`, 5864.196671190685`, 93.64613598950203`,
363.75742889587553`, 0, 74.57391425692403`, 0.7709229304701313`, 39.204040723089555`, 49.85338517454888`,
2263.5963342263913`, 2.9238558863396724`, -0.9987083139152817`, 41.15136157908181`, 3484.098325720771`,
1.5038573306174965`, 1.000000000000171`, 0.02942311779662669`, -0.9705768822033783`, 0.2832326289115518`},
{0.20770433175112651`, 3.109204400403325`, 0.20099665267948374`, 0.8742255636364782`, 0.024813473089767113`,
0.240282569360604`, 0.18576559892192723`, 0.1317848182037604`, 1411.5851094618392`, 158.08107833990675`,
0.17690068584227342`, 3.904436541828723`, 1.357207699254508`, 37313.591743681936`, 21.182120918846262`,
173.94322762124457`, 0, 2.0495690792199155`, 0.08398835178537288`, 536.5664804018825`, 6387.58209496771`,
9404.033849904617`, 253.91080407640473`, -0.9729997990087009`, 3.7305278993386306`, 27903.693807991647`,
1.7647530204123223`, 1.000000000001685`, 3.6135448898221436`, 2.6135448898215348`, 0.04496026736238624`},

{0.09448668201105037`, 2.410919641885151`, 1.350132874851214`, 1.3762843812070134`, 0.004651491449426448`, 0.6365872610465713`, 0.23322630347732265`, 0.08615671221466674`, 2950.762127886288`, 316.0892657764441`, 0.16180448088816285`, 6.47689574973119`, 0.7706785449987734`, 10931.407896987113`, 25.08585044133976`, 151.68561358138692`, 0, 30.103822725976293`, 1.0314798391928706`, 170.17270549962538`, 163.9791872158984`, 4623.669332461291`, 10.775982276057404`, −0.9976693873412611`, 35.52592863598045`, 6241.073913435922`, 1.160911292702464`, 1.000000000000049`, 0.09500495864286303`, −0.9049950413571416`, 0.20834441328645845`},
{0.0849153683065022`, 0.6319892236147719`, 2.114551707925985`, 1.2461670457408607`, 0.7306014894102357`, 0.32449846710553176`, 0.08929492495989536`, 0.016408600115246787`, 558.733384495335`, 217.00633714400976`, 0.13070090260521539`, 5.821164045556291`, 0.7435891013457101`, 11663.312668329194`, 181.0120732822622`, 240.6220503276592`, 0, 77.29601144327721`, 1.7687327612854291`, 220.85339259135765`, 123.86532585671796`, 5227.217440927377`, 7.402574043613004`, −0.9985838404223147`, 15.968857779811223`, 6341.015631636005`, 3.4597633545746125`, 1.0000000000000004`, 0.07201241286269632`, −0.9279875871373037`, 0.26906644429783827`},
{0.20859643515675713`, 1.8877506327350426`, 3.610942473179815`, 0.9188330116771175`, 0.07225013193132535`, 0.31512009685680187`, 0.2084254211369836`, 0.13036688863188475`, 1423.8303839771024`, 148.24888484193133`, 0.09090215154618603`, 2.653159557091028`, 1.1769997259400542`, 30538.69936224344`, 15.77644609837419`, 129.98806888470622`, 0, 25.535726665966354`, 1.6708163676380563`, 710.4484153715424`, 424.2103517371363`, 7654.980981389374`, 18.343697377348168`, −0.9976036913191626`, 45.05835221704007`, 22811.453484437192`, 1.167669598105258`, 1.0000000000000024`, 0.24225915085950098`, −0.7577408491404996`, 0.05644809366910221`},
{0.2775711100993279`, 3.8796646995160193`, 2.028692966768544`, 0.959772508161437`, 0.14615379499145553`, 0.5344882702156523`, 0.050540537283472214`, 0.008717405363258768`, 605.4303926477064`, 86.02465982441038`, 0.19867732233067387`, 8.175417405675173`, 0.7557300476034672`, 62752.474593603285`, 159.08659861668394`, 534.3492252089459`, 0, 110.13236275641974`, 0.24181349115498163`, 123.1643839031978`, 508.3362794396779`, 12613.252555175173`, 22.756913614535833`, −0.9981957933915151`, 13.402218078581575`, 50015.35019575932`, 7.72889912530285`, 1.0000000000000937`, 0.2875531900641945`, −0.7124468099358324`, 0.08980010642698051`},
{0.2734564636981751`, 2.078794252055899`, 5.58118723900359`, 1.49140438303907`, 0.8827845137379815`, 0.5395409262732532`, 0.10413442884619478`, 0.18779130214107398`, 2950.6303080099433`, 94.68533320113221`, 0.033296934697099045`, 2.835225178912273`, 0.8590623879542898`, 129867.90248681772`, 30.60313588937996`, 321.2065516029762`, 0, 32.39708872119856`, 2.791635666130564`, 3081.8553144029706`, 1102.9604314393487`, 26444.361039105162`, 76.62089912109722`, −0.9971025619031675`, 82.90337395123508`, 103305.4493501643`, 0.9079087754515748`, 1.0000000000000322`, 0.6237366578328067`, −0.37626334216721335`, 0.029038540449978983`},
{0.06619581350761466`, 2.7881066711576734`, 2.8537509705176394`, 0.9891677990616923`, 0.8373344986239073`,

0.19790897524975626`, 0.09185213371202688`, 0.00857321041500942`, 2055.3736975529255`, 31.88640228370963`,
0.03523831218645268`, 5.696097023837108`, 0.29187148351753467`, 211744.85380983056`, 41.83132180398387`,
1767.7558026716686`, 0, 107.19519051633432`, 0.3211279214749472`, 565.8501757552502`, 1761.0709315972547`,
108767.30185144061`, 80.83321721586192`, -0.9992568242859764`, 12.790555716561395`, 102856.28612977715`,
0.866242445030646`, 1.000000000000001`, 0.9899950110684763`, -0.010004988931524794`, 0.014653945064054597`},
{0.1298132756954506`, 2.8585402154144006`, 6.437713761400808`, 1.4980481550734701`, 0.9611669791610145`,
0.5357765761577084`, 0.16040271618955604`, 0.016100163586007913`, 522.564427621146`, 43.6121866921153`,
0.20871550454007498`, 5.59776191182581`, 0.8806650157270011`, 96811.6099258385`, 426.7601518271345`,
2019.2769730979123`, 0, 220.53768917971712`, 1.2051044867649907`, 432.7136568563894`, 358.06733532954934`,
33820.76721008133`, 25.246832260138387`, -0.9992535109536896`, 49.21199484562959`, 62719.779686770824`,
6.864149940089099`, 1.000000000000026`, 0.20417141983015225`, -0.795828580169853`, 0.10233765213828833`},
{0.1843088049905594`, 0.4280411676285585`, 1.9800464046244353`, 1.4120188565898317`, 0.7240044582523009`,
0.296746547744528`, 0.10234889391033763`, 0.005447859153693297`, 2517.6110832821096`, 241.50069071394864`,
0.10993322637089764`, 3.2673971617867004`, 1.4127349426750984`, 19812.7165653923`, 20.86684339434545`,
125.71668359676093`, 0, 45.07849900275239`, 0.4883919107015752`, 201.0739335537637`, 410.7061096792551`,
5440.188574441717`, 27.013065437441124`, -0.9950345350960167`, 2.9864549102434954`, 14323.923643980677`,
1.0557284542260066`, 0.9999999999999994`, 0.23274498018996337`, -0.7672550198100365`, 0.05607409941944544`},
{0.17276893398546517`, 1.4696753313802207`, 3.9973169318549147`, 0.8654667209129807`, 0.10876030003077308`,
0.19704558070570188`, 0.12440236076545855`, 0.1569376119224937`, 3938.384639642497`, 57.46421942479361`,
0.12445676420469665`, 2.5408037017095726`, 0.5238442802739391`, 55461.201389090544`, 4.7063821614034`,
281.529902668244`, 0, 23.222670062273437`, 2.3613187042235335`, 1743.727917286573`, 737.4551327898615`,
15970.01223161678`, 29.615433530048193`, -0.9981455597465719`, 49.57674070177192`, 39416.02841416101`,
0.28307528937899895`, 1.0000000000006728`, 0.41501440252440275`, -0.5849855974758764`, 0.0042795214975034695`},
{0.16473183239015682`, 1.8712094759941964`, 3.0571210356797724`, 1.4644853700911185`, 0.18431094252367552`,
0.5500426776194254`, 0.09375157274845106`, 0.031874339239305535`, 3596.1155435077626`, 188.36890996702562`,
0.22217643971583478`, 4.716624148360237`, 1.2836160756903663`, 6445.753152613684`, 16.023924324517214`,
158.70940488613206`, 0, 74.91870634291972`, 1.2142111040441972`, 72.72365413358577`, 58.893748205203885`,
1889.8188811586685`, 4.10119463584436`, -0.9978298477823812`, 32.45776176778381`, 4447.333245411199`,
1.5593246868845165`, 0.9999999999999848`, 0.033973739967536686`, -0.9660262600324628`, 0.09332044926027643`},
{0.24923018426453097`, 3.1125598045900347`, 2.019868693874246`, 1.2491757763779443`, 0.3111545672890146`,
0.6578188710626947`, 0.147672270757542 8`, 0.3114765600131322`, 2384.9574981509613`, 297.0737195350481`,

0.08236658901774402`, 8.424499891492555`, 1.0114329054147206`, 18493.655250311076`, 54.53213571105593`,
226.1019008268320 6`, 0, 24.637553608786856`, 2.308399910345838`, 332.12512895123234`, 142.87677259157158`,
4026.826664174222`, 8.505314700368233`, −0.9978878368974662`, 102.6433253408818`, 14337.23930733527`,
2.2603569478587975`, 1.0000000000020495`, 0.08259791461527896`, −0.9174020853848903`, 0.2625966360689463`},
{0.13234258341034721`, 3.856159276277494`, 1.0641752125958632`, 1.2393517449503635`, 0.10012444404217513`,
0.25632211545729044`, 0.16888027141449713`, 0.016585410322055873`, 915.4984496215893`, 322.7924808984179`,
0.249680065848771`, 3.0405535188651083`, 0.4380264905508511`, 7846.803735762121`, 26.70263290391044`,
65.76611097892155`, 0, 19.954770614361536`, 0.08331511398145605`, 26.798125530330115`, 320.6478289436744`,
2706.0624259657257`, 18.738570576297644`, −0.9930753369188775`, 4.589662137624411`, 5116.104176028228`,
0.9129949005758756`, 0.9999999999999999`, 0.184306474351257`, −0.815693525648743`, 0.09425843114201833`},
{0.20144727254246125`, 3.9392724652693136`, 7.1335815410177625`, 0.970870453628196`, 0.39841924348884183`,
0.5432660762964611`, 0.1877336667874709`, 0.10436699241700248`, 3471.7993430199795`, 68.39740948767945`,
0.03176666869513867`, 6.4702458499294195`, 1.1303015452765406`, 208935.19866832008`, 28.174727851866013`,
871.0230257606944`, 0, 138.5257304600687`, 3.584226693580837`, 2183.4485901287417`, 608.1826150503216`,
53790.91424990663`, 27.5768918088145`, −0.9994873317884002`, 201.70350747580454`, 154800.47090298744`,
2.411800684972319`, 1.0000000000000493`, 0.3440303758956768`, −0.6559696241043401`, 0.05396050323912067`},
{0.05157825200138594`, 0.5074659893035784`, 0.4819934303065594`, 1.4763707530169474`, 0.8893682022937035`,
0.32243152253555507`, 0.2150347661898181`, 0.2715592826957914`, 590.6719840967398`, 259.49776063587854`, 0.04318917041730147`,
4.420302579747371`, 0.3690391195033085`, 68710.21014655166`, 169.1912977940964`, 291.8862639545607`, 0, 3.563046658114056`,
1.5781440875421684`, 6622.034909904955`, 4195.090180978493`, 39550.49081260528`, 296.6727354821854`, −0.9924988861228586`,
11.440777866402561`, 29142.0740273007`, 1.9398136311244896`, 1.`, 2.450991197602146`, 1.450991197602146`, 0.2399475213663721`},
{0.06436950144190184`, 1.0629347664880244`, 4.764095504810017`, 0.9320606212428386`, 0.7789254637724665`,
0.15639368410950716`, 0.08900209182216046`, 0.013774341985629202`, 2340.6363425632753`, 63.84867242722913`,
0.2371034531227812`, 2.23106662065706`, 0.9122412114799938`, 16673.034820507615`, 11.291992844613786`,
312.8291448967664`, 0, 65.9585817860483`, 0.7986739949703013`, 169.38214524623478`, 211.0792041720768`,
8644.686159146877`, 9.169175565433829`, −0.9989393281147944`, 12.127690804911625`, 7949.344831228494`,
0.7369745997846981`, 1.0000000000016098`, 0.11921619368545394`, −0.880783806314738`, 0.011442360103841922`},
{0.18481151841666188`, 0.509983650803119`, 2.442367304149805`, 1.4454403198705184`, 0.8862858075507192`,
0.20520962400760967`, 0.08831882715621944`, 0.028416610934367554`, 2887.1745513215255`, 96.99849051015605`,
0.012592247838918769`, 7.919739870540438`, 0.6672717811625197`, 745879.2039604629`, 46.09727170063247`,
838.0726467850207`, 0, 102.01513942052961`, 4.986118116254259`, 25992.895622712407`, 5212.052522357644`,

204863.22719095656`, 349.3243913292118`, -0.9982948409232878`, 36.32626743232732`, 540872.6297842617`,
1.5524782655750053`, 1.0000000000000095`, 2.9306246375202676`, 1.9306246375202396`, 0.02466132477592058`},
{0.1929089042890706`, 2.044772709758891`, 3.6659878682330156`, 0.820711070285316`, 0.988447409153429`,
0.31657524049709074`, 0.23150398070670547`, 0.04424265567984524`, 3720.3695113921785`, 173.54302693598845`,
0.04329067746612414`, 6.852489239335057`, 0.8460112403464835`, 28338.53467068611`, 51.38438838621834`,
857.5771963604986`, 0, 115.33517395424555`, 2.4000366027354914`, 363.2658982823223`, 150.35848256159198`,
7495.174160389785`, 5.807872098366575`, -0.9992251184596804`, 70.10756210994256`, 20655.51192480782`,
1.1054786684451712`, 1.000000000000281`, 0.08573370585955245`, -0.9142662941404717`, 0.08417312473542105`},
{0.2302575021554365`, 0.7722117915953741`, 6.903211159271258`, 0.7585731891993218`, 0.822213946918968`, 0.5878727883839281`,
0.2427267432025807`, 0.09976117360743081`, 2459.4926457112624`, 40.66955655789843`, 0.10301492463748846`, 3.6967733258702205`,
1.4430046145797544`, 167773.8510368169`, 44.758856767621985`, 1497.1769569560681`, 0, 78.5464969611097l`, 9.260726461434949`,
8194.532394334205`, 883.8692841170975`, 39069.37124783146`, 31.284084949752057`, -0.9991992682771549`, 102.16060246084844`,
128514.51191870145`, 2.736843099718176`, 1.`, 0.499647692087607`, -0.500352307912393`, 0.0282909324286541`},
{0.12214842072252496`, 1.1315944174857293`, 0.8186389490806132`, 1.4945075781385797`, 0.27658322947733693`,
0.6814994349474557`, 0.172591676587236`, 0.06293351186433137`, 2301.8386453341027`, 260.5275220937808`,
0.06414760022752092`, 1.8028968417694637`, 0.30689034634076684`, 44275.7140560154`, 11.691694249854823`,
54.635069429335495`, 0, 5.921483976860416`, 0.30773421103000465`, 1221.1055386983794`, 3967.052608162306`,
16125.636706687748`, 276.63714072617415`, -0.9828448857085162`, 4.9747187895847045`, 28138.87224095827`,
0.2595113626229092`, 1.00000000000029`, 2.279900452248826`, 1.2799004522481647`, 0.04985763260280618`},
{0.08360100641365104`, 1.0333139170716557`, 1.9968821416355915`, 0.9506164337764446`, 0.3637549658684145`,
0.6013653723888159`, 0.09205612227237822`, 0.02498916600861499`, 2612.089754066442`, 275.94814368199616`,
0.0659809292763155 5`, 5.08476480449065`, 0.8697872625899152`, 7517.111801764629`, 29.837626959237948`,
129.27175820375865`, 0, 57.36286512833428`, 1.2794750082995536`, 125.46645886107655`, 97.06089063656181`,
3390.4032531109715`, 4.412619021014547`, -0.998698497290266`, 18.88713332316145`, 4049.1589158313413`,
1.3159637888050044`, 0.9999999999999988`, 0.05626538557121871`, -0.9437346144287813`, 0.17391096454891358`},
{0.14651792469342528`, 3.8182021256883862`, 6.121567760696932`, 0.9268713362782599`, 0.31845465027744546`,
0.3641583988116113`, 0.18264126532755154`, 0.03701030981443568`, 3175.4599520184383`, 92.90395359335434`,
0.14653570379145064`, 5.086657320457199`, 0.6916416664989691`, 12757.395889868814`, 18.06163513056803`,
463.73541529851053`, 0, 151.9564123698959`, 1.440977885286708`, 79.43434863812077`, 54.12530723003837`,
4049.44297573457`, 2.3826407022357623`, -0.9994116127288337`, 78.59921178102388`, 8475.94258527137`,
0.9393337841964033`, 0.9999999999999998`, 0.031137266449731`, -0.968862733550269`, 0.04375934374770596`},

{0.19531388174641623`, 1.6812423772528433`, 1.9285683163805027`, 0.9563894243653364`, 0.3037850003884799`,
0.25494604947946875`, 0.19624000618521847`, 0.006277091862366441`, 3495.8037135332197`, 160.73634765492886`,
0.21386501651912365`, 6.171699440629407`, 0.5167732609586244`, 16790.25465679264`, 16.916919541272488`,
332.49263564161004`, 0, 81.04124008309181`, 0.2899722963426697`, 55.2297939293823`, 189.46576043980235`,
4406.596291312762`, 8.453681121732929`, −0.9980815848417068`, 6.964481612008829`, 12295.277527795082`,
0.677960935086831`, 1.000000000000117`, 0.10720895919211047`, −0.8927910408079021`, 0.03141656030233646`},
{0.0992057581918559`, 1.9152747962131755`, 1.1385439717492112`, 0.8734081572386667`, 0.11569399031757222`,
0.6355935198684373`, 0.242859485933678`, 0.028089883137919644`, 3591.070191292653 4`, 270.70715346890586`,
0.15233109233560077`, 2.1031889712747507`, 0.926279804309432`, 10647.946861111614`, 7.177219751352795`,
61.15158851035912`, 0, 12.967261694475193`, 0.18217229870479837`, 107.51498565811185`, 589.1829554905866`,
4397.525223863771`, 24.248279584162084`, −0.994485925981146`, 4.98442874682169`, 6232.283200017378`,
0.3907863611309032`, 1.0000000000018054`, 0.3379491539009887`, −0.6620508460996215`, 0.05351863294865678`},
{0.271295688290387`, 3.0581992057736915`, 1.212750414175316`, 1.3253429727133483`, 0.8276664257363009`,
0.6720258079110331`, 0.1884616475047557`, 0.2223467966408117`, 2524.6082917357207`, 148.45022765754868`,
0.13442291461399386`, 4.368234255778699`, 0.5211143399174365`, 69784.31886344489`, 58.15760257655526`,
424.4647845488239`, 0, 9.502372337675403`, 0.6708452080086763`, 1187.66516889107`, 1769.401211356211`,
14304.71812105982`, 108.77243521867838`, −0.9923960448365257`, 29.30826117613163`, 55440.11926361288`,
0.8135893436602601`, 1.0000000000000002`, 1.001138318458343`, 0.0011383184583426686`, 0.03843346761056682`},
{0.0730994714406894`, 2.8447930433832704`, 4.637114941779341`, 0.8747237021104985`, 0.8742653369550875`,
0.4602914635394686`, 0.058259665223879015`, 0.01073521329241528`, 2525.027865177576`, 85.21306353882699`,
0.10502971061086047`, 4.901287944789633`, 0.3817190308545939`, 12012.322742050113`, 54.74396740961853`,
471.44486052946394`, 0, 147.11984538730454`, 0.5396903986884178`, 38.35879788311175`, 70.07556105562223`,
5793.045037137634`, 2.9010052681211045`, −0.9994992261842393`, 21.932964168136508`, 6049.55043209815`,
0.9413172494330243`, 1.0000000000004958`, 0.04016821249323876`, −0.9598317875067811`, 0.05251547534110265`},
{0.21011190738341773`, 1.8519766611225768`, 0.8172862439758575`, 1.0025174016275906`, 0.4801024655035677`,
0.5883298648617231`, 0.199167083104676`, 0.2995026554507827`, 1201.2334950278255`, 265.10269406466296`,
0.16527637611813623`, 2.4641537957227406`, 1.185487228781699`, 22330.04165342388`, 35.29657767772614`,
92.36517754580426`, 0, 2.9822939161420625`, 0.4488715321039252`, 659.2081636040238`, 1467.5898223801823`,
5576.454957369012`, 68.92315774614963`, −0.987640327363342`, 11.875708589982871`, 16738.279821864555`,
1.0025986977941204`, 1.000000000000156`, 0.8426769723211188`, −0.15732302767901274`, 0.07152567705920478`},
{0.13575534771417241`, 3.107967888976927`, 0.2262387959194534`, 1.3197095272988466`, 0.6342015998603714`,

0.3957050275427402`, 0.21335490357997616`, 0.008750656688013836`, 1614.3899114822752`, 338.63558488350077`,
0.10329242712159703`, 6.6095853898508246`, 0.24958476272321328`, 28282.999029085597`, 70.7247047016965`,
243.04799088873335`, 0, 10.044209219402665`, 0.027513283587537612`, 69.2246001053394`, 2515.042837456715`,
9618.310292144815`, 154.19481127118638`, -0.98396861750269`, 1.2215771701483227`, 18653.38654475598`,
1.0733708788696246`, 1.0000000000000038`, 1.4374665669236184`, 0.4374665669236131`, 0.1531317292309467`},
{0.06252530163633607`, 1.277236277016332`, 7.142814422389127`, 1.3604161019492071`, 0.8603611689699608`,
0.477237284782289`, 0.23913503177709117`, 0.03184426252613322`, 3236.2308449191105`, 49.142351256039206`,
0.06457622252014433`, 7.960619025437833`, 0.9912633563842763`, 156004.21962918845`, 70.16910649998678`,
2881.8361501779445`, 0, 291.2958362927321`, 6.865310526823733`, 2534.4588524533924`, 368.1688587939184`,
82177.44314951586`, 23.42564691305049`, -0.9997149382359046`, 125.26605225487702`, 73402.42029466183`,
2.593589585062264`, 1.000000000000207`, 0.20851328510485997`, -0.7914867148951832`, 0.0647593111949024`},
{0.05927125459941385`, 2.080731116332318`, 1.403515084093046`, 1.4338880552058586`, 0.836296749585115`,
0.24670417168146108`, 0.17625476342874957`, 0.3553142034940628`, 3624.424617443623`, 371.6887670168725`,
0.06984462665929475`, 7.678587831183411`, 0.5475538898722119`, 9663.14142322163`, 37.00329580109272`,
294.0566953529251`, 0, 13.73462306617252`, 2.190760030339117`, 262.7440799081508`, 118.93284351982611`,
5188.636799141869`, 8.05914375232606`, -0.9984467704978581`, 65.11975090776721`, 4393.38589636893`,
0.7910436216182437`, 1.000000000000635`, 0.06814492016865455`, -0.9318550798313887`, 0.1390598996321286`},
{0.272219924072492`, 3.699843372922853`, 0.8152026115407764`, 0.8049313533010627`, 0.8541230874020713`,
0.3759570960402523`, 0.18901354163393813`, 0.05512718200659539`, 2420.333744013932`, 22.324560553991944`,
0.136832191006448`, 5.284550340747209`, 0.8839580209731261`, 333036.53014934174`, 51.52886492451052`,
3564.5778139342324`, 0, 17.83829001498452`, 0.26063486297448263`, 2724.461876222068`, 10452.175162866855`,
68115.04594694368`, 388.433631794786`, -0.9942973886841778`, 13.775831007554272`, 264889.60908387613`,
2.088470728860619`, 1.0000000000000728`, 5.875324575519532`, 4.8753245755191035`, 0.00801015988185487`},
{0.2192562547626593`, 1.328996578641208`, 4.408276687847465`, 1.066557441081671`, 0.8788419772651497`,
0.420358813032901`, 0.18871280922209183`, 0.12866248281712975`, 2376.751601415135`, 21.932515037813403`,
0.11160054269697284`, 8.38604448753847`, 0.8324170692270734`, 436418.6681431768`, 95.843589810574`,
6030.021497104598`, 0, 96.50477671007404`, 8.861820088702801`, 14142.323787519646`, 1594.8712370552998`,
105547.08247986711`, 78.98776150594406`, -0.9992516348187928`, 168.2475511202847`, 330597.9715094424`,
3.195852969350114`, 1.0000000000000244`, 0.8972033871276544`, -0.10279661287236752`, 0.015320793636208061`},
{0.05422626465899324`, 1.41058877238651`, 8.629501826749408`, 1.1478067099640077`, 0.4503418362410423`,
0.2944196840300318`, 0.21119418614347968`, 0.01982525406608443`, 1227.9750083569616`, 89.64728336361748`,

0.015407425530496982`, 1.3479803050296402`, 1.4374405820845841`, 3724.9198269790413`, 12.258825763192185`,
144.39059776147369`, 0, 69.54336245384395`, 0.9184987284329444`, 38.868772481682974`, 41.3177205133394`,
2048.774199903545`, 2.3049547717741157`, -0.9988749590990151`, 18.508914196839903`, 1587.105314149989`,
0.9575017284131802`, 0.9999999999999823`, 0.024336675427395378`, -0.9756633245726042`, 0.04930443717807061`},
{0.16726133018607092`, 1.930958758925592`, 3.0083121233683894`, 1.210686290991208`, 0.7910212105934074`,
0.3434585725088355`, 0.1642660591272488`, 0.14345094159717983`, 3630.780310378209`, 105.55549533534378`,
0.20790081387395154`, 3.589057052449082`, 0.2001887825986941`, 2523.200646511847`, 19.279101869018422`,
427.70039949201714`, 0, 26.5872358492485`, 1.8912436873908618`, 59.32216847649362`, 30.366750288184072`,
720.6342386194924`, 1.7720187483530956`, -0.9975410289250928`, 52.17019376328746`, 1721.9177332731824`,
0.2653435061374191`, 1.000000000000536`, 0.017760783633589957`, -0.9822392163664195`, 0.03986254137593107`},
{0.13700552002384053`, 3.065949790291736`, 4.950454801095971`, 1.0391958372853642`, 0.9776337698753284`,
0.6909746470667117`, 0.057475002733817115`, 0.00904662991314958`, 1580.3042677972962`, 74.3483640784055`,
0.03877364360478597`, 4.297289530434117`, 1.330917817336955`, 141784.71733262957`, 140.80814486393734`,
500.5508414554062`, 0, 141.11872827253845`, 0.4033248220796779`, 362.2111594571783`, 897.063148182887`,
47891.37352962356`, 43.44267805206987`, -0.9990928913737419`, 17.665337909637763`, 93734.03621545792`,
4.0330405462440515`, 1.0000000000000178`, 0.5065278152676421`, -0.49347218473236687`, 0.059179086486968686`},
{0.1031747291007129`, 1.0753817850602383`, 2.3901827825214443`, 0.9494324054212877`, 0.8655243169054956`,
0.5875365461047798`, 0.09875542522403158`, 0.020636236338079122`, 3668.9306940550296`, 32.944367358953286`,
0.21955795791262667`, 5.455907508975919`, 0.3138297355806803`, 45363.74877918646`, 49.42988267080978`,
1740.933134942887`, 0, 74.62842674040822`, 1.3434337080696466`, 631.2266616687074`, 468.8606696237394`,
18297.6896301533`, 20.71129410489552`, -0.9988680923917976`, 20.638630558486167`, 26969.416725142815`,
0.6086980356233643`, 1.`, 0.26437745400419627`, -0.7356225459958037`, 0.01661149456380435`},
{0.2492174803391337`, 3.8186304877300854`, 0.6593165104089422`, 1.3669144880495308`, 0.40531282588620576`,
0.25181467777873967`, 0.15220457771402102`, 0.006740465790302422`, 796.5961892440373`, 51.67728195440782`,
0.07309861388864569`, 3.0773122735176646`, 1.432188704910093`, 469910.41339711193`, 40.16771912361044`,
506.2117339674609З`, 0, 13.763033117368309`, 0.023778204438811068`, 742.9881897449746`, 31245.606179071307`,
103041.57545375846`, 1949.92159405025`, -0.9810763608236434`, 1.2971453773360424`, 366853.74006800476`,
3.4283006423290656`, 1.0000000000000002`, 17.60204557856539`, 16.602045578565388`, 0.0224061282019231`},
{0.27928795263916784`, 0.5798885963915357`, 3.438729573463075`, 0.7859704525714069`, 0.13732274355578267`,
0.6009735798147406`, 0.18539967791007028`, 0.1369068620522905`, 2588.2655297569117`, 78.78627673888747`,
0.13662571551540947`, 7.147737978252394`, 0.16582779201112174`, 11421.504435574754`, 34.19428690603585`,

```
683.343810573242`, 0, 62.48342568172516`, 13.090359561776316`, 814.0156572985338`, 61.184361969357155`,
2252.0793781637217`, 2.2906104198912`, -0.9989828909042456`, 108.44214617912843`, 8985.409124403395`,
0.737849566050369`, 1.0000000000001894`, 0.03531589831148912`, -0.9646841016885176`, 0.09577097976351072`},
{0.158309183474712`, 2.284367344540179`, 3.1130137774580398`, 0.8245076604975312`, 0.2939127991946775`,
0.5448027938340341`, 0.15596740404331277`, 0.060580071785009876`, 1917.5874264102922`, 102.41199828758255`,
0.15653233667838135`, 8.020771066756815`, 0.16368718495509893`, 7228.055683558272`, 57.02310506220705`,
625.8741338703402`, 0, 101.20577068673056`, 2.5886478644778737`, 99.7006947960262`, 37.514583680594846`,
2158.40977302579`, 1.4974372861867495`, -0.9993062312333364`, 84.4774664018132`, 4881.372696736444`,
1.1209549120839422`, 1.0000000000000029`, 0.022000771906003604`, -0.9779992280939964`, 0.16613994271485275`},
{0.09855067343586066`, 0.42248858314270876`, 5.888441909786696`, 1.374275487614058`, 0.713481343784103`,
0.39177356523031326`, 0.12647140495570885`, 0.021778948867459624`, 3561.4755687032084`, 75.36389346411136`,
0.14783328914983274`, 7.192274801142693`, 1.4077827751177328`, 37810.215351567706`, 38.4709948604879`,
992.3772176577374`, 0, 241.34237414649746`, 10.607823046051715`, 1275.2779762908058`, 119.22051751376742`,
15571.523972905274`, 7.698860942030008`, -0.9995055807668263`, 64.02405898507097`, 21922.63105646375`,
3.4260958782048063`, 1.0000000000001419`, 0.0678509723363943`, -0.9321490276636153`, 0.07392935257343049`},
{0.09888970514692547`, 1.0773269521808775`, 4.509787207697677`, 1.034834313165084`, 0.46524158243265124`,
0.6370595817977545`, 0.17795346839566295`, 0.052840345624928245`, 1394.9195521303727`, 145.8914281907807`,
0.14189384893594092`, 6.214985461828465`, 0.5648213653786103`, 5247.909614584016`, 79.74835700269983`,
406.43944052091473`, 0, 123.21156924231298`, 5.601336558820972`, 137.72786352384136`, 23.58839280188366`,
2085.9886362211287`, 1.2157418385801606`, -0.9994171867394338`, 86.2067263293417`, 2946.8971596535034`,
2.072955988992506`, 0.9999999999999969`, 0.014229963016584418`, -0.9857700369834156`, 0.24139656925220732`},
{0.2613599171041266`, 2.58453322148865`, 6.473133758347821`, 1.1092209897073517`, 0.7935873547661896`,
0.22081691696159222`, 0.21037162231016598`, 0.22559388527799015`, 2971.40901940996`, 103.45193855805343`,
0.031105461375102783`, 7.240063151509036`, 0.6982732456981431`, 48551.436545424214`, 37.246972244069156`,
1040.0905618445931`, 0, 83.25215415740813`, 7.003304864019305`, 977.1108538791376`, 138.5213935208239`,
10182.83210429194`, 7.20909977966509`, -0.9992920339149434`, 258.57534401815525`, 38019.773638042476`,
1.1927136171469992`, 1.0000000000001383`, 0.0787333519257199`, -0.921266648074291`, 0.0546323822136633`},
{0.14168212332279645`, 3.629529656644613`, 0.31240616809545685`, 0.7793905839088386`, 0.6288942527555839`,
0.6686461551742169`, 0.051179205682700984`, 0.015333271439627172`, 3119.547847449303`, 332.77374740974926`,
0.18323666728985671`, 8.115016271373861`, 1.3496472408131814`, 10194.19003037813`, 60.93362138325542`,
170.8156633229224`, 0, 15.60384145176738`, 0.06404463380270323`, 34.65937127084973`, 540.1752587675318`,
```

3364.7788551021304`, 19.56116195201547`, -0.9941864940329276`, 3.3207413962265098`, 6810.41446717881`,
2.4392269323349445`, 1.0000000000000018`, 0.30560279535363066`, -0.6943972046463699`, 0.11656208958740022`},
{0.22123407652153293`, 2.082927160793078`, 3.494558969518348`, 1.0455662143345417`, 0.1785350961948371`,
0.6180201085084245`, 0.0648475297068132`, 0.006469955463562222`, 2336.7510217065665`, 120.4669332891844`,
0.020699881273580856`, 5.403351657201977`, 1.2197076183004314`, 197545.03965499974`, 29.98554160994682`,
262.92468541275355`, 0, 129.16449228560455`, 0.38539370250362`, 543.4320975627493`, 1409.0699986337863`,
47447.305443987236`, 68.57369686653198`, -0.998554739911469`, 11.467814436334224`, 149956.582904794`,
2.8993908418141245`, 1.0000000000000004`, 0.7951043234262846`, -0.20489567657371577`, 0.06454589268159407`},
{0.09707681532069068`, 2.1085331709443667`, 7.06540706713491`, 0.9411343900767416`, 0.9036401467365582`,
0.2916719417534003`, 0.09652690802667035`, 0.03828184000699648`, 3174.8977687655497`, 60.92568989682479`,
0.05469741369744979`, 2.8213654015691247`, 1.0633604979013946`, 64300.36440319607`, 18.78814805422754`,
485.10397182717634`, 0, 95.99395324603339`, 1.677584771182131`, 730.4398758927015`, 434.41160389646836`,
26877.754385476943`, 19.045158525327885`, -0.9992914155605344`, 50.53204481583759`, 37274.38283876906`,
1.07714813975594`, 1.0000000000010951`, 0.24514921209454626`, -0.7548507879057222`, 0.018259680230534925`},
{0.07690717877213288`, 1.853650900512208`, 4.705122089204835`, 1.177168847413763`, 0.20514199762323693`,
0.5853606940036812`, 0.13349096224239904`, 0.025824642502794638`, 3833.38590479347`, 77.1112215680152`,
0.1481478527297927`, 3.8687163896946233`, 0.3394641684298805`, 14185.631897286317`, 12.91535929161814`,
350.59689902386395`, 0, 99.12937515551224`, 1.3277248249985474`, 139.11615843654897`, 103.77785442981468`,
6694.690026935928`, 5.7544383590442365`, -0.9991404473790584`, 35.159118821299614`, 7355.281753222543`,
0.4010164077517141`, 1.000000000000265`, 0.05922764819136298`, -0.9407723518086527`, 0.033406720328728774`},
{0.2792305556244907`, 3.773985442432103`, 1.91428975676104`, 1.3562902020721337`, 0.4175948252900008`,
0.22518067870989456`, 0.1671047483185617`, 0.02746479862850403`, 1101.9393721601946`, 344.3881723084337`,
0.08960284517652922`, 9.994170351350675`, 1.3158131558054151`, 16060.338653642963`, 92.79862179898906`,
263.9872605170865`, 0, 105.28957990593808`, 0.7398088525333634`, 82.4137419988413`, 110.39869672636105`,
3189.893214495866`, 7.132024467358357`, -0.9977641808086402`, 39.88611199490442`, 12724.50935237819`,
3.558883377365687`, 1.000000000000027`, 0.06379899944711705`, -0.9362010005528847`, 0.3305657000514901`},
{0.22478599831633878`, 3.8226352096828684`, 0.3445246491273739`, 1.3626729827827502`, 0.6328825985865549`,
0.4911616627377584`, 0.2351834938983145`, 0.020644881197453675`, 1711.2523897617402`, 90.3557844360617`,
0.22332245485704472`, 4.143025581982037`, 1.4109410839085532`, 86530.84101859828`, 47.16199653584685`,
594.3196999564491`, 0, 8.2383780451267`, 0.04353390938411032`, 354.4005295229256`, 8139.792649609368`,
20545.1159296915`, 505.85072444655606`, -0.9753785413224405`, 2.3773464975263345`, 65975.06298577909`,

2.4687119552777426`, 1.0000000000000002`, 4.607301179249813`, 3.607301179249812`, 0.04617961306783235`},
{0.05455278033197386`, 2.653355923086507`, 2.8901946723921075`, 1.0586559640275577`, 0.4813227217769469`,
0.39307761087509174`, 0.2243292319383302`, 0.14625278281931525`, 3083.4776957133126`, 151.39135551576533`,
0.22240872343945634`, 6.123940268706669`, 0.19893741817415211`, 939.0638365127197`, 27.697283904559793`,
431.53545971963234`, 0, 43.40844955347903`, 2.306183035926932`, 16.32417287803158`, 6.078437671132356`,
452.93773225705024`, 0.35239116964476397`, -0.9992219876054734`, 87.4160631156906`, 352.985894455447`,
0.5215984036629125`, 1.0000000000004499`, 0.0040322798558596155`, -0.9959677201441421`, 0.11180561029287356`},
{0.1261136314023228`, 2.7924651112189345`, 3.1510744750802027`, 1.10573405830265`, 0.8251438792845518`,
0.38426336320599863`, 0.09970139546376985`, 0.07482975828652592`, 1939.6449909308958`, 136.19904432267293`,
0.18392510915657945`, 2.6983520820510374`, 0.2768728179957576`, 5084.736516476803`, 30.715871758503077`,
196.38892970848963`, 0, 31.059547400840234`, 0.8042254208628764`, 64.24551692407655`, 78.88496167547862`,
1792.1003419740405`, 4.141835005203091`, -0.9976888375565841`, 32.0824489902136`, 3228.6897423384353`,
0.4525211343446359`, 1.0000000000008091`, 0.045449044988017655`, -0.954550955012019l`, 0.048810709785868704`},
{0.08906555841605851`, 3.099177869846099`, 0.23414284508699804`, 0.9801911457566903`, 0.5103708245250258`,
0.6482190821775309`, 0.09673419046052228`, 0.05503363591579261`, 1161.2636269890509`, 33.10846443598513`,
0.20242548439112923`, 4.392158138354276`, 0.5844178562263633`, 88437.80538320878`, 72.79268954561743`,
1046.442031288759`, 0, 4.293056616992735`, 0.0742609282354573`, 859.7867107696484`, 11576.914943960084`,
38915.44733220055`, 522.0926665444877`, -0.9865839222638851`, 3.2878260768794116`, 49514.657823616515`,
2.6288010645746316`, 1.0000000000002076`, 6.535708735518346`, 5.535708735516989`, 0.03340571170509742`},
{0.0485781063525863`, 1.7849942773456657`, 1.173858124805676`, 1.3582711053956302`, 0.42211815922461504`,
0.5511485692462849`, 0.057310028881033426`, 0.193275446076273`, 3997.3620434488857`, 116.86597951838672`,
0.12079571732839778`, 1.998996511553914`, 0.9355040158846233`, 35183.134650513544`, 7.645720031419541`,
105.67802930872963`, 0, 4.7290602608002095`, 0.4823072674937298`, 974.6671373434853`, 2019.8427345668315`,
20758.837435626876`, 127.07240786217797`, -0.9938786356289832`, 12.298795891407583`, 14406.07161005622 2`,
0.6342429928398561`, 0.9999999999999999`, 1.139516127810565`, 0.13951612781056522`, 0.020300048409452272`},
{0.042020095209803626`, 0.8125545855681908`, 0.8820021130226205`, 0.8679479110418857`, 0.336031336984445`,
0.27953459644666345`, 0.05504645570926081`, 0.01654003058638047`, 2110.5518526782043`, 28.793440121823267`,
0.16487272926817176`, 7.902234236019242`, 1.1698954211202324`, 82122.38334347398`, 38.61823651310428`,
1695.9581792736178`, 0, 41.833727116685004`, 0.7805262014809571`, 1244.9080438646795`, 1593.959966113389`,
51283.08260495151`, 64.12329715313105`, -0.9987496208516347`, 9.060287773849614`, 30784.571624461278`,
6.725434234061408`, 1.000000000000198`, 0.8954794272823872`, -0.10452057271763049`, 0.03144602691951818`},

```
{0.06217291008845033`, 1.247503477484723`, 2.5904087629086465`, 1.4683499815329046`, 0.08458788439332876`,
  0.3930546684373296`, 0.18769435268404766`, 0.1379057398065497`, 1008.6694100163231`, 278.1009416145228`,
  0.21639096057090101`, 7.204492217199894`, 0.5358436507882129`, 1747.350945151064`, 68.78525682077775`,
  188.9982852424403`, 0, 50.54284616956075`, 5.0803218835072235`, 61.58036689588803`, 11.121351423775483`,
  847.9995203699301`, 0.862295966336415`, -0.9989831409739945`, 90.5388459488147`, 753.1799705001247`,
  2.374244008638594`, 1.0000000000002633`, 0.007115772495320295 4`, -0.9928842275046815`, 0.4410299334722293`},
{0.11128373013292153`, 3.3734167013736274`, 6.971024005221597`, 0.8270211193396084`, 0.0351306115056782`,
  0.4871945686666914`, 0.14931850539545372`, 0.02878252625021986`, 527.2114941578984`, 74.82432219823664`,
  0.24191356032713884`, 9.568158477220692`, 1.3339247715553384`, 14829.128329327828`, 186.73785722440502`,
  853.3440946197896`, 0, 362.7476511863767`, 2.985640211657711`, 87.83606326374475`, 28.419507052718757`,
  5529.2403125607625`, 1.154098605192919`, -0.9997912735674427`, 143.8829793471256`, 8790.206954044039`,
  12.12824357851539`, 1.0000000000000209`, 0.01689679276630817`, -0.9831032072336922`, 0.3336191408692054`},
{0.22883543071148366`, 3.066706533284199`, 1.1439898434307647`, 0.7535281320523861`, 0.1149711120814263`,
  0.31581178336786275`, 0.15168936854496579`, 0.1415060368713317`, 2986.3979531848126`, 146.71341498099025`,
  0.14475182625551103`, 7.74072484577729`, 0.18905729960113726`, 14766.12435511747`, 23.369032049887267`,
  369.92168759759517`, 0, 21.874100170774213`, 0.9817241228542736`, 220.46663495131764`, 223.57086448108348`,
  3443.427490855988`, 7.870386010253807`, -0.9977143744042372`, 43.00942544914149`, 11256.831614197052`,
  0.6734503510399388`, 1.000000000000031`, 0.12672908959694903`, -0.8732709104030549`, 0.052529755443632795`},
{0.089875647559861`, 1.0302327249870382`, 2.0596378930749815`, 1.3241139107745634`, 0.7453299280714754`, 0.5095722380183404`,
  0.17917298341587806`, 0.222730051899884`, 744.7092202123449`, 118.59827741373601`, 0.0989210230322109`, 4.9882080789644085`,
  0.660240574996108`, 58896.59715327873`, 157.23812779156611`, 531.5215606169223`, 0, 19.12779574265142`, 3.7619583851476484`,
  2851.1180111962526`, 756.8813265060486`, 25753.027069499272`, 47.56652910544818`, -0.998152973280497`, 55.367037691693135`,
  33065.28549282687`, 2.865067090232365`, 1.`, 0.4356807393112678`, -0.5643192606887322`, 0.15623402206466921`},
{0.16322971965863348`, 1.170728402327721`, 4.129867013138943`, 0.9550069661931267`, 0.7632521383792994`,
  0.23062906697550656`, 0.15191938086081902`, 0.036668793904547074`, 984.9683279569181`, 143.85195072672764`,
  0.06218474573819438`, 6.894986630118397`, 0.7118840534242801`, 24296.94210179531`, 105.05574096003002`,
  573.0429440807337`, 0, 141.42061322142752`, 4.131313411599043`, 471.22918022791674`, 113.0628011675167`,
  7227.894153611617`, 5.112193358961252`, -0.9992927133062115`, 69.0949421410923`, 16854.387663090027`,
  2.8487526147628466`, 0.9999999999999998`, 0.06484786997832534`, -0.9351521300216746`, 0.14243770284591784`},
{0.0673211374947203`, 3.556048182108581`, 2.056072368577791`, 0.8749285572736749`, 0.3104681364634243`,
  0.18652482922479574`, 0.0820102065725382 7`, 0.044529186328014586`, 1689.51074710815`, 162.90358092109705`,
```

0.0729615101290263`, 8.407666834293693`, 0.4031543367979451`, 17891.111979198937`, 42.898826054268085`,
346.3601253195717`, 0, 79.16618131242582`, 0.9636341223994327`, 134.28589174214054`, 138.35360799363448`,
9054.230019689441`, 5.681311235286162`, -0.999372524088417`, 48.953276702518686`, 8707.72948663341`,
1.706146126859274`, 1.0000000000010079`, 0.07865693795931858`, -0.9213430620407607`, 0.09117470211748833`},
{0.04130707589470009`, 3.0971581646939166`, 5.095324578907563`, 1.2851498582955627`, 0.14534319212364366`,
0.3324535366896153`, 0.10589589728908583`, 0.27538535378439405`, 3967.498675941073`, 98.17019510308785`,
0.19069934275540157`, 9.378576326653985`, 0.907790255687607`, 9252.803216163345`, 22.36790359379976`,
623.8807588652744`, 0, 73.34661288041137`, 6.250739801515133`, 163.05665748653558`, 25.085977446543506`,
5594.795324431349`, 1.5617982109757427`, -0.9997208480166995`, 276.5647115948540з`, 3301.4947868799804`,
2.219344601775325`, 0.9999999999999967`, 0.014715605838282029`, -0.9852843941617179`, 0.08395383994382918`},
{0.15433792416794267`, 1.8940009891686929`, 3.062826353150152`, 1.3594047846621364`, 0.2865379799536014`,
0.5165321095788333`, 0.13923535270760634`, 0.41888186557593104`, 1933.0228901580695`, 124.09667777854662`,
0.10026068856071241`, 1.584303992606234l`, 0.6139086768148141`, 43033.801801317684`, 10.416962687156445`,
92.91530863535512`, 0, 5.333174253304456`, 1.1068660775585117`, 1314.1764617456654`, 1186.2949116341445`,
13416.451654267346`, 75.26268842810495`, -0.9943902687262197`, 29.94864922532979`, 29580.961400273973`,
0.4384048524753169`, 1.000000000001182`, 0.6740039096658308`, -0.3259960903349658`, 0.02399716105509026`},
{0.2448264512865554`, 1.018619142628224`, 0.3220150078672237`, 1.1814459661349885`, 0.2096682202943383`,
0.4340268291716256`, 0.23122304821991574`, 0.028723954302408285`, 3147.8296084170843`, 26.23322055856977`,
0.011843614667667979`, 1.6271943864377292`, 0.8914600074049073`, 4.311885886713754`*^6, 5.919891083371849`,
539.6520828774846`, 0, 2.7501204459967656`, 0.07251378492107487`, 80669.80679377935`, 1.1124744676863388`*^6,
958724.209333067`, 57383.13088033896`, -0.940146362925103`, 1.0551989917861808`, 3.3531577990503456`*^6,
0.5022713319551044`, 1.0000000000005058`, 625.4106907806579`, 624.4106907803415`, 0.003035540134896684`},
{0.26599408746951475`, 1.69869318027059`, 1.7982317117152924`, 1.282444106635544`, 0.20521633822817087`,
0.28537099789283193`, 0.17587118168619353`, 0.016831793401851286`, 735.9167536628461`, 185.12449867405962`,
0.08818428944304713`, 9.590487549937748`, 0.2554550886680036`, 60778.671008121`, 121.91231011789202`,
406.7282959733328`, 0, 104.51809158670798`, 0.9890147577863672`, 466.2816871387952`, 470.46079820126874`,
12635.454713140787`, 28.3109464930384`, -0.9977594042212351`, 24.000466060552473`, 48013.660659775225`,
3.305658942879598`, 1.0000000000000009`, 0.2678418609434057`, -0.7321581390565945`, 0.16229678289112007`},
{0.26593765918786066`, 1.469820509215408`, 3.318163900631456`, 0.910737040027697`, 0.772245323565576`,
0.6475514852601838`, 0.05903920363599993`, 0.25524741893447345`, 3882.180697561048`, 173.8977279389328`,
0.22560057599213157`, 8.523060233161317`, 0.6568135548380971`, 3090.014053511762`, 64.67015889508639`,

388.29228556013265`, 0, 46.67219135855951`, 7.5265679659056515`, 110.23915996629233`, 13.646670363658579`,
599.648063033092`, 0.6241113282773342`, -0.9989592039618698`, 158.03862800416886`, 2278.128603136497`,
1.7886223818399842`, 0.999999999987838`, 0.008303872170966146`, -0.9916961278290237`, 0.13590128549292765`},
{0.10144857451882727`, 3.09793869586695`, 1.9042542140912566`, 1.128489317749993`, 0.9516046831815148`,
0.5718665343974156`, 0.24712737605206275`, 0.10884528767737062`, 614.5820043578747`, 372.33327243103304`,
0.18531918510116352`, 6.894885736301427`, 1.4197916184504975`, 3571.0958627875825`, 462.4955261893786`,
393.70557255144394`, 0, 43.37485979418784`, 1.416266017583714`, 48.02362175662228`, 32.90861685614345`,
1414.1487403279605`, 1.9244053005036745`, -0.9986391775874599`, 62.67864713591417`, 2049.476769483811`,
3.4169362859041983`, 1.0000000000013543`, 0.020671076878055965`, -0.9793289231219721`, 0.7602977544304027`},
{0.10212230421521268`, 2.1055462656508395`, 0.1549942156465125`, 1.1640680269346606`, 0.2898972915158997`,
0.471231711737255`, 0.20995913095460078`, 0.12219762483818075`, 1171.3965983974595`, 79.93077709858363`,
0.21373183331424755`, 1.9880929888308274`, 0.4174963770861413`, 44393.09067278057`, 21.821309197560765`,
222.454416520487`, 0, 0.843145222522877`, 0.04703417044637621`, 908.1547244375527`, 19307.4031421994`,
18053.177563652403`, 994.8310426344464`, -0.9448944076948868`, 1.4147517420193165`, 26337.60130295092`,
0.6176352525965214`, 1.0000000000000062`, 10.949058842562863`, 9.949058842562795`, 0.02490182782153062`},
{0.08578994575352372`, 0.9866526872309773`, 0.8038090794361139`, 0.9523570781926003`, 0.948882623737388`,
0.4631164799606274`, 0.2441006263954396`, 0.07831609826614802`, 1671.5327042927038`, 210.47342650267535`,
0.12673136991162653`, 7.353061586513307`, 1.0620625305759188`, 23128.211128322382`, 151.87260885622555`,
734.6970370514715`, 0, 21.351093185922423`, 1.5609934065183986`, 809.0410762531961`, 517.286030469316`,
10371.848157722161`, 23.34356965916386`, -0.9977493336477565`, 22.002261989874484`, 12711.432725925244`,
2.5030449650464894`, 1.`, 0.2973955902253344`, -0.7026044097746655`, 0.2127635053991033`},
{0.09782332022288404`, 3.741906492613495`, 0.4566213801268671`, 0.9419423117532648`, 0.9082792827328243`,
0.61004571220758`, 0.08136955531902801`, 0.05934353731887512`, 1613.3231664795385`, 279.2732289747655`,
0.2228587683695682`, 2.342091864078169`, 0.3301362991730612`, 7935.419910834372`, 54.410635937224846`,
80.97183783170513`, 0, 4.447097490654997`, 0.06836721068006808`, 66.85815844638898`, 976.9272516946628`,
3306.4973043007244`, 43.30496169324084`, -0.9869030706188949`, 3.654624421794603`, 4620.750637353064`,
0.5119345910058614`, 1.0000000000000007`, 0.5639275828213066`, -0.4360724171786937`, 0.08035158797378938`},
{0.1509673631351663`, 1.3366819546761866`, 1.080587080528149`, 1.0668100793723834`, 0.024191324402023318`,
0.40903296900163466`, 0.12044262077141116`, 0.013316823826732663`, 3093.793000213601`, 297.44428012608614`,
0.05031916378077894`, 4.50357864911024`, 1.4241445207811716`, 41445.137231853645`, 13.506398824876664`,
93.15643070635652`, 0, 30.766295183345292`, 0.2887107411400315`, 329.64284564346747`, 1140.775482068341`,

13117.76764510288`, 56.79154847260424`, -0.9956706392422032`, 5.513063397186693`, 28290.782737157057`,
1.2211077093657445`, 1.0000000000000298`, 0.6472464124727692`, -0.35275358752725006`, 0.08032181308585948`},
{0.2168252075927255`, 1.2147794793098745`, 1.3294046517319291`, 0.8204909797710571`, 0.5261154919084852`,
0.32065750745864985`, 0.23412216559654364`, 0.026797285872998095`, 2049.1492401808873`, 336.0554255621465`,
0.1855137474935905`, 1.9365416597785146`, 1.1594140358846108`, 8491.108876647051`, 12.163160154464155`,
63.58920424311334`, 0, 14.11978102662532`, 0.29189738194823384`, 128.07415152775752`, 437.7643036499405`,
2067.5100733261993`, 16.851219113237928`, -0.991849510514777`, 5.0655849950713`, 6404.118583557185`,
0.47642870274199794`, 1.0000000000000282`, 0.2506651531778178`, -0.7493348468221892`, 0.047446512064921 2`},
{0.06878866730089028`, 3.1245944973527457`, 4.360637333709242`, 0.9232062619650272`, 0.8528374363112068`,
0.38941545287751145`, 0.1888087204525729`, 0.06440167068123348`, 613.447059996161`, 135.8084051425272 5`,
0.06946957961295136`, 9.642980612869511`, 1.3375272307560842`, 21678.66438510157`, 384.79191652111206`,
1084.539973888157`, 0, 172.12514154413876`, 3.3770634770774235`, 227.6183596580739`, 66.40126775912998`,
10769.355238269549`, 2.987270180437818`, -0.9997226138320869`, 150.74219939552881`, 10582.994207577516`,
7.9791993076574625`, 1.0000000000001195`, 0.0391818422640818`, -0.9608181577359229`, 0.4939523870399271`},
{0.05526353896520764`, 2.7240387277783293`, 3.0534884167300707`, 1.3987200069968335`, 0.586176455097533`,
0.6957346200792549`, 0.06800542073977661`, 0.01575809860169522`, 1863.0130158242628`, 26.33179097220551`,
0.24642894997981157`, 6.726887116499235`, 1.3266658102153124`, 102675.06613566946`, 80.49953669302113`,
1887.4659982723028`, 0, 124.86431708966455`, 0.7000953345016961`, 472.6713507850587`, 674.1528363226273`,
57291.00683087416`, 43.8607869173933`, -0.9992344210838033`, 27.24409720456488`, 45230.05411934287`,
9.372817937686383`, 1.`, 0.37989901438316603`, -0.620100985616834`, 0.050265782842964166`},
{0.16680821963704395`, 0.5163225257189996`, 0.5139271367473146`, 0.8080955581319136`, 0.922329371415127`,
0.31540072696317234`, 0.14858531583274454`, 0.09051887795629471`, 1828.24438708676`, 386.07575250791444`,
0.1874671503356095`, 9.751925633231235`, 0.9375414879403592`, 9533.331757909566`, 129.98269051268483`,
366.7909469025148`, 0, 16.586334592369774`, 2.523485259405578`, 641.8288192246589`, 253.34221057262903`,
2806.881461614852`, 9.61731813395385`, -0.9965736643084241`, 18.61331832501365`, 6688.727133488528`,
2.2849517612702113`, 1.`, 0.14456606766166846`, -0.8554339323383315`, 0.1851552767218669`},
{0.19224907453000317`, 0.9379671824303957`, 0.1768927357330181`, 1.2227110146173203`, 0.42649904659884763`,
0.4545029045501904`, 0.23904198572269159`, 0.01364047530387634`, 3771.0454255220884`, 186.69997665609355`,
0.04831449314300046`, 2.795709036233411`, 0.30562138724668775`, 105020.49463301543`, 10.861373183208922`,
160.69055669877486`, 0, 3.0780049609371334`, 0.04158848269715436`, 1189.4295352116278`, 28598.97427347868`,
28031.27953362995`, 1540.5338100504673`, -0.9450423300084377`, 0.5572661705286456`, 76985.53640331636`,

0.2260324587019598`, 1.0000000000000235`, 16.139336737234725`, 15.139336737234345`, 0.019215380812152582`},
{0.250195686307457`, 1.9873181983813915`, 2.436986915389067`, 1.2297238031002529`, 0.8787886927027242`,
0.17801285885202778`, 0.10899785398220965`, 0.042181056020100846`, 2975.5972306347185`, 176.4908556989327`,
0.20657495695660671`, 8.247111235919043`, 1.3291652831072591`, 20378.805811651575`, 42.153576812326`,
533.2935270559263`, 0, 93.58993864308661`, 1.9056169132795562`, 260.71932770969306`, 135.8162330491686`,
4422.433906381909`, 7.814334723249425`, -0.9982330239662887`, 54.10095958434052`, 15806.769805094182`,
2.4000486183382415`, 1.000000000000009`, 0.0770614672198706`, -0.92293853278013`, 0.06288825701386061`},
{0.04941033680222237`, 1.4372745957329531`, 3.017421402893529`, 1.227554635491979`, 0.5953141676696632`,
0.39465993131783816`, 0.0709805192571406`, 0.011053419404823772`, 580.3765952495469`, 188.40590324275774`,
0.07356671197341247`, 3.331653425139695`, 1.2649663092466472`, 8471.807289083954`, 90.71221551354309`,
129.67653118796815`, 0, 67.68480316617959`, 0.48251035811337284`, 53.65460927181`, 110.1988755673574`,
4920.401304625204`, 6.485996742085868`, -0.9986818154982625`, 9.907141141348033`, 3473.1240809089372`,
2.5095790094395216`, 1.0000000000000664`, 0.0640462750751991`, -0.9359537249248051`, 0.17671468208632393`},
{0.08087154180018502`, 0.9611156520127571`, 3.21224132650018`, 1.479145162275284`, 0.656808362332749`,
0.6575245079334169`, 0.06402086777278035`, 0.03166366563194078`, 3830.357327788698`, 29.178148219068248`,
0.1565085846563346`, 2.9278897142269216`, 0.2225569823133069`, 48416.65437074023`, 17.972474018448448`,
733.6380182026467`, 0, 48.10884025677779`, 1.4752820455592213`, 1023.7192331262378`, 692.9142492838962`,
22431.486065322937`, 47.70266602003558`, -0.9978734058955737`, 20.255952358862366`, 25915.269471029`,
0.2805538477099798`, 1.0000000000001896`, 0.39124201701882666`, -0.6087579829812475`, 0.015311380855147456`},
{0.05254670070328532`, 1.9573141803351248`, 1.2965014076065788`, 1.332575014963222`, 0.16006264378785184`,
0.5916179072285757`, 0.24512530080047995`, 0.22325909451251788`, 1444.5445370368107`, 345.46000244130437`,
0.09820743687290096`, 1.9309038402941605`, 1.3978069307443537`, 12178.356333290965`, 16.341454128295684`,
45.51763585294383`, 0, 4.955670881635361`, 0.5137187123200626`, 320.1277548423588`, 622.1576681265784`,
6944.981059162468`, 40.46098848938582`, -0.9941740678419841`, 14.364413147536492`, 5213.369158654219`,
0.6917937637419728`, 1.0000000000000515`, 0.36971632760410894`, -0.6302836723959101`, 0.14263966631823208`},
{0.1997594423184546`, 2.9770319172231083`, 0.9793144795904194`, 1.1541884780655445`, 0.8809204935983468`,
0.3405913744561432`, 0.1996665175564089`, 0.005381059121025975`, 3350.374432264831`, 76.29678229001865`,
0.1902613732440958`, 8.131907933048556`, 0.2784170059108251`, 46467.10745689307`, 57.08824557894052`,
1750.3749809128522`, 0, 54.745847509105566`, 0.09660412225508362`, 76.20302363731679`, 787.8175148064837`,
12042.457604382467`, 42.13187257762867`, -0.9965013891714016`, 4.108479361267237`, 34365.63735992955`,
0.7100137750019845`, 1.0000000000000124`, 0.44344994182352765`, -0.5565500581764778`, 0.024542195109450937`},

{0.20820380555279266`, 1.947139162442057`, 0.8103918093452425`, 0.8157066692238308`, 0.08411468051466398`,
0.597917518430226`, 0.19620399803346056`, 0.2948101237919289`, 3907.9815148798407`, 119.17289873329406`,
0.10826075744997776`, 6.209951432179045`, 1.4468389844056118`, 70860.31267102694`, 18.750382694440546`,
384.8058173967723`, 0, 7.2760678446384075`, 1.0492341913481547`, 1987.1962291067396`, 1892.9491731139672`,
17820.015441359443`, 71.81648519080761`, -0.9959698977014282`, 29.18578549353165`, 53002.78614143656`,
2.3253026055725123`, 1.0000000000001426`, 1.070122289576655`, 0.07012228957650235`, 0.05576849869235012`},
{0.18623015008520377`, 1.9857475843613202`, 5.839525692796151`, 1.2062427816326777`, 0.7227315679666446`,
0.4380351155851495`, 0.06781424675520398`, 0.3047046595907428`, 3879.8004023641743`, 31.750244384294604`,
0.1136575036642325`, 7.396681541568938`, 0.3865663286027079`, 142664.64893699327`, 39.50880819812861`,
1875.8967884758138`, 0, 59.84664487357435`, 8.84548063800028`, 3950.3413548528265`, 445.5943136975641`,
38887.508284857046`, 24.99661218610778`, -0.9993572071524098`, 250.92702584891276`, 103457.52149040751`,
0.9533370242856138`, 1.0000000000000535`, 0.2510241295754444`, -0.748975870424569`, 0.01187467468615967`},
{0.11240037278862203`, 0.6491092968888421`, 0.8347611214383086`, 1.4549390021490687`, 0.14670428342309072`,
0.37243292165179553`, 0.23972369902467655`, 0.01185953233820889`, 3372.5164268352446`, 237.4368160205147`,
0.1138398904730133`, 5.916126824166563`, 0.20110182675101163`, 18231.635642557874`, 17.31844760528657`,
205.90239634340364`, 0, 31.617700140632287`, 0.5197324103364599`, 257.1147986664836`, 493.7061094381911`,
6982.583930159443`, 33.5587577968645`, -0.9951939342036523`, 4.819473420626318`, 11212.071953968065`,
0.44949916615897606`, 1.0000000000002212`, 0.2805689042071634`, -0.7194310957928987`, 0.08024042955463337`},
{0.1783157725484701`, 2.833766552573177`, 0.3970480956539326`, 0.7937141247518862`, 0.3426969414627741`,
0.17660776442515302`, 0.06749542877910886`, 0.2962004793965935`, 2934.9500228426696`, 381.96465886346436`,
0.2370505433342167`, 2.5471608872904294`, 0.782904324178523`, 6765.013729774736`, 7.354270449992673`,
42.13239500141911`, 0, 1.4688591085803449`, 0.14603199372781211`, 131.9082238190015`, 902.2830440216013`,
1904.929732018615`, 32.75905776090355`, -0.9828030098904543`, 5.911722563306417`, 4852.557383077839`,
0.4125805221859478`, 1.000000000000241`, 0.5083733547629542`, -0.49162664523716826`, 0.018864930412685627`},
{0.24765743502672427`, 0.8633830393481601`, 4.686988270445781`, 1.3325105661709873`, 0.9808367367869013`,
0.6497982062118903`, 0.12182607994660094`, 0.10862851464254074`, 1618.8129303586047`, 29.218901620977476`,
0.12364149245529477`, 6.112534098964166`, 0.4592681454217531`, 313153.9415385345`, 189.83689206883247`,
2822.8893085086606`, 0, 83.295640127247`, 9.676598850015212`, 14282.001409474016`, 1474.9319499383364`,
68960.8075326035`, 91.31906667760633`, -0.9986757831013735`, 119.35159036684391`, 243980.81015565907`,
2.0226451272812724`, 1.0000000000000528`, 0.8307231497407781`, -0.16927685025926575`, 0.019215183473095882`},
{0.05305812976899127`, 2.2171177144674177`, 7.758771838981232`, 0.9284694801023683`, 0.19067769039866356`,

0.644253374243589`, 0.22681045533276267`, 0.012934901517196042`, 1447.005934926009`, 36.93966570702122`,
0.23490962595246417`, 9.29802294373275`, 0.16252108168662027`, 4529.398655226197`, 87.10371791429012`,
2126.303854529301`, 0, 458.6330927682963`, 2.586169252203909`, 21.621004260230155`, 7.360243337440092`,
2267.3423682888783`, 0.36591993792007455`, -0.9998386128433722`, 81.91202373817508`, 1718.5849372486205`,
1.6997704368704216`, 1.0000000000002467`, 0.0047716715792718205`, -0.9952283284207294`, 0.18282395803957824`},
{0.15871988201336973`, 0.69579697553139`, 0.8043899133503648`, 1.3164539868322986`, 0.019311018286041204`,
0.4418003624772314`, 0.15298309339567961`, 0.16062079917918642`, 1028.6861638399246`, 387.4645099104921`,
0.1109630441095813`, 9.931289248375212`, 0.6360820981063933`, 21246.666643984914`, 94.12234183588787`,
171.24590255232397`, 0, 18.989791903541803`, 3.8645093792257925`, 1352.6284159656773`, 349.0129727299717`,
6483.817391235517`, 21.90694506929743`, -0.9966212890111757`, 38.41305625682863`, 14701.581876187636`,
3.0384856109874088`, 1.0000000000000264`, 0.20213063913328297`, -0.7978693608667223`, 0.3820524711690226`},
{0.2570743120023581`, 3.9645616544323667`, 0.778960090135616`, 1.0710848615453759`, 0.4119983463981953`,
0.5200874369461032`, 0.1117389487686139`, 0.10363949916728431`, 1639.730583125267`, 312.26786162753615`,
0.028234231304594515`, 5.869357124529651`, 1.28422648371814`, 125896.19933140976`, 53.84861863486312`,
145.91846414836684`, 0, 13.98812835981963`, 0.35372149206185977`, 1303.7402812140176`, 3684.781922988202`,
26936.776596027106`, 183.89910778616647`, -0.9931729356283376`, 20.033580911100785`, 98925.04729978414`,
2.034946271875073`, 1.`, 2.0927650279276766`, 1.0927650279276766`, 0.10464797819895809`},
{0.2562944092973924`, 3.075529570162291`, 2.568785124728583`, 0.8870148603223287`, 0.9606731370217558`,
0.2809776665243148`, 0.1016902992050977`, 0.08251508016369583`, 3903.9002781741165`, 210.26921647621452`,
0.17009591440353533`, 5.238706912116847`, 0.8143837239091778`, 6876.904377937949`, 31.69526518229395`,
291.9966987972128`, 0, 46.3005608409018`, 1.2029916890865602`, 82.7976083181031`, 67.82641756317692`,
1453.7736505490823`, 2.840261081087578`, -0.9980462838351659`, 52.85480732064557`, 5322.772271708419`,
0.87543775916292`, 0.9999999999999847`, 0.03883780692533717`, -0.9611621930746622`, 0.049987598634597216`},
{0.19549413117658376`, 0.48089214427271987`, 1.3626169663716503`, 0.9415198179325406`, 0.6690196233409793`,
0.21285229920071647`, 0.06541758209495863`, 0.01678513797168908`, 1726.2120585826779`, 324.36081581294445`,
0.2027319827154383`, 9.808516157446391`, 0.15828659811715773`, 1738.7498079791606`, 73.46216859794144`,
235.26617490221673`, 0, 81.729396645004`, 2.461254768455667`, 42.73149751961344`, 16.36167180548548`,
431.77162774343657`, 0.7676880163952285`, -0.9982220044878646`, 16.908544045772846`, 1205.8402747486098`,
1.4265309008646232`, 1.0000000000000515`, 0.009887206012281715`, -0.9901127939877188`, 0.24080213748889207`},
{0.13514798653424465`, 2.2171967161009567`, 6.79713748604177`, 1.0120008311676625`, 0.6388353844515477`,
0.24746546435416983`, 0.06301863255527632`, 0.010147071600538154`, 1724.1318426660982`, 61.023906450601714`,

0.20149638211275833`, 9.77881328490188`, 0.3825890641127949`, 12865.235595679364`, 76.24671555017035`,
1207.3956085153202`, 0, 431.253832152915`, 1.9072913643961464`, 49.85001932034329`, 25.136551683138325`,
4221.3084513192125`, 1.229385070659321`, -0.9997087668231694`, 60.412002139812316`, 8150.019110511187`,
2.2995986714140444`, 1.`, 0.014710218634362828`, -0.9852897813656372`, 0.05698902136305081`},
{0.1787587102327428`, 2.3010027450563504`, 2.4341725113616057`, 1.2732377481963428`, 0.4302268260704325`,
0.6620369180993946`, 0.07196824748401134`, 0.018432445015582038`, 2032.846726882145`, 239.6521240526355`,
0.16901168551833623`, 7.60355154430442`, 1.2541734909464242`, 6090.928045823267`, 66.01102985676056`,
219.28840273533618`, 0, 111.97894703438867`, 0.8556382264140857`, 37.48182827832592`, 42.80569628756465`,
1674.2913138041065`, 2.6265293947252855`, -0.9984312590210137`, 28.126084396485115`, 4275.630797278657`,
3.101874565975476`, 0.999999999999985`, 0.025010893865137232`, -0.9749891061348623`, 0.24091029748354345`},
{0.040040522389580085`, 2.419089216699528`, 0.6029402922994809`, 0.9540354124914854`, 0.5857842300398015`,
0.29335248867278896`, 0.08466193409101153`, 0.02752786168327366`, 1658.0955376199636`, 315.19357733141317`,
0.05596420932223484`, 9.548403397933097`, 0.8186383788896436`, 20510.900587173757`, 79.2437946350975`,
244.40372663390386`, 0, 31.478409153123415`, 0.3423312683434501`, 162.81187636572028`, 474.597444409829`,
13019.236478996816`, 21.30382791105766`, -0.9983636653389447`, 11.830426854124477`, 7447.100424750129`,
2.6197463425031873`, 1.0000000000000124`, 0.27076545567349997`, -0.7292345443265034`, 0.25621916354739793`},
{0.2738861805350336`, 1.7653732412133003`, 3.204285049736129`, 0.8904691615720403`, 0.006777008376177118`,
0.6422126566237496`, 0.21196430882173833`, 0.02384817430133364`, 3509.269393393407`, 210.0509220408095`,
0.16655185586266846`, 5.6829810466015855`, 1.030785881009633`, 7463.326399499966`, 18.561193746207547`,
193.45924857329717`, 0, 102.80249545173193`, 1.3263389757900848`, 72.33281859351816`, 53.535695560352764`,
1491.1923791801457`, 2.287771484311319`, -0.9984658106383503`, 33.44976195197257`, 5834.528359665717`,
1.3211367956792508`, 1.000000000000126`, 0.031148610387569747`, -0.9688513896124342`, 0.13138739345208314`},
{0.07916527150572594`, 1.5183903543815438`, 4.168943084064299`, 0.867458547779902`, 0.553589800680331`,
0.48176316193720536`, 0.2283008873811488`, 0.05557602912612958`, 764.3466604125074`, 98.1402416311065`,
0.08270787403012858`, 8.344308234825018`, 0.19844893248168471`, 22202.43997817072`, 187.33975350468359`,
993.3658288119424`, 0, 148.63151742744347`, 5.150397344701415`, 433.0342735868929`, 83.07783800067124`,
10294.511593289617`, 3.4746530619521176`, -0.9996624751907398`, 111.71876642038545`, 11642.397218594499`,
2.8760872379851805`, 1.0000000000003408`, 0.048506282591087135`, -0.9514937174089294`, 0.2778255380848331`},
{0.1629622003008503`, 2.9652820153229094`, 3.631698402387231`, 1.3160868122315574`, 0.8459043277254139`,
0.3376309499507988`, 0.22909924766435485`, 0.29198486983597477`, 2152.5623501482423`, 151.56102061804546`,
0.24318844331582645`, 4.2450714851638445`, 1.1792634690726134`, 13820.006389401451`, 42.0899158844314`,

464.03578665340444`, 0, 22.943713087958734`, 2.1484123514507836`, 257.08525254993015`, 118.6629000835548`,
4117.721021480078`, 7.393451835828002`, -0.9982044796630806`, 91.00926438935177`, 9586.183969792277`,
1.4468406101014126`, 0.9999999999999957`, 0.06812822172104688`, -0.9318717782789528`, 0.08327472748805863`},
{0.21708195818330828`, 2.190522036446822`, 0.6621285433489934`, 1.031682155458868`, 0.05602003593078142`,
0.3042918074708675`, 0.06460489028881022`, 0.10032641034223391`, 967.4633500940654`, 32.783765325462525`,
0.23824254150550733`, 7.5221087110888885`, 0.8625924583250821`, 145099.75455789085`, 66.20589057262717`,
1293.2024519811205`, 0, 15.206687864927051`, 0.6718812795778157`, 2646.6057556616843`, 3938.097331785623`,
35371.08222399397`, 187.69114800289097`, -0.994693655489128`, 21.025296411304204`, 109691.76846067756`,
6.928221199757594`, 1.0000000000000013`, 2.2141057224817264`, 1.2141057224817233`, 0.022081960683798574`},
{0.23286963035122604`, 0.6749345188436879`, 0.20207668159083703`, 1.2334071076981241`, 0.15415172519231857`,
0.5502451893184188`, 0.06023408333501554`, 0.2884596823004714`, 1454.378698010155`, 160.31978742859337`,
0.13928726255657442`, 2.5761586107644754`, 0.6603399169512614`, 62462.92963487824`, 22.07171373787987`,
94.87891829453761`, 0, 0.7703323741034096`, 0.292685817837999`, 4723.317023110421`, 16136.84042561559`,
14435.693385083718`, 872.922492699993`, -0.9395302692143644`, 2.8220537376408688`, 48023.35117782979`,
1.0157271222791913`, 1.0000000000011586`, 9.118998451735001`, 8.118998451724435`, 0.026265914253772133`},
{0.16797762422450352`, 3.393739269293537`, 1.1723349699849432`, 0.7876651455122983`, 0.44858157260876874`,
0.38511915317210543`, 0.11126403235021692`, 0.0942998855830809`, 1121.8751632200092`, 393.3700502870249`,
0.18309808816527456`, 7.014749603006731`, 1.3459972330025094`, 5066.701369012587`, 83.60459525768759`,
142.06959985579581`, 0, 27.2995405735397`, 0.7199348050949663`, 58.76950857918809`, 80.631709098209`,
1471.837787604005`, 3.0404600758030944`, -0.9979342424135254`, 34.90387170545662`, 3531.9402115081425`,
2.504431084772431`, 1.000000000000129`, 0.04682470036644391`, -0.9531752996335622`, 0.2539299478309531`},
{0.217831918966031`, 1.9579162032970752`, 8.666870397376897`, 1.3889143705395077`, 0.46152386514296384`,
0.5340210525452818`, 0.23464141923209442`, 0.08443497471803191`, 2881.1816355963792`, 40.761442588478076`,
0.026544318054481375`, 2.7988265733593654`, 0.6550641924577596`, 739279.5080132923`, 15.32966680593877`,
715.1786796829259`, 0, 81.60672859943229`, 3.3918922684652903`, 13951.902071384357`, 4112.309317367291`,
179747.176032861`, 265.3551790626691`, -0.9985237310264382`, 94.8720118895179`, 559352.4611994725`,
0.6060720754632819`, 1.0000000000006877`, 2.3162421986655977`, 1.3162421986640047`, 0.010362401795446126`},
{0.05909313438823349`, 3.998253006220927`, 2.7606012432005738`, 1.485525656051693`, 0.006754191626425765`,
0.414858609675688`, 0.1539104163421624`, 0.018873368421200944`, 1163.2065814066136`, 134.3441176716297`,
0.2419809034541145`, 8.466048197729386`, 1.4640823465517014`, 16123.378567191101`, 67.88099528049129`,
415.2622866172925`, 0, 140.67313497278658`, 0.6436439717783644`, 58.91473301815641`, 90.53310774491865`,

8646.14788307223`, 6.429178460489017`, -0.9992564112310551`, 36.76359207284021`, 7298.971125641826`,
5.556125952496123`, 0.9999999999999999`, 0.05243064827132726`, -0.9475693517286727`, 0.2731403378909622`},
{0.07096987014316519`, 2.2631739720341697`, 4.701888958284682`, 1.1268609277641355`, 0.5889482309057459`,
0.3638885919169875`, 0.05633232701579177`, 0.3132395508309391`, 2469.4656057395405`, 63.55428754000428`,
0.06344769284580837`, 7.209878698655041`, 1.262286526290143`, 91466.73771630006`, 44.72521338313976`,
793.441409324752`, 0, 47.3173984351371`, 6.150108967571809`, 2242.954051099513`, 363.7015138961152`,
45293.318702491415`, 19.07980258149392`, -0.9995787501749028`, 198.8395220083209`, 45920.87066669711`,
4.786686631463984`, 1.0000000000000102`, 0.2050578763901423`, -0.7949421236098597`, 0.05421119776150546`},
{0.13424044754942377`, 3.4568739781145084`, 0.7268053299269095`, 0.8378137298915767`, 0.4654803229022981`,
0.5071605573546728`, 0.16764031212635366`, 0.06824087778957048`, 600.6265082502678`, 271.9873255003124`,
0.09465386647177981`, 2.8029066087185885`, 0.5029743243735594`, 23385.678163238463`, 72.29265453924974`,
94.08817970502018`, 0, 8.0973336449192`, 0.1535369614378166`, 229.12168187182013`, 1491.2900630973852`,
8009.623728922196`, 59.928644784026545`, -0.9925179200905995`, 7.582256095330827`, 15360.22105818572`,
1.3273105652501762`, 1.`, 0.8724425326435865`, -0.1275574673564135`, 0.14730917829803994`},
{0.25116849017091947`, 1.0524160237641933`, 0.8392172051883035`, 1.4326662099306988`, 0.4925377955188637`,
0.4563121318749678`, 0.10417530701605077`, 0.012130927300066367`, 2850.5603662771573`, 179.7348471844905`,
0.14797581172978125`, 6.731564570008534`, 0.3667284512812037`, 47794.14401656854`, 36.45967137242655`,
302.17501338385387`, 0, 35.798227519735654`, 0.38612114076867426`, 440.25687193661236`, 1139.2040071159192`,
10407.774247697367`, 75.54253397079277`, -0.9927417205472625`, 5.8051439379866085`, 37344.35634048456`,
0.9045519276581837`, 1.0000000000000118`, 0.6429806231892374`, -0.3570193768107701`, 0.04482627527698816`},
{0.2598662442063618`, 1.900211126429065`, 0.6320497826451614`, 1.3904132145962274`, 0.3261450588717545`,
0.21444284573744743`, 0.1520608211075556`, 0.47681332398299126`, 3867.6546770468185`, 328.5593273663678`,
0.12810041704772773`, 2.5338343017479055`, 0.3750039812454511`, 25906.29265383803`, 5.943894363853591`,
61.66529518665966`, 0, 1.5433423158843922`, 0.36781230258429715`, 802.6012548200581`, 2181.094642242462`,
5494.978088092383`, 137.6666927782427`, -0.9749468167895762`, 9.984586140116805`, 20399.41882498315`,
0.20174018744356717`, 1.0000000000671796`, 1.2293891109843076`, 0.22938911090171765`, 0.013736892413259807`},
{0.15997673085841746`, 3.7261077489541483`, 1.1512593893886542`, 1.376812544747029`, 0.7263504373210632`,
0.29998090848320735`, 0.07358394021576772`, 0.4410919571058908`, 3522.7326806197016`, 234.3351737610849`,
0.05138796494631709`, 5.103095752677643`, 0.43578634794289095`, 53657.03869478016`, 23.865050633410085`,
179.54346039170994`, 0, 6.1071846196728155`, 0.6938884741662413`, 853.9182625170652`, 1229.6275349840787`,
16318.742828031023`, 78.5640034833406`, -0.9951856583370877`, 36.935760292868544`, 37294.55899068077`,

0.6062323290074187`, 1.000000000001635`, 0.694118133378465`, -0.3058818666226699`, 0.03932078136285248`},
{0.25099622201635424`, 3.95450763611092`, 4.239563708075597`, 1.1463399035908803`, 0.8567090221336071`,
0.6807931178480928`, 0.18419051557334198`, 0.2564924471830997`, 698.4202789859287`, 154.98713283086113`,
0.08058524622619823`, 2.246868522171841`, 1.4084793726849045`, 26052.309500665702`, 113.98950597305468`,
208.28418301686932`, 0, 16.705738921680485`, 1.0020373664309263`, 355.9860300101022`, 354.2622306671649`,
5665.049715692064`, 19.614713831957243`, -0.9965375919336374`, 56.60806310313701`, 20312.943945336072`,
1.7810764698354182`, 1.0000000000019482`, 0.2078552485942884`, -0.7921447514061166`, 0.12789979361382334`},
{0.24225332438192398`, 3.651195248984296`, 0.388765132686828`, 0.8400870426253413`, 0.1821318445823885`,
0.5573875510223865`, 0.23605316030342982`, 0.08377442811214376`, 1289.2099511812157`, 318.7461100660206`,
0.19657856943178847`, 4.498486658412734`, 0.6713610627619064`, 16679.3534952134`, 43.281645873070126`,
119.1462050644325`, 0, 6.058456399487112`, 0.13503919278680876`, 170.93151681254287`, 1264.7919029655236`,
3736.1592451804604`, 49.63288646635113`, -0.9867155323932254`, 7.043635130426711`, 12929.957108074643`,
1.2549312390233938`, 1.`, 0.7248348420408769`, -0.2751651579591231`, 0.11092986234995002`},
{0.16862146125981442`, 2.3151392610028543`, 3.2020316316267774`, 1.3239310858987743`, 0.7072607224053895`,
0.3500350043589193`, 0.17362573378910773`, 0.060358617251082186`, 2001.4962431618678`, 114.46542957104901`,
0.20499895995123107`, 5.257752800260672`, 0.4338865062331345`, 24226.969462121375`, 45.95666008208979`,
547.7912767473758`, 0, 67.51005408461461`, 1.7010738509951984`, 329.21751126783124`, 192.53510788213305`,
7070.2140040330105`, 11.940749188234209`, -0.9983111191285833`, 56.26032654720398`, 17031.283096852185`,
0.9692005978687218`, 1.0000000000000002`, 0.10936715201431102`, -0.890632847985689`, 0.046409312130933786`},
{0.12291711454694165`, 1.1877487218897347`, 9.942019369479574`, 0.7752358604533577`, 0.7160618607453235`,
0.17825216453319837`, 0.07028904237235806`, 0.009021701114390435`, 805.4612461749266`, 63.6125839558365`,
0.06956787912807044`, 9.33154348593904`, 1.458279492320873`, 13286.365711191082`, 144.08909403760782`,
1202.5515273610133`, 0, 617.0660503983631`, 4.364791717822204`, 84.42137436255767`, 18.341443949742324`,
4568.40950635979`, 0.6969030334070876`, -0.9998474517154303`, 74.06108263083179`, 8021.938779865211`,
10.399383543453068`, 1.0000000000000766`, 0.01088407950371651`, -0.9891159204962843`, 0.14800038168746177`},
{0.08216873811896613`, 1.3179095560013607`, 1.6483112503948085`, 1.3628139186636958`, 0.6342779575022122`,
0.2115034893488278`, 0.10646590409334977`, 0.055264018212609206`, 510.50583604116036`, 394.4203282160594`,
0.13986107835869105`, 2.2752923009835655`, 0.9070556531583542`, 1978.981088158196`, 52.683870676059705`,
49.968510117557855`, 0, 16.92733635644876`, 0.639194509832781`, 44.034659059768`, 67.89085932744926`,
896.7443996243201`, 4.492380475105502`, -0.9949903445430074`, 12.034293609317515`, 1052.6336533197182`,
1.0818394821725976`, 0.9999999999997691`, 0.04010318041714796`, -0.9598968195828428`, 0.16027832791136667`},

```
{0.0476259458526086`, 3.971655945581807`, 1.7442422392857733`, 1.129286089919653`, 0.5285852533702986`,
  0.39600384447582293`, 0.2215904518169189`, 0.024542351730056703`, 3543.890003102313`, 364.0135445894731`,
  0.06778311290485467`, 8.573488767539896`, 0.9317909345539408`, 6862.22404685655`, 36.15536298895538`,
  264.83914867544394`, 0, 84.71244856914132`, 0.50873126601246`, 30.85271359631898`, 59.64638770514122`,
  4015.7615287328103`, 3.250736884242145`, -0.9991905054966579`, 28.864365105167767`, 2732.2063017773894`,
  1.3688653033339915`, 0.9999999999999993`, 0.0348749909118367`, -0.9651250090881632`, 0.28626490627859125`},
{0.07405512950518717`, 1.967002000369666`, 3.1335932173998042`, 1.0217576314214762`, 0.5481985257231932`,
  0.23482039961813828`, 0.1719074884408075`, 0.010212529243490897`, 3198.221153713561`, 38.498265037806334`,
  0.12854240418059937`, 5.86125181611254`, 1.042171003495977`, 108843.34630564722`, 21.334192120769437`,
  1541.407409887598`, 0, 118.81429572337292`, 0.59466590443923`, 476.2111638729947`, 799.8045531415861`,
  52823.417703373816`, 37.986380904890126`, -0.9992808799097741`, 16.710128908337108`, 55883.50055614192`,
  1.9374458226128037`, 1.0000000000000004`, 0.45038350751944156`, -0.5496164924805587`, 0.022624564166001632`},
{0.08682640281398363`, 0.4802834262657396`, 6.166420960796351`, 1.3554629143634556`, 0.6132401723597833`,
  0.3572005740934433`, 0.19494034836925994`, 0.012289451288465592`, 600.315756452052`, 42.934435033309114`,
  0.23123185628174636`, 1.349114625805871`, 1.1058635186946257`, 63854.76560858458`, 33.47086316538659`,
  341.60733186452734`, 0, 53.3128429510063`, 1.1825061414847458`, 1213.0906307212326`, 1024.8641271816866`,
  28473.813166038613`, 65.16703948813462`, -0.9977113343018679`, 8.113401445893908`, 35318.26816577961`,
  1.6199922176056596`, 1.0000000000000004`, 0.5833442046139202`, -0.41665579538608`, 0.026326080975873842`},
{0.14493124545339742`, 3.446306300213565`, 2.261950562129204`, 1.3227848229460633`, 0.5758774796201231`,
  0.35943404017729164`, 0.23613553647425112`, 0.03288786445085886`, 1106.0434527992074`, 293.5700758832494`,
  0.23807677975419816`, 3.6561682593343647`, 0.5615502445855836`, 4007.5244898250467`, 48.619730102133026`,
  147.80475762862085`, 0, 43.69774819328735`, 0.4025114519377973`, 25.501107789084813`, 62.354986960782576`,
  1284.3741297827844`, 4.011828069037673`, -0.9968764334503404`, 19.816825038876274`, 2659.227746536317`,
  1.0704405021782206`, 1.0000000000017808`, 0.03682820013798496`, -0.9631717998620806`, 0.1716044250105655`},
{0.15370210593846179`, 1.1469734999246084`, 8.184859582737346`, 0.8081018637833421`, 0.7671904423744005`,
  0.5546779173711273`, 0.13880357299862195`, 0.3920406444124731`, 2732.468857403337`, 28.150193121997006`,
  0.06240913374814305`, 6.929933598007185`, 0.8840553716357653`, 284250.15035551105`, 66.1032389446229`,
  2821.0945221667107`, 0, 64.10140063017641`, 20.496123524219843`, 13872.940869439086`, 675.856814072462`,
  88814.89995905061`, 25.417074229301857`, -0.9997138197054658`, 335.8358647637363`, 195014.8166059984`,
  3.031851256834608`, 1.0000000000002747`, 0.3808675646269288`, -0.6191324353731759`, 0.023051636334796802`},
{0.10991493466216079`, 3.7381599633374956`, 1.5402547174363423`, 0.8574896025687118`, 0.9021341382697974`,
```

0.5521862271024119`, 0.19447159277653675`, 0.017387026381523858`, 877.7862619046055`, 398.7215907021463`,
0.023981889033480308`, 9.30826908769696`, 0.3374448683929794`, 21432.16815483367`, 374.52801484376323`,
390.1502705450047`, 0, 92.90144606544155`, 0.41661398049811893`, 78.38398102277777`, 187.145344832305`,
8293.692658555503`, 8.039661114554182`, -0.9990306294860998`, 22.24813860092482`, 13022.866952473876`,
2.9134214680655215`, 1.`, 0.11361043511467318`, -0.8863895648853268`, 0.8644671865103419`},
{0.2669155508949231`, 0.8202069836030885`, 1.7120733781356066`, 0.7716732548944238`, 0.42105306969825285`,
0.4443694926042744`, 0.19913166298690133`, 0.07350737572131678`, 3667.631913873951`, 382.69157822195143`,
0.19936982959712085`, 3.809394225029287`, 0.38364950845782375`, 1057.0194411841014`, 14.292487305830784`,
94.30825711619718`, 0, 24.276114816141067`, 1.982177562966878`, 42.5742213514009`, 20.47851037506286`,
209.3328025649743`, 0.7823685401059933`, -0.9962625611919415`, 23.225655426954038`, 798.2025759572617`,
0.3522485478649553`, 1.0000000000017253`, 0.012317378132002505`, -0.9876826218680187`, 0.10378958856356546`},
{0.045935603836807504`, 2.302483508903091`, 2.46282437804547`, 1.0004914316838749`, 0.17651744242150835`,
0.27731852138484747`, 0.16499475989489315`, 0.14699844290776085`, 3995.605017809292`, 299.6167282278376`,
0.2482978016224947`, 6.149511597608322`, 0.9467803390801968`, 284.8267017407449`, 13.7131176842289`,
152.31321975922168`, 0, 37.566477868645926`, 2.270572880118738`, 5.692875097497585`, 1.5072417394503015`,
102.8244147187055`, 0.11804428673689736`, -0.998851981924139`, 74.68509446051424`, 67.47573684671471`,
0.9544276928577764`, 0.9999999999931928`, 0.001429289569679364 3`, -0.9985707104303109`, 0.1488436482081973`},
{0.048513291302257844`, 0.4457687353343176`, 2.3881995464771766`, 1.1956444252802232`, 0.6250208921524281`,
0.23033921635096732`, 0.16695818246187422`, 0.0058846456882393735`, 1714.5960674937287`, 267.0960709959734`,
0.21708290835986865`, 1.1611050275443535`, 0.4203748665144109`, 371.536263407861`, 8.072532492886786`,
44.30128803849542`, 0, 19.440385996804526`, 0.21947544003097785`, 3.910305104906373`, 16.816595352785047`,
206.99384274538292`, 1.0011484354066533`, -0.9951633902625879`, 1.3976469905650284`, 143.4564655840073`,
0.2117389803638373`, 0.999999999999292`, 0.010182928262521831`, -0.9898170717374709`, 0.034266745136007484`},
{0.16008991075718454`, 0.9889641902273629`, 4.070194520482062`, 1.048412343563395`, 0.9623075048370111`,
0.2561461923913073`, 0.06710665317178915`, 0.4051510354125659`, 2424.3911699885257`, 364.2049627898059`,
0.11376488110702476`, 6.677727754478127`, 0.46263232179329394`, 156.32991643139223`, 61.32021353754079`,
172.62016798875143`, 0, 23.869852411347363`, 8.74014326561774`, 8.811384129312842`, 0.008150994958555424`,
0.07260529115116648`, 0.03782752788752401`, -0.47899764207589335`, 123.48126724503942`, 0.1660482082986243`,
1.0269478002611503`, 0.7714243147376799`, 0.00043713120840670395`, -0.9994333453067844`, 0.10822134502839033`},
{0.23888202110078255`, 0.8810916509901707`, 5.093732295047118`, 1.4600046563601987`, 0.26112133308478946`,
0.18414540656565503`, 0.10719043869683506`, 0.019052269075044592`, 1361.794327483607`, 157.34664623392825`,

0.2248991829845473`, 7.770022547327725`, 1.3856793740595026`, 303.4473391024908`, 47.02938615353104`,
344.5333262086874`, 0, 236.02150988592172`, 4.650601227488466`, 4.778160357620555`, 0.027428524591212033`,
0.939533809024789`, 0.0702035004473599`, -0.9252783670230779`, 58.537227337496`, 3.2062533598909195`,
3.891918368827484`, 0.9999634477382617`, 0.0005826912952043511`, -0.9994172874053324`, 0.1982638815817234`},
{0.15821344070119114`, 2.676629891143981`, 7.114918370652138`, 1.231756200149393`, 0.7440882968059128`,
0.609542391688829`, 0.20113875027795403`, 0.008293436499309746`, 3321.626865604231`, 300.9780595191306`,
0.1294374982810616`, 4.573476150462291`, 0.6252568014280515`, 184.61955333786025`, 36.20278448216762`,
203.17221829309`, 0, 164.76184754791248`, 0.495494036125151`, 0.4984077213070531`, 0.005880363777307274`,
0.08217583688627902`, 0.04384024126123368`, -0.4665069085709095`, 18.946487828230776`, 0.18573316994682562`,
0.664411018699979`, 0.7572652446782872`, 0.00043119622265744707`, -0.9994305875970306`, 0.097729364750055`},
{0.05919792771600385`, 1.1566416574477563`, 7.068615334313819`, 0.9063681130718225`, 0.7973057249073783`,
0.32056833480557556`, 0.14802374216985142`, 0.006390669590825526`, 3472.301841329897`, 218.2462028286681 7`,
0.09503155541006275`, 6.650147058357586`, 0.752916478623388`, 331.7883078897081`, 36.80586922834271`,
372.5485566129848`, 0, 303.6171684438913`, 1.5762085323266075`, 1.5839718371808609`, 0.00492530315312667`,
0.1265982938634435`, 0.04015376306104685`, -0.6828254012304533`, 26.044406418764865`, 0.10706223784428275`,
1.0608465322880174`, 0.9476278360472721`, 0.0005364250768958909`, -0.9994339285355595`, 0.07039022444910314`},
{0.12780221907252537`, 3.8001966548501374`, 9.893992295428603`, 0.9054601762352339`, 0.3495759940365448`,
0.1520403910810577`, 0.10896185913129744`, 0.3816734295429438`, 1447.6496724709095`, 224.52426056149727`,
0.14587747065522566`, 4.530340760708304`, 0.2310603840049561`, 103.078391484516`, 25.340311886488394`,
146.6646402741003`, 0, 16.63568863643608`, 1.5626840006611251`, 1.5754333102132627`, 0.00815859735349167`,
0.015645966203600474`, 0.013067789036220776`, -0.16478222781705898`, 84.83580731286091`, 0.028565560004583043`,
0.7966738221656027`, 0.31059546317312137`, 0.00017477478942099354`, -0.9994372912352439`, 0.07369841518243606`},
{0.052097538451470415`, 0.8314166561245999`, 5.481254262333325`, 1.3932692734662013`, 0.3669026419040864`,
0.3620022667552303`, 0.11591227570051149`, 0.01952536971625568`, 3813.0335975189155`, 109.62712481259092`,
0.17741871372248502`, 1.8758946193559272`, 0.37818057550442363`, 77.26049926884558`, 5.641914881430802`,
124.31571716322335`, 0, 59.8607588584299`, 1.2924367362080666`, 1.3085282845403512`, 0.01245055009771412`,
0.4207482794236092`, 0.06562713160541679`, -0.8440228164561466`, 15.350763278152913`, 0.31314213808086`,
0.19292946490877846`, 0.9988591272638007`, 0.000571061282622572`, -0.9994282864649924`, 0.025869398969298813`},
{0.2134810376991253`, 1.4110142375655572`, 5.989091842275013`, 1.4582774889459504`, 0.06026935559352742`,
0.21384589376924967`, 0.11902027597851936`, 0.012348918184910978`, 2514.3629410926587`, 346.5120508621949`,
0.0374109599718197`, 6.153474798173264`, 0.7147791109959445`, 98.67894752368512`, 18.225307820177722`,

112.03110267851086`, 0, 83.88345434633628`, 0.690898283019059`, 0.6994140146385779`, 0.012325593837499 76`,
0.03035317576110248`, 0.02448892737643538`, −0.1932004885031542`, 13.926675914992712`, 0.09256896369918746`,
1.0503320464867587`, 0.3604538318875694`, 0.000203496386490699`, −0.9994354439640021`, 0.1562604187795328 4`},
{0.183290589228506`, 0.5116934633594044`, 9.423512566077996`, 1.207136012185702`, 0.9255825329486453`,
0.5109486403531394`, 0.061891644358164966`, 0.06517375803184387`, 1490.0042360738662`, 338.5269107534542`,
0.2295202271938529`, 6.23442268470835`, 0.9091680440302747`, 162.28028032208988`, 145.76147563703694`,
161.90980293203245`, 0, 84.97698112044496`, 9.291059425943365`, 9.41564295651069`, 0.013408969295735096`,
0.02614907071677659`, 0.02092345540809491 3`, −0.19983942700223967`, 67.91677679912858`, 0.0684696939920655 7`,
2.0559504710206467`, 0.3717040524647953`, 0.00020992795929545736`, −0.99943522822013`, 0.20770987902014423`},
{0.2691641473228049`, 2.5948088236594433`, 9.806966931633422`, 1.0098220223982928`, 0.036933113005692464`,
0.496552488389817`, 0.11856800340468354`, 0.008793318254398408`, 2701.46341207771`, 37.07745796913986`,
0.05351989235558863`, 6.680707066067022`, 0.2241309195665182`, 452.82905625621356`, 25.511480940315543`,
1123.3353634082928`, 0, 401.53593770334766`, 1.3224284614197108`, 1.3274851441912736`, 0.003823785496974263 5`,
0.12457122139863226`, 0.043796720330890604`, −0.6484202383250336`, 49.020700576432134`, 0.47900152269608703`,
0.7718396427171867`, 0.9260835723395966`, 0.0005252798460351031`, −0.9994327943376556`, 0.06792215879515873`},
{0.2094783821158741`, 2.6664932858575137`, 7.297360825644024`, 0.9801370202263215`, 0.5993287287749127`,
0.5949313991226747`, 0.07854776414147877`, 0.007471189820589976`, 3737.8249085720936`, 225.90376084120953`,
0.12230719137003099`, 4.645596524735494`, 0.773898586938345`, 145.24317429300464`, 25.37143643987266`,
158.4940749917159`, 0, 131.0744201364753`, 0.355230365560724`, 0.3583017502389588`, 0.00864617717403382`,
0.03918886513877418`, 0.026344124681331037`, −0.32776505295465475`, 13.531705495719706`, 0.11727457237468698`,
0.9699662892573255`, 0.574986432866013`, 0.00032561320950667334`, −0.9994337027955883`, 0.09414525378353117`},
{0.04064294172797539`, 1.2711785498005437`, 6.414222157609632 5`, 1.3607991978128624`, 0.4152034158458744`,
0.43390213144020495`, 0.1732390453420793`, 0.044490756173200585`, 3317.76480386853`, 267.9877730423784`,
0.04731961656359201 5`, 9.540745693864437`, 1.14427781856239`, 298.77929241051686`, 39.65179956617213`,
326.469834033415`, 0, 183.42894497294438`, 6.014383720773742`, 6.059841973426154`, 0.0075582561344396115`,
0.0679575731487076`, 0.04170091406553705`, −0.38636840408816`, 109.21936537310249`, 0.03945708122072665`,
2.1738397070425215`, 0.6566957087905123`, 0.00037105455645059464`, −0.9994349672892884`, 0.24554969047716235`},
{0.09357862879768009`, 0.6953322280042711`, 5.170280682368036`, 1.0934248327219471`, 0.7339854764578926`,
0.4916807813635117`, 0.08077481700228195`, 0.04710573802150511`, 2462.550423949988`, 223.30073476186828`,
0.13718177246897728`, 6.470496132991109`, 0.9927113806256207`, 238.6057419439037`, 59.45480656036568`,
250.6420846810697`, 0, 143.2038745312356`, 8.691631108193642`, 8.778585953681818`, 0.010004433506871191`,

0.15727150751581837`, 0.04893652189094297`, -0.688840511139496`, 86.3367317635932`, 0.21024645746102438`,
2.0018713686334384`, 0.9495810649826323`, 0.0005420542829799354`, -0.9994291648149177`, 0.14499911404797267`},
{0.18983638851163714`, 1.3904426162746537`, 6.462224486154522`, 1.3121224928907784`, 0.33702569938499116`,
0.4827790093546729`, 0.17642716165084854`, 0.14070582325373504`, 1445.2238937860711`, 169.08245719423826`,
0.08107423780437323`, 3.772942227886274`, 0.5974646397153294`, 164.88004598097837`, 34.2523236613152`,
187.6466325996898`, 0, 55.35310371797766`, 5.22453578200281`, 5.277365526204909`, 0.010111854221399597`,
0.1414397248562396`, 0.055636415678020655`, -0.6066422235014248`, 103.77738859355034`, 0.3835772365541959`,
1.152348778019642`, 0.8923582333974464`, 0.0005139483739578717`, -0.9994240559959859`, 0.13224874947757065`},
{0.07643772187175424`, 1.9519546589360584`, 6.900928196416569`, 0.876872363876414`, 0.7662923029927724`,
0.15407458175108746`, 0.15169600745126946`, 0.11514584917672349`, 3018.1218065404273`, 170.6089829682045`,
0.07107865878327907`, 5.366481527317603`, 0.4272921221427337`, 273.2434482844035`, 21.281102016818423`,
373.4228533693074`, 0, 103.49904188738267`, 5.861633875808671`, 5.883160435079084`, 0.003672450331511534`,
0.20588636515351325`, 0.04060477742272622`, -0.8027806387642502`, 163.45205075517396`, 0.2248212102398069 7`,
0.5703091451609`, 0.9931634921070284`, 0.000560746023268308`, -0.9994353940436547`, 0.02883869514960390 3`},
{0.14382797385454654`, 3.902431027294017`, 8.163450688536429`, 0.8614076883883874`, 0.9648536276916164`,
0.1955756537598935`, 0.13123090751766925`, 0.17894545684886398`, 1385.50217697701`, 120.46271140783404`,
0.09423544317816807`, 8.260526259890764`, 0.9048289425944`, 506.9904572416273`, 110.04949912247456`,
962.3685316187962`, 0, 144.36768199184493`, 6.356876778410168`, 6.4218170877755245`, 0.01021575714443812`,
0.6142306795145123`, 0.04064843544866171`, -0.9338221993717568`, 354.389616810752`, 1.2620507730561734`,
3.2544889408353512`, 0.9999945416718813`, 0.000571389015772143`, -0.9994286078653821`, 0.12464826936052532`},
{0.26055110138040244`, 2.367351112459766`, 4.465438967228517`, 1.1966516268556373`, 0.9350425579579571`,
0.17322311915607413`, 0.07783685162029783`, 0.005169562389067373`, 1562.7816595558625`, 214.0110363941617`,
0.12855831417818353`, 8.432278535705368`, 1.4776941418404932`, 286.70732893128843`, 87.43930157160808`,
395.55075637162946`, 0, 263.670191849169`, 0.5516180612487395`, 0.5662790539152477`, 0.026578159230898013`,
0.747157145025088`, 0.05714939289066655 5`, -0.923510879510163`, 18.655337585001455`, 2.781037386293226`,
3.588590886857461`, 0.999983214077736`, 0.0005786864125645113`, -0.9994213038734873`, 0.16361314191744086`},
{0.1718184288134474`, 1.149839439352271`, 8.637641060035964`, 0.8458363550999337`, 0.6513629677556381`,
0.2037277594433513`, 0.1742817105699909 2`, 0.14466402071943818`, 1236.767232462591`, 142.28311915163187`,
0.08823953882269175`, 7.82350558871963`, 1.0042870130174983`, 508.95914923339683`, 77.70646496275542`,
626.4072268007968`, 0, 167.6792926587859`, 19.501069701797014`, 19.68246859076718`, 0.00930199685166233`,
0.4193886017916396`, 0.0399832432418272`, -0.9046630188063822`, 320.32998646691203`, 1.029409866030394`,

3.2578239243152676`, 0.9995322407322959`, 0.0005724004521023038`, -0.9994273316769823`, 0.1423561988771582`},
{0.09592803445036208`, 0.8366316591486305`, 5.0931225791851045`, 1.1660530096752404`, 0.2059334355486433`,
0.4873422492046261`, 0.1598589026416986`, 0.029544379859417352`, 2582.9507509173`, 296.85131956979524`,
0.07927444657096727`, 2.8630420963700924`, 1.2775475419622428`, 65.71166200260419`, 12.80376281727538`,
70.53139726730839`, 0, 46.351820000206914`, 1.4849460774369891`, 1.5058015659955333`, 0.014044610020143455`,
0.05025045898096847`, 0.032950009775061156`, -0.34428440170983454`, 17.747898578748`, 0.06886325371816662`,
0.8139318674939384`, 0.5996076611210122`, 0.0003424870711397801`, -0.9994288147177781`, 0.13040858468349986`},
{0.06735695081703474`, 1.6218831494512376`, 8.01632413374589`, 1.2380602139197665`, 0.3870710635961401`,
0.2680531526020482`, 0.07829277488965869`, 0.011053339337592866`, 594.5995475908121`, 283.2125249310004`,
0.06462100682727939`, 4.113525656200814`, 1.255132039019542`, 98.1622654489261`, 71.97731559479051`,
97.85662102850388`, 0, 84.98035226919959`, 0.5404421373823602`, 0.5538534159876637`, 0.024815382957112053`,
0.029537213750576916`, 0.023126995233789756`, -0.217021773648571`, 12.52191422676944`, 0.02842195219814254`,
2.292210818616929`, 0.4004935911873144`, 0.00022623963186433753`, -0.9994350979969652`, 0.19541740865666923`},
{0.16614727413225283`, 2.422977153037391`, 4.380699036993274`, 1.2650547043993527`, 0.7289628812438445`,
0.5253372649543967`, 0.1282487061525488`, 0.005846875030812566`, 1525.6199865357175`, 306.5080581733838`,
0.2463161390095024`, 8.0298979658821`, 0.3034478973618291`, 257.5404324976677`, 124.01665930686386`,
279.5074579430823`, 0, 236.93547576437922`, 0.5518982251771798`, 0.5574568823772115`, 0.010071888160624543`,
0.2052362946654679`, 0.05810236285506737`, -0.7169001567204605`, 19.10338272008846`, 0.48713501302389145`,
1.6243549192538882`, 0.9586169317794406`, 0.0005564236347152824`, -0.9994195558035028`, 0.3020058795863103`},
{0.09439363668230882`, 0.5823127951342895`, 9.668824509749783`, 1.1792283442388243`, 0.7110022446147217`,
0.31000063243129616`, 0.126665851525797`, 0.01012852201633408`, 3883.1686659534244`, 167.0440580830566`,
0.14786211145387196`, 1.794206476801321`, 0.758471056166536`, 91.13538140793409`, 7.302627022975878`,
105.84183178597655`, 0, 79.4603070602828`, 1.229188831023966`, 1.236198187376536`, 0.005702424375863435`,
0.06724669439149468`, 0.03885321489304998`, -0.42222862782138315`, 10.225319770591636`, 0.09068085769253438`,
0.2903285575458205`, 0.7051548375096057`, 0.0003992588117050666`, -0.999433799797623`, 0.01876971258260699`},
{0.10782261295184936`, 0.4917827154911696`, 7.249268882781941`, 1.3611009578260718`, 0.7853661815311994`,
0.41488889777264726`, 0.055591995773018654`, 0.052207153417698195`, 693.7280684674711`, 321.57606910090226`,
0.24632437610376973`, 5.428213742379803`, 0.6782641447302435`, 134.89205915269662`, 172.7256940261541`,
129.31790814396965`, 0, 78.25855784277948`, 7.044034172451037`, 7.180413209429382`, 0.01936092779216181`,
0.039206887628989474`, 0.029275831722839256`, -0.25329875709917915`, 49.48763219057946`, 0.06039127242671387`,
2.4752639161374557`, 0.4598698756015752`, 0.0002605246540825342`, -0.9994334818001686`, 0.2866590325192061`},

{0.05850570936819943`, 2.3562511218485964`, 4.849470518860397`, 1.0037692866900862`, 0.20135980488529004`,
0.6258574217265285`, 0.20434104039710765`, 0.046458478403025076`, 1143.066165281296`, 382.63411260362705`,
0.03938722953573187`, 7.245742033238452`, 0.7325066156705251`, 149.90176189284617`, 84.97208117850828`,
154.2560564211639`, 0, 90.93727667042342`, 1.6989691497366837`, 1.7252931519647137`, 0.015494102545717148`,
0.039852897158015016`, 0.026874702214663652`, -0.3256524837301886`, 57.18854235790312`, 0.0333088859801392`,
2.323338213671667`, 0.5703585502566495`, 0.00032422249105829075`, -0.9994315461898268`, 0.6427053241098817`},
{0.11828518723170395`, 1.6435868292536613`, 7.819044307747686`, 1.2773743957591923`, 0.9657225148907191`,
0.25540329193006406`, 0.2479438125086047`, 0.3343373077742329`, 1140.611649524565`, 164.9221880908642`,
0.023651248717273365`, 8.003232493785013`, 0.21949174382120273`, 468.94965881072284`, 157.9519247512417`,
1068.0778568075198`, 0, 82.38044238067447`, 15.737211062157465`, 15.8401457990648`, 0.006540849995642306`,
0.4926969645709606`, 0.06007569737531182`, -0.878067652745485`, 369.5067547278147`, 0.8325536100406208`,
1.7229814053424446`, 0.9996773558310104`, 0.0005695353813995084`, -0.9994302808020233`, 0.12034944762526084`},
{0.26141584699667636`, 0.7473599798954296`, 9.058762118324648`, 0.765952368266298`, 0.21904751242478016`,
0.26721271327445206`, 0.22245249424068714`, 0.03093590173170892`, 2551.7003206225927`, 385.7759782876328`,
0.1131827887828083`, 7.852734975537449`, 1.3811110977409826`, 158.6726267741283`, 28.147162774574614`,
173.6595211385866`, 0, 110.51367996506876`, 4.117789938413026`, 4.164892477944104`, 0.011438791253453484`,
0.012757460718386949`, 0.010722552471432325`, -0.1595073104181126`, 43.9638772226565`, 0.047642891417531495`,
1.7874759298562293`, 0.30129992333487443`, 0.00016952189864404387`, -0.9994373649459724`, 0.31305086285895245`},
{0.2597492463712146`, 3.976490463784595`, 9.94323613361071`, 0.955277937303862`, 0.36781597694318213`,
0.3161337854088553`, 0.14241883254664206`, 0.0371161903088566`, 1242.5354975091168`, 278.37240293523723`,
0.07851375810023631`, 8.744407246757977`, 1.3989634873529022`, 248.04203867631824`, 79.10493400656651`,
257.8917711204431`, 0, 163.23157230682355`, 1.4651191191532984`, 1.486801825440134`, 0.014799278777664293`,
0.021596533495696783`, 0.017103263799327872`, -0.20805513520325924`, 83.22903150887952`, 0.08013833285340007`,
3.5834403506659926`, 0.3851477996707205`, 0.00021681072203417204`, -0.999437071373502`, 0.35016895975672185`},
{0.057509555667969614`, 0.5897063506624876`, 7.194630155059524`, 1.2755298225691563`, 0.689564930155854`,
0.36119278217271744`, 0.2498841160696511`, 0.4009780889195491`, 1320.9907894027438`, 256.63876030059`,
0.13571670185918522`, 7.16857463714371`, 1.3676255250557143`, 382.49560006041344`, 94.98543044537928`,
397.8797792559164`, 0, 59.127630086786816`, 34.26140016516431`, 34.55363627734383`, 0.008529602140330717`,
0.26111473497648335`, 0.05937124234779418`, -0.7726239296562819`, 288.6309322855171`, 0.2145227483835312`,
2.7860386619867494`, 0.9804144941338424`, 0.0005636360348682209`, -0.9994251043428666`, 0.23290773036785895`},
{0.14523765830527252`, 1.8881182449998724`, 9.138446692610067`, 0.8999286193853917`, 0.5310683312029048`,

0.6925914130374311`, 0.2344374703010791`, 0.005837765173497995 5`, 3489.658152730246`, 207.59827142911195`,
0.0635989978100086`, 4.4743246714218365`, 0.16854984601616874`, 129.69663050337812`, 26.094520011893618`,
244.02115587761648`, 0, 119.55320344782803`, 0.3554043533478247`, 0.3569019445244494 5`, 0.004213767114886968`,
0.023329433191347868`, 0.017913298969020896`, −0.2321588432047994`, 9.586363484405853`, 0.048404460661440045`,
0.3406437956879851`, 0.42565697368372324`, 0.00024106957825385485`, −0.9994336529337988`, 0.10643447866434341`},
{0.23258902546586485`, 2.08539598507119`, 3.699686103649381`, 1.0931833498928993`, 0.05064531761576041`,
0.520823194388518`, 0.08776225589270567`, 0.3513344393005501`, 3745.4841551781074`, 189.61513791631432`,
0.22360193983114673`, 3.043950615318625`, 0.6185359661549543`, 78.239265251336`, 8.54005423686738`,
92.01703580652752`, 0, 13.33367855553737`, 2.0896743811714447`, 2.1187977244916674`, 0.013936785358825476`,
0.129929652566627 6`, 0.04755496831507571`, −0.633994493361016`, 62.25426520858649`, 0.4317173038512548`,
0.5274039380190312`, 0.9158594970460019`, 0.0005280023738139254`, −0.9994234897650601`, 0.08114482392359276`},
{0.25987401963349727`, 2.104611334162005`, 6.476348965574324`, 1.4421427522116774`, 0.618645235230914`,
0.3805640132205469`, 0.11350606280121234`, 0.0053044995482471255`, 2985.7971643892297`, 372.29941068623043`,
0.13537534314965316`, 9.556057499862177`, 1.2376769397336673`, 227.2978708828447`, 52.696199698126534`,
236.8176069143273`, 0, 211.01591061570434`, 0.5094679848613854`, 0.5153891250688427`, 0.011622202735797504`,
0.04582770082546174`, 0.03322909363773564`, −0.2749124865702699`, 15.317601361884991`, 0.17013469748677743`,
2.1716137478425894`, 0.4943871020250971 7`, 0.00027912578909238 5`, −0.9994354104547852`, 0.2353927748853919`},
{0.12870736792434306`, 1.8011116706209283`, 5.964705884730831`, 1.0934961970990227`, 0.1765898735142728`,
0.17954421546189758`, 0.07865767771997476`, 0.015380472377885535`, 1239.7199915121791`, 204.53201197549618`,
0.09598785640324625`, 4.188391285134756`, 0.8112464540523976`, 113.13325170738234`, 25.440187587848772`,
123.49287556342261`, 0, 92.87013988868931`, 0.7511805708393293`, 0.7624295552001363`, 0.014975073634077019`,
0.04792435410964814`, 0.031063887231956397`, −0.3518141702883668`, 19.328001326891563`, 0.08811739252750404`,
1.5138536157155602`, 0.6090620395553928`, 0.00034416569630213255`, −0.9994349250586141`, 0.09821542410760642`},
{0.21477552815648338`, 3.895561319530766`, 6.525902778767705`, 1.1779397940067482`, 0.3665320870360802`,
0.26560577062434`, 0.16739034513753454`, 0.18566064627492787`, 2084.753342798819`, 127.5970184006917`,
0.1877200023200773`, 6.725934789039004`, 0.6926175903352805`, 331.7738620986929`, 33.30064650793632`,
453.6067293365148`, 0, 91.15897087225436`, 4.194199569576031`, 4.252962114529771`, 0.014010431306128668`,
0.7398346421391744`, 0.0558595728093276`, −0.9244972192058837`, 233.41088013761455`, 2.269976800197709`,
1.5886603353010222`, 0.9999943990854605`, 0.0005744635911383895`, −0.9994255331913221`, 0.10742251876065623`},
{0.16557107707187058`, 3.9808920811325805`, 5.098629354330779`, 1.349285917396553`, 0.636892795916012`,
0.22358004297978562`, 0.184200177687603`, 0.010072170136625726`, 2379.9993388287503`, 250.88159080337186`,

0.22088678734814615`, 3.6464862549045485`, 1.4194198069422335`, 141.38596105074203`, 18.917322392712567`,
163.21864062304766`, 0, 122.43112578143537`, 0.30181643152154736`, 0.30732303183357446`, 0.018244865875150174`,
0.4139429891516101`, 0.064319111742886 3`, -0.8446184295216342`, 17.164266317140275`, 0.9790998080025591`,
1.0514090944547523`, 0.9975058609275463`, 0.0005779521153738931`, -0.9994206027873996`, 0.0898553604857702`},
{0.07456457809354955`, 0.9644871227153224`, 4.441895089579111`, 1.073622161985627`, 0.23632853710586565`,
0.6486715280931723`, 0.07294638203638015`, 0.02103243083607793`, 1904.5570203562393`, 282.8945937517137`,
0.14375820330010797`, 5.555062182602162`, 0.9932669340579485`, 118.22662339650165`, 41.00154899601797`,
121.31585404075494`, 0, 91.23880940452908`, 1.8169173187477163`, 1.848338742778388`, 0.017293810624430694`,
0.05148423974206441`, 0.03227659955500972`, -0.37307805812584194`, 25.034190813866097`, 0.054841437354737115`,
1.923988181210185`, 0.6385583740159471`, 0.0003641427407775054`, -0.999429742439227`, 0.2688034188116122`},
{0.2103616483258971`, 1.9343221969844642`, 6.714766349656301`, 1.0369410555646754`, 0.10623162247280038`,
0.4483194849728549`, 0.05288545252588117`, 0.039734874146343004`, 3533.119265437819`, 182.57420336612938`,
0.1697677503867407`, 5.3789812588786425`, 1.1987739832103812`, 149.67847691339438`, 15.730233982309967`,
161.4026400574431`, 0, 96.03384271820299`, 1.8677570518705184`, 1.8928536246883254`, 0.01343674370961323`,
0.039403275990807195`, 0.026958285258791556`, -0.3158364480892163`, 51.61205605724875`, 0.1184134012408803`,
1.8602504712498678`, 0.5574245372272953`, 0.00031495028889496295`, -0.9994349902670924`, 0.10590802729065922`},
{0.20281939839069102`, 1.9616090683543987`, 9.233817142633974`, 0.9596707111380289`, 0.0428378421346598`,
0.4996155069836299`, 0.1873396325631559`, 0.010300259610186683`, 2982.0763963636246`, 256.635560614439`,
0.10323853661350679`, 1.5583642397770507`, 1.1284223856531295`, 36.61400125445325`, 5.2559077936160845`,
41.03846627420804`, 0, 31.88102409767368`, 0.1597272816594257`, 0.1618215329463986`, 0.013111418820976883`,
0.01866048708475405`, 0.015305032227593343`, -0.17981603813022506`, 4.476035488096093`, 0.05406726806010573`,
0.3979232977806063`, 0.33996370368800366`, 0.00019337954792871388`, -0.9994311758995714`, 0.06845040993103445`},
{0.2497495903654815`, 1.9922654552033938`, 4.7803148098485195`, 1.4608470462421037`, 0.7453342000999874`,
0.579116855343716`, 0.20607471500549013`, 0.11029005763163223`, 2995.828190949672`, 372.8602258250605`,
0.24417612053219007`, 5.214690665497957`, 0.479039467804907`, 171.95301214670457`, 44.52491978653581`,
190.84039946351044`, 0, 66.86605946478583`, 3.540770329076356`, 3.570490213046775`, 0.008393620937896529`,
0.16912424855661695`, 0.06315727900736014`, -0.6265628403592447`, 100.77363444897065`, 0.6034101685412805`,
0.663838639499948`, 0.9090299440148863`, 0.0005242498690804951`, -0.9994232864686887`, 0.13874919991761514`},
{0.07327234901270369`, 0.8531314502177398`, 8.241277338171692`, 1.2515317235008665`, 0.44747974375408006`,
0.36044147530449333`, 0.1678069040859092`, 0.08333627347055737`, 3911.155878788726`, 119.81486891303797`,
0.14940939146949633`, 5.454641004053358`, 0.6006995465954545`, 326.74341820453753`, 17.859557764172177`,

416.00235709719266`, 0, 149.34518967436492`, 13.417881217719977`, 13.465748930910511`, 0.0035674569191535`,
0.21906564416172314`, 0.05708944799133419`, -0.7393957039233936`, 163.5316637446114`, 0.22930649051012816`,
0.7194974714258079`, 0.9749048362444757`, 0.0005524077184528419`, -0.9994333726760647`, 0.05026752403158727`},
{0.1930703354969151`, 1.921406348468734`, 8.333479032313342`, 1.3125156750016385`, 0.34274795388496426`,
0.18757059400837783`, 0.06254344682622745`, 0.48499736430114065`, 1025.3342142153256`, 313.5826206578507`,
0.06795986160544415`, 8.852485488110243`, 1.3326254900136894`, 178.14120211881036`, 76.64497862073068`,
180.7240089584147`, 0, 25.618873390382916`, 5.358138825867175`, 5.4720617450361875`, 0.02126165873475183`,
0.024067077285527587`, 0.01988924120214771`, -0.1735913353256301`, 147.07374222854293`, 0.06638055265631948`,
3.667449743605586`, 0.326071247837875`, 0.00018352126765523182`, -0.9994371743326892`, 0.257683125937168`},
{0.075070301444424`, 1.1158533918226947`, 4.504617389718993`, 1.302858138181639`, 0.7006793741520845`,
0.20393211135209022`, 0.13347728349830462`, 0.12945769800166135`, 2084.9216842923897`, 207.91665221421215`,
0.1979670492115873`, 1.2145592445361792`, 1.330809319256201`, 41.82686038149478`, 7.113535345271648`,
57.669930190172806`, 0, 14.63376001678337`, 1.553121273808646`, 1.6025049672169656`, 0.03179641811693079`,
0.42560616165952747`, 0.06233837251714497`, -0.8535303805892409`, 24.757937732733744`, 0.4564340407483538`,
0.4096801347896363`, 0.9989147906964425`, 0.0005810215571592816`, -0.999418347227841`, 0.034664940104430324`},
{0.04793776202493774`, 3.0088158689008297`, 5.170365665010708`, 1.3304854892588214`, 0.4098476660063537`,
0.28021047255972975`, 0.1484797686973009`, 0.06051575982799722`, 541.2425249553703`, 199.10653072626633`,
0.20919118463500602`, 1.1760815484955796`, 1.3182149861572752`, 46.37344271003044`, 22.707270233192244`,
47.436577501373215`, 0, 25.02268846864709`, 0.4757995752115936`, 0.4888652830947066`, 0.027460528684378538`,
0.2512376712417531`, 0.06355357847898302`, -0.7470380211499864`, 20.45133303304165`, 0.1720538813669451`,
0.8289900504831961`, 0.9692332632402618`, 0.0005795944732706373`, -0.9994020072409268`, 0.0729035629793527`},
{0.27316321779569186`, 1.2038217889581082`, 3.7625718380424993`, 0.7524163201956243`, 0.8286278988606164`,
0.5838413652579414`, 0.1289047793447199`, 0.04536097191427075`, 2261.212930013986`, 291.3114882787695`,
0.1550429747126918`, 5.46528595832512`, 1.2275549298228752`, 159.69321446087852`, 73.52502687873006`,
218.95701158619445`, 0, 96.2451198787026`, 3.3664453447956357`, 3.450636998089951`, 0.025009065845810197`,
0.4452678886258658`, 0.036642184500814405`, -0.9177075521572075`, 57.89428939145112`, 1.7375829891162056`,
1.7148730428389152`, 0.9999554470122989`, 0.0005902042931814776`, -0.9994097694102824`, 0.21212646771723723`},
{0.12542240143398464`, 0.5428740326561194`, 7.689007709078183`, 0.7695505557683522`, 0.47414990819814995`,
0.5751602267111138`, 0.1894118267745437`, 0.4188796421855669`, 2595.8116503362608`, 121.7056153778716`,
0.02093687945319478`, 2.468481199752018`, 0.4696061183497453`, 142.0611366485916`, 15.842105119541863`,
193.96116487322854`, 0, 20.612996132333638`, 13.80346551639696`, 13.902388014685302`, 0.00716649729525054`,

0.21279166661677876`, 0.0361349106870534`, -0.8301864388696689`, 107.05061413594413`, 0.3812691690315691`,
0.4199190749266494`, 0.9968075640507139`, 0.0005688382048684914`, -0.9994293399996315`, 0.04730889500332113`},
{0.15904117838601922`, 2.05514897407154`, 8.990862054448137`, 1.399223458930086`, 0.16448814747402052`,
0.679414685909332`, 0.20987970122910526`, 0.011549865816715998`, 3644.479968128845`, 344.7039952488134`,
0.14067273235487315`, 5.192736401046902`, 1.265673086788253`, 109.22384396831376`, 19.195313903582942`,
119.32319285898598`, 0, 93.78712292465713`, 0.503664511155642`, 0.5095417860638634`, 0.01166902725533725`,
0.024618440385267202`, 0.020538220309818195`, -0.16573836569642308`, 14.787222905396584`, 0.05593351098426953`,
1.170968466929593`, 0.3139565378827526`, 0.0001778006882020936`, -0.9994336773828596`, 0.2545190465533683`},
{0.15956630372935293`, 0.48645969647201426`, 2.666967707997488`, 1.3223736190803834`, 0.3256325807266449`,
0.3482049071188411`, 0.2077761075845453`, 0.13946170160498525`, 1352.5873448025677`, 303.93582335946746`,
0.20467290406100863`, 9.626982891792906`, 0.24128691898319654`, 201.16510353214503`, 80.56816029051765`,
289.0010076759628`, 0, 67.49345035394028`, 16.45075293739531`, 16.848735133180664`, 0.024192339238204186`,
0.8835557652429453`, 0.0649773125222231`, -0.9264592965398661`, 114.32326115230589`, 2.0140818228368325`,
1.8778492605886052`, 0.9999972557592637`, 0.0005954677975158662`, -0.9994045305683726`, 0.4665172894490428`},
{0.1706421742509121`, 2.0231510661206737`, 3.9336130035151675`, 1.4585682836966742`, 0.11643260032474756`,
0.3849941275209635`, 0.09390422638545698`, 0.012272074152593612`, 1400.0032271122882`, 356.7106633003026`,
0.22727785977132925`, 4.752121167794142`, 0.5739518122089307`, 77.24008389391977`, 32.89247422096266`,
81.39289412743169`, 0, 65.7041479185864`, 0.3780449056963888`, 0.3853059610459086`, 0.019206859397151055`,
0.05478370479189014`, 0.037773114726366464`, -0.31050455842923985`, 10.926313628587685`, 0.13354872141726642`,
1.2671964039379435`, 0.549208796665812`, 0.0003140343770979235`, -0.9994282058499346`, 0.26608654273500953`},
{0.15918053794813153`, 2.206413285621343`, 5.933314299085708`, 1.101458457677796`, 0.578828119858223`,
0.5102982514637783`, 0.14381010711428127`, 0.005244970313706263`, 866.7690619050336`, 266.3421407794259`,
0.02684631402674248`, 2.069701061730642`, 0.9768437925368372`, 72.36268951686326`, 42.263211524349494`,
72.32687352945604`, 0, 67.0751012913239`, 0.1523413832645265`, 0.15510549629683476`, 0.01814420332201294`,
0.0785137522017812`, 0.040672520897195336`, -0.4819694670474223`, 4.80182931406834`, 0.17854087588293732`,
0.9688431066188739`, 0.7730423547478433`, 0.0004478404478771759`, -0.9994206779937391`, 0.11998729509857713`},
{0.04928925392103686`, 1.1598100203546071`, 3.5959706980197943`, 1.0567798767912857`, 0.5147503710857468`,
0.3483622087425814`, 0.2380152845766259`, 0.2996884511067484`, 3369.979145995473`, 363.0241772367476`,
0.01463432097258610I`, 4.5222312521830705`, 0.7343151280843321`, 120.69207501983729`, 18.114361432229266`,
139.74207876832804`, 0, 23.267863549013253`, 5.524539865187503`, 5.568944506799652`, 0.008037708604831106`,
0.2142717493577496 7`, 0.049205733627509414`, -0.7703582773977583`, 91.5345241927567`, 0.150875638031324`,

0.6036681784688609`, 0.9817794175603561`, 0.0005640575739443999`, −0.9994254742319348`, 0.11203432296907546`},
{0.21304011543754336`, 1.277940262843642`, 8.381125715629981`, 0.9676658592923792`, 0.3190964277032775`,
0.4221745616471033`, 0.15115883673524522`, 0.016195119502937567`, 3029.1645990840216`, 191.62516925020475`,
0.15425858593294461`, 3.4086567800829783`, 0.2418820655638112`, 88.29454944469944`, 13.497231012496432`,
139.22361028923953`, 0, 71.65917952850415`, 0.8570011927941963`, 0.8615148539468408`, 0.005266808483577412`,
0.024221127564639976`, 0.018759601030941955`, −0.22548605630033558`, 15.645661851096104`, 0.07371531160572314`,
0.3319266035052809`, 0.4149115361070388`, 0.00023488855594927833`, −0.9994338828026977`, 0.06687815903603742`},
{0.18459742862139494`, 2.6783696392861316`, 5.280110904616961`, 0.799376053447836`, 0.38050898992691184`,
0.5204372051986464`, 0.12691110515834225`, 0.11946707964132822`, 2456.6727054183984`, 281.1798234579603`,
0.015729828687073455`, 5.211513473652531`, 0.2697983327311402`, 114.78763769596205`, 30.5561510463887`,
145.19267698649068`, 0, 42.785271237919325`, 1.83103504403348`, 1.8434548375390023`, 0.006782935993493444`,
0.030653323915338856`, 0.02092061920767845`, −0.31750895056409145`, 70.05983814868888`, 0.08083606819251246`,
0.6734388287272405`, 0.5587684583650117`, 0.00031703105141634737`, −0.9994326253626699`, 0.17278271207531837`},
{0.10846037232055339`, 2.4223111603013434`, 3.931890423132195`, 1.3804697934424115`, 0.1958381176894204`,
0.37535535595877956`, 0.06969589452072714`, 0.03328599418804682`, 502.38138813825617`, 240.4375056150992`,
0.057245528883930086`, 5.6090889313070385`, 0.20778481434400486`, 140.41333638122273`, 114.66175870420382`,
140.01325277097266`, 0, 95.84370582249832`, 1.242761247173569`, 1.2709376209437597`, 0.02267239490632056`,
0.12160995634757807`, 0.05512261728803469`, −0.54672611566041`, 43.00506340883643`, 0.18842658776208634`,
2.904493490509507`, 0.8369552888541034`, 0.00048386946813340075`, −0.999421869394247`, 0.5150460603894683`},
{0.06691193258644623`, 0.6501044545968662`, 4.921653023328441`, 1.039296561059753`, 0.3007307833256643`,
0.63941363731021`, 0.1869993853971682`, 0.22083355826369067`, 1064.9317102210284`, 220.88788456016823`,
0.19206991947921326`, 8.047969449780908`, 0.8982344488011158`, 297.1307961447968`, 113.25296718141817`,
311.4368170836702`, 0, 74.07631375186963`, 21.642898340969253`, 21.931622532616636`, 0.013340366299315676`,
0.20937736660814354`, 0.048929763430814247`, −0.7663082489599372`, 201.0020660264462`, 0.2001406319945128`,
3.618518127222012`, 0.9766110779523651`, 0.0005701169421370117`, −0.9994162292902387`, 0.5062827490896461`},
{0.05561357895854885`, 2.541859626964298`, 6.751848587656245`, 1.0550493742938256`, 0.47524313475951674`,
0.6531938104590875`, 0.23105177416584038`, 0.08329104995794483`, 3011.985435651468`, 344.16286415130503`,
0.16793335434061596`, 8.98503885095289`, 0.954537509095668`, 261.53831659796634`, 55.33150625205841`,
283.7642005888341`, 0, 120.34402324104768`, 3.7820640260759673`, 3.808597426834878`, 0.007015587408349511`,
0.04270052702035099`, 0.02854983126906531`, −0.33139393676666995`, 137.3353693496645`, 0.03392470187236546
6`,
1.7887701481915714`, 0.57932413428437`, 0.00032764705028698457`, −0.9994344322445815`, 0.32415493587641636`},

{0.2794959518803075`, 2.632356308938026`, 9.83032757153688`, 1.3936963350666467`, 0.29701793223992223`,
0.20626280407223174`, 0.16564637606336619`, 0.029273176379366037`, 3072.6434786295413`, 349.76316755112407`,
0.019513472485920585`, 8.521067400493664`, 0.14808884776404962`, 100.74402641558086`, 24.542818523460927`,
199.47662631792193`, 0, 71.17860442545881`, 0.7640462468255574`, 0.7695071598909305`, 0.007147359322897007`,
0.011474160454794059`, 0.010517734627318564`, -0.08335475447146001`, 28.73202797359533`, 0.04581401997627193`,
0.6512003215431229`, 0.16241836869659187`, 0.00009140520060073185`, -0.9994372237491717`, 0.1633934979087995`},
{0.23109541414596008`, 0.5235141892941444`, 4.716797836648434`, 1.149537093670705`, 0.6317810219681328`,
0.3294586442905981`, 0.09670796472340015`, 0.006726344420984968`, 700.9172696415767`, 217.90397850913655`,
0.1081952457359675`, 4.152209762717746`, 1.275784993485201`, 152.20779914557608`, 89.46846642803209`,
161.70408201586758`, 0, 137.24296422301114`, 1.5266343487873222`, 1.5737597531970373`, 0.03086882228684895`,
0.43705976578881855`, 0.055997509830065004`, -0.8718767678626308`, 11.41735347791413`, 1.442892965450039`,
2.5310657453652405`, 0.9981241247536483`, 0.0005906414895935597`, -0.9994082484583374`, 0.18088774507724462`},
{0.2685717150273603`, 3.803415502373147`, 9.096737964614093`, 1.4671460946906174`, 0.7887407820119334`,
0.32631565166991583`, 0.10844882100921999`, 0.25610077987407076`, 3027.035564030042`, 145.16929800361856`,
0.22414767286679493`, 5.11102243526882`, 0.8167747408960464`, 320.37192822422185`, 32.09293604415167`,
360.4669497662974`, 0, 69.49565547322727`, 4.518248388606924`, 4.546921666389684`, 0.006346104799165308 6`,
0.17805898476804202`, 0.06340763809738063`, -0.6438953182846574`, 245.49679949714377`, 0.6831658130743887`,
1.1646265738027963`, 0.9240721499625332`, 0.0005236903547723872`, -0.9994332797987758`, 0.05203807529720777`},
{0.26187896590333215`, 2.5078884075536303`, 9.466913557279852`, 1.4296579631390225`, 0.32008183029716575`,
0.24032929426056993`, 0.06161020998156652`, 0.06072142573048648`, 3034.015450440843`, 187.9292507538172`,
0.24599280561665748`, 9.218911969505061`, 0.5310393366539319`, 258.4449042583786`, 29.142678470123563`,
310.04256483760076`, 0, 138.98353999306568`, 3.2394400989097147`, 3.2598460040700843`, 0.006299207436259557`,
0.03391317700553771`, 0.026565837461142692`, -0.21665146686773895`, 116.059346728858`, 0.12687353892422754`,
1.471044176079969`, 0.3997132313871198`, 0.00022505749796617646`, -0.9994369525942758`, 0.10184229463645955`},
{0.19280830132567833`, 3.755171648384974`, 8.021128419894612`, 1.335462575824002`, 0.48264871431934253`,
0.18420836982360544`, 0.06051840370015368`, 0.009206053536078937`, 2566.2923313365445`, 129.64164707934253`,
0.24657184473865845`, 2.9358400550970494`, 1.228808415909655`, 135.02654124032787`, 10.96905199579468`,
147.83310317535918`, 0, 119.0712750864033`, 0.2838580725933176`, 0.2871979767958917`, 0.011766106110919772`,
0.09171841074977384`, 0.04800937486032129`, -0.4765568388303144`, 15.227654090966139`, 0.2526295853850441`,
1.136160294935399`, 0.7706728404468803`, 0.00043577757 16217102`, -0.9994345492033052`, 0.03282011694025315`},
{0.16230945707171968`, 3.49945541680664`, 3.914597683293998`, 1.1051912745206218`, 0.5225762284594742`,

0.37657675843017935`, 0.10709361805155937`, 0.30774950760646624`, 1978.9594818216538`, 231.32180409655837`,
0.1878700890542328`, 3.1200520265606095`, 0.5684357627808438`, 92.39046043395422`, 22.152575304207726`,
108.5364970401756`, 0, 17.5147401312492`, 1.4502764235743135`, 1.4761030220914375`, 0.017808052380436923`,
0.2780919230974687`, 0.05238666798466007`, -0.811621037385185`, 72.50253837634388`, 0.6448135579142301`,
0.6999168291414798`, 0.9937640734711153`, 0.0005748992645778399`, -0.9994214932095806`, 0.09176614575893197`},
{0.2783243544518275`, 1.6065452524928183`, 9.36513522454521`, 1.0091499802528594`, 0.9718534638380145`,
0.5642633711867375`, 0.20737635968923002`, 0.08580910729338392`, 3111.9739304829964`, 94.14000266759376`,
0.09401444314711727`, 7.942384951366636`, 0.4018498645586557`, 561.262045986913`, 111.1508544213112`,
1642.9620318556488`, 0, 250.13401203245886`, 12.750884997608331`, 12.905920319587388`, 0.012158789135666703`,
1.152656840202926`, 0.04778543536138279`, -0.9585432249263621`, 292.641053685566`, 4.583035299341647`,
1.007840149221834`, 0.9999999959486273`, 0.0005734747254459216`, -0.9994265252722307`, 0.09212344052702003`},
{0.13125352185182698`, 1.2184353998248145`, 9.320259419097251`, 0.7781699346988293`, 0.39678231153919463`,
0.199523418988783`, 0.19269759399037872`, 0.23378140134846623`, 1538.2313512668088`, 153.62701919814924`,
0.16196850129753104`, 1.3309378497731819`, 0.3512246763428848`, 55.27255660563233`, 7.734121306646269`,
76.35953020756449`, 0, 12.903950590709147`, 2.2966854894938984`, 2.31096687792184`, 0.006218260398853648`,
0.03314996209203132`, 0.02174352379438913`, -0.344086013310528`, 39.9766128951907`, 0.0621578467690304`,
0.24848005664686465`, 0.5978664808128766`, 0.00033863930869930484`, -0.9994335870640567`, 0.01923802145848475`},
{0.1755298706210477`, 1.1941209557460892`, 3.229216030497655`, 1.0124753772100286`, 0.8865998145070868`,
0.346236517225073`, 0.10475625859225324`, 0.48133977507102055`, 1150.8999752212085`, 275.6916176605181`,
0.02747195653647755`, 4.902957385276519`, 0.7900769747759009`, 124.33512100255034`, 100.51449898735049`,
196.07258156144735`, 0, 16.984481850332052`, 5.79590249848278`, 6.010846763345996`, 0.03708555568688099`,
0.7649697897397324`, 0.049846550623765286`, -0.9348385370346132`, 98.8715518699915`, 1.918214974600394`,
1.7658623940028866`, 0.9999878934134707`, 0.000596832130474405`, -0.9994031606438383`, 0.22428569084562383`},
{0.08731955025115129`, 0.716566084545263`, 3.9517372388296312`, 0.9979934558679373`, 0.7980323468748562`,
0.2894395727404939`, 0.23900619972839288`, 0.013550707634954815`, 3303.670456871152`, 218.2198703581039`,
0.21020148991020782`, 6.102509356835768`, 0.9882047778905934`, 190.72870590128636`, 33.25700226265034`,
448.06464645390605`, 0, 153.10729788132656`, 2.61753189418614`, 2.752901854736074`, 0.05171664225013162`,
3.611587553759175`, 0.04952413557871138`, -0.9862874332017333`, 26.794779722704416`, 4.505174298384379`,
1.166071580905663`, 0.9999999986322267`, 0.0006010811946051045`, -0.9993989188045728`, 0.16060947312010168`},
{0.22902394998753428`, 3.040671713374585`, 4.278745423137005`, 1.1872158647718087`, 0.928367976025805`,
0.6049768179185504`, 0.23137362815653145`, 0.010091922508071186`, 2258.092758703583`, 198.0963189618003`,

```
0.016671766650619035`, 7.17480421825168`, 0.31956308961413415`, 245.27862100432452`, 128.3481770366565`,
 704.3872595592933`, 0, 204.77919780475028`, 0.6609725986172275`, 0.6954865641777457`, 0.05221693854287213`,
 2.567405646069927 3`, 0.05948889060911686 4`, -0.9768291813566041`, 28.711438341872753`, 8.399962604046214`,
 1.0423623138107256`, 0.999999999214167`, 0.0006072210042472587`, -0.9993927789952756`, 0.24479051803506072`},
{0.25718420980427975`, 3.2208936021473447`, 9.13093887998258`, 0.7880508822382197`, 0.19579212797696832`,
 0.6844641096337076`, 0.10728371238227297`, 0.15509978165260505`, 1971.6454958829345`, 171.21565981007905`,
 0.07048499237249867`, 2.8101083115304704`, 0.7477956360854239`, 96.6509454149429`, 19.59495107185513`,
 106.14316431166327`, 0, 30.647831522707932`, 1.4013658323884435`, 1.4165320914831347`, 0.010822483854085796`,
 0.02589401004348559`, 0.01861889156940596`, -0.2809575829260136`, 64.48071776868291`, 0.09513615016731859`,
 0.9454173586920033`, 0.5041952173503894`, 0.0002862735368161597`, -0.9994322168736138`, 0.10771111150981864`},
{0.22344776749508172`, 2.0217933679260236`, 8.515379660775892`, 1.0649182548208418`, 0.4042870031976431`,
 0.16656241643092373`, 0.13003071453691661`, 0.03138725525414384`, 1808.0816946786463`, 137.22272185069306`,
 0.15677750076118624`, 3.7329055172229837`, 0.8930251877812045`, 188.91519798897366`, 17.924159855255905`,
 216.0620448833551`, 0, 130.53013349474486`, 1.9419690083895378`, 1.957114025533975`, 0.007798794460163405`,
 0.07989878695801626`, 0.03969836125640112`, -0.5031418777701709`, 56.089429455426256`, 0.2550457938762329`,
 1.1243901823451132`, 0.7991564146594715`, 0.0004516785602202529`, -0.9994348058128111`, 0.0495669983065623`},
{0.232764074403509`, 1.2733235890477337`, 2.798704761143199`, 1.2089738970000061`, 0.2230756746500473`,
 0.581248946739561`, 0.23598452021555733`, 0.3774029175060033`, 1879.298545402161`, 390.18642709154335`,
 0.06147319592641398`, 6.399283876049893`, 0.6943722912689665`, 132.32428164227917`, 44.69168804341732`,
 142.79382821559153`, 0, 21.71709771841737`, 5.721177630535252`, 5.816522135996312`, 0.016665188815705445`,
 0.1886287358117407`, 0.05597522579634023`, -0.7032518637446321`, 104.07014905846788`, 0.6272284728160331`,
 1.40493739622043`, 0.9491853732848676`, 0.0005615192825286087`, -0.9994084197899243`, 0.42986962203289525`},
{0.1797514088363001`, 3.6024179997471064`, 3.854320723033281`, 1.3651410466183755`, 0.3139114488968535`,
 0.24530825544465074`, 0.2138602169809023`, 0.009505828045846924`, 3546.9761969830042`, 314.2947763723504`,
 0.014606932674341683`, 5.351501403328509`, 1.3913390486692032`, 137.9799044131377`, 14.289237738093975`,
 152.7771673876927`, 0, 121.07479167967254`, 0.31060293529682836`, 0.31445084149288294`, 0.01238850557666904`,
 0.14325922091462892`, 0.05724659896378173`, -0.6003985042059098`, 15.984594355536855`, 0.3678720969742295`,
 1.1110120681919422`, 0.8895722382222677`, 0.0005084004641203992`, -0.999428488837358`, 0.1568355971513982`},
{0.08593348279794505`, 1.5299819772683403`, 7.853142207396741`, 0.8569688383589438`, 0.17350357661082394`,
 0.3599516000206078`, 0.22245867865147873`, 0.35987771942812175`, 2188.9020302076506`, 267.66973385037716`,
 0.2117450466607785`, 1.9987769240506328`, 0.6930609981274307`, 50.53690593240434`, 8.780655444651597`,
```

59.00533138529461`, 0, 8.54179449616477`, 1.8351003580302228`, 1.8509795559745`, 0.008653040622433217`,
0.022639789178475984`, 0.017308426818922012`, -0.2354863959874055`, 40.109578203784416`, 0.027793084770259668`,
0.3974517688661377`, 0.43145201287999646`, 0.0002446890037544909`, -0.9994328708721947`, 0.0689166228731781`},
{0.16049167400816333`, 2.9496377174467003`, 2.796051273230102`, 0.7713172859306066`, 0.5895053698909607`,
0.5330035027095973`, 0.21946432427500168`, 0.0063958696081320284`, 3721.6840074196953`, 354.1366035930697`,
0.05846005834212181`, 3.723454931340358`, 1.352543221697399`, 82.56927142328126`, 18.737988417976716`,
122.14447776980205`, 0, 73.70670857546754`, 0.15440311373635845`, 0.16024754084081078`, 0.037851743808946736`,
0.645403864089954`, 0.03834589120189756`, -0.9405862075896201`, 6.506189256685351`, 1.4797420937019252`,
0.814650151678194`, 0.9999994437701527`, 0.0006027549867814473`, -0.9993972446779481`, 0.18139181517187108`},
{0.2459634383839392`, 2.317311476187519`, 5.114630918885933`, 1.230187162908409`, 0.6324239129659683`,
0.3459102702870417`, 0.21279405046832994`, 0.012571031115264336`, 3926.1200815627817`, 399.1203840176114`,
0.14512335124342485`, 1.859263107025912`, 0.81357096327371`, 49.348780593911634`, 7.185337238043029`,
54.658055821343126`, 0, 41.59055497542612`, 0.21777787793319367`, 0.220296995137251`, 0.011567369596787502`,
0.0674147253457897`, 0.03993249796627617`, -0.4076591165883894`, 7.209416797062201`, 0.23687939491084598`,
0.23508074390235775`, 0.6876523412874955`, 0.00039439814766612034`, -0.9994264570568789`, 0.04264133981424875`},
{0.25338042742565964`, 2.965005843458032`, 9.015448508781162`, 0.9648345670574521`, 0.4738085996714536`,
0.5217539624890015`, 0.09898738245676192`, 0.10703385809743371`, 584.7617542091971`, 399.01325318858073`,
0.22656752967920823`, 7.539332683081733`, 0.5787011055505342`, 158.44138108606305`, 208.7611437203211`,
147.51369292402487`, 0, 64.61385174258024`, 2.162252648785132`, 2.209387913500953`, 0.021799147635362726`,
0.016934983568193788`, 0.014113854738076385`, -0.16658586167251255`, 91.58702483829336`, 0.06129990535646153`,
3.383272275673347`, 0.3140442114201353`, 0.00017715848872227395`, -0.9994358804197627`, 0.7200109330139924`},
{0.13715202199195703`, 2.280611332139774`, 9.546074571903652`, 1.3294680551764548`, 0.96176455508361`,
0.20271717396909816`, 0.1365163915603838`, 0.01317998381974412`, 622.5257559162319`, 109.88086672718458`,
0.09246427533408269`, 2.547747841165865`, 0.5344581094207239`, 184.82703380962403`, 73.69201699601584`,
317.53570044665986`, 0, 154.23627196257803`, 0.8570679626105939`, 0.872125035869509`, 0.01756812051759793`,
0.5797524147755426`, 0.06336427559674532`, -0.8907045939234639`, 27.923412970623826`, 1.1359173705883636`,
1.3788583943224415`, 0.9998893298635944`, 0.0005775609487944809`, -0.9994223751253819`, 0.054762834821954265`},
{0.26021306080280165`, 2.6412569822545393`, 9.393216711347169`, 0.7980583185168122`, 0.12902542313562004`,
0.41288706652580376`, 0.11050318145828913`, 0.08290531025525745`, 2885.381565930523`, 235.35959645539515`,
0.24979107480174895`, 7.498842240586485`, 1.2653883281677927`, 190.88224023553656`, 26.53449404430071`,
206.6557294871179`, 0, 88.54305613321527`, 2.640159958037325`, 2.672099713542578`, 0.012097659237661151`,

0.016892993226526935`, 0.013572788024954268`, -0.19654333350225783`, 99.61915604907048`, 0.06279682105139849`,
2.2775909682982127`, 0.36574148775969306`, 0.00020595415426143384`, -0.999436885994195`, 0.2162204151316696`},
{0.21577611209111464`, 2.7972286676872242`, 9.151464868370521`, 1.0125832913995234`, 0.03171655307538246`,
0.37863990617192567`, 0.08961735731262133`, 0.24231024163547266`, 1129.2395603552968`, 145.3261697746342`,
0.1783001304868151`, 2.7363213922238323`, 0.4255887662905571`, 91.1498296239212`, 21.501416629529267`,
106.76524185613711`, 0, 21.10642956990857`, 1.7068672922908141`, 1.7265418720680348`, 0.011526719075397596`,
0.031700239679404656`, 0.023115441981587846`, -0.27081175993108564`, 68.20711602762184`, 0.09771649243410015`,
0.9385183019005802`, 0.4878863259029014`, 0.0002766379329906288`, -0.999432986910468`, 0.09716454357109637`},
{0.18088366879997198`, 3.821865573482567`, 4.692160635426452`, 1.0482928849004183`, 0.06532272769142566`,
0.687087343414349`, 0.24770159954715665`, 0.017305980256248125`, 3393.812324444456`, 259.70338252927684`,
0.20399575556521798`, 4.91689758771607`, 1.287491266139321`, 132.36622300311623`, 18.00654691721507`,
147.83246800899246`, 0, 106.12931215237884`, 0.4664659554114304`, 0.4719213185281515`, 0.0116950938293221`,
0.07129628258224864`, 0.03772030207743458`, -0.4709359210430113`, 25.468145374122898`, 0.18423333093247307`,
1.3127146902515439`, 0.7621423033925188`, 0.0004360404052885346`, -0.999427875341196`, 0.2546322769017392`},
{0.05949240242631926`, 3.2421313428658216`, 6.819444043498855`, 1.0897919998234773`, 0.4914757475836029`,
0.6217813654383784`, 0.14811683608778792`, 0.1990515558123701`, 2177.5035606095144`, 392.178441681785`,
0.16794023089571636`, 3.103361276547407`, 0.452347159579072`, 64.96015653986953`, 25.551721493830943`,
70.0998305775464`, 0, 17.275092111117235`, 1.0067424666050566`, 1.0157050718247913`, 0.008902579872247074`,
0.026960881308380893`, 0.020988088905609718`, -0.22153550302951375`, 46.628447216775676`, 0.0229138228651643`,
0.5343789289787165`, 0.40933128315470924`, 0.00023328098917527735`, -0.9994300924488904`, 0.14932993045713813`},
{0.06601763360963414`, 2.324714740822664`, 5.47799549721827`, 1.4572263706661284`, 0.6058468419307841`,
0.40657776744209273`, 0.16352063484885299`, 0.154889405316064`, 3091.1261645885334`, 170.4797745198008`,
0.16879539576852792`, 3.0092390791565844`, 1.1781015424249213`, 125.40347386013983`, 15.812749492900197`,
180.47524468707854`, 0, 39.25898938976733`, 2.482346762189318`, 2.5246811415981356`, 0.017054176335735027`,
0.6293402698059396`, 0.06952056909391895`, -0.8895342115714986`, 82.43925871278458`, 0.5935365049691813`,
0.8882407570526304`, 0.9997883040721965`, 0.0005781556784528233`, -0.9994217219024287`, 0.08110059523038914`},
{0.09129438454580396`, 0.5458728552745402`, 8.578700312104903`, 0.7708504139325052`, 0.9169389731376072`,
0.22022269715135345`, 0.2062604041361082`, 0.19807010574894265`, 1283.9010613736064`, 230.53840695892347`,
0.015448919626635749`, 5.258490435173719`, 1.2826107951025567`, 339.2196811344456`, 74.52593556152239`,
396.8693716628985`, 0, 89.88865240671076`, 28.265888538095084`, 28.487777356166283`, 0.007850056359351765`,
0.2788598422634652`, 0.03638181818247307`, -0.8695336772510266`, 220.42258975945512`, 0.36369053820002095`,

2.001867635328114`, 0.9979637621628461`, 0.0005714944920230939`, -0.9994273394348663`, 0.1024935243970807`},
{0.14843032745543416`, 2.891906313088681`, 7.801883637284866`, 1.3483733856378388`, 0.31815406883941244`,
0.21186149946857102`, 0.16733027048593607`, 0.016108958562959286`, 2806.829919143951`, 114.3420493392897`,
0.021367639016282636`, 6.332693333253493`, 0.6091184877547111`, 358.2582347249456`, 20.330617635293017`,
457.9078397090219`, 0, 290.72260292531104`, 1.5757594251375864`, 1.5839520557528723`, 0.005199163326959466`,
0.2305846942703935`, 0.06134928017644588`, -0.7339403624747742`, 65.09926613520531`, 0.48893945252524423`,
0.998929819556356`, 0.9725233932033762`, 0.0005510891338292175`, -0.9994333409996299`, 0.05481658847995699`},
{0.2503454420826467`, 0.6816060830582531`, 6.557163545095662`, 1.251247380535385`, 0.5924064529900261`,
0.3249563797350232`, 0.13275565091305608`, 0.008288831069377075`, 3729.443509243899`, 228.98969063119478`,
0.16939843347227213`, 6.2896214012738305`, 1.016677469632238`, 236.63333630645275`, 24.33771536119123`,
260.3167208569051`, 0, 211.19018874598834`, 2.315082149830697`, 2.33333534908566`, 0.00788447151056726`,
0.08616457916314349`, 0.04503222124097972`, -0.4773696839426792`, 22.542486801488288`, 0.3081558523208798`,
1.2600931227832546`, 0.7707120695720183`, 0.00043606275996847614`, -0.9994342079523801`, 0.09470593732282039`},
{0.21895003474025426`, 1.413342329996616`, 6.660357061219696`, 1.3885224416718682`, 0.6085057775930085`,
0.3063420766693563`, 0.18696649922653613`, 0.1721695479130944`, 1066.3987466436151`, 245.09142596090578`,
0.1673351585832708`, 3.1429897838085274`, 0.7494357676944992`, 131.84659306768904`, 41.193545032101454`,
140.09624161855953`, 0, 38.738952620910744`, 4.370563666596771`, 4.423462702933939`, 0.012103481466581467`,
0.119372663641271`, 0.05480985104370318`, -0.5408509002663016`, 88.24432337066341`, 0.3733806978753975`,
1.0970129484625`, 0.8349936615037234`, 0.00047867640719828186`, -0.9994267304899821`, 0.1025800070620124`},
{0.1254259042141826`, 0.6960838019109872`, 5.192144520750629`, 0.8756261738238285`, 0.8328612976967942`,
0.22677152638905884`, 0.14617978450286806`, 0.02130952691785612`, 2632.7908352385166`, 212.41036911499543`,
0.027687267864634157`, 4.124602621711544`, 1.0784411017781297`, 161.70234827239986`, 25.235442488567468`,
241.2943899561976`, 0, 123.3560247439031`, 3.4029881167393325`, 3.4482266399919475`, 0.013293764685831766`,
0.38084183030151714`, 0.041478040372192736`, -0.8910885384114605`, 33.839498659397414`, 0.6823918704021776`,
1.0160583358364246`, 0.9998393304188886`, 0.0005738534980474023`, -0.9994260542863352`, 0.07205672542563314`},
{0.21183190072031832`, 2.1405207408150053`, 6.713672348966824`, 1.4852810672850318`, 0.9707621809425402`,
0.24869479497663283`, 0.14712840909677755`, 0.010718332694701214`, 2858.107536317915`, 341.2444641156959`,
0.09413983850760904`, 3.9282483342993455`, 1.4253439441659714`, 163.46590135941238`, 29.82395573893237`,
170.7817803166572`, 0, 141.36028232754148`, 0.6809554001472978`, 0.6873622391741344`, 0.009408603009023464`,
0.1233832460731881`, 0.05771412622104457`, -0.532236927963381`, 20.822845108361033`, 0.3733786790389249`,
0.9192574867104506`, 0.8294035840458374`, 0.00047106592305199503`, -0.9994320425759988`, 0.06232881605355378`},

{0.18422677214256195`, 0.6743311410267894`, 3.1036416049630393`, 1.492376851920162`, 0.6594088757028673`,
0.5950695833857709`, 0.1860537678137455`, 0.02943690821344356`, 3269.4748824588514`, 385.0414868746369`,
0.02235734672967704`, 2.542522241338192`, 0.26953310124704477`, 59.40154124093528`, 17.18063283634961`,
75.55595352862153`, 0, 41.24685073544425`, 1.621798040774066`, 1.6537663296978937`, 0.019711633705371767`,
0.24848515178314629`, 0.07022542403926167`, -0.7173858335787098`, 15.623270333574084`, 0.6539659634052001`,
0.23933642312662065`, 0.9695920837814005`, 0.0005721039943018586`, -0.9994099539343694`, 0.101950115001121`},
{0.12205814528256814`, 3.0220310797867835`, 4.8913469029191035`, 0.8430601347747492`, 0.1108026510072466 2`,
0.5338876009745465`, 0.22020963395963772`, 0.11433363332445597`, 1824.128984278892`, 211.3967074697807 2`,
0.11084187323527839`, 8.781693512702038`, 0.22047341418167488`, 227.26706170936922`, 55.003557128494066`,
326.9141293065958`, 0, 87.06315354115344`, 3.171016491203628`, 3.1912447103231156`, 0.006379096159102593`,
0.04889756798381896`, 0.027993982350722462`, -0.4274974501802187`, 136.89871987048278`, 0.08526209224193546`,
1.3436267795677985`, 0.7088242245467699`, 0.00040210858805445824`, -0.9994327104321081`, 0.3500242347321348`},
{0.15978649721954924`, 2.191594266371557`, 3.0757440378000283`, 1.4265108854971194`, 0.1706212563060796`,
0.5071304116694296`, 0.24651071826762905`, 0.09495214929797174`, 590.7999831566981`, 288.39017916639295`,
0.04539479043869582`, 1.2808934229971172`, 0.510577419948266`, 36.299997356537894`, 24.443146685348577`,
36.13353131634994`, 0, 14.427499497549757`, 0.5790624470561149`, 0.646750480383288`, 0.11689245895894484`,
0.9638068384098911`, 0.08190547791948401`, -0.915018783167576`, 18.12957055484659`, 2.200047410082367`,
0.6513158432782553`, 0.9999920659932457`, 0.0007002562588088636`, -0.9992997381853091`, 0.19313493384375696`},
{0.06137349343986376`, 1.2320478370611792`, 8.061209423245938`, 0.9154821233959622`, 0.94847137331962`,
0.5807992742846495`, 0.08922764994064156`, 0.006685723224911482`, 1682.5088546386705`, 136.82405088198965`,
0.1751207515405141`, 5.423583339438931`, 0.7354883118939113`, 330.41205349779165`, 132.08422627178751`,
425.54412580046545`, 0, 300.8748361789859`, 1.5340053180853135`, 1.5486770324366805`, 0.009564317788467447`,
0.37117979044472477`, 0.04342454405744344`, -0.8830094062895644`, 26.999541916962272`, 0.32543714905529725`,
2.112555681058083`, 0.9995450365801325`, 0.00057435836759154`, -0.9994253802014198`, 0.12121789903876347`},
{0.17433851186752986`, 3.075048744775078`, 5.850960000178599`, 1.4590538879216268`, 0.970972084241031`,
0.3933384319595665`, 0.19496970022116206`, 0.08582933681764099`, 1421.4342985094609`, 163.5857848008759`,
0.22598131859497045`, 6.6523561809248974`, 0.7554779380569299`, 303.93064195735064`, 149.200744185414`,
750.8592212370442`, 0, 134.48458190829112`, 3.6085172100442335`, 3.7259041722518353`, 0.032530525801805155`,
2.0964640527810183`, 0.0714676925399681`, -0.9659103658633382`, 158.51951881779698`, 5.221348902080239`,
2.2623870147871075`, 0.9999999820383417`, 0.0005934518067574852`, -0.9994065481825831`, 0.2207395663875397`},
{0.07998121844244105`, 2.715580846021229`, 9.746524688173917`, 0.8568934646193223`, 0.7996741613090028`,

0.42485864684277896`, 0.060688942211416824`, 0.012691467663490894`, 3356.3929343624186`, 81.65136803572034`,
0.10607621946860207`, 3.6515832472165357`, 0.6293869420871867`, 265.326282613934`, 24.714589820170275`,
351.67214369029176`, 0, 224.075375080164`, 1.0170160903259953`, 1.0233252547186924`, 0.006203603318286621`,
0.29741259532162767`, 0.04009208609880423`, -0.8651970806567628`, 39.45413449978097`, 0.3398203107707473`,
0.8542295158825238`, 0.999429046074376`, 0.0005665821283092038`, -0.9994330941945957`, 0.030662556651774146`},
{0.24304694498168755`, 1.1442280451992772`, 8.167052671305406`, 1.1537428575449382`, 0.8133574376235939`,
0.588378131385339`, 0.08683207301011828`, 0.2314931244773344`, 1071.7662116086185`, 394.7462751939248`,
0.23166609859748916`, 7.333555001844963`, 0.7455281507224547`, 183.61723729514455`, 204.2226332707931`,
177.135807039989`, 0, 44.483886253399874`, 8.013636218644718`, 8.12777950351074`, 0.01424363194830991`,
0.028598687529711612`, 0.022193761730948565`, -0.22395873209597017`, 130.9918186485428`, 0.0992974804939124`,
2.466830263334288`, 0.4120362766584226`, 0.00023299528416326085`, -0.9994345272555785`, 0.38221039356137043`},
{0.1801027988172071`, 1.3432512708969133`, 4.741465237867114`, 1.304640592667152`, 0.29820882042399743`,
0.18296919601651807`, 0.12795168258022568`, 0.15240389940354152`, 1270.873559520909`, 206.3480725013959`,
0.08783057128294997`, 2.6716194652601057`, 1.4440291039953363`, 88.56162690281789`, 17.196792001904782`,
93.91378197989496`, 0, 28.505234927706695`, 2.945940933718596`, 3.005923494670319`, 0.020361087442445136`,
0.16230388922959302`, 0.05713080333753139`, -0.6480010207474765`, 56.530555760067756`, 0.4175912101309108`,
1.2142068125698895`, 0.9233597841125251`, 0.0005313393846041871`, -0.9994245586674376`, 0.08638731589640856`},
{0.23177860104219328`, 0.964191776963677`, 8.537582892707828`, 1.3413871161177928`, 0.724114448343743`,
0.6184241033984961`, 0.21927672383789093`, 0.15745254433360553`, 952.8150091975604`, 129.0382057619487`,
0.13010975974299993`, 9.838389368601312`, 0.45129901177253107`, 648.5213702244475`, 269.83761587553977`,
1069.4951078087568`, 0, 200.88418625362826`, 30.002267884392364`, 30.386461885397754`, 0.012805498653828629`,
1.0156587688877112`, 0.0649126396267417`, -0.9360107292394584`, 413.25628549132256`, 3.3629709798426655`,
3.8397743546343603`, 0.999997078937573`, 0.0005867849199227752`, -0.9994132133660368`, 0.38702141951877056`},
{0.20049846933924464`, 3.760252667630848`, 8.551337156929279`, 1.0134432778283617`, 0.24009952242243937`,
0.3670355852238165`, 0.13721671891900983`, 0.05322626702251803`, 601.1668980568775`, 144.18193811625173`,
0.13298602138197724`, 6.629891741121696`, 0.6106305978341504`, 313.73617840255065`, 116.96830133524378`,
337.5870067068193`, 0, 179.7714579749108`, 2.4436409853177317`, 2.476611781122527`, 0.01349248764564659`,
0.06457881023127897`, 0.035193175821962734`, -0.4550352399506301`, 131.26725048247218`, 0.18497075147319647`,
4.042654340867356`, 0.7432133005739627`, 0.0004205694549601615`, -0.9994341201178244`, 0.30745679461789815`},
{0.062486454467917896`, 1.2059427569006989`, 6.5765672576808525`, 1.393503279917707`, 0.48537073645078954`,
0.6632121477086064`, 0.05231057461071709`, 0.07259823309572629`, 1438.7234727065425`, 224.83427991463657`,

0.2172117922601684`, 1.0640862319201538`, 0.7673574870722413`, 28.44834588979572`, 13.354480982439583`,
28.554897519071453`, 0, 14.14107115321117`, 0.7797031693553607`, 0.7945766079411516`, 0.01907577033204566`,
0.05017647835810461`, 0.035480173762671534`, -0.2928923088333739`, 13.432534137380218`, 0.04479071757547716`,
0.44515530509146833`, 0.5304454815880719`, 0.0003089544977842037`, -0.9994175565472643`, 0.06054287506705123`},
{0.05859295952350296`, 0.6393158910447498`, 7.38865195012022`, 1.4291671399819033`, 0.009131117329247873`,
0.22529958337877898`, 0.1303909880662386`, 0.09497257153796428`, 2573.212680919086`, 234.8157611604919`,
0.05429505063598533`, 9.846003813495852`, 0.7602792516330825`, 229.3226857624181`, 28.375229461922107`,
266.5632221569962 3`, 0, 98.32991276362723`, 12.920551259563117`, 13.028422178441692`, 0.008348786109163564`,
0.03683028841284905`, 0.02827675825802415`, -0.23224173698951578`, 118.00448201852807`, 0.030828508545719346`,
1.8878325412176133`, 0.4254360998909651`, 0.00023957163838427752`, -0.9994368798547052`, 0.1995249192134674`},
{0.22902870315185453`, 2.2080085479773457`, 7.247023111118531`, 1.0318589243161904`, 0.282713068412896`,
0.6376211846150526`, 0.2313240848611663`, 0.02849242642499769`, 1791.1158452651816`, 374.3905300694138`,
0.08265635018766693`, 3.8730202440010704`, 1.4480774422437706`, 88.26684131749356`, 31.27481775796266`,
92.26745829091008`, 0, 62.986955654750076`, 0.7730024159224528`, 0.7846335512074607`, 0.015046699784408979`,
0.026379872855232345`, 0.02035184064718897`, -0.2285087665556258`, 24.38279917091309`, 0.08631068670492596`,
1.236948206425817`, 0.4205847529358664`, 0.00023900212609678494`, -0.9994317384916395`, 0.2702091712358791`},
{0.11087514755950806`, 2.820953169055314`, 7.453143180044123`, 0.7876974831919437`, 0.32208463912551366`,
0.4854106516260327`, 0.2381966627750387`, 0.019508031907783826`, 3675.7660426974826`, 393.486383654267`,
0.19475280794369443`, 3.2333842106996737`, 1.137763300389826`, 68.79693851560599`, 11.34823846401575`,
76.17165625588599`, 0, 53.87273290160419`, 0.3597709939320311`, 0.36305928260104275`, 0.009139949369105782`,
0.017782562156577555`, 0.014109926420884206`, -0.2065301784610879`, 14.498530363810607`, 0.02816634575853248`,
0.5778805473267912`, 0.383483382541029`, 0.00021696312476754694`, -0.9994342307003503`, 0.12578679251520622`},
{0.1489453640293268`, 3.0321837029004524`, 5.620984907920828`, 0.8681722630051818`, 0.8115372327820141`,
0.35816484450213293`, 0.1426190893322286`, 0.05255336055473145`, 2844.388518993981`, 198.5585650329739`,
0.018535462639950012`, 9.846692622248703`, 0.39135970086161165`, 414.46185385934336`, 74.15763632278423`,
612.7724915283267`, 0, 236.66982037455108`, 3.9884649565478982`, 4.010424651167547`, 0.00550580106855314`,
0.3291813934975992`, 0.04080159098562233`, -0.8760513449678926`, 172.7679777262014`, 0.7004291783741687`,
1.2357690544387774`, 0.999627199879353`, 0.0005691088736180074`, -0.9994306788834011`, 0.12271839494827633`},
{0.24690212899704622`, 2.8546371760166815`, 5.1085465841448325`, 0.8777130205154242`, 0.2272138406192541`,
0.3209103618486969`, 0.13407179467911823`, 0.026501037209412173`, 2259.637457560987`, 234.32947168493797`,
0.12630638676582567`, 7.901213702591074`, 0.632382460130227`, 212.66992677443614`, 34.7337884999784`,

245.25080758526136`, 0, 154.50067182227076`, 1.3859974467438902`, 1.3978578704760438`, 0.008557320044143779`,
0.050548669041519355`, 0.029014981867847695`, -0.4259990931904547`, 56.52171196199034`, 0.17829391434730524`,
1.5787095907600017`, 0.7082033357403098`, 0.0004004317522571178`, -0.9994345808159198`, 0.18554357477217046`},
{0.08931462102812832`, 1.1252555996419966`, 6.092718311835377`, 1.0218956744375698`, 0.19346465860989204`,
0.1765746718654928`, 0.12857456324762098`, 0.36563376111510176`, 3469.1850778098988`, 184.0525308849526`,
0.10979923715549866`, 4.959384809772031`, 1.0536081443626233`, 162.0100933563563`, 10.890990972086833`,
186.42765114353375`, 0, 26.847884008053246`, 7.907618895293116`, 7.963501853140279`, 0.007066976619273468`,
0.06106713467910682`, 0.03438136285700141`, -0.4369907309771902`, 127.1156063109062`, 0.07791697130200928`,
1.0236081889584765`, 0.722389919458184`, 0.00040748981427837967`, -0.9994359143126139`, 0.0686904405437037`},
{0.06880098426216452`, 3.7509374271202`, 6.144272737202943`, 0.9237943864650779`, 0.699854200915176`,
0.3107815967047757`, 0.08743219256910584`, 0.007090772506686245`, 1964.75988825503`, 142.8027500827095`,
0.1044689728863506`, 3.2094136485070344`, 0.6936760287011401`, 148.39228372305817`, 25.939423275174565`,
190.58717523368878`, 0, 134.1238458646159`, 0.2471931627287718`, 0.2500521467818495`, 0.011565789366976498`,
0.32435926640131435`, 0.04361746637772594`, -0.8655272998312923`, 13.245801225822353`, 0.3188033826137749`,
0.9223006513149418`, 0.9993618810983657`, 0.0005719113695707347`, -0.9994277234499457`, 0.05405087647167275`},
{0.05355871135675744`, 3.309279473250874`, 4.916547265237652`, 0.9710244117509734`, 0.2158289016549566`,
0.1820848416187855 8`, 0.142360576114893`, 0.05203968288186598`, 866.5149045887983`, 189.9142460402237`,
0.14005711660330905`, 2.6475969017133205`, 0.76310092982505`, 89.42830818133262`, 23.418823952980325`,
98.6917485648539`, 0, 51.648893177581435`, 0.778390926126678`, 0.7899189188085912`, 0.014810029632895683`,
0.11381857882678544`, 0.041846331957336134`, -0.6323418163477521`, 36.79875877136792`, 0.08708537729172248`,
1.1299152812051683`, 0.9115856524756306`, 0.0005221274477415561`, -0.9994272316086551`, 0.08659030661841319`},
{0.1293133825399687`, 3.5858476083752784`, 9.218617588443664`, 0.956805767292237`, 0.24196147957252068`,
0.1873204590192723`, 0.13102150029404855`, 0.057479405562115365`, 1692.2136201181738`, 281.3785219692511`,
0.06648388834980773`, 9.468482191548883`, 1.1484858817527992`, 228.57602060998408`, 45.97605224392732`,
246.90978239354112`, 0, 126.72065899515741`, 1.9491671520733629`, 1.9733897217987415`, 0.012427138277808725`,
0.01941013920267499`, 0.01572701741810916`, -0.18975246628103748`, 99.84880529408436`, 0.035857010798319634`,
2.8184029524361893`, 0.3539132892814846`, 0.00019902062854205472`, -0.9994376570912438`, 0.2580736660743622`},
{0.19221768216079177`, 1.1625228381265575`, 8.172090892620595`, 0.9088617496226665`, 0.5295960957742345`,
0.666157439310989`, 0.15242811761548303`, 0.007090627883866567`, 1515.1410756583437`, 333.01297852270045`,
0.2052004181173977`, 5.919585120700083`, 0.3172585313240366`, 156.4842499582333`, 77.98250799012574`,
168.32715287858227`, 0, 141.99391254893433`, 0.809846066803805`, 0.8174417377994493`, 0.009379154023210656`,

```
0.02419924984520221`, 0.018449106114201672`, -0.2376166107537656`, 13.449493543234146`, 0.06645033878963176`,
  1.239268518630961`, 0.43439240730339457`, 0.00024584408671083117`, -0.9994340506818777`, 0.31798852789676163`},
{0.16098880364150353`, 3.127450251430191`, 2.20024435719991`, 0.7637976091034757`, 0.9064557867648941`,
  0.5784333391778734`, 0.10152394954071559`, 0.028389209793756094`, 1613.7605012073036`, 376.897672127746`,
  0.043498602143453025`, 2.961300492740582`, 0.7808989235332793`, 53.904162099104376`, 65.46469120456905`,
  85.16464550601404`, 0, 36.84009195590534`, 0.31960730416772565`, 0.3405041232040056`, 0.06538279558627846`,
  0.7449963401749936`, 0.039574638789615146`, -0.9468794185212784`, 14.279370625404024`, 1.7133724217438522`,
  0.8442740536766634`, 0.9999997801042264`, 0.0006287565136067687`, -0.9993712433481323`, 0.21503359821143175`},
{0.23820794255869648`, 2.736768610725435`, 5.161003241600229`, 1.3463074049058292`, 0.830070705025693`,
  0.6819229399748419`, 0.14093440819681458`, 0.012428436330344567`, 3231.77982743126`, 163.97686949725403`,
  0.16644222788576973`, 5.029839035337567`, 1.3104845535759155`, 217.49415691015574`, 53.06421073453003`,
  375.3895149335204`, 0, 168.34387772562235`, 0.7370892251611744`, 0.8178590305727975`, 0.10957941407156202`,
  4.425668416056481`, 0.07071387358067217`, -0.9840218771645612`, 28.817752210357572`, 15.060419540511772`,
  1.8855740879445264`, 0.9999999938001878`, 0.0006367558847938349`, -0.9993632441112584`, 0.1703800331411842`},
{0.10397296284216978`, 2.835686168016201`, 8.434918863116089`, 0.8374208138967196`, 0.6614675061514772`,
  0.2543464777963602`, 0.10409619202490922`, 0.05685431008257157`, 2232.998807497718`, 265.245542499567`,
  0.1684824275948374`, 9.30545987088001`, 0.3476653622341219`, 264.5263591292683`, 58.317018285584176`,
  323.0486942181233`, 0, 146.5251013602755`, 2.841254563256283`, 2.8561200276379175`, 0.005232007217472745`,
  0.02363790725041952`, 0.017715150153622277`, -0.25056182148663464`, 115.09866092626804`, 0.03511004646015473`,
  1.3223556874036817`, 0.4552076500751798`, 0.00025613197408198173`, -0.9994373293725629`, 0.13277053678497988`},
{0.14550969692078547`, 0.6504460416509024`, 3.203391452599939`, 0.9809775787785509`, 0.8932996308568291`,
  0.28864614416923273`, 0.12555971991665899`, 0.04147738167353124`, 2412.5327079966373`, 332.6211608259723`,
  0.10031126168945415`, 7.026514484853989`, 1.271775072831928`, 172.17068886566972`, 61.9975173089058`,
  263.29723272467515`, 0, 107.78950746650877`, 6.101521371440396`, 6.224154593067655`, 0.02009879408130444`,
  0.5121136558819414`, 0.047068009405600325`, -0.9080906965377799`, 56.69586320145418`, 1.0645357550910692`,
  1.8120005982507115`, 0.9999344053113624`, 0.0005813301544986014`, -0.9994186317108295`, 0.198779617030302`},
{0.16309305365471205`, 3.729196744300981`, 9.307515882091003`, 0.8570302037138485`, 0.8937228771832244`,
  0.5152206015210095`, 0.1870688649861051`, 0.010071822941243438`, 3256.3985186156888`, 304.0187763152717`,
  0.14293680908473783`, 5.902796930663761`, 1.3773483940741578`, 298.8720250241222`, 58.52153731949297`,
  306.9813985026297`, 0, 261.1307625172301`, 0.6883958319380425`, 0.6926596487265979`, 0.006193844574205798`,
  0.05270745153458436 5`, 0.02925979875526779`, -0.4448640959984801`, 36.67376421791015`, 0.12280313173052593`,
```

1.4614061762077108`, 0.7320317335006659`, 0.00041343499045289316`, −0.9994352225845787`, 0.11036275921421411`},
{0.16894031536983917`, 1.5924806965086606`, 5.6931408636729035`, 0.9867223743305529`, 0.8224539897688026`,
0.597939456714043`, 0.09503923786081717`, 0.017883705208976936`, 748.6938025465079`, 285.65032417980353`,
0.22927316540826853`, 3.9293139418745917`, 0.9067902983749052`, 139.97516361608834`, 158.60533827642251`,
134.6873368766289`, 0, 111.62125139173978`, 1.179949545718371`, 1.1993378955332876`, 0.01643150750408795`,
0.08294791209341565`, 0.038724886748192466`, −0.5331421156860388`, 26.843526777295228`, 0.20018923469047947`,
1.941447745285963`, 0.8260585073985683`, 0.00047554970580977507`, −0.999424314740965`, 0.21613157261436677`},
{0.1623342771322262`, 3.665527787592313`, 5.80147646237554`, 0.8352024744734798`, 0.7018868526857065`,
0.24394785423193066`, 0.08747688957871178`, 0.16089532027636136`, 3759.8814706106723`, 381.8961690598002`,
0.2313099688547675`, 3.097651818220701`, 0.4382846191648839`, 57.91989679624034`, 11.446324030649732`,
68.83276048571678`, 0, 17.74830860553438`, 0.7513989760831156`, 0.7563139828327137`, 0.006541141132796024 5`,
0.022120780264513917`, 0.016883062091279245`, −0.2367781837079681`, 39.34676894858686`, 0.051299441054763924`,
0.3045831563833299`, 0.43345808207267766`, 0.0002449459349275496`, −0.9994349028313043`, 0.04273984073363935`},
{0.22071774239833053`, 2.103280372140615`, 9.912636083572693`, 1.1033554615041679`, 0.6309736957880776`,
0.6000918403254052`, 0.08729060724669563`, 0.08702471997827647`, 909.0462708578816`, 192.7187476296187 5`,
0.022259580341630003`, 7.880477191609103`, 0.5653775848064009`, 336.7790920251503`, 189.06302125231198`,
341.25851330016167`, 0, 152.02856526719643`, 5.9447164026185835`, 6.0117652114108475`, 0.011278722860981194`,
0.04466918088586068`, 0.02985259952058097`, −0.33169583752026355`, 178.6200761081434`, 0.14084686799862056`,
3.6159118168375133`, 0.5796177553442072`, 0.0003276551776437486`, −0.9994347047263085`, 0.35152527369117004`},
{0.21944864620047738`, 1.5851568289063627`, 2.8253271766672086`, 1.4978148427385942`, 0.6625588078225084`,
0.3414024356763743`, 0.10952337049286448`, 0.007987153739989428`, 796.8444419321349`, 241.79415763040436`,
0.07683828049172353`, 5.204625250868856`, 0.2239996119446115`, 174.76858802224157`, 105.7182289037189`,
198.77450671453042`, 0, 103.88434979607341`, 0.4928428988229401`, 0.7569919147762332`, 0.5359700151593172`,
14.301187677930326`, 0.11146528142331395`, −0.9922058724118887`, 11.160475523531273`, 44.833946785439366`,
1.7551389816895189`, 0.999999999527448`, 0.000903272437674127`, −0.999096727561899`, 0.34453396541376824`},
{0.22738531013884145`, 1.6748591792805003`, 7.320466644196401`, 1.2670610367241493`, 0.9156886696389757`,
0.27273514068006444`, 0.2379234232685784`, 0.022570855753759948`, 3790.0561229040195`, 356.22460568492386`,
0.13302881383052462`, 2.695355303099337`, 1.0187081757900263`, 132.36757305245166`, 14.650032216505942`,
141.50727658813713`, 0, 99.7336177689084`, 1.285053378780036`, 1.2947711396889023`, 0.00756214572043068`,
0.1348456125843976`, 0.05327265434434961`, −0.6049359462028685`, 30.746906390216676`, 0.43802730626236336`,
0.3911967396081380 6`, 0.8950201704124349`, 0.0005098476362162036`, −0.9994303506746879`, 0.03553506208901852`},

{0.12775499399576257`, 3.9034479686029915`, 9.751449972499827`, 0.7624853991106708`, 0.7599399040180521`,
0.19562916289510757`, 0.12110783011333992`, 0.024828285786588924`, 1328.3636666047569`, 234.8344441201524`,
0.018565368539142812`, 9.598538649304729`, 1.107753026473853`, 421.1574245521442`, 99.57791059703924`,
439.75295320630414`, 0, 311.6704055848313`, 1.9258490284040253`, 1.9426018888289165`, 0.008698947932992862`,
0.03318284663523971`, 0.021535854480429554`, -0.3509943641315335`, 107.39216396799668`, 0.06056106246639217`,
3.47900628008695`, 0.6080170864797108`, 0.0003419666378609728`, -0.999437570677757`, 0.16992384989559295`},
{0.14782514342208763`, 2.652895873833227`, 4.391872450348963`, 1.4876628412878459`, 0.36986838675832656`,
0.39309495169020825`, 0.13298297698236788`, 0.014228765232101433`, 3735.496122883863`, 215.5408051024042`,
0.11408789381411022`, 7.212929997484123`, 0.10464527448884264`, 88.09971951326636`, 23.991542311378637`,
267.29078771475565`, 0, 73.17664959054285`, 0.37998665089834527`, 0.3826029476470497`, 0.006885233316799688`,
0.03244063874581519`, 0.025950308342805147`, -0.20006789798019275`, 14.400928832570374`, 0.06850774393291353`,
0.4647543589049308`, 0.3726092303917211`, 0.00021136188911584678`, -0.9994327518701197`, 0.20967683586965166`},
{0.09639258202167578`, 1.466780969423465`, 4.239504689160903`, 1.0209716259894424`, 0.025576954982881928`,
0.3026304446672493`, 0.21752127412681987`, 0.031317816620157334`, 525.4375097331736`, 358.0480696321463`,
0.16662257108917217`, 7.934540763419077`, 1.3681027859387838`, 161.41328060481115`, 125.070531133007`,
163.25709788637516`, 0, 112.64087275264957`, 2.2154789104859614`, 2.27264177260004`, 0.025801582602986706`,
0.049391572224027486`, 0.030742737116678364`, -0.37757119823525354`, 46.42317577228347`, 0.06801401681123274`,
4.146675687135103`, 0.6439418571075936`, 0.0003646833545219032`, -0.9994336703686884`, 0.7875557166085899`},
{0.16556454843244983`, 1.858436546204688`, 5.85841964381955`, 1.4381472842647658`, 0.6188369558385702`,
0.43793021380482466`, 0.22826919863757877`, 0.3543109226514421`, 3912.388498432202`, 125.48108540961005`,
0.039979729590999546`, 4.62864467759934`, 0.44406393527696175`, 212.16255939169744`, 20.67369175638443`,
454.2957251437215`, 0, 33.78897211985043`, 6.122964707595576`, 6.430752006021838`, 0.050267691081813615`,
2.8798993593951674`, 0.07056260697883716`, -0.975498238593429`, 162.55916262453053`, 6.811560528130834`,
0.4644947652669158`, 0.9999999751258305`, 0.0005946316450126307`, -0.9994053683401964`, 0.06591837983983903`},
{0.06965658134691888`, 0.9261903057977481`, 7.316991447780513`, 1.3759095728452715`, 0.2908565323439223`,
0.2820661078343176`, 0.18803658000898693`, 0.00943996945527825`, 744.7814906580452`, 341.56345427898043`,
0.1305415452351143`, 4.644709496928025`, 0.9315609895678807`, 109.59002831502661`, 61.01688812496366`,
113.55802777991248`, 0, 96.65581542504512`, 0.8981341881371634`, 0.9150783671021612`, 0.01886597703194215`,
0.036484623788339755`, 0.027840230691445967`, -0.23693249920961168`, 11.883473976545305`, 0.036305630926058326`,
1.929921662332569`, 0.4333021033768111`, 0.0002450719056087903`, -0.999434408686921`, 0.2878036722527424`},
{0.14063443384893737`, 0.6174318191344224`, 9.816767595769278`, 1.0438016034352748`, 0.579595650707708`,

0.4622649825890621`, 0.24429017498983885`, 0.01619284881329759`, 2900.32749785945`, 335.82024218042955`,
0.09977744332778332`, 6.127347054973281`, 1.0166995269478085`, 206.76696164385933`, 36.349998446262035`,
224.18180366480294`, 0, 168.005817452624`, 3.929298630584342`, 3.955501755131837`, 0.006668651841206241`,
0.030238150877964467`, 0.022518473318220378`, -0.25529595347609924`, 34.65820002005832`, 0.06075036041941872`,
1.2064953099984597`, 0.4630111159220601`, 0.0002612484660690515`, -0.9994357619999062`, 0.16744681452285573`},
{0.18537094617034594`, 1.96648991280105`, 7.838266997084801`, 1.0125623241231316`, 0.014033931484803919`,
0.3423492209947736`, 0.1167129177170042`, 0.32838459574903894`, 3257.498914359846`, 126.9547671220904`,
0.1116209985614412`, 2.857468681315895`, 0.2336256655095057`, 71.28934728521887`, 7.36737182106995`,
134.0880731104634`, 0, 12.721008522543006`, 2.00985747985815`, 2.0198571423893164`, 0.0049753092601732`,
0.026350802819627935`, 0.020212433222427342`, -0.2329481055745406`, 56.46234943298273`, 0.06978104644324092`,
0.2602652446692609`, 0.42721839477034285`, 0.00024190046248753142`, -0.9994337779799544`, 0.04488898885400686`},
{0.24032952463671997`, 3.1818327111380986`, 8.060728243579248`, 1.022907398702028`, 0.8948393046349232`,
0.3515250656278254`, 0.11210328340390646`, 0.4812285429766681`, 3331.6694795416133`, 199.3192679768665`,
0.1656921504225664`, 6.971086437067735`, 1.1039536289023264`, 381.0664163622659`, 51.34240761098081`,
408.9695366708799`, 0, 49.8695203551996`, 7.118325329579959`, 7.161272527797946`, 0.00603332894037889`,
0.11658433939149025`, 0.04352767200230786`, -0.6266422040087054`, 323.56171974543463`, 0.4002665552292763`,
1.774476262692037 2`, 0.9116012513666858`, 0.0005153941924929791`, -0.9994346275943343`, 0.0818578393734169`},
{0.16505190858637997`, 0.8247371132573029`, 2.6996089778841377`, 0.7893797405853178`, 0.33037619005051444`,
0.49339259475674924`, 0.09174509005830872`, 0.3021633567730715`, 3738.8764636919877`, 330.53731575160793`,
0.1571748109049641`, 2.9654306879318377`, 0.993830283737029`, 56.534017280076114`, 10.400678454581726`,
60.4429576935098`, 0, 11.053546495799177`, 3.531620403388558`, 3.5910431325430285`, 0.016825910592614957`,
0.10108877343034028`, 0.0349243663508133`, -0.6545178543008092`, 41.60940595158956`, 0.23835564273334872`,
0.6080995126404539`, 0.9282833815333509`, 0.00053670395823324`, -0.999421831771515`, 0.09947974424664527`},
{0.041347104778859584`, 0.6250053944652225`, 5.297803080507409`, 1.3565417270883027`, 0.7676292422012339`,
0.6702284155028233`, 0.05669620363458108`, 0.027633397705997945`, 3343.0533516535834`, 245.48920969891526`,
0.16311205424117575`, 9.389474878516673`, 1.0487847358699702`, 287.6587517177128`, 84.11818618220181`,
298.576727580039`, 0, 206.89150412040732`, 8.115383662669197`, 8.200179144549487`, 0.010448733591037707`,
0.094943873694546`, 0.04923552224612717`, -0.48142496898190623`, 72.45940810461697`, 0.05608077562506684`,
2.792980270441396`, 0.7748446535673464`, 0.00043949513089581484`, -0.9994327958141385`, 0.21298402957033752`},
{0.1215137090416612`, 1.1969038422236755`, 9.326465300388158`, 1.005636980391936`, 0.6897518274648109`,
0.6108427950530713`, 0.10731717880916097`, 0.23857365224573474`, 2004.0983212715491`, 148.7170844059682`,

0.23834887248578368`, 9.94205781935025`, 0.30611051261382083`, 516.6090907157466`, 121.06016239303418`,
639.8130646133907`, 0, 118.53928912421195`, 21.984634819051845`, 22.068868616330157`, 0.003831484942625307 3`,
0.0651012840394285 4`, 0.035168366344421415`, -0.4597899739869689 7`, 375.90705549725175`, 0.11300997838567528`,
1.807611839230639`, 0.7490073081528726`, 0.00042343063241262143`, -0.9994346775688253`, 0.196413722958002`},
{0.279562619019907`, 2.2124846272464014`, 7.584786512534812`, 1.3700002017675512`, 0.23921169075395055`,
0.3169220525365052`, 0.1334424504019662 3`, 0.14640355547821746`, 3971.2444101007122`, 129.66148944966505`,
0.11735939166830095`, 3.485518254759493`, 0.4332823244894972`, 136.20379195482093`, 8.570801889756154`,
191.52444906008506`, 0, 44.171788688822296`, 2.810958642851088`, 2.825963898432387`, 0.00533812748169038`,
0.07515194060034025`, 0.04424021971009104 6`, -0.4113229897101187`, 88.84575407333504`, 0.3001381905522246`,
0.35169362820374017`, 0.6907170725142979`, 0.00039155436338874543`, -0.9994331190309754`, 0.03534626301078295 4`},
{0.2002208994690245`, 3.902908071048812`, 5.625275625663315`, 1.4917900472855687`, 0.70165150687693`,
0.17917645896398615`, 0.1075286064543329`, 0.022727063680328688`, 1296.438202913817`, 261.4435185272306`,
0.04322514446110226`, 5.849567498164653`, 1.0949838652058799`, 203.7804143669919 2`, 54.504889961214644`,
212.42252546804835`, 0, 153.9398925618265`, 0.8689364601005255`, 0.88001238898122`, 0.01274653486103361`,
0.12415731072420233`, 0.05793604603984596`, -0.5333658106646448`, 48.44827319078454`, 0.3551269775550783`,
2.05365266373039`, 0.8297485764160515`, 0.00047066127816099287`, -0.9994327663926897`, 0.12104088064366668`},
{0.13038773573510337`, 1.934638714312162`, 3.5362126783730226`, 1.036300296330771`, 0.1950167370197864`,
0.2191580432274216`, 0.06372829025507237`, 0.4487751055567468`, 2715.7233289184524`, 201.32035054817607`,
0.24618537045557576`, 1.5657139002447844`, 1.056666818669949`, 39.664841548366226`, 4.580178079869235`,
43.69270592352921`, 0, 5.673463744893042`, 1.1715968133431927`, 1.1981667975950037`, 0.022678436770404486`,
0.1535429092873963`, 0.04645209474939614`, -0.697465060646671`, 32.380236466549974`, 0.28600160400236213`,
0.4468819779185703`, 0.9523018549628649`, 0.0005444632007082712`, -0.9994282661554519`, 0.03230605077447095`},
{0.25257809880572757`, 1.2556801180516963`, 6.596575825508853`, 1.3398305186381605`, 0.284033409354278`,
0.1515369008103068`, 0.1400005745311825`, 0.02101007059419062`, 611.632102430428`, 356.19419891984376`,
0.010524324923865458`, 2.8673905561599544`, 0.6614014808678237`, 57.32746955549392`, 35.629591470825126`,
59.62673829325813`, 0, 44.236618101927`, 0.6825206817454882`, 0.698811639124869`, 0.02386881132703267`,
0.03263612705755651`, 0.025441418073802236`, -0.2204522911393808`, 12.243252146098579`, 0.11775958463684882`,
1.19685069113736`, 0.4067550311596453`, 0.00023034084474503414`, -0.9994337111354509`, 0.13647289611569582`},
{0.2042789431730157`, 3.7869781485826124`, 7.097464150909694`, 1.0298616729281707`, 0.4360801596581312`,
0.5374359749706832`, 0.19209770462574638`, 0.13005052253847288`, 2094.968893303633`, 292.0969799908029`,
0.013655036785018004`, 7.349385967933083`, 0.6499659559020465`, 219.6651069178736`, 55.19621616000367`,

242.54794142945454`, 0, 77.79756106775956`, 2.5718883043593492`, 2.591697734883173`, 0.007702290371726761`,
0.0401453442312276`, 0.027214352647341247`, -0.32210438922647977`, 139.1383544172004`, 0.11715497846993206`,
1.5554023611397199`, 0.565674833445361`, 0.00032005189649987`, -0.9994342122407134`, 0.25848745583668653`},
{0.2595298239660976`, 3.319555998413656`, 5.7932515179865565`, 0.881744759172532`, 0.6648616448212201`,
0.6517725519271407`, 0.19583264477028905`, 0.005149860640734175`, 3979.7041048852343`, 156.14061322234556`,
0.24304909070853137`, 9.19022988333834`, 0.6081568865700206`, 405.72865794424314`, 56.26392813139535`,
724.0254576365425`, 0, 372.71452642165303`, 0.5642580736707083`, 0.574193416217743`, 0.01760779864858919`,
1.1183285968435734`, 0.04216387031426318`, -0.9622974227492094`, 26.75837533009913`, 4.146280341072319`,
1.3289276975885465`, 0.9999999867461333`, 0.0005791626114671024`, -0.9994208373808567`, 0.17205064177602059`},
{0.12106147370268983`, 1.1502892713218662`, 9.893056355486525`, 1.0779684733916626`, 0.2654952298373383`,
0.5544611241456747`, 0.21486298948769583`, 0.008770181568324542`, 1487.6775848537964`, 210.36931291761118`,
0.08815050293390886`, 9.314054867228961`, 1.3778011559930095`, 363.5311949548578`, 84.02347271818466`,
388.79906044207445`, 0, 323.1681695125447`, 2.293309422804477`, 2.321793917404863`, 0.01242069400541368`,
0.03809564258903868`, 0.02669405738677369`, -0.2992884337261603`, 37.68527464104755`, 0.06588449476403385`,
4.228509311350363`, 0.5315449094544515`, 0.00029983753671428674`, -0.9994359130689032`, 0.4660113213296359`},
{0.04600515107837966`, 1.2443774572294632`, 7.3914675121007605`, 0.8524218043851708`, 0.25925086237615913`,
0.25962408201227716`, 0.17007776716368422`, 0.3647699508957847`, 2650.181739077888`, 363.5928673248461`,
0.07845934701674573`, 6.64885092299725`, 0.9963780523907508`, 132.15486115235558`, 23.331495669396794`,
145.64209385323306`, 0, 22.317802188326084`, 5.848031460046529`, 5.905009699566452`, 0.009743148597813667`,
0.01787340370398694`, 0.014388159121518387`, -0.19499613169321062`, 103.95940740072318`, 0.0117466948240581`,
1.281988676814027`, 0.362976107478294`, 0.00020437109276995062`, -0.9994369571755293`, 0.19268372075773058`},
{0.1217700466706656`, 2.558519459791139`, 5.216939277492001`, 1.0963651311366025`, 0.09816261776323953`,
0.6044055975062239`, 0.09115244000304745`, 0.06193230544868139`, 3735.1363049157717`, 184.7914603929239`,
0.05165113964049522`, 2.282481339681045`, 0.8427745163468003`, 64.12973590867173`, 7.1619144501206105`,
72.2611466413348`, 0, 34.177566344944964`, 0.7929122472681491`, 0.8017009354811145`, 0.011084061626296382`,
0.06486249661834165`, 0.037010728049880426`, -0.4293971095862099`, 28.981163064889802`, 0.11283298914842438`,
0.541823957678809`, 0.7148906962042499`, 0.00040930780509463064`, -0.999427454004832`, 0.06623278741024667`},
{0.07220853239716318`, 1.7719009634055194`, 4.958541757826833`, 0.8715174582622381`, 0.41441036947741816`,
0.32423173571278785`, 0.1998499231948091`, 0.007134676741262494`, 2969.140628850977`, 169.59950979095402`,
0.08473674334867182`, 5.398547675716223`, 1.090541691203971`, 204.56127624991728`, 21.359806686686667`,
302.757900600032`, 0, 183.75545405871344`, 0.7064012536280705`, 0.7190843327919512`, 0.017954496964353472`,

1.002924995703717`, 0.04179376292395275`, -0.958328127125172`, 17.88104374077778`, 1.0345677434885243`,
1.3965695764834525`, 0.9999999432652391`, 0.0005807725759753755`, -0.9994192273910746`, 0.13803701650697286`},
{0.16122326429150147`, 3.7922162994918844`, 7.367254304672895`, 0.921371724322694`, 0.6381223018466633`,
0.5967903533432476`, 0.1832909738697283`, 0.43512505909917726`, 2133.3461489698684`, 214.82179755577738`,
0.22692868851616532`, 5.899255886059763`, 0.6939454361655824`, 286.56502798116634`, 60.31434444243885`,
312.94324708177544`, 0, 40.87753457304435`, 4.447349189915617`, 4.474039533128278`, 0.006001404898266616`,
0.09298375061990109`, 0.038123034152839394`, -0.5900032651008162`, 240.9330012504292`, 0.21415919715708154`,
1.4200792213755307`, 0.8803017022413097`, 0.0005011109411302655`, -0.9994307509120403`, 0.1499213284571238`},
{0.2570676551725277`, 1.8968814761831432`, 5.846591463128114`, 1.1172410815007583`, 0.43583764365067834`,
0.15357951833619232`, 0.128960566744062`, 0.20068004601330075`, 2555.9384884114397`, 300.14317051223054`,
0.1562959949020017`, 5.731951708372693`, 0.952499750656465`, 141.92392132112116`, 19.66818504152704`,
157.24350781767902`, 0, 37.458415718445394`, 3.7105469042056614`, 3.744754938484735`, 0.00921913538953012`,
0.04396466355478878`, 0.029675353536839397`, -0.32501806820702805`, 100.5495384156631`, 0.16145561386372406`,
1.1072784488169922`, 0.5705405249791691`, 0.00032185771480477705`, -0.999435872298788`, 0.09442990035727185`},
{0.05513596268515280 6`, 2.9220194449851506`, 9.20311067851182`, 0.9951015075863325`, 0.9088587426511963`,
0.6564149766220422`, 0.21100197083140715`, 0.41165435784921034`, 2658.942850929282`, 386.04298432332337`,
0.012398563555194947`, 5.099390369220371`, 1.471358614723604`, 229.5095886134606`, 77.34660139739466`,
230.11213016333127`, 0, 34.45268198976779`, 4.561385497608497`, 4.589144008391059`, 0.006085543700946827`,
0.049780722437007166`, 0.030544649805280117`, -0.38641610024982054`, 190.40653028693288`, 0.039210115067264247`,
1.3023944506870138`, 0.6567207693840778`, 0.00037167343584302193`, -0.9994340464727626`, 0.13380027033874486`},
{0.13155432244432247`, 2.613811333361477`, 8.357167885800742`, 0.8056721180002253`, 0.6603026707260147`,
0.5801438627936167`, 0.11636775006500844`, 0.26451755332760135`, 2521.6309343959783`, 242.4992509443806`,
0.160383814941372`, 4.821776617001756`, 1.0110903713708876`, 178.20157883417608`, 42.189945840760885`,
187.0892916529765`, 0, 38.358678586694644`, 3.6448753486222287`, 3.6753033489382987`, 0.008348159376032394`,
0.03396227754611702`, 0.02235990261595166`, -0.34162534931323785`, 136.10023564169504`, 0.06382692016075425`,
1.392267797733479`, 0.594473920659689`, 0.0003360925815162055`, -0.9994346386446301`, 0.12375850726084496`},
{0.10755292243344333`, 3.7450945369693454`, 9.057610815009983`, 1.4262360104426115`, 0.8847828962958162`,
0.32323944244008773`, 0.10925879484006157`, 0.04648694210521617`, 2597.7878756158707`, 114.3810122188126`,
0.2151089301912223`, 9.739939811090224`, 0.6433246757255959`, 658.5821669968592`, 85.91934839370788`,
983.8505522935309`, 0, 395.69439648700984`, 4.7959042967999475`, 4.82232456725155`, 0.005508923618269712`,
0.5866713811945787`, 0.0668255924565024 5`, -0.8860936554968263`, 256.58735688247555`, 0.9014031650799202`,

2.1095935501490573`, 0.9998181026698257`, 0.0005673533582031212`, -0.9994325434229605`, 0.0915947295242353`},
{0.11067094408083894`, 3.800544284934695`, 7.350733993347461`, 1.2140806206228738`, 0.3865719277011892`,
0.3640208182074153`, 0.2468843803041872`, 0.02524798025663777`, 519.3604951232796`, 258.54462558963803`,
0.248859794496116`, 2.6642578190064192`, 1.225620094151966`, 99.66844436437835`, 60.737298076178185`,
102.07110911530218`, 0, 73.53125070383913`, 0.4693610861759286`, 0.47780924191541135`, 0.01799926749001135`,
0.0648564565098574`, 0.0387512208544045`, -0.40250789297261735`, 25.48325133766667`, 0.10253893245259556`,
1.627412263678968`, 0.6764901455791107`, 0.00038678027351801865`, -0.9994282543861938`, 0.20094837316999772`},
{0.17620161354602198`, 3.207637663312279`, 8.038302489204167`, 0.9527508465697585`, 0.2777263075882186`,
0.3259017504461914`, 0.20696733159344116`, 0.041067631602668214`, 3666.144435165166`, 184.14344105031228`,
0.011349261156917284`, 2.0150600100651097`, 0.6578483910293456`, 73.02190999630994`, 5.530031871790882`,
91.24818910902312`, 0, 46.025731648981385`, 0.573387866900867`, 0.5766369042006667`, 0.0056663865549870795`,
0.04129047768318009`, 0.02693020617129978`, -0.3477865192567159`, 26.274578822250135`, 0.1039349827409079`,
0.2686621999717262`, 0.6041293088508786`, 0.00034262137311931744`, -0.9994328674869772`, 0.0316213206752307`},
{0.098793524136433`, 2.9572290906746908`, 8.636235866475928`, 1.1358825426984174`, 0.44571351797486836`,
0.28917170815364945`, 0.20954073160539777`, 0.05617013174448901`, 938.6734643463747`, 177.03868080567804`,
0.11564637982987985`, 4.570821554383135`, 0.26856807485341383`, 199.17438298593177`, 55.6943098678135`,
256.13724809942624`, 0, 110.98587451703688`, 2.035818201106879`, 2.050383594200119`, 0.007154564727499091`,
0.06020370765389744`, 0.03596517481064441`, -0.4026086396970252`, 86.00544010911834`, 0.08496766350297336`,
1.2826056725980914`, 0.6775499420999616`, 0.0003834672783603818`, -0.9994340383571255`, 0.1209909906844287`},
{0.1873960215776312`, 2.8261178368849595`, 9.525044121358242`, 1.3550980694568904`, 0.08983672188035041`,
0.6607774222729332`, 0.06657454788578898`, 0.12572829008110223`, 835.4208642240601`, 103.25606657618908`,
0.08845188667521903`, 5.652525212839048`, 0.4964020494140271`, 285.4575233768848`, 84.2107841881027`,
309.9607841924893`, 0, 104.0865445398955`, 4.3768002569596165`, 4.4320302124937605`, 0.012618797361456435`,
0.07862918976203813`, 0.04507893318215154`, -0.42668958794338896`, 176.7050467810895`, 0.2104971048759799`,
3.3940606495281025`, 0.7078670768823021`, 0.0004029448241307983`, -0.9994307620211615`, 0.27573354977115627`},
{0.261714977559417`, 0.5482487201542185`, 3.431220669456936`, 0.9478503018778801`, 0.34449029105022966`,
0.1671252432139082`, 0.1374901654337824`, 0.05820678844321773`, 3721.854350957653`, 371.28139834560955`,
0.08783171860573763`, 6.880528123303602`, 0.4952942413378081`, 118.99291884066895`, 15.64558978999501`,
146.72534714244145`, 0, 65.12384970542197`, 6.07222470638906`, 6.116719261184661`, 0.007327554059187724`,
0.05007482425412364`, 0.02995678454642806`, -0.4017595669552224`, 47.55842033952325`, 0.18721902151371317`,
0.6340872184997836`, 0.6775334927401357`, 0.000383057778997489`, -0.9994346290137653`, 0.08843264458460606`},

{0.19384041765838167`, 0.8310343395086068`, 9.704183649775683`, 1.2913450048559905`, 0.5670495339238457`,
0.6409270851330959`, 0.14192751733077513`, 0.022909401085561467`, 1113.6891447077023`, 383.5158989221919`,
0.1816390006173258`, 4.168222848202584`, 1.1878448557299315`, 103.43322425572882`, 76.18473439589869`,
102.03907048472615`, 0, 78.21496380134744`, 1.9500002225701027`, 1.9813939622501342`, 0.01609935184451139`,
0.025333853614509814`, 0.020715128279722477`, −0.18231436105488463`, 23.15024495721689`, 0.0701532109361634`,
1.5451170901595233`, 0.3425758931250823`, 0.0001943406801883438`, −0.9994327076595626`, 0.26076605159207683`},
{0.21619628547349445`, 3.9792600826840445`, 2.630502577782165`, 1.4259463002457018`, 0.22306595661200035`,
0.38072127406591705`, 0.08501836292019685`, 0.035809155258912144`, 1510.787355159533`, 321.0088965168211`,
0.03982099495880792`, 2.097097341241465`, 0.5759181842297432`, 38.463384178147415`, 14.388710009634922`,
40.25873605811438`, 0, 25.21282657123565`, 0.21814343230225458`, 0.22503013950286216`, 0.03156962888099013`,
0.15389139433649848`, 0.06200754362717785`, −0.5970694534640948`, 12.400706463715014`, 0.47529639745554525`,
0.5586684547040213`, 0.886910955557963`, 0.0005299120113548436`, −0.9994025194885303`, 0.12000805403491922`},
{0.12757698942849355`, 3.9475071221290925`, 7.046874473492651`, 0.9016631589117734`, 0.33636145019883923`,
0.46329966420746505`, 0.22687767004632875`, 0.06279665921261046`, 3797.4437504167317`, 319.7090357145597`,
0.1993551083304292`, 8.931980068156673`, 0.8508236103120903`, 232.12816870506026`, 30.818930146488885`,
265.28136677983474`, 0, 122.9359108881633`, 1.9010951850023068`, 1.9125756408590413`, 0.0060388643069024805`,
0.02886041658831505`, 0.02087088353479553`, −0.2768336011045075`, 107.20838260917074`, 0.05259892945708203`,
1.3491857377219858`, 0.49699941771139455`, 0.0002802832578048327`, −0.9994360491223602`, 0.2349292181564715`},
{0.16572747960623446`, 1.3999561572838486`, 8.592203336105698`, 1.0149199248092395`, 0.9204720561931288`,
0.6093468506279616`, 0.11483304130851824`, 0.012170616273068445`, 1337.06098163951`, 286.8021266598324`,
0.027824790783174902`, 6.4485130480990716`, 1.1250259465267032`, 273.55204911607956`, 190.82275846513033`,
273.1160717515755`, 0, 233.12827624656157`, 1.909336837001666`, 1.9294327958095523`, 0.010525098776936526`,
0.05164435784004907`, 0.031445799574446515`, −0.3911087117814642`, 38.18554087556215`, 0.12226984658157827`,
2.5175340899036853`, 0.662725644528609`, 0.00037522298627329576`, −0.9994338185199696`, 0.20871107906658304`},
{0.15460498360139963`, 1.7922898385073154`, 5.637575606203491`, 1.3053191224828369`, 0.530866536081585`,
0.39279997373545783`, 0.23407598158461806`, 0.3220260634134779`, 3258.312065268822`, 390.4363804447645`,
0.21449854536638602`, 8.936127787959748`, 0.6328205146062444`, 232.38087887847973`, 40.960793950788606`,
264.74807571195686`, 0, 42.3669686710533`, 7.135152530873939`, 7.175088097995252`, 0.005597016594741433`,
0.05899984645761963`, 0.037780865002364175`, −0.35964468942300265`, 182.68944824693034`, 0.1303095756294811`,
1.1210984627044351`, 0.619685600387323`, 0.00035056796874151434`, −0.9994342809183844`, 0.2034570789157225`},
{0.08442779942280981`, 0.8533376706252112`, 5.086343297092757`, 1.4765588559370955`, 0.4420393122680226`,

0.47437344679325344`, 0.24643808934122824`, 0.005941536592530679`, 2172.43229526953`, 242.96995635142855`,
0.157510417696612`, 9.228156626391328`, 0.9577175035728991`, 358.5068645852647`, 63.21182816251446`,
411.49537140082504`, 0, 329.228829975735`, 2.103109625861503`, 2.1264459939147047`, 0.011096125359438824`,
0.5349242529220118`, 0.0707720225377651`, -0.8676971138414935`, 25.63803813145877`, 0.6451782504585514`,
2.4007199276300804`, 0.9975950111847315`, 0.0005804842517101602`, -0.9994181163245586`, 0.3180051516699377`},
{0.2040419029224893`, 1.8168800092917534`, 6.174811187872505`, 0.9288754688257587`, 0.6581669676988375`,
0.5754364926136093`, 0.20349981430348302`, 0.3349223421963805`, 3304.529174698706`, 164.1808894290948`,
0.03840187502351833`, 7.840532439325425`, 0.2974189092407089`, 363.0298511672356`, 52.59252027146905 6`,
590.9904857428097`, 0, 63.18138760715137`, 11.078217589664082`, 11.139571200063623`, 0.005538220377327097`,
0.3142772565892687 5`, 0.04370680102068863`, -0.8609291633285149`, 287.53988681778395`, 0.9160818497110564`,
0.7718256962307579`, 0.9992180369887774`, 0.0005698842986336824`, -0.9994296697241865`, 0.1211832995993509`},
{0.1166411660555075`, 0.7450165479373432`, 8.310812790636028`, 1.063932072189387`, 0.9753313471385676`,
0.15156075882067166`, 0.057187743785721765`, 0.12429446652524245`, 3685.6740694089303`, 232.00827662364395`,
0.20692272239979642`, 9.093961104192626`, 0.5500507674166981`, 290.65159766580825`, 38.8209954632563`,
348.4293127965795`, 0, 105.38904323382624`, 15.903498711322`, 15.96966075376141`, 0.004160219310252122`,
0.03623897724283878`, 0.025680937322574822`, -0.2913448646608907`, 169.26242442907292`, 0.06038509374667048`,
1.1089913734451262`, 0.519663159666544`, 0.00029227368484241684`, -0.9994375708968287`, 0.05021331849873584`},
{0.15032567895129462`, 2.925200495412599`, 9.469770009612255`, 1.2491630117650723`, 0.5515247294638286`,
0.2864898115312603`, 0.05595545885252959`, 0.4795641847786323`, 1954.175399450165`, 168.25938072536803`,
0.17272189335079902`, 2.4562744379868438`, 1.1923215617571556`, 90.66591651696919`, 15.731113824485663`,
95.80457625179253`, 0, 12.453617117069474`, 1.8246977251801437`, 1.8490323802026276`, 0.013336266432886434`,
0.0432333968922936`, 0.030535806871444762`, -0.293698643492716`, 76.25152413821696`, 0.09284413915996717`,
1.0668455533642465`, 0.5239643083362366`, 0.00029623838282311164`, -0.9994346210646222`, 0.04405951439203434`},
{0.26351647839883513`, 0.8673354775463569`, 5.854038424853368`, 0.9424071816766871`, 0.9232227163729747`,
0.413019745414411`, 0.17796832053957906`, 0.014103729537635504`, 3690.4717118898934`, 307.30915173008475`,
0.17184547983019266`, 6.1008526886318215`, 1.0313875805176784`, 268.6690151474691`, 48.486252334511576`,
317.86242707339807`, 0, 222.52143706798503`, 3.331499761690546`, 3.364645274790819`, 0.009949126661036756`,
0.29573896918750636`, 0.04447240211327358 6`, -0.8496227864881855`, 41.27897052502063`, 1.1133155955084715`,
1.102265049703587`, 0.9979095008413676`, 0.000571722215846088`, -0.9994270800955758`, 0.09858516710323016`},
{0.22251678082176873`, 2.4705119403336626`, 3.219964315804703`, 1.347383920776866`, 0.9659234897125795`,
0.3313519776076561`, 0.0865111078215019`, 0.112897975300224`, 1782.1419072767694`, 367.3360449099217`,

0.11075042639430366`, 6.566322730486396`, 1.1397550110996595`, 161.89414697532314`, 101.01348124535997`,
193.415994746943`, 0, 62.39141969671743`, 2.6886018045626146`, 2.7535007746113065`, 0.024138557795563953`,
0.460709367892465`, 0.06466167260593172`, -0.859647584546133`, 94.88889801392249`, 1.4645080776836439`,
1.8930377531867446`, 0.9986534245025631`, 0.0005819309517774558`, -0.9994172843776435`, 0.19342156517836726`},
{0.1726869732489258`, 1.0689003029764699`, 9.291822504855592`, 1.1990435589159916`, 0.8172577994876133`,
0.22377486544985759`, 0.217477012217474`, 0.16181368479198038`, 1626.097345224801 8`, 102.05318105921833`,
0.2209045520339541`, 2.2233716047935985`, 1.1920782532980323`, 160.61597653683904`, 20.88518028781105`,
327.06325675172684`, 0, 47.721303017325866`, 6.63897677463279`, 6.890920688480024`, 0.037949208500005494`,
1.4071397001801922`, 0.05845015051810537`, -0.9584617287746053`, 101.37720408369623`, 3.471352796607331`,
0.9975852872457128`, 0.9999998799979424`, 0.0005908665095333223`, -0.9994091334195615`, 0.05197920204348817`},
{0.25925369048850816`, 0.417309499595206`, 6.855645959180599`, 1.1773438518732138`, 0.2888562944151887`,
0.22102634108263253`, 0.2071754486228008`, 0.04320874178397733`, 1948.065661484895`, 384.2385254384834`,
0.1833279639586075`, 5.15481721625838`, 0.9762665296404283`, 105.43994232615796`, 23.515347601945177`,
116.34213454686093`, 0, 65.52509533385397`, 5.71401139108128`, 5.780827078863792`, 0.011693306717379226`,
0.02816219488104618`, 0.022031443903896577`, -0.21769435951441918`, 34.064446204191995`, 0.10430218507381579`,
1.1141512344580944`, 0.4016848917745921`, 0.0002267551190259749`, -0.9994354900478726`, 0.1776797356258755`},
{0.22057802576642993`, 2.653890334277995`, 3.4863828466021776`, 1.174291282966625 2`, 0.8455831985967934`,
0.6315585043048466`, 0.21227533056536496`, 0.0900482536994353`, 1462.898116977769`, 366.27377580582504`,
0.07912574234824876`, 7.4931206124945415`, 1.2283305207046857`, 208.34475221876755`, 173.0944273987836`,
329.5559328026428`, 0, 91.03003010974287`, 2.927528402976816`, 3.0124187526030464`, 0.028997276180108278`,
0.818221432851183`, 0.05872666209869198 4`, -0.9282264436730134`, 110.99056188549227`, 2.578309547115938`,
2.482702040673397`, 0.9999855308704791`, 0.0006061196431077447`, -0.9993938715867418`, 0.4853766350207399`},
{0.18767448152183636`, 2.9367397535675925`, 4.254981609198115`, 0.9658072683472699`, 0.6065533427003664`,
0.6047075695535695`, 0.22956691915882899`, 0.016808738900599973`, 3085.080662132773`, 300.13204027351287`,
0.16815220633174727`, 7.212084588140897`, 0.5334832326025793`, 233.84133171211516`, 49.27597636912892`,
296.91144923278574`, 0, 187.64933382853556`, 1.0435409589025715`, 1.0541323948857249`, 0.01014951631059291`,
0.34830132611739834`, 0.04617008352085607`, -0.8674421253702204`, 43.78011740693178`, 0.9338181541779405`,
0.9876377172963465`, 0.9991521183565495`, 0.0005791304081129075`, -0.9994203781411528`, 0.20319599161619917`},
{0.16674052895366065`, 1.1207156804364002`, 9.241855378678228`, 1.116892231935736`, 0.24719875970191896`,
0.3952404123967743`, 0.24464165205494265`, 0.025889397470345094`, 2185.868573954095`, 178.37862169115658`,
0.08745488697820436`, 7.480105474877458`, 0.1628128610930062`, 191.7041836389824`, 37.91332098269115`,

378.5946962347414`, 0, 140.0882491218748`, 3.0277108953976533`, 3.04279652319289`, 0.004982519241902628`,
0.024336613509807998`, 0.01943793788258614`, −0.20128830271506848`, 48.47432966143261`, 0.057969997279531574`,
0.8457028739387067`, 0.3737748631102784`, 0.00021075519721031337`, −0.9994361440053604`, 0.18126298112647285`},
{0.18395565451029489`, 3.44119115260109`, 6.476610943613181`, 1.4097688201668712`, 0.3016652649845466`,
0.23377636743924812`, 0.22702955380114842`, 0.044315995698336386`, 2035.6758637667072`, 215.7759126319412`,
0.23319048350186838`, 1.2339772874754598`, 0.4032881579686409`, 37.531144491494096`, 5.333788525162513`,
49.729036609113656`, 0, 22.90806509734157`, 0.286832199252264`, 0.29006069471371`, 0.011255693990640747`,
0.06462965144111739`, 0.041396778296251664`, −0.35947699897519436`, 14.100634662114356`, 0.16984271188030864`,
0.1847303864894244`, 0.6222423259847167`, 0.00035690318610508663`, −0.9994264241257772`, 0.026119643452159037`},
{0.22900819014324658`, 0.8599246184787699`, 4.575509667907129`, 1.1011546323816885`, 0.08315194396691528`,
0.18390636972505037`, 0.1408377449166481`, 0.3122650757195923`, 2947.0026584149073`, 167.82607804795214`,
0.10792455151802871`, 5.931199053322571`, 0.9798220386267653`, 201.98102140471138`, 14.489874346255709`,
235.58660844462923`, 0, 37.82854283481756`, 12.272865400517558`, 12.419944395028468`, 0.011984079488454924`,
0.2603066565266972`, 0.051395815920249506`, −0.8025566591111037`, 150.767701388305`, 0.851605089905853`,
1.308731055545126`, 0.9899843557617302`, 0.0005656426908936706`, −0.9994286347177088`, 0.09567208938472316`},
{0.2044811200034678`, 2.892822767813837`, 7.595852124568353`, 1.216932964982977`, 0.7343890780610787`,
0.5330606684550935`, 0.058989793379298994`, 0.012080434529966445`, 2521.088434314187`, 248.82760985623736`,
0.0448064575868322 7`, 4.109426945013643`, 0.10374011787051507`, 81.05714318090341`, 38.401494562545025`,
124.24726490709979`, 0, 69.0686551239276`, 0.2798999521181449`, 0.2818555357693296`, 0.006986723778928283`,
0.024327464435722482`, 0.019820141598768697`, −0.18527713189604855`, 11.567156488533879`, 0.07106438820945465`,
0.4225154691967837`, 0.3476248166449286`, 0.00019735453928384852`, −0.9994322771855341`, 0.14280857771516436`},
{0.04803156621959867`, 0.5827754735791681`, 9.214605590518953`, 1.1720588669143222`, 0.8133320484998057`,
0.6073432395586391`, 0.13364149823347654`, 0.026188730330046147`, 821.8332545613489`, 133.84484048922508`,
0.12502450253620295`, 2.6224761178996694`, 0.4958664794043681`, 185.53927482643863`, 94.41347387662846`,
222.81237544200005`, 0, 134.8835385208375`, 5.360071326016513`, 5.41701848820627`, 0.010624329178857916`,
0.37995912272611`, 0.05646732766708556`, −0.8513857826022261`, 44.624544363391344`, 0.26071473948509866`,
1.2802888651598314`, 0.9980167007364463`, 0.0005835272666022609`, −0.9994153131243478`, 0.10526811014385354`},
{0.06724626093304326`, 3.981403022919509`, 8.288988323065759`, 1.0703538801483565`, 0.1653330791428167`,
0.4148194823615189`, 0.14440389123820263`, 0.19094336267448156`, 2003.8545802245517`, 246.03939081881197`,
0.139046230518015`, 4.691983788055152`, 0.20050055393273314`, 91.72472897402064`, 24.405379532719014`,
133.67770331463902`, 0, 25.054828648813533`, 1.151274948773478`, 1.1596777943501146`, 0.0072987304949081455`,

0.0190476215436208`, 0.01584279742961144`, -0.1682532439375708`, 65.48127944654595`, 0.01829830469273734`,
0.6416996967042738`, 0.31723899647701614`, 0.00017924291525525047`, -0.999434990914592`, 0.1682397903199964`},
{0.21881875684613517`, 0.5898239021275495`, 9.93413148158805`, 0.7544358459883748`, 0.3693391180254211`,
0.6579008857369009`, 0.12709107324060326`, 0.48252891951701626`, 2900.587783201154`, 140.00284155203332`,
0.2319315634448451`, 3.003524790772248`, 0.5189129901116001`, 134.70012770182666`, 16.60686013653917`,
163.5452247985029`, 0, 17.52893090923771`, 12.416192385711172`, 12.47976296806163`, 0.005119974012614215`,
0.032834660839217863`, 0.021368203949038912`, -0.3492180700853582`, 104.61952917866478`, 0.10264056666157106`,
0.5612155764059995`, 0.6053428100063114`, 0.0003430998080486815`, -0.9994332140361176`, 0.06208077466012295`},
{0.18529128706683434`, 3.034817908847505`, 4.618773666141445`, 1.078127573609324`, 0.45431233294938433`,
0.23204854612180326`, 0.21276533477653692`, 0.005983047100273517`, 2838.235019182518`, 133.97625538243244`,
0.13415379883362671`, 8.373997227624333`, 0.3370984440619733`, 497.58622476550426`, 31.426267449939147`,
641.6290457133032`, 0, 265.6833607755031`, 0.5103115288132388`, 0.8833564204805308`, 0.7310140386889379`,
57.302443950442786`, 0.08702120508682236`, -0.998481370093707`, 22.124322381910957`, 151.6806227378952`,
0.8688583418421023`, 0.9999999999991742`, 0.0009777378937572425`, -0.9990222621062419`, 0.08540623959390653`},
{0.09989873104684788`, 3.3669591438562216`, 5.451558876338645`, 1.2627430306397467`, 0.36748912342215223`,
0.31108122243153336`, 0.12542210883620514`, 0.007371687908480407`, 3955.966637225736`, 182.23968139905298`,
0.15451142826141562`, 8.671574224679677`, 1.2389048546471688`, 337.6069868837003`, 24.448858550506912`,
374.42059874309433`, 0, 304.96806205338584`, 0.6494374353361563`, 0.655118653408964`, 0.008747906670743655`,
0.18873026562256287`, 0.05659188986517526`, -0.7001440670975793`, 31.237561589537236`, 0.2693416292261603`,
2.212785802502552`, 0.9565724748440527`, 0.0005427581651193453`, -0.9994326011050989`, 0.14270836549553653`},
{0.22609069450991742`, 1.379608652743129`, 6.641039030693791`, 1.0528553454528264`, 0.9110900221670273`,
0.41585330034333745`, 0.15736472782730937`, 0.24498648200968076`, 2031.8230399130634`, 206.889154659356`,
0.10264734406954457`, 2.177599971086371`, 1.0407277908428236`, 109.98236164157815`, 29.629441039968555`,
147.06403508747815`, 0, 24.665384817387892`, 4.0521747380238855`, 4.130670125690766`, 0.019371175416082798`,
0.33138769662138484`, 0.05017206361971487`, -0.8486001015389621`, 79.86307615721249`, 1.0703382068738874`,
0.6896407965904171`, 0.9984274941210403`, 0.0005779928932020261`, -0.9994210967780781`, 0.05474244106165511 4`},
{0.14149819408460695`, 3.9050575954333624`, 9.383356210665369`, 0.960335311543541`, 0.3424013679631459`,
0.21333414365316794`, 0.05126386183615583`, 0.4919218322625912`, 3186.224620232545`, 108.82685651649962`,
0.06233434212075295`, 5.950211096913752`, 0.2482752929278060 3`, 186.6018628186002`, 17.18122258610968`,
329.72833275629074`, 0, 23.755129725767794`, 2.8662743780050355`, 2.876674880054221`, 0.0036285786625998018`,
0.02678783936889807 3`, 0.020143261879095733`, -0.24804454731489112`, 159.89952186320832`, 0.05414901277321783`,

0.5966413508943156`, 0.45121094566416675`, 0.00025400308438762323`, -0.9994370635579025`, 0.0435980994459464`},
{0.08341672306903047`, 2.823299860683674`, 4.834884605952357`, 1.4354641770214007`, 0.9014467152160488`,
0.6047529781600969`, 0.2470533033127953`, 0.006202153420058688`, 2417.2420994663553`, 158.0041607308176`,
0.0735259731187195`, 6.516775962062823`, 0.4133308952289383`, 260.9923915367041`, 100.76361797765395`,
797.6522933458414`, 0, 220.19558014144388`, 0.46980618826754617`, 0.5139344159375571`, 0.09392857900134932`,
9.648268716003976`, 0.07460231723665015`, -0.9922678027081787`, 18.94862494120133`, 11.497527993978583`,
1.0274528907632094`, 0.9999999999119054`, 0.0006295388482637193`, -0.9993704611516808`, 0.20411999226682373`},
{0.16837082644708545`, 0.688574652311627`, 5.390405754713122`, 1.3217406774272815`, 0.6156990878136903`,
0.18352461332079384`, 0.0882858818891156`, 0.4733971203783309`, 2234.460265184529`, 202.49554378641346`,
0.1503105450782266`, 3.806358767200937`, 0.9148594831472519`, 137.98624265931497`, 18.70524091765933`,
151.72245582920888`, 0, 18.560937358218588`, 10.975093790272789`, 11.092866376544196`, 0.010730895655378214`,
0.14407557243492067`, 0.055728370740446356`, -0.6132004211496782`, 107.95959129606544`, 0.3465446171672261`,
0.9820052534546881`, 0.9005957674771334`, 0.0005111145024406534`, -0.9994324706811887`, 0.05275041209483772`},
{0.10718623369591462`, 2.960462485410858`, 9.423477882136847`, 1.1211033662754974`, 0.7860054376705241`,
0.5141091538304853`, 0.05867214344346472`, 0.09405923440011967`, 1537.0957508113852`, 239.00629492907444`,
0.09357298190053748`, 9.410898004520863`, 0.8654487723896875`, 309.2432771481713`, 158.6070244887968`,
314.74944093462796`, 0, 133.95462473451695`, 4.0470142374413305`, 4.095341796405675`, 0.011941534209896743`,
0.033148789294034`, 0.024538023463986057`, -0.25976109575735484`, 171.15762611241`, 0.0507584839429135`,
3.5835828341201434`, 0.4700204616627339`, 0.00026501568417898615`, -0.9994361613891415`, 0.2683079997635519`},
{0.18162340679997874`, 2.5029500310491875`, 3.7850875297449385`, 0.8714265661124054`, 0.04795600587846183`,
0.17826165391264504`, 0.1376006876951453`, 0.011710367976498486`, 1897.0378575605146`, 378.5017307178937`,
0.04683765268043216`, 7.517716852692461`, 0.3647662687926059`, 106.14536253343056`, 27.81384021112243`,
129.79266940534777`, 0, 90.95016480078901`, 0.4089320073138758`, 0.41386727605300233`, 0.012068678046368797`,
0.0283380304555362`, 0.020393560771474365`, -0.2803465715984429`, 14.621948291475324`, 0.07352642333338098`,
1.1237665584892769`, 0.5025516681301242`, 0.00028349977959376676`, -0.9994358793382407`, 0.1968379606591197`},
{0.1295394448875809`, 2.022900103755206`, 6.745994489858977`, 0.9580215588674443`, 0.9679692042597987`,
0.6904939360195417`, 0.05882320965699680 5`, 0.04912899750957061`, 1189.5399921293747`, 163.6733664974323`,
0.16033185329150101`, 5.9616048020309655`, 1.2439833727010554`, 306.12151066194275`, 253.85476295305983`,
321.076022484709`, 0, 180.92275143568176`, 4.155630574878008`, 4.22244307693144`, 0.016077584580624915`,
0.3315892910077956`, 0.045761266477557856`, -0.8619941363652723`, 120.09179315841503`, 0.6136270383974044`,
3.896609491254629`, 0.9967458862054872`, 0.0005784932221568399`, -0.9994196181492566`, 0.18904767383549734`},

{0.18194781074756367`, 2.220335509721994`, 7.082070220373439`, 1.2346660258162963`, 0.911357302435295`,
0.2993417896543701`, 0.10379984911373669`, 0.06373968962233406`, 1006.0135021400088`, 377.69931275745535`,
0.06750680684166793`, 5.51898040870112`, 1.3240328384144195`, 165.44898314186977`, 122.95833896916115`,
164.9350169637921`, 0, 87.48351410337325`, 2.3775113462236597`, 2.4099757312869228`, 0.013654776081228004`,
0.048512173202580174`, 0.0327874627680105`, -0.32413947668156284`, 75.41246952553334`, 0.12609548155435885`,
2.057085379458262`, 0.5689301951281369`, 0.0003217055946510895`, -0.9994345429414612`, 0.16608031010104732`},
{0.1644023253774144`, 3.882290753800083`, 5.67814834147805`, 1.1217171634204655`, 0.4202214546941414`,
0.4110647435892903 5`, 0.06359886932295966`, 0.24993914606767362`, 1680.5799994030467`, 230.96461331819444`,
0.016083788677699107`, 8.870452591991157`, 0.6584854072019537`, 241.41093732075382`, 71.06269132026577`,
256.6988536365437`, 0, 54.85380116203973`, 3.300946755127602`, 3.3402040349283153`, 0.011892733422534718`,
0.054172639293247636`, 0.03373709497817064`, -0.37722999251439815`, 183.07478666026034`, 0.1272301124521504`,
2.6964826495707386`, 0.6440344640730872`, 0.00036426548361635596`, -0.9994344006354682`, 0.2575258947583881`},
{0.11290442193886385`, 3.342282901193297`, 2.898098788178677`, 1.2208920487711645`, 0.15571856198486667`,
0.6450091875601396`, 0.07200970199664747`, 0.18140557939535418`, 3133.8759234972686`, 359.44606615721364`,
0.05885138914392884 5`, 2.4968280987996607`, 0.44903712610868673`, 36.31857905999528`, 10.204365575213874`,
39.98118346449747`, 0, 10.333157229019355`, 0.529746491586097`, 0.5375979295112603`, 0.014821123027460859`,
0.06197020992008163`, 0.038364284753528315`, -0.3809237567049736`, 25.293752011362102`, 0.09995301040651676`,
0.38108485679901244`, 0.6526666338696017`, 0.00038145262910300657`, -0.9994155475256312`, 0.13830477326680884`},
{0.057179701065231625`, 2.855281586486381`, 5.482300889649739`, 1.2083595069262243`, 0.795518738527804`,
0.6366469580580449`, 0.11918231638825821`, 0.16920403900000133`, 799.767703151836`, 247.48605975500072`,
0.10228761215396959`, 9.288149547399101`, 1.2673098981919697`, 394.36132354854726`, 355.2405520709499`,
414.2865797155583`, 0, 118.79460385154283`, 6.571622825298859`, 6.675240160150261`, 0.015767389213590333`,
0.5175599045974225`, 0.05873595684629316`, -0.8865137033905666`, 268.0547663772786`, 0.42277029468829164`,
5.198336351123804`, 0.9970457769232616`, 0.0005885940138281614`, -0.9994096619960174`, 0.4697707962081789`},
{0.0875578111208043`, 1.1766586937430805`, 5.077956330142905`, 0.8750465005858246`, 0.8596423212250299`,
0.6288939270221594`, 0.1488852290513452`, 0.012846012396330703`, 3117.450697007702`, 283.31782716202474`,
0.08644475242948957`, 6.632814251236063`, 1.2827414734862095`, 255.72561308551565`, 73.76769902650184`,
308.89860900056226`, 0, 215.58759238622167`, 2.202815876487132`, 2.2264934702204298`, 0.010748784765005848`,
0.34245990982429747`, 0.0416948414862204`, -0.8782489853845598`, 37.028035025483895`, 0.4283577157262948`,
1.8090461129073847`, 0.9991007953042073`, 0.000577033495654893`, -0.9994224471661248`, 0.17852216724622472`},
{0.1753150595055662`, 3.713574756709245`, 6.279606102538107`, 0.9857257532666506`, 0.18592289869330236`,

0.21496347741152755`, 0.07049642727707597`, 0.017218370675811997`, 2472.055030254328`, 256.95308160663865`,
0.22538544536854038`, 4.255341860945123`, 0.22574651763666997`, 66.65514840292124`, 13.886160565210373`,
98.0989747962423 5`, 0, 53.51939509032805`, 0.24136744605278584`, 0.2434520882322909`, 0.00863679926019989`,
0.018787392914517344`, 0.015423885384396669`, -0.17903003069263723`, 12.804800782185763`, 0.04705304152519327`,
0.48684942166033535`, 0.33593558079528874`, 0.00018957472446486415`, -0.9994356813171856`, 0.07986945744529236`},
{0.22967780600547444`, 3.4707114805627004`, 7.357111611957344`, 0.9509066717740751`, 0.7301577279137197`,
0.34891003518552344`, 0.14994464399131946`, 0.18785696851097627`, 1448.9102951912027`, 296.3879865990905`,
0.13500832540748392`, 3.275227564043599`, 0.3389151549206586`, 109.20774214062158`, 40.21515534455434`,
123.25511528540363`, 0, 30.08029918276036`, 1.5608297394602277`, 1.5729298933490579`, 0.007752385531182071`,
0.041622129949291035`, 0.027027463131890424`, -0.3506468033995749`, 77.38842422783286`, 0.1365668498289273`,
0.6659536931196002`, 0.606951187671528`, 0.0003444436601819312`, -0.9994325018762986`, 0.08566346047803716`},
{0.10636965549911737`, 2.776846234427638`, 9.809572024338209`, 1.0633435610610082`, 0.685773597384219`,
0.389828683015383`, 0.18173035709309476`, 0.0701248233933049`, 611.7373128577356`, 348.65549321322396`,
0.0858335193643106`, 8.943700469577841`, 1.0373885202439106`, 316.1823899962558`, 267.8493775251145`,
310.9121265675433`, 0, 159.9712011841429`, 3.8389521464925895`, 3.890705956057945`, 0.013481233313272778`,
0.03294786661755755`, 0.024053811171014003`, -0.26994328797616307`, 152.2882830305121`, 0.05006647459357891`,
4.163389131825682`, 0.4860201710635157`, 0.0002738949115766722`, -0.9994364536126611`, 0.46581114775970217`},
{0.09051269700635717`, 3.510668586088882`, 4.372912512921815`, 1.3267853376570602`, 0.8120222411850102`,
0.239383728318748`, 0.21506169431109157`, 0.20950664990140405`, 1339.6563194330593`, 329.7735045463686`,
0.2280878170659803`, 2.1716129406234614`, 0.869217412898017`, 72.34045845114191`, 25.603875456937278`,
97.4642287903841`, 0, 18.347798055816902`, 1.0387020449416862`, 1.0600365454188097`, 0.02053957685076213`,
0.37528769463664413`, 0.06332559130023035`, -0.8312612105186593`, 52.09340913547227`, 0.4852614484982519`,
0.5982740318747789`, 0.996969097728199`, 0.0005789507746847795`, -0.999419289147473`, 0.0743085279945231`},
{0.10777187590532172`, 0.739057715374912`, 1.4330240793355227`, 0.7755224108621632`, 0.12555368221137964`,
0.545351416919415`, 0.13748218086765884`, 0.05919866095169625`, 3976.4209480252466`, 374.54745407708265`,
0.062136934556040746`, 7.297702295605297`, 0.10338190129278124`, 62.92996051061395`, 21.39231271323534`,
133.4075198281132`, 0, 34.21973519400601`, 2.4711753142820756`, 2.494766284745336`, 0.009546457641801842`,
0.05824932927289376`, 0.029263224063135508`, -0.4976212700057113`, 26.09058831520267`, 0.08968056408527914`,
0.45994180981142135`, 0.7869376996365535`, 0.00045733182137495324`, -0.9994188462166875`, 0.41924595850127533`},
{0.05012776858713164`, 2.0872339482718107`, 6.705710827191493`, 1.4141058091423697`, 0.06890952245028781`,
0.47612787079844376`, 0.08471180395825834`, 0.016954072038827265`, 2788.124252935484`, 108.68029812587577`,

0.08106980932549152`, 8.734393861782642`, 0.5389481104734093`, 388.09052381766577`, 32.67941178779199`,
478.6302161475129`, 0, 312.4587524446412`, 2.441435306444001`, 2.455375577638834`, 0.005709867125308854`,
0.1459336904689994`, 0.05880951133175511`, -0.5970121008880538`, 72.79780934456181`, 0.10450471806979246`,
1.8442524979288044`, 0.8874838482270831`, 0.0005035806542976174`, -0.9994325748515833`, 0.1411450463725533`},
{0.1308447304032681`, 2.6119158907935276`, 7.388927202269429`, 1.1426009806290098`, 0.6170224096703181`,
0.5032614025378099`, 0.21866608861569725`, 0.005956991186122966`, 1137.1164610175856`, 368.0725112372012`,
0.24678418452608802`, 7.5428083328821245`, 0.14982171102850428`, 214.6398669674025`, 127.09826951151855`,
250.48194579808265`, 0, 197.63157554303072`, 0.435466167272794`, 0.43936198875021243`, 0.008946324123908056`,
0.03890364774562513`, 0.02761552762280427`, -0.29015582797349004`, 16.24858574575375`, 0.07271910429971941`,
1.5939825195100472`, 0.5173286413141774`, 0.0002926911040201911`, -0.999434225982005`, 0.3848907386507499`},
{0.06751611881159836`, 2.2474089970429993`, 6.402097457423925`, 0.9363574880786951`, 0.3836841636235191`,
0.3647284912476121`, 0.21335232520714842`, 0.22248453220280073`, 2502.3638711494295`, 397.5911497982886`,
0.16288128573213767`, 9.005290951575024`, 1.0427120513238162`, 201.6278767845655`, 43.958913410096116`,
218.7487432099531`, 0, 49.593996907901975`, 4.590708056680412`, 4.6327924837567185`, 0.009167306340699621`,
0.027803556859381493`, 0.020554571288878953`, -0.2607215187310318`, 147.38855127687634`, 0.02681697497575588`,
1.8262858639047528`, 0.47148855056828853`, 0.0002657933672671042`, -0.9994362676104293`, 0.3206331546191099`},
{0.04551056845008081`, 1.6215675270048893`, 9.700566391026626`, 1.40945752023491`, 0.4723808944761221`,
0.3499440053674635`, 0.2064578553856145`, 0.09474747167065985`, 3852.6928203016614`, 220.886718735037`,
0.22402828336678815`, 8.267759522981173`, 1.1674803222752286`, 344.85474895031246`, 28.152299787298514`,
385.5559053695072`, 0, 147.4642898330536`, 8.16446952007299`, 8.207176682476693`, 0.005230855758442621`,
0.05690209837777409`, 0.03804060241840695`, -0.3314727663318374`, 189.13198069959387`, 0.03699495490243142`,
1.7368491989745498`, 0.5797877246023423`, 0.0003267870777291873 3`, -0.9994363677189728`, 0.15170995051063982`},
{0.04140464424456583`, 0.7619598093834323`, 9.994885078827323`, 1.3154910694140878`, 0.5846504333106828`,
0.5277457306392407`, 0.1974778280215821 5`, 0.20923520620939917`, 3747.8984478066395`, 346.71554418059986`,
0.04757978117327022`, 1.731006219467508`, 0.3938580217562748`, 43.58597074358899`, 8.35759113599478`,
53.416498234137656`, 0, 11.038608532569826`, 2.735106414680545`, 2.7479384696105527`, 0.00469161084962999`,
0.025283896093013177`, 0.020789859767110765`, -0.1777430309541681`, 29.772016605334063`, 0.014955296040689334`,
0.16492262886019388`, 0.33629343798165623`, 0.00019146256906746493`, -0.9994306681384669`, 0.04205895909764186`},
{0.11628555922147576`, 0.9133810470221562`, 6.60919827925733`, 0.784993620588668`, 0.15558932911031897`,
0.27453121261635205`, 0.2030077151739278`, 0.01885747776005145`, 1720.9600172356268`, 220.92402081631303`,
0.1375226538362544`, 8.707812842669803`, 0.5031177384459613`, 256.04033547859956`, 44.59525706427541`,

311.5053886097685`, 0, 201.93303365494484`, 3.839487512705216`, 3.8678909076979924`, 0.007397704745434552`,
0.03349853713197704`, 0.021920015811664136`, −0.34564259551681376`, 50.098787491188325`, 0.055648516049916406`,
1.7484347114210563`, 0.6000593061423851`, 0.00033811474658593905`, −0.9994365311176331`, 0.21450596393199894`},
{0.16627919709086636`, 3.258899380742358`, 5.159743357401544`, 1.0084152828841422`, 0.293274671001003`,
0.35878951423125527`, 0.20447475990027797`, 0.06840907951998164`, 1127.403994426183`, 328.5526444815032`,
0.06047838915389364`, 6.7509258891150505`, 0.42840334463359087`, 161.15421679104998`, 65.99792730236479`,
179.41297405174174`, 0, 82.27713871756345`, 1.6553641510713568`, 1.6748974133316603`, 0.011799979024350371`,
0.04577581650358364`, 0.029271600970175157`, −0.36054442703641165`, 77.0666458118506`, 0.10873665734857377`,
1.7733203546924272`, 0.620564833696931`, 0.0003516121106169256`, −0.9994333998777819`, 0.33674907566304024`},
{0.11413129353935564`, 2.5718486613546734`, 2.678541647274315`, 1.0538897286852023`, 0.5347432665041771`,
0.651301492741162`, 0.13507333994249482`, 0.03374013401687532`, 1792.985239436316`, 324.1256033282515`,
0.22051507650373564`, 9.850878408710493`, 0.1276954587958865`, 206.84983593530643`, 110.27084142159853`,
280.5361541451156`, 0, 139.38765382955657`, 1.7622312810481486`, 1.7825646262102512`, 0.011538408936883964`,
0.356536007103788`, 0.05183388981070966`, −0.8546180784606678`, 64.7456023023003`, 0.5813130812015326`,
1.4230114886766678`, 0.9964495759795039`, 0.0005957930202742408`, −0.9994020841248403`, 0.6116946053710749`},
{0.2202391290111929`, 2.272383906087442`, 9.526635756893253`, 1.2801331448227915`, 0.9676407538336758`,
0.2750641951622783`, 0.1295591726514962`, 0.005511573366300065 4`, 1435.4738327945734`, 244.53753727132892`,
0.22242949998676909`, 3.5195011696028082`, 0.5942454573278351`, 178.46667356738746`, 56.86542249119429`,
194.43985529303131`, 0, 165.04843276328825`, 0.386198597061557`, 0.3892773784925531`, 0.007972016093329648`,
0.07503315878634431`, 0.042734966259627714`, −0.4304522566974045`, 12.537021093089015`, 0.23607482197232657`,
0.9118761476396706`, 0.7139819883915085`, 0.00040452935935604074`, −0.9994334179770173`, 0.056328290200658535`},
{0.08672713507238722`, 2.273561509630496`, 5.582415438341933`, 0.8483312100313163`, 0.348511515665392 6`,
0.5097094533881305`, 0.23367094415518952`, 0.14443826868757056`, 1562.9150740851455`, 249.7667060966911`,
0.16191416277041454`, 3.437993337535003`, 0.32781925870094497`, 105.91636297648358`, 29.971381003203433`,
129.78462017824003`, 0, 34.97821791912976`, 2.1152957188850707`, 2.1309081214119874`, 0.007380718633111849`,
0.05324220969534336`, 0.02937343559379227`, −0.44830547488788186`, 68.703641827761`, 0.06596491874000937`,
0.6720870503656937`, 0.7333597141660084`, 0.00041942032038281673`, −0.9994280837735138`, 0.1415789966832421`},
{0.18381668769060344`, 3.1958936164482523`, 3.6493637720914887`, 1.44172169143792`, 0.026838260836304473`,
0.6302019111021357`, 0.18006792550977113`, 0.04278842418986425`, 2871.506610396247`, 235.17217671272113`,
0.15357027364724346`, 9.443732417373614`, 1.0754484669415296`, 254.450683359263`, 38.29569911383381`,
280.93877323375017`, 0, 158.29712507436383`, 2.0414098724530185`, 2.06900429064665`, 0.013517333567351297`,

0.24859107679587492`, 0.0667627201436331`, -0.7314355728123989`, 93.20183971324323`, 0.6527884046579937`,
2.606104219622805`, 0.9669252316385607`, 0.0005611208214596722`, -0.9994196854078274`, 0.4320845869511773`},
{0.18865219826812762`, 2.4805712334398518`, 8.790023396255851`, 1.3488199759969297`, 0.9395739613202174`,
0.6166147449809938`, 0.13773668752267337`, 0.19466267340323914`, 3969.2421406756894`, 297.90931549743027`,
0.10386024186718207`, 2.094376919169571`, 0.2651891384942813`, 70.92669097806005`, 21.41782075829836`,
93.44813854126625`, 0, 18.84056750257942`, 1.4248725554741788`, 1.4317271806764944`, 0.004810693543068734`,
0.04558125355486601`, 0.032569004167767514`, -0.2854737062339826`, 50.49282674895954`, 0.12284290975639979`,
0.17138798721658238`, 0.5128890255953891`, 0.0002928049516265409`, -0.9994291066156336`, 0.04013710071876566`},
{0.13375040892255174`, 3.276165649558605`, 4.292971358510812`, 1.2543962009506533`, 0.622136912433882`,
0.38876133504362986`, 0.059362281046229176`, 0.37593623792715036`, 1950.8385805745784`, 382.0130407968936`,
0.20132818276194275`, 9.006278307672524`, 1.186932922075334`, 170.10016139049702`, 77.14760905018653`,
172.09010685842634`, 0, 28.88911941039723`, 2.950869583198962`, 2.999830762878282`, 0.016592119136028538`,
0.052389521873881115`, 0.03462596398135796`, -0.3390669976963314`, 138.10767949719664`, 0.10010171391246579`,
2.647869845239842`, 0.5907568631972966`, 0.00033436049827422214`, -0.9994340133494775`, 0.2727114574984836`},
{0.08974721760036025`, 3.2713030704465336`, 9.414991051375608`, 1.4492271123735618`, 0.9934682041726086`,
0.2039523558527424`, 0.14566778076348777`, 0.062238114816719925`, 3000.2912643397094`, 293.40777590216123`,
0.11936140149668095`, 5.192024217841877`, 0.8616976591715506`, 245.42108088428114`, 34.029098707510464`,
271.93684774222504`, 0, 130.22394771058853`, 2.40969442819186`, 2.4198799146948002`, 0.004226878887122032`,
0.07622649644542984`, 0.04565942487491602`, -0.4010032337298439`, 112.61201116831356`, 0.0977302280486054`,
0.8580640025520925`, 0.676587313155106`, 0.0003815652675798312`, -0.9994360443062397`, 0.047925778435807524`},
{0.19365605952074066`, 1.0097214328963666`, 6.254195026060383`, 0.7786863431193308`, 0.8378299450565547`,
0.5381078852313175`, 0.13430741341317382`, 0.39183098626736546`, 3848.3805063541085`, 285.46142645085683`,
0.147680954768134 7`, 3.2123723043853616`, 0.9596643052107194`, 124.9867859823265`, 24.01829847933813`,
132.87636043492782`, 0, 19.42923371141099`, 6.8274962468788285`, 6.87489590985033`, 0.006942466353338617`,
0.06536784098307125`, 0.030384681863601937`, -0.5351738499138917`, 98.48384704990075`, 0.18084112148801337`,
0.6185189005814123`, 0.8317913717682035`, 0.00047280319196990985`, -0.9994315843996255`, 0.060068080465153065`},
{0.08593805782066072`, 2.847179937660589`, 9.336517990063154`, 1.1546615909588782`, 0.6233557238718435`,
0.5408319922909341`, 0.12713218439588464`, 0.009396030605633099`, 2805.654341504175`, 67.07123229055907`,
0.22248402654525073`, 4.783547942175421`, 0.25670135456607057`, 334.09165879567774`, 34.01558155063026`,
674.7817761934123`, 0, 293.6221668269117`, 0.9439040666023379`, 0.9487375437714629`, 0.005120729256441692`,
0.40015390131040873`, 0.05420651096733808`, -0.864535842859898`, 38.39235316437745`, 0.4912635586854172`,

```
  0.5657168944782165`, 0.9995221566775296`, 0.0005685179165238982`, -0.9994312102911118`, 0.05502908355009389`},
 {0.13317608837170147`, 1.503932075956504`, 4.603993052764295`, 1.367556919883856`, 0.822788205819833`,
  0.29931793037743193`, 0.1910982001847455`, 0.009186595448818861`, 2039.1477227581072`, 222.71270432048289`,
  0.13206186826480382`, 1.3638292654387778`, 0.18903235795918816`, 47.13878662983394`, 12.185721210091218`,
  84.35694952497907`, 0, 40.99553243570957`, 0.23829090755516188`, 0.24321668996842105`, 0.020671298220301892`,
  0.25865918130149285`, 0.06419957370185742`, -0.7517985892523704`, 5.11961913258562`, 0.49210311410228147`,
  0.16815839302870855`, 0.9836493877657282`, 0.0005705547517567029`, -0.9994199612597201`, 0.030579959290949393`},
 {0.27769905250843735`, 3.334803063409833`, 9.639698328103112`, 0.972586344049939`, 0.15282290468559845`,
  0.3020316347056351`, 0.21057051005264293`, 0.06155018310530056`, 2190.880276553241`, 205.62596489641203`,
  0.17875345007490812`, 2.046885089160499`, 1.0703002508596509`, 66.63942692083647`, 8.200202080436071`,
  76.07670537265776`, 0, 35.64620659704236`, 0.6343884195208701`, 0.6410253270533491`, 0.010461898938022296`,
  0.027402845434444528`, 0.02059010921472741`, -0.24861418993933093`, 30.222292068713113`, 0.10871063161683958`,
  0.578244873563573`, 0.4531073431840442`, 0.00025667758268793433`, -0.9994335170538525`, 0.06552496871924597`},
 {0.2349663788193207`, 3.197269954964022`, 9.218845167290574`, 1.0446779264109838`, 0.588347422519365`,
  0.31448476179540896`, 0.13233876836756175`, 0.2796850376475317`, 2984.1524918506084`, 161.29090180593857`,
  0.1933086585832205`, 6.384613193914876`, 0.9299276109407706`, 333.6576932770157`, 30.200119234694387`,
  373.41981916246544`, 0, 67.93767428118264`, 5.6863895983107025`, 5.717502291009132`, 0.005471431768880652`,
  0.070274769203431`, 0.03716078186338892`, -0.4712073439072264`, 259.7274659271248`, 0.23588868631580232`,
  1.5262168754596666`, 0.7634876078465475`, 0.00043082770481268245`, -0.9994357109396603`, 0.08047766395565205`},
 {0.23753660977363267`, 1.5829476505005262`, 6.77431038963988`, 1.365304095754423`, 0.13883005311148944`,
  0.18235912783176345`, 0.11037102150981828`, 0.0059073701404183405`, 3040.16542258953`, 172.94160910300627`,
  0.10069032496396058`, 7.204527027300948`, 0.264985624786922`, 164.52087835364765`, 17.77472723190549`,
  271.4014425820212`, 0, 151.46234777608316`, 0.5389112355021224`, 0.5422133398923978`, 0.006127362305220485`,
  0.03849060723764714`, 0.028871414053341527`, -0.2499101436595994`, 12.18668962952027`, 0.13061326216228342`,
  0.6672013735849402`, 0.4544413674560014`, 0.0002561911040576506`, -0.9994362504771698`, 0.07180035674072714`},
 {0.23896625246574088`, 1.021768300475304`, 5.961217623879941`, 0.8744606875234477`, 0.07851707385676243`,
  0.3191621351693026`, 0.12686157250224162`, 0.07080906253325867`, 754.026010583138`, 128.7873645709799`,
  0.07054179811818284`, 9.522685613917606`, 0.14468703962562834`, 315.77685145677907`, 111.61348526408477`,
  485.7348933477372`, 0, 158.39462522943813`, 10.074722783087664`, 10.18136688095561`, 0.010585313379239514`,
  0.05640904450489068`, 0.030525584432065666`, -0.45885301373223786`, 147.05760536907627`, 0.19256939957878094`,
  3.136581758819522`, 0.7479532175562487`, 0.00042276542199998137`, -0.9994347702341849`, 0.39816698001393314`},
```

```
{0.0664141372741357`, 2.373389311008811`, 6.163630213717491`, 0.7749555716943826`, 0.011151563708291068`,
  0.26661102099162604`, 0.06978956594786417`, 0.06307180805406282`, 3305.773752462622`, 398.92218816248055`,
  0.15362130283700076`, 6.399110469992867`, 0.5018401596166369`, 76.69973833472842`, 15.043431032741319`,
  88.7735203794044`, 0, 40.705580932377195`, 1.0305621601897377`, 1.0414741042711233`, 0.010588341492546727`,
  0.010968081447207760`, 0.009458740096032462`, -0.1376121574625565`, 34.9417887903496`, 0.010406223812472432`,
  0.8011294790852977`, 0.26244523348637927`, 0.00014779212432952486`, -0.9994368648941868`, 0.15239697024224924`},
{0.07724629482189549`, 2.6662607156107567`, 3.4543142766524646`, 1.1527116922070777`, 0.6194059290185789`,
  0.17356203127759262`, 0.12523844349086252`, 0.006404139963734165`, 681.0439615527839`, 333.4912680980369`,
  0.21335410013399275`, 5.078097228348419`, 1.1402121008441597`, 134.63516096100602`, 80.8589206376935`,
  143.05860274056351`, 0, 122.68401894003583`, 0.2818898719112805`, 0.28869747269847884`, 0.02414985945057624`,
  0.3824013180222748`, 0.055496140555705946`, -0.8548746096307301`, 10.737027022937102`, 0.42198692788898884`,
  2.2536397968952913`, 0.9962544647797801`, 0.0005833231693345673`, -0.9994144837589626`, 0.20051785526607974`},
{0.2790792381180839`, 1.4178392715488872`, 7.586791613535141`, 1.4555087381759333`, 0.0017991755342297022`,
  0.24850929517479325`, 0.05953650221890522`, 0.3815756253123188`, 2337.3041910345446`, 253.88772622846432`,
  0.0832002701070979`, 3.446598610267717`, 0.1790778319506987`, 45.2738571448028`, 10.923741376838823`,
  71.41874840985471`, 0, 7.339997156404705`, 1.78050270610873`, 1.7998638365210602`, 0.010873968540404011`,
  0.017956811499293`, 0.015793575056346383`, -0.12046885066604329`, 36.06380942600037`, 0.07159104674636423`,
  0.40509720348447775`, 0.2325033510625859`, 0.0001315867693315436`, -0.9994340435579523`, 0.09185977081879727`},
{0.11080721322368536`, 2.0431071845443514`, 5.511023662772233`, 1.3452026921196156`, 0.11486345745135074`,
  0.24454012333479547`, 0.160320934401384`, 0.03396351864461054`, 2929.9466481691034`, 234.80413085200098`,
  0.15939227470850165`, 4.957656716995926`, 0.9862870867437366`, 129.2194958871509`, 13.629380746229321`,
  148.01402385622634`, 0, 87.21503483446641`, 1.3857041484849697`, 1.3984156099573715`, 0.009173286726679386`,
  0.06338208486928071`, 0.03991017329991776`, -0.37032406898213355`, 40.444887163179324`, 0.10033131703807471`,
  1.03629019969`, 0.6350832206076251`, 0.0003594453811473816`, -0.9994340184569772`, 0.11519161199293759`},
{0.2269507001135535`, 1.5304419335739787`, 3.489177129657852`, 0.8382349859440477`, 0.3185553593952979`,
  0.48806107220778994`, 0.1765391455298561`, 0.025680745838524602`, 3051.647362946771`, 356.25991793699154`,
  0.11555959688444262`, 5.191309509924922`, 1.4793748585613802`, 114.9869306173817`, 22.208556334987506`,
  122.09184633448908`, 0, 84.22548448368622`, 1.330896808023026`, 1.349862958702091`, 0.01425065456970942`,
  0.07386344984896555`, 0.033365879223691546`, -0.5482761867754972`, 29.098004060831336`, 0.23947659508607577`,
  1.32460574622781`, 0.843372166700586`, 0.00048246091245260277`, -0.9994279383035131`, 0.22358264970456254`},
{0.08130128725263741`, 1.139181654994668`, 8.356523528104287`, 1.0901388323360164`, 0.43395806919787105`,
```

0.6945552692578585`, 0.15512850330366812`, 0.013156059374009372`, 1072.341203797791`, 380.15089775887463`,
0.2428753710582479`, 6.517091771782727`, 0.9223679840610632`, 152.5326956462683`, 110.34379821834334`,
151.24319358267647`, 0, 128.65220422012726`, 1.3748510233013012`, 1.397015935004058`, 0.016121682514760316`,
0.0240283321547026`, 0.019163308976550705`, -0.20247028161710168`, 22.374358058506992`, 0.027907633353007066`,
2.3485886076878875`, 0.376176052556508`, 0.00021286147184173033`, -0.9994341440121052`, 0.4971575771589105`},
{0.11639393264339765`, 3.920728456674687`, 7.161326334970468`, 1.2230707245468682`, 0.4475871708897683`,
0.6126582241159237`, 0.06411943719695506`, 0.012576022333314589`, 2883.638838241297`, 313.1192616144217`,
0.21454888313615755`, 9.840193495130112`, 0.5865096618206191`, 200.61378248401098`, 59.03884613896867`,
214.3164178288007`, 0, 170.19270375380628`, 0.5304078405232723`, 0.5359150696521167`, 0.010383008523047632`,
0.027544583881073424`, 0.02185306804862683`, -0.2066292181802527`, 29.70835877118515`, 0.045800320584927015`,
1.9100360953809816`, 0.38311820144559405`, 0.0002163601409824355`, -0.9994352653041123`, 0.317362328213779`},
{0.15100737716165968`, 3.554370686110622`, 4.587082662015709`, 1.0501596960456492`, 0.32405591591236504`,
0.16453196956775862`, 0.1064831105262615`, 0.33127320693075046`, 902.0301791506135`, 373.05242738162826`,
0.0458010200387457`, 5.548602584093444`, 0.22333879786319444`, 93.11223916497407`, 50.57988521650585`,
106.44337586366683`, 0, 17.414912662619432`, 1.459971455085928`, 1.4846304053902089`, 0.01689002221131064`,
0.03318943844356009`, 0.02409961288019622`, -0.2738770521478231`, 74.13256775022421`, 0.07159785784039356`,
1.463775042898485`, 0.4924032216008243`, 0.0002780354525466684`, -0.9994353500538791`, 0.2007292747558256`},
{0.06860802282414658`, 3.9707655631721437`, 5.6945082231101`, 1.3132775124268627`, 0.833710937956144`,
0.4055938689007862`, 0.09163259509867216`, 0.3768554443970733`, 2744.3744850106896`, 240.03583759728133`,
0.035389680502848464`, 1.2976415449147964`, 1.3504035222444002`, 48.17178301306154`, 10.732593165883886`,
50.24327609539747`, 0, 7.864616338968582`, 0.6939682965014727`, 0.7037766397720773`, 0.014133705127527652`,
0.12508996273126705`, 0.053629407396106776`, -0.5712732962330498`, 39.36550590973261`, 0.12260250025906574`,
0.42509354411635575`, 0.8651363428768719`, 0.0004954448836988546`, -0.9994273216149361`, 0.032226309054989064`},
{0.05446295287328684`, 1.6696297250378036`, 9.103903568630106`, 0.7607067601908715`, 0.16998909961411246`,
0.4015279687740475`, 0.15306183541311225`, 0.23729003810245272`, 1974.2834194249153`, 302.21506503791556`,
0.16971647699912568`, 4.841445731177878`, 1.0533590220242193`, 106.6162887708526`, 25.291045063795572`,
114.70353741515794`, 0, 25.29749152958546`, 3.2711423191529985`, 3.3128044888201718`, 0.01273627546659628`,
0.013975189954095208`, 0.01154988424044231`, -0.1735436671429429`, 78.02280644124203`, 0.010873287312378991`,
1.3672072951192302`, 0.32615458941994346`, 0.000183837640048017`, -0.9994363481428393`, 0.20467894881402957`},
{0.07612701991269089`, 1.8994519379164023`, 6.640792134140813`, 1.254560879057461`, 0.12469411598778724`,
0.4203442966564226`, 0.10535108099169138`, 0.03365653592102131`, 2960.4487643491357`, 231.61182906412773`,

0.044585724001347915`, 3.1581032850809603`, 1.1020659040461283`, 77.34900912889589`, 10.711951273978247`,
84.60541174545325`, 0, 52.44871053176951`, 0.8818387852485904`, 0.8926477837408123`, 0.012257340766855584`,
0.03998493535159693`, 0.029025727277744779`, -0.27408342610748904`, 23.928719851003972`, 0.043484770995987104`,
0.8752606009508528`, 0.49414613530005624`, 0.00028029937605035483`, -0.9994327601573163`, 0.09479241722730752`},
{0.05971802623282291`, 2.1738091632492136`, 8.683813711430187`, 0.7864060587963683`, 0.5208725203898663`,
0.5102800946784368`, 0.10321808603446814`, 0.07803262912760096`, 1116.648121119908`, 221.4014904292125 7`,
0.07733870760092837`, 7.219382522940123`, 0.8997811624944188`, 259.76612789938434`, 109.44321601755571`,
266.3850486164721`, 0, 124.47702197175639`, 4.218864052670972`, 4.271824780846809`, 0.01255331471093668`,
0.029949578698962493`, 0.02045401724474919`, -0.3170515869240673`, 131.01436194569837`, 0.02555042466245734`,
3.215841905218052`, 0.5581605154127571`, 0.00031490935403926556`, -0.9994358086153651`, 0.28897089276393145`},
{0.25957147156308197`, 2.9787560040757644`, 6.8514986634875745`, 1.2875526348358215`, 0.5271393050674782`,
0.38366555469751695`, 0.09532745198151626`, 0.00993086596705298`, 3516.3450563644556`, 352.0852345112979`,
0.03547806916020174`, 5.814047961801249`, 0.6954919748641395`, 119.1102270446553`, 23.99619164739186`,
130.84769376602964`, 0, 104.22079754785383`, 0.33663989091284274`, 0.3397793852105024`, 0.009325972299796259`,
0.03086299189496562`, 0.02414672922122104`, -0.2176154112537657`, 14.325258518114882`, 0.11444503175735413`,
0.8815185534898345`, 0.401985216830939`, 0.00022724715632355672`, -0.9994346877775381`, 0.12453581297613055`},
{0.07058107847879669`, 1.480448796537483`, 8.054675740834305`, 1.3102914246603354`, 0.04592614768203762`,
0.23013975911080142`, 0.1465551863327878`, 0.10936312847944579`, 1149.9199910201796`, 342.3645344528402`,
0.16288013724428108`, 8.990901684554295`, 0.299529341502206`, 149.02404763577906`, 59.711986962178166`,
175.18511488405403`, 0, 59.368158486058704`, 4.046005647190975`, 4.102369000661506`, 0.013930616609411528`,
0.01982293345276771`, 0.01692376768248592`, -0.1462531152207951`, 85.57005987382486`, 0.019987486024416804`,
2.067555231436397`, 0.2778109969522842`, 0.00015639347768932514`, -0.9994370508028658`, 0.38155908247325704`},
{0.12936768312046054`, 1.4931741189869152`, 8.533444012750714`, 1.3526677837265888`, 0.34592179565846926`,
0.5020921142394136`, 0.1702518668235985`, 0.4026569331476235`, 2469.5593778381244`, 155.39036984038785`,
0.09801542749760322`, 2.524614796651056`, 1.2701205529096362`, 122.37163296102538`, 13.679507756560152`,
133.95305015469165`, 0, 18.893349123846807`, 4.622307566715786`, 4.668319678952531`, 0.009954359715928174`,
0.09032713244012351`, 0.048182387123563916`, -0.4665790242427623`, 98.59871469453421`, 0.16693445495280157`,
0.976938879024579`, 0.7577220196455521`, 0.00043169287519726184`, -0.9994302753991507`, 0.07641939741250357`},
{0.10764603393952993`, 1.2107066619029974`, 4.216520809210639`, 1.0534038454487438`, 0.2705194689594228`,
0.49749815429385036`, 0.21553478853143437`, 0.04863912466742637`, 3262.748108283069`, 369.9540993093576`,
0.034536512984739975`, 2.033964341592309`, 0.4918523584430632`, 38.73557098540996`, 7.667257911778504`,

46.33530198371953`, 0, 22.883272053295805`, 0.8601240844708467`, 0.8679928551544904`, 0.0091484133809423`, 0.04524127974493137`, 0.029811332792436505`, −0.34105902926460807`, 14.876542273315287`, 0.06957206192700804`, 0.2420870797180119`, 0.5955719471583107`, 0.0003432621920865096`, −0.9994236427794755`, 0.07903870085315859`},
{0.16173577525354788`, 1.3192464264843462`, 3.8155846300226686`, 1.0837420201848613`, 0.1916516348586692`, 0.5385828520739745`, 0.08577245096999242`, 0.02449629028204095`, 2335.082397263821`, 344.7269386138737`, 0.14212635984223515`, 7.933933586100813`, 1.1885980026661422`, 141.1313235429736`, 41.76806750456974`, 146.00258320237302`, 0, 104.98932805432048`, 1.811468710390653`, 1.843434565391427`, 0.01764637435767824`, 0.04969801804031134`, 0.03169566275919544`, −0.36223487356203476`, 34.13962318387248`, 0.1148278210902116 3`, 2.3217757251457196`, 0.6236798254340463`, 0.0003543132186950921`, −0.9994318988618102`, 0.34559827357872286`},
{0.2639188435968321`, 3.196402084598291`, 5.8733763940010615`, 1.001723636198023`, 0.8720114445540239`, 0.5139297111136746`, 0.13829965727943472`, 0.009850038986012472`, 3721.325240000034`, 357.3898095475904`, 0.10886509504864544`, 4.342058559354168`, 0.9364053798077125`, 146.17366910357427`, 35.38474830899742`, 153.46771876073038`, 0, 127.86040666890864`, 0.3832920538305002`, 0.38642068065963614`, 0.008162514192166181`, 0.06838918834736762`, 0.03600361903496605`, −0.47354808698571327`, 17.50222171248242`, 0.2578456500451985`, 0.7561106684932906`, 0.7661783240363065`, 0.00043561139412735806`, −0.9994314490759377`, 0.08628385885456667`},
{0.21291226343526198`, 2.8179245900150987`, 9.921791649942335`, 1.3352776575768062`, 0.9588380301695891`, 0.6936227079381379`, 0.0795524915712694`, 0.4777153216598742`, 2688.6717707047637`, 396.8627131234574`, 0.23818107150054235`, 1.6319345031256631`, 1.1969051802794537`, 39.695719598557005`, 28.59350696174632`, 39.86075856634459`, 0, 5.4338143160380605`, 0.828046530719587`, 0.8392090354795667`, 0.013480528383204904`, 0.02473357670752947`, 0.02050868503038198`, −0.17081604197832556`, 33.33389543702021`, 0.07522973999498843`, 0.4286257151743758`, 0.32637599017523933`, 0.00018632213155109686`, −0.9994291181423883`, 0.055596655191412665`},
{0.08637419772835031`, 2.5843437924117936`, 9.303756737243045`, 0.9248602316926738`, 0.9539236366945725`, 0.30709475006465425`, 0.15355620403975512`, 0.05658228330400466`, 3850.542613273592`, 202.84343087793332`, 0.1503135323154285`, 6.227303197646734`, 1.09139924606997 1`, 423.817311194808`, 40.51484061703827`, 465.797970841371`, 0, 234.72107727946485`, 4.976164802953811`, 4.99605376300193`, 0.003996845127860915`, 0.17896755833776853`, 0.04246414604575381`, −0.7627271308825117`, 183.7160088361679`, 0.22083113244046038`, 1.2855269224053072`, 0.9821102518765922`, 0.0005559476852232277`, −0.9994339253824498`, 0.056994122445724056`},
{0.09166372218259045`, 1.1041359694939814`, 7.465464940650059`, 0.8285398481134653`, 0.01938900145906275`, 0.6417891529291755`, 0.14382119055783732`, 0.023209894299314995`, 3535.4335241265126`, 296.7460220437812`, 0.08660924945570686`, 6.289282036011649`, 0.7877277513120826`, 121.58629415800685`, 20.604575529871035`, 136.84864840017167`, 0, 91.53730052788896`, 1.7876678507252766`, 1.8049925599282546`, 0.009691234977431185`,

0.016548441019941084`, 0.013466420199548142`, -0.1862423666784725`, 28.1975482213396`, 0.021669881431523638`,
1.1562735995108357`, 0.3484902248921473`, 0.00019680688301558277`, -0.9994352585267593`, 0.24623723529923033`},
{0.06057989660476104`, 0.9664690813204819`, 5.824893628794225`, 1.1149397320321297`, 0.7552676734434385`,
0.19045278243945962`, 0.155017124958911 8`, 0.20977491640661988`, 1881.802129389779`, 160.06890201789201`,
0.2135476446394321`, 7.516581972853506`, 0.39758727016160034`, 326.76819019437795`, 53.48002631771335`,
563.8774975526519`, 0, 82.48319376880069`, 16.442732589714506`, 16.54726581923755`, 0.006357412245968863`,
0.44084677397124866`, 0.05222475874654695`, -0.8815353500807223`, 227.01989514828287`, 0.38152074265308444`,
1.2475093018063732`, 0.9997320241298281`, 0.0005672114225070792`, -0.9994326365377755`, 0.08246572255174814`},
{0.22678054201622128`, 1.767805615425421`, 3.537786208995465`, 1.13577377357013`, 0.9133787654245775`,
0.32400922076880223`, 0.1547055518280115`, 0.1014618010767026`, 3393.1210765994138`, 223.6969184131283`,
0.01741141735821 8523`, 7.612407230565935`, 0.356714236328328`, 206.72300194940212`, 54.123458758898`,
495.09349774879837`, 0, 82.83515462653342`, 4.532023403963828`, 4.659319101014326`, 0.028088049355429412`,
1.15630480119321`, 0.05467175029301487`, -0.9527185650041423`, 114.45337746809514`, 3.7461061364366857`,
0.7460463630662024`, 0.999999989669764`, 0.000583376321337313`, -0.9994166236726363`, 0.12279614383970021`},
{0.17952419657415108`, 2.390230853640494`, 9.385595862371645`, 1.2510854246897463`, 0.45025706246945174`,
0.39156066611114926`, 0.09061933973189976`, 0.007387745401806454`, 1060.2070144427744`, 157.30384712261866`,
0.017455537733710474`, 7.390923217745433`, 0.4592263583377443`, 316.04590254159956`, 94.1551629350242 1`,
350.8345926617623`, 0, 285.7021192901003`, 0.8499098505637035`, 0.8584441978139002`, 0.010041473509850851`,
0.05544560102288391`, 0.03577045873874697`, -0.35485488336606663`, 29.021153537576375`, 0.1421975282457806`,
2.7011820088441665`, 0.6129630688626657`, 0.0003462607106974939`, -0.9994351034698714`, 0.21541784207423098`},
{0.17085484316695765`, 3.896251009939033`, 5.375947987134143`, 1.4639220165256899`, 0.9541878933608088`,
0.33462154790480914`, 0.21681254773335146`, 0.0069834922249469925`, 3376.9159214269603`, 211.30439423571625`,
0.23015330461401873`, 6.0128254119306135`, 1.4535070849014953`, 248.87550430260364`, 47.682478564226436`,
549.1974995348337`, 0, 222.5886317103122`, 0.38896076909329935`, 0.3984511119179801`, 0.024399228865171985`,
1.1703121088999258`, 0.07069113594286447`, -0.9395963389549878`, 21.649839848663273`, 2.8564784546069824`,
1.6797922499892244`, 0.999999647223783`, 0.0005851431067870766`, -0.9994148566867883`, 0.1628767510402205`},
{0.10845833265331112`, 2.205454065801659`, 8.04431016182664`, 0.9250832587897069`, 0.5103893648573781`,
0.6279318303072325`, 0.0552625938696442 7`, 0.03668517084277843`, 3275.5705391236816`, 196.31559723816565`,
0.1402871557364922`, 7.884261458348763`, 1.2767651696233133`, 257.26411045844145`, 45.48048644104072`,
269.39280447798075`, 0, 169.7630073997532`, 2.6886450264800974`, 2.7214205527589272`, 0.012190350885307621`,
0.034076146575994906`, 0.023552228136428235`, -0.3088353436940634`, 84.70975864497068`, 0.05279774344112389`,

```
3.029712746645581`, 0.5464600813390137`, 0.00030827831140441435`, -0.9994358630722869`, 0.16738158008872658`},
{0.1701034994591067`, 3.2342889044936154`, 4.148994458467392`, 1.1940097836786847`, 0.6849042746613065`,
0.28365434424986613`, 0.24647764422374546`, 0.16243634058501621`, 3080.6851495717774`, 370.74406137436483`,
0.0951116860303981`, 9.02678426482985`, 1.2346863975759255`, 284.067932267944`, 44.79014567611737`,
344.99778653474607`, 0, 86.27915741567064`, 4.15771562777846`, 4.205536490594899`, 0.011501715628875475`,
0.4453104866874272`, 0.056817130847939004`, -0.8724100766847196`, 192.1036217566222`, 1.082126744733652`,
1.714443235230245`, 0.9987052919138186`, 0.0005764476650480504`, -0.9994228050359647`, 0.2280856689028294`},
{0.06598975858427436`, 1.9097424631845499`, 9.748507937422985`, 0.8697722369253212`, 0.5875961327091277`,
0.6558947995694533`, 0.20328697625166237`, 0.09192814399763598`, 849.932255943002`, 347.93153422311434`,
0.24798460225057473`, 8.434300711569456`, 1.1653187620032228`, 281.04688652192687`, 214.65463278515017`,
278.2337311050637`, 0, 123.48538360007609`, 5.5693584648174355`, 5.6414958636364005`, 0.012952550868231771`,
0.02501302594354141`, 0.018662259895537846`, -0.2538983512965727`, 151.9434336136881`, 0.023580050621081722`,
3.609604752311777`, 0.4604818470349712`, 0.0002597796576266605`, -0.9994358525546677`, 0.5551245825653957`},
{0.24510065816515997`, 2.793418227932695`, 5.458971845554931`, 1.0315954512213719`, 0.7555698096939896`,
0.6374757156959183`, 0.22964026454409542`, 0.029915287766295595`, 3671.7453130653475`, 246.1074690301475`,
0.07031820961864549`, 2.73413282232541`, 0.7379537484131258`, 114.39531228930815`, 20.352772396357178`,
159.53065523669855`, 0, 79.11185721778907`, 0.8219948660815268`, 0.8373031485172793`, 0.018623330956709427`,
0.3657040291866806`, 0.04954078759975747`, -0.8645331097118746`, 32.80250631684606`, 1.2804899749615761`,
0.4343266810229741`, 0.9994975285815707`, 0.0005824938676495848`, -0.9994172132986899`, 0.07109293321880887`},
{0.20946691214847096`, 2.8105866650441875`, 8.539169469770648`, 1.3728902130481506`, 0.8085940550353596`,
0.2919084226376192`, 0.2093509791170406`, 0.014346546703503377`, 2081.7407715487734`, 169.23962157045707`,
0.03811656206153746`, 8.571585131217248`, 0.31122812087193474`, 544.8802849212221`, 76.67249749719875`,
770.6220085188266`, 0, 450.9837298483141`, 2.2401805948837388`, 2.2521549056034713`, 0.005345243480405104`,
0.3653811605296144`, 0.06413264847034654`, -0.8244774077092885`, 89.94602438958567`, 1.0933609064766412`,
1.1822151612039848`, 0.9961421098812594`, 0.0005657673891679486`, -0.9994320414893058`, 0.08189520851018493`},
{0.07543371305305874`, 3.820737964468253`, 7.006759966869079`, 1.4837946743911272`, 0.9807341557825149`,
0.31098343284147656`, 0.07258154131712924`, 0.2086007075373027`, 2270.591159595845`, 320.3654657559956`,
0.09813455813826927`, 7.706001493171778`, 0.25408983910049043`, 200.74614660513356`, 91.99608601819017`,
240.56796554553745`, 0, 51.171659875787014`, 2.689266172056638`, 2.7042499916163867`, 0.005571713099819187`,
0.04802241221890318`, 0.03462120889146597`, -0.27906143627999724`, 146.78544800195758`, 0.05175012662033418`,
1.013222533030606`, 0.5004187178848359`, 0.0002825624718282701`, -0.9994353479161958`, 0.13294168007382942`},
```

```
{0.19210055303112722`, 1.6234167219204663`, 6.9088164888327235`, 1.007010553449088`, 0.1818811973938239`,
  0.28265941449906173`, 0.05224493104477926`, 0.18359878228489843`, 3879.878639699238`, 252.77022062868934`,
  0.19702879614057178`, 8.129123272011793`, 1.4896800872011862`, 173.2806225291551`, 19.03652197082293`,
  184.16066934427622`, 0, 49.52899667969385`, 5.111562851386999`, 5.185693307845138`, 0.014502503170439107`,
  0.0252109329657725`, 0.019497405253717216`, -0.2266289676709794`, 118.54566582984432`, 0.06918620235931183`,
  2.426308488181294`, 0.4164406067641644`, 0.00023448562586367354`, -0.9994369290072702`, 0.1370074213179641`},
{0.25397209407460797`, 0.7718183137480485`, 8.774125702249133`, 0.8493367748387377`, 0.629933366847081`,
  0.44793398136834006`, 0.1722010399919054`, 0.007968172925746744`, 1049.3506020599693`, 93.83027794564788`,
  0.034976329114162485`, 3.5404695212710173`, 1.4516591802815526`, 294.3388306777891`, 60.19799149869615`,
  406.8031280893948`, 0, 214.66570341189248`, 2.0035352210880637`, 2.5013163619877576`, 0.2484514051264659`,
  11.965733075646673`, 0.050482525811284124`, -0.9957810753848396`, 22.090931083928762`, 43.41374694799691`,
  2.9822821757217852`, 0.9999999990818431`, 0.0007201060180971669`, -0.9992798939812416`, 0.15040803952049886`},
{0.045030174726766375`, 2.4891362382491593`, 7.362757592309742`, 1.3044006364876828`, 0.9149255196562065`,
  0.40547780792622723`, 0.18724836348467627`, 0.040102604909543316`, 2100.7064218407268`, 212.58570231059025`,
  0.17762315543370177`, 6.925880601095635`, 0.30534617107023765`, 381.12454358349663`, 93.88691440261263`,
  535.1471259175289`, 0, 242.2781944853812`, 3.781109238133036`, 3.7970519606146946`, 0.004216414146640757`,
  0.36229665144791307`, 0.06118866756121899`, -0.8311089343037552`, 134.4528003630804`, 0.23306116453738723`,
  0.9901157536712821`, 0.9969366253660104`, 0.0005680218043955796`, -0.9994302327851712`, 0.09988552287935766`},
{0.2769245298620152`, 1.0085946471087137`, 4.542179183090196`, 1.0124324963510387`, 0.5561370973292907`,
  0.2204460879068223`, 0.09699841861743363`, 0.4013041825941674`, 2543.8306625681216`, 129.04963561861496`,
  0.06920814067274877`, 7.689149017779954`, 0.3201001440714819`, 268.7835775275385`, 34.76856413855763`,
  489.8447798532344`, 0, 39.48041606556726`, 14.327881043933019`, 14.853024649324908`, 0.03665186804536291`,
  1.7215430144432875`, 0.04887175133474327`, -0.9716116583061113`, 206.44320179030396`, 6.8105355701705435`,
  0.9738292368550245`, 0.9999999271509146`, 0.0005848894423213907`, -0.9994151105150699`, 0.08096121948365888`},
{0.09132185718513763`, 1.6890298787004205`, 2.765293267846525`, 1.131503269745704`, 0.5764776999808194`,
  0.5819767311690106`, 0.1370413367124192`, 0.18256196348098624`, 2411.1512688147986`, 324.527748891113`,
  0.027976143287822552`, 9.606504565586533`, 1.4766385859888795`, 207.73535091443463`, 79.10351757707278`,
  284.5148467634269`, 0, 59.222174315703256`, 5.830405890779882`, 5.975545867715133`, 0.024893631704916652`,
  0.8682280756579587`, 0.0555156265885948`, -0.93605870606462`, 140.68185363540246`, 1.1326885761335859`,
  3.0647099169539107`, 0.9999881124440917`, 0.0005943159836137412`, -0.9994056769513378`, 0.49807537322721734`},
{0.2668975721045642`, 1.1689144747314444`, 8.555782168057934`, 1.3239106462159618`, 0.5762520317144466`,
```

0.46812793372285433`, 0.21778203282682917`, 0.19497129015379866`, 2321.6608050102914`, 177.91119737865392`,
0.06980210378448115`, 6.741021070773617`, 0.3841763241252334`, 347.09439393884264`, 50.24404376011543`,
440.6752532245899`, 0, 92.29196981332437`, 14.368827198019968`, 14.43032649600987`, 0.004280049940219133`,
0.10217255458072974`, 0.04998537212418332`, -0.510774959779553`, 239.94185852400517`, 0.3895658107615839`,
0.9953987384946648`, 0.8058545989514097`, 0.00045737854447352O3`, -0.9994324304346359`, 0.11140338006705798`},
{0.23978880653880486`, 1.194922909654343`, 4.719390315894781`, 1.228676410453662`, 0.2690436202211013`,
0.15286621224443908`, 0.10420745578996382`, 0.13476606932746013`, 2057.7110605479693`, 394.7187402510973`,
0.20560140619917516`, 7.632899208080902`, 0.8870899800709835`, 123.77284417676921`, 28.8845783438613`,
134.44511290355854`, 0, 43.254665029396115`, 4.447115738039381`, 4.5079793742259495`, 0.013686092238607062`,
0.03555287351282301`, 0.026478859182182457`, -0.2552259053650836`, 75.91372110382322`, 0.12178830155227205`,
1.5433130016881986`, 0.4630450837712684`, 0.000261134070430057`, -0.9994360504417772`, 0.17692918354204298`},
{0.14115594725086872`, 1.0548987126390301`, 4.221995326207644`, 1.1742416630307742`, 0.9684670314689854`,
0.2982166752619343`, 0.232586446320313`, 0.2439177749163077`, 3034.8452920870423`, 398.0846179891778`,
0.1346647166413732`, 6.263806688157171`, 1.2365678782206118`, 200.50309095206626`, 52.22570303168065`,
328.815773791622`, 0, 45.320963120796414`, 9.571749407625127`, 9.694674553877856`, 0.012842495244892671`,
0.4522740677433973`, 0.055844525243675176`, -0.8765250337647942`, 144.24608754010285`, 0.9120167778470236`,
1.180566486312421`, 0.9994690214194936`, 0.0005762070530892668`, -0.9994234868307665`, 0.15520660155586272`},
{0.1729303989394022`, 1.7633209846264775`, 4.719997292494861`, 1.358984906012456`, 0.8429652151291751`,
0.587790420447317`, 0.06264894796386378`, 0.017353544405872348`, 3524.820728982824`, 361.1436357327882`,
0.017184717238444003`, 5.167166487729965`, 1.3598724575284078`, 116.87278149032608`, 46.1853408772872`,
118.91741297896097`, 0, 93.71210617242042`, 0.874114273012942`, 0.8865037369706231`, 0.014173734876764543`,
0.06512054621745815`, 0.04083476467163246`, -0.3729357776688139`, 22.019200579503384`, 0.16087602909341586`,
1.3688189956829997`, 0.6402120078512423`, 0.0003641939843428482`, -0.9994311353428605`, 0.13375996535122836`},
{0.1806934944296955`, 0.9977416627132909`, 2.873305835487411`, 0.9197375741494748`, 0.03558129425516987`,
0.4888154998532678`, 0.06242566822825624`, 0.4327688666695781`, 577.8384758076331`, 373.1652197475112`,
0.03824901809490494`, 6.041786978778951`, 0.10163020529956279`, 101.0251138026971`, 106.76029225746541`,
88.85192519193521`, 0, 16.116397864735067`, 5.551417533391013`, 5.700971012782686`, 0.0269396921582874`,
0.06434283016227418`, 0.03356233457355342`, -0.47838268088443736`, 79.12686514544671`, 0.1660904403358789`,
2.612607177946049`, 0.7663314655304104`, 0.0004422783461003842`, -0.9994228628654909`, 1.0177143203971561`},
{0.05413140542514305`, 2.0680722669723384`, 4.8344183696420355`, 0.8194669559280681`, 0.2769337929060782`,
0.48015527013462056`, 0.18187780015894756`, 0.00828099754016471 9`, 3109.753891098221`, 77.01250512455277`,

0.1133305062638244`, 4.98724945291187`, 0.14526723437594202`, 145.33964731728278`, 19.892758804441684`,
529.8978181816296`, 0, 129.80329006347299`, 0.5011615173845291`, 0.5033685364807423`, 0.004403807993341724`,
0.0633084939154804`, 0.03100052855557445`, -0.5103259193472566`, 14.806260505381747`, 0.04895682501421353`,
0.4084953867176612`, 0.8040976471284902`, 0.00045813383935164954`, -0.99943025098881`, 0.09690840559387486`},
{0.04855602650750329`, 0.6081046972703708`, 9.327171063477682`, 0.9416092665673466`, 0.16765715967906902`,
0.2590243352245839`, 0.2187512491754251`, 0.05460948171436536`, 1404.1776553137433`, 151.91892927077328`,
0.22495478161340088`, 3.6643606235348707`, 1.0355618722545916`, 168.2102695871551`, 22.136995559785266`,
192.15770338954843`, 0, 95.05309186934275`, 7.543364095351194`, 7.609756481240238`, 0.008801429315861808`,
0.047731625076735104`, 0.029085132879497873`, -0.3906527835006759`, 65.53078770862444`, 0.03310940074976717`,
1.442120441999167`, 0.6622030767735718`, 0.0003740399149169005`, -0.9994351582950365`, 0.11158236293162235`},
{0.25495798287734545`, 3.119749211986244`, 7.11103782773306`, 1.024996658386143`, 0.715060033499787`,
0.6413092223890626`, 0.1635833958172549`, 0.00714360523367534`, 2176.4539355050265`, 202.8994026859108`,
0.2178929688734338`, 6.062772582072755`, 0.23204754406386696`, 266.16918460703795`, 72.43412814793733`,
350.26551456861483`, 0, 241.02963612583238`, 0.5379338751320272`, 0.5408997625599764`, 0.005513479565162793`,
0.0841565552560957 1`, 0.03970071342104835`, -0.5282516816398244`, 23.97455404348352`, 0.30651979391430445`,
0.832914437613221`, 0.8225470737860561`, 0.0004692098273672307`, -0.9994295647722536`, 0.14546655923574323`},
{0.13839247434786828`, 2.7262203960757274`, 9.158826513860994`, 1.4613001862440893`, 0.08417876673230018`,
0.5490480248818964`, 0.16559733844524815`, 0.07310698628337725`, 1278.2933939526329`, 222.03326298760976`,
0.23267447938843522`, 2.718547094516405`, 0.8865837550934321`, 77.63152970470998`, 23.493469554418432`,
83.30691675757029`, 0, 38.44838585766494`, 0.9781501294840098`, 0.9928024265247295`, 0.014979599346829353`,
0.03687462560826226`, 0.02857248036204663`, -0.22514520782972836`, 38.09504047747739`, 0.07290243826542958`,
1.0907902873843631`, 0.4156707175840261`, 0.0002369600007713983`, -0.9994299333805646`, 0.16758682369281683`},
{0.21091599252169352`, 2.726329060939385`, 6.86461568422051`, 1.1885210188613522`, 0.09348887154977148`,
0.3177710803820454`, 0.16213356559574865`, 0.08883572559161597`, 2040.1598914696724`, 141.3083228416025`,
0.09021913069336979`, 2.444490167264778`, 1.4613873909313537`, 106.07179738659735`, 10.313598055484002`,
117.32788910338819`, 0, 47.17268587818817`, 1.4640167407717424`, 1.4850776240126695`, 0.014385684708649649`,
0.10345160428570739`, 0.04708159410619142`, -0.5448925666134297`, 57.01987694382506`, 0.3117085399411921`,
1.1394850586809073`, 0.8411555608638477`, 0.0004804066355018473 3`, -0.9994288730196249`, 0.08090625663138402`},
{0.0736068126313521`, 3.668322450041937`, 9.040034492942976`, 0.8406840844419607`, 0.9489073810268334`,
0.18492930812213693`, 0.07953103883609952`, 0.21231255359096723`, 2489.458988929943`, 66.10862660280799`,
0.025206551238390773`, 7.73776692806604`, 0.6102159281560899`, 527.6390476604055`, 53.58273493760965`,

1197.2554242779117`, 0, 130.07552801973367`, 7.292005603881221`, 7.4302545522521255`, 0.0189589745100196`,
3.9662253936053764`, 0.0398052150425525`, -0.9899639553751209`, 382.13468375070147`, 4.170588705726324`,
1.6813062361469062`, 0.9999999981356724`, 0.0005732773568574784`, -0.9994267226420738`, 0.04605618099232648`},
{0.24859051354510608`, 1.2059184673508376`, 9.814154373421665`, 0.999806271433672`, 0.5018863929556321`,
0.5028910758235472`, 0.0547445799608962 7`, 0.010244417191277673`, 1992.5389939670486`, 81.33759966126644`,
0.05634540009721645`, 5.237660071402415`, 0.5456697726165687`, 359.03779584007026`, 42.75039861002982`,
422.3546679402722`, 0, 312.70080390462664`, 2.496566250909809`, 2.514708876900084`, 0.007267031661452261`,
0.1294350017116797`, 0.0438175115645659 7`, -0.6614709237446392`, 43.00936209909972`, 0.459661622088847`,
1.76047056795387`, 0.9347119223320315`, 0.000530839910687591`, -0.9994320818018848`, 0.07435502011085154`},
{0.23828594722598612`, 2.796383207017321`, 7.808028439446116`, 0.842565467286883`, 0.02113404997076329`,
0.6079237099359636`, 0.11031479903153951`, 0.30382867676630676`, 3734.3994148280126`, 266.6055228534507`,
0.0826889096493309`, 9.854969604997791`, 0.9421568133509814`, 207.1236135532621`, 30.022351031643403`,
227.73024064811642`, 0, 40.448610231531305`, 4.068472084895615`, 4.11101964653529`, 0.010457872329426854`,
0.01767894184333095`, 0.014234043986527483`, -0.19485882624264594`, 162.5286716631547`, 0.06018062004414938`,
2.1523646329137347`, 0.362945361078441`, 0.00020457216010941103`, -0.9994363554902547`, 0.3185093908675365`},
{0.25363168051984797`, 1.914628055312483`, 6.622889612666614`, 0.7775608926930352`, 0.30452546646747747`,
0.5186902253252116`, 0.23177406235661663`, 0.009523543254042525`, 2339.914222113791`, 312.39264418675145`,
0.06116037168819993`, 6.862836561311433`, 1.3371389516738463`, 188.2440245542742`, 39.21863083041139`,
203.14371801286788`, 0, 165.68760409609735`, 0.7860031466222447`, 0.7951388058549752`, 0.011622929592572051`,
0.029549203010817947`, 0.020207473371999102`, -0.3161415093124117`, 21.498623944097744`, 0.10706591453793794`,
1.9612532022112958`, 0.5570303246747309`, 0.0003147629585851572`, -0.9994349267092972`, 0.3206814246574988`},
{0.2006523908963831`, 1.4235542095429166`, 8.760081949765812`, 0.898891491102243`, 0.20916515630212307`,
0.606680748263352`, 0.1734284929050921`, 0.008390530446632872`, 1251.1518948117655`, 294.61074552228456`,
0.1942512237263747`, 3.244161217546491`, 0.31687231038469954`, 74.14687090810536`, 33.82710429223264`,
83.1881955781802`, 0, 66.17871318098861`, 0.36740633997159045`, 0.3719595316517079`, 0.012392795618250796`,
0.018083985096763226`, 0.014712258959106295`, -0.18644818161570054`, 7.47175488399018`, 0.05183706923715713`,
0.8098407659814655`, 0.34942257157607504`, 0.00019828772182290815`, -0.9994325274382575`, 0.2338600401357046`},
{0.2188265776716416`, 1.1127955725409375`, 6.7824679934503616`, 1.4242069861528708`, 0.19844568020247255`,
0.5897838311529561`, 0.14866055702548336`, 0.11878670475760818`, 3389.027367705158`, 288.45426002597424`,
0.06947315366014212`, 7.544154049880284`, 0.26623655067609353`, 141.36583388690264`, 28.85256874055941`,
192.12493142008188`, 0, 52.81149167567875`, 5.233282047993112`, 5.264161904531764`, 0.005900667354723277`,

0.03081689908373319`, 0.024684789489070217`, -0.19898528979185415`, 83.19390132663852`, 0.09633652229927259`,
0.7185991526695875`, 0.37060848494769516`, 0.00020997120983665626`, -0.999433441978895`, 0.24517175221933288`},
{0.09304029298502164`, 1.718583593389087`, 3.3604210127127847`, 1.2870363565175618`, 0.488105058647589`,
0.628165881843018`, 0.2385374614927307`, 0.005700260097503202`, 2964.7790422043363`, 284.02452311663967`,
0.049929771885406815`, 9.399697265119372`, 0.1891315432188958`, 239.15529269920245`, 58.48492277542652`,
370.1740051103994`, 0, 220.3329516570372`, 0.6994740835418949`, 0.7048003236436736`, 0.007614635376922640 5`,
0.3037921422248347`, 0.061140325827842255`, -0.7987428990754057`, 17.172924056799513`, 0.403784427416403`,
0.854334017314594`, 0.9905623051871792`, 0.0005754214282363989`, -0.9994190961787833`, 0.3089718331480282`},
{0.1372470868668202`, 0.5235381747902137`, 5.631494663469729`, 0.937247593354749`, 0.03802747675264895`,
0.555726088455389`, 0.17904217377421067`, 0.005332568756650943`, 2773.350295110703`, 213.09690601297643`,
0.061598067845519855`, 6.563446448883308`, 1.299385122835837`, 194.08895057486416`, 25.83224751462325`,
214.1095017988333`, 0, 180.1586243773652`, 1.6000281599088297`, 1.6203315269340306`, 0.012689381058366811`,
0.053488733874589744`, 0.030882898461468`, -0.4226279774377091`, 11.966797463594467`, 0.10487389863545638`,
2.1578377245789704`, 0.7039040696630678`, 0.00039907891336835174`, -0.9994330492881518`, 0.2735870256088968`},
{0.22025109593540232`, 0.8195100379779809`, 7.427283096510775`, 1.0066916952384835`, 0.042788382562981786`,
0.22140131473144997`, 0.09976052534424382`, 0.3169961605914291`, 1982.7909761773044`, 250.35193827375224`,
0.1491805579320653`, 4.623227908408321`, 0.5222292355158549`, 93.17843756201702`, 17.203532073122155`,
109.72938519976313`, 0, 17.548229877992924`, 5.944761246252566`, 6.0063406030089705`, 0.01035859207890355`,
0.02132545237533903`, 0.01713675107290964`, -0.19641793424618026`, 69.59702163837828`, 0.0670993465283761`,
0.8878948722016714`, 0.3656804550711046`, 0.0002062308991629941`, -0.9994360352151638`, 0.10411557663530203`},
{0.18899782585068592`, 2.4715170036459453`, 7.1946744158464355`, 0.9247708579288002`, 0.021153717979273567`,
0.5975157085148236`, 0.06082076754336166`, 0.3491165783662039`, 1598.0596114559885`, 238.85974939012692`,
0.11421989314530723`, 3.1171043316464786`, 1.3648370017589126`, 68.21407742366462`, 21.471628713374148`,
69.10138471175253`, 0, 12.589478716544612`, 1.5297407466689754`, 1.5650011562609214`, 0.023049925073072464`,
0.022624863124545563`, 0.017642212482428643`, -0.22022898501919674`, 54.011146665177414`, 0.06108642772443969`,
1.498820080948371`, 0.40719750647659314`, 0.00023116661735328 1`, -0.9994322985438847`, 0.14764038860385886`},
{0.10674642605203749`, 3.74860921502051`, 5.369830496673435`, 1.1859139787437987`, 0.18962703041335294`,
0.5643238513377844`, 0.22288847642351867`, 0.016526944643235637`, 1801.300442728153`, 341.7838883552571`,
0.21833110166851838`, 9.013661833764864`, 0.39778302570712243`, 191.06619161146133`, 63.074586118666126`,
221.05894457029083`, 0, 154.7329025911597`, 0.6627695645035415`, 0.6689873024099918`, 0.00938144754897996`,
0.04184735648330794`, 0.029378507442589484`, -0.29796025576172347`, 35.49234424190146`, 0.0638150820616939 8`,

1.6598904538269756`, 0.5292776784118809`, 0.00030000991082184303`, -0.9994331710497937`, 0.49209091801107`},
{0.08668509537236802`, 3.2156402873225405`, 9.589080153119365`, 1.1094271452742452`, 0.38296738962607746`,
0.3187881217442111`, 0.09471255988041424`, 0.04715614001519508`, 3816.5009707519202`, 190.70162191213433`,
0.12608220202713782`, 5.338078693011617`, 0.15622194317981242`, 88.52770369451888`, 15.791280807106174`,
200.53660296570206`, 0, 53.02171489993522`, 0.7557509108420968`, 0.7591549329300242`, 0.004504158763281474`,
0.01370603326741516`, 0.012042231878851683`, -0.12139189772134984`, 34.717472515493625`, 0.016972982870934187`,
0.3670326206506046`, 0.2332303943585079`, 0.00013144100496386l7`, -0.9994364327800183`, 0.0883011663075137`},
{0.2778234194471715`, 3.6898619438208646`, 9.09826349254168`, 0.7771496857479276`, 0.47494449837227326`,
0.40530279224610744`, 0.24790715727254958`, 0.03660245395755471`, 1572.9621965794513`, 202.7146387762226`,
0.11027468006890989`, 9.901732606435996`, 0.25331571472189185`, 403.29816562107527`, 89.6857629711084`,
549.22095075161`, 0, 265.27699734954444`, 2.5660777232546956`, 2.5780740587835216`, 0.0046749696706811505`,
0.033503629280056894`, 0.021835720988318548`, -0.34825804076944267`, 135.2638933703426`, 0.13297275500691447`,
1.702695478024144`, 0.6038048347871707`, 0.00034022806455274686`, -0.9994365264321332`, 0.22472049448893175`},
{0.055450231563250685`, 1.3594142714077613`, 6.005951115471895`, 1.0656701550312766`, 0.9603365707697988`,
0.4169951955673704`, 0.09937287247557686`, 0.03650907654126198`, 3150.102571227402`, 288.6133211175843`,
0.0673017768034384`, 1.580996224465487`, 0.5598870619846228`, 53.80217065065473`, 15.45716320590283`,
60.2103697232662`, 0, 35.35644589479824`, 0.8969644440980777`, 0.9031376606375529`, 0.006882342527726903 5`,
0.07038696496176437`, 0.03786039159456774`, -0.46211075282001046`, 17.419232375032188`, 0.0557567643738009`,
0.23828197199939902`, 0.7541382344686519`, 0.0004306265119854343`, -0.9994289819925536`, 0.02973975557266041`},
{0.18890773944419526`, 3.611362633522033`, 5.813075251756651`, 1.273103869446512`, 0.8898144869747651`,
0.6114578055859192`, 0.07139582948563947`, 0.027880925348167697`, 2889.9541028194217`, 350.19982759883226`,
0.08313976867192174`, 6.993039414663205`, 0.8874083489329938`, 179.66334275326858`, 88.70840983566373`,
183.95270603648578`, 0, 128.7850792980372`, 0.9632120870955563`, 0.9731012559362614`, 0.010266865390492041`,
0.05345974726686876`, 0.035298711706589755`, -0.3397142053369593`, 49.69297342133808`, 0.14427085724912908`,
1.6077050123182395`, 0.5921425151832702`, 0.00033589314980024754`, -0.9994327494797494`, 0.1860561706050303`},
{0.09328585740614403`, 2.929301480876771`, 5.799770741738055`, 0.9079806695024137`, 0.4314055581005425`,
0.32164171745260006`, 0.17247524633063527`, 0.16386897475111406`, 712.2588807999496`, 70.83959017853539`,
0.20999817663531567`, 4.513003403554821`, 0.1126618170824738`, 200.9911776273738`, 75.06617621545819`,
576.7772120126691`, 0, 60.854929215603995`, 3.2437204775977975`, 3.286843587615765`, 0.01329433603042851`,
0.4938684793792195`, 0.04375016664447377`, -0.9114133246578793`, 135.740502836822`, 0.6581563506391332`,
1.497060772378793`, 0.9999730227110896`, 0.0005835984090130638`, -0.9994163858466593`, 0.1642249495794424 2`},

{0.06022915767996573`, 2.387138635828979`, 4.788528842745`, 0.9281538320395417`, 0.2752953410792893`,
0.5017610843522332`, 0.23773129300939827`, 0.14096915249034483`, 1499.4959807822306`, 322.9162482919975`,
0.15516909665873135`, 4.726500227282653`, 1.1616754765054393`, 124.17731254411503`, 39.88555723612112`,
131.89307130613918`, 0, 42.16511056136575`, 2.3333431673599137`, 2.3638274213983608`, 0.01306462524024643`,
0.05764030874108836`, 0.03195476549355244`, -0.4456177249659008`, 79.5716232207488`, 0.04959467491262408`,
1.558276396566899`, 0.7308138686103258`, 0.00041697887961260777`, -0.9994294321748362`, 0.2919012634487765`},
{0.24946839754186306`, 0.6156533776470687`, 5.962248269725286`, 1.0862299419007784`, 0.6836705094026099`,
0.5738298940987708`, 0.24726310480002844`, 0.11371634775304798`, 3850.890468269413`, 155.80403877473225`,
0.1865059666282849`, 5.974825484126367`, 0.19524283912722917`, 264.1643170577135`, 35.53337775396924`,
536.1702872271123`, 0, 100.517095123924`, 16.60204952364744`, 16.693318262776646`, 0.005497438072281646`,
0.22554023509761562`, 0.050524794698719996`, -0.7759832312099326`, 146.01582664424961`, 0.8037880147285492`,
0.41737434738401186`, 0.9873962752061024`, 0.0005635759667847236`, -0.9994292302078341`, 0.0845541823650016`},
{0.16738121319303478`, 0.840466372179935`, 6.567452802957117`, 1.3673648738160649`, 0.9708045560189591`,
0.43291645558985303`, 0.10721701325461769`, 0.4069236600033958`, 2609.682510998089`, 166.81040184723918`,
0.052553234892402045`, 3.550175474274951`, 0.3845611655536718`, 173.789261744847`, 45.83932681573903`,
256.10246658942793`, 0, 25.788580973489122`, 11.287513557074574`, 11.400427638498543`, 0.01000345034830108`,
0.3502641633726413`, 0.0639060310611711`, -0.8175490451383052`, 135.52536528923272`, 0.837537722904982 7`,
0.5338874669208378`, 0.9960134865702251`, 0.0005664867428527698`, -0.9994312459113345`, 0.05524143342147787`},
{0.27699847669745875`, 2.1853512734955096`, 5.84751172204828`, 0.9661484791489172`, 0.9868204791467026`,
0.40527218684546984`, 0.18624243293429998`, 0.043237932216039034`, 919.7606571301644`, 386.4267934374768`,
0.13084528987555377`, 5.85403217789433`, 0.6639489535590795`, 260.44486912866387`, 216.0479886585115`,
276.46606083134856`, 0, 160.89554495575098`, 3.048452899983783`, 3.0865451824608607`, 0.01249561129098642`,
0.2665844271053356`, 0.04610790071889308`, -0.8270420323514455`, 95.17057753100916`, 1.0549068602777427`,
1.885965648718203`, 0.9919004943588925`, 0.0005782334241559923`, -0.9994170449279495`, 0.1996327818713528`},
{0.11555514134387701`, 3.2236896162066904`, 2.732079118025373`, 1.164987224676954`, 0.23114456633378633`,
0.6224082143049414`, 0.17910660716870297`, 0.49555341188081165`, 1154.204177573869`, 364.61723023266256`,
0.21380198259541727`, 1.1657297203283505`, 0.5170897309434226`, 23.83942654057379`, 13.40141156634526`,
24.091456178239607`, 0, 3.202355026163156`, 0.430069013284255`, 0.44394382280173944`, 0.032261820984330836`,
0.15133594258708294`, 0.05441433314206752`, -0.6404401214155984`, 19.805843033952957`, 0.24982351765802077`,
0.35715770281721027`, 0.90399830102199`, 0.0005688768431610946`, -0.9993707102739928`, 0.14058190172838653`},
{0.27279971271031717`, 1.1118456280045157`, 7.947060215794888`, 0.8164028833969883`, 0.5079045986266995`,

0.311953435377873`, 0.24456124910810323`, 0.015036338564254712`, 1857.2767207919096`, 90.80006961498503`,
0.07729824929242363`, 4.7437001414515265`, 0.238261523479399`, 283.1732268830037`, 32.40588565084819`,
590.1457681616845`, 0, 231.73990803188897`, 2.939697276105736`, 2.9656986562867886`, 0.008844917601684932`,
0.3425326764928088 3`, 0.03847397878911979`, -0.8876779313931309`, 46.69270805849917`, 1.3348973677305156`,
0.6764677177085264`, 0.9999047891830247`, 0.0005708763475212922`, -0.9994290692936997`, 0.05587420515539963`},
{0.13280526554371425`, 1.6805260743084913`, 5.020140137415806`, 1.1878313111770886`, 0.7515564764604186`,
0.6825215664361661`, 0.0545109419053262 4`, 0.03202911588893443`, 2354.572550424513`, 215.4436338815351`,
0.06241668215114659 6`, 4.270435440493394`, 0.24902957081957822`, 127.34308048764554`, 52.18093400133016`,
146.56636672644274`, 0, 87.39656507499836`, 1.5868115469546777`, 1.5985478285450745`, 0.007396140778607618`,
0.08710297584444829`, 0.04425148337464528`, -0.49196358740175283`, 38.09540256673039`, 0.16525334052383375`,
0.674692752295276`, 0.7843203249604462`, 0.00045144094084484696`, -0.9994244176435596`, 0.1461429128839746`},
{0.09633016509755776`, 2.8964637462473926`, 5.167447635911598`, 1.1556815734309027`, 0.8460405566030094`,
0.6768234428979001`, 0.20660641079063646`, 0.37899350311781294`, 2084.615204649709`, 251.12222995868854`,
0.0719093058983113`, 5.055969069135582`, 0.9365067275794634`, 200.81315816429523`, 85.30127424428147`,
314.46111567351437`, 0, 32.34259909531726`, 3.947474175333305`, 3.9998423052475354`, 0.013266237494716115`,
0.49840686579391924`, 0.055729680662425625`, -0.8881843640463238`, 163.3387976871537`, 0.6858802238238314`,
1.4504129622389754`, 0.9995662243166386`, 0.0005841511900069323`, -0.999415595309449`, 0.20812329355247441`},
{0.06896509664813616`, 2.160626722426797`, 4.988265718811416`, 1.2639821322192157`, 0.4392795364272064`,
0.4617851413606676`, 0.11584839231589822`, 0.2654092351920312`, 1506.9815785286419`, 346.2179045294025`,
0.0968394930023978`, 3.8794768609952577`, 0.5989898263674145`, 84.27511549235446`, 36.8147176043394`,
87.87625622508894`, 0, 18.341815328633746`, 2.065855975389666`, 2.0927218554147493`, 0.013004720728421404`,
0.05164984548192623 5`, 0.03452926438207915`, -0.3314740042317855`, 63.76490893017132`, 0.05088623693597083`,
1.0000612787013239`, 0.5794797309757892`, 0.00033097119880266 82`, -0.9994288476695374`, 0.18830478844318765`},
{0.16407676745668215`, 2.743210330542553`, 9.218463902446366`, 1.4442040388312085`, 0.8033045111109769`,
0.5898809766156388`, 0.14591263450208874`, 0.06903241777052908`, 3856.2015344843503`, 358.8528117340675`,
0.03175828336990505`, 3.8731976390854896`, 1.2881241509575583`, 125.47787576547368`, 28.854938592209756`,
129.25873848443808`, 0, 63.51552471376426`, 1.5381966602320851`, 1.5502438009242374`, 0.007831989890248803`,
0.04299966088739847`, 0.031836260060950965`, -0.2596160201281741`, 60.279956696495866`, 0.10078921943046473`,
0.8354802325006744`, 0.47136935467600105`, 0.0002670868660888142`, -0.9994333809284814`, 0.09303158091053422`},
{0.19253541459312512`, 0.9166337410286696`, 9.734161690686161`, 0.9309028594902833`, 0.7413366163896413`,
0.3490367975563471`, 0.060904155020362505`, 0.013080541604234533`, 2740.950745040873`, 120.34372569041233`,

0.17782424959038423`, 7.718689374811543`, 1.0556383976941355`, 462.01999000404913`, 52.367200883818825`,
502.4152973835503`, 0, 389.30250121265993`, 5.121599823954986`, 5.160945232219385`, 0.0076822496127813`,
0.07666449088456463`, 0.035926880304531686`, −0.5313752183050748`, 67.0661600954806`, 0.21086613624327039`,
2.9888522293334305`, 0.8289340042522475`, 0.0004673392302752017`, −0.9994362166012277`, 0.0812570154475887`},
{0.2344407889708734`, 1.4871626351148812`, 6.830413107063742`, 1.1276656092803836`, 0.7850884426482851`,
0.26642284093684543`, 0.1715246889391343`, 0.04980986785514341`, 2193.834713356332`, 311.9394292836438`,
0.1837203477628585`, 1.994457399163636`, 0.8876922575805768`, 75.16358082794952`, 14.689918568571782`,
79.97962067663646`, 0, 43.909451941049326`, 1.391130590628213`, 1.404734757222939`, 0.009779216046555739`,
0.07053355758311922`, 0.038972545578095`, −0.44746094038758244`, 29.55482049925112`, 0.23622775555289952`,
0.42199175659480637`, 0.7356199283762157`, 0.00041932361284207845`, −0.9994299724672119`, 0.03929354056060646`},
{0.1378028697180832`, 3.3038928286894196`, 8.205199135932347`, 1.1443333838214729`, 0.43666320558690463`,
0.2863993034286605`, 0.21178222433889116`, 0.37748074045027574`, 3064.1095742596744`, 356.28526904181854`,
0.15861739359359306`, 8.78386257360005`, 0.18434483317647987`, 150.75721950402234`, 32.97994821726236`,
248.7933028754912`, 0, 24.163967096257288`, 2.625412475756126`, 2.6385499855810335`, 0.005003979354186505`,
0.017648188455318112`, 0.015022793921341603`, −0.1487628342491646`, 123.91544930003441`, 0.034742443063989624`,
0.7041640002129361`, 0.28232045234844017`, 0.00015897068774810999`, −0.9994369140229631`, 0.1663483904147807`},
{0.24756545924701062`, 1.5758450559815111`, 8.280763168619`, 0.7891779726588277`, 0.6872262965666405`,
0.4388878534659798`, 0.12835670237954216`, 0.07541218293771562`, 709.0120611967568`, 337.23881046719544`,
0.12174060791630043`, 8.605194748975368`, 1.4619068526165075`, 267.33984759822516`, 238.9199317898913`,
262.329400651287`, 0, 130.76354633956532`, 5.80375459843774`, 5.896760746151512`, 0.016025168903386566`,
0.025900144010589498`, 0.01858205702282761`, −0.2825500500989425`, 130.65454271540068`, 0.0915997292363593`,
4.135704255022433`, 0.5058210790426849`, 0.0002851321710996777`, −0.9994362983613904`, 0.4187061352643039`},
{0.15289027955876405`, 3.4056340118853177`, 8.85602064850179`, 1.1900353929127663`, 0.49460846965690886`,
0.2120137239983716`, 0.13358418114947193`, 0.3517341695519152`, 1451.4695761117755`, 341.1785262828431`,
0.2218032559662697`, 9.743566224950836`, 0.41975629001252956`, 224.63520730385932`, 71.82304687038265`,
253.81374877740114`, 0, 38.90196485087137`, 3.7391701959676658`, 3.773267516798602`, 0.009118953950720599`,
0.023957355966179313`, 0.0194439546619654`, −0.18839313113623646`, 181.91778850878976`, 0.05232638358793939`,
1.9757707324618778`, 0.3516123193949594`, 0.00019784945358599396`, −0.9994373079591568`, 0.23050338899271253`},
{0.10700117050359564`, 1.3986545196260032`, 5.134806342648034`, 1.3393940232844244`, 0.8110706849311933`,
0.31760122726745454`, 0.23195078300551503`, 0.016714409489937344`, 2707.277188066014`, 260.0392856094112`,
0.179990816902801`, 9.35690893256919`, 0.5478079740525017`, 361.60734608166064`, 68.36169153862481`,

585.7646688036432`, 0, 290.915875983791`, 3.2961064803014297`, 3.322120685444224`, 0.007892404355946514`,
0.554094827673465`, 0.06325868385261073`, -0.8858341917425552`, 65.85877465488802`, 0.8469827875863059`,
1.2941565560038815`, 0.9998309697579386`, 0.0005719978089885929`, -0.9994279054897378`, 0.15286980267502356`},
{0.27215562413942285`, 2.0660135805917337`, 5.081795444601147`, 0.8142657507592871`, 0.26635598111633185`,
0.328163277836687`, 0.07966098389627496`, 0.17309045811215876`, 992.4120776836976`, 330.82978884166914`,
0.11630419854588847`, 1.9092525381665606`, 1.4920575883264906`, 35.48383373945988`, 19.217168028012573`,
35.55441086117641`, 0, 10.76714901016617`, 0.8059094686064736`, 0.8279989411546025`, 0.027409372154820977`,
0.025527450159487257`, 0.018647572415146838`, -0.2695090070240924`, 23.785998669549148`, 0.09924913044063047`,
0.8400977916537276`, 0.48729633311121934`, 0.0002779480117508552`, -0.9994296119365884`, 0.09645522088337691`},
{0.16724732499606915`, 3.8236591860031757`, 7.535496783883705`, 0.9286997421550199`, 0.15670002022620788`,
0.2590202034037111`, 0.17595862302525594`, 0.2753459074292645`, 1491.5245062190434`, 377.6558909388741`,
0.10962242795283667`, 4.774971062285175`, 0.9122667174222503`, 86.92843239380696`, 27.14813487724858`,
93.66355338809932`, 0, 18.57004857672926`, 1.2279059453857477`, 1.246023591825983`, 0.014754913850135054`,
0.017027019513212317`, 0.014077945186970555`, -0.1731996797180736`, 67.07276925174506`, 0.04068176380341143`,
1.2509936171758207`, 0.32561165465134434`, 0.0001836085104822661`, -0.9994361119822972`, 0.2169041306367033`},
{0.25659351647641615`, 2.0548774277019515`, 7.952450585873037`, 1.1700596392648208`, 0.24509849180489507`,
0.5476063706170384`, 0.0511776409060387`, 0.047107635368495424`, 1627.113553842868`, 255.2038828951103 8`,
0.18601650100638534`, 1.4517697640535214`, 0.19929851500937135`, 26.37935140304867`, 11.19469090492552`,
31.17464834465072`, 0, 15.823262586007056`, 0.34467945288593305`, 0.3495701678917001`, 0.014189168994026335`,
0.01984254752780434`, 0.016734365500387947`, -0.15664228713883932`, 10.118200393256611`, 0.07273527208586769`,
0.2849310044716464`, 0.3019016774055471`, 0.00017365167595627927`, -0.9994248071840852`, 0.08942468573393039`},
{0.11981560320968349`, 3.640754447753876`, 5.349606530517274`, 1.2002498449288461`, 0.9084970459884618`,
0.2543242557071712`, 0.166873467304054`, 0.28421934758961104`, 1487.006076738341`, 292.7577364926251`,
0.15406116741524772`, 6.480379092988072`, 1.4335012083087681`, 262.57458253412483`, 87.05103796549606`,
333.4497907086179`, 0, 53.468492716821814`, 3.9225264181866684`, 3.9743843716805274`, 0.013220549198450504`,
0.43143941471252206`, 0.057038666896311575`, -0.8677944922247827`, 204.01365004921718`, 0.7384739103178687`,
2.261915721516179`, 0.9982668180783411`, 0.0005756307998709933`, -0.9994233697950824`, 0.1707884770915537`},
{0.1768569128079565`, 2.3909839842824203`, 9.326880330280886`, 1.4539501621465738`, 0.2185806450894794`,
0.20927947571084793`, 0.1254436846064726`, 0.4971843180024683`, 563.0454599592058`, 164.61314507136376`,
0.20371869506435597`, 2.3340090949236654`, 0.42101883446758315`, 86.46490015787369`, 33.32315500921454`,
96.479621640613`, 0, 11.67422446093449`, 2.122155086217517`, 2.1558686847983766`, 0.0158864914255394 4`,

0.05168036275828031`, 0.03620194217525975`, -0.29950293993516086`, 72.4862689044222`, 0.1305718487173801`,
1.1962811244400924`, 0.5320139305753189`, 0.0003018662002235644`, -0.9994325971880151`, 0.08835889653526978`},
{0.2133813191703517`, 1.451835237764156`, 4.809264415807901`, 1.4381107471220305`, 0.9545564566791402`,
0.6082425656359232`, 0.08609247081289534`, 0.07332616905477864`, 1657.6679333430675`, 311.00628516031566`,
0.15574013896641808`, 2.719528414156583`, 0.38330836635142496`, 88.43938080705325`, 69.55754457429003`,
90.18234927166462`, 0, 43.21001192835031`, 2.052933477434135`, 2.081179792234151`, 0.013759001502240409`,
0.14755856825684274`, 0.06088032523452857`, -0.5874158583013673`, 42.57887376177969`, 0.4498034564219731`,
0.6038895191569995`, 0.8775128023495522`, 0.0005139099535845964`, -0.9994143561755354`, 0.11513559978604627`},
{0.07679027731361865`, 1.6374208448422314`, 5.731071459295233`, 1.355982875935464`, 0.05181959233087374`,
0.15382155253913876`, 0.1281658546365993`, 0.05032482400738273`, 1230.193557333262`, 218.81347681784587`,
0.015802664299199992`, 1.3298614212710635`, 0.20143294852402427`, 24.246400796474656`, 6.892701196660307`,
36.874501899895776`, 0, 14.171516637695778`, 0.4100086762769088`, 0.41574953327365277`, 0.014001793934884432`,
0.035110784948936`, 0.027018821794689712`, -0.2304694459555655`, 9.59081075859972`, 0.038516670184673855`,
0.2650055676591805`, 0.42433534359495617`, 0.0002417790467715626`, -0.9994302170431452`, 0.035678263782726434`},
{0.2370685516334804`, 3.237122537432753`, 3.9976792452307883`, 1.4537281576817178`, 0.8596265155383829`,
0.4350695278152106`, 0.13391759982092372`, 0.21967358388020922`, 1928.121818584993`, 246.10473403948492`,
0.1821805288264413`, 9.36511897288407`, 0.6656126516266203`, 289.4157280441301`, 130.27978586235983`,
479.00821494588286`, 0, 70.52878662657841`, 4.533639532926327`, 4.645568034800618`, 0.02468844315067198`,
1.070719942428846`, 0.07047629943460418`, -0.9341785870965156`, 209.65638155188466`, 3.626200370809942`,
2.284718261821487`, 0.9999983087568209`, 0.0005875089360637282`, -0.9994124900703141`, 0.30023364523705953`},
{0.14216552227340323`, 2.5649444615040133`, 6.744792074075217`, 0.7596927543658916`, 0.15436598317484496`,
0.17272955179378358`, 0.11731963299504705`, 0.1434376311009391`, 3629.5744652923077`, 39.43043872717277`,
0.05961813000749189`, 7.187412396441442`, 0.1404254814851862`, 130.97477046247772`, 14.750346116264245`,
1216.3569170783123`, 0, 43.25453480625754`, 2.329087550766934`, 2.3383328578298523`, 0.003969497436828284`,
0.01602578241950232`, 0.012881848539836665`, -0.19617974320178566`, 85.34257448139415`, 0.03254733896451791`,
0.47183193401138224`, 0.3649611718378698`, 0.00020533364091203602`, -0.9994373822292437`, 0.06319024565400666`},
{0.09737410253356232`, 2.2163561335231643`, 6.975749882171211`, 0.9583917748679426`, 0.07040378529617852`,
0.6807220990959502`, 0.09176157414171682`, 0.09133640049629771`, 816.8955105959267`, 131.23291821104817`,
0.07570904922141436`, 6.1606408812314815`, 0.7251058774037329`, 268.5512299502334`, 94.01037177946847`,
280.81389648525436`, 0, 118.63467514822625`, 4.583944412203484`, 4.6547122204513665`, 0.015438190755429515`,
0.08138139358274701`, 0.037586444047485165`, -0.5381445021672183`, 145.13790448166299`, 0.11320628804373763`,

4.062831997953843`, 0.8317147293328593`, 0.00047492381105121906`, -0.9994289823249467`, 0.3673968443358577`},
{0.16605942412072794`, 0.6402554572286978`, 3.356342996009028`, 1.3219512159205855`, 0.7993093802210203`,
0.15372675446550776`, 0.10521895558502489`, 0.005367878364442133`, 806.3427040773236`, 321.0178159818362`,
0.15297743964506222`, 6.095134085467343`, 0.509077325139712`, 156.73753824200898`, 94.34390622238328`,
194.8053922616763`, 0, 143.982685682644`, 1.0724235899856516`, 1.1002030850009463`, 0.025903472540795613`,
0.5070404784949708`, 0.06349920346395749`, -0.8747650214191187`, 9.808929370701504`, 1.2028407123539007`,
2.0726450691441847`, 0.999384136349221`, 0.0005822868912938736`, -0.9994173542783849`, 0.17731389800392447`},
{0.27402590763234025`, 2.048044129965374`, 8.367988840463223`, 1.356112416883951`, 0.549124019732967`,
0.6110139101654402`, 0.05091107534805153`, 0.12010047430936349`, 1627.3034033398817`, 297.4011443552333`,
0.1966061654681946`, 8.76675255073281`, 1.4917606486762143`, 198.21849806877287`, 105.78125649462716`,
197.92476520968904`, 0, 75.30359402625673`, 4.057619341074393`, 4.138745110479684`, 0.019993440139658647`,
0.028558448210233206`, 0.022990878701942545`, -0.19495350263098887`, 118.71692316505865`, 0.11179649559111775`,
3.784431026862729`, 0.36355939337644194`, 0.00020535152511694515`, -0.9994351637480473`, 0.3223786446233027`},
{0.10823031336368771`, 0.8296257610813722`, 5.332629740125888`, 0.8124935731916766`, 0.40472412917038403`,
0.20010405592172198`, 0.09023994109265432`, 0.01008956636997111`, 1875.5688641041388`, 260.72528052100415`,
0.14449594942054067`, 1.5456312543093684`, 1.2237716091504276`, 38.091352901660876`, 7.466981050506643`,
40.52698038165148`, 0, 33.249747592626655`, 0.3674278322545059`, 0.3733869318628549`, 0.016218421919168335`,
0.03665843364532761`, 0.023536311059749102`, -0.35795644496259105`, 4.354679928237479`, 0.05667933944079532`,
0.4941991775100615`, 0.6186721817047409`, 0.00035123086131736927`, -0.9994322827634669`, 0.03810574259397599`},
{0.20273952773619414`, 0.8775156867465963`, 8.853127710010899`, 1.0632724405144907`, 0.5470610681912369`,
0.35564991449731365`, 0.24613003734690775`, 0.2102067712155018`, 2109.2588977466867`, 152.77073389943882`,
0.07849302645811645`, 3.0982028200464633`, 0.790674694279 4358`, 202.73221424636782`, 20.591603427835743`,
236.19700467672692`, 0, 51.00609424156075`, 11.148622650152726`, 11.235760434018557`, 0.007816013385710585`,
0.2102135739936723`, 0.04929153083174162`, -0.7655168983843766`, 139.75844658753456`, 0.6088371530744163`,
0.7613632136147505`, 0.9813721770627453`, 0.0005617327982036298`, -0.9994276047239439`, 0.05649867427302374`},
{0.16207753282118065`, 2.4074661619125743`, 6.439531987652202`, 1.216188209247578`, 0.5635250461042756`,
0.41870015151777173`, 0.15568766166521003`, 0.14566116758733688`, 2407.082097704033`, 343.2271284427226`,
0.14229137239354006`, 4.642019464618446`, 0.8431314645967629`, 123.51530437278372`, 30.50221978063873`,
132.0890131585702`, 0, 40.683432004377956`, 2.3360848551696236`, 2.357126639638024`, 0.009007286024664829`,
0.045932314401627546`, 0.031522151570093186`, -0.31372603404072663`, 80.34350343110417`, 0.10635137421410712`,
0.9642253183206347`, 0.55375927668566`, 0.00031403142011914915`, -0.9994329098701539`, 0.13683395840172105`},

{0.22160418402095905`, 3.3706460558066524`, 3.148818263883834`, 0.8683984407887995`, 0.063094397851442`,
0.5589539148331857`, 0.058740794885626`, 0.0138702175197992`, 2238.370585799582`, 370.4864263128153`,
0.21378440885525551`, 5.817374569517135`, 0.5948516756530484`, 82.16118166235404`, 28.977246489080848`,
86.22568333342932`, 0, 68.61668110134666`, 0.27160219468712066`, 0.27643957775303196`, 0.01781054483555944`,
0.03720236608671669`, 0.0244228387071504`, -0.34351383322711004`, 13.078212375293916`, 0.11777428543280076`,
1.2893211343765343`, 0.5971278571475055`, 0.0003408832369664496`, -0.9994291285645341`, 0.3058051724022367`},
{0.09473876647477247`, 3.4861652630821096`, 6.982828746666236`, 1.2832000030310025`, 0.6886348717794659`,
0.4656186082808249`, 0.150625912685666`, 0.03239358546984584`, 1673.775609656389`, 245.14838945542726`,
0.016860445211711483`, 6.847475541460803`, 0.5191626673367391`, 281.7732235070947`, 83.12528891828873`,
307.17222486918655`, 0, 192.96221966052263`, 1.7433153193613244`, 1.7546968424385525`, 0.006528665784568233`,
0.09396239171810308`, 0.04745513317171611`, -0.4949560956889387`, 86.82121869937606`, 0.12716972980560218`,
1.593212200329654`, 0.7882941356591033`, 0.00044786944218402636`, -0.9994318498363437`, 0.17816808043316607`},
{0.1760690989521051`, 0.7026153943847899`, 3.261997951042007`, 0.993553588623698`, 0.646826501995915`,
0.3736019899194698`, 0.21533349413201053`, 0.25320537109656943`, 1549.1302534082051`, 389.4106290690562`,
0.0763719271638561`, 2.133344446029941`, 1.4125782678502792`, 54.74422994072687`, 22.343288032123997`,
65.99923370820483`, 0, 12.117885636093137`, 3.7579860330753103`, 3.8856830997978093`, 0.03398018662086377`,
0.32660029145484926`, 0.048914982859666306`, -0.8502298248364284`, 37.7202691245963`, 0.8214888433421783`,
0.6568084874992854`, 0.9979214365236648`, 0.0005978707104944992`, -0.9994008839888466`, 0.12125034016792896`},
{0.06662682417897325`, 3.243608945481448`, 8.237889478852154`, 1.2035188774949521`, 0.1279184849350048`,
0.6784394756208973`, 0.18789313451307382`, 0.086116484822983`, 685.6358133562785`, 181.08244593249674`,
0.08115584053395464`, 7.371846442202106`, 0.7077387695529302`, 293.6336700352119`, 141.04942792618095`,
306.0132888741719`, 0, 133.8855712980883`, 3.372244445782028`, 3.4216861304533706`, 0.0146613584709685`,
0.058943264411654865`, 0.03652449666085201`, -0.3803448617000925`, 156.2606035812677`, 0.05610289306412625`,
4.3639979145943215`, 0.6473061606045438`, 0.00036747449716658287`, -0.9994323018696077`, 0.620132543985479`},
{0.044118696420245185`, 1.9220692229229748`, 8.936584527376546`, 1.198231045412435`, 0.8133241613996403`,
0.3641703381601298`, 0.06715507154740682`, 0.009124556845180784`, 2953.448531772523`, 220.86580819426717`,
0.0755192290605344`3, 5.11062195030733`, 1.275640621281854`, 184.84486693355007`, 36.78842616997562`,
193.96515801964333`, 0, 163.53533576803164`, 0.7414460894875748`, 0.7490339449312933`, 0.01023385995462256`,
0.043986425982867235`, 0.030437516256479172`, -0.30802479227717605`, 20.358724415152274`, 0.02772319677926324`,
1.6642045181877536`, 0.5453707536800645`, 0.0003075924446256234`, -0.9994359938765509`, 0.08000580424798547`},
{0.08620550491180146`, 0.4949942611001066`, 5.984234002717921`, 0.7531376788437371`, 0.21717148283204812`,

0.22262766010657997`, 0.11267502779798616`, 0.05995928028643471`, 1301.4568875111263`, 314.2464318877011`,
0.04884398840792192`, 1.2952896203900384`, 0.6666662921648308`, 24.425693925288567`, 8.022665462390247`,
26.599289436807602`, 0, 13.294348079768179`, 1.3737569382821357`, 1.3962076970389774`, 0.016342598993469792`,
0.01930286068916417`, 0.014879154331425093`, -0.22917361467683206`, 9.714311437087305`, 0.023771612170737776`,
0.35841010257987066`, 0.42162195293107996`, 0.00023948871299913046`, -0.9994319823450031`, 0.04858537895762061`},
{0.18748668988618272`, 2.2339195778761853`, 8.488097011441944`, 1.47468717511323`, 0.23541263239484844`,
0.20987853326618222`, 0.07676134969948037`, 0.061849152465116755`, 2541.5254126664167`, 387.5680307693225`,
0.09509361772923897`, 9.161664648477608`, 0.10551879857006874`, 80.23168230621658`, 30.64547987483623`,
150.40365433495853`, 0, 42.9672079925`, 1.1310426243258667`, 1.1434917448813922`, 0.011006765163201093`,
0.010278422016906402`, 0.009547597551460852`, -0.07110278836999084`, 36.09511802705731`, 0.02752953316005937`,
0.8472556466996249`, 0.13931116021231826`, 0.00007841059790060415`, -0.9994371549430706`, 0.27230924050983013`},
{0.13868488683280167`, 1.226702016867053`, 6.040570905634304`, 0.759875582646631`, 0.27881848833013434`,
0.595446367026503`, 0.23094105751878413`, 0.2051375441364418`, 1528.2920750506055`, 270.40251576187586`,
0.23385239775529226`, 1.5893916227857634`, 0.2397907655262943`, 39.9003720625578`, 14.129222527331965`,
50.79638203711839`, 0, 10.34361610631199`, 1.5909482020794332`, 1.6059428809792973`, 0.009424995031431793`,
0.02869002312166004`, 0.019796610889747 61`, -0.3099827488531437`, 27.880276688883626`, 0.05684103728362771`,
0.29600190417418565`, 0.5481904414775758`, 0.00031585054564931334`, -0.9994238306220773`, 0.09343583489797695`},
{0.26277447700446094`, 1.2458735546481199`, 6.447910682321009`, 1.49065802757454`, 0.9176757405073024`,
0.4907046225412167`, 0.15937178447602146`, 0.008327156523724668`, 782.8427424300617`, 244.66740068612717`,
0.09440964708909416`, 9.330818440145894`, 0.2131775359264001`, 465.0087727042175`, 399.77562265257495`,
572.9289522865931`, 0, 413.09259738086604`, 2.5946141914601486`, 2.6298404108040345`, 0.013576669494766769`,
0.5828579907201158`, 0.07227740735091931`, -0.8759948246370934`, 46.17944579507016`, 2.188002909704968`,
3.0714347535363675`, 0.9986331220628222`, 0.0005874027651885505`, -0.9994117932279523`, 0.39750829383208774`},
{0.13025097964415272`, 1.0189597502038827`, 8.886210776319402`, 0.9111028640704337`, 0.2284753731773581`,
0.5590994693246798`, 0.2266682667598287`, 0.0061069720799473886`, 2065.306144054668`, 113.92755603976332`,
0.14827318077057916`, 4.714739506212668`, 0.4593044246185465`, 270.59034396723763`, 29.245672208907873`,
352.5979186604657`, 0, 248.48266456300215`, 1.3882800554380696`, 1.3957254498587117`, 0.005363034923304921`,
0.08584118283929003`, 0.03692827144141075`, -0.5698070527459175`, 20.20859283574585`, 0.1597271165518656`,
0.9348277997524743`, 0.8629027513038884`, 0.0004908531407193122`, -0.9994311605334696`, 0.09993441878037002`},
{0.09142437343902587`, 2.458407615666487`, 5.031014986619411`, 1.0519598677125233`, 0.487417438430646`,
0.4280564058665117`, 0.2026543236563062`, 0.1589331435911828`, 3989.0362964026026`, 249.7709598067779`,

0.24152899778887077`, 6.187241064247978`, 0.924037240334326`, 224.73384628550465`, 22.787606434365237`,
254.4459719339861`, 0, 69.30701433302038`, 4.292442493935871`, 4.319418029197547`, 0.006284425545545647`,
0.16623219865269956`, 0.047656895690967954`, -0.7133112833901989`, 150.75104738431955`, 0.217109637245918`,
1.0384542595693043`, 0.9615431496643586`, 0.000548693011618435`, -0.9994293620501483`, 0.12484532170090587`},
{0.05362058570834477`, 1.4723855364180096`, 9.67162648709779`, 1.3845527897944452`, 0.7224375135481673`,
0.2670471970549346`, 0.10991701130195836`, 0.006877132758778975`, 886.1772845072878`, 273.92553716604675`,
0.12489313995376372`, 6.85687873202156`, 0.7259468075744833`, 239.34558154877314`, 119.2317842129614`,
245.57929422599588`, 0, 217.90353119999537`, 0.9597611460296671`, 0.971719104890564`, 0.012459307099859762`,
0.04297865564545208`, 0.03136401160644817`, -0.2702421437938335`, 20.18769185471509`, 0.03292200983804865`,
2.6578715114692644`, 0.48655683737800803`, 0.0002742919248172929`, -0.9994362592327437`, 0.20187054243024302`},
{0.26384722865028404`, 3.0959478641925884`, 9.455492188682882`, 1.339136584268803`, 0.7399277527624639`,
0.3172867286222486`, 0.06239618913281497`, 0.23953121953891282`, 1016.1140721617635`, 88.38662693497645`,
0.16528145715165937`, 7.22137987568804`, 1.361790640282516`, 518.0494728746964`, 124.32642543986617`,
643.7463295665209`, 0, 121.23905480884555`, 8.677583436135723`, 8.86386604592335`, 0.021467106730647778`,
0.9104539116001865`, 0.0637470372219234`, -0.9299832353843331`, 383.79065579224897`, 3.4317248769925977`,
6.5626064322247535`, 0.9999609319115067`, 0.0005766077786341489`, -0.999423369693522`, 0.14517638570252706`},
{0.17041949742674112`, 0.566658425910223`, 8.176154533792225`, 1.187496781729836`, 0.9612002380635534`,
0.5389795298813838`, 0.1929826430211613`, 0.013244827072013646`, 716.151240381786`, 217.26337969751137`,
0.13800797665502906`, 7.448468008162791`, 0.8606351443610007`, 470.1994775581754`, 420.70482286433923`,
620.5694325034558`, 0, 394.10152054668396`, 8.116781448178624`, 8.225507980103425`, 0.013395276516801724`,
0.5911360913479703`, 0.05753593150684599`, -0.9026688907191464`, 65.70632284117433`, 1.4391587942619226`,
3.9153781382761004`, 0.9995263875456997`, 0.0005867859228798562`, -0.9994129360363154`, 0.31499721150217097`},
{0.22961450923300392`, 3.8785966005678736`, 7.959504018056748`, 1.2921046945277759`, 0.1917613861571974`,
0.3981097458569077`, 0.1102143743054892`, 0.20929581166220373`, 1753.2557317520059`, 231.02169920905055`,
0.10007865476065686`, 4.452819250691443`, 1.402721807760341`, 121.2486618865712`, 26.542136530168364`,
127.34203624739558`, 0, 31.69279262216626`, 1.5848153299674237`, 1.6107507475425078`, 0.016364946176799622`,
0.037095746411235246`, 0.027723720792822152`, -0.2526442119405522`, 87.81227644770706`, 0.12168173724068931`,
1.8493095485867554`, 0.4593932632280454`, 0.0002599976806431305`, -0.9994340411550309`, 0.16976101428427676`},
{0.12232907903298573`, 1.3289405346143397`, 5.1200974038340075`, 1.1450551183026931`, 0.6675798963164832`,
0.5719242545987869`, 0.08761535581144905`, 0.11107132883791111`, 3964.0786866225717`, 279.32313661047124`,
0.2162046831451349`, 8.020270743802456`, 1.0641453807394075`, 233.4959214299155`, 45.381459198924055`,

246.55534213250874`, 0, 91.3039302012771`, 7.104375134524988`, 7.166883811121431`, 0.00879861710745966`,
0.07715836984194703`, 0.04085571110802823`, -0.47049540844735316`, 134.8756012768063`, 0.1348387474634315`,
1.8418262800432423`, 0.7624433680185396`, 0.00043214116564363773`, -0.9994332153917652`, 0.17121362092013784`},
{0.08231211479941364`, 2.387884906790136`, 8.578001165552415`, 0.9642461558703308`, 0.8238919598475782`,
0.2020584877079309`, 0.19955393113038733`, 0.1095787053029616`, 2239.622315346988`, 371.5987310561687 3`,
0.17019241214364111`, 3.55759780947197`, 0.4666380334441058`, 121.70915053106295`, 23.41272235620549`,
140.05970297704337`, 0, 47.70494865503648`, 2.105476777446631`, 2.1151007405493014`, 0.004570918665909751`,
0.034625169727263864`, 0.02413259136485697`, -0.3030332687191144`, 71.82337454945625`, 0.04071529922199414`,
0.47985527562366265`, 0.5373296159183069`, 0.00030310553956084725`, -0.9994359039022206`, 0.0439639474676950 3`},
{0.17519323235207396`, 1.2618694118815563`, 6.2707952492510515`, 1.261446257781183`, 0.5333912181747638`,
0.18672075814011158`, 0.09419037155450471`, 0.010359125778073429`, 2462.605666629158`, 256.6607054858181`,
0.10840920502450685`, 9.870044854094623`, 1.435459480935227`, 293.3824115223875`, 42.17826574140466`,
310.6536706473716`, 0, 255.58867993036694`, 1.9650315523127655`, 1.987734483182423`, 0.01155346887073505`,
0.059879854948211776`, 0.037522317180696554`, -0.37337327865692993`, 35.42304584636588`, 0.14986493344499666`,
2.9676992985742077`, 0.6394422970403263`, 0.00036025160084201 91`, -0.9994366159346834`, 0.17021444236658376`},
{0.23749378089306467`, 0.4157158512844461`, 5.854529102702003`, 1.3110653325714712`, 0.14508930723778435`,
0.1573757215384779`, 0.06204301966356823`, 0.16029852320715812`, 2190.57271228382`, 132.40666721155492`,
0.12799517810137861`, 9.84238745503276`, 1.14600445718771`, 393.2082249786656`, 32.67290272445369`,
435.0538754959596`, 0, 121.87352792885245`, 39.006621844708505`, 39.443616269749285`, 0.011203083076010145`,
0.15380545518533703`, 0.056131319558989874`, -0.635049878488698`, 231.6524429414784`, 0.5218262724847724`,
3.747161399214052`, 0.9180347298195513`, 0.0005186134302644595`, -0.9994350829947726`, 0.11721032294026335`},
{0.2211475447831816`, 0.6764621696057107`, 7.070732773871981`, 1.484318092913942`, 0.39213098277018266`,
0.49361758237854214`, 0.08219155305388848`, 0.007529395154295265`, 1570.1936066911949`, 345.9142373695621`,
0.2485313792002623`, 1.2269777403584339`, 0.7159550405850554`, 22.802858432502592`, 10.710529885384396`,
23.266585672282698`, 0, 20.49252901139621`, 0.20321270100510286`, 0.20766974165452337`, 0.021932884250717155`,
0.029722688896001076`, 0.024383859611169695`, -0.1796213425882095`, 1.9637957801906933`, 0.0939014239100715`,
0.3521591709455008`, 0.34524999855255845`, 0.00019975689338710476`, -0.9994214137748745`, 0.06992682999140118`},
{0.1605397117399036`, 2.9861451600751723`, 5.985367280571584`, 0.9013600575793626`, 0.4265711037361093`,
0.6369377322655665`, 0.2099395794597424`, 0.0440322954061731`, 1331.4501176292097`, 178.64680303201567`,
0.0465601985112431`, 1.120746116836358`, 1.298216136515848`, 53.07481173090489`, 13.833301202449599`,
58.53759636474258`, 0, 32.24405653891251`, 0.4562354805427182`, 0.4694139407589044`, 0.02888521559197832`,

0.27637410561739684`, 0.04464851211153759`, -0.8384490037089527`, 19.462648172531605`, 0.6338431321170096`,
0.5878659879318842`, 0.9964497552558135`, 0.0006013177262043258`, -0.9993965398425433`, 0.06995290012687708`},
{0.07579699484940727`, 2.2173315933991757`, 7.471866642915501`, 0.8771923938554881`, 0.8171799463467477`,
0.18907166793764196`, 0.16741258135296538`, 0.034704832899603626`, 2559.9885855263465`, 365.24466351817625`,
0.21912261270377126`, 5.637070181749923`, 1.0559796296250288`, 194.50241524838788`, 31.447687415677308`,
205.50810070683278`, 0, 130.32098289572997`, 1.9611912936076354`, 1.9729548173214173`, 0.005998152119135014`,
0.0398287385546296`, 0.02542390293786412`, -0.36166939098529627`, 62.123020228794324`, 0.043127124158408775`,
1.066901462285025`, 0.6229495066315045`, 0.0003509587196056689`, -0.9994366177099916`, 0.08258102587753356`},
{0.06741497368958249`, 1.0249716767004005`, 9.449217531688483`, 1.0872407203117702`, 0.5615736740328037`,
0.5521415654810891`, 0.18288037923286848`, 0.005326201838481523`, 3978.4896243421163`, 351.6470055302269`,
0.09021448343705185`, 1.426476556917251`, 0.13651689047774385`, 21.30479021756226`, 6.4290213802314256`,
40.67797300418529`, 0, 19.761993095160648`, 0.095682596756253`, 0.09630392043809728`, 0.006493591341663496`,
0.012062355635070738`, 0.010777899919604867`, -0.10648464979190098`, 1.4010278804043548`, 0.011616905539610372`,
0.09071076696810673`, 0.21040585967753955`, 0.0001201241613529827`, -0.9994290835743022`, 0.04835434218320168`},
{0.09804650260585268`, 0.6377788755200506`, 9.103001426868161`, 0.9608072109179064`, 0.12522756138173952`,
0.16039700353140673`, 0.1476329494772725`, 0.03181180155196076`, 3994.864550131647`, 201.1651941507272`,
0.22810874694301092`, 5.8973245769190275`, 0.5910947751289481`, 157.49172312557963`, 10.47465857019683`,
203.0651991459402`, 0, 108.42009574727894`, 4.846342776458324`, 4.8668540263889355`, 0.004232315144988563`,
0.022527844061336278`, 0.017691326058088756`, -0.21469067302131506`, 44.15564351934733`, 0.031553947449454556`,
0.6019920101512272`, 0.39630522544359226`, 0.00022296242796198264`, -0.9994373972038536`, 0.05210021175083620 6`},
{0.18148677226867904`, 2.435655182727153`, 9.932013575043552`, 1.3523612276244852`, 0.270501525518553`,
0.3129668058670274`, 0.09479199345181186`, 0.24780135341453666`, 2571.145269598781`, 147.08034915528242`,
0.05706779714237542`, 9.481295153852962`, 0.5023669027433282`, 357.6084895984909`, 37.80535478215227`,
440.5809147086804`, 0, 80.0244704998048`, 7.750275649762204`, 7.789910839827594`, 0.005114036178391457`,
0.04518323990114235 5`, 0.032231319287402874`, -0.2866532068545182`, 269.6714150558195`, 0.11714514814715551`,
1.6797221463809875`, 0.5123188741253708`, 0.00028861336724234885`, -0.9994366528702753`, 0.12369228018248805`},
{0.2282743224553333`, 3.8004017582328853`, 8.466984049452861`, 1.390127484711968`, 0.35687133565138884`,
0.6519422926243725`, 0.17290926755870883`, 0.3014446142578311`, 2372.838415127524`, 111.84440462244766`,
0.08570923572829736`, 3.872670068176556`, 0.42950313779436455`, 227.59061707046465`, 25.843459315814194`,
293.84094746900337`, 0, 43.32397440766237`, 3.319636136019157`, 3.340487171874636`, 0.006281120882267288`,
0.16876895634887754`, 0.06050630823742944`, -0.6414843727992761`, 180.22787154315188`, 0.5503659880290345`,

0.7116664772700536`, 0.9203121748944492`, 0.0005275075806324308`, −0.9994268166878343`, 0.08332399935964235`},
{0.049399735586138194`, 2.5477631442089104`, 4.041803486797548`, 1.3304361527101167`, 0.993281383326639`,
0.3001497243237138`, 0.21152425523664786`, 0.4874448677287525`, 780.8123462023354`, 361.74617623884797`,
0.046928831923920444`, 5.346338091727542`, 1.4411222226599376`, 167.7239316324237`, 183.14775021727928`,
299.16765633168785`, 0, 22.92076917083917`, 3.84498313710963`, 3.921807800557643`, 0.019980494246267133`,
0.5943553271437777`, 0.06478797261222378`, −0.8909945454286283`, 139.94437609760948`, 0.4194428000731931`,
2.2729565485557766`, 0.9991059706061154`, 0.0005898981213167789`, −0.9994095740205027`, 0.2919848680691693`},
{0.20618269294314817`, 1.8587454568359414`, 4.34452965291473`, 0.8960498853155586`, 0.6640333303615127`,
0.5390503581736582`, 0.2329294063957692`, 0.4180316503514536`, 633.3353428142332`, 244.55036900145114`,
0.12942298196931046`, 3.095308470158429`, 1.4194968692598873`, 112.48322334848848`, 102.41979900215996`,
163.8287082111389`, 0, 17.205524582602077`, 3.3096060570041614`, 3.4968101449732614`, 0.0565638582794219`,
1.0357320524934899`, 0.04697645216716631`, −0.9546442035330739`, 87.88164603390261`, 3.0507146250091575`,
1.9468557317198287`, 0.9999966721660881`, 0.0006357035599202816`, −0.9993642943245569`, 0.29799147598738446`},
{0.09869674443849347`, 1.9183657949501018`, 3.7344926730748456`, 1.0943839787542378`, 0.42780632576683675`,
0.5861386946846783`, 0.14629063580280066`, 0.03398060371034166`, 2435.986502306805`, 302.9804614286604`,
0.13730947981168928`, 9.171543304681595`, 0.26064124405682665`, 212.044374335447`, 61.93859448191102`,
263.19522317547944`, 0, 142.96722192892543`, 2.423603632504137`, 2.4395362010692008`, 0.0065739167706238`,
0.09025203285972731`, 0.042514367668591985`, −0.5289372846075476`, 66.4194044158963`, 0.1272511688887954`,
1.170684815305317`, 0.8219699109898426`, 0.00047050724418313834`, −0.9994275858058885`, 0.3382285306748128`},
{0.06272312309945755`, 3.461529579186063`, 7.564878788392824`, 1.3521314303721368`, 0.32784603357531017`,
0.6503539369626794`, 0.11129074624350982`, 0.1957733709006163`, 2103.0882520472996`, 262.6269091211209`,
0.16330910334057364`, 4.05183701830078`, 1.3383335426462928`, 107.22670468623951`, 29.54162667278792`,
110.83493598966515`, 0, 29.31466881879757`, 1.5428263343998283`, 1.565598170747652`, 0.014759818289387772`,
0.039975516971979874`, 0.02967994645220061`, −0.25754690119444257`, 76.29341417246015`, 0.03581984674254129`,
1.523834824303648`, 0.4679773731670127`, 0.00026586588271381627`, −0.9994318830397406`, 0.18052795895620816`},
{0.1334851760745519`, 3.2697077297961004`, 8.047111995239536`, 0.842079209613761`, 0.8088703615622166`,
0.5692447244516433`, 0.16235903984142924`, 0.09701377075706732`, 2643.3784916344785`, 292.46187756743905`,
0.08266433823104813`, 7.391813212062153`, 0.547699130297068`, 303.6701431772931`, 80.76909107738538`,
330.7440724671764`, 0, 127.92300297920613`, 3.6808617829842882`, 3.697198568711418`, 0.00443830458471739`,
0.04566342709485526`, 0.026982053179514034`, −0.4091102027128838`, 171.93346034478287`, 0.08707700865609402`,
1.2147383968777636`, 0.6864183641730207`, 0.00038799368490038835`, −0.9994347562577702`, 0.148829028951822`},

```
{0.09401709418411008`, 0.8110971536952984`, 8.245292807864445`, 1.0264842776394885`, 0.12930370633004684`,
  0.1740236808265636`, 0.07252752048975858`, 0.09884601796368213`, 3866.984141926244`, 274.6428268585172`,
  0.20040469873166988`, 4.952717873499136`, 0.15734837909658306`, 48.38364739148174`, 9.288938552801362`,
  105.10674777868473`, 0, 20.237793603726395`, 2.2345382200568027`, 2.25030404974417`, 0.007055520261795456`,
  0.008314298421209908`, 0.007632447970543638`, -0.08200937903874828`, 25.89182271587765`, 0.01116694539627698`,
  0.32264806050419353`, 0.15997605257330982`, 0.00009004560579926765`, -0.9994371307183118`, 0.07482531937947029`},
{0.16503822022515502`, 3.799827999093722`, 2.226332530621681`, 1.4474524276394465`, 0.4523982192034186`,
  0.1981242269935809`, 0.06722881851519197`, 0.02132348079412978`, 3790.4878202500677`, 323.4156208714485`,
  0.10032103134111592`, 5.6566553483532305`, 0.498005589518143`, 95.36116821130314`, 14.684418035595485`,
  118.84101314717677`, 0, 72.12181202554234`, 0.3940123766252919`, 0.40276672464132995`, 0.02221845945809830 5`,
  0.4270475747044383`, 0.06864333036586588`, -0.8392606949860931`, 21.38827515271781`, 1.006845309724118`,
  0.6188804036224295`, 0.9985180741818669`, 0.0005754868497949156`, -0.9994236590556796`, 0.08689231967556645`},
{0.2175565696926961`, 2.150769749820693`, 7.849181160138611`, 1.1076816333069537`, 0.23775832216164305`,
  0.4289452843039179`, 0.23138213560150006`, 0.007541689166030151`, 1830.5976676958226`, 333.35844770130507`,
  0.13740519457686395`, 6.97796965521683`, 1.0770120195809052`, 168.66163534703347`, 43.46369065605549`,
  183.26174235283813`, 0, 152.20340925657746`, 0.5106293256140151`, 0.5170638615192092`, 0.012601187559012406`,
  0.027633800914231316`, 0.021402995017544692`, -0.22547770087891805`, 15.689230098599502`, 0.08588449906383806`,
  1.9145204222066203`, 0.41448706666459545`, 0.00023403266060202302`, -0.9994353680019854`, 0.34998529225938824`},
{0.12052689532243871`, 2.1969784256629277`, 8.110278600920065`, 0.9177559121464718`, 0.6615840119954921`,
  0.34704707074624996`, 0.07379725368855697`, 0.00573204546098014`, 2320.908483262354`, 384.0513502933301`,
  0.016004877265817924`, 9.641293645315589`, 0.17473869996337155`, 161.03858609199114`, 68.89582811937123`,
  202.53590118320292`, 0, 148.7485427403951`, 0.37329522564343043`, 0.3763661365622583 7`, 0.008226493959398562`,
  0.013898450478811934`, 0.011863754107758756`, -0.14639735373055118`, 11.716022244879868`, 0.023930529800056444`,
  1.1156752148279736`, 0.2780537808852832`, 0.00015652094364441863`, -0.999437083922592`, 0.26686323877262885`},
{0.2044156153713736`, 2.9629304518198207`, 4.386784427445061`, 1.111833791211073`, 0.9591350877217695`,
  0.43840802875011997`, 0.2469979148221132`, 0.01642865649440784`, 2743.650369521729`, 173.03541841863364`,
  0.08406686391515611`, 5.337297453020938`, 0.28953137005492313`, 184.97631954121064`, 65.17903092102092`,
  658.5210691071982`, 0, 143.0004355859457`, 0.771459058450364`, 0.8115139867734266`, 0.05192100330446725`,
  2.1638434480194584`, 0.055081303383363193`, -0.9745446910987121`, 32.65399338021186`, 6.318905571345912`,
  0.5790830695988661`, 0.9999999995722662`, 0.0006004295061692786`, -0.9993995704935739`, 0.11448111353961643`},
{0.09180583003794474`, 1.0245902356282572`, 8.242121052091868`, 1.338438298182017`, 0.7912235553845766`,
```

0.4563538789330911`, 0.0736594669866128`, 0.007188071678387363`, 2109.8793350532806`, 273.6588416089354`,
0.0378211628956065`, 4.589600480914077`, 0.9767739503530284`, 139.00339267297292`, 51.411315078958566`,
143.43364308394416`, 0, 125.98769010522247`, 0.8181685598278816`, 0.8278164996758037`, 0.011792117567988258`,
0.042517683880815324`, 0.03086100910594565`, −0.27416062473076896`, 11.975535964252595`, 0.05576244657098101`,
1.396249548928249`, 0.49349668699842486`, 0.00027927053785252215`, −0.9994340984545386`, 0.11397194165107763`},
{0.10187608814553512`, 3.02527275016552`, 8.112504151515616`, 1.0430646419869034`, 0.02000177204588316`,
0.406606876656344`, 0.10224573377309781`, 0.465955444901958`, 2596.0557042261407`, 108.81287726186605`,
0.13773652673586545`, 3.785014828823544`, 0.37908929500916355`, 146.65087545516647`, 13.339723230901736`,
203.7839107142125`, 0, 19.642326486141382`, 2.8695378976334824`, 2.883364049342962`, 0.004818250255862466`,
0.050012609402254896`, 0.03125857576952636`, −0.37498610564165`, 124.01621153254045`, 0.0727869857693305`,
0.5914477301174181`, 0.6410038879945756`, 0.00036297418169965777`, −0.9994337410607051`, 0.06082442461538256`},
{0.27609243280065304`, 2.4334000704041427`, 3.768968853050694`, 0.9897634681270504`, 0.04043037685648332`,
0.4200152717945378`, 0.22935860430417082`, 0.1525987200218085`, 3069.7266486703747`, 262.01073398400206`,
0.24155932723410417`, 8.399632627499091`, 0.7132175733358337`, 204.4533153219116`, 26.327634938971173`,
243.32131196839003`, 0, 65.03867440468315`, 3.885490895423395`, 3.919134473461056`, 0.008658771553753608`,
0.09271123763670384`, 0.0400907942364133`, −0.567573519042943`, 135.07076883539912`, 0.3656695878153391`,
1.3568642364203245`, 0.8606624068240566`, 0.0004908230041371544`, −0.9994297148333127`, 0.2626738147146062`},
{0.0978918800897613`, 2.777864894021529`, 4.606090716083992`, 1.4880029918673265`, 0.6064208129951822`,
0.3601049854666406`, 0.18842765361648817`, 0.044059072442558106`, 799.5331038282079`, 182.92623376411473`,
0.07894991488107306`, 2.020622256243092`, 0.5601073680228721`, 75.99584468151129`, 37.96036380247329`,
119.0620576740567`, 0, 44.874387501376326`, 0.6806128071602224`, 0.7215626068084311`, 0.060166072717712904`,
1.4297761884420779`, 0.07609479608602009`, −0.9467785261069881`, 27.009291763311797`, 1.9994782742023933`,
0.8878950119407226`, 0.9999998603105386`, 0.0006205629164078637`, −0.999379436996906`, 0.12204560486791488`},
{0.08900054818761971`, 2.823318859242785`, 7.144670702377984`, 0.8315383304970898`, 0.2795052665240947`,
0.4576393886321606`, 0.05117478065859138`, 0.06552086595628574`, 2102.911960214631`, 101.67438536233578`,
0.08762396482794987`, 9.096737685205667`, 0.25171195782463274`, 370.84843088631555`, 53.27522599811497`,
533.7273623082874`, 0, 192.2444574919397`, 4.317925004774309`, 4.3378212158329665`, 0.004607817652381385`,
0.056781699895009516`, 0.029782062752956147`, −0.47549892292721485`, 174.1554156967897`, 0.07219432025240703`,
1.5900190135080166`, 0.767418763544598`, 0.0004336527947125543`, −0.9994349202608631`, 0.15770622534565146`},
{0.1517146567463153`, 0.8404448307598287`, 8.427449210104406`, 1.2489405079089722`, 0.7937040652491076`,
0.6477616643753492`, 0.08512282603978563`, 0.016388604299130645`, 3405.4110812811105`, 399.1826202269507`,

0.20314112870963702`, 6.737758928823055`, 0.8484255316811282`, 151.81368643238335`, 60.481655147020746`,
156.46155342873104`, 0, 123.1407939000037`, 2.193655756977992`, 2.2143223499705953`, 0.009421073897699417`,
0.025947890209353862`, 0.020954356688466609`, −0.19244468357923272`, 26.33780916312417`, 0.05623821794863669`,
1.2778621996936192`, 0.35949143361537184`, 0.000203194948209577`, −0.9994347710982538`, 0.18644294347800153`},
{0.07742729394690523`, 3.317040912283317`, 9.880999371532482`, 0.8010465312053925`, 0.28743469962710155`,
0.6365305604148241`, 0.19411226430840994`, 0.47831260845896817`, 3458.518042369643`, 116.53097484036505`,
0.09815493609620851`, 4.832652127106721`, 0.5129478012500222`, 268.9105755564252`, 20.37163699828287`,
349.73691122893763`, 0, 34.91427523370904`, 4.833695970809141`, 4.8480721698805676`, 0.002974162867967811`,
0.05300925462893492`, 0.028314315993119833`, −0.46586089181369916`, 229.05096132446945`, 0.05863375914358626`,
0.698535570816615`, 0.7563935795767155`, 0.00042799142593925415`, −0.9994341683516421`, 0.0731494014738828`},
{0.1007555201432343`, 3.8180664304949197`, 4.270424159744305`, 1.4627535709622805`, 0.3807188794187142`,
0.17571212182398588`, 0.053627366653205444`, 0.4983255094785715`, 1310.607317823291`, 380.8287865986265`,
0.23700173797672958`, 3.1928180922211973`, 0.4025308666594032`, 47.225906923722384`, 21.073203453941264`,
50.73444786296926`, 0, 6.4401339740625705`, 0.7324261583535393`, 0.7455223021344018`, 0.017880497073318846`,
0.042648971202489575`, 0.03175532165906575`, −0.2554258458358294`, 39.949310400371445`, 0.0613874182440003`,
0.7349231630719953`, 0.4636570979750012`, 0.0002632380772410772`, −0.9994322569882123`, 0.09500684269931167`},
{0.11012573052984026`, 2.7973646534087315`, 6.317504258164328`, 1.4230817420807518`, 0.4665277158140053`,
0.4964083914255707`, 0.11975389672549558`, 0.008384974576684757`, 3433.4182170381464`, 104.6436908016745`,
0.1542234498742236`, 1.795119919919351`, 0.21223525603288929`, 66.84348949785524`, 7.874854500227617`,
135.07055842840575`, 0, 59.42860572767692`, 0.17332184417231372`, 0.17455936555373944`, 0.007140019697663646 5`,
0.10190334029961227`, 0.05255252928101255`, −0.4842904155398673`, 6.926348579303521`, 0.16031685419893704`,
0.1584294662023804`, 0.7813662815089575`, 0.00044795428626033523`, −0.9994267038431768`, 0.033914519418849876`},
{0.08998066206695893`, 2.280866979574526`, 7.329281823836337`, 0.8411001090310725`, 0.6029746925458184`,
0.27522739162036525`, 0.10930338132980638`, 0.008444888139321425`, 599.859329243076`, 286.89260187390653`,
0.08925071767070175`, 9.77708834471905`, 0.3030303902445597`, 299.72091885848835`, 221.49784791716863`,
305.9587729204112`, 0, 267.6136622599224`, 0.9467535584457812`, 0.9607630512023316`, 0.014797401743647853`,
0.03253093963322669`, 0.02208227544500783`, −0.3211915888696534`, 30.84884184648086`, 0.041816506940807706`,
4.097890769457849`, 0.5644311409390905`, 0.0003178774253483534`, −0.9994368180592951`, 0.44220256971013705`},
{0.09290951514101703`, 0.41749240574692914`, 4.773370270407071`, 0.8850952467526404`, 0.9766517437912483`,
0.4208978561725222`, 0.06525352787617444`, 0.011206616294756987`, 2604.3347674109446`, 377.7615808940852`,
0.1923606467616938`, 6.325030382869231`, 0.9625675284230475`, 152.23669612754233`, 82.96563031497529`,

156.66907343420513`, 0, 131.25579645380716`, 2.9832741203063198`, 3.0167034727418507`, 0.011205591939401849`,
0.046804267645997766`, 0.028006498313103256`, -0.40162511408298696`, 17.792775564131993`, 0.06212231162173162`,
1.4676672640154964`, 0.6770477336073508`, 0.00038321738462214364`, -0.9994339876413435`, 0.13925600967565704`},
{0.12849833171545855`, 1.198455475456262`, 3.2539226138784088`, 1.3648636137864978`, 0.5280448025145577`,
0.6072554649082684`, 0.24978634440506725`, 0.03994982606054573`, 2204.2740688947933`, 354.6777076623247`,
0.08439759959738258`, 4.855199768823253`, 0.40974365814152147`, 123.27628778473348`, 41.31358370492168`,
159.73519130350044`, 0, 77.91147551027325`, 2.43037471004824`, 2.471853379989691`, 0.017066779772666463`,
0.46507681694623904`, 0.06675906107244621`, -0.8564558398958784`, 41.6099411238249`, 0.8537370728160654`,
0.7520889565878913`, 0.9990128306487988`, 0.0005930329392112979`, -0.9994063810583632`, 0.2434635997539952`},
{0.27774537273159505`, 2.913102479760642`, 7.942633412240518`, 1.0326494422606074`, 0.869193199676557`,
0.6849829313653006`, 0.16756021109529395`, 0.031844382721063484`, 1157.358808485019`, 382.85201159938254`,
0.04020804442348902`, 7.095647344980355`, 1.3824063653570637`, 273.4320565088204`, 232.6841345732932`,
266.25937956234054`, 0, 188.55907921581667`, 1.9850490000299836`, 2.0062038075406305`, 0.010657070687084 99`,
0.050867100256258936`, 0.031435535703431945`, -0.38200653182379973`, 82.6093023490531`, 0.20183002457780422`,
2.6135452231477805`, 0.6503225748259922`, 0.0003687283074545978`, -0.9994330070618366`, 0.3015263226847255`},
{0.11633291490443043`, 1.4450658892853943`, 9.421948807050416`, 1.0073582836381636`, 0.9376244508422855`,
0.2923945304421177`, 0.08314225478001569`, 0.015377391816799622`, 1342.5955234153207`, 117.3592016068073 5`,
0.24930648416353973`, 5.058158894639092`, 1.4288639894036779`, 358.7232607929495`, 87.17869086014393`,
439.63576507222444`, 0, 293.64236760546305`, 2.946380119251762`, 2.9851479129282894`, 0.013157770588803963`,
0.4350796693692892`, 0.04758609679218615`, -0.8906267055383924`, 60.82447724570506`, 0.7230583736197739`,
3.3466171680745616`, 0.9995310330605276`, 0.0005720578147515642`, -0.9994276737831741`, 0.08710306800844064`},
{0.16308341937411774`, 2.9768832237222593`, 6.137404711598409`, 1.4978818887932133`, 0.6270064524377599`,
0.3856550031514199`, 0.07688210862674477`, 0.2679284542888144`, 1679.641743573421`, 154.41066935622916`,
0.011847012076814933`, 5.409061271016132`, 0.714419786721034`, 248.29735544089507`, 53.14749547783482`,
274.47787357953734`, 0, 52.67063569634126`, 4.467589004635176`, 4.522810598753797`, 0.012360491097396897`,
0.35029686276161565`, 0.07026786140917225`, -0.7994048223692172`, 189.99272511977813`, 0.8161087167883562`,
1.8748845162924157`, 0.989595034597909`, 0.0005684326600851757`, -0.9994255906302965`, 0.11549065226976969`},
{0.10408875617257995`, 0.7208211774242166`, 9.395809932215364`, 0.8438330742384919`, 0.9431300864694563`,
0.42081055780340804`, 0.06365903598523207`, 0.007548231362222756`, 2388.75487627468`, 266.1557043275724`,
0.07149291165582927`, 4.419002029188226`, 0.6654732054016175`, 138.81213326819974`, 57.0921529838827`,
147.6164469162356`, 0, 125.23925532118413`, 1.185613852134955`, 1.1959705450121105`, 0.008735300164135262`,

0.023525065670778028`, 0.017705611621677116`, -0.24737248900986597`, 12.208793898091127`, 0.03498135463642844`,
1.0131691729542156`, 0.4503450495343311`, 0.00025408708857640088`, -0.9994357946449303`, 0.08308308082805008`},
{0.24431991434407568`, 1.8546418038040455`, 9.014242525232781`, 1.0012178057162573`, 0.9513564875697376`,
0.5540264355265693`, 0.16546833659186994`, 0.010160023644511053`, 3069.21926399773`, 161.6333371189296`,
0.2033203481299034`, 5.464378114605116`, 1.4999257720510482`, 372.91591625211225`, 70.7912894081629`,
534.9136477391231`, 0, 323.8322354724031`, 1.6981546316009306`, 1.7190793644201523`, 0.012322042074280892`,
0.4769058377352096`, 0.04756022440306142`, -0.9002733440443477`, 44.992408129864955`, 1.6645370489379039`,
2.140416577360974`, 0.9998099885184839`, 0.0005753796930910003`, -0.9994245109573834`, 0.1100724707129578`},
{0.19404987421370834`, 1.8913121151690095`, 6.875162123920855`, 0.9378067865957597`, 0.9620319910139619`,
0.17047428599364534`, 0.1676311609323027`, 0.36688161374531547`, 2436.6727196810125`, 312.9444240665532`,
0.08367109576690585`, 6.310397829770168`, 0.3372839322361041`, 252.6037033115109`, 42.7364504135267`,
328.89559059609036`, 0, 41.02918155608893`, 7.542135823400406`, 7.570999581952219`, 0.0038270006305456405`,
0.06716214958761159`, 0.034262126603721854`, -0.4898595888592332`, 203.77904081496231`, 0.18618295256277154`,
0.7104433668158565`, 0.7843565976338066`, 0.0004425003537402111`, -0.9994358428869278`, 0.04726154143090353`},
{0.14433043129139256`, 1.3202167920086731`, 8.463992130027801`, 1.1010419605014736`, 0.14350706526043644`,
0.4137152347701779`, 0.23975541502065495`, 0.00799616367743015`, 1911.0537077498657`, 153.77127625620255`,
0.2417426282164734`, 8.21507395149112`, 1.2501529132389022`, 392.0065391407034`, 44.56042824385376`,
442.7597310385029`, 0, 351.6517941084015`, 2.004359592144795`, 2.0235654469085502`, 0.009582040487656984`,
0.0731227937917448`, 0.038958430540986515`, -0.467219063703434`, 37.802702725331585`, 0.1507692052170762`,
3.3236338520788498`, 0.7581198739770645`, 0.00042846975951966033`, -0.9994348258445305`, 0.2940000178856479`},
{0.047715308213800045`, 1.0917315932828888`, 7.945801231718313`, 1.3183453869548736`, 0.37495408831467225`,
0.3679377030871549`, 0.15114689724124963`, 0.44212694961805754`, 1904.8096035348635`, 21.765700565880422`,
0.02920214850379993`, 1.0562137220921812`, 0.16322301362739666`, 60.750814869752546`, 6.355994129288801`,
380.1407596207104`, 0, 8.37378196271396`, 3.132032247733031`, 3.1601937157824707`, 0.008991436173692913`,
0.23626031509877285`, 0.05981296486802583`, -0.7468344827906458`, 48.84769365758534`, 0.16104619648030477`,
0.14469723980253787`, 0.9793394885885093`, 0.0005501437680736099`, -0.9994382501936417`, 0.01850480961574455`},
{0.20298433403939725`, 3.2126236854576584`, 5.763393946680621`, 1.2592915169856764`, 0.03637647134670963`,
0.17490854387375587`, 0.13159008302912958`, 0.006425245135071102`, 1770.122374418922`, 374.53287469529414`,
0.12888233713533293`, 8.436918800144038`, 0.31480413938383744`, 116.64701187585986`, 33.13876630619161`,
145.11817853469074`, 0, 106.76342234268286`, 0.20649703608976136`, 0.20912377998063478`, 0.012720491977093573`,
0.02289379363789656`, 0.01895333390686241`, -0.17211912509386074`, 9.477103844553879`, 0.06638687793176373`,

```
  1.2895140927077835`, 0.3236268839542823`, 0.00018231610539830507`, -0.9994366472180226`, 0.2251741841565337`},
 {0.05881255333180735`, 2.3801271276267855`, 7.362434757054917`, 1.0646703054096496`, 0.806093766428617`,
  0.17381221060809304`, 0.15459898392254903`, 0.11200780858178885`, 3179.4524228830724`, 78.98698912374886`,
  0.05455878974952594`, 9.203168125761906`, 0.21263297657280256`, 502.039584003915`, 39.529158263495155`,
  1490.9932593871174`, 0, 193.2987452800977`, 8.803270208231693`, 8.826848283401283`, 0.0026783314168343786`,
  0.33168528877154885`, 0.049515210669174`, -0.850716289370078`, 299.3271747777269`, 0.2786751247893442`,
  0.7317950168735735`, 0.9988660093050908`, 0.0005631093495176431`, -0.9994362513647758`, 0.03822360196336204`},
 {0.06579519568062725`, 3.2190545867000324`, 6.479421382941614`, 1.2315548176863993`, 0.37078258595806113`,
  0.3204788641456453`, 0.18121938321856745`, 0.0745089189861041`, 2845.013880184243`, 202.75756338852523`,
  0.11614494172591355`, 2.5591762204175446`, 0.6923322117865267`, 91.63805672573898`, 9.852967488198749`,
  108.67359858955102`, 0, 44.539548422960394`, 0.999173598930731`, 1.005579566629433`, 0.006411265975759806`,
  0.07766416494741697`, 0.04268976029838805`, -0.4503287284773946`, 45.94849080782216`, 0.07299898471556901`,
  0.4410122802055096`, 0.7384923387935479`, 0.00042000363277067257`, -0.9994312688017092`, 0.04942445404054823`},
 {0.11289150267992315`, 0.6299562074506553`, 2.6857393547101953`, 0.7515616774015286`, 0.8067525150133392`,
  0.6856411274749656`, 0.203218351458197`, 0.13643075142532088`, 665.9502014092905`, 389.69992150044357`,
  0.0982534706706813`, 2.604584539279596`, 0.7279001379553152`, 60.576338840790875`, 124.6834537061381`,
  95.58001436431347`, 0, 20.753211101874125`, 3.817195734418963`, 4.003760308962664`, 0.04887477287619335`,
  0.632883697856614`, 0.04078719266913789`, -0.9355534155686553`, 34.35237354216272`, 1.0206741667522141`,
  1.1751457563760934`, 0.9999958512470862`, 0.0006581339469505215`, -0.999341863322603`, 0.383286246395501`},
 {0.1447096604875236`, 0.781557395333436`, 5.079515305869995`, 0.8829448417099249`, 0.5606054450578024`,
  0.17333438670886359`, 0.13484876640308333`, 0.34281542799085973`, 2132.4017848523063`, 228.9439898899625`,
  0.07892443810945204`, 8.583015855961246`, 1.0651655314559392`, 321.69868098648186`, 41.90868935532836`,
  351.6464000759475`, 0, 56.290732481968504`, 21.769319212371354`, 21.96027803176784`, 0.00877192426339013`,
  0.18970560657912486`, 0.04088598858326854`, -0.7844766461015726`, 243.05674888290122`, 0.3921747702950107 4`,
  2.2372371024952815`, 0.986217206147797`, 0.0005607905470983926`, -0.9994313721727804`, 0.13645633907930005`},
 {0.08073679542779827`, 3.436765213609185`, 9.447896083410843`, 1.2321734810402918`, 0.28274585136082786`,
  0.4537000076509107`, 0.2378954237232333`, 0.016202557156482104`, 1882.6285138697513`, 143.19709501896392`,
  0.04435960951477524 4`, 6.7684345441443945`, 0.3648612319782283`, 322.65526736855435`, 43.902979978027176`,
  433.30454403401126`, 0, 262.01245580949507`, 1.2051921664175969`, 1.210580077943261`, 0.004470582929259725 4`,
  0.06533723824777526`, 0.03902090461424572`, -0.40277695138768155`, 59.17089304654696`, 0.07535884626047751`,
  1.1860230806252585`, 0.677892478860429`, 0.0003834678959050035`, -0.9994343234246387`, 0.13995342470328287`},
```

```
{0.17570347686215215`, 3.233960190352999`, 8.02035493411961`, 1.3345169115484334`, 0.9514022176165366`,
  0.3640798512190224`, 0.2136248279814617`, 0.18054342141341007`, 1968.2648913603198`, 103.08834326532372`,
  0.05518740857004362`, 6.915277843565254`, 1.0364954394923056`, 517.391185133362`, 100.3488229877761`,
  1280.916518803755`, 0, 119.14760510979697`, 6.364363240521176`, 7.89916024223973`, 0.24115484043190305`,
  27.87688598333264`, 0.07811471388770938`, -0.9971978680138659`, 294.0299622398793`, 69.97236844801947`,
  2.6828125913501433`, 0.9999999995780156`, 0.0007089725092996828`, -0.9992910274904011`, 0.1704231653862012`},
{0.11925732953940865`, 3.6343980885034854`, 9.962959338328435`, 1.011868751364569`, 0.06493978743288742`,
  0.17742779913292284`, 0.1769046107233871`, 0.005362579496616395`, 3376.132332358329`, 358.9394383628304`,
  0.05158147181807593`, 6.474636554195383`, 0.334042905285048`, 91.21467835508821`, 13.520518367865936`,
  127.05968122879956`, 0, 84.64837516273562`, 0.12176991326358858`, 0.12264874481374105`, 0.0072171485270760005`,
  0.009964088011474564`, 0.008980338713349665`, -0.09872948703303519`, 6.322290571463178`, 0.016975578964911142`,
  0.5372541757972948`, 0.19106572395573806`, 0.00010746686270292795`, -0.9994375398136411`, 0.10012849550943985`},
{0.04791496352556143`, 3.7363958898231298`, 7.859362293932024`, 1.2955394392367978`, 0.21077880799821336`,
  0.6986917555356797`, 0.24146054265338857`, 0.25080131253737453`, 3174.4582657930478`, 214.6472473246855`,
  0.12886781736255348`, 2.1496769931608686`, 0.9494229236050717`, 73.58910844805564`, 9.49951295090009`,
  84.16433298335035`, 0, 16.413416981137786`, 1.0498312260683011`, 1.0580137068243012`, 0.007794091614748533`,
  0.0527934557652493`, 0.035397679659158 9`, -0.3295062968304676`, 56.036929686993645`, 0.0361370929626978`,
  0.5032578666526646`, 0.5786579286005878`, 0.0003310294376270936`, -0.9994279358818644`, 0.08714513295834246`},
{0.07782021540679757`, 2.7209102520090163`, 8.736874384750735`, 1.114706684991148`, 0.7103121173081006`,
  0.20678262886709498`, 0.11036600245925934`, 0.19154162173345113`, 2448.698607159915`, 243.62539193659143`,
  0.13554193141415577`, 3.0074065943997237`, 0.3291541909818123`, 88.72024659300452`, 15.752924500560878`,
  116.37432659120621`, 0, 23.998650136643242`, 1.6216417685285922`, 1.6296609424859787`, 0.004945095836217161 5`,
  0.031491728955669034`, 0.0236164212067852`, -0.25007543282142175`, 63.03345304393539`, 0.03500990186951515`,
  0.37137970842793305`, 0.4546823703947769`, 0.00025661857747160766`, -0.9994356091324834`, 0.03409240840980249`},
{0.17004475261494956`, 0.4834545237544501`, 3.4349181823258377`, 0.9765473132257132`, 0.7016074781874622`,
  0.38572946598569846`, 0.23641719832548008`, 0.044465090938447804`, 728.0925200892948`, 360.1524759911083`,
  0.2343727375251467`, 4.114001391225679`, 0.6989801734218899`, 113.94096765979592`, 103.0243159057653`,
  157.58268916577984`, 0, 69.09927229578183`, 5.4212381130419764`, 5.598119038791633`, 0.0326274039364054`,
  0.5759371006441785`, 0.04935940242380423`, -0.9142972342490241`, 37.441744144288265`, 1.3990725971545033`,
  1.6330239230841108`, 0.999927346507445`, 0.0006127914040866305`, -0.9993871640712427`, 0.3145686056121116`},
{0.14843112442757472`, 3.2599821678006826`, 5.742419985764322`, 1.482241642317249`, 0.2702489297613082`,
```

0.3314664434605483`, 0.1749862727560041`, 0.21934886574956672`, 1289.3717845828933`, 179.7867019868338`,
0.03877340906995197`, 2.6701245702655445`, 1.4405073568802078`, 109.80753601557892`, 21.038401000272273`,
117.02397787317423`, 0, 27.41787754249792`, 1.7185282047810773`, 1.7498542932439098`, 0.018228440112696864`,
0.20421912489537278`, 0.0665160066811 4559`, -0.6742910013191781`, 80.03387574926907`, 0.43303534768328367`,
1.3688148789394319`, 0.9378553970761286`, 0.0005442760986482325`, -0.9994196588297672`, 0.11819216447335312`},
{0.0976585079740021`, 1.3728751665341834`, 5.874627728896462`, 1.1995783460582101`, 0.28537590201313856`,
0.6491235286522472`, 0.13987394486973953`, 0.02287954128834537`, 1880.8681942009343`, 204.39831340676108`,
0.24472299006607134`, 8.011571455649513`, 0.7571200668542781`, 275.6244101441159`, 63.37326330201056`,
300.50184146872857`, 0, 208.11841212070982`, 3.262092015801254`, 3.2939288180072404`, 0.009759627273471017`,
0.08629842955706894`, 0.04408643637460424`, -0.48913976070155496`, 63.97778742061396`, 0.1203967981578655`,
2.471395435242166`, 0.7817396209935753`, 0.0004450750079393513`, -0.9994306608031793`, 0.31870323711277715`},
{0.1434955367081326`, 3.5028874653292856`, 9.961555440173449`, 1.4583790499213787`, 0.7878184950293752`,
0.3804561019338192`, 0.248272531578832`, 0.013284854321714896`, 2386.8492620887737`, 259.97790040425207`,
0.11312758713523102`, 1.6001299135943265`, 0.42901490870982695`, 79.66424879318025`, 13.5082539736143`,
94.86572469232024`, 0, 66.76547926892088`, 0.24742975860259406`, 0.2489619252266776`, 0.006192329624119308`,
0.07513834211627637`, 0.04567877959680903`, -0.3920709678938451`, 12.381694285121089`, 0.15402881041907365`,
0.22062987546430143`, 0.6658033719282772`, 0.0003798347171270316`, -0.9994295091717139`, 0.026921387302082486`},
{0.2174892525974506`, 1.155561627178 4261`, 6.330891972593395`, 0.8691848701006539`, 0.5548185878553895`,
0.5753044260463678`, 0.11961066111713925`, 0.21454325341169517`, 3637.70292743186`, 299.3308064827128`,
0.20558605918965206`, 2.653266548572333`, 0.34524692314089034`, 61.67834938083598`, 13.512556803689833`,
75.68902085031952`, 0, 15.3499398790531`, 2.6396811626628507`, 2.6562087161406716`, 0.006261193098468176`,
0.027467892958685552`, 0.020009823118699893`, -0.2715195465193977`, 43.57591799369889`, 0.08534245014300136`,
0.27415480953737553`, 0.48994576921094934`, 0.0002790619023031955`, -0.9994304228756733`, 0.06835714010841185`},
{0.06749293860797512`, 3.7228921901379612`, 3.4260664843308817`, 1.4192699415393606`, 0.03614877922192594`,
0.4559586129932802`, 0.13413544415463274`, 0.029486897805871767`, 2795.273468589703`, 381.34485667532954`,
0.1616868776536713`, 3.6141605923908813`, 1.2368738510133124`, 56.1006967068942`, 12.62301179076042`,
59.653409912887625`, 0, 39.6305774024739`, 0.30161532019627574`, 0.30687322117440524`, 0.01743247317380292`,
0.06147777077054575`, 0.04031250615174071`, -0.34427508274170227`, 16.041161714066757`, 0.05927593440527228`,
0.873845332869604`, 0.6002034520979805`, 0.00034433297467593565`, -0.9994263062408716`, 0.18791727027847038`},
{0.17265939500359245`, 0.9179315961122354`, 9.1852760379758`, 1.297645317836566`, 0.4142675534047153`,
0.41824524574451727`, 0.0852150150076 7603`, 0.21771628238910337`, 2996.1049031716466`, 204.18324706241947`,

0.02572954244273534`, 1.6360785031153569`, 0.4558745456460018`, 44.87289803894464`, 7.048370914338896`,
54.29485910219759`, 0, 11.07760260690024`, 2.386976752404936`, 2.4059231618851817`, 0.00793740846497859`,
0.030911340329076047`, 0.024280161300916325`, -0.21452253307574165`, 31.30116257454089`, 0.07624476171381339`,
0.24944194189186167`, 0.3987628977691703`, 0.00022693493637047833`, -0.9994309025798538`, 0.034669723661665555`},
{0.11391904455060164`, 3.7004347183812403`, 4.98869805344277`, 0.9900086895027544`, 0.7790608041506635`,
0.5499151960612685`, 0.1741544112916672`, 0.15121348451654992`, 2279.078015486494`, 277.9770114196665`,
0.21022773210729895`, 8.293592982203787`, 1.3768230125107617`, 316.11351028109743`, 96.99535297609322`,
393.1460773158572`, 0, 101.46888277199584`, 3.9631234361027694`, 4.011701364342553`, 0.01225748554719619`,
0.44816279150431587`, 0.04746832642164414`, -0.8940824019274098`, 209.50399365978652`, 0.72934681444675`,
2.664548284845697`, 0.9992690639397661`, 0.0005806664221689069`, -0.9994189088373461`, 0.2761164291063623`},
{0.11443345192899396`, 3.850377768547795`, 8.00437153143687`, 1.3020216404842988`, 0.3359559076583225`,
0.49678442177055226`, 0.2301468632075337`, 0.14684067776603849`, 2776.269241624055`, 290.27051916223877`,
0.07166092983110212`, 1.5123971688855153`, 0.6362818855430454`, 40.36851896973048`, 7.085652134541958 5`,
47.09403236189929`, 0, 13.163474385008689`, 0.4840719758287284`, 0.4881109640750232`, 0.008343776231582112`,
0.03582918757327489`, 0.027209411511860862`, -0.24057972410860773`, 26.626571058684824`, 0.05857225162603691`,
0.24639777933566961`, 0.44257812334857993`, 0.00025343308738701636`, -0.9994273709566358`, 0.05370442161603038`},
{0.1069158661028563`, 2.29284029360078 8`, 3.2223682955272235`, 0.954682665552236`, 0.20354287774001567`,
0.3896645344037619`, 0.21864993682483302`, 0.034752231866091665`, 2419.249949369646`, 316.3106467196254`,
0.1946047812364281`, 6.8989000582764195`, 0.6984200141969727`, 158.74669162913744`, 30.14196739992648`,
182.064879613249`, 0, 106.2470574550683`, 1.5449090624661304`, 1.5602269352520601`, 0.00991506436079681`,
0.1344687465082292`, 0.042769683855452816`, -0.6819358775473124`, 50.60328211959087`, 0.205383464238465`,
1.2472922290497204`, 0.9425256031512304`, 0.0005427042791369058`, -0.9994242020828692`, 0.2526155932159017`},
{0.2686042849529644`, 0.7627258559442236`, 7.922182847008894`, 1.344754730879767`, 0.5223454903809039`,
0.3059455193661864`, 0.17153078163225305`, 0.02722979062605091`, 1945.288646450902`, 327.63559196356823`,
0.15374403181997548`, 2.2340529482759806`, 0.8539303526410174`, 60.31060175137575`, 14.352050194724317`,
65.21428095173985`, 0, 43.42163478833879`, 1.4034077342496882`, 1.4198242959967406`, 0.011697642350410264`,
0.03824590203902547`, 0.028702561479591264`, -0.24952583285122532`, 15.291648076347649`, 0.1467573309938944`,
0.5107199650994567`, 0.4553310345988306`, 0.00025898161225247087`, -0.9994312234559618`, 0.06729744318123726`},
{0.25819223047450574`, 1.7095391280955443`, 6.520962454670094`, 1.1430189535372957`, 0.9365205495994724`,
0.41508933167510853`, 0.1765660191827808`, 0.05380792374101585`, 3754.7043853290033`, 182.2517379088373`,
0.07048991272145094`, 9.71641095911452`, 1.30627739480544`, 480.58068515594096`, 79.9852403327084`,

881.4462486852605`, 0, 270.6229838779728`, 8.102202086552998`, 8.215889784057172`, 0.014031703515869909`,
0.8483310267926204`, 0.05439948591389576`, -0.9358746949058672`, 197.8718784385669`, 3.129035428404606`,
2.6700405936380105`, 0.9999975773581568`, 0.0005764509107298248`, -0.9994235476927327`, 0.1868908716303164`},
{0.11085289703164392`, 3.6133570849476584`, 4.542500201931212`, 1.3471228260354866`, 0.6367711489528869`,
0.3976210616429948`, 0.050489910733311105`, 0.00824863073511752`, 1898.7553106453079`, 276.59295465999946`,
0.21653605876058957`, 5.75252417538405`, 0.6232368998851006`, 136.35563903999218`, 51.6560481785979`,
142.95585788913746`, 0, 121.86733208212597`, 0.2695428152983946`, 0.27316964277503275`, 0.013455478205283145`,
0.07566375428005598`, 0.04409476805064631`, -0.4172273307053025`, 13.913634876502764`, 0.119822090889069`,
1.5165841751206328`, 0.6969689050954667`, 0.0003965822638436669`, -0.9994309900184294`, 0.16280404799508982`},
{0.21896388001044664`, 3.129900208151482`, 6.123839398439741`, 0.8969815671266188`, 0.011595810869147183`,
0.37111266340117355`, 0.1718245052376185`, 0.005208778420460518`, 3863.605974823663`, 250.7012639800747`,
0.15570381237665487`, 7.7211234554844195`, 1.4785494708568354`, 188.093209116885`, 17.778573181053858`,
209.11795689481616`, 0, 174.89048490392864`, 0.28156156404765875`, 0.2849719209397991`, 0.012112295595726685`,
0.032075480204355376`, 0.022390410385624865`, -0.3019462142741489`, 12.589422827431761`, 0.1003338799820467`,
2.0216872694509`, 0.5359484028184001`, 0.00030233081814257735`, -0.9994358956635514`, 0.25483879321543923`},
{0.1576166709633643`, 1.3075085634970804`, 7.656001845154428`, 0.9231504863095856`, 0.8454152618498512`,
0.25647657664732126`, 0.07864463104098307`, 0.005296200134401313`, 3012.7436478007676`, 197.2045635665247`,
0.12709746544272688`, 9.860728262025162`, 1.31076068876941`, 445.2085019695612`, 59.90045236771202`,
472.23721067268116`, 0, 413.469285097379`, 1.5777480139768283`, 1.5913236237201744`, 0.008604422013581248`,
0.06828488054675062`, 0.03416958518617278`, -0.4996024755029207`, 29.470271990217356`, 0.15375479355583455`,
3.0914371078385225`, 0.795675032774044`, 0.0004481939824238283`, -0.9994367122707606`, 0.11193333846027455`},
{0.17154635825703612`, 1.53373134752687`, 8.885182514407113`, 0.952500439197914`, 0.18392969628669165`,
0.39214328491936956`, 0.11223174543931136`, 0.01936642254266069`, 1934.647469413505`, 216.7923390085249`,
0.0451455537258712`, 2.0009219573176864`, 0.3777871026684809`, 48.28358703801988`, 10.440261637376441`,
59.254601265875394`, 0, 37.83348431509711`, 0.4524725655748532`, 0.4567104815810057`, 0.009366128089486114`,
0.020139702242817057`, 0.01622137324308528`, -0.19455744441948108`, 9.913876538829422`, 0.04935560823051193`,
0.3724476148886073`, 0.3636911200079651`, 0.00020640329013639751`, -0.9994324764098396`, 0.062378755531182314`},
{0.0855854522942322`, 1.8098534994044577`, 9.52981117766399`, 1.2278891902893156`, 0.1610609466726174`,
0.1996687073144625`, 0.062022895674954254`, 0.00706028348998438`, 1475.3823287257756`, 92.05986706201287`,
0.09971055550215713`, 4.508838235939827`, 0.938398866245965`, 249.37555804105705`, 23.62637883107007`,
280.18090191570246`, 0, 226.35108446091067`, 0.8418101638142271`, 0.8507842801192693`, 0.010660498875875613`,

```
0.0871954403308072`, 0.04472127138881278`, -0.4871145644870123`, 21.765043868763126`, 0.10660944569579224`,
  2.242795732078179`, 0.7816165502661949`, 0.0004410811793677675`, -0.9994356808601129`, 0.0590022796554568`},
{0.1989224969181887`, 2.0656981604697133`, 7.019145766333462`, 1.189741191045774`, 0.9813366885674477`,
  0.4707046727829801`, 0.138351198132442`, 0.006338022742977132`, 1353.111767359248`, 264.1038821274834`,
  0.2333484554805464`, 8.079661388491221`, 1.0209030718005039`, 427.587607589874`, 229.38175261399897`,
  454.1131455253719`, 0, 390.7883725597692`, 1.1509476806039016`, 1.1635797717750584`, 0.010975382620805885`,
  0.33192436943918036`, 0.05628642596759042`, -0.8304239424701114`, 33.964435808862284`, 0.9432460622405414`,
  2.94961668526772`, 0.9934592670969478`, 0.0005730087851727369`, -0.9994232186420212`, 0.2055813688245668`},
{0.252908269598038`, 1.3368065237315108`, 7.681913157962232`, 0.9670562213906954`, 0.8885087458364935`,
  0.6712630307685601`, 0.1982792702108667 8`, 0.0287236993413668`, 1511.0621870028344`, 393.3001097237484`,
  0.20663414848444228`, 4.051732672816565`, 0.5729449981349937`, 165.56321588319113`, 103.48338388804642`,
  165.53841760173464`, 0, 117.4685114833943`, 2.379126338122919`, 2.396880992392909`, 0.007462678204806172`,
  0.05632055505572121 4`, 0.0322654681060617`, -0.42711026064747437`, 45.43473727977408`, 0.20348477317056843`,
  0.9481258860477866`, 0.708423295712889`, 0.0004042660480218731`, -0.9994293439381958`, 0.15081289564294748`},
{0.19935758638978301`, 1.4210929413876547`, 6.276637411024748`, 1.1091985064517424`, 0.2725861619363459`,
  0.30993881233332166`, 0.061372778664464`, 0.007027369768062639`, 3344.583850960579`, 315.8180746154343`,
  0.0888647235789749`, 8.724591660226011`, 0.9460844323713795`, 157.8293118124209`, 26.623284546148927`,
  170.21168812437378`, 0, 143.37600295391275`, 0.6672516639583925`, 0.6759720297014813`, 0.013069080549543033`,
  0.025391823971817433`, 0.020060211362650987`, -0.2099735968193558`, 13.546094711149125`, 0.07231503915792781`,
  1.819971445439052`, 0.38865728199413163`, 0.000219042861555283`, -0.9994364112761984`, 0.17585766533451488`},
{0.15752424015374422`, 1.1744034635310063`, 9.19368700774531`, 1.4814874364707953`, 0.3259429279011785`,
  0.169821918628328 3`, 0.0532987651599028 8`, 0.11291488360711258`, 1392.2159030202329`, 251.1254711586031`,
  0.20643313217875114`, 1.2559120984693628`, 1.2401551704457958`, 27.190448314685447`, 7.1522186373535686`,
  28.417507190513163`, 0, 10.677752776656426`, 0.9240788229087944`, 0.9441811880045897`, 0.02175395063433827`,
  0.026202369202565367`, 0.02184504652336994`, -0.16629498827032851`, 15.503448145710637`, 0.05896440426945134`,
  0.5006829052140264`, 0.3156396405725411`, 0.00017897031480452438`, -0.9994329916404673`, 0.029988389069811303`},
{0.12243829123563332`, 2.3703577767539246`, 4.839202410108355`, 1.1566976539300236`, 0.5361220364781598`,
  0.5850654781694432`, 0.18441510087344481`, 0.008912199456594784`, 1964.6638548143164`, 274.09302664051813`,
  0.22153291468521247`, 9.911211328491756`, 1.3136185654470256`, 362.498207975259`, 95.10317909855516`,
  379.54539438870944`, 0, 320.977411560505`, 1.1600002021448845`, 1.174332691277818`, 0.012355591926994647`,
  0.29316970316405466`, 0.0548614948970923 1`, -0.8128677885027145`, 39.28022143128926`, 0.5127885356780782`,
```

3.3843488884052264`, 0.9898723580720404`, 0.0005744059529122793`, -0.9994197171501676`, 0.39483396533211174`},
{0.06243550103024703`, 1.4478067318733245`, 4.568123699237523`, 1.4686635133498154`, 0.7846213517525722`,
0.6840252274506751`, 0.19851402241018223`, 0.005300678072293084`, 2514.1415778711344`, 343.58199097307386`,
0.07822848450027337`, 7.993810681409931`, 0.8901709005463876`, 279.54086922764003`, 99.5953229843944`,
331.6006601955318`, 0, 258.8276979308051`, 0.8977741638162383`, 0.9075267154032336`, 0.010863034357704526`,
0.5036117004005608`, 0.07054912961473321`, -0.8599136406905954`, 18.568621115358468`, 0.44918926913147994`,
1.7508130105042552`, 0.9984244232422053`, 0.0005817912555198657`, -0.9994172906411578`, 0.2887867517009347`},
{0.1313087568846084`, 3.644656164823556`, 9.460965509789986`, 0.8885448984294761`, 0.9355716622019723`,
0.25119735021109135`, 0.1694476044963361`, 0.3877738779001962`, 921.248806442728`, 163.92044778354148`,
0.11679660196129243`, 3.129177349018949`, 1.1599711195943847`, 223.38542622267468`, 68.74129136901948`,
293.40060020833926`, 0, 35.42732324433572`, 3.5249057850451133`, 3.565839555751712`, 0.011612727602611672`,
0.31421484952202183`, 0.04206844685883913`, -0.86611566282487`, 183.52956571266924`, 0.5894165897918924`,
1.6632579317151481`, 0.998197784919787`, 0.0005734595522187394`, -0.9994255050843808`, 0.07279912230424977`},
{0.08032938050984856`, 3.6316655074823947`, 4.889233151652448`, 1.047924531280927`, 0.6480389250926271`,
0.41632199836938133`, 0.08565204097388288`, 0.014661199276299969`, 1480.2883480287883`, 317.22766308099494`,
0.1835562909337356`, 7.402391506079066`, 0.616770981275718`, 189.22810009128725`, 89.58540054862539`,
195.24220156375026`, 0, 156.63109616153108`, 0.6124031900745501`, 0.6196858468698819`, 0.011891931514016463`,
0.05934163831679993`, 0.034384008354561776`, -0.42057534422962695`, 31.77205060094179`, 0.06809824349188148`,
2.030318469828585`, 0.7005832335430843`, 0.00039736208203701234`, -0.9994328124582322`, 0.2572850618417254`},
{0.21878401484644522`, 2.7819817431568543`, 8.543045035400766`, 0.9200940784159304`, 0.4855794665787383`,
0.5527693274070447`, 0.20427128258242377`, 0.013404369082852467`, 3643.8303336510735`, 176.1607363330378`,
0.03966906929471847`, 8.016907827776176`, 0.33840217640400705`, 339.50741848795576`, 37.39849669388815`,
472.13451294774006`, 0, 284.75794428423455`, 1.3348081741275486`, 1.3392391706628395`, 0.0033195755174237895`,
0.047242271729540826`, 0.02862424273022709`, -0.3940968187537812`, 53.04874244341965`, 0.14765505542077415`,
0.7593740578762336`, 0.6668422178560777`, 0.00037674133676657317`, -0.999435036764802`, 0.11180951711747686`},
{0.155933613154269`, 0.7900752087685747`, 5.426020030668559`, 1.1675266200556353`, 0.3526751580238494`,
0.32689871232148693`, 0.08872571343828128`, 0.47047660149359233`, 1973.8960115130649`, 259.15398868988336`,
0.014745308659178585`, 4.1998835891931225`, 0.8551070030738948`, 103.60286597566774`, 23.45473384291949`,
110.94665355903537`, 0, 14.390740251862653`, 7.247325609332343`, 7.339973841921077`, 0.012783782264375043`,
0.05135895394827355`, 0.03328836503399692`, -0.3518488505913987`, 81.79903276867282`, 0.1144083893854061`,
1.1626938943861869`, 0.6091034141078163`, 0.0003455148486977973`, -0.9994327484615992`, 0.11660118659529908`},

{0.1460076457981399`, 2.5733113885431225`, 9.325775684390766`, 1.4910714614077438`, 0.8342963441408033`,
0.1882740945287651`, 0.13611188525292933`, 0.04396527811717692`, 667.8083571719217`, 108.22134685664759`,
0.1548306029558182`, 2.291019500853446`, 1.424632992657926`, 164.3480954215385`, 48.39517829622044`,
247.64254016966026`, 0, 100.25751269223734`, 1.6351355813078352`, 1.6790037207747066`, 0.02682844160958453`,
0.758336705129494`, 0.07190941947092266`, -0.9051748135300356`, 60.11018590273628`, 1.581756529118062`,
1.95411122494797`, 0.9999333959168785`, 0.0005846076807257461`, -0.9994153533794221`, 0.06827954545314056`},
{0.26317109341632255`, 3.059519748021583`, 7.63492936446163`, 1.3537697529302852`, 0.126934299410153`,
0.5548921430733772`, 0.20372329915496434`, 0.22074667207931783`, 2265.8574749330373`, 181.2073565626807 3`,
0.09466585200925526`, 2.880276570782346`, 0.773128569372235`, 102.93192565582018`, 14.66547510939779`,
119.22785218547254`, 0, 25.251733164139367`, 1.7309102987959233`, 1.748033912336901`, 0.00989283705394195`,
0.06211676916555562`, 0.039708246935231006`, -0.36074835396864735`, 75.65363201742969`, 0.2335334008682816`,
0.7445338013351385`, 0.6223565168360619`, 0.00035573326197892266`, -0.9994284091957655`, 0.10620933063798066`},
{0.11472120130152375`, 1.4874886419048003`, 7.95738176456699`, 0.7931641460551735`, 0.8816884572777486`,
0.3579341724415851`, 0.08277045109409453`, 0.048113870328459145`, 3701.2844061656924`, 204.25872179904388`,
0.20301688272576546`, 3.1735817377996653`, 1.31887862582745`, 139.4222518813849`, 20.178140313014353`,
148.02094183092322`, 0, 82.92949047179334`, 2.532919914921594`, 2.552552231347363`, 0.007750863463986235`,
0.05026112725282332`, 0.027477203884728504`, -0.45331103008269624`, 53.824137204290615`, 0.0823716699601267`,
0.939199409623012`, 0.7428747450886182`, 0.0004195906505702893`, -0.9994351798155149`, 0.04189713477081495`},
{0.1767902158998531`, 3.597571059730683`, 4.894388517781371`, 1.2037647529410567`, 0.8088893202611276`,
0.4862113706827499`, 0.053623813047437074`, 0.369409668420823`, 1883.086591548903`, 266.54822065935684`,
0.18383750630418133`, 6.949409820987145`, 0.17339220023403645`, 174.287395462389`, 95.44065950271633`,
201.72167143826883`, 0, 28.62868213950449`, 2.7756731598397435`, 2.796559939353251`, 0.007524941990905365`,
0.06536474632140618`, 0.03869406396677207`, -0.40802854528787136`, 142.6525918730093`, 0.16508353734859535`,
1.0773644622755878`, 0.6839524710554955`, 0.0003894334102091885`, -0.9994306133442165`, 0.229090144471731`},
{0.07711195041153773`, 1.310646675677738`, 7.287601935184053`, 0.9421992854061032`, 0.6629854601936369`,
0.19600775055309494`, 0.11597851857342667`, 0.34647099332821635`, 1165.7488565303056`, 392.06574230549916`,
0.08156422577601757`, 9.98958158861084`, 1.355669623216047`, 242.79979405110853`, 105.50026085936771`,
246.45357240681707`, 0, 43.52475817137395`, 10.100625874642693`, 10.24108391958934`, 0.0139058754071133`,
0.026217960634845088`, 0.019724598100576952`, -0.24766848286583`, 189.11931035521368`, 0.028881686862484077`,
3.3019622513445896`, 0.4505365089496076`, 0.00025347198477887613`, -0.9994373996785081`, 0.2850448881021524`},
{0.07030228204141997`, 2.841321925057506`, 9.523175071752728`, 0.957721444616676`, 0.5285225389857424`,

0.687368450677392`, 0.09615328133104123`, 0.49849774355724874`, 811.2137274256684`, 346.8639129623606`,
0.02062867223765208`, 4.642931209436085`, 0.8694461988489701`, 111.05136437969988`, 115.61779961677617`,
105.51617993008246`, 0, 15.42182561561434`, 2.298435515386821`, 2.3413490270843447`, 0.018670748607146237`,
0.018403150358311568`, 0.01509711323627945`, -0.1796451725744328`, 93.29421747427726`, 0.018482620956320847`,
2.035685877456976`, 0.33727070712971396`, 0.0001908813392726106`, -0.9994340411567401`, 0.3607732390896129`},
{0.15160173019169382`, 2.757072174383392`, 6.0862251898613255`, 0.9273854095685228`, 0.9702354173159373`,
0.3952766424689479`, 0.139970431273921`, 0.019384819518267777`, 1328.4246699458445`, 112.51314475175559`,
0.06030008858760033`, 1.0538552754157138`, 0.12506996939343384`, 48.943408585312646`, 23.629902825576043`,
127.32360292799598`, 0, 37.81259775440715`, 0.2579363025573392`, 0.26281693734767714`, 0.018921860715022776`,
0.22092330914180103`, 0.04429986921820025`, -0.7994785186303447`, 10.159271464916804`, 0.47846222722244014`,
0.19507435148262672`, 0.9943562250198984`, 0.0005799343231634248`, -0.9994167740809871`, 0.03046028274094313`},
{0.22937140077917728`, 2.9197558499797838`, 5.991654392537161`, 0.7609979028883141`, 0.7071044023774187`,
0.2974885161887877`, 0.18825283303648666`, 0.032338266898960494`, 2797.371157028768`, 202.1761455304635`,
0.23680881860212954`, 3.62202546942657`, 0.8089322407730246`, 163.80582968142028`, 20.345236163329673`,
219.4919768775447`, 0, 111.39757723042014`, 1.1974614840076547`, 1.211909954298012`, 0.012065916510317631`,
0.29203896792652656`, 0.0360270776560023`, -0.876636060208697`, 49.94707390081173`, 0.9569341022201718`,
0.6931030807963772`, 0.9996166993336256`, 0.0005736469535590755`, -0.9994261330828692`, 0.05808751155522679`},
{0.21166318477480112`, 1.2271964963163153`, 9.968674707787947`, 1.4226030031673544`, 0.5017683729752407`,
0.4896306570856972`, 0.14845091745225086`, 0.13950989408153797`, 3248.3492688454417`, 221.1597513183293`,
0.152837589460797`, 8.879252520814859`, 1.0157863292829834`, 340.605083902022`, 44.38631153012762`,
372.4939719883037`, 0, 115.12218333708967`, 12.157113980838991`, 12.238941831885978`, 0.006730861549538503`,
0.0484168000029555`, 0.034353255590052104`, -0.29046827572340417`, 213.13096689433863`, 0.14640077264604592`,
2.118179158943989`, 0.5183897167368428`, 0.00029244950219698296`, -0.9994358501089916`, 0.18666996028650887`},
{0.20922455243011878`, 3.6942310466303763`, 5.573446425852463`, 1.1411804023081409`, 0.9621527847139406`,
0.28451282132289957`, 0.08975759414482132`, 0.051227081701645576`, 3447.2439359977625`, 280.22849496162496`,
0.2438675699856881`, 2.418161847018231`, 0.8016087345021319`, 82.99789333995739`, 16.28601560573895`,
91.24242767134707`, 0, 47.88594489270214`, 0.6460573356440836`, 0.6522120255349423`, 0.00952653820534799`,
0.09251705712231854`, 0.04402662912297326`, -0.5241241940417016`, 34.095500960566746`, 0.2765262838366548`,
0.41355852687782185`, 0.8223234285184269`, 0.0004680276014373058`, -0.9994308472977834`, 0.0321409756690976`},
{0.2765607651857069`, 1.2704530487690837`, 6.006554660027694`, 1.1061086441305394`, 0.12440018829651067`,
0.36025788190000807`, 0.19633101014307436`, 0.019373612586789046`, 1432.0769734472487`, 313.8129593237045`,

0.16870756948717214`, 9.609773835891207`, 1.2876583132286008`, 220.46482577600156`, 64.66489123145168`,
234.9067683338674`, 0, 173.1726653037675`, 2.456613063224512`, 2.495669322815031`, 0.015898417286462907`,
0.0372224712270125`, 0.026507152336397267`, -0.28787231307842565`, 44.58587936885059`, 0.14706107320919898`,
3.350718021027535`, 0.5141689444132397`, 0.00029027811986414095`, -0.9994354421381957`, 0.5334241792811034`},
{0.08169573554069165`, 2.8299360298510665`, 4.320933911813235`, 1.1825738775709533`, 0.3027516653278022`,
0.5305834377007563`, 0.1475083038117065`, 0.007523165551877513`, 585.1893929943135`, 275.5228279325412`,
0.17684956635249888`, 4.120555621767931`, 1.4111853521392486`, 117.30959605513561`, 94.00709066893859`,
115.48776055006726`, 0, 105.84643378689343`, 0.26828687613482066`, 0.274797186648287`, 0.024266228028965386`,
0.1143376155466905`, 0.0488719110685822 6`, -0.5725648918345241`, 10.84620995871597`, 0.13344136574367624`,
2.5048357894874993`, 0.8608699984744338`, 0.0005007416624579484`, -0.9994183306848359`, 0.3541051466425126`},
{0.18526653849571717`, 2.309967457429205`, 7.235178488173702`, 0.8111816500462055`, 0.21148492456283652`,
0.18475826854386967`, 0.15626027102897042`, 0.057689140810915564`, 766.0020232731708`, 185.02183046070866`,
0.06106549734909339`, 7.0384168617832215`, 0.11685665088837416`, 170.62938847246016`, 72.79558537680839`,
285.5129488381206`, 0, 94.41446408086986`, 2.2391646138584766`, 2.2673625112699005`, 0.012593043511362811`,
0.02270588404530961`, 0.01705738846782644`, -0.24876792139921056`, 73.89139128343028`, 0.060094864865104174`,
2.0702273186434685`, 0.452354245964642`, 0.00025464971361479835`, -0.9994370568732659`, 0.23133955430210315`},
{0.10801077582204055`, 0.4767848668332846`, 7.25138663495552`, 1.4325801889738408`, 0.33847640924280453`,
0.5718972515878133`, 0.105744974897243`, 0.11534684356295588`, 2594.1807156101713`, 318.6618729662216`,
0.06179469905331658`, 5.34696732616077`, 1.3364569926001173`, 116.27203430777665`, 29.910787048045535`,
120.76190421474388`, 0, 44.86036872627769`, 9.130985393871203`, 9.263547760745839`, 0.014517859919435772`,
0.034444267093692554`, 0.026954587354765077`, -0.21744343459405446`, 62.193080786765044`, 0.05314788587709391`,
1.6150573851651309`, 0.4022596929814749`, 0.0002279083679897061`, -0.9994334297669735`, 0.21122665947614802`},
{0.23792597358173623`, 2.688465494136551`, 6.845829046034535`, 1.1002091615985592`, 0.37282035219472753`,
0.3011297207106588`, 0.2473335682094463`, 0.01991156261653569`, 2038.6789052153845`, 210.83183249328954`,
0.08037072870463724`, 9.093415216874622`, 0.3344692931868205`, 324.99741836898056`, 49.352384940568335`,
438.37338121688845`, 0, 253.02269512220585`, 1.81720258002731`, 1.8264180637193046`, 0.005071247307967264`,
0.06230538745485477`, 0.03604237069873096`, -0.42152079986908775`, 69.79266331799053`, 0.21177242813692454`,
1.2868192682178525`, 0.702495043550234`, 0.0003967714656332597`, -0.9994351967757266`, 0.15568002355756133`},
{0.22993078275515533`, 2.525474685703524`, 6.125260191496192`, 1.4929402058003913`, 0.486733717931074`,
0.6225356403190192`, 0.23641243849582955`, 0.0803729054195917`, 1157.473735647006`, 169.73954640708985`,
0.04795903068483898`, 8.28333454337778`, 1.445122239815786`, 405.63288137200686`, 130.71558497289834`,

540.8860515102126`, 0, 187.73448101973975`, 5.661118590618307`, 5.847287826920358`, 0.03288559201189911`,
1.865647492173004`, 0.07487302528302324`, -0.9598675389658873`, 204.24302419103063`, 6.128139831721795`,
4.954923789581232`, 0.9999995643347526`, 0.0006075989314136556`, -0.9993924008038765`, 0.5220310292014275`},
{0.1869769669715985`, 1.485119860176268`, 7.03335904463459`, 0.8235833311873434`, 0.33587109510820534`,
0.6579510066594008`, 0.17782007925307802`, 0.007365768570817107`, 1161.9126891061214`, 206.93000870629214`,
0.23913461974132133`, 1.154555664614577`, 0.17860914505549097`, 34.93368022050941`, 15.060243508639793`,
45.194006162277844`, 0, 31.497452881822085`, 0.147920245091103`, 0.14970894084942493`, 0.012092298503293408`,
0.032570742895395695`, 0.022191334691478118`, -0.31867274987408534`, 3.1382756243848298`, 0.08699969597989876`,
0.27415208589571116`, 0.5620833747011118`, 0.0003269491917768333`, -0.9994183261656677`, 0.08061796887659703`},
{0.2594386598277771`, 0.4907291838119914`, 8.253740230346917`, 1.2562319857439164`, 0.630751008353968`,
0.6003942152343791`, 0.16893163054122257`, 0.12552255641031881`, 2555.8077872611675`, 287.20210893930584`,
0.11105710767727667`, 7.093346066656089`, 1.3316012208831718`, 260.6276191449383`, 60.378428952789`,
270.65425530429303`, 0, 94.55846899354147`, 20.700231770099546`, 20.887680957356384`, 0.009055414902532588`,
0.05347274265462844`, 0.03508619477507427`, -0.34384897738105236`, 145.1172548751429`, 0.19818423845163946`,
2.115446969520992`, 0.5978387814243871`, 0.0003383234371196296`, -0.9994340891764941`, 0.2210033712120714`},
{0.12452706981171563`, 3.242843098104845`, 5.361835103883211`, 1.0740271680209217`, 0.6460165689100792`,
0.29441767681046616`, 0.22857190710285924`, 0.07930287454728857`, 1257.5807309824504`, 295.430402570252`,
0.10895057078510323`, 6.841864249157172`, 1.163933956705701`, 278.6746143553853`, 79.90920171764309`,
298.1664018061183`, 0, 131.4626682066796`, 3.0912488003130267`, 3.1276297956416688`, 0.011769028531432912`,
0.32894646301691133`, 0.05119483767325717`, -0.8443672651053092`, 143.20621195171393`, 0.5851819880631379`,
2.3448867145646433`, 0.9936392330180416`, 0.0005773286292956776`, -0.9994189756099484`, 0.23138554259486646`},
{0.2557093973805171`, 0.4051303627282947`, 4.69346256564500 5`, 0.8860437827795489`, 0.9194156940466172`,
0.45815570325126265`, 0.1510910941798535`, 0.07870004480420398`, 3132.5620218229606`, 302.90817331129847`,
0.08637897198804784`, 9.737653549202872`, 0.46859993602327443`, 344.27398437036226`, 99.43219117652139`,
468.5991510399556`, 0, 162.01089658364452`, 26.632437410183346`, 26.844377752335564`, 0.007957977667908844`,
0.32082988058544065`, 0.04187829607926642`, -0.8694688412349617`, 154.13727183323098`, 1.171988791802469`,
1.227405355971969`, 0.9993176536336449`, 0.0005724929208017618`, -0.9994271161740013`, 0.16744347651103667`},
{0.06140104313465666`, 2.460539185106426`, 5.961918171032632`, 1.1102491165917632`, 0.7798976124509112`,
0.16029666400283926`, 0.13508017803518807`, 0.4919794640607069`, 2590.2148343601484`, 305.84622942181363`,
0.11973020837325932`, 3.9806430906660957`, 1.1111030420450256`, 136.83000162026013`, 18.86879184828117`,
146.02212050237392`, 0, 17.750100307880547`, 3.289968670858684`, 3.313544707953537`, 0.007166036960680078`,

0.07761314342282467`, 0.0401648657902482`, −0.48249917450919255`, 115.64424046314701`, 0.06807897095899607`,
0.8321177762641524`, 0.7756998767444974`, 0.00043818315042539743`, −0.9994351125176604`, 0.05521758284814368`},
{0.1711775238741562`, 0.5019625567967068`, 9.586274753416383`, 0.9484474385429034`, 0.980521223518446`,
0.4330626846988278`, 0.21438996930373572`, 0.2245552941607962`, 3058.4173866521887`, 152.6790489040933`,
0.0944629158119748`, 2.577057545046525`, 1.1465158474128474`, 186.21537319288706`, 28.793793766711087`,
327.4920417381706`, 0, 44.49866861575011`, 17.18317395135661`, 17.37410486689181`, 0.01111504549491169`,
0.38161603550832546`, 0.04484535680631734`, −0.8824856593183202`, 123.21871329293609`, 0.9332012575569659`,
0.7322890168680021`, 0.9997424897243887`, 0.0005728687544067347`, −0.9994269836880049`, 0.045417128480549664`},
{0.26691735492143615`, 2.5320514430618486`, 8.550940885105003`, 1.4373682402592758`, 0.4771646947972308`,
0.18844330768909645`, 0.11201955955138962`, 0.11789932012031278`, 2886.771279587132`, 93.01623349049612`,
0.010902609553686937`, 2.9431133951513715`, 0.705430973142406`, 188.73593479343594`, 9.824373947360769`,
249.7259779404756`, 0, 69.82248746890639`, 3.1379208676891466`, 3.185831852588948`, 0.01526838531625696`,
0.4716565521008937`, 0.06755788516238768`, −0.8567646630552137`, 113.50538658923149`, 1.7984759902586935`,
0.5708740717083125`, 0.9992701715539082`, 0.0005699638211120317`, −0.9994296198992654`, 0.022608821653667812`},
{0.08289232854140277`, 1.1468625434643034`, 6.113671903882498`, 1.141476131790856`, 0.8113048716462563`,
0.5114785013510522`, 0.1437446486191194`, 0.02760266646248284`, 3012.3201335015347`, 281.64171273471936`,
0.03564353737323955`, 9.384615831703261`, 0.7944935894043699`, 387.0123863088327`, 84.56282467583826`,
412.6321744785477`, 0, 277.8347681256067`, 6.2614466536036915`, 6.2960872348157695`, 0.005532360671337999`,
0.12616705945954435`, 0.04832565862234719`, −0.616970873147417`, 102.58598049882835`, 0.14940401919746132`,
1.8037264200428842`, 0.9029604354435461`, 0.000512648663933535`, −0.9994322578888168`, 0.17493544624192794`},
{0.09040683538793876`, 3.340658966022863`, 2.160986355031987`, 1.2703609168223777`, 0.7359849646797021`,
0.4196403007867846`, 0.14182087201107557`, 0.34521905027534144`, 2988.2505759920587`, 354.16133220107486`,
0.21419902011703607`, 5.4345853130842805`, 0.9728978889757345`, 105.52422178241872`, 37.188623997565934`,
170.76081902170702`, 0, 16.14459605747323`, 1.5226195052268283`, 1.8006503282273862`, 0.1826003292655436`,
6.629626880449358`, 0.07154984694141607`, −0.9892075605110726`, 72.665035742533`, 8.562336943774774`,
1.107919478771892`, 0.9999999645725981`, 0.0006828845936788906`, −0.9993171153821283`, 0.24187211744136142`},
{0.10421417760908869`, 3.9806755529248097`, 3.6408344459764717`, 1.322172627166737`, 0.1000592332348238`,
0.48996158640872345`, 0.07063639655536227`, 0.07096628857497574`, 3218.761349505553`, 296.2823833741422`,
0.08840787957878266`, 1.0269425117351112`, 0.10667022544527116`, 9.47411217405095`, 3.305388840340097`,
18.744519868918125`, 0, 4.715005161978408`, 0.08116637607922628`, 0.0824225280398085`, 0.015476260260235186`,
0.025009644811486577`, 0.0209175022520407`, −0.16362257802103675`, 4.615671556829666`, 0.0372337080903571`,

0.08485038118211431`, 0.3254132495941151`, 0.00019257728114354677`, −0.9994082070063719`, 0.07059863338254257`},
{0.2754934613307095`, 2.4772150697863067`, 4.699383051224052`, 0.8551969359601641`, 0.3181125413370589`,
0.21381447461486702`, 0.12130217582978334`, 0.022512766834479486`, 795.9986377450518`, 357.5238954990325`,
0.24036872560514078`, 2.0345694437060526`, 0.6072647478635216`, 39.20739997758763`, 22.335485375469272`,
40.7589786606412 3`, 0, 29.714275874114076`, 0.25633423426669416`, 0.2621969716343798`, 0.022871456808948754`,
0.03175982996482389`, 0.021955432560750707`, −0.308704341771735`, 9.071357543251256`, 0.12499464983264172`,
0.7274176627976501`, 0.546647062446108`, 0.00031165081827331175`, −0.999429886594691`, 0.10099698257423023`},
{0.20556043625089965`, 1.8192901890738584`, 4.292636640368679`, 1.2838668900928696`, 0.08760791600423734`,
0.6818222490294337`, 0.142672004987712`, 0.006686505560345256`, 1995.540026133921`, 283.4866335300326`,
0.2085798635478609`, 3.280310512987043`, 0.6015416808762613`, 69.69322986917942`, 20.58081694953185`,
76.16856476205429`, 0, 63.39262800518685`, 0.22168840973029638`, 0.22514910473094976`, 0.015610626666787253`,
0.06419179518196343`, 0.039803125488430416`, −0.3799343767283474`, 5.761650697910198`, 0.18850419173332336`,
0.7897612563200189`, 0.6488513963130078`, 0.0003761534939792651`, −0.9994202779001221`, 0.2077962306648337`},
{0.24058104513431994`, 2.1873687346811277`, 5.659026523633111`, 1.004047709044065`, 0.8328913078837221`,
0.623891871350166`, 0.08766340991931373`, 0.008430134039147183`, 2601.7165814407754`, 249.12373989835567`,
0.030474235896864155`, 8.991227710673165`, 1.2833381452421007`, 345.8089667017886`, 112.967580476706`,
354.04447819534346`, 0, 308.27756542315336`, 1.1396834266354532`, 1.1526124526004908`, 0.011344401140592542`,
0.11380801400007762`, 0.04281681887226735`, −0.6237802825358314`, 35.6129699265235`, 0.3911435850400102`,
3.103323985934854`, 0.9084666534634034`, 0.0005165223476953953`, −0.9994314349946405`, 0.21433597254492984`},
{0.14879926745595878`, 1.0145913123947308`, 5.175666788640903`, 1.4216396007747538`, 0.30528652163260794`,
0.41852006684217935`, 0.08636270796505932`, 0.024569495844874063`, 1528.3687993879967`, 399.50130012547027`,
0.11493939543886256`, 2.7975290190897475`, 0.9743823827588485`, 44.932627749787045`, 21.479697981684037`,
45.63018322256206`, 0, 33.38047230320228`, 0.7378669555735878`, 0.753423058424443`, 0.021082530845635272`,
0.03555513745216882`, 0.027643793077843867`, −0.22250917704840345`, 10.694762897544436`, 0.07557969153113514`,
0.8277997471384234`, 0.4124754908421502`, 0.00023584925095918198`, −0.9994282102665599`, 0.14836115399106686`},
{0.1829951377410336`, 1.361564914411157`, 4.694817997056893`, 1.2880900199343872`, 0.9454435675757777`,
0.5028590377428427`, 0.06468894028667527`, 0.1666609451608123`, 604.7693853692326`, 275.5852017799763`,
0.22566362443421`, 2.928657758289848`, 1.3281605404183678`, 97.31833701396096`, 171.20003674719035`,
93.9316161690456`, 0, 29.84015785444088`, 3.271185005364941`, 3.3511118148333034`, 0.024433594962461003`,
0.1603147594281334`, 0.05711556739862603 4`, −0.6437285774412427`, 63.62758188361107`, 0.41909744976386243`,
1.744761253162673`, 0.9179673924451094`, 0.0005379239581847699`, −0.9994140053746877`, 0.1501813033954964`},

{0.2341957938761366`, 2.5114017057248956`, 4.373268682378841`, 1.4631865500968817`, 0.20458772158961103`,
0.577553240998902`, 0.11818082574498356`, 0.03355766009617581`, 3153.579024983129`, 307.79686137602005`,
0.09194856938832352`, 3.1157373823819228`, 0.10640897514406333`, 34.61705649468107`, 12.509890621754069`,
67.93587884059397`, 0, 23.394358541077786`, 0.3009256593700678`, 0.30418796399076153`, 0.010840898803786692`,
0.02821309196943124`, 0.023268883637290474`, -0.175245178284528`, 10.796360203405378`, 0.0943912495925862l`,
0.25293978701865677`, 0.3345798553771361`, 0.00019307974832585896`, -0.9994229187883765`, 0.18461860268674393`},
{0.20513991416113786`, 2.033431069067677`, 9.422225723793833`, 1.0288902090438978`, 0.6375890014689067`,
0.2390905203768514`, 0.1852902306229682З`, 0.013541725424023602`, 2549.6747377089105`, 150.27313290555605`,
0.19604710287572352`, 2.636313809911791`, 0.4621075910698911`, 150.78162634910464`, 13.066803148124883`,
195.3429260099746`, 0, 126.06915575033702`, 0.8092842712090532`, 0.8133711084810441`, 0.005049940320581259`,
0.08120998339724343`, 0.0391693488652057`, -0.5176781569624695`, 23.508911154061167`, 0.23799155747338685`,
0.3626810835443128`, 0.8146553099703082`, 0.0004613296912731125`, -0.9994337117973368`, 0.023341709235527357`},
{0.11204311855852472`, 1.7013572433137565`, 3.058168037173486`, 1.4867504783146572`, 0.8996771100732928`,
0.6819517798967545`, 0.19493702157072262`, 0.24346814437911918`, 2914.354454850107`, 387.06404549612205`,
0.01985945025481517`, 5.249142162935986`, 1.0430073575453092`, 125.658061910877`, 73.17307483893188`,
221.467754685356`, 0, 28.516147355817264`, 3.764907213213888`, 3.856834223995575`, 0.02441680646445854`,
0.7192710774159098`, 0.07295343157977381`, -0.8985731056476369`, 91.50645939436633`, 1.151276780037719`,
1.1272237807119996`, 0.9999140913478398`, 0.0005949141099652866`, -0.9994050347773743`, 0.273104831677745`},
{0.2428184603969214`, 2.139771549805288`, 8.962038430839804`, 1.2513955518769984`, 0.15789830342021016`,
0.6821214869737191`, 0.08731003079523225`, 0.005960116741721571`, 2057.9200252572427`, 141.81337749372574`,
0.11756230704325321`, 2.6769215386493905`, 0.895060254520057`, 104.23119415541953`, 17.232471221922367`,
113.52307941071585`, 0, 95.8334156129739`, 0.2555535526368062`, 0.2590702346516335`, 0.01376103747548063`,
0.05085413599579812`, 0.03408301120589954`, -0.3297888059937606`, 7.811803162627225`, 0.17640461439022034`,
1.1166820403723328`, 0.5790381473637654`, 0.0003301937599994793`, -0.9994297547380897`, 0.0931889788362596`},
{0.16708285607632617`, 2.514917463798218`, 5.621232009809386`, 1.0053867061169064`, 0.033964338040640696`,
0.565944967748532`, 0.21275159258566828`, 0.33969022847136165`, 3900.0636957226443`, 236.24950114350304`,
0.17313930544506606`, 5.330368544786425`, 0.772540585340217`, 138.21861625862638`, 14.944513644493819`,
164.0165282811315`, 0, 24.265457495199293`, 3.0817143260882482`, 3.1034542574353754`, 0.007054492742266216`,
0.045317208611382824`, 0.02909540202772262`, -0.35796129286713374`, 110.71795967309265`, 0.10816755205998`,
0.8556261013451438`, 0.6176128612213575`, 0.0003505834294235981`, -0.9994323573043309`, 0.1638046434869408`},
{0.26506128276311264`, 1.6499267057635905`, 8.348095275849516`, 0.7847265312910929`, 0.6385591297525388`,

0.153373190400086`, 0.07053246573144985`, 0.21097306215358952`, 2309.981041141632`, 237.73247710249848`,
0.1662652738138321`, 1.110923557283094`, 0.6469777658769382`, 29.37997803937559`, 4.756396602261252`,
33.60779171935273`, 0, 7.4538539263117425`, 0.8884342919733508`, 0.8978460756320278`, 0.010593674449206558`,
0.02025546690444725`, 0.015567487329445582`, -0.23144268148034564`, 20.94073520918571`, 0.07669914343795725`,
0.21224075866908432`, 0.4254086588150846`, 0.00024076507328901788`, -0.9994340381459098`, 0.013584946490857142`},
{0.08790421115050512`, 0.939015726321446`, 6.470926650783501`, 0.9066579200686449`, 0.8639128923505324`,
0.5386650165495622`, 0.10434830834744135`, 0.024067546956899128`, 1142.301427947522`, 369.2550313603065`,
0.13202765600512467`, 1.8671579613852387`, 0.37302887352364733`, 53.74705602167503 6`, 48.97855965453898 4`,
52.32876035619161`, 0, 40.05200393212561`, 0.9433832325682703`, 0.9539965293359017`, 0.011250249528749645`,
0.03819071470914974`, 0.025293707461508767`, -0.3377000756822994`, 12.65502416185096`, 0.04795892356831136`,
0.5028767570889305`, 0.5885832330017191`, 0.0003380809774750413`, -0.9994256020924163`, 0.09610855444225283`},
{0.2034753024853403`, 2.7863395292250592`, 3.7168431579559567`, 1.0150680060811876`, 0.5649574956583596`,
0.4443788859910486`, 0.06710359282875367`, 0.04727087216660339`, 1537.3049723874492`, 264.87714036516047`,
0.039914831594556555`, 2.4435728162566726`, 1.470321086577068`, 64.13779283572866`, 25.621783341090772`,
64.53362921247144`, 0, 38.42116908728207`, 0.6180596822783667`, 0.6335861579188182`, 0.025121320943013004`,
0.12677529074942173`, 0.04487908025544339`, -0.6459950516370786`, 24.601773202178542`, 0.36850915189865874`,
1.085037697163716`, 0.923129246650015`, 0.0005366956588057145`, -0.9994186126582455`, 0.09436672501613481`},
{0.24480551289378455`, 2.7380941448464746`, 6.271736317361168`, 0.9720924783254401`, 0.03239591203313119`,
0.6916885160816673`, 0.2288933066843994`, 0.006623283304233729`, 2475.282531422873`, 397.0207555711145`,
0.19280932100568626`, 5.189856377538095`, 0.933411035896893`, 89.24490835074657`, 25.48061376875532`,
97.19323178901861`, 0, 81.46169862139507`, 0.18961179718403934`, 0.1923013788894568`, 0.014184674927198548`,
0.020812566653484816`, 0.016718797163494323`, -0.19669700321680605`, 7.416785023763363`, 0.07278615791775854`,
1.145049946343388`, 0.3671733620490104`, 0.00020837898086644392`, -0.9994324779452856`, 0.36739243581183173`},
{0.14578019322843683`, 1.8234631063666207`, 5.479118994270218`, 1.0733190277156792`, 0.6408034729339063`,
0.35566594296423915`, 0.17953675903981858`, 0.2394318445417401`, 2582.269870386629`, 303.8771057624848`,
0.24431388570309442`, 8.496328107267644`, 1.3584441574118422`, 305.26798106307075`, 53.567936901086874`,
320.43076936459573`, 0, 70.69576079369315`, 8.65978123210284`, 8.732515237096303`, 0.008399058018212724`,
0.10695673627597646`, 0.04416696035887757`, -0.5870577029864186`, 225.5827369420805`, 0.22274533830584575`,
2.2423400310171724`, 0.878951160499538`, 0.0004983710870306195`, -0.9994329934250871`, 0.2121464861842721`},
{0.09189072955199334`, 0.5859834843121772`, 9.928586025072377`, 1.1393198615050828`, 0.6662151934740392`,
0.2733768925781913`, 0.07354107739402921`, 0.00787232194478042`, 1945.927425117371`, 381.272657803446`,

0.10334632248372438`, 1.3094920948276538`, 0.643540323804628`, 24.244555629737448`, 9.2325707114852`,
25.661843313661624`, 0, 21.7712008848393`, 0.2579782288343374`, 0.26154058175691325`, 0.013808734708630865`,
0.015301276750615092`, 0.0133211146862782`, -0.12941155804252036`, 2.15958544870041`, 0.020086364052731214`,
0.2741023733199173`, 0.24993121903171434`, 0.00014172208620834973`, -0.9994329556477282`, 0.03561358459481868`},
{0.10029172339419551`, 3.9666265224186326`, 5.94698722721138`, 1.3749912659713441`, 0.4161641564514196`,
0.2013069921355808`, 0.080115115934907 7`, 0.014791692046983768`, 2279.1361385342816`, 360.73732873948904`,
0.013735250572300073`, 8.514487693936715`, 0.49310119875702574`, 147.61363048406673`, 36.2220588156515`,
167.27696015867048`, 0, 121.97535089436577`, 0.4421138744326521`, 0.446550404329858 5`, 0.010034812643913593`,
0.03195246728415929`, 0.025154400380874256`, -0.21275561736214443`, 25.05286600362596`, 0.045779543008938245`,
1.3366002496056784`, 0.3931712603715446`, 0.00022155651540885525`, -0.9994364885286898`, 0.16177366011963595`},
{0.22600110400418738`, 1.9843308027955775`, 5.115418048953915`, 1.1683391486965746`, 0.6057502933993939`,
0.6602202205935461`, 0.11123798530174628`, 0.4580159171910898`, 500.42496785799995`, 188.28582504079975`,
0.18938415994147217`, 1.0587108294757588`, 0.29531889665054534`, 45.1018225021162 1`, 45.024438779943296`,
47.07704529271664`, 0, 6.345149873645536`, 1.2729121758962392`, 1.3330772283369063`, 0.04726567439604046`,
0.3310271131973142`, 0.06073619589262151`, -0.8165219902805403`, 36.083983426920675`, 1.0687499005415566`,
0.644481991891336`, 0.9923678730268315`, 0.0006329205499843412`, -0.9993622117692567`, 0.12993885146681378`},
{0.0717975612264255`, 0.4997816218149955`, 9.589567616976403`, 0.9450897781833669`, 0.6186434889471488`,
0.2554640898762033`, 0.06713444953336731`, 0.20940488388432735`, 1744.4191807628713`, 383.9973692232637`,
0.15609378466736462`, 9.862078934227451`, 0.38202361199438517`, 178.82776526071916`, 75.4036804807058`,
195.34524036938737`, 0, 46.14146033726351`, 16.297831212205622`, 16.465592596293135`, 0.010293479046578513`,
0.01295257189003728`, 0.011217820812986763`, -0.13393101322099854`, 116.36223593290293`, 0.01328518676166072`,
1.8110138561794946`, 0.25553195006550766`, 0.00014370803189290467`, -0.9994376122756623`, 0.24782671004229123`},
{0.26375231265942045`, 3.5837756528213704`, 7.490302795205832`, 1.1022035672057404`, 0.22095637191261486`,
0.15013919278259136`, 0.12602227786755316`, 0.062932127995949`, 2037.490516700962`, 396.7515615806642`,
0.09025977921836642`, 2.8179238772656654`, 0.8710125696526174`, 44.27355135244942`, 10.016708948393898`,
48.79866951717266`, 0, 23.527413024074104`, 0.3959007449424324`, 0.4015530995633139`, 0.01427720127605081`,
0.017017538954191953`, 0.014491802414378356`, -0.14841961264859804`, 20.26884929512188`, 0.0641202179277213`,
0.5545347620936079`, 0.2823769653329411`, 0.00015941505036062153`, -0.9994354530647617`, 0.07350114595370964`},
{0.14659244900446955`, 3.2113358110509322`, 7.247014044575871`, 1.1315336622196723`, 0.22569695706644644`,
0.6068766796842944`, 0.10140090760989762`, 0.07092703696530009`, 1627.6855361153575`, 309.5759062179927`,
0.23915839319989673`, 9.196051471747733`, 0.7212967644528518`, 193.25179972109325`, 76.59283288843133`,

200.97527593291045`, 0, 97.2722280659997`, 2.0457768595952466`, 2.074858122427413`, 0.014215266291515372`,
0.02609453104418374`, 0.02059256696203171`, -0.21084740219458264`, 93.85252129482187`, 0.054646588734109006`,
2.7190165515619955`, 0.39011502144931454`, 0.00022038226538210665`, -0.9994350838771515`, 0.5000391564845541`},
{0.09472256798275819`, 0.9080295101270028`, 6.479473262813432`, 1.475083725476`, 0.4311907138108162`,
0.6630526571829747`, 0.09230911068175318`, 0.22906599144409456`, 1061.3916072436969`, 256.8481876635019`,
0.14505807910429386`, 3.6658722632692093`, 0.8887817463762948`, 103.80674392857824`, 60.027417173693095`,
103.19955541675678`, 0, 25.508273826279027`, 5.593838884405253`, 5.690694037315233`, 0.01731461254273836`,
0.060425256704344045`, 0.040434318366828405`, -0.3308374581729906`, 72.56243974194136`, 0.08176622122922848`,
1.6619591266438056`, 0.5792246850083058`, 0.00033214072635669704`, -0.9994265770521297`, 0.22941648509775164`},
{0.04636398495503513`, 3.7018219205829457`, 3.897884943302158`, 1.3406897199868504`, 0.4374398316223367`,
0.6128218100825888`, 0.1104350576389786`, 0.15302416148791886`, 2822.82344897813`, 307.2546204697094`,
0.07105282376647054`, 2.3240488998943984`, 1.1737510413014665`, 55.099845497000445`, 13.615443334741812`,
57.23515298949648`, 0, 17.67559184195593`, 0.6913774410878645`, 0.7016254505753314`, 0.014822597438733265`,
0.10265341398443008`, 0.051116050467661996`, -0.5020521141613954`, 36.562230954508564`, 0.06799173345085449`,
0.6467006281030435`, 0.7982333737770767`, 0.00046226359880939067`, -0.9994208916665285`, 0.10237858980460728`},
{0.24799856399906778`, 3.665797094880114`, 6.286133411580671`, 1.3307944188010161`, 0.12338821047601245`,
0.6316889254705509`, 0.13912514557691014`, 0.04125136326689172`, 2913.271077872927`, 274.142281421658`,
0.16386049841393313`, 5.467517906165242`, 0.14206080539009291`, 79.12963133196885`, 23.45037664187552`,
135.48337504804874`, 0, 49.90316431999069`, 0.5456233490797633`, 0.5497773788811798`, 0.007613365169255815`,
0.02293017293920604`, 0.01921410460163154`, -0.16206019672973182`, 28.573492685076555`, 0.08123785658807546`,
0.5059295391602454`, 0.3077525590429502`, 0.00017497617827974847`, -0.9994314387544855`, 0.2760609109497168`},
{0.06486156480593103`, 2.9105746808401234`, 9.680954493261392`, 1.2128101831918736`, 0.9623327252795202`,
0.40985068402002933`, 0.2170918483208174`, 0.22870254244919774`, 3443.315648780802`, 310.5579520138933`,
0.21496605031805494`, 3.304326571943408`, 1.3905287372116648`, 194.77493635927956`, 30.116577678231423`,
203.61073813123878`, 0, 46.166597458350225`, 3.4857562418810444`, 3.5023792936686213`, 0.00476885089893897`,
0.0965947800415056`, 0.046339231662198825`, -0.5202718858898232`, 144.93648373142`, 0.08950412265114484`,
0.7396963771116838`, 0.8166725850050852`, 0.0004627490351882253`, -0.9994333726346583`, 0.05338229236016878`},
{0.19444530138658833`, 2.174470015755692`, 3.8344558373626527`, 1.2294570245280045`, 0.29239376697066977`,
0.28694291912822245`, 0.19808018049330495`, 0.005960265699843253`, 3278.3081237334245`, 293.3280672381975`,
0.14107283306589785`, 3.3795768777624478`, 1.417577504179854`, 88.66079807075364`, 10.09618028321883`,
97.33372987102447`, 0, 81.20396969209858`, 0.21357415054739082`, 0.2169676641711356`, 0.015889158941038595`,

0.1379926041604749`, 0.05268972943337826`, -0.6181699029891188`, 6.634436950082777`, 0.383314478644339`,
0.8007137285308161`, 0.9032718145350417`, 0.0005199710424865645`, -0.9994243470967217`, 0.11251474807305568`},
{0.2440718787450452`, 1.76561180349694`, 4.95225717475204`, 1.4224475381097175`, 0.8297144603659123`,
0.6870441812099664`, 0.08604894661393966`, 0.28377285612513553`, 3055.531967355695`, 255.76266261457465`,
0.07376742267862452`, 5.8076740025606295`, 0.30546127533463907`, 186.16633665568156`, 65.3860166213598 4`,
216.66786803934662`, 0, 37.302706835662384`, 5.654275487367979`, 5.694619287467388`, 0.007135096298 2861255`,
0.13181460481734492`, 0.05772322201729277`, -0.5620878119137127`, 142.6179362960045`, 0.45960340348304957`,
0.730472543255289`, 0.8556131648585138`, 0.0004919368147815298`, -0.999425047632521`, 0.15414794638984014`},
{0.13811436070524757`, 1.6573442876198152`, 8.270384729474788`, 0.8471995866439316`, 0.9496826491555777`,
0.3970545948223342`, 0.06715784938015576`, 0.0832034730885496`, 1059.2542132237509`, 302.271366930348`,
0.038752991973303785`, 7.756453819144459`, 1.2408044408349754`, 241.3351475140859`, 223.92718215625504`,
240.74613934789653`, 0, 112.057912280632`, 5.2355402471378145`, 5.311443332578267`, 0.014497660577043048`,
0.027918410142425715`, 0.020004767940928276`, -0.28345604785967426`, 123.95846744567848`, 0.05508476241055649`,
3.3690717591740906`, 0.5072770334885544`, 0.0002859204669532875`, -0.9994363622871175`, 0.22884370728010883`},
{0.04766948135443208`, 3.0149795060421347`, 8.932446832206747`, 1.1136088500069867`, 0.021594711979614933`,
0.15138413756352542`, 0.08685938210991187`, 0.005007564365564 6575`, 1362.2649899849648`, 276.8149684326663`,
0.20202028836193903`, 3.9701409072362193`, 0.8085370569094716`, 74.44765584834802`, 18.811622823186667`,
81.47755159143661`, 0, 69.45156397698383`, 0.11088179765714765`, 0.11277640885578355`, 0.01708676481323068`,
0.01724688547189499`, 0.014671908643434293`, -0.14930097568380107`, 4.775804964705867`, 0.011745001220352557`,
1.1698368483339197`, 0.2833568748542198`, 0.000159546859152298`, -0.9994369402216396`, 0.10494292503155828`},
{0.18773746739616126`, 0.4299564973161223`, 3.9318971489849446`, 1.0624860251059998`, 0.544093066833734`,
0.43356539469874333`, 0.16330330819292005`, 0.040590232235511496`, 2399.820966791748`, 233.03213128875973`,
0.16697913284338012`, 7.094059719533927`, 0.9913896416500063`, 215.89702736066215`, 47.057304068541846`,
302.47168984720423`, 0, 135.32827627167134`, 10.832821603756141`, 11.094743137132648`, 0.024178514421920205`,
0.8659005180829897`, 0.05158386093624547`, -0.9404274973175365`, 66.53774332573438`, 2.3223138611703718`,
1.912339096924777`, 0.9999989290372476`, 0.0005882518014398908`, -0.9994117475685637`, 0.24180386663498968`},
{0.11479403264996152`, 3.823640197501849`, 7.042467218439598`, 0.8671713775083026`, 0.2915521094222331`,
0.3428757834262214`, 0.11149570618715388`, 0.0062188234292322`, 2055.9308370344506`, 383.5883910698325`,
0.2126209830320115`, 4.650760077469522`, 0.9718780818420139`, 80.91371402417963`, 24.127923255354673`,
85.5487589927382`, 0, 74.28970305602137`, 0.11690435397482156`, 0.11868896564221654`, 0.015265570585842658`,
0.0163380247288 8082`, 0.013443456491771187`, -0.1771675759550594`, 6.38571695887303`, 0.026792967773759238`,

1.1217488065912302`, 0.332710105210159`, 0.0001877530317854603`, -0.9994356858152329`, 0.17385768311712405`},
{0.1495588092693269`, 2.9562247150199887`, 9.2229842597602`, 1.0688736942010013`, 0.2958780650578341`,
0.6794653079911299`, 0.137246604211619`, 0.025056259065215164`, 2160.2080931306828`, 208.1776104802966`,
0.2306160093069518`, 8.141052229334282`, 1.0329085826709994`, 280.10096532637834`, 58.18726173571833`,
300.3704731060498`, 0, 206.87637162153501`, 1.6901089123953985`, 1.7078392241427172`, 0.010490632655258514`,
0.03475702808046918`, 0.025025942091060658`, -0.27997462748768953`, 71.37631054141173`, 0.07426028190660311`,
2.835762764058317`, 0.5019881884105197`, 0.0002835150967643905`, -0.9994352156020601`, 0.3072544348202311`},
{0.15518171148576348`, 2.3499047639493327`, 4.360850342323632`, 0.8133940186135149`, 0.676314303847662`,
0.37760044288930084`, 0.07941455326257985`, 0.04333405133254413`, 3294.797232932802`, 200.86295390407247`,
0.24389739709186076`, 4.759995129088669`, 0.4543298663517916`, 155.42962888105674`, 25.14847329868073 8`,
193.2241324224549`, 0, 95.81898910005798`, 1.7041304667577024`, 1.7191052051011475`, 0.00878731918450848`,
0.2155929399956457`, 0.038206452323293665`, -0.8227843067399827`, 57.20777574607328`, 0.4779440201824927`,
0.6589768876662504`, 0.9959153903619355`, 0.0005690752677631109`, -0.999428590748501`, 0.0710461134787618`},
{0.11690059278696574`, 0.8783363086389482`, 5.7539466958675`, 1.1072974777335425`, 0.9679537377157941`,
0.4053342552984798`, 0.06890031093641119`, 0.23033316548333493`, 3511.029547228316`, 369.67514830597565`,
0.18966854703112757`, 4.698501468560957`, 0.7333804491317486`, 112.63940628826683`, 42.811552329780966`,
120.05070713884942`, 0, 26.821115016798643`, 6.3261908192608205`, 6.376453497002716`, 0.007945172565592795`,
0.04239654578736346`, 0.028943734800402042`, -0.31730912830571`, 79.37890131335934`, 0.07080259049505254`,
0.775864792578772`, 0.5593640172280137`, 0.00031664945415730417`, -0.9994339116489357`, 0.0844200827516529`},
{0.104439705960304`, 1.3659305120838559`, 9.102673754552008`, 1.4903475140374016`, 0.6812753308651178`,
0.23307188642089138`, 0.0933185162696803`, 0.15281827719772503`, 2644.042120762095`, 355.84712203695506`,
0.219313267777153`, 8.034413215209995`, 1.3985960311786072`, 193.01059106268926`, 41.440463336438`,
200.84930868390364`, 0, 61.981575866512536`, 6.383810670709939`, 6.453127473186333`, 0.010858217145196969`,
0.030512055993644763`, 0.024684838082675683`, -0.19098083433587065`, 124.56916826413169`, 0.04552385937458573`,
2.0123805213498356`, 0.35624957402347635`, 0.00020057259402630082`, -0.9994369885365446`, 0.15397185150375167`},
{0.12963957180850916`, 3.3365224852422513`, 8.071825011712662`, 0.9114075438466118`, 0.39364722797662766`,
0.55978745554174`, 0.07369835156013937`, 0.12865812035500993`, 1440.5511589977796`, 134.4701033570321`,
0.035366553272213386`, 5.503562356456834`, 0.5009896530595863`, 246.60195549236585`, 58.179829850017796`,
275.2736611973459`, 0, 88.062698255032`, 3.2543750951084083`, 3.28181561050748`, 0.00843188464670752 1`,
0.05833421493595795`, 0.031750982843219915`, -0.45570566299593407`, 155.11850971773717`, 0.10803460923019526`,
1.9298743051425706`, 0.7438446943411983`, 0.00042188538291131566`, -0.9994328313610075`, 0.16115187976301618`},

{0.08241763840187705`, 2.142561606833244`, 5.369049459923637`, 0.9852636250226738`, 0.24182715902115692`,
0.28976691554543155`, 0.24065607285215418`, 0.27404651530612695`, 2847.588493754236`, 186.1353278207099`,
0.17363556899862892`, 9.029927532666129`, 0.11169436731877491`, 128.6516175713218`, 30.659763015580115`,
435.7632168841241`, 0, 26.736355236521007`, 3.2229489803057483`, 3.2420897986322412`, 0.005938914467295442`,
0.020307291646763512`, 0.016409244019358435`, -0.1919531021275469`, 98.64809637122066`, 0.023909700283717757`,
0.7241816931304369`, 0.35782109670598594`, 0.00020167272830317484`, -0.9994363867022942`, 0.2163301968998771`},
{0.05241322492447886`, 1.041476241641588`, 8.16095761491868`, 1.1603610389656813`, 0.26866448983520885`,
0.3320545378754204`, 0.11976413567707805`, 0.00713319584941315`, 967.2404027877401`, 264.4900675349543`,
0.2287367563952014`, 6.379237490331231`, 0.83403545199883`, 165.54806813956918`, 68.50872159503166`,
173.09482512755613`, 0, 150.28960322755313`, 0.948305619582691`, 0.9641948237638349`, 0.01675536225139762`,
0.03023485828247858`, 0.02315923851475921`, -0.23402192600385907`, 14.109111037293953`, 0.022638663253132116`,
2.5871260844306496`, 0.4283700064291117`, 0.00024167282374131457`, -0.999435831593916`, 0.2951347515627514`},
{0.18364277582298344`, 0.43366671305956395`, 6.703835279617622`, 1.4870472910528665`, 0.4047782007506182`,
0.5684293651673247`, 0.22561255273502645`, 0.1213725120163492`, 2694.706223544171`, 135.0368961894007`,
0.022200225329951195`, 8.985808481864087`, 0.4546405408559282`, 455.5012664277439`, 52.48460100928635`,
670.1012123653221`, 0, 166.54983963872212`, 39.806561770711205`, 40.14996417398974`, 0.008626778802363289`,
0.6992454410645264`, 0.07055263481130575`, -0.8991017593137298`, 246.6111543043837`, 1.8344481968375022`,
1.3728017491789728`, 0.999952199803701`, 0.000574675507889225`, -0.9994252970211955`, 0.17803466611200122`},
{0.17373830102452775`, 0.5396236269330235`, 8.716548818231772`, 1.2781997926114008`, 0.2994029165619403`,
0.6311279153766021`, 0.2004572873058531`, 0.029829077121486105`, 1206.6484556475298`, 212.29803760588413`,
0.13764513912866333`, 6.42052885239311`, 0.6565129788897008`, 241.5216774649244`, 78.66650036568984`,
262.98366239761754`, 0, 169.87332849160816`, 8.203030312863433`, 8.286576168940647`, 0.01018475525394602`,
0.05172348263240134`, 0.03461352969499847`, -0.3307966143541273`, 63.23641384669847`, 0.12837642850904143`,
2.3043844409908805`, 0.5782070735226741`, 0.000327985270113206`, -0.9994327546563638`, 0.3426492031473719`},
{0.06373356464631308`, 2.4033771734928857`, 7.841805250818684`, 1.218475494212359`, 0.1766490969017389`,
0.5314535158814123`, 0.18470611278961951`, 0.013035445084968872`, 3902.45780595225`, 188.60058905321694`,
0.11342781551209663`, 2.042548476018199`, 0.8129410978823182`, 67.80435946656323`, 6.108572743647684`,
80.41544939925379`, 0, 57.10937317589976`, 0.2991093020750964`, 0.3012504162913586`, 0.007158300331711172`,
0.047583837413609206`, 0.03235289042956222`, -0.3200865632516401`, 10.269606699809628`, 0.043324108256014945`,
0.36758527394346246`, 0.5650071998648878`, 0.00032172350016127974`, -0.9994305851319448`, 0.05248339618353854`},
{0.0964110511384016`, 2.2365286327741067`, 5.8526191264605`, 1.4618699408667182`, 0.8182028658978528`,

```
  0.387343137712196`, 0.24955267111553703`, 0.1017336334795354`, 1300.5591312646511`, 204.02951635694023`,
  0.010778216795093748`, 2.376136268071548`, 0.7014041691125024`, 107.7437495629868 9`, 39.839762606355215`,
  191.03004065471043`, 0, 43.70897646207576`, 1.8950902354739112`, 1.9370483995600485`, 0.022140457114246503`,
  0.6691438987212733`, 0.070993985779567`, -0.8939032606958895`, 60.54890819040036`, 0.9216123805510236`,
  0.7459499819861413`, 0.9999151455896251`, 0.000588822059666451`, -0.9994111279719449`, 0.09371504028934664`},
 {0.22055192958492525`, 2.063754319806727`, 9.746892660619345`, 1.1263743900346668`, 0.3824619170868737`,
  0.3377396468040734`, 0.07248726297246674`, 0.022971440771960824`, 3783.69159517103`, 308.71982775738024`,
  0.16114587515785966`, 9.879452049970482`, 0.6820463020022816`, 194.6427554512164`, 30.79907367502361`,
  217.7232135124382`, 0, 146.83210608017544`, 1.5648484109665808`, 1.5777222291066217`, 0.008226878750567934`,
  0.016683689453938253`, 0.014289360235219576`, -0.1435131734697619`, 46.13518097107098`, 0.05256599859515442`,
  1.4969286828167185`, 0.27295190650348844`, 0.00015362178082161217`, -0.9994371837046698`, 0.1710518472623681`},
 {0.22700143296888314`, 1.2844277954779395`, 7.271913694665059`, 1.375337551210531`, 0.5655186709000168`,
  0.37705025836034534`, 0.20454803556589107`, 0.08200704828690611`, 929.9638116909423`, 312.52970459059486`,
  0.17194018780473708`, 2.7869672276628847`, 0.6271449892742724`, 92.67461357954767`, 44.238962657447324`,
  96.80445116019284`, 0, 43.063871619269094`, 2.5506726538816507`, 2.583987572986102`, 0.013061228791453328`,
  0.06075127968430173`, 0.039411987770925916`, -0.35125666527959465`, 46.802212197301124`, 0.1970089649003771`,
  0.9521780663827808`, 0.6078929630797478`, 0.00034753119877453285`, -0.9994283019875508`, 0.1329969775645709`},
 {0.18044570897271706`, 0.9524926761671173`, 6.511131206394843`, 1.1225857366708927`, 0.011276794855722416`,
  0.6477197715834044`, 0.16181965045393948`, 0.013358577208209321`, 2629.2386884676316`, 244.22468112889408`,
  0.24315714767663638`, 3.893544737577824`, 0.17056295407521116`, 64.25473279603885`, 16.97015634889428`,
  104.15072584279032`, 0, 53.92926289483713`, 0.699309807266662`, 0.7049411578572302`, 0.00805272646265176`,
  0.021746671250641763`, 0.01784491384494106`, -0.1794186043799949`, 9.515535282761906`, 0.05605847873741906`,
  0.4153923933856015`, 0.3382092199014537`, 0.00019262697211679174`, -0.9994304502633816`, 0.2008947187107132`},
 {0.2574834517331688`, 1.8047481810399715`, 6.678550158926733`, 0.8506612742210977`, 0.9197441161866675`,
  0.28136838946825016`, 0.14128624992883748`, 0.0064270584722317895`, 1966.6222967379854`, 284.61250325943354`,
  0.23333938304045732`, 6.6167222434451745`, 0.3934211369713194`, 275.56435171321044`, 73.7884663423755`,
  320.2566654713368`, 0, 251.93035866568974`, 0.8600079723847167`, 0.8652532513797971`, 0.006099105082172374`,
  0.07257576728537729`, 0.033283753575253254`, -0.5413930183558757`, 22.17282605487413`, 0.26695798675465376`,
  1.062024792621861`, 0.8375590416727359`, 0.00047392708620077836`, -0.999434156802541`, 0.0884179432589972`},
 {0.2641034060728411`, 0.6388918081456714`, 7.451950258224173`, 1.0512911558430225`, 0.698530834423349`,
  0.4711872720679928`, 0.06135082806583303`, 0.2926679041436665`, 1310.8742778358974`, 123.75694501020632`,
```

0.09712150854448914`, 7.74080995928431`, 0.8193975183528215`, 436.06340972251894`, 123.24680456607639`,
479.1721433559594`, 0, 87.08474446704946`, 34.27840726285552`, 34.74831130309952`, 0.013708456073838526`,
0.3855826004118916`, 0.04986575205957785`, -0.870674267961496`, 312.8599085217068`, 1.4547668298745096`,
4.073318701130587`, 0.9975870054699573`, 0.0005745222939772809`, -0.9994240880335981`, 0.17671463362587045`},
{0.05972716054183652`, 2.4084080668442445`, 4.924113152287115`, 1.0973018723863064`, 0.45846865431577455`,
0.17436075418224495`, 0.13306789950554873`, 0.08269850672896895`, 1061.1497496191419`, 231.6428562776456`,
0.027554419387727458`, 5.529015160121926`, 1.014914954734902`, 189.5310296402236`, 48.819523981047865`,
201.84576364974575`, 0, 87.92463433768239`, 2.862448258679441`, 2.899134910534125`, 0.0128165292572342`,
0.1396789315570367`, 0.04793472109197502`, -0.6568221094073785`, 98.48490681611179`, 0.11918037099177045`,
2.170445763975724`, 0.9295632249295755`, 0.0005290760583482308`, -0.9994308337032284`, 0.1475793257711124`},
{0.20906782584649525`, 2.253200985640894`, 8.714537859819917`, 1.139406043052919`, 0.18448556976959418`,
0.3412765002806809`, 0.08047235865027924`, 0.007526938093912886`, 3981.3819787959537`, 150.99126827565385`,
0.12762508996370686`, 7.834813706411341`, 1.133986173662005`, 289.02981128798774`, 19.351839055353157`,
323.0572344779211`, 0, 260.79508871191746`, 0.8378617589271731`, 0.8455116739409477`, 0.009130283047611432`,
0.04485993804449307`, 0.030268433005894133`, -0.3252680604268058`, 26.9695848720788`, 0.13398242449390096`,
2.2291305944488076`, 0.5709898945107781`, 0.00032172380482212447`, -0.9994365507902766`, 0.11002948248418339`},
{0.11363747349097014`, 3.3902353384962547`, 6.9199640132703095`, 0.8644073024825804`, 0.6214378859880609`,
0.26046821126038044`, 0.13701105918453266`, 0.15359214599837653`, 2561.5661019345407`, 185.76122258296698`,
0.15708434183781178`, 8.31641484873343`, 0.7597169041059111`, 393.2053403540493`, 43.64224433482965`,
445.3471903660348`, 0, 124.17730817050568`, 5.436968989572688`, 5.4641709069860545`, 0.005003140070420686`,
0.10221212452370373`, 0.037069233304903505`, -0.6373303707594211`, 263.3229200393944`, 0.16593039415739183`,
1.7042280733312858`, 0.918830852776613`, 0.0005192697442870075`, -0.9994348581757808`, 0.10023433223357714`},
{0.26969936534459843`, 3.0645680107332938`, 7.896848185396699`, 1.1676096938151472`, 0.03246672129411676`,
0.3430750433397468`, 0.18007472085753745`, 0.00937848460863108`, 691.2033005272847`, 393.60922326457455`,
0.10952344673101261`, 8.268833108688174`, 1.2664329385455138`, 145.38130780872692`, 104.79542010740711`,
146.8347030614722`, 0, 128.43627071359282`, 0.3739466131730565`, 0.3835824957469777`, 0.02576807018562821`,
0.01938840800855133`, 0.01631153719575977`, -0.1586964134154023`, 16.371211835031527`, 0.07470059049928071`,
3.630233372270742`, 0.30013493773439504`, 0.00016920926855029252`, -0.9994362226876098`, 0.7442327577210783`},
{0.14835712091109915`, 0.7730242131483069`, 3.477772408678769`, 0.9950646362476758`, 0.43363684394770785`,
0.4723829080128319`, 0.247934187727751`, 0.09606686055299227`, 3641.8956482526974`, 352.02514132734564`,
0.05990938105658994`, 4.715269674310706`, 0.9126567361088749`, 124.26376511812028`, 18.72735460942508 5`,

141.4595460740006`, 0, 52.4837549100302`, 5.897436415605701`, 5.968883776581394`, 0.012114986231412495`,
0.2423384666732727`, 0.047281963343629725`, -0.8048928674316631`, 65.12658778236815`, 0.5136091028807775`,
0.7432369698628076`, 0.989591413268493`, 0.0005759044139847044`, -0.9994180381859998`, 0.1555238191810636`},
{0.20246997439609643`, 2.796989949233529`, 8.601219291155875`, 1.0746358846435033`, 0.23672394588378642`,
0.5308972062471274`, 0.07979598008259464`, 0.06626312934080632`, 1425.6928357201414`, 284.5057677770119`,
0.012297934961132934`, 4.043474061276271`, 1.0687107503597373`, 86.85854473261764`, 35.50918749158596`,
88.58521930822452`, 0, 45.33005274158373`, 1.0119973691836652`, 1.0312833397158643`, 0.019057332676424066`,
0.02048818864195676`, 0.016836607078836375`, -0.17822861878782637`, 40.43637814653551`, 0.0592606147108403`,
1.587344750830773`, 0.33525074386563136`, 0.00018980666533104245`, -0.9994338367063934`, 0.20207106113348128`},
{0.19898919294397377`, 1.094863215028309`, 5.141348317963875`, 1.122536541475456`, 0.25105837287153987`,
0.22994429074756872`, 0.06388318517905306`, 0.28661130713234945`, 1651.4164658316613`, 309.756100189421`,
0.22912307516081604`, 6.461389927282495`, 0.7712732446594246`, 119.18989517446721`, 34.468681908754604`,
126.89223345221336`, 0, 24.750006925711492`, 5.667198893843663`, 5.756529801467634`, 0.01576279733556074`,
0.03450195762201819`, 0.025264239671705754`, -0.26774474803763537`, 88.64010858740764`, 0.09807881003131713`,
1.749293725077939`, 0.48293315918055557`, 0.00027267797001201465`, -0.9994353712002823`, 0.1717515450854139`},
{0.12731801883579802`, 0.6615371738972167`, 6.357864878818987`, 1.255082973105248`, 0.4534404170796209`,
0.35401897300932406`, 0.1793246308628349`, 0.24542217350407658`, 3327.8256670682013`, 361.04151598602084`,
0.1826398340506512`, 5.639198085050134`, 1.1435146635553615`, 135.97016428464661`, 21.672434301600198`,
147.46225323590784`, 0, 30.972234362250205`, 10.035875060958725`, 10.123410322448136`, 0.008722235077431195`,
0.041757031355365334`, 0.029863563576781754`, -0.28482551063954875`, 94.8443489344599`, 0.0759488929232885`,
1.0811273631783698`, 0.5097858859016294`, 0.0002882229578079793`, -0.9994346195809282`, 0.15536497308582436`},
{0.19766525289867687`, 1.5539440876458963`, 7.208959079796127`, 1.2770132142294717`, 0.17265151827851555`,
0.3201118036294218`, 0.21136859719947498`, 0.44059120086782566`, 1120.1312471078827`, 306.58844853012556`,
0.02781674233524073`, 4.752053599009571`, 1.1633837773022284`, 117.19129865559454`, 39.67529696660062`,
123.50824335818906`, 0, 17.525764659164068`, 4.2900304946728385`, 4.358979512438159`, 0.01607191786886797`,
0.0366791057626394`, 0.027401325888839555`, -0.2529445492438914`, 95.23525032882067`, 0.1035740673809696`,
1.7871070359480377`, 0.45934956389696857`, 0.00025998905675619704`, -0.9994340060877591`, 0.2760973879201368`},
{0.16821931653812278`, 3.9393436886276305`, 3.179019625415814`, 1.1903697350607159`, 0.6888287521080438`,
0.2381411552279049`, 0.2058483354518103`, 0.007287906278596642`, 1880.9980419826588`, 370.4098828230061`,
0.17633160606015752`, 7.349472380855945`, 1.3310916733121423`, 181.02536997885247`, 54.282064515870346`,
255.47032651404265`, 0, 161.9396516932446`, 0.2908665855613917`, 0.29802687841708164`, 0.02461710354893487`,

0.6666285981784198`, 0.05783924862762908`, -0.9132361726069413`, 16.368906400913357`, 1.6019972452906324`,
1.9338322947440065`, 0.9999460478598909`, 0.0005888486244335524`, -0.9994111196042088`, 0.28001046476391983`},
{0.27267841809763693`, 2.4698162119019047`, 8.674593338753226`, 0.9507011512806018`, 0.410886345842024`,
0.5761365147011471`, 0.24420414171291205`, 0.043929586623096185`, 2067.2152285645698`, 299.4614654667555`,
0.08071123306974876`, 3.4242661195281348`, 0.9268436590103244`, 106.55924392049582`, 25.857609975754013`,
116.2091782896755`, 0, 65.70761659099954`, 1.1218890165172513`, 1.1324198523071964`, 0.009386700141370996`,
0.02913820792692407`, 0.02139019319235606`, -0.2659056711380231`, 39.58370972784279`, 0.11350514919594921`,
0.8707246248742727`, 0.48045510545889625`, 0.00027263906856260604`, -0.999432539969989`, 0.15050803835979273`},
{0.09734540105037814`, 1.0928119036432262`, 3.3746924724651315`, 0.9148152167379004`, 0.5520222973975617`,
0.5701876550674219`, 0.07525801655581627`, 0.11060319201619584`, 3458.3887900851914`, 360.58656442125186`,
0.07476322979210304`, 5.145406927076468`, 0.3705928304372572`, 93.39702129477001`, 27.768641261774626`,
105.64673858204908`, 0, 36.52035929143659`, 3.4160927804226207`, 3.4432125741434443`, 0.007938834061020028`,
0.05433352120926901`, 0.03086685503149896`, -0.4319003380507347`, 53.330669348507435`, 0.07555883446570429`,
0.6135025596413296`, 0.7147038016251432`, 0.00040878286975353276`, -0.999428038763997`, 0.16505467095087484`},
{0.2386999966275894`, 2.722739872786626`, 1.3062557112348774`, 1.1073558597851454`, 0.32667496326027057`,
0.18792754690523328`, 0.11880359556139461`, 0.03176567183554873`, 2751.8624934955715`, 349.72147263095553`,
0.1223537595062607`, 3.6928606229815575`, 0.1168338292618869`, 35.76602810079658`, 11.505291038426357`,
77.50931701896425`, 0, 24.17937409219017`, 0.2715131092162754`, 0.2798304744027935`, 0.030633383450715312`,
0.1702765548115262`, 0.05121987325396257`, -0.699195973804754`, 10.560850977820325`, 0.5806430437038436`,
0.311639008140795`, 0.9589531800879064`, 0.0005634990480835657`, -0.9994123810632424`, 0.1113554547829968 7`},
{0.11566857496325095`, 2.3986647351668298`, 8.513377414925483`, 0.9662333740036599`, 0.8541931022288194`,
0.36101996809007564`, 0.11530341629473645`, 0.14705784731340077`, 1645.8753074989063`, 310.3763160081718`,
0.036104725588746445`, 9.667079447216771`, 1.4942192927137747`, 354.84974000469805`, 136.84383106361074`,
359.0173883998134`, 0, 116.72870940712517`, 6.7490714793728745`, 6.816997440644581`, 0.010064489830831969`,
0.039195183410354786`, 0.026149755148743483`, -0.33283243313424304`, 231.26799646702707`, 0.06476644300710879`,
3.4431121968630314`, 0.5816781019483852`, 0.0003276882800677101`, -0.9994366501352379`, 0.23173147884991907`},
{0.12899904957253838`, 3.8001456108425966`, 4.68277328679647`, 0.7500997606167263`, 0.7705050389454449`,
0.5067773892687756`, 0.08071179965828676`, 0.006247983675383929`, 2714.029884924169`, 310.5929142878384`,
0.10714318122808536`, 8.497633727984809`, 1.3195673297775237`, 239.92825914554936`, 77.77453193949998`,
245.55191015205327`, 0, 220.11007306101087`, 0.35127088968378034`, 0.3556015622209167`, 0.012328583621133316`,
0.05833603163489966`, 0.028384181645508547`, -0.5134365357048447`, 19.069721852122736`, 0.10750418052481635`,

2.5345324667372395`, 0.8094625179946792`, 0.000458240906177841`, -0.9994338948425765`, 0.2059112452242447`},
{0.19490440880029264`, 2.2807650240422737`, 4.164948405329779`, 0.8920125211738439`, 0.9916182528214048`,
0.24033665404408822`, 0.09749261388251534`, 0.009353219207928497`, 558.6138953254322`, 192.81883910349768`,
0.12112913056266378`, 4.477996891709701`, 1.3978148126017071`, 155.5541216875668`, 177.47719450091026`,
272.04103383413553`, 0, 127.46206334795495`, 0.4942697820726491`, 0.5459676438851125`, 0.10459442128077479`,
3.009181104586404`, 0.04665401506916501`, -0.9844961092577452`, 16.10447473417565`, 8.378609488034636`,
3.236677913603544`, 0.9999999902033692`, 0.0006338721949541649`, -0.999366127798836`, 0.21643943496065735`},
{0.16009076205688888`, 0.5349813484836639`, 5.374724113132956`, 1.4400444163269621`, 0.6922562033282762`,
0.5453216758418981`, 0.13981434114543434`, 0.007280151675390544`, 3925.9979560926067`, 341.46046547891854`,
0.19348925902086372`, 8.443436699305352`, 1.4876822395937013`, 256.76591005178705`, 48.8137716507635`,
265.58049659367396`, 0, 232.27748817457348`, 2.7706259887617173`, 2.798761425773278`, 0.010154902583634273`,
0.09584735557328303`, 0.05111833522098067`, -0.466669321075879`, 21.174760394451834`, 0.21920394564090448`,
2.028123174608541`, 0.7581198510867209`, 0.0004300070017845454`, -0.9994327981239798`, 0.21307342834777887`},
{0.1933638890845395`, 3.103067052973441`, 5.111571620050322`, 0.8661544577982099`, 0.8149222692447449`,
0.1595760565881339`, 0.15190282082017698`, 0.005535189167289717`, 2999.703437637353`, 300.1128617408108`,
0.10100183998523121`, 2.2676395814752386`, 1.3326114208284863`, 85.50230069765172`, 9.45283364194059`,
91.91197283685018`, 0, 78.62511765315718`, 0.13630386926218316`, 0.13822281636602418`, 0.014078449234261159`,
0.1624152821736568`, 0.040040729284790494`, -0.7534669844554507`, 6.042286370004`, 0.44864643725514713`,
0.48824899350363`, 0.9785054771785955`, 0.0005606126768048978`, -0.9994270725204101`, 0.034391109650348264`},
{0.22725673950089992`, 2.3639145368218726`, 8.344676289930643`, 1.325883986841482`, 0.6505898944316628`,
0.43112151917918207`, 0.20810105395654238`, 0.010151436710313634`, 754.9801668003361`, 190.32738043229529`,
0.07958968362345098`, 7.5054987393450965`, 0.6093046711635028`, 463.17679556878113`, 182.5482850625461`,
488.55501876670763`, 0, 403.74334566860097`, 1.6697391218510282`, 1.6871578194690917`, 0.01043198748242391`,
0.2502817564918668`, 0.06152355222249542`, -0.7541828334399807`, 56.38743689776897`, 0.8125459419557427`,
3.429518256686306`, 0.9742776342672934`, 0.0005620453197425046`, -0.9994231158553022`, 0.27498683367744436`},
{0.2309391357161658`, 2.323989179094352`, 2.3593321069217`, 1.3993677346710833`, 0.7027057115205266`,
0.26166872252110007`, 0.14152733686240188`, 0.2848639929776201`, 2532.106437595179`, 386.9846027360387`,
0.07114341876102281`, 5.5959659257370316`, 0.47197407419590953`, 102.70895000517731`, 32.65105448199968`,
155.81710423095606`, 0, 20.231181975958712`, 2.3179211707648686`, 2.4107921518737596`, 0.04006649677316054`,
0.7455512857157446`, 0.06814885582566163`, -0.9085926654123635`, 76.95462455501816`, 2.459670993646622`,
0.7547701275413359`, 0.9999895390733425`, 0.000591231742192433`, -0.999408762072911`, 0.1548562069570183`},

{0.18315330325553064`, 2.869805671153377`, 9.468498073936939`, 1.1725531828202505`, 0.3267495326537466`,
0.6971808886096422`, 0.09399727517157247`, 0.4622104919604099`, 2265.8497866660555`, 116.1383358572474`,
0.02715190911437171`, 5.561797604651474`, 0.6165440590050792`, 289.01416438359024`, 39.422503389796134`,
331.2791548278614`, 0, 39.39823917524596`, 5.937330174932671`, 5.976609929110071`, 0.006615726769455987`,
0.07314874142749027`, 0.04037932752309898`, -0.4479832908249616`, 243.41405439331237`, 0.1913919088777806`,
1.6683642772702736`, 0.7351489712922481`, 0.00041709640261218203`, -0.9994326368955139`, 0.12938425515068247`},
{0.1197189938163688`, 2.6914481530927663`, 7.902408409631434`, 0.8148212240990508`, 0.32365266714619123`,
0.3812630996449249`, 0.2044952740314684`, 0.28795554719398225`, 1263.0479831599341`, 345.1145303739841`,
0.1466603335899901`, 5.094081228711747`, 0.9032476272618188`, 123.6364589925561`, 46.700436698795365`,
130.89062274999654`, 0, 25.39388599673507`, 2.4889604001342844`, 2.5211811810517353`, 0.012945477523753413`,
0.020266858127028414`, 0.01570569568024596`, -0.22505523146183026`, 95.69868388660633`, 0.03466182661121731`,
1.574179018851996`, 0.41362836849458795`, 0.0002334182526519041`, -0.9994356812287768`, 0.26672695165161264`},
{0.17809194642727977`, 1.7697866199706649`, 7.1763151221741985`, 0.9277024880907543`, 0.02661598115385022`,
0.6083929126896732`, 0.174056840727485`, 0.0393026670208333`, 1347.608160247999`, 208.03482783091147`,
0.17902076650274934`, 4.82657960035886`, 0.6328054071662224`, 141.62575060546408`, 40.34030544616048`,
157.2649600638452`, 0, 91.17224071209453`, 1.9147365727788868`, 1.9375997576777229`, 0.011940642500839926`,
0.03448702037645132`, 0.023799860226133916`, -0.3098893448508787`, 48.409645246750905`, 0.08774086550452179`,
1.6297834272248113`, 0.547455048667367`, 0.0003107526514900637`, -0.9994323686442449`, 0.2845735553157551`},
{0.17570694085504118`, 3.3716783189083603`, 9.273626081950546`, 1.268846725094217`, 0.2584124599965636`,
0.4223453277058432`, 0.22832672513046204`, 0.20979915181246073`, 1007.8354949521076`, 148.0706036186794`,
0.04379179030226882`, 1.2158335564871514`, 0.28231812649411747`, 52.091738435922856`, 13.31706028063578`,
68.9609602858697 9`, 0, 13.236405409781717`, 0.7862324684048816`, 0.7944269152833483`, 0.010422422384936292`,
0.05662867903936542`, 0.03671147398802629`, -0.35171586887085415`, 37.87032810489356`, 0.14214359940959176`,
0.3462460931963716`, 0.6088884779150139`, 0.00035112256224542816`, -0.9994233384684044`, 0.04983014903672207 4`},
{0.2345875520938262`, 0.8974705100407228`, 2.4400268875162734`, 1.1692882451601165`, 0.6149116315758598`,
0.4838775739193003`, 0.16902409799301954`, 0.4305314166593524`, 3073.906515744251`, 376.94525437674156`,
0.0327953725790982`, 6.842916385098416`, 0.9809907681036996`, 135.16329435432905`, 41.49916974103216`,
195.85555523511619`, 0, 19.08803496094565`, 7.90266625165965`, 8.411870512110267`, 0.06443448884655578`,
1.5748234077399184`, 0.05931132857604675 5`, -0.962337790837662`, 101.32014159369407`, 5.27762811716805`,
1.335955463815419`, 0.9999986419248871`, 0.0006152285987342985`, -0.9993847705657379`, 0.3044636665443496`},
{0.13345787440765705`, 3.0774883151503953`, 6.064298363382008`, 0.9302596892930706`, 0.6158208110956915`,

0.2585099500502801`, 0.21374523912969606`, 0.4331393141373296`, 3227.8457633864045`, 202.63915585725056`,
0.17639348124623005`, 1.682515063867495`, 1.3735184213990381`, 76.43471174486858`, 6.587603389320726`,
95.34527305684396`, 0, 10.909544621601352`, 1.440997948568805`, 1.463402850672314`, 0.015548184593712655`,
0.2518334189694486`, 0.043677378839035295`, −0.8265624196432243`, 63.35220498394563`, 0.4801307542925155`,
0.4429511708448082`, 0.9953935800236154`, 0.0005693308308404552`, −0.9994280344556502`, 0.03866933234823202`},
{0.24299864830682322`, 0.6348382188885924`, 8.738994620341877`, 1.2356500801881907`, 0.5770135193458605`,
0.3643052268917296`, 0.09417548384960592`, 0.18321093257407522`, 2194.4027446042355`, 392.84971084704546`,
0.021287183084439776`, 6.026738164007792`, 0.24540222372747866`, 106.09299054826107`, 41.256525579470456`,
125.48040118351318`, 0, 29.87473107199416`, 7.5614219237899345`, 7.623672281427478`, 0.008232625855950415`,
0.018207749778772493`, 0.015616209963583556`, −0.14233169099293552`, 68.57542323377128`, 0.06320655121359199`,
0.7926685663131374`, 0.27127923040478397`, 0.00015309340699862285`, −0.9994356611570662`, 0.18305216837131916`},
{0.23128596410211183`, 3.0231614800775644`, 9.282448241767675`, 0.9502905953068014`, 0.1244248471921543`,
0.513834937748406`, 0.07158073583305624`, 0.006593831926001459`, 1596.9261281907548`, 180.24662525069675`,
0.024392561873633367`, 8.693689234388824`, 0.44515269072814956`, 252.2400234718569`, 61.705581161343666`,
285.673113386978`, 0, 230.41866212969825`, 0.4861356200041556`, 0.49142078406783213`, 0.010871789365344942`,
0.024201982082324706`, 0.018641840396290927`, −0.22973910430643973`, 20.995235435574095`, 0.07996541084418025`,
2.4381490303911693`, 0.42132586903824243`, 0.00023755323134169448`, −0.9994361769623025`, 0.3066810133856683`},
{0.14763336174315628`, 0.9609083924153881`, 5.767031511187355`, 0.7806215764240099`, 0.6823220865642827`,
0.65198969122125`, 0.1566301693296936`, 0.37595000066945006`, 3860.5140609631935`, 333.3928376138639`,
0.19492955999326989`, 4.361121532997624`, 0.15723055392755292`, 90.63310141229265`, 27.803504956912697`,
143.29293291322216`, 0, 14.468009908215466`, 5.166621926883488`, 5.188104646793184`, 0.004157981794238763`,
0.024088679570257267`, 0.017649687832831766`, −0.26730364022841013`, 70.92357671399576`, 0.050804182070133416`,
0.30272477124641745`, 0.4821719392415573`, 0.00027387344197139926`, −0.9994320004552687`, 0.12127979229039353`},
{0.08020873136305112`, 2.3560872112839712`, 7.656859171402218`, 1.2445222240766278`, 0.028603428928333008`,
0.3751343060067356`, 0.17582324568527535`, 0.011898009060677216`, 1487.4044642092413`, 223.64331235141128`,
0.06070604179895395`, 4.4126063398287325`, 0.25472858441799273`, 99.73741651191187`, 26.469084471206966`,
133.90782254682873`, 0, 85.26267094471065`, 0.41415839495029083`, 0.41807162949090115`, 0.009448642327001489`,
0.029346579784936273`, 0.02305274498276071`, −0.21446570088573713`, 13.939904254118245`, 0.03362645620560968`,
0.8361950118702937`, 0.39634719489655923`, 0.000224349890954491`, −0.9994339561529809`, 0.17667310887473922`},
{0.2593624262161626`, 2.295431745726197`, 3.3398768253362086`, 1.233697621211021`, 0.17962106789139365`,
0.6119977215212877`, 0.1479404509305488`, 0.2392187562484209`, 1384.473686906931`, 390.9701237284811`,

0.07023942191875382`, 2.3245474291144017`, 1.4493220736835069`, 41.78830130010848`, 21.345422172608412`,
41.545785861320454`, 0, 10.011331349701049`, 0.9305121271914312`, 0.9566911035676647`, 0.02813394432080063`,
0.07081855036832169`, 0.041493872239991327`, -0.41408187509932104`, 30.513243950548834`, 0.26239530063772043`,
0.8439049287783036`, 0.6952303980257134`, 0.00040907171126007596`, -0.9994116026680914`, 0.20173469010858516`},
{0.24870831012490324`, 2.875980304064811`, 9.212538429441743`, 1.2722063248788578`, 0.3282913490792485`,
0.2805639294283834`, 0.1572124446820367`, 0.027187541841276704`, 1491.5273167119358`, 343.00060613142807`,
0.23102970098548903`, 4.50321802361168`, 1.2980803493745015`, 100.64121937273006`, 30.450714028189378`,
105.86655823981083`, 0, 72.7927577170037`, 0.6588319011661047`, 0.66896506142505`, 0.01538049423686072`,
0.023425353064028022`, 0.019358594157185875`, -0.17360502083902685`, 27.06839387776129`, 0.08322971392333899`,
1.4413454645187047`, 0.3266132451606364`, 0.00018436870107250896`, -0.9994355137037328`, 0.1882894852617396`},
{0.044603778808110894`, 0.8401502429199104`, 7.1121926093305605`, 1.2733813165569137`, 0.08797724114115923`,
0.30964677383308725`, 0.08962339338921654`, 0.02592380473764512`, 3666.957163896842`, 393.9775757725391`,
0.24527553411695868`, 5.578947990675762`, 0.6585937869939971`, 74.84756773692959`, 13.184735701294594`,
84.75552676109723`, 0, 54.78739102383128`, 1.539691804198738`, 1.5557327274138892`, 0.01041826888433639`,
0.01738198598871486`, 0.015075102858486655`, -0.1327168904477275`, 18.479606333133763`, 0.011075746546946234`,
0.7497212433582724`, 0.25413128674756047`, 0.00014336246575646574`, -0.9994358724280223`, 0.14056503607086984`},
{0.07858821042838116`, 1.6977143802882821`, 8.212860235255583`, 1.2964521187700828`, 0.2987911497011755`,
0.506126935243553`, 0.11440340360512286`, 0.05206277291990468`, 1339.867027105688`, 383.7096297624307`,
0.2078889107072941`, 8.822910683955193`, 0.960825500308768`, 165.5716655675822`, 86.93178799592657`,
167.95884002335995`, 0, 96.09200893657402`, 2.7493673076757896`, 2.7965077447359152`, 0.0171459218739225`,
0.022212234182763614`, 0.01859327192606623`, -0.16292653079921382`, 66.68057735622385`, 0.024937424771934644`,
2.8222038598565518`, 0.3077462498370518`, 0.00017365740017521382`, -0.9994357123758059`, 0.5184211770734581`},
{0.22377870799620725`, 3.1928985468211346`, 8.059299312075755`, 1.3921709162211404`, 0.8873395666266357`,
0.17674042984905347`, 0.1675175234386073`, 0.03730263939446273`, 3395.3924465278315`, 247.66374801748043`,
0.05539393372492046`, 2.5087357156089176`, 1.4318151504319032`, 135.42240462114998`, 10.819240367798091`,
143.41379059769338`, 0, 88.13466205279002`, 1.0009864461357996`, 1.0099831952882028`, 0.008987883089860205`,
0.14800257548369825`, 0.058434168439862676`, -0.6051814081688132`, 45.657830989352135`, 0.47314035888366757`,
0.5786717845531774`, 0.8953748975772725`, 0.000509040006102782`, -0.9994314783589755`, 0.03124784217306769`},
{0.1736316989567453`, 1.5454802872078899`, 7.071248146205292`, 1.1599254463866382`, 0.1492821504834294`,
0.5667749351814715`, 0.17572322700248577`, 0.00782459398724836`, 1916.2311636952181`, 145.94896290337653`,
0.12610631603338446`, 7.247312880006737`, 1.199936026275772`, 336.4355601477547`, 45.87002706553747`,

370.74963099140444`, 0, 302.3108538537636`, 1.4490674230571003`, 1.46569368971314`, 0.011473770227311775`,
0.11516153825744392`, 0.04778232993856738`, -0.5850843027838872`, 31.992930530998358`, 0.28565276488733016`,
3.26265855290155`, 0.8767150068625382`, 0.0004989331916352929`, -0.9994309060667037`, 0.271430113990466`},
{0.08130667751836484`, 0.5260337564871658`, 8.449122941731599`, 1.4306920647099228`, 0.5994021973571051`,
0.5116121891099925`, 0.1394200086694753`, 0.006254931094025137 5`, 1045.8518042423143`, 239.77908991527943`,
0.20556349167963178`, 7.684382915701004`, 1.0762288196903413`, 308.9990584064868`, 138.68036069151805`,
313.98894251906984`, 0, 283.53040630959845`, 2.938455044943126`, 2.9767265157243314`, 0.013024351298845938`,
0.06821866107131556`, 0.04273424385025651`, -0.3735695896232517`, 22.081807793715637`, 0.07923760966370398`,
3.5332864919974574`, 0.6387871170914663`, 0.00036170134276442863`, -0.9994337685700185`, 0.3214000319294397`},
{0.09526052730311735`, 2.7416128013280456`, 5.368501678201278`, 1.47877684019711`, 0.27293015978313817`,
0.15337029979855754`, 0.05033287162982811`, 0.0696637301808707`, 1979.7812280561757`, 208.2796492673417`,
0.10966372378753608`, 9.228791958515647`, 0.5867218918142831`, 229.60542156358417`, 36.650196048742046`,
262.9789485717359`, 0, 116.01240321246405`, 2.8241105194796092`, 2.8513317812076613`, 0.009638879760650365`,
0.06750799664794303`, 0.04300489782203982`, -0.36296587134244074`, 110.60882217957814`, 0.0918692479696911`,
2.053891252842835`, 0.624844049432296`, 0.00035220683665500746`, -0.9994363284135057`, 0.11460165914720014`},
{0.2246406125901032`, 2.0919939936953575`, 9.60889385739615`, 1.3369466676292021`, 0.09066206605235938`,
0.26041948811653903`, 0.1688935708724063`, 0.00631072521856942 6`, 2524.013414165627`, 287.3446683034351`,
0.19227735577509142`, 2.226723678369055`, 0.32009387356875707`, 38.69036627237492`, 6.98390543910818`,
52.939248355129976`, 0, 35.41088028884907`, 0.10261818775483016`, 0.10359530841307196`, 0.009521905225770455`,
0.017334060421551372`, 0.015152485262359383`, -0.12585482605562193`, 3.0668090346715307`, 0.055627627882445393`,
0.258737261953702`, 0.24265595651673844`, 0.0001374284695270996`, -0.9994336488932732`, 0.05332922321599092`},
{0.2242164690648932 3`, 0.8944001471401766`, 8.80542685575837`, 1.1224114425628389`, 0.07399275239021863`,
0.6541781208595552`, 0.1361819449849342`, 0.011426236224829198`, 1677.1018012951417`, 95.2905099154865`,
0.2261399510764292`, 3.273626323923104`, 0.6313176099847766`, 187.13715130141787`, 23.58043778884421`,
221.17950456185628`, 0, 160.56087428016792`, 1.888499068577733`, 1.9060734136266437`, 0.009305985552932494`,
0.10574040763774424`, 0.045699322668887356`, -0.5678159022665343`, 24.12962635442871`, 0.33869629768597675`,
1.201783919566512`, 0.8612543143240508`, 0.0004933942549100569`, -0.999427121296574`, 0.10088033617470656`},
{0.04830398547205017`, 1.275316218137875`, 4.614953899177735`, 1.2829927355973278`, 0.16612061807564849`,
0.1639575953122483`, 0.12272200896549845`, 0.011345722370418136`, 3563.2586080627198`, 299.98688385512116`,
0.24385149927488797`, 1.0836535164777654`, 1.0916200214657223`, 20.37963869670357`, 2.113535622311134`,
23.01987068048485`, 0, 17.484154635730576`, 0.14587074462072838`, 0.14789979586479163`, 0.01390992586854134`,

0.04708975960621404 6`, 0.03273448310190362`, -0.30484922039007556`, 2.6575903766666134`, 0.0324946151985866 2`,
0.19233000411587353`, 0.5434954585468432`, 0.00030970788394094 44`, -0.9994301555255511`, 0.02456570262081358 6` },
{0.0990512599695320 7`, 1.8406681837351115`, 7.615683537936391`, 1.104455595350767`, 0.8507777546248461`,
0.6055060761025552`, 0.22355503841638658`, 0.019159603171833028`, 3698.4676016336716`, 125.34182227908332`,
0.18683452817866786`, 1.0248116987499856`, 1.4496781158120586`, 61.521083790530376`, 8.628227633026386`,
127.37114122614238`, 0, 46.74180298655649`, 0.4647103276028991`, 0.48434534352202785`, 0.04225216172924639 6`,
0.8600127722105201`, 0.05453956172938101`, -0.9365828467998258`, 12.219678781025364`, 1.2169335525334617`,
0.4002267327062011`, 0.999999604797999`, 0.00059907970378451 86`, -0.9994009200594579`, 0.03615697429328431` },
{0.2122053342005913`, 3.2723497606887317`, 8.074911535839025`, 0.761570924685792`, 0.7803544809949507`,
0.2686687403920138`, 0.24235001021375163`, 0.4921495003944174`, 553.2367270947411`, 320.67673970953626`,
0.04374240634138288`, 8.97316011873557`, 0.13752746723967268`, 402.24129159303493`, 275.04181413570865`,
446.4349388269953`, 0, 53.12568753122195`, 7.307511571284933`, 7.375271551851547`, 0.00927264772769964 6`,
0.04901655721800158`, 0.026535145370074467`, -0.45864934470898944`, 341.6104820217774`, 0.14859392722659506`,
3.514337117689015`, 0.7482826790922352`, 0.00042190731207902 85`, -0.9994361658717119`, 0.31409586258297617` },
{0.202676870171640 5`, 2.320679359442215`, 5.20658497151164`, 0.8289159455996751`, 0.9558590658000565`,
0.5980005929117111`, 0.20724756852094034`, 0.0547372318523302`, 1071.5776650859361`, 208.90678381552232`,
0.10872472503561953`, 4.389182379894066`, 1.1547524475482653`, 182.86552923201523`, 174.59601196081223`,
388.80896159639997`, 0, 101.37959943698881`, 2.2707933127035886`, 2.349607305869559`, 0.03470769123947037`,
1.0089798682087465`, 0.041604840070344457`, -0.9587654408365888`, 75.28261671929471`, 2.921384025067781`,
2.204439034434538`, 0.9999999139463286`, 0.00060814739662683 41`, -0.9993918525510398`, 0.2720348491943002` },
{0.2780417648717225`, 2.452933782766964`, 5.240377582277642`, 1.0300585799728414`, 0.04659296483118158`,
0.5270409461030353`, 0.07214810969171709`, 0.4018520591476553`, 1664.9812973970193`, 368.4059321600538`,
0.19070038794765692`, 6.66208935797424`, 0.8907914326786526`, 101.26494953336142`, 42.84916086930728`,
102.48492775337655`, 0, 16.656801311791437`, 2.3442212220962095`, 2.392829101467631`, 0.02073519295587478`,
0.023719739432960275`, 0.018740079352221304`, -0.2099373854764769`, 82.14599185655821`, 0.094215403060562`,
2.011225044507218`, 0.3891264091903722`, 0.00022044472405351 67`, -0.9994334881446053`, 0.379134815164016` },
{0.07805827059301479`, 2.683721229977123`, 9.002208120629334`, 1.281556673983075`, 0.9431208960986435`,
0.16472833391173414`, 0.07811354421183636`, 0.31224015378681164`, 1280.994389531913`, 98.15018684851424`,
0.09634892070904322`, 1.4706754835946878`, 0.8821078383157976`, 98.47612422310405`, 16.98858084853399`,
141.21989130031184`, 0, 18.443471932296145`, 2.014100188870932`, 2.044850018423405`, 0.015267279017391333`,
0.37833205097568295`, 0.05976792278285974`, -0.8420225761240057`, 77.21833480248344`, 0.4218849372725186`,

0.681279495263404`, 0.9980161917151896`, 0.0005652995476755297`, -0.9994335767772424`, 0.01900489660260804`},
{0.18436703585318248`, 1.635211707023542`, 8.369994731341517`, 1.0460883726353645`, 0.04194749676902032`,
0.5545482251392689`, 0.12087395792220468`, 0.0196688212581193`, 3332.658025818686`, 221.69628027119882`,
0.14765622082279756`, 3.2016279616467553`, 0.8091642391531453`, 77.78051622837076`, 10.345299704612975`,
88.45463648498598`, 0, 60.77808279995679`, 0.6934493348921494`, 0.700369828254495`, 0.009979811089474877`,
0.024092970513005886`, 0.01904494536280063`, -0.20952273807334088`, 16.199092437761866`, 0.06345642226258824`,
0.6849472527384588`, 0.3891709564895366`, 0.00022059129214481264`, -0.9994331763754042`, 0.09958931739263624`},
{0.23462399893919866`, 1.1910919878969217`, 8.83980004261467`, 0.9529401154166846`, 0.0219549211086527`,
0.5092860902949421`, 0.15815979544916958`, 0.02056729012396142`, 2175.860445178102`, 128.66287522198218`,
0.01279382216028177`, 3.236783194651748`, 0.9208763725654232`, 141.79486265378398`, 15.108853263592536`,
163.3625199447715`, 0, 109.64797495198007`, 1.770542255979749`, 1.787382373427633`, 0.009511276780324707`,
0.04866865964014384`, 0.02962089911889315`, -0.3913763120268744`, 30.126838504720276`, 0.16312622211117794`,
1.136196445565297`, 0.6636898039382744`, 0.0003766327112955998`, -0.9994325169543653`, 0.09706228032981683`},
{0.17738230405709043`, 3.796341718770906`, 2.6191444768927727`, 0.8810359048502269`, 0.8432004845871259`,
0.5364474114112706`, 0.12890044163052178`, 0.09199163668005597`, 2908.5735595435463`, 327.07488963843366`,
0.2488393941661181`, 4.302397057177101`, 1.240771752535033`, 91.21360197902183`, 43.40549404677515`,
154.60788177045433`, 0, 38.15769670845899`, 0.8810541269343193`, 0.9378063367298757`, 0.06441398781369889`,
1.2428316774026913`, 0.04469446437706809`, -0.9640381998707404`, 47.782607693943234`, 3.1493763784689666`,
1.1325159278700447`, 0.9999999214457937`, 0.0006151814834433397`, -0.9993848184682316`, 0.19710488606688326`},
{0.16610260493961904`, 1.5013302413059524`, 6.689821314230944`, 1.0482229687817899`, 0.1182529778116388`,
0.3276703522967399`, 0.18950977903448152`, 0.011602315706762772`, 1976.8505440085055`, 128.55302132767815`,
0.1206362258416388`, 2.990415653764348`, 0.8762156912964318`, 144.02994408485648`, 13.509526837112668`,
169.39498579166272`, 0, 123.15144391001643`, 0.9026775507000422`, 0.9122745484392306`, 0.010631700912187014`,
0.15711929412825018`, 0.04732623672333074`, -0.6987878733422757`, 19.360244357342374`, 0.37282748629963897`,
0.9260962225438852`, 0.9542070901071289`, 0.0005471675192767508`, -0.9994265736180861`, 0.07399706590248893`},
{0.07362676393672318`, 0.7355538194163032`, 3.988559823436466`, 1.1003960033820657`, 0.2330826487371216`,
0.30236238420511485`, 0.2034701501720217`, 0.007403410519656421`, 3383.0372997062996`, 216.63011163159672`,
0.15749031565529237`, 1.8803990102478743`, 0.9980728680407593`, 57.349137376096664`, 5.198564659371774`,
69.3679549342544`, 0, 51.202822219082016`, 0.46717690285142066`, 0.4766527506327998`, 0.02028321118519183`,
0.3510160316679863`, 0.052724556515083`, -0.8497944488046822`, 4.909053646220603`, 0.36920249288034174`,
0.3729271299781259`, 0.9987963610785904`, 0.0005811314210547281`, -0.9994181682636216`, 0.05349785387691844`},

{0.19113040953496924`, 3.008225434073468`, 6.355992034495486`, 1.3442852038382949`, 0.5652481966732263`,
0.17677420590456394`, 0.15942860908355244`, 0.011393875146253816`, 1546.892426029899`, 241.3481933910041`,
0.08634784635015025`, 8.231809963935362`, 0.7429779031641539`, 310.0118356785577`, 56.06413404006201`,
343.742118871018`, 0, 266.41830938344066`, 0.9785655517148123`, 0.9868601743844331`, 0.00847630764754137`,
0.10026739683020396`, 0.0499750157931265`, -0.5015825944124608`, 42.05351116538045`, 0.27377355170234124`,
2.0601021320962447`, 0.7969392891313625`, 0.0004503116542148576`, -0.9994349486085625`, 0.1410926008948358`},
{0.1657364273353646`, 1.7016560013922657`, 2.864207314541927`, 1.4862862793345373`, 0.7297929998129427`,
0.24571335694321284`, 0.16930689708064994`, 0.03637877544295533`, 2361.9950805518038`, 368.27061311393015`,
0.19309626758691123`, 5.461234510584882`, 0.819553318889934`, 120.82072911737184`, 34.14285930748782`,
179.3464921638975`, 0, 78.3025553750268`, 1.5874200862109837`, 1.6324999696304343`, 0.02839820650565894`,
0.6930667117954503`, 0.07192837313529776`, -0.896217244442514`, 38.58918452045071`, 1.640948581686411`,
1.000562764606836`, 0.9999427957610062`, 0.0005870292873408736`, -0.9994129371301744`, 0.15953157657877556`},
{0.243670782866162`, 2.468817897966531`, 5.276425534951404`, 1.2860325806374826`, 0.9986905753378303`,
0.15094415892422752`, 0.11186418412899801`, 0.017007046631466297`, 3882.4128529746977`, 364.42553589609224`,
0.22618030191028127`, 8.141676219478274`, 0.7328485154782189`, 259.62195713235053`, 33.91495078064327`,
294.57960962222063`, 0, 208.6169453001219`, 1.391248866346156`, 1.3992083347767978`, 0.005721096076466869 5`,
0.10080233435219618`, 0.04875254913952969`, -0.5163549589120451`, 49.067715739443386`, 0.3508940532333664`,
0.9299746871290894`, 0.813563139508922`, 0.0004593052093235124`, -0.9994354399959654`, 0.059561729439982156`},
{0.047521269126133275`, 3.6122781353018008`, 6.744377644847237`, 0.8245850034533604`, 0.6257570737736955`,
0.5030346499913471`, 0.1805220904232982`, 0.04172896692907123`, 2236.066689248032`, 158.14626584756797`,
0.19953621789908887`, 3.2116522076145895`, 1.150911515892405`, 164.9399854022696`, 27.2160032500319`,
222.02535206354074`, 0, 103.38671761579502`, 1.1582701252868546`, 1.171060284689467`, 0.011042466798878214`,
0.36768378424634796`, 0.03940396684239604`, -0.8928319155462254`, 59.771340690671046`, 0.24961142949277978`,
1.187665707531772`, 0.999682361752938`, 0.0005787208840213276`, -0.9994210952336835`, 0.09485613079304361`},
{0.25961180109703935`, 3.304460665024532`, 9.731602575575781`, 0.7572846132780213`, 0.5757688453852501`,
0.6201294851486256`, 0.16223330053532697`, 0.09572577856146514`, 1068.913375906082`, 270.60830273991644`,
0.06817614592711113`, 6.616513224635819`, 1.43430723538359`, 247.6047828415589`, 126.39031410638759`,
248.75199279973899`, 0, 106.3308436597036`, 2.9280777721000866`, 2.9668223271384013`, 0.013232078535443614`,
0.02574979264709624`, 0.0182758177375683`, -0.29025379007744234`, 138.2245403148202`, 0.0954992863851546`,
3.162892993952629`, 0.5177434201495019`, 0.0002922530634343034`, -0.9994355252967775`, 0.33212324152645883`},
{0.05227980057052345`, 2.0688773882101916`, 3.6600804041449706`, 1.1141893430147094`, 0.9026814754625292`,

0.2868484255785748`, 0.07257484506693682`, 0.008111547996607639`, 2542.960019998747`, 267.5073034447911`,
0.05331315230928124`, 5.17983990011182`, 0.732439763493062`, 142.78336490492123`, 43.44671906031177`,
179.33705178573345`, 0, 127.39944461332762`, 0.47899328440543454`, 0.4852642091940556`, 0.013091884568705359`,
0.36168048764412747`, 0.0526469243075291`, -0.8544380299571742`, 14.156833931584792`, 0.2701226252040676`,
1.0946709399566519`, 0.9989053410890713`, 0.0005723797256444245`, -0.9994269930271668`, 0.0957574295195311`},
{0.16735387248889688`, 2.2901749191726726`, 8.076219774814714`, 0.8654381827857112`, 0.8324588302247302`,
0.6842201049055578`, 0.20618844268782105`, 0.08330233274804327`, 3386.0149520877076`, 231.00380607589523`,
0.24136440782861118`, 8.110578461374818`, 0.1918239477431709`, 351.10716101266024`, 83.40097481070718`,
546.2496678937277`, 0, 160.5977451898308`, 5.645086653499106`, 5.658508025313032`, 0.0023775315841443323`,
0.05159042165639401`, 0.02911500196572466`, -0.4356510175544145`, 184.68908386285776`, 0.12334081210773844`,
0.660541748299396`, 0.7196169934436327`, 0.00040737401257055924`, -0.9994339016222767`, 0.13101246722984805`},
{0.15392813884203022`, 1.030461564252417`, 8.114572747609092`, 0.8272078545061425`, 0.12582813602684628`,
0.5315104372547351`, 0.15396277604540914`, 0.15739980098951944`, 2790.789894250489`, 293.11851381168844`,
0.07517370467239265`, 9.08396266539139`, 0.493350448003097`, 187.46473445449158`, 37.6057384379116`,
220.4466897835582`, 0, 58.61122662481523`, 8.193039508212303`, 8.253855113429177`, 0.007422838026829526`,
0.016167949655614265`, 0.01320439586596594`, -0.18329805898543483`, 120.60874725163262`, 0.035552891419710804`,
1.3753629296225396`, 0.3429752105089462`, 0.00019328233989577778`, -0.9994364539069487`, 0.3137510342011374`},
{0.21281579254928695`, 2.151234091423703`, 4.99405590028015`, 1.327714565612129`, 0.5224256111223291`,
0.6816332648036347`, 0.12550320306228152`, 0.04036971585137323`, 3491.0437940299144`, 389.9199715703103`,
0.034574092345333096`, 7.184915600941272`, 1.1300147670199618`, 155.79999582520963`, 41.47338230969908`,
161.9411761428872`, 0, 99.22433523730905`, 1.775824224021711`, 1.7958783687963584`, 0.011292865872293723`,
0.05408086809292846`, 0.036158703233345656`, -0.3313956578653049`, 54.574480158450726`, 0.164418040070639`,
1.5405871457907108`, 0.5804186876712153`, 0.0003299939393696949`, -0.999431455350458`, 0.2609732319340238`},
{0.1743683640254448`, 2.5950167332919714`, 4.4795399396250115`, 1.4920254306676737`, 0.7406209345210579`,
0.5005424929143194`, 0.1556663350770055`, 0.009754157595577621`, 589.2836300166855`, 248.33057190508907`,
0.03897335434322141`, 4.862373453735419`, 0.5620446056385968`, 176.11427034452961`, 189.656960400544`,
228.48694705090637`, 0, 152.29051572066376`, 0.5480831317850916`, 0.565755889329585`, 0.03224466603621545`,
0.8121007884858166`, 0.07562418512599561`, -0.9068783256977266`, 20.318355688819743`, 2.0229240844577867`,
2.553193782480168`, 0.999869093719104`, 0.000614506406924971`, -0.9993854131397949`, 0.3911429904764247 6`},
{0.14295588103954704`, 1.412350123248217`, 3.177274858880626`, 1.1913477441560478`, 0.681260929106482`,
0.5789699232051367`, 0.09066128493810405`, 0.04793649880497841`, 2544.3651836876206`, 337.2728729655139`,

```
0.011905134502266851`, 4.430427622941885`, 0.74003827479563`, 106.86707465280617`, 39.65129838352222`,
113.0342550610454`, 0, 63.35266009714286`, 2.01749455210697`, 2.0512058554691666`, 0.01670948916664483`,
0.2592470903020445`, 0.05668964989993813`, -0.7813296579958141`, 40.70583827601273`, 0.5294413743009183`,
0.9707104435865801`, 0.985616690250828`, 0.0005769751133365698`, -0.9994146049686012`, 0.16788260166689298`},
{0.11809903796554244`, 3.7153066081905903`, 9.394589088557098`, 0.7843066157779603`, 0.3510759725555237`,
0.22803969658113876`, 0.10165008188335536`, 0.005399145823047646`, 3902.7306906909416`, 388.8592048663462`,
0.07729944387610005`, 2.9718049006587925`, 0.2891160085661364`, 38.87703545474008`, 7.090405750389786`,
53.624917430212655`, 0, 36.05271834551893`, 0.051103277342071686`, 0.0514812103850760 8`, 0.007395475645810556`,
0.007587321174418573`, 0.006853277832049942`, -0.09674604850570101`, 2.7123477715599376`, 0.012800790449059893`,
0.22593216634520327`, 0.18783379887950202`, 0.0001058299641998181`, -0.9994365765648614`, 0.04779539878196332`},
{0.16202429237235855`, 1.84055090570393`, 9.090225524744397`, 1.4016003618243056`, 0.18111565798783458`,
0.3854966323949548`, 0.18149685472874316`, 0.020436792872453445`, 1570.1908216352922`, 182.31609839273892`,
0.10515949401254626`, 5.892117812074294`, 0.4606489730839265`, 204.1054437668955 2`, 38.55241027909658`,
247.6603591867031`, 0, 158.11996456938942`, 1.677139857530992`, 1.6899023310006547`, 0.007609665593691917`,
0.0469791258712131`, 0.03351347419692513`, -0.28663052844368`, 44.09801833958329`, 0.10873942322222985`,
1.3645009996586248`, 0.5122089154749278`, 0.00028961569951027274`, -0.9994345750517799`, 0.17159370560885026`},
{0.23592081280505794`, 2.903542817229688`, 5.375766226090379`, 1.0406060233325234`, 0.5645881743757346`,
0.6023633607842607`, 0.10170656063773648`, 0.2230968616100032`, 1009.1076309440446`, 206.38966170547087`,
0.05554284619714861`, 3.437370706600392`, 0.2602834274636374`, 126.67312917869677`, 66.23002868446201`,
136.07468681594563`, 0, 30.929474602073952`, 2.242054314754703`, 2.2698159071826782`, 0.012382212261888181`,
0.11509944569616753`, 0.04471536239218147`, -0.6115067095091113`, 92.99858144921185`, 0.3879193540295302`,
1.0628415225746544`, 0.8921500943700836`, 0.0005209923486035608`, -0.9994160261239771`, 0.19223625561670946`},
{0.17119714315024748`, 2.9315531156526395`, 7.9309025517292255`, 1.4075914943739267`, 0.478244345688974`,
0.6490520691087696`, 0.11764936739953108`, 0.10875560486489166`, 3501.9685138343757`, 327.0147307274891`,
0.208688235529143`, 1.76928881617237`, 0.15623787817751156`, 27.816705048350183`, 9.03264550965156`,
42.680712423985135`, 0, 10.950271915093419`, 0.3917249942432718`, 0.3946841378640519`, 0.007554135335419598`,
0.02017756179693932`, 0.017480053744023213`, -0.13368850409494393`, 16.4051803907554`, 0.04934772764810054`,
0.14122244881824356`, 0.2619605190504869`, 0.00015065998915997345`, -0.9994248752075081`, 0.07676301339911809`},
{0.05150190326045606`, 3.439128676554682`, 6.3456448543065065`, 1.3220565831174902`, 0.02053589536141165`,
0.5629905593040019`, 0.12931144501098696`, 0.09719268107904352`, 992.6848015373967`, 364.3637294024504`,
0.08014172250870272`, 4.69481466986781`, 1.4428291086778402`, 80.72461777305354`, 50.87593508331862`,
```

80.27982969382828`, 0, 34.8935557373086`, 0.913270952143277`, 0.9360974106235387`, 0.02499417990541808`,
0.027880983989823475`, 0.022478898918705 72`, -0.19375517998538028`, 44.86937601400496`, 0.02051319628923944`,
2.0159690821556406`, 0.36238425747490377`, 0.00020593272878508537`, -0.9994317282703724`, 0.4125704462274414`},
{0.1814998220796576`, 0.5457041309396096`, 6.628442116729424`, 1.4816442478897651`, 0.04014018359229943`,
0.36141091722571295`, 0.13153411394886588`, 0.008451051131756494`, 2397.6906312548626`, 255.11228411318268`,
0.24580682176341695`, 6.012737749288213`, 0.971372885437352`, 135.91958705859696`, 22.39800044671929`,
150.46216696003302`, 0, 121.20684835203704`, 1.643035188778585`, 1.6646386787805083`, 0.01314852545427403`,
0.04237038785883841`, 0.031695777392944324`, -0.2519356325332145`, 12.80872985422307`, 0.10986025511179284`,
1.5508170178977805`, 0.4581023393359469`, 0.0002591862202990859`, -0.9994342176451777`, 0.20140750808044924`},
{0.10627844972153494`, 1.9122914449389148`, 4.879880104768988`, 1.1152938555639593`, 0.36058806344071925`,
0.25105589966667485`, 0.133980532681923`, 0.018281043179813147`, 2529.9927880778405`, 195.84606271036728`,
0.10858226709292867`, 2.0748738252120926`, 0.9066780951872615`, 71.3024280978474`, 7.934565025486043`,
80.68989788265371`, 0, 56.33033138938615`, 0.5150223613181099`, 0.521396811215797`, 0.012377035205564324`,
0.14467737967678057`, 0.04922211946917843`, -0.659780128869184`, 14.069612221440833`, 0.2196583945974579`,
0.4916837854112693`, 0.9334943510195826`, 0.000535201186756734`, -0.9994266690674966`, 0.043336653438469826`},
{0.2706982412362611`, 0.6287160520084307`, 4.14855201722539`, 0.8172328386793793`, 0.4851216922484758`,
0.20982300489352756`, 0.08395856094354753`, 0.16391690500747624`, 3554.6452712204655`, 185.42012094476195`,
0.05154754434614739`, 8.513250180668177`, 0.37417348469464895`, 252.90632042645808`, 25.17917427194304`,
344.1715338195005`, 0, 76.09193247358944`, 17.652841367886754`, 17.754937121974272`, 0.005783530931923853`,
0.12528246011574906`, 0.036713836571284034`, -0.7069515035275972`, 158.5517818792 6967`, 0.4844820230155462`,
0.8487503781941739`, 0.9604214504945109`, 0.0005442285727256472`, -0.9994333440049206`, 0.07097894561107848`},
{0.18294446021192495`, 2.0751042203060504`, 7.88640633654303`, 1.253927303712154`, 0.8981192233254209`,
0.26591266597763197`, 0.15456768387072084`, 0.011263961326455277`, 2103.388409363515`, 330.13115223762657`,
0.1896947651115678`, 4.4078344078613245`, 0.7904477019841809`, 175.09313795474068`, 41.78969814719652`,
186.25667694244765`, 0, 150.649012545947`, 0.7863697798624665`, 0.7919758353287022`, 0.007129032180275496`,
0.06616630003756882`, 0.03961913548931271`, -0.40121881581987806`, 23.311417841624927`, 0.17292511492275328`,
0.8916923659161785`, 0.6766305144799525`, 0.00038280561365841104`, -0.9994342471918332`, 0.07033824643392965`},
{0.15110272633071126`, 1.2120684691778738`, 9.821466182804322`, 0.999249111664473`, 0.8884716643403618`,
0.5717874967013771`, 0.23862826359871653`, 0.2965485968483864`, 799.8579275077459`, 260.7684224687745`,
0.20976611603443018`, 5.031757425833598`, 0.8119791706180317`, 356.03608144629686`, 215.8984383308125`,
353.034517198484`, 0, 69.67682788635925`, 15.608884921892058`, 15.726973146262246`, 0.007565449099093957`,

0.15146149661980848`, 0.045177358673386896`, -0.7017238065012061`, 270.27196075501865`, 0.32694635819083384`,
2.203587124687442`, 0.9524981549231506`, 0.0005475346182929574`, -0.999425159392212`, 0.18307100020172873`},
{0.15717196764210273`, 1.1630073112625743`, 2.634220279774457`, 1.0045604176965792`, 0.8286996726157703`,
0.4235785617926241`, 0.10102087300007184`, 0.09118767410472639`, 1523.7756565066265`, 301.9435543713025`,
0.21266930821707325`, 6.460470764249699`, 0.28210830372520523`, 133.66837823845182`, 103.5581903188853`,
221.44889563734165`, 0, 57.856613132083744`, 4.182990213141879`, 4.293044369014525`, 0.026309924304122045`,
0.656602901394554`, 0.04947544032232832`, -0.9246493729813746`, 69.49783144034`, 1.4742795710241066`,
1.3025262321511568`, 0.9999973474152692`, 0.0005969335887365242`, -0.9994030648278424`, 0.31832661868354517`},
{0.1799797654068389`, 2.619455956239074`, 7.697999886354548`, 1.3015491336074843`, 0.555451962044029`,
0.4433835581064074`, 0.0862654213077988`, 0.03372825256230826`, 2391.7036278961978`, 159.9832142066033`,
0.13737033538391902`, 3.425775682450297`, 0.8987360464358476`, 150.50828590006748`, 22.96004787720416`,
163.78506593163783`, 0, 101.70599054112186`, 1.261711334852186`, 1.2737693451111929`, 0.009556869250461064`,
0.08801351969030471`, 0.0465829559425888`, -0.47072954125114674`, 47.21424673047024`, 0.22629503752127572`,
1.1106881263601986`, 0.7631776600762953`, 0.00043374769450102256`, -0.9994316556718161`, 0.06866272818366578`},
{0.12274664777380989`, 1.2488875899911855`, 5.516183439796784`, 1.1021378510059003`, 0.9773171588432767`,
0.5961273492690635`, 0.12759486552792382`, 0.1532767587236556`, 2513.3632749376284`, 182.05354083120335`,
0.05801301004138587`, 1.8616328305112617`, 0.5158476130411485`, 78.45321286882705`, 32.61900877682983`,
134.96483663036182`, 0, 24.513712608493663`, 2.8065580854751837`, 2.858470937780639`, 0.01849698125761967`,
0.3851189032312433`, 0.05276331735605615`, -0.8629947350977613`, 50.07250805056252`, 0.6753150623708637`,
0.3646317216781246`, 0.9995213327282735`, 0.0005806194722401185`, -0.9994191024711245`, 0.053757838230757554`},
{0.15063755404470214`, 2.71723210471837`, 7.148609543518862`, 1.1908848946871171`, 0.5800141434913164`,
0.29138799856290376`, 0.0950097459950863`, 0.009655692674775408`, 856.5663047299263`, 347.96462878162436`,
0.12103625881441793`, 4.831460173736216`, 0.8148377888737439`, 112.72914424895467`, 75.14701375243729`,
113.23340717699175`, 0, 99.12618281459878`, 0.337083368066674`, 0.34318455190360814`, 0.018099925463327393`,
0.032098371154866405`, 0.024385573373887012`, -0.24028626698118483`, 13.084767852533798`, 0.06907457313697585`,
1.8425208288653534`, 0.4388438056638244`, 0.0002480560489402738`, -0.9994347509393119`, 0.20603507268160243`},
{0.25325667629893317`, 2.6359345155712095`, 6.284411921985339`, 1.14262610260638`, 0.592877633639628`,
0.19560008128978879`, 0.18445820883756597`, 0.009163632115762594`, 1288.7476768868028`, 192.8490635634654`,
0.16457035981631868`, 2.082802800802952`, 1.1518950127144034`, 100.17138330137978`, 17.504634523333014`,
113.82922751658909`, 0, 87.24424693640255`, 0.29270163268104665`, 0.2999620842252545`, 0.02480495745003064`,
0.3332777718095294`, 0.05475962286174804`, -0.8356937440969103`, 11.022033376400234`, 1.205783153897081`,

0.816926738283963`, 0.9966015951631014`, 0.000581626876317367`, -0.9994163897798878`, 0.05543549669866915`},
{0.14655389849752304`, 3.5234321911845736`, 9.93741755620114`, 1.0550925690751938`, 0.4448537382022568`,
0.18176809875202415`, 0.07434797690036096`, 0.27672257287602875`, 2966.99983242307`, 232.89159682919671`,
0.13795973482450868`, 7.950903042528848`, 1.2964183380427592`, 223.26375627484367`, 25.526939475484763`,
240.19311051715945`, 0, 46.7868672060998`, 3.436346462866708`, 3.4711172467064344`, 0.010118532637922506`,
0.023570275525402563`, 0.018712393731444697`, -0.20610203680997896`, 172.9676249618257 8`, 0.04934736809853651`,
2.1903620377436264`, 0.38185336806075443`, 0.00021475427772245142`, -0.9994376001478968`, 0.11020953060704042`},
{0.2776663951441912`, 0.8310897457250155`, 2.0994479814067635`, 1.100340126975314 4`, 0.30863525720948326`,
0.2434882924277243`, 0.08746325015577566`, 0.02243947015640089`, 2140.6294222376637`, 299.3219451566707`,
0.02431460260750562`, 6.100643757524728`, 0.33277870666165055`, 130.37596438649624`, 26.928712147873664`,
137.57542848783004`, 0, 73.69823739461789`, 1.812818093721165`, 2.458570560507003`, 0.35621470737877736`,
6.7086882598630675`, 0.07013019683051802`, -0.9895463622523505`, 21.52306469366328`, 26.611104075176232`,
0.9020327028467833`, 0.9999999982513406`, 0.0007736247267029555`, -0.9992263752719442`, 0.16332686648397693`},
{0.11907642171566374`, 1.274849235584174`, 6.899983100552333`, 1.1682344315973607`, 0.7800022001471016`,
0.41036075345185574`, 0.1345484896240624`, 0.007510763802150046`, 2156.4467856375795`, 343.9093505673661`,
0.08426365817638315`, 2.310160002798696`, 0.6194154558015803`, 69.29376944862142`, 22.639640378052935`,
73.7100301021381`, 0, 62.452306495653254`, 0.34630044183852393`, 0.349494796722794`, 0.009224229883482371`,
0.04758749281154641`, 0.03184328520020079`, -0.3308475963147509`, 6.306869336575779`, 0.08095069089169057`,
0.4442163165926073`, 0.5797043182570392`, 0.0003303829993837508`, -0.9994300835978295`, 0.06095951824553901`},
{0.17937371459880497`, 2.8718008248848648`, 2.8198656683422243`, 0.9976096277706823`, 0.8604494338327144`,
0.3675397910291335`, 0.21060856912564696`, 0.007211517100138 7954`, 3962.8369792288295`, 287.0010951200238`,
0.23748423652768247`, 9.572841373703643`, 0.6693837986608342`, 252.96361536269018`, 57.698976832853496`,
551.2891551393486`, 0, 184.98525211982974`, 0.4506467321145251`, 0.562195527151254`, 0.24753046474634255`,
13.714888576219666`, 0.05864267401307252`, -0.9957241596468561`, 18.48810938597375`, 35.144150131789225`,
1.1609015791034796`, 0.9999999999093413`, 0.0007122006558694647`, -0.999287799344066`, 0.23086138016721378`},
{0.1446481422691977`, 3.7473728388010814`, 7.773074810909127`, 0.7768035182896058`, 0.045349053147926144`,
0.5946440249560018`, 0.18594588773391896`, 0.007600014271866709`, 2201.8419670169405`, 172.3692269613906`,
0.22887729014375824`, 6.4667868323956865`, 0.5687524909215391`, 220.56299966606784`, 33.37744303851937`,
264.8595589606935`, 0, 198.83628810943048`, 0.3931395356984341`, 0.39611266385620125`, 0.007562526502162159`,
0.03235073960521541`, 0.02139224881647751`, -0.3387400387894449`, 21.046291684788276`, 0.06684963407040781`,
1.4235330226349578`, 0.5898873183997508`, 0.0003334762114809065`, -0.9994346781138039`, 0.21481835385926343`},

{0.2743682788249912`, 2.567007165847575`, 8.880180070850319`, 1.3334617648385514`, 0.053478262105743024`,
0.5870310036210435`, 0.14269834247006347`, 0.006777940660941963`, 3288.224802818225`, 343.8350462827967`,
0.07407598386826336`, 1.0311140447256584`, 1.1022538897878755`, 17.196393310136475`, 3.4356317123010784`,
18.613815752376674`, 0, 15.603458245463434`, 0.03953239071032237`, 0.04029817724957611`, 0.019371116330027194`,
0.017872377187885587`, 0.01565177672181481`, -0.12424762765056041`, 1.4497132890926252`, 0.07005161953644543`,
0.23457162167843712`, 0.24793917855986491`, 0.00014273237327694935`, -0.9994243250538055`, 0.05585589512390169`},
{0.04351054041483948`, 3.010882988409519`, 9.401958493592993`, 1.3186120593482111`, 0.49329539098927055`,
0.4698069940559316`, 0.20226774754753019`, 0.03712775492888272`, 1947.5927514696796`, 172.9604174112643`,
0.17909729794724766`, 1.3306147942525488`, 0.46866213033580717`, 62.03084619417367`, 10.203035842434474`,
75.41521796098039`, 0, 40.549623991172936`, 0.4855252563290129`, 0.4884579997785175`, 0.006040351992558701`,
0.06681888389820084`, 0.041002608590464146`, -0.3863619653849355`, 20.883710496059933`, 0.04153322497609587`,
0.26396483049227104`, 0.6578657334444156`, 0.0003768344687784547`, -0.9994271863548729`, 0.03459368860771306`},
{0.13996819046085934`, 1.009532661617322`, 6.145072622160212`, 1.3450345414096851`, 0.2868040480361447`,
0.18183470956578074`, 0.13804571299234253`, 0.12631256619855588`, 3863.110841624364`, 279.40163171428946`,
0.18548475334584474`, 9.210069253579718`, 1.2809334264760701`, 228.05358193990597`, 20.049948826408706`,
253.14405855977654`, 0, 82.39783181168502`, 9.435190855414385`, 9.514754993343997`, 0.008432700424279682`,
0.049081220602165146`, 0.03401020706999774`, -0.3070627288250971`, 136.07333338762714`, 0.09814013761804638`,
1.804381887813754`, 0.5436538221176186`, 0.0003062390540897856`, -0.999436702104113`, 0.16399098159970885`},
{0.0871063251493665`, 2.6634842127081404`, 9.193238345408428`, 1.4387089146023326`, 0.7548565000722143`,
0.6823132747448253`, 0.19549587941758378`, 0.13611301540454773`, 3578.7577781208274`, 129.49687931265402`,
0.027673492175817715`, 9.652606809319298`, 1.320301893923864`, 670.6970685778498`, 79.78870964558926`,
1020.5935677126732`, 0, 229.49301129542522`, 11.252419480619361`, 11.342325763714815`, 0.007989951250066962`,
0.8018304384520374`, 0.06825118234444523`, -0.9148807789384915`, 428.1520234485606`, 0.9977786126632188`,
3.532158328529256`, 0.9999007108246225`, 0.0005744164447469303`, -0.9994255265162546`, 0.2127078261413211`},
{0.04655003226957066`, 2.717305676329323`, 6.261066689781977`, 1.313518883642772`, 0.22988974709922583`,
0.48948284819450716`, 0.1924496553607991`, 0.40105381179739646`, 1594.1735712767286`, 296.74834364325125`,
0.10951121773307326`, 3.7043174616791976`, 0.46788624174476623`, 88.04675376239354`, 27.65134404225796`,
99.60743994901847`, 0, 13.748832461931249`, 1.8640838775072055`, 1.8826037312700405`, 0.009935096798112664`,
0.04361146821363219`, 0.03128666626362835`, -0.28260460963227363`, 72.36122430720418`, 0.029001646466663086`,
0.8210034956268214`, 0.5061631254318391`, 0.0002885163385788714`, -0.9994299933675873`, 0.18702195487582135`},
{0.24058904599557956`, 2.305486806402752`, 6.870865029896366`, 1.1663266064905513`, 0.291316248043183`,

```
0.4756853450103681`, 0.11657670703629042`, 0.014427542030819147`, 3856.0018830548897`, 333.80293042286075`,
0.20384852921840946`, 8.723427410033644`, 0.10055245356998821`, 85.6142824694147`, 28.716893498660014`,
190.98913696359887`, 0, 71.0020072201342`, 0.42791192012601265`, 0.43112165827441284`, 0.007500931844700798`,
0.011602053514445O7`, 0.010453354190164181`, -0.09900827666849732`, 14.093504087899875`, 0.03987609980899685`,
0.5559234447647075`, 0.19236868724805822`, 0.00010855785276411 95`, -0.9994356781536688`, 0.35195910572877986`},
{0.09672875865983738`, 3.3181066447219294`, 8.772587658219397`, 1.2949486969635013`, 0.026680322952838287`,
0.3340762083993216`, 0.2420609904328586`, 0.3944381790098702`, 1841.1432974548734`, 89.15662847720563`,
0.1287997244865557`, 3.057938101959733`, 0.8332397440047616`, 217.07736874896082`, 13.957772647286477`,
254.8753393563051`, 0, 31.29952320554249`, 3.5284009513012062`, 3.808961197840461`, 0.0795148426756287`,
6.296755453331231`, 0.06573860835489352`, -0.989559923544416`, 167.25158059651056`, 8.701104836932135`,
0.9995636286811739`, 0.9999999580429081`, 0.000614995211457604`, -0.999385004762739`, 0.06776865029640251`},
{0.06413533732712456`, 2.7505505433928237`, 9.582115463692716`, 1.419324092949555`, 0.0693862133505021`,
0.4085416515074848`, 0.14517410092691557`, 0.08408491973612985`, 1524.8762411053558`, 131.37866475928564`,
0.011227169364072448`, 3.5456702127620083`, 1.1107372198382217`, 159.48088397183247`, 22.104930429794425`,
176.6709270664162`, 0, 73.3009671570961`, 2.135660035578029`, 2.159885680239577`, 0.011343399351007255`,
0.06596140897937382`, 0.04180569404192817`, -0.3662098082986547`, 83.91772673373535`, 0.06043510307808467`,
1.7357108104032974`, 0.6292359892754766`, 0.0003567487728154132`, -0.9994330445510179`, 0.12178432358559076`}};
PatientsTrainingSet = result[[All, 1 ;; 10]];
```

In[ ]:=

```
(* The scripts hidden in this group of cells have to be initialized to run the following codes *)
```

In[ ]:=

```
CreateResultArray[Njj_] := ( (* argument is how many random sets will be chosen *)
    result = {};
    (* Table that will contatin the results *)
    result = Array[f, {Njj + 1, 31}];

    result[[1, 1]] = "κc";
    result[[1, 2]] = "κp";
    result[[1, 3]] = "γ";
    result[[1, 4]] = "V";
```

```
result⟦1, 5⟧ = "k_s";
result⟦1, 6⟧ = "ρ_0";
result⟦1, 7⟧ = "ρ_m";
result⟦1, 8⟧ = "ω";
result⟦1, 9⟧ = "α_0";
result⟦1, 10⟧ = "α_m";
result⟦1, 11⟧ = "k_f";
result⟦1, 12⟧ = "N_0";
result⟦1, 13⟧ = "k_D";

(* minimal curative dose *)
result⟦1, 14⟧ = "A_cur^sim"; (* simulation result *)
result⟦1, 15⟧ = "A_min^est"; (* analytical estimation for most radiosensitive population *)
result⟦1, 16⟧ = "A_max^est"; (* analytical estimation for most radioresistant population *)
result⟦1, 17⟧ = "M_m"; (* minimal curative number of radioconjugate molecules of cancer cell *)

(* paths of activity *)
result⟦1, 18⟧ = "Canc"; (* released in cancer *)
result⟦1, 19⟧ = "Bl_Frg^sim"; (* released in blood from fragments, simulation result *)
result⟦1, 20⟧ = "Bl_Frg^est"; (* analytical estimation *)
result⟦1, 21⟧ = "Err"; (* error in estimation *)
result⟦1, 22⟧ = "Bl_Ab^sim"; (* released in blood from antibodies, simulation result *)
result⟦1, 23⟧ = "Bl_Ab^est"; (* analytical estimation *)
result⟦1, 24⟧ = "Err"; (* error in estimation *)
result⟦1, 25⟧ = "Cld_Frg^sim"; (* cleared in form of fragments,
simulation result *) (* no need to estimate -- error will be the same as for release *)
result⟦1, 26⟧ = "Cld_Ab^sim"; (* cleared in form of fragments, simulation result *)

result⟦1, 27⟧ = "ToVbl"; (* How much activity affects viable cells *)

(* receptors occupancy *)
```

```
    result[[1, 28]] = "fD^sim_Max"; (* maximum occupancy of receptors on damaged cancer cells, simulation result *)
    result[[1, 29]] = "fD^est_Max"; (* analytical estimation *)
    result[[1, 30]] = "Err"; (* error in estimation *)
    result[[1, 31]] = "Nnew"; (*number of newborn cells until Ncur is achieved*)
  )

WriteDownMeasures[] := (

    result[[ij, 1]] = kappac;
    result[[ij, 2]] = kappap;
    result[[ij, 3]] = gamma * 10^7 / Nnor;
    result[[ij, 4]] = V;
    result[[ij, 5]] = ks;
    result[[ij, 6]] = rhomax;
    result[[ij, 7]] = rhomin;
    result[[ij, 8]] = omega;
    result[[ij, 9]] = alphamax;
    result[[ij, 10]] = alphamin;
    result[[ij, 11]] = kf;
    result[[ij, 12]] = N0 * Nnor / 10^7;
    result[[ij, 13]] = kD;

    (* minimal curative dose *)
    result[[ij, 14]] = DA1 / nCpm; (* simulation result *)
```

$$\text{result}[[ij, 15]] = -\frac{\text{kappac} + \text{lambda} + \text{kon} * \text{gamma} * \text{N0} / V}{\text{kon} * \text{gamma} * \text{N0} / V} * \text{N0} * \frac{\frac{\text{rhomax}}{\text{lambda}} * \frac{\text{nu}}{\text{alphamax}} * \text{ProductLog}\left[-1, -E^{-2} * \left(\frac{\text{Ncur}}{\text{N0} * \text{Nnor}}\right)^{\frac{\text{lambda}}{\text{rhomax}}}\right]}{1 - \left(1 - \frac{\text{ProductLog}\left[-1, -E^{-2} * \left(\frac{\text{Ncur}}{\text{N0} * \text{Nnor}}\right)^{\frac{\text{lambda}}{\text{rhomax}}}\right]}{\text{ProductLog}\left[-1, -E^{-1} * \left(\frac{\text{Ncur}}{\text{N0} * \text{Nnor}}\right)^{\frac{\text{lambda}+\text{rhomax}}{\text{rhomax}}}\right]}\right) * \text{ks}} \Big/ \text{nCpm};;$$

(* analytical estimation for most radiosensitive population *)

$$\text{result}[[ij, 16]] = -\frac{\text{kappac} + \text{lambda} + \text{kon} * \text{gamma} * \text{N0} / V}{\text{kon} * \text{gamma} * \text{N0} / V} * \text{N0} * \frac{\frac{\text{rhomin}}{\text{lambda}} * \frac{\text{nu}}{\text{alphamin}} * \text{ProductLog}\left[-1, -E^{-2} * \left(\frac{\text{Ncur}}{\text{N0} * \text{Nnor}}\right)^{\frac{\text{lambda}}{\text{rhomin}}}\right]}{1 - \left(1 - \frac{\text{ProductLog}\left[-1, -E^{-2} * \left(\frac{\text{Ncur}}{\text{N0} * \text{Nnor}}\right)^{\frac{\text{lambda}}{\text{rhomin}}}\right]}{\text{ProductLog}\left[-1, -E^{-1} * \left(\frac{\text{Ncur}}{\text{N0} * \text{Nnor}}\right)^{\frac{\text{lambda}+\text{rhomin}}{\text{rhomin}}}\right]}\right) * \text{ks}} \Big/ \text{nCpm};;$$

(* analytical estimation for most radioresistant population *)

result[[ij, 17]] = 0; (* minimal curative number of radioconjugate molecules of cancer cell *)

(* paths of activity *)

result[[ij, 18]] = ActTumorpw[[Npw]] [tEnd] / nCpm; (* released in cancer *)

result[[ij, 19]] = ActBloodFragpw[[Npw]] [tEnd] / nCpm; (* released in blood from fragments, simulation result *)

$$\text{result}[[ij, 20]] = \frac{\text{lambda} * \text{omega} * \text{DA1}}{(\text{lambda} + \text{omega}) * (\text{lambda} + \text{kappap})} \Big/ \text{nCpm};$$ (* analytical estimation *)

result[[ij, 21]] = result[[ij, 20]] / result[[ij, 19]] - 1; (* error in estimation *)

result[[ij, 22]] = (ActBloodpw[[Npw]] [tEnd] - ActBloodFragpw[[Npw]] [tEnd]) / nCpm;

(* released in blood from antibodies, simulation result *)

$$\text{result}[[ij, 23]] = \frac{\text{lambda} * \text{DA1}}{\text{lambda} + \text{kappac} + \text{kon} * \text{gamma} * \text{N0} / V} \Big/ \text{nCpm};$$ (* analytical estimation *)

result[[ij, 24]] = result[[ij, 23]] / result[[ij, 22]] - 1; (* error in estimation *)

result[[ij, 25]] = ActOutFragpw[[Npw]] [tEnd] / nCpm; (* cleared in form of fragments,
simulation result *) (* no need to estimate -- error will be the same as for release *)

result[[ij, 26]] = (ActOutpw[[Npw]] [tEnd] - ActOutFragpw[[Npw]] [tEnd]) / nCpm; (* cleared in form of fragments, simulation result *)

result[[ij, 27]] = (SDpw[[Npw]] [tEnd] + CFNpw[[Npw]] [tEnd] + CFDpw[[Npw]] [tEnd] + UNpw[[Npw]] [tEnd]) / nCpm;

```
(* How much activity affects viable cells *)

(* receptors occupancy *)
result[[ij, 28]] = 1 - If[Ainj[[1, 1]] == 0
    , Min[Table[NMinimize[{dFpw[[nn]][t] / DDpw[[nn]][t], t > tB[[nn]], t < 10}, t][[1]], {nn, 1, npw}]]
    , Min[Table[NMinimize[{dFpw[[nn]][t] / DDpw[[nn]][t], t > tB[[nn]], t < 10}, t][[1]], {nn, 2, npw}]]];
(* maximum occupancy of receptors on damaged cancer cells, simulation result *)
result[[ij, 29]] = DA1 / (gamma * N0); (* analytical estimation *)
result[[ij, 30]] = result[[ij, 29]] / result[[ij, 28]] - 1; (* error in estimation *)

result[[ij, 31]] = NewCellspw[[npw]][tminNn] * Nnor / 10^7; (*number of newborn cells until Ncur is achieved*)

)
```

In[ ]:=

```
FullSystemSolutionHetMDStopNmin[TMIN_] := (

  (* maximum time of simulation-- where little radioactivity of the last dose remains, namely 0.17% of the last dose *)
  tEnd = Ainj[[Length[Ainj], 1]] + (-Log[0.0017] / lambda);
  lAinj = Length[Ainj]; (* number of injections*)
  If[Ainj[[1, 1]] == 0, Npw = lAinj, Npw = lAinj + 1];
  (* the injections are treated as new initial conditions for a new system, which as well takes the actual vaules of other parameters *)
  (* therefore the number of injections has to me remembered and logic differs whether the first injection is made at t=0 or t>0 *)

  apw = Array[ff, Npw]; bpw = Array[ff, Npw]; NNpw = Array[ff, Npw]; DDpw = Array[ff, Npw];
  papw = Array[ff, Npw]; pbpw = Array[ff, Npw]; fFNpw = Array[ff, Npw]; fANpw = Array[ff, Npw];
```

```
dFpw = Array[ff, Npw];
dApw = Array[ff, Npw];
rhopw = Array[ff, Npw]; (* cancer cell proliferation rate -- is now an explicit variable,
while variables alpha and omega can be expressed through it *)
(* inert antibody fragments are as well accounted for as pb *)

(* times of beginning and end for solution of separate systems *)
tB = Array[ff, Npw]; tE = Array[ff, Npw];

(* for monitoring the paths of activity *)
ActBloodpw = Array[ff, Npw]; ActBloodFragpw = Array[ff, Npw];
ActOutpw = Array[ff, Npw]; ActOutFragpw = Array[ff, Npw]; ActTumorpw = Array[ff, Npw];

(* for monitoring influence of self-dose, croos-fire and decays in blood *)
SDpw = Array[ff, Npw]; CFNpw = Array[ff, Npw]; CFDpw = Array[ff, Npw]; UNpw = Array[ff, Npw];

(* for monitoring the number of new cancer cells appearing during treatment *)
NewCellspw = Array[ff, Npw];

Clear[a, b, NN, DD, pa, pb, fFN, fAN, dF, dA, rho, ActBlood, ActBloodFrag, ActOut, ActOutFrag, ActTumor, SD, CFN, CFD, UN, NewCells];

(* EQUATIONS *)

(* Radiation damage function *)
RD[NN_, DD_, fAN_, dA_, a_, pa_, rho_] :=
```

$$\left(\frac{rho - rhomin}{rhomax - rhomin} * (alphamax - alphamin) + alphamin\right) (*alpha*) * \left(ks * \frac{lambda * gamma * fAN}{nu} (*self-dose*) + (1 - ks) * \frac{lambda * gamma * (fAN * NN + dA)}{nu * (NN + DD)} (*cross-fire*) + kf * lambda * (a + pa) (*dose from unanchored nuclides*)\right);$$

```
(* Active antibodies *)
Fa[t_] := (*injections are considered as initial conditions*)
  - lambda * a[t] (*decay*) - kon * gamma/V * (fFN[t] * NN[t] + dF[t]) * a[t] (*binding*) - kappac * a[t] (*clearance*);

(* Inert antibodies *)
Fb[t_] := (*injections are considered as initial conditions*)
  + lambda * a[t] (*decay of a*) - kon * gamma/V * (fFN[t] * NN[t] + dF[t]) * b[t] (*binding*) - kappac * b[t] (*clearance*);

(* Viable cells *)
FNN[t_] := rho[t] * NN[t] (*proliferation*) - RD[NN[t], DD[t], fAN[t], dA[t], a[t], pa[t], rho[t]] * NN[t] (*damage*);

(* Damaged cells *)
FDD[t_] := RD[NN[t], DD[t], fAN[t], dA[t], a[t], pa[t], rho[t]] * NN[t] (*damage*) - (* rho[t]/rhomax **) omega * DD[t] (*death*);

(* Active fragments *)
Fpa[t_] := (* rho[t]/rhomax **) omega * (gamma * dA[t])/V (*release*) - lambda * pa[t] (*decay*) - kappap * pa[t] (*clearance*);

(* Inert fragments *)
Fpb[t_] := (* rho[t]/rhomax **)
  omega * (gamma * (DD[t] - dF[t] - dA[t]))/V (*release*) + lambda * pa[t] (*decay*) - kappap * pb[t] (*clearance*);

(* Free receptors of viable cells *) FfFN[t_] := (1 - fFN[t]) * rho[t] - kon * (a[t] + b[t]) * fFN[t];

(* Active receptors of viable cells *) FfAN[t_] := kon * a[t] * fFN[t] - (lambda + rho[t]) * fAN[t];

(* Free receptors of damaged cells *)
FdF[t_] := RD[NN[t], DD[t], fAN[t], dA[t], a[t], pa[t], rho[t]] * fFN[t] * NN[t] -
```

```
     kon * (a[t] + b[t]) * dF[t] - (* rho[t]/rhomax **) omega * dF[t];

   (* Active receptors of damaged cells *)
   FdA[t_] := RD[NN[t], DD[t], fAN[t], dA[t], a[t], pa[t], rho[t]] * fAN[t] * NN[t] +
     kon * a[t] * dF[t] - lambda * dA[t] - (* rho[t]/rhomax **) omega * dA[t];

   (* Cancer cell proliferation rate *)
   Frho[t_] := -kD * rho[t] * Log[rho[t] / rhomin] * RD[NN[t], DD[t], fAN[t], dA[t], a[t], pa[t], rho[t]];

   (* Initial conditions *)

   If[Ainj⟦1, 1⟧ == 0
    , a0 = Ainj⟦1, 2⟧ / V; b0 = Ainj⟦1, 3⟧ / V
    , a0 = 0; b0 = 0]; (*complexes in blood*)
   NN0 = N0;
   DD0 = 0;
   pa0 = 0; pb0 = 0; fFN0 = 1; fAN0 = 0;
   dF0 = 0; dA0 = 0; rho0 = rhomax;
   ActBlood0 = 0; ActBloodFrag0 = 0; ActOut0 = 0; ActOutFrag0 = 0; ActTumor0 = 0; NewCells0 = 0;
   SD0 = 0; CFN0 = 0; CFD0 = 0; UN0 = 0;

   (* SOLVER *)

   tB⟦1⟧ = 0; If[Ainj⟦1, 1⟧ == 0, If[lAinj > 1, tE⟦1⟧ = Ainj⟦2, 1⟧, tE⟦1⟧ = tEnd], tE⟦1⟧ = Ainj⟦1, 1⟧];

   For[npw = 1, npw ≤ Npw, npw++,

    Clear[a, b, NN, DD, pa, pb, fFN, fAN, dF, dA, rho, ActBlood, ActBloodFrag, ActOut, ActOutFrag, ActTumor, SD, CFN, CFD, UN, NewCells];
    (*fFD,fAD,*)
```

```
sol = NDSolve[{
  (*INITIAL CONDITIONS*)
  a[tB[[npw]]] == a0, b[tB[[npw]]] == b0, NN[tB[[npw]]] == NN0, DD[tB[[npw]]] == DD0, pa[tB[[npw]]] == pa0, pb[tB[[npw]]] == pb0,
  fFN[tB[[npw]]] == fFN0, fAN[tB[[npw]]] == fAN0, dF[tB[[npw]]] == dF0, dA[tB[[npw]]] == dA0, rho[tB[[npw]]] == rho0,
  ActBlood[tB[[npw]]] == ActBlood0, ActBloodFrag[tB[[npw]]] == ActBloodFrag0, ActOut[tB[[npw]]] == ActOut0,
  ActOutFrag[tB[[npw]]] == ActOutFrag0, ActTumor[tB[[npw]]] == ActTumor0, SD[tB[[npw]]] == SD0,
  CFN[tB[[npw]]] == CFN0, CFD[tB[[npw]]] == CFD0, UN[tB[[npw]]] == UN0, NewCells[tB[[npw]]] == NewCells0,

  a'[t] == Fa[t], b'[t] == Fb[t], NN'[t] == FNN[t], DD'[t] == FDD[t], pa'[t] == Fpa[t],
  pb'[t] == Fpb[t], fFN'[t] == FfFN[t], fAN'[t] == FfAN[t], dF'[t] == FdF[t], dA'[t] == FdA[t], rho'[t] == Frho[t],

  ActBlood'[t] == V * lambda * (a[t] + pa[t]),
  ActBloodFrag'[t] == V * lambda * pa[t],
  ActOut'[t] == V * (kappac * a[t] + kappap * pa[t]),
  ActOutFrag'[t] == V * kappap * pa[t],
  ActTumor'[t] == (lambda * gamma) * (fAN[t] * NN[t] + dA[t]),

  SD'[t] == ks * (lambda * gamma) * (fAN[t] * NN[t]),
  CFN'[t] == (1 - ks) * (lambda * gamma) * (fAN[t] * NN[t]) * NN[t] / (NN[t] + DD[t]),
  CFD'[t] == (1 - ks) * (lambda * gamma) * dA[t] * NN[t] / (NN[t] + DD[t]),
  UN'[t] == kf * lambda * (a[t] + pa[t]) * nu * NN[t],
  NewCells'[t] == If[t > Ainj[[1, 1]], rho[t] * NN[t], 0] (* start counting new cells from the moment of the first injection *)

  (* The simulations run until the injected activity decays to negligible amounts
    If viable cancer cell number becomes too small (cure) or too great (host death) it just stays there to allow assessment of activity paths
    You can make simulations just stop there by using commented functions *)
  , WhenEvent[t > TMIN, NN[t] → 0]
  , WhenEvent[NN[t] > 10^9 / Nnor, NN[t] → 0.99 * 10^9 / Nnor (*tE[[npw]]=t;
```

```
          tEnd=t;
          "StopIntegration"*) ] (* cancer wins *)
        (*,WhenEvent[NN[t]<0.01/Nnor,NN[t]→0 (*tE[[npw]]=t;
          tEnd=t;
          "StopIntegration"*) ] (* treatment wins *) *)
      }
    , {a, b, NN, DD, pa, pb, fFN, fAN, dF, dA, rho, ActBlood,
       ActBloodFrag, ActOut, ActOutFrag, ActTumor, SD, CFN, CFD, UN, NewCells}, {t, tB[[npw]], tE[[npw]]}
    , AccuracyGoal → 10, PrecisionGoal → 10];

  apw[[npw]] = First[a /. sol]; bpw[[npw]] = First[b /. sol]; NNpw[[npw]] = First[NN /. sol]; DDpw[[npw]] = First[DD /. sol];
  papw[[npw]] = First[pa /. sol]; pbpw[[npw]] = First[pb /. sol]; fFNpw[[npw]] = First[fFN /. sol]; fANpw[[npw]] = First[fAN /. sol];
  dFpw[[npw]] = First[dF /. sol]; dApw[[npw]] = First[dA /. sol]; rhopw[[npw]] = First[rho /. sol];
  ActBloodpw[[npw]] = First[ActBlood /. sol];
  ActBloodFragpw[[npw]] = First[ActBloodFrag /. sol];
  ActOutpw[[npw]] = First[ActOut /. sol];
  ActOutFragpw[[npw]] = First[ActOutFrag /. sol];
  ActTumorpw[[npw]] = First[ActTumor /. sol];
  SDpw[[npw]] = First[SD /. sol];
  CFNpw[[npw]] = First[CFN /. sol];
  CFDpw[[npw]] = First[CFD /. sol];
  UNpw[[npw]] = First[UN /. sol];
  NewCellspw[[npw]] = First[NewCells /. sol];

  If[npw < Npw,
    (*renew initial conditions*)
   If[Ainj[[1, 1]] == 0
    , a0 = apw[[npw]][tE[[npw]]] + Ainj[[npw + 1, 2]] / V; b0 = bpw[[npw]][tE[[npw]]] + Ainj[[npw + 1, 3]] / V
    , a0 = apw[[npw]][tE[[npw]]] + Ainj[[npw, 2]] / V; b0 = bpw[[npw]][tE[[npw]]] + Ainj[[npw, 3]] / V];
```

```
      NN0 = NNpw[[npw]] [tE[[npw]]] ;
      DD0 = DDpw[[npw]] [tE[[npw]]] ;
      pa0 = papw[[npw]] [tE[[npw]]] ;
      pb0 = pbpw[[npw]] [tE[[npw]]] ;
      fFN0 = fFNpw[[npw]] [tE[[npw]]] ; fAN0 = fANpw[[npw]] [tE[[npw]]] ;
      dF0 = dFpw[[npw]] [tE[[npw]]] ;
      dA0 = dApw[[npw]] [tE[[npw]]] ;
      rho0 = rhopw[[npw]] [tE[[npw]]] ;
      ActBlood0 = ActBloodpw[[npw]] [tE[[npw]]] ;
      ActBloodFrag0 = ActBloodFragpw[[npw]] [tE[[npw]]] ;
      ActOut0 = ActOutpw[[npw]] [tE[[npw]]] ;
      ActOutFrag0 = ActOutFragpw[[npw]] [tE[[npw]]] ;
      ActTumor0 = ActTumorpw[[npw]] [tE[[npw]]] ;
      SD0 = SDpw[[npw]] [tE[[npw]]] ;
      CFN0 = CFNpw[[npw]] [tE[[npw]]] ;
      CFD0 = CFDpw[[npw]] [tE[[npw]]] ;
      UN0 = UNpw[[npw]] [tE[[npw]]] ;
      NewCells0 = NewCellspw[[npw]] [tE[[npw]]] ;

      (*renew time frame*)
      tB[[npw + 1]] = tE[[npw]];

      If [Ainj[[1, 1]] == 0, If [Npw > npw + 1, tE[[npw + 1]] = Ainj[[npw + 2, 1]], tE[[npw + 1]] = tEnd] ,
        If [Npw > npw + 1, tE[[npw + 1]] = Ainj[[npw + 1, 1]], tE[[npw + 1]] = tEnd] ];]
];
npw --;
(* It will be convenient to have estimation of minimal viable cell number here *)
TableNN = If [Ainj[[1, 1]] == 0, Table [NMinimize [ {Nnor * (NNpw[[nn]] [t] ) , t > tB[[nn]], t < tE[[nn]]}, t] , {nn, 1, npw} ] ,
      Table [NMinimize [ {Nnor * (NNpw[[nn]] [t] ) , t > tB[[nn]], t < tE[[nn]]}, t] [[1]], {nn, 2, npw} ] ];
```

```
Nn = Min[TableNN[[All, 1]]];

tminNn = t /. TableNN[[Position[TableNN[[All, 1]], Nn][[1, 1]]]][[2]];

Return[Nn])
```

In[ ]:=

```
(* the same training set that was used for the sweep under pure radioconjugates *)
PatientsTrainingSet = {{"\!\(\*SubscriptBox[\(κ\), \(c\)]\)", "\!\(\*SubscriptBox[\(κ\), \(p\)]\)", "γ", "V",
    "\!\(\*SubscriptBox[\(k\), \(s\)]\)", "\!\(\*SubscriptBox[\(ρ\), \(0\)]\)", "\!\(\*SubscriptBox[\(ρ\), \(m\)]\)",
    "ω", "\!\(\*SubscriptBox[\(α\), \(0\)]\)", "\!\(\*SubscriptBox[\(α\), \(m\)]\)",
    "\!\(\*SubscriptBox[\(k\), \(f\)]\)", "\!\(\*SubscriptBox[\(N\), \(0\)]\)", "\!\(\*SubscriptBox[\(k\), \(D\)]\)"},
   {0.08287723093332094`, 3.0858822376571258`, 1.0544020640762781`, 0.8913551057392846`, 0.07800980208832753`,
    0.18650785858589003`, 0.06335580187071355`, 0.005963202911440555`, 1833.7547379259959`, 302.5435916235979`,
    0.054836866330129685`, 9.998403612912536`, 1.2206910575241197`}, {0.16008991075718454`, 0.9889641902273629`,
    4.070194520482062`, 1.048412343563395`, 0.9623075048370111`, 0.2561461923913073`, 0.06710665317178915`, 0.4051510354125659`,
    2424.3911699885257`, 364.2049627898059`, 0.11376488110702476`, 6.677727754478127`, 0.46263232179329394`},
   {0.23888202110078255`, 0.8810916509901707`, 5.093732295047118`, 1.4600046563601987`, 0.26112133308478946`,
    0.18414540656565503`, 0.10719043869683506`, 0.019052269075044592`, 1361.794327483607`, 157.34664623392825`,
    0.2248991829845473`, 7.770022547327725`, 1.3856793740595026`}, {0.20921512222542155`, 0.8329034625019238`,
    5.512850623987205`, 1.0082829806829832`, 0.6432484314226474`, 0.16609066867476052`, 0.06366860478505312`,
    0.09015760505638287`, 3121.845933283612`, 31.743667621759982`, 0.23453419786488217`, 5.669698519018798`, 0.2162114279190297`},
   {0.15821344070119114`, 2.676629891143981`, 7.114918370652138`, 1.231756200149393`, 0.7440882968059128`, 0.609542391688829`,
    0.20113875027795403`, 0.008293436499309746`, 3321.626865604231`, 300.9780595191306`, 0.1294374982810616`,
    4.573476150462291`, 0.6252568014280515`}, {0.04416264589453098`, 1.237497547847834`, 1.3522815348119845`,
    1.466732288470342`, 0.6840124021467311`, 0.46728396632651026`, 0.07378117717995297`, 0.04606361653599369`,
    2651.0081518614816`, 102.21078555052515`, 0.21006656956644504`, 2.3728441322292344`, 0.992859548694442`},
   {0.2369260528945452`, 2.0701500057376476`, 2.467879519617215`, 1.3729060512118858`, 0.7839988344536148`,
    0.6604044950304033`, 0.16113998364121102`, 0.05466214114010759`, 886.5316249876132`,
    108.21460952627575`, 0.04090907280605699`, 1.8765499412863633`, 0.31190921443437514`},
   {0.10845014170633277`, 2.595808559087966`, 3.825660598104694`, 0.9092167031122353`, 0.4998129490409222`,
    0.3952109813060457`, 0.14370479224814003`, 0.06482861565134085`, 2096.8410035229635`, 61.50816633658093`,
```

0.11793078229478832`, 9.815002337220978`, 0.4969144428042134`}, {0.1958274434061072`, 1.1625250352222158`, 4.38654338158619`,
1.0335502224347561`, 0.757936701785614`, 0.28764737226179815`, 0.053841290852541135`, 0.15446581430564627`,
1541.8645934819597`, 81.13506069010731`, 0.032171888494962775`, 6.764038142553707`, 0.3026234451737826`},
{0.11706495920321314`, 3.996131916383794`, 3.4445284668439964`, 1.0745666843500223`, 0.4349463063881305`,
0.49843595508242333`, 0.15623150182584739`, 0.02045656151514831`, 3837.5091768132115`, 201.1196769174046`,
0.10740962694701128`, 6.186844269751422`, 0.8954099169722194`}, {0.15007898341668874`, 1.7157935178042445`,
2.2029422939279666`, 1.1981889493296447`, 0.6780839054962491`, 0.6847631092713093`, 0.13190149021010728`,
0.01567939294607179`, 3588.049208356785`, 265.194158460061`, 0.020899950425706137`, 5.763952406784453`, 0.2759848394189104`},
{0.05919792771600385`, 1.1566416574477563`, 7.068615334313819`, 0.9063681130718225`, 0.7973057249073783`,
0.32056833480557556`, 0.14802374216985142`, 0.006390669590825526`, 3472.301841329897`, 218.2462028286681 7`,
0.09503155541006275`, 6.650147058357586`, 0.752916478623388`}, {0.12780221907252537`, 3.8001966548501374`,
9.893992295428603`, 0.9054601762352339`, 0.3495759940365448`, 0.1520403910810577`, 0.10896185913129744`, 0.3816734295429438`,
1447.6496724709095`, 224.52426056149727`, 0.14587747065522566`, 4.530340760708304`, 0.2310603840049561`},
{0.052097538451470415`, 0.8314166561245999`, 5.481254262333325`, 1.3932692734662013`, 0.3669026419040864`,
0.3620022667552303`, 0.11591227570051149`, 0.01952536971625568`, 3813.0335975189155`, 109.62712481259092`,
0.17741871372248502`, 1.8758946193559272`, 0.37818057550442363`}, {0.16758241286506004`, 2.4053214221448718`,
3.775183470319785`, 1.3879685907097887`, 0.849157385981911`, 0.1600626918561956`, 0.12028733740307626`, 0.23825099986429926`,
3824.577207611347`, 166.9670016449728`, 0.011876010376545704`, 5.836418598900732`, 0.8350403039197589`},
{0.2134810376991253`, 1.4110142375655572`, 5.989091842275013`, 1.4582774889459504`, 0.06026935559352742`,
0.21384589376924967`, 0.11902027597851936`, 0.012348918184910978`, 2514.3629410926587`, 346.5120508621949`,
0.0374109599718197`, 6.153474798173264`, 0.7147791109959445`}, {0.183290589228506`, 0.5116934633594044`,
9.423512566077996`, 1.207136012185702`, 0.9255825329486453`, 0.5109486403531394`, 0.061891644358164966`,
0.06517375803184387`, 1490.0042360738662`, 338.5269107534542`, 0.2295202271938529`, 6.23442268470835`, 0.9091680440302747`},
{0.07608249414500318`, 1.6334157835018592`, 2.1569194913061693`, 1.197668491595115`, 0.09742648797035525`,
0.6759576090965576`, 0.07148505885394518`, 0.13535119072972454`, 1569.9807697844644`, 192.45980341337224`,
0.19113163797167387`, 2.99326845694066`, 0.7712705061277614`}, {0.2761380591152234`, 0.5948415416580923`, 4.492630890025399`,
1.4137462343952987`, 0.19259719440314438`, 0.2905045944613839`, 0.092594223894082`, 0.0064155840660979225`,
2524.964006213205`, 55.97986174003478`, 0.1939779490397887`, 7.138405092365562`, 0.3980368971508512`},
{0.2691641473228049`, 2.5948088236594433`, 9.806966931633422`, 1.0098220223982928`, 0.036933113005692464`,
0.496552488389817`, 0.11856800340468354`, 0.008793318254398408`, 2701.46341207771`, 37.07745796913986`, 0.05351989235558863`,
6.680707066067022`, 0.2241309195665182`}, {0.18781219936172783`, 1.4023238769545223`, 1.2203108039534882`,

1.4529300029349537`, 0.39441104236838154`, 0.21312489114159694`, 0.1140711924706900 3`, 0.05613185190396572 5`,
1464.0480188461024`, 295.31541235105215`, 0.1586938998074992`, 9.00673891342328`, 0.8723355545572158`},
{0.2094783821158741`, 2.6664932858575137`, 7.297360825644024`, 0.9801370202263215`, 0.5993287287749127`,
0.5949313991226747`, 0.07854776414147877`, 0.007471189820589976`, 3737.8249085720936`, 225.90376084120953`,
0.12230719137003099`, 4.645596524735494`, 0.773898586938345`}, {0.04064294172797539`, 1.2711785498005437`,
6.4142221576096325`, 1.3607991978128624`, 0.4152034158458744`, 0.43390213144020495`, 0.1732390453420793`,
0.044490756173200585`, 3317.76480386853`, 267.9877730423784`, 0.047319616563592015`, 9.540745693864437`, 1.14427781856239`},
{0.09357862879768009`, 0.6953322280042711`, 5.170280682368036`, 1.0934248327219471`, 0.7339854764578926`,
0.4916807813635117`, 0.08077481700228195`, 0.04710573802150511`, 2462.550423949988`, 223.30073476186828`, 0.13718177246897728`,
6.470496132991109`, 0.9927113806256207`}, {0.18983638851163714`, 1.3904426162746537`, 6.462224486154522`,
1.3121224928907784`, 0.33702569938499116`, 0.4827790093546729`, 0.17642716165084854`, 0.14070582325373504`,
1445.2238937860711`, 169.08245719423826`, 0.08107423780437323`, 3.772942227886274`, 0.5974646397153294`},
{0.14369943000217644`, 1.7114006053749717`, 7.290050955062047`, 0.8291375841791329`, 0.041053311258442804`,
0.588971030507233`, 0.22011431905536544`, 0.14621434360249197`, 2238.8977574406254`, 92.23431071779362`, 0.19794610644205557`,
8.103641190648325`, 1.3952824759429032`}, {0.07643772187175424`, 1.9519546589360584`, 6.900928196416569`,
0.876872363876414`, 0.7662923029927724`, 0.15407458175108746`, 0.15169600745126946`, 0.11514584917672349`,
3018.1218065404273`, 170.6089829682045`, 0.07107865878327907`, 5.366481527317603`, 0.4272921221427337`},
{0.2710591123734774`, 3.4140873238389613`, 0.9957406410201042`, 1.2040917290884572`, 0.20012464577442923`,
0.17413508978502545`, 0.1542723564673828`, 0.3932031693142617`, 919.8622664894237`, 351.88082244581324`, 0.07678285799586054`,
9.51148011674945`, 0.10358667667962984`}, {0.10385665451428133`, 0.45328132552019573`, 1.5503843984121968`,
1.4694004919600818`, 0.4251249704277895`, 0.18123604867037413`, 0.08236798590439715`, 0.07631033842692926`,
1502.3783558556506`, 20.10790624452443`, 0.14290440434604346`, 4.641987001123601`, 1.0114789952636625`},
{0.08203524638521087`, 2.7438300558988873`, 0.5228963098315518`, 1.1830383771323514`, 0.45293779314745897`,
0.15188403574429454`, 0.10479303433979437`, 0.2038448791081171`, 983.5363393912057`, 168.65049566992798`,
0.019976684129921263`, 1.733382066252318`, 1.4812211807580051`}, {0.04913245961465748`, 2.276131341102663`,
7.044266329747086`, 0.9173667594455086`, 0.41644326647059726`, 0.25990231144478826`, 0.18555590900866242`,
0.210063875037094`, 1773.4485121819462`, 75.91056730044647`, 0.184796166571025`, 2.2167031467842424`, 0.9244440644631444`},
{0.15077968496954103`, 0.971820860662203`, 0.23396787648709783`, 0.9304382139491995`, 0.8434070020798108`,
0.5328351579449485`, 0.0913866934528598`, 0.005065303834986521`, 1783.900832424436`, 398.4573153532763`,
0.17963340543276235`, 6.010950437900332`, 0.19621378169468162`}, {0.15622813333517427`, 1.117114824910261`,
4.688994947991285`, 1.188191128841129`, 0.5951306703894546`, 0.30511551468851006`, 0.2217863487164401`, 0.33567754538344835`,

2568.8944572362634`, 80.78669839983178`, 0.03729030447311804`, 7.7879419731274115`, 0.9647929000978697`},
{0.11331162134690587`, 1.2483595201539153`, 4.922077543835222`, 1.0559046192245036`, 0.27438155190999924`,
0.25039971454185184`, 0.17415638432949032`, 0.1908750894543732`, 1293.217041147549`, 78.02731777513316`, 0.14932153866568026`,
5.182262423333571`, 0.7834976909752784`}, {0.13914220002486866`, 3.6703502896291074`, 6.379985611157895`,
1.2081851433506285`, 0.7677675288971362`, 0.4439146253901465`, 0.18717370496505287`, 0.22151595381283107`,
1066.171533351664`, 40.74945145753361`, 0.011595132472834446`, 8.264527016617006`, 0.7438205368393507`},
{0.22919129490265872`, 2.16424636204603`, 7.737422782052235`, 1.4860287946100363`, 0.8497692228491351`,
0.6288301911078309`, 0.10881904848202179`, 0.012090233236638177`, 2725.277273810464`, 77.99221256357112`,
0.11691550532917167`, 1.34040213575469`, 0.8482131070094612`}, {0.14382797385454654`, 3.902431027294017`,
8.163450688536429`, 0.8614076883883874`, 0.9648536276916164`, 0.1955756537598935`, 0.13123090751766925`,
0.17894545684886398`, 1385.50217697701`, 120.46271140783404`, 0.09423544317816807`, 8.260526259890764`, 0.9048289425944`},
{0.26055110138040244`, 2.367351112459766`, 4.465438967228517`, 1.1966516268556373`, 0.9350425579579571`,
0.17322311915607413`, 0.07783685162029783`, 0.005169562389067373`, 1562.7816595558625`,
214.0110363941617`, 0.12855831417818353`, 8.432278535705368`, 1.4776941418404932`},
{0.14680250287088714`, 2.9795896823389043`, 0.5962523026989057`, 1.2784383052923718`, 0.4427855138400896`,
0.39715884806633817`, 0.14338219057113172`, 0.008545850364486081`, 3591.485740200138`,
175.10126732731499`, 0.10771590512275864`, 1.6757545718316624`, 0.6195088999107756`},
{0.1718184288134474`, 1.149839439352271`, 8.637641060035964`, 0.8458363550999337`, 0.6513629677556381`,
0.2037277594433513`, 0.17428171056999092`, 0.14466402071943818`, 1236.767232462591`, 142.28311915163187`,
0.08823953882269175`, 7.82350558871963`, 1.0042870130174983`}, {0.06783774432411926`, 1.4183998935859234`,
4.514906377341504`, 1.3074717506903661`, 0.44121610557851443`, 0.3859898733629271`, 0.18885629375508112`,
0.08215446401079224`, 1996.0572117605661`, 32.84560309478019`, 0.14544575809597998`, 5.16703571475392`, 0.8091181446129996`},
{0.09592803445036208`, 0.8366316591486305`, 5.0931225791851045`, 1.1660530096752404`, 0.2059334355486433`,
0.4873422492046261`, 0.1598589026416986`, 0.029544379859417352`, 2582.9507509173`, 296.85131956979524`, 0.07927444657096727`,
2.8630420963700924`, 1.2775475419622428`}, {0.06735695081703474`, 1.6218831494512376`, 8.01632413374589`,
1.2380602139197665`, 0.3870710635961401`, 0.2680531526020482`, 0.07829277488965869`, 0.011053339337592866`,
594.5995475908121`, 283.2125249310004`, 0.06462100682727939`, 4.113525656200814`, 1.255132039019542`},
{0.25318695512908856`, 3.592770590245955`, 0.3860629768890025`, 0.8375102060070931`, 0.25324293087688066`,
0.22639700269651497`, 0.18616406291052912`, 0.3042902902243334`, 535.9279722175538`,
147.98665712717872`, 0.04054733608151545`, 8.866857733517588`, 0.9845499556289559`},
{0.10623464305011832`, 2.9795950019626645`, 8.394466481175808`, 1.3150375502652678`, 0.3977517440977254`,

0.1685394428801441`, 0.14812837191515202`, 0.06701796399112063`, 3705.0856036562427`,
41.40776885031511`, 0.10405528061386266`, 6.850682270825037`, 0.7771134705080516`},
{0.16614727413225283`, 2.422977153037391`, 4.380699036993274`, 1.2650547043993527`, 0.7289628812438445`,
0.5253372649543967`, 0.1282487061525488`, 0.0058468750308125665`, 1525.6199865357175`, 306.5080581733838`,
0.2463161390095024`, 8.0298979658821`, 0.3034478973618291`}, {0.09439363668230882`, 0.5823127951342895`, 9.668824509749783`,
1.1792283442388243`, 0.7110022446147217`, 0.31000063243129616`, 0.126665851525797`, 0.01012852201633408`,
3883.1686659534244`, 167.0440580830566`, 0.14786211145387196`, 1.794206476801321`, 0.758471056166536`},
{0.10782261295184936`, 0.4917827154911696`, 7.249268882781941`, 1.3611009578260718`, 0.7853661815311994`,
0.41488889777264726`, 0.055591995773018654`, 0.052207153417698195`, 693.7280684674711`,
321.57606910090226`, 0.24632437610376973`, 5.428213742379803`, 0.6782641447302435`},
{0.05850570936819943`, 2.3562511218485964`, 4.849470518860397`, 1.0037692866900862`, 0.20135980488529004`,
0.6258574217265285`, 0.20434104039710765`, 0.046458478403025076`, 1143.066165281296`,
382.63411260362705`, 0.03938722953573187`, 7.245742033238452`, 0.7325066156705251`},
{0.26935136701037576`, 2.6617924377127427`, 1.8653214881187097`, 1.3783772798670109`, 0.352129512501022`,
0.17316862578533354`, 0.07359966841424637`, 0.03044640192221252`, 2578.790502033272`,
266.15340429326216`, 0.24670889410575075`, 9.827796845989266`, 0.8840863683155358`},
{0.2583950802004026`, 3.7896744268241553`, 0.3341722318602649`, 0.884753511926686`, 0.6013312124575798`,
0.2239296898773392`, 0.10126995172624759`, 0.005456842095783797 5`, 755.0573605362365`,
345.1189599908564`, 0.23686856237319676`, 4.65826036739878`, 1.1534719871772796`},
{0.11828518723170395`, 1.6435868292536613`, 7.819044307747686`, 1.2773743957591923`, 0.9657225148907191`,
0.25540329193006406`, 0.2479438125086047`, 0.3343373077742329`, 1140.611649524565`,
164.9221880908642`, 0.023651248717273365`, 8.003232493785013`, 0.21949174382120273`},
{0.26141584699667636`, 0.7473599798954296`, 9.058762118324648`, 0.765952368266298`, 0.21904751242478016`,
0.26721271327445206`, 0.22245249424068714`, 0.03093590173170892`, 2551.7003206225927`,
385.7759782876328`, 0.1131827887828083`, 7.852734975537449`, 1.3811110977409826`},
{0.2597492463712146`, 3.976490463784595`, 9.94323613361071`, 0.955277937303862`, 0.36781597694318213`,
0.3161337854088553`, 0.14241883254664206`, 0.0371161903088566`, 1242.5354975091168`, 278.37240293523723`,
0.07851375810023631`, 8.744407246757977`, 1.3989634873529022`}, {0.057509555667969614`, 0.5897063506624876`,
7.194630155059524`, 1.2755298225691563`, 0.689564930155854`, 0.36119278217271744`, 0.2498841160696511`,
0.4009780889195491`, 1320.9907894027438`, 256.63876030059`, 0.13571670185918522`, 7.16857463714371`, 1.3676255250557143`},
{0.1397208239381057`, 1.3512320351631422`, 4.315748656362622`, 1.0234149323507424`, 0.4387218679676288`,

0.5908017035553538`, 0.24436343171483993`, 0.05514077662518975`, 1030.1549542852385`,
113.10980109596312`, 0.2358198015899574`, 7.7106342544090705`, 0.46099446073278916`},
{0.04583854888632438`, 2.3264689225741337`, 6.981580565170444`, 1.2372980328075218`, 0.8253651804903572`,
0.45495452249062196`, 0.0835295837141499`, 0.03439901257655977`, 1013.5313447757526`,
64.02695030885803`, 0.1777154613277634`, 6.347498471152688`, 0.28284114950471473`},
{0.08906331353528191`, 1.5482338770119695`, 5.590038221637837`, 1.3588168835620769`, 0.3085034668822382`,
0.2676722221657748`, 0.07831428730380899`, 0.019104385555027914`, 2856.0098758390477`,
29.221123010272038`, 0.07226770477125283`, 4.247245472589608`, 0.2276638271182967`},
{0.14523765830527252`, 1.8881182449998724`, 9.138446692610067`, 0.8999286193853917`, 0.5310683312029048`,
0.6925914130374311`, 0.2344374703010791`, 0.0058377651734979955`, 3489.658152730246`,
207.59827142911195`, 0.0635989978100086`, 4.4743246714218365`, 0.16854984601616874`},
{0.23258902546586485`, 2.08539598507119`, 3.699686103649381`, 1.0931833498928993`, 0.05064531761576041`,
0.520823194388518`, 0.08776225589270567`, 0.3513344393005501`, 3745.4841551781074`,
189.61513791631432`, 0.22360193983114673`, 3.043950615318625`, 0.6185359661549543`},
{0.1820955139503701`, 2.1301463589452796`, 1.0837335286589127`, 0.8319946959595101`, 0.024409178193170122`,
0.6747387048956719`, 0.18033910089153304`, 0.00804054909792445`, 2554.5651932014816`,
147.37665368444442`, 0.18624166889237875`, 4.7992891492518055`, 0.5892958331460565`},
{0.11714865623011333`, 0.7803444845933583`, 3.7286455817586397`, 0.8712432490902873`, 0.9005891902672722`,
0.6478151883640173`, 0.15581100010071458`, 0.01574292115738763`, 1037.936039940223`,
180.67525214379884`, 0.029753801751762954`, 7.189499669067537`, 0.709240932965888`},
{0.25987401963349727`, 2.104611334162005`, 6.476348965574324`, 1.4421427522116774`, 0.618645235230914`,
0.3805640132205469`, 0.11350606280121234`, 0.0053044995482471255`, 2985.7971643892297`,
372.29941068623043`, 0.13537534314965316`, 9.556057499862177`, 1.2376769397336673`},
{0.14562691763665958`, 0.7916589364478996`, 6.589457190185791`, 1.2275476972045025`, 0.23387470631242246`,
0.5473498244385164`, 0.05668225254693815`, 0.02480814693768771`, 3264.7917840803066`,
42.76592076077594`, 0.07262816395108834`, 7.451596380699327`, 1.1772112101747596`},
{0.12870736792434306`, 1.8011116706209283`, 5.964705884730831`, 1.0934961970990227`, 0.1765898735142728`,
0.17954421546189758`, 0.07865767771997476`, 0.015380472377885535`, 1239.7199915121791`,
204.53201197549618`, 0.09598785640324625`, 4.188391285134756`, 0.8112464540523976`},
{0.14650986127607446`, 2.5172437701709836`, 0.1805184658738579`, 0.7600994183135076`, 0.9075051465461681`,
0.2801821328714206`, 0.17102217375046636`, 0.35873020461861416`, 1565.8031681921566`,

125.39398648792553`, 0.18475404806650503`, 5.639231846073234`, 1.0926853341214517`},
{0.21477552815648338`, 3.895561319530766`, 6.525902778767705`, 1.1779397940067482`, 0.3665320870360802`,
0.26560577062434`, 0.16739034513753454`, 0.18566064627492787`, 2084.753342798819`,
127.5970184006917`, 0.1877200023200773`, 6.725934789039004`, 0.6926175903352805`},
{0.16557107707187058`, 3.9808920811325805`, 5.098629354330779`, 1.349285917396553`, 0.636892795916012`,
0.22358004297978562`, 0.184200177687603`, 0.010072170136625726`, 2379.9993388287503`,
250.88159080337186`, 0.22088678734814615`, 3.6464862549045485`, 1.4194198069422335`},
{0.2183191832780194`, 0.7656891998585413`, 0.2734070058927056`, 0.9147050043440094`, 0.3441094983626165`,
0.18539360977555885`, 0.1738037138598677`, 0.02857315375757566`, 2216.527228285475`,
152.12573952853177`, 0.143511762959395`, 2.048281105987993`, 0.3150624921161962`},
{0.07967342618536455`, 2.504619914171559`, 1.5706511952625795`, 1.0567028274669426`, 0.8710362851311728`,
0.17476539269891578`, 0.1454017836571002`, 0.013152148214347218`, 996.5768638290242`,
179.4306604453801`, 0.15492643832143987`, 3.5116876584841332`, 0.7677856536659751`},
{0.07456457809354955`, 0.9644871227153224`, 4.441895089579111`, 1.073622161985627`, 0.23632853710586565`,
0.6486715280931723`, 0.07294638203638015`, 0.02103243083607793`, 1904.5570203562393`,
282.8945937517137`, 0.14375820330010797`, 5.555062182602162`, 0.9932669340579485`},
{0.2103616483258971`, 1.9343221969844642`, 6.714766349656301`, 1.0369410555646754`, 0.10623162247280038`,
0.4483194849728549`, 0.05288545252588117`, 0.039734874146343004`, 3533.119265437819`,
182.57420336612938`, 0.1697677503867407`, 5.3789812588786425`, 1.1987739832103812`},
{0.20281939839069102`, 1.9616090683543987`, 9.233817142633974`, 0.9596707111380289`, 0.0428378421346598`,
0.4996155069836299`, 0.1873396325631559`, 0.010300259610186683`, 2982.0763963636246`,
256.635560614439`, 0.10323853661350679`, 1.5583642397770507`, 1.1284223856531295`},
{0.09628613710357908`, 1.5426640827734222`, 0.6350126000666823`, 1.1824558202170816`, 0.4624355823492319`,
0.315548754668174`, 0.24429500934345866`, 0.021157750682009126`, 1240.1379477304627`,
114.8299639556875`, 0.022101718613827426`, 8.73583411100617`, 1.006336055790952`},
{0.2497495903654815`, 1.9922654552033938`, 4.7803148098485195`, 1.4608470462421037`, 0.7453342000999874`,
0.579116855343716`, 0.20607471500549013`, 0.11029005763163223`, 2995.828190949672`,
372.8602258250605`, 0.24417612053219007`, 5.214690665497957`, 0.479039467804907`},
{0.1826369436867583`, 3.4688854117825922`, 2.4438258950850287`, 1.4614655744939826`, 0.6869584196330984`,
0.32705660778535306`, 0.23164888048001758`, 0.012304471968752876`, 769.9733124793984`,
249.22075665117416`, 0.24824092697008104`, 9.063288482525959`, 1.0083702284112093`},

{0.07327234901270369`, 0.8531314502177398`, 8.241277338171692`, 1.2515317235008665`, 0.44747974375408006`,
0.36044147530449333`, 0.1678069040859092`, 0.08333627347055737`, 3911.155878788726`,
119.81486891303797`, 0.14940939146949633`, 5.454641004053358`, 0.6006995465954545`},
{0.1930703354969151`, 1.921406348468734`, 8.333479032313342`, 1.3125156750016385`, 0.34274795388496426`,
0.18757059400837783`, 0.06254344682622745`, 0.48499736430114065`, 1025.3342142153256`,
313.5826206578507`, 0.06795986160544415`, 8.852485488110243`, 1.3326254900136894`},
{0.075070301444424`, 1.1158533918226947`, 4.504617389718993`, 1.302858138181639`, 0.7006793741520845`,
0.20393211135209022`, 0.13347728349830462`, 0.12945769800166135`, 2084.9216842923897`,
207.91665221421215`, 0.1979670492115873`, 1.2145592445361792`, 1.330809319256201`},
{0.04793776202493774`, 3.0088158689008297`, 5.170365665010708`, 1.3304854892588214`, 0.4098476660063537`,
0.28021047255972975`, 0.1484797686973009`, 0.06051575982799722`, 541.2425249553703`,
199.10653072626633`, 0.20919118463500602`, 1.1760815484955796`, 1.3182149861572752`},
{0.27316321779569186`, 1.2038217889581082`, 3.7625718380424993`, 0.7524163201956243`, 0.8286278988606164`,
0.5838413652579414`, 0.1289047793447199`, 0.04536097191427075`, 2261.212930013986`,
291.3114882787695`, 0.1550429747126918`, 5.46528595832512`, 1.2275549298228752`},
{0.2494292406608673`, 1.335820360094238`, 3.8994390375054255`, 0.9165975453056769`, 0.13563044706422311`,
0.5269987018012078`, 0.205844612334116`, 0.012716247145242394`, 1091.290454779412`,
128.1406782295989`, 0.24389684971209813`, 1.43576099827777`, 0.4903794001833075`},
{0.12542240143398464`, 0.5428740326561194`, 7.689007709078183`, 0.7695505557683522`, 0.47414990819814995`,
0.5751602267111138`, 0.1894118267745437`, 0.4188796421855669`, 2595.8116503362608`,
121.7056153778716`, 0.02093687945319478`, 2.468481199752018`, 0.4696061183497453`},
{0.09391968152154795`, 3.06447396666384`, 0.887078247635884`, 1.3969498711006763`, 0.9012814324673109`,
0.6019227650187913`, 0.056809355713011156`, 0.11429079373151174`, 3926.488447769728`,
52.970576122622276`, 0.24789590005226048`, 5.914871844531001`, 0.869797459980445`},
{0.15904117838601922`, 2.05514897407154`, 8.990862054448137`, 1.399223458930086`, 0.16448814747402052`,
0.679414685909332`, 0.20987970122910526`, 0.011549865816715998`, 3644.479968128845`,
344.7039952488134`, 0.14067273235487315`, 5.192736401046902`, 1.265673086788253`},
{0.10596194132792924`, 2.903583061514107`, 0.8170701309155923`, 1.3631505832711714`, 0.6107209987788129`,
0.24870490908317233`, 0.18241426717129616`, 0.018338650769170847`, 1588.8736918382`,
58.688447621427315`, 0.2068253327055226`, 8.58249867744179`, 0.3849797974029039`},
{0.18968282299824824`, 0.519816328948473`, 1.9818995109191384`, 0.8631954007357836`, 0.033894385168637076`,

0.36152588787936835`, 0.13701549203954322`, 0.2296501432463657`, 2230.7628620359865`,
54.21442465485268`, 0.025809495734432863`, 5.0872858624540225`, 0.22420423998520422`},
{0.1333252885737945`, 0.6199239307530391`, 1.8571978251971686`, 0.9095514210206579`, 0.11191699009299061`,
0.2478793840048047`, 0.08625348528814925`, 0.24475415564717562`, 2619.34383923971`,
229.53625600516762`, 0.07038297161992796`, 1.9415562105060538`, 1.123956994156356`},
{0.15956630372935293`, 0.48645969647201426`, 2.666967707997488`, 1.3223736190803834`, 0.3256325807266449`,
0.3482049071188411`, 0.2077761075845453`, 0.13946170160498525`, 1352.5873448025677`,
303.93582335946746`, 0.20467290406100863`, 9.626982891792906`, 0.24128691898319654`},
{0.1706421742509121`, 2.0231510661206737`, 3.9336130035151675`, 1.4585682836966742`, 0.11643260032474756`,
0.3849941275209635`, 0.09390422638545698`, 0.012272074152593612`, 1400.0032271122882`,
356.7106633003026`, 0.22727785977132925`, 4.752121167794142`, 0.5739518122089307`},
{0.15918053794813153`, 2.206413285621343`, 5.933314299085708`, 1.101458457677796`, 0.578828119858223`,
0.5102982514637783`, 0.14381010711428127`, 0.005244970313706263`, 866.7690619050336`,
266.3421407794259`, 0.02684631402674248`, 2.069701061730642`, 0.9768437925368372`},
{0.05059760434947658`, 2.210267681954983`, 2.4323928454491863`, 1.1361578898617037`, 0.6967300845119604`,
0.5269320202068383`, 0.09927434886429598`, 0.24328677204756152`, 3807.4700659446307`,
207.19795070674797`, 0.16480436384127967`, 4.45009968475919`, 0.7729577502629965`},
{0.04928925392103686`, 1.1598100203546071`, 3.5959706980197943`, 1.0567798767912857`, 0.5147503710857468`,
0.3483622087425814`, 0.2380152845766259`, 0.2996884511067484`, 3369.979145995473`,
363.0241772367476`, 0.014634320972586101`, 4.5222312521830705`, 0.7343151280843321`},
{0.21304011543754336`, 1.277940262843642`, 8.381125715629981`, 0.9676658592923792`, 0.3190964277032775`,
0.4221745616471033`, 0.15115883673524522`, 0.016195119502937567`, 3029.1645990840216`,
191.62516925020475`, 0.15425858593294461`, 3.4086567800829783`, 0.2418820655638112`},
{0.24406698410388555`, 1.4917825523241879`, 1.8330046648415304`, 0.8025451323745518`, 0.3882804043356616`,
0.6182505929388649`, 0.23260014303933024`, 0.008919285535719272`, 1172.1683108026946`,
354.24325796754124`, 0.11125790867650998`, 4.298846537988302`, 1.0733397741970694`},
{0.18459742862139494`, 2.6783696392861316`, 5.280110904616961`, 0.799376053447836`, 0.38050898992691184`,
0.5204372051986464`, 0.12691110515834225`, 0.11946707964132822`, 2456.6727054183984`,
281.1798234579603`, 0.015729828687073455`, 5.211513473652531`, 0.2697983327311402`},
{0.10846037232055339`, 2.4223111603013434`, 3.931890423132195`, 1.3804697934424115`, 0.1958381176894204`,
0.37535535595877956`, 0.06969589452072714`, 0.03328599418804682`, 502.38138813825617`,

240.4375056150992`, 0.057245528883930086`, 5.6090889313070385`, 0.20778481434400486`},
{0.06691193258644623`, 0.6501044545968662`, 4.921653023328441`, 1.039296561059753`, 0.3007307833256643`,
0.63941363731021`, 0.1869993853971682`, 0.22083355826369067`, 1064.9317102210284`,
220.88788456016823`, 0.19206991947921326`, 8.047969449780908`, 0.8982344488011158`},
{0.05561357895854885`, 2.541859626964298`, 6.751848587656245`, 1.0550493742938256`, 0.47524313475951674`,
0.6531938104590875`, 0.23105177416584038`, 0.08329104995794483`, 3011.985435651468`,
344.16286415130503`, 0.16793335434061596`, 8.98503885095289`, 0.954537509095668`},
{0.2794959518803075`, 2.632356308938026`, 9.83032757153688`, 1.3936963350666467`, 0.29701793223992223`,
0.20626280407223174`, 0.16564637606336619`, 0.029273176379366037`, 3072.6434786295413`,
349.76316755112407`, 0.019513472485920585`, 8.521067400493664`, 0.14808884776404962`},
{0.08563187236977793`, 0.7699393339104481`, 2.55010147552081`, 0.886086358774756`, 0.5489407388189`,
0.6967816713320905`, 0.17330539558613361`, 0.06157221588457279`, 1722.9132401400711`,
240.8998329943014`, 0.21060998547292448`, 8.988787778446675`, 1.3846037250141348`},
{0.23109541414596008`, 0.5235141892941444`, 4.716797836648434`, 1.149537093670705`, 0.6317810219681328`,
0.3294586442905981`, 0.09670796472340015`, 0.006726344420984968`, 700.9172696415767`,
217.90397850913655`, 0.1081952457359675`, 4.152209762717746`, 1.275784993485201`},
{0.23775769207863484`, 1.2489503941392437`, 0.7600016230767555`, 1.095753134818721`, 0.8325305358876416`,
0.5578220865599602`, 0.11931867110919586`, 0.014230310160314848`, 1755.4498344511894`,
146.4106779572221`, 0.028427482579488184`, 4.979696715264749`, 1.3071286627299754`},
{0.2685717150273603`, 3.803415502373147`, 9.096737964614093`, 1.4671460946906174`, 0.7887407820119334`,
0.32631565166991583`, 0.10844882100921999`, 0.25610077987407076`, 3027.035564030042`,
145.16929800361856`, 0.22414767286679493`, 5.11102243526882`, 0.8167747408960464`},
{0.26187896590333215`, 2.5078884075536303`, 9.466913557279852`, 1.4296579631390225`, 0.32008183029716575`,
0.24032929426056993`, 0.06161020998156652`, 0.06072142573048648`, 3034.015450440843`,
187.9292507538172`, 0.24599280561665748`, 9.218911969505061`, 0.5310393366539319`},
{0.2642492231861682`, 0.678656463776123`, 5.229669084224717`, 1.4960336643896621`, 0.09351401012737992`,
0.6251695134859938`, 0.20442619944666085`, 0.006818687130271267`, 2270.7823160691296`,
72.58428376873474`, 0.07107729542645586`, 9.843971184435041`, 0.8047286671187344`},
{0.08663733907452292`, 1.1055276206514`, 6.650123879097649`, 1.442255346530818`, 0.5561115773214398`,
0.5670470763741954`, 0.06364195502160275`, 0.013792669120976587`, 1277.4869438309242`,
50.05278631474647`, 0.06972164372518636`, 6.513399093256677`, 1.399747572725158`},

```
{0.19280830132567833`, 3.755171648384974`, 8.021128419894612`, 1.335462575824002`, 0.48264871431934253`,
  0.18420836982360544`, 0.06051840370015368`, 0.009206053536078937`, 2566.2923313365445`,
  129.64164707934253`, 0.24657184473865845`, 2.9358400550970494`, 1.228808415909655`},
{0.16230945707171968`, 3.49945541680664`, 3.914597683293998`, 1.1051912745206218`, 0.5225762284594742`,
  0.37657675843017935`, 0.10709361805155937`, 0.30774950760646624`, 1978.9594818216538`,
  231.32180409655837`, 0.1878700890542328`, 3.1200520265606095`, 0.5684357627808438`},
{0.1178483592983135`, 1.9540292922377533`, 2.158901124676431`, 0.8036721080520579`, 0.022324771726093706`,
  0.23728547762141228`, 0.05703389546493082`, 0.19717089259438486`, 1678.2311544802042`,
  81.55285235418881`, 0.08629691407172851`, 9.548029647858971`, 0.23903934985577635`},
{0.07697798125264199`, 2.659703661710245`, 6.212164355972867`, 1.1315710739609641`, 0.8617717680117074`,
  0.1678172363037963`, 0.11812638083909416`, 0.3038591454145657`, 1784.188819853568`,
  30.892067935544162`, 0.199805435190976`, 6.1761313696591404`, 0.7335007728106389`},
{0.2783243544518275`, 1.6065452524928183`, 9.36513522454521`, 1.0091499802528594`, 0.9718534638380145`,
  0.5642633711867375`, 0.20737635968923002`, 0.08580910729338392`, 3111.9739304829964`,
  94.14000266759376`, 0.09401444314711727`, 7.942384951366636`, 0.4018498645586557`},
{0.13125352185182698`, 1.2184353998248145`, 9.320259419097251`, 0.7781699346988293`, 0.39678231153919463`,
  0.199523418988783`, 0.19269759399037872`, 0.23378140134846623`, 1538.2313512668088`,
  153.62701919814924`, 0.16196850129753104`, 1.3309378497731819`, 0.3512246763428848`},
{0.19506025087199597`, 3.126229772881034`, 2.308505447241318`, 0.9330443187011872`, 0.44656066575469633`,
  0.6840356045619456`, 0.09118120944389022`, 0.034391869601605236`, 2773.7056708459277`,
  224.4415538260822`, 0.0593475599896775`, 3.3310563178619557`, 0.5039179573441945`},
{0.06456964840349072`, 3.9537241675412282`, 3.641109216568884`, 1.3108140470595528`, 0.011724209788321671`,
  0.438559457390715`, 0.1826458897676254`, 0.0058767891936883`, 1354.5450008176776`,
  86.5151446772075`, 0.010849475740387471`, 9.258377423570199`, 0.4300240953856598`},
{0.1755298706210477`, 1.1941209557460892`, 3.229216030497655`, 1.0124753772100286`, 0.8865998145070868`,
  0.346236517225073`, 0.10475625859225324`, 0.48133977507102055`, 1150.8999752212085`,
  275.6916176605181`, 0.02747195653647755`, 4.902957385276519`, 0.7900769747759009`},
{0.23831756341954657`, 2.9118296516681834`, 0.7702789156400254`, 0.809756039261601`, 0.15598009967599835`,
  0.15072565384210967`, 0.12556989454649928`, 0.015474985134933292`, 1269.344776285695`,
  338.73985440309866`, 0.16681668263344573`, 4.906919687762464`, 0.7266912138912884`},
{0.1708861337350846`, 3.1382784382473305`, 0.22397208377012315`, 1.1376251902182828`, 0.5276068694742282`,
```

0.6173335030361042`, 0.09813160076689689`, 0.04549667808286232`, 3051.102513690643`,
71.6500993647141`, 0.0653128233426199`, 7.970153906640311`, 0.19805272219502368`},
{0.08731955025115129`, 0.716566084545263`, 3.9517372388296312`, 0.9979934558679373`, 0.7980323468748562`,
0.2894395727404939`, 0.23900619972839288`, 0.013550707634954815`, 3303.670456871152`,
218.2198703581039`, 0.21020148991020782`, 6.102509356835768`, 0.9882047778905934`},
{0.22902394998753428`, 3.040671713374585`, 4.278745423137005`, 1.1872158647718087`, 0.928367976025805`,
0.6049768179185504`, 0.23137362815653145`, 0.010091922508071186`, 2258.092758703583`,
198.0963189618003`, 0.016671766650619035`, 7.17480421825168`, 0.31956308961413415`},
{0.06329193275668865`, 1.9001497619691943`, 5.252505884188258`, 1.1790665711161483`, 0.6185584326542477`,
0.29903601211054487`, 0.18805549054580545`, 0.08613649661622771`, 1994.6987223768147`,
102.68040788991027`, 0.2346504641795843`, 5.384687813670801`, 1.1000521056786416`},
{0.1794793632102442`, 3.220429652904432`, 3.9791446112229796`, 1.2467997677613296`, 0.11361466513971896`,
0.5165426921925539`, 0.22271304495125288`, 0.2987687877435581`, 2176.453438414389`,
103.4889254155421`, 0.20600780354143933`, 7.995100635365489`, 1.3485017278608633`},
{0.25718420980427975`, 3.2208936021473447`, 9.13093887998258`, 0.7880508822382197`, 0.19579212797696832`,
0.6844641096337076`, 0.10728371238227297`, 0.15509978165260505`, 1971.6454958829345`,
171.21565981007905`, 0.07048499237249867`, 2.8101083115304704`, 0.7477956360854239`},
{0.0634556155295738`, 3.680544991233944`, 1.898464422832438`, 1.0496696027333048`, 0.16836359340314266`,
0.3732784807189835`, 0.2175697213065485`, 0.48318139091353535`, 714.7371038686642`,
368.95513713314926`, 0.1959410717243747`, 1.9243643979934077`, 1.1366603632987364`},
{0.22344776749508172`, 2.0217933679260236`, 8.515379660775892`, 1.0649182548208418`, 0.4042870031976431`,
0.16656241643092373`, 0.13003071453691661`, 0.03138725525414384`, 1808.0816946786463`,
137.22272185069306`, 0.15677750076118624`, 3.7329055172229837`, 0.8930251877812045`},
{0.21641932432281485`, 3.6568426450830875`, 1.306554690894977`, 1.4626425107711718`, 0.8835071341302185`,
0.5676582474171099`, 0.11158412214739238`, 0.023181801672309155`, 527.0796987655522`,
75.44193071736254`, 0.13812060798969733`, 3.0114637760169054`, 0.9949014306145822`},
{0.27575001701682567`, 3.4736287001446966`, 8.538624919070354`, 1.4033945242686854`, 0.5325831427167897`,
0.4946990317811615`, 0.09780340245600855`, 0.03288516063918166`, 2535.414355248974`,
43.33184327775365`, 0.19548398944691353`, 1.2011151230670372`, 1.3252105639060514`},
{0.232764074403509`, 1.2733235890477337`, 2.798704761143199`, 1.2089738970000061`, 0.2230756746500473`,
0.581248946739561`, 0.23598452021555733`, 0.3774029175060033`, 1879.298545402161`,

```
390.18642709154335`, 0.06147319592641398`, 6.399283876049893`, 0.6943722912689665`},
{0.1797514088363001`, 3.6024179997471064`, 3.854320723033281`, 1.3651410466183755`, 0.3139114488968535`,
 0.24530825544465074`, 0.2138602169809023`, 0.009505828045846924`, 3546.9761969830042`,
 314.2947763723504`, 0.014606932674341683`, 5.351501403328509`, 1.3913390486692032`},
{0.08593348279794505`, 1.5299819772683403`, 7.853142207396741`, 0.8569688383589438`, 0.17350357661082394`,
 0.3599516000206078`, 0.22245867865147873`, 0.35987771942812175`, 2188.9020302076506`,
 267.66973385037716`, 0.21174504666607785`, 1.9987769240506328`, 0.6930609981274307`},
{0.06256964883486332`, 3.548483703832706`, 5.540385857803061`, 1.459008903547168`, 0.4922186967390012`,
 0.21082467659119386`, 0.05317469777445105`, 0.051461677692841074`, 2370.1608114198016`,
 100.46792579677714`, 0.24298196986117648`, 3.038179651692209`, 1.2632718479945817`},
{0.23779270937803498`, 3.159033522410793`, 8.923446211238858`, 1.0790485164876151`, 0.3460136054742138`,
 0.6482331083335031`, 0.1636040824670958`, 0.011344127288819656`, 1091.512574756292`,
 48.569342600520315`, 0.22296455933807102`, 5.246566496412501`, 0.7138062053370233`},
{0.22757425351270916`, 2.581225270499978`, 0.37748773050037343`, 0.887385361142591`, 0.4937063046079888`,
 0.23328592019488925`, 0.08261142866202484`, 0.006732946168949427 4`, 1345.0595989079266`,
 159.82616980913542`, 0.14361492663679165`, 1.2675214995529185`, 1.4755532059142284`},
{0.07923961132457574`, 1.4534207730102917`, 0.3049651947786387`, 0.809144094297255`, 0.9651646059166545`,
 0.27855980457998597`, 0.18910876827885104`, 0.18936230186753644`, 1265.284678743018`,
 224.5662215566969 7`, 0.05882269816842833`, 4.935952740691619`, 1.3773991041706521`},
{0.18119347985627088`, 3.338563344897951`, 0.31616309891789207`, 0.8233375529098934`, 0.07175467184830686`,
 0.559491101800496`, 0.06295447855153882`, 0.018102651459346017`, 3519.8202135278207`,
 127.22666926323683`, 0.07035715001733955`, 6.64501017919611`, 1.131299832263819`},
{0.16049167400816333`, 2.9496377174467003`, 2.796051273230102`, 0.7713172859306066`, 0.5895053698909607`,
 0.5330035027095973`, 0.21946432427500168`, 0.006395869608132028 4`, 3721.6840074196953`,
 354.1366035930697`, 0.05846005834212181`, 3.723454931340358`, 1.352543221697399`},
{0.2459634383839392`, 2.317311476187519`, 5.114630918885933`, 1.230187162908409`, 0.6324239129659683`,
 0.3459102702870417`, 0.21279405046832994`, 0.012571031115264336`, 3926.1200815627817`,
 399.1203840176114`, 0.14512335124342485`, 1.859263107025912`, 0.81357096327371`},
{0.07535246017332142`, 2.911051338058316`, 5.442775177543007`, 0.8607203087442552`, 0.8022890490197512`,
 0.40209854436599723`, 0.144612694452116`, 0.016940102043156918`, 3925.350442074646`,
 91.64171206007711`, 0.21807597998523465`, 2.8164983201304956`, 1.3696264396607019`},
```

{0.2204293092455124`, 2.4745010079982768`, 1.6220990910883482`, 1.0541684584544448`, 0.6868676732591923`,
0.6458136221345077`, 0.24561205720827334`, 0.010048142648758356`, 2710.1004320631428`,
99.89140315660762`, 0.194718215544868 5`, 4.969891992321378`, 0.16288064769797317`},
{0.25338042742565964`, 2.965005843458032`, 9.015448508781162`, 0.9648345670574521`, 0.4738085996714536`,
0.5217539624890015`, 0.09898738245676192`, 0.10703385809743371`, 584.7617542091971`,
399.01325318858073`, 0.22656752967920823`, 7.539332683081733`, 0.5787011055505342`},
{0.13715202199195703`, 2.280611332139774`, 9.546074571903652`, 1.3294680551764548`, 0.96176455508361`,
0.2027171739690981 6`, 0.1365163915603838`, 0.01317998381974412`, 622.5257559162319`,
109.88086672718458`, 0.09246427533408269`, 2.547747841165865`, 0.5344581094207239`},
{0.26021306080280165`, 2.6412569822545393`, 9.393216711347169`, 0.7980583185168122`, 0.12902542313562004`,
0.41288706652580376`, 0.11050318145828913`, 0.08290531025525745`, 2885.381565930523`,
235.35959645539515`, 0.24979107480174895`, 7.498842240586485`, 1.2653883281677927`},
{0.21577611209111464`, 2.7972286676872242`, 9.151464868370521`, 1.0125832913995234`, 0.03171655307538246`,
0.37863990617192567`, 0.08961735731262133`, 0.24231024163547266`, 1129.2395603552968`,
145.3261697746342`, 0.1783001304868151`, 2.7363213922238323`, 0.4255887662905571`},
{0.18983832850361748`, 0.47273123425625707`, 0.543837247642804`, 1.0885560496178721`, 0.427736490811788`,
0.6656708549524757`, 0.12908120643315885`, 0.022867246439630798`, 898.0605548746116`,
139.83397705936972`, 0.09941082510128124`, 1.7064291808810135`, 1.0668304792785985`},
{0.17497599862759233`, 3.3657454284285517`, 2.034553169278075`, 1.2437246851025225`, 0.5208306933739273`,
0.3059877216844632`, 0.14568158867858877`, 0.07100111598759662`, 1376.7566751341965`,
41.042975981802954`, 0.07580837625359882`, 2.9604840388353644`, 0.18481744658999433`},
{0.18088366879997198`, 3.821865573482567`, 4.692160635426452`, 1.0482928849004183`, 0.06532272769142566`,
0.687087343414349`, 0.24770159954715665`, 0.017305980256248125`, 3393.812324444456`,
259.70338252927684`, 0.20399575556521798`, 4.91689758771607`, 1.287491266139321`},
{0.05949240242631926`, 3.2421313428658216`, 6.819444043498855`, 1.0897919998234773`, 0.4914757475836029`,
0.6217813654383784`, 0.14811683608778792`, 0.1990515558123701`, 2177.5035606095144`,
392.178441681785`, 0.16794023089571636`, 3.103361276547407`, 0.452347159579072`},
{0.22260435016590752`, 0.807488655841027`, 7.62907904124237`, 1.0059991047708425`, 0.2817863964260634`,
0.32047133388609383`, 0.11883206718041817`, 0.029206686321015127`, 2029.3046388765315`,
71.8362522665995`, 0.05374037941301346`, 8.93317694888524`, 1.0534684445303992`},
{0.06601763360963414`, 2.324714740822664`, 5.47799549721827`, 1.4572263706661284`, 0.6058468419307841`,

0.40657776744209273`, 0.16352063484885299`, 0.154889405316064`, 3091.1261645885334`,
170.4797745198008`, 0.16879539576852792`, 3.0092390791565844`, 1.1781015424249213`},
{0.09129438454580396`, 0.5458728552745402`, 8.578700312104903`, 0.7708504139325052`, 0.9169389731376072`,
0.22022269715135345`, 0.2062604041361082`, 0.19807010574894265`, 1283.9010613736064`,
230.53840695892347`, 0.015448919626635749`, 5.258490435173719`, 1.2826107951025567`},
{0.14843032745543416`, 2.891906313088681`, 7.801883637284866`, 1.3483733856378388`, 0.31815406883941244`,
0.21186149946857102`, 0.16733027048593607`, 0.016108958562959286`, 2806.829919143951`,
114.3420493392897`, 0.021367639016282636`, 6.332693333253493`, 0.6091184877547111`},
{0.19262113260207286`, 0.4100732369307054`, 0.6039207257051533`, 0.8273370736078748`, 0.5063544182124549`,
0.2807655019410843`, 0.08795158701010541`, 0.19422202595284532`, 2504.9035989294416`,
81.69837632668111`, 0.1499476760788805`, 6.632628072950256`, 0.568480342643902`},
{0.17039397430438102`, 1.0518028751020552`, 7.3508889662987045`, 1.2231850486496643`, 0.08959309467655463`,
0.438007460954018`, 0.13336650260220756`, 0.015835766380232735`, 635.4248848043253`,
46.431191145002344`, 0.019547600805230075`, 2.463817783746629`, 1.1305047170660831`},
{0.2503454420826467`, 0.6816060830582531`, 6.557163545095662`, 1.251247380535385`, 0.5924064529900261`,
0.3249563797350232`, 0.13275565091305608`, 0.008288831069377075`, 3729.443509243899`,
228.98969063119478`, 0.16939843347227213`, 6.2896214012738305`, 1.016677469632238`},
{0.21895003474025426`, 1.413342329996616`, 6.660357061219696`, 1.3885224416718682`, 0.6085057775930085`,
0.3063420766693563`, 0.18696649922653613`, 0.1721695479130944`, 1066.3987466436151`,
245.09142596090578`, 0.1673351585832708`, 3.1429897838085274`, 0.7494357676944992`},
{0.1254259042141826`, 0.6960838019109872`, 5.192144520750629`, 0.8756261738238285`, 0.8328612976967942`,
0.22677152638905884`, 0.14617978450286806`, 0.02130952691785612`, 2632.7908352385166`,
212.41036911499543`, 0.027687267864634157`, 4.124602621711544`, 1.0784411017781297`},
{0.21183190072031832`, 2.1405207408150053`, 6.713672348966824`, 1.4852810672850318`, 0.9707621809425402`,
0.24869479497663283`, 0.14712840909677755`, 0.010718332694701214`, 2858.107536317915`,
341.2444641156959`, 0.09413983850760904`, 3.9282483342993455`, 1.4253439441659714`},
{0.0808570843788532`, 1.9944028866868502`, 1.847446007042576`, 1.1962230350350178`, 0.9881944572508863`,
0.5065109438103407`, 0.13986962480807952`, 0.21324716376368485`, 3595.9260786344357`,
95.54926882555071`, 0.18522194155873545`, 5.182795178847357`, 1.4775938218806233`},
{0.18422677214256195`, 0.6743311410267894`, 3.1036416049630393`, 1.492376851920162`, 0.6594088757028673`,
0.5950695833857709`, 0.1860537678137455`, 0.02943690821344356`, 3269.4748824588514`,

385.0414868746369`, 0.02235734672967704`, 2.542522241338192`, 0.26953310124704477`},
{0.12205814528256814`, 3.0220310797867835`, 4.8913469029191035`, 0.8430601347747492`, 0.11080265100724662`,
0.5338876009745465`, 0.22020963395963772`, 0.11433363332445597`, 1824.128984278892`,
211.39670746978072`, 0.11084187323527839`, 8.781693512702038`, 0.22047341418167488`},
{0.1379604951115434`, 1.4163155012457889`, 4.400902234814488`, 0.7945346450123854`, 0.8193909852858012`,
0.6724735717160395`, 0.06698309689845514`, 0.09275337016012092`, 3209.7522511906463`,
80.79361285288883`, 0.15292409302459276`, 2.596140589311666`, 1.3399665214161485`},
{0.2598992397477407`, 1.4339452918245774`, 1.0784655091863726`, 1.4648755135291642`, 0.5315691563517717`,
0.48571691620677493`, 0.23359094834009136`, 0.11846678481017199`, 3222.0061450547964`,
66.19766765214632`, 0.2128172489806378`, 7.890656547448669`, 1.1120638442139867`},
{0.15978649721954924`, 2.191594266371557`, 3.0757440378000283`, 1.4265108854971194`, 0.1706212563060796`,
0.5071304116694296`, 0.24651071826762905`, 0.09495214929797174`, 590.7999831566981`,
288.39017916639295`, 0.04539479043869582`, 1.2808934229971172`, 0.510577419948266`},
{0.07381047173055871`, 2.1206143008004066`, 2.7168272133152236`, 1.2500057406362022`, 0.5621616976632486`,
0.5554267209678074`, 0.23180667473506628`, 0.07059925918611996`, 921.2671327671924`,
62.71094421988312`, 0.17012421692254281`, 1.3889827292164867`, 1.4926610431972103`},
{0.06137349343986376`, 1.2320478370611792`, 8.061209423245938`, 0.9154821233959622`, 0.94847137331962`,
0.5807992742846495`, 0.08922764994064156`, 0.006685723224911482`, 1682.5088546386705`,
136.82405088198965`, 0.1751207515405141`, 5.423583339438931`, 0.7354883118939113`},
{0.17433851186752986`, 3.075048744775078`, 5.850960000178599`, 1.4590538879216268`, 0.970972084241031`,
0.3933384319595665`, 0.19496970022116206`, 0.08582933681764099`, 1421.4342985094609`,
163.5857848008759`, 0.22598131859497045`, 6.6523561809248974`, 0.7554779380569299`},
{0.07998121844244105`, 2.715580846021229`, 9.746524688173917`, 0.8568934646193223`, 0.7996741613090028`,
0.42485864684277896`, 0.060688942211416824`, 0.012691467663490894`, 3356.3929343624186`,
81.65136803572034`, 0.10607621946860207`, 3.6515832472165357`, 0.6293869420871867`},
{0.24304694498168755`, 1.1442280451992772`, 8.167052671305406`, 1.1537428575449382`, 0.8133574376235939`,
0.588378131385339`, 0.08683207301011828`, 0.2314931244773344`, 1071.7662116086185`,
394.7462751939248`, 0.23166609859748916`, 7.333555001844963`, 0.7455281507224547`},
{0.1801027988172071`, 1.3432512708969133`, 4.741465237867114`, 1.304640592667152`, 0.29820882042399743`,
0.18296919601651807`, 0.12795168258022568`, 0.15240389940354152`, 1270.873559520909`,
206.3480725013959`, 0.08783057128294997`, 2.6716194652601057`, 1.4440291039953363`},

{0.23177860104219328`, 0.964191776963677`, 8.537582892707828`, 1.3413871161177928`, 0.724114448343743`, 0.6184241033984961`, 0.21927672383789093`, 0.15745254433360553`, 952.8150091975604`, 129.0382057619487`, 0.13010975974299993`, 9.838389368601312`, 0.45129901177253107`},
{0.18229092854924844`, 2.666175072988888`, 1.1843338363668607`, 1.0835916247483197`, 0.8465995494931746`, 0.28296103301830944`, 0.11340515234724796`, 0.021054051155640643`, 2912.6266504267696`, 38.65103872844048`, 0.22568389644671255`, 7.751508465891021`, 0.13543768169398884`},
{0.20049846933924464`, 3.760252667630848`, 8.551337156929279`, 1.0134432778283617`, 0.24009952242243937`, 0.3670355852238165`, 0.13721671891900983`, 0.05322626702251803`, 601.1668980568775`, 144.18193811625173`, 0.13298602138197724`, 6.629891741121696`, 0.6106305978341504`},
{0.062486454467917896`, 1.2059427569006989`, 6.5765672576808525`, 1.393503279917707`, 0.48537073645078954`, 0.6632121477086064`, 0.05231057461071709`, 0.07259823309572629`, 1438.7234727065425`, 224.83427991463657`, 0.2172117922601684`, 1.0640862319201538`, 0.7673574870722413`},
{0.19627224659336223`, 0.5402822985479045`, 2.0302490383203153`, 0.7794095357324428`, 0.7903414997496967`, 0.18384078811354698`, 0.12435150563892122`, 0.011407347781693793`, 1336.2245441274245`, 314.05659164167207`, 0.0727358338086812`, 6.3699550531919975`, 1.0324129890894564`},
{0.12533935047015737`, 1.521368835274294`, 3.474516335185184`, 0.949787360467496`, 0.3211564169863861`, 0.652599038806982`, 0.05499170112558746`, 0.016940628240370355`, 2778.039504369459`, 65.83823943133149`, 0.237336473550759`, 8.791957984840526`, 0.6376701649045213`},
{0.05859295952350296`, 0.6393158910447498`, 7.38865195012022`, 1.4291671399819033`, 0.009131117329247873`, 0.22529958337877898`, 0.1303909880662386`, 0.09497257153796428`, 2573.212680919086`, 234.8157611604919`, 0.05429505063598533`, 9.846003813495852`, 0.7602792516330825`},
{0.22902870315185453`, 2.2080085479773457`, 7.247023111118531`, 1.0318589243161904`, 0.282713068412896`, 0.6376211846150526`, 0.2313240848611663`, 0.02849242642499769`, 1791.1158452651816`, 374.3905300694138`, 0.08265635018766693`, 3.8730202440010704`, 1.4480774422437706`},
{0.11087514755950806`, 2.820953169055314`, 7.453143180044123`, 0.7876974831919437`, 0.32208463912551366`, 0.485410651626032 7`, 0.2381966627750387`, 0.019508031907783826`, 3675.7660426974826`, 393.486383654267`, 0.19475280794369443`, 3.2333842106996737`, 1.137763300389826`},
{0.14807057579619815`, 1.4220065645386768`, 3.077692541817422`, 1.3463149673717403`, 0.07642859383354339`, 0.6424799174126323`, 0.13735279371122205`, 0.011944441122857653`, 3609.93932375683`, 78.77118206979054`, 0.05838602009446581`, 3.355260507755876`, 1.0164328161645106`},
{0.1489453640293268`, 3.0321837029004524`, 5.620984907920828`, 0.8681722630051818`, 0.8115372327820141`,

0.3581648445021329̀3`, 0.1426190893322286`, 0.05255336055473145`, 2844.388518993981`,
198.5585650329739`, 0.018535462639950012`, 9.846692622248703`, 0.39135970086161165`},
{0.24690212899704622`, 2.8546371760166815`, 5.1085465841448325`, 0.8777130205154242`, 0.2272138406192541`,
0.3209103618486969`, 0.13407179467911823`, 0.026501037209412173`, 2259.637457560987`,
234.32947168493797`, 0.12630638676582567`, 7.901213702591074`, 0.632382460130227`},
{0.20217859061685106`, 2.487845857605743`, 0.8626257496221044`, 1.199588786641617`, 0.6707739359709337`,
0.41572085938323444`, 0.16212565365900633`, 0.02422500357599282`, 546.6195403678685`,
211.4653119641191`, 0.06380462964198375`, 1.3445609170340642`, 0.9773390980954635`},
{0.08931462102812832`, 1.1252555996419966`, 6.092718311835377`, 1.0218956744375698`, 0.19346465860989204`,
0.1765746718654928`, 0.12857456324762098`, 0.36563376111510176`, 3469.1850778098988`,
184.0525308849526`, 0.10979923715549866`, 4.959384809772031`, 1.0536081443626233`},
{0.06880098426216452`, 3.7509374271202`, 6.144272737202943`, 0.9237943864650779`, 0.699854200915176`,
0.3107815967047757`, 0.08743219256910584`, 0.0070907725066866245`, 1964.75988825503`,
142.8027500827095`, 0.1044689728863506`, 3.2094136485070344`, 0.6936760287011401`},
{0.2326571783338366`, 3.2198726087577585`, 0.7879059046396755`, 0.9520817800309282`, 0.3381503807716493`,
0.6953568861265582`, 0.09096644213287763`, 0.03053119099726608`, 3051.5436637428375`,
193.8478952968402`, 0.1252944454366426`, 7.5675708168133315`, 0.1396895662891593`},
{0.20686587279815516`, 2.3834956380504195`, 3.228648073866996`, 1.4616470372504198`, 0.35618390382684884`,
0.6609631833857008`, 0.09990239078555083`, 0.2797108933201619`, 2467.4094286971804`,
85.00316013652997`, 0.14363808446123288`, 3.440698993285203`, 1.2812874699661427`},
{0.14277465652344312`, 2.2128169765977077`, 1.5395722189285141`, 1.2588391498483595`, 0.06601455592248096`,
0.5924077255302849`, 0.2026072042186735`, 0.006704812631657588`, 2107.7558344556182`,
393.22260119726275`, 0.1318126775401366`, 3.5561906346112124`, 0.787606393777565`},
{0.16055207404865252`, 0.7029113592886698`, 3.6403774574439822`, 1.1854100604530788`, 0.5916505631446303`,
0.6508026186847373`, 0.196607205292044`, 0.08269873784662057`, 2208.609406288604`,
140.20841727601464`, 0.08922686090021398`, 7.843962027709463`, 0.25680184922543736`},
{0.10433679528385242`, 1.2124558701074761`, 1.611384270831014`, 0.8947495690924911`, 0.7004462194859642`,
0.22790601678310374`, 0.1107678931866184`, 0.03745668981933727`, 3402.687823353078`,
290.01464659756004`, 0.06742303128382326`, 2.428172996695036`, 0.7057307677392819`},
{0.05355871135675744`, 3.309279473250874`, 4.916547265237652`, 0.9710244117509734`, 0.2158289016549566`,
0.18208484161878558`, 0.142360576114893`, 0.05203968288186598`, 866.5149045887983`,

```
189.9142460402237`, 0.14005711660330905`, 2.6475969017133205`, 0.76310092982505`},
{0.1293133825399687`, 3.5858476083752784`, 9.218617588443664`, 0.956805767292237`, 0.24196147957252068`,
 0.1873204590192723`, 0.13102150029404855`, 0.057479405562115365`, 1692.2136201181738`,
 281.3785219692511`, 0.06648388834980773`, 9.468482191548883`, 1.1484858817527992`},
{0.19221768216079177`, 1.1625228381265575`, 8.172090892620595`, 0.9088617496226665`, 0.5295960957742345`,
 0.666157439310989`, 0.15242811761548303`, 0.007090627883866567`, 1515.1410756583437`,
 333.01297852270045`, 0.2052004181173977`, 5.919585120700083`, 0.3172585313240366`},
{0.16098880364150353`, 3.127450251430191`, 2.20024435719991`, 0.7637976091034757`, 0.9064557867648941`,
 0.5784333391778734`, 0.10152394954071559`, 0.028389209793756094`, 1613.7605012073036`,
 376.897672127746`, 0.043498602143453025`, 2.961300492740582`, 0.7808989235332793`},
{0.23820794255869648`, 2.736768610725435`, 5.161003241600229`, 1.3463074049058292`, 0.830070705025693`,
 0.6819229399748419`, 0.14093440819681458`, 0.012428436330344567`, 3231.77982743126`,
 163.97686949725403`, 0.16644222788576973`, 5.029839035337567`, 1.3104845535759155`},
{0.2556095337978518`, 3.423935597729149`, 0.44377078820861904`, 1.3028637036663855`, 0.63065566823863`,
 0.2812042846574222`, 0.0670032727620068`, 0.017466077681157405`, 3127.9987163099076`,
 356.3279534853283`, 0.04932332227468639`, 8.912956605460284`, 0.5977251839966713`},
{0.17842055683347624`, 1.0601527835649565`, 1.9863486015726688`, 1.4771465685197964`, 0.5130989252089981`,
 0.50009800158305`, 0.10443547318598384`, 0.006507994811629916 5`, 919.6368613396894`,
 101.38339784479837`, 0.013661722290118639`, 4.3887706359180285`, 0.5048436819791595`},
{0.09313560387857911`, 0.7038695534977393`, 3.6724738159211565`, 0.9475746331523089`, 0.19159788938387368`,
 0.6119542230262267`, 0.1716716908175685`, 0.15713357297550534`, 2263.7776933944124`,
 51.55094049564468`, 0.21456002491809933`, 4.890757314915582`, 1.3381695004184304`},
{0.14593842634629578`, 1.5677173946402423`, 2.2260911107394623`, 1.0410408322166074`, 0.5774262288207519`,
 0.5605768734730473`, 0.06528103792572038`, 0.18580384429276964`, 1821.6991564326672`,
 281.50492694809225`, 0.09810886942125424`, 9.103899808381804`, 0.814497273403965`},
{0.10172713838830055`, 2.2418772278235775`, 3.04935119913241`, 1.1880003837277453`, 0.642679290083189`,
 0.5307574217574084`, 0.20278574850964748`, 0.28989270792503147`, 1645.7112772203664`,
 50.385249878477794`, 0.23495391386029668`, 2.9089393035407407`, 0.6416895284655368`},
{0.10397296284216978`, 2.835686168016201`, 8.434918863116089`, 0.8374208138967196`, 0.6614675061514772`,
 0.2543464777963602`, 0.10409619202490922`, 0.05685431008257157`, 2232.998807497718`,
 265.245542499567`, 0.1684824275948374`, 9.30545987088001`, 0.3476653622341219`},
```

```
{0.19614726189645104`, 0.5421409973222318`, 7.311205144883296`, 1.4430957843086838`, 0.5642408555529124`,
  0.4388312833001765`, 0.24379751153424895`, 0.027088524601856054`, 3097.2470344285757`,
  24.388568117880652`, 0.033832395593974274`, 8.563316908406161`, 1.3116329767787245`},
{0.14550969692078547`, 0.6504460416509024`, 3.203391452599939`, 0.9809775787785509`, 0.8932996308568291`,
  0.28864614416923273`, 0.12555971991665899`, 0.04147738167353124`, 2412.5327079966373`,
  332.6211608259723`, 0.10031126168945415`, 7.026514484853989`, 1.271775072831928`},
{0.26324845548994974`, 0.6443701830080291`, 0.83901460272339`, 1.148634249041292`, 0.7108758830917159`,
  0.6261909633849789`, 0.1815221463640686`, 0.007774421269715129`, 2091.205586215532`,
  76.86431957676541`, 0.07691551140505637`, 2.5668632188750475`, 1.1962425546812079`},
{0.16309305365471205`, 3.729196744300981`, 9.307515882091003`, 0.8570302037138485`, 0.8937228771832244`,
  0.5152206015210095`, 0.1870688649861051`, 0.010071822941243438`, 3256.3985186156888`,
  304.0187763152717`, 0.14293680908473783`, 5.902796930663761`, 1.3773483940741578`},
{0.16894031536983917`, 1.5924806965086606`, 5.6931408636729035`, 0.9867223743305529`, 0.8224539897688026`,
  0.597939456714043`, 0.09503923786081717`, 0.017883705208976936`, 748.6938025465079`,
  285.65032417980353`, 0.22927316540826853`, 3.9293139418745917`, 0.9067902983749052`},
{0.1623342771322262`, 3.665527787592313`, 5.80147646237554`, 0.8352024744734798`, 0.7018868526857065`,
  0.24394785423193066`, 0.08747688957871178`, 0.16089532027636136`, 3759.8814706106723`,
  381.8961690598002`, 0.2313099688547675`, 3.097651818220701`, 0.4382846191648839`},
{0.22071774239833053`, 2.103280372140615`, 9.912636083572693`, 1.1033554615041679`, 0.6309736957880776`,
  0.6000918403254052`, 0.08729060724669563`, 0.08702471997827647`, 909.0462708578816`,
  192.71874762961875`, 0.022259580341630003`, 7.880477191609103`, 0.5653775848064009`},
{0.06066901211156295`, 0.5118142470076563`, 2.3575039706529495`, 1.153198866823781`, 0.34052857777406675`,
  0.48310824315452683`, 0.15644429107129876`, 0.01838618195497249`, 3533.672368817396`,
  112.99427366098905`, 0.18169108406320317`, 9.689478468414666`, 0.6768155401525213`},
{0.17215213474108`, 3.1043854956757873`, 4.4538232156171205`, 1.2105616525577976`, 0.07070121186202716`,
  0.43467897429752544`, 0.1858388910260701`, 0.10113399844294489`, 1450.8531111854936`,
  101.3426083924827`, 0.1798803264840549`, 9.89915477207009`, 1.3540588713189834`},
{0.24067020849458426`, 3.9616105393925194`, 6.770909075338004`, 0.8588077802255987`, 0.4748383175874904`,
  0.6078443186142359`, 0.2469104749167066`, 0.3360706312022538`, 1507.4555524848456`,
  50.50450753574768`, 0.19906071527290714`, 6.968998972407538`, 1.347049135136762`},
{0.21944864620047738`, 1.5851568289063627`, 2.8253271766672086`, 1.4978148427385942`, 0.6625588078225084`,
```

0.3414024356763743`, 0.10952337049286448`, 0.007987153739989428`, 796.8444419321349`,
241.79415763040436`, 0.07683828049172353`, 5.204625250868856`, 0.2239996119446115`},
{0.22738531013884145`, 1.6748591792805003`, 7.320466644196401`, 1.2670610367241493`, 0.9156886696389757`,
0.27273514068006444`, 0.2379234232685784`, 0.022570855753759948`, 3790.0561229040195`,
356.22460568492386`, 0.13302881383052462`, 2.695355303099337`, 1.0187081757900263`},
{0.12775499399576257`, 3.9034479686029915`, 9.751449972499827`, 0.7624853991106708`, 0.7599399040180521`,
0.19562916289510757`, 0.12110783011333992`, 0.024828285786588924`, 1328.3636666047569`,
234.8344441201524`, 0.018565368539142812`, 9.598538649304729`, 1.107753026473853`},
{0.09993396962410062`, 1.749743490015244`, 1.6240016651924947`, 1.3301699142654`, 0.8818156710905265`,
0.15681061125193507`, 0.1352532563629385`, 0.07239358889803704`, 2734.4752174906116`,
304.1441804766208`, 0.060678774409857306`, 8.403610161636486`, 0.537751994770234`},
{0.14782514342208763`, 2.652895873833227`, 4.391872450348963`, 1.4876628412878459`, 0.36986838675832656`,
0.39309495169020825`, 0.13298297698236788`, 0.014228765232101433`, 3735.496122883863`,
215.5408051024042`, 0.11408789381411022`, 7.212929997484123`, 0.10464527448884264`},
{0.2706278159985153`, 3.6076079533892003`, 4.017828860181465`, 0.931912448165567`, 0.07202782033095079`,
0.3509804409724484`, 0.12413238666840237`, 0.15319152742338107`, 1515.3910453914696`,
86.92889855061799`, 0.057359319076296045`, 7.405405977315304`, 0.4162338517487447`},
{0.10989408866872064`, 2.7814923653363133`, 7.842262663464009`, 1.0963577256835755`, 0.7584107639763666`,
0.4703739490649106`, 0.21862941653484513`, 0.05112895789567924`, 3757.6555119131663`,
58.131608822022315`, 0.16080796994998975`, 8.15898170434339`, 1.4928178868639161`},
{0.09639258202167578`, 1.466780969423465`, 4.239504689160903`, 1.0209716259894424`, 0.025576954982881928`,
0.3026304446672493`, 0.21752127412681987`, 0.031317816620157334`, 525.4375097331736`,
358.0480696321463`, 0.16662257108917217`, 7.934540763419077`, 1.3681027859387838`},
{0.07632945356776594`, 2.5680095036868336`, 1.636586934722592`, 1.0974626755451315`, 0.20070740407760934`,
0.2792825326359145`, 0.08134798544013919`, 0.2967486253326174`, 1516.758023227967`,
278.84396411497187`, 0.08326880329861536`, 5.936954264251678`, 0.30521315193193943`},
{0.16556454843244983`, 1.858436546204688`, 5.85841964381955`, 1.4381472842647658`, 0.6188369558385702`,
0.43793021380482466`, 0.22826919863757877`, 0.3543109226514421`, 3912.388498432202`,
125.48108540961005`, 0.039979729590999546`, 4.62864467759934`, 0.44406393527696175`},
{0.17960696499390383`, 2.6986368926848217`, 0.39096283943110777`, 1.4614976941662214`, 0.0584358271913763`,
0.28608789101372956`, 0.23241827692103179`, 0.025712440835301015`, 1101.1873067230754`,

```
291.87610702538757`, 0.09533779650969992`, 6.256274031322354`, 0.1260305021418897`},
{0.06965658134691888`, 0.9261903057977481`, 7.316991447780513`, 1.3759095728452715`, 0.2908565323439223`,
 0.2820661078343176`, 0.18803658000898693`, 0.00943996945527825`, 744.7814906580452`,
 341.56345427898043`, 0.1305415452351143`, 4.644709496928025`, 0.9315609895678807`},
{0.14063443384893737`, 0.6174318191344224`, 9.816767595769278`, 1.0438016034352748`, 0.579595650707708`,
 0.4622649825890621`, 0.24429017498983885`, 0.01619284881329759`, 2900.32749785945`,
 335.82024218042955`, 0.09977744332778332`, 6.127347054973281`, 1.0166995269478085`},
{0.18537094617034594`, 1.96648991280105`, 7.838266997084801`, 1.0125623241231316`, 0.014033931484803919`,
 0.3423492209947736`, 0.1167129177170042`, 0.32838459574903894`, 3257.498914359846`,
 126.9547671220904`, 0.1116209985614412`, 2.857468681315895`, 0.2336256655095057`},
{0.24032952463671997`, 3.1818327111380986`, 8.060728243579248`, 1.022907398702028`, 0.8948393046349232`,
 0.3515250656278254`, 0.11210328340390646`, 0.4812285429766681`, 3331.6694795416133`,
 199.3192679768665`, 0.1656921504225664`, 6.971086437067735`, 1.1039536289023264`},
{0.06861147798506062`, 2.729021236857605`, 1.928866504736478`, 1.4151352288745787`, 0.45478865294711146`,
 0.6487874608126984`, 0.19220035768006005`, 0.027739198253996934`, 956.7552237848031`,
 40.992278320550554`, 0.10039930252063617`, 4.815840989066018`, 1.4694203679389397`},
{0.16505190858637997`, 0.8247371132573029`, 2.6996089778841377`, 0.7893797405853178`, 0.33037619005051444`,
 0.49339259475674924`, 0.09174509005830872`, 0.3021633567730715`, 3738.8764636919877`,
 330.53731575160793`, 0.1571748109049641`, 2.9654306879318377`, 0.993830283737029`},
{0.07254416598733587`, 2.8849957402027044`, 0.27388492744414883`, 1.0617374480096804`, 0.016195895901701673`,
 0.5364706306281042`, 0.056724271897686834`, 0.010490294664589572`, 1785.3192582331621`,
 96.17787055857661`, 0.24599567314403442`, 2.275980053816065`, 0.5487867290449304`},
{0.24080755705319862`, 2.3759884700193172`, 5.524917589968679`, 1.2325829370203099`, 0.2181690975782502`,
 0.6636754905333937`, 0.21732363084691986`, 0.11964660140986366`, 3828.961095769544`,
 112.06990257277658`, 0.03236755171123323`, 3.877460599831684`, 0.7029311637647315`},
{0.15494186822563605`, 2.0029420912170632`, 3.8996790997983233`, 1.319513511447182`, 0.5220998731870559`,
 0.2887737008742528`, 0.22727260445012404`, 0.040774601815574976`, 1826.996357882992`,
 128.81708545308533`, 0.21865899508635123`, 9.155723124997508`, 0.5134933136755975`},
{0.22396249520436795`, 1.9570085864611029`, 1.4759242627722013`, 1.3116517086556398`, 0.6599681888830524`,
 0.6953504264310013`, 0.09804476293632919`, 0.007430663549575122`, 1204.900095750464`,
 62.93764873891854`, 0.10119557783088817`, 5.061320104116531`, 1.1487439857805883`},
```

{0.10601932444652468`, 3.342699233573309`, 6.832537694215926`, 1.4208647912460013`, 0.5802751516427387`,
0.2570998642455964`, 0.07303385433227993`, 0.04213475658223818`, 3584.7181661863006`,
57.24897785837004`, 0.15446538158110595`, 5.395898684860962`, 0.7872614775312905`},
{0.041347104778859584`, 0.6250053944652225`, 5.297803080507409`, 1.3565417270883027`, 0.7676292422012339`,
0.6702284155028233`, 0.05669620363458108`, 0.027633397705997945`, 3343.0533516535834`,
245.48920969891526`, 0.16311205424117575`, 9.389474878516673`, 1.0487847358699702`},
{0.04498655055054335`, 1.4491138993771555`, 1.16394731374956`, 1.169713814519905`, 0.8457893355464972`,
0.3136806500838595`, 0.06778013214591205`, 0.09196917164932833`, 3408.380878728516`,
174.30204224938495`, 0.10551969253057247`, 1.6463871196433963`, 0.4159425601435516`},
{0.1215137090416612`, 1.1969038422236755`, 9.326465300388158`, 1.005636980391936`, 0.6897518274648109`,
0.6108427950530713`, 0.10731717880916097`, 0.23857365224573474`, 2004.0983212715491`,
148.7170844059682`, 0.23834887248578368`, 9.94205781935025`, 0.30611051261382083`},
{0.279562619019907`, 2.2124846272464014`, 7.584786512534812`, 1.3700002017675512`, 0.23921169075395055`,
0.3169220525365052`, 0.13344245040196623`, 0.14640355547821746`, 3971.2444101007122`,
129.66148944966505`, 0.11735939166830095`, 3.485518254759493`, 0.4332823244894972`},
{0.21456925154594686`, 2.7215826602358453`, 4.860741945426051`, 1.4762193210184287`, 0.8476298137931575`,
0.5583227752271869`, 0.16730998643348832`, 0.023150536959116674`, 1148.3854317955947`,
98.18795037270098`, 0.13536301841717513`, 8.630410561575445`, 0.520586144335875`},
{0.2192605474178046`, 3.4277372937593142`, 0.317243264743297`, 1.207905605629452`, 0.354638331302892`,
0.2232326751046343`, 0.19168786121654457`, 0.1758842660002451`, 1819.052685961844`,
205.84067214106688`, 0.24408767199328762`, 9.526826970263976`, 0.6180461674628193`},
{0.2002208994690245`, 3.902908071048812`, 5.625275625663315`, 1.4917900472855687`, 0.70165150687693`,
0.17917645896398615`, 0.1075286064543329`, 0.022727063680328688`, 1296.438202913817`,
261.4435185272306`, 0.04322514446110226`, 5.849567498164653`, 1.0949838652058799`},
{0.09281080427028687`, 3.9541950243176203`, 2.7903763295535473`, 1.1746304733040271`, 0.7021084302247975`,
0.18301687365086206`, 0.15380914186275987`, 0.31491382108322413`, 1955.6345018254533`,
45.589098202841626`, 0.16844466131119568`, 4.505740157817099`, 0.739135067608975`},
{0.07553841622273444`, 3.998538946411479`, 5.0568584828370255`, 0.9418368972909419`, 0.5147743656673969`,
0.5745728154097487`, 0.1277108500152948`, 0.02699422488607613`, 3283.474706944715`,
85.73668262235145`, 0.1900835508559061`, 1.7563639079857047`, 1.1017643436886124`},
{0.17556485477494027`, 3.9790149464214117`, 7.860200999240184`, 1.4870239255992979`, 0.43953202100784705`,

```
  0.2354118710730827`, 0.08492480553651033`, 0.015229986606251729`, 2122.168041719343`,
  48.98316603866812`, 0.24455585463944812`, 1.202710250977347`, 0.4184441960486769`},
{0.13038773573510337`, 1.934638714312162`, 3.5362126783730226`, 1.036300296330771`, 0.1950167370197864`,
  0.2191580432274216`, 0.06372829025507237`, 0.4487751055567468`, 2715.7233289184524`,
  201.32035054817607`, 0.24618537045557576`, 1.5657139002447844`, 1.056666818669949`},
{0.24054054866212005`, 3.45017251549384`, 2.434688830833908`, 1.0572260280057717`, 0.42678683298038966`,
  0.6433017075143015`, 0.14198529935191645`, 0.0642120318165855`, 1461.85471205871`,
  46.12110813109672`, 0.14351244735387614`, 4.149492178713276`, 0.5419589373402318`},
{0.2576647285795562`, 1.7400215170432691`, 5.671933058359681`, 1.159126105619701`, 0.5426396846396173`,
  0.16289934098722736`, 0.12398680737225279`, 0.2145763112631837`, 795.8175406593573`,
  85.6025153828084`, 0.04739051555756513`, 6.248380217555781`, 0.8502018161731435`},
{0.25257809880572757`, 1.2556801180516963`, 6.596575825508853`, 1.3398305186381605`, 0.284033409354278`,
  0.1515369008103068`, 0.1400005745311825`, 0.02101007059419062`, 611.632102430428`,
  356.19419891984376`, 0.010524324923865458`, 2.8673905561599544`, 0.6614014808678237`},
{0.2042789431730157`, 3.7869781485826124`, 7.097464150909694`, 1.0298616729281707`, 0.4360801596581312`,
  0.5374359749706832`, 0.19209770462574638`, 0.13005052253847288`, 2094.968893303633`,
  292.0969799908029`, 0.013655036785018004`, 7.349385967933083`, 0.6499659559020465`},
{0.06006756967417276`, 3.885393698216971`, 2.4182637836940106`, 1.1497042341120063`, 0.852306387023593`,
  0.3634054456246256`, 0.23851842406334756`, 0.1905044887986902`, 1969.8862529979606`,
  336.32300959810993`, 0.04225162262081883`, 6.207670246795658`, 1.4904811957625173`},
{0.2595298239660976`, 3.319555998413656`, 5.7932515179865565`, 0.881744759172532`, 0.6648616448212201`,
  0.6517725519271407`, 0.19583264477028905`, 0.005149860640734175`, 3979.7041048852343`,
  156.14061322234556`, 0.24304909070853137`, 9.19022988333834`, 0.6081568865700206`},
{0.12106147370268983`, 1.1502892713218662`, 9.893056355486525`, 1.0779684733916626`, 0.2654952298373383`,
  0.5544611241456747`, 0.21486298948769583`, 0.008770181568324542`, 1487.6775848537964`,
  210.36931291761118`, 0.08815050293390886`, 9.314054867228961`, 1.3778011559930095`},
{0.08502696111412816`, 3.6275157197896295`, 7.363159164909369`, 1.488436793334436`, 0.26568493648971336`,
  0.6629645451225747`, 0.17264441279507348`, 0.30453583199957635`, 1224.7690817107496`,
  42.94433873275722`, 0.17512531432720302`, 7.6510105838564435`, 0.2548403509235575`},
{0.04600515107837966`, 1.2443774572294632`, 7.3914675121007605`, 0.8524218043851708`, 0.25925086237615913`,
  0.25962408201227716`, 0.17007776716368422`, 0.3647699508957847`, 2650.181739077888`,
```

363.5928673248461`, 0.07845934701674573`, 6.64885092299725`, 0.9963780523907508`},
{0.1217700466706656`, 2.558519459791139`, 5.216939277492001`, 1.0963651311366025`, 0.09816261776323953`,
0.6044055975062239`, 0.09115244000304745`, 0.06193230544868139`, 3735.1363049157717`,
184.7914603929239`, 0.05165113964049522 5`, 2.282481339681045`, 0.8427745163468003`},
{0.07220853239716318`, 1.7719009634055194`, 4.958541757826833`, 0.8715174582622381`, 0.41441036947741816`,
0.32423173571278785`, 0.1998499231948091`, 0.007134676741262494 5`, 2969.140628850977`,
169.59950979095402`, 0.08473674334867182`, 5.398547675716223`, 1.090541691203971`},
{0.16122326429150147`, 3.7922162994918844`, 7.367254304672895`, 0.921371724322694`, 0.6381223018466633`,
0.5967903533432476`, 0.1832909738697283`, 0.43512505909917726`, 2133.3461489698684`,
214.82179755577738`, 0.22692868851616532`, 5.899255886059763`, 0.6939454361655824`},
{0.14827779601188723`, 0.9768409840676426`, 0.6891894673357726`, 0.9389444267785162`, 0.30176378998265774`,
0.1751647596247058`, 0.07879547499477868`, 0.023619248417657335`, 3252.235532426287`,
167.1595777298993`, 0.05956658968902967`, 2.6817550689507357`, 0.5206583529379527`},
{0.2570676551725277`, 1.8968814761831432`, 5.846591463128114`, 1.1172410815007583`, 0.43583764365067834`,
0.15357951833619232`, 0.128960566744062`, 0.20068004601330075`, 2555.9384884114397`,
300.14317051223054`, 0.1562959949020017`, 5.731951708372693`, 0.952499750656465`},
{0.055135962685152806`, 2.9220194449851506`, 9.20311067851182`, 0.9951015075863325`, 0.9088587426511963`,
0.6564149766220422`, 0.21100197083140715`, 0.41165435784921034`, 2658.942850929282`,
386.04298432332337`, 0.012398563555194947`, 5.099390369220371`, 1.471358614723604`},
{0.09951701144549052`, 3.3599674683318668`, 5.87127293585465`, 1.0675363096846835`, 0.6922846316607385`,
0.4471508361318377`, 0.09256546388382819`, 0.022405598476994366`, 2159.6702917097355`,
22.498867590732686`, 0.07537929727214032`, 4.117708447722572`, 0.7807622026379022`},
{0.13155432244432247`, 2.613811333361477`, 8.357167885800742`, 0.8056721180002253`, 0.6603026707260147`,
0.5801438627936167`, 0.11636775006500844`, 0.26451755332760135`, 2521.6309343959783`,
242.4992509443806`, 0.160383814941372`, 4.821776617001756`, 1.0110903713708876`},
{0.10755292243344333`, 3.7450945369693454`, 9.057610815009983`, 1.4262360104426115`, 0.8847828962958162`,
0.32323944244008773`, 0.10925879484006157`, 0.04648694210521617`, 2597.7878756158707`,
114.3810122188126`, 0.2151089301912223`, 9.739939811090224`, 0.6433246757255959`},
{0.11067094408083894`, 3.800544284934695`, 7.350733993347461`, 1.2140806206228738`, 0.3865719277011892`,
0.3640208182074153`, 0.2468843803041872`, 0.02524798025663777`, 519.3604951232796`,
258.54462558963803`, 0.248859794496116`, 2.6642578190064192`, 1.225620094151966`},

{0.17620161354602198`, 3.207637663312279`, 8.038302489204167`, 0.9527508465697585`, 0.2777263075882186`,
0.3259017504461914`, 0.20696733159344116`, 0.041067631602668214`, 3666.144435165166`,
184.14344105031228`, 0.011349261156917284`, 2.0150600100651097`, 0.6578483910293456`},
{0.098793524136433`, 2.9572290906746908`, 8.636235866475928`, 1.1358825426984174`, 0.44571351797486836`,
0.28917170815364945`, 0.20954073160539777`, 0.05617013174448901`, 938.6734643463747`,
177.03868080567804`, 0.11564637982987985`, 4.570821554383135`, 0.26856807485341383`},
{0.1873960215776312`, 2.8261178368849595`, 9.525044121358242`, 1.3550980694568904`, 0.08983672188035041`,
0.6607774222729332`, 0.06657454788578898`, 0.12572829008110223`, 835.4208642240601`,
103.25606657618908`, 0.08845188667521903`, 5.652525212839048`, 0.4964020494140271`},
{0.261714977559417`, 0.5482487201542185`, 3.431220669456936`, 0.9478503018778801`, 0.34449029105022966`,
0.1671252432139082`, 0.1374901654337824`, 0.05820678844321773`, 3721.854350957653`,
371.28139834560955`, 0.08783171860573763`, 6.880528123303602`, 0.4952942413378081`},
{0.19384041765838167`, 0.8310343395086068`, 9.704183649775683`, 1.2913450048559905`, 0.5670495339238457`,
0.6409270851330959`, 0.14192751733077513`, 0.022909401085561467`, 1113.6891447077023`,
383.5158989221919`, 0.1816390006173258`, 4.168222848202584`, 1.1878448557299315`},
{0.24542600753982147`, 1.0072788201913916`, 4.873030125775312`, 1.3215761420654866`, 0.934997683525973`,
0.5336155406906149`, 0.153057029286203`, 0.37775895009481864`, 1381.366911360472`,
23.478593484050236`, 0.23171577878109345`, 1.0189498874051495`, 0.9824058311213721`},
{0.21619628547349445`, 3.9792600826840445`, 2.630502577782165`, 1.4259463002457018`, 0.22306595661200035`,
0.38072127406591705`, 0.08501836292019685`, 0.035809155258912144`, 1510.787355159533`,
321.0088965168211`, 0.03982099495880792`, 2.097097341241465`, 0.5759181842297432`},
{0.12757698942849355`, 3.9475071221290925`, 7.046874473492651`, 0.9016631589117734`, 0.33636145019883923`,
0.46329966420746505`, 0.22687767004632875`, 0.06279665921261046`, 3797.4437504167317`,
319.7090357145597`, 0.1993551083304292`, 8.931980068156673`, 0.8508236103120903`},
{0.07009559015680356`, 2.7849471367228507`, 3.0258579570105155`, 1.1102521989309375`, 0.4599988219148854`,
0.6809018065475554`, 0.21885987895906978`, 0.1581032784122877`, 1010.998941039169`,
191.1453687810041`, 0.0813026067841443`, 7.337454751758624`, 0.7004457216179918`},
{0.16572747960623446`, 1.3999561572838486`, 8.592203336105698`, 1.0149199248092395`, 0.9204720561931288`,
0.6093468506279616`, 0.11483304130851824`, 0.012170616273068445`, 1337.06098163951`,
286.8021266598324`, 0.027824790783174902`, 6.4485130480990716`, 1.1250259465267032`},
{0.1546049836013996 3`, 1.7922898385073154`, 5.637575606203491`, 1.3053191224828369`, 0.530866536081585`,

0.3927999737354578`, 0.2340759815846180`, 0.3220260634134779`, 3258.312065268822`,
390.4363804447645`, 0.21449854536638602`, 8.936127787959748`, 0.6328205146062444`},
{0.0818655292269848`, 3.5534207813275085`, 2.793085272759267`, 1.44138898302728`, 0.2645186976244649`,
0.667000460134499`, 0.1031352647835832`, 0.11738652278758988`, 1616.7923697759452`,
22.69493932260025`, 0.19900500778151703`, 7.38154340093187`, 0.5255971444464016`},
{0.08442779942280981`, 0.8533376706252112`, 5.086343297092757`, 1.4765588559370955`, 0.4420393122680226`,
0.47437344679325344`, 0.24643808934122824`, 0.005941536592530679`, 2172.43229526953`,
242.96995635142855`, 0.157510417696612`, 9.228156626391328`, 0.9577175035728991`},
{0.16851133973676596`, 2.4752237313475174`, 2.42575793497873`, 1.0578005973522087`, 0.5315655422073631`,
0.41472938652046143`, 0.24177717448224295`, 0.0988063583504681`, 1028.5760265584995`,
54.93252411146938`, 0.04650878688675153`, 2.5043219440736237`, 0.71806339741822`},
{0.2040419029224893`, 1.8168800092917534`, 6.174811187872505`, 0.9288754688257587`, 0.6581669676988375`,
0.5754364926136093`, 0.20349981430348302`, 0.3349223421963805`, 3304.529174698706`,
164.1808894290948`, 0.03840187502351833`, 7.840532439325425`, 0.2974189092407089`},
{0.17393597413635692`, 1.5761991979266723`, 3.500768537824096`, 1.241966650800916`, 0.7553239598200872`,
0.5647220891577981`, 0.0698456460365596`, 0.07461556744160115`, 1637.6446967775864`,
102.27592905310792`, 0.14712502478326317`, 2.669212259273973`, 0.5885270492629138`},
{0.06303562103190802`, 2.7937374046310888`, 1.991826202033483`, 1.3853239568680058`, 0.8642226718049046`,
0.42627609641461395`, 0.1739341576099066`, 0.013248801141587125`, 3504.113575209887`,
145.60993330770657`, 0.09071202532973738`, 5.892751585058276`, 1.3807549697256851`},
{0.2432575073739432`, 0.6270765712575339`, 1.9550603903514805`, 1.0314330185313336`, 0.13493745232200305`,
0.4469694845616632`, 0.19582833943170452`, 0.02468113928377954`, 1814.663806970997`,
145.9448155579372`, 0.17557852480928543`, 9.315910864758589`, 1.2079900473113105`},
{0.11357589384964273`, 2.240672872189519`, 7.052253737321249`, 0.7646938475078471`, 0.009142046869567766`,
0.5509926958854778`, 0.24090899686155176`, 0.02638287046938754`, 2863.3930915889323`,
84.10264867474814`, 0.16678159718247448`, 7.7951222229657064`, 0.5053738528157279`},
{0.1116182977145373`, 2.360842887008661`, 0.9627021602560966`, 1.1405981578601385`, 0.14103896539205096`,
0.5791003705327337`, 0.05180942465934579`, 0.03555380982336255`, 762.58124752924`,
235.4407827441829`, 0.05499220801683008`, 8.486879923307693`, 1.2577154495282135`},
{0.1166411660555075`, 0.7450165479373432`, 8.310812790636028`, 1.063932072189387`, 0.9753313471385676`,
0.15156075882067166`, 0.057187743785721765`, 0.12429446652524245`, 3685.6740694089303`,

```
  232.00827662364395`, 0.20692272239979642`, 9.093961104192626`, 0.5500507674166981`},
 {0.12701256602307204`, 3.4510594830271417`, 0.17236039015202387`, 1.4218046895736245`, 0.5977738378490018`,
  0.6174455495269762`, 0.06547152715546964`, 0.021505928148282425`, 2080.7788466921183`,
  285.32441857492176`, 0.13527159522812743`, 2.0719517072723157`, 0.8493272554882347`},
 {0.16525167732730567`, 1.567211428071798`, 0.46961269510991777`, 1.0926048049265726`, 0.5982927168612024`,
  0.4872752592438997`, 0.13461015322956665`, 0.008651638441061493`, 1318.8282746769155`,
  92.92105735217046`, 0.15473787356946228`, 9.951552143172052`, 0.8370529835607092`},
 {0.26512296149188996`, 3.233300402128563`, 3.292718317720548`, 1.2400916214421978`, 0.5963811820900797`,
  0.3451658167692204`, 0.10292393665820598`, 0.09694787300120739`, 3191.5882949078978`,
  195.13476182743136`, 0.1886240896532882`, 4.4133830990517655`, 1.1858146199794088`},
 {0.15032567895129462`, 2.925200495412599`, 9.469770009612255`, 1.2491630117650723`, 0.5515247294638286`,
  0.2864898115312603`, 0.05595545885252959`, 0.4795641847786323`, 1954.175399450165`,
  168.25938072536803`, 0.17272189335079902`, 2.4562744379868438`, 1.1923215617571556`},
 {0.051008388371518276`, 3.802328793778008`, 0.48994620522738513`, 1.2897891346361594`, 0.07123389103998612`,
  0.5215810111010959`, 0.05373739522257526`, 0.03556482649077374`, 3356.8546057655267`,
  95.53240615712758`, 0.03711021868869718`, 1.106351331760008`, 0.48227443908576983`},
 {0.10228292810378975`, 0.7288905389375508`, 3.433004371974512`, 1.274497031997786`, 0.6101427090150058`,
  0.660255061516275`, 0.2170030685035811`, 0.012721245103120268`, 2195.647120536849`,
  85.90708396049939`, 0.06296262837152478`, 5.578028384550223`, 0.44836886971981804`},
 {0.19207820059415281`, 3.655701792168804`, 2.510935445862126`, 1.2073260918388902`, 0.9635406057316576`,
  0.42073159663388215`, 0.061393125488842015`, 0.03366936773322557`, 1456.9751171045727`,
  203.989380011923`, 0.13700463901391435`, 8.26369238323722`, 0.48805011827851597`},
 {0.2627532955765636`, 1.639112549779572`, 2.4635443376968276`, 1.095970586814591`, 0.7703245554604254`,
  0.3899598370308347`, 0.22903116483622232`, 0.3600410344547181`, 1714.6905884118041`,
  141.27158792075448`, 0.22143418867904247`, 4.170924599810817`, 1.4302741156765273`},
 {0.23274157877277596`, 1.9065983093139076`, 3.8223892292706183`, 1.0506044130310364`, 0.03733185970169073`,
  0.2700778128853568`, 0.09296420307934006`, 0.03280802595849235`, 1773.9605198177533`,
  179.0899731082768`, 0.06001861030669786`, 8.285893379373807`, 1.1764377117465887`},
 {0.26351647839883513`, 0.8673354775463569`, 5.854038424853368`, 0.9424071816766871`, 0.9232227163729747`,
  0.413019745414411`, 0.17796832053957906`, 0.014103729537635504`, 3690.4717118898934`,
  307.30915173008475`, 0.17184547983019266`, 6.1008526886318215`, 1.0313875805176784`},
```

```
{0.22251678082176873`, 2.4705119403336626`, 3.219964315804703`, 1.347383920776866`, 0.9659234897125795`,
  0.3313519776076561`, 0.0865111078215019`, 0.112897975300224`, 1782.1419072767694`,
  367.3360449099217`, 0.11075042639430366`, 6.566322730486396`, 1.1397550110996595`},
{0.1726869732489258`, 1.0689003029764699`, 9.291822504855592`, 1.1990435589159916`, 0.8172577994876133`,
  0.22377486544985759`, 0.217477012217474`, 0.16181368479198038`, 1626.0973452248018`,
  102.05318105921833`, 0.2209045520339541`, 2.2233716047935985`, 1.1920782532980323`},
{0.25925369048850816`, 0.417309499595206`, 6.855645959180599`, 1.1773438518732138`, 0.2888562944151887`,
  0.22102634108263253`, 0.2071754486228008`, 0.04320874178397733`, 1948.065661484895`,
  384.2385254384834`, 0.1833279639586075`, 5.15481721625838`, 0.9762665296404283`},
{0.16707182228455675`, 3.9273258352780225`, 7.016188766085528`, 0.799128142527102`, 0.012996916454743213`,
  0.30869623835953575`, 0.08235696915123317`, 0.07054168097616643`, 791.6946431232782`,
  51.289432144121236`, 0.13635302362949547`, 6.005214476564535`, 0.8946308584625005`},
{0.22880237101805018`, 3.9696692691682696`, 1.9380469146031594`, 1.4177879339964377`, 0.6814855489346618`,
  0.5439318492089463`, 0.21630816993687996`, 0.03709761261377448`, 3976.9551811029105`,
  217.4437376330418`, 0.19674001267675278`, 8.838870490399184`, 0.20990582924569878`},
{0.22057802576642993`, 2.653890334277995`, 3.4863828466021776`, 1.1742912829666252`, 0.8455831985967934`,
  0.6315585043048466`, 0.21227533056536496`, 0.0900482536994353`, 1462.898116977769`,
  366.27377580582504`, 0.07912574234824876`, 7.4931206124945415`, 1.2283305207046857`},
{0.18767448152183636`, 2.9367397535675925`, 4.254981609198115`, 0.9658072683472699`, 0.6065533427003664`,
  0.6047075695535695`, 0.22956691915882899`, 0.016808738900599973`, 3085.080662132773`,
  300.13204027351287`, 0.16815220633174727`, 7.212084588140897`, 0.5334832326025793`},
{0.16674052895366065`, 1.1207156804364002`, 9.241855378678228`, 1.116892231935736`, 0.24719875970191896`,
  0.3952404123967743`, 0.24464165205494265`, 0.025889397470345094`, 2185.868573954095`,
  178.37862169115658`, 0.08745488697820436`, 7.480105474877458`, 0.1628128610930062`},
{0.1343775710472654`, 3.3862425535357383`, 3.6167615926265064`, 1.4522710413875881`, 0.41930411990329275`,
  0.6633621503450218`, 0.2054877494749729`, 0.014727056691440865`, 2132.348768069086`,
  153.41895938212008`, 0.07798120175302098`, 2.3819365486087474`, 1.4841799853609898`},
{0.18395565451029489`, 3.44119115260109`, 6.476610943613181`, 1.4097688201668712`, 0.3016652649845466`,
  0.23377636743924812`, 0.22702955380114842`, 0.044315995698336386`, 2035.6758637667072`,
  215.7759126319412`, 0.23319048350186838`, 1.2339772874754598`, 0.4032881579686409`},
{0.1877085328273454`, 2.4698348389855864`, 6.723740484115233`, 0.7818601707856943`, 0.1041858328478078`,
```

```
  0.45810434108336306`, 0.1358172536153655`, 0.022077708334214 59`, 1185.161236523103`,
  108.74647947804544`, 0.020759548694208763`, 9.734619845644367`, 1.2692223563341272`},
 {0.22900819014324658`, 0.8599246184787699`, 4.575509667907129`, 1.1011546323816885`, 0.08315194396691528`,
  0.18390636972505037`, 0.1408377449166481`, 0.3122650757195923`, 2947.0026584149073`,
  167.82607804795214`, 0.10792455151802871`, 5.931199053322571`, 0.9798220386267653`},
 {0.1416858046718097`, 2.2139997848909125`, 1.7657175272923222`, 0.989271832961955`, 0.13788431085704778`,
  0.5209143755701833`, 0.21392682943087216`, 0.022178375354938 98`, 1932.3838117923324`,
  368.90142938726285`, 0.22169599764103476`, 7.130639713263575`, 1.0904656940263826`},
 {0.10996511298893497`, 0.7862218358455948`, 0.8377076631006055`, 1.462919598856414`, 0.4311311224143921`,
  0.3856327187176355`, 0.0881986225258021`, 0.45328063368772087`, 3501.1649053241954`,
  360.18740869055375`, 0.12284319478126604`, 4.367675405932559`, 1.4276764859907565`},
 {0.2044811200034678`, 2.892822767813837`, 7.595852124568353`, 1.216932964982977`, 0.7343890780610787`,
  0.5330606684550935`, 0.058989793379298994`, 0.012080434529966445`, 2521.088434314187`,
  248.82760985623736`, 0.04480645758683227`, 4.109426945013643`, 0.10374011787051507`},
 {0.04803156621959867`, 0.5827754735791681`, 9.214605590518953`, 1.1720588669143222`, 0.8133320484998057`,
  0.6073432395586391`, 0.13364149823347654`, 0.026188730330046147`, 821.8332545613489`,
  133.8448404892250 8`, 0.12502450253620295`, 2.6224761178996694`, 0.4958664794043681`},
 {0.1676455194581572`, 2.242553618614152`, 0.4702874552580045`, 1.338910822252204`, 0.5222950299457514`,
  0.5002656056336167`, 0.18155773122559443`, 0.023205215351886358`, 1812.861489403048`,
  208.09707961592778`, 0.04482084976511336`, 4.8128979171410045`, 1.2921971759159723`},
 {0.18135996247125585`, 0.9876459274110312`, 2.54354039452296`, 1.3578342543318755`, 0.8930073147262432`,
  0.19298717253815956`, 0.057956724513287594`, 0.0261548554577047`, 3458.0576394641494`,
  159.9558341984582`, 0.04888106360623701`, 2.7801567336765522`, 1.2025006457139424`},
 {0.06724626093304326`, 3.981403022919509`, 8.288988323065759`, 1.0703538801483565`, 0.1653330791428167`,
  0.4148194823615189`, 0.14440389123820263`, 0.19094336267448156`, 2003.8545802245517`,
  246.03939081881197`, 0.139046230518015`, 4.691983788055152`, 0.20050055393273314`},
 {0.21881875684613517`, 0.5898239021275495`, 9.93413148158805`, 0.7544358459883748`, 0.3693391180254211`,
  0.6579008857369009`, 0.12709107324060326`, 0.48252891951701626`, 2900.587783201154`,
  140.00284155203332`, 0.2319315634448451`, 3.003524790772248`, 0.5189129901116001`},
 {0.18529128706683434`, 3.034817908847505`, 4.618773666141445`, 1.078127573609324`, 0.45431233294938433`,
  0.23204854612180326`, 0.21276533477653692`, 0.005983047100273517`, 2838.235019182518`,
```

```
133.97625538243244`, 0.13415379883362671`, 8.373997227624333`, 0.3370984440619733`},
{0.11830138320073424`, 2.557281635675193`, 6.588149241112692`, 0.9721130031371765`, 0.10225884860358048`,
 0.23082972097158927`, 0.158140431907085`, 0.066545959983725`, 1023.3689587458948`,
 34.93006289140902`, 0.04839524009283808`, 8.085053919685542`, 0.4877285564517453`},
{0.09989873104684788`, 3.3669591438562216`, 5.451558876338645`, 1.2627430306397467`, 0.36748912342215223`,
 0.31108122243153336`, 0.12542210883620514`, 0.007371687908480407`, 3955.966637225736`,
 182.23968139905298`, 0.15451142826141562`, 8.671574224679677`, 1.2389048546471688`},
{0.22609069450991742`, 1.379608652743129`, 6.641039030693791`, 1.0528553454528264`, 0.9110900221670273`,
 0.41585330034333745`, 0.15736472782730937`, 0.24498648200968076`, 2031.8230399130634`,
 206.889154659356`, 0.10264734406954457`, 2.177599971086371`, 1.0407277908428236`},
{0.14149819408460695`, 3.9050575954333624`, 9.383356210665369`, 0.960335311543541`, 0.3424013679631459`,
 0.21333414365316794`, 0.05126386183615583`, 0.4919218322625912`, 3186.224620232545`,
 108.82685651649962`, 0.06233434212075295`, 5.950211096913752`, 0.24827529292780603`},
{0.1828220078605639`, 2.600312322938115`, 2.7331064358556674`, 1.452306554796921`, 0.9684456895629803`,
 0.28153635898957774`, 0.13708089241679355`, 0.10257553866825626`, 1556.8951227385596`,
 164.52245666603233`, 0.13014520838075316`, 8.353642416878472`, 1.085154972102886`},
{0.21889388250937136`, 0.5333175361369764`, 2.4121127215145144`, 1.3818810352832893`, 0.021571596232064838`,
 0.5244501966414251`, 0.09076416020812753`, 0.010775282992960485`, 1502.4651132234349`,
 214.3639418846526`, 0.02290218457939025`, 5.386530044496094`, 1.4845771171901991`},
{0.08341672306903047`, 2.823299860683674`, 4.834884605952357`, 1.4354641770214007`, 0.9014467152160488`,
 0.6047529781600969`, 0.2470533033127953`, 0.006202153420058688`, 2417.2420994663553`,
 158.0041607308176`, 0.0735259731187195`, 6.516775962062823`, 0.4133308952289383`},
{0.16837082644708545`, 0.688574652311627`, 5.390405754713122`, 1.3217406774272815`, 0.6156990878136903`,
 0.18352461332079384`, 0.0882858818891156`, 0.4733971203783309`, 2234.460265184529`,
 202.49554378641346`, 0.1503105450782266`, 3.806358767200937`, 0.9148594831472519`},
{0.10718623369591462`, 2.960462485410858`, 9.423477882136847`, 1.1211033662754974`, 0.7860054376705241`,
 0.5141091538304853`, 0.05867214344346472`, 0.09405923440011967`, 1537.0957508113852`,
 239.00629492907444`, 0.09357298190053748`, 9.410898004520863`, 0.8654487723896875`},
{0.08165938583300264`, 3.5663918013786944`, 1.9719703539316173`, 1.0863058189118182`, 0.9278717656325091`,
 0.29627245453078765`, 0.07723831560352282`, 0.005496209135881868`, 2965.7691899558304`,
 36.38856085381832`, 0.1944071056697382`, 4.620185477920922`, 1.4555441950836703`},
```

{0.18162340679997874`, 2.5029500310491875`, 3.7850875297449385`, 0.8714265661124054`, 0.04795600587846183`,
0.17826165391264504`, 0.1376006876951453`, 0.011710367976498486`, 1897.0378575605146`,
378.5017307178937`, 0.04683765268043216`, 7.517716852692461`, 0.3647662687926059`},
{0.10569564926832292`, 3.94925758524324`, 2.123437162636053`, 0.9889632230650253`, 0.6091744144977718`,
0.44858710373952704`, 0.06967757399007424`, 0.06579434912735219`, 2048.3556961935665`,
192.929988723693`, 0.1607054226404262`, 3.065879080542288`, 0.48218127318000126`},
{0.1295394448875809`, 2.022900103755206`, 6.745994489858977`, 0.9580215588674443`, 0.9679692042597987`,
0.6904939360195417`, 0.05882320965696805`, 0.04912899750957061`, 1189.5399921293747`,
163.6733664974323`, 0.16033185329150101`, 5.9616048020309655`, 1.2439833727010554`},
{0.24619035501818215`, 2.0773850626788413`, 3.018461478313549`, 0.8871662168885301`, 0.552617674236112`,
0.602322011487733`, 0.20434748314233808`, 0.039023258548983815`, 3150.0536016394335`,
203.54823250165975`, 0.24910387895313074`, 1.7034902327529498`, 0.8084130559906435`},
{0.18194781074756367`, 2.220335509721994`, 7.082070220373439`, 1.2346660258162963`, 0.911357302435295`,
0.2993417896543701`, 0.10379984911373669`, 0.06373968962233406`, 1006.0135021400088`,
377.69931275745535`, 0.06750680684166793`, 5.51898040870112`, 1.3240328384144195`},
{0.1644023253774144`, 3.882290753800083`, 5.67814834147805`, 1.1217171634204655`, 0.4202214546941414`,
0.41106474358929035`, 0.06359886932295966`, 0.24993914606767362`, 1680.5799994030467`,
230.96461331819444`, 0.016083788677699107`, 8.870452591991157`, 0.6584854072019537`},
{0.11290442193886385`, 3.342282901193297`, 2.898098788178677`, 1.2208920487711645`, 0.15571856198486667`,
0.6450091875601396`, 0.0720097019966474`, 0.18140557939535418`, 3133.8759234972686`,
359.44606615721364`, 0.058851389143928845`, 2.4968280987996607`, 0.44903712610868673`},
{0.057179701065231625`, 2.855281586486381`, 5.482300889649739`, 1.2083595069262243`, 0.795518738527804`,
0.6366469580580449`, 0.11918231638825821`, 0.16920403900000133`, 799.767703151836`,
247.48605975500072`, 0.10228761215396959`, 9.288149547399101`, 1.2673098981919697`},
{0.0875578111208043`, 1.1766586937430805`, 5.077956330142905`, 0.8750465005858246`, 0.8596423212250299`,
0.6288939270221594`, 0.1488852290513452`, 0.012846012396330703`, 3117.450697007702`,
283.31782716202474`, 0.08644475242948957`, 6.632814251236063`, 1.2827414734862095`},
{0.1753150595055662`, 3.713574756709245`, 6.279606102538107`, 0.9857257532666506`, 0.18592289869330236`,
0.21496347741152755`, 0.07049642727707597`, 0.017218370675811997`, 2472.055030254328`,
256.95308160663865`, 0.22538544536854038`, 4.255341860945123`, 0.22574651763666997`},
{0.16802857593346404`, 0.7065678176512091`, 9.645249981168057`, 1.3116063027060454`, 0.32088080529355767`,

0.3691390904550721`, 0.21874840715793664`, 0.07094779858961066`, 1630.2542224830704`,
63.80576916127296`, 0.10975436657116389`, 7.114567585043865`, 0.902530053910509`},
{0.22967780600547444`, 3.4707114805627004`, 7.357111611957344`, 0.9509066717740751`, 0.7301577279137197`,
0.34891003518552344`, 0.14994464399131946`, 0.18785696851097627`, 1448.9102951912027`,
296.3879865990905`, 0.13500832540748392`, 3.275227564043599`, 0.3389151549206586`},
{0.10636965549911737`, 2.776846234427638`, 9.809572024338209`, 1.0633435610610082`, 0.685773597384219`,
0.389828683015383`, 0.18173035709309476`, 0.0701248233933049`, 611.7373128577356`,
348.65549321322396`, 0.0858335193643106`, 8.943700469577841`, 1.0373885202439106`},
{0.12945851018082827`, 1.6530391126563773`, 0.25296383859783056`, 1.0004082847943978`, 0.6153536990324371`,
0.46726548359691056`, 0.22184757314007908`, 0.029263173246622916`, 2804.4069174712213`,
201.7772707782526`, 0.15804840166726702`, 6.079299069162072`, 0.5497928673982018`},
{0.17704121544802504`, 3.3101767206813077`, 0.17894892947703944`, 0.9519221706044084`, 0.3716159634368834`,
0.17251563798408087`, 0.06428454684265117`, 0.050202794311224846`, 1196.331297175113`,
92.7477589491034`, 0.08675697489654427`, 7.03036379115748`, 0.9556798715945236`},
{0.09051269700635717`, 3.510668586088882`, 4.372912512921815`, 1.3267853376570602`, 0.8120222411850102`,
0.239383728318748`, 0.21506169431109157`, 0.20950664990140405`, 1339.6563194330593`,
329.7735045463686`, 0.2280878170659803`, 2.1716129406234614`, 0.869217412898017`},
{0.10777187590532172`, 0.739057715374912`, 1.4330240793355227`, 0.7755224108621632`, 0.12555368221137964`,
0.545351416919415`, 0.13748218086765884`, 0.05919866095169625`, 3976.4209480252466`,
374.54745407708265`, 0.062136934556040746`, 7.297702295605297`, 0.10338190129278124`},
{0.05012776858713164`, 2.0872339482718107`, 6.705710827191493`, 1.4141058091423697`, 0.06890952245028781`,
0.47612787079844376`, 0.08471180395825834`, 0.016954072038827265`, 2788.124252935484`,
108.68029812587577`, 0.08106980932549152`, 8.734393861782642`, 0.5389481104734093`},
{0.27321591675550233`, 0.6886264672292262`, 5.359177927011217`, 1.2203089609351563`, 0.741856408018519`,
0.5849406177035197`, 0.07433546983180528`, 0.21690743690508418`, 1384.5673397625906`,
70.87997184926286`, 0.033379798700165386`, 1.435341337958821`, 1.463005840477979`},
{0.08919135792007882`, 1.9079775156637941`, 0.7718276581060107`, 1.2887666627276828`, 0.6966500412335945`,
0.2943209647073368`, 0.16567285203320248`, 0.2074475031655441`, 1406.902570296439`,
161.54627762916846`, 0.02754527329468942`, 1.9201358824343482`, 0.9977637383308959`},
{0.1308447304032681`, 2.6119158907935276`, 7.388927202269429`, 1.1426009806290098`, 0.6170224096703181`,
0.5032614025378099`, 0.21866608861569725`, 0.005956991186122966`, 1137.1164610175856`,

```
  368.0725112372012`, 0.24678418452608802`, 7.5428083328821245`, 0.14982171102850428`},
 {0.06751611881159836`, 2.2474089970429993`, 6.402097457423925`, 0.9363574880786951`, 0.3836841636235191`,
  0.3647284912476121`, 0.21335232520714842`, 0.22248453220280073`, 2502.3638711494295`,
  397.5911497982886`, 0.16288128573213767`, 9.005290951575024`, 1.0427120513238162`},
 {0.09177879720493187`, 2.6455642646824806`, 3.5115323646466643`, 1.0647038743499257`, 0.023437829577520386`,
  0.20767277287096642`, 0.13833050574986264`, 0.16097998475232683`, 3330.8307600954595`,
  36.94740532139957`, 0.0838397051953012`, 4.654426462364112`, 1.1418435912898404`},
 {0.04551056845008081`, 1.6215675270048893`, 9.700566391026626`, 1.40945752023491`, 0.4723808944761221`,
  0.3499440053674635`, 0.2064578553856145`, 0.09474747167065985`, 3852.6928203016614`,
  220.886718735037`, 0.22402828336678815`, 8.267759522981173`, 1.1674803222752286`},
 {0.04140464424456583`, 0.7619598093834323`, 9.994885078827323`, 1.3154910694140878`, 0.5846504333106828`,
  0.5277457306392407`, 0.19747782802158215`, 0.20923520620939917`, 3747.8984478066395`,
  346.71554418059986`, 0.04757978117327022`, 1.731006219467508`, 0.3938580217562748`},
 {0.2669155508949231`, 0.8202069836030885`, 1.7120733781356066`, 0.7716732548944238`, 0.42105306969825285`,
  0.4443694926042744`, 0.19913166298690133`, 0.07350737572131678`, 3667.631913873951`,
  382.69157822195143`, 0.19936982959712085`, 3.809394225029287`, 0.38364950845782375`},
 {0.11628555922147576`, 0.9133810470221562`, 6.60919827925733`, 0.784993620588668`, 0.15558932911031897`,
  0.27453121261635205`, 0.2030077151739278`, 0.01885747776005145`, 1720.9600172356268`,
  220.92402081631303`, 0.1375226538362544`, 8.707812842669803`, 0.5031177384459613`},
 {0.16627919709086636`, 3.258899380742358`, 5.159743357401544`, 1.0084152828841422`, 0.293274671001003`,
  0.35878951423125527`, 0.20447475990027797`, 0.06840907951998164`, 1127.403994426183`,
  328.5526444815032`, 0.06047838915389364`, 6.7509258891150505`, 0.42840334463359087`},
 {0.27348098404578464`, 2.2019037456007027`, 4.59402198031265`, 0.8385696628288007`, 0.6096935872297715`,
  0.3604633388375672`, 0.23592549449411898`, 0.025613961789364897`, 724.0273482822054`,
  51.59152478645882`, 0.07558496433041467`, 2.104114801800101`, 0.41664957185729956`},
 {0.11413129353935564`, 2.5718486613546734`, 2.678541647274315`, 1.0538897286852023`, 0.5347432665041771`,
  0.651301492741162`, 0.13507333994249482`, 0.03374013401687532`, 1792.985239436316`,
  324.1256033282515`, 0.22051507650373564`, 9.850878408710493`, 0.1276954587958865`},
 {0.1382612217370991`, 3.544859312958268`, 4.625085964177936`, 0.9631685672533119`, 0.6192785632371416`,
  0.30258401315557804`, 0.17357832021845082`, 0.08489498345439296`, 3734.2868382787856`,
  118.6130866776067`, 0.14202527245998875`, 6.035316827283889`, 1.23738828709505`},
```

```
{0.20062439708640356`, 3.062479927466259`, 1.8588498709761279`, 1.1971327198257382`, 0.2717889530441755`,
  0.5772443937125967`, 0.13761254703825482`, 0.02383075282424653`, 748.4785019436172`,
  123.78948146072491`, 0.21845089918107502`, 7.7975595199904255`, 0.4961018196149929`},
{0.2202391290111929`, 2.272383906087442`, 9.526635756893253`, 1.2801331448227915`, 0.9676407538336758`,
  0.2750641951622783`, 0.1295591726514962`, 0.0055115733663000654`, 1435.4738327945734`,
  244.53753727132892`, 0.22242949998676909`, 3.5195011696028082`, 0.5942454573278351`},
{0.08672713507238722`, 2.273561509630496`, 5.582415438341933`, 0.8483312100313163`, 0.3485115156653926`,
  0.5097094533881305`, 0.23367094415518952`, 0.14443826868757056`, 1562.9150740851455`,
  249.7667060966911`, 0.16191416277041454`, 3.437993337535003`, 0.32781925870094497`},
{0.18381668769060344`, 3.1958936164482523`, 3.6493637720914887`, 1.44172169143792`, 0.026838260836304473`,
  0.6302019111021357`, 0.18006792550977113`, 0.04278842418986425`, 2871.506610396247`,
  235.17217671272113`, 0.15357027364724346`, 9.443732417373614`, 1.0754484669415296`},
{0.18865219826812762`, 2.4805712334398518`, 8.790023396255851`, 1.3488199759969297`, 0.9395739613202174`,
  0.6166147449809938`, 0.13773668752267337`, 0.19466267340323914`, 3969.2421406756894`,
  297.90931549743027`, 0.10386024186718207`, 2.094376919169571`, 0.2651891384942813`},
{0.1825851308210345`, 2.4396329783561983`, 0.6407630961927655`, 1.1714042800046478`, 0.8170667200239692`,
  0.31301546820459225`, 0.11111924655180566`, 0.011382861853813236`, 3697.837667320504`,
  70.61298960423318`, 0.08120950744691857`, 1.4309282478289982`, 0.7664004986892703`},
{0.13375040892255174`, 3.276165649558605`, 4.292971358510812`, 1.2543962009506533`, 0.622136912433882`,
  0.38876133504362986`, 0.059362281046229176`, 0.37593623792715036`, 1950.8385805745784`,
  382.0130407968936`, 0.20132818276194275`, 9.006278307672524`, 1.186932922075334`},
{0.11761221086988144`, 1.0914541886641524`, 0.5591025160362655`, 1.4775466222869675`, 0.9603313566507792`,
  0.2704038317127577`, 0.19659054677243826`, 0.04563469543817772`, 3422.6083976447926`,
  269.42977341830294`, 0.0784511002461219`, 5.408577374365102`, 0.46841691657751894`},
{0.2429320475534284`, 3.9794232967059395`, 1.0019515993916865`, 1.0030250425538783`, 0.21362795630084253`,
  0.32541860695965363`, 0.056903912665728335`, 0.007999914248720421`, 1861.2936578099834`,
  133.12444614515988`, 0.12956497529754557`, 5.59967766310799`, 0.3532739838839296`},
{0.08974721760036025`, 3.2713030704465336`, 9.414991051375608`, 1.4492271123735618`, 0.9934682041726086`,
  0.2039523558527424`, 0.14566778076348777`, 0.062238114816719925`, 3000.2912643397094`,
  293.40777590216123`, 0.11936140149668095`, 5.192024217841877`, 0.8616976591715506`},
{0.26359879599023367`, 1.2163041539701664`, 3.7777909348817094`, 1.254691553657424`, 0.9061229415337297`,
```

```
  0.533372724047131`, 0.10691633026129371`, 0.27923770769686623`, 2206.202484705579`,
  127.40873012168657`, 0.13182990312478088`, 9.420656570983855`, 1.2733451287842463`},
 {0.19365605952074066`, 1.0097214328963666`, 6.254195026060383`, 0.7786863431193308`, 0.8378299450565547`,
  0.5381078852313175`, 0.13430741341317382`, 0.39183098626736546`, 3848.3805063541085`,
  285.46142645085683`, 0.1476809547681347`, 3.2123723043853616`, 0.9596643052107194`},
 {0.11992752605735896`, 1.1695863474317374`, 5.859787436657632`, 1.088943542117163`, 0.7084196598983477`,
  0.2068540131141825`, 0.10134897101245235`, 0.042086980108994226`, 642.019354817593`,
  42.643211093372884`, 0.14904119135720395`, 5.991168965595664`, 0.5811778237079277`},
 {0.08593805782066072`, 2.847179937660589`, 9.336517990063154`, 1.1546615909588782`, 0.6233557238718435`,
  0.5408319922909341`, 0.12713218439588464`, 0.009396030605633099`, 2805.654341504175`,
  67.07123229055907`, 0.22248402654525073`, 4.783547942175421`, 0.25670135456607057`},
 {0.13317608837170147`, 1.503932075956504`, 4.603993052764295`, 1.367556919883856`, 0.822788205819833`,
  0.29931793037743193`, 0.1910982001847455`, 0.009186595448818861`, 2039.1477227581072`,
  222.71270432048289`, 0.13206186826480382`, 1.3638292654387778`, 0.18903235795918816`},
 {0.09391708894580503`, 3.041674977657679`, 1.8104005667515946`, 0.8959187121202952`, 0.2181847991244159`,
  0.2824961199777095`, 0.12004816256362977`, 0.10420303973300599`, 1895.0150961574109`,
  50.54358082712821`, 0.23566298754715342`, 8.257703012553833`, 1.009710413761872`},
 {0.27769905250843735`, 3.334803063409833`, 9.639698328103112`, 0.972586344049939`, 0.15282290468559845`,
  0.3020316347056351`, 0.21057051005264293`, 0.06155018310530056`, 2190.880276553241`,
  205.62596489641203`, 0.17875345007490812`, 2.046885089160499`, 1.0703002508596509`},
 {0.16775949346003155`, 3.734350305060132`, 8.90335371268981`, 0.9286973988343983`, 0.35313792052011217`,
  0.3940821550050888`, 0.14910438190884368`, 0.1880509002120729`, 2527.5769345457447`,
  29.455979238374994`, 0.037167259903154726`, 1.2969361348709503`, 1.0523609025190432`},
 {0.2349663788193207`, 3.197269954964022`, 9.218845167290574`, 1.0446779264109838`, 0.588347422519365`,
  0.31448476179540896`, 0.13233876836756175`, 0.2796850376475317`, 2984.1524918506084`,
  161.29090180593857`, 0.1933086585832205`, 6.384613193914876`, 0.9299276109407706`},
 {0.23753660977363267`, 1.5829476505005262`, 6.77431038963988`, 1.365304095754423`, 0.13883005311148944`,
  0.18235912783176345`, 0.11037102150981828`, 0.0059073701404183405`, 3040.16542258953`,
  172.94160910300627`, 0.10069032496396058`, 7.204527027300948`, 0.264985624786922`},
 {0.23896625246574088`, 1.021768300475304`, 5.961217623879941`, 0.8744606875234477`, 0.07851707385676243`,
  0.3191621351693026`, 0.12686157250224162`, 0.07080906253325867`, 754.026010583138`,
```

128.7873645709799`, 0.07054179811818284`, 9.522685613917606`, 0.14468703962562834`},
{0.0664141372741357`, 2.373389311008811`, 6.163630213717491`, 0.7749555716943826`, 0.011151563708291068`,
0.26661102099162604`, 0.06978956594786417`, 0.06307180805406282`, 3305.773752462622`,
398.92218816248055`, 0.15362130283700076`, 6.399110469992867`, 0.5018401596166369`},
{0.07724629482189549`, 2.6662607156107567`, 3.4543142766524646`, 1.1527116922070777`, 0.6194059290185789`,
0.17356203127759262`, 0.12523844349086252`, 0.006404139963734165`, 681.0439615527839`,
333.4912680980369`, 0.21335410013399275`, 5.078097228348419`, 1.1402121008441597`},
{0.11115827720243887`, 2.3474129584418977`, 0.7793271183465329`, 1.2896581551764172`, 0.2400074156115546`,
0.5586724719427854`, 0.16881638153305045`, 0.3542961312162605`, 3281.9581897651888`,
363.45984424300025`, 0.19399862158515324`, 1.0793406751665469`, 0.15013258901534354`},
{0.2790792381180839`, 1.4178392715488872`, 7.586791613535141`, 1.4555087381759333`, 0.0017991755342297022`,
0.24850929517479325`, 0.05953650221890522`, 0.3815756253123188`, 2337.3041910345446`,
253.88772622846432`, 0.0832002701070979`, 3.446598610267717`, 0.1790778319506987`},
{0.05958430298107065`, 0.7208194464934747`, 0.8555730299303229`, 1.0890886690114123`, 0.12206612248038917`,
0.22763156357860503`, 0.13379446389365296`, 0.0940380442991097`, 890.0674874512752`,
363.7513660796561`, 0.17126775622478085`, 2.343574670731165`, 0.6210533219689349`},
{0.17145251727538108`, 2.2285999256796796`, 4.099234007029899`, 0.9794004497404906`, 0.5456157460051374`,
0.6134582400670898`, 0.07617057839260499`, 0.06280998418842913`, 3845.79457797157`,
146.34600196120715`, 0.1780830585354753`, 4.861786782312107`, 1.4658755518344249`},
{0.12996780445602385`, 1.481230978947499`, 0.6236753706365583`, 1.469443592582019`, 0.8723955727041814`,
0.3094468996181755`, 0.06710955059959622`, 0.0369322761528374`, 2374.524656049569`,
217.63840782204932`, 0.06713489355669272`, 5.581729605257106`, 1.404891625361591`},
{0.05377365231121939`, 0.5209314028947571`, 8.011482854018304`, 0.8336514898133974`, 0.5477465381895592`,
0.6097956289357849`, 0.1415470974234781`, 0.1739421756208861`, 1218.4890478272073`,
28.334007654588277`, 0.1640483947776385`, 1.719700868912403`, 0.7776819970376057`},
{0.11080721322368536`, 2.0431071845443514`, 5.511023662772233`, 1.3452026921196156`, 0.11486345745135074`,
0.24454012333479547`, 0.160320934401384`, 0.03396351864461054`, 2929.9466481691034`,
234.80413085200098`, 0.15939227470850165`, 4.957656716995926`, 0.9862870867437366`},
{0.0760667810432224`, 2.1066017580574803`, 2.855976527079802`, 0.8406874141346559`, 0.5880407566565378`,
0.5900461865444994`, 0.0836613609935555`, 0.006020335814040566`, 1435.8256232073982`,
78.12343431322768`, 0.05191171052641791`, 2.750551419426905`, 0.6067313927910662`},

{0.1959001110368495`, 2.209062410088075`, 0.3560695839737882`, 0.9080944676346985`, 0.9265730083719161`,
0.5325736257413008`, 0.19563779045692792`, 0.10606554062225873`, 521.6446274837854`,
359.26470542430604`, 0.157717920696867 9`, 8.108669567071114`, 0.6211999644108912`},
{0.2577200305567735`, 3.2042225311100276`, 2.603156269860186`, 0.8353734407000886`, 0.2430563561244512`,
0.34928665438367135`, 0.16249163015987889`, 0.02250334329978988`, 1913.3488368943863`,
296.02734369523876`, 0.11827389650606279`, 8.829720137683108`, 1.3154105657657276`},
{0.2269507001135535`, 1.5304419335739787`, 3.489177129657852`, 0.8382349859440477`, 0.3185553593952979`,
0.48806107220778994`, 0.1765391455298561`, 0.025680745838524602`, 3051.647362946771`,
356.25991793699154`, 0.11555959688444262`, 5.191309509924922`, 1.4793748585613802`},
{0.08130128725263741`, 1.139181654994668`, 8.356523528104287`, 1.0901388323360164`, 0.43395806919787105`,
0.6945552692578585`, 0.15512850330366812`, 0.013156059374009372`, 1072.341203797791`,
380.15089775887463`, 0.2428753710582479`, 6.517091771782727`, 0.9223679840610632`},
{0.2434557780636919`, 0.7224758340812225`, 2.1790233830774692`, 1.461101906273748`, 0.9519880988578835`,
0.24073725238447052`, 0.12794532446798884`, 0.029247247168962882`, 1555.7186853348103`,
187.90922583014026`, 0.22271376685703087`, 9.305754826562229`, 1.0292202961269128`},
{0.11639393264339765`, 3.920728456674687`, 7.161326334970468`, 1.2230707245468682`, 0.4475871708897683`,
0.6126582241159237`, 0.06411943719695506`, 0.012576022333314589`, 2883.638838241297`,
313.1192616144217`, 0.21454888313615755`, 9.840193495130112`, 0.5865096618206191`},
{0.15100737716165968`, 3.554370686110622`, 4.587082662015709`, 1.0501596960456492`, 0.32405591591236504`,
0.16453196956775862`, 0.1064831105262615`, 0.33127320693075046`, 902.0301791506135`,
373.05242738162826`, 0.0458010200387457`, 5.548602584093444`, 0.22333879786319444`},
{0.25418798006115284`, 2.1642591478158595`, 6.996499815725443`, 1.0633791975130336`, 0.9920570228042984`,
0.41215457570127223`, 0.23951743090069744`, 0.250242517415804`, 977.2894626743205`,
66.34773026593149`, 0.24515846775975497`, 7.886389497471068`, 0.4453335711584776`},
{0.17142178078611126`, 3.468749463314624`, 4.1220489747329765`, 1.0943061437323611`, 0.026879199595517766`,
0.33123915944031324`, 0.10298062238993438`, 0.021389796005587176`, 1913.2712703835568`,
144.71515384537201`, 0.0624079843624904`, 5.28189397954334`, 0.5834593874886529`},
{0.20869037066000085`, 0.5770116927483819`, 5.389618587282298`, 1.0412482369515845`, 0.4332354730171122`,
0.23498113287901135`, 0.16276058881271271`, 0.005129384861959153`, 3721.998380233862`,
47.9738718293973`, 0.14995337445020457`, 8.81275872972273`, 0.9157809024114361`},
{0.06860802282414658`, 3.9707655631721437`, 5.6945082231101`, 1.3132775124268627`, 0.833710937956144`,

```
0.4055938689007862`, 0.09163259509867216`, 0.3768554443970733`, 2744.3744850106896`,
240.03583759728133`, 0.035389680502848464`, 1.2976415449147964`, 1.3504035222444002`},
{0.05446295287328684`, 1.6696297250378036`, 9.103903568630106`, 0.7607067601908715`, 0.16998909961411246`,
0.4015279687740475`, 0.15306183541311225`, 0.23729003810245272`, 1974.2834194249153`,
302.21506503791556`, 0.16971647699912568`, 4.841445731177878`, 1.0533590220242193`},
{0.07612701991269089`, 1.8994519379164023`, 6.640792134140813`, 1.254560879057461`, 0.12469411598778724`,
0.4203442966564226`, 0.10535108099169138`, 0.03365653592102131`, 2960.4487643491357`,
231.61182906412773`, 0.044585724001347915`, 3.1581032850809603`, 1.1020659040461283`},
{0.08639685731709784`, 1.2263080297925724`, 1.8023918869870366`, 1.4078333023480911`, 0.09921808861838555`,
0.1733433235749129`, 0.14800205989677365`, 0.20963109628072585`, 1801.4315718325315`,
163.03870519568818`, 0.04013342775966272`, 4.222103734481822`, 0.21608307503029467`},
{0.05971802623282291`, 2.1738091632492136`, 8.683813711430187`, 0.7864060587963683`, 0.5208725203898663`,
0.5102800946784368`, 0.10321808603446814`, 0.07803262912760096`, 1116.648121119908`,
221.40149042921257`, 0.07733870760092837`, 7.219382522940123`, 0.8997811624944188`},
{0.25957147156308197`, 2.9787560040757644`, 6.8514986634875745`, 1.2875526348358215`, 0.5271393050674782`,
0.38366555469751695`, 0.09532745198151626`, 0.00993086596705298`, 3516.3450563644556`,
352.0852345112979`, 0.03547806916020174`, 5.814047961801249`, 0.6954919748641395`},
{0.07058107847879669`, 1.480448796537483`, 8.054675740834305`, 1.3102914246603354`, 0.04592614768203762`,
0.23013975911080142`, 0.1465551863327878`, 0.10936312847944579`, 1149.9199910201796`,
342.3645344528402`, 0.16288013724428108`, 8.990901684554295`, 0.299529341502206`},
{0.2559888963315523`, 2.7036918392629854`, 1.376199541747745`, 1.1184454818096095`, 0.29882869252546684`,
0.4809279853463262`, 0.1762427890412958`, 0.033675551797332184`, 2802.532681882846`,
74.90505814957066`, 0.08607671060416239`, 7.246865433053017`, 1.1485805723085236`},
{0.12936768312046054`, 1.4931741189869152`, 8.533444012750714`, 1.3526677837265888`, 0.34592179565846926`,
0.5020921142394136`, 0.1702518668235985`, 0.4026569331476235`, 2469.5593778381244`,
155.39036984038785`, 0.09801542749760322`, 2.524614796651056`, 1.2701205529096362`},
{0.10764603393952993`, 1.2107066619029974`, 4.216520809210639`, 1.0534038454487438`, 0.2705194689594228`,
0.49749815429385036`, 0.21553478853143437`, 0.04863912466742637`, 3262.748108283069`,
369.9540993093576`, 0.034536512984739975`, 2.033964341592309`, 0.4918523584430632`},
{0.16173577525354788`, 1.3192464264843462`, 3.8155846300226686`, 1.0837420201848613`, 0.1916516348586692`,
0.5385828520739745`, 0.0857724509699924 2`, 0.02449629028204095`, 2335.082397263821`,
```

```
344.7269386138737`, 0.14212635984223515`, 7.933933586100813`, 1.1885980026661422`},
{0.2639188435968321`, 3.196402084598291`, 5.8733763940010615`, 1.001723636198023`, 0.8720114445540239`,
 0.5139297111136746`, 0.13829965727943472`, 0.009850038986012472`, 3721.325240000034`,
 357.3898095475904`, 0.10886509504864544`, 4.342058559354168`, 0.9364053798077125`},
{0.19137997577853977`, 1.0693425445674762`, 5.859392287604482`, 1.4113333162411321`, 0.03799318259045559`,
 0.31471592998942965`, 0.1330917250869928`, 0.057621317595080414`, 1667.5088619944472`,
 46.210982838293944`, 0.016645464987740083`, 9.646205098992475`, 0.9349271114716617`},
{0.19721869082033683`, 3.2840461091147555`, 6.111568583801249`, 1.2835967155205092`, 0.47111924671273675`,
 0.3724666179342665`, 0.13247758167737783`, 0.03750893971444233`, 2173.6667113114927`,
 83.61542153296074`, 0.1890720712372439`, 6.476185684583786`, 0.7126657781022314`},
{0.21291226343526198`, 2.8179245900150987`, 9.921791649942335`, 1.3352776575768062`, 0.9588380301695891`,
 0.6936227079381379`, 0.0795524915712694`, 0.4777153216598742`, 2688.6717707047637`,
 396.8627131234574`, 0.23818107150054235`, 1.6319345031256631`, 1.1969051802794537`},
{0.08637419772835031`, 2.5843437924117936`, 9.303756737243045`, 0.9248602316926738`, 0.9539236366945725`,
 0.30709475006465425`, 0.15355620403975512`, 0.05658228330400466`, 3850.542613273592`,
 202.84343087793332`, 0.1503135323154285`, 6.227303197646734`, 1.091399246069971`},
{0.09166372218259045`, 1.1041359694939814`, 7.465464940650059`, 0.8285398481134653`, 0.01938900145906275`,
 0.6417891529291755`, 0.14382119055783732`, 0.023209894299314995`, 3535.4335241265126`,
 296.7460220437812`, 0.08660924945570686`, 6.289282036011649`, 0.7877277513120826`},
{0.06057989660476104`, 0.9664690813204819`, 5.824893628794225`, 1.1149397320321297`, 0.7552676734434385`,
 0.19045278243945962`, 0.1550171249589118`, 0.20977491640661988`, 1881.802129389779`,
 160.06890201789201`, 0.2135476446394321`, 7.516581972853506`, 0.39758727016160034`},
{0.22678054201622128`, 1.767805615425421`, 3.537786208995465`, 1.13577377357013`, 0.9133787654245775`,
 0.32400922076880223`, 0.1547055518280115`, 0.1014618010767026`, 3393.1210765994138`,
 223.6969184131283`, 0.017411417358218523`, 7.612407230565935`, 0.356714236328328`},
{0.21988733193649235`, 2.7975541724377093`, 2.3611483960904547`, 1.1600939571587892`, 0.24697322096821828`,
 0.5139658559575447`, 0.06350156651392477`, 0.17257512128303623`, 3203.836504260581`,
 102.69376441927619`, 0.05643237763666792`, 2.2035776970401866`, 1.4380067287288245`},
{0.17952419657415108`, 2.390230853640494`, 9.385595862371645`, 1.2510854246897463`, 0.45025706246945174`,
 0.39156066611114926`, 0.09061933973189976`, 0.007387745401806454`, 1060.2070144427744`,
 157.30384712261866`, 0.017455537733710474`, 7.390923217745433`, 0.4592263583377443`},
```

```
{0.17085484316695765`, 3.896251009939033`, 5.375947987134143`, 1.4639220165256899`, 0.9541878933608088`,
  0.33462154790480914`, 0.21681254773335146`, 0.0069834922249469925`, 3376.9159214269603`,
  211.30439423571625`, 0.23015330461401873`, 6.0128254119306135`, 1.4535070849014953`},
{0.13727970757875596`, 2.8452993900541435`, 0.2811962573637228`, 1.4853711561890859`, 0.35668909434089957`,
  0.6882050716830825`, 0.1434517751263107`, 0.09344751133387784`, 2265.9177865516995`,
  139.6613455165895`, 0.057526142554834014`, 2.484713339974709`, 0.10336153105397905`},
{0.10845833265331112`, 2.205454065801659`, 8.04431016182664`, 0.9250832587897069`, 0.5103893648573781`,
  0.6279318303072325`, 0.0552625938696427`, 0.03668517084277843`, 3275.5705391236816`,
  196.31559723816565`, 0.1402871557364922`, 7.884261458348763`, 1.2767651696233133`},
{0.13079509007818008`, 3.451126694395393`, 8.265071576900024`, 0.9978113975551193`, 0.28001559296341805`,
  0.46031518977081587`, 0.08917008131877008`, 0.017138702372490412`, 1442.9762283162472`,
  33.814218463353484`, 0.17998121143599194`, 7.358113266662068`, 1.1154000745850698`},
{0.15200365596867`, 1.2321714037993603`, 1.2826182456063666`, 1.2525135629617612`, 0.10189417734334105`,
  0.42182447183087945`, 0.16343163967002294`, 0.04597060352203497`, 2744.3901597051326`,
  303.2449917542002`, 0.2131773205429714`, 4.894467965266408`, 0.9895276491017069`},
{0.1701034994591067`, 3.2342889044936154`, 4.148994458467392`, 1.1940097836786847`, 0.6849042746613065`,
  0.28365434424986613`, 0.24647764422374546`, 0.16243634058501621`, 3080.6851495717774`,
  370.74406137436483`, 0.0951116860303981`, 9.02678426482985`, 1.2346863975759255`},
{0.06598975858427436`, 1.9097424631845499`, 9.748507937422985`, 0.8697722369253212`, 0.5875961327091277`,
  0.6558947995694533`, 0.20328697625166237`, 0.09192814399763598`, 849.932255943002`,
  347.93153422311434`, 0.24798460225057473`, 8.434300711569456`, 1.1653187620032228`},
{0.24510065816515997`, 2.793418227932695`, 5.458971845554931`, 1.0315954512213719`, 0.7555698096939896`,
  0.6374757156959183`, 0.22964026454409542`, 0.029915287766295595`, 3671.7453130653475`,
  246.1074690301475`, 0.07031820961864549`, 2.73413282232541`, 0.7379537484131258`},
{0.22754574199703576`, 2.0500842079624535`, 1.4317719989732147`, 1.4394511204103708`, 0.9096762268457756`,
  0.3629999302632213`, 0.106795983345218`, 0.12347919331546342`, 3709.9742200770725`,
  60.75950393256113`, 0.07245771005729762`, 9.46479696180289`, 0.2919149083841388`},
{0.20503801915719277`, 1.0262506067723134`, 1.0800304574674584`, 1.0922656655058383`, 0.19306135916753409`,
  0.5113898504084636`, 0.20945291149465461`, 0.029409960652989255`, 1028.4817812589972`,
  23.515618103058102`, 0.10367816050153927`, 1.7590551256620532`, 0.7974605289147969`},
{0.20946691214847096`, 2.8105866650441875`, 8.539169469770648`, 1.3728902130481506`, 0.8085940550353596`,
```

0.2919084226376192`, 0.2093509791170406`, 0.01434654670350337 7`, 2081.7407715487734`,
169.23962157045707`, 0.0381165620615374 6`, 8.571585131217248`, 0.31122812087193474`},
{0.07543371305305874`, 3.820737964468253`, 7.006759966869079`, 1.4837946743911272`, 0.9807341557825149`,
0.31098343284147656`, 0.07258154131712924`, 0.2086007075373027`, 2270.591159595845`,
320.3654657559956`, 0.09813455813826927`, 7.706001493171778`, 0.25408983910049043`},
{0.21236403141675547`, 1.9573734849300308`, 2.6535583097467583`, 1.4800706545848268`, 0.3008051623864796`,
0.20266843878015717`, 0.18796743731817545`, 0.01387997496279432`, 631.4288286008941`,
122.32978434555201`, 0.20929941217323872`, 7.603405617879708`, 1.123897133765297`},
{0.19210055303112722`, 1.6234167219204663`, 6.9088164888327235`, 1.007010553449088`, 0.1818811973938239`,
0.28265941449906173`, 0.05224493104477926`, 0.18359878228489843`, 3879.878639699238`,
252.77022062868934`, 0.19702879614057178`, 8.129123272011793`, 1.4896800872011862`},
{0.25397209407460797`, 0.7718183137480485`, 8.774125702249133`, 0.8493367748387377`, 0.629933366847081`,
0.44793398136834006`, 0.17220103999190545`, 0.007968172925746744`, 1049.3506020599693`,
93.83027794564788`, 0.034976329114162485`, 3.5404695212710173`, 1.4516591802815526`},
{0.04503017472676 6375`, 2.4891362382491593`, 7.362757592309742`, 1.3044006364876828`, 0.9149255196562065`,
0.40547780792622723`, 0.18724836348467627`, 0.040102604909543316`, 2100.7064218407268`,
212.58570231059025`, 0.17762315543370177`, 6.925880601095635`, 0.30534617107023765`},
{0.1552219340650659`, 0.8853243438823553`, 3.704192180484896`, 1.4225984400522793`, 0.30727783518964036`,
0.6899910723821459`, 0.23852460823207766`, 0.04807056691157154`, 2363.417831098286`,
24.86201270145591`, 0.056442694072345256`, 9.159332366722428`, 1.3338948458352062`},
{0.20103480965042153`, 1.3452512500000138`, 4.3677490431720685`, 0.877487125235695`, 0.3607930851168655`,
0.3984625684159562`, 0.053314676617049916`, 0.01650783971749592`, 3182.8108727507833`,
127.76026016049212`, 0.014515185076648118`, 8.964504972667974`, 1.3475969548800526`},
{0.2769245298620152`, 1.0085946471087137`, 4.542179183090196`, 1.0124324963510387`, 0.5561370973292907`,
0.2204460879068223`, 0.09699841861743363`, 0.4013041825941674`, 2543.8306625681216`,
129.04963561861496`, 0.06920814067274877`, 7.689149017779954`, 0.3201001440714819`},
{0.19504311625596543`, 2.4976887333066733`, 0.9780959245602254`, 1.4635414205075887`, 0.2583014895103719`,
0.4579159763055559`, 0.20087332417307124`, 0.20355826875644964`, 1388.181044794952`,
55.982899470557015`, 0.20280310820910474`, 7.420374461676447`, 0.562614521298161`},
{0.13592819841871134`, 2.9064950846732005`, 2.3581026922159136`, 0.7780848542747187`, 0.30526997799339717`,
0.3770830944311493`, 0.13314104598243304`, 0.28747750393724975`, 817.0224638364293`,

```
  99.04079463539466`, 0.1276940051281772`, 7.752046366250018`, 0.9291873586736861`},
 {0.09132185718513763`, 1.6890298787004205`, 2.765293267846525`, 1.131503269745704`, 0.5764776999808194`,
  0.5819767311690106`, 0.1370413367124192`, 0.18256196348098624`, 2411.1512688147986`,
  324.527748891113`, 0.027976143287822552`, 9.606504565586533`, 1.4766385859888795`},
 {0.2668975721045642`, 1.1689144747314444`, 8.555782168057934`, 1.3239106462159618`, 0.5762520317144466`,
  0.46812793372285433`, 0.21778203282682917`, 0.19497129015379866`, 2321.6608050102914`,
  177.91119737865392`, 0.06980210378448115`, 6.741021070773617`, 0.3841763241252334`},
 {0.24059659553061208`, 3.474305761400969`, 0.3551751508042571`, 0.7879508982528726`, 0.7115917578613755`,
  0.16553560182957539`, 0.059563521780316875`, 0.007818446748999992`, 3031.006140812062`,
  311.2165043893773`, 0.22179274663929038`, 7.521418478036052`, 0.4828099783575506`},
 {0.2655286560784189`, 1.7978740647108253`, 3.744260697890588`, 1.4979569598721256`, 0.2121339839068337`,
  0.5203547954080169`, 0.20358958391513882`, 0.3001328269718666`, 1853.7326098612984`,
  25.1210343423665`, 0.20564102192348216`, 6.431952930060138`, 0.9412816530341135`},
 {0.11579761780826803`, 3.5947224028634324`, 1.7024337214669405`, 0.9403811808729834`, 0.8023022872362131`,
  0.2690829028689966`, 0.10714511128523063`, 0.11270721526081176`, 1656.7229648447174`,
  66.45258485414047`, 0.04987530781728505`, 9.865292743118054`, 1.1705893328455934`},
 {0.23978880653880486`, 1.194922909654343`, 4.719390315894781`, 1.228676410453662`, 0.2690436202211013`,
  0.15286621224443908`, 0.10420745578996382`, 0.13476606932746013`, 2057.7110605479693`,
  394.7187402510973`, 0.20560140619917516`, 7.632899208080902`, 0.8870899800709835`},
 {0.14115594725086872`, 1.0548987126390301`, 4.221995326207644`, 1.1742416630307742`, 0.9684670314689854`,
  0.2982166752619343`, 0.232586446320313`, 0.2439177749163077`, 3034.8452920870423`,
  398.0846179891778`, 0.1346647166413732`, 6.263806688157171`, 1.2365678782206118`},
 {0.19091547503659084`, 3.8724415822010574`, 1.7819835666756205`, 0.970240031108671`, 0.6126075063757175`,
  0.3126984772797464`, 0.17470342972606634`, 0.006235187142203773`, 3131.6216914066945`,
  328.479620734891`, 0.10630619937563857`, 8.167239786634365`, 0.8931338365031225`},
 {0.1729303989394022`, 1.7633209846264775`, 4.719997292494861`, 1.358984906012456`, 0.8429652151291751`,
  0.587790420447317`, 0.06264894796386378`, 0.017353544405872348`, 3524.820728982824`,
  361.1436357327882`, 0.017184717238444003`, 5.167166487729965`, 1.3598724575284078`},
 {0.1806934944296955`, 0.9977416627132909`, 2.873305835487411`, 0.9197375741494748`, 0.03558129425516987`,
  0.4888154998532678`, 0.06242566822825624`, 0.4327688666695781`, 577.8384758076331`,
  373.1652197475112`, 0.03824901809490494`, 6.041786978778951`, 0.10163020529956279`},
```

{0.05413140542514305`, 2.0680722669723384`, 4.8344183696420355`, 0.8194669559280681`, 0.2769337929060782`,
0.48015527013462056`, 0.18187780015894756`, 0.008280997540164719`, 3109.753891098221`,
77.01250512455277`, 0.1133305062638244`, 4.98724945291187`, 0.14526723437594202`},
{0.100462842594787`, 2.3014533321484603`, 1.3291106382168039`, 0.7767037741986953`, 0.6186202165209542`,
0.5591414823289833`, 0.13230502890488538`, 0.011503939527627875`, 2830.1764339684587`,
75.11206729916319`, 0.04992405177453235`, 4.899377436291939`, 1.144111391119885`},
{0.04855602650750329`, 0.6081046972703708`, 9.327171063477682`, 0.9416092665673466`, 0.16765715967906902`,
0.2590243352245839`, 0.2187512491754251`, 0.05460948171436536`, 1404.1776553137433`,
151.91892927077328`, 0.22495478161340088`, 3.6643606235348707`, 1.0355618722545916`},
{0.07918907579486661`, 1.9928853739387806`, 6.231134938054945`, 1.0170500506380848`, 0.5386394257050193`,
0.6572554793818131`, 0.08180903968242625`, 0.09102806961295826`, 3292.3299295864044`,
25.797137029704572`, 0.14701504075704036`, 8.245305783801324`, 0.46404074549033125`},
{0.08056415815759815`, 3.9535505406039233`, 0.5752240632800181`, 1.4853820342325343`, 0.15510043342374935`,
0.6600536355917208`, 0.1525299204594313`, 0.16813894995214293`, 3147.186246353149`,
370.3183465224665`, 0.19963532109395127`, 2.5689014931104612`, 0.8619285687304634`},
{0.2470446862474004`, 0.7616885134704638`, 0.987661776479948`, 1.418205334084091`, 0.19365902643767297`,
0.457070951657317 5`, 0.06398975270684754`, 0.008457878153676336`, 2687.6783620781935`,
271.5689565049082`, 0.13340639067828225`, 6.091940716408539`, 0.7457858481599575`},
{0.21332713902898792`, 1.1214424929874225`, 9.162265965223703`, 1.195968506251218`, 0.6194628623567042`,
0.6911884704772038`, 0.19705041057272926`, 0.35280397622564785`, 1556.9191271276404`,
81.28832183992904`, 0.22397741498275942`, 6.620834183658268`, 1.366777947793718`},
{0.25495798287734545`, 3.119749211986244`, 7.11103782773306`, 1.024996658386143`, 0.715060033499787`,
0.6413092223890626`, 0.1635833958172549`, 0.00714360523367534`, 2176.4539355050265`,
202.8994026859108`, 0.2178929688734338`, 6.062772582072755`, 0.23204754406386696`},
{0.13839247434786828`, 2.7262203960757274`, 9.158826513860994`, 1.4613001862440893`, 0.08417876673230018`,
0.5490480248818964`, 0.16559733844524815`, 0.07310698628337725`, 1278.2933939526329`,
222.03326298760976`, 0.23267447938843522`, 2.718547094516405`, 0.8865837550934321`},
{0.21091599252169352`, 2.726329060939385`, 6.86461568422051`, 1.1885210188613522`, 0.09348887154977148`,
0.3177710803820454`, 0.16213356559574865`, 0.08883572559161597`, 2040.1598914696724`,
141.3083228416025`, 0.09021913069336979`, 2.444490167264778`, 1.4613873909313537`},
{0.0736068126313521`, 3.668322450041937`, 9.040034492942976`, 0.8406840844419607`, 0.9489073810268334`,

```
  0.18492930812213693`, 0.07953103883609952`, 0.21231255359096723`, 2489.458988929943`,
  66.10862660280799`, 0.025206551238390773`, 7.73776692806604`, 0.6102159281560899`},
 {0.24859051354510608`, 1.2059184673508376`, 9.814154373421665`, 0.999806271433672`, 0.5018863929556321`,
  0.5028910758235472`, 0.05474457996089627`, 0.010244417191277673`, 1992.5389939670486`,
  81.33759966126644`, 0.05634540009721645`, 5.237660071402415`, 0.5456697726165687`},
 {0.15548452904006277`, 0.7941888445067193`, 0.96068281269703`, 0.8969171753329834`, 0.8390319554716188`,
  0.17764885192591462`, 0.10783704631792568`, 0.4257693977926573`, 1410.5075470475076`,
  21.97142523719117`, 0.06053827716094551`, 9.545575914809223`, 1.320581285010927`},
 {0.23828594722598612`, 2.796383207017321`, 7.808028439446116`, 0.842565467286883`, 0.02113404997076329`,
  0.6079237099359636`, 0.11031479903153951`, 0.30382867676630676`, 3734.3994148280126`,
  266.6055228534507`, 0.0826889096493309`, 9.854969604997791`, 0.9421568133509814`},
 {0.25363168051984797`, 1.914628055312483`, 6.622889612666614`, 0.7775608926930352`, 0.3045254664674747`,
  0.5186902253252116`, 0.23177406235661663`, 0.009523543254042525`, 2339.914222113791`,
  312.39264418675145`, 0.06116037168819993`, 6.862836561311433`, 1.3371389516738463`},
 {0.11287267839860893`, 0.6274623554262155`, 1.4782831154053753`, 1.2121235395747008`, 0.42002460109961204`,
  0.37694632007071904`, 0.10874502522899515`, 0.13201309589288884`, 1491.5919687682626`,
  61.86932965722724`, 0.04486960703076642`, 6.490904378609384`, 0.9946688583085637`},
 {0.2430762813238787`, 2.3310866531539265`, 1.0183842540902877`, 1.0634798069102733`, 0.11637048805058381`,
  0.6527039790628004`, 0.1653633422639308`, 0.011869416806071493`, 3558.3378742172754`,
  124.12326765089796`, 0.15620828250230562`, 4.139014546068248`, 0.5423323870297714`},
 {0.12740998379951485`, 2.0965772202079593`, 9.26399010812127`, 1.4391840070681714`, 0.39059961846309776`,
  0.502473290684485`, 0.2058431543840883`, 0.008446302531916432`, 3264.970078645587`,
  50.15257581792508`, 0.04320568862225377`, 1.5793394493087352`, 0.8400374654569864`},
 {0.2006523908963831`, 1.4235542095429166`, 8.760081949765812`, 0.898891491102243`, 0.20916515630212307`,
  0.606680748263352`, 0.1734284929050921`, 0.008390530446632872`, 1251.1518948117655`,
  294.61074552228456`, 0.1942512237263747`, 3.244161217546491`, 0.31687231038469954`},
 {0.2188265776716416`, 1.1127955725409375`, 6.7824679934503616`, 1.4242069861528708`, 0.19844568020247255`,
  0.5897838311529561`, 0.14866055702548336`, 0.11878670475760818`, 3389.027367705158`,
  288.45426002597424`, 0.06947315366014212`, 7.544154049880284`, 0.26623655067609353`},
 {0.09304029298502164`, 1.718583593389087`, 3.3604210127127847`, 1.2870363565175618`, 0.488105058647589`,
  0.628165881843018`, 0.2385374614927307`, 0.005700260097503202`, 2964.7790422043363`,
```

284.02452311663967`, 0.04992977188540681`, 9.399697265119372`, 0.1891315432188958`},
{0.1372470868668202`, 0.5235381747902137`, 5.631494663469729`, 0.937247593354749`, 0.03802747675264895`,
0.555726088455389`, 0.17904217377421067`, 0.005332568756650943`, 2773.350295110703`,
213.09690601297643`, 0.061598067845519855`, 6.563446448883308`, 1.299385122835837`},
{0.1326843363181519`, 3.8211461387770695`, 8.543417929499473`, 1.102470057077619`, 0.6181175624574178`,
0.32965325202173645`, 0.07539452320036058`, 0.007386442292018944`, 562.3263637037148`,
31.555160873736668`, 0.05487832008430005`, 6.002761669320297`, 1.041478797158169`},
{0.22025109593540232`, 0.8195100379779809`, 7.427283096510775`, 1.0066916952384835`, 0.042788382562981786`,
0.22140131473144997`, 0.09976052534424382`, 0.3169961605914291`, 1982.7909761773044`,
250.35193827375224`, 0.1491805579320653`, 4.623227908408321`, 0.5222292355158549`},
{0.1573792753543678`, 0.7169949173367489`, 0.7718194209824034`, 0.9372436231934692`, 0.09272278999885297`,
0.4028207043118923`, 0.14589558430857152`, 0.11971411886775803`, 3682.2389249814178`,
277.8795147586992`, 0.21886799519974814`, 6.424836122129667`, 1.423509413643259`},
{0.18899782585068592`, 2.4715170036459453`, 7.1946744158464355`, 0.9247708579288002`, 0.021153717979273567`,
0.5975157085148236`, 0.06082076754336166`, 0.3491165783662039`, 1598.0596114559885`,
238.85974939012692`, 0.11421989314530723`, 3.1171043316464786`, 1.3648370017589126`},
{0.26630725498764907`, 1.3537243132764054`, 3.4681976762260653`, 0.8182294329126494`, 0.5922927850334725`,
0.35114377894258253`, 0.24401965334161962`, 0.12295914082921328`, 3688.719544583101`,
187.1594487192641`, 0.18710725308822346`, 1.0160874849867394`, 1.302279127254418`},
{0.10674642605203749`, 3.74860921502051`, 5.369830496673435`, 1.1859139787437987`, 0.18962703041335294`,
0.5643238513377844`, 0.22288847642351867`, 0.016526944643235637`, 1801.300442728153`,
341.7838883552571`, 0.21833110166851838`, 9.013661833764864`, 0.39778302570712243`},
{0.08668509537236802`, 3.2156402873225405`, 9.589080153119365`, 1.1094271452742452`, 0.38296738962607746`,
0.3187881217442111`, 0.09471255988041424`, 0.04715614001519508`, 3816.5009707519202`,
190.70162191213433`, 0.12608220202713782`, 5.338078693011617`, 0.15622194317981242`},
{0.2778234194471715`, 3.6898619438208646`, 9.09826349254168`, 0.7771496857479276`, 0.47494449837227326`,
0.40530279224610744`, 0.24790715727254958`, 0.03660245395755471`, 1572.9621965794513`,
202.7146387762226`, 0.11027468006890989`, 9.901732606435996`, 0.25331571472189185`},
{0.055450231563250685`, 1.3594142714077613`, 6.005951115471895`, 1.0656701550312766`, 0.9603365707697988`,
0.4169951955673704`, 0.09937287247557686`, 0.03650907654126198`, 3150.102571227402`,
288.6133211175843`, 0.0673017768034384`, 1.580996224465487`, 0.5598870619846228`},

{0.18890773944419526`, 3.611362633522033`, 5.813075251756651`, 1.273103869446512`, 0.8898144869747651`,
0.6114578055859192`, 0.07139582948563947`, 0.027880925348167697`, 2889.9541028194217`,
350.19982759883226`, 0.08313976867192174`, 6.993039414663205`, 0.8874083489329938`},
{0.09328585740614403`, 2.929301480876771`, 5.799770741738055`, 0.9079806695024137`, 0.4314055581005425`,
0.32164171745260006`, 0.17247524633063527`, 0.16386897475111406`, 712.2588807999496`,
70.83959017853539`, 0.20999817663531567`, 4.513003403554821`, 0.1126618170824738`},
{0.06022915767996573`, 2.387138635828979`, 4.788528842745`, 0.9281538320395417`, 0.2752953410792893`,
0.5017610843522332`, 0.23773129300939827`, 0.14096915249034483`, 1499.4959807822306`,
322.9162482919975`, 0.15516909665873135`, 4.726500227282653`, 1.1616754765054393`},
{0.24946839754186306`, 0.6156533776470687`, 5.962248269725286`, 1.0862299419007784`, 0.6836705094026099`,
0.5738298940987708`, 0.24726310480002844`, 0.11371634775304798`, 3850.890468269413`,
155.80403877473225`, 0.1865059666282849`, 5.974825484126367`, 0.19524283912722917`},
{0.16738121319303478`, 0.840466372179935`, 6.567452802957117`, 1.3673648738160649`, 0.9708045560189591`,
0.43291645558985303`, 0.10721701325461769`, 0.4069236600033958`, 2609.682510998089`,
166.81040184723918`, 0.052553234892402045`, 3.550175474274951`, 0.3845611655536718`},
{0.27699847669745875`, 2.1853512734955096`, 5.84751172204828`, 0.9661484791489172`, 0.9868204791467026`,
0.40527218684546984`, 0.18624243293429998`, 0.043237932216039034`, 919.7606571301644`,
386.4267934374768`, 0.13084528987555377`, 5.85403217789433`, 0.6639489535590795`},
{0.19754063204381567`, 1.2678132216826858`, 8.502246050318615`, 1.3049855200762384`, 0.9149570338409301`,
0.5843100223668893`, 0.12148336049510372`, 0.006360783777788973`, 2748.553955173861`,
51.9861836865702`, 0.0949023054382408`, 9.034485905171376`, 0.4708246470769286`},
{0.04026554170616964`, 0.5302615276235345`, 1.524346923969097`, 1.4195352782408457`, 0.35050648466256606`,
0.19736961374591044`, 0.19256070262690145`, 0.16778953261543247`, 3042.5326446416893`,
157.0549085671795`, 0.10950358517591147`, 5.218120753168542`, 0.25613055846451904`},
{0.11555514134387701`, 3.2236896162066904`, 2.732079118025373`, 1.164987224676954`, 0.23114456633378633`,
0.6224082143049414`, 0.17910660716870297`, 0.49555341188081165`, 1154.204177573869`,
364.61723023266256`, 0.21380198259541727`, 1.1657297203283505`, 0.5170897309434226`},
{0.27279971271031717`, 1.1118456280045157`, 7.947060215794888`, 0.8164028833969883`, 0.5079045986266995`,
0.311953435377873`, 0.24456124910810323`, 0.015036338564254712`, 1857.2767207919096`,
90.80006961498503`, 0.07729824929242363`, 4.7437001414515265`, 0.238261523479399`},
{0.13808283652405223`, 3.3382876893654982`, 1.764700397616487`, 1.2575917791512339`, 0.24597284032716238`,

0.16023118424518423`, 0.11783424678280677`, 0.22421613327193346`, 2741.9690444157613`,
110.60741736270495`, 0.06748966623215114`, 5.495223824255797`, 0.7719457903315097`},
{0.2720572435314704`, 2.092047115388646`, 0.9775412869939437`, 1.361730496090756`, 0.8643513713503745`,
0.6446371862205038`, 0.07237861696827474`, 0.06459834622446187`, 2040.0072337232305`,
288.27046449548277`, 0.2127097516522497`, 3.2460958268505635`, 0.7031503645327328`},
{0.13280526554371425`, 1.6805260743084913`, 5.020140137415806`, 1.1878313111770886`, 0.7515564764604186`,
0.6825215664361661`, 0.0545109419053262`, 0.03202911588893443`, 2354.572550424513`,
215.4436338815351`, 0.062416682151146596`, 4.270435440493394`, 0.24902957081957822`},
{0.04136924877145362`, 3.2505996769323806`, 7.3279282523448686`, 0.890763406913394`, 0.44633950095226127`,
0.48929728316730614`, 0.184351500605828`, 0.4646336778944382`, 1146.503881369189`,
33.19464868615876`, 0.20060766821265197`, 6.423062520789202`, 1.3180525740121252`},
{0.12443960147958688`, 1.4681483834231432`, 1.2961197051090405`, 1.2383341395061327`, 0.20461077825222174`,
0.2180798495866123`, 0.13955102593271534`, 0.036090000549476184`, 981.8387463156896`,
155.4019202626156`, 0.2259516918970782`, 6.485966100174822`, 0.46818640769970843`},
{0.09633016509755776`, 2.8964637462473926`, 5.167447635911598`, 1.1556815734309027`, 0.8460405566030094`,
0.6768234428979001`, 0.20660641079063646`, 0.37899350311781294`, 2084.615204649709`,
251.12222995868854`, 0.0719093058983113`, 5.055969069135582`, 0.9365067275794634`},
{0.0668112344511611`, 2.714918834733731`, 2.085154017953151`, 1.4476779676976927`, 0.2539160749138807`,
0.4870680566357908`, 0.17330271142201725`, 0.24053269905137525`, 735.8247906168481`,
182.42768732112722`, 0.23388978376591213`, 1.775892168476009`, 0.1844693395246435`},
{0.18113629662628528`, 0.6738122810255058`, 2.859320071162905`, 1.31555093037265`, 0.36681457284995544`,
0.471103526437621`, 0.08814722965023356`, 0.04506210537052006`, 2186.7720797416923`,
139.59007135140308`, 0.07150479816488481`, 8.93352349425783`, 1.2069106988965403`},
{0.06896509664813616`, 2.160626722426797`, 4.988265718811416`, 1.2639821322192157`, 0.4392795364272064`,
0.4617851413606676`, 0.11584839231589822`, 0.2654092351920312`, 1506.9815785286419`,
346.2179045294025`, 0.0968394930023978`, 3.8794768609952577`, 0.5989898263674145`},
{0.1192842170105507`, 2.0587406052635338`, 2.982429260158481`, 0.9607554862496793`, 0.6846341105753171`,
0.4253089041287377`, 0.1448155559009277`, 0.03303667191414802`, 3625.499229568628`,
138.38440688618937`, 0.05121864534109544`, 6.262852463653789`, 0.9148148813580201`},
{0.19942002949430754`, 3.5293725272813257`, 4.475631886088314`, 1.3690551967616487`, 0.1535320198116885`,
0.586863827463982`, 0.20957264255775987`, 0.0070808196203240945`, 2259.133238896783`,

93.9809316080586`, 0.18155601470027005`, 6.2714522403203645`, 0.9597826662416344`},
{0.19290552010423068`, 2.5311022785006196`, 4.590275609347875`, 1.0183350360370953`, 0.6768490777669534`,
0.27037924456465456`, 0.13002648835247615`, 0.039251036869391874`, 2833.896145381659`,
40.558338114360765`, 0.02944272635318218`, 3.3740003853748863`, 0.42763776068514936`},
{0.16407676745668215`, 2.743210330542553`, 9.218463902446366`, 1.4442040388312085`, 0.8033045111109769`,
0.5898809766156388`, 0.14591263450208874`, 0.06903241777052908`, 3856.2015344843503`,
358.8528117340675`, 0.03175828336990505`, 3.8731976390854896`, 1.2881241509575583`},
{0.19253541459312512`, 0.9166337410286696`, 9.734161690686161`, 0.9309028594902833`, 0.7413366163896413`,
0.3490367975563471`, 0.060904155020362505`, 0.013080541604234533`, 2740.950745040873`,
120.34372569041233`, 0.17782424959038423`, 7.718689374811543`, 1.0556383976941355`},
{0.2344407889708734`, 1.4871626351148812`, 6.830413107063742`, 1.1276656092803836`, 0.7850884426482851`,
0.26642284093684543`, 0.1715246889391343`, 0.04980986785514341`, 2193.834713356332`,
311.9394292836438`, 0.1837203477628585`, 1.994457399163636`, 0.8876922575805768`},
{0.1574181424723891`, 2.4334976996958355`, 7.454513230702137`, 1.3609349886556783`, 0.33361805963053826`,
0.5460996944909747`, 0.19878306773728516`, 0.24115086657234458`, 1578.7144043122416`,
80.91704790184991`, 0.12351463962730552`, 9.357025478355425`, 1.447433410261179`},
{0.137802869718083`, 3.3038928286894196`, 8.205199135932347`, 1.1443333838214729`, 0.43666320558690463`,
0.2863993034286605`, 0.21178222433889116`, 0.37748074045027574`, 3064.1095742596744`,
356.28526904181854`, 0.15861739359359306`, 8.78386257360005`, 0.18434483317647987`},
{0.24756545924701062`, 1.5758450559815111`, 8.280763168619`, 0.7891779726588277`, 0.6872262965666405`,
0.4388878534659798`, 0.12835670237954216`, 0.07541218293771562`, 709.0120611967568`,
337.23881046719544`, 0.12174060791630043`, 8.605194748975368`, 1.4619068526165075`},
{0.13084693466492658`, 3.6481233497064993`, 4.2504273066825995`, 1.0067280367884681`, 0.5994607748865513`,
0.5813901581577328`, 0.20650373563828406`, 0.1827740239544238`, 3288.755010061308`,
116.07278762719005`, 0.07676913646394057`, 7.439949797465348`, 1.2495449290646898`},
{0.15289027955876405`, 3.4056340118853177`, 8.85602064850179`, 1.1900353929127663`, 0.49460846965690886`,
0.2120137239983716`, 0.13358418114947193`, 0.3517341695519152`, 1451.4695761117755`,
341.1785262828431`, 0.2218032559662697`, 9.743566224950836`, 0.41975629001252956`},
{0.10700117050359564`, 1.3986545196260032`, 5.134806342648034`, 1.3393940232844244`, 0.8110706849311933`,
0.31760122726745454`, 0.23195078300551503`, 0.016714409489937344`, 2707.277188066014`,
260.0392856094112`, 0.17990816902801`, 9.35690893256919`, 0.5478079740525017`},

{0.27215562413942285`, 2.0660135805917337`, 5.081795444601147`, 0.8142657507592871`, 0.26635598111633185`, 0.328163277836687`, 0.07966098389627496`, 0.17309045811215876`, 992.4120776836976`, 330.82978884166914`, 0.11630419854588847`, 1.9092525381665606`, 1.4920575883264906`},
{0.16724732499606915`, 3.8236591860031757`, 7.535496783883705`, 0.9286997421550199`, 0.15670002022620788`, 0.2590202034037111`, 0.17595862302525594`, 0.2753459074292645`, 1491.5245062190434`, 377.6558909388741`, 0.10962242795283667`, 4.774971062285175`, 0.9122667174222503`},
{0.1513970595453768`, 3.565269862926928`, 2.882233750328341`, 1.46147567899756`, 0.43028383944211446`, 0.5449527314944854`, 0.05024950045158358`, 0.013582696865047886`, 1199.944383613747`, 41.76183563022494`, 0.15753768337487067`, 1.280336274118845`, 0.8861263254663858`},
{0.054023975296500326`, 3.9030377038282893`, 2.6328389166852855`, 1.06539261997144`, 0.07270428031369969`, 0.6221832003428629`, 0.16473607550076202`, 0.3329139837617463`, 3016.8950283011136`, 162.89707817078545`, 0.17967527091419977`, 4.813593384225332`, 0.9924030037454137`},
{0.25659351647641615`, 2.0548774277019515`, 7.952450585873037`, 1.1700596392648208`, 0.24509849180489507`, 0.5476063706170384`, 0.05117766409060387`, 0.047107635368495424`, 1627.113553842868`, 255.20388289511038`, 0.18601650100638534`, 1.4517697640535214`, 0.19929851500937135`},
{0.1846136822622525`, 2.83222539763279`, 6.185815327147518`, 0.9985231745146829`, 0.7838598641352239`, 0.6970792682180826`, 0.05665196048496565`, 0.0105368043370621`, 1139.0438628747534`, 42.309712199780904`, 0.09405173581505571`, 1.6109366539180776`, 0.8793784843352515`},
{0.10141299379366919`, 1.6007716008532835`, 0.6398899685364796`, 1.0164701283406912`, 0.8225647972224088`, 0.6292458866768325`, 0.13571266692666845`, 0.07872745868282192`, 1791.1473855018166`, 192.97393158172054`, 0.14848279636744627`, 1.8821154571770222`, 1.1130294782612764`},
{0.11981560320968349`, 3.640754447753876`, 5.349606530517274`, 1.2002498449288461`, 0.9084970459884618`, 0.2543242557071712`, 0.166873467304054`, 0.28421934758961104`, 1487.006076738341`, 292.7577364926251`, 0.15406116741524772`, 6.480379092988072`, 1.4335012083087681`},
{0.1768569128079565`, 2.3909839842824203`, 9.326880330280886`, 1.4539501621465738`, 0.2185806450894794`, 0.20927947571084793`, 0.1254436846064726`, 0.4971843180024683`, 563.0454599592058`, 164.61314507136376`, 0.20371869506435597`, 2.3340090949236654`, 0.42101883446758315`},
{0.2133813191703517`, 1.451835237764156`, 4.809264415807901`, 1.4381107471220305`, 0.9545564566791402`, 0.6082425656359232`, 0.08609247081289534`, 0.07332616905477864`, 1657.6679333430675`, 311.00628516031566`, 0.15574013896641808`, 2.719528414156583`, 0.38330836635142496`},
{0.07679027731361865`, 1.6374208448422314`, 5.731071459295233`, 1.355982875935464`, 0.05181959233087374`,

0.15382155253913876`, 0.1281658546365993`, 0.05032482400738273`, 1230.193557333262`,
218.81347681784587`, 0.015802664299199992`, 1.3298614212710635`, 0.20143294852402427`},
{0.2370685516334804`, 3.237122537432753`, 3.9976792452307883`, 1.4537281576817178`, 0.8596265155383829`,
0.4350695278152106`, 0.13391759982092372`, 0.21967358388020922`, 1928.121818584993`,
246.10473403948492`, 0.1821805288264413`, 9.36511897288407`, 0.6656126516266203`},
{0.21885531656429402`, 2.687374858822965`, 1.1296170207345284`, 1.325784614121181`, 0.40511738093112615`,
0.5937209983207841`, 0.0879699618250398`, 0.34321707574230476`, 3195.5291529724673`,
92.2297421582175`, 0.21288285544599406`, 8.464472481658461`, 0.25864431062726`},
{0.14216552227340323`, 2.5649444615040133`, 6.744792074075217`, 0.7596927543658916`, 0.15436598317484496`,
0.17272955179378358`, 0.11731963299504705`, 0.1434376311009391`, 3629.5744652923077`,
39.43043872717277`, 0.05961813000749189`, 7.187412396441442`, 0.1404254814851862`},
{0.20003331751651454`, 0.9418735138273977`, 0.3780377423788224`, 1.1513921169618064`, 0.6645698632364119`,
0.4730945551961576`, 0.051565459379669804`, 0.1952333288925963`, 2322.1212203613577`,
230.2978627277754`, 0.10438353064420558`, 8.70346208169968`, 0.8526709835745097`},
{0.045463415265626916`, 1.0604864030915095`, 1.5457624129661234`, 0.9218745066798075`, 0.3077029567700027`,
0.3372897124352183`, 0.13368077049637672`, 0.019951884664535027`, 2805.850760096928`,
274.4324431552926`, 0.0964209850306606`, 7.937294323580787`, 0.5250555034364572`},
{0.09737410253356232`, 2.2163561335231643`, 6.975749882171211`, 0.9583917748679426`, 0.07040378529617852`,
0.6807220990959502`, 0.09176157414171682`, 0.09133640049629771`, 816.8955105959267`,
131.23291821104817`, 0.07570904922141436`, 6.1606408812314815`, 0.7251058774037329`},
{0.12981111976782828`, 1.1084267930609286`, 4.931023641639941`, 1.2438579943673111`, 0.8299266795906743`,
0.296336649166421`, 0.21441842262722777`, 0.013448184708505154`, 893.4222083111035`,
95.2241911833517`, 0.18109496842042927`, 2.912115357106975`, 1.136648183382977`},
{0.04881288196111078`, 1.5001688796472123`, 0.7565632194730121`, 1.206042774752989`, 0.6805558920404622`,
0.3640356697656889`, 0.08453170981106164`, 0.19820032009109562`, 3391.2112266676813`,
283.705794296156`, 0.20144392981632914`, 6.84055461750941`, 1.1851088923020137`},
{0.16605942412072794`, 0.6402554572286978`, 3.356342996009028`, 1.3219512159205855`, 0.7993093802210203`,
0.15372675446550776`, 0.10521895558502489`, 0.005367878364442133`, 806.3427040773236`,
321.0178159818362`, 0.15297743964506222`, 6.095134085467343`, 0.509077325139712`},
{0.27402590763234025`, 2.0480444129965374`, 8.367988840463223`, 1.356112416883951`, 0.549124019732967`,
0.6110139101654402`, 0.05091107534805153`, 0.12010047430936349`, 1627.3034033398817`,

297.4011443552333`, 0.1966061654681946`, 8.76675255073281`, 1.4917606486762143`},
{0.10823031336368771`, 0.8296257610813722`, 5.332629740125888`, 0.8124935731916766`, 0.40472412917038403`,
0.20010405592172198`, 0.09023994109265432`, 0.01008956636997111`, 1875.5688641041388`,
260.72528052100415`, 0.14449594942054067`, 1.5456312543093684`, 1.2237716091504276`},
{0.20273952773619414`, 0.8775156867465963`, 8.853127710010899`, 1.0632724405144907`, 0.5470610681912369`,
0.35564991449731365`, 0.24613003734690775`, 0.2102067712155018`, 2109.2588977466867`,
152.77073389943882`, 0.07849302645811645`, 3.0982028200464633`, 0.7906746942794358`},
{0.18840966187391878`, 3.686049426661911`, 6.507340210098313`, 1.3405973621357103`, 0.6314264717273861`,
0.6528641596796794`, 0.05949520733205324`, 0.005267163480527638`, 1036.4467237949607`,
79.36725884036872`, 0.16755847890105047`, 9.771579716601678`, 1.20892036427258`},
{0.1000404296450419`, 3.096045401845493`, 7.6799040120684445`, 0.803333640129631`, 0.6198933615423707`,
0.29060269426384966`, 0.18836459448376747`, 0.04739137692785461`, 3452.9247864535073`,
66.50695651270411`, 0.1918335051763443`, 8.136439035690735`, 0.9349024413134766`},
{0.16207753282118065`, 2.4074661619125743`, 6.439531987652202`, 1.216188209247578`, 0.5635250461042756`,
0.41870015151777173`, 0.15568766166521003`, 0.14566116758733688`, 2407.082097704033`,
343.2271284427226`, 0.14229137239354006`, 4.642019464618446`, 0.8431314645967629`},
{0.22160418402095905`, 3.3706460558066524`, 3.148818263883834`, 0.8683984407887995`, 0.063094397851442`,
0.5589539148331857`, 0.058740794885626`, 0.0138702175197992`, 2238.370585799582`,
370.4864263128153`, 0.21378440885525551`, 5.817374569517135`, 0.5948516756530484`},
{0.09473876647477247`, 3.4861652630821096`, 6.982828746666236`, 1.2832000030310025`, 0.6886348717794659`,
0.4656186082808249`, 0.150625912685666`, 0.03239358546984584`, 1673.775609656389`,
245.14838945542726`, 0.016860445211711483`, 6.847475541460803`, 0.5191626673367391`},
{0.1760690989521051`, 0.7026153943847899`, 3.261997951042007`, 0.993553588623698`, 0.646826501995915`,
0.3736019899194698`, 0.21533349413201053`, 0.25320537109656943`, 1549.1302534082051`,
389.4106290690562`, 0.0763719271638561`, 2.133344446029941`, 1.4125782678502792`},
{0.06662682417897325`, 3.243608945481448`, 8.237889478852154`, 1.2035188774949521`, 0.1279184849350048`,
0.6784394756208973`, 0.18789313451307382`, 0.086116484822983`, 685.6358133562785`,
181.08244593249674`, 0.08115584053395464`, 7.371846442202106`, 0.7077387695529302`},
{0.13921193952574745`, 0.46815880576040936`, 4.3864048931388595`, 0.7514079591567522`, 0.40824219063798206`,
0.5617438808850261`, 0.24339108461093562`, 0.020519348847045586`, 1920.4537040361229`,
81.58086121632874`, 0.1022235991904683`, 4.414511511279496`, 0.3646779989849891`},

{0.22961340343556502`, 1.6030194574854644`, 2.9811826554982517`, 1.0274834008587208`, 0.6451263943126482`,
0.5097595717714782`, 0.19295611967128257`, 0.020325995630318172`, 1782.352038334574`,
109.5991443386025`, 0.16872014474364577`, 5.851396867706905`, 0.6578614287996216`},
{0.26748619026781384`, 2.88696602814122`, 2.7118514018243634`, 0.9885856963040458`, 0.34646533154127157`,
0.2537466476440833`, 0.09945272546945017`, 0.07388745663205695`, 3603.2051356484208`,
34.51441620104788`, 0.19141821235831952`, 6.868891191349718`, 0.1593219725686645 4`},
{0.10551271069457996`, 0.6900129952362093`, 8.232394419645587`, 1.130400293700848`, 0.7046770328984258`,
0.2711667378009396`, 0.05003959656184298`, 0.014784496484154912`, 540.5369479440305`,
45.69515764102175`, 0.16942738870919372`, 8.057748743914669`, 1.27779344817754`},
{0.044118696420245185`, 1.9220692229229748`, 8.936584527376546`, 1.198231045412435`, 0.8133241613996403`,
0.3641703381601298`, 0.06715507154740682`, 0.009124556845180784`, 2953.448531772523`,
220.86580819426717`, 0.07551922906053443`, 5.11062195030733`, 1.275640621281854`},
{0.08620550491180146`, 0.4949942611001066`, 5.984234002717921`, 0.7531376788437371`, 0.21717148283204812`,
0.22262766010657997`, 0.11267502779798616`, 0.05995928028643471`, 1301.4568875111263`,
314.2464318877011`, 0.04884398840792192`, 1.2952896203900384`, 0.6666662921648308`},
{0.18748668988618272`, 2.2339195778761853`, 8.488097011441944`, 1.47468717511323`, 0.23541263239484844`,
0.20987853326618222`, 0.07676134969948037`, 0.061849152465116755`, 2541.5254126664167`,
387.5680307693225`, 0.09509361772923897`, 9.161664648477608`, 0.10551879857006874`},
{0.08696471520703919`, 3.613913666113315`, 7.0626231358127285`, 1.3208938988261034`, 0.28024078243683714`,
0.37947312403263656`, 0.23429714063319607`, 0.06281923284932987`, 2203.521948663617`,
29.0776175939651`, 0.14152777414422596`, 8.483647124571803`, 0.7235011997593546`},
{0.09172278717373272`, 1.2157183406525425`, 2.2812242417200608`, 0.9103021242940673`, 0.7524885408365471`,
0.5328408138361465`, 0.11958813531559015`, 0.005068237864616913`, 874.3804057883599`,
319.73415041813746`, 0.21547584642190498`, 9.166843855230969`, 0.9284914101950568`},
{0.13868488683280167`, 1.226702016867053`, 6.040570905634304`, 0.759875582646631`, 0.27881848833013434`,
0.595446367026503`, 0.23094105751878413`, 0.2051375441364418`, 1528.2920750506055`,
270.40251576187586`, 0.23385239775529226`, 1.5893916227857634`, 0.2397907655262943`},
{0.1306914623455918`, 0.7537584653459817`, 0.21965733160577017`, 1.2102680433379889`, 0.2532343966945656`,
0.6654185660627909`, 0.08400179279005554`, 0.3103404088337731`, 3637.61631387517`,
133.45404753770885`, 0.037164269643724435`, 2.2620412269050973`, 0.956581036605332`},
{0.26277447700446094`, 1.2458735546481199`, 6.447910682321009`, 1.49065802757454`, 0.9176757405073024`,

0.4907046225412167`, 0.1593717844760214 6`, 0.008327156523724668`, 782.8427424300617`,
244.66740068612717`, 0.09440964708909416`, 9.330818440145894`, 0.2131775359264001`},
{0.13025097964415272`, 1.0189597502038827`, 8.886210776319402`, 0.9111028640704337`, 0.2284753731773581`,
0.5590994693246798`, 0.2266682667598287`, 0.006106972079947388 6`, 2065.306144054668`,
113.92755603976332`, 0.14827318077057916`, 4.714739506212668`, 0.4593044246185465`},
{0.09142437343902587`, 2.458407615666487`, 5.031014986619411`, 1.0519598677125233`, 0.487417438430646`,
0.4280564058665117`, 0.2026543236563062`, 0.1589331435911828`, 3989.0362964026026`,
249.7709598067779`, 0.24152899778887077`, 6.187241064247978`, 0.924037240334326`},
{0.05362058570834477`, 1.4723855364180096`, 9.67162648709779`, 1.3845527897944452`, 0.7224375135481673`,
0.2670471970549346`, 0.10991701130195836`, 0.006877132758778975`, 886.1772845072878`,
273.92553716604675`, 0.12489313995376372`, 6.85687873202156`, 0.7259468075744833`},
{0.15232520315695658`, 1.456608465380997`, 9.96731462021549`, 1.4944244110861535`, 0.5157767046786654`,
0.3722073718864378`, 0.22976562023274222`, 0.07428975878835796`, 1050.2078985192002`,
33.60662262776219`, 0.220518601124694`, 7.093566043209907`, 0.6560171292141301`},
{0.0694009118290565`, 3.678178592599034`, 1.755843032698575`, 1.2142042066341803`, 0.3177757036060529`,
0.6847455380724545`, 0.08295711309717257`, 0.10240501118816868`, 536.5981094108779`,
211.10249067816517`, 0.18136782974370297`, 8.788955479280464`, 0.4777460308714221`},
{0.16483058262413092`, 2.446957095238485`, 8.164140625789859`, 1.1581574304294189`, 0.8637023907646861`,
0.5910929885256712`, 0.17429603155004098`, 0.036201628416469234`, 3040.5552834584214`,
70.89960300369222`, 0.19751315642431794`, 1.918185462218652`, 0.5717895645919415`},
{0.26384722865028404`, 3.0959478641925884`, 9.455492188682882`, 1.339136584268803`, 0.7399277527624639`,
0.3172867286222486`, 0.06239618913281497`, 0.23953121953891282`, 1016.1140721617635`,
88.38662693497645`, 0.16528145715165937`, 7.22137987568804`, 1.361790640282516`},
{0.17041949742674112`, 0.566658425910223`, 8.176154533792225`, 1.187496781729836`, 0.9612002380635534`,
0.5389795298813838`, 0.1929826430211613`, 0.013244827072013646`, 716.151240381786`,
217.26337969751137`, 0.13800797665502906`, 7.448468008162791`, 0.8606351443610007`},
{0.04627776053028976`, 2.3407537273567067`, 0.9761597154152462`, 1.37527944112939`, 0.3584342070422313`,
0.22640865017403333`, 0.13240363624591456`, 0.038325642841679504`, 1249.254134864137`,
143.93359215534173`, 0.13242235789061851`, 6.269547025830331`, 1.0591127938814697`},
{0.1264814146333596`, 1.0630284736090054`, 7.0368315732194056`, 0.7618799049872292`, 0.05995838274087717`,
0.17364177343291576`, 0.06194914835661871 6`, 0.006022075969931666`, 3448.0521144594068`,

47.239369625587926`, 0.19227473651596427`, 8.592453879074284`, 1.3325684049534696`},
{0.04081744499533296`, 2.3413945804849527`, 2.3401462602509717`, 1.006052431149826`, 0.9376889728762128`,
0.63499912288976`, 0.07105146864527681`, 0.02628017367597553`, 775.1156602545625`,
104.41750831638893`, 0.12180266898464448`, 8.067006250284603`, 0.5522058225859034`},
{0.22961450923300392`, 3.8785966005678736`, 7.959504018056748`, 1.2921046945277759`, 0.1917613861571974`,
0.3981097458569077`, 0.1102143743054892`, 0.20929581166220373`, 1753.2557317520059`,
231.02169920905055`, 0.10007865476065686`, 4.452819250691443`, 1.402721807760341`},
{0.12232907903298573`, 1.3289405346143397`, 5.1200974038340075`, 1.1450551183026931`, 0.6675798963164832`,
0.5719242545987869`, 0.08761535581144905`, 0.11107132883791111`, 3964.0786866225717`,
279.32313661047124`, 0.2162046831451349`, 8.020270743802456`, 1.0641453807394075`},
{0.11461934044260397`, 2.0495668840197947`, 1.6301061314690006`, 1.1441247153256733`, 0.9293434036316242`,
0.32161099339559507`, 0.24691379277403247`, 0.09383957046935675`, 3183.5471916851793`,
210.97634406977602`, 0.19279849145037242`, 7.844330233274125`, 0.7023420045147568`},
{0.10880423027814301`, 3.010977164978767`, 0.4477158763471074`, 1.3560425240667602`, 0.3025579540978818`,
0.35180253133086303`, 0.2109756008586861`, 0.03552671363577697`, 2668.2716874123935`,
170.59395733846873`, 0.23332643955483107`, 9.954767028855915`, 1.4592401570775992`},
{0.22749339290790638`, 1.7311095241107948`, 1.2544972871049804`, 1.3263167066853254`, 0.7333948539753357`,
0.4493142943490447`, 0.2441212061859469`, 0.014870392763137733`, 1580.7600138635335`,
264.3754143437418`, 0.09646148923631659`, 4.446033883263464`, 0.6510175252381905`},
{0.10680853367942073`, 2.671856809998241`, 2.225956166276376`, 0.9564945044055834`, 0.04023151222298815`,
0.66439395337897`, 0.05090336466790368`, 0.0058123339013462704`, 3923.759723431366`,
155.48006966286152`, 0.1590662885693253`, 5.07114362920246`, 0.8193607123659179`},
{0.1306588579678935`, 3.206018430436262`, 4.178329357094787`, 1.096081964467313`, 0.37321063526655895`,
0.6088846209535219`, 0.18229329291576957`, 0.013520486536436158`, 782.6656351062597`,
123.9415631086822`, 0.023548806194033767`, 9.649188633359959`, 0.8429625600097688`},
{0.06490925302735506`, 3.8934906583752236`, 0.9790395136780409`, 1.48284195083516`, 0.9349692905507263`,
0.3703362249698605`, 0.1491469107421124`, 0.13667677484236057`, 1004.3258994396238`,
82.66219089427364`, 0.1407191619667938`, 7.134069953930672`, 0.6810640411318756`},
{0.09335758271301797`, 3.886141513910534`, 2.1262193060875347`, 1.2374865217449704`, 0.20774186356921476`,
0.6885354117013798`, 0.06828062526335338`, 0.04634612342186317`, 2337.7355280609045`,
265.47200855531526`, 0.06367324855715212`, 6.746431099417454`, 1.2698799762745048`},

```
{0.08231211479941364`, 2.387884906790136`, 8.578001165552415`, 0.9642461558703308`, 0.8238919598475782`,
  0.2020584877079309`, 0.19955393113038733`, 0.1095787053029616`, 2239.622315346988`,
  371.5987310561687 3`, 0.17019241214364111`, 3.55759780947197`, 0.4666380334441058`},
{0.17519323235207396`, 1.2618694118815563`, 6.2707952492510515`, 1.261446257781183`, 0.5333912181747638`,
  0.18672075814011158`, 0.09419037155450471`, 0.010359125778073429`, 2462.605666629158`,
  256.6607054858181`, 0.10840920502450685`, 9.870044854094623`, 1.435459480935227`},
{0.23749378089306467`, 0.4157158512844461`, 5.854529102702003`, 1.3110653325714712`, 0.14508930723778435`,
  0.1573757215384779`, 0.06204301966356823`, 0.16029852320715812`, 2190.57271228382`,
  132.40666721155492`, 0.12799517810137861`, 9.84238745503276`, 1.14600445718771`},
{0.06958654560186628`, 1.2810573894719157`, 1.5604323721050744`, 1.2865370396261684`, 0.41858142719439173`,
  0.3987961369914276`, 0.21356300651181537`, 0.0062459309627175864`, 2941.8823280388497`,
  383.1127710935082`, 0.10442332143534439`, 6.649327249023878`, 0.3474394649692636`},
{0.12162705978453553`, 2.1459364566550487`, 0.8994451186914905`, 1.339976475017456`, 0.23917524394620604`,
  0.5256296133560587`, 0.21370599200931129`, 0.027381167717735945`, 996.433258066003`,
  270.7208781713538`, 0.090253513817738`, 8.675427486273929`, 0.7193288794908814`},
{0.2211475447831816`, 0.6764621696057107`, 7.070732773871981`, 1.484318092913942`, 0.39213098277018266`,
  0.49361758237854214`, 0.08219155305388848`, 0.007529395154295265`, 1570.1936066911949`,
  345.9142373695621`, 0.2485313792002623`, 1.2269777403584339`, 0.7159550405850554`},
{0.1091983534434775`, 0.4123168888384443`, 1.8612158499296871`, 0.8333258155688079`, 0.39517409251864644`,
  0.5848465884265365`, 0.12470223152921811`, 0.040839984644619944`, 3265.644855437202`,
  125.19293803224065`, 0.0879734848590002`, 8.526937482297015`, 1.0338521843728818`},
{0.1605397117399036`, 2.9861451600751723`, 5.985367280571584`, 0.9013600575793626`, 0.4265711037361093`,
  0.6369377322655665`, 0.2099395794597424`, 0.0440322954061731`, 1331.4501176292097`,
  178.64680303201567`, 0.0465601985112431`, 1.120746116836358`, 1.298216136515848`},
{0.2316510553565545`, 1.3345287101431156`, 2.181207240226371`, 1.1295787756210554`, 0.4108038804094787`,
  0.55044788544399`, 0.15705941808893342`, 0.0693266889389808`, 2764.047895868124`,
  88.55030846959835`, 0.19804190063549815`, 3.6671215326087623`, 1.3965489185868663`},
{0.23613672937320296`, 2.4419271800403806`, 3.4588792688353487`, 1.4108864845746094`, 0.7134807082114705`,
  0.592154370313409`, 0.22091066377388413`, 0.019900714899270838`, 1522.4223604141134`,
  78.60644958620696`, 0.18909869538832608`, 2.793726035246588`, 0.7950281959201368`},
{0.0484675208754865`, 0.6825441464113089`, 2.084792805230027`, 1.4119489784121344`, 0.5877996575498201`,
```

```
  0.6759892639478722`, 0.22086781309234377`, 0.0387732952545585`, 2548.5220980600216`,
  370.2048073952212`, 0.03857632786812504`, 3.327588495854709`, 1.4128853163273263`},
{0.15820801401395784`, 2.3900304014986444`, 6.76991723319799`, 1.147995471223545`, 0.5011235356714243`,
  0.39186104654424503`, 0.19387948138295608`, 0.05551860155117706`, 723.5959050648971`,
  29.172220754504224`, 0.17373406698962968`, 6.019570931242669`, 1.3786373610317848`},
{0.07579699484940727`, 2.2173315933991757`, 7.471866642915501`, 0.8771923938554881`, 0.8171799463467477`,
  0.18907166793764196`, 0.16741258135296538`, 0.034704832899603626`, 2559.9885855263465`,
  365.24466351817625`, 0.21912261270377126`, 5.637070181749923`, 1.0559796296250288`},
{0.06741497368958249`, 1.0249716767004005`, 9.449217531688483`, 1.0872407203117702`, 0.5615736740328037`,
  0.5521415654810891`, 0.18288037923286848`, 0.005326201838481523`, 3978.4896243421163`,
  351.6470055302269`, 0.09021448343705185`, 1.426476556917251`, 0.13651689047774385`},
{0.05715322142426921`, 2.4704612263538817`, 0.36234457179092416`, 0.9684061618557824`, 0.863166347709152`,
  0.23804186668051197`, 0.13749867219091239`, 0.1430597139428923`, 3758.60599725621`,
  74.20395287068635`, 0.1109041971133704`, 1.6071731513826233`, 0.773390027344468`},
{0.060904719608809554`, 1.0227281808325657`, 0.3338146022683226`, 1.4161209446138403`, 0.8561719858721175`,
  0.4775008498746448`, 0.07550639206271559`, 0.17875509724795088`, 3800.334358301937`,
  331.7811845820245`, 0.1313746491970903`, 6.20009884490432`, 1.2664955877170798`},
{0.09804650260585268`, 0.6377788755200506`, 9.103001426868161`, 0.9608072109179064`, 0.12522756138173952`,
  0.16039700353140673`, 0.1476329494772725`, 0.03181180155196076`, 3994.864550131647`,
  201.1651941507272`, 0.22810874694301092`, 5.8973245769190275`, 0.5910947751289481`},
{0.10576408085084482`, 2.4399851863533764`, 2.261754328254943`, 0.8217420902241768`, 0.7416397192385102`,
  0.2463678861595856`, 0.2027535408226292`, 0.2140679841559756`, 1564.164909651323`,
  70.02516408668095`, 0.19509596638498505`, 2.4981390117767504`, 1.4295314398093057`},
{0.18148677226867904`, 2.435655182727153`, 9.932013575043552`, 1.3523612276244852`, 0.270501525518553`,
  0.3129668058670274`, 0.09479199345181186`, 0.24780135341453666`, 2571.145269598781`,
  147.08034915528242`, 0.05706779714237542`, 9.481295153852962`, 0.5023669027433282`},
{0.045935603836807504`, 2.302483508903091`, 2.46282437804547`, 1.0004914316838749`, 0.17651744242150835`,
  0.27731852138484747`, 0.16499475989489315`, 0.14699844290776085`, 3995.605017809292`,
  299.6167282278376`, 0.2482978016224947`, 6.149511597608322`, 0.9467803390801968`},
{0.24610403760574778`, 2.8350518587284927`, 2.483700295848834`, 1.2187293428205228`, 0.8051620910181194`,
  0.287775756384263`, 0.0943064533423209`, 0.11494360430719369`, 621.5470695356762`,
```

130.28571465739265`, 0.12477806190504986`, 6.74453372259727`, 1.1595275933030642`},
{0.2282743224553333`, 3.8004017582328853`, 8.466984049452861`, 1.390127484711968`, 0.35687133565138884`,
0.6519422926243725`, 0.17290926755870883`, 0.3014446142578311`, 2372.838415127524`,
111.84440462244766`, 0.08570923572829736`, 3.872670068176556`, 0.42950313779436455`},
{0.11402356544669451`, 0.8158491547123496`, 4.200202309736193`, 0.971641321517407`, 0.6807220168616288`,
0.6359800608186865`, 0.13923173061454314`, 0.037851089653330894`, 2281.1440386375243`,
126.7093813407077`, 0.11913552316776838`, 8.934189056957887`, 0.5820810660938738`},
{0.15189623977997108`, 2.7915541399359975`, 9.913479700081336`, 0.7741019472122475`, 0.15478570831213023`,
0.4331484807185151`, 0.23125927282289244`, 0.006529023247839043`, 823.7432504407011`,
34.92144293228938`, 0.20656993466421963`, 9.240164591756866`, 0.7039592626126989`},
{0.19262437700106766`, 3.938833744371137`, 3.6810076480536082`, 1.109072783031779`, 0.7645216483353034`,
0.616702884118477`, 0.13777110888715882`, 0.010064854238404494`, 3438.969205276325`,
98.49201035779356`, 0.20668453166808726`, 2.7590741497101465`, 0.5477570556056697`},
{0.15161686088493992`, 2.6676538201668016`, 4.769091008698259`, 0.9792911695535791`, 0.8799626502245399`,
0.5483041230008869`, 0.08973546330141513`, 0.2940210093980764`, 2792.4321626291066`,
123.07394825008441`, 0.21143511654972236`, 3.6673259692190108`, 1.1506144153300002`},
{0.15928964461807182`, 2.676103796250932`, 3.0276164230907305`, 1.1887325681254923`, 0.14578762490346686`,
0.4353007161971356`, 0.22317821296952212`, 0.2148623762619366`, 3792.9286804177964`,
76.64814683698182`, 0.1595894948400946`, 1.5825099373005287`, 0.48932638994153477`},
{0.16818423551071837`, 1.2425750232039041`, 0.8451308809778482`, 1.0511110147264935`, 0.32337126733921173`,
0.6302171747780823`, 0.06186616786607688`, 0.012735137580066362`, 2086.5412121340623`,
307.75958728157127`, 0.20485938116823293`, 3.379450004526051`, 0.9974668662640704`},
{0.049399735586138194`, 2.5477631442089104`, 4.041803486797548`, 1.3304361527101167`, 0.993281383326639`,
0.3001497243237138`, 0.21152425523664786`, 0.4874448677287525`, 780.8123462023354`,
361.74617623884797`, 0.046928831923920444`, 5.346338091727542`, 1.4411222226599376`},
{0.20618269294314817`, 1.8587454568359414`, 4.34452965291473`, 0.8960498853155586`, 0.6640333303615127`,
0.5390503581736582`, 0.2329294063957692`, 0.4180316503514536`, 633.3353428142332`,
244.55036900145114`, 0.12942298196931046`, 3.095308470158429`, 1.4194968692598873`},
{0.09869674443849347`, 1.9183657949501018`, 3.7344926730748456`, 1.0943839787542378`, 0.42780632576683675`,
0.5861386946846783`, 0.14629063580280066`, 0.03398060371034166`, 2435.986502306805`,
302.9804614286604`, 0.13730947981168928`, 9.171543304681595`, 0.26064124405682665`},

{0.1902308570197676`, 3.972029979384046`, 4.634110792119705`, 1.2197488970650956`, 0.8492525180509605`, 0.5006542712905349`, 0.18652832755791343`, 0.005076576077217329`, 1943.0673920285199`, 83.26055229551639`, 0.05802581167056625`, 7.38223678334383`, 0.9931576433164864`},
{0.06272312309945755`, 3.461529579186063`, 7.564878788392824`, 1.3521314303721368`, 0.32784603357531017`, 0.6503539369626794`, 0.11129074624350982`, 0.1957733709006163`, 2103.0882520472996`, 262.6269091211209`, 0.16330910334057364`, 4.05183701830078`, 1.3383335426462928`},
{0.1334851760745519`, 3.2697077297961004`, 8.047111995239536`, 0.842079209613761`, 0.8088703615622166`, 0.5692447244516433`, 0.16235903984142924`, 0.09701377075706732`, 2643.3784916344785`, 292.46187756743905`, 0.08266433823104813`, 7.391813212062153`, 0.547699130297068`},
{0.09401709418411008`, 0.8110971536952984`, 8.245292807864445`, 1.0264842776394885`, 0.12930370633004684`, 0.1740236808265636`, 0.07252752048975858`, 0.09884601796368213`, 3866.984141926244`, 274.6428268585172`, 0.20040469873166988`, 4.952717873499136`, 0.15734837909658306`},
{0.16503822022515502`, 3.799827999093722`, 2.226332530621681`, 1.4474524276394465`, 0.4523982192034186`, 0.1981242269935809`, 0.06722881851519197`, 0.02132348079412978`, 3790.4878202500677`, 323.4156208714485`, 0.10032103134111592`, 5.6566553483532305`, 0.498005589518143`},
{0.2175565696926961`, 2.150769749820693`, 7.849181160138611`, 1.1076816333069537`, 0.23775832216164305`, 0.4289452843039179`, 0.23138213560150006`, 0.007541689166030151`, 1830.5976676958226`, 333.35844770130507`, 0.13740519457686395`, 6.97796965521683`, 1.0770120195809052`},
{0.04261251981259187`, 1.6956083841270555`, 8.388949518118906`, 0.8816084343593602`, 0.6157741880137009`, 0.15816307961776666`, 0.1228282175848856`, 0.05282284640298743`, 1555.166107990478`, 26.393755355375106`, 0.19116960681111228`, 7.474128794459224`, 0.8297590847908434`},
{0.12052689532243871`, 2.1969784256629277`, 8.110278600920065`, 0.9177559121464718`, 0.6615840119954921`, 0.34704707074624996`, 0.07379725368855697`, 0.00573204546098014`, 2320.908483262354`, 384.0513502933301`, 0.016004877265817924`, 9.641293645315589`, 0.17473869996337155`},
{0.2044156153713736`, 2.9629304518198207`, 4.386784427445061`, 1.111833791211073`, 0.9591350877217695`, 0.43840802875011997`, 0.2469979148221132`, 0.01642865649440784`, 2743.650369521729`, 173.03541841863364`, 0.08406686391515611`, 5.337297453020938`, 0.28953137005492313`},
{0.12469439922923026`, 3.2167561610705597`, 1.2966549629505941`, 1.3380513288544775`, 0.9647032506590352`, 0.3520228082837078`, 0.24731936603055854`, 0.03725013837412559`, 3227.904692724528`, 130.80077454497865`, 0.06370555142915824`, 7.153312146366998`, 1.3824196402345823`},
{0.0769260990011068`, 2.196578391656532`, 0.8056525760561577`, 0.8210657722273409`, 0.8826879394098923`,

0.3537183279427859`, 0.2409594091636174`, 0.049834595534127876`, 2188.2120164663`,
216.0992703121101`, 0.032694389865458584`, 6.1007061352710465`, 0.6801916687054326`},
{0.09180583003794474`, 1.0245902356282572`, 8.242121052091868`, 1.338438298182017`, 0.7912235553845766`,
0.4563538789330911`, 0.0736594669866128`, 0.007188071678387363 5`, 2109.8793350532806`,
273.6588416089354`, 0.0378211628956065`, 4.589600480914077`, 0.9767739503530284`},
{0.25211089910042056`, 2.9465650077307215`, 6.640985006189094`, 1.09304531251437`, 0.4582346106456783`,
0.5101745978688277`, 0.23290591035097613`, 0.10611238144646964`, 2453.147250432534`,
22.888736398204685`, 0.22056464944244836`, 3.2852146146779617`, 1.410915433494099`},
{0.10187608814553512`, 3.02527275016552`, 8.112504151515616`, 1.0430646419869034`, 0.02000177204588316`,
0.406606876656344`, 0.10224573377309781`, 0.465955444901958`, 2596.0557042261407`,
108.81287726186605`, 0.13773652673586545`, 3.785014828823544`, 0.37908929500916355`},
{0.27609243280065304`, 2.4334000704041427`, 3.768968853050694`, 0.9897634681270504`, 0.04043037685648332`,
0.4200152717945378`, 0.22935860430417082`, 0.1525987200218085`, 3069.7266486703747`,
262.01073398400206`, 0.24155932723410417`, 8.399632627499091`, 0.7132175733358337`},
{0.0978918800897613`, 2.777864894021529`, 4.606090716083992`, 1.4880029918673265`, 0.6064208129951822`,
0.3601049854666406`, 0.18842765361648817`, 0.044059072442558106`, 799.5331038282079`,
182.92623376411473`, 0.07894991488107306`, 2.020622256243092`, 0.5601073680228721`},
{0.08900054818761971`, 2.823318859242785`, 7.144670702377984`, 0.8315383304970898`, 0.2795052665240947`,
0.4576393886321606`, 0.05117478065859138`, 0.06552086595628574`, 2102.911960214631`,
101.67438536233578`, 0.08762396482794987`, 9.096737685205667`, 0.25171195782463274`},
{0.1517146567463153`, 0.8404448307598287`, 8.427449210104406`, 1.2489405079089722`, 0.7937040652491076`,
0.6477616643753492`, 0.08512282603978563`, 0.016388604299130645`, 3405.4110812811105`,
399.1826202269507`, 0.20314112870963702`, 6.737758928823055`, 0.8484255316811282`},
{0.07742729394690523`, 3.317040912283317`, 9.880999371532482`, 0.8010465312053925`, 0.28743469962710155`,
0.6365305604148241`, 0.19411226430840994`, 0.47831260845896817`, 3458.518042369643`,
116.53097484036505`, 0.09815493609620851`, 4.832652127106721`, 0.5129478012500222`},
{0.25118009579444556`, 0.4969745065700919`, 2.29184449492171`, 0.9917654079551637`, 0.683514928780494`,
0.655379840437561`, 0.10467228571183546`, 0.22778373737657998`, 3254.986072380726`,
170.41822686556145`, 0.16061682605470035`, 9.463970581786636`, 1.0777227394408708`},
{0.2292340810710673`, 2.064324787257691`, 0.2897872149067009`, 0.9514880626424775`, 0.10199246953503383`,
0.4890381533823508`, 0.20734257158694452`, 0.0515148990758617`, 1032.7042553842448`,

333.02101571525463`, 0.23061763375632705`, 3.9940977361306764`, 0.21053286147363237`},
{0.25996807730167304`, 3.8602372982487374`, 0.7583354986548105`, 0.984076713917565`, 0.09643958841060729`,
0.4475162082476123`, 0.14281046726777719`, 0.2769302537407711`, 2666.244743594094`,
250.78576435414914`, 0.15930752740741816`, 6.009785296615535`, 0.38944531154385054`},
{0.1007555201432343`, 3.8180664304949197`, 4.270424159744305`, 1.4627535709622805`, 0.3807188794187142`,
0.17571212182398588`, 0.053627366653205444`, 0.4983255094785715`, 1310.607317823291`,
380.8287865986265`, 0.23700173797672958`, 3.1928180922211973`, 0.4025308666594032`},
{0.11012573052984026`, 2.7973646534087315`, 6.317504258164328`, 1.4230817420807518`, 0.4665277158140053`,
0.4964083914255707`, 0.11975389672549558`, 0.008384974576684757`, 3433.4182170381464`,
104.6436908016745`, 0.1542234498742236`, 1.795119919919351`, 0.21223525603288929`},
{0.08998066206695893`, 2.280866979574526`, 7.329281823836337`, 0.8411001090310725`, 0.6029746925458184`,
0.27522739162036525`, 0.10930338132980638`, 0.008444888139321425`, 599.859329243076`,
286.89260187390653`, 0.08925071767070175`, 9.77708834471905`, 0.3030303902445597`},
{0.21313668431881322`, 1.7635041678835899`, 2.1912925923575113`, 0.8252025033116523`, 0.016757328021256512`,
0.6998740735780284`, 0.1801084581445288`, 0.26779805577049626`, 1220.0176309294266`,
152.79020888909633`, 0.1629579286674429`, 2.280125156684093`, 0.10691523188617968`},
{0.09290951514101703`, 0.41749240574692914`, 4.773370270407071`, 0.8850952467526404`, 0.9766517437912483`,
0.4208978561725222`, 0.06525352787617444`, 0.011206616294756987`, 2604.3347674109446`,
377.7615808940852`, 0.1923606467616938`, 6.325030382869231`, 0.9625675284230475`},
{0.1404288473454146`, 2.9064356368530344`, 5.23236147204817`, 1.4484340156797024`, 0.10732611967199035`,
0.27136542485299375`, 0.18681354316538268`, 0.3953864116976934`, 677.7158408842279`,
88.88656461439052`, 0.043948668205834374`, 4.975328880565433`, 0.3586361093450223`},
{0.12849833171545855`, 1.198455475456262`, 3.2539226138784088`, 1.3648636137864978`, 0.5280448025145577`,
0.6072554649082684`, 0.24978634440506725`, 0.03994982606054573`, 2204.2740688947933`,
354.6777076623247`, 0.08439759959738258`, 4.855199768823253`, 0.40974365814152147`},
{0.17543069017848928`, 1.9027743233680443`, 8.420650851113926`, 1.2989717187578058`, 0.4754143700193414`,
0.44151569893640996`, 0.17410928694054217`, 0.39942064454570464`, 1461.6707316679758`,
62.00370069117048`, 0.1325925609245337`, 5.299407195683747`, 0.7461186052859023`},
{0.27774537273159505`, 2.913102479760642`, 7.942633412240518`, 1.0326494422606074`, 0.869193199676557`,
0.6849829313653006`, 0.16756021109529395`, 0.031844382721063484`, 1157.358808485019`,
382.85201159938254`, 0.04020804442348902`, 7.095647344980355`, 1.3824063653570637`},

{0.11633291490443043`, 1.4450658892853943`, 9.421948807050416`, 1.0073582836381636`, 0.9376244508422855`,
0.2923945304421177`, 0.08314225478001569`, 0.015377391816799622`, 1342.5955234153207`,
117.35920160680735`, 0.24930648416353973`, 5.058158894639092`, 1.4288639894036779`},
{0.09982764185325876`, 2.300171702541898`, 0.4858627002636364`, 0.9919442401423758`, 0.5310842871377108`,
0.5692781547371908`, 0.20674425403353813`, 0.29388576255746934`, 1499.3990415110002`,
267.8323361172651`, 0.11758456647217047`, 5.8552111813994445`, 0.6586971862135473`},
{0.16308341937411774`, 2.9768832237222593`, 6.137404711598409`, 1.4978818887932133`, 0.6270064524377599`,
0.3856550031514199`, 0.07688210862674477`, 0.2679284542888144`, 1679.641743573421`,
154.41066935622916`, 0.011847012076814933`, 5.409061271016132`, 0.714419786721034`},
{0.10408875617257995`, 0.7208211774242166`, 9.395809932215364`, 0.8438330742384919`, 0.9431300864694563`,
0.42081055780340804`, 0.06365903598523207`, 0.007548231362222756`, 2388.75487627468`,
266.1557043275724`, 0.07149291165582927`, 4.419002029188226`, 0.6654732054016175`},
{0.1859503570691094`, 3.4941122484776246`, 1.6297837870350822`, 1.1031963489157173`, 0.4293650493894907`,
0.20959825245659025`, 0.17774273230533655`, 0.18327500412709075`, 1189.2239199460373`,
282.6299270365462`, 0.11232193397712892`, 9.90920375985121`, 0.8278911236090769`},
{0.2642909093671957`, 3.7936229586154386`, 9.112438874043047`, 1.343319643044862`, 0.7298819945671444`,
0.241631702945693 7`, 0.21308855511768615`, 0.0296357748833522`, 1462.8216839780898`,
69.98981659671449`, 0.07124306277278569`, 2.5551101499765654`, 0.4563514754130473`},
{0.24431991434407568`, 1.8546418038040455`, 9.014242525232781`, 1.0012178057162573`, 0.9513564875697376`,
0.5540264355265693`, 0.16546833659186994`, 0.010160023644511053`, 3069.219263999773`,
161.6333371189296`, 0.2033203481299034`, 5.464378114605116`, 1.4999257720510482`},
{0.2550662278820944`, 2.7952392159553643`, 1.7242958226447735`, 0.9892662747315195`, 0.6466530652803972`,
0.41633012667926605`, 0.09373409721262951`, 0.04014553436100187`, 3927.541477581917`,
214.6071511864991`, 0.12072625793793595`, 4.810615700541705`, 1.0908243473679682`},
{0.07397988502541059`, 0.707910258007638`, 2.664085400294777`, 0.9518019482057067`, 0.7875244424943906`,
0.2321255676355145`, 0.14195245680232`, 0.005701405567921862`, 2118.0076336028906`,
265.7950362273598`, 0.09877063847703516`, 6.01326524592864`, 1.001781869102726`},
{0.2536234425791481`, 3.084120548082856`, 3.3542268996076317`, 1.2342076675511184`, 0.07971619232511307`,
0.37753168173048746`, 0.24305535176309745`, 0.09244094904749854`, 1429.525330090045`,
199.04655556460716`, 0.17472732471161218`, 3.7784584150910736`, 1.055845732825249`},
{0.23487265302124444`, 2.745668437703702`, 1.8560463908813354`, 1.0225932800667423`, 0.519798686110498`,

0.6167124420851566`, 0.13872112580125617`, 0.02304360370209753`, 1950.6949460047217`,
63.792561577309925`, 0.17121277633362675`, 1.4754433886909606`, 1.2090151658939319`},
{0.19404987421370834`, 1.8913121151690095`, 6.875162123920855`, 0.9378067865957597`, 0.9620319910139619`,
0.17047428599364534`, 0.1676311609323027`, 0.36688161374531547`, 2436.6727196810125`,
312.9444240665532`, 0.08367109576690585`, 6.310397829770168`, 0.3372839322361041`},
{0.14433043129139256`, 1.3202167920086731`, 8.463992130027801`, 1.1010419605014736`, 0.14350706526043644`,
0.4137152347701779`, 0.23975541502065495`, 0.00799616367743015`, 1911.0537077498657`,
153.77127625620255`, 0.2417426282164734`, 8.21507395149112`, 1.2501529132389022`},
{0.047715308213800045`, 1.0917315932828888`, 7.945801231718313`, 1.3183453869548736`, 0.37495408831467225`,
0.3679377030871549`, 0.15114689724124963`, 0.44212694961805754`, 1904.8096035348635`,
21.765700565880422`, 0.02920214850379993`, 1.0562137220921812`, 0.16322301362739666`},
{0.20298433403939725`, 3.2126236854576584`, 5.763393946680621`, 1.2592915169856764`, 0.03637647134670963`,
0.17490854387375587`, 0.13159008302912958`, 0.006425245135071102`, 1770.122374418922`,
374.53287469529414`, 0.12888233713533293`, 8.436918800144038`, 0.31480413938383744`},
{0.05881255333180735`, 2.3801271276267855`, 7.362434757054917`, 1.0646703054096496`, 0.806093766428617`,
0.17381221060809304`, 0.15459898392254903`, 0.11200780858178885`, 3179.4524228830724`,
78.98698912374886`, 0.05455878974952594`, 9.203168125761906`, 0.21263297657280256`},
{0.11153574406001432`, 1.6124096945608146`, 2.7742957521629403`, 1.2244678342329687`, 0.3686430365196074`,
0.6752573668077164`, 0.2389841389881603`, 0.023498980178372084`, 1283.4055152922938`,
282.1703009016943`, 0.019235563557756297`, 3.0024732562806733`, 0.7791774112114904`},
{0.06579519568062725`, 3.2190545867000324`, 6.479421382941614`, 1.2315548176863993`, 0.37078258595806113`,
0.3204788641456453`, 0.18121938321856745`, 0.0745089189861041`, 2845.013880184243`,
202.75756338852523`, 0.11614494172591355`, 2.5591762204175446`, 0.6923322117865267`},
{0.11289150267992315`, 0.6299562074506553`, 2.6857393547101953`, 0.7515616774015286`, 0.8067525150133392`,
0.6856411274749656`, 0.203218351458197`, 0.13643075142532088`, 665.9502014092905`,
389.69992150044357`, 0.0982534706706813`, 2.604584539279596`, 0.7279001379553152`},
{0.07532861497660431`, 3.2456716657650855`, 3.170566131100358`, 0.7667747544642987`, 0.6612666356256824`,
0.6262282997594197`, 0.0817137209363257`, 0.09276751126759113`, 725.6513719895092`,
116.55380786905249`, 0.24676733030978965`, 3.599391507892623`, 0.6435082289815082`},
{0.1447096604875236`, 0.781557395333436`, 5.079515305869995`, 0.8829448417099249`, 0.5606054450578024`,
0.17333438670886359`, 0.13484876640308333`, 0.3428154279908597З`, 2132.4017848523063`,

228.9439898899625`, 0.07892443810945204`, 8.583015855961246`, 1.0651655314559392`},
{0.08073679542779827`, 3.436765213609185`, 9.447896083410843`, 1.2321734810402918`, 0.28274585136082786`,
0.4537000076509107`, 0.2378954237232333`, 0.016202557156482104`, 1882.6285138697513`,
143.19709501896392`, 0.044359609514775244`, 6.7684345441443945`, 0.3648612319782283`},
{0.17570347686215215`, 3.233960190352999`, 8.02035493411961`, 1.3345169115484334`, 0.9514022176165366`,
0.3640798512190224`, 0.2136248279814617`, 0.18054342141341007`, 1968.2648913603198`,
103.08834326532372`, 0.05518740857004362`, 6.915277843565254`, 1.0364954394923056`},
{0.11925732953940865`, 3.6343980885034854`, 9.962959338328435`, 1.011868751364569`, 0.06493978743288742`,
0.17742779913292284`, 0.1769046107233871`, 0.005362579496616395`, 3376.132332358329`,
358.9394383628304`, 0.05158147181807593`, 6.474636554195383`, 0.334042905285048`},
{0.1849591682194595`, 2.435795929316444`, 3.5195053556656077`, 1.1867529613387038`, 0.18511861536763652`,
0.2725329941056882`, 0.1913335103508631`, 0.007193119734412014`, 888.0411283184822`,
208.53516984841042`, 0.12216322134268065`, 5.549467591217473`, 0.7979574318926956`},
{0.04791496352556143`, 3.7363958898231298`, 7.859362293932024`, 1.2955394392367978`, 0.21077880799821336`,
0.6986917555356797`, 0.24146054265338857`, 0.25080131253737453`, 3174.4582657930478`,
214.6472473246855`, 0.12886781736255348`, 2.1496769931608686`, 0.9494229236050717`},
{0.2320051986600553`, 0.8210990691375057`, 3.0745548516676084`, 1.0653538281011379`, 0.9016142110480403`,
0.45929895353677397`, 0.05711960119403317`, 0.10928247272113006`, 3933.7984981846193`,
65.47103817619023`, 0.11710947379300074`, 4.724190385949658`, 0.932323733177236`},
{0.2740885790936093`, 3.0208727150533035`, 3.9044562082788996`, 0.9239050692909432`, 0.13393679798267133`,
0.2935571665601744`, 0.17832264013603433`, 0.12993009746044476`, 1616.3784196961215`,
45.86996911978616`, 0.06338312332920809`, 5.191606790783375`, 1.0490428175043491`},
{0.07782021540679757`, 2.7209102520090163`, 8.736874384750735`, 1.114706684991148`, 0.7103121173081006`,
0.20678262886709498`, 0.11036600245925934`, 0.19154162173345113`, 2448.698607159915`,
243.62539193659143`, 0.13554193141415577`, 3.0074065943997237`, 0.3291541909818123`},
{0.20127067113693758`, 0.7134036992984543`, 1.5848778254201719`, 0.9599985271802891`, 0.5544064241430855`,
0.6980173556057399`, 0.23861657897987476`, 0.010226664436870918`, 3481.525245131613`,
209.51927751371306`, 0.18791735173124585`, 4.5381928864523005`, 1.4066869656787473`},
{0.17004475261494956`, 0.4834545237544501`, 3.4349181823258377`, 0.9765473132257132`, 0.7016074781874622`,
0.38572946598569846`, 0.23641719832548008`, 0.044465090938447804`, 728.0925200892948`,
360.1524759911083`, 0.2343727375251467`, 4.114001391225679`, 0.6989801734218899`},

{0.14843112442757472`, 3.2599821678006826`, 5.742419985764322`, 1.482241642317249`, 0.2702489297613082`,
0.3314664434605483`, 0.1749862727560041`, 0.21934886574956672`, 1289.3717845828933`,
179.7867019868338`, 0.03877340906995197`, 2.6701245702655445`, 1.4405073568802078`},
{0.0976585079740021`, 1.3728751665341834`, 5.874627728896462`, 1.1995783460582101`, 0.28537590201313856`,
0.6491235286522472`, 0.13987394486973953`, 0.02287954128834537`, 1880.8681942009343`,
204.39831340676108`, 0.24472299006607134`, 8.011571455649513`, 0.7571200668542781`},
{0.1434955367081326`, 3.5028874653292856`, 9.961555440173449`, 1.4583790499213787`, 0.7878184950293752`,
0.3804561019338192`, 0.248272531578832`, 0.013284854321714896`, 2386.8492620887737`,
259.97790040425207`, 0.11312758713523102`, 1.6001299135943265`, 0.42901490870982695`},
{0.14734788675597893`, 0.461750217153563`, 2.7608760404738693`, 0.8246000601521355`, 0.6486099382776269`,
0.46153306705501707`, 0.07562875863771559`, 0.011120543224542678`, 2174.875101604558`,
76.28988785327772`, 0.1816491692635701`, 7.465741450424737`, 1.3681893457620165`},
{0.2174892525974506`, 1.1555616271784261`, 6.330891972593395`, 0.8691848701006539`, 0.5548185878553895`,
0.5753044260463678`, 0.11961066111713925`, 0.21454325341169517`, 3637.70292743186`,
299.3308064827128`, 0.20558605918965206`, 2.653266548572333`, 0.34524692314089034`},
{0.06749293860797512`, 3.7228921901379612`, 3.4260664843308817`, 1.4192699415393606`, 0.03614877922192594`,
0.4559586129932802`, 0.13413544415463274`, 0.029486897805871767`, 2795.273468589703`,
381.34485667532954`, 0.1616868776536713`, 3.6141605923908813`, 1.2368738510133124`},
{0.17265939500359245`, 0.9179315961122354`, 9.1852760379758`, 1.297645317836566`, 0.4142675534047153`,
0.41824524574451727`, 0.08521501500767603`, 0.21771628238910337`, 2996.1049031716466`,
204.18324706241947`, 0.025729542442735343`, 1.6360785031153569`, 0.4558745456460018`},
{0.11391904455060164`, 3.7004347183812403`, 4.98869805344277`, 0.9900086895027544`, 0.7790608041506635`,
0.5499151960612685`, 0.1741544112916672`, 0.15121348451654992`, 2279.078015486494`,
277.9770114196665`, 0.21022773210729895`, 8.293592982203787`, 1.3768230125107617`},
{0.11443345192899396`, 3.850377768547795`, 8.00437153143687`, 1.3020216404842988`, 0.3359559076583225`,
0.49678442177055226`, 0.2301468632075337`, 0.14684067776603849`, 2776.269241624055`,
290.27051916223877`, 0.07166092983110212`, 1.5123971688855153`, 0.6362818855430454`},
{0.07628805291838442`, 0.7253635030519439`, 0.2787614932535387`, 1.2938825783187968`, 0.6322486251684503`,
0.6415725594877175`, 0.11902418817624266`, 0.020293492548644005`, 3744.3615927821575`,
331.54916368743454`, 0.0324650937333269`, 2.843278699811469`, 1.2796927021756468`},
{0.26704012645507424`, 1.8838541538663058`, 7.9577608030460505`, 1.250964933852727`, 0.37177913789440953`,

```
0.5400109924589773`, 0.2090957264740766`, 0.02799412229122407`, 2680.237536243104`,
  60.33735070739806`, 0.08963423619120237`, 8.220004781923546`, 0.7126427956038843`},
{0.14352951682222453`, 2.7122840992738713`, 1.912849383145993`, 1.4933758944571824`, 0.4317056886375634`,
  0.3933320053063153`, 0.10505217994551203`, 0.4954512847212043`, 1346.891754031455`,
  320.6507144731578`, 0.20904379157314928`, 1.5308675559916907`, 0.6151100951642487`},
{0.1069158661028563`, 2.292840293600788`, 3.2223682955272235`, 0.954682665552236`, 0.20354287774001567`,
  0.3896645344037619`, 0.21864993682483302`, 0.034752231866091665`, 2419.249949369646`,
  316.3106467196254`, 0.1946047812364281`, 6.8989000582764195`, 0.6984200141969727`},
{0.2686042849529644`, 0.7627258559442236`, 7.922182847008894`, 1.344754730879767`, 0.5223454903809039`,
  0.3059455193661864`, 0.17153078163225305`, 0.02722979062605091`, 1945.288646450902`,
  327.63559196356823`, 0.15374403181997548`, 2.2340529482759806`, 0.8539303526410174`},
{0.25819223047450574`, 1.7095391280955443`, 6.520962454670094`, 1.1430189535372957`, 0.9365205495994724`,
  0.41508933167510853`, 0.1765660191827808`, 0.05380792374101585`, 3754.7043853290033`,
  182.2517379088373`, 0.07048991272145094`, 9.71641095911452`, 1.30627739480544`},
{0.11085289703164392`, 3.6133570849476584`, 4.542500201931212`, 1.3471228260354866`, 0.6367711489528869`,
  0.3976210616429948`, 0.050489910733311105`, 0.00824863073511752`, 1898.7553106453079`,
  276.59295465999946`, 0.21653605876058957`, 5.75252417538405`, 0.6232368998851006`},
{0.0604034128862494`, 3.158158875209411`, 7.876995843447027`, 1.2883875500461601`, 0.12376268888818709`,
  0.6326665048379214`, 0.11422725471154377`, 0.15234673981455935`, 2211.579578858029`,
  40.246357658710394`, 0.057745413831265746`, 5.692997033958765`, 0.2028088406309343`},
{0.17311414162710254`, 0.8702497549340356`, 0.34304718385733324`, 1.0485386108632935`, 0.3716017788116761`,
  0.6371600148503609`, 0.055104118812441344`, 0.2168377629398712`, 703.8776101728622`,
  164.53996042397466`, 0.027022709136328538`, 3.1628771308577264`, 0.32716182080568834`},
{0.20458649602921813`, 3.3198040898334424`, 1.372039361136828`, 1.1755123934413545`, 0.5154858348933944`,
  0.2152893723924586`, 0.1607481890336261`, 0.022174783215048698`, 2483.3920145693382`,
  114.03490520349692`, 0.026176842372398812`, 2.8250322344315557`, 1.2559643496212312`},
{0.1578531250336248`, 0.5854218989222861`, 2.149284744894146`, 1.0306646027288366`, 0.9441735295761224`,
  0.33751540864617946`, 0.1297018205371923`, 0.0054893062071085874`, 3639.0053252131047`,
  207.4000118567045`, 0.1305358640736498`, 4.5222314256344625`, 0.9549981466481912`},
{0.21896388001044664`, 3.129900208151482`, 6.123839398439741`, 0.8969815671266188`, 0.011595810869147183`,
  0.37111266340117355`, 0.1718245052376185`, 0.005208778420460518`, 3863.605974823663`,
```

250.7012639800747`, 0.15570381237665487`, 7.7211234554844195`, 1.4785494708568354`},
{0.17565226905516895`, 1.7038906647375853`, 4.512441985203527`, 1.1172179676607337`, 0.956149515870736`,
0.40808719408253036`, 0.2318723848780873`, 0.25398457087869114`, 3825.126366136904`,
43.499586017725164`, 0.07242686582534269`, 9.792971274976665`, 0.9137108964160303`},
{0.06615318131527015`, 2.492376536317514`, 9.394184914213039`, 1.4348602118065803`, 0.7341697528786502`,
0.24813924799413356`, 0.2232148533154487`, 0.026418827644963606`, 737.3840226181687`,
59.19942623358838`, 0.013447593432626304`, 4.186359481235684`, 0.5021329151763947`},
{0.1576166709633643`, 1.3075085634970804`, 7.656001845154428`, 0.9231504863095856`, 0.8454152618498512`,
0.25647657664732126`, 0.07864463104098307`, 0.005296200134401313`, 3012.7436478007676`,
197.2045635665247`, 0.12709746544272688`, 9.860728262025162`, 1.31076068876941`},
{0.17154635825703612`, 1.53373134752687`, 8.885182514407113`, 0.952500439197914`, 0.18392969628669165`,
0.39214328491936956`, 0.11223174543931136`, 0.01936642254266069`, 1934.647469413505`,
216.7923390085249`, 0.0451455537258712`, 2.0009219573176864`, 0.3777871026684809`},
{0.0855854522942322`, 1.8098534994044577`, 9.52981117766399`, 1.2278891902893156`, 0.1610609466726174`,
0.1996687073144625`, 0.06202289567495425`, 0.00706028348998438`, 1475.3823287257756`,
92.05986706201287`, 0.09971055550215713`, 4.508838235939827`, 0.938398866245965`},
{0.1989224969181887`, 2.0656981604697133`, 7.019145766333462`, 1.189741191045774`, 0.9813366885674477`,
0.4707046727829801`, 0.138351198132442`, 0.006338022742977132`, 1353.111767359248`,
264.1038821274834`, 0.2333484554805464`, 8.079661388491221`, 1.0209030718005039`},
{0.252908269598038`, 1.3368065237315108`, 7.681913157962232`, 0.9670562213906954`, 0.8885087458364935`,
0.6712630307685601`, 0.19827927021086678`, 0.0287236993413668`, 1511.0621870028344`,
393.3001097237484`, 0.20663414848444228`, 4.051732672816565`, 0.5729449981349937`},
{0.19935758638978301`, 1.4210929413876547`, 6.276637411024748`, 1.1091985064517424`, 0.2725861619363459`,
0.30993881233332166`, 0.061372778664464`, 0.007027369768062639`, 3344.583850960579`,
315.8180746154343`, 0.0888647235789749`, 8.724591660226011`, 0.9460844323713795`},
{0.15752424015374422`, 1.1744034635310063`, 9.19368700774531`, 1.4814874364707953`, 0.3259429279011785`,
0.1698219186283283`, 0.05329876515990288`, 0.11291488360711258`, 1392.2159030202329`,
251.1254711586031`, 0.20643313217875114`, 1.2559120984693628`, 1.2401551704457958`},
{0.12243829123563332`, 2.3703577767539246`, 4.839202410108355`, 1.1566976539300236`, 0.5361220364781598`,
0.5850654781694432`, 0.18441510087344481`, 0.008912199456594784`, 1964.6638548143164`,
274.09302664051813`, 0.22153291468521247`, 9.911211328491756`, 1.3136185654470256`},

{0.06243550103024703`, 1.4478067318733245`, 4.568123699237523`, 1.4686635133498154`, 0.7846213517525722`,
0.6840252274506751`, 0.19851402241018223`, 0.005300678072293084`, 2514.1415778711344`,
343.58199097307386`, 0.07822848450027337`, 7.993810681409931`, 0.8901709005463876`},
{0.14543352811569443`, 1.222289804727784`, 2.326184459246356`, 1.2918343464415338`, 0.9224834580379948`,
0.39580748300164703`, 0.1846442329251613`, 0.21500168948406848`, 3817.4733751688063`,
116.03894445631153`, 0.16529510077911613`, 3.062979779789736`, 1.0702400368818767`},
{0.1313087568846084`, 3.644656164823556`, 9.460965509789986`, 0.8885448984294761`, 0.9355716622019723`,
0.25119735021109135`, 0.1694476044963361`, 0.3877738779001962`, 921.248806442728`,
163.92044778354148`, 0.11679660196129243`, 3.129177349018949`, 1.1599711195943847`},
{0.08032938050984856`, 3.6316655074823947`, 4.889233151652448`, 1.047924531280927`, 0.6480389250926271`,
0.41632199836938133`, 0.08565204097388288`, 0.014661199276299969`, 1480.2883480287883`,
317.22766308099494`, 0.1835562909337356`, 7.402391506079066`, 0.616770981275718`},
{0.13052094951744303`, 1.9253311736310001`, 1.1697698857489094`, 1.0141116955010334`, 0.850854427903335`,
0.33601527543931153`, 0.2494690024453417`, 0.2360086443642378`, 1519.2042074690844`,
278.4809507811633`, 0.016312333738175505`, 8.02616595157577`, 1.4482171660091545`},
{0.1763567523046698`, 0.4764578027902213`, 1.019702924150577`, 1.411009603642741`, 0.10601942889051807`,
0.5663043206905454`, 0.11523580705445918`, 0.20578529268229545`, 886.8032845661132`,
265.22978093426616`, 0.0734753688299642`, 5.606338149623012`, 0.8756860344077033`},
{0.21878401484644522`, 2.7819817431568543`, 8.543045035400766`, 0.9200940784159304`, 0.4855794665787383`,
0.5527693274070447`, 0.20427128258242377`, 0.013404369082852467`, 3643.8303336510735`,
176.1607363330378`, 0.03966906929471847`, 8.016907827776176`, 0.33840217640400705`},
{0.155933613154269`, 0.7900752087685747`, 5.426020030668559`, 1.1675266200556353`, 0.3526751580238494`,
0.32689871232148693`, 0.08872571343828128`, 0.47047660149359233`, 1973.8960115130649`,
259.15398868988336`, 0.014745308659178585`, 4.1998835891931225`, 0.8551070030738948`},
{0.2479337375923964`, 3.025341092699729`, 2.1453632749289397`, 0.9396124783291141`, 0.0672772492322784`,
0.28991645499385843`, 0.23601049551242942`, 0.03982040313739142`, 3865.9337586257416`,
194.53557465876634`, 0.20896839632361264`, 7.896823118164437`, 0.4853457216015631`},
{0.1460076457981399`, 2.5733113885431225`, 9.325775684390766`, 1.4910714614077438`, 0.8342963441408033`,
0.1882740945287651`, 0.13611188525292933`, 0.04396527811717692`, 667.8083571719217`,
108.22134685664759`, 0.1548306029558182`, 2.291019500853446`, 1.424632992657926`},
{0.26317109341632255`, 3.059519748021583`, 7.63492936446163`, 1.3537697529302852`, 0.126934299410153`,

```
0.5548921430733772`, 0.20372329915496434`, 0.22074667207931783`, 2265.8574749330373`,
  181.20735656268073`, 0.09466585200925526`, 2.880276570782346`, 0.773128569372235`},
{0.1645690290583518`, 3.8533306281399318`, 1.0369585072362497`, 1.3664278344740945`, 0.6261111939893407`,
  0.20406169893014847`, 0.18309667446609262`, 0.4646249368336043`, 2770.959571622493`,
  166.0796256998841`, 0.048578133356614095`, 3.3069773434670147`, 1.4087558492193972`},
{0.22921820594790826`, 0.8201682437489284`, 5.405881273282434`, 1.1219214796772738`, 0.17914795775514958`,
  0.5820849501977261`, 0.054142164266046844`, 0.17169981881854485`, 1492.1967102291455`,
  65.66187445337869`, 0.1524226111094261 2`, 1.916490304748569`, 0.9747174266139518`},
{0.22532476518940014`, 1.9533397508457995`, 5.9287214686276055`, 1.441991810272455`, 0.5762935247594674`,
  0.4183646366318182`, 0.09350661705723778`, 0.3294355875363928`, 558.5643880556918`,
  49.17304258267865`, 0.1200905527225728`, 2.331392728849849`, 0.39194349265472495`},
{0.22156365739259543`, 2.454378375997047`, 0.8895152478568865`, 1.026128317479294`, 0.516725505225655`,
  0.686434221522195`, 0.23046056758531142`, 0.005492504017628765`, 1555.2392746607857`,
  206.29023226314905`, 0.041365082475744275`, 1.4870135325767126`, 0.5063457915321476`},
{0.11472120130152375`, 1.4874886419048003`, 7.95738176456699`, 0.7931641460551735`, 0.8816884572777486`,
  0.3579341724415851`, 0.08277045109409453`, 0.048113870328459145`, 3701.2844061656924`,
  204.2587217990438 8`, 0.20301688272576546`, 3.1735817377996653`, 1.31887862582745`},
{0.06425688602740343`, 2.6618634637938463`, 2.643754911431923`, 0.8785376073839924`, 0.5804636394156946`,
  0.5001986382462079`, 0.14138114151328934`, 0.3139131008244074`, 2397.9696216564225`,
  78.15653575226656`, 0.17684868004389004`, 9.538390690744457`, 0.6033965244651656`},
{0.1767902158998531`, 3.597571059730683`, 4.894388517781371`, 1.2037647529410567`, 0.8088893202611276`,
  0.4862113706827499`, 0.053623813047437074`, 0.369409668420823`, 1883.086591548903`,
  266.54822065935684`, 0.18383750630418133`, 6.949409820987145`, 0.17339220023403645`},
{0.07711195041153773`, 1.310646675677738`, 7.287601935184053`, 0.9421992854061032`, 0.6629854601936369`,
  0.19600775055309494`, 0.11597851857342667`, 0.34647099332821635`, 1165.7488565303056`,
  392.06574230549916`, 0.08156422577601757`, 9.98958158861084`, 1.355669623216047`},
{0.07030228204141997`, 2.841321925057506`, 9.523175071752728`, 0.957721444616676`, 0.5285225389857424`,
  0.687368450677392`, 0.09615328133104123`, 0.49849774355724874`, 811.2137274256684`,
  346.8639129623606`, 0.02062867223765208`, 4.642931209436085`, 0.8694461988489701`},
{0.15160173019169382`, 2.757072174383392`, 6.0862251898613255`, 0.9273854095685228`, 0.9702354173159373`,
  0.3952766424689479`, 0.139970431273921`, 0.019384819518267777`, 1328.4246699458445`,
```

```
112.51314475175559`, 0.06030008858760033`, 1.0538552754157138`, 0.12506996939343384`},
{0.07720185370449029`, 1.8816151498237366`, 1.2505047699374146`, 1.3773873945814676`, 0.6708624603801276`,
0.176415527179277`, 0.09395893068196878`, 0.03821422736625399`, 2260.6042381201514`,
369.60131923785855`, 0.06295297192499111`, 8.500378555792118`, 1.404314834211414`},
{0.22937140077917728`, 2.9197558499797838`, 5.991654392537161`, 0.7609979028883141`, 0.7071044023774187`,
0.2974885161887877`, 0.18825283303648666`, 0.032338266898960494`, 2797.371157028768`,
202.1761455304635`, 0.23680881860212954`, 3.62202546942657`, 0.8089322407730246`},
{0.2310975321060973`, 1.6649278018384956`, 6.117367353219549`, 1.3711053926671974`, 0.5776666118144214`,
0.34300872993879494`, 0.05030597587094107`, 0.013880459047725028`, 1272.690119313539`,
46.12165613423167`, 0.07701873188143649`, 9.481774546264408`, 1.4810089454694788`},
{0.21166318477480112`, 1.2271964963163153`, 9.968674707787947`, 1.4226030031673544`, 0.5017683729752407`,
0.4896306570856972`, 0.14845091745225086`, 0.13950989408153797`, 3248.3492688454417`,
221.1597513183293`, 0.152837589460797`, 8.879252520814859`, 1.0157863292829834`},
{0.20922455243011878`, 3.6942310466303763`, 5.573446425852463`, 1.1411804023081409`, 0.9621527847139406`,
0.28451282132289957`, 0.08975759414482132`, 0.051227081701645576`, 3447.2439359977625`,
280.22849496162496`, 0.2438675699856881`, 2.418161847018231`, 0.8016087345021319`},
{0.2765607651857069`, 1.2704530487690837`, 6.006554660027694`, 1.1061086441305394`, 0.12440018829651067`,
0.36025788190000807`, 0.19633101014307436`, 0.019373612586789046`, 1432.0769734472487`,
313.8129593237045`, 0.16870756948717214`, 9.609773835891207`, 1.2876583132286008`},
{0.21142405672597675`, 2.9545989174954075`, 0.2974772339667986`, 0.9658276187146195`, 0.64106654096473`,
0.6263249493059542`, 0.09733527159806632`, 0.007468085156923343`, 3517.602581345549`,
75.36708608662542`, 0.23543132772166975`, 9.603866416931954`, 0.19450738989435057`},
{0.08169573554069165`, 2.8299360298510665`, 4.320933911813235`, 1.1825738775709533`, 0.3027516653278022`,
0.5305834377007563`, 0.1475083038117065`, 0.007523165551877513`, 585.1893929943135`,
275.5228279325412`, 0.17684956635249888`, 4.120555621767931`, 1.4111853521392486`},
{0.1982041419042297`, 2.2268219215065645`, 0.4137546724148198`, 1.378661149527917`, 0.7206026710685052`,
0.68793041191814`, 0.15316897603405166`, 0.07890511093433691`, 2517.4293419356973`,
86.78917960165899`, 0.20777476794521893`, 3.6952651421831053`, 0.25322177655756106`},
{0.22157133430375964`, 2.1006687367628096`, 5.865585434381025`, 0.862163364473078`, 0.7961242271327948`,
0.1679554170546661`, 0.16778560934705797`, 0.21616705792607036`, 1509.8006246813002`,
104.18673597092118`, 0.04226318913943389`, 2.3850476310310498`, 1.1811985793876256`},
```

{0.18526653849571717`, 2.309967457429205`, 7.235178488173702`, 0.8111816500462055`, 0.21148492456283652`,
0.18475826854386967`, 0.15626027102897042`, 0.057689140810915564`, 766.0020232731708`,
185.02183046070866`, 0.06106549734909339`, 7.0384168617832215`, 0.11685665088837416`},
{0.10801077582204055`, 0.4767848668332846`, 7.25138663495552`, 1.4325801889738408`, 0.33847640924280453`,
0.5718972515878133`, 0.105744974897243`, 0.11534684356295588`, 2594.1807156101713`,
318.6618729662216`, 0.06179469905331658`, 5.34696732616077`, 1.3364569926001173`},
{0.23792597358173623`, 2.688465494136551`, 6.845829046034535`, 1.1002091615985592`, 0.37282035219472753`,
0.3011297207106588`, 0.2473335682094463`, 0.01991156261653569`, 2038.6789052153845`,
210.83183249328954`, 0.08037072870463724`, 9.093415216874622`, 0.3344692931868205`},
{0.22993078275515533`, 2.525474685703524`, 6.125260191496192`, 1.4929402058003913`, 0.486733717931074`,
0.6225356403190192`, 0.23641243849582955`, 0.0803729054195917`, 1157.473735647006`,
169.73954640708985`, 0.04795903068483898`, 8.28333454337778`, 1.445122239815786`},
{0.1869769669715985`, 1.485119860176268`, 7.03335904463459`, 0.8235833311873434`, 0.33587109510820534`,
0.6579510066594008`, 0.17782007925307802`, 0.007365768570817107`, 1161.9126891061214`,
206.93000870629214`, 0.23913461974132133`, 1.154555664614577`, 0.17860914505549097`},
{0.2594386598277771`, 0.4907291838119914`, 8.253740230346917`, 1.2562319857439164`, 0.630751008353968`,
0.6003942152343791`, 0.16893163054122257`, 0.12552255641031881`, 2555.8077872611675`,
287.20210893930584`, 0.11105710767727667`, 7.093346066656089`, 1.3316012208831718`},
{0.24168594197714738`, 2.487527173714957`, 2.5005074702928987`, 1.3162259499649314`, 0.4221537034288636`,
0.3099363412058589`, 0.20921038714233392`, 0.06741194388477754`, 2733.252958969397`,
173.34117039752516`, 0.11909087418011138`, 8.486444686364809`, 0.9620364764733642`},
{0.12452706981171563`, 3.242843098104845`, 5.361835103883211`, 1.0740271680209217`, 0.6460165689100792`,
0.29441767681046616`, 0.22857190710285924`, 0.07930287454728857`, 1257.5807309824504`,
295.430402570252`, 0.10895057078510323`, 6.841864249157172`, 1.163933956705701`},
{0.2557093973805171`, 0.4051303627282947`, 4.693462565645005`, 0.8860437827795489`, 0.9194156940466172`,
0.45815570325126265`, 0.1510910941798535`, 0.07870004480420398`, 3132.5620218229606`,
302.90817331129847`, 0.08637897198804784`, 9.737653549202872`, 0.46859993602327443`},
{0.1829582147550864`, 1.5256866045998043`, 2.03189641777327`, 1.1222778010348193`, 0.03811462288929479`,
0.5618740829076075`, 0.11263433834449038`, 0.2791855460572071`, 3277.6533486117487`,
172.65623022385375`, 0.030075112520539238`, 2.59232430828043`, 0.783030452394347`},
{0.06140104313465666`, 2.460539185106426`, 5.961918171032632`, 1.1102491165917632`, 0.7798976124509112`,

0.16029666400283926`, 0.13508017803518807`, 0.4919794640607069`, 2590.2148343601484`,
305.84622942181363`, 0.11973020837325932`, 3.9806430906660957`, 1.1111030420450256`},
{0.1711775238741562`, 0.5019625567967068`, 9.586274753416383`, 0.9484474385429034`, 0.980521223518446`,
0.4330626846988278`, 0.21438996930373572`, 0.2245552941607962`, 3058.4173866521887`,
152.6790489040933`, 0.0944629158119748`, 2.577057545046525`, 1.1465158474128474`},
{0.26691735492143615`, 2.5320514430618486`, 8.550940885105003`, 1.4373682402592758`, 0.4771646947972308`,
0.18844330768909645`, 0.11201955955138962`, 0.11789932012031278`, 2886.771279587132`,
93.01623349049612`, 0.010902609553686937`, 2.9431133951513715`, 0.705430973142406`},
{0.08703489710225831`, 1.1696029176126457`, 2.8929571896991018`, 0.8551088804080895`, 0.7972556998184932`,
0.6510731694726453`, 0.11258011739373719`, 0.045162705810826695`, 3535.7555380745443`,
62.84409202124414`, 0.17813120427323514`, 8.03275953302387`, 1.2552854307001606`},
{0.13518231998646707`, 3.6948829381267645`, 2.131072716483498`, 1.4937759276297344`, 0.7767923162254364`,
0.5165458326040475`, 0.16227719412689456`, 0.04519981251511891`, 3983.684815901678`,
191.70315936858992`, 0.13930377370940616`, 6.306826705642012`, 1.032935178026166`},
{0.10774309849896646`, 3.736554195863712`, 2.2341742787936347`, 1.2045029497948032`, 0.9303574820449074`,
0.4582352004403937`, 0.19359404123783952`, 0.039983935436586286`, 2352.3551825652967`,
320.72717703493936`, 0.06514512285730684`, 6.779792172665335`, 0.8797330310810398`},
{0.20770433175112651`, 3.109204400403325`, 0.20099665267948374`, 0.8742255636364782`, 0.024813473089767113`,
0.240282569360604`, 0.18576559892192723`, 0.1317848182037604`, 1411.5851094618392`,
158.08107833990675`, 0.17690068584227342`, 3.904436541828723`, 1.357207699254508`},
{0.09448668201105037`, 2.410919641885151`, 1.350132874851214`, 1.3762843812070134`, 0.004651491449426448`,
0.6365872610465713`, 0.23322630347732265`, 0.08615671221466674`, 2950.762127886288`,
316.0892657764441`, 0.16180448088816285`, 6.47689574973119`, 0.7706785449987734`},
{0.08289232854140277`, 1.1468625434643034`, 6.113671903882498`, 1.141476131790856`, 0.8113048716462563`,
0.5114785013510522`, 0.1437446486191194`, 0.02760266646248284`, 3012.3201335015347`,
281.64171273471936`, 0.03564353737323955`, 9.384615831703261`, 0.7944935894043699`},
{0.0849153683065022`, 0.6319892236147719`, 2.114551707925985`, 1.2461670457408607`, 0.7306014894102357`,
0.32449846710553176`, 0.08929492495989536`, 0.016408600115246787`, 558.733384495335`,
217.00633714400976`, 0.13070090260521539`, 5.821164045556291`, 0.7435891013457101`},
{0.09040683538793876`, 3.340658966022863`, 2.160986355031987`, 1.2703609168223777`, 0.7359849646797021`,
0.4196403007867846`, 0.14182087201107557`, 0.34521905027534144`, 2988.2505759920587`,

```
354.16133220107486`, 0.21419902011703607`, 5.4345853130842805`, 0.9728978889757345`},
{0.20859643515675713`, 1.8877506327350426`, 3.610942473179815`, 0.9188330116771175`, 0.07225013193132535`,
 0.31512009685680187`, 0.2084254211369836`, 0.13036688863188475`, 1423.8303839771024`,
 148.24888484193133`, 0.09090215154618603`, 2.653159557091028`, 1.1769997259400542`},
{0.2775711100993279`, 3.8796646995160193`, 2.028692966768544`, 0.959772508161437`, 0.14615379499145553`,
 0.5344882702156523`, 0.050540537283472214`, 0.008717405363258768`, 605.4303926477064`,
 86.02465982441038`, 0.19867732233067387`, 8.175417405675173`, 0.7557300476034672`},
{0.2734564636981751`, 2.078794252055899`, 5.58118723900359`, 1.49140438303907`, 0.8827845137379815`,
 0.5395409262732532`, 0.10413442884619478`, 0.18779130214107398`, 2950.6303080099433`,
 94.68533320113221`, 0.033296934697099045`, 2.835225178912273`, 0.8590623879542898`},
{0.1042141776090886 9`, 3.9806755529248097`, 3.6408344459764717`, 1.322172627166737`, 0.1000592332348238`,
 0.48996158640872345`, 0.07063639655536227`, 0.07096628857497574`, 3218.761349505553`,
 296.2823833741422`, 0.08840787957878266`, 1.0269425117351112`, 0.10667022544527116`},
{0.2754934613307095`, 2.4772150697863067`, 4.699383051224052`, 0.8551969359601641`, 0.3181125413370589`,
 0.21381447461486702`, 0.12130217582978334`, 0.022512766834479486`, 795.9986377450518`,
 357.5238954990325`, 0.24036872560514078`, 2.0345694437060526`, 0.6072647478635216`},
{0.06619581350761466`, 2.7881066711576734`, 2.8537509705176394`, 0.9891677990616923`, 0.8373344986239073`,
 0.19790897524975626`, 0.09185213371202688`, 0.00857321041500942`, 2055.3736975529255`,
 31.88640228370963`, 0.03523831218645268`, 5.696097023837108`, 0.29187148351753467`},
{0.1298132756954506`, 2.8585402154144006`, 6.437713761400808`, 1.4980481550734701`, 0.9611669791610145`,
 0.5357765761577084`, 0.16040271618955604`, 0.016100163586007913`, 522.564427621146`,
 43.6121866921153`, 0.20871550454007498`, 5.59776191182581`, 0.8806650157270011`},
{0.20556043625089965`, 1.8192901890738584`, 4.292636640368679`, 1.2838668900928696`, 0.08760791600423734`,
 0.6818222490294337`, 0.142672004987712`, 0.006686505560345256`, 1995.540026133921`,
 283.4866335300326`, 0.2085798635478609`, 3.280310512987043`, 0.6015416808762613`},
{0.24058104513431994`, 2.1873687346811277`, 5.659026523633111`, 1.004047709044065`, 0.8328913078837221`,
 0.623891871350166`, 0.08766340991931373`, 0.008430134039147183`, 2601.7165814407754`,
 249.12373989835567`, 0.030474235896864155`, 8.991227710673165`, 1.2833381452421007`},
{0.1843088049905594`, 0.4280411676285585`, 1.9800464046244353`, 1.4120188565898317`, 0.7240044582523009`,
 0.296746547744528`, 0.10234889391033763`, 0.005447859153693297`, 2517.6110832821096`,
 241.50069071394864`, 0.10993322637089764`, 3.2673971617867004`, 1.4127349426750984`},
```

{0.14879926745595878`, 1.0145913123947308`, 5.175666788640903`, 1.4216396007747538`, 0.30528652163260794`,
0.41852006684217935`, 0.08636270796505932`, 0.024569495844874063`, 1528.3687993879967`,
399.50130012547027`, 0.11493939543886256`, 2.7975290190897475`, 0.9743823827588485`},
{0.1829951377410336`, 1.361564914411157`, 4.694817997056893`, 1.2880900199343872`, 0.9454435675757777`,
0.5028590377428427`, 0.06468894028667527`, 0.1666609451608123`, 604.7693853692326`,
275.5852017799763`, 0.22566362443421`, 2.928657758289848`, 1.3281605404183678`},
{0.2341957938761366`, 2.5114017057248956`, 4.373268682378841`, 1.4631865500968817`, 0.20458772158961103`,
0.577553240998902`, 0.11818082574498356`, 0.03355766009617581`, 3153.579024983129`,
307.79686137602005`, 0.09194856938832352`, 3.1157373823819228`, 0.10640897514406333`},
{0.20513991416113786`, 2.033431069067677`, 9.422225723793833`, 1.0288902090438978`, 0.6375890014689067`,
0.2390905203768514`, 0.18529023062296823`, 0.013541725424023602`, 2549.6747377089105`,
150.27313290555605`, 0.19604710287572352`, 2.636313809911791`, 0.4621075910698911`},
{0.17276893398546517`, 1.4696753313802207`, 3.9973169318549147`, 0.8654667209129807`, 0.10876030003077308`,
0.19704558070570188`, 0.12440236076545855`, 0.1569376119224937`, 3938.384639642497`,
57.46421942479361`, 0.12445676420469665`, 2.5408037017095726`, 0.5238442802739391`},
{0.11204311855852472`, 1.7013572433137565`, 3.058168037173486`, 1.4867504783146572`, 0.8996771100732928`,
0.6819517798967545`, 0.19493702157072262`, 0.24346814437911918`, 2914.354454850107`,
387.06404549612205`, 0.01985945025481517`, 5.249142162935986`, 1.0430073575453092`},
{0.16473183239015682`, 1.8712094759941964`, 3.0571210356797724`, 1.4644853700911185`, 0.18431094252367552`,
0.5500426776194254`, 0.09375157274845106`, 0.031874339239305535`, 3596.1155435077626`,
188.36890996702562`, 0.22217643971583478`, 4.716624148360237`, 1.2836160756903663`},
{0.24923018426453097`, 3.1125598045900347`, 2.019868693874246`, 1.2491757763779443`, 0.3111545672890146`,
0.6578188710626947`, 0.1476722707575428`, 0.3114765600131322`, 2384.9574981509613`,
297.0737195350481`, 0.08236658901774402`, 8.424499891492555`, 1.0114329054147206`},
{0.2428184603969214`, 2.139771549805288`, 8.962038430839804`, 1.2513955518769984`, 0.15789830342021016`,
0.6821214869737191`, 0.08731003079523225`, 0.005960116741721571`, 2057.9200252572427`,
141.81337749372574`, 0.11756230704325321`, 2.6769215386493905`, 0.895060254520057`},
{0.16708285607632617`, 2.514917463798218`, 5.621232009809386`, 1.0053867061169064`, 0.033964338040640696`,
0.565944967748532`, 0.21275159258566828`, 0.33969022847136165`, 3900.0636957226443`,
236.24950114350304`, 0.17313930544506606`, 5.330368544786425`, 0.772540585340217`},
{0.26506128276311264`, 1.6499267057635905`, 8.348095275849516`, 0.7847265312910929`, 0.6385591297525388`,

0.153373190400086`, 0.07053246573144985`, 0.21097306215358952`, 2309.981041141632`,
237.73247710249848`, 0.1662652738138321`, 1.110923557283094`, 0.6469777658769382`},
{0.13234258341034721`, 3.856159276277494`, 1.0641752125958632`, 1.2393517449503635`, 0.10012444404217513`,
0.25632211545729044`, 0.16888027141449713`, 0.016585410322055873`, 915.4984496215893`,
322.7924808984179`, 0.249680065848771`, 3.0405535188651083`, 0.4380264905508511`},
{0.08790421115050512`, 0.939015726321446`, 6.470926650783501`, 0.9066579200686449`, 0.8639128923505324`,
0.5386650165495622`, 0.10434830834744135`, 0.024067546956899128`, 1142.301427947522`,
369.2550313603065`, 0.13202765600512467`, 1.8671579613852387`, 0.37302887352364733`},
{0.20144727254246125`, 3.9392724652693136`, 7.1335815410177625`, 0.970870453628196`, 0.39841924348884183`,
0.5432660762964611`, 0.1877336667874709`, 0.10436699241700248`, 3471.7993430199795`,
68.39740948767945`, 0.03176666869513867`, 6.4702458499294195`, 1.1303015452765406`},
{0.05157825200138594`, 0.5074659893035784`, 0.4819934303065594`, 1.4763707530169474`, 0.8893682022937035`,
0.32243152253555507`, 0.2150347661898181`, 0.2715592826957914`, 590.6719840967398`,
259.49776063587854`, 0.04318917041730147`, 4.420302579747371`, 0.3690391195033085`},
{0.2034753024853403`, 2.7863395292250592`, 3.7168431579559567`, 1.0150680060811876`, 0.5649574956583596`,
0.4443788859910486`, 0.06710359282875367`, 0.04727087216660339`, 1537.3049723874492`,
264.87714036516047`, 0.039914831594556555`, 2.4435728162566726`, 1.470321086577068`},
{0.06436950144190184`, 1.0629347664880244`, 4.764095504810017`, 0.9320606212428386`, 0.7789254637724665`,
0.15639368410950716`, 0.08900209182216046`, 0.013774341985629202`, 2340.6363425632753`,
63.84867242722913`, 0.2371034531227812`, 2.23106662065706`, 0.9122412114799938`},
{0.24480551289378455`, 2.7380941448464746`, 6.271736317361168`, 0.9720924783254401`, 0.03239591203313119`,
0.6916885160816673`, 0.2288933066843994`, 0.006623283304233729`, 2475.282531422873`,
397.0207555711145`, 0.19280932100568626`, 5.189856377538095`, 0.933411035896893`},
{0.14578019322843683`, 1.8234631063666207`, 5.479118994270218`, 1.0733190277156792`, 0.6408034729339063`,
0.35566594296423915`, 0.17953675903981858`, 0.2394318445417401`, 2582.269870386629`,
303.8771057624848`, 0.24431388570309442`, 8.496328107267644`, 1.3584441574118422`},
{0.18481151841666188`, 0.509983650803119`, 2.442367304149805`, 1.4454403198705184`, 0.8862858075507192`,
0.20520962400760967`, 0.08831882715621944`, 0.028416610934367554`, 2887.1745513215255`,
96.99849051015605`, 0.012592247838918769`, 7.919739870540438`, 0.6672717811625197`},
{0.09189072955199334`, 0.5859834843121772`, 9.928586025072377`, 1.1393198615050828`, 0.6662151934740392`,
0.273376892578191 3`, 0.07354107739402921`, 0.00787232194478042`, 1945.927425117371`,

381.272657803446`, 0.10334632248372438`, 1.3094920948276538`, 0.643540323804628`},
{0.1929089042890706`, 2.044772709758891`, 3.6659878682330156`, 0.820711070285316`, 0.988447409153429`,
0.31657524049709074`, 0.23150398070670547`, 0.04424265567984524`, 3720.3695113921785`,
173.54302693598845`, 0.04329067746612414`, 6.852489239335057`, 0.8460112403464835`},
{0.10029172339419551`, 3.9666265224186326`, 5.94698722721138`, 1.3749912659713441`, 0.4161641564514196`,
0.2013069921355808`, 0.0801151159349077`, 0.014791692046983768`, 2279.1361385342816`,
360.73732873948904`, 0.013735250572300073`, 8.514487693936715`, 0.49310119875702574`},
{0.2302575021554365`, 0.7722117915953741`, 6.903211159271258`, 0.7585731891993218`, 0.822213946918968`,
0.5878727883839281`, 0.2427267432025807`, 0.09976117360743081`, 2459.4926457112624`,
40.66955655789843`, 0.10301492463748846`, 3.6967733258702205`, 1.4430046145797544`},
{0.22600110400418738`, 1.9843308027955775`, 5.115418048953915`, 1.1683391486965746`, 0.6057502933993939`,
0.6602202205935461`, 0.11123798530174628`, 0.4580159171910898`, 500.42496785799995`,
188.28582504079975`, 0.18938415994147217`, 1.0587108294757588`, 0.29531889665054534`},
{0.0717975612264255`, 0.4997816218149955`, 9.589567616976403`, 0.9450897781833669`, 0.6186434889471488`,
0.2554640898762033`, 0.06713444953336731`, 0.20940488388432735`, 1744.4191807628713`,
383.9973692232637`, 0.15609378466736462`, 9.862078934227451`, 0.38202361199438517`},
{0.26375231265942045`, 3.5837756528213704`, 7.490302795205832`, 1.1022035672057404`, 0.22095637191261486`,
0.15013919278259136`, 0.12602227786755316`, 0.062932127995949`, 2037.490516700962`,
396.7515615806642`, 0.09025977921836642`, 2.8179238772656654`, 0.8710125696526174`},
{0.048513291302257844`, 0.4457687353343176`, 2.3881995464771766`, 1.1956444252802232`, 0.6250208921524281`,
0.23033921635096732`, 0.16695818246187422`, 0.0058846456882393735`, 1714.5960674937287`,
267.0960709959734`, 0.21708290835986865`, 1.1611050275443535`, 0.4203748665144109`},
{0.14659244900446955`, 3.2113358110509322`, 7.247014044575871`, 1.1315336622196723`, 0.22569695706644644`,
0.6068766796842944`, 0.10140090760989762`, 0.07092703696530009`, 1627.6855361153575`,
309.5759062179927`, 0.23915839319989673`, 9.196051471747733`, 0.7212967644528518`},
{0.12214842072252496`, 1.1315944174857293`, 0.8186389490806132`, 1.4945075781385797`, 0.27658322947733693`,
0.6814994349474557`, 0.172591676587236`, 0.06293351186433137`, 2301.8386453341027`,
260.5275220937808`, 0.06414760022752092`, 1.8028968417694637`, 0.30689034634076684`},
{0.09472256798275819`, 0.9080295101270028`, 6.479473262813432`, 1.475083725476`, 0.4311907138108162`,
0.6630526571829747`, 0.09230911068175318`, 0.22906599144409456`, 1061.3916072436969`,
256.8481876635019`, 0.14505807910429386`, 3.6658722632692093`, 0.8887817463762948`},

```
{0.08360100641365104`, 1.0333139170716557`, 1.9968821416355915`, 0.9506164337764446`, 0.3637549658684145`,
  0.6013653723888159`, 0.09205612227237822`, 0.02498916600861499`, 2612.089754066442`,
  275.94814368199616`, 0.06598092927631555`, 5.08476480449065`, 0.8697872625899152`},
{0.14651792469342528`, 3.8182021256883862`, 6.121567760696932`, 0.9268713362782599`, 0.31845465027744546`,
  0.3641583988116113`, 0.18264126532755154`, 0.03701030981443568`, 3175.4599520184383`,
  92.90395359335434`, 0.14653570379145064`, 5.086657320457199`, 0.6916416664989691`},
{0.19531388174641623`, 1.6812423772528433`, 1.9285683163805027`, 0.9563894243653364`, 0.3037850003884799`,
  0.25494604947946875`, 0.19624000618521847`, 0.006277091862366441`, 3495.8037135332197`,
  160.73634765492886`, 0.21386501651912365`, 6.171699440629407`, 0.5167732609586244`},
{0.0992057581918559`, 1.9152747962131755`, 1.1385439717492112`, 0.8734081572386667`, 0.11569399031757222`,
  0.6355935198684373`, 0.242859485933678`, 0.028089883137919644`, 3591.0701912926534`,
  270.70715346890586`, 0.15233109233560077`, 2.1031889712747507`, 0.926279804309432`},
{0.04636398495503513`, 3.7018219205829457`, 3.897884943302158`, 1.3406897199868504`, 0.4374398316223367`,
  0.6128218100825888`, 0.1104350576389786`, 0.15302416148791886`, 2822.82344897813`,
  307.2546204697094`, 0.07105282376647054`, 2.3240488998943984`, 1.1737510413014665`},
{0.24799856399906778`, 3.665797094880114`, 6.286133411580671`, 1.3307944188010161`, 0.12338821047601245`,
  0.6316889254705509`, 0.13912514557691014`, 0.04125136326689172`, 2913.271077872927`,
  274.142281421658`, 0.16386049841393313`, 5.467517906165242`, 0.14206080539009291`},
{0.06486156480593103`, 2.9105746808401234`, 9.680954493261392`, 1.2128101831918736`, 0.9623327252795202`,
  0.40985068402002933`, 0.2170918483208174`, 0.22870254244919774`, 3443.315648780802`,
  310.5579520138933`, 0.21496605031805494`, 3.304326571943408`, 1.3905287372116648`},
{0.271295688290387`, 3.0581992057736915`, 1.212750414175316`, 1.3253429727133483`, 0.8276664257363009`,
  0.6720258079110331`, 0.1884616475047557`, 0.2223467966408117`, 2524.6082917357207`,
  148.45022765754868`, 0.13442291461399386`, 4.368234255778699`, 0.5211143399174365`},
{0.19444530138658833`, 2.174470015755692`, 3.8344558373626527`, 1.2294570245280045`, 0.29239376697066977`,
  0.28694291912822245`, 0.19808018049330495`, 0.005960265699843253`, 3278.3081237334245`,
  293.3280672381975`, 0.14107283306589785`, 3.3795768777624478`, 1.417577504179854`},
{0.2440718787450452`, 1.76561180349694`, 4.95225717475204`, 1.4224475381097175`, 0.8297144603659123`,
  0.6870441812099664`, 0.08604894661393966`, 0.28377285612513553`, 3055.531967355695`,
  255.76266261457465`, 0.07376742267862452`, 5.8076740025606295`, 0.30546127533463907`},
{0.0730994714406894`, 2.8447930433832704`, 4.637114941779341`, 0.8747237021104985`, 0.8742653369550875`,
```

0.4602914635394686`, 0.05825966522387901 5`, 0.01073521329241528`, 2525.027865177576`,
85.21306353882699`, 0.10502971061086047`, 4.901287944789633`, 0.3817190308545939`},
{0.21011190738341773`, 1.8519766611225768`, 0.8172862439758575`, 1.0025174016275906`, 0.4801024655035677`,
0.5883298648617231`, 0.199167083104676`, 0.2995026554507827`, 1201.2334950278255`,
265.10269406466296`, 0.16527637611813623`, 2.4641537957227406`, 1.185487228781699`},
{0.13811436070524757`, 1.6573442876198152`, 8.270384729474788`, 0.8471995866439316`, 0.9496826491555777`,
0.3970545948223342`, 0.06715784938015576`, 0.0832034730885496`, 1059.2542132237509`,
302.271366930348`, 0.038752991973303785`, 7.756453819144459`, 1.2408044408349754`},
{0.13575534771417241`, 3.107967888976927`, 0.2262387959194534`, 1.3197095272988466`, 0.6342015998603714`,
0.3957050275427402`, 0.21335490357997616`, 0.008750656688013836`, 1614.3899114822752`,
338.63558488350077`, 0.10329242712159703`, 6.6095853898508246`, 0.24958476272321328`},
{0.04766948135443208`, 3.0149795060421347`, 8.932446832206747`, 1.1136088500069867`, 0.021594711979614933`,
0.15138413756352542`, 0.08685938210991187`, 0.005007564365564 6575`, 1362.2649899849648`,
276.8149684326663`, 0.20202028836193903`, 3.9701409072362193`, 0.8085370569094716`},
{0.18773746739616126`, 0.4299564973161223`, 3.9318971489849446`, 1.0624860251059998`, 0.544093066833734`,
0.43356539469874333`, 0.16330330819292005`, 0.040590232235511496`, 2399.820966791748`,
233.03213128875973`, 0.16697913284338012`, 7.094059719533927`, 0.9913896416500063`},
{0.11479403264996152`, 3.823640197501849`, 7.042467218439598`, 0.8671713775083026`, 0.2915521094222331`,
0.3428757834262214`, 0.11149570618715388`, 0.0062188234292322`, 2055.9308370344506`,
383.5883910698325`, 0.2126209830320115`, 4.650760077469522`, 0.9718780818420139`},
{0.1495588092693269`, 2.9562247150199887`, 9.2229842597602`, 1.0688736942010013`, 0.2958780650578341`,
0.6794653079911299`, 0.137246604211619`, 0.025056259065215164`, 2160.2080931306828`,
208.1776104802966`, 0.2306160093069518`, 8.141052229334282`, 1.0329085826709994`},
{0.15518171148576348`, 2.3499047639493327`, 4.360850342323632`, 0.8133940186135149`, 0.676314303847662`,
0.37760044288930084`, 0.07941455326257985`, 0.04333405133254413`, 3294.797232932802`,
200.86295390407247`, 0.24389739709186076`, 4.759995129088669`, 0.4543298663517916`},
{0.11690059278696574`, 0.8783363086389482`, 5.7539466958675`, 1.1072974777335425`, 0.9679537377157941`,
0.4053342552984798`, 0.06890031093641119`, 0.23033316548333493`, 3511.029547228316`,
369.67514830597565`, 0.18966854703112757`, 4.698501468560957`, 0.7333804491317486`},
{0.104439705960304`, 1.3659305120838559`, 9.102673754552008`, 1.4903475140374016`, 0.6812753308651178`,
0.23307188642089138`, 0.0933185162696803`, 0.15281827719772503`, 2644.042120762095`,

355.84712203695506`, 0.219313267777153`, 8.034413215209995`, 1.3985960311786072`},
{0.06252530163633607`, 1.277236277016332`, 7.142814422389127`, 1.3604161019492071`, 0.8603611689699608`,
0.477237284782289`, 0.23913503177709117`, 0.03184426252613322`, 3236.2308449191105`,
49.142351256039206`, 0.06457622252014433`, 7.960619025437833`, 0.9912633563842763`},
{0.05927125459941385`, 2.080731116332318`, 1.403515084093046`, 1.4338880552058586`, 0.836296749585115`,
0.24670417168146108`, 0.17625476342874957`, 0.3553142034940628`, 3624.424617443623`,
371.6887670168725`, 0.06984462665929475`, 7.678587831183411`, 0.5475538898722119`},
{0.272219924072492`, 3.699843372922853`, 0.8152026115407764`, 0.8049313533010627`, 0.8541230874020713`,
0.3759570960402523`, 0.18901354163393813`, 0.05512718200659539`, 2420.333744013932`,
22.324560553991944`, 0.136832191006448`, 5.284550340747209`, 0.8839580209731261`},
{0.2192562547626593`, 1.328996578641208`, 4.408276687847465`, 1.066557441081671`, 0.8788419772651497`,
0.420358813032901`, 0.18871280922209183`, 0.12866248281712975`, 2376.751601415135`,
21.932515037813403`, 0.11160054269697284`, 8.38604448753847`, 0.8324170692270734`},
{0.05422626465899324`, 1.41058877238651`, 8.629501826749408`, 1.1478067099640077`, 0.4503418362410423`,
0.2944196840300318`, 0.21119418614347968`, 0.01982525406608443`, 1227.9750083569616`,
89.64728336361748`, 0.015407425530496982`, 1.3479803050296402`, 1.4374405820845841`},
{0.12963957180850916`, 3.3365224852422513`, 8.071825011712662`, 0.9114075438466118`, 0.39364722797662766`,
0.55978745554174`, 0.07369835156013937`, 0.12865812035500993`, 1440.5511589977796`,
134.4701033570321`, 0.035366553272213386`, 5.503562356456834`, 0.5009896530595863`},
{0.16726133018607092`, 1.930958758925592`, 3.0083121233683894`, 1.210686290991208`, 0.7910212105934074`,
0.3434585725088355`, 0.1642660591272488`, 0.14345094159717983`, 3630.780310378209`,
105.55549533534378`, 0.20790081387395154`, 3.589057052449082`, 0.2001887825986941`},
{0.08241763840187705`, 2.142561606833244`, 5.369049459923637`, 0.9852636250226738`, 0.24182715902115692`,
0.28976691554543155`, 0.24065607285215418`, 0.27404651530612695`, 2847.588493754236`,
186.1353278207099`, 0.17363556899862892`, 9.029927532666129`, 0.11169436731877491`},
{0.13700552002384053`, 3.065949790291736`, 4.950454801095971`, 1.0391958372853642`, 0.9776337698753284`,
0.6909746470667117`, 0.057475002733817115`, 0.00904662991314958`, 1580.3042677972962`,
74.3483640784055`, 0.03877364360478597`, 4.297289530434117`, 1.330917817336955`},
{0.05241322492447886`, 1.041476241641588`, 8.16095761491868`, 1.1603610389656813`, 0.26866448983520885`,
0.3320545378754204`, 0.11976413567707805`, 0.00713319584941315`, 967.2404027877401`,
264.4900675349543`, 0.2287367563952014`, 6.379237490331231`, 0.83403545199883`},

{0.18364277582298344`, 0.43366671305956395`, 6.703835279617622`, 1.4870472910528665`, 0.4047782007506182`,
0.5684293651673247`, 0.22561255273502645`, 0.1213725120163492`, 2694.706223544171`,
135.0368961894007`, 0.022200225329951195`, 8.985808481864087`, 0.4546405408559282`},
{0.17373830102452775`, 0.5396236269330235`, 8.716548818231772`, 1.2781997926114008`, 0.2994029165619403`,
0.6311279153766021`, 0.2004572873058531`, 0.029829077121486105`, 1206.6484556475298`,
212.29803760588413`, 0.13764513912866333`, 6.42052885239311`, 0.6565129788897008`},
{0.06373356464631308`, 2.4033771734928857`, 7.841805250818684`, 1.218475494212359`, 0.1766490969017389`,
0.5314535158814123`, 0.18470611278961951`, 0.013035445084968872`, 3902.45780595225`,
188.60058905321694`, 0.11342781551209663`, 2.042548476018199`, 0.8129410978823182`},
{0.0964110511384016`, 2.2365286327741067`, 5.8526191264605`, 1.4618699408667182`, 0.8182028658978528`,
0.387343137712196`, 0.24955267111553703`, 0.1017336334795354`, 1300.5591312646511`,
204.02951635694023`, 0.010778216795093748`, 2.376136268071548`, 0.7014041691125024`},
{0.22055192958492525`, 2.063754319806727`, 9.746892660619345`, 1.1263743900346668`, 0.3824619170868737`,
0.3377396468040734`, 0.07248726297246674`, 0.022971440771960824`, 3783.69159517103`,
308.71982775738024`, 0.16114587515785966`, 9.879452049970482`, 0.6820463020022816`},
{0.22700143296888314`, 1.2844277954779395`, 7.271913694665059`, 1.375337551210531`, 0.5655186709000168`,
0.37705025836034534`, 0.20454803556589107`, 0.08200704828690611`, 929.9638116909423`,
312.52970459059486`, 0.17194018780473708`, 2.7869672276628847`, 0.6271449892742724`},
{0.18044570897271706`, 0.9524926761671173`, 6.511131206394843`, 1.1225857366708927`, 0.011276794855722416`,
0.6477197715834044`, 0.16181965045393948`, 0.013358577208209321`, 2629.2386884676316`,
244.22468112889408`, 0.24315714767663638`, 3.8935447375778236`, 0.17056295407521116`},
{0.1031747291007129`, 1.0753817850602383`, 2.3901827825214443`, 0.9494324054212877`, 0.8655243169054956`,
0.5875365461047798`, 0.09875542522403158`, 0.020636236338079122`, 3668.9306940550296`,
32.944367358953286`, 0.21955795791262667`, 5.455907508975919`, 0.3138297355806803`},
{0.2492174803391337`, 3.8186304877300854`, 0.6593165104089422`, 1.3669144880495308`, 0.40531282588620576`,
0.25181467777873967`, 0.15220457771402102`, 0.006740465790302422`, 796.5961892440373`,
51.67728195440782`, 0.07309861388864569`, 3.0773122735176646`, 1.432188704910093`},
{0.27928795263916784`, 0.5798885963915357`, 3.438729573463075`, 0.7859704525714069`, 0.13732274355578267`,
0.6009735798147406`, 0.18539967791007028`, 0.1369068620522905`, 2588.2655297569117`,
78.78627673888747`, 0.13662571551540947`, 7.147737978252394`, 0.16582779201112174`},
{0.158309183474712`, 2.284367344540179`, 3.1130137774580398`, 0.8245076604975312`, 0.2939127991946775`,

0.5448027938340341`, 0.15596740404331277`, 0.060580071785009876`, 1917.5874264102922`,
102.41199828758255`, 0.15653233667838135`, 8.020771066756815`, 0.16368718495509893`},
{0.09855067343586066`, 0.42248858314270876`, 5.888441909786696`, 1.374275487614058`, 0.713481343784103`,
0.39177356523031326`, 0.12647140495570885`, 0.021778948867459624`, 3561.4755687032084`,
75.36389346411136`, 0.14783328914983274`, 7.192274801142693`, 1.4077827751177328`},
{0.09888970514692547`, 1.0773269521808775`, 4.509787207697677`, 1.034834313165084`, 0.46524158243265124`,
0.6370595817977545`, 0.17795346839566295`, 0.052840345624928245`, 1394.9195521303727`,
145.8914281907807`, 0.14189384893594092`, 6.214985461828465`, 0.5648213653786103`},
{0.2574834517331688`, 1.8047481810399715`, 6.678550158926733`, 0.8506612742210977`, 0.9197441161866675`,
0.28136838946825016`, 0.14128624992883748`, 0.0064270584722317895`, 1966.6222967379854`,
284.61250325943354`, 0.23333938304045732`, 6.6167222434451745`, 0.3934211369713194`},
{0.264103406072841`, 0.6388918081456714`, 7.451950258224173`, 1.0512911558430225`, 0.698530834423349`,
0.4711872720679928`, 0.06135082806583303`, 0.2926679041436665`, 1310.8742778358974`,
123.75694501020632`, 0.09712150854448914`, 7.74080995928431`, 0.8193975183528215`},
{0.2613599171041266`, 2.58453322148865`, 6.473133758347821`, 1.1092209897073517`, 0.7935873547661896`,
0.22081691696159222`, 0.21037162231016598`, 0.22559388527799015`, 2971.40901940996`,
103.45193855805343`, 0.031105461375102783`, 7.240063151509036`, 0.6982732456981431`},
{0.05972716054183652`, 2.4084080668442445`, 4.924113152287115`, 1.0973018723863064`, 0.45846865431577455`,
0.17436075418224495`, 0.13306789950554873`, 0.08269850672896895`, 1061.1497496191419`,
231.6428562776456`, 0.027554419387727458`, 5.529015160121926`, 1.014914954734902`},
{0.20906782584649525`, 2.253200985640894`, 8.714537859819917`, 1.139406043052919`, 0.18448556976959418`,
0.3412765002806809`, 0.08047235865027924`, 0.007526938093912886`, 3981.3819787959537`,
150.99126827565385`, 0.12762508996370686`, 7.834813706411341`, 1.133986173662005`},
{0.14168212332279645`, 3.629529656644613`, 0.31240616809545685`, 0.7793905839088386`, 0.6288942527555839`,
0.6686461551742169`, 0.051179205682700984`, 0.015333271439627172`, 3119.547847449303`,
332.77374740974926`, 0.18323666728985671`, 8.115016271373861`, 1.3496472408131814`},
{0.11363747349097014`, 3.3902353384962547`, 6.9199640132703095`, 0.8644073024825804`, 0.6214378859880609`,
0.26046821126038044`, 0.13701105918453266`, 0.15359214599837653`, 2561.5661019345407`,
185.76122258296698`, 0.15708434183781178`, 8.31641484873343`, 0.7597169041059111`},
{0.22123407652153293`, 2.082927160793078`, 3.494558969518348`, 1.0455662143345417`, 0.1785350961948371`,
0.6180201085084245`, 0.0648475297068132`, 0.006469955463562222`, 2336.7510217065665`,

120.4669332891844`, 0.020699881273580856`, 5.403351657201977`, 1.2197076183004314`},
{0.26969936534459843`, 3.0645680107332938`, 7.896848185396699`, 1.1676096938151472`, 0.03246672129411676`,
0.3430750433397468`, 0.18007472085753745`, 0.00937848460863108`, 691.2033005272847`,
393.60922326457455`, 0.10952344673101261`, 8.268833108688174`, 1.2664329385455138`},
{0.09707681532069068`, 2.1085331709443667`, 7.06540706713491`, 0.9411343900767416`, 0.9036401467365582`,
0.2916719417534003`, 0.09652690802667035`, 0.03828184000699648`, 3174.8977687655497`,
60.92568989682479`, 0.05469741369744979`, 2.8213654015691247`, 1.0633604979013946`},
{0.14835712091109915`, 0.7730242131483069`, 3.477772408678769`, 0.9950646362476758`, 0.43363684394770785`,
0.4723829080128319`, 0.247934187727751`, 0.09606686055299227`, 3641.8956482526974`,
352.02514132734564`, 0.059909381056589994`, 4.715269674310706`, 0.9126567361088749`},
{0.07690717877213288`, 1.853650900512208`, 4.705122089204835`, 1.177168847413763`, 0.20514199762323693`,
0.5853606940036812`, 0.13349096224239904`, 0.025824642502794638`, 3833.38590479347`,
77.1112215680152`, 0.1481478527297927`, 3.868716389694623`, 0.3394641684298805`},
{0.2792305556244907`, 3.773985442432103`, 1.91428975676104`, 1.3562902020721337`, 0.4175948252900008`,
0.22518067870989456`, 0.1671047483185617`, 0.02746479862850403`, 1101.9393721601946`,
344.3881723084337`, 0.08960284517652922`, 9.994170351350675`, 1.3158131558054151`},
{0.22478599831633878`, 3.8226352096828684`, 0.3445246491273739`, 1.3626729827827502`, 0.6328825985865549`,
0.4911616627377584`, 0.2351834938983145`, 0.020644881197453675`, 1711.2523897617402`,
90.3557844360617`, 0.22332245485704472`, 4.143025581982037`, 1.4109410839085532`},
{0.20246997439609643`, 2.796989949233529`, 8.601219291155875`, 1.0746358846435033`, 0.23672394588378642`,
0.5308972062471274`, 0.07979598008259464`, 0.06626312934080632`, 1425.6928357201414`,
284.5057677770119`, 0.012297934961132934`, 4.043474061276271`, 1.0687107503597373`},
{0.05455278033197386`, 2.653355923086507`, 2.8901946723921075`, 1.0586559640275577`, 0.4813227217769469`,
0.39307761087509174`, 0.2243292319383302`, 0.14625278281931525`, 3083.4776957133126`,
151.39135551576533`, 0.22240872343945634`, 6.123940268706669`, 0.19893741817415211`},
{0.1261136314023228`, 2.7924651112189345`, 3.1510744750802027`, 1.10573405830265`, 0.8251438792845518`,
0.38426336320599863`, 0.09970139546376985`, 0.07482975828652592`, 1939.6449909308958`,
136.19904432267293`, 0.18392510915657945`, 2.6983520820510374`, 0.2768728179957576`},
{0.19898919294397377`, 1.094863215028309`, 5.141348317963875`, 1.122536541475456`, 0.25105837287153987`,
0.22994429074756872`, 0.06388318517905306`, 0.28661130713234945`, 1651.4164658316613`,
309.756100189421`, 0.22912307516081604`, 6.461389927282495`, 0.7712732446594246`},

{0.08906555841605851`, 3.099177869846099`, 0.23414284508699804`, 0.9801911457566903`, 0.5103708245250258`,
0.6482190821775309`, 0.09673419046052228`, 0.05503363591579261`, 1161.2636269890509`,
33.10846443598513`, 0.20242548439112923`, 4.392158138354276`, 0.5844178562263633`},
{0.12731801883579802`, 0.6615371738972167`, 6.357864878818987`, 1.255082973105248`, 0.4534404170796209`,
0.35401897300932406`, 0.1793246308628349`, 0.24542217350407658`, 3327.8256670682013`,
361.04151598602084`, 0.1826398340506512`, 5.639198085050134`, 1.1435146635553615`},
{0.19766525289867687`, 1.5539440876458963`, 7.208959079796127`, 1.2770132142294717`, 0.17265151827851555`,
0.3201118036294218`, 0.21136859719947498`, 0.44059120086782566`, 1120.1312471078827`,
306.58844853012556`, 0.02781674233524073`, 4.752053599009571`, 1.1633837773022284`},
{0.16821931653812278`, 3.9393436886276305`, 3.179019625415814`, 1.1903697350607159`, 0.6888287521080438`,
0.2381411552279049`, 0.2058483354518103`, 0.007287906278596642`, 1880.9980419826588`,
370.4098828230061`, 0.17633160606015752`, 7.349472380855945`, 1.3310916733121423`},
{0.27267841809763693`, 2.4698162119019047`, 8.674593338753226`, 0.9507011512806018`, 0.410886345842024`,
0.5761365147011471`, 0.24420414171291205`, 0.043929586623096185`, 2067.2152285645698`,
299.4614654667555`, 0.08071123306974876`, 3.4242661195281348`, 0.9268436590103244`},
{0.09734540105037814`, 1.0928119036432262`, 3.3746924724651315`, 0.9148152167379004`, 0.5520222973975617`,
0.5701876550674219`, 0.07525801655581627`, 0.11060319201619584`, 3458.3887900851914`,
360.58656442125186`, 0.07476322979210304`, 5.145406927076468`, 0.3705928304372572`},
{0.0485781063525863`, 1.7849942773456657`, 1.173858124805676`, 1.3582711053956302`, 0.42211815922461504`,
0.5511485692462849`, 0.057310028881033426`, 0.193275446076273`, 3997.3620434488857`,
116.86597951838672`, 0.12079571732839778`, 1.998996511553914`, 0.9355040158846233`},
{0.2386999966275894`, 2.722739872786626`, 1.3062557112348774`, 1.1073558597851454`, 0.32667496326027057`,
0.18792754690523328`, 0.11880359556139461`, 0.03176567183554873`, 2751.8624934955715`,
349.72147263095553`, 0.1223537595062607`, 3.6928606229815575`, 0.1168338292618869`},
{0.11566857496325095`, 2.3986647351668298`, 8.513377414925483`, 0.9662333740036599`, 0.8541931022288194`,
0.36101996809007564`, 0.11530341629473645`, 0.14705784731340077`, 1645.8753074989063`,
310.3763160081718`, 0.036104725588746445`, 9.667079447216771`, 1.4942192927137747`},
{0.12899904957253838`, 3.8001456108425966`, 4.68277328679647`, 0.7500997606167263`, 0.7705050389454449`,
0.5067773892687756`, 0.08071179965828676`, 0.006247983675383929`, 2714.029884924169`,
310.5929142878384`, 0.10714318122808536`, 8.497633727984809`, 1.3195673297775237`},
{0.04202009520980362 6`, 0.8125545855681908`, 0.8820021130226205`, 0.8679479110418857`, 0.336031336984445`,

```
0.27953459644666345`, 0.05504645570926081`, 0.01654003058638047`, 2110.5518526782043`,
  28.793440121823267`, 0.16487272926817176`, 7.902234236019242`, 1.1698954211202324`},
{0.1949044088002926 4`, 2.2807650240422737`, 4.164948405329779`, 0.8920125211738439`, 0.9916182528214048`,
  0.24033665404408822`, 0.09749261388251534`, 0.009353219207928497`, 558.6138953254322`,
  192.81883910349768`, 0.12112913056266378`, 4.477996891709701`, 1.3978148126017071`},
{0.16009076205688888`, 0.5349813484836639`, 5.374724113132956`, 1.4400444163269621`, 0.6922562033282762`,
  0.5453216758418981`, 0.13981434114543434`, 0.007280151675390544`, 3925.9979560926067`,
  341.46046547891854`, 0.19348925902086372`, 8.443436699305352`, 1.4876822395937013`},
{0.1933638890845395`, 3.103067052973441`, 5.111571620050322`, 0.8661544577982099`, 0.8149222692447449`,
  0.1595760565881339`, 0.15190282082017698`, 0.005535189167289717`, 2999.703437637353`,
  300.1128617408108`, 0.10100183998523121`, 2.2676395814752386`, 1.3326114208284863`},
{0.06217291008845033`, 1.247503477484723`, 2.5904087629086465`, 1.4683499815329046`, 0.08458788439332876`,
  0.3930546684373296`, 0.18769435268404766`, 0.1379057398065497`, 1008.6694100163231`,
  278.10094161452287`, 0.21639096057090101`, 7.204492217199894`, 0.5358436507882129`},
{0.22725673950089992`, 2.3639145368218726`, 8.344676289930643`, 1.325883986841482`, 0.6505898944316628`,
  0.43112151917918207`, 0.20810105395654238`, 0.010151436710313634`, 754.9801668003361`,
  190.32738043229529`, 0.07958968362345098`, 7.5054987393450965`, 0.6093046711635028`},
{0.2309391357161658`, 2.323989179094352`, 2.3593321069217`, 1.3993677346710833`, 0.7027057115205266`,
  0.26166872252110007`, 0.14152733686240188`, 0.2848639929776201`, 2532.106437595179`,
  386.9846027360387`, 0.07114341876102281`, 5.5959659257370316`, 0.47197407419590953`},
{0.11128373013292153`, 3.3734167013736274`, 6.971024005221597`, 0.8270211193396084`, 0.0351306115056782`,
  0.4871945686666914`, 0.14931850539545372`, 0.02878252625021986`, 527.2114941578984`,
  74.82432219823664`, 0.24191356032713884`, 9.568158477220692`, 1.3339247715553384`},
{0.18315330325553064`, 2.869805671153377`, 9.468498073936939`, 1.1725531828202505`, 0.3267495326537466`,
  0.6971808886096422`, 0.09399727517157247`, 0.4622104919604099`, 2265.8497866660555`,
  116.1383358572474`, 0.02715190911437171`, 5.561797604651474`, 0.6165440590050792`},
{0.22883543071148366`, 3.066706533284199`, 1.1439898434307647`, 0.7535281320523861`, 0.11497111120814263`,
  0.31581178336786275`, 0.15168936854496579`, 0.1415060368713317`, 2986.3979531848126`,
  146.71341498099025`, 0.14475182625551103`, 7.74072484577729`, 0.18905729960113726`},
{0.1197189938163688`, 2.6914481530927663`, 7.902408409631434`, 0.8148212240990508`, 0.32365266714619123`,
  0.3812630996449249`, 0.2044952740314684`, 0.28795554719398225`, 1263.0479831599341`,
```

```
345.11453037398417`, 0.1466603335899901`, 5.094081228711747`, 0.9032476272618188`},
{0.089875647559861`, 1.0302327249870382`, 2.0596378930749815`, 1.3241139107745634`, 0.7453299280714754`,
 0.5095722380183404`, 0.17917298341587806`, 0.222730051899884`, 744.7092202123449`,
 118.59827741373601`, 0.0989210230322109`, 4.9882080789644085`, 0.660240574996108`},
{0.17809194642727977`, 1.7697866199706649`, 7.1763151221741985`, 0.9277024880907543`, 0.02661598115385022`,
 0.6083929126896732`, 0.174056840727485`, 0.0393026670208333`, 1347.608160247999`,
 208.03482783091147`, 0.17902076650274934`, 4.82657960035886`, 0.6328054071662224`},
{0.16322971965863348`, 1.170728402327721`, 4.129867013138943`, 0.9550069661931267`, 0.7632521383792994`,
 0.23062906697550656`, 0.15191938086081902`, 0.036668793904547074`, 984.9683279569181`,
 143.85195072672764`, 0.06218474573819438`, 6.894986630118397`, 0.7118840534242801`},
{0.0673211374947203`, 3.556048182108581`, 2.056072368577791`, 0.8749285572736749`, 0.3104681364634243`,
 0.18652482922479574`, 0.08201020657253827`, 0.044529186328014586`, 1689.51074710815`,
 162.90358092109705`, 0.0729615101290263`, 8.407666834293693`, 0.4031543367979451`},
{0.04130707589470009`, 3.0971581646939166`, 5.095324578907563`, 1.2851498582955627`, 0.14534319212364366`,
 0.3324535366896153`, 0.10589589728908583`, 0.27538535378439405`, 3967.498675941073`,
 98.17019510308785`, 0.19069934275540157`, 9.378576326653985`, 0.907790255687607`},
{0.15433792416794267`, 1.8940009891686929`, 3.062826353150152`, 1.3594047846621364`, 0.2865379799536014`,
 0.5165321095788333`, 0.13923535270760634`, 0.41888186557593104`, 1933.0228901580695`,
 124.09667777854662`, 0.10026068856071241`, 1.5843039926062341`, 0.6139086768148141`},
{0.2448264512865554`, 1.018619142628224`, 0.3220150078672237`, 1.1814459661349885`, 0.2096682202943383`,
 0.4340268291716256`, 0.23122304821991574`, 0.028723954302408285`, 3147.8296084170843`,
 26.23322055856977`, 0.011843614667667979`, 1.6271943864377292`, 0.8914600074049073`},
{0.17570694085504118`, 3.3716783189083603`, 9.273626081950546`, 1.268846725094217`, 0.2584124599965636`,
 0.4223453277058432`, 0.22832672513046204`, 0.20979915181246073`, 1007.8354949521076`,
 148.0706036186794`, 0.04379179030226882`, 1.2158335564871514`, 0.28231812649411747`},
{0.2345875520938262`, 0.8974705100407228`, 2.4400268875162734`, 1.1692882451601165`, 0.6149116315758598`,
 0.4838775739193003`, 0.16902409799301954`, 0.4305314166593524`, 3073.906515744251`,
 376.94525437674156`, 0.0327953725790982`, 6.842916385098416`, 0.9809907681036996`},
{0.26599408746951475`, 1.69869318027059`, 1.7982317117152924`, 1.282444106635544`, 0.20521633822817087`,
 0.28537099789283193`, 0.17587118168619353`, 0.016831793401851286`, 735.9167536628461`,
 185.12449867405962`, 0.08818428944304713`, 9.590487549937748`, 0.2554550886680036`},
```

{0.26593765918786066`, 1.469820509215408`, 3.318163900631456`, 0.910737040027697`, 0.772245323565576`,
0.6475514852601838`, 0.05903920363599993`, 0.25524741893447345`, 3882.180697561048`,
173.8977279389328`, 0.22560057599213157`, 8.523060233161317`, 0.6568135548380971`},
{0.13345787440765705`, 3.0774883151503953`, 6.064298363382008`, 0.9302596892930706`, 0.6158208110956915`,
0.2585099500502801`, 0.21374523912969606`, 0.4331393141373296`, 3227.8457633864045`,
202.63915585725056`, 0.17639348124623005`, 1.682515063867495`, 1.3735184213990381`},
{0.24299864830682322`, 0.6348382188885924`, 8.738994620341877`, 1.2356500801881907`, 0.5770135193458605`,
0.3643052268917296`, 0.09417548384960592`, 0.18321093257407522`, 2194.4027446042355`,
392.84971084704546`, 0.021287183084439776`, 6.026738164007792`, 0.24540222372747866`},
{0.23128596410211183`, 3.0231614800775644`, 9.282448241767675`, 0.9502905953068014`, 0.1244248471921543`,
0.513834937748406`, 0.07158073583305624`, 0.006593831926001459`, 1596.9261281907548`,
180.2466252506967 5`, 0.024392561873633367`, 8.693689234388824`, 0.44515269072814956`},
{0.14763336174315628`, 0.9609083924153881`, 5.767031511187355`, 0.7806215764240099`, 0.6823220865642827`,
0.65198969122125`, 0.1566301693296936`, 0.37595000066945006`, 3860.5140609631935`,
333.3928376138639`, 0.19492955999326989`, 4.361121532997624`, 0.15723055392755292`},
{0.08020873136305112`, 2.3560872112839712`, 7.656859171402218`, 1.2445222240766278`, 0.028603428928333008`,
0.3751343060067356`, 0.17582324568527535`, 0.011898009060677216`, 1487.4044642092413`,
223.64331235141128`, 0.06070604179895395`, 4.4126063398287325`, 0.25472858441799273`},
{0.2593624262161626`, 2.295431745726197`, 3.3398768253362086`, 1.233697621211021`, 0.17962106789139365`,
0.6119977215212877`, 0.1479404509305488`, 0.2392187562484209`, 1384.473686906931`,
390.9701237284811`, 0.07023942191875382`, 2.3245474291144017`, 1.4493220736835069`},
{0.10144857451882727`, 3.09793869586695`, 1.9042542140912566`, 1.128489317749993`, 0.9516046831815148`,
0.5718665343974156`, 0.24712737605206275`, 0.10884528767737062`, 614.5820043578747`,
372.33327243103304`, 0.18531918510116352`, 6.894885736301427`, 1.4197916184504975`},
{0.10212230421521268`, 2.1055462656508395`, 0.1549942156465125`, 1.1640680269346606`, 0.2898972915158997`,
0.471231711737255`, 0.20995913095460078`, 0.12219762483818075`, 1171.3965983974595`,
79.93077709858363`, 0.21373183331424755`, 1.9880929888308272`, 0.4174963770861413`},
{0.24870831012490324`, 2.875980304064811`, 9.212538429441743`, 1.2722063248788578`, 0.3282913490792485`,
0.2805639294283834`, 0.1572124446820367`, 0.027187541841276704`, 1491.5273167119358`,
343.00060613142807`, 0.23102970098548903`, 4.50321802361168`, 1.2980803493745015`},
{0.044603778808110894`, 0.8401502429199104`, 7.1121926093305605`, 1.273381316556913 7`, 0.08797724114115923`,

0.30964677383308725`, 0.08962339338921654`, 0.02592380473764512`, 3666.957163896842`,
393.9775757725391`, 0.24527553411695868`, 5.578947990675762`, 0.6585937869939971`},
{0.08578994575352372`, 0.9866526872309773`, 0.8038090794361139`, 0.9523570781926003`, 0.948882623737388`,
0.46311647996062744`, 0.2441006263954396`, 0.07831609826614802`, 1671.5327042927038`,
210.47342650267535`, 0.12673136991162653`, 7.353061586513307`, 1.0620625305759188`},
{0.07858821042838116`, 1.6977143802882821`, 8.212860235255583`, 1.2964521187700828`, 0.2987911497011755`,
0.506126935243553`, 0.11440340360512286`, 0.05206277291990468`, 1339.867027105688`,
383.7096297624307`, 0.2078889107072941`, 8.822910683955193`, 0.960825500308768`},
{0.22377870799620725`, 3.1928985468211346`, 8.059299312075755`, 1.3921709162211404`, 0.8873395666266357`,
0.17674042984905347`, 0.1675175234386073`, 0.03730263939446273`, 3395.3924465278315`,
247.66374801748043`, 0.05539393372492046`, 2.5087357156089176`, 1.4318151504319032`},
{0.1736316989567453`, 1.5454802872078899`, 7.071248146205292`, 1.1599254463866382`, 0.1492821504834294`,
0.5667749351814715`, 0.17572322700248577`, 0.00782459398724836`, 1916.2311636952181`,
145.94896290337653`, 0.12610631603338446`, 7.247312880006737`, 1.199936026275772`},
{0.09782332022288404`, 3.741906492613495`, 0.4566213801268671`, 0.9419423117532648`, 0.9082792827328243`,
0.61004571220758`, 0.08136955531902801`, 0.05934353731887512`, 1613.3231664795385`,
279.2732289747655`, 0.2228587683695682`, 2.342091864078169`, 0.3301362991730612`},
{0.08130667751836484`, 0.5260337564871658`, 8.449122941731599`, 1.4306920647099228`, 0.5994021973571051`,
0.5116121891099925`, 0.1394200086694753`, 0.0062549310940251375`, 1045.8518042423143`,
239.77908991527943`, 0.20556349167963178`, 7.684382915701004`, 1.0762288196903413`},
{0.1509673631351663`, 1.3366819546761866`, 1.080587080528149`, 1.0668100793723834`, 0.024191324402023318`,
0.40903296900163466`, 0.12044262077141116`, 0.013316823826732663`, 3093.793000213601`,
297.44428012608614`, 0.05031916378077894`, 4.50357864911024`, 1.4241445207811716`},
{0.09526052730311735`, 2.7416128013280456`, 5.368501678201278`, 1.47877684019711`, 0.27293015978313817`,
0.1533702997985575 4`, 0.05033287162982811`, 0.0696637301808707`, 1979.7812280561757`,
208.2796492673417`, 0.10966372378753608`, 9.228791958515647`, 0.5867218918142831`},
{0.2168252075927255`, 1.2147794793098745`, 1.3294046517319291`, 0.8204909797710571`, 0.5261154919084852`,
0.32065750745864985`, 0.23412216559654364`, 0.026797285872998095`, 2049.1492401808873`,
336.0554255621465`, 0.1855137474935905`, 1.9365416597785146`, 1.1594140358846108`},
{0.2246406125901032`, 2.0919939936953575`, 9.60889385739615`, 1.3369466676292021`, 0.09066206605235938`,
0.26041948811653903`, 0.1688935708724063`, 0.006310725218569426`, 2524.013414165627`,

```
287.3446683034351`, 0.19227735577509142`, 2.226723678369055`, 0.32009387356875707`},
{0.06878866730089028`, 3.1245944973527457`, 4.360637333709242`, 0.9232062619650272`, 0.8528374363112068`,
 0.38941545287751145`, 0.1888087204525729`, 0.06440167068123348`, 613.447059996161`,
 135.80840514252725`, 0.06946957961295136`, 9.642980612869511`, 1.3375272307560842`},
{0.05526353896520764`, 2.7240387277783293`, 3.0534884167300707`, 1.3987200069968335`, 0.586176455097533`,
 0.6957346200792549`, 0.06800542073977661`, 0.01575809860169522`, 1863.0130158242628`,
 26.33179097220551`, 0.24642894997981157`, 6.726887116499235`, 1.3266658102153124`},
{0.22421646906489323`, 0.8944001471401766`, 8.80542685575837`, 1.1224114425628389`, 0.07399275239021863`,
 0.6541781208595552`, 0.1361819449849342`, 0.011426236224829198`, 1677.1018012951417`,
 95.2905099154865`, 0.2261399510764292`, 3.273626323923104`, 0.6313176099847766`},
{0.04830398547205017`, 1.275316218137875`, 4.614953899177735`, 1.2829927355973278`, 0.16612061807564849`,
 0.1639575953122483`, 0.12272200896549845`, 0.011345722370418136`, 3563.2586080627198`,
 299.98688385512116`, 0.24385149927488797`, 1.0836535164777654`, 1.0916200214657223`},
{0.16680821963704395`, 0.5163225257189996`, 0.5139271367473146`, 0.8080955581319136`, 0.922329371415127`,
 0.31540072696317234`, 0.14858531583274454`, 0.09051887795629471`, 1828.24438708676`,
 386.07575250791444`, 0.1874671503356095`, 9.751925633231235`, 0.9375414879403592`},
{0.19224907453000317`, 0.9379671824303957`, 0.1768927357330181`, 1.2227110146173203`, 0.42649904659884763`,
 0.4545029045501904`, 0.23904198572269159`, 0.01364047530387634`, 3771.0454255220884`,
 186.69997665609355`, 0.04831449314300046`, 2.795709036233411`, 0.30562138724668775`},
{0.09905125996953207`, 1.8406681837351115`, 7.615683537936391`, 1.104455595350767`, 0.8507777546248461`,
 0.6055060761025552`, 0.22355503841638658`, 0.019159603171833028`, 3698.4676016336716`,
 125.34182227908332`, 0.18683452817866786`, 1.0248116987499856`, 1.4496781158120586`},
{0.2122053342005913`, 3.2723497606887317`, 8.074911535839025`, 0.761570924685792`, 0.7803544809949507`,
 0.2686687403920138`, 0.24235001021375163`, 0.4921495003944174`, 553.2367270947411`,
 320.67673970953626`, 0.04374240634138288`, 8.97316011873557`, 0.13752746723967268`},
{0.250195686307457`, 1.9873181983813915`, 2.436986915389067`, 1.2297238031002529`, 0.8787886927027242`,
 0.17801285885202778`, 0.10899785398220965`, 0.042181056020100846`, 2975.5972306347185`,
 176.4908556989327`, 0.20657495695660671`, 8.247111235919043`, 1.3291652831072591`},
{0.2026768701716405`, 2.320679359442215`, 5.20658497151164`, 0.8289159455996751`, 0.9558590658000565`,
 0.5980005929117111`, 0.20724756852094034`, 0.0547372318523302`, 1071.5776650859361`,
 208.90678381552232`, 0.10872472503561953`, 4.389182379894066`, 1.1547524475482653`},
```

{0.2780417648717225`, 2.452933782766964`, 5.240377582277642`, 1.0300585799728414`, 0.04659296483118158`,
0.5270409461030353`, 0.07214810969171709`, 0.4018520591476553`, 1664.9812973970193`,
368.4059321600538`, 0.19070038794765692`, 6.66208935797424`, 0.8907914326786526`},
{0.07805827059301479`, 2.683721229977123`, 9.002208120629334`, 1.281556673983075`, 0.9431208960986435`,
0.16472833391173414`, 0.07811354421183636`, 0.31224015378681164`, 1280.994389531913`,
98.15018684851424`, 0.09634892070904322`, 1.4706754835946878`, 0.8821078383157976`},
{0.04941033680222237`, 1.4372745957329531`, 3.017421402893529`, 1.227554635491979`, 0.5953141676696632`,
0.39465993131783816`, 0.0709805192571406`, 0.011053419404823772`, 580.3765952495469`,
188.40590324275774`, 0.07356671197341247`, 3.331653425139695`, 1.2649663092466472`},
{0.08087154180018502`, 0.9611156520127571`, 3.21224132650018`, 1.479145162275284`, 0.656808362332749`,
0.6575245079334169`, 0.06402086777278035`, 0.03166366563194078`, 3830.357327788698`,
29.178148219068248`, 0.1565085846563346`, 2.9278897142269216`, 0.2225569823133069`},
{0.05254670070328532`, 1.9573141803351248`, 1.2965014076065788`, 1.332575014963222`, 0.16006264378785184`,
0.5916179072285757`, 0.24512530080047995`, 0.22325909451251788`, 1444.5445370368107`,
345.46000244130437`, 0.09820743687290096`, 1.9309038402941605`, 1.3978069307443537`},
{0.18436703585318248`, 1.635211707023542`, 8.369994731341517`, 1.0460883726353645`, 0.04194749676902032`,
0.5545482251392689`, 0.12087395792220468`, 0.0196688212581193`, 3332.658025818686`,
221.69628027119882`, 0.14765622082279756`, 3.2016279616467553`, 0.8091642391531453`},
{0.23462399893919866`, 1.1910919878969217`, 8.83980004261467`, 0.9529401154166846`, 0.02195492110865227`,
0.5092860902949421`, 0.15815979544916958`, 0.02056729012396142`, 2175.860445178102`,
128.66287522198218`, 0.01279382216028177`, 3.236783194651748`, 0.9208763725654232`},
{0.17738230405709043`, 3.796341718770906`, 2.6191444768927727`, 0.8810359048502269`, 0.8432004845871259`,
0.5364474114112706`, 0.12890044163052178`, 0.09199163668005597`, 2908.5735595435463`,
327.07488963843366`, 0.2488393941661181`, 4.302397057177101`, 1.240771752535033`},
{0.1997594423184546`, 2.9770319172231083`, 0.9793144795904194`, 1.1541884780655445`, 0.8809204935983468`,
0.3405913744561432`, 0.1996665175564089`, 0.005381059121025975`, 3350.374432264831`,
76.29678229001865`, 0.1902613732440958`, 8.131907933048556`, 0.2784170059108251`},
{0.20820380555279266`, 1.947139162442057`, 0.8103918093452425`, 0.8157066692238308`, 0.08411468051466398`,
0.597917518430226`, 0.19620399803346056`, 0.2948101237919289`, 3907.9815148798407`,
119.17289873329406`, 0.10826075744997776`, 6.209951432179045`, 1.4468389844056118`},
{0.16610260493961904`, 1.5013302413059524`, 6.689821314230944`, 1.0482229687817899`, 0.1182529778116388`,

0.3276703522967399`, 0.18950977903448152`, 0.011602315706762772`, 1976.8505440085055`,
128.55302132767815`, 0.1206362258416388`, 2.990415653764348`, 0.8762156912964318`},
{0.18623015008520377`, 1.9857475843613202`, 5.839525692796151`, 1.2062427816326777`, 0.7227315679666446`,
0.4380351155851495`, 0.06781424675520398`, 0.3047046595907428`, 3879.8004023641743`,
31.750244384294604`, 0.1136575036642325`, 7.396681541568938`, 0.3865663286027079`},
{0.11240037278862203`, 0.6491092968888421`, 0.8347611214383086`, 1.4549390021490687`, 0.14670428342309072`,
0.37243292165179553`, 0.23972369902467655`, 0.01185953233820889`, 3372.5164268352446`,
237.4368160205147`, 0.1138398904730133`, 5.916126824166563`, 0.20110182675101163`},
{0.07362676393672318`, 0.7355538194163032`, 3.988559823436466`, 1.1003960033820657`, 0.2330826487371216`,
0.30236238420511485`, 0.203470150172021 7`, 0.007403410519656421`, 3383.0372997062996`,
216.63011163159672`, 0.15749031565529237`, 1.8803990102478743`, 0.9980728680407593`},
{0.1783157725484701`, 2.833766552573177`, 0.3970480956539326`, 0.7937141247518862`, 0.3426969414627741`,
0.17660776442515302`, 0.06749542877910886`, 0.2962004793965935`, 2934.9500228426696`,
381.96465886346436`, 0.2370505433342167`, 2.5471608872904294`, 0.782904324178523`},
{0.19113040953496924`, 3.008225434073468`, 6.355992034495486`, 1.3442852038382949`, 0.5652481966732263`,
0.17677420590456394`, 0.15942860908355244`, 0.011393875146253816`, 1546.892426029899`,
241.3481933910041`, 0.08634784635015025`, 8.231809963935362`, 0.7429779031641539`},
{0.1657364273353646`, 1.7016560013922657`, 2.864207314541927`, 1.4862862793345373`, 0.7297929998129427`,
0.24571335694321284`, 0.16930689708064994`, 0.03637877544295533`, 2361.9950805518038`,
368.27061311393015`, 0.19309626758691123`, 5.461234510584882`, 0.819553318889934`},
{0.243670782866162`, 2.468817897966531`, 5.276425534951404`, 1.2860325806374826`, 0.9986905753378303`,
0.15094415892422752`, 0.11186418412899801`, 0.017007046631466297`, 3882.4128529746977`,
364.42553589609224`, 0.22618030191028127`, 8.141676219478274`, 0.7328485154782189`},
{0.24765743502672427`, 0.8633830393481601`, 4.686988270445781`, 1.3325105661709873`, 0.9808367367869013`,
0.6497982062118903`, 0.12182607994660094`, 0.10862851464254074`, 1618.8129303586047`,
29.218901620977476`, 0.12364149245529477`, 6.112534098964166`, 0.4592681454217531`},
{0.05305812976899127`, 2.2171177144674177`, 7.758771838981232`, 0.9284694801023683`, 0.19067769039866356`,
0.644253374243589`, 0.22681045533276267`, 0.012934901517196042`, 1447.005934926009`,
36.93966570702122`, 0.23490962595246417`, 9.29802294373275`, 0.16252108168662027`},
{0.15871988201336973`, 0.69579697553139`, 0.8043899133503648`, 1.3164539868322986`, 0.019311018286041204`,
0.4418003624772314`, 0.15298309339567961`, 0.16062079917918642`, 1028.6861638399246`,

```
  387.4645099104921`, 0.1109630441095813`, 9.931289248375212`, 0.6360820981063933`},
 {0.047521269126133275`, 3.6122781353018008`, 6.744377644847237`, 0.8245850034533604`, 0.6257570737736955`,
  0.5030346499913471`, 0.1805220904232982`, 0.04172896692907123`, 2236.066689248032`,
  158.14626584756797`, 0.19953621789908887`, 3.2116522076145895`, 1.150911515892405`},
 {0.25961180109703935`, 3.304460665024532`, 9.731602575575781`, 0.7572846132780213`, 0.5757688453852501`,
  0.6201294851486256`, 0.16223330053532697`, 0.09572577856146514`, 1068.913375906082`,
  270.60830273991644`, 0.06817614592711113`, 6.616513224635819`, 1.43430723538359`},
 {0.2570743120023581`, 3.9645616544323667`, 0.778960090135616`, 1.0710848615453759`, 0.4119983463981953`,
  0.5200874369461032`, 0.1117389487686139`, 0.10363949916728431`, 1639.730583125267`,
  312.26786162753615`, 0.028234231304594515`, 5.869357124529651`, 1.28422648371814`},
 {0.2562944092973924`, 3.075529570162291`, 2.568785124728583`, 0.8870148603223287`, 0.9606731370217558`,
  0.2809776665243148`, 0.1016902992050977`, 0.08251508016369583`, 3903.9002781741165`,
  210.26921647621452`, 0.17009591440353533`, 5.238706912116847`, 0.8143837239091778`},
 {0.19549413117658376`, 0.48089214427271987`, 1.3626169663716503`, 0.9415198179325406`, 0.6690196233409793`,
  0.21285229920071647`, 0.06541758209495863`, 0.01678513797168908`, 1726.2120585826779`,
  324.36081581294445`, 0.2027319827154383`, 9.808516157446391`, 0.15828659811715773`},
 {0.05227980057052345`, 2.0688773882101916`, 3.6600804041449706`, 1.1141893430147094`, 0.9026814754625292`,
  0.2868484255785748`, 0.07257484506693682`, 0.008111547996607639`, 2542.960019998747`,
  267.5073034447911`, 0.05331315230928124`, 5.17983990011182`, 0.732439763493062`},
 {0.16735387248889688`, 2.2901749191726726`, 8.076219774814714`, 0.8654381827857112`, 0.8324588302247302`,
  0.6842201049055578`, 0.20618844268782105`, 0.08330233274804327`, 3386.0149520877076`,
  231.00380607589523`, 0.24136440782861118`, 8.110578461374818`, 0.1918239477431709`},
 {0.13514798653424465`, 2.2171967161009567`, 6.79713748604177`, 1.0120008311676625`, 0.6388353844515477`,
  0.24746546435416983`, 0.06301863255527632`, 0.010147071600538154`, 1724.1318426660982`,
  61.023906450601714`, 0.20149638211275833`, 9.77881328490188`, 0.3825890641127949`},
 {0.1787587102327428`, 2.3010027450563504`, 2.4341725113616057`, 1.2732377481963428`, 0.4302268260704325`,
  0.6620369180993946`, 0.07196824748401134`, 0.018432445015582038`, 2032.846726882145`,
  239.6521240526355`, 0.16901168551833623`, 7.60355154430442`, 1.2541734909464242`},
 {0.040040522389580085`, 2.419089216699528`, 0.6029402922994809`, 0.9540354124914854`, 0.5857842300398015`,
  0.29335248867278896`, 0.08466193409101153`, 0.02752786168327366`, 1658.0955376199636`,
  315.19357733141317`, 0.05596420932223484`, 9.548403397933097`, 0.8186383788896436`},
```

{0.2738861805350336`, 1.7653732412133003`, 3.204285049736129`, 0.8904691615720403`, 0.006777008376177118`,
0.6422126566237496`, 0.21196430882173833`, 0.02384817430133364`, 3509.269393393407`,
210.0509220408095`, 0.1665518558626684 6`, 5.6829810466015855`, 1.030785881009633`},
{0.15392813884203022`, 1.030461564252417`, 8.114572747609092`, 0.8272078545061425`, 0.12582813602684628`,
0.5315104372547351`, 0.15396277604540914`, 0.15739980098951944`, 2790.789894250489`,
293.11851381168844`, 0.07517370467239265`, 9.08396266539139`, 0.493350448003097`},
{0.07916527150572594`, 1.5183903543815438`, 4.168943084064299`, 0.867458547779902`, 0.553589800680331`,
0.48176316193720536`, 0.2283008873811488`, 0.05557602912612958`, 764.3466604125074`,
98.1402416311065`, 0.08270787403012858`, 8.344308234825018`, 0.19844893248168471`},
{0.21281579254928695`, 2.151234091423703`, 4.99405590028015`, 1.327714565612129`, 0.5224256111223291`,
0.6816332648036347`, 0.12550320306228152`, 0.04036971585137323`, 3491.0437940299144`,
389.9199715703103`, 0.034574092345333096`, 7.184915600941272`, 1.1300147670199618`},
{0.1629622003008503`, 2.9652820153229094`, 3.631698402387231`, 1.3160868122315574`, 0.8459043277254139`,
0.3376309499507988`, 0.22909924766435485`, 0.29198486983597477`, 2152.5623501482423`,
151.56102061804546`, 0.24318844331582645`, 4.2450714851638445`, 1.1792634690726134`},
{0.1743683640254448`, 2.5950167332919714`, 4.4795399396250115`, 1.4920254306676737`, 0.7406209345210579`,
0.5005424929143194`, 0.1556663350770055`, 0.009754157595577621`, 589.2836300166855`,
248.33057190508907`, 0.03897335434322141`, 4.862373453735419`, 0.5620446056385968`},
{0.21708195818330828`, 2.190522036446822`, 0.6621285433489934`, 1.031682155458868`, 0.05602003593078142`,
0.3042918074708675`, 0.06460489028881022`, 0.10032641034223391`, 967.4633500940654`,
32.783765325462525`, 0.23824254150550733`, 7.5221087110888885`, 0.8625924583250821`},
{0.23286963035122604`, 0.6749345188436879`, 0.20207668159083703`, 1.2334071076981241`, 0.15415172519231857`,
0.5502451893184188`, 0.06023408333501554`, 0.2884596823004714`, 1454.378698010155`,
160.31978742859337`, 0.13928726255657442`, 2.5761586107644754`, 0.6603399169512614`},
{0.14295588103954704`, 1.412350123248217`, 3.177274858880626`, 1.1913477441560478`, 0.681260929106482`,
0.5789699232051367`, 0.09066128493810405`, 0.04793649880497841`, 2544.3651836876206`,
337.2728729655139`, 0.011905134502266851`, 4.430427622941885`, 0.74003827479563`},
{0.11809903796554244`, 3.7153066081905903`, 9.394589088557098`, 0.7843066157779603`, 0.3510759725555237`,
0.22803969658113876`, 0.10165008188335536`, 0.005399145823047646`, 3902.7306906909416`,
388.8592048663462`, 0.07729944387610005`, 2.9718049006587925`, 0.2891160085661364`},
{0.16202429237235855`, 1.84055090570393`, 9.090225524744397`, 1.4016003618243056`, 0.18111565798783458`,

0.3854966323949548`, 0.18149685472874316`, 0.020436792872453445`, 1570.1908216352922`,
182.31609839273892`, 0.10515949401254626`, 5.892117812074294`, 0.4606489730839265`},
{0.23592081280505794`, 2.903542817229688`, 5.375766226090379`, 1.0406060233325234`, 0.5645881743757346`,
0.6023633607842607`, 0.10170656063773648`, 0.2230968616100032`, 1009.1076309440446`,
206.38966170547087`, 0.05554284619714861`, 3.437370706600392`, 0.2602834274636374`},
{0.16797762422450352`, 3.393739269293537`, 1.1723349699849432`, 0.7876651455122983`, 0.44858157260876874`,
0.38511915317210543`, 0.11126403235021692`, 0.0942998855830809`, 1121.8751632200092`,
393.3700502870249`, 0.18309808816527456`, 7.014749603006731`, 1.3459972330025094`},
{0.17119714315024748`, 2.9315531156526395`, 7.9309025517292255`, 1.4075914943739267`, 0.478244345688974`,
0.6490520691087696`, 0.11764936739953108`, 0.10875560486489166`, 3501.9685138343757`,
327.0147307274891`, 0.208688235529143`, 1.76928881617237`, 0.15623787817751156`},
{0.217831918966031`, 1.9579162032970752`, 8.666870397376897`, 1.3889143705395077`, 0.46152386514296384`,
0.5340210525452818`, 0.23464141923209442`, 0.08443497471803191`, 2881.1816355963792`,
40.761442588478076`, 0.026544318054481375`, 2.7988265733593654`, 0.6550641924577596`},
{0.05909313438823349`, 3.998253006220927`, 2.7606012432005738`, 1.485525656051693`, 0.006754191626425765`,
0.414858609675688`, 0.1539104163421624`, 0.018873368421200944`, 1163.2065814066136`,
134.3441176716297`, 0.2419809034541145`, 8.466048197729386`, 1.4640823465517014`},
{0.05150190326045606`, 3.439128676554682`, 6.3456448543065065`, 1.3220565831174902`, 0.020535895361411 65`,
0.5629905593040019`, 0.12931144501098696`, 0.09719268107904352`, 992.6848015373967`,
364.3637294024504`, 0.08014172250870272`, 4.69481466986781`, 1.4428291086778402`},
{0.07096987014316519`, 2.2631739720341697`, 4.701888958284682`, 1.1268609277641355`, 0.5889482309057459`,
0.3638885919169875`, 0.05633232701579177`, 0.3132395508309391`, 2469.4656057395405`,
63.55428754000428`, 0.06344769284580837`, 7.209878698655041`, 1.262286526290143`},
{0.13424044754942377`, 3.4568739781145084`, 0.7268053299269095`, 0.8378137298915767`, 0.4654803229022981`,
0.5071605573546728`, 0.16764031212635366`, 0.06824087778957048`, 600.6265082502678`,
271.9873255003124`, 0.09465386647177981`, 2.8029066087185885`, 0.5029743243735594`},
{0.1814998220796576`, 0.5457041309396096`, 6.628442116729424`, 1.4816442478897651`, 0.04014018359229943`,
0.36141091722571295`, 0.13153411394886588`, 0.008451051131756494`, 2397.6906312548626`,
255.11228411318268`, 0.24580682176341695`, 6.012737749288213`, 0.971372885437352`},
{0.25116849017091947`, 1.0524160237641933`, 0.8392172051883035`, 1.4326662099306988`, 0.4925377955188637`,
0.4563121318749678`, 0.10417530701605077`, 0.012130927300066367`, 2850.5603662771573`,

```
  179.7348471844905`, 0.14797581172978125`, 6.731564570008534`, 0.3667284512812037`},
 {0.10627844972153494`, 1.9122914449389148`, 4.879880104768988`, 1.1152938555639593`, 0.36058806344071925`,
  0.2510558996667485`, 0.133980532681923`, 0.018281043179813147`, 2529.9927880778405`,
  195.84606271036728`, 0.10858226709292867`, 2.0748738252120926`, 0.9066780951872615`},
 {0.2706982412362611`, 0.6287160520084307`, 4.14855201722539`, 0.8172328386793793`, 0.4851216922484758`,
  0.20982300489352756`, 0.08395856094354753`, 0.16391690500747624`, 3554.6452712204655`,
  185.42012094476195`, 0.05154754434614739`, 8.513250180668177`, 0.37417348469464895`},
 {0.2598662442063618`, 1.900211126429065`, 0.6320497826451614`, 1.3904132145962274`, 0.3261450588717545`,
  0.21444284573744743`, 0.1520608211075556`, 0.47681332398299126`, 3867.6546770468185`,
  328.5593273663678`, 0.12810041704772773`, 2.5338343017479055`, 0.3750039812454511`},
 {0.15997673085841746`, 3.7261077489541483`, 1.1512593893886542`, 1.376812544747029`, 0.7263504373210632`,
  0.29998090848320735`, 0.07358394021576772`, 0.4410919571058908`, 3522.7326806197016`,
  234.3351737610849`, 0.05138796494631709`, 5.103095752677643`, 0.43578634794289095`},
 {0.25099622201635424`, 3.95450763611092`, 4.239563708075597`, 1.1463399035908803`, 0.8567090221336071`,
  0.6807931178480928`, 0.18419051557334198`, 0.2564924471830997`, 698.4202789859287`,
  154.98713283086113`, 0.08058524622619823`, 2.246868522171841`, 1.4084793726849045`},
 {0.24225332438192398`, 3.651195248984296`, 0.388765132686828`, 0.8400870426253413`, 0.1821318445823885`,
  0.5573875510223865`, 0.23605316030342982`, 0.08377442811214376`, 1289.2099511812157`,
  318.7461100660206`, 0.19657856943178847`, 4.498486658412734`, 0.6713610627619064`},
 {0.18294446021192495`, 2.0751042203060504`, 7.88640633654303`, 1.253927303712154`, 0.8981192233254209`,
  0.26591266597763197`, 0.15456768387072084`, 0.011263961326455277`, 2103.388409363515`,
  330.13115223762657`, 0.1896947651115678`, 4.4078344078613245`, 0.7904477019841809`},
 {0.15110272633071126`, 1.2120684691778738`, 9.821466182804322`, 0.999249111664473`, 0.8884716643403618`,
  0.5717874967013771`, 0.23862826359871653`, 0.2965485968483864`, 799.8579275077459`,
  260.7684224687745`, 0.20976611603443018`, 5.031757425833598`, 0.8119791706180317`},
 {0.15717196764210273`, 1.1630073112625743`, 2.634220279774457`, 1.0045604176965792`, 0.8286996726157703`,
  0.4235785617926241`, 0.10102087300007184`, 0.09118767410472639`, 1523.7756565066265`,
  301.9435543713025`, 0.21266930821707325`, 6.460470764249699`, 0.28210830372520523`},
 {0.1799797654068389`, 2.619455956239074`, 7.697999886354548`, 1.3015491336074843`, 0.555451962044029`,
  0.4433835581064074`, 0.0862654213077988`, 0.03372825256230826`, 2391.7036278961978`,
  159.9832142066033`, 0.13737033538391902`, 3.425775682450297`, 0.8987360464358476`},
```

{0.16862146125981442`, 2.3151392610028543`, 3.2020316316267774`, 1.3239310858987743`, 0.7072607224053895`,
0.3500350043589193`, 0.17362573378910773`, 0.060358617251082186`, 2001.4962431618678`,
114.46542957104901`, 0.20499895995123107`, 5.257752800260672`, 0.4338865062331345`},
{0.12274664777380989`, 1.2488875899911855`, 5.516183439796784`, 1.1021378510059003`, 0.9773171588432767`,
0.5961273492690635`, 0.12759486552792382`, 0.1532767587236556`, 2513.3632749376284`,
182.05354083120335`, 0.05801301004138587`, 1.8616328305112617`, 0.5158476130411485`},
{0.12291711454694165`, 1.1877487218897347`, 9.942019369479574`, 0.7752358604533577`, 0.7160618607453235`,
0.17825216453319837`, 0.07028904237235806`, 0.009021701114390435`, 805.4612461749266`,
63.6125839558365`, 0.06956787912807044`, 9.33154348593904`, 1.458279492320873`},
{0.08216873811896613`, 1.3179095560013607`, 1.6483112503948085`, 1.3628139186636958`, 0.6342779575022122`,
0.2115034893488278`, 0.10646590409334977`, 0.055264018212609206`, 510.50583604116036`,
394.4203282160594`, 0.13986107835869105`, 2.2752923009835655`, 0.9070556531583542`},
{0.0476259458526086`, 3.971655945581807`, 1.7442422392857733`, 1.129286089919653`, 0.5285852533702986`,
0.39600384447582293`, 0.2215904518169189`, 0.024542351730056703`, 3543.890003102313`,
364.0135445894731`, 0.06778311290485467`, 8.573488767539896`, 0.9317909345539408`},
{0.15063755404470214`, 2.71723210471837`, 7.148609543518862`, 1.1908848946871171`, 0.5800141434913164`,
0.29138799856290376`, 0.0950097459950863`, 0.009655692674775408`, 856.5663047299263`,
347.96462878162436`, 0.12103625881441793`, 4.831460173736216`, 0.8148377888737439`},
{0.25325667629893317`, 2.6359345155712095`, 6.284411921985339`, 1.14262610260638`, 0.592877633639628`,
0.19560008128978879`, 0.18445820883756597`, 0.009163632115762594`, 1288.7476768868028`,
192.8490635634654`, 0.16457035981631868`, 2.082802800802952`, 1.1518950127144034`},
{0.14655389849752304`, 3.5234321911845736`, 9.93741755620114`, 1.0550925690751938`, 0.4448537382022568`,
0.18176809875202415`, 0.07434797690036096`, 0.27672257287602875`, 2966.99983242307`,
232.89159682919671`, 0.13795973482450868`, 7.950903042528848`, 1.2964183380427592`},
{0.07405512950518717`, 1.967002000369666`, 3.1335932173998042`, 1.0217576314214762`, 0.5481985257231932`,
0.23482039961813828`, 0.1719074884408075`, 0.010212529243490897`, 3198.221153713561`,
38.498265037806334`, 0.12854240418059937`, 5.86125181611254`, 1.042171003495977`},
{0.2776663951441912`, 0.8310897457250155`, 2.0994479814067635`, 1.1003401269753144`, 0.30863525720948326`,
0.2434882924277243`, 0.08746325015577566`, 0.02243947015640089`, 2140.6294222376637`,
299.3219451566707`, 0.02431460260750562`, 6.100643757524728`, 0.33277870666165055`},
{0.11907642171566374`, 1.274849235584174`, 6.899983100552333`, 1.1682344315973607`, 0.7800022001471016`,

```
  0.41036075345185574`, 0.1345484896240624`, 0.007510763802150046`, 2156.4467856375795`,
  343.9093505673661`, 0.08426365817638315`, 2.310160002798696`, 0.6194154558015803`},
 {0.17937371459880497`, 2.8718008248848648`, 2.8198656683422243`, 0.9976096277706823`, 0.8604494338327144`,
  0.3675397910291335`, 0.21060856912564696`, 0.0072115171001387954`, 3962.8369792288295`,
  287.0010951200238`, 0.23748423652768247`, 9.572841373703643`, 0.6693837986608342`},
 {0.1446481422691977`, 3.7473728388010814`, 7.773074810909127`, 0.7768035182896058`, 0.045349053147926144`,
  0.5946440249560018`, 0.18594588773391896`, 0.007600014271866709`, 2201.8419670169405`,
  172.3692269613906`, 0.22887729014375824`, 6.4667868323956865`, 0.5687524909215391`},
 {0.2743682788249912`, 2.567007165847575`, 8.880180070850319`, 1.3334617648385514`, 0.053478262105743024`,
  0.5870310036210435`, 0.14269834247006347`, 0.006777940660941963`, 3288.224802818225`,
  343.8350462827967`, 0.07407598386826336`, 1.0311140447256584`, 1.1022538897878755`},
 {0.08682640281398363`, 0.4802834262657396`, 6.166420960796351`, 1.3554629143634556`, 0.6132401723597833`,
  0.3572005740934433`, 0.19494034836925994`, 0.012289451288465592`, 600.315756452052`,
  42.934435033309114`, 0.23123185628174636`, 1.349114625805871`, 1.1058635186946257`},
 {0.04351054041483948`, 3.010882988409519`, 9.401958493592993`, 1.3186120593482111`, 0.49329539098927055`,
  0.4698069940559316`, 0.20226774754753019`, 0.03712775492888272`, 1947.5927514696796`,
  172.9604174112643`, 0.17909729794724766`, 1.3306147942525488`, 0.46866213033580717`},
 {0.13996819046085934`, 1.009532661617322`, 6.145072622160212`, 1.3450345414096851`, 0.2868040480361447`,
  0.18183470956578074`, 0.13804571299234253`, 0.12631256619855588`, 3863.110841624364`,
  279.40163171428946`, 0.18548475334584474`, 9.210069253579718`, 1.2809334264760701`},
 {0.0871063251493665`, 2.6634842127081404`, 9.193238345408428`, 1.4387089146023326`, 0.7548565000722143`,
  0.6823132747448253`, 0.19549587941758378`, 0.13611301540454773`, 3578.7577781208274`,
  129.49687931265402`, 0.027673492175817715`, 9.652606809319298`, 1.320301893923864`},
 {0.04655003226957066`, 2.717305676329323`, 6.261066689781977`, 1.313518883642772`, 0.22988974709922583`,
  0.48948284819450716`, 0.1924496553607991`, 0.40105381179739646`, 1594.1735712767286`,
  296.74834364325125`, 0.10951121773307326`, 3.7043174616791976`, 0.46788624174476623`},
 {0.24058904599557956`, 2.305486806402752`, 6.870865029896366`, 1.1663266064905513`, 0.291316248043183`,
  0.4756853450103681`, 0.11657670703629042`, 0.014427542030819147`, 3856.0018830548897`,
  333.80293042286075`, 0.20384852921840946`, 8.723427410033644`, 0.10055245356998821`},
 {0.09672875865983738`, 3.3181066447219294`, 8.772587658219397`, 1.2949486969635013`, 0.026680322952838287`,
  0.3340762083993216`, 0.2420609904328586`, 0.3944381790098702`, 1841.1432974548734`,
```

```
89.15662847720563`, 0.1287997244865557`, 3.057938101959733`, 0.8332397440047616`},
{0.14493124545339742`, 3.446306300213565`, 2.261950562129204`, 1.3227848229460633`, 0.5758774796201231`,
0.35943404017729164`, 0.23613553647425112`, 0.03288786445085886`, 1106.0434527992074`,
293.5700758832494`, 0.23807677975419816`, 3.6561682593343647`, 0.5615502445855836`},
{0.15370210593846179`, 1.1469734999246084`, 8.184859582737346`, 0.8081018637833421`, 0.7671904423744005`,
0.5546779173711273`, 0.13880357299862195`, 0.3920406444124731`, 2732.468857403337`,
28.150193121997006`, 0.06240913374814305`, 6.929933598007185`, 0.8840553716357653`},
{0.06413533732712456`, 2.7505505433928237`, 9.582115463692716`, 1.419324092949555`, 0.0693862133505021`,
0.4085416515074848`, 0.14517410092691557`, 0.08408491973612985`, 1524.8762411053558`,
131.37866475928564`, 0.011227169364072448`, 3.5456702127620083`, 1.1107372198382217`},
{0.10991493466216079`, 3.7381599633374956`, 1.5402547174363423`, 0.8574896025687118`, 0.9021341382697974`,
0.5521862271024119`, 0.19447159277653675`, 0.017387026381523858`, 877.7862619046055`,
398.7215907021463`, 0.023981889033480308`, 9.30826908769696`, 0.3374448683929794`}};

(* The outcome of the global parameter sweep was obtained by the code hidden here.
   It uses randomization and will generate a new array of data.
   It takes a long time, at least half a day. *)

Npar = 1000; (*how many sets will be tested*)
SetBasicParameterValuesHet[];
eta = 1780;
CreateResultArray[Npar];
Nnres = Array[f, {Npar + 1, 2}];
(* The minimal viable cell number achieved in simulations can be monitored.
   Numerical acurracy can be adjusted if desired. *)
Nnres[[1, 1]] = "#";
Nnres[[1, 2]] = "Nn";
Quiet[For[ij = 2, ij ≤ Npar + 1, ij++,
```

```
NotebookDelete[pr]; (* To see the code running *)
pr = PrintTemporary["Set " <> ToString[ij - 1] <> " of " <> ToString[Npar]];

kappac = PatientsTrainingSet[[ij, 1]];
kappap = PatientsTrainingSet[[ij, 2]];
gamma = PatientsTrainingSet[[ij, 3]] * Nnor / 10^7;
V = PatientsTrainingSet[[ij, 4]];
ks = PatientsTrainingSet[[ij, 5]];
rhomax = PatientsTrainingSet[[ij, 6]];
rhomin = PatientsTrainingSet[[ij, 7]];
omega = PatientsTrainingSet[[ij, 8]];
alphamax = PatientsTrainingSet[[ij, 9]];
alphamin = PatientsTrainingSet[[ij, 10]];
kf = PatientsTrainingSet[[ij, 11]];
N0 = PatientsTrainingSet[[ij, 12]] * 10^7 / Nnor;
kD = PatientsTrainingSet[[ij, 13]];

ACurSim = FindCurDoseHet[]; (* find curative dose *)
DA1 = ACurSim * nCpm; (* set curative dose *)
Ainj = {{t1, DA1, eta * DA1}}; (* set schedule *)
FullSystemSolutionHetMD[]; (* find time of achieving of minimal N *)
FullSystemSolutionHetMDStopNmin[tminNn]; (* solve it with cell proliferation stopping at this moment*)

Nnres[[ij, 1]] = ij;
Nnres[[ij, 2]] = Nn;

WriteDownMeasures[];

check = 11; OkFlg = 1; (* to avoid possible bugs *)
While[check < 31, If[ ! NumericQ[result[[ij, check]]], OkFlg = 0]; check++];
If[OkFlg == 0, ij--];
```

```
]];

In[•]:=
(* Figure S.22, left plot: scatter plot of the minimal single curative dose with respect to cancer binding capacity *)
LethalTox = Select[result, #[[19]] + #[[22]] ≥ Abld / nCpm &];
Ill = Select[result, Abld / nCpm > #[[19]] + #[[22]] ≥ 100 &];
Cured = Select[result, #[[19]] + #[[22]] < 100 &];

Show[ListLogPlot[{Thread[{Cured[[1 ;;, 3]] * Cured[[1 ;;, 12]], Cured[[1 ;;, 14]]}], Thread[{Ill[[1 ;;, 3]] * Ill[[1 ;;, 12]], Ill[[1 ;;, 14]]}],
    Thread[{LethalTox[[1 ;;, 3]] * LethalTox[[1 ;;, 12]], LethalTox[[1 ;;, 14]]}]}, PlotStyle → {Darker[Green], Darker[Yellow], Darker[Red]},
  AxesLabel → {"γN0" (*"Cancer binding capacity, γN0 (pmol)"*), "Acur" (*"Minimal single curative dose, Acur (nCi)"*)},
  PlotRange → {{0, 100}, {1.5, Automatic}}],
 LogPlot[{gN0/(eta + 1) / nCpm}, {gN0, 0, 100}, PlotStyle → Directive[Darker[Blue], Dashed]],
 LogPlot[{(gN0 + 2) * 1.5/(eta + 1) / nCpm}, {gN0, 0.1, 100}, PlotStyle → Darker[Blue]],
 LogPlot[(*non-toxic maximum dose*){(((Abld - gN0/(eta + 1) * (lambda * 0.5)/((lambda + 0.5) * (lambda + 0.4))) * (lambda + 0.04)/lambda + gN0/(eta + 1)) / nCpm},
  {gN0, 0.1, 100}, PlotStyle → {Darker[Orange]}] (*,LogPlot[(*non-toxic maximum dose under eta=inf*)
  {((Abld) * (lambda+0.04)/lambda) / nCpm},{gN0,0.1,100},PlotStyle→{Directive[Dashed,Darker[Orange]]}]*)]
```

Out[∘]=

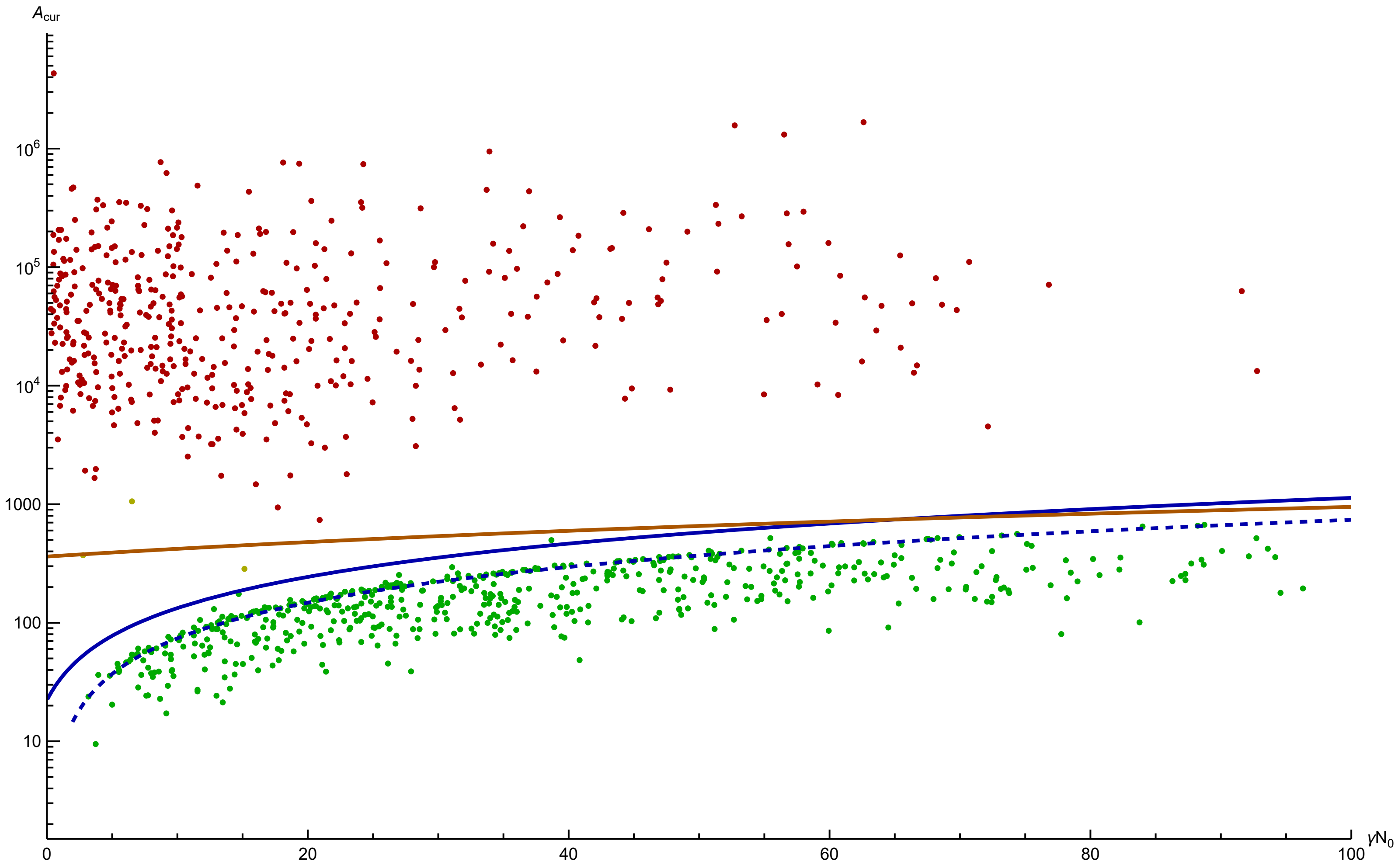

(* Figure S.22, right plots: in silico trial *)

In[∘]:=

(* The results of the trial are saved here *)

In[∘]:=

```
Npar = 1000;
Patients = {{"κc", "κp", "γ", "V", "ks", "ρ0", "ρm", "ω", "α0", "αm", "kf", "N0", "kD"}, {0.19106794235726526`, 3.422211425984277`,
    0.043657305888837719`, 0.8061975142942569`, 0.8339298566036648`, 0.5953772058253635`, 0.2237160252367293`, 0.008443468845115031`,
    2880.2762677101127`, 85.74785636777187`, 0.126766968040477`, 683.4468278824766`, 0.6294729165525801`}, {0.1606889138703409`,
    2.2231673786561217`, 0.008877852281252067`, 0.9039296731847397`, 0.34590644767728174`, 0.568784316236284`, 0.13226557086208773`,
```

0.046818349482260095`, 1216.1172793574287`, 64.70541101587469`, 0.03002574443244105`, 825.3955274980484`, 0.4936711220217507`},
{0.22940474241593012`, 3.465573558480557`, 0.05403006151569757`, 1.4833996144759156`, 0.42019962663308985`,
0.562859662433015`, 0.17302228714555912`, 0.006000155249706144`, 567.4301692358536`, 252.27194074911688`, 0.20525487785742114`,
308.12203149981394`, 0.2833278045039913`}, {0.14142574638887473`, 3.814113738544756`, 0.0035922301635856967`,
1.0658038562942458`, 0.47759091456026126`, 0.16373712203284474`, 0.21882964803654442`, 0.007631679116598651 5`,
2665.869331956139`, 22.562737753752515`, 0.06601460261723141`, 987.2000969897812`, 1.1538087284669323`}, {0.05313904785907647`,
2.1608731959555607`, 0.010753202055824768`, 1.0302113673860718`, 0.8874099687289569`, 0.5973785513169955`, 0.05555059571079654`,
0.019065046650065063`, 659.0888422771477`, 246.0781847522136`, 0.2452972409541973`, 579.6629619714057`, 0.8366484674191468`},
{0.1461217338635024`, 3.780489655710582`, 0.02457505680564415`, 1.4362626519898651`, 0.6945407380647459`, 0.1784909952189706`,
0.18394686464768112`, 0.00547849813416339`, 1279.4163297709756`, 29.98768886541717`, 0.03751217598877543`, 376.5115418614892`,
0.9748513761561566`}, {0.1656346104979341`, 2.6753846842450333`, 0.01200592731844207`, 1.4354057372844258`, 0.6384290136403854`,
0.39125592829873357`, 0.18923488815384854`, 0.3268243142117359`, 2495.818527440937`, 398.9965282206566`, 0.033737429558096`,
516.2253597010539`, 0.5233734407747033`}, {0.24578356986766597`, 1.6941685982352883`, 0.050192966959191024`,
0.8686928603280664`, 0.5845718432711879`, 0.6173129507981705`, 0.2432660657233256`, 0.12596652963694252`, 1269.7519551052783`,
52.54130944389664`, 0.1649468254558416`, 343.904295875501`, 0.7296417516214699`}, {0.18006942483414456`, 2.7787480334278314`,
0.022984023966977105`, 0.9120974032589063`, 0.9056603278473274`, 0.5260534950664542`, 0.1346014979743776`, 0.2528057174651332`,
1274.5680192011396`, 93.48967900074439`, 0.1614720428897813`, 384.50133673145785`, 1.378599273049681`}, {0.20536060152333618`,
3.137317923463736`, 0.08428112694808328`, 1.0250620366088086`, 0.31812111401327137`, 0.20390370486415277`, 0.24220064658208773`,
0.006549777177560355`, 2912.6312791206938`, 66.52226765631491`, 0.14740404961579828`, 906.2339428539893`, 0.6560001828426458`},
{0.0800858655120334`, 0.6618913176745953`, 0.058567217568644914`, 1.1697269178410128`, 0.2539379145085754`, 0.41116028173405084`,
0.24791380614440833`, 0.06726706041032536`, 1951.0780160530967`, 20.18075527430335`, 0.1633048585703344`, 704.7266306618028`,
1.346819900728074`}, {0.1427440001421425`, 0.8731762273275221`, 0.0759119130421918`, 1.1726204598213206`, 0.8290497873757678`,
0.15592023629523832`, 0.1215542147628661`, 0.021543427940341548`, 1026.4896183114438`, 384.9750041656`, 0.168767653354427`,
939.8175471142928`, 1.2011209588855243`}, {0.2722109131430544`, 1.9964798849422287`, 0.015776125144579895`, 1.4544794409980366`,
0.2483828941377093`, 0.38074384103321446`, 0.06471878888587654`, 0.029192043361603578`, 3894.8098440052436`, 95.96109875168708`,
0.18990156851113926`, 672.4986464354188`, 0.5319935854898028`}, {0.1746670814348818`, 1.8393237859656457`, 0.08972365387779192`,
0.8751901755923585`, 0.00455090829985938`, 0.2601220508561798`, 0.1778920604706679`, 0.07966080501698186`, 2001.5885793038442`,
367.22994182217144`, 0.02859406885364424`, 783.4683428680175`, 0.6132740649066617`}, {0.08518927112077468`, 1.3677033668075627`,
0.06369308205098861`, 0.9115110882307769`, 0.9890654418709195`, 0.5578286233550702`, 0.05812686346309367 5`, 0.16253245674476674`,
2213.0272209784544`, 378.3215567528058`, 0.18233278053877572`, 537.4265813435059`, 1.4377252399021003`}, {0.0631381406281086 3`,
0.6582560847354748`, 0.0580879963028560 9`, 1.1791238901044823`, 0.716121654202388`, 0.5608443897225928`, 0.2324904118378157`,

0.32816385732706316`, 2702.412453193848`, 230.2702036642254`, 0.01736953811318198`, 960.1501952092772`, 0.5352548623519049`},
{0.17087620961977168`, 0.6596222937811591`, 0.015344712827605349`, 1.477447946096491`, 0.21721015176978087`,
0.27000732803763594`, 0.1401635267711841`, 0.06649380761400499`, 2125.832528472877`, 340.30036658006145`, 0.19763533905387054`,
630.3760061021453`, 0.46969823042952785`}, {0.1876699600027345`, 3.8375456575428206`, 0.04459718618546079`, 1.1043408190126014`,
0.7199310275373116`, 0.5112746407765627`, 0.19810264462937666`, 0.06545782504636853`, 3217.6732260327844`, 88.76578226522383`,
0.011721578459465504`, 696.9325207849971`, 0.7314214621885999`}, {0.2392549464281612`, 1.795193779291644`, 0.02776223431068825`,
0.9669315761268646`, 0.35933464809639304`, 0.3601166401184539`, 0.2417906387916794`, 0.08773992594474914`, 2871.3040859090306`,
58.7576499050067`, 0.2470604406017366`, 774.4748276597932`, 1.156313724422814`}, {0.1737971526085056`, 2.1870614592779765`,
0.09297040304867042`, 1.0062942133144444`, 0.055734902893130656`, 0.18118559595398542`, 0.22637373107900532`,
0.02261978764585558`, 2828.1937650901555`, 343.25250266407613`, 0.039115615095715484`, 772.9224453590211`, 0.775643343789234`},
{0.1879201082088256`, 2.714479296405634`, 0.04257291731461232`, 1.4645111514641003`, 0.7687558780767825`, 0.2498446975340901`,
0.08261692546503524`, 0.0052842850968332486`, 3569.5765109754357`, 143.14849824565044`, 0.1911551734077982`, 112.2456475873558 9`,
0.37450152118548674`}, {0.2029349790797736`, 0.5073425453271465`, 0.015399499619165926`, 0.8256325749105065`,
0.9715600306910885`, 0.5206336989814279`, 0.21540780167219908`, 0.030197837754120722`, 3500.639786384246`, 319.3092203462264`,
0.04647124323802765`, 148.4765417210717`, 0.936551900132419`}, {0.04363481768554994`, 3.686454443265511`, 0.0489492286612927 5`,
1.2631580091216332`, 0.5608693599746899`, 0.44670208525318733`, 0.21575866488430306`, 0.005475611299859268`,
2400.7804245689213`, 248.43411656857631`, 0.23097164842437257`, 402.6740755979805`, 0.24962090491699085`},
{0.26962394763575037`, 2.334727661200981`, 0.016049433749389514`, 1.0790379549510207`, 0.7758702352052684`,
0.6658857330523145`, 0.18772701883383996`, 0.09410322494954683`, 1180.8014899627397`, 134.26933546127094`, 0.18231382698109116`,
593.2793153438197`, 0.8852861599320581`}, {0.09034620915848934`, 1.2320672901921554`, 0.034354715993540556`,
0.8941070575583449`, 0.22513695864254468`, 0.43256452639054477`, 0.18780716928400432`, 0.006305139251426374`,
3704.1505361172276`, 122.3021132078274`, 0.2250895667982365`, 468.817178096915`, 0.7172002637422072`}, {0.1342106021790008`,
2.0596254872356985`, 0.012319515048170882`, 0.8837406623067632`, 0.3983823545830507`, 0.45323264007273933`, 0.18657204998538657`,
0.1296958596077897`, 1593.2330856154304`, 51.32683078246629`, 0.19183307397259208`, 576.5411026673484`, 0.630449487035553`},
{0.06170586289679392`, 3.2295657795353785`, 0.07966363375087564`, 1.1377981654875684`, 0.20162887542316899`,
0.4342814897693673`, 0.21448328533213668`, 0.11228022856240194`, 926.8710155427257`, 265.4832564824155`, 0.21687753083833983`,
379.962720094481`, 0.4273787501515345`}, {0.04764308581161522`, 0.9371880740680609`, 0.006947147194552059`,
0.9428927176685253`, 0.429512812646198`, 0.24188106349689364`, 0.06557983813391796`, 0.08662819961453128`, 2315.856153795171`,
138.98267731009662`, 0.047723900638965644`, 334.42576088327644`, 0.3916206088780101`}, {0.26589329062888045`,
1.0759041046690623`, 0.09098290107321436`, 1.492194715545446`, 0.30880295716195594`, 0.5693439000482624`, 0.207603238579945`,
0.1295860607277565 8`, 3161.4311861316437`, 237.8866444957921`, 0.15242003401285342`, 884.4715990402623`, 0.4433218526714149`},

{0.26905358294868803`, 3.3919802122267706`, 0.04147589572903964`, 1.0037652369740533`, 0.4298649501981706`, 0.5934902973807605`, 0.20287355499227328`, 0.1009646066318723`, 2939.153636739231`, 266.90335488489507`, 0.16710797194793942`, 716.160191473655`, 0.24291273546189984`}, {0.16827328193675278`, 1.5842746769537523`, 0.07834710847821182`, 0.893011821677676 6`, 0.0164489448310301`, 0.26719414260532226`, 0.10897410587741868`, 0.05546032500060332`, 3729.9356370320866`, 366.03838308957233`, 0.2467467940947815`, 280.8997278664284`, 1.3976570053406192`}, {0.11812198404504082`, 1.7143688556745076`, 0.0832917161855492`, 0.887920151886646`, 0.2844937165953241`, 0.6435150704879566`, 0.06700362084802414`, 0.01408673031095068`, 543.1573996923576`, 295.45176643681475`, 0.2057051011256828`, 765.2807166448698`, 0.9517400306023305`}, {0.12444099438125777`, 1.1171724928457651`, 0.028511177234083772`, 0.933134368676857`, 0.10182189281611165`, 0.6889449181372187`, 0.203919061786128 5`, 0.005988982231713841`, 3688.035286123303`, 286.957420534039`, 0.10085972566220996`, 615.3946099330735`, 0.21076251183699046`}, {0.08027446845496083`, 2.479731306841286`, 0.06522289260526304`, 0.9982362164597688`, 0.6124472785915616`, 0.4582687396145567`, 0.15229603458418023`, 0.01658267281569019`, 856.3394753774232`, 154.71665498391894`, 0.2377250397334606`, 930.1720290102619`, 0.690848688153989`},
{0.10872364340348423`, 3.9186011831831573`, 0.060170610355699275`, 1.054054619879691`, 0.43607078275253386`, 0.38921767264411744`, 0.2349661515048571`, 0.01754083242559743`, 564.7500488612209`, 325.2917995830418`, 0.10088425624691544`, 834.1393864508678`, 0.4927364959774134`}, {0.24492098634381376`, 1.4756640495798008`, 0.09039482330566831`, 1.268925416092852`, 0.3024040327706343`, 0.45764515914975745`, 0.23166238972905379`, 0.023210902825104126`, 807.1632437981157`, 29.522505236809934`, 0.10690662787620342`, 584.5058809301456`, 0.31432520762302896`}, {0.16732793105199517`, 3.2880731171627424`, 0.005770995891697268`, 0.7766975281116707`, 0.8788326498971`, 0.6167159630096988`, 0.1750480395448365`, 0.008479198492855647`, 1944.938075118327`, 136.8787169124431`, 0.12682672878274887`, 984.6088525906439`, 1.1445848145284443`}, {0.1419174087829056`, 2.110310772914495`, 0.0994020641570776`, 1.3292823679611436`, 0.45306482985145036`, 0.21599849849197728`, 0.1648221116866192`, 0.07781846472882653`, 3300.405330206996`, 281.8673090646507`, 0.046295872099867075`, 992.9571861332284`, 1.3857559155067012`},
{0.2591125569646212`, 3.335687221959671`, 0.07513802464873895`, 1.3406828203408132`, 0.6265837134202594`, 0.6120418692219203`, 0.0866367153439846`, 0.4809505230456383`, 3114.6522079089373`, 221.56125745782288`, 0.20534271172029817`, 335.1014901728946 7`, 0.40335210531180654`}, {0.07780736685783007`, 0.7679598820377875`, 0.012045588711308569`, 1.0389144587186114`, 0.7195684766363588`, 0.6734059322739894`, 0.21686849930491836`, 0.026573266211939978`, 2294.210357887281`, 267.5185327523642`, 0.08203009983541742`, 991.821304407524`, 0.38912660665464593`}, {0.1904519050799695`, 2.657952961985268`, 0.02710161526517439`, 0.985954788155796`, 0.5251143181731064`, 0.42073644688984846`, 0.10648893239143287`, 0.14163998379464854`, 1085.3227825202925`, 308.1879161440921`, 0.104664110853609`, 817.9277612317442`, 0.5436958127562483`}, {0.22716916445595814`, 2.656016676501846`, 0.009517481576770397`, 1.0137664230208285`, 0.8043856154499402`, 0.41138192198228407`, 0.1738550532424621`, 0.17302082087067405`, 3751.1096105640627`, 111.27351598762436`, 0.17188158545785143`, 817.8255794182604`, 1.2270335604376563`}, {0.19526765651905897`, 1.170106032470911`, 0.029545567906451495`, 1.258518526673052`, 0.7747990582331867`, 0.39576632700890235`, 0.18305339088151568`, 0.02643870862927767`, 1884.0495794074404`, 234.1335611016765`, 0.14281646420476163`, 909.6515286624651`, 0.6684124803614486`},

{0.12012929518089455`, 3.420184120937985`, 0.056897087005522734`, 1.3634536151823533`, 0.9491877687797128`, 0.253864371133531`, 0.18455925227026615`, 0.0073008740876704245`, 1907.7035456100348`, 109.41207280054618`, 0.13804126708123865`, 663.8249108849926`, 1.1745809822043474`}, {0.18004368965987544`, 2.493068178401411`, 0.04357556910203431`, 0.951982651317441`, 0.9190753490675412`, 0.29262792363727297`, 0.09335139651065807`, 0.12797729680258604`, 1075.3049250913018`, 20.346664166353094`, 0.159816697988900 8`, 490.7079602487705`, 0.8287540344274262`}, {0.07137650827315256`, 1.1644074423925526`, 0.07883616833848947`, 0.8111924042178744`, 0.7891311048320933`, 0.6650273782867975`, 0.06584916483850378`, 0.0154742297073125 9`, 3718.0909095476036`, 105.99409648698133`, 0.04964251380425461`, 308.95839707694853`, 0.9535513403748106`}, {0.24819121040136383`, 3.408632253507159`, 0.0700354423290172`, 1.0457922524763448`, 0.8494854436109764`, 0.36211596804590307`, 0.1544281929321608`, 0.032194517398342386`, 3459.1706232839124`, 58.43681524538766`, 0.09104985650854264`, 998.8365909239153`, 0.1324215402910296`},
{0.15327298096484765`, 0.553292143287722`, 0.019171700626543782`, 1.325625468275524`, 0.33653648627901345`, 0.5671780635228321`, 0.22338008800803133`, 0.017390374940727985`, 655.4779546995996`, 163.54545479728597`, 0.24475065397030032`, 457.0274116092612`, 0.2002087095075782`}, {0.050260260457999084`, 1.8101909082706271`, 0.04907960598453585`, 1.1804193672613088`, 0.4898614729559376`, 0.6091870752278385`, 0.13261124288899945`, 0.008276897081376874`, 679.5489395787226`, 147.0687581264096`, 0.24156476674196764`, 901.2241966388324`, 0.860083408938559`}, {0.19155975200910907`, 2.828268698998448`, 0.08099748515383133`, 1.4129358104809766`, 0.25202365917372016`, 0.2610333538076903`, 0.06470236085760778`, 0.13231447243327385`, 3657.1471742138483`, 99.53336672122356`, 0.2186876840562944`, 379.7898678366399`, 0.7460712792648501`}, {0.05722787116135330 5`, 1.9542133082270539`, 0.0941850856470937`, 1.3433539531804848`, 0.0049225534478287 7`, 0.6704519586766744`, 0.13588991963793728`, 0.006982623939918504 4`, 1401.1270191432368`, 94.15848734626655`, 0.1246744536984335`, 421.38564228842944`, 0.3803864024276935`},
{0.24480323160774858`, 0.9770653379881473`, 0.08685669997057631`, 1.4498617889181338`, 0.8073264607502391`, 0.6305694929755865`, 0.15063383948553194`, 0.015705201239965556`, 1946.4630777758775`, 52.187741013416485`, 0.24545737962314446`, 375.39510854009063`, 0.30204852732475596`}, {0.25970237019928477`, 0.7550490603863071`, 0.07885325551164271`, 0.8868418649202492`, 0.7758690712841101`, 0.563476812268233 4`, 0.06146029828390087`, 0.015372908427161824`, 3422.5495435058256`, 153.29144336305944`, 0.0813754580903866`, 895.3227772185351`, 1.2107198292075951`}, {0.2686290577973279`, 3.724121776944381`, 0.00932776140836893`, 0.7623127577577206`, 0.9985793499221969`, 0.5715705858701519`, 0.2272124180083509`, 0.04939653222624373`, 905.2797899069656`, 146.3324973074445`, 0.21968505846594472`, 216.11092810747544`, 1.010000147661278`}, {0.20047049725128124`, 1.8814364277396898`, 0.0432403590269178 5`, 0.9877836628945245`, 0.27807821948644573`, 0.197236404912554`, 0.20529415261546086`, 0.03166209667272855 4`, 3398.9894713721287`, 298.02691616284164`, 0.20797152315104522`, 150.16494944733464`, 1.1654851111421038`}, {0.2512194361806632`, 2.004375758234323`, 0.06837051526374249`, 1.073034631418757`, 0.21230174877842556`, 0.3870339378328487`, 0.21465201801423972`, 0.005579499304206773`, 2769.5850515783122`, 309.0781466707855`, 0.15837012698169045`, 539.2786269004328`, 0.5433968371424678`},
{0.043360600224958024`, 1.3764651985145262`, 0.02535710065613106`, 0.7591390204905761`, 0.8399268718247683`, 0.661542343998672 5`, 0.10574369241628473`, 0.015785884267018505`, 3509.29815185808`, 249.52395393584789`, 0.0945856486341341 6`,

828.1689637928107`, 1.4849521371547798`}, {0.16502493462469858`, 1.3193937325633494`, 0.005297674820664434`,
1.399627234646531`, 0.5986438437218671`, 0.36462467617622896`, 0.09886817064468989`, 0.35916760941583314`, 1973.0646905944786`,
277.8296512971136`, 0.08186881694392967`, 886.8974898406602`, 0.5811884147183604`}, {0.1604191127844457`, 1.5411338204142337`,
0.021119033843873786`, 1.1537986057644793`, 0.5877124731937753`, 0.665052160653417 2`, 0.0766372821312167`, 0.02864858324767506`,
2894.8860727589745`, 252.3542717045484`, 0.13159415019762655`, 617.6188772907143`, 0.4037929053946674`}, {0.06587503044357917`,
3.2873531409049876`, 0.05992464214689152`, 1.2716013551535004`, 0.11049481999056887`, 0.18967571479034684`, 0.07897597966338432`,
0.031055517834585124`, 714.9987378291867`, 325.8642447438518`, 0.03072933349161089`, 224.31009981954944`, 0.43056971700983326`},
{0.08558513745915747`, 2.154255807582154`, 0.021216449329710264`, 1.1918995320957213`, 0.34196392851080737`,
0.5573515514335343`, 0.22558987429406974`, 0.007944290685435238`, 834.0182642868863`, 31.379704803286472`, 0.1195562672794239 8`,
325.50235011549694`, 0.8406624234657869`}, {0.09862932260986751`, 1.1425955151993277`, 0.037698072695789724`,
1.491905389525704`, 0.1342994271915916`, 0.6637616641871509`, 0.09223732011612629`, 0.012475844688280356`, 3939.0226775789997`,
397.333620535426`, 0.0508506095131005`, 831.5131725802565`, 1.4572445176898348`}, {0.05563390759261477`, 2.6211334188133373`,
0.07830706285006298`, 0.9848703558242382`, 0.48848418735092713`, 0.33622964561630175`, 0.1772757464686212`,
0.023318256256132857`, 790.324142248483`, 191.62274927661304`, 0.07022775577916623`, 872.1463768535212`, 1.1475901866726725`},
{0.0450044292247927`, 2.6444119917698954`, 0.010486623507702646`, 1.292800283920526`, 0.66423417236758`, 0.28389618486603263`,
0.24677009065733846`, 0.11220695982262166`, 2672.977850751955`, 87.96122153798507`, 0.06709118435712969`, 216.5522211221999`,
0.2429094015064066`}, {0.11879404706782437`, 3.789618206602083`, 0.03251088649203727`, 0.987991532044179`, 0.7585217141302756`,
0.15964902929688196`, 0.19822464964072078`, 0.022073028942100684`, 3475.3716666593464`, 312.0884469031125`,
0.029626743583721027`, 805.4291209783966`, 1.0998242858520388`}, {0.12364670575648518`, 1.704353415774131`, 0.0326234494972551`,
0.8760531935907923`, 0.2626377881683686`, 0.1839738057589828`, 0.20952406506824728`, 0.008408761066587262`, 3045.189867661229`,
108.60098428908321`, 0.18692953913456606`, 601.961477498206`, 1.2642444482354516`}, {0.09132207920746305`, 0.9774272699831279`,
0.07760198412056862`, 1.4107783121842268`, 0.827750409442384`, 0.1863375069777602`, 0.1905810815314894`, 0.0145029178274559 3`,
1184.0795378614466`, 136.3054300863837`, 0.012949339041693986`, 982.7817718255617`, 0.5665175077608942`}, {0.06846656167634407`,
2.2042129590064485`, 0.09505039831429671`, 0.904118439136535`, 0.9720546451470755`, 0.26602028349422535`, 0.175976078993885`,
0.027849754372599213`, 1160.278822015648`, 112.03824475491439`, 0.19795078753251916`, 790.5510580545088`, 0.3510630630037874`},
{0.13838711463128356`, 1.8990755409776376`, 0.06089762564101539`, 0.9260876837712111`, 0.5467956393288698`,
0.4154234813941261`, 0.2406735358249083`, 0.027516661825209305`, 3787.9517541333744`, 81.14252736489732`, 0.13645656815038215`,
789.4958810976195`, 0.4146652577798402`}, {0.12164280733643401`, 0.7306319855226859`, 0.025198568646703345`,
1.1551991645011488`, 0.941354796240917`, 0.4224489649921713`, 0.15396125676390515`, 0.01354882579488883`, 2221.510085308023`,
119.64078489855603`, 0.2054303088204294`, 342.7773232112294`, 0.1247118086329031`}, {0.2519565498080063`, 2.4410449134923757`,
0.07024102883952417`, 0.8833407539406914`, 0.6421794902187536`, 0.40467426968810916`, 0.22319490531900443`,

0.17538572536288508`, 1331.429944069846`, 221.4890821215995`, 0.044389279091602174`, 257.0119508141362`, 1.22479074870354`},
{0.16374213023614953`, 3.1390042086612384`, 0.03373731800873852`, 1.089029132536505`, 0.11724704085340676`,
0.5296870432761128`, 0.17071485848976026`, 0.04786607306752958`, 3763.1992478782404`, 370.00427398775923`,
0.02376010964377334`, 754.8700401368384`, 1.0384956689422715`}, {0.27122054006063806`, 1.2075664765675027`,
0.06711442538391144`, 0.832211218210569`, 0.7308431867527925`, 0.5097342933249054`, 0.2090747753642715`, 0.005222739578099166`,
2533.5566401070446`, 119.27730732188166`, 0.058785202842960294`, 383.8097092659238`, 0.5852236453173183`},
{0.16099754801819155`, 2.803686505264608`, 0.09789640807817002`, 1.3395731410540108`, 0.4720461431978873`,
0.23606773074102838`, 0.1779301572715749`, 0.030480382589919204`, 1594.2767443042094`, 324.25356531314105`,
0.02788920553651708`, 332.281231054637`, 0.7320097263460532`}, {0.240959142326446`, 2.8908037187383364`,
0.04591139951525085`, 1.215044016749563`, 0.07255726424776343`, 0.19896968608124577`, 0.21759225069112315`,
0.15361311860074922`, 1484.973331409603`, 357.4955424369882`, 0.16732946138615118`, 895.1709812867172`, 0.6260218339469641`},
{0.22713051804101841`, 3.448857536632832`, 0.08883733223194078`, 1.2204276076702472`, 0.7015080733875199`,
0.270638009373773`, 0.13556684761509336`, 0.3005936600252856`, 3190.493089374653`, 36.46706287700698`, 0.24892935837310498`,
534.3147865770438`, 0.43339592656925885`}, {0.2140370203656632`, 1.072785551410031`, 0.00827132039380027`,
1.134741501596982`, 0.29397729694226404`, 0.5140551637859047`, 0.20750141402425348`, 0.019004772000016268`,
796.0111470790175`, 373.32691405016396`, 0.140213863005466`, 967.0314809727088`, 0.9637483615615969`},
{0.06933966577055967`, 1.297247792971909`, 0.007707212207521685`, 0.8555001879754492`, 0.7807562676133215`,
0.38716914713624506`, 0.17206101135561241`, 0.03526726108815429`, 3749.4603311841947`, 67.01515585258915`,
0.19434986214621586`, 923.9400195972745`, 0.690132140613763`}, {0.17542394645598242`, 3.965818546215888`,
0.062056547597429186`, 0.9850779413260611`, 0.601386510044081`, 0.21330649482025787`, 0.21801131247804295`,
0.17027605823047298`, 2205.5939454134923`, 152.8778401480049`, 0.2188200840890871`, 568.3054509854786`, 0.6522343084950992`},
{0.19829561009020058`, 1.2861272130118886`, 0.058996877896024404`, 1.4424762353484448`, 0.9372114462493188`,
0.2471431841445586`, 0.21652042075160782`, 0.12965531261684238`, 2812.5526828528127`, 227.17172512233492`,
0.05073826076510812`, 464.49251636449384`, 1.4100125857183432`}, {0.19600081418559695`, 1.651187867615155`,
0.05197207893997586`, 0.960020754108465`, 0.332478690083134`, 0.5694189798263312`, 0.06838425247663296`,
0.0978359611909278`, 2995.29109873983`, 20.115670869343433`, 0.045198099172925776`, 342.0653919059017`, 0.821807385463202`},
{0.2209947483457645`, 1.4020678629806715`, 0.03675625043919015`, 0.903776827294768`, 0.49803899572957544`,
0.46330846135999904`, 0.11400511310118877`, 0.12115905075499117`, 3966.2098327086014`, 347.14465435247064`,
0.2411656213466012`, 151.30146041465161`, 1.2357696533454088`}, {0.2664601042578329`, 2.18004849132239`,
0.009471850733786091`, 1.0512732444703214`, 0.9038030522442144`, 0.4969898978734798`, 0.06499880462130786`,
0.20013158620669227`, 624.4692722865911`, 220.73124435648106`, 0.19588614661648518`, 921.2734663026869`, 0.5276604059320618`},

```
{0.23976279601123018`, 1.933451518140278`, 0.005134271002718638`, 1.0027078459657732`, 0.43722636012098826`,
  0.3010780106798514`, 0.08240022711392475`, 0.0262387390141533`, 3025.6772834268304`, 226.6780468733184`,
  0.18217341414743382`, 977.0168952880347`, 0.7193529184389784`}, {0.06448481048103188`, 2.7661388978360613`,
  0.06036532707564378`, 1.1892973874707082`, 0.31578555851567147`, 0.17260886383835816`, 0.21319401531953114`,
  0.273114108684512 1`, 3100.677481816945`, 182.28886327848545`, 0.20650995565351316`, 560.889122370509`, 1.3474365375160953`},
{0.26693532515941354`, 1.7001452942796655`, 0.08394024954597865`, 1.2334662437552246`, 0.369356041021468`,
  0.5993145483164963`, 0.18461564545025727`, 0.005657971329960341`, 1103.9625789879192`, 291.3790867616192`,
  0.081591574459291`, 297.2691458866644`, 0.4442469777086968`}, {0.23404224237305243`, 3.5382279785554323`,
  0.028098777229809004`, 1.412175491351071`, 0.7362290924867472`, 0.40542344017673515`, 0.20694731909844316`,
  0.23054626236103598`, 920.765611404483`, 327.87645985335644`, 0.22708849822784044`, 623.0758473260137`, 1.2589680512376185`},
{0.2526103539382635`, 2.547931288585443`, 0.057545435658846955`, 1.0799280292862183`, 0.26803824891659467`,
  0.5422641865936386`, 0.10670620310283474`, 0.015048566202955036`, 1669.9795813049495`, 284.59210787610255`,
  0.09939470761594038`, 910.7354264879077`, 0.23029390043835596`}, {0.20498132103380212`, 2.6211424090556026`,
  0.07762208530125073`, 1.1832827633152303`, 0.8568091758085628`, 0.5894723992960115`, 0.10142392423494573`,
  0.04372655638432287`, 2437.946032478544`, 340.39303612646665`, 0.13764877471768105`, 791.0767860370837`, 0.7906970719763469`},
{0.052884163020471886`, 1.2361602008046733`, 0.03233906970393402`, 1.0838607244245748`, 0.616587109974311`,
  0.559374990666015`, 0.21087375474914583`, 0.15514427385108334`, 3747.5731722542832`, 95.47537824900917`, 0.12993350623396865`,
  167.54234393212286`, 0.8619709575029382`}, {0.05627650922310734`, 1.0565799018388518`, 0.008366765457386034`,
  1.1073611344737277`, 0.9044686671773263`, 0.28690930410443183`, 0.11591556118584567`, 0.14689559685446202`,
  3731.6698914895933`, 392.47642718052816`, 0.020651712276451295`, 939.1244138538231`, 0.4613670273277768`},
{0.25614611903098866`, 0.501588304413537`, 0.053511219958544645`, 0.7857092689071256`, 0.9876895393221807`,
  0.3456392946361567`, 0.05590901092977035`, 0.07203517315346229`, 3132.724710868225`, 278.8991477003925`,
  0.06005350450423286`, 876.9439935865144`, 1.1055280583211329`}, {0.11069127843446674`, 1.642484243592409`,
  0.070110700887708`, 1.3832777517420403`, 0.3622860207207357`, 0.3836224055728722`, 0.15916492136080163`, 0.2154097044793371`,
  1404.2072290030046`, 257.494317292554`, 0.20162401744322056`, 747.9948616680554`, 0.20055495528518263`},
{0.23635975177699187`, 1.4278635712323373`, 0.08207702715017469`, 1.1483776113925963`, 0.7632552719315546`,
  0.34524913679570324`, 0.1634249476654709`, 0.10058801454224892`, 1725.004535969757`, 398.158696129433`, 0.08864513579051425`,
  559.4954423618487`, 1.2953970347315642`}, {0.12468954023606449`, 0.9209324164026818`, 0.05160306109706056`,
  1.1837667083763086`, 0.4183823607204986`, 0.4893599168660857`, 0.14817154950922268`, 0.15638710068945372`,
  970.123512566352`, 184.69812342202283`, 0.03928600117573128`, 437.0106178512586`, 0.32224911388261734`},
{0.1136723932042572`, 0.6199365119920177`, 0.09273319896843067`, 1.3537320494338452`, 0.2761893692481572`,
```

0.5396058982956279`, 0.13344673281450065`, 0.24254228219402654`, 2160.7922483974116`, 146.95731784842445`,
0.13691723926446808`, 873.6178269165389`, 1.1324154624202296`}, {0.07079805010219775`, 0.7955834961587143`,
0.007286880406056398`, 0.7759447063653185`, 0.606913248117499`, 0.5727355449238691`, 0.19664851603655176`,
0.42356259711811844`, 1870.233792468227`, 180.8680005717208`, 0.1477041570904561`, 461.25839258459234`, 1.0396002694794824`},
{0.19864755422159153`, 0.6568431508163588`, 0.02264315477057977`, 0.9605923376704882`, 0.136079878466844`,
0.6198125035939634`, 0.16879028253624867`, 0.05139387826366887`, 676.394422841598`, 97.16228902400195`, 0.24446258606357268`,
760.0176740418274`, 1.3140607416703007`}, {0.048007848200414927`, 0.7820168097881699`, 0.030878021770607837`,
0.7769318586855605`, 0.47601163678872327`, 0.424435753843303`, 0.12914087194572849`, 0.016126797460090613`,
2391.7736550154577`, 303.654219877969`, 0.16983739394093017`, 503.48158273443585`, 0.9290908627601415`},
{0.12397022314680178`, 1.9134640235338276`, 0.06105428774566038`, 0.9122496903611289`, 0.09299571956919839`,
0.161077191984759`, 0.055841000655506595`, 0.07262178518496949`, 2160.0662597146666`, 336.44931344871543`, 0.21806383201402385`,
384.7611898720537`, 0.17507287029723262`}, {0.05301501605726672`, 0.8291187641227715`, 0.02984653658407648`,
1.3077528187466787`, 0.13819653878699478`, 0.2536166465348916`, 0.11265850752223971`, 0.024480317080495372`,
1936.8617125045566`, 40.54026079230806`, 0.022084887619422844`, 553.7793764313719`, 1.0440991529785322`},
{0.11658183035395936`, 2.762592184376561`, 0.07346205541959992`, 1.2295614203440737`, 0.6271055790007973`,
0.15303051240019472`, 0.16138486241435002`, 0.4267612908647562`, 2508.650704386575`, 116.70178119956302`,
0.19761081967588084`, 185.23351154279322`, 0.1847796860241131`}, {0.08409076068914684`, 1.662698628529669`,
0.06628429377752397`, 0.7857144090679992`, 0.7795509023890674`, 0.6018604557050868`, 0.16480473412686047`,
0.005962382001557943 5`, 3977.0829039157243`, 254.5962936913628`, 0.2261876908515416`, 844.2141194077337`, 0.3894981951227854`},
{0.19761181149741824`, 3.1650013068192457`, 0.031231235090574804`, 0.9829735619308844`, 0.24011624161395817`,
0.60475867563176`, 0.16895126677030392`, 0.0254175286260218`, 2860.9641481420167`, 373.1607187297708`, 0.21925704745794167`,
724.4947911904533`, 0.19223966948354976`}, {0.23958674048112422`, 1.786152171196827`, 0.07603602807686496`,
1.4947444743530112`, 0.3888221968373877`, 0.6727816783460017`, 0.2201181168504901`, 0.009098542684181752`,
1162.307159476678`, 306.8901763683342`, 0.24475348803407654`, 207.76249392712708`, 0.18624447218509665`},
{0.18497933722186266`, 2.878239385109943`, 0.029671802277615955`, 1.3950519293368966`, 0.6535847580099059`,
0.28583055261465407`, 0.06281293655903902`, 0.07015923191349795`, 1497.206779557504`, 321.6169217231677`,
0.15100076776551224`, 636.282418356698`, 0.8836299614756897`}, {0.04699074995172342`, 3.527089706612217`,
0.017172205617879382`, 1.4397369738427286`, 0.9722510580056485`, 0.2850441231724531`, 0.13610254491128737`,
0.08132269772335915`, 1613.1773056053416`, 297.0949282222699`, 0.19498003445021045`, 975.592345021716`, 0.956187941848555`},
{0.059279771048519225`, 2.9476055156258996`, 0.004006435879316967`, 1.4801548316000854`, 0.9862355491643009`,
0.3236904880428353`, 0.24593445029925698`, 0.030703436579741963`, 1164.618584565893`, 311.9485451244826`,

0.0814613161233278`, 737.5914531965469`, 1.4171378647573056`}, {0.16816786444307136`, 2.6137473348331444`,
0.07076297903903203`, 1.019152659019317`, 0.7897292343970541`, 0.2904237309856543`, 0.10265957086458505`,
0.1242098206583793`, 2557.127925783334`, 190.75663203257477`, 0.05154733155834185`, 585.9278503003982`, 1.2031505305537111`},
{0.23589393150994414`, 3.5954827944340595`, 0.002589326464734434`, 1.07666069673867 7`, 0.8710534665945737`,
0.23092928246977074`, 0.12104038352615137`, 0.0077806152647714585`, 910.81794445578`, 376.7646826896379`,
0.07194257432745349`, 933.120412953463`, 0.7007462820744532`}, {0.2629227685105871`, 2.0851399811253684`,
0.010355337716732258`, 1.4331274522563435`, 0.10755222661429853`, 0.25750736007418285`, 0.12851049495463712`,
0.009614286629076997`, 3636.164423132087`, 74.30061463023867`, 0.21872899173554`, 459.74174064208455`, 1.468160327237476`},
{0.2400384480490722`, 2.9922092033533563`, 0.013305871192182402`, 1.3480217683521585`, 0.38725368404928284`,
0.2680931235316556`, 0.0674084697086222 2`, 0.02772094895950137`, 1170.441062614942`, 333.2109706670877 4`,
0.2381521821307621`, 651.4066045789472`, 1.3386613831968286`}, {0.23548158630131782`, 2.9033781654727733`,
0.040491371210406905`, 1.0601239180195892`, 0.5817670983808367`, 0.30028661587590033`, 0.08280278767937144`,
0.10236121879115082`, 3883.801004485351`, 392.989204422178`, 0.20957385541449597`, 347.54389194472566`, 0.8839469187754787`},
{0.20072665864872835`, 1.7342789598853416`, 0.05336214159587758 5`, 1.378831363330126`, 0.279817121833563`,
0.34851340561961164`, 0.08527007162399383`, 0.06116979696227779`, 1664.8045106480495`, 262.24794736057106`,
0.04157087012689414 4`, 341.35985722177725`, 0.32442612870117826`}, {0.2064601139553282`, 3.6644497503093163`,
0.09227260017585742`, 0.8929880867590039`, 0.8344392301716108`, 0.4832434995094098`, 0.06907401919384712`,
0.027035044919824103`, 818.4391870586455`, 84.77315819631156`, 0.1009530170840407`, 942.900883429499`, 0.23598889511113552`},
{0.12719627740719097`, 2.9733761028292323`, 0.09742374634691622`, 1.0211425931985967`, 0.02439902153255047`,
0.30128340099205786`, 0.15718838379835864`, 0.008843851547334546`, 3743.8680513165464`, 384.0747269940448`,
0.07782562134332088`, 518.1528362520605`, 1.128939238370243`}, {0.13039302867873082`, 2.200528521622667`,
0.06961777298816149`, 1.1216120236963125`, 0.1086112390088314`, 0.38366092046384237`, 0.14322107097123632`,
0.029824170310659256`, 540.97845551605`, 72.3153302707558`, 0.09297610907651943`, 660.4680774293762`, 0.7233496998045528`},
{0.222500359307434`, 3.4535374937263983`, 0.0868016212877675`, 1.2214174899802255`, 0.1849311348992897`,
0.38498253497979107`, 0.07795469796553028`, 0.012086727936026123`, 1996.9337288084698`, 257.81863994754974`,
0.2027153487601394`, 801.5525359394233`, 0.16016525271905224`}, {0.23199034328555274`, 2.9010062530942484`,
0.07097241474964218`, 1.107108199321086`, 0.873726298695783`, 0.47987435777888476`, 0.16697752582401082`, 0.0170507556931864`,
1312.7250285609325`, 127.59443469719008`, 0.11073198777184767`, 966.2268440720261`, 0.49952262939015823`},
{0.27187802880187073`, 2.559403666206004`, 0.015056024621818676`, 0.8768651042380317`, 0.33835916752750217`,
0.37330120529306376`, 0.22568341034057088`, 0.11794794428375593`, 574.9694511173925`, 131.3345851146771`, 0.0451009876673527 1`,
686.8278432194975`, 1.2020385103664943`}, {0.08548511765374378`, 2.482049024745182`, 0.05017645784062221`,

1.4219912237224814`, 0.327294454480884`, 0.44998663061238187`, 0.16726475567233134`, 0.07135147808523594`,
2405.6932893616913`, 248.75296743820104`, 0.08635212705734746`, 605.2402671665627`, 1.4361998566697811`},
{0.05380409197821412`, 1.276263456449561`, 0.07912154448734558`, 1.1181973957516564`, 0.4435123745041323`,
0.2876791269674245`, 0.0815154189573789`, 0.03197775022271448`, 3764.7314930595203`, 335.9941138330631`,
0.24571269437936372`, 541.8080427411584`, 0.6765713726878688`}, {0.07857283998988374`, 0.669747297741146`,
0.011991652027119848`, 0.9047813258408326`, 0.4948664512450338`, 0.17188789698209417`, 0.0900702309740816`,
0.2080497456783535`, 2273.237712249512`, 359.1085328387985`, 0.03304978297554606`, 553.0436427851484`, 0.8835277923418241`},
{0.19809356044953158`, 3.7647853678969785`, 0.0332317595401717`, 1.3237859397513285`, 0.6778361086222637`,
0.6132054544815579`, 0.1518752674209206 7`, 0.0050323756891086395`, 2069.4978256728464`, 253.4252647654863`,
0.1206154998771164`, 149.1444221608047`, 1.3671390039165248`}, {0.13270662662012006`, 0.8703343401570143`,
0.039532238995681195`, 0.9479511542299632`, 0.8591559471078247`, 0.6561464954754304`, 0.2379173574170802`,
0.03579291774606323`, 1412.3774879786724`, 242.77013846996567`, 0.18116091643157523`, 444.063662360095`, 0.20120251434399794`},
{0.25803359644246004`, 1.2353751816929144`, 0.09902849131166033`, 1.180498222807844`, 0.44682972346841376`,
0.48656656102578644`, 0.15527816738494515`, 0.08437074120626252`, 700.3732308035255`, 253.29612906847433`,
0.21261468015460605`, 453.5061063590302`, 1.1999377578332049`}, {0.2088571736926273`, 3.421969080688669`,
0.04711762344238785`, 0.7993735597478425`, 0.86925247504024`, 0.5169439523806889`, 0.2129542352076002`, 0.05976643836187778`,
2468.6210696135768`, 366.7374727303529`, 0.1596803267376969`, 362.4665660686645`, 1.2048830771569192`},
{0.15904124805346231`, 0.8421361165303543`, 0.07852907662318188`, 1.0244687656177145`, 0.9219933836774745`,
0.6275063005535417`, 0.15329665745162913`, 0.03526321220206451`, 772.9495223843433`, 352.25276435700846`, 0.0684297413361667`,
992.7590246601259`, 0.2973530726589353`}, {0.19315853039683828`, 0.6782299469051556`, 0.028518227285774637`,
1.4855260404103439`, 0.891204704658816 5`, 0.18558970744865766`, 0.08069447427281715`, 0.12476168901657161`,
1742.1251150345388`, 272.67731901981506`, 0.1910899454655819`, 949.8684711151548`, 0.4837978027326524`},
{0.07464756304849529`, 1.7543433367968868`, 0.03656075229685295`, 1.3819446788540803`, 0.057518379114474216`,
0.6501262336964491`, 0.10580150704729113`, 0.005673334483094171`, 819.0317130299181`, 299.572756300115`, 0.014603264528849358`,
224.71087044681948`, 0.4006548472176015`}, {0.26113780857221375`, 3.8494229849133816`, 0.04682022604226681 5`,
1.4883637799915408`, 0.7900669158347569`, 0.47083751171330124`, 0.15155564758462187`, 0.010825659962383764`,
1933.6936843245703`, 39.837827547197264`, 0.21005212004250612`, 183.95998799833927`, 0.790480798301477`},
{0.21280526601523658`, 3.8175300011873547`, 0.01178825474982185`, 1.1796390965639145`, 0.24464914256183867`,
0.49607974193777327`, 0.1108318563283811`, 0.2585069615095704`, 3052.7117223008136`, 57.29215081006703`, 0.24724367689310922`,
312.73630886871297`, 1.3222876920267037`}, {0.09386137633395408`, 1.2417629817905382`, 0.018140024857634492`,
0.7528874360062701`, 0.05161017537699197 4`, 0.316465636653249`, 0.11563751015075924`, 0.015502118210496327`,

2537.1028911949325`, 215.50008562226128`, 0.15953203596048937`, 891.0958178177601`, 0.6303896441449477`},
{0.2101066749755645`, 1.7460285662456823`, 0.003613265851717014`, 1.4978040204528609`, 0.19417651500666167`,
0.3353888430719626`, 0.2135354574732401`, 0.10872497203058913`, 617.8241320162933`, 165.6178357259961`, 0.22482396009863348`,
680.5494065665993`, 1.0930861852892142`}, {0.1112672619459268`, 3.3162320425795633`, 0.07058367566893697`,
0.7877852731870432`, 0.1707310681645522`, 0.4444865161263436`, 0.1532321738843465`, 0.009348450270298528`,
1667.1750774394905`, 85.72117290696644`, 0.24866619285478148`, 935.622061742448`, 0.5890122601053216`},
{0.17891338225865921`, 1.3879814267612094`, 0.07063552789068005`, 0.9858124782156832`, 0.8454867069502239`,
0.4777267419001211`, 0.10862340741986154`, 0.3419967187627656`, 2373.3715516757165`, 161.58290450862694`,
0.19591978902087276`, 919.4258466096794`, 0.2933881153059088`}, {0.1653106759173461`, 1.1481925036530471`,
0.05458967966350432`, 1.0227892012277866`, 0.4058064301041644`, 0.672612684002786`, 0.1985086141744311`,
0.19183978047011319`, 3909.583942777279`, 132.8561148586229`, 0.14248766584355943`, 442.3662361173358`, 1.1775737029395668`},
{0.12322909436108842`, 2.545795936253934`, 0.027456591872337 22`, 0.8619092164627277`, 0.9512446297491959`,
0.4576923053450873`, 0.1604872340439737`, 0.036926357884616644`, 998.0536777311845`, 182.18168740905003`, 0.15873837567158589`,
961.4975425542667`, 0.7170267800258272`}, {0.10156778306976194`, 3.380566814142899`, 0.055374039877145745`,
0.8608219403463572`, 0.3813483945767739 4`, 0.3969718766428616`, 0.051242342442559236`, 0.007002420383387194`,
3131.553993471214`, 52.59105780167522`, 0.17297174385532188`, 241.4930948000597`, 0.47202529784999103`},
{0.2260909402095127`, 3.8238840861300467`, 0.07923635586820367`, 1.2367862807328076`, 0.6970149893575608`,
0.20215590379516502`, 0.14750251801994407`, 0.007161130667253309`, 3485.8168945203815`, 274.78793653602065`,
0.10044398401394355`, 915.7360879520926`, 1.4627232261949978`}, {0.08584999254388637`, 1.9101901010955737`,
0.09041390636656661`, 0.8302117370443965`, 0.84040110051092`, 0.20746283103435226`, 0.1285682776782044`, 0.12189846448305729`,
3556.093558726954`, 327.42869357012637`, 0.06996987029918372`, 538.4353024258736`, 0.6622106824474181`},
{0.20751789924653252`, 2.2951480032161156`, 0.07400046237618799`, 0.7618091811998637`, 0.9517376623194758`,
0.27093883228026916`, 0.09398949537881615`, 0.4975497728011761`, 2798.0174576575673`, 52.48361179447795`, 0.21376841245369815`,
598.2572265284546`, 0.454584804818660 7`}, {0.2024822707517489`, 1.7059989916581548`, 0.04472552255456291`,
1.4640452073679922`, 0.4668935216729655`, 0.48904553328366107`, 0.16370228894592265`, 0.010180900951853148`,
2341.121484744116`, 250.25527127935698`, 0.05318961727659599`, 247.03741386093049`, 1.1190750190618344`},
{0.18669432201917052`, 2.5970038426197393`, 0.00843993439268539`, 1.0017524404675315`, 0.6105032460519033`,
0.21067883770363727`, 0.05604408280610981`, 0.15653694810083688`, 1273.0121205063797`, 85.95148300395982`,
0.08357781824026594`, 112.3381773345919`, 1.227830064153364`}, {0.04570033535669746`, 2.7338860841068247`,
0.09388959288790343`, 0.947529890157045`, 0.48030136336821094`, 0.44858407570092773`, 0.13981884569514474`,
0.06620904711884006`, 3194.685855722214`, 70.46406678630393`, 0.06909845501541895`, 198.2639069782989`, 0.6900054697141409`},

{0.16503709054938737`, 2.985233481583232`, 0.04417914375147241`, 1.2197735426680427`, 0.48666273106149527`,
0.694079227497234`, 0.08593979314068356`, 0.006480267921513794`, 1566.7640781678692`, 149.07891763813257`, 0.1490457956326493`,
464.73140132085683`, 0.7009674729323847`}, {0.18220446781920863`, 2.814454939286408`, 0.09750314667838876`,
0.9093299494928886`, 0.06192145030337337`, 0.4883655013376432`, 0.12548846908172778`, 0.11684488174176547`,
3141.7328488106486`, 343.39575247392884`, 0.10580341279824401`, 128.64172706549476`, 0.959547687627015`},
{0.17646753913480967`, 3.877605681938035`, 0.09673714555241926`, 0.9042768181739222`, 0.365906303865281`,
0.6579349674050132`, 0.21683106282535208`, 0.2703020165533033`, 2777.880663361223`, 45.4262687502511`, 0.05013008108119932`,
614.3604658216427`, 0.5334638599548847`}, {0.16148732336077443`, 0.4799441143455345`, 0.0222410600426918`,
0.7782926647413204`, 0.8449913962552762`, 0.5970388010087131`, 0.08998502266715866`, 0.18927880707875058`,
2550.9022991164866`, 230.16675065693698`, 0.19878594858548299`, 587.7876082919338`, 0.36374785686229094`},
{0.1627935422045057`, 1.3141683749643658`, 0.08143788325592968`, 0.9081601913698019`, 0.2980586396556304`,
0.4842631358009777`, 0.2462507021866155`, 0.20525933721902084`, 948.9464583554395`, 51.04858375707653`, 0.22197754088545613`,
815.2987590412856`, 0.9614493065272702`}, {0.15563246762508942`, 2.38625639329363`, 0.020266594620626854`,
0.9899448700876087`, 0.2834248447542558`, 0.48771257554615854`, 0.07052369980147624`, 0.041549048685807256`,
665.3536423870837`, 68.25659678022646`, 0.029094743006415624`, 438.4007943223818`, 1.3976068032780908`},
{0.13605062789280092`, 1.049131974960007`, 0.008828380206619038`, 0.8948704515081785`, 0.15652561650035524`,
0.17037511582071851`, 0.10373637464922081`, 0.07221417238539751`, 918.9971540109341`, 196.5200060886334`, 0.22594618285207502`,
486.24136163538213`, 1.0741284858756952`}, {0.24918116632300125`, 1.9948280446343052`, 0.05902075373048836 6`,
0.7731813144396198`, 0.5501836661952755`, 0.2617436780708874`, 0.16575494358645082`, 0.07563890453756453`,
1281.1452227639593`, 398.48774672543414`, 0.21879906450996234`, 595.9215324563207`, 1.3915332165855423`},
{0.11985037949008298`, 3.5818856832003307`, 0.020526087402207464`, 1.4737066057441737`, 0.7202328924581087`,
0.6189826280066466`, 0.2385876293236635`, 0.033085223313669816`, 1598.5757323227162`, 94.60718661733506`, 0.11382615657286799`,
841.8509580517654`, 0.6679937196172974`}, {0.24088059008180213`, 3.631907750030397`, 0.0168352920389297`,
1.3538676630895496`, 0.23385332493594202`, 0.26686562095439126`, 0.09580773502822287`, 0.057761677223265265`,
1601.4182343541897`, 244.02622564292437`, 0.10876591504607291`, 374.12466078896693`, 0.6544983346049063`},
{0.08760069706420665`, 1.5227527422959213`, 0.00629045950066 4963`, 1.1503135082898694`, 0.26645256698643016`,
0.39511245013747087`, 0.05345219446473912`, 0.14421660292805977`, 2834.3627724481303`, 254.19956673554077`,
0.11041365471948272`, 798.3015591654827`, 1.23271065670275`}, {0.23133617480401286`, 2.6640802032796778`, 0.08978974948273911`,
1.0116020807932957`, 0.8318488887924349`, 0.6675616164544869`, 0.20426690479035364`, 0.009865509541424233`,
3844.447222155466`, 229.58899935707439`, 0.06687157142421862`, 250.43258115681581`, 1.0696487653145943`},
{0.0473053484356106`, 2.528271455014294`, 0.04379578478698365`, 1.3398428537278833`, 0.08517028315925956`,

0.30213055509057296`, 0.07620648505482527`, 0.008295633953604362`, 2015.7868739005626`, 387.64466778029384`,
0.0666771763644267`, 417.4538391282507`, 0.5826339539547711`}, {0.18116484695073853`, 3.4640449810960954`,
0.06839891630037272`, 1.2399097480371752`, 0.04817783768661066`, 0.4014034395872152`, 0.21465050782080902`,
0.05325165527509632`, 1924.9662741805496`, 105.86732031533398`, 0.1393718767482346`, 199.83243525714568`, 0.9697594233167135`},
{0.17722553403205998`, 3.0007942819058586`, 0.08426092627271814`, 1.4743379967270709`, 0.3388445160459277`,
0.49501989043280803`, 0.23485706445345372`, 0.1699025165729446`, 3271.48111705866`, 267.7251421531412`, 0.010606754344912706`,
293.72103804583566`, 0.3210723928217516`}, {0.2609069655363017`, 2.768625994878656`, 0.016519950760961476`,
0.9887870303489649`, 0.6799726637663899`, 0.1521729338333606`, 0.1829476921720294`, 0.024347967927797087`,
1511.523258009417`, 216.7008643159378`, 0.20491614279750514`, 766.9812474491696`, 1.1622260241747542`},
{0.23282671028013624`, 2.5250507958220236`, 0.08166038762353982`, 1.475559688752855`, 0.31100019275894764`,
0.34731406308855295`, 0.10202086873040123`, 0.015443655656521078`, 1784.4495502725722`, 134.46976371037226`,
0.1076961354527613`, 202.15134815632805`, 0.27496454456166486`}, {0.2534621586512541`, 1.2520346038792995`,
0.06988065973168091`, 1.0828761112021472`, 0.4217604177964145`, 0.6558920896364391`, 0.0795985746179077`,
0.05023618324124778`, 2156.02809766098`, 203.64825237901084`, 0.027694941681807916`, 715.8959308344329`, 0.9783462091491826`},
{0.2686823247228523`, 2.3272174691261194`, 0.05681759674940757`, 0.7993266208566256`, 0.2458779194644325`,
0.5770429526834894`, 0.12131467155627579`, 0.4868472618681081`, 1679.8842844618148`, 375.7402625387416`, 0.12867203143383737`,
784.5750629299616`, 0.2872196054138809`}, {0.06875580312098611`, 3.247874145057774`, 0.004291910991276619`,
0.9003362880741821`, 0.15011045285970526`, 0.48387884467664743`, 0.20834263351493287`, 0.005600038363098535`,
1867.8976114757097`, 330.54959320440037`, 0.17341156809005037`, 891.1128985995628`, 1.1328075701946863`},
{0.2604474947725116`, 2.680469039151512`, 0.09347507864427006`, 1.3406525567757532`, 0.7753893981722564`,
0.47003775861879526`, 0.18433459175258887`, 0.02710838514478081`, 1097.303846652012`, 59.989846637326195`,
0.2396324272435308`, 520.7105066211856`, 1.2154616346683937`}, {0.1854944480743531`, 0.6033066796677575`,
0.0861209712751036`, 1.1120331271465362`, 0.3293546265134706`, 0.33872219273946513`, 0.2061923652390631`,
0.2584587801912725`, 1825.5334120480466`, 212.5256504914529`, 0.10510444444692912`, 667.6505780665874`, 0.7924319167221996`},
{0.2145355745809865`, 2.3949467945293534`, 0.06000787597004747`, 1.4175461979943185`, 0.7493920190402923`,
0.5088093754665587`, 0.09502350553034591`, 0.1173542986001807`, 2030.7163641144753`, 103.79286114917784`, 0.01605177667938798`,
133.56275054572927`, 0.43414320931006234`}, {0.05893414206311359`, 0.9939048106210828`, 0.03472063380974756`,
0.8895006767925664`, 0.8467819165039143`, 0.4431527902122355`, 0.10041539174164343`, 0.010243718017719164`,
1440.4416841569873`, 311.7369831199235`, 0.1489953036256355`, 194.67837557930937`, 1.0922234313628207`},
{0.2420420742334215`, 3.327651376005319`, 0.09221878812979487`, 1.023812330120501`, 0.31791734944482886`,
0.573412342531516`, 0.13067974780311492`, 0.34870113408913816`, 2647.793584539433`, 238.25324829318913`, 0.0829648683841314 2`,

306.7121434559024`, 1.1586558107989333`}, {0.15335352445527894`, 3.9441098821753853`, 0.06075944734347927`,
1.3845316610599772`, 0.6757829236078086`, 0.5696541876954208`, 0.06003577474489652`, 0.023263365343848087`,
589.7802750625278`, 88.19982568392368`, 0.23736578827870863`, 221.98499600792357`, 1.3342293846904383`},
{0.2057321001535352`, 3.477779477099153`, 0.01565475102216789`, 0.9687332995474296`, 0.6480125448543947`,
0.39386770090672185`, 0.22315396933933046`, 0.02640444330093665`, 3417.147962775489`, 232.91214784925864`,
0.15089237419732604`, 192.7292075164047`, 1.3944865179171138`}, {0.1382457721974787`, 3.0544648625958937`,
0.07716919901266764`, 0.9188595143416286`, 0.3462722986119262`, 0.4678136009242426`, 0.06900012532030372`,
0.15987243160058234`, 2251.784182537266`, 42.776493852806254`, 0.04481823583577049`, 305.38626913967073`, 0.6807011616001419`},
{0.1360635203544221`, 1.5230456533832566`, 0.08773264634483066`, 0.8971993717897444`, 0.41889764927618045`,
0.6459655706147649`, 0.1320560311967482`, 0.007435436409474443`, 2317.5252104356077`, 185.8479807677138`,
0.1368848203143725`, 819.4789596432643`, 0.4626308298490758`}, {0.1861397800048244`, 3.666291125612447`,
0.021920815316383013`, 1.1713857749035106`, 0.8058121631951614`, 0.45419771290609245`, 0.21685242650840164`,
0.0407532476934853`, 3146.7299686184115`, 28.54292237930042`, 0.2264320092753036`, 761.8535421939166`, 0.9373607827575752`},
{0.08830406143986902`, 3.7526081288669237`, 0.07494143823437988`, 1.4154058952186817`, 0.7895172172836553`,
0.45020465431213974`, 0.16056190593714087`, 0.4153876934131835`, 2210.0649284405526`, 85.44530773747346`,
0.014324232063731318`, 620.5737905469`, 1.0673781193481333`}, {0.2717606248255099`, 2.190734766375872`, 0.04820408141221305`,
1.481116016638827`, 0.9487591167057607`, 0.5004805959988711`, 0.09470907151001362`, 0.022749914009150274`,
1463.5914480732572`, 44.86936007523616`, 0.1535785872152154`, 621.355498213895`, 0.12539551874383625`},
{0.18220583824801023`, 1.0625585799582478`, 0.028083263945712977`, 1.1645964084188172`, 0.8719186901479166`,
0.48268958474301904`, 0.22994397150784385`, 0.005060591603207206`, 1871.4173689923691`, 234.5250185404907`,
0.11727853548632389`, 753.2774350271709`, 0.8971842414567273`}, {0.2289641503471393`, 0.8435829149195362`,
0.07341508670809282`, 1.1800630307579887`, 0.9099879849290238`, 0.1842380631394942`, 0.1353990590747029`,
0.2460479957202637`, 2538.6411130243523`, 376.4472190407379`, 0.19536635931849855`, 899.4524708620066`, 1.3395813518411068`},
{0.2558849246941212`, 1.4676778621227573`, 0.030798125829003525`, 1.1689139590956696`, 0.7691883746673562`,
0.5388694505073812`, 0.1361154351943364`, 0.3831441538414148`, 2417.3225094387635`, 266.7425967228331`, 0.1876794743998071`,
155.9147602627455`, 1.3939349559367282`}, {0.19420894747739698`, 3.1162153286102505`, 0.04127640851578526`,
0.8173502169159728`, 0.5947055633011988`, 0.6348016129519378`, 0.2364866641651267`, 0.18344513627477504`,
2242.3390284544057`, 237.08174389397698`, 0.15698549004184387`, 728.9985780985606`, 1.44622587134397`},
{0.27545009548871136`, 3.127893266073621`, 0.03400458772020659`, 1.4808519645039966`, 0.4800503155758624`,
0.47449829993405623`, 0.18073165759115012`, 0.006704497641205637`, 1459.9183193368299`, 312.7211386030251`,
0.1398281732233812`, 869.0388163641735`, 0.4615911595033746`}, {0.07114801259115294`, 0.8039187253193969`,

0.07087753760270328`, 1.1368343592838785`, 0.9290711881578881`, 0.4252536504419058`, 0.2382414790697724`, 0.025836595824633303`, 1711.2456032707296`, 72.7364695982946`, 0.12118216604343984`, 768.6671479810769`, 0.632180119061315`},
{0.26598042670604183`, 3.0385166887298514`, 0.02079658973946371`, 1.443191517710432`, 0.665685095273779`, 0.6943006407620043`, 0.2025102580873962`, 0.11372405284116688`, 2673.104118847503`, 370.06247369983964`, 0.053592563905555446`, 563.6719393230547`, 1.295392633466629`}, {0.08434213498518345`, 2.884776026053059`, 0.01784506804909295`, 0.89070743084921`, 0.017758504513472495`, 0.5944904408038866`, 0.08169223645971949`, 0.48395975236571387`, 3126.8084615370162`, 225.92436096103188`, 0.13569185682385082`, 922.819555797193`, 0.48539825293850236`},
{0.20082698180802527`, 1.3695058228039958`, 0.05769047845200127`, 1.3351750243018488`, 0.6007433563354125`, 0.2798384815932218`, 0.1254244694071216`, 0.2088178298818577`, 1463.6183018216052`, 66.70452998585449`, 0.10122328952997744`, 994.0096743149159`, 0.1952649260331527`}, {0.16711464800943515`, 3.1644124525346893`, 0.01768189665496589`, 1.2178309320247656`, 0.26119802448805807`, 0.6220273840786965`, 0.22306661457801585`, 0.25320367273032`, 1041.2898981285657`, 239.33192762256772`, 0.2494618690750568`, 586.6116774613973`, 1.4555919851154595`},
{0.09893505527682533`, 2.3064339565147867`, 0.018529297996236507`, 0.9171685163059136`, 0.6085941812161881`, 0.5418084179199836`, 0.142836160371682`, 0.04294641438387997`, 3725.1095010195586`, 72.31557731486487`, 0.13173976875752064`, 305.64284849893346`, 0.5400156051874061`}, {0.06381696121235875`, 0.8477768355753303`, 0.07631684653785538`, 1.4174211538900077`, 0.9458906699158072`, 0.3776304180991159`, 0.1653305931284833`, 0.32073149440027426`, 1049.1771967953623`, 394.3231592531165`, 0.11611437981360967`, 589.0094824399774`, 1.3089227640655174`},
{0.2378006147252607`, 3.570542408692237`, 0.04070723728792734`, 1.4668920719251455`, 0.3850691388242016`, 0.38663377846347213`, 0.12291876071159141`, 0.024967204088222464`, 1019.054575714878`, 399.34037923434903`, 0.20844637146033518`, 738.592358680102`, 0.5493427805486055`}, {0.1881362818832813`, 0.6539887289428306`, 0.08252169555407118`, 0.7826144603056664`, 0.15958844477801626`, 0.3639290894981396`, 0.09993133748219013`, 0.2800958710382864`, 1510.4921914959268`, 179.86986002714605`, 0.04524685145077617`, 958.5854189778906`, 0.7527910327031067`},
{0.12173609366240618`, 3.3383924267851466`, 0.02357436557391788`, 0.7942780764348314`, 0.4482107224237768`, 0.5589424659276201`, 0.22838421145596588`, 0.00607374801023532`, 2214.1972066261615`, 150.29964512667806`, 0.13288910211093596`, 661.9252088811149`, 0.6211587857736114`}, {0.17746743672659737`, 0.7310287150615178`, 0.0016819543630418644`, 1.3266640182173695`, 0.4546571462430635`, 0.22307471049184446`, 0.19166301141702335`, 0.1797900377452938`, 3977.2901389622366`, 241.80336568037205`, 0.2450277902502666`, 864.1156776348996`, 0.3320454806697619`},
{0.15879665197931575`, 3.743865441720499`, 0.07993719497571487`, 1.3863263907663648`, 0.11252221546195584`, 0.21875561456961523`, 0.18302533012196398`, 0.045789791272496695`, 3439.5627007746716`, 199.28734529643611`, 0.07897689570997235`, 459.6929995870351`, 1.3839396717519366`}, {0.252949973166296`, 1.3581534604691656`, 0.08688814005768553`, 1.0419555520781258`, 0.12611664359116936`, 0.6288841132737231`, 0.057758397286019114`,

0.023916306725018766`, 2170.169755491919`, 250.99206088874678`, 0.1368133923489449`, 653.4734602865017`, 1.3170221020371793`},
{0.2661154401694392`, 1.7363251483805628`, 0.07998433430000482`, 1.4648159539236127`, 0.9171376526207291`,
0.376137994473548`, 0.20184227650680636`, 0.00641223261337026`, 2641.087277658683`, 325.4103605263397`, 0.1804146068978643 7`,
970.2568641507999`, 0.4610617170014743 6`}, {0.20475120614685927`, 0.7105407398192498`, 0.051404829387709794`,
0.9439007980440538`, 0.24798083084742606`, 0.4041219508364089`, 0.1761513697864865`, 0.033528565901712194`,
2574.619737539824`, 91.43827843964641`, 0.08690149322351048`, 212.67135692362783`, 0.8521245521141365`},
{0.27856029419349126`, 2.6124347520486566`, 0.023393407033533063`, 1.2557352787748788`, 0.4729425013672317`,
0.24817450935476848`, 0.22640315695606628`, 0.06515877133364438`, 1870.640565324531`, 193.0827657804283`,
0.16771466854670142`, 247.94062164522063`, 0.7229974552247493`}, {0.17763585118778763`, 1.69130586852142`,
0.08311087769484558`, 0.9826749568318511`, 0.45337328335392657`, 0.21474822792200154`, 0.1555340321416117`,
0.027521931192543606`, 1271.747175675947`, 161.7079968021975`, 0.0336230930745573`, 828.6518012704438`, 0.7903403405022158`},
{0.08560372119248377`, 2.0326212469188967`, 0.09774899638415875`, 1.3147409174316498`, 0.6069928334747081`,
0.27045589535840175`, 0.16338469644902603`, 0.011937107311501327`, 2319.466961118257`, 167.71081942469993`,
0.12882822755255752`, 991.7237911197067`, 0.6456894832624427`}, {0.23095619026180086`, 1.9650930385120509`,
0.03720210640921742`, 0.7846831883983061`, 0.9323485081814453`, 0.5442302420451045`, 0.1134332998976597 1`,
0.02000268586804057`, 3625.4316547741264`, 165.86198259310902`, 0.0747214722536606`, 140.5400587090387`, 1.046207575686258`},
{0.042361467332411695`, 2.351508973377748`, 0.05020066669402127`, 1.3381475201559838`, 0.9502939867746623`,
0.22339810946446648`, 0.1776730690645264`, 0.13884699714056517`, 2483.907451252583`, 345.80151663214974`,
0.23295220871879369`, 657.2301375881013`, 1.3539198362038034`}, {0.05222246181345991`, 3.1124125730133345`,
0.04638638954750201`, 1.2048079021028357`, 0.8628303915909414`, 0.6316299274473753`, 0.23206262863735339`,
0.050479713416653006`, 865.7107858703316`, 328.65142874629487`, 0.2112604095528537`, 780.6058977111785`, 1.1251591915825423`},
{0.1054769380389059`, 1.963024130505957`, 0.010929860014988755`, 0.7527221292531261`, 0.7877911595800804`,
0.4427274809590327`, 0.09186197295869336`, 0.009361896500265338`, 2543.2513249608983`, 305.1962245654714`,
0.08555047317346959`, 568.9996155589185`, 1.3900372047516214`}, {0.07150643414925262`, 0.6878857567351409`,
0.09888398952360124`, 0.7656655956357804`, 0.37210334287103475`, 0.260310628036696`, 0.15951251879346917`,
0.13194679873906393`, 2993.6941520930577`, 66.43420963359301`, 0.11946732539702587`, 368.75788109109317`, 1.4741065859337095`},
{0.2368181024692313`, 1.4647672481223086`, 0.024071231381867193`, 1.0003538218119317`, 0.22124294930227162`,
0.6642920859190384`, 0.10241519659664405`, 0.00779505302627578`, 3581.4210404838586`, 180.93440745772705`,
0.02603549662345339`, 977.9183918821726`, 0.8927799153406495`}, {0.1572164510688962`, 1.9956296501593913`,
0.06995463553791044`, 0.9021878757660133`, 0.7121036009099331`, 0.18660249768107195`, 0.09628060679677442`,
0.00513309669467921 9`, 2072.960715167388`, 254.38971334836992`, 0.15086193745266063`, 399.2707619797427`, 0.8748167284203028`},

{0.24534142614141574`, 1.687688545866143`, 0.00893897841660829`, 0.8574829133339625`, 0.3235975392976549`,
0.1572853430672102`, 0.10830928575061027`, 0.009622672584254309`, 2235.2506682077483`, 237.6189385519175`,
0.13315245055978842`, 331.6379641189695`, 0.8138167399569669`}, {0.20273322123229126`, 3.807783630168501`,
0.0528348210896372l`, 1.298494789242956`, 0.7546619106198402`, 0.5096454237368658`, 0.15643304361162286`, 0.009752697085812 88`,
2036.7744469965892`, 269.6432683433537`, 0.16633213445960715`, 144.13948334867771`, 0.5936203262679156`},
{0.1090686535015345`, 1.7307875384792988`, 0.08370587136016067`, 0.786342057604364`, 0.23934091881167352`,
0.5532152340977895`, 0.20719531069983066`, 0.014423232394765291`, 584.8364488330963`, 51.11463783438455`,
0.16497190924113214`, 560.5075303992755`, 0.1945748226515868 8`}, {0.10034378100957048`, 0.568705426381547`,
0.02631549043317051`, 1.3259763203127861`, 0.7842317195053146`, 0.6667962285287576`, 0.22117939688046728`,
0.16765946430887244`, 1204.8776783456187`, 272.401683001617`, 0.0894333154234141`, 568.3487666754199`, 0.9949483973277107`},
{0.1986541919808587`, 2.0148542695905025`, 0.06460882456504251`, 1.2994940254215859`, 0.7635937084174482`,
0.6424396356854862`, 0.06586018518510109`, 0.006013522908555186`, 2692.9607759270366`, 314.9662870765974`,
0.19049409999496447`, 364.1958358496419`, 1.3259548914455555`}, {0.17744677367592238`, 2.8145985178919455`,
0.06458677929717328`, 1.0151544396603582`, 0.973904623984277`, 0.3578214916805402`, 0.20505450389578128`,
0.01490278868680736`, 773.6258181122789`, 369.6787024916995`, 0.08787090717618273`, 688.925001559185`, 1.4879745833927323`},
{0.23763644337528228`, 1.1258000565699833`, 0.08655539028822659`, 1.4421367371812033`, 0.8420701889414131`,
0.32987565806156993`, 0.11212886969804808`, 0.3762796311178309`, 3215.55980605459`, 111.34208665632849`, 0.22009588575654682`,
825.3201480137325`, 0.19638937037165594`}, {0.17540678056874115`, 0.6434355263587648`, 0.013010071844994845`,
1.2433841886466697`, 0.5062551328846527`, 0.5669844668553855`, 0.1092457434005506`, 0.15931564416611965`,
550.8900308572843`, 156.55271966553812`, 0.17731364157704432`, 452.20755081083723`, 0.19674489194717237`},
{0.21458195958779397`, 3.778821411488644`, 0.0544123586305583`, 1.3813871613779953`, 0.28817264717842317`,
0.3905647665571196`, 0.16940925423969155`, 0.011645502955259427`, 1453.9648482104221`, 230.24670037474345`,
0.2325812549220277`, 256.2011663046178`, 1.0224258251262421`}, {0.17907151518430287`, 2.0902683059142593`,
0.047597680087910064`, 1.0095534558784067`, 0.9743952128768429`, 0.24863451857193952`, 0.21511207630279083`,
0.04723644399643398`, 563.527055433814`, 396.8635580795858`, 0.023114505576187605`, 501.60344826144365`, 1.2934975273868083`},
{0.21664580336020506`, 2.7904192580156826`, 0.08609886107928513`, 1.1647177582250994`, 0.3698516199203479`,
0.5070272569314359`, 0.14483524067955328`, 0.005288959493555501`, 1100.3297047123851`, 291.3784227167082`,
0.09697922502086948`, 197.3920629001695`, 0.1833113229910892`}, {0.15091066558189192`, 0.8205016700103087`,
0.05142439889969195`, 1.4884443510354735`, 0.9443666397570984`, 0.6117943969814366`, 0.2452931392292768`,
0.010625687103423183`, 1748.06559934296`, 379.6595004784634`, 0.024553189607445347`, 653.9613250240327`, 1.486022535990486`},
{0.26237441466241135`, 2.440069093193034`, 0.0543466670723198 9`, 1.3909450384568052`, 0.22886475429539455`,

0.3333613822349881`, 0.18766974606672882`, 0.08759548462627104`, 529.6923329004185`, 399.324801020412`, 0.13863879997995981`,
387.3632140613029`, 0.25314232161811057`}, {0.22245717731971576`, 2.985856947787706`, 0.09874590330326534`,
0.7839091846681046`, 0.2998538430686788`, 0.2434540627112538`, 0.23827626072331998`, 0.34056910820256414`,
935.8921807628012`, 318.7028533368011`, 0.18600268736607273`, 921.2986949779363`, 1.0491943194466424`},
{0.17705759438035373`, 1.2612299197876062`, 0.01214557400592165`, 1.4661769906251096`, 0.22314672171436967`,
0.16128242220657885`, 0.23663971605845535`, 0.007691191670947209`, 3484.1943522580896`, 354.38023616202486`,
0.24655636450459506`, 425.6786427946139`, 0.3751516455152999`}, {0.055848576504706315`, 1.9472210920598059`,
0.03173532459024104`, 1.325134260655478`, 0.08790998918380089`, 0.5687863813936657`, 0.10245756490130259`,
0.45067638130329446`, 576.6807124823881`, 55.58610306925942`, 0.24818565712266133`, 162.07773977499951`, 1.063344668080981`},
{0.16698983479065355`, 1.0522389386501594`, 0.035194198126175545`, 1.0890628330087604`, 0.1283893530701905`,
0.5323384931873707`, 0.16572710881656028`, 0.06132443296308713`, 1884.016222150257`, 247.6559916293744`, 0.23240265758402334`,
912.2413207261773`, 0.48590491632061616`}, {0.19213360041078048`, 2.590075898572982`, 0.0637115522696601 7`,
1.3285805805974358`, 0.6305535106016507`, 0.17188728405564424`, 0.13311332712928986`, 0.062401537051158744`,
1961.9440455293789`, 177.47431796919864`, 0.19646251883328636`, 293.2613347892044`, 1.2835678539821682`},
{0.2559724012887107`, 3.9171177081392896`, 0.08950823703715674`, 0.8753902886317178`, 0.5807718075416117`,
0.45909018772831434`, 0.24658626134849687`, 0.005965266679061545`, 2471.9794700586917`,
216.13259055465608`, 0.19693571594639458`, 129.4059181062192`, 0.9306846243350071`},
{0.18110313832110714`, 2.4479809284572847`, 0.03899788404590981`, 1.0633093307444568`, 0.1618649881048959`,
0.290943879867789`, 0.07419912462575726`, 0.33289891181756515`, 3153.780133233505`, 293.70004990298673`, 0.08144484935001611`,
106.7702121498634`, 0.32495369359064674`}, {0.130000547849555`, 0.41521332414928436`, 0.012969290047647597`,
1.0493157846583636`, 0.04283335297082713`, 0.4007810804065476`, 0.11394600749611428`, 0.11578287004988093`,
1796.8591049770039`, 167.87252198040312`, 0.20418110664782435`, 727.5932811583257`, 1.2803096926850688`},
{0.19877977882502146`, 1.0832793701616295`, 0.05483303847758306`, 0.9311449051195431`, 0.023003174517098435`,
0.49049424918949713`, 0.12198662583039671`, 0.46882390469238466`, 2948.680187076294`, 221.39677048954547`,
0.2485915935899095`, 570.0809377814049`, 0.5879175980044202`}, {0.0741392858728453`, 3.4806374070586843`,
0.010742699898884 07`, 1.2595082140234737`, 0.6445584019601798`, 0.15229840921907478`, 0.17159475345202657`,
0.05062649216284031`, 3263.5780984266157`, 155.64419895290098`, 0.1549608958054639`, 259.1282244794769`, 0.2745307433995672`},
{0.1612737866024479`, 2.749056795295459`, 0.0902866160682542`, 1.187443722965291`, 0.9773938246087577`, 0.6575443587455145`,
0.18631663384855063`, 0.10452486858949339`, 3326.5992601038115`, 184.7910857322314`, 0.2287875695437117`,
533.6745676036653`, 0.38021040879624235`}, {0.25655997868932134`, 0.5390964756507546`, 0.01345728258515793`,
1.3430828379841862`, 0.4968714378885024`, 0.5549382199645455`, 0.14032147925430888`, 0.11510578618840167`,

3208.997077456527`, 66.83628322986237`, 0.23931361191280243`, 959.9765746023068`, 0.9065373285911056`},
{0.2732923370028608`, 3.191754856678056`, 0.08671034382587475`, 1.254274144916288`, 0.6141422039702695`,
0.6138269440818178`, 0.1312335840480907`, 0.36491894902365596`, 3421.4842108873227`, 23.16093507468986`,
0.24396898979330917`, 507.21752620619895`, 0.16630140622414458`}, {0.04707952880948485`, 0.7465233529742044`,
0.04706998709939844`, 1.3662809192553709`, 0.37778692891819765`, 0.4702166758808979`, 0.09708477572911622`,
0.1347044186557228`, 3991.9105022265494`, 258.2663726134549`, 0.24796704003286507`, 901.9630957104425`, 0.4922414925301777`},
{0.19262163688901013`, 1.3436980923041064`, 0.013792753701933726`, 1.2928623056534811`, 0.019658757972596197`,
0.20917723178587588`, 0.06033608018289768`, 0.1922745773151402`, 1054.9440070441665`,
31.33370933741969`, 0.014040105584901569`, 372.30121099360576`, 0.37448991574388635`},
{0.14563719191991992`, 1.181742783414987`, 0.0819250069285285`, 1.467868385361502`, 0.9444960221302114`, 0.5553246728511431`,
0.22472303268716215`, 0.01264460342880809`, 915.7026359876504`, 186.56527666655563`, 0.12457605924490184`,
318.1531387348837`, 0.7541970118335879`}, {0.10210428290433804`, 3.826490675391449`, 0.080346705846139`,
1.3008168616087474`, 0.16591420773860333`, 0.32805892491350064`, 0.23447289661071624`, 0.2837454098366554`,
2683.0314652244433`, 189.01176571184033`, 0.13052585222400032`, 267.90388654231714`, 0.20917194903453495`},
{0.12052810058893842`, 2.795812984745673`, 0.09515810041066104`, 0.9315452703676583`, 0.2705434252164278`,
0.4167987044691702`, 0.1906828711296492`, 0.029894324474092116`, 2292.2942574099197`, 264.5322169631228`,
0.2203622175006481`, 840.0009516539466`, 1.2395096491075774`}, {0.22795980832341722`, 3.404863594634805`,
0.02719613349386277`, 0.7595996750409736`, 0.8219783106306704`, 0.1726658660400514`, 0.19985944434733172`,
0.2894757551974026`, 2187.731451178087`, 207.77973657236976`, 0.1052337903600648`, 641.9055616662746`, 1.3020724270827007`},
{0.13720254863293707`, 2.3915859466098954`, 0.0919105989230366`, 1.0940464279088769`, 0.03132830155840516`,
0.5703183689284286`, 0.19751876872853957`, 0.011623152338057673`, 1748.3509365100372`,
170.55954378355295`, 0.01335539854821316`, 114.79133761281375`, 0.19460280871282887`},
{0.09439533592668731`, 0.6507098913433871`, 0.005269549199355091`, 1.0850876192066536`, 0.6416369653597627`,
0.62380203725431`, 0.21782081104308287`, 0.030530446580309525`, 689.3655232735896`, 108.38669463356308`,
0.22081299461896486`, 770.936917166393`, 1.2350213442139077`}, {0.1621728529863754`, 2.62731206702155`, 0.0868580220947588`,
1.1899093090480013`, 0.27135894166672614`, 0.42180303316476253`, 0.07951824390093176`, 0.017797934747156557`,
1311.122346565584`, 311.8655925483372`, 0.11734675218317075`, 642.6024484733432`, 0.8698728553338388`},
{0.18405459487807496`, 2.5443763633285634`, 0.08628379610209035`, 1.2294279796845355`, 0.6362691694521727`,
0.6439995368484592`, 0.2101068931673229`, 0.005831602612256186`, 2767.2360340443865`, 235.90491299628115`,
0.19685553147203227`, 433.6773778219662`, 0.9066991169945369`}, {0.24729818009539917`, 2.8593998276797707`,
0.06711603931458107`, 1.4216447849719087`, 0.22262651692613722`, 0.326634644955593`, 0.10319932160331718`,

```
0.00794799105736181 8`, 693.8093018984014`, 385.3842338220428`, 0.13651121260701127`, 996.1351869238076`, 0.7139242262978014`},
{0.12505367012471102`, 1.453024212479912`, 0.008251581952681341`, 1.404228527369547`, 0.39480637487252124`,
0.2468615579015107 6`, 0.2134208863515915`, 0.007363155270613886`, 2628.2439094207602`,
25.17006992107042`, 0.05777712357041542`, 400.57243878499804`, 1.3387107844228217`},
{0.08567551388035011`, 3.464918929604984`, 0.08802313872508816`, 0.8370245282845814`, 0.24100029770289266`,
0.6029152058249727`, 0.16858156068718322`, 0.04508221291688602`, 2362.8576847811073`,
226.099135663967`, 0.19216608111047973`, 192.05855595303058`, 0.41397055564515006`},
{0.2507958759088808`, 1.0809218131003409`, 0.01427635398027098`, 1.0897975256896224`, 0.2982930370148591`,
0.5122364652910176`, 0.19218108861924366`, 0.007800612537408604`, 1683.3624934079508`,
199.62318079466206`, 0.19321234491500755`, 155.2174298378196`, 1.2171745788711106`},
{0.1331763145468211`, 0.9363058039413881`, 0.06508163345642771`, 1.0054503590807204`, 0.6800457362729408`,
0.30829814187463367`, 0.15011476715645322`, 0.3149036800209024`, 2368.4541991406722`,
57.579334768474155`, 0.10662323591696854`, 965.8057986376197`, 1.1607184632847534`},
{0.1814991268012232`, 1.0587693064986254`, 0.08251980636818647`, 0.7918362425044922`, 0.00040981792220384783`,
0.656542189112322`, 0.07166411362313155`, 0.016162360132857555`, 2520.3966209277296`, 187.9298688193668`,
0.12169384514616288`, 864.4026212517676`, 1.0124583071874809`}, {0.0570375888171617`, 2.983765376156457`,
0.035721128762554545`, 1.247671545439212`, 0.8701774499685375`, 0.6021938640855395`, 0.14511231254960394`,
0.09415882262648916`, 2396.908389438371`, 131.332950526094`, 0.10566511719156679`, 870.4532941591235`, 1.0030180176548633`},
{0.19802885711816837`, 1.697809568333927`, 0.08239867751944935`, 0.9543366145673586`, 0.596442677587373`,
0.28741947199407214`, 0.09111396607137803`, 0.0192474188223014`, 898.9528852923713`, 108.61992317486363`,
0.24393048739993817`, 745.6562645006805`, 0.8624057124808657`}, {0.08235339735163255`, 2.04901458998253`,
0.08274380950614318`, 1.3352377456168298`, 0.04240317686192241`, 0.393909136430005`, 0.19534240597655483`,
0.09049932561873943`, 755.5408016924339`, 123.97101820275657`, 0.12979502259555054`, 981.2814332796322`, 0.981556072779636`},
{0.13259019690992763`, 2.977265161565919`, 0.00530648337965463`, 1.3624008940040682`, 0.3850479851659516`,
0.5471181175764608`, 0.16732987155111434`, 0.04673053188976421`, 3935.3957008871193`,
54.56774637029497`, 0.011500428375237126`, 978.4391753399174`, 1.3258009401711512`},
{0.1017980356042581`, 3.0130928286191994`, 0.0670922248821713`, 1.3177452427145766`, 0.9972137316419376`,
0.4917433851632499`, 0.24362584210374416`, 0.04249401043520963`, 1526.3944932079658`, 396.91773501745604`,
0.06915640077930263`, 100.50184972013483`, 1.2530434948898224`}, {0.12274259637222268`, 2.2135947166114356`,
0.002323008478056394`, 1.2379425640649502`, 0.8899842440859225`, 0.4879062825569156`, 0.2207845474252344`,
0.018491490641248397`, 2578.825700078766`, 134.4349608893425`, 0.24442230387433622`, 521.5687684536549`, 0.527389113190134`},
```

{0.15866154261273802`, 2.3869138952741666`, 0.06042642751510929`, 0.848895333807981`, 0.934843242794851`,
0.5842556575575175`, 0.20518241138115345`, 0.40889745342028183`, 2125.073558317614`, 177.04753680177294`,
0.049126150350459585`, 167.20015380479526`, 0.8102237358134827`}, {0.0585830238212548`, 1.377881287434195`,
0.07124000935189038`, 1.0850336387140695`, 0.2992905052348962`, 0.4727992964459681`, 0.23922864395175558`,
0.05243119362224866`, 1784.0351214606744`, 218.5479927009584`, 0.12414817227943536`, 871.428315424253`, 1.4940940994940308`},
{0.1374815155453828`, 1.832494798550922`, 0.014678314171590632`, 0.8577781493036968`, 0.7065388854676318`,
0.35241990107026966`, 0.22478247528073847`, 0.14081196832155904`, 1564.2316558396888`,
63.33175535259653`, 0.1822685539351926`, 505.04572359046716`, 0.6746373869675295`},
{0.24907355276388254`, 2.751578500929239`, 0.08256944464960672`, 0.8833669189132862`, 0.7371567866852367`,
0.23897440464314357`, 0.16501436679241233`, 0.01958796305805405`, 2915.5330263604083`,
134.0428963037141`, 0.07767346073952919`, 929.0516462117552`, 0.9988643418119487`},
{0.23189720878591213`, 2.7231900873151442`, 0.031417708884032156`, 1.1861683753173395`, 0.9551934586691826`,
0.37227454560407613`, 0.07127190671645484`, 0.012276183183919116`, 3516.4021798452222`,
55.7061542721662`, 0.13600912350457384`, 803.6520907403464`, 1.4980631550215575`},
{0.25323525051238344`, 3.8701603777982685`, 0.05716138648097892 5`, 1.2330934547823138`, 0.4437780175794521`,
0.21244417226639944`, 0.10049546251977226`, 0.14156994275348248`, 2985.8281985310914`,
188.5768875307433`, 0.16554356776660822`, 752.0830220079755`, 0.48064358318558353`},
{0.07400495176845395`, 1.371778024263044`, 0.06366073117599395`, 0.9113217976134347`, 0.6450439371549741`,
0.2028695853666771`, 0.134893293356957`, 0.010129621335087692`, 1619.093037443351`, 95.10382898978872`, 0.21460298593776378`,
449.7726020715618`, 1.0380133088295604`}, {0.09174097232490325`, 0.5195079257804815`, 0.09671751166466962`,
1.3737488638206528`, 0.5227074578569586`, 0.2710165188583308`, 0.11856204039867918`, 0.06811453385903786`,
2434.6303851805524`, 299.9843115022044`, 0.1678462883337608`, 186.8371930733158`, 0.4784242625831241`},
{0.11242118513218191`, 3.134251656842568`, 0.09650309966311003`, 1.3449356906576169`, 0.3883840820004587`,
0.6476616921712703`, 0.0873739057627867`, 0.25937474579892167`, 1160.5159523366365`, 274.5065784906882`, 0.20165149906752045`,
983.2491591733699`, 0.5244507425864533`}, {0.18675484299553718`, 1.6680998726059926`, 0.06980886233291318`,
0.9554678238435506`, 0.14384208534779463`, 0.44531424976479517`, 0.12023694180999711`, 0.43169366437755463`,
1135.6653148798032`, 239.65712822052774`, 0.2065769214271021`, 935.9859640723332`, 0.5534954020424208`},
{0.23767823194930537`, 3.602590605886231`, 0.060852474647757`, 0.7714576975128256`, 0.508592316287158`, 0.5398400739227621`,
0.14769420291278085`, 0.01528621033732573`, 1647.1971434084526`, 233.51361896017966`, 0.061100070503878445`,
300.710012065689`, 1.2671164264297592`}, {0.16213543978297962`, 3.414345886884365`, 0.004779084954917607`,
0.9921063364864315`, 0.1785001821110408`, 0.6724004321070518`, 0.1269026207013904`, 0.04822311475039711`,

723.8518877561896`, 30.49297773826595`, 0.08363274585873043`, 747.9812681511243`, 1.4783809272902158`},
{0.15893018843067214`, 3.2697131207533676`, 0.08335905478077443`, 1.0052424631748718`, 0.008139916906689137`,
0.4429455955042595`, 0.1767129250787733`, 0.0935032817730384`, 3498.572089064376`, 207.67897478841815`, 0.11675101583991215`,
487.67250951072293`, 0.8073799631161638`}, {0.2682381172670912`, 1.9224360686126598`, 0.08405641837269681`,
1.0744188845946134`, 0.13427407777422062`, 0.3593169543749103`, 0.12950520232599422`, 0.3443164693786831`,
2590.304101144844`, 106.66275736164647`, 0.025299699910539764`, 980.4883790730589`, 0.687217667589437`},
{0.15864725855906098`, 2.1894087109409286`, 0.020607078237198755`, 0.8053646594139596`, 0.7295897192525798`,
0.1613992782799614`, 0.2251057243729967`, 0.031334294944303724`, 1285.0188074984508`,
178.0125916551774`, 0.23488122465202665`, 172.494267692295`, 0.47309832904681937`},
{0.2463676264622125`, 3.8431989079059443`, 0.0830636330061051`, 1.4506553887775853`, 0.4157101189501755`,
0.5706880235467402`, 0.09082539124587127`, 0.005952661726265365`, 2360.5016458005193`,
281.4673092408842`, 0.014113705835521767`, 564.6585348365634`, 0.8269660235540686`},
{0.11019554333046278`, 3.5051367646262026`, 0.02618769243692467 6`, 0.9701173772624384`, 0.828045505344766`,
0.3329586916028372`, 0.1726056537041083`, 0.011410895915187734`, 1714.8868152567811`, 160.9310995211573`,
0.017867320810878384`, 709.7816452283784`, 0.6538676800616441`}, {0.12429394005150873`, 2.8200040902174734`,
0.04409372643357232`, 1.4825479254137954`, 0.6438802520792373`, 0.5253997805968301`, 0.11516898024351507`,
0.2090633248975017`, 2369.8391266859117`, 150.7153439217572`, 0.2339441457923686`, 454.4014097364425`, 0.7454584507599868`},
{0.1327687684875733`, 1.2523070928491569`, 0.0034972949986995694`, 0.961734854335881`, 0.7173342396101765`,
0.5124389557647466`, 0.1819823354846934`, 0.039690987585360354`, 1268.0722955113333`,
354.50985731736694`, 0.061401586587447154`, 289.9872408410486`, 0.8372867806717847`},
{0.25119415291363933`, 3.699957407218835`, 0.03691761530494933`, 0.8519313476704744`, 0.4260845773680011`,
0.45282452720541466`, 0.10272915361876822`, 0.06885052434013854`, 3045.5649926855003`,
308.5910379858059`, 0.1655679250488603`, 274.90436712317177`, 0.3516531460623591`},
{0.24181327853377987`, 3.924471184361728`, 0.06442172021207551`, 1.1468756919134357`, 0.4683765678963312`,
0.2392409003220175`, 0.10655262173137836`, 0.01050989827281461`, 3011.569604707088`,
157.96617642901117`, 0.06447038330316018`, 832.8395648582813`, 0.7391131914586526`},
{0.20316931352002288`, 0.878955926140808`, 0.08609000563691026`, 1.4265202894817164`, 0.5787311896010581`,
0.4937633929020542`, 0.15053209007715584`, 0.005433330383769161`, 550.1238876941347`,
242.96229200526432`, 0.18930501383788928`, 820.2173201760484`, 0.13083350361507562`},
{0.09452847068050013`, 1.6173995428510484`, 0.08378889374586894`, 1.0570110909031192`, 0.6217186731824058`,
0.657332460023951`, 0.19095038645292134`, 0.15943889654124058`, 3237.8559151922873`,

210.3037282348174`, 0.05358306931452622`, 183.74246730078988`, 0.10353004412483036`},
{0.19488414343020988`, 3.5208927551118876`, 0.061263993543847414`, 1.4769849645204522`, 0.853114197633267`,
0.24334616405333398`, 0.2468260970715581l`, 0.04406892072139569`, 3024.5419739897516`,
278.7766239000307`, 0.15826763252336173`, 111.29790453335673`, 1.1260358248330853`},
{0.16041791996208227`, 3.9329082889470177`, 0.06107867473368609`, 1.0707912315920178`, 0.35078802407426135`,
0.3302878127385429`, 0.1742518226406985`, 0.22436340469211272`, 948.9315242302914`, 295.89794785347067`,
0.12200395983956575`, 443.3288106621854`, 1.1360821716382503`}, {0.16423899229758965`, 1.3138086325207086`,
0.0691507383076826`, 1.3781985631312954`, 0.5199406702032707`, 0.561438424219397`, 0.1622498281593202`, 0.04944015525612536`,
1993.9060826675595`, 161.30849993643335`, 0.1869658221748846`, 409.76120701126143`, 0.8598128840536354`},
{0.1728408486961382`, 1.146440967871185`, 0.003590671465871154`, 1.3869212879713686`, 0.9384256838276226`,
0.5023045119953624`, 0.09620780540560417`, 0.010488565432974887`, 1931.4866228221326`,
267.1499190279868`, 0.21494802212466085`, 145.85413549694212`, 0.30471824098222555`},
{0.25476405918451717`, 0.9501786024371341`, 0.006743742440571125`, 1.1773804132629142`, 0.9261742704739533`,
0.6907632538002968`, 0.05916705117458520 6`, 0.388385802512907`, 2327.9366084758294`,
382.0620458738822`, 0.11500638439043326`, 799.3983434362978`, 0.4350856867848527`},
{0.10950678316275353`, 3.6760115538957567`, 0.029402183746122842`, 0.9326374985587951`, 0.6852863701908152`,
0.3870953214392294`, 0.24335109527629128`, 0.01218488500325287`, 2796.7295109467823`,
323.29792928925644`, 0.09155413630983544`, 728.6990873013968`, 0.26376126165483504`},
{0.163551248798117`, 1.6633993319421343`, 0.034275010847829`, 1.1128795027660672`, 0.1382201854632803`, 0.4960737020500119`,
0.16086028129627217`, 0.43317574700485156`, 3166.4551967087828`, 291.68472752217326`, 0.011653808348350336`,
743.571123288983`, 1.4870562515146286`}, {0.20636484294348667`, 1.3331885283006724`, 0.06418620324523439`,
0.8156849902245289`, 0.846478078478194`, 0.3634936294690221`, 0.231181984574578`, 0.28258725445236976`,
2798.3260469955667`, 290.1913294212843`, 0.10937544276935934`, 437.63721142598655`, 0.4015128699093473`},
{0.20547386544073876`, 0.7900704392690665`, 0.0812168824235561`, 1.188826721473041`, 0.976250607947333`,
0.6908772615190524`, 0.12722535124330514`, 0.011647380698834212`, 3575.475972236056`,
90.69833367636664`, 0.19307016139248817`, 423.36994924313416`, 1.3519738992435562`},
{0.10948479528861432`, 3.829378672251859`, 0.06039700201416281`, 1.1603289915277062`, 0.6158500210093096`,
0.603657210616175`, 0.1476615217089668`, 0.01972537688204371`, 2051.8187549303293`, 316.16486862359227`,
0.1312558371888642`, 434.8554057837925`, 0.3549078089064188`}, {0.21736048342421987`, 3.70578817107093`,
0.05644933691564836`, 0.9655179559877884`, 0.22623410346541428`, 0.2474639255809764`, 0.13317885031923654`,
0.3624237848221962`, 1753.527377862224`, 323.7167834415719`, 0.23631201802334267`, 387.1411803187586`, 0.7949245514701109`},

{0.11614168249963264`, 2.4432349434757095`, 0.028151150404223255`, 1.3022567438958985`, 0.9605717973157235`, 0.4833016704249957`, 0.22807903763828796`, 0.04984000778611073`, 3280.400046570974`, 305.78807538220474`, 0.12991183855122485`, 820.9216145921939`, 0.42008587023650845`},
{0.2553791241982973`, 3.361620807208541`, 0.06677443347624383`, 0.8670734265419993`, 0.11040911028180322`, 0.6801218730423624`, 0.12541925369547308`, 0.31131291968557195`, 1902.9188966521297`, 96.06061314019757`, 0.010389852626860707`, 642.2281487245083`, 1.1001994189973279`},
{0.12704207222892605`, 1.1628273966458673`, 0.08527328785857773`, 0.8365181177679062`, 0.7223756255303246`, 0.22901818615931202`, 0.1203046430318036`, 0.042517213825228134`, 878.0558283062142`, 305.4388353984085`, 0.03167829108798248`, 573.1363514229927`, 0.4578463399808892`},
{0.21019250612187246`, 3.3723264243216624`, 0.05080814437299916`, 0.9812850379872056`, 0.09150102750252853`, 0.3338288248427723`, 0.06484477712339956`, 0.0689228824965226`, 612.8201590193585`, 124.84621243046996`, 0.19453763033805516`, 118.3212191068728`, 0.25449019892751523`},
{0.05454250212049144`, 3.2024915989826592`, 0.028233465868114517`, 0.9216789997773293`, 0.9497061314253168`, 0.6744717310680077`, 0.15700855458335805`, 0.028986910337379608`, 2317.6812128111524`, 303.84007023121455`, 0.03000869351136215`, 190.10718599412007`, 1.2944308041726935`},
{0.21303256407260035`, 2.8786914372271166`, 0.013104389096981191`, 1.0555107506813834`, 0.956903194093049`, 0.2520046794033417`, 0.0891713794077007`, 0.13656202727058825`, 1008.7620556429179`, 177.7176029228849`, 0.20057020979132534`, 618.3016937975689`, 1.4173393410661035`},
{0.20865719895059348`, 2.2435235464600973`, 0.06928974554725371`, 0.8985003634483657`, 0.22474759207779793`, 0.26859677109763735`, 0.2381780586776946`, 0.020421240745008753`, 2998.003490602676`, 324.2321548929817`, 0.22795801338780824`, 185.8288223184566`, 0.29311387456910776`},
{0.06445455118485005`, 2.5014637187974866`, 0.009712848934901146`, 1.113241000173885`, 0.43392912531343164`, 0.6501742115306579`, 0.24685508411573603`, 0.009411016718828982`, 2675.354793315116`, 130.1817572042242`, 0.17737280777592673`, 697.9780988605116`, 1.498582373444357`},
{0.19737221690406592`, 1.8631921948394359`, 0.011182660188463754`, 1.0502698943037188`, 0.08609592166751923`, 0.3607950150039636`, 0.21433649366936036`, 0.3689042688491969`, 1147.4521148009344`, 260.76660193682267`, 0.051623276246451855`, 999.176577419826`, 1.1548661107354268`},
{0.08906427686597185`, 1.129852324347059`, 0.09140290776617871`, 1.4976153584339291`, 0.10862942012833243`, 0.21137366073893882`, 0.16932042326838176`, 0.005800987576377906`, 1379.3305547868067`, 108.85277960508245`, 0.12372140825532296`, 139.91399972124032`, 0.15370688120787235`},
{0.1261654821003177`, 1.5947971506666327`, 0.05593249923129049`, 1.0033615240392035`, 0.6183404043465164`,

0.21416994093354402`, 0.08633015203983824`, 0.38698283237677483`, 974.3819470194694`,
293.5131814195212`, 0.108873074350402`, 552.0807660403041`, 0.9190091346479994`},
{0.1355226761948981`, 1.344272961945551`, 0.07513196510449792`, 1.1712401710495086`, 0.9680307353832129`,
0.6233308142667433`, 0.24083815300463013`, 0.14765064691825197`, 1323.7182013070742`,
348.7232130621037`, 0.21849425287384605`, 327.9490632909029`, 0.23857872384438972`},
{0.1550857600606415`, 3.4504174955438254`, 0.08400943853903374`, 1.1551511289452336`, 0.8759297897135132`,
0.35050080971023223`, 0.12164531860088268`, 0.048277789120459075`, 831.3621060993951`,
389.31756123419245`, 0.12950267374759572`, 639.51708793359`, 0.6322774532267426`},
{0.06454506498499168`, 0.9204135574994012`, 0.036358021707619696`, 0.8427468163749889`, 0.7709527632773838`,
0.29059160814456453`, 0.11648625849142763`, 0.08232561817979563`, 1840.3054444549834`,
161.68135285764447`, 0.054714472579224405`, 679.4503841543531`, 0.46429629473894396`},
{0.1536916832442427`, 3.43119334536589`, 0.004793268707920184`, 1.3168956371094804`, 0.07372937129901525`,
0.20953493396084777`, 0.10284092256638205`, 0.20873209585464722`, 1462.7121406620445`,
191.090169255751`, 0.01060695667647657`, 537.0729625339702`, 1.427971393264623`},
{0.09768048595099643`, 0.6757273754077091`, 0.03934875882573964`, 1.0701701611082628`, 0.07148535549576684`,
0.6142784144411337`, 0.10336763328669979`, 0.3174771550017104`, 3543.1590511081704`,
179.42221499979325`, 0.059937790163951954`, 585.4906918962374`, 0.1792745769069808`},
{0.11093744843988207`, 1.139281584231001`, 0.03792596719465658`, 1.4712179350850514`, 0.09531459753873972`,
0.4482130481346429`, 0.2411832829588788`, 0.36731215858298116`, 944.6277677947814`,
200.25811469302596`, 0.0168796929850501`, 248.89634029012325`, 1.1048774013448766`},
{0.2561934904697367`, 1.9350150602392846`, 0.05338199696709335`, 0.7754194094915202`, 0.09467921904454579`,
0.16803953903191093`, 0.10131723911729429`, 0.012814846859783775`, 623.1704638270421`,
52.174205283986566`, 0.06361479325154268`, 176.76164123415177`, 0.9999546673314952`},
{0.2052128459234911`, 1.1897795773061866`, 0.08317677987785423`, 1.4749920280768674`, 0.19904740525622588`,
0.17984962141830496`, 0.2052680263678649`, 0.00741647127595457`, 2588.6593918279505`, 340.9517444127914`,
0.2046076143969877`, 352.2129035501141`, 1.3485807934223022`}, {0.17935227991336467`, 1.5853882677551843`,
0.06569015691066475`, 1.159680524153029`, 0.4013565084570856`, 0.390767904995891`, 0.23486419101050904`,
0.02761033126218625`, 3365.179341343847`, 209.853227362256`, 0.15688720840408887`, 213.8719546746717`, 0.4665754403259277`},
{0.24420658938233086`, 2.3631667105844176`, 0.011636058656798091`, 0.7699037767738292`, 0.2334071597209253`,
0.1772360051588755`, 0.22736713920513163`, 0.0051651255057250725`, 3387.5742500946835`,
67.8941314260141`, 0.10329046386479473`, 644.550133807103`, 0.46391586011260877`},

```
{0.1565799871778506`, 1.2583377161922806`, 0.024964491740953373`, 1.1702004879227101`, 0.7783275467928219`,
  0.5012890313214394`, 0.23530757482014591`, 0.34452851290714537`, 1633.380422182493`,
  138.45317506651554`, 0.08276316750883178`, 526.8498338681917`, 0.8787386694394961`},
{0.12762919436902181`, 1.0284069551450923`, 0.020915383194947134`, 1.3322952223445226`, 0.2829156904285557`,
  0.5149241136029713`, 0.1689585489061317`, 0.15408082498053616`, 2969.7185950133517`,
  154.81241829398527`, 0.1924782550248358`, 919.0147237575549`, 0.5539322070467121`},
{0.041454706797677654`, 0.4235388809185028`, 0.0711166894367517`, 1.0177515404715998`, 0.3959583445976351`,
  0.5095994657063209`, 0.16041563555046656`, 0.008204389355143152`, 3956.172943281231`,
  146.6955360862866`, 0.23629828054244195`, 959.9747641936272`, 0.658565318156795`},
{0.10527476096646576`, 0.41594398566453084`, 0.06777087482346833`, 1.4252254286591717`, 0.5851359194260937`,
  0.6005705290816346`, 0.21624616394741747`, 0.4756618413610822`, 2536.0574640922323`,
  263.78246053884095`, 0.0363778983789938`, 218.66286913847796`, 0.6480151044493636`},
{0.13448672535543155`, 0.45587768978852417`, 0.09169485354759886`, 0.9984333604381879`, 0.22545114225043816`,
  0.2261223957931866`, 0.2016309939821746`, 0.25762662882773885`, 3983.0852337416372`, 211.21067976049187`,
  0.0772172358070719`, 548.630989759385`, 0.22933239495994617`}, {0.08077460873547221`, 3.1578375162776995`,
  0.015253020463429757`, 1.4167842986517556`, 0.18887233138438875`, 0.225095926719601`, 0.18845443951350754`,
  0.48008903041412254`, 2107.486640599107`, 58.27596934178075`, 0.0329628858857266`, 721.0920138161233`, 1.3047638238164008`},
{0.1627210388204795`, 2.3486652860563124`, 0.07334147889748355`, 1.297802663033296`, 0.8896488444500756`,
  0.6367608859636733`, 0.13844266845989056`, 0.028105720298914772`, 1278.9617102086122`,
  103.86032737516456`, 0.13398816426568466`, 401.36714882545874`, 1.4876475604632269`},
{0.1991733888062892`, 2.232280796966574`, 0.02878565083642366`, 1.1378784024996818`, 0.9331355769640246`,
  0.5978968544848732`, 0.10707936186453992`, 0.04500082893686236`, 1216.2749142243183`,
  96.74942698117707`, 0.209253733883974`, 180.23985384162967`, 1.450345068539872`},
{0.07006975226908452`, 0.5088993200730623`, 0.06645083916640811`, 1.2192344369115269`, 0.809141944131883`,
  0.6050714872296197`, 0.11290910204391225`, 0.4205963617287652`, 2860.7340509641726`,
  237.4532029608671`, 0.23737915616168098`, 410.38572416543605`, 0.7213651518381718`},
{0.13014952175806743`, 1.2383651907296915`, 0.033466528386360626`, 0.7608179457351143`, 0.035887935204249155`,
  0.30066555241820714`, 0.22194754482894746`, 0.010014737909855631`, 3447.0059811378305`,
  115.24150997756686`, 0.05902709128611322`, 419.3031011752403`, 1.1372885455851796`},
{0.22839849743494078`, 1.206288301576035`, 0.013326065846012636`, 1.3845248483797399`, 0.7626327581902952`,
  0.3754386638058326`, 0.16053945324506902`, 0.014116556581987155`, 1794.3782905834978`,
```

```
159.38642300652236`, 0.0712833656764853`, 823.5469375082665`, 0.7207830721959967`},
{0.04337108923026292`, 1.888268909664844`, 0.08288225028973259`, 0.855814919343177`, 0.17165426591642308`,
 0.5184298574192848`, 0.18334741750742567`, 0.02904567352634542`, 3958.112424596885`,
 386.76795326406966`, 0.2461352187724518`, 371.03988163750364`, 0.6490702952773506`},
{0.2612721349522831`, 3.840978760137725`, 0.002842556189941217`, 0.97686328730745`, 0.44880391224027494`,
 0.5727559276417925`, 0.18956148497729763`, 0.03276257748967842`, 2211.176414289902`,
 358.93604732927395`, 0.18442709982307676`, 464.98052505632313`, 0.2540849694991574`},
{0.24789805361095096`, 3.4057245361540636`, 0.05900845832164460 7`, 1.3299018476154343`, 0.8312504959647706`,
 0.29504869999910865`, 0.23129555899840143`, 0.11045893049148961`, 2449.0374944139776`,
 372.4740944416101`, 0.23674150267599486`, 707.1501409070081`, 1.0357095360429422`},
{0.20407523893303542`, 2.523301559184878`, 0.0630284853489859`, 0.9928756541538545`, 0.615801951486598`,
 0.23887170760351184`, 0.13817398611171328`, 0.03995692517332042`, 2340.374866629386`,
 363.0619692672575`, 0.08780428805507734`, 643.8318915603425`, 0.1377149565480864`},
{0.24650823881149114`, 1.4921303332816969`, 0.08398739918085261`, 1.4432990795744263`, 0.5407394698528241`,
 0.24235787305657475`, 0.17078866800370185`, 0.12166224207759718`, 561.5315929420126`,
 388.70070975042916`, 0.06571895249922433`, 798.4108672871722`, 0.18089590787223675`},
{0.130403433861568`, 2.564945939086962`, 0.03938991087643217`, 0.876261720902835`, 0.04823811429373581`,
 0.6679978536003199`, 0.21552343838945165`, 0.007100380217954227`, 2671.511757568389`,
 51.97056323428569`, 0.09050840826397427`, 832.0124908826144`, 0.16778476814117793`},
{0.19301359386940037`, 1.916835285100226`, 0.013026709781399361`, 0.7610233489687097`, 0.2807825707044036`,
 0.2725248315709231`, 0.18510532276589875`, 0.006549584153913358`, 3482.023140031519`,
 223.38456749033946`, 0.07222327127355554`, 745.5573161559128`, 0.5028516505918408`},
{0.2684665003313201`, 3.537796754706533`, 0.0579292923787218`, 1.3116248162305904`, 0.938103305079736`,
 0.303548243902228`, 0.17710993143610637`, 0.022253686893995598`, 2385.9279516499173`,
 87.51735857414678`, 0.13636461232998032`, 182.26020483570852`, 0.6067332169603943`},
{0.1163329056504609`, 0.840362468819281`, 0.09011457674854512`, 1.4046094230031378`, 0.24754903085374202`,
 0.35558618615105175`, 0.09603602219488144`, 0.4400291135028793`, 2379.874563964334`,
 291.08707065265946`, 0.0813431778790557`, 873.9667979217803`, 1.4071371793368979`},
{0.08845067382827221`, 2.8968609262973715`, 0.026295018770535618`, 0.924672187140021`, 0.8266765998939338`,
 0.16453043420958635`, 0.1436301429404348`, 0.03203292742039931`, 3358.876899764703`,
 340.03971511585826`, 0.21800949324859614`, 758.9780281076877`, 0.3147697227657398`},
```

{0.22753897832657932`, 2.3626010404022777`, 0.06605301101645089`, 0.9861469601905177`, 0.715699676979547`,
0.27811071822727396`, 0.12640216601889592`, 0.1307699593246265`, 2086.15260815056`,
86.00779248052135`, 0.12306333462763464`, 220.79674426988075`, 0.19597696533701958`},
{0.22568007090069508`, 2.690166184833209`, 0.03809980637132476`, 1.033282327047553`, 0.2322901923352989`,
0.25190177549821213`, 0.106961778903361`, 0.33025539017011785`, 1095.6591402639856`,
384.7567493155469`, 0.15562925209926093`, 402.44719645639793`, 0.33551373738348733`},
{0.21019754310189576`, 2.2821279974051283`, 0.015593946545040663`, 1.4973542862533429`, 0.654617568369658`,
0.21608426449113316`, 0.09011313521803482`, 0.04944367661242139`, 594.5748264592376`,
46.176949539178736`, 0.16057971105621394`, 409.0668080421482`, 0.2959994600539888`},
{0.26032271813421254`, 3.1506419827011234`, 0.04944216171812114`, 1.485970749729839`, 0.5400908938035165`,
0.5796967425656009`, 0.24293350926289264`, 0.006484214563558236`, 3415.105165914886`,
97.70247652208917`, 0.2350556387074887`, 911.1914546951435`, 0.9596772406097074`},
{0.11107187448428635`, 3.1480231762768742`, 0.0962726511045031`, 0.9564838072230462`, 0.7241222355765891`,
0.1536273802849054`, 0.10639405078602365`, 0.2722757429659684`, 2244.4463871942344`,
218.19000080498938`, 0.11119753112694813`, 343.8491134497958`, 0.19660089317154927`},
{0.2510397690396396`, 0.46935952347382814`, 0.021738320051900236`, 0.8968830827424275`, 0.5854694493208272`,
0.17068057197218844`, 0.11419385594295173`, 0.006642770597936426`, 1450.2248224479126`,
347.4259504787941`, 0.22780237490723132`, 126.52823830865731`, 1.1776653684396283`},
{0.10508178381516031`, 3.241817080522851`, 0.09317370421720714`, 0.8241478588549098`, 0.2544360789616906`,
0.34970027433888684`, 0.06395982551620327`, 0.03349866959593217`, 1692.667446698536`,
234.2549082570206`, 0.06949624322824821`, 252.083004521546`, 0.6444183458615718`},
{0.1738021137341914`, 2.6655766880839264`, 0.010882627149841291`, 1.3612559957480876`, 0.9827783188881223`,
0.5963630292797856`, 0.23063728087455293`, 0.024370830019574876`, 1313.4321507111035`,
140.47707600099756`, 0.12474321231605512`, 490.98604406828963`, 0.4920136259822776`},
{0.13500993604969308`, 2.3512168951422705`, 0.02556888715768917`, 1.2552325322390168`, 0.18881397547730505`,
0.3494719033874386`, 0.06997757940288904`, 0.1847493739286579`, 3396.2424502847934`,
99.81103419296869`, 0.07140799292026223`, 263.6581807978814`, 0.8331423395780275`},
{0.2295473478278719`, 1.920919876240534`, 0.06400297143770356`, 1.1088034744610789`, 0.12850843681594637`,
0.245655090976371 4`, 0.05707535554246118`, 0.10243105349922502`, 3852.5231597722195`,
305.6356345865926`, 0.09928555868031341`, 154.48888788275852`, 0.17108593589139942`},
{0.15475239915831557`, 1.334283212055679`, 0.020788763317006236`, 0.8779580647205354`, 0.6802987006095416`,

0.22422834935670477`, 0.20406615878595552`, 0.054905866643000394`, 1860.1823145158332`,
184.50838739049425`, 0.0879081741512493`, 304.0210105388518`, 0.31886861005440137`},
{0.08592688510555363`, 1.876394026229402`, 0.06920545098642468`, 1.431675430178669`, 0.8239775274642684`,
0.6607725052527167`, 0.068117656179428`, 0.0053062583905724705`, 806.7259545120805`,
270.2452939192209`, 0.05283774936657276`, 196.8753086866249`, 0.1063772549155293`},
{0.2594352113058643`, 2.897375522885567`, 0.03798263176479136`, 1.100293780244943`, 0.4422676107149168`,
0.21248505456127442`, 0.16565138674519075`, 0.06093024787068363`, 3848.7820191353094`,
47.13186957311484`, 0.09572270042081266`, 451.4558891839364`, 1.2537888752120385`},
{0.16006869287790637`, 0.9138642167640691`, 0.05539778023151806`, 1.4518040129667544`, 0.5826185074798902`,
0.3656462712405105`, 0.06170788162217322`, 0.1216822876868403`, 3863.404448884796`,
323.90265345199316`, 0.03177599643936252`, 199.64726882578051`, 0.3443630619149598`},
{0.2574986064149328`, 1.513208530344186`, 0.01017284770938657`, 0.8841213615409358`, 0.24579912631567602`,
0.6494279555535813`, 0.11031141135597855`, 0.13918991049121004`, 521.3701852206909`,
316.7378393572294`, 0.09988669353502583`, 903.5356229915028`, 1.114184557918394`},
{0.25538400934228883`, 2.4151203872804157`, 0.07378895262994443`, 1.339399865761794`, 0.2988750039664314`,
0.6363626186066054`, 0.1427420291093046`, 0.009517618526644634`, 2204.7304069719194`,
107.49385335999716`, 0.18572660657525258`, 767.4875104948669`, 1.2366495703195204`},
{0.20371950494257662`, 3.26435038453044`, 0.0673224206633365`, 0.942711931684787`, 0.6977520454648647`,
0.29162189123352034`, 0.15729926402190736`, 0.06608992057090111`, 3190.672922367533`,
77.27617660983225`, 0.03600244325410762`, 291.28372784345834`, 0.7447676275285735`},
{0.08966772423850533`, 0.9742810639432404`, 0.04636739498086445`, 0.9405444099771487`, 0.6773239698111666`,
0.6031171967734776`, 0.17649265874576214`, 0.4579425421803454`, 1962.566342842545`,
234.35593295121691`, 0.04690692255255746`, 561.6464610333816`, 0.31865403879003873`},
{0.06773406570474322`, 1.920419703381559`, 0.013765933996251185`, 1.4291947009047403`, 0.2664080740368808`,
0.19005312331140156`, 0.1451294803024616`, 0.08200925278026623`, 2093.336077426793`,
255.07422606817795`, 0.03483849095781699`, 137.75493828635064`, 1.1450754886684913`},
{0.06852495934684488`, 1.6642300409302244`, 0.011861922047950363`, 1.1071027134964053`, 0.34977020992191044`,
0.46476334405193176`, 0.1053167552908732`, 0.22857351767455047`, 523.4371079295597`, 279.1517454574988`,
0.13658074480507632`, 855.7256793246814`, 0.49296675730852035`}, {0.180663173623006`, 1.9629040357204355`,
0.034104181571869584`, 1.2207915880351414`, 0.9040776922893723`, 0.2647119813436606`, 0.14573565454181786`,
0.1248340048237434`, 1930.558235074679`, 301.1110773057741`, 0.2254857692904556`, 114.5985274899342`, 1.481468950655068`},

{0.21204160464602817`, 2.2751102033296142`, 0.09262730518205146`, 1.0948515354420605`, 0.40769495621258334`,
0.1936053798977645`, 0.11749125587531761`, 0.20275504351255905`, 1244.474686551528`,
113.18426815292582`, 0.013466672709863459`, 598.8748104034764`, 1.4984857870137662`},
{0.27473277223474885`, 2.2636221638925837`, 0.047212242125489697`, 1.2743856962833575`, 0.7257539495394636`,
0.30322378247677906`, 0.1124921444643237`, 0.03703552128793425`, 1459.2672419267528`,
75.1854249648913`, 0.19487973279008403`, 319.77189547608606`, 0.7014138972285817`},
{0.1312962905221532`, 0.44177139115229735`, 0.08719755024715678`, 0.7629548047135822`, 0.22337797682067917`,
0.41864954310442715`, 0.09512746105381009`, 0.00534189753945476`, 2680.170847801335`,
368.9804281348463`, 0.18778908189950588`, 838.9486671386724`, 0.4092729826415049`},
{0.16046919678502158`, 0.8683151525644242`, 0.07975042054708346`, 0.7670045102156273`, 0.5892690356019428`,
0.35545909362996564`, 0.1380495644461121`, 0.43971124374733395`, 3165.758795241846`, 34.8086561244931`,
0.16730492888906962`, 570.1510915304169`, 0.7841806284310633`}, {0.22370413346274232`, 2.5025504584039506`,
0.055442737148399125`, 0.9745429729054862`, 0.26334885202676994`, 0.292995553155592`, 0.0899069350018546`,
0.052090218054286645`, 3713.96646787573`, 60.9651090846437`, 0.02697699350525906`, 424.9206374115019`, 0.991124433973821`},
{0.23837993756501574`, 3.674781782586927`, 0.07566951539032658`, 0.9544498006759445`, 0.21717792475949826`,
0.5392603150983969`, 0.2497203185975594`, 0.49633440743176716`, 3853.533625842738`,
31.158368614461892`, 0.23280130783000508`, 447.50809469316425`, 0.1807281782040251`},
{0.13591646380273686`, 1.372083575202705`, 0.09870297459922632`, 0.7922579751372021`, 0.07042511191463574`,
0.3209190140872761`, 0.16294463965647366`, 0.024890970634302487`, 540.294684826667`,
68.22489062767903`, 0.20470533563711102`, 386.65875028452723`, 0.5513768530944945`},
{0.20558874695968898`, 3.999693650069309`, 0.0617766069558904`, 0.9666829242876398`, 0.6453728580477212`,
0.5190085928841239`, 0.24227801042920738`, 0.1275209673550862`, 2460.1178704636586`,
269.8811755836872`, 0.19886738747036248`, 315.3714262314404`, 0.5965893549921157`},
{0.187643306528258`, 1.676756825470198`, 0.08634508127467523`, 1.437101734700365`, 0.5607700413655154`,
0.24605314035549708`, 0.16914792084289304`, 0.012003126342315035`, 2066.524232462435`,
98.64843735664022`, 0.12649811562148994`, 706.7509818275873`, 1.4945161401194085`},
{0.2578658816681165`, 2.654120731507229`, 0.0612275421807386`, 1.3430831855485592`, 0.394847238666296`,
0.6632051318878478`, 0.09069571897643935`, 0.006885619319489394`, 1520.653894509599`,
208.630006341382`, 0.125001492130195`, 783.5023156108674`, 0.7537696978021406`},
{0.26694056526639104`, 2.589928525387398`, 0.04440846617064281`, 1.0510310992262157`, 0.7103641006398789`,
0.5608274401504179`, 0.18485075823814467`, 0.048167265529310214`, 3832.809795627375`,

```
164.40955199605082`, 0.21122610122000635`, 283.2936506989295`, 1.1502434619314381`},
{0.0670413893388308`, 3.4807202196030387`, 0.06807104247513276`, 1.4249364441609478`, 0.23907494438810772`,
 0.24691068350496204`, 0.2269901340944389`, 0.47429378337981265`, 1136.8587227875369`,
 219.92936797845755`, 0.15803291099968242`, 664.4972109143304`, 1.1398702056762398`},
{0.12297654692881094`, 2.388794442755981`, 0.0150896895702037`, 0.8824912867140063`, 0.36012595607696074`,
 0.6662707672943755`, 0.0843157973909674`, 0.03688714998355043`, 1553.50488341044`,
 112.75357357278085`, 0.08204472096069687`, 482.0486491990756`, 0.3111282075755535`},
{0.22811270210861684`, 2.743295920339892`, 0.028247961503946453`, 1.0901415242176606`, 0.15194202610393526`,
 0.4369220152696016`, 0.07582059949983239`, 0.008612431410101781`, 1024.7594376340194`,
 314.07680269266916`, 0.09214933733104858`, 919.7491213280941`, 0.3722904259830171`},
{0.09327165407041943`, 2.773412634419051`, 0.009118016580451976`, 0.830261374296958`, 0.4560243809595399`,
 0.1902390609255734`, 0.23616473748847938`, 0.006188193236419718`, 692.5485171642076`,
 33.478422197960015`, 0.1399053023180536`, 363.91360651079674`, 0.7741685958851368`},
{0.10912104096356073`, 0.9480201542588675`, 0.022647180395832134`, 0.9467779105888361`, 0.06837267408246017`,
 0.28743337490819176`, 0.06613923338342581`, 0.29845131831850175`, 899.6086678018696`,
 94.81889996328516`, 0.012252709235090031`, 536.0650359615496`, 0.9779826770128248`},
{0.19340977815283472`, 2.2087055887009654`, 0.02290210768142595`, 1.1160344159380808`, 0.996916189065836`,
 0.4376804904125826`, 0.07420586783174699`, 0.4518046167809544`, 1202.166850235947`,
 80.55043612489692`, 0.24085119373649283`, 502.4542476242761`, 1.4927287209404438`},
{0.11789604389328145`, 0.5542617488275599`, 0.06446168785511634`, 0.9041263109674751`, 0.9924077505306126`,
 0.34277484870218033`, 0.18917489318085307`, 0.008441976929008489`, 3262.9353299457944`,
 139.44631421970422`, 0.18013950654590932`, 876.859213008694`, 0.19494037114810836`},
{0.13962660699497859`, 2.3927609600987836`, 0.08159301834299441`, 1.049401860055105`, 0.3113357406541495`,
 0.2537093830800228`, 0.18465806989501898`, 0.026302317707551127`, 823.7938459482134`,
 331.7337743967473`, 0.15672554077357326`, 867.8617573038789`, 0.4132481015981111`},
{0.15850810956754724`, 3.864543709669153`, 0.03598427061543385`, 1.4143483334223004`, 0.0357565050013704`,
 0.24682256570049188`, 0.09573276039768144`, 0.4393218504526066`, 1718.854818898987`,
 353.6724420241661`, 0.040402408241165366`, 712.0976142213683`, 1.3036789332708847`},
{0.21401575132547984`, 0.5018329407507416`, 0.06759356384183143`, 1.3297025310268573`, 0.001671825527291615`,
 0.6101055589611741`, 0.05875815827421124`, 0.011374646113462002`, 3970.128947518484`,
 156.96142232322563`, 0.0917136113709433`, 174.4977122967498`, 0.8959358505359223`},
```

```
{0.2792670899179436`, 3.983609524746483`, 0.015373325478506353`, 1.284162269414171`, 0.4144911988592501`,
  0.20973140148557545`, 0.2064253934923826`, 0.009708052401557348`, 2919.154274329003`,
  95.27049280385836`, 0.21631060910216127`, 657.0100718456624`, 0.42802658479066547`},
{0.08586486032312168`, 2.8745367333362415`, 0.017404871090795117`, 1.448481174666579`, 0.6550029259108587`,
  0.261096174497212`, 0.23623350301395302`, 0.006860074140603398`, 2403.509696068202`,
  302.2271343454148`, 0.08284221943674058`, 398.07784106649467`, 1.4110981615707505`},
{0.2531456244398701`, 1.5087481268574274`, 0.03956539541907127`, 1.3535866527268379`, 0.2663063430466912`,
  0.20762559577200923`, 0.05003859635226954`, 0.007766233551706456`, 3087.5888209434943`,
  52.266750933306696`, 0.06893185306099098`, 260.4004829709748`, 0.2264732381031589`},
{0.2390474413923262`, 2.7248848026910784`, 0.009644862426693273`, 1.0874671448098052`, 0.6223997807258579`,
  0.5980328455683533`, 0.10837162649139648`, 0.4472152431247179`, 3219.0050585265553`,
  170.04718420413622`, 0.20891374897438736`, 164.1258515073261`, 0.8600811658710139`},
{0.21006044037871285`, 3.8919509170249027`, 0.08418011196002813`, 0.9312675467657101`, 0.6762175846842489`,
  0.4074230201065292`, 0.18199286155069172`, 0.021147386898532253`, 2607.6500201914723`,
  346.50735501537986`, 0.16390942823137622`, 607.4012838312752`, 1.368359255207702`},
{0.21943719574173853`, 2.5234564852413968`, 0.05055626190914204`, 0.9875820321088962`, 0.2097707513494178`,
  0.4801611549454008`, 0.19441475035195993`, 0.05616938099512998`, 858.8205138152148`, 233.32849688618205`,
  0.194623719628982`, 240.03028172830233`, 1.269314148567283`}, {0.09724652458006494`, 1.6560889107841694`,
  0.0926155371922556`, 1.200826968404316`, 0.912706191483829`, 0.6859460319928354`, 0.08081449329505674`,
  0.19679014827941896`, 2935.15729330425`, 348.5478797151012`, 0.2287430956619509`, 796.3888990239035`, 0.6558444261025023`},
{0.21365795624647405`, 0.7682682208004006`, 0.022129944265977457`, 1.2082880538899952`, 0.4112409396201824`,
  0.3618211174445499`, 0.13028475533646633`, 0.017015124211224277`, 3760.370110811533`,
  65.89323742117233`, 0.21000791268881402`, 770.7333101786915`, 0.28462856805174597`},
{0.05283758132341515`, 1.4513913955588515`, 0.03949385232321362`, 0.7816256445170693`, 0.670006928262793`,
  0.18787106688989696`, 0.1399790307009185`, 0.007130550575690688`, 712.8040594777108`,
  286.29932138009247`, 0.14267142443174463`, 324.4682549417618`, 0.2993842375308353`},
{0.14890285999467656`, 2.4613222981483354`, 0.0806996970710636`, 0.9018953208894718`, 0.6300890474947529`,
  0.4504343459036029`, 0.20016359339659373`, 0.04025945776569903`, 2428.0414141322`, 85.24312607515157`,
  0.24248272499227708`, 143.38723937703347`, 0.5760944223721347`}, {0.20861720873598616`, 2.6604903550530903`,
  0.09042249345613346`, 1.4955989354725137`, 0.9954085892674707`, 0.4195820037469886`, 0.15274210463791832`,
  0.2045663841333299`, 956.756336979041`, 181.3851188327484`, 0.2476969365005231`, 943.2766808778183`, 0.4133268509253283`},
```

{0.2516798051365456`, 1.7935819651644245`, 0.006315894541399629`, 0.826849246644094`, 0.9906199127255553`,
0.300089310250239`, 0.05007861990514026`, 0.01533408656074374`, 3514.6399243004853`,
79.59405459380781`, 0.04497739996201494`, 878.7321570232773`, 1.373207002825759`},
{0.10841439790592017`, 0.6102575719274435`, 0.009125164133908204`, 1.0416429904529543`, 0.7566608127159209`,
0.38353734297442355`, 0.13156167944900465`, 0.04092468970744303`, 1536.4656962874878`,
240.32485913254902`, 0.19407881741659705`, 120.61468769589077`, 0.49517351370464313`},
{0.2643920277502225`, 2.3626902926699884`, 0.09960802720169255`, 0.8766300951710877`, 0.3664961055638398`,
0.180398126898761`, 0.19738190980086878`, 0.03872122266168245`, 2686.7355080693733`,
155.54709120703637`, 0.09039353043320764`, 108.36041929997222`, 0.47183924277102496`},
{0.2453822421254454`, 2.3621384482944885`, 0.0982471001582772`, 0.9257058216864319`, 0.47067825691870246`,
0.15079473657289633`, 0.12580272591118963`, 0.09112866174294972`, 761.0414023448257`,
78.77983892336437`, 0.11325833367121868`, 763.9949127516898`, 0.2744994590952936`},
{0.24403606580027765`, 0.739488342971601`, 0.030397415475841037`, 1.4288670285184657`, 0.6556115197581618`,
0.6539469634758848`, 0.06126135856953269`, 0.18457615230963131`, 2435.0905899038917`,
235.07141862508888`, 0.23879195594661445`, 484.38612253237886`, 0.16790604938463582`},
{0.19591792902131888`, 2.024706659152229`, 0.07020896928631384`, 1.1059302966977391`, 0.592776934634698`,
0.4179645299177581`, 0.16647650099279782`, 0.050854165122599174`, 2586.680067618023`,
250.3462757241216`, 0.19153306543388898`, 459.0700108781757`, 0.48904985597436235`},
{0.08070672361047693`, 1.7186916452862029`, 0.09416691564224372`, 1.3772469524721833`, 0.2924931568991078`,
0.29526464742643277`, 0.1600761827380135`, 0.3950934072579848`, 2576.3570009489285`,
247.90622579591388`, 0.24561337656248805`, 983.5039323063746`, 1.1761948860881364`},
{0.21660951849397125`, 2.3196374704256426`, 0.05342316811356318`, 0.8289468724889826`, 0.7971305382411904`,
0.46074817573447346`, 0.15068573986203698`, 0.20580718911973747`, 3818.821611211164`,
350.52312835824`, 0.09235300295154902`, 475.32889171724156`, 1.125863530197082`},
{0.17940300375660923`, 3.9177984343955865`, 0.07105365044247063`, 0.757021558275959`, 0.4559636481482827`,
0.18005474947746414`, 0.1392439975021591`, 0.009941379445590972`, 3056.4734665715378`,
123.06533488976527`, 0.10926800518663438`, 552.2328964138499`, 1.1889079071247783`},
{0.07802804586155354`, 0.5096185174034713`, 0.06664862576684673`, 1.1862702021967564`, 0.5213561633865729`,
0.41191318863008`, 0.22922533735314032`, 0.011665818207521975`, 3087.392092968994`,
281.58204064030156`, 0.1505904968540086`, 820.8287357874727`, 0.778268239775108`},
{0.14330880389332618`, 3.8501494289978577`, 0.09965690584166872`, 1.4089363867233466`, 0.39603629386572115`,

0.23158254874830175`, 0.24447411517512563`, 0.025315604779868724`, 3144.4956641217395`,
290.98059796752693`, 0.08543923299299833`, 938.4948454802362`, 0.6704851572608315`},
{0.1489257482346233`, 2.722416995606439`, 0.06213054624480915`, 0.8777939506713979`, 0.1811171065388173`,
0.38320061953446705`, 0.23534509902122153`, 0.049135530364634236`, 2618.5458764724017`,
253.8337977374607`, 0.07977492200738517`, 162.53919437009188`, 0.5373036298341383`},
{0.04532633880903228`, 2.0324704362392225`, 0.0478747614499228`, 1.4391357348359777`, 0.9416481271911004`,
0.2259990869074704`, 0.15840015286996068`, 0.3064132356034908`, 3560.1936405958113`,
152.6935465427623`, 0.21666243725505485`, 629.7119635807428`, 0.5036546688652173`},
{0.24129706716051202`, 3.0237855327987955`, 0.01988991036728214`, 1.129952209632555`, 0.5037225250618498`,
0.4224710675380836`, 0.07151857427735692`, 0.29929961505888664`, 1212.4551300172916`,
61.72989641181027`, 0.2179175128149733`, 842.3144251917861`, 0.969988975926587`},
{0.19002631068475484`, 3.8981908536005667`, 0.04307499183239271`, 0.9439953626562146`, 0.3518967865217828`,
0.5065457313244797`, 0.20348514359156689`, 0.4339804168113216`, 3191.7067441455547`,
90.12627487658898`, 0.19751874488156212`, 129.95933135853423`, 0.9986012808462785`},
{0.24717589012682145`, 1.0611664579859363`, 0.028540101402321376`, 1.4095368803165638`, 0.02130872190433397`,
0.21476295322020744`, 0.06341239067086171`, 0.005484011620995269`, 2681.63382319244`,
30.775925014323946`, 0.14914898332909388`, 184.54132902320913`, 0.2631242685176223`},
{0.12664655845246947`, 3.3935154954902327`, 0.006424635474681555`, 1.1463241134491313`, 0.17303071665670777`,
0.34654411791927886`, 0.18826037991294525`, 0.10828265131904012`, 2142.2239368780365`,
24.541155239218995`, 0.0814573388695572`, 477.1918953461185`, 0.6588399203016164`},
{0.06601269200185123`, 3.5782541070610865`, 0.0030681981325742847`, 0.9576275035920703`, 0.060588745220000684`,
0.6334120545565287`, 0.07995909997137443`, 0.025755772461025783`, 3877.3085423122357`,
115.37536384282828`, 0.08751981998343811`, 907.0482227139778`, 1.3355928389571075`},
{0.16151618904036913`, 2.2149912104770015`, 0.08050850339564136`, 0.8827049481666495`, 0.051091377520918746`,
0.6580988952508544`, 0.0981301462416955`, 0.15480179725872822`, 1701.7948249970304`,
276.37405818080936`, 0.23536451876512487`, 528.9381965463817`, 0.1961657892657085`},
{0.1442876286012253`, 3.070982393215818`, 0.08937873284832687`, 1.070431449143425`, 0.13269426266093953`,
0.4740102536905647`, 0.10282615146639285`, 0.35398957314171764`, 2945.724737378385`,
261.8552762801802`, 0.06715670701416887`, 705.2153179842652`, 1.3308975837743504`},
{0.16233623314495849`, 3.4553734453458063`, 0.008560200072148874`, 0.756137361995231`, 0.4827879889988891`,
0.4086251145453882`, 0.10730998584391388`, 0.015110506255319556`, 2878.0507375058914`,

```
  100.5084019104537`, 0.019894403773317942`, 521.5989633304663`, 1.450978212968487`},
 {0.10273797318741995`, 2.8544639047986253`, 0.08958124507002775`, 0.9841213601810538`, 0.36130309554582407`,
  0.5645536454765842`, 0.1712872481474244`, 0.16697305717409527`, 2810.6559941255537`,
  276.0618364859972`, 0.21782828728104364`, 439.9844268945197`, 1.4183015382740192`},
 {0.2182252943577736`, 2.4525789695843985`, 0.08318031794797971`, 1.193151503041143`, 0.23942220316332286`,
  0.4717144601343749`, 0.10371136475589693`, 0.2886617598698014`, 3334.600721689477`,
  112.41766341052562`, 0.16176010228405946`, 276.39802210908914`, 1.2933253830267075`},
 {0.1440748438152863`, 0.6777087607077816`, 0.09956862493461496`, 1.058294823400781`, 0.4649271637113346`,
  0.48063929676562156`, 0.16901013660063097`, 0.014362989299119928`, 503.6123640405335`,
  135.33166094031424`, 0.22196065262851217`, 983.5049108437765`, 0.7474803631589975`},
 {0.10845289564241622`, 0.8013900369852482`, 0.03922867226190281`, 1.3344776272203909`, 0.7828666058285663`,
  0.37119544038343244`, 0.24230551417716817`, 0.29545998381667377`, 923.6039988475495`,
  188.90122910207378`, 0.12317009730359191`, 164.97831479135226`, 0.5066550281996964`},
 {0.21318749760763323`, 0.8567503382014827`, 0.005568400588553697`, 1.4991587396348072`, 0.3028967861438274`,
  0.6508875728460157`, 0.11049488532380414`, 0.11619977958091687`, 3080.242915147459`,
  137.87981351792547`, 0.18138540752237242`, 302.3680238908369`, 1.0949953256351836`},
 {0.13496427485957063`, 2.7283006816793147`, 0.09237967487945227`, 1.4490038235264517`, 0.6302201809907408`,
  0.5877346006467078`, 0.06697736219228653`, 0.04922914190160384`, 663.8668620490516`,
  335.7933027107945`, 0.17582981098551087`, 654.18454202242`, 0.44374000148338677`},
 {0.17388014932548607`, 3.111180280919621`, 0.056014288473038645`, 1.015429518897542`, 0.39480427055863765`,
  0.6750686722055181`, 0.18700426668370929`, 0.1677642663239631`, 2545.734774511843`,
  397.4387033305651`, 0.09752373896011335`, 461.2777465382832`, 0.42402931497807383`},
 {0.07202769047499952`, 3.0640046854127183`, 0.08529672567482578`, 1.416153278933504`, 0.15539095009660953`,
  0.2508085901716265`, 0.12590994490201673`, 0.023836815965361607`, 2166.6928497013578`,
  120.01295741599779`, 0.026350693255496882`, 262.1932299001767`, 0.23550805714597023`},
 {0.2344719470115761`, 2.9213412168639046`, 0.06561037985592968`, 1.1399590705602594`, 0.46229855871728676`,
  0.540651938593169`, 0.24897147736747038`, 0.3063021311471059`, 2018.6462740085035`,
  197.31014883549676`, 0.02963537679288497`, 904.7501462673378`, 0.4612050545983406`},
 {0.09930853473494639`, 3.190683422605318`, 0.012588493276496528`, 0.8564698401985231`, 0.4241100438819636`,
  0.6117864501697707`, 0.06508170484272746`, 0.13534529866674969`, 576.3943815459502`,
  120.1163163068245`, 0.22806489011803205`, 792.8443253465151`, 0.6109252634175233`},
```

{0.155129520302198`, 1.8997585971647313`, 0.09737531096993497`, 1.3718308115164255`, 0.2333867181594349`,
0.48744217462028383`, 0.08399258512123511`, 0.03493071638637842`, 3323.246065873769`,
72.24661825083996`, 0.02096715576053787`, 677.5312586033781`, 0.6843380214744161`},
{0.20872983082106922`, 1.4633663739045595`, 0.013542009986806623`, 0.8714987441953177`, 0.5529534354796966`,
0.4878202660569759`, 0.17957502696846167`, 0.03950369806184579`, 3257.000000221464`,
214.50731020891828`, 0.02998159248988419`, 645.3074036689163`, 0.39549000609068097`},
{0.10453533148836885`, 1.418825586034628`, 0.040458261900160476`, 0.9371914808994821`, 0.7246736327614698`,
0.3651812208685298`, 0.192570960580907 6`, 0.04654956137182639`, 2766.350618333946`,
387.896616661376`, 0.06593124133195366`, 425.67253166152136`, 1.2555868175020377`},
{0.26220787506554066`, 2.9036259356594245`, 0.05879931050249061`, 1.1619363844601243`, 0.5219505001215548`,
0.6851905987493814`, 0.1794345477135204`, 0.014698485336420037`, 2680.020930185922`,
162.04607832711383`, 0.07571498102789304`, 176.54986617784854`, 0.9815944787661288`},
{0.17324817711018847`, 3.2376920995940512`, 0.07031010978129959`, 1.190416797012486`, 0.41801250088798736`,
0.4917082587143309`, 0.24772103681045993`, 0.07801449579558445`, 3009.8872028258465`,
299.3732730635486`, 0.19869329332516927`, 778.948706607488`, 0.4858664724840105`},
{0.05291908765428205`, 1.7294651427062249`, 0.05370126877502191`, 1.30146380854426`, 0.7418716727362076`,
0.23982678531015622`, 0.10080584601371567`, 0.007763151721376845`, 2031.3352691765976`,
210.78013371277189`, 0.07571162903253476`, 453.6682306444499`, 1.2336222936143058`},
{0.15708387938144003`, 1.9802596236541152`, 0.013259493315333853`, 1.0870360407426616`, 0.612480783932406`,
0.16750611392071513`, 0.1388565149570245`, 0.1909076709632498`, 838.8231853193456`,
271.73638049287683`, 0.15673541364345672`, 387.4787546434236`, 1.3227017748674101`},
{0.05075120527662519`, 1.658278662109228`, 0.055498212859945543`, 1.3814012915228837`, 0.16895148133986182`,
0.6812130381253805`, 0.07487704834522335`, 0.2508499028960761`, 713.8345694730342`,
123.64359741991814`, 0.11002289441557239`, 593.2971800331226`, 1.1500560474759731`},
{0.11202714811938802`, 1.7105899624358942`, 0.010381126706145594`, 1.0054068550209911`, 0.04934819528640788`,
0.615934259966066`, 0.1303494604977316`, 0.022112608705146197`, 2447.2612219369903`,
182.25321606642694`, 0.15717700981930294`, 236.45309549520482`, 1.3903493664382807`},
{0.12318352600646293`, 1.8288595812397084`, 0.03904823634202577`, 1.288806694323773`, 0.28292033724427323`,
0.6005905709171615`, 0.08373236539480303`, 0.12316387476289713`, 3523.5763591478762`,
75.40245579153373`, 0.056877072228675996`, 238.56592030593868`, 0.9375070479990169`},
{0.26931410967264824`, 2.69719087509666`, 0.08384388287111115`, 1.3796710324585384`, 0.2247919494212578`,

```
0.5887008550241606`, 0.11861962923873237`, 0.09148625928011728`, 1858.9754660241542`,
  166.10069990529155`, 0.19956103343932746`, 261.19414739024336`, 0.29522995885208503`},
{0.2403519413161186`, 0.452036439834286`, 0.03701297667153169`, 1.3340099176775428`, 0.49480439352224015`,
  0.39695386943771294`, 0.07107593226259559`, 0.05909142255746949`, 1909.9388905399783`,
  359.8121801518196`, 0.20502341768749782`, 102.24778063046696`, 0.3220358024816872`},
{0.10136617197464448`, 1.3130610717671125`, 0.02524859191046721`, 1.0853590110134903`, 0.42585172943012717`,
  0.6058525421180085`, 0.06958276092152879`, 0.05333443623231907`, 3398.9438983697855`,
  218.11163628107204`, 0.10985443518440224`, 140.2825003305976`, 0.4953393370012593`},
{0.16976252678442283`, 0.9647527047931614`, 0.08818886283730552`, 1.1392397746835041`, 0.4909778159033493`,
  0.4052861545938551`, 0.2077452574645165`, 0.45700489341954625`, 1657.6598329177532`,
  111.7589470678767`, 0.11414078165899233`, 633.9306281485486`, 1.0429247511158057`},
{0.05433908662691467`, 0.5078628204594482`, 0.055140488777023114`, 1.46833618333674`, 0.3259802807830994`,
  0.18873243131534212`, 0.2473502217885819`, 0.04633122891956912`, 1367.5690438607025`,
  143.44749559285043`, 0.08373331435281339`, 164.03836627166015`, 1.1182352855015014`},
{0.2771550373474385`, 2.8977996458788846`, 0.04239677623574016`, 1.0862002053990911`, 0.7908823723095428`,
  0.2697599682406717`, 0.10523521905774536`, 0.07238010622330943`, 3342.328635212777`,
  213.7504722906981`, 0.09524809623281333`, 270.96224459882023`, 0.9837646835478662`},
{0.1893295304154176`, 3.14219389183255`, 0.0015318981770392171`, 1.2930405146043173`, 0.31336242030620154`,
  0.3119472692840014`, 0.06362255996299637`, 0.010422709629382633`, 3023.7033413150557`,
  387.7732632982362`, 0.04061983641833472`, 739.7659274371783`, 0.4080765279707703`},
{0.17269271604904424`, 1.2834103890518715`, 0.0292767583135827`, 1.42270454907134`, 0.3615631980089622`,
  0.5532153863959471`, 0.12756623192818806`, 0.009152848137910454`, 1397.3190500397777`,
  395.33971393158765`, 0.21618308365952243`, 382.3795589307066`, 1.1034685682245162`},
{0.20748958090090247`, 0.6715365495569787`, 0.015270235730757165`, 1.2682090350907567`, 0.027917510241186827`,
  0.20294481531761766`, 0.20177509042694025`, 0.009106216844212263`, 986.7596644119567`,
  118.56143578777386`, 0.22712867569734158`, 111.56126792730028`, 0.322813589733014`},
{0.24083821335054179`, 3.6137797892784924`, 0.09336457200141268`, 1.0797740860311573`, 0.8199325513846139`,
  0.3539746867824163`, 0.12909549075417193`, 0.013662226735187627`, 1502.8143729950698`,
  250.56209048302856`, 0.049724628431015716`, 313.37642550425426`, 0.8392567585896558`},
{0.10000136890870276`, 1.1502087303411992`, 0.0944571459501206`, 1.1724977882376677`, 0.7996243506337819`,
  0.4962372074444592`, 0.0603727086003252`, 0.2639783844121682`, 2353.019737184237`,
```

```
127.74505004467323`, 0.09299005369814217`, 636.4052006283524`, 1.4271122966454026`},
{0.26406026561562296`, 1.706584857095037`, 0.03857658038618588`, 1.3287538083826989`, 0.5875972821517326`,
 0.23557312384629447`, 0.2421177513607941`, 0.2483474524422114`, 3000.1065962870443`,
 195.48114834089677`, 0.0674624940467825`, 669.3899302934673`, 1.4374620149910742`},
{0.20839853050458745`, 2.7683735045576725`, 0.07111401913454113`, 1.0122896675899635`, 0.16255435538219554`,
 0.5442170854022602`, 0.10447485141638024`, 0.016148101637118748`, 839.8752571489258`,
 224.44173429653904`, 0.033345756578954444`, 278.2812855948505`, 1.0178351002123138`},
{0.16891108017836104`, 3.7602156504468685`, 0.04267190544380434`, 1.3854834135835425`, 0.002545924582557646`,
 0.4570980168340113`, 0.1462590472263144`, 0.006653775010572637`, 1699.4425819778526`,
 160.32367419748755`, 0.032194593135707994`, 529.1649178846573`, 0.6716364867154336`},
{0.21096886156797762`, 3.803640125981465`, 0.06521785381396392`, 1.326400035688831`, 0.8325668340030914`,
 0.5716861768165042`, 0.15927299065753547`, 0.005902169706458335`, 3675.449116653418`,
 108.34151688685841`, 0.2421241867129867`, 164.51905561663835`, 0.7715866897465053`},
{0.23651600428102237`, 1.4286853683508793`, 0.02309572718354245`, 1.1119738301172222`, 0.8465878092164822`,
 0.45147130714420547`, 0.1259070857351121`, 0.20545395650898168`, 3451.654229458929`,
 260.1995622832778`, 0.1709613073307505`, 104.75381439645605`, 1.258589701616009`},
{0.10683757402937272`, 1.1691573286214876`, 0.09108430019301114`, 1.147070602611124`, 0.7662425775131751`,
 0.19507974400431394`, 0.1412781136285035`, 0.12318156847184114`, 836.8870989043648`,
 108.75193009869707`, 0.033679963527427303`, 835.996133826864`, 1.008683790648051`},
{0.16702967737299357`, 1.583805930522023`, 0.0795867672037231`, 0.7916262230690996`, 0.5482471426227531`,
 0.48920029746235116`, 0.0860526223129423`, 0.17416184853885908`, 3039.2710655331775`,
 314.91056543765546`, 0.06675361535041735`, 938.139281468398`, 0.9367311641527709`},
{0.10101526583500386`, 1.0895476748270756`, 0.020956692803068347`, 1.332338835863128`, 0.19224704842980378`,
 0.33946531175262473`, 0.2201012607708301`, 0.028436436854709174`, 3198.5457536687663`,
 342.6564227239878`, 0.07165216011817538`, 876.3555875387123`, 0.19036344281374684`},
{0.22801599865413047`, 2.4265177747549664`, 0.08045128457087845`, 1.280649056234088`, 0.5066227908773708`,
 0.45824885394528636`, 0.24935541865503402`, 0.09727040619128513`, 1793.5136088892068`,
 328.8750039461519`, 0.07777336570646293`, 258.5972563822235`, 1.286726638810186`},
{0.050131284999999415`, 2.5381966344131737`, 0.023566099986165413`, 0.9596441184824318`, 0.9890834507163451`,
 0.1880469111415669`, 0.24775985220046265`, 0.26922657004927647`, 3696.1433902885665`,
 40.634439387065925`, 0.22672385255648603`, 395.0946393419086`, 1.2906364332577072`},
```

{0.2740243661897643`, 3.1257366757798284`, 0.050771525925798214`, 1.1713543432340343`, 0.7435201337788511`,
0.2700186888748983`, 0.09233432661262192`, 0.06892858981294737`, 1956.0905636839561`,
293.9850525436867`, 0.1723709598044405`, 653.2617911720198`, 0.27712013083411513`},
{0.18537873247815345`, 3.1629325551816736`, 0.026298767066633533`, 0.7598525902012292`, 0.4236052605709584`,
0.2871483002255688`, 0.13552853446532648`, 0.1466987068019017`, 3571.961515908004`,
53.27465426829667`, 0.23780002151321356`, 570.2776946725376`, 0.5238143093332714`},
{0.21755401348296644`, 0.6826495148112177`, 0.03194628124413461`, 1.0017035210812666`, 0.700005093034884`,
0.46496814946435916`, 0.15767061291551837`, 0.21115232253313693`, 769.9714893193427`,
126.61943813215908`, 0.22480665451604215`, 684.8786066716252`, 1.391937184736154`},
{0.1299920449583697`, 1.124226653583758`, 0.0658560947382249`, 0.9366547036945532`, 0.09148120632051326`,
0.19271089809377107`, 0.06382341479251963`, 0.10215983519338719`, 1581.7372347006003`,
361.29640929342713`, 0.21184368887336869`, 963.4483854626012`, 0.9569916000759902`},
{0.06482146162037378`, 1.7386138242962055`, 0.006294913040326762`, 1.0543666852020932`, 0.7177918395662721`,
0.22028694120443182`, 0.197584671199816`, 0.07237611244935063`, 3559.6285090884303`,
20.05129970254461`, 0.06364798504398156`, 718.8036114020285`, 1.3398072812316473`},
{0.05920116869427322`, 1.0172330817296027`, 0.0520081189210668`, 1.169182438451001`, 0.6565906322943122`,
0.516171699692693`, 0.22384468810478442`, 0.1500035925656519`, 1986.348417598957`,
257.4719146110266`, 0.03749420639904766`, 269.4194085678575`, 0.9066351682404119`},
{0.24156398222703768`, 0.9716874669019102`, 0.008355837963237267`, 0.8784594618342041`, 0.8325209917457579`,
0.30385027059438907`, 0.0898462551158559`, 0.024480976299738097`, 3115.4858141935565`,
339.5869917094344`, 0.12265925236237346`, 779.2258785326308`, 1.055542691022442`},
{0.27482759886213576`, 2.2237739085115047`, 0.0023360105049110304`, 1.0871333264565135`, 0.18614640387495784`,
0.41252671893025217`, 0.10894654104390089`, 0.1539856737307203`, 1385.3366685720175`,
108.28639013375192`, 0.15631867882599226`, 860.9546854908384`, 1.1596655274085768`},
{0.22350381929765023`, 3.0829702562280312`, 0.09526002466589784`, 1.1840553348219054`, 0.020732679221296957`,
0.2132053686951818`, 0.2396115026671829`, 0.019984126705045537`, 1417.4408529012408`,
335.37725312028135`, 0.20386421139463362`, 345.99876592619694`, 0.3311932335564971`},
{0.1834380884768096`, 2.3945858338449204`, 0.09188172171123846`, 1.20037534355039`, 0.5939425509277743`,
0.3151701873017214`, 0.06919333220778115`, 0.006775158119597426`, 3733.3645444976655`,
377.01571703421394`, 0.01900909995792402`, 378.89409901393145`, 0.7550088851163079`},
{0.20773065983007594`, 3.039477008822284`, 0.08563803566541378`, 1.0527825166308218`, 0.12439650890480225`,

0.3282184620674399`, 0.10951824158846224`, 0.03987551109575542`, 3738.826098867432`,
196.0942924487914`, 0.10223276133596665`, 431.17440346293245`, 0.5939575461779609`},
{0.203112152669082б`, 3.3378093228399655`, 0.01358023564229974`, 0.8713522068690513`, 0.7157456674387634`,
0.460349069545651`, 0.21359788923260142`, 0.11895634803249971`, 2277.1129157276755`,
333.61099176856544`, 0.17846540968641372`, 450.0560523707627`, 1.3096077945002254`},
{0.20649696052842004`, 2.4110320135660483`, 0.02585371934070478`, 0.9884162307676314`, 0.17804442332168846`,
0.35556951419685934`, 0.07197094167333543`, 0.005456911078868312`, 818.8745660422469`,
344.91636431903476`, 0.11333027928697625`, 186.38787601125753`, 1.2460759895970748`},
{0.2100602676019933 7`, 3.133045087271854`, 0.0876771062125723`, 1.2111283838857314`, 0.4844486583550667`,
0.16793248727501464`, 0.1508305322597191`, 0.017207598934169146`, 1788.093322209058`,
57.89910558347589`, 0.21084051675410503`, 239.6388034926808`, 0.7210215862133578`},
{0.18874466055450428`, 1.876011387622853`, 0.06984198915759844`, 1.2919282198778506`, 0.9771602268090784`,
0.6882416012532946`, 0.17797992613055436`, 0.007288834726300062`, 3759.800408670101`,
337.91531788494206`, 0.05643378283259548`, 351.68048029955565`, 0.47781282816385207`},
{0.15697374199207237`, 0.6819096706133223`, 0.0179178152588838 9`, 1.198212074619125`, 0.9648774654110843`,
0.24792803454210777`, 0.24405075083280214`, 0.15299629544038454`, 2443.3927159348796`,
170.3155411474246`, 0.018728493413308622`, 659.0430828684646`, 1.498985711786688`},
{0.2766745814490056`, 0.5937141839493281`, 0.09437880969315848`, 1.3681515960176152`, 0.505818014736998`,
0.24799065686502586`, 0.24428267106140578`, 0.005934267598295972`, 1032.498478023751`,
194.54063662431383`, 0.2222385443758933`, 642.2369952604342`, 1.1009771777833475`},
{0.24755278827398247`, 0.4128549531995316`, 0.06859391692173589`, 1.0675609281065503`, 0.5732617258880361`,
0.561679119769882б`, 0.07077205928868102`, 0.02330351136736182`, 3729.403380267335`,
91.66702716990835`, 0.07585185853205106`, 972.478000732956`, 1.0182049598996858`},
{0.17041133886834853`, 1.8887112028505824`, 0.08964149308071669`, 1.1129443572568858`, 0.2070982961763257`,
0.4550805206354912`, 0.09254514787915327`, 0.0391000384422241`, 678.0575403119929`,
166.00223501604398`, 0.18497674769202788`, 556.755225562403`, 1.241022800576844`},
{0.25040457906720115`, 3.426224862124318`, 0.09832750203012389`, 1.125026362498485`, 0.02803644444885167 2`,
0.5348225731389479`, 0.16049726914416734`, 0.209317498850674`, 772.8662222964022`,
225.16799959483228`, 0.24482779326545456`, 539.3358513259806`, 0.15462060582476123`},
{0.26491460150494606`, 2.684712497550426`, 0.0815749679009846`, 1.3265140586844715`, 0.5588145573430325`,
0.5550950789966788`, 0.08570950279707723`, 0.29264718586434774`, 1531.3233704677932`,

```
193.50265609380517`, 0.15524738739170274`, 225.53345036467823`, 0.29870791676030284`},
{0.09083504852579721`, 0.8574578170463196`, 0.06232773212544137`, 1.393770145638282`, 0.12904257223584725`,
 0.4916695202036764`, 0.13359362373726208`, 0.15784559729372594`, 612.2653946460396`,
 53.75382506481952`, 0.21663747484532092`, 585.8420383833167`, 1.3456582052570978`},
{0.1416458309769676`, 2.6805466759930185`, 0.07044468571948123`, 1.0253774066579173`, 0.9344509727620742`,
 0.18791655895950587`, 0.1629164499308236`, 0.005277495364907988`, 3972.00852143462`,
 29.738049027025113`, 0.04663858939744209`, 116.81181536808118`, 1.2492927547565982`},
{0.11463650597403963`, 1.2200891172733863`, 0.0019395481291827289`, 0.9627893116044163`, 0.7623014931044829`,
 0.5201742926932665`, 0.19018703995921898`, 0.052867709445707047`, 753.2112504763372`,
 77.89195679132797`, 0.12790905268981173`, 980.9311321533947`, 0.841767267407671`},
{0.11928614803493165`, 3.3342483606620608`, 0.04393402593143259`, 1.173333473146248`, 0.8826521182531568`,
 0.20855848214800976`, 0.18068194333027965`, 0.030415200846726265`, 3988.1527317930513`,
 235.181873505142`, 0.043450888179495384`, 304.31835650127556`, 1.333581175618054`},
{0.1862362974207981`, 0.42420218048419667`, 0.06637372171349537`, 1.010452464753652`, 0.26011634643177173`,
 0.4937176428085319`, 0.14164516353138884`, 0.20913728466945072`, 804.0547464718138`,
 173.96731837220352`, 0.19535392466045437`, 231.27158494293775`, 0.5706224957073267`},
{0.10041286781732989`, 3.583471762671337`, 0.0817721928594316`, 1.1898666702221108`, 0.827848014309974`,
 0.4003529226230641`, 0.09889664321010594`, 0.014409376687062665`, 615.0026252520665`,
 51.63755018873172`, 0.15230198669571815`, 774.359933386204`, 0.6621996699941299`},
{0.24398113139209332`, 2.8068625546493813`, 0.006122635019790632`, 0.906165488321155`, 0.8564709779616588`,
 0.4954773362854056`, 0.0735074168040917`, 0.00916440400714602`, 2813.6943291903117`,
 265.03905907443516`, 0.034734794181952244`, 278.2698048959309`, 1.0858859694461658`},
{0.2044841788485734`, 2.3645508871940253`, 0.034156689117083465`, 0.9944973328242781`, 0.8293663166545069`,
 0.20452426051473516`, 0.19020429519981563`, 0.028330524126455137`, 2762.4423710997`,
 143.14422591386642`, 0.01581210039830841`, 769.1047770547148`, 0.4799501196881659`},
{0.17196429276531555`, 1.8665391951183032`, 0.01239607279962085`, 1.4991018573013593`, 0.42320416637437575`,
 0.6125569452805888`, 0.11199347298979728`, 0.10600099095787566`, 792.7750535232199`,
 316.87646195399816`, 0.16982913946643552`, 171.15064383889126`, 1.0255469486964395`},
{0.04152141451882724`, 3.432330722354058`, 0.029207988122312418`, 0.7909746992129041`, 0.8303913233359719`,
 0.6932521825436264`, 0.2233256431027394`, 0.3096420684428805`, 543.5686485359438`,
 302.57844618242586`, 0.1144122561136649`, 915.7150227867241`, 1.2544376124090229`},
```

{0.26828575516054964`, 2.721186816659044`, 0.0759533363763515`, 1.158020747451974`, 0.6868605274298898`,
0.4168339437642369`, 0.20181287935629966`, 0.05848291442309303`, 1339.4631296152347`,
91.65207136341775`, 0.0944288641703086`, 416.313127587053`, 0.31988842714419174`},
{0.17503743466253052`, 3.9335316991022555`, 0.06003565836252969`, 1.3980809326266879`, 0.32535999975753316`,
0.5418889614120084`, 0.15398597658420166`, 0.028272517455802684`, 1276.0446170684936`,
264.29150337395083`, 0.10046418411993568`, 157.0712699559408`, 0.4175068100947137`},
{0.08377205105521557`, 1.4790039309566367`, 0.0842194368043219`, 1.0252350364797664`, 0.10712139945808596`,
0.6626670944446229`, 0.1713316209501882`, 0.47668638848197165`, 3979.2541734581173`,
228.70388218899905`, 0.23221381787506684`, 894.8847990203216`, 0.3775459136098127`},
{0.05601692844288059`, 3.229968306481971`, 0.06933319107526313`, 0.8422022950409469`, 0.5303692096451496`,
0.1755095509974488`, 0.22808427924258645`, 0.033400142295722635`, 753.1407429143319`,
285.1540865148073`, 0.15699902112047975`, 313.44371227642273`, 0.9623754562438389`},
{0.2741234252601426`, 2.1582642451161087`, 0.001949128731223322`, 1.1124294378939896`, 0.6738167664177599`,
0.3154043249710131`, 0.22091670528572266`, 0.05102831240663123`, 3983.2154474422505`,
351.44075887939755`, 0.24343237436829407`, 113.14983972704766`, 0.361200341772409`},
{0.18323520079728134`, 3.094716322277824`, 0.022588033818458354`, 1.0061600134517525`, 0.13652211984507567`,
0.6952521189278127`, 0.08049715523453915`, 0.1395113839460471`, 3192.37026116423`,
66.96851814556055`, 0.13602884654286274`, 543.2294069576474`, 0.49694998213573305`},
{0.27510406861011016`, 2.3745048825525004`, 0.08975895520003017`, 0.8874614251284896`, 0.6181898851454295`,
0.480578824225393`, 0.1194816825065107`, 0.0050026468684324955`, 1547.3095563962843`,
398.0180799984753`, 0.07049482349556163`, 244.44729151599844`, 0.43947862609073907`},
{0.21464134084400444`, 2.924636187759581`, 0.04848394078892399`, 0.8703567108637134`, 0.6190505136014375`,
0.6353957114473807`, 0.13368245462930878`, 0.030631194683636915`, 2876.498085157249`,
378.9971369370453`, 0.13151655670563506`, 909.8457201958183`, 0.8245011301944629`},
{0.20237010004014644`, 0.5027267604868988`, 0.010913395548778198`, 0.8067127940258948`, 0.34250228239034874`,
0.537283631188114`, 0.05230287930974917`, 0.005649299471258621`, 3275.753477363538`,
63.89856154763055`, 0.0950573018245281`, 108.14153325995335`, 0.963038018360606`},
{0.20111461541710635`, 3.235733343602166`, 0.05461210779534966`, 1.1121005004873767`, 0.6170771797656427`,
0.5487602962835693`, 0.11944192847933494`, 0.12918460112920302`, 3981.650657604958`,
309.3446236530506`, 0.21360854247348138`, 603.6248717339404`, 0.7458033887005944`},
{0.11050956520445593`, 1.8945252075605188`, 0.05867105494468937`, 0.8052571835648545`, 0.029637091807201754`,

0.4733667914073866`, 0.2135066163598513`, 0.011964135527872498`, 2365.808074476904`,
23.208105423993686`, 0.10565773815744062`, 235.51831883145002`, 0.7153231377653682`},
{0.10698667432090436`, 3.912592611233639`, 0.09987161673257852`, 1.2229665646526566`, 0.46969468166711437`,
0.4166924099655738`, 0.18024437629765055`, 0.22632138134499458`, 780.5662641213426`,
211.0858727024007`, 0.1693256978398835`, 190.2435132122905`, 0.9592117502549766`},
{0.05200773937474429`, 1.001370093078111`, 0.07827281768861905`, 0.9165117379583676`, 0.6975843792560903`,
0.3460474014697589`, 0.09712290823226866`, 0.047486861172818966`, 3177.2807033235713`,
377.6167961339943`, 0.20939204920981647`, 493.77316200042145`, 0.1509454252279645`},
{0.04187623523900166`, 3.38646598055613`, 0.05859457856327694`, 1.3428771756245013`, 0.8954772787619216`,
0.6344488750516974`, 0.09602398640162021`, 0.011698632241471723`, 911.333436862355`,
270.87429664142564`, 0.037675691766878905`, 465.25463589219953`, 0.15840496206016108`},
{0.25980807031436626`, 3.9712259630362077`, 0.08510159117237868`, 0.9955932490099979`, 0.6885908828855853`,
0.572932153846583`, 0.2138506214325791`, 0.06740137051817302`, 837.595825653787`,
195.527290553454`, 0.23240575623638543`, 418.14273041797634`, 0.4363054103798196`},
{0.2655396740638951`, 3.9714529830910656`, 0.08874076072721454`, 1.1035875733346898`, 0.9765193222129274`,
0.5662265485594603`, 0.20247185305527654`, 0.013935286866009542`, 3738.0787829268875`,
194.75018625673079`, 0.07859798708147653`, 209.9734253562536`, 0.726358072526724`},
{0.18401430969763982`, 0.834428946364218`, 0.04481238388476491`, 1.1126380724066056`, 0.9444669663018621`,
0.5746023883330807`, 0.08356535776483942`, 0.3156799788963399`, 1493.52635093563`,
155.17530295117228`, 0.17004347748467463`, 775.377703667726`, 0.5249257652039196`},
{0.05832428017904451`, 3.262173706397699`, 0.09314635141678405`, 1.2855684711413076`, 0.023278777366840142`,
0.23811465791289144`, 0.17801096226514213`, 0.35620387724034486`, 2846.2273088042257`,
38.82943668968096`, 0.09787537933584767`, 560.7776858076173`, 0.9863847695071732`},
{0.24157720380670267`, 1.9121901802321215`, 0.07641159687586214`, 0.8946235718345144`, 0.33812653929846204`,
0.40728986932840183`, 0.055239293605974266`, 0.1133476110179573`, 3726.6434000841564`,
383.8063058413302`, 0.22574622323913468`, 695.9404902040998`, 0.6458539986537395`},
{0.05980031999007035`, 1.5781942539557097`, 0.03881331427397477`, 1.3464648221754443`, 0.19875919773692297`,
0.6909041176814996`, 0.13585420315356544`, 0.020142367653338888`, 2916.900272742476`,
48.31624325405903`, 0.09927102732483634`, 715.2861110865044`, 0.6419849231180266`},
{0.13020255300958`, 0.8955861384561823`, 0.0674443730419138`, 1.0790974450064994`, 0.702124269236061`,
0.49408516237585054`, 0.13712856781521454`, 0.005980282421149676`, 3052.6598309618203`,

376.65624623443193`, 0.09424944781198791`, 348.3609852092831`, 0.38899187799217927`},
{0.08427108981256215`, 2.5087840127539227`, 0.05560923448958375 6`, 1.2336432619736746`, 0.8673120496998292`,
0.4546269580797774`, 0.1601299814573277`, 0.06536369007559495`, 1449.9805606027303`,
349.643844362192`, 0.2204512503835499`, 529.0163698091421`, 0.6027390941774509`},
{0.17499946900036517`, 1.7232873290327664`, 0.05919529281857301`, 0.7764458583115545`, 0.5500563628834825`,
0.39161009943267555`, 0.07267777245500653`, 0.05205831170538842`, 3601.5623447533017`,
347.59325532326716`, 0.03602041148604612`, 715.1891588936643`, 0.8267665075819255`},
{0.2534223348499394`, 3.7224669071701957`, 0.007976010635987567`, 0.991565837401734`, 0.5232050743962777`,
0.6520248651649048`, 0.060751794626500905`, 0.10316704198675233`, 2638.550216871454`,
290.064985416788`, 0.021007154119888072`, 298.9696551672612`, 1.393891460140742`},
{0.23942509862761246`, 2.637951466655549`, 0.087396652918138`, 1.096181616085685`, 0.32229644437555716`,
0.5430930438053733`, 0.15536605150257066`, 0.036099935600693564`, 2985.2212254258275`,
221.12101433502164`, 0.10855284731338688`, 683.5524075494795`, 0.49544928546683775`},
{0.13413358137083242`, 1.1073003960448755`, 0.010194162739922064`, 0.8689668369140942`, 0.802185063393402`,
0.2758106798897956`, 0.2205990013542981`, 0.010406549227361166`, 1957.7265053573155`,
137.92990419360092`, 0.03524688148783328`, 307.30963518936215`, 0.7766811715120236`},
{0.13353396767847725`, 1.3849072243916227`, 0.037625198545465394`, 1.3513536910825439`, 0.7380955553030428`,
0.18309283444874924`, 0.09948540900118083`, 0.005779683863655912`, 714.6798783816498`,
47.88802610077255`, 0.06939772440799813`, 708.5385394581679`, 0.46598294195988577`},
{0.15328466707871513`, 1.1304815732127782`, 0.01938200589375258`, 0.860713691368932`, 0.8686087342835431`,
0.19549566133114904`, 0.13389173766876322`, 0.20092224091352398`, 2454.6572820858282`,
229.62547898433456`, 0.023595815931105768`, 451.99647185998356`, 0.5542635415039234`},
{0.10913486741948175`, 2.5618917874655365`, 0.03632731532740589`, 1.317449133242918`, 0.5461890252207326`,
0.5290476062296563`, 0.24573868945288957`, 0.05221162803231191`, 2894.2346649184556`,
65.69305898751048`, 0.22867084061262016`, 437.3007308314914`, 0.9981270389103005`},
{0.07182443987408843`, 2.118402704654513`, 0.024683401322780175`, 1.271671281027994`, 0.8434134491740564`,
0.3327390098363363`, 0.2204781036429838`, 0.008681247976891265`, 2792.2729873568005`,
132.69980501716213`, 0.10908117786406141`, 118.91594308888287`, 0.6182606769605985`},
{0.08649263503146493`, 3.536681251374949`, 0.0569050133761948`, 0.8562794648493628`, 0.7659191608193836`,
0.6565822189966053`, 0.05288084888103428`, 0.019635489626894872`, 1655.9616779394137`,
61.32397635416885`, 0.1955430223924139`, 311.9843964912535`, 0.4576213820146753`},

{0.22773072194859834`, 0.49466962054525876`, 0.08082350237169326`, 0.9137135215130885`, 0.1823775005015713`,
0.5726101004151865`, 0.23337703520122455`, 0.4544647688324883`, 2384.785999976277`,
326.5754925428238`, 0.12798818019626473`, 753.143535233797`, 1.42500442376395`},
{0.11881375923670412`, 3.165876232555499`, 0.04491722695227754`, 1.078828907225097`, 0.9645951067529017`,
0.6063187671697836`, 0.16341215475471582`, 0.14733335269134448`, 3402.0680117124375`,
291.6157501983695`, 0.21567924545289535`, 577.8927612762207`, 0.8760438785713225`},
{0.06677317944646227`, 2.5557029853612327`, 0.08299647520509826`, 1.1714916172842476`, 0.53834026321508`,
0.36412225606774273`, 0.09630887876106681`, 0.09653408014583209`, 1348.4769260577114`,
308.1653739279909`, 0.12635145179170415`, 237.77573672838983`, 0.849398167671354`},
{0.051058235561583065`, 1.0883505901422357`, 0.019385660796646675`, 1.3306544101357418`, 0.10573256336501768`,
0.5915195698551534`, 0.11302175114137919`, 0.08230316146493033`, 2381.2495058503437`,
87.36698150886224`, 0.23585950584558818`, 170.69179515230317`, 0.11549443721954855`},
{0.10669841380848516`, 0.46480131413400017`, 0.027804589336698892`, 0.9780646667676929`, 0.33292032627220314`,
0.3515080327600074`, 0.06213130143895429`, 0.40821633351803516`, 1959.990643885535`,
392.14924626881907`, 0.021646162368732513`, 391.34090041594743`, 0.3301573268664859`},
{0.23913138005417744`, 3.4658538531888485`, 0.09560668931994744`, 0.9478899351606546`, 0.1851833909530347`,
0.6284190548860538`, 0.13451252654827872`, 0.017357623205987157`, 2581.944301351283`,
107.40224160177524`, 0.09991048108069184`, 599.2743983948739`, 0.974328914098896`},
{0.22826012109835803`, 3.836861574888042`, 0.05509279461777915`, 1.4912092979517328`, 0.4642963440191781`,
0.22029114513577774`, 0.11383547791557092`, 0.007050421082781396`, 1758.7013456922487`,
154.10277336705235`, 0.16403778051781875`, 394.8039655006175`, 0.27233621390934215`},
{0.17224897941223827`, 1.1547458219129014`, 0.06848734964800933`, 1.0798606791815302`, 0.6793934308354268`,
0.5195769723171072`, 0.24421982225968486`, 0.018918189073712316`, 2277.1640113753156`,
124.76910867447333`, 0.059001381540061015`, 441.76755933414836`, 0.6913643466412176`},
{0.25196912428211926`, 3.701308573426246`, 0.07155044386562917`, 0.9205593843664102`, 0.6966808730428866`,
0.5317486364764509`, 0.08330537331859847`, 0.007174482199638971`, 1492.4760285107968`,
182.15469349472028`, 0.05728669754062743`, 653.9194953278023`, 0.706760474752985`},
{0.14877264747459418`, 2.0670620933767996`, 0.01601966805926528`, 1.0570603479826162`, 0.5104296265028303`,
0.6668501765015311`, 0.14239666349567554`, 0.20970833064118982`, 708.0907065311021`,
355.8809496419981`, 0.08554411428635944`, 938.3956157489915`, 0.658083822136255`},
{0.18383849863705426`, 1.9811742485431596`, 0.043876132749369495`, 1.0843252250015878`, 0.6952167897227755`,

0.5851222098181931`, 0.2132041724254265`, 0.052788607633891496`, 1122.7916764961756`,
104.18817509926055`, 0.10490421519475424`, 520.2958268116344`, 1.226091401235227`},
{0.23537400847528311`, 0.9117048603091051`, 0.09040927006257299`, 1.1840523722046592`, 0.6011281057721607`,
0.6661129533205559`, 0.12634486819219382`, 0.07559113550945733`, 2884.655587473878`,
75.41906586131233`, 0.15876518957644842`, 352.18639639607864`, 0.27889485514921253`},
{0.04046724154519818`, 0.6197081144874961`, 0.06892771141270096`, 1.3289596481673351`, 0.9868497712795179`,
0.629939664681324`, 0.1697696238363825`, 0.02895794002617104`, 3718.638687383367`,
333.9168843600603`, 0.10907724447996336`, 761.2481971218346`, 0.31158444317050904`},
{0.15495819869077576`, 1.363644267830967`, 0.03988247618826955`, 1.2787205752737487`, 0.8253792043193915`,
0.5133151421919353`, 0.1934247455215985`, 0.21880942396018985`, 3282.8435923366296`,
217.90221507458887`, 0.1289200640318704`, 313.8563005906939`, 1.3475356201670712`},
{0.25034739366283343`, 1.765550736773882`, 0.03379971130909553`, 0.8173618594977419`, 0.961387781286206`,
0.4964284021069221`, 0.17664239319657843`, 0.022734185984243344`, 3628.182612586047`,
238.82174366066783`, 0.012747210205689724`, 742.3288298341786`, 1.3628660007193019`},
{0.2290754791009489`, 1.5694999130287757`, 0.08695096665206335`, 1.2629680203810834`, 0.34324142040142713`,
0.27057006908504144`, 0.19252050244778507`, 0.11818787777998406`, 2800.6917880913597`,
348.0998144900143`, 0.19674767591467895`, 859.7939250506549`, 1.2779861391154372`},
{0.15063618155264874`, 3.5735854135931975`, 0.031211761407245894`, 1.2180797028458579`, 0.9446129993864278`,
0.661178554146082`, 0.14871704306041994`, 0.46124095718859154`, 1841.5707342457372`,
323.9598317358344`, 0.14832479826481065`, 283.10272982736836`, 1.0736336212979056`},
{0.1796949813664332`, 0.6901508326930648`, 0.05099330294125327`, 0.8184486057542235`, 0.6476048151425755`,
0.46556945976992514`, 0.22507021011234668`, 0.1328547608240361`, 1724.8924220370582`,
146.4544425373445`, 0.0930275887140457`, 548.1000661718926`, 0.33221757232993765`},
{0.24145972187532339`, 2.140644861368135`, 0.0685565178904361`, 0.9880842725652856`, 0.7666053447146475`,
0.5569802031571479`, 0.20933119638050102`, 0.19839884114022932`, 3998.354424724157`,
37.45811677666467`, 0.028554067192584076`, 879.3654138253781`, 0.41994018196137617`},
{0.04768021071772133`, 0.4663305014644865`, 0.048598424831649306`, 1.392231670691952`, 0.5367124626846249`,
0.40287577042798994`, 0.1728227629667849`, 0.21925196434148`, 1705.651302071452`,
55.26509416726611`, 0.11465700418078695`, 792.6590097658584`, 0.9651009874859775`},
{0.16754673986618684`, 3.9381045648629653`, 0.09679687219899162`, 1.3078206979084968`, 0.9771079672155749`,
0.16886102141132375`, 0.17790850404386188`, 0.05642636769681439`, 1000.2506649578745`,

90.56122033650217`, 0.20756398154627204`, 585.3088604483944`, 0.10715962910201982`},
{0.1795057572674727`, 2.1092645760301263`, 0.010074314215989534`, 0.964625794774524`, 0.6050543370936221`,
0.6687661147192752`, 0.0928779368536034`, 0.014730882375678251`, 2873.7309814919727`,
188.76851356319332`, 0.1756121580394589`, 319.03583024241317`, 0.45092963095842364`},
{0.25387127566134227`, 1.9399729078614856`, 0.056502389879184135`, 1.039890481285489`, 0.4361537108493585`,
0.297966293840626`, 0.13895505571131983`, 0.09194126229211623`, 2206.5559116496224`,
59.92555126676399`, 0.20109643319254233`, 327.594142755293`, 0.32857422242857415`},
{0.15037235350121642`, 3.946253956137225`, 0.0874365325257077`, 0.9780133448544013`, 0.85222293184821`,
0.33076558351361784`, 0.15932635247024757`, 0.010721743599637545`, 3463.7803477985426`,
332.0784562602478`, 0.04131798533024522`, 941.4901525911389`, 0.4378832875716143`},
{0.09205001774674731`, 2.5177700057707106`, 0.049346106054516725`, 0.9143142417490343`, 0.49868925471019887`,
0.4793926103788798`, 0.12588748832490998`, 0.024098501628388422`, 2554.2220368928865`,
68.27478255532003`, 0.11273611136671119`, 996.3345849547857`, 0.4144436918388672`},
{0.15693412801059092`, 3.700899504120283`, 0.07435255814337495`, 1.4026080477695813`, 0.3689911684888476`,
0.5704364955563762`, 0.11356719415593713`, 0.021460592892593465`, 3294.4594758977264`,
364.9747390444064`, 0.10812391295347468`, 321.77320911336903`, 1.1048051376114976`},
{0.16855801495796496`, 2.237670840363541`, 0.03527018973681164`, 1.4676623787802505`, 0.14577610492892124`,
0.4218267027484024`, 0.06206806417614308`, 0.19753672807453063`, 3067.5040328857795`,
170.67749843156867`, 0.07990323306768399`, 534.3400571811429`, 0.9236974994482514`},
{0.15762013685903437`, 1.4038303947744808`, 0.011232057855739495`, 0.7680552916195039`, 0.5652087744910961`,
0.3718522384040487`, 0.1764820843724627`, 0.08001290271077666`, 869.1719452376124`,
242.1101277861785`, 0.18956168693591746`, 255.4637417771586`, 0.7781964612704693`},
{0.08990334086152668`, 3.09583014761472`, 0.0494920375014657`, 0.8178371553346289`, 0.7639710373000737`,
0.5584444064197014`, 0.16183093026509648`, 0.2174446942930795`, 1822.0540123230248`,
362.0352307073209`, 0.05956106644578513`, 345.2088277183874`, 0.6734195226750912`},
{0.06149762320975155`, 2.409648279749617`, 0.09509423014631586`, 1.0957799482440618`, 0.481557333512314`,
0.3029197769210944`, 0.06005452575628312`, 0.4071052512682893 4`, 2670.7351490418996`,
342.46537265562074`, 0.08873767286982714`, 836.658041974486`, 0.47632675411509395`},
{0.1699434251396082`, 3.407884131739408`, 0.0877637127661945`, 0.9021855334337681`, 0.43810861304224935`,
0.6113586783700802`, 0.17432426650981642`, 0.12676156567074337`, 1024.2531200418712`,
355.83104912437625`, 0.193565453878392 2`, 751.5567515422491`, 1.2175093407155568`},

{0.06386876492158416`, 2.202470363165676`, 0.021659672591390233`, 1.4968330484774348`, 0.42676841314327163`,
0.1836548058899573`, 0.08314879736029188`, 0.4058072228591992`, 833.4948356554346`,
176.3211220001566`, 0.16203634232573438`, 644.9255487274186`, 0.34648482646844214`},
{0.04726401693260365`, 3.5383387491219978`, 0.08803212425018757`, 0.9643054181107631`, 0.37798156216549383`,
0.35680530341059313`, 0.1819331573591999`, 0.04804439512512987`, 740.8039744031075`,
99.74610762277933`, 0.13183741771972846`, 786.6064261802112`, 0.7295145055440901`},
{0.26983186617354693`, 0.913348202871922`, 0.009721705368217233`, 0.8584821966256463`, 0.7211222193350622`,
0.4221586517250291`, 0.24170736194123216`, 0.013071548897295462`, 3196.6333373494945`,
310.8739162562979`, 0.17293958263124531`, 680.915438777651`, 0.8965392196471194`},
{0.23108816023443868`, 0.6666677584662737`, 0.06859959092166783`, 1.005949051672165`, 0.9484631104197723`,
0.6018346423764072`, 0.2121269137196204`, 0.0074937913365613375`, 1285.5603006478423`,
293.27130380586834`, 0.21210289629599277`, 854.7325900609192`, 0.809578100628189`},
{0.20175614818661275`, 2.701625495629883`, 0.08653175554973223`, 0.9163028370819354`, 0.3628373709561381`,
0.3162154692661757`, 0.1536607003671867`, 0.060343138882665154`, 951.7544335137281`,
204.1424143962746`, 0.11961477756869299`, 480.1931017217365`, 0.7047056593067129`},
{0.2252231084744657`, 1.532245374881045`, 0.04840401406127875`, 1.48300949428943`, 0.9003690049532742`,
0.31956547342412045`, 0.09770192136178546`, 0.21979165597690545`, 1892.7302501902905`,
218.5730147728798`, 0.013430117867281793`, 964.2530487425447`, 0.24857847638154196`},
{0.09570293927564222`, 3.352630115507343`, 0.08185052547198762`, 0.8127331866760581`, 0.19447918997017277`,
0.27430912859699885`, 0.20651591994416552`, 0.272541085695813`, 3756.6484578458203`,
381.6771428051378`, 0.060936973966651986`, 826.1833188329095`, 0.35465172563434266`},
{0.15663982012884625`, 3.492141820716843`, 0.07084161455644694`, 0.973965026079981`, 0.541800635756025`,
0.46391898477079574`, 0.10827517968482281`, 0.2329275319281203`, 2476.6045590540934`,
212.14557435122367`, 0.12210891625276166`, 717.6200949431407`, 0.1625983249722769`},
{0.2690768341477545`, 0.6797714832119492`, 0.09067542002391918`, 1.4566543690074854`, 0.4018724589543907`,
0.1806547502336413`, 0.08872066198877215`, 0.2941590564386127`, 3290.0544765513923`,
348.6405349191908`, 0.1309148435711281`, 298.929866316441`, 0.8252758294906357`},
{0.09803635169062763`, 3.43679709117008`, 0.05631791249463285`, 1.2679547562370437`, 0.6534863264872288`,
0.3163581865497872`, 0.23769369973279741`, 0.006385292852697121`, 2697.817181775471`,
63.56019603436363`, 0.19981078151319925`, 649.6989098012755`, 1.3989348107667925`},
{0.2798654762709178`, 3.4901022139081705`, 0.05116885519764173`, 0.8129985747348281`, 0.09581913784553886`,

0.28378674129553305`, 0.21934769025527562`, 0.01706425564447086`, 2167.5547064305565`,
64.77955617624622`, 0.014128949817443504`, 731.3826858406161`, 1.3717618311417867`},
{0.08847759659263299`, 1.0504313027603605`, 0.08367496054202356`, 0.9266702833833269`, 0.06351030238705624`,
0.5076804210603109`, 0.17428777472341178`, 0.011934484410327208`, 2107.0152196441804`,
147.88531123365203`, 0.14362378885099236`, 570.8011060729483`, 1.066082920317022`},
{0.16712651403373163`, 0.9036163299524826`, 0.08723582692416074`, 0.7725748607088503`, 0.5590447128660234`,
0.553010672615067`, 0.2106944736836217`, 0.23196717041815604`, 3504.135992477057`,
345.46778835361613`, 0.14466895574208005`, 449.3071744773598`, 0.9373325611868881`},
{0.09977059511053005`, 3.654429382574282`, 0.05808010745114982 5`, 1.3767727047263745`, 0.4973311450651643`,
0.478335308276144`, 0.07137234822917637`, 0.0776742690082331`, 3650.4647678457313`,
161.0199474191338`, 0.20248247048151535`, 468.10197225554384`, 0.21420934147564208`},
{0.2732049031860878`, 2.2115264359985387`, 0.04033914222298785`, 1.431920347313945`, 0.4635034149119637`,
0.2433548322704291`, 0.1931025087098862`, 0.006254733657129221`, 2323.9500225093207`,
347.08289749840674`, 0.20247939325168302`, 703.9477533486487`, 0.44373441772179323`},
{0.20521894957573278`, 0.840258882931634`, 0.08391110356152752`, 1.4989691651761248`, 0.41978850017981073`,
0.36231741728451783`, 0.2302442259566324`, 0.32821922110443635`, 2525.315172001321`,
247.87056061090937`, 0.12246047234680596`, 368.10104086833013`, 1.212069360646515`},
{0.0806556692378968`, 2.5015449163166883`, 0.01565893553791968`, 1.1718167667497388`, 0.6778479341246784`,
0.39778996379524356`, 0.2050963890003198`, 0.326207141760844`, 2970.291345097916`,
139.28537502278596`, 0.1458086372395309`, 924.4017220588397`, 0.6417661331381843`},
{0.18406472272233038`, 0.5788412216158938`, 0.01504375110909681`, 1.252033621725871`, 0.7304470092974189`,
0.4869703273021193`, 0.1348186931663269`, 0.005306817201377391 5`, 3442.570945895269`,
237.60457831157294`, 0.2437381300950216`, 917.1982298271836`, 0.8267158808986377`},
{0.07330573614714225`, 1.0919681834525266`, 0.099983721668732`, 0.8877582007572096`, 0.2699891834685879`,
0.32168710898191144`, 0.24656149832334956`, 0.13112832741619704`, 2277.9376027515864`,
118.56008953794134`, 0.2132339733546027`, 850.6855810487803`, 0.6121523564060969`},
{0.20536176065122608`, 3.0245843463205677`, 0.03823241728257237`, 1.4092527833044808`, 0.3539417585422069`,
0.6661223521098298`, 0.22524266544185734`, 0.10224020864798576`, 2574.9858312538963`,
24.040110924595524`, 0.2179031480355273`, 718.6427350069159`, 1.4775900673`},
{0.11844830431630282`, 0.572454401397466`, 0.07336092747215574`, 0.9178774482483805`, 0.11050443206394278`,
0.6062126915998407`, 0.18924440894141614`, 0.17751692455247325`, 1489.593938732396`,

131.53690489226068`, 0.10236390991206162`, 807.825633861843`, 0.8442256131342014`},
{0.052661771227287435`, 2.408882179980335`, 0.08700590795525553`, 1.100759517775085`, 0.009764392451289794`,
0.5271147228690322`, 0.13908991680762323`, 0.2933393370691795`, 2892.0588962515585`,
191.6699895746367`, 0.06931402088983452`, 678.4592514468841`, 0.888774996107458`},
{0.07977487479310064`, 0.6144204943798766`, 0.09424228086283336`, 1.2775428015699744`, 0.6767638412484815`,
0.5816419361753261`, 0.15698565577379914`, 0.369345501502071`, 1597.886185636543`,
116.59757565327084`, 0.05456927055006355`, 686.0167593354774`, 0.6493502506076234`},
{0.11381624462274387`, 3.2619424782291704`, 0.09692952579260582`, 0.9063291873551669`, 0.2692494018233931`,
0.19021625083351945`, 0.08348910726180397`, 0.3034738877311782`, 2094.7472269826194`,
304.11240807458546`, 0.09080827131735081`, 148.3350919983124`, 1.3616309719625361`},
{0.20555474556702025`, 1.243227025764214`, 0.005452291496766079`, 1.0028181389697215`, 0.4262225401606001`,
0.25423493482979376`, 0.2357351060940912`, 0.304508097031852`, 1442.1971734148965`,
133.35578434728035`, 0.1905528736862201`, 472.11876551422324`, 0.6916175696879465`},
{0.15025677548534122`, 0.8715754624872867`, 0.046775603311489125`, 1.3665267154940866`, 0.23928236082045418`,
0.2581285397803651`, 0.0759316214605753`, 0.025825089224507897`, 2985.40046755803`,
158.92340451009602`, 0.06763363037434023`, 532.016425146908`, 1.053167239001454`},
{0.08775073136015715`, 0.41017145441885106`, 0.06517183357890968`, 0.8212051993436631`, 0.42021671795786575`,
0.190510010996208`, 0.10055235599888329`, 0.21068761111108875`, 2024.1627530057158`,
388.609685286714`, 0.22547430485894593`, 282.28651403913113`, 0.5913677443569922`},
{0.1422509126185048`, 2.2066826119346636`, 0.028836373159347117`, 1.3974933493835435`, 0.1770723260801481`,
0.3294683993505656`, 0.1900742320092279`, 0.3712551877342868`, 1382.505103632153`,
370.91434372962544`, 0.2245006002094218`, 338.99432500611687`, 0.187504361874558`},
{0.07408769187996272`, 1.5987096367534441`, 0.09728678420585812`, 1.245671613860702`, 0.9273828722420925`,
0.4634095358027954`, 0.23919329005926998`, 0.2859258629777347`, 2977.6019243027486`,
298.4245885922551`, 0.19957132639438402`, 412.30128466820764`, 0.10687867224607484`},
{0.07342121519421135`, 3.397784251352663`, 0.06992350715887767`, 1.1430158444328629`, 0.5184959355660546`,
0.3233976520214229`, 0.09356972082625967`, 0.04780554372430901`, 2561.789683204537`,
171.37563950728975`, 0.23147234439029735`, 929.8880358743162`, 0.7791013990903939`},
{0.14045636776301584`, 3.0775874681954534`, 0.08805790649816672`, 1.4244507230598402`, 0.3537336581155126`,
0.35054479393495386`, 0.21012549414970255`, 0.05474733920200264`, 1794.2873321746529`,
151.0281969911186`, 0.1584599860438842`, 811.0824570076709`, 1.0066029107223646`},

{0.0627117248797564`, 1.4496909143257044`, 0.05702679749520348`, 1.4073871754768068`, 0.8835988887333364`,
0.41818877169257096`, 0.15847798860856022`, 0.03579255985858823`, 2988.649547410595`,
176.22575971335903`, 0.197471843005935`, 298.08853404857666`, 1.0498863998053283`},
{0.2307582908335536`, 1.4887728274677698`, 0.05819262706407674`, 1.362401278473583`, 0.2806886465177003`,
0.650750309301428`, 0.18043418693563984`, 0.01592038251646597`, 1639.2068126619315`,
307.06631711369926`, 0.23208019530409113`, 480.71761542554975`, 1.245525405955219`},
{0.15010224762509766`, 0.5884279973356987`, 0.014597252591713463`, 0.9059023657574754`, 0.3395866340461027`,
0.1814894530762421`, 0.11123211074057754`, 0.013431373090630761`, 2318.3376899340465`,
260.8104060052091`, 0.20755411915631972`, 876.1627116455188`, 1.3936880176307933`},
{0.21430562593126373`, 0.5692934407956964`, 0.05324116006713125`, 0.8834743517913386`, 0.7177700679252177`,
0.43002166839845024`, 0.1951444797143625`, 0.39670010134941197`, 2878.3097085584714`,
283.7837492962392`, 0.22423361832710048`, 140.3352401910123`, 1.311190149506114`},
{0.18678817447103918`, 2.874523857008006`, 0.09020594271662528`, 1.0736138311553896`, 0.8620284759446812`,
0.1673361289648747`, 0.10337138967936071`, 0.38010441870360445`, 925.3135939961285`,
368.3042050232999`, 0.08307034917614708`, 124.63710866133172`, 0.5135522150558252`},
{0.27276277342987304`, 0.5245850392146525`, 0.08556299171725443`, 0.894639184477342`, 0.9300208692390719`,
0.3364253728189047`, 0.10919526956202819`, 0.007322369238274647`, 2520.565010172062`,
60.82982777400633`, 0.01746471551362877`, 415.53089965542006`, 0.12417649143512866`},
{0.05545885650589821`, 2.3027708761902757`, 0.08432810271750882`, 1.160732562978869`, 0.6481332641844062`,
0.5998265073788811`, 0.07942388820240143`, 0.01666014225847401`, 3543.4233035414063`,
362.04468010705796`, 0.050083529213518274`, 704.0152536358094`, 1.3557294791110333`},
{0.08669036979582162`, 3.4944122700871167`, 0.047903481999836135`, 1.4024670927538987`, 0.436399824105137`,
0.6793646290585225`, 0.1697632359200183`, 0.06444132208346684`, 2507.809711790901`,
43.076413302799324`, 0.1955622469478828`, 218.57744831874868`, 0.7700973940438778`},
{0.0876548286067993`, 3.2736552275570014`, 0.09479718730625386`, 1.3128191834345158`, 0.7466389347206845`,
0.3601382642749472`, 0.11662791421342883`, 0.010928218533259938`, 1843.54787559731`,
203.88304575574648`, 0.1852072372041924`, 543.6450007282846`, 1.304102697159209`},
{0.19673128986865657`, 2.4052168641966745`, 0.017519659627304823`, 0.8240129601650297`, 0.1774242659102987`,
0.30692081334647836`, 0.24258185239932817`, 0.0148893162998064`, 1785.448102831053`,
391.14545111605526`, 0.05311488627173433`, 520.487546858836`, 0.689130249539381`},
{0.18485372826983115`, 3.5125521146584084`, 0.028828586884183684`, 1.2401537420455744`, 0.6009794218438815`,

0.5005801901350211`, 0.1121471366711167`, 0.00879525170164682`, 3378.5177429959467`,
53.519609785196906`, 0.10630727805990942`, 930.5976248724736`, 1.3765475584506879`},
{0.05067172121697561`, 3.2344932860225466`, 0.07244314662322726`, 1.0719945913259104`, 0.6253613096528405`,
0.6472961830110899`, 0.22310618665014892`, 0.06955381236115103`, 2095.6331184642186`,
379.9306020238764`, 0.2440957013693878`, 110.53139823690898`, 0.7134266809309353`},
{0.27191488554980203`, 1.3271333047971599`, 0.013966861879408016`, 1.4399763856935377`, 0.09423065544528719`,
0.6916460109733984`, 0.17320148472810265`, 0.05067688919295299`, 981.5112057683373`,
118.47049357238319`, 0.09683908456886242`, 286.54050574263385`, 0.8055235195742512`},
{0.07537053635292423`, 2.2841497454439903`, 0.021876083828669176`, 1.0793889621049275`, 0.5764979028803272`,
0.56027729170804`, 0.06557769178553285`, 0.20616175044131754`, 2504.0401677491473`,
387.12284353904465`, 0.03456676225290817`, 495.63500064184257`, 0.6524093728751728`},
{0.25132199929384896`, 1.0877201972617367`, 0.044139235426797256`, 1.3628858399880959`, 0.08162600665706332`,
0.5377453312450597`, 0.13722172704521313`, 0.030338518132330662`, 1161.4328259640579`,
27.77533061224733`, 0.027423631124412373`, 999.8640953200606`, 0.2860208123975949`},
{0.17385693860421964`, 1.7814219098759274`, 0.06481450939854622`, 1.272318871305831`, 0.15690900552419706`,
0.5063936315108339`, 0.18915528803835074`, 0.039387512819403274`, 1449.1462845720043`,
223.59001485447902`, 0.2197929313597805`, 800.2966775847274`, 1.0640062347288946`},
{0.20133067868053667`, 3.742519754506324`, 0.05737108940865847`, 1.221412902898203`, 0.9668148605376667`,
0.3035743337320249`, 0.14522842555919147`, 0.012157098988733935`, 2564.808909930338`,
126.90418310844473`, 0.24703618310040487`, 293.3552994614871`, 0.5458232471571232`},
{0.08924020517948811`, 1.0270326429684689`, 0.07676353347146375`, 1.131003758089916`, 0.7748479553024716`,
0.200564520773445`, 0.11148117168647947`, 0.337451823400795`, 2463.8847447647013`,
110.14096179360945`, 0.14254974667173514`, 846.7044720732453`, 0.7620006790740845`},
{0.08864792694658014`, 3.4474127480510797`, 0.06915192916650409`, 0.9259578556695667`, 0.44741297448842765`,
0.18949515222444357`, 0.1873833189826145`, 0.29819714981746315`, 1906.2013932789632`,
236.5674392722292`, 0.048729938462062417`, 683.8816457197021`, 1.260203007534988`},
{0.1656099906780112`, 1.4670089939329651`, 0.0518819029485268`, 0.8325595002033203`, 0.24156826254784813`,
0.42946465742331585`, 0.08760703374347478`, 0.07111204792904867`, 3998.1898760428303`,
138.31796802155804`, 0.014314122524196726`, 446.7480204023895`, 1.0255690658934498`},
{0.10500200584207342`, 1.7983084098660198`, 0.08377159957079307`, 1.0937443778983704`, 0.4146074446951571`,
0.31523749676266755`, 0.17876860378713083`, 0.018938893962850055`, 3788.0955938411444`,

300.53321385646257`, 0.08619275897605883`, 844.6773265112824`, 1.4734438237094047`},
{0.26449812940354733`, 2.8111722130938945`, 0.0694601955698758`, 1.0528664245885755`, 0.8592186810402287`,
0.6083116174527485`, 0.13147904972554314`, 0.05308740991258193`, 810.3693962980747`,
144.66403995182003`, 0.20598208418947245`, 248.554822802744`, 0.7835491171036311`},
{0.217784662211506`, 2.985014472578972`, 0.05230766877036651`, 1.1281213963352716`, 0.7915863081492618`,
0.6194911725400838`, 0.1262366501339806`, 0.06492499346063356`, 1911.610005338608`,
22.582605326314535`, 0.02973232322161412`, 817.1797630072301`, 0.6241302309034371`},
{0.16407952617221688`, 1.9311600813766496`, 0.003186259037403243`, 1.4176269891459357`, 0.6668604418782187`,
0.45431913271435664`, 0.20211798752943044`, 0.110602168678852`, 701.780217760851`,
206.86535950329358`, 0.10130069168392819`, 973.8704347803445`, 1.430310820431341`},
{0.14852662822324864`, 3.8872570761058904`, 0.08430277514641259`, 1.402909597630153`, 0.32034543090238654`,
0.5184949771086632`, 0.11287270717215081`, 0.11648206557894379`, 2989.4142567483023`,
178.5190400367801`, 0.13741218491440566`, 702.8660784415425`, 0.5844818999668677`},
{0.08509838803775677`, 3.4869927579766937`, 0.0699908986014718`, 1.2591039838882583`, 0.35789792210830584`,
0.46675450148760345`, 0.19285224441116428`, 0.13397566606718236`, 1429.2693314937715`,
336.90369041209703`, 0.13568711108595238`, 592.4752523836762`, 1.0487133858887376`},
{0.18835913419814282`, 1.0324501434606566`, 0.013872676347317566`, 1.157187288153083`, 0.972872790494185`,
0.2176901976011053`, 0.11294521185855727`, 0.010459818560081168`, 507.5274817455811`,
342.25106614281435`, 0.04629903582546069`, 725.033666977789`, 0.9872991463446792`},
{0.10761358360420281`, 0.9416588851840011`, 0.05176325383423576`, 0.9745645686696686`, 0.11996269558618744`,
0.20970159498882923`, 0.09734814071007147`, 0.09276060347112786`, 558.1529023471949`,
274.054827831687`, 0.03982765944326161`, 864.0718044278809`, 0.47581292100480166`},
{0.26617455578526183`, 2.2106538112685366`, 0.07973436740817678`, 1.4169239857667657`, 0.04238005915333187`,
0.6441569423522808`, 0.19229611942946145`, 0.00982322102868099`, 1382.1306912143564`,
129.76262194582898`, 0.10774617054670926`, 436.73846977639295`, 0.18708549973416932`},
{0.23531808327158626`, 0.981274658571575`, 0.0996126212636975`, 1.0430158381121901`, 0.21788542243555886`,
0.23345901412878056`, 0.20326832391990562`, 0.013107687687960806`, 3740.894093737673`,
177.01173128487028`, 0.12167342189254288`, 311.27473089807154`, 0.8336415777230792`},
{0.19345775875800608`, 2.2375394120214125`, 0.09196016495864537`, 1.2844146132663097`, 0.3626610343795176`,
0.1590510582415654`, 0.19983558903582405`, 0.006151041104400663`, 1996.7428249410377`,
65.44708192246623`, 0.17817174698330063`, 536.1198168253698`, 0.2578767535458877`},

{0.0940734770621976`, 0.684803443691588`, 0.0073408921719101804`, 0.892829864535689`, 0.9731944881516741`,
0.4891776011844119`, 0.22415830104835244`, 0.005121184741123514`, 1719.5384074845915`,
380.8213684625457`, 0.11957621798296758`, 376.9012473422597`, 1.367534878959134`},
{0.0614327441767758`, 2.703684160515885`, 0.025419219635842847`, 1.3132662471349392`, 0.16274374036741257`,
0.21532675266624846`, 0.20483453827921239`, 0.22376282475719544`, 3024.0914896406166`,
397.57763305420883`, 0.06113496246240635`, 558.1856500724314`, 1.3887120139014781`},
{0.1775326567342782`, 2.094650427988209`, 0.07114564176840332`, 1.0125667561037248`, 0.15375021984854387`,
0.3764651870882101`, 0.1731929872572361`, 0.01072302025208973`, 3195.6053839936712`,
39.57988472026659`, 0.1061334168929704`, 438.08809518083297`, 0.5446779021889512`},
{0.15634457328329354`, 3.4193660741644845`, 0.07563592951267598`, 1.2509928075009493`, 0.4150940971879922`,
0.2927532085074789`, 0.08918084911931298`, 0.06964624181475114`, 2936.306601916581`,
337.84439623809976`, 0.091084627687114`, 646.7789210140498`, 1.0462054433708614`},
{0.1403070008916965`, 0.7648020503609967`, 0.05621259527520252`, 0.8919537575547567`, 0.8304314331444413`,
0.625382806561833`, 0.1279202347529816`, 0.01681698485579172`, 2819.1849429614485`,
393.93211794332706`, 0.1620755372820416`, 121.32757433231376`, 1.0341950090458973`},
{0.17466875716377034`, 0.7192137738026902`, 0.05184365938465096`, 1.0833090804522032`, 0.22764180155965308`,
0.19030662643181673`, 0.24190727957410718`, 0.032538241709817714`, 2050.6119484822757`,
228.1073976904795`, 0.22638254826085685`, 522.9150783144646`, 0.975049326428358`},
{0.17478160619585154`, 1.031188031398707`, 0.0576976913194488`, 1.3405398719724155`, 0.43474786371942153`,
0.3179557100985815`, 0.17878222846578956`, 0.013615083230319978`, 2259.2113798203063`,
77.79716697376443`, 0.018552500311960624`, 795.0857768738224`, 0.793637666032919`},
{0.04301908400428475`, 2.1294578636637382`, 0.0228424408571518`, 1.36102068953005`, 0.0022599924510029457`,
0.2905241462361252`, 0.1328788570787381`, 0.010670983207295094`, 1262.4341869513219`,
379.9596392159698`, 0.03636129559333251`, 141.66530644460113`, 0.18668462204456304`},
{0.18894016053578305`, 1.4715923805440783`, 0.019885788559893404`, 1.2997863067854758`, 0.3581426667585348`,
0.4676572673292877`, 0.20313480908165227`, 0.05980996382224595`, 990.6383273102883`,
396.57842847459983`, 0.1909349492938665`, 655.4313171630283`, 1.2844477045801628`},
{0.13014268795429035`, 2.494723970000373`, 0.016359305580955434`, 0.7711967270475593`, 0.25539285155520686`,
0.453714683019137`, 0.08890633002331283`, 0.027816439578619304`, 837.9683682388286`,
112.45738365915167`, 0.18785648474606642`, 258.33574385313085`, 1.3632769861710168`},
{0.10054465227190196`, 3.310496186673542`, 0.00750320414979921`, 1.4866665839305195`, 0.898075043381193`,

0.6764187311799383`, 0.08146991740983911`, 0.006007819039248795`, 1042.0722797417648`,
213.68783370335075`, 0.12950980822629182`, 521.1378640592093`, 1.09180681814545`},
{0.20867159318320344`, 1.1373369899634742`, 0.009872313285350423`, 1.4792202680526125`, 0.9935963817974927`,
0.37233935738056234`, 0.15597241325824251`, 0.02744516737224626`, 3990.3359791597904`,
254.1465081277163`, 0.0629260491779966`, 831.6658192410579`, 1.1944105499977882`},
{0.20224442635867973`, 0.5241454524024682`, 0.03896232512512681`, 1.162721989382709`, 0.4876087959739268`,
0.38540881948341144`, 0.21676672666317276`, 0.05197770557989867`, 3192.7466977047143`,
274.5516667945552`, 0.0681836129362326`, 854.4240525352062`, 0.9758313247266246`},
{0.07416542875062498`, 1.2069172297197222`, 0.0022085328185865016`, 1.3626003754696674`, 0.030009709042837507`,
0.4523284620761664`, 0.13222679903557488`, 0.27823112461866`, 2711.3058702485396`,
362.2761010348281`, 0.23333223754579197`, 770.1419216605747`, 0.9880409119053444`},
{0.05000686357088241`, 0.8474227766129117`, 0.015931834179246582`, 1.3835882619607132`, 0.22582526049816165`,
0.5022902361686673`, 0.17203885652394435`, 0.007403997652535603 5`, 1737.3995761016859`,
20.08458762033723`, 0.12181751617048181`, 123.8391726714406`, 0.5766930040179512`},
{0.22612738808242866`, 1.7787747226620336`, 0.0377778336624985`, 1.0017914100637972`, 0.895144187315148`,
0.4424679794538967`, 0.05844674752208181 6`, 0.01787395711783792`, 563.426039119553`,
318.3846088085469`, 0.16815887233019144`, 689.4595688976403`, 1.3216269495748754`},
{0.05854328762990285`, 1.6817249013785789`, 0.06397042936609594`, 1.0424907757118325`, 0.3056786692377855`,
0.5220103924855791`, 0.07017743755598088`, 0.4282286368716264`, 2278.574519349251`,
281.02639560176374`, 0.15764134289520088`, 977.7119953432973`, 0.9144986369174108`},
{0.18731162930151107`, 0.5589912392796332`, 0.08263189808974207`, 0.8092055438463432`, 0.1360300234755596`,
0.4257699185123174`, 0.21267928512199308`, 0.007355731333931366`, 3627.4241155903474`,
295.69240017733466`, 0.1255446410548438`, 514.868309587726`, 0.1669525692880891`},
{0.05550445137300852`, 2.3292893386580618`, 0.07970144411383022`, 1.4963213396850843`, 0.8423666102137801`,
0.2696772669658447`, 0.15799141576952752`, 0.010160791485693446`, 2698.2585841988794`,
349.75055306544834`, 0.11800399326710415`, 779.3123667782133`, 0.8433204242427768`},
{0.1265607245823615`, 0.5017039605352203`, 0.019655450839343676`, 0.8702042649342335`, 0.14394338861472344`,
0.16157495690642731`, 0.08109066108562163`, 0.022901666965866513`, 1464.4135092988427`,
29.121324685416994`, 0.11830730931774475`, 712.5882392797087`, 1.0478193311089337`},
{0.14536564055338874`, 2.293682126796403`, 0.03493360119994513`, 1.101749307821256`, 0.2952692982581917`,
0.5400236852037054`, 0.20994973514394843`, 0.05797620998940398`, 572.1970109030017`,

181.65223274304992`, 0.149163863457287`, 529.3354874154545`, 0.9016759497382885`},
{0.09742817247800262`, 1.964828903796203`, 0.0047037433678341145`, 0.9453747171262818`, 0.40796443210263966`,
0.5944864474979785`, 0.21308333023908277`, 0.11862056477176541`, 1423.3901416845429`,
155.68468206220814`, 0.22844381229028082`, 476.7938097214161`, 0.6623468167840558`},
{0.24283182159455097`, 1.603100908958103`, 0.05285289504817564`, 1.1682758325883023`, 0.935264341081167`,
0.18122621646634884`, 0.14901817546799717`, 0.007722329904883057`, 3951.852486693314`,
377.25452197144887`, 0.16773700308197348`, 251.95256246454764`, 0.5431948192215346`},
{0.13336383893154985`, 2.180435493246489`, 0.07158315591410591`, 0.8060538474934296`, 0.09026648216568645`,
0.4800876981972235`, 0.11791366871814568`, 0.012733405933773284`, 2450.8177706650013`,
208.08772742013343`, 0.23094460017932056`, 263.2586358816642`, 1.1454791364403092`},
{0.05384327330799882`, 1.6621464748581287`, 0.005969047367170006`, 0.7979749951675864`, 0.7063953508249934`,
0.24499487541468168`, 0.18766533488676096`, 0.21413058173591698`, 618.8272511172145`,
174.99492689953763`, 0.2251646566457952`, 196.31954322071152`, 1.1477926608639715`},
{0.15807260919458554`, 3.663648747663368`, 0.04732313807630286`, 0.8796847898373553`, 0.5697420979089782`,
0.2561173621880035`, 0.10099872400397214`, 0.1627573663804833`, 2126.2908815590417`,
23.99885634601037`, 0.1766668406334702`, 926.199284420499`, 0.5117040237769424`},
{0.22974561562779744`, 2.5413619293768583`, 0.046352603532486325`, 1.0604230919336857`, 0.762314631152764`,
0.3932762433737357`, 0.23311877473927634`, 0.21038063032334042`, 3154.920175405916`,
140.6370367610425`, 0.1530914264041`, 550.0519139504759`, 0.8273451555880165`},
{0.11161620923890908`, 3.7039707333619107`, 0.05116839889505817`, 1.0975304731925206`, 0.46404170674715206`,
0.667166760292226`, 0.23121989785245994`, 0.4992623840736095`, 3841.9320755673343`,
149.45978460235324`, 0.07342398948726425`, 719.946210624367`, 0.9166471080707068`},
{0.12006085342307354`, 1.336225258280848`, 0.09905562939094605`, 0.8223800577302112`, 0.06966857675102323`,
0.29458617584491154`, 0.24895630516439893`, 0.013857427856894573`, 657.5708782723063`,
273.724404391484`, 0.025033943082609922`, 379.3155667581619`, 0.154926328057732`},
{0.2502886350178106`, 3.0052560785276246`, 0.0467987716534058`, 0.8778578631800616`, 0.6921028317126472`,
0.20549432727538663`, 0.05290408999928786`, 0.319972046277131`, 2254.0036568067053`,
108.4857770734987`, 0.0882725905767554`, 195.09483107947557`, 0.1714507431394312`},
{0.04802155814720732`, 3.1570324757660755`, 0.025083253742492373`, 0.9661705225245113`, 0.3037226989619122`,
0.19279302108042473`, 0.22598572164615194`, 0.28257712689719744`, 2851.3618689079713`,
288.4464169633019`, 0.12914694499111795`, 287.11263813957794`, 0.3513846628801711`},

```
{0.17288830542045353`, 2.434158174303456`, 0.058219081477261535`, 1.3621928030965615`, 0.9440211333667559`,
  0.48515110575880005`, 0.2397410066575077`, 0.019723716791185127`, 2652.818717896923`,
  217.7437938157317`, 0.030650125567323883`, 725.6362756844544`, 1.157208376795479`},
{0.19947405793715844`, 0.9196811294683878`, 0.09402085479351147`, 1.4789340479755062`, 0.7588132167755843`,
  0.5898448435822807`, 0.05124352425758871`, 0.03978381975178661`, 1116.2343972855797`,
  321.3549022959937`, 0.030855778963666758`, 328.5430856721781`, 0.18400788517528288`},
{0.04840582516744904`, 3.549022423939368`, 0.02210665201355962`, 1.3517211192950607`, 0.8863669402621561`,
  0.31242940629823013`, 0.2242975603155371`, 0.07117001080991502`, 3465.7907171300903`,
  59.91508161769508`, 0.23259403953376656`, 109.76260335115455`, 0.4948885033216808`},
{0.22077031875404196`, 0.9174090930413614`, 0.02288657391929915`, 1.2692862821699495`, 0.3850365599434735`,
  0.6450323289435635`, 0.2215489376959433`, 0.03779540281909232`, 1321.4428078633655`,
  174.35243214457842`, 0.08153714067237927`, 178.27283265496004`, 0.8282755310506298`},
{0.08112270845960284`, 1.1050261763245794`, 0.09269833680404535`, 0.8404989955370767`, 0.7632468745248251`,
  0.43143529999238117`, 0.16471746806791576`, 0.015755834419509625`, 1475.0972555416893`,
  246.46926533747626`, 0.11990013871507182`, 914.1228979682971`, 1.360979352127512`},
{0.13123417516663272`, 2.306554816524983`, 0.06893336273137883`, 0.8106550723335249`, 0.11616358281026073`,
  0.5557972218405267`, 0.06580258469239769`, 0.020691100593992522`, 737.9589931329506`,
  327.9054513019988`, 0.013776534818911362`, 217.5584374245096`, 1.2891116873553963`},
{0.04707948855003952`, 2.8156058954613696`, 0.013494136493520267`, 1.4651324062254065`, 0.8597380714628`,
  0.3948141586168282`, 0.1298745913067923`, 0.020885446437694175`, 3862.5443602527475`,
  101.76734782102716`, 0.05235929943919165`, 162.81512877490502`, 0.9324236865867443`},
{0.13022676180037496`, 1.2995273111109826`, 0.025292116318853`, 0.7667784909973042`, 0.4085195698733748`,
  0.5488633511957021`, 0.11142700791539295`, 0.45491143146611357`, 1009.4124405190137`,
  95.25879259356469`, 0.18965649593752032`, 592.3079633957238`, 0.8088186049272146`},
{0.15979886522930659`, 2.470143292857272`, 0.06628985540091048`, 1.0410518253426442`, 0.04574609880224534`,
  0.5444829065944847`, 0.0515572270933472`, 0.006444710619215029`, 3922.6080751566415`,
  209.99907738622835`, 0.20280170081937393`, 700.5258132946823`, 0.29837052140157283`},
{0.1783615302907124`, 1.0671710143810182`, 0.09299127243044521`, 1.1291712374166352`, 0.5541154884696773`,
  0.5225463323517774`, 0.17275207904587242`, 0.011042773489281243`, 1214.0400229686757`,
  395.5842597201831`, 0.09362557505446806`, 964.8880276082249`, 1.4961679823922784`},
{0.2445840178295805`, 2.7365103712818213`, 0.09105195193157574`, 1.0553426617811257`, 0.11692968298408002`,
```

0.6995186804276932`, 0.2044510942635801`, 0.1973645633242339`, 3833.681426463623`,
198.20896247551912`, 0.10973562821269262`, 898.9967289535186`, 1.3105385578503905`},
{0.1430243356352418`, 2.877833600533017`, 0.047363325113895856`, 1.0097690270880397`, 0.14459998911317373`,
0.5795367878532327`, 0.24842436563308823`, 0.1316841602798839`, 3153.7951431926067`,
91.20404904063724`, 0.20822959248980527`, 377.472081358205`, 1.2093276261125783`},
{0.1534054225050746`, 3.3094512569579946`, 0.037650754787147335`, 0.8621710157306695`, 0.8633013860555436`,
0.45573016458758864`, 0.08151393130926812`, 0.4900162368345317`, 1890.3983471964139`,
166.43716733468727`, 0.012387756715024095`, 699.4703893982227`, 0.6503442939495674`},
{0.05779448034464035`, 3.500681856350031`, 0.02306669523829964`, 0.8234246828188458`, 0.2923878618710869`,
0.5146413725175935`, 0.1477100927803886`, 0.0357185966336039`, 3853.3526912365296`,
355.89855551951825`, 0.1801389850005799`, 374.930002647617`, 0.657459999302608`},
{0.07520117051755226`, 0.5348378809957639`, 0.007001830149721453`, 0.8476231139074708`, 0.7587937471238555`,
0.3267071970651595`, 0.17249778831698842`, 0.0148256980232596`, 1911.0330895320967`,
346.15589461050536`, 0.2448723364840062`, 916.4340489647959`, 0.6492603206300078`},
{0.18805588895659647`, 1.0212929901976855`, 0.0685964445285019`, 1.467638163463905`, 0.14943065624479224`,
0.34652799496134357`, 0.22894228445830533`, 0.245357059895091`, 525.6565771257192`,
108.86287287999363`, 0.06157819615262983`, 421.09691099484985`, 0.3930443766302789`},
{0.2423050473507231`, 3.6875635681191623`, 0.05599977965059413`, 0.8387713631408364`, 0.07013194661998368`,
0.40727183676263834`, 0.14496533351812652`, 0.31002668011906354`, 1417.1775137012846`,
204.9018435179862`, 0.017839357740137785`, 267.4454058150507`, 0.3467251205895048`},
{0.2152073330578984`, 3.4542218284695965`, 0.05126315438250687`, 1.089229081217571`, 0.2735465270373023`,
0.23328061087184981`, 0.178181663117721`, 0.005958955292137833`, 2394.3883232906155`,
321.0424909694008`, 0.012316111150270953`, 868.1570075551506`, 0.3116189546022836`},
{0.24248957546163674`, 2.514971284967592`, 0.03810488317636157`, 1.2037013806849715`, 0.049257733433564876`,
0.4029965766449014`, 0.21185448594701367`, 0.009388580578881478`, 2796.5258913232537`,
255.79583133987603`, 0.15699986437057706`, 345.52508771711297`, 0.11511240255378863`},
{0.06444784908803713`, 0.497863838605451`, 0.009032642675356596`, 0.9566408425061232`, 0.861464880252155`,
0.6233509819285923`, 0.21042453011758416`, 0.11738678022526211`, 1374.5825305991384`,
117.3002199698276`, 0.0744893029214378`, 536.0472732902684`, 0.4136074016916129`},
{0.12130400595359758`, 2.76172743206038`, 0.07655125006568177`, 1.171972819137396`, 0.7232733164937175`,
0.4974869098901661`, 0.12156821845508575`, 0.07411688982222371`, 1718.8766721447118`,

77.45580912331013`, 0.022912924617915387`, 639.440197865433`, 0.2534607552566033`},
{0.2722480292821207`, 0.8983930508251685`, 0.07825341396573578`, 1.0425282706352832`, 0.036416599481725376`,
0.18493281693678576`, 0.07940460860991741`, 0.029201945878282665`, 3235.673513818516`,
373.6060298298661`, 0.028535738832318025`, 883.8721990588838`, 1.2405068570013098`},
{0.24180361404150308`, 3.5250596331711863`, 0.09588765993741348`, 1.4923584939978196`, 0.8354415457125168`,
0.5641131195221286`, 0.11592185783429804`, 0.1464121261697459`, 3046.3514230765077`,
347.85064643474016`, 0.15888340207214824`, 928.7953812954008`, 1.4848234296355156`},
{0.0873923709042464`, 0.8352169347600631`, 0.09859339305118354`, 0.9474511957824416`, 0.18115914066793803`,
0.6691381776034326`, 0.23738115098813173`, 0.022909648195529254`, 2830.740904385917`,
58.295495914215735`, 0.10110496933519186`, 869.5543889668901`, 0.2667089738942936`},
{0.23618579956296948`, 3.335540308460586`, 0.0013869936998743171`, 1.4201459041181137`, 0.5063658746372321`,
0.17751153024367672`, 0.14758651543530382`, 0.03193814618077176`, 2472.005493081676`,
71.84821837270778`, 0.07318415254499161`, 886.5492775169994`, 1.4358663611619202`},
{0.07326005057687957`, 2.813528425221647`, 0.023844157069030862`, 1.3274622296329985`, 0.4794638501948938`,
0.20244840808573306`, 0.1242468347168455`, 0.02712536224456867`, 3125.498558382639`,
241.20055803608932`, 0.23579032214153867`, 207.56946695330106`, 1.2793883150097018`},
{0.21285674785237985`, 0.9563878535471098`, 0.07125312010716922`, 0.8970241393659186`, 0.6448363728322131`,
0.48565150183572015`, 0.18919360722486928`, 0.005685166597944712`, 2115.5591312196975`,
160.0403862977406`, 0.08379707307903328`, 487.32074649476937`, 0.9368465978587537`},
{0.163787990903001`, 3.0907216491378913`, 0.08151395631745227`, 0.881812775476595`, 0.15816582262820744`,
0.5810986514232814`, 0.055637381694556204`, 0.08137428284401542`, 3899.809687277211`,
123.08540478701798`, 0.03789214319448464`, 371.3885334055345`, 0.39899756224149807`},
{0.20347203403511765`, 1.2008245121943535`, 0.009786939785735456`, 1.2450250784257453`, 0.6487956168907103`,
0.15670289825865413`, 0.14653051618333596`, 0.1295957345669408`, 1297.4955140902184`,
193.8283516109342`, 0.08661868775162623`, 322.66806857619395`, 1.343304641390601`},
{0.1074118361891333`, 1.3516612971051885`, 0.013094718077518178`, 1.287532165218197`, 0.8818422525174148`,
0.6421405007051253`, 0.11039199644924791`, 0.15578284299088804`, 2327.7475893890796`,
157.79572353444826`, 0.13830626806034524`, 344.51560915512493`, 1.2601410683305891`},
{0.18859760053832475`, 3.0991467844722376`, 0.0627122700729491`, 0.8444578613011013`, 0.34332780763538295`,
0.6880300960731556`, 0.18043055707836286`, 0.35919916843728106`, 922.6180349948272`,
46.74510147614933`, 0.010982098798781731`, 352.25197846832117`, 1.3530882358514238`},

```
{0.09587841359076754`, 2.7393809840640655`, 0.018214710918511567`, 1.2224675313888498`, 0.7200419959639157`,
  0.1848236167906918`, 0.16074887671040433`, 0.1171871307664133`, 3931.560076402542`,
  374.2997780287128`, 0.1556782352741259`, 769.0117617677013`, 0.2214929832094279`},
{0.12052325403848685`, 1.7426995200528097`, 0.05308490755525904`, 1.3604905436707253`, 0.28945326828820983`,
  0.3702830825929113`, 0.15508895850532967`, 0.4934843297125574`, 869.7357154438155`,
  167.62622034482172`, 0.2322243307694563`, 779.8890634969338`, 0.39686847589427643`},
{0.20534141484995183`, 1.4599872724292524`, 0.004934761919204647`, 0.9170782643018778`, 0.45676458671134657`,
  0.6757439027995402`, 0.23137092973028983`, 0.00705053768432672`, 1528.402619995768`,
  297.4875360079079`, 0.10716266992268952`, 462.432136217919`, 0.2106564513430802`},
{0.0947490376193943`, 0.5504476894830996`, 0.040084006255007694`, 1.4001817527243008`, 0.13717525827551658`,
  0.4212015684835967`, 0.19410681566258958`, 0.16363824432612178`, 795.4890862128677`,
  279.6576427840944`, 0.24258158323548706`, 715.0862568713405`, 1.3048014188030517`},
{0.11720061458965603`, 1.8793202785259666`, 0.0056047916240591`, 1.2815939112785486`, 0.47328089722234923`,
  0.48537365954177514`, 0.07193231789895638`, 0.4837822255277002`, 1006.8936520392858`,
  266.29409262263346`, 0.10734222501155061`, 667.4662182771683`, 0.5466023157081943`},
{0.22323827676343333`, 2.0999832775811553`, 0.08295636110784234`, 1.2177777376879173`, 0.8051871676261451`,
  0.2413630891404912`, 0.21613359971135926`, 0.021641589982308404`, 1479.6000918673717`,
  237.04330869179228`, 0.1806769105225065`, 237.89168962937327`, 0.3680741022087848`},
{0.16833323748982054`, 2.279291473034756`, 0.04872797388068765`, 1.3782147829415425`, 0.5235128700441671`,
  0.6353815130498364`, 0.23818130745015742`, 0.031997765770809365`, 1681.4203193205176`,
  341.27124612504224`, 0.19521609328636874`, 322.97787286956157`, 0.7920708320888563`},
{0.273099990190753`, 1.1962767380974908`, 0.029992857458039757`, 1.0647957006119935`, 0.1631245377809365`,
  0.45216746044437406`, 0.06824479229224134`, 0.08335040600236426`, 2072.2213944725363`,
  264.8564314656236`, 0.05858236261134292`, 481.87788126624014`, 1.3763526534904549`},
{0.07538304099433674`, 0.5981387414467765`, 0.035302807249782284`, 0.7769529517440688`, 0.8475122140578524`,
  0.4220960486351526`, 0.13407475386141487`, 0.01190768572644493`, 3292.9191524313455`,
  111.35090909251323`, 0.2135134717624977`, 125.63373704662091`, 1.3311738059077673`},
{0.2787437349209606`, 1.9582288830242849`, 0.0853453841961642`, 0.8581388969818479`, 0.7266903072059603`,
  0.19787744820816988`, 0.10533314062765764`, 0.15251427790346472`, 2466.6746962804873`,
  42.763613941111714`, 0.030092083437008366`, 496.4379154919019`, 1.3322844995399565`},
{0.1317043174519385`, 3.704889949293934`, 0.09971187989611252`, 0.9874741223559969`, 0.3422574133020093`,
```

```
0.6572392966332523`, 0.12828958704603438`, 0.03190147915800032`, 2199.228416413893`,
 88.86823113961168`, 0.045671534113712886`, 745.568696003622`, 1.2154056441400982`},
{0.24268972416802936`, 3.630246373820083`, 0.078158736221378`, 1.1557790010974371`, 0.49593151659359`,
 0.6187782509524469`, 0.22284512392898798`, 0.008554629585849361`, 1207.3921767832107`,
 383.6800829249721`, 0.1711220510619066`, 855.0741040620348`, 0.3059380047747071`},
{0.09074543263758239`, 1.5956215466959787`, 0.04915642911467069`, 0.9084611406931549`, 0.23724163053664404`,
 0.5052586308068423`, 0.08999063354829462`, 0.40131196701053695`, 3350.701552733175`,
 105.43319386779535`, 0.19122502477222536`, 652.3243036110294`, 0.16381385531216885`},
{0.06742095447495844`, 0.9402149425738457`, 0.09952228082525785`, 1.2561890137114702`, 0.4928486745345546`,
 0.30437934430107383`, 0.1631625606401702`, 0.014864320540401724`, 1181.9273790226398`,
 145.08668977178723`, 0.15170763252488906`, 450.8565940630259`, 0.3249408619151928`},
{0.24504727510909707`, 2.5310330321332666`, 0.0318300682470702`, 1.1273370412795622`, 0.16190949887357586`,
 0.24494520229297967`, 0.07932293214912559`, 0.039266767566225394`, 1428.2913444808219`,
 383.23844614926327`, 0.07928778825191907`, 802.5429842164205`, 0.19779963659414745`},
{0.15808741547209754`, 3.1301080493421356`, 0.01718263381854655`, 0.8408773418426974`, 0.7945003737499969`,
 0.5606271012390185`, 0.09243895010941977`, 0.1332935295046631`, 3770.221664075196`,
 142.7746655645762`, 0.15162807905653314`, 168.7501928066892`, 0.5574221586247512`},
{0.21749447923539056`, 1.3236277579587323`, 0.03853411681158381`, 0.7855342958153004`, 0.10191765603309633`,
 0.46214476159348195`, 0.19776651227614178`, 0.009160615369931551`, 2107.430970422496`,
 313.0283721663086`, 0.14260348249510701`, 577.5672050808926`, 1.3452444611665975`},
{0.2517687253100037`, 0.7106511941617053`, 0.060692191709689376`, 1.0347016624317908`, 0.04270276896851399`,
 0.23195763084753873`, 0.07758622145024907`, 0.11904739047616211`, 1586.4680549998602`,
 156.05253570543368`, 0.0623877257340304`, 172.38377111304715`, 1.0056198882975504`},
{0.06710969562151936`, 0.8479205678467574`, 0.03616206298751045`, 1.092800168199752`, 0.18715471024523445`,
 0.37274878450809534`, 0.09425293775920374`, 0.0875635159816428`, 2193.0302797760787`,
 249.59898372333964`, 0.03678946141084355`, 780.0397747149573`, 1.3026869340517515`},
{0.11349056586987222`, 3.129162659113181`, 0.08848003351297055`, 0.9345043834540654`, 0.6523467884321008`,
 0.15897700282460103`, 0.21697439928384604`, 0.023350034208549766`, 999.8041562532949`,
 187.28175375712487`, 0.027209319071962057`, 405.96875479857636`, 0.7853538898753998`},
{0.20628458604312377`, 2.2581549505153387`, 0.04993801227667913`, 1.1383015917485786`, 0.8208008988568332`,
 0.19149450090611586`, 0.17964993010780744`, 0.33652837660783486`, 1265.3821888367647`,
```

318.7342528832547`, 0.23703582181566174`, 854.4150961260996`, 1.133760010540752`},
{0.1271807238393557`, 2.6924918944125427`, 0.07229885554391988`, 1.4491561934822368`, 0.13474507994451623`,
0.43215914676291`, 0.17229148862703447`, 0.17884993838529148`, 1844.5022325089094`,
70.74425563388434`, 0.05799179206302968`, 301.03807587014376`, 0.784660036151664`},
{0.2433350096398842`, 1.9526092703436149`, 0.005290750625255143`, 1.1678864431463098`, 0.35471020990659197`,
0.17030248641252488`, 0.21077957089492727`, 0.018111586736183335`, 2773.232159794804`,
331.24828804439994`, 0.2127740250864072`, 719.6978805137825`, 1.3025743958182376`},
{0.2778812981095553`, 2.521281554699769`, 0.045274185957400716`, 1.1543617675491402`, 0.39926623351571666`,
0.26082438225176674`, 0.0625091058899934`, 0.04700114810792583`, 556.3926620617749`,
392.900360676403`, 0.05885687264275341`, 861.48276971577`, 0.886981998718182`},
{0.1716649333073833`, 1.6364207461773592`, 0.07172631922538393`, 1.109395446901821`, 0.5043905378139939`,
0.29187969395101954`, 0.0849491707445037`, 0.017760080386729146`, 987.8547996498728`,
91.2640190841791`, 0.23613341712959413`, 837.4584267910773`, 0.6157698411691688`},
{0.1666340221561945`, 2.396796957453212`, 0.09580621273720485`, 1.3719480323945639`, 0.99956285839998`,
0.2664681089706301`, 0.22186783120466347`, 0.1580382504314607`, 2895.5718543083367`,
142.49632353984464`, 0.05357527562362774`, 145.1743518458759`, 1.1291035431771692`},
{0.2518675718431633`, 2.9941043162888645`, 0.06584041024944166`, 0.7776274963381491`, 0.5607925985711462`,
0.27743717594180006`, 0.2445569124091585`, 0.04026532075598932`, 2240.72055548992`,
39.47505147264894`, 0.03602368791749283`, 790.048922600031`, 1.2030687322402196`},
{0.17492734142801014`, 0.4177034717007251`, 0.0028610469376033443`, 1.179341336608955`, 0.2648821071435268`,
0.5252771933795577`, 0.2057622479033312`, 0.19804286109034952`, 985.3182671454169`,
300.50593232768756`, 0.101181892756623 7`, 662.7856775741117`, 0.8793831836779726`},
{0.2459932890741956`, 0.8586292925170431`, 0.08749050529508676`, 0.8055948955265257`, 0.9012574698185647`,
0.41853698576562415`, 0.09469809414636007`, 0.11713850779527407`, 2034.5907705678792`,
282.7567654196798`, 0.22000053605017417`, 980.4382550992017`, 1.314398408925928`},
{0.12371488180059548`, 1.2366640465892695`, 0.09488231757636065`, 1.4847761416688645`, 0.3124294107285137`,
0.616175072264525`, 0.06524388043275703`, 0.0705979498919378`, 2867.889553158305`,
197.04826146755283`, 0.1268973457740542`, 299.43576728668563`, 0.6718748999110407`},
{0.13850317811811458`, 2.736073689480241`, 0.09027292799694919`, 1.1349648026127952`, 0.06991128954169956`,
0.6209297427354297`, 0.06524943493994803`, 0.01678377384881713`, 1141.742593279414`,
89.89236003028913`, 0.11782185651197419`, 939.9336034552384`, 0.43089662229831616`},

{0.12855643329130917`, 1.7433715374370253`, 0.08183288445560344`, 1.3786914733726912`, 0.6859926518858188`,
0.6772178810442473`, 0.2175322455167097`, 0.14623385815598697`, 902.4306709432485`,
330.73899832990105`, 0.23341002225709423`, 531.3931915253991`, 1.3780625228413514`},
{0.20212919870052765`, 3.2955733797914846`, 0.03172829491606887`, 1.315752307897653`, 0.04609865401242308`,
0.5605950585048131`, 0.10938169005256959`, 0.17389738721072828`, 3390.744440448758`,
76.9999007590693`, 0.14653256376270601`, 966.3094060874915`, 0.7918997118307547`},
{0.07036852489057943`, 1.3947849056068868`, 0.05658582729782806`, 1.1795564424767362`, 0.5030542104944831`,
0.45917722862921206`, 0.08125069032070675`, 0.006499195467650178`, 2022.8385066393803`,
164.1114663290521 8`, 0.027422777508066043`, 703.4980132526266`, 1.3803584134731208`},
{0.2608954596524286`, 1.8536850596011103`, 0.07838965550140334`, 1.3589533212123983`, 0.10024028116278183`,
0.4205769223810475`, 0.22785159239407005`, 0.017096040942865168`, 3993.95074455929`,
258.41008030007765`, 0.1573631998647665`, 608.145030411683`, 0.22129204704988514`},
{0.20137026738573705`, 1.2236991588600663`, 0.006890636730831243`, 1.1020308879431522`, 0.4576033714121108`,
0.4217969201513676`, 0.08818053669186957`, 0.4780958438842435`, 3911.4260586606297`,
79.18325268982579`, 0.21177883740067838`, 768.9604703495736`, 0.6595855038096903`},
{0.11954683452324327`, 3.1026940396302045`, 0.08633148752342802`, 1.1393044564633368`, 0.7254865592501778`,
0.4833596463995181`, 0.10658246121405845`, 0.01065441653834488`, 3629.47195637029`,
221.58046145149956`, 0.21340584277613434`, 962.8753584799746`, 0.4572009757006883`},
{0.18575234158089682`, 3.7738553468103033`, 0.0772095134201085`, 1.4457622099088967`, 0.1561179301918303`,
0.2748139321875236`, 0.05136378195005617`, 0.007489516874652134`, 888.8440716717027`,
25.341672299498384`, 0.1304041347295058`, 849.5241983647115`, 1.2243295919264692`},
{0.11751671153876497`, 2.0965587450416505`, 0.09811204143062575`, 1.1158724018136064`, 0.04812415624581168`,
0.3357765421260823`, 0.24042619823928923`, 0.01741625056698872`, 1095.2975408771795`,
114.29489638855244`, 0.08375660851279204`, 875.2391096819657`, 0.13573086934280565`},
{0.12647229068431487`, 3.5505406534654664`, 0.07329051507792227`, 1.1460069757106344`, 0.5254947596957511`,
0.6565137790152902`, 0.17604334648098396`, 0.006131485299743147`, 2568.029052792832`,
373.62123483502035`, 0.03814772407837863`, 358.7086448082507`, 0.4816375540787621`},
{0.14950821906797657`, 0.8411934789816247`, 0.06135045133721484`, 0.9520819747853632`, 0.7692887227567426`,
0.33928306988506296`, 0.077049022293331`, 0.005497154256964613`, 2383.2910724678595`,
261.78575725104076`, 0.15813616835600214`, 156.94520695192313`, 0.8601115737357534`},
{0.27252745234259734`, 0.8844641996533626`, 0.08281449173977258`, 1.3019473657901404`, 0.9007700278570128`,

0.5491847873001705`, 0.1756559177184771`, 0.1973475257954469`, 3820.3954969214456`,
264.4132016347096`, 0.01603736658113844`, 303.439145500556`, 0.8192347365278416`},
{0.22201282741508022`, 2.2865110105605897`, 0.02732141679639163`, 1.2088326190954195`, 0.9155616249059464`,
0.2897174556022799`, 0.051011741486320705`, 0.03750348264787712`, 677.4649363050203`,
244.1570264446632`, 0.17836226061979`, 249.9426689722469`, 0.18292827867706296`},
{0.11901746724303741`, 3.647987419943229`, 0.05084170051223323`, 1.1466390768234165`, 0.512508006375221`,
0.48025658340888344`, 0.062300084411450546`, 0.2659660790493203`, 3369.068028339053`,
307.36086330541`, 0.10289668378579803`, 669.4620554149892`, 0.35847029922000684`},
{0.12215675882759947`, 2.253462636192223`, 0.00860084809687967`, 1.3155680281694941`, 0.45255021152683006`,
0.6984938355012964`, 0.09125072116122349`, 0.27166164908808715`, 2778.7035754910257`,
157.85561129866164`, 0.010348228055683611`, 676.5634640450587`, 0.9582637172565875`},
{0.16878636593085306`, 0.979943447781241`, 0.033774106149647806`, 1.0285690549281143`, 0.43958302496115875`,
0.23046960852651432`, 0.10362926673447892`, 0.007171599555435339`, 1163.9359887405535`,
150.72831296727452`, 0.18642719855656936`, 476.5632411996181`, 1.4261829604266003`},
{0.2607739761973086`, 1.5236620116980548`, 0.002941057719636788`, 1.2187708630124812`, 0.4491656925334122`,
0.5405655313135616`, 0.24818041849913597`, 0.03089080101184376`, 1437.4090837992044`,
219.78630621374782`, 0.08228486677073948`, 260.21980864730114`, 0.3535950731028925`},
{0.1257746501265688`, 1.2855538911240263`, 0.03176524876045283`, 1.2793441287766227`, 0.4331676201171979`,
0.2200833521759865`, 0.1898050000470356`, 0.10683156444635543`, 2769.340849250989`,
311.9982825870983`, 0.04454079812848932`, 246.6478434300784`, 1.4300643280227212`},
{0.05938847538424702`, 1.4214850773927568`, 0.0852876787756453`, 1.2412710476979203`, 0.6114346587637478`,
0.17527864998699128`, 0.1725346931524498`, 0.008163129788201664`, 3803.241262513511`,
113.21336353047991`, 0.1285711966326999`, 914.6849616294649`, 1.030365639718188`},
{0.27760108056352295`, 3.642538228153872`, 0.09407003747174592`, 1.140712786683659`, 0.2314104630626561`,
0.5770158776914208`, 0.23914023803758194`, 0.14952850154089545`, 3691.778424015968`,
126.97066141512789`, 0.19447768743371685`, 584.268924597396`, 0.25284792691108104`},
{0.051328722971227814`, 2.2089568088739044`, 0.008222968815243804`, 1.0361795422375173`, 0.8781568546599507`,
0.510116616474858`, 0.19565717872054916`, 0.23373733471304647`, 1379.830706691615`,
25.02255129483575`, 0.041815424243916655`, 182.59763233600557`, 0.33942739001683586`},
{0.11927607247345928`, 2.748929565225363`, 0.06035545841420539`, 0.7813641217870153`, 0.22576952890593227`,
0.5831384770942369`, 0.08732270385933963`, 0.1634243179759573`, 3799.726291954219`,

157.2796238233659`, 0.11576601984763729`, 420.4713062563755`, 0.8298533216759214`},
{0.0459615105148177`, 0.71290868377918`, 0.05357476875715964`, 0.9510782333639749`, 0.09416225873000883`,
0.29545951249788127`, 0.15341864047339965`, 0.013205652363295179`, 628.373478034679`,
58.974683376526116`, 0.19388248932889862`, 623.0753968088891`, 0.7014824035335501`},
{0.041699960469811864`, 3.685309567736942`, 0.0024983475914295106`, 1.241106147926668`, 0.23194965201972684`,
0.5723908197871082`, 0.21487240017584658`, 0.009855615104424566`, 570.129465344653`,
356.71107059450856`, 0.21603453424530678`, 316.30136857557005`, 1.2925110176591588`},
{0.04248714289993419`, 1.5523198077435492`, 0.049464612748991804`, 0.8473408690906996`, 0.8858285680999842`,
0.6817894649254013`, 0.20122355649983875`, 0.013635533365215913`, 636.283253618331`,
314.5797931986465`, 0.10344513516887804`, 848.2195062462374`, 0.575701711837691`},
{0.05944325065716416`, 1.885421933855735`, 0.07796792309126112`, 0.8202056467894248`, 0.9730026227590733`,
0.4488135650512052`, 0.14473508644669597`, 0.01128042264569159`, 1713.106109215185`,
294.0613236333512`, 0.17386924429788225`, 821.9836694856166`, 1.459740205985688`},
{0.1768991602445048`, 3.3816133067731506`, 0.023047114587396837`, 1.0651837429933015`, 0.8920250241508236`,
0.5655790113307488`, 0.2121632249886347`, 0.16850604512690834`, 1653.7103845536312`,
103.7948543871903`, 0.21450842811826587`, 575.0341077430492`, 1.3306278343148783`},
{0.20688954673669746`, 1.5420319094946917`, 0.02318214965779122`, 1.1535691189168953`, 0.7713522311993926`,
0.6287128727958715`, 0.11692826993079436`, 0.2409467705896142`, 2091.6424199307103`,
323.56021263407024`, 0.11894699326354469`, 427.17263937490395`, 0.6051683941020936`},
{0.10099740489066411`, 3.428960947150115`, 0.042929674633651146`, 0.9490168791905045`, 0.42904121779075055`,
0.624368140740833`, 0.1435149253333201`, 0.05641817422036388`, 3627.209673974323`,
220.7684874387745`, 0.14314016357909792`, 846.8664463152276`, 1.31297189497734`},
{0.08823806111956839`, 1.7382450985757734`, 0.04561063531492142`, 1.3407883145880533`, 0.6309335894734245`,
0.5914912783213876`, 0.14110534360336235`, 0.30547119743728557`, 2517.5729980151254`,
38.90059714806284`, 0.22979848052462665`, 274.4688663972855`, 0.6700859181925467`},
{0.18301395644765756`, 2.094312123871233`, 0.08931909610543215`, 1.082182555225455`, 0.11440899502078294`,
0.49023558510915777`, 0.06407223467314624`, 0.21909531662234039`, 1107.9461726795425`,
52.077781802277514`, 0.048720222088439624`, 503.797630480376`, 1.0186461011128185`},
{0.06258714579377739`, 2.0725894256763375`, 0.02619673484704304`, 1.3665329983040115`, 0.05917066224715262`,
0.29304519504057835`, 0.21999969054275503`, 0.04607334967195291`, 1619.547974336816`,
350.7234368471078`, 0.05599111671238714`, 682.5248066526665`, 1.2619253051740573`},

```
{0.0821514233789194`, 3.48394675409775`, 0.08036194863535116`, 1.1713772966871077`, 0.23227820295353196`,
  0.16986083803984497`, 0.09693595871976121`, 0.27421366766856714`, 2508.1716674077097`,
  208.85641498812367`, 0.23938632089274536`, 559.4060491059312`, 0.41088627841022407`},
{0.12695625585077036`, 0.9055805406752917`, 0.07136624073326925`, 1.0533863988394403`, 0.2688455845226885`,
  0.6583064256594597`, 0.22869092198316376`, 0.010546436632144591`, 1370.5041602465626`,
  56.87760753172677`, 0.014084785316324344`, 188.37677108656266`, 1.1807915572628231`},
{0.08950639967435958`, 3.127797093993925`, 0.004304878148216176`, 0.9994495038910538`, 0.6607701647707902`,
  0.6677060930551793`, 0.21419196332913165`, 0.09060882425984546`, 904.7767716940707`,
  352.4458159828937`, 0.033444370824501135`, 610.10549985863`, 1.3624403325203964`},
{0.08046882667284722`, 3.1895427332511526`, 0.09332522884303238`, 1.0774096474310326`, 0.8456805674398595`,
  0.2080983211380557`, 0.1489383009609127`, 0.00881812370501745`, 765.3238188441901`,
  44.92494549461372`, 0.17682389813125493`, 217.9987999975758`, 0.24873412254426808`},
{0.13023125778612638`, 3.0617930520558385`, 0.08248596221112592`, 1.2913467592991585`, 0.30796838311543073`,
  0.5430948784062183`, 0.05819933270842659`, 0.0706807516809647`, 3265.5015268092357`,
  307.2503808691964`, 0.1981765891140455`, 877.6961801975868`, 1.226125486370198`},
{0.18926427299639786`, 2.2044151043851157`, 0.0786699581457643`, 0.9100485031870047`, 0.24520532363890601`,
  0.36172535128495464`, 0.1253898270167032`, 0.009282056134193287`, 2072.955568031791`,
  222.29337378468983`, 0.21611928034970213`, 526.401015569487`, 1.092415006408931`},
{0.14404817712733708`, 3.434829434098533`, 0.008441883520571078`, 1.27304850087542`, 0.1574156460229048`,
  0.267867624866853`, 0.22099653559330107`, 0.07614096807454392`, 2595.26366879535`,
  349.2501997001442`, 0.029318640375964433`, 102.51124241937663`, 1.1512883555331084`},
{0.2019360251387704`, 2.468147046811403`, 0.04087788711663661`, 1.0307105123920455`, 0.9335628247977914`,
  0.27959624121200743`, 0.20680629887239194`, 0.005041733529338272`, 1699.7394769038638`,
  207.15056075880227`, 0.12456205306306928`, 294.2891683569076`, 0.45158082182728276`},
{0.11807505670419094`, 3.6982669960702674`, 0.08969669959640002`, 1.0260598684674993`, 0.6730903200693736`,
  0.31494041927113203`, 0.07288613418514214`, 0.00811547347787327`, 2471.949198622903`,
  216.38244182455435`, 0.012347735759937528`, 473.39979695944106`, 0.6376299724873731`},
{0.21379327373675244`, 2.4356205764274987`, 0.02308591924309587`, 1.1769370290426453`, 0.048393911997163475`,
  0.1928171126719566 7`, 0.16529207657122197`, 0.015244159836885984`, 3170.716827973676`,
  324.4969972283328`, 0.23795389565791775`, 897.0462703053165`, 1.4305563244094728`},
{0.16939725274340062`, 1.704386777590262`, 0.09055445850114723`, 1.0583937721501466`, 0.5585380165071265`,
```

0.6597274999874374`, 0.24981293724850306`, 0.010429168410045223`, 1700.9181960570158`,
75.40130766073861`, 0.12215272513035352`, 888.253720852617`, 0.6706216443455744`},
{0.26277862254572243`, 2.5111273948956763`, 0.018424085952828247`, 0.8892523028684387`, 0.13256487083402124`,
0.6547129974987742`, 0.19589869791351094`, 0.005314390746980977 5`, 2233.45199914323`,
346.72371836700427`, 0.2069478132366927`, 534.0501380189867`, 0.6586938015848474`},
{0.06071591835913792`, 1.5344770394964593`, 0.08488857292258317`, 1.16085277269633`, 0.012314997915542136`,
0.1970572753969776`, 0.1815597686617646`, 0.009011864678263348`, 1601.1468131654856`,
93.61760562605889`, 0.19589445883215478`, 155.9873325124464`, 1.3875071308830398`},
{0.19232587353152963`, 1.795714865178108`, 0.05248646647141419`, 1.276771524837172`, 0.4794003449897233`,
0.6282630693132611`, 0.06782898446947824`, 0.006269315415684664`, 1172.9196117695392`,
24.66645837468286`, 0.16462472181311094`, 383.7216664769239`, 1.1123949205179273`},
{0.061322042469276916`, 1.346774816093621`, 0.023755098326839832`, 0.8190780893402302`, 0.8530375648244182`,
0.6734229332311457`, 0.09231001517446433`, 0.018840592481257717`, 2241.9124104356215`,
142.6261265578928`, 0.03627895025754296`, 137.15797558199742`, 0.4762561990314458`},
{0.20284748714232304`, 2.1182728347217266`, 0.07257367834001659`, 1.1214580287717397`, 0.6657596601957971`,
0.6001118400119587`, 0.052319201621775974`, 0.006847463132495197`, 2312.796301550129`,
335.959925090792`, 0.18135866243258514`, 664.2801658787187`, 1.0785445180007343`},
{0.0875325235875436`, 1.8899135155056905`, 0.027310154167180568`, 1.0916038575219362`, 0.9475094753790501`,
0.388669681768962`, 0.15744724906456786`, 0.007000692325086661`, 942.6478265180972`,
234.35897075283265`, 0.10603930431325287`, 669.817984807659`, 0.27807555242626236`},
{0.1683196029405799`, 2.610388685276223`, 0.022446760857976925`, 1.0115269486106233`, 0.7274633946868387`,
0.5555381137003065`, 0.21329639632284214`, 0.11034566775287691`, 3869.8828138625267`,
252.9795244499428`, 0.09058882702972271`, 741.7880319430171`, 0.12417150091727724`},
{0.1304693689913462`, 3.878544887977264`, 0.010025746613633668`, 1.215041004546161`, 0.11429765766642963`,
0.40628812415887794`, 0.06971345696682255`, 0.039454429819743085`, 1608.001351012168`,
41.325173632177496`, 0.19968983632430443`, 595.1317927437989`, 1.01501596817595`},
{0.23953608209480404`, 2.295256494232315`, 0.059959480254970485`, 0.9091672695294781`, 0.16299145371489643`,
0.6267054452627416`, 0.1928279966051275`, 0.007676275168194312`, 3854.303826189119`,
391.47078568539644`, 0.1496326525454318`, 482.8696032318236`, 0.5184649593117545`},
{0.2049276569020297`, 1.569843134258913`, 0.01785397326549715`, 1.2002257155317748`, 0.030566860976887877`,
0.6108783713397288`, 0.22701086353094413`, 0.14087464075209163`, 3366.0585217081443`,

227.22574154179028`, 0.22809591645212163`, 477.4032422395823`, 0.13336101217599405`},
{0.16255786292436286`, 2.8985550610399473`, 0.05441337942038972`, 0.9164164347014612`, 0.8364706687403434`,
0.24404613407823117`, 0.17873477306429308`, 0.02244245203636072`, 576.1215774493044`,
313.8277507549394`, 0.0859246757503036`, 971.3632668842642`, 1.3953479203342374`},
{0.21465893365256106`, 0.7188829728406123`, 0.01243231981652297`, 1.3567309219359105`, 0.33356996007380135`,
0.39527327672477164`, 0.19560443814335465`, 0.007580107017714151`, 1185.1397768181087`,
96.26891368922156`, 0.24342110720025`, 854.361279799415`, 0.6145514030943262`},
{0.06474115211257891`, 1.450233029670005`, 0.011882593331705672`, 1.029493568856084`, 0.33530925461163985`,
0.39646467578552214`, 0.23530293546654102`, 0.12132704244191478`, 612.3108649763481`,
154.10097494122363`, 0.02237955917940654`, 657.0860116279679`, 1.292690105277087`},
{0.2682885379534571`, 1.7178523970472162`, 0.0027281926211814955`, 0.9635327837961776`, 0.8446815644640695`,
0.5827592119201297`, 0.20620718727884557`, 0.007427541178923538`, 3555.433091862509`,
305.26368651008795`, 0.24346518468152906`, 360.19887099622815`, 0.36316340800069447`},
{0.13482653118967497`, 0.7667147511920009`, 0.06409996826329589`, 0.8039414582256676`, 0.5663312860722476`,
0.5579672090711701`, 0.07227231769503673`, 0.03046683225920074`, 3382.97144208772`,
279.0297524590417`, 0.13421880227756655`, 183.57110498357372`, 0.5477994218249109`},
{0.13993018040025812`, 1.068109634718776`, 0.05415775535373877`, 1.1727902476567227`, 0.43298113599666155`,
0.6746158440453967`, 0.08297701187594433`, 0.4384059064333589`, 794.109706649243`,
48.63009083535184`, 0.12421389575223252`, 340.4273269522202`, 0.8230576885672689`},
{0.25019621362492483`, 3.9314857540987758`, 0.028994965154607328`, 1.494980999944956`, 0.8984941885326381`,
0.2707914703557329`, 0.18912153571304913`, 0.39984518730142565`, 3123.9449960141983`,
45.45655083634858`, 0.1469915195667563`, 603.6608411706435`, 0.46874010186610304`},
{0.19847617490577518`, 2.7391911900194934`, 0.07281425929562818`, 0.7823924176107713`, 0.8133119005196725`,
0.36876559170774414`, 0.08048524432855414`, 0.014674062774926`, 2731.5225136110766`,
271.7260678057189`, 0.209208050335906`, 153.71767621509366`, 1.369468138587889`},
{0.1882288487278972`, 2.68377882167474`, 0.013972093662718219`, 1.3753091516437448`, 0.013152963262650363`,
0.6674754952573481`, 0.21260354595781955`, 0.1637730139399825`, 1368.0597579881814`,
87.97160254764486`, 0.01456493337113629`, 201.76949127327077`, 1.4325948017705734`},
{0.15895878747539166`, 3.2892426978978415`, 0.04018352493584093`, 1.043536259421726`, 0.8602258871459734`,
0.18478458098960793`, 0.15137320476739347`, 0.010712136755484629`, 1963.56933289267`,
192.8851946899789`, 0.08139628887153044`, 668.4101880437114`, 1.1842165997662324`},

{0.15124327084346628`, 1.4174139686202176`, 0.086885906049279`, 1.4209445213910399`, 0.19825351175053907`,
0.6013570139408189`, 0.07155844209360457`, 0.006000425159947759`, 2881.1613860024254`,
234.7326263690693`, 0.16648184890501122`, 607.4262567565064`, 0.33628728359292825`},
{0.1576458398600452`, 3.175642747252656`, 0.004395217595715871`, 1.0540148889434962`, 0.7643771427701391`,
0.5381065127181593`, 0.06994145932025692`, 0.005880617018259763`, 898.6669162180524`,
83.67797042387457`, 0.14609763278199983`, 286.7987331621912`, 1.205330382625795`},
{0.16629563986384754`, 2.100958571590546`, 0.017627003762802625`, 0.9402363202437876`, 0.963466078127361`,
0.26777872099985656`, 0.17106948695040214`, 0.05962920226054498`, 2247.5346915803448`,
321.229677189393`, 0.1421366323331762`, 880.0119535113491`, 1.2274047682874625`},
{0.21649106843438393`, 1.642999904879039`, 0.0764702943643911`, 0.8041360501896018`, 0.32900194165160634`,
0.6494366959724371`, 0.23995463391653205`, 0.009054706103175676`, 3799.570031222298`,
367.4767984694778`, 0.2090296810243552`, 901.7331691802276`, 0.33862252189821085`},
{0.16023148675513704`, 1.5874665005890938`, 0.022713779018727464`, 0.7723962850070947`, 0.7584914511404672`,
0.48760614422787285`, 0.05206217237167621`, 0.018014318333328904`, 1127.087244541527`,
363.3185765837975`, 0.18018422508962034`, 755.4447212608605`, 0.7723569881930747`},
{0.19295290634414808`, 1.6743921652546883`, 0.050527180792512304`, 1.3668291018737078`, 0.9725304594734514`,
0.23215309335820766`, 0.13057924179381342`, 0.0065053406814003945`, 3583.5125548453852`,
244.33059798965974`, 0.17209662454772445`, 250.9092595219318`, 1.3511527430421606`},
{0.2535220446470122`, 1.94078502963045`, 0.045010119130647425`, 1.0524636163534082`, 0.5723380281066421`,
0.2632426836131684`, 0.18436530689114122`, 0.006824542562337267`, 2040.8560611260673`,
57.16935279832637`, 0.15871365117602193`, 423.5357760794054`, 0.4032888673499688`},
{0.14106917548004944`, 1.4111346193454857`, 0.06668958606452816`, 1.3853632941553151`, 0.2560062226777571`,
0.41421950826120557`, 0.20735729832157795`, 0.03046562835253217`, 2539.4186525685755`,
232.24532561451088`, 0.2082239924946942`, 729.0483825812547`, 0.5698471937280565`},
{0.19276310010255365`, 0.9460553592690832`, 0.04469676712644795`, 0.9508097051090971`, 0.20681880048978218`,
0.199326519275018`, 0.18871882817483498`, 0.0173110078078557`, 1640.8112077974602`,
140.40973531518557`, 0.23963176956433574`, 322.6954261563856`, 0.24930116269419744`},
{0.214367213040549`, 2.8550240935340305`, 0.09733961023138392`, 1.1310187472108582`, 0.1157319359093334`,
0.658878820958219`, 0.2175085107470872`, 0.3666046643260637`, 886.2408133428294`,
174.81565681280813`, 0.13981930903085987`, 490.5009569739224`, 1.3956423386660108`},
{0.08235436479510316`, 2.7500744906534242`, 0.05205144696340524`, 1.0091743736098246`, 0.8018227353790188`,

0.6634862769661276`, 0.2324846052365857`, 0.19072331103694154`, 1787.2105677992495`,
132.23390012578204`, 0.14176512858162799`, 771.3871520680126`, 0.8490483034075753`},
{0.16534446916605516`, 0.4394021469774003`, 0.007472952564921673`, 1.432910975457304`, 0.3489473101803855`,
0.514195750030394`, 0.19860714212768454`, 0.1002781120611743`, 992.9992041395913`,
339.0906370351388`, 0.22454321777282166`, 952.4559080246348`, 1.1706013334924248`},
{0.2799011130410289`, 1.9427743062232334`, 0.0931591255317306`, 1.2511039419021233`, 0.2834276556528106`,
0.6705418273452337`, 0.06054608170542347`, 0.4634439937005361`, 2924.949862807881`,
293.46460920400125`, 0.06207484018040582`, 194.63074278138777`, 0.25704946086716807`},
{0.046904366313107154`, 3.3676410123906093`, 0.08198411470642239`, 1.28739837533766`, 0.04766654264267167`,
0.22292986195489484`, 0.2382310219278428`, 0.2975605095007606`, 1222.9931622113372`,
323.5243675219401`, 0.07913973133419283`, 632.9944818267026`, 0.8965431924142191`},
{0.1793519013210338`, 3.79991911899058`, 0.05038309784624635`, 1.2522219349866446`, 0.19876178268515088`,
0.5001398090746884`, 0.19853190676822136`, 0.0061119320852519865`, 1009.3441889665614`,
337.1705403878706`, 0.03225605123583991`, 322.5742499604372`, 0.3783065819561162`},
{0.21449584780532938`, 2.02618697774013`, 0.09815924536927831`, 1.199185099638896`, 0.6773751390977416`,
0.3981434210226825`, 0.2403499457472954`, 0.03942102687941344`, 2984.052694528219`,
98.02272932814492`, 0.22732752043860166`, 537.8562292856776`, 0.6042553960644044`},
{0.25114702541297323`, 0.937656791418509`, 0.01657525933924745`, 0.9431642919586363`, 0.05079394682556293`,
0.19182378975846615`, 0.08346488243454997`, 0.05268453809726009`, 3724.8164163388883`,
197.49864461513357`, 0.17840261866020518`, 421.3962540881912`, 1.496091577855418`},
{0.13072217304047706`, 3.6947985059562924`, 0.0390656831799542`, 1.2809807038070122`, 0.2731833971270681`,
0.4107039594124198`, 0.09185840248486382`, 0.03806090866464301`, 2005.1488340788264`,
83.32706515594339`, 0.21163600041699526`, 498.4711432942616`, 0.8178390980003436`},
{0.24205926533524386`, 1.328055389202607`, 0.02327440139060226`, 1.3808700415025879`, 0.4506945244840761`,
0.17328546967798097`, 0.10141678818222832`, 0.06965940959932373`, 1687.183981368078`,
235.8440413165166`, 0.059961745345995576`, 386.499319080281`, 0.5181088176903803`},
{0.08721442242369265`, 3.5171147224186257`, 0.005763083465305083`, 1.0932552668633062`, 0.6105527358219478`,
0.6081825530322815`, 0.23393208141681016`, 0.07072473014339693`, 1870.5451604169084`,
162.0338260203672`, 0.0425117945021809`, 122.7947800833288`, 0.9547129756979542`},
{0.11666921571941458`, 3.403960361409056`, 0.07023671001506276`, 1.4138556197846848`, 0.6402859773939595`,
0.5473417950090529`, 0.21935634026822765`, 0.21057613850603782`, 1030.9406946316417`,

```
  44.64461322353708`, 0.06622728247466253`, 921.7989627661123`, 0.5856729337597959`},
 {0.12052224806819772`, 2.1859059713021347`, 0.0825603630236072`, 1.1899431467623498`, 0.9290479641044416`,
  0.606806779992755`, 0.1535294746607575`, 0.012262721471671982`, 531.3257310153786`,
  74.96912508440238`, 0.2359957691241606`, 248.61353150189984`, 0.33300904443154655`},
 {0.05000635757691613`, 0.5942918545802862`, 0.016428498876090884`, 1.3698044550524862`, 0.48286655015562263`,
  0.2465497415969773`, 0.17716865417176203`, 0.026787989137474672`, 1323.4894671376014`,
  264.0791628485521`, 0.2372467819820393`, 917.4565619849908`, 0.8992603400161796`},
 {0.2055655720712738`, 2.0620424182981267`, 0.0525464389432920 1`, 0.79919347070743`, 0.2645816965112293`,
  0.6196338453192136`, 0.07084646532156291`, 0.008837949713102242`, 2521.523327327026`,
  43.90197972831635`, 0.018289313043999084`, 707.5217270886153`, 0.8091579245076588`},
 {0.15746452557644974`, 1.5644535452235493`, 0.05463699718150849 5`, 1.284737526295929`, 0.732730603834779`,
  0.6525735613164507`, 0.13843510246727037`, 0.005550406546213277`, 1303.1733483391`,
  237.6854625822482`, 0.0524817172360809 7`, 508.1884183252752`, 0.48521892735022254`},
 {0.13429794970835918`, 2.8397309358751333`, 0.032896451821273605`, 1.2286658089643048`, 0.549054642783976`,
  0.30082933211865237`, 0.2084797368797613`, 0.3291428681159996`, 626.4243260382923`,
  116.22455976139463`, 0.11089105437552388`, 568.5188353841722`, 0.6425564216320787`},
 {0.083323377302916`, 1.6071812650562443`, 0.06496124525357874`, 1.4515556814313988`, 0.0018586520847039267`,
  0.34689546766601886`, 0.16350843033593565`, 0.20854467743130406`, 1857.1723991389872`,
  204.6758535417963`, 0.013660804731299298`, 130.86684079851273`, 0.6429467481903259`},
 {0.2541973370857226`, 1.059652703500329`, 0.02813642069983322`, 1.358381082150844`, 0.9943388842711929`,
  0.6409121766817665`, 0.2315138780928253`, 0.016119845259862913`, 3035.5494191312637`,
  388.0116892361733`, 0.16353487999919275`, 992.6949273065819`, 0.42749656382049217`},
 {0.12694832669943912`, 3.9909308964771038`, 0.08736969035407079`, 0.7679433585939324`, 0.6211296076517474`,
  0.6707444280465436`, 0.18030809900164502`, 0.027585373279948283`, 2330.998702349789`,
  229.47673941680046`, 0.055648252322398006`, 300.2827018876246`, 0.8155157915599447`},
 {0.06701179206935765`, 3.1402345998710137`, 0.028679015139681213`, 0.9724946079554642`, 0.4390581371257649`,
  0.6663961791027206`, 0.18389066756590433`, 0.016057196091602898`, 2162.6157151948482`,
  290.19041740098066`, 0.13574751513776562`, 508.30721447397417`, 1.378273199277404`},
 {0.2551478726592332`, 0.9710991048496993`, 0.0502782066307244`, 1.3759271828004023`, 0.04597053923079142`,
  0.5137530485657317`, 0.2163260048798143`, 0.005382450868157514`, 935.1168316155349`,
  294.2393547847511`, 0.06973594035716213`, 637.6312365701453`, 0.21562633084527905`},
```

```
{0.04825767890451898`, 3.0997378012337604`, 0.04725403789237719`, 1.4652492583832208`, 0.3655051085575758`,
  0.32427093426556386`, 0.22534086262026876`, 0.17716451921209012`, 3929.1236383937567`,
  185.5825568895683`, 0.06300473176508586`, 634.1083033306166`, 0.20247901493103915`},
{0.1698758724224145`, 0.43231144259603127`, 0.002409187141779281`, 0.9162019651756379`, 0.822227858745906`,
  0.2391813610919995`, 0.05176655466468527`, 0.42746797016977933`, 3974.021638163792`,
  108.66633602052758`, 0.22882422273192393`, 741.3278135128533`, 0.9018865049056357`},
{0.04660475583469445`, 2.3855216774760954`, 0.056897014703218236`, 0.8912545650013179`, 0.9756349874685741`,
  0.6123093487163376`, 0.20422585918719693`, 0.016798428134429014`, 1781.884916444099`,
  202.76047976009897`, 0.12566417336300922`, 750.1608446702237`, 0.4875771496585619`},
{0.06101525389417878`, 1.4858763807694029`, 0.004281304768480041`, 1.2961605209852705`, 0.2795918934896324`,
  0.3250617967882581`, 0.09201788875836953`, 0.008265630650431816`, 684.838324591567`,
  273.6718180911174`, 0.20864513891362757`, 424.90286856021095`, 0.6144091102688116`},
{0.2114266557127773`, 3.9510767790041346`, 0.04955949580037189`, 0.8796510262737004`, 0.7245803628691674`,
  0.2034219604427765`, 0.14817629501942436`, 0.1308358930575406`, 1884.03227418395`,
  115.34989915938382`, 0.12548919180203416`, 458.9171943736833`, 1.2601422668751887`},
{0.25876582213338`, 1.7913786184559761`, 0.011001101731588712`, 0.9650444872020401`, 0.32140872524621455`,
  0.34059129995706905`, 0.19470083208722305`, 0.006844495461692708`, 3762.299116572869`,
  380.72736491787214`, 0.20525756765393416`, 214.40624780816665`, 0.3466113002989706`},
{0.27288935760198135`, 3.6868906104982395`, 0.09367295953911044`, 1.1960739739092252`, 0.5641013299613014`,
  0.6123502409113553`, 0.06802741681481622`, 0.29275015082960376`, 1430.171308765005`,
  255.97728417501946`, 0.1353380260804738`, 868.0107492040826`, 0.5771652706916472`},
{0.05594504491086682 6`, 2.1796171720551456`, 0.017082325431853018`, 1.498202820965981`, 0.1563620665310197`,
  0.29252823150447926`, 0.1092310684629964`, 0.16611284267001641`, 3665.2677617559457`,
  335.4207385817559`, 0.0477423160634477`, 743.4127289601747`, 0.5070732684644526`},
{0.23772330215861104`, 3.0851011770644803`, 0.08472956433878671`, 1.3229388882031805`, 0.005960160138338555`,
  0.1969419905339791`, 0.057048637960360554`, 0.25066884731535677`, 1535.9921025203785`,
  94.0792154218787`, 0.13672779802451274`, 384.0284417915331`, 0.6623107015701892`},
{0.1265405926702166`, 2.0280961897653746`, 0.01858613984823812`, 1.4995550564273865`, 0.1817737571651301`,
  0.6189216862120657`, 0.099008949988757`, 0.38464022260866426`, 3025.5338291582675`,
  307.1040113041719`, 0.16858454334032053`, 991.5309397422028`, 0.48961810090644686`},
{0.19723522755077538`, 0.5115046849320883`, 0.09151685418462237`, 0.8852342302160248`, 0.7816521461923478`,
```

0.5142650038939364`, 0.1517127726049825 6`, 0.032012599995280215`, 2211.905098555595`,
347.4582850699088`, 0.03992309967667379`, 216.76777190034107`, 1.2737480763900084`},
{0.22013625727212333`, 3.0836415525004277`, 0.044513221040964446`, 0.7585693520182641`, 0.8969064860978653`,
0.1656825405042367`, 0.18822199973974063`, 0.06691736404999271`, 3917.5498531852427`,
151.53623705292762`, 0.019182875619929513`, 884.6870086975908`, 1.2149241337350047`},
{0.04990295227896396`, 3.287718861968469`, 0.015321167824674084`, 1.2579596789375718`, 0.3578901479711274`,
0.20122321075161909`, 0.09420130968723223`, 0.046425521640662576`, 1604.8487981325925`,
236.07244037306964`, 0.23470256214534602`, 396.9561498244922`, 0.17534809527440665`},
{0.04505461266119529`, 3.9121314943722654`, 0.05417989573741018`, 1.3212460484453303`, 0.07910763162436996`,
0.3021999801273072`, 0.17686908949574404`, 0.37965138855994596`, 1050.7598687909544`,
252.49697896657278`, 0.06284129003676986`, 198.2088277330142`, 0.8023977830447109`},
{0.253543688032733`, 0.6212935455063744`, 0.0056714907542881575`, 0.9133383768427127`, 0.3733612701371345`,
0.47234247648396743`, 0.1624817092864591`, 0.037530881033610755`, 2012.7028580629776`,
318.07903716953786`, 0.17919210216351872`, 522.7892460974715`, 0.12925094345920107`},
{0.09144904658452618`, 2.8422457636855416`, 0.09254061586043004`, 1.311816831266331`, 0.995039390694886`,
0.2603311881764725`, 0.1829345435312656`, 0.020882966214961527`, 1970.8884320862662`,
226.5578980017866`, 0.053904058430067014`, 272.9098019142313`, 0.9162552477096924`},
{0.2662505830175855`, 2.1360912407660955`, 0.09465014849159573`, 0.9897432528529452`, 0.8565117410457146`,
0.47014250403951685`, 0.14357629162457725`, 0.08740999739230045`, 1468.3302820747294`,
172.47227765663683`, 0.04073835904318762`, 693.3935559697125`, 0.4887486514820367`},
{0.1592534919550468`, 1.0602809656697758`, 0.007242387790533425`, 0.9757707290584382`, 0.9420802481437847`,
0.4275135788115605`, 0.07774419101684499`, 0.048710161823605276`, 773.9449969094794`,
283.01334246478496`, 0.18041897531853707`, 758.3007442810434`, 0.30937239228422553`},
{0.2261024582304404`, 1.4647123602415633`, 0.0413326992928363`, 1.1137902572114895`, 0.7842433302533265`,
0.6835941051890313`, 0.1477212605527568`, 0.04833709227345784`, 3279.9047777282995`,
303.39493301919686`, 0.07092250334616551`, 301.3932243620919`, 1.2412502906105978`},
{0.1927002578828958`, 3.4319676543608937`, 0.0015006893541665712`, 1.293980421423588`, 0.030376015480821472`,
0.37598862971795843`, 0.21135657050395712`, 0.238703482802058`, 1866.9171759939145`,
339.36466688227597`, 0.19328733632865874`, 771.7553953263039`, 1.039449037583735`},
{0.27919536258398936`, 3.051301487514108`, 0.08192425799391025`, 0.8082104089659077`, 0.24067118654408226`,
0.2824681871862217`, 0.07319200257625871`, 0.31638488754466454`, 913.3093194468702`,

```
  37.86490845076713`, 0.08360043181938548`, 110.79582857254877`, 0.5690367506628553`},
 {0.25067638618328086`, 1.8058646518958632`, 0.0967058272966451`, 0.8287710803215935`, 0.0557673573651718`,
  0.41160216930056925`, 0.1262674554654139`, 0.00908472185395493`, 2114.9134257702635`,
  42.170104655757825`, 0.04993759328062314`, 987.7508811085783`, 0.3660947317126473`},
 {0.20280563799966256`, 2.170449398415938`, 0.08903457092675997`, 0.7702147043135429`, 0.7717585892579155`,
  0.21757068756076314`, 0.16840693839930398`, 0.4915615398858017`, 1255.793920152315`,
  182.76330350495914`, 0.11099408275148614`, 339.4266703294745`, 1.061882429556499`},
 {0.04023578648795795`, 3.273707983807552`, 0.03955467525616028`, 0.9702950801066533`, 0.029438949864620145`,
  0.33528685061470376`, 0.08732366401888822`, 0.371840089245749`, 544.4224854571298`,
  72.2019719679943`, 0.15067629300973023`, 474.65260453385537`, 1.1087090962284143`},
 {0.09957487782476543`, 3.210506657187307`, 0.08542676152501041`, 1.0797421686761655`, 0.3265457205797211`,
  0.6518494493489448`, 0.08353986333150965`, 0.018408232185589193`, 1931.1793189838627`,
  136.44435044845414`, 0.14008057000914625`, 895.6427708703583`, 1.439905266685154`},
 {0.15335825161328226`, 1.6569191653476727`, 0.004594308480296725`, 1.0254890198096218`, 0.15484705027123336`,
  0.20252889672592156`, 0.19554959531372568`, 0.22509744241743374`, 2035.8770692870276`,
  50.56992386118708`, 0.1744111980623378`, 994.0326590715831`, 0.1726840478076943`},
 {0.07108782063333186`, 2.3378063321638898`, 0.02778511825143033`, 0.8210529259634597`, 0.40321192995442723`,
  0.5037627791090605`, 0.13008627675772605`, 0.22754577917693372`, 3969.553010296944`,
  387.1830947735623`, 0.08272258121594372`, 483.5387417707407`, 0.40085673814097467`},
 {0.25413386280928835`, 2.3687859393975863`, 0.09429627414104236`, 1.4650074734538618`, 0.42879990732973416`,
  0.1601166258544381`, 0.10441473638394894`, 0.1122487054463002`, 792.9231425385933`,
  319.01627570616427`, 0.220123695294615`, 629.1978029642925`, 1.0229488956382289`},
 {0.20423398159922745`, 1.7043433000703967`, 0.09815735393861971`, 0.8078800927020084`, 0.12146100624318734`,
  0.5482980695676744`, 0.2078334357067212`, 0.01579498437692769`, 3768.8216846988653`,
  135.36495166756907`, 0.21138066170339564`, 936.1846599403652`, 1.098780795897763`},
 {0.22043472255825064`, 1.9082177547958645`, 0.08765904186240878`, 1.3175260999559186`, 0.16416016341675066`,
  0.35700594738419944`, 0.16596780118940468`, 0.22629163202805042`, 3107.4496580418418`,
  298.84699964961226`, 0.17965868903167787`, 998.2763603956375`, 0.9169210069510552`},
 {0.17164803196261041`, 3.7963591226151854`, 0.010515743513855806`, 1.225912705397515`, 0.7138736730174926`,
  0.5786295813848837`, 0.1651616868046183`, 0.21436545354379566`, 3331.6547510344944`,
  175.39298944131383`, 0.07947843909448465`, 352.7620949739909`, 0.8583056103861026`},
```

{0.14720878775659768`, 1.886295870436359`, 0.07118845852578286`, 1.0013909835719`, 0.33545002848688776`,
0.5106247643702192`, 0.205659315447689`, 0.13454033872255697`, 952.5967538756531`,
283.3679118848729`, 0.20361595373953167`, 457.4999710669589`, 1.4438191416840445`},
{0.22444226386071825`, 1.932705332772655`, 0.025958484111454416`, 0.9760598096887098`, 0.08906396387301618`,
0.4075766651350454`, 0.10211908837029643`, 0.018483903618285842`, 889.4299688241804`,
140.9680866013084`, 0.1561853144922712`, 131.6038358972806`, 0.7564520246842394`},
{0.05738079595216994`, 0.838221889562937`, 0.0830896911590868`, 0.768281868451993`, 0.8227065979664494`,
0.2581871068601813`, 0.14376704023099218`, 0.010236230590069182`, 3518.8225156903463`,
327.18055983790043`, 0.1075504816417247`, 505.5671506121936`, 0.6295022332903608`},
{0.06462462270429015`, 2.12781949412771`, 0.01470095808159531`, 1.4060576196231425`, 0.7556365878023952`,
0.6166131760403777`, 0.22642887077514828`, 0.008297744085251716`, 977.557223698087`,
252.32932728865592`, 0.0695623790828252`, 923.7569772206889`, 1.205231128740317`},
{0.15310044070189893`, 3.292549648182284`, 0.07395039189708363`, 0.8145812262363505`, 0.8268155309484735`,
0.6895147061518268`, 0.14286943772614014`, 0.005330292153155717`, 983.4596543080652`,
119.32129447754829`, 0.025714447103420413`, 865.6456959821181`, 0.35119487290449736`},
{0.2553565633191542`, 0.4852766539597013`, 0.039298992653587654`, 1.4954849186586032`, 0.6707975889995446`,
0.48435267987553765`, 0.13847313803513883`, 0.014881431295667331`, 1017.0627921600253`,
381.815007677293`, 0.05173590583345733`, 711.4101586997037`, 0.9759965026441928`},
{0.08289519215290081`, 1.0609125218807032`, 0.09007483638557279`, 0.981508856314536`, 0.6610987974406086`,
0.24420188998206283`, 0.17425985579431763`, 0.03686872574133253`, 1551.6188909943276`,
358.0694811689174`, 0.017779169522654625`, 158.83186255451955`, 0.8495721159520282`},
{0.1294604175134954`, 1.292721032112544`, 0.09389791542367519`, 1.344762561570708`, 0.31323487103500214`,
0.6064404065198115`, 0.08775709065257703`, 0.035137707022568715`, 2159.099276841379`,
218.3195423957203`, 0.14616575449161084`, 206.86411074670926`, 1.4428316510696142`},
{0.21498871151315552`, 3.907170852024813`, 0.08435970233792822`, 1.2500208945993787`, 0.10273638815842046`,
0.18371302977537207`, 0.23542514842600448`, 0.40538184702673075`, 3766.4900595213985`,
236.30715576447142`, 0.23687330465657053`, 950.0610567890757`, 1.2167521681615692`},
{0.24914943062618045`, 3.005946431898553`, 0.049783763294016355`, 0.9229662997644423`, 0.8600773268419926`,
0.2663020406921677`, 0.07794164504084955`, 0.028208424701322544`, 2845.792514350296`,
95.07677463443162`, 0.22121458272450006`, 947.7608782127451`, 0.12593704599618816`},
{0.18806940419866192`, 2.153252025400028`, 0.01971140551745492`, 0.9447143753143707`, 0.6059631192633228`,

0.42997123041690943`, 0.1644736568564774`, 0.02534710230326659`, 741.1039498095388`,
373.71340174575187`, 0.22177860547925293`, 460.7102222572536`, 0.37864796640028575`},
{0.19271366677108503`, 2.333600217592526`, 0.05327029790085312`, 0.8074380085002821`, 0.45647958152228063`,
0.6352733257828436`, 0.17986160851896765`, 0.02001568781909359`, 3441.271831347881`,
165.33775121358667`, 0.24110463310064223`, 241.33443206076404`, 1.4852567665341567`},
{0.27395745685850337`, 1.4702736974926776`, 0.02523565636445774`, 0.8868230141825463`, 0.03287286718932947`,
0.5681911729812511`, 0.23462147101272757`, 0.08726475038388391`, 1157.5731579040548`,
62.8501123131262`, 0.16009532420859435`, 628.061822667461`, 1.428585504058412`},
{0.2535045089543946`, 2.971422356142389`, 0.05681320075005312`, 1.234499186723072`, 0.8102628118146575`,
0.6630684168330143`, 0.0843225776488441`, 0.42981264987661166`, 3615.9984687792085`,
171.33827256677534`, 0.07239394734362886`, 474.8577641995018`, 0.4969541838396545`},
{0.26680978483065515`, 3.753692630660738`, 0.0727183187872879`, 0.9443215667858356`, 0.1472888240484238`,
0.2852597162823993`, 0.19383248243003076`, 0.1119861674763849`, 3816.2889008611055`,
369.4399368079453`, 0.23325943204963862`, 502.93592854646613`, 0.30635569919383254`},
{0.0831668174354972`, 3.202396827511703`, 0.01943252669011601`, 1.0264469849572777`, 0.6450702462590181`,
0.37507347971667615`, 0.19857225859965633`, 0.014377612102999638`, 3915.697201216317`,
148.9303720251349`, 0.0959480274061818`, 597.0603177349946`, 1.0235360159986202`},
{0.1761184580941187`, 1.0880606012202536`, 0.056035413143063394`, 0.9133031965291323`, 0.6553369682459182`,
0.18147108567866932`, 0.22793475723716022`, 0.050442445436625453`, 2269.6335579336182`,
340.7196289964704`, 0.16497191830499675`, 996.411637505058`, 0.6490520504204338`},
{0.19180913858025628`, 0.46576442091479153`, 0.09113165038154353`, 0.9682223438671578`, 0.6850638819650454`,
0.2967682908372965`, 0.22756086094605277`, 0.2414449867798 6875`, 3015.4965257592585`,
80.71914021386806`, 0.23250406382137212`, 660.2115068347056`, 1.0521918676066413`},
{0.1327009994934153`, 0.5691124201803208`, 0.08083755441414134`, 1.2106458970258838`, 0.8969101543918885`,
0.30665628588088056`, 0.10204743942971378`, 0.12587566174763262`, 1808.6765638244442`,
360.1100204597151`, 0.11342721366807024`, 531.5749878133441`, 1.4143000405747657`},
{0.09096675408381416`, 1.617429791388009`, 0.021959269593061777`, 1.0499582680829807`, 0.44344364311571494`,
0.1636319827439292`, 0.058805897319389316`, 0.015383944267490367`, 3363.4536198312953`,
82.82063566141363`, 0.045134833345098435`, 272.45601049285995`, 0.9795277603779553`},
{0.04871290768419401`, 2.303270966264968`, 0.04917429964582876`, 1.2514848643355008`, 0.4139364608290057`,
0.26416554854680063`, 0.1466649862599524 5`, 0.008702282623435703`, 2710.289073206067`,

```
165.27247317882438`, 0.2479455063256154`, 449.2859559731945`, 1.0627869410308968`},
{0.10726217183005304`, 2.8659630286429767`, 0.05837581233373151`, 0.9321211035972367`, 0.8093349795541211`,
 0.4265641896687735`, 0.08420892689622417`, 0.1509093384785956`, 1734.826333986327`,
 49.534695979129424`, 0.056496227308578784`, 346.53654899414494`, 0.8911980914503808`},
{0.19459644061234482`, 2.8839426568558233`, 0.04573240729455296`, 1.1531783998190988`, 0.9593481726431483`,
 0.6554940707062837`, 0.18680284013665382`, 0.11183732587493084`, 1905.590340808838`,
 85.87882895446239`, 0.15757969746577954`, 929.3802349785484`, 0.28538221574821887`},
{0.04181485421993228`, 3.9603428763376254`, 0.0925276216776887 6`, 0.786809091452586 9`, 0.8309570427208837`,
 0.3567422429005209`, 0.24881357800860981`, 0.037364994409719854`, 2485.6171983851864`,
 179.4502782786251`, 0.10287303709932949`, 321.29896914471897`, 0.16326302864721187`},
{0.21278416042953557`, 1.00093561648755`, 0.09199784378572812`, 1.2638064310407802`, 0.456013854987239`,
 0.4517808871182415`, 0.12526924845738036`, 0.1469656318045671`, 3840.1462518105336`,
 60.51362472301514`, 0.10205000436163664`, 109.41300786603065`, 0.9741360996683417`},
{0.26984436049680055`, 2.864702857686769`, 0.03126989223638253`, 1.0210333783430154`, 0.6324349427669569`,
 0.4018935710383199`, 0.11774752235151603`, 0.09476733200960193`, 1018.0428378206293`,
 399.86188390225027`, 0.017647024801586753`, 731.8612442279936`, 0.41421396907720975`},
{0.23935972948044087`, 2.5381964736540885`, 0.07038086738769866`, 1.0952226344883353`, 0.42436102076201676`,
 0.17068516965401004`, 0.08490089576078583`, 0.47391516626034874`, 1644.3725470342197`,
 89.91841083916285`, 0.18824668200329076`, 618.9686544088639`, 0.6238588065096458`},
{0.2614200113031115`, 2.313437098854811`, 0.07488478430908806`, 1.3832897312094579`, 0.7283181993799872`,
 0.4528815613995919`, 0.13570146007510925`, 0.3589686491912747`, 654.9604933558062`,
 252.3341131285125`, 0.17054218234721713`, 588.6190190687697`, 0.8967543741687383`},
{0.08869420771566178`, 3.152863463145354`, 0.0858870052279904`, 1.2133807791849687`, 0.7760132172555274`,
 0.45493367629976955`, 0.16073453944235905`, 0.052470605503318814`, 1744.4405424035513`,
 371.93933122912324`, 0.13507595399837985`, 958.2542953294002`, 0.2723176988644096`},
{0.11709374740411904`, 2.45035218779289`, 0.020760935610916357`, 1.329337624714733`, 0.3201664343997752`,
 0.6240254857335847`, 0.22283064854574797`, 0.22208200243300433`, 3170.167918261848`,
 164.8114615746979`, 0.08294234934097972`, 666.3205975970773`, 1.0593645948163495`},
{0.18522830328431206`, 0.5883198346630394`, 0.019565794723225487`, 1.023693028654033`, 0.3896270564713873`,
 0.45889050818569543`, 0.22713026776570683`, 0.00919982303844724`, 1841.233266766917`,
 122.89348776080823`, 0.19082938107230524`, 495.3605505762605`, 1.317502982849899`},
```

{0.21230665184513414`, 3.2419047452471714`, 0.039601676444948664`, 1.4171967944286763`, 0.5083150713318949`,
0.6264608542822363`, 0.09271177860298965`, 0.005911865310459946`, 3910.5469739831724`,
316.55202184722236`, 0.16793978180994706`, 931.2094944072218`, 0.8708955190379366`},
{0.07551733133991706`, 2.843113736310138`, 0.0614720793793248`, 0.7825848859720709`, 0.753911134511744`,
0.5301051123908531`, 0.1470264431848624`, 0.011463249749326136`, 1910.9939980619774`,
136.3178521015276`, 0.07846586871729211`, 605.3673271133318`, 1.3084219498355365`},
{0.2658880592721208`, 2.589498619431751`, 0.06516087534117801`, 0.7758907664154213`, 0.504824325913263`,
0.22079686939730403`, 0.177706814995653`, 0.039261837542442`, 2398.3618390656766`,
242.25270765488256`, 0.1383332945193519`, 758.6481550412612`, 0.9463395383920883`},
{0.06887366412945373`, 1.2421956952121516`, 0.03253271308652717`, 1.2748787757100968`, 0.9578353592678488`,
0.44371299797785235`, 0.21837633046380062`, 0.17183051803296134`, 3966.7023303967035`,
392.27839739096396`, 0.056535182358574354`, 637.67185561579`, 0.3005366771454052`},
{0.19932158075601353`, 0.4521519349416465`, 0.0383460083718602`, 1.3640765288960315`, 0.03347160674537797`,
0.4087237326483594`, 0.08226474410564774`, 0.06028893375686413`, 582.8537545471304`,
197.92620375100046`, 0.13965374207522357`, 887.1314139499823`, 0.5949015879226918`},
{0.24918529198520062`, 0.4393468753599614`, 0.03567046175854587`, 1.125868660695242`, 0.8151418009574027`,
0.3339933056421456`, 0.23040412134982702`, 0.06526803883154646`, 3315.148247857058`,
160.21008066129787`, 0.24355166257564564`, 250.64393737739596`, 1.0418856340816274`},
{0.10828692823259789`, 2.0131192710851664`, 0.09684409481585946`, 1.3688065216924983`, 0.29083855972294503`,
0.692021178461353`, 0.11016223469300951`, 0.07494246253210811`, 1847.9903596713293`,
150.9099240355182`, 0.11874828554507061`, 137.68820370064674`, 0.9807227597820849`},
{0.2157552798021256`, 3.4246276265628692`, 0.016346803340960926`, 1.4263250372930059`, 0.03576034189550903`,
0.6751019070004078`, 0.06784659551328931`, 0.01886806104905562`, 1760.570907838951`,
249.8448620976094`, 0.04413438567228828`, 635.616014912297`, 0.38044802523323806`},
{0.1034926507865987`, 3.6029810186406976`, 0.07026547904038093`, 1.1580843341848976`, 0.8972849001632752`,
0.3648275054646687`, 0.14990936813690808`, 0.05874430932646101`, 1808.6962719844605`,
309.2043267183793`, 0.11735186728685665`, 743.6581170182778`, 0.8225101717350425`},
{0.19211087552008244`, 1.7217332265793912`, 0.04381461857079728`, 1.137554571583389`, 0.9112812261712655`,
0.5752303646722726`, 0.18334554856806273`, 0.06904251568645374`, 1558.9115074230922`,
316.6799040011855`, 0.053593168580480466`, 878.0684257857686`, 1.0887871634772481`},
{0.21816588781232243`, 2.626691755041903`, 0.02892639211395599`, 1.0392235959095848`, 0.12801121433755847`,

0.6306389281813851`, 0.2316966814305887`, 0.015510833883817432`, 967.341586064023`,
262.64302987012104`, 0.0675458107238735`, 956.8633692424413`, 0.1937891037769437`},
{0.0995733920497558`, 2.9481594951784498`, 0.03243515004975547`, 1.2521617479500935`, 0.6991862071266612`,
0.5499894406540011`, 0.2183790697300324`, 0.011007191077888412`, 1662.4943985861892`,
297.3376985062954`, 0.16809390353412246`, 238.48560390262145`, 0.5126845907210813`},
{0.2312105097961264`, 1.7081001769816204`, 0.04432263702800036`, 1.4476341606622412`, 0.6701171613688006`,
0.654758182156157`, 0.24595939785661447`, 0.38416569410089263`, 1903.2549784079793`,
159.86869313207023`, 0.11974677931426836`, 883.967611763973`, 0.358654356132899`},
{0.0585717034355423`, 0.4835243907679039`, 0.04546478598573502`, 0.8818411506370951`, 0.6976613591625698`,
0.19919853669539989`, 0.23928966869207724`, 0.03865767298041177`, 2715.6246721981024`,
204.22160499218182`, 0.2099546011205627`, 570.681988730437`, 0.6004012450066176`},
{0.1723862419015113`, 1.8928060256780403`, 0.05298967214109956`, 0.9412055443353899`, 0.5502673874985677`,
0.24299530894245747`, 0.12838114854932603`, 0.0343351450578303`, 3450.341201430593`,
112.91217594634026`, 0.07378162659094861`, 325.6726820197704`, 1.127366874406185`},
{0.0574661976037385`, 3.3236481202539983`, 0.025610779836561744`, 1.4053111254550474`, 0.3713522524171269`,
0.34333892840473035`, 0.22358983160593737`, 0.05987380432449144`, 3113.3879175080965`,
91.7427421029817`, 0.08515699097126012`, 779.4439557053674`, 1.3021534134824582`},
{0.15644254226044835`, 2.904822998267117`, 0.033286495503675496`, 0.9889340723971365`, 0.22262261036978903`,
0.39221982525214016`, 0.2305607458035509`, 0.1650221174142012`, 2966.3545380818796`,
391.0053982344714`, 0.20713963115671252`, 333.6034170157532`, 0.44492733930504613`},
{0.2539183817572606`, 2.6030580156501806`, 0.06328337578833751`, 1.3620447628355437`, 0.8353471933708569`,
0.32142623870011033`, 0.23840936405273394`, 0.01752691148331343`, 3972.2457291523033`,
42.80037625415753`, 0.016803857959632407`, 904.4400053243962`, 0.9562777329819383`},
{0.15271984239218678`, 1.6021991673869955`, 0.04008054421934018`, 1.4000425441579867`, 0.09748284038847976`,
0.20173946232407625`, 0.22075354418195442`, 0.030862041221293046`, 1388.8297805268667`,
238.79874816898928`, 0.01796740516715775`, 358.48526317792613`, 0.8448467366664789`},
{0.232351439804047`, 3.3272867583583876`, 0.011562598529594155`, 1.1348281518037249`, 0.8419381151476175`,
0.4029550743524166`, 0.20806510385376886`, 0.28748588253620927`, 807.8660171600686`,
109.09980191672719`, 0.23010513232054303`, 682.5505365107897`, 0.8627607283448464`},
{0.09625012094475777`, 3.116977597619565`, 0.017106519598542872`, 1.207630153999634`, 0.30599260627033353`,
0.6066404338501987`, 0.22690921468244707`, 0.1593550602164506`, 3523.747223837997`,

38.877392472027225`, 0.04048036375447872`, 875.4605974335324`, 1.4375148517361915`},
{0.22649777459941334`, 3.1340345685408675`, 0.04776498170234476`, 1.1510633521098381`, 0.44536866343993675`,
0.5630028887964033`, 0.14192500455265017`, 0.18948727513923766`, 1065.6299504242902`,
155.38553555932117`, 0.12321539349733784`, 776.5011200637042`, 1.2629601996464541`},
{0.06379914937598441`, 0.9073978730635979`, 0.09890654314321375`, 1.1808364000799703`, 0.9929413050184941`,
0.5685402007387017`, 0.15001647853628997`, 0.09940844038806868`, 1827.553222775543`,
98.41096311058413`, 0.18497146191852015`, 994.3704081824361`, 0.9700313130352076`},
{0.10820412743998448`, 2.8366008329426755`, 0.005893852941580722`, 1.2554045241636351`, 0.5447542567848354`,
0.2701750757499882`, 0.12287149444164497`, 0.4999937327999362`, 3191.730418133502`,
394.7987660065088`, 0.033231627960763954`, 626.9707268127556`, 0.9973585143734787`},
{0.13923441833638273`, 2.1246530891886177`, 0.008275180483557864`, 1.3916896084398624`, 0.8952405517164088`,
0.41136032766533337`, 0.09539692051433327`, 0.005949637655091856`, 2740.016315284098`,
28.470886988302425`, 0.14197612010553723`, 552.0302810775945`, 1.1013578184328043`},
{0.20310721220279843`, 1.327363489445358`, 0.018096995853178886`, 0.9967778756558304`, 0.8018609140722133`,
0.5893307073801274`, 0.07216611763138306`, 0.07828688522893962`, 3889.1382115508313`,
274.22056364275807`, 0.19350724596590868`, 969.2656029384289`, 0.5876263180430124`},
{0.06275464440627121`, 1.277574105625555`, 0.01347582193530167`, 1.0457495003670847`, 0.09154461891678212`,
0.29084690943080904`, 0.2148582342459503`, 0.007325723145254006`, 843.8431912081396`,
97.16323627162507`, 0.024104919550110515`, 623.6355511981506`, 0.14755011408917773`},
{0.12359515481358774`, 2.0305790499090204`, 0.06708472304949722`, 0.9057233291174347`, 0.22960130018181424`,
0.6285607599838292`, 0.08817828601741587`, 0.4748546331077217`, 3260.431131614655`,
179.43068603520248`, 0.1890102375415254`, 362.3974796892316`, 0.9622158225617059`},
{0.06948728972594465`, 0.51090278124415`, 0.035936562894876956`, 0.8667074657182937`, 0.8085779702858387`,
0.382742596468321`, 0.12566425611349363`, 0.007737234004365364`, 2048.8634514818114`,
306.8418541643264`, 0.17136334570338607`, 848.3129834153345`, 0.3704642619799363`},
{0.050049496102907765`, 1.3151733384496236`, 0.08960678655774754`, 1.003607455388209`, 0.2419867265185358`,
0.4154436014149323`, 0.125439303079254`, 0.2924251328627742`, 3385.8016445242683`,
305.79961205315897`, 0.16498282923627505`, 266.33868597078384`, 1.1090348362413631`},
{0.11826273972178508`, 2.788085877881148`, 0.09054001235451012`, 0.9896795010763948`, 0.8061614577132081`,
0.5581707521312328`, 0.17530270390580582`, 0.19290329785210641`, 755.3772246989865`,
304.1021412587852`, 0.17820434228297638`, 604.9961085215052`, 0.15427573247017112`},

{0.2712509585118122`, 1.0581790279996168`, 0.05659762154346131`, 0.8024669973276888`, 0.9282363346360583`,
0.4127619682763257`, 0.23291152894285283`, 0.2946176607588353`, 1146.4804544229783`,
149.90785558597548`, 0.10428627648934868`, 874.3202223507158`, 0.7409866753784651`},
{0.2374512887093062`, 2.3072284463283577`, 0.06301706038718162`, 1.3403420265449104`, 0.6149895430099195`,
0.15466183699199576`, 0.17490550568927749`, 0.38540862196456355`, 1263.6992928345717`,
72.86174435764508`, 0.17358058164049028`, 846.1569833003423`, 0.1463173092850527`},
{0.2583672781430382`, 2.6268623993777895`, 0.06205601788158825`, 0.8235050717722767`, 0.08727687481867474`,
0.6357636820328836`, 0.05851949319043051`, 0.010999057505063525`, 1473.1674122992072`,
304.2864656373356`, 0.012909768358259105`, 823.8314624062951`, 0.4751823031250133`},
{0.043577758347122936`, 1.721562842235091`, 0.0234352564488443`, 0.9132551445382597`, 0.37311775072636233`,
0.6528441635985238`, 0.16226121956597517`, 0.034989394292024543`, 936.6771189752785`,
115.68607475497669`, 0.12474087420473307`, 195.06474478744042`, 0.7881998071633989`},
{0.1701792225175116`, 1.179302589093206`, 0.03123477236887025`, 1.4414320519466277`, 0.9903024362640889`,
0.4389604463449559`, 0.09164999807100366`, 0.008846932361855247`, 724.0592612271148`,
338.9152684852804`, 0.21260781082553598`, 491.09110101763173`, 0.9542275020448274`},
{0.130317181547839`, 3.8262873731024536`, 0.053854592324169226`, 1.2067434665942098`, 0.7894425338521145`,
0.6114818372735824`, 0.051330900152700254`, 0.014707230943787104`, 3691.352269042867`,
211.00316682971982`, 0.24430109987491683`, 925.7659428871058`, 1.1915286425790357`},
{0.25486658794939426`, 3.961862391500823`, 0.0016915807450080856`, 1.0039710384043325`, 0.4439256488497578`,
0.2640519340353227`, 0.24794830048559635`, 0.3022308978535489`, 1015.0154463245726`,
25.96591275867155`, 0.17823539573078245`, 990.4987865010767`, 0.7497663925877376`},
{0.0910636880593409`, 3.3345224118514185`, 0.014290038687226652`, 1.2474917024555112`, 0.14234098176829657`,
0.2745492574730136`, 0.07470894503449785`, 0.006486531838891606`, 2792.8473416264023`,
243.79029268269937`, 0.10689498421398869`, 679.8105890776037`, 0.8841737722770295`},
{0.2393830566829872`, 1.8914518565313667`, 0.03911146011956786`, 0.9233045602346788`, 0.858165921623284`,
0.18581781744440107`, 0.1750287663309945`, 0.005150798631758578`, 2361.3990338282965`,
68.02155429252394`, 0.13800946518149532`, 553.39899933643`, 0.4912240353089825`},
{0.15447178914554444`, 3.070388186516799`, 0.0062453635675441535`, 0.7518341283463481`, 0.27071624004025363`,
0.4837242717578115`, 0.06531649316964588`, 0.012250492736785322`, 2884.162728669763`,
192.2692876855201`, 0.09709059821437976`, 462.7696019529214`, 0.9343366853672381`},
{0.17686834391516937`, 1.6232487343434094`, 0.02597802435280336`, 1.3041196293171433`, 0.5862495437437565`,

0.1782074866868083`, 0.0989259569866783`, 0.011138815336252416`, 658.7384470635911`,
368.738090259161`, 0.21057216388771816`, 739.5771513523569`, 0.2828426309471912`},
{0.18233417743723207`, 1.5274224716614926`, 0.017609190501484892`, 0.9437186173664076`, 0.49839057078460836`,
0.3075805446682879`, 0.12248140184331402`, 0.07404001497316069`, 1029.4971705842381`,
79.7877480194478`, 0.12205068650277201`, 408.695360998691`, 1.2545477425718192`},
{0.0913911007727759`, 3.1324380514820316`, 0.06772259991696085`, 0.7767602765726433`, 0.9921348110546835`,
0.3782679882791412`, 0.21535665611030852`, 0.045252490515844346`, 950.4778542730078`,
31.83451272704201`, 0.2008451902109652`, 482.6845627433862`, 1.032433145428839`},
{0.22583895811127175`, 2.94982287382856`, 0.0434035992390066`, 0.8931297204139108`, 0.15348196318747953`,
0.15016270158232614`, 0.12402319088371505`, 0.08945799240051626`, 2079.2308353195403`,
248.5371068974273`, 0.24356928033627223`, 261.96208097999244`, 1.4956168641039076`},
{0.06545431670352686`, 0.4657917114529746`, 0.02591732993793956`, 1.241489040181722`, 0.5503077758546375`,
0.2892452368315044`, 0.09999791256972682`, 0.05344187790454679`, 3407.2442488021343`,
153.8341671789367`, 0.08087533994590407`, 571.6195777438754`, 1.2008592165803909`},
{0.08904825805621058`, 1.5141292954415215`, 0.06283954851421164`, 1.4241380428011252`, 0.4632426196285373`,
0.5196751057452885`, 0.14485491709730686`, 0.015349239215653421`, 1749.315967626415`,
347.80513464061664`, 0.2377891756121447`, 945.3930853411337`, 0.9452322400256437`},
{0.13401777192846137`, 3.0684632480446883`, 0.05480331995921842`, 1.3261489424010848`, 0.8062164301516244`,
0.6331041572170104`, 0.1452796385295831`, 0.02441368064936862`, 3400.367428155201`,
306.9644208004196`, 0.1315358526151355`, 990.4612003567611`, 1.4560233572454293`},
{0.20554956037907224`, 3.192391881337918`, 0.009901186722592747`, 1.2891569369922138`, 0.8039655252473428`,
0.6642128613008436`, 0.12450893357747278`, 0.015265735622223034`, 2949.532841272806`,
202.30535192051218`, 0.09343744224172695`, 365.64046592453076`, 0.24198475882975234`},
{0.2552568406196402`, 1.1312646889353166`, 0.09815902094474993`, 1.1736607390155205`, 0.7216800093462903`,
0.674131109397206`, 0.07364063660564513`, 0.01792744548437268`, 2421.1681901255743`,
183.1682448584395`, 0.18416278003826436`, 261.8150303320675`, 0.10121194291250091`},
{0.26707630790371234`, 1.5643754810872466`, 0.09172629825385457`, 0.9188308071288775`, 0.3603608499780673`,
0.36307263833991177`, 0.23799649846270937`, 0.08941354043858946`, 3337.2360773081746`,
356.8922745809465`, 0.07860074428455693`, 274.44074186760423`, 0.8821726606791338`},
{0.20406125637688227`, 3.9557949493292535`, 0.090998308424541`, 1.1030821178156798`, 0.5820095387187791`,
0.2895144642365983`, 0.12406381054373888`, 0.28129889230260957`, 3863.992547399419`,

212.9714354931541`, 0.24237606989974275`, 394.1999775797596`, 1.1306818753973285`},
{0.1555958543239555`, 3.0567996079781343`, 0.044044975729054074`, 0.8330047095021981`, 0.6345639623145571`,
0.32277062952533075`, 0.16206923321189637`, 0.02124616216655347`, 2789.6328263694513`,
70.02394417944924`, 0.20557043596463515`, 903.1422472563104`, 1.3562882271726817`},
{0.04649250921545231`, 3.697996652317366`, 0.039776895647662534`, 1.026428180982236`, 0.6003109026474431`,
0.28791567223252323`, 0.05566301641401358`, 0.18332868170152372`, 2810.7066875164637`,
247.60325123184555`, 0.22674424459040515`, 780.3137731007916`, 0.11308185478509825`},
{0.20300605710941072`, 2.3083402980344108`, 0.03591405338895173`, 1.1801458520300514`, 0.5008186442555631`,
0.2073943068996874`, 0.07984435403291612`, 0.016582171849056596`, 721.7673192147868`,
334.85992757725705`, 0.12562557623146764`, 224.91754831531773`, 0.11484469479390524`},
{0.23873235642254892`, 1.5902805585544497`, 0.05156788053090951`, 0.9363571173263263`, 0.19878836474219752`,
0.5063532483760987`, 0.21175056156026556`, 0.01580179842666557`, 3131.68119461093`,
[truncated: 557,709 more chars]
