# Supplementary material for: Mathematical Modeling Unveils Optimization Strategies for Targeted Radionuclide Therapy of Blood Cancers
Source: Cancer Res Commun. 2024 Nov 14;4(11):2955–67. doi: 10.1158/2767-9764.CRC-24-0306 (PMC11562018; doi:10.1158/2767-9764.CRC-24-0306)
Supplement: Computational codes — Designed in Wolfram Mathematica, version 13.3.1.0. [file crc-24-0306_computational_codes_suppscc.zip › CodesPDF/05-Multi-dose.pdf]

```
(* This file contains simulations for the paper
 "Mathematical Modeling Unveils Optimization Strategies
for Targeted Radionuclide Therapy of Blood Cancers"
 by Maxim Kuznetsov, Vikram Adhikarla, Enrico Caserta,
 Flavia Pichiorri, John E.Shively, Xiuli Wang and Russell C.Rockne *)

(******************************************************************************)
```

In[∘]:=

```
(* The scripts hidden in this cell have to be initialized at first *)
```

In[∘]:=

```
(* The script for the solution of full system *)

FullSystemSolutionMD[] := (Quiet[

    tEnd = Ainj[[Length[Ainj], 1]] + (-Log[0.0017] / lambda);
    lAinj = Length[Ainj];
    If[Ainj[[1, 1]] == 0, Npw = lAinj, Npw = lAinj + 1];

    apw = Array[ff, Npw]; bpw = Array[ff, Npw]; NNpw = Array[ff, Npw]; DDpw = Array[ff, Npw];
    papw = Array[ff, Npw]; pbpw = Array[ff, Npw]; fFNpw = Array[ff, Npw]; fANpw = Array[ff, Npw];
    dFpw = Array[ff, Npw]; dApw = Array[ff, Npw];

    tB = Array[ff, Npw]; tE = Array[ff, Npw];

    (* for monitoring the paths of activity *)
    ActBloodpw = Array[ff, Npw]; ActBloodFragpw = Array[ff, Npw];
    ActOutpw = Array[ff, Npw]; ActOutFragpw = Array[ff, Npw]; ActTumorpw = Array[ff, Npw];

    (* for monitoring influence of self-dose, croos-fire and decays in blood *)
    SDpw = Array[ff, Npw]; CFNpw = Array[ff, Npw]; CFDpw = Array[ff, Npw]; UNpw = Array[ff, Npw];

    (* for monitoring the number of new cancer cells appearing during treatment *)
    NewCellspw = Array[ff, Npw];
```

```
Clear[a, b, NN, DD, pa, pb, fFN, fAN, dF, dA, ActBlood, ActBloodFrag, ActOut, ActOutFrag, ActTumor, SD, CFN, CFD, UN, NewCells];

(* EQUATIONS *)

(* Radiation damage function *)

RD[NN_, DD_, fAN_, dA_, a_, pa_] := alpha * (ks * (lambda * gamma * fAN)/nu (*self-dose*) +
    (1 - ks) * (lambda * gamma * (fAN * NN + dA))/(nu * (NN + DD)) (*cross-fire*) + kf * lambda * (a + pa) (*dose from unanchored nuclides*));

(* Active antibodies *)
Fa[t_] := (*injections are considered as initial conditions*)
  - lambda * a[t] (*decay*) - kon * gamma/V * (fFN[t] * NN[t] + dF[t]) * a[t] (*binding*) - kappac * a[t] (*clearance*);

(* Inert antibodies *)
Fb[t_] := (*injections are considered as initial conditions*)
  + lambda * a[t] (*decay of a*) - kon * gamma/V * (fFN[t] * NN[t] + dF[t]) * b[t] (*binding*) - kappac * b[t] (*clearance*);

(* Viable cells *)
FNN[t_] := rho * NN[t] (*proliferation*) - RD[NN[t], DD[t], fAN[t], dA[t], a[t], pa[t]] * NN[t] (*damage*);

(* Damaged cells *) FDD[t_] := RD[NN[t], DD[t], fAN[t], dA[t], a[t], pa[t]] * NN[t] (*damage*) - omega * DD[t] (*death*);

(* Active fragments *)
Fpa[t_] := omega * (gamma * dA[t])/V (*release*) - lambda * pa[t] (*decay*) - kappap * pa[t] (*clearance*);
```

```
(* Inert fragments *)
Fpb[t_] :=
  omega * (gamma * (DD[t] - dF[t] - dA[t]))/V (*release*) + lambda * pa[t] (*decay*) - kappap * pb[t] (*clearance*);

(* Free receptors of viable cells *) FfFN[t_] := (1 - fFN[t]) * rho - kon * (a[t] + b[t]) * fFN[t];

(* Active receptors of viable cells *) FfAN[t_] := kon * a[t] * fFN[t] - (lambda + rho) * fAN[t];

(* Free receptors of damaged cells *)
FdF[t_] := RD[NN[t], DD[t], fAN[t], dA[t], a[t], pa[t]] * fFN[t] * NN[t] - kon * (a[t] + b[t]) * dF[t] - omega * dF[t];

(* Active receptors of damaged cells *)
FdA[t_] :=
  RD[NN[t], DD[t], fAN[t], dA[t], a[t], pa[t]] * fAN[t] * NN[t] + kon * a[t] * dF[t] - lambda * dA[t] - omega * dA[t];

(* Initial conditions *)

If[Ainj[[1, 1]] == 0
  , a0 = Ainj[[1, 2]]/V; b0 = Ainj[[1, 3]]/V (*eta*Ainj[[1,2]]/V*)
  , a0 = 0; b0 = 0]; (*complexes in blood*)
NN0 = N0;
DD0 = 0;
pa0 = 0; pb0 = 0; fFN0 = 1; fAN0 = 0;
dF0 = 0; dA0 = 0;
ActBlood0 = 0; ActBloodFrag0 = 0; ActOut0 = 0; ActOutFrag0 = 0; ActTumor0 = 0; NewCells0 = 0;
SD0 = 0; CFN0 = 0; CFD0 = 0; UN0 = 0;

(* SOLVER *)

tB[[1]] = 0; If[Ainj[[1, 1]] == 0, If[lAinj > 1, tE[[1]] = Ainj[[2, 1]], tE[[1]] = tEnd], tE[[1]] = Ainj[[1, 1]]];
```

```
For[npw = 1, npw ≤ Npw, npw++,

  Clear[a, b, NN, DD, pa, pb, fFN, fAN, dF, dA, ActBlood, ActBloodFrag, ActOut, ActOutFrag, ActTumor, SD, CFN, CFD, UN, NewCells];

  sol = NDSolve[{
      (*INITIAL CONDITIONS*)
      a[tB[[npw]]] == a0, b[tB[[npw]]] == b0, NN[tB[[npw]]] == NN0, DD[tB[[npw]]] == DD0, pa[tB[[npw]]] == pa0,
      pb[tB[[npw]]] == pb0, fFN[tB[[npw]]] == fFN0, fAN[tB[[npw]]] == fAN0, dF[tB[[npw]]] == dF0, dA[tB[[npw]]] == dA0,
      ActBlood[tB[[npw]]] == ActBlood0, ActBloodFrag[tB[[npw]]] == ActBloodFrag0, ActOut[tB[[npw]]] == ActOut0,
      ActOutFrag[tB[[npw]]] == ActOutFrag0, ActTumor[tB[[npw]]] == ActTumor0, SD[tB[[npw]]] == SD0,
      CFN[tB[[npw]]] == CFN0, CFD[tB[[npw]]] == CFD0, UN[tB[[npw]]] == UN0, NewCells[tB[[npw]]] == NewCells0,

      a'[t] == Fa[t], b'[t] == Fb[t], NN'[t] == FNN[t], DD'[t] == FDD[t], pa'[t] == Fpa[t],
      pb'[t] == Fpb[t], fFN'[t] == FfFN[t], fAN'[t] == FfAN[t], dF'[t] == FdF[t], dA'[t] == FdA[t],

      ActBlood'[t] == V * lambda * (a[t] + pa[t]),
      ActBloodFrag'[t] == V * lambda * pa[t],
      ActOut'[t] == V * (kappac * a[t] + kappap * pa[t]),
      ActOutFrag'[t] == V * kappap * pa[t],
      ActTumor'[t] == (lambda * gamma) * (fAN[t] * NN[t] + dA[t]),

      SD'[t] == ks * (lambda * gamma) * (fAN[t] * NN[t]),
      CFN'[t] == (1 - ks) * (lambda * gamma) * (fAN[t] * NN[t]) * NN[t] / (NN[t] + DD[t]),
      CFD'[t] == (1 - ks) * (lambda * gamma) * dA[t] * NN[t] / (NN[t] + DD[t]),
      UN'[t] == kf * lambda * (a[t] + pa[t]) * nu * NN[t],
      NewCells'[t] == If[t > Ainj[[1, 1]], rho * NN[t], 0] (* start counting new cells from the moment of the first injection *)
```

```
        , WhenEvent[NN[t] > Cd / Nnor, tEnd = t; "StopIntegration"]
      }
      , {a, b, NN, DD, pa, pb, fFN, fAN, dF, dA, ActBlood,
        ActBloodFrag, ActOut, ActOutFrag, ActTumor, SD, CFN, CFD, UN, NewCells}, {t, tB[[npw]], tE[[npw]]}
      , AccuracyGoal → 10, PrecisionGoal → 10];

apw[[npw]] = First[a /. sol]; bpw[[npw]] = First[b /. sol]; NNpw[[npw]] = First[NN /. sol]; DDpw[[npw]] = First[DD /. sol];
papw[[npw]] = First[pa /. sol]; pbpw[[npw]] = First[pb /. sol]; fFNpw[[npw]] = First[fFN /. sol];
fANpw[[npw]] = First[fAN /. sol]; dFpw[[npw]] = First[dF /. sol]; dApw[[npw]] = First[dA /. sol];
ActBloodpw[[npw]] = First[ActBlood /. sol];
ActBloodFragpw[[npw]] = First[ActBloodFrag /. sol];
ActOutpw[[npw]] = First[ActOut /. sol];
ActOutFragpw[[npw]] = First[ActOutFrag /. sol];
ActTumorpw[[npw]] = First[ActTumor /. sol];
SDpw[[npw]] = First[SD /. sol];
CFNpw[[npw]] = First[CFN /. sol];
CFDpw[[npw]] = First[CFD /. sol];
UNpw[[npw]] = First[UN /. sol];
NewCellspw[[npw]] = First[NewCells /. sol];

If[npw < Npw,
   (*renew initial conditions*)

  If[Ainj[[1, 1]] == 0
    , a0 = apw[[npw]][tE[[npw]]] + Ainj[[npw + 1, 2]] / V; b0 = bpw[[npw]][tE[[npw]]] + Ainj[[npw + 1, 3]] / V
    , a0 = apw[[npw]][tE[[npw]]] + Ainj[[npw, 2]] / V; b0 = bpw[[npw]][tE[[npw]]] + Ainj[[npw, 3]] / V];

  NN0 = NNpw[[npw]][tE[[npw]]];
  DD0 = DDpw[[npw]][tE[[npw]]];
  pa0 = papw[[npw]][tE[[npw]]];
```

```
      pb0 = pbpw⟦npw⟧[tE⟦npw⟧];
      fFN0 = fFNpw⟦npw⟧[tE⟦npw⟧]; fAN0 = fANpw⟦npw⟧[tE⟦npw⟧];
      dF0 = dFpw⟦npw⟧[tE⟦npw⟧];
      dA0 = dApw⟦npw⟧[tE⟦npw⟧];
      ActBlood0 = ActBloodpw⟦npw⟧[tE⟦npw⟧];
      ActBloodFrag0 = ActBloodFragpw⟦npw⟧[tE⟦npw⟧];
      ActOut0 = ActOutpw⟦npw⟧[tE⟦npw⟧];
      ActOutFrag0 = ActOutFragpw⟦npw⟧[tE⟦npw⟧];
      ActTumor0 = ActTumorpw⟦npw⟧[tE⟦npw⟧];
      SD0 = SDpw⟦npw⟧[tE⟦npw⟧];
      CFN0 = CFNpw⟦npw⟧[tE⟦npw⟧];
      CFD0 = CFDpw⟦npw⟧[tE⟦npw⟧];
      UN0 = UNpw⟦npw⟧[tE⟦npw⟧];
      NewCells0 = NewCellspw⟦npw⟧[tE⟦npw⟧];

      (*renew time frame*)
      tB⟦npw + 1⟧ = tE⟦npw⟧;

      If[Ainj⟦1, 1⟧ == 0, If[Npw > npw + 1, tE⟦npw + 1⟧ = Ainj⟦npw + 2, 1⟧, tE⟦npw + 1⟧ = tEnd],
        If[Npw > npw + 1, tE⟦npw + 1⟧ = Ainj⟦npw + 1, 1⟧, tE⟦npw + 1⟧ = tEnd]];]
   ];
   npw--;
   (* It will be convenient to have estimation of minimal viable cell number here *)
   TableNN = If[Ainj⟦1, 1⟧ == 0,
      Table[If[NumberQ[NMinimize[{Nnor * (NNpw⟦nn⟧[t]), t > tB⟦nn⟧, t < Min[tEnd, tE⟦nn⟧]}, t]⟦1⟧],
         NMinimize[{Nnor * (NNpw⟦nn⟧[t]), t > tB⟦nn⟧, t < Min[tEnd, tE⟦nn⟧]}, t], {Cd, t → 0}], {nn, 1, npw}],
      Table[If[NumberQ[NMinimize[{Nnor * (NNpw⟦nn⟧[t]), t > tB⟦nn⟧, t < Min[tEnd, tE⟦nn⟧]}, t]⟦1⟧],
         NMinimize[{Nnor * (NNpw⟦nn⟧[t]), t > tB⟦nn⟧, t < Min[tEnd, tE⟦nn⟧]}, t], {Cd, t → 0}], {nn, 2, npw}]];

   Nn = Max[0, Min[TableNN⟦All, 1⟧]];
```

```
tminNn = t /. TableNN[[Position[TableNN[[All, 1]], Nn][[1, 1]]]][[2]];];

Return[Nn])
```

In[ ]:=

```
SetBasicParameterValues[] := (
  Nnor = 10^5; (* due to numerical pecularities, it is better to use this normalization number of cells *)

  lambda = 0.07; (* radionuclide decay rate *)
  kon = 11.15; (* antibody-receptor association rate *)
  kappac = 0.1; (* antibody clearance rate (0.04-0.28) *)
  kappap = 1; (* antibody fragments clearance rate (0.4-4.) *)
  gamma = 2.1 * Nnor / 10^7; (* number of receptors of Nnor cancer cells (0.13-10) *Nnor/10^7 *)
  V = 1.; (* volume of drug distribution (0.75-1.5) *)
  nu = 0.015 * Nnor / 10^7; (* volume of lesion with Nnor cancer cells *)
  ks = 0.3; (* relative significance of self-dose (0.1-0.5) *)

  rho = 0.34; (* cancer cells proliferation rate (0.15-0.7) *)
  omega = 0.05; (* damaged cells death rate (0.005-0.5) *)
  alpha = 500; (* cancer cells radiosensitivity (50-5000) *)
  eta = 1780; (* coefficient of drug impurity (0-10^5) *)
  kf = 0.05; (* significance of unanchored nuclides decays (0.01-0.25) *)
  N0 = 3. * 10^7 / Nnor; (* initial number of viable cancer cells, xNnor (1-10) *Nnor/10^7 *)
  D0 = 0;
  (*0.1/Nnor; *)
  (* initial number of damaged cancer cells -- use non-zero value if considering fraction of receptors of damaged cells, to avoid division by zero*)
  nCpm = 0.000076; (* convenient parameter of conversion from nCi to pmol *)
  DA1 = 50 * nCpm; (* injected amount of radionuclides *)
  t1 = 0; (* moment of their injection *)
  Ncur = 0.01; (* number of viable cancer cells (in cells), corresponding to cancer cure *)
```

```
Abld = 0.0175; (* lethal amount of decays in blood *)
Cd = 10^11; (* critical number of cacner cells, leading to death of tumor burden *)
Ainj = {{t1, DA1, eta * DA1}}; (* Schedule of injections *)
dayInit = 20; (* initial interval between two doses for numericla optimization *)

(* maximum time of simulation-- where little radioactivity of the last dose remains, namely 0.17% *)
tEnd = -Log[0.0017] / lambda;

kGy = 2500; (* coefficient of conversion of nM of 225-Ac decay in bone marrow into Gy *)
)
```

In[∘]:=

```
(* Script for finding curative dose *)

FindCurDose[] := (Quiet[DA1 = -N0 * rho/lambda * nu/alpha * ProductLog[-1, -E^-2 * (Ncur/(N0 * Nnor))^(lambda/rho)]/2.;
    (* half the curative dose on cancer cells for cross-fire only, real value is hardly below it *)

    Ainj = {{t1, DA1, eta * DA1}};

    While[FullSystemSolutionMD[] > Ncur, DA1 = 2 * DA1; Ainj = {{t1, DA1, eta * DA1}}; ];
    Dmax = DA1; Dmin = DA1 / 2;

    While[Abs[Dmin - Dmax] / Dmax > 0.000001,
      DA1 = (Dmin + Dmax) / 2; Ainj = {{t1, DA1, eta * DA1}};

      If[FullSystemSolutionMD[] > Ncur, Dmin = DA1, Dmax = DA1]];
    Dcur = Dmax;

    Return[Dcur / nCpm]]);
```

In[∘]:=

```
FullSystemSolutionToxicity[] := (
```

```
(* maximum time of simulation-- where little radioactivity of the last dose remains, namely 0.17% of the last dose *)
tEnd = Ainj[[Length[Ainj], 1]] + (-Log[0.0017] / lambda);
lAinj = Length[Ainj]; (* number of injections*)
If[Ainj[[1, 1]] == 0, Npw = lAinj, Npw = lAinj + 1];

apw = Array[ff, Npw]; bpw = Array[ff, Npw]; NNpw = Array[ff, Npw]; DDpw = Array[ff, Npw];
papw = Array[ff, Npw]; pbpw = Array[ff, Npw]; fFNpw = Array[ff, Npw]; fANpw = Array[ff, Npw];
dFpw = Array[ff, Npw]; dApw = Array[ff, Npw];
(* inert antibody fragments are as well accounted for as pb *)

(* times of beginning and end for solution of separate systems *)
tB = Array[ff, Npw]; tE = Array[ff, Npw];

(* for monitoring the paths of activity *)
ActBloodpw = Array[ff, Npw]; ActBloodFragpw = Array[ff, Npw];
ActOutpw = Array[ff, Npw]; ActOutFragpw = Array[ff, Npw]; ActTumorpw = Array[ff, Npw];

(* for monitoring influence of self-dose, croos-fire and decays in blood *)
SDpw = Array[ff, Npw]; CFNpw = Array[ff, Npw]; CFDpw = Array[ff, Npw]; UNpw = Array[ff, Npw];

(* for monitoring the number of new cancer cells appearing during treatment *)
NewCellspw = Array[ff, Npw];

Clear[a, b, NN, DD, pa, pb, fFN, fAN, dF, dA, ActBlood, ActBloodFrag, ActOut, ActOutFrag, ActTumor, SD, CFN, CFD, UN, NewCells];

(* EQUATIONS *)

(* Radiation damage function *)
RD[NN_, DD_, fAN_, dA_, a_, pa_] := alpha * (ks * (lambda * gamma * fAN)/nu (*self-dose*) +
```

```
(1 - ks) * (lambda * gamma * (fAN * NN + dA)) / (nu * (NN + DD)) (*cross-fire*) + kf * lambda * (a + pa) (*dose from unanchored nuclides*) );


(* Active antibodies *)
Fa[t_] := (*injections are considered as initial conditions*)
  - lambda * a[t] (*decay*) - kon * gamma / V * (fFN[t] * NN[t] + dF[t]) * a[t] (*binding*) - kappac * a[t] (*clearance*);

(* Inert antibodies *)
Fb[t_] := (*injections are considered as initial conditions*)
  + lambda * a[t] (*decay of a*) - kon * gamma / V * (fFN[t] * NN[t] + dF[t]) * b[t] (*binding*) - kappac * b[t] (*clearance*);

(* Viable cells *) FNN[t_] := rho * NN[t] (*proliferation*) - RD[NN[t], DD[t], fAN[t], dA[t], a[t], pa[t]] * NN[t] (*damage*);

(* Damaged cells *) FDD[t_] := RD[NN[t], DD[t], fAN[t], dA[t], a[t], pa[t]] * NN[t] (*damage*) - omega * DD[t] (*death*);

(* Active fragments *) Fpa[t_] := omega * (gamma * dA[t]) / V (*release*) - lambda * pa[t] (*decay*) - kappap * pa[t] (*clearance*);

(* Inert fragments *)
Fpb[t_] := omega * (gamma * (DD[t] - dF[t] - dA[t])) / V (*release*) + lambda * pa[t] (*decay*) - kappap * pb[t] (*clearance*);

(* Free receptors of viable cells *) FfFN[t_] := (1 - fFN[t]) * rho - kon * (a[t] + b[t]) * fFN[t];

(* Active receptors of viable cells *) FfAN[t_] := kon * a[t] * fFN[t] - (lambda + rho) * fAN[t];

(* Free receptors of damaged cells *)
FdF[t_] := RD[NN[t], DD[t], fAN[t], dA[t], a[t], pa[t]] * fFN[t] * NN[t] - kon * (a[t] + b[t]) * dF[t] - omega * dF[t];
```

```
(* Active receptors of damaged cells *)
FdA[t_] :=
  RD[NN[t], DD[t], fAN[t], dA[t], a[t], pa[t]] * fAN[t] * NN[t] + kon * a[t] * dF[t] - lambda * dA[t] - omega * dA[t];

(* Initial conditions *)

If[Ainj⟦1, 1⟧ == 0
  , a0 = Ainj⟦1, 2⟧ / V; b0 = Ainj⟦1, 3⟧ / V
  , a0 = 0; b0 = 0]; (*complexes in blood*)
NN0 = N0;
DD0 = 0;
pa0 = 0; pb0 = 0; fFN0 = 1; fAN0 = 0;
dF0 = 0; dA0 = 0;
ActBlood0 = 0; ActBloodFrag0 = 0; ActOut0 = 0; ActOutFrag0 = 0; ActTumor0 = 0; NewCells0 = 0;
SD0 = 0; CFN0 = 0; CFD0 = 0; UN0 = 0;

(* SOLVER *)

tB⟦1⟧ = 0; If[Ainj⟦1, 1⟧ == 0, If[lAinj > 1, tE⟦1⟧ = Ainj⟦2, 1⟧, tE⟦1⟧ = tEnd], tE⟦1⟧ = Ainj⟦1, 1⟧];

For[npw = 1, npw ≤ Npw, npw++,

  Clear[a, b, NN, DD, pa, pb, fFN, fAN, dF, dA, ActBlood, ActBloodFrag, ActOut, ActOutFrag, ActTumor, SD, CFN, CFD, UN, NewCells];

  sol = NDSolve[{
      (*INITIAL CONDITIONS*)
      a[tB⟦npw⟧] == a0, b[tB⟦npw⟧] == b0, NN[tB⟦npw⟧] == NN0, DD[tB⟦npw⟧] == DD0, pa[tB⟦npw⟧] == pa0,
      pb[tB⟦npw⟧] == pb0, fFN[tB⟦npw⟧] == fFN0, fAN[tB⟦npw⟧] == fAN0, dF[tB⟦npw⟧] == dF0, dA[tB⟦npw⟧] == dA0,
      ActBlood[tB⟦npw⟧] == ActBlood0, ActBloodFrag[tB⟦npw⟧] == ActBloodFrag0, ActOut[tB⟦npw⟧] == ActOut0,
```

```
        ActOutFrag[tB[[npw]]] == ActOutFrag0, ActTumor[tB[[npw]]] == ActTumor0, SD[tB[[npw]]] == SD0,
        CFN[tB[[npw]]] == CFN0, CFD[tB[[npw]]] == CFD0, UN[tB[[npw]]] == UN0, NewCells[tB[[npw]]] == NewCells0,

        a'[t] == Fa[t], b'[t] == Fb[t], NN'[t] == FNN[t], DD'[t] == FDD[t],
        pa'[t] == Fpa[t], pb'[t] == Fpb[t], fFN'[t] == FfFN[t], fAN'[t] == FfAN[t], dF'[t] == FdF[t], dA'[t] == FdA[t],

        ActBlood'[t] == V * lambda * (a[t] + pa[t]),
        ActBloodFrag'[t] == V * lambda * pa[t],
        ActOut'[t] == V * (kappac * a[t] + kappap * pa[t]),
        ActOutFrag'[t] == V * kappap * pa[t],
        ActTumor'[t] == (lambda * gamma) * (fAN[t] * NN[t] + dA[t]),

        SD'[t] == ks * (lambda * gamma) * (fAN[t] * NN[t]),
        CFN'[t] == (1 - ks) * (lambda * gamma) * (fAN[t] * NN[t]) * NN[t] / (NN[t] + DD[t]),
        CFD'[t] == (1 - ks) * (lambda * gamma) * dA[t] * NN[t] / (NN[t] + DD[t]),
        UN'[t] == kf * lambda * (a[t] + pa[t]) * nu * NN[t],
        NewCells'[t] == If[t > Ainj[[1, 1]], rho * NN[t], 0] (* start counting new cells from the moment of the first injection *)

        , WhenEvent[NN[t] < Ncur / Nnor, NN[t] → 0 (*tE[[npw]]=t;
          tEnd=t;
          "StopIntegration"*) ] (* treatment wins *)
      }
      , {a, b, NN, DD, pa, pb, fFN, fAN, dF, dA, ActBlood,
        ActBloodFrag, ActOut, ActOutFrag, ActTumor, SD, CFN, CFD, UN, NewCells}, {t, tB[[npw]], tE[[npw]]}
      , AccuracyGoal → 10, PrecisionGoal → 10];

  apw[[npw]] = First[a /. sol]; bpw[[npw]] = First[b /. sol]; NNpw[[npw]] = First[NN /. sol]; DDpw[[npw]] = First[DD /. sol];
  papw[[npw]] = First[pa /. sol]; pbpw[[npw]] = First[pb /. sol]; fFNpw[[npw]] = First[fFN /. sol];
```

```
fANpw[[npw]] = First[fAN /. sol]; dFpw[[npw]] = First[dF /. sol]; dApw[[npw]] = First[dA /. sol];
ActBloodpw[[npw]] = First[ActBlood /. sol];
ActBloodFragpw[[npw]] = First[ActBloodFrag /. sol];
ActOutpw[[npw]] = First[ActOut /. sol];
ActOutFragpw[[npw]] = First[ActOutFrag /. sol];
ActTumorpw[[npw]] = First[ActTumor /. sol];
SDpw[[npw]] = First[SD /. sol];
CFNpw[[npw]] = First[CFN /. sol];
CFDpw[[npw]] = First[CFD /. sol];
UNpw[[npw]] = First[UN /. sol];
NewCellspw[[npw]] = First[NewCells /. sol];

If[npw < Npw,
  (*renew initial conditions*)
  If[Ainj[[1, 1]] == 0
    , a0 = apw[[npw]][tE[[npw]]] + Ainj[[npw + 1, 2]]/V; b0 = bpw[[npw]][tE[[npw]]] + Ainj[[npw + 1, 3]]/V
    , a0 = apw[[npw]][tE[[npw]]] + Ainj[[npw, 2]]/V; b0 = bpw[[npw]][tE[[npw]]] + Ainj[[npw, 3]]/V];
  NN0 = NNpw[[npw]][tE[[npw]]];
  DD0 = DDpw[[npw]][tE[[npw]]];
  pa0 = papw[[npw]][tE[[npw]]];
  pb0 = pbpw[[npw]][tE[[npw]]];
  fFN0 = fFNpw[[npw]][tE[[npw]]]; fAN0 = fANpw[[npw]][tE[[npw]]];
  dF0 = dFpw[[npw]][tE[[npw]]];
  dA0 = dApw[[npw]][tE[[npw]]];
  ActBlood0 = ActBloodpw[[npw]][tE[[npw]]];
  ActBloodFrag0 = ActBloodFragpw[[npw]][tE[[npw]]];
  ActOut0 = ActOutpw[[npw]][tE[[npw]]];
  ActOutFrag0 = ActOutFragpw[[npw]][tE[[npw]]];
  ActTumor0 = ActTumorpw[[npw]][tE[[npw]]];
  SD0 = SDpw[[npw]][tE[[npw]]];
  CFN0 = CFNpw[[npw]][tE[[npw]]];
```

```
    CFD0 = CFDpw[[npw]] [tE[[npw]]] ;
    UN0 = UNpw[[npw]] [tE[[npw]]] ;
    NewCells0 = NewCellspw[[npw]] [tE[[npw]]] ;

    (*renew time frame*)
    tB[[npw + 1]] = tE[[npw]];

    If [Ainj[[1, 1]] == 0, If [Npw > npw + 1, tE[[npw + 1]] = Ainj[[npw + 2, 1]], tE[[npw + 1]] = tEnd],
      If [Npw > npw + 1, tE[[npw + 1]] = Ainj[[npw + 1, 1]], tE[[npw + 1]] = tEnd] ];]
 ];
 npw--;
 (* It will be convenient to have estimation of minimal viable cell number here *)
 TableNN = If [Ainj[[1, 1]] == 0, Table [NMinimize [ {Nnor * (NNpw[[nn]] [t]) , t > tB[[nn]], t < tE[[nn]]}, t], {nn, 1, npw} ],
    Table [NMinimize [ {Nnor * (NNpw[[nn]] [t]) , t > tB[[nn]], t < tE[[nn]]}, t] [[1]], {nn, 2, npw} ] ];

 Nn = Min [TableNN[[All, 1]]] ;

 tminNn = t /. TableNN[[Position [TableNN[[All, 1]], Nn] [[1, 1]]]][[2]];

 Return [ActBloodpw[[npw]] [tEnd] ] )
```

In[ ]:=

```
(* Script for finding toxic dose *)
FindToxDose[] := (Quiet[DA1 = Abld;
    Ainj = {{t1, DA1, eta * DA1}};

    While[FullSystemSolutionToxicity[] < Abld, DA1 = 2 * DA1; Ainj = {{t1, DA1, eta * DA1}}; ];
    Dmax = DA1; Dmin = DA1 / 2;

    While[Abs[Dmin - Dmax] / Dmax > 0.000001,
      DA1 = (Dmin + Dmax) / 2; Ainj = {{t1, DA1, eta * DA1}};
      If[FullSystemSolutionToxicity[] < Abld, Dmin = DA1, Dmax = DA1]];
    Dtox = Dmax;];
  Return[Dtox / nCpm]);
```

In[ ]:=

```
FindCurativeSecondDose[day_] := (
  Dmin = 0; Dmax = Ainj[[1, 2]];
  IC2 = FullSystemSolutionGetValues[day][[2]];
  While[FullSystemSolutionIC[Join[IC2, {Dmax, eta * Dmax}]][[2, 1]] > Ncur, Dmax = 2 * Dmax];

  While[Abs[Dmin - Dmax] / Dmax > 0.000001,
    DA2 = (Dmin + Dmax) / 2;
    If[FullSystemSolutionIC[Join[IC2, {DA2, eta * DA2}]][[2, 1]] > Ncur, Dmin = DA2, Dmax = DA2]];
  DA2 = Dmax; Return[DA2]);
```

In[ ]:=

```
FullSystemSolutionIC[{ai_, bi_, NNi_, DDi_, pai_, pbi_, fFNi_, fANi_, dFi_, dAi_, Ai_, Bi_}] := (
  Quiet[tEnd = -Log[0.0017] / lambda;

    (* SOLVER *)
    ClearAll[a, b, NN, DD, pa, pb, fFN, fAN, dF, dA];

    sol = NDSolve[{
        (*INITIAL CONDITIONS*)
        a[0] == ai + Ai / V, b[0] == bi + Bi / V, NN[0] == NNi,
        DD[0] == DDi, pa[0] == pai, pb[0] == pbi, fFN[0] == fFNi, fAN[0] == fANi, dF[0] == dFi, dA[0] == dAi,

        a'[t] == Fa[t], b'[t] == Fb[t], NN'[t] == FNN[t], DD'[t] == FDD[t],
        pa'[t] == Fpa[t], pb'[t] == Fpb[t], fFN'[t] == FfFN[t], fAN'[t] == FfAN[t], dF'[t] == FdF[t], dA'[t] == FdA[t]

        , WhenEvent[NN[t] > 10^9 / Nnor, tEnd = t; "StopIntegration"]
      }
      , {a, b, NN, DD, pa, pb, fFN, fAN, dF, dA}, {t, 0, tEnd}, AccuracyGoal → 10, PrecisionGoal → 10];

    NN = First[NN /. sol];
    tminNn = t /. NMinimize[{Nnor * (NN[t]), t > 0, t < tEnd}, t][[2]];
    Nmin = NMinimize[{Nnor * (NN[t]), t > 0, t < tEnd}, t][[1]];
    If[Nmin < 0, Nmin = Cd]; (*bug*)
    Return[
     {{"Nmin", "tmin"}, {(*minimal viable cell number*) Nmin,
        tminNn (*moment when it is achieved*)}}]])
```

In[∘]:=

```
FullSystemSolutionGetNNIC[{ai_, bi_, NNi_, DDi_, pai_, pbi_, fFNi_, fANi_, dFi_, dAi_, Ai_, Bi_, Interval_}] := (
  Quiet[tEnd = Interval;

   (* SOLVER *)
   ClearAll[a, b, NN, DD, pa, pb, fFN, fAN, dF, dA];

   sol = NDSolve[{
       (*INITIAL CONDITIONS*)
       a[0] == ai + Ai / V, b[0] == bi + Bi / V, NN[0] == NNi,
       DD[0] == DDi, pa[0] == pai, pb[0] == pbi, fFN[0] == fFNi, fAN[0] == fANi, dF[0] == dFi, dA[0] == dAi,

       a'[t] == Fa[t], b'[t] == Fb[t], NN'[t] == FNN[t], DD'[t] == FDD[t],
       pa'[t] == Fpa[t], pb'[t] == Fpb[t], fFN'[t] == FfFN[t], fAN'[t] == FfAN[t], dF'[t] == FdF[t], dA'[t] == FdA[t]

       , WhenEvent[NN[t] > 10^9 / Nnor, tEnd = t; "StopIntegration"]
      }
     , {a, b, NN, DD, pa, pb, fFN, fAN, dF, dA}, {t, 0, tEnd}, AccuracyGoal → 10, PrecisionGoal → 10];

   NN = First[NN /. sol];
   Return[NN[tEnd]]])
```

In[∘]:=

```
FullSystemSolutionGetValues[ttE_] := (
   lAinj = Length[Ainj]; (* number of injections*)
   tEnd = ttE;
   If[Ainj⟦1, 1⟧ == 0, Npw = lAinj, Npw = lAinj + 1];
   apw = Array[ff, Npw]; bpw = Array[ff, Npw]; NNpw = Array[ff, Npw]; DDpw = Array[ff, Npw];
   papw = Array[ff, Npw]; pbpw = Array[ff, Npw]; fFNpw = Array[ff, Npw]; fANpw = Array[ff, Npw];
   dFpw = Array[ff, Npw]; dApw = Array[ff, Npw];
   tB = Array[ff, Npw]; tE = Array[ff, Npw];
   Clear[a, b, NN, DD, pa, pb, fFN, fAN, dF, dA];
```

```
(* Initial conditions *)
If[Ainj[[1, 1]] == 0
  , a0 = Ainj[[1, 2]] / V; b0 = Ainj[[1, 3]] / V
  , a0 = 0; b0 = 0]; (*complexes in blood*)
NN0 = N0;
DD0 = 0;
pa0 = 0; pb0 = 0; fFN0 = 1; fAN0 = 0;
dF0 = 0; dA0 = 0;

(* SOLVER *)
tB[[1]] = 0; If[Ainj[[1, 1]] == 0, If[lAinj > 1, tE[[1]] = Ainj[[2, 1]], tE[[1]] = tEnd], tE[[1]] = Ainj[[1, 1]]];

For[npw = 1, npw ≤ Npw, npw++,
  Clear[a, b, NN, DD, pa, pb, fFN, fAN, dF, dA];
  sol = NDSolve[{
        a[tB[[npw]]] == a0, b[tB[[npw]]] == b0, NN[tB[[npw]]] == NN0, DD[tB[[npw]]] == DD0, pa[tB[[npw]]] == pa0,
        pb[tB[[npw]]] == pb0, fFN[tB[[npw]]] == fFN0, fAN[tB[[npw]]] == fAN0, dF[tB[[npw]]] == dF0, dA[tB[[npw]]] == dA0,

        a'[t] == Fa[t], b'[t] == Fb[t], NN'[t] == FNN[t], DD'[t] == FDD[t],
        pa'[t] == Fpa[t], pb'[t] == Fpb[t], fFN'[t] == FfFN[t], fAN'[t] == FfAN[t], dF'[t] == FdF[t], dA'[t] == FdA[t]
      }
    , {a, b, NN, DD, pa, pb, fFN, fAN, dF, dA}, {t, tB[[npw]], tE[[npw]]}, AccuracyGoal → 10, PrecisionGoal → 10];

  apw[[npw]] = First[a /. sol]; bpw[[npw]] = First[b /. sol]; NNpw[[npw]] = First[NN /. sol]; DDpw[[npw]] = First[DD /. sol];
  papw[[npw]] = First[pa /. sol]; pbpw[[npw]] = First[pb /. sol]; fFNpw[[npw]] = First[fFN /. sol];
  fANpw[[npw]] = First[fAN /. sol]; dFpw[[npw]] = First[dF /. sol]; dApw[[npw]] = First[dA /. sol];
  If[npw < Npw,
    (*renew initial conditions*)
    If[Ainj[[1, 1]] == 0
      , a0 = apw[[npw]][tE[[npw]]] + Ainj[[npw + 1, 2]] / V; b0 = bpw[[npw]][tE[[npw]]] + Ainj[[npw + 1, 3]] / V
```

```
      , a0 = apw[[npw]] [tE[[npw]]] + Ainj[[npw, 2]] / V; b0 = bpw[[npw]] [tE[[npw]]] + Ainj[[npw, 3]] / V];
    NN0 = NNpw[[npw]] [tE[[npw]]];
    DD0 = DDpw[[npw]] [tE[[npw]]];
    pa0 = papw[[npw]] [tE[[npw]]];
    pb0 = pbpw[[npw]] [tE[[npw]]];
    fFN0 = fFNpw[[npw]] [tE[[npw]]]; fAN0 = fANpw[[npw]] [tE[[npw]]];
    dF0 = dFpw[[npw]] [tE[[npw]]]; dA0 = dApw[[npw]] [tE[[npw]]];
    (*renew time frame*)
    tB[[npw + 1]] = tE[[npw]];
    If [Ainj[[1, 1]] == 0, If [Npw > npw + 1, tE[[npw + 1]] = Ainj[[npw + 2, 1]], tE[[npw + 1]] = tEnd],
      If [lAinj > npw, tE[[npw + 1]] = Ainj[[npw + 1, 1]], tE[[npw + 1]] = tEnd] ];]
];
npw--;

Return [
  {{"a", "b", "NN", "DD", "pa", "pb", "fFN", "fAN", "dF", "dA"},
   {apw[[npw]] [tEnd], bpw[[npw]] [tEnd], NNpw[[npw]] [tEnd], DDpw[[npw]] [tEnd], papw[[npw]] [tEnd],
    pbpw[[npw]] [tEnd], fFNpw[[npw]] [tEnd], fANpw[[npw]] [tEnd], dFpw[[npw]] [tEnd], dApw[[npw]] [tEnd]}}]);
```

In[ ]:=

```
f1crit = 0.95;
f1lowest = 0.05;

Move[dir_] := (
    If[dir == 1, f1 = f1 + f1step];
    If[dir == 2, f1 = f1 + f1step; dday = dday + ddaystep];
    If[dir == 3, dday = dday + ddaystep];
    If[dir == 4, f1 = f1 - f1step; dday = dday + ddaystep];
    If[dir == 5, f1 = f1 - f1step];
    If[dir == 6, f1 = f1 - f1step; dday = dday - ddaystep];
    If[dir == 7, dday = dday - ddaystep];
    If[dir == 0, f1 = f1 + f1step; dday = dday - ddaystep];

    df = f1step; dd = ddaystep; (* step that actually was taken, accounting for boundaries *)
    If[f1 > f1crit, df = f1step - (f1 - f1crit); f1 = f1crit];
    If[f1 < f1lowest, df = f1step - (f1lowest - f1); f1 = f1lowest];
    If[dday < 1, dd = ddaystep - (1 - dday); dday = 1];
  )

MoveBack[dir_] := (
    If[dir == 1, f1 = f1 - df];
    If[dir == 2, f1 = f1 - df; dday = dday - dd];
    If[dir == 3, dday = dday - dd];
    If[dir == 4, f1 = f1 + df; dday = dday - dd];
    If[dir == 5, f1 = f1 + df];
    If[dir == 6, f1 = f1 + df; dday = dday + dd];
    If[dir == 7, dday = dday + dd];
    If[dir == 0, f1 = f1 - df;
      dday = dday + dd];)
```

In[ ]:=

```
OptimizeTwoDose[] := (
```

```
prCur = PrintTemporary["Single curative dose is " <> ToString[ASC / nCpm]];
Ainj = {{0, ASC, eta * ASC}};
FullSystemSolutionMD[];
FullSystemSolutionMDStopNmin[tminNn]; (* solve it with cell proliferation stopping *)

f1step1 = 0.1; f1step2 = 0.03; f1step3 = 0.01; (*final accuracy is 1% of initial curative dose*)
ddaystep1 = Floor[dayInit / 3]; ddaystep2 = 3; ddaystep3 = 1; (*final accuracy is 1 day*)
Steps = {{f1step1, ddaystep1}, {f1step2, ddaystep2}, {f1step3, ddaystep3}};

(* select initial two-dose setting *)
f1 = 0.8; dday = dayInit;

resultDD = Array[f, {2, 5}];
resultDD⟦1, 1⟧ = "1st / Acur";
resultDD⟦1, 2⟧ = "Interval";
resultDD⟦1, 3⟧ = "TotalDose";
resultDD⟦1, 4⟧ = "2nd Dose";
resultDD⟦1, 5⟧ = "TotalTox";

ii = 2; iiAll = 2;
resultDD⟦ii, 1⟧ = f1; resultDD⟦ii, 2⟧ = dday;

Ainj = {{0, f1 * ASC, eta * f1 * ASC}};
FindCurativeSecondDose[dday];

resultDD⟦ii, 3⟧ = DA2 / nCpm + f1 * ASC / nCpm;
resultDD⟦ii, 4⟧ = DA2 / nCpm;
Ainj = {{0, f1 * ASC, eta * f1 * ASC}, {dday, DA2, eta * DA2}};

FullSystemSolutionMDStopNmin[tminNn];
```

```
resultDD[[ii, 5]] = ActBloodpw[[npw]][tEnd] / nCpm;

NotebookDelete[pr]; pr = PrintTemporary[MatrixForm[resultDD]];
ii++; iiAll++;

For[istep = 1, istep ≤ 3, istep++, (* three levels from rough to fine *)

  f1step = Steps[[istep, 1]]; ddaystep = Steps[[istep, 2]];

  Dir = 1; CantMakeStep = 0; (*rotate while you can't make a step in any direction *)
  While[CantMakeStep ≤ 8, NotebookDelete[pr2]; pr2 = PrintTemporary[CantMakeStep];
    Stop = 0; MovedFlg = 0;
    While[Stop == 0, (* move by increasing the first fraction while it's profitable *)

      resultDD = Join[resultDD, {{"", "", "", "", ""}}];
      Move[Dir];
      resultDD[[ii, 1]] = f1;
      resultDD[[ii, 2]] = dday;

      Ainj = {{0, f1 * ASC, eta * f1 * ASC}};
      FindCurativeSecondDose[dday];

      resultDD[[ii, 3]] = DA2 / nCpm + f1 * ASC / nCpm; resultDD[[ii, 4]] = DA2 / nCpm;
      Ainj = {{0, f1 * ASC, eta * f1 * ASC}, {dday, DA2, eta * DA2}};
      FullSystemSolutionMDStopNmin[tminNn];
      resultDD[[ii, 5]] = ActBloodpw[[npw]][tEnd] / nCpm;

      NotebookDelete[pr]; pr = PrintTemporary[MatrixForm[resultDD]];
```

```
            If[resultDD[[ii, 5]] ≥ resultDD[[ii - 1, 5]], Stop = 1;
              ii--;
              MoveBack[Dir];
              resultDD = resultDD[[1 ;; Length[resultDD] - 1]];
              If[MovedFlg == 0, CantMakeStep++],
              MovedFlg = 1; CantMakeStep = 0];

            If[0 ≤ Dir ≤ 2 && f1 == f1crit, Stop = 1];
            If[4 ≤ Dir ≤ 6 && f1 == 0, Stop = 1]; (* hardly, but let it be *)
            If[(6 ≤ Dir ≤ 7 || Dir == 0) && dday == 1, Stop = 1];

            ii++; iiAll++;];
          Dir = Mod[Dir + 1, 8]]];
      NotebookDelete[prCur];
      NotebookDelete[prTox];
      NotebookDelete[pr];
      NotebookDelete[pr2];

      resultDDOpt = resultDD[[Length[resultDD]]];)
```

In[ ]:=

```
  FullSystemSolutionMDStopNmin[TMIN_] := (

      (* maximum time of simulation-- where little radioactivity of the last dose remains, namely 0.17% of the last dose *)
      tEnd = Ainj[[Length[Ainj], 1]] + (-Log[0.0017] / lambda);

      lAinj = Length[Ainj]; (* number of injections*)
      If[Ainj[[1, 1]] == 0, Npw = lAinj, Npw = lAinj + 1];

      apw = Array[ff, Npw]; bpw = Array[ff, Npw]; NNpw = Array[ff, Npw]; DDpw = Array[ff, Npw];
      papw = Array[ff, Npw]; pbpw = Array[ff, Npw]; fFNpw = Array[ff, Npw]; fANpw = Array[ff, Npw];
      dFpw = Array[ff, Npw]; dApw = Array[ff, Npw];
```

```
(* inert antibody fragments are as well accounted for as pb *)

(* times of beginning and end for solution of separate systems *)
tB = Array[ff, Npw]; tE = Array[ff, Npw];

(* for monitoring the paths of activity *)
ActBloodpw = Array[ff, Npw]; ActBloodFragpw = Array[ff, Npw];
ActOutpw = Array[ff, Npw]; ActOutFragpw = Array[ff, Npw]; ActTumorpw = Array[ff, Npw];

(* for monitoring influence of self-dose, croos-fire and decays in blood *)
SDpw = Array[ff, Npw]; CFNpw = Array[ff, Npw]; CFDpw = Array[ff, Npw]; UNpw = Array[ff, Npw];

(* for monitoring the number of new cancer cells appearing during treatment *)
NewCellspw = Array[ff, Npw];

Clear[a, b, NN, DD, pa, pb, fFN, fAN, dF, dA, ActBlood, ActBloodFrag, ActOut, ActOutFrag, ActTumor, SD, CFN, CFD, UN, NewCells];

(* EQUATIONS *)

(* Radiation damage function *)
RD[NN_, DD_, fAN_, dA_, a_, pa_] := alpha * (ks * (lambda * gamma * fAN)/nu (*self-dose*) +
    (1 - ks) * (lambda * gamma * (fAN * NN + dA))/(nu * (NN + DD)) (*cross-fire*) + kf * lambda * (a + pa) (*dose from unanchored nuclides*));


(* Active antibodies *)
Fa[t_] := (*injections are considered as initial conditions*)
  - lambda * a[t] (*decay*) - kon * gamma/V * (fFN[t] * NN[t] + dF[t]) * a[t] (*binding*) - kappac * a[t] (*clearance*);
```

```
(* Inert antibodies *)
Fb[t_] := (*injections are considered as initial conditions*)
  + lambda*a[t] (*decay of a*) - kon*gamma/V*(fFN[t]*NN[t] + dF[t])*b[t] (*binding*) - kappac*b[t] (*clearance*);

(* Viable cells *) FNN[t_] := rho*NN[t] (*proliferation*) - RD[NN[t], DD[t], fAN[t], dA[t], a[t], pa[t]]*NN[t] (*damage*);

(* Damaged cells *) FDD[t_] := RD[NN[t], DD[t], fAN[t], dA[t], a[t], pa[t]]*NN[t] (*damage*) - omega*DD[t] (*death*);

(* Active fragments *) Fpa[t_] := omega*(gamma*dA[t])/V (*release*) - lambda*pa[t] (*decay*) - kappap*pa[t] (*clearance*);

(* Inert fragments *)
Fpb[t_] := omega*(gamma*(DD[t] - dF[t] - dA[t]))/V (*release*) + lambda*pa[t] (*decay*) - kappap*pb[t] (*clearance*);

(* Free receptors of viable cells *) FfFN[t_] := (1 - fFN[t])*rho - kon*(a[t] + b[t])*fFN[t];

(* Active receptors of viable cells *) FfAN[t_] := kon*a[t]*fFN[t] - (lambda + rho)*fAN[t];

(* Free receptors of damaged cells *)
FdF[t_] := RD[NN[t], DD[t], fAN[t], dA[t], a[t], pa[t]]*fFN[t]*NN[t] - kon*(a[t] + b[t])*dF[t] - omega*dF[t];

(* Active receptors of damaged cells *)
FdA[t_] :=
  RD[NN[t], DD[t], fAN[t], dA[t], a[t], pa[t]]*fAN[t]*NN[t] + kon*a[t]*dF[t] - lambda*dA[t] - omega*dA[t];

(* Initial conditions *)

If[Ainj[[1, 1]] == 0
  , a0 = Ainj[[1, 2]]/V; b0 = Ainj[[1, 3]]/V
```

```
    , a0 = 0; b0 = 0]; (*complexes in blood*)
NN0 = N0;
DD0 = 0;
pa0 = 0; pb0 = 0; fFN0 = 1; fAN0 = 0;
dF0 = 0; dA0 = 0;
ActBlood0 = 0; ActBloodFrag0 = 0; ActOut0 = 0; ActOutFrag0 = 0; ActTumor0 = 0; NewCells0 = 0;
SD0 = 0; CFN0 = 0; CFD0 = 0; UN0 = 0;

(* SOLVER *)

tB[[1]] = 0; If[Ainj[[1, 1]] == 0, If[lAinj > 1, tE[[1]] = Ainj[[2, 1]], tE[[1]] = tEnd], tE[[1]] = Ainj[[1, 1]]];

For[npw = 1, npw ≤ Npw, npw++,

  Clear[a, b, NN, DD, pa, pb, fFN, fAN, dF, dA, ActBlood, ActBloodFrag, ActOut, ActOutFrag, ActTumor, SD, CFN, CFD, UN, NewCells];

  sol = NDSolve[{
      (*INITIAL CONDITIONS*)
      a[tB[[npw]]] == a0, b[tB[[npw]]] == b0, NN[tB[[npw]]] == NN0, DD[tB[[npw]]] == DD0, pa[tB[[npw]]] == pa0,
      pb[tB[[npw]]] == pb0, fFN[tB[[npw]]] == fFN0, fAN[tB[[npw]]] == fAN0, dF[tB[[npw]]] == dF0, dA[tB[[npw]]] == dA0,
      ActBlood[tB[[npw]]] == ActBlood0, ActBloodFrag[tB[[npw]]] == ActBloodFrag0, ActOut[tB[[npw]]] == ActOut0,
      ActOutFrag[tB[[npw]]] == ActOutFrag0, ActTumor[tB[[npw]]] == ActTumor0, SD[tB[[npw]]] == SD0,
      CFN[tB[[npw]]] == CFN0, CFD[tB[[npw]]] == CFD0, UN[tB[[npw]]] == UN0, NewCells[tB[[npw]]] == NewCells0,

      a'[t] == Fa[t], b'[t] == Fb[t], NN'[t] == FNN[t], DD'[t] == FDD[t],
      pa'[t] == Fpa[t], pb'[t] == Fpb[t], fFN'[t] == FfFN[t], fAN'[t] == FfAN[t], dF'[t] == FdF[t], dA'[t] == FdA[t],

      ActBlood'[t] == V * lambda * (a[t] + pa[t]),
      ActBloodFrag'[t] == V * lambda * pa[t],
      ActOut'[t] == V * (kappac * a[t] + kappap * pa[t]),
```

```
      ActOutFrag'[t] == V * kappap * pa[t],
      ActTumor'[t] == (lambda * gamma) * (fAN[t] * NN[t] + dA[t]),

      SD'[t] == ks * (lambda * gamma) * (fAN[t] * NN[t]),

                                                                  NN[t]
      CFN'[t] == (1 - ks) * (lambda * gamma) * (fAN[t] * NN[t]) * ─────────────── ,
                                                              NN[t] + DD[t]

                                                     NN[t]
      CFD'[t] == (1 - ks) * (lambda * gamma) * dA[t] * ─────────────── ,
                                                 NN[t] + DD[t]

      UN'[t] == kf * lambda * (a[t] + pa[t]) * nu * NN[t],
      NewCells'[t] == If[t > Ainj[[1, 1]], rho * NN[t], 0] (* start counting new cells from the moment of the first injection *)

      , WhenEvent[t > TMIN, NN[t] → 0]
    }
    , {a, b, NN, DD, pa, pb, fFN, fAN, dF, dA, ActBlood,
      ActBloodFrag, ActOut, ActOutFrag, ActTumor, SD, CFN, CFD, UN, NewCells}, {t, tB[[npw]], tE[[npw]]}
    , AccuracyGoal → 10, PrecisionGoal → 10];

apw[[npw]] = First[a /. sol]; bpw[[npw]] = First[b /. sol]; NNpw[[npw]] = First[NN /. sol]; DDpw[[npw]] = First[DD /. sol];
papw[[npw]] = First[pa /. sol]; pbpw[[npw]] = First[pb /. sol]; fFNpw[[npw]] = First[fFN /. sol];
fANpw[[npw]] = First[fAN /. sol]; dFpw[[npw]] = First[dF /. sol]; dApw[[npw]] = First[dA /. sol];
ActBloodpw[[npw]] = First[ActBlood /. sol];
ActBloodFragpw[[npw]] = First[ActBloodFrag /. sol];
ActOutpw[[npw]] = First[ActOut /. sol];
ActOutFragpw[[npw]] = First[ActOutFrag /. sol];
ActTumorpw[[npw]] = First[ActTumor /. sol];
SDpw[[npw]] = First[SD /. sol];
CFNpw[[npw]] = First[CFN /. sol];
CFDpw[[npw]] = First[CFD /. sol];
UNpw[[npw]] = First[UN /. sol];
NewCellspw[[npw]] = First[NewCells /. sol];
```

```
If[npw < Npw,
  (*renew initial conditions*)
  If[Ainj⟦1, 1⟧ == 0
    , a0 = apw⟦npw⟧[tE⟦npw⟧] + Ainj⟦npw + 1, 2⟧/V; b0 = bpw⟦npw⟧[tE⟦npw⟧] + Ainj⟦npw + 1, 3⟧/V
    , a0 = apw⟦npw⟧[tE⟦npw⟧] + Ainj⟦npw, 2⟧/V; b0 = bpw⟦npw⟧[tE⟦npw⟧] + Ainj⟦npw, 3⟧/V];
  NN0 = NNpw⟦npw⟧[tE⟦npw⟧];
  DD0 = DDpw⟦npw⟧[tE⟦npw⟧];
  pa0 = papw⟦npw⟧[tE⟦npw⟧];
  pb0 = pbpw⟦npw⟧[tE⟦npw⟧];
  fFN0 = fFNpw⟦npw⟧[tE⟦npw⟧]; fAN0 = fANpw⟦npw⟧[tE⟦npw⟧];
  dF0 = dFpw⟦npw⟧[tE⟦npw⟧];
  dA0 = dApw⟦npw⟧[tE⟦npw⟧];
  ActBlood0 = ActBloodpw⟦npw⟧[tE⟦npw⟧];
  ActBloodFrag0 = ActBloodFragpw⟦npw⟧[tE⟦npw⟧];
  ActOut0 = ActOutpw⟦npw⟧[tE⟦npw⟧];
  ActOutFrag0 = ActOutFragpw⟦npw⟧[tE⟦npw⟧];
  ActTumor0 = ActTumorpw⟦npw⟧[tE⟦npw⟧];
  SD0 = SDpw⟦npw⟧[tE⟦npw⟧];
  CFN0 = CFNpw⟦npw⟧[tE⟦npw⟧];
  CFD0 = CFDpw⟦npw⟧[tE⟦npw⟧];
  UN0 = UNpw⟦npw⟧[tE⟦npw⟧];
  NewCells0 = NewCellspw⟦npw⟧[tE⟦npw⟧];

  (*renew time frame*)
  tB⟦npw + 1⟧ = tE⟦npw⟧;

  If[Ainj⟦1, 1⟧ == 0, If[Npw > npw + 1, tE⟦npw + 1⟧ = Ainj⟦npw + 2, 1⟧, tE⟦npw + 1⟧ = tEnd],
    If[Npw > npw + 1, tE⟦npw + 1⟧ = Ainj⟦npw + 1, 1⟧, tE⟦npw + 1⟧ = tEnd]];]
];
```

```
npw--;
(* It will be convenient to have estimation of minimal viable cell number here *)
Nn = If[Ainj[[1, 1]] == 0
   , Min[Table[NMinimize[{Nnor * (NNpw[[nn]][t]), t > tB[[nn]], t < tE[[nn]]}, t][[1]], {nn, 1, npw}]]
   , Min[Table[NMinimize[{Nnor * (NNpw[[nn]][t]), t > tB[[nn]], t < tE[[nn]]}, t][[1]], {nn, 2, npw}]]];
Return[Nn])
```

In[∘]:=

```
tT = 40;
PlotPlots[] := (IS = 320;
   GraphicsRow[
    {(*cells*) Quiet[Show[LogPlot[{Piecewise[Table[{Nnor * NNpw[[nn]][t], tB[[nn]] ≤ t < tE[[nn]]}, {nn, 1, npw}]],
          Piecewise[Table[{Nnor * (NNpw[[nn]][t] + DDpw[[nn]][t]), tB[[nn]] ≤ t < tE[[nn]]}, {nn, 1, npw}]]}, {t, 0, tE[[npw]]}, Filling →
           {1 → Axis, 2 → {1}}, PlotStyle → {Directive[Lighter[Gray], Thickness[0.002]], Directive[Darker[Gray], Thickness[0.002]]},
         PlotRange → {{0, tT}, {0.001, Max[Table[NMaximize[{Nnor * (NNpw[[nn]][t] + DDpw[[nn]][t]), t > tB[[nn]], t < tE[[nn]]}, t][[1]],
              {nn, 1, npw}]]}}, AxesLabel → {"days", "cells"},
         PlotLegends → Placed[{"N", "+D"}, Above], ImageSize → IS],

        ListLogPlot[{{tminNn, Nn}}, PlotStyle → Lighter[Gray]]]],

     (*occupied receptors, viable*) Quiet[Show[Plot[{Piecewise[Table[{fANpw[[nn]][t], tB[[nn]] ≤ t < tE[[nn]]}, {nn, 1, npw}]],
          Piecewise[Table[{1 - fFNpw[[nn]][t], tB[[nn]] ≤ t < tE[[nn]]}, {nn, 1, npw}]], 1}, {t, 0, tE[[npw]]}, Filling → {1 → Axis, 2 → {1},
           3 → {2}}, PlotStyle → {Directive[Lighter[Red], Thickness[0.002]], Directive[Lighter[Gray], Thickness[0.002]],
           Directive[Lighter[Green], Thickness[0.002]]}, PlotRange → {{0, tT}, {0, 1}}, AxesLabel → {"days", "fraction"},
         PlotLegends → Placed[{"fAN", "+fBN", "+fFN"}, Above], ImageSize → IS],
        Plot[Piecewise[Table[{1000 * fANpw[[nn]][t], tB[[nn]] ≤ t < tE[[nn]]}, {nn, 1, npw}]], {t, 0, tE[[npw]]},
         PlotStyle → {Directive[Lighter[Red], Dashed, Thickness[0.002]]}, PlotRange → {{0, 40}, {0, 1}}]
       ]],
```

```
(*occupied receptors, damaged*) Quiet[Show[Plot[{Piecewise[Table[{dApw[[nn]][t]/DDpw[[nn]][t], tB[[nn]] < t < tE[[nn]]}, {nn, 1, npw}]],
    Piecewise[Table[{1 - dFpw[[nn]][t]/DDpw[[nn]][t], tB[[nn]] < t < tE[[nn]]}, {nn, 1, npw}]], 1}, {t, 0, tE[[npw]]}, Filling -> {1 -> Axis, 2 -> {1},
     3 -> {2}}, PlotStyle -> {Directive[Darker[Red], Thickness[0.002]], Directive[Darker[Gray], Thickness[0.002]],
     Directive[Darker[Green], Thickness[0.002]]}, PlotRange -> {{0, tT}, {0, 1}}, AxesLabel -> {"days", "fraction"},
   PlotLegends -> Placed[{"fAD", "+fBD", "+fFD"}, Above], ImageSize -> IS],
  Plot[Piecewise[Table[{1000 * dApw[[nn]][t]/DDpw[[nn]][t], tB[[nn]] ≤ t < tE[[nn]]}, {nn, 1, npw}]], {t, 0, tE[[npw]]},
   PlotStyle -> {Directive[Lighter[Red], Dashed, Thickness[0.002]]}, PlotRange -> {{0, 40}, {0, 1}}]]]
 , ImageSize -> 3.3 * IS]];
```

In[ ]:=

```
SolutionConsta[OccRec_, Nelimin_] := ( (* a is const *)

   CureFlg = 0;
   ClearAll[a, b, NN, DD, pa, pb, fFN, fAN, dF, dA, ActBlood];

   (* Radiation damage function *)
   RD[NN_, DD_, fAN_, dA_, a_, pa_] := alpha * (ks * (lambda * gamma * fAN)/nu (*self-dose*) +
        (1 - ks) * (lambda * gamma * (fAN * NN + dA))/(nu * (NN + DD)) (*cross-fire*) + kf * lambda * (a + pa) (*dose from unanchored nuclides*));

   (* Active antibodies *)
   Fa[t_] := 0;

   (* Inert antibodies *)
   Fb[t_] :=
```

```
eta * (lambda * a[t] (*decay*) + kon * gamma/V * (fFN[t] * NN[t] + dF[t]) * a[t] (*binding*) + kappac * a[t] (*clearance*)) +
   lambda * a[t] (*decay of a*) - kon * gamma/V * (fFN[t] * NN[t] + dF[t]) * b[t] (*binding*) - kappac * b[t] (*clearance*);


(* Viable cells *)
FNN[t_] := rho * NN[t] * (1 - NN[t]/(Cd / Nnor)) (*proliferation*) - RD[NN[t], DD[t], fAN[t], dA[t], a[t], pa[t]] * NN[t] (*damage*);


(* Damaged cells *) FDD[t_] := RD[NN[t], DD[t], fAN[t], dA[t], a[t], pa[t]] * NN[t] (*damage*) - omega * DD[t] (*death*);


(* Active fragments *)
Fpa[t_] := omega * (gamma * dA[t])/V (*release*) - lambda * pa[t] (*decay*) - kappap * pa[t] (*clearance*);


(* Inert fragments *)
Fpb[t_] :=
  omega * (gamma * (DD[t] - dF[t] - dA[t]))/V (*release*) + lambda * pa[t] (*decay*) - kappap * pb[t] (*clearance*);


(* Free receptors of viable cells *) FfFN[t_] := (1 - fFN[t]) * rho - kon * (a[t] + b[t]) * fFN[t];


(* Active receptors of viable cells *) FfAN[t_] := kon * a[t] * fFN[t] - (lambda + rho) * fAN[t];


(* Free receptors of damaged cells *)
FdF[t_] := RD[NN[t], DD[t], fAN[t], dA[t], a[t], pa[t]] * fFN[t] * NN[t] - kon * (a[t] + b[t]) * dF[t] - omega * dF[t];


(* Active receptors of damaged cells *)
FdA[t_] :=
  RD[NN[t], DD[t], fAN[t], dA[t], a[t], pa[t]] * fAN[t] * NN[t] + kon * a[t] * dF[t] - lambda * dA[t] - omega * dA[t];
```

```
NN0 = N0;
DD0 = 0; a0 = aconst; b0 = eta * aconst;
pa0 = 0; pb0 = 0;

If [ OccRec == 0,

   fFN0 = 1; fAN0 = 0;
   dF0 = DD0; dA0 = 0,

   fFN0 = 0; fAN0 = 1 / (eta + 1) ;
   dF0 = 0; dA0 = DD0 / (eta + 1)
 ];

ActBlood0 = 0;

ClearAll [ a, b, NN, DD, pa, pb, fFN, fAN, dF, dA, ActBlood ] ;

sol = NDSolve [ {
          (*INITIAL CONDITIONS*)
         a [ 0 ] == a0, b [ 0 ] == b0, NN [ 0 ] == NN0, DD [ 0 ] == DD0, pa [ 0 ] == pa0, pb [ 0 ] == pb0,
         fFN [ 0 ] == fFN0, fAN [ 0 ] == fAN0, dF [ 0 ] == dF0, dA [ 0 ] == dA0, ActBlood [ 0 ] == ActBlood0, (*kinj [ 0 ] ==1,*)

         a' [ t ] == 0,

         b' [ t ] == Fb [ t ],

         NN' [ t ] == FNN [ t ], DD' [ t ] == FDD [ t ], pa' [ t ] == Fpa [ t ], pb' [ t ] == Fpb [ t ], fFN' [ t ] == FfFN [ t ],
         fAN' [ t ] == FfAN [ t ], dF' [ t ] == FdF [ t ], dA' [ t ] == FdA [ t ], ActBlood' [ t ] == V * lambda * (a [ t ] + pa [ t ] )

         , WhenEvent [ NN [ t ] < Ncur / Nnor, {tEnd = t, CureFlg = 1} ]
```

```
    }
    , {a, b, NN, DD, pa, pb, fFN, fAN, dF, dA, ActBlood}, {t, 0, tEnd}
    , AccuracyGoal → 10, PrecisionGoal → 10];


If[tEnd < tEndInit,

  a1 = First[a /. sol]; b1 = First[b /. sol]; NN1 = First[NN /. sol]; DD1 = First[DD /. sol];
  pa1 = First[pa /. sol]; pb1 = First[pb /. sol]; fFN1 = First[fFN /. sol];
  fAN1 = First[fAN /. sol]; dF1 = First[dF /. sol]; dA1 = First[dA /. sol]; ActBlood1 = First[ActBlood /. sol];

  If[Nelimin == 1, NN0 = 0, NN0 = NN1[tEnd]];
  DD0 = DD1[tEnd]; a0 = a1[tEnd]; b0 = b1[tEnd];
  pa0 = pa1[tEnd]; pb0 = pb1[tEnd]; fFN0 = fFN1[tEnd]; fAN0 = fAN1[tEnd];
  dF0 = dF1[tEnd]; dA0 = dA1[tEnd]; ActBlood0 = ActBlood1[tEnd];

  ClearAll[a, b, NN, DD, pa, pb, fFN, fAN, dF, dA, ActBlood, sol];

  sol = NDSolve[{
      (*INITIAL CONDITIONS*)
      a[tEnd] == a0, b[tEnd] == b0, NN[tEnd] == NN0, DD[tEnd] == DD0, pa[tEnd] == pa0, pb[tEnd] == pb0,
      fFN[tEnd] == fFN0, fAN[tEnd] == fAN0, dF[tEnd] == dF0, dA[tEnd] == dA0, ActBlood[tEnd] == ActBlood0,

      a'[t] ==
        - lambda * a[t] (*decay*) - kon * gamma/V * (fFN[t] * NN[t] + dF[t]) * a[t] (*binding*) - kappac * a[t] (*clearance*),

      b'[t] == lambda * a[t] (*decay of a*) -
          kon * gamma/V * (fFN[t] * NN[t] + dF[t]) * b[t] (*binding*) - kappac * b[t] (*clearance*),
```

```
        NN'[t] == FNN[t], DD'[t] == FDD[t], pa'[t] == Fpa[t], pb'[t] == Fpb[t], fFN'[t] == FfFN[t],
        fAN'[t] == FfAN[t], dF'[t] == FdF[t], dA'[t] == FdA[t], ActBlood'[t] == V * lambda * (a[t] + pa[t])

        , WhenEvent[NN[t] > Cd / Nnor, {tEndInit = t, "RestartIntegration"}]
      }
      , {a, b, NN, DD, pa, pb, fFN, fAN, dF, dA, ActBlood}, {t, tEnd, tEndInit}
      , AccuracyGoal → 10, PrecisionGoal → 10, Method → "StiffnessSwitching"];

  a2 = First[a /. sol]; b2 = First[b /. sol]; NN2 = First[NN /. sol]; DD2 = First[DD /. sol];
  pa2 = First[pa /. sol]; pb2 = First[pb /. sol]; fFN2 = First[fFN /. sol];
  fAN2 = First[fAN /. sol]; dF2 = First[dF /. sol]; dA2 = First[dA /. sol]; ActBlood2 = First[ActBlood /. sol];

  a = Piecewise[{{a1, 0 ≤ t < tEnd}, {a2, tEnd ≤ t < tEndInit}}];
  b = Piecewise[{{b1, 0 ≤ t < tEnd}, {b2, tEnd ≤ t < tEndInit}}];
  NN = Piecewise[{{NN1, 0 ≤ t < tEnd}, {NN2, tEnd ≤ t < tEndInit}}];
  DD = Piecewise[{{DD1, 0 ≤ t < tEnd}, {DD2, tEnd ≤ t < tEndInit}}];
  pa = Piecewise[{{pa1, 0 ≤ t < tEnd}, {pa2, tEnd ≤ t < tEndInit}}];
  pb = Piecewise[{{pb1, 0 ≤ t < tEnd}, {pb2, tEnd ≤ t < tEndInit}}];
  fFN = Piecewise[{{fFN1, 0 ≤ t < tEnd}, {fFN2, tEnd ≤ t < tEndInit}}];
  fAN = Piecewise[{{fAN1, 0 ≤ t < tEnd}, {fAN2, tEnd ≤ t < tEndInit}}];
  dF = Piecewise[{{dF1, 0 ≤ t < tEnd}, {dF2, tEnd ≤ t < tEndInit}}];
  dA = Piecewise[{{dA1, 0 ≤ t < tEnd}, {dA2, tEnd ≤ t < tEndInit}}];
  ActBlood = Piecewise[{{ActBlood1, 0 ≤ t < tEnd}, {ActBlood2, tEnd ≤ t < tEndInit}}]
  ,
  a = First[a /. sol]; b = First[b /. sol]; NN = First[NN /. sol]; DD = First[DD /. sol];
  pa = First[pa /. sol]; pb = First[pb /. sol]; fFN = First[fFN /. sol];
  fAN = First[fAN /. sol]; dF = First[dF /. sol]; dA = First[dA /. sol]; ActBlood = First[ActBlood /. sol];
];
```

```
);

SolutionOccRec[Nm_, purity_] := ( (* receptors are fully occupied, kf=0 *)

  If[purity == 0, etast = eta * (lambda + kappac)/kappac, etast = eta];
  ClearAll[NN, DD, fAN, dA, pa];

  tEnd = tEndInit;
  tCure = 999999999;

  (* Radiation damage function *)
  RD[NN_, DD_, fAN_, dA_] :=
   alpha * (ks * (lambda * gamma * fAN)/nu (*self-dose*) + (1 - ks) * (lambda * gamma * (fAN * NN + dA))/(nu * (NN + DD)) (*cross-fire*));

  (* Viable cells *) FNN[t_] := rho * NN[t] (*proliferation*) - RD[NN[t], DD[t], fAN[t], dA[t]] * NN[t] (*damage*);

  (* Damaged cells *) FDD[t_] := RD[NN[t], DD[t], fAN[t], dA[t]] * NN[t] (*damage*) - omega * DD[t] (*death*);

  (* Active receptors of viable cells *) FfAN[t_] := 1/(etast + 1) * rho - (lambda + rho) * fAN[t];
  (* Active receptors of damaged cells *)
  FdA[t_] := RD[NN[t], DD[t], fAN[t], dA[t]] * fAN[t] * NN[t] - lambda * dA[t] - omega * dA[t];

  (* Active fragments *)
  Fpa[t_] := omega * (gamma * dA[t])/V (*release*) - lambda * pa[t] (*decay*) - kappap * pa[t] (*clearance*);

  NN0 = N0;
```

```
DD0 = 0;
fAN0 = 1/(eta + 1);
dA0 = 0;
pa0 = 0;

ClearAll[NN, DD, fAN, dA, pa];

sol = NDSolve[{
    (*INITIAL CONDITIONS*)
    NN[0] == NN0, DD[0] == DD0, fAN[0] == fAN0, dA[0] == dA0, pa[0] == pa0,


    NN'[t] == FNN[t], DD'[t] == FDD[t], fAN'[t] == FfAN[t], dA'[t] == FdA[t], pa'[t] == Fpa[t]

    , WhenEvent[NN[t] < Ncur / Nnor, {NN[t] → Nm / Nnor, tCure = t}]
   }
   , {NN, DD, fAN, dA, pa}, {t, 0, tEnd}
   , AccuracyGoal → 10, PrecisionGoal → 10];

NN = First[NN /. sol]; DD = First[DD /. sol]; fAN = First[fAN /. sol]; dA = First[dA /. sol]; pa = First[pa /. sol];

);
```

In[ ]:=

```
SetBasicParameterValues[];
```

In[ ]:=

```
(* Here is the script for the solution of full system -- which yields outcome type (CURE, TOX, BUR) and time of it *)

TOC0 = 365; (* one year *)
```

```
PatientTEST[] := (
  Outcome = "";
  TOC = TOC0; (* maximum accountable overall survival *)

  FullSystemSolutionMD[]; (*find minimal N and time of achieving it *)

  If[Nn < Ncur, (* tumor cure is achieved, but toxicity can still be lethal, then it will be death of toxicity rather than cure *)
   FullSystemSolutionMDStopNmin[tminNn]; (* solve it with cell proliferation stopping at this moment*)

   If[Abs[ActBloodpw⟦npw⟧[tEnd]] > Total[Ainj⟦All, 2⟧], (* it's a rare bug when activity in blood spikes *)

    ttt = t /. NMinimize[Abs[ActBloodpw⟦npw⟧[t] - Ainj⟦1, 2⟧], t]⟦2⟧;
    If[ActBloodpw⟦npw⟧[ttt - 1] > Abld, (*in that case that's a much more realistic actual toxicity, and yes, that's toxic*)
     Outcome = "TOX"; Tox = ActBloodpw⟦npw⟧[ttt - 1] / nCpm;
     TOC = t /. NMinimize[Abs[ActBloodpw⟦npw⟧[t] - Abld], {t, tB⟦npw⟧, tE⟦npw⟧}]⟦2⟧ (* time of death *),
     (* otherwise it is cure *)
     Outcome = "CURE"; Tox = ActBloodpw⟦npw⟧[ttt - 1] / nCpm;],

    If[ActBloodpw⟦npw⟧[tEnd] > Abld (*yes, that's toxic*),
     Outcome = "TOX"; Tox = ActBloodpw⟦npw⟧[tEnd] / nCpm;
     TOC = t /. NMinimize[Abs[ActBloodpw⟦npw⟧[t] - Abld], {t, tB⟦npw⟧, tE⟦npw⟧}]⟦2⟧ (* time of death *),
     (* otherwise it is cure *)
     Outcome = "CURE"; Tox = ActBloodpw⟦npw⟧[tEnd] / nCpm;]],

   (* tumor cure is not achieved, so what is the reason of death and when it happens? Need to run simulation until 10^11 cells *)
   Quiet[FullSystemSolutionTBUR[]]; TOC = tEnd; Tox = ActBloodpw⟦npw⟧[tEnd] / nCpm;
   If[ActBloodpw⟦npw⟧[tEnd] > Abld (*yes, that's toxic*),
    Outcome = "TOX";
    TOC = t /. NMinimize[Abs[ActBloodpw⟦npw⟧[t] - Abld], {t, tB⟦npw⟧, tE⟦npw⟧}]⟦2⟧ (* time of death *),
    (* otherwise it is death of tumor burden *)
```

```
        Outcome = "TBUR";] ];
    Return[ {Outcome, Min[365, TOC], Tox} ] )
```

In[ ]:=

```
(* Here is the script for the solution of full system -- which yields outcome type (CURE, TOX, BUR ) and time of it *)

TOC0 = 365; (* one year *)

PatientTEST2[ ] := (
    Outcome = "";
    TOC = TOC0; (* maximum accountable overall survival *)

    FullSystemSolutionMD[ ]; (*find minimal N and time of achieving it *)

    If[Nn < Ncur, (* tumor cure is achieved, but toxicity can still be lethal, then it will be death of toxicity rather than cure *)
      NNall[t_] := Piecewise[Table[ {Nnor * NNpw[[nn]][t], tB[[nn]] ≤ t < tE[[nn]]}, {nn, 1, npw} ] ];
      tminNn = t /. NMinimize[Abs[Ncur - NNall[t] ], {t, 0, tEnd} ][[2]];

      FullSystemSolutionMDStopNmin[tminNn]; (* solve it with cell proliferation stopping at this moment*)

      If[Abs[ActBloodpw[[npw]][tEnd] ] > Total[Ainj[[All, 2]]], (* it's a rare bug when activity in blood spikes *)

        ttt = t /. NMinimize[Abs[ActBloodpw[[npw]][t] - Ainj[[1, 2]]], t][[2]];
        If[ActBloodpw[[npw]][ttt - 1] > Abld, (*in that case that's a much more realistic actual toxicity, and yes, that's toxic*)
          Outcome = "TOX"; Tox = ActBloodpw[[npw]][ttt - 1] / nCpm;
          TOC = t /. NMinimize[Abs[ActBloodpw[[npw]][t] - Abld], {t, tB[[npw]], tE[[npw]]} ][[2]] (* time of death *),
          (* otherwise it is cure *)
          Outcome = "CURE"; Tox = ActBloodpw[[npw]][ttt - 1] / nCpm;],

        If[ActBloodpw[[npw]][tEnd] > Abld (*yes, that's toxic*),
          Outcome = "TOX"; Tox = ActBloodpw[[npw]][tEnd] / nCpm;
```

```
        TOC = t /. NMinimize[Abs[ActBloodpw[[npw]][t] - Abld], {t, tB[[npw]], tE[[npw]]}][[2]] (* time of death *),
        (* otherwise it is cure *)
        Outcome = "CURE"; Tox = ActBloodpw[[npw]][tEnd] / nCpm;]],

    (* tumor cure is not achieved, so what is the reason of death and when it happens? Need to run simulation until 10^11 cells *)
    CCall[t_] := Piecewise[Table[{Nnor * (NNpw[[nn]][t] + DDpw[[nn]][t]), tB[[nn]] ≤ t < tE[[nn]]}, {nn, 1, npw}]];
    tBB = t /. NMinimize[{Abs[Cd - CCall[t]], t > 0, t < tEnd}, t][[2]];
    Ainj = Select[Ainj, #[[1]] < tBB &];
    Quiet[FullSystemSolutionTBUR[]]; TOC = tEnd; Tox = ActBloodpw[[npw]][tEnd] / nCpm;
    If[ActBloodpw[[npw]][tEnd] > Abld (*yes, that's toxic*),
      Outcome = "TOX";
      TOC = t /. NMinimize[Abs[ActBloodpw[[npw]][t] - Abld], {t, tB[[npw]], tE[[npw]]}][[2]] (* time of death *),
      (* otherwise it is death of tumor burden *)
      Outcome = "TBUR";]];
  Return[{Outcome, Min[365, TOC], Tox}])
```

In[ ]:=

```
FullSystemSolutionMDStopNmin[TMIN_] := (

  (* maximum time of simulation-- where little radioactivity of the last dose remains, namely 0.17% of the last dose *)
  tEnd = Ainj[[Length[Ainj], 1]] + (-Log[0.0017] / lambda);

  krho = 1; (*cells do proliferate yet*)
  lAinj = Length[Ainj]; (* number of injections*)
  If[Ainj[[1, 1]] == 0, Npw = lAinj, Npw = lAinj + 1];

  apw = Array[ff, Npw]; bpw = Array[ff, Npw]; NNpw = Array[ff, Npw]; DDpw = Array[ff, Npw];
  papw = Array[ff, Npw]; pbpw = Array[ff, Npw]; fFNpw = Array[ff, Npw]; fANpw = Array[ff, Npw];
  dFpw = Array[ff, Npw]; dApw = Array[ff, Npw];
  (* inert antibody fragments are as well accounted for as pb *)

  (* times of beginning and end for solution of separate systems *)
```

```
tB = Array[ff, Npw]; tE = Array[ff, Npw];

(* for monitoring the paths of activity *)
ActBloodpw = Array[ff, Npw]; ActBloodFragpw = Array[ff, Npw];
ActOutpw = Array[ff, Npw]; ActOutFragpw = Array[ff, Npw]; ActTumorpw = Array[ff, Npw];

(* for monitoring influence of self-dose, croos-fire and decays in blood *)
SDpw = Array[ff, Npw]; CFNpw = Array[ff, Npw]; CFDpw = Array[ff, Npw]; UNpw = Array[ff, Npw];

(* for monitoring the number of new cancer cells appearing during treatment *)
NewCellspw = Array[ff, Npw];

Clear[a, b, NN, DD, pa, pb, fFN, fAN, dF, dA, ActBlood, ActBloodFrag, ActOut, ActOutFrag, ActTumor, SD, CFN, CFD, UN, NewCells];

(* EQUATIONS *)

(* Radiation damage function *)
RD[NN_, DD_, fAN_, dA_, a_, pa_] := alpha * (ks * (lambda * gamma * fAN)/nu (*self-dose*) +
    (1 - ks) * (lambda * gamma * (fAN * NN + dA))/(nu * (NN + DD)) (*cross-fire*) + kf * lambda * (a + pa) (*dose from unanchored nuclides*));

(* Active antibodies *)
Fa[t_] := (*injections are considered as initial conditions*)
  - lambda * a[t] (*decay*) - kon * gamma/V * (fFN[t] * NN[t] + dF[t]) * a[t] (*binding*) - kappac * a[t] (*clearance*);

(* Inert antibodies *)
Fb[t_] := (*injections are considered as initial conditions*)
```

```
  + lambda * a[t] (*decay of a*) - kon * gamma/V * (fFN[t] * NN[t] + dF[t]) * b[t] (*binding*) - kappac * b[t] (*clearance*);

(* Viable cells *)
FNN[t_] := krho * rho * NN[t] (*proliferation*) - RD[NN[t], DD[t], fAN[t], dA[t], a[t], pa[t]] * NN[t] (*damage*);

(* Damaged cells *) FDD[t_] := RD[NN[t], DD[t], fAN[t], dA[t], a[t], pa[t]] * NN[t] (*damage*) - omega * DD[t] (*death*);

(* Active fragments *) Fpa[t_] := omega * (gamma * dA[t])/V (*release*) - lambda * pa[t] (*decay*) - kappap * pa[t] (*clearance*);

(* Inert fragments *)
Fpb[t_] := omega * (gamma * (DD[t] - dF[t] - dA[t]))/V (*release*) + lambda * pa[t] (*decay*) - kappap * pb[t] (*clearance*);

(* Free receptors of viable cells *) FfFN[t_] := krho * (1 - fFN[t]) * rho - kon * (a[t] + b[t]) * fFN[t];

(* Active receptors of viable cells *) FfAN[t_] := kon * a[t] * fFN[t] - (lambda + krho * rho) * fAN[t];

(* Free receptors of damaged cells *)
FdF[t_] := RD[NN[t], DD[t], fAN[t], dA[t], a[t], pa[t]] * fFN[t] * NN[t] - kon * (a[t] + b[t]) * dF[t] - omega * dF[t];

(* Active receptors of damaged cells *)
FdA[t_] :=
  RD[NN[t], DD[t], fAN[t], dA[t], a[t], pa[t]] * fAN[t] * NN[t] + kon * a[t] * dF[t] - lambda * dA[t] - omega * dA[t];

(* Initial conditions *)

If[Ainj[[1, 1]] == 0
  , a0 = Ainj[[1, 2]]/V; b0 = eta * Ainj[[1, 2]]/V
  , a0 = 0; b0 = 0]; (*complexes in blood*)
```

```
NN0 = N0;
DD0 = 0;
pa0 = 0; pb0 = 0; fFN0 = 1; fAN0 = 0;
dF0 = 0; dA0 = 0;
ActBlood0 = 0; ActBloodFrag0 = 0; ActOut0 = 0; ActOutFrag0 = 0; ActTumor0 = 0; NewCells0 = 0;
SD0 = 0; CFN0 = 0; CFD0 = 0; UN0 = 0;

(* SOLVER *)

tB[[1]] = 0; If[Ainj[[1, 1]] == 0, If[lAinj > 1, tE[[1]] = Ainj[[2, 1]], tE[[1]] = tEnd], tE[[1]] = Ainj[[1, 1]]];

For[npw = 1, npw ≤ Npw, npw++,

 Clear[a, b, NN, DD, pa, pb, fFN, fAN, dF, dA, ActBlood, ActBloodFrag, ActOut, ActOutFrag, ActTumor, SD, CFN, CFD, UN, NewCells];

 sol = NDSolve[{
    (*INITIAL CONDITIONS*)
    a[tB[[npw]]] == a0, b[tB[[npw]]] == b0, NN[tB[[npw]]] == NN0, DD[tB[[npw]]] == DD0, pa[tB[[npw]]] == pa0,
    pb[tB[[npw]]] == pb0, fFN[tB[[npw]]] == fFN0, fAN[tB[[npw]]] == fAN0, dF[tB[[npw]]] == dF0, dA[tB[[npw]]] == dA0,
    ActBlood[tB[[npw]]] == ActBlood0, ActBloodFrag[tB[[npw]]] == ActBloodFrag0, ActOut[tB[[npw]]] == ActOut0,
    ActOutFrag[tB[[npw]]] == ActOutFrag0, ActTumor[tB[[npw]]] == ActTumor0, SD[tB[[npw]]] == SD0,
    CFN[tB[[npw]]] == CFN0, CFD[tB[[npw]]] == CFD0, UN[tB[[npw]]] == UN0, NewCells[tB[[npw]]] == NewCells0,

    a'[t] == Fa[t], b'[t] == Fb[t], NN'[t] == FNN[t], DD'[t] == FDD[t],
    pa'[t] == Fpa[t], pb'[t] == Fpb[t], fFN'[t] == FfFN[t], fAN'[t] == FfAN[t], dF'[t] == FdF[t], dA'[t] == FdA[t],

    ActBlood'[t] == V * lambda * (a[t] + pa[t]),
    ActBloodFrag'[t] == V * lambda * pa[t],
    ActOut'[t] == V * (kappac * a[t] + kappap * pa[t]),
    ActOutFrag'[t] == V * kappap * pa[t],
```

```
ActTumor'[t] == (lambda * gamma) * (fAN[t] * NN[t] + dA[t]),

SD'[t] == ks * (lambda * gamma) * (fAN[t] * NN[t]),

CFN'[t] == (1 - ks) * (lambda * gamma) * (fAN[t] * NN[t]) * NN[t] / (NN[t] + DD[t]),

CFD'[t] == (1 - ks) * (lambda * gamma) * dA[t] * NN[t] / (NN[t] + DD[t]),

UN'[t] == kf * lambda * (a[t] + pa[t]) * nu * NN[t],
NewCells'[t] == If[t > Ainj[[1, 1]], krho * rho * NN[t], 0] (* start counting new cells from the moment of the first injection *)

, WhenEvent[t > TMIN, NN[t] → 0]
, WhenEvent[NN[t] > 10^9 / Nnor, NN[t] → 0.99 * 10^9 / Nnor (*tE[[npw]]=t;
  tEnd=t;
  "StopIntegration"*)] (* cancer wins *)
 (*,WhenEvent[NN[t]<0.01/Nnor,NN[t]→0(*tE[[npw]]=t;
  tEnd=t;
  "StopIntegration"*)] (* treatment wins *) *)
}
, {a, b, NN, DD, pa, pb, fFN, fAN, dF, dA, ActBlood,
  ActBloodFrag, ActOut, ActOutFrag, ActTumor, SD, CFN, CFD, UN, NewCells}, {t, tB[[npw]], tE[[npw]]}
, AccuracyGoal → 10, PrecisionGoal → 10];

apw[[npw]] = First[a /. sol]; bpw[[npw]] = First[b /. sol]; NNpw[[npw]] = First[NN /. sol]; DDpw[[npw]] = First[DD /. sol];
papw[[npw]] = First[pa /. sol]; pbpw[[npw]] = First[pb /. sol]; fFNpw[[npw]] = First[fFN /. sol];
fANpw[[npw]] = First[fAN /. sol]; dFpw[[npw]] = First[dF /. sol]; dApw[[npw]] = First[dA /. sol];
ActBloodpw[[npw]] = First[ActBlood /. sol];
ActBloodFragpw[[npw]] = First[ActBloodFrag /. sol];
ActOutpw[[npw]] = First[ActOut /. sol];
ActOutFragpw[[npw]] = First[ActOutFrag /. sol];
ActTumorpw[[npw]] = First[ActTumor /. sol];
```

```
SDpw[[npw]] = First[SD /. sol];
CFNpw[[npw]] = First[CFN /. sol];
CFDpw[[npw]] = First[CFD /. sol];
UNpw[[npw]] = First[UN /. sol];
NewCellspw[[npw]] = First[NewCells /. sol];

If[npw < Npw,
  (*renew initial conditions*)
  If[Ainj[[1, 1]] == 0
    , a0 = apw[[npw]][tE[[npw]]] + Ainj[[npw + 1, 2]]/V; b0 = bpw[[npw]][tE[[npw]]] + eta * Ainj[[npw + 1, 2]]/V
    , a0 = apw[[npw]][tE[[npw]]] + Ainj[[npw, 2]]/V; b0 = bpw[[npw]][tE[[npw]]] + eta * Ainj[[npw, 2]]/V];
  NN0 = NNpw[[npw]][tE[[npw]]];
  DD0 = DDpw[[npw]][tE[[npw]]];
  pa0 = papw[[npw]][tE[[npw]]];
  pb0 = pbpw[[npw]][tE[[npw]]];
  fFN0 = fFNpw[[npw]][tE[[npw]]]; fAN0 = fANpw[[npw]][tE[[npw]]];
  dF0 = dFpw[[npw]][tE[[npw]]];
  dA0 = dApw[[npw]][tE[[npw]]];
  ActBlood0 = ActBloodpw[[npw]][tE[[npw]]];
  ActBloodFrag0 = ActBloodFragpw[[npw]][tE[[npw]]];
  ActOut0 = ActOutpw[[npw]][tE[[npw]]];
  ActOutFrag0 = ActOutFragpw[[npw]][tE[[npw]]];
  ActTumor0 = ActTumorpw[[npw]][tE[[npw]]];
  SD0 = SDpw[[npw]][tE[[npw]]];
  CFN0 = CFNpw[[npw]][tE[[npw]]];
  CFD0 = CFDpw[[npw]][tE[[npw]]];
  UN0 = UNpw[[npw]][tE[[npw]]];
  NewCells0 = NewCellspw[[npw]][tE[[npw]]];

  (*renew time frame*)
  tB[[npw + 1]] = tE[[npw]];
```

```
    If[Ainj[[1, 1]] == 0, If[Npw > npw + 1, tE[[npw + 1]] = Ainj[[npw + 2, 1]], tE[[npw + 1]] = tEnd],
      If[Npw > npw + 1, tE[[npw + 1]] = Ainj[[npw + 1, 1]], tE[[npw + 1]] = tEnd]];]
  ];
  npw--;
  (* It will be convenient to have estimation of minimal viable cell number here *)
  Nn = If[Ainj[[1, 1]] == 0
    , Min[Table[NMinimize[{Nnor* (NNpw[[nn]][t]), t > tB[[nn]], t < tE[[nn]]}, t][[1]], {nn, 1, npw}]]
    , Min[Table[NMinimize[{Nnor* (NNpw[[nn]][t]), t > tB[[nn]], t < tE[[nn]]}, t][[1]], {nn, 2, npw}]]];
  Return[Nn])
```

In[ ]:=

```
FullSystemSolutionTBUR[] := (

  tEnd = 365; (* it will stop before this time*)
  lAinj = Length[Ainj]; (* number of injections*)
  If[Ainj[[1, 1]] == 0, Npw = lAinj, Npw = lAinj + 1];

  apw = Array[ff, Npw]; bpw = Array[ff, Npw]; NNpw = Array[ff, Npw]; DDpw = Array[ff, Npw];
  papw = Array[ff, Npw]; pbpw = Array[ff, Npw]; fFNpw = Array[ff, Npw]; fANpw = Array[ff, Npw];
  dFpw = Array[ff, Npw]; dApw = Array[ff, Npw];
  (* inert antibody fragments are as well accounted for as pb *)

  (* times of beginning and end for solution of separate systems *)
  tB = Array[ff, Npw]; tE = Array[ff, Npw];

  (* for monitoring the paths of activity *)
  ActBloodpw = Array[ff, Npw]; ActBloodFragpw = Array[ff, Npw];
  ActOutpw = Array[ff, Npw]; ActOutFragpw = Array[ff, Npw]; ActTumorpw = Array[ff, Npw];

  (* for monitoring influence of self-dose, croos-fire and decays in blood *)
```

```
SDpw = Array[ff, Npw]; CFNpw = Array[ff, Npw]; CFDpw = Array[ff, Npw]; UNpw = Array[ff, Npw];

(* for monitoring the number of new cancer cells appearing during treatment *)
NewCellspw = Array[ff, Npw];

Clear[a, b, NN, DD, pa, pb, fFN, fAN, dF, dA, ActBlood, ActBloodFrag, ActOut, ActOutFrag, ActTumor, SD, CFN, CFD, UN, NewCells];

(* EQUATIONS *)

(* Radiation damage function *)
RD[NN_, DD_, fAN_, dA_, a_, pa_] := alpha * (ks * (lambda * gamma * fAN)/nu (*self-dose*) +
    (1 - ks) * (lambda * gamma * (fAN * NN + dA))/(nu * (NN + DD)) (*cross-fire*) + kf * lambda * (a + pa) (*dose from unanchored nuclides*));


(* Active antibodies *)
Fa[t_] := (*injections are considered as initial conditions*)
  - lambda * a[t] (*decay*) - kon * gamma/V * (fFN[t] * NN[t] + dF[t]) * a[t] (*binding*) - kappac * a[t] (*clearance*);

(* Inert antibodies *)
Fb[t_] := (*injections are considered as initial conditions*)
  + lambda * a[t] (*decay of a*) - kon * gamma/V * (fFN[t] * NN[t] + dF[t]) * b[t] (*binding*) - kappac * b[t] (*clearance*);

(* Viable cells *) FNN[t_] := rho * NN[t] (*proliferation*) - RD[NN[t], DD[t], fAN[t], dA[t], a[t], pa[t]] * NN[t] (*damage*);

(* Damaged cells *) FDD[t_] := RD[NN[t], DD[t], fAN[t], dA[t], a[t], pa[t]] * NN[t] (*damage*) - omega * DD[t] (*death*);
```

```
(* Active fragments *) Fpa[t_] := omega * (gamma * dA[t])/V (*release*) - lambda * pa[t] (*decay*) - kappap * pa[t] (*clearance*);

(* Inert fragments *)
Fpb[t_] := omega * (gamma * (DD[t] - dF[t] - dA[t]))/V (*release*) + lambda * pa[t] (*decay*) - kappap * pb[t] (*clearance*);

(* Free receptors of viable cells *) FfFN[t_] := (1 - fFN[t]) * rho - kon * (a[t] + b[t]) * fFN[t];

(* Active receptors of viable cells *) FfAN[t_] := kon * a[t] * fFN[t] - (lambda + rho) * fAN[t];

(* Free receptors of damaged cells *)
FdF[t_] := RD[NN[t], DD[t], fAN[t], dA[t], a[t], pa[t]] * fFN[t] * NN[t] - kon * (a[t] + b[t]) * dF[t] - omega * dF[t];

(* Active receptors of damaged cells *)
FdA[t_] :=
  RD[NN[t], DD[t], fAN[t], dA[t], a[t], pa[t]] * fAN[t] * NN[t] + kon * a[t] * dF[t] - lambda * dA[t] - omega * dA[t];

(* Initial conditions *)

If[Ainj[[1, 1]] == 0
  , a0 = Ainj[[1, 2]]/V; b0 = eta * Ainj[[1, 2]]/V
  , a0 = 0; b0 = 0]; (*complexes in blood*)
NN0 = N0;
DD0 = 0;
pa0 = 0; pb0 = 0; fFN0 = 1; fAN0 = 0;
dF0 = 0; dA0 = 0;
ActBlood0 = 0; ActBloodFrag0 = 0; ActOut0 = 0; ActOutFrag0 = 0; ActTumor0 = 0; NewCells0 = 0;
SD0 = 0; CFN0 = 0; CFD0 = 0; UN0 = 0;

(* SOLVER *)
```

```
tB⟦1⟧ = 0; If[Ainj⟦1, 1⟧ == 0, If[lAinj > 1, tE⟦1⟧ = Ainj⟦2, 1⟧, tE⟦1⟧ = tEnd], tE⟦1⟧ = Ainj⟦1, 1⟧];

For[npw = 1, npw ≤ Npw, npw++,

  Clear[a, b, NN, DD, pa, pb, fFN, fAN, dF, dA, ActBlood, ActBloodFrag, ActOut, ActOutFrag, ActTumor, SD, CFN, CFD, UN, NewCells];

  sol = NDSolve[{
      (*INITIAL CONDITIONS*)
      a[tB⟦npw⟧] == a0, b[tB⟦npw⟧] == b0, NN[tB⟦npw⟧] == NN0, DD[tB⟦npw⟧] == DD0, pa[tB⟦npw⟧] == pa0,
      pb[tB⟦npw⟧] == pb0, fFN[tB⟦npw⟧] == fFN0, fAN[tB⟦npw⟧] == fAN0, dF[tB⟦npw⟧] == dF0, dA[tB⟦npw⟧] == dA0,
      ActBlood[tB⟦npw⟧] == ActBlood0, ActBloodFrag[tB⟦npw⟧] == ActBloodFrag0, ActOut[tB⟦npw⟧] == ActOut0,
      ActOutFrag[tB⟦npw⟧] == ActOutFrag0, ActTumor[tB⟦npw⟧] == ActTumor0, SD[tB⟦npw⟧] == SD0,
      CFN[tB⟦npw⟧] == CFN0, CFD[tB⟦npw⟧] == CFD0, UN[tB⟦npw⟧] == UN0, NewCells[tB⟦npw⟧] == NewCells0,

      a'[t] == Fa[t], b'[t] == Fb[t], NN'[t] == FNN[t], DD'[t] == FDD[t],
      pa'[t] == Fpa[t], pb'[t] == Fpb[t], fFN'[t] == FfFN[t], fAN'[t] == FfAN[t], dF'[t] == FdF[t], dA'[t] == FdA[t],

      ActBlood'[t] == V*lambda*(a[t] + pa[t]),
      ActBloodFrag'[t] == V*lambda*pa[t],
      ActOut'[t] == V*(kappac*a[t] + kappap*pa[t]),
      ActOutFrag'[t] == V*kappap*pa[t],
      ActTumor'[t] == (lambda*gamma)*(fAN[t]*NN[t] + dA[t]),

      SD'[t] == ks*(lambda*gamma)*(fAN[t]*NN[t]),
      CFN'[t] == (1 - ks)*(lambda*gamma)*(fAN[t]*NN[t])*NN[t]/(NN[t] + DD[t]),
      CFD'[t] == (1 - ks)*(lambda*gamma)*dA[t]*NN[t]/(NN[t] + DD[t]),
      UN'[t] == kf*lambda*(a[t] + pa[t])*nu*NN[t],
```

```
      NewCells'[t] == If[t > Ainj⟦1, 1⟧, rho * NN[t], 0] (* start counting new cells from the moment of the first injection *)

      , WhenEvent[NN[t] + DD[t] > Cd / Nnor, "StopIntegration"; tEnd = t; tE⟦npw⟧ = t; NN[t] → 0]
    }
    , {a, b, NN, DD, pa, pb, fFN, fAN, dF, dA, ActBlood,
      ActBloodFrag, ActOut, ActOutFrag, ActTumor, SD, CFN, CFD, UN, NewCells}, {t, tB⟦npw⟧, tE⟦npw⟧}
    , AccuracyGoal → 10, PrecisionGoal → 10];

apw⟦npw⟧ = First[a /. sol]; bpw⟦npw⟧ = First[b /. sol]; NNpw⟦npw⟧ = First[NN /. sol]; DDpw⟦npw⟧ = First[DD /. sol];
papw⟦npw⟧ = First[pa /. sol]; pbpw⟦npw⟧ = First[pb /. sol]; fFNpw⟦npw⟧ = First[fFN /. sol];
fANpw⟦npw⟧ = First[fAN /. sol]; dFpw⟦npw⟧ = First[dF /. sol]; dApw⟦npw⟧ = First[dA /. sol];
ActBloodpw⟦npw⟧ = First[ActBlood /. sol];
ActBloodFragpw⟦npw⟧ = First[ActBloodFrag /. sol];
ActOutpw⟦npw⟧ = First[ActOut /. sol];
ActOutFragpw⟦npw⟧ = First[ActOutFrag /. sol];
ActTumorpw⟦npw⟧ = First[ActTumor /. sol];
SDpw⟦npw⟧ = First[SD /. sol];
CFNpw⟦npw⟧ = First[CFN /. sol];
CFDpw⟦npw⟧ = First[CFD /. sol];
UNpw⟦npw⟧ = First[UN /. sol];
NewCellspw⟦npw⟧ = First[NewCells /. sol];

If[npw < Npw,
  (*renew initial conditions*)
  If[Ainj⟦1, 1⟧ == 0
    , a0 = apw⟦npw⟧[tE⟦npw⟧] + Ainj⟦npw + 1, 2⟧/V; b0 = bpw⟦npw⟧[tE⟦npw⟧] + eta * Ainj⟦npw + 1, 2⟧/V
    , a0 = apw⟦npw⟧[tE⟦npw⟧] + Ainj⟦npw, 2⟧/V; b0 = bpw⟦npw⟧[tE⟦npw⟧] + eta * Ainj⟦npw, 2⟧/V];
  NN0 = NNpw⟦npw⟧[tE⟦npw⟧];
  DD0 = DDpw⟦npw⟧[tE⟦npw⟧];
  pa0 = papw⟦npw⟧[tE⟦npw⟧];
```

```
        pb0 = pbpw⟦npw⟧[tE⟦npw⟧];
        fFN0 = fFNpw⟦npw⟧[tE⟦npw⟧]; fAN0 = fANpw⟦npw⟧[tE⟦npw⟧];
        dF0 = dFpw⟦npw⟧[tE⟦npw⟧];
        dA0 = dApw⟦npw⟧[tE⟦npw⟧];
        ActBlood0 = ActBloodpw⟦npw⟧[tE⟦npw⟧];
        ActBloodFrag0 = ActBloodFragpw⟦npw⟧[tE⟦npw⟧];
        ActOut0 = ActOutpw⟦npw⟧[tE⟦npw⟧];
        ActOutFrag0 = ActOutFragpw⟦npw⟧[tE⟦npw⟧];
        ActTumor0 = ActTumorpw⟦npw⟧[tE⟦npw⟧];
        SD0 = SDpw⟦npw⟧[tE⟦npw⟧];
        CFN0 = CFNpw⟦npw⟧[tE⟦npw⟧];
        CFD0 = CFDpw⟦npw⟧[tE⟦npw⟧];
        UN0 = UNpw⟦npw⟧[tE⟦npw⟧];
        NewCells0 = NewCellspw⟦npw⟧[tE⟦npw⟧];

        (*renew time frame*)
        tB⟦npw + 1⟧ = tE⟦npw⟧;

        If[Ainj⟦1, 1⟧ == 0, If[Npw > npw + 1, tE⟦npw + 1⟧ = Ainj⟦npw + 2, 1⟧, tE⟦npw + 1⟧ = tEnd],
          If[Npw > npw + 1, tE⟦npw + 1⟧ = Ainj⟦npw + 1, 1⟧, tE⟦npw + 1⟧ = tEnd]];
      ];
      npw--;)
```

(* Subsection S.5.1 Optimization of two-dose treatment *)

(* S.5.1.1 Algorithm for optimization of two-dose treatment *)

In[ ]:=

```
(* Figure S.23: The work of the algorithm for the basic set of parameters *)
(* Two-dose optimization for the basic parameter set, ks=0.3, it takes several minutes to perform *)
SetBasicParameterValues[]; ks = 0.3;
ASC = FindCurDose[] * nCpm;
ASC03 = ASC / nCpm; (* remember single curative dose *)
Ainj = {{0, ASC, eta * ASC}};
ToxASC = FullSystemSolutionToxicity[] / nCpm;
ToxASC03 = ToxASC; (* remember toxicity due to single curative dose *)
OptimizeTwoDose[];
ToxDD03 = resultDD[[Length[resultDD], 5]]; (* remember optimized two-dose toxicity *)
AinjOpt03 = {{0, resultDDOpt[[1]] * ASC, eta * resultDDOpt[[1]] * ASC}, {resultDDOpt[[2]], resultDDOpt[[4]] * nCpm, eta * resultDDOpt[[4]] * nCpm}};
(* remember optimal two-dose schedule *)
MatrixForm[resultDD]

(* Illustration *)
resultDD[[1]] = {1, 0, ASC / nCpm, 0, ToxASC};
ListPlot[Style[Labeled[#, Round[resultDD[[Position[resultDD[[All, {1, 2}]], #][[1]], 5]][[1]], 0.01]] & /@ resultDD[[All, {1, 2}]],
  ColorData["DarkRainbow"][1]], PlotRange → {{0.7, 1}, {0, Max[resultDD[[All, 2]]]}}, ImageSize → 300]
```

Out[ ]//MatrixForm=

1st / Acur	Interval	TotalDose	2nd Dose	TotalTox
0.8	20	63.3454	18.163	7.24293
0.9	20	55.9551	5.12479	3.09309
0.95	20	55.8506	2.19637	2.53493
0.85	14	53.604	5.5976	2.3331
0.75	8	53.668	11.3094	2.14297
0.85	8	54.1233	6.11687	2.06769
0.88	8	54.3763	4.67562	2.06518
0.85	5	54.8463	6.83996	2.06288
0.86	6	54.6126	6.04145	2.05568

Out[ ]=

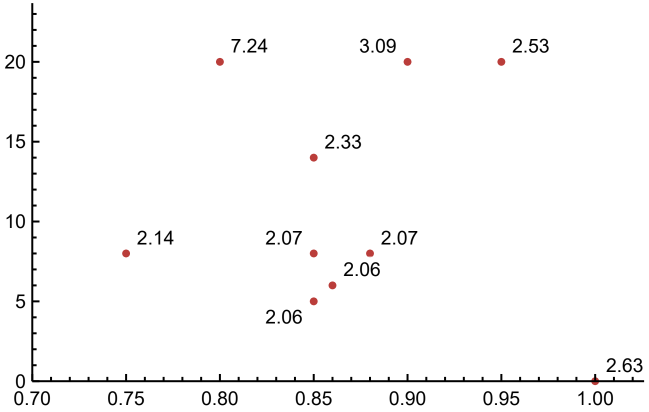

(* S.5.1.2 Optimized two-dose schedules under variation of particle range *)

```
(* Two-dose optimization for the basic parameter set and ks=0 & ks=1 are hidden here, each takes several minutes to perform *)

SetBasicParameterValues[]; ks = 0.;
ASC = FindCurDose[] * nCpm;
ASC0 = ASC / nCpm; (* remember single curative dose *)
Ainj = {{0, ASC, eta * ASC}};
ToxASC = FullSystemSolutionToxicity[] / nCpm;
ToxASC0 = ToxASC; (* remember toxicity due to single curative dose *)
OptimizeTwoDose[];
ToxDD0 = resultDD⟦Length[resultDD], 5⟧; (* remember optimized two-dose toxicity *)
AinjOpt0 = {{0, resultDDOpt⟦1⟧ * ASC, eta * resultDDOpt⟦1⟧ * ASC}, {resultDDOpt⟦2⟧, resultDDOpt⟦4⟧ * nCpm, eta * resultDDOpt⟦4⟧ * nCpm}};
(* remember optimal two-dose schedule *)

SetBasicParameterValues[]; ks = 1.;
ASC = FindCurDose[] * nCpm;
ASC1 = ASC / nCpm; (* remember single curative dose *)
Ainj = {{0, ASC, eta * ASC}};
ToxASC = FullSystemSolutionToxicity[] / nCpm;
ToxASC1 = ToxASC; (* remember toxicity due to single curative dose *)
OptimizeTwoDose[];
ToxDD1 = resultDD⟦Length[resultDD], 5⟧; (* remember optimized two-dose toxicity *)
AinjOpt1 = {{0, resultDDOpt⟦1⟧ * ASC, eta * resultDDOpt⟦1⟧ * ASC}, {resultDDOpt⟦2⟧, resultDDOpt⟦4⟧ * nCpm, eta * resultDDOpt⟦4⟧ * nCpm}};
(* remember optimal two-dose schedule *)
```

In[ ]:=

```
(* The results of two-dose optimization for these cases are hidden here, run this group of cells to upload them *)
```

In[ ]:=

```
AinjOpt0 = {{0, 0.0028925490736092015`, 5.148737351024379`}, {11, 0.0008098743485199973`, 1.441576340365595`}};
AinjOpt03 = {{0, 0.003691408413506022`, 6.57070697604072`}, {6, 0.0004591501121861741`, 0.81728719969139`}};
AinjOpt1 = {{0, 0.004207596174525871`, 7.489521190656051`}, {16, 0.00006804790695473014`, 0.12112527437941965`}};
ASC0 = 50.746474975600016`; (* minimal single curative dose *)
ToxASC0 = 1.5553654432952029`; (* toxic decays for treatment with minimal single curative dose *)
ToxDD0 = 1.464242269720281`; (* toxic decays for optimal two-dose treatment *)
ASC03 = 56.47809690186692`;
ToxASC03 = 2.6344196232982453`;
ToxDD03 = 2.0556768602168307`;
ASC1 = 63.63575581557579`;
ToxASC1 = 5.597978146160257`;
ToxDD1 = 2.4893977220747927`;
```

In[ ]:=

```
(* Figure S.24: Simulations of treatments by optimal two-dose schedules *)
(*ks=0*) SetBasicParameterValues[]; ks = 0;
Ainj = {{0, ASC0 * nCpm, eta * ASC0 * nCpm}}; FullSystemSolutionMD[]; NNsd = NNpw; tminNnsd = tminNn;
Ainj = AinjOpt0; FullSystemSolutionMD[]; PlotPlots[]
(*ks=0.3*) ks = 0.3;
Ainj = {{0, ASC03 * nCpm, eta * ASC03 * nCpm}}; FullSystemSolutionMD[]; NNsd = NNpw; tminNnsd = tminNn;
Ainj = AinjOpt03; FullSystemSolutionMD[]; PlotPlots[]
(*ks=1*) ks = 1;
Ainj = {{0, ASC1 * nCpm, eta * ASC1 * nCpm}}; FullSystemSolutionMD[]; NNsd = NNpw; tminNnsd = tminNn;
Ainj = AinjOpt1; FullSystemSolutionMD[]; PlotPlots[]
```

Out[ ]=

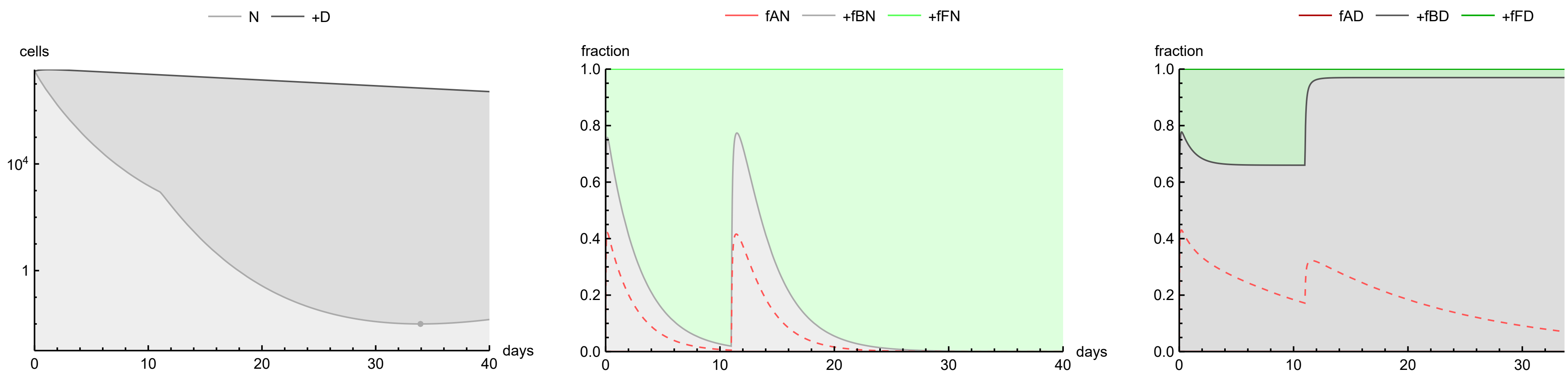

N
+D
cells
10^4
1
days
fAN
+fBN
+fFN
fraction
days
fAD
+fBD
+fFD
fraction

Out[ ]=

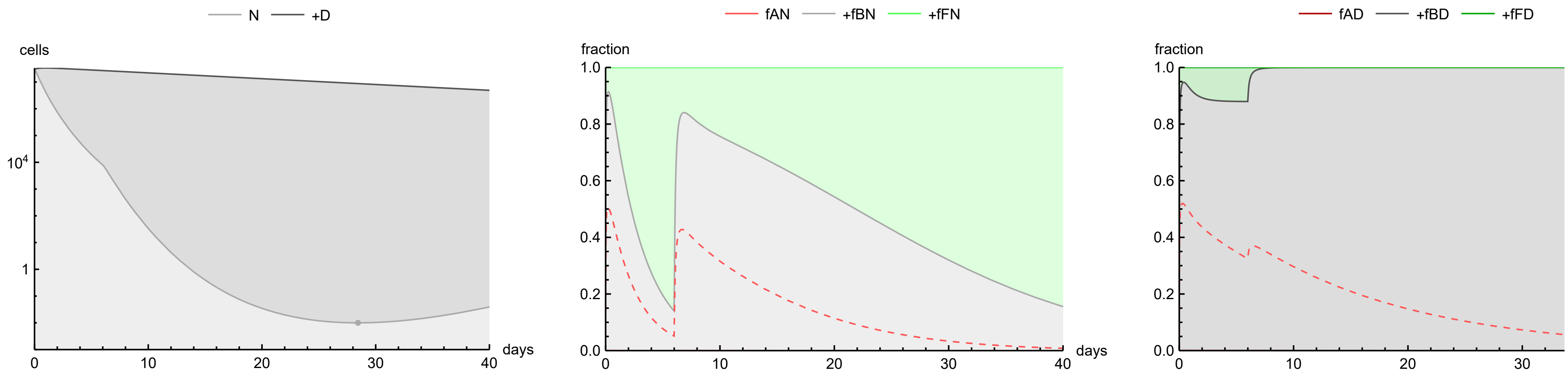

N
+D
cells
10^4
1
days
fAN
+fBN
+fFN
fraction
days
fAD
+fBD
+fFD
fraction

Out[ ]=

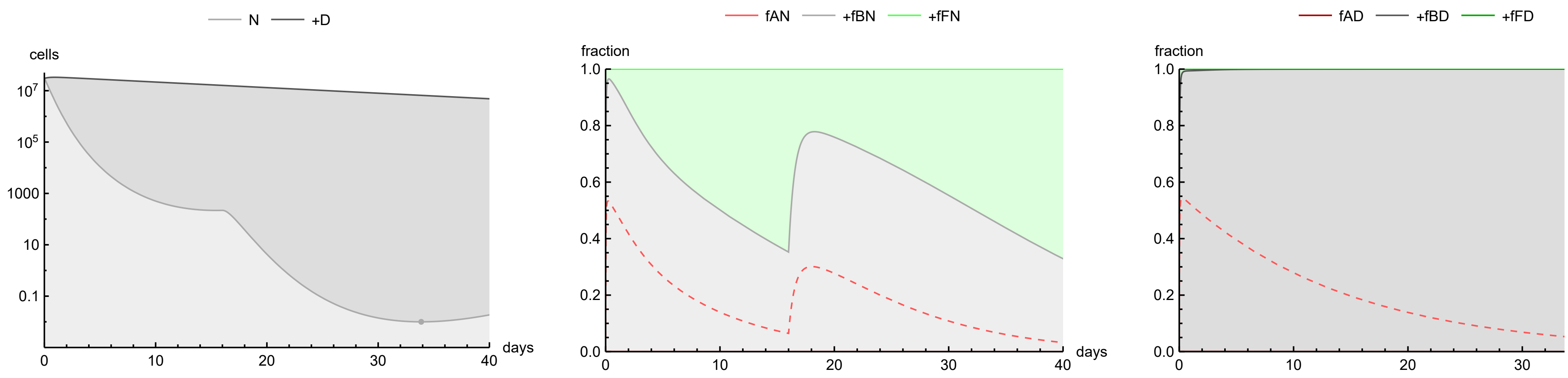

In[ ]:=

```
(* Optimal schedules in the form {day, dose in nCi} *)
Thread[{AinjOpt0[[All, 1]], AinjOpt0[[All, 2]]/nCpm}] (*ks=0*)
Thread[{AinjOpt03[[All, 1]], AinjOpt03[[All, 2]]/nCpm}] (*ks=0.3*)
Thread[{AinjOpt1[[All, 1]], AinjOpt1[[All, 2]]/nCpm}] (*ks=1*)
```

Out[ ]=

{{0, 38.0599}, {11, 10.6562}}

Out[ ]=

{{0, 48.5712}, {6, 6.04145}}

Out[ ]=

{{0, 55.3631}, {16, 0.895367}}

In[ ]:=

```
(* Optimal schedules in the form {day, amount of antibodies in pmol} *)
Thread[{AinjOpt0[[All, 1]], AinjOpt0[[All, 3]]}] (*ks=0*)
Thread[{AinjOpt03[[All, 1]], AinjOpt03[[All, 3]]}] (*ks=0.3*)
Thread[{AinjOpt1[[All, 1]], AinjOpt1[[All, 3]]}] (*ks=1*)
```

Out[ ]=

{{0, 5.14874}, {11, 1.44158}}

Out[ ]=

{{0, 6.57071}, {6, 0.817287}}

Out[ ]=

{{0, 7.48952}, {16, 0.121125}}

In[ ]:=

```
(* Reduction of the amount of toxic decays *)
PercentForm[1 - ToxDD0 / ToxASC0] (*ks=0*)
PercentForm[1 - ToxDD03 / ToxASC03] (*ks=0.3*)
PercentForm[1 - ToxDD1 / ToxASC1] (*ks=1*)
```

Out[ ]//PercentForm=

5.859%

Out[ ]//PercentForm=

21.97%

Out[ ]//PercentForm=

55.53%

In[ ]:=

```
(* amount of free receptors at the moments of second injection *)
(*ks=0*)
SetBasicParameterValues[]; ks = 0; Ainj = AinjOpt0; FullSystemSolutionMD[];
rV = fFNpw[[1]][AinjOpt0[[2, 1]]] * gamma * NNpw[[1]][AinjOpt0[[2, 1]]]; (*on viable cells*)
rD = dFpw[[1]][AinjOpt0[[2, 1]]] / DDpw[[1]][AinjOpt0[[2, 1]]] * gamma * DDpw[[1]][AinjOpt0[[2, 1]]]; (*on damaged cells*)
rD + rV

(*ks=0.3*)
SetBasicParameterValues[]; ks = 0.3; Ainj = AinjOpt03; FullSystemSolutionMD[];
rV = fFNpw[[1]][AinjOpt03[[2, 1]]] * gamma * NNpw[[1]][AinjOpt03[[2, 1]]]; (*on viable cells*)
rD = dFpw[[1]][AinjOpt03[[2, 1]]] / DDpw[[1]][AinjOpt03[[2, 1]]] * gamma * DDpw[[1]][AinjOpt03[[2, 1]]]; (*on damaged cells*)
rD + rV

(*ks=1*)
SetBasicParameterValues[]; ks = 1; Ainj = AinjOpt1; FullSystemSolutionMD[];
rV = fFNpw[[1]][AinjOpt1[[2, 1]]] * gamma * NNpw[[1]][AinjOpt1[[2, 1]]]; (*on viable cells*)
rD = dFpw[[1]][AinjOpt1[[2, 1]]] / DDpw[[1]][AinjOpt1[[2, 1]]] * gamma * DDpw[[1]][AinjOpt1[[2, 1]]]; (*on damaged cells*)
rD + rV
```

Out[ ]=
1.57002

Out[ ]=
0.679937

Out[ ]=
0.000293399

```
(* Subsection S.5.2 Optimization of multidose treatment without predefined number of doses *)

(* S.5.2.1 Analytical estimation of cancer curability
under neglect of irradiation from unanchored nuclides *)

(* Figure S.25: Regions of potentially curable cancers *)
SetBasicParameterValues[];

nn = 40;
CSDAr = Array[f, {(nn + 1) * (nn + 1), 3}];
CLTAr = Array[f, {(nn + 1) * (nn + 1), 3}];
C0Ar = Array[f, {(nn + 1) * (nn + 1), 3}];
Pic = Array[f, 3];

For[kk = 1, kk ≤ 3, kk++,

  If[kk == 1, ks = 0.1,
    If[kk == 2, ks = 0.3,
      If[kk == 3, ks = 0.8]]];

  ii = 0; jj = 0;
  For[omega = 0.005, omega ≤ 0.5, omega = omega * 10^(2/nn),
    ii++; jj = 0;
    For[alpha = 50, alpha ≤ 5000, alpha = alpha * 10^(2/nn),
      jj++;

      (*CSD*)
      CSDAr[[(nn + 1) * (ii - 1) + jj, 1]] = Log10[omega];
      CSDAr[[(nn + 1) * (ii - 1) + jj, 2]] = Log10[alpha];
```

```
CSDAr[[(nn + 1) * (ii - 1) + jj, 3]] = (alpha * ks * lambda * gamma)/(nu * (eta * (lambda + kappac)/kappac + 1)) - lambda - rho;

(*CLT*)
chi = alpha * lambda * gamma / nu;

FANC = 1/(eta * (lambda + kappac)/kappac + 1) * rho/(lambda + rho);

Aa = rho + omega + lambda;

Bb = (rho + omega + lambda * (1 + ks)) - (rho + omega)/(chi * FANC) * (rho + omega + lambda);

Cc = lambda * ks - (rho + omega)/(chi * FANC) * lambda;

kDD = yC /. Solve[{Aa * yC^2 + Bb * yC + Cc == 0}, yC][[2]];
CLTAr[[(nn + 1) * (ii - 1) + jj, 1]] = Log10[omega];
CLTAr[[(nn + 1) * (ii - 1) + jj, 2]] = Log10[alpha];
CLTAr[[(nn + 1) * (ii - 1) + jj, 3]] = omega / rho - kDD;

(*C0*)
C0Ar[[(nn + 1) * (ii - 1) + jj, 1]] = Log10[omega];
C0Ar[[(nn + 1) * (ii - 1) + jj, 2]] = Log10[alpha];

C0Ar[[(nn + 1) * (ii - 1) + jj, 3]] = gamma/(eta + 1) - rho/lambda nu/alpha (-ProductLog[-1, -E^-2 * (Ncur/(N0 * Nnor))^(lambda/rho)])/(1 - ks * (1 - (-ProductLog[-1, -E^-2 * (Ncur/(N0 * Nnor))^(lambda/rho)])/(-ProductLog[-1, -E^-1 * (Ncur/(N0 * Nnor))^((lambda + rho)/rho)])))]];

Pic[[kk]] = Show[
  ListContourPlot[C0Ar, PlotRange -> {{Min[C0Ar[[All, 1]]], Max[C0Ar[[All, 1]]]}, {Min[C0Ar[[All, 2]]], Max[C0Ar[[All, 2]]]}},
   Contours -> {0}, ContourShading -> {Opacity[0], Opacity[0.2, Lighter[Green]]},
   ContourStyle -> Directive[Thick, Darker[Green]], FrameLabel -> {"cancer cell death rate, ω", "cancer cell radiosensitivity, α"},
   FrameTicks -> {{{{2, 10^2}, {Log10[300], 10^Log10[300]}, {3, 10^3}, {Log10[3000], 10^Log10[3000]}}, None},
```

```
{{{-2, 0.01}, {Log10[0.03], 0.03}, {-1, 0.1}, {Log10[0.3], 0.3}}, None}}],

ListContourPlot[CSDAr, PlotRange → {{Min[C0Ar⟦All, 1⟧], Max[C0Ar⟦All, 1⟧]}, {Min[C0Ar⟦All, 2⟧], Max[C0Ar⟦All, 2⟧]}}, Contours → {0},
  ContourShading → {Opacity[0], Opacity[0.2, Lighter[Yellow]]}, ContourStyle → Directive[Thick, Darker[Yellow]]],

ListContourPlot[CLTAr, PlotRange → {{Min[C0Ar⟦All, 1⟧], Max[C0Ar⟦All, 1⟧]}, {Min[C0Ar⟦All, 2⟧], Max[C0Ar⟦All, 2⟧]}}, Contours → {0},
  ContourShading → {Opacity[0], Opacity[0.2, Lighter[Red]]}, ContourStyle → Directive[Thick, Darker[Red]]]

]]

GraphicsGrid[{{Pic⟦1⟧, Pic⟦2⟧, Pic⟦3⟧}}]
```

Out[•]=

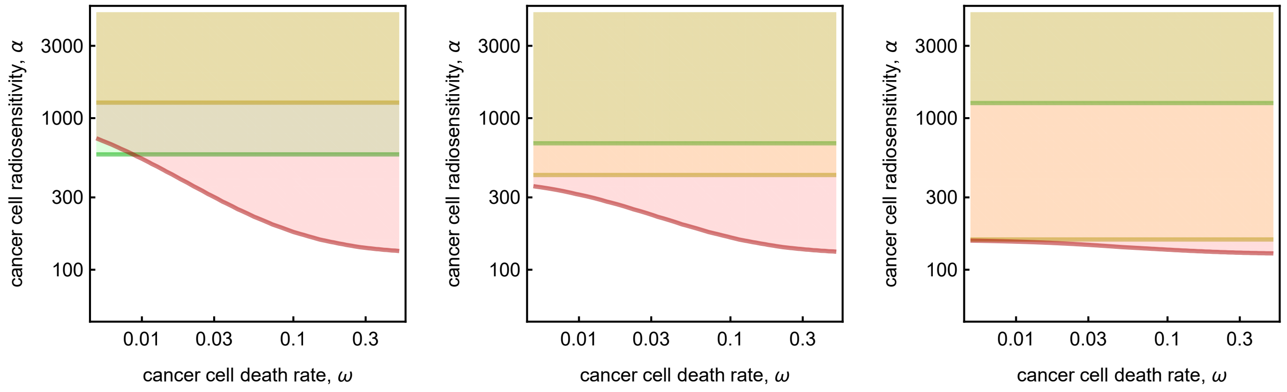

In[∘]:=

```
(* "Upon its decrease [of cancer cell radiosensitivity]
   to α = 450 the level of toxic decays accompanying treatment by minimal single curative dose reaches 564 nCi·day" *)
SetBasicParameterValues[];
alpha = 450;
Acur = FindCurDose[];
Ainj = {{0, Acur * nCpm, eta * Acur * nCpm}};
FullSystemSolutionToxicity[] / nCpm
```

Out[∘]=

```
564.091
```

```
(* Figure S.26: Simulations of system, corresponding to the limiting case of immediate binding of all receptors by antibodies,
under variation of cancer cell radiosensitivity *)

SetBasicParameterValues[];
tEndInit = 300;

CurPar1 = Array[f, 3];
CurPar2 = Array[f, 3];
NDPic = Array[f, 3];

alphaArray = {450, 400, 150};

For[ii = 1, ii ≤ 3, ii++,
   alpha = alphaArray[[ii]];


   (* Analytical parameter estimating cancer curability by self-damage only, should be positive for that *)
```

$$\text{CurPar1}[[\text{ii}]] = \frac{\text{alpha} * \text{ks} * \text{lambda} * \text{gamma}}{\text{nu} * \left(\text{eta} * \frac{\text{lambda}+\text{kappac}}{\text{kappac}} + 1\right)} - \text{lambda} - \text{rho};$$

```
(* Analytical parameter estimating cancer curability by self−damage and cross−fire in long term, should be positive for that *)

chi = alpha * lambda * gamma / nu;
FANC = 1/(eta * (lambda+kappac)/kappac + 1) * rho/(lambda + rho);
Aa = rho + omega + lambda;
Bb = (rho + omega + lambda * (1 + ks)) − (rho + omega)/(chi * FANC) * (rho + omega + lambda);
Cc = lambda * ks − (rho + omega)/(chi * FANC) * lambda;
Quiet[kDD = y /. Solve[{Aa * y^2 + Bb * y + Cc == 0, y > 0}, y]〚1〛];
CurPar2〚ii〛 = If[NumberQ[kDD], omega / rho − kDD, 10];

SolutionOccRec[0, 0];

If[ (*cure is not achieved*)
  NN[tEnd] * Nnor > Ncur && (*but N decreases*) (Log10[NN[tEnd − 1] * Nnor] − Log10[NN[tEnd] * Nnor]) > 0,

  (*then increase timeframe of simulation*)
  tAdd = (Log10[NN[tEnd] * Nnor] − Log10[Ncur]) / (Log10[NN[tEnd − 1] * Nnor] − Log10[NN[tEnd] * Nnor]);
  tEndInit = 300 + 2 * tAdd;
  SolutionOccRec[0, 0];];

tt = If[ii > 1, tEndInit, 2];

NDPic〚ii〛 = Quiet[Show[LogPlot[{Nnor * NN[t], Nnor * (NN[t] + DD[t])}, {t, 0, tEndInit}, Filling → {1 → Axis, 2 → {1}},
      PlotStyle → {Directive[Lighter[Gray], Thickness[0.002]], Directive[Darker[Gray], Thickness[0.002]]}, PlotRange →
        {{0, tEndInit}, {10^(−4), NMaximize[{Nnor * (NN[t] + DD[t]), t > 0, t < tEndInit}, t]〚1〛}}, AxesLabel → {"days", "cells"},
      PlotLegends → Placed[{"N", "+D"}, Above], ImageSize → IS],
    LogPlot[kDD * Nnor * DD[t], {t, 1, tt}, PlotStyle → Directive[Darker[Red], Dashed]],
```

```
ListLogPlot[{{tCure, Ncur}}]]];

GraphicsRow[{NDPic[[1]], NDPic[[2]], NDPic[[3]]}, ImageSize → 1050]
```

Out[•]=

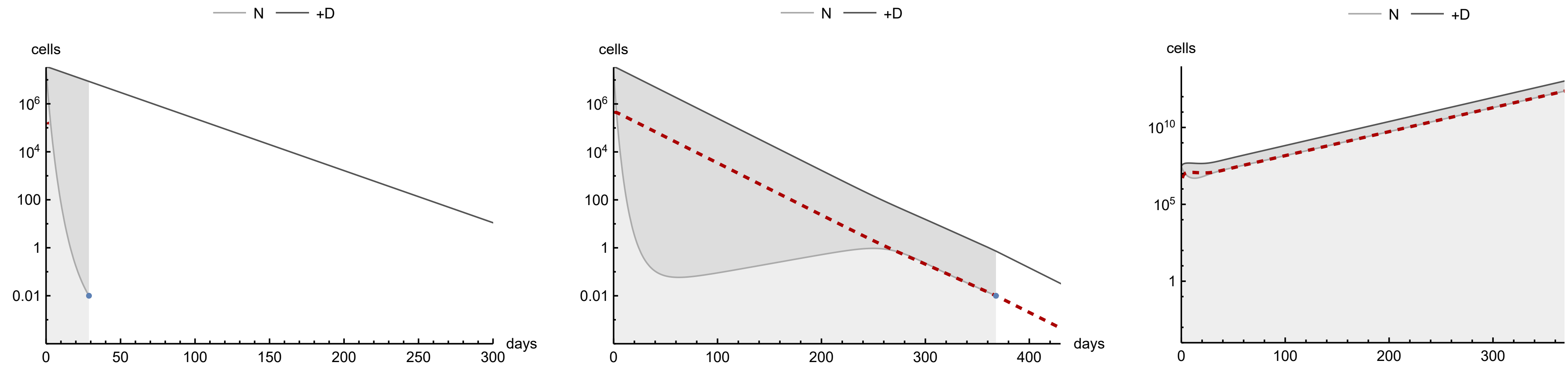

In[•]:=

```
(* values of C_SD *)
CurPar1
```

Out[•]=

{0.0270664, −0.0214965, −0.264311}

In[•]:=

```
(* values of C_LT *)
CurPar2
```

Out[•]=

{0.142289, 0.133256, −0.135585}

In[•]:=

```
(* values of stationary state of N / D *)
−(CurPar2 − omega / rho)
```

Out[•]=

{0.00476994, 0.0138031, 0.282644}

In[•]:=

```
(* Figure S.27: Curability by single dose, but not in long term *)
```

```
SetBasicParameterValues[];
tEndInit = 300;

alpha = 600;
ks = 0.1; omega = 0.006;

(* Analytical parameter estimating cancer curability by self-damage only, should be positive for that *)
```

$$CurPar1 = \frac{alpha * ks * lambda * gamma}{nu * \left(eta * \frac{lambda + kappac}{kappac} + 1\right)} - lambda - rho$$

```
(* Analytical parameter estimating cancer curability by self-damage and cross-fire in long term, should be positive for that *)

chi = alpha * lambda * gamma / nu;
```

$$FANC = \frac{1}{eta * \frac{lambda + kappac}{kappac} + 1} \frac{rho}{lambda + rho};$$

Aa = rho + omega + lambda;

$$Bb = (rho + omega + lambda * (1 + ks)) - \frac{rho + omega}{chi * FANC} * (rho + omega + lambda);$$

$$Cc = lambda * ks - \frac{rho + omega}{chi * FANC} * lambda;$$

```
Quiet[kDD = y /. Solve[{Aa * y^2 + Bb * y + Cc == 0, y > 0}, y][[1]]];
CurPar2 = If[NumberQ[kDD], omega / rho - kDD, 10]
- (CurPar2 - omega / rho)
```

$$C0 = gamma * N0 - (eta + 1) * N0 * \frac{rho}{lambda} \frac{nu}{alpha} \frac{-ProductLog\left[-1, -E^{-2} * \left(\frac{Ncur}{N0 * Nnor}\right)^{\frac{lambda}{rho}}\right]}{1 - ks * \left(1 - \frac{-ProductLog\left[-1, -E^{-2} * \left(\frac{Ncur}{N0 * Nnor}\right)^{\frac{lambda}{rho}}\right]}{-ProductLog\left[-1, -E^{-1} * \left(\frac{Ncur}{N0 * Nnor}\right)^{\frac{lambda + rho}{rho}}\right]}\right)}$$

```
SolutionOccRec[Ncur, 0];

If[(*cure is not achieved*) NN[tEnd] * Nnor > Ncur && (*but N decreases*) (Log10[NN[tEnd - 1] * Nnor] - Log10[NN[tEnd] * Nnor]) > 0,

  (*then increase timeframe of simulation*)
  tAdd = (Log10[NN[tEnd] * Nnor] - Log10[Ncur]) / (Log10[NN[tEnd - 1] * Nnor] - Log10[NN[tEnd] * Nnor]);
  tEndInit = 300 + 2 * tAdd;
  SolutionOccRec[Ncur, 0];];

tt = 365;

Quiet[Show[LogPlot[{Nnor * NN[t], Nnor * (NN[t] + DD[t])}, {t, 0, tt}, Filling → {1 → Axis, 2 → {1}},
    PlotStyle → {Directive[Lighter[Gray], Thickness[0.002]], Directive[Darker[Gray], Thickness[0.002]]},
    PlotRange → {{0, tt}, {10^(-7), 10^8}}, AxesLabel → {"days", "cells"},
    PlotLegends → Placed[{"N", "+D"}, Above], ImageSize → 320],
  LogPlot[kDD * Nnor * DD[t], {t, 1, tt}, PlotStyle → Directive[Darker[Red], Dashed]],
  ListLogPlot[{{tCure, Ncur}}]]]
```

Out[•]=

-0.215748

Out[•]=

-0.00575365

Out[•]=

0.0234007

Out[•]=

0.2532

Out[•]=

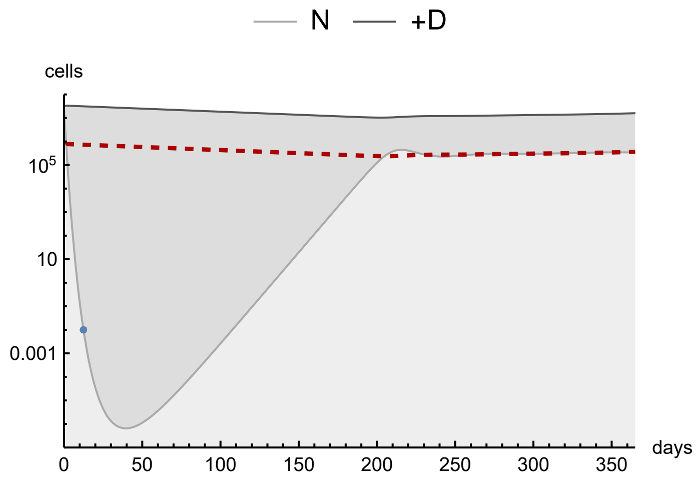

(* Subsection S.5.3 Multi-dose optimization under parameter sweep *)

(* S.5.3.1 Comparison of results of single-dose and multi-dose personalized numerical optimization *)

In[•]:=

(* The outcome of multidose optimization for the training set is saved here in a closed cell *)

In[•]:=

```
Npar = 1000;
resultMD =
  {{"κc", "κp", "γ", "V", "ks", "ρ", "ω", "α", "kf", "N0", "SD-Acur", "SD-Tox", "MD-TotalTox", "MD-TotalDose", "CurFlg", "MD-Schedule"},
   {0.172487255493398`, 1.2765963995094278`, 0.2324603897081641`, 0.9331633444796544`, 0.5595329534566904`, 0.18253234530133355`,
    0.2215389709063929`, 78.80610179612091`, 0.19190036455619353`, 1.548441048845115`, 67743.09410144144`,
    19555.049438130354`, 230.26322157036012`, 819.2175940463417`, 0.`, {{0, 0.06226053714752197`, 110.8237561225891`}}},
   {0.14090572053825962`, 0.941193557649755`, 3.8052282638940014`, 1.32622706664442`, 0.616835667267962`,
    0.48175991442091237`, 0.08306143231635953`, 874.0744302472489`, 0.12491077781228116`,
    5.511666182290989`, 116.46970588923584`, 4.39935659461148`, 2.972054999026616`, 79.20843537392956`, 1.`,
    {{0, 0.004248814545348463 5`, 7.562889890720266`}, {20, 0.0017710265430701839`, 3.1524272466649275`}}},
   {0.19800522656535097`, 1.47676715957736`, 1.6845843495593211`, 1.0829526404841552`, 0.6930225215765629`,
    0.2749384033948816`, 0.06680029700083288`, 3207.8452923633004`, 0.18818451872370445`, 6.655704647717006`, 31.887329398445925`,
    0.7173316633754113`, 0.5677913458030442`, 25.127263720508385`, 1.`, {{0, 0.001148133851407738`, 2.043678255505773 3`},
     {9, 0.0006134100101931511`, 1.0918698181438087`}, {18, 0.00014812818115774828`, 0.26366816246079194`}}},
```

```
{0.2711930593993829`, 3.0021543969280486`, 4.914701090552741`, 1.464602766667476`, 0.8471176050384048`,
  0.28257943270869035`, 0.04953192991909177`, 1933.8633413872876`, 0.19773521340575795`,
  9.992985154010565`, 105.13570106511645`, 1.0047590058577736`, 0.6472049955355441`, 66.42178597358607`,
  1.`, {{0, 0.003675519559409394`, 6.5424248157487215`}, {35, 0.0013725361745831476`, 2.443114390758003`}}},
{0.27990766836391096`, 3.477149477100016`, 9.931945787531824`, 0.8846854018858359`, 0.8967333599095253`,
  0.5903168559967787`, 0.24882968868487906`, 980.8551320933655`, 0.02004206946214948`, 1.1376503486553808`, 42.727467045547805`,
  0.6810979755489913`, 0.6810979755489913`, 42.727467045547805`, 1.`, {{0, 0.0032472874954616333`, 5.780171741921707`}}},
{0.11737985888146019`, 1.1225833279930164`, 5.309079794927442`, 0.8606501327539497`, 0.8514188342619913`,
  0.5642039473140849`, 0.008986110654413243`, 2018.9822529242097`, 0.06040743860925685`,
  4.536035630396375`, 73.55385408728918`, 0.508476867815065`, 0.5013342403085895`, 72.57375415003304`, 1.`,
  {{0, 0.004527977083638434`, 8.059799208876413`}, {4, 0.0009876282317640773`, 1.7579782525400576`}}},
{0.17121040978700802`, 0.4138167442315588`, 6.781135564610604`, 1.3665406840239216`, 0.9405996253397015`,
  0.6832439228870748`, 0.04185895434111264`, 62.7113536738392`, 0.18142736225630413`, 8.46259785026923`,
  126972.27674519586`, 36735.71940739372`, 228.3960133560378`, 2188.722452826318`, 0.`,
  {{0, 0.07478081190509338`, 133.10984519106623`}, {3, 0.02420447841686193`, 43.083971582014236`},
   {6, 0.012612915599626136`, 22.450989767334523`}, {9, 0.00682037091778674`, 12.140260233660399`},
   {12, 0.003843764418599704`, 6.841900665107474`}, {15, 0.0022827236554921475`, 4.063248106776022`},
   {18, 0.001447650922358986`, 2.576818641798995`}, {21, 0.0009917147311051856`, 1.7652522213672306`},
   {24, 0.0007370580126330141`, 1.311963262486765`}, {27, 0.03862141783524298`, 68.74612374673251`}}},
{0.07382619562964193`, 0.625700255424527`, 9.939999934198667`, 1.4556453073248168`, 0.9580351575689623`,
  0.4163162212955882`, 0.006430521616962508`, 92.19524127790851`, 0.19149999891406427`,
  4.303065608360628`, 10092.366546722247`, 4736.37843573128`, 70.52241663152961`, 521.5199239251207`, 1.`,
  {{0, 0.03835099860951311`, 68.26477752493334`}, {34, 0.0012845156087960604`, 2.2864377836569876`}}},
{0.1627307868596377`, 1.5327585946689206`, 6.648641489637967`, 1.0898921981857375`, 0.08271984399349908`,
  0.5169005126785295`, 0.006231976105310307`, 2017.0154068595314`, 0.2464092033815996`, 1.6313338993270357`, 8.432667214624587`,
  0.03496655164625874`, 0.03496655164625874`, 8.432667214624587`, 1.`, {{0, 0.0006408827083114687`, 1.1407712207944143`}}},
{0.08926805346894312`, 1.228740834290055`, 5.100082063336529`, 1.0507919032716577`, 0.3229790419061651`,
  0.45218339761418835`, 0.024533377777917375`, 814.1470590464116`, 0.1414263079606694`, 5.247711540674161`, 79.62930256895696`,
  1.1186179181883982`, 1.1186179181883982`, 79.62930256895696`, 1.`, {{0, 0.006051826995240729`, 10.772252051528497`}}},
{0.17431851399024534`, 2.020213173041662`, 9.224968841892476`, 1.3485881267881612`, 0.8890825947176819`,
  0.5177484922558413`, 0.03701694525181502`, 131.5752033573906`, 0.228949740900746`, 4.795606274383001`, 443.4587427958742`,
```

```
19.308319192170078`, 6.076235138020227`, 408.57187042332464`, 1.`, {{0, 0.027317048236165088`, 48.62434586037386`},
   {2, 0.0032553301132664863`, 5.794487601614345`}, {4, 0.00026661012661018287`, 0.4745660253661254`},
   {6, 0.000050846713524055461`, 0.09050715007281872`}, {8, 0.000038874076470262471`, 0.06919585611706719`},
   {10, 0.000038352355305037285`, 0.06826719244296635`}, {12, 0.000038332151485854999`, 0.06823122964482188`},
   {14, 0.000038331279800146115`, 0.06822967804426008`}, {16, 7.737099545555471`*^-6, 0.013772037191088738`}}},
{0.25439204271280724`, 0.8534844729419486`, 2.8150623547133353`, 0.8024992452769948`, 0.43579385025887163`,
  0.4594767236090769`, 0.057877631906885676`, 1195.6419878079198`, 0.17833211044546654`,
  5.195270679096961`, 61.19257325377502`, 2.0934655528170314`, 1.6674144721634536`, 48.04569362819225`,
  1.`, {{0, 0.0033484560170481837`, 5.960251710345767`}, {47, 0.000303016698694427`, 0.53936972367608`}}},
{0.129274073243755`, 2.8469118022753515`, 4.121632588773114`, 1.2196787674185057`, 0.8228855513020883`,
  0.4828002917324088`, 0.013782155470303842`, 388.66064646424104`, 0.2255512748456478`, 1.0022663108918284`, 37.4176094379522`,
  0.9172122873435897`, 0.5659070377184102`, 38.03723723489609`, 1.`, {{0, 0.002003853940810287`, 3.566860014642311`},
   {5, 0.000678900071338035`, 1.2084421269817023`}, {10, 0.00015789269193489016`, 0.2810489916441045`},
   {15, 0.000041004643669296880`, 0.07298826573134845`}, {20, 9.17868209959381`*^-6, 0.016338054137276983`}}},
{0.06343574194222029`, 3.9924293708982113`, 9.079106395846292`, 1.3161791897123671`, 0.10938228479468126`,
  0.3240611741083924`, 0.010321331270591745`, 76.94152103093134`, 0.06038801289350482`,
  9.425890207979073`, 70205.26592740085`, 36460.37688751812`, 227.96229549445366`, 3109.2099534914464`,
  0.`, {{0, 0.05805689208543195`, 103.34126791206887`}, {11, 0.00041937435116902493`, 0.7464863450808644`},
   {22, 0.0004188052245751153`, 0.7454732997437052`}, {33, 0.0004188581975321326`, 0.745567591607196`},
   {44, 0.00041942716598504043`, 0.746580355453372`}, {55, 0.0004257509262112402`, 0.7578366486560074`},
   {66, 0.0005121390692787315`, 0.911607543316142`}, {77, 0.0019203212710493416`, 3.418171862467828`},
   {88, 0.005351620584583996`, 9.525884640559513`}, {99, 0.16835676758953336`, 299.6750463093693`}}},
{0.110036074735397`, 2.490203037930516`, 9.729597307787852`, 1.4397421355398403`, 0.8533380591052817`,
  0.37040757295392324`, 0.047916566872126407`, 665.4588326486067`, 0.16916230254726894`, 1.9013228378216027`, 67.32591358102374`,
  0.785058864865475`, 0.703666196878499`, 60.29559738250721`, 1.`, {{0, 0.002591359278219956`, 4.612619515231521`},
   {5, 0.0016461169540192089`, 2.9300881781541914`}, {10, 0.0003449891688313835`, 0.6140807205198626`}}},
{0.21512225225566667`, 1.75750084110844`, 0.8044386006474246`, 1.3520587790631349`, 0.026316391204000755`,
  0.16031186333306224`, 0.13761957869990635`, 493.8180531489964`, 0.06250485252841559`, 2.6863460670624115`, 31526.667301351725`,
  7736.426664784584`, 3.2886124157089536`, 30.62210408525064`, 1.`, {{0, 0.00192738361647891`, 3.4307428373324598`},
   {29, 0.00012829422911458198`, 0.22836372782395592`}, {58, 0.000038446745527670880`, 0.06843520703925417`},
   {87, 0.000034973615276886650`, 0.06225303519285823`}, {116, 0.000034309548423001740`, 0.061070996192943094`},
```

```
{145, 0.00003423026592671799`, 0.06092987334955802`}, {174, 0.00003422010068971649 4`, 0.0609117792276953 6`},
  {203, 0.000034218768413197936`, 0.060909407775492315`}, {232, 0.00006120302062836493`, 0.10894137671848958`}}},
{0.15874286665935028`, 2.268211451796655`, 1.8981682437883747`, 1.375959688601855`, 0.24814706074480242`,
 0.5134877163486878`, 0.3316652449509965`, 685.632474632321`, 0.05319219913715695`, 1.7896119638851753`,
 31.96808009735873`, 1.3426042498073825`, 0.8574778706205547`, 21.78028761519868`, 1.`,
 {{0, 0.0015549279501936623`, 2.767771751344719`}, {20, 0.00010037390856143756`, 0.17866555723935887`}}},
{0.07432908705186819`, 3.0619407712521785`, 1.7905302646779082`, 0.9549392645174688`, 0.353226782755657`,
 0.6119520610188782`, 0.0496137109124893`, 317.5186768305259`, 0.07812208014742161`, 6.620973402603631`,
 20840.6317126216`, 10059.119246951217`, 228.38714874481042`, 4937.801336818378`, 0.`,
 {{0, 0.012031044591275023`, 21.415259372469542`}, {9, 0.0017535353859072257`, 3.1212929869148613`},
  {18, 0.0006793650895686698`, 1.2092698594322322`}, {27, 0.001319350980203518`, 2.3484447447622623`},
  {36, 0.0027231901812622295`, 4.847278522646769`}, {45, 0.002801797130132536`, 4.987198891635914`},
  {54, 0.002154576190903892`, 3.835145619808927 5`}, {63, 0.0020073876366172995`, 3.5731499931787924`},
  {72, 0.002164021510218747`, 3.8519582881893695`}, {81, 0.3476386329021076`, 618.7967665657516`}}},
{0.1228443083266203`, 2.0512729485932484`, 1.9256217622349043`, 1.4810122884336692`, 0.07741458536098089`,
 0.4641958329015734`, 0.007197139507585793`, 240.76714600918612`, 0.03301937102418029`, 9.328377628444095`, 131731.62537204823`,
 47764.06607762351`, 230.2448203786112`, 858.6768100136206`, 0.`, {{0, 0.06525943756103517`, 116.16179885864261`}}},
{0.2606519449147342`, 3.818605783771848`, 8.595720359336667`, 1.112914365339814`, 0.820058745442926`, 0.4441576207168524`,
 0.053270363092160024`, 1117.1606910609416`, 0.20810873381921946`, 6.830482236754131`, 158.217216712755`,
 1.2434347050188215`, 1.2434347050188215`, 158.217216712755`, 1.`, {{0, 0.01202450847016938`, 21.4036250769015`}}},
{0.24750930586460296`, 3.1846993652287674`, 3.026718658979185`, 1.4845570571034035`, 0.869864794311124`,
 0.6669561203654737`, 0.2363643381944309`, 1924.496556700219`, 0.06437651322808385`, 2.548184450019704`,
 51.48595308643028`, 0.9757842970686094`, 0.5660213707510338`, 29.015190600878185`, 1.`,
 {{0, 0.00148047650131468`, 2.63524817234013`}, {4, 0.000563553233111273`, 1.0031247549380657`},
  {8, 0.00014252043683027357`, 0.253686377557887`}, {12, 0.000018604314410515655`, 0.033115679650717864`}}},
{0.24908529437358162`, 3.626300618072209`, 2.0230682918259397`, 1.4602193573319078`, 0.40130475942739396`,
 0.6650543311625685`, 0.019582320105998748`, 125.14663606859848`, 0.03716084844005629`, 8.149858668846527`, 485275.3769879387`,
 106429.87948799224`, 229.6219838279999`, 1648.313497242175`, 0.`, {{0, 0.1252718257904053`, 222.98384990692142`}}},
{0.2795730004018654`, 2.183215118244356`, 0.7910515142573491`, 1.2847699393543137`, 0.70345738565049`,
 0.2632113724473232`, 0.1394636484917589`, 223.91272255356017`, 0.16150366940434352`, 9.859315316872873`,
 53076.817736040095`, 10617.626409581217`, 229.19862475246333`, 8005.333037062093`, 0.`,
```

```
{{0, 0.025477958308998464`, 45.35076579001727`}, {11, 0.015888177374833612`, 28.28095572720383`},
  {22, 0.013997156666115111`, 24.9149388656849`}, {33, 0.012708823338629131`, 22.62170554275985`},
  {44, 0.011594006888857906`, 20.637332262167075`}, {55, 0.01059471335062146`, 18.8585897641062`},
  {66, 0.009697638039628169`, 17.261795710538138`}, {77, 0.00889246983395413`, 15.82859630443835`},
  {88, 0.008169485660248063`, 14.541684475241553`}, {99, 0.4913848813548331`, 874.6650888116029`}}},
{0.08969576723701717`, 0.49356011207206274`, 0.2529879905685366`, 1.1041769014657603`, 0.7443718717514693`,
 0.6180257213599576`, 0.03574805853679543`, 3205.3226496399375`, 0.061225880931137455`,
 7.560768798013438`, 2171.9947059835604`, 945.4490635557612`, 229.68115671270223`, 561.8580133015874`,
 0.`, {{0, 0.016904408506187817`, 30.08984714101431`}, {35, 0.0027953668272540093`, 4.975752952512136`},
  {70, 0.0027953234810351745`, 4.975675796242611`}, {105, 0.0027953150986639638`, 4.975660875621855`},
  {140, 0.0027953122824852863`, 4.9756558628238094`}, {175, 0.0027953112957081606`, 4.975654106360526`},
  {210, 0.0027953109487773463`, 4.975653488823676`}, {245, 0.0027953108267734766`, 4.975653271656789`},
  {280, 0.002795310783869965`, 4.975653195288538`}, {315, 0.0034342389601654546`, 6.112945349094509`}}},
{0.10298476113266081`, 3.435925289367793`, 4.954566778741102`, 0.9299871443466801`, 0.6352691398464292`,
 0.5470174017743717`, 0.005507148986589441`, 973.5524715904572`, 0.1328513609688533`, 5.618259356059234`,
 117.49696230705861`, 0.20530441110451222`, 0.20458133854416566`, 117.51947171090909`, 1.`,
 {{0, 0.008126085091965381`, 14.464431463698379`}, {3, 0.0008053947580637102`, 1.4336026693534043`}}},
{0.14277679649227548`, 3.0428582592918065`, 7.414614443890604`, 1.1046799505745009`, 0.2872163059276376`,
 0.23677108379761724`, 0.04608733451503689`, 2695.430951291806`, 0.1274330436692639`, 8.066834406130887`, 27.508200949789227`,
 0.2432312351431745`, 0.2432312351431745`, 27.508200949789227`, 1.`, {{0, 0.0020906232721839813`, 3.721309424487487`}}},
{0.18738335776550036`, 1.508373316422973`, 6.602438992729994`, 0.7532357689229783`, 0.8880958875806098`,
 0.4853414284787141`, 0.10975729606650726`, 2118.598035369477`, 0.05093222572935868`, 5.0448517662608`, 76.70717437665523`,
 2.075588819207914`, 2.075588819207914`, 76.70717437665523`, 1.`, {{0, 0.005829745252625798`, 10.376946549673919`}}},
{0.061980185747317895`, 2.54906136842855`, 8.170240160100523`, 1.0602973698223939`, 0.7221801534586956`,
 0.6067985789160708`, 0.38301062006579967`, 81.55317476370394`, 0.23749102694434165`,
 4.188403663256118`, 22951.475716120684`, 12020.796006830042`, 229.02911179513242`, 815.662092123973`,
 0.`, {{0, 0.033043982300796304`, 58.81828849541741`}, {4, 0.004270394677615477`, 7.601302526155549`},
  {8, 0.0005746948021371916`, 1.022956747804201`}, {12, 0.00011340062716394836`, 0.20185311635182807`},
  {16, 0.00004503304388815782`, 0.08015881812092092`}, {20, 0.00003261957856176928`, 0.05806284983994932`},
  {24, 0.000029610254574828756`, 0.052706253143195186`}, {28, 0.000028596220527084043`, 0.0509012725382096`},
  {32, 0.000028139930198497558`, 0.05008907575332569`}, {36, 0.02382384756595869 2`, 42.40644866740647`}}},
```

{0.2562298689236346`, 3.2913375042385793`, 9.509882785943699`, 1.013989937500057`, 0.7792587778840148`,
0.49077497767429623`, 0.021163604915718004`, 145.96678185214677`, 0.05845136191000838`,
7.439112841768198`, 627.823017294315`, 7.654048938333821`, 3.585396203335648`, 641.2931556551023`, 1.`,
{{0, 0.035627614034272344`, 63.417152981004776`}, {2, 0.010499432277189491`, 18.688989453397298`},
{4, 0.0020662299452216767`, 3.677889302494585`}, {6, 0.0003892474099078845`, 0.6928603896360345`},
{8, 0.00008484836703367901`, 0.15103009331994866`}, {10, 0.00003143714723591271`, 0.05595812207992462`},
{12, 0.000022663021677909l6`, 0.04034017858667831`}, {14, 0.000016807627248878512`, 0.02991757650300375`}}},
{0.2313093023364764`, 2.988629640967633`, 6.855539828479465`, 1.0799498768994156`, 0.4522471358822151`,
0.2285321965248518`, 0.04827992453162874`, 1204.6728692817303`, 0.13073517219860892`, 1.9378468299164582`,
16.257879702552408`, 0.15741530693290215`, 0.10659914097931827`, 10.404522628701468`, 1.`,
{{0, 0.0006301523134968638`, 1.1216711180244174`}, {45, 0.00016059140628444773`, 0.28585270318631695`}}},
{0.2663151909473739`, 2.2417238355718707`, 0.9398485531379794`, 1.0477480676389026`, 0.7226958973603346`,
0.5716146939488362`, 0.007506086251562624`, 76.16131590346896`, 0.024911856209502148`, 4.070747059506875`, 954570.6446215768`,
198676.31622242514`, 230.10240752666618`, 1454.7861249823318`, 0.`, {{0, 0.11056374549865722`, 196.80346698760985`}}},
{0.19929196929676601`, 1.9362608032362312`, 0.5353176928827672`, 0.7753335837033051`, 0.8607728477687919`,
0.15482089410819944`, 0.02026432754563482`, 192.89182731397838`, 0.1831217833074511`, 5.333726168282188`, 25098.2949487175`,
6518.616127894766`, 230.26292392333698`, 923.4778504622612`, 0.`, {{0, 0.07018431663513186`, 124.9280836105347`}}},
{0.10696417975467426`, 1.9440197080088568`, 2.0936739600677434`, 1.1829890119130342`, 0.8864433537218006`,
0.5974069860908109`, 0.31351620960128274`, 84.46268853549806`, 0.02353560759327844`, 4.656274919049821`, 572513.3395312632`,
226433.78938795067`, 230.13693652129615`, 1104.5119636937193`, 0.`, {{0, 0.08394290924072267`, 149.41837844848635`}}},
{0.21459986298539707`, 1.6769788549349993`, 9.356964926187835`, 1.2601407238694713`, 0.895239496536256`,
0.38446102196425924`, 0.012005779035619358`, 146.09102757400478`, 0.02879957197754268`,
2.3826604675291154`, 193.3328311433502`, 3.0051272329820184`, 1.5980886196141846`, 195.7536591781014`,
1.`, {{0, 0.010746193496689548`, 19.128224424107394`}, {3, 0.0032163868960182044`, 5.725168674912404`},
{6, 0.0006954570706542276`, 1.2379135857645251`}, {9, 0.00015715631349416545`, 0.27973823801961456`},
{12, 0.000046738011095316186`, 0.08319365974966281`}, {15, 0.000015346309584245838`, 0.02731643105995759`}}},
{0.07383925157057758`, 2.6358268091067414`, 6.251616293116599`, 1.1087155777827833`, 0.9205185682116173`,
0.19659269091732745`, 0.29632431074176147`, 531.6045072192474`, 0.12599923353908937`, 2.5100327097656248`,
79.86268730333829`, 1.707997182802082`, 0.45247700830123394`, 20.537140309001337`, 1.`,
{{0, 0.0010707349791007664`, 1.9059082627993642`}, {8, 0.0004171230815626617`, 0.7424790851815378`},
{16, 0.000059783903872871975`, 0.1064153488937121`}, {24, 0.000013180698947801 66`, 0.023461644127086956`}}},

```
{0.0817054148315825`, 2.796991931647562`, 7.6919124752432`, 0.9355088496592321`, 0.8578766380044134`,
  0.4976427721124693`, 0.008010560795693933`, 67.84344037448854`, 0.080482215315478`,
  7.8347514420046025`, 92958.46968521253`, 42660.8296238346`, 229.5495752533822`, 1267.4953028220164`,
  0.`, {{0, 0.07289310954689024`, 129.74973499346464`}, {4, 0.0027097651981073576`, 4.823382052631096`},
   {8, 0.0004960032198322301`, 0.8828857313013695`}, {12, 0.0002930622586983115`, 0.5216508204829944`},
   {16, 0.0002664227925798093`, 0.4742325707920605`}, {20, 0.0002609407558429203`, 0.46447454540039806`},
   {24, 0.00025931051095175466`, 0.46157270949412327`}, {28, 0.000258679970034066`, 0.4604503466606375`},
   {32, 0.0002583859711785202`, 0.4599270286977659 7`}, {36, 0.018633962790358043`, 33.16845376683732`}}},
{0.23618801128357925`, 0.8353216061115365`, 4.620412867513723`, 1.4504705173961072`, 0.3108708881036355`,
  0.3400688454787608`, 0.014061322572830477`, 2071.8308884477033`, 0.0364017192015193`, 3.610034284058532`, 18.55464388397803`,
  0.24539479092054442`, 0.24539479092054442`, 18.55464388397803`, 1.`, {{0, 0.0014101529351823305`, 2.5100722246245484`}}},
{0.1662441272521787`, 3.725772450959033`, 9.814227303865422`, 1.3148509050359762`, 0.25298305094984697`,
  0.32303330970203903`, 0.055784478318004704`, 213.77275876328028`, 0.04990125708904308`,
  9.833393949415544`, 466.6166735795884`, 3.800152829506548`, 2.6009004820322037`, 314.8797958898342`, 1.`,
  {{0, 0.01950459361878954`, 34.71817664144538`}, {39, 0.0044262708688378605`, 7.8787621465313915`}}},
{0.22831788422124638`, 2.590756298379799`, 9.795768388114066`, 1.125355926333821`, 0.3650031672541203`,
  0.1680578019226482`, 0.4143448211959925`, 831.1374036828946`, 0.21412864715242685`, 3.6048538714465157`,
  36.91913379485488`, 0.8180111878790336`, 0.27568593593280144`, 13.069797965677543`, 1.`,
  {{0, 0.0006651506077833032`, 1.18396808185428`}, {8, 0.00028515855703305573`, 0.5075822315188392`},
   {16, 0.0000373731899122874`, 0.06652427804387159`}, {24, 5.622290662846925`*^-6, 0.010007677379867526`}}},
{0.23359626472155715`, 2.56674003123695`, 1.1969232842409259`, 1.0442579072771299`, 0.8882810964774697`,
  0.3176999965402193`, 0.007340467537817918 5`, 1908.0313626467632`, 0.2040761417499009`,
  9.648978920291999`, 91.99309767384098`, 0.6025242927439644`, 0.3468744949584973`, 87.44001946112931`,
  1.`, {{0, 0.0038077763657982347`, 6.777841931120858`}, {4, 0.0018535340738794343`, 3.299290651505393`},
   {8, 0.0007634272500983332`, 1.358900505175033`}, {12, 0.00022070378926982543`, 0.39285274490028926`}}},
{0.2534162537452666`, 1.6838285982829593`, 0.8034274134000761`, 0.870316935261816`, 0.7856829676536639`,
  0.46427070430809425`, 0.12261242146523575`, 210.18201198829036`, 0.1377493870208059`, 1.4006481974514937`, 42825.83088445403`,
  9267.503051852329`, 230.24025041440913`, 1117.643808063708`, 0.`, {{0, 0.08494092941284181`, 151.19485435485845`}}},
{0.25695579183074796`, 3.423363059474246`, 4.134350766656727`, 1.2838485736639544`, 0.4149036036442244`,
  0.6493708976181658`, 0.12875465899507466`, 1172.2742202486922`, 0.012611679323919878`,
  3.4839902382375243`, 48.702991447874965`, 0.65748433814564`, 0.6208637109448831`, 45.888646326306215`,
```

```
1.`, {{0, 0.002903532131703635`, 5.168287194432469 5`}, {5, 0.0005840049890956372`, 1.039528880590234`}}},
{0.17682432929033887`, 2.45185381012344`, 3.9338825987195882`, 1.2879615271399696`, 0.02702036645869277`,
  0.6694394004648141`, 0.11330549796901213`, 154.96108716979026`, 0.11583322861965717`,
  8.805457336592767`, 62075.72329942178`, 17528.050441664243`, 230.08726130902008`, 2259.9382011819957`,
  0.`, {{0, 0.07013092813738786`, 124.83305208455039`}, {6, 0.0024280205613064643`, 4.321876599125507`},
    {12, 0.0009277338341657874`, 1.6513662248151015`}, {18, 0.0013685847224634243`, 2.4360808059848953`},
    {24, 0.0030501711705164694`, 5.429304683519316`}, {30, 0.003170963051620341`, 5.644314231884207`},
    {36, 0.0018154617237414966`, 3.2315218682598643`}, {42, 0.0012367617538607543`, 2.2014359218721427`},
    {48, 0.0010822956692640496`, 1.9264862912900083`}, {54, 0.08654438266550503`, 154.04900114459898`}}},
{0.2509750819824499`, 3.8883567883501247`, 6.384989889678234`, 1.4613334209290016`, 0.7183189010632474`,
  0.4965470763778975`, 0.3859108436384297`, 748.6242458418322`, 0.020020198926280153`,
  5.690775103224375`, 170.7024644094458`, 2.5944458826572196`, 1.1980749561052297`, 77.71224737395839`,
  1.`, {{0, 0.004423110346586026`, 7.873136416923127`}, {5, 0.001310193195639694 8`, 2.332143888238657`},
    {10, 0.00015366960723483603`, 0.27353190087800816`}, {15, 0.000019157650960281436`, 0.03410061870930096`}}},
{0.1441623498715573`, 3.343288457669903`, 1.621696793707331`, 1.3375455077897458`, 0.06814809058713811`,
  0.4010236514346772`, 0.3002310191555227`, 155.99799531014958`, 0.10001900063604485`, 4.2763953522223215`,
  79261.33786662415`, 25889.777658093553`, 229.48715740079373`, 2184.1793761854055`, 0.`,
  {{0, 0.009010023154098771`, 16.037841214295813`}, {6, 0.0052932580224703905`, 9.421999279997296`},
    {12, 0.004202940373138667`, 7.481233864186826`}, {18, 0.0035321523963320767`, 6.287231265471096`},
    {24, 0.002995939208331463`, 5.332771790830004`}, {30, 0.0025466902372256357`, 4.533108622261631`},
    {36, 0.002171176053084226`, 3.864693374489922`}, {42, 0.0018580332866064307`, 3.3072992501594465`},
    {48, 0.0015969624775966367`, 2.842593210122013`}, {54, 0.13279045738120654`, 236.36701413854766`}}},
{0.2657479684896014`, 3.193863728015164`, 2.3963245604206165`, 1.4113571594678591`, 0.26744798738201037`,
  0.235299669124016`, 0.018584060295236755`, 466.949287196137`, 0.23679723903863448`, 8.690245518267893`, 169.65722705089624`,
  1.3970498550038997`, 0.9433627119388714`, 161.01687037064448`, 1.`, {{0, 0.010412789016913233`, 18.534764450105556`},
    {9, 0.0016362360747052237`, 2.912500212975298`}, {18, 0.00018825705655052397`, 0.3350975606599327`}}},
{0.18653936584035707`, 1.0683088955449334`, 5.185702152338592`, 0.9522710688882444`, 0.206752787597013`,
  0.5240389817239608`, 0.06412446666117125`, 220.00685924183844`, 0.2055887216336732`, 7.885811409920794`, 991.1698661262881`,
  181.83591850922107`, 12.766468639581527`, 376.5855950629068`, 1.`, {{0, 0.027124541008529707`, 48.2816829951828 8`},
    {2, 0.0010955293257127361`, 1.9500421997686699`}, {4, 0.00007091605251815847`, 0.12623057348232206`},
    {6, 0.000046692228987539027`, 0.0831121675978194 8`}, {8, 0.00004611600576922433`, 0.0820864902692193`},
```

```
      {10, 0.0000460922479420568`, 0.0820442013368611`}, {12, 0.0000460903971497471 65`, 0.08204090692654996`},
      {14, 0.0000460901696307035 55`, 0.08204050194265232`}, {16, 0.000046090130177899 82`, 0.08204043171666167`},
      {18, 0.000046090121154604 5`, 0.082040415655196`}, {20, 6.257537208541714`*^-6, 0.011138416231204252`}}},
  {0.07587231017464335`, 3.3343312881276663`, 8.762311039358057`, 0.8284399428795388`, 0.17449762015817138`,
    0.34311440561675743`, 0.03578987408308253`, 1364.0076205471958`, 0.21103062426335195`, 8.577574022027257`,
    60.89652107606923`, 0.41878329266776515`, 0.39661758530306784`, 57.60750923312915`, 1.`,
    {{0, 0.003702506502015658`, 6.590461573587872`}, {32, 0.0006756641997021577`, 1.2026822754698405`}}},
  {0.1141532151024588`, 1.4516849606811304`, 7.634928187688176`, 0.8394863515309923`, 0.0282734940185132`,
    0.6576494992828068`, 0.009900377337816624`, 932.466930942293`, 0.19488805826375527`, 9.750042758265863`, 122.34910866029524`,
    0.6936239398495962`, 0.6936239398495962`, 122.34910866029524`, 1.`, {{0, 0.009298532258182439`, 16.551387419564744`}}},
  {0.21903027892555205`, 2.3035966499415492`, 1.6904861150255108`, 0.7741880968122689`, 0.5538208564897962`,
    0.4216122395122083`, 0.4439804577081556`, 61.55285726796139`, 0.16334632191055676`, 6.147879562247702`, 109561.838109268`,
    26517.053038735598`, 230.25954709705326`, 1322.8686232315865`, 0.`, {{0, 0.10053801536560059`, 178.95766735076904`}}},
  {0.19219532042344978`, 0.8633096904694177`, 0.1660296606087659`, 1.0595398890946446`, 0.3981268836303198`,
    0.35447506211304225`, 0.06527455092271564`, 2352.6780619798324`, 0.04729158696423241`,
    8.334762180963512`, 8886.551170717703`, 2369.9829198023567`, 229.3020127509575`, 3685.9255954851656`,
    0.`, {{0, 0.011292096713756371`, 20.09993215048634`}, {25, 0.002522807554041273 5`, 4.490597446193466`},
      {50, 0.0026940950948014394`, 4.795489268746561`}, {75, 0.001822629487605105`, 3.244280487937087`},
      {100, 0.001415587562836463`, 2.5197458618489037`}, {125, 0.0011203535165675803`, 1.9942292594902928`},
      {150, 0.0009245450540672426`, 1.6456901962396917`}, {175, 0.0007916608536446208`, 1.409156319487425`},
      {200, 0.0006998582275618997`, 1.2457476450601814`}, {225, 0.25684671119199054`, 457.18714592174314`}}},
  {0.05942123092712986 4`, 0.7565633878530198`, 4.881127251124937`, 0.9039794772130322`, 0.669150670013211 3`,
    0.3437226952926379`, 0.013854681683978944`, 1303.847591809871`, 0.1331369696675042`, 3.817043920573575`,
    48.02279776226351`, 0.6805113141180831`, 0.6788759886979309`, 47.90497741670923`, 1.`,
    {{0, 0.003284762007597346`, 5.846876373523276`}, {7, 0.00035601627607255567`, 0.6337089714091491`}}},
  {0.27625614155976197`, 2.681313109654546`, 6.389652815751472`, 1.3504817156087858`, 0.7587234585548364`,
    0.5970419449613528`, 0.3005699072645848`, 854.9062988397701`, 0.165124447314083`, 9.6053618622676`,
    311.00243135268454`, 6.433961145493596`, 3.4234773070261384`, 165.61700548604708`, 1.`,
    {{0, 0.009503469681226971`, 16.91617603258401`}, {5, 0.00265749201775924`, 4.730335791611447`},
      {10, 0.0004018004236601599`, 0.7152047541150846`}, {15, 0.00002413029429320805`, 0.04295192384191033`}}},
  {0.09922116949114601`, 0.5509823607034723`, 3.2884104382793122`, 1.3318058680427458`, 0.6719251293345294`,
```

0.6187329358641112`, 0.3259483616243979`, 1099.7736795288956`, 0.13645709285092156`,
2.0261792473532125`, 42.609141387369384`, 4.001881453185266`, 2.60265408230275`, 27.72385954595262`,
1.`, {{0, 0.0014069763087879302`, 2.5044178296425157`}, {3, 0.000550616632952633`, 0.9800976066556867`},
{6, 0.0001232447129145821`, 0.21937558898795614`}, {9, 0.000026175670837253656`, 0.046592694090311505`}}},
{0.11800216420621701`, 3.285091391336728`, 1.6522276383131942`, 0.9670398406334181`, 0.4307591958110746`,
0.30003613158270603`, 0.29799743711056653`, 701.4335436984502`, 0.043710117716904806`,
7.7162513520402545`, 106.9250381098324`, 2.540775371732082`, 1.2856449060562174`, 61.390570679875125`,
1.`, {{0, 0.004388203990120266`, 7.811003102414074`}, {18, 0.0002774793815502433`, 0.4939132991594331`}}},
{0.19357111334803345`, 1.327860467490665`, 5.552252240997824`, 1.1813151963757291`, 0.7103742334207412`,
0.3776068940225674`, 0.2456309648230094`, 3934.634788049085`, 0.12355866435459295`, 9.745686147306856`, 47.12550835881893`,
1.8222966332217296`, 1.8222966332217296`, 47.12550835881893`, 1.`, {{0, 0.0035815386352702388`, 6.375138770781025`}}},
{0.11447070818541266`, 1.7335861954191927`, 8.736387174086214`, 1.0335531844510784`, 0.08646802454747982`,
0.4443642965951369`, 0.015374273787766714`, 123.60141703755143`, 0.21867059554150092`,
6.82672203062274`, 961.1969955767904`, 170.50360316392167`, 5.580713995277018`, 532.3014599037986`,
1.`, {{0, 0.03759806033277844`, 66.92454739234562`}, {3, 0.0025940299999038375`, 4.617373399828831`},
{6, 0.00011847376631999542`, 0.21088330404959185`}, {9, 0.000026984284337738363`, 0.04803202612117428`},
{12, 0.000024506901039656644`, 0.04362228385058846`}, {15, 0.0000244625794301620 9`, 0.04354339138568852`},
{18, 0.000024461919159737494`, 0.04354221610433274`}, {21, 0.00004393116971911758`, 0.07819748210002929`}}},
{0.22987433852030326`, 3.2498283820092855`, 3.7918096781351274`, 0.808040368560279`, 0.9416246029763107`,
0.49895559050949523`, 0.02048494990466312`, 278.1506433463176`, 0.24538025309113942`, 1.1463070343242556`, 698.8582853330865`,
154.33220393387447`, 1.0606237333188373`, 46.9868808385639`, 1.`, {{0, 0.002680107359293269`, 4.7705910995420195`},
{4, 0.0006922070657577445`, 1.2321285770487853`}, {8, 0.00010642392367718474`, 0.18943458414538883`},
{12, 0.0000258906120413403`, 0.046085289433585724`}, {16, 0.000017155913671833887`, 0.03053752633586432`},
{20, 0.000016404053104805066`, 0.029199214526553018`}, {24, 0.00003281401618467883`, 0.05840894880872831 6`}}},
{0.2774701327316953`, 3.217064080543545`, 9.848999257942918`, 1.1587780417232185`, 0.0062851183500154395`,
0.6835477959342324`, 0.37074868705631564`, 2643.6047855840716`, 0.14999893314602414`, 6.1386041394007655`, 27.694623011240807`,
0.49019559268481394`, 0.49019559268481394`, 27.694623011240807`, 1.`, {{0, 0.0021047913488543013`, 3.7465286009606564`}}},
{0.07518728576930783`, 0.8073344777020095`, 0.2079911159294223`, 1.2717171333972301`, 0.7961690644438275`,
0.349516332980527`, 0.00930168975466583 7`, 660.9000723394831`, 0.24128632812714051`, 8.034216989913379`, 5976.864073579396`,
2875.29409732162`, 230.22464414838888`, 532.5981817747418`, 0.`, {{0, 0.04047746181488038`, 72.04988203048707`}}},
{0.19386888157639426`, 1.1721624963279647`, 7.488800420822528`, 1.2206737749782337`, 0.39882649538868`,

0.6168747383393647`, 0.005894727770540639`, 784.8706404426375`, 0.21917965839925868`, 7.12627169471982`, 142.94961223057967`,
0.641458456149524`, 0.641458456149524`, 142.94961223057967`, 1.`, {{0, 0.010864170529524056`, 19.33822354255282`}}},
{0.051543962131442966`, 1.0824196854643793`, 1.5789464925861978`, 0.9324716268311393`, 0.16692765658832065`,
0.33113273649509023`, 0.16543741477462187`, 443.37752852110856`, 0.07774169596012481`,
8.031423814374019`, 6198.047736321502`, 3513.2861156844347`, 229.19647689070572`, 506.466344314055`,
0.`, {{0, 0.007283845530641766`, 12.965245044542343`}, {2, 0.0084738850056049`, 15.083515309976724`},
{4, 0.007640726090945576`, 13.600492441883127`}, {6, 0.005657945454899781`, 10.07114290972161`},
{8, 0.003650817790538052 3`, 6.498455667157733`}, {10, 0.002147388158930296`, 3.822350922895927`},
{12, 0.0011911066146388992`, 2.1201697740572403`}, {14, 0.000638293663900969`, 1.136162721743725`},
{16, 0.0003359245848222787`, 0.5979457609836559`}, {18, 0.00147150927294566`, 2.6192865058432746`}}},
{0.04886198458272409`, 2.4240564285395276`, 3.0585768323132374`, 1.3030342316587755`, 0.5506088802661759`,
0.40370465638646613`, 0.05636145780930632`, 1567.8212350001168`, 0.20179368484919008`,
9.505728478640599`, 93.72953893448614`, 1.1911568943712976`, 1.1360547078656151`, 89.36351511143803`,
1.`, {{0, 0.005443834702396229`, 9.690025770265285`}, {7, 0.001347792446073062`, 2.3990705540100503`}}},
{0.26828907890664055`, 1.4916419764625646`, 9.30244345383333`, 0.8462433381711008`, 0.9150350508953056`,
0.16353772752060636`, 0.1124240025000419`, 2042.0256023360328`, 0.08362962700521354`, 9.175430964325553`,
69.61149000933557`, 1.9017063360182092`, 0.7119546006653314`, 26.533820189835506`, 1.`,
{{0, 0.0016400459664503806`, 2.9192818202816775`}, {28, 0.00037652436797711784`, 0.6702133749992698`}}},
{0.11853219177887636`, 2.436539187916523`, 4.07490711978506`, 0.8391678158796785`, 0.8274679746994464`, 0.4783994312610165`,
0.02880518630114325`, 249.91551468654188`, 0.20172418525520536`, 8.758671893830364`, 2309.3944678355565`,
745.2535155289736`, 4.395271770732933`, 335.4787857082667`, 1.`, {{0, 0.023207082842327094`, 41.308607459342234`},
{3, 0.002065033102448729`, 3.675758922358738`}, {6, 0.00011518333547179144`, 0.20502633713978877`},
{9, 0.000022989207070118318`, 0.04092078858481061`}, {12, 0.0000198900918910789`, 0.03540436356612045`},
{15, 0.000019812641755206356`, 0.03526650232426732`}, {18, 0.00001981033974219117`, 0.03526240474110028`},
{21, 0.000019810201598136034`, 0.035262158844682145`}, {24, 6.775951523920837`*^-6, 0.01206119371257909`}}},
{0.15380346197105899`, 2.5002802569502807`, 7.104691399911929`, 0.895619363352984`, 0.7056556726651881`,
0.2904087856211036`, 0.27890663528622706`, 1570.4796825528501`, 0.2402855906015`, 2.015690994359673`, 19.903873302171796`,
0.4355426274059869`, 0.4355426274059869`, 19.903873302171796`, 1.`, {{0, 0.0015126943709650566`, 2.6925959803178006`}}},
{0.07681933116098377`, 1.701376646381478`, 3.4749545233581878`, 0.9897106512386663`, 0.21660819673269338`,
0.4456181241675946`, 0.020856154503598144`, 170.86262737853005`, 0.11272605112949713`,
7.4521875824230435`, 22464.06171742289`, 10608.902720460776`, 228.84450805394775`, 2122.595281036927`,

```
0.`, {{0, 0.021230974327562935`, 37.79113430306202`}, {6, 0.00036453291462096294`, 0.648868588025314`},
   {12, 0.00006284666867112769`, 0.1118670702346073`}, {18, 0.000051613951596299035`, 0.09187283384141227`},
   {24, 0.00005164257630112814`, 0.09192378581600809`}, {30, 0.00005688483256558713`, 0.1012550019667451`},
   {36, 0.0000793992209600094`, 0.14133061330881672`}, {42, 0.00018541989275726762`, 0.33004740910793634`},
   {48, 0.0005526550029531773`, 0.9837259052566556`}, {54, 0.138681271970818`, 246.85266410805602`}}},
{0.19680750576810457`, 2.9845730901840906`, 8.034687795284743`, 1.0668124507997105`, 0.6731645050394575`,
  0.24725877139168673`, 0.05097134738484948`, 549.1786460489706`, 0.029127445194553014`,
  6.0096318557240025`, 153.44629044382324`, 1.486558513168845`, 0.9244276421176474`, 94.28049069925059`,
  1.`, {{0, 0.005131261067440104`, 9.133644700043385`}, {35, 0.0020340562257029414`, 3.6206200817512357`}}},
{0.2481156970490977`, 1.2202113043036036`, 9.306028403345813`, 0.8009108612869359`, 0.02681732734233533`,
  0.15549305073805075`, 0.021015785720259927`, 53.28601671876581`, 0.06540227633756851`,
  2.0322216725641393`, 134925.1646269719`, 29659.573348960883`, 228.51226986924146`, 1225.396540339537`,
  0.`, {{0, 0.036002747925741`, 64.08489130781898`}, {31, 0.0007336458558119779`, 1.305889623345321`},
   {62, 0.0008278846203003793`, 1.473634624134675`}, {93, 0.0022208806107071317`, 3.9531674870586944`},
   {124, 0.0016374034342814736`, 2.914578113021023`}, {155, 0.0014442143736141794`, 2.5707015850332393`},
   {186, 0.0012500674468259255`, 2.225120055350147`}, {217, 0.0011197976128063162`, 1.9932397507952426`},
   {248, 0.0010225444097523581`, 1.8201290493591975`}, {279, 0.04687095077596408`, 83.43029238121606`}}},
{0.05969602287151182`, 2.177557565950017`, 1.7675183816518665`, 0.8372040785938111`, 0.9188853493106184`,
  0.1878023064060833`, 0.1313271602899384`, 239.3049490553974`, 0.14060221077790058`, 6.359907705361997`,
  8945.507931814971`, 4781.960324165878`, 229.23578245318248`, 522.3946248209257`, 0.`,
  {{0, 0.026866379232427684`, 47.82215503372128`}, {11, 0.009534237887929732`, 16.970943440514922`},
   {22, 0.001970154980282215`, 3.5068758649023426`}, {33, 0.00040090083307668705`, 0.7136034828765028`},
   {44, 0.00009838162369649086`, 0.17511929017975375`}, {55, 0.00003886433308908785`, 0.06917851289857636`},
   {66, 0.000027044491743140918`, 0.04813919530279084`}, {77, 0.00002451059803514703`, 0.04362886450256171`},
   {88, 0.000023801766382700988`, 0.04236714416120776`}, {99, 0.0007177157397274711`, 1.2775340167148985`}}},
{0.1932263333480873`, 0.7780345756067408`, 9.679599745167298`, 1.1108495595363916`, 0.27977393862884203`,
  0.2181645563751744`, 0.006193313005348089`, 412.49802062580375`, 0.21651680739670837`, 9.973248740719807`, 215.44831285761188`,
  1.428496321465079`, 1.428496321465079`, 215.44831285761188`, 1.`, {{0, 0.016374071777178503`, 29.145847763377738`}}},
{0.05080144024762184`, 3.462591354860418`, 9.637683194306994`, 0.7734436451051352`, 0.6551811041034619`,
  0.18020194036251547`, 0.05139770733113868`, 3391.683612113084`, 0.04739051831745644`,
  1.6399754771823183`, 5.39612108586466`, 0.046106316483623`, 0.0442242597357994`, 5.1620040485660175`, 1.`,
```

```
{{0, 0.00037729716673457533`, 0.6715889567875442`}, {26, 0.000015015140956442019`, 0.02672695090246679`}}},
{0.17269587719558416`, 2.280528270589695`, 6.304702452448467`, 0.855238720891303`, 0.7302844321830138`,
 0.25273349413775115`, 0.28098261768807636`, 2514.837947344666`, 0.04999285469609732`, 2.363210664893213`, 14.044368065944093`,
 0.33515630479376113`, 0.33515630479376113`, 14.044368065944093`, 1.`, {{0, 0.0010673719730117512`, 1.899922111960917`}}},
{0.12416213981002666`, 0.8389921874064035`, 3.610730817659622`, 1.0777639636353653`, 0.18253050124358539`,
 0.3654692817582218`, 0.277997956001176`, 563.1802062075279`, 0.17509802310960038`, 1.6234053595359104`, 28.236683392130647`,
 1.7415286884938095`, 1.1210576477374425`, 18.147179969300336`, 1.`, {{0, 0.0012074472637196455`, 2.149256129420969`},
  {8, 0.00016315584266418602`, 0.29041739994225113`}, {16, 8.582571282994045`*^-6, 0.0152769768837294`}}},
{0.05389681485101466`, 0.42474703837564753`, 9.702496123506663`, 1.2875902192251218`, 0.839862878766310 7`,
 0.4145702753216558`, 0.012323091180371398`, 1207.1917708220153`, 0.0487427592688896`, 1.997348446926472`,
 41.061008317886824`, 0.8811331522058605`, 0.8757491959302175`, 40.81146613826966`, 1.`,
 {{0, 0.002652541815220023`, 4.721524431091641`}, {5, 0.000449129611288471`, 0.7994507080934783`}}},
{0.06391261825702438`, 1.7835225604286613`, 8.062142041499534`, 1.3025605866836996`, 0.9222349035188417`,
 0.39598365567854665`, 0.09958099473376647`, 75.95083350193653`, 0.043061298974276296`, 9.739451519751867`, 145277.60685188547`,
 75612.55829201033`, 146.4995559423053`, 1127.8024435077423`, 1.`, {{0, 0.057969609058401844`, 103.18590412395528`},
  {7, 0.0034911293658283537`, 6.214210271174469`}, {14, 0.003466994083950115`, 6.171249469431204`},
  {21, 0.00346656432286579`, 6.170484494701107`}, {28, 0.0034665395580506333`, 6.170440413330128`},
  {35, 0.0034665369882142566`, 6.170435839021377`}, {42, 0.003466536617796391`, 6.170435179677575`},
  {49, 0.003466536552895103`, 6.170435064153283`}, {56, 0.0034525391585859344`, 6.1455197022829635`}}},
{0.08850946488241984`, 0.9673836199236003`, 8.299835087678265`, 0.9900607622617293`, 0.0809660102577392`, 0.6707110673061827`,
 0.0056133585030711565`, 223.20635429959376`, 0.07233923327715214`, 4.067688416849283`, 220.78650248063363`,
 1.1788606043992216`, 1.1788606043992216`, 220.78650248063363`, 1.`, {{0, 0.016779774188528156`, 29.867998055580117`}}},
{0.056027023765889306`, 2.0857485685464603`, 9.366491292380005`, 1.196524731276077`, 0.9516747873190254`,
 0.6119693625054259`, 0.014912996736033244`, 220.0557248806415`, 0.0820080753991021`, 2.040285256013867`, 162.60731154020272`,
 1.9181020476199566`, 1.4123267796442491`, 177.30934607956937`, 1.`, {{0, 0.007749403470126346`, 13.793938176824897`},
  {2, 0.003814673446341092`, 6.790118734487143`}, {4, 0.0012922501663736228`, 2.3002052961450485`},
  {6, 0.00042060522977515686`, 0.7486773089997791`}, {8, 0.0001378076761371829`, 0.24529766352418556`},
  {10, 0.00004682854695477733`, 0.08335481357950363`}, {12, 0.000013941766339093848`, 0.024816344083587048`}}},
{0.04516470805711162`, 3.292123721374173`, 5.263943679637629`, 1.138536155221258`, 0.48426832878752823`,
 0.5662939024082725`, 0.09160607397723192`, 3360.7159646360847`, 0.026694430856043894`, 4.180658858897127`, 20.557542556718097`,
 0.24644547562175345`, 0.24644547562175345`, 20.557542556718097`, 1.`, {{0, 0.0015623732343105754`, 2.781024357072824`}}},
```

```
{0.2782184518736612`, 3.845021085593168`, 1.137061402050021`, 1.4385887178277776`, 0.42032329684699743`,
  0.5854257024714923`, 0.03249096611383817`, 328.6688300716124`, 0.02617032692156479`,
  8.869579059594898`, 244319.42508432153`, 49098.24054252135`, 229.23045365138677`, 71228.43881345209`,
  0.`, {{0, 0.046282516340458`, 82.38287908601524`}, {6, 0.012703238832022927`, 22.611765121000808`},
    {12, 0.009693655186639808`, 17.254706232218858`}, {18, 0.010470936392888703`, 18.638266779341894`},
    {24, 0.01318743325856878`, 23.47363120025243`}, {30, 0.016795377403096924`, 29.895771777512525`},
    {36, 0.020037480972849225`, 35.66671613167162`}, {42, 0.022429742317210972`, 39.92494132463553`},
    {48, 0.024385299856115837`, 43.405833743886184`}, {54, 5.237375669262508`, 9322.528691287262`}}},
{0.09319840750005726`, 2.6994281444799046`, 8.85475415324603`, 1.038403514470577`, 0.3378909888092929`, 0.6279203689803818`,
  0.23307552493819164`, 1846.0011275272348`, 0.034946033021005685`, 7.6621208156759835`, 62.210714593531755`,
  1.1979364634740302`, 1.1979364634740302`, 62.210714593531755`, 1.`, {{0, 0.004728014309108413`, 8.415865470212976`}}},
{0.06034484465157863`, 2.8968090048449424`, 3.142651522085412`, 1.307236858746047`, 0.3617717997572676`,
  0.29303713371207274`, 0.18131081790115888`, 4578.414121282968`, 0.17962394399488657`, 3.3138716244433386`, 7.5985320742277365`,
  0.1328316761739567`, 0.1328316761739567`, 7.5985320742277365`, 1.`, {{0, 0.000577488437641308`, 1.0279294190015282`}}},
{0.20102830374342656`, 1.9832552045818916`, 0.5122029117897267`, 0.8414910669557318`, 0.023679729667421823`,
  0.562606520598186`, 0.21796137676857033`, 2537.351175593395`, 0.0373739667120736`, 9.865525950238812`,
  44.28800566628175`, 1.3466436610435104`, 0.8888435019133337`, 29.017771099883166`, 1.`,
  {{0, 0.0019858752154254507`, 3.5348578834573026`}, {21, 0.00021947538816566978`, 0.39066619093489224`}}},
{0.04583409051630541`, 3.6747726707008503`, 0.7420872691712788`, 1.2157743693615881`, 0.8915453306179026`,
  0.4926202034522773`, 0.4217762472968932`, 73.4269782954483`, 0.10619296232023001`, 3.9517710932009376`, 114367.17200270195`,
  69097.16537760061`, 230.24401562086646`, 776.0019678818553`, 0.`, {{0, 0.05897614955902101`, 104.97754621505739`}}},
{0.10119631213999619`, 1.851973173732965`, 1.6003758779141855`, 0.9328064269865228`, 0.39319494883808903`,
  0.3867811485974866`, 0.006630825974655251`, 2020.948278622179`, 0.24307528382326266`, 8.411215463490773`, 52.12703977141673`,
  0.19294049823542175`, 0.19294049823542175`, 52.12703977141673`, 1.`, {{0, 0.003961655022627672`, 7.051745940277256`}}},
{0.19796477344593338`, 2.2434362012846876`, 1.2309355399833084`, 1.3299187291217778`, 0.059794768114105334`,
  0.6215799505741797`, 0.02273921518352973`, 1004.7174087228818`, 0.138969497102004`, 8.58482448486276`, 215.95651753218183`,
  31.890930363626634`, 3.1912484884836423`, 107.4808871734523`, 1.`, {{0, 0.007538371507208598`, 13.418301282831305`},
    {3, 0.00025575387770884734`, 0.45524190232174827`}, {6, 0.00006483590481723973`, 0.11540791057468673`},
    {9, 0.0000631942908323835`, 0.11248583768164262`}, {12, 0.00006318489058357535`, 0.11246910523876412`},
    {15, 0.00006318477819319671`, 0.11246890518389016`}, {18, 0.00012002217583853444`, 0.2136394729925913`}}},
{0.18841727652913837`, 3.2954166443999204`, 4.985214271603974`, 1.3919313698468052`, 0.8063258092579206`,
```

```
  0.6389441864140679`, 0.00880414284139521`, 1247.8494355462315`, 0.09671339442963328`, 8.749674730462491`, 227.245975107258`,
  0.6014078886366768`, 0.5834127146172289`, 224.10709375990342`, 1.`, {{0, 0.00976503525729211`, 17.381762757979953`},
   {2, 0.005382657504200114`, 9.581130357476201`}, {4, 0.0018844463642604368`, 3.3543145283835774`}}},
{0.08822838789215687`, 3.6314565393637377`, 8.720964691808945`, 1.4591613734708808`, 0.09172379783619267`,
  0.17510896742376914`, 0.2630216511777198`, 431.756995427934`, 0.16850915581941245`, 3.8927198296202143`, 63.86231170544817`,
  0.9453195274927054`, 0.4039004752826626`, 27.630096845399276`, 1.`, {{0, 0.0017756338415112728`, 3.1606282378900654`},
   {13, 0.0003098478677418893`, 0.5515292045805629`}, {26, 0.000014405650997183134`, 0.025642058774985977`}}},
{0.22004819658675462`, 1.2435615798896285`, 4.521096993231973`, 1.3090616735635763`, 0.5905184190150714`,
  0.6892934240599866`, 0.09109967416767972`, 239.35075160225182`, 0.045734190683186515`, 5.8358773331232445`, 37853.08680514885`,
  9086.500693941229`, 107.79359143415641`, 673.3993947432447`, 1.`, {{0, 0.045984200091459815`, 81.8518761627984`},
   {21, 0.0024239970266149895`, 4.314714707374681`}, {42, 0.0027701568824117892`, 4.930879250692985`}}},
{0.18183828174362843`, 3.2786199220563104`, 2.2240542093570888`, 0.9831747738881791`, 0.39067106215615355`,
  0.5999409005287338`, 0.32534468961787655`, 53.98091103084368`, 0.10022266621367804`, 9.980846637487563`, 249893.91414954638`,
  69412.99819740932`, 230.07967423343845`, 1721.3081058702974`, 0.`, {{0, 0.1308194160461426`, 232.85856056213385`}}},
{0.21252199352031792`, 1.818911285509734`, 0.45179175410691036`, 0.8473192883797733`, 0.5752582261250987`,
  0.4244690624430477`, 0.010390335454685632`, 819.8846878599306`, 0.22518903335941576`, 6.3452240038645735`, 4949.901295132101`,
  1220.9203984111116`, 230.26174918898093`, 956.5832740382147`, 0.`, {{0, 0.07270032882690432`, 129.40658531188967`}}},
{0.13408565703158576`, 3.9481177636984475`, 4.409802796026355`, 1.1882851460555317`, 0.7016940523769313`,
  0.6437617694336766`, 0.472695817526219`, 268.88735252515323`, 0.02238688650951909`, 7.198883746978469`, 15489.633381362073`,
  5218.421108903513`, 7.22686752815782`, 304.7853498582594`, 1.`, {{0, 0.021731965263457626`, 38.68289816895459`},
   {3, 0.0011913691924984383`, 2.12063716264722`}, {6, 0.000057580209456250915`, 0.10249277283212664`},
   {9, 0.000032558204496244024`, 0.05795360400331436`}, {12, 0.000032066570108375 36`, 0.05707849479290814`},
   {15, 0.000032049921059147054`, 0.05704885948528175`}, {18, 0.0000320487747671368`, 0.057046819085503506`},
   {21, 0.00003204864942152745`, 0.05704659597031886`}, {24, 0.000021999803962965 87`, 0.03915965105407924 5`}}},
{0.11550511139863262`, 2.7266825017818803`, 9.715437717848832`, 1.4226217916614743`, 0.7434913376024772`,
  0.6527594414247178`, 0.026462900292485443`, 517.125461393156`, 0.19263485762362964`,
  9.20218742349427`, 502.3896339203605`, 3.515177318766205`, 3.3743189500637563`, 483.3336592808018`, 1.`,
  {{0, 0.025581681652683836`, 45.53539334177723`}, {7, 0.011151676452657099`, 19.849984085729638`}}},
{0.1537432379066465`, 3.3010385217103515`, 7.8154151393713`, 1.099357680158021`, 0.2670513361076359`, 0.6099579487912872`,
  0.01626886763741749`, 3071.882113963599`, 0.04550777584421739`, 6.952411393791335`, 30.85937412244917`,
  0.1229036553246634`, 0.1229036553246634`, 30.85937412244917`, 1.`, {{0, 0.002345312433306137`, 4.174656131284924`}}},
```

{0.08915960893105551`, 3.2848427178141772`, 0.6302374569507307`, 1.4630290564093034`, 0.8347103436436674`,
0.36812644478584344`, 0.040963141333673035`, 3229.889147199789`, 0.1995027638419964`,
3.7582834934312945`, 19.868013913202574`, 0.52683038073008`, 0.3569491460004263`, 17.869912603663604`,
1.`, {{0, 0.0008747250272112735`, 1.557010548436067`}, {6, 0.00035048131218503795`, 0.6238567356893676`},
{12, 0.00010904599360745416`, 0.19410186862126844`}, {18, 0.000023861024874668833`, 0.04247262427690963`}}},
{0.2095156599086041`, 1.856403794983298`, 1.6647511783688493`, 0.8963074241076089`, 0.5257557556911894`,
0.2962680347723602`, 0.005638948645619698`, 398.77430140016673`, 0.1712593265312492`,
6.243180864475704`, 6593.453678424819`, 1630.762732475924`, 41.10980261158486`, 255.4097616119235`,
1.`, {{0, 0.018186397693354495`, 32.371787894171`}, {9, 0.00031268096193115064`, 0.5565721122374481`},
{18, 0.0001240146045812662`, 0.22074599615465382`}, {27, 0.00012343580718134612`, 0.21971573678279607`},
{36, 0.00012342798656135906`, 0.21970181607921913`}, {45, 0.000123427037362214`, 0.2197001265047409`},
{54, 0.00012342683264687635`, 0.2196997621114399`}, {63, 0.00012342677534367324`, 0.2196996601117384`},
{72, 0.00012342675690574906`, 0.21969962729223336`}, {81, 0.000047477426638055335`, 0.0845098194157385`}}},
{0.08606673974307905`, 3.001046395083865`, 6.871125535025986`, 1.29871337654279`, 0.0013879217267305233`,
0.4709543850320145`, 0.028486772088755836`, 255.05656064251065`, 0.15733152617600732`, 4.419059383126843`, 166.6965921491207`,
1.153845417542925`, 1.153845417542925`, 166.6965921491207`, 1.`, {{0, 0.012668941003333174`, 22.55071498593305`}}},
{0.2794012033077364`, 3.8964244114394253`, 7.589043584575673`, 0.9660750334911808`, 0.6823441866078543`,
0.315494435212184`, 0.013950850602606538`, 1092.7384181067544`, 0.06831145072823913`, 6.790315469699049`,
100.97260273736335`, 0.30578518348582706`, 0.30543645414613546`, 100.84401996067788`, 1.`,
{{0, 0.006983291480244503`, 12.430258834835215`}, {7, 0.0006808540367670168`, 1.21192018544529`}}},
{0.16053897675716755`, 1.21110484978606`, 6.017303762103152`, 1.2353982330406967`, 0.20086989110627473`,
0.3272106220983775`, 0.02355461265150193`, 77.09093986775292`, 0.04848901873161415`,
3.5142859649597167`, 223526.18527287157`, 67821.92773453232`, 229.28475474824307`, 4125.337318875974`,
0.`, {{0, 0.052104050823616445`, 92.74521046603726`}, {16, 0.0007077381809547466`, 1.259773962099449`},
{32, 0.0009625377060465823`, 1.7133171167629162`}, {48, 0.003889322989808828`, 6.922994921859714`},
{64, 0.00662692199364081`, 11.795921148680641`}, {80, 0.004241390014649394`, 7.549674226075922`},
{96, 0.003792393441132731`, 6.7504603252162605`}, {112, 0.0038717491206253386`, 6.891713434713102`},
{128, 0.003547449544960772`, 6.314460190030174`}, {144, 0.23378208241913842`, 416.13210670606634`}}},
{0.06315507597420161`, 1.4395182613839497`, 3.0858504661132873`, 1.1448617905347693`, 0.30610701790846395`,
0.20244634599341438`, 0.2950980917537417`, 2179.6214593356067`, 0.03473000454292019`, 1.333335366603153`, 5.165641791158565`,
0.1979350027402686`, 0.08841645970775135`, 2.202599550762626`, 1.`, {{0, 0.00013104866085033134`, 0.23326661631358978`},

```
{11, 0.00003358077673912248`, 0.05977378259563801`}, {22, 2.768128268505756`*^-6, 0.004927268317940246`}}},
{0.27116635506122466`, 2.640185082938654`, 3.1282641056246216`, 0.9199164829096633`, 0.32218318348593566`,
 0.5777637686875948`, 0.006117113694401221`, 324.8095638375007`, 0.04342122620091826`, 2.609798810681127`, 18981.386036116564`,
 3880.2957110144807`, 26.450043792766564`, 205.4983663037971`, 1.`, {{0, 0.013690719659223475`, 24.369480993417785`},
  {4, 0.0002856557691008459`, 0.5084672689995058`}, {8, 0.0002447456282172967`, 0.43564721822678815`},
  {12, 0.0002445501818268614`, 0.4352993236518134`}, {16, 0.0002445449305120609`, 0.43528997631146843`},
  {20, 0.00024454452733174454`, 0.4352892586505052`}, {24, 0.0002445444663307172`, 0.4352891500686765`},
  {28, 0.0002445444516964449`, 0.43528912401967196`}, {32, 0.00017402622484913218`, 0.30976668023145526`}}},
{0.08076786811690995`, 2.5389877544798445`, 7.211847948605141`, 1.1500644274940979`, 0.7690232347129535`,
 0.1924810447357974`, 0.3170033067881963`, 2456.45541532025`, 0.12977198817972552`, 1.000471570970415`, 5.333962766938453`,
 0.12071298321351967`, 0.12071298321351967`, 5.333962766938453`, 1.`, {{0, 0.00040538117028732245`, 0.7215784831114339`}}},
{0.14196490482109098`, 3.5800560378690767`, 8.191314882292886`, 0.8521719014833709`, 0.20577614391452248`,
 0.2973229046898421`, 0.032689134381726236`, 2192.8696238585753`, 0.20870222148197032`, 8.620659635124824`, 36.87870251732632`,
 0.22294148311780343`, 0.22294148311780343`, 36.87870251732632`, 1.`, {{0, 0.0028027813913168004`, 4.988950876543905`}}},
{0.16225217210911858`, 2.1337475700719883`, 4.607583982995607`, 1.274907826520427`, 0.760916097212121`,
 0.6512132808308`, 0.1988962707427588`, 88.66654318682235`, 0.17629703311441042`, 5.991536547797288`,
 81943.04021995429`, 24633.999746672544`, 228.36279073884253`, 1540.9072684387938`, 0.`,
 {{0, 0.029468521872069695`, 52.45396893228405`}, {2, 0.009632312477724862`, 17.145516210350255`},
  {4, 0.006012015053149862`, 10.701386794606755`}, {6, 0.003937706105929049`, 7.009116868553709`},
  {8, 0.002688173087279081`, 4.784948095356764`}, {10, 0.0019098696095417203`, 3.3995679049842624`},
  {12, 0.0014115231540437114`, 2.512511214197806`}, {14, 0.001084452767155327`, 1.9303259255364822`},
  {16, 0.000864814352021594`, 1.5393695465984374`}, {18, 0.06009956392243344`, 106.97722378193151`}}},
{0.09998370398747453`, 1.3561107931887042`, 7.055733599099842`, 0.9997997594446719`, 0.4231050232131661`,
 0.35289870383552235`, 0.01863838099230359`, 585.7262791471302`, 0.12303242645253354`, 2.5487735356675643`, 52.08286953695101`,
 0.5502911270520651`, 0.5502911270520651`, 52.08286953695101`, 1.`, {{0, 0.003958298084808277`, 7.045770590958734`}}},
{0.0790420625297461`, 0.7951762600254768`, 1.0975467432818782`, 0.8295886125705915`, 0.21974762785224522`,
 0.4003083061581494`, 0.12181590550696897`, 256.3718289629029`, 0.07504947990766975`, 1.4404680202816778`, 26896.800412169825`,
 12627.053456620704`, 230.2542789078192`, 528.5286275964036`, 0.`, {{0, 0.040168175697326676`, 71.49935274124148`}}},
{0.09310243320239603`, 1.7939569577957926`, 0.5112771848209494`, 1.3548866082012778`, 0.9609312900127882`,
 0.31815651234043585`, 0.047992777894275945`, 1572.364241162158`, 0.10220690607690369`,
 1.3696469849079624`, 1079.3676768922296`, 460.8164468365005`, 1.4991208829946963`, 9.702090870001877`, 1.`,
```

```
{{0, 0.0005223241866042924`, 0.9297370521556404`}, {8, 0.00010869930381973231`, 0.19348476079912352`},
  {16, 0.000024244607039312203`, 0.043155400529975727`}, {24, 0.000017494836930111191`, 0.031140809735599198`},
  {32, 0.000017178795839737312`, 0.030578256594732418`}, {40, 0.000047417175886956526`, 0.08440257307878261`}}},
{0.20315703606221613`, 1.4806269686939117`, 9.851530344798562`, 0.807291927894971`, 0.7692364729235686`,
 0.5064599484352303`, 0.07616257600145751`, 550.4721115636684`, 0.18512304424700965`, 4.821016411964738`,
 218.39630892442125`, 5.127347106143511`, 3.5754703424162377`, 155.71219289758454`, 1.`,
 {{0, 0.007137193292249268`, 12.704204060203697`}, {17, 0.004696933367967157`, 8.360541394981539`}}},
{0.22897905358022103`, 2.219649931841208`, 8.024983925513396`, 1.3490395658771779`, 0.7992198564081268`,
 0.5097552623833034`, 0.017934168491060075`, 2636.2345048980164`, 0.04871567850920905`, 1.38033814014487`, 13.596944980969587`,
 0.09501555395462136`, 0.09501555395462136`, 13.596944980969587`, 1.`, {{0, 0.0010333678185536887`, 1.8393947170255658`}}},
{0.14728957561352346`, 3.8290117168547324`, 5.577517394641557`, 1.2037071314600827`, 0.7629976630105226`,
 0.33391252098357616`, 0.00623505541513591`, 1551.1825745003182`, 0.05716501203669799`, 3.4524118308532703`,
 41.86753105286014`, 0.08001806651121557`, 0.07985753896844178`, 41.846528912425526`, 1.`,
 {{0, 0.0028637383542707623`, 5.097454270601957`}, {4, 0.0003165978430735775`, 0.563544160670968`}}},
{0.1747499923357959`, 1.9364726507678487`, 4.539322141325334`, 1.0358217348301002`, 0.687467494258686`,
 0.22118436656891216`, 0.06970356230137446`, 4108.781309212477`, 0.15897963252631786`, 3.216561469360766`,
 10.347680597824837`, 0.18184122770159203`, 0.12029501485871506`, 6.7789943919918`, 1.`,
 {{0, 0.0004325328765851717`, 0.7699085203216056`}, {27, 0.00008267069720620516`, 0.14715384102704518`}}},
{0.061449808274615936`, 2.451738473864639`, 8.032153511575046`, 1.3988277328315561`, 0.653844119276888`,
 0.6033333182307286`, 0.12509363159734996`, 3162.271766392656`, 0.18189469469951425`, 1.0210674887190017`, 6.918144332950094`,
 0.12941274763074606`, 0.12941274763074606`, 6.918144332950094`, 1.`, {{0, 0.0005257789693042072`, 0.9358865653614887`}}},
{0.1951885853322612`, 0.6765436415231281`, 7.0581513804142055`, 1.4397439961417247`, 0.9899034195557828`,
 0.3660842570171081`, 0.2551485659861665`, 53.30479560198744`, 0.15667683142074817`,
 8.392793083340557`, 167811.20606479805`, 44206.793487562834`, 227.9607434004859`, 1440.80380245777`,
 0.`, {{0, 0.07300701547022401`, 129.95248753699875`}, {14, 0.02126593948111036`, 37.85337227637644`},
  {28, 0.006394557235312674`, 11.382311878856559`}, {42, 0.001993842864555619`, 3.5490402989090017`},
  {56, 0.0006642626583511386`, 1.1823875318650268`}, {70, 0.00026015545788190945`, 0.4630767150297988`},
  {84, 0.0001361898531896717`, 0.24241793867761566`}, {98, 0.00009624213300148852`, 0.17131099674264957`},
  {112, 0.00008151648470767784`, 0.14509934277966655`}, {126, 0.005601367348455971`, 9.970433880251628`}}},
{0.15955516943400022`, 0.8482855893464007`, 9.331572022970409`, 1.1667938097447013`, 0.29910958141522603`,
 0.1963171731712634`, 0.010305064592809629`, 1013.0765277256257`, 0.20900313016467104`, 3.301983133021727`, 27.457654288105932`,
```

```
  0.26852615935122764`, 0.26852615935122764`, 27.457654288105932`, 1.`, {{0, 0.002086781725896051`, 3.7144714720949703`}}},
{0.17746707158856556`, 0.44139712602022296`, 2.8621670830493073`, 0.8100971992338867`, 0.0373958036205273`,
  0.5075452637360592`, 0.09067013663296572`, 114.80525128927701`, 0.16719225120884412`,
  9.820606806768879`, 45629.05944977913`, 12860.34067541259`, 230.10683477704328`, 4904.557928777284`,
  0.`, {{0, 0.053141838477027664`, 94.59247248910926`}, {5, 0.013836455198493232`, 24.62889025331795`},
    {10, 0.010488131309552864`, 18.668873731004098`}, {15, 0.011361519295547708`, 20.223504346074918`},
    {20, 0.013624853003788498`, 24.252238346743525`}, {25, 0.0155394407144941`, 27.6602044717995`},
    {30, 0.0165162483806953`, 29.398922117637788`}, {35, 0.01700201405526311`, 30.263585018368335`},
    {40, 0.017476631813916427`, 31.108404628771236`}, {45, 0.2037592703382946`, 362.6915012021644`}}},
{0.25772135278013303`, 3.070426405636976`, 1.2338255709842532`, 1.3135003325072887`, 0.5149580362176238`,
  0.41732865316044054`, 0.04496826136197767`, 318.8324074107286`, 0.010846019130009599`,
  7.957536959758443`, 459343.15708679357`, 98098.12262310713`, 228.82752818649735`, 6230.320093402143`,
  0.`, {{0, 0.036304699168364964`, 64.62236451968964`}, {16, 0.006774150196656145`, 12.057987350047938`},
    {32, 0.00679810004151662`, 12.100618073899582`}, {48, 0.008585083517577637`, 15.281448661288193`},
    {64, 0.007836868935265591`, 13.949626704772752`}, {80, 0.00688317270674158`, 12.252047418000013`},
    {96, 0.0063788839125387495`, 11.354413364318974`}, {112, 0.005928184472664673`, 10.55216836134312`},
    {128, 0.005471153517302044`, 9.738653260797639`}, {144, 0.382544030629935`, 680.9283745212841`}}},
{0.08988753363169116`, 3.536295514824336`, 7.275260820034448`, 0.8993825689374235`, 0.025163868977214232`,
  0.47083049518550046`, 0.018253871395130763`, 416.18519037331805`, 0.11499620973251179`, 2.265482484941062`, 52.34014899122266`,
  0.228452147397505`, 0.228452147397505`, 52.34014899122266`, 1.`, {{0, 0.003977851323332922`, 7.080575355532602`}}},
{0.14192760186457132`, 2.3958894382433478`, 3.253512770748353`, 0.8655063905996925`, 0.8143036151546204`,
  0.5834364577519011`, 0.17553594321370639`, 62.22207407824173`, 0.16384862691545943`, 3.0979894918131596`, 90045.54264929114`,
  29716.589543776136`, 230.0705414225763`, 1135.2220334504784`, 0.`, {{0, 0.08627687454223637`, 153.57283668518073`}}},
{0.14248286143558947`, 2.096310454690764`, 9.38011512102738`, 0.9675972750885355`, 0.0803295271719453`, 0.6033546111821413`,
  0.13762578908199044`, 1262.8186365484712`, 0.18785001372865495`, 4.9706998985924695`, 45.47802637953347`,
  0.9617980250909921`, 0.9617980250909921`, 45.47802637953347`, 1.`, {{0, 0.0034563300048445437`, 6.152267408623287`}}},
{0.10446764528219044`, 3.442117541556258`, 1.7911289531773882`, 0.8226507378787467`, 0.06660293233402026`,
  0.33605831499521144`, 0.1966635974545483`, 110.27400709595466`, 0.23027159794407875`, 2.5346674177346316`, 24914.904403613527`,
  9982.349116398846`, 230.26285249684662`, 680.0736251630282`, 0.`, {{0, 0.051685595512390145`, 92.00036001205446`}}},
{0.0959624592169791`, 0.7155878562202682`, 5.1612847015717165`, 1.2701047018452214`, 0.05672968500006248`,
  0.6450160655606327`, 0.04218472167952095`, 1614.7615476312947`, 0.23305782159027644`, 5.6652476983851425`,
```

```
   41.181567675359915`, 1.3656949421986688`, 1.3168301620045595`, 39.64189817604284`, 1.`,
   {{0, 0.00219086282279276`, 3.8997358245711133`}, {29, 0.0008219214385864956`, 1.4630201606839621`}}},
  {0.24311048293765425`, 1.2773072105524381`, 8.674158333323465`, 1.236248375623142`, 0.5430857314344402`,
   0.29169516048698574`, 0.10383247735445839`, 522.6667880889086`, 0.0459109896778586 7`, 9.694591495258663`, 242.4184657661748`,
   7.427759163268731`, 5.057058033609398`, 166.27424975187117`, 1.`, {{0, 0.008642977852784388`, 15.384500577956212`},
    {10, 0.0033796945663087034`, 6.015856328029492`}, {20, 0.0006141705620491156`, 1.0932236004474258`}}},
  {0.08713889377857453`, 2.2859880795657075`, 9.600148060687761`, 1.2921363299986606`, 0.6294865567710719`,
   0.5590186325118135`, 0.25992443653112657`, 633.6168560418654`, 0.16781120217455497`, 6.04666254822`, 195.42311188862982`,
   4.561521495356281`, 2.930176391528049`, 125.90920050651653`, 1.`, {{0, 0.006846873407944163`, 12.18743466614061`},
    {5, 0.0023102921686644507`, 4.112320060222722`}, {10, 0.0004119336618866424`, 0.7332419181582235`}}},
  {0.26758004713434824`, 2.448981429729897`, 4.582999027119474`, 1.4554483602004895`, 0.3942207099215642`,
   0.5288182096517661`, 0.017684947331647518`, 53.19217741492592`, 0.1488296927599813`, 5.857737808697124`, 280035.4092206887`,
   58024.95083935424`, 229.6497786026346`, 1762.4639209948084`, 0.`, {{0, 0.13394725799560545`, 238.4261192321777`}}},
  {0.13431037279286218`, 0.7397108148766112`, 9.99887072123514`, 1.436439642978205`, 0.2003363652340402`, 0.6779870054162531`,
   0.009538236593206559`, 4558.309876478542`, 0.14371659865116293`, 1.1480275619139793`, 3.3042548635671993`,
   0.036197515652239456`, 0.036197515652239456`, 3.3042548635671993`, 1.`, {{0, 0.00025112336963110715`, 0.44699959794337074`}}},
  {0.27730778316543614`, 1.7365428506012295`, 3.277328223559843`, 1.1643406154035905`, 0.2621424262474452`,
   0.5836632043576286`, 0.19147730163610321`, 594.1704278677969`, 0.14363610143643862`, 8.32222051121584`,
   189.14428731602885`, 5.396532575587932`, 3.3975651359149106`, 118.68059659517839`, 1.`,
   {{0, 0.008337478695575181`, 14.840712078123822`}, {19, 0.0006822466456583762`, 1.2143990292719096`}}},
  {0.05614793436458493`, 1.997529172364887`, 6.840549262003957`, 1.2853232683568476`, 0.15723714782247722`,
   0.6374125080636333`, 0.1308231427534364`, 1207.3035504224565`, 0.15724403465210185`, 1.842303075231806`,
   18.954068087070414`, 0.4236038654591067`, 0.40284407530551847`, 18.00414313509485`, 1.`,
   {{0, 0.0011679109987897342`, 2.078881577845727`}, {6, 0.00020040387947747442`, 0.3567189054699045`}}},
  {0.0647068787712346`, 0.8380824418888393`, 1.0963289034750066`, 0.857562051048008`, 0.6907626550964823`,
   0.2999007220652139`, 0.27885748113518277`, 1238.4520803651262`, 0.1379529162857775`,
   8.96709675960637`, 80.42072928246569`, 5.322409164827887`, 2.955886448463175`, 45.67393253934753`, 1.`,
   {{0, 0.0029948680149921216`, 5.330865066685976`}, {13, 0.00047635085799829107`, 0.8479045272369581`}}},
  {0.20132622112515353`, 1.9086557669063273`, 0.7030652496772074`, 0.7755069309180247`, 0.32762519329281337`,
   0.20767575421456186`, 0.011318073987463645`, 431.8155255411275`, 0.17436258525110954`,
   5.81443415452358`, 9566.819329129976`, 2460.2197690892676`, 228.16860578318`, 1113.8944400519952`, 0.`,
```

{{0, 8.449103442328318`*^-6, 0.015039404127344407`}, {1, 8.80444161713884`*^-6, 0.015671906078507134`},
{2, 9.178452271466315`*^-6, 0.01633764504321004`}, {3, 9.452766637742635`*^-6, 0.016825924615181887`},
{4, 9.571001899127223`*^-6, 0.017036383380446454`}, {5, 9.519298286100664`*^-6, 0.016944350949259182`},
{6, 9.309406748787438`*^-6, 0.01657074401284164`}, {7, 8.966453422308741`*^-6, 0.01596028709170956`},
{8, 8.520835269177252`*^-6, 0.01516708677913551`}, {9, 0.08457420568435746`, 150.5420861181563`}}},
{0.045022613849116516`, 3.5058573906861508`, 8.47131465431961`, 1.2697383949459176`, 0.5022287267438617`,
0.23847623822456332`, 0.06077359284826833`, 243.2833936812314`, 0.1613793589669978`, 8.022277670460284`, 373.1013242752542`,
3.4105927832195526`, 2.805824331085938`, 307.6831845766793`, 1.`, {{0, 0.016054244037098257`, 28.576554386034896`},
{9, 0.006078276057409472`, 10.819331382188858`}, {18, 0.0012514019333198978`, 2.227495441309418`}}},
{0.17418182442148117`, 3.0892110646487794`, 3.4522019380421036`, 1.2965827481879664`, 0.41818871776887856`,
0.6556019601202148`, 0.075063644002762`, 2886.574837344593`, 0.15315141138676647`, 2.6062865811306466`,
14.839837611470706`, 0.18225213339011284`, 0.1548476574977275`, 12.138445093070532`, 1.`,
{{0, 0.0007105296481891985`, 1.2647427737767736`}, {20, 0.00021199217888416186`, 0.37734607841380813`}}},
{0.045831059772204225`, 2.899702133961444`, 5.925065562245003`, 0.8929739121804446`, 0.5933600691515191`,
0.4490635153302144`, 0.16447888961080792`, 1912.79886244215`, 0.11499785968845799`, 4.757985376452911`, 42.357168547527465`,
0.7008229700102899`, 0.7008229700102899`, 42.357168547527465`, 1.`, {{0, 0.0032191448096120876`, 5.7300777611095155`}}},
{0.2429697761558342`, 1.8449858081251405`, 7.508754000794074`, 0.9127320360969664`, 0.44886430542069955`,
0.35316747155912975`, 0.02193269408167387`, 161.73721618998414`, 0.16775422018188862`,
9.221971574736251`, 588.9519172690613`, 7.784759488801926`, 5.398856022479488`, 586.4278292649898`,
1.`, {{0, 0.03826530553712193`, 68.11224385607703`}, {2, 0.0055483592043746475`, 9.876079383786873`},
{4, 0.0005805289881916317`, 1.0333415989811046`}, {6, 0.00007780365093157309`, 0.13849049865820012`},
{8, 0.00003208004514311679`, 0.05710248035474789`}, {10, 0.00002844909778420388`, 0.0506393940558829`},
{12, 0.000028204557117958655`, 0.050204111669966404`}, {14, 7.783943474163172`*^-6, 0.013855419384010446`}}},
{0.160442241636698`, 3.55050251528807`, 3.9052606138637995`, 1.2025377341211225`, 0.791383919489673`,
0.40672925070026333`, 0.35477124416027767`, 105.50559352550128`, 0.10792398319932495`,
9.162379917819035`, 89514.77688574418`, 27109.089399729866`, 228.89197276776972`, 1276.2489916862048`,
0.`, {{0, 0.04149459160048902`, 73.86037304887046`}, {17, 0.01717942879184894`, 30.579383249491112`},
{34, 0.006966232674964839`, 12.399894161437413`}, {51, 0.0028623826778786696`, 5.095041166624031`},
{68, 0.0011995809209107543`, 2.135254039221143`}, {85, 0.0005238205300125356`, 0.9324005434223134`},
{102, 0.0002486623591853209`, 0.44261899934987115`}, {119, 0.00013613156429087483`, 0.2423141844377572`},
{136, 0.00008941056179677804`, 0.1591507999982649`}, {153, 0.026294681686773846`, 46.80453340245745`}}},

```
{0.20468313725879644`, 1.5880039947408164`, 1.8181726149942818`, 1.4361114186612707`, 0.6452252370101204`,
  0.23877202353940497`, 0.026067763377395026`, 956.3204095901076`, 0.08759161290164996`,
  6.530440575770296`, 91.112966981299`, 1.2998352988418567`, 1.0202660318051826`, 75.69120689306092`, 1.`,
  {{0, 0.004293241473846522`, 7.641969823446808`}, {22, 0.0014592902500261086`, 2.5975366450464734`}}},
{0.07364401078716826`, 2.5859680523819737`, 7.8227327965838285`, 0.8181378301360093`, 0.36855652079172274`,
  0.32445369334254925`, 0.005391697017678555`, 139.66827785366002`, 0.0644557011853657`,
  2.5433237558513593`, 168.49700533080954`, 1.3887139972045723`, 0.5814558657672043`, 177.77439618959107`,
  1.`, {{0, 0.008522845650213228`, 15.170665257379545`}, {5, 0.003557540944483986`, 6.332422881181495`},
    {10, 0.0010290685679888753`, 1.831742051020198`}, {15, 0.00029555422614747`, 0.5260886522542496`},
    {20, 0.00008619869236974135`, 0.15343367241813963`}, {25, 0.000019644832738344688`, 0.03496780227425354`}}},
{0.2506656721970057`, 1.5258727034335298`, 1.8333712411976357`, 1.2495957919641723`, 0.1010075460664761`,
  0.5024718466416268`, 0.012345305326089913`, 1052.1669408921373`, 0.03320454993831845`, 2.130087729732141`, 21.908887159312037`,
  0.2127226947914407`, 0.2127226947914407`, 21.908887159312037`, 1.`, {{0, 0.001665075424107715`, 2.9638342549117325`}}},
{0.10121864512314288`, 0.9521305504715327`, 3.6368221202357223`, 1.491518490019038`, 0.3322534458031634`,
  0.2564348282281962`, 0.03507970829425946`, 4627.905596988043`, 0.07121256842175683`, 7.352385124666178`,
  15.635940940148682`, 0.3552600376157034`, 0.2631970282687649`, 11.528259997492706`, 1.`,
  {{0, 0.0006416992586781915`, 1.1422246804471807`}, {48, 0.0002344485011312541`, 0.4173183320136323`}}},
{0.1942542932466596`, 3.1177323303054916`, 7.932133913996736`, 1.3085296181137158`, 0.8325654731630694`,
  0.19060678658466512`, 0.044940459115954866`, 2391.994107548152`, 0.24452644883302188`, 1.4227474861405387`,
  8.535177468325813`, 0.07854032617435734`, 0.05217489848368566`, 5.212785029014432`, 1.`,
  {{0, 0.0002919022038102313`, 0.5195859227822117`}, {41, 0.00010426945839486552`, 0.1855996359428606`}}},
{0.12644890469661946`, 3.0196313965941393`, 8.688658870512302`, 1.3655010622962382`, 0.5847686349152417`,
  0.30770354208104556`, 0.013061738242062998`, 1213.9477293174991`, 0.0486468587160932`, 6.312801306430787`, 72.48326340118683`,
  0.26658516523397136`, 0.26658516523397136`, 72.48326340118683`, 1.`, {{0, 0.0055087280184902`, 9.805535872912555`}}},
{0.11280111274715143`, 3.2009360918012444`, 2.9222221030043283`, 0.9684721327188957`, 0.03329493891954294`,
  0.1886848273472841`, 0.39445850876705457`, 50.69043630972767`, 0.07560282132089569`,
  3.828945975174229`, 193146.62062500234`, 73930.0503397643`, 228.36069859508817`, 1145.746488353264`,
  0.`, {{0, 0.010483949731169218`, 18.661430521481208`}, {6, 0.0035090403035628473`, 6.2460917403418685`},
    {12, 0.002430051444645526`, 4.325491571469037`}, {18, 0.0017851325927563761`, 3.1775360151063494`},
    {24, 0.0013312541909950883`, 2.3696324599712573`}, {30, 0.0010013269855785926`, 1.7823620343298947`},
    {36, 0.0007602412839726924`, 1.3532294854713927`}, {42, 0.0005838970206370874`, 1.0393366967340159`},
```

```
    {48, 0.0004548610582850612`, 0.809652683747409`}, {54, 0.06473697850324558`, 115.23182173577716`}}},
{0.21704875939335333`, 0.6532232080519234`, 2.918554590657344`, 1.428534955684801`, 0.6202310312510246`,
  0.3415578035649972`, 0.1858190740142067`, 1428.9901262383053`, 0.23526262585558444`, 9.378643714384769`,
  102.55197063756951`, 7.146752487834662`, 3.7806441502730106`, 54.82326899549946`, 1.`,
  {{0, 0.0029991600191231734`, 5.338504834039249`}, {8, 0.0009824354000310606`, 1.748735012055288`},
    {16, 0.00017207419770102087`, 0.3062920719078172`}, {24, 0.000012898826802704442`, 0.022959911708813868`}}},
{0.23408001184633387`, 2.4049572740765104`, 0.41973647277501946`, 0.9771854975111727`, 0.20272636213110817`,
  0.5074761434023957`, 0.2724896408017817`, 4254.760257351377`, 0.16397942354351142`, 7.3871482427729305`,
  21.437258244144928`, 0.5569513126875294`, 0.3719106769822289`, 12.977742513205094`, 1.`,
  {{0, 0.0008960771791581628`, 1.5950173789015298`}, {14, 0.00009023125184544244`, 0.16061162828485542`}}},
{0.1483945695425406`, 2.699298050205127`, 5.571982823617271`, 0.9775705576725262`, 0.05426683476548022`,
  0.5307838109252759`, 0.3096243181655647`, 323.46321359362`, 0.06725520461051537`, 9.792268234153859`,
  330.6252128903389`, 6.754103973517194`, 4.202907248477701`, 207.23921291095746`, 1.`,
  {{0, 0.014825240085228147`, 26.388927351706098`}, {14, 0.000924940096004621`, 1.6463933708882252`}}},
{0.11663741077922185`, 0.4431208542421814`, 9.780884572399291`, 1.0122403254522139`, 0.996318833483786`,
  0.22155768960679945`, 0.39993089984673347`, 139.93164861672463`, 0.10490063957337303`,
  5.3884722727325105`, 418.0089597902204`, 48.34450646724173`, 19.107566876489493`, 174.13848805538277`,
  1.`, {{0, 0.010067235607514897`, 17.919679381376515`}, {7, 0.0028248320993941463`, 5.028201136921581`},
    {14, 0.00029945733052751615`, 0.5330340483389787`}, {21, 0.000026814740030727965`, 0.04773023725469579`},
    {28, 4.701588215842848`*^-6, 0.008368827024200269`}, {35, 0.000011483726525961034`, 0.02044103321621064`}}},
{0.11314041374373823`, 3.32806970136921`, 5.495643049849155`, 1.495187492234928`, 0.8329498208958137`,
  0.41734162088096116`, 0.32389371604169326`, 602.537083528763`, 0.025714962476490244`, 1.3686646556271693`,
  54.67461567681306`, 1.1045608000501135`, 0.48276883323466785`, 22.242896445053248`, 1.`,
  {{0, 0.0011919575471130567`, 2.121684433861241`}, {5, 0.0004173276584068728`, 0.7428432319642336`},
    {10, 0.00006520330750088782`, 0.11606188735158032`}, {15, 0.00001597161680322955`, 0.0284294779097486`}}},
{0.09294922428523011`, 0.8101676653238759`, 3.5463022431515436`, 0.9341830117643879`, 0.15981251792750606`,
  0.6527933768073686`, 0.016252799814697117`, 738.0588634619963`, 0.024900612098687713`, 6.915149516601594`, 120.57807445002786`,
  1.820920375535185`, 1.820920375535185`, 120.57807445002786`, 1.`, {{0, 0.009163933658202117`, 16.31180191159977`}}},
{0.2696435798732645`, 3.282053008859009`, 8.218787637608994`, 1.4011858805306123`, 0.38871702639887196`,
  0.49514225831047376`, 0.01812451162452924`, 173.50575962443355`, 0.20495943261505556`,
  3.6349041099248875`, 263.54632725302497`, 2.698481727407224`, 1.7138134527174693`, 262.3956773829294`,
```

```
1.`, {{0, 0.016424212398852136`, 29.2350980699568`}, {6, 0.0035178590822504984`, 6.261789166405888`}}},
{0.13023501639399904`, 3.4827106844829796`, 7.180310384726493`, 1.0417379014779535`, 0.3157954886073613`,
 0.3090830033379006`, 0.027522996818882862`, 1326.2576636786057`, 0.24748434887668458`,
 4.853044482161593`, 37.76653376783778`, 0.2132402850848749`, 0.1882570458229858`, 32.72285690534322`, 1.`,
 {{0, 0.001750859625730687`, 3.116530133800623`}, {48, 0.0007360774990753981`, 1.3102179483542087`}}},
{0.0474731410927452`, 0.7132410347452449`, 0.7493200461382123`, 1.2237616503945397`, 0.9506868210145907`,
 0.6554164129507942`, 0.030282495208288053`, 4252.97050681489`, 0.14327214907032687`, 7.932983145622682`, 49.19049856623758`,
 1.7394653874433654`, 1.564181958549412`, 49.56611347734799`, 1.`, {{0, 0.0023973608821059333`, 4.2673023701485615`},
  {2, 0.000959802687854463`, 1.7084487843809442`}, {4, 0.0003002287851417602`, 0.5344072375523331`},
  {6, 0.00009498229320131352`, 0.1690684818983381`}, {8, 0.000014649975974977364`, 0.02607695723545971`}}},
{0.16300334800486183`, 3.0919140294206544`, 3.354218048792589`, 0.9673340843818878`, 0.9052111330146941`,
 0.3760205832601582`, 0.15433250667793078`, 4163.373290035795`, 0.2440496776808055`, 4.495712049614825`, 29.572134404917954`,
 0.46008960718557185`, 0.46008960718557185`, 29.572134404917954`, 1.`, {{0, 0.0022474822147737646`, 4.000518342297301`}}},
{0.2283283078987099`, 0.42840256075325867`, 9.446993924606975`, 1.3839314736668495`, 0.8927356525671915`,
 0.3909626915031965`, 0.018767685887155043`, 53.5712236843488`, 0.10749676377570666`, 1.5496335657505222`,
 198159.6868324399`, 46472.447177061586`, 228.86382349853784`, 1409.0487831003384`, 0.`,
 {{0, 0.03196751095447501`, 56.90216949896552`}, {32, 0.009073138603663202`, 16.1501867145205`},
  {64, 0.0052867615586971554`, 9.410435574480937`}, {96, 0.004020259272258902`, 7.156061504620847`},
  {128, 0.0031435653765694107`, 5.595546370293551`}, {160, 0.002453676461136773`, 4.367544100823456`},
  {192, 0.0019168910164923626`, 3.4120660093564057`}, {224, 0.0015052815939966657`, 2.679401237314065`},
  {256, 0.001191486925524047`, 2.120846727432804`}, {288, 0.04652913575281219`, 82.82186164000571`}}},
{0.1380652378345807`, 3.317739091147252`, 0.7336293987943634`, 0.9484386065310282`, 0.5023326788118547`,
 0.2130184622271264`, 0.06305960906666651`, 1932.031257216769`, 0.01203085244100871`, 4.258055262793977`, 23.697070191819076`,
 0.3706949566064180 5`, 0.255400118893372`, 17.01558788140166`, 1.`, {{0, 0.0007138507581050385`, 1.2706543494269686`},
  {8, 0.0003793965946264886 7`, 0.6753259384351499`}, {16, 0.00013688395752858634`, 0.24365344440088368`},
  {24, 0.00005001426180211209`, 0.08902538600775954`}, {32, 0.000013039106924300582`, 0.023209610325255037`}}},
{0.0926632627436077`, 3.578083963812018`, 1.8944161244777453`, 1.1381278842914715`, 0.7382269863407194`,
 0.4777843546205588`, 0.04528998830707099`, 1233.5473532765689`, 0.08357615053600725`, 2.9903558138075255`, 46.48512135694232`,
 0.6673336697388551`, 0.48401881423904974`, 43.983374708883666`, 1.`, {{0, 0.00196722877342876`, 3.5016672167031926`},
  {3, 0.0009242017638126424`, 1.6450791395865032`}, {6, 0.0003269647187289486`, 0.5819971993375286`},
  {9, 0.00011658885292733067`, 0.20752815821064857`}, {12, 7.752368977477002`*^-6, 0.013799216779909062`}}},
```

{0.2706310445313141`, 2.5166396966047158`, 0.5049158559505837`, 1.3168707464723388`, 0.5754269054535`,
0.5968444469720801`, 0.1025449755078217`, 681.183394170123`, 0.05243619315919962`, 2.183793658787195`,
51157.04603679401`, 10511.152459592155`, 228.51776194086398`, 35451.27771827777`, 0.`,
{{0, 0.014707864064423587`, 26.179998034673986`}, {8, 0.00920292217341309`, 16.3812014686753`},
{16, 0.009187803000214252`, 16.354289340381364`}, {24, 0.010010559104224439`, 17.818795205519503`},
{32, 0.010873188218360863`, 19.354275028682338`}, {40, 0.011638807891306687`, 20.717078046525902`},
{48, 0.012360420492486717`, 22.001548476626354`}, {56, 0.013074030721287143`, 23.27177468389111`},
{64, 0.013785129264545318`, 24.537530090890662`}, {72, 2.5894563816588487`, 4609.23235935275`}}},
{0.20804007909247985`, 0.9673945938397814`, 0.48619022305575754`, 1.3068640855075515`, 0.047796616526516456`,
0.6050138674479171`, 0.4706922223098622`, 348.3137462309647`, 0.24685340199219946`, 5.551550038883448`, 21419.69842270605`,
5388.483264355175`, 230.2303187076905`, 1050.58795527408`, 0.`, {{0, 0.0798446846008301`, 142.12353858947756`}}},
{0.048451644236667674`, 3.3925416461261833`, 7.486002650281495`, 1.2431466818882033`, 0.1294136137122328`,
0.6416005560424389`, 0.04082335443875802`, 58.92112932799084`, 0.09857305139503486`, 6.047753646247408`, 120598.42779870231`,
71035.63385602889`, 229.89691483327317`, 1252.182408383018`, 0.`, {{0, 0.09516586303710936`, 169.39523620605468`}}},
{0.09431215650771135`, 0.5526170445339527`, 7.677473256451986`, 0.7715239445968243`, 0.7622192109377914`,
0.1566986505563851`, 0.018040482265180933`, 824.8087273379117`, 0.09073506826067207`, 9.996434684615583`,
151.78796140332793`, 3.4491128634017123`, 2.6667749116501063`, 117.68302231365837`, 1.`,
{{0, 0.005883296657683187`, 10.472268050676073`}, {69, 0.0030606130381548493`, 5.447891207915631`}}},
{0.26113782676147984`, 1.4684069653405487`, 4.54225535313282`, 1.1193463339337828`, 0.6236318874525932`,
0.1751148412910668`, 0.053183289402392066`, 554.4897241223483`, 0.22550063278101773`, 9.001504879035508`,
183.26146960123302`, 3.5785028955315044`, 1.8885456865811672`, 96.30842248749182`, 1.`,
{{0, 0.005710426812620591`, 10.164559726464653`}, {42, 0.0016090132964287885`, 2.8640436676432435`}}},
{0.23311836154945775`, 2.5262146716446363`, 6.3107841154355`, 1.4422657044398717`, 0.3118898255230633`,
0.2567889403043644`, 0.03481026984978327`, 1604.0167510242688`, 0.16315782107376747`, 5.777943122601165`,
34.574880388189655`, 0.3116832248617571`, 0.2375048358281744`, 25.97102125011014`, 1.`,
{{0, 0.0013926793568013018`, 2.478969255106317`}, {44, 0.0005811182582070693`, 1.0343904996085833`}}},
{0.22640126428063923`, 3.7808015365334118`, 7.033092553948691`, 1.0460349638379132`, 0.8860375200295565`,
0.499376231890995`, 0.3693111113992884`, 210.995030019894`, 0.19589698445197923`, 2.861160479579169`,
180.13242090714255`, 4.611053799843468`, 2.748897784459581`, 151.92972117416`, 1.`,
{{0, 0.010151762434587507`, 18.070137133565762`}, {4, 0.0012629821591135814`, 2.2481082432221746`},
{8, 0.00008161133482738616`, 0.14526817599274736`}, {12, 0.000021230263472805183`, 0.03778986898159323`},

{16, 0.00001880738685925838`, 0.03347714860947993`}, {20, 0.00001026523037562292`, 0.01827211006860879`}}},
{0.19770244273120569`, 3.3508846225196347`, 6.757833537731408`, 0.8466672464019638`, 0.28373063232656337`,
0.6609960258126362`, 0.006803814757890119`, 1256.448281122042`, 0.0187925047862397`, 9.149585751239048`, 105.78945537743523`,
0.19898591180210362`, 0.19898591180210362`, 105.78945537743523`, 1.`, {{0, 0.008039998608685078`, 14.31119752345944`}}},
{0.06481064744600706`, 2.3696983632913335`, 9.923821775061434`, 1.1006879887228784`, 0.22746584913410017`,
0.6483433064071293`, 0.08284957522863273`, 281.66193979187017`, 0.13350656503173153`, 1.0702403000648566`,
49.97201871535207`, 0.8129969609790598`, 0.6753931521795572`, 39.518290079866766`, 1.`,
{{0, 0.00262053343200524`, 4.664549508969327`}, {27, 0.00038285661406463415`, 0.6814847730350487`}}},
{0.1537084817657926`, 0.5942996643982652`, 0.8160795607483013`, 1.042756674430172`, 0.7722126796580249`,
0.49669902434825364`, 0.00611858149596996`, 1518.932458333751`, 0.21666908673557295`, 1.634333111202272`,
20.73434981571264`, 2.8648247152695214`, 0.7584070203147868`, 15.925821260407334`, 1.`,
{{0, 0.0006402206460263543`, 1.1395927499269105`}, {3, 0.00034601866063501953`, 0.6159132159303347`},
{6, 0.00011753106708965125`, 0.20920529941957922`}, {9, 0.00004017832682983254`, 0.07151742175710193`},
{12, 0.0000175876161654072`, 0.03130595677442482`}, {15, 0.000011459727115112691`, 0.020398314264900592`},
{18, 9.949987984325547`*^-6, 0.017710978612099477`}, {21, 0.000027416383945254228`, 0.04880116342255252`}}},
{0.13754450751212471`, 1.8598194811531465`, 9.485701362019345`, 1.4994328167980717`, 0.8397969618927028`,
0.642370724162252`, 0.08665379792235217`, 239.43302754758346`, 0.1633413998793405`,
9.552795266495377`, 758.8743360705054`, 16.946774747594866`, 14.3582631902185`, 685.9459012280564`, 1.`,
{{0, 0.043832572029786666`, 78.02197821302026`}, {11, 0.00829931646354562`, 14.772783305111204`}}},
{0.07858197291799773`, 2.783199788795301`, 0.9642830041255817`, 1.3105090636097434`, 0.9572373854271048`,
0.41778523295843695`, 0.00823451224525384`, 90.59081205787001`, 0.24323880940762027`, 7.348654585309644`, 49788.50884527534`,
23429.360862693688`, 230.11682727062862`, 889.5880297610631`, 0.`, {{0, 0.0676086902618408`, 120.34346866607663`}}},
{0.09118935700706149`, 1.2770460819223013`, 0.666402509206284`, 1.1661439001587546`, 0.35202394044239993`,
0.23733544361366754`, 0.06689005296661862`, 1909.3731761645724`, 0.030837584446851807`,
5.2718694661097025`, 27.123683538190996`, 0.8552134936669606`, 0.6185270479951679`, 16.563876920527527`,
1.`, {{0, 0.0008657879514184848`, 1.541102553524903`}, {26, 0.0003930666945416072`, 0.6996587162840607`}}},
{0.26692387934872025`, 2.295217680243936`, 3.3610155473383347`, 0.802490311252646`, 0.6241394164828811`,
0.45771836786665954`, 0.015176154328102385`, 1461.3995866509204`, 0.02843534740955445`,
8.762751383914615`, 109.91228007536021`, 0.5980734391685734`, 0.5959052980692934`, 109.55953065590585`,
1.`, {{0, 0.007518002011091198`, 13.382043579742332`}, {6, 0.0008085223187576461`, 1.4391697273886102`}}},
{0.16789560992918506`, 3.010819021450227`, 9.549236085030234`, 1.3823009139889233`, 0.2184000924395355`,

```
0.5213598249132101`, 0.3210433649481226`, 558.1245473691072`, 0.20748907999743366`, 5.921770487462836`, 129.16341927127607`,
2.3890901770771436`, 1.6454161723924543`, 89.39703697160495`, 1.`, {{0, 0.005498963137868487`, 9.788154385405907`},
 {6, 0.0012303670168746605`, 2.190053290036896`}, {12, 0.0000648446550988288`, 0.11542348607591527`}}},
{0.05001315370963505`, 3.027404779172768`, 3.8220563025552714`, 0.9962642247459573`, 0.44362718458195727`,
0.3510033113401648`, 0.04839212642640241`, 1615.957133832461`, 0.048401134986770955`, 4.739296636982807`,
36.471937352834274`, 0.3456171032670265`, 0.2601130530231295`, 26.599599842452427`, 1.`,
{{0, 0.0015245264031281752`, 2.7136569975681515`}, {36, 0.0004970431848982093`, 0.8847368691188126`}}},
{0.0729686004745061`, 0.5044823312287607`, 5.5024178276878075`, 1.2230366941116102`, 0.012758742281838265`,
0.5648708708999995`, 0.03232052448858605`, 634.5000769880222`, 0.22553164736796405`, 5.75812027698643`, 96.42441715249049`,
3.663244816584558`, 3.663244816584558`, 96.42441715249049`, 1.`, {{0, 0.007328255703589278`, 13.044295152388914`}}},
{0.25149333924897926`, 1.871859550111978`, 9.811462629766478`, 1.3386558973788294`, 0.5189637829018348`,
0.6377508608690272`, 0.024369302194970535`, 194.42061666753636`, 0.14619611626015583`,
6.325307886810732`, 535.7933383860712`, 6.665859175313074`, 5.363481812042018`, 518.5085114887554`, 1.`,
{{0, 0.03298343511194064`, 58.71051449925435`}, {14, 0.006423211761204773`, 11.433316934944495`}}},
{0.1922243891646514`, 1.6850594067594091`, 0.2630953725218035`, 1.4711962401085972`, 0.15977146838462364`,
0.6883258791888056`, 0.09212778533128511`, 774.9062808250895`, 0.24229201708328568`, 1.1723783184073415`, 9959.795383571882`,
2658.1197865626714`, 230.18978045734488`, 897.9247745714691`, 0.`, {{0, 0.06824228286743166`, 121.47126350402836`}}},
{0.2327924107307146`, 3.0114739814321467`, 6.475065538974626`, 1.3312648289930094`, 0.6507217418486848`,
0.3841095338158853`, 0.3176950069808959`, 737.689863101484`, 0.19812174358831114`, 7.321794649353192`,
171.9341475044876`, 3.200100897289971`, 1.4077198318763657`, 75.83377425438007`, 1.`,
{{0, 0.004491774644868488`, 7.9953588678659075`}, {7, 0.001154770654809748`, 2.055491765561351`},
 {14, 0.00010733361400390818`, 0.19105383292695655`}, {21, 9.48792965074156`*^-6, 0.016888514778319975`}}},
{0.20811447549122308`, 0.49053077700921355`, 2.218461283385089`, 1.0634384889669943`, 0.30011342208125913`,
0.5589173637133283`, 0.16385051847701637`, 124.79731876547663`, 0.15435702825422665`,
9.111119072715418`, 71178.68437728196`, 17888.641764540287`, 229.41222852416388`, 3848.6560586216146`,
0.`, {{0, 0.03133410905838044`, 55.77471412391718`}, {3, 0.015693325917600934`, 27.93412013332966`},
 {6, 0.014386467895325444`, 25.60791285367929`}, {9, 0.014172545696825484`, 25.227131340349363`},
 {12, 0.014547904099677303`, 25.8952692974256`}, {15, 0.015234455829310097`, 27.117331376171975`},
 {18, 0.01606609934639256`, 28.59765683657876`}, {21, 0.016951771683875162`, 30.17415359729779`},
 {24, 0.01785105188436268`, 31.774872354165566`}, {27, 0.1362601290434926`, 242.54302969741684`}}},
{0.19580212756702065`, 2.2297474474451375`, 8.070495694463702`, 1.2202670372174012`, 0.07482300382970508`,
```

```
  0.34257881980750315`, 0.24674110677687536`, 1287.6149126957`, 0.12354587287124019`, 3.0573978018103922`, 20.978376512665147`,
  0.4918148098256799`, 0.4918148098256799`, 20.978376512665147`, 1.`, {{0, 0.0015943566149625513`, 2.837954774633341`}}},
{0.18591817623779283`, 1.5316284759435828`, 4.0240186618411435`, 1.0506796825025935`, 0.8560461598734517`,
  0.5496710897949223`, 0.05348974137997375`, 3910.687147405355`, 0.18912146822955533`, 8.614502889074807`,
  72.92031233700214`, 1.3869589927342942`, 1.0658864608761385`, 55.65203460344592`, 1.`,
  {{0, 0.002992647095670581`, 5.326911830293634`}, {26, 0.0012369075341913094`, 2.201695410860531`}}},
{0.2552337642427184`, 2.0636949211593727`, 3.6788035742608756`, 1.2716726068122697`, 0.4767575621154567`,
  0.33698161388823356`, 0.0079039809444615`, 742.5948236907665`, 0.22094334214516276`, 6.7847431915852425`,
  117.10515594649874`, 0.4409861312695163`, 0.43936919912798944`, 116.9815244493711`, 1.`,
  {{0, 0.00809901594533203`, 14.416248382691014`}, {7, 0.0007915799128201731`, 1.4090122448199083`}}},
{0.2297403387592854`, 3.933034881515791`, 9.92502862171417`, 1.2059622632705849`, 0.1421363362803283`,
  0.1853832242974326`, 0.06367011009221722`, 57.09955187350921`, 0.13182928467731042`,
  9.7382206276645`, 90501.93218328858`, 20967.24631520538`, 22.72299494215657`, 777.4570333655893`, 1.`,
  {{0, 0.05134308966980217`, 91.39069961224787`}, {43, 0.0011096321101495697`, 1.9751451560662343`},
   {86, 0.0011141410523395315`, 1.9831710731643661`}, {129, 0.0011120986134426584`, 1.979535531927932`},
   {172, 0.0011095281692678916`, 1.9749601412968474`}, {215, 0.0011093165646480678`, 1.9745834850735609`},
   {258, 0.001109293489858537`, 1.974542411948196`}, {301, 0.00107963486627636`, 1.9217500619719208`}}},
{0.04776006202628119`, 3.695464511472898`, 0.268839094050918`, 1.3548340559244303`, 0.4311024896438589`,
  0.6662260908047339`, 0.1893860365797585`, 146.80096070953766`, 0.0868075345269877`, 5.3982731410948785`, 88985.20892200657`,
  52886.915709516936`, 230.00519893352217`, 829.1389440235338`, 0.`, {{0, 0.06301455974578857`, 112.16591634750365`}}},
{0.16250916383331093`, 2.090868942469317`, 5.046057727155544`, 1.3781211464947924`, 0.9692295654960448`,
  0.507149644697747`, 0.012667564467634538`, 467.92228322579444`, 0.2269164868867607`,
  2.233796179341983`, 93.88057303980034`, 1.110196015050939`, 0.8040479579245496`, 94.03473061856774`,
  1.`, {{0, 0.005438215495939916`, 9.68002358277283`}, {4, 0.0013997250115395027`, 2.4915105205403147`},
   {8, 0.0002929887872215583`, 0.5215200412543738`}, {12, 0.000015710232310295523`, 0.02796421351232603`}}},
{0.12952636821033042`, 2.4189205073691173`, 1.5337482028150813`, 1.4058710509218408`, 0.9636406844740666`,
  0.3882701702830901`, 0.27400686262252805`, 4688.706549038708`, 0.0765768774664311`, 6.843480813486918`,
  47.08218834930365`, 1.1038956470689463`, 0.3912072322370629`, 15.458363102719844`, 1.`,
  {{0, 0.000797618432694015`, 1.4197608101953467`}, {6, 0.00030551189861908323`, 0.5438111795419681`},
   {12, 0.000055398210964647804`, 0.0986088155170731`}, {18, 0.000016307053528962192`, 0.029026555281552704`}}},
{0.08153578837307857`, 2.0915987061965353`, 5.850951169546045`, 1.037700922521303`, 0.7897818160030217`,
```

0.3237340771928561`, 0.17831164332408336`, 1464.5863160958056`, 0.2436610405225158`,
7.1217069723220146`, 96.11741179649037`, 2.228899497590422`, 0.9887235045691608`, 42.85754431891809`,
1.`, {{0, 0.0023286849422660045`, 4.145059197233488`}, {9, 0.0007742962626064108`, 1.3782473474394115`},
{18, 0.00013809989360681702`, 0.2458178106201343`}, {27, 0.000016092269758542470`, 0.028644240170205598`}}},
{0.2624813864915975`, 2.8946734437676342`, 8.625458110929756`, 1.37764442375384`, 0.19776637799413987`,
0.31049273319228277`, 0.046660769123637466`, 609.6700244678286`, 0.017643025422342112`,
4.098918143681717`, 62.57851701665736`, 0.5942911489984278`, 0.44002989097069045`, 45.562100272331435`,
1.`, {{0, 0.002710907177122318`, 4.825414775277726`}, {41, 0.0007518124435748715`, 1.3382261495632715`}}},
{0.22181257821621975`, 3.747903553799671`, 8.070266666229205`, 0.9808557386469257`, 0.7823522438549146`,
0.2263615830500163`, 0.06976335847986787`, 287.144692837758`, 0.1821266272405107`, 3.59762935889718`,
192.2889886815009`, 1.832769392311514`, 1.0491517101092849`, 104.46429653994423`, 1.`,
{{0, 0.007160847899298300`, 12.746309260750975`}, {37, 0.000778438637737461`, 1.3856207751726808`}}},
{0.21058778737677059`, 1.3147483391864796`, 0.4651324593397846`, 0.8516412222118703`, 0.046686774964893374`,
0.4819914326166439`, 0.10586616607566471`, 1877.0848108087591`, 0.011164572002709261`,
8.732010177522003`, 16818.565577651105`, 4188.426453678885`, 30.694608870417966`, 158.7119625997208`, 1.`,
{{0, 0.010954776227929251`, 19.499501685714073`}, {57, 0.00030353420066861047`, 0.5402908771901267`},
{114, 0.00023565795864530650`, 0.4194711663886456`}, {171, 0.00023488478238432392`, 0.41809491264409654`},
{228, 0.00023486779187318854`, 0.4180646695342756`}, {285, 0.000098388196078100030`, 0.17513098901901805`}}},
{0.19996482365829993`, 1.5454344843054368`, 8.94969050535013`, 1.027667483044064`, 0.3164796074442535`,
0.5223314119072894`, 0.012949156080520758`, 231.31648814801656`, 0.19929090705614294`,
9.341058456545078`, 538.4992188076072`, 3.6761549002447507`, 3.675672828361052`, 538.796487341043`,
1.`, {{0, 0.04002523974725114`, 71.24492675010704`}, {3, 0.0009232932906681264`, 1.643462057389265`}}},
{0.10440009635624109`, 1.532425369535546`, 0.308138348960842`, 1.2734376519411275`, 0.20147673343032046`,
0.35081245482125123`, 0.05722019474177449`, 85.42879625764479`, 0.08356786949647793`, 8.411462154648635`, 173222.50849639316`,
69519.48231775036`, 230.09501351022027`, 820.4965214980275`, 0.`, {{0, 0.062357735633850095`, 110.99676942825317`}}},
{0.1446017732242294`, 0.797427468093673`, 5.032298069921831`, 1.3706636028120882`, 0.10121695844960277`,
0.6068141920969097`, 0.25341251132298065`, 197.97452899503492`, 0.2275401984407136`, 3.68386367767849`, 7643.8237417228265`,
2450.3618689212753`, 13.602513029976947`, 180.06647696834693`, 1.`, {{0, 0.013186734834077929`, 23.47238800465871`},
{21, 0.00013515146210750083`, 0.24056960255135146`}, {42, 0.00013535702434077406`, 0.24093550332657782`},
{63, 0.0001350854984840202`, 0.2404521873015559`}, {84, 0.000092723430584143540`, 0.16504770643977548`}}},
{0.1841264162344997`, 3.4194642457460365`, 0.8344796094826652`, 1.4377258087185927`, 0.5867256025093719`,

0.6103981120475732`, 0.3542274534468216`, 522.9821129731852`, 0.2244081861480518`, 8.361737950667973`,
12814.08333505623`, 3514.955256595798`, 228.75573458132033`, 990.9750385893225`, 0.`,
{{0, 0.011303718270332023`, 20.120618521191002`}, {5, 0.0029468204019667543`, 5.2453403155008225`},
{10, 0.0012247698016205732`, 2.18009024688462`}, {15, 0.0006425749540027806`, 1.143783418124949`},
{20, 0.00041349316121182126`, 0.7360178269570418`}, {25, 0.00031434288606588663`, 0.5595303371972782`},
{30, 0.0002677603534522039`, 0.4766134291449229`}, {35, 0.00024383048205599042`, 0.43401825805966293`},
{40, 0.0002303390771561127`, 0.41000355733788063`}, {45, 0.05772645354492437`, 102.75308730996538`}}},
{0.17849930658201318`, 3.2992561971358683`, 5.635580909426066`, 1.1878114797167083`, 0.48988207998590316`,
0.27890916833354473`, 0.03715549210344845`, 144.67131391199885`, 0.0866110192158086`,
7.798176718489761`, 19657.06600454017`, 5439.370970064462`, 191.73265423502815`, 1044.3950585190435`,
1.`, {{0, 0.07469675943089307`, 132.96023178698965`}, {26, 0.004677265016554225`, 8.32553172946652`}}},
{0.09510173640093084`, 0.7659692827065738`, 0.5464490010035838`, 1.4050402850247132`, 0.11782959453955089`,
0.3589214817200733`, 0.08175234954735425`, 225.11260306454756`, 0.1010344837489014`, 6.4310165595390805`, 52018.17631537765`,
22044.04215266731`, 229.99910783688435`, 709.2452049255371`, 0.`, {{0, 0.05390263557434082`, 95.94669132232667`}}},
{0.06416093472533757`, 1.2573296539610634`, 9.92323628294643`, 1.30190007637989`, 0.030494015633026894`,
0.4401773012522794`, 0.02586427825345657`, 3143.6595843648015`, 0.20592664064058636`, 7.935954028868563`, 24.301225123239742`,
0.34058352166565437`, 0.34058352166565437`, 24.301225123239742`, 1.`, {{0, 0.0018468931093662206`, 3.2874697346718724`}}},
{0.25237835724682106`, 2.979406809596097`, 0.9138356130609361`, 0.7932538461611548`, 0.3933816374531405`,
0.6211446249652819`, 0.0749645015318215`, 95.73291003138912`, 0.06307891905410445`, 9.368280708056208`, 227658.0785614993`,
49418.86930142859`, 229.47979141605285`, 1606.9486266688302`, 0.`, {{0, 0.12212809562683109`, 217.38801021575932`}}},
{0.05665762575451494`, 1.7380994050720693`, 0.46391281319797173`, 1.3490794355447004`, 0.9653780951392605`,
0.17540498059526055`, 0.4972377260445833`, 285.101267300382`, 0.1881031714219547`, 6.261558552266614`,
14307.785124377497`, 7895.649386809754`, 229.5327864040123`, 1646.4669417853204`, 0.`,
{{0, 0.0030214022619649396`, 5.378096026297593`}, {10, 0.0016472402572258159`, 2.932087657861952`},
{20, 0.0014099625540889608`, 2.50973334627835`}, {30, 0.0012327708281690746`, 2.194332074140952`},
{40, 0.0010837512681670359`, 1.9290772573373238`}, {50, 0.0009569255512916432`, 1.7033274812991248`},
{60, 0.0008488223820174923`, 1.5109038399911363`}, {70, 0.0007566312839542233`, 1.3468036854385175`},
{80, 0.0006779826302615332`, 1.2068090818655293`}, {90, 0.11349599855854363`, 202.0228774342077`}}},
{0.25642346088610446`, 3.010945889122482`, 9.963520677018519`, 1.467899384826252`, 0.4946077322511526`,
0.6868780403912886`, 0.012314696297102353`, 88.3173980921876`, 0.10491074891333174`,
1.4353196490865765`, 96124.6612060573`, 20587.89424393893`, 229.36922711348586`, 3918.6794666235796`,

```
0.`, {{0, 0.04996198626132382`, 88.93233554515639`}, {17, 0.003271353700108422`, 5.823009586192992`},
   {34, 0.003271186040557335`, 5.822711152192056`}, {51, 0.003271180459273232`, 5.822701217506353`},
   {68, 0.003271180542029397`, 5.822701364812326`}, {85, 0.003271181993745757`, 5.8227039488674475`},
   {102, 0.0032711842098830544`, 5.822707893591837`}, {119, 0.0032711871074530083`, 5.822713051266356`},
   {136, 0.00327119075834845`, 5.822719549860241`}, {153, 0.22168800839066957`, 394.60465493539186`}}},
{0.09174036922136902`, 1.4204870656163582`, 7.877654658381974`, 0.9368390881044215`, 0.3909649395372681`,
  0.6267838157099552`, 0.00737791814963061`, 223.00641924802295`, 0.22589847441224364`,
  3.7895604923910007`, 257.84855811434767`, 2.020208654165159`, 1.3492397990267042`, 261.06117646905324`,
  1.`, {{0, 0.015388386416181118`, 27.391327820802392`}, {3, 0.0037391959495610308`, 6.6557687902186355`},
   {6, 0.0006085012339640471`, 1.083132196456004`}, {9, 0.00010456581194184987`, 0.18612714525649277`}}},
{0.2260021855376967`, 2.1215536107061057`, 4.2648042233683015`, 1.1043329178413561`, 0.5641485347029902`,
  0.48705118822040194`, 0.49950773055219905`, 4133.317514629252`, 0.08759305230550185`, 2.0466954881219834`, 8.360890332626482`,
  0.2374808226607802`, 0.2374808226607802`, 8.360890332626482`, 1.`, {{0, 0.0006354276652796127`, 1.1310612441977106`}}},
{0.1540246000020472`, 3.8655612430526176`, 1.2043257775886698`, 1.4535724207675174`, 0.7982285789403216`,
  0.6724657214579106`, 0.417253246623641`, 114.93402398229541`, 0.10143088124264565`, 7.877832682736377`, 157235.21671098113`,
  49107.510837106645`, 230.17243727765006`, 1353.6050445155095`, 0.`, {{0, 0.10287398338317873`, 183.11569042205812`}}},
{0.18967559668177286`, 1.3751140072639014`, 7.146984940201236`, 1.0524950668750344`, 0.5379883166421491`,
  0.5342982154057455`, 0.18359081743318598`, 57.97987210615365`, 0.21651827946513758`, 4.096495164957771`,
  83944.59661895152`, 22573.112874185812`, 229.84711051281988`, 1317.7530453830009`, 0.`,
  {{0, 0.046438679341417544`, 82.66084922772323`}, {3, 0.008635625275900066`, 15.371412991102117`},
   {6, 0.0035194944090451707`, 6.264700048100403`}, {9, 0.001771794485501084`, 3.1537941841919297`},
   {12, 0.0010844759646673l`, 1.9303672171078115`}, {15, 0.0007808463425595391`, 1.3899064897559794`},
   {18, 0.0006323922626570956`, 1.1256582275296303`}, {21, 0.0005524465583517095`, 0.983354873866043`},
   {24, 0.0005044915553541964`, 0.8979949685304695`}, {27, 0.036228985253654346`, 64.48759375150473`}}},
{0.11610573160351356`, 2.8105133752566074`, 0.4045001364193191`, 0.9020454264062931`, 0.06323256453719583`,
  0.49478351534124754`, 0.06895293734611843`, 538.3487824750697`, 0.04516006162070901`, 5.298889101080752`, 33913.91032449796`,
  12749.686753941694`, 230.17474726596961`, 724.0573983443411`, 0.`, {{0, 0.05502836227416993`, 97.95048484802246`}}},
{0.06094054623730827`, 3.5785656752883286`, 2.8105466781089756`, 1.0666569400098407`, 0.8956485783622179`,
  0.6416539064413922`, 0.4241071070816547`, 1544.2747045672372`, 0.05890410014619657`, 6.674766669452417`, 146.73981626915102`,
  2.691160264164061`, 1.2099304836126816`, 67.32462084822015`, 1.`, {{0, 0.0033930524742646715`, 6.039633404191115`},
   {3, 0.001384104347927493`, 2.463705739310937`}, {6, 0.00027469607536898693`, 0.4889590141567968`},
```

```
{9, 0.000051535858475628476`, 0.0917338280866186 7`}, {12, 0.000013282428427951389`, 0.02364272260175347`}}},
{0.06400872221443815`, 3.352604720184938`, 3.3612644751655854`, 0.9279864636339992`, 0.6811335659068025`,
 0.5445450422212498`, 0.04222211989978537`, 4877.834369436828`, 0.09303122695369598`, 6.110250683676178`,
 27.638691796772214`, 0.21913316989062903`, 0.1907990715756629`, 23.475899725573164`, 1.`,
 {{0, 0.001281328357560166`, 2.2807644764570956`}, {32, 0.0005028400215833946`, 0.8950552384184425`}}},
{0.1838955211549798`, 0.6066893455768754`, 3.1997125487162617`, 1.2441392193895697`, 0.8430620231566095`,
 0.17975264196625795`, 0.008385470075528607`, 217.99247821227752`, 0.10887259656335785`, 8.65519228817503`, 13476.826750153878`,
 3658.061947843039`, 3.2804814705330125`, 235.10158474797205`, 1.`, {{0, 0.015159367329571447`, 26.983673846637174`},
  {6, 0.002323124845740629`, 4.135162225418319`}, {12, 0.00027410629942896885`, 0.48790921298356454`},
  {18, 0.000050818557471057015`, 0.0904570322984815`}, {24, 0.000029232261385200777`, 0.05203342526565738`},
  {30, 0.000027623367983032127`, 0.04916959500979719`}, {36, 3.4477792655412328`*^-6, 0.006137047092663394`}}},
{0.10877154933938238`, 0.6652486439511347`, 7.420477343235575`, 1.1719120037873472`, 0.8277401239831172`,
 0.4096056229223566`, 0.3960687102590752`, 4083.2161783729553`, 0.09124503977845877`, 8.628655226336182`, 52.90970298330367`,
 4.250536441991451`, 4.250536441991451`, 52.90970298330367`, 1.`, {{0, 0.004021137426731079`, 7.157624619581321`}}},
{0.27429237480005003`, 0.4974953244631517`, 8.109325428142139`, 1.1022725368505206`, 0.3620164170986986`,
 0.5133215689169995`, 0.013475856804577652`, 252.124017519504`, 0.17376346726865255`,
 1.283750502496817`, 68.04699729848504`, 1.421968348410269`, 1.4172455314033343`, 68.04272262671432`, 1.`,
 {{0, 0.0048612782825177454`, 8.653075342881586`}, {5, 0.00030996863711254294`, 0.5517441740603265`}}},
{0.2348606786833104`, 2.9248975834862243`, 4.089597609027345`, 1.0125076012045389`, 0.4800911130241774`,
 0.44584481805285503`, 0.013867879702002976`, 69.31755646766315`, 0.08239556859020553`,
 3.2481286029667444`, 221236.72662704941`, 50775.21213268807`, 229.86258287737735`, 132858.678168598`,
 0.`, {{0, 0.04918077355949568`, 87.54177693590232`}, {6, 0.014767376541570069`, 26.28593024399472`},
  {12, 0.010991577050898515`, 19.565007150599357`}, {18, 0.010800994324082725`, 19.22576989686725`},
  {24, 0.01240001927300131`, 22.072034305942335`}, {30, 0.01527450590684335`, 27.18862051418116`},
  {36, 0.019017357309548552`, 33.85089601099642`}, {42, 0.023154411040239315`, 41.21485165162597`},
  {48, 0.027335786794164887`, 48.65770049361349`}, {54, 9.914336739013605`, 17647.519395444215`}}},
{0.2276305764129738`, 0.759063573478838`, 9.75608531797917`, 0.9651630732227953`, 0.2616315835146603`,
 0.25394525967231996`, 0.42721777606241185`, 115.33714629065052`, 0.15474162363619287`,
 5.678602403105034`, 450.4943651944117`, 32.70882953475571`, 14.438857265099651`, 210.03248491819812`,
 1.`, {{0, 0.012669339222792423`, 22.551423816570512`}, {7, 0.0030769821293362043`, 5.477028190218444`},
  {14, 0.00019103599779952252`, 0.340044076083 1501`}, {21, 0.000025111503854905996`, 0.04469847686173267`}}},
```

```
{0.22740101487152548`, 3.662274246882813`, 5.673159128278421`, 0.7501649913658239`, 0.6663016096811909`,
  0.46449661159333955`, 0.15875103883050143`, 79.3840483030526`, 0.23925351496597358`,
  9.512843911813135`, 42809.697532988925`, 9980.876550247307`, 229.6440036363801`, 1702.832928239124`,
  0.`, {{0, 0.06942741146452247`, 123.58079240685001`}, {16, 0.021028526942020644`, 37.430777956796746`},
   {32, 0.008131334301119734`, 14.473775055993128`}, {48, 0.0034712834052209505`, 6.178884461293292`},
   {64, 0.0014744936596087347`, 2.6245987141035476`}, {80, 0.0006405604895361905`, 1.1401976713744193`},
   {96, 0.0002932846244871928`, 0.5220466315872031`}, {112, 0.00014823449432322265`, 0.2638573998953363`},
   {128, 0.00008721209459706894`, 0.1552375283827827`}, {144, 0.024712961070737227`, 43.98907070591226`}}},
{0.18907356960866467`, 0.8903250429215461`, 0.8912625465714132`, 1.4194566043771595`, 0.25430531352108066`,
  0.6668345122251143`, 0.01430802337144644`, 354.71023908442675`, 0.05435127807039547`, 4.360013356436832`, 88116.18412159599`,
  23800.235827767923`, 229.77646875750528`, 1017.6467895507815`, 0.`, {{0, 0.07734115600585939`, 137.66725769042972`}}},
{0.22841094141655494`, 2.082125305118022`, 3.0130589699894585`, 0.8668572537043583`, 0.35284672466002664`,
  0.48298470604644195`, 0.055294539730325123`, 1324.8059697322965`, 0.2155561741975654`, 1.2621538558570524`,
  12.277535646914766`, 0.19430371590346768`, 0.19231579964139497`, 12.131291126442067`, 1.`,
  {{0, 0.0008204519757556778`, 1.4604045168451065`}, {7, 0.00010152614985391 93`, 0.18071654673997636`}}},
{0.1257670047049273`, 2.665283038938149`, 6.579106420165356`, 1.3213793494994266`, 0.07362490185046933`,
  0.671746727583086`, 0.11039555694375124`, 54.48594040704261`, 0.2029696416579515`, 8.437004538119528`, 112676.07140636715`,
  40130.690585659984`, 229.4276873814344`, 1834.6917001824634`, 0.`, {{0, 0.1394365692138672`, 248.19709320068364`}}},
{0.07898342764241062`, 2.336170261443863`, 9.558440542813312`, 0.8746675798923161`, 0.3314927429594914`,
  0.5846930166673705`, 0.14173550706968557`, 4831.453144750826`, 0.22811466814427805`, 5.138718102637142`, 15.064003340588322`,
  0.29054640371960017`, 0.29054640371960017`, 15.064003340588322`, 1.`, {{0, 0.0011448642538847125`, 2.0378583719147882`}}},
{0.2360815041275312`, 3.0069216843113518`, 1.9005978173203566`, 1.189378383663731`, 0.3594173887197991`,
  0.5615883220885535`, 0.16515958276300607`, 91.7495984293873`, 0.10675594880460115`, 2.979677734696839`, 176997.05695890484`,
  40469.15867402111`, 230.14713023673937`, 1291.3240884479724`, 0.`, {{0, 0.0981406307220459`, 174.6903226852417`}}},
{0.19503604064380992`, 2.260639826798138`, 3.7301326124020027`, 1.0003156010412233`, 0.94917046396899`,
  0.3935362271642996`, 0.08733555022372319`, 2234.7260448390443`, 0.16743068067899064`,
  7.659400521016185`, 108.52857909107487`, 1.8288981238216353`, 1.310208066300985`, 77.64062775689192`,
  1.`, {{0, 0.0028881852707802196`, 5.140969781988791`}, {5, 0.0018654308387871625`, 3.3204668930411496`},
   {10, 0.0009687228157811465`, 1.724326612090441`}, {15, 0.00017834878417525787`, 0.317460835831959`}}},
{0.09766567347807259`, 2.1751916900809825`, 2.9967711374909367`, 1.239918427231443`, 0.884426059472291`,
  0.5758255537556012`, 0.011950287911566193`, 133.4222213549303`, 0.18860770237483848`,
```

```
7.181211491841234`, 36544.24702647398`, 15182.664878012227`, 228.96030371338637`, 1247.7495156125788`,
 0.`, {{0, 0.0251436845383972`, 44.75575847834701`}, {3, 0.005826464041932692`, 10.371105994640192`},
  {6, 0.0026525789957100167`, 4.72159061236383`}, {9, 0.0013552595955140803`, 2.4123620800150625`},
  {12, 0.0007940640084414896`, 1.4134339350258516`}, {15, 0.0005396960254721605`, 0.9606589253404457`},
  {18, 0.0004184169946625629 4`, 0.744782250499362`}, {21, 0.0003574566213234996`, 0.6362727859558294`},
  {24, 0.0003254469626309157`, 0.5792955934830298`}, {27, 0.057415895402471384`, 102.20029381639907`}}},
{0.14345253817386028`, 1.510087082683639`, 7.446143698111681`, 1.3871400228991382`, 0.2507990125704793`,
 0.592096235116642`, 0.03860344741119924`, 206.9907110306594`, 0.033941806565597976`,
 5.68471806318026`, 365.65526130683713`, 6.744120572268356`, 5.87513498843834`, 345.667216228804`, 1.`,
 {{0, 0.02167604544643477`, 38.583360894653886`}, {15, 0.004594662986954339`, 8.178500116778723`}}},
{0.23351662183055255`, 2.7403756212365034`, 1.6128399905233286`, 1.1394774719326448`, 0.2292996808057013`,
 0.38219770472020054`, 0.028922618161783233`, 3147.1184318469313`, 0.11734182225292555`, 5.055074464096112`,
 16.961401026417935`, 0.13773329541246648`, 0.1346887805997339`, 15.622507081345088`, 1.`,
 {{0, 0.0008121092028287607`, 1.445554381035194`}, {40, 0.000375201335353466`, 0.6678583769291695`}}},
{0.05894445024535572`, 1.7190376171466255`, 3.4445271438587763`, 1.1005021934401353`, 0.8920369053637744`,
 0.4553589712941485`, 0.02602138727445485`, 2996.975539830326`, 0.01616140506923186`, 6.007209949084906`,
 62.548788659013965`, 0.6849169888032782`, 0.6349748928145044`, 57.23006547009661`, 1.`,
 {{0, 0.0025670172033936765`, 4.569290622040745`}, {25, 0.001782467772333666`, 3.1727926347539257`}}},
{0.1969133083502746`, 1.0306356019031098`, 0.4532514022896326`, 0.7704659952544493`, 0.6922371279082573`,
 0.35856337468106037`, 0.02342746544122668`, 625.7704338271294`, 0.24611682235411125`, 7.40783802504553`, 5445.851291335578`,
 1421.7322447870085`, 230.26003270482258`, 909.6529609278629`, 0.`, {{0, 0.06913362503051758`, 123.0578525543213`}}},
{0.23578418763137027`, 1.109904439772956`, 9.416464917532476`, 0.9968768007328397`, 0.9319056056422497`,
 0.20705868768300972`, 0.023381736885244357`, 73.41723955051782`, 0.19857821780967083`,
 6.130556036938069`, 24173.921788739648`, 5436.7529322039245`, 7.8685074119650045`, 496.8562647822905`,
 1.`, {{0, 0.03233152378118332`, 57.55011233050631`}, {3, 0.004661555957664181`, 8.297569604642243`},
  {6, 0.0005113797120467348`, 0.9102558874431878`}, {9, 0.00007433472417652274`, 0.13231580903421047`},
  {12, 0.00003503712612986061`, 0.06236608451115189`}, {15, 0.000032128393322824554`, 0.05718854011462770 6`},
  {18, 0.00003194825669590047`, 0.05686789691870284`}, {21, 0.000031937538660918824`, 0.0568488188164355`},
  {24, 0.00003193681011805928 6`, 0.05684752201014553`}, {27, 0.000019293823455753885`, 0.03434300575124191`}}},
{0.06254010222908407`, 2.833030713124539`, 1.1523226094346608`, 0.8553536481119535`, 0.24975724964580737`,
 0.6445417390978168`, 0.03834946206048674`, 4173.282831948493`, 0.23424935668510183`, 4.880796947048751`, 16.2004543965820 1`,
```

```
  0.15498756456538104`, 0.15498756456538104`, 16.20045439658201`, 1.`, {{0, 0.0012312345341402328`, 2.1915974707696146`}}},
{0.1868109134318776`, 3.0098056229686154`, 1.9105557354746436`, 0.9827601516958456`, 0.5236893411586465`,
  0.474044891890932`, 0.006475636749568416`, 1404.5093361888864`, 0.13430982294875038`, 1.0667742763750176`,
  12.064364390599708`, 0.08554855219582914`, 0.08183169501247116`, 12.304004645212004`, 1.`,
  {{0, 0.000678499636256515`, 1.2077293525365966`}, {3, 0.0002566047167795973`, 0.4567563958676832`}}},
{0.06031809352972184`, 0.6708570664744204`, 0.23575165127461165`, 0.8219834374256857`, 0.8758699419986131`,
  0.18852573702588948`, 0.186745146986022`, 107.40963970974032`, 0.12527667123697356`, 3.1788633087725113`, 40101.04512569063`,
  21537.264291087085`, 230.26249659081168`, 486.0666551088034`, 0.`, {{0, 0.036941065788269056`, 65.75509710311893`}}},
{0.1880120590539 3248`, 2.0266227725882997`, 1.7626950623641149`, 1.1991174158264077`, 0.011000047841808414`,
  0.3353078609658676`, 0.05399718652686698`, 93.79336861840014`, 0.2334529633377767`, 8.960462961249508`, 64931.84912393902`,
  17584.74268095962`, 230.1391951586414`, 1175.2640573601975`, 0.`, {{0, 0.08932006835937502`, 158.98972167968753`}}},
{0.14122716980793654`, 1.0036898987844207`, 5.592205787770849`, 0.8071832042112255`, 0.9861798903854433`,
  0.6717138208444808`, 0.19154806886518252`, 64.00473270059831`, 0.09434989637003327`,
  6.618037610942842`, 132572.20946399213`, 43846.59430462496`, 229.3680919896021`, 2785.0521086812423`,
  0.`, {{0, 0.05397993644980394`, 96.08428688065102`}, {3, 0.02543094277941889`, 45.267078147365616`},
    {6, 0.01853048888834956`, 32.984270221262214`}, {9, 0.013633517224681318`, 24.267660659932748`},
    {12, 0.010097411454449434`, 17.97339238891999`}, {15, 0.007530041172763439`, 13.403473287518922`},
    {18, 0.005658004499842187`, 10.071248009719092`}, {21, 0.004287855753396232`, 7.632383241045294`},
    {24, 0.00328186857491323`, 5.841726063345549`}, {27, 0.06923389346215618`, 123.236330362638`}}},
{0.14179341245484034`, 1.2991893501557028`, 6.1750864357305275`, 1.3220469832389021`, 0.954868801179809`,
  0.5061480199077149`, 0.3454995627472`, 2784.154142185204`, 0.062422742009026755`, 1.1334761108568099`, 15.166297898994495`,
  0.6594122705250538`, 0.6594122705250538`, 15.166297898994495`, 1.`, {{0, 0.0011526386403235816`, 2.0516967797759755`}}},
{0.17346773507380248`, 2.4886801471142252`, 4.186844167829875`, 0.9059139514582338`, 0.8823714489891827`,
  0.3118060351580627`, 0.18611640479783054`, 317.2758158326019`, 0.18366106179531033`, 4.660916164387663`, 162.61028755708978`,
  4.185925186244971`, 2.6274710266954386`, 122.97361308090954`, 1.`, {{0, 0.007879750978269573`, 14.025956741319838`},
    {7, 0.0013187384300306837`, 2.347354405454617`}, {14, 0.00012174469616236494`, 0.21670555916900963`},
    {21, 0.000017911888766929188`, 0.031883162005133955`}, {28, 7.848600919575249`*^-6, 0.013970509636843943`}}},
{0.17801738501831404`, 3.953347877342175`, 5.116473955634309`, 1.3026800881545206`, 0.2660443419983707`,
  0.5551592463622865`, 0.021882498869656555`, 368.98117336094424`, 0.13387054033002443`,
  7.8289062285527375`, 277.6692222090391`, 1.283014374251992`, 1.2679508970111388`, 278.84945790294023`,
  1.`, {{0, 0.019203614426943162`, 34.18243367995883`}, {5, 0.001988944373680299`, 3.5403209851509323`}}},
```

```
{0.1502521637125906`, 3.604808817430471`, 9.516864148680163`, 1.170214417719616`, 0.46260070316916013`,
  0.4333225308578431`, 0.19319459383231874`, 1379.7428965277768`, 0.03462365036850307`, 7.002259791633362`, 72.21085203551476`,
  1.002884840452641`, 1.002884840452641`, 72.21085203551476`, 1.`, {{0, 0.005488024754699122`, 9.768684063364438`}}},
{0.23041123856532575`, 3.7235663376199373`, 7.422983674409959`, 1.0720190294684302`, 0.9567615568260381`,
  0.6795713708474191`, 0.42619421138851304`, 252.0789228698587`, 0.23264645009848384`, 7.716645878625323`, 504.6156747600694`,
  10.414133444976592`, 7.968418834015453`, 472.61435548790183`, 1.`, {{0, 0.031088817315007337`, 55.33809482071306`},
    {2, 0.0043943215293933075`, 7.821892322320086`}, {4, 0.0003342938652267579`, 0.595043080103629`},
    {6, 0.000042999067866157`, 0.07653983408017595`}, {8, 0.000025680763923600099`, 0.045711759784009764`},
    {10, 0.000024806821264443227`, 0.044156141850708944`}, {12, 7.770815478485108`*^-6, 0.013832051551703493`}}},
{0.17190873580866728`, 2.1300233762526144`, 7.263444695009253`, 1.2209987713639263`, 0.8761821538663868`,
  0.2004596237918942`, 0.1361627927608223`, 3975.405902408578`, 0.13971132873354192`, 8.98211109187212`, 38.025600682596114`,
  0.7937102493727904`, 0.7937102493727904`, 38.025600682596114`, 1.`, {{0, 0.002889945651877305`, 5.144103260341603`}}},
{0.25963938649205515`, 2.4653900827356168`, 4.997042605235823`, 1.1014911256242188`, 0.41576920876875767`,
  0.18114027248931974`, 0.22217255314368375`, 81.71471228407572`, 0.2378841388493836`, 9.715142052575505`,
  52650.08568113075`, 11109.455790580594`, 24.570072153460888`, 508.94397939091175`, 1.`,
  {{0, 0.03720669841011636`, 66.22792317000713`}, {14, 0.0010358375428599167`, 1.8437908262906513`},
    {28, 0.00007886487467289201`, 0.14037947691774777`}, {42, 0.00005762296441210828`, 0.10256887665355273`},
    {56, 0.00005520411527239711`, 0.09826332518486687`}, {70, 0.00005477006245136794`, 0.09750307111634349`},
    {84, 0.00005472657809070396`, 0.09741330900145304`}, {98, 0.00005472134430193981 6`, 0.09740399285745288`},
    {112, 0.00005472078051486128`, 0.09740298931645307`}, {126, 0.000026568817222977 95`, 0.04729249465690074 6`}}},
{0.2762626642080059`, 0.4739284043667089`, 1.351212961630882`, 1.24608482511035`, 0.19517067645291641`,
  0.24775742659101618`, 0.08503066911676711`, 2141.7873291111496`, 0.17338810608691535`,
  4.319189194420735`, 17.40648154681566`, 1.2179315872358787`, 0.7834100538442579`, 11.262442943157241`, 1.`,
  {{0, 0.0005423859994070884`, 0.9654470789446175`}, {23, 0.000313559664272862`, 0.5581362024056944`}}},
{0.1906362991887024`, 1.590121005227628`, 9.42412383550555`, 1.4957920989499214`, 0.709065663948995`,
  0.3417182681188283`, 0.37536592261162544`, 505.4667159946151`, 0.11728445265057086`,
  5.855972486658924`, 205.02681117245638`, 7.242681103334082`, 2.7911316319949875`, 80.06773945377371`,
  1.`, {{0, 0.005278149556190699`, 9.395106210019444`}, {8, 0.0007694244676976506`, 1.369575552501818`},
    {16, 0.000035164767001393216`, 0.06259328526247991`}, {24, 2.409407597059366`*^-6, 0.004288745522765672`}}},
{0.2658746234670645`, 3.614932865157483`, 8.460828570764`, 0.8159863776033582`, 0.2726846463148733`,
  0.34564016673603704`, 0.10623048732883861`, 3778.1279822475276`, 0.23711150436988554`,
```

5.459482199196464`, 15.113367034537486`, 0.171194155414627`, 0.11134587060642222`, 9.890466516538229`, 1.`,
{{0, 0.0005743081570265141`, 1.0222685195071952`}, {18, 0.0001773672982303913`, 0.3157137908500966`}}},
{0.26863157332159515`, 2.9396265733275415`, 6.862183762414347`, 0.9732277188028977`, 0.5002013826688112`,
0.5818631486183298`, 0.034332997185588514`, 711.1213518313073`, 0.15904422112946637`, 1.3044149622459462`,
30.670437836883494`, 0.2582729298116462`, 0.25817296794716144`, 30.65342216432659`, 1.`,
{{0, 0.002214404437422049`, 3.941639898611248`}, {7, 0.00011525564706677164`, 0.20515505177885354`}}},
{0.27209369466384475`, 3.5584095462332135`, 0.9720509149176574`, 0.9873292487031791`, 0.8069894772531618`,
0.17871886216847`, 0.10218788035646292`, 96.95116373019758`, 0.02386269594633994`, 2.5608838628408552`, 607526.4899707471`,
124309.82921418153`, 230.2631977901921`, 1172.4119437368295`, 0.`, {{0, 0.08910330772399905`, 158.6038877487183`}}},
{0.14444262093504412`, 3.863449844229998`, 8.726684391435349`, 0.8553749492129846`, 0.451984917853794`,
0.5228822441273482`, 0.008115157266947242`, 403.9888714010655`, 0.1027695319041006`, 9.711355034259547`, 372.50117745610584`,
0.7142040269254306`, 0.7142040269254306`, 372.50117745610584`, 1.`, {{0, 0.028310089486664046`, 50.391959286262`}}},
{0.2538137652575522`, 0.7540411224449701`, 8.794714857317246`, 0.9184514413182489`, 0.11359389239638507`,
0.27911589396705727`, 0.11507441894157476`, 65.76953791858952`, 0.10155553687026442`,
2.859221903044178`, 91049.2654400193`, 19650.09081939116`, 26.791704377844756`, 299.65194076791033`,
1.`, {{0, 0.021863534383009 82`, 38.91709120175748`}, {27, 0.00013068830534873995`, 0.2326251835207571`},
{54, 0.00014889421882786037`, 0.26503170951359145`}, {81, 0.00012739949861045158`, 0.22677110752660382`},
{108, 0.00012608570844857 55`, 0.22443256103846443`}, {135, 0.00012587550123225601`, 0.2240583921934157`},
{162, 0.0001258566726737801`, 0.22402487735932858`}, {189, 0.00012521321020970305`, 0.22287951417327143`}}},
{0.07943689831455408`, 1.7727348695278575`, 3.5998215792473456`, 1.4458682286750966`, 0.6331999429153172`,
0.478090570864118`, 0.46084046549589247`, 472.0739644000896`, 0.10830302137693731`, 2.3782715249335666`,
72.31348620505452`, 2.9369476728471184`, 1.7957006414612484`, 44.729052818112564`, 1.`,
{{0, 0.0030227043084195947`, 5.380413668986878`}, {8, 0.00037670370575696`, 0.6705325962473887`}}},
{0.05556808280528502 4`, 1.296674173505255`, 4.7380956025626375`, 1.4538691472242138`, 0.09550221320066177`,
0.19041481571096486`, 0.007131815235409 4295`, 86.20076273581975`, 0.04910104396213488`,
4.3238451034891945`, 120296.3074315137`, 66970.85429599139`, 229.06100885398152`, 23306.543297670225`,
0.`, {{0, 0.023709494376988043`, 42.20289999103871`}, {20, 0.0006367426550514127`, 1.1334019259915147`},
{40, 0.0006855242138426032`, 1.2202331006398337`}, {60, 0.0018395641886163152`, 3.274424255737041`},
{80, 0.005618750353206335`, 10.001375628707278`}, {100, 0.004453377115761492`, 7.927011266055455`},
{120, 0.0044086806229492745`, 7.8474515088497085`}, {140, 0.005119913988476543`, 9.113446899488247`},
{160, 0.005453513343850435`, 9.707253752053775`}, {180, 1.7193717297641946`, 3060.4816789802667`}}},

{0.1596260086552564`, 2.5471852815710525`, 0.22684585875990315`, 1.2716791760964523`, 0.8208442532787252`,
0.3888937433082962`, 0.04289548126799573`, 69.96603930346842`, 0.07461229317798801`, 1.378327695847128`, 284870.1840749884`,
86840.10113274284`, 230.2438776609239`, 849.1867466976769`, 0.`, {{0, 0.06453819274902345`, 114.87798309326175`}}},
{0.14264033419885946`, 0.8035889732308119`, 6.673404894657215`, 1.4157343373387477`, 0.18185291353504507`,
0.4765318357304136`, 0.03778101114485503`, 210.869852474353`, 0.22822304787397907`, 1.0663935058760412`,
55.67351992410398`, 1.7379793679303903`, 1.6896160201658237`, 55.035691495128326`, 1.`,
{{0, 0.003596510535138539`, 6.4017887525466`}, {10, 0.000586202018491214`, 1.043439592914361`}}},
{0.2209992311467341`, 3.548991401043337`, 2.677760813541523`, 1.1645638964766105`, 0.17856966244183803`,
0.2409694046188614`, 0.16792458477559433`, 53.365358865447796`, 0.019626895089088575`,
3.834874658630479`, 1.36730593164984`*^6, 328888.2072713738`, 229.12593512453657`, 12737.001697252896`,
0.`, {{0, 0.023787364561494353`, 42.341508919459955`}, {7, 0.011293646994874658`, 20.10269165087689`},
{14, 0.011000731960508233`, 19.581302889704656`}, {21, 0.011195032363140029`, 19.92715760638925`},
{28, 0.011414588052293855`, 20.317966733083065`}, {35, 0.011608005121340182`, 20.662249115985528`},
{42, 0.011788098302164074`, 20.98281497785205`}, {49, 0.011962965293325654`, 21.294078222119666`},
{56, 0.012134704074934504`, 21.599773253383418`}, {63, 0.8518269922671444`, 1516.2520462355171`}}},
{0.08986394714808477`, 2.7800808748945913`, 9.536673819459821`, 0.8308776528106754`, 0.5315183679881144`,
0.4614339535602666`, 0.046951751845033245`, 1052.1471359283046`, 0.24213976319820152`,
2.8230201755788666`, 41.81085216188932`, 0.4155144369645898`, 0.3378678670007217`, 33.57563861191257`, 1.`,
{{0, 0.0019383510927112586`, 3.4502649450260403`}, {34, 0.0006133974417940964`, 1.0918474463934915`}}},
{0.08237232438525194`, 0.904854561594771`, 7.190948808493694`, 0.9264355987892083`, 0.7428148600467364`,
0.6232177046085758`, 0.0730718251800762`, 62.25627183567972`, 0.1453325784006102`, 9.786938339680962`,
70111.33250513591`, 31955.47523445797`, 228.84363069800244`, 1880.0679253693643`, 0.`,
{{0, 0.06860048553567122`, 122.10886425349477`}, {2, 0.016007539685984503`, 28.493420641052417`},
{4, 0.007825252724237784`, 13.928949849143255`}, {6, 0.004086926549769404`, 7.274729258589539`},
{8, 0.0022711490000238746`, 4.0426452200424965`}, {10, 0.0013458807816141979`, 2.395667791273272`},
{12, 0.0008540352549003795`, 1.5201827537226755`}, {14, 0.0005823868858070561`, 1.03664865673656`},
{16, 0.0004270357762214547`, 0.7601236816741894`}, {18, 0.04088447013384182`, 72.77435683823843`}}},
{0.14754836278222488`, 0.5816922789151606`, 9.91636448422577`, 0.7971447359984003`, 0.9834799375197849`,
0.6545637466726528`, 0.017670578507760985`, 406.58618651198583`, 0.1328908282216244`,
3.7962615345853075`, 298.7116857186342`, 6.808419650449969`, 6.577080778287982`, 298.2656699627115`,
1.`, {{0, 0.01848042385239934`, 32.89515445727083`}, {1, 0.003525079887267345`, 6.274642199335874`},

```
      {2, 0.0005742889902319206`, 1.0222344026128187`}, {3, 0.00008839818726747159`, 0.15734877333609942`}}},
  {0.06758332214646862`, 3.0558788120173555`, 4.64800500980858`, 1.4828314301436276`, 0.7395538908104162`,
   0.3075642133576061`, 0.04417074894369504`, 145.23097869725217`, 0.1743358052899487`, 7.770691560418564`, 15237.204425877404`,
   7604.110878462426`, 229.51517325288714`, 769.2507394860237`, 0.`, {{0, 0.03529830355846217`, 62.83098033406266`},
      {5, 0.0029102802544482714`, 5.1802988529179235`}, {10, 0.002893737884433627`, 5.1508534342918555`},
      {15, 0.0028934673157599835`, 5.1503718220527706`}, {20, 0.0028934544687933147`, 5.150348954452101`},
      {25, 0.0028934533124597684`, 5.150346896178388`}, {30, 0.002893453156451445`, 5.150346618483572`},
      {35, 0.00289345312861708`, 5.150346568938402`}, {40, 0.002893453121512149`, 5.150346556291626`}}},
  {0.16631267310517406`, 2.413572311283116`, 5.471184235465843`, 1.4579537524645423`, 0.9497208653345599`,
   0.439033675828699`, 0.21085287275356013`, 3128.2027597762994`, 0.18409987492192065`, 5.612876798830806`, 61.677985584717064`,
   1.31717195858983`, 1.31717195858983`, 61.677985584717064`, 1.`, {{0, 0.004687526904438497`, 8.343797889900525`}}},
  {0.18539796406610926`, 1.3871953304353832`, 7.757698949197568`, 1.3585428901946988`, 0.4532718222049952`,
   0.29058122288435795`, 0.010738744574908157`, 486.55354471220693`, 0.20251048022954932`, 4.51278311919668`, 106.72598666736846`,
   0.7016849041262303`, 0.7016849041262303`, 106.72598666736846`, 1.`, {{0, 0.008111174986720003`, 14.437891476361607`}}},
  {0.04144083471615087`, 3.41458508027812`, 2.4600046087844927`, 1.2787341279549589`, 0.569252466493285`,
   0.6736151572579898`, 0.4762197736813673`, 718.8382183063302`, 0.07598079948793823`,
   4.331845758841565`, 94.40191581040338`, 2.506221329634898`, 1.736668513259571`, 68.70395068707144`, 1.`,
   {{0, 0.004735197215143071`, 8.428651042954666`}, {8, 0.0004863030370743587`, 0.8656194059923584`}}},
  {0.13724293356211342`, 3.6916535362348286`, 5.489803233715362`, 1.3457045887873154`, 0.0754297582883523`,
   0.6293482685634375`, 0.017573021108899616`, 778.5577282359714`, 0.17674997296512807`, 2.6916234726152264`, 40.13379743236351`,
   0.17494325831601434`, 0.17494325831601434`, 40.13379743236351`, 1.`, {{0, 0.0030501686048596266`, 5.429300116650135`}}},
  {0.08176385784973572`, 2.023736863649704`, 9.823987283528833`, 1.0905304980935506`, 0.6417627833690904`,
   0.2509575610218163`, 0.3071390754059633`, 168.49990583950182`, 0.0669392059366426`,
   7.784078345121018`, 600.1270112577715`, 16.87354602434372`, 6.079018530867088`, 230.4715405348727`,
   1.`, {{0, 0.013904793853794882`, 24.75053305975489`}, {8, 0.003339914129563598`, 5.945047150623204`},
      {16, 0.00024712103179303803`, 0.43987543659160766`}, {24, 0.000024008065498808 85`, 0.04273435658787975`}}},
  {0.20636275093165019`, 3.041433098250965`, 7.709885134336993`, 1.0647262222072884`, 0.28984076250310165`,
   0.17246518959666646`, 0.007377660819744997`, 464.8055142552055`, 0.19020267666710844`, 9.452984008311837`, 168.195936249229`,
   0.3698257793363428`, 0.3698257793363428`, 168.195936249229`, 1.`, {{0, 0.012782891154941405`, 22.7535462557957`}}},
  {0.2140607830978551`, 1.6917207171996171`, 1.9218182027339432`, 0.9997605714664014`, 0.5513493876922198`,
   0.4464250883384161`, 0.2328414126003874`, 611.4769203082982`, 0.08477583973629349`, 2.188379571162489`,
```

```
50.27417320276394`, 4.443088188307459`, 1.5630771815978148`, 36.12244335295506`, 1.`,
 {{0, 0.0022518296095206626`, 4.0082567049467785`}, {4, 0.0003852960616851482`, 0.6858269897995637`},
  {8, 0.000044682859640765984`, 0.07953549016056345`}, {12, 0.000019294072584207232`, 0.0343434491998887`},
  {16, 0.000017798508690401158`, 0.031681345468914816`}, {20, 0.000026404582703398987`, 0.0470001572120502`}}},
{0.17696168772261972`, 1.6101558302339125`, 0.7107550695951144`, 1.133554468522512`, 0.8128396324743541`,
 0.15732538361458315`, 0.06392981831267872`, 277.86150200910555`, 0.15413566109625954`,
 1.907309832357937`, 23455.448721662666`, 6645.5971399408`, 229.26755095976435`, 5961.677884234886`, 0.`,
 {{0, 0.017820689091049297`, 31.720826582067748`}, {34, 0.007113207114374583`, 12.661508663586755`},
  {68, 0.00377729863109521`, 6.723591563349473`}, {102, 0.0022409766092961535`, 3.9889383645471526`},
  {136, 0.001472082968943412`, 2.620307684719272`}, {170, 0.001081553446001763`, 1.9251651338831381`},
  {204, 0.0008767149013172967`, 1.5605525243447878`}, {238, 0.0007635110643328163`, 1.3590496945124129`},
  {272, 0.0006969205423635266`, 1.2405185654070772`}, {306, 0.41724456483307726`, 742.6953254028774`}}},
{0.04328176764500202`, 2.9602821723444555`, 0.6131528539518083`, 1.0058908218647606`, 0.19964040264767258`,
 0.6669684114614443`, 0.23851965705730427`, 2287.3379976105803`, 0.08593310169788088`, 8.660468891635798`, 50.06694733360331`,
 1.7038514630463655`, 1.3549717374894787`, 46.11188147863593`, 1.`, {{0, 0.0027299293587394716`, 4.85927425855626`},
  {3, 0.0006718627401268078`, 1.1959156774257178`}, {6, 0.00007427018283377204`, 0.13220092544411421`},
  {9, 0.000011541977347773307`, 0.020544719679036063`}, {12, 0.000016898733328505735`, 0.03007974532474021`}}},
{0.08735803097627126`, 3.963951857586263`, 7.965851341925532`, 1.189504619761999`, 0.40822475538796854`,
 0.4952091412789442`, 0.01984161894285721`, 1116.2764923646498`, 0.2379409272833778`, 1.293491554272407`, 15.845252533906509`,
 0.07240475589567427`, 0.07240475589567427`, 15.845252533906509`, 1.`, {{0, 0.0012042391925768947`, 2.143545762786873`}}},
{0.15281896372135534`, 0.55010519971598`, 2.817048022996838`, 1.3198176246010052`, 0.16238216376752557`,
 0.6925657414172184`, 0.11807724637466313`, 76.78768290686035`, 0.04193274426603899`, 1.2341700274455292`, 454294.9909386107`,
 142712.62658394687`, 227.70810922888228`, 989.152004844264`, 0.`, {{0, 0.07517555236816406`, 133.81248321533204`}}},
{0.19086658543964008`, 1.9905232804946325`, 4.078173781767919`, 0.8519485127716493`, 0.08904379736388202`,
 0.23536266216215151`, 0.24991927271295455`, 192.66677656391008`, 0.127316992739278`, 7.8449864029309175`, 7092.509972562512`,
 1841.058563620366`, 5.2842146713214735`, 199.19492593748078`, 1.`, {{0, 0.015081334494880127`, 26.84477540088663`},
  {34, 0.000031296485663479 85`, 0.05570774448099413`}, {68, 0.000026183390704933402`, 0.04660643545478146`}}},
{0.06664208120921677`, 2.003234095381499`, 6.199949723153024`, 0.888873086526756`, 0.04784068730663105`,
 0.37023377411027647`, 0.049828770496122325`, 349.42476257926376`, 0.05267583263583803`,
 7.0912634633886915`, 183.49474747556806`, 2.549346847276688`, 2.2973881161225513`, 165.22083505424752`,
 1.`, {{0, 0.010738092607712394`, 19.11380484172806`}, {26, 0.001818690856410418`, 3.237269724410544`}}},
```

```
{0.22983292033798441`, 0.7814702114508636`, 3.2012106727008174`, 1.142026804432239`, 0.1064467335224153`,
  0.4404014420960507`, 0.006695571873889409`, 4217.329674971789`, 0.15347806495470107`, 2.7989551297088675`, 6.6341998642552`,
  0.051917072293360914`, 0.051917072293360914`, 6.6341998642552`, 1.`, {{0, 0.0005041991896833952`, 0.8974745576364435`}}},
{0.13211782844464454`, 0.44983284117427225`, 1.0762780754680463`, 1.3187859174208114`, 0.8445651695343355`,
  0.2965729998791703`, 0.0542223541794765`, 616.1259401245338`, 0.10515194159293045`, 5.24820204317008`, 7264.328790209427`,
  2502.723991592556`, 6.020269543567927`, 60.09084754512251`, 1.`, {{0, 0.004176029722701665`, 7.433332906408965`},
    {8, 0.0001349666055466363`, 0.2402405578730126`}, {16, 0.000047873454536395 47`, 0.08521474907478394`},
    {24, 0.000047332899804697 78`, 0.0842525616523620 4`}, {32, 0.000047327652570741 06`, 0.0842432215759190 8`},
    {40, 0.000047327410532203 38`, 0.0842427907473220 4`}, {48, 0.000066046667736971 22`, 0.1175630685718087 7`}}},
{0.17817224333688036`, 1.8900871904197354`, 9.98947566566499`, 0.792780936871982`, 0.9109839945193492`,
  0.4314201285170226`, 0.03598067399007004`, 128.67700477089366`, 0.059019163579936806`,
  2.366574414393561`, 208.9501876429785`, 4.596800939641771`, 2.916239190537959`, 209.02971039114937`,
  1.`, {{0, 0.012836537566148899`, 22.849036867745042`}, {2, 0.0025794230304602658`, 4.591372994219274`},
    {4, 0.0003569271602458058`, 0.6353303452375344`}, {6, 0.000056106260566478544`, 0.09986914380833181`},
    {8, 0.000020427230868765606`, 0.03636047094640278`}, {10, 0.00001671959767914007`, 0.029760883868869326`},
    {12, 0.000016392878266618338`, 0.02917932331458064`}, {14, 3.7242654913814303`*^-6, 0.006629192574658946`}}},
{0.2509149027719233`, 2.580879402706512`, 1.8893709427148198`, 0.8255744895957449`, 0.1652280455910582`,
  0.6768016323195194`, 0.009322737452992792`, 2112.3599589856663`, 0.08696925005081763`, 4.203193192682905`, 26.11496607326686`,
  0.10144177262637238`, 0.10144177262637238`, 26.11496607326686`, 1.`, {{0, 0.0019847374215682815`, 3.5328326103915413`}}},
{0.08555200390318413`, 3.7277336286219658`, 7.5570643278825855`, 1.1117862222014783`, 0.20420751548200644`,
  0.3245432768958507`, 0.00950175247006993`, 144.2017035489447`, 0.14385517513119161`, 5.452203080835416`, 347.82159403096705`,
  2.4422972925698057`, 1.0687654844446477`, 352.8355664668367`, 1.`, {{0, 0.020304080757489334`, 36.141263748331006`},
    {5, 0.005294625066240246`, 9.424432617907637`}, {10, 0.0009926712515202613`, 1.7669548277060652`},
    {15, 0.00018912759131367596`, 0.3366471125383432`}, {20, 0.000034998384916075744`, 0.06229712515061483`}}},
{0.07297367338130328`, 1.9451098216604938`, 8.396918429735464`, 1.2137718043700492`, 0.7314346040557373`,
  0.4669601248496963`, 0.016599550382003152`, 192.8440550768832`, 0.21285961816083276`,
  7.382641950639707`, 519.8715870231912`, 5.258577266039362`, 3.9763947851158328`, 528.1503303886279`,
  1.`, {{0, 0.033579640933371555`, 59.77176086140136`}, {2, 0.005739806340086514`, 10.216855285353995`},
    {4, 0.0006984176969133092`, 1.2431835005056902`}, {6, 0.00009090581965427589`, 0.1618123589846111`},
    {8, 0.000022290452089253188`, 0.03967700471887067`}, {10, 8.36386742081908`*^-6, 0.014887684009057961`}}},
{0.07358197980029185`, 1.0291227850237563`, 2.532589152827489`, 1.0117020834715413`, 0.8098748594005145`,
```

```
    0.5301759885595309`, 0.00813301998886755`, 830.3082383512614`, 0.02407211472876364`, 4.5236701681781`, 95.69019050420202`,
    1.1763325494939245`, 0.9001329258467724`, 98.4888122866639`, 1.`, {{0, 0.005258880856252013`, 9.360807924128585`},
      {3, 0.0017035524306541646`, 3.032323326564413`}, {6, 0.0004144506127333533`, 0.7377220906653689`},
      {9, 0.00010273279373261257`, 0.18286437284405035`}, {12, 5.5330404143133154`*^-6, 0.009848811937477702`}}},
  {0.18797192534273177`, 0.513697145142666`, 2.3563599620417435`, 0.8181944603817968`, 0.26394673984920813`,
    0.3209514271347109`, 0.025430853857263652`, 265.9518711391782`, 0.07720757078553087`,
    7.134393115551401`, 22006.71583029791`, 5939.336851364693`, 227.79359955892062`, 1449.3597604415118`,
    0.`, {{0, 0.04503909123050394`, 80.1695823902970 1`}, {30, 0.0016182957007790358`, 2.880566347386684`},
      {60, 0.0016210719132527523`, 2.885508005589899`}, {90, 0.0016639778090198562`, 2.9618805000553445`},
      {120, 0.001901538203941076`, 3.3847380030151153`}, {150, 0.0018357373657143304`, 3.2676125109715084`},
      {180, 0.001737712055564 5408`, 3.093127458904883`}, {210, 0.0016972609151584533`, 3.0211244289820467`},
      {240, 0.0016674820213499432`, 2.9681179980028984`}, {270, 0.05136917457827097`, 91.43713074932232`}}},
  {0.2528075291703835`, 2.1409422046846034`, 2.3989238084434295`, 1.1058748574151904`, 0.4698643832575349`,
    0.20099009902051257`, 0.02857754945135185`, 57.682491948215834`, 0.240427409232489`, 4.4402688370357115`, 106670.808020232`,
    23114.47782084567`, 230.25739607196272`, 1216.3337908293074`, 0.`, {{0, 0.09244136810302737`, 164.5456352233887`}}},
  {0.07054141508315104`, 0.5799884401515247`, 2.99755260715291`, 1.1960365141205305`, 0.29917509086343896`,
    0.44670601886119`, 0.13984458882947357`, 3612.976166750159`, 0.22678215214973713`, 7.656934137991781`, 25.730362813570387`,
    1.8220680468959274`, 1.8220680468959274`, 25.730362813570387`, 1.`, {{0, 0.0019555075738313496`, 3.480803481419802`}}},
  {0.13968170289558773`, 2.9225254745826437`, 6.832387664740558`, 1.248628328449276`, 0.2500405375269379`,
    0.6548966838897512`, 0.01310969508256717`, 2778.8012693248406`, 0.21649543926111503`, 9.334771326724606`, 47.06514159535508`,
    0.1769016576362463`, 0.1769016576362463`, 47.06514159535508`, 1.`, {{0, 0.003576950761246986`, 6.3669723550196355`}}},
  {0.14258976979051796`, 0.734197214461469`, 7.171919374129239`, 0.945223896823731`, 0.9937893052327855`,
    0.2333579308191156`, 0.10311334331996398`, 86.76571756149787`, 0.17032024165228216`, 9.316237353631934`, 24735.381002245496`,
    7997.5752250000805`, 33.45150317578429`, 593.8538313288369`, 1.`, {{0, 0.04407788323418956`, 78.45863215685742`},
      {4, 0.0007014798273642567`, 1.248634092708377`}, {8, 0.00006794232985802522`, 0.1209373471472849`},
      {12, 0.0000552103975260135`, 0.09827450759630404`}, {16, 0.00005483239789575 2804`, 0.09760166825444`},
      {20, 0.0000548127492307598`, 0.09756669363075245`}, {24, 0.00005481125559482991 6`, 0.09756403495879726`},
      {28, 0.00005481111496400932`, 0.09756378463593661`}, {32, 0.000011107874368391706`, 0.019772016375737236`}}},
  {0.06222401125326871`, 0.8031654209648247`, 7.912640972265759`, 1.0061100735669513`, 0.06612689335476452`,
    0.2257508005074531`, 0.2688428052003874`, 1016.6634644345198`, 0.08792308855751246`, 8.5639571694838`,
    65.07309714397097`, 4.0337876597750055`, 1.8645429420490969`, 31.291739780093646`, 1.`,
```

```
{{0, 0.001825440695403661`, 3.249284437818517`}, {10, 0.0005137109341366556`, 0.9144054627632472`},
   {20, 0.0000374392619817333`, 0.06664188632748529`}, {30, 1.5813317650668202`*^-6, 0.00281477054181894`}}},
{0.04567594599148308 6`, 1.972555523342196`, 9.234608119853927`, 1.10067888297902`, 0.060950291683742996`,
  0.5641176167348642`, 0.005654158004083115`, 79.62566643520867`, 0.10418567571675635`,
  3.9217157633863025`, 40611.54441421846`, 24382.787578422045`, 229.08822678936252`, 79920.25746480835`,
  0.`, {{0, 0.02952804506911629`, 52.559920223027`}, {8, 0.00017576171505064145`, 0.3128558527901418`},
   {16, 0.00016268648836381021`, 0.2895819492875822`}, {24, 0.0001683825739712292`, 0.29972098166878797`},
   {32, 0.0002791809647973948 3`, 0.4969421173393628`}, {40, 0.0039025202344710025`, 6.946486017358384`},
   {48, 0.014100081113628772`, 25.098144382259214`}, {56, 0.008375071941194702`, 14.907628055326567`},
   {64, 0.006039804663320913`, 10.750852300711225`}, {72, 6.01120803256152`, 10699.950297959505`}}},
{0.19725457740267938`, 0.673691333497294`, 1.5958254230238804`, 0.9055402555445284`, 0.8526577157919533`,
  0.18700866613724199`, 0.221308084367558`, 137.27532297707575`, 0.16306687928789393`, 6.444987143866487`,
  41177.728449479386`, 10770.355413888878`, 229.33682922782094`, 1029.031900336364`, 0.`,
  {{0, 0.05567535092314153`, 99.10212464319193`}, {24, 0.002826253463342751`, 5.030731164750096`},
   {48, 0.002440759829338247`, 4.34455249622208`}, {72, 0.002411966209543928`, 4.293299852988191`},
   {96, 0.002408786093501572`, 4.287639246432798`}, {120, 0.0024084196511301686`, 4.2869869790117`},
   {144, 0.0024083772167659907`, 4.286911445843463`}, {168, 0.0024083722999105207`, 4.286902693840727`},
   {192, 0.002408371730187603`, 4.286901679733933`}, {216, 0.0028097670087013533`, 5.001385275488409`}}},
{0.17905209129143224`, 1.6872990209581902`, 6.743325221637608`, 0.8692054788625995`, 0.5776472609105132`,
  0.5432377341075647`, 0.12670077461750193`, 462.43790721394`, 0.057341353016641616`,
  6.31060148160045`, 253.65003828437148`, 6.49321281036121`, 5.05707154079146`, 197.7599005762567`, 1.`,
  {{0, 0.01387972104732453`, 24.705903464237664`}, {15, 0.001150031396470979`, 2.0470558857183425`}}},
{0.21834559506325857`, 2.5253820753047362`, 6.659656299000567`, 1.4243771911650907`, 0.9747752944821462`,
  0.1772255451247845`, 0.01986842431027196`, 102.11368670749582`, 0.09272454748376258`,
  4.914431231863471`, 42988.39881764499`, 10375.375693047159`, 2.368795118057256`, 277.2706293824633`,
  1.`, {{0, 0.017095706169832123`, 30.43035698230118`}, {7, 0.003352853839549649 7`, 5.968079834398376`},
   {14, 0.00047992993019099447`, 0.8542752757399703`}, {21, 0.00008584302040504901`, 0.15280057632098723`},
   {28, 0.0000355592349013476`, 0.06329543812439874`}, {35, 0.000022675638188052692`, 0.040362635974733795`}}},
{0.2429292029568641`, 1.5636324867576725`, 8.233163527811005`, 0.9303215118662402`, 0.8597394087604655`,
  0.6215210972087029`, 0.12468068919290341`, 61.92974770793832`, 0.07970211447796205`, 9.85507721294049`,
  222607.84358287588`, 49667.11365010005`, 229.30124309946626`, 2143.8657765255907`, 0.`,
```

```
{{0, 0.11632072285187818`, 207.05088667634317`}, {8, 0.026843834893863095`, 47.782026111076306`},
  {16, 0.007806854315277539`, 13.896200681194019`}, {24, 0.002839086602169l255`, 5.053574151861043`},
  {32, 0.0012207846208074646`, 2.1729966250372867`}, {40, 0.000601976105899569`, 1.0715174685012328`},
  {48, 0.00033899582171494904`, 0.6034125626526093`}, {56, 0.00021896436500327423`, 0.3897565697058281`},
  {64, 0.00016068890480800137`, 0.28602625055824243`}, {72, 0.006581890534523704`, 11.715765151452194`}}},
{0.09149398523540997`, 2.310449584405898`, 9.73345399143432`, 1.4402850137921024`, 0.7807243420630963`,
 0.37700223940662914`, 0.04452259966898517`, 785.475395176315`, 0.18361612690246515`, 4.855709318281368`,
 130.85907622438398`, 1.5117647290984322`, 1.1248241188238637`, 95.88986200869023`, 1.`,
 {{0, 0.005271002613808421`, 9.38238465257899`}, {34, 0.0020166268988520367`, 3.5895958799566254`}}},
{0.11249424084619364`, 2.9716096914630903`, 4.4521238184847185`, 1.4266434477040648`, 0.9997833218301952`,
 0.5867347171724102`, 0.06910846060462165`, 1820.2194505805683`, 0.24722705290784003`,
 7.621221876581703`, 207.3438173544296`, 2.4703423773132482`, 1.9512118637231262`, 165.3320509348539`,
 1.`, {{0, 0.005717763532513398`, 10.177619087873849`}, {2, 0.003933562194243109`, 7.001740705752733`},
  {4, 0.002322578855974899`, 4.13419036363532`}, {6, 0.000591331288317491`, 1.052569693205134`}}},
{0.09866336490626898`, 1.7258477745644702`, 0.8848358466481533`, 0.910425763108387`, 0.9172877053292023`,
 0.36334488527428765`, 0.3804604060347452`, 1006.1834168170927`, 0.013687075322010439`, 8.165252701558408`, 9257.29364849209`,
 3818.8190471709113`, 2.886508800464856З`, 62.10899182143077`, 1.`, {{0, 0.004145579021250463`, 7.379130657825824`},
  {5, 0.00046453121542095426`, 0.8268655634492987`}, {10, 0.000031934508708707655`, 0.05684342550149963`},
  {15, 0.000012569257992967916`, 0.02237327922748289`}, {20, 0.000011940352325707501`, 0.02125382713975935`},
  {25, 0.000011918289282577964`, 0.021214554922988776`}, {30, 0.00001191689607219872`, 0.02121207500851372`},
  {35, 0.0000119167480969281`, 0.021211811612532015`}, {40, 0.000017977089278232648`, 0.03199921891525411`}}},
{0.22803797815686577`, 0.698146546972529`, 1.035170425484365`, 0.9822431866703154`, 0.6417091531359376`, 0.21874316930074555`,
 0.005531062831453447 5`, 1727.3746181384265`, 0.11140836748423671`, 9.157656749061658`, 68.60746280959205`,
 0.5836516422463038`, 0.4948490446813869`, 63.038598324895965`, 1.`, {{0, 0.00313169373218742`, 5.574414843293607`},
  {6, 0.001276571863018305`, 2.2722979161725836`}, {12, 0.0003826678774863685`, 0.6811488219257359`}}},
{0.12680960193351426`, 3.133735649340868`, 7.511849069758438`, 1.0897808931740574`, 0.04885314856347578`,
 0.24060421848165847`, 0.3693592758602631`, 883.7388687037887`, 0.08961301811176398`, 2.076054864701238`, 17.752106102751814`,
 0.3250738487462817`, 0.16444893940624775`, 8.948020540211113`, 1.`, {{0, 0.0005946744356589176`, 1.0585204954728733`},
  {10, 0.00008288051996961495`, 0.1475273255459146`}, {20, 2.494605427512035`*^-6, 0.004440397660971422`}}},
{0.11645318221252937`, 1.153640861264721`, 2.619716900319183`, 0.8529577052637052`, 0.5619295219152276`,
 0.5231720769226155`, 0.0460981579608882 2`, 565.9088521347549`, 0.20476637224893374`,
```

8.831648882728363`, 201.75684757988182`, 5.684389401143838`, 4.892850002791681`, 192.2138773273918`, 1.`,
{{0, 0.012113482659719677`, 21.561999134301026`}, {11, 0.002494772017162101`, 4.440694190548539`}}},
{0.042446348872052314`, 3.5353553586920903`, 5.120248896798044`, 1.1724129507972807`, 0.0738809680757258`,
0.47979821691937163`, 0.43439767413472635`, 83.35178002891637`, 0.15043407845955598`,
2.47226753883535`, 42440.865848042566`, 26353.56211800002`, 229.64044235856403`, 616.1778766142294`,
0.`, {{0, 0.011551206916195166`, 20.561148310827395`}, {3, 0.0017044193466978883`, 3.0338664371222417`},
{6, 0.0005821120985781354`, 1.036159535469081`}, {9, 0.0002677894039563092`, 0.4766651390422304`},
{12, 0.00015846176226445272`, 0.28206193683072583`}, {15, 0.00011339039724891694`, 0.20183490710307214`},
{18, 0.00009242554909555856`, 0.1645174773900942 3`}, {21, 0.00008188440790339528`, 0.1457542460680436`},
{24, 0.0000763103722606162`, 0.13583246262389684`}, {27, 0.032201518368481`, 57.31870269589618`}}},
{0.1455833195103322`, 2.7843188408171127`, 7.76885376753495`, 1.3211895319342402`, 0.9135586666939064`,
0.6453968687054246`, 0.1333226247130129`, 368.7414592704895`, 0.16919558147748776`, 3.2843355334407627`, 209.7611336671 0553`,
4.024482339641899`, 3.455695687474116`, 198.9382899742153`, 1.`, {{0, 0.011170781985086906`, 19.88399193345469`},
{2, 0.0031434927442246597`, 5.5954170847198945`}, {4, 0.0006551264008510233`, 1.1661249935148217`},
{6, 0.00014260803128300113`, 0.253842295683742`}, {8, 7.3008765947735785`*^-6, 0.01299556033869697`}}},
{0.19543798537508206`, 1.2324134940956721`, 7.418056367639444`, 1.0725732268441615`, 0.3282068086166905`,
0.15859490968399015`, 0.32000646124266346`, 1339.7132456219883`, 0.07635813740352487`, 1.32927343177637`, 7.774764526594433`,
0.338503930313826`, 0.338503930313826`, 7.774764526594433`, 1.`, {{0, 0.0005908821040211769`, 1.051770145157695`}}},
{0.14288798158791094`, 1.8492145343896036`, 4.320087837799633`, 1.416387968755192`, 0.03040014321801321`, 0.26264512496919146`,
0.017396141813896236`, 476.32655268366966`, 0.1492295937607505`, 1.5210123574307455`, 24.129362571981456`,
0.21349349728742642`, 0.21349349728742642`, 24.129362571981456`, 1.`, {{0, 0.0018338315554705907`, 3.2642201687376513`}}},
{0.08126639977214484`, 1.4848577063399988`, 1.4511591326569544`, 1.4690309046886825`, 0.09088454373979671`,
0.2634538760346167`, 0.006391735651833628`, 757.569692438266`, 0.1906091612906891`, 5.0187956659047614`,
54.5659135160035`, 0.4168413557556116`, 0.38186834705414074`, 56.243689166899195`, 1.`,
{{0, 0.0031517266311966987`, 5.610073403530124`}, {3, 0.0011227937454876395`, 1.9985728669679985`}}},
{0.15406825481222286`, 3.5722547377798985`, 5.518246507815627`, 1.3255687223204002`, 0.2843400333432804`,
0.5740150104722689`, 0.007989301755943026`, 636.0707307721444`, 0.19470033172956325`, 2.3727553064621407`, 49.23941847690839`,
0.13795230804107514`, 0.13795230804107514`, 49.23941847690839`, 1.`, {{0, 0.003742195804245038`, 6.6611085315561676`}}},
{0.1284209869933174`, 2.657038147126583`, 3.485135675760706`, 0.826438046300177`, 0.2940359222573743`, 0.4455502493814366`,
0.0077922251797686165`, 642.5517685154364`, 0.18333530742444448`, 4.125506851960305`, 76.29055054462059`,
0.2398014100407063`, 0.2398014100407063`, 76.29055054462059`, 1.`, {{0, 0.005798081841391165`, 10.320585677676274`}}},

```
{0.1519197321396482`, 1.414313316427302`, 9.693328908271443`, 1.0879341282517354`, 0.5935096502675883`,
  0.32269331092414044`, 0.04853394307287227`, 323.7551755246228`, 0.012675975614693441`,
  6.5738064876305415`, 293.69172923458626`, 5.635388736829623`, 3.8437374811437435`, 199.7583991974518`,
  1.`, {{0, 0.01160669289229439`, 20.659913348284014`}, {37, 0.0035749454467119473`, 6.363402895147266`}}},
{0.10245812606330185`, 3.8066698620788078`, 2.784482506819291`, 0.8153557037427375`, 0.41559745180116914`,
  0.2970790998891699`, 0.019454402362339196`, 101.06708659388384`, 0.03906278599147861`, 1.0136093018296712`, 129608.872405188`,
  52598.60688128732`, 230.26228025161`, 608.4435864498741`, 0.`, {{0, 0.04624171257019044`, 82.31024837493898`}}},
{0.08994858347063645`, 1.3905867780774148`, 6.975873482712982`, 0.9748960320168515`, 0.19705721946727817`,
  0.428438510479348`, 0.008778830773896138`, 53.85788205383214`, 0.2055785802889155`, 2.165498620130503`, 53759.61969917746`,
  23473.55075553633`, 230.2294307642852`, 744.1530729594984`, 0.`, {{0, 0.056555633544921886`, 100.66902770996096`}}},
{0.2555268425086469`, 0.6701554458004759`, 6.763443756394938`, 0.9242821290525386`, 0.9506231196396431`,
  0.49497463337651615`, 0.1535425304369218`, 4370.366828894948`, 0.17262313668823565`,
  9.906703002274519`, 87.38994679506919`, 5.652448580898638`, 2.849953660152137`, 44.23965840713793`, 1.`,
  {{0, 0.002523810637153069`, 4.492382934132463`}, {15, 0.000838403401789414`, 1.492358055185157`}}},
{0.13340663060001784`, 0.4096518827228479`, 7.81563732077705`, 1.2681845828171425`, 0.264399527619257`,
  0.43786671579755854`, 0.11528071789135624`, 3397.5276395199044`, 0.15041338875391808`,
  8.11261999427796`, 27.78841443647035`, 2.4798199854819156`, 1.7794559719553353`, 20.115561933763193`, 1.`,
  {{0, 0.0011193173233351114`, 1.9923848355364981`}, {14, 0.00040946538363089124`, 0.7288483828629865`}}},
{0.09831375749458693`, 3.5924209186692435`, 1.6118282365623244`, 0.8326891410736474`, 0.6715734708323489`,
  0.5976781170151362`, 0.03815316295555065`, 115.73199628594814`, 0.0304507712440647`, 4.7148191409051226`, 216972.37346790274`,
  90210.68650850531`, 229.72283870708026`, 1017.2392192639805`, 0.`, {{0, 0.07731018066406252`, 137.6121215820313`}}},
{0.09108239111305882`, 3.309852207951435`, 3.0566194803884894`, 1.4370488086380533`, 0.21629452377334557`,
  0.2421124982368058`, 0.4670454509414495`, 208.22129409387404`, 0.23659860207487343`, 3.442603557657883`, 7466.42313325171`,
  3209.3080096200765`, 2.5337503499757768`, 84.0557601947292`, 1.`, {{0, 0.0062240075715902935`, 11.078733477430722`},
    {15, 0.00008179364564230211`, 0.14559268924329774`}, {30, 0.000023794163220990268`, 0.04235361053336268`},
    {45, 0.000023657713129489963`, 0.04211072937049213`}, {60, 0.00003498468121634436`, 0.06227273256509297`}}},
{0.05787448179355209`, 1.5695297669455615`, 8.686108197389068`, 1.3362839901345183`, 0.8882645350301348`,
  0.2220019613784866`, 0.20455164120837535`, 127.50819012666335`, 0.2481722521034202`,
  6.402394955038988`, 448.8377815216411`, 15.755971198879578`, 8.48246250423914`, 269.055774772679`,
  1.`, {{0, 0.014479554780982685`, 25.77360751014918`}, {7, 0.005026325004077259`, 8.946858507257524`},
    {14, 0.0008015835752991465`, 1.426818764032481`}, {21, 0.00011034865751678448`, 0.19642061037987635`},
```

```
    {28, 0.00001706559656167787 5`, 0.03037676187978661 7`}, {35, 0.0000133612682860511 3`, 0.02378305754917101`}}},
  {0.2051260145065577`, 0.6712958473867032`, 3.046620158397806`, 1.125533167922526`, 0.5107051094055373`,
   0.47305603590798795`, 0.035994055865640424`, 73.71677428550161`, 0.039721863923625866`, 8.822427071575206`, 491269.438664817`,
   124945.78372703417`, 227.8039688305845`, 1490.6321073833267`, 0.`, {{0, 0.11328804016113282`, 201.6527114868164`}}},
  {0.04365375531995927`, 0.9346121184769336`, 9.554451222092258`, 0.75459846537052`, 0.7382972174511151`,
   0.44670601219095474`, 0.02981461788584879`, 1443.346811017674`, 0.026466349135542522`,
   4.5970910839514385`, 68.83632808882238`, 1.4267308280192466`, 1.2951385955833676`, 62.36424150216906`,
   1.`, {{0, 0.003138938335381736 7`, 5.587310236979492`}, {36, 0.0016007440187831123`, 2.84932435343394`}}},
  {0.18832502219499725`, 2.144648019015553 7`, 7.011391892939212`, 1.2684842496371584`, 0.8800953133738048`,
   0.3019976503972389`, 0.01607749175819828`, 253.61560940445747`, 0.0505443969728126`,
   8.205874646962478`, 459.3330175848634`, 3.747699924420079`, 2.9526605813487223`, 456.8048243923683`,
   1.`, {{0, 0.02815521113818526`, 50.116275825969765`}, {3, 0.005519332419364366`, 9.824411706468572`},
    {6, 0.0009188038437505351`, 1.6354708418759523`}, {9, 0.0001238192525198311`, 0.22039826948529936`}}},
  {0.2789832227001883`, 3.864947879576305`, 9.015175334965505`, 1.1939022970453932`, 0.8102757584298972`,
   0.6575626089292559`, 0.013706864097109712`, 131.89661477643062`, 0.1558047394747739`,
   7.390258992479394`, 9326.543438691775`, 1753.283718230961`, 5.775073205575813`, 663.5341941266996`,
   1.`, {{0, 0.04641725709366048`, 82.62271762671564`}, {3, 0.003583594081637627`, 6.378379746531497`},
    {6, 0.00017969325303207 6`, 0.3198539903970953`}, {9, 0.0000574490004960071`, 0.10225922088289265`},
    {12, 0.0000542832262288817 7`, 0.09662414268740954`}, {15, 0.0000542088968118225 5`, 0.09649183632504414`},
    {18, 0.0000542062830065770 4`, 0.09648718375170713`}, {21, 0.0000281415922956673 5`, 0.050092034168628785`}}},
  {0.10740283086494345`, 3.8162968757181996`, 6.158389397925204`, 0.8038652030060122`, 0.9187954678523191`,
   0.31270681581700654`, 0.027540968538212463`, 2177.2729976761966`, 0.021577241346914133`,
   9.805063749007854`, 112.67195629309921`, 0.5789560729980795`, 0.48649175775932635`, 93.78653116967105`,
   1.`, {{0, 0.004452800932852413`, 7.925985660477296`}, {36, 0.002674975436042587`, 4.761456276155805`}}},
  {0.14667176624268824`, 3.777226248423828`, 0.6973504720596608`, 0.7758342249413661`, 0.494379064234173`,
   0.5228383904297224`, 0.014800119213367281`, 81.50804211451084`, 0.12794308526059`, 9.842771813142875`, 91934.67769669769`,
   29683.642891247717`, 230.03945053579923`, 1213.5985023096987`, 0.`, {{0, 0.0922334861755371`, 164.17560539245605`}}},
  {0.15361999046758062`, 1.6832845721068805`, 5.017027003357642`, 1.2257765840301957`, 0.8758480691873987`,
   0.30492603190517764`, 0.17353190337485183`, 1218.2701158582597`, 0.01908888394356914`, 4.8706184705887665`, 88.4270901347789`,
   2.526885166117563`, 0.9939696265601635`, 34.66802175983304`, 1.`, {{0, 0.0016793034745330852`, 2.9891601846688918`},
    {8, 0.0007434659295958465`, 1.3233693546806067`}, {16, 0.00016749479733548723`, 0.2981407392571673`},
```

```
{24, 0.000036720913732328 75`, 0.06536322644354517`}, {32, 7.784538550563225`*^-6, 0.01385647862000254`}}},
{0.1285936813753743`, 1.2517058162042831`, 2.0098881407856393`, 1.434144339335301`, 0.06632650483661995`,
 0.16162194262920127`, 0.05011873598676769`, 354.0526180115314`, 0.1584833535532481`,
 2.894273123117289`, 3009.490192742772`, 1045.5460189364628`, 229.5264675110446`, 695.2328047548373`,
 0.`, {{0, 0.04036877149291295`, 71.85641325738507`}, {31, 0.0008047285908657821`, 1.432416891741092`},
  {62, 0.0007915186225938364`, 1.4089031482170289`}, {93, 0.0007924384336118413`, 1.4105404118290774`},
  {124, 0.0008047678543642436`, 1.4324867807683535`}, {155, 0.0007965437265711527`, 1.4178478332966518`},
  {186, 0.0007929089460209584`, 1.411377923917306`}, {217, 0.0007919776323699056`, 1.409720185618432`},
  {248, 0.0007916430971938942`, 1.4091247130051316`}, {279, 0.006102394764863069`, 10.862262681456262`}}},
{0.05871769486491246`, 0.822876240174752`, 3.5461717275684297`, 1.3634413021309426`, 0.14962847564878934`,
 0.2425078078443763`, 0.045889110192962865`, 1618.8495608622088`, 0.1824696213391762`, 5.484504386708753`,
 27.885896118678033`, 0.8565975782168966`, 0.5760875026781517`, 18.71679203978185`, 1.`,
 {{0, 0.001102050512382029`, 1.9616499120400117`}, {44, 0.0003204256826413919`, 0.5703577151016777`}}},
{0.21414990207917795`, 3.2291184159683555`, 3.7200101298578847`, 0.893435725539721`, 0.18550851463991336`,
 0.6299970912008048`, 0.007788531351629846`, 1185.7534950662077`, 0.10596189074334122`, 9.85351453889281`, 107.90952971362401`,
 0.2460298139094877`, 0.2460298139094877`, 107.90952971362401`, 1.`, {{0, 0.008201124258235425`, 14.598001179659056`}}},
{0.14399581513021464`, 3.6650427925648215`, 8.960857802594514`, 0.9993788071734813`, 0.7678347002262149`,
 0.3917750513543251`, 0.06821121121423936`, 446.58045234253916`, 0.09781275073737189`, 4.158206599173292`,
 196.1648640455883`, 1.8522388269208039`, 1.2692497680282349`, 132.64117561530554`, 1.`,
 {{0, 0.007454269170116519`, 13.268599122807405`}, {20, 0.0026264601766467035`, 4.675099114431132`}}},
{0.21762093156371237`, 1.4799568210517782`, 6.880501462590445`, 1.1700220578418317`, 0.49477438305707566`,
 0.30056004401666714`, 0.03776191240235276`, 139.28017196074`, 0.13055880095851463`,
 4.30304009869343`, 571.5957497424812`, 82.48349582490346`, 4.569739095273646`, 255.3825943748081`, 1.`,
 {{0, 0.016049588284497887`, 28.568267146406242`}, {3, 0.0028379040633541974`, 5.051469232770471`},
  {6, 0.0003538021433467008`, 0.6297678151571274`}, {9, 0.00006195213600290194`, 0.11027480208516545`},
  {12, 0.000031439828589867456`, 0.055962894889964074`}, {15, 0.000028731929439448016`, 0.051142834402217466`},
  {18, 0.000028531795716938044`, 0.05078659637614972`}, {21, 0.000017126991537472318`, 0.030486044936700728`}}},
{0.20676336326895633`, 3.023915070136316`, 2.1344025288266977`, 0.7647942269583019`, 0.38268253024987975`,
 0.6058201621166657`, 0.06287157366362177`, 52.11423962493933`, 0.23050267576131828`, 4.129012828530946`, 92316.29930931194`,
 23331.86381098574`, 229.6054173996737`, 1425.4726861652575`, 0.`, {{0, 0.10833592414855958`, 192.83794498443606`}}},
{0.14056217319026204`, 2.827702131488536`, 4.822127390400377`, 0.9317030787852092`, 0.5881617813064273`,
```

```
  0.663416624487535`, 0.01875862506453023`, 1486.513173237306`, 0.1539754165327772`, 8.90838886614388`, 127.34888668402371`,
  0.6651868390521599`, 0.6651868390521599`, 127.34888668402371`, 1.`, {{0, 0.009678515387985802`, 17.227757390614727`}}},
{0.15432054500385795`, 3.142590945055387`, 0.6412922856028498`, 0.9338764110697222`, 0.4036132844000331`,
  0.253764192888976`, 0.009287914173605602`, 800.3088033129984`, 0.0773663480819351`, 1.9942494398991926`,
  7595.197344877993`, 2366.979277674336`, 230.1790368014995`, 2004.3964421032658`, 0.`,
  {{0, 0.004054676381144608`, 7.217323958437403`}, {31, 0.00026336919479576064`, 0.46879716673645394`},
    {62, 0.000266593388580515`, 0.47453623167331677`}, {93, 0.00029152688914711077`, 0.5189178626818571`},
    {124, 0.0003879261844354418`, 0.6905086082950864`}, {155, 0.00044089299738474805`, 0.7847895353448516`},
    {186, 0.000403143306910424`, 0.7175950863005548`}, {217, 0.0003906066704336048`, 0.6952798733718164`},
    {248, 0.00038334424036626675`, 0.6823527478519549`}, {279, 0.1454520503466497 4`, 258.9046496170365`}}},
{0.1558313627948012`, 2.6183453593149384`, 1.5210778217010041`, 1.2213020905618333`, 0.8957140746385861`,
  0.2067404945185849`, 0.06533654021268133`, 3401.5830280519913`, 0.07866132934502523`, 1.2687008259414867`,
  6.28614533852348`, 0.10838774401076817`, 0.07802815617928208`, 3.804048619466861`, 1.`,
  {{0, 0.00015155462031396096`, 0.26976722415885046`}, {11, 0.00008778979693207564`, 0.15626583853909462`},
    {22, 0.000037116535607038276`, 0.06606743338052813`}, {33, 0.00001264674222640654`, 0.022511201163003643`}}},
{0.27749339228385056`, 3.960153029334033`, 7.7991060624230615`, 1.1159035815659704`, 0.47604804800745515`,
  0.18775668040836846`, 0.02760276902429692`, 890.1551806101527`, 0.14097999756547475`, 3.493022856148759`,
  38.071550460823495`, 0.19357192577812563`, 0.18306448616353`, 35.860314938093474`, 1.`,
  {{0, 0.0018877419791271043`, 3.3601807228462457`}, {15, 0.0008376419561679997`, 1.4910026819790394`}}},
{0.1388142019807993`, 3.4960143968183592`, 0.8496516336862268`, 1.4057148799798922`, 0.27289215927666`,
  0.4161162296591887`, 0.1071416651953492`, 563.7099077527477`, 0.24491201094978954`, 8.47264522499269`,
  7216.9781578825605`, 2400.5070871877256`, 219.4960173845712`, 937.7954128859501`, 0.`,
  {{0, 0.04237803479960282`, 75.43290194329302`}, {22, 0.0018118702540804387`, 3.225129052263181`},
    {44, 0.00155265137698008`, 2.763719451024542`}, {66, 0.0008589937571674401`, 1.5290088877580432`},
    {88, 0.0007298188044954466`, 1.299077472001895`}, {110, 0.0006687573344066075`, 1.1903880552437613`},
    {132, 0.0006417498021517183`, 1.1423146478300585`}, {154, 0.0006273293861453739`, 1.1166463073387656`},
    {176, 0.0006190680590307984`, 1.101941145074821`}, {198, 0.02138417780527148`, 38.06383649338323`}}},
{0.0729917462714273`, 3.1893255155706086`, 3.1041281178047555`, 1.014074092539919`, 0.28719491349307913`,
  0.2514291775131975`, 0.05245081359414425`, 423.2923229706584`, 0.11450677404966558`, 9.081189649788715`,
  203.0642495691459`, 1.9758391277026222`, 1.4618835522240405`, 139.8582507360827`, 1.`,
  {{0, 0.00709916160758055 9`, 12.636507661493397`}, {27, 0.0035300654483617264`, 6.2835164980838725`}}},
```

```
{0.11420284690084648`, 0.577787691297051`, 2.567084026939444`, 1.4491737131438955`, 0.8025258647238394`,
  0.3690270257872911`, 0.13672661029124697`, 86.53603740064875`, 0.011299238359278513`, 6.563498155623039`,
  1.2392422190728874`*^6, 470889.9852414057`, 228.27488347887643`, 1871.6605000986322`, 0.`,
  {{0, 0.019121536550371975`, 34.036335059662115`}, {4, 0.008730504347578407`, 15.540297738689567`},
   {8, 0.006895348940194253`, 12.273721113545772`}, {12, 0.00568442182605632`, 10.118270850380249`},
   {16, 0.0047970765790974505`, 8.538796310793463`}, {20, 0.004109414882889025`, 7.314758491542466`},
   {24, 0.0035563644007195073`, 6.330328633280724`}, {28, 0.0031000457593192334`, 5.518081451588235`},
   {32, 0.0027170561196373385`, 4.836359892954462`}, {36, 0.08353442860163256`, 148.69128291090598`}}},
{0.26373133325063186`, 3.0221428803953465`, 7.660698925394574`, 1.4577816176348994`, 0.5393449604126412`,
  0.22813670918251328`, 0.05400872825934766`, 78.22373236540282`, 0.11174025693188905`, 6.564224566850031`,
  102095.0625583668`, 21336.212617439414`, 229.08372612691534`, 1506.7923491713966`, 0.`,
  {{0, 0.11119746697015095`, 197.93149120686869`}, {31, 0.00048134015187573884`, 0.8567854703388151`},
   {62, 0.0002600712965188516`, 0.46292690780355583`}, {93, 0.00025963451525744404`, 0.46214943715825046`},
   {124, 0.0002594960697621533`, 0.46190300417663294`}, {155, 0.00025942514248368444`, 0.46177675362095827`},
   {186, 0.00025938731766701403`, 0.461709425447285`}, {217, 0.0002593668859508322`, 0.46167305699248135`},
   {248, 0.0002593561452296394`, 0.46165393850875813`}, {279, 0.001020674042129838`, 1.8167997949911119`}}},
{0.21289488654178568`, 3.397179033451687`, 0.7632571166797604`, 1.0142699830327386`, 0.0835760348267307`,
  0.5184383444209231`, 0.049731529265758516`, 2070.206130759874`, 0.06133590421436391`, 5.427522099525574`,
  29.086836908647165`, 0.3381904360055326`, 0.3296904328157547`, 28.828390132309213`, 1.`,
  {{0, 0.0017905861598253485`, 3.1872433644891207`}, {9, 0.0004003714902301515`, 0.7126612526096697`}}},
{0.22999162513252186`, 2.379033562263336`, 2.5591649989178986`, 1.3870393830660817`, 0.8120421313192066`,
  0.6084139253527632`, 0.017274035559015792`, 82.84440150538262`, 0.15048886650658344`, 7.465486944171833`, 163958.07412768368`,
  38223.2110663672`, 229.3798619007311`, 1638.7127575121426`, 0.`, {{0, 0.12454216957092284`, 221.68506183624265`}}},
{0.1769680798340355`, 1.1633029664887262`, 4.7984019847292405`, 1.1781683757559547`, 0.8107109545176789`,
  0.3886685867136115`, 0.009172138674078711`, 82.52474973299203`, 0.0366989899628154`,
  4.7780773811523165`, 340862.7226664043`, 96562.79876869603`, 230.1263228241041`, 1122.8387966870253`,
  0.`, {{0, 0.05973433248898331`, 106.32711183039031`}, {5, 0.006636869298343455`, 11.81362735105135`},
   {10, 0.0015429704952564895`, 2.7464874815565516`}, {15, 0.0006077392704536171`, 1.0817759014074386`},
   {20, 0.00040059218271207487`, 0.7130540852274933`}, {25, 0.00034402969336425065`, 0.6123728541883661`},
   {30, 0.00032429667752835125`, 0.5772480860004653`}, {35, 0.0003156988674882852`, 0.5619439841291476`},
   {40, 0.0003112545473045357`, 0.5540330942020736`}, {45, 0.015117965026779557`, 26.90997774766761`}}},
```

```
{0.06792516420354372`, 2.678804428347224`, 7.00137481313343`, 1.1889039591838686`, 0.130109850192629`, 0.17336690021836265`,
  0.006561591278144 7905`, 559.1859620197067`, 0.12479016185335001`, 7.679232726268854`, 100.64931860232065`,
  0.2293253839911767`, 0.2293253839911767`, 100.64931860232065`, 1.`, {{0, 0.007649348213776369`, 13.615839820521938`}}},
{0.04696109785508959`, 0.9059167168392257`, 1.3656449005023852`, 0.8785005151977332`, 0.7117341492461746`,
  0.37541668809892415`, 0.06884837024201017`, 2425.2572297139236`, 0.025009444315193585`,
  7.4432891311871785`, 58.154526164970484`, 2.101084016183464`, 1.3599778479351898`, 36.93443540064826`,
  1.`, {{0, 0.00203308141930644`, 3.618884926365464`}, {23, 0.0007739356711428276`, 1.377605494634233`}}},
{0.18481744149515084`, 2.8908169503663625`, 0.7719563125670259`, 1.477735031707543`, 0.05143123515478387`,
  0.4060758128174351`, 0.19176878359926766`, 114.34865924476063`, 0.02384868002335655`, 4.461346915500394`, 686710.1283072006`,
  188636.7440173377`, 230.22875504564598`, 1056.775293852154`, 0.`, {{0, 0.0803149223327637`, 142.9605617523194`}}},
{0.11940413672862621`, 0.6385297463968325`, 0.6629513696361364`, 1.2353159995947312`, 0.3854086082897601`,
  0.4772941268081121`, 0.0318040184551439`, 4110.432043833155`, 0.1129636900773972`, 2.562436110087784`,
  8.7154545791285`, 0.3192779264161698`, 0.3180030845145246`, 8.689162420441953`, 1.`,
  {{0, 0.0005828555552762622`, 1.0374828883917468`}, {7, 0.00007752078867 73262`, 0.13798700384564064`}}},
{0.16756813285689298`, 1.9469615336157506`, 0.3903678609266308`, 1.2604526712473199`, 0.11212576188863221`,
  0.45563235469719776`, 0.0065229352440185`, 190.16738409260836`, 0.045949495779464666`, 5.424569346408827`, 179836.78862903337`,
  52984.42555850293`, 230.16730822082104`, 997.0161789341979`, 0.`, {{0, 0.07577322959899904`, 134.8763486862183`}}},
{0.07183551197561577`, 3.34536856927257`, 9.136834569708476`, 1.4150518758492396`, 0.289745011670542`, 0.5142172775325705`,
  0.41137464999480466`, 1635.4988121892525`, 0.15390110584062433`, 5.743187227898911`, 45.403468842338704`,
  0.7899421396441832`, 0.7899421396441832`, 45.403468842338704`, 1.`, {{0, 0.0034506636320177416`, 6.142181264991581`}}},
{0.2384422062087977`, 2.1587544451320504`, 5.065398759698054`, 0.8914719118393273`, 0.7773656949632277`,
  0.1997211683380481`, 0.4677952481930321`, 402.45383708693856`, 0.19717109492345014`, 2.7966746550751953`,
  97.80449009759113`, 2.7255119309861584`, 0.7648580759453699`, 27.626977172196703`, 1.`,
  {{0, 0.0017782801105232534`, 3.165338596731391`}, {9, 0.0002977952202567255`, 0.5300754920569715`},
   {18, 0.0000153722179954912`, 0.027362548031974337`}, {27, 8.202716311479155`*^-6, 0.014600835034432897`}}},
{0.049151474922052546`, 1.7029958107544303`, 5.984640650536118`, 1.1787547055685845`, 0.01700706976856159`,
  0.4330742864041065`, 0.015091472025893045`, 551.6808300976855`, 0.14911096385124378`, 2.965493287771624`, 49.79231323763358`,
  0.366113839712773`, 0.366113839712773`, 49.79231323763358`, 1.`, {{0, 0.0037842158060601523`, 6.735904134787071`}}},
{0.05913727892151971`, 3.1040367182848003`, 9.835708161805986`, 1.4919208308945082`, 0.5501557876127892`,
  0.5378623906029458`, 0.015011170514212647`, 280.8434087480442`, 0.0269165475419888`, 2.8649095039964387`, 177.74352047826127`,
  0.8099459818717041`, 0.7887593137633028`, 176.4855763911425`, 1.`, {{0, 0.010987149426767607`, 19.557125979646344`},
```

```
{3, 0.002219496630587888`, 3.950704002446441`}, {6, 0.00020625774837133523`, 0.3671387921009767`}}},
{0.1028902787116574`, 3.248894721912677`, 2.8223976230891363`, 1.4617310236348016`, 0.21163710737184238`,
 0.2736720564831455`, 0.052563491644450414`, 157.16268136885162`, 0.03718011423514023`,
 9.1247890605319`, 132273.22917826028`, 53473.86134630606`, 230.03325500279172`, 1278.5773906708685`,
 0.`, {{0, 0.05440706390356071`, 96.84457374833806`}, {11, 0.0005096170178920219`, 0.907118291847799`},
  {22, 0.0003642742974182395`, 0.6484082494044664`}, {33, 0.0003838364490157074`, 0.6832288792479592`},
  {44, 0.0005102027865325368`, 0.9081609600279156`}, {55, 0.000903586057051836`, 1.6083831815522678`},
  {66, 0.000982960342727959`, 1.7496694100557673`}, {77, 0.0006597803292627511`, 1.174408986087697`},
  {88, 0.0005229368056848457`, 0.9308275141190254`}, {99, 0.03792762370183939`, 67.51117018927413`}}},
{0.13701717915337458`, 3.8023949322113495`, 2.868993121081161`, 1.1378721602373125`, 0.9948229422967421`,
 0.24001294614371227`, 0.1498271577324817`, 115.7171521166015`, 0.23683719126671393`,
 3.874936558168838`, 28805.699328342518`, 9711.936987847152`, 228.6350215168592`, 790.888505796149`,
 0.`, {{0, 0.04943271163130551`, 87.99022670372382`}, {27, 0.005548478597937392`, 9.876291904328559`},
  {54, 0.0007250813323008997`, 1.2906447714956015`}, {81, 0.0002350018539420009`, 0.41830330001676164`},
  {108, 0.00017812869223446048`, 0.3170690721773397`}, {135, 0.00016615930175284264`, 0.2957635571200599`},
  {162, 0.00016176649467435398`, 0.28794436052035005`}, {189, 0.00015971330615542034`, 0.28428968495664825`},
  {216, 0.0001586506757576973`, 0.2823982028487012`}, {243, 0.00334183455444675`, 5.948465506915214`}}},
{0.12767680767420947`, 3.2442504564252905`, 9.68431286349912`, 1.3341607174516774`, 0.1954172777492651`,
 0.4247465971362525`, 0.25891115414709387`, 128.98950675665216`, 0.14024381160911475`,
 6.508167926731167`, 541.0361674867996`, 10.886822705412685`, 5.3690276211588035`, 314.4648588533543`,
 1.`, {{0, 0.022615311197160936`, 40.255253930946466`}, {18, 0.001284018075693991`, 2.285552174735304`}}},
{0.15001338826271665`, 1.2298087626140388`, 4.614349177257099`, 1.406777099587714`, 0.7364706782176744`,
 0.4119762663671258`, 0.007028619260401152`, 151.01374639678102`, 0.013332718449329678`,
 9.705143720051169`, 337276.5667751779`, 107194.04092087985`, 49.79524005093154`, 608.9310658739143`,
 1.`, {{0, 0.03092376838271807`, 55.04430772123816`}, {4, 0.0017669723866614174`, 3.1452108482573227`},
  {8, 0.0017009157116310476`, 3.0276299667032647`}, {12, 0.0016990897308024784`, 3.0243797208284118`},
  {16, 0.0016989837806786724`, 3.024191129608037`}, {20, 0.0016989741583688172`, 3.0241740018964944`},
  {24, 0.0016989729992304622`, 3.0241719386302224`}, {28, 0.001698972830851374`, 3.0241716389154454`},
  {32, 0.0016989728029785925`, 3.0241715893018943`}, {36, 0.0016931382224965618`, 3.01378603604388`}}},
{0.20002396942953493`, 1.8945790659495865`, 6.47237980815893`, 1.0186003524309086`, 0.9948715481686918`,
 0.5599748214378268`, 0.06647091233379371`, 636.3545963390636`, 0.214859543893563`, 8.583745959022096`,
```

```
435.5091992219396`, 7.971146010554413`, 7.4920845297874274`, 424.5867797391667`, 1.`,
 {{0, 0.025701940411583434`, 45.74945393261851`}, {1, 0.005406062922530087`, 9.622792002103552`},
  {2, 0.0009916420283376335`, 1.7651228104409875`}, {3, 0.00016894989772551691`, 0.3007308179514201`}}},
{0.0426995949672023`, 2.62335318941125`, 9.6559263809094`, 1.1610440596461276`, 0.9636197200326426`, 0.24951659449471042`,
 0.04756766795277323`, 104.67802103676537`, 0.10920782191363448`, 6.683384921443938`, 532.8192282341329`,
 8.913068529453856`, 6.004322363527325`, 532.2272153959419`, 1.`, {{0, 0.033656853374940655`, 59.90919900739437`},
  {3, 0.0059072887804335955`, 10.514974029171801`}, {6, 0.0007525952601612414`, 1.3396195630870098`},
  {9, 0.0000971316194594542`, 0.1728942826378285`}, {12, 0.000019634593452840088`, 0.03494957634605354`},
  {15, 0.000011615519160437284`, 0.02067562410557837`}, {18, 4.149222483357872`*^-6, 0.007385616020377012`}}},
{0.2109009326123063`, 1.0923929642765158`, 8.593806993622852`, 1.1682615459081633`, 0.4499643089978256`,
 0.499787017067712`, 0.035780106156421375`, 2693.2423996223015`, 0.018435796880638622`,
 3.8866756448846873`, 21.33556390806201`, 0.4327824485320539`, 0.39446092745130085`, 19.37507496565752`,
 1.`, {{0, 0.0010539804093991178`, 1.8760851287304297`}, {36, 0.000418525287990854`, 0.74497501262372`}}},
{0.1340526977662379`, 3.5323141681247634`, 7.368159769503098`, 1.0026662265470512`, 0.9539160554997537`,
 0.16176440546396775`, 0.3999369934637664`, 62.1244000754222`, 0.21257466381414752`, 3.7256546669386505`,
 34792.509861961975`, 11866.046086593118`, 4.2692664408419105`, 214.45348416710385`, 1.`,
 {{0, 0.014980091839188048`, 26.664563473754722`}, {12, 0.0011257087275317154`, 2.0037615350064533`},
  {24, 0.00007303758995833576`, 0.13000691012583765`}, {36, 0.000019850773226311282`, 0.03533437634283408`},
  {48, 0.000016941129691949257`, 0.030155210851669677`}, {60, 0.000016727122404498586`, 0.029774277880007485`},
  {72, 0.000016702825417360767`, 0.029731029242902163`}, {84, 0.000016699445613635205`, 0.029725013192270666`},
  {96, 0.000016698947174059056`, 0.029724125969825113`}, {108, 0.000016006396493979924`, 0.028491385759283047`}}},
{0.138783517590022`, 3.0993312847332284`, 6.04052192414912`, 1.482957943290502`, 0.5742828550914962`,
 0.6307262387789596`, 0.006158597883984412`, 223.4781142247734`, 0.1875211630254222`, 1.5541408452833192`, 95.9704023462689`,
 3.5834500694329443`, 1.3948645502339263`, 93.99740850339165`, 1.`, {{0, 0.005686332223867728`, 10.121671358484557`},
  {4, 0.0012300891466233243`, 2.1895586809895176`}, {8, 0.00015474140029725928`, 0.27543969252912154`},
  {12, 0.000032061857478536435`, 0.05707010631179485`}, {16, 0.000040578417990917544`, 0.07222958402383323`}}},
{0.13089267345973798`, 3.445236909656975`, 9.841043091432308`, 1.0552044625838208`, 0.6603410104708771`,
 0.19921054981100283`, 0.006557758988700662`, 85.91513533777795`, 0.010838310480980468`, 8.608032245025406`, 40476.67770227611`,
 13867.062829681665`, 1.6237039722455913`, 719.5997295820805`, 1.`, {{0, 0.03735210746725839`, 66.48675129171993`},
  {5, 0.012846770937024292`, 22.867252267903236`}, {10, 0.003332852019580206`, 5.9324765948527665`},
  {15, 0.0008545583223247068`, 1.521113813737978`}, {20, 0.00022306434855218378`, 0.3970545404228871`},
```

```
{25, 0.00006265653748634461`, 0.11152863672569341`}, {30, 0.000017569816011996`, 0.031274272501352875`}}},
{0.0796809200271959`, 1.3022197759162255`, 4.457724373908043`, 0.819757860237272`, 0.9902524891863433`,
 0.3732869275715256`, 0.027666886465779635`, 3268.657024391763`, 0.02176038735057556`, 2.8954452621820828`,
 27.87668098845641`, 0.41283644867958724`, 0.38345725983632084`, 25.629905113002682`, 1.`,
 {{0, 0.0010593138303923103`, 1.8855786180983125`}, {22, 0.0008885589581958935`, 1.5816349455886904`}}},
{0.24784589915696786`, 0.6840125293527293`, 8.78766727614829`, 1.2765252846824708`, 0.05554020049431552`,
 0.46845321895484016`, 0.4169012658954577`, 507.69507152027995`, 0.17834533234287903`, 5.09521235383073`, 102.54709769645665`,
 7.994982119504308`, 5.1572816837848885`, 67.01703505956863`, 1.`, {{0, 0.004549115967612908`, 8.097426422350976`},
  {7, 0.0005354832731855023`, 0.953160226270194`}, {14, 8.695423728804904`*^-6, 0.01547785423727273`}}},
{0.1280056031133443`, 3.9743049299675812`, 6.787496113248967`, 1.4777257411519527`, 0.41697417826757177`,
 0.16856234271449722`, 0.016472885834375787`, 675.9035984651438`, 0.06538866409107441`, 1.0273997992144341`,
 12.90960733752032`, 0.060912857840721285`, 0.06085634323523742`, 12.864486476274628`, 1.`,
 {{0, 0.0008732063204119452`, 1.5543072503332624`}, {10, 0.00010449465178492657`, 0.18600048017716928`}}},
{0.2694840025057293`, 1.4398402455219186`, 1.997018914620883`, 1.3694289195380824`, 0.1761008172166878`, 0.6152080706831848`,
 0.020267602026759375`, 203.92916364743616`, 0.23224028188307977`, 5.1018397742421016`, 39622.275687718065`,
 8153.9655981178585`, 230.10970880621792`, 1287.9194711384023`, 0.`, {{0, 0.09788187980651858`, 174.22974605560307`}}},
{0.05785779964873633`, 3.866745902394764`, 0.7980363800640653`, 1.2385898080722695`, 0.5722218009229685`,
 0.20306189134783081`, 0.040832709866995026`, 2800.195372434598`, 0.18949558604342587`, 8.332936748246446`,
 34.127750333283466`, 0.28531609951459647`, 0.22207578324757887`, 22.33714906493113`, 1.`,
 {{0, 0.001193105847615732`, 2.123728408756003`}, {35, 0.0005045174813190338`, 0.8980411167478801`}}},
{0.2528189510796667`, 3.3250798883848782`, 4.1415523484020405`, 0.9119401620778833`, 0.8401965817654944`,
 0.5070162083757728`, 0.10337827675659322`, 381.96079877048203`, 0.13245695994197593`,
 7.95834379608233`, 287.51497260134045`, 5.333221335894053`, 3.8822772851423757`, 282.3270338679911`,
 1.`, {{0, 0.01880939126860364`, 33.48071645811448`}, {2, 0.002345544461649496`, 4.1750691417361026`},
  {4, 0.00020725549427270918`, 0.36891477980542225`}, {6, 0.000040787100667608 32`, 0.07260103918834279`},
  {8, 0.000029627313228211095`, 0.052736617546215746`}, {10, 0.000024248935545658977`, 0.043163105271272977`}}},
{0.20018502874097505`, 2.5781656282009475`, 6.942068190225046`, 0.9929389842661138`, 0.15941471595895984`,
 0.3421827829774996`, 0.4839576109293149`, 375.34729484852807`, 0.1596957174670693`, 8.895509004013046`,
 231.55234637931534`, 5.26173365271212`, 2.8085826325504297`, 125.77476693240457`, 1.`,
 {{0, 0.00843946069398716`, 15.022240035297147`}, {7, 0.0010880551670456589`, 1.936738197341273`},
  {14, 0.00003087146802415642`, 0.05495121308299843`}, {21, 4.949578057720166`*^-7, 0.0008810248942741895`}}},
```

```
{0.272440775669993`, 3.035804235517027`, 9.484814840956616`, 1.224288286053278`, 0.8025814347303761`,
  0.5890268382574878`, 0.3843203491912798`, 336.978314494752`, 0.04553466081406238`, 4.10287356038395`,
  311.86392382124046`, 6.384881042337917`, 3.3668907180504326`, 172.39530822172964`, 1.`,
  {{0, 0.010532173746005178`, 18.747269267889216`}, {4, 0.0022870173704072636`, 4.0708909193249285`},
   {8, 0.00025119629819210764`, 0.44712941078195156`}, {12, 0.00003165601024690232`, 0.056347698239486134`}}},
{0.20623021381347656`, 2.305665489644068`, 6.160210330863352`, 1.4997848102690652`, 0.31029755609374554`,
  0.2932546230651458`, 0.047029701289319936`, 605.575343672007`, 0.11131413350838143`, 4.271934365443833`,
  70.80595675888081`, 0.8507625892455735`, 0.6241925550413577`, 50.699136970010095`, 1.`,
  {{0, 0.0027444395859043316`, 4.88510246290971`}, {36, 0.001108694823816436`, 1.9734767863932563`}}},
{0.27799817940399246`, 2.8456068500128753`, 7.4650958706635215`, 1.2794446296629622`, 0.3185515090611555`,
  0.5440861077528716`, 0.2050159519579307`, 96.61991147082354`, 0.24522478882121745`, 9.22867476531573`,
  47930.32433071013`, 9539.051565914551`, 228.32347453050167`, 1791.5789400256065`, 0.`,
  {{0, 0.06871234370238855`, 122.30797179025161`}, {14, 0.04797596762088632`, 85.39722236517765`},
   {28, 0.010342128198791501`, 18.408988193848874`}, {42, 0.0025042612590056444`, 4.457585041030047`},
   {56, 0.0008304982522162406`, 1.4782868889449081`}, {70, 0.0002766964695508l863`, 0.49251971580045717`},
   {84, 0.00009184044744512169`, 0.1634759964523166`}, {98, 0.00003679054078925521`, 0.06548716260487428`},
   {112, 0.00002057461684422863`, 0.036622817982726964`}, {126, 0.005368898334028418`, 9.556639034570583`}}},
{0.18731205279881885`, 3.3920364906001828`, 9.53367346846489`, 1.150711712471592`, 0.269617044263482`,
  0.4407414224913243`, 0.0689482800442352`, 54.451263142554176`, 0.1525802464986004`, 2.2696676207606785`,
  105385.61397125013`, 28623.682756717877`, 228.25125169483317`, 1352.9804117700266`, 0.`,
  {{0, 0.06507896596734344`, 115.84055942187133`}, {13, 0.0008608149485598354`, 1.5322506084365068`},
   {26, 0.0009208580245265443`, 1.6391272836572488`}, {39, 0.002108217231352264 5`, 3.752626671807031`},
   {52, 0.0018667718109013388`, 3.3228538234043823`}, {65, 0.0010664360115396389`, 1.8982561005405574`},
   {78, 0.0008973461654022675`, 1.5972761744160362`}, {91, 0.0007928267140494653`, 1.4112315510080482`},
   {104, 0.00069502299618721 8`, 1.237140933213248`}, {117, 0.02853925142466002`, 50.79986753589484`}}},
{0.07598141369986516`, 3.9958506473293216`, 3.287957942109602`, 1.3569966945049934`, 0.38975760386481206`,
  0.6382110342097327`, 0.02530287278972352`, 4836.578585308585`, 0.06124631354128618`, 7.15493409138095`, 23.53519249599506`,
  0.11512591908703132`, 0.11512591908703132`, 23.53519249599506`, 1.`, {{0, 0.0017886746296956245`, 3.183840840858212`}}},
{0.21782025688272888`, 3.800225088388993`, 1.7719223148490801`, 1.3823014458300409`, 0.9034484926621247`,
  0.21119490910082483`, 0.008011320454875006`, 549.8645476667474`, 0.16991323645757794`,
  1.3522541901702763`, 33.4745444670415`, 3.4545364941737593`, 0.4235816604049635`, 22.069128182261682`,
```

```
1.`, {{0, 0.0009340881623308515`, 1.6626769289489156`}, {9, 0.0004653359948626457`, 0.8282980708555092`},
   {18, 0.0001705843226254502`, 0.3036400942733099`}, {27, 0.0000658599826586238`, 0.11723076913235035`},
   {36, 0.00002968416413894393`, 0.05283781216732019`}, {45, 0.000011701115235367918`, 0.020827985118954895`}}},
{0.12917865835123116`, 3.4713877505357775`, 5.802428296169513`, 1.4965547140183602`, 0.061568597458759644`,
 0.5580721907461164`, 0.2777380762847274`, 381.024601401567`, 0.014984965589617844`, 6.51082587242481`,
 190.56984665272216`, 3.0255243254216286`, 1.9514864433138248`, 122.08518573225435`, 1.`,
 {{0, 0.008834816130774591`, 15.725972712778772`}, {15, 0.0004436579848767394`, 0.7897112130805961`}}},
{0.12408328322582662`, 2.9809522601952096`, 4.3074275514408615`, 1.1040930179447719`, 0.6796918144407815`,
 0.5931470332615022`, 0.005369942238542098`, 502.88367879292525`, 0.06875474145528054`,
 6.831128450148093`, 246.37891169750367`, 1.2213672474058272`, 0.7997289757449559`, 249.68688979314146`,
 1.`, {{0, 0.01572882794593813`, 27.997313743769872`}, {7, 0.0032473756783406196`, 5.780328707446303`}}},
{0.2301374553102561`, 3.6285008777535515`, 6.8583997394674485`, 1.291013232269163`, 0.6431022470858925`,
 0.6246309281545763`, 0.18498728864660263`, 132.58216720365365`, 0.011374938240932164`,
 6.052136677369937`, 453621.9727944937`, 105719.75889089226`, 84.19170871000557`, 755.5663338445595`,
 1.`, {{0, 0.03891048398155535`, 69.26066148716852`}, {16, 0.014657287495846653`, 26.08997174260704`},
   {32, 0.002991798420536178`, 5.325401188554397`}, {48, 0.0008634714742483397`, 1.5369792241620446`}}},
{0.18974259222357154`, 1.3104397818774611`, 5.6865397453703626`, 1.3109561369338216`, 0.42925562092874125`,
 0.6235284138933523`, 0.06332638490149935`, 1635.1348002386078`, 0.08473820825835521`, 3.2599591654117823`,
 32.271097197560664`, 0.7816333756394138`, 0.6408796886606892`, 26.223964492618762`, 1.`,
 {{0, 0.0015451428606542`, 2.7503542919644763`}, {26, 0.0004478784407848256`, 0.7972236245969896`}}},
{0.2309824769455583`, 1.2471345787782342`, 1.0215019422726535`, 1.0312798793045614`, 0.7898876098277747`,
 0.6152586670568927`, 0.006979047924720161`, 310.31208829749596`, 0.24081833704073502`,
 8.344184598629436`, 18896.563466806674`, 4379.240374494906`, 229.18957218327873`, 53100.54455426177`,
 0.`, {{0, 0.035463875243398064`, 63.12569793324855`}, {5, 0.017717071031703877`, 31.5363864364329`},
   {10, 0.01433337871260535`, 25.513414108437527`}, {15, 0.01288224063115974`, 22.93038832346434`},
   {20, 0.01246532212782791`, 22.18827338753368`}, {25, 0.012680341601811725`, 22.571008051224872`},
   {30, 0.01332173156223584`, 23.712682180779797`}, {35, 0.014270524755569723`, 25.40153406491411`},
   {40, 0.015449456435887042`, 27.500032455878934`}, {45, 3.887057444021695`, 6918.962250358618`}}},
{0.1769680396277803`, 1.6046472005597128`, 8.695674701006173`, 0.7703180616996756`, 0.764260429383415`,
 0.1992432648214062`, 0.012440799720035065`, 274.53159119005693`, 0.16375790457626532`,
 6.298702841855883`, 324.10454017424814`, 2.069701351489611`, 1.838270048978943`, 288.47616638394175`,
```

```
  1.`, {{0, 0.014532838836375381`, 25.868453128748175`}, {26, 0.007391349808804191`, 13.15660265967146`}}},
{0.23450402912552432`, 3.0762297015464597`, 2.593937576427738`, 1.1039402177647473`, 0.4424612211889203`,
  0.6666998367628838`, 0.44528706596344714`, 463.5224063697932`, 0.20661121111075748`, 4.223015851637719`,
  1155.1398207121988`, 244.56164517111125`, 4.08500835976621`, 112.20331798220302`, 1.`,
  {{0, 0.007760464836841428`, 13.813627409577741`}, {3, 0.00045955084312543934`, 0.818000500763282`},
   {6, 0.00005077861227676967`, 0.09038592985265002`}, {9, 0.00004349662552851103`, 0.07742399344074963`},
   {12, 0.000043386613586881216`, 0.07722817218464857`}, {15, 0.00004338326948475712`, 0.07722221968286769`},
   {18, 0.00004338301212494317`, 0.07722176158239885`}, {21, 0.00008300835367870175`, 0.1477548695480891`}}},
{0.2755523363386182`, 3.945462181815995`, 5.876565708554855`, 1.4395306041819111`, 0.28572441613120825`,
  0.3101513953075886`, 0.39757529919045964`, 3302.499033894636`, 0.07285156297216239`, 3.794286848749824`, 11.653610063142143`,
  0.17367995126498226`, 0.17367995126498226`, 11.653610063142143`, 1.`, {{0, 0.0008856743647988029`, 1.576500369341869`}}},
{0.1912671247263708`, 2.190434692724648`, 6.310276417856439`, 0.9527360176604049`, 0.6104292936966895`,
  0.2522020081398627`, 0.010518470607815496`, 284.3254609055096`, 0.1005439259073278`, 6.418961425083908`,
  295.60125963965584`, 1.334370721944137`, 1.1723486018986922`, 271.72715719153047`, 1.`,
  {{0, 0.01662461056758136`, 29.59180681029482`}, {13, 0.004026653378974955`, 7.16744301457542`}}},
{0.2597914621776059`, 0.5051242650756058`, 1.0118962727972`, 0.8968589895031354`, 0.07276014875602654`, 0.6898744359309281`,
  0.03193322527994425`, 3177.5504656788207`, 0.10860322045075349`, 3.7076953543063436`, 14.58393856542346`,
  0.5703817529608101`, 0.5703817529608101`, 14.58393856542346`, 1.`, {{0, 0.001108379330972183`, 1.972915209130486`}}},
{0.058009311425788745`, 1.2821997162065424`, 2.0542232518025845`, 1.4132652568351216`, 0.6228832317992945`,
  0.16342794118309045`, 0.044680537911227475`, 201.38656694501756`, 0.06413530627116476`,
  9.590029577588982`, 38611.37494832676`, 21032.588839353055`, 230.11910782025458`, 586.7558460631279`,
  0.`, {{0, 0.025610719360597903`, 45.58708046186426`}, {26, 0.0020396734655307542`, 3.630618768644742`},
   {52, 0.0020394621917419866`, 3.630242701300736`}, {78, 0.00203944341932315`, 3.630209286395207`},
   {104, 0.0020394390669620567`, 3.6302015391924605`}, {130, 0.0020394378652230966`, 3.630199400097112`},
   {156, 0.0020394375219903874`, 3.6301987891428893`}, {182, 0.002039437423297973`, 3.630198613470392`},
   {208, 0.0020394373948813735`, 3.630198562888845`}, {234, 0.002666956591249042`, 4.747182732423294`}}},
{0.19641852899773504`, 2.1398264375306333`, 5.320272455292722`, 1.1437489346098513`, 0.5956525384016933`,
  0.542078693849333`, 0.2264882318745723`, 868.9198508977777`, 0.040131295366706066`, 7.0792614425236255`, 156.24157045842674`,
  3.77834556720008`, 2.68790905774112`, 111.32684037599364`, 1.`, {{0, 0.006319907164820471`, 11.249434753380438`},
   {5, 0.0018792019445971722`, 3.344979461382966`}, {10, 0.0002617307591578729`, 0.46588075130101375`}}},
{0.09296068013545083`, 1.2991493564864323`, 0.26387265122439096`, 0.9093728692378444`, 0.35146944109549483`,
```

```
0.5215346752572301`, 0.009103351916553084`, 3690.6422305701226`, 0.14178477746644713`,
  8.458943719263925`, 330.9853641115859`, 133.94357152293685`, 48.637314463291034`, 133.5413190792638`,
  1.`, {{0, 0.005118235171472737 5`, 9.110458605221474`}, {8, 0.0013094918477720555`, 2.330895489034259`},
   {16, 0.001309479041611848`, 2.3308726940690896`}, {24, 0.0024119341891674076`, 4.293242856717986`}}},
{0.09381338426095137`, 2.303475789921615`, 0.696644313515133`, 0.9364121618865129`, 0.9663355677423924`,
  0.5212308135336106`, 0.24626013703252375`, 1385.562539836289`, 0.20934361709679128`,
  7.7725134925150865`, 644.4886236714975`, 256.3426912021232`, 6.231402692202652`, 62.98992274078501`, 1.`,
  {{0, 0.00440829994605495 7`, 7.846773903977823`}, {20, 0.0003789341822447049`, 0.6745028443955746`}}},
{0.10039832860995906`, 2.055462616090492`, 1.0012272143748433`, 1.102551063992137`, 0.7631597204325553`,
  0.3864879566262683 4`, 0.021413905310258614`, 653.3550083645581`, 0.055519462287367316`,
  4.402154187880063`, 11462.535829451072`, 4694.144499481846`, 229.8682276325651`, 620.6342243145833`,
  0.`, {{0, 0.033056614413829156`, 58.8407736566159`}, {31, 0.0004513053962542659`, 0.8033236053325933`},
   {62, 0.0004441841926023 6025`, 0.7906478628322012`}, {93, 0.00044361699113351475`, 0.7896382442176564`},
   {124, 0.0004432807697835402`, 0.7890397702147016`}, {155, 0.0004430104633845512`, 0.7885586248245011`},
   {186, 0.0004427829927075596 7`, 0.7881537270194564`}, {217, 0.000442588194434448`, 0.7878069860933175`},
   {248, 0.0004424185976109483 4`, 0.7875051037474881`}, {279, 0.010558399036167994`, 18.79395028437903`}}},
{0.16980453046164407`, 1.1980162751260872`, 9.435065425338543`, 1.2054727390347812`, 0.15783339617816905`,
  0.5987514193964574`, 0.2639813183074236`, 624.8721767770594`, 0.14884913893972124`, 1.0597319526281854`,
  20.34133516162648`, 0.8865357031902038`, 0.73612577161772`, 16.894279749921154`, 1.`,
  {{0, 0.0011088870009500346`, 1.973818861691061`}, {6, 0.00017507826004397312`, 0.3116393028782722`}}},
{0.1106187483985549`, 0.9718711331456742`, 7.0205608914526`, 1.3199794157480607`, 0.2870266036717726`, 0.2842604073783652`,
  0.12440193317594331`, 1067.250206889352`, 0.20077332983402157`, 1.344770962154577`, 11.869506742128625`,
  0.5090076740217098`, 0.3601646081895556`, 8.406438198545201`, 1.`, {{0, 0.0004401757057245553`, 0.7835127561897085`},
   {10, 0.00017889949673849457`, 0.3184411041945204`}, {20, 0.000019814100626385463`, 0.035269099114966125`}}},
{0.22322153760577584`, 3.532203149750069`, 4.201686494998453`, 0.809746094501941`, 0.17157339217602185`,
  0.16416014932043588`, 0.005086092661785311`, 81.42460105493491`, 0.09946867199032089`,
  6.2584574953677805`, 88862.3611754758`, 21166.30589216803`, 228.25902175429835`, 107235.61418157646`,
  0.`, {{0, 0.038122411160817875`, 67.85789186625583`}, {19, 0.0008070604565450507`, 1.4365676126501905`},
   {38, 0.00082775653113722`, 1.4734066254242515`}, {57, 0.001030045225147157`, 1.8334805007619395`},
   {76, 0.001870613249406775`, 3.3296915839440597`}, {95, 0.003665696851941795`, 6.524940396456395`},
   {114, 0.004813419607449154`, 8.567886901259493`}, {133, 0.0047138853165290115`, 8.390715863421642`},
```

{152, 0.004566400999790864`, 8.128193779627738`}, {171, 8.089489388401047`, 14399.291111353865`}}},
{0.11344753764961812`, 0.6515213816912864`, 6.9223785093110415`, 0.7705690533378804`, 0.053758347821018404`,
0.22122664443377937`, 0.23238889135484875`, 1612.9665239639735`, 0.23659636795633343`, 7.8050806983048435`, 36.58172375869865`,
2.6556328223964183`, 2.6556328223964183`, 36.58172375869865`, 1.`, {{0, 0.0027802110056610978`, 4.948775590076754`}}},
{0.05086592503936643`, 0.6345548618632657`, 4.1212243894392255`, 0.9757969272512688`, 0.4720155756440034`,
0.3491598005177542`, 0.10619345643493772`, 163.13358888497802`, 0.24650090837908084`,
9.412849245254243`, 6601.556914453258`, 3655.1627103657056`, 229.50548375304552`, 720.0738711075254`,
0.`, {{0, 0.05169677728719346`, 92.02026357120437`}, {7, 0.00022809560906709062`, 0.4060101841394213`},
{14, 0.00006916509211758054`, 0.12311386396929337`}, {21, 0.00006831452283325233`, 0.12159985064318915`},
{28, 0.00006826642333233274`, 0.12151423353155229`}, {35, 0.00006825870355423345`, 0.12150049232653555`},
{42, 0.00006825826166614502`, 0.12149970576573815`}, {49, 0.00006826143865390593`, 0.12150536080395255`},
{56, 0.00006826875128129787`, 0.12151837728071022`}, {63, 0.002321948114472626`, 4.133067643761275`}}},
{0.18344840265344153`, 3.595124583701854`, 4.199071914809606`, 1.0537057639813663`, 0.12507220232647875`,
0.4903855977737973`, 0.3568003256791643`, 83.2177314580599`, 0.1899680886834239`, 7.625578775230776`,
70364.39573615063`, 19367.190175423224`, 228.5350544998478`, 1608.3478282917897`, 0.`,
{{0, 0.03795264670948308`, 67.5557111428799`}, {3, 0.008630121887994344`, 15.361616960629933`},
{6, 0.004509951011968413`, 8.027712801303775`}, {9, 0.00273949037411507`, 4.876292865924825`},
{12, 0.0018270437788200783`, 3.25213792629974`}, {15, 0.0012935193763776466`, 2.302464489952211`},
{18, 0.0009603770021077172`, 1.7094710637517367`}, {21, 0.0007475555683082734`, 1.3306489115887268`},
{24, 0.000610784728309644`, 1.0871968163911663`}, {27, 0.06296294451269177`, 112.07404123259136`}}},
{0.11456761033436458`, 3.133847301219358`, 1.1215010618012613`, 1.2799949994814193`, 0.7250649174618937`,
0.2748176967060689`, 0.05347527364447488`, 4312.248498236981`, 0.1363057898672484`, 6.512229405589068`, 24.422370873582945`,
0.2625099849060634`, 0.21922233566128282`, 19.867754655068005`, 1.`, {{0, 0.000856677614464915`, 1.5248861537475487`},
{9, 0.0004969357299616261`, 0.8845455993316945`}, {18, 0.00015633600935862718`, 0.2782780966583564`}}},
{0.2733102228285163`, 0.9906705811868095`, 1.8602414047947509`, 1.4451100284824412`, 0.2848844825831507`,
0.554129635350794`, 0.011612796259976983`, 302.8864567264163`, 0.1580490958422761`, 2.3921629765885086`, 31773.060719865047`,
6471.474625651964`, 230.25394573640904`, 1178.0740085401033`, 0.`, {{0, 0.08953362464904785`, 159.36985187530516`}}},
{0.13611751569452646`, 1.612649572698647`, 9.438389550358693`, 0.8529700096304889`, 0.38386891649316235`,
0.16702474363312747`, 0.015283436679942941`, 2091.084776788581`, 0.04888707786432367`, 3.7550559614266916`,
15.362296473715713`, 0.11406299951985402`, 0.10182222569814636`, 13.602946605483456`, 1.`,
{{0, 0.0006538343535463969`, 1.1638251493125864`}, {70, 0.00037998958847034587`, 0.6763814674772156`}}},

```
{0.06405507608053002`, 1.3528358541887275`, 2.745226236199782`, 1.0639243234460365`, 0.36966523495796855`,
  0.3262944894947615`, 0.12612633222162115`, 59.71942118372549`, 0.2416255104540047`, 1.4175051543133999`, 45852.432199162926`,
  23927.30288934075`, 230.2533367179935`, 574.8080579858077`, 0.`, {{0, 0.04368541240692139`, 77.76003408432008`}}},
{0.20954125488723696`, 2.353113320872523`, 8.807677395871629`, 1.2953396401945112`, 0.754037675145256`,
  0.6760987806408354`, 0.0257504108557459`, 115.6397082887867`, 0.08726278776335833`, 1.3938676616131378`, 39748.23335577215`,
  9927.122256000317`, 38.66263909762041`, 272.985119457459`, 1.`, {{0, 0.01903927448969614`, 33.88990859165912`},
    {4, 0.0008927126487111918`, 1.5890285147059213`}, {8, 0.00013585242980441545`, 0.24181732505185946`},
    {12, 0.00012190884841783799`, 0.21699775018375161`}, {16, 0.00012153277995539729`, 0.21632834832060718`},
    {20, 0.00012150778671460236`, 0.2162838603519922`}, {24, 0.00012150496877847046`, 0.2162788444256774`},
    {28, 0.00012150454330939297`, 0.21627808709071947`}, {32, 0.00007107058337943732`, 0.12650563841539844`}}},
{0.12907099769038483`, 2.604139615282878`, 9.661547795493739`, 1.0824039436985886`, 0.5892380332813292`,
  0.2973399722037613`, 0.047844179127263436`, 189.25086236267023`, 0.09074457722059731`,
  6.471849996577652`, 471.91695910904076`, 5.198177803260762`, 3.6633979207109637`, 321.7695932081626`,
  1.`, {{0, 0.020443437613958212`, 36.38931895284562`}, {34, 0.004011051469862145`, 7.139671616354618`}}},
{0.2477279255184573`, 2.8408395942947395`, 2.128090335748766`, 1.4989746812981888`, 0.49854461135120975`,
  0.5556994499234343`, 0.04795352432490085`, 88.91629411642595`, 0.17336384521962678`, 2.1291108959892835`, 142269.56679491166`,
  31336.44149286737`, 230.023323718808`, 1269.401374616121`, 0.`, {{0, 0.0964745044708252`, 171.72461795806885`}}},
{0.17138396941106926`, 2.571547212239655`, 8.060362653989774`, 1.424545143219825`, 0.3585647265624352`,
  0.4155041928809974`, 0.0058496443892025185`, 1444.9053577030656`, 0.14315469959013916`, 8.6363710850225`, 74.42149826534792`,
  0.15959185013186267`, 0.15959185013186267`, 74.42149826534792`, 1.`, {{0, 0.005656033868166442`, 10.067740285336267`}}},
{0.046024454977220214`, 1.8018961949102152`, 1.8889771640586375`, 1.2101342547143499`, 0.33571961358008684`,
  0.4325808303075249`, 0.005213559292121167`, 454.61857984803544`, 0.13129751322022398`,
  8.467405558196546`, 3466.722922722436`, 2008.9773008731768`, 229.01488382291737`, 1265.0188890170023`,
  0.`, {{0, 0.024176980615619324`, 43.0350254958024`}, {15, 0.0018844967982371934`, 3.354404300862204`},
    {30, 0.0018844942862212993`, 3.354399829473913`}, {45, 0.0018844952039971782`, 3.3544014631149777`},
    {60, 0.001884504977989145`, 3.354418860820678`}, {75, 0.0018846234742296633`, 3.354629784128801`},
    {90, 0.0018861989982098789`, 3.357434216813584`}, {105, 0.0019078737364938956`, 3.396015250959134`},
    {120, 0.002183872720845553`, 3.8872934431050847`}, {135, 0.05656389475344904`, 100.68373266113929`}}},
{0.15552548139256261`, 1.3158122598456092`, 2.9803095987090753`, 0.857616115693088４`, 0.19622947720693906`,
  0.41449590928153224`, 0.03892742585126438`, 2129.9557657820133`, 0.1528564450859799`, 8.904626117418289`,
  44.784464050096815`, 0.8020597327934813`, 0.6817009455745405`, 37.91976599655222`, 1.`,
```

```
{{0, 0.0020421786920470195`, 3.635078071843695`}, {38, 0.0008397235236909496`, 1.49470787216989`}}},
{0.20226341603934178`, 2.23359139800782`, 8.92211343859698`, 1.0176324123098368`, 0.17770526936314845`,
 0.5466941503204505`, 0.02261985699694341`, 208.46398724865384`, 0.0356723302826476`, 4.800574849057675`, 272.62929382791367`,
 2.0742704271559353`, 2.0742704271559353`, 272.62929382791367`, 1.`, {{0, 0.02071982633092144`, 36.881290869040164`}}},
{0.1612087208534252`, 3.1252528746197825`, 0.891851633192701`, 1.468881451365178`, 0.6554353298599058`,
 0.19493335173588566`, 0.018954452797492544`, 551.070999166768`, 0.11474062580409572`,
 7.406826770535428`, 12565.975767021757`, 3789.1423323508475`, 101.87319683404178`, 392.8602631195093`,
 1.`, {{0, 0.02592091490836619`, 46.13922853689182`}, {31, 0.000551965550060503 3`, 0.9824986791076958`},
  {62, 0.00024036392303221335`, 0.4278477829973397`}, {93, 0.0002387603668659475`, 0.4249934530213865`},
  {124, 0.00023847887340977273`, 0.4244923946693955`}, {155, 0.00023837116213214113`, 0.42430066859521115`},
  {186, 0.0002383287271773691`, 0.42422513437571696`}, {217, 0.000238311941076446 3`, 0.42419525511607437`},
  {248, 0.00023830529438616875`, 0.42418342400738035`}, {279, 0.0017135792505759532`, 3.0501710660251966`}}},
{0.2365245631846375`, 1.6915599352668007`, 9.677850457164503`, 0.7567859979545984`, 0.725618873672359`,
 0.2424250415266589`, 0.36826031218846694`, 892.5435235500784`, 0.17475643237365474`, 6.493737892117458`, 108.87173098858135`,
 3.593289437652701`, 1.1004808963696124`, 34.60075836823942`, 1.`, {{0, 0.0020230943636887207`, 3.6011079673659223`},
  {8, 0.0005606623384577634`, 0.9979789624548188`}, {16, 0.00004258596659740502`, 0.07580302054338094`},
  {24, 2.70649312663511`*^-6, 0.004817557765410496`}, {32, 6.084741156718943`*^-7, 0.0010830839258959717`}}},
{0.22790261965108521`, 1.8196334440146895`, 4.716201039891137`, 1.2810805872690838`, 0.6237465626441163`,
 0.5616811859186371`, 0.03042013590776336`, 637.6118251230974`, 0.08011306001688712`, 9.216145754556873`,
 297.65408263271866`, 3.4435924355889447`, 3.2254077197652085`, 279.69791738488937`, 1.`,
 {{0, 0.016740075361578857`, 29.79733414361037`}, {15, 0.004516966359672732`, 8.040200120217463`}}},
{0.10184647957852327`, 3.0801202872185955`, 8.412464860766345`, 0.9942252517730743`, 0.7984635108014702`,
 0.4565736536432896`, 0.009191281945386102`, 103.82351882247931`, 0.22274711417837167`,
 2.5201460619086546`, 8657.49900539858`, 3452.9184606595545`, 128.1066915953676`, 508.92095177715817`,
 1.`, {{0, 0.032898596865815415`, 58.55950242115144`}, {25, 0.005779395469248605`, 10.287323935262517`}}},
{0.18768732765242752`, 1.4966861207841546`, 1.129759551095228`, 1.2240080138702374`, 0.5362083693873154`,
 0.686948995479657`, 0.005867661202712241`, 74.92355531972449`, 0.165025723744549`, 3.2590978632956187`, 141080.67716032866`,
 38316.12545784039`, 229.9399089134957`, 1293.518292276483`, 0.`, {{0, 0.09830739021301271`, 174.98715457916265`}}},
{0.14949493319923252`, 0.867900380757324`, 9.54711835147964`, 0.8271983961134595`, 0.23831574478972195`,
 0.375815029784779`, 0.0451684556906921 8`, 2094.687766706356`, 0.0279158592619283`, 5.743304871768913`, 28.82201261093747`,
 0.8292396437558637`, 0.8292396437558637`, 28.82201261093747`, 1.`, {{0, 0.0021904729584312477`, 3.899041866007621`}}},
```

{0.10785599050108607`, 1.35490623294936`, 7.749733046704847`, 1.2972878372630243`, 0.6070878493023009`,
0.6937073302769028`, 0.28374543116039413`, 66.83074779041495`, 0.1939262594152683`, 9.425365274493942`,
75173.25433684074`, 29365.66695444302`, 229.82747690333053`, 1765.384753263916`, 0.`,
{{0, 0.08157838144377229`, 145.20951896991468`}, {3, 0.013879048124225577`, 24.704705661121526`},
{6, 0.004503554239647066`, 8.016326546571777`}, {9, 0.0017828953242725214`, 3.173553677205088`},
{12, 0.000867456612176433`, 1.5440727696740506`}, {15, 0.0005180240429751678`, 0.9220827964957987`},
{18, 0.00036857621353954164`, 0.6560656601003843`}, {21, 0.00029700231626366786`, 0.5286641229493287`},
{24, 0.0002583085422078689`, 0.4597892051300066`}, {27, 0.03011599438897749`, 53.60647001237994`}}},
{0.13969386881974227`, 3.165109549305347`, 4.62533417349867`, 1.0389272094346946`, 0.7061162924631639`,
0.5973647953915502`, 0.19864442086151357`, 3440.0036010322765`, 0.16071761832429643`, 1.3360604448248414`, 9.20219692500833`,
0.15607929252218125`, 0.15607929252218125`, 9.20219692500833`, 1.`, {{0, 0.0006993669663006331`, 1.244873200015127`}}},
{0.18701505616348296`, 1.1471835668231432`, 1.9652898147298057`, 1.007866470439728`, 0.8673024418570234`,
0.4964521059357`, 0.11361709646832255`, 189.11029986824036`, 0.17527157137077032`, 1.608206453868661`,
29410.57255772462`, 8004.147886617053`, 228.8215260421501`, 912.6133348635481`, 0.`,
{{0, 0.004915672824935782`, 8.749897628385693`}, {8, 0.002226612230645810 4`, 3.9633697705495425`},
{16, 0.0012369473570921974`, 2.2017662956241115`}, {24, 0.0007713149268560854`, 1.372940569803832`},
{32, 0.0005232602321286967`, 0.9314032131890801`}, {40, 0.00037867834910616576`, 0.6740474614089751`},
{48, 0.0002893861952608089`, 0.5151074275642399`}, {56, 0.00023225500958603913`, 0.4134139170631496`},
{64, 0.00019483114188711037`, 0.34679943255905643`}, {72, 0.058589655182130954`, 104.28958622419309`}}},
{0.12078071855653999`, 3.7816423804380825`, 3.0527531818157634`, 1.3792123087306463`, 0.18684955879429088`,
0.29398025647621684`, 0.011105429740027619`, 471.0240529628659`, 0.1734264666127333`, 9.864280424563177`,
194.00178108156413`, 0.5982490569955066`, 0.590268159681205`, 195.13860787831928`, 1.`,
{{0, 0.012974823465895891`, 23.095185769294687`}, {6, 0.0018557107328563748`, 3.3031651044843473`}}},
{0.12022941283008587`, 2.2264210098571606`, 6.2451953049416655`, 1.3438290154028227`, 0.5358944653344513`,
0.32618163573980585`, 0.199782178892696`, 3338.755251465298`, 0.1788085018354036`, 4.512919273292992`, 18.095061850982948`,
0.4073810956226061`, 0.4073810956226061`, 18.095061850982948`, 1.`, {{0, 0.001375224700674704`, 2.447899967200973`}}},
{0.04681963491488872`, 2.902738250264786`, 8.529727895043578`, 1.3985345888875074`, 0.35876551940164103`,
0.2520539039703089`, 0.05124631524713002`, 163.50426816944002`, 0.24665810410586203`, 8.118758344754049`,
498.79726428600173`, 5.0753168845019845`, 3.6650900136267595`, 342.5182679134838`, 1.`,
{{0, 0.020091569586623136`, 35.762993864189184`}, {30, 0.005939818774801632`, 10.572877419146904`}}},
{0.1911559295611578`, 2.175586072104755`, 6.8117639016300915`, 1.0586708066957606`, 0.34517099105389626`,

0.196211531611845`, 0.1489787018319715`, 3704.576077864889`, 0.10408111144660032`, 4.8300445149655875`, 11.585309266571299`,
0.24218990208485922`, 0.24218990208485922`, 11.585309266571299`, 1.`, {{0, 0.0008804835042594188`, 1.5672606375817655`}}},
{0.17192712556630163`, 3.7260903741599414`, 4.118781130521407`, 1.2245874304759414`, 0.6062180312792598`,
0.5331783629983038`, 0.1203111914746069`, 226.51013650082294`, 0.017839169449302028`, 7.150760371208259`,
96985.85234012778`, 27992.572669250974`, 228.65985600749167`, 1058.4230460232186`, 0.`,
{{0, 0.07861749346555294`, 139.93913836868424`}, {27, 0.0006800990147546101`, 1.2105762462632057`},
{54, 0.00035914287443262913`, 0.6392743164900798`}, {81, 0.00009317607480107482`, 0.16585341314591318`},
{108, 0.00007625909015420806`, 0.13574118047449035`}, {135, 0.00007201455436366682`, 0.12818590676732694`},
{162, 0.00007122154823480898`, 0.12677435585795999`}, {189, 0.00007104440357954007`, 0.12645903837158134`},
{216, 0.00007100588924872833`, 0.12639048286273644`}, {243, 0.000328694582642413`, 0.5850763571034951`}}},
{0.1575695043509842`, 3.8758817205894927`, 3.3547063374262347`, 1.3334178116995439`, 0.4921394422884451`,
0.402330971011554`, 0.12204131883407823`, 201.57298534982124`, 0.22899817129952033`,
2.4884430439924596`, 13254.481155256848`, 4056.772415350327`, 172.3026285854497`, 636.6360081090488`,
1.`, {{0, 0.04101014683099802`, 72.99806135917648`}, {31, 0.003059440194853924`, 5.445803546839984`},
{62, 0.0006214255142462348`, 1.106137415358298`}, {93, 0.0001531401858173825`, 0.27258953075494086`},
{124, 0.00009219863600462298`, 0.16411357208822888`}, {155, 0.00007915230265019042`, 0.14089109871733893`},
{186, 0.00007460261826752016`, 0.13279266051618588`}, {217, 0.00007249394715170316`, 0.12903922593003161`},
{248, 0.0000713168211930756`, 0.12694394172367454`}, {279, 0.003150419565105037`, 5.6077468258869665`}}},
{0.06573776532431397`, 2.613579102769201`, 3.806271127858438`, 1.1314163600990936`, 0.4483602258230772`,
0.6823320235871475`, 0.18768855278686034`, 189.85674956999534`, 0.0631967400861071`,
1.3499467761035964`, 36717.984603400604`, 18912.528429782837`, 229.7575055391861`, 1342.768787617225`,
0.`, {{0, 0.017212874070146808`, 30.638915844861323`}, {5, 0.0009460294433935883`, 1.683932409240587`},
{10, 0.0001879793817231475`, 0.3346032994672025`}, {15, 0.0001448658129369067`, 0.2578611470276939`},
{20, 0.00014061221234089815`, 0.25028973796679865`}, {25, 0.00014399719269137713`, 0.2563150029906513`},
{30, 0.0001529139960797522`, 0.2721869130219591`}, {35, 0.0001602542849759128`, 0.28525262725712475`},
{40, 0.0001583761427043158`, 0.28190953401368213`}, {45, 0.08280252532191638`, 147.38849507301114`}}},
{0.06757463956251891`, 2.3005441553217736`, 6.868151131987258`, 1.3860241166029201`, 0.025379963517129678`,
0.6856836713524472`, 0.1183550729914282`, 3863.192203814942`, 0.1108156231672533`, 2.823244077255117`,
8.59241485568045`, 0.16028049249456805`, 0.15022896655322654`, 8.043039776910419`, 1.`,
{{0, 0.0005550701120053112`, 0.988024799369454`}, {10, 0.00005620091103988064`, 0.10003762165098753`}}},
{0.06632069369203508`, 0.7568152865276287`, 0.22726671156421221`, 1.362987063673106`, 0.9228910129465389`,

```
  0.1868567265073786`, 0.1296811990167267`, 123.29501155053357`, 0.19348527358813117`, 9.470702921306387`, 40466.119973540954`,
  20771.37897756188`, 230.2566567298926`, 551.6349641900315`, 0.`, {{0, 0.041924257278442394`, 74.62517795562746`}}},
 {0.1352416913789251`, 3.40347330811998`, 4.693938060979022`, 1.2778213334701187`, 0.7052646641955478`,
  0.6043325609148968`, 0.013064813980624626`, 333.49561209931903`, 0.11153764285740131`,
  8.005884287110216`, 334.87069873308224`, 3.5502324151301963`, 1.872308935732637`, 341.1775376890767`,
  1.`, {{0, 0.02167589060428523`, 38.58308527562771`}, {3, 0.0037575041260732773`, 6.688357344410433`},
   {6, 0.0004106740790294589`, 0.7309998606724368`}, {9, 0.00005689307864168057`, 0.10126967998219141`},
   {12, 0.000022588975783934463`, 0.04020837689540364`}, {15, 5.942000556248625`*^-6, 0.010576760990122553`}}},
 {0.11675242158451127`, 2.833886804623358`, 0.47584343623935504`, 1.0607616820427617`, 0.7666611109645796`,
  0.4737758025893787`, 0.1681420982868212`, 120.33220123866614`, 0.17915094289032796`, 3.5814785888826783`, 50878.84686836332`,
  19065.874044117078`, 230.17849069949685`, 844.1347197482464`, 0.`, {{0, 0.06415423870086673`, 114.19454488754279`}}},
 {0.24323887781179143`, 3.4699698183729026`, 8.39097272098094`, 1.1938324247391086`, 0.7463001660827029`,
  0.6306644967356683`, 0.2992521819376612`, 322.6100644812281`, 0.10289378547186823`,
  7.076414822100832`, 486.63939727132947`, 8.58357024732422`, 5.45783562750253`, 328.5589748893577`, 1.`,
  {{0, 0.0244098257162847 5`, 43.44948977498685`}, {12, 0.0005606563753064381`, 0.9979683480454599`}}},
 {0.24390696598302103`, 0.8206355382610386`, 2.839705408503791`, 1.0872176805395473`, 0.8466198387041834`,
  0.5785697848880403`, 0.1356337036504287`, 2343.8028799398503`, 0.22246042363346175`, 4.459217822696726`, 62.83767603591975`,
  3.2789800675303953`, 2.4607964618091214`, 47.18839327696716`, 1.`, {{0, 0.002221020748957598`, 3.9534169331445246`},
   {4, 0.0010591929133892863`, 1.8853633858329297`}, {8, 0.0003061042267026202`, 0.544865523530664`}}},
 {0.06769491456810739`, 3.362305338073525`, 1.8871867183871915`, 1.4803795481758868`, 0.5363168854717104`,
  0.37458837250617005`, 0.011183256466341666`, 407.888606379481`, 0.051902827430772014`,
  9.719761204172809`, 16144.112966006198`, 8129.534711127395`, 229.1129551302731`, 648.5265618565245`, 0.`,
  {{0, 0.025311044037172385`, 45.053658386166845`}, {11, 0.0001314461649274001`, 0.23397417357077216`},
   {22, 0.00010931324346555348`, 0.1945775733686852`}, {33, 0.00010930143049135693`, 0.19455654627461533`},
   {44, 0.00010930058193984194`, 0.19455503585291864`}, {55, 0.00010930043286475884`, 0.19455477049927072`},
   {66, 0.0001093004433605998`, 0.1945547891818676`}, {77, 0.0001093005249464851`, 0.19455493440474347`},
   {88, 0.00010930065625066004`, 0.1945551681261749`}, {99, 0.02308041118567682`, 41.08313191050473`}}},
 {0.21105892687940375`, 0.7047276416914965`, 5.768010388957135`, 0.810198720595394`, 0.17857267890229922`,
  0.19525448649651245`, 0.036176279358565294`, 67.82203673017167`, 0.07478241594027568`,
  5.650997388906681`, 128879.69321226129`, 32043.778704423217`, 228.5511589149654`, 1663.0153386952832`,
  0.`, {{0, 0.07881992125150342`, 140.2994598276761`}, {28, 0.001303221451346696`, 2.319734183397119`},
```

```
{56, 0.00199118644248467`, 3.544311867622712`}, {84, 0.0025572858894926413`, 4.551968883296901`},
{112, 0.0017770361995033171`, 3.163124435115905`}, {140, 0.0015442136393237847`, 2.7487002779963365`},
{168, 0.0014007380488122846`, 2.4933137268858667`}, {196, 0.0013177959955833088`, 2.3456768721382897`},
{224, 0.0012703280141114863`, 2.2611838651184457`}, {252, 0.034407438808679934`, 61.24524107945029`}}},
{0.13030085884608883`, 3.691040694570585`, 3.5546925177950874`, 1.392417125140739`, 0.5227226143537942`,
0.3153665172319682`, 0.008712610924073824`, 695.2883528301467`, 0.04802018879975545`, 9.078480195703946`,
172.6046088439054`, 0.4314355501621877`, 0.42232054011311176`, 170.71406592177888`, 1.`,
{{0, 0.010887890065931053`, 19.380444317357274`}, {7, 0.0020863789441241424`, 3.7137545205409737`}}},
{0.21250061023000932`, 2.8149517595358526`, 2.295631419336827`, 1.0543549080196901`, 0.6519014079329188`,
0.6534060058028184`, 0.09088447710322001`, 650.6798329466749`, 0.04016188412840693`,
1.3861729584655489`, 35.94675041263064`, 2.1358757630756697`, 1.0209119278763652`, 32.25520973982862`,
1.`, {{0, 0.001806955161001833`, 3.2163801865832626`}, {3, 0.000493019415906506`, 0.8775745603135806`},
{6, 0.00008173992124738679`, 0.14549705982034847`}, {9, 0.00002447651100633778`, 0.043568189591281246`},
{12, 0.000017741024709180735`, 0.031579023982341706`}, {15, 0.000027463906355731167`, 0.048885753313201476`}}},
{0.14347475236734758`, 3.69539554202769`, 4.673626861854084`, 0.8876695712812951`, 0.05519972772145398 4`,
0.3291461786147223`, 0.031312636638690675`, 836.9629099625735`, 0.07687709551472927`, 5.335882334514919`,
54.829959766225926`, 0.3215530909691539`, 0.2830032959301143`, 46.93800793718517`, 1.`,
{{0, 0.0026669303821566796`, 4.74713608023889`}, {49, 0.0009003582210693933`, 1.60263763350352`}}},
{0.0805761857877269`, 3.094619489710217`, 9.945877040804334`, 1.11372690786775`, 0.6430409751953907`, 0.42229007304684385`,
0.05506378209236767`, 302.8456666998151`, 0.050941087889112335`, 7.632080993355595`, 453.37242002750685`,
4.446786026547152`, 4.318148650517267`, 441.0808864137011`, 1.`, {{0, 0.029368067868941614`, 52.275160806716066`},
{2, 0.003802435948593307`, 6.768335988496087`}, {4, 0.00035164354990636253`, 0.6259255188333253`}}},
{0.26865716137877294`, 2.5539808428845117`, 7.725254140854183`, 1.421576189719117`, 0.2484256166411507`,
0.1545757521332899`, 0.008556741498662016`, 1177.1024036480305`, 0.08015420001442447`, 4.125195480316323`, 26.423863208177067`,
0.08235183332333182`, 0.08235183332333182`, 26.423863208177067`, 1.`, {{0, 0.0020082136038214573`, 3.5746202148021937`}}},
{0.05207035897383483`, 1.5461440288878858`, 9.695008671418819`, 0.9618476339297223`, 0.8208653770038725`,
0.48583250041037973`, 0.22651174945218666`, 56.560953214157614`, 0.1695178841867513`, 7.246656846935364`,
43198.164833436254`, 24447.260685794765`, 229.29206841217686`, 1177.3520565528033`, 0.`,
{{0, 0.06366596356755791`, 113.3254151502531`}, {7, 0.0005538920085467948`, 0.9859277752132948`},
{14, 0.00035644099664873703`, 0.6344649740347519`}, {21, 0.00035000724708947534`, 0.6230128998192661`},
{28, 0.0003491808213486847`, 0.6215418620006588`}, {35, 0.0003489790957103199`, 0.6211827903643693`},
```

```
{42, 0.00034891589046099367`, 0.6210702850205686`}, {49, 0.0003488767305020218`, 0.6210005802935988`},
{56, 0.00034882300424914905`, 0.6209049475634854`}, {63, 0.022807676935898972`, 40.59766494590017`}}},
{0.09188932948313594`, 3.928289836924776`, 1.4719659367359732`, 1.3955743442040527`, 0.5432280807299197`,
0.32698938665455834`, 0.013007122973082902`, 2762.880112969401`, 0.13947463148509326`, 4.274432647648432`,
21.04301641057549`, 0.09481649215093979`, 0.09446900417927186`, 21.05905522020263`, 1.`,
{{0, 0.0014073567441248254`, 2.505095004542189`}, {5, 0.0001931314526105746`, 0.34377398564682277`}}},
{0.04429611977972214`, 3.507177302954225`, 7.234549676890296`, 0.7623401767303408`, 0.010148640019661537`,
0.662687845406686`, 0.08775344792279212`, 3965.4644576584396`, 0.17480873042969614`, 5.118348118050843`,
14.840432833946723`, 0.160429806805405`, 0.14981443817883183`, 13.86067295057423`, 1.`,
{{0, 0.0008233447718388603`, 1.4655369387317 12`}, {11, 0.00023006637240478107`, 0.4095181428805103`}}},
{0.14748247662042147`, 2.024427515527907`, 9.506929645646245`, 1.2214601071407813`, 0.2054881053340767`,
0.2020185622482833`, 0.07806490597707526`, 302.23580657852216`, 0.2425913704635762`, 6.045088630499148`,
161.50635741738552`, 2.7987098119519116`, 1.4667302801541355`, 85.27117052621904`, 1.`,
{{0, 0.005400779803841752`, 9.613388050838319`}, {37, 0.001079829156150894`, 1.9220958979485911`}}},
{0.05191368478403385`, 1.8292747620439185`, 5.888828307542417`, 1.141375509610469`, 0.9869968200599144`,
0.28747330669734017`, 0.24632331590197062`, 4082.2304223282904`, 0.0447948152020462`, 8.998917183247265`, 57.792533896672`,
1.6537748454077743`, 1.6537748454077743`, 57.792533896672`, 1.`, {{0, 0.004392232576147072`, 7.818173985541788`}}},
{0.22928260338606776`, 2.222139650206395`, 4.336407604983492`, 1.4545262441104416`, 0.17670484975018264`,
0.15215120204412502`, 0.00931582374755147`, 107.9598634817244`, 0.18384173269782222`,
2.0618261552271964`, 43912.86751160214`, 10255.111630467081`, 229.90183120207132`, 1771.9428951123161`,
0.`, {{0, 0.015256620131665125`, 27.15678383436392`}, {31, 0.0004196935505295989`, 0.7470545199426861`},
{62, 0.0004209746620227895`, 0.7493348984005653`}, {93, 0.00043657104064535 9`, 0.7770964523487391`},
{124, 0.0005543530824097727`, 0.9867484866893954`}, {155, 0.0009295472378593043`, 1.654594083389562`},
{186, 0.0009552812908212064`, 1.7004006976617474`}, {217, 0.000821262396622874`, 1.4618470659887155`},
{248, 0.0007941434726532388`, 1.413575381322765`}, {279, 0.11407921316330676`, 203.06099943068605`}}},
{0.13057179539780978`, 1.32287055831243`, 2.838629919542937`, 0.8498204532586893`, 0.8949438289681946`,
0.46229782752490833`, 0.06831034580553909`, 355.6715560557054`, 0.046330937271765626`,
4.510601406855825`, 5133.779070705771`, 1755.649544665958`, 4.076322692130164`, 121.55814611629032`,
1.`, {{0, 0.00836450903233895`, 14.88882607756333`}, {3, 0.0007145751020562052`, 1.2719436816600451`},
{6, 0.00005149130224085021`, 0.09165451798871337`}, {9, 0.00002274249033117823`, 0.04048163278949725`},
{12, 0.000021894599460804527`, 0.03897238704023206`}, {15, 0.000021873219636261058`, 0.038934330952544685`},
```

{18, 0.000021872431297599214`, 0.038932927709726596`}, {21, 0.000019460927476214607`, 0.034640450907662004`}}},
{0.16617324858328203`, 1.7665378156673919`, 1.5537022391595983`, 1.3738499573440288`, 0.09625830296842142`,
0.5757224393125404`, 0.011187699121044243`, 1140.5955024024072`, 0.09128758443232887`, 2.6133507581824666`,
26.706628066836902`, 0.24742650616504958`, 0.24457291525505376`, 27.46176613117568`, 1.`,
{{0, 0.001745545245444746`, 3.107070536891648`}, {1, 0.00034154898052460574`, 0.6079571853337982`}}},
{0.150610971367461`, 1.9051770945291775`, 1.9970914506574022`, 1.332029884280907`, 0.017627751946745374`,
0.6342635790195508`, 0.03224365795250487`, 84.16237057378729`, 0.025181518731154096`, 6.093120594420567`, 745302.1534159952`,
236454.3723120988`, 228.73060795723134`, 1364.939714732923`, 0.`, {{0, 0.10373541831970215`, 184.64904460906985`}}},
{0.170193722625894`, 0.42908710938865147`, 8.009796665724636`, 0.9108642019460496`, 0.48256370773751156`,
0.1504864882770205`, 0.015501266854459406`, 231.18780020236528`, 0.059845835174358764`,
2.951447491236335`, 113.43542295065046`, 2.856663886412237`, 2.373783519176844`, 93.58129004676354`,
1.`, {{0, 0.0045691807627337485`, 8.133141757666072`}, {69, 0.002542997280820281`, 4.5265351598601`}}},
{0.14155286072340578`, 2.768731185287475`, 2.866796585793665`, 0.9268420036617817`, 0.38841014261523243`,
0.6739227770046636`, 0.08018336457681292`, 85.63532436040691`, 0.10664760400309609`, 4.590937221386067`, 110072.15334528571`,
36386.75376131949`, 229.3699148548497`, 1258.7948849326685`, 0.`, {{0, 0.09566841125488282`, 170.2897720336914`}}},
{0.17254585725985527`, 2.4767354176461422`, 1.5265879737476418`, 1.3533294324013416`, 0.010476993931212819`,
0.3024537953894164`, 0.005382899126762437`, 1067.886819263142`, 0.025600178888030733`, 4.688973100729083`,
35.70334589010506`, 0.13582099415323615`, 0.13544712618423352`, 36.23651713092898`, 1.`,
{{0, 0.0024149748859071396`, 4.298655296914708`}, {1, 0.00033900041604346314`, 0.6034207405573644`}}},
{0.12080218784009655`, 2.1868434076988192`, 8.77616791832854`, 0.9616886370191489`, 0.26020855267635734`,
0.6201783502287406`, 0.03811904516021991`, 117.06014479510438`, 0.17593156125676795`,
4.349098586723594`, 9884.244212671638`, 3505.7643145341494`, 131.6604382967754`, 1357.069496843839`,
0.`, {{0, 0.04179083501572555`, 74.38768632799147`}, {22, 0.003572906433942544`, 6.359773452417729`},
{44, 0.00357291901554233`, 6.359795847665347`}, {66, 0.003574459932959975`, 6.362538680668755`},
{88, 0.00370158292265462`, 6.588817602325235`}, {110, 0.003770938838527046`, 6.712271132578142`},
{132, 0.0036538695218247407`, 6.503887748848038`}, {154, 0.003633911824108049`, 6.4683630469123266`},
{176, 0.003609558573043412`, 6.425014260017272`}, {198, 0.032256299681803484`, 57.41621343361021`}}},
{0.06629025039264891`, 1.530609759106781`, 1.900084106095834`, 1.2729265107514505`, 0.009336607275947317`,
0.20692029297881853`, 0.07757288734346537`, 56.094679581136695`, 0.10338780030561462`, 4.34093728430811`, 139732.2680952435`,
71735.8099343002`, 230.25723729370466`, 640.0113984158165`, 0.`, {{0, 0.04864086627960206`, 86.58074197769167`}}},
{0.11054921486908309`, 1.9601623754484976`, 4.1405236153603`, 1.0982919546164496`, 0.2371613718862391`, 0.581139907733862 7`,

```
0.014126785714663546`, 63.169894546693705`, 0.15558848051416968`, 8.651696062244092`, 102818.72606315033`,
  39750.25146239912`, 229.08826257744093`, 1542.320627915232`, 0.`, {{0, 0.11721636772155765`, 208.6451345443726`}}},
{0.09844925238529889`, 2.9547765664281824`, 3.97533240344332`, 0.8901779978265776`, 0.6485331039194935`,
  0.172046787329591`, 0.2843834249851245`, 265.56383421685405`, 0.07856132944640065`, 2.1823933339229455`, 69.3512138766139`,
  1.7033671684593807`, 0.6411904753268423`, 28.455307952130124`, 1.`, {{0, 0.0015539684312676119`, 2.766063807656349`},
   {10, 0.0005276924946412216`, 0.9392926404613745`}, {20, 0.00006476715273412251`, 0.11528553186673808`},
   {30, 7.058231528471488`*^-6, 0.012563652120679248`}, {40, 9.117094190461817`*^-6, 0.016228427659022035`}}},
{0.07379407071323735`, 1.0020834251379886`, 3.8959201685323244`, 1.3275435329440872`, 0.5495795554378471`,
  0.5203295123605114`, 0.006236204073249997`, 495.6126936444482`, 0.05339932410900344`, 4.300380134475608`, 139.97973413292246`,
  1.3297593826183745`, 1.0156244930537361`, 138.02382312796638`, 1.`, {{0, 0.00938729070463417`, 16.709377454248823`},
   {5, 0.00104662590432925`, 1.862994109706065`}, {10, 0.00005589394876202498`, 0.09949122879640446`}}},
{0.137830658132162`, 0.964244066849937`, 5.678313655870262`, 1.056362681888576`, 0.27898051740088525`,
  0.6674603903550571`, 0.022240144180942223`, 3206.233069292432`, 0.13749704640301397`, 7.378694929535063`, 33.32057608226024`,
  0.5407800240294687`, 0.5407800240294687`, 33.32057608226024`, 1.`, {{0, 0.0025323637822517783`, 4.507607532408166`}}},
{0.09412349267697989`, 2.412142947637907`, 8.593900106949853`, 1.0545990130363978`, 0.8443965196041454`,
  0.4441557500447094`, 0.020813982545198062`, 67.68562305269263`, 0.21037364591803787`,
  1.9282456472076213`, 33026.472906715855`, 14027.82655406001`, 229.80372781814376`, 775.3535237157879`,
  0.`, {{0, 0.040545638872487584`, 72.1712371930279`}, {6, 0.007874142035548912`, 14.015972823277062`},
   {12, 0.0010505776143507144`, 1.8700281535442718`}, {18, 0.00019928397757074356`, 0.35472548007592347`},
   {24, 0.00008324237207279701`, 0.14817142228957866`}, {30, 0.0000643711958709558`, 0.11458072865030132`},
   {36, 0.00006016977185872394`, 0.10710219390852861`}, {42, 0.00005881591534965492`, 0.10469232932238576`},
   {48, 0.00005823929820472589`, 0.10366595080441209`}, {54, 0.008932386749085072`, 15.89964841337143`}}},
{0.059604065321211375`, 2.060924190389435`, 4.151577648882975`, 1.0218595307135387`, 0.8259153199816331`,
  0.67511248087556`, 0.012173840559063499`, 163.32402156550611`, 0.22896057989768043`, 4.41755081858776`,
  12073.803854849582`, 6434.010498545403`, 228.29075658908496`, 683.5258640048322`, 0.`,
  {{0, 0.01890324523122268`, 33.64777651157637`}, {17, 0.001318163176511957`, 2.3463304541912833`},
   {34, 0.0013173267944813541`, 2.34484169417681`}, {51, 0.0013181114304026544`, 2.3462383461167238`},
   {68, 0.00132120922900474`3`, 2.3517524276284423`}, {85, 0.0013321048627861144`, 2.3711466557592837`},
   {102, 0.0013665595957037839`, 2.432476080352735`}, {119, 0.0014431441659523857`, 2.568796615395246`},
   {136, 0.0015082398448268552`, 2.684666923791802`}, {153, 0.02211986133347472`, 39.373353173584995`}}},
{0.06230382839069248`4, 3.237012523552033`, 1.9717708145626371`, 0.9726411613979222`, 0.1873520229034391`,
```

```
0.5560570337810927`, 0.026607710207102397`, 62.963998927305475`, 0.022649002664224704`, 8.659052494024476`, 551979.9411245878`,
  291967.5233098147`, 229.33170894147332`, 1277.7047408254523`, 0.`, {{0, 0.09710556030273437`, 172.8478973388672`}}},
{0.058207327156306`, 1.429925878818433`, 8.458349879741764`, 1.1245084153106533`, 0.19874114947102073`,
  0.3371100826465334`, 0.04401493286992449`, 375.7204818983131`, 0.2159694260931322`, 8.59363725607883`,
  224.38273198488443`, 3.983022346366739`, 2.9838107140054766`, 168.4267712018572`, 1.`,
  {{0, 0.010061306829231231`, 17.90912615603159`}, {42, 0.0027391277821099172`, 4.875647452155652`}}},
{0.0526423548412821`, 1.6513512325831732`, 4.083783641771872`, 1.373155154639566`, 0.8939370398042139`,
  0.2364887781328291`, 0.0490949664593259`, 1618.3201075393572`, 0.07451928539997066`, 4.7651781050102695`,
  55.91013911539432`, 0.9574522235324223`, 0.5975318987076149`, 33.89006849238964`, 1.`,
  {{0, 0.0017846495889077385`, 3.1766762682557745`}, {33, 0.0007909956165138741`, 1.407972197394696`}}},
{0.21322884859448837`, 1.5615738832323176`, 8.40996483053398`, 1.2929614343326012`, 0.3765546169235392`,
  0.671739728089668`, 0.1608794324574438`, 485.1832614690647`, 0.0705178455433636`, 2.1513131242884285`,
  70.01350433462389`, 2.102705540113824`, 1.990224061662053`, 66.28962648695152`, 1.`,
  {{0, 0.004217839415474184`, 7.507754159544047`}, {4, 0.0008201721975341313`, 1.4599065116107537`}}},
{0.11682922382752631`, 0.5721136495795571`, 9.687079392836907`, 0.91254998083727`, 0.43543762812336806`,
  0.4740169657662334`, 0.10620954791167687`, 4372.038997558794`, 0.0729116423313943`, 5.645356891940546`, 18.442711478826446`,
  1.195429498620114`, 1.195429498620114`, 18.442711478826446`, 1.`, {{0, 0.00140164607239081`, 2.4949300088556416`}}},
{0.26956767736567855`, 0.8444303847788461`, 1.0020073721955711`, 0.7502641425367418`, 0.966405616850982`,
  0.6715103286393609`, 0.23328928938153737`, 246.8438332486948`, 0.10178810353613177`, 2.531792031152129`, 46725.70937092259`,
  9629.245644818157`, 230.1099262203983`, 1226.9060235274467`, 0.`, {{0, 0.09324485778808596`, 165.975846862793`}}},
{0.07372356616920961`, 2.107710372997479`, 6.08076458707294`, 0.8379230467338923`, 0.9571077593283461`,
  0.37371168094765217`, 0.1939664465355383`, 131.4329681577719`, 0.026286944156709513`, 5.373906305884013`, 47586.938025175004`,
  23049.58521453898`, 32.559059234494725`, 393.70922076164373`, 1.`, {{0, 0.014239999793477355`, 25.347199632389692`},
   {3, 0.001981714501135058`, 3.5274518120204035`}, {6, 0.0019577839529364067`, 3.4848554362268045`},
   {9, 0.001957146891679881`, 3.483721467190188`}, {12, 0.001957115803158658`, 3.483666129622411`},
   {15, 0.0019571135289657383`, 3.4836620815590145`}, {18, 0.001957113308661122`, 3.483661689416798`},
   {21, 0.001957113282675028`, 3.48366164316155`}, {24, 0.001956799715195676`, 3.4831034930483034`}}},
{0.2128059772734779`, 3.088861612111426`, 5.437928143232815`, 1.2368766361188581`, 0.5161736795080103`,
  0.22159582314348258`, 0.17105793552921358`, 1293.4067318655482`, 0.22754002195492767`,
  9.689789287743999`, 83.94591454059119`, 1.3087448876614043`, 0.557383469236617`, 36.27238869649826`,
  1.`, {{0, 0.002064480256171733`, 3.674774855985685`}, {12, 0.0006112186064926608`, 1.0879691195569363`},
```

```
{24, 0.00007456661524767509`, 0.13272857514086164`}, {36, 6.436063021798843`*^-6, 0.011456192178801 94`}}},
{0.09487232790202699`, 3.2902977287485546`, 3.9754892015026773`, 1.2274952505878522`, 0.2657953162834845`,
 0.47376077240276926`, 0.26204707729007887`, 80.73499841507218`, 0.18195262450329797`,
 6.708419132160676`, 61736.54428786671`, 26121.965329469243`, 229.11282702793324`, 1491.1360990346911`,
 0.`, {{0, 0.028927746069262655`, 51.491388003287526`}, {3, 0.00824479695228216`, 14.675738575062244`},
  {6, 0.004843924147894142`, 8.622184983251572`}, {9, 0.003217252181543743`, 5.726708883147862`},
  {12, 0.002315424376630646`, 4.12145539040255`}, {15, 0.0017480478284185746`, 3.111525134585063`},
  {18, 0.0013554028924468102`, 2.412617148555322`}, {21, 0.0010683207936848568`, 1.9016110127590449`},
  {24, 0.0008540070441782879`, 1.5201325386373523`}, {27, 0.06075142124029466`, 108.1375298077245`}}},
{0.05710457017724263`, 2.2468049124614753`, 4.035987116327698`, 0.7517191945594467`, 0.40903626729229714`,
 0.6582116219387326`, 0.04046385674463665`, 815.0779000126262`, 0.186726177475597`, 7.464159626289564`,
 151.31900803907894`, 1.6913653827565691`, 1.6720276122412772`, 149.0295841806289`, 1.`,
 {{0, 0.009315194939024359`, 16.58104699146336`}, {20, 0.0020110534587034377`, 3.579675156492119`}}},
{0.1356890149134694`, 1.6504364529320803`, 8.903330095009764`, 0.8652149931542472`, 0.7882939470826769`,
 0.5853867881019097`, 0.12191584954894137`, 2002.2565687817998`, 0.19189449537551667`, 8.0829058846114`, 117.04286954479666`,
 3.01263703155388`, 3.01263703155388`, 117.04286954479666`, 1.`, {{0, 0.008895258085404546`, 15.833559392020094`}}},
{0.21947794079961902`, 1.5730764365956098`, 2.118289006802259`, 0.973502067608673`, 0.7638917093126882`,
 0.33981639014260245`, 0.009615294802656809`, 1765.6534921210653`, 0.16626255675053375`,
 4.010994756897377`, 43.75467344861621`, 0.2738991110392781`, 0.25784514626206717`, 41.69282833024699`,
 1.`, {{0, 0.0024275074256297287`, 4.320963217620917`}, {8, 0.0007411475274690425`, 1.3192425988948957`}}},
{0.2345658346849942`, 2.081301191128982`, 3.6234436876627587`, 1.1134590239676974`, 0.8739003578896458`,
 0.6696041669913848`, 0.06590896300137702`, 558.8606273551264`, 0.09113830356870467`, 1.2137876330314246`, 41.63461180591715`,
 1.4099272842698602`, 1.0419861156362902`, 40.314479471253684`, 1.`, {{0, 0.002488565993080148`, 4.429647467682663`},
  {3, 0.00047076706166219507`, 0.8379653697587073`}, {6, 0.00007283347614659932`, 0.12964358754094676`},
  {9, 0.000027631536319875687`, 0.04918413464937872`}, {12, 4.1023726064619055`*^-6, 0.0073022232395021916`}}},
{0.23053748957807368`, 3.8668800248110458`, 5.535000453565214`, 1.1361194466757834`, 0.24488877583427326`,
 0.5300195072451362`, 0.08816752471869817`, 3094.9084693615328`, 0.16131439142801796`, 1.2619255962975515`, 4.93320059035842`,
 0.053021790115783986`, 0.053021790115783986`, 4.93320059035842`, 1.`, {{0, 0.00037492324486723997`, 0.6673633758636872`}}},
{0.05839671607004826`, 2.466861519533315`, 8.278076935325572`, 0.9179604454629582`, 0.7485509623778881`,
 0.3739704677333707`, 0.3976055017684991`, 334.0137006202725`, 0.18218149392519362`, 2.0150765691128605`, 116.7443927237742`,
 2.818057068671489`, 1.149748916 4628067`, 47.181037293334754`, 1.`, {{0, 0.003279474350029803`, 5.837464343053049`},
```

```
    {8, 0.0002956143625498418`, 0.5261935653387184`}, {16, 0.000010670121713796583`, 0.018992816650557916`}}},
  {0.14569114559043927`, 3.1555485829295717`, 1.7894848948555018`, 1.1727674941432193`, 0.06666495623301039`,
   0.21747003985461844`, 0.032197916321861565`, 1115.0145850241458`, 0.11404115123479613`,
   9.530247146566754`, 65.05622278157867`, 0.4710915976323946`, 0.37052780018937426`, 47.587223108406796`,
   1.`, {{0, 0.002669910608553349`, 4.752440883224962`}, {52, 0.0009467183476855679`, 1.685158658880311`}}},
  {0.24150266620923067`, 1.7188165992139313`, 4.322636691993072`, 1.2029528995785472`, 0.9471494725848191`,
   0.6615414605337746`, 0.19861497081852103`, 2170.4118977268995`, 0.144713056075588`, 4.793357249969096`, 107.47949593117515`,
   3.1542315207735587`, 3.1542315207735587`, 107.47949593117515`, 1.`, {{0, 0.008168441690769312`, 14.539826209569377`}}},
  {0.20918490948785712`, 3.1569386485962374`, 8.450247389660383`, 1.207442014040422`, 0.02962131605799323`,
   0.6403349881706148`, 0.07844599973162336`, 325.7491474109025`, 0.10080604275363181`, 2.998503110312266`,
   104.59496326856535`, 1.2192712878902452`, 0.9996383751928009`, 83.58003958463655`, 1.`,
   {{0, 0.005484958153040078`, 9.763225512411339`}, {29, 0.0008671248553923011`, 1.543482242598296`}}},
  {0.20782035028778872`, 3.1560227942933192`, 4.024085032034362`, 1.1076699632387197`, 0.7825797887689212`,
   0.681476671590229`, 0.005917686238352288 6`, 2392.206038919967`, 0.17125268254719206`, 3.7066097570560648`,
   48.38473282362777`, 0.11032255919395578`, 0.10965287535931391`, 48.30904386352173`, 1.`,
   {{0, 0.003199197508794287`, 5.694571565653831`}, {3, 0.0004722898248333642`, 0.8406758882033882`}}},
  {0.16569005768607342`, 0.9318724619299896`, 5.387727961962419`, 1.4204768501331082`, 0.162422581490264`,
   0.6283948310518737`, 0.42435650593933405`, 403.29853257093646`, 0.17732127106821444`, 7.752705227642123`,
   248.6620777557737`, 14.747770121450847`, 11.199212822846262`, 189.25042159943544`, 1.`,
   {{0, 0.014362729388136162`, 25.565658310882366`}, {20, 0.000020302653420931308`, 0.03613872308925773`}}},
  {0.25387146604005345`, 2.177421517695631`, 2.9195785593706507`, 1.205984878491204`, 0.22933817566590875`,
   0.5142965710820098`, 0.00715658453704801`, 291.5834795916249`, 0.18546405096886748`, 3.1957985304348036`,
   11155.114683384925`, 2394.5051126607223`, 230.11197865360305`, 7346.185920522666`, 0.`,
   {{0, 0.00018275892603032706`, 0.3253108883339822`}, {1, 0.0002082416908595283`, 0.3706702097299603`},
    {2, 0.0002109876315831826`, 0.375557984218065`}, {3, 0.00018940096539455298`, 0.33713371840230427`},
    {4, 0.00015475725560 6007`, 0.2754679149786925`}, {5, 0.00011826671887055866`, 0.21051475958959437`},
    {6, 0.00008643292592885644`, 0.15385060815336446`}, {7, 0.00006141664550356895`, 0.10932162899635274`},
    {8, 0.000042918532402681306`, 0.07639498767677272`}, {9, 0.5570549486675435`, 991.5578086282273`}}},
  {0.27254750481249423`, 3.728211063877054`, 5.204302820921312`, 1.137745914342209`, 0.04515932299981973`,
   0.21786268187051538`, 0.17709327429647695`, 87.47051100453898`, 0.05078893394192652`, 2.761931237968355`,
   216228.26157717768`, 44165.34555449934`, 12.443017455691656`, 183.68877427461516`, 1.`,
```

```
{{0, 0.013384387642623058`, 23.824210003869045`}, {23, 0.0002753515935351431`, 0.49012583649255465`},
  {46, 0.000058188051452454174`, 0.10357473158536844`}, {69, 0.00005014737697092772`, 0.08926233100825133`},
  {92, 0.00004885623833030644`, 0.08696410422794545`}, {115, 0.000048783115367575116`, 0.08683394535428371`},
  {138, 0.000048776836223754394`, 0.08682276847828284`}, {161, 0.000045855990367533388`, 0.0816236628542103`}}},
{0.24124989455736606`, 1.138551887899828`, 6.0414451617780465`, 1.0864227463934355`, 0.8606911125263128`,
 0.6167713883354349`, 0.008616924398628963`, 4204.638992315412`, 0.042475052828396476`, 4.683417206387297`, 40.026024938586524`,
 0.2627897103941579`, 0.2627897103941579`, 40.026024938586524`, 1.`, {{0, 0.003041977895332576`, 5.414720653691985`}}},
{0.16291183507690532`, 0.9001170306894437`, 5.6406094859134015`, 1.3830538570390154`, 0.30535931966424634`,
 0.523800888605104`, 0.2170932130058295`, 52.61233898698406`, 0.0847713214076296 6`, 2.26771827115102`,
 309692.89217789605`, 93050.43284391065`, 229.08009901668007`, 4529.511370604914`, 0.`,
 {{0, 0.02825574321537451`, 50.29522292336663`}, {5, 0.015836359499888878`, 28.1887199098022`},
  {10, 0.013414588566460436`, 23.877967648299574`}, {15, 0.0123542384590942`, 21.990544457187678`},
  {20, 0.011634549951619697`, 20.70949891388306`}, {25, 0.010976154999287568`, 19.537555898731874`},
  {30, 0.010343104843637228`, 18.410726621674268`}, {35, 0.009744804834129935`, 17.345752604751286`},
  {40, 0.009186709291091342`, 16.352342538142587`}, {45, 0.22249661050538969`, 396.0439666995936`}}},
{0.24307985322679526`, 3.5312268091205707`, 7.143117328957406`, 1.2474301004946668`, 0.43966589306292936`,
 0.28635789146968393`, 0.04752738776816154`, 125.46273060513494`, 0.15229886979218882`,
 3.763239253417467`, 5018.292514045951`, 1074.0299537343824`, 2.533764213958453`, 232.33569727784322`,
 1.`, {{0, 0.013143388221913594`, 23.395231035006198`}, {4, 0.0036090385518991477`, 6.424088622380484`},
  {8, 0.0006587635148902966`, 1.172599056504728`}, {12, 0.0001289850423083005`, 0.22959337530877488`},
  {16, 0.000039450855444369173`, 0.07022252269097713`}, {20, 0.000025293777067143372`, 0.04502292317951521`},
  {24, 0.000023322436972256317`, 0.04151393781061625`}, {28, 0.000029270592620979 16`, 0.05210165486534290 6`}}},
{0.08579390683558069`, 2.8983719603389604`, 3.99564346069112`, 1.2420354947694623`, 0.9313337583971846`,
 0.4737657288125191`, 0.010571420568870461`, 66.04207510362154`, 0.1244519483642591`, 8.73466885165751`,
 114907.61684658368`, 51501.11156825629`, 230.08691462567384`, 3583.5446757955906`, 0.`,
 {{0, 0.042551109331565265`, 75.74097461018617`}, {3, 0.017643675804597907`, 31.405742932184275`},
  {6, 0.013490827762774705`, 24.013673417738975`}, {9, 0.010588503955255181`, 18.847537040354222`},
  {12, 0.008448211537248403`, 15.037816536302156`}, {15, 0.006835608825192275`, 12.16738370884225`},
  {18, 0.005603763885367132`, 9.974699715953495`}, {21, 0.004652064747267413`, 8.280675250135994`},
  {24, 0.003909400839001749`, 6.958733493423113`}, {27, 0.15862622867219486`, 282.3546870365069`}}},
{0.20336933942425872`, 1.2934536281273639`, 5.591224704743386`, 1.058733783245077`, 0.09184020179750241`,
```

0.3092776948325172`, 0.4547775021552O385`, 95.84900263920687`, 0.1497141413596374 5`, 8.98848136080165`,
53395.708942902056`, 13588.794970292527`, 24.102748307077043`, 477.8451940854613`, 1.`,
{{0, 0.0351837583067579`, 62.62708978602907`}, {7, 0.0006936402827531319`, 1.2346797033005747`},
{14, 0.00006882990165702412`, 0.12251722494950293`}, {21, 0.00005608835630206144`, 0.09983727421766934`},
{28, 0.00005484129181649414 5`, 0.09761749943335958`}, {35, 0.00005459380986677609`, 0.09717698156286143`},
{42, 0.00005455760254599213`, 0.097112532531866`}, {49, 0.00005455320132081695 5`, 0.09710469835105419`},
{56, 0.00005455266207397035`, 0.09710373849166723`}, {63, 0.00004081933540089356`, 0.07265841701359053`}}},
{0.12808268874911022`, 3.6824385947617753`, 2.1347222010883335`, 0.9228913029818868`, 0.1301948165557758`, 0.29194798379239884`,
0.27062051931711223`, 4508.281780436825`, 0.033597809391257005`, 7.5117374932700365`, 14.752932769981127`,
0.21891564583611628`, 0.21891564583611628`, 14.752932769981127`, 1.`, {{0, 0.0011212228905185656`, 1.995776745123047`}}},
{0.14553068051711526`, 1.8082756931498238`, 2.368533159601707`, 1.1602558844471123`, 0.9691057704573378`,
0.4792380149128871`, 0.010639214796165341`, 788.9564435352116`, 0.18893878705870149`, 9.465562662789438`, 188.0051174376203 8`,
1.963546649191763`, 1.3301792893709443`, 193.53033233051093`, 1.`, {{0, 0.010584079433975538`, 18.839661392476458`},
{3, 0.0032004982408462947`, 5.696886868706405`}, {6, 0.0007186305096O4796`, 1.2791623070965368`},
{9, 0.00016558260142101482`, 0.29473703052940636`}, {12, 0.00003951447127118738 6`, 0.07033575886271355`}}},
{0.24858998623664108`, 2.4028729322353692`, 5.907867944453356`, 0.899298954014168`, 0.6845394050547509`,
0.4831163766793648`, 0.006208316855544933`, 932.6458115441258`, 0.22545041645445363`, 9.209981899602862`,
206.52376409082336`, 0.5006740746907912`, 0.498834200914221`, 205.95235692445797`, 1.`,
{{0, 0.01412622810227922`, 25.14468602205701`}, {4, 0.0015261510239795873`, 2.7165488226836656`}}},
{0.2519271524040908`, 1.2045387595495214`, 4.5140841978362705`, 1.0152142452788597`, 0.29419718036266773`,
0.20432622138273293`, 0.02287101962327847`, 2390.149329793605`, 0.07799608049040802`,
9.888014794979117`, 36.46856987462557`, 0.4867504777613409`, 0.3893757391718832`, 29.12964815855507`, 1.`,
{{0, 0.0015521062768652052`, 2.7627491728200653`}, {63, 0.0006617469831849802`, 1.1779096300692649`}}},
{0.11736070154865536`, 2.38096944376945`, 5.3494850274310295`, 1.276899864236343`, 0.07495782665645989`,
0.3912082450614217`, 0.41113333692039317`, 80.12914367323096`, 0.2186956786299285`, 8.297333470694408`,
49120.19641657008`, 18226.490087193673`, 229.38412788119075`, 1132.1680766322115`, 0.`,
{{0, 0.03307699644502299`, 58.87705367214092`}, {6, 0.01783317280279292`, 31.743047588971397`},
{12, 0.006414837091049752`, 11.418410022068558`}, {18, 0.002265048556890014`, 4.0317864312642255`},
{24, 0.0008323965830994209`, 1.481665917916969`}, {30, 0.00031825238970036924`, 0.5664892536666574`},
{36, 0.0001291341070058844`, 0.22985871047047426`}, {42, 0.000059158228342323015`, 0.10530164644933497`},
{48, 0.00003327836587970948`, 0.05923549126588288`}, {54, 0.025082499254264687`, 44.64684867259113`}}},

```
{0.16035901956271875`, 2.606522752147513`, 4.69883411815465`, 1.3021358666964324`, 0.6150695298380762`,
  0.6434499333892203`, 0.12329290515746044`, 1079.0196669558486`, 0.2419176548365854`,
  8.070742396729585`, 163.20465155503854`, 2.745433324563681`, 2.574788677942377`, 153.107544480356`, 1.`,
  {{0, 0.009787873502352272`, 17.42241483418704`}, {4, 0.0018482998781547866`, 3.2899737831155202`}}},
{0.16449617597041116`, 3.191202853555228`, 6.979734889593845`, 0.755053893640484`, 0.8431581165413089`,
  0.5308423930900061`, 0.0662197921799213`, 915.9947229465104`, 0.1651520479769521`, 4.094114041787371`, 136.4721946884852`,
  1.4492036040417706`, 1.291312538787242`, 121.838154068165`, 1.`, {{0, 0.005254567604438628`, 9.353130335900758`},
   {3, 0.0030390864812201964`, 5.409573936571949`}, {6, 0.000966045623521716`, 1.7195612098686546`}}},
{0.11360527105324508`, 0.4683409445122746`, 6.198135239531911`, 1.4397350406163711`, 0.8287446034488006`,
  0.16487005319303316`, 0.2956243647100192`, 339.82112374821816`, 0.14870620145140367`, 8.492417686013223`, 347.2523030444991`,
  36.07880818135753`, 8.307571863095795`, 85.47523469117989`, 1.`, {{0, 0.004414480186172575`, 7.857774731387183`},
   {9, 0.001780699563168618`, 3.1696452224401406`}, {18, 0.0002635061096501167`, 0.4690408751772077`},
   {27, 0.000028166921976397054`, 0.05013712111798676`}, {36, 9.265055561965836`*^-6, 0.016491798900299188`}}},
{0.08657280459787114`, 2.8376273381057535`, 2.18201840556476`, 0.9732923844552535`, 0.8927467229995099`,
  0.4599530868891304`, 0.0428939946393510 7`, 1129.687177458504`, 0.08547871234303578`, 2.1820677830816138`,
  38.931711207022914`, 0.6594126992347105`, 0.4915489081995819`, 37.256702877178945`, 1.`,
  {{0, 0.0017553777765816042`, 3.124572442315255`}, {3, 0.000746282252919083`, 1.3283824101959678`},
   {6, 0.0002484141122049255`, 0.4421771197247674`}, {9, 0.00008143527695998696`, 0.14495479298877678`}}},
{0.14288327643386806`, 3.0416391272131467`, 7.259522365277451`, 0.8919048885163637`, 0.7100314935930478`,
  0.32120037052790307`, 0.014548712666776912`, 690.6861027330066`, 0.2370448478751857`, 7.1501615854828415`,
  177.67594007321128`, 0.7045585395303291`, 0.6913730138068538`, 174.38812361937212`, 1.`,
  {{0, 0.010397593936602239`, 18.507717207151988`}, {10, 0.002855903458470043`, 5.083508156076676`}}},
{0.05887079706837223`, 1.3439289639420835`, 4.010009055307599`, 1.087305166401462`, 0.6661788396152433`,
  0.35567096782369767`, 0.008830796062004187`, 96.96443168903161`, 0.0354538286259683`,
  6.082802068311153`, 154167.19762556435`, 83632.13466144334`, 228.9500540514794`, 870.8483625662344`,
  0.`, {{0, 0.022248152418298514`, 39.60171130457135`}, {5, 0.0011172699481005746`, 1.9887405076190225`},
   {10, 0.00033258106625383015`, 0.5919942979318178`}, {15, 0.00022416967848745315`, 0.3990220277076666`},
   {20, 0.0002015940616519574`, 0.35883742974048416`}, {25, 0.00019525678607295148`, 0.3475570792098537`},
   {30, 0.00019361074905734906`, 0.34462713332208134`}, {35, 0.00019412043936342988`, 0.3455343820669052`},
   {40, 0.00019626347037141176`, 0.34934897726111297`}, {45, 0.04128145693737635`, 73.48099334852992`}}},
{0.275808383383374`, 3.614253930768469`, 8.690573450897112`, 0.8084062901760818`, 0.7881641108236563`,
```

```
0.5461332704061045`, 0.16300280176413603`, 213.49162259077596`, 0.11620699031214832`,
  3.4918574760984407`, 258.9594454251696`, 4.420332175532701`, 3.1633765313208695`, 207.99297692940775`,
  1.`, {{0, 0.013383022229630961`, 23.82177956874311`}, {10, 0.002424444017004029`, 4.3155103502671714`}}},
{0.16345104545617634`, 3.976575006158881`, 6.050721111049688`, 0.8077170991659299`, 0.4737043917748365`,
  0.42206087766889466`, 0.011122815850097148`, 819.7636995837653`, 0.0134560852671817`, 8.084495823489597`, 140.41931321801366`,
  0.34614779897715536`, 0.34614779897715536`, 140.41931321801366`, 1.`, {{0, 0.010671867804569038`, 18.99592469213289`}}},
{0.27078615466317746`, 3.6662669410444693`, 7.922278334055675`, 1.4466898496109064`, 0.8715287168554611`,
  0.2219561421798496`, 0.02446106939657988`, 188.339766844502`, 0.03692414185938048`, 1.663076961237424`,
  106.79079116375168`, 1.1713365305406822`, 0.6788789953689655`, 98.04638962162194`, 1.`,
  {{0, 0.004970307100399716`, 8.847146638711495`}, {6, 0.0018278988757316308`, 3.2536599988023025`},
   {12, 0.0005638818346041301`, 1.0037096655953515`}, {18, 0.00008943780050779053`, 0.15919928490386714`}}},
{0.24286662289220456`, 2.6814172670718373`, 2.6369699156223714`, 0.8218929956416956`, 0.8808977613854625`,
  0.43575724866517207`, 0.024751792159432087`, 472.6370271533013`, 0.14270041872793765`, 9.280134575613399`, 233.98565963541557`,
  6.927052895182722`, 1.9257157610911688`, 223.7031461138357`, 1.`, {{0, 0.012143414684134754`, 21.61527813775986`},
   {2, 0.003775010493457009`, 6.719518678353475`}, {4, 0.000805550791995962`, 1.4338804097528124`},
   {6, 0.0001659066365407871`, 0.295313813042601`}, {8, 0.000043049005775333595`, 0.07662723028009799`},
   {10, 0.000020745567261836573`, 0.0369271097260691`}, {12, 0.000017024012959895843`, 0.030302743068614604`},
   {14, 0.000016464204966554617`, 0.029306284840467215`}, {16, 0.000014273707559376788`, 0.025407199455690685`}}},
{0.042342161544045354`, 1.3013804906014936`, 7.13634807471312`, 1.1105942412761596`, 0.2407240140528013`,
  0.6802475696307242`, 0.0062232822378432`, 4037.4772502763867`, 0.18461914595887624`, 5.716947148449252`, 19.849475888450513`,
  0.08483376179400899`, 0.08483376179400899`, 19.849475888450513`, 1.`, {{0, 0.001508560167522239`, 2.6852370981895857`}}},
{0.14720397501884225`, 0.5316247300014076`, 6.683828314747185`, 1.481587653959343`, 0.013428589995876372`,
  0.17720084833285243`, 0.005004553323761107`, 364.08088787611194`, 0.035796657483315286`, 8.51611862140901`, 160.522092861328`,
  1.2395514504525742`, 1.2395514504525742`, 160.522092861328`, 1.`, {{0, 0.012199679057460928`, 21.715428722280453`}}},
{0.1926486177557311`, 2.6336525621782014`, 9.444109966651418`, 1.4788802142070587`, 0.6654095862145473`,
  0.16653655494047714`, 0.03828733327035071`, 2433.4281263020134`, 0.12844440731237916`, 4.149230973672752`, 19.155383302174474`,
  0.17667273474205503`, 0.17667273474205503`, 19.155383302174474`, 1.`, {{0, 0.00145580913096526`, 2.591340253118163`}}},
{0.055762854118976635`, 2.4026093631268655`, 6.857734870811047`, 0.7509249944976302`, 0.22671157472868875`,
  0.4788061070906606`, 0.1564030997457896`, 55.02740807629177`, 0.03469990388231514`, 7.492235364197862`,
  219607.0192408077`, 122000.07868975631`, 229.20068495650597`, 1625.4761552359648`, 0.`,
  {{0, 0.05158412898187777`, 91.81974958774245`}, {3, 0.009335408981297902`, 16.61702798671027`},
```

```
    {6, 0.004115004231003997`, 7.324707531187116`}, {9, 0.0022987944039046444`, 4.091854038950268`},
    {12, 0.0015570199108677525`, 2.7714954413446`}, {15, 0.001218393173617545`, 2.16873984903923`},
    {18, 0.001048271262030774`, 1.8659228464147777`}, {21, 0.0009454813241910144`, 1.6829567570600057`},
    {24, 0.0008585043155479605`, 1.5281376816753698`}, {27, 0.050575181213593966`, 90.02382256019726`}}},
 {0.05296847355619744`, 1.6490117913903575`, 9.992115392915188`, 0.8066960527418563`, 0.1291629696144665`,
   0.33931322884091664`, 0.26435083615790894`, 660.4050522121521`, 0.10186522527726305`, 2.8696181659920086`, 39.684763508085595`,
   1.2554726084092678`, 0.7534089240607152`, 24.1493541811178`, 1.`, {{0, 0.0015337913973563233`, 2.7301486872942555`},
    {9, 0.0002905051663439081`, 0.5170991960921564`}, {18, 0.000011054354064721369`, 0.019676750235204037`}}},
 {0.21365538200916506`, 3.0547845209089406`, 0.35609106729359574`, 0.8256521864420123`, 0.9506371395328554`,
   0.271042805312945`, 0.12766968769895812`, 68.7970381277751`, 0.05673347586404265`, 3.4610065999125617`, 288137.8627021797`,
   71103.97827093807`, 230.25960636973454`, 1020.7378236871017`, 0.`, {{0, 0.07757607460021973`, 138.08541278839112`}}},
 {0.22385277301638717`, 3.365536903395271`, 8.935305056074029`, 1.445762062116566`, 0.43390325179453604`,
   0.6187135527847307`, 0.01939946413637202`, 453.1242214832122`, 0.13325485950688953`, 8.825528492983036`, 321.8021318833832`,
   1.4565434430283026`, 1.4565434430283026`, 321.8021318833832`, 1.`, {{0, 0.024456962023137123`, 43.53339240118409`}}},
 {0.26129739321020845`, 3.355909888714172`, 1.041845959949887`, 1.206440545734901`, 0.09347792890054829`,
   0.5333101115239811`, 0.015503351594481202`, 579.4832207194656`, 0.24065061389170217`,
   7.475999528371727`, 8747.97885270187`, 1835.382469762018`, 227.96273970814775`, 24812.499726481907`,
   0.`, {{0, 0.01700052532327559`, 30.260935075430552`}, {14, 0.004431257537773516`, 7.887638417236858`},
    {28, 0.005501877910288768`, 9.793342680314007`}, {42, 0.011188482189628021`, 19.91549829753788`},
    {56, 0.009052789143537408`, 16.113964675496586`}, {70, 0.01122323532400561`, 19.977358876729983`},
    {84, 0.01295147853992011`, 23.053631801057797`}, {98, 0.01443027530534967`, 25.685890043522413`},
    {112, 0.016815889681097936`, 29.932283632354327`}, {126, 1.783154168257748`, 3174.0144194987915`}}},
 {0.14172561423935526`, 2.161658858177276`, 2.5726304321806133`, 1.1364876244324849`, 0.9181535957758225`,
   0.5861373014149901`, 0.010418800942482771`, 101.70999367656657`, 0.1500483081169689`,
   9.64604938537483`, 78537.11429852412`, 25899.46294304563`, 228.31989182809394`, 48944.909044944216`,
   0.`, {{0, 0.04175847199729758`, 74.33008015518969`}, {3, 0.025492831622241814`, 45.37724028759044`},
    {6, 0.02549976529661426`, 45.389582227973385`}, {9, 0.025933529197155376`, 46.16168197093657`},
    {12, 0.0266402510676405`, 47.41964690040009`}, {15, 0.027578695310666392`, 49.090077652986174`},
    {18, 0.028730305978843216`, 51.13994464234093`}, {21, 0.03008449290321733`, 53.550397367726845`},
    {24, 0.03163523606035405`, 56.310720187430206`}, {27, 3.4564595079817297`, 6152.497924207479`}}},
 {0.06568219588241164`, 1.0305120784213448`, 8.720293141386708`, 1.4160313463775192`, 0.752508918071946`,
```

```
0.31681781371940887`, 0.13417309715525413`, 209.13734568668048`, 0.22402731709711876`,
  4.416848552940577`, 306.50031128237816`, 13.345214165454443`, 7.3369892287033`, 172.59821096645274`, 1.`,
  {{0, 0.010948191670821197`, 19.48778117406173`}, {18, 0.002169272362629212`, 3.8613048054799974`}}},
{0.27044630440719625`, 2.4698840490073675`, 6.955264919348561`, 1.0249819835130385`, 0.106623120735865`,
  0.6358905088319218`, 0.012311596840628138`, 346.23093318344644`, 0.07401305589494472`, 1.2937187987733016`, 44.23096306646051`,
  0.2278991062245313`, 0.2278991062245313`, 44.23096306646051`, 1.`, {{0, 0.003361553193050999`, 5.983564683630778`}}},
{0.08403118541616988`, 3.378337092807227`, 2.524635889786053`, 0.8671019090417934`, 0.46085963082622694`,
  0.5847463933658021`, 0.04733165775893332`, 1030.6526695312073`, 0.10944158368617352`,
  3.930070348109142`, 62.54081465868531`, 0.5761667931674175`, 0.5427282248556635`, 56.25636229610529`, 1.`,
  {{0, 0.0030895153218636236`, 5.499337272917249`}, {20, 0.0011859682126403785`, 2.1110234184998737`}}},
{0.11975601650409934`, 1.1540823534574054`, 5.101398975621729`, 0.9964575213162732`, 0.8596230582994027`,
  0.5017711629445061`, 0.09812020963113087`, 54.445260820005984`, 0.07947868464718594`,
  4.273753987333478`, 200201.01107563608`, 73793.2337170358`, 229.30369556141358`, 3260.3609061777015`,
  0.`, {{0, 0.03207261833450716`, 57.08926063542275`}, {4, 0.015958276034880387`, 28.405731342087083`},
   {8, 0.012512013308336848`, 22.271383688839588`}, {12, 0.0101785518245383`, 18.11782247678173`},
   {16, 0.00849082408148491`, 15.113666865043138`}, {20, 0.007218449894244028`, 12.84884081175437`},
   {24, 0.006225843614930039`, 11.08200163457547`}, {28, 0.005428889023433914`, 9.663422461712367`},
   {32, 0.004773798470573067`, 8.49736127762006`}, {36, 0.14492816428257665`, 257.9721324229865`}}},
{0.26353274820980593`, 3.2715371213501925`, 8.419438906869026`, 0.9426613525835754`, 0.1294979869931494`,
  0.5387601140015186`, 0.040849691062558216`, 744.2937861154428`, 0.1766059692159136`, 7.8822033078787435`,
  120.81442853553429`, 0.9269715371201283`, 0.8606683697475057`, 111.85012456697717`, 1.`,
  {{0, 0.005968218997364339`, 10.623429815308523`}, {29, 0.002532390469725926`, 4.507655036112148`}}},
{0.15837784563387913`, 1.590657770632209`, 9.4421417173358`, 0.8199017044479102`, 0.629826423052569`,
  0.2661387442145513`, 0.049173845467465206`, 187.50695430622352`, 0.06908425180315825`,
  9.722920562989135`, 713.5642589271415`, 12.756720963643456`, 8.20238491549149`, 459.2196549539174`, 1.`,
  {{0, 0.029826976518394385`, 53.09201820274201`}, {36, 0.005073717258103342`, 9.031216719423949`}}},
{0.18489841859634304`, 3.5401138358224227`, 7.262892362437768`, 0.8851173674042263`, 0.05104398233694862`,
  0.6826543300991057`, 0.13384148697112022`, 730.4506518091381`, 0.22533987085622137`, 8.188627124718394`,
  136.4777320365579`, 1.7211054460868436`, 1.6792902099163376`, 133.2176411366313`, 1.`,
  {{0, 0.009156571827119587`, 16.298697852272863`}, {5, 0.0009679688992643925`, 1.7229846406906186`}}},
{0.22773890477301867`, 2.1437195677585157`, 6.152335866192775`, 1.1180903919170035`, 0.02705103803804154`,
```

0.3333465628704615`, 0.00917020779025847 5`, 2321.6700090659583`, 0.14789384405564093`, 7.237611958554762`, 26.590895556777824`,
0.09921542601518282`, 0.09921542601518282`, 26.590895556777824`, 1.`, {{0, 0.0020209080623151145`, 3.5972163509209043`}}},
{0.17605039997850958`, 2.727076411741753`, 8.728585319721596`, 1.1320082850991182`, 0.7355770210071702`,
0.5402004706661186`, 0.09745706159326789`, 177.96581560566344`, 0.01922037387361028`,
4.980887347403458`, 379.80400571840204`, 7.575390139846118`, 5.922882730733103`, 348.68468610155685`,
1.`, {{0, 0.02222613197812132`, 39.56251492105595`}, {9, 0.004273904165597003`, 7.607549414762666`}}},
{0.06947420594938447`, 0.8077718165326004`, 3.821443319804267`, 1.3891202366711977`, 0.6756008025235485`,
0.23550127195203518`, 0.0653335798785588`, 483.3383501610001`, 0.09612644227220152`, 2.381497911922425`,
65.84106637907664`, 2.6709157644082158`, 1.589052580380182`, 37.147107493057824`, 1.`,
{{0, 0.0023518419060297576`, 4.186278592732969`}, {31, 0.0004713382634426372`, 0.8389821089278942`}}},
{0.23569362997389992`, 1.2270876586626818`, 0.9845589085102358`, 1.1982217261670929`, 0.43286649637666286`,
0.634774301600445`, 0.09976341665112172`, 228.04937393006952`, 0.12724994607105566`, 3.0426000813607676`, 59653.73018148092`,
13655.206591996344`, 229.82876181309572`, 1163.9232384531124`, 0.`, {{0, 0.08845816612243655`, 157.4555369793705`}}},
{0.12274586224015488`, 2.0471402784080235`, 1.4612976870222134`, 0.9456822010625827`, 0.8327879461898879`,
0.22053943969717293`, 0.006767755909702022`, 174.3096643655477`, 0.1850505728558421`,
1.2537872990507584`, 19731.476101653963`, 7160.777223932612`, 229.4867025469556`, 697.5803746314814`,
0.`, {{0, 0.0020698040790678588`, 3.6842512607407882`}, {12, 0.000657985576549847`, 1.1712143262587276`},
{24, 0.00024904204301823626`, 0.44329483657246055`}, {36, 0.00013121668197753656`, 0.2335656939200151`},
{48, 0.00008776431403992442`, 0.15622047899106548`}, {60, 0.00006860493150095518`, 0.12211677807170022`},
{72, 0.00005906975318980896`, 0.10514416067785995`}, {84, 0.00005388641373881676`, 0.09591781645509383`},
{96, 0.00005086790317968315 5`, 0.090544867659836`}, {108, 0.049587866775729926`, 88.26640286079927`}}},
{0.13491824188855928`, 1.669720674179887`, 1.3334401828831126`, 0.968437561771696`, 0.34828727193756603`,
0.3135712369052611`, 0.22891096239253717`, 1910.612731597179`, 0.10930115833371806`, 4.270908360205038`, 24.203493543643585`,
0.7654971814236254`, 0.44219181478083563`, 13.671008616623874`, 1.`, {{0, 0.0008600236037000025`, 1.5308420145860044`},
{9, 0.00016470787139730945`, 0.2931800110872108`}, {18, 0.0000142651797661025`, 0.02539201998366245`}}},
{0.04199865921066254`, 2.754174936868967`, 5.53210979521042`, 1.3844956478579449`, 0.47136731805345633`,
0.24068198696313148`, 0.022972136801125642`, 58.648848297948106`, 0.2240134714991286`,
4.479259216921056`, 43127.7408143082`, 26830.872461700073`, 230.15558451452543`, 811.1849112598231`,
0.`, {{0, 0.020024942260906307`, 35.644397224413225`}, {6, 0.0009978437587015196`, 1.7761618904887047`},
{12, 0.00023981383520405968`, 0.4268686266632262`}, {18, 0.00013043573405570813`, 0.23217560661916045`},
{24, 0.00010654680010556148`, 0.1896533041878994`}, {30, 0.00010109054674354431`, 0.17994117320350886`},

```
      {36, 0.00010262014215797353`, 0.18266385304119287`}, {42, 0.00010953419431528523`, 0.1949708658812077`},
      {48, 0.00012327527806542835`, 0.21942999495646245`}, {54, 0.03971395070549117`, 70.69083225577427`}}},
  {0.18074106663079648`, 0.9554759541683486`, 8.202625386518609`, 1.4769540911095849`, 0.5922233662884384`,
    0.5544055394726339`, 0.15090969809745627`, 4632.951795907658`, 0.16872394703920557`, 7.933658625009354`,
    33.040934066417904`, 1.527853541254256`, 0.929308311208837`, 20.226457386422396`, 1.`,
    {{0, 0.0012304442776345417`, 2.190190814189484`}, {15, 0.00030676648373356035`, 0.5460443410457374`}}},
  {0.22308930012837075`, 1.8877770902637643`, 5.727481376105105`, 1.290937410786533`, 0.5385704303274257`,
    0.17837528465279628`, 0.11591468148922064`, 2665.0058694088225`, 0.1309938954283596`, 2.3264420617360244`, 8.71333110105202`,
    0.19524061833786588`, 0.19524061833786588`, 8.71333110105202`, 1.`, {{0, 0.0006622131636799535`, 1.1787394313503172`}}},
  {0.16528939075200133`, 0.44637388983991233`, 6.000554224390708`, 0.7620462868306999`, 0.45157540745748825`,
    0.6293554847029814`, 0.08179042637541929`, 626.5470719450981`, 0.11922182895709121`,
    4.5474321331423475`, 121.81520506316748`, 8.80834886003703`, 8.344264366076557`, 97.82920566841726`, 1.`,
    {{0, 0.005554770682732638`, 9.887491815264097`}, {29, 0.0018802489480670734`, 3.346843127559391`}}},
  {0.12057640522465035`, 1.6899679349832946`, 5.872613897455656`, 0.8685022109314445`, 0.5819827862907108`,
    0.45575489869095254`, 0.005408595604133131`, 1249.3305456098487`, 0.22090492300977427`, 9.765554151588912`, 134.239724991178`,
    0.3930412049448029`, 0.3930412049448029`, 134.239724991178`, 1.`, {{0, 0.01020221909932953`, 18.1599499680656`}}},
  {0.26839288378136816`, 1.20431418513886`, 3.4264235210106797`, 1.1464020290887187`, 0.9129873318113406`,
    0.17958510842302844`, 0.03476022275277427`, 85.14929883968256`, 0.03637248443077806`,
    6.177611921122705`, 422073.38356184616`, 87279.80080230112`, 229.3776243202297`, 1301.1542707076958`,
    0.`, {{0, 0.09253241191166466`, 164.7076932027631`}, {23, 0.0024175452019817717`, 4.303230459527554`},
      {46, 0.0005439882272169962`, 0.9682990444462533`}, {69, 0.00048606715944772647`, 0.8651995438169532`},
      {92, 0.0004815889109726255`, 0.8572282615312734`}, {115, 0.0004810922900579046`, 0.8563442763030702`},
      {138, 0.00048103461185235505`, 0.8562416090971919`}, {161, 0.00048102787296841547`, 0.8562296138837796`},
      {184, 0.00048102708492270236`, 0.8562282111624103`}, {207, 0.0005019413026997409`, 0.8934555188055389`}}},
  {0.19312844950593372`, 3.9129912832786564`, 0.42277195731844414`, 0.8655327062249413`, 0.5847393470300801`,
    0.16029168895981472`, 0.1525854675774217`, 2234.1231120850057`, 0.03649943574663328`, 5.234397199033289`,
    20.984268224598885`, 1.123451124855433`, 0.30613688326148736`, 11.421537214968119`, 1.`,
    {{0, 0.0004852722169705667`, 0.8637845462076088`}, {6, 0.00025472941613062066`, 0.4534183607125048`},
      {12, 0.00007618245531627462`, 0.1356047704629688`}, {18, 0.000022266995162479207`, 0.03963525138921299`},
      {24, 8.52993353910533`*^-6, 0.015183281699607488`}, {30, 5.17106669589546`*^-6, 0.00920449871869391`},
      {36, 4.383866948142577`*^-6, 0.007803283167693789`}, {42, 0.000011500877574492425`, 0.020471562082596515`}}},
```

{0.05831506452479063`, 1.4447664488109728`, 9.399667906542401`, 0.8838396243177019`, 0.4494233408794772`,
0.5909311609620335`, 0.2085880113243902`, 309.3416067223959`, 0.04816991376818669`, 2.147582543132131`, 110.97250965331297`,
3.839239949766759`, 3.4933367292018107`, 101.14041099770185`, 1.`, {{0, 0.006292821493189316`, 11.201222257876982`},
{3, 0.001260807485687092`, 2.2442373245230236`}, {6, 0.00013304225694893245`, 0.23681521736909975`}}},
{0.23965113012644185`, 0.9128974489725401`, 8.261297062012524`, 0.788417407034967`, 0.8016641172773833`, 0.35686628470413617`,
0.030635478621033968`, 346.1316581378353`, 0.043365488693742626`, 1.9545026373639716`, 116.95354517929417`,
2.6140998540385865`, 2.3559305543889417`, 106.8382345186106`, 1.`, {{0, 0.006703734332826475`, 11.932647112431125`},
{2, 0.001235026593418932 2`, 2.1983473362857`}, {4, 0.0001809448971689992`, 0.3220819169608185 6`}}},
{0.20520507007680205`, 1.512442754504547`, 9.386452795492342`, 1.0402158263719963`, 0.9284696718653804`,
0.3818637796049891`, 0.053241178903524394`, 3017.2611079460976`, 0.15062298010038172`,
2.3864513615272553`, 22.46099795120003`, 0.4326055589848879`, 0.31680737848030693`, 16.2788522619648`, 1.`,
{{0, 0.0008535173289495435`, 1.5192608455301875`}, {27, 0.00038367544295978136`, 0.6829422884684109`}}},
{0.20853248950244857`, 2.7801356391066916`, 9.335557605849111`, 0.8182981643225358`, 0.24140252954455388`,
0.3825831953385048`, 0.013327430792399009`, 77.96529661289064`, 0.07323986754942219`,
3.7008101112034457`, 68157.41842395031`, 17059.776366121343`, 228.14200798370405`, 3093.042186795802`,
0.`, {{0, 0.0649917172021832`, 115.6852566198861`}, {15, 0.0012991403487436926`, 2.3124698207637726`},
{30, 0.001299000696077915`, 2.312221239018689`}, {45, 0.0012990258280533083`, 2.3122659739348888`},
{60, 0.0012991761948125333`, 2.3125336267663092`}, {75, 0.001299974663990539`, 2.313954901903159`},
{90, 0.0013043309800921672`, 2.3217091445640574`}, {105, 0.001328141403360043`, 2.3640916979808764`},
{120, 0.0014493691954885239`, 2.5798771679695727`}, {135, 0.15950132968367906`, 283.9123668369487`}}},
{0.13576335201419282`, 2.033104701519708`, 4.843203210944571`, 1.3428754702553487`, 0.2671111595179354`,
0.18962864690949777`, 0.12183777475102511`, 1231.483599580866`, 0.14101782654161166`, 2.1508880203421494`, 14.062304512997493`,
0.30133962822324134`, 0.16237133774875553`, 7.436153498270738`, 1.`, {{0, 0.0004403790419583306`, 0.7838746946858283`},
{16, 0.00011325685077337115`, 0.20159719437660062`}, {32, 0.000011511773136874358`, 0.020490956183636357`}}},
{0.25963449652183795`, 2.4481737185281203`, 5.334705083491999`, 0.8989699854247848`, 0.7130966522497681`,
0.695912989145927`, 0.04220235704717508`, 140.4631283114693`, 0.11660454588716207`, 7.56536482611441`,
51517.39459822761`, 10872.8365086451`, 229.63407209106788`, 1605.0463391123828`, 0.`,
{{0, 0.10271241250759498`, 182.82809426351906`}, {11, 0.0029797788005178263`, 5.304006264921731`},
{22, 0.000452432414832878`, 0.8053296984025229`}, {33, 0.00030390721741338766`, 0.5409548469958301`},
{44, 0.0003094909402720857`, 0.5508938736843125`}, {55, 0.00035553723838502034`, 0.6328562843253362`},
{66, 0.0004124617659238569`, 0.7341819433444653`}, {77, 0.00044831152477250595`, 0.7979945140950606`},

```
    {88, 0.0004377965361116435`, 0.7792778342787254`}, {99, 0.01357139282671691`, 24.1570792315561`}}},
  {0.17509339161516935`, 3.0306683644318824`, 1.9789343166711684`, 0.8960196738550836`, 0.2152496986108292`,
   0.6250130758758401`, 0.02087454134280109`, 59.51435674676293`, 0.2018230747082378`, 3.546682887992379`, 97708.20265429444`,
   27890.038848900374`, 229.75153162873764`, 1302.3732837877778`, 0.`, {{0, 0.09898036956787112`, 176.1850578308106`}}},
  {0.10593857456933181`, 2.955002391424368`, 8.212267239719637`, 0.932578721051657`, 0.3205115590912122`,
   0.6246255275862143`, 0.01773467921096997`, 102.27924186693049`, 0.015490300413002644`,
   6.09864277306975`, 227762.3423639426`, 90447.83707953537`, 228.73428577091337`, 1304.5066946226764`,
   0.`, {{0, 0.03997774492659391`, 71.16038596933716`}, {7, 0.0005058385837636261`, 0.9003926790992545`},
    {14, 0.0004992922571985587`, 0.8887402178134346`}, {21, 0.00049916328776129`, 0.8885106522150963`},
    {28, 0.0004991725458195254`, 0.8885271315587552`}, {35, 0.000499264628130814`, 0.8886910380728489`},
    {42, 0.00049965609008568 1`, 0.8893878403525121`}, {49, 0.0005014730349325142`, 0.8926220021798754`},
    {56, 0.000510704322024058`, 0.9090536932028233`}, {63, 0.05515019911501342`, 98.1673544247239`}}},
  {0.10583476133380088`, 3.0591620947997713`, 7.753926172578531`, 1.2866948671665401`, 0.7481949494801159`,
   0.21582592530952183`, 0.02844379415675546`, 101.39583001744663`, 0.09278999192622717`, 1.6976300926057526`, 8561.00645836453 3`,
   3366.2134324464582`, 1.3083674718177083`, 112.40975567619273`, 1.`, {{0, 0.005837288854658519`, 10.390374161292165`},
    {6, 0.0020400101382465033`, 3.6312180460787755`}, {12, 0.00048807400198656463`, 0.868771723536085`},
    {18, 0.00011743152029820209`, 0.20902810613079967`}, {24, 0.000033052895822710 47`, 0.058834154564424625`},
    {30, 0.000014683154090189345`, 0.026136014280537037`}, {36, 0.000012600866287959494`, 0.0224295419925679`}}},
  {0.23086787628538824`, 0.6622997055352342`, 7.183940408138384`, 1.353990838360309`, 0.25975387914696335`,
   0.6772244715959861`, 0.055054708296352145`, 683.0312933373987`, 0.1367743885435413`, 6.244797760097583`, 130.76856833688336`,
   5.445117312601971`, 5.445117312601971`, 130.76856833688336`, 1.`, {{0, 0.009938411193603136`, 17.690371924613583`}}},
  {0.20388536464835005`, 2.2276602475557015`, 5.0708546023033385`, 0.8827779974574592`, 0.1554825605038257`,
   0.2618026330626365`, 0.006155066765134505`, 1063.8834649875878`, 0.12744889948308613`, 2.149276150894316`, 16.705175788430296`,
   0.0494331073858992`, 0.0494331073858992`, 16.705175788430296`, 1.`, {{0, 0.0012695933599207025`, 2.2598761806588503`}}},
  {0.10738337044420926`, 3.7501964356372497`, 1.0776334746380822`, 0.9682422375974065`, 0.09998974406674432`,
   0.504170653500009`, 0.22038799040453166`, 2051.555691372483`, 0.09521018879793502`, 2.016584418866474`,
   10.745437156692924`, 0.19029526861361393`, 0.1452270688122808`, 7.006451749345438`, 1.`,
   {{0, 0.00048182536904587433`, 0.8576491569016562`}, {14, 0.000050664963904379`, 0.0901836357497946`}}},
  {0.2588804970943782`, 0.5492314132783744`, 9.644794872912858`, 0.9613308813426055`, 0.2817033035077452`,
   0.232857138958534`, 0.012136672860729404`, 78.78277994386292`, 0.023082056754425706`,
   4.302644367094656`, 137772.13272659492`, 29258.978388750067`, 8.09377820044302`, 360.90470093830186`,
```

1.`, {{0, 0.025944168309507982`, 46.18061959092421`}, {4, 0.0010518846652108169`, 1.872354704075254`},
{8, 0.0000742226823123579`, 0.13211637451599706`}, {12, 0.0000526465391596112`, 0.09371083970410794`},
{16, 0.0000523165930185082l`, 0.09312353557294463`}, {20, 0.00005231046840250822 6`, 0.09311263375646464`},
{24, 0.0000523100693173012 26`, 0.09311192338479618`}, {28, 0.0000523100011721090 8`, 0.09311180208635417`},
{32, 0.0000523099835659048 05`, 0.09311177074731056`}, {36, 0.0000442779596438408 8`, 0.07881476816603677`}}},
{0.08494913534264076`, 3.4349155581389494`, 7.0407634051690415`, 1.119369452553694`, 0.9329533821244145`,
0.5630698272627359`, 0.4838120213175008`, 71.45974855722541`, 0.09271025542437755`, 2.443770975165119`,
95890.81008474117`, 43256.031528884465`, 230.08379007330387`, 759.4480827877715`, 0.`,
{{0, 0.030349502647287942`, 54.02211471217254`}, {3, 0.0037843817314938476`, 6.736199482059048`},
{6, 0.0010397774690226519`, 1.8508038948603203`}, {9, 0.0004176122044733752`, 0.7433497239626078`},
{12, 0.000266562740040237`, 0.4744816772716219`}, {15, 0.00022495102055654682`, 0.4004128165906533`},
{18, 0.00021122967018539644`, 0.37598881293000563`}, {21, 0.0002057994901616406`, 0.36632309248772027`},
{24, 0.00020328881989558763`, 0.36185409941414604`}, {27, 0.021014948498753405`, 37.406608327781065`}}},
{0.2312360951475228`, 2.6472972630846083`, 4.924168333426426`, 0.8758299082609615`, 0.33581096057577486`,
0.16094039113701897`, 0.009823651093858611`, 148.81624524939704`, 0.12101804849376735`,
7.0744188173362765`, 9248.687097685755`, 2086.5835663460634`, 1.4293883364736226`, 292.38021963052176`,
1.`, {{0, 0.014926618429645872`, 26.569380804769654`}, {5, 0.00526189744107993`, 9.366177445122277`},
{10, 0.001432452032570021 2`, 2.549764617974638`}, {15, 0.00038912545383766264`, 0.6926433078310396`},
{20, 0.00011147412327743021`, 0.1984239394338258`}, {25, 0.00003806607304605811`, 0.06775761002198344`},
{30, 0.000019184313820698314`, 0.034148078600843`}, {35, 0.000014671356924883401`, 0.026115015326292453`},
{40, 0.000013700729211818161`, 0.024387297997036327`}, {45, 0.000013706738505282783`, 0.024397994539403355`}}},
{0.2359361250604497`, 3.266770315195857`, 7.411396165562348`, 1.2275925063587856`, 0.15344120088912327`,
0.536013355113413`, 0.3013372435966537`, 712.0245065436816`, 0.039861802547139946`, 9.042443794820379`, 149.79165608831158`,
2.5225446697720937`, 1.886259023099888`, 112.59656947916369`, 1.`, {{0, 0.006434950484543647`, 11.454211862487693`},
{5, 0.0019406093605597995`, 3.4542846617964433`}, {10, 0.0001817794353129946`, 0.3235673948571304`}}},
{0.26580884355261647`, 3.957059703696273`, 5.486829732050921`, 1.3210508471658353`, 0.3282573265157698`,
0.6164824430541005`, 0.042206581416411024`, 114.97331259087073`, 0.22266675421103638`,
9.071401748075406`, 55556.658656069565`, 11499.557465532658`, 230.08724359080458`, 1869.576895696268`,
0.`, {{0, 0.052362311519042595`, 93.20491450389582`}, {5, 0.0016614832670845707`, 2.9574402154105353`},
{10, 0.0004004831602978107`, 0.7128600253301031`}, {15, 0.0003018411383351793`, 0.5372772262366192`},
{20, 0.0002909803879873182 5`, 0.5179450906174264`}, {25, 0.0003049166933984467 5`, 0.5427517142492352`},

```
{30, 0.0003530115588087958`, 0.6283605746796564`}, {35, 0.00048441166084140506`, 0.862252756297701`},
  {40, 0.0007980941472166765`, 1.4206075820456843`}, {45, 0.08513031053990355`, 151.53195276102832`}}},
{0.24965572729559277`, 1.088568489897936`, 8.460543344934337`, 1.1505850947024536`, 0.8975822933983539`,
 0.5110185880609677`, 0.06613687599714729`, 1096.4114980052268`, 0.1003107894781316`, 4.101976455241168`, 127.8628755202702`,
 3.762126473887197`, 3.762126473887197`, 127.8628755202702`, 1.`, {{0, 0.009717578539540535`, 17.297289800382153`}}},
{0.1942611669549973`, 1.9123387865104826`, 2.4307010631404715`, 1.2953041584793583`, 0.36588469810004653`,
 0.5550679926918506`, 0.01452356295191177`, 246.06581986793796`, 0.04070230178651557`, 1.4358727366404163`, 97040.48025496164`,
 25697.558464652077`, 230.26011695530718`, 909.0934301677505`, 0.`, {{0, 0.06909110069274904`, 122.9821592330933`}}},
{0.20522561065351508`, 3.886130079640968`, 5.862633336878224`, 1.1071510042707493`, 0.46587070079395265`,
 0.3092363340879616`, 0.06237106286442892`, 3704.443052142306`, 0.23732711327647033`, 5.5948716983231535`,
 18.24073513341799`, 0.1533205952946215`, 0.13294500599881778`, 15.758861063535505`, 1.`,
 {{0, 0.0010397229803466395`, 1.8507069050170182`}, {20, 0.00015795046048205908`, 0.2811518196580652`}}},
{0.24537724051627174`, 3.881922170050893`, 2.190905545388226`, 1.4415825883984728`, 0.23029515887672103`,
 0.5607538040686315`, 0.02143037658176177`, 612.4231659367224`, 0.18957440226421673`, 2.1066205948825285`,
 44.96270739213868`, 0.9443872102968023`, 0.6355170684730753`, 44.26633809876663`, 1.`,
 {{0, 0.0026131634387261972`, 4.651430920932631`}, {6, 0.0006202348986491448`, 1.1040181195954777`},
  {12, 0.00010102802763995095`, 0.17982988919911266`}, {18, 0.000029815330490971123`, 0.053071288273928596`}}},
{0.06117754469042569`, 1.77753347893454`, 8.494748688583062`, 1.4528026068281314`, 0.13374021962928717`,
 0.2747744172973582`, 0.02769785108344557`, 761.9216006517844`, 0.1674213538626742`, 9.223974549251146`,
 104.37122598019772`, 1.106937317790655`, 0.9253595839595733`, 87.05869063379347`, 1.`,
 {{0, 0.004600675044050828`, 8.189201578410474`}, {50, 0.0020157854441174763`, 3.5880980905291078`}}},
{0.2543463901897882`, 3.8184481545424687`, 6.435093510144283`, 1.0179149591357712`, 0.6456387658124723`,
 0.39719113288117625`, 0.03560663844887868`, 836.6099873310699`, 0.05172688927212099`,
 4.4940967912431145`, 92.50705857694102`, 0.580524101062166`, 0.491790343419445`, 76.04783154184503`, 1.`,
 {{0, 0.004077716420335571`, 7.258335228197316`}, {36, 0.0017019187768446502`, 3.0294154227834773`}}},
{0.25340300959745826`, 3.7481040027914663`, 6.564583042000866`, 0.9665595115932711`, 0.6900722172611409`,
 0.5320596915161446`, 0.007953381223150922`, 557.3521763213881`, 0.16633866213427612`,
 7.2685179596650595`, 289.9526955762029`, 0.6124033654618907`, 0.5865056780384655`, 282.12724053488074`,
 1.`, {{0, 0.01696802035886134`, 30.20307623877319`}, {5, 0.004473649921789595`, 7.963096860785479`}}},
{0.21905085798311597`, 3.1393917093979224`, 6.344370247647537`, 0.9036844621009067`, 0.45657431516696256`,
 0.6738956741194089`, 0.01713634873689236`, 595.7124001741428`, 0.1327223790362297`, 6.393427116779321`, 189.5667522528146`,
```

```
0.8438091800899522`, 0.8438091800899522`, 189.5667522528146`, 1.`, {{0, 0.01440707317121391`, 25.64459024476076`}}},
{0.26756748312672074`, 2.582420366138944`, 0.22100971269994044`, 1.2986139421663903`, 0.40694219440887625`,
 0.4206092167171217`, 0.07540978051777308`, 286.7043124505423`, 0.12484831726628037`, 7.450517421109604`, 61364.44550581311`,
 12722.421392849294`, 230.17513478357588`, 1228.9921860945853`, 0.`, {{0, 0.0934034061431885`, 166.2580629348755`}}},
{0.1649279191778415`, 1.2798961157951103`, 8.443353077105307`, 1.1073246765756934`, 0.5543130008347743`,
 0.4677559431287117`, 0.04549043803104127`, 88.19757502008439`, 0.08859698434873292`, 4.4693006714200685`,
 66968.59336970124`, 19867.382305666528`, 228.78739091971033`, 1127.358035313329`, 0.`,
 {{0, 0.07852961095535806`, 139.78270750053733`}, {16, 0.00025949969651916597`, 0.4619094598041154`},
  {32, 0.0002506548202853745`, 0.44616558010796664`}, {48, 0.00025051872736619107`, 0.44592333471182005`},
  {64, 0.00025052257044005466`, 0.4459301753832972`}, {80, 0.00025056171057759324`, 0.445999844828116`},
  {96, 0.0002506224835066749`, 0.44610802064188126`}, {112, 0.0002507030213232532`, 0.4462513779553906`},
  {128, 0.00025080319544079594`, 0.44642968788461673`}, {144, 0.005135713502995852`, 9.141570035332617`}}},
{0.24671033429293737`, 0.7439602453864245`, 2.8830481632165945`, 1.0433060628070765`, 0.2605457162416671`,
 0.35613575939670206`, 0.02699949405773168`, 552.8129873973498`, 0.15941770646667952`,
 8.472285283746757`, 162.91448578185557`, 3.917260182187165`, 3.727155808562145`, 150.77437071811968`,
 1.`, {{0, 0.007552728634804629`, 13.44385696995224`}, {33, 0.003906123539772467`, 6.952899900794991`}}},
{0.15869950794106824`, 3.1033559606377414`, 9.144330738839969`, 1.4178115100024233`, 0.3048291795319098`,
 0.2869274730275907`, 0.17534043095376647`, 52.531250426358234`, 0.14088030152015352`,
 4.046496400392222`, 118882.02386303202`, 36302.28283380921`, 228.91998490750933`, 1088.0209781796827`,
 0.`, {{0, 0.07813989830173135`, 139.08901897708182`}, {23, 0.0035040794202391384`, 6.237261368025665`},
  {46, 0.00027750721965883775`, 0.49396285099273113`}, {69, 0.00009232165066851001`, 0.1643325381899478`},
  {92, 0.00007144910322251059`, 0.12717940373606884`}, {115, 0.00006879826770065968`, 0.12246091650717422`},
  {138, 0.00006820740903051683`, 0.12140918807431995`}, {161, 0.00006806532419421761`, 0.12115627706570735`},
  {184, 0.00006803034059670318`, 0.12109400626213165`}, {207, 0.0003312373046134263`, 0.5896024022118989`}}},
{0.2764621058346162`, 2.9381918751992178`, 0.9446276562373105`, 1.4121699137634485`, 0.5065538844804354`,
 0.6085910850890284`, 0.12806524333884275`, 90.5764532701865`, 0.169606806379912`, 4.122343563126204`, 163538.67889342218`,
 33035.97402988522`, 229.96106427086733`, 1470.4266347383202`, 0.`, {{0, 0.11175242424011234`, 198.91931514739997`}}},
{0.23050133288232844`, 1.3238077162346595`, 3.1108482993453865`, 1.1555015486294062`, 0.6747942262463269`,
 0.6285668584610278`, 0.027391537177886824`, 109.70658223761104`, 0.027210691652561736`,
 1.9248854769177905`, 486392.0716893193`, 113291.54937011692`, 228.82060799528549`, 36342.47545313132`,
 0.`, {{0, 0.03822140263259417`, 68.03409668601763`}, {5, 0.017449480733084603`, 31.060075704890593`},
```

```
    {10, 0.014095438260600876`, 25.089880103869557`}, {15, 0.013271185134148494`, 23.62270953878432`},
    {20, 0.013714268247218183`, 24.411397480048365`}, {25, 0.014908718464894622`, 26.53751886751243`},
    {30, 0.01655625757439023`, 29.47013848241461`}, {35, 0.018440995353699967`, 32.82497172958595`},
    {40, 0.0204135411421291`, 36.3361032329898`}, {45, 2.5949568468952204`, 4619.023187473493`}}},
{0.16511179970471734`, 1.257711050374506`, 7.3270141910702415`, 1.3219934358839476`, 0.5519839493462388`,
  0.6927265248357952`, 0.057669326830527015`, 282.0308127392517`, 0.18961708461999577`,
  6.119113848125975`, 386.1275084674433`, 10.304976175248559`, 8.470619225884345`, 328.22516521623504`,
  1.`, {{0, 0.02142235137750017`, 38.1317854519503`}, {19, 0.0035227611789336935`, 6.2705148985019745`}}},
{0.18051596911200918`, 3.1154638731836117`, 5.271301524292921`, 0.9821224785779399`, 0.579876513557519`,
  0.2578243252832043`, 0.01324703728258807`, 298.19257336110485`, 0.04626579120349833`, 3.252038865320179`,
  134.57577658022095`, 0.7669676539278052`, 0.5334015828477452`, 124.12750873044041`, 1.`,
  {{0, 0.006221134651771846`, 11.073619680153886`}, {5, 0.0023699078893841072`, 4.218436043103711`},
    {10, 0.0007517670046252754`, 1.3381452682329904`}, {15, 0.0000908811177322429`, 0.16176838956339237`}}},
{0.2700603276661351`, 3.8874233694867435`, 9.935191315459868`, 0.9369398631417749`, 0.9680175802467335`,
  0.2817307366148821`, 0.03116132832319949`, 267.3428425281449`, 0.2310886914635361`, 4.719763737676903`,
  363.02233411688366`, 2.384158600524495`, 1.6993976017738093`, 289.0691485926092`, 1.`,
  {{0, 0.016277924885004645`, 28.97470629530827`}, {22, 0.005691330408033655`, 10.130568126299906`}}},
{0.2505990837413898`, 3.9386610341401305`, 4.197732331385515`, 1.4863985983426793`, 0.6450186838764853`,
  0.5983146588433161`, 0.0058599277111039`, 4347.60789332823`, 0.1797443484893408`, 5.002549103667693`, 25.07650818288479`,
  0.045454901500411246`, 0.045454901500411246`, 25.07650818288479`, 1.`, {{0, 0.0019058146218992442`, 3.3923500269806546`}}},
{0.15385799276986173`, 3.328452062918277`, 1.1907303286310018`, 0.9499432416831306`, 0.24618506024325426`,
  0.6220480490433855`, 0.08397939385457624`, 160.09510109398894`, 0.21511864519397922`, 7.34323009363505`, 32608.290024555277`,
  10175.047107730481`, 229.78192556745918`, 1141.094032086824`, 0.`, {{0, 0.08672314643859863`, 154.36720066070555`}}},
{0.10621732051626104`, 1.365898117434102`, 6.864395407461604`, 0.9825773772303991`, 0.8367483775504436`,
  0.5624371522540671`, 0.07041397408927531`, 60.72834180417396`, 0.20758521960363197`,
  6.929584578355666`, 58409.78224203836`, 23054.55896982592`, 229.44534969609307`, 1501.5250533374176`,
  0.`, {{0, 0.05345564646260932`, 95.1510507034446`}, {3, 0.009866536315142848`, 17.56243464095427`},
    {6, 0.0037803092852819083`, 6.728950527801796`}, {9, 0.0016409202230207682`, 2.9208379969769678`},
    {12, 0.0008317629352156704`, 1.4805380246838933`}, {15, 0.0005067324963468355`, 0.9019838434973672`},
    {18, 0.0003675857051333633`, 0.6543025551373867`}, {21, 0.0003036309998986804`, 0.5404631798196511`},
    {24, 0.0002721054846649122`, 0.48434776270354374`}, {27, 0.04309067414632943`, 76.7013999804664`}}},
```

```
{0.2268285366732684`, 3.377330494378361`, 0.792106071900994`, 1.4321394301198889`, 0.4771428999999012`,
  0.6107585820948693`, 0.0427110994331284`, 2178.207920314213`, 0.06460930790004932`, 5.987666522418898`, 44.45312816329743`,
  1.0151094388342128`, 0.7451010722652529`, 42.86392301334045`, 1.`, {{0, 0.002824397944976279`, 5.027428342057776`},
    {6, 0.00038249571765741034`, 0.6808423774301904`}, {12, 0.000050764486380185046`, 0.09036078575672939`}}},
{0.27738294555080567`, 1.5184858354681863`, 2.5078605202307482`, 1.4658418288357904`, 0.7951974935096349`,
  0.3102987260459962`, 0.028082255793227213`, 4738.743053997216`, 0.039514889508313555`, 1.270204126939964`,
  5.0477582232509945`, 0.07786306149120237`, 0.07380920704709575`, 4.41645625178013`, 1.`,
  {{0, 0.00019948741217790973`, 0.3550875936766793 6`}, {30, 0.00013616326295738017`, 0.2423706080641367`}}},
{0.12722141666194153`, 2.144800891916389`, 0.9267204145449971`, 1.474327309271881`, 0.07507296673368935`,
  0.4110764361383803`, 0.01396212306727472`, 341.96044988390173`, 0.08796076167353961`, 8.378052186889676`, 40703.317562712655`,
  14425.054601162854`, 230.2343957580028`, 777.0424140127083`, 0.`, {{0, 0.059055223464965834`, 105.11829776763918`}}},
{0.14199823373416764`, 1.4769393574961134`, 9.82023825383516`, 0.8826223667346553`, 0.41403084809983204`,
  0.6165765653923829`, 0.060760206852004485`, 76.65984772713675`, 0.1884299459763023`, 7.821991164404671`,
  34702.494111446664`, 11257.138448159352`, 228.46632743085576`, 1509.456632778878`, 0.`,
  {{0, 0.06737189362231324`, 119.92197064771757`}, {14, 0.0015234559857578308`, 2.711751654648939`},
    {28, 0.001522423368617232`, 2.7099135961386733`}, {42, 0.0015235331255738346`, 2.7118889635214254`},
    {56, 0.001530551212267486 8`, 2.724381157836126`}, {70, 0.001567260460393457 3`, 2.789723619500354`},
    {84, 0.0016146985382595395`, 2.87416339810198`}, {98, 0.001571954332025753`, 2.7980787110058403`},
    {112, 0.0015442829031186063`, 2.748823567551119`}, {126, 0.034948650542867735`, 62.20859796630457`}}},
{0.2704769900712498`, 1.6373927417773038`, 3.0886177841931914`, 1.4817168381054997`, 0.05399471731043759`,
  0.4806448506103316`, 0.008090658829532001`, 56.76295821829396`, 0.22108849119145935`, 4.3771215003685064`, 185341.5555474955`,
  38083.78034056347`, 229.9383912143852`, 1538.6068193536062`, 0.`, {{0, 0.11693411827087406`, 208.1427305221558`}}},
{0.15635355369607173`, 1.2350233440589857`, 3.2938536959067304`, 0.7696047462951592`, 0.9490314349758091`,
  0.5590905433465692`, 0.008743912631106248`, 94.37874467585033`, 0.17474749461052458`,
  8.353315585312398`, 48722.418553545765`, 14999.643760905892`, 230.1856103642279`, 2859.1993735499846`,
  0.`, {{0, 0.03747811903048946`, 66.71105187427125`}, {3, 0.015556703814273615`, 27.69093278940704`},
    {6, 0.011122265340141992`, 19.797632305452744`}, {9, 0.00813686248160267 3`, 14.48361521725276`},
    {12, 0.00605182518671535`, 10.772248832353322`}, {15, 0.004572770853855737`, 8.139532119863214`},
    {18, 0.003511380792185058`, 6.250257810089403`}, {21, 0.0027418674811998897`, 4.880524116535804`},
    {24, 0.0021787191322336025`, 3.878120055375812`}, {27, 0.12594863827710145`, 224.1885761332406`}}},
{0.16339273559725687`, 2.0059391082075457`, 1.9960227683476044`, 0.8832597488866586`, 0.2201232888636404`,
```

```
  0.41940637033719763`, 0.260701203173729`, 668.3313383270491`, 0.16081430237854888`, 8.573990161395482`,
  142.88418368381315`, 4.134531799989724`, 2.221413920402483`, 80.75729182086233`, 1.`,
  {{0, 0.005755372453872358`, 10.244562967892797`}, {18, 0.0003821817245131801`, 0.6802834696334605`}}},
 {0.17131668518162624`, 1.9273467762184806`, 5.930228637778157`, 0.8979121960709446`, 0.761163722566164`,
  0.6467717629026779`, 0.01019608706680365`, 260.442341207733`, 0.11039361670332914`, 7.204688135978646`, 387.7453116017390 5`,
  4.699201787910189`, 2.458491329195802`, 394.41795731786414`, 1.`, {{0, 0.025261490707080683`, 44.96545345860361`},
   {2, 0.004181035963636641`, 7.442244015273221`}, {4, 0.00042114910815263 29`, 0.7496454125116867`},
   {6, 0.00005283615136235458`, 0.09404834942499116`}, {8, 0.000021789820991855646`, 0.03878588136550305`},
   {10, 0.00001958408153481695`, 0.03485966513197418`}, {12, 0.00001787892339868917`, 0.031824483649666724`}}},
 {0.11942926109779373`, 3.6640895220352094`, 2.8978096019149504`, 1.1395608488550686`, 0.02531057599926112`,
  0.3982329172386545`, 0.26228943719870584`, 89.66215495367044`, 0.22730124409898161`,
  8.643250664580492`, 47645.08297986628`, 17535.064347488933`, 230.1212528360634`, 2569.1056546589816`,
  0.`, {{0, 0.038533010093216954`, 68.58875796592618`}, {4, 0.012699193643410217`, 22.604564685270187`},
   {8, 0.008515934193136879`, 15.158362863783646`}, {12, 0.006451501174244872`, 11.483672090155872`},
   {16, 0.005114715689655442`, 9.104193927586687`}, {20, 0.0040974396635638875`, 7.29344260114372`},
   {24, 0.0032890889263663646`, 5.854578288932129`}, {28, 0.002650008276179687`, 4.717014731599843`},
   {32, 0.002150119507314529`, 3.827212723019862`}, {36, 0.1117510185869938`, 198.91681308484894`}}},
 {0.06995397995088021`, 2.4465420953300523`, 1.0825256418482638`, 1.4045898231548657`, 0.8050454938859057`,
  0.5373350569389552`, 0.3667462640330922`, 406.36701228872533`, 0.024784828878876453`,
  8.741309230400322`, 82872.9610250695`, 41411.78331037222`, 228.3457688461868`, 625.1217678496429`, 0.`,
  {{0, 0.010309885011171364`, 18.35159531988503`}, {4, 0.0015863641204007838`, 2.8237281343133946`},
   {8, 0.0005119489168594062`, 0.9112690720097432`}, {12, 0.00024635985298866763`, 0.4385205383198284`},
   {16, 0.0001718128949258949`, 0.30582695296809287`}, {20, 0.00014713965556921384`, 0.2619085869132006`},
   {24, 0.00013739938766795998`, 0.24457091004896875`}, {28, 0.00013299780678600702`, 0.23673609607909252`},
   {32, 0.0001308925513995346`, 0.23298874149117163`}, {36, 0.03413445415880403`, 60.759328402671166`}}},
 {0.25135395656732756`, 1.8293827828852027`, 0.6280251816297326`, 0.776655925877336`, 0.712342015777852`,
  0.6529270906121196`, 0.026921744082008515`, 2433.232128346219`, 0.014846384799260759`,
  4.779446799164496`, 35.958359007037274`, 2.231465687822763`, 0.767915022104513`, 32.73703357108716`,
  1.`, {{0, 0.001398624046374028`, 2.4895508025457698`}, {2, 0.0007130612903649202`, 1.2692490968495578`},
   {4, 0.0002249680900119271 4`, 0.4004432002212303`}, {6, 0.00006832167280661728`, 0.12161257759577876`},
   {8, 0.000025387975784831398`, 0.04519059689699988 4`}, {10, 0.000014243370948877 33`, 0.025353200289001645`},
```

```
    {12, 0.0000115218500945767`, 0.020508893168346594`}, {14, 0.000010903264331680471`, 0.019407810510391237`},
    {16, 0.000010771357474542102`, 0.019173016304684938`}, {18, 0.000010211633210623573`, 0.01817670711490996`}}},
  {0.22686431298761822`, 3.544454161776195`, 7.257561779040259`, 1.4835566223233323`, 0.41530778886586006`,
   0.5693420006079292`, 0.0281206594086524`, 300.82869963510313`, 0.2233455291441253`, 1.8496714258079727`, 93.02073939466582`,
   0.6632223524814925`, 0.6391921809764639`, 92.55612538630156`, 1.`, {{0, 0.005967776731409778`, 10.622642581909405`},
    {4, 0.0009920971414236522`, 1.7659329117341012`}, {8, 0.00007439165652548808`, 0.1324171486153688`}}},
  {0.11505780978488611`, 2.035276516222745`, 0.3473218826840405`, 1.3302263404025527`, 0.8863987944255065`,
   0.20525449010517516`, 0.166947674879067`, 1822.5924091420022`, 0.2368153750944742`, 8.635263957438244`, 911.616911200983`,
   336.4008973640811`, 1.8029725440221993`, 26.75023291086213`, 1.`, {{0, 0.0016644928179858714`, 2.9627972160148506`},
    {10, 0.00023250064883094753`, 0.41385115491908664`}, {20, 0.000027020376644309777`, 0.048096270426871396`},
    {30, 0.000016237836449761`, 0.028903348880574584`}, {40, 0.000015860555807649428`, 0.028231789337615967`},
    {50, 0.000015847056150131866`, 0.02820775994723472`}, {60, 0.000015846087160760188`, 0.028206035146153115`},
    {70, 0.00001584596079356609`, 0.028205810212538398`}, {80, 0.000029366361402529942`, 0.0522721232965033`}}},
  {0.26450836027154073`, 2.000822664278666`, 6.699190267572199`, 1.2017845913999912`, 0.3438445280396565`,
   0.6571515800951808`, 0.025420529665258214`, 670.8719376728277`, 0.0689542698910992`, 7.858447976519758`, 180.07809198342397`,
   1.631566021536522`, 1.631566021536522`, 180.07809198342397`, 1.`, {{0, 0.013685934990740223`, 24.360964283517596`}}},
  {0.1895156330025849`, 2.3307833365396586`, 5.573081915828208`, 1.037167544233136`, 0.6437280244785568`, 0.698584836099976`,
   0.020283589241000377`, 257.16539910388553`, 0.09483519874959423`, 5.443513900146426`, 297.2872756457915`,
   7.29127410601314`, 2.9270885667880653`, 293.0006806026297`, 1.`, {{0, 0.01848653404786665`, 32.90603060520264`},
    {2, 0.003293252645000096`, 5.861989708100171`}, {4, 0.00033748701519934285`, 0.6007268870548302`},
    {6, 0.000048521846183422396`, 0.08636888620649187`}, {8, 0.000025588847966886205`, 0.045548149381057446`},
    {10, 0.00002409899075285957`, 0.04289620354009003`}, {12, 0.000024019404875962717`, 0.042754540679213644`},
    {14, 0.000024015677327279136`, 0.042747905642556865`}, {16, 4.5332506273581535`*^-6, 0.008069186116697514`}}},
  {0.05605442916152037`, 2.091827585999483`, 5.134093728882359`, 0.7852275512421112`, 0.9528052242020535`,
   0.26916872175155115`, 0.16382211865694157`, 830.5018741809612`, 0.0523186960509337`, 8.350099727317033`, 236.07075787885512`,
   5.356581636495559`, 1.7809659686254897`, 79.26239146868267`, 1.`, {{0, 0.004082483294189026`, 7.266820263656467`},
    {9, 0.0015645174459230824`, 2.784841053743087`}, {18, 0.00030568981807719685`, 0.5441278761774104`},
    {27, 0.000057104003340748096`, 0.10164512594653162`}, {36, 0.000014147190089829198`, 0.02518199835989597`}}},
  {0.160097742020257`, 2.0916642582432887`, 7.779572730473025`, 1.4293729759208196`, 0.262481852877007`, 0.6066507214539851`,
   0.010299825795425889`, 166.88640436294955`, 0.11119499215436468`, 1.1831617215189443`, 89.48839484073358`,
   2.0011699408449672`, 1.0993326533679624`, 90.23547651735272`, 1.`, {{0, 0.005260079195208545 5`, 9.36294096747121`},
```

{4, 0.0013313073595440138`, 2.369727099988345`}, {8, 0.0001981723647700915`, 0.35274680929076285`},
{12, 0.0000402364791869711`, 0.07162093295280857`}, {16, 0.0000281008166091851`, 0.050019453564349475`}}},
{0.096645259209576`, 1.264318941007299`, 4.097121191163998`, 1.119171761441141`, 0.3848067085465605`,
0.38298305619587747`, 0.22103546881327146`, 186.61459180270506`, 0.035388155328102466`,
2.6745014933563738`, 41233.10616888702`, 17285.14102119014`, 229.82692036773113`, 928.3793645238734`,
0.`, {{0, 0.01866964126987034`, 33.2319614603692`}, {14, 0.013077118239698603`, 23.277270466663516`},
{28, 0.003141045570305957 7`, 5.591061115144605`}, {42, 0.0005476581557449048`, 0.9748315172259306`},
{56, 0.0001007910796410416 6`, 0.17940812176105414`}, {70, 0.000027666893605942788`, 0.049247070618578165`},
{84, 0.000014782502524470487`, 0.026312854493557466`}, {98, 0.000012276033839650294`, 0.021851340234577524`},
{112, 0.000011713151669812924`, 0.020849409972267006`}, {126, 0.034954138806913664`, 62.218367076306315`}}},
{0.08994789013179427`, 1.57009542864993`, 6.7950768142652755`, 1.2471941772808341`, 0.17502080201658954`,
0.4646155607499266`, 0.031711372215814826`, 510.77004725104206`, 0.22094423048581935`,
5.498912040996139`, 117.8921968426536`, 1.5691336101840652`, 1.4916555585615898`, 110.98948468063912`,
1.`, {{0, 0.0061822809368498035`, 11.00446006759265`}, {40, 0.002252919898878769`, 4.010197420004209`}}},
{0.09559160148361812`, 1.3237772094663507`, 8.951678422145957`, 0.7806574543205589`, 0.8827653082752778`,
0.45488871998545977`, 0.011151733542884613`, 3151.7776331194414`, 0.22534056788365686`, 6.821338260162536`, 66.03533983615444`,
0.45800672470875603`, 0.45800672470875603`, 66.03533983615444`, 1.`, {{0, 0.005018685827547737`, 8.933260773034972`}}},
{0.20804533970301103`, 1.865488871682949`, 2.4561594295175926`, 0.8614398462681598`, 0.7232950760420711`,
0.3427980526897182`, 0.02113398228875948`, 356.75904569388376`, 0.23964513558905903`, 4.163032360670947`, 1984.185422153315`,
478.9426612210035`, 1.5969382942952957`, 96.61178349554054`, 1.`, {{0, 0.00574534286304715`, 10.226710296223928`},
{4, 0.0012918870260470625`, 2.2995589063637714`}, {8, 0.0001864121192948937`, 0.33181357234491077`},
{12, 0.000036727868792738376`, 0.0653756064510743`}, {16, 0.000019276386271926645`, 0.034311967564029426`},
{20, 0.000017591188915061408`, 0.03131231626880931`}, {24, 0.000017457967032256635`, 0.031075181317416815`},
{28, 0.000017448654836230677`, 0.031058605608490608`}, {32, 0.000010351471423761413`, 0.018425619134295315`}}},
{0.23422673966465207`, 3.962742244147649`, 9.344278992351082`, 0.9436428211653483`, 0.6202800294740252`,
0.15583995298414144`, 0.0468134491479599`, 1145.0934679847246`, 0.11614669946806178`, 8.273469182440888`, 76.81217943247714`,
0.5301581170214313`, 0.5301581170214313`, 76.81217943247714`, 1.`, {{0, 0.005837725636868262`, 10.391151633625508`}}},
{0.23911413452544644`, 3.701379132317136`, 3.3596187018850023`, 1.2070387760959833`, 0.14815711868787962`,
0.5552188532365824`, 0.010060549211211993`, 324.2801725236705`, 0.014653386006449387`,
6.641284984634726`, 40898.704017999575`, 9218.031781586853`, 229.8537663901108`, 1244.893029505707`,
0.`, {{0, 0.010286044205442019`, 18.309158685686793`}, {1, 0.0010161057448829544`, 1.8086682258916587`},

```
{2, 0.00017206089552454233`, 0.3062683940336854`}, {3, 0.00006556454606442448`, 0.11670489199467558`},
{4, 0.0000507574296083814`, 0.0903482247029189`}, {5, 0.00004840344526861025`, 0.08615813257812624`},
{6, 0.000047966094552870675`, 0.08537964830410981`}, {7, 0.000047871674623993024`, 0.08521158083070758`},
{8, 0.000047848326324793386`, 0.08517002085813223`}, {9, 0.08282924788014115`, 147.43606122665125`}}},
{0.16379493488608782`, 3.773301948253276`, 6.66686177778092`, 1.3720180958436647`, 0.9115747518300852`, 0.36809943749279594`,
 0.006089940921734969`, 188.41929546062013`, 0.11766502525697531`, 3.8904832664355844`, 225.71959389388627`,
 2.99474919975301`, 0.8780063626743505`, 244.40359955602173`, 1.`, {{0, 0.010377948287346443`, 18.47274795147667`},
 {4, 0.0053657276064697 7`, 9.550995139516191`}, {8, 0.0018723105140790964`, 3.3327127150607923`},
 {12, 0.0006307214494778799`, 1.1226841800706262`}, {16, 0.00021531574544292998`, 0.3832620268884154`},
 {20, 0.00007723640975527041`, 0.13748080936438134`}, {24, 0.00003541355368626165`, 0.06303612556154574`}}},
{0.27173801863077446`, 2.1930338431922545`, 7.869758966833125`, 1.0214571559787395`, 0.457397629072652`,
 0.6590886785471264`, 0.009120521470678952`, 4004.0778102470904`, 0.1596904202504179`, 8.692703920363371`, 38.1367075420029`,
 0.1378590388075727`, 0.1378590388075727`, 38.1367075420029`, 1.`, {{0, 0.0028983897731922207`, 5.159133796282152`}}},
{0.2361473928644357`, 1.7414292069064263`, 7.944932185587515`, 1.1578150203530142`, 0.6270752096378571`,
 0.30275674150027165`, 0.11839453780639704`, 3486.2324040163485`, 0.1006900893181421`, 6.697770970769804`,
 28.187080331557592`, 0.6783964220846059`, 0.35426780712525585`, 14.865461825269685`, 1.`,
 {{0, 0.0008354646616606067`, 1.48712709775588`}, {20, 0.0002943104370598895`, 0.5238725779666034`}}},
{0.08448906159522324`, 2.163318965633623`, 2.5852320656449397`, 0.9870547358990118`, 0.27911648709335135`,
 0.1950863385031143`, 0.04472304557991584`, 1858.3083947017237`, 0.05117109151128901`,
 9.531470241589851`, 43.9374515470728`, 0.5361959836978123`, 0.3439027367682504`, 27.986200852042955`, 1.`,
 {{0, 0.001469266920886273 4`, 2.6152951191775666`}, {38, 0.0006576843438689913`, 1.1706781320868045`}}},
{0.1142146683211071`, 3.422389653131356`, 1.8177299602895634`, 1.2969315528452774`, 0.1391750354375907`,
 0.5170758706651055`, 0.01897326911835137`, 87.52693577556593`, 0.11000871045324723`, 3.4664980752399295`, 128654.38482185059`,
 48868.20603545219`, 230.00163865143017`, 970.0866749412138`, 0.`, {{0, 0.07372658729553225`, 131.23332538604743`}}},
{0.14347242790835574`, 2.011431525472254`, 0.8946353742157331`, 1.3818589873326788`, 0.974530392330166`,
 0.4363768778472087`, 0.006586014840232095`, 142.93650600689625`, 0.2189385420017279`, 6.889239014732425`, 48131.69261379855`,
 15766.952534271968`, 230.12910474207135`, 986.6407043055483`, 0.`, {{0, 0.07498469352722167`, 133.4727544784546`}}},
{0.07350179944910268`, 2.001951652427108`, 6.856579719843172`, 1.3883617808830775`, 0.83650706130597`,
 0.6246458892820506`, 0.03443555932639553`, 159.6804633773726`, 0.22561794329975743`, 6.769156049603524`,
 4954.5855265563205`, 2217.361469048306`, 38.700549007943756`, 509.5899766917724`, 1.`,
 {{0, 0.03765390512136205 4`, 67.02395111602446`}, {21, 0.001074933107212648`, 1.9133809308385135`}}},
```

```
{0.2038713609791254`, 2.9048730428426977`, 6.578839990238183`, 1.1970767733981913`, 0.3047670438201835`,
  0.4988995484689318`, 0.027219703475022124`, 126.68486054839573`, 0.1069997024432573`,
  5.709865936189522`, 40974.57930763331`, 10395.121495215124`, 223.11607130971092`, 2509.0930419001957`,
  0.`, {{0, 0.06215275342889786`, 110.6319011034382`}, {24, 0.006430135579506069`, 11.445641331520802`},
    {48, 0.006430137499095197`, 11.44564474838945`}, {72, 0.00643017075617713`, 11.445703945995291`},
    {96, 0.006430407772505147`, 11.446125835059162`}, {120, 0.006432090783514113`, 11.449121594655121`},
    {144, 0.006443072815241331`, 11.46866961112957`}, {168, 0.006477188246634259`, 11.529395079008983`},
    {192, 0.006473047845512551`, 11.522025165012339`}, {216, 0.07699206645733123`, 137.0458782940495`}}},
{0.10518373092933642`, 1.253339618555911`, 0.9435760784539173`, 1.312750209379657`, 0.30938419913876847`,
  0.5412461470886345`, 0.010398682812166098`, 560.2137041073427`, 0.038740677468453555`, 9.486718448691846`, 38653.28260921861 5`,
  15415.719046408201`, 230.23918529319297`, 688.6642230184457`, 0.`, {{0, 0.052338480949401876`, 93.16249608993535`}}},
{0.21800976245075693`, 1.265555352818649`, 7.66080710349685`, 0.9976040672991493`, 0.42066904207729006`,
  0.6052137627060103`, 0.1434637338398527`, 296.0621032273413`, 0.0508815854649306`, 9.05489394101457`,
  493.94820755870967`, 17.28719118697359`, 10.875597739177893`, 313.92155641067933`, 1.`,
  {{0, 0.02102244439504822`, 37.41995102318583`}, {20, 0.002835593892163409`, 5.0473571280508684`}}},
{0.14099839999887143`, 0.709118554874764`, 1.895334494340693`, 1.0269112445912816`, 0.6721979672345315`,
  0.244304954855444`, 0.0716581941489167`, 50.83934571528565`, 0.0295628703988986`, 3.2095234353654245`, 655663.7336615705`,
  217506.89315498224`, 230.2201138800017`, 858.4146123183403`, 0.`, {{0, 0.06523951053619387`, 116.12632875442509`}}},
{0.16175267254619935`, 2.3816849613140647`, 5.017466007223192`, 1.1449585227321033`, 0.0709392075018902`,
  0.21629140918390133`, 0.24748736884470657`, 967.6089395954832`, 0.2384053881049194`, 4.566103496741098`, 35.429388262638646`,
  0.7782996425559533`, 0.3651875874516589`, 16.86422245561216`, 1.`, {{0, 0.0011348826564351428`, 2.0200911284545535`},
    {14, 0.00014248249157002593`, 0.25361883499464616`}, {28, 4.315758621355583`*^-6, 0.007682050346012937`}}},
{0.15219039437367055`, 3.36973706117323`, 6.4489465173864`, 1.4529455229052564`, 0.15905243014518256`,
  0.19120675078037952`, 0.09107249936889779`, 1785.557342737041`, 0.07266026496560668`, 8.481021831493972`, 36.83199429513763`,
  0.4187335753951166`, 0.4187335753951166`, 36.83199429513763`, 1.`, {{0, 0.0027992315664304604`, 4.98263218824622`}}},
{0.1147282820108807`, 1.275793939396646`, 2.8627986987042053`, 1.2289286563020643`, 0.9300053983658252`,
  0.5333937455451996`, 0.40966617997380966`, 55.29536930365472`, 0.09207723861615985`, 9.133686457942215`, 242169.74999445686`,
  91695.05662141072`, 230.16735667012227`, 1421.602525209126`, 0.`, {{0, 0.10804179191589358`, 192.3143896102906`}}},
{0.22214055647639447`, 3.77956922277194`, 8.844837651533133`, 1.3011494481281731`, 0.35962071678441987`,
  0.36854620389209614`, 0.030699018263752917`, 153.2818434470183`, 0.18511332787655327`,
  9.937679092387768`, 735.0843607721722`, 6.320045006462561`, 4.190566001261678`, 662.9220656396021`, 1.`,
```

```
{{0, 0.039665148963937566`, 70.60396515580887`}, {19, 0.010716928024672195`, 19.07613188391651`}}},
{0.11158828176697494`, 2.207019844443386`, 8.337407964588238`, 0.903037166955644`, 0.0492331295750732`,
  0.5332115600383506`, 0.10225753888572825`, 270.6598424430625`, 0.11536791182647016`,
  7.224089837931905`, 285.305710775736`, 5.142862292277078`, 3.594091968186049`, 199.42119547116036`, 1.`,
  {{0, 0.013660437356669943`, 24.3155784948725`}, {28, 0.0014955734991382442`, 2.6621208284660747`}}},
{0.11228066784233581`, 2.8383409912516155`, 8.777526606092515`, 0.8243326677721828`, 0.7215210925398621`,
  0.47575335568293875`, 0.01179892034723288`, 1881.3977477117185`, 0.1350202375088484`, 6.891832227766253`, 80.58248619015855`,
  0.28446090672132424`, 0.28446090672132424`, 80.58248619015855`, 1.`, {{0, 0.00612426895045205`, 10.90119873180465`}}},
{0.18415971316226493`, 3.627641157385548`, 4.072786596426541`, 1.1318163513169097`, 0.431227341960456`,
  0.35638232246082124`, 0.12315140656794354`, 98.11903649760767`, 0.20630818704214687`,
  2.4221856850573946`, 46780.71874464393`, 12863.638422235004`, 228.49737352742005`, 976.5569521909572`,
  0.`, {{0, 0.053088975666602124`, 94.49837668655177`}, {20, 0.001827105844705189`, 3.2522484035752366`},
    {40, 0.0016028051780965584`, 2.852993217011874`}, {60, 0.0013645286271160143`, 2.4288609562665058`},
    {80, 0.0012958940667284911`, 2.3066914387767143`}, {100, 0.0012767157701460805`, 2.2725540708600236`},
    {120, 0.001270733670710373`, 2.261905933864464`}, {140, 0.0012688735422092415`, 2.25859490513245`},
    {160, 0.0012682924267186002`, 2.2575605195591084`}, {180, 0.009954403573480083`, 17.718838360794546`}}},
{0.2708373028347817`, 0.6873063866109219`, 4.963135106708849`, 1.480469510663712`, 0.1951402154410362`,
  0.5673083531043152`, 0.10423026212852673`, 2550.63829867399`, 0.08286966503737198`, 2.6901867167370597`, 12.85023651233897`,
  0.7048581298633668`, 0.7048581298633668`, 12.85023651233897`, 1.`, {{0, 0.0009766179749377618`, 1.738379995389216`}}},
{0.2248876472394919`, 0.40722939312005924`, 9.89488647226247`, 1.363579526182362`, 0.9039905300116995`,
  0.6204673017769224`, 0.16140635189013572`, 124.79693831214993`, 0.1938029988590731`, 3.2922153328074497`, 607.7271815578437`,
  103.36413621052058`, 34.19227278819086`, 317.8450368414178`, 1.`, {{0, 0.023571592645368192`, 41.95743490875538`},
    {6, 0.0004873121653970479`, 0.8674156544067452`}, {12, 0.00009731798918251566`, 0.17322602074487786`}}},
{0.23989116476268346`, 3.6056249656972357`, 9.989783431378257`, 1.4358686490541388`, 0.7020019529209951`,
  0.17871498413227982`, 0.15143540034751335`, 1116.6227738463333`, 0.03611482374706981`, 5.098351562607867`,
  55.847101211837916`, 0.7252345537570063`, 0.28556504656229087`, 22.123375427386787`, 1.`,
  {{0, 0.0013157571374664888`, 2.34204770469035`}, {22, 0.00036561939501490715`, 0.6508025231265347`}}},
{0.10260002089245757`, 1.5297774189947768`, 6.3178804183723365`, 1.026166177715942`, 0.11867434322817161`,
  0.5793673959868294`, 0.011727656957059851`, 115.5782430006307`, 0.20678944231462698`, 5.80216317155719`,
  19681.456005117754`, 7856.744057599795`, 228.15781342942586`, 18417.025888359614`, 0.`,
  {{0, 0.030406984126999707`, 54.124431746059486`}, {7, 0.00041739161424129094`, 0.7429570733494979`},
```

```
      {14, 0.000394414669232754`, 0.7020581112343021`}, {21, 0.00039519641991658843`, 0.7034496274515274`},
      {28, 0.00040823209831820527`, 0.7266531350064054`}, {35, 0.0005513084870399891`, 0.9813291069311805`},
      {42, 0.00252975219476351`, 4.50295890667905`}, {49, 0.007625785186211364`, 13.573897631456227`},
      {56, 0.005810833343620945`, 10.343283351645281`}, {63, 1.3511540693749866`, 2405.054243487476`}}},
  {0.15334449354379004`, 1.7605906069617427`, 2.299359928311352`, 0.8222860702278779`, 0.9712651951139977`,
    0.6108690739001781`, 0.14744839931335676`, 2744.2788694525566`, 0.1958145599491105`,
    2.932280984329149`, 48.72012410108711`, 1.3558444898565878`, 0.7944985872548053`, 28.84678432005307`,
    1.`, {{0, 0.0012745431316942`, 2.268686774415676`}, {4, 0.0006291433888890883`, 1.1198752322225771`},
      {8, 0.00022911392297173637`, 0.4078227828896907`}, {12, 0.0000595551647690091`, 0.1060081932888362`}}},
  {0.19466496130115485`, 2.7024357312795884`, 5.257508792652555`, 1.2867770062917014`, 0.4348182682381361`,
    0.22799121214772633`, 0.23891047775514818`, 1782.3644894856986`, 0.18533305313279363`, 5.119329477376578`, 29.475101309884298`,
    0.5727814965168042`, 0.5727814965168042`, 29.475101309884298`, 1.`, {{0, 0.0022401076995512068`, 3.987391705201148`}}},
  {0.1432020102164725`, 0.6405991610607145`, 3.208206356969493`, 1.3290628649451517`, 0.9481170196997712`,
    0.4490165359974049`, 0.04841648328555665`, 413.10755212325614`, 0.04750626711264738`,
    5.496555870600891`, 159.61465056709488`, 8.867815934414379`, 6.907322206693801`, 156.36084236329557`,
    1.`, {{0, 0.010186627816268544`, 18.13219751295801`}, {2, 0.0014216476502728471`, 2.5305328174856676`},
      {4, 0.0001487019806708158`, 0.26468952559405207`}, {6, 0.000036968518075913 07`, 0.06580396217512527`},
      {8, 0.00002915771649937735`, 0.051900735368891684`}, {10, 0.000028726160592674747`, 0.051132565854961044`},
      {12, 0.000028706748488694 06`, 0.05109801230987543`}, {14, 2.8874287415972167`*^-6, 0.005139623160043046`}}},
  {0.2767015708016651`, 1.3448440647834508`, 8.399713367158164`, 0.7521061389207052`, 0.4173808724493866`,
    0.513702172075632`, 0.342860965595718`, 77.17931109285855`, 0.021177395775830554`, 7.103551277505744`,
    437287.0815572779`, 88212.28596674583`, 229.30810969345038`, 1649.8068384152298`, 0.`,
    {{0, 0.0955822576850984`, 170.13641867947516`}, {14, 0.024924202193612483`, 44.36507990463022`},
      {28, 0.003633827995940699`, 6.468213832774444`}, {42, 0.0005699127065444043`, 1.0144446176490396`},
      {56, 0.00011040855288673024`, 0.19652722413837984`}, {70, 0.000040275521359029045`, 0.0716904280190717`},
      {84, 0.000028646994881155 22`, 0.05099165088845629`}, {98, 0.0000259424744431638`, 0.04617760450883156`},
      {112, 0.000024988569679475138`, 0.04447965402946574`}, {126, 0.000444857025111922`, 0.7918455046992212`}}},
  {0.2397400236888016`, 3.6266796947004982`, 2.6465237726626594`, 1.459013465973801`, 0.6743470274003898`,
    0.6381986185617847`, 0.04750041433271958`, 90.95305912243636`, 0.11079011330617267`, 9.85823320912452`, 219618.26699193037`,
    49587.30203745857`, 229.16394953979494`, 1809.0499074835525`, 0.`, {{0, 0.13748779296875`, 244.72827148437497`}}},
  {0.10671756328879578`, 1.1279363098789865`, 5.068949642759208`, 1.0745694995138004`, 0.5897325137042213`,
```

```
0.3714383645112781`, 0.14529484820960328`, 91.27351773968599`, 0.0653436372538082`, 9.206661650127103`,
107108.53746142687`, 42292.06730382224`, 229.22902328142158`, 1043.6973958967255`, 0.`,
{{0, 0.05632384303549764`, 100.25644060318578`}, {4, 0.015453722415530413`, 27.50762589964414`},
 {8, 0.0035113214504152177`, 6.250152181739087`}, {12, 0.0009304324874904726`, 1.6561698277330412`},
 {16, 0.0003227731142703451`, 0.5745361434012142`}, {20, 0.00014936897938722316`, 0.26587678330925724`},
 {24, 0.00008995787383678942`, 0.16012501542948515`}, {28, 0.0000664043786543072`, 0.1181997940046668`},
 {32, 0.00005594452980863292`, 0.09958126305936658`}, {36, 0.0024172338232601003`, 4.302676205402977`}}},
{0.2358453438636856`, 3.7679680180462416`, 4.640233719273176`, 1.297156455219631`, 0.3876479327909328`,
0.3193535012332763`, 0.027047144947079417`, 401.3110605008155`, 0.0397014782942956`, 9.539243086069693`,
272.5452215368195`, 1.4635183030252048`, 1.3521097772209403`, 240.13930737028332`, 1.`,
{{0, 0.01180665028361052`, 21.015837504826727`}, {34, 0.006443937076531014`, 11.470207996225206`}}},
{0.07226742189042767`, 2.8421547517397343`, 4.74887948268691`, 0.9400141356818887`, 0.26271764199006165`,
0.24510878200647013`, 0.011565327673117277`, 79.9069988164106`, 0.11613362579809078`, 3.4810370415732024`,
45756.333802778565`, 22449.056965639138`, 229.05018353980557`, 3742.6669057481276`, 0.`,
{{0, 0.01276661549458061`, 22.724575580353488`}, {8, 0.00047022757367856134`, 0.8370050811478393`},
 {16, 0.00007565229579291868`, 0.13466108651139527`}, {24, 0.00005146882217838196`, 0.0916145034775199`},
 {32, 0.00005295076577947148`, 0.09425236308745923`}, {40, 0.00006849234834296071`, 0.12191638005047008`},
 {48, 0.00011277762238035261`, 0.20074416783702767`}, {56, 0.0002093921417297349`, 0.3727180122789282`},
 {64, 0.00036588867610892864`, 0.6512818434738931`}, {72, 0.2702692190962858`, 481.0792099913888`}}},
{0.19794910251471837`, 2.250622511000005`, 5.810822485387694`, 0.8325726726239642`, 0.42888216834124404`,
0.3003167364214152`, 0.2221971352698835`, 492.4997381844193`, 0.06654773222193389`, 7.816458771254976`,
184.15306461881525`, 4.175990300970388`, 2.1621955832495066`, 96.74314205209818`, 1.`,
{{0, 0.005495015849434104`, 9.781128211992703`}, {8, 0.0016301822585066848`, 2.901724420141899`},
 {16, 0.00020857778461868835`, 0.37126845662126523`}, {24, 0.000018702903399984985`, 0.033291168051973276`}}},
{0.23749910377351813`, 2.442156143626838`, 7.627688188064528`, 0.9684475529781722`, 0.7107198130457415`,
0.2572554624659992`, 0.4475955253238462`, 132.3529645103848`, 0.18259209603279808`, 1.6799945249508887`,
119.28324170816055`, 5.687589236879086`, 1.6459560166660272`, 61.137085925902824`, 1.`,
{{0, 0.0033414990598659875`, 5.947868326561458`}, {6, 0.001104977793934444`, 1.9668604732033101`},
 {12, 0.00014465362351130776`, 0.2574834498501278`}, {18, 0.000018671987559865147`, 0.03323613785655996`},
 {24, 7.331384293540206`*^-6, 0.013049864042501566`}, {30, 6.478223518894448`*^-6, 0.011531237863632117`},
 {36, 6.416472797250729`*^-6, 0.011421321579106298`}, {42, 0.000016389984887325343`, 0.0291741730994391`}}},
```

{0.06924882755772316`, 2.8414116897854322`, 0.5206442594268132`, 0.9917884594202446`, 0.6526760474283868`,
0.5988068411525457`, 0.47108712727434415`, 720.0807499233221`, 0.11578003411625926`,
8.400733392823671`, 8026.80557412179`, 4017.059229987359`, 229.96911184755328`, 1398.045539631518`,
0.`, {{0, 0.005436617222960317`, 9.677178656869364`}, {5, 0.003473902058335157`, 6.18354566383658`},
{10, 0.002651237659971113`, 4.719203034748582`}, {15, 0.0020604823544800727`, 3.6676585909745296`},
{20, 0.0016144808084247093`, 2.8737758389959827`}, {25, 0.001274426864787327`, 2.2684798193214424`},
{30, 0.0010143008463436062`, 1.8054555064916191`}, {35, 0.0008149011460753945`, 1.450524040014202`},
{40, 0.0006618122331283112`, 1.1780257749683938`}, {45, 0.08724929981748936`, 155.30375367513105`}}},
{0.06824086699485615`, 3.3015665983523297`, 9.09230580657946`, 1.0780523992371156`, 0.9189494010575767`,
0.47997916674377217`, 0.09047840499022508`, 3815.186560881826`, 0.152329057733407`, 1.5202594192596113`, 13.174949957237954`,
0.15997113266615204`, 0.15997113266615204`, 13.174949957237954`, 1.`, {{0, 0.0010012961967500846`, 1.7823072302151506`}}},
{0.23257418904935773`, 1.8011483887717636`, 0.9612178256197179`, 0.8025101750942898`, 0.31352490932039023`,
0.2355458085864931`, 0.2680725991098069`, 2738.209295799684`, 0.0662676878204258`, 1.8613360761320532`, 6.289638016238291`,
0.2024577297892045`, 0.10763593678298188`, 3.00905596093503`, 1.`, {{0, 0.0001752448911724092`, 0.31193590628688833`},
{10, 0.000047042466237702I5`, 0.08373558990310982`}, {20, 6.400895620950948`*^-6, 0.011393594205292689`}}},
{0.13474024315490335`, 3.3704964680175626`, 9.170639775134308`, 0.7502511885050491`, 0.6264710065934431`, 0.21904625510680875`,
0.040564870634629845`, 2527.7664803980215`, 0.19403763861714918`, 6.378419170081072`, 31.377266380232548`,
0.23289238810289375`, 0.23289238810289375`, 31.377266380232548`, 1.`, {{0, 0.0023846722448976738`, 4.244716595917859`}}},
{0.2693026298173556`, 1.772983767924213`, 1.344226083033309`, 1.4113484218782142`, 0.12593618066908396`,
0.656520191233777`, 0.018659842469751033`, 117.56506476136576`, 0.1712196470744854`, 4.023445632685666`, 118587.99350201279`,
24456.8139026606`, 229.56996144283545`, 1463.5067237050912`, 0.`, {{0, 0.11122651100158694`, 197.98318958282474`}}},
{0.17866994815918202`, 2.442046867777872`, 1.8194825512463648`, 1.2285671890230836`, 0.7081083864311548`,
0.17566313743095552`, 0.006280075202364708`, 2604.6083474637735`, 0.1295692924324935`, 1.862340891527415`,
8.533108465462853`, 0.04228510409907325`, 0.04185118210381829`, 8.492435032284801`, 1.`,
{{0, 0.0005058423508015835`, 0.9003993844268187`}, {7, 0.00013958271165206136`, 0.2484572267406692`}}},
{0.26090114261485403`, 3.9086029270457496`, 1.920295645800332`, 1.1838686314265923`, 0.4729800090210985`,
0.36383922429133153`, 0.014536748208445867`, 79.30674088085132`, 0.030053561477188684`, 7.670997166386538`, 751917.9713923598`,
159039.63879875856`, 230.1488053742728`, 1460.7204888996325`, 0.`, {{0, 0.11101475715637207`, 197.6062677383423`}}},
{0.19544602681310375`, 0.41028512203974055`, 4.6003645589109095`, 1.2671666166106164`, 0.8219059973725353`,
0.22911191370711304`, 0.03702179299429459`, 318.28074751099626`, 0.09079865705400514`,
1.4662081657512314`, 55.00755654891589`, 3.1157160924661134`, 2.4200895730411163`, 42.80853780188165`,

```
1.`, {{0, 0.00259195549357970 1`, 4.613680778571867`}, {29, 0.0006614933793633045`, 1.177458215266682`}}},
{0.26537062770714803`, 1.229239774996202`, 8.744773690909977`, 1.151653436583517`, 0.3537059210373261`,
 0.2894924335103778`, 0.13068544785366612`, 312.2140250313751`, 0.146894495801125`, 5.715731252046433`,
 191.9126361577726`, 6.631411174350536`, 4.190660113398283`, 122.44387487832637`, 1.`,
 {{0, 0.008459511373778624`, 15.057930245325949`}, {19, 0.0008462231169741801`, 1.5062771482140405`}}},
{0.18712917295151626`, 0.630329154174047`, 6.232729389529142`, 0.7582043174026267`, 0.7607866373637777`,
 0.2530331438958473`, 0.06090476948059027`, 64.21018866863231`, 0.08418997914362486`,
 2.694793972266142`, 105602.6313301362`, 28719.283837931172`, 229.4177625555607`, 983.9045751115544`,
 0.`, {{0, 0.07094289314728525`, 126.27834980216772`}, {24, 0.0007435775066460891`, 1.3235679618300387`},
  {48, 0.0002663047800438859`, 0.47402250847811694`}, {72, 0.0002474944973589269`, 0.4405402052988899`},
  {96, 0.00024335301575067035`, 0.43316836803619324`}, {120, 0.00024198677397759837`, 0.4307364576801251`},
  {144, 0.00024150064789217067`, 0.4298711532480638`}, {168, 0.00024132670412231987`, 0.4295615333377294`},
  {192, 0.00024126571234364666`, 0.4294529679716911`}, {216, 0.0013670449230575757`, 2.4333399630424846`}}},
{0.19413873235593193`, 0.8072205389170897`, 9.684031161687852`, 0.8518820755271745`, 0.2120317281227424`,
 0.1532879757233685`, 0.02372050181467318`, 987.8329384880758`, 0.08094704090521981`, 8.693316755778081`,
 66.0282686364779`, 1.3009422934460457`, 0.9217764877677543`, 47.235490658662776`, 1.`,
 {{0, 0.0025592749583453565`, 4.555509425854734`}, {68, 0.0010306223317130144`, 1.8345077504491658`}}},
{0.0816546741022599`, 0.4036812381177306`, 0.7902893788690406`, 1.4273906025641667`, 0.1897112398644374`,
 0.6362443390402239`, 0.007446156154142883`, 176.61944749055874`, 0.10188842005948545`, 9.51311671217389`, 73461.04814503502`,
 33879.41211920117`, 227.59281793897026`, 1057.757327431127`, 0.`, {{0, 0.08038955688476565`, 143.09341125488285`}}},
{0.2491419431624458`, 3.7694376618574204`, 4.74673147811253`, 0.8416284883026488`, 0.188731240527098`,
 0.3192559789027146`, 0.07121130413051815`, 671.9000833119555`, 0.02760758509345429`,
 2.5790410082926702`, 35.5148908973705`, 0.3376065027443952`, 0.3098773212756588`, 32.46297247787505`, 1.`,
 {{0, 0.0019222351731649557`, 3.421578608233621`}, {10, 0.0005449507351535478`, 0.9700123085733151`}}},
{0.11168227968802413`, 2.173584861364678`, 5.640951633995167`, 0.9377704673639422`, 0.3315982783471285`,
 0.5266083888319896`, 0.005432939117039627`, 621.842609802462`, 0.09631877509870435`, 9.43919460268938`, 206.3367253174613`,
 0.48791746484751225`, 0.48791746484751225`, 206.3367253174613`, 1.`, {{0, 0.015681591124127058`, 27.913232200946165`}}},
{0.15970197279447784`, 3.9587696847183347`, 4.0238996390694854`, 0.8027865138486989`, 0.03588289413016432`,
 0.35386710298208024`, 0.016574217538633067`, 2019.1576160982734`, 0.07473946383041635`,
 3.5352801788382404`, 15.128961218806715`, 0.054779114843042744`, 0.054779114843042744`,
 15.128961218806715`, 1.`, {{0, 0.0011498010526293104`, 2.0466458736801725`}}},
```

```
{0.2064322311255224`, 0.43645383124552994`, 3.8213880668877356`, 0.8257710590869434`, 0.08868496943554072`,
  0.17616539751645888`, 0.04042707492740236`, 2037.3062213977416`, 0.05324630165142419`,
  4.6107951341494555`, 15.98315551573246`, 0.7891528871664529`, 0.4931824512477613`, 10.129596029182625`, 1.`,
  {{0, 0.0005952154642065848`, 1.059483526287721`}, {50, 0.00017463383401129478`, 0.3108482245401047`}}},
{0.1394617695133732`, 3.387614469631334`, 8.352661875580207`, 1.2219251402369191`, 0.6325442693826859`,
  0.17624447718949754`, 0.021853560156019736`, 288.68541357569393`, 0.164910064915528`,
  5.7766720481305995`, 225.3227622093112`, 1.1209550001700646`, 0.8676223032924237`, 167.93766892186042`,
  1.`, {{0, 0.00856226404308949 9`, 15.24082999669931`}, {56, 0.004200998794971893`, 7.47777785504997`}}},
{0.10599692192016741`, 2.4119661618453145`, 1.4892081676893856`, 1.3031849980783952`, 0.42063329504650815`,
  0.5860601104625098`, 0.3077592160131421`, 52.52666428206092`, 0.19647413521024198`, 3.2052596491028216`, 129035.76649976932`,
  51307.13577554338`, 230.14423341003337`, 1056.0980596040426`, 0.`, {{0, 0.08026345252990724`, 142.8689455032349`}}},
{0.24388484229914548`, 3.2519594625966253`, 4.312340316913895`, 0.9914403572709836`, 0.29358995419342615`,
  0.1835819110057716`, 0.13612159308161606`, 1157.8864188899545`, 0.08218618541111161`, 5.046821007659727`,
  36.346239182681884`, 0.5042957362335848`, 0.23960990196039741`, 17.306985163564182`, 1.`,
  {{0, 0.0009551902277133453`, 1.700238605329755`}, {14, 0.00031310251787777403`, 0.5573224818224378`},
    {28, 0.000043728609824 31927`, 0.07783692548728831`}, {42, 3.3095170154394583`*^-6, 0.005890940287482236`}}},
{0.23494551018662208`, 3.3331099682410468`, 0.9744758817090952`, 0.9989991912838305`, 0.20118476247466943`,
  0.3741832660739852`, 0.07385095150097958`, 91.01998469145919`, 0.19994684540705993`, 6.360950891328324`, 81250.82668148632`,
  18640.527664251713`, 230.16939317597667`, 1242.3181533813477`, 0.`, {{0, 0.09441617965698243`, 168.06079978942873`}}},
{0.23944024111665796`, 0.7499363805955559`, 7.775454975750338`, 1.268235612825383`, 0.3786721827881383`,
  0.6740341016595366`, 0.11977329505957622`, 117.53909816274609`, 0.2108164269301916`,
  1.8777741577958924`, 26931.64335491816`, 6070.771476408655`, 230.01566680406904`, 1132.3550154405932`,
  0.`, {{0, 0.08010477230319338`, 142.5864946996842`}, {22, 0.0010328137893678973`, 1.8384085450748573`},
    {44, 0.0018775375103363768`, 3.3420167683987505`}, {66, 0.00031341233301684316`, 0.5578739527699809`},
    {88, 0.00026013875894002295`, 0.46304699091324086`}, {110, 0.00020093417270313857`, 0.3576628274115866`},
    {132, 0.00019158888628872966`, 0.34102821759393875`}, {154, 0.0001874595645038794`, 0.3336780248169053`},
    {176, 0.0001861754151769544`, 0.33139223901497883`}, {198, 0.00170414843995786`, 3.033384223124991`}}},
{0.24513403447625742`, 2.0133471925570054`, 9.940759001187548`, 1.083812287959604`, 0.5683273089881642`,
  0.5900825394859205`, 0.005339720116331014`, 58.9416557608406`, 0.03145644437596545`, 6.28807827028038`,
  599847.2882128722`, 133131.50937740185`, 228.1666197761728`, 2168.5831291933528`, 0.`,
  {{0, 0.07508029586778023`, 133.64292664464878`}, {10, 0.0018270129145260847`, 3.2520829878564306`},
```

```
{20, 0.0017138849145644144`, 3.0507151479246573`}, {30, 0.0017059332836671037`, 3.0365612449274444`},
{40, 0.0017042672561605428`, 3.033595715965766`}, {50, 0.0017038851536702567`, 3.032915573533056`},
{60, 0.0017039606947451319`, 3.033050036646334`}, {70, 0.0017042668838367742`, 3.0335950532294578`},
{80, 0.0017047227081534338`, 3.034406420513112`}, {90, 0.07596408814159086`, 135.2160768920317`}}},
{0.15958822823144275`, 2.509825654597928`, 3.728427574062339`, 0.9294074209823535`, 0.01651867581778177`, 0.5367648350921979`,
 0.015785937236593408`, 685.9822133189093`, 0.020860236554209932`, 1.2753268301578835`, 18.923628836260693`,
 0.12411788080185998`, 0.12411788080185998`, 18.923628836260693`, 1.`, {{0, 0.0014381957915558128`, 2.559988508969347`}}},
{0.06806026871424425`, 1.205607754446362`, 0.7536813502622586`, 1.4624982795129167`, 0.7076540801030038`,
 0.15470161284122685`, 0.268116863806763`, 324.06126360879364`, 0.036033823716353997`,
 3.8267580932938614`, 61566.02055294689`, 31204.999380432353`, 228.05872703198756`, 707.7733273963837`,
 0.`, {{0, 0.02813926047315891`, 50.08788364222287`}, {29, 0.0015095052199391578`, 2.686919291491701`},
  {58, 0.001393225643328633`, 2.479941645124967`}, {87, 0.001380237669353972`, 2.456823051450071`},
  {116, 0.0013762784427656296`, 2.449775628122821`}, {145, 0.001374773937670009`, 2.447097609052616`},
  {174, 0.0013741701977635276`, 2.4460229520190793`}, {203, 0.001373923910939139`, 2.4455845614716676`},
  {232, 0.0013738228566389683`, 2.445404684817364`}, {261, 0.014495574530567224`, 25.802122664409662`}}},
{0.15319733389695195`, 3.558603911914262`, 4.579050417046558`, 0.9321187332487358`, 0.6642338797631109`,
 0.569667348599446`, 0.2858050889038288`, 341.1212503566884`, 0.20343887540165378`, 9.501626804091558`,
 381.0996592898564`, 7.7800818778923535`, 5.270975429880085`, 296.01819156287723`, 1.`,
 {{0, 0.019984874167141918`, 35.57307601751261`}, {9, 0.002512508391636755`, 4.472264937113423`}}},
{0.15071537712057487`, 1.2850575528534174`, 6.479674501252795`, 1.2297249014764047`, 0.051196968811160426`,
 0.17856837950014437`, 0.01824538414392949`, 77.70419036862091`, 0.224107560352308`, 8.31176551046438`,
 34586.80095434776`, 10841.15223327196`, 228.02236313328953`, 2410.7025998098356`, 0.`,
 {{0, 0.06738882026922198`, 119.95210007921511`}, {31, 0.0016421427024683481`, 2.92301401039366`},
  {62, 0.0020509205541598776`, 3.6506385864045825`}, {93, 0.005864856818221928 5`, 10.439445136435033`},
  {124, 0.004090210365545525`, 7.280574450671034`}, {155, 0.0038538594190279715`, 6.859869765869789`},
  {186, 0.0033865903332601036`, 6.028130793202985`}, {217, 0.003084621356251764`, 5.49062601412814`},
  {248, 0.0028318715393031635`, 5.040731339959631`}, {279, 0.08901950422808683`, 158.45471752599457`}}},
{0.24584690511060947`, 2.473478204800995`, 6.676188280246508`, 1.4832363218386881`, 0.14420161768506268`,
 0.523836460034543`, 0.04402629271492787`, 360.52310897688045`, 0.0850887320320231`, 2.528306265858081`,
 78.43126692580904`, 0.880734935014093`, 0.8246656494371566`, 69.80646325861798`, 1.`,
 {{0, 0.00405331830919025`, 7.214906590358645`}, {34, 0.001251972898464716`, 2.228511759267195`}}},
```

```
{0.14137397451874373`, 3.652676161056461`, 8.39527364337399`, 1.0080745646578813`, 0.09190129083286003`,
  0.15081167091134762`, 0.35462309792221747`, 1217.6311173423508`, 0.09724487810479643`, 6.223920941944753`, 35.30083569932627`,
  0.542182478545574`, 0.542182478545574`, 35.30083569932627`, 1.`, {{0, 0.0026828635131487966`, 4.775497053404858`}}},
{0.04933630800724648`, 1.8511857532828166`, 5.885062761777403`, 1.1138733840213622`, 0.4607664856084661`, 0.43439208322360623`,
  0.018761076496074794`, 3507.5800668673905`, 0.21191062303011793`, 3.3573407631201917`, 13.305766777384259`,
  0.10573195828468424`, 0.10573195828468424`, 13.305766777384259`, 1.`, {{0, 0.0010112382750812037`, 1.8000041296445426`}}},
{0.2339008817663396`, 3.669675022809618`, 9.160989469851096`, 1.2207724907901325`, 0.9659294978123167`,
  0.3234992643572032`, 0.05037324961884807`, 93.86107259786391`, 0.14430488254373952`,
  3.9894219201696473`, 18463.93102232838`, 4186.142066331362`, 3.465593681596775`, 334.9735685153462`,
  1.`, {{0, 0.01939407953216679`, 34.521461567256885`}, {3, 0.004939459788992219`, 8.79223842440615`},
   {6, 0.0008282659122013632`, 1.4743133237184263`}, {9, 0.00013981766960407754`, 0.24887545189525803`},
   {12, 0.000040912078129942576`, 0.0728234990712978`}, {15, 0.000028354533161357464`, 0.05047106902721628`},
   {18, 0.000026999280803648773`, 0.04805871983049481`}, {21, 0.00002687216358151966`, 0.04783245117510499`},
   {24, 0.000026861180139096154`, 0.04781290064759115`}, {27, 6.369068386293852`*^-6, 0.011336941727603056`}}},
{0.11569404259913263`, 3.2009969772206803`, 2.2264401820433353`, 0.7539773410821206`, 0.3237735314376855`,
  0.22021748910844674`, 0.21956604535044472`, 1445.3927245947305`, 0.06533194774972478`, 1.3139082141801541`, 8.036716264969558`,
  0.14265022935780536`, 0.07668847701944398`, 3.850008362755986`, 1.`, {{0, 0.0002313204178001266`, 0.41175034368422536`},
   {11, 0.000055481988167687576`, 0.09875793893848389`}, {22, 5.798229601640785`*^-6, 0.010320848690920597`}}},
{0.1426983247121278`, 1.4581176174915065`, 6.531549332871599`, 0.9310047864665083`, 0.893336243428055`,
  0.18377286371920176`, 0.08056837661087574`, 664.5727162634348`, 0.04347044784294363`, 7.864371974772275`, 191.70224383722768`,
  4.6678616491060065`, 1.9315954680107723`, 80.39953651717464`, 1.`, {{0, 0.003713718541803317`, 6.610419004409904`},
   {14, 0.0017366804757006182`, 3.0912912467471`}, {28, 0.00048899887988553 9`, 0.8704180061962594`},
   {42, 0.00013557774398364084`, 0.24132838429088066`}, {56, 0.00003538913393215834`, 0.06299265839924185`}}},
{0.05831687901068116 6`, 0.732484500007835`, 9.716813224212896`, 0.9062398770267781`, 0.045816647832010116`,
  0.25112700202789096`, 0.0489500536916562`, 2141.8470035057708`, 0.06373448098002471`,
  7.679073247267311`, 27.96932262473565`, 0.9792718651779404`, 0.6580860309313615`, 19.05000289670098`, 1.`,
  {{0, 0.0010628361345187685`, 1.891848319443408`}, {38, 0.0003849640856305062`, 0.685236072422301`}}},
{0.1624041267001165`, 2.3409738007594383`, 7.927758092789709`, 1.1108935833128895`, 0.2655878375527121`,
  0.22381693809974978`, 0.10055816102060769`, 86.2207746546923`, 0.23778527923179793`, 1.5984483564142007`, 13018.994692477769`,
  3892.517251943087`, 2.9308675496084535`, 105.11640267637266`, 1.`, {{0, 0.007694564501101273`, 13.696324811960267`},
   {28, 0.00014141638509719906`, 0.2517211654730143`}, {56, 0.000019421316503170012`, 0.03456994337564262`},
```

```
{84, 0.0000203197137381355`, 0.03616909045388119`}, {112, 0.000018639620719933338`, 0.03317852488148134`},
{140, 0.00001838702273670474`, 0.032728900471334435`}, {168, 0.000018370277328468413`, 0.0326990936446737`},
{196, 0.0000183662330392494`, 0.03269189480986393`}, {224, 0.000039361533140188536`, 0.07006352898953559`}}},
{0.05796853524365786`, 2.115435321939346`, 7.135826377560314`, 1.1217856002992745`, 0.9949497841824515`,
 0.6493572179052653`, 0.16432568991023785`, 205.88622896743587`, 0.07308989915497993`, 5.091714109271411`, 332.08050399628326`,
 11.023914507444946`, 8.984346162940836`, 331.5973314976113`, 1.`, {{0, 0.023870566005741993`, 42.48960749022075`},
 {2, 0.001194372968955112`, 2.125983884740099`}, {4, 0.00005268910439677329`, 0.09378660582625646`},
 {6, 0.00002312794658729144`, 0.04116774492537876`}, {8, 0.000022591662639461003`, 0.04021315949824058`},
 {10, 0.000022582384945178013`, 0.04019664520241686`}, {12, 0.000015467120552653126`, 0.027531474583722564`}}},
{0.09343457742759681`, 3.0751406184941388`, 6.999926605820257`, 1.4764339149831611`, 0.27064650694064607`,
 0.6662559245780291`, 0.012835205507442616`, 1884.4110587763687`, 0.01681160009525748`, 8.661215052575166`, 66.18109341738075`,
 0.2355104236606273`, 0.2355104236606273`, 66.18109341738075`, 1.`, {{0, 0.005029763099720938`, 8.952978317503268`}}},
{0.23943678973010468`, 2.920807454124679`, 1.1099817114095707`, 1.3932739733707113`, 0.44392785132951373`,
 0.17254202331513768`, 0.008938503858702969`, 2712.044809794402`, 0.1652368227234951`, 2.8738798121545877`,
 9.787950706974414`, 0.05851710563289111`, 0.05829434500058225`, 9.800267359559017`, 1.`,
 {{0, 0.0006323018823072228`, 1.1254973505068566`}, {6, 0.00011251843701926267`, 0.20028281789428756`}}},
{0.176569287716667`, 3.4304813087836825`, 0.9837478487319995`, 1.1028599055297788`, 0.8616178142897495`,
 0.24038775568738768`, 0.03874939820930588`, 2380.99712756261`, 0.18505877859540681`, 3.338709015344154`,
 24.805716106707905`, 0.33738450779879336`, 0.2122687165839013`, 18.453214299955906`, 1.`,
 {{0, 0.0007448148621614686`, 1.3257704546474143`}, {7, 0.000417895341359561`, 0.7438537076200186`},
 {14, 0.0001902637560257418`, 0.3386694857258204`}, {21, 0.000049470327249877484`, 0.08805718250478192`}}},
{0.23886394100929725`, 3.974888040813318`, 7.275822510559582`, 1.2870566617016084`, 0.12273034821485096`,
 0.6651538290269186`, 0.16897919530659566`, 150.6876048176701`, 0.1338282848735754`, 2.432758352876487`, 14917.350560072266`,
 3347.1655171464`, 18.067954493580135`, 242.17279351496396`, 1.`, {{0, 0.017803501126458864`, 31.690232005096778`},
 {17, 0.00009760127292248695`, 0.1737302658020268`}, {34, 0.00009752472692863406`, 0.17359401393296864`},
 {51, 0.00010175441680213114`, 0.18112286190779342`}, {68, 0.00009614838078133828`, 0.17114411779078215`},
 {85, 0.00009609836198823447`, 0.17105508433905736`}, {102, 0.00011250402125557589`, 0.2002571578349251`}}},
{0.06973130535662914`, 1.4096929441217387`, 8.65071450995816`, 1.181254696978707`, 0.5406204719849208`,
 0.2725259583108167`, 0.12003736669178822`, 4806.091595362366`, 0.20844978312325513`, 8.8784124085225`, 23.229834666551877`,
 0.6848353884846621`, 0.6848353884846621`, 23.229834666551877`, 1.`, {{0, 0.0017654674346579428`, 3.142532033691138`}}},
{0.04409294567306185`, 1.3468266911815716`, 8.46560672493381`, 1.1221333539921368`, 0.8074609652543419`,
```

```
  0.33934404303057597`, 0.1090535991677299`, 2100.6487939981484`, 0.1955313854386307`, 6.974828032327105`, 69.83561426610376`,
  2.0894427933297344`, 2.0894427933297344`, 69.83561426610376`, 1.`, {{0, 0.005307506684223886`, 9.447361897918517`}}},
 {0.08301734541584582`, 0.4950033643125962`, 0.3447103403996028`, 1.3973171280993089`, 0.5174429014299606`,
  0.6090072510200681`, 0.013328621450736109`, 78.08408450957157`, 0.15235476028821748`, 7.41363923833616`, 117207.01329950699`,
  53608.64988482157`, 229.34385056228658`, 973.5325763100075`, 0.`, {{0, 0.07398847579956057`, 131.69948692321782`}}},
 {0.07508111728102967`, 2.9328515590120503`, 2.9247828964602327`, 1.0997610261500241`, 0.15381049135387959`,
  0.44875941041801015`, 0.005374403719665632`, 955.806314296473`, 0.04180941570568808`, 8.210271230243798`, 91.90359966838545`,
  0.186409169366441`, 0.186409169366441`, 91.90359966838545`, 1.`, {{0, 0.006984673574797295`, 12.432718963139186`}}},
 {0.1868411586163749`, 1.4148901359883457`, 3.2886483363036496`, 1.1024292265597047`, 0.35071605335062417`,
  0.20216786873751635`, 0.033694215571138546`, 50.84055962824541`, 0.13756443231160648`,
  9.762651891575405`, 170456.0745443864`, 46393.25040786791`, 228.84772464683047`, 12525.457490403474`,
  0.`, {{0, 0.05265796921094479`, 93.7311851954817`}, {8, 0.011506318689670603`, 20.48124726761367`},
   {16, 0.008628826369937342`, 15.359310938488466`}, {24, 0.008340557455945776`, 14.84619227158348`},
   {32, 0.008962375214496414`, 15.953027881803614`}, {40, 0.00984345594994084`, 17.521351590894696`},
   {48, 0.010617396744709313`, 18.89896620558258`}, {56, 0.011150802800188632`, 19.848428984335765`},
   {64, 0.011483124889571532`, 20.439962303437323`}, {72, 0.8187439419452588`, 1457.3642166625605`}}},
 {0.25728291653412544`, 1.1620186239014583`, 0.48798465750503445`, 1.2217305465551163`, 0.36537296293338284`,
  0.2112385814144826`, 0.1783844678719564`, 175.70497466833672`, 0.14885286931859365`, 6.164090300210196`, 68143.10139680833`,
  14570.635441052931`, 230.26306609017027`, 1134.8900042082137`, 0.`, {{0, 0.08625164031982424`, 153.52791976928717`}}},
 {0.13759840983832694`, 1.6824227355669867`, 8.881204796952819`, 1.07728991023014`, 0.8275568317133928`,
  0.2913709338376398`, 0.47392581861361016`, 1028.758263171597`, 0.1629375199726112`, 7.630724789672264`, 147.135753648158`,
  5.0834473138027185`, 5.0834473138027185`, 147.135753648158`, 1.`, {{0, 0.011182317277260007`, 19.90452475352281`}}},
 {0.22999415874686646`, 3.253135821240728`, 9.26678168881324`, 0.8937302072810551`, 0.5038748821521797`,
  0.2035592770142407`, 0.010501376095491947`, 229.44271937909937`, 0.14963151143698977`,
  8.069758108498657`, 372.3238558562111`, 1.0482823486453843`, 1.0220970074054951`, 363.61477513608787`,
  1.`, {{0, 0.02207139316380066`, 39.28707983156517`}, {13, 0.005563329746542018`, 9.902726948844792`}}},
 {0.09925066924077935`, 1.4815445119155024`, 7.881183919866995`, 0.7663286317492166`, 0.2976933875506338`,
  0.33062028037546887`, 0.05105854284999101`, 168.2571992844445`, 0.22488226539851913`,
  5.574589576514775`, 347.7521027408701`, 6.870569355211341`, 5.096473426592013`, 251.908594822632`, 1.`,
  {{0, 0.01585749792807718`, 28.22634631197738`}, {30, 0.0032875552784428536`, 5.851848395628279`}}},
 {0.20983327896511272`, 1.8859920859197894`, 0.6151282764886545`, 0.8412663476815937`, 0.027122934645615437`,
```

```
0.6844735714100607`, 0.01254007978155685`, 549.5308557472557`, 0.10371695352022153`, 7.780558961111437`, 19165.672067231193`,
  4784.756425722391`, 230.13091195206613`, 1051.9257344697653`, 0.`, {{0, 0.07994635581970216`, 142.30451335906986`}}},
{0.2225389666164696`, 1.2827559929647245`, 1.2157460525678818`, 1.1336603904726232`, 0.21910751958531538`,
  0.6021275716391656`, 0.016887660465617623`, 151.92995003475355`, 0.056506174885467386`, 4.721491520685097`, 190388.5285192307`,
  45546.47135435988`, 229.48761608865587`, 1281.776051772268`, 0.`, {{0, 0.09741497993469236`, 173.3986642837524`}}},
{0.23629660338151143`, 1.375829581220887`, 0.7878883261410508`, 0.9498849347602389`, 0.6605330073956415`,
  0.33733697402858687`, 0.028977316405205664`, 446.06430846982937`, 0.16303388369839966`,
  6.718018447521894`, 14557.75897704643`, 3318.14147254034`, 229.66600956346934`, 2453.718168076093`,
  0.`, {{0, 0.05182517002340216`, 92.24880264165583`}, {19, 0.002824633415357201`, 5.027847479335817`},
    {38, 0.002155937505661658`, 3.837568760077751`}, {57, 0.002139374267040848`, 3.8080861953327103`},
    {76, 0.0021591638621182934`, 3.843311674570562`}, {95, 0.002126788004719308`, 3.7856826484003676`},
    {114, 0.0020466520593871488`, 3.6430406657091243`}, {133, 0.0019573484224218746`, 3.4840801919109365`},
    {152, 0.0018819790173200456`, 3.349922650829681`}, {171, 0.11736553419635455`, 208.9106508695111`}}},
{0.2240884195389573`, 3.0168075094859823`, 9.22253837134286`, 1.0534395436787098`, 0.7253455428103461`,
  0.1646055270291652`, 0.006115122680716056`, 258.28718729642685`, 0.11665954601209721`, 2.992281031623701`,
  136.92517641644926`, 0.2985641799747155`, 0.27929900382338474`, 129.51089342681166`, 1.`,
  {{0, 0.007284459109006315`, 12.966337214030915`}, {13, 0.002558368791431555`, 4.553896448748168`}}},
{0.04820076342563051`, 1.9965427255473651`, 1.4646173572921608`, 1.475676204663891`, 0.346581356777814`,
  0.35258214744905925`, 0.16114708977216574`, 53.64321165141119`, 0.1945121008436112`, 8.557828905511698`, 96135.59216903089`,
  56873.37885210987`, 230.15170193281023`, 903.9803555137231`, 0.`, {{0, 0.06870250701904296`, 122.29046249389646`}}},
{0.1395588171486265`, 3.3167971972296737`, 5.120579942113229`, 1.4414502422836475`, 0.46235265068089726`,
  0.21590338795839814`, 0.018401804903068468`, 321.8138540224943`, 0.09243860589675884`, 1.0657915725386573`, 32.76146072165554`,
  0.24125328366539545`, 0.22497567431784085`, 31.299296788674862`, 1.`, {{0, 0.0015008942278776256`, 2.6715917256221737`},
    {10, 0.0007116042788050374`, 1.2666556162729667`}, {20, 0.00016624804925662666`, 0.29592152767679547`}}},
{0.2085065587668823`, 0.6294064238759702`, 7.1150002800734775`, 0.8698405330489325`, 0.07589384997930004`,
  0.6271818704466325`, 0.014863365323890152`, 3717.018925299029`, 0.09558183779388002`, 3.993723504265942`, 12.489992847490445`,
  0.2170462535984263`, 0.2170462535984263`, 12.489992847490445`, 1.`, {{0, 0.0009492394564092738`, 1.6896462324085073`}}},
{0.11579616742063126`, 1.4637518716217537`, 6.091821149634162`, 0.8701973607843327`, 0.580668178059593`,
  0.4892081276931839`, 0.43069272205186815`, 58.104542288448926`, 0.09639134486345691`,
  1.0613512257562903`, 112608.65838155973`, 42408.052496827055`, 228.71462654869583`, 744.1601293521567`,
  0.`, {{0, 0.007125422685199724`, 12.683252379655508`}, {4, 0.002267070395982922`, 4.035385304849601`},
```

```
    {8, 0.0011252112162892156`, 2.002875964994804`}, {12, 0.0006061197438918068`, 1.078893144127416`},
    {16, 0.00034972863007187407`, 0.6225169615279359`}, {20, 0.00021908528152311745`, 0.38997180111114904`},
    {24, 0.00015157632065114696`, 0.2698058507590416`}, {28, 0.00011612586267675675`, 0.20670403556462705`},
    {32, 0.00009701485301067501`, 0.17268643835900152`}, {36, 0.044498814841466665`, 79.20789041781066`}}},
 {0.07650954660329923`, 2.451622188741575`, 3.5124331635719983`, 0.9208132913481966`, 0.22417245589301404`,
  0.5962277485405788`, 0.1631058588288524`, 149.68967360762312`, 0.15724921240628176`,
  3.991974201007654`, 21862.27748609377`, 10390.196210246908`, 229.53161770456344`, 695.5827351831643`,
  0.`, {{0, 0.012774628015125326`, 22.738837866923078`}, {3, 0.002914960371394225`, 5.188629461081721`},
    {6, 0.0008256456071577609`, 1.4696491807408143`}, {9, 0.00032648223079719414`, 0.5811383708190055`},
    {12, 0.00018280259972845424`, 0.3253886275166485`}, {15, 0.00013531601152105428`, 0.24086250050747662`},
    {18, 0.00012186678623411159`, 0.21692287949671862`}, {21, 0.0001237999233511914`, 0.2203638635651207`},
    {24, 0.00013133687759355`, 0.23377964211651903`}, {27, 0.03532744945101763`, 62.882860022811386`}}},
 {0.27715040216901043`, 3.1093705724520717`, 5.602120685872263`, 1.4185083740786066`, 0.20116695432127574`,
  0.3482455682148583`, 0.014646070094414987`, 268.87083253973395`, 0.19497961893886057`,
  8.247252623819538`, 306.22565384499995`, 1.282967003319471`, 1.272842189979067`, 306.931822814469`, 1.`,
  {{0, 0.021178597656832724`, 37.69790382916225`}, {7, 0.002148220877066922`, 3.8238331611791208`}}},
 {0.10373802184314534`, 1.9484823746249935`, 4.344672894707308`, 0.8659474233939041`, 0.34328087466446`,
  0.6599529078817681`, 0.006604951530776707`, 74.02272771702236`, 0.12108236246583282`, 3.25882057231545`, 81784.74233045777`,
  32903.63131021203`, 229.93251702169206`, 1055.8020441155686`, 0.`, {{0, 0.08024095535278322`, 142.82890052795412`}}},
 {0.08995654352927362`, 1.6673082382989595`, 4.04257302618344`, 1.1477945472560402`, 0.1500209798917984`,
  0.1761016570773115`, 0.012888472951564884`, 1050.2473441342943`, 0.2022489804478793`, 4.465566609444316`, 31.237520689619966`,
  0.2040793014905706`, 0.2040793014905706`, 31.237520689619966`, 1.`, {{0, 0.0023740515724111175`, 4.225811798891789`}}},
 {0.18428208667431017`, 2.663013532673764`, 4.442362759588184`, 1.3723983485913218`, 0.7325310003414629`,
  0.5418182340232052`, 0.027460635126683182`, 53.281000517524745`, 0.08334630855570291`, 6.274365305120075`, 372683.15922425175`,
  102534.04783237082`, 229.37289513784563`, 1586.9372769405968`, 0.`, {{0, 0.12060723304748536`, 214.68087482452395`}}},
 {0.1326476876438774`, 2.8731759222440596`, 6.672951038295707`, 1.243076119232064`, 0.9225218753338189`,
  0.23536778742386455`, 0.07924576221140303`, 2119.223051638056`, 0.014542489730864222`, 5.05029094558817`, 47.256296859171584`,
  0.6022989758128595`, 0.6022989758128595`, 47.256296859171584`, 1.`, {{0, 0.0035914785612970405`, 6.392831839108733`}}},
 {0.16449021574582717`, 1.5264131018768046`, 3.118813817198866`, 1.0873075186223824`, 0.9114527670749883`,
  0.32623274128336255`, 0.005473446059082923`, 2600.919647538226`, 0.12372800503579795`, 1.4807994816594086`,
  13.699394480882559`, 0.06823748329309721`, 0.06600791048592435`, 13.372121509764927`, 1.`,
```

```
{{0, 0.0007392191150553554`, 1.3158100247985325`}, {5, 0.00027706211968677905`, 0.4931705730424667`}}},
{0.1059475440552839`, 2.4614128361434053`, 6.764865578702089`, 0.897041430377437`, 0.5452264302150955`,
 0.6377616415175531`, 0.031109305885935246`, 290.7153568287518`, 0.12025877488242465`, 1.854625189562679`, 107.9942079391424`,
 1.413292747031753`, 1.1442344571583651`, 107.00258673180238`, 1.`, {{0, 0.007247700476677486`, 12.900906848485926`},
  {4, 0.0008193589764479941`, 1.4584589780774295`}, {8, 0.00006513713849150037`, 0.11594410651487065`}}},
{0.09376889465512256`, 2.694562234128588`, 9.901749913963968`, 1.2671081853876889`, 0.9355728957697818`,
 0.41426328356338693`, 0.1676359905682518`, 324.1423914220847`, 0.048114260882103244`, 6.992923371816153`, 539.5368707617577`,
 10.149578952208063`, 5.357775796346885`, 297.90784863740777`, 1.`, {{0, 0.014175440018657292`, 25.232283233209984`},
  {5, 0.006203576339154303`, 11.04236588369466`}, {10, 0.001695054462864361`, 3.0171969438985626`},
  {15, 0.0004564998226676282`, 0.8125696843483782`}, {20, 0.0001104258530994039`, 0.19655801851693894`}}},
{0.07408607058446992`, 2.173441202450359`, 5.83110243938874`, 1.2671639264693393`, 0.15034130588796946`,
 0.26151521374964837`, 0.00796428041791113`, 3349.7895146720416`, 0.06475249420724477`, 9.113413370060233`, 23.308505588366984`,
 0.07599399909550364`, 0.07599399909550364`, 23.308505588366984`, 1.`, {{0, 0.0017714464247158908`, 3.1531746359942856`}}},
{0.2682300613650819`, 3.6108652762368214`, 9.881761872038165`, 0.9825580258076282`, 0.9296545706074566`,
 0.25219322618587503`, 0.024464898005065273`, 1815.4892808719587`, 0.08994453092924143`,
 7.425857575199714`, 87.5716937286244`, 0.43494041530216465`, 0.36536475201761914`, 72.90375718377214`,
 1.`, {{0, 0.0033942920982232673`, 6.041839934837416`}, {41, 0.002146393447743416`, 3.8205803369832805`}}},
{0.04962174488386037`, 1.9694465660050104`, 4.880585401279637`, 1.2957318091391512`, 0.6289429156960646`,
 0.36977064682826033`, 0.006935918860683708`, 414.38904513183815`, 0.2065379649808241`, 4.444261705692702`, 170.58419588601262`,
 0.8896132970731725`, 0.6405211274362136`, 159.96294347812469`, 1.`, {{0, 0.00938359101459877`, 16.70279200598581`},
  {4, 0.0023491811466503742`, 4.181542441037666`}, {8, 0.00042441154308833057`, 0.7554525466972284`}}},
{0.2594824517719198`, 0.5971319663647825`, 9.453687965953478`, 0.8401327653868795`, 0.7997988949991743`,
 0.15899752713279824`, 0.005075100457461465`, 409.49724454586783`, 0.2091478816442816`, 4.600741868044251`,
 144.37239454971117`, 1.0289752630460813`, 0.9985140145754567`, 140.17532115785284`, 1.`,
 {{0, 0.007900065005928799`, 14.062115710553261`}, {12, 0.002753259402068016`, 4.9008017356810685`}}},
{0.1975048020318434`, 2.1175350807064808`, 9.527018017184652`, 0.8875453366861839`, 0.2770809005507884`,
 0.4437813967164592`, 0.08906290963263506`, 98.18685485298634`, 0.18494734685711195`,
 2.236429925751402`, 9252.548489974435`, 2376.9115070878424`, 201.2078327232124`, 948.7966580725665`,
 1.`, {{0, 0.07020676164344959`, 124.96803572534027`}, {30, 0.0005452538960244157`, 0.9705519349234597`},
  {60, 0.0006978363092650211`, 1.2421486304917375`}, {90, 0.00011374801456188899`, 0.20247146592016402`},
  {120, 0.00007316081462766992`, 0.13022625003725244`}, {150, 0.000057065748391193774`, 0.10157703213632492`},
```

```
{180, 0.00005545040113692313`, 0.09870171402372317`}, {210, 0.00005459872625335214`, 0.09718573273096683`},
{240, 0.00005451957438621824`, 0.09704484240746847`}, {270, 0.0002501508854187748`, 0.44526857604541914`}}},
{0.18592803424857857`, 3.7511626605606008`, 0.2948073517622305`, 1.1421602142936857`, 0.24567142070681736`,
0.41248813426514863`, 0.25834870652665815`, 931.542828982051`, 0.012674565140645466`,
9.754712584362995`, 107008.11656396952`, 29262.372867084086`, 228.47491226389562`, 2035.749314762999`,
0.`, {{0, 0.00511642755743482`, 9.10724105223398`}, {8, 0.0030382962306124196`, 5.408167290490107`},
{16, 0.0023248932866942133`, 4.1383100503157`}, {24, 0.0018747968252934588`, 3.3371383490223567`},
{32, 0.0015262455098429973`, 2.716717007520536`}, {40, 0.0012528940757847561`, 2.230151454896866`},
{48, 0.0010394280981062514`, 1.850182014629127 5`}, {56, 0.0008727578257888746`, 1.5535089299041969`},
{64, 0.0007424996612588665`, 1.3216493970407823`}, {72, 0.13692870885117128`, 243.7331017550849`}}},
{0.058165051218229624`, 1.2475983837798745`, 4.326003024744654`, 1.2604302116068957`, 0.7278275017755875`,
0.49389546693051767`, 0.021556729129120156`, 452.84942671713645`, 0.038214009527072046`,
8.73167788977904`, 311.9395115847039`, 4.848793818638901`, 4.276346971284773`, 312.69085188774903`,
1.`, {{0, 0.021108215570966662`, 37.572623716320656`}, {3, 0.0024234215319828`, 4.313690326929384`},
{6, 0.00021907184553991992`, 0.3899478850610575`}, {9, 0.000013795794979544982`, 0.024556515063590068`}}},
{0.17310243018283944`, 0.49256885761049496`, 5.2474038118867075`, 1.2167083563026317`, 0.588616706923442`,
0.41089714710891945`, 0.1821711177021871`, 4451.29023370465`, 0.12001647220652961`, 2.269196213787966`, 8.083272027047439`,
0.7206383792464865`, 0.7206383792464865`, 8.083272027047439`, 1.`, {{0, 0.0006143286740556054`, 1.0935050398189776`}}},
{0.1805022145737788`, 3.870623878161763`, 2.6853201865429206`, 1.4540056051610473`, 0.3465395939248448`,
0.3116685990581043`, 0.3430495408207368`, 123.77189026179846`, 0.21554545019710575`, 7.5674497363872035`,
50603.35160539281`, 14098.433317572255`, 228.78729565483306`, 1055.5099173917113`, 0.`,
{{0, 0.060517688830850014`, 107.72148611891302`}, {9, 0.010656128289324026`, 18.967908354996766`},
{18, 0.002392740038850336`, 4.259077269153598`}, {27, 0.0007239484681777303`, 1.28862827335636`},
{36, 0.0003356862928618933 4`, 0.5975216012941701`}, {45, 0.00024118587594993709`, 0.429310859190888`},
{54, 0.00021440255335238232`, 0.38163654496724053`}, {63, 0.00020472708062329716`, 0.36441420350946896`},
{72, 0.0002004574983688544`, 0.35681434709656085`}, {81, 0.004731788793411597`, 8.422584052272642`}}},
{0.12919486377057743`, 2.6304051581320387`, 9.030615531469042`, 1.1852097760732927`, 0.8025656477879668`,
0.2709665069296936`, 0.01610528402445943`, 631.0560544240994`, 0.23139307171140971`, 4.580191033116536`,
129.3234760021952`, 0.6492873467201221`, 0.6191077157656223`, 121.43349376348422`, 1.`,
{{0, 0.005209139784236734`, 9.272268815941386`}, {38, 0.004019805741788066`, 7.1552542203827585`}}},
{0.11792807938525018`, 2.9992967519426994`, 5.197929087471108`, 1.1316540273093707`, 0.8248866470317724`,
```

0.3561695895739371`, 0.01696020529134022`, 1283.8412020113915`, 0.1325959329361287`, 7.000673815586042`,
122.9084651155277`, 0.5730732050730232`, 0.5506812197861342`, 118.0921699618674`, 1.`,
{{0, 0.0065387195796164305`, 11.638920851717247`}, {10, 0.0024362853374854916`, 4.336587900724175`}}},
{0.049303290582256715`, 1.1925070924339343`, 0.7957633600668377`, 1.4128683276782472`, 0.000022730879762677958`,
0.4285104885078085`, 0.00878130985829771`, 1257.460569712898`, 0.24085716752239034`,
7.587289884781604`, 327.1525055128447`, 161.5612301758804`, 161.26865739457747`, 327.1260486786488`,
1.`, {{0, 0.015415379035824765`, 27.439374683768083`}, {1, 0.009446200663752544`, 16.81423718147953`}}},
{0.13128664635800402`, 2.4046868210274015`, 6.858470528577773`, 1.007040533285536`, 0.4840752461507001`,
0.3489790633820432`, 0.006590730128885638`, 3596.8950880067596`, 0.16587148750288666`, 3.462016809878005`, 12.368163834688406`,
0.032967111255973375`, 0.032967111255973375`, 12.368163834688406`, 1.`, {{0, 0.000939980451436319`, 1.6731652035566476`}}},
{0.04846841194802434`, 1.828640157997615`, 8.053204702491172`, 0.7912000348817253`, 0.19137275290127498`,
0.26044015409759047`, 0.137444226583514`, 395.2194426771421`, 0.10623448877689068`,
6.754668978902`, 150.15138902721569`, 3.597289374817706`, 1.909995453244897`, 81.3260886204246`, 1.`,
{{0, 0.005135176142618174`, 9.14061353386035`}, {20, 0.0010456065925340961`, 1.8611797347106913`}}},
{0.22606436547954117`, 2.759207160262239`, 4.797641172785626`, 1.1197512107663665`, 0.8744312540778343`,
0.4079143329219078`, 0.021680363867763237`, 112.017364386689`, 0.04841847187742204`, 6.247097164626069`,
179011.64375117325`, 42269.70503819758`, 228.62863976620557`, 1578.1569745876823`, 0.`,
{{0, 0.04573634801077244`, 81.41069945917495`}, {33, 0.01053103119048332`, 18.74523551906031`},
{66, 0.006470281958662622`, 11.517101886419468`}, {99, 0.005021438286268526`, 8.938160149557975`},
{132, 0.003801418287030407`, 6.766524550914124`}, {165, 0.0028186482871015678`, 5.017193951040791`},
{198, 0.002085821861123733`, 3.7127629128002444`}, {231, 0.0015508549617734642`, 2.7605218319567664`},
{264, 0.001160931979988692`, 2.0664589243798717`}, {297, 0.04076315524545908`, 72.55841633691715`}}},
{0.21439187995731207`, 1.4871413304129364`, 0.5160724591468409`, 1.1806787917625174`, 0.8679222077666104`,
0.6556391626343723`, 0.05962099501823598`, 325.40523317512117`, 0.12955288081431815`, 2.055442432220964`, 39489.35959638253`,
9717.94786347691`, 229.88604677083794`, 1061.0328222575943`, 0.`, {{0, 0.08063849449157717`, 143.53652019500737`}}},
{0.09778347249929609`, 1.5399588911598938`, 7.050045797549721`, 0.9082485710483456`, 0.7454636238668619`,
0.6248569918637821`, 0.04029077002834171`, 1056.397985521477`, 0.20938181714809317`,
3.6320484036778673`, 92.52153069881075`, 1.485050987110705`, 1.453822365788269`, 90.61226526880658`, 1.`,
{{0, 0.005713811374015267`, 10.170584245747175`}, {4, 0.001172720786414034`, 2.0874429998169806`}}},
{0.12927232662770405`, 2.951438179906213`, 0.40510823994438105`, 1.0187470674786288`, 0.2032238445066854`,
0.5456409554372916`, 0.01881027549673071`, 134.69705409101317`, 0.12921233603333943`, 7.23494261394152`, 67746.06365450741`,

```
  23789.474558493246`, 230.02786144707235`, 1012.0690496344315`, 0.`, {{0, 0.0769172477722168`, 136.9127010345459`}}},
{0.20182779178318416`, 0.6881028184372244`, 1.057422534977741`, 1.0568090274497894`, 0.12452101430667706`,
  0.6254257177994904`, 0.38702446915906646`, 3365.5277260432094`, 0.12211162516890145`, 5.63708838473557`, 21.067396734074475`,
  1.640212255468917 1`, 1.2828146444794872`, 16.512483126369194`, 1.`, {{0, 0.00100869839061457`, 1.7954831352939347`},
   {4, 0.0002274960744786865`, 0.4049430125720619`}, {8, 0.000018754252510802436`, 0.03338256946922834`}}},
{0.21480237208045305`, 2.4248480984277982`, 7.5563453916242835`, 0.9609967545999567`, 0.4216733331369118`,
  0.3788510295397969`, 0.02865689876021866`, 142.8377853769303`, 0.053409606561145295`,
  2.2123406251771502`, 159.10785880878555`, 5.059638625536616`, 1.642756756229928`, 150.24182331826964`,
  1.`, {{0, 0.008345745434273927`, 14.85542687300759`}, {3, 0.0024367432009112316`, 4.337402897621992`},
   {6, 0.00047050846287965335`, 0.8375050639257828`}, {9, 0.00009589660229905653`, 0.17069595209232058`},
   {12, 0.00002936630386171854`, 0.052272020873859`}, {15, 0.000018304359311783827`, 0.03258175957497521`},
   {18, 0.000016676572625153 96`, 0.029684299272774047`}, {21, 5.137636025966445`*^-6, 0.009144992126220273`}}},
{0.19117013383013431`, 1.4594963084730592`, 8.271393984038092`, 0.8911461626518757`, 0.4023315908892062`,
  0.6430024305607471`, 0.425955961175158`, 811.9982424604128`, 0.08470327958141488`, 1.5517888018340713`, 30.304227280128735`,
  1.1883184000334581`, 0.8288892494971217`, 21.17930667074597`, 1.`, {{0, 0.0012188364953248318`, 2.1695289616782003`},
   {4, 0.0003516112030858578`, 0.6258679414928269`}, {8, 0.000039179608566004094`, 0.06973970324748728`}}},
{0.16704561027767095`, 0.7136544792609025`, 7.482780404676742`, 1.2150283597546259`, 0.9046207606306917`,
  0.271745131902447`, 0.2695234426940548`, 220.6020580904624`, 0.17006503868594403`, 3.14899802359108`,
  188.1924029274620 7`, 13.655240890566922`, 6.283718339238313`, 90.33029438287217`, 1.`,
  {{0, 0.004631584288609813`, 8.244220033725467`}, {6, 0.001848061413838219`, 3.28954931663203`},
   {12, 0.0003174474350597064`, 0.5650564344062773`}, {18, 0.000047061080589181354`, 0.08376872344874281`},
   {24, 9.640641161024232`*^-6, 0.01716034126662313 2`}, {30, 0.000011307513840341 83`, 0.020127374635808458`}}},
{0.16595765366147208`, 2.092996799011412`, 4.948615364795604`, 0.8929763621996465`, 0.8391454185145462`,
  0.5892008264762102`, 0.044299676614507844`, 130.42998143434642`, 0.19085435393011602`,
  9.39386568753341`, 26573.72469699617`, 7773.945361438284`, 228.97362841164554`, 1297.5824888585598`,
  0.`, {{0, 0.0752309678681851`, 133.91112280536947`}, {5, 0.015461932162498455`, 27.52223924924725`},
   {10, 0.0025948694288307613`, 4.618867583318755`}, {15, 0.0005582303183360467`, 0.9936499666381631`},
   {20, 0.00017446089301016967`, 0.31054038955810204`}, {25, 0.00008750655151018631`, 0.15576166168813163`},
   {30, 0.0000643607544780255`, 0.1145621429708854`}, {35, 0.00005689278740805139`, 0.10126916158633147`},
   {40, 0.000053904095498014984`, 0.09594928998646667`}, {45, 0.004333144293495733 5`, 7.712996842422404`}}},
{0.27224148961405453`, 3.064354031176171`, 9.703480945324245`, 1.4628863651144104`, 0.3875440410966342`,
```

```
0.15502532019017645`, 0.01538110765065082`, 96.32001554152914`, 0.17185939304406467`,
  6.478173160029735`, 508.2653260559073`, 4.433122664486242`, 2.2332220374275304`, 489.0505336258501`,
  1.`, {{0, 0.030317538636829275`, 53.9652187735561`}, {8, 0.005793040561701206`, 10.311612199828145`},
   {16, 0.0009505304513447579`, 1.6919442033936691`}, {24, 0.00010673090568937173`, 0.18998101212708168`}}},
{0.17789655356032075`, 0.42086448766877327`, 8.212577079102342`, 1.145177796821741`, 0.7675840121122328`,
  0.5713332044931656`, 0.08476669830762888`, 407.4176887081235`, 0.039911846147414665`, 5.425104426537898`,
  350.12885558368856`, 27.405241796064693`, 18.98103641226656`, 244.26322514093596`, 1.`,
  {{0, 0.011974403670591768`, 21.314438533653348`}, {14, 0.006589601440119365`, 11.729490563412469`}}},
{0.25890879804354505`, 0.827757285125033`, 6.146318214990161`, 1.0400613992204162`, 0.4812180568243971`,
  0.29251954183586915`, 0.03603273141312643`, 1733.9600457555907`, 0.08508345454030858`, 2.745915615562448`,
  18.586470565912578`, 0.49046162767961676`, 0.37912381380744364`, 14.287944585768319`, 1.`,
  {{0, 0.0008051685200521667`, 1.433199965692857`}, {45, 0.00028071526846622555`, 0.49967317786988147`}}},
{0.13429919704858378`, 3.97301958069563`, 0.23275296336668916`, 1.4075463260369605`, 0.23061890929775308`,
  0.5504417933404041`, 0.22771166040596233`, 272.6044063125219`, 0.22898591641919652`, 6.483734227937328`, 26385.39698790132`,
  9036.523208331322`, 230.1868920708533`, 893.0392014352899`, 0.`, {{0, 0.06787097930908204`, 120.81034317016602`}}},
{0.12030391986517214`, 0.7748656879950588`, 5.112522469071294`, 1.1531030157403883`, 0.37283988475313556`,
  0.569214715358989З`, 0.013539004333470354`, 948.0585012448981`, 0.020895787868669358`, 3.48210387762453`, 53.13242829537899`,
  0.7288635487967867`, 0.7288635487967867`, 53.13242829537899`, 1.`, {{0, 0.004038064550448804`, 7.187754899798871`}}},
{0.1321541010040706`, 0.831080421127889`, 5.684518793971316`, 1.0145204035612485`, 0.08667418093385804`,
  0.2219847293666507`, 0.022523926566952547`, 3181.1487538157703`, 0.21535243439086593`, 6.5479272590953945`,
  15.757187434428145`, 0.2924794085961605`, 0.24893842566285088`, 13.42037523428861`, 1.`,
  {{0, 0.0006945840058040657`, 1.236359530331237`}, {55, 0.00032536451200186866`, 0.5791488313633262`}}},
{0.10292542985717545`, 0.9480363128916887`, 1.8187023134383153`, 1.095683835227761`, 0.3789693754557397`,
  0.6741099730044744`, 0.007927682110189524`, 599.0709679738629`, 0.23730403059109434`,
  8.347995806673719`, 1577.4614793503047`, 584.3066189289428`, 202.61075716220904`, 645.3528392052004`,
  1.`, {{0, 0.014386699357584562`, 25.60832485650052`}, {12, 0.034660116422010674`, 61.695007231179005`}}},
{0.11768271904228267`, 0.8044018805503832`, 4.992994745146397`, 0.8839437991548327`, 0.9627780258088621`,
  0.4036610370791115`, 0.010677704183731065`, 75.90134138634313`, 0.09047277628943856`,
  3.892249177500955`, 90358.61297984415`, 33643.98872514561`, 228.0541990041228`, 963.2901468928435`,
  0.`, {{0, 0.04880652066398899`, 86.87560678190039`}, {4, 0.009225327370831185`, 16.42108272007951`},
   {8, 0.0028754614664453144`, 5.118321410272659`}, {12, 0.001006018785673867З`, 1.7907134384994838`},
```

```
    {16, 0.00042642740773437393`, 0.7590407857671856`}, {20, 0.0002427983644442941`, 0.43218108871084343`},
    {24, 0.00018187426219365204`, 0.32373618670470067`}, {28, 0.000159542435321939`, 0.2839855348730514`},
    {32, 0.00015016074924895128`, 0.2672861336631333`}, {36, 0.010135919657973542`, 18.041936991192905`}}},
 {0.23810204192481904`, 3.5212069305389937`, 6.24449843698252`, 1.12580298554529`, 0.9906238590423209`,
  0.651194539832239`, 0.008821192031195845`, 3794.027232128564`, 0.08728181755971864`, 9.039220988882679`,
  128.21203881894698`, 0.29735077572027796`, 0.2920467305444724`, 126.05878856540755`, 1.`,
  {{0, 0.007307938657919575`, 13.008130811096844`}, {2, 0.0022725292730513998`, 4.045102106031492`}}},
 {0.05170854445494355`, 0.9544694556169109`, 2.4941099826351163`, 0.9097774283707087`, 0.0255473932825480 7`,
  0.22408928016637408`, 0.36587983814629343`, 578.5087062785725`, 0.14318945676945632`,
  8.489550606354296`, 110.81059570015968`, 6.227635032220989`, 2.8955715284104206`, 53.04580491209655`,
  1.`, {{0, 0.0034028073216222383`, 6.056997032487584`}, {9, 0.0006044353995498025`, 1.0758950111986485`},
    {18, 0.000022122388733399843`, 0.03937785194545172`}, {27, 2.1160634138977204`*^-6, 0.0037665928767379423`}}},
 {0.2758391104750425`, 2.3950700125014723`, 3.0825274454463827`, 1.2324094107661792`, 0.33578451389793784`,
  0.4521239993250691`, 0.02669897011071422`, 4321.015062663416`, 0.06443072522161825`, 4.390191809873814`, 12.709093420348216`,
  0.10534248124175283`, 0.10534248124175283`, 12.709093420348216`, 1.`, {{0, 0.0009658910999464645`, 1.7192861579047067`}}},
 {0.04136033639560718`, 3.5342644851920078`, 2.1080294445485137`, 0.9950381948590621`, 0.26813425336643104`,
  0.30392774702749736`, 0.047958977131448324`, 92.66270711931676`, 0.1731864034169307`, 4.57325203890367`, 32961.012784380415`,
  20669.425770601043`, 230.2445290036974`, 580.7301245237652`, 0.`, {{0, 0.04413548946380616`, 78.5611712455749 7`}}},
 {0.1958059683987035`, 3.200106783136203`, 7.203120771344906`, 1.1276577663917136`, 0.37865874140280775`,
  0.19330414092595893`, 0.01322903896050458`, 235.27978791284824`, 0.1213328646575434`, 2.0980953870537427`,
  78.94411939152164`, 0.32471451524222644`, 0.31755968369028176`, 77.63770466816136`, 1.`,
  {{0, 0.004739787665975871`, 8.43682204543705`}, {12, 0.0011606778888043922`, 2.066006642071818`}}},
 {0.2141293518430885`, 1.3445267096173987`, 9.440612710104876`, 1.3031822475396773`, 0.08631549676886618`,
  0.2421378295292581`, 0.053475196461594834`, 50.8320364740117`, 0.167124755886029`, 6.69448094138391`,
  113801.9853730022`, 27926.50354717801`, 229.15536518245426`, 1502.9702194649115`, 0.`,
  {{0, 0.0704094395987141`, 125.32880248571108`}, {31, 0.0022141359995364934`, 3.9411620791749584`},
    {62, 0.0031740183638618185`, 5.649752687674037`}, {93, 0.0023235050786826537`, 4.1358390400551235`},
    {124, 0.0021399320075706336`, 3.8090789734757275`}, {155, 0.0020548188848189507`, 3.6575776149777326`},
    {186, 0.002021950656579726`, 3.5990721687119125`}, {217, 0.002008767836910112`, 3.5756067496999995`},
    {248, 0.0020034712589133133`, 3.5661788408656974`}, {279, 0.02587569699374548`, 46.05874064886695`}}},
 {0.14124099610833857`, 1.6304240145491873`, 4.431235349124869`, 0.7694928939095955`, 0.9477250896887188`,
```

```
    0.5434618086833787`, 0.13057745213792676`, 1388.4740941330226`, 0.07045603839931946`, 8.981021929620123`, 270.06412862421405`,
    7.281119715008524`, 4.538897832698657`, 169.2807383598099`, 1.`, {{0, 0.00592913286579925`, 10.553856501122667`},
      {3, 0.0039061197191762377`, 6.952893100133703`}, {6, 0.0019141064185385387`, 3.407109424998599`},
      {9, 0.0009331486948810812`, 1.6610046768883244`}, {12, 0.00018282841695044488`, 0.3254345821717919`}}},
  {0.08317638924124338`, 3.4814117090049965`, 4.537378351353009`, 1.1287962009049006`, 0.37603042712104306`,
    0.6772825294518297`, 0.08278526718675269`, 52.354988641883`, 0.1936331782676135`, 8.933135500909255`, 97609.87806211805`,
    44452.810745293835`, 228.76549363854298`, 1757.5572666368985`, 0.`, {{0, 0.13357435226440428`, 237.76234703063963`}}},
  {0.14458191325048447`, 2.576522592570102`, 7.664240990513161`, 1.1334692879511565`, 0.1460097548297743`,
    0.5479004940247297`, 0.17641432391033549`, 874.6457178122505`, 0.14737345925317297`, 7.125853291619361`,
    95.38030714927152`, 1.7862928487935592`, 1.5437329952013341`, 82.60645257294593`, 1.`,
    {{0, 0.005352240774422926`, 9.526988578472809`}, {7, 0.000925849621120964`, 1.6480123255953159`}}},
  {0.21964890100642098`, 3.948215647629967`, 1.3239539515383179`, 0.7541520677887659`, 0.2631388544858304`,
    0.4680193255276971`, 0.07455346059859487`, 734.1983096733243`, 0.22794453971709777`, 5.810951908142973`, 1081.510384640808`,
    246.19250033208806`, 52.38904964115036`, 282.92833105255784`, 1.`, {{0, 0.019189550013929167`, 34.15739902479391`},
      {4, 0.0003189451321822512`, 0.5677223352844071`}, {8, 0.0002783131464702631`, 0.4953974007170683`},
      {12, 0.00027815536505055487`, 0.49511654978998765`}, {16, 0.0002781516554150722`, 0.49510994663882857`},
      {20, 0.00027815139281570843`, 0.49510947921196097`}, {24, 0.00027815135482817013`, 0.4951094115941428`},
      {28, 0.0002781513459294294`, 0.49510939575438434`}, {32, 0.00032498375337378094`, 0.5784710810053301`}}},
  {0.24357090033802437`, 2.585687351965424`, 7.1434831024545`, 1.2789397105658484`, 0.15335660895486725`,
    0.5235228651937761`, 0.12790639986488742`, 56.5359989413313`, 0.05251799125371648`, 7.0717564062965526`,
    528577.8097152971`, 117914.13527095078`, 228.46303703031936`, 2888.0429990565676`, 0.`,
    {{0, 0.07849768226398733`, 139.72587442989743`}, {4, 0.012788448774234056`, 22.763438818136617`},
      {8, 0.005563354319591639`, 9.902770688873117`}, {12, 0.0037383340525230725`, 6.654234613491069`},
      {16, 0.0033214539753773143`, 5.91218807617162`}, {20, 0.003359000045197576`, 5.9790200804516855`},
      {24, 0.003397629898996801`, 6.047781220214306`}, {28, 0.0031898754740649266`, 5.677978343835568`},
      {32, 0.0027773244056126063`, 4.943637441990439`}, {36, 0.10285816471871383`, 183.0875331993106`}}},
  {0.09627432586752127`, 2.332762155680175`, 5.679853879558527`, 1.2640734140093113`, 0.8156330491360497`,
    0.2895792785477985`, 0.02307681035132743`, 658.139699058721`, 0.029189068125338835`, 7.800914789874877`,
    229.73736831393308`, 1.7107469762391414`, 1.4205735756122182`, 186.91424371852492`, 1.`,
    {{0, 0.009079214967785682`, 16.161002642658513`}, {38, 0.00512626755482221`, 9.124756247583534`}}},
  {0.07523258003696548`, 2.5308231598738997`, 0.19036315944607196`, 0.7501464923569574`, 0.1153260012566284`,
```

```
0.48261384042066857`, 0.014254428894325608`, 71.6138994625911`, 0.24976914139283035`, 2.597638512857568`, 36879.97968610944`,
 17773.636090166478`, 230.2226522703035`, 675.6184603038587`, 0.`, {{0, 0.05134700298309326`, 91.397665309906`}}},
{0.20487865745542216`, 2.4577449266837927`, 0.3620012570001929`, 1.1621484663425825`, 0.45716579542364766`,
 0.40055848752773193`, 0.09344580216885054`, 2051.8175124763634`, 0.1493227569977858`, 9.69569860745337`,
 1986.8538965457685`, 499.17025527709814`, 32.952138955613705`, 159.9275809015442`, 1.`,
 {{0, 0.007614555962308703`, 13.55390961290949`}, {9, 0.0005130636672937177`, 0.9132533277828173`},
  {18, 0.0005120907232047699`, 0.9115214873044905`}, {27, 0.0005120829758973351`, 0.9115076970972564`},
  {36, 0.0005120824574619592`, 0.9115067742822874`}, {45, 0.0005120823696355041`, 0.9115066179511974`},
  {54, 0.0005120823454075576`, 0.9115065748254526`}, {63, 0.0005120823366061809`, 0.911506559159002`},
  {72, 0.0005120823328646198`, 0.9115065524990232`}, {81, 0.00044229097783701066`, 0.787277940549879`}}},
{0.1890789829788747`, 2.8864588443110275`, 0.3020897698703884`, 0.7861583937331655`, 0.3631295895274096`,
 0.49565973888016235`, 0.15528595628066338`, 2271.132408212757`, 0.21092746640771154`,
 4.683826377759731`, 1042.0690091087292`, 278.6051553524747`, 143.788288710238`, 544.1544094403495`,
 1.`, {{0, 0.032291797376458216`, 57.47939933009563`}, {33, 0.0016183906632301764`, 2.880735380549714`},
  {66, 0.0014649299428551104`, 2.6075752982820966`}, {99, 0.0014539239491057204`, 2.587984629408182`},
  {132, 0.00145318670996209`, 2.586672343732732`}, {165, 0.00145314163650378`, 2.5865921129767413`},
  {198, 0.0014531388778867324`, 2.5865872026383836`}, {231, 0.0001672259614646116`, 0.29766221140700866`}}},
{0.13636211858102731`, 3.2225156772879746`, 1.985405604082965`, 0.8074403105824717`, 0.51683495700684`,
 0.17846947550569658`, 0.2866421049071916`, 363.9511542941515`, 0.043491080354586376`,
 7.48314403026421`, 8549.383571778442`, 2862.1419794083`, 1.6399596568818566`, 76.30817835955234`, 1.`,
 {{0, 0.004901040466287979`, 8.723852029992601`}, {11, 0.0008215405127818032`, 1.4623421127516096`},
  {22, 0.00004546939210548665`, 0.08093551794776623`}, {33, 6.531390735664555`*^-6, 0.011625875509482908`},
  {44, 5.210569607747678`*^-6, 0.009274813901790867`}, {55, 5.16538162476507`*^-6, 0.009194379292081823`},
  {66, 5.1622496223051226`*^-6, 0.00918880432770312`}, {77, 9.301592560225993`*^-6, 0.016556834757202266`}}},
{0.1760012024892597`, 3.3901909690471133`, 5.258617572133415`, 0.8627686224489919`, 0.7422533085216929`, 0.41138487941188706`,
 0.007592509582269234`, 424.62789093038504`, 0.09162929479721776`, 1.3722835015951313`, 58.438503856773174`,
 0.378218613718889`, 0.22321110364660784`, 55.245804872928645`, 1.`, {{0, 0.003177614112104377`, 5.656153119545791`},
  {6, 0.0009191125278347919`, 1.6360202995459294`}, {12, 0.00010195453040340817`, 0.18147906411806655`}}},
{0.1813951042683553`, 2.693386252451666`, 9.617741712202033`, 1.407583958403493`, 0.6407257633308476`,
 0.4405499114150466`, 0.008535253338252974`, 83.84476850180724`, 0.06822985382579094`,
 7.163997237119144`, 101706.29625766253`, 28162.851503653405`, 60.68595098166223`, 902.9842891560263`,
```

```
1.`, {{0, 0.04355997372459042`, 77.53675322977094`}, {3, 0.0028924776640809123`, 5.148610242064024`},
   {6, 0.0027782983209139332`, 4.945371011226801`}, {9, 0.002774877098935716`, 4.939281236105575`},
   {12, 0.002774687159571302`, 4.938943144036918`}, {15, 0.002774671135462876`, 4.93891462112392`},
   {18, 0.002774669323854189`, 4.9389113964604565`}, {21, 0.002774669070399758`, 4.93891094531157`},
   {24, 0.0027746690286993363`, 4.938910871084818`}, {27, 0.002747813449349562`, 4.89110793984222`}}},
{0.17566594009418735`, 3.2466526484278377`, 9.344725381698979`, 1.0138972604548542`, 0.5838549167343343`,
  0.6073333607184574`, 0.10710975925951867`, 945.005957021721`, 0.208189974443157`, 1.4109192016259353`,
  28.749701412650325`, 0.3784419743692973`, 0.2748872057565413`, 20.379216069358296`, 1.`,
  {{0, 0.0011798871205515068`, 2.100199074581682`}, {17, 0.0003689333007197237`, 0.6567012752811081`}}},
{0.22088610125498404`, 1.594941397589448`, 0.6585367344105002`, 1.4859166690390868`, 0.411769154740657`,
  0.4715175396654723`, 0.024538179644211296`, 3156.4892991025154`, 0.15641735671760065`, 8.553654142069323`, 39.198446573157874`,
  0.5625784496314697`, 0.5404928107134863`, 38.80871508356419`, 1.`, {{0, 0.0022859141264512336`, 4.068927145083196`},
   {5, 0.0005847794737392971`, 1.0409074632559487`}, {10, 0.00007876874616034778`, 0.14020836816541907`}}},
{0.08792198919034061`, 0.7563929024204028`, 9.775416388823775`, 0.7563974438325178`, 0.8271569221820632`,
  0.32401969363676375`, 0.0333282008681181`, 102.10380045307154`, 0.1014972556908379`, 6.35713244492311`, 554.8359448931674`,
  27.33665827390643`, 15.26658223411166`, 532.0958639038806`, 1.`, {{0, 0.038215814193509064`, 68.02414926444614`},
   {2, 0.0020074715673172004`, 3.573299389824617`}, {4, 0.00009873163427321258`, 0.17574230900631838`},
   {6, 0.000022771510514872803`, 0.04053328871647359`}, {8, 0.00002021715829418401`, 0.035986541763647545`},
   {10, 0.000020128460786832316`, 0.035828660200561524`}, {12, 0.000020124008454387336`, 0.035820735048809456`},
   {14, 0.000020123669799663216`, 0.035820132243400526`}, {16, 0.000013903453745512137`, 0.024748147667011604`}}},
{0.13210283054552296`, 3.2703864704797683`, 0.8879166279114746`, 1.2876213813677582`, 0.37930173682375035`,
  0.2978584700900475`, 0.018740615394798876`, 111.76209976448992`, 0.22677958819730465`, 8.664567998658026`, 50581.820234339546`,
  17498.86349643624`, 230.19703528395843`, 905.8557058635511`, 0.`, {{0, 0.06884503364562988`, 122.54415988922119`}}},
{0.19507506176309325`, 3.126733009164254`, 1.2939386659529877`, 0.8626822462482099`, 0.4036124839823858`,
  0.4778725494331981`, 0.06798274218289758`, 2442.14562500692`, 0.025650848833468687`, 3.9818096584261493`,
  22.673448332103852`, 0.27445333972959485`, 0.22425602142498846`, 16.585108943999792`, 1.`,
  {{0, 0.001051141295242984`, 1.8710315055325115`}, {30, 0.00020932698450100022`, 0.3726020324117804`}}},
{0.08607083224139878`, 0.4430857620402713`, 5.378081455692687`, 1.015604011959475`, 0.6736258822863768`,
  0.30157456176441344`, 0.06550214653497834`, 2724.6632477522535`, 0.20587133389175544`, 1.8687253311000447`,
  10.318810318580342`, 0.6770865792303281`, 0.42191625724534493`, 6.427241187364826`, 1.`,
  {{0, 0.0003921156806431666`, 0.6979659115448366`}, {32, 0.00009635464959656016`, 0.17151127628187707`}}},
```

```
{0.14471266481637884`, 3.5830029087352937`, 5.919067097135953`, 1.3383183012471727`, 0.9997339519904831`,
  0.40166871745176214`, 0.015827643464025123`, 2493.129738188907`, 0.10618580931649058`, 1.661604036688603`,
  22.29895088566018 7`, 0.10053912404096749`, 0.09812502124612989`, 21.819403724053476`, 1.`,
  {{0, 0.0011863239430199726`, 2.1116566185755516`}, {3, 0.00047195074000809154`, 0.840072317214403`}}},
{0.15884129765285837`, 1.9554524456621927`, 0.3975714737116185`, 1.180621526159508`, 0.13357193827486524`,
  0.6393982147520227`, 0.07953097836767822`, 3991.2803311425578`, 0.2365716915141447`, 8.584420428066604`,
  28.506128337321066`, 0.6975869662610875`, 0.6537411083442448`, 27.20960484659943 6`, 1.`,
  {{0, 0.001646514763807895`, 2.930796279578053`}, {8, 0.00042141520453366227`, 0.7501190640699189`}}},
{0.04917531951043441`, 2.0521955455867618`, 5.720052321648424`, 1.1771597571169652`, 0.4322660150443707`,
  0.1629991214278883`, 0.005091310116027936`, 189.05888707261903`, 0.1735533969163952`, 4.172239042736072`,
  187.91675973261079`, 1.0240271382802288`, 0.5479949980244643`, 180.20092586122948`, 1.`,
  {{0, 0.008546336009828312`, 15.212478097494392`}, {8, 0.0035762366865662687`, 6.365701302087958`},
    {16, 0.0012564471721619595`, 2.2364759664482876`}, {24, 0.00031625049689690016`, 0.5629258844764823`}}},
{0.27542629055144846`, 0.7843233583874856`, 9.783752583368699`, 1.3481471177954405`, 0.23017739141462767`,
  0.5420497851091893`, 0.2059235624279975`, 3468.61907862177`, 0.15188055880627876`, 9.878186090289375`, 35.8690582981044`,
  2.158267556578268`, 2.158267556578268`, 35.8690582981044`, 1.`, {{0, 0.0027260484306559345`, 4.852366206567563`}}},
{0.242913589428772`, 2.017077890959616`, 6.7411875410249`, 1.4794325227990526`, 0.8119526659059784`,
  0.6637027966074835`, 0.028536028992749693`, 2430.705278612085`, 0.11516348105007651`, 2.1124785829469186`,
  28.31791319247922`, 0.2935605812309376`, 0.2922455326396928`, 28.180017289633533`, 1.`,
  {{0, 0.0018508585519167095`, 3.294528222411743`}, {5, 0.000290822762095439`, 0.5176645165298814`}}},
{0.27369802527373327`, 0.5072292829799574`, 6.3938654238644155`, 0.9166058122252008`, 0.8588715137395462`,
  0.6363372429100727`, 0.4973592888199021`, 125.12883357781175`, 0.0982596068118865`, 5.59867632569453`,
  60166.060284302344`, 12224.772888953192`, 82.86790101579484`, 596.5304981341321`, 1.`,
  {{0, 0.03759001841982912`, 66.91023278729584`}, {18, 0.006425189015942819`, 11.43683644837822`},
    {36, 0.001100286077482822`, 1.9585092179194232`}, {54, 0.00022082434493928433`, 0.3930673339919261`}}},
{0.18540637687565897`, 1.1775430389730683`, 2.7268491301469933`, 1.4767982648221127`, 0.8902187555583527`,
  0.5282942786301189`, 0.1497900469847022`, 304.29028125771407`, 0.023895791395609434`, 5.874748340307197`, 94821.83147001214`,
  25956.43124383153`, 68.47328995041131`, 385.8663223410875`, 1.`, {{0, 0.024722839062550354`, 44.00665353133963`},
    {25, 0.003968629551376031`, 7.064160601449336`}, {50, 0.0006343718839962624`, 1.129181953513347`}}},
{0.2722155439547531`, 0.9139371325579537`, 7.770199974150746`, 0.8847133947824111`, 0.9018075103619831`,
  0.1996028715400288 7`, 0.008582048560181034`, 1921.873640365849`, 0.18448070830995128`,
```

```
2.8786799978881135`, 25.034955966091786`, 0.19843900885504995`, 0.198068877642215`, 24.98397823719116`,
1.`, {{0, 0.0016362815704771003`, 2.9125811954492384`}, {6, 0.0002625007755494278`, 0.4672513804779815`}}},
{0.06964367539322192`, 3.707453741922901`, 4.6834761045808655`, 1.337177597600478`, 0.13817299390105364`,
0.15072857839162668`, 0.030325540973499845`, 324.16896723003435`, 0.11870351893768993`,
8.4259642987084`, 184.69188829640746`, 1.0659696094590083`, 0.7408700923793374`, 121.12394006007298`,
1.`, {{0, 0.006597237795003305`, 11.743083275105882`}, {56, 0.002608181649562241`, 4.642563336220789`}}},
{0.17261439973382386`, 3.0972705794401207`, 5.672236578564393`, 1.4223285072729144`, 0.7931550721000631`,
0.3896544494671119`, 0.01712882834694823`, 1350.492916562551`, 0.2427963854280547`, 3.05685280243325`,
49.605842097918476`, 0.24496579865513127`, 0.24060209297167218`, 48.73424376321656`, 1.`,
{{0, 0.002902932826883564`, 5.167220431852744`}, {7, 0.0008008696991208951`, 1.4255480644351932`}}},
{0.11394032550346922`, 3.2108067304960466`, 9.096957387649113`, 1.399541009089664`, 0.15147624678363414`,
0.6188818627652795`, 0.08261238136824677`, 151.04115640404754`, 0.09860367843525403`,
6.06148281050898`, 500.3812667100334`, 8.275150946950316`, 5.965613066192502`, 464.24322851154386`, 1.`,
{{0, 0.029662606734079902`, 52.79943998666223`}, {8, 0.005619878632797431`, 10.003383966379426`}}},
{0.20354065160019702`, 3.5209980508829766`, 0.4053576884306178`, 0.7576791600370161`, 0.6570403323895382`,
0.46832222917239164`, 0.005063986272754102`, 161.28160618619455`, 0.12637144234319758`, 8.874326085906798`, 53801.02557794975`,
13760.660522826178`, 230.21134983607266`, 1156.5834597537391`, 0.`, {{0, 0.08790034294128418`, 156.46261043548583`}}},
{0.24345495866234085`, 1.8344199239977046`, 3.71003568577021`, 1.0568366445286865`, 0.9797132100793247`,
0.5295146440336447`, 0.23321770204647818`, 2779.737816031833`, 0.01572768263207122`, 9.835586906627377`, 155.3449258885264`,
4.407784489993092`, 4.407784489993092`, 155.3449258885264`, 1.`, {{0, 0.011806214367528007`, 21.01506157419985`}}},
{0.18352727434547023`, 0.4163243700753796`, 8.057415014521496`, 1.2522528089453002`, 0.16203828635471806`,
0.6604646928880291`, 0.25830735718962067`, 2461.4350174740794`, 0.12685187153570027`, 8.600866037504808`, 45.8502551707053`,
5.1098383783224985`, 5.1098383783224985`, 45.8502551707053`, 1.`, {{0, 0.0034846193929736033`, 6.202622519493014`}}},
{0.23920496896525717`, 1.1838719961494162`, 9.218551103388815`, 1.265080848919387`, 0.9542453823473309`,
0.22929853041342196`, 0.4982471338661781`, 90.20981971435951`, 0.0856312153630287`,
3.6772130334476696`, 15280.30366185599`, 3410.191501752564`, 9.068372100723312`, 187.45149335909412`,
1.`, {{0, 0.0122797975752165`, 21.858039683885373`}, {8, 0.0017535919237167438`, 3.121393624215804`},
  {16, 0.00012018434363164611`, 0.21392813166433008`}, {24, 0.000021004057555722413`, 0.0373872224491859`},
  {32, 0.00001548449236834536`, 0.02756239641565474`}, {40, 0.000015160395599050014`, 0.026985504166309026`},
  {48, 0.000015137205829013755`, 0.02694422637564448`}, {56, 0.000025953501374131808`, 0.04619723244595462`}}},
{0.10156561975861944`, 0.5164671469302382`, 5.401464431301745`, 0.8562900606586841`, 0.5357330343495086`,
```

```
  0.16718344164197263`, 0.02721990257094452`, 330.98847924494044`, 0.2190497368128987`,
  7.446609110605853`, 225.6291338421051`, 7.4363605283874215`, 5.149753903486025`, 155.7209029763867`, 1.`,
  {{0, 0.009945724113150952`, 17.703388921408695`}, {73, 0.001889064513054436`, 3.362534833236896`}}},
{0.2533884831264572`, 2.217775528299157`, 1.87005833593021`, 1.0852798553697922`, 0.3630118105229163`, 0.49869259108707253`,
  0.006826900671537389`, 2180.4237702051914`, 0.21841135731377453`, 2.8539212819504436`, 17.65882355869093`,
  0.07585909427905235`, 0.07585909427905235`, 17.65882355869093`, 1.`, {{0, 0.0013420705904605107`, 2.388885651019709`}}},
{0.04019446235159935`, 2.9685609440334373`, 3.1967275735949383`, 1.2384363485833663`, 0.006837582253808039`,
  0.6265805854044944`, 0.10123843723710083`, 64.97108080441517`, 0.1585540441972691`, 8.616308543170305`, 82754.54501528785`,
  52417.65356194783`, 228.98152996725062`, 1370.0765057613976`, 0.`, {{0, 0.10412581443786623`, 185.3439496994019`}}},
{0.18466310913340017`, 2.870831467521074`, 5.7568671895307055`, 1.0469133934587485`, 0.5974897879057268`,
  0.6447436436670198`, 0.1911914248698421`, 363.92035372259653`, 0.11435325769039895`, 2.2759611974712186`,
  112.51918288110635`, 2.454325316847649`, 1.6177697391561434`, 76.25585463017067`, 1.`,
  {{0, 0.005130873162969076`, 9.132954230084955`}, {14, 0.0006645717889238957`, 1.1829377842845343`}}},
{0.22919090442152185`, 1.3485088294529417`, 3.7111141569564556`, 1.1683364276022934`, 0.8698699225738455`,
  0.45530089098812654`, 0.06782162054416256`, 403.6677278730082`, 0.1398561461848175`,
  8.195868200542787`, 264.7668887685776`, 8.311706080876128`, 6.849231671087748`, 259.9857086523036`,
  1.`, {{0, 0.0182583450849103`, 32.499854251140334`}, {2, 0.0012824419272668853`, 2.2827466305350557`},
    {4, 0.00009645904994275542`, 0.17169710889810463`}, {6, 0.00004768047655771356`, 0.08487124827273014`},
    {8, 0.00004615986610259153`, 0.08216456166261293`}, {10, 0.000027827452794827003`, 0.04953286597479206`}}},
{0.12718220564190125`, 0.7794468104625536`, 3.4523481867286847`, 1.042660599955479`, 0.8212977484948716`,
  0.42153700480377276`, 0.05083556365712203`, 907.9591078654141`, 0.03221255361915021`, 1.403008654938164`,
  36.433778707561316`, 1.3913035995720187`, 1.0308238080665721`, 26.754141081211817`, 1.`,
  {{0, 0.0016613802684022126`, 2.9572568777559387`}, {23, 0.00037193445376988563`, 0.6620433277103964`}}},
{0.24829534536640735`, 0.6361539074945153`, 1.4367506745172012`, 1.2157272327840285`, 0.5920355341003019`, 0.22834833308349511`,
  0.008858258633323783`, 152.73617223659593`, 0.05098711045238086`, 6.308971873547048`, 194460.44354841363`,
  42751.573375306914`, 230.25211443465173`, 1157.9765771564682`, 0.`, {{0, 0.0880062198638916`, 156.65107135772703`}}},
{0.24634814165734203`, 1.0199939463339236`, 6.874973257490155`, 1.1669106806380989`, 0.9547234223304992`,
  0.2467804242930165`, 0.010102520966714356`, 1373.9164154017546`, 0.22192762621195788`,
  8.148592815397581`, 130.46850608034433`, 1.0658684312215678`, 1.036130867461657`, 126.8649306804241`,
  1.`, {{0, 0.006742617667892332`, 12.001859448848352`}, {7, 0.002899117063819899`, 5.16042837359942`}}},
{0.11968292559735771`, 0.4405556662519543`, 0.9343787484302961`, 1.4969299043499453`, 0.5720179190625709`,
```

```
0.5743610731089337`, 0.058376899138915264`, 4861.966479565238`, 0.13911792619470742`,
 8.404040973858844`, 33.48884913923602`, 2.112641088991093`, 2.0662513842058448`, 32.65965470133787`, 1.`,
 {{0, 0.0023669920864448656`, 4.2132459138718605`}, {23, 0.00011514167085681219`, 0.20495217412512567`}}},
{0.0912885213280808`, 1.1352603856466574`, 9.64216394840902`, 1.0423303168159856`, 0.8496114679763396`,
 0.3948758352214041`, 0.050073459411100744`, 1020.4170701645861`, 0.08585808288581304`,
 1.5724283740137839`, 37.42979637377136`, 0.9172660357076834`, 0.6784090564126155`, 27.27338042897581`,
 1.`, {{0, 0.0015076730615155842`, 2.68365804949774`}, {30, 0.000565103851086577`, 1.005884854934107`}}},
{0.23601424519793518`, 0.7493625485076763`, 8.159569145163797`, 1.0789985894921148`, 0.9135595458313224`,
 0.43990663477596414`, 0.03553380189426036`, 63.68276264433202`, 0.11799293833856772`, 7.92015369426547`,
 139480.46948593995`, 31802.993182151746`, 229.52831910579428`, 1656.2973861816984`, 0.`,
 {{0, 0.08507927211237143`, 151.44110436002111`}, {24, 0.01885857722200225`, 33.56826745516401`},
  {48, 0.0063875394642153565`, 11.369820246303336`}, {72, 0.0029643317567697824`, 5.276510527050213`},
  {96, 0.0015206302629758746`, 2.706721868097057`}, {120, 0.000816834177071285`, 1.4539648351868875`},
  {144, 0.00045813721267552493`, 0.8154842385624345`}, {168, 0.00027247304074934126`, 0.4850020125338274`},
  {192, 0.00017536645917547527`, 0.31215229733234595`}, {216, 0.009345439641802764`, 16.634882562408922`}}},
{0.09505824539478602`, 0.994707682773301`, 2.092747921993542`, 1.2334327544777528`, 0.1649122243213208`,
 0.435178801412204`, 0.4947441968705133`, 1030.121381906519`, 0.19720224785958157`, 8.531247013968503`,
 90.45835009258096`, 5.164654190894186`, 3.143660059363912`, 55.492877711986985`, 1.`,
 {{0, 0.0036031887559494933`, 6.413675985590099`}, {5, 0.0005817384071960853`, 1.0354943648090318`},
  {10, 0.00003086654435908953`, 0.054942448959179366`}, {15, 1.6649986063428471`*^-6, 0.002963697519290268`}}},
{0.13338172675277454`, 1.0224437122231844`, 3.1176391252125253`, 1.1808523949493024`, 0.5954558720182712`,
 0.3646371291004906`, 0.024821319877175146`, 118.84942434665426`, 0.15904005586434156`,
 2.0945755856004613`, 44395.625014980316`, 15262.94067357032`, 228.53791083942048`, 810.0560154504376`,
 0.`, {{0, 0.006211326701280833`, 11.056161528279883`}, {6, 0.0011001655836825144`, 1.9582947389548753`},
  {12, 0.0003169355468639554`, 0.5641452734178406`}, {18, 0.00015862576971061392`, 0.2823538700848928`},
  {24, 0.00011595314643038119`, 0.20639660064607848`}, {30, 0.0001039378913775418`, 0.1850094466520244`},
  {36, 0.00010366066635095974`, 0.18451598610470835`}, {42, 0.00010976153518043349`, 0.19537553262117158`},
  {48, 0.00011995210930775418`, 0.21351475456780242`}, {54, 0.05322393822404827`, 94.73861003880593`}}},
{0.2663723170815805`, 0.6117369492537685`, 4.996758576680092`, 1.4798016834758696`, 0.3454705507334743`,
 0.5628818087325649`, 0.10659789380555212`, 347.2598858446038`, 0.128819557084905`, 4.579853140224845`,
 188.09878980109036`, 11.79335511099655`, 8.33722734162322`, 129.41646872354798`, 1.`,
```

```
{{0, 0.00843435081531609`, 15.01314445126264`}, {20, 0.0014013008076735557`, 2.4943154376589294`}}},
{0.19858704302380392`, 3.77240735130054`, 4.552319154599175`, 1.490234534684174`, 0.9267739623702391`,
 0.4663128979271708`, 0.016623264635311285`, 190.7315614324941`, 0.03981298108873932`,
 1.7245962595124684`, 59538.529850613384`, 15500.102427884609`, 4.536365103938697`, 91.60216782192656`,
 1.`, {{0, 0.00595630797687311`, 10.602228198834137`}, {6, 0.0007445945093200385`, 1.3253782265896688`},
  {12, 0.00007554440167755028`, 0.13446903498603952`}, {18, 0.00004124341662286516`, 0.07341328158869999`},
  {24, 0.000040124161838184974`, 0.07142100807196926`}, {30, 0.00010395028813466934`, 0.18503151287971142`}}},
{0.16356962246540357`, 2.7590365436019733`, 9.315311335363422`, 1.411146654012818`, 0.2509435276133807`,
 0.3090283790000361`, 0.243114119684150 2`, 313.55839056266797`, 0.2467169299716112`, 8.038445579591425`,
 255.0347181232233`, 4.834212775913408`, 2.6394297405941582`, 141.36634245206852`, 1.`,
 {{0, 0.008025860391286672`, 14.286031496490276`}, {8, 0.0024261901991117023`, 4.31861855441883`},
  {16, 0.00027444414708292753`, 0.488510581807611`}, {24, 0.000017347288875906013`, 0.030878174199112703`}}},
{0.25383472083693576`, 3.9769524497483806`, 7.011565744250568`, 1.066285473960517`, 0.7734216029556533`,
 0.6850552501893354`, 0.013843595247453381`, 297.6124430270534`, 0.10716201039628298`, 2.5950386184147853`, 159.74436524213615`,
 1.4335083760851326`, 0.8387108210967673`, 164.40887917495738`, 1.`, {{0, 0.009167955667267401`, 16.31896108773598`},
  {3, 0.002670148680788578`, 4.75286465180367`}, {6, 0.0005266483924996159`, 0.9374341386493164`},
  {9, 0.00011202385538140971`, 0.1994024625789093`}, {12, 0.00001829822135975621`, 0.03257083402036605`}}},
{0.17343366236722435`, 1.3936719133530833`, 0.599503921563171`, 0.8621470436054358`, 0.43567440743840313`,
 0.22823781097601215`, 0.018669299661272933`, 3618.675535403083`, 0.22611694811187344`, 3.1306846738065635`,
 8.932755013182055`, 0.12573270703814995`, 0.12285003529067938`, 8.731347837940765`, 1.`,
 {{0, 0.0005159558319426054`, 0.9184013808578376`}, {11, 0.0001476266037408928`, 0.26277535465878915`}}},
{0.27302715407014244`, 1.5486379048502288`, 9.868645627334566`, 1.0694698241775202`, 0.6816384789594119`,
 0.4416154581373778`, 0.05961824305163796`, 87.63839207083008`, 0.05267766985704242`,
 3.1260333504958955`, 91650.78842242043`, 18655.777329715973`, 8.383013405035161`, 303.25672634401957`,
 1.`, {{0, 0.02098185496365314`, 37.347701835302594`}, {3, 0.0015779246789807667`, 2.808705928585765`},
  {6, 0.00011894021349026604`, 0.21171358001267357`}, {9, 0.00005707232318597605`, 0.10158873527103737`},
  {12, 0.000054774920285720806`, 0.09749935810858303`}, {15, 0.00005465934777052087`, 0.09729363903152716`},
  {18, 0.00005464929343981091`, 0.0972757423228634`}, {21, 0.00005464801588551554`, 0.09727346827621765`},
  {24, 0.00005464781295305703`, 0.09727310705644152`}, {27, 0.00003833963250071612`, 0.0682445458512747`}}},
{0.12081145422022538`, 2.0244942048813837`, 8.652229532190159`, 0.835020699056165`, 0.9214042127932665`,
 0.46285611589460973`, 0.11861503789539068`, 2689.091322411565`, 0.07096583948487911`, 5.509201411600676`, 69.0594168629277 8`,
```

```
1.450570833487345`, 1.450570833487345`, 69.05941686292778`, 1.`, {{0, 0.005248515681582511`, 9.34235791321687`}}},
{0.27038445563951496`, 3.3688963138915424`, 3.4133330216440854`, 1.1975198070883244`, 0.31492494684053174`,
0.45427916274833`, 0.0070970717881839104`, 3067.136751023867`, 0.17569148508387616`, 1.2036801730392703`, 4.720745024840074`,
0.017760086249794187`, 0.017760086249794187`, 4.720745024840074`, 1.`, {{0, 0.00035877662188784563`, 0.6386223869603652`}}},
{0.1767050761987432`, 2.85608165701536`, 6.140634667151749`, 1.3554192221984884`, 0.6330624847112656`,
0.5906276578639604`, 0.04152902973081543`, 1510.3499876585004`, 0.15541682752363528`, 8.81551919212286`,
125.0655075646142`, 1.1257739611990036`, 0.9906659373256477`, 108.72468829865319`, 1.`,
{{0, 0.005988145644013343`, 10.658899246343752`}, {32, 0.002274930666684299`, 4.0493765866980524`}}},
{0.16171803894826015`, 2.2156907324583015`, 1.0134587159780288`, 0.8322559746700846`, 0.34733770749260806`,
0.6849579573613365`, 0.018772440750507276`, 1617.8437562512436`, 0.21218330633531807`, 7.516753350682977`, 69.77474506785634`,
1.0286636734145835`, 0.7147405888694356`, 71.1085578770052`, 1.`, {{0, 0.004164534189943776`, 7.412870858099922`},
{3, 0.0010350963020609736`, 1.8424714176685328`}, {6, 0.00016062701937730794`, 0.2859160944916081`},
{9, 0.000031273889157101175`, 0.05566752269964009`}, {12, 0.000012718998113236715`, 0.022639816641561353`}}},
{0.24108871826638628`, 1.1722622038849657`, 2.5945274526654174`, 1.1873482780450624`, 0.3399058776037083`,
0.3423735862363967`, 0.09173881369912114`, 1455.6792358614214`, 0.2278375557130481`, 4.818602627144232`, 36.755851350699345`,
1.1761734073688008`, 1.015162128517092`, 31.737277397477587`, 1.`, {{0, 0.0015134455965173804`, 2.693933161800937`},
{7, 0.0007572607640285794`, 1.3479241599708713`}, {14, 0.00014132672166233703`, 0.2515615645589599`}}},
{0.2202280085702742`, 2.180975046803316`, 5.6134680006393936`, 1.2506485034427453`, 0.4748208651226249`,
0.39757955628981323`, 0.017520218948128618`, 280.3947437474927`, 0.12359899846366301`, 9.521190146527957`, 443.13414131294326`,
4.0012691767464945`, 2.9265927317913025`, 436.63416417833344`, 1.`, {{0, 0.02723144197071806`, 48.471966707878146`},
{3, 0.005091350774097999`, 9.06260437789444`}, {6, 0.0007360945672869921`, 1.310248329770846`},
{9, 0.00012229486843845454`, 0.2176848658204491`}, {12, 3.0142970118326007`*^-6, 0.005365448681062029`}}},
{0.12315072752895034`, 2.8609501087269207`, 1.79674111361204`, 1.4514496090822089`, 0.3032919079034333`,
0.2763824218936657`, 0.028981582964232436`, 188.78817200372555`, 0.08856946510090113`,
4.546561013073182`, 58633.29990505694`, 21226.46006365395`, 228.36963469946718`, 6321.334166879353`,
0.`, {{0, 0.028467204189925453`, 50.67162345806731`}, {19, 0.0018315556410310446`, 3.2601690410352595`},
{38, 0.0033160711098204915`, 5.902606575480474`}, {57, 0.00602197842391446`, 10.71912159456774`},
{76, 0.004904161920839694`, 8.729408219094656`}, {95, 0.004252547249482463`, 7.569534104078784`},
{114, 0.004214613938685208`, 7.502012810859672`}, {133, 0.003971407141555028`, 7.069104711967951`},
{152, 0.003703611309387587`, 6.592428130709905`}, {171, 0.41973824575818947`, 747.1340774495773`}}},
{0.14162785770391484`, 1.92013166099325`, 8.540643644714823`, 1.2350689068338556`, 0.301574102817308`,
```

```
0.40858591883894513`, 0.080613435450821`, 585.1346359267128`, 0.15211712109726616`, 7.686797097032363`,
 153.20803973682908`, 2.8523818830050782`, 1.9282209070527614`, 104.81873458109281`, 1.`,
 {{0, 0.006171220383429845`, 10.984772282505125`}, {24, 0.001795003444733209`, 3.1951061316251117`}}},
{0.22524615675817938`, 2.031565502127312`, 0.245821316301889`, 1.298167355078078`, 0.689325845538592`, 0.661487626561178`,
 0.00610096551453922`, 373.63064864758115`, 0.20592315239272913`, 2.994117316721619`, 26349.97598308941`,
 6245.9383793900615`, 230.18905065739318`, 1107.5792814555923`, 0.`, {{0, 0.08417602539062502`, 149.83332519531254`}}},
{0.06014178921319169`, 2.215935435785701`, 1.3525069683978295`, 1.0400202244696481`, 0.697929353581739`,
 0.692932654029565`, 0.014666911144259826`, 1018.9785490283374`, 0.16122419934759502`,
 4.978072859014281`, 70.28298129569727`, 4.474533428080961`, 1.7007627655137836`, 69.50677419984144`,
 1.`, {{0, 0.004121997861506609`, 7.337156193481762`}, {3, 0.0009757731768622059`, 1.7368762548147263`},
  {6, 0.000122877238853223`, 0.21872148515873693`}, {9, 0.000019933362933261007`, 0.03548138602120459`},
  {12, 9.876593417034333`*^-6, 0.01758033628232111`}, {15, 0.000032056605615617 42`, 0.05706075799579901`}}},
{0.11674773262981253`, 1.910335393882857`, 4.221858661435528`, 1.4404651869296854`, 0.4563596481936356`,
 0.5439902833298367`, 0.005611633628996954`, 451.6526207878965`, 0.24247834364497922`,
 9.182241064841566`, 320.8342668292945`, 1.5754092532936728`, 1.080615310968751`, 317.66137329462447`,
 1.`, {{0, 0.019994385541920314`, 35.59000626461816`}, {5, 0.004147878828471146 5`, 7.383224314678641`}}},
{0.12898119467887553`, 3.729766126068422`, 2.3744826976889595`, 1.2465399522360021`, 0.1415631230326866`,
 0.3607064691421532`, 0.03995461220531779`, 1238.1663657022145`, 0.14506115517150608`,
 8.953141814946814`, 69.96367527499407`, 0.49133419730561056`, 0.4132138689482232`, 55.8685193755646`,
 1.`, {{0, 0.0032435141043262923`, 5.7734551057008`}, {42, 0.0010024933682166175`, 1.784438195425579`}}},
{0.2498624462217614`, 2.827129073229683`, 2.690968559152097`, 1.0446525535360744`, 0.06936419216205603`,
 0.5607634545552321`, 0.008891348377622028`, 196.01664126075448`, 0.10064633302983284`, 7.405206717033214`, 58885.82980967864`,
 12852.241876421574`, 230.24084550712385`, 1297.2725065130937`, 0.`, {{0, 0.09859271049499513`, 175.4950246810913`}}},
{0.25890580889310233`, 3.9576703566675278`, 2.211552265086926`, 0.8006241502346895`, 0.26522568617457876`,
 0.4892067360859801`, 0.02025985822061832`, 94.72915936196773`, 0.06766512891079995`, 5.4984531961033785`, 187331.1336026766`,
 39849.14644132568`, 230.0673154914951`, 1439.3511571382223`, 0.`, {{0, 0.10939068794250491`, 194.71542453765872`}}},
{0.2032139210380186`, 1.0205320875718096`, 3.2697243543529257`, 1.0866214178514029`, 0.5111053959745286`,
 0.4079750171889772`, 0.04834589720060758 5`, 145.20906840025864`, 0.23728279570010086`,
 2.517042827760779`, 26575.78611810111`, 6793.849374524416`, 230.16089293087612`, 1597.1222620897918`,
 0.`, {{0, 0.04953781587387166`, 88.17731225549156`}, {24, 0.002787351331169623`, 4.961485369481928`},
  {48, 0.002907414019995403 4`, 5.175196955591818`}, {72, 0.0030469122964951714`, 5.423503887761404`},
```

{96, 0.0028790718131973493`, 5.124747827491281`}, {120, 0.002765580419863198`, 4.922733147356492`},
{144, 0.002717730167869411`, 4.83755969880755`}, {168, 0.002692939591049738`, 4.793432472068533`},
{192, 0.002680163635824442`, 4.7706912717675065`}, {216, 0.04936631276948819`, 87.87203672968897`}}},
{0.15288075349234037`, 1.8405591021591183`, 8.732063156275487`, 0.8366527628406224`, 0.7247685787231319`,
0.43308603413296065`, 0.06703535093550567`, 1294.178726539122`, 0.16108367938164575`, 4.274431762914247`,
68.15273844370063`, 1.2195005013817808`, 0.8210963301166182`, 45.724067896295225`, 1.`,
{{0, 0.002745189567573441`, 4.886437430280725`}, {29, 0.0007298395925449958`, 1.2991144747300927`}}},
{0.1609254870534111`, 1.384768951726243`, 6.196355509527487`, 0.9770497723863997`, 0.43819861370403546`,
0.17710326003602894`, 0.2243750211158665`, 120.51310647409781`, 0.10277566384729958`, 6.21125463242891`, 11256.15117516689`,
3330.5560042757675`, 7.474877762836798`, 201.76833482407199`, 1.`, {{0, 0.014110713236449504`, 25.11706956088012`},
{14, 0.001147647172072024`, 2.0428119662882027`}, {28, 0.00003193430422438741`, 0.05684306151940959`},
{42, 9.125901746256528`*^-6, 0.01624410510833662`}, {56, 8.783707407065752`*^-6, 0.015634999184577038`},
{70, 8.7767473368866`*^-6, 0.01562261025965815`}, {84, 0.00001741237739334638`, 0.030994031760156558`}}},
{0.21184155187533854`, 1.0400436907537394`, 2.77253033361667`, 1.25783985819887`, 0.5916706672066951`,
0.18670557332607218`, 0.05860949172916328`, 177.69964385957368`, 0.1825191510166627`,
3.5990512292385053`, 23476.583376099206`, 5813.84084994689`, 26.353717636447445`, 182.98353739478335`,
1.`, {{0, 0.012728576192256884`, 22.656865622217254`}, {12, 0.0003361152313957123`, 0.598285111884368`},
{24, 0.00011303083344145004`, 0.2011948835257811`}, {36, 0.00011158250290748608`, 0.19861685517532524`},
{48, 0.00011154921049009732`, 0.19855759467237324`}, {60, 0.00011154451526734125`, 0.19854923717586745`},
{72, 0.00011154352720124991`, 0.19854747841822487`}, {84, 0.00011154329088938307`, 0.1985470577831019`},
{96, 0.0001115432312839896`, 0.19854695168550146`}, {108, 0.00005972030686994`, 0.1063021462284932`}}},
{0.15826860039724194`, 2.914200187526596`, 3.057619613104965`, 0.9127068016590583`, 0.36895217716385176`,
0.3294645201729436`, 0.006405724718526674`, 799.6829736219844`, 0.2321605158663716`, 7.540832982633661`,
106.7202092065175`, 0.24690559797161593`, 0.24689694955995262`, 106.94005632772262`, 1.`,
{{0, 0.007705201941103779`, 13.715259455164729`}, {5, 0.00042224233980313954`, 0.7515913648495883`}}},
{0.2486148170283075`, 0.5446418582356878`, 7.121193243885838`, 1.048132429671941`, 0.7615900023009023`,
0.5400927540260018`, 0.07717023450494316`, 388.33902457321517`, 0.04098404938849237`,
2.529691537294738`, 145.99404495397135`, 8.925554713277112`, 6.437429714399523`, 103.36047434541696`, 1.`,
{{0, 0.00687924042441745`, 12.245047955463061`}, {23, 0.0009761556258342382`, 1.7375570139849439`}}},
{0.1973186549669898`, 3.31823274961833`, 5.404057922234477`, 1.0748992098641554`, 0.1837120164888255`,
0.5027766473922404`, 0.30055211808677745`, 460.171808421071`, 0.10833443978919949`, 3.5562239100468123`, 88.9845536613686`,

```
  1.508606189576967`, 1.1350065918484682`, 66.79142510707189`, 1.`, {{0, 0.00408264709754465`, 7.267111833629477`},
    {5, 0.0009045891349940053`, 1.6101686602893295`}, {10, 0.00008891207559880749`, 0.15826349456587735`}}},
 {0.16177424691159015`, 1.9234518490470327`, 6.336020872512709`, 1.465202064627133`, 0.3679027675625519`,
  0.6088093981583469`, 0.02752967040620688`, 1523.605195139083`, 0.023927731291662258`, 6.9520681394676025`, 68.97739749780388`,
  0.6894525907707301`, 0.6894525907707301`, 68.97739749780388`, 1.`, {{0, 0.005242282209833095`, 9.33126233350291`}}},
 {0.12468946686374066`, 2.602287301847274`, 9.387923649086364`, 1.237964958422611`, 0.6279283704003791`,
  0.5225362832845872`, 0.15386398985407668`, 4877.253164044286`, 0.10729269282887094`, 6.155025385024989`, 24.79324834329096`,
  0.44483555089978855`, 0.44483555089978855`, 24.79324834329096`, 1.`, {{0, 0.001884286874090113`, 3.354030635880401`}}},
 {0.1891437323001075`, 2.037539508644585`, 6.753647851102151`, 1.0264835122983769`, 0.08414675462879395`,
  0.15050525073770327`, 0.01724785647741675`, 835.2003740810117`, 0.09305345804576048`, 5.34515441362371`,
  43.22750720053642`, 0.2833296073986218`, 0.23657145754898026`, 35.65347803969223`, 1.`,
  {{0, 0.0017083626612874082`, 3.0408855370915866`}, {69, 0.0010013016697292014`, 1.7823169721179783`}}},
 {0.11951180040558451`, 2.9137311414381974`, 6.217108794108201`, 1.4729889111648695`, 0.6540356010640074`,
  0.40115742558392786`, 0.08157249569611574`, 391.2473393910366`, 0.0369147403900103`, 8.338813449127404`, 381.22059843425484`,
  4.996720658631816`, 4.452216303441097`, 345.8386194279569`, 1.`, {{0, 0.02212065657607823`, 39.37476870541925`},
    {3, 0.0037194746593741685`, 6.62066489368602`}, {6, 0.00044360384107233309`, 0.789614837108749`}}},
 {0.17883917330505905`, 1.5543060950462326`, 9.905917854358197`, 1.2462903035005124`, 0.3202858713356645`,
  0.3166477244063972`, 0.12099723319057126`, 400.41544183552224`, 0.027102889569123156`, 3.023770086067156`, 78.29135256141872`,
  2.116784333005153`, 1.5979758669282`, 59.36696725496117`, 1.`, {{0, 0.002971472120563716`, 5.289220374603413`},
    {8, 0.0012984183842752323`, 2.3111847240099133`}, {16, 0.0002419990065381007`, 0.43075823163781923`}}},
 {0.25477439586299494`, 1.0781629016953422`, 9.675717925989822`, 1.4583691370753404`, 0.484439918991886`,
  0.6784957922502513`, 0.09307366059264305`, 217.6033516010123`, 0.05467318278554256`, 4.774031073697932`,
  393.88283325884083`, 14.421476973242328`, 10.461918250454785`, 285.6598299451639`, 1.`,
  {{0, 0.019158450622136548`, 34.10204210740306`}, {20, 0.002551696453695908`, 4.542019687578716`}}},
 {0.26738035849024727`, 1.6336260676101997`, 9.900661691242231`, 0.846806505065853`, 0.2415106216321452`,
  0.502680613934997`, 0.14320441138979886`, 77.88432317464672`, 0.12860121706274624`, 9.963544099078408`,
  61516.02042546346`, 12619.197495003178`, 230.21749943437422`, 1955.7860381962944`, 0.`,
  {{0, 0.10741490266179524`, 191.19852673799554`}, {12, 0.036983008250876914`, 65.8297546865609`},
    {24, 0.0023521465739343756`, 4.1868209016031885`}, {36, 0.0003960071537188958`, 0.7048927336196347`},
    {48, 0.0007102630554041556`, 1.264268238619397`}, {60, 0.0004592509917512606`, 0.8174667653172439`},
    {72, 0.00008081213001077252`, 0.14384559141917508`}, {84, 0.000021691488567337153`, 0.038610849649860134`},
```

```
{96, 0.000018914049356271795`, 0.033667007854163794`}, {108, 0.00020274254750317 2`, 0.3608817345556462`}}},
{0.0966033889391959`, 0.8191052864079662`, 6.963587929197736`, 1.39989556064551`, 0.208903818004208`,
 0.4044327200024739`, 0.345646875315088`, 51.22809362991988`, 0.05669448608759026`, 3.5983266970468653`,
 313231.1273348109`, 131534.30218807794`, 228.3318304881135`, 948.5009851040065`, 0.`,
 {{0, 0.02967389375055995`, 52.81953087599671`}, {6, 0.0025198095291294395`, 4.485260961850402`},
  {12, 0.0009759872776760629`, 1.7372573542633918`}, {18, 0.0006323642694475729`, 1.12560839961668`},
  {24, 0.0005198028355368523`, 0.925249047255597`}, {30, 0.0004753542518183628`, 0.8461305682366856`},
  {36, 0.0004553171778315349 3`, 0.8104645765401322`}, {42, 0.0004450252003366385 4`, 0.7921448565992166`},
  {48, 0.0004390726461214289`, 0.7815493100961435`}, {54, 0.03594944792944667`, 63.99001731441507`}}},
{0.07268626506253911`, 2.0017174568948795`, 2.5547427204964173`, 0.8446935149578232`, 0.0630154383873418`,
 0.49766282606547807`, 0.00568323780270915`, 197.50095618865632`, 0.04182941385906325`, 9.915201734242256`, 54749.04327797868`,
 26755.105774355114`, 230.24034171485545`, 782.6078565497148`, 0.`, {{0, 0.05947819709777833`, 105.87119083404544`}}},
{0.14445086415814334`, 1.1396367789378896`, 8.108045316850049`, 1.1452867101498132`, 0.43349891091368953`,
 0.5412309783014353`, 0.17300117449492944`, 151.5195914284387`, 0.22841440999808194`, 1.401560283159844`, 110.15771793878415`,
 6.813611101913338`, 4.835366601075164`, 103.94935797078084`, 1.`, {{0, 0.006248747828554546`, 11.122771134827094`},
  {2, 0.0013524257211833066`, 2.4073177837062865`}, {4, 0.0001748164397245708`, 0.3111732627097361`},
  {6, 0.000033367138161559966`, 0.05939350592757674`}, {8, 0.000019897207714771 69`, 0.03541702973229361`},
  {10, 0.000018832776233444846`, 0.03352234169553183`}, {12, 0.0000187618576053016 2`, 0.03339610653743688`},
  {14, 0.000018757656546542715`, 0.03338862865284603`}, {16, 0.000014544580055301905`, 0.025889352498437393`}}},
{0.2737062633152032`, 1.87379900045931`, 2.9341815386065377`, 1.0257953180476898`, 0.5994241695021645`,
 0.2666028641788709`, 0.16092630392754248`, 2757.7643970777704`, 0.13082540497870587`, 6.7306102844961035`,
 32.471263536437455`, 0.8126475683787923`, 0.39512608161094803`, 15.852867351421184`, 1.`,
 {{0, 0.00083165960581559 2`, 1.4803540983517538`}, {10, 0.0003096739933837671`, 0.5512197082231055`},
  {20, 0.0000576249726122206`, 0.10257245124975269`}, {30, 5.859346896430275`*^-6, 0.01042963747564589`}}},
{0.14450675367477284`, 1.4907097867645147`, 8.628071674125838`, 0.8645742960679661`, 0.06677853457714877`,
 0.6218432064874626`, 0.019848227343967184`, 449.15101395266043`, 0.20772413403212947`, 3.059722840773212`, 77.90011327461615`,
 0.7767020932033593`, 0.7767020932033593`, 77.90011327461615`, 1.`, {{0, 0.005920408608870827`, 10.538327323790073`}}},
{0.05092006104579416`, 0.9079056727720332`, 9.25200153286255`, 1.1365283513840736`, 0.03327106644526534`,
 0.582893635885294`, 0.1249724078419535`, 82.97595681905067`, 0.15395748273911747`, 7.1070379565760815`,
 28619.623157717273`, 16258.817070521458`, 228.89453189851463`, 1048.1825127107277`, 0.`,
 {{0, 0.05411226715805024 5`, 96.31983554132943`}, {6, 0.0002490442058838842`, 0.4432986864733139`},
```

```
{12, 0.0001941082568318016`, 0.34551269716060695`}, {18, 0.00019289501915188955`, 0.3433531340903634`},
 {24, 0.00019643776907292694`, 0.34965922894980994`}, {30, 0.00022505739994220354`, 0.40060217189712233`},
 {36, 0.000403536023247243`, 0.7182941213800926`}, {42, 0.0004991763950136842`, 0.8885339831243579`},
 {48, 0.00030606967401340326`, 0.5448040197438578`}, {54, 0.023283279064808017`, 41.44423673535827`}}},
{0.2364459713820966`, 3.884359137525399`, 0.954741626535867`, 0.8976843312448166`, 0.4180287949880024`,
 0.15414803582881131`, 0.32241293356212986`, 4897.582666581565`, 0.05447310320870352`, 4.86045092756919`,
 8.678563707036247`, 0.13451346219967578`, 0.053100645906626574`, 2.9799566753694617`, 1.`,
 {{0, 0.00014974237673022912`, 0.2665414305798078`}, {9, 0.00006479097603851468`, 0.11532793734855616`},
 {18, 9.193593994518662`*^-6, 0.016364597310243218`}, {27, 2.749760564816654`*^-6, 0.004894573805373645`}}},
{0.042914457849633436`, 3.519672647432988`, 3.392121169492343`, 1.4941116772804357`, 0.33812708289181614`, 0.3684690007615431`,
 0.017148240098985187`, 3621.3180321373566`, 0.22166064632630456`, 1.0781698542512608`, 3.2964433019883033`,
 0.021188431633903347`, 0.021188431633903347`, 3.2964433019883033`, 1.`, {{0, 0.00025052969095111104`, 0.44594284989297767`}}},
{0.1470328346746822`, 2.9686959435849998`, 9.643655350717612`, 1.0921756097932163`, 0.41500029005274564`,
 0.5183995564120613`, 0.04078998201684905`, 4770.787596114335`, 0.08630863643009795`, 3.8345772490574745`,
 11.611914838569161`, 0.09916033800370502`, 0.09296200791761723`, 10.825031908092205`, 1.`,
 {{0, 0.0006177506053328196`, 1.099596077492419`}, {26, 0.00020495181968218797`, 0.36481423903429455`}}},
{0.10223589016255652`, 0.4809064129783538`, 6.158635896611097`, 0.9490677758592585`, 0.9235714285317382`,
 0.43663660593873277`, 0.45300992250929206`, 1331.4948553442105`, 0.21785131418517045`, 5.425736978481032`, 130.2181377673123`,
 14.263266286175892`, 4.12012606455869`, 38.300159802833875`, 1.`, {{0, 0.002134577612450266`, 3.799548150161474`},
 {5, 0.0006882273029936867`, 1.2250445993287622`}, {10, 0.00007838711356508868`, 0.13952906214585786`},
 {15, 7.888168389275749`*^-6, 0.014040939732910832`}, {20, 1.7319476170575404`*^-6, 0.003082866758362422`}}},
{0.08719902184880185`, 3.645892777856403`, 2.8891082836443758`, 1.428386787110956`, 0.151537003432892`,
 0.34505159105650784`, 0.041961341889936235`, 290.12993265609543`, 0.15015043093723618`,
 6.608503242578238`, 5360.4407545880495`, 2317.1123789087073`, 229.12745823862804`, 685.4613929967691`,
 0.`, {{0, 0.014904708654750264`, 26.53038140545547`}, {32, 0.002135224794993405`, 3.800700135088261`},
 {64, 0.002135991378915372`, 3.802064654469362`}, {96, 0.0021789089971242536`, 3.8784580148811716`},
 {128, 0.0021580096726612603`, 3.841257217337044`}, {160, 0.002146927939786283`4`, 3.821531732819585`},
 {192, 0.0021407200597080215`, 3.8104817062802785`}, {224, 0.0021378849740904668`, 3.805435253881031`},
 {256, 0.0021365050292569262`, 3.8029789520773285`}, {288, 0.020020184366468196`, 35.635928172313385`}}},
{0.14456990131617375`, 0.9947424361823325`, 3.150441801621305`, 1.0015653051761273`, 0.8447006281771343`,
 0.5703016332732033`, 0.008271730569805881`, 52.80371817926736`, 0.13650904531369146`, 3.8670961625783473`, 153936.2750796065`,
```

```
50187.5719095843`, 229.34728317846555`, 1271.5867946022433`, 0.`, {{0, 0.0966405963897705`, 172.0202615737915`}}},
{0.14643438827373756`, 2.4493630197222025`, 5.7044459384485755`, 0.7643715149180945`, 0.8231136724552808`,
 0.4724466281569806`, 0.012687117803733845`, 1217.9307406282053`, 0.17452698640833697`,
 5.969503699935295`, 132.7426642836786`, 0.5873997016031466`, 0.5693306196837659`, 128.83861819870194`,
 1.`, {{0, 0.007667216316926176`, 13.647645044128593`}, {6, 0.002124518666175171`, 3.7816432257918047`}}},
{0.045156311345747796`, 1.2463805103118828`, 6.638333254697063`, 1.0774632518381033`, 0.7965921624862937`,
 0.19987812094053103`, 0.1230725004501963`, 3016.4870397653995`, 0.18711874545495605`, 6.497285772809487`, 31.870694821839166`,
 1.0703325436252242`, 1.0703325436252242`, 31.870694821839166`, 1.`, {{0, 0.0024221728064597768`, 4.311467595498403`}}},
{0.21753427776894996`, 2.5707121359144134`, 7.635927852201033`, 1.3489791075447177`, 0.07755529833527275`,
 0.4139475400754866`, 0.049678130502208456`, 727.9927710408293`, 0.24367647097486184`, 3.0823691783926943`,
 40.2670998924629`, 0.44880980098093815`, 0.36532592264790986`, 32.11438476324045`, 1.`,
 {{0, 0.0019279888135920512`, 3.4318200881938514`}, {37, 0.0005127044284142228`, 0.9126138825773167`}}},
{0.08480900190064672`, 0.6294563083477476`, 5.454864363444388`, 0.7943923084343845`, 0.9968128471314037`,
 0.29575569030654725`, 0.01722905088446216`, 309.5962534203848`, 0.1957861782089137`,
 7.847856100229725`, 341.2031653600431`, 7.410300986862198`, 6.770433670006571`, 332.31297879552807`,
 1.`, {{0, 0.019448573102085556`, 34.61846012171229`}, {14, 0.005807213286374578`, 10.33683964974675`}}},
{0.12061650440369748`, 3.919019990284525`, 1.2611661330191772`, 1.4204820377968523`, 0.21263599304474745`,
 0.23829484446556248`, 0.008404906276562513`, 547.3513122366178`, 0.16414531089750106`,
 3.5940701557610755`, 3715.5206049358126`, 1351.1511599184096`, 228.64750462483283`, 676.1278054078939`,
 0.`, {{0, 0.003875710998051851`, 6.898765576532295`}, {31, 0.00059626985338694`, 1.061360339028753`},
  {62, 0.0005962706714028766`, 1.0613617950971204`}, {93, 0.0005962780625927655`, 1.0613749514151227`},
  {124, 0.0005963382653466341`, 1.0614821123170086`}, {155, 0.0005968259577159068`, 1.0623502047343143`},
  {186, 0.0006006137794340964`, 1.0690925273926917`}, {217, 0.0006213405955681616`, 1.1059862601113275`},
  {248, 0.000647604450145371`, 1.1527359212587605`}, {279, 0.04265846057735534`, 75.9320598276925`}}},
{0.2541152986933387`, 3.9531330061598675`, 6.8389480578926864`, 1.4090336327472661`, 0.5067749551810137`,
 0.5768128332044816`, 0.18877117253369932`, 176.17163988983094`, 0.11435366361110944`, 3.2749545355180913`,
 436.43178457413836`, 52.975967158665526`, 3.9943312469254617`, 209.62977191923898`, 1.`,
 {{0, 0.013725332340101004`, 24.431091565379788`}, {3, 0.0018657320396385903`, 3.321003030556691`},
  {6, 0.000147545371134556`, 0.2626307606195097`}, {9, 0.00004411118517860679`, 0.07851790961792009`},
  {12, 0.000039382372219642434`, 0.07010062255096354`}, {15, 0.000039209906994596267`, 0.06979363445038136`},
  {18, 0.00003920422398845635`, 0.0697835186994523`}, {21, 0.000031345226606706644`, 0.055794503359937825`}}},
```

{0.26839792559441034`, 1.5919359357927423`, 0.607262674381337`, 1.065158649344713`, 0.7342994823745972`,
0.6373140564402076`, 0.019311239061898532`, 131.67334362372645`, 0.026592393453336638`, 2.002632914376134`, 522742.8241948918`,
108131.13173975951`, 229.95496665683189`, 1327.9448057475843`, 0.`, {{0, 0.1009238052368164`, 179.6443733215332`}}},
{0.0804335350475921`, 1.0990801178785707`, 6.42097149017635`, 1.215697698043305`, 0.5864093543250795`,
0.4707288466973014`, 0.04708688161958605`, 3199.4245739234016`, 0.17424214472199046`, 5.845755402883285`,
31.705913645576064`, 0.7606266593191873`, 0.6071105856371353`, 25.23868527323334`, 1.`,
{{0, 0.0013734979064129657`, 2.444826273415079`}, {32, 0.000544642174352768`, 0.9694630703479271`}}},
{0.04545514913582513`, 3.0084108739176516`, 7.583320976280454`, 0.795641140955456 7`, 0.21703974982634144`,
0.40384085540761094`, 0.39906200752611665`, 3808.370230770943`, 0.07445295911170413`, 7.244769365152968`, 20.688207038340703`,
0.39429098900015497`, 0.39429098900015497`, 20.688207038340703`, 1.`, {{0, 0.0015723037349138936`, 2.7987006481467303`}}},
{0.21811178043156876`, 3.634131336571894`, 2.2696382083853486`, 1.2012104146171985`, 0.07478503245860835`,
0.304565647018059`, 0.049403646487048036`, 242.22074286172042`, 0.22363734402413937`,
5.499597099973035`, 15686.16133302832`, 3788.028446140847`, 227.96806223806306`, 1071.0379555430948`,
0.`, {{0, 0.04835840615074192`, 86.07796294832062`}, {29, 0.0011677929401067795`, 2.0786714333900678`},
{58, 0.003221733564620741`, 5.734685745024919`}, {87, 0.0015387891340398552`, 2.7390446585909425`},
{116, 0.0012889667994816915`, 2.294360903077411`}, {145, 0.0011073255398717677`, 1.9710394609717465`},
{174, 0.0010145866950250545`, 1.8059643171445972`}, {203, 0.0009695364950691508`, 1.7257749612230886`},
{232, 0.0009449360838314177`, 1.6819862292199232`}, {261, 0.02178681121848683`, 38.78052396890656`}}},
{0.22643881069864102`, 1.8950339409059218`, 4.49788713237294`, 0.781319815193253`, 0.8852654232409718`,
0.19866746146736047`, 0.009828863939504507`, 672.8810907026934`, 0.061975672509268276`,
2.025268353742364`, 48.51080213629765`, 0.25452132390832916`, 0.22434127068794915`, 43.42199987910346`,
1.`, {{0, 0.002285836776526309`, 4.06878946221683`}, {13, 0.0010142352142855542`, 1.8053386814282866`}}},
{0.13526256211607002`, 2.462351571487578`, 7.058879409696349`, 0.9886974127254741`, 0.579411758510507`,
0.519067819756009`, 0.20147771873022094`, 163.35469430255884`, 0.08074145629003388`,
7.486187513545234`, 873.2249300900974`, 146.5526111649307`, 11.409624242172539`, 480.14510176519127`,
1.`, {{0, 0.03414461516810293`, 60.77741499922321`}, {2, 0.002049893559786353`, 3.6488105364197074`},
{4, 0.00010309081146209179`, 0.1835016444025234`}, {6, 0.000033742700296599 62`, 0.060062006527947326`},
{8, 0.00003166067199968545`, 0.056355996159440105`}, {10, 0.000031586601568506515`, 0.0562241507919416`},
{12, 0.00003158230035174977`, 0.056216494626114576`}, {14, 0.00003158191022137675`, 0.056215800194050605`},
{16, 0.00003158186193781099`, 0.05621571424930355`}, {18, 1.6921484274323121`*^-6, 0.0030120242008295156`}}},
{0.20702996858127698`, 3.5459212947722705`, 9.044651158282573`, 1.2898647778788441`, 0.1120438185942787`,

```
0.3399243113730348`, 0.005774130262077124`, 68.71847405774774`, 0.13094328364017765`,
7.770082768301062`, 90101.20345696728`, 22626.393878521205`, 228.49544852808762`, 23785.253339532872`,
0.`, {{0, 0.07885369462135297`, 140.3595764260083`}, {13, 0.002056310873037148`, 3.6602333540061243`},
 {26, 0.002054764067576131`, 3.657480040285513`}, {39, 0.002064099797965822`, 3.674097640379163`},
 {52, 0.0021960178948739546`, 3.9089118528756392`}, {65, 0.004344437525001576`, 7.733098794502804`},
 {78, 0.014834511551156592`, 26.405430561058733`}, {91, 0.014256214643924238`, 25.376062066185142`},
 {104, 0.010543757382835979`, 18.767888141448044`}, {117, 1.6764754454467738`, 2984.126292895258`}}},
{0.10369509204579469`, 2.7722566029085502`, 8.491805279999415`, 1.0043154825901828`, 0.1196997608855428`,
 0.46660526882559294`, 0.07137717728073698`, 124.06613686120853`, 0.02382042507485488`,
 7.245869706736816`, 20549.77479733502`, 8071.871379475704`, 230.1738090045454`, 1102.7347743907123`,
 0.`, {{0, 0.050461411892601636`, 89.82131316883091`}, {3, 0.003723646699938822`, 6.628091125891103`},
 {6, 0.003703036231552 7685`, 6.591404492163929`}, {9, 0.0037028269893382617`, 6.591032041022106`},
 {12, 0.0037028205995135698`, 6.591020667134154`}, {15, 0.003702820146327859`, 6.5910198604635895`},
 {18, 0.003702820085446949`, 6.591019752095568`}, {21, 0.0037028200719691606`, 6.591019728105106`},
 {24, 0.003702820068502558`, 6.591019721934554`}, {27, 0.003702820068502558`, 6.591019721934554`}}},
{0.12495211269478451`, 3.2710300653782634`, 8.31741576802569`, 0.8301159562153473`, 0.45240597007929817`,
 0.45987625913811714`, 0.011306277140564175`, 1464.8239131751898`, 0.06894180622066476`, 6.472250284276024`, 63.56073112134365`,
 0.18897961574484248`, 0.18897961574484248`, 63.56073112134365`, 1.`, {{0, 0.004830615565222117`, 8.59849570609537`}}},
{0.2548094999525184`, 1.991565099208616`, 8.435964917534815`, 1.3597221193616629`, 0.25512389762112764`,
 0.2087626588648025`, 0.2568272531538555`, 1822.5061165805678`, 0.026223328458720774`, 8.894025776493041`, 42.13888951048874`,
 1.1017974153110277`, 1.1017974153110277`, 42.13888951048874`, 1.`, {{0, 0.0032025556027971444`, 5.7005489729789165`}}},
{0.1329827943052238`, 0.6587055670228517`, 4.6853626661703505`, 1.028170006221078`, 0.5310002897488229`, 0.3139925260456 5075`,
 0.010724910779551164`, 62.931190858884165`, 0.12157208629491184`, 4.054529845871336`, 104722.53821266741`,
 36064.36696530927`, 230.21431407968154`, 913.6917716578433`, 0.`, {{0, 0.0694405746459961`, 123.60422286987306`}}},
{0.05388606525951978`, 3.0261209146571506`, 4.311810802135282`, 1.2867466894982391`, 0.14121116952696844`,
 0.17229324040800142`, 0.239982541075217`, 290.98679707372355`, 0.1893837871763393`, 8.200911880146922`,
 209.93122960746882`, 3.6664122960766172`, 1.5557593043633766`, 89.968237136789`, 1.`,
 {{0, 0.00504454118633353`, 8.979283311673685`}, {10, 0.0016238369041234291`, 2.890429689339704`},
 {20, 0.00015250952793418866`, 0.27146695972285584`}, {30, 0.000016698404004816724`, 0.02972315912857377`}}},
{0.18877028807710305`, 3.0713928282220317`, 2.350050214349242`, 0.9003573816536171`, 0.8295355664150708`,
 0.3697046533237569`, 0.07836282343745943`, 203.60143886304388`, 0.1119456128362123`, 8.250251130667593`,
```

```
    32442.650373910943`, 8736.276225371186`, 229.61498995178468`, 1045.3581608486095`, 0.`,
    {{0, 0.058665253406223625`, 104.42415106307804`}, {24, 0.009928881268360671`, 17.673408657681993`},
     {48, 0.0027424135121416073`, 4.881496051612061`}, {72, 0.0010149894118311271`, 1.806681153059406`},
     {96, 0.0004012687398041141`, 0.7142583568513232`}, {120, 0.00019157923358750003`, 0.3410110357857501`},
     {144, 0.00012061234476688047`, 0.21468997368504725`}, {168, 0.0000941151374964672`, 0.16752494474371166`},
     {192, 0.00008244687281612538`, 0.14675543361270318`}, {216, 0.006205660297466212`, 11.046075329489858`}}},
  {0.1549731585118843`, 1.0794091822629426`, 5.872175042913531`, 1.1954059661912195`, 0.1582784842110334`,
    0.47191465043849123`, 0.04704948286374014`, 132.48832982484745`, 0.0611350826684427`,
    8.646340597834747`, 65632.70263228439`, 20300.489810435334`, 230.11143514320668`, 1596.19884619084`,
    0.`, {{0, 0.07583801393144507`, 134.99166479797225`}, {13, 0.0005944217132373082`, 1.0580706495624086`},
     {26, 0.0005939891802653417`, 1.057300740872308`}, {39, 0.0005994830513285364`, 1.067079831364795`},
     {52, 0.0007676572973663787`, 1.3664299893121543`}, {65, 0.0019244499637874884`, 3.425520935541729`},
     {78, 0.001093767619221925`, 1.9469063622150267`}, {91, 0.000861345584880459`, 1.5331951410872169`},
     {104, 0.0008890453525622961`, 1.5825007275608873`}, {117, 0.03814893861640904`, 67.9051107372081`}}},
  {0.0973630018497127`, 2.6509059407435345`, 2.661281949905481`, 1.0384603558144696`, 0.8313691963066465`,
    0.6553290382276395`, 0.19978363792296042`, 613.3149478611653`, 0.10086553584387609`, 7.337446850354657`,
    176.24752619494032`, 5.068867044569248`, 3.9492477543830375`, 173.70990759209215`, 1.`,
    {{0, 0.011048031056845806`, 19.665495281185535`}, {2, 0.0018952893182531483`, 3.373614986490604`},
     {4, 0.00018765437589510876`, 0.33402478909329353`}, {6, 0.000027104377704889792`, 0.048245791147038294`},
     {8, 0.000014741483814303063`, 0.02623984118945945`}, {10, 0.000013964377778426236`, 0.024856592445598697`},
     {12, 0.000013924929211031339`, 0.024786373995635783`}, {14, 1.243058152284093`*^-6, 0.0022126435110656857`}}},
  {0.25760446309632484`, 3.1710442921339457`, 1.104410531583154`, 1.067160019670409`, 0.29416815684722497`,
    0.2106415408174861`, 0.1640132441258384`, 295.52940308175954`, 0.22278260496644214`, 4.791464741488767`,
    17326.968891317352`, 3694.2555943164675`, 118.31770947664386`, 602.1103369207432`, 1.`,
    {{0, 0.040009777402441195`, 71.21740377634532`}, {30, 0.001429867349314271`, 2.545163881779402`},
     {60, 0.0006208710270057392`, 1.1051504280702156`}, {90, 0.0005537641593045922`, 0.985700203562174`},
     {120, 0.0005465483298887554`, 0.9728560272019846`}, {150, 0.0005456478144013652`, 0.9712531096344299`},
     {180, 0.0005455339669299941`, 0.9710504611353896`}, {210, 0.000545519542366269`, 0.9710247854119588`},
     {240, 0.0005455177142993709`, 0.9710215314528801`}, {270, 0.00041733830002493584`, 0.7428621740443858`}}},
  {0.08814664727699922`, 0.49325198699724604`, 7.4393784579313085`, 1.082856668773565`, 0.36684849391494345`,
    0.47651248778047417`, 0.010016127241926027`, 222.2306387901171`, 0.10060123761752315`,
```

```
2.9576363340294876`, 174.6579785741582`, 2.956707233005326`, 2.7982065761789467`, 172.23973131137933`,
  1.`, {{0, 0.011681123908024841`, 20.792400556284218`}, {6, 0.0014090956716399897`, 2.5081902955191815`}}},
{0.09405288750913654`, 1.8996899418118014`, 4.321657830772438`, 1.3285146456969388`, 0.4687194709250446`,
  0.37672748824359026`, 0.1617154754485949`, 124.22044587269936`, 0.08001764444851872`,
  7.0616286930304`, 64535.99375880696`, 27436.518024658355`, 229.20889555069405`, 838.7209379425223`,
  0.`, {{0, 0.03177322817114974`, 56.55634614464653`}, {7, 0.019538465073865318`, 34.77846783148026`},
   {14, 0.005768278387543847`, 10.267535529828045`}, {21, 0.0015342381132915305`, 2.7309438416589242`},
   {28, 0.0005243049395816157`, 0.9332627924552759`}, {35, 0.0002588627962871193`, 0.46077577739107234`},
   {42, 0.00016140536912151796`, 0.2873015570363019`}, {49, 0.00010469749679246664`, 0.1863615442905906`},
   {56, 0.00006489323110394291`, 0.11550995136501838`}, {63, 0.004014417704894606`, 7.145663514712399`}}},
{0.26673333380428393`, 2.6775026015459398`, 7.186300359062184`, 1.3779081516185778`, 0.2197224262472608`,
  0.44634915685926935`, 0.2549448072449753`, 573.3906335364269`, 0.19327206597748986`, 8.09151224456528`, 160.50042410240889`,
  3.175172442072823`, 2.142002793680495`, 108.96925767733957`, 1.`, {{0, 0.006627314330817879`, 11.796619508855825`},
   {7, 0.0015503428005845183`, 2.7596101850404424`}, {14, 0.00010400645207541082`, 0.18513148469423127`}}},
{0.06695152315589192`, 3.739348796633019`, 8.531551488563299`, 1.3873826920360777`, 0.5359483678583272`,
  0.39821450250245216`, 0.041894311644931556`, 1681.9385322117223`, 0.03536815464018617`,
  7.504735436115399`, 66.65528863153285`, 0.461749612715838`, 0.36639133954843417`, 52.22967729515151`, 1.`,
  {{0, 0.0029381597824250463`, 5.229924412716582`}, {38, 0.001031295692006469`, 1.8357063317715148`}}},
{0.2432492145111665`, 1.524425096090667`, 0.7030547277601631`, 1.1448142947983793`, 0.5481222164790474`,
  0.4314400790527364`, 0.005980753513921293`, 1722.4066531232882`, 0.11386175120469438`,
  3.8888258531503404`, 37.99101118815494`, 3.283772005058157`, 0.6505463913238074`, 27.90389189023268`,
  1.`, {{0, 0.0014117655414822299`, 2.512942663838369`}, {5, 0.000508163637374582`, 0.9045312745267561`},
   {10, 0.00012384591805110218`, 0.22044573413096186`}, {15, 0.00003806189470029551`, 0.06775017256652602`},
   {20, 0.000020049084998655203`, 0.035687371297606255`}, {25, 0.000018809707050818666`, 0.033481278550457225`}}},
{0.24077340164047406`, 3.026302168091572`, 3.348461380748672`, 1.1251559242118545`, 0.33184191297746213`,
  0.3141742813991918`, 0.0703872676061783`, 173.09096476750136`, 0.24564160017502346`, 6.392098114270558`,
  19415.596295889234`, 4337.2267839746955`, 228.1406371509195`, 1203.4236803689773`, 0.`,
  {{0, 0.04451617572946601`, 79.23879279844948`}, {31, 0.005053071405495053 5`, 8.994467101781195`},
   {62, 0.005053038199285431`, 8.994407994728068`}, {93, 0.005053075039803358`, 8.994473570849978`},
   {124, 0.005053094672845633 5`, 8.994508517665228`}, {155, 0.005053004225910421`, 8.99434752212055`},
   {186, 0.005052946397954006`, 8.994244588358129`}, {217, 0.0050529381741913956`, 8.994229950060685`},
```

{248, 0.005052937084682047`, 8.994228010734044`}, {279, 0.00651991877840894`, 11.60545542556791`}}},
{0.09741803900657164`, 2.8226058170826276`, 6.285345102913118`, 0.8413213607246539`, 0.2506059249236361`,
0.26188997247349455`, 0.05425664970500174`, 4955.988737338467`, 0.11641107051911387`, 2.1128007380367517`, 3.7979111683690276`,
0.040687277353183136`, 0.040687277353183136`, 3.7979111683690276`, 1.`, {{0, 0.0002886412487960461`, 0.5137814228569622`}}},
{0.14314599793313348`, 2.5518611864266925`, 6.295888200856282`, 1.4241645467251498`, 0.07884119203240614`,
0.5217209274223922`, 0.006381498715981013`, 359.4043872790348`, 0.24023808698930982`, 4.143010259943262`, 122.94540160771717`,
0.3341742334760208`, 0.3341742334760208`, 122.94540160771717`, 1.`, {{0, 0.009343850522186505`, 16.63205392949198`}}},
{0.225655266424848`, 1.5867432872088365`, 3.0858574789264335`, 1.3244931698407223`, 0.6079478177713131`,
0.6933074034991797`, 0.013957254253711016`, 59.76089956157712`, 0.13530140261382512`, 4.737681248427657`, 247220.22263676152`,
58505.31574689696`, 228.86520545164228`, 1662.651363172029`, 0.`, {{0, 0.1263615036010742`, 224.9234764099121`}}},
{0.1122606973400958`, 1.7187290694442563`, 9.979465273640976`, 1.2098446125137294`, 0.9761419756238592`,
0.37158902094365565`, 0.02080861609061981`, 179.99091662757314`, 0.030562090142117915`,
6.57677031568597`, 531.2202130337728`, 6.055303002874999`, 5.1568924682676105`, 531.7895582664783`,
1.`, {{0, 0.03645943621947393`, 64.8977964706636`}, {3, 0.0036341790745496806`, 6.4688387526984314`},
{6, 0.00030163596807152433`, 0.5369120231673132`}, {9, 0.000020755166157211003`, 0.03694419575983559`}}},
{0.24459409433338708`, 0.8193086201254509`, 4.65991702304226`, 0.8806460188595719`, 0.2108783317674836`,
0.532516488691834`, 0.10515966693757768`, 2682.0355154099257`, 0.13211135275557007`, 8.414844442987327`, 38.24452045357361`,
1.780037666479099`, 1.780037666479099`, 38.24452045357361`, 1.`, {{0, 0.0029065835544715944`, 5.173718726959438`}}},
{0.06530542208683338`, 0.9596039403304868`, 4.254394351359874`, 1.2566656605190776`, 0.7704919647854709`,
0.333532652338865`, 0.010126143082696715`, 1215.361514466369`, 0.2378411046297244`, 9.6572715838455`,
156.18001988518674`, 1.3680951143253164`, 1.3324649687492145`, 152.23333947888946`, 1.`,
{{0, 0.009139663760350462`, 16.268601493423823`}, {8, 0.002430070040045136`, 4.325524671280342`}}},
{0.1695388010672208`, 1.173836844893498`, 5.733937529803059`, 0.8972303818101501`, 0.03459597061008757`,
0.45955942513269943`, 0.02896845637601857`, 512.9632938088884`, 0.14318523913512782`, 1.8784414403823462`, 35.00221682664215`,
0.5860831831217672`, 0.5860831831217672`, 35.00221682664215`, 1.`, {{0, 0.002660168478824803 2`, 4.73509989230815`}}},
{0.21476635225191587`, 0.5109603344620304`, 9.116157263242219`, 1.0856571152809298`, 0.22597102427026194`, 0.39468057790613453`,
0.008122876676631087`, 1537.5523384133612`, 0.22468288668284858`, 1.5725594082493757`, 10.517775320361492`,
0.13388902438990782`, 0.13388902438990782`, 10.517775320361492`, 1.`, {{0, 0.0007993509243474735`, 1.4228446453385026`}}},
{0.06813064867410118`, 0.8082337111165119`, 3.599754755102856`, 0.8970806442820121`, 0.12954010392326953`,
0.2841840584225793`, 0.09462247566679993`, 471.57171291886044`, 0.0960155452146989`, 9.92783198210315`,
184.09040682748852`, 8.277305362702343`, 4.660280883552349`, 104.61337692988918`, 1.`,

```
  {{0, 0.0069954353355744`, 12.451874897322432`}, {32, 0.0009551813110971778`, 1.7002227337529767`}}},
{0.12903273933048381`, 3.2486278014701124`, 1.90816116411313`, 1.4556940559632274`, 0.8634695831402321`,
  0.3517692054558007`, 0.07017000005506284`, 82.66475099620249`, 0.22883036061603934`,
  2.0030440452333433`, 69303.4093640065`, 24363.71668460711`, 229.25109270236135`, 793.0763725472199`,
  0.`, {{0, 0.030191577833979195`, 53.74100854448296`}, {1, 0.030082226479609512`, 53.54636313370492`}}},
{0.25243522618186365`, 1.7361695497710992`, 4.779805866927312`, 1.3770344402735035`, 0.053441804379047`,
  0.21192069167526972`, 0.3494903419308112`, 922.4310224013639`, 0.09853668667446419`, 9.287299936208932`,
  76.1616549187417`, 2.4062740501310174`, 1.0620690140041815`, 34.904440200584894`, 1.`,
  {{0, 0.0020269361214898547`, 3.607946296251942`}, {9, 0.0005866140864673607`, 1.044173073911902`},
   {18, 0.00003646429520865736`, 0.0649064454714101`}, {27, 2.722952078579142`*^-6, 0.004846854699870872 5`}}},
{0.1954776887577972`, 2.556119761332786`, 4.179140500485959`, 0.8918497427192082`, 0.9985940433520333`,
  0.19919828622889002`, 0.10341194686720168`, 185.89024422047143`, 0.23152045430083634`, 6.38817726005194`, 3298.7595801783145`,
  817.0515581055768`, 3.5308852491286804`, 205.5333856693607`, 1.`, {{0, 0.012688052394346006`, 22.584733261935888`},
   {6, 0.002525496705987 2447`, 4.495384136657295`}, {12, 0.00032190705216495743`, 0.5729945528536242`},
   {18, 0.00004875227389763536`, 0.08677904753779093`}, {24, 0.000017797582446054385`, 0.031679696753976805`},
   {30, 0.000014731899842859061`, 0.026222781720289128`}, {36, 3.7994021866586038`*^-6, 0.006762935892252315`}}},
{0.21581636372683116`, 2.527066218755916`, 0.7074271020081202`, 1.2545848439034097`, 0.6959200155465519`,
  0.5313070485281961`, 0.086636650755472`, 1874.5635156658839`, 0.10607902266846381`, 5.753646937249579`,
  53.559567860711624`, 4.838480999053522`, 1.3717182395846221`, 39.832418538158024`, 1.`,
  {{0, 0.0024496550352835146`, 4.360385962804656`}, {4, 0.0004379725289618539`, 0.7795911015520999`},
   {8, 0.00006111434880112314`, 0.1087835408659992`}, {12, 0.000027581692240251096`, 0.04909541218764696`},
   {16, 0.000025182878969517 67`, 0.04482552456574145`}, {20, 0.000025757324643749377`, 0.045848037865873895`}}},
{0.1492413868080179`, 2.613673396032418`, 3.0904979954224796`, 1.2072285951382535`, 0.18001792552689877`,
  0.24732759197473053`, 0.00562211589804969`, 2528.5611678534033`, 0.0881107110472133`, 9.594393869960811`, 32.67014173050535`,
  0.07072668332887602`, 0.07072668332887602`, 32.67014173050535`, 1.`, {{0, 0.002482930771518407`, 4.419616773302764`}}},
{0.07601227699098417`, 2.712563755157329`, 4.823883660189505`, 1.3951422907519642`, 0.1445858824844255`,
  0.4724433117860949`, 0.00914361266793413`, 231.39818056782485`, 0.14481529205028987`,
  4.810561016418756`, 218.95355784799887`, 7.034192334156138`, 2.295704360844113`, 210.56105582346208`,
  1.`, {{0, 0.014890719489711837`, 26.50548069168707`}, {5, 0.0010051549926547517`, 1.7891758869254577`},
   {10, 0.0000566356875939476`, 0.10081152391722674`}, {15, 0.00005013007262258269 4`, 0.08923152926819719`}}},
{0.12871665135347787`, 2.8014401685833805`, 7.283786002739568`, 0.7524429330430051`, 0.8818706538097647`,
```

```
  0.37826653388239995`, 0.007972118927365966`, 65.78330734043783`, 0.1996049007295676`,
  1.2990854064714608`, 32877.13968270576`, 11554.431559362985`, 228.58583897078702`, 767.3410864606523`,
  0.`, {{0, 0.04417701838821738`, 78.63509273102693`}, {7, 0.008452395145033431`, 15.04526335815951`},
    {14, 0.0013228365752142063`, 2.3546491038812873`}, {21, 0.00030850795089925297`, 0.5491441526006703`},
    {28, 0.00014592649331174188`, 0.25974915809490057`}, {35, 0.00011541084952451563`, 0.20543131215363783`},
    {42, 0.00010775563442732146`, 0.19180502928063223`}, {49, 0.00010503344103276334`, 0.18695952503831875`},
    {56, 0.00010376729839839934`, 0.18470579114915087`}, {63, 0.0034792707949505633`, 6.193102015012004`}}},
{0.09952335410600305`, 3.347751049949922`, 0.6305859630946937`, 1.1305375150416004`, 0.9039729545790072`,
  0.47386245025326623`, 0.16157270551479597`, 562.0685567880952`, 0.16407167074796686`,
  8.59213583452907`, 9020.334132459173`, 3706.952617912802`, 228.13400664500787`, 704.2261928214914`,
  0.`, {{0, 0.005489833942820321`, 9.77190441822017`}, {4, 0.002070758616640581 7`, 3.685950337620235`},
    {8, 0.00110499749742536`, 1.9668955454171404`}, {12, 0.0006285841518479559`, 1.1188797902893617`},
    {16, 0.00038015026324345156`, 0.6766674685733437`}, {20, 0.00024652550040086743`, 0.43881539071354403`},
    {24, 0.00017281607674607435`, 0.3076126166080123`}, {28, 0.00013116122928918153`, 0.23346698813474315`},
    {32, 0.00010699971562473818`, 0.19045949381203395`}, {36, 0.04318936366039482`, 76.87706731550277`}}},
{0.09189223639403032`, 1.6732139429664894`, 9.692701238874825`, 1.282228547207058`, 0.15855153018111912`,
  0.4131843305852153`, 0.03830010741000983`, 3674.584524793622`, 0.10140311695331994`, 4.468652597668594`,
  12.385367741288915`, 0.17447237396359844`, 0.15684074075439094`, 11.10344540984229`, 1.`,
  {{0, 0.0005365379466043371`, 0.95503754495572`}, {32, 0.000307323904543677`, 0.5470365500877451`}}},
{0.15046133692934927`, 3.1811544489323884`, 8.294233740646352`, 1.3617577237735539`, 0.4847724596901488`,
  0.37433619970600795`, 0.06464979105054385`, 793.4336208513492`, 0.16357169207826316`,
  3.060886752086996`, 51.2377712642987`, 0.5406781523100124`, 0.40342832273628587`, 37.55408800416902`,
  1.`, {{0, 0.002336443067159685`, 4.15886865954424`}, {27, 0.0005176676211571601`, 0.921448365659745`}}},
{0.2766206750277398`, 2.8724354428398637`, 5.189326643025524`, 0.8952441889736982`, 0.9323758122824941`,
  0.4464935529399745`, 0.01922331430839979`, 81.97107113159659`, 0.06878282991185858`, 7.425462926136905`,
  198681.53433538997`, 40064.11282545068`, 228.98819686103795`, 1866.1731456319371`, 0.`,
  {{0, 0.0552318205931489`, 98.31264065580505`}, {15, 0.021350710199598888`, 38.00426415528602`},
    {30, 0.009351039557578513`, 16.644850412489753`}, {45, 0.004894844990873027 5`, 8.71282408375399`},
    {60, 0.0028334117155466346`, 5.04347285367301`}, {75, 0.0017404964426739127`, 3.0980836679595645`},
    {90, 0.0011140656121124924`, 1.983036789560237`}, {105, 0.000738533347737097`, 1.3145893589720326`},
    {120, 0.0005071986230961664`, 0.9028135491111763`}, {135, 0.04406703798566160 4`, 78.43932761447766`}}},
```

{0.23204393359414766`, 1.3807564884192223`, 0.49288335289722696`, 0.9255887528096592`, 0.5628667572225596`,
0.43401537857521066`, 0.010225716982610468`, 1119.882052780943`, 0.015435359995479236`,
2.904839645045426`, 45206.247438694016`, 10474.162404581424`, 218.36771178353564`, 1165.298195225054`, 0.`,
{{0, 0.03357476867204392`, 59.76308823623818`}, {29, 0.0028953599396275456`, 5.15374069253703`}, {58, 0.005902293915227264`,
10.50608316910453`}, {87, 0.008325352075161562`, 14.81912669378758`}, {116, 0.007422577433499373`, 13.212187831628883`},
{145, 0.00753084560681091`, 13.40490518012342`}, {174, 0.00762309295979217`, 13.569105468430061`},
{203, 0.00762755841737468`, 13.577053982926932`}, {232, 0.007660813817566687`, 13.636248595268702`}}},
{0.06896766630182821`, 3.238145856087148`, 5.061610251787275`, 0.9979492539875935`, 0.9640249344246659`,
0.343776595701085`, 0.005657576047359979`, 3386.0339854064414`, 0.07870221044254616`, 4.290344560110242`,
36.07799034501086`, 0.06833807174524678`, 0.06787811437625957`, 35.88676993817426`, 1.`,
{{0, 0.002220951539899406`, 3.953293741020943`}, {3, 0.0005064429754018378`, 0.9014684962152713`}}},
{0.14163054804702563`, 1.8677837362731973`, 0.4370922457889872`, 1.166715795330906`, 0.13182109027382305`,
0.4473367986720722`, 0.3453878303019619`, 2024.858554151485`, 0.17866806603084778`, 3.146330044100834`,
741.4972791914795`, 241.74381892302915`, 3.192376481158719`, 21.376018415879955`, 1.`,
{{0, 0.0015124714396190485`, 2.692199162521906`}, {22, 0.000037734727191102999`, 0.06716781440016333`},
{44, 0.0000372051163905047`, 0.06622510717509834`}, {66, 0.00003716611640622048`, 0.06615568720307245`}}},
{0.14346666218024207`, 3.6942323053195354`, 6.0819497997897844`, 0.8206348775106591`, 0.4021755195313119`,
0.4030089388647059`, 0.4636571249048107`, 744.6939263196219`, 0.08354527905403086`, 5.555269667846608`, 95.98575252739228`,
1.542846306848636`, 0.7963745669934121`, 49.79960259013104`, 1.`, {{0, 0.003380384709916461`, 6.017084783651301`},
{7, 0.0003921885157512023`, 0.6980955580371401`}, {14, 0.000012196571182295726`, 0.021709896704486392`}}},
{0.09149267062932814`, 2.234522341263885`, 6.944559583117852`, 1.114683357002141`, 0.5085103250363343`,
0.6658564606426403`, 0.006987139923383322`, 1505.8338370139704`, 0.21635495075213407`, 1.8879751532316735`, 22.94382407295265`,
0.07620868526885341`, 0.07620868526885341`, 22.94382407295265`, 1.`, {{0, 0.0017437306295444016`, 3.1038405205890345`}}},
{0.22068432056467163`, 3.569728941728428`, 4.540468856326312`, 1.2038410371742398`, 0.3148195694928344`,
0.5227498853534928`, 0.10369270254334745`, 4010.634501486441`, 0.16206884164220114`, 3.771999452670874`, 12.326676862690043`,
0.1446260728643782`, 0.1446260728643782`, 12.326676862690043`, 1.`, {{0, 0.0009368274415644433`, 1.667552845984709`}}},
{0.2097350071754976`, 1.1102722915344074`, 3.321403610485124`, 1.0935329934230222`, 0.7183578411763796`,
0.40562405317483374`, 0.006176942100465586`, 446.96867612346506`, 0.08354129713872654`,
7.284441181467236`, 205.63259620740783`, 2.2475357113184176`, 1.3456683779707554`, 212.46535814223535`,
1.`, {{0, 0.01095315151887485`, 19.496609703597237`}, {3, 0.003858116657399794`, 6.867447650171633`},
{6, 0.00099006126961436`, 1.7622109059913562`}, {9, 0.0002565862609022334`, 0.45672354440597546`},

```
    {12, 0.00007342517286713454`, 0.1306968077034995`}, {15, 0.000016081481804438662`, 0.028625037611900817`}}},
{0.15395695699718487`, 1.7653707587036722`, 8.640858401956272`, 1.0488086821127276`, 0.2563370285663329`,
  0.39045184386637677`, 0.03142873441769787`, 98.7771934627432`, 0.2047745102794421`, 8.897923651735105`,
  14362.54073416313`, 4297.204914315321`, 224.11035098661918`, 2088.2192815775097`, 0.`,
  {{0, 0.0824819144375462`, 146.81780769883224`}, {31, 0.005113242205854775`, 9.1015711264215`},
    {62, 0.005113242759690078`, 9.101572112248338`}, {93, 0.005113247570293487`, 9.101580675122408`},
    {124, 0.005113283080871238`, 9.101643883950803`}, {155, 0.005113543131271097`, 9.102106773662554`},
    {186, 0.0051152732732367735`, 9.105186426361456`}, {217, 0.005119492327578435`, 9.112696343089615`},
    {248, 0.005117038209934505`, 9.108328013683417`}, {279, 0.03530438840361416`, 62.841811358433205`}}},
{0.2495726529131425`, 0.6471319987804813`, 4.234303245586215`, 1.3697781718226154`, 0.5893758025334812`,
  0.16113556190203493`, 0.016137549539750166`, 661.2554849181507`, 0.08255896742665592`,
  6.142572860377916`, 96.35706052546873`, 1.7668012407882054`, 1.4656924468594874`, 78.90631012753373`, 1.`,
  {{0, 0.003881268023817715`, 6.908657082395532`}, {68, 0.0021156115458748482`, 3.7657885516572303`}}},
{0.18703143603461198`, 3.5026925577041474`, 3.736145496532833`, 0.8654363814884891`, 0.7031489482980493`,
  0.15906583747765657`, 0.008377691039792691`, 131.85234583624222`, 0.16088793676433022`, 8.514675246308464`, 22834.21164833812`,
  6152.582547769346`, 39.47406098413678`, 411.15356622171663`, 1.`, {{0, 0.026884350390667226`, 47.85414369538766`},
    {12, 0.00380828900905026 5`, 6.778754436109472`}, {24, 0.0003618576452118732`, 0.6441066084771342`},
    {36, 0.000052078593009690 77`, 0.09269989555724957`}, {48, 0.00002766131535053029`, 0.04923714132394392`},
    {60, 0.000026292379188135584`, 0.04680043495488134`}, {72, 0.000026235260984978524`, 0.04669876455326177`},
    {84, 0.000026232813630248 16`, 0.04669440826184172`}, {96, 0.000034673625757517334`, 0.061719053848380855`}}},
{0.2617533772269884`, 3.6583043975291023`, 1.4938470678500337`, 1.229216471284039`, 0.8448795594977094`,
  0.4744971024115313`, 0.027729197093825105`, 163.20786376161018`, 0.21172311156121143`,
  7.676981105027913`, 52647.37130250868`, 11090.104555750093`, 228.10822983068155`, 29029.527772571542`,
  0.`, {{0, 0.03969888075884036`, 70.66400775073583`}, {5, 0.02148802500430348`, 38.248684507660194`},
    {10, 0.018636625134674054`, 33.17319273971981`}, {15, 0.017043174512184768`, 30.336850631688883`},
    {20, 0.016170947288501887`, 28.78428617353336`}, {25, 0.015754032354111935`, 28.04217759031924`},
    {30, 0.01563630079877569`, 27.832615421820726`}, {35, 0.01571938833835843`, 27.98051124227801`},
    {40, 0.015938312455798388`, 28.370196171321133`}, {45, 2.0301584240698882`, 3613.6819948444013`}}},
{0.2277950960946843`, 2.381865480846251`, 9.991476447433318`, 0.832979598782657`, 0.5926733949589658`,
  0.27276410945547525`, 0.04327492387940349`, 669.5530400360332`, 0.024018555255208573`,
  9.681708943673282`, 194.43401795257486`, 2.09960465413912`, 1.4093295980568734`, 130.8297077478833`, 1.`,
```

```
{{0, 0.007240734844120121`, 12.888508022533815`}, {41, 0.0027023229447190097`, 4.810134841599837`}}},
{0.15963824410704142`, 2.654604852862681`, 7.469701098598773`, 1.491664105533315`, 0.25955793675814864`,
 0.27946634303609463`, 0.06966019051655106`, 86.7013584077103`, 0.017186465149371527`,
 9.819694766966123`, 432015.65701375157`, 131519.50072307783`, 228.065869824973`, 1373.331290185775`,
 0.`, {{0, 0.06090280033814885`, 108.40698460190495`}, {31, 0.004389480515377529`, 7.813275317372002`},
  {62, 0.004389491655338623`, 7.81329514650275`}, {93, 0.0043895531141322445`, 7.813404543155396`},
  {124, 0.004389697198094012`, 7.8136610126073425`}, {155, 0.004389599057791495`, 7.813486322868862`},
  {186, 0.004389481949891766`, 7.813277870807343`}, {217, 0.004389467076528161`, 7.813251396220127`},
  {248, 0.004389464795599096`, 7.813247336166391`}, {279, 0.008354142353217117`, 14.870373388726469`}}},
{0.13626056470386483`, 2.820076398995975`, 2.87943303050835`, 0.9523411928557022`, 0.8935766203190545`,
 0.356125796963648`, 0.009331421764352204`, 68.56313381348697`, 0.147099680293568`, 8.670054262776382`,
 89913.6527658248`, 30448.152077360977`, 228.31359859348828`, 14286.011444490188`, 0.`,
 {{0, 0.050631528758883375`, 90.12412119081239`}, {5, 0.023890637066419335`, 42.525333978226406`},
  {10, 0.019657871543048884`, 34.991011346627005`}, {15, 0.01681905930008699`, 29.937925554154837`},
  {20, 0.014767380409930246`, 26.285937129675833`}, {25, 0.013230646918645244`, 23.55055151518853`},
  {30, 0.012048154735507847`, 21.44571542920397`}, {35, 0.011116918191546012`, 19.7881143809519`},
  {40, 0.010368385008343542`, 18.455725314851506`}, {45, 0.913206287848843`, 1625.5071923709402`}}},
{0.22962572899038486`, 1.387681996287987`, 7.569216994527352`, 0.873789488954491`, 0.15662470794012862`,
 0.483300190980835`, 0.02173528342644958`, 88.10021701991921`, 0.04718506749066664`,
 8.783666403079792`, 163469.5500264975`, 38071.14839551498`, 229.24105879812458`, 4310.74082296236`,
 0.`, {{0, 0.07891510023659665`, 140.46887842114202`}, {8, 0.0006527601046834504`, 1.1619129863365416`},
  {16, 0.0006042121189877227`, 1.0754975717981463`}, {24, 0.0006048128075744377`, 1.07656679748249`},
  {32, 0.0006197517719221338`, 1.103158154021398`}, {40, 0.0007266210770189874`, 1.2933855170937973`},
  {48, 0.0016002087899550052`, 2.8483716461199093`}, {56, 0.005277204693742763`, 9.393424354862116`},
  {64, 0.006888129642823967`, 12.260870764226661`}, {72, 0.23172750130183423`, 412.47495231726487`}}},
{0.16187203397799577`, 1.769575035055972`, 1.1614771267268384`, 0.8287067492253789`, 0.7900895875825826`,
 0.2962983919698179`, 0.014442973603799043`, 137.47659064397268`, 0.2186302306878819`, 9.341476742915006`, 29236.76291243101`,
 8801.363588098673`, 230.20319842345634`, 968.0874724137158`, 0.`, {{0, 0.0735746479034424`, 130.96287326812748`}}},
{0.09685210682031803`, 1.7960713074535244`, 5.69551378974773`, 0.7869885608747933`, 0.9421312080797839`,
 0.5036667090451988`, 0.01697045527462802`, 52.99769310364423`, 0.21425647446502405`,
 9.869621253243459`, 55208.485035568345`, 22970.899241307397`, 229.7804359527208`, 2576.192356273686`,
```

```
0.`, {{0, 0.056794508463013`, 101.09422506416315`}, {2, 0.017858919559733847`, 31.788876816326248`},
    {4, 0.012592365775286031`, 22.414411080009135`}, {6, 0.009101512443053033`, 16.200692148634396`},
    {8, 0.006677214595532045 4`, 11.885441980047041`}, {10, 0.004959783535563328`, 8.828414693302724`},
    {12, 0.0037299913975895097`, 6.6393846877093265`}, {14, 0.002842738382170457`, 5.060074320263414`},
    {16, 0.0021985902099491217`, 3.9134905737094368`}, {18, 0.07903499471490978`, 140.6822905925394`}}},
{0.16013522976515993`, 1.0007721789830901`, 6.380859482894074`, 1.440495462142086`, 0.5398127028451485`,
  0.19075782788209084`, 0.04652110166049526`, 84.8416541425776`, 0.16547472912452094`,
  6.4422585881915015`, 47027.6331692644`, 14215.185434804762`, 229.1568264268649`, 1075.0244413232717`,
  0.`, {{0, 0.07891495421098581`, 140.4686184955547`}, {31, 0.001175490630640689`, 2.0923733225404257`},
    {62, 0.0001128474095954026`, 0.20086838907981658`}, {93, 0.00010774269969671884`, 0.19178200546015953`},
    {124, 0.0001082262891299038`, 0.19264279465122874`}, {155, 0.00010815967423258203`, 0.192524220133996`},
    {186, 0.0001067151775580088`, 0.18995301605325562`}, {217, 0.00010515671989444338`, 0.1871789614121092`},
    {248, 0.00010452505963570766`, 0.1860546061515596`}, {279, 0.0008580396691993716`, 1.5273106111748813`}}},
{0.1912199176775608`, 2.2121001603415005`, 9.917361836602723`, 1.435922264503217`, 0.9790995032802718`,
  0.6767636439858111`, 0.07238714810610002`, 269.421010262202`, 0.015607565070540702`,
  2.5093255880098546`, 208.89881766280894`, 4.12582814686209`, 3.686218401598009`, 201.73661387014096`,
  1.`, {{0, 0.01301858300228318`, 23.173077744064063`}, {6, 0.0023133996518475333`, 4.117851380288609`}}},
{0.24179572266521915`, 2.5600514990256196`, 5.593167080478407`, 1.0641609773948193`, 0.6297315486340287`,
  0.26623731831221253`, 0.08310036534342866`, 636.0105501064265`, 0.05032454414330245`, 1.4627567207550134`, 30.402343053601534`,
  0.4640831114809422`, 0.3327179788885418`, 21.27050852628339`, 1.`, {{0, 0.0010747211241657372`, 1.913003601015012`},
    {11, 0.000444626527992834 8`, 0.791435219827246`}, {22, 0.0000972109958389657`, 0.17303557259335894`}}},
{0.20288510482307215`, 0.7288550347693712`, 9.019074578521877`, 1.2840716127441223`, 0.5874481378002983`,
  0.6980893050148065`, 0.008225735950864725`, 1276.121996072157`, 0.1607915330045036`, 9.69181498105582`, 166.12595428875255`,
  1.5314264133857123`, 1.5314264133857123`, 166.12595428875255`, 1.`, {{0, 0.012625572525945195`, 22.473519096182446`}}},
{0.1862496077639137`, 1.6905827743030857`, 2.538407600056317`, 0.9208769830081895`, 0.38109944279089136`,
  0.3320011945053185`, 0.011054373793833769`, 1065.4164452217328`, 0.07943719416592698`, 4.231039339853934`, 45.055776493899415`,
  0.2742904915684351`, 0.2742904915684351`, 45.055776493899415`, 1.`, {{0, 0.0034242390135363556`, 6.0951454440947135`}}},
{0.05689751321611025`, 3.0250332600067678`, 2.529462845718742`, 1.466915953431471`, 0.22269501402715108`,
  0.6695459886571014`, 0.14207393160159743`, 917.9363525294286`, 0.02546682059293326`,
  3.574551553390743`, 53.95020469379218`, 0.904344577640405`, 0.6838023601189388`, 37.7792684448821`, 1.`,
  {{0, 0.00254213429967244 9`, 4.524999053416958`}, {19, 0.00032909010213859064`, 0.5857803818066913`}}},
```

```
{0.1967142498798619`, 3.0620830451218577`, 7.9498840213828466`, 1.4844762448061533`, 0.6042217303737281`,
  0.6207898959804532`, 0.0334891282473782`, 576.7285784287807`, 0.16199601914875666`, 9.348325121692032`,
  341.0713575055191`, 2.5061800669007175`, 2.3852519339867593`, 319.57246968892133`, 1.`,
  {{0, 0.016589719744507042`, 29.52970114522254`}, {30, 0.007697787951850981`, 13.702062554294747`}}},
{0.26434225760699476`, 2.569764850665571`, 5.652188171834926`, 0.8392687504503539`, 0.3834838466329171`,
  0.17354269300981617`, 0.03414034179009787`, 716.202171206297`, 0.028057924329779993`, 1.7997126741152787`,
  21.329250289769735`, 0.1932861088220418`, 0.13642391972689907`, 14.230828285272953`, 1.`,
  {{0, 0.0007943064570525613`, 1.413865493553559`}, {52, 0.0002872364926281831`, 0.511280956878166`}}},
{0.19233187457454282`, 2.1451040206615035`, 4.677354160755579`, 0.8253029687642099`, 0.9809911268657268`,
  0.22566854695954142`, 0.015470928716805988`, 111.90002994139898`, 0.12242650367871766`,
  3.4296984602053584`, 34044.88615793034`, 9051.746285092602`, 22.99547409882565`, 225.5922879572955`,
  1.`, {{0, 0.01415485334503047`, 25.195638954154234`}, {7, 0.0025193124008551873`, 4.484376073522234`},
    {14, 0.0002806409496618591`, 0.4995408903981092`}, {21, 0.00004736045664021979`, 0.08430161281959124`},
    {28, 0.000028349917414869508`, 0.05046285299846773`}, {35, 0.000027233179790133795`, 0.04847506002643816`},
    {42, 0.00002717829042037964`, 0.04837735694827577`}, {49, 0.000027175035737143825`, 0.04837156361211601`},
    {56, 0.000027174751091417283`, 0.04837105694272277`}, {63, 5.735558112778346`*^-6, 0.010209293440745455`}}},
{0.21830260718528288`, 1.0813728060954029`, 9.236753498274854`, 1.3292857287875703`, 0.7084275620345744`,
  0.4530838967889582`, 0.022838882777727113`, 80.81382094438914`, 0.07293079158751065`,
  5.841787640828064`, 128890.30204091553`, 31194.286857340987`, 228.9143017044015`, 1433.7713148712344`,
  0.`, {{0, 0.09908017333065368`, 176.36270852856356`}, {17, 0.002811991880025248`, 5.005345546444942`},
    {34, 0.0001620158687764667`, 0.2883882464221107`}, {51, 0.00011586190903652008`, 0.20623419808500576`},
    {68, 0.00011826855944828888`, 0.21051803581795422`}, {85, 0.0001269002558171151`, 0.2258824553544649`},
    {102, 0.00013702751131110813`, 0.2439089701337725`}, {119, 0.00014609819486858058`, 0.2600547868660734`},
    {136, 0.00015224972320488018`, 0.27100450730468667`}, {153, 0.006116032697071938`, 10.886538200788051`}}},
{0.27637635892023465`, 3.838779152844408`, 0.15938000205200176`, 1.3392032462951913`, 0.02123906289427646`,
  0.6059474038465393`, 0.007164747514275051`, 100.75472745890974`, 0.10946913978259332`, 8.952098728322259`, 231916.74584535134`,
  46866.34116375629`, 230.23717427080922`, 1404.1885576750103`, 0.`, {{0, 0.1067183303833008`, 189.9586280822754`}}},
{0.15064788979833238`, 0.9109936464272579`, 0.5800958535668599`, 1.4999646884179314`, 0.9779856120080317`,
  0.34948818201364285`, 0.011067508252105101`, 2641.6705923333384`, 0.011474828296826944`,
  3.667140614388831`, 20.30259376241613`, 1.0275815096029397`, 0.6043041193149595`, 19.30928171688587`,
  1.`, {{0, 0.0010342211194418424`, 1.8409135926064792`}, {7, 0.00032411526929323255`, 0.576925179341954`},
```

{14, 0.00008270535531416076`, 0.14721553245920616`}, {21, 0.000026463666434090625`, 0.047105326252681316`}}},
{0.0419185722234717`, 1.709255049022068`, 2.3539768873704308`, 1.3792050115017793`, 0.22715212217109637`,
0.2926929451571887`, 0.00505223249889438`, 88.91455127714036`, 0.1107140298924858`, 2.282618721837757`, 70255.40262284165`,
43912.690814495785`, 230.25324278759345`, 489.2310343290631`, 0.`, {{0, 0.0371815586090088`, 66.18317432403566`}}},
{0.24924491240080032`, 1.373280333190027`, 8.919496594562187`, 1.4037493221091317`, 0.24632944507518628`,
0.3548949637369072`, 0.18998328780525742`, 2947.236236962295`, 0.1153856006596437`, 7.477168052601716`, 26.5311566718809`,
0.9243776901506614`, 0.9243776901506614`, 26.53115667188809`, 1.`, {{0, 0.002016367907063495`, 3.589134874573021`}}},
{0.20687871564233656`, 0.44181655503120965`, 8.570924850704476`, 1.0055678634725929`, 0.5549096280819519`,
0.326726210951258`, 0.20129490353773463`, 306.54002765810475`, 0.1699967418388691`,
1.7003969931174137`, 74.07810128302826`, 7.431170026369797`, 4.13138502509764`, 41.72500209611239`, 1.`,
{{0, 0.0022539459981439935`, 4.012023876696308`}, {7, 0.0007681696791911457`, 1.3673420289602392`},
{14, 0.0001316129155731354`, 0.234270989720181`}, {21, 0.000017371566396267487`, 0.030921388185356127`}}},
{0.20196179343132148`, 2.101313615300616`, 3.7695608295023266`, 0.8255643571631567`, 0.9698552359083252`,
0.3038610819528902`, 0.02519738143348337`, 88.57713238465558`, 0.03933307441687739`, 1.9858938348907018`,
218759.52169416408`, 56291.75490525618`, 229.45626031211282`, 983.1427922117493`, 0.`,
{{0, 0.016074318617529088`, 28.612287139201776`}, {19, 0.005753022225101857`, 10.240379560681305`},
{38, 0.002043517450639547`, 3.6374610621383936`}, {57, 0.0008069952086048266`, 1.4364514713165915`},
{76, 0.0003564008869646012`, 0.6343935787969901`}, {95, 0.00018399126427743654`, 0.3275044504138371`},
{114, 0.00011582969934290312`, 0.20617686483036754`}, {133, 0.00008754060194488146`, 0.15582227146188898`},
{152, 0.00007469101880403947`, 0.13295001347119026`}, {171, 0.04922545234883765`, 87.6161305180931`}}},
{0.10010100507466935`, 2.4192735538683836`, 9.823330355933038`, 1.1133108806457672`, 0.011014651746178439`,
0.5998442212295849`, 0.013751847561218578`, 3832.28466694177`, 0.1060300423959204`, 2.751621101527464`, 7.718266370888943`,
0.03690915641649215`, 0.03690915641649215`, 7.718266370888943`, 1.`, {{0, 0.0005865882441875597`, 1.0441270746538562`}}},
{0.21311625184066546`, 1.4247782113917893`, 5.690068626185788`, 0.8615233428628426`, 0.12086241410351461`,
0.29764577871833753`, 0.005395932799405541`, 882.4049045614522`, 0.2481341739836153`, 1.9572384227218778`, 18.721375600773758`,
0.07123487609292699`, 0.07123487609292699`, 18.721375600773758`, 1.`, {{0, 0.0014228245456588057`, 2.532627691272674`}}},
{0.09607643000919447`, 2.545123365745101`, 1.6292494594570959`, 0.9689766494611143`, 0.9239031581606687`,
0.5340288721519064`, 0.06266594590032853`, 79.64776165873695`, 0.19537727971057256`, 3.908080115643834`, 57144.5511487094`,
24064.514972483616`, 229.7541694028561`, 961.1280340897409`, 0.`, {{0, 0.07304573059082031`, 130.02140045166016`}}},
{0.25410295729391263`, 0.5246172329407881`, 1.519575137014602`, 1.3035504391628365`, 0.641505202173666`,
0.18662868599370885`, 0.02992528952684035`, 1308.2457641643925`, 0.1567967571574565`, 7.528281484333206`,

```
68.38848774654484`, 2.4498897253725382`, 1.6815503510779377`, 45.481141066020434`, 1.`,
 {{0, 0.0024948120366255202`, 4.440765425193426`}, {50, 0.0009617546843920333`, 1.7119233382178194`}}},
{0.20247384111644234`, 3.9381206949664582`, 3.525512124148932`, 0.8160399146038965`, 0.003770588899372651`,
 0.4372678593782203`, 0.005846407125549171`, 81.38331905868344`, 0.05740074457617078`, 6.792038617151225`, 201901.8125728869`,
 51821.289467345785`, 230.20978999314536`, 1343.6020048041091`, 0.`, {{0, 0.1021137523651123`, 181.76247920989988`}}},
{0.15622647656935051`, 2.478038073204126`, 4.93355773626655`, 1.0681326166577831`, 0.57283306272417`,
 0.24473580012035412`, 0.09417540555806864`, 340.729792091258`, 0.1606536419587833`, 9.486285960325056`,
 347.4636591568219`, 5.586013696260242`, 2.8060696517523662`, 175.32287488004926`, 1.`,
 {{0, 0.010298844751638132`, 18.331943657915875`}, {25, 0.003025693739245612`, 5.385734855857189`}}},
{0.2472522705346833`, 1.1701552284997945`, 3.914120438755436`, 1.4973295978551664`, 0.1631737194969931`,
 0.4864210576866177`, 0.39314364156002357`, 172.17497479862377`, 0.10619847023829598`, 4.473646521439088`, 63299.723146061595`,
 13942.214135774`, 50.394207127691026`, 370.71123947816193`, 1.`, {{0, 0.026430835982641263`, 47.04688804910145`},
  {8, 0.00040140494096845355`, 0.7145007949238473`}, {16, 0.00019702660640654148`, 0.35070735940364384`},
  {24, 0.00019366768530226748`, 0.3447284798380361`}, {32, 0.0001925991736918387`, 0.3428265291714729`},
  {40, 0.0001923294627799054`, 0.3423464437482316`}, {48, 0.00019230349112419226`, 0.3423002142010622`},
  {56, 0.00019230101780311028`, 0.3422958116895363`}, {64, 0.0001815858396227369`, 0.3232227945284717`}}},
{0.1494891985884202`, 3.3979182405498527`, 4.585626936341594`, 1.2118171179612203`, 0.6586829775426448`,
 0.36431745127605797`, 0.33277789805879787`, 51.20287439819706`, 0.10641801334172246`,
 7.126742388333813`, 213930.34361840397`, 68149.02385169019`, 228.23992837778027`, 1916.5233586421177`,
 0.`, {{0, 0.035893760360742565`, 63.890893442121765`}, {3, 0.01124501700754251`, 20.01613027342567`},
  {6, 0.00779392412795059`, 13.873184947752051`}, {9, 0.005662414666495034`, 10.079098106361158`},
  {12, 0.004218585857552782`, 7.509082826443952`}, {15, 0.003196208081l243146`, 5.68925038440128`},
  {18, 0.002457191033703986 3`, 4.373800039993096`}, {21, 0.0019180696454226002`, 3.414163968852229`},
  {24, 0.0015231296647517498`, 2.7111708032581148`}, {27, 0.07174747481151482`, 127.71050516449638`}}},
{0.2014310029114028`, 3.9869430882246055`, 0.7136208449188892`, 1.3208947167282403`, 0.46844054682093383`,
 0.39516715199771224`, 0.14154388607116256`, 2748.591928872397`, 0.05354911946309293`, 6.211900336444712`,
 31.788658869757267`, 0.5010615275525613`, 0.349228371815562`, 17.759046904056103`, 1.`,
 {{0, 0.0011354913254371381`, 2.021174559278106`}, {21, 0.00021419623927112583`, 0.381269305902604`}}},
{0.13228384635594964`, 2.6408616938406997`, 3.2993392931538836`, 0.952940068464383`, 0.8367144819205878`,
 0.6111943245350302`, 0.13146835864980566`, 2246.7842161758563`, 0.024436379332296754`, 9.73655688789367`, 147.94801617198212`,
 2.5184950060936804`, 1.941499365702518`, 114.01480283220111`, 1.`, {{0, 0.005360474603638795`, 9.541644794477055`},
```

{4, 0.0025980548477380918`, 4.624537628973803`}, {8, 0.0007065955638703985`, 1.2577401036893092`}}},
{0.23113058156717692`, 2.2605125411397546`, 4.002625660970853`, 1.3839850516092966`, 0.507564008077771`,
0.4982541689482688`, 0.0228570122346136`, 138.3250229234806`, 0.07006503343759235`, 8.064244271550876`,
137295.50474742881`, 31857.076659929677`, 229.13687822347592`, 2388.3800904903737`, 0.`,
{{0, 0.05596952971090401`, 99.62576288540915`}, {13, 0.003198825411072998`, 5.693909231709936`},
{26, 0.0031669651637119245`, 5.637197991407226`}, {39, 0.003164785830151558`, 5.633318777669773`},
{52, 0.003165270189849801`, 5.634180937932647`}, {65, 0.003166916897018944`, 5.637112076693721`},
{78, 0.003169649479161922`, 5.641976072908221`}, {91, 0.0031736765297707584`, 5.64914422299195`},
{104, 0.003179288673219763`, 5.659133838331179`}, {117, 0.10016197899240674`, 178.288322606484`}}},
{0.17570245022422137`, 3.187809299248391`, 9.693328941627644`, 1.4591302419173326`, 0.2844948752823342`,
0.5066677812093952`, 0.2998214113578054`, 349.3700788988757`, 0.18995614940943706`, 5.5658132930392785`, 202.53782306525252`,
3.513592772128769`, 2.491364958498298`, 143.9950372493169`, 1.`, {{0, 0.009238407263779785`, 16.444364929528017`},
{6, 0.001628756603336371`, 2.89918675393874`}, {12, 0.00007645896383192896`, 0.13609695562083354`}}},
{0.2497541467888837`, 1.085030464715203`, 1.0043906551890043`, 0.864213346242746`, 0.8846254264305906`,
0.601718469375484`, 0.012654582561683364`, 62.423429857566624`, 0.12690014020771856`, 6.991109163451483`, 187560.33432596203`,
41048.71318679938`, 229.4835993105773`, 1560.4039242393094`, 0.`, {{0, 0.11859069824218751`, 211.0914428710938`}}},
{0.22558722869536918`, 1.12607467611575`, 0.7911165205665469`, 1.2733751455833207`, 0.6009341950827205`,
0.6641796486046712`, 0.175358669032309`, 3424.3638773504586`, 0.14969161390071423`, 8.91095466688921`,
57.22783854061539`, 2.5746024268619747`, 1.5963420755275664`, 34.40371113386621`, 1.`,
{{0, 0.0023051370536268387`, 4.103143955455773`}, {16, 0.0003095449925469934`, 0.5509900867336482`}}},
{0.050627269144448594`, 3.276563036391985`, 7.788230793264948`, 0.8177022370463224`, 0.523932893406847`,
0.34959907144360225`, 0.11342446098213065`, 1447.0777642274236`, 0.2385428599947077`, 6.8056995790460695`, 64.71978671862897`,
0.8310686649717774`, 0.8310686649717774`, 64.71978671862897`, 1.`, {{0, 0.004918703790615802`, 8.755292747296128`}}},
{0.060006217186970356`, 3.9647259629353027`, 0.34014645604634985`, 0.9146780185619144`, 0.0343140190064282`,
0.5737721437004861`, 0.026766328146322068`, 169.17748604048344`, 0.08721098377832243`, 3.968142168145161`, 51646.57184929879`,
27802.06337517515`, 230.06912709580916`, 751.7128869106898`, 0.`, {{0, 0.05713017940521243`, 101.69171934127812`}}},
{0.15350947465095238`, 2.9043684969368213`, 4.5670292387828155`, 1.3486905179026798`, 0.8383532909017835`,
0.5087310077763043`, 0.053857845014854355`, 402.8647291760491`, 0.025030223476360525`,
5.400937139619534`, 208.85862250688703`, 3.25095922866471`, 2.619672209944673`, 207.08791361278486`,
1.`, {{0, 0.014033074517729564`, 24.978872641558628`}, {3, 0.0015407086565632719`, 2.742461408682624`},
{6, 0.00013973614804208042`, 0.24873034351490309`}, {9, 0.000025162112236733680`, 0.044788559781385955`}}},

```
{0.2587691832069661`, 1.4843472048382624`, 4.771777259704052`, 1.1550607981645458`, 0.08010207781888057`,
  0.5149452814008634`, 0.01659115431008023`, 1531.656314816295`, 0.15778107864815993`, 1.1951975901128993`, 8.1238810035257`,
  0.07941103611289003`, 0.07941103611289003`, 8.1238810035257`, 1.`, {{0, 0.0006174149562679532`, 1.0989986221569568`}}},
{0.1547206763399963`, 3.3076918921708103`, 2.0706333626337727`, 1.4168143839445906`, 0.37770487420755305`,
  0.5386802863405199`, 0.00701518941055165`, 122.30089778268855`, 0.134914758355765`, 2.269217335741727`, 87748.79668642554`,
  27321.73641760942`, 230.2035546299879`, 929.1776857878032`, 0.`, {{0, 0.07061750411987304`, 125.69915733337402`}}},
{0.1424711737630725`, 2.7520949793854754`, 4.596768650339829`, 0.8362726667059688`, 0.00009639230672808807`,
  0.36579283504807614`, 0.010146114868681232`, 3412.101956455065`, 0.24682488221918303`, 8.269337606653004`, 21.123256610483015`,
  0.06767989976191276`, 0.06767989976191276`, 21.123256610483015`, 1.`, {{0, 0.0016053675023967092`, 2.8575541542661425`}}},
{0.10882832688629612`, 2.4842578785622536`, 4.2235021418772565`, 1.4458161330640105`, 0.36880250968204553`,
  0.4736058884190225`, 0.023130853597073738`, 1306.5861668001219`, 0.20634842182153856`, 4.016571058814693`, 40.5440424045921 8`,
  0.2969762356020966`, 0.2969762356020966`, 40.54404240459218`, 1.`, {{0, 0.003081347222749006`, 5.484798056493231`}}},
{0.18639271747297592`, 2.4942906430499407`, 3.445345566170081`, 1.148752163319608`, 0.12885074455456724`,
  0.17966201189701259`, 0.0741834712240346`, 1169.2351269821615`, 0.09106655178874168`, 4.423161126411237`, 27.641819441051528`,
  0.3914588852612661`, 0.2634755468003807`, 18.458434484277443`, 1.`, {{0, 0.000947120125981874`, 1.6858738242477356`},
    {16, 0.00038860726176927847`, 0.6917209259493157`}, {32, 0.00006711363305393327`, 0.11946226683600122`}}},
{0.09766181155973352`, 3.6268090588227118`, 4.041820185573664`, 1.3253596170020208`, 0.4793934216010949`,
  0.49032555462271876`, 0.006759043878711846`, 262.0748117860937`, 0.11178249583803845`,
  2.0681460387230555`, 1470.4489382075433`, 583.0447070066657`, 2.5507329025509216`, 83.43329381310959`,
  1.`, {{0, 0.005484757970750338 4`, 9.762869187935603`}, {4, 0.0006986750975407447`, 1.2436416736225255`},
    {8, 0.00006262002781872705`, 0.11146364951733416`}, {12, 0.000022795740785641075`, 0.04057641859844112`},
    {16, 0.000021053858671838924`, 0.03747586843587328 4`}, {20, 0.00005102763422903949`, 0.09082918892769029`}}},
{0.07951891239009712`, 2.8897027315487103`, 1.5411848578052467`, 0.863635998632232`, 0.29085460372381067`,
  0.43469992564602655`, 0.009145988318489378`, 180.1504573795744`, 0.0803419958474173`, 8.628584719983632`, 41939.599247497405`,
  19583.982492955023`, 230.19257144951334`, 783.1181977924548`, 0.`, {{0, 0.059516983032226564`, 105.94022979736329`}}},
{0.07234637915492992`, 1.3584521644778373`, 7.973775020047323`, 0.7807691250526536`, 0.9100932527758132`,
  0.18771974645581058`, 0.009648266694560257`, 1956.923094358971`, 0.1277442569760165`, 5.404137919151016`,
  45.78956320934368`, 0.27381989642652166`, 0.27286840892563174`, 45.62239391849991`, 1.`,
  {{0, 0.0028187889018887`, 5.017444245361886`}, {7, 0.0006485130359172935`, 1.1543532039327824`}}},
{0.26334607729010473`, 1.8094502738990368`, 2.2460302448509974`, 1.2023222076508349`, 0.5513938303777435`,
  0.6812297944975665`, 0.00739497505047526`, 1157.7965975362908`, 0.15524857884582421`, 1.7991882609973402`, 31.848849297753326`,
```

```
0.2756134849831372`, 0.23956679479557635`, 32.05951904390279`, 1.`, {{0, 0.0016917640657313473`, 3.0113400370017986`},
   {4, 0.0006036336309349599`, 1.0744678630642286`}, {8, 0.00014112575067030473`, 0.2512038361931424`}}},
{0.22679920860671537`, 1.574644711817462`, 3.814781873556772`, 0.9990271813793515`, 0.3871644432916528`,
  0.6894928310515707`, 0.054622933309625116`, 69.61156300085906`, 0.09745707491156125`, 5.357011938436007`, 210478.88519243314`,
  49605.48864221299`, 228.08456671587325`, 1638.0205907319719`, 0.`, {{0, 0.12448956489562987`, 221.59142551422119`}}},
{0.054135826493752204`, 3.1195801041969027`, 7.2615169638158825`, 1.0984819310653413`, 0.10801447255588448`,
  0.6366822484935735`, 0.01066160509264612`, 211.97043506851273`, 0.24501368432397974`,
  8.92838638172081`, 517.0449016496841`, 1.8984941508850781`, 1.6897411046245898`, 528.7965006646828`,
  1.`, {{0, 0.03457991936187761`, 61.552256464142154`}, {1, 0.005608614688638275`, 9.98333414577613`}}},
{0.14590720393667378`, 1.5766246964498798`, 5.158062459855282`, 1.1478320866243528`, 0.3287329300123256`,
  0.4460050972404973`, 0.032458645789603134`, 482.4725013461066`, 0.10279090509715616`,
  8.020019312365584`, 207.5348979371129`, 2.8046299297175974`, 2.5610381483554336`, 186.2818335465696`,
  1.`, {{0, 0.010252248440542754`, 18.249002224166098`}, {40, 0.003905170908996537`, 6.951204218013836`}}},
{0.14965591009740292`, 1.460367057362724`, 7.337474105676788`, 0.8826427720923014`, 0.44478408083773613`,
  0.631222883334907`, 0.365159753458356`, 157.97713196457462`, 0.16784289093699323`, 1.7957284623930878`,
  1663.810184214276`, 496.2652722539276`, 6.530235410281181`, 129.51421541144654`, 1.`,
  {{0, 0.009352773053202725`, 16.64793603470085`}, {3, 0.000304445537300237`, 0.5419130563944218`},
   {6, 0.000035833324957611594`, 0.06378331842454864`}, {9, 0.00003288477922948907`, 0.058534907028490554`},
   {12, 0.00003284500357645957`, 0.05846410636609804`}, {15, 0.00003284346748403794`, 0.05846137212158754`},
   {18, 0.000032843334137813254`, 0.0584611347653076`}, {21, 0.000018611871381562943`, 0.03312913105918204`}}},
{0.0810328468371882`, 1.4659479028658335`, 3.234238812997848`, 1.4263751048195057`, 0.08355223050311089`,
  0.5582345497973803`, 0.12450549955799516`, 121.80826494117302`, 0.16010939682803116`,
  9.339681585379608`, 50113.962035273005`, 23116.05796974019`, 229.1672567198335`, 2133.354854272422`,
  0.`, {{0, 0.03159712353729983`, 56.24287989639369`}, {3, 0.008222452133920241`, 14.635964798378028`},
   {6, 0.004214759244062585`, 7.5022714544314`}, {9, 0.0028164811064455672`, 5.01333636947311`},
   {12, 0.00233240219373979`, 4.151675904856827`}, {15, 0.0022386328737808714`, 3.984766515329951`},
   {18, 0.002319254305588568`, 4.128272663947651`}, {21, 0.00243143487264874`, 4.327954073314757`},
   {24, 0.0024702157603457607`, 4.396984053415453`}, {27, 0.10349221289687216`, 184.21613895643245`}}},
{0.15633465648304357`, 2.148716979150252`, 1.5640986685796054`, 1.3949237088061017`, 0.3567264406830608`,
  0.23012843132782612`, 0.08829396925964683`, 2593.62904544209`, 0.24948663022889017`, 4.657794257043932`, 17.211142276573653`,
  0.3195315382646666`, 0.22585753237455944`, 11.792491830175715`, 1.`, {{0, 0.0005855043934895961`, 1.042197820411481`},
```

```
{12, 0.00025523249517648904`, 0.4543138414141505`}, {24, 0.000055492490427269274`, 0.0987766329605393`}}},
{0.24418896051468997`, 0.7850580710666257`, 9.594718039690303`, 1.3796447707658`, 0.3888924613344984`,
 0.22950540339413805`, 0.26843428081063014`, 169.97373853516677`, 0.16535615066521753`,
 5.752488029735448`, 334.8105392571657`, 21.36154545012821`, 9.161325134624034`, 148.1428629569330 4`,
 1.`, {{0, 0.008977802866480802`, 15.980489102335827`}, {9, 0.0021097397645640013`, 3.7553367809239226`},
  {18, 0.00015294275106520405`, 0.2722380968960632`}, {27, 0.000018372202616903635`, 0.03270252065808847`}}},
{0.161324838944309`, 0.8594526582032156`, 2.9743924517148237`, 1.2482811580906663`, 0.28485670242217753`,
 0.27691573296739147`, 0.12631097975241765`, 80.56658004023716`, 0.24230111347476613`, 7.982613747504004`,
 60463.398414618045`, 18249.445681317182`, 228.53669342540337`, 1243.1843018650404`, 0.`,
 {{0, 0.02495761937611151`, 44.42456248947849`}, {5, 0.005383038599249481`, 9.581808706664077`},
  {10, 0.0027309532573092524`, 4.861096798010469`}, {15, 0.0017952869421678803`, 3.1956107570588275`},
  {20, 0.0013796781403169792`, 2.455827089764223`}, {25, 0.0011421692996349242`, 2.033061353350165`},
  {30, 0.0009645317867677677`, 1.7168665804466268`}, {35, 0.0008099843822916475`, 1.4417722004791325`},
  {40, 0.000674586181248381`, 1.2007634026221181`}, {45, 0.05464415897664525`, 97.26660297842854`}}},
{0.26772168843838734`, 1.7983273472260084`, 5.340026177070573`, 1.4142901992580086`, 0.4537298076184495`,
 0.2865003384437863`, 0.010579448173665554`, 2804.399167772796`, 0.15280115052346555`, 9.9218473198287`, 41.42050852053327`,
 0.20713529912223858`, 0.20713529912223858`, 41.42050852053327`, 1.`, {{0, 0.0031479586475605284`, 5.603366392657741`}}},
{0.18205384870834052`, 1.3062696845921566`, 5.075170850216699`, 0.8732096494236974`, 0.8323770222253974`,
 0.3345416157316672`, 0.015614629383397784`, 819.3644184370967`, 0.04873823503517111`,
 3.447951588629733`, 90.28689949452875`, 0.8754646299918332`, 0.8124642833601854`, 84.2044361980461`,
 1.`, {{0, 0.004713658687435209`, 8.390312463634674`}, {5, 0.0016858784636162943`, 3.000863665237004`}}},
{0.15466361671507556`, 2.9485738979940246`, 0.2621130142172472`, 1.4016672383633995`, 0.3670414989298545`,
 0.40252781093021794`, 0.06082671195258038`, 80.30981146445014`, 0.15720245773080782`, 9.014567082907547`, 137194.02505755573`,
 42740.89075885058`, 230.1698734349559`, 987.9863889593829`, 0.`, {{0, 0.0750869655609131`, 133.6547986984253`}}},
{0.07129651605023579`, 1.2229228380041173`, 7.261778729610821`, 1.077075564756948`, 0.8974689473036932`,
 0.5583016490383189`, 0.012773781923945723`, 355.9690714870232`, 0.15455215759656415`, 2.741649121941279`, 162.51193480243643`,
 1.904546783816667`, 1.6137857062938301`, 165.2753988554905`, 1.`, {{0, 0.009081559815249992`, 16.16517647114499`},
  {2, 0.00269483952590699`, 4.796814356114442`}, {4, 0.0006323916258003054`, 1.1256570939245436`},
  {6, 0.00015066724130763135`, 0.26818768952758376`}, {8, 1.4721047523603493`*^-6, 0.0026203464592014216`}}},
{0.11901300846945845`, 0.5315793015902206`, 2.6671039936143597`, 1.3880864279120932`, 0.8420811291146328`,
 0.5566538571030129`, 0.2079188053795858`, 171.97845991450583`, 0.13095338568755877`, 2.9760745568663016`,
```

```
  44126.950750833035`, 16323.360292806978`, 229.86061653960076`, 742.9598978033326`, 0.`,
  {{0, 0.018081685429519965`, 32.18540006454554`}, {4, 0.002875376675347078`, 5.118170482117799`},
   {8, 0.0009708313921341475`, 1.7280798779987825`}, {12, 0.0005170448520252693`, 0.9203398366049793`},
   {16, 0.00039154105583079444`, 0.6969430793788141`}, {20, 0.0003495238487525192`, 0.6221524507794841`},
   {24, 0.0003323493150277505`, 0.5915817807493958`}, {28, 0.0003239071187387976`, 0.5765546713550597`},
   {32, 0.0003189569861615784`, 0.5677434353676095`}, {36, 0.03230373555951538`, 57.50064929593737`}}},
{0.23041310070356935`, 3.6790901828404454`, 2.4540732736358937`, 1.23507526691096`, 0.821599531342406`,
  0.33616875277136504`, 0.035023029642451846`, 3607.9630264867633`, 0.21590997641376625`,
  4.226227139273737`, 24.95325756361989`, 0.17558718404337376`, 0.14888770766036`, 19.586650509725548`, 1.`,
  {{0, 0.0009671883380661217`, 1.7215952417576965`}, {32, 0.00052139710067302`, 0.9280868391979756`}}},
{0.07203443304476603`, 0.5285696054477942`, 8.334423467085383`, 0.8854719875782417`, 0.3459640631982801`,
  0.2633909150103768`, 0.03987288196821301`, 568.5739309534505`, 0.056948467212013576`, 6.873946140568169`,
  121.32092100878576`, 5.0543041952161465`, 3.5410185820749294`, 85.64741623554242`, 1.`,
  {{0, 0.00497900619284827`, 8.862631023269921`}, {47, 0.0015301974410529542`, 2.7237514450742584`}}},
{0.16979941581750402`, 2.100179291019132`, 5.184869462011486`, 1.1997264241396737`, 0.9307785088479614`,
  0.3430074594655337`, 0.013577650230569586`, 4088.01556659017`, 0.029357726641530413`,
  9.133680945256565`, 61.4034768838668`, 0.32972354060703907`, 0.3278547699795958`, 61.05065217132644`,
  1.`, {{0, 0.003733313764110284`, 6.645298500116305`}, {5, 0.0009065358009105254`, 1.6136337256207354`}}},
{0.21619782984172958`, 1.7733623067535955`, 0.5980153626590834`, 0.9798687198034648`, 0.7538483940425484`,
  0.5789929247394512`, 0.006264547120133815`, 61.29286164809138`, 0.15848957716004353`, 2.3238471342304834`, 153388.4206672678`,
  37513.99517177614`, 230.18687720563662`, 1196.0923044305098`, 0.`, {{0, 0.09090301513671875`, 161.80736694335937`}}},
{0.04816719755022911`, 1.0519290863610147`, 3.0181083809828984`, 0.7801564927089879`, 0.4325460609784135`,
  0.2938523943485086`, 0.04249246462941157`, 626.2418769059092`, 0.2424260119832079`, 9.623530958234195`,
  177.58049556924993`, 4.169825922956149`, 3.2662688384687613`, 133.9791967104193`, 1.`,
  {{0, 0.009312316637554344`, 16.575923614846733`}, {56, 0.0008701023124375233`, 1.5487821161387914`}}},
{0.2279802782152826`, 2.528677960359964`, 2.539878173580604`, 0.7527299875368043`, 0.8436281080694634`,
  0.27514078202436143`, 0.005327161585143342`, 920.5142066248217`, 0.04689927589992776`,
  5.404864622946203`, 106.74849217596841`, 0.4289355766219013`, 0.24677700164945174`, 95.3277626996755`,
  1.`, {{0, 0.003674131169609434`, 6.539953481904792`}, {4, 0.00213762126672947`, 3.8049658547784566`},
   {8, 0.0010616432859983733`, 1.8897250490771045`}, {12, 0.0003715142428380613`, 0.6612953522517492`}}},
{0.18595171467721877`, 2.938499684754029`, 3.6306522502244434`, 1.1726699653227697`, 0.9888859131507246`,
```

```
0.3480894711653968`, 0.38543337220237905`, 80.27026890715871`, 0.03443629355490174`, 9.328937618149844`,
439769.12189084693`, 120204.63639375265`, 229.2655492254026`, 1347.6172342849165`, 0.`,
{{0, 0.05549198487515628`, 98.77573307777817`}, {5, 0.017864450071331247`, 31.79872112696961 8`},
  {10, 0.008514881698308273`, 15.156489422988722`}, {15, 0.004190202879828544`, 7.458561126094809`},
  {20, 0.0021073337729443026`, 3.751054115840858`}, {25, 0.0010986713941203634`, 1.9556350815342467`},
  {30, 0.0006099351041347777`, 1.0856844853599041`}, {35, 0.0003729491872387465`, 0.6638495532849689`},
  {40, 0.0002573606502112678`, 0.4581019573760567`}, {45, 0.011911140172379861`, 21.20182950683615`}}},
{0.2088003110428162`, 1.665360213541189`, 8.163801498355127`, 1.2665930603692175`, 0.5382347880039544`,
  0.33255923205592086`, 0.03591580004997877`, 88.64295388071032`, 0.08847100949056225`,
  8.845547783450662`, 75417.26309473606`, 18797.836330755854`, 229.2573202224973`, 1542.1102191619152`,
  0.`, {{0, 0.11442641647379825`, 203.67902132336087`}, {24, 0.00022347368512743023`, 0.3977831595268258`},
  {48, 0.00018115895618638433`, 0.32246294201176406`}, {72, 0.00018111775766483062`, 0.32238960864339855`},
  {96, 0.00018110503507388136`, 0.32236696243150886`}, {120, 0.0001810985929533474`, 0.3223554954569584`},
  {144, 0.00018109500660648783`, 0.32234911175954833`}, {168, 0.00018109297344169728`, 0.32234549272622115`},
  {192, 0.00018109181684285166`, 0.322343433980276`}, {216, 0.0012827263586104019`, 2.283252918326516`}}},
{0.21556335034608287`, 3.553132086531601`, 5.919169032409666`, 1.028261766481214`, 0.01873638959650825`,
  0.38505243852661264`, 0.12008970777658311`, 72.3007614977653`, 0.1189730390969882`, 5.747570490343056`,
  115832.83713928123`, 28331.492669762632`, 229.79844139807904`, 1548.5919415254039`, 0.`,
  {{0, 0.07977749032472932`, 142.0039327780182`}, {16, 0.003113020279977139`, 5.541176098359307`},
  {32, 0.0033953320732841544`, 6.043691090445795`}, {48, 0.001825096258797347`, 3.248671340659278`},
  {64, 0.0014653296849185618`, 2.60828683915504`}, {80, 0.0013010253634143268`, 2.3158251468775015`},
  {96, 0.0012293695216058058`, 2.188277748458334`}, {112, 0.0011956751325776227`, 2.128301735988168`},
  {128, 0.001179181035019342`, 2.0989422423344286`}, {144, 0.02321146788160708`, 41.31641282926059`}}},
{0.2061330284551588`, 3.7571620320677477`, 5.463889035312752`, 1.195880973038654`, 0.4606689625278244`,
  0.6539206160067124`, 0.07294517832285098`, 79.70328938032284`, 0.04960591713533524`,
  2.0731473047959454`, 326312.9778560789`, 82698.00464290603`, 229.91300137524453`, 11713.821079496696`,
  0.`, {{0, 0.04223324868369197`, 75.17518265697171`}, {6, 0.011943156737520378`, 21.258818992786274`},
  {12, 0.007125481305119273`, 12.683356723112308`}, {18, 0.006553521679783765`, 11.665268590015103`},
  {24, 0.007271729878170264`, 12.943679183143072`}, {30, 0.008088781600021428`, 14.398031248038142`},
  {36, 0.008366038521530552`, 14.891548568324382`}, {42, 0.008168095629657765`, 14.539210220790823`},
  {48, 0.007828525721341434`, 13.934775783987751`}, {54, 0.7826718222849122`, 1393.1558436671437`}}},
```

```
{0.13099744861910317`, 3.0069806711745155`, 9.6713740346189`, 0.7856145676036781`, 0.3887426527541791`,
  0.16465925249784186`, 0.029006890169876405`, 3080.757337617379`, 0.22037025279153283`, 7.1587428033756435`,
  20.28849897556103`, 0.1334753320498062`, 0.12561706719117674`, 19.104014989183487`, 1.`,
  {{0, 0.0011718661844692235`, 2.085921808355218`}, {19, 0.0002800389547087215`, 0.49846933938152427`}}},
{0.20740821458680525`, 2.460110895835281`, 0.6082454159142117`, 0.8064214695794831`, 0.18139487742338534`,
  0.30057404623847583`, 0.2202140246268176`, 4056.9433443728003`, 0.07208592746284093`, 2.251460510548089`, 5.154319536228488`,
  0.12870452786513434`, 0.08824667774964709`, 3.1054332777366236`, 1.`, {{0, 0.00018476650597000477`, 0.32888438062660846`},
    {9, 0.000045634755333387816`, 0.08122986449430311`}, {18, 5.611667804100467`*^-6, 0.009988768691298831`}}},
{0.20705709543566436`, 1.1783515457685674`, 5.677465659011105`, 1.0594933834891802`, 0.45712361519708566`,
  0.4929063565541889`, 0.07712216778297526`, 129.94158279886685`, 0.033100299542402456`,
  5.957665662265306`, 149139.72924688712`, 37618.9547622294`, 229.45684443881999`, 1229.9765084805053`,
  0.`, {{0, 0.08802713597834848`, 156.6883020414603`}, {16, 0.00030634138717018254`, 0.5452876691629249`},
    {32, 0.00022507039825583284`, 0.40062530889538245`}, {48, 0.00028195423358615726`, 0.5018785357833598`},
    {64, 0.00041748218456401766`, 0.7431182885239515`}, {80, 0.00029515507962736304`, 0.5253760417367063`},
    {96, 0.00023412263273720418`, 0.41673828627222337`}, {112, 0.00022283813556696526`, 0.39665188130919815`},
    {128, 0.00021551752136745757`, 0.3836211880340744`}, {144, 0.0032525970932947294`, 5.789622826064619`}}},
{0.04662251177018917`, 1.087376542683649`, 6.170097112149202`, 0.7648803529340509`, 0.9868309112238562`,
  0.551889656411283`, 0.011192508637080182`, 2374.3826064993154`, 0.12804730636711914`, 7.114439815654695`,
  136.4379886532796`, 1.1507409355626528`, 1.1048519601584392`, 131.07875488355862`, 1.`,
  {{0, 0.007154841575360873`, 12.735618004142353`}, {3, 0.002807143795789583`, 4.996715956505457`}}},
{0.061725502446144886`, 3.13849642385113`, 9.540008859499451`, 1.4660101376451562`, 0.45012796893408935`,
  0.4212144239280121`, 0.02511250628464842`, 120.07361261472948`, 0.1571099365418398`,
  6.445559562130283`, 538.8634237080685`, 7.7259527315725816`, 4.707384594305264`, 543.3821982042989`,
  1.`, {{0, 0.03481058119605968`, 61.962834528986235`}, {4, 0.006486465867467034`, 11.54590924409132`}}},
{0.047554055600075584`, 3.265459078802472`, 8.714860138547323`, 0.8369196863770172`, 0.4962846406178121`,
  0.46335229122842925`, 0.007763546254085084`, 1888.52152221599`, 0.22218896292653229`, 2.633616973587875`, 20.753454841274323`,
  0.04786342918552461`, 0.04786342918552461`, 20.753454841274323`, 1.`, {{0, 0.0015772625679368487`, 2.8075273709275907`}}},
{0.12567467788677716`, 3.0158399320010547`, 4.901658754742801`, 1.351978052591392`, 0.7221310556231506`,
  0.5024953321675816`, 0.09886516020555723`, 193.78321929587645`, 0.11023982026692764`, 3.349767411033996`,
  12011.429039086428`, 4247.9362979664775`, 185.63415666114705`, 665.2971123543801`, 1.`,
  {{0, 0.04564336780948647`, 81.24519470088592`}, {26, 0.004919212729446428`, 8.756198658414641`}}},
```

{0.19691840575768232`, 1.6501723246031572`, 9.011299029553381`, 1.1479337267630148`, 0.646058584475099`,
0.36485695404700413`, 0.027042804382441818`, 3550.5063392884595`, 0.2269993732427253`, 4.86925454673478`,
22.58052204958477`, 0.2564488496255294`, 0.23180996747676472`, 20.280972748157247`, 1.`,
{{0, 0.001029664142968337`, 1.83280217448364`}, {42, 0.0005116897858916139`, 0.9108078188870727`}}},
{0.22383158609367865`, 3.396592529608882`, 7.0059346900996395`, 1.1116596498970173`, 0.7389255639756462`,
0.40169511261271784`, 0.008944445083981989`, 1191.861359234809`, 0.06327031154858104`, 2.975039312230072`,
50.09786658408003`, 0.13332515564748135`, 0.13315079147395895`, 50.070317979574114`, 1.`,
{{0, 0.0035028309911201093`, 6.235039164193795`}, {4, 0.00030251317532752336`, 0.5384734520829916`}}},
{0.22089456196581753`, 2.0637610394456862`, 7.594372198729954`, 1.2863006630967566`, 0.5843792508368972`,
0.43898418015861007`, 0.0169422428941798`, 186.99955924932385`, 0.09805501720710208`,
5.47194276574208`, 357.82707975079154`, 4.244795348928369`, 2.9592171641571476`, 356.3495008408816`,
1.`, {{0, 0.022843676171294524`, 40.66174358490425`}, {7, 0.00423888589261248`, 7.545216888850216`}}},
{0.09910547662807062`, 2.346178866312991`, 7.421049922557871`, 1.3147434313884303`, 0.2750558853148206`,
0.21685738835966661`, 0.006760522552087531`, 4988.988013498636`, 0.18492351030582366`, 2.8590709623133`, 4.8884755885299205`,
0.014073959384580264`, 0.014073959384580264`, 4.8884755885299205`, 1.`, {{0, 0.000371524144728274`, 0.6613129776163276`}}},
{0.20816183484471307`, 3.9485705583569546`, 6.550493120626773`, 0.7774564054459185`, 0.5499519895881679`,
0.2003723071725072`, 0.34567679795977113`, 1808.1918719614162`, 0.23441319529101012`, 4.082991759791533`, 24.46088933504238`,
0.3520750523604996`, 0.3520750523604996`, 24.46088933504238`, 1.`, {{0, 0.0018590275894632211`, 3.3090691092445335`}}},
{0.16112203008102222`, 2.4895492142589735`, 0.44352326574872514`, 1.2469876196885359`, 0.9780986797319231`,
0.6188841396088389`, 0.029743069155058133`, 112.64100571320267`, 0.13296296295840093`, 7.528063016828725`, 112098.87372591226`,
33943.45439379531`, 229.83017814093927`, 1188.7797556425396`, 0.`, {{0, 0.09034726142883301`, 160.81812534332275`}}},
{0.17781253097781763`, 1.7213960002432414`, 4.693599924413801`, 1.2010501070587627`, 0.4002263609998786`,
0.5127527245385753`, 0.08027502692564248`, 156.85332391803223`, 0.06371789077354856`, 6.562151621871855`,
68421.52213659421`, 19260.434488323568`, 228.46072772202234`, 1112.6458424755206`, 0.`,
{{0, 0.06994607664384397`, 124.50401642604228`}, {27, 0.0012695015805902665`, 2.2597128134506743`},
{54, 0.0012857196034869937`, 2.288580894206849`}, {81, 0.0013013668773571006`, 2.316433041695639`},
{108, 0.0012726037391476105`, 2.265234655682747`}, {135, 0.0012687783628048197`, 2.2584254857925794`},
{162, 0.0012679352693054215`, 2.25692477936365`}, {189, 0.0012677713725901339`, 2.2566330432104382`},
{216, 0.001267738917365845`, 2.256575272911204`}, {243, 0.004413591661647413`, 7.8561931577323945`}}},
{0.11782384895680592`, 1.1169968183756591`, 9.741344822271138`, 0.8926206302886914`, 0.732042643272727`,
0.38545027561144396`, 0.005148133378435907`, 521.4771678665235`, 0.04124959681193141`,

```
8.98600497767481`, 343.69395222225114`, 1.4034784827892934`, 1.386523614561914`, 339.6991252095682`, 1.`,
 {{0, 0.021941425527126048`, 39.05573743828436`}, {6, 0.0038757079888011364`, 6.8987602200660225`}}},
{0.1669047046062308`, 1.025805255669411`, 7.1552343684117155`, 0.8176510429563385`, 0.8282389242605148`,
 0.29891546072913033`, 0.009889011750027802`, 94.42085507888534`, 0.16558325818795072`,
 8.472484566593426`, 22505.774134398995`, 6510.395603853672`, 24.60141712984409`, 641.8151492142495`,
 1.`, {{0, 0.03728646099388444`, 66.36990056911431`}, {4, 0.0016810034326064267`, 2.9921861100394396`},
  {8, 0.001636811376944175`, 2.9135242509606316`}, {12, 0.0016359059538773137`, 2.9119125979016185`},
  {16, 0.0016358692581128621`, 2.9118472794408947`}, {20, 0.00163586707135643 9`, 2.9118433870144615`},
  {24, 0.0016358669082143238`, 2.9118430966214963`}, {28, 0.0016301663452869806`, 2.9016960946108257`}}},
{0.1009959491070086`, 1.5581491170930981`, 4.3701504080039815`, 1.3889561652621691`, 0.5506040634320337`, 0.37952236600496847`,
 0.006611879537937531`, 361.09151974058886`, 0.21353768992235467`, 8.563496620010564`, 307.91252075697554`,
 2.1957450296001118`, 1.5378557461546398`, 306.0900375747318`, 1.`, {{0, 0.021445350557369156`, 38.1727239921171`},
  {6, 0.0017437174138672758`, 3.103816996683751`}, {12, 0.00007377488444318238`, 0.13131929430886463`}}},
{0.23246941421316458`, 3.062147704374147`, 7.181406357346838`, 1.3012169805011569`, 0.348059834110821`,
 0.639530677915989`, 0.09989941363865933`, 83.25264127475623`, 0.015153685907020209`, 6.841473199486905`,
 1.0484676335373272`*^6, 242557.57234810587`, 228.00444043626575`, 1647.3339805675685`, 0.`,
 {{0, 0.07256427276569793`, 129.16440552294233`}, {4, 0.003992265398090591`, 7.106232408601252`},
  {8, 0.0009648006044439511`, 1.717345075910233`}, {12, 0.0005880393361226486`, 1.0467100182983144`},
  {16, 0.0005150741681007913`, 0.9168320192194086`}, {20, 0.0005068045804136042`, 0.9021121531362157`},
  {24, 0.0005308143730017773`, 0.9448495839431635`}, {28, 0.000591172391505519`, 1.052286856879824`},
  {32, 0.0006961779275018094`, 1.239196710953221`}, {36, 0.04424796097825656`, 78.76137054129669`}}},
{0.05240567594084544`, 2.6092929572780967`, 9.364433651132018`, 1.4357965076882815`, 0.3985784064329525`,
 0.6640850131228939`, 0.14135898686373455`, 340.71602063464445`, 0.17921846525178464`, 4.7134535629319085`, 225.40676807168447`,
 3.9612669438832118`, 3.912726167337091`, 222.85358994910567`, 1.`, {{0, 0.014883244828406632`, 26.492175794563803`},
  {2, 0.001902283865690872`, 3.386065280929752`}, {4, 0.00015134414203452605`, 0.26939257282145634`}}},
{0.186251758126929`, 2.4933710973488976`, 3.5328550575209547`, 1.325175018028979`, 0.37317622556186136`,
 0.25977278720819974`, 0.09748704597661054`, 108.88467391318876`, 0.2369656062609477`, 9.08676709988832`,
 43221.349195109644`, 11742.505198842806`, 229.25250358599354`, 1250.1228243385247`, 0.`,
 {{0, 0.06761246026805334`, 120.35017927713494`}, {31, 0.003025158224899525`, 5.384781640321153`},
  {62, 0.0029807123125388164`, 5.305667916319092`}, {93, 0.002927227599535608`, 5.210465127173381`},
  {124, 0.0029209953740170903`, 5.199371765750421`}, {155, 0.00292024105604162 4`, 5.1980290797540905`},
```

{186, 0.0029201491270727208`, 5.197865446189443`}, {217, 0.0029201379654774226`, 5.197845578549812`},
{248, 0.0029201366092188917`, 5.197843164409627`}, {279, 0.0038621161128728435`, 6.874566680913661`}}},
{0.2432348147394116`, 1.8751950266382789`, 2.5453519666394815`, 1.0050719073546701`, 0.45557496345664417`,
0.6934059109337196`, 0.0796473645979629`, 116.90111969840831`, 0.13836004707768368`, 5.732847922404471`, 91128.56424834066`,
20340.861847055665`, 229.29851100804942`, 1479.4968303881194`, 0.`, {{0, 0.11244175910949708`, 200.1463312149048`}}},
{0.10016679782659832`, 2.355527283731478`, 1.9202752019167662`, 1.4361517107451491`, 0.549370310329528`,
0.5953848202767148`, 0.3151585002444098`, 3628.217577021284`, 0.16393396333788363`, 2.9174291385165`, 15.074600454037883`,
0.37952204834744635`, 0.25669843746637105`, 9.78889544124877`, 1.`, {{0, 0.0005663580390931807`, 1.0081173095858618`},
{5, 0.00015965649012067567`, 0.2841885524148028`}, {10, 0.000017941524321050014`, 0.031935913291469026`}}},
{0.14701675506563494`, 1.5420515267654622`, 0.49939909194610793`, 1.0246394569387973`, 0.7670831420663697`,
0.6948893607838256`, 0.005182980912963712`, 1858.6105410956102`, 0.23965764027041103`,
6.073448972699818`, 1000.3214305472883`, 314.21780749176884`, 23.6774974150652`, 104.30705069108326`, 1.`,
{{0, 0.006362729754854313`, 11.325658963640677`}, {6, 0.00020674382327362135`, 0.36800400542704603`},
{12, 0.00017517561248183255`, 0.31181259021766194`}, {18, 0.00017482127919151748`, 0.31118187696090116`},
{24, 0.00017480210719747054`, 0.3111477508114976 5`}, {30, 0.00017479992205280098`, 0.3111438612539858`},
{36, 0.00017479955231192027`, 0.3111432031152181`}, {42, 0.00017479947345611392`, 0.3111430627518828`},
{48, 0.00017479945406428027`, 0.3111430282344189`}, {54, 0.00013386487363845734`, 0.23827947507645408`}}},
{0.19166603750475464`, 2.2499911524034095`, 2.4198836269597095`, 0.7671801018390442`, 0.14979093568204394`,
0.302546021842622`, 0.008837119919208427`, 4450.048280356515`, 0.1035437468879743`, 7.585339524339819`, 15.357066200668918`,
0.05490306997154907`, 0.05490306997154907`, 15.357066200668918`, 1.`, {{0, 0.0011671370312508378`, 2.077503915626491`}}},
{0.14239569401382052`, 2.8387649854604886`, 1.4027026171382353`, 1.2048713691971291`, 0.21617980215068333`,
0.2290581611576099`, 0.32444520029725066`, 2595.236024076599`, 0.07657553404881084`, 7.455893283173221`, 24.916355486371895`,
0.5016837388867641`, 0.23265443315679787`, 11.206963419250137`, 1.`, {{0, 0.0007084641147620543`, 1.2610661242764565`},
{10, 0.0001351347993130553`, 0.24053994277723847`}, {20, 8.130305787900784`*^-6, 0.014471944302463395`}}},
{0.14874493002083228`, 3.1275671408493704`, 8.341714492380408`, 1.302209514279003`, 0.6258906648056601`,
0.45549735048188655`, 0.04029116024037646`, 765.0281459953038`, 0.12017823562381152`, 2.4241463017742984`,
56.116400371856514`, 0.47095260067659495`, 0.40826730131123257`, 46.79642625253653`, 1.`,
{{0, 0.0025589078990216904`, 4.55485606025861`}, {33, 0.0009976204961710858`, 1.775764483184533`}}},
{0.06222670957040222`, 3.9065456744185374`, 1.0946055811329174`, 0.8246557540237`, 0.5907991824162977`,
0.2630274030573405`, 0.006980770676502568`, 3176.3819350381427`, 0.13217715504568522`, 5.816838705721813`,
23.875326558918164`, 0.06418393742570058`, 0.06338880158906722`, 23.81101328588761`, 1.`,

```
{{0, 0.001469764143591412`, 2.616180175592713`}, {6, 0.0003398728661360465`, 0.6049737017221628`}}},
{0.04971186392349858`, 0.9916432478771275`, 0.9981695561230755`, 0.8524884405534577`, 0.1203482174371421 9`,
 0.2529703866830304`, 0.3237478827730366`, 360.7437887352434`, 0.010006209625245316`, 7.274213959868414`,
 100108.64796647927`, 58505.180580586086`, 230.04276853547165`, 614.9653370970806`, 0.`,
 {{0, 0.025506383770070855`, 45.40136311072612`}, {7, 0.0008841849257814014`, 1.5738491678908944`},
  {14, 0.00027231618405207733`, 0.4847228076126977`}, {21, 0.00021083053269002456`, 0.37527834818824374`},
  {28, 0.00019605526611452315`, 0.34897837368385126`}, {35, 0.0001906860844374233`, 0.3394212302986135`},
  {42, 0.00018848210071634744`, 0.3354981392750984`}, {49, 0.00018745576431940638`, 0.33367126048854334`},
  {56, 0.00018691022126579308`, 0.33270019385311167`}, {63, 0.01891406076993028`, 33.667028170475895`}}},
{0.1940949007109463 7`, 1.638712293913553`, 0.39703017050116074`, 0.7643187357680644`, 0.5335846868412271`,
 0.21830616448247264`, 0.0963055671350066`, 635.9574479204244`, 0.22613729069446092`, 5.479828782022301`,
 5226.009218756005`, 1381.147498706492`, 228.77372117678382`, 2753.3411277442647`, 0.`,
 {{0, 0.010890033938492728`, 19.384260410517054`}, {31, 0.005341222298576901 6`, 9.507375691466883`},
  {62, 0.0033143840477690384`, 5.899603605028888`}, {93, 0.00210225733473619`, 3.742018055830418`},
  {124, 0.001362069325351616`, 2.424483399125876`}, {155, 0.0009113206597615843`, 1.6221507743756203`},
  {186, 0.0006356205508566069`, 1.1314045805247603`}, {217, 0.0004662421893216262`, 0.8299110969924947`},
  {248, 0.00036146917018978447`, 0.6434151229378163`}, {279, 0.18386930619350803`, 327.2873650244443`}}},
{0.27029725962327555`, 2.240924310463286`, 1.2051421639323703`, 1.1356504301287609`, 0.773380004303778`,
 0.6607812449412671`, 0.04949916223108672`, 204.2592199369304`, 0.19899441283798802`, 7.18276351221955`, 45644.00349640439`,
 9375.75189592115`, 229.67696749216054`, 1422.2841513784313`, 0.`, {{0, 0.10809359550476078`, 192.40659999847418`}}},
{0.14266269539818904`, 3.072017618366117`, 3.4103252419872643`, 0.7755852439360265`, 0.43949005760262017`,
 0.1924018543570919`, 0.0671210280508097 7`, 656.0250616869308`, 0.04537130588160604`, 5.285505421294628`,
 77.40683502854972`, 0.8561621617523025`, 0.5825167742323624`, 52.31210159978514`, 1.`,
 {{0, 0.002349068345507347`, 4.181341655003077`}, {12, 0.0011709244820895758`, 2.084245578119445`},
  {24, 0.0003853458640056788`, 0.6859156379301082`}, {36, 0.00007038102998106872`, 0.12527823336630234`}}},
{0.10769494377900635`, 0.832286650700361`, 9.122967245453143`, 0.9815088864732582`, 0.8421400260693508`,
 0.5448142839433918`, 0.07976087110962524`, 4710.110387525812`, 0.13361893143992154`, 5.1516712920505725`,
 34.095338229861866`, 1.4035979413318018`, 0.9408315974768962`, 22.86360908624493`, 1.`,
 {{0, 0.0013474483935928116`, 2.3984581405952046`}, {24, 0.00039018589696180315`, 0.6945308965920096`}}},
{0.21491444350519606`, 1.295137699490157`, 7.08600395884199`, 0.8977823469890502`, 0.4181882258302472`,
 0.21987671758091132`, 0.14871331978208877`, 664.8642422386233`, 0.19734382780045878`,
```

```
6.556011365474522`, 98.52364269830204`, 3.3760123178322097`, 1.636681854290492`, 48.82963648918074`,
1.`, {{0, 0.002838241769628316 4`, 5.052070349938403`}, {13, 0.0007743642965200791`, 1.378368447805741`},
  {26, 0.00009800626227397612`, 0.1744511468476775`}, {39, 4.4004475536430776`*^-7, 0.0007832796645484678`}}},
{0.1490469101160943 3`, 3.9586033442731177`, 2.425615791755316`, 1.0187686401741827`, 0.6625310044158794`,
0.43390068486512956`, 0.00854220404180854`, 96.98019438566024`, 0.2008209408788944`, 6.643325956478872`,
52483.96610627175`, 16731.214973438313`, 228.03663828647242`, 1100.8298843137977`, 0.`,
{{0, 0.04237879742503249`, 75.43425941655785`}, {1, 0.04128427378281614`, 73.48600733341273`}}},
{0.21251186737978806`, 3.785250570140386`, 5.706304857162573`, 0.8638958511126991`, 0.620802282746526`,
0.4325048599697877`, 0.008982590727965567`, 699.3542650472853`, 0.09331259312392975`, 7.885660147589962`,
198.6373289803386`, 0.4409929321893687`, 0.43788866212379146`, 197.65723067892523`, 1.`,
{{0, 0.013284876675153535`, 23.647080481773294`}, {5, 0.0017370728564447828`, 3.091989684471714`}}},
{0.24515641699904833`, 3.817676488470438`, 5.668256418323743`, 1.3090245110359662`, 0.8705848781731611`,
0.5675799494003652`, 0.13511363154659006`, 1804.7556662922755`, 0.20417175811210386`, 6.336735207604568`, 122.94450041578052`,
1.4851355655917682`, 1.4851355655917682`, 122.94450041578052`, 1.`, {{0, 0.00934378203159932`, 16.63193201624679`}}},
{0.16393709899357184`, 1.7364683461504136`, 6.294465981846198`, 0.7908159510076651`, 0.7611075592144951`, 0.36561813704297086`,
0.008326625223173748`, 2834.6413370290525`, 0.20899318399811945`, 1.1709155007061636`, 7.919504357196832`,
0.037888008888923595`, 0.037888008888923595`, 7.919504357196832`, 1.`, {{0, 0.0006018823311469592`, 1.0713505494415876`}}},
{0.25878729889995966`, 3.1361195765858056`, 8.880297290408144`, 1.0867236136347516`, 0.0902130745148968`,
0.5352173770607672`, 0.11164972929109021`, 57.41366920823615`, 0.17534323232121918`,
1.541035034614442`, 112794.8560829351`, 23992.48058428929`, 230.08015622659116`, 1509.327483895863`,
0.`, {{0, 0.06628313488775199`, 117.98398010019852`}, {15, 0.00361201270938453`, 6.4293826227044635`},
  {30, 0.005372797480801107`, 9.56357951582597`}, {45, 0.002385064407541456`, 4.245414645423791`},
  {60, 0.0016712336701663183`, 2.974795932896046`}, {75, 0.0012370881940936653`, 2.202016985486724`},
  {90, 0.001028515547709069`, 1.8307576749221428`}, {105, 0.0009161564068534283`, 1.6307584041991023`},
  {120, 0.0008511245919865262`, 1.5150017737360166`}, {135, 0.031351760879797505`, 55.806134366039565`}}},
{0.11203607225462164`, 0.6218658244264867`, 6.440483663444866`, 1.39680689202378`, 0.16534679246130501`,
0.5081267228820908`, 0.03214478110427463`, 1906.9275025607635`, 0.21932209633484323`, 1.251120855111619`, 7.230030948388517`,
0.23325550069144407`, 0.23325550069144407`, 7.230030948388517`, 1.`, {{0, 0.0005494823520775273`, 0.9780785866979986`}}},
{0.24357827172423074`, 3.9240029450424014`, 2.804272198831022`, 0.75931848861878`, 0.2959071515485636`,
0.6138549707668646`, 0.10713705928623629`, 339.1202760730188`, 0.035220064551167996`,
7.297254233929856`, 30837.35402677289`, 6847.042064452546`, 229.07530859434863`, 1216.8302672118807`,
```

```
  0.`, {{0, 0.08006857264480424`, 142.52205930775156`}, {16, 0.007940888155415266`, 14.134780916639174`},
    {32, 0.00037426999825486266`, 0.6662005968936555`}, {48, 0.002303642322915345`, 4.100483334789314`},
    {64, 0.00083423925476626 7`, 1.4849458734839551`}, {80, 0.0000791738339744187`, 0.14092942447446527`},
    {96, 0.00014367257757302054`, 0.2557371880799766`}, {112, 0.00007439628163125812`, 0.13242538130363943`},
    {128, 0.000033617785332890576`, 0.05983965789254522`}, {144, 0.0006266274534353754`, 1.1153968671149683`}}},
{0.24204908128005126`, 1.6039606621918692`, 7.422941916054803`, 1.4757185212808246`, 0.48127457855983957`,
  0.4526414340963527`, 0.017845029630840916`, 286.8959659308558`, 0.1351657578784522`, 4.5748150305594475`,
  234.98183715576994`, 2.1319845144633116`, 2.0587432010099183`, 229.88807483005522`, 1.`,
  {{0, 0.015001233547577451`, 26.70219571468786`}, {8, 0.0024702601395067457`, 4.397063048322008`}}},
{0.0765658571842559`, 1.8453576022836256`, 0.8953985896867352`, 1.0948080634821278`, 0.11609992165592442`,
  0.45437290836767896`, 0.20627344098654643`, 689.3327160072474`, 0.18998846953804188`,
  8.643335566773064`, 3592.048018278957`, 1686.288163530658`, 229.05649463338997`, 563.8628480403461`,
  0.`, {{0, 0.03382596151987901`, 60.21021150538463`}, {6, 0.0037723658608185225`, 6.71481123225697`},
    {12, 0.0002521993223274497`, 0.4489147937428605`}, {18, 0.00011052396259859596`, 0.1967326534255008`},
    {24, 0.00010436460998142283`, 0.18576900576693264`}, {30, 0.00011139847354020521`, 0.19828928290156528`},
    {36, 0.00012037892542110477`, 0.2142744872495665`}, {42, 0.00011403649853245934`, 0.2029849673877776`},
    {48, 0.00010341121864961701`, 0.18407196919631827`}, {54, 0.00433893605931791`, 7.723306185585879`}}},
{0.2258389851785662`, 0.888336357939937`, 0.32961763645801767`, 0.9210788231382718`, 0.8596897387491109`,
  0.5585019604624183`, 0.04381971178906294`, 415.715019082411`, 0.24817184929202513`, 7.285653344604734`, 13479.357265858447`,
  3185.4443040570727`, 229.87446225536527`, 1074.1102068047774`, 0.`, {{0, 0.08163237571716309`, 145.3056287765503`}}},
{0.19210976086993148`, 0.9419570933782317`, 5.851922457612897`, 1.40657571618961`, 0.8492777725664675`,
  0.5465312705852027`, 0.10025464283588155`, 725.5272924080149`, 0.05639485661927951`,
  6.120948580338062`, 268.9483672482861`, 11.102901501101766`, 8.871210042798086`, 217.28534569725494`,
  1.`, {{0, 0.009584128249882572`, 17.059748284790977`}, {2, 0.004640330451346838`, 8.259788203397372`},
    {4, 0.0018460774044764365`, 3.286017779968057`}, {6, 0.0004431501672855294`, 0.7888072977682423`}}},
{0.04040676752094921`, 0.7630185090261814`, 4.034875094487921`, 0.9393317585048341`, 0.5565744536568444`,
  0.3001585479016161`, 0.008819784570716005`, 3975.779851508773`, 0.23610962920225392`, 2.0975088246946054`, 6.788814298312004`,
  0.0676539477951859`, 0.0676539477951859`, 6.788814298312004`, 1.`, {{0, 0.0005159498866717123`, 0.918390798275648`}}},
{0.2323130275502685`, 0.49681822670142894`, 1.2539687147478098`, 0.9468502150118296`, 0.13096405194857463`,
  0.3219849827616931`, 0.04046745571036271`, 95.23490611137812`, 0.01783356001323902`, 6.936606555558118`, 780592.5082509076`,
  180732.15672202612`, 229.66119011526806`, 1216.180073587518`, 0.`, {{0, 0.09242968559265138`, 164.52484035491946`}}},
```

```
{0.1110467064554368`, 3.3883763096282147`, 5.159131249779884`, 1.2482205179674581`, 0.8201347270890553`,
  0.46628896390017405`, 0.2764030660881924`, 2912.733505822841`, 0.03458542948115101`, 2.6312047625809307`,
  23.575177885540555`, 0.3924725086266436`, 0.19614618131244313`, 11.31816847362382`, 1.`,
  {{0, 0.0005912654485034429`, 1.0524524983361285`}, {10, 0.0002689153554919673`, 0.47866933277570184`}}},
{0.1632243866872038`, 2.5373481239797906`, 9.958152546995578`, 1.2439631103015851`, 0.6072525242529792`,
  0.6395983432777625`, 0.3899581087433961`, 56.26697227139985`, 0.18832987989003086`, 4.752251898931052`,
  97167.93252372758`, 29054.657674408238`, 228.21268642197884`, 1384.8163676827085`, 0.`,
  {{0, 0.06588514594118686`, 117.2755597753126`}, {4, 0.022474277322836304`, 40.00421363464863`},
    {8, 0.0074531640832760345`, 13.266632068231342`}, {12, 0.002729271509511436`, 4.858103286930357`},
    {16, 0.00109898299653 2879`, 1.9561897338285248`}, {20, 0.0004834003906112014`, 0.8604526952879386`},
    {24, 0.00023820841288479358`, 0.4240109749349326`}, {28, 0.00013823978659090717`, 0.24606682013181475`},
    {32, 0.00009675697178207352`, 0.1722274097720909`}, {36, 0.004648596528673346`, 8.274501821038555`}}},
{0.16346002078838484`, 3.218794874634134`, 9.025936619626364`, 1.1259348004332868`, 0.7415896232848811`,
  0.4862549245509259`, 0.364081252118343`, 137.81289300851844`, 0.05819144446151425`, 8.24717117891944`,
  672.8629758010734`, 18.81059138823567`, 12.198559182500398`, 556.7304030963317`, 1.`,
  {{0, 0.037330454531714276`, 66.4482090664514`}, {7, 0.00498105610360693`, 8.866279864420335`}}},
{0.19514620721272602`, 2.87732243599938`, 0.26739185721478975`, 1.4891953183216857`, 0.05289250725423611`,
  0.4217001319921505`, 0.03447210696714538`, 218.70480172461387`, 0.0713049851341147`, 6.944425315128572`, 131416.31876315633`,
  34690.870523593`, 230.13792638331623`, 1048.6370638797162`, 0.`, {{0, 0.07969641685485843`, 141.859622001648`}}},
{0.06305409129179324`, 2.891634576752894`, 4.887865973915014`, 1.4252999692587962`, 0.37958790888649707`,
  0.5644849529569385`, 0.3706753829912659`, 1888.9507831786343`, 0.129560920022812`, 6.557119455026179`, 51.592938499520955`,
  1.0264744353915818`, 1.0264744353915818`, 51.592938499520955`, 1.`, {{0, 0.003921063325963593`, 6.9794927202151955`}}},
{0.07134729442997761`, 1.925040188749243`, 1.1575324502817335`, 0.9987470940766925`, 0.48237853230783223`,
  0.6023915002525595`, 0.11093155711801116`, 121.08543179928522`, 0.23351841944906743`, 5.375810156733433`, 29915.823911628922`,
  14790.360380110285`, 229.46257141678765`, 913.8577862789757`, 0.`, {{0, 0.06945319175720216`, 123.62668132781985`}}},
{0.1360789534218197`, 0.4455509561284261`, 2.009215820725272`, 0.8936493225292423`, 0.96227260378033`, 0.5587588217121244`,
  0.041826495849356216`, 90.5779416890061`, 0.15671264889903508`, 7.812316525319764`, 68111.03518287199`,
  23098.686410980947`, 224.83714129742526`, 1209.6712463780455`, 0.`, {{0, 0.09193501472473146`, 163.644326210022`}}},
{0.15849386520968983`, 3.640724074855333`, 1.8861164484754733`, 1.1569149894265711`, 0.5885808127893892`,
  0.1748767871381529`, 0.4650269726463 2115`, 193.59851086045592`, 0.1503277806325949`, 4.212693145809347`,
  26164.56895608105`, 7997.862451153615`, 14.354764035133297`, 115.59561713701977`, 1.`,
```

```
{{0, 0.008044397619308225`, 14.319027762368641`}, {26, 0.0002908024691234178`, 0.5176283950396837`},
  {52, 0.00006247948949775323`, 0.11121349130600075`}, {78, 0.00005474472882278718 6`, 0.0974456173045612`},
  {104, 0.000054171807424780654`, 0.09642581721610957`}, {130, 0.0000541079999423 2209`, 0.09631223989733333`},
  {156, 0.00005410044570491884`, 0.09629879335475554`}, {182, 0.00005409954494584818`, 0.09629719000360978`},
  {208, 0.00005409943744676603`, 0.09629699865524353`}, {234, 0.00006226336019668534`, 0.1108287811500999`}}},
{0.2646664636377358`, 0.7835752608143567`, 7.19251403558616`, 1.494052846937745`, 0.8510899770532778`,
 0.5885640033353514`, 0.48048609718479995`, 154.5393756420333`, 0.18357560397946`, 4.027566835631127`,
 8249.340663343237`, 1689.9856855812072`, 21.682731417307522`, 284.49970249458795`, 1.`,
 {{0, 0.019687938158513746`, 35.04452992215447`}, {2, 0.0012656088417068736`, 2.2527837382382345`},
  {4, 0.0001388928674937568`, 0.2472293041388871`}, {6, 0.00008651894566870003`, 0.15400372329028605`},
  {8, 0.00008392527142048142`, 0.1493869831284569`}, {10, 0.00008375867606406887`, 0.14909044339404257`},
  {12, 0.00008374459076835808`, 0.14906537156767738`}, {14, 0.00008374313988575817`, 0.14906278899664951`},
  {16, 0.00008374296904674508`, 0.14906248490320623`}, {18, 0.000024103929020197295`, 0.04290499365595118 5`}}},
{0.12495672263621771`, 3.9102582538632182`, 1.1196234634441904`, 1.2648993449094799`, 0.05034343178950351`,
 0.5637162084123208`, 0.0377865951253564`, 87.92444212100203`, 0.05653305553347371`, 1.253921938823833`, 264111.2768128192`,
 94826.15245960628`, 230.0845101012478`, 885.2092843306689`, 0.`, {{0, 0.06727590560913084`, 119.7511119842529`}}},
{0.2203214214659513 5`, 2.68397072068061`, 2.6883455170430324`, 1.1162234719994681`, 0.40997309115380154`,
 0.5558969738438672`, 0.02165064984593792`, 1526.6454168541877`, 0.24054798278325668`, 4.966530798126495`, 49.03536488580379`,
 0.32433737596842843`, 0.32433737596842843`, 49.03536488580379`, 1.`, {{0, 0.003726687731321088`, 6.633504161751537`}}},
{0.10964967041383433`, 0.7460930180313401`, 6.531951119775776`, 1.1455194328360876`, 0.1524629724176152`,
 0.28792352560938195`, 0.09608079533773954`, 1051.3203547256705`, 0.17626541392472717`,
 8.294356744338359`, 70.12448646374816`, 3.4068172121712728`, 1.929976975113108`, 40.83651398418684`, 1.`,
 {{0, 0.0025048480841775595`, 4.458629589836056`}, {28, 0.0005987269786206401`, 1.0657340219447395`}}},
{0.07988018646299105`, 2.6019950195572488`, 9.606629629213668`, 1.0662798233306474`, 0.7693895670648874`,
 0.32124913017390155`, 0.04062772179058327`, 1822.0454711227774`, 0.21556365885863799`,
 7.4522296585239225`, 77.67789447755827`, 0.7468666325011134`, 0.5449053258710589`, 56.36255279601884`,
 1.`, {{0, 0.003187930248167922`, 5.674515841738901`}, {41, 0.0010956237643295098`, 1.9502103005065277`}}},
{0.1682679068810577`, 1.5454861727740674`, 1.469740171862469`, 0.9427424173065856`, 0.7350490973518766`,
 0.3805201167376455`, 0.012952191039290932`, 4650.232844583341`, 0.019462289964433777`, 1.394906478790226`,
 5.782774890399873`, 0.05841464781507852`, 0.058014509647637054`, 5.759461754780917`, 1.`,
 {{0, 0.0003691723482467881`, 0.6571267798792828`}, {5, 0.00006854674511656162`, 0.12201320630747971`}}},
```

```
{0.1878353522665755`, 2.6490780379134238`, 9.484290061448991`, 1.1885927795104771`, 0.9576534469556572`,
  0.2804046795618802`, 0.13562182147346188`, 68.8621810881709`, 0.06230339869012863`, 8.081807561887658`, 129865.38196915698`,
  35102.31071357749`, 15.283757613265806`, 702.8376509951507`, 1.`, {{0, 0.050029352732192`, 89.05224786330176`},
    {4, 0.002932182921314458`, 5.219285599939735`}, {8, 0.0001623568238866624`, 0.288995146518259`},
    {12, 0.000052059870500807855`, 0.09266656949143798`}, {16, 0.000048106480220114472`, 0.08562953479180421`},
    {20, 0.00004793224562669648`, 0.08531939721551975`}, {24, 0.00004791989267053275`, 0.08529740895354829`},
    {28, 0.000047918690831047964`, 0.08529526967926536`}, {32, 0.00004783181838913873`, 0.08514063673266693`}}},
{0.195165600718623`, 2.3826254740878197`, 7.662410402528582`, 0.9519993324167042`, 0.5246436284800724`,
  0.22761517290878341`, 0.02889008666683709`, 101.1296763305189`, 0.18152629399038878`, 2.6385617746928904`,
  8075.8965636638795`, 2090.2918376079683`, 2.0070309717260346`, 175.57865023892322`, 1.`,
  {{0, 0.009439305525466396`, 16.801963835330184`}, {4, 0.002987906251950709`, 5.318473128472261`},
    {8, 0.0006611092451633885`, 1.1767744563908316`}, {12, 0.00014656242983786166`, 0.2608811251113937`},
    {16, 0.000040021363474916074`, 0.07123802698535062`}, {20, 0.000018875651755036206`, 0.033598660123964445`},
    {24, 0.000015078174727839543`, 0.026839151015554383`}, {28, 0.000014484350221602289`, 0.025782143394452076`},
    {32, 0.000014401489045747117`, 0.025634650501429868`}, {36, 6.23293651466816`*^-6, 0.011094626996109325`}}},
{0.21914596002979703`, 3.4188550030486784`, 4.710500615641845`, 1.4527326168664971`, 0.03624911304432854`,
  0.28384065291200533`, 0.4254237737178384`, 3505.5630456480862`, 0.12312118964896007`, 9.273808474100267`, 21.994509170033602`,
  0.3738262415484731`, 0.3738262415484731`, 21.994509170033602`, 1.`, {{0, 0.0016715826969225538`, 2.975417200522146`}}},
{0.1814951436259516`, 1.11858432682377`, 5.7029405020905966`, 0.816783193035002`, 0.6766309251156815`,
  0.4321734260024682`, 0.2931263656368919`, 87.29527865588257`, 0.044724422492498916`, 2.4174522941648533`,
  160375.63566669732`, 44612.514293739536`, 230.17034559615922`, 960.6890058582703`, 0.`,
  {{0, 0.05585074463898289`, 99.41432545738954`}, {20, 0.008638964266920486`, 15.377356395118467`},
    {40, 0.001292166746911452`, 2.3000568095023843`}, {60, 0.00027841708026535083`, 0.4955824028723245`},
    {80, 0.0001331180903557479 2`, 0.23695020083323132`}, {100, 0.00010686250869304748`, 0.19021526547362452`},
    {120, 0.00009898871649783746`, 0.17619991536615065`}, {140, 0.00009542199186654153`, 0.16985114552244396`},
    {160, 0.00009340603198474442`, 0.16626273693284505`}, {180, 0.006424274372750444`, 11.435208383495791`}}},
{0.22203693974513244`, 0.9431525918127432`, 4.196712511517475`, 1.3261224346879132`, 0.8036004422541301`,
  0.6271444136150295`, 0.10733581071086257`, 369.9451385562586`, 0.16273465181192748`, 5.731309489564488`,
  225.90071966344982`, 12.121876874434493`, 9.999434966762738`, 218.19995226876827`, 1.`,
  {{0, 0.015665018696391354`, 27.88373327957661`}, {2, 0.0006713976559359684`, 1.1950878275660237`},
    {4, 0.000074687934202293`, 0.13294452288008152`}, {6, 0.00006360428035002288`, 0.11321561902304073`},
```

```
{8, 0.0000634644676954835`, 0.11296675249796062`}, {10, 0.00004502333785126848`, 0.08014154137525789`}}},
{0.18924733350115397`, 2.8150904404250037`, 2.4574844917932026`, 0.8737719488383665`, 0.6977173227044986`,
 0.3482841124888072`, 0.48079795163551986`, 94.66261771766344`, 0.16471072416530214`, 1.3078470560939532`, 57313.14013302701`,
 15468.970992231965`, 230.26324482030645`, 913.1603491933721`, 0.`, {{0, 0.06940018653869628`, 123.53233203887937`}}},
{0.07907817004881895`, 1.0481324229512046`, 3.8137728667258273`, 1.2920968808020492`, 0.04181350128653394`,
 0.5803710922764521`, 0.02851300126208627`, 266.99553906648964`, 0.22548933562284668`,
 2.0140176417924156`, 2720.1059443241925`, 1246.0977957015787`, 228.80795012889305`, 619.7135497934252`,
 0.`, {{0, 0.001975239872374726`, 3.5159269728270126`}, {2, 0.00196001525867780 9`, 3.4888271604465007`},
  {4, 0.0011730593045438055`, 2.0880455620879737`}, {6, 0.0005551708624719077`, 0.9882041351999957`},
  {8, 0.0002459671990444626`, 0.4378216142991434`}, {10, 0.00010803512876786698`, 0.19230252920680324`},
  {12, 0.0000480575056685467`, 0.08554236009001312`}, {14, 0.000022200703828874808`, 0.039517252815397155`},
  {16, 0.000011174568115659744`, 0.019890731245874343`}, {18, 0.04099930938080665`, 72.97877069783584`}}},
{0.22302087067747528`, 1.8201511620752422`, 1.875184623833773`, 0.7813352529024251`, 0.8705057770695381`,
 0.22074020090969038`, 0.1367588752741694`, 187.45561858905666`, 0.07793294287132913`,
 8.923384483271061`, 51526.866471983536`, 12281.710464838616`, 228.9616900600276`, 1105.5115319361055`,
 0.`, {{0, 0.07138067003642234`, 127.05759266483179`}, {31, 0.00935713315535197`, 16.655697016526506`},
  {62, 0.001268539768643422`, 2.2580007881852913`}, {93, 0.00026992443284677573`, 0.4804654904672607`},
  {124, 0.00014015925467587454`, 0.2494834733230567`}, {155, 0.00011916788451322887`, 0.2121188344335474`},
  {186, 0.00011400642773114805`, 0.20293144136144353`}, {217, 0.00011240383691980339`, 0.20007882971725`},
  {248, 0.00011186432333964842`, 0.19911849554457417`}, {279, 0.0011450073066997966`, 2.038113005925638`}}},
{0.21429795289589076`, 2.896671823601787`, 8.636702505614757`, 0.9811380648364925`, 0.20610104158853804`,
 0.1778381348410949`, 0.007936414694970003`, 185.13169573811163`, 0.014685687390926405`, 3.2353159752463547`,
 132.5848029001317`, 0.35573564909215344`, 0.35571482424227807`, 132.70534146292533`, 1.`,
 {{0, 0.009572586779519795`, 17.039204467545236`}, {8, 0.0005130191716625312`, 0.9131741255593055`}}},
{0.17507336986882482`, 0.7983306290353371`, 2.750450064120228`, 0.8050075785267179`, 0.7818959542274213`,
 0.6526925477724106`, 0.09653256947066893`, 72.78219945862553`, 0.07700161447215975`, 3.655530763820291`, 178673.84361342076`,
 51014.7612697059`, 227.70993478320165`, 1263.6049170243111`, 0.`, {{0, 0.09603397369384765`, 170.94047317504882`}}},
{0.1470766374450453`, 1.2533511161361544`, 4.593526904612924`, 1.479552314271993`, 0.7400477950348978`,
 0.5768087033937906`, 0.10619660427114413`, 242.62879808709684`, 0.045931800134058565`,
 3.771378090721337`, 10744.69302168057`, 3419.650218114266`, 7.743102998565684`, 171.80888221006063`,
 1.`, {{0, 0.01197149887482627`, 21.30926799719076`}, {3, 0.0008027456320706625`, 1.4288872250857794`},
```

```
{6, 0.00006571135006985667`, 0.11696620312434486`}, {9, 0.00004498700656416582 5`, 0.08007687168421516`},
{12, 0.00004457393155690575 3`, 0.07934159817129224`}, {15, 0.00004456339310492418`, 0.07932283972676504`},
{18, 0.00004456281844727168 4`, 0.0793218168361436`}, {21, 0.00003883204132455347`, 0.06912103355770517`}}},
{0.0788585810797619`, 2.548378832414274`, 3.111482597871378`, 0.9346713900878518`, 0.19963135196405957`,
0.5058944800586684`, 0.03749384602431879`, 1118.4164778142415`, 0.22872744797764172`, 1.4303214452036404`, 14.58640832427198`,
0.15771399637545064`, 0.15771399637545064`, 14.58640832427198`, 1.`, {{0, 0.0011085670326446706`, 1.9732493181075135`}}},
{0.24182502946725698`, 1.8945200327623395`, 6.300131593939071`, 0.7669603268824658`, 0.6959163653444247`,
0.5105300710554361`, 0.2329066367569749`, 178.13280663193103`, 0.21420184435482204`, 3.590310973659246 2`, 684.3658492283569`,
113.922042944103`, 6.511496840528573`, 208.77561908191177`, 1.`, {{0, 0.014424733835485098`, 25.67602622716347`},
{2, 0.0011596158303861684`, 2.0641161780873793`}, {4, 0.00008628981093153648`, 0.15359586345813492`},
{6, 0.00003536878038240698`, 0.06295642908068443`}, {8, 0.00003336239906128416 3`, 0.05938507032908581`},
{10, 0.0000332759762627805`, 0.05923123774774928 5`}, {12, 0.00003327066566845162`, 0.0592217848898438 8`},
{14, 0.00003327019775420278`, 0.05922095200248095`}, {16, 0.00002775955429336512 3`, 0.04941200664218992`}}},
{0.05758087297506348`, 2.9919516996565125`, 9.945704556467732`, 1.1770757830874041`, 0.6136122924727272`,
0.31603398268032157`, 0.21009959511709828`, 118.22678602178958`, 0.2258513747270965`, 6.274152149731609`,
518.6124611616934`, 10.939539558820295`, 6.2670342757067745`, 335.56246225077314`, 1.`,
{{0, 0.022466294512313686`, 39.99000423191836`}, {15, 0.003036452618745075`, 5.404885661366234`}}},
{0.12816326660379745`, 2.7095777054297967`, 7.985014815061977`, 0.8582103259977079`, 0.28527001685481057`,
0.4561351167228034`, 0.41006088059385215`, 4928.696897400591`, 0.12257221770331383`, 5.545857987270676`, 13.653307289162441`,
0.29013769700094194`, 0.29013769700094194`, 13.653307289162441`, 1.`, {{0, 0.0010376513539763455`, 1.8470194100778952`}}}};
PatientsTrainingSet = resultMD[[All, 1 ;; 10]];
```

In[ ]:=

```
(* The scripts hidden in this group of cells have to be initialized to run the following codes *)
```

In[ ]:=

```
(* Results for parameter sweep for a single dose in a training set *)
result = {{"\!\(\*SubscriptBox[\(κ\), \(c\)]\)", "\!\(\*SubscriptBox[\(κ\), \(p\)]\)",
    "γ", "V", "\!\(\*SubscriptBox[\(k\), \(s\)]\)", "ρ", "ω", "α", "\!\(\*SubscriptBox[\(k\), \(f\)]\)",
    "\!\(\*SubscriptBox[\(N\), \(0\)]\)", "\!\(\*SubsuperscriptBox[\(A\), \(cur\), \(sim\)]\)",
    "\!\(\*SubsuperscriptBox[\(A\), \(cur\), \(est\)]\)", "Err", "\!\(\*SubscriptBox[\(M\), \(m\)]\)", "Canc",
    "\!\(\*SubsuperscriptBox[\(Bl\), \(Frg\), \(sim\)]\)", "\!\(\*SubsuperscriptBox[\(Bl\), \(Frg\), \(est\)]\)", "Err",
```

```
"\!\(\*SubsuperscriptBox[\(Bl\),\(Ab\),\(sim\)]\)", "\!\(\*SubsuperscriptBox[\(Bl\),\(Ab\),\(est\)]\)", "Err",
"\!\(\*SubsuperscriptBox[\(Cld\),\(Frg\),\(sim\)]\)", "\!\(\*SubsuperscriptBox[\(Cld\),\(Ab\),\(sim\)]\)", "ToVbl",
"\!\(\*SubsuperscriptBox[\(fD\),\(Max\),\(sim\)]\)", "\!\(\*SubsuperscriptBox[\(fD\),\(Max\),\(est\)]\)", "Err", "Nnew"},
{0.172487255493398`, 1.2765963995094278`, 0.2324603897081641`, 0.9331633444796544`, 0.5595329534566904`,
 0.18253234530133355`, 0.2215389709063929`, 78.80610179612091`, 0.19190036455619353`, 1.548441048845115`,
 67743.09410144144`, 0, 0, 0, 0.6852674042781265`, 0.10718857420740713`, 0, 0, 19554.94224955615`, 0, 0,
 1.9548078271675062`, 48185.40457082622`, 4.008069782940419`, 1.0000000000003644`, 0, 0, 0.05191808718578425`},
{0.14090572053825962`, 0.941193557649755`, 3.8052282638940014`, 1.32622706664442`, 0.616835667267962`,
 0.48175991442091237`, 0.08306143231635953`, 874.0744302472489`, 0.12491077781228116`, 5.511666182290989`,
 116.46970588923584`, 0, 0, 0, 53.87426457434697`, 4.315726531299664`, 0, 0, 0.08363006331181534`, 0, 0,
 58.02762868339093`, 0.16834220470863487`, 1.3599387416101831`, 0.7310244216325208`, 0, 0, 0.5110180678336491`},
{0.19800522656535097`, 1.47676715957736`, 1.6845843495593211`, 1.0829526404841552`, 0.6930225215765629`,
 0.2749384033948816`, 0.06680029700083288`, 3207.8452923633004`, 0.18818451872370445`, 6.655704647717006`,
 31.887329398445925`, 0, 0, 0, 16.478864419030586`, 0.6931194467673891`, 0, 0, 0.02421221660802215`, 0, 0,
 14.622514809293001`, 0.06848779193029836`, 0.43414946439205965`, 0.3789034695609528`, 0, 0, 0.40056255200378343`},
{0.2711930593993829`, 3.0021543969280486`, 4.914701090552741`, 1.464602766667476`, 0.8471176050384048`,
 0.28257943270869035`, 0.04953192991909177`, 1933.8633413872876`, 0.19773521340575795`, 9.992985154010565`,
 105.13570106511645`, 0, 0, 0, 61.94141836278736`, 0.9815746806714228`, 0, 0, 0.02318432518635069`, 0, 0,
 42.09769633558496`, 0.08982040110563455`, 1.0647436647159723`, 0.28815869775973646`, 0, 0, 0.43962980242854593`},
{0.27990766836391096`, 3.477149477100016`, 9.931945787531824`, 0.8846854018858359`, 0.8967333599095253`,
 0.5903168559967787`, 0.24882968868487906`, 980.8551320933655`, 0.02004206946214948`, 1.1376503486553808`,
 42.727467045547805`, 0, 0, 0, 9.53729452918055`, 0.6521216712025372`, 0, 0, 0.02897630434645414`, 0, 0,
 32.39320754324975`, 0.11586699696319083`, 0.24177553336415214`, 0.49835109389883314`, 0, 0, 0.06389331109327692`},
{0.11737985888146019`, 1.1225833279930164`, 5.309079794927442`, 0.8606501327539497`, 0.8514188342619913`,
 0.5642039473140849`, 0.008986110654413243`, 2018.9822529242097`, 0.06040743860925685`, 4.536035630396375`,
 73.55385408728918`, 0, 0, 0, 65.13966916603187`, 0.4872861326413754`, 0, 0, 0.02119073517368962`, 0, 0,
 7.814561263791457`, 0.03553379291831031`, 0.46864576046452`, 0.40857714960977276`, 0, 0, 0.25798090396825835`},
{0.17121040978700802`, 0.4138167442315588`, 6.781135564610604`, 1.3665406840239216`, 0.9405996253397015`,
 0.6832439228870748`, 0.04185895434111264`, 62.7113536738392`, 0.18142736225630413`, 8.46259785026923`,
 126972.27674519586`, 0, 0, 0, 298.1254945315542`, 25.354892784275624`, 0, 0, 36710.36451460944`, 0, 0,
 149.88970260470268`, 89788.52217109627`, 29.778277025817353`, 1.000000000000286`, 0, 0, 0.9990782166212182`},
```

```
{0.07382619562964193`, 0.625700255424527`, 9.939999934198667`, 1.4556453073248168`, 0.9580351575689623`,
  0.4163162212955882`, 0.006430521616962508`, 92.19524127790851`, 0.19149999891406427`, 4.303065608360628`,
  10092.366546722247`, 0, 0, 0, 336.4548319381863`, 3.0304371986770398`, 0, 0, 4733.347998532603`, 0, 0,
  27.08779041800309`, 4992.072504612018`, 10.739730236128622`, 0.9999999999999984`, 0, 0, 0.7137046293076434`},
{0.1627307868596377`, 1.5327585946689206`, 6.648641489637967`, 1.0898921981857375`, 0.08271984399349908`,
  0.5169005126785295`, 0.006231976105310307`, 2017.0154068595314`, 0.2464092033815996`, 1.6313338993270357`,
  8.432667214624587`, 0, 0, 0, 7.733066884282825`, 0.029388353758957216`, 0, 0, 0.005578197887301527`, 0, 0,
  0.643503597245891`, 0.012967779020847576`, 0.190737104776319`, 0.10304664105276695`, 0, 0, 0.3179124752554388`},
{0.08926805346894312`, 1.228740834290055`, 5.100082063336529`, 1.0507919032716577`, 0.3229790419061651`,
  0.45218339761418835`, 0.024533377777917375`, 814.1470590464116`, 0.1414263079606694`, 5.247711540674161`,
  79.62930256895696`, 0, 0, 0, 59.267347626410526`, 1.0936076543521438`, 0, 0, 0.025010263836254435`, 0, 0,
  19.196576879923473`, 0.031894536705833865`, 1.4402240475823056`, 0.39845435605455803`, 0, 0, 0.6932296020886753`},
{0.17431851399024534`, 2.020213173041662`, 9.224968841892476`, 1.3485881267881612`, 0.8890825947176819`,
  0.5177484922558413`, 0.03701694525181502`, 131.5752033573906`, 0.228949740900746`, 4.795606274383001`,
  443.45874279587423`, 0, 0, 0, 259.0825396937692`, 4.43540653733214`, 0, 0, 14.872912654837938`, 0, 0,
  128.0066673501924`, 37.03748618140094`, 8.661545697586357`, 0.9999999995956493`, 0, 0, 0.9785932185552205`},
{0.25439204271280724`, 0.8534844729419486`, 2.8150623547133353`, 0.8024992452769948`, 0.43579385025887163`,
  0.4594767236090769`, 0.057877631906885676`, 1195.6419878079198`, 0.17833211044546654`, 5.195270679096961`,
  61.19257325377502`, 0, 0, 0, 33.83687275287343`, 2.06271165727129`, 0, 0, 0.03075389554574142`, 0, 0,
  25.149891023391465`, 0.11176494727508911`, 0.9581936401948361`, 0.5558870737465285`, 0, 0, 0.6093891684973874`},
{0.129274073243755`, 2.8469118022753515`, 4.121632588773114`, 1.2196787674185057`, 0.8228855513020883`,
  0.4828002917324088`, 0.013782155470303842`, 388.66064646424104`, 0.2255512748456478`, 1.0022663108918284`,
  37.4176094379522`, 0, 0, 0, 29.4879073769192`, 0.13654781137290922`, 0, 0, 0.7806644759706806`, 0, 0,
  5.5534225110343485`, 1.441709666363287`, 0.5920060413350425`, 0.9999924873036613`, 0, 0, 0.16352024139151572`},
{0.06343574194222029`, 3.9924293708982113`, 9.079106395846292`, 1.3161791897123671`, 0.10938228479468126`,
  0.3240611741083924`, 0.010321331270591745`, 76.94152103093134`, 0.06038801289350482`, 9.425890207979073`,
  70205.26592740085`, 0, 0, 0, 617.0349251028783`, 1.5271193117917539`, 0, 0, 36458.849768206324`, 0, 0,
  87.09879990376076`, 33039.91693437305`, 27.081181575773556`, 1.000000000000078`, 0, 0, 1.1313574553864445`},
{0.110036074735397`, 2.490203037930516`, 9.729597307787852`, 1.4397421355398403`, 0.8533380591052817`,
  0.37040757295392324`, 0.047916566872126407`, 665.4588326486067`, 0.16916230254726894`, 1.9013228378216027`,
  67.32591358102374`, 0, 0, 0, 40.13822181606069`, 0.7401561002292918`, 0, 0, 0.04490276463618321`, 0, 0,
```

26.330556704768394`, 0.07058462807615701`, 0.5937917974429145`, 0.4840972551092325`, 0, 0, 0.1007400788020962`},
{0.21512225225566667`, 1.75750084110844`, 0.8044386006474246`, 1.3520587790631349`, 0.026316391204000755`,
0.16031186333306224`, 0.13761957869990635`, 493.8180531489964`, 0.06250485252841559`, 2.6863460670624115`,
31526.667301351725`, 0, 0, 0, 5.7374860567496535`, 0.41344711426966924`, 0, 0, 7736.013217670315`, 0, 0,
10.380480729754288`, 23774.122669497756`, 1.1123862754205833`, 1.0000000000000093`, 0, 0, 0.0968571533989443`},
{0.15874286665935028`, 2.268211451796655`, 1.8981682437883747`, 1.375959688601855`, 0.24814706074480242`,
0.5134877163486878`, 0.3316652449509965`, 685.632474632321`, 0.05319219913715695`, 1.7896119638851753`,
31.96808009735873`, 0, 0, 0, 5.723056881609258`, 0.7253203465303132`, 0, 0, 0.6172839032770692`, 0, 0,
23.50257023173107`, 1.3998488049839315`, 0.6308164743113699`, 0.9990402409674988`, 0, 0, 0.40176322274149523`},
{0.07432908705186819`, 3.0619407712521785`, 1.7905302646779082`, 0.9549392645174688`, 0.353226782755657`,
0.6119520610188782`, 0.0496137109124893`, 317.5186768305259`, 0.07812208014742161`, 6.620973402603631`,
20840.6317126216`, 0, 0, 0, 60.48673906551274`, 0.9317428431738233`, 0, 0, 10058.187504108044`, 0, 0,
40.75630571194792`, 10680.227065383699`, 4.815192177506494`, 1.0000000000000064`, 0, 0, 1.125521406931183`},
{0.1228443083266203`, 2.0512729485932484`, 1.9256217622349043`, 1.4810122884336692`, 0.07741458536098089`,
0.4641958329015734`, 0.007197139507585793`, 240.76714600918612`, 0.03301937102418029`, 9.328377628444095`,
131731.62537204823`, 0, 0, 0, 133.12817715678864`, 0.44449474733410377`, 0, 0, 47763.62158287618`,
0, 0, 13.025429299974743`, 83821.27223604081`, 8.468469846397717`, 1.`, 0, 0, 1.0021975742950489`},
{0.2606519449147342`, 3.818605783771848`, 8.595720359336667`, 1.112914365339814`, 0.820058745442926`,
0.4441576207168524`, 0.053270363092160024`, 1117.1606910609416`, 0.20810873381921946`, 6.830482236754131`,
158.217216712755`, 0, 0, 0, 90.32808338603802`, 1.2200679224930844`, 0, 0, 0.023366782525737022`, 0, 0,
66.55654893466564`, 0.08700853302474741`, 1.273332899934598`, 0.3626997465843119`, 0, 0, 0.37693957444604737`},
{0.24750930586460296`, 3.1846993652287674`, 3.026718658979185`, 1.4845570571034035`, 0.869864794311124`,
0.6669561203654737`, 0.2363643381944309`, 1924.496556700219`, 0.06437651322808385`, 2.548184450019704`,
51.48595308643028`, 0, 0, 0, 11.838152098414339`, 0.8394163885406494`, 0, 0, 0.13636790852796`, 0, 0,
38.18984056782889`, 0.48217609117090143`, 0.2806487892452064`, 0.8351464246350968`, 0, 0, 0.188360875388165`},
{0.24908529437358162`, 3.626300618072209`, 2.0230682918259397`, 1.4602193573319078`, 0.40130475942739396`,
0.6650543311625685`, 0.019582320105998748`, 125.14663606859848`, 0.03716084844005629`, 8.149858668846527`,
485275.3769879387`, 0, 0, 0, 103.34858566354733`, 0.541837019969642`, 0, 0, 106429.33765097226`, 0, 0,
28.069484577290076`, 378714.0414111101`, 13.938126391391329`, 1.000000000000063`, 0, 0, 0.6879769444359256`},
{0.2795730004018654`, 2.183215118244356`, 0.7910515142573491`, 1.2847699393543137`, 0.70345738565049`,
0.2632113724473232`, 0.1394636484917589`, 223.91272255356017`, 0.16150366940434352`, 9.859315316872873`,

```
53076.817736040095`, 0, 0, 0, 20.24333656208575`, 1.2211784841523583`, 0, 0, 10616.405231097064`, 0, 0,
  38.08707612394481`, 42400.86091342664`, 8.984599844368203`, 1.000000000001575`, 0, 0, 0.33363349472311576`},
{0.08969576723701717`, 0.4935601120720627 4`, 0.2529879905685366`, 1.1041769014657603`, 0.7443718717514693`,
  0.6180257213599576`, 0.03574805853679543`, 3205.3226496399375`, 0.061225880931137455`, 7.560768798013438`,
  2171.9947059835604`, 0, 0, 0, 11.112362854328696`, 0.6868508337658673`, 0, 0, 944.7622127219953`, 0, 0,
  4.842888207003865`, 1210.5881646663056`, 0.5500948044981915`, 0.9999999999999967`, 0, 0, 1.3729366406278294`},
{0.10298476113266081`, 3.435925289367793`, 4.954566778741102`, 0.9299871443466801`, 0.6352691398464292`,
  0.5470174017743717`, 0.00550714898658944 1`, 973.5524715904572`, 0.132851360968853 3`, 5.618259356059234`,
  117.49696230705861`, 0, 0, 0, 108.82106480134435`, 0.16901447170440292`, 0, 0, 0.0362899394001093`, 0, 0,
  8.29601567997564`, 0.05339015343770032`, 1.2448752933815868`, 0.5637226440483438`, 0, 0, 0.5222944997497027`},
{0.14277679649227548`, 3.0428582592918065`, 7.414614443890604`, 1.1046799505745009`, 0.2872163059276376`,
  0.23677108379761724`, 0.04608733451503689`, 2695.430951291806`, 0.1274330436692639`, 8.066834406130887`,
  27.508200949789227`, 0, 0, 0, 16.827455730939224`, 0.2399406828065002`, 0, 0, 0.00329055233667431 1`, 0, 0,
  10.43007840596963`, 0.00671163601886364`, 0.6414452374245303`, 0.062102275311818`, 0, 0, 0.6932870847812999`},
{0.18738335776550036`, 1.508373316422973`, 6.602438992729994`, 0.7532357689229783`, 0.8880958875806098`,
  0.4853414284787141`, 0.10975729606650726`, 2118.598035369477`, 0.05093222572935868`, 5.0448517662608`,
  76.70717437665523`, 0, 0, 0, 30.152081431348936`, 2.062569830305099`, 0, 0, 0.013018988902814879`, 0, 0,
  44.44464707558958`, 0.034850597933199036`, 0.4926114049094028`, 0.30970139788639983`, 0, 0, 0.24295238136663233`},
{0.061980185747317895`, 2.54906136842855`, 8.170240160100523`, 1.0602973698223939`, 0.7221801534586956`,
  0.6067985789160708`, 0.3830106200657996 7`, 81.55317476370394`, 0.23749102694434165`, 4.188403663256118`,
  22951.475716120684`, 0, 0, 0, 50.828471892691006`, 6.645904161706482`, 0, 0, 12014.150102668335`, 0, 0,
  242.01167938406448`, 10637.703642279172`, 12.050133884605344`, 0.9999999999999992`, 0, 0, 0.7907448228697149`},
{0.2562298689236346`, 3.2913375042385793`, 9.509882785943699`, 1.013989937500057`, 0.7792587778840148`,
  0.49077497767429623`, 0.021163604915718004`, 145.96678185214677`, 0.05845136191000838`, 7.439112841768198`,
  627.823017294315`, 0, 0, 0, 467.54872550802116`, 2.870264203324775`, 0, 0, 4.783784735009045`, 0, 0,
  134.95726027823258`, 17.510693365860277`, 11.695328836932138`, 0.9999999978600449`, 0, 0, 1.2103433598707598`},
{0.2313093023364764`, 2.988629640967633`, 6.855539828479465`, 1.0799498768994156`, 0.4522471358822151`,
  0.2285321965248518`, 0.04827992453162874`, 1204.6728692817303`, 0.13073517219860892`, 1.9378468299164582`,
  16.257879702552408`, 0, 0, 0, 9.734941782863014`, 0.14838698613263554`, 0, 0, 0.009028320800266611`, 0, 0,
  6.33533921556925`, 0.029833351222570893`, 0.3416993786251959`, 0.16396532992320656`, 0, 0, 0.14777542398238708`},
{0.2663151909473739`, 2.2417238355718707`, 0.9398485531379794`, 1.0477480676389026`, 0.7226958973603346`,
```

0.5716146939488362`, 0.007506086251562624`, 76.16131590346896`, 0.024911856209502148`, 4.070747059506875`,
954570.6446215768`, 0, 0, 0, 27.240681578816293`, 0.0876924791752946`, 0, 0, 198676.22852994598`, 0, 0,
2.808318868109256`, 755864.2533950963`, 11.263422543948309`, 1.0000000000000069`, 0, 0, 0.2756279026641679`},
{0.1992919692967660 1`, 1.9362608032362312`, 0.5353176928827672`, 0.7753335837033051`, 0.8607728477687919`,
0.15482089410819944`, 0.02026432754563482`, 192.89182731397838`, 0.1831217833074511`, 5.333726168282188`,
25098.2949487175`, 0, 0, 0, 16.832505736651342`, 0.16752429125285953`, 0, 0, 6518.448603603513`, 0, 0,
4.633867410612032`, 18558.206556741323`, 5.600408897991077`, 1.000000000001345`, 0, 0, 0.13965757044260615`},
{0.10696417975467426`, 1.9440197080088568`, 2.0936739600677434`, 1.1829890119130342`, 0.8864433537218006`,
0.5974069860908109`, 0.31351620960128274`, 84.46268853549806`, 0.02353560759327844`, 4.656274919049821`,
572513.3395312632`, 0, 0, 0, 15.609951394631532`, 2.2684509853665826`, 0, 0, 226431.5209369653`, 0, 0,
62.998763174353535`, 346000.8843946555`, 12.23863480062775`, 1.000000000000286`, 0, 0, 0.5811789555379678`},
{0.21459986298539707`, 1.6769788549349993`, 9.356964926187835`, 1.2601407238694713`, 0.895239496536256`,
0.38446102196425924`, 0.012005779035619358`, 146.09102757400478`, 0.02879957197754268`, 2.3826604675291154`,
193.3328311433502`, 0, 0, 0, 158.73957240461607`, 1.0659101113960512`, 0, 0, 1.9392171215859675`, 0, 0,
25.535838829608362`, 5.9450818370183605`, 3.6383279149492043`, 0.9999999665113224`, 0, 0, 0.3104099907350998`},
{0.07383925157057758`, 2.6358268091067414`, 6.251616293116599`, 1.1087155777827833`, 0.9205185682116173`,
0.19659269091732745`, 0.29632431074176147`, 531.6045072192474`, 0.12599923353908937`, 2.5100327097656248`,
79.86268730333829`, 0, 0, 0, 16.01828676867377`, 1.6484978580661422`, 0, 0, 0.05949932473593963`, 0, 0,
62.07364070065391`, 0.0627626515351894`, 0.9671079186658935`, 0.680115980217955`, 0, 0, 0.0948365295512122`},
{0.0817054148315825`, 2.796991931647562`, 7.6919124752432`, 0.9355088496592321`, 0.8578766380044134`,
0.4976427721124693`, 0.008010560795693933`, 67.84344037448854`, 0.080482215315478`, 7.8347514420046025`,
92958.46968521253`, 0, 0, 0, 454.23945465571876`, 1.2453927234059647`, 0, 0, 42659.58423111119`, 0, 0,
49.762191415700954`, 49793.12894493963`, 25.94960471701653`, 1.0000000000000016`, 0, 0, 1.0851682251877146`},
{0.23618801128357925`, 0.8353216061115365`, 4.620412867513723`, 1.4504705173961072`, 0.3108708881036355`,
0.3400688454787608`, 0.014061322572830477`, 2071.8308884477033`, 0.0364017192015193`, 3.610034284058532`,
18.55464388397803`, 0, 0, 0, 15.46537846505077`, 0.2344826609461253`, 0, 0, 0.010912129974419132`, 0, 0,
2.798120470668914`, 0.036818775393231105`, 0.38298554235939963`, 0.14841974544056524`, 0, 0, 0.4102708513984332`},
{0.1662441272521787`, 3.725772450959033`, 9.814227303865422`, 1.3148509050359762`, 0.25298305094984697`,
0.32303330970203903`, 0.055784478318004704`, 213.77275876328028`, 0.04990125708904308`, 9.833393949415544`,
466.6166735795884`, 0, 0, 0, 263.83326207866`, 3.7355284106241533`, 0, 0, 0.06462441888239463`, 0, 0,
198.82469774397546`, 0.15347757308971366`, 10.077154442444469`, 0.6512817269992872`, 0, 0, 1.0813263206266797`},

```
{0.22831788422124638`, 2.590756298379799`, 9.795768388114066`, 1.125355926333821`, 0.3650031672541203`,
  0.1680578019226482`, 0.4143448211959925`, 831.1374036828946`, 0.21412864715242685`, 3.6048538714465157`,
  36.91913379485488`, 0, 0, 0, 6.0942373753121775`, 0.8100593739778663`, 0, 0, 0.007951813901167277`, 0, 0,
  29.980948931353613`, 0.02593630465196313`, 0.9110259063453554`, 0.14102749767699185`, 0, 0, 0.2315638436862914`},
 {0.23359626472155715`, 2.56674003123695`, 1.1969232842409259`, 1.0442579072771299`, 0.8882810964774697`,
  0.3176999965402193`, 0.007340467537817918 5`, 1908.0313626467632`, 0.2040761417499009`, 9.648978920291999`,
  91.99309767384098`, 0, 0, 0, 81.80779411021146`, 0.22480149934642626`, 0, 0, 0.37772279339753806`, 0, 0,
  8.24295724935086`, 1.2604947662550834`, 1.0623402223119554`, 0.9984625645942601`, 0, 0, 0.6210452897612784`},
 {0.2534162537452666`, 1.6838285982829593`, 0.8034274134000761`, 0.870316935261816`, 0.7856829676536639`,
  0.46427070430809425`, 0.12261242146523575`, 210.18201198829036`, 0.1377493870208059`, 1.4006481974514937`,
  42825.83088445403`, 0, 0, 0, 3.2648574074127836`, 0.2226653540419275`, 0, 0, 9267.280386498287`, 0, 0,
  5.35614701403706`, 33549.70682790549`, 1.3999479013669103`, 1.0000000000000007`, 0, 0, 0.09018742377791931`},
 {0.25695579183074796`, 3.423363059474246`, 4.134350766656727`, 1.2838485736639544`, 0.4149036036442244`,
  0.6493708976181658`, 0.12875465899507466`, 1172.2742202486922`, 0.012611679323919878`, 3.4839902382375243`,
  48.702991447874965`, 0, 0, 0, 17.511948122187576`, 0.6216523980056863`, 0, 0, 0.03583194013995375`, 0, 0,
  30.402026502374998`, 0.1315317793070353`, 0.6763770526054163`, 0.44344726123802747`, 0, 0, 0.5333666313252329`},
 {0.17682432929033887`, 2.45185381012344`, 3.9338825987195882`, 1.2879615271399696`, 0.02702036645869277`,
  0.6694394004648141`, 0.11330549796901213`, 154.96108716979026`, 0.11583322861965717`, 8.805457336592767`,
  62075.72329942178`, 0, 0, 0, 112.03221704654264`, 4.883048713426663`, 0, 0, 17523.167392950818`, 0,
  0, 171.03602275762168`, 44264.60459001253`, 12.638706545489008`, 1.`, 0, 0, 1.1176483827463262`},
 {0.2509750819824499`, 3.8883567883501247`, 6.384989889678234`, 1.4613334209290016`, 0.7183189010632474`,
  0.4965470763778975`, 0.3859108436384297`, 748.6242458418322`, 0.020020198926280153`, 5.690775103224375`,
  170.7024644094458`, 0, 0, 0, 27.51216914856034`, 2.5266988536944233`, 0, 0, 0.06774702896279644`, 0, 0,
  140.35295199827402`, 0.24289737354303692`, 1.6132276002711672`, 0.6253733053317605`, 0, 0, 0.4282447957611487`},
 {0.1441623498715573`, 3.343288457669903`, 1.621696793707331`, 1.3375455077897458`, 0.06814809058713811`,
  0.4010236514346772`, 0.3002310191555227`, 155.99799531014958`, 0.10001900063604485`, 4.2763953522223215`,
  79261.33786662415`, 0, 0, 0, 11.038068702257304`, 0.9081687320150765`, 0, 0, 25888.86948936154`, 0, 0,
  43.375286276610275`, 53317.1465872061`, 5.848386997227198`, 1.0000000000005629`, 0, 0, 0.34611033087063475`},
 {0.2657479684896014`, 3.193863728015164`, 2.3963245604206165`, 1.4113571594678591`, 0.26744798738201037`,
  0.235299669124016`, 0.018584060295236755`, 466.949287196137`, 0.23679723903863448`, 8.690245518267893`,
  169.65722705089624`, 0, 0, 0, 132.32820284668716`, 0.730937257935614`, 0, 0, 0.6661125970682856`, 0, 0,
```

33.35019993679177`, 2.5288295636604`, 4.02174804841339`, 0.9953683071925011`, 0, 0, 0.8247132317345214`},
{0.18653936584035707`, 1.0683088955449334`, 5.185702152338592`, 0.9522710688882444`, 0.206752787597013`,
0.5240389817239608`, 0.06412446666117125`, 220.00685924183844`, 0.2055887216336732`, 7.885811409920794`,
991.1698661262881`, 0, 0, 0, 193.33399449579008`, 10.43390802795337`, 0, 0, 171.4020104812677`, 0, 0,
159.23766802229005`, 456.7603191276858`, 8.541748380285116`, 0.9999999999975049`, 0, 0, 1.6356876412557833`},
{0.07587231017464335`, 3.3343312881276663`, 8.762311039358057`, 0.8284399428795388`, 0.17449762015817138`,
0.34311440561675743`, 0.03578987408308253`, 1364.0076205471958`, 0.21103062426335195`, 8.577574022027257`,
60.89652107606923`, 0, 0, 0, 40.733300373690845`, 0.41432231599909114`, 0, 0, 0.004460976668674021`, 0, 0,
19.73554088007551`, 0.004835208649842515`, 1.3951068425481417`, 0.10944570623616079`, 0, 0, 1.0639702272693774`},
{0.1141532151024588`, 1.4516849606811304`, 7.634928187688176`, 0.8394863515309923`, 0.0282734940185132`,
0.6576494992828068`, 0.009900377337816624`, 932.466930942293`, 0.19488805826375527`, 9.750042758265863`,
122.34910866029524`, 0, 0, 0, 107.37265105301785`, 0.6838380596098153`, 0, 0, 0.009785880239781032`, 0, 0,
14.181677523956232`, 0.015958424171120936`, 2.5196697274032465`, 0.22155497504055477`, 0, 0, 2.15413464441722`},
{0.21903027892555205`, 2.3035966499415492`, 1.6904861150255108`, 0.7741880968122689`, 0.5538208564897962`,
0.4216122395122083`, 0.4439804577081556`, 61.55285726796139`, 0.16334632191055676`, 6.147879562247702`,
109561.838109268`, 0, 0, 0, 11.718227588417857`, 2.051820308996737`, 0, 0, 26515.0012184266`, 0, 0,
67.52237700124147`, 82965.5444654762`, 20.883070045574417`, 1.0000000000004252`, 0, 0, 0.3648777850749606`},
{0.19219532042344978`, 0.8633096904694177`, 0.1660296606087659`, 1.0595398890946446`, 0.3981268836303198`,
0.35447506211304225`, 0.06527455092271564`, 2352.6780619798324`, 0.04729158696423241`, 8.334762180963512`,
8886.551170717703`, 0, 0, 0, 5.6980565214198675`, 0.3893273780910939`, 0, 0, 2369.593592424266`, 0, 0,
4.801572832444199`, 6506.06856813336`, 0.7453522133390061`, 1.0000000000000784`, 0, 0, 0.5500117445726479`},
{0.05942123092712986 4`, 0.7565633878530198`, 4.881127251124937`, 0.9039794772130322`, 0.669150670013211 3`,
0.3437226952926379`, 0.013854681683978944`, 1303.847591809871`, 0.1331369696675042`, 3.817043920573575`,
48.02279776226351`, 0, 0, 0, 40.14221392635678`, 0.662586202741585`, 0, 0, 0.0179251113764981`, 0, 0,
7.161263747012019`, 0.015216174035677002`, 0.6181848373081742`, 0.34563058142731384`, 0, 0, 0.2667847249431114`},
{0.27625614155976197`, 2.681313109654546`, 6.389652815751472`, 1.3504817156087858`, 0.7587234585548364`,
0.5970419449613528`, 0.3005699072645848`, 854.9062988397701`, 0.165124447314083`, 9.6053618622676`,
311.00243135268454`, 0, 0, 0, 60.58970821109794`, 6.362049740068461`, 0, 0, 0.07191140542513516`, 0, 0,
243.694962461712`, 0.2837995342410335`, 2.3775107428227904`, 0.6771290063903772`, 0, 0, 0.6928859478967527`},
{0.09922116949114601`, 0.5509823607034723`, 3.2884104382793122`, 1.3318058680427458`, 0.6719251293345294`,
0.6187329358641112`, 0.3259483616243979`, 1099.7736795288956`, 0.13645709285092156`, 2.0261792473532125`,

42.609141387369384`, 0, 0, 0, 7.813566728750504`, 3.8925148948596706`, 0, 0, 0.10936655832559596`, 0, 0,
30.63867208347441`, 0.15502111171840008`, 0.4042271621398462`, 0.7962636709403171`, 0, 0, 0.2262499476133617`},
{0.11800216420621701`, 3.285091391336728`, 1.6522276383131942`, 0.9670398406334181`, 0.4307591958110746`,
0.30003613158270603`, 0.29799743711056653`, 701.4335436984502`, 0.043710117716904806`, 7.7162513520402545`,
106.9250381098324`, 0, 0, 0, 21.872269761199796`, 1.7290395441317221`, 0, 0, 0.8117358276003599`, 0, 0,
81.14361316725574`, 1.3683797774365105`, 2.431710273119533`, 0.9999469609404865`, 0, 0, 0.9258766300280641`},
{0.19357111334803345`, 1.327860467490665`, 5.552252240997824`, 1.1813151963757291`, 0.7103742334207412`,
0.3776068940225674`, 0.2456309648230094`, 3934.634788049085`, 0.12355866435459295`, 9.745686147306856`,
47.12550835881893`, 0, 0, 0, 10.846474446758554`, 1.815434142335734`, 0, 0, 0.006862490885995592`, 0, 0,
34.43776041343489`, 0.018976857159180027`, 0.5204426963736446`, 0.11737129694225745`, 0, 0, 0.6295903412810214`},
{0.11447070818541266`, 1.7335861954191927`, 8.736387174086214`, 1.0335531844510784`, 0.08646802454747982`,
0.4443642965951369`, 0.015374273787766714`, 123.60141703755143`, 0.21867059554150092`, 6.82672203062274`,
961.1969955767904`, 0, 0, 0, 429.4682789069383`, 3.552858427494491`, 0, 0, 166.95074473642717`, 0, 0,
87.98837605975957`, 273.01385688658587`, 12.95781749730508`, 0.9999999999987393`, 0, 0, 1.2880746327407837`},
{0.22987433852030326`, 3.2498283820092855`, 3.7918096781351274`, 0.808040368560279`, 0.9416246029763107`,
0.49895559050949523`, 0.02048494990466312`, 278.1506433463176`, 0.24538025309113942`, 1.1463070343242556`,
698.8582853330865`, 0, 0, 0, 29.962424189665473`, 0.1793802431060896`, 0, 0, 154.15282369076837`, 0, 0,
8.327928645969925`, 506.225405385031`, 0.9795853002849119`, 0.9999999999937034`, 0, 0, 0.23421919287576345`},
{0.2774701327316953`, 3.217064080543545`, 9.848999257942918`, 1.1587780417232185`, 0.0062851183500154395`,
0.6835477959342324`, 0.37074868705631564`, 2643.6047855840716`, 0.14999893314602414`, 6.1386041394007655`,
27.694623011240807`, 0, 0, 0, 4.820181153813061`, 0.4867619896063718`, 0, 0, 0.003433603078442154`, 0, 0,
22.37063589337957`, 0.013610318598898311`, 0.5629739621586601`, 0.061617378941259404`, 0, 0, 1.4020178749278014`},
{0.07518728576930783`, 0.8073344777020095`, 0.2079911159294223`, 1.2717171333972301`, 0.7961690644438275`,
0.349516332980527`, 0.009301689754665837`, 660.9000723394831`, 0.24128632812714051`, 8.034216989913379`,
5976.864073579396`, 0, 0, 0, 11.890254730517627`, 0.12370842595109272`, 0, 0, 2875.170388895669`, 0, 0,
1.4267725350366163`, 3088.2322523621597`, 2.618206837112195`, 1.0000000000001361`, 0, 0, 0.7330066576239155`},
{0.19386888157639426`, 1.1721624963279647`, 7.488800420822528`, 1.2206737749782337`, 0.39882649538868`,
0.6168747383393647`, 0.005894727770540639`, 784.8706404426375`, 0.21917965839925868`, 7.12627169471982`,
142.94961223057967`, 0, 0, 0, 131.77909561765668`, 0.6160474402646072`, 0, 0, 0.025411015884916784`, 0, 0,
10.315824363385909`, 0.07037721756184355`, 2.049136252502275`, 0.35948095808665914`, 0, 0, 1.0224831794428593`},
{0.05154396213144296`, 1.0824196854643793`, 1.5789464925861978`, 0.9324716268311393`, 0.16692765658832065`,

0.33113273649509023`, 0.16543741477462187`, 443.37752852110856`, 0.07774169596012481`, 8.031423814374019`,
6198.047736321502`, 0, 0, 0, 33.035885005246634`, 4.389950224756196`, 0, 0, 3508.8961654596783`, 0, 0,
67.88240773549808`, 2583.7487296516933`, 4.031117223316372`, 0.9999999999999973`, 0, 0, 1.0242639311582824`},
{0.04886198458272409`, 2.4240564285395276`, 3.0585768323132374`, 1.3030342316587755`, 0.5506088802661759`,
0.40370465638646613`, 0.05636145780930632`, 1567.8212350001168`, 0.20179368484919008`, 9.505728478640599`,
93.72953893448614`, 0, 0, 0, 52.458835543814736`, 1.1566623361110004`, 0, 0, 0.03449455826029714`, 0, 0,
40.05449673570605`, 0.02407817962712711`, 1.303773242633816`, 0.4315933904993403`, 0, 0, 0.8509606245398961`},
{0.26828907890664055`, 1.4916419764625646`, 9.30244345383333`, 0.8462433381711008`, 0.9150350508953056`,
0.16353772752060636`, 0.1124240025000419`, 2042.0256023360328`, 0.08362962700521354`, 9.175430964325553`,
69.61149000933557`, 0, 0, 0, 27.266190971873012`, 1.8971174428545485`, 0, 0, 0.004588893163660724`, 0, 0,
40.42600017201668`, 0.01758785600112448`, 0.915500733032626`, 0.11026287284073621`, 0, 0, 0.29658044489400576`},
{0.11853219177887636`, 2.436539187916523`, 4.07490711978506`, 0.8391678158796785`, 0.8274679746994464`,
0.4783994312610165`, 0.02880518630114325`, 249.91551468654188`, 0.20172418525520536`, 8.758671893830364`,
2309.3944678355565`, 0, 0, 0, 220.68233102569042`, 2.4602298994467726`, 0, 0, 742.7932856295268`, 0, 0,
85.63495087551426`, 1257.7845169185823`, 8.133091284430767`, 1.0000000000000067`, 0, 0, 1.5397622918616254`},
{0.15380346197105899`, 2.5002802569502807`, 7.104691399911929`, 0.895619363352984`, 0.7056556726651881`,
0.2904087856211036`, 0.27890663528622706`, 1570.4796825528501`, 0.2402855906015`, 2.015690994359673`,
19.903873302171796`, 0, 0, 0, 4.200637461128517`, 0.4269168838008991`, 0, 0, 0.008625743605087818`, 0, 0,
15.248740798944638`, 0.018952417550531554`, 0.2690035569380948`, 0.1863912047871622`, 0, 0, 0.12479641821661798`},
{0.07681933116098377`, 1.701376646381478`, 3.4749545233581878`, 0.9897106512386663`, 0.21660819673269338`,
0.4456181241675946`, 0.020856154503598144`, 170.86262737853005`, 0.11272605112949713`, 7.4521875824230435`,
22464.06171742289`, 0, 0, 0, 167.8768014369435`, 1.9294998962362573`, 0, 0, 10606.97322056454`, 0, 0,
46.89722946645518`, 11640.294120660501`, 9.77855768554486`, 1.00000000000004`, 0, 0, 1.0131487131480696`},
{0.19680750576810457`, 2.9845730901840906`, 8.034687795284743`, 1.0668124507997105`, 0.6731645050394575`,
0.24725877139168673`, 0.05097134738484948`, 549.1786460489706`, 0.029127445194553014`, 6.0096318557240025`,
153.44629044382324`, 0, 0, 0, 89.68107054491801`, 1.4587882745630063`, 0, 0, 0.02777023860583877`, 0, 0,
62.19800326481193`, 0.07807701992281582`, 2.282305451404751`, 0.42830304262427976`, 0, 0, 0.3408947948902159`},
{0.2481156970490977`, 1.2202113043036036`, 9.306028403345813`, 0.8009108612869359`, 0.02681732734233533`,
0.15549305073805075`, 0.021015785720259927`, 53.28601671876581`, 0.06540227633756851`, 2.0322216725641393`,
134925.1646269719`, 0, 0, 0, 112.09006009549734`, 1.7866195185451472`, 0, 0, 29657.786729442338`, 0, 0,
31.143619043117823`, 105122.3203901292`, 7.820222867439965`, 1.0000000000002116`, 0, 0, 0.07910152952873042`},

```
{0.05969602287151182`, 2.177557565950017`, 1.7675183816518665`, 0.8372040785938111`, 0.9188853493106184`,
  0.1878023064060833`, 0.1313271602899384`, 239.3049490553974`, 0.14060221077790058`, 6.359907705361997`,
  8945.507931814971`, 0, 0, 0, 32.20457042436641`, 1.759047425688187`, 0, 0, 4780.201276740189`, 0, 0,
  54.720386152459994`, 4076.5572106673208`, 5.610960761035375`, 1.0000000000000235`, 0, 0, 0.44326106995985115`},
{0.1932263333480873`, 0.7780345756067408`, 9.679599745167298`, 1.1108495595363916`, 0.27977393862884203`,
  0.2181645563751744`, 0.006193313005348089`, 412.49802062580375`, 0.21651680739670837`, 9.973248740719807`,
  215.44831285761188`, 0, 0, 0, 198.0866882354231`, 1.409980010648914`, 0, 0, 0.018516310816165008`, 0, 0,
  15.671617131417346`, 0.05111983516296866`, 5.158159519688026`, 0.3015175352037487`, 0, 0, 0.8072529272876969`},
{0.05080144024762184`, 3.462591354860418`, 9.637683194306994`, 0.7734436451051352`, 0.6551811041034619`,
  0.18020194036251547`, 0.05139770733113868`, 3391.683612113084`, 0.04739051831745644`, 1.6399754771823183`,
  5.39612108586466`, 0, 0, 0, 3.151862179612429`, 0.04441137925976248`, 0, 0, 0.0016949372238605162`, 0, 0,
  2.196835112604007`, 0.0012300750300230639`, 0.10038633590585623`, 0.04601875058141702`, 0, 0, 0.0851165552354477`},
{0.17269587719558416`, 2.280528270589695`, 6.304702452448467`, 0.855238720891303`, 0.7302844321830138`,
  0.25273349413775115`, 0.28098261768807636`, 2514.837947344666`, 0.04999285469609732`, 2.363210664893213`,
  14.044368065944093`, 0, 0, 0, 2.952718466163068`, 0.32975791665742826`, 0, 0, 0.005398388136332847`, 0, 0,
  10.743175019828943`, 0.0133182767807025`, 0.19568386875840552`, 0.12664251265206772`, 0, 0, 0.13016297821683165`},
{0.12416213981002666`, 0.8389921874064035`, 3.610730817659622`, 1.0777639636353653`, 0.18253050124358539`,
  0.3654692817582218`, 0.277997956001176`, 563.1802062075279`, 0.17509802310960038`, 1.6234053595359104`,
  28.236683392130647`, 0, 0, 0, 6.143203032394316`, 1.6904783065025228`, 0, 0, 0.05105038199128661`, 0, 0,
  20.26140131622321`, 0.09055035237366182`, 0.6510595353265616`, 0.6239536701215939`, 0, 0, 0.2343581146888573`},
{0.05389681485101466`, 0.42474703837564753`, 9.702496123506663`, 1.2875902192251218`, 0.8398628787663107`,
  0.4145702753216558`, 0.012323091180371398`, 1207.1917708220153`, 0.0487427592688896`, 1.997348446926472`,
  41.061008317886824`, 0, 0, 0, 34.91631110925429`, 0.8610474066360961`, 0, 0, 0.020085745569764373`, 0, 0,
  5.224676226710198`, 0.015465110144553222`, 0.3447579457197174`, 0.2830285743373264`, 0, 0, 0.11134226014408562`},
{0.06391261825702438`, 1.7835225604286613`, 8.062142041499534`, 1.3025605866836996`, 0.9222349035188417`,
  0.39598365567854665`, 0.09958099473376647`, 75.95083350193653`, 0.043061298974276296`, 9.73945151975186 7`,
  145277.60685188547`, 0, 0, 0, 277.85405077509415`, 14.221882690681829`, 0, 0, 75598.33640931966`,
  0, 0, 362.35783758001156`, 69024.10879707066`, 28.63062760846588`, 1.`, 0, 0, 1.278116636017239`},
{0.08850946488241984`, 0.9673836199236003`, 8.299835087678265`, 0.9900607622617293`, 0.0809660102577392`,
  0.6707110673061827`, 0.005613358503071156 5`, 223.20635429959376`, 0.07233923327715214`, 4.067688416849283`,
  220.78650248063363`, 0, 0, 0, 204.2828868526055`, 1.0838069424294456`, 0, 0, 0.09505366196977594`, 0, 0,
```

```
14.977958332367512`, 0.12018783937226953`, 4.399246643194375`, 0.859670992152177`, 0, 0, 0.9074732622930282`},
{0.056027023765889306`, 2.0857485685464603`, 9.366491292380005`, 1.196524731276077`, 0.9516747873190254`,
 0.6119693625054259`, 0.014912996736033244`, 220.0557248806415`, 0.0820080753991021`, 2.040285256013867`,
 162.60731154020272`, 0, 0, 0, 132.85338966225322`, 0.9045827456389447`, 0, 0, 1.013519301981012`, 0, 0,
 26.953316669260772`, 0.8112067145611457`, 2.092262077478548`, 0.9999906660420418`, 0, 0, 0.2924802700680639`},
{0.04516470805711162`, 3.292123721374173`, 5.263943679637629`, 1.138536155221258`, 0.48426832878752823`,
 0.5662939024082725`, 0.09160607397723192`, 3360.7159646360847`, 0.026694430856043894`, 4.180658858897127`,
 20.557542556718097`, 0, 0, 0, 9.050559156410635`, 0.23933360590074976`, 0, 0, 0.0071118697210037`, 0, 0,
 11.255940590112516`, 0.004588650281279936`, 0.2759218443158702`, 0.12489322922752011`, 0, 0, 0.5176355411971597`},
{0.2782184518736612`, 3.845021085593168`, 1.137061402050021`, 1.4385887178277776`, 0.42032329684699743`,
 0.5854257024714923`, 0.03249096611383817`, 328.6688300716124`, 0.02617032692156479`, 8.869579059594898`,
 244319.42508432153`, 0, 0, 0, 54.87429782919783`, 0.44991090360616615`, 0, 0, 49097.79063161775`, 0, 0,
 24.713098728628445`, 195141.58999922717`, 5.725676371215893`, 1.0000000000000016`, 0, 0, 0.6651344896556899`},
{0.09319840750005726`, 2.6994281444799046`, 8.85475415324603`, 1.038403514470577`, 0.3378909888092929`,
 0.6279203689803818`, 0.23307552493819164`, 1846.0011275272348`, 0.034946033021005685`, 7.6621208156759835`,
 62.210714593531755`, 0, 0, 0, 15.053766335273249`, 1.1915622822113954`, 0, 0, 0.0063741812626346706`, 0, 0,
 45.950525150030565`, 0.00848662203992161`, 0.94597778630427`, 0.12352797921180092`, 0, 0, 1.185677890021089`},
{0.06034484465157863`, 2.8968090048449424`, 3.142651522085412`, 1.307236858746047`, 0.3617717997572676`,
 0.29303713371207274`, 0.18131081790115888`, 4578.414121282968`, 0.17962394399488657`, 3.3138716244433386`,
 7.5985320742277365`, 0, 0, 0, 2.2228502333596034`, 0.126560317559128`, 0, 0, 0.006271358614828696`, 0, 0,
 5.2374438223045425`, 0.00540634516236525`, 0.1568797747276815`, 0.09729072250039572`, 0, 0, 0.3253152950828148`},
{0.20102830374342656`, 1.9832552045818916`, 0.5122029117897267`, 0.8414910669557318`, 0.023679729667421823`,
 0.562606520598186`, 0.21796137676857033`, 2537.351175593395`, 0.0373739667120736`, 9.865525950238812`,
 44.28800566628175`, 0, 0, 0, 11.202085414994313`, 1.0947180008851187`, 0, 0, 0.2519256601583918`, 0, 0,
 31.01578818292707`, 0.7234884018726097`, 0.9450317378892474`, 0.9789355903716794`, 0, 0, 2.2837194990574226`},
{0.04583409051630541`, 3.6747726707008503`, 0.7420872691712788`, 1.2157743693615881`, 0.8915453306179026`,
 0.4926202034522773`, 0.4217762472968932`, 73.4269782954483`, 0.10619296232023001`, 3.9517710932009376`,
 114367.17200270195`, 0, 0, 0, 3.89224527544947`, 0.3894214933840609`, 0, 0, 69096.77595610723`, 0, 0,
 20.443363732446596`, 45242.68405081549`, 12.1542648667526`, 1.0000000000000009`, 0, 0, 0.5699803508650577`},
{0.10119631213999619`, 1.851973173732965`, 1.6003758779141855`, 0.9328064269865228`, 0.39319494883808903`,
 0.3867811485974866`, 0.006630825974655251`, 2020.948278622179`, 0.24307528382326266`, 8.411215463490773`,
```

52.12703977141673`, 0, 0, 0, 47.57719670408545`, 0.1611015653340528`, 0, 0, 0.03183893290136895`, 0, 0,
4.262225389215066`, 0.04602832274415613`, 0.911600990046119`, 0.5150454262856234`, 0, 0, 0.9230966925265243`},
{0.19796477344593338`, 2.2434362012846876`, 1.2309355399833084`, 1.3299187291217778`, 0.059794768114105334`,
0.6215799505741797`, 0.022739215183529730`, 1004.7174087228818`, 0.138969497102004`, 8.58482448486276`,
215.95651753218183`, 0, 0, 0, 73.37016952277021`, 0.7010189282799196`, 0, 0, 31.189911435346716`, 0, 0,
22.467017735556684`, 88.20719644424484`, 2.097845322381845`, 0.9999999999081356`, 0, 0, 2.09440021157553`},
{0.18841727652913837`, 3.2954166443999204`, 4.985214271603974`, 1.3919313698468052`, 0.8063258092579206`,
0.6389441864140679`, 0.008804142841395211`, 1247.8494355462315`, 0.09671339442963328`, 8.749674730462491`,
227.245975107258`, 0, 0, 0, 201.612301031144`, 0.523587332669309`, 0, 0, 0.07782055596736777`, 0, 0,
24.649120155362876`, 0.20946767447652606`, 1.4760031678352414`, 0.6923198851548567`, 0, 0, 0.5822625190339311`},
{0.08822838789215687`, 3.6314565393637377`, 8.720964691808945`, 1.4591613734708808`, 0.09172379783619267`,
0.17510896742376914`, 0.2630216511777198`, 431.756995427934`, 0.16850915581941245`, 3.8927198296202143`,
63.86231170544817`, 0, 0, 0, 14.880630958346147`, 0.9254671524391951`, 0, 0, 0.019852375053510344`, 0, 0,
48.0113391813094`, 0.025022043525710358`, 1.9262817081036043`, 0.25347591193871777`, 0, 0, 0.3211538936786713`},
{0.22004819658675462`, 1.2435615798896285`, 4.521096993231973`, 1.3090616735635763`, 0.5905184190150714`,
0.6892934240599866`, 0.09109967416767972`, 239.35075160225182`, 0.045734190683186515`, 5.8358773331232445`,
37853.08680514885`, 0, 0, 0, 102.5230522980113`, 6.855128268692879`, 0, 0, 9079.645565672536`, 0, 0,
121.78248771659709`, 28542.280462473827`, 5.724965984041423`, 1.000000000001392`, 0, 0, 1.1068488847597557`},
{0.18183828174362843`, 3.2786199220563104`, 2.2240542093570888`, 0.9831747738881791`, 0.39067106215615355`,
0.5999409005287338`, 0.32534468961787655`, 53.98091103084368`, 0.10022266621367804`, 9.980846637487563`,
249893.91414954638`, 0, 0, 0, 32.976362884384315`, 3.040397266226465`, 0, 0, 69409.9578001431`, 0, 0,
142.4043864002231`, 180305.5351753684`, 39.73711627550715`, 1.0000000000012097`, 0, 0, 0.8873851930990582`},
{0.21252199352031792`, 1.818911285509734`, 0.45179175410691036`, 0.8473192883797733`, 0.5752582261250987`,
0.4244690624430477`, 0.010390335454685632`, 819.8846878599306`, 0.22518903335941576`, 6.3452240038645735`,
4949.901295132101`, 0, 0, 0, 19.75409747980972`, 0.10731120285943542`, 0, 0, 1220.8130872082522`, 0, 0,
2.78842225632359`, 3706.4233001313096`, 1.6359579812829674`, 1.0000000000006541`, 0, 0, 0.4506802867295168`},
{0.13408565703158576`, 3.9481177636984475`, 4.409802796026355`, 1.1882851460555317`, 0.7016940523769313`,
0.6437617694336766`, 0.472695817526219`, 268.88735252515323`, 0.02238688650951909`, 7.198883746978469`,
15489.633381362073`, 0, 0, 0, 42.155269566803945`, 4.278558158295536`, 0, 0, 5214.142550745218`, 0, 0,
241.3178781111923`, 9987.738996757467`, 6.514768190160185`, 1.000000000000201`, 0, 0, 1.676592693604853`},
{0.11550511139863262`, 2.7266825017818803`, 9.715437717848832`, 1.4226217916614743`, 0.7434913376024772`,

0.6527594414247178`, 0.026462900292485443`, 517.125461393156`, 0.19263485762362964`, 9.20218742349427`,
502.3896339203605`, 0, 0, 0, 365.35893319721896`, 3.421687781801913`, 0, 0, 0.09348953696429224`, 0, 0,
133.28366001714468`, 0.1542645625950983`, 3.7848400088157717`, 0.7515144416125528`, 0, 0, 0.7144807331574318`},
{0.1537432379066465`, 3.3010385217103515`, 7.8154151393713`, 1.099357680158021`, 0.2670513361076359`,
0.6099579487912872`, 0.01626886763741749`, 3071.882113963599`, 0.04550777584421739`, 6.952411393791335`,
30.85937412244917`, 0, 0, 0, 25.111673702980585`, 0.11883184273865889`, 0, 0, 0.004071812586004499`, 0, 0,
5.603835578373423`, 0.008943052158872993`, 0.5215921738342812`, 0.07642922341960157`, 0, 0, 1.1657549331107344`},
{0.08915960893105551`, 3.2848427178141772`, 0.6302374569507307`, 1.4630290564093034`, 0.8347103436436674`,
0.36812644478584344`, 0.040963141333673035`, 3229.889147199789`, 0.1995027638419964`, 3.7582834934312945`,
19.868013913202574`, 0, 0, 0, 12.077800309351087`, 0.14438483605991506`, 0, 0, 0.382445544670165`, 0, 0,
6.7754496756315`, 0.48712421714594945`, 0.2550380347307239`, 0.9957934256626366`, 0, 0, 0.4153557159184469`},
{0.2095156599086041`, 1.856403794983298`, 1.6647511783688493`, 0.8963074241076089`, 0.5257557556911894`,
0.2962680347723602`, 0.005638948645619698`, 398.77430140016673`, 0.1712593265312492`, 6.243180864475704`,
6593.453678424819`, 0, 0, 0, 76.43225147621729`, 0.2197271123677149`, 0, 0, 1630.5430053635562`, 0, 0,
5.827174932287811`, 4880.347053972915`, 3.3270199047394544`, 1.0000000000011917`, 0, 0, 0.47891799706932303`},
{0.08606673974307905`, 3.001046395083865`, 6.871125535025986`, 1.29871337654279`, 0.0013879217267305233`,
0.4709543850320145`, 0.028486772088755836`, 255.05656064251065`, 0.15733152617600732`, 4.419059383126843`,
166.6965921491207`, 0, 0, 0, 119.40940315014575`, 1.0732556166827885`, 0, 0, 0.08058980086013642`, 0, 0,
46.012712849276966`, 0.09908716309399489`, 4.061449217501498`, 0.7283190974727068`, 0, 0, 0.8294969124190619`},
{0.2794012033077364`, 3.8964244114394253`, 7.589043584575673`, 0.9660750334911808`, 0.6823441866078543`,
0.315494435212184`, 0.013950850602606538`, 1092.7384181067544`, 0.06831145072823913`, 6.790315469699049`,
100.97260273736335`, 0, 0, 0, 84.30978117087255`, 0.2919985211745414`, 0, 0, 0.013786662311285643`, 0, 0,
16.25357380012422`, 0.05502871484816052`, 1.3036658771644114`, 0.264181100867383`, 0, 0, 0.4274845256277511`},
{0.16053897675716755`, 1.21110484978606`, 6.017303762103152`, 1.2353982330406967`, 0.20086989110627473`,
0.3272106220983775`, 0.02355461265150193`, 77.09093986775292`, 0.04848901873161415`, 3.5142859649597167`,
223526.18527287157`, 0, 0, 0, 126.3090452450785`, 2.276012972571607`, 0, 0, 67819.65172155976`, 0, 0,
39.378433560820845`, 155538.535591524`, 9.686780526974268`, 1.000000000000008`, 0, 0, 0.2693133127647481`},
{0.06315507597420161`, 1.4395182613839497`, 3.0858504661132873`, 1.1448617905347693`, 0.30610701790846395`,
0.20244634599341438`, 0.2950980917537417`, 2179.6214593356067`, 0.03473000454292019`, 1.333335366603153`,
5.165641791158565`, 0, 0, 0, 1.0898227658467072`, 0.18814168177364365`, 0, 0, 0.009793320966624948`, 0, 0,
3.869048380580692`, 0.00883568470980081`, 0.13106667615880044`, 0.16609509668667566`, 0, 0, 0.11408838441204643`},

{0.27116635506122466`, 2.640185082938654`, 3.1282641056246216`, 0.9199164829096633`, 0.32218318348593566`, 0.5777637686875948`, 0.006117113694401221`, 324.8095638375007`, 0.04342122620091826`, 2.609798810681127`, 18981.386036116564`, 0, 0, 0, 64.66497911307027`, 0.14322591872378398`, 0, 0, 3880.152485095757`, 0, 0, 5.402041915781699`, 15030.954378073846`, 1.8534847415306202`, 1.0000000000000082`, 0, 0, 0.44048434744888754`},
{0.08076786811690995`, 2.5389877544798445`, 7.211847948605141`, 1.1500644274940979`, 0.7690232347129535`, 0.1924810447357974`, 0.3170033067881963`, 2456.45541532025`, 0.12977198817972552`, 1.000471570970415`, 5.333962766938453`, 0, 0, 0, 1.0312960187375153`, 0.11511868054806304`, 0, 0, 0.005594302665456636`, 0, 0, 4.175498860334408`, 0.006454855712706904`, 0.08433524697997714`, 0.09876437295911666`, 0, 0, 0.04916835237627691`},
{0.14196490482109098`, 3.5800560378690767`, 8.191314882292886`, 0.8521719014833709`, 0.20577614391452248`, 0.2973229046898421`, 0.032689134381726236`, 2192.8696238585753`, 0.20870222148197032`, 8.620659635124824`, 36.87870251732632`, 0, 0, 0, 25.39270028592754`, 0.22004552627608914`, 0, 0, 0.002895956841714297`, 0, 0, 11.253933070725603`, 0.00587320339143145`, 0.8603157758293198`, 0.07055087334483057`, 0, 0, 0.937912713988889`},
{0.16225217210911858`, 2.1337475700719883`, 4.607583982995607`, 1.274907826520427`, 0.760916097212121`, 0.6512132808308`, 0.1988962707427588`, 88.66654318682235`, 0.17629703311441042`, 5.991536547797288`, 81943.04021995429`, 0, 0, 0, 61.17626920176433`, 5.290666137242431`, 0, 0, 24628.7090805353`, 0, 0, 161.27065734861714`, 57086.59349372028`, 14.912828567794431`, 1.0000000000003275`, 0, 0, 0.7079595205338512`},
{0.09998370398747453`, 1.3561107931887042`, 7.055733599099842`, 0.9997997594446719`, 0.4231050232131661`, 0.35289870383552235`, 0.01863838099230359`, 585.7262791471302`, 0.12303242645253354`, 2.5487735356675643`, 52.08286953695101`, 0, 0, 0, 41.26730008165286`, 0.5273304248952306`, 0, 0, 0.02296070215683445`, 0, 0, 10.215978296817251`, 0.03279565782562263`, 0.9473463991230855`, 0.3874903477631825`, 0, 0, 0.2626472513160205`},
{0.0790420625297461`, 0.7951762600254768`, 1.0975467432818782`, 0.8295886125705915`, 0.21974762785224522`, 0.4003083061581494`, 0.12181590550696897`, 256.3718289629029`, 0.07504947990766975`, 1.4404680202816778`, 26896.800412169825`, 0, 0, 0, 4.885755968122807`, 0.6556713732704874`, 0, 0, 12626.397785247435`, 0, 0, 7.448204434328501`, 14257.378903813871`, 1.2358829528174902`, 1.0000000000001126`, 0, 0, 0.16488288439216547`},
{0.09310243320239603`, 1.7939569577957926`, 0.5112771848209494`, 1.3548866082012778`, 0.9609312900127882`, 0.31815651234043585`, 0.047992777894275945`, 1572.364241162158`, 0.10220690607690369`, 1.3696469849079624`, 1079.3676768922296`, 0, 0, 0, 3.532221426690215`, 0.08711057723517644`, 0, 0, 460.7293362592653`, 0, 0, 2.2324660875521816`, 612.7860321923214`, 0.19518149935578233`, 0.9999999999999161`, 0, 0, 0.18529482759872745`},
{0.20315703606221613`, 1.4806269686939117`, 9.851530344798562`, 0.807291927894971`, 0.7692364729235686`, 0.5064599484352303`, 0.07616257600145751`, 550.4721115636684`, 0.18512304424700965`, 4.821016411964738`, 218.39630892442125`, 0, 0, 0, 105.4785177847609`, 5.091037737463172`, 0, 0, 0.036309368680338586`, 0, 0,

107.68468246752009`, 0.10537862460550501`, 1.8438604772878473`, 0.6174815323864675`, 0, 0, 0.3216186925687856`},
{0.22897905358022103`, 2.219649931841208`, 8.024983925513396`, 1.3490395658771779`, 0.7992198564081268`,
0.5097552623833034`, 0.017934168491060075`, 2636.2345048980164`, 0.04871567850920905`, 1.38033814014487`,
13.596944980969587`, 0, 0, 0, 10.80439802079283`, 0.0837669196304792`, 0, 0, 0.011248634324142153`, 0, 0,
2.6561891064048724`, 0.03679573773731852`, 0.110542313086496`, 0.1619857979004531`, 0, 0, 0.09616685899135761`},
{0.14728957561352346`, 3.8290117168547324`, 5.577517394641557`, 1.2037071314600827`, 0.7629976630105226`,
0.33391252098357616`, 0.00623505541513591`, 1551.1825745003182`, 0.05716501203669799`, 3.4524118308532703`,
41.86753105286014`, 0, 0, 0, 38.38881982061887`, 0.060649530868368474`, 0, 0, 0.019368535642847098`, 0, 0,
3.3175394902389415`, 0.04075404850129067`, 0.46510681771226386`, 0.2910049940817847`, 0, 0, 0.20301383083622274`},
{0.1747499923357959`, 1.9364726507678487`, 4.539322141325334`, 1.0358217348301002`, 0.687467494258686`,
0.22118436656891216`, 0.06970356230137446`, 4108.781309212477`, 0.15897963252631786`, 3.216561469360766`,
10.347680597824837`, 0, 0, 0, 5.256901644327983`, 0.17701232425632085`, 0, 0, 0.0048289034452711896`, 0, 0,
4.896850353874509`, 0.01205501200073839`, 0.16294550377269001`, 0.09516713398030718`, 0, 0, 0.1756095626756004`},
{0.061449808274615936`, 2.451738473864639`, 8.032153511575046`, 1.3988277328315561`, 0.653844119276888`,
0.6033333182307286`, 0.12509363159734996`, 3162.271766392656`, 0.18189469469951425`, 1.0210674887190017`,
6.918144332950094`, 0, 0, 0, 2.521378800731132`, 0.12164317268642931`, 0, 0, 0.007769574944316761`, 0, 0,
4.260532093689695`, 0.006820555581478194`, 0.07053103876401944`, 0.1103103854102856`, 0, 0, 0.10899329614135286`},
{0.1951885853322612`, 0.6765436415231281`, 7.0581513804142055`, 1.4397439961417247`, 0.9899034195557828`,
0.3660842570171081`, 0.2551485659861665`, 53.30479560198744`, 0.15667683142074817`, 8.392793083340557`,
167811.20606479805`, 0, 0, 0, 104.67478435154212`, 33.904624031634974`, 0, 0, 44172.888863531196`,
0, 0, 327.6851115262117`, 123172.0526758837`, 33.13489491286899`, 1.`, 0, 0, 0.5562008416856223`},
{0.15955516943400022`, 0.8482855893464007`, 9.331572022970409`, 1.1667938097447013`, 0.29910958141522603`,
0.1963171731712634`, 0.010305064592809629`, 1013.0765277256257`, 0.20900313016467104`, 3.301983133021727`,
27.457654288105932`, 0, 0, 0, 23.984816466844165`, 0.26158176901850494`, 0, 0, 0.006944390332722704`, 0, 0,
3.1699435013448096`, 0.015828762516476767`, 0.6930193543426566`, 0.12011236804260406`, 0, 0, 0.25523051757683335`},
{0.17746707158856556`, 0.44139712602022296`, 2.8621670830493073`, 0.8100971992338867`, 0.0373958036205273`,
0.5075452637360592`, 0.09067013663296572`, 114.80525128927701`, 0.16719225120884412`, 9.820606806768879`,
45629.05944977913`, 0, 0, 0, 99.65064733179511`, 17.23092962768157`, 0, 0, 12843.109745784908`, 0, 0,
108.65261166164836`, 32560.415381071565`, 18.354586224025393`, 1.`, 0, 0, 0.8558878109151827`},
{0.25772135278013303`, 3.070426405636976`, 1.2338255709842532`, 1.3135003325072887`, 0.5149580362176238`,
0.41732865316044054`, 0.04496826136197767`, 318.8324074107286`, 0.010846019130009599`, 7.957536959758443`,

```
459343.15708679357`, 0, 0, 0, 47.168078434389265`, 0.6647065937046605`, 0, 0, 98097.45791651343`, 0, 0,
  29.156181104454166`, 361168.7079790835`, 5.2318539397853865`, 1.0000000000004765`, 0, 0, 0.4940916165349102`},
{0.08988753363169116`, 3.536295514824336`, 7.275260820034448`, 0.8993825689374235`, 0.025163868977214232`,
  0.47083049518550046`, 0.018253871395130763`, 416.18519037331805`, 0.11499620973251179`, 2.265482484941062`,
  52.34014899122266`, 0, 0, 0, 41.69995458934051`, 0.2051682386651946`, 0, 0, 0.02328390873231041`, 0, 0,
  10.364793173944816`, 0.02989904470361`, 1.2763544091891301`, 0.42339300346023445`, 0, 0, 0.42592985017809504`},
{0.14192760186457132`, 2.3958894382433478`, 3.253512770748353`, 0.8655063905996925`, 0.8143036151546204`,
  0.5834364577519011`, 0.17553594321370639`, 62.22207407824173`, 0.16384862691545943`, 3.0979894918131596`,
  90045.54264929114`, 0, 0, 0, 24.198372486978617`, 1.6542053842798152`, 0, 0, 29714.935338391857`, 0, 0,
  56.61847441259001`, 60248.13588769615`, 10.897746739193282`, 1.0000000000080536`, 0, 0, 0.3376179128684148`},
{0.14248286143558947`, 2.096310454690764`, 9.38011512102738`, 0.9675972750885355`, 0.0803295271719453`,
  0.6033546111821413`, 0.13762578908199044`, 1262.8186365484712`, 0.18785001372865495`, 4.9706998985924695`,
  45.47802637953347`, 0, 0, 0, 15.890021577043804`, 0.9554551914470057`, 0, 0, 0.006342833643986323`, 0, 0,
  28.613295811698936`, 0.012910644102888783`, 0.9341359328225532`, 0.1312185423988348`, 0, 0, 1.0061643785996222`},
{0.10446764528219044`, 3.442117541556258`, 1.7911289531773882`, 0.8226507378787467`, 0.06660293233402026`,
  0.33605831499521144`, 0.1966635974545483`, 110.27400709595466`, 0.23027159794407875`, 2.5346674177346316`,
  24914.904403613527`, 0, 0, 0, 9.932913382555805`, 0.5245732241588901`, 0, 0, 9981.824543174687`, 0, 0,
  25.794895667257563`, 14896.824366363391`, 4.909941662234955`, 1.0000000000000004`, 0, 0, 0.2086232219382652`},
{0.0959624592169791`, 0.7155878562202682`, 5.1612847015717165`, 1.2701047018452214`, 0.05672968500006248`,
  0.6450160655606327`, 0.04218472167952095`, 1614.7615476312947`, 0.23305782159027644`, 5.6652476983851425`,
  41.181567675359915`, 0, 0, 0, 25.963024646163475`, 1.3532871364464931`, 0, 0, 0.01240780575217576`, 0, 0,
  13.834226297431567`, 0.01700976504951979`, 0.8436295375600448`, 0.1882151431782655`, 0, 0, 1.2368723602894067`},
{0.24311048293765425`, 1.2773072105524381`, 8.674158333323465`, 1.236248375623142`, 0.5430857314344402`,
  0.29169516048698574`, 0.10383247735445839`, 522.6667880889086`, 0.0459109896778S867`, 9.694591495258663`,
  242.4184657661748`, 0, 0, 0, 99.87292501721628`, 7.399439451013098`, 0, 0, 0.02831971225563329`, 0, 0,
  135.01939092607407`, 0.09835455604390633`, 3.929376215925806`, 0.3889029487845639`, 0, 0, 0.7110976988438057`},
{0.08713889377857453`, 2.2859880795657075`, 9.600148060687761`, 1.2921363299986606`, 0.6294865567710719`,
  0.5590186325118135`, 0.25992443653112657`, 633.6168560418654`, 0.16781120217455497`, 6.04666254822`,
  195.42311188862982`, 0, 0, 0, 43.037367386660655`, 4.525189693422276`, 0, 0, 0.03633180193400465`, 0, 0,
  147.77899567052762`, 0.04522732899292192`, 2.0597426342625944`, 0.4519340572539954`, 0, 0, 0.5657815572465027`},
{0.26758004713434824`, 2.448981429729897`, 4.582999027119474`, 1.4554483602004895`, 0.3942207099215642`,
```

0.5288182096517661`, 0.017684947331647518`, 53.19217741492592`, 0.1488296927599813`, 5.857737808697124`,
280035.4092206887`, 0, 0, 0, 169.19417872092365`, 1.1755451280054454`, 0, 0, 58023.77529422624`, 0, 0,
41.12697411849718`, 221800.06468774052`, 23.198738608694565`, 1.0000000000003841`, 0, 0, 0.3944854198378658 4`},
{0.1343103727928621 8`, 0.7397108148766112`, 9.99887072123514`, 1.436439642978205`, 0.2003363652340402`,
0.6779870054162531`, 0.009538236593206559`, 4558.309876478542`, 0.14371659865116293`, 1.1480275619139793`,
3.3042548635671993`, 0, 0, 0, 2.9058272412855133`, 0.033576384391287004`, 0, 0, 0.0026211312609524524`, 0, 0,
0.35481163798127474`, 0.005029215954250556`, 0.06024629459660911`, 0.03782638590927245`, 0, 0, 0.24338692288349578`},
{0.2773077831654361 4`, 1.7365428506012295`, 3.277328223559843`, 1.164340615403590 5`, 0.2621424262474452`,
0.5836632043576286`, 0.19147730163610321`, 594.1704278677969`, 0.14363610143643862`, 8.32222051121584`,
189.14428731602885`, 0, 0, 0, 52.67644380842103`, 5.2619975039848`, 0, 0, 0.1345350716031323`, 0, 0,
130.53834493466164`, 0.5329660352037449`, 3.225739805626592`, 0.8989569376745145`, 0, 0, 1.3887514389808886`},
{0.05614793436458493`, 1.997529172364887`, 6.840549262003957`, 1.2853232683568476`, 0.15723714782247722`,
0.6374125080636333`, 0.1308231427534364`, 1207.3035504224565`, 0.15724403465210185`, 1.842303075231806`,
18.954068087070414`, 0, 0, 0, 6.815332129818864`, 0.4101588765104715`, 0, 0, 0.013444988948635227`, 0, 0,
11.704347444772463`, 0.01078440510029886`, 0.3635979515998539`, 0.19836003372301791`, 0, 0, 0.3818272884502662`},
{0.0647068787712346`, 0.8380824418888393`, 1.0963289034750066`, 0.857562051048008`, 0.6907626550964823`,
0.2999007220652139`, 0.27885748113518277`, 1238.4520803651262`, 0.1379529162857775`, 8.96709675960637`,
80.42072928246569`, 0, 0, 0, 17.181032990215837`, 4.796910696398284`, 0, 0, 0.5254984684296022`, 0, 0,
57.43152328514522`, 0.4857623670163112`, 1.5756204122831978`, 0.9997765796433502`, 0, 0, 0.9196438205903477`},
{0.20132622112515353`, 1.9086557669063273`, 0.7030652496772074`, 0.7755069309180247`, 0.32762519329281337`,
0.20767575421456186`, 0.011318073987463645`, 431.8155255411275`, 0.17436258525110954`, 5.81443415452358`,
9566.819329129976`, 0, 0, 0, 27.051129901055535`, 0.15245891809875264`, 0, 0, 2460.067310171169`, 0, 0,
4.15702276064977`, 7075.3722181469075`, 2.762757576156191`, 1.0000000000001021`, 0, 0, 0.23010736097895226`},
{0.045022613849116516`, 3.5058573906861508`, 8.47131465431961`, 1.2697383949459176`, 0.5022287267438617`,
0.23847623822456332`, 0.06077359284826833`, 243.2833936812314`, 0.1613793589669978`, 8.022277670460284`,
373.1013242752542`, 0, 0, 0, 202.8153647572864`, 3.3308556035968717`, 0, 0, 0.07973717962268075`, 0, 0,
166.8214962168352`, 0.05128537496531917`, 6.9578220967984805`, 0.7388769358637977`, 0, 0, 0.5541833641433178`},
{0.17418182442148117`, 3.0892110646487794`, 3.4522019380421036`, 1.2965827481879664`, 0.41818871776887856`,
0.6556019601202148`, 0.075063644002762`, 2886.574837344593`, 0.15315141138676647`, 2.6062865811306466`,
14.839837611470706`, 0, 0, 0, 7.237474343856993`, 0.16729190847943057`, 0, 0, 0.014960224910682267`, 0, 0,
7.3828573528695705`, 0.037225703838533325`, 0.2063022009564729`, 0.21513548224898793`, 0, 0, 0.4109475802970207`},

{0.045831059772204225`, 2.899702133961444`, 5.925065562245003`, 0.8929739121804446`, 0.5933600691515191`,
0.4490635153302144`, 0.16447888961080792`, 1912.79886244215`, 0.11499785968845799`, 4.757985376452911`,
42.357168547527465`, 0, 0, 0, 13.008327945211114`, 0.6914263896813574`, 0, 0, 0.00939658032893252`, 0, 0,
28.64186539480406`, 0.0061522176387047035`, 0.5350533783819423`, 0.20194493552601112`, 0, 0, 0.42749205835811055`},
{0.2429697761558342`, 1.8449858081251405`, 7.508754000794074`, 0.9127320360969664`, 0.44886430542069955`,
0.35316747155912975`, 0.02193269408167387`, 161.73721618998414`, 0.16775422018188862`, 9.221971574736251`,
588.9519172690613`, 0, 0, 0, 441.58024391633523`, 4.912626465773639`, 0, 0, 2.8721330230282875`, 0, 0,
129.48180157103303`, 9.969164538499692`, 12.71568676488441`, 0.9999997760947584`, 0, 0, 1.1981339747015711`},
{0.160442241636698`, 3.55050251528807`, 3.9052606138637995`, 1.2025377341211225`, 0.791383919489673`,
0.40672925070026333`, 0.35477124416027767`, 105.50559352550128`, 0.10792398319932495`, 9.162379917819035`,
89514.77688574418`, 0, 0, 0, 50.175942566805766`, 4.555296957208729`, 0, 0, 27104.534102772657`, 0, 0,
231.05133292076724`, 62124.46014238817`, 18.55023837225004`, 1.000000000000064`, 0, 0, 0.7538665588460479`},
{0.20468313725879644`, 1.5880039947408164`, 1.8181726149942818`, 1.4361114186612707`, 0.6452252370101204`,
0.23877202353940497`, 0.026067763377395026`, 956.3204095901076`, 0.08759161290164996`, 6.530440575770296`,
91.112966981299`, 0, 0, 0, 65.94955046636952`, 1.014495655327166`, 0, 0, 0.28533964351469066`, 0, 0,
23.01461647581059`, 0.8343459059841843`, 1.4358700521906775`, 0.9691988786357222`, 0, 0, 0.4268620632545388`},
{0.07364401078716826`, 2.5859680523819737`, 7.8227327965838285`, 0.8181378301360093`, 0.36855652079172274`,
0.32445369334254925`, 0.005391697017678555`, 139.66827785366002`, 0.0644557011853657`, 2.5433237558513593`,
168.49700533080954`, 0, 0, 0, 154.51058847848495`, 0.3054342988856176`, 0, 0, 1.0832796983189548`, 0, 0,
11.283476271712788`, 1.1396723112645946`, 4.085108711444888`, 0.9999857456835558`, 0, 0, 0.3475140781100951`},
{0.2506656721970057`, 1.5258727034335298`, 1.8333712411976357`, 1.2495957919641723`, 0.1010075460664761`,
0.5024718466416268`, 0.012345305326089913`, 1052.1669408921373`, 0.03320454993831845`, 2.130087729732141`,
21.908887159312037`, 0, 0, 0, 18.392971997680284`, 0.1388435726167236`, 0, 0, 0.07387912217471712`, 0, 0,
3.0265373929007087`, 0.2645565688749998`, 0.48192013005600964`, 0.6879687778596334`, 0, 0, 0.4390184448731985`},
{0.10121864512314288`, 0.9521305504715327`, 3.6368221202357223`, 1.491518490019038`, 0.3322534458031634`,
0.2564348282281962`, 0.03507970829425946`, 4627.905596988043`, 0.07121256842175683`, 7.352385124666178`,
15.635940940148682`, 0, 0, 0, 10.516518919511546`, 0.3495690673077044`, 0, 0, 0.005690970307999049`, 0, 0,
4.754791264050073`, 0.008229032914452606`, 0.34123838344021146`, 0.07861634866660128`, 0, 0, 0.6489912684494356`},
{0.1942542932466596`, 3.1177323303054916`, 7.932133913996736`, 1.3085296181137158`, 0.8325654731630694`,
0.19060678658466512`, 0.044940459115954866`, 2391.994107548152`, 0.24452644883302188`, 1.4227474861405387`,
8.535177468325813`, 0, 0, 0, 5.230586013405013`, 0.07202019927314394`, 0, 0, 0.006520126901213391 5`, 0, 0,

3.2077100529846394`, 0.018093752043911296`, 0.12249927208082094`, 0.10123333433501236`, 0, 0, 0.061874702325715125`},
{0.12644890469661946`, 3.0196313965941393`, 8.688658870512302`, 1.3655010622962382`, 0.5847686349152417`,
0.30770354208104556`, 0.013061738242062998`, 1213.9477293174991`, 0.0486468587160932`, 6.312801306430787`,
72.48326340118683`, 0, 0, 0, 61.194223760578666`, 0.25412154163856593`, 0, 0, 0.012463623595405437`, 0, 0,
10.96219122403884`, 0.02251445074557796`, 1.1010717976956128`, 0.1781171472619033`, 0, 0, 0.459526311705594`},
{0.11280111274715143`, 3.2009360918012444`, 2.9222221030043283`, 0.9684721327188957`, 0.03329493891954294`,
0.1886848273472841`, 0.39445850876705457`, 50.69043630972767`, 0.07560282132089569`, 3.828945975174229`,
193146.62062500234`, 0, 0, 0, 14.210065098056779`, 1.5454248303912013`, 0, 0, 73928.50491493392`, 0, 0,
70.66865881092846`, 119131.68025911167`, 15.598834636997408`, 1.0000000000013987`, 0, 0, 0.1773268098610338`},
{0.21704875939335333`, 0.6532232080519234`, 2.918554590657344`, 1.428534955684801`, 0.6202310312510246`,
0.3415578035649972`, 0.1858190740142067`, 1428.9901262383053`, 0.23526262585558444`, 9.378643714384769`,
102.55197063756951`, 0, 0, 0, 29.003478163782347`, 7.100205082309487`, 0, 0, 0.0465474055251751`, 0, 0,
66.25741059561103`, 0.14432938031736794`, 1.3907016321762191`, 0.50062560456789`, 0, 0, 0.6903373846548709`},
{0.23408001184633387`, 2.4049572740765104`, 0.41973647277501946`, 0.9771854975111727`, 0.20272636213110817`,
0.5074761434023957`, 0.2724896408017817`, 4254.760257351377`, 0.16397942354351142`, 7.3871482427729305`,
21.437258244144928`, 0, 0, 0, 4.602951849244492`, 0.46480918345204486`, 0, 0, 0.09214212923548448`, 0, 0,
15.9692318114365`, 0.308123295756984`, 0.4060309503930818`, 0.8265741755072029`, 0, 0, 1.3658444699284693`},
{0.1483945695425406`, 2.699298050205127`, 5.571982823617271`, 0.9775705576725262`, 0.05426683476548022`,
0.5307838109252759`, 0.3096243181655647`, 323.46321359362`, 0.06725520461051537`, 9.792268234153859`,
330.6252128903389`, 0, 0, 0, 66.22111118081338`, 6.677331233677972`, 0, 0, 0.07677273983922155`, 0, 0,
257.4872454234386`, 0.1627522525862059`, 7.079466335526397`, 0.8090245549289123`, 0, 0, 1.8101295527336911`},
{0.11663741077922185`, 0.4431208542421814`, 9.780884572399291`, 1.0122403254522139`, 0.996318833483786`,
0.22155768960679945`, 0.39993089984673347`, 139.93164861672463`, 0.10490063957337303`, 5.3884722727325105`,
418.0089597902204`, 0, 0, 0, 68.74064004340796`, 47.24860992548619`, 0, 0, 1.0958965417555337`, 0, 0,
299.09777702767224`, 1.8260362158891166`, 8.12735928254823`, 0.9999397292701419`, 0, 0, 0.3737199637059701`},
{0.11314041374373823`, 3.32806970136921`, 5.495643049849155`, 1.495187492234928`, 0.8329498208958137`,
0.41734162088096116`, 0.32389371604169326`, 602.537083528763`, 0.025714962476490244`, 1.3686646556271693`,
54.67461567681306`, 0, 0, 0, 10.018687540369724`, 0.9093902182404022`, 0, 0, 0.19517058180971128`, 0, 0,
43.23591474353447`, 0.315452576807908`, 0.47997048008743576`, 0.9167141087138199`, 0, 0, 0.09661541331170055`},
{0.09294922428523011`, 0.8101676653238759`, 3.5463022431515436`, 0.9341830117643879`, 0.15981251792750606`,
0.6527933768073686`, 0.016252799814697117`, 738.0588634619963`, 0.024900612098687713`, 6.915149516601594`,

```
120.57807445002786`, 0, 0, 0, 98.11442412363415`, 1.7740689832765024`, 0, 0, 0.046851392258682595`, 0, 0,
20.532761804351775`, 0.06221143667327454`, 2.2180372146778127`, 0.6521487564441385`, 0, 0, 1.3791967483701655`},
{0.2696435798732645`, 3.282053008859009`, 8.218787637608994`, 1.4011858805306123`, 0.38871702639887196`,
0.49514225831047376`, 0.01812451162452924`, 173.50575962443355`, 0.20495943261505556`, 3.6349041099248875`,
263.54632725302497`, 0, 0, 0, 204.0059578879462`, 1.0773345536458587`, 0, 0, 1.6211471737613652`, 0, 0,
50.51241590487376`, 6.244741820491991`, 4.811886094534686`, 0.9999407587317961`, 0, 0, 0.5952049815402674`},
{0.13023501639399904`, 3.4827106844829796`, 7.180310384726493`, 1.0417379014779535`, 0.3157954886073613`,
0.3090830033379006`, 0.027522996818882862`, 1326.2576636786057`, 0.24748434887668458`, 4.853044482161593`,
37.76653376783778`, 0, 0, 0, 27.305129466395933`, 0.205587795719306`, 0, 0, 0.007652489365568896`, 0, 0,
10.22861161072758`, 0.014237458256860094`, 0.7965391564730171`, 0.1459855640397396`, 0, 0, 0.4994640667146315`},
{0.0474731410927452`, 0.7132410347452449`, 0.7493200461382123`, 1.2237616503945397`, 0.9506868210145907`,
0.6554164129507942`, 0.030282495208288053`, 4252.97050681489`, 0.14327214907032687`, 7.932983145622682`,
49.19049856623758`, 0, 0, 0, 33.93649674214015`, 1.2963265124262529`, 0, 0, 0.44313887501711247`, 0, 0,
13.208475187008498`, 0.300531347676694`, 0.4097127113454254`, 0.9991336353640207`, 0, 0, 0.895663659961093`},
{0.16300334800486183`, 3.0919140294206544`, 3.354218048792589`, 0.9673340843818878`, 0.9052111330146941`,
0.3760205832601582`, 0.15433250667793078`, 4163.373290035795`, 0.2440496776808055`, 4.495712049614825`,
29.572134404917954`, 0, 0, 0, 9.36601223745691`, 0.4463181954241059`, 0, 0, 0.01377141176146594`, 0, 0,
19.713964143107205`, 0.032068374626748664`, 0.22272556767703097`, 0.2622219260482481`, 0, 0, 0.20255566316874402`},
{0.2283283078987099`, 0.42840256075325867`, 9.446993924606975`, 1.3839314736668495`, 0.8927356525671915`,
0.3909626915031965`, 0.018767685887155043`, 53.5712236843488`, 0.10749676377570666`, 1.5496335657505222`,
198159.6868324399`, 0, 0, 0, 92.09012113511595`, 3.411762452567349`, 0, 0, 46469.03541460902`, 0, 0,
20.880111019452496`, 151574.23179861237`, 6.151915103242879`, 1.0000000000000002`, 0, 0, 0.12016557744657524`},
{0.1380652378345807`, 3.317739091147252`, 0.7336293987943634`, 0.9484386065310282`, 0.5023326788118547`,
0.2130184622271264`, 0.0630596090666651`, 1932.031257216769`, 0.01203085244100871`, 4.258055262793977`,
23.697070191819076`, 0, 0, 0, 12.455350344108883`, 0.22322489164024273`, 0, 0, 0.1474700649661753`, 0, 0,
10.580027844456323`, 0.29086413704335534`, 0.46939097175441147`, 0.9412860110318996`, 0, 0, 0.33679342038715754`},
{0.0926632627436077`, 3.578083963812018`, 1.8944161244777453`, 1.1381278842914715`, 0.7382269863407194`,
0.4777843546205588`, 0.04528998830707099`, 1233.5473532765689`, 0.08357615053600725`, 2.9903558138075255`,
46.48512135694232`, 0, 0, 0, 27.968871779570687`, 0.34070381507501224`, 0, 0, 0.32662985466384287`, 0, 0,
17.4152408161354`, 0.4323798291802552`, 0.529766776343631`, 0.9948824558579736`, 0, 0, 0.32067237225679696`},
{0.2706310445313141`, 2.5166396966047158`, 0.5049158559505837`, 1.316870746472338`, 0.5754269054535`,
```

```
  0.5968444469720801`, 0.1025449755078217`, 681.183394170123`, 0.05243619315919962`, 2.183793658787195`,
  51157.04603679401`, 0, 0, 0, 3.6169021855438985`, 0.14033685249194672`, 0, 0, 10511.012122739663`, 0, 0,
  5.045389912539885`, 40637.23128369062`, 0.6842433788530087`, 1.000000000001069`, 0, 0, 0.17775552395470823`},
 {0.20804007909247985`, 0.9673945938397814`, 0.48619022305575754`, 1.3068640855075515`, 0.047796616526516456`,
  0.6050138674479171`, 0.4706922223098622`, 348.3137462309647`, 0.24685340199219946`, 5.551550038883448`,
  21419.69842270605`, 0, 0, 0, 2.972683060165869`, 1.2596076362932673`, 0, 0, 5387.223656718882`, 0, 0,
  17.40768025299164`, 16010.834794752536`, 3.416581371681877`, 1.0000000000168994`, 0, 0, 0.4779974944021405`},
 {0.04845164423666767 4`, 3.3925416461261833`, 7.486002650281495`, 1.2431466818882033`, 0.1294136137122328`,
  0.6416005560424389`, 0.04082335443875802`, 58.92112932799084`, 0.09857305139503486`, 6.047753646247408`,
  120598.42779870231`, 0, 0, 0, 253.0996638138065`, 2.9039282512539923`, 0, 0, 71032.72992777763`, 0, 0,
  140.738536139165`, 49166.46513742825`, 24.11192991834449`, 1.0000000000000069`, 0, 0, 1.1504705369624977`},
 {0.09431215650771135`, 0.5526170445339527`, 7.677473256451986`, 0.7715239445968243`, 0.7622192109377914`,
  0.1566986505563851`, 0.018040482265180933`, 824.8087273379117`, 0.09073506826067207`, 9.996434684615583`,
  151.78796140332793`, 0, 0, 0, 121.13091265837993`, 3.437968266246487`, 0, 0, 0.011144597155224957`, 0, 0,
  27.141140892780708`, 0.015015299873121384`, 2.4848385843199945`, 0.2673985951106643`, 0, 0, 0.387782505054708`},
 {0.26113782676147984`, 1.4684069653405487`, 4.54225535313282`, 1.1193463339337828`, 0.6236318874525932`,
  0.1751148412910668`, 0.053183289402392066`, 554.4897241223483`, 0.22550063278101773`, 9.001504879035508`,
  183.26146960123302`, 0, 0, 0, 105.44569117764044`, 3.530237184427758`, 0, 0, 0.04826571110374622`, 0, 0,
  74.05464101311325`, 0.1800571843532451`, 3.3625616259722904`, 0.6034346837064544`, 0, 0, 0.44532485524450693`},
 {0.23311836154945775`, 2.5262146716446363`, 6.3107841154355`, 1.4422657044398717`, 0.3118898255230633`,
  0.2567889403043644`, 0.03481026984978327`, 1604.0167510242688`, 0.16315782107376747`, 5.777943122601165`,
  34.574880388189655`, 0, 0, 0, 23.312714609764967`, 0.3025157966923146`, 0, 0, 0.009167428169442505`, 0, 0,
  10.917426342976992`, 0.03052994049262022`, 0.7753309645965217`, 0.12761741945452254`, 0, 0, 0.5231857433840987`},
 {0.22640126428063923`, 3.7808015365334118`, 7.033092553948691`, 1.0460349638379132`, 0.8860375200295565`,
  0.499376231890995`, 0.3693111113992884`, 210.995030019894`, 0.19589698445197923`, 2.861160479579169`,
  180.13242090714255`, 0, 0, 0, 29.92734128038333`, 2.5736081185385844`, 0, 0, 2.037445681304884`, 0, 0,
  139.00430755722257`, 6.589718259293563`, 3.1017370774371567`, 0.9999998819133887`, 0, 0, 0.45472513705278866`},
 {0.19770244273120569`, 3.3508846225196347`, 6.757833537731408`, 0.8466672464019638`, 0.28373063232656337`,
  0.6609960258126362`, 0.006803814757890119`, 1256.448281122042`, 0.0187925047862397`, 9.149585751239048`,
  105.78945537743523`, 0, 0, 0, 96.43320825096124`, 0.18865798749804952`, 0, 0, 0.010327924304054102`, 0, 0,
  9.031016417467352`, 0.02916936947506787 4`, 1.6833439707750741`, 0.23041424358826057`, 0, 0, 1.5665897521176189`},
```

```
{0.06481064744600706`, 2.3696983632913335`, 9.923821775061434`, 1.1006879887228784`, 0.22746584913410017`,
  0.6483433064071293`, 0.08284957522863273`, 281.66193979187017`, 0.13350656503173153`, 1.0702403000648566`,
  49.97201871535207`, 0, 0, 0, 23.29286694258288`, 0.762699479064042`, 0, 0, 0.05029748191501782`, 0, 0,
  25.819538674588813`, 0.04656874811165457`, 0.9013993123248479`, 0.6107874374737551`, 0, 0, 0.21613508709895082`},
{0.1537084817657926`, 0.5942996643982652`, 0.8160795607483013`, 1.042756674430172`, 0.7722126796580249`,
  0.49669902434825364`, 0.00611858149596996`, 1518.932458333751`, 0.21666908673557295`, 1.634333111202272`,
  20.73434981571264`, 0, 0, 0, 10.947282428171848`, 0.09846066038647738`, 0, 0, 2.766364054883044`, 0, 0,
  0.8359305346302165`, 6.0744802698219305`, 0.2585379762415023`, 0.9999999955570568`, 0, 0, 0.3553556240625911 4`},
{0.13754450751212471`, 1.8598194811531465`, 9.485701362019345`, 1.4994328167980717`, 0.8397969618927028`,
  0.642370724162252`, 0.08665379792235217`, 239.43302754758346`, 0.1633413998793405`, 9.552795266495377`,
  758.8743360705054`, 0, 0, 0, 341.3179763565046`, 14.928916577178994`, 0, 0, 2.017858170415871`, 0, 0,
  396.6441411821089`, 3.9649329754164824`, 8.87704512997872`, 0.999988009077999`, 0, 0, 1.2161908818259288`},
{0.07858197291799773`, 2.783199788795301`, 0.9642830041255817`, 1.3105090636097434`, 0.9572373854271048`,
  0.41778523295843695`, 0.00823451224525384`, 90.59081205787001`, 0.24323880940762027`, 7.348654585309644`,
  49788.50884527534`, 0, 0, 0, 51.58966451677703`, 0.14634945298953278`, 0, 0, 23429.2145132407`, 0, 0,
  5.8188538092967965`, 26301.627148134878`, 17.64436861644863`, 1.000000000000011`, 0, 0, 0.7499950018352105`},
{0.09118935700706149`, 1.2770460819223013`, 0.666402509206284`, 1.1661439001587546`, 0.35202394044239993`,
  0.23733544361366754`, 0.06689005296661862`, 1909.3731761645724`, 0.030837584446851807`, 5.2718694661097025`,
  27.123683538190996`, 0, 0, 0, 13.929523775667976`, 0.6625859751579776`, 0, 0, 0.19262751850898308`, 0, 0,
  12.087897478745202`, 0.2509368507814187`, 0.5989964379471928`, 0.944370426473294`, 0, 0, 0.5229163738441659`},
{0.26692387934872025`, 2.295217680243936`, 3.3610155473383347`, 0.802490311252646`, 0.6241394164828811`,
  0.45771836786665954`, 0.015176154328102385`, 1461.3995866509204`, 0.02843534740955544 5`, 8.762751383914615`,
  109.91228007536021`, 0, 0, 0, 90.41188715602163`, 0.5720141726090834`, 0, 0, 0.026059266559490025`, 0, 0,
  18.75567203317822`, 0.09936915032915507`, 1.2835669431208339`, 0.5003832139050478`, 0, 0, 0.7413232310110921`},
{0.16789560992918506`, 3.010819021450227`, 9.549236085030234`, 1.3823009139889233`, 0.2184000924395355`,
  0.5213598249132101`, 0.3210433649481226`, 558.1245473691072`, 0.20748907999743366`, 5.921770487462836`,
  129.16341927127607`, 0, 0, 0, 24.975959523334478`, 2.365441180517958`, 0, 0, 0.02364899655918565`, 0, 0,
  101.74164714893097`, 0.056722324307415174`, 2.4264307882846885`, 0.30688577555059493`, 0, 0, 0.9397658130838289`},
{0.05001315370963505`, 3.027404779172768`, 3.8220563025552714`, 0.9962642247459573`, 0.44362718458195727`,
  0.3510033113401648`, 0.04839212642640241`, 1615.957133832461`, 0.04840113498677095 5`, 4.739296636982807`,
  36.471937352834274`, 0, 0, 0, 21.801267891922844`, 0.3309655069022828`, 0, 0, 0.01465159636474366 2`, 0, 0,
```

14.313807961961519`, 0.010468179158308255`, 0.6353943783091293`, 0.26986387779801857`, 0, 0, 0.46300509483497754`},
{0.0729686004745061`, 0.5044823312287607`, 5.5024178276878075`, 1.2230366941116102`, 0.012758742281838265`,
0.5648708708999995`, 0.03232052448858605`, 634.5000769880222`, 0.22553164736796405`, 5.75812027698643`,
96.42441715249049`, 0, 0, 0, 66.53598103487538`, 3.6332799044768884`, 0, 0, 0.02996491210766959`, 0, 0,
26.184650231672965`, 0.031235681426263758`, 2.1630845313954663`, 0.40672546976271684`, 0, 0, 1.195764719316445`},
{0.25149333924897926`, 1.871859550111978`, 9.811462629766478`, 1.3386558973788294`, 0.5189637829018348`,
0.6377508608690272`, 0.024369302194970535`, 194.42061666753636`, 0.14619611626015583`, 6.325307886810732`,
535.7933383860712`, 0, 0, 0, 393.0388255897867`, 4.840151932106971`, 0, 0, 1.8257072432061032`, 0, 0,
129.42978025867703`, 6.559331586927809`, 7.409988490809702`, 0.9998173735714251`, 0, 0, 0.9740084095383307`},
{0.1922243891646514`, 1.6850594067594091`, 0.2630953725218035`, 1.4711962401085972`, 0.15977146838462364`,
0.6883258791888056`, 0.09212778533128511`, 774.9062808250895`, 0.24229201708328568`, 1.1723783184073415`,
9959.795383571882`, 0, 0, 0, 1.104007458284433`, 0.05664314016401417`, 0, 0, 2658.0631434225074`, 0, 0,
1.363529373739483`, 7299.2080586494785`, 0.3311366550878065`, 1.0000000000032747`, 0, 0, 0.12772787681008008`},
{0.2327924107307146`, 3.0114739814321467`, 6.475065538974626`, 1.3312648289930094`, 0.6507217418486848`,
0.3841095338158853`, 0.3176950069808959`, 737.689863101484`, 0.19812174358831114`, 7.321794649353192`,
171.9341475044876`, 0, 0, 0, 32.710268949662066`, 3.1585861898354866`, 0, 0, 0.04151470745448477`, 0, 0,
135.88571612572107`, 0.1380615547013875`, 2.103451330939696`, 0.48687732533321215`, 0, 0, 0.5401252884119456`},
{0.20811447549122308`, 0.49053077700921355`, 2.218461283385089`, 1.0634384889669943`, 0.30011342208125913`,
0.5589173637133283`, 0.16385051847701637`, 124.79731876547663`, 0.15435702825422665`, 9.111119072715418`,
71178.68437728196`, 0, 0, 0, 49.456052098965436`, 14.006844082770115`, 0, 0, 17874.634920457516`,
0, 0, 98.15411587668724`, 53142.43244383021`, 15.60793996680812`, 1.`, 0, 0, 0.7578815161476723`},
{0.19580212756702065`, 2.2297474474451375`, 8.070495694463702`, 1.2202670372174012`, 0.07482300382970508`,
0.34257881980750315`, 0.24674110677687536`, 1287.6149126957`, 0.12354587287124019`, 3.0573978018103922`,
20.978376512665147`, 0, 0, 0, 5.020952067600417`, 0.4849168071680647`, 0, 0, 0.0068980026576152065`, 0, 0,
15.446314471517717`, 0.019294908518888747`, 0.5342538896590189`, 0.1141165002048583`, 0, 0, 0.42802946207427683`},
{0.18591817623779283`, 1.5316284759435828`, 4.0240186618411435`, 1.0506796825025935`, 0.8560461598734517`,
0.5496710897949223`, 0.05348974137997375`, 3910.687147405355`, 0.18912146822955533`, 8.614502889074807`,
72.92031233700214`, 0, 0, 0, 41.49802472664708`, 1.3706835905964023`, 0, 0, 0.016275402137891946`, 0, 0,
29.991114555229206`, 0.04322704404309801`, 0.4582535159371218`, 0.2821837688527753`, 0, 0, 0.4654001979332382`},
{0.2552337642427184`, 2.0636949211593727`, 3.6788035742608756`, 1.2716726068122697`, 0.4767575621154567`,
0.33698161388823356`, 0.00790398094444615`, 742.5948236907665`, 0.22094334214516276`, 6.7847431915852425`,

```
117.10515594649874`, 0, 0, 0, 105.08576355356632`, 0.3821615602392608`, 0, 0, 0.05882457103025551`, 0, 0,
  11.266641013258624`, 0.21448595277163646`, 1.9667777381175904`, 0.625341391854903`, 0, 0, 0.615219579118226`},
{0.2297403387592854`, 3.933034881515791`, 9.92502862171417`, 1.2059622632705849`, 0.1421363362803283`,
  0.1853832242974326`, 0.06367011009221722`, 57.09955187350921`, 0.13182928467731042`, 9.7382206276645`,
  90501.93218328858`, 0, 0, 0, 396.5921028493311`, 6.114559400864283`, 0, 0, 20961.131755804516`, 0, 0,
  343.5539344099945`, 68794.53586223634`, 35.2071389216039`, 1.0000000000008784`, 0, 0, 0.4473594399020187`},
{0.04776006202628119`, 3.695464511472898`, 0.268839094050918`, 1.3548340559244303`, 0.4311024896438589`,
  0.6662260908047339`, 0.1893860365797585`, 146.80096070953766`, 0.0868075345269877`, 5.3982731410948785`,
  88985.20892200657`, 0, 0, 0, 3.5416873487739853`, 0.16824788063027388`, 0, 0, 52886.7474616363`, 0, 0,
  8.882201028567225`, 36083.91913051457`, 8.548430795357481`, 1.0000000000000318`, 0, 0, 0.9599773198076297`},
{0.16250916383331093`, 2.090868942469317`, 5.046057727155544`, 1.3781211464947924`, 0.9692295654960448`,
  0.507149644697747`, 0.012667564467634538`, 467.92228322579444`, 0.2269164868867607`, 2.233796179341983`,
  93.88057303980034`, 0, 0, 0, 77.75460109631932`, 0.4497083624470351`, 0, 0, 0.6604876526039039`, 0, 0,
  13.432589260131987`, 1.5333613735269624`, 1.04246345785151`, 0.9998447039015927`, 0, 0, 0.23768270212437162`},
{0.12952636821033042`, 2.4189205073691173`, 1.5337482028150813`, 1.4058710509218408`, 0.9636406844740666`,
  0.3882701702830901`, 0.27400686262252805`, 4688.706549038708`, 0.0765768774664311`, 6.843480813486918`,
  47.08218834930365`, 0, 0, 0, 9.784867407741704`, 1.044187252753108`, 0, 0, 0.059708394315838374`, 0, 0,
  36.08294227454165`, 0.1104830209628453`, 0.3005961757784642`, 0.5892337231703326`, 0, 0, 0.2975159870004854`},
{0.08153578837307857`, 2.0915987061965353`, 5.850951169546045`, 1.037700922521303`, 0.7897818160030217`,
  0.3237340771928561`, 0.17831164332408336`, 1464.5863160958056`, 0.2436610405225158`, 7.1217069723220146`,
  96.11741179649037`, 0, 0, 0, 27.805448942445867`, 2.210915547262076`, 0, 0, 0.017983950328345953`, 0, 0,
  66.06211568804503`, 0.020947650972525936`, 1.0100343715982412`, 0.3107172815679229`, 0, 0, 0.37332446051993295`},
{0.2624813864915975`, 2.8946734437676342`, 8.625458110929756`, 1.37764442375384`, 0.19776637799413987`,
  0.31049273319228277`, 0.046660769123637466`, 609.6700244678286`, 0.017643025422342112`, 4.098918143681717`,
  62.57851701665736`, 0, 0, 0, 38.06326729617098`, 0.5768451672782727`, 0, 0, 0.017445981720155074`, 0, 0,
  23.853976955515883`, 0.06541779243733258`, 1.4821557032848367`, 0.23784890556697125`, 0, 0, 0.4794531041521771`},
{0.22181257821621975`, 3.747903553799671`, 8.070266666229205`, 0.9808557386469257`, 0.7823522438549146`,
  0.2263615830500163`, 0.06976335847986787`, 287.144692837758`, 0.1821266272405107`, 3.59762935889718`,
  192.2889886815009`, 0, 0, 0, 97.38339440838759`, 1.7323788485053793`, 0, 0, 0.10039054380613478`, 0, 0,
  92.7541263262954`, 0.31811264785958154`, 2.5911718020083234`, 0.8823543991386094`, 0, 0, 0.17217954673598396`},
{0.21058778737677059`, 1.3147483391864796`, 0.4651324593397846`, 0.8516412222118703`, 0.046686774964893374`,
```

```
0.4819914326166439`, 0.1058661660756647l`, 1877.0848108087591`, 0.011164572002709261`, 8.732010177522003`,
16818.565577651105`, 0, 0, 0, 13.755371371141498`, 1.010181814744984`, 0, 0, 4187.41627186414`, 0, 0,
18.97335518874785`, 12597.410393105127`, 1.0345747733561375`, 1.0000000000006053`, 0, 0, 1.107378426574178`},
{0.19996482365829993`, 1.5454344843054368`, 8.94969050535013`, 1.027667483044064`, 0.3164796074442535`,
0.5223314119072894`, 0.012949156080520758`, 231.31648814801656`, 0.19929090705614294`, 9.341058456545078`,
538.4992188076072`, 0, 0, 0, 455.231420556362`, 3.5797159644074874`, 0, 0, 0.09643893583726341`, 0, 0,
79.03166422020023`, 0.2754913542642124`, 9.10507114499417`, 0.8612913511533439`, 0, 0, 1.3301171578937585`},
{0.10440009635624109`, 1.532425369535546`, 0.308138348960842`, 1.2734376519411275`, 0.20147673343032046`,
0.35081245482125123`, 0.05722019474177449`, 85.42879625764479`, 0.08356786949647793`, 8.411462154648635`,
173222.50849639316`, 0, 0, 0, 11.438635560231702`, 0.398407193261451`, 0, 0, 69519.08391055709`, 0, 0,
8.721847005132823`, 103682.8436979968`, 20.886878485239617`, 1.000000000000095`, 0, 0, 0.6291983404868714`},
{0.1446017732242294`, 0.797427468093673`, 5.032298069921831`, 1.3706636028120882`, 0.10121695844960277`,
0.6068141920969097`, 0.25341251132298065`, 197.97452899503492`, 0.2275401984407136`, 3.68386367767849`,
7643.8237417228265`, 0, 0, 0, 37.50991816617386`, 10.094522771703554`, 0, 0, 2440.267346149572`, 0, 0,
114.99499622076387`, 5040.956934205909`, 4.376097820550947`, 0.999999999999969`, 0, 0, 0.7056729080551883`},
{0.1841264162344997`, 3.4194642457460365`, 0.8344796094826652`, 1.4377258087185927`, 0.5867256025093719`,
0.6103981120475732`, 0.3542274534468216`, 522.9821129731852`, 0.2244081861480518`, 8.361737950667973`,
12814.08333505623`, 0, 0, 0, 9.898660579326544`, 0.9426973968903923`, 0, 0, 3514.0125591989076`, 0, 0,
46.050286331779304`, 9243.179130404564`, 3.4867208508325045`, 1.0000000000000107`, 0, 0, 0.8772914240519025`},
{0.17849930658201318`, 3.2992561971358683`, 5.635580909426066`, 1.1878114797167083`, 0.48988207998590316`,
0.27890916833354473`, 0.03715549210344845`, 144.67131391199885`, 0.0866110192158086`, 7.798176718489761`,
19657.06600454017`, 0, 0, 0, 235.46408357564417`, 2.5092451491940118`, 0, 0, 5436.8617249152685`, 0, 0,
118.26632298016388`, 13863.943541138045`, 11.700968488881811`, 0.9999999999999984`, 0, 0, 0.7786486752925036`},
{0.09510173640093084`, 0.7659692827065738`, 0.5464490010035838`, 1.4050402850247132`, 0.11782959453955089`,
0.3589214817200733`, 0.08175234954735425`, 225.11260306454756`, 0.1010344837489014`, 6.4310165595390805`,
52018.17631537765`, 0, 0, 0, 13.193627452652244`, 1.249249204076487`, 0, 0, 22042.792903463233`, 0,
0, 13.669807382403246`, 29947.255432078142`, 6.113349086179084`, 1.`, 0, 0, 0.5416892021024865`},
{0.06416093472533757`, 1.2573296539610634`, 9.92323628294643`, 1.30190007637989`, 0.030494015633026894`,
0.4401773012522794`, 0.02586427825345657`, 3143.6595843648015`, 0.20592664064058636`, 7.935954028868563`,
24.301225123239742`, 0, 0, 0, 17.88297508105898`, 0.33800947148945903`, 0, 0, 0.002574050176195318`, 0, 0,
6.071276168905765`, 0.00235933521906514 6`, 0.582369920595796`, 0.04162524395206724`, 0, 0, 1.3399614037805696`},
```

```
{0.25237835724682106`, 2.979406809596097`, 0.9138356130609361`, 0.7932538461611548`, 0.3933816374531405`,
  0.6211446249652819`, 0.0749645015318215`, 95.73291003138912`, 0.06307891905410445`, 9.368280708056208`,
  227658.0785614993`, 0, 0, 0, 33.029373971486436`, 0.7992331873107785`, 0, 0, 49418.07006824128`, 0, 0,
  34.017725724698906`, 178172.16203044305`, 20.7303843084989`, 1.0000000000000486`, 0, 0, 0.6869252432764058`},
{0.05665762575451494`, 1.7380994050720693`, 0.46391281319797173`, 1.3490794355447004`, 0.9653780951392605`,
  0.17540498059526055`, 0.4972377260445833`, 285.101267300382`, 0.1881031714219547`, 6.261558552266614`,
  14307.785124377497`, 0, 0, 0, 3.216975351542537`, 0.752818555879761`, 0, 0, 7894.896568253875`, 0, 0,
  18.692478344311795`, 6390.087073353325`, 4.583280481933906`, 0.9999999999999957`, 0, 0, 0.35905008933353394`},
{0.25642346088610446`, 3.010945889122482`, 9.963520677018519`, 1.467899384826252`, 0.4946077322511526`,
  0.6868780403912886`, 0.012314696297102353`, 88.3173980921876`, 0.10491074891333174`, 1.4353196490865765`,
  96124.6612060573`, 0, 0, 0, 103.28809299713838`, 0.40648992930296063`, 0, 0, 20587.487754009624`, 0, 0,
  17.484559737206283`, 75415.92658333486`, 3.6887068125091527`, 1.0000000000004474`, 0, 0, 0.21528375893815935`},
{0.09174036922136902`, 1.4204870656163582`, 7.877654658381974`, 0.9368390881044215`, 0.3909649395372681`,
  0.6267838157099552`, 0.00737791814963061`, 223.00641924802295`, 0.22589847441224364`, 3.7895604923910007`,
  257.84855811434767`, 0, 0, 0, 231.56215194067548`, 1.1270708698377105`, 0, 0, 0.8931377843274487`, 0, 0,
  22.871279894820677`, 1.1705255728536461`, 3.9168445344992424`, 0.9981994870729389`, 0, 0, 0.6360789789437301`},
{0.2260021855376967`, 2.1215536107061057`, 4.2648042233683015`, 1.1043329178413561`, 0.5641485347029902`,
  0.48705118822040194`, 0.49950773055219905`, 4133.317514629252`, 0.08759305230550185`, 2.0466954881219834`,
  8.360890332626482`, 0, 0, 0, 1.116542878674277`, 0.23043926620057903`, 0, 0, 0.007041556460201166`, 0, 0,
  6.9841322465186195`, 0.022734387851329182`, 0.10802373411491849`, 0.12644066280729738`, 0, 0, 0.2155529001093871`},
{0.1540246000020472`, 3.8655612430526176`, 1.2043257775886698`, 1.4535724207675174`, 0.7982285789403216`,
  0.6724657214579106`, 0.417253246623641`, 114.93402398229541`, 0.10143088124264565`, 7.877832682736377`,
  157235.21671098113`, 0, 0, 0, 11.83407280657064`, 1.171023658397862`, 0, 0, 49106.33981344825`, 0, 0,
  64.6666238371495`, 108051.20496187091`, 15.008225850847673`, 1.0000000000020113`, 0, 0, 0.861943416869752`},
{0.18967559668177286`, 1.3751140072639014`, 7.146984940201236`, 1.0524950668750344`, 0.5379883166421491`,
  0.5342982154057455`, 0.18359081743318598`, 57.97987210615365`, 0.21651827946513758`, 4.096495164957771`,
  83944.59661895152`, 0, 0, 0, 67.25645226526096`, 8.205619702826532`, 0, 0, 22564.907254482987`, 0,
  0, 161.19517988053374`, 61143.03210804171`, 15.27443884183193`, 1.`, 0, 0, 0.3905955607541936`},
{0.11610573160351356`, 2.8105133752566074`, 0.4045001364193191`, 0.9020454264062931`, 0.06323256453719583`,
  0.49478351534124754`, 0.06895293734611843`, 538.3487824750697`, 0.04516006162070901`, 5.298889101080752`,
  33913.91032449796`, 0, 0, 0, 8.912403155582608`, 0.20782549056893054`, 0, 0, 12749.478928451126`, 0, 0,
```

8.344233156617799`, 21146.96540787701`, 2.1456350018169905`, 1.0000000000006162`, 0, 0, 0.5536175243755074`},
{0.06094054623730827`, 3.5785656752883286`, 2.8105466781089756`, 1.0666569400098407`, 0.8956485783622179`,
0.6416539064413922`, 0.4241071070816547`, 1544.2747045672372`, 0.0589041001461965 7`, 6.674766669452417`,
146.73981626915102`, 0, 0, 0, 21.490677135964585`, 2.392255407237095`, 0, 0, 0.2989048569269657`, 0, 0,
122.29775838373718`, 0.2602203607725147`, 0.9130961011371358`, 0.9923308088130209`, 0, 0, 0.4695941553486991`},
{0.06400872221443815`, 3.352604720184938`, 3.3612644751655854`, 0.9279864636339992`, 0.6811335659068025`,
0.5445450422212498`, 0.04222211989978537`, 4877.834369436828`, 0.09303122695369598`, 6.110250683676178`,
27.638691796772214`, 0, 0, 0, 17.328451193523115`, 0.210509149333481`, 0, 0, 0.008624020557148046`, 0, 0,
10.082199538536349`, 0.007885893374482018`, 0.2681175936702911`, 0.1801409209337259`, 0, 0, 0.5161042656531725`},
{0.1838955211549798`, 0.6066893455768754`, 3.1997125487162617`, 1.2441392193895697`, 0.8430620231566095`,
0.17975264196625795`, 0.008385470075528607`, 217.99247821227752`, 0.10887259656335785`, 8.65519228817503`,
13476.826750153878`, 0, 0, 0, 194.39320467282874`, 2.3475642758867514`, 0, 0, 3655.7143835671523`, 0, 0,
20.346317631962638`, 9603.850025140517`, 8.33400229865434`, 0.9999999999999976`, 0, 0, 0.5496734408260051`},
{0.10877154933938238`, 0.6652486439511347`, 7.420477343235575`, 1.1719120037873472`, 0.8277401239831172`,
0.4096056229223566`, 0.3960687102590752`, 4083.2161783729553`, 0.09124503977845877`, 8.628655226336182`,
52.90970298330367`, 0, 0, 0, 8.315169314202365`, 4.244096288133143`, 0, 0, 0.006440153858308299`, 0, 0,
40.333990006837325`, 0.010007221616446018`, 0.4388207223872889`, 0.11142691863069842`, 0, 0, 0.449797465255998`},
{0.27429237480005003`, 0.4974953244631517`, 8.109325428142139`, 1.1022725368505206`, 0.3620164170986986`,
0.5133215689169995`, 0.013475856804577652`, 252.124017519504`, 0.17376346726865255`, 1.283750502496817`,
68.04699729848504`, 0, 0, 0, 56.80138748752961`, 1.322504345288549`, 0, 0, 0.09946400312171998`, 0, 0,
9.399138976617904`, 0.38974596604823875`, 1.1574505136285569`, 0.8332963415776767`, 0, 0, 0.19480960462239175`},
{0.2348606786833104`, 2.9248975834862243`, 4.089597609027345`, 1.0125076012045389`, 0.4800911130241774`,
0.44584481805285503`, 0.013867879702002976`, 69.31755646766315`, 0.08239556859020553`, 3.2481286029667444`,
221236.72662704941`, 0, 0, 0, 87.49473985025338`, 0.4003859484353412`, 0, 0, 50774.81174673964`, 0, 0,
16.729827043433843`, 170357.23924080888`, 9.871402377271412`, 1.0000000000001361`, 0, 0, 0.2187962959994254`},
{0.2276305764129738`, 0.759063573478838`, 9.75608531797917`, 0.9651630732227953`, 0.2616315835146603`,
0.25394525967231996`, 0.42721777606241185`, 115.33714629065052`, 0.1547162363619287`, 5.678602403105034`,
450.4943651944117`, 0, 0, 0, 71.80891222427346`, 31.561521418145315`, 0, 0, 1.14730811661039`, 0, 0,
342.2457318869473`, 3.7308915415322`, 10.677702148543808`, 0.9994255127085235`, 0, 0, 0.5611784520254767`},
{0.22740101487152548`, 3.662274246882813`, 5.673159128278421`, 0.7501649913658239`, 0.6663016096811909`,
0.46449661159333955`, 0.15875103883050143`, 79.3840483030526`, 0.23925351496597358`, 9.512843911813135`,

```
42809.697532988925`, 0, 0, 0, 134.2548508466297 5`, 5.519322280268204`, 0, 0, 9975.357227967039`, 0, 0,
288.76102638961123`, 32405.80510493875`, 25.4417178317641`, 1.0000000000003584`, 0, 0, 0.720133314631578`},
{0.1890735696086646 7`, 0.8903250429215461`, 0.8912625465714132`, 1.4194566043771595`, 0.2543053135210806 6`,
0.6668345122251143`, 0.01430802337144644`, 354.71023908442675`, 0.0543512780703954 7`, 4.360013356436832`,
88116.18412159599`, 0, 0, 0, 26.391195442974286`, 0.38845564458667997`, 0, 0, 23799.847372123335`,
0, 0, 4.940739834853598`, 64284.6014112676`, 2.684756127514098`, 1.`, 0, 0, 0.46502649181855615`},
{0.22841094141655494`, 2.082125305118022`, 3.0130589699894585`, 0.8668572537043583`, 0.35284672466002664`,
0.48298470604644195`, 0.055294539730325123`, 1324.8059697322965`, 0.2155561741975654`, 1.2621538558570524`,
12.277535646914766`, 0, 0, 0, 6.89677809527377`, 0.1719039615029102`, 0, 0, 0.022399754400557467`, 0, 0,
5.113222689932044`, 0.07309069985900915`, 0.21631121818460575`, 0.4148111905953411`, 0, 0, 0.18980280387838153`},
{0.1257670047049273`, 2.665283038938149`, 6.579106420165356`, 1.3213793494994266`, 0.07362490185046933`,
0.671746727583086`, 0.11039555694375124`, 54.48594040704261`, 0.2029696416579515`, 8.437004538119528`,
112676.07140636715`, 0, 0, 0, 183.38666864370958`, 7.175656712849308`, 0, 0, 40123.51492894714`, 0, 0,
273.21651614285537`, 72088.77558353017`, 34.621127010653645`, 1.0000000000000357`, 0, 0, 1.1205764339721322`},
{0.07898342764241062`, 2.336170261443863`, 9.558440542813312`, 0.8746675798923161`, 0.3314927429594914`,
0.5846930166673705`, 0.1417355070696855 7`, 4831.453144750826`, 0.22811466814427805`, 5.138718102637142`,
15.064003340588322`, 0, 0, 0, 5.132196552800319`, 0.2888284902461183`, 0, 0, 0.0017179134734818908`, 0, 0,
9.639321851010195`, 0.0019383813503999257`, 0.24178962811783522`, 0.04131636828392471`, 0, 0, 0.7801378780016732`},
{0.2360815041275312`, 3.0069216843113518`, 1.9005978173203566`, 1.189378383663731`, 0.3594173887197991`,
0.5615883220885535`, 0.16515958276300607`, 91.7495984293873`, 0.10675594880460115`, 2.979677734696839`,
176997.05695890484`, 0, 0, 0, 13.678774503264352`, 0.7129661159426484`, 0, 0, 40468.44570790517`, 0, 0,
30.626189631531346`, 136483.59332036562`, 6.901670403051124`, 1.0000000000063616`, 0, 0, 0.22866481863958865`},
{0.19503604064380992`, 2.260639826798138`, 3.7301326124020027`, 1.0003156010412233`, 0.94917046396899`,
0.3935362271642996`, 0.08733555022372319`, 2234.7260448390443`, 0.16743068067899064`, 7.659400521016185`,
108.52857909107487`, 0, 0, 0, 48.61887181320319`, 1.7955770959430126`, 0, 0, 0.03332102787862274`, 0, 0,
57.98790135964726`, 0.09284001925187925`, 0.7034320656853185`, 0.5090423085480824`, 0, 0, 0.30528781388373716`},
{0.09766567347807259`, 2.1751916900809825`, 2.9967711374909367`, 1.239918427231443`, 0.884426059472291`,
0.5758255537556012`, 0.011950287911566193`, 133.4222213549303`, 0.18860770237483848`, 7.181211491841234`,
36544.24702647398`, 0, 0, 0, 154.2889768634669`, 0.8074226286474061`, 0, 0, 15181.857455383579`, 0,
0, 25.089985603102495`, 21182.090471830536`, 12.070751786596034`, 1.`, 0, 0, 0.9786881964279124`},
{0.14345253817386028`, 1.510087082683639`, 7.446143698111681`, 1.3871400228991382`, 0.2507990125704793`,
```

0.592096235116642`, 0.03860344741119924`, 206.9907110306594`, 0.033941806565597976`, 5.68471806318026`,
365.65526130683713`, 0, 0, 0, 235.65634306470935`, 5.604308842293485`, 0, 0, 1.1398117299748707`, 0, 0,
120.89991985881568`, 2.3358412243603066`, 6.417555468705951`, 0.9980544716029628`, 0, 0, 1.0457120768565946`},
{0.23351662183055255`, 2.7403756212365034`, 1.6128399905233286`, 1.1394774719326448`, 0.2292996808057013`,
0.38219770472020054`, 0.028922618161783233`, 3147.1184318469313`, 0.11734182225292555`, 5.055074464096112`,
16.961401026417935`, 0, 0, 0, 12.047660307847641`, 0.1204736154468713`, 0, 0, 0.017259679965595177`, 0, 0,
4.716327982468962`, 0.05757745942060456`, 0.35974189430627834`, 0.27433617180483083`, 0, 0, 0.6811539582579005`},
{0.05894445024535572`, 1.7190376171466255`, 3.4445271438587763`, 1.1005021934401353`, 0.8920369053637744`,
0.4553589712941485`, 0.02602138727445485`, 2996.975539830326`, 0.01616140506923186`, 6.007209949084906`,
62.548788659013965`, 0, 0, 0, 45.66727535236215`, 0.6582052206564115`, 0, 0, 0.026711768146866675`, 0, 0,
16.16399334443806`, 0.022493006978555646`, 0.41468509797900915`, 0.40375450287784576`, 0, 0, 0.2896489379384584`},
{0.1969133083502746`, 1.0306356019031098`, 0.4532514022896326`, 0.7704659952544493`, 0.6922371279082573`,
0.35856337468106037`, 0.02342746544122668`, 625.7704338271294`, 0.24611682235411125`, 7.40783802504553`,
5445.851291335578`, 0, 0, 0, 19.774486855349103`, 0.4146926543015613`, 0, 0, 1421.317552132707`, 0, 0,
6.105671619584095`, 3998.2334486680697`, 2.478980409799793`, 1.0000000000000004`, 0, 0, 0.451943386583717`},
{0.23578418763137027`, 1.109904439772956`, 9.416464917532476`, 0.9968768007328397`, 0.9319056056422497`,
0.20705868768300972`, 0.023381736885244357`, 73.41723955051782`, 0.1985782178096708 3`, 6.130556036938069`,
24173.921788739648`, 0, 0, 0, 341.8477452892159`, 6.595777361811057`, 0, 0, 5430.157154842113`, 0, 0,
104.58117968040035`, 18290.645620930336`, 17.53365329854135`, 1.0000000000000513`, 0, 0, 0.39162424840818966`},
{0.06254010222908407`, 2.833030713124539`, 1.1523226094346608`, 0.8553536481119535`, 0.24975724964580737`,
0.6445417390978168`, 0.03834946206048674`, 4173.282831948493`, 0.23424935668510183`, 4.880796947048751`,
16.20045439658201`, 0, 0, 0, 10.532746464941145`, 0.13576569965935592`, 0, 0, 0.01922186490602511`, 0, 0,
5.494691384625678`, 0.017173391375092033`, 0.2745794414669904`, 0.37377940303342216`, 0, 0, 0.9250845773142755`},
{0.1868109134318776`, 3.0098056229686154`, 1.9105557354746436`, 0.9827601516958456`, 0.5236893411586465`,
0.474044891890932`, 0.006475636749568416`, 1404.5093361888864`, 0.13430982294875038`, 1.0667742763750176`,
12.064364390599708`, 0, 0, 0, 10.834631853286757`, 0.022431382115654343`, 0, 0, 0.0631171700801748`, 0, 0,
0.9644871431807739`, 0.16844251708446542`, 0.17066044061510274`, 0.7122700503846873`, 0, 0, 0.147676247587264`},
{0.06031809352972184`, 0.6708570664744204`, 0.23575165127461165`, 0.8219834374256857`, 0.8758699419986131`,
0.18852573702588948`, 0.186745146986022`, 107.40963970974032`, 0.12527667123697356`, 3.1788633087725113`,
40101.04512569063`, 0, 0, 0, 1.6812026979041739`, 0.3941713975163883`, 0, 0, 21536.87011968957`, 0, 0,
3.777609534656665`, 18558.042088813025`, 6.18255790553413`, 1.0000000000001872`, 0, 0, 0.18573936562784168`},

```
{0.18801205905393248`, 2.0266227725882997`, 1.7626950623641149`, 1.1991174158264077`, 0.011000047841808414`,
  0.3353078609658676`, 0.05399718652686698`, 93.79336861840014`, 0.2334529633377767`, 8.960462961249508`,
  64931.84912393902`, 0, 0, 0, 70.01211334794534`, 1.7691686934272184`, 0, 0, 17582.973512266195`, 0, 0,
  51.22053660928405`, 47225.872204741885`, 19.887971272865396`, 1.0000000000005884`, 0, 0, 0.4906934676634336`},
{0.14122716980793654`, 1.0036898987844207`, 5.592205787770849`, 0.8071832042112255`, 0.9861798903854433`,
  0.6717138208444808`, 0.19154806886518252`, 64.00473270059831`, 0.09434989637003327`, 6.618037610942842`,
  132572.20946399213`, 0, 0, 0, 84.91643919137049`, 14.514772575389655`, 0, 0, 43832.07953204957`, 0,
  0, 208.11900881531022`, 88432.57913011049`, 22.990056621251085`, 1.`, 0, 0, 0.837422711326799`},
{0.14179341245484034`, 1.2991893501557028`, 6.1750864357305275`, 1.3220469832389021`, 0.954868801179809`,
  0.5061480199077149`, 0.3454995627472`, 2784.154142185204`, 0.062422742009026755`, 1.1334761108568099`,
  15.166297898994495`, 0, 0, 0, 2.614599630672807`, 0.6384675112274864`, 0, 0, 0.02094475929756748`, 0, 0,
  11.849859871530986`, 0.042426127054953994`, 0.08450220156369434`, 0.28316715757522837`, 0, 0, 0.05854423369861831`},
{0.17346773507380248`, 2.4886801471142252`, 4.186844167829875`, 0.9059139514582338`, 0.8823714489891827`,
  0.3118060351580627`, 0.18611640479783054`, 317.2758158326019`, 0.18366106179531033`, 4.660916164387663`,
  162.61028755708978`, 0, 0, 0, 45.7278074463258`, 3.0937273164090153`, 0, 0, 1.0921978698359556`, 0, 0,
  109.98996789902978`, 2.7065870104696126`, 3.2065237690168535`, 0.9999993413330208`, 0, 0, 0.49366955711919047`},
{0.17801738501831404`, 3.953347877342175`, 5.116473955634309`, 1.3026800881545206`, 0.2660443419983707`,
  0.5551592463622865`, 0.021882498869656555`, 368.98117336094424`, 0.13387054033002443`, 7.8289062285527375`,
  277.6692222090391`, 0, 0, 0, 212.16490043682845`, 1.1290530614994065`, 0, 0, 0.15396131275258548`, 0, 0,
  63.764850344076635`, 0.391539861288485`, 4.861625812655857`, 0.9063581285176536`, 0, 0, 1.2599256495474198`},
{0.1502521637125906`, 3.604808817430471`, 9.516864148680163`, 1.170214417719616`, 0.46260070316916013`,
  0.4333225308578431`, 0.19319459383231874`, 1379.7428965277768`, 0.03462365036850307`, 7.002259791633362`,
  72.21085203551476`, 0, 0, 0, 19.98645631083161`, 0.9942868170342185`, 0, 0, 0.008598023418422506`, 0, 0,
  51.203055501426086`, 0.018455308889587296`, 1.1069384503358126`, 0.14607946503836033`, 0, 0, 0.7360118923636092`},
{0.23041123856532575`, 3.7235663376199373`, 7.422983674409959`, 1.0720190294684302`, 0.9567615568260381`,
  0.6795713708474191`, 0.42619421138851304`, 252.0789228698587`, 0.23264645009848384`, 7.716645878625323`,
  504.6156747600694`, 0, 0, 0, 75.58550755126718`, 7.701802013502826`, 0, 0, 2.7123314314737663`, 0, 0,
  409.6881530927504`, 8.927880636078946`, 7.061694911598791`, 0.9999999626448`, 0, 0, 1.3025500217070463`},
{0.17190873580866728`, 2.1300233762526144`, 7.263444695009253`, 1.2209987713639263`, 0.8761821538663868`,
  0.2004596237918942`, 0.1361627927608223`, 3975.405902408578`, 0.13971132873354192`, 8.98211109187212`,
  38.025600682596114`, 0, 0, 0, 13.210233568722787`, 0.789060151511953`, 0, 0, 0.004650097860837522`, 0, 0,
```

24.010236685569897`, 0.011419892066288522`, 0.46239697544673436`, 0.07871941631301738`, 0, 0, 0.33151494749550514`},
{0.25963938649205515`, 2.4653900827356168`, 4.997042605235823`, 1.1014911256242188`, 0.41576920876875767`,
0.18114027248931974`, 0.22217255314368375`, 81.71471228407572`, 0.2378841388493836`, 9.715142052575505`,
52650.08568113075`, 0, 0, 0, 92.15729729999626`, 7.6802858203528235`, 0, 0, 11101.77550476024`, 0, 0,
270.49857848675396`, 41177.974014692285`, 24.239509655421934`, 1.000000000001409`, 0, 0, 0.32052903927949106`},
{0.2762626642080059`, 0.4739284043667089`, 1.351212961630882`, 1.24608482511035`, 0.19517067645291641`,
0.24775742659101618`, 0.08503066911676711`, 2141.7873291111496`, 0.17338810608691535`, 4.319189194420735`,
17.40648154681566`, 0, 0, 0, 8.025343503096861`, 1.1886445588186125`, 0, 0, 0.02928702841726609`, 0, 0,
8.047605987429652`, 0.1155844642469887`, 0.439595610400643`, 0.3911876956057405`, 0, 0, 0.45118036948652146`},
{0.1906362991887024`, 1.590121005227628`, 9.42412383550555`, 1.4957920989499214`, 0.709065663948995`,
0.3417182681188283`, 0.37536592261162544`, 505.4667159946151`, 0.11728445265057086`, 5.855972486658924`,
205.02681117245638`, 0, 0, 0, 34.22523152354299`, 7.194365850342365`, 0, 0, 0.04831525299171719`, 0, 0,
163.4273179703106`, 0.13158058606752904`, 2.430973522723896`, 0.49924207469114446`, 0, 0, 0.3698269438953232`},
{0.2658746234670645`, 3.614932865157483`, 8.460828570764`, 0.8159863776033582`, 0.2726846463148733`,
0.34564016673603704`, 0.10623048732883861`, 3778.1279822475276`, 0.23711150436988554`, 5.459482199196464`,
15.113367034537486`, 0, 0, 0, 6.183307702575298`, 0.16948175248232072`, 0, 0, 0.0017124029323062509`, 0, 0,
8.752359387040366`, 0.006504064069301846`, 0.3179863937494972`, 0.04414126941704333`, 0, 0, 0.6275771492296697`},
{0.26863157332159515`, 2.9396265733275415`, 6.862183762414347`, 0.9732277188028977`, 0.5002013826688112`,
0.5818631486183298`, 0.034332997185588514`, 711.1213518313073`, 0.15904422112946637`, 1.3044149622459462`,
30.670437836883494`, 0, 0, 0, 20.616838098286436`, 0.23067443037194965`, 0, 0, 0.02759849943969653`, 0, 0,
9.687095504408244`, 0.10591183322572847`, 0.41001738643907304`, 0.44811696955123526`, 0, 0, 0.17288377063682137`},
{0.27209369466384475`, 3.5584095462332135`, 0.9720509149176574`, 0.9873292487031791`, 0.8069894772531618`,
0.17871886216847`, 0.10218788035646292`, 96.95116373019758`, 0.02386269594633994`, 2.5608838628408552`,
607526.4899707471`, 0, 0, 0, 7.761980674233945`, 0.21367397770549934`, 0, 0, 124309.61554020383`, 0, 0,
10.861993172126759`, 483198.03677966207`, 5.341267617848818`, 1.0000000000000053`, 0, 0, 0.06284950370544327`},
{0.14444262093504412`, 3.863449844229998`, 8.726684391435349`, 0.8553749492129846`, 0.451984917853794`,
0.5228822441273482`, 0.008115157266947242`, 403.9888714010655`, 0.1027695319041006`, 9.711355034259547`,
372.50117745610584`, 0, 0, 0, 333.9655873736296`, 0.6784327923306188`, 0, 0, 0.03577123459481179`, 0, 0,
37.444158083575104`, 0.07381272684217945`, 5.307321387838847`, 0.5920163781553305`, 0, 0, 1.152078773428255`},
{0.2538137652575522`, 0.7540411224449701`, 8.794714857317246`, 0.9184514413182489`, 0.11359389239638507`,
0.27911589396705727`, 0.11507441894157476`, 65.76953791858952`, 0.10155553687026442`, 2.859221903044178`,

```
91049.2654400193`, 0, 0, 0, 76.35280168178367`, 10.26330912866354`, 0, 0, 19639.827510262498`, 0, 0,
  110.55653050538653`, 71212.26527698334`, 9.115385281388017`, 1.0000000000012783`, 0, 0, 0.17831913269501568`},
{0.07943689831455408`, 1.7727348695278575`, 3.5998215792473456`, 1.4458682286750966`, 0.6331999429153172`,
  0.478090570864118`, 0.46084046549589247`, 472.0739644000896`, 0.10830302137693731`, 2.3782715249335666`,
  72.31348620505452`, 0, 0, 0, 10.332462955265331`, 2.303069693682795`, 0, 0, 0.6338779791643236`, 0, 0,
  58.32474218491888`, 0.7193328653532274`, 1.1380746351948783`, 0.9987319883415517`, 0, 0, 0.3438225272557363`},
{0.055568082805285024`, 1.296674173505255`, 4.7380956025626375`, 1.4538691472242138`, 0.09550221320066177`,
  0.19041481571096486`, 0.0071318152354094295`, 86.20076273581975`, 0.04910104396213488`, 4.3238451034891945`,
  120296.3074315137`, 0, 0, 0, 147.29444538194028`, 0.7480883672879537`, 0, 0, 66970.1062076241`, 0, 0,
  13.857526648028617`, 53162.86296034293`, 10.618865280747494`, 1.0000000000010396`, 0, 0, 0.3139499499033247`},
{0.1596260086552564`, 2.5471852815710525`, 0.22684585875990315`, 1.2716791760964523`, 0.8208442532787252`,
  0.3888937433082962`, 0.04289548126799573`, 69.96603930346842`, 0.07461229317798801`, 1.378327695847128`,
  284870.1840749884`, 0, 0, 0, 1.5408905589215078`, 0.024789390269905245`, 0, 0, 86840.07634335257`, 0, 0,
  0.9020452862089052`, 198027.63968581587`, 4.157310378218687`, 1.000000000001114`, 0, 0, 0.09541639508677296`},
{0.14264033419885946`, 0.8035889732308119`, 6.673404894657215`, 1.4157343373387477`, 0.18185291353504507`,
  0.4765318357304136`, 0.03778101114485503`, 210.869852474353`, 0.22822304787397907`, 1.0663935058760412`,
  55.67351992410398`, 0, 0, 0, 36.104345828067714`, 1.513058629106873`, 0, 0, 0.22492073882351732`, 0, 0,
  17.369674717171616`, 0.4583252764863029`, 1.187321608900792`, 0.9334175239895807`, 0, 0, 0.20214951318998842`},
{0.2209992311467341`, 3.548991401043337`, 2.677760813541523`, 1.1645638964766105`, 0.17856966244183803`,
  0.2409694046188614`, 0.16792458477559433`, 53.365358865447796`, 0.019626895089088575`, 3.834874658630479`,
  1.36730593164984`*^6, 0, 0, 0, 23.73654763818811`, 1.0631658604876244`, 0, 0, 328887.1441055133`, 0, 0,
  53.902378525048384`, 1.0383400854480534`*^6, 14.719437257513663`, 1.`, 0, 0, 0.1450328050962076`},
{0.08986394714808477`, 2.7800808748945913`, 9.536673819459821`, 0.8308776528106754`, 0.5315183679881144`,
  0.4614339535602666`, 0.046951751845033245`, 1052.1471359283046`, 0.24213976319820152`, 2.8230201755788666`,
  41.81085216188932`, 0, 0, 0, 25.240682662126332`, 0.4064412749836607`, 0, 0, 0.009073161980929055`, 0, 0,
  16.141994504997765`, 0.011647859267474288`, 0.5839724069515665`, 0.20869686132376963`, 0, 0, 0.2900659622935604`},
{0.08237232438525194`, 0.904854561594771`, 7.190948808493694`, 0.9264355987892083`, 0.7428148600467364`,
  0.6232177046085758`, 0.0730718251800762`, 62.25627183567972`, 0.1453325784006102`, 9.786938339680962`,
  70111.33250513591`, 0, 0, 0, 297.8249530014896`, 21.654976028603954`, 0, 0, 31933.820258429365`, 0, 0,
  279.9229120101098`, 37578.04287410966`, 35.85536105202004`, 1.0000000000002305`, 0, 0, 1.5229819255557968`},
{0.14754836278222488`, 0.5816922789151606`, 9.91636448422577`, 0.7971447359984003`, 0.9834799375197849`,
```

0.6545637466726528`, 0.017670578507760985`, 406.58618651198583`, 0.1328908282216244`, 3.7962615345853075`,
298.7116857186342`, 0, 0, 0, 237.79234590030978`, 6.393896457161015`, 0, 0, 0.4145231932889547`, 0, 0,
53.132574304479334`, 0.8737459786435651`, 1.9746860794833625`, 0.9993786787050523`, 0, 0, 0.2716691106028291`},
{0.06758332214646862`, 3.0558788120173555`, 4.64800500980858`, 1.4828314301436276`, 0.7395538908104162`,
0.3075642133576061`, 0.04417074894369504`, 145.23097869725217`, 0.1743358052899487`, 7.770691560418564`,
15237.204425877404`, 0, 0, 0, 184.3143912870575`, 2.509473027088446`, 0, 0, 7601.601405435337`, 0, 0,
109.55207789726657`, 7339.163951608383`, 11.761375816465602`, 1.000000000000001`, 0, 0, 0.8837634520336762`},
{0.16631267310517406`, 2.413572311283116`, 5.471184235465843`, 1.4579537524645423`, 0.9497208653345599`,
0.439033675828699`, 0.21085287275356013`, 3128.2027597762994`, 0.18409987492192065`, 5.612876798830806`,
61.677985584717064`, 0, 0, 0, 15.631106174578136`, 1.2958080511211751`, 0, 0, 0.021363907468654995`, 0, 0,
44.67894904176853`, 0.05075840798692196`, 0.3688171121388909`, 0.2689619128817412`, 0, 0, 0.23271241439608115`},
{0.18539796406610926`, 1.3871953304353832`, 7.757698949197568`, 1.3585428901946988`, 0.4532718222049952`,
0.29058122288435795`, 0.010738744574908157`, 486.55354471220693`, 0.20251048022954932`, 4.51278311919668`,
106.72598666736846`, 0, 0, 0, 92.62360919403208`, 0.6682787533658657`, 0, 0, 0.03340615076036453`, 0, 0,
13.243330944261544`, 0.08847760483224389`, 1.9868498302723758`, 0.4094292875846528`, 0, 0, 0.385208275896955`},
{0.04144083471615087`, 3.41458508027812`, 2.4600046087844927`, 1.2787341279549589`, 0.569252466493285`,
0.6736151572579898`, 0.4762197736813673`, 718.8382183063302`, 0.07598079948793823`, 4.331845758841565`,
94.40191581040338`, 0, 0, 0, 13.134825083273519`, 1.6036667872693122`, 0, 0, 0.902554542365586`, 0, 0,
78.2265240792479`, 0.534323051606878`, 1.4243966133655426`, 0.9998637306805985`, 0, 0, 0.8559685758826732`},
{0.13724293356211342`, 3.6916535362348286`, 5.489803233715362`, 1.3457045887873154`, 0.0754297582883523`,
0.6293482685634375`, 0.017573021108899616`, 778.5577282359714`, 0.17674997296512807`, 2.6916234726152264`,
40.13379743236351`, 0, 0, 0, 32.15350230222615`, 0.14668913500733366`, 0, 0, 0.028254123308680688`, 0, 0,
7.736078056815016`, 0.05539541097299154`, 0.8328739550983658`, 0.35754090318291465`, 0, 0, 0.593808163959279`},
{0.08176385784973572`, 2.023736863649704`, 9.823987283528833`, 1.0905304980935506`, 0.6417627833690904`,
0.2509575610218163`, 0.3071390754059633`, 168.49990583950182`, 0.0669392059366426`, 7.784078345121018`,
600.1270112577715`, 0, 0, 0, 118.91723434942925`, 16.026942624528672`, 0, 0, 0.84660339981505`, 0, 0,
463.34735144082043`, 0.9888794291084954`, 9.703472982868652`, 0.9988644238651814`, 0, 0, 0.5000953611894512`},
{0.20636275093165019`, 3.041433098250965`, 7.709885134336993`, 1.0647262222072884`, 0.28984076250310165`,
0.17246518959666646`, 0.007377660819744997`, 464.8055142552055`, 0.19020267666710844`, 9.452984008311837`,
168.195936249229`, 0, 0, 0, 152.35919830019034`, 0.35134785365373683`, 0, 0, 0.01847792568260599`, 0, 0,
15.265728444313037`, 0.0544736510767613`, 4.285843041890438`, 0.3117558483111462`, 0, 0, 0.6402389552490766`},

```
{0.2140607830978551`, 1.6917207171996171`, 1.9218182027339432`, 0.9997605714664014`, 0.5513493876922198`,
  0.4464250883384161`, 0.2328414126003874`, 611.4769203082982`, 0.08477583973629349`, 2.188379571162489`,
  50.27417320276394`, 0, 0, 0, 9.121864531105887`, 1.0953497898928768`, 0, 0, 3.3477383984145823`, 0, 0,
  26.47179903060029`, 10.237421473876893`, 0.8396101195026512`, 0.9999999545671305`, 0, 0, 0.4128582945988603`},
{0.17696168772261972`, 1.6101558302339125`, 0.7107550695951144`, 1.133554468522512`, 0.8128396324743541`,
  0.15732538361458315`, 0.06392981831267872`, 277.86150200910555`, 0.15413566109625954`, 1.907309832357937`,
  23455.448721662666`, 0, 0, 0, 5.471051765267195`, 0.20272701584411584`, 0, 0, 6645.394412924956`, 0, 0,
  4.663172664390376`, 16799.71729848097`, 1.3989749076613207`, 1.0000000000003626`, 0, 0, 0.062211265570376845`},
{0.04328176764500202`, 2.9602821723444555`, 0.6131528539518083`, 1.0058908218647606`, 0.19964040264767258`,
  0.6669684114614443`, 0.23851965705730427`, 2287.3379976105803`, 0.08593310169788088`, 8.660468891635798`,
  50.06694733360331`, 0, 0, 0, 11.748199222905255`, 0.8533751044364636`, 0, 0, 0.850476358609902`, 0, 0,
  36.08901582836925`, 0.5258588591559813`, 0.9401618062871069`, 0.999978733694613`, 0, 0, 2.2352347480605093`},
{0.08735803097627126`, 3.963951857586263`, 7.965851341925532`, 1.189504619761999`, 0.40822475538796854`,
  0.4952091412789442`, 0.01984161894285721`, 1116.2764923646498`, 0.2379409272833778`, 1.293491554272407`,
  15.845252533906509`, 0, 0, 0, 12.374994498402648`, 0.059643218262354136`, 0, 0, 0.012761537633320138`, 0, 0,
  3.377469226049734`, 0.01592603999823442`, 0.259779674398017`, 0.20328661058968045`, 0, 0, 0.17577056159951326`},
{0.15281896372135534`, 0.55010519971598`, 2.817048022996838`, 1.3198176246010052`, 0.1623821637675257`,
  0.6925657414172184`, 0.11807724637466313`, 76.78768290686035`, 0.04193274426603899`, 1.2341700274455292`,
  454294.9909386107`, 0, 0, 0, 10.944462818426269`, 2.022821644362139`, 0, 0, 142710.60376230252`, 0, 0,
  15.896638638023441`, 311555.5225572018`, 3.5699321946107685`, 1.0000000000000147`, 0, 0, 0.15473927486259167`},
{0.19086658543964008`, 1.9905232804946325`, 4.078173781767919`, 0.8519485127716493`, 0.08904379736388202`,
  0.23536266216215151`, 0.2499192727129545`, 192.66677656391008`, 0.127316992739278`, 7.8449864029309175`,
  7092.509972562512`, 0, 0, 0, 60.69279583556107`, 6.643790057121158`, 0, 0, 1834.4147735632448`, 0, 0,
  188.92312542026292`, 5001.835487286369`, 8.731385021822419`, 0.9999999999999238`, 0, 0, 0.6784024881171183`},
{0.06664208120921677`, 2.003234095381499`, 6.199949723153024`, 0.888873086526756`, 0.04784068730663105`,
  0.37023377411027647`, 0.049828770496122325`, 349.42476257926376`, 0.05267583263583803`, 7.0912634633886915`,
  183.49474747556806`, 0, 0, 0, 108.93215738744479`, 2.515134803670689`, 0, 0, 0.03421204360599895`, 0, 0,
  71.97719704562552`, 0.032570882690229154`, 4.597842486210418`, 0.5613856038483607`, 0, 0, 1.0488433015379324`},
{0.22983292033798441`, 0.7814702114508636`, 3.2012106727008174`, 1.142026804432239`, 0.1064467335224153`,
  0.4404014420960507`, 0.006695571873889409`, 4217.329674971789`, 0.15347806495470107`, 2.7989551297088675`,
  6.6341998642552`, 0, 0, 0, 6.040177560275248`, 0.046375877536743555`, 0, 0, 0.00554119475661736`, 0, 0,
```

0.51773381178369`, 0.018193556758212506`, 0.15312703442338224`, 0.09790367119486842`, 0, 0, 0.47303328205692763`},
{0.13211782844464454`, 0.44983284117427225`, 1.0762780754680463`, 1.3187859174208114`, 0.8445651695343355`,
0.2965729998791703`, 0.054223541794765`, 616.1259401245338`, 0.10515194159293045`, 5.24820204317008`,
7264.328790209427`, 0, 0, 0, 26.05843805054559`, 2.624163748275537`, 0, 0, 2500.0998278442808`, 0, 0,
16.86335763704736`, 4718.682287851659`, 1.838514004902742`, 1.0000000000000717`, 0, 0, 0.49109588439143365`},
{0.17817224333688036`, 1.8900871904197354`, 9.98947566566499`, 0.792780936871982`, 0.9109839945193492`,
0.4314201285170226`, 0.03598067399007004`, 128.67700477089366`, 0.059019163579936806`, 2.366574414393561`,
208.9501876429785`, 0, 0, 0, 134.25215469954733`, 2.38745966340107`, 0, 0, 2.2093412762407003`, 0, 0,
64.46438467768824`, 5.623475592636604`, 4.197184645127444`, 0.9999999922172493`, 0, 0, 0.3698367400625289`},
{0.2509149027719233`, 2.580879402706512`, 1.8893709427148198`, 0.8255744895957449`, 0.1652280455910582`,
0.6768016323195194`, 0.009322737452992792`, 2112.3599589856663`, 0.08696925005081763`, 4.203193192682905`,
26.11496607326686`, 0, 0, 0, 22.99062886459954`, 0.07932150775683831`, 0, 0, 0.02212026486953407`, 0, 0,
2.9245606508749864`, 0.07929005870039847`, 0.4756950947512479`, 0.4291046464776467`, 0, 0, 0.8879924472674233`},
{0.08555200390318413`, 3.7277336286219658`, 7.5570643278825855`, 1.1117862222014783`, 0.20420751548200644`,
0.3245432768958507`, 0.00950175247006993`, 144.2017035489447`, 0.14385517513119161`, 5.452203080835416`,
347.82159403096705`, 0, 0, 0, 303.7020958110105`, 0.7388545679221763`, 0, 0, 1.7034427246476294`, 0, 0,
39.346471707206405`, 2.081899123255883`, 8.472313691387646`, 0.9998560459682716`, 0, 0, 0.7378553948090492`},
{0.07297367338130328`, 1.9451098216604938`, 8.396918429735464`, 1.2137718043700492`, 0.7314346040557373`,
0.4669601248496963`, 0.016599550382003152`, 192.8440550768832`, 0.21285961816083276`, 7.382641950639707`,
519.8715870231912`, 0, 0, 0, 418.5881191473881`, 3.3780919815434816`, 0, 0, 1.8804852844958806`, 0, 0,
93.86799845389687`, 1.9603702707021893`, 8.539009282811891`, 0.9999899670096274`, 0, 0, 0.9606237444246684`},
{0.07358197980029185`, 1.0291227850237563`, 2.532589152827489`, 1.0117020834715413`, 0.8098748594005145`,
0.5301759885595309`, 0.008133019998886755`, 830.3082383512614`, 0.02407211472876364`, 4.5236701681781`,
95.69019050420202`, 0, 0, 0, 84.7599321183816`, 0.6182647388006959`, 0, 0, 0.5580678106932286`, 0, 0,
9.089576141093662`, 0.5866247767660383`, 1.2066786581972506`, 0.9998101363542926`, 0, 0, 0.5527003372804556`},
{0.18797192534273177`, 0.513697145142666`, 2.3563599620417435`, 0.8181944603817968`, 0.26394673984920813`,
0.3209514271347109`, 0.025430853857263652`, 265.9518711391782`, 0.07720757078553087`, 7.134393115551401`,
22006.71583029791`, 0, 0, 0, 98.77975918658576`, 4.211206977815588`, 0, 0, 5935.125644386877`, 0, 0,
30.904071458696343`, 15937.67135037741`, 5.716528734236558`, 1.0000000000005265`, 0, 0, 0.5685692194572877`},
{0.2528075291703835`, 2.1409422046846034`, 2.3989238084434295`, 1.1058748574151904`, 0.4698643832575349`,
0.20099009902051257`, 0.02857754945135185`, 57.682491948215834`, 0.240427409232489`, 4.4402688370357115`,

106670.808020232`, 0, 0, 0, 57.70391870437626`, 0.7356931903117923`, 0, 0, 23113.742127655358`, 0, 0,
22.501094297679735`, 83476.11481677076`, 15.633138012423817`, 1.0000000000023601`, 0, 0, 0.1286401765036652`},
{0.07054141508315104`, 0.5799884401515247`, 2.99755260715291`, 1.1960365141205305`, 0.29917509086343896`,
0.44670601886119`, 0.13984458882947357`, 3612.976166750159`, 0.22678215214973713`, 7.656934137991781`,
25.730362813570387`, 0, 0, 0, 8.877677695843058`, 1.81296951242969`, 0, 0, 0.009098534466237384`, 0, 0,
15.021447993662413`, 0.00916890709186834`, 0.4739066492349142`, 0.15015708939027084`, 0, 0, 1.018280569757712`},
{0.13968170289558773`, 2.9225254745826437`, 6.832387664740558`, 1.248628328449276`, 0.2500405375269379`,
0.6548966838897512`, 0.01310969508256717`, 2778.8012693248406`, 0.21649543926111503`, 9.33477132672460`,
47.06514159535508`, 0, 0, 0, 39.719979516895776`, 0.17081787277185007`, 0, 0, 0.006083784864396233`, 0, 0,
7.131708352710705`, 0.012139906141558486`, 0.7805135882251287`, 0.09928244707312583`, 0, 0, 1.6542822326989568`},
{0.14258976979051796`, 0.734197214461469`, 7.171919374129239`, 0.945223896823731`, 0.9937893052327855`,
0.2333579308191156`, 0.10311334331996398`, 86.76571756149787`, 0.17032024165228216`, 9.316237353631934`,
24735.381002245496`, 0, 0, 0, 219.83002813840054`, 26.852061247322418`, 0, 0, 7970.723163752758`, 0, 0,
281.63869386189856`, 16236.336871192198`, 22.715317525872422`, 1.0000000000004348`, 0, 0, 0.6697108264379853`},
{0.06222401125326871`, 0.8031654209648247`, 7.912640972265759`, 1.0061100735669513`, 0.06612689335476452`,
0.2257508005074531`, 0.2688428052003874`, 1016.6634644345198`, 0.08792308855751246`, 8.5639571694838`,
65.07309714397097`, 0, 0, 0, 14.825208214017687`, 4.027293650722222`, 0, 0, 0.006494009052783631`, 0, 0,
46.20832857616125`, 0.005772618462559186`, 1.8267779452166237`, 0.12973715512977857`, 0, 0, 0.8459493654220915`},
{0.045675945991483086`, 1.972555523342196`, 9.234608119853927`, 1.10067888297902`, 0.060950291683742996`,
0.5641176167348642`, 0.005654158004083115`, 79.62566643520867`, 0.10418567571675635`, 3.9217157633863025`,
40611.54441421846`, 0, 0, 0, 295.2522704564025`, 0.7996396907627535`, 0, 0, 24381.987938731283`, 0, 0,
22.533338409967357`, 15909.576632206876`, 11.634072913337993`, 1.0000000000000002`, 0, 0, 0.7718963370398129`},
{0.19725457740267938`, 0.673691333497294`, 1.5958254230238804`, 0.9055402555445284`, 0.8526577157919533`,
0.18700866613724199`, 0.221308084367558`, 137.27532297707575`, 0.16306687928789393`, 6.444987143866487`,
41177.728449479386`, 0, 0, 0, 19.59093416776736`, 5.547098003118837`, 0, 0, 10764.808315885759`, 0, 0,
53.38616929659013`, 30334.395931012907`, 9.580071822118246`, 1.0000000000042728`, 0, 0, 0.2182239551681294`},
{0.17905209129143224`, 1.6872990209581902`, 6.743325221637608`, 0.8692054788625995`, 0.5776472609105132`,
0.5432377341075647`, 0.12670077461750193`, 462.43790721394`, 0.057341353016641616`, 6.31060148160045`,
253.65003828437148`, 0, 0, 0, 92.05097404757947`, 6.427850610123717`, 0, 0, 0.06536220023749234`, 0, 0,
154.93865773324632`, 0.16718912348473539`, 2.9677773808653445`, 0.7949588139241099`, 0, 0, 0.6429565089349959`},
{0.21834559506325857`, 2.5253820753047362`, 6.659656299000567`, 1.4243771911650907`, 0.9747752944821462`,

0.1772255451247845`, 0.01986842431027196`, 102.11368670749582`, 0.09272454748376258`, 4.914431231863471`,
42988.39881764499`, 0, 0, 0, 200.64256545594384`, 1.4928206051360497`, 0, 0, 10373.882872442024`, 0, 0,
53.85631996937347`, 32358.451841427155`, 10.073664898893513`, 1.000000000000125`, 0, 0, 0.29744134836411473`},
{0.2429292029568641`, 1.5636324867576725`, 8.233163527811005`, 0.9303215118662402`, 0.8597394087604655`,
0.6215210972087029`, 0.12468068919290341`, 61.92974770793832`, 0.07970211447796205`, 9.85507721294049`,
222607.84358287588`, 0, 0, 0, 239.38145025002552`, 17.795110265235756`, 0, 0, 49649.318539834814`, 0, 0,
397.5001788022503`, 172303.84828905124`, 34.333638267034566`, 1.0000000000007385`, 0, 0, 0.9180421450623234`},
{0.09149398523540997`, 2.310449584405898`, 9.73345399143432`, 1.4402850137921024`, 0.7807243420630963`,
0.37700223940662914`, 0.04452259966898517`, 785.475395176315`, 0.18361612690246515`, 4.855709318281368`,
130.85907622438398`, 0, 0, 0, 80.43720482414446`, 1.4804773164693015`, 0, 0, 0.03128741262913068`, 0, 0,
48.865260007983586`, 0.04089442955925364`, 1.29075587666227`, 0.3721330609862755`, 0, 0, 0.2811660598216434`},
{0.11249424084619364`, 2.9716096914630903`, 4.4521238184847185`, 1.4266434477040648`, 0.9997833218301952`,
0.5867347171724102`, 0.06910846060462165`, 1820.2194505805683`, 0.24722705290784003`, 7.621221876581703`,
207.3438173544296`, 0, 0, 0, 104.61399742519369`, 2.3574484833935996`, 0, 0, 0.1128938939196488`, 0, 0,
100.07738229110542`, 0.18142732703798678`, 0.859686294467838`, 0.8064175001367722`, 0, 0, 0.3072050590815158`},
{0.09866336490626898`, 1.7258477745644702`, 0.8848358466481533`, 0.910425763108387`, 0.9172877053292023`,
0.36334488527428765`, 0.3804604060347452`, 1006.1834168170927`, 0.013687075322010439`, 8.165252701558408`,
9257.29364849209`, 0, 0, 0, 10.603370033810643`, 1.9501860891169016`, 0, 0, 3816.8688610817944`, 0, 0,
48.08177602698558`, 5379.787503432669`, 1.8255941961394857`, 0.9999999999999732`, 0, 0, 1.1416191699944227`},
{0.22803797815686577`, 0.698146546972529`, 1.035170425484365`, 0.9822431866703154`, 0.6417091531359376`,
0.21874316930074555`, 0.005531062831453475`, 1727.3746181384265`, 0.11140836748423671`, 9.157656749061658`,
68.60746280959205`, 0, 0, 0, 63.05593122111219`, 0.4460889693692715`, 0, 0, 0.1375626728770324`, 0, 0,
4.449078194395586`, 0.44813591132477004`, 1.108479249915986`, 0.9345181075361613`, 0, 0, 0.5437884520623447`},
{0.12680960193351426`, 3.133735649340868`, 7.511849069758438`, 1.0897808931740574`, 0.04885314856347578`,
0.24060421848165847`, 0.3693592758602631`, 883.7388687037887`, 0.08961301811176398`, 2.076054864701238`,
17.752106102751814`, 0, 0, 0, 3.2363477476383697`, 0.31664411271944704`, 0, 0, 0.008429736026834661`, 0, 0,
14.175413488326283`, 0.015271020999561645`, 0.5152084613497765`, 0.15277261482881144`, 0, 0, 0.23084783414278584`},
{0.11645318221252937`, 1.153640861264721`, 2.619716900319183`, 0.8529577052637052`, 0.5619295219152276`,
0.5231720769226155`, 0.04609815796088822`, 565.9088521347549`, 0.20476637224893374`, 8.831648882728363`,
201.75684757988182`, 0, 0, 0, 121.03225856359961`, 4.425884341397462`, 0, 0, 1.2585050597463754`, 0, 0,
72.94115747811156`, 2.093670271971972`, 3.590680739169312`, 0.9999989204659508`, 0, 0, 1.4638077301961343`},

{0.042446348872052314`, 3.5353553586920903`, 5.120248896798044`, 1.1724129507972807`, 0.07388809680757258`,
0.47979821691937163`, 0.43439767413472635`, 83.35178002891637`, 0.15043407845955598`, 2.47226753883535`,
42440.865848042566`, 0, 0, 0, 16.932845710996283`, 1.7773363803811608`, 0, 0, 26351.784781619637`, 0, 0,
89.7645099511278`, 15979.100717740946`, 6.813498339632957`, 0.9999999999999994`, 0, 0, 0.40517968409536254`},
{0.1455833195103322`, 2.7843188408171127`, 7.76885376753495`, 1.3211895319342402`, 0.9135586666939064`,
0.6453968687054246`, 0.1333226247130129`, 368.7414592704895`, 0.16919558147748776`, 3.2843355334407627`,
209.76113366710553`, 0, 0, 0, 72.72623243809846`, 3.3064441658129824`, 0, 0, 0.7180381738289169`, 0, 0,
131.51706838546983`, 1.4933482983021409`, 1.9439028191082217`, 0.9997600974855981`, 0, 0, 0.3474388111136395`},
{0.19543798537508206`, 1.2324134940956721`, 7.418056367639444`, 1.0725732268441615`, 0.3282068086166905`,
0.15859490968399015`, 0.32000646124266346`, 1339.7132456219883`, 0.07635813740352487`, 1.32927343177637`,
7.774764526594433`, 0, 0, 0, 1.5593816481774654`, 0.3329152880753311`, 0, 0, 0.005588642238494854`, 0, 0,
5.861275620211226`, 0.015603328286769962`, 0.20905300170778526`, 0.10572036360969439`, 0, 0, 0.08975335270303055`},
{0.14288798158791094`, 1.8492145343896036`, 4.320087837799633`, 1.416387968755192`, 0.03040014321801321`,
0.26264512496919146`, 0.017396141813896236`, 476.32655268366966`, 0.1492295937607505`, 1.5210123574307455`,
24.129362571981456`, 0, 0, 0, 19.34541060822146`, 0.1692700180116515`, 0, 0, 0.044223479275774906`, 0, 0,
4.471665393479082`, 0.09027148132157664`, 0.7119632630914821`, 0.48085250573791394`, 0, 0, 0.1972311452823421`},
{0.08126639977214484`, 1.4848577063399988`, 1.4511591326569544`, 1.4690309046886825`, 0.09088454373979671`,
0.2634538760346167`, 0.006391735651833628`, 757.569692438266`, 0.1906091612906891`, 5.0187956659047614`,
54.5659135160035`, 0, 0, 0, 49.6376873683472`, 0.19826690112351747`, 0, 0, 0.21857445463209413`, 0, 0,
4.205687657791512`, 0.25375370014442983`, 1.471040573520449`, 0.9333098728755304`, 0, 0, 0.6275774939711962`},
{0.15406825481222286`, 3.5722547377798985`, 5.518246507815627`, 1.3255687223204002`, 0.2843400333432804`,
0.5740150104722689`, 0.007989301755943026`, 636.0707307721444`, 0.19470033172956325`, 2.3727553064621407`,
49.23941847690839`, 0, 0, 0, 44.11847846991745`, 0.09499733429187215`, 0, 0, 0.042954973749202985`, 0, 0,
4.847923964294297`, 0.09454282630063339`, 0.861771145071176`, 0.4926279495693625`, 0, 0, 0.404524864694873`},
{0.1284209869933174`, 2.657038147126583`, 3.485135675760706`, 0.826438046300177`, 0.2940359222573743`,
0.4455502493814366`, 0.0077922251797686165`, 642.5517685154364`, 0.18333530742444448`, 4.125506851960305`,
76.29055054462059`, 0, 0, 0, 68.6069832167959`, 0.19211554008130477`, 0, 0, 0.047685869959401515`, 0, 0,
7.292261695026472`, 0.0874838069403041`, 1.4425994420496933`, 0.7017117720252724`, 0, 0, 0.5710135843551346`},
{0.1519197321396482`, 1.414313316427302`, 9.693328908271443`, 1.0879341282517354`, 0.5935096502675883`,
0.32269331092414044`, 0.04853394307287227`, 323.7551755246228`, 0.012675975614693441`, 6.5738064876305415`,
293.69172923458626`, 0, 0, 0, 175.07708225714228`, 5.586199257760622`, 0, 0, 0.04918947906900626`, 0, 0,

```
  112.86622854953073`, 0.1067550354894853`, 4.302788707021305`, 0.6204183086822823`, 0, 0, 0.4843140864042932`},
 {0.10245812606330185`, 3.806669862078807 8`, 2.784482506819291`, 0.8153557037427375`, 0.41559745180116914`,
  0.2970790998891699`, 0.019454402362339196`, 101.06708659388384`, 0.03906278599147861`, 1.0136093018296712`,
  129608.872405188`, 0, 0, 0, 17.71607485126992`, 0.0869126942767891`, 0, 0, 52598.51996859304`, 0,
  0, 4.726399056221741`, 76987.79699550293`, 2.142599016557059`, 1.`, 0, 0, 0.083558317113368`},
 {0.08994858347063645`, 1.3905867780774148`, 6.975873482712982`, 0.9748960320168515`, 0.19705721946727817`,
  0.428438510479348`, 0.008778830773896138`, 53.85788205383214`, 0.2055785802889155`, 2.165498620130503`,
  53759.61969917746`, 0, 0, 0, 110.77461530327123`, 0.6531175596242664`, 0, 0, 23472.897637976705`, 0, 0,
  12.974523470624176`, 30162.19846410374`, 8.871127221120604`, 1.00000000000024`, 0, 0, 0.2552540074158636`},
 {0.2555268425086469`, 0.6701554458004759`, 6.763443756394938`, 0.9242821290525386`, 0.9506231196396431`,
  0.49497463337651615`, 0.1535425304369218`, 4370.366828894948`, 0.17262313668823565`, 9.906703002274519`,
  87.38994679506919`, 0, 0, 0, 27.67219742361427`, 5.644132332456548`, 0, 0, 0.008316248442089915`, 0, 0,
  54.03494313448996`, 0.030357495798890292`, 0.46531991502527636`, 0.17586298846708914`, 0, 0, 0.396965231479573`},
 {0.13340663060001784`, 0.4096518827228479`, 7.81563732077705`, 1.2681845828171425`, 0.264399527619257`,
  0.43786671579755854`, 0.11528071789135624`, 3397.5276395199044`, 0.15041338875391808`, 8.11261999427796`,
  27.78841443647035`, 0, 0, 0, 10.810443009228429`, 2.47622732225379`, 0, 0, 0.003592663228125407`, 0, 0,
  14.491302637300299`, 0.006846929944943989`, 0.5346952025666641`, 0.059070602022710195`, 0, 0, 1.091698102642102`},
 {0.09831375749458693`, 3.5924209186692435`, 1.6118282365623244`, 0.8326891410736474`, 0.6715734708323489`,
  0.5976781170151362`, 0.03815316295555065`, 115.73199628594814`, 0.0304507712440647`, 4.7148191409051226`,
  216972.37346790274`, 0, 0, 0, 41.29392194519502`, 0.4213925671086129`, 0, 0, 90210.26511593821`, 0, 0,
  21.625992472181707`, 126698.71611615321`, 9.08823103305409`, 1.0000000000012792`, 0, 0, 0.6142964101707995`},
 {0.09108239111305882`, 3.309852207951435`, 3.0566194803884894`, 1.4370488086380533`, 0.21629452377334557`,
  0.2421124982368058`, 0.4670454509414495`, 208.22129409387404`, 0.23659860207487343`, 3.442603557657883`,
  7466.42313325171`, 0, 0, 0, 12.774100120545762`, 1.4888313870709642`, 0, 0, 3207.8191782330055`, 0, 0,
  70.3973121966317`, 4173.940585882697`, 3.561703129277759`, 1.0000000000514209`, 0, 0, 0.3149501130744804`},
 {0.05787448179355209`, 1.5695297669455615`, 8.686108197389068`, 1.3362839901345183`, 0.8882645350301348`,
  0.2220019613784866`, 0.20455164120837535`, 127.50819012666335`, 0.2481722521034202`, 6.402394955038988`,
  448.8377815216411`, 0, 0, 0, 121.5453153777918`, 13.823049179231537`, 0, 0, 1.9329220196480417`, 0, 0,
  309.9383879536612`, 1.5980980033494923`, 10.778788828533717`, 0.9999947975004293`, 0, 0, 0.5611499131644467`},
 {0.2051260145065577`, 0.6712958473867032`, 3.046620158397806`, 1.125533167922526`, 0.5107051094055373`,
  0.47305603590798795`, 0.035994055865640424`, 73.71677428550161`, 0.03972186392362586 6`, 8.822427071575206`,
```

```
491269.438664817`, 0, 0, 0, 141.10422104616686`, 6.75052543136971`, 0, 0, 124939.0332016028`, 0, 0,
  64.73713842509723`, 366117.79909924744`, 25.30836598669515`, 1.0000000000005151`, 0, 0, 0.6302051961389624`},
{0.04365375531995927`, 0.9346121184769336`, 9.554451222092258`, 0.75459846537052`, 0.7382972174511151`,
  0.44670601219095474`, 0.02981461788584879`, 1443.346811017674`, 0.026466349135542522`, 4.5970910839514385`,
  68.83632808882238`, 0, 0, 0, 48.45851422789191`, 1.4183960108791893`, 0, 0, 0.008334817140057182`, 0, 0,
  18.937858579529056`, 0.005197800972400321`, 0.671101132807143`, 0.2112609837489674`, 0, 0, 0.31064276204320623`},
{0.18832502219499725`, 2.1446480190155537`, 7.011391892939212`, 1.2684842496371584`, 0.8800953133738048`,
  0.3019976503972389`, 0.01607749175819828`, 253.61560940445747`, 0.0505443969728126`, 8.205874646962478`,
  459.3330175848634`, 0, 0, 0, 371.3653600555775`, 2.646249474138071`, 0, 0, 1.101450450282008`, 0, 0,
  81.07533846473083`, 2.963295435657895`, 6.854008473153046`, 0.9999154103291084`, 0, 0, 0.6014286027580846`},
{0.2789832227001883`, 3.864947879576305`, 9.015175334965505`, 1.1939022970453932`, 0.8102757584298972`,
  0.6575626089292559`, 0.013706864097109712`, 131.89661477643062`, 0.1558047394747739`, 7.390258992479394`,
  9326.543438691775`, 0, 0, 0, 498.31187433571523`, 1.698031410079824`, 0, 0, 1751.5856868208812`, 0, 0,
  93.75432711202822`, 6980.90028206876`, 13.401234184628784`, 1.0000000000001823`, 0, 0, 1.5654666173210223`},
{0.10740283086494345`, 3.8162968757181996`, 6.158389397925204`, 0.8038652030060122`, 0.9187954678523191`,
  0.31270681581700654`, 0.027540968538212463`, 2177.2729976761966`, 0.021577241346914133`, 9.805063749007854`,
  112.67195629309921`, 0, 0, 0, 81.08802088437051`, 0.5681113861480389`, 0, 0, 0.010844686850040584`, 0, 0,
  30.972595828809855`, 0.016639286679127386`, 0.9233101057432938`, 0.25191613179752026`, 0, 0, 0.3804469398889166`},
{0.14667176624268824`, 3.777226248423828`, 0.6973504720596608`, 0.7758342249413661`, 0.494379064234173`,
  0.5228383904297224`, 0.014800119213367281`, 81.50804211451084`, 0.12794308526059`, 9.842771813142875`,
  91934.67769669769`, 0, 0, 0, 45.57292670276536`, 0.17304996882245732`, 0, 0, 29683.469841278893`, 0, 0,
  9.3378412075016`, 62196.099283313684`, 25.926643445505526`, 1.0000000000000078`, 0, 0, 0.864259942503491`},
{0.1536199904675806 2`, 1.6832845721068805`, 5.017027003357642`, 1.2257765840301957`, 0.8758480691873987`,
  0.30492603190517764`, 0.17353190337485183`, 1218.2701158582597`, 0.01908888394356914`, 4.8706184705887665`,
  88.4270901347789`, 0, 0, 0, 25.96794117075366`, 2.4888321497265715`, 0, 0, 0.03805301639099156`, 0, 0,
  59.84875371711911`, 0.08351005736062264`, 0.8243409445840376`, 0.4846190095228925`, 0, 0, 0.21768255053554647`},
{0.1285936813753743`, 1.2517058162042831`, 2.0098881407856393`, 1.434144339335301`, 0.06632650483661995`,
  0.16162194262920127`, 0.05011873598676769`, 354.0526180115314`, 0.1584833535532481`, 2.894273123117289`,
  3009.490192742772`, 0, 0, 0, 27.370525650810837`, 0.9878621827889326`, 0, 0, 1044.558156753674`, 0, 0,
  17.664469140073848`, 1918.9082683947126`, 1.7316842912613932`, 0.9999999999998307`, 0, 0, 0.212137487924534`},
{0.05871769486491246`, 0.822876240174752`, 3.5461717275684297`, 1.3634413021309426`, 0.14962847564878934`,
```

```
  0.2425078078443763`, 0.045889110192962865`, 1618.8495608622088`, 0.1824696213391762`, 5.484504386708753`,
  27.885896118678033`, 0, 0, 0, 17.107389856656585`, 0.8429945785541966`, 0, 0, 0.0136029996270007`, 0, 0,
  9.909717275548203`, 0.011410525477769011`, 0.7365941226932841`, 0.19245044943228562`, 0, 0, 0.5571666471303832`},
 {0.21414990207917795`, 3.2291184159683555`, 3.7200101298578847`, 0.893435725539721`, 0.18550851463991336`,
  0.6299970912008048`, 0.007788531351629846`, 1185.7534950662077`, 0.10596189074334122`, 9.85351453889281`,
  107.90952971362401`, 0, 0, 0, 97.12715568321971`, 0.22504945458037526`, 0, 0, 0.02098035932911245`, 0, 0,
  10.381590546987482`, 0.0641848842273687`, 1.9449681086712902`, 0.39465494154336356`, 0, 0, 1.8314993075330654`},
 {0.14399581513021464`, 3.6650427925648215`, 8.960857802594514`, 0.9993788071734813`, 0.7678347002262149`,
  0.3917750513543251`, 0.0682112112142393 6`, 446.58045234253916`, 0.09781275073737189`, 4.158206599173292`,
  196.1648640455883`, 0, 0, 0, 100.21770377574656`, 1.7948871046332944`, 0, 0, 0.05735172228750947`, 0, 0,
  93.97625780434001`, 0.1179772571415514`, 1.9499962161068367`, 0.7047383856138275`, 0, 0, 0.2539997093759647`},
 {0.21762093156371237`, 1.4799568210517782`, 6.880501462590445`, 1.1700220578418317`, 0.49477438305707566`,
  0.30056004401666714`, 0.03776191240235276`, 139.28017196074`, 0.13055880095851463`, 4.30304009869343`,
  571.5957497424812`, 0, 0, 0, 163.74827429672212`, 3.821763543195586`, 0, 0, 78.66173228170787`, 0, 0,
  80.80064320284755`, 244.54913510800776`, 6.953162845979232`, 0.999999999941123`, 0, 0, 0.6037110015464655`},
 {0.20676336326895633`, 3.023915070136316`, 2.1344025288266977`, 0.7647942269583019`, 0.38268253024987975`,
  0.6058201621166657`, 0.06287157366362177`, 52.11423962493933`, 0.23050267576131828`, 4.129012828530946`,
  92316.29930931194`, 0, 0, 0, 37.50372427725364`, 0.7491832875300305`, 0, 0, 23331.114627698207`, 0, 0,
  32.36380904937607`, 68914.56756052027`, 16.97394192424923`, 1.0000000000008284`, 0, 0, 0.3528800016323853`},
 {0.14056217319026204`, 2.827702131488536`, 4.822127390400377`, 0.9317030787852092`, 0.5881617813064273`,
  0.663416624487535`, 0.01875862506453023`, 1486.513173237306`, 0.1539754165327772`, 8.90838886614388`,
  127.34888668402371`, 0, 0, 0, 100.62143918348326`, 0.6430961079209907`, 0, 0, 0.022090731131169255`, 0, 0,
  25.978346216002386`, 0.04435887393088312`, 1.312465615972908`, 0.39772530066950007`, 0, 0, 0.9766647680453755`},
 {0.15432054500385795`, 3.142590945055387`, 0.6412922856028498`, 0.9338764110697222`, 0.4036132844000331`,
  0.253764192888976`, 0.009287914173605602`, 800.3088033129984`, 0.0773663480819351`, 1.9942494398991926`,
  7595.197344877993`, 0, 0, 0, 8.936745842428042`, 0.02531232212004141`, 0, 0, 2366.953965352216`, 0, 0,
  1.1363753470395328`, 5218.137513317069`, 0.526748637759796`, 1.0000000000005027`, 0, 0, 0.14166252556869594`},
 {0.1558313627948012`, 2.6183453593149384`, 1.5210778217010041`, 1.2213020905618333`, 0.8957140746385861`,
  0.2067404945185849`, 0.06533654021268133`, 3401.5830280519913`, 0.07866132934502523`, 1.2687008259414867`,
  6.28614533852348`, 0, 0, 0, 3.235871917647214`, 0.07676698794608382`, 0, 0, 0.031620756064684355`, 0, 0,
  2.871464094817349`, 0.07039293585945267`, 0.07740560265261512`, 0.4178958091816912`, 0, 0, 0.06536053561186543`},
```

```
{0.27749339228385056`, 3.960153029334033`, 7.7991060624230615`, 1.1159035815659704`, 0.47604804800745515`,
  0.18775668040836846`, 0.02760276902429692`, 890.1551806101527`, 0.14097999756547475`, 3.493022856148759`,
  38.071550460823495`, 0, 0, 0, 27.49114202214165`, 0.18274611856373807`, 0, 0, 0.010825807214387 56`, 0, 0,
  10.338608500417473`, 0.042915570973305464`, 0.8233694782474501`, 0.18820255665758512`, 0, 0, 0.22047195240840403`},
{0.1388142019807993`, 3.4960143968183592`, 0.8496516336862268`, 1.4057148799798922`, 0.27289215927666`,
  0.4161162296591887`, 0.1071416651953492`, 563.7099077527477`, 0.24491201094978954`, 8.47264522499269`,
  7216.9781578825605`, 0, 0, 0, 23.484448851095387`, 0.68050297047411`, 0, 0, 2399.8265842172514`, 0, 0,
  33.986402597930606`, 4759.000174006078`, 3.2482082982318086`, 1.0000000000000568`, 0, 0, 0.8046605905950109`},
{0.0729917462714273`, 3.1893255155706086`, 3.1041281178047555`, 1.014074092539919`, 0.28719491349307913`,
  0.2514291775131975`, 0.05245081359414425`, 423.2923229706584`, 0.11450677404966558`, 9.081189649788715`,
  203.0642495691459`, 0, 0, 0, 117.82037554138317`, 1.8240447851928925`, 0, 0, 0.15179434250972965`, 0, 0,
  83.10675107084582`, 0.1582819161985703`, 4.621687301078058`, 0.9487384642673123`, 0, 0, 0.8308557708574072`},
{0.11420284690084648`, 0.577787691297051`, 2.567084026939444`, 1.4491737131438955`, 0.8025258647238394`,
  0.3690270257872911`, 0.13672661029124697`, 86.53603740064875`, 0.011299238359278513`, 6.563498155623039`,
  1.2392422190728874`*^6, 0, 0, 0, 46.844699996295624`, 9.482359965814343`, 0, 0, 470880.5028814399`, 0, 0,
  78.2684410385064`, 768227.0568451777`, 16.20629520472433`, 1.0000000000004172`, 0, 0, 0.5421399241064936`},
{0.26373133325063186`, 3.0221428803953465`, 7.660698925394574`, 1.4577816176348994`, 0.5393449604126412`,
  0.22813670918251328`, 0.05400872825934766`, 78.22373236540282`, 0.11174025693188905`, 6.564224566850031`,
  102095.0625583668`, 0, 0, 0, 223.19343325927366`, 3.7948670748955826`, 0, 0, 21332.41775036452`, 0, 0,
  163.83757874917694`, 80371.81392518697`, 17.438554599918582`, 1.0000000000006353`, 0, 0, 0.3472601621576046`},
{0.21289488654178568`, 3.397179033451687`, 0.7632571166797604`, 1.0142699830327386`, 0.0835760348267307`,
  0.5184383444209231`, 0.049731529265758516`, 2070.206130759874`, 0.06133590421436391`, 5.427522099525574`,
  29.086836908647165`, 0, 0, 0, 17.002229893389224`, 0.23559833954197962`, 0, 0, 0.10259209646355301`, 0, 0,
  11.433853420114936`, 0.3120190390956047`, 0.624704428217243`, 0.84821873083187`, 0, 0, 1.1250303833672726`},
{0.22999162513252186`, 2.379033562263336`, 2.5591649989178986`, 1.3870393830660817`, 0.8120421313192066`,
  0.6084139253527632`, 0.017274035559015792`, 82.84440150538262`, 0.15048886650658344`, 7.465486944171833`,
  163958.07412768368`, 0, 0, 0, 122.57310065814526`, 0.8552947545961453`, 0, 0, 38222.3557716126`, 0, 0,
  29.068213240171676`, 125583.16743295104`, 19.23656853077887`, 1.0000000000001046`, 0, 0, 0.60896598065567`},
{0.1769680798340355`, 1.1633029664887262`, 4.7984019847292405`, 1.1781683757559547`, 0.8107109545176789`,
  0.3886685867136115`, 0.009172138674078711`, 82.52474973299203`, 0.0366989899628154`, 4.7780773811523165`,
  340862.7226664043`, 0, 0, 0, 161.23356183885593`, 1.181885740320605`, 0, 0, 96561.6168829557`, 0, 0,
```

```
19.641302682366916`, 244118.9132206653`, 12.304064836429516`, 1.`, 0, 0, 0.3665646354493819`},
{0.06792516420354372`, 2.678804428347224`, 7.00137481313343`, 1.1889039591838686`, 0.130109850192629`,
 0.17336690021836265`, 0.006561591278144790 5`, 559.1859620197067`, 0.12479016185335001`, 7.679232726268854`,
 100.64931860232065`, 0, 0, 0, 92.14990768111805`, 0.21322619563774586`, 0, 0, 0.01609918835343084`, 0, 0,
 8.159875387343213`, 0.015622000177859596`, 2.9216775572623934`, 0.2527739675872085`, 0, 0, 0.5984900600379364`},
{0.04696109785508959`, 0.9059167168392257`, 1.3656449005023852`, 0.8785005151977332`, 0.7117341492461746`,
 0.37541668809892415`, 0.06884837024201017`, 2425.2572297139236`, 0.025009444315193585`, 7.4432891311871785`,
 58.154526164970484`, 0, 0, 0, 29.586516172274628`, 2.0420162168063456`, 0, 0, 0.05906779937711862`, 0, 0,
 26.427094669452302`, 0.03962698152335503`, 0.6477431088653635`, 0.7522627709673673`, 0, 0, 0.5161587358744965`},
{0.18481744149515084`, 2.8908169503663625`, 0.7719563125670259`, 1.477735031707543`, 0.05143123515478387`,
 0.4060758128174351`, 0.19176878359926766`, 114.34865924476063`, 0.02384868002335655`, 4.461346915500394`,
 686710.1283072006`, 0, 0, 0, 7.443185993988482`, 0.4639587050561051`, 0, 0, 188636.28005863266`, 0, 0,
 19.16028126923157`, 498046.78076570353`, 8.197513756815376`, 1.0000000000001192`, 0, 0, 0.28801828230870624`},
{0.11940413672862621`, 0.6385297463968325`, 0.6629513696361364`, 1.2353159995947312`, 0.3854086082897601`,
 0.4772941268081121`, 0.0318040184551439`, 4110.432043833155`, 0.1129636900773972`, 2.562436110087784`,
 8.7154545791285`, 0, 0, 0, 5.924453013748718`, 0.2597394660142647`, 0, 0, 0.05953846040190511 5`, 0, 0,
 2.3693053623333844`, 0.10155912094916161`, 0.1438490604163631`, 0.6072809310888855`, 0, 0, 0.4333916138007292`},
{0.16756813285689298`, 1.9469615336157506`, 0.3903678609266308`, 1.2604526712473199`, 0.11212576188863221`,
 0.45563235469719776`, 0.0065229352440185`, 190.16738409260836`, 0.045949495779464666`, 5.424569346408827`,
 179836.78862903337`, 0, 0, 0, 15.366215348119287`, 0.04912031397985624`, 0, 0, 52984.37643818895`, 0, 0,
 1.3662194548272593`, 126835.61471905855`, 6.049850066115853`, 1.0000000000003624`, 0, 0, 0.404550424563747`},
{0.07183551197561577`, 3.34536856927257`, 9.136834569708476`, 1.4150518758492396`, 0.289745011670542`,
 0.5142172775325705`, 0.41137464999480466`, 1635.4988121892525`, 0.15390110584062433`, 5.743187227898911`,
 45.403468842338704`, 0, 0, 0, 7.243022768729974`, 0.7817820466251569`, 0, 0, 0.008160093019026265`, 0, 0,
 37.36212981144821`, 0.008374063711275748`, 0.7937241119503211`, 0.11636017107492314`, 0, 0, 0.8340293074342983`},
{0.2384422062087977`, 2.1587544451320504`, 5.065398759698054`, 0.8914719118393273`, 0.7773656949632277`,
 0.1997211683380481`, 0.4677952481930321`, 402.45383708693856`, 0.19717109492345014`, 2.7966746550751953`,
 97.80449009759113`, 0, 0, 0, 13.912185980322105`, 2.6203021949812744`, 0, 0, 0.10520973600488423`, 0, 0,
 80.80841444293007`, 0.35837773668061224`, 1.436396598727791`, 0.909785788439908`, 0, 0, 0.132280847794653`},
{0.049151474922052546`, 1.7029958107544303`, 5.984640650536118`, 1.1787547055685845`, 0.01700706976856159`,
 0.4330742864041065`, 0.015091472025893045`, 551.6808300976855`, 0.14911096385124378`, 2.965493287771624`,
```

49.79231323763358`, 0, 0, 0, 41.1106361419491`, 0.34016215005816713`, 0, 0, 0.02595168965460587`, 0, 0,
8.275638807518265`, 0.018222340332053208`, 1.2491790203797886`, 0.3739266237730092`, 0, 0, 0.5261905158243115`},
{0.05913727892151971`, 3.1040367182848003`, 9.835708161805986`, 1.4919208308945082`, 0.5501557876127892`,
0.5378623906029458`, 0.015011170514212647`, 280.8434087480442`, 0.0269165475419888`, 2.8649095039964387`,
177.74352047826127`, 0, 0, 0, 146.4857952566367`, 0.6824594161440132`, 0, 0, 0.12748656572769088`, 0, 0,
30.262558377860287`, 0.10770297994553507`, 2.2401464199504217`, 0.825890430329675`, 0, 0, 0.32268430419064315`},
{0.1028902787116574`, 3.248894721912677`, 2.8223976230891363`, 1.4617310236348016`, 0.21163710737184238`,
0.2736720564831455`, 0.052563491644450414`, 157.16268136885162`, 0.03718011423514023`, 9.1247890605319`,
132273.22917826028`, 0, 0, 0, 118.48503867324101`, 1.8181008262900569`, 0, 0, 53472.04324547977`,
0, 0, 84.3831168348405`, 78596.47761156005`, 12.345754966518049`, 1.`, 0, 0, 0.7060235077094906`},
{0.13701717915337458`, 3.8023949322113495`, 2.868993121081161`, 1.1378721602373125`, 0.9948229422967421`,
0.24001294614371227`, 0.1498271577324817`, 115.7171521166015`, 0.23683719126671393`, 3.874936558168838`,
28805.699328342518`, 0, 0, 0, 28.590752718562594`, 1.0530396152551995`, 0, 0, 9710.883948231896`, 0, 0,
57.20103566377387`, 19007.970366750324`, 6.995121739717677`, 1.0000000000001523`, 0, 0, 0.2263049794518096`},
{0.12767680767420947`, 3.2442504564252905`, 9.68431286349912`, 1.3341607174516774`, 0.1954172777492651`,
0.4247465971362525`, 0.25891115414709387`, 128.98950675665216`, 0.14024381160911475`, 6.508167926731167`,
541.0361674867996`, 0, 0, 0, 122.79114494641446`, 8.703493732480348`, 0, 0, 2.183328972932337`, 0, 0,
403.37590734420064`, 3.982292476665336`, 11.517310723644972`, 0.9994621695091643`, 0, 0, 1.0189340367057684`},
{0.15001338826271665`, 1.2298087626140388`, 4.614349177257099`, 1.406777099587714`, 0.7364706782176744`,
0.4119762663671258`, 0.007028619260401152`, 151.01374639678102`, 0.013332718449329678`, 9.705143720051169`,
337276.5667751779`, 0, 0, 0, 332.90751921478255`, 1.7677204003518183`, 0, 0, 107192.2732004795`, 0, 0,
31.056543402917974`, 229718.2299769535`, 14.06025857915104`, 1.000000000000174`, 0, 0, 1.0528707192618085`},
{0.20002396942953493`, 1.8945790659495865`, 6.47237980815893`, 1.0186003524309086`, 0.9948715481686918`,
0.5599748214378268`, 0.06647091233379371`, 636.3545963390636`, 0.214859543893563`, 8.583745959022096`,
435.5091992219396`, 0, 0, 0, 223.7750990181799`, 7.476216100890313`, 0, 0, 0.49492990966410005`, 0, 0,
202.3468931037431`, 1.4142549302916265`, 2.8188586656617667`, 0.9990490923393271`, 0, 0, 0.5048114078864476`},
{0.0426995949672023`, 2.62335318941125`, 9.6559263809094`, 1.1610440596461276`, 0.9636197200326426`,
0.24951659449471042`, 0.04756766795277323`, 104.67802103676537`, 0.10920782191363448`, 6.683384921443938`,
532.8192282341329`, 0, 0, 0, 319.5152868754587`, 5.396272893877545`, 0, 0, 3.5167956355763117`, 0, 0,
202.23328152981622`, 2.145224988879019`, 13.98366578549268`, 0.9999999696849449`, 0, 0, 0.7331128841026799`},
{0.2109009326123063`, 1.0923929642765158`, 8.593806993622852`, 1.1682615459081633`, 0.4499643089978256`,

0.499787017067712`, 0.035780106156421375`, 2693.2423996223015`, 0.018435796880638622`, 3.8866756448846873`,
21.33556390806201`, 0, 0, 0, 14.209031402753126`, 0.42789584099596756`, 0, 0, 0.004886607536086354`, 0, 0,
6.67757723067399`, 0.014722715523861868`, 0.31908587580155295`, 0.08575461212279667`, 0, 0, 0.46749411258398094`},
{0.1340526977662379`, 3.5323141681247634`, 7.368159769503098`, 1.0026662265470512`, 0.9539160554997537`,
0.16176440546396775`, 0.3999369934637664`, 62.1244000754222`, 0.21257466381414752`, 3.7256546669386505`,
34792.509861961975`, 0, 0, 0, 35.25959313580443`, 3.445521389276077`, 0, 0, 11862.600565203842`, 0, 0,
173.86662885595365`, 22717.337261269735`, 12.419086304555119`, 1.0000000000010818`, 0, 0, 0.18342198418845482`},
{0.138783517590022`, 3.0993312847332284`, 6.04052192414912`, 1.482957943290502`, 0.5742828550914962`,
0.6307262387789596`, 0.006158597883984412`, 223.4781142247734`, 0.1875211630254222`, 1.5541408452833192`,
95.9704023462689`, 0, 0, 0, 78.85662286688681`, 0.14994212270634252`, 0, 0, 3.4335079467266016`, 0, 0,
6.638861597186808`, 6.807347293142964`, 1.7086853677184761`, 0.9999999936590418`, 0, 0, 0.38058499399205803`},
{0.13089267345973798`, 3.445236909656975`, 9.841043091432308`, 1.0552044625838208`, 0.6603410104708771`,
0.19921054981100283`, 0.006557758988700662`, 85.91513533777795`, 0.010838310480980468`, 8.608032245025406`,
40476.67770227611`, 0, 0, 0, 625.533965574402`, 1.1295252334627681`, 0, 0, 13865.933304448203`, 0, 0,
55.592600353069216`, 25927.8440033377`, 21.64001286424822`, 1.000000000000019`, 0, 0, 0.8119379033413446`},
{0.0796809200271959`, 1.3022197759162255`, 4.457724373908043`, 0.819757860237272`, 0.9902524891863433`,
0.3732869275715256`, 0.027666886465779635`, 3268.657024391763`, 0.02176038735057556`, 2.895445262182028`,
27.87668098845641`, 0, 0, 0, 20.008468586281868`, 0.3997483953763486`, 0, 0, 0.013088053303238634`, 0, 0,
7.4365752264265685`, 0.01489811612238699`, 0.18162266299817412`, 0.28893731394469435`, 0, 0, 0.11244327072180114`},
{0.24784589915696786`, 0.6840125293527293`, 8.78766727614829`, 1.2765252846824708`, 0.05554020049431552`,
0.46845321895484016`, 0.4169012658954577`, 507.69507152027995`, 0.17834533234287903`, 5.09521235383073`,
102.54709769645665`, 0, 0, 0, 16.5647415472258`, 7.973082777686043`, 0, 0, 0.021899341818264504`, 0, 0,
77.90983596433904`, 0.07753802948418609`, 2.3206097453311005`, 0.30752240064196834`, 0, 0, 0.8741424424367815`},
{0.1280056031133443`, 3.9743049299675812`, 6.787496113248967`, 1.4777257411519527`, 0.41697417826757177`,
0.16856234271449722`, 0.016472885834375787`, 675.9035984651438`, 0.06538866409107441`, 1.0273997992144341`,
12.90960733752032`, 0, 0, 0, 10.461392548266884`, 0.041330315794753276`, 0, 0, 0.01958254204596801`, 0, 0,
2.3465611117172154`, 0.035809644358376386`, 0.3205264052434416`, 0.24604180304524892`, 0, 0, 0.07026795290380472`},
{0.2694840025057293`, 1.4398402455219186`, 1.997018914620883`, 1.3694289195380824`, 0.1761008172166878`,
0.6152080706831848`, 0.020267602026759375`, 203.92916364743616`, 0.23224028188307977`, 5.1018397742421016`,
39622.275687718065`, 0, 0, 0, 63.329259377245364`, 0.8406115673772749`, 0, 0, 8153.1249865504815`, 0, 0,
17.29066236515808`, 31387.667918644107`, 5.349216653625247`, 1.000000000000169`, 0, 0, 0.42519072379818446`},

```
{0.0578577996487363`, 3.866745902394764`, 0.7980363800640653`, 1.2385898080722695`, 0.5722218009229685`,
  0.20306189134783081`, 0.040832709866995026`, 2800.195372434598`, 0.18949558604342587`, 8.332936748246446`,
  34.127750333283466`, 0, 0, 0, 21.70326270991887`, 0.21873410618963213`, 0, 0, 0.06658199332496435`, 0, 0,
  12.082702983182028`, 0.05503268042869859`, 0.6238529560723276`, 0.6751761754936392`, 0, 0, 0.5180747339177854`},
{0.2528189510796667`, 3.3250798883848782`, 4.1415523484020405`, 0.9119401620778833`, 0.8401965817654944`,
  0.5070162083757728`, 0.10337827675659322`, 381.96079877048203`, 0.13245695994197593`, 7.95834379608233`,
  287.51497260134045`, 0, 0, 0, 115.2194257089084`, 3.365280716876808`, 0, 0, 1.967940619017245`, 0, 0,
  159.85467472080717`, 7.107609758385419`, 4.717912899513644`, 0.9999999868683452`, 0, 0, 1.1720822916263298`},
{0.20018502874097505`, 2.5781656282009475`, 6.942068190225046`, 0.9929389842661138`, 0.15941471595895984`,
  0.3421827829774996`, 0.4839576109293149`, 375.34729484852807`, 0.1596957174670693`, 8.895509004013046`,
  231.55234637931534`, 0, 0, 0, 33.60180050731654`, 5.22918334056198`, 0, 0, 0.03255031215014048`, 0, 0,
  192.59572503139864`, 0.09308693104716526`, 5.252500153148418`, 0.5051722856236773`, 0, 0, 1.0934837307023828`},
{0.272440775669993`, 3.035804235517027`, 9.484814840956616`, 1.224288286053278`, 0.8025814347303761`,
  0.5890268382574878`, 0.3843203491912798`, 336.978314494752`, 0.04553466081406238`, 4.10287356038395`,
  311.86392382124046`, 0, 0, 0, 49.83742101068079`, 5.8462854462366804`, 0, 0, 0.5385955961012368`, 0, 0,
  253.5454017103833`, 2.09622002820353`, 2.602199314362214`, 0.9966059513995549`, 0, 0, 0.3400091204347524`},
{0.20623021381347656`, 2.305665489644068`, 6.160210330863352`, 1.4997848102690652`, 0.31029755609374554`,
  0.2932546230651458`, 0.047029701289319936`, 605.575343672007`, 0.11131413350838143`, 4.271934365443833`,
  70.80595675888081`, 0, 0, 0, 42.87102662673742`, 0.8194197054081458`, 0, 0, 0.03134288383742769`, 0, 0,
  26.990110518483856`, 0.09234070907607139`, 1.5333724234143722`, 0.36035597585138723`, 0, 0, 0.43283313290464415`},
{0.27799817940399246`, 2.8456068500128753`, 7.4650958706635215`, 1.2794446296629622`, 0.3185515090611555`,
  0.5440861077528716`, 0.2050159519579307`, 96.61991147082354`, 0.24522478882121745`, 9.22867476531573`,
  47930.32433071013`, 0, 0, 0, 146.3294876622195`, 9.856276432073933`, 0, 0, 9529.195289482477`, 0, 0,
  400.67268186757246`, 37844.27059516039`, 20.6482538457012`, 1.0000000000027915`, 0, 0, 0.8794896932498582`},
{0.18731205279881885`, 3.3920364906001828`, 9.53367346846489`, 1.150711712471592`, 0.269617044263482`,
  0.4407414224913243`, 0.0689482800442352`, 54.451263142554176`, 0.1525802464986004`, 2.2696676207606785`,
  105385.61397125013`, 0, 0, 0, 89.09724362518111`, 1.7279368148326135`, 0, 0, 28621.954819903043`, 0, 0,
  83.73178184805226`, 76589.10160615832`, 8.99493436646208`, 1.000000000000081`, 0, 0, 0.2119124408473703`},
{0.07598141369986516`, 3.9958506473293216`, 3.287957942109602`, 1.3569966945049934`, 0.38975760386481206`,
  0.6382110342097327`, 0.02530287278972352`, 4836.578585308585`, 0.06124631354128618`, 7.15493409138095`,
  23.53519249599506`, 0, 0, 0, 17.354575053706704`, 0.10601233936017307`, 0, 0, 0.009113579726858247`, 0, 0,
```

6.051563926532053`, 0.009892323878763142`, 0.33586610040336695`, 0.13336152332993223`, 0, 0, 1.0755760392851568`},
{0.21782025688272888`, 3.800225088388993`, 1.7719223148490801`, 1.3823014458300409`, 0.9034484926621247`,
0.21119490910082483`, 0.008011320454875006`, 549.8645476667474`, 0.16991323645757794`, 1.3522541901702763`,
33.4745444670415`, 0, 0, 0, 17.4611858204885`, 0.03503862855065179`, 0, 0, 3.4194978656231076`, 0, 0,
1.9022096468704206`, 10.640512907142412`, 0.5355162831507864`, 0.9999999802528862`, 0, 0, 0.13968366611240923`},
{0.12917865835123116`, 3.4713877505357775`, 5.802428296169513`, 1.4965547140183602`, 0.061568597458759644`,
0.5580721907461164`, 0.2777380762847274`, 381.024601401567`, 0.014984965589617844`, 6.51082587242481`,
190.56984665272216`, 0, 0, 0, 41.26129040692625`, 2.946847067501354`, 0, 0, 0.07867725792027475`, 0, 0,
146.1378401832348`, 0.14519175172776075`, 4.028495239016292`, 0.6697089404146246`, 0, 0, 1.2662834782395656`},
{0.12408328322582662`, 2.9809522601952096`, 4.3074275514408615`, 1.1040930179447719`, 0.6796918144407815`,
0.5931470332615022`, 0.005369942238542098`, 502.88367879292525`, 0.06875474145528054`, 6.831128450148093`,
246.37891169750367`, 0, 0, 0, 226.66992812820945`, 0.39367117986740807`, 0, 0, 0.8276960675384191`, 0, 0,
16.76449990570666`, 1.467189222475327`, 3.0198730487402288`, 0.9997549305017908`, 0, 0, 0.8633388844216997`},
{0.2301374553102561`, 3.6285008777535515`, 6.8583997394674485`, 1.291013232269163`, 0.6431022470858925`,
0.6246309281545763`, 0.18498728864660263`, 132.58216720365365`, 0.011374938240932164`, 6.052136677369937`,
453621.9727944937`, 0, 0, 0, 99.61267405757036`, 4.739280898632772`, 0, 0, 105715.01960999363`, 0, 0,
245.66407000870942`, 347556.93715882447`, 10.30509003597132`, 1.000000000000469`, 0, 0, 0.8703039197089103`},
{0.18974259222357154`, 1.3104397818774611`, 5.6865397453703626`, 1.3109561369338216`, 0.42925562092874125`,
0.6235284138933523`, 0.06332638490149935`, 1635.1348002386078`, 0.08473820825835521`, 3.2599591654117823`,
32.271097197560664`, 0, 0, 0, 17.116202473802712`, 0.7654221094356638`, 0, 0, 0.01621126620375002`, 0, 0,
14.329136887615118`, 0.04394239532464371`, 0.450479782875668`, 0.23094526565212126`, 0, 0, 0.4721230292218956`},
{0.2309824769455583`, 1.2471345787782342`, 1.0215019422726535`, 1.0312798793045614`, 0.7898876098277747`,
0.6152586670568927`, 0.006979047924720161`, 310.31208829749596`, 0.24081833704073502`, 8.344184598629436`,
18896.563466806674`, 0, 0, 0, 62.13220203619788`, 0.3259709537517354`, 0, 0, 4378.914403541155`, 0, 0,
5.8075664014444195`, 14449.321360893158`, 5.763202513999704`, 1.0000000000002685`, 0, 0, 0.7169985686514875`},
{0.1769680396277803`, 1.6046472005597128`, 8.695674701006173`, 0.7703180616996756`, 0.764260429383415`,
0.1992432648214062`, 0.012440799720035065`, 274.53159119005693`, 0.16375790457626532`, 6.298702841855883`,
324.10454017424814`, 0, 0, 0, 275.58189049825705`, 2.012282495862444`, 0, 0, 0.05741885562716724`, 0, 0,
46.12862105315697`, 0.14516146168592725`, 4.729543503042238`, 0.797046192977441`, 0, 0, 0.2799032835304817`},
{0.23450402912552432`, 3.0762297015464597`, 2.593937576427738`, 1.1039402177647473`, 0.4424612211889203`,
0.6666998367628838`, 0.44528706596344714`, 463.5224063697932`, 0.20661121111075748`, 4.223015851637719`,

```
1155.1398207121988`, 0, 0, 0, 15.183794907417532`, 1.8745354388017086`, 0, 0, 242.68710973230955`, 0, 0,
  82.37859419204685`, 813.0157864150681`, 2.220095410416822`, 1.0000000000681724`, 0, 0, 0.9909082489344998`},
{0.2755523363386182`, 3.945462181815995`, 5.876565708554855`, 1.4395306041819111`, 0.28572441613120825`,
  0.3101513953075886`, 0.39757529919045964`, 3302.499033894636`, 0.07285156297216239`, 3.794286848749824`,
  11.653610063142143`, 0, 0, 0, 1.9464590417096055`, 0.1688011653733941`, 0, 0, 0.004878785891588168`, 0, 0,
  9.514265917531343`, 0.019205155013191477`, 0.2510041861396807`, 0.0700416206328971`, 0, 0, 0.4056813025834673`},
{0.1912671247263708`, 2.190434692724648`, 6.310276417856439`, 0.9527360176604049`, 0.6104292936966895`,
  0.2522020081398627`, 0.010518470607815496`, 284.3254609055096`, 0.100543925907327 8`, 6.418961425083908`,
  295.60125963965584`, 0, 0, 0, 256.90778739305284`, 1.1736607143279418`, 0, 0, 0.16071000761619517`, 0, 0,
  36.72610208788453`, 0.43912201530715933`, 4.74006816006764`, 0.9641058741362747`, 0, 0, 0.40949703536864224`},
{0.2597914621776059`, 0.5051242650756058`, 1.0118962727972`, 0.8968589895031354`, 0.07276014875602654`,
  0.6898744359309281`, 0.03193322527994425`, 3177.5504656788207`, 0.10860322045075349`, 3.7076953543063436`,
  14.58393856542346`, 0, 0, 0, 9.999643943644074`, 0.5408669738051731`, 0, 0, 0.02951477915563695`, 0, 0,
  3.902929037814356`, 0.10953839475274281`, 0.2871582036875228`, 0.4871921638461789`, 0, 0, 0.9154337115723002`},
{0.05800931142578874 5`, 1.2821997162065424`, 2.0542232518025845`, 1.4132652568351216`, 0.6228832317992945`,
  0.16342794118309045`, 0.044680537911227475`, 201.38656694501756`, 0.06413530627116476`, 9.590029577588982`,
  38611.37494832676`, 0, 0, 0, 95.56841612174067`, 3.0352448133882213`, 0, 0, 21029.553594539666`, 0, 0,
  55.597000547768026`, 17427.284623013817`, 9.973467375988829`, 1.0000000000000646`, 0, 0, 0.5864836912865944`},
{0.19641852899773504`, 2.1398264375306333`, 5.320272455292722`, 1.1437489346098513`, 0.5956525384016933`,
  0.542078693849333`, 0.2264882318745723`, 868.9198508977777`, 0.040131295366706066`, 7.0792614425236255`,
  156.24157045842674`, 0, 0, 0, 38.16942137925954`, 3.7348974122046164`, 0, 0, 0.04344815499546371`, 0, 0,
  114.17188891571686`, 0.12191460988384738`, 1.7659999268132076`, 0.554516169046393 7`, 0, 0, 0.6956013501919427`},
{0.09296068013545083`, 1.2991493564864323`, 0.26387265122439096`, 0.9093728692378444`, 0.35146944109549483`,
  0.5215346752572301`, 0.009103351916553084`, 3690.6422305701226`, 0.14178477746644713`, 8.458943719263925`,
  330.9853641115859`, 0, 0, 0, 17.220687160908497`, 0.11189052149119676`, 0, 0, 133.83168100144565`, 0, 0,
  2.0766071284602856`, 177.72977270807232`, 0.5349567409842193`, 0.9999999999996935`, 0, 0, 1.5443482434126155`},
{0.09381338426095137`, 2.303475789921615`, 0.696644313515133`, 0.9364121618865129`, 0.9663355677423924`,
  0.5212308135336106`, 0.24626013703252375`, 1385.562539836289`, 0.20934361709679128`, 7.7725134925150865`,
  644.4886236714975`, 0, 0, 0, 11.157243471735109`, 1.0593559606970693`, 0, 0, 255.28333524142613`, 0, 0,
  34.86001154821206`, 342.1284803488745`, 1.3079983957754966`, 0.9999999999991838`, 0, 0, 1.4098753105391066`},
{0.10039832860995906`, 2.055462616090492`, 1.0012272143748433`, 1.102551063992137`, 0.7631597204325553`,
```

0.38648795662626834`, 0.021413905310258614`, 653.3550083645581`, 0.055519462287367316`, 4.402154187880063`,
11462.535829451072`, 0, 0, 0, 28.051611093764738`, 0.27556377341025895`, 0, 0, 4693.868935708436`, 0, 0,
8.091586208480264`, 6732.237083704767`, 1.4913150440428338`, 0.9999999999999998`, 0, 0, 0.5345937887034802`},
{0.16980453046164407`, 1.1980162751260872`, 9.435065425338543`, 1.2054727390347812`, 0.15783339617816905`,
0.5987514193964574`, 0.2639813183074236`, 624.8721767770594`, 0.14884913893972124`, 1.0597319526281854`,
20.34133516162648`, 0, 0, 0, 4.543195001535352`, 0.8687651526828858`, 0, 0, 0.017770550507317986`, 0, 0,
14.868497031092831`, 0.04310742835629348`, 0.40242251052055666`, 0.26686138964363315`, 0, 0, 0.21434537439221504`},
{0.1106187483985549`, 0.9718711331456742`, 7.0205608914526`, 1.3199794157480607`, 0.2870266036717726`,
0.2842604073783652`, 0.12440193317594331`, 1067.250206889352`, 0.20077332983402157`, 1.344770962154577`,
11.869506742128625`, 0, 0, 0, 4.433007669792648`, 0.49766896861431764`, 0, 0, 0.0113387054073921`, 0, 0,
6.909572920837651`, 0.017918191437512467`, 0.2755041481884452`, 0.16719802914414617`, 0, 0, 0.1449904589460031`},
{0.22322153760577584`, 3.532203149750069`, 4.201686494998453`, 0.809746094501941`, 0.17157339217602185`,
0.16416014932043588`, 0.005086092661785311`, 81.42460105493491`, 0.09946867199032089`, 6.2584574953677805`,
88862.3611754758`, 0, 0, 0, 186.75805318078113`, 0.2600243039333528`, 0, 0, 21166.045867864097`, 0, 0,
13.120838076642306`, 67495.96148084292`, 15.651746909591054`, 1.0000000000000053`, 0, 0, 0.19869122846706128`},
{0.11344753764961812`, 0.6515213816912864`, 6.9223785093110415`, 0.7705690533378804`, 0.053758347821018404`,
0.22122664443377937`, 0.23238889135484875`, 1612.9665239639735`, 0.23659636795633343`, 7.8050806983048435`,
36.58172375869865`, 0, 0, 0, 9.23531707623484`, 2.6521994671586975`, 0, 0, 0.003433355237720941`, 0, 0,
24.68520944805899`, 0.005564367108506654`, 1.0493798300273212`, 0.09148072588520051`, 0, 0, 0.7708329572476157`},
{0.05086592503936643`, 0.6345548618632657`, 4.1212243894392255`, 0.9757969272512688`, 0.4720155756440034`,
0.3491598005177542`, 0.10619345643493772`, 163.13358888497802`, 0.24650090837908084`, 9.412849245254243`,
6601.556914453258`, 0, 0, 0, 132.65832405728287`, 18.898796930826887`, 0, 0, 3636.2639134348788`, 0, 0,
171.31890679746854`, 2642.3132520590343`, 12.869139863976867`, 0.9999999999999989`, 0, 0, 1.224054879995296`},
{0.18344840265344153`, 3.595124583701854`, 4.199071914809606`, 1.0537057639813663`, 0.12507220232647875`,
0.4903855977737973`, 0.3568003256791643`, 83.2177314580599`, 0.1899680886834239`, 7.625578775230776`,
70364.39573615063`, 0, 0, 0, 44.42963655615566`, 4.049098427552748`, 0, 0, 19363.14107699567`, 0, 0,
207.957332838907`, 50744.81858468717`, 19.624285309079713`, 1.0000000000027942`, 0, 0, 0.6487363216170323`},
{0.11456761033436458`, 3.133847301219358`, 1.1215010618012613`, 1.2799949994814193`, 0.7250649174618937`,
0.2748176967060689`, 0.05347527364447488`, 4312.248498236981`, 0.1363057898672484`, 6.512229405589068`,
24.422370873582945`, 0, 0, 0, 13.92807428814374`, 0.2272485963022845`, 0, 0, 0.0352613886037789`, 0, 0,
10.173748574682868`, 0.057711614705801204`, 0.3158661537910846`, 0.44109972728729896`, 0, 0, 0.3890435552190303`},

```
{0.2733102228285163`, 0.9906705811868095`, 1.8602414047947509`, 1.4451100284824412`, 0.2848844825831507`,
  0.554129635350794`, 0.011612796259976983`, 302.8864567264163`, 0.1580490958422761`, 2.3921629765885086`,
  31773.060719865047`, 0, 0, 0, 30.72739190069384`, 0.33238103290326315`, 0, 0, 6471.142244619061`, 0, 0,
  4.704001586310689`, 25266.133269026574`, 1.6983727755740867`, 1.0000000000010982`, 0, 0, 0.2142018621633714`},
{0.13611751569452646`, 1.612649572698647`, 9.438389550358693`, 0.8529700096304889`, 0.38386891649316235`,
  0.16702474363312747`, 0.015283436679942941`, 2091.084776788581`, 0.04888707786432367`, 3.7550559614266916`,
  15.362296473715713`, 0, 0, 0, 12.664293099919938`, 0.11167305858781539`, 0, 0, 0.0023899409320386313`, 0, 0,
  2.5727072887655904`, 0.004647326033228428`, 0.3768350068312319`, 0.058536234373103735`, 0, 0, 0.23744670127518488`},
{0.06405507608053002`, 1.3528358541887275`, 2.745226236199782`, 1.0639243234460365`, 0.36966523495796855`,
  0.3262944894947615`, 0.12612633222162115`, 59.71942118372549`, 0.2416255104540047`, 1.4175051543133999`,
  45852.432199162926`, 0, 0, 0, 11.652011567390522`, 0.9794310356758635`, 0, 0, 23926.323458305073`, 0, 0,
  18.928706025250122`, 21894.320992130044`, 5.153115884539112`, 1.0000000000000004`, 0, 0, 0.141716409655376`},
{0.20954125488723696`, 2.353113320872523`, 8.807677395871629`, 1.2953396401945112`, 0.754037675145256`,
  0.6760987806408354`, 0.0257504108557459`, 115.6397082887867`, 0.08726278776335833`, 1.3938676616131378`,
  39748.23335577215`, 0, 0, 0, 79.49913694701168`, 0.8256886661106985`, 0, 0, 9926.296567334206`, 0, 0,
  27.756271415979374`, 29713.837701458255`, 2.845168467556828`, 1.0000000000017244`, 0, 0, 0.27313889461412155`},
{0.12907099769038483`, 2.604139615282878`, 9.661547795493739`, 1.0824039436985886`, 0.5892380332813292`,
  0.2973399722037613`, 0.047844179127263436`, 189.25086236267023`, 0.09074457722059731`, 6.471849996577652`,
  471.91695910904076`, 0, 0, 0, 282.76155621044995`, 4.931310740779148`, 0, 0, 0.26686706248161385`, 0, 0,
  183.45459507618506`, 0.49206854293139074`, 7.236684017523637`, 0.9872253620888142`, 0, 0, 0.4671967053963582`},
{0.2477279255184573`, 2.8408395942947395`, 2.128090335748766`, 1.4989746812981888`, 0.49854461135120975`,
  0.5556994499234343`, 0.04795352432490085`, 88.91629411642595`, 0.17336384521962678`, 2.1291108959892835`,
  142269.56679491166`, 0, 0, 0, 21.47371149641406`, 0.34858182735582166`, 0, 0, 31336.092911040014`, 0, 0,
  14.14664367148624`, 110897.50415293692`, 5.081844664215574`, 1.0000000000017248`, 0, 0, 0.16030707181555431`},
{0.17138396941106926`, 2.571547212239655`, 8.060362653989774`, 1.424545143219825`, 0.3585647265624352`,
  0.4155041928809974`, 0.005849644389202518 5`, 1444.9053577030656`, 0.14315469959013916`, 8.6363710850225`,
  74.42149826534792`, 0, 0, 0, 68.67817664860193`, 0.14927910684165038`, 0, 0, 0.010312743290212272`, 0, 0,
  5.483975300632453`, 0.025249126865630384`, 1.3175371200479251`, 0.14405489406005345`, 0, 0, 1.0091255643037393`},
{0.046024454977220214`, 1.8018961949102152`, 1.8889771640586375`, 1.2101342547143499`, 0.33571961358008684`,
  0.4325808303075249`, 0.005213559292121167`, 454.61857984803544`, 0.13129751322022398`, 8.467405558196546`,
  3466.722922722436`, 0, 0, 0, 127.90781913024439`, 0.34759017560528727`, 0, 0, 2008.6297106975715`, 0, 0,
```

```
  8.947448783019166`, 1320.6583955129654`, 4.28960643808751`, 0.9999999999999962`, 0, 0, 1.4132773636097318`},
{0.15552548139256261`, 1.3158122598456092`, 2.9803095987090753`, 0.857616115693088 4`, 0.19622947720693906`,
  0.41449590928153224`, 0.03892742585126438`, 2129.9557657820133`, 0.1528564450859799`, 8.904626117418289`,
  44.784464050096815`, 0, 0, 0, 29.07407713059654`, 0.791771587248902`, 0, 0, 0.010288145544579375`, 0, 0,
  14.883182307136016`, 0.022858125549429776`, 0.9402292107354331`, 0.2267718586017171`, 0, 0, 1.2421168018088982`},
{0.20226341603934178`, 2.23359139800782`, 8.92211343859698`, 1.0176324123098368`, 0.17770526936314845`,
  0.5466941503204505`, 0.02261985699694341`, 208.46398724865384`, 0.0356723302826476`, 4.800574849057675`,
  272.62929382791367`, 0, 0, 0, 206.94663050587897`, 1.9833540034646806`, 0, 0, 0.09091642369125472`, 0, 0,
  63.28574916204422`, 0.26270094899813723`, 5.343825993576406`, 0.8442607161080874`, 0, 0, 0.8436678724539768`},
{0.1612087208534252`, 3.1252528746197825`, 0.891851633192701`, 1.468881451365178`, 0.6554353298599058`,
  0.19493335173588566`, 0.018954452797492544`, 551.070999166768`, 0.11474062580409572`, 7.406826770535428`,
  12565.975767021757`, 0, 0, 0, 40.53443895017065`, 0.23516582556795626`, 0, 0, 3788.9071665252795`, 0, 0,
  10.499323890979825`, 8725.783967827332`, 2.790348587034135`, 0.9999999999999997`, 0, 0, 0.38408616225973197`},
{0.2365245631846375`, 1.6915599352668007`, 9.677850457164503`, 0.7567859979545984`, 0.725618873672359`,
  0.2424250415266589`, 0.36826031218846694`, 892.5435235500784`, 0.17475643237365474`, 6.493737892117458`,
  108.87173098858135`, 0, 0, 0, 18.64089377618501`, 3.583917772033314`, 0, 0, 0.009371665619386774`, 0, 0,
  86.60588163517447`, 0.03166613024208092`, 1.5096143402310391`, 0.2340109875634127 6`, 0, 0, 0.33307105455648356`},
{0.22790261965108521`, 1.8196334440146895`, 4.716201039891137`, 1.2810805872690838`, 0.6237465626441163`,
  0.5616811859186371`, 0.03042013590776336`, 637.6118251230974`, 0.08011306001688712`, 9.216145754556873`,
  297.65408263271866`, 0, 0, 0, 207.9626080422809`, 3.2984764022637556`, 0, 0, 0.14511603332518916`, 0, 0,
  85.7431139407485`, 0.47246177354544755`, 3.1270065061625925`, 0.8990107964062035`, 0, 0, 0.8858569627281917`},
{0.10184647957852327`, 3.0801202872185955`, 8.412464860766345`, 0.9942252517730743`, 0.7984635108014702`,
  0.4565736536432896`, 0.009191281945386102`, 103.82351882247931`, 0.22274711417837167`, 2.5201460619086546`,
  8657.49900539858`, 0, 0, 0, 161.10276100632785`, 0.45875492389757266`, 0, 0, 3452.459705735657`, 0, 0,
  20.186004970833398`, 5023.155241655466`, 5.583815314642994`, 1.0000000000000049`, 0, 0, 0.4171594681587733`},
{0.18768732765242752`, 1.4966861207841546`, 1.129759551095228`, 1.2240080138702374`, 0.5362083693873154`,
  0.686948995479657`, 0.005867661202712241`, 74.92355531972449`, 0.165025723744549`, 3.2590978632956187`,
  141080.67716032866`, 0, 0, 0, 27.591686970782444`, 0.10229924736224338`, 0, 0, 38316.02315859303`, 0, 0,
  2.1872837670533545`, 102734.74275578395`, 9.449466071537145`, 1.0000000000000004`, 0, 0, 0.3280397203672199`},
{0.14949493319923252`, 0.867900380757324`, 9.54711835147964`, 0.8271983961134595`, 0.23831574478972195`,
  0.375815029784779`, 0.0451684556906921 8`, 2094.687766706356`, 0.0279158592619283`, 5.743304871768913`,
```

28.82201261093747`, 0, 0, 0, 17.73956249528244`, 0.8264104170606028`, 0, 0, 0.002829226695260828 7`, 0, 0,
10.246313080410241`, 0.006042215083050599`, 0.6093642380555557`, 0.07093905797246414`, 0, 0, 0.7239293917339223`},
{0.10785599050108607`, 1.35490623294936`, 7.749733046704847`, 1.2972878372630243`, 0.6070878493023009`,
0.6937073302769028`, 0.28374543116039413`, 66.83074779041495`, 0.1939262594152683`, 9.425365274493942`,
75173.25433684074`, 0, 0, 0, 129.87317939297245`, 24.198238149793852`, 0, 0, 29341.468716293224`, 0, 0,
468.37633850783646`, 45209.33101646309`, 32.17684020950471`, 1.0000000000006573`, 0, 0, 1.4700063471580047`},
{0.13969386881974227`, 3.165109549305347`, 4.62533417349867`, 1.0389272094346946`, 0.7061162924631639`,
0.5973647953915502`, 0.19864442086151357`, 3440.0036010322765`, 0.16071761832429643`, 1.3360604448248414`,
9.20219692500833`, 0, 0, 0, 2.4506398422056974`, 0.14539493799792594`, 0, 0, 0.010684354524255302`, 0, 0,
6.574155809684221`, 0.02132198313335193`, 0.08394459582059134`, 0.19427593949172262`, 0, 0, 0.12703943031868117`},
{0.18701505616348296`, 1.1471835668231432`, 1.9652898147298057`, 1.007866470439728`, 0.8673024418570234`,
0.4964521059357`, 0.11361709646832255`, 189.11029986824036`, 0.17527157137077032`, 1.608206453868661`,
29410.57255772462`, 0, 0, 0, 9.901403178399516`, 0.8961572245259516`, 0, 0, 8003.251729392527`, 0, 0,
14.686526303800148`, 21381.836738040605`, 1.8339323610279963`, 1.000000000001476`, 0, 0, 0.1489920782748668`},
{0.12078071855653999`, 3.7816423804380825`, 3.0527531818157634`, 1.3792123087306463`, 0.18684955879429088`,
0.29398025647621684`, 0.011105429740027619`, 471.0240529628659`, 0.1734264666127333`, 9.864280424563177`,
194.00178108156413`, 0, 0, 0, 167.63488142568585`, 0.4706835810073993`, 0, 0, 0.12756547598810727`, 0, 0,
25.427956824484863`, 0.2201064264692886`, 4.595801171774258`, 0.8535193529426245`, 0, 0, 1.1036995761291348`},
{0.12022941283008587`, 2.2264210098571606`, 6.2451953049416655`, 1.3438290154028227`, 0.5358944653344513`,
0.32618163573980585`, 0.199782178892696`, 3338.755251465298`, 0.1788085018354036`, 4.512919273292992`,
18.095061850982948`, 0, 0, 0, 4.9005914963462445`, 0.401728437005387`, 0, 0, 0.005652658617219108`, 0, 0,
12.777380462941041`, 0.009708797521092152`, 0.2887065849709432`, 0.08627281655787244`, 0, 0, 0.3709452364340069`},
{0.04681963491488872`, 2.902738250264786`, 8.529727895043578`, 1.3985345888875074`, 0.35876551940164103`,
0.2520539039703089`, 0.05124631524713002`, 163.50426816944002`, 0.24665810410586203`, 8.118758344754049`,
498.79726428600173`, 0, 0, 0, 292.1282634787895`, 4.857756541844558`, 0, 0, 0.2175603426574267`, 0, 0,
201.43993892123146`, 0.14551565450261067`, 10.630769194416908`, 0.9563551728448765`, 0, 0, 0.6880007520947627`},
{0.1911559295611578`, 2.175586072104755`, 6.8117639016300915`, 1.0586708066957606`, 0.34517099105389626`,
0.196211531611845`, 0.1489787018319715`, 3704.576077864889`, 0.10408111144660032`, 4.8300445149655875`,
11.585309266571299`, 0, 0, 0, 3.8837907020579663`, 0.239795273425023`, 0, 0, 0.002394628659836233`, 0, 0,
7.452789386000446`, 0.006539249534643027`, 0.2760266485777854`, 0.04749500907942272`, 0, 0, 0.3509348642477312`},
{0.17192712556630163`, 3.7260903741599414`, 4.118781130521407`, 1.2245874304759414`, 0.6062180312792598`,

0.5331783629983038`, 0.1203111914746069`, 226.51013650082294`, 0.017839169449302028`, 7.150760371208259`,
96985.85234012778`, 0, 0, 0, 94.9118504195885`, 2.870771012165869`, 0, 0, 27989.701898238807`, 0, 0,
152.8107462121237`, 68745.55704031169`, 7.196003678634697`, 1.000000000000098`, 0, 0, 1.1077430257378866`},
{0.1575695043509842`, 3.875881720589427`, 3.3547063374262347`, 1.3334178116995439`, 0.4921394422884451`,
0.402330971011554`, 0.12204131883407823`, 201.57298534982124`, 0.22899817129952033`, 2.4884430439924596`,
13254.481155256848`, 0, 0, 0, 25.678919192334924`, 0.7579965018288155`, 0, 0, 4056.014418848498`, 0, 0,
41.97006836727274`, 9130.059737405745`, 2.707978591595361`, 1.0000000000000036`, 0, 0, 0.27722395716804016`},
{0.06573776532431397`, 2.613579102769201`, 3.806271127858438`, 1.131416360099093 6`, 0.4483602258230772`,
0.6823320235871475`, 0.18768855278686034`, 189.85674956999534`, 0.0631967400861071`, 1.3499467761035964`,
36717.984603400604`, 0, 0, 0, 13.097048208541445`, 0.858312699341934`, 0, 0, 18911.670117083493`, 0, 0,
32.046687637735744`, 17760.156172109626`, 1.7075283451989283`, 1.0000000000001865`, 0, 0, 0.292594518225327`},
{0.06757463956251891`, 2.3005441553217736`, 6.868151131987258`, 1.3860241166029201`, 0.025379963517129678`,
0.6856836713524472`, 0.11835507299142825`, 3863.192203814942`, 0.1108156231672533`, 2.823244077255117`,
8.59241485568045`, 0, 0, 0, 3.2906599403374575`, 0.15632649154012412`, 0, 0, 0.003954000954443936`, 0, 0,
5.1376570919227404`, 0.0038170027046631853`, 0.17873073971424458`, 0.05888130082232235`, 0, 0, 0.6751433383592685`},
{0.06632069369203508`, 0.7568152865276287`, 0.22726671156421221`, 1.362987063673106`, 0.9228910129465389`,
0.1868567265073786`, 0.1296811990167267`, 123.29501155053357`, 0.19348527358813117`, 9.470702921306387`,
40466.119973540954`, 0, 0, 0, 6.067626277988171`, 0.9028770522232125`, 0, 0, 20770.476100509655`,
0, 0, 9.761587928250451`, 19678.748332851952`, 15.968892332303048`, 1.`, 0, 0, 0.504979886807201`},
{0.1352416913789251`, 3.40347330811998`, 4.693938060979022`, 1.2778213334701187`, 0.7052646641955478`,
0.6043325609148968`, 0.013064813980624626`, 333.49561209931903`, 0.11153764285740131`, 8.005884287110216`,
334.87069873308224`, 0, 0, 0, 276.6816484255834`, 1.0196898343797367`, 0, 0, 2.53054258075046`, 0, 0,
49.57838762675298`, 4.889069410386926`, 5.611612451201835`, 0.9999999875475801`, 0, 0, 1.47611956968482`},
{0.11675242158451127`, 2.833886804623358`, 0.47584343623935504`, 1.0607616820427617`, 0.7666611109645796`,
0.4737758025893787`, 0.1681420982868212`, 120.33220123866614`, 0.17915094289032796`, 3.5814785888826783`,
50878.84686836332`, 0, 0, 0, 4.172391937108239`, 0.2314646482954453`, 0, 0, 19065.642579468782`, 0, 0,
9.37063732201787`, 31799.427717396287`, 6.434804116296943`, 1.0000000000001792`, 0, 0, 0.3417130831629521`},
{0.24323887781179143`, 3.4699698183729026`, 8.39097272098094`, 1.1938324247391086`, 0.7463001660827029`,
0.6306644967356683`, 0.2992521819376612`, 322.6100644812281`, 0.10289378547186823`, 7.076414822100832`,
486.63939727132947`, 0, 0, 0, 95.31948256003494`, 7.655948565796998`, 0, 0, 0.9276216815272216`, 0, 0,
379.5130064904408`, 3.223337954979814`, 4.778077265952935`, 0.9992864250858389`, 0, 0, 0.7336279766231871`},

```
{0.24390696598302103`, 0.8206355382610386`, 2.839705408503791`, 1.0872176805395473`, 0.8466198387041834`,
  0.5785697848880403`, 0.1356337036504287`, 2343.8028799398503`, 0.22246042363346175`, 4.459217822696726`,
  62.83767603591975`, 0, 0, 0, 21.568665604801854`, 3.224282783827755`, 0, 0, 0.0546972837026407`, 0, 0,
  37.7994433973184`, 0.19058640736334442`, 0.399036715841267`, 0.6490365577580033`, 0, 0, 0.2794500928886442`},
{0.06769491456810739`, 3.362305338073525`, 1.8871867183871915`, 1.4803795481758868`, 0.5363168854717104`,
  0.37458837250617005`, 0.011183256466341666`, 407.888606379481`, 0.051902827430772014`, 9.719761204172809`,
  16144.112966006198`, 0, 0, 0, 132.7533869186642`, 0.4215754866689822`, 0, 0, 8129.113135640726`, 0, 0,
  20.249507274686604`, 7861.423131881135`, 5.3446267167496195`, 0.9999999999999998`, 0, 0, 1.3256346686714973`},
{0.21105892687940375`, 0.7047276416914965`, 5.768010388957135`, 0.810198720595394`, 0.17857267890229922`,
  0.19525448649651245`, 0.036176279358565294`, 67.82203673017167`, 0.07478241594027568`, 5.650997388906681`,
  128879.69321226129`, 0, 0, 0, 166.21041009393582`, 7.601388209212898`, 0, 0, 32036.177316214005`, 0, 0,
  76.52726266085908`, 96593.16008112034`, 17.106822412961694`, 1.0000000000000009`, 0, 0, 0.22744827144041332`},
{0.13030085884608883`, 3.691040694570585`, 3.5546925177950874`, 1.392417125140739`, 0.5227226143537942`,
  0.3153665172319682`, 0.008712610924073824`, 695.2883528301467`, 0.04802018879975545`, 9.078480195703946`,
  172.6046088439054`, 0, 0, 0, 153.47391406433508`, 0.3492003336964452`, 0, 0, 0.0822352164657 4247`, 0, 0,
  18.41303774616009`, 0.15307599046969142`, 2.785792982284983`, 0.7137214073388354`, 0, 0, 0.7352955029424042`},
{0.21250061023000932`, 2.8149517595358526`, 2.295631419336827`, 1.0543549080196901`, 0.6519014079329188`,
  0.6534060058028184`, 0.09088447710322001`, 650.6798329466749`, 0.04016188412840693`, 1.3861729584655489`,
  35.94675041263064`, 0, 0, 0, 12.86448296657865`, 0.3890069460211892`, 0, 0, 1.7468688170544804`, 0, 0,
  15.643368388200162`, 5.303009851655024`, 0.5210382549318701`, 0.9999999803118867`, 0, 0, 0.3315744072654034`},
{0.14347475236734758`, 3.69539554202769`, 4.673626861854084`, 0.8876695712812951`, 0.055199727721453984`,
  0.3291461786147223`, 0.031312636638690675`, 836.9629099625735`, 0.07687709551472927`, 5.335882334514919`,
  54.829959766225926`, 0, 0, 0, 38.26368744020944`, 0.3070484562025946`, 0, 0, 0.014504634766559311`, 0, 0,
  16.20950708910785`, 0.02972926973300662`, 1.4303929484425626`, 0.2955424915819511`, 0, 0, 0.7298592792533447`},
{0.0805761857877269`, 3.094619489710217`, 9.945877040804334`, 1.11372690786775`, 0.6430409751953907`,
  0.42229007304684385`, 0.05506378209236767`, 302.8456666998151`, 0.050941087889112335`, 7.632080993355595`,
  453.37242002750685`, 0, 0, 0, 255.98007983870508`, 4.362086054288794`, 0, 0, 0.08469997225835843`, 0, 0,
  192.84280741993217`, 0.09749715286997629`, 5.363688034141614`, 0.8011129444596202`, 0, 0, 0.5980587129055255`},
{0.26865716137877294`, 2.5539808428845117`, 7.725254140854183`, 1.421576189719117`, 0.2484256166411507`,
  0.1545757521332899`, 0.008556741498662016`, 1177.1024036480305`, 0.08015420001442447`, 4.125195480316323`,
  26.423863208177067`, 0, 0, 0, 23.57183069912218`, 0.07452023945075942`, 0, 0, 0.007831593872572404`, 0, 0,
```

2.718903770920085`, 0.03005733969823982`, 0.738074439826507`, 0.11172825222410165`, 0, 0, 0.27666978853355695`},
{0.05207035897383483`, 1.5461440288878858`, 9.695008671418819`, 0.9618476339297223`, 0.8208653770038725`,
0.48583250041037973`, 0.22651174945218666`, 56.560953214157614`, 0.1695178841867513`, 7.246656846935364`,
43198.164833436254`, 0, 0, 0, 150.12924572576497`, 19.43175361598606`, 0, 0, 24427.828932178778`, 0, 0,
429.204140345391`, 18170.940306428245`, 29.262426872894498`, 1.000000000000123`, 0, 0, 1.1392376577518215`},
{0.09188932948313594`, 3.928289836924776`, 1.4719659367359732`, 1.3955743442040527`, 0.5432280807299197`,
0.32698938665455834`, 0.013007122973082902`, 2762.880112969401`, 0.13947463148509326`, 4.274432647648432`,
21.04301641057549`, 0, 0, 0, 17.71279560860649`, 0.05656260235784133`, 0, 0, 0.03825388979309847`, 0, 0,
3.174204228461792`, 0.050216061188703365`, 0.33246428912045956`, 0.4369244646708216`, 0, 0, 0.379599481699878`},
{0.04429611977972214`, 3.507177302954225`, 7.234549676890296`, 0.7623401767303408`, 0.010148640019661537`,
0.662687845406686`, 0.08775344792279212`, 3965.4644576584396`, 0.17480873042969614`, 5.118348118050843`,
14.840432833946723`, 0, 0, 0, 6.739464549255401`, 0.158460720923232`, 0, 0, 0.0019690858821729993`, 0, 0,
7.939283483310325`, 0.0012460409156287184`, 0.313197576799703`, 0.053923357346165934`, 0, 0, 1.1743194353465891`},
{0.14748247662042147`, 2.024427515527907`, 9.506929645646245`, 1.2214601071407813`, 0.2054881053340767`,
0.2020185622482833`, 0.07806490597707526`, 302.23580657852216`, 0.2425913704635762`, 6.045088630499148`,
161.50635741738552`, 0, 0, 0, 78.49369444112942`, 2.77163111762892`, 0, 0, 0.027078694322991405`, 0, 0,
80.1566613917336`, 0.05705189860558325`, 4.2761002307941`, 0.37915361502965805`, 0, 0, 0.5030237567882169`},
{0.05191368478403385`, 1.8292747620439185`, 5.888828307542417`, 1.141375509610469`, 0.9869968200599144`,
0.28747330669734017`, 0.24632331590197062`, 4082.2304223282904`, 0.0447948152020462`, 8.998917183247265`,
57.792533896672`, 0, 0, 0, 13.135972552796542`, 1.64532775379547`, 0, 0, 0.00844709161230426`, 0, 0,
42.99652193297801`, 0.006264566447198061`, 0.4498584994608277`, 0.1471392290177299`, 0, 0, 0.3056414954641059`},
{0.22928260338606776`, 2.222139650206395`, 4.336407604983492`, 1.4545262441104416`, 0.17670484975018264`,
0.15215120204412502`, 0.00931582374755147`, 107.9598634817244`, 0.18384173269782222`, 2.0618261552271964`,
43912.86751160214`, 0, 0, 0, 60.562614366910516`, 0.24152309583198886`, 0, 0, 10254.87010737125`, 0, 0,
7.667114966983765`, 33589.47593720064`, 3.9127625852013166`, 1.0000000000008717`, 0, 0, 0.07844183971651292`},
{0.13057179539780978`, 1.32287055831243`, 2.838629919542937`, 0.8498204532586893`, 0.8949438289681946`,
0.46229782752490833`, 0.06831034580553909`, 355.6715560557054`, 0.046330937271765626`, 4.510601406855825`,
5133.779070705771`, 0, 0, 0, 57.41302597358992`, 2.6937957107438972`, 0, 0, 1752.9557489552142`, 0, 0,
50.90775765502015`, 3269.8082770570754`, 2.9498096145602735`, 0.9999999999999893`, 0, 0, 0.8051596976277798`},
{0.16617324858328203`, 1.7665378156673919`, 1.5537022391595983`, 1.3738499573440288`, 0.09625830296842142`,
0.5757224393125404`, 0.011187699121044243`, 1140.5955024024072`, 0.09128758443232887`, 2.6133507581824666`,

26.706628066836902`, 0, 0, 0, 22.75723009031861`, 0.1355161731446444`, 0, 0, 0.11191033302040516`, 0, 0,
3.419920635636349`, 0.2656643369719646`, 0.5571413835871438`, 0.7834152280290758`, 0, 0, 0.6063788881816633`},
{0.150610971367461`, 1.9051770945291775`, 1.9970914506574022`, 1.332029884280907`, 0.017627751946745374`,
0.6342635790195508`, 0.03224365795250487`, 84.16237057378729`, 0.025181518731154096`, 6.093120594420567`,
745302.1534159952`, 0, 0, 0, 68.44502184959798`, 1.1000369788888436`, 0, 0, 236453.2722751199`, 0, 0,
29.93950364734405`, 508749.3860052945`, 15.83275989872226`, 1.0000000000001636`, 0, 0, 0.6583273234411555`},
{0.170193722625894`, 0.42908710938865147`, 8.009796665724636`, 0.9108642019460496`, 0.48256370773751156`,
0.1504864882770205`, 0.015501266854459406`, 231.18780020236528`, 0.059845835174358764`, 2.951447491236335`,
113.43542295065046`, 0, 0, 0, 93.18201426414895`, 2.8125828358140397`, 0, 0, 0.04408105059819751`, 0, 0,
17.240614841936864`, 0.10717597283669834`, 2.657633668119249`, 0.6452443111872204`, 0, 0, 0.1615757502316108`},
{0.14155286072340578`, 2.768731185287475`, 2.866796585793665`, 0.9268420036617817`, 0.38841014261523243`,
0.6739227770046636`, 0.08018336457681292`, 85.63532436040691`, 0.10664760400309609`, 4.590937221386067`,
110072.15334528571`, 0, 0, 0, 51.44947424047499`, 1.4187763073561648`, 0, 0, 36385.33498501213`, 0, 0,
56.11728867320014`, 73577.83222154103`, 11.876741428061052`, 1.000000000000012`, 0, 0, 0.562210410217698`},
{0.17254585725985527`, 2.4767354176461422`, 1.5265879737476418`, 1.3533294324013416`, 0.010476993931212819`,
0.3024537953894164`, 0.005382899126762437`, 1067.886819263142`, 0.025600178888030733`, 4.688973100729083`,
35.70334589010506`, 0, 0, 0, 32.96645371893008`, 0.06771953558970577`, 0, 0, 0.06810145856353038`, 0, 0,
2.3960481751653284`, 0.16786606497843357`, 0.9891132169028817`, 0.6472677490422285`, 0, 0, 0.6627491342713744`},
{0.12080218784009655`, 2.1868434076988192`, 8.77616791832854`, 0.9616886370191489`, 0.26020855267635734`,
0.6201783502287406`, 0.03811904516021991`, 117.06014479510438`, 0.17593156125676795`, 4.349098586723594`,
9884.244212671638`, 0, 0, 0, 221.26735494983524`, 3.6299212112516193`, 0, 0, 3502.1343933228977`, 0, 0,
113.40098958988196`, 6043.792811763627`, 8.833052043478956`, 0.9999999999999991`, 0, 0, 0.889826393742482`},
{0.06629025039264891`, 1.530609759106781`, 1.900084106095834`, 1.2729265107514505`, 0.009336607275947317`,
0.20692029297881853`, 0.07757288734346537`, 56.094679581136695`, 0.10338780030561462`, 4.34093728430811`,
139732.2680952435`, 0, 0, 0, 31.333913547664746`, 1.457517852349525`, 0, 0, 71734.35241644784`, 0, 0,
31.869872126836263`, 67932.68833486906`, 16.227797018482672`, 1.`, 0, 0, 0.27121429193054364`},
{0.11054921486908309`, 1.9601623754484976`, 4.1405236153603`, 1.0982919546164496`, 0.2371613718862391`,
0.5811399077338627`, 0.014126785714663546`, 63.169894546693705`, 0.15558848051416968`, 8.651696062244092`,
102818.72606315033`, 0, 0, 0, 247.0464506036112`, 1.6925238285493798`, 0, 0, 39748.558938570575`, 0, 0,
47.394593261036135`, 62773.88546909241`, 30.310313057993852`, 1.0000000000000175`, 0, 0, 1.0494469612270647`},
{0.09844925238529889`, 2.9547765664281824`, 3.97533240344332`, 0.8901779978265776`, 0.6485331039194935`,

0.172046787329591`, 0.2843834249851245`, 265.56383421685405`, 0.07856132944640065`, 2.1823933339229455`,
69.3512138766139`, 0, 0, 0, 14.865826727383386`, 1.2348186256928588`, 0, 0, 0.4685485427665218`, 0, 0,
52.123044842661606`, 0.6589750534511492`, 1.7420629555512792`, 0.999525598522091`, 0, 0, 0.1615931850007642`},
{0.07379407071323735`, 1.0020834251379886`, 3.8959201685323244`, 1.3275435329440872`, 0.5495795554378471`,
0.5203295123605114`, 0.006236204073249997`, 495.6126936444482`, 0.05339932410900344`, 4.300380134475608`,
139.97973413292246`, 0, 0, 0, 127.43094036165337`, 0.730091385289095`, 0, 0, 0.5996679973292796`, 0, 0,
10.451606800489074`, 0.6321706085626099`, 1.9338999791045584`, 0.9968350766784568`, 0, 0, 0.5558444478979311`},
{0.137830658132162`, 0.964244066849937`, 5.678313655870262`, 1.056362681888576`, 0.27898051740088525`,
0.6674603903550571`, 0.022240144180942223`, 3206.233069292432`, 0.13749704640301397`, 7.378694929535063`,
33.32057608226024`, 0, 0, 0, 25.388644891537986`, 0.5352126553697917`, 0, 0, 0.00556736865967698`, 0, 0,
7.372508963476018`, 0.010962201234684988`, 0.5337426376831993`, 0.10681480727225168`, 0, 0, 1.291908435020394`},
{0.09412349267697989`, 2.412142947637907`, 8.593900106949853`, 1.0545990130363978`, 0.8443965196041454`,
0.4441557500447094`, 0.020813982545198062`, 67.68562305269263`, 0.21037364591803787`, 1.9282456472076213`,
33026.472906715855`, 0, 0, 0, 107.37599113448101`, 0.879041980594993`, 0, 0, 14026.947512079414`,
0, 0, 30.291070202426575`, 18860.93273476561`, 6.381233546593703`, 1.`, 0, 0, 0.2601377140780715`},
{0.059604065321211375`, 2.060924190389435`, 4.151577648882975`, 1.0218595307135387`, 0.8259153199816331`,
0.67511248087556`, 0.012173840559063499`, 163.32402156550611`, 0.22896057989768043`, 4.41755081858776`,
12073.803854849582`, 0, 0, 0, 138.91000642285542`, 0.7774714131251298`, 0, 0, 6433.233027132277`, 0, 0,
22.890137752083408`, 5477.812022510951`, 6.432896470909497`, 1.0000000000000506`, 0, 0, 0.9057248942928413`},
{0.062303828390692484`, 3.237012523552033`, 1.9717708145626371`, 0.9726411613979222`, 0.1873520229034391`,
0.5560570337810927`, 0.026607710207102397`, 62.963998927305475`, 0.022649002664224704`, 8.659052494024476`,
551979.9411245878`, 0, 0, 0, 104.7133167342808`, 0.8247189434873022`, 0, 0, 291966.6985908712`, 0, 0,
38.137507835414226`, 259866.32978289493`, 30.991413625726278`, 1.0000000000000002`, 0, 0, 1.2277537360017368`},
{0.058207327156306`, 1.429925878818433`, 8.458349879741764`, 1.1245084153106533`, 0.19874114947102073`,
0.3371100826465334`, 0.04401493286992449`, 375.7204818983131`, 0.2159694260931322`, 8.593637256078832`,
224.38273198488443`, 0, 0, 0, 139.5814254504947`, 3.9548434790934164`, 0, 0, 0.028178867273322677`, 0, 0,
80.78761482045729`, 0.023431664946639983`, 5.056053017153806`, 0.41628812663516135`, 0, 0, 1.031320304290618`},
{0.0526423548412821`, 1.6513512325831732`, 4.083783641771872`, 1.373155154639566`, 0.8939370398042139`,
0.2364887781328291`, 0.0490949664593259`, 1618.3201075393572`, 0.07451928539997066`, 4.7651781050102695`,
55.91013911539432`, 0, 0, 0, 33.07791563872257`, 0.9262177604831087`, 0, 0, 0.03123446304931371`, 0, 0,
21.850154863060066`, 0.02348936695886498`, 0.6045708521773702`, 0.3851496240014458`, 0, 0, 0.1919936733912312`},

{0.21322884859448837`, 1.5615738832323176`, 8.40996483053398`, 1.2929614343326012`, 0.3765546169235392`,
0.671739728089668`, 0.1608794324574438`, 485.1832614690647`, 0.0705178455433636`, 2.1513131242884285`,
70.01350433462389`, 0, 0, 0, 21.846711813760933`, 2.058915583098246`, 0, 0, 0.043789957015578126`, 0, 0,
45.930697176375105`, 0.1333897444918938`, 1.0200350816573338`, 0.5088971414437666`, 0, 0, 0.3562002119957909`},
{0.11682922382752631`, 0.5721136495795571`, 9.687079392836907`, 0.91254998083727`, 0.43543762812336806`,
0.4740169657662334`, 0.10620954791167687`, 4372.038997558794`, 0.0729116423313943`, 5.645356891940546`,
18.442711478826446`, 0, 0, 0, 7.489820480334162`, 1.193454026190729`, 0, 0, 0.0019754724293849207`, 0, 0,
9.754161978991371`, 0.0032970415802321316`, 0.28449693319519503`, 0.04547478639457747`, 0, 0, 0.6567236545839082`},
{0.26956767736567855`, 0.8444303847788461`, 1.0020073721955711`, 0.7502641425367418`, 0.966405616850982`,
0.6715103286393609`, 0.23328928938153737`, 246.8438332486948`, 0.10178810353613177`, 2.531792031152129`,
46725.70937092259`, 0, 0, 0, 4.814491318797165`, 1.1864800868138503`, 0, 0, 9628.059164731343`, 0, 0,
14.31285480343803`, 37077.336379656605`, 2.1956610508315406`, 1.0000000000090319`, 0, 0, 0.21426750454322224`},
{0.07372356616920961`, 2.107710372997479`, 6.08076458707294`, 0.8379230467338923`, 0.9571077593283461`,
0.37371168094765217`, 0.1939664465355383`, 131.4329681577719`, 0.026286944156709513`, 5.373906305884013`,
47586.938025175004`, 0, 0, 0, 77.38285970985962`, 6.339007807753926`, 0, 0, 23043.246206731226`, 0, 0,
190.86846444164263`, 24269.004092504798`, 9.209290301661785`, 1.0000000000000153`, 0, 0, 0.7588091139952156`},
{0.2128059772734779`, 3.088861612111426`, 5.437928143232815`, 1.2368766361188581`, 0.5161736795080103`,
0.22159582314348258`, 0.17105793552921358`, 1293.4067318655482`, 0.22754002195492767`, 9.689789287743999`,
83.94591454059119`, 0, 0, 0, 25.45840700517334`, 1.2948305306896501`, 0, 0, 0.013914356971754104`, 0, 0,
57.13646172053037`, 0.04230083333580931`, 1.5735096092818872`, 0.2149147492092967 6`, 0, 0, 0.6218295149899081`},
{0.09487232790202699`, 3.2902977287485546`, 3.9754892015026773`, 1.2274952505878522`, 0.2657953162834845`,
0.47376077240276926`, 0.26204707729007887`, 80.73499841507218`, 0.18195262450329797`, 6.708419132160676`,
61736.54428786671`, 0, 0, 0, 48.838017432236036`, 3.551613102868963`, 0, 0, 26118.413716366373`, 0, 0,
166.94092179661936`, 35398.78157685593`, 18.28888138114523`, 1.000000000000014`, 0, 0, 0.7727817705744372`},
{0.05710457017724263`, 2.2468049124614753`, 4.035987116327698`, 0.7517191945594467`, 0.40903626729229714`,
0.6582116219387326`, 0.04046385674463665`, 815.0779000126262`, 0.186726177475597`, 7.464159626289564`,
151.31900803907894`, 0, 0, 0, 96.56429622121492`, 1.6520006700260201`, 0, 0, 0.03936471273054896`, 0, 0,
53.02461744005885`, 0.032112928580334134`, 2.074210058089379`, 0.6700006913081767`, 0, 0, 1.101763784992707`},
{0.1356890149134694`, 1.6504364529320803`, 8.903330095009764`, 0.8652149931542472`, 0.7882939470826769`,
0.5853867881019097`, 0.12191584954894137`, 2002.2565687817998`, 0.19189449537551667`, 8.0829058846114`,
117.04286954479666`, 0, 0, 0, 43.21494823074433`, 3.0026708064874197`, 0, 0, 0.009966225066460556`, 0, 0,

70.79596221688303`, 0.019318675166615493`, 0.8490625335195947`, 0.21927900759277041`, 0, 0, 0.5306353613699298`},
{0.21947794079961902`, 1.5730764365956098`, 2.118289006802259`, 0.973502067608673`, 0.7638917093126882`,
0.33981639014260245`, 0.009615294802656809`, 1765.6534921210653`, 0.16626255675053375`, 4.010994756897377`,
43.75467344861621`, 0, 0, 0, 38.309366499549526`, 0.22141465726191958`, 0, 0, 0.052484453777358546`, 0, 0,
4.975745429365973`, 0.1645597119863978`, 0.47620107993397254`, 0.6756518774405146`, 0, 0, 0.24908948282009444`},
{0.2345658346849942`, 2.081301191128982`, 3.6234436876627587`, 1.1134590239676974`, 0.8739003578896458`,
0.6696041669913848`, 0.06590896300137702`, 558.8606273551264`, 0.09113830356870467`, 1.2137876330314246`,
41.63461180591715`, 0, 0, 0, 19.85727601983152`, 0.5929306412713616`, 0, 0, 0.8169966429984985`, 0, 0,
17.62953214192793`, 2.7377071357111866`, 0.5061750624869119`, 0.9999988242076193`, 0, 0, 0.21947771564398794`},
{0.23053748957807368`, 3.8668800248110458`, 5.535000453565214`, 1.1361194466757834`, 0.24488877583427326`,
0.5300195072451362`, 0.08816752471869817`, 3094.9084693615328`, 0.16131439142801796`, 1.2619255962975515`,
4.93320059035842`, 0, 0, 0, 2.222627288365176`, 0.0477966238532895`, 0, 0, 0.00522516626249449`, 0, 0,
2.6403401433098903`, 0.0172085244683358`, 0.09443597353094543`, 0.09251285560973999`, 0, 0, 0.21891258912196637`},
{0.05839671607004826`, 2.466861519533315`, 8.278076935325572`, 0.9179604454629582`, 0.7485509623778881`,
0.3739704677333707`, 0.3976055017684991`, 334.0137006202725`, 0.18218149392519362`, 2.0150765691128605`,
116.7443927237742`, 0, 0, 0, 18.516523340505156`, 2.7046779233354408`, 0, 0, 0.11337914533604813`, 0, 0,
95.31522702582123`, 0.0945852822636001`, 1.270438374202759`, 0.9139132116197723`, 0, 0, 0.1349321340430081`},
{0.14569114559043927`, 3.1555485829295717`, 1.7894848948555018`, 1.1727674941432193`, 0.06666495623301039`,
0.21747003985461844`, 0.032197916321861565`, 1115.0145850241458`, 0.11404115123479613`, 9.530247146566754`,
65.05622278157867`, 0, 0, 0, 45.03050107674059`, 0.4318373715121046`, 0, 0, 0.03925422612029003`, 0, 0,
19.466911510443612`, 0.08169990246757111`, 1.8525231753413671`, 0.5104837081002765`, 0, 0, 0.9354106781918841`},
{0.24150266620923067`, 1.7188165992139313`, 4.322636691993072`, 1.2029528995785472`, 0.9471494725848191`,
0.6615414605337746`, 0.19861497081852103`, 2170.4118977268995`, 0.144713056075588`, 4.793357249969096`,
107.47949593117515`, 0, 0, 0, 28.26893340697244`, 3.088164096731122`, 0, 0, 0.06606742404243655`, 0, 0,
75.82839586511372`, 0.22793512936890128`, 0.4565971182447191`, 0.682385296622298`, 0, 0, 0.22772901119473937`},
{0.20918490948785712`, 3.1569386485962374`, 8.450247389660383`, 1.207442014040422`, 0.02962131605799323`,
0.6403349881706148`, 0.07844599973162336`, 325.7491474109025`, 0.10080604275363181`, 2.998503110312266`,
104.59496326856535`, 0, 0, 0, 50.294581157526`, 1.1739835277089419`, 0, 0, 0.04528776018130331`, 0, 0,
52.945628163424416`, 0.1353359430634095`, 2.230421343585763`, 0.5470981410156657`, 0, 0, 0.682723335965693`},
{0.20782035028778872`, 3.1560227942933192`, 4.024085032034362`, 1.1076699632387197`, 0.7825797887689212`,
0.681476671590229`, 0.005917686238352288 6`, 2392.206038919967`, 0.17125268254719206`, 3.7066097570560648`,

```
48.38473282362777`, 0, 0, 0, 44.487175177316985`, 0.08100477290852295`, 0, 0, 0.029317786285432832`, 0, 0,
 3.652184424940749`, 0.0870404659357284`, 0.329490635377137`, 0.42764177304802853`, 0, 0, 0.2869849830791269`},
{0.16569005768607342`, 0.9318724619299896`, 5.387727961962419`, 1.4204768501331082`, 0.162422581490264`,
 0.6283948310518737`, 0.42435650593933405`, 403.29853257093646`, 0.17732127106821444`, 7.752705227642123`,
 248.6620777557737`, 0, 0, 0, 38.73811378442789`, 14.642433220362216`, 0, 0, 0.10533690108863092`, 0, 0,
 194.92686133863432`, 0.24933253168333894`, 4.512113856617136`, 0.7861273539339508`, 0, 0, 1.4672545190065696`},
{0.25387146604005345`, 2.177421517695631`, 2.9195785593706507`, 1.205984878491204`, 0.22933817566590875`,
 0.5142965710820098`, 0.00715658453704801`, 291.5834795916249`, 0.18546405096886748`, 3.1957985304348036`,
 11155.114683384925`, 0, 0, 0, 70.25760311384663`, 0.22005258298296912`, 0, 0, 2394.285060077739`, 0, 0,
 6.844960417308839`, 8683.437975996185`, 2.4334786419334304`, 1.0000000000007003`, 0, 0, 0.39931646496624223`},
{0.27254750481249423`, 3.728211063877054`, 5.204302820921312`, 1.137745914342209`, 0.04515932299981973`,
 0.21786268187051538`, 0.17709327429647695`, 87.47051100453898`, 0.05078893394192652`, 2.761931237968355`,
 216228.26157717768`, 0, 0, 0, 32.35781243075348`, 1.442369770094732`, 0, 0, 44163.90318472925`, 0, 0,
 76.82084192955718`, 171953.73736826418`, 6.495627498322657`, 1.000000000001427`, 0, 0, 0.11682643634412401`},
{0.24124989455736606`, 1.138551887899828`, 6.0414451617780465`, 1.0864227463934355`, 0.8606911125263128`,
 0.6167713883354349`, 0.008616924398628963`, 4204.638992315412`, 0.042475052828396476`, 4.683417206387297`,
 40.026024938586524`, 0, 0, 0, 35.59440928144155`, 0.25212368497122845`, 0, 0, 0.01066602542292943`, 0, 0,
 4.1007985358322045`, 0.036759678694687686`, 0.23230230364491455`, 0.18931702336638623`, 0, 0, 0.2654359057390758`},
{0.16291183507690532`, 0.9001170306894437`, 5.6406094859134015`, 1.3830538570390154`, 0.30535931966424634`,
 0.523800888605104`, 0.2170932130058295`, 52.61233898698406`, 0.08477132140762966`, 2.26771827115102`,
 309692.89217789605`, 0, 0, 0, 26.20586440063368`, 5.589977380177901`, 0, 0, 93044.84286653047`, 0, 0,
 71.88048344381279`, 216544.37279755337`, 9.344408341695722`, 1.0000000000014535`, 0, 0, 0.22321304588624663`},
{0.24307985322679526`, 3.5312268091205707`, 7.143117328957406`, 1.2474301004946668`, 0.43966589306292936`,
 0.28635789146968393`, 0.04752738776816154`, 125.46273060513494`, 0.15229886979218882`, 3.763239253417467`,
 5018.292514045951`, 0, 0, 0, 134.69299746925606`, 1.7014033459723223`, 0, 0, 1072.3285503884101`, 0, 0,
 85.82915869178385`, 3723.7352377045218`, 6.6468646002410585`, 0.9999999999995505`, 0, 0, 0.4620252472713346`},
{0.08579390683558069`, 2.8983719603389604`, 3.99564346069112`, 1.2420354947694623`, 0.9313337583971846`,
 0.4737657288125191`, 0.010571420568870461`, 66.04207510362154`, 0.1244519483642591`, 8.73466885165751`,
 114907.61684658368`, 0, 0, 0, 249.93135236172873`, 0.8746263278761671`, 0, 0, 51500.236941928415`, 0, 0,
 36.21417749272142`, 63120.093288659285`, 29.129820703613195`, 1.0000000000000286`, 0, 0, 1.0125531043213005`},
{0.20336933942425872`, 1.2934536281273639`, 5.591224704743386`, 1.058733783245077`, 0.09184020179750241`,
```

0.3092776948325172`, 0.45477750215520385`, 95.84900263920687`, 0.14971414135963745`, 8.98848136080165`,
53395.708942902056`, 0, 0, 0, 57.49520095974479`, 17.351921744895332`, 0, 0, 13571.443048547631`, 0, 0,
320.6272305131`, 39428.7915402439`, 19.733292679774443`, 1.000000000000003`, 0, 0, 0.5946951790822039`},
{0.12808268874911022`, 3.6824385947617753`, 2.1347222010883335`, 0.9228913029818868`, 0.1301948165557758`,
0.29194798379239884`, 0.27062051931711223`, 4508.281780436825`, 0.033597809391257005`, 7.5117374932700365`,
14.752932769981127`, 0, 0, 0, 3.305893181408903`, 0.21323958300618914`, 0, 0, 0.00567606282992713`, 0, 0,
11.217738148469923`, 0.0103857912681027`, 0.3668711584043515`, 0.12351643950964764`, 0, 0, 0.8683192084193201`},
{0.14553068051711526`, 1.8082756931498238`, 2.368533159601707`, 1.1602558844471123`, 0.9691057704573378`,
0.4792380149128871`, 0.010639214796165341`, 788.9564435352116`, 0.18893878705870149`, 9.465562662789438`,
188.00511743762038`, 0, 0, 0, 160.58781416128048`, 0.8946462835875092`, 0, 0, 1.068900365604254`, 0, 0,
23.110958979687386`, 2.222254251591198`, 2.6493946163246567`, 0.9999980343343114`, 0, 0, 1.1250725744804597`},
{0.24858998623664108`, 2.4028729322353692`, 5.907867944453356`, 0.899298954014168`, 0.6845394050547509`,
0.4831163766793648`, 0.006208316855544933`, 932.6458115441258`, 0.22545041645445363`, 9.209981899602862`,
206.52376409082336`, 0, 0, 0, 189.55932233240006`, 0.4706899927433083`, 0, 0, 0.029984081947482824`, 0, 0,
16.15726061481366`, 0.10648203598060986`, 2.0987087134326847`, 0.5104686165742491`, 0, 0, 0.7072676837125372`},
{0.2519271524040908`, 1.2045387595495214`, 4.5140841978362705`, 1.0152142452788597`, 0.29419718036266773`,
0.20432622138273293`, 0.02287101962327847`, 2390.149329793605`, 0.07799608049040802`, 9.888014794979117`,
36.46856987462557`, 0, 0, 0, 27.673047257425146`, 0.4812387425510709`, 0, 0, 0.005511735210270012`, 0, 0,
8.281010257137709`, 0.019836510804686222`, 0.878870819077891`, 0.11026928930348223`, 0, 0, 0.7551898698837941`},
{0.11736070154865536`, 2.38096944376945`, 5.3494850274310295`, 1.276899864236343`, 0.07495782665645989`,
0.3912082450614217`, 0.41113333692039317`, 80.12914367323096`, 0.2186956786299285`, 8.297333470694408`,
49120.19641657008`, 0, 0, 0, 56.85487492792499`, 8.617128103237475`, 0, 0, 18217.872959090437`, 0, 0,
293.10169581222044`, 30543.74787433048`, 22.381710603597867`, 1.0000000000012923`, 0, 0, 0.789364873997676`},
{0.16035901956271875`, 2.606522752147513`, 4.69883411815465`, 1.3021358666964324`, 0.6150695298380762`,
0.6434499333892203`, 0.12329290515746044`, 1079.0196669558486`, 0.2419176548365854`, 8.070742396729585`,
163.20465155503854`, 0, 0, 0, 60.05769959740915`, 2.693135764488597`, 0, 0, 0.05229756007508371`, 0, 0,
100.28170921088166`, 0.11980550655947625`, 1.6299058646677747`, 0.5731747600102901`, 0, 0, 0.8400067196747673`},
{0.16449617597041116`, 3.191202853555228`, 6.979734889593845`, 0.755053893640484`, 0.8431581165413089`,
0.5308423930900061`, 0.0662197921799213`, 915.9947229465104`, 0.1651520479769521`, 4.094114041787371`,
136.4721946884852`, 0, 0, 0, 70.51812604110641`, 1.4130579962360044`, 0, 0, 0.036145607805766235`, 0, 0,
64.41935299753389`, 0.0849402037452669`, 0.9324955089514776`, 0.638583292006481`, 0, 0, 0.23363485189352354`},

```
{0.11360527105324508`, 0.4683409445122746`, 6.198135239531911`, 1.4397350406163711`, 0.8287446034488006`,
  0.16487005319303316`, 0.2956243647100192`, 339.82112374821816`, 0.14870620145140367`, 8.492417686013223`,
  347.2523030444991`, 0, 0, 0, 70.52987107076412`, 35.93185528131616`, 0, 0, 0.1469529000413674`, 0, 0,
  240.40512915042828`, 0.238494629160585`, 5.117295463840134`, 0.8840239261869494`, 0, 0, 0.31833879669708753`},
{0.08657280459787114`, 2.8376273381057535`, 2.18201840556476`, 0.9732923844552535`, 0.8927467229995099`,
  0.4599530868891304`, 0.04289399463935107`, 1129.687177458504`, 0.08547871234303578`, 2.1820677830816138`,
  38.931711207022914`, 0, 0, 0, 23.862436046417827`, 0.3458705222240545`, 0, 0, 0.3135421770106559`, 0, 0,
  14.020737847255624`, 0.38777465176473414`, 0.41862005171926614`, 0.9980751094502535`, 0, 0, 0.21400805863886838`},
{0.14288327643386806`, 3.0416391272131467`, 7.259522365277451`, 0.8919048885163637`, 0.7100314935930478`,
  0.32120037052790307`, 0.014548712666776912`, 690.6861027330066`, 0.2370448478751857`, 7.1501615854828415`,
  177.67594007321128`, 0, 0, 0, 147.34027328806292`, 0.6788603221831261`, 0, 0, 0.025698217347203043`, 0, 0,
  29.4978302552103`, 0.052454935615417905`, 2.1667295295262483`, 0.4613404294188883`, 0, 0, 0.43225695260395974`},
{0.05887079706837223`, 1.3439289639420835`, 4.010009055307599`, 1.087305166401462`, 0.6661788396152433`,
  0.35567096782369767`, 0.008830796062004187`, 96.96443168903161`, 0.0354538286259683`, 6.082802068311153`,
  154167.19762556435`, 0, 0, 0, 178.1341783005994`, 1.0878488749984485`, 0, 0, 83631.04681256834`, 0,
  0, 20.885594450031792`, 70334.66265026089`, 13.791541253544137`, 1.`, 0, 0, 0.6927953288508171`},
{0.275808383383374`, 3.614253930768469`, 8.690573450897112`, 0.8084062901760818`, 0.7881641108236563`,
  0.5461332704061045`, 0.16300280176413603`, 213.49162259077596`, 0.11620699031214832`, 3.4918574760984407`,
  258.9594454251696`, 0, 0, 0, 78.719045742071`, 3.3213781361140535`, 0, 0, 1.098954039418648`, 0, 0,
  171.49005691455196`, 4.33001052892405`, 3.6618776047914134`, 0.9999972841573727`, 0, 0, 0.4691621670628258`},
{0.16345104545617634`, 3.976575006158881`, 6.050721111049688`, 0.8077170991659299`, 0.4737043917748365`,
  0.42206087766889466`, 0.011122815850097148`, 819.7636995837653`, 0.0134560852671817`, 8.084495823489597`,
  140.41931321801366`, 0, 0, 0, 121.32406389196018`, 0.3277530427130408`, 0, 0, 0.018394756264114583`, 0, 0,
  18.619065112074313`, 0.04295203060402728`, 2.144708962761351`, 0.3866908487528976`, 0, 0, 0.823487250352661`},
{0.27078615466317746`, 3.6662669410444693`, 7.922278334055675`, 1.4466898496109064`, 0.8715287168554611`,
  0.2219561421798496`, 0.02446106939657988`, 188.339766844502`, 0.03692414185938048`, 1.663076961237424`,
  106.79079116375168`, 0, 0, 0, 77.15648129416783`, 0.4929714433127562`, 0, 0, 0.6783650872279261`, 0, 0,
  25.81949864995045`, 2.62416962040284`, 1.861741219255553`, 0.99967756660701`, 0, 0, 0.11349856965297846`},
{0.24286662289220456`, 2.6814172670718373`, 2.6369699156223714`, 0.8218929956416956`, 0.8808977613854625`,
  0.43575724866517207`, 0.024751792159432087`, 472.6370271533013`, 0.14270041872793765`, 9.280134575613399`,
  233.98565963541557`, 0, 0, 0, 155.6198350286499`, 1.359698976289721`, 0, 0, 5.5673539188930015`, 0, 0,
```

52.084575900616606`, 19.316063496103215`, 4.5001958457373945`, 0.999999999317221`, 0, 0, 1.4964586355645568`},
{0.042342161544045354`, 1.3013804906014936`, 7.13634807471312`, 1.1105942412761596`, 0.2407240140528013`,
0.6802475696307242`, 0.0062232822378432`, 4037.4772502763867`, 0.18461914595887624`, 5.716947148449252`,
19.849475888450513`, 0, 0, 0, 18.231208207755326`, 0.08133206011704014`, 0, 0, 0.0035017016769688454`, 0, 0,
1.5120565185249113`, 0.0021181374012179837`, 0.3316740572033398`, 0.0653361200388406`, 0, 0, 1.0679602522873475`},
{0.1472039750188425`, 0.5316247300014076`, 6.683828314747185`, 1.481587653959343`, 0.013428589995876372`,
0.17720084833285243`, 0.005004553323761107`, 364.08088787611194`, 0.035796657483315286`, 8.51611862140901`,
160.522092861328`, 0, 0, 0, 149.87481767916253`, 1.2065648207600534`, 0, 0, 0.03298662969252070 4`, 0, 0,
9.163424243796566`, 0.06936804304592022`, 5.013438930470866`, 0.38037873517589815`, 0, 0, 0.7320543545577588`},
{0.1926486177557311`, 2.6336525621782014`, 9.444109966651418`, 1.4788802142070587`, 0.6654095862145473`,
0.16653655494047714`, 0.03828733327035071`, 2433.4281263020134`, 0.12844440731237916`, 4.149230973672752`,
19.155383302174474`, 0, 0, 0, 12.494114596367076`, 0.1719843920601078`, 0, 0, 0.004688342681947243`, 0, 0,
6.4706733543394686`, 0.012902896246324676`, 0.3523811932402043`, 0.06592613828015015`, 0, 0, 0.19540684877755854`},
{0.055762854118976635`, 2.4026093631268655`, 6.857734870811047`, 0.7509249944976302`, 0.22671157472868875`,
0.4788061070906606`, 0.1564030997457896`, 55.02740807629177`, 0.03469990388231514`, 7.492235364197862`,
219607.0192408077`, 0, 0, 0, 139.19080493791822`, 8.312296054817251`, 0, 0, 121991.76639370149`, 0,
0, 285.30286186266255`, 97180.1296161177`, 30.722267489891045`, 1.`, 0, 0, 1.0733028380482137`},
{0.05296847355619744`, 1.6490117913903575`, 9.992115392915188`, 0.8066960527418563`, 0.1291629696144665`,
0.33931322884091664`, 0.26435083615790894`, 660.4050522121521`, 0.10186522527726305`, 2.8696181659920086`,
39.684763508085595`, 0, 0, 0, 9.03042688716153`, 1.2477230354825688`, 0, 0, 0.00774957292669902`, 0, 0,
29.39299997000185`, 0.00586404355196638`, 0.9710940607810559`, 0.18640820724971063`, 0, 0, 0.3797133165338059`},
{0.21365538200916506`, 3.0547845209089406`, 0.35609106729359574`, 0.8256521864420123`, 0.9506371395328554`,
0.271042805312945`, 0.12766968769895812`, 68.7970381277751`, 0.05673347586404265`, 3.4610065999125617`,
288137.8627021797`, 0, 0, 0, 3.413262972618208`, 0.1356298075538366`, 0, 0, 71103.84264113051`, 0, 0,
5.918854809847409`, 217024.55231157603`, 10.328867006966568`, 1.0000000000001048`, 0, 0, 0.13934372488759988`},
{0.22385277301638717`, 3.365536903395271`, 8.935305056074029`, 1.445762062116566`, 0.43390325179453604`,
0.6187135527847307`, 0.01939946413637202`, 453.1242214832122`, 0.13325485950688953`, 8.825528492983036`,
321.8021318833832`, 0, 0, 0, 252.63027700486515`, 1.402884928477957`, 0, 0, 0.05365851455034574`, 0, 0,
67.44944282870857`, 0.17159438968623245`, 4.366020775831812`, 0.5473727380916782`, 0, 0, 1.1981103175274894`},
{0.26129739321020845`, 3.355909888714172`, 1.041845959949887`, 1.206440545734901`, 0.09347792890054829`,
0.5333101115239811`, 0.015503351594481202`, 579.4832207194656`, 0.24065061389170217`, 7.475999528371727`,

8747.97885270187`, 0, 0, 0, 51.288326879864684`, 0.229134290959011`, 0, 0, 1835.1533354710589`, 0, 0,
10.985057612469378`, 6850.296895708696`, 2.7695752133077964`, 1.0000000000003404`, 0, 0, 0.6556071215526034`},
{0.14172561423935526`, 2.161658858177276`, 2.5726304321806133`, 1.1364876244324849`, 0.9181535957758225`,
0.5861373014149901`, 0.010418800942482771`, 101.70999367656657`, 0.1500483081169689`, 9.64604938537483`,
78537.11429852412`, 0, 0, 0, 176.6420690322347`, 0.8138487974358283`, 0, 0, 25898.649094248194`, 0, 0,
25.132335174201177`, 52435.74215502642`, 20.72391833480457`, 1.0000000000000324`, 0, 0, 1.0336206151941896`},
{0.06568219588241164`, 1.0305120784213448`, 8.720293141386708`, 1.4160313463775192`, 0.752508918071946`,
0.3168178137194088 7`, 0.1341730971552541 3`, 209.13734568668048`, 0.22402731709711876`, 4.416848552940577`,
306.50031128237816`, 0, 0, 0, 107.49654833824754`, 12.561338987324715`, 0, 0, 0.783875178129728`, 0, 0,
184.9230221083295`, 0.7355234713895394`, 4.487528554212731`, 0.9988533989911941`, 0, 0, 0.3382678214604524`},
{0.27044630440719625`, 2.4698840490073675`, 6.955264919348561`, 1.0249819835130385`, 0.106623120735865`,
0.6358905088319218`, 0.012311596840628138`, 346.23093318344644`, 0.07401305589494472`, 1.2937187987733016`,
44.23096306646051`, 0, 0, 0, 37.509931434721466`, 0.17784682045980724`, 0, 0, 0.05005228576472407`, 0, 0,
6.275157500290781`, 0.1933779387457598`, 0.9036713139082971`, 0.6333491836326076`, 0, 0, 0.2915346112652073`},
{0.08403118541616988`, 3.378337092807227`, 2.524635889786053`, 0.8671019090417934`, 0.46085963082622694`,
0.5847463933658021`, 0.0473316577589333 2`, 1030.6526695312073`, 0.10944158368617352`, 3.930070348109142`,
62.54081465868531`, 0, 0, 0, 37.5488121118119`, 0.5040790110190088`, 0, 0, 0.07208778214840868`, 0, 0,
24.32784029473001`, 0.08653745411360485`, 0.8561619380460329`, 0.8116038857990568`, 0, 0, 0.540784627048789`},
{0.11975601650409934`, 1.1540823534574054`, 5.101398975621729`, 0.9964575213162732`, 0.8596230582994027`,
0.5017711629445061`, 0.09812020963113087`, 54.445260820005984`, 0.07947868464718594`, 4.273753987333478`,
200201.01107563608`, 0, 0, 0, 75.58911673300778`, 5.867610349450755`, 0, 0, 73787.36610668634`, 0, 0,
96.73865087521635`, 126235.44333237651`, 17.1656433809397`, 1.0000000000000033`, 0, 0, 0.4614915342077035`},
{0.26353274820980593`, 3.2715371213501925`, 8.419438906869026`, 0.9426613525835754`, 0.1294979869931494`,
0.5387601140015186`, 0.040849691062558216`, 744.2937861154428`, 0.1766059692159136`, 7.8822033078787435`,
120.81442853553429`, 0, 0, 0, 77.08988711707805`, 0.914621791906871`, 0, 0, 0.012349745213257298`, 0, 0,
42.745987774559524`, 0.046493747081979314`, 2.4609830141891633`, 0.2452900320628374`, 0, 0, 1.3983814768053315`},
{0.15837784563387913`, 1.590657770632209`, 9.4421417173358`, 0.8199017044479102`, 0.629826423052569`,
0.2661387442145513`, 0.049173845467465206`, 187.50695430622352`, 0.06908425180315825`, 9.722920562989135`,
713.5642589271415`, 0, 0, 0, 422.501346385137`, 12.190388376125343`, 0, 0, 0.5663325875181128`, 0, 0,
277.01051425011906`, 1.281350501762617`, 10.88510334272081`, 0.9990327945760411`, 0, 0, 0.6183111519610864`},
{0.18489841859634304`, 3.5401138358224227`, 7.262892362437768`, 0.8851173674042263`, 0.0510439823369486 2`,

0.6826543300991057`, 0.13384148697112022`, 730.4506518091381`, 0.22533987085622137`, 8.188627124718394`,
136.4777320365579`, 0, 0, 0, 48.44545983722825`, 1.7058671920142472`, 0, 0, 0.015238254072596432`, 0, 0,
86.27091497893132`, 0.04025041543134584`, 2.702943695165952`, 0.3087439051276356`, 0, 0, 1.8148322615798451`},
{0.22773890477301867`, 2.1437195677585157`, 6.152335866192775`, 1.1180903919170035`, 0.02705103803804154`,
0.3333465628704615`, 0.009170207790258475`, 2321.6700090659583`, 0.14789384405564093`, 7.237611958554762`,
26.590895556777824`, 0, 0, 0, 23.552954671219293`, 0.09485138678969592`, 0, 0, 0.00436403922548691`, 0, 0,
2.904782484144325`, 0.014198021622844413`, 0.7010275881105487`, 0.08047210632316648`, 0, 0, 1.0086653256988045`},
{0.17605039997850958`, 2.727076411741753`, 8.728585319721596`, 1.1320082850991182`, 0.7355770210071702`,
0.5402004706661186`, 0.09745706159326789`, 177.96581560566344`, 0.01922037387361028`, 4.980887347403458`,
379.80400571840204`, 0, 0, 0, 159.1789846245606`, 5.323276605109517`, 0, 0, 2.2521135347366013`, 0, 0,
207.38545804244146`, 5.664078408391555`, 6.419737802060431`, 0.9999999301745343`, 0, 0, 0.8077482825940543`},
{0.06947420594938447`, 0.8077718165326004`, 3.821443319804267`, 1.3891202366711977`, 0.6756008025235485`,
0.23550127195203518`, 0.0653335798785588`, 483.3383501610001`, 0.09612644227220152`, 2.381497911922425`,
65.84106637907664`, 0, 0, 0, 34.35487077800581`, 2.4806897793547913`, 0, 0, 0.19022598505342425`, 0, 0,
28.626161276046734`, 0.1887971323218744`, 1.0352004261899022`, 0.9273736309072381`, 0, 0, 0.15361922394466737`},
{0.23569362997389992`, 1.2270876586626818`, 0.9845589085102358`, 1.1982217261670929`, 0.43286649637666286`,
0.634774301600445`, 0.09976341665112172`, 228.04937393006952`, 0.12724994607105566`, 3.0426000813607676`,
59653.73018148092`, 0, 0, 0, 10.033694085350785`, 0.7555851383189621`, 0, 0, 13654.451006858026`, 0,
0, 13.245274261430442`, 45975.244615816526`, 2.8619804171297307`, 1.`, 0, 0, 0.2642757759108095`},
{0.12274586224015488`, 2.0471402784080235`, 1.4612976870222134`, 0.9456822010625827`, 0.8327879461898879`,
0.22053943969717293`, 0.006767755909702022`, 174.3096643655477`, 0.1850505728558421`, 1.2537872990507584`,
19731.476101653963`, 0, 0, 0, 13.048767717499132`, 0.04093556089332934`, 0, 0, 7160.736288371719`, 0, 0,
1.1971547931994144`, 12556.439285579327`, 1.5013468104966172`, 1.0000000000007732`, 0, 0, 0.07215561243513452`},
{0.13491824188855928`, 1.669720674179887`, 1.3334401828831126`, 0.968437561771696`, 0.34828727193756603`,
0.3135712369052611`, 0.22891096239253717`, 1910.612731597179`, 0.10930115833371806`, 4.270908360205038`,
24.203493543643585`, 0, 0, 0, 6.0034635105778085`, 0.727870727981357`, 0, 0, 0.03762645344226842`, 0, 0,
17.362011466297684`, 0.07252135638474559`, 0.4881510498180058`, 0.556359446007006`, 0, 0, 0.45460753543937366`},
{0.04199865921066254`, 2.754174936868967`, 5.53210979521042`, 1.3844956478579449`, 0.47136731805345633`,
0.24068198696313148`, 0.022972136801125642`, 58.648848297948106`, 0.2240134714991286`, 4.479259216921056`,
43127.7408143082`, 0, 0, 0, 151.08597035688362`, 1.1908474661016089`, 0, 0, 26829.68161423397`, 0, 0,
46.854317782442386`, 16097.295069239897`, 16.43621562230337`, 1.000000000000079`, 0, 0, 0.4048307084464748`},

{0.18074106663079648`, 0.9554759541683486`, 8.202625386518609`, 1.4769540911095849`, 0.5922233662884384`,
0.5544055394726339`, 0.15090969809745627`, 4632.951795907658`, 0.16872394703920557`, 7.933658625009354`,
33.040934066417904`, 0, 0, 0, 10.712289054718704`, 1.5229848792360225`, 0, 0, 0.0048686620182349`, 0, 0,
20.788220438171454`, 0.012570959517735585`, 0.371401827893485`, 0.06831783768644684`, 0, 0, 0.7844876547957236`},
{0.22308930012837075`, 1.8877770902637643`, 5.727481376105105`, 1.290937410786533`, 0.5385704303274257`,
0.17837528465279628`, 0.11591468148922064`, 2665.0058694088225`, 0.1309938954283596`, 2.3264420617360244`,
8.71333110105202`, 0, 0, 0, 3.384167289328283`, 0.1897163558382017`, 0, 0, 0.00552426249966417 6`, 0, 0,
5.1163170028526626`, 0.01760576935392956`, 0.182447820449347`, 0.08768868794201012`, 0, 0, 0.1370954690716077`},
{0.16528939075200133`, 0.44637388983991233`, 6.000554224390708`, 0.7620462868306999`, 0.45157540745748825`,
0.6293554847029814`, 0.08179042637541929`, 626.5470719450981`, 0.11922182895709121`, 4.5474321331423475`,
121.81520506316748`, 0, 0, 0, 56.96845531464038`, 8.77583428321263`, 0, 0, 0.032514576824400516`, 0, 0,
55.96147550840125`, 0.07677592276950779`, 1.6293386138365742`, 0.5958716845127731`, 0, 0, 0.6249117123050363`},
{0.12057640522465035`, 1.6899679349832946`, 5.872613897455656`, 0.8685022109314445`, 0.5819827862907108`,
0.45575489869095254`, 0.005408595604133131`, 1249.3305456098487`, 0.22090492300977427`, 9.765554151588912`,
134.239724991178`, 0, 0, 0, 124.56135027301646`, 0.3777152917742554`, 0, 0, 0.015325913170547462`, 0, 0,
9.118953309305013`, 0.026399193098426074`, 1.6809714823287245`, 0.31547891981786536`, 0, 0, 0.8748968798843233`},
{0.26839288378136816`, 1.20431418513886`, 3.4264235210106797`, 1.1464020290887187`, 0.9129873318113406`,
0.17958510842302844`, 0.03476022275277427`, 85.14929883968256`, 0.03637248443077806`, 6.177611921122705`,
422073.38356184616`, 0, 0, 0, 107.99032373008933`, 2.8989640494591113`, 0, 0, 87276.90183825167`, 0, 0,
49.87519324244567`, 334635.70531245373`, 14.726337697245086`, 1.0000000000001266`, 0, 0, 0.17567054885659564`},
{0.19312844950593372`, 3.9129912832786564`, 0.42277195731844414`, 0.8655327062249413`, 0.5847393470300801`,
0.16029168895981472`, 0.1525854675774217`, 2234.1231120850057`, 0.03649943574663328`, 5.234397199033289`,
20.984268224598885`, 0, 0, 0, 5.854653886374717`, 0.20523903248764888`, 0, 0, 0.9182120923677841`, 0, 0,
11.472836358753073`, 2.53332682452272`, 0.5000619720221265`, 0.9999994424011686`, 0, 0, 0.4260828490366689`},
{0.05831506452479063`, 1.4447664488109728`, 9.399667906542401`, 0.8838396243177019`, 0.4494233408794772`,
0.5909311609620335`, 0.2085880113243902`, 309.3416067223959`, 0.04816991376818669`, 2.147582543132131`,
110.97250965331297`, 0, 0, 0, 28.97934176361099`, 3.7844020277743318`, 0, 0, 0.05483792199242731`, 0, 0,
78.10824397915084`, 0.04568395656261662`, 1.5584563011583141`, 0.7264990652800153`, 0, 0, 0.2950340307009241`},
{0.23965113012644185`, 0.9128974489725401`, 8.261297062012524`, 0.788417407034967`, 0.8016641172773833`,
0.35686628470413617`, 0.030635478621033968`, 346.1316581378353`, 0.043365488693742626`, 1.9545026373639716`,
116.95354517929417`, 0, 0, 0, 81.34456664304592`, 2.4987652523750543`, 0, 0, 0.1153346016635322`, 0, 0,

32.58737749249156`, 0.39485810901931506`, 1.1805178344034986`, 0.9418974839675771`, 0, 0, 0.11581131430142279`},
{0.20520507007680205`, 1.512442754504547`, 9.386452795492342`, 1.0402158263719963`, 0.9284696718653804`,
0.3818637796049891`, 0.053241178903524394`, 3017.2611079460976`, 0.15062298010038172`, 2.3864513615272553`,
22.46099795120003`, 0, 0, 0, 12.812062765049124`, 0.4255908782004235`, 0, 0, 0.007014680784464398`, 0, 0,
9.195454858820835`, 0.02056354374202737`, 0.16286751528836196`, 0.13455568328463563`, 0, 0, 0.1033786649093968`},
{0.20853248950244857`, 2.7801356391066916`, 9.335557605849111`, 0.8182981643225358`, 0.24140252954455388`,
0.3825831953385048`, 0.013327430792399009`, 77.96529661289064`, 0.07323986754942219`, 3.7008101112034457`,
68157.41842395031`, 0, 0, 0, 236.0548590551081`, 1.0823845957489935`, 0, 0, 17058.693981525594`, 0,
0, 42.988228426598084`, 50818.45605182816`, 10.304819183106764`, 1.`, 0, 0, 0.36984252950704843`},
{0.13576335201419282`, 2.033104701519708`, 4.843203210944571`, 1.3428754702553487`, 0.2671111595179354`,
0.18962864690949777`, 0.12183777475102511`, 1231.483599580866`, 0.14101782654161166`, 2.1508880203421494`,
14.062304512997493`, 0, 0, 0, 5.347448423874563`, 0.2888437794400696`, 0, 0, 0.0124958487831717`, 0, 0,
8.389280656918954`, 0.024235404529519552`, 0.3724242128799225`, 0.1804585200032418`, 0, 0, 0.1728591538078442`},
{0.25963449652183795`, 2.4481737185281203`, 5.334705083491999`, 0.8989699854247848`, 0.7130966522497681`,
0.695912989145927`, 0.04220235704717508`, 140.4631283114693`, 0.11660454588716207`, 7.56536482611441`,
51517.39459822761`, 0, 0, 0, 208.87380364420574`, 3.442295227607988`, 0, 0, 10869.394213417492`, 0, 0,
120.39052439492362`, 40315.28134425745`, 11.877411001165987`, 1.000000000000663`, 0, 0, 0.8875492589395017`},
{0.17509339161516935`, 3.0306683644318824`, 1.9789343166711684`, 0.8960196738550836`, 0.2152496986108292`,
0.6250130758758401`, 0.02087454134280109`, 59.51435674676293`, 0.2018230747082378`, 3.546682887992379`,
97708.20265429444`, 0, 0, 0, 43.93160268359486`, 0.2919679650464629`, 0, 0, 27889.746880935327`, 0, 0,
12.640829644198089`, 69761.57675245129`, 12.915017213597034`, 1.0000000000000742`, 0, 0, 0.34770386581547696`},
{0.10593857456933181`, 2.955002391424368`, 8.212267239719637`, 0.932578721051657`, 0.3205115590912122`,
0.6246255275862143`, 0.01773467921096997`, 102.27924186693049`, 0.015490300413002644`, 6.09864277306975`,
227762.3423639426`, 0, 0, 0, 348.4297661045804`, 2.000016737823238`, 0, 0, 90445.83706279755`, 0, 0,
84.42934633080581`, 136881.47220232585`, 13.879182444493322`, 1.`, 0, 0, 1.0937553363123036`},
{0.10583476133380088`, 3.0591620947997713`, 7.753926172578531`, 1.2866948671665401`, 0.7481949494801159`,
0.21582592530952183`, 0.02844379415675546`, 101.39583001744663`, 0.09278999192622717`, 1.6976300926057526`,
8561.006458364533`, 0, 0, 0, 76.9999401704853`, 0.6711330953265338`, 0, 0, 3365.5422993511315`, 0, 0,
29.330070368408165`, 5088.448085866253`, 3.6480650121362896`, 1.0000000000048883`, 0, 0, 0.1765227141574361`},
{0.23086787628538824`, 0.6622997055352342`, 7.183940408138384`, 1.353990838360309`, 0.25975387914696335`,
0.6772244715959861`, 0.055054708296352145`, 683.0312933373987`, 0.1367743885435413`, 6.244797760097583`,

130.76856833688336`, 0, 0, 0, 73.99648015698148`, 5.413766956174858`, 0, 0, 0.03135035642711215`, 0, 0,
51.22194658444273`, 0.10339700298737761`, 2.1333186171942065`, 0.38973108966027525`, 0, 0, 1.1379606702416871`},
{0.20388536464835005`, 2.2276602475557015`, 5.0708546023033385`, 0.8827779974574592`, 0.1554825605038257`,
0.2618026330626365`, 0.006155066765134505`, 1063.8834649875878`, 0.12744889948308613`, 2.149276150894316`,
16.705175788430296`, 0, 0, 0, 15.34018138969411`, 0.039960950176043446`, 0, 0, 0.009472157209855754`, 0, 0,
1.271706002310371`, 0.02758906038197066`, 0.4430659186650144`, 0.20511098268605288`, 0, 0, 0.2390010750085391`},
{0.10738337044420926`, 3.7501964356372497`, 1.0776334746380822`, 0.9682422375974065`, 0.09998974406674432`,
0.504170653500009`, 0.22038799040453166`, 2051.555691372483`, 0.09521018879793502`, 2.016584418866474`,
10.745437156692924`, 0, 0, 0, 2.7270488003687032`, 0.14481444957865908`, 0, 0, 0.04548081903495484`, 0, 0,
7.758323323409392`, 0.0697697662648037`, 0.23494318021597405`, 0.6032609361887338`, 0, 0, 0.425543942667319`},
{0.2588804970943782`, 0.5492314132783744`, 9.644794872912858`, 0.9613308813426055`, 0.2817033035077452`,
0.232857138958534`, 0.012136672860729404`, 78.78277994386292`, 0.023082056754425706`, 4.302644367094656`,
137772.13272659492`, 0, 0, 0, 282.3215864311667`, 5.393619556181917`, 0, 0, 29253.584769193883`, 0, 0,
42.319218450395255`, 108188.3223834497`, 11.63145159048731`, 1.0000000000005516`, 0, 0, 0.3402366293837788`},
{0.08494913534264076`, 3.4349155581389494`, 7.0407634051690415`, 1.119369452553694`, 0.9329533821244145`,
0.5630698272627359`, 0.4838120213175008`, 71.45974855722541`, 0.09271025542437755`, 2.443770975165119`,
95890.81008474117`, 0, 0, 0, 20.627754674663144`, 2.5143886386789522`, 0, 0, 43253.517140245785`, 0, 0,
123.38160934580272`, 52490.698308457`, 7.800929113162356`, 1.0000000000001543`, 0, 0, 0.38065468704163996`},
{0.2312360951475228`, 2.6472972630846083`, 4.924168333426426`, 0.8758299082609615`, 0.33581096057577486`,
0.16094039113701897`, 0.009823651093858611`, 148.81624524939704`, 0.12101804849376735`, 7.0744188173362765`,
9248.687097685755`, 0, 0, 0, 240.04361169803227`, 0.8432575827842137`, 0, 0, 2085.7403087632792`, 0, 0,
31.890764156856875`, 6889.977778431549`, 9.96795948882698`, 0.9999999999999839`, 0, 0, 0.4414456588569006`},
{0.2359361250604497`, 3.266770315195857`, 7.411396165562348`, 1.2275925063587856`, 0.15344120088912327`,
0.536013355113413`, 0.3013372435966537`, 712.0245065436816`, 0.039861802547139946`, 9.042443794820379`,
149.79165608831158`, 0, 0, 0, 30.432918836211947`, 2.502074871248999`, 0, 0, 0.020469798523094447`, 0, 0,
116.76719879705296`, 0.06899378491893797`, 2.9323484758205605`, 0.30073232914685155`, 0, 0, 1.5362696871862067`},
{0.26580884355261647`, 3.957059703696273`, 5.486829732050921`, 1.3210508471658353`, 0.3282573265157698`,
0.6164824430541005`, 0.042206581416411024`, 114.97331259087073`, 0.22266675421103638`, 9.071401748075406`,
55556.658656069565`, 0, 0, 0, 252.70336144734682`, 2.6069030186974453`, 0, 0, 11496.95056251396`, 0, 0,
147.3667269533114`, 43657.01619147745`, 17.070419450393373`, 1.0000000000003424`, 0, 0, 0.8726544584105114`},
{0.24965572729559277`, 1.088568489897936`, 8.460543344934337`, 1.1505850947024536`, 0.8975822933983539`,

0.5110185880609677`, 0.06613687599714729`, 1096.4114980052268`, 0.1003107894781316`, 4.101976455241168`,
127.8628755202702`, 0, 0, 0, 66.03446139642045`, 3.7254938768137342`, 0, 0, 0.03663259707346264`, 0, 0,
57.93507490867333`, 0.13065053807286173`, 0.7743986168113812`, 0.4928551527107755`, 0, 0, 0.19992525819466803`},
{0.1942611669549973`, 1.9123387865104826`, 2.4307010631404715`, 1.2953041584793583`, 0.36588469810004653`,
0.5550679926918506`, 0.01452356295191177`, 246.06581986793796`, 0.04070230178651557`, 1.4358727366404163`,
97040.48025496164`, 0, 0, 0, 23.86095189089513`, 0.17207659089975044`, 0, 0, 25697.386388061175`, 0, 0,
4.7009819861155515`, 71314.34667768893`, 1.2860653886072495`, 1.0000000000013012`, 0, 0, 0.16750805287136414`},
{0.20522561065351508`, 3.886130079640968`, 5.862633336878224`, 1.1071510042707493`, 0.46587070079395265`,
0.3092363340879616`, 0.06237106286442892`, 3704.443052142306`, 0.23732711327647033`, 5.5948716983231535`,
18.24073513341799`, 0, 0, 0, 9.786440843885856`, 0.14930982802442025`, 0, 0, 0.004010767270201246`, 0, 0,
8.289105912453135`, 0.01175874517453023`, 0.32380663667984183`, 0.0748705453448042`, 0, 0, 0.4827127466802505`},
{0.24537724051627174`, 3.881922170050893`, 2.190905545388226`, 1.4415825883984728`, 0.23029515887672103`,
0.5607538040686315`, 0.02143037658176177`, 612.4231659367224`, 0.18957440226421673`, 2.1066205948825285`,
44.96270739213868`, 0, 0, 0, 31.930028431004782`, 0.16876761013321223`, 0, 0, 0.7756196001635901`, 0, 0,
9.359181819466041`, 2.718848531121083`, 0.83486117459084`, 0.9996389277788165`, 0, 0, 0.48390691267912467`},
{0.06117754469042569`, 1.77753347893454`, 8.494748688583062`, 1.4528026068281314`, 0.13374021962928717`,
0.2747744172973582`, 0.02769785108344557`, 761.9216006517844`, 0.1674213538626742`, 9.223974549251146`,
104.37122598019772`, 0, 0, 0, 75.46905412178918`, 1.0935522749806401`, 0, 0, 0.013385042810014852`, 0, 0,
27.768939710615967`, 0.011698057924200568`, 2.6509876076003303`, 0.1797848348361325`, 0, 0, 1.0099053738560961`},
{0.2543463901897882`, 3.8184481545424687`, 6.435093510144283`, 1.0179149591357712`, 0.6456387658124723`,
0.39719113288117625`, 0.03560663844887868`, 836.6099873310699`, 0.05172688927212099`, 4.4940967912431145`,
92.50705857694102`, 0, 0, 0, 61.6106448630882`, 0.5538624651478468`, 0, 0, 0.026661635914319946`, 0, 0,
30.21278725591626`, 0.09687558359092514`, 1.142432810634279`, 0.42882045936404467`, 0, 0, 0.3484747525300642`},
{0.25340300959745826`, 3.7481040027914663`, 6.564583042000866`, 0.9665595115932711`, 0.6900722172611409`,
0.5320596915161446`, 0.007953381223150922`, 557.3521763213881`, 0.16633866213427612`, 7.2685179596650595`,
289.9526955762029`, 0, 0, 0, 260.1185272102084`, 0.5361207656716079`, 0, 0, 0.07628259979028275`, 0, 0,
28.706234111334048`, 0.27614629095393467`, 2.7826107197815406`, 0.8100390294346935`, 0, 0, 0.5893456868060367`},
{0.21905085798311597`, 3.1393917093979224`, 6.344370247647537`, 0.9036844621009067`, 0.45657431516696256`,
0.6738956741194089`, 0.01713634873689236`, 595.7124001741428`, 0.1327223790362297`, 6.393427116779321`,
189.5667522528146`, 0, 0, 0, 152.54896031016338`, 0.8021561218626119`, 0, 0, 0.04165305822734033`, 0, 0,
35.9754611231182`, 0.1303448306044758`, 2.4147933341625163`, 0.6244288343564561`, 0, 0, 0.8950860382912293`},

{0.26756748312672074`, 2.582420366138944`, 0.22100971269994044`, 1.2986139421663903`, 0.40694219440887625`, 0.4206092167171217`, 0.07540978051777308`, 286.7043124505423`, 0.12484831726628037`, 7.450517421109604`, 61364.44550581311`, 0, 0, 0, 6.200064112029521`, 0.1734064473586258`, 0, 0, 12722.247986401935`, 0, 0, 6.397262018267335`, 48629.426763365314`, 5.386502904980735`, 1.0000000000018558`, 0, 0, 0.37410626848781486`},
{0.1649279191778415`, 1.2798961157951103`, 8.443353077105307`, 1.1073246765756934`, 0.5543130008347743`, 0.4677559431287117`, 0.04549043803104127`, 88.19757502008439`, 0.08859698434873292`, 4.4693006714200685`, 66968.59336970124`, 0, 0, 0, 191.38166699056654`, 6.279472528682782`, 0, 0, 19861.102833137844`, 0, 0, 114.81532141004523`, 46795.00518352212`, 11.23371082872364`, 1.`, 0, 0, 0.5506820011070802`},
{0.24671033429293737`, 0.7439602453864245`, 2.8830481632165945`, 1.0433060628070765`, 0.2605457162416671`, 0.35613575939670206`, 0.02699949405773168`, 552.8129873973498`, 0.15941770646667952`, 8.472285283746757`, 162.91448578185557`, 0, 0, 0, 118.10149297053503`, 3.8100428607022123`, 0, 0, 0.10721732148495285`, 0, 0, 40.49314887972586`, 0.3778803032222056`, 3.388528707302103`, 0.8785474011840592`, 0, 0, 1.0187096172125372`},
{0.15869950794106824`, 3.1033559606377414`, 9.144330738839969`, 1.4178115100024233`, 0.3048291795319098`, 0.2869274730275907`, 0.17534043095376647`, 52.531250426358234`, 0.14088030152015352`, 4.046496400392222`, 118882.02386303202`, 0, 0, 0, 86.72360529670013`, 4.535941489561502`, 0, 0, 36297.74689231964`, 0, 0, 201.0948722676384`, 82291.9224454366`, 16.278288552748755`, 1.`, 0, 0, 0.28610578650798163`},
{0.2764621058346162`, 2.9381918751992178`, 0.9446276562373105`, 1.4121699137634485`, 0.5065538844804354`, 0.6085910850890284`, 0.12806524333884275`, 90.5764532701865`, 0.169606806379912`, 4.122343563126204`, 163538.67889342218`, 0, 0, 0, 11.02146588671046`, 0.4594339939708134`, 0, 0, 33035.51459589125`, 0, 0, 19.28436040393394`, 130472.39903586148`, 9.611199253124855`, 1.0000000000021017`, 0, 0, 0.28843535938144205`},
{0.23050133288232844`, 1.3238077162346595`, 3.1108482993453865`, 1.1555015486294062`, 0.6747942262463269`, 0.6285668584610278`, 0.027391537177886824`, 109.70658223761104`, 0.027210691652561736`, 1.9248854769177905`, 486392.0716893193`, 0, 0, 0, 34.90003051867164`, 0.6767899247721898`, 0, 0, 113290.87258019214`, 0, 0, 12.799138924047147`, 373052.8161876614`, 3.7909521704860194`, 1.0000000000003244`, 0, 0, 0.1823027494501815`},
{0.16511179970471734`, 1.257711050374506`, 7.3270141910702415`, 1.3219934358839476`, 0.5519839493462388`, 0.6927265248357952`, 0.057669326830527015`, 282.0308127392517`, 0.18961708461999577`, 6.119113848125975`, 386.1275084674433`, 0, 0, 0, 211.46831373242944`, 8.972245900066515`, 0, 0, 1.3327302751820447`, 0, 0, 161.20704021701454`, 3.143564203660761`, 4.952134299030402`, 0.9996861392736609`, 0, 0, 0.957268756867683`},
{0.18051596911200918`, 3.1154638731836117`, 5.271301524292921`, 0.9821224785779399`, 0.579876513557519`, 0.2578243252832043`, 0.01324703728258807`, 298.19257336110485`, 0.04626579120349833`, 3.252038865320179`, 134.57577658022095`, 0, 0, 0, 112.5394751444557`, 0.458476026729155`, 0, 0, 0.30849162719865014`, 0, 0,

```
  20.40522139993495`, 0.7955380720957889`, 2.311269900478183`, 0.9900243657705059`, 0, 0, 0.2399256338833419`},
 {0.2700603276661351`, 3.8874233694867435`, 9.935191315459868`, 0.9369398631417749`, 0.9680175802467335`,
  0.2817307366148821`, 0.03116132832319949`, 267.3428425281449`, 0.2310886914635361`, 4.719763737676903`,
  363.02233411688366`, 0, 0, 0, 250.8215835988255`, 1.9463754754179983`, 0, 0, 0.4377831251064967`, 0, 0,
  108.09122155622579`, 1.6889693458994506`, 3.6407035554075304`, 0.9982322319737682`, 0, 0, 0.21170419265869264`},
 {0.2505990837413898`, 3.9386610341401305`, 4.197732331385515`, 1.4863985983426793`, 0.6450186838764853`,
  0.5983146588433161`, 0.0058599277111039`, 4347.60789332823`, 0.1797443484893408`, 5.002549103667693`,
  25.07650818288479`, 0, 0, 0, 23.084273303790066`, 0.033386861391585934`, 0, 0, 0.012068040108825314`, 0, 0,
  1.8785647145039597`, 0.04320342562608318`, 0.24923273258062129`, 0.15866413215865827`, 0, 0, 0.48751962110478475`},
 {0.15385799276986173`, 3.328452062918277`, 1.1907303286310018`, 0.9499432416831306`, 0.24618506024325426`,
  0.6220480490433855`, 0.08397939385457624`, 160.09510109398894`, 0.21511864519397922`, 7.34323009363505`,
  32608.290024555277`, 0, 0, 0, 32.83289982363894`, 0.7926586587382974`, 0, 0, 10174.254449071743`, 0, 0,
  37.69037639810738`, 22362.71953520019`, 10.005929227107977`, 1.0000000000007632`, 0, 0, 0.7730645504252401`},
 {0.10621732051626104`, 1.365898117434102`, 6.864395407461604`, 0.9825773772303991`, 0.8367483775504436`,
  0.5624371522540671`, 0.07041397408927531`, 60.72834180417396`, 0.20758521960363197`, 6.929584578355666`,
  58409.78224203836`, 0, 0, 0, 200.48360499742128`, 9.556215528057246`, 0, 0, 23045.002754297864`, 0, 0,
  186.46881142239891`, 34968.263483591145`, 25.45969464168525`, 1.0000000000002776`, 0, 0, 0.9041156194737352`},
 {0.2268285366732684`, 3.377330494378361`, 0.792106071900994`, 1.4321394301198889`, 0.4771428999999012`,
  0.6107585820948693`, 0.0427110994331284`, 2178.207920314213`, 0.06460930790004932`, 5.987666522418898`,
  44.45312816329743`, 0, 0, 0, 26.001806075000907`, 0.3142910404330502`, 0, 0, 0.7008183984011627`, 0, 0,
  15.163781642349205`, 2.270937311186252`, 0.6488609793179442`, 0.9997842411559313`, 0, 0, 1.1733277088559524`},
 {0.27738294555080567`, 1.5184858354681863`, 2.5078605202307482`, 1.4658418288357904`, 0.7951974935096349`,
  0.3102987260459962`, 0.028082255793227213`, 4738.743053997216`, 0.039514889508313555`, 1.270204126939964`,
  5.0477582232509945`, 0, 0, 0, 3.561836097843187`, 0.061976471395522405`, 0, 0, 0.015886590095679956`, 0, 0,
  1.3444341992342788`, 0.0629524165071266`, 0.05642032085155145`, 0.20302857666984309`, 0, 0, 0.08442698543933684`},
 {0.12722141666194153`, 2.144800891916389`, 0.9267204145449971`, 1.474327309271881`, 0.07507296673368935`,
  0.4110764361383803`, 0.01396212306727472`, 341.96044988390173`, 0.08796076167353961`, 8.378052186889676`,
  40703.317562712655`, 0, 0, 0, 52.1193032669522`, 0.32317263433548166`, 0, 0, 14424.73142852852`, 0, 0,
  9.902013633795866`, 26216.210961506204`, 5.264910612610111`, 1.0000000000000517`, 0, 0, 0.7439299038135906`},
 {0.14199823373416764`, 1.4769393574961134`, 9.82023825383516`, 0.8826223667346553`, 0.41403084809983204`,
  0.6165765653923829`, 0.060760206852004485`, 76.65984772713675`, 0.1884299459763023`, 7.821991164404671`,
```

34702.494111446664`, 0, 0, 0, 352.6424061133488`, 13.480607772427225`, 0, 0, 11243.657840386924`, 0, 0,
284.4291454580839`, 22808.279343518156`, 23.13840294977153`, 1.0000000000009193`, 0, 0, 1.1651935011061372`},
{0.2704769900712498`, 1.6373927417773038`, 3.0886177841931914`, 1.4817168381054997`, 0.05399471731043759`,
0.4806448506103316`, 0.008090658829532001`, 56.76295821829396`, 0.22108849119145935`, 4.3771215003685064`,
185341.5555474955`, 0, 0, 0, 94.8985104549962`, 0.4455467403882427`, 0, 0, 38083.33479382308`, 0, 0,
10.421928554774903`, 147152.36809869987`, 16.13816173716126`, 1.0000000000006162`, 0, 0, 0.2641975271580191`},
{0.15635355369607173`, 1.2350233440589857`, 3.2938536959067304`, 0.7696047462951592`, 0.9490314349758091`,
0.5590905433465692`, 0.008743912631106248`, 94.37874467585033`, 0.17474749461052458`, 8.353315585312398`,
48722.418553545765`, 0, 0, 0, 198.80115788512518`, 1.3149849045993227`, 0, 0, 14998.328776001294`, 0, 0,
23.200529346647773`, 33500.600051855`, 19.22882263139428`, 1.0000000000000067`, 0, 0, 0.8416312746893391`},
{0.16339273559725687`, 2.0059391082075457`, 1.9960227683476044`, 0.8832597488866586`, 0.2201232888636404`,
0.41940637033719763`, 0.260701203173729`, 668.3313383270491`, 0.16081430237854888`, 8.573990161395482`,
142.88418368381315`, 0, 0, 0, 32.15249716324679`, 3.6830814509823795`, 0, 0, 0.45145034900734454`, 0, 0,
105.54338744627644`, 1.0537672501521091`, 2.906524164501829`, 0.9954233907123432`, 0, 0, 1.2681171757710217`},
{0.17131668518162624`, 1.9273467762184806`, 5.930228637778157`, 0.8979121960709446`, 0.761163722566164`,
0.6467717629026779`, 0.01019608706680365`, 260.442341207733`, 0.11039361670332914`, 7.204688135978646`,
387.7453116017390 5`, 0, 0, 0, 329.8809395731619`, 1.6506287710266334`, 0, 0, 3.048573016883556`, 0, 0,
45.44762915102363`, 7.4610203398090915`, 6.551975816389322`, 0.9999999981344533`, 0, 0, 1.4411322673776423`},
{0.11942926109779373`, 3.6640895220352094`, 2.8978096019149504`, 1.1395608488550686`, 0.02531057599926112`,
0.3982329172386545`, 0.26228943719870584`, 89.66215495367044`, 0.22730124409898161`, 8.643250664580492`,
47645.08297986628`, 0, 0, 0, 44.48934368346081`, 2.9303698592837346`, 0, 0, 17532.133977629648`, 0,
0, 153.38767852984697`, 29912.140091654583`, 20.672779457443163`, 1.`, 0, 0, 0.7481501765293876`},
{0.06995397995088021`, 2.4465420953300523`, 1.0825256418482638`, 1.4045898231548657`, 0.8050454938859057`,
0.5373350569389552`, 0.3667462640330922`, 406.36701228872533`, 0.024784828878876453`, 8.741309230400322`,
82872.9610250695`, 0, 0, 0, 13.941054607322318`, 1.8390597231893664`, 0, 0, 41409.94425064903`, 0, 0,
64.27624326584012`, 41382.72014109954`, 4.874167506966916`, 1.0000000000001976`, 0, 0, 1.2942419330446062`},
{0.25135395656732756`, 1.8293827828852027`, 0.6280251816297326`, 0.776655925877336`, 0.712342015777852`,
0.6529270906121196`, 0.026921744082008515`, 2433.232128346219`, 0.014846384799260759`, 4.779446799164496`,
35.958359007037274`, 0, 0, 0, 19.5985766740256`, 0.27110396238749407`, 0, 0, 1.960361725435269`, 0, 0,
7.085041730909113`, 7.039209657018692`, 0.4742029272475706`, 0.999999997941037`, 0, 0, 1.0667052482680945`},
{0.22686431298761822`, 3.544454161776195`, 7.257561779040259`, 1.4835566223233323`, 0.41530778886586006`,

0.5693420006079292`, 0.0281206594086524`, 300.82869963510313`, 0.2233455291441253`, 1.8496714258079727`,
93.02073939466582`, 0, 0, 0, 66.25819263823034`, 0.505074282199308`, 0, 0, 0.15814807028218458`, 0, 0,
25.57446630782091`, 0.5125450473555149`, 1.397168661979499`, 0.8715748770394933`, 0, 0, 0.27989129610532903`},
{0.11505780978488611`, 2.035276516222745`, 0.3473218826840405`, 1.3302263404025527`, 0.8863987944255065`,
0.20525449010517516`, 0.166947674879067`, 1822.5924091420022`, 0.2368153750944742`, 8.635263957438244`,
911.616911200983`, 0, 0, 0, 7.379620742389669`, 0.5431642183314624`, 0, 0, 335.85773314574965`, 0, 0,
15.792705400321557`, 552.0436453580962`, 1.0026142414284305`, 0.9999999999995264`, 0, 0, 0.6233453797553505`},
{0.2645083602715407 3`, 2.000822664278666`, 6.699190267572199`, 1.2017845913999912`, 0.3438445280396565`,
0.6571515800951808`, 0.02542052966525821 4`, 670.8719376728277`, 0.0689542698910992`, 7.858447976519758`,
180.07809198342397`, 0, 0, 0, 132.63612120991124`, 1.597069022061446`, 0, 0, 0.03449699947507593`, 0, 0,
45.64931279654147`, 0.13035349664919996`, 2.681825195741119`, 0.4582561505051592`, 0, 0, 1.2573024367259216`},
{0.1895156330025849`, 2.3307833365396586`, 5.573081915828208`, 1.037167544233136`, 0.6437280244785568`,
0.6985848360999776`, 0.020283589241000377`, 257.16539910388553`, 0.09483519874959423`, 5.443513900146426`,
297.2872756457915`, 0, 0, 0, 215.75479832314957`, 1.7792133335823193`, 0, 0, 5.512060772430821`, 0, 0,
59.24229700089792`, 14.923166949084864`, 5.187641248734884`, 0.9999999991788738`, 0, 0, 1.3158340515296152`},
{0.05605442916152037`, 2.091827585999483`, 5.134093728882359`, 0.7852275512421112`, 0.9528052242020535`,
0.26916872175155115`, 0.16382211865694157`, 830.5018741809612`, 0.0523186960509337`, 8.350099727317033`,
236.07075787885512`, 0, 0, 0, 72.08490530847557`, 5.306970864413325`, 0, 0, 0.04961077208223399 5`, 0, 0,
158.58954360393372`, 0.03972719299100841`, 2.055474885950989`, 0.7407769595263233`, 0, 0, 0.2990840519882315`},
{0.160097742020257`, 2.0916642582432887`, 7.779572730473025`, 1.4293729759208196`, 0.262481852877007`,
0.6066507214539851`, 0.010299825795425889`, 166.88640436294955`, 0.11119499215436468`, 1.1831617215189443`,
89.48839484073358`, 0, 0, 0, 73.4242197789048`, 0.34171599536022135`, 0, 0, 1.6594539454847457`, 0, 0,
10.210787628071511`, 3.7953547094101823`, 1.7430679583449078`, 0.9999994370652392`, 0, 0, 0.29070295651371664`},
{0.096645259209576`, 1.264318941007299`, 4.097121191163998`, 1.119171761441141`, 0.3848067085465605`,
0.38298305619587747`, 0.22103546881327146`, 186.61459180270506`, 0.035388155328102466`, 2.6745014933563738`,
41233.10616888702`, 0, 0, 0, 23.586631923732092`, 3.578762721753753`, 0, 0, 17281.562258468384`, 0, 0,
64.63853563548703`, 23859.729485944317`, 3.217690631044048`, 1.0000000000031333`, 0, 0, 0.3678698275363588`},
{0.08994789013179427`, 1.57009542864993`, 6.7950768142652755`, 1.2471941772808341`, 0.17502080201658954`,
0.4646155607499266`, 0.031711372215814826`, 510.77004725104206`, 0.22094423048581935`, 5.498912040996139`,
117.8921968426536`, 0, 0, 0, 81.79460035858277`, 1.537041688784525`, 0, 0, 0.03209192139954003`, 0, 0,
34.47574470292791`, 0.04123715171665078`, 2.4579960392072966`, 0.42291179336679585`, 0, 0, 0.8621397757292277`},

{0.09559160148361812`, 1.3237772094663507`, 8.951678422145957`, 0.7806574543205589`, 0.8827653082752778`,
0.4548887199854597`, 0.011151733542884613`, 3151.7776331194414`, 0.22534056788365686`, 6.821338260162536`,
66.03533983615444`, 0, 0, 0, 56.97534787323443`, 0.452281129700052`, 0, 0, 0.005725595008703993`, 0, 0,
8.55313502526606`, 0.007818839947550634`, 0.4472120082534556`, 0.14592939415636963`, 0, 0, 0.3201932876358072`},
{0.20804533970301103`, 1.865488871682949`, 2.4561594295175926`, 0.8614398462681598`, 0.7232950760420711`,
0.3427980526897182`, 0.02113398228875948`, 356.75904569388376`, 0.23964513558905903`, 4.163032360670947`,
1984.185422153315`, 0, 0, 0, 65.34725459711909`, 0.6934759746045214`, 0, 0, 478.249185246399`, 0, 0,
18.48102447720318`, 1421.3930601039315`, 2.5848339324844942`, 0.9999999999998133`, 0, 0, 0.5092596014670594`},
{0.23422673966465207`, 3.962742244147649`, 9.344278992351082`, 0.9436428211653483`, 0.6202800294740252`,
0.15583995298414144`, 0.0468134491479599`, 1145.0934679847246`, 0.11614669946806178`, 8.273469182440888`,
76.81217943247714`, 0, 0, 0, 46.60407659757578`, 0.523840442245332`, 0, 0, 0.0063176747760993425`, 0, 0,
29.654923566836562`, 0.02113954807246444`, 1.4917014446928603`, 0.13430322141815954`, 0, 0, 0.3810945602927282`},
{0.23911413452544644`, 3.701379132317136`, 3.3596187018850023`, 1.2070387760959833`, 0.14815711868787962`,
0.5552188532365824`, 0.010060549211211993`, 324.2801725236705`, 0.014653386006449387`, 6.641284984634726`,
40898.704017999575`, 0, 0, 0, 170.5122422821959`, 0.4448402950946854`, 0, 0, 9217.586941291758`, 0, 0,
23.521751221103845`, 31486.504626857717`, 4.790120164660258`, 0.9999999999999992`, 0, 0, 1.228976049617983`},
{0.16379493488608782`, 3.773301948253276`, 6.66686177778092`, 1.3720180958436647`, 0.9115747518300852`,
0.36809943749279594`, 0.006089940921734969`, 188.41929546062013`, 0.11766502525697531`, 3.8904832664355844`,
225.71959389388627`, 0, 0, 0, 199.575274552741`, 0.30891095199023727`, 0, 0, 2.6858382477627725`, 0, 0,
16.651632814021976`, 6.284667155812326`, 4.628680627825107`, 0.999999989715336`, 0, 0, 0.5283226316289775`},
{0.27173801863077446`, 2.1930338431922545`, 7.869758966833125`, 1.0214571559787395`, 0.457397629072652`,
0.6590886785471264`, 0.009120521470678952`, 4004.0778102470904`, 0.1596904202504179`, 8.692703920363371`,
38.1367075420029`, 0, 0, 0, 33.753326790727044`, 0.13414750630735928`, 0, 0, 0.003711532500213427`, 0, 0,
4.20271459016979`, 0.014408064109883373`, 0.4865355154945623`, 0.07509136504173985`, 0, 0, 1.177801458927541`},
{0.2361473928644357`, 1.7414292069064263`, 7.944932185587515`, 1.1578150203530142`, 0.6270752096378571`,
0.30275674150027165`, 0.11839453780639704`, 3486.2324040163485`, 0.1006900893181421`, 6.697770970769804`,
28.187080331557592`, 0, 0, 0, 10.7176384718845`, 0.6744063118203748`, 0, 0, 0.003990110264231114`, 0, 0,
16.777583553229217`, 0.013460773373488557`, 0.40472902945737355`, 0.07144044041278341`, 0, 0, 0.45119161414259945`},
{0.08448906159522324`, 2.163318965633623`, 2.5852320656449397`, 0.9870547358990118`, 0.27911648709335135`,
0.1950863385031143`, 0.04472304557991584`, 1858.3083947017237`, 0.05117109151128901`, 9.531470241589851`,
43.9374515470728`, 0, 0, 0, 27.20392295454003`, 0.5235725920666632`, 0, 0, 0.01262339163114912`, 0, 0,

16.18077883291099`, 0.015236264472349768`, 1.0885483474044169`, 0.24025321327724714`, 0, 0, 0.717679656428075`},
{0.1142146683211071`, 3.422389653131356`, 1.8177299602895634`, 1.2969315528452774`, 0.1391750354375907`,
0.5170758706651055`, 0.01897326911835137`, 87.52693577556593`, 0.11000871045324723`, 3.4664980752399295`,
128654.38482185059`, 0, 0, 0, 40.661462706846976`, 0.2172512043204479`, 0, 0, 48867.98878424787`, 0, 0,
10.621689625666033`, 79734.87329303497`, 8.663006262889986`, 1.0000000000004021`, 0, 0, 0.3752836561740276`},
{0.14347242790835574`, 2.011431525472254`, 0.8946353742157331`, 1.3818589873326788`, 0.974530392330166`,
0.4363768778472087`, 0.006586014840232095`, 142.93650600689625`, 0.2189385420017279`, 6.889239014732425`,
48131.69261379855`, 0, 0, 0, 44.90838995974941`, 0.1402704636246576 7`, 0, 0, 15766.812263808344`, 0, 0,
4.030634751817778`, 32315.754798054648`, 10.273017823465416`, 1.0000000000012867`, 0, 0, 0.5505999805201837`},
{0.07350179944910268`, 2.001951652427108`, 6.856579719843172`, 1.3883617808830775`, 0.83650706130597`,
0.6246458892820506`, 0.03443555932639553`, 159.6804633773726`, 0.22561794329975743`, 6.769156049603524`,
4954.5855265563205`, 0, 0, 0, 283.1279495553944`, 4.565203579291466`, 0, 0, 2212.7962654690145`, 0, 0,
130.5616692746957 6`, 2323.4929618032425`, 10.254907506313257`, 0.999999999999996`, 0, 0, 1.5275530894221712`},
{0.2038713609791254`, 2.9048730428426977`, 6.578839990238183`, 1.1970767733981913`, 0.3047670438201835`,
0.4988995484689318`, 0.027219703475022124`, 126.68486054839573`, 0.1069997024432573`, 5.709865936189522`,
40974.57930763331`, 0, 0, 0, 226.06330895187114`, 2.0240191376584638`, 0, 0, 10393.097476077466`, 0, 0,
83.99312330259689`, 30269.356103380353`, 10.02578431383393`, 1.0000000000000617`, 0, 0, 0.7253838425013082`},
{0.10518373092933642`, 1.253339618555911`, 0.9435760784539173`, 1.312750209379657`, 0.30938419913876847`,
0.5412461470886345`, 0.010398682812166098`, 560.2137041073427`, 0.038740677468453555`, 9.486718448691846`,
38653.282609218615`, 0, 0, 0, 65.2084102817794`, 0.5035938328773518`, 0, 0, 15415.215452575325`, 0, 0,
9.016772891508706`, 23163.28391973476`, 3.7870910129018425`, 1.0000000000002616`, 0, 0, 1.2626216594838782`},
{0.21800976245075693`, 1.265555352818649`, 7.66080710349685`, 0.9976040672991493`, 0.42066904207729006`,
0.6052137627060103`, 0.1434637338398527`, 296.0621032273413`, 0.0508815854649306`, 9.05489394101457`,
493.94820755870967`, 0, 0, 0, 166.21017956480406`, 17.147494254154815`, 0, 0, 0.13969693281877602`, 0, 0,
310.0157591538936`, 0.43507564484206285`, 6.863216638882216`, 0.939856592738258`, 0, 0, 1.2402603819361724`},
{0.14099839999887143`, 0.709118554874764`, 1.895334494340693`, 1.0269112445912816`, 0.6721979672345315`,
0.244304954855444`, 0.0716581941489167`, 50.83934571528565`, 0.0295628703988986`, 3.2095234353654245`,
655663.7336615705`, 0, 0, 0, 23.621931809191572`, 2.1142737290270897`, 0, 0, 217504.7788812532`, 0,
0, 21.41815330481962`, 438111.7973480704`, 13.082745302398354`, 1.`, 0, 0, 0.16040883736982242`},
{0.16175267254619935`, 2.3816849613140647`, 5.017466007223192`, 1.1449585227321033`, 0.0709392075018902`,
0.21629140918390133`, 0.24748736884470657`, 967.6089395954832`, 0.2384053881049194`, 4.566103496741098`,

35.429388262638646`, 0, 0, 0, 8.564661952052175`, 0.7658602146194249`, 0, 0, 0.012439427936528372`, 0, 0,
26.057682223254886`, 0.028744438766845865`, 1.0229335383745985`, 0.2080131004415453`, 0, 0, 0.4488813299605002`},
{0.15219039437367055`, 3.36973706117323`, 6.4489465173864`, 1.4529455229052564`, 0.15905243014518256`,
0.19120675078037952`, 0.09107249936889779`, 1785.557342737041`, 0.07266026496560668`, 8.481021831493972`,
36.83199429513763`, 0, 0, 0, 16.55151862672847`, 0.4122997081041004`, 0, 0, 0.006433867291016219`, 0, 0,
19.84773723870415`, 0.013988182862385986`, 1.0141345217055322`, 0.09088795337634381`, 0, 0, 0.6936745696539156`},
{0.1147282820108807`, 1.275793939396646`, 2.8627986987042053`, 1.2289286563020643`, 0.9300053983658252`,
0.5333937455451996`, 0.40966617997380966`, 55.29536930365472`, 0.09207723861615985`, 9.133686457942215`,
242169.74999445686`, 0, 0, 0, 33.420501392465006`, 9.363771984092077`, 0, 0, 91685.69284942663`, 0,
0, 170.66062210281106`, 150270.60036559936`, 36.07134541671736`, 1.`, 0, 0, 0.9721772722707703`},
{0.22214055647639447`, 3.77956922277194`, 8.844837651533133`, 1.3011494481281731`, 0.35962071678441987`,
0.36854620389209614`, 0.030699018263752917`, 153.2818434470183`, 0.18511332787655327`, 9.937679092387768`,
735.0843607721722`, 0, 0, 0, 508.36947740236917`, 3.9405772854692014`, 0, 0, 2.3794677209933597`, 0, 0,
212.76692325876556`, 7.551089766558006`, 14.339139975146336`, 0.9996366044087849`, 0, 0, 1.1984986068070864`},
{0.11158828176697494`, 2.207019844443386`, 8.337407964588238`, 0.903037166955644`, 0.0492331295750732`,
0.5332115600383506`, 0.10225753888572825`, 270.6598424430625`, 0.11536791182647016`, 7.224089837931905`,
285.305710775736`, 0, 0, 0, 119.29410569337824`, 5.100107165789035`, 0, 0, 0.042755126488042486`, 0, 0,
160.80053890977572`, 0.06815673002179955`, 6.268021685978404`, 0.63629750307533`, 0, 0, 1.3715190903481698`},
{0.11228066784233581`, 2.8383409912516155`, 8.777526606092515`, 0.8243326677721828`, 0.7215210925398621`,
0.47575335568293875`, 0.01179892034723288`, 1881.3977477117185`, 0.1350202375088484`, 6.891832227766253`,
80.58248619015855`, 0, 0, 0, 69.0124915017005`, 0.27686700739819575`, 0, 0, 0.00759389932312848`, 0, 0,
11.226328231763752`, 0.01218068696468851`, 0.7742526611713688`, 0.1796712976070458`, 0, 0, 0.48866565198440703`},
{0.18415971316226493`, 3.627641157385548`, 4.072786596426541`, 1.1318163513169097`, 0.431227341960456`,
0.35638232246082124`, 0.12315140656794354`, 98.11903649760767`, 0.20630818704214687`, 2.4221856850573946`,
46780.71874464393`, 0, 0, 0, 28.96562433074999`, 0.930472962822017`, 0, 0, 12862.707949272182`, 0, 0,
48.22031451096596`, 33839.8943775422`, 5.227483232810534`, 1.0000000000000322`, 0, 0, 0.1765867816192133`},
{0.2708373028347817`, 0.6873063866109219`, 4.963135106708849`, 1.480469510663712`, 0.1951402154410362`,
0.5673083531043152`, 0.10423026212852673`, 2550.63829867399`, 0.08286966503737198`, 2.6901867167370597`,
12.85023651233897`, 0, 0, 0, 5.281037722028744`, 0.6953741438742828`, 0, 0, 0.009483985989084084`, 0, 0,
6.827644145269933`, 0.036694531220102376`, 0.24632656232512776`, 0.12691534682763816`, 0, 0, 0.49314910108862897`},
{0.2248876472394919`, 0.40722939312005924`, 9.89488647226247`, 1.363579526182362`, 0.9039905300116995`,

0.6204673017769224`, 0.16140635189013572`, 124.79693831214993`, 0.1938029988590731`, 3.2922153328074497`,
607.7271815578437`, 0, 0, 0, 94.60010477672807`, 29.82360613522872`, 0, 0, 73.54053007529187`, 0, 0,
173.50070038715603`, 236.262239791105`, 6.48167985222469`, 0.9999999998845347`, 0, 0, 0.8061799251175958`},
{0.23989116476268346`, 3.6056249656972357`, 9.989783431378257`, 1.4358686490541388`, 0.7020019529209951`,
0.17871498413227982`, 0.15143540034751335`, 1116.6227738463333`, 0.03611482374706981`, 5.098351562607867`,
55.847101211837916`, 0, 0, 0, 18.27951553422904`, 0.7145506304896654`, 0, 0, 0.010683923267340962`, 0, 0,
36.80573703640343`, 0.03661398281199109`, 0.9428165209860951`, 0.14793545244619133`, 0, 0, 0.23568512626486382`},
{0.10260002089245757`, 1.5297774189947768`, 6.3178804183723365`, 1.026166177715942`, 0.11867434322817161`,
0.5793673959868294`, 0.011727656957059851`, 115.5782430006307`, 0.20678944231462698`, 5.80216317155719`,
19681.456005117754`, 0, 0, 0, 269.3042838635489`, 1.9361477435117007`, 0, 0, 7854.807909856284`, 0, 0,
42.31250139802688`, 11512.906509392828`, 11.510384197310032`, 1.000000000000001`, 0, 0, 0.9382767375968091`},
{0.15334449354379004`, 1.7605906069617427`, 2.299359928311352`, 0.8222860702278779`, 0.9712651951139977`,
0.6108690739001781`, 0.14744839931335676`, 2744.2788694525566`, 0.195814559949110 5`, 2.932280984329149`,
48.72012410108711`, 0, 0, 0, 15.723860461195601`, 1.2486687801329581`, 0, 0, 0.10717570972362964`, 0, 0,
31.405636078692456`, 0.23478292753951266`, 0.2223144863322389`, 0.9245430295453997`, 0, 0, 0.15885810180664878`},
{0.19466496130115485`, 2.7024357312795884`, 5.257508792652555`, 1.2867770062917014`, 0.4348182682381361`,
0.22799121214772633`, 0.23891047775514818`, 1782.3644894856986`, 0.18533305313279363`, 5.119329477376578`,
29.475101309884298`, 0, 0, 0, 7.131566233544068`, 0.5632302123113518`, 0, 0, 0.009551284205452419`, 0, 0,
21.744192152662627`, 0.02656143386047796`, 0.6086823375567861`, 0.1473319050615819`, 0, 0, 0.37746569564297744`},
{0.1432020102164725`, 0.6405991610607145`, 3.208206356969493`, 1.3290628649451517`, 0.9481170196997712`,
0.4490165359974049`, 0.04841648328555665`, 413.10755212325614`, 0.04750626711264738`, 5.496555870600891`,
159.61465056709488`, 0, 0, 0, 90.34063160931055`, 5.947569486258707`, 0, 0, 2.9202464481556722`, 0, 0,
54.42868604639467`, 5.974073738620095`, 3.0529416034413766`, 0.9999999950670907`, 0, 0, 0.8934898423739168`},
{0.2767015708016651`, 1.3448440647834508`, 8.399713367158164`, 0.7521061389207052`, 0.4173808724493866`,
0.513702172075632`, 0.342860965595718`, 77.17931109285855`, 0.021177395775830554`, 7.103551277505744`,
437287.0815572779`, 0, 0, 0, 85.42046867657348`, 19.45261763785817`, 0, 0, 88192.83334910797`, 0,
0, 373.7248196396493`, 348615.6503021081`, 19.741930901909846`, 1.`, 0, 0, 0.6163697660060206`},
{0.2397400236888016`, 3.6266796947004982`, 2.6465237726626594`, 1.459013465973801`, 0.6743470274003898`,
0.6381986185617847`, 0.04750041433271958`, 90.95305912243636`, 0.11079011330617267`, 9.85823320912452`,
219618.26699193037`, 0, 0, 0, 124.92514346083301`, 1.5829887360105777`, 0, 0, 49585.71904872256`, 0, 0,
82.01418722613113`, 169824.02084809987`, 23.159876470010182`, 1.0000000000000209`, 0, 0, 0.814505742434381`},

{0.10671756328879578`, 1.1279363098789865`, 5.068949642759208`, 1.0745694995138004`, 0.5897325137042213`,
0.3714383645112781`, 0.14529484820960328`, 91.27351773968599`, 0.0653436372538082`, 9.206661650127103`,
107108.53746142687`, 0, 0, 0, 126.96000441564674`, 14.647772843633108`, 0, 0, 42277.4195309786`, 0, 0,
236.02506927418943`, 64453.474206916515`, 21.841682006662396`, 1.0000000000000264`, 0, 0, 0.8940785781827851`},
{0.2358453438636856`, 3.7679680180462416`, 4.640233719273176`, 1.297156455219631`, 0.3876479327909328`,
0.3193535012332763`, 0.027047144947079417`, 401.3110605008155`, 0.0397014782942956`, 9.539243086069693`,
272.54522153681955`, 0, 0, 0, 197.61318104854269`, 1.3574941614147087`, 0, 0, 0.10602414161049617`, 0, 0,
73.07135121278748`, 0.3572185733712653`, 5.141070210655502`, 0.821251811127323`, 0, 0, 0.914143162858279 7`},
{0.07226742189042767`, 2.8421547517397343`, 4.74887948268691`, 0.940014135681888 7`, 0.26271764199006165`,
0.24510878200647013`, 0.011565327673117277`, 79.9069988164106`, 0.11613362579809078`, 3.4810370415732024`,
45756.333802778565`, 0, 0, 0, 113.47371635999882`, 0.43962673026437993`, 0, 0, 22448.617338908873`, 0, 0,
17.849817148753004`, 23175.767144109996`, 9.298519820330046`, 1.0000000000000004`, 0, 0, 0.2835814749894877`},
{0.19794910251471837`, 2.250622511000005`, 5.810822485387694`, 0.8325726726239642`, 0.42888216834124404`,
0.3003167364214152`, 0.2221971352698835`, 492.4997381844193`, 0.06654773222193389`, 7.816458771254976`,
184.15306461881525`, 0, 0, 0, 46.61054240736927`, 4.145337327955961`, 0, 0, 0.030652973014427026`, 0, 0,
133.27985008551875`, 0.0866818356802916`, 3.4048755082554574`, 0.5460542692018426`, 0, 0, 0.6798360649642274`},
{0.23749910377351813`, 2.442156143626838`, 7.627688188064528`, 0.9684475529781722`, 0.7107198130457415`,
0.2572554624659992`, 0.4475955253238462`, 132.3529645103848`, 0.18259209603279808`, 1.6799945249508887`,
119.28324170816055`, 0, 0, 0, 16.627877284230525`, 2.466119136762668`, 0, 0, 3.2214701001164188`, 0, 0,
86.03782858229536`, 10.929946594440343`, 2.7945497611718975`, 0.9999992541615398`, 0, 0, 0.19399799922562555`},
{0.06924882755772316`, 2.8414116897854322`, 0.5206442594268132`, 0.9917884594202446`, 0.6526760474283868`,
0.5988068411525457`, 0.47108712727434415`, 720.0807499233221`, 0.11578003411625926`, 8.400733392823671`,
8026.80557412179`, 0, 0, 0, 5.332485067283718`, 0.768568665266216`, 0, 0, 4016.2906613220925`, 0, 0,
31.197428427002937`, 3973.191706108386`, 2.6646309287199847`, 1.0000000000001283`, 0, 0, 1.320930425866904`},
{0.06824086699485615`, 3.3015665983523297`, 9.09230580657946`, 1.0780523992371156`, 0.9189494010575767`,
0.47997916674377217`, 0.09047840499022508`, 3815.186560881826`, 0.152329057733407`, 1.5202594192596113`,
13.174949957237954`, 0, 0, 0, 5.787335543593501`, 0.15309872078907125`, 0, 0, 0.006872411877080788`, 0, 0,
7.220937468680971`, 0.00669970492625373`, 0.08248532272884376`, 0.12710874738012135`, 0, 0, 0.0742048589969552`},
{0.23257418904935773`, 1.8011483887717636`, 0.9612178256197179`, 0.8025101750942898`, 0.31352490932039023`,
0.2355458085864931`, 0.2680725991098069`, 2738.209295799684`, 0.0662676878204258`, 1.8613360761320532`,
6.289638016238291`, 0, 0, 0, 1.3950524003995872`, 0.17937468933767398`, 0, 0, 0.023083040451530534`, 0, 0,

4.615434752671237`, 0.076693134482977`, 0.1474966472961464`, 0.4498127210230807`, 0, 0, 0.1849724918743638`},
{0.13474024315490335`, 3.3704964680175626`, 9.170639775134308`, 0.7502511885050491`, 0.6264710065934431`,
0.21904625510680875`, 0.040564870634629845`, 2527.7664803980215`, 0.19403763861714918`, 6.378419170081072`,
31.377266380232548`, 0, 0, 0, 20.050446815952935`, 0.2302707920316805`, 0, 0, 0.002621596071213253`, 0, 0,
11.087527017576951`, 0.005046207029843317`, 0.5260400685184886`, 0.07249011938201366`, 0, 0, 0.35876019070297793`},
{0.2693026298173556`, 1.772983767924213`, 1.344226083033309`, 1.4113484218782142`, 0.12593618066908396`,
0.656520191233777`, 0.018659842469751033`, 117.56506476136576`, 0.1712196470744854`, 4.023445632685666`,
118587.99350201279`, 0, 0, 0, 34.055538749978965`, 0.3414516700750393 5`, 0, 0, 24456.472450990525`, 0, 0,
8.648403836766564`, 94088.46210153509`, 7.2835317610097885`, 1.0000000000000433`, 0, 0, 0.3150852104471957`},
{0.17866994815918202`, 2.442046867777872`, 1.8194825512463648`, 1.2285671890230836`, 0.7081083864311548`,
0.17566313743095552`, 0.006280075202364708`, 2604.6083474637735`, 0.1295692924324935`, 1.862340891527415`,
8.533108465462853`, 0, 0, 0, 7.759442369725082`, 0.01902909987710558`, 0, 0, 0.023256004221967667`, 0, 0,
0.6638564821645417`, 0.05935927241040969`, 0.14866371014559718`, 0.3299359212345786`, 0, 0, 0.09952686100136685`},
{0.26090114261485403`, 3.9086029270457496`, 1.920295645800332`, 1.1838686314265923`, 0.4729800090210985`,
0.36383922429133153`, 0.014536748208445867`, 79.30674088085132`, 0.030053561477188684`, 7.670997166386538`,
751917.9713923598`, 0, 0, 0, 94.43651665584603`, 0.341479354332147`, 0, 0, 159039.29731940423`, 0, 0,
19.067245769547466`, 592764.7770185155`, 19.988165776266673`, 1.0000000000000033`, 0, 0, 0.3606640159881867`},
{0.19544602681310375`, 0.41028512203974055`, 4.6003645589109095`, 1.2671666166106164`, 0.8219059973725353`,
0.22911191370711304`, 0.03702179299429459`, 318.28074751099626`, 0.09079865705400514`, 1.4662081657512314`,
55.00755654891589`, 0, 0, 0, 35.112762094527525`, 2.6314481266734586`, 0, 0, 0.4842679657926547`, 0, 0,
15.42348593990667`, 1.352117854671968`, 0.9777978065224117`, 0.9991813872096696`, 0, 0, 0.11061053367004757`},
{0.26537062770714803`, 1.229239774996202`, 8.744773690909977`, 1.151653436583517`, 0.3537059210373261`,
0.2894924335103778`, 0.13068544785366612`, 312.2140250313751`, 0.146894495801125`, 5.715731252046433`,
191.9126361577726`, 0, 0, 0, 69.36755138039021`, 6.592362913904827`, 0, 0, 0.039048260445709165`, 0, 0,
115.76563864259505`, 0.14803230550499288`, 3.9520949796148996`, 0.5166560445132857`, 0, 0, 0.5360261192196613`},
{0.18712917295151626`, 0.630329154174047`, 6.232729389529142`, 0.7582043174026267`, 0.7607866373637777`,
0.2530331438958473`, 0.06090476948059027`, 64.21018866863231`, 0.08418997914362486`, 2.694793972266142`,
105602.6313301362`, 0, 0, 0, 70.85427819049693`, 5.998700718402652`, 0, 0, 28713.28513721277`, 0, 0,
54.016513571056855`, 76758.47572068109`, 8.743410964880452`, 1.0000000000009288`, 0, 0, 0.1497141049342933`},
{0.19413873235593193`, 0.8072205389170897`, 9.684031161687852`, 0.8518820755271745`, 0.2120317281227424`,
0.1532879757233685`, 0.02372050181467318`, 987.8329384880758`, 0.08094704090521981`, 8.693316755778081`,

```
66.0282686364779`, 0, 0, 0, 49.75059239023731`, 1.2965098172749694`, 0, 0, 0.004432476171076335`, 0, 0,
  14.95099076302855`, 0.012293075786430543`, 1.8516576066540358`, 0.10604855253639223`, 0, 0, 0.5742670127337486`},
{0.0816546741022599`, 0.4036812381177306`, 0.7902893788690406`, 1.4273906025641667`, 0.1897112398644374`,
  0.6362443390402239`, 0.007446156154142883`, 176.61944749055874`, 0.10188842005948545`, 9.51311671217389`,
  73461.04814503502`, 0, 0, 0, 57.25246238249253`, 0.8864170176175891`, 0, 0, 33878.52570218356`, 0, 0,
  5.111855988007074`, 39519.14250395467`, 12.140761517949105`, 1.0000000000000064`, 0, 0, 1.3513092260928705`},
{0.2491419431624458`, 3.7694376618574204`, 4.74673147811253`, 0.8416284883026488`, 0.188731240527098`,
  0.3192559789027146`, 0.07121130413051815`, 671.9000833119555`, 0.02760758509345429`, 2.5790410082926702`,
  35.5148908973705`, 0, 0, 0, 17.969358898467316`, 0.3182789815223071`, 0, 0, 0.019327521222088105`, 0, 0,
  17.139039713254384`, 0.06878994562547462`, 0.8511523607942953`, 0.38720459895078796`, 0, 0, 0.31853165989150006`},
{0.11168227968802413`, 2.173584861364678`, 5.640951633995167`, 0.9377704673639422`, 0.3315982783471285`,
  0.5266083888319896`, 0.005432939117039627`, 621.842609802462`, 0.09631877509870435`, 9.43919460268938`,
  206.3367253174613`, 0, 0, 0, 191.4333235520565`, 0.4556817186483322`, 0, 0, 0.03223574619918009`, 0, 0,
  14.149469789352203`, 0.0514308803281233`, 3.4164579680323603`, 0.5207944496748775`, 0, 0, 1.3249343698668`},
{0.15970197279447784`, 3.9587696847183347`, 4.0238996390694854`, 0.8027865138486989`, 0.03588289413016432`,
  0.35386710298208024`, 0.016574217538633067`, 2019.1576160982734`, 0.07473946383041635`, 3.5352801788382404`,
  15.128961218806715`, 0, 0, 0, 12.283447768473755`, 0.04901243860734586`, 0, 0, 0.005766676235696886`, 0, 0,
  2.7718422304697032`, 0.01315642244725667`, 0.3971858656542986`, 0.1425595473542709`, 0, 0, 0.5280895418121334`},
{0.2064322311255224`, 0.43645383124552994`, 3.8213880668877356`, 0.8257710590869434`, 0.08868496943554072`,
  0.17616539751645888`, 0.04042707492740236`, 2037.3062213977416`, 0.05324630165142419`, 4.6107951341494555`,
  15.98315551573246`, 0, 0, 0, 10.28927657296144`, 0.7841464650309774`, 0, 0, 0.005006422135475574`, 0, 0,
  4.889196127434406`, 0.014764098448320669`, 0.48387933913581194`, 0.12218313808458448`, 0, 0, 0.3839775018519923`},
{0.1394617695133732`, 3.387614469631334`, 8.352661875580207`, 1.2219251402369191`, 0.6325442693826859`,
  0.17624447718949754`, 0.021853560156019736`, 288.68541357569393`, 0.164910064915528`, 5.7766720481305995`,
  225.3227622093112`, 0, 0, 0, 172.52252727715234`, 1.0644553355065969`, 0, 0, 0.056499664663467605`, 0, 0,
  51.51377566912032`, 0.11256490301262276`, 4.1466022255087545`, 0.6290435451390459`, 0, 0, 0.28845008379986015`},
{0.10599692192016741`, 2.4119661618453145`, 1.4892081676893856`, 1.3031849980783952`, 0.42063329504650815`,
  0.5860601104625098`, 0.3077592160131421`, 52.52666428206092`, 0.19647413521024198`, 3.2052596491028216`,
  129035.76649976932`, 0, 0, 0, 7.720498582551073`, 0.8951847493056616`, 0, 0, 51306.24059079407`, 0, 0,
  30.845076056074948`, 77690.05111313886`, 13.498367162978887`, 1.0000000000010505`, 0, 0, 0.38712965019929807`},
{0.24388484229914548`, 3.2519594625966253`, 4.312340316913895`, 0.9914403572709836`, 0.29358995419342615`,
```

0.1835819110057716`, 0.13612159308161606`, 1157.8864188899545`, 0.08218618541111161`, 5.046821007659727`,
36.346239182681884`, 0, 0, 0, 12.919089338988677`, 0.49254419134349264`, 0, 0, 0.011751544890092116`, 0, 0,
22.88191062552108`, 0.04094319531844243`, 0.9228455705924611`, 0.22480299646863522`, 0, 0, 0.36716744955080993`},
{0.23494551018662208`, 3.3331099682410468`, 0.9744758817090952`, 0.9989991912838305`, 0.20118476247466943`,
0.3741832660739852`, 0.07385095150097958`, 91.01998469145919`, 0.19994684540705993`, 6.360950891328324`,
81250.82668148632`, 0, 0, 0, 23.620189652633865`, 0.5032749682830447`, 0, 0, 18640.02438928343`, 0, 0,
23.963868765005948`, 62562.7148575896`, 14.490537505334727`, 1.000000000000303`, 0, 0, 0.32162020264735247`},
{0.23944024111665796`, 0.7499363805955559`, 7.775454975750338`, 1.268235612825383`, 0.3786721827881383`,
0.6740341016595366`, 0.11977329505957622`, 117.53909816274609`, 0.2108164269301916`, 1.8777741577958924`,
26931.64335491816`, 0, 0, 0, 47.06108450848899`, 6.620557741651935`, 0, 0, 6064.150918667003`, 0, 0,
70.92853014711945`, 20742.882259048933`, 3.6510383152917876`, 1.000000000000002`, 0, 0, 0.2965339153783451`},
{0.24513403447625742`, 2.0133471925570054`, 9.940759001187548`, 1.083812287959604`, 0.5683273089881642`,
0.5900825394859205`, 0.005339720116331014`, 58.9416557608406`, 0.03145644437596545`, 6.28807827028038`,
599847.2882128722`, 0, 0, 0, 469.9609134515975`, 1.1910664614842499`, 0, 0, 133130.31831094038`, 0, 0,
34.25757594825888`, 466211.0291238492`, 23.09250033229727`, 1.0000000000000018`, 0, 0, 0.6082134403641165`},
{0.15958822823144275`, 2.509825654597928`, 3.728427574062339`, 0.9294074209823535`, 0.01651867581778177`,
0.5367648350921979`, 0.015785937236593408`, 685.9822133189093`, 0.020860236554209932`, 1.2753268301578835`,
18.923628836260693`, 0, 0, 0, 15.42345318456745`, 0.09189605988777752`, 0, 0, 0.03222182091408246`, 0, 0,
3.2949012666115958`, 0.07346033300099397`, 0.44845843926197926`, 0.5101549205219732`, 0, 0, 0.2833547020988764`},
{0.06806026871424425`, 1.205607754446362`, 0.7536813502622586`, 1.4624982795129167`, 0.7076540801030038`,
0.15470161284122685`, 0.268116863806763`, 324.06126360879364`, 0.036033823716353997`, 3.8267580932938614`,
61566.02055294689`, 0, 0, 0, 5.025767250554875`, 0.9530842027643552`, 0, 0, 31204.04629622959`, 0, 0,
16.414938649900478`, 30339.368227044306`, 2.4523962948868805`, 1.0000000000000036`, 0, 0, 0.19985689494618974`},
{0.15319733389695195`, 3.558603911914262`, 4.579050417046558`, 0.9321187332487358`, 0.6642338797631109`,
0.569667348599446`, 0.2858050889038288`, 341.1212503566884`, 0.20343887540165378`, 9.501626804091558`,
381.0996592898564`, 0, 0, 0, 78.79705715365046`, 5.704073264340943`, 0, 0, 2.076008613551411`, 0, 0,
289.9791061761318`, 4.543414067760561`, 6.4303567910422`, 0.9999998158853786`, 0, 0, 1.612265364837121`},
{0.15071537712057487`, 1.2850575528534174`, 6.479674501252795`, 1.2297249014764047`, 0.051196968811160426`,
0.17856837950014437`, 0.01824538414392949`, 77.70419036862091`, 0.224107560352308`, 8.31176551046438`,
34586.80095434776`, 0, 0, 0, 332.75982968984044`, 4.37632232920313`, 0, 0, 10836.775910942757`, 0,
0, 80.34037232662202`, 23332.410974127135`, 22.180094645424653`, 1.`, 0, 0, 0.4205650608587388`},

```
{0.24584690511060947`, 2.473478204800995`, 6.676188280246508`, 1.4832363218386881`, 0.14420161768506268`,
  0.523836460034543`, 0.04402629271492787`, 360.52310897688045`, 0.0850887320320231`, 2.528306265858081`,
  78.43126692580904`, 0, 0, 0, 48.54753395264296`, 0.8140979146830841`, 0, 0, 0.06663702033100886`, 0, 0,
  28.766477836322128`, 0.23403578877388082`, 1.6393182772190993`, 0.6084773889835442`, 0, 0, 0.46615515280872266`},
{0.14137397451874373`, 3.652676161056461`, 8.39527364337399`, 1.0080745646578813`, 0.09190129083286003`,
  0.15081167091134762`, 0.35462309792221747`, 1217.6311173423508`, 0.09724487810479643`, 6.223920941944753`,
  35.30083569932627`, 0, 0, 0, 6.691745722622908`, 0.5377016647434605`, 0, 0, 0.004480813802113485`, 0, 0,
  28.05785789384006`, 0.009049577946907262`, 1.0807872515535566`, 0.09128280821770851`, 0, 0, 0.44381335176285086`},
{0.04933630800724648`, 1.8511857532828166`, 5.885062761777403`, 1.1138733840213622`, 0.4607664856084661`,
  0.43439208322360623`, 0.018761076496074794`, 3507.5800668673905`, 0.21191062303011793`, 3.3573407631201917`,
  13.305766777384259`, 0, 0, 0, 10.526452263209032`, 0.10080954876560111`, 0, 0, 0.004922409519083124`, 0, 0,
  2.665960006710717`, 0.003469335888164013`, 0.20975641139939036`, 0.0902160253868346`, 0, 0, 0.3703954360060874`},
{0.2339008817663396`, 3.669675022809618`, 9.160989469851096`, 1.2207724907901325`, 0.9659294978123167`,
  0.3234992643572032`, 0.05037324961884807`, 93.86107259786391`, 0.14430488254373952`, 3.9894219201696473`,
  18463.93102232838`, 0, 0, 0, 177.18560333043416`, 2.29920195108497`, 0, 0, 4183.842864380277`, 0, 0,
  120.53319960416631`, 13980.06478786231`, 9.345938589766687`, 1.0000000000001037`, 0, 0, 0.4551585047962883`},
{0.11569404259913263`, 3.2009969772206803`, 2.2264401820433353`, 0.7539773410821206`, 0.3237735314376855`,
  0.22021748910844674`, 0.21956604535044472`, 1445.3927245947305`, 0.06533194774972478`, 1.3139082141801541`,
  8.036716264969558`, 0, 0, 0, 2.0765556131152243`, 0.12663973561022038`, 0, 0, 0.016010493747584988`, 0, 0,
  5.791048726919161`, 0.02646169636667127`, 0.1955759043936973`, 0.36138722123021594`, 0, 0, 0.1183573333921082`},
{0.1426983247121278`, 1.4581176174915065`, 6.531549332871599`, 0.9310047864665083`, 0.893336243428055`,
  0.18377286371920176`, 0.08056837661087574`, 664.5727162634348`, 0.04347044784294363`, 7.864371974772275`,
  191.70224383722768`, 0, 0, 0, 90.37142028860633`, 4.637529207844169`, 0, 0, 0.03033244126183768`, 0, 0,
  96.60090056555751`, 0.0618341221785563`, 2.4177014740680196`, 0.503627821132505`, 0, 0, 0.27643610073896374`},
{0.05831687901068116`, 0.732484500007835`, 9.716813224212896`, 0.9062398770267781`, 0.04581664783201011 6`,
  0.2511270020278909 6`, 0.0489500536916562`, 2141.8470035057708`, 0.06373448098002471`, 7.679073247267311`,
  27.96932262473565`, 0, 0, 0, 16.763355141072694`, 0.9770842164410961`, 0, 0, 0.002187648736844265`, 0, 0,
  10.224272053505807`, 0.0018225263814781038`, 0.7856335098588071`, 0.05065566847948377`, 0, 0, 0.8466411016503986`},
{0.1624041267001165`, 2.3409738007594383`, 7.927758092789709`, 1.1108935833128895`, 0.2655878375527121`,
  0.22381693809974978`, 0.10055816102060769`, 86.2207746546923`, 0.23778527923179793`, 1.5984483564142007`,
  13018.994692477769`, 0, 0, 0, 42.946919595295476`, 1.692059512715228`, 0, 0, 3890.8251924303718`, 0, 0,
```

```
  56.58667126560196`, 9026.943821706702`, 3.964513310999989`, 0.9999999999999974`, 0, 0, 0.1334569163178338`},
 {0.05796853524365786`, 2.115435321939346`, 7.135826377560314`, 1.1217856002992745`, 0.9949497841824515`,
  0.6493572179052653`, 0.16432568991023785`, 205.88622896743587`, 0.07308989915497993`, 5.091714109271411`,
  332.08050399628326`, 0, 0, 0, 101.25823649647742`, 7.1674751456162635`, 0, 0, 3.8564393618286825`, 0, 0,
  216.6047156022712`, 3.1936020151603746`, 5.9404639725448485`, 0.9999999926286909`, 0, 0, 1.1051218350872967`},
 {0.09343457742759681`, 3.0751406184941388`, 6.999926605820257`, 1.4764339149831611`, 0.27064650694064607`,
  0.6662559245780291`, 0.012835205507442616`, 1884.4110587763687`, 0.01681160009525748`, 8.661215052575166`,
  66.18109341738075`, 0, 0, 0, 56.029605787779936`, 0.224586473136842`, 0, 0, 0.01092395052378532`, 0, 0,
  9.866214084392068`, 0.01458106715757844`, 1.0665839074693682`, 0.14662865995612395`, 0, 0, 1.5221896675528477`},
 {0.23943678973010468`, 2.920807454124679`, 1.1099817114095707`, 1.3932739733707113`, 0.44392785132951373`,
  0.17254202331513768`, 0.008938503858702969`, 2712.044809794402`, 0.1652368227234951`, 2.8738798121545877`,
  9.787950706974414`, 0, 0, 0, 8.566402892630526`, 0.02494538644613259`, 0, 0, 0.03357171918675852`, 0, 0,
  1.0408667239697842`, 0.1148329238256872 6`, 0.223691243093998`, 0.3976535306318262`, 0, 0, 0.1998676088931596`},
 {0.1765669287716667`, 3.4304813087836825`, 0.9837478487319995`, 1.1028599055297788`, 0.8616178142897495`,
  0.24038775568738768`, 0.03874939820930588`, 2380.99712756261`, 0.18505877859540681`, 3.338709015344154`,
  24.805716106707905`, 0, 0, 0, 15.6914611999434`, 0.1704787055909662`, 0, 0, 0.1669058022078272`, 0, 0,
  8.354628758220658`, 0.4210006412858177`, 0.2925356485496698`, 0.9516096995449252`, 0, 0, 0.190358783017881`},
 {0.23886394100929725`, 3.974888040813318`, 7.275822510559582`, 1.2870566617016084`, 0.12273034821485096`,
  0.6651538290269186`, 0.16897919530659566`, 150.6876048176701`, 0.1338282848735754`, 2.432758352876487`,
  14917.350560072266`, 0, 0, 0, 47.87680507119167`, 1.8855419069742936`, 0, 0, 3345.2799752394258`, 0, 0,
  107.06882823549186`, 11415.239409502452`, 3.8358742822286747`, 1.0000000000103144`, 0, 0, 0.4958697686548741`},
 {0.06973130535662914`, 1.4096929441217387`, 8.65071450995816`, 1.181254696978707`, 0.5406204719849208`,
  0.2725259583108167`, 0.12003736669178822`, 4806.091595362366`, 0.20844978312325513`, 8.8784124085225`,
  23.229834666551877`, 0, 0, 0, 8.797274846909731`, 0.6825465497483377`, 0, 0, 0.002288838736324298`, 0, 0,
  13.745443645926741`, 0.0022800530404710416`, 0.39042570562004664`, 0.04085177733975354`, 0, 0, 0.6287908848363538`},
 {0.04409294567306185`, 1.3468266911815716`, 8.46560672493381`, 1.1221333539921368`, 0.8074609652543419`,
  0.33934404303057597`, 0.1090535991677299`, 2100.6487939981484`, 0.1955313854386307`, 6.974828032327105`,
  69.83561426610376`, 0, 0, 0, 27.71330858421421`, 2.0803715846721462`, 0, 0, 0.009071208657588313`, 0, 0,
  40.02714254017356`, 0.005713947293302169 5`, 0.6889531565684387`, 0.15955078879193518`, 0, 0, 0.3578982973123104`},
 {0.08301734541584582`, 0.4950033643125962`, 0.3447103403996028`, 1.3973171280993089`, 0.5174429014299606`,
  0.6090072510200681`, 0.013328621450736109`, 78.08408450957157`, 0.15235476028821748`, 7.41363923833616`,
```

```
117207.01329950699`, 0, 0, 0, 17.967930437067338`, 0.41714676748455837`, 0, 0, 53608.23273805409`, 0, 0,
 2.9498436188140094`, 63577.33106211575`, 21.225238399199775`, 1.0000000000000187`, 0, 0, 0.9836175383384149`},
{0.07508111728102967`, 2.9328515590120503`, 2.9247828964602327`, 1.0997610261500241`, 0.15381049135387959`,
 0.44875941041801015`, 0.005374403719665632`, 955.806314296473`, 0.04180941570568808`, 8.210271230243798`,
 91.90359966838545`, 0, 0, 0, 85.32099745530205`, 0.1494224207608528`, 0, 0, 0.03698674860558821`, 0, 0,
 6.260482566854589`, 0.03967152014142433`, 1.95871108424927`, 0.5109077100055246`, 0, 0, 1.2752807767321597`},
{0.1868411586163749`, 1.4148901359883457`, 3.2886483363036496`, 1.1024292265597047`, 0.35071605335062417`,
 0.20216786873751635`, 0.033694215571138546`, 50.84055962824541`, 0.13756443231160648`, 9.762651891575405`,
 170456.0745443864`, 0, 0, 0, 166.63133851049434`, 3.7160067651892734`, 0, 0, 46389.53440110273`, 0, 0,
 75.11059024760391`, 123821.06221680332`, 39.2399479544543`, 1.0000000000000002`, 0, 0, 0.3452514064088697`},
{0.25728291653412544`, 1.1620186239014583`, 0.48798465750503445`, 1.2217305465551163`, 0.36537296293338284`,
 0.2112385814144826`, 0.1783844678719564`, 175.70497466833672`, 0.14885286931859365`, 6.164090300210196`,
 68143.10139680833`, 0, 0, 0, 6.588168592351783`, 0.9238754078222671`, 0, 0, 14569.7115656451`, 0, 0,
 15.336577572200326`, 53550.541209573785`, 7.121035852929336`, 1.0000000000011686`, 0, 0, 0.17533025216466067`},
{0.13759840983832694`, 1.6824227355669867`, 8.881204796952819`, 1.07728991023014`, 0.8275568317133928`,
 0.2913709338376398`, 0.47392581861361016`, 1028.758263171597`, 0.1629375199726112`, 7.630724789672264`,
 147.135753648158`, 0, 0, 0, 20.256905107375353`, 5.0660801274887675`, 0, 0, 0.017367186313950803`, 0, 0,
 121.761262667017`, 0.03413853171663882`, 1.5316475770837685`, 0.29298599874487097`, 0, 0, 0.3527970574949527`},
{0.22999415874686646`, 3.253135821240728`, 9.26678168881324`, 0.8937302072810551`, 0.5038748821521797`,
 0.2035592770142407`, 0.010501376095491947`, 229.44271937909937`, 0.14963151143698977`, 8.069758108498657`,
 372.3238558562111`, 0, 0, 0, 324.3156632310892`, 1.0019293622537002`, 0, 0, 0.04635298639168399`, 0, 0,
 46.56303283857702`, 0.15229880157939596`, 7.372596533453699`, 0.6717582822563974`, 0, 0, 0.5009574538719916`},
{0.09925066924077935`, 1.4815445119155024`, 7.881183919866995`, 0.7663286317492166`, 0.2976933875506338`,
 0.33062028037546887`, 0.05105854284999101`, 168.2571992844445`, 0.22488226539851913`, 5.574589576514775`,
 347.7521027408701`, 0, 0, 0, 203.4946333774482`, 6.463719180179649`, 0, 0, 0.4068501750316917`, 0, 0,
 136.80410968511598`, 0.5768593164660593`, 7.274014436773202`, 0.9977976482091094`, 0, 0, 0.6265305946710429`},
{0.20983327896511272`, 1.8859920859197894`, 0.6151282764886545`, 0.8412663476815937`, 0.027122934645615437`,
 0.6844735714100607`, 0.01254007978155685`, 549.5308557472557`, 0.10371695352022153`, 7.780558961111437`,
 19165.672067231193`, 0, 0, 0, 33.009064603371236`, 0.20931210005051928`, 0, 0, 4784.5471136223405`, 0, 0,
 5.639442345464739`, 14342.245845920552`, 3.0756587142349034`, 1.0000000000003735`, 0, 0, 0.7829658773852107`},
{0.2225389666164696`, 1.2827559929647245`, 1.2157460525678818`, 1.1336603904726232`, 0.21910751958531538`,
```

0.6021275716391656`, 0.016887660465617623`, 151.92995003475355`, 0.056506174885467386`, 4.721491520685097`,
190388.5285192307`, 0, 0, 0, 37.04579097617488`, 0.4573929166311305`, 0, 0, 45546.01396144325`, 0, 0,
8.38176435640278`, 144796.61257826933`, 6.644090290314435`, 1.0000000000002238`, 0, 0, 0.3929858632090125`},
{0.23629660338151143`, 1.375829581220887`, 0.7878883261410508`, 0.9498849347602389`, 0.6605330073956415`,
0.33733697402858687`, 0.028977316405205664`, 446.06430846982937`, 0.16303388369839966`, 6.718018447521894`,
14557.75897704643`, 0, 0, 0, 29.268939187275095`, 0.5778513283606073`, 0, 0, 3317.5636212119794`, 0, 0,
11.357499301518674`, 11198.985931349358`, 3.134404728247765`, 1.0000000000003124`, 0, 0, 0.36592168072365977`},
{0.2240884195389573`, 3.0168075094859823`, 9.22253837134286`, 1.0534395436787098`, 0.7253455428103461`,
0.1646055270291652`, 0.006115122680716056`, 258.28718729642685`, 0.11665954601209721`, 2.992281031623701`,
136.92517641644926`, 0, 0, 0, 125.7835193453226`, 0.24447694609430887`, 0, 0, 0.054087233880406635`, 0, 0,
10.536284098192988`, 0.17314746796421857`, 2.3881702267353617`, 0.6665827607579404`, 0, 0, 0.13301007412346386`},
{0.04820076342563051`, 1.9965427255473651`, 1.4646173572921608`, 1.475676204663891`, 0.346581356777814`,
0.35258214744905925`, 0.16114708977216574`, 53.64321165141119`, 0.1945121008436112`, 8.557828905511698`,
96135.59216903089`, 0, 0, 0, 32.272576739845086`, 2.3681800136294133`, 0, 0, 56871.010672096236`, 0, 0,
67.5453225571211`, 39160.373302603024`, 34.817674062913134`, 1.0000000000000762`, 0, 0, 0.9053458477045645`},
{0.1395588171486265`, 3.3167971972296737`, 5.120579942113229`, 1.4414502422836475`, 0.46235265068089726`,
0.21590338795839814`, 0.018401804903068468`, 321.8138540224943`, 0.09243860589675884`, 1.0657915725386573`,
32.76146072165554`, 0, 0, 0, 25.829661082200175`, 0.13657641227015474`, 0, 0, 0.10467687139524069`, 0, 0,
6.471375163219072`, 0.20869400506769054`, 0.7071376013769992`, 0.7722462791480589`, 0, 0, 0.08721292479858984`},
{0.2085065587668823`, 0.6294064238759702`, 7.1150002800734775`, 0.8698405330489325`, 0.07589384997930004`,
0.6271818704466325`, 0.014863365323890152`, 3717.018925299029`, 0.09558183779388002`, 3.993723504265942`,
12.489992847490445`, 0, 0, 0, 10.330576406600908`, 0.2145781642312358`, 0, 0, 0.002468089367190499 5`, 0, 0,
1.9293839284378973`, 0.007351611724029095`, 0.25704215804423863`, 0.05898588024473084`, 0, 0, 0.8471246980566874`},
{0.11579616742063126`, 1.4637518716217537`, 6.091821149634162`, 0.8701973607843327`, 0.580668178059593`,
0.4892081276931839`, 0.43069272205186815`, 58.104542288448926`, 0.09639134486345691`, 1.0613512257562903`,
112608.65838155973`, 0, 0, 0, 8.149657742336753`, 2.063597394581422`, 0, 0, 42405.98889943247`, 0,
0, 43.15135069417614`, 70149.29986051611`, 4.035225452980323`, 1.`, 0, 0, 0.12660441227673397`},
{0.07650954660329923`, 2.451622188741575`, 3.5124331635719983`, 0.9208132913481966`, 0.22417245589301404`,
0.5962277485405788`, 0.1631058588288524`, 149.68967360762312`, 0.15724921240628176`, 3.991974201007654`,
21862.27748609377`, 0, 0, 0, 37.55343022983086`, 2.3010771133082377`, 0, 0, 10387.8951331336`, 0, 0,
80.59102441417001`, 11353.902097123853`, 6.137372679041324`, 1.0000000000043676`, 0, 0, 0.6627791208363929`},

{0.27715040216901043`, 3.1093705724520717`, 5.602120685872263`, 1.4185083740786066`, 0.20116695432127574`,
0.3482455682148583`, 0.014646070094414987`, 268.87083253973395`, 0.19497961893886057`, 8.247252623819538`,
306.22565384499995`, 0, 0, 0, 253.6541386251208`, 1.1386666729645585`, 0, 0, 0.14430033035491266`, 0, 0,
50.57909492497004`, 0.5713270655855781`, 6.808118539478593`, 0.8785974021016731`, 0, 0, 1.0273035095609577`},
{0.10373802184314534`, 1.9484823746249935`, 4.344672894707308`, 0.8659474233939041`, 0.34328087466446`,
0.6599529078817681`, 0.006604951530776707`, 74.02272771702236`, 0.12108236246583282`, 3.25882057231545`,
81784.74233045777`, 0, 0, 0, 109.43739834180306`, 0.3528434402071802`, 0, 0, 32903.27846677182`, 0, 0,
9.821560346367704`, 48761.72886138687`, 9.965693721850466`, 1.0000000000001068`, 0, 0, 0.4787977901243346`},
{0.08995654352927362`, 1.6673082382989595`, 4.04257302618344`, 1.1477945472560402`, 0.1500209798917984`,
0.1761016570773115`, 0.012888472951564884`, 1050.2473441342943`, 0.2022489804478793`, 4.465566609444316`,
31.237520689619966`, 0, 0, 0, 26.475200729954675`, 0.18991012756568387`, 0, 0, 0.014169173924886718`, 0, 0,
4.523410288952453`, 0.018208713013523472`, 0.9064173078374559`, 0.23274062339121815`, 0, 0, 0.35770940281109737`},
{0.18428208667431017`, 2.663013532673764`, 4.442362759588184`, 1.3723983485913218`, 0.7325310003414629`,
0.5418182340232052`, 0.027460635126683182`, 53.281000517524745`, 0.08334630855570291`, 6.274365305120075`,
372683.15922425175`, 0, 0, 0, 161.25858862569763`, 1.597264083915644`, 0, 0, 102532.4505682869`, 0, 0,
60.76479815315875`, 269927.05632220715`, 25.26666000649568`, 1.0000000000005085`, 0, 0, 0.5465482001651858`},
{0.1326476876438774`, 2.8731759222440596`, 6.672951038295707`, 1.243076119232064`, 0.9225218753338189`,
0.23536778742386455`, 0.07924576221140303`, 2119.223051638056`, 0.014542489730864222`, 5.05029094558817`,
47.256296859171584`, 0, 0, 0, 22.406571200779076`, 0.5901862265277513`, 0, 0, 0.01211274928510822`, 0, 0,
24.22441222428019`, 0.022953259766856658`, 0.48788589167022295`, 0.18888552342481701`, 0, 0, 0.1883334657890097`},
{0.16449021574582717`, 1.5264131018768046`, 3.118813817198866`, 1.0873075186223824`, 0.9114527670749883`,
0.32623274128336255`, 0.005473446059082923`, 2600.919647538226`, 0.12372800503579795`, 1.4807994816594086`,
13.699394480882559`, 0, 0, 0, 12.622550885856187`, 0.042871614294049634`, 0, 0, 0.02536586899904758`, 0, 0,
0.9348541965292323`, 0.05960624663190951`, 0.11804461600631633`, 0.3877520648235925`, 0, 0, 0.07479163086644577`},
{0.1059475440552839`, 2.4614128361434053`, 6.764865578702089`, 0.897041430377437`, 0.5452264302150955`,
0.6377616415175531`, 0.031109305885935246`, 290.7153568287518`, 0.12025877488242465`, 1.854625189562679`,
107.9942079391424`, 0, 0, 0, 74.30036710395441`, 0.8954231460812143`, 0, 0, 0.5178696009505386`, 0, 0,
31.485800364917388`, 0.783814462308494`, 1.4579651073335045`, 0.9987988189675591`, 0, 0, 0.2928959005420834`},
{0.09376889465512256`, 2.694562234128588`, 9.901749913963968`, 1.2671081853876889`, 0.9355728957697818`,
0.41426328356338693`, 0.1676359905682518`, 324.1423914220847`, 0.048114260882103244`, 6.992923371816153`,
539.5368707617577`, 0, 0, 0, 161.6705190861599`, 9.53111457583444`, 0, 0, 0.6184643763736231`, 0, 0,

366.8883055027995`, 0.828467442230212`, 4.4934411217208075`, 0.9984173369459198`, 0, 0, 0.38675138256817326`},
{0.07408607058446992`, 2.173441202450359`, 5.83110243938874`, 1.2671639264693393`, 0.15034130588796946`,
0.26151521374964837`, 0.00796428041791113`, 3349.7895146720416`, 0.06475249420724477`, 9.113413370060233`,
23.308505588366984`, 0, 0, 0, 20.96143951678631`, 0.07240018871521363`, 0, 0, 0.0035938103802900146`, 0, 0,
2.2479650459832414`, 0.003803589850017114`, 0.5929516986788032`, 0.05918426606795424`, 0, 0, 0.9503211054995397`},
{0.2682300613650819`, 3.6108652762368214`, 9.881761872038165`, 0.9825580258076282`, 0.9296545706074566`,
0.25219322618587503`, 0.024464898005065273`, 1815.4892808719587`, 0.08994453092924143`, 7.425857575199714`,
87.5716937286244`, 0, 0, 0, 65.06775665335743`, 0.4269184477622557`, 0, 0, 0.008021967539908956`, 0, 0,
22.02207141156649`, 0.030739040649965233`, 0.8366693699996218`, 0.1611778359278765`, 0, 0, 0.2702280762585307`},
{0.04962174488386037`, 1.9694465660050104`, 4.880585401279637`, 1.2957318091391512`, 0.6289429156960646`,
0.36977064682826033`, 0.006935918860683708`, 414.38904513183815`, 0.2065379649808241`, 4.444261705692702`,
170.58419588601262`, 0, 0, 0, 154.6929284529902`, 0.5183506272848223`, 0, 0, 0.3712626697883502`, 0, 0,
14.583769469894841`, 0.26318144978770197`, 2.294972360502477`, 0.985966745021521`, 0, 0, 0.37419608100879637`},
{0.2594824517719198`, 0.5971319663647825`, 9.453687965953478`, 0.8401327653868795`, 0.7997988949991743`,
0.15899752713279824`, 0.005075100457461465`, 409.49724454586783`, 0.2091478816442816`, 4.600741868044251`,
144.37239454971117`, 0, 0, 0, 134.52173828750722`, 1.0057684011817902`, 0, 0, 0.02320686186429102`, 0, 0,
8.579663758646374`, 0.0860253344925436`, 2.303008290014094`, 0.4479837389201414`, 0, 0, 0.17750758088118113`},
{0.1975048020318434`, 2.1175350807064808`, 9.527018017184652`, 0.8875453366861839`, 0.2770809005507884`,
0.4437813967164592`, 0.08906290963263506`, 98.18685485298634`, 0.18494734685711195`, 2.236429925751402`,
9252.548489974435`, 0, 0, 0, 81.91996397719407`, 3.1815892316519747`, 0, 0, 2373.7299178561902`, 0, 0,
96.24466872030031`, 6697.472250046448`, 5.19689945275396`, 1.0000000000002145`, 0, 0, 0.348923950681847`},
{0.18592803424857857`, 3.7511626605606008`, 0.2948073517622305`, 1.142160214293685`, 0.24567142070681736`,
0.41248813426514863`, 0.25834870652665815`, 931.542828982051`, 0.012674565140645466`, 9.754712584362995`,
107008.11656396952`, 0, 0, 0, 5.035692623044833`, 0.3237080395756408`, 0, 0, 29262.049159044513`, 0, 0,
17.346878728277446`, 77723.36111752044`, 2.2105784935090513`, 1.0000000000008644`, 0, 0, 0.6788133737550534`},
{0.05816505121822962`, 1.2475983837798745`, 4.326003024744654`, 1.2604302116068957`, 0.7278275017755875`,
0.49389546693051767`, 0.021556729129120156`, 452.84942671713645`, 0.03821400952707204`, 8.73167788977904`,
311.9395115847039`, 0, 0, 0, 237.9972229147099`, 3.8246808928361964`, 0, 0, 1.0241129258027049`, 0, 0,
68.16665286251737`, 0.8509654397509611`, 4.241499192854593`, 0.9998443136388088`, 0, 0, 1.0001751996514265`},
{0.17310243018283944`, 0.49256885761049496`, 5.2474038118867075`, 1.2167083563026317`, 0.588616706923442`,
0.41089714710891945`, 0.1821711177021871`, 4451.29023370465`, 0.12001647220652961`, 2.269196213787966`,

8.083272027047439`, 0, 0, 0, 2.3163803178288997`, 0.7152347127693163`, 0, 0, 0.005403666477170222`, 0, 0,
5.032890648459328`, 0.01336268284422703`, 0.10979295189601518`, 0.09029459825975683`, 0, 0, 0.20698661270069899`},
{0.1805022145737788`, 3.870623878161763`, 2.6853201865429206`, 1.4540056051610473`, 0.3465395939248448`,
0.3116685990581043`, 0.3430495408207368`, 123.77189026179846`, 0.21554545019710575`, 7.5674497363872035`,
50603.35160539281`, 0, 0, 0, 28.57963517275441`, 2.3157148154763387`, 0, 0, 14096.117602756778`, 0, 0,
128.0465865685096`, 36348.292059857435`, 12.785406941445546`, 0.9999999999999999`, 0, 0, 0.45041845533278396`},
{0.12919486377057743`, 2.6304051581320387`, 9.030615531469042`, 1.1852097760732927`, 0.8025656477879668`,
0.2709665069296936`, 0.01610528402445943`, 631.0560544240994`, 0.2313930717140971`, 4.580191033116536`,
129.3234760021952`, 0, 0, 0, 105.30290918390429`, 0.6191108680704439`, 0, 0, 0.030176478649678245`, 0, 0,
23.264463154687178`, 0.0556949435459957`, 1.502011776126406`, 0.42088431527835124`, 0, 0, 0.22226733530551915`},
{0.11792807938525018`, 2.9992967519426994`, 5.197929087471108`, 1.1316540273093707`, 0.8248866470317724`,
0.3561695895739371`, 0.01696020529134022`, 1283.8412020113915`, 0.1325959329361287`, 7.000673815586042`,
122.90846511155277`, 0, 0, 0, 99.05158720680863`, 0.5411114808104002`, 0, 0, 0.03196172426262296`, 0, 0,
23.185055811907674`, 0.053845496516179876`, 1.1310307815473633`, 0.453713416070862`, 0, 0, 0.3564500373476507`},
{0.049303290582256715`, 1.1925070924339343`, 0.7957633600668377`, 1.4128683276782472`, 0.000022730879762677958`,
0.4285104885078085`, 0.00878130985829771`, 1257.460569712898`, 0.24085716752239034`, 7.587289884781604`,
327.1525055128447`, 0, 0, 0, 46.601512089468855`, 0.3154072769727649`, 0, 0, 161.2458228989076 5`, 0, 0,
5.373220211361368`, 113.57070945085594`, 1.4036528399642243`, 0.9999999999986575`, 0, 0, 1.3555698221262484`},
{0.13128664635800402`, 2.4046868210274015`, 6.858470528577773`, 1.007040533285536`, 0.4840752461507001`,
0.3489790633820432`, 0.006590730128885638`, 3596.8950880067596`, 0.16587148750288666`, 3.462016809878005`,
12.368163834688406`, 0, 0, 0, 11.301768401220224`, 0.02955982512676542`, 0, 0, 0.003407286129207959`, 0, 0,
1.015458884488675`, 0.006390445272656781 5`, 0.2076032080652111`, 0.07002086406109953`, 0, 0, 0.32139978522227547`},
{0.04846841194802434`, 1.828640157997615`, 8.053204702491172`, 0.7912000348817253`, 0.19137275290127498`,
0.26044015409759047`, 0.137444226583514`, 395.2194426771421`, 0.10623448877689068`, 6.754668978902`,
150.15138902721569`, 0, 0, 0, 53.01673350461782`, 3.580138073100756`, 0, 0, 0.017151301716950042`, 0, 0,
93.52548930926064`, 0.01187566224374669`, 3.7132526625558286`, 0.3726344190246683`, 0, 0, 0.680915905229137`},
{0.22606436547954117`, 2.759207160262239`, 4.797641172785626`, 1.1197512107663665`, 0.8744312540778343`,
0.4079143329219078`, 0.021680363867763237`, 112.017364386689`, 0.04841847187742204`, 6.247097164626069`,
179011.64375117325`, 0, 0, 0, 182.45224449733476`, 1.376164573490534`, 0, 0, 42268.32887362409`, 0, 0,
54.24461635534751`, 136505.1849528057`, 11.848911477775355`, 1.000000000001059`, 0, 0, 0.4778075987249561`},
{0.21439187995731207`, 1.4871413304129364`, 0.5160724591468409`, 1.1806787917625174`, 0.8679222077666104`,

0.6556391626343723`, 0.05962099501823598`, 325.40523317512117`, 0.12955288081431815`, 2.055442432220964`,
39489.35959638253`, 0, 0, 0, 4.6617489289387155`, 0.1755116702201063`, 0, 0, 9717.772351806689`, 0, 0,
3.728723696487519`, 29763.021192871664`, 1.3639250863187127`, 1.0000000000008105`, 0, 0, 0.19328814838030428`},
{0.09778347249929609`, 1.5399588911598938`, 7.050045797549721`, 0.9082485710483456`, 0.7454636238668619`,
0.6248569918637821`, 0.04029077002834171`, 1056.397985521477`, 0.20938181714809317`, 3.6320484036778673`,
92.52153069881075`, 0, 0, 0, 58.941298252668055`, 1.4569379816013033`, 0, 0, 0.028113005509401733`, 0, 0,
32.051779980506836`, 0.03927124715861237`, 0.732325049198392`, 0.48230875639429804`, 0, 0, 0.2876602453651809`},
{0.12927232662770405`, 2.951438179906213`, 0.40510823994438105`, 1.0187470674786288`, 0.2032238445066854`,
0.5456409554372916`, 0.01881027549673071`, 134.69705409101317`, 0.12921233603333943`, 7.23494261394152`,
67746.06365450741`, 0, 0, 0, 18.781520967460814`, 0.11524046831733357`, 0, 0, 23789.35931802493`, 0, 0,
4.858930258029305`, 43932.94040033616`, 11.647641841241272`, 1.0000000000000449`, 0, 0, 0.714166163244508`},
{0.20182779178318416`, 0.6881028184372244`, 1.057422534977741`, 1.0568090274497894`, 0.12452101430667706`,
0.6254257177994904`, 0.38702446915906646`, 3365.5277260432094`, 0.12211162516890145`, 5.63708838473557`,
21.067396734074475`, 0, 0, 0, 3.518761421252774`, 1.6092728464837467`, 0, 0, 0.030939408985170485`, 0, 0,
15.819216875713723`, 0.08920617992220255`, 0.3994165723752473`, 0.4535026004451209`, 0, 0, 1.173765862356064`},
{0.21480237208045305`, 2.4248480984277982`, 7.5563453916242835`, 0.9609967545999567`, 0.4216733331369118`,
0.3788510295397969`, 0.02865689876021866`, 142.8377853769303`, 0.053409606561145295`, 2.2123406251771502`,
159.10785880878555`, 0, 0, 0, 102.58668293562981`, 1.1376216675718818`, 0, 0, 3.9220169579647344`, 0, 0,
39.40799624774194`, 12.035122084436953`, 3.561158525373078`, 0.999999988273511`, 0, 0, 0.3649105613384747`},
{0.19117013383013431`, 1.4594963084730592`, 8.271393984038092`, 0.8911461626518757`, 0.4023315908892062`,
0.6430024305607471`, 0.425955961175158`, 811.9982424604128`, 0.08470327958141488`, 1.5517888018340713`,
30.304227280128735`, 0, 0, 0, 4.625278141582771`, 1.1725478777446567`, 0, 0, 0.01577052228880131`, 0, 0,
24.44756141537506`, 0.04306932652166065`, 0.43558099353041796`, 0.31267344909970585`, 0, 0, 0.24024560532698871`},
{0.16704561027767095`, 0.7136544792609025`, 7.482780404676742`, 1.2150283597546259`, 0.9046207606306917`,
0.271745131902447`, 0.2695234426940548`, 220.6020580904624`, 0.17006503868594403`, 3.14899802359108`,
188.19240292746207`, 0, 0, 0, 40.71278068257978`, 12.9647706933667`, 0, 0, 0.6904701972002221`, 0, 0,
132.17666682730876`, 1.647714506712291`, 3.0238056640481537`, 0.9996754334558481`, 0, 0, 0.2307612584358348`},
{0.16595765366147208`, 2.092996799011412`, 4.948615364795604`, 0.8929763621996465`, 0.8391454185145462`,
0.5892008264762102`, 0.044299676614507844`, 130.42998143434642`, 0.19085435393011602`, 9.39386568753341`,
26573.72469699617`, 0, 0, 0, 237.82303019615898`, 4.769167192238285`, 0, 0, 7769.176194246045`,
0, 0, 142.59788096149938`, 18419.34645828063`, 15.970104514385284`, 1.`, 0, 0, 1.1599018538288`},

```
{0.27224148961405453`, 3.064354031176171`, 9.703480945324245`, 1.4628863651144104`, 0.3875440410966342`,
  0.15502532019017645`, 0.01538110765065082`, 96.32001554152914`, 0.17185939304406467`, 6.478173160029735`,
  508.2653260559073`, 0, 0, 0, 409.08053665195206`, 1.9379518525833948`, 0, 0, 2.495170811902847`, 0, 0,
  84.8367224527036`, 9.704128838199233`, 14.218417285847924`, 0.9999974563972014`, 0, 0, 0.4607189233507776`},
{0.17789655356032075`, 0.42086448766877327`, 8.212577079102342`, 1.145177796821741`, 0.7675840121122328`,
  0.5713332044931656`, 0.08476669830762888`, 407.4176887081235`, 0.03991184614741466 5`, 5.425104426537898`,
  350.12885558368856`, 0, 0, 0, 159.28670802717232`, 27.02110491540723`, 0, 0, 0.38413688065746515`, 0, 0,
  162.4603353781003`, 0.9762375309197382`, 2.828516317260325`, 0.9909462018174414`, 0, 0, 0.413659316105187`},
{0.25890879804354505`, 0.827757285125033`, 6.146318214990161`, 1.0400613992204162`, 0.4812180568243971`,
  0.29251954183586915`, 0.03603273141312643`, 1733.9600457555907`, 0.08508345454030858`, 2.745915615562448`,
  18.586470565912578`, 0, 0, 0, 12.358016916321917`, 0.4827069567414854`, 0, 0, 0.0077546709381313824`, 0, 0,
  5.708060000332828`, 0.0286821790259254`, 0.33905577222489813`, 0.14763958638999786`, 0, 0, 0.2328241413892715`},
{0.13429919704858378`, 3.97301958069563`, 0.23275296336668916`, 1.4075463260369605`, 0.23061890929775308`,
  0.5504417933404041`, 0.22771166040596233`, 272.6044063125219`, 0.22898591641919652`, 6.483734227937328`,
  26385.39698790132`, 0, 0, 0, 2.980489802199025`, 0.16013254043634617`, 0, 0, 9036.363075790885`, 0, 0,
  9.088710266573386`, 17336.80436168836`, 5.155024561799034`, 1.000000000001482`, 0, 0, 0.6363633459151411`},
{0.12030391986517214`, 0.7748656879950588`, 5.112522469071294`, 1.1531030157403883`, 0.37283988475313556`,
  0.5692147153589893`, 0.013539004333470354`, 948.0585012448981`, 0.020895787868669358`, 3.48210387762453`,
  53.13242829537899`, 0, 0, 0, 44.56512270245526`, 0.7014268579029871`, 0, 0, 0.02743669089379966`, 0, 0,
  7.764451497531577`, 0.047153449466466296`, 0.8319904517512042`, 0.39603877971667767`, 0, 0, 0.5143592684528563`},
{0.1321541010040706`, 0.831080421127889`, 5.684518793971316`, 1.0145204035612485`, 0.08667418093385804`,
  0.219847293666507`, 0.022523926566952547`, 3181.1487538157703`, 0.21535243439086593`, 6.5479272590953945`,
  15.757187434428145`, 0, 0, 0, 12.01634769376629`, 0.28970588100611155`, 0, 0, 0.002773527590048969`, 0, 0,
  3.4395555084255034`, 0.005236186361040101`, 0.4461508780723974`, 0.05711754936780533`, 0, 0, 0.6430524464019814`},
{0.10292542985717545`, 0.9480363128916887`, 1.8187023134383153`, 1.095683835227761`, 0.3789693754557397`,
  0.6741099730044744`, 0.007927682110189524`, 599.0709679738629`, 0.23730403059109434`, 8.347995806673719`,
  1577.4614793503047`, 0, 0, 0, 122.60668025426332`, 0.9352006574482559`, 0, 0, 583.3714182714946`, 0, 0,
  12.665774044301811`, 857.7679141711956`, 3.356830464462308`, 0.999999999999997`, 0, 0, 1.8409678845119857`},
{0.11768271904228267`, 0.8044018805503832`, 4.992994745146397`, 0.8839437991548327`, 0.9627780258088621`,
  0.4036610370791115`, 0.010677704183731065`, 75.90134138634313`, 0.09047277628943856`, 3.892249177500955`,
  90358.61297984415`, 0, 0, 0, 136.76330634924594`, 1.6407763187997662`, 0, 0, 33642.34794882681`, 0, 0,
```

```
18.854907948643824`, 56558.89973692154`, 11.11015996031324`, 1.0000000000000067`, 0, 0, 0.3803494470776358 5`},
{0.23810204192481904`, 3.5212069305389937`, 6.24449843698252`, 1.12580298554529`, 0.9906238590423209`,
 0.651194539832239`, 0.008821192031195845`, 3794.027232128564`, 0.08728181755971864`, 9.039220988882679`,
 128.21203881894698`, 0, 0, 0, 113.75649262887852`, 0.27822016872763866`, 0, 0, 0.01913060699263932`, 0, 0,
 13.995296947706994`, 0.06507195126011893`, 0.4882626809160267`, 0.3051887356989189`, 0, 0, 0.3467097554612438`},
{0.05170854445494355`, 0.9544694556169109`, 2.4941099826351163`, 0.9097774283707087`, 0.02554739328254807`,
 0.22408928016637408`, 0.36587983814629343`, 578.5087062785725`, 0.14318945676945632`, 8.489550606354296`,
 110.81059570015968`, 0, 0, 0, 20.33343793956698`, 6.175992534439358`, 0, 0, 0.05164249778163143 6`, 0, 0,
 84.21137474629195`, 0.038147977032861574`, 3.1914329859086092`, 0.7012068103906186`, 0, 0, 0.8648930201421581`},
{0.2758391104750425`, 2.3950700125014723`, 3.0825274454463827`, 1.2324094107661792`, 0.33578451389793784`,
 0.4521239993250691`, 0.02669897011071422`, 4321.015062663416`, 0.06443072522161825`, 4.390191809873814`,
 12.709093420348216`, 0, 0, 0, 9.230995290645087`, 0.09762993553985712`, 0, 0, 0.007712545701895708`, 0, 0,
 3.340436156199478`, 0.03039173922727425`, 0.22719035037849017`, 0.12478063072199175`, 0, 0, 0.5837190166235212`},
{0.04136033639560718`, 3.5342644851920078`, 2.1080294445485137`, 0.9950381948590621`, 0.26813425336643104`,
 0.30392774702749736`, 0.047958977131448324`, 92.66270711931676`, 0.1731864034169307`, 4.57325203890367`,
 32961.012784380415`, 0, 0, 0, 47.25297300364927`, 0.606508126819986`, 0, 0, 20668.819262474222`, 0, 0,
 30.622287608574418`, 12212.418822799145`, 10.758740270066053`, 1.0000000000000737`, 0, 0, 0.47788022176082323`},
{0.1958059683987035`, 3.200106783136203`, 7.203120771344906`, 1.1276577663917136`, 0.37865874140280775`,
 0.19330414092595893`, 0.01322903896050458`, 235.27978791284824`, 0.1213328646575434`, 2.0980953870537427`,
 78.94411939152164`, 0, 0, 0, 66.45333288588871`, 0.26136212076785414`, 0, 0, 0.06335239447437228`, 0, 0,
 11.9483813646296`, 0.1772110992918677`, 1.8899825731810644`, 0.6960758182400872`, 0, 0, 0.15492308962211748`},
{0.2141293518430885`, 1.3445267096173987`, 9.440612710104876`, 1.3031822475396773`, 0.08631549676886618`,
 0.2421378295292581`, 0.053475196461594834`, 50.8320364740117`, 0.167124755886029`, 6.69448094138391`,
 113801.9853730022`, 0, 0, 0, 281.1255249589892`, 10.368359031053972`, 0, 0, 27916.135188146956`, 0, 0,
 199.15050931649802`, 85395.19905431359`, 27.335245089536187`, 1.0000000000001608`, 0, 0, 0.3456836817320885`},
{0.14124099610833857`, 1.6304240145491873`, 4.431235349124869`, 0.7694928939095955`, 0.9477250896887188`,
 0.5434618086833787`, 0.13057745213792676`, 1388.4740941330226`, 0.07045603839931946`, 8.981021929620123`,
 270.06412862421405`, 0, 0, 0, 95.02431479727532`, 7.19502949786867`, 0, 0, 0.08609021713985418`, 0, 0,
 167.5849839816406`, 0.1737066860573192`, 1.3306710992149524`, 0.9002848300622284`, 0, 0, 0.38016337207409434`},
{0.08317638924124338`, 3.4814117090049965`, 4.537378351353009`, 1.1287962009049006`, 0.37603042712104306`,
 0.6772825294518297`, 0.08278526718675269`, 52.354988641883`, 0.1936331782676135`, 8.933135500909255`,
```

97609.87806211805`, 0, 0, 0, 160.39917969128118`, 3.6302763505867897`, 0, 0, 44449.18046894325`, 0, 0,
180.54980848366776`, 52816.03337341575`, 38.83461992112134`, 1.0000000000003122`, 0, 0, 1.3683406706881718`},
{0.14458191325048447`, 2.576522592570102`, 7.664240990513161`, 1.1334692879511565`, 0.1460097548297743`,
0.5479004940247297`, 0.17641432391033549`, 874.6457178122505`, 0.14737345925317297`, 7.125853291619361`,
95.38030714927152`, 0, 0, 0, 28.336698108603443`, 1.7721429349018143`, 0, 0, 0.01414991389174492`, 0, 0,
65.22809012911448`, 0.029226023182826067`, 1.8914501777516688`, 0.23493229381703784`, 0, 0, 1.2561806214165052`},
{0.21964890100642098`, 3.948215647629967`, 1.3239539515383179`, 0.7541520677887659`, 0.2631388544858304`,
0.4680193255276971`, 0.07455346059859487`, 734.1983096733243`, 0.22794453971709777`, 5.810951908142973`,
1081.510384640808`, 0, 0, 0, 32.20685495470932`, 0.5744434635542539`, 0, 0, 245.6180568685338`, 0, 0,
32.400381021195095`, 770.7105179786574`, 1.7969216403604862`, 0.9999999999995876`, 0, 0, 0.8734853697697074`},
{0.24357090033802437`, 2.585687351965424`, 7.1434831024545`, 1.2789397105658484`, 0.15335660895486725`,
0.5235228651937761`, 0.12790639986488742`, 56.5359989413313`, 0.05251799125371648`, 7.0717564062965526`,
528577.8097152971`, 0, 0, 0, 144.62179647282983`, 6.780873064319313`, 0, 0, 117907.35439788646`,
0, 0, 250.4745388241922`, 410268.5781023958`, 26.60427320508903`, 1.`, 0, 0, 0.5490126870635812`},
{0.09627432586752127`, 2.332762155680175`, 5.679853879558527`, 1.2640734140093113`, 0.8156330491360497`,
0.2895792785477985`, 0.02307681035132743`, 658.139699058721`, 0.029189068125338835`, 7.800914789874877`,
229.73736831393308`, 0, 0, 0, 173.2208752119352`, 1.64021322920117`, 0, 0, 0.07053374703797155`, 0, 0,
54.66039069037804`, 0.09700841352847822`, 2.451610902476585`, 0.6956868835533483`, 0, 0, 0.37416434980298635`},
{0.07523258003696548`, 2.5308231598738997`, 0.19036315944607196`, 0.7501464923569574`, 0.1153260012566284`,
0.48261384042066857`, 0.014254428894325608`, 71.6138994625911`, 0.24976914139283035`, 2.597638512857568`,
36879.97968610944`, 0, 0, 0, 3.3983333824590742`, 0.018286157970731857`, 0, 0, 17773.617804008507`, 0, 0,
0.6611290299634422`, 19102.21605695006`, 8.009699929426743`, 1.0000000000000036`, 0, 0, 0.3086311083813645`},
{0.20487865745542216`, 2.4577449266837927`, 0.3620012570001929`, 1.1621484663425825`, 0.45716579542364766`,
0.40055848752773193`, 0.09344580216885054`, 2051.8175124763634`, 0.1493227569977858`, 9.69569860745337`,
1986.8538965457685`, 0, 0, 0, 12.427268872178908`, 0.44326359366186296`, 0, 0, 498.72699168343627`, 0, 0,
15.563269264372448`, 1459.6930927554854`, 1.0243488474576605`, 0.9999999999999862`, 0, 0, 0.9533043743209016`},
{0.1890789829788747`, 2.8864588443110275`, 0.3020897698703884`, 0.7861583937331655`, 0.3631295895274096`,
0.49565973888016235`, 0.15528595628066338`, 2271.132408212757`, 0.21092746640771154`, 4.683826377759731`,
1042.0690091087292`, 0, 0, 0, 3.718471226732954`, 0.18677285776151895`, 0, 0, 278.41838249471317`, 0, 0,
7.7016023880426285`, 752.0437800674795`, 0.4527175609913666`, 1.0000000000006106`, 0, 0, 0.5256266812684689`},
{0.13636211858102731`, 3.2225156772879746`, 1.985405604082965`, 0.8074403105824717`, 0.51683495700684`,

0.1784694755056958`, 0.2866421049071916`, 363.9511542941515`, 0.043491080354586376`, 7.48314403026421`,
8549.383571778442`, 0, 0, 0, 25.484467054882078`, 1.9555899030677855`, 0, 0, 2860.186389505232`, 0, 0,
90.0274160140287`, 5571.729651565042`, 4.351443504723342`, 1.000000000001897`, 0, 0, 0.5410012632124653`},
{0.1760012024892597`, 3.3901909690471133`, 5.258617572133415`, 0.8627686224489919`, 0.7422533085216929`,
0.41138487941188706`, 0.007592509582269234`, 424.62789093038504`, 0.09162929479721776`, 1.3722835015951313`,
58.438503856773174`, 0, 0, 0, 51.9005049167311`, 0.11236509224947686`, 0, 0, 0.2658535214694121`, 0, 0,
5.441987442576034`, 0.6684362780660096`, 0.6949849042159959`, 0.9942589291505001`, 0, 0, 0.12294036857108155`},
{0.1813951042683553`, 2.693386252451666`, 9.617741712202033`, 1.407583958403493`, 0.6407257633308476`,
0.4405499114150466`, 0.008535253338252974`, 83.84476850180724`, 0.06822985382579094`, 7.163997237119144`,
101706.29625766253`, 0, 0, 0, 507.72808128870975`, 1.5387923109838262`, 0, 0, 28161.31271134242`, 0, 0,
59.208029368316964`, 72976.06079439593`, 18.88770460302187`, 1.000000000000163`, 0, 0, 0.8597545450760986`},
{0.17566594009418735`, 3.2466526484278377`, 9.344725381698979`, 1.0138972604548542`, 0.5838549167343343`,
0.6073333607184574`, 0.10710975925951867`, 945.005957021721`, 0.208189974443157`, 1.4109192016259353`,
28.749701412650325`, 0, 0, 0, 11.533936074182584`, 0.36214193280720125`, 0, 0, 0.01630004156209602`, 0, 0,
16.796415217932505`, 0.040905173208271056`, 0.328659339791929`, 0.28874284923544025`, 0, 0, 0.1627116381665004`},
{0.22088610125498404`, 1.594941397589448`, 0.6585367344105002`, 1.4859166690390868`, 0.411769154740657`,
0.4715175396654723`, 0.024538179644211296`, 3156.4892991025154`, 0.15641735671760065`, 8.553654142069323`,
39.198446573157874`, 0, 0, 0, 28.715936453755408`, 0.4145579169470008`, 0, 0, 0.148020532684469`, 0, 0,
9.445651191960271`, 0.4670811195765569`, 0.6094365177253301`, 0.8470116440783818`, 0, 0, 1.192990415473792`},
{0.08792198919034061`, 0.7563929024204028`, 9.775416388823775`, 0.7563974438325178`, 0.8271569221820632`,
0.32401969363676375`, 0.0333282008681181`, 102.10380045307154`, 0.1014972556908379`, 6.35713244492311`,
554.8359448931674`, 0, 0, 0, 359.9218592559756`, 13.947843007568355`, 0, 0, 13.388815266338074`, 0, 0,
150.71499221426754`, 16.81673244454918`, 14.103063731070058`, 0.9999999997631528`, 0, 0, 0.9387480084713695`},
{0.13210283054552296`, 3.2703864704797683`, 0.8879166279114746`, 1.2876213813677582`, 0.37930173682375035`,
0.2978584700900475`, 0.018740615394798876`, 111.76209976448992`, 0.22677958819730465`, 8.664567998658026`,
50581.820234339546`, 0, 0, 0, 47.5984003591553`, 0.2626713419276714`, 0, 0, 17498.600825094312`, 0, 0,
12.271954326043147`, 33023.06713687394`, 16.206351360554418`, 1.0000000000000409`, 0, 0, 0.5124616822293998`},
{0.19507506176309325`, 3.126733009164254`, 1.2939386659529877`, 0.8626822462482099`, 0.4036124839823858`,
0.4778725494331981`, 0.06798274218289758`, 2442.14562500692`, 0.025650848833468687`, 3.9818096584261493`,
22.673448332103852`, 0, 0, 0, 11.607816949515968`, 0.23939960480946418`, 0, 0, 0.03505373492013066`, 0, 0,
10.693409239123248`, 0.09768727863673615`, 0.36479898880883427`, 0.5682376625318122`, 0, 0, 0.5320444321820464`},

{0.08607083224139878`, 0.4430857620402713`, 5.378081455692687`, 1.015604011959475`, 0.6736258822863768`,
0.30157456176441344`, 0.06550214653497834`, 2724.6632477522535`, 0.20587133389175544`, 1.8687253311000447`,
10.318810318580342`, 0, 0, 0, 5.3916228342984915`, 0.6700725916949846`, 0, 0, 0.007013987535343537`, 0, 0,
4.241423213049599`, 0.008624282064257527`, 0.14455979334977806`, 0.13714482471883516`, 0, 0, 0.1269169005221545`},
{0.14471266481637884`, 3.5830029087352937`, 5.919067097135953`, 1.3383183012471727`, 0.9997339519904831`,
0.40166871745176214`, 0.015827643464025123`, 2493.129738188907`, 0.10618580931649058`, 1.661604036688603`,
22.298950885660187`, 0, 0, 0, 18.14844126164094`, 0.07803838962128709`, 0, 0, 0.0225007344196804`, 0, 0,
3.994453957229851`, 0.04651630340281911`, 0.13713169823774385`, 0.299666406309293`, 0, 0, 0.07062429700073451`},
{0.15884129765285837`, 1.9554524456621927`, 0.3975714737116185`, 1.180621526159508`, 0.13357193827486524`,
0.6393982147520227`, 0.07953097836767822`, 3991.2803311425578`, 0.2365716915141447`, 8.584420428066604`,
28.506128337321066`, 0, 0, 0, 13.30795609370322`, 0.5032991119871744`, 0, 0, 0.19428785427391315`, 0, 0,
14.059678277641874`, 0.4408704984436621`, 0.5284524182546331`, 0.9243864028096954`, 0, 0, 2.1022017370864705`},
{0.04917531951043441`, 2.0521955455867618`, 5.720052321648424`, 1.1771597571169652`, 0.4322660150443707`,
0.1629991214278883`, 0.005091310116027936`, 189.05888707261903`, 0.1735533969163952`, 4.172239042736072`,
187.91675973261079`, 0, 0, 0, 174.32167332886735`, 0.40714712410293846`, 0, 0, 0.6168800141772903`, 0, 0,
11.936364492607282`, 0.4333610256681225`, 4.642040709529168`, 0.9976480410290857`, 0, 0, 0.2743641473059501`},
{0.27542629055144846`, 0.7843233583874856`, 9.783752583368699`, 1.3481471177954405`, 0.23017739141462767`,
0.5420497851091893`, 0.2059235624279975`, 3468.61907862177`, 0.15188055880627876`, 9.878186090289375`,
35.8690582981044`, 0, 0, 0, 9.551626553766976`, 2.1550485005378857`, 0, 0, 0.003219056040381982`, 0, 0,
24.146498248996995`, 0.012665895203992434`, 0.6512265995433762`, 0.05004342769274028`, 0, 0, 1.5666894163102356`},
{0.242913589428772`, 2.017077890959616`, 6.7411875410249`, 1.4794325227990526`, 0.8119526659059784`,
0.6637027966074835`, 0.028536028992749693`, 2430.705278612085`, 0.11516348105007651`, 2.1124785829469186`,
28.31791319247922`, 0, 0, 0, 20.099992908699434`, 0.2723204769720097`, 0, 0, 0.021240104258927896`, 0, 0,
7.847023047940268`, 0.07370728521967662`, 0.18411731042493443`, 0.2613939988066486`, 0, 0, 0.1550316729767205`},
{0.27369802527373327`, 0.5072292829799574`, 6.3938654238644155`, 0.9166058122252008`, 0.8588715137395462`,
0.6363372429100727`, 0.4973592888199021`, 125.12883357781175`, 0.0982596068118865`, 5.59867632569453`,
60166.060284302344`, 0, 0, 0, 39.33952795771803`, 31.002922939288496`, 0, 0, 12193.769966013904`, 0, 0,
224.65129103968738`, 47677.29657628805`, 9.864159729400342`, 1.0000000000120837`, 0, 0, 0.6550637356796396`},
{0.18540637687565897`, 1.1775430389730683`, 2.7268491301469933`, 1.4767982648221127`, 0.8902187555583527`,
0.5282942786301189`, 0.1497900469847022`, 304.29028125771407`, 0.023895791395609434`, 5.874748340307197`,
94821.83147001214`, 0, 0, 0, 44.081064980288524`, 5.044130903510678`, 0, 0, 25951.387112928016`, 0, 0,

84.8525890442562`, 68736.46656436627`, 4.323054414527168`, 1.0000000000001628`, 0, 0, 0.7902664422089832`},
{0.2722155439547531`, 0.9139371325579537`, 7.770199974150746`, 0.8847133947824111`, 0.9018075103619831`,
0.19960287154002887`, 0.008582048560181034`, 1921.873640365849`, 0.18448070830995128`, 2.8786799978881135`,
25.034955966091786`, 0, 0, 0, 22.287636217222975`, 0.19171343975345823`, 0, 0, 0.006725569101591714`, 0, 0,
2.503057591444253`, 0.026154349305643667`, 0.30671968036813596`, 0.1507511159284114`, 0, 0, 0.10800077803892284`},
{0.06964367539322192`, 3.707453741922901`, 4.6834761045808655`, 1.337177597600478`, 0.13817299390105364`,
0.15072857839162668`, 0.030325540973499845`, 324.16896723003435`, 0.11870351893768993`, 8.4259642987084`,
184.69188829640746`, 0, 0, 0, 130.3730752099109`, 1.0039128503671308`, 0, 0, 0.062056759091877564`, 0, 0,
53.170863623687126`, 0.06174086837369313`, 5.4866135160238105`, 0.6301746432723709`, 0, 0, 0.5855575466092724`},
{0.17261439973382386`, 3.0972705794401207`, 5.672236578564393`, 1.4223285072729144`, 0.7931550721000631`,
0.3896544494671119`, 0.01712882834694823`, 1350.492916562551`, 0.2427963854280547`, 3.05685280243325`,
49.605842097918476`, 0, 0, 0, 39.84275544462721`, 0.21292618948008946`, 0, 0, 0.03203960917504181`, 0, 0,
9.421286032413906`, 0.07900711293509091`, 0.47369977021571125`, 0.3804935903856288`, 0, 0, 0.1844366656727454`},
{0.11394032550346922`, 3.2108067304960466`, 9.096957387649113`, 1.399541009089664`, 0.15147624678363414`,
0.6188818627652795`, 0.08261238136824677`, 151.04115640404754`, 0.09860367843525403`, 6.06148281050898`,
500.3812667100334`, 0, 0, 0, 231.4261452985217`, 5.587806721664983`, 0, 0, 2.687344225285333`, 0, 0,
256.30524900904294`, 4.374241082411589`, 9.65195816417009`, 0.9994282565464863`, 0, 0, 1.3249213320763602`},
{0.20354065160019702`, 3.5209980508829766`, 0.4053576884306178`, 0.7576791600370161`, 0.6570403323895382`,
0.46832222917239164`, 0.005063986272754102`, 161.28160618619455`, 0.12637144234319758`, 8.874326085906798`,
53801.02557794975`, 0, 0, 0, 26.390964160181678`, 0.036849636771129145`, 0, 0, 13760.623673189406`, 0, 0,
1.8535357035270212`, 40012.09012665813`, 11.576408172642804`, 1.0000000000001452`, 0, 0, 0.5854532593989298`},
{0.24345495866234085`, 1.8344199239977046`, 3.71003568577021`, 1.0568366445286865`, 0.9797132100793247`,
0.5295146440336447`, 0.23321770204647818`, 2779.737816031833`, 0.01572768263207122`, 9.835586906627377`,
155.3449258885264`, 0, 0, 0, 36.37701545868119`, 4.3659740951357415`, 0, 0, 0.04181039485735065`, 0, 0,
114.41471239678411`, 0.1454135421662062 5`, 0.7255311809278353`, 0.5694640953129739`, 0, 0, 0.382798509919777`},
{0.18352727434547023`, 0.4163243700753796`, 8.057415014521496`, 1.2522528089453002`, 0.16203828635471806`,
0.6604646928880291`, 0.25830735718962067`, 2461.4350174740794`, 0.12685187153570027`, 8.600866037504808`,
45.8502551707053`, 0, 0, 0, 10.367791013052232`, 5.104398274735264`, 0, 0, 0.00544010358723522`, 0, 0,
30.3583628049002`, 0.014262962621744734`, 0.8234820243969442`, 0.08904307529459743`, 0, 0, 1.6690012518064845`},
{0.23920496896525717`, 1.1838719961494162`, 9.218551103388815`, 1.265080848919387`, 0.9542453823473309`,
0.22929853041342196`, 0.4982471338661781`, 90.20981971435951`, 0.0856312153630287`, 3.6772130334476696`,

```
  15280.30366185599`, 0, 0, 0, 40.26490004056679`, 13.078212214431058`, 0, 0, 3397.113289538133`, 0, 0,
  221.18470286234566`, 11608.662557077618`, 8.843968393369343`, 1.00000000000102`, 0, 0, 0.3650384598913887`},
 {0.10156561975861944`, 0.5164671469302382`, 5.401464431301745`, 0.8562900606586841`, 0.5357330343495086`,
  0.16718344164197263`, 0.02721990257094452`, 330.98847924494044`, 0.2190497368128987`, 7.446609110605853`,
  225.6291338421051`, 0, 0, 0, 163.62625890888182`, 7.380027002325344`, 0, 0, 0.056333526062077435`, 0, 0,
  54.45059271655836`, 0.08173642125263166`, 4.677522590094146`, 0.755374277545537`, 0, 0, 0.3976350740360762`},
 {0.2533884831264572`, 2.217775528299157`, 1.87005833593021`, 1.0852798553697922`, 0.3630118105229163`,
  0.49869259108707253`, 0.006826900671537389`, 2180.4237702051914`, 0.21841135731377453`, 2.8539212819504436`,
  17.65882355869093`, 0, 0, 0, 15.977957581466667`, 0.04683481065823965`, 0, 0, 0.029024283620812698`, 0, 0,
  1.483844242148122`, 0.10506313143585203`, 0.2962478099856194`, 0.4261442072144227`, 0, 0, 0.4188706039894201`},
 {0.04019446235159935`, 2.9685609440334373`, 3.1967275735949383`, 1.2384363485833663`, 0.006837582253808039`,
  0.6265805854044944`, 0.10123843723710083`, 64.97108080441517`, 0.1585540441972691`, 8.616308543170305`,
  82754.54501528785`, 0, 0, 0, 100.41526749813409`, 3.2105641297246907`, 0, 0, 52414.44299781811`, 0, 0,
  136.15364691164908`, 30096.719367940746`, 30.94071411782546`, 1.0000000000000282`, 0, 0, 1.5689664200372246`},
 {0.18466310913340017`, 2.870831467521074`, 5.7568671895307055`, 1.0469133934587485`, 0.5974897879057268`,
  0.6447436436670198`, 0.1911914248698421`, 363.92035372259653`, 0.11435325769039895`, 2.2759611974712186`,
  112.51918288110635`, 0, 0, 0, 30.6425909987059`, 1.9009736670474748`, 0, 0, 0.553351649800174`, 0, 0,
  77.96250031841166`, 1.4597662299457475`, 1.4203160083692783`, 0.9987543809470352`, 0, 0, 0.34290711880219377`},
 {0.22919090442152185`, 1.3485088294529417`, 3.7111141569564556`, 1.1683364276022934`, 0.8698699225738455`,
  0.45530089098812654`, 0.06782162054416256`, 403.6677278730082`, 0.1398561461848175`, 8.195868200542787`,
  264.7668887685776`, 0, 0, 0, 131.88846145002162`, 6.088203301192917`, 0, 0, 2.2235027796832103`, 0, 0,
  117.28565581661694`, 7.280094472277014`, 4.568242137518486`, 0.999999989191112`, 0, 0, 1.1473282172917645`},
 {0.12718220564190125`, 0.7794468104625536`, 3.4523481867286847`, 1.042660599955479`, 0.8212977484948716`,
  0.42153700480377276`, 0.05083556365712203`, 907.9591078654141`, 0.03221255361915021`, 1.403008654938164`,
  36.433778707561316`, 0, 0, 0, 20.990633597951664`, 1.2366676997873356`, 0, 0, 0.154635899784683`, 0, 0,
  13.77023848859002`, 0.28095621151481726`, 0.3277128232701847`, 0.9408696273065174`, 0, 0, 0.10457721619743707`},
 {0.24829534536640735`, 0.6361539074945153`, 1.4367506745172012`, 1.2157272327840285`, 0.5920355341003019`,
  0.22834833308349511`, 0.008858258633323783`, 152.73617223659593`, 0.05098711045238086`, 6.308971873547048`,
  194460.44354841363`, 0, 0, 0, 61.495969878706035`, 0.7626058633979217`, 0, 0, 42750.81076944352`, 0, 0,
  6.930495712554487`, 151640.3903527569`, 8.432621206318899`, 1.0000000000002724`, 0, 0, 0.21672397195787066`},
 {0.24634814165734203`, 1.0199939463339236`, 6.874973257490155`, 1.1669106806380989`, 0.9547234223304992`,
```

0.2467804242930165`, 0.010102520966714356`, 1373.9164154017546`, 0.2219276262119578 8`, 8.148592815397581`,
130.46850608034433`, 0, 0, 0, 114.008326888658`, 1.045407171815537`, 0, 0, 0.020461259406030788`, 0, 0,
15.23298552437023`, 0.07200847472349382`, 1.2112985224193757`, 0.3140788603434711`, 0, 0, 0.2834700023117419`},
{0.11968292559735771`, 0.4405556662519543`, 0.9343787484302961`, 1.4969299043499453`, 0.5720179190625709`,
0.5743610731089337`, 0.058376899138915264`, 4861.966479565238`, 0.13911792619470742`, 8.404040973858844`,
33.48884913923602`, 0, 0, 0, 18.343940845431863`, 2.054985851555301`, 0, 0, 0.05765523743579236`, 0, 0,
12.933366585289802`, 0.09857639274751538`, 0.38109727202797605`, 0.5464929604647888`, 0, 0, 0.9838953149377347`},
{0.0912885213280808`, 1.1352603856466574`, 9.64216394840902`, 1.0423303168159856`, 0.8496114679763396`,
0.3948758352214041`, 0.050073459411100744`, 1020.4170701645861`, 0.08585808288581304`, 1.5724283740137839`,
37.42979637377136`, 0, 0, 0, 21.927178186410725`, 0.8977156114227023`, 0, 0, 0.01955042428498116`, 0, 0,
14.559156731782346`, 0.025496133204457847`, 0.32063974532357453`, 0.3295391528033339`, 0, 0, 0.08531539328667118`},
{0.23601424519793518`, 0.7493625485076763`, 8.159569145163797`, 1.0789985894921148`, 0.9135595458313224`,
0.43990663477596414`, 0.03553380189426036`, 63.68276264433202`, 0.11799293833856772`, 7.92015369426547`,
139480.46948593995`, 0, 0, 0, 342.37571951254813`, 14.600366994556234`, 0, 0, 31788.39281515719`, 0, 0,
156.2995460026859`, 107178.76480463958`, 26.414047573532336`, 1.0000000000006088`, 0, 0, 0.6025852195383089`},
{0.09505824539478602`, 0.994707682773301`, 2.092747921993542`, 1.2334327544777528`, 0.1649122243213208`,
0.435178801412204`, 0.4947441968705133`, 1030.121381906519`, 0.19720224785958157`, 8.531247013968503`,
90.45835009258096`, 0, 0, 0, 12.740088806256784`, 5.099552492760274`, 0, 0, 0.0651016981339128`, 0, 0,
72.46520061791965`, 0.08840647424030458`, 1.8774255151938661`, 0.6691537334855536`, 0, 0, 1.2675655622685091`},
{0.13338172675277454`, 1.0224437122231844`, 3.1176391252125253`, 1.1808523949493024`, 0.5954558720182712`,
0.3646371291004906`, 0.024821319877175146`, 118.84942434665426`, 0.15904005586434156`, 2.0945755856004613`,
44395.625014980316`, 0, 0, 0, 38.86754582382741`, 0.8650631723589228`, 0, 0, 15262.07561039796`, 0, 0,
12.635405732203093`, 29081.171410661183`, 3.781652835272766`, 1.0000000000005274`, 0, 0, 0.18262387950562123`},
{0.2663723170815805`, 0.6117369492537685`, 4.996758576680092`, 1.4798016834758696`, 0.3454705507334743`,
0.5628818087325649`, 0.10659789380555212`, 347.2598858446038`, 0.128819557084905`, 4.579853140224845`,
188.09878980109036`, 0, 0, 0, 75.46206065539009`, 11.343405823556367`, 0, 0, 0.44994928744018414`, 0, 0,
99.13114960928293`, 1.7122004894948653`, 3.0197858586805846`, 0.9856496709206579`, 0, 0, 0.7333093564182788`},
{0.19858704302380392`, 3.77240735130054`, 4.552319154599175`, 1.490234534684174`, 0.9267739623702391`,
0.4663128979271708`, 0.016623264635311285`, 190.7315614324941`, 0.03981298108873932`, 1.7245962595124684`,
59538.529850613384`, 0, 0, 0, 53.68049898485968`, 0.22693461879264332`, 0, 0, 15499.875493265816`, 0, 0,
12.229854631399334`, 43972.49202063981`, 2.041441918143971`, 1.0000000000011595`, 0, 0, 0.2481985915763493`},

{0.16356962246540357`, 2.7590365436019733`, 9.315311335363422`, 1.411146654012818`, 0.2509435276133807`,
0.3090283790000361`, 0.243114119684150 2`, 313.55839056266797`, 0.2467169299716112`, 8.038445579591425`,
255.0347181232233`, 0, 0, 0, 61.1578029158479`, 4.793842183492308`, 0, 0, 0.04037059242109976`, 0, 0,
188.94836812165667`, 0.09433432229988671`, 5.600862075136447`, 0.45890709938303065`, 0, 0, 0.859620731978695`},
{0.25383472083693576`, 3.9769524497483806`, 7.011565744250568`, 1.066285473960517`, 0.7734216029556533`,
0.6850552501893354`, 0.013843595247453381`, 297.6124430270534`, 0.10716201039628298`, 2.5950386184147853`,
159.74436524213615`, 0, 0, 0, 129.7848271206461`, 0.437177468257577`, 0, 0, 0.9963309078275555`, 0, 0,
24.837628619453753`, 3.6129053978517573`, 1.9933286552524427`, 0.9999957921392091`, 0, 0, 0.41070774282119205`},
{0.17343366236722435`, 1.3936719133530833`, 0.599503921563171`, 0.8621470436054358`, 0.43567440743840313`,
0.22823781097601215`, 0.018669299661272933`, 3618.675535403083`, 0.22611694811187344`, 3.1306846738065635`,
8.932755013182055`, 0, 0, 0, 6.98086317638967`, 0.08672814155854232`, 0, 0, 0.03900456547960763`, 0, 0,
1.7267224998207276`, 0.09663863771672004`, 0.1856807031200472`, 0.6053454901077919`, 0, 0, 0.2737009644632621`},
{0.27302715407014244`, 1.5486379048502288`, 9.868645627334566`, 1.0694698241775202`, 0.6816384789594119`,
0.4416154581373778`, 0.05961824305163796`, 87.63839207083008`, 0.05267766985704242`, 3.1260333504958955`,
91650.78842242043`, 0, 0, 0, 139.47199081894604`, 4.980990699028664`, 0, 0, 18650.796339016943`, 0, 0,
110.19644286031756`, 72745.34065119475`, 7.882684135404812`, 1.000000000002026`, 0, 0, 0.3741505155050053`},
{0.12081145422022538`, 2.0244942048813837`, 8.652229532190159`, 0.835020699056165`, 0.9214042127932665`,
0.46285611589460973`, 0.11861503789539068`, 2689.091322411565`, 0.07096583948487911`, 5.509201411600676`,
69.05941686292778`, 0, 0, 0, 25.885982306069312`, 1.4421282691263906`, 0, 0, 0.008442564360954488`, 0, 0,
41.70829033631434`, 0.014570835397052683`, 0.42183349684365856`, 0.19525044163516647`, 0, 0, 0.2381658300652869`},
{0.27038445563951496`, 3.3688963138915424`, 3.4133330216440854`, 1.1975198070883244`, 0.31492494684053174`,
0.45427916274833`, 0.0070970717881839104`, 3067.136751023867`, 0.17569148508387616`, 1.2036801730392703`,
4.720745024840074`, 0, 0, 0, 4.249725916190107`, 0.00859534249416999`, 0, 0, 0.009164743755624196`, 0, 0,
0.41366882350349443`, 0.03540006073485821`, 0.08922750082613147`, 0.14791726775127545`, 0, 0, 0.18292942604020812`},
{0.1767050761987432`, 2.85608165701536`, 6.140634667151749`, 1.3554192221984884`, 0.6330624847112656`,
0.5906276578639604`, 0.04152902973081543`, 1510.3499876585004`, 0.15541682752363528`, 8.81551919212286`,
125.0655075646142`, 0, 0, 0, 78.90153610038297`, 1.1022707403464223`, 0, 0, 0.023503220852581476`, 0, 0,
44.973932036687906`, 0.0593305490238638 6`, 1.261127873658465`, 0.310108021728718`, 0, 0, 0.8351861156519741`},
{0.16171803894826015`, 2.2156907324583015`, 1.0134587159780288`, 0.8322559746700846`, 0.34733770749260806`,
0.6849579573613365`, 0.018772440750507276`, 1617.8437562512436`, 0.21218330633531807`, 7.516753350682977`,
69.77474506785634`, 0, 0, 0, 53.67414246720448`, 0.43193967532335276`, 0, 0, 0.5967239980912309`, 0, 0,

```
  13.67206765135714`, 1.3785862109240026`, 1.1121787409691533`, 0.9998061050075341`, 0, 0, 1.5998592388966126`},
 {0.24108871826638628`, 1.1722622038849657`, 2.5945274526654174`, 1.1873482780450624`, 0.3399058776037083`,
  0.3423735862363967`, 0.09173881369912114`, 1455.6792358614214`, 0.2278375557130481`, 4.818602627144232`,
  36.755851350699345`, 0, 0, 0, 16.25069512971041`, 1.148513712931908`, 0, 0, 0.02765969443689275`, 0, 0,
  19.233703090195114`, 0.09526343256332741`, 0.7253125369770169`, 0.3903071419461721`, 0, 0, 0.5308975319968847`},
 {0.2202280085702742`, 2.180975046803316`, 5.6134680006393936`, 1.2506485034427453`, 0.4748208651226249`,
  0.39757955628981323`, 0.017520218948128618`, 280.3947437474927`, 0.12359899846366301`, 9.521190146527957`,
  443.13414131294326`, 0, 0, 0, 351.39428838542267`, 2.6774415703015553`, 0, 0, 1.3238276064449392`, 0, 0,
  83.42047505859398`, 4.164913106538831`, 7.45493695113794`, 0.9991506751179341`, 0, 0, 1.0697905438924589`},
 {0.12315072752895034`, 2.8609501087269207`, 1.79674111361204`, 1.4514496090822089`, 0.3032919079034333`,
  0.2763824218936657`, 0.028981582964232436`, 188.78817200372555`, 0.08856946510090113`, 4.546561013073182`,
  58633.29990505694`, 0, 0, 0, 45.86430021621891`, 0.44328938231496273`, 0, 0, 21226.016774271637`,
  0, 0, 18.117554379021193`, 37342.84868990359`, 5.080524084297705`, 1.`, 0, 0, 0.3130961184834628`},
 {0.14162785770391484`, 1.92013166099325`, 8.540643644714823`, 1.2350689068338556`, 0.301574102817308`,
  0.40858591883894513`, 0.080613435450821`, 585.1346359267128`, 0.15211712109726616`, 7.686797097032363`,
  153.20803973682908`, 0, 0, 0, 72.66492137584426`, 2.8306727422462665`, 0, 0, 0.02170914075881159`, 0, 0,
  77.64663363282335`, 0.043923129975259614`, 2.9133697817508404`, 0.3143702490925293`, 0, 0, 0.950418308024325`},
 {0.22524615675817938`, 2.031565502127312`, 0.245821316301889`, 1.298167355078078`, 0.689325845538592`,
  0.661487626561178`, 0.00610096551453922`, 373.63064864758115`, 0.20592315239272913`, 2.994117316721619`,
  26349.97598308941`, 0, 0, 0, 5.4339895307532196`, 0.01562982934487665`, 0, 0, 6245.9227495607165`, 0, 0,
  0.4536146014455515`, 20098.144210671828`, 1.7200689956793889`, 1.0000000000000422`, 0, 0, 0.2620804767691623`},
 {0.06014178921319169`, 2.215935435785701`, 1.3525069683978295`, 1.040020224469648`, 0.697929353581739`,
  0.692932654029565`, 0.014666911144259826`, 1018.9785490283374`, 0.16122419934759502`, 4.978072859014281`,
  70.28298129569727`, 0, 0, 0, 51.91608648711861`, 0.3253370616875185`, 0, 0, 4.149196366393443`, 0, 0,
  10.298941765253865`, 3.5648584753110324`, 1.2217713060141497`, 0.9999999991299203`, 0, 0, 1.329718155499555`},
 {0.11674773262981253`, 1.910335393882857`, 4.221858661435528`, 1.4404651869296854`, 0.4563596481936356`,
  0.5439902833298367`, 0.005611633628996954`, 451.6526207878965`, 0.24247834364497922`, 9.182241064841566`,
  320.8342668292945`, 0, 0, 0, 295.19591681894025`, 0.8238098441184336`, 0, 0, 0.751599409175239`, 0, 0,
  22.48218718640802`, 1.2535360981016483`, 4.542903941439494`, 0.9956044256299839`, 0, 0, 1.2136189232695411`},
 {0.12898119467887553`, 3.729766126068422`, 2.3744826976889595`, 1.2465399522360021`, 0.1415631230326866`,
  0.3607064691421532`, 0.03995461220531779`, 1238.1663657022145`, 0.14506115517150608`, 8.953141814946814`,
```

```
69.96367527499407`, 0, 0, 0, 45.03223950605033`, 0.4574587477165292`, 0, 0, 0.033875449589081355`, 0, 0,
  24.374487732954165`, 0.06241851368973307`, 1.6192034419924282`, 0.4395389726750034`, 0, 0, 1.2047306080235658`},
{0.2498624462217614`, 2.827129073229683`, 2.690968559152097`, 1.0446525535360744`, 0.06936419216205603`,
  0.5607634545552321`, 0.008891348377622028`, 196.01664126075448`, 0.10064633302983284`, 7.405206717033214`,
  58885.82980967864`, 0, 0, 0, 141.993093689474`, 0.43089469922865736`, 0, 0, 12851.810981722347`, 0, 0,
  17.40278473842709`, 45874.07043246908`, 8.108321936456687`, 1.0000000000000375`, 0, 0, 0.647581184193504`},
{0.25890580889310233`, 3.9576703566675278`, 2.211552265086926`, 0.8006241502346895`, 0.26522568617457876`,
  0.4892067360859801`, 0.02025985822061832`, 94.72915936196773`, 0.06766512891079995`, 5.4984531961033785`,
  187331.1336026766`, 0, 0, 0, 74.22665409389836`, 0.3692943214246534`, 0, 0, 39848.77714700426`, 0, 0,
  20.87921698268574`, 147386.85543780113`, 12.18508168897698`, 1.0000000000000089`, 0, 0, 0.34991172717359315`},
{0.2032139210380186`, 1.0205320875718096`, 3.2697243543529257`, 1.0866214178514029`, 0.5111053959745286`,
  0.4079750171889772`, 0.048345897200607585`, 145.20906840025864`, 0.23728279570010086`, 2.517042827760779`,
  26575.78611810111`, 0, 0, 0, 39.16650708934835`, 1.6995154689572323`, 0, 0, 6792.149859055458`, 0, 0,
  24.777286705650017`, 19717.9915019498`, 3.6976022221948006`, 1.0000000000075226`, 0, 0, 0.20337906887486398`},
{0.15288075349234037`, 1.8405591021591183`, 8.732063156275487`, 0.8366527628406224`, 0.7247685787231319`,
  0.43308603413296065`, 0.06703535093550567`, 1294.178726539122`, 0.16108367938164575`, 4.274431762914247`,
  68.15273844370063`, 0, 0, 0, 35.132887270352306`, 1.2085048468673847`, 0, 0, 0.010995654514396071`, 0, 0,
  31.776065655788315`, 0.02401462781856319`, 0.6968624896001716`, 0.245802846289621`, 0, 0, 0.2950155922816745`},
{0.1609254870534111`, 1.384768951726243`, 6.196355509527487`, 0.9770497723863997`, 0.43819861370403546`,
  0.17710326003602894`, 0.2243750211158665`, 120.51310647409781`, 0.10277566384729958`, 6.21125463242891`,
  11256.15117516689`, 0, 0, 0, 78.6196360060992`, 10.881244302821855`, 0, 0, 3319.6747599729456`, 0, 0,
  215.2572752385117`, 7631.718251536588`, 10.925472507475028`, 1.0000000000007818`, 0, 0, 0.45983603687035696`},
{0.21184155187533854`, 1.0400436907537394`, 2.77253033361667`, 1.25783985819887`, 0.5916706672066951`,
  0.18670557332607218`, 0.05860949172916328`, 177.69964385957368`, 0.18251915101666627`, 3.5990512292385053`,
  23476.583376099206`, 0, 0, 0, 42.4984779751775`, 2.1781735697397084`, 0, 0, 5811.66267637715`, 0, 0,
  32.362795408204676`, 17587.880576281685`, 4.180742713074167`, 1.0000000000000488`, 0, 0, 0.16505899389759981`},
{0.15826860039724194`, 2.914200187526596`, 3.057619613104965`, 0.9127068016590583`, 0.36895217716385176`,
  0.3294645201729436`, 0.006405724718526674`, 799.6829736219844`, 0.2321605158663716`, 7.540832982633661`,
  106.7202092065175`, 0, 0, 0, 97.72249819120857`, 0.20551011053949034`, 0, 0, 0.04139548743212558`, 0, 0,
  8.55568003818276`, 0.09359436940920825`, 2.048692550041607`, 0.6192692417945318`, 0, 0, 0.7599100742754853`},
{0.2486148170283075`, 0.5446418582356878`, 7.121193243885838`, 1.048132429671941`, 0.7615900023009023`,
```

0.5400927540260018`, 0.07717023450494316`, 388.33902457321517`, 0.04098404938849237`, 2.529691537294738`,
145.99404495397135`, 0, 0, 0, 69.30859999182236`, 8.526752085903013`, 0, 0, 0.39880262737409977`, 0, 0,
66.34323001116063`, 1.4164034605003268`, 1.402697339474069`, 0.9961293151219387`, 0, 0, 0.23023385552895392`},
{0.1973186549669898`, 3.31823274961833`, 5.404057922234477`, 1.0748992098641554`, 0.1837120164888255`,
0.5027766473922404`, 0.30055211808677745`, 460.171808421071`, 0.10833443978919949`, 3.5562239100468123`,
88.9845536613686`, 0, 0, 0, 18.123103029081093`, 1.4601564893105565`, 0, 0, 0.048449700266410455`, 0, 0,
69.216272605686`, 0.13657185271596653`, 1.7786260472090378`, 0.6131787961064874`, 0, 0, 0.5907088185314855`},
{0.16177424691159015`, 1.9234518490470327`, 6.336020872512709`, 1.465202064627133`, 0.3679027675625519`,
0.6088093981583469`, 0.02752967040620688`, 1523.605195139083`, 0.023927731291662258`, 6.9520681394676025`,
68.97739749780388`, 0, 0, 0, 49.73952159093552`, 0.6733197124040651`, 0, 0, 0.016132878366665`, 0, 0,
18.5014006546202`, 0.03728406068978142`, 1.034694979242523`, 0.20984356706905294`, 0, 0, 1.0353628667243167`},
{0.12468946686374066`, 2.602287301847274`, 9.387923649086364`, 1.237964958422611`, 0.6279283704003791`,
0.5225362832845872`, 0.15386398985407668`, 4877.253164044286`, 0.10729269282887094`, 6.155025385024989`,
24.79324834329096`, 0, 0, 0, 7.932831964781891`, 0.4414051073451314`, 0, 0, 0.0034304435546571783`, 0, 0,
16.409470083068083`, 0.0061105739705188036`, 0.27156383047247096`, 0.057776850604039476`, 0, 0, 0.5557014098537689`},
{0.1891437323001075`, 2.037539508644585`, 6.753647851102151`, 1.0264835122983769`, 0.08414675462879395`,
0.15050525073770327`, 0.01724785647741675`, 835.2003740810117`, 0.09305345804576048`, 5.34515441362371`,
43.22750720053642`, 0, 0, 0, 34.903621306023695`, 0.2749211539464209`, 0, 0, 0.00840845345220087`, 0, 0,
8.002324470399909`, 0.022720089554583198`, 1.3553696582182078`, 0.16162662237745373`, 0, 0, 0.39033883476851244`},
{0.11951180040558451`, 2.9137311414381974`, 6.217108794108201`, 1.4729889111648695`, 0.6540356010640074`,
0.40115742558392786`, 0.08157249569611574`, 391.2473393910366`, 0.0369147403900103`, 8.338813449127404`,
381.22059843425484`, 0, 0, 0, 178.15304424797108`, 4.748289814215446`, 0, 0, 0.24843084441636978`, 0, 0,
197.64628428933335`, 0.42414882132141396`, 4.530011147261324`, 0.9603483043844986`, 0, 0, 0.6411175830137548`},
{0.17883917330505905`, 1.5543060950462326`, 9.905917854358197`, 1.2462903035005124`, 0.32028587133565645`,
0.3166477244063972`, 0.12099723319057126`, 400.41544183552224`, 0.027102889569123156`, 3.023770086067156`,
78.29135256141872`, 0, 0, 0, 29.66684422372291`, 2.091636304859099`, 0, 0, 0.025148028146053714`, 0, 0,
46.443472246606795`, 0.06424932234132459`, 1.6487180455104837`, 0.3508624167247224`, 0, 0, 0.3211022161499714`},
{0.25477439586299494`, 1.0781629016953422`, 9.675717925989822`, 1.4583691370753404`, 0.484439918991886`,
0.6784957922502513`, 0.09307366059264305`, 217.6033516010123`, 0.05467318278554256`, 4.774031073697932`,
393.88283325884083`, 0, 0, 0, 169.7770591487085`, 13.363882133780361`, 0, 0, 1.0575948394619668`, 0, 0,
205.83488484673114`, 3.8492583755959857`, 4.9821157218364736`, 0.9972392685356379`, 0, 0, 0.7188693284828338`},

{0.26738035849024727`, 1.6336260676101997`, 9.900661691242231`, 0.846806505065853`, 0.2415106216321452`,
0.502680613934997`, 0.14320441138979886`, 77.88432317464672`, 0.12860121706274624`, 9.963544099078408`,
61516.02042546346`, 0, 0, 0, 269.37810200237897`, 21.808366883288663`, 0, 0, 12597.38912811989`, 0, 0,
508.9530947506785`, 48118.491730254755`, 27.757174486568182`, 1.000000000000218`, 0, 0, 0.9898228926455391`},
{0.0966033889391959`, 0.8191052864079662`, 6.963587929197736`, 1.39989556064551`, 0.208903818004208`,
0.4044327200024739`, 0.345646875315088`, 51.22809362991988`, 0.05669448608759026`, 3.5983266970468653`,
313231.1273348109`, 0, 0, 0, 37.18778545383081`, 13.16211286421061`, 0, 0, 131521.14007521374`, 0, 0,
154.01651753390342`, 181505.54069160466`, 15.332350873656974`, 1.00000000000002`, 0, 0, 0.3812719754534076`},
{0.07268626506253911`, 2.0017174568948795`, 2.5547427204964173`, 0.8446935149578232`, 0.0630154383873418`,
0.49766282606547807`, 0.00568323780270915`, 197.50095618865632`, 0.04182941385906325`, 9.915201734242256`,
54749.04327797868`, 0, 0, 0, 197.13157167331536`, 0.5307609469389022`, 0, 0, 26754.575013408175`, 0, 0,
15.177620756080799`, 27781.287586573842`, 11.309149088071935`, 1.0000000000000007`, 0, 0, 1.4015415771737663`},
{0.14445086415814334`, 1.1396367789378896`, 8.108045316850049`, 1.1452867101498132`, 0.43349891091368953`,
0.5412309783014353`, 0.17300117449492944`, 151.5195914284387`, 0.22841440999808194`, 1.401560283159844`,
110.15771793878415`, 0, 0, 0, 30.91393956198547`, 4.1056446167251375`, 0, 0, 2.7079664851882006`, 0, 0,
66.84205152097633`, 5.5881156985244695`, 2.23051886079269`, 0.999999937715034`, 0, 0, 0.31080740399649354`},
{0.2737062633152032`, 1.87379900045931`, 2.9341815386065377`, 1.0257953180476898`, 0.5994241695021645`,
0.2666028641788709`, 0.16092630392754248`, 2757.7643970777704`, 0.13082540497870587`, 6.7306102844961035`,
32.471263536437455`, 0, 0, 0, 10.177959892055263`, 0.8007155805340579`, 0, 0, 0.011931987844734341`, 0, 0,
21.43400077795595`, 0.04665514009859763`, 0.5135737022803337`, 0.2207290294779013`, 0, 0, 0.4453870918623241`},
{0.14450675367477284`, 1.4907097867645147`, 8.628071674125838`, 0.8645742960679661`, 0.06677853457714877`,
0.6218432064874626`, 0.019848227343967184`, 449.15101395266043`, 0.20772413403212947`, 3.059722840773212`,
77.90011327461615`, 0, 0, 0, 60.9520306350899`, 0.7563567893063481`, 0, 0, 0.020345303897011217`, 0, 0,
16.107263830067982`, 0.04200048312403961`, 1.6336771716973972`, 0.3946127594529154`, 0, 0, 0.6580338014691158`},
{0.05092006104579416`, 0.9079056727720332`, 9.25200153286255`, 1.1365283513840736`, 0.03327106644526534`,
0.582893635885294`, 0.1249724078419535`, 82.97595681905067`, 0.15395748273911747`, 7.1070379565760815`,
28619.623157717273`, 0, 0, 0, 214.67789576773436`, 26.017656138801264`, 0, 0, 16232.799414382656`, 0, 0,
337.4511085807112`, 11808.216244635694`, 20.197265086659513`, 1.000000000000002`, 0, 0, 1.38412484938077`},
{0.2364459713820966`, 3.884359137525399`, 0.954741626535867`, 0.8976843312448166`, 0.4180287949880024`,
0.15414803582881131`, 0.32241293356212986`, 4897.582666581565`, 0.05447310320870352`, 4.86045092756919`,
8.678563707036247`, 0, 0, 0, 1.706388198902468`, 0.12248953215129474`, 0, 0, 0.012023930048381034`, 0, 0,

6.797047620901318`, 0.04061442600170851`, 0.20758305483707606`, 0.24866198113237914`, 0, 0, 0.29060194238737924`},
{0.042914457849633436`, 3.519672647432988`, 3.392121169492343`, 1.4941116772804357`, 0.33812708289181614`,
0.3684690007615431`, 0.017148240098985187`, 3621.3180321373566`, 0.22166064632630456`, 1.0781698542512608`,
3.2964433019883033`, 0, 0, 0, 2.647949240267482`, 0.01234458246926532`, 0, 0, 0.008843849164638028`, 0, 0,
0.6206984180150567`, 0.005421842745776806`, 0.06657366871535743`, 0.1163432902647008`, 0, 0, 0.14332457690728992`},
{0.1470328346746822`, 2.9686959435849998`, 9.643655350717612`, 1.0921756097932163`, 0.41500029005274564`,
0.5183995564120613`, 0.04078998201684905`, 4770.787596114335`, 0.08630863643009795`, 3.8345772490574745`,
11.611914838569161`, 0, 0, 0, 7.395385715289845`, 0.09696466183875081`, 0, 0, 0.0021956761649542106`, 0, 0,
4.112265689597008`, 0.004611949865154065`, 0.1790621782083117`, 0.042212457263193226`, 0, 0, 0.493712020172387`},
{0.10223589016255652`, 0.4809064129783538`, 6.158635896611097`, 0.9490677758592585`, 0.9235714285317382`,
0.43663660593873277`, 0.45300992250929206`, 1331.4948553442105`, 0.21785131418517045`, 5.425736978481032`,
130.2181377673123`, 0, 0, 0, 18.142705133465405`, 14.230402405701376`, 0, 0, 0.03286388047451553`, 0, 0,
97.76416823091942`, 0.047998115350071445`, 0.8394750717483217`, 0.5227791698604188`, 0, 0, 0.23756384221838825`},
{0.08719902184880185`, 3.645892777856403`, 2.8891082836443758`, 1.428386787110956`, 0.151537003432892`,
0.34505159105650784`, 0.041961341889936235`, 290.12993265609543`, 0.15015043093723618`, 6.608503242578238`,
5360.4407545880495`, 0, 0, 0, 101.02554303495141`, 1.0988913801069782`, 0, 0, 2316.0134875286003`, 0, 0,
57.23485923400981`, 2885.058724301796`, 5.095762145215109`, 1.0000000000001024`, 0, 0, 0.8822246072597989`},
{0.14456990131617375`, 0.9947424361823325`, 3.150441801621305`, 1.0015653051761273`, 0.8447006281771343`,
0.5703016332732033`, 0.008271730569805881`, 52.80371817926736`, 0.13650904531369146`, 3.8670961625783473`,
153936.2750796065`, 0, 0, 0, 88.7506952957576`, 0.6806885193864544`, 0, 0, 50186.891221064914`, 0, 0,
9.67299651508323`, 103650.19873135575`, 15.947648274902237`, 1.000000000000216`, 0, 0, 0.39951916117024533`},
{0.14643438827373756`, 2.4493630197222025`, 5.7044459384485755`, 0.7643715149180945`, 0.8231136724552808`,
0.4724466281569806`, 0.012687117803733845`, 1217.9307406282053`, 0.17452698640833697`, 5.969503699935295`,
132.7426642836786`, 0, 0, 0, 112.40163275387134`, 0.5609096906508442`, 0, 0, 0.02649001095230237`, 0, 0,
19.626735052628547`, 0.05541497927378731`, 1.0221107017333109`, 0.5234113864521879`, 0, 0, 0.3377871911494979`},
{0.045156311345747796`, 1.2463805103118828`, 6.638333254697063`, 1.0774632518381033`, 0.7965921624862937`,
0.19987812094053103`, 0.1230725004501963`, 3016.4870397653995`, 0.18711874545495605`, 6.497285772809487`,
31.870694821839166`, 0, 0, 0, 11.832923792060136`, 1.0650704233520012`, 0, 0, 0.005262120273223023`, 0, 0,
18.96404311107948`, 0.0033945420199473077`, 0.4431287262546541`, 0.09975923789638685`, 0, 0, 0.2752873306460161`},
{0.21753427776894996`, 2.5707121359144134`, 7.635927852201033`, 1.3489791075447177`, 0.07755529833527275`,
0.4139475400754866`, 0.049678130502208456`, 727.9927710408293`, 0.24367647097486184`, 3.0823691783926943`,

40.2670998924629`, 0, 0, 0, 23.886497922146752`, 0.43241109359009555`, 0, 0, 0.016398707390842614`, 0, 0,
15.8800635142297`, 0.050961156694467526`, 0.971147999221201`, 0.22873074370837254`, 0, 0, 0.4997070195356858`},
{0.08480900190064672`, 0.6294563083477476`, 5.454864363444388`, 0.7943923084343845`, 0.9968128471314037`,
0.29575569030654725`, 0.01722905088446216`, 309.5962534203848`, 0.1957861782089137`, 7.847856100229725`,
341.2031653600431`, 0, 0, 0, 273.33634169390234`, 6.6002299675293035`, 0, 0, 0.8100710193328942`, 0, 0,
59.35080556581665`, 0.9814473516895452`, 5.380142062382915`, 0.9999608966324525`, 0, 0, 0.5915478162872025`},
{0.12061650440369748`, 3.919019990284525`, 1.2611661330191772`, 1.4204820377968523`, 0.21263599304474745`,
0.23829484446556248`, 0.008404906276562513`, 547.3513122366178`, 0.16414531089750106`, 3.5940701557610755`,
3715.5206049358126`, 0, 0, 0, 32.556641202034974`, 0.06679912253209264`, 0, 0, 1351.0843607958775`, 0, 0,
3.739815664810516`, 2328.0438964814725`, 1.411527662845774`, 0.9999999999999819`, 0, 0, 0.3204442061326845`},
{0.2541152986933387`, 3.9531330061598675`, 6.8389480578926864`, 1.4090336327472661`, 0.5067749551810137`,
0.5768128332044816`, 0.18877117253369932`, 176.17163988983094`, 0.11435366361110944`, 3.2749545355180913`,
436.43178457413836`, 0, 0, 0, 58.135884950319976`, 2.5169902623987497`, 0, 0, 50.458976896266776`, 0, 0,
142.14281832102122`, 183.17711408221587`, 4.521670620470263`, 0.999999999452333`, 0, 0, 0.7611021153733701`},
{0.26839792559441034`, 1.5919359357927423`, 0.607262674381337`, 1.065158649344713`, 0.7342994823745972`,
0.6373140564402076`, 0.019311239061898532`, 131.67334362372645`, 0.026592393453336638`, 2.002632914376134`,
522742.8241948918`, 0, 0, 0, 7.589338503743456`, 0.08731591247029656`, 0, 0, 108131.04442384704`, 0, 0,
1.9857334118285486`, 414602.11451025127`, 3.231198490359931`, 1.000000000000285`, 0, 0, 0.1530458253701156`},
{0.0804335350475921`, 1.0990801178785707`, 6.42097149017635`, 1.215697698043305`, 0.5864093543250795`,
0.4707288466973014`, 0.04708688161958605`, 3199.4245739234016`, 0.17424214472199046`, 5.845755402883285`,
31.705913645576064`, 0, 0, 0, 19.10121589579792`, 0.7537949001462523`, 0, 0, 0.006831759172934952`, 0, 0,
11.835442681557252`, 0.007850036298207736`, 0.3940882141045094`, 0.1135329063930014`, 0, 0, 0.5425792150529647`},
{0.04545514913582513`, 3.0084108739176516`, 7.583320976280454`, 0.7956411409554567`, 0.21703974982634144`,
0.40384085540761094`, 0.39906200752611665`, 3808.370230770943`, 0.07445295911170413`, 7.244769365152968`,
20.688207038340703`, 0, 0, 0, 3.4300136092210907`, 0.3923617906128052`, 0, 0, 0.0019291983873497725`, 0, 0,
16.86264967699096`, 0.0012527428629896813`, 0.4250215594980936`, 0.05082593819317116`, 0, 0, 0.9563372061393004`},
{0.21811178043156876`, 3.634131336571894`, 2.2696382083853486`, 1.2012104146171985`, 0.07478503245860835`,
0.304565647018059`, 0.049403646487048036`, 242.22074286172042`, 0.22363734402413937`, 5.499597099973035`,
15686.16133302832`, 0, 0, 0, 58.135121905636204`, 0.7570453643442391`, 0, 0, 3787.2714007765026`, 0, 0,
39.30288973956982`, 11800.692974298934`, 4.776519189369514`, 1.0000000000004723`, 0, 0, 0.3623945011712749`},
{0.22643881069864102`, 1.8950339409059218`, 4.49788713237294`, 0.781319815193253`, 0.8852654232409718`,

```
0.19866746146736047`, 0.009828863939504507`, 672.8810907026934`, 0.061975672509268276`, 2.025268353742364`,
  48.51080213629765`, 0, 0, 0, 42.41558729040904`, 0.20906659655604495`, 0, 0, 0.04545472735228421`, 0, 0,
  5.6598328054769995`, 0.14703877717546313`, 0.6182741826480198`, 0.7078956060983954`, 0, 0, 0.08259167973104065`},
{0.13526256211607002`, 2.462351571487578`, 7.058879409696349`, 0.9886974127254741`, 0.579411758510507`,
  0.519067819756009`, 0.20147771873022094`, 163.35469430255884`, 0.08074145629003388`, 7.486187513545234`,
  873.2249300900974`, 0, 0, 0, 129.55235625178602`, 9.443263842462681`, 0, 0, 137.10934732246804`, 0, 0,
  332.1805080351399`, 264.93945155570304`, 10.972788786912462`, 0.9999999999895204`, 0, 0, 1.5955923810279335`},
{0.20702996858127698`, 3.5459212947722705`, 9.044651158282573`, 1.2898647778788441`, 0.1120438185942787`,
  0.3399243113730348`, 0.005774130262077124`, 68.71847405774774`, 0.13094328364017765`, 7.770082768301062`,
  90101.20345696728`, 0, 0, 0, 516.3716874344561`, 0.8115193047036483`, 0, 0, 22625.5823592165`, 0, 0,
  41.108336909534955`, 66916.76578516724`, 24.049782210728402`, 1.`, 0, 0, 0.6034165297477406`},
{0.10369509204579469`, 2.7722566029085502`, 8.491805279999415`, 1.0043154825901828`, 0.1196997608855428`,
  0.46660526882559294`, 0.07137717728073698`, 124.06613686120853`, 0.02382042507485488`, 7.245869706736816`,
  20549.77479733502`, 0, 0, 0, 271.9805668838826`, 6.520690717299001`, 0, 0, 8065.350688758405`, 0, 0,
  258.24325566509464`, 11947.67545789167`, 13.67099544590684`, 1.0000000000002363`, 0, 0, 1.347742093915066`},
{0.12495211269478451`, 3.2710300653782634`, 8.31741576802569`, 0.8301159562153473`, 0.45240597007929817`,
  0.45987625913811714`, 0.011306277140564175`, 1464.8239131751898`, 0.06894180622066476`, 6.472250284276024`,
  63.56073112134365`, 0, 0, 0, 54.80311389634417`, 0.1822826569283911`, 0, 0, 0.006696958816451369 4`, 0, 0,
  8.517886445854272`, 0.01195427361064159`, 0.9690429351109626`, 0.15921168759320858`, 0, 0, 0.7197678555720566`},
{0.2548094999525184`, 1.991565099208616`, 8.435964917534815`, 1.3597221193616629`, 0.25512389762112764`,
  0.2087626588648025`, 0.2568272531538555`, 1822.5061165805678`, 0.026223328458720774`, 8.894025776493041`,
  42.13888951048874`, 0, 0, 0, 9.813527909790823`, 1.096815834716801`, 0, 0, 0.004981580594226629`, 0, 0,
  31.205430524019103`, 0.018133629431257518`, 1.0402265456018427`, 0.07584211330834767`, 0, 0, 0.7114583852315919`},
{0.1329827943052238`, 0.6587055670228517`, 4.6853626661703505`, 1.028170006221078`, 0.5310002897488229`,
  0.31399252604565075`, 0.010724910779551164`, 62.931190858884165`, 0.12157208629491184`, 4.054529845871336`,
  104722.53821266741`, 0, 0, 0, 130.46290351306445`, 1.88760669793842`, 0, 0, 36062.47935861134`, 0, 0,
  17.762529146880848`, 68509.84678117985`, 13.624646693421196`, 1.0000000000000357`, 0, 0, 0.28970737890384785`},
{0.05388606525951978`, 3.0261209146571506`, 4.311810802135282`, 1.2867466894982391`, 0.14121116952696844`,
  0.17229324040800142`, 0.239982541075217`, 290.98679707372355`, 0.1893837871763393`, 8.200911880146922`,
  209.93122960746882`, 0, 0, 0, 51.85482077676696`, 3.5700850589807347`, 0, 0, 0.09632723709588271`, 0, 0,
  154.33584377266502`, 0.07415279692039099`, 5.990275733843488`, 0.796253938336295`, 0, 0, 0.6307499467856054`},
```

```
{0.18877028807710305`, 3.0713928282220317`, 2.350050214349242`, 0.9003573816536171`, 0.8295355664150708`,
  0.3697046533237569`, 0.07836282343745943`, 203.60143886304388`, 0.1119456128362123`, 8.250251130667593`,
  32442.650373910943`, 0, 0, 0, 73.56303787726057`, 1.7856596241637053`, 0, 0, 8734.490565747023`, 0, 0,
  78.34945947574326`, 23554.46143289725`, 8.59020352273772`, 1.0000000000000226`, 0, 0, 0.6112363772708249`},
 {0.1549731585118843`, 1.0794091822629426`, 5.872175042913531`, 1.1954059661912195`, 0.1582784842110334`,
  0.47191465043849123`, 0.04704948286374014`, 132.48832982484745`, 0.0611350826684427`, 8.646340597834747`,
  65632.70263228439`, 0, 0, 0, 254.85550317068217`, 10.150241604995161`, 0, 0, 20290.33956883034`, 0,
  0, 156.518057008844`, 44920.828718004246`, 14.506171383574891`, 1.`, 0, 0, 1.0912780582955397`},
 {0.0973630018497127`, 2.6509059407435345`, 2.661281949905481`, 1.0384603558144696`, 0.8313691963066465`,
  0.6553290382276395`, 0.19978363792296042`, 613.3149478611653`, 0.10086553584387609`, 7.337446850354657`,
  176.24752619494032`, 0, 0, 0, 46.66588019074762`, 3.2199867285294985`, 0, 0, 1.8488803160397491`, 0, 0,
  121.94117068248852`, 2.5716076804354686`, 2.827117152601293`, 0.9999999782970291`, 0, 0, 1.4477134106739136`},
 {0.25760446309632484`, 3.1710442921339457`, 1.104410531583154`, 1.067160019670409`, 0.29416815684722497`,
  0.2106415408174861`, 0.1640132441258384`, 295.52940308175954`, 0.22278260496644214`, 4.791464741488767`,
  17326.968891317352`, 0, 0, 0, 12.415794054421802`, 0.6055029082248164`, 0, 0, 3693.6500914082426`, 0, 0,
  27.42966487138294`, 13592.867838041591`, 3.312332845059602`, 1.0000000000062113`, 0, 0, 0.16825345202109734`},
 {0.08814664727699922`, 0.49325198699724604`, 7.4393784579313085`, 1.082856668773565`, 0.36684849391494345`,
  0.47651248778047417`, 0.010016127241926027`, 222.2306387901171`, 0.10060123761752315`, 2.9576363340294876`,
  174.6579785741582`, 0, 0, 0, 152.46532560555116`, 2.659480111580967`, 0, 0, 0.29722712142435875`, 0, 0,
  18.739912134528147`, 0.37427963190499064`, 2.992737840622153`, 0.9847424815463162`, 0, 0, 0.41209241338031916`},
 {0.09405288750913654`, 1.8996899418118014`, 4.321657830772438`, 1.3285146456969388`, 0.4687194709250446`,
  0.37672748824359026`, 0.1617154754485949`, 124.22044587269936`, 0.08001764444851872`, 7.0616286930304`,
  64535.99375880696`, 0, 0, 0, 78.55498382665232`, 6.080543946380513`, 0, 0, 27430.437480711975`, 0, 0,
  165.01640250976706`, 36855.883581425776`, 12.44925439829093`, 1.000000000000054`, 0, 0, 0.7737277641777537`},
 {0.26673333380428393`, 2.6775026015459398`, 7.186300359062184`, 1.3779081516185778`, 0.2197224262472608`,
  0.44634915685926935`, 0.2549448072449753`, 573.3906335364269`, 0.19327206597748986`, 8.09151224456528`,
  160.50042410240889`, 0, 0, 0, 36.90128826719276`, 3.1453665833535247`, 0, 0, 0.02980585871929862`, 0, 0,
  120.31038871063888`, 0.11357451518707325`, 3.180852683136688`, 0.3709825903773226`, 0, 0, 1.149434841394638`},
 {0.06695152315589192`, 3.739348796633019`, 8.531551488563299`, 1.3873826920360777`, 0.5359483678583272`,
  0.39821450250245216`, 0.041894311644931556`, 1681.9385322117223`, 0.03536815464018617`, 7.504735436115399`,
  66.65528863153285`, 0, 0, 0, 42.03677029429763`, 0.4519883971636132`, 0, 0, 0.009761215552224778`, 0, 0,
```

24.144889557512098`, 0.00933611784390311`, 0.9600598190060812`, 0.14029993583719857`, 0, 0, 0.676723352504752`},
{0.2432492145111665`, 1.524425096090667`, 0.7030547277601631`, 1.1448142947983793`, 0.5481222164790474`,
0.4314400790527364`, 0.005980753513921293`, 1722.4066531232882`, 0.11386175120469438`, 3.8888258531503404`,
37.99101118815494`, 0, 0, 0, 21.810915697875622`, 0.07985967367945009`, 0, 0, 3.203912331378707`, 0, 0,
1.7391441531795004`, 11.133559399578742`, 0.5251823350138729`, 0.9999999936055809`, 0, 0, 0.6943670574735994`},
{0.24077340164047406`, 3.026302168091572`, 3.348461380748672`, 1.1251559242118545`, 0.33184191297746213`,
0.3141742813991918`, 0.0703872676061783`, 173.09096476750136`, 0.24564160017502346`, 6.392098114270558`,
19415.596295889234`, 0, 0, 0, 85.06176206728789`, 1.8821065135428534`, 0, 0, 4335.344677461153`, 0, 0,
81.3689003216286`, 14911.938361089275`, 7.765363217900851`, 1.0000000000001408`, 0, 0, 0.4180805565956372`},
{0.09741803900657164`, 2.8226058170826276`, 6.285345102913118`, 0.8413213607246539`, 0.2506059249236361`,
0.26188997247349455`, 0.05425664970500174`, 4955.988737338467`, 0.11641107051911387`, 2.1128007380367517`,
3.7979111683690276`, 0, 0, 0, 2.1763703659193636`, 0.03915059493898787`, 0, 0, 0.001536682414195269`, 0, 0,
1.578667100243323`, 0.0021385798195290655`, 0.09268473438796926`, 0.03842322633200457`, 0, 0, 0.21454472896756727`},
{0.14314599793313348`, 2.5518611864266925`, 6.295888200856282`, 1.4241645467251498`, 0.07884119203240614`,
0.5217209274223922`, 0.006381498715981013`, 359.4043872790348`, 0.24023808698930982`, 4.143010259943262`,
122.94540160771717`, 0, 0, 0, 112.5889783816885`, 0.26799097100893765`, 0, 0, 0.06618326246708316`, 0, 0,
9.769653674721567`, 0.13534098789028898`, 2.7069726361918436`, 0.6240483691659555`, 0, 0, 0.786338805655986`},
{0.225655266424848`, 1.5867432872088365`, 3.0858574789264335`, 1.3244931698407223`, 0.6079478177713131`,
0.6933074034991797`, 0.013957254253711016`, 59.76089956157712`, 0.13530140261382512`, 4.737681248427657`,
247220.22263676152`, 0, 0, 0, 98.42544811278613`, 0.8207468506331641`, 0, 0, 58504.49500004633`, 0, 0,
18.604493653428037`, 188597.82008981446`, 17.094656922225912`, 1.0000000000000417`, 0, 0, 0.43838560723796505`},
{0.1122606973400958`, 1.7187290694442563`, 9.979465273640976`, 1.2098446125137294`, 0.9761419756238592`,
0.37158902094365565`, 0.02080861609061981`, 179.99091662757314`, 0.030562090142117915`, 6.57677031568597`,
531.2202130337728`, 0, 0, 0, 408.3868654022719`, 4.659335332735464`, 0, 0, 1.3959676701395354`, 0, 0,
114.40192972373106`, 2.2387472016297063`, 7.863723528712906`, 0.999983487024757`, 0, 0, 0.5945836367901125`},
{0.24459409433338708`, 0.8193086201254509`, 4.65991702304226`, 0.8806460188595719`, 0.2108783317674836`,
0.532516488691834`, 0.10515966693757768`, 2682.0355154099257`, 0.13211135275557007`, 8.414844442987327`,
38.24452045357361`, 0, 0, 0, 15.677571526373264`, 1.774266476741619`, 0, 0, 0.00577118973747996`, 0, 0,
20.766740268486064`, 0.020165698958067763`, 0.7195959158535113`, 0.13120909501002143`, 0, 0, 1.3637300268808157`},
{0.06530542208683338`, 0.9596039403304868`, 4.254394351359874`, 1.2566656605190776`, 0.7704919647854709`,
0.333532652338865`, 0.010126143082696715`, 1215.361514466369`, 0.2378411046297244`, 9.6572715838455`,

156.18001988518674`, 0, 0, 0, 136.48670396052668`, 1.3261171338977242`, 0, 0, 0.041977980427592106`, 0, 0,
18.179246100400388`, 0.039162710431086746`, 1.654416075041823`, 0.5106920416564533`, 0, 0, 0.5303441248919486`},
{0.1695388010672208`, 1.173836844893498`, 5.733937529803059`, 0.8972303818101501`, 0.03459597061008757`,
0.45955942513269943`, 0.02896845637601857`, 512.9632938088884`, 0.14318523913512782`, 1.8784414403823462`,
35.00221682664215`, 0, 0, 0, 24.92593118455567`, 0.5622262997322247`, 0, 0, 0.02385688338954254`, 0, 0,
9.428027797054582`, 0.05778096295804812`, 0.857351470963138`, 0.43026116551087645`, 0, 0, 0.3498270554838933`},
{0.21476635225191587`, 0.5109603344620304`, 9.116157263242219`, 1.0856571152809298`, 0.22597102427026194`,
0.39468057790613453`, 0.008122876676631087`, 1537.5523384133612`, 0.22468288668284858`, 1.5725594082493757`,
10.517775320361492`, 0, 0, 0, 9.420063981381597`, 0.12864966477355708`, 0, 0, 0.005239359616350735`, 0, 0,
0.9390696534446409`, 0.0160748307562795`, 0.23028272816036896`, 0.09796646344463233`, 0, 0, 0.22140411191316786`},
{0.06813064867410118`, 0.8082337111165119`, 3.599754755102856`, 0.8970806442820121`, 0.12954010392326953`,
0.2841840584225793`, 0.09462247566679993`, 471.57171291886044`, 0.0960155452146989`, 9.92783198210315`,
184.09040682748852`, 0, 0, 0, 80.76473218731564`, 8.227831154108255`, 0, 0, 0.04947420859408715`, 0, 0,
95.00015011607087`, 0.04815299891650146`, 4.623608434668151`, 0.6918601878782531`, 0, 0, 1.1193402967047292`},
{0.12903273933048381`, 3.2486278014701124`, 1.90816116411313`, 1.4556940559632274`, 0.8634695831402321`,
0.3517692054558007`, 0.07017000005506284`, 82.66475099620249`, 0.22883036061603934`, 2.0030440452333433`,
69303.4093640065`, 0, 0, 0, 15.364517957258593`, 0.31600134302788424`, 0, 0, 24363.40068326408`, 0, 0,
14.665296403746904`, 44909.66185096797`, 5.145152666505382`, 1.0000000000006786`, 0, 0, 0.15192462866503972`},
{0.25243522618186365`, 1.7361695497710992`, 4.779805866927312`, 1.3770344402735035`, 0.053441804379047`,
0.21192069167526972`, 0.3494903419308112`, 922.4310224013639`, 0.09853668667446419`, 9.287299936208932`,
76.1616549187417`, 0, 0, 0, 14.430988870255728`, 2.389429865360067`, 0, 0, 0.01684418477095051`, 0, 0,
59.26364819359689`, 0.06074379417868068`, 2.17524311314453`, 0.23120942670803402`, 0, 0, 0.8790261594157454`},
{0.1954776887577972`, 2.556119761332786`, 4.179140500485959`, 0.8918497427192082`, 0.9985940433520333`,
0.19919828622889002`, 0.10341194686720168`, 185.89024422047143`, 0.23152045430083634`, 6.38817726005194`,
3298.7595801783145`, 0, 0, 0, 89.00621002019965`, 3.2930762836467826`, 0, 0, 813.75848182193`, 0, 0,
120.24996234579832`, 2272.451817622927`, 7.324998891859227`, 0.9999999999998067`, 0, 0, 0.5108282957161648`},
{0.21581636372683116`, 2.527066218755916`, 0.7074271020081202`, 1.2545848439034097`, 0.6959200155465519`,
0.5313070485281961`, 0.086636650755472`, 1874.5635156658839`, 0.10607902266846381`, 5.753646937249579`,
53.559567860711624`, 0, 0, 0, 16.464245736439246`, 0.5251503983204138`, 0, 0, 4.313330600733108`, 0, 0,
18.95842616231043`, 13.298390368598415`, 0.7287770096600079`, 0.9999999969380268`, 0, 0, 1.1681229507010087`},
{0.1492413868080179`, 2.613673396032418`, 3.0904979954224796`, 1.2072285951382535`, 0.18001792552689877`,

0.24732759197473053`, 0.00562211589804969`, 2528.5611678534033`, 0.0881107110472133`, 9.594393869960811`,
32.67014173050535`, 0, 0, 0, 30.242924684209555`, 0.061702296670529705`, 0, 0, 0.009024386658346314`, 0, 0,
2.303852161169472`, 0.019240171142618567`, 0.8221591079959655`, 0.14832530444716785`, 0, 0, 0.9385960764533205`},
{0.07601227699098417`, 2.712563755157329`, 4.823883660189505`, 1.3951422907519642`, 0.1445858824844255`,
0.4724433117860949`, 0.00914361266793413`, 231.39818056782485`, 0.1448152920502898 7`, 4.810561016418756`,
218.95355784799887`, 0, 0, 0, 182.18486247231112`, 0.582561577881569`, 0, 0, 6.451630756274569`, 0, 0,
22.574791732983012`, 7.005759201278408`, 4.948174875201186`, 0.9999999977628957`, 0, 0, 0.9907655451519464`},
{0.12871665135347787`, 2.8014401685833805`, 7.283786002739568`, 0.7524429330430051`, 0.8818706538097647`,
0.37826653388239995`, 0.007972118927365966`, 65.78330734043783`, 0.199604900729567 6`, 1.2990854064714608`,
32877.13968270576`, 0, 0, 0, 69.06298869753316`, 0.18817354573257253`, 0, 0, 11554.243385817254`, 0,
0, 7.530813281142713`, 21246.050250649536`, 4.292157424174587`, 1.`, 0, 0, 0.13149963710279522`},
{0.09952335410600305`, 3.347751049949922`, 0.6305859630946937`, 1.1305375150416004`, 0.9039729545790072`,
0.47386245025326623`, 0.16157270551479597`, 562.0685567880952`, 0.16407167074796686`, 8.59213583452907`,
9020.334132459173`, 0, 0, 0, 13.908811369912382`, 0.6263747683566765`, 0, 0, 3706.3262431444455`, 0, 0,
29.956382691831458`, 5269.514558983394`, 3.3594202001127123`, 1.0000000000004197`, 0, 0, 0.9748681160617912`},
{0.09189223639403032`, 1.6732139429664894`, 9.692701238874825`, 1.282228547207058`, 0.15855153018111912`,
0.4131843305852153`, 0.03830010741000983`, 3674.584524793622`, 0.10140311695331994`, 4.468652597668594`,
12.385367741288915`, 0, 0, 0, 8.092754585194593`, 0.17212855253955478`, 0, 0, 0.0023438214240436594`, 0, 0,
4.1143984870260395`, 0.0030768427480536422`, 0.2753905177711895`, 0.03849067822160057`, 0, 0, 0.658539126869065`},
{0.15046133692934927`, 3.1811544489323884`, 8.294233740646352`, 1.3617577237735539`, 0.4847724596901488`,
0.37433619970600795`, 0.06464979105054385`, 793.4336208513492`, 0.16357169207826316`, 3.060886752086996`,
51.2377712642987`, 0, 0, 0, 26.994507228780897`, 0.5206110672169495`, 0, 0, 0.02006708509306293`, 0, 0,
23.659203037723433`, 0.04313314930536999`, 0.8358720463045203`, 0.270260119377845`, 0, 0, 0.2993721025888442`},
{0.2766206750277398`, 2.8724354428398637`, 5.189326643025524`, 0.8952441889736982`, 0.9323758122824941`,
0.4464935529399745`, 0.01922331430839979`, 81.97107113159659`, 0.06878282991185858`, 7.425462926136905`,
198681.53433538997`, 0, 0, 0, 238.0351316654172`, 1.536626142531753`, 0, 0, 40062.57619930815`, 0, 0,
63.055134202892994`, 158316.24102289797`, 19.02367258825559`, 1.0000000000000315`, 0, 0, 0.4754505596290809`},
{0.23204393359414766`, 1.3807564884192223`, 0.49288335289722696`, 0.9255887528096592`, 0.5628667572225596`,
0.43401537857521066`, 0.010225716982610468`, 1119.882052780943`, 0.015435359995479236`, 2.904839645045426`,
45206.247438694016`, 0, 0, 0, 9.996568207598092`, 0.06947344680857712`, 0, 0, 10474.092931134615`, 0, 0,
1.3703701779112956`, 34720.71035101622`, 0.5544844393676486`, 1.0000000000003586`, 0, 0, 0.24134336230560097`},

{0.06896766630182821`, 3.238145856087148`, 5.061610251787275`, 0.9979492539875935`, 0.9640249344246659`,
0.343776595701085`, 0.005657576047359979`, 3386.0339854064414`, 0.07870221044254616`, 4.290344560110242`,
36.07799034501086`, 0, 0, 0, 33.344029279150526`, 0.056579666075244416`, 0, 0, 0.011758405670002367`, 0, 0,
2.617331589147821`, 0.011584997121287155`, 0.2597374055467094`, 0.2231176370715645`, 0, 0, 0.16568584631771738`},
{0.14163054804702563`, 1.8677837362731973`, 0.4370922457889872`, 1.166715795330906`, 0.13182109027382305`,
0.4473367986720722`, 0.3453878303019619`, 2024.858554151485`, 0.17866806603084778`, 3.146330044100834`,
741.4972791914795`, 0, 0, 0, 2.163443790390036`, 0.343533459157471`, 0, 0, 241.40028546387168`, 0, 0,
9.166374398285662`, 488.42363898509177`, 0.35352970502126907`, 0.9999999999964051`, 0, 0, 0.48063137018636065`},
{0.14346666218024207`, 3.6942323053195354`, 6.0819497997897844`, 0.8206348775106591`, 0.4021755195313119`,
0.4030089388647059`, 0.4636571249048107`, 744.6939263196219`, 0.08354527905403086`, 5.555269667846608`,
95.98575252739228`, 0, 0, 0, 13.954350785834414`, 1.524418228087694`, 0, 0, 0.018428078760942008`, 0, 0,
80.45078664313618`, 0.03776878500308928`, 1.6328854590147057`, 0.38208456836865523`, 0, 0, 0.60579641065929`},
{0.09149267062932814`, 2.234522341263885`, 6.944559583117852`, 1.114683357002141`, 0.5085103250363343`,
0.6658564606426403`, 0.006987139923383322`, 1505.8338370139704`, 0.21635495075213407`, 1.8879751532316735`,
22.94382407295265`, 0, 0, 0, 20.838291568192695`, 0.06235574681046822`, 0, 0, 0.013852938458385188`, 0, 0,
1.9905044193455068`, 0.018106319080306358`, 0.28139637149892627`, 0.23129667189769`, 0, 0, 0.25895481556777494`},
{0.22068432056467163`, 3.569728941728428`, 4.540468856326312`, 1.2038410371742398`, 0.3148195694928344`,
0.5227498853534928`, 0.10369270254334745`, 4010.634501486441`, 0.16206884164220114`, 3.771999452670874`,
12.326676862690043`, 0, 0, 0, 5.078924093600477`, 0.13893575517307366`, 0, 0, 0.005690317691304539`, 0, 0,
7.0851855183173855`, 0.017939484192903137`, 0.21325828581232759`, 0.0958899685134581`, 0, 0, 0.5615258116188699`},
{0.2097350071754976`, 1.1102722915344074`, 3.321403610485124`, 1.0935329934230222`, 0.7183578411763796`,
0.40562405317483374`, 0.00617694210046558 6`, 446.96867612346506`, 0.08354129713872654`, 7.284441181467236`,
205.63259620740783`, 0, 0, 0, 184.29012616068303`, 0.9455701100311007`, 0, 0, 1.301965601287317`, 0, 0,
14.997718469581024`, 3.900968067546396`, 3.6195028513121805`, 0.9999991114553949`, 0, 0, 0.9124344348098669`},
{0.1539569569971848 7`, 1.7653707587036722`, 8.640858401956272`, 1.0488086821127276`, 0.2563370285663329`,
0.39045184386637677`, 0.03142873441769787`, 98.7771934627432`, 0.2047745102794421`, 8.897923651735105`,
14362.54073416313`, 0, 0, 0, 443.9780791258702`, 7.388530740795001`, 0, 0, 4289.816383574525`, 0, 0,
186.33565885118063`, 9434.958235597227`, 20.013806786423853`, 0.9999999999999994`, 0, 0, 1.11840851797613`},
{0.2495726529131425`, 0.6471319987804813`, 4.234303245586215`, 1.3697781718226154`, 0.5893758025334812`,
0.16113556190203493`, 0.016137549539750166`, 661.2554849181507`, 0.08255896742665592`, 6.142572860377916`,
96.35706052546873`, 0, 0, 0, 78.46778549841127`, 1.7228016551819012`, 0, 0, 0.043999585606304285`, 0, 0,

15.92685826600269`, 0.15687276152633195`, 1.9252331395222115`, 0.4976113518082417`, 0, 0, 0.307653405691378`},
{0.18703143603461198`, 3.5026925577041474`, 3.736145496532833`, 0.8654363814884891`, 0.7031489482980493`,
0.15906583747765657`, 0.008377691039792691`, 131.85234583624222`, 0.16088793676433022`, 8.514675246308464`,
22834.21164833812`, 0, 0, 0, 218.66408445879256`, 0.5029357810874591`, 0, 0, 6152.079611988259`, 0, 0,
25.166134534545247`, 16437.604063277427`, 13.2708273584082`, 1.0000000000005123`, 0, 0, 0.35092599490052223`},
{0.2617533772269884`, 3.6583043975291023`, 1.4938470678500337`, 1.229216471284039`, 0.8448795594977094`,
0.4744971024115313`, 0.027729197093825105`, 163.20786376161018`, 0.21172311156121143`, 7.676981105027913`,
52647.37130250868`, 0, 0, 0, 64.60949989845876`, 0.47484058678741925`, 0, 0, 11089.629715163306`, 0, 0,
24.815877239567328`, 41467.82900201101`, 9.857539681722647`, 1.0000000000004021`, 0, 0, 0.47441407855301654`},
{0.2277950960946843`, 2.381865480846251`, 9.991476447433318`, 0.832979598782657`, 0.5926733949589658`,
0.27276410945547525`, 0.04327492387940349`, 669.5530400360332`, 0.024018555255208573`, 9.681708943673282`,
194.43401795257486`, 0, 0, 0, 121.26258138141593`, 2.0873445013092673`, 0, 0, 0.01226015282985287`, 0, 0,
71.02534020432522`, 0.039897181314510276`, 3.0413147034371812`, 0.2716001225143515`, 0, 0, 0.6356533514728432`},
{0.15963824410704142`, 2.654604852862681`, 7.469701098598773`, 1.491664105533315`, 0.25955793675814864`,
0.27946634303609463`, 0.06966019051655106`, 86.7013584077103`, 0.017186465149371527`, 9.819694766966123`,
432015.65701375157`, 0, 0, 0, 297.93417269011394`, 7.348088052837702`, 0, 0, 131512.15263502498`, 0, 0,
278.66100291893196`, 299919.5589341797`, 24.124187662842356`, 1.0000000000004412`, 0, 0, 0.7777444277594865`},
{0.13626056470386483`, 2.820076398995975`, 2.87943303050835`, 0.9523411928557022`, 0.8935766203190545`,
0.356125796963648`, 0.009331421764352204`, 68.56313381348697`, 0.147099680293568`, 8.670054262776382`,
89913.6527658248`, 0, 0, 0, 174.34072542995494`, 0.5546794277342522`, 0, 0, 30447.597397933245`, 0, 0,
22.346262330885054`, 59268.6687902614`, 26.735693028574303`, 1.000000000000001`, 0, 0, 0.617565759976627`},
{0.22962572899038486`, 1.387681996287987`, 7.569216994527352`, 0.873789488954491`, 0.15662470794012862`,
0.483300190980835`, 0.02173528342644958`, 88.10021701991921`, 0.04718506749066664`, 8.783666403079792`,
163469.5500264975`, 0, 0, 0, 411.1288288304322`, 6.031557456884641`, 0, 0, 38065.1168380581`, 0, 0,
119.56976703564796`, 124867.57432918876`, 21.53379113664193`, 1.0000000000000608`, 0, 0, 0.8284674051571311`},
{0.16187203397799577`, 1.769575035055972`, 1.1614771267268384`, 0.8287067492253789`, 0.7900895875825826`,
0.2962983919698179`, 0.014442973603799043`, 137.47659064397268`, 0.2186302306878819`, 9.341476742915006`,
29236.76291243101`, 0, 0, 0, 70.11789996064272`, 0.5426042686214169`, 0, 0, 8800.820983830052`, 0, 0,
13.716842395246612`, 20351.525618982752`, 14.133004590107186`, 1.0000000000002414`, 0, 0, 0.5028401783971442`},
{0.09685210682031803`, 1.7960713074535244`, 5.69551378974773`, 0.7869885608747933`, 0.9421312080797839`,
0.5036667090451988`, 0.0169704552746280 2`, 52.99769310364423`, 0.21425647446502405`, 9.869621253243459`,

55208.485035568345`, 0, 0, 0, 373.77954444001483`, 3.338928721786358`, 0, 0, 22967.56031258561`, 0, 0,
85.67077249761334`, 31777.951497094753`, 41.04548596216295`, 1.000000000000003`, 0, 0, 1.1519801623080321`},
{0.16013522976515993`, 1.0007721789830901`, 6.380859482894074`, 1.440495462142086`, 0.5398127028451485`,
0.19075782788209084`, 0.04652110166049526`, 84.8416541425776`, 0.16547472912452094`, 6.4422585881915015`,
47027.6331692644`, 0, 0, 0, 194.68541143606416`, 8.197104778590825`, 0, 0, 14206.988330026172`, 0, 0,
117.19192015175778`, 32500.562007138244`, 15.812702103507656`, 1.000000000000001`, 0, 0, 0.35505881457246236`},
{0.1912199176775608`, 2.2121001603415005`, 9.917361836602723`, 1.435922264503217`, 0.9790995032802718`,
0.6767636439858111`, 0.07238714810610002`, 269.421010262202`, 0.01560756507054070 2`, 2.5093255880098546`,
208.89881766280894`, 0, 0, 0, 101.99095719511912`, 3.1697903099670017`, 0, 0, 0.9560378368950885`, 0, 0,
100.16990932752765`, 2.611621092396032`, 2.0602893672280396`, 0.9999718786712853`, 0, 0, 0.30310644831137346`},
{0.24179572266521915`, 2.5600514990256196`, 5.593167080478407`, 1.0641609773948193`, 0.6297315486340287`,
0.26623731831221253`, 0.08310036534342866`, 636.0105501064265`, 0.05032454414330245`, 1.4627567207550134`,
30.402343053601534`, 0, 0, 0, 14.09138579167949`, 0.43009314564244494`, 0, 0, 0.033989965838497276`, 0, 0,
15.729437174608345`, 0.11740897647551443`, 0.4856820426414173`, 0.4914017623060243`, 0, 0, 0.10233105609565801`},
{0.20288510482307215`, 0.7288550347693712`, 9.019074578521877`, 1.2840716127441223`, 0.5874481378002983`,
0.6980893050148065`, 0.008225735950864725`, 1276.121996072157`, 0.1607915330045036`, 9.69181498105582`,
166.12595428875255`, 0, 0, 0, 148.64669031657846`, 1.5137483139833343`, 0, 0, 0.017678099402377915`, 0, 0,
15.76147257172002`, 0.051237472147459134`, 1.6666229904361092`, 0.2557661930715356`, 0, 0, 1.0840439236266957`},
{0.1862496077639137`, 1.6905827743030857`, 2.538407600056317`, 0.9208769830081895`, 0.38109944279089136`,
0.3320011945053185`, 0.011054373793833769`, 1065.4164452217328`, 0.07943719416592698`, 4.231039339853934`,
45.055776493899415`, 0, 0, 0, 38.88907297895234`, 0.23891577540419542`, 0, 0, 0.0353747161642397`, 0, 0,
5.770098491537104`, 0.09412181443356371`, 0.8652557027839695`, 0.5562533761787571`, 0, 0, 0.4397061137083344`},
{0.05689751321611025`, 3.0250332600067678`, 2.529462845718742`, 1.466915953431471`, 0.22269501402715108`,
0.6695459886571014`, 0.14207393160159743`, 917.9363525294286`, 0.02546682059293326`, 3.574551553390743`,
53.95020469379218`, 0, 0, 0, 18.32259539883518`, 0.8015716525159937`, 0, 0, 0.1027729251244113`, 0, 0,
34.639727273420995`, 0.08353605522185008`, 0.9281772354583665`, 0.7487557855632081`, 0, 0, 0.7422870861411103`},
{0.1967142498798619`, 3.0620830451218577`, 7.9498840213828466`, 1.4844762448061533`, 0.6042217303737281`,
0.6207898959804532`, 0.0334891282473782`, 576.7285784287807`, 0.16199601914875666`, 9.348325121692032`,
341.0713575055191`, 0, 0, 0, 231.6265489181281`, 2.439739120022417`, 0, 0, 0.06644094687830014`, 0, 0,
106.72405419915941`, 0.18671258609253605`, 3.527711677656057`, 0.6145430616184154`, 0, 0, 0.9599710603191095`},
{0.2643422576069476`, 2.569764850665571`, 5.652188171834926`, 0.8392687504503539`, 0.3834838466329171`,

0.17354269300981617`, 0.03414034179009787`, 716.202171206297`, 0.028057924329779993`, 1.7997126741152787`,
21.329250289769735`, 0, 0, 0, 14.46423143368313`, 0.18036247198599592`, 0, 0, 0.012923636836045893`, 0, 0,
6.621273441268088`, 0.04880376196763115`, 0.5297078431352612`, 0.2810720884802882`, 0, 0, 0.12246872518103848`},
{0.19233187457454282`, 2.1451040206615035`, 4.677354160755579`, 0.8253029687642099`, 0.9809911268657268`,
0.22566854695954142`, 0.015470928716805988`, 111.90002994139898`, 0.12242650367871766`, 3.4296984602053584`,
34044.88615793034`, 0, 0, 0, 102.88466272570214`, 0.7040496990334464`, 0, 0, 9051.042235393568`, 0,
0, 21.575140573459542`, 24868.627428379958`, 6.397761857387997`, 1.`, 0, 0, 0.19757757407967005`},
{0.21830260718528288`, 1.0813728060954029`, 9.236753498274854`, 1.3292857287875703`, 0.7084275620345744`,
0.4530838967889582`, 0.022838882777727113`, 80.81382094438914`, 0.07293079158751065`, 5.841787640828064`,
128890.30204091553`, 0, 0, 0, 333.212858742036`, 6.48300512448861`, 0, 0, 31187.803852216497`, 0, 0,
100.15064919141612`, 97262.55561888675`, 15.770634688955711`, 1.0000000000001597`, 0, 0, 0.6156042831168366`},
{0.27637635892023465`, 3.838779152844408`, 0.15938000205200176`, 1.3392032462951913`, 0.02123906289427646`,
0.6059474038465393`, 0.007164747514275051`, 100.75472745890974`, 0.10946913978259332`, 8.952098728322259`,
231916.74584535134`, 0, 0, 0, 10.184564070349696`, 0.018522214618547757`, 0, 0, 46866.32264154167`, 0, 0,
1.015752733459872`, 185039.19439500396`, 18.689642474347835`, 1.0000000000000935`, 0, 0, 0.5887898962821706`},
{0.15064788979833238`, 0.9109936464272579`, 0.5800958535668599`, 1.4999646884179314`, 0.9779856120080317`,
0.34948818201364285`, 0.011067508252105101`, 2641.6705923333384`, 0.011474828296826944`, 3.667140614388831`,
20.30259376241613`, 0, 0, 0, 15.224711251918981`, 0.1682552867877102`, 0, 0, 0.8593262228152295`, 0, 0,
2.189707103448573`, 1.849366887364084`, 0.3104284428353334`, 0.9999946752072961`, 0, 0, 0.48777780870995335`},
{0.0419185722234717`, 1.709255049022068`, 2.3539768873704308`, 1.3792050115017793`, 0.22715212217109637`,
0.2926929451571887`, 0.00505223249889438`, 88.91455127714036`, 0.1107140298924858`, 2.282618721837757`,
70255.40262284165`, 0, 0, 0, 40.81629223213463`, 0.11314806457043461`, 0, 0, 43912.577666431214`, 0, 0,
2.762841437915573`, 26296.46512041589`, 5.596329491971364`, 1.0000000000000846`, 0, 0, 0.23852991542372498`},
{0.24924491240080032`, 1.373280333190027`, 8.919496594562187`, 1.4037493221091317`, 0.24632944507518628`,
0.3548949637369072`, 0.18998328780525742`, 2947.236236962295`, 0.1153856006596437`, 7.477168052601716`,
26.53115667188809`, 0, 0, 0, 7.529858291801952`, 0.920778822750132`, 0, 0, 0.0035988674005294373`, 0, 0,
18.064106407151744`, 0.012814276999812177`, 0.5601649695263842`, 0.05363466200215994`, 0, 0, 0.8875869994780539`},
{0.20687871564233656`, 0.44181655503120965`, 8.570924850704476`, 1.0055678634725929`, 0.5549096280819519`,
0.326726210951258`, 0.20129490353773463`, 306.54002765810475`, 0.1699967418388691`, 1.7003969931174137`,
74.07810128302826`, 0, 0, 0, 19.92331411155454`, 7.377705921149648`, 0, 0, 0.053464105220149334`, 0, 0,
46.565608773081294`, 0.1580083631559221`, 1.1878526509842307`, 0.673979387240365`, 0, 0, 0.1444998893281906`},

{0.20196179343132148`, 2.101313615300616`, 3.7695608295023266`, 0.8255643571631567`, 0.9698552359083252`, 0.3038610819528902`, 0.02519738143348337`, 88.57713238465558`, 0.03933307441687739`, 1.9858938348907018`, 218759.52169416408`, 0, 0, 0, 43.19072416120458`, 0.49271271675191297`, 0, 0, 56291.26219253943`, 0, 0, 14.790627716322149`, 162409.77524168516`, 4.682919920048927`, 1.000000000000478`, 0, 0, 0.11575756301601851`},
{0.10010100507466935`, 2.4192735538683836`, 9.823330355933038`, 1.1133108806457672`, 0.011014651746178439`, 0.5998442212295849`, 0.013751847561218578`, 3832.28466694177`, 0.1060300423959204`, 2.751621101527464`, 7.718266370888943`, 0, 0, 0, 6.469187209468058`, 0.03487982926791932`, 0, 0, 0.0020293271485728303`, 0, 0, 1.2054835501617376`, 0.002901966959964656`, 0.173075892169175`, 0.03825695374211602`, 0, 0, 0.6089777442105473`},
{0.21311625184066546`, 1.4247782113917893`, 5.690068626185788`, 0.8615233428628426`, 0.12086241410351461`, 0.29764577871833753`, 0.005395932799405541`, 882.4049045614522`, 0.2481341739836153`, 1.9572384227218778`, 18.721375600773758`, 0, 0, 0, 17.35827413912524`, 0.06097490765173577`, 0, 0, 0.01025996844119122`, 0, 0, 1.2410817123402813`, 0.031236657402714522`, 0.49301127510035087`, 0.2247398732313297`, 0, 0, 0.24694178572782563`},
{0.09607643000919447`, 2.545123365745101`, 1.6292494594570959`, 0.9689766494611143`, 0.9239031581606687`, 0.5340288721519064`, 0.06266594590032853`, 79.64776165873695`, 0.19537727971057256`, 3.908080115643834`, 57144.5511487094`, 0, 0, 0, 28.06069446946635`, 0.6551528323044373`, 0, 0, 24063.859819651312`, 0, 0, 23.82063973760139`, 33028.13919591121`, 10.834184689154943`, 1.0000000000002474`, 0, 0, 0.4640405945096687`},
{0.25410295729391263`, 0.5246172329407881`, 1.519575137014602`, 1.3035504391628365`, 0.641505202173666`, 0.18662868599370885`, 0.02992528952684035`, 1308.2457641643925`, 0.1567967571574565`, 7.528281484333206`, 68.38848774654484`, 0, 0, 0, 47.943377827407296`, 2.353200970285775`, 0, 0, 0.0966887550867633`, 0, 0, 17.636139736927166`, 0.3509842657801947`, 1.1964761275982982`, 0.7876247990839995`, 0, 0, 0.40249496795844947`},
{0.20247384111644234`, 3.9381206949664582`, 3.525512124148932`, 0.8160399146038965`, 0.003770588899372651`, 0.4372678593782203`, 0.005846407125549171`, 81.38331905868344`, 0.05740074457617078`, 6.792038617151225`, 201901.8125728869`, 0, 0, 0, 174.66950935384705`, 0.2518855596529265`, 0, 0, 51821.037581786135`, 0, 0, 14.170796217605705`, 149891.49328319664`, 17.63777785112771`, 1.0000000000000917`, 0, 0, 0.48076062694864474`},
{0.15622647656935051`, 2.478038073204126`, 4.93355773626655`, 1.0681326166577831`, 0.57283306272417`, 0.24473580012035412`, 0.09417540555806864`, 340.729792091258`, 0.1606536419587833`, 9.486285960325056`, 347.4636591568219`, 0, 0, 0, 151.16691938236076`, 5.373846588014266`, 0, 0, 0.2121671082459761`, 0, 0, 190.23709206653447`, 0.4735159966453461`, 5.852471491570005`, 0.9769046165582984`, 0, 0, 0.6172117732844979`},
{0.2472522705346833`, 1.1701552284997945`, 3.914120438755436`, 1.4973295978551664`, 0.1631737194969931`, 0.4864210576866177`, 0.39314364156002357`, 172.17497479862377`, 0.10619847023829598`, 4.473646521439088`, 63299.723146061595`, 0, 0, 0, 22.872887614750162`, 6.696724915020107`, 0, 0, 13935.51741085898`, 0, 0,

111.94582390193754`, 49222.690298721005`, 5.616904327480769`, 1.0000000000070322`, 0, 0, 0.42627797586870914`},
{0.1494891985884202`, 3.3979182405498527`, 4.585626936341594`, 1.2118171179612203`, 0.6586829775426448`,
0.36431745127605797`, 0.33277789805879787`, 51.20287439819706`, 0.10641801334172246`, 7.126742388333813`,
213930.34361840397`, 0, 0, 0, 47.57694881685964`, 4.254058174500717`, 0, 0, 68144.76979351569`, 0, 0,
206.49916953566088`, 145527.24320607205`, 29.420016490471124`, 1.0000000000331681`, 0, 0, 0.5056306207189001`},
{0.2014310029114028`, 3.9869430882246055`, 0.7136208449188892`, 1.3208947167282403`, 0.46844054682093383`,
0.39516715199771224`, 0.14154388607116256`, 2748.591928872397`, 0.05354911946309293`, 6.211900336444712`,
31.788658869757267`, 0, 0, 0, 10.657059879675359`, 0.35482868777391824`, 0, 0, 0.1462328397786430l`, 0, 0,
20.209739774628986`, 0.420797536788503`, 0.49947122723703136`, 0.8724252628266254`, 0, 0, 0.7438354229766668`},
{0.13228384635594964`, 2.6408616938406997`, 3.2993392931538836`, 0.952940068464383`, 0.8367144819205878`,
0.6111943245350302`, 0.13146835864980566`, 2246.7842161758563`, 0.024436379332296754`, 9.73655688789367`,
147.94801617198212`, 0, 0, 0, 51.949607417873466`, 2.4756806508753795`, 0, 0, 0.042814355218300924`, 0, 0,
93.3990028154198`, 0.08090925125033074`, 0.9061167900452572`, 0.6145463557655078`, 0, 0, 0.578410884412367`},
{0.23113058156717692`, 2.2605125411397546`, 4.002625660970853`, 1.3839850516092966`, 0.507564008077771`,
0.4982541689482688`, 0.0228570122346136`, 138.3250229234806`, 0.07006503343759235`, 8.064244271550876`,
137295.50474742881`, 0, 0, 0, 195.74584601858857`, 1.8918096938838496`, 0, 0, 31855.184850235793`, 0, 0,
61.09227912106`, 105181.53429092707`, 12.499286470668954`, 1.0000000000000084`, 0, 0, 0.6958404411750952`},
{0.17570245022422137`, 3.187809299248391`, 9.693328941627644`, 1.4591302419173326`, 0.2844948752823342`,
0.5066677812093952`, 0.2998214113578054`, 349.3700788988757`, 0.18995614940943706`, 5.5658132930392785`,
202.53782306525252`, 0, 0, 0, 41.07142374538911`, 3.465796575574633`, 0, 0, 0.047796196554136174`, 0, 0,
157.83283647028728`, 0.11997012637058864`, 3.6035267605028602`, 0.5030884043840982`, 0, 0, 0.812939813038693`},
{0.2497541467888837`, 1.085030464715203`, 1.0043906551890043`, 0.864213346242746`, 0.8846254264305906`,
0.601718469375484`, 0.012654582561683364`, 62.423429857566624`, 0.12690014020771856`, 6.991109163451483`,
187560.33432596203`, 0, 0, 0, 47.11209169611373`, 0.5114201149434897`, 0, 0, 41048.20176668444`, 0,
0, 7.92723435688339`, 146456.55156366044`, 23.696543146107683`, 1.`, 0, 0, 0.5036035005808313`},
{0.22558722869536918`, 1.12607467611575`, 0.7911165205665469`, 1.2733751455833207`, 0.6009341950827205`,
0.6641796486046712`, 0.175358669032309`, 3424.3638773504586`, 0.14969161390071423`, 8.91095466688921`,
57.22783854061539`, 0, 0, 0, 16.43487670169562`, 2.325948836409714`, 0, 0, 0.24865359045226107`, 0, 0,
37.41702975174107`, 0.8013296339325525`, 0.5821637459340423`, 0.9569931237793909`, 0, 0, 1.1896505459277373`},
{0.050627269144448594`, 3.276563036391985`, 7.788230793264948`, 0.8177022370463224`, 0.523932893406847`,
0.34959907144360225`, 0.11342446098213065`, 1447.0777642274236`, 0.2385428599947077`, 6.8056995790460695`,

64.71978671862897`, 0, 0, 0, 25.303502878784084`, 0.8242228767588446`, 0, 0, 0.006845788212932835`, 0, 0, 38.5802601676671`, 0.00495119374800163`, 1.0070808144581005`, 0.16479355125170647`, 0, 0, 0.5780766391722235`},
{0.060006217186970356`, 3.9647259629353027`, 0.34014645604634985`, 0.9146780185619144`, 0.03431401900644282`, 0.5737721437004861`, 0.026766328146322068`, 169.17748604048344`, 0.08721098377832243`, 3.968142168145161`, 51646.57184929879`, 0, 0, 0, 8.315211731129057`, 0.05398393427736835`, 0, 0, 27802.00939124087`, 0, 0, 3.0575929401553594`, 23832.763053785588`, 5.318433214578619`, 1.0000000000001288`, 0, 0, 0.5906375843184641`},
{0.15350947465095238`, 2.9043684969368213`, 4.5670292387828155`, 1.3486905179026798`, 0.8383532909017835`, 0.5087310077763043`, 0.053857845014854355`, 402.8647291760491`, 0.025030223476360525`, 5.400937139619534`, 208.85862250688703`, 0, 0, 0, 117.19885746613353`, 2.068217992472714`, 0, 0, 1.182741236191996`, 0, 0, 85.81238831622383`, 2.5937426545121385`, 2.989216626187583`, 0.9999925367114894`, 0, 0, 0.7005959257596613`},
{0.2587691832069661`, 1.4843472048382624`, 4.771777259704052`, 1.1550607981645458`, 0.08010207781888057`, 0.5149452814008634`, 0.01659115431008023`, 1531.656314816295`, 0.15778107864815993`, 1.1951975901128993`, 8.1238810035257`, 0, 0, 0, 6.554559005034133`, 0.06815470789790856`, 0, 0, 0.011256328214981475`, 0, 0, 1.4452178594975522`, 0.041611297972863726`, 0.1852413343438131`, 0.18488799067050732`, 0, 0, 0.24234777909679678`},
{0.1547206763399963`, 3.3076918921708103`, 2.0706333626337727`, 1.4168143839445906`, 0.37770487420755305`, 0.5386802863405199`, 0.00701518941055165`, 122.30089778268855`, 0.134914758355765`, 2.269217335741727`, 87748.79668642554`, 0, 0, 0, 34.71290293126618`, 0.07115864719453859`, 0, 0, 27321.665258962224`, 0, 0, 3.3624411483316954`, 60388.95039430905`, 4.032860276088764`, 1.0000000000000004`, 0, 0, 0.22977633343481327`},
{0.1424711737630725`, 2.7520949793854754`, 4.596768650339829`, 0.8362726667059688`, 0.00009639230672808807`, 0.36579283504807614`, 0.010146114868681232`, 3412.101956455065`, 0.24682488221918303`, 8.269337606653004`, 21.123256610483015`, 0, 0, 0, 18.49327890745746`, 0.06465067228689285`, 0, 0, 0.0030292274750199043`, 0, 0, 2.5417827230664822`, 0.006165394199446895`, 0.5508532876716289`, 0.07491316965601957`, 0, 0, 1.2538048030851756`},
{0.10882832688629612`, 2.4842578785622536`, 4.2235021418772565`, 1.4458161330640105`, 0.36880250968204553`, 0.4736058884190225`, 0.023130853597073738`, 1306.5861668001219`, 0.20634842182153856`, 4.016571058814693`, 40.54404240459218`, 0, 0, 0, 30.581178343460607`, 0.27098167095373243`, 0, 0, 0.025994564648364176`, 0, 0, 9.616976443039643`, 0.040413499697425255`, 0.687047942331`, 0.3171667318637328`, 0, 0, 0.5317114515826485`},
{0.18639271747297592`, 2.4942906430499407`, 3.445345566170081`, 1.148752163319608`, 0.12885074455456724`, 0.17966201189701259`, 0.0741834712240346`, 1169.2351269821615`, 0.09106655178874168`, 4.423161126411237`, 27.641819441051528`, 0, 0, 0, 13.794438796240481`, 0.3765094501475352`, 0, 0, 0.014949435113730923`, 0, 0, 13.416057121755346`, 0.03980665479332093`, 0.808434380414435`, 0.24348933401805084`, 0, 0, 0.36610491222599256`},
{0.09766181155973352`, 3.6268090588227118`, 4.041820185573664`, 1.3253596170020208`, 0.4793934216010949`,

0.49032555462271876`, 0.006759043878711846`, 262.0748117860937`, 0.11178249583803845`, 2.0681460387230555`,
1470.4489382075433`, 0, 0, 0, 67.80350216838771`, 0.12071041243812188`, 0, 0, 582.9239965942276`, 0, 0,
6.254194533211518`, 813.277335843322`, 1.882401285500198`, 0.9999999999997817`, 0, 0, 0.4313924395028549`},
{0.07951891239009712`, 2.8897027315487103`, 1.5411848578052467`, 0.863635998632232`, 0.29085460372381067`,
0.43469992564602655`, 0.009145988318489378`, 180.1504573795744`, 0.0803419958474173`, 8.628584719983632`,
41939.599247497405`, 0, 0, 0, 96.61599165662427`, 0.29315710944773204`, 0, 0, 19583.689335845575`, 0, 0,
12.101955713486229`, 22246.766808171247`, 10.516603353420026`, 1.0000000000000193`, 0, 0, 0.9705745295865424`},
{0.07234637915492992`, 1.3584521644778373`, 7.973775020047323`, 0.7807691250526536`, 0.9100932527758132`,
0.18771974645581058`, 0.009648266694560257`, 1956.923094358971`, 0.1277442569760165`, 5.404137919151016`,
45.78956320934368`, 0, 0, 0, 40.27251661763253`, 0.26820004690719834`, 0, 0, 0.00561984951932333`, 0, 0,
5.204813346202008`, 0.005808225201693241`, 0.5641052555458007`, 0.14355025235689023`, 0, 0, 0.18900913336922961`},
{0.26334607729010473`, 1.8094502738990368`, 2.2460302448509974`, 1.2023222076508349`, 0.5513938303777435`,
0.6812297944975665`, 0.00739497505047526`, 1157.7965975362908`, 0.15524857884582421`, 1.7991882609973402`,
31.848849297753326`, 0, 0, 0, 28.09754757298095`, 0.10918509025011523`, 0, 0, 0.16642839473302196`, 0, 0,
2.8223570208394593`, 0.6261180700375718`, 0.35596149311936565`, 0.9063923399512008`, 0, 0, 0.2889421686136274`},
{0.22679920860671537`, 1.574644711817462`, 3.814781873556772`, 0.9990271813793515`, 0.3871644432916528`,
0.6894928310515707`, 0.054622933309625116`, 69.61156300085906`, 0.09745707491156125`, 5.357011938436007`,
210478.88519243314`, 0, 0, 0, 93.37590625530791`, 3.0535671509361575`, 0, 0, 49602.43507506206`, 0,
0, 68.68976237715918`, 160711.32885700068`, 16.625754689537448`, 1.`, 0, 0, 0.5068683718005674`},
{0.054135826493752204`, 3.1195801041969027`, 7.2615169638158825`, 1.0984819310653413`, 0.10801447255588448`,
0.6366822484935735`, 0.01066160509264612`, 211.97043506851273`, 0.24501368432397974`, 8.92838638172081`,
517.0449016496841`, 0, 0, 0, 448.98049096578944`, 1.4697413175523817`, 0, 0, 0.4287528333326963`, 0, 0,
65.49965389360777`, 0.3315841284857715`, 10.039874474639161`, 0.9948179346369059`, 0, 0, 1.8542735254217175`},
{0.14590720393667378`, 1.5766246964498798`, 5.158062459855282`, 1.1478320866243528`, 0.3287329300123256`,
0.4460050972404973`, 0.032458645789603134`, 482.4725013461066`, 0.10279090509715616`, 8.020019312365584`,
207.5348979371129`, 0, 0, 0, 142.7693337532617`, 2.744587346216255`, 0, 0, 0.060042583501342395`, 0, 0,
61.81691702297697`, 0.12515207822584565`, 3.7008519378375038`, 0.670950040392353`, 0, 0, 1.0268144326021957`},
{0.14965591009740292`, 1.460367057362724`, 7.337474105676788`, 0.8826427720923014`, 0.44478408083773613`,
0.631222883334907`, 0.365159753458356`, 157.97713196457462`, 0.16784289093699323`, 1.7957284623930878`,
1663.810184214276`, 0, 0, 0, 21.61101656584831`, 4.536729165085953`, 0, 0, 491.72854308884166`, 0, 0,
94.64699744097449`, 1051.2868948118705`, 2.799520016159948`, 1.0000000000294`, 0, 0, 0.44505626331488635`},

```
{0.0810328468371882`, 1.4659479028658335`, 3.234238812997848`, 1.4263751048195057`, 0.08355223050311089`,
  0.5582345497973803`, 0.12450549955799516`, 121.80826494117302`, 0.16010939682803116`, 9.339681585379608`,
  50113.962035273005`, 0, 0, 0, 93.96404491669944`, 7.301620699239646`, 0, 0, 23108.756349040952`, 0, 0,
  152.9113650224588`, 26750.975911853486`, 17.275201949111285`, 1.000000000000055`, 0, 0, 1.321914454940688`},
{0.15633465648304357`, 2.148716979150252`, 1.5640986685796054`, 1.3949237088061017`, 0.3567264406830608`,
  0.23012843132782612`, 0.08829396925964683`, 2593.62904544209`, 0.24948663022889017`, 4.657794257043932`,
  17.211142276573653`, 0, 0, 0, 7.784357935792154`, 0.2948997926295885`, 0, 0, 0.024631745635078115`, 0, 0,
  9.05223130815835`, 0.05501136417767677`, 0.3844577919524369`, 0.31254119319872775`, 0, 0, 0.3943862841890921 6`},
{0.24418896051468997`, 0.7850580710666257`, 9.594718039690303`, 1.3796447707658`, 0.3888924613344984`,
  0.22950540339413805`, 0.26843428081063014`, 169.97373853516677`, 0.16535615066521753`, 5.752488029735448`,
  334.8105392571657`, 0, 0, 0, 74.71862856394286`, 21.25259469548302`, 0, 0, 0.10895075464519142`, 0, 0,
  238.3502999542387`, 0.38006530748705736`, 7.19622826380652`, 0.81307792198309`, 0, 0, 0.44490573345677314`},
{0.161324838944309`, 0.8594526582032156`, 2.9743924517148237`, 1.2482811580906663`, 0.28485670242217753`,
  0.27691573296739147`, 0.12631097975241765`, 80.56658004023716`, 0.24230111347476613`, 7.982613747504004`,
  60463.398414618045`, 0, 0, 0, 67.39440710736584`, 8.838201347173214`, 0, 0, 18240.607479970007`, 0,
  0, 108.5145091651895`, 42038.04377075036`, 20.610770072639117`, 1.`, 0, 0, 0.4307851549407836`},
{0.26772168843838734`, 1.7983273472260084`, 5.340026177070573`, 1.4142901992580086`, 0.4537298076184495`,
  0.2865003384437863`, 0.010579448173665554`, 2804.399167772796`, 0.15280115052346555`, 9.9218473198287`,
  41.42050852053327`, 0, 0, 0, 36.02506154489168`, 0.19981106415955072`, 0, 0, 0.00732423496268786`, 0, 0,
  5.133224299377865`, 0.028012236439007115`, 0.7552739836201043`, 0.10535865963210866`, 0, 0, 0.8098073190982712`},
{0.18205384870834052`, 1.3062696845921566`, 5.075170850216699`, 0.8732096494236974`, 0.8323770222253974`,
  0.3345416157316672`, 0.015614629383397784`, 819.3644184370967`, 0.04873823503517111`, 3.447951588629733`,
  90.28689949452875`, 0, 0, 0, 73.80719436253649`, 0.8275020348292288`, 0, 0, 0.047962595162604406`, 0, 0,
  15.442011743367763`, 0.12473964347703752`, 0.8733481116044234`, 0.6873516025088179`, 0, 0, 0.1777062749558821`},
{0.15466361671507556`, 2.9485738979940246`, 0.2621130142172472`, 1.4016672383633995`, 0.3670414989298545`,
  0.40252781093021794`, 0.06082671195258038`, 80.30981146445014`, 0.15720245773080782`, 9.014567082907547`,
  137194.02505755573`, 0, 0, 0, 10.05548881632298`, 0.1985564589204616`, 0, 0, 42740.69220239166`, 0, 0,
  8.363691315014227`, 94434.71481325316`, 23.646104115945164`, 1.0000000000000278`, 0, 0, 0.607104691956466`},
{0.07129651605023579`, 1.2229228380041173`, 7.261778729610821`, 1.077075564756948`, 0.8974689473036932`,
  0.5583016490383189`, 0.012773781923945723`, 355.9690714870232`, 0.15455215759656415`, 2.741649121941279`,
  162.51193480243643`, 0, 0, 0, 136.64256135478362`, 1.3334672559830523`, 0, 0, 0.5710795278336147`, 0, 0,
```

```
23.296108015319348`, 0.5816568674593063`, 1.6732530410986546`, 0.9996866757969151`, 0, 0, 0.27619096530157305`},
{0.11901300846945845`, 0.5315793015902206`, 2.6671039936143597`, 1.3880864279120932`, 0.8420811291146328`,
 0.5566538571030129`, 0.2079188053795858`, 171.97845991450583`, 0.13095338568755877`, 2.9760745568663016`,
 44126.950750833035`, 0, 0, 0, 17.37644146358779`, 5.678267687605614`, 0, 0, 16317.682025119371`, 0, 0,
 43.12070816599573`, 27743.09184367804`, 3.8617966118017297`, 1.0000000000009217`, 0, 0, 0.388931004135622`},
{0.23041310070356935`, 3.6790901828404454`, 2.4540732736358937`, 1.23507526691096`, 0.821599531342406`,
 0.33616875277136504`, 0.035023029642451846`, 3607.9630264867633`, 0.21590997641376625`, 4.226227139273737`,
 24.95325756361989`, 0, 0, 0, 16.647604714070773`, 0.15325394185737695`, 0, 0, 0.022333242185996798`, 0, 0,
 8.054786756701088`, 0.07351245115485214`, 0.24369361127118797`, 0.3189581637108555`, 0, 0, 0.22857639418276876`},
{0.07203443304476603`, 0.5285696054477942`, 8.334423467085383`, 0.8854719875782417`, 0.3459640631982801`,
 0.2633909150103768`, 0.03987288196821301`, 568.5739309534505`, 0.056948467212013576`, 6.873946140568169`,
 121.32092100878576`, 0, 0, 0, 78.18601442173417`, 5.040444419553298`, 0, 0, 0.013859775662848373`, 0, 0,
 38.060367401783054`, 0.014262586885741294`, 2.595903677996945`, 0.2858913518481274`, 0, 0, 0.6042671977926947`},
{0.16979941581750402`, 2.100179291019132`, 5.184869462011486`, 1.1997264241396737`, 0.9307785088479614`,
 0.3430074594655337`, 0.013577650230569586`, 4088.01556659017`, 0.029357726641530413`, 9.133680945256565`,
 61.4034768838668`, 0, 0, 0, 51.44631265924629`, 0.3190029448264792`, 0, 0, 0.010720595780559873`, 0, 0,
 9.570905407124151`, 0.026005012867924754`, 0.4582510414917612`, 0.1746742451163822`, 0, 0, 0.357895174020099`},
{0.21619782984172958`, 1.7733623067535955`, 0.5980153626590834`, 0.9798687198034648`, 0.7538483940425484`,
 0.5789929247394512`, 0.006264547120133815`, 61.29286164809138`, 0.15848957716004353`, 2.3238471342304834`,
 153388.4206672678`, 0, 0, 0, 10.163243997014836`, 0.03420582691301811`, 0, 0, 37513.96096594923`,
 0, 0, 0.8665617731269122`, 115863.3849943653`, 8.077633797354013`, 1.`, 0, 0, 0.1846663980593181`},
{0.04816719755022911`, 1.0519290863610147`, 3.0181083809828984`, 0.7801564927089879`, 0.4325460609784135`,
 0.2938523943485086`, 0.04249246462941157`, 626.2418769059092`, 0.2424260119832079`, 9.623530958234195`,
 177.58049556924993`, 0, 0, 0, 111.64333174177102`, 4.10695093964694`, 0, 0, 0.06287498330920893`, 0, 0,
 61.71744499531883`, 0.043264453457440094`, 3.293773275042274`, 0.8179156950240566`, 0, 0, 0.8274760829463038`},
{0.2279802782152826`, 2.528677960359964`, 2.539878173580604`, 0.7527299875368043`, 0.8436281080694634`,
 0.27514078202436143`, 0.005327161585143342`, 920.5142066248217`, 0.04689927589992776`, 5.404864622946203`,
 106.74849217596841`, 0, 0, 0, 98.27228643520625`, 0.19895377884495197`, 0, 0, 0.2299817977769493`, 0, 0,
 7.187000509936569`, 0.7490187748805713`, 1.21969261815306`, 0.9929501622576247`, 0, 0, 0.2837069174699126`},
{0.18595171467721877`, 2.938499684754029`, 3.6306522502244434`, 1.1726699653227697`, 0.9888859131507246`,
 0.3480894711653968`, 0.38543337220237905`, 80.27026890715871`, 0.03443629355490174`, 9.328937618149844`,
```

439769.12189084693`, 0, 0, 0, 43.37353810877927`, 5.165971824180637`, 0, 0, 120199.47042192846`, 0,
0, 216.86009395432828`, 319304.2518321612`, 24.346574113603307`, 1.`, 0, 0, 0.5728797095607162`},
{0.2088003110428162`, 1.665360213541189`, 8.163801498355127`, 1.2665930603692175`, 0.5382347880039544`,
0.33255923205592086`, 0.03591580004997877`, 88.64295388071032`, 0.08847100949056225`, 8.845547783450662`,
75417.26309473606`, 0, 0, 0, 385.9628193699226`, 7.789382385142075`, 0, 0, 18790.046948370713`, 0,
0, 185.31610731820322`, 56048.10924755633`, 21.41128498718074`, 1.`, 0, 0, 0.7707805923164529`},
{0.21556335034608287`, 3.553132086531601`, 5.919169032409666`, 1.028261766481214`, 0.01873638959650825`,
0.38505243852661264`, 0.1200897077765831 1`, 72.3007614977653`, 0.1189730390969882`, 5.747570490343056`,
115832.83713928123`, 0, 0, 0, 100.79158600590245`, 3.2380519640350864`, 0, 0, 28328.254617798597`, 0, 0,
164.36037616099662`, 87236.19249813685`, 16.76799906638972`, 1.000000000001483`, 0, 0, 0.39494762468019895`},
{0.2061330284551588`, 3.7571620320677477`, 5.463889035312752`, 1.195880973038654`, 0.4606689625278244`,
0.6539206160067124`, 0.07294517832285098`, 79.70328938032284`, 0.04960591713533524`, 2.0731473047959454`,
326312.9778560789`, 0, 0, 0, 45.93079411421758`, 0.8568811943128917`, 0, 0, 82697.14776171172`, 0, 0,
45.99202127521791`, 243523.0501817906`, 5.69846732734016`, 1.0000000000000213`, 0, 0, 0.2280647711096057`},
{0.13099744861910317`, 3.0069806711745155`, 9.6713740346189`, 0.7856145676036781`, 0.3887426527541791`,
0.16465925249784186`, 0.029006890169876405`, 3080.757337617379`, 0.22037025279153283`, 7.1587428033756435`,
20.28849897556103`, 0, 0, 0, 14.479404703592769`, 0.1320011672267636`, 0, 0, 0.001474164823042568`, 0, 0,
5.670356548905044`, 0.002758740438034927`, 0.48640347535316536`, 0.03962038146282865`, 0, 0, 0.4336119827347677`},
{0.20740821458680525`, 2.460110895835281`, 0.6082454159142117`, 0.8064214695794831`, 0.18139487742338534`,
0.30057404623847583`, 0.2202140246268176`, 4056.9433443728003`, 0.07208592746284093`, 2.251460510548089`,
5.154319536228488`, 0, 0, 0, 1.3092926534853337`, 0.10363027069279064`, 0, 0, 0.025074257172343697`, 0, 0,
3.6420279724242106`, 0.07429438446009008`, 0.12454185628995643`, 0.46944406974608177`, 0, 0, 0.30851971613096696`},
{0.20705709543566436`, 1.1783515457685674`, 5.677465659011105`, 1.0594933834891802`, 0.45712361519708566`,
0.4929063565541889`, 0.07712216778297526`, 129.94158279886685`, 0.033100299542402456`, 5.957665662265306`,
149139.72924688712`, 0, 0, 0, 133.96327144298098`, 8.035921990346752`, 0, 0, 37610.91884023905`, 0,
0, 135.2734442714388`, 111251.53731037713`, 10.057200863812247`, 1.`, 0, 0, 0.6637005727324241`},
{0.04662251177018917`, 1.087376542683649`, 6.170097112149202`, 0.7648803529340509`, 0.9868309112238562`,
0.551889656411283`, 0.011192508637080182`, 2374.3826064993154`, 0.12804730636711914`, 7.114439815654695`,
136.4379886532796`, 0, 0, 0, 117.61443152414277`, 1.1314154784376593`, 0, 0, 0.019325457124993433`, 0, 0,
17.575352161175807`, 0.012871447889642049`, 0.6139363437111349`, 0.4181363509438931`, 0, 0, 0.27134243175649775`},
{0.061725502446144886`, 3.13849642385113`, 9.540008859499451`, 1.4660101376451562`, 0.45012796893408935`,

```
0.4212144239280121`, 0.02511250628464842`, 120.07361261472948`, 0.1571099365418398`, 6.445559562130283`,
 538.8634237080685`, 0, 0, 0, 393.20601754475183`, 2.980969711499458`, 0, 0, 4.744983020073123`, 0, 0,
 133.65375398785125`, 4.1840923001777846`, 12.41903557016007`, 0.9999999859366913`, 0, 0, 1.1098460005447526`},
{0.047554055600075584`, 3.265459078802472`, 8.714860138547323`, 0.8369196863770172`, 0.4962846406178121`,
 0.46335229122842925`, 0.007763546254085084`, 1888.52152221599`, 0.22218896292653229`, 2.633616973587875`,
 20.753454841274323`, 0, 0, 0, 18.68777698167536`, 0.04280693493719975`, 0, 0, 0.005056494248324852`, 0, 0,
 1.9969184903769368`, 0.003435097266091797`, 0.3052767349223153`, 0.12145934137856074`, 0, 0, 0.28742622701749027`},
{0.12567467788677716`, 3.0158399320010547`, 4.901658754742801`, 1.351978052591392`, 0.7221310556231506`,
 0.5024953321675816`, 0.09886516020555723`, 193.78321929587645`, 0.11023982026692764`, 3.349767411033996`,
 12011.429039086428`, 0, 0, 0, 60.609392735669736`, 1.8489569633778267`, 0, 0, 4246.0873410031`, 0, 0,
 79.65940346723234`, 7623.223697995529`, 4.012160186580301`, 1.0000000000001423`, 0, 0, 0.5895117975509551`},
{0.19691840575768232`, 1.6501723246031572`, 9.011299029553381`, 1.1479337267630148`, 0.646058584475099`,
 0.36485695404700413`, 0.027042804382441818`, 3550.5063392884595`, 0.2269993732427253`, 4.86925454673478`,
 22.58052204958477`, 0, 0, 0, 16.354919048714876`, 0.25261082887501735`, 0, 0, 0.00383802075051203`, 0, 0,
 5.95501998149454`, 0.01079681324936135`, 0.2905037808245897`, 0.06932242737702965`, 0, 0, 0.3566853989851077`},
{0.22383158609367865`, 3.396592529608882`, 7.0059346900996395`, 1.1116596498970173`, 0.7389255639756462`,
 0.40169511261271784`, 0.008944445083981989`, 1191.861359234809`, 0.06327031154858104`, 2.975039312230072`,
 50.09786658408003`, 0, 0, 0, 44.372202948996076`, 0.11314270896401002`, 0, 0, 0.020182446683471328`, 0, 0,
 5.489995429240973`, 0.06453527217732427`, 0.5256841562762193`, 0.3214045835549837`, 0, 0, 0.1994433628605833`},
{0.22089456196581753`, 2.0637610394456862`, 7.594372198729954`, 1.2863006630967566`, 0.5843792508368972`,
 0.43898418015861007`, 0.0169422428941798`, 186.99955924932385`, 0.09805501720710208`, 5.47194276574208`,
 357.82707975079154`, 0, 0, 0, 282.3981844529872`, 2.1901744231794797`, 0, 0, 2.0546209257488885`, 0, 0,
 64.57138063068908`, 6.483636991415819`, 6.594581984295816`, 0.9999991397368484`, 0, 0, 0.7761891195743341`},
{0.09910547662807062`, 2.346178866312991`, 7.421049922557871`, 1.3147434313884303`, 0.2750558853148206`,
 0.21685738835966661`, 0.006760522552087531`, 4988.988013498636`, 0.18492351030582366`, 2.8590709623133`,
 4.8884755885299205`, 0, 0, 0, 4.460058061569598`, 0.012146242782543755`, 0, 0, 0.0019277166020365089`, 0, 0,
 0.407103687450155`, 0.002729246752124255`, 0.12281248140309119`, 0.03099176083660571`, 0, 0, 0.24532636095835267`},
{0.20816183484471307`, 3.9485705583569546`, 6.550493120626773`, 0.7774564054459185`, 0.5499519895881679`,
 0.2003723071725072`, 0.34567679795977113`, 1808.1918719614162`, 0.23441319529101012`, 4.082991759791533`,
 24.46088933504238`, 0, 0, 0, 4.503180104720139`, 0.34731653632355614`, 0, 0, 0.004758516036943434`, 0, 0,
 19.59148356796721`, 0.014150591848402418`, 0.4723040860547257`, 0.12336178499405681`, 0, 0, 0.24402471641989038`},
```

{0.16112203008102222`, 2.489549214258973 5`, 0.44352326574872514`, 1.2469876196885359`, 0.9780986797319231`,
0.6188841396088389`, 0.029743069155058133`, 112.64100571320267`, 0.13296296295840093`, 7.528063016828725`,
112098.87372591226`, 0, 0, 0, 19.04086908848619`, 0.21816831846543355`, 0, 0, 33943.236225476845`, 0, 0,
7.759153797311746`, 78128.61611669339`, 14.463714757787711`, 1.0000000000000018`, 0, 0, 0.7265878230892805`},
{0.17781253097781763`, 1.7213960002432414`, 4.693599924413801`, 1.2010501070587627`, 0.4002263609998786`,
0.5127527245385753`, 0.08027502692564248`, 156.85332391803223`, 0.06371789077354856`, 6.562151621871855`,
68421.52213659421`, 0, 0, 0, 120.61289793872835`, 5.238852438796462`, 0, 0, 19255.19563588477`, 0, 0,
128.8305662001256`, 48911.64386413825`, 9.258953059410686`, 1.0000000000000129`, 0, 0, 0.7961560338308824`},
{0.11782384895680592`, 1.1169968183756591`, 9.741344822271138`, 0.8926206302886914`, 0.732042643272727`,
0.38545027561144396`, 0.005148133378435907`, 521.4771678665235`, 0.04124959681193141`, 8.98600497767481`,
343.69395222225114`, 0, 0, 0, 319.9749285794344`, 1.372137203622895`, 0, 0, 0.0313412791663986`, 0, 0,
21.895327011737844`, 0.05275357346590926`, 3.613258040826765`, 0.5296294827285093`, 0, 0, 0.5607683108553674`},
{0.1669047046062308`, 1.025805255669411`, 7.1552343684117155`, 0.8176510429563385`, 0.8282389242605148`,
0.29891546072913033`, 0.009889011750027802`, 94.42085507888534`, 0.16558325818795072`, 8.472484566593426`,
22505.774134398995`, 0, 0, 0, 425.7778382110164`, 3.763664847385679`, 0, 0, 6506.631939006286`, 0, 0,
55.15410258609217`, 15514.106882304386`, 19.210325169895096`, 0.9999999999999999`, 0, 0, 0.7177157674836364`},
{0.1009959491070086`, 1.5581491170930981`, 4.3701504080039815`, 1.3889561652621691`, 0.5506040634320337`,
0.37952236600496847`, 0.006611879537937531`, 361.09151974058886`, 0.21353768992235467`, 8.563496620010564`,
307.91252075697554`, 0, 0, 0, 279.1034006794144`, 1.112509482972122`, 0, 0, 1.0832355466279897`, 0, 0,
24.76365240929587`, 1.5628914591163634`, 5.1868348263164`, 0.999562585126199`, 0, 0, 0.9259752027204486`},
{0.23246941421316458`, 3.062147704374147`, 7.181406357346838`, 1.3012169805011569`, 0.348059834110821`,
0.639530677915989`, 0.09989941363865933`, 83.25264127475623`, 0.015153685907020209`, 6.841473199486905`,
1.0484676335373272`*^6, 0, 0, 0, 167.62545353424275`, 5.21392807374731`, 0, 0, 242552.3584200321`, 0, 0,
228.08311259710254`, 805514.352541805`, 17.93530935190787`, 1.0000000000001024`, 0, 0, 0.7238803093118942`},
{0.05240567594084544`, 2.6092929572780967`, 9.364433651132018`, 1.4357965076882815`, 0.3985784064329525`,
0.6640850131228939`, 0.14135898686373455`, 340.71602063464445`, 0.17921846525178464`, 4.7134535629319085`,
225.40676807168447`, 0, 0, 0, 76.61117490911195`, 3.883945125550361`, 0, 0, 0.07732181833285072`, 0, 0,
144.77643803647373`, 0.05788717363840836`, 3.148349305270226`, 0.678429105185048`, 0, 0, 0.7215245422381906`},
{0.186251758126929`, 2.4933710973488976`, 3.5328550575209547`, 1.325175018028979`, 0.37317622556186136`,
0.25977278720819974`, 0.09748704597661054`, 108.88467391318876`, 0.2369656062609477`, 9.08676709988832`,
43221.349195109644`, 0, 0, 0, 106.16372972632715`, 3.9119675352634515`, 0, 0, 11738.593231307543`, 0,

0, 139.34266837418696`, 31233.337532398396`, 17.334417287675652`, 1.`, 0, 0, 0.4763249286660489`},
{0.2432348147394116`, 1.8751950266382789`, 2.5453519666394815`, 1.0050719073546701`, 0.45557496345664417`,
0.6934059109337196`, 0.0796473645979629`, 116.90111969840831`, 0.13836004707768368`, 5.732847922404471`,
91128.56424834066`, 0, 0, 0, 55.5435613472234`, 2.23372192930428`, 0, 0, 20338.62812512636`, 0, 0,
59.83806075320364`, 70672.32062955583`, 10.568308096110375`, 1.0000000000000322`, 0, 0, 0.526750592915759`},
{0.10016679782659832`, 2.355527283731478`, 1.9202752019167662`, 1.4361517107451491`, 0.5493703103299528`,
0.5953848202767148`, 0.3151585002444098`, 3628.217577021284`, 0.16393396333788363`, 2.9174291385165`,
15.074600454037883`, 0, 0, 0, 2.869231081060213`, 0.35018517424205464`, 0, 0, 0.029336874105391708`, 0, 0,
11.783867604077447`, 0.04197972481969596`, 0.1792149104581407`, 0.3439826164832661`, 0, 0, 0.3770730160732839`},
{0.14701675506563494`, 1.5420515267654622`, 0.49939909194610793`, 1.0246394569387973`, 0.7670831420663697`,
0.6948893607838256`, 0.005182980912963712`, 1858.6105410956102`, 0.23965764027041103`, 6.073448972699818`,
1000.3214305472883`, 0, 0, 0, 24.592864341531794`, 0.07777240799842297`, 0, 0, 314.14003508377044`, 0, 0,
1.71327229277421`, 659.7692656317222`, 0.7624438247256285`, 0.9999999999999779`, 0, 0, 1.1064540858062712`},
{0.19166603750475464`, 2.2499911524034095`, 2.4198836269597095`, 0.7671801018390442`, 0.14979093568204394`,
0.302546021842622`, 0.008837119919208427`, 4450.048280356515`, 0.1035437468879743`, 7.585339524339819`,
15.357066200668918`, 0, 0, 0, 13.651128846968827`, 0.05063691779449218`, 0, 0, 0.004266152177056893`, 0, 0,
1.6276088146083723`, 0.011681092616697648`, 0.37593848350013465`, 0.11250716005659456`, 0, 0, 0.8909121142029123`},
{0.14239569401382052`, 2.8387649854604886`, 1.4027026171382353`, 1.2048713691971291`, 0.21617980215068333`,
0.2290581611576099`, 0.32444520029725066`, 2595.236024076599`, 0.07657553404881084`, 7.455893283173221`,
24.916355486371895`, 0, 0, 0, 4.901582087772632`, 0.4800821150157779`, 0, 0, 0.021601623870986162`, 0, 0,
19.469147117894384`, 0.043942546041893064`, 0.618873519742012`, 0.31774957006196847`, 0, 0, 0.6817958397275017`},
{0.14874493002083228`, 3.1275671408493704`, 8.341714492380408`, 1.302209514279003`, 0.6258906648056601`,
0.45549735048188655`, 0.04029116024037646`, 765.0281459953038`, 0.12017823562381152`, 2.4241463017742984`,
56.116400371856514`, 0, 0, 0, 35.80308866297329`, 0.4427051742036021`, 0, 0, 0.02824742647299284`, 0, 0,
19.77985937033116`, 0.06002373534278167`, 0.6814573933958684`, 0.36963063377596417`, 0, 0, 0.2172797362209177`},
{0.06222670957040222`, 3.9065456744185374`, 1.0946055811329174`, 0.8246557540237`, 0.5907991824162977`,
0.2630274030573405`, 0.006980770676502568`, 3176.3819350381427`, 0.13217715504568522`, 5.816838705721813`,
23.875326558918164`, 0, 0, 0, 21.679208704937462`, 0.037388526492123396`, 0, 0, 0.026795410933577186`, 0, 0,
2.086571234866963`, 0.023819860771187706`, 0.3866164256211813`, 0.4973208101601343`, 0, 0, 0.40523787477390716`},
{0.04971186392349858`, 0.9916432478771275`, 0.9981695561230755`, 0.8524884405534577`, 0.12034821743714219`,
0.2529703866830304`, 0.3237478827730366`, 360.7437887352434`, 0.010006209625245316`, 7.274213959868414`,

```
100108.64796647927`, 0, 0, 0, 11.434237231669137`, 3.091776412961654`, 0, 0, 58502.08880417312`, 0, 0,
  43.799131483702745`, 41546.39825533525`, 4.336206779965715`, 1.0000000000000007`, 0, 0, 0.6513683314428592`},
 {0.1940949007109463 7`, 1.638712293913553`, 0.39703017050116074`, 0.7643187357680644`, 0.5335846868412271`,
  0.21830616448247264`, 0.0963055671350066`, 635.9574479204244`, 0.22613729069446092`, 5.479828782022301`,
  5226.009218756005`, 0, 0, 0, 7.154763952661755`, 0.391660763886526`, 0, 0, 1380.7558379426055`, 0, 0,
  9.168847268920347`, 3828.538103878989`, 1.769490141067366`, 1.0000000000011615`, 0, 0, 0.22179475266256654`},
 {0.27029725962327555`, 2.240924310463286`, 1.2051421639323703`, 1.1356504301287609`, 0.773380004303778`,
  0.6607812449412671`, 0.04949916223108672`, 204.2592199369304`, 0.19899441283798802`, 7.18276351221955`,
  45644.00349640439`, 0, 0, 0, 40.63839933517644`, 0.8590091506957326`, 0, 0, 9374.892886770454`, 0, 0,
  27.499635552921195`, 36200.1122365112`, 7.492991062991821`, 1.000000000000215`, 0, 0, 0.5718333929470829`},
 {0.14266269539818904`, 3.072017618366117`, 3.4103252419872643`, 0.7755852439360265`, 0.43949005760262017`,
  0.1924018543570919`, 0.06712102805080977`, 656.0250616869308`, 0.04537130588160604`, 5.285505421294628`,
  77.40683502854972`, 0, 0, 0, 40.281376566182836`, 0.8249895124044744`, 0, 0, 0.03117264934782816`, 0, 0,
  36.2054616724831`, 0.06353105969525863`, 1.6948837400391388`, 0.5768867023958195`, 0, 0, 0.34805137842462436`},
 {0.10769494377900635`, 0.832286650700361`, 9.122967245453143`, 0.9815088864732582`, 0.842140026069350 8`,
  0.5448142839433918`, 0.07976087110962524`, 4710.110387525812`, 0.13361893143992154`, 5.1516712920505725`,
  34.095338229861866`, 0, 0, 0, 16.051813465867586`, 1.3988999701173515`, 0, 0, 0.004697971214450261`, 0, 0,
  16.6326538684829`, 0.007227824940264355`, 0.22804169057407198`, 0.09766966655157483`, 0, 0, 0.29044315729841297`},
 {0.21491444350519606`, 1.295137699490157`, 7.08600395884199`, 0.8977823469890502`, 0.4181882258302472`,
  0.21987671758091132`, 0.14871331978208877`, 664.8642422386233`, 0.19734382780045878`, 6.556011365474522`,
  98.52364269830204`, 0, 0, 0, 32.90190402783381`, 3.36194115265057`, 0, 0, 0.014071165181639967`, 0, 0,
  62.20252471807389`, 0.04320138049232362`, 2.0866375434861983`, 0.2861988332477583`, 0, 0, 0.47313079471238595`},
 {0.1490469101160943 3`, 3.9586033442731177`, 2.425615791755316`, 1.0187686401741827`, 0.6625310044158794`,
  0.43390068486512956`, 0.00854220404180854`, 96.98019438566024`, 0.2008209408788944`, 6.643325956478872`,
  52483.96610627175`, 0, 0, 0, 114.78564282202817`, 0.24008429416116647`, 0, 0, 16730.974889144152`, 0, 0,
  13.5771212824835`, 35624.28729224145`, 14.63168268159128`, 1.0000000000002283`, 0, 0, 0.5461900967007979`},
 {0.21251186737978806`, 3.785250570140386`, 5.706304857162573`, 0.8638958511126991`, 0.620802282746526`,
  0.4325048599697877`, 0.008982590727965567`, 699.3542650472853`, 0.09331259312392975`, 7.885660147589962`,
  198.6373289803386`, 0, 0, 0, 176.05378144752902`, 0.40467521826501923`, 0, 0, 0.03631771392434948`, 0, 0,
  21.882815723704994`, 0.11025636007184565`, 2.408988118185892`, 0.5930261941528048`, 0, 0, 0.6503361689121229`},
 {0.24515641699904833`, 3.817676488470438`, 5.668256418323743`, 1.3090245110359662`, 0.8705848781731611`,
```

0.5675799494003652`, 0.13511363154659006`, 1804.7556662922755`, 0.20417175811210386`, 6.336735207604568`,
122.94450041578052`, 0, 0, 0, 42.37887253035392`, 1.4475898739438986`, 0, 0, 0.03754569164786958`, 0, 0,
78.9489975243359`, 0.13149381768778998`, 0.7300113159985043`, 0.4572283927833528`, 0, 0, 0.33855789540392367`},
{0.16393709899357184`, 1.7364683461504136`, 6.294465981846198`, 0.7908159510076651`, 0.7611075592144951`,
0.36561813704297086`, 0.008326625223173748`, 2834.6413370290525`, 0.20899318399811945`, 1.1709155007061636`,
7.919504357196832`, 0, 0, 0, 7.064031553154856`, 0.03216234658749174`, 0, 0, 0.005725662301431857`, 0, 0,
0.7978413826728311`, 0.01340926382162346`, 0.08685105087970829`, 0.1429835920922108`, 0, 0, 0.07645516728134889`},
{0.25878729889995966`, 3.1361195765858056`, 8.880297290408144`, 1.0867236136347516`, 0.0902130745148968`,
0.5352173770607672`, 0.11164972929109021`, 57.41366920823615`, 0.17534323232121918`, 1.541035034614442`,
112794.8560829351`, 0, 0, 0, 43.08512356784165`, 1.4600612640362003`, 0, 0, 23991.020523025254`, 0, 0,
65.41323875940788`, 88693.87712867449`, 5.767269864311419`, 1.0000000000003577`, 0, 0, 0.13664026560732145`},
{0.11203607225462164`, 0.6218658244264867`, 6.440483663444866`, 1.39680689202378`, 0.16534679246130501`,
0.5081267228820908`, 0.03214478110427463`, 1906.9275025607635`, 0.21932209633484323`, 1.251120855111619`,
7.230030948388517`, 0, 0, 0, 4.9842356547041815`, 0.22496891340940184`, 0, 0, 0.008286587282042231`, 0, 0,
1.9985782686809808`, 0.013262809878214552`, 0.15329074286559266`, 0.11758722719969783`, 0, 0, 0.22993843541931833`},
{0.24357827172423074`, 3.9240029450424014`, 2.804272198831022`, 0.75931848861878`, 0.2959071515485636`,
0.6138549707668646`, 0.10713705928623629`, 339.1202760730188`, 0.035220064551167996`, 7.297254233929856`,
30837.35402677289`, 0, 0, 0, 69.78639293923663`, 1.8058014240124127`, 0, 0, 6845.236263028533`, 0, 0,
101.22814437123526`, 23819.29740703586`, 4.865557457489675`, 0.9999999999999998`, 0, 0, 1.0627923210308656`},
{0.24204908128005126`, 1.6039606621918692`, 7.422941916054803`, 1.4757185212808246`, 0.48127457855983957`,
0.4526414340963527`, 0.017845029630840916`, 286.8959659308558`, 0.1351657578784522`, 4.5748150305594475`,
234.98183715576994`, 0, 0, 0, 187.26312816812273`, 1.9600785885382555`, 0, 0, 0.1719059259250563`, 0, 0,
44.912699297427466`, 0.5944238776679516`, 3.4964399531920964`, 0.9031196227966126`, 0, 0, 0.5076318600774292`},
{0.0765658571842559`, 1.8453576022836256`, 0.8953985896867352`, 1.0948080634821278`, 0.11609992165592442`,
0.45437290836767896`, 0.20627344098654643`, 689.3327160072474`, 0.18998846953804188`, 8.643335566773064`,
3592.048018278957`, 0, 0, 0, 17.345435709906592`, 1.739333284595913`, 0, 0, 1684.5488302460622`, 0, 0,
45.85274142334337`, 1842.5560736646466`, 2.8181815731141935`, 1.0000000000031193`, 0, 0, 1.199499208143396`},
{0.2258389851785662`, 0.888336357939937`, 0.32961763645801767`, 0.9210788231382718`, 0.8596897387491109`,
0.5585019604624183`, 0.04381971178906294`, 415.715019082411`, 0.24817184929202513`, 7.285653344604734`,
13479.357265858447`, 0, 0, 0, 11.778751967467588`, 0.5310834627150206`, 0, 0, 3184.913220594358`, 0, 0,
6.739724986148474`, 10275.393851726076`, 3.716559314200005`, 1.0000000000007299`, 0, 0, 0.5424963508098691`},

{0.19210976086993148`, 0.9419570933782317`, 5.851922457612897`, 1.40657571618961`, 0.8492777725664675`,
0.5465312705852027`, 0.10025464283588155`, 725.5272924080149`, 0.05639485661927951`, 6.120948580338062`,
268.9483672482861`, 0, 0, 0, 111.21428982042713`, 10.843810876877289`, 0, 0, 0.259090624224477`, 0, 0,
145.9200653532371`, 0.7110548266200625`, 1.7651244610569592`, 0.9644667140043115`, 0, 0, 0.36765767549999695`},
{0.04040676752094921`, 0.7630185090261814`, 4.034875094487921`, 0.9393317585048341`, 0.5565744536568444`,
0.3001585479016161`, 0.008819784570716005`, 3975.779851508773`, 0.23610962920225392`, 2.0975088246946054`,
6.788814298312004`, 0, 0, 0, 6.029930060408326`, 0.06266963828245461`, 0, 0, 0.0049843095127312794`, 0, 0,
0.6831156280498365`, 0.0028771405104775585`, 0.11248539374733864`, 0.1071123233232455`, 0, 0, 0.16839146411271355`},
{0.2323130275502685`, 0.49681822670142894`, 1.2539687147478098`, 0.9468502150118296`, 0.13096405194857463`,
0.3219849827616931`, 0.04046745571036271`, 95.23490611137812`, 0.01783356001323902`, 6.936606555558118`,
780592.5082509076`, 0, 0, 0, 42.771465308589285`, 3.008943985450297`, 0, 0, 180729.14777804067`, 0,
0, 21.35568878707635`, 599796.2212413768`, 15.025906674358811`, 1.`, 0, 0, 0.3137468959580257`},
{0.1110467064554368`, 3.3883763096282147`, 5.159131249779884`, 1.2482205179674581`, 0.8201347270890553`,
0.46628896390017405`, 0.2764030660881924`, 2912.733505822841`, 0.03458542948115101`, 2.6312047625809307`,
23.575177885540555`, 0, 0, 0, 4.9060892178991695`, 0.3770692916092532`, 0, 0, 0.015403217017390449`, 0, 0,
18.25218078252979`, 0.024435378837167555`, 0.18923684824037684`, 0.23072981895037614`, 0, 0, 0.16152421504556433`},
{0.1632243866872038`, 2.5373481239797906`, 9.958152546995578`, 1.2439631103015851`, 0.6072525242529792`,
0.6395983432777625`, 0.3899581087433961`, 56.26697227139985`, 0.18832987989003086`, 4.752251898931052`,
97167.93252372758`, 0, 0, 0, 64.02807892790582`, 8.853199293487346`, 0, 0, 29045.80447511475`, 0, 0,
320.9092659792758`, 67728.33744695803`, 18.80636744414252`, 1.0000000000012554`, 0, 0, 0.6091800407089617`},
{0.16346002078838484`, 3.218794874634134`, 9.025936619626364`, 1.1259348004332868`, 0.7415896232848811`,
0.4862549245509259`, 0.364081252118343`, 137.81289300851844`, 0.05819144446151425`, 8.24717117891944`,
672.8629758010734`, 0, 0, 0, 116.17157154370891`, 11.316886329969748`, 0, 0, 7.493705058265925`, 0, 0,
520.3819387960533`, 17.498874065801665`, 13.976733839643085`, 0.9999999740883458`, 0, 0, 1.5121110567605833`},
{0.19514620721272602`, 2.87732243599938`, 0.26739185721478975`, 1.4891953183216857`, 0.05289250725423611`,
0.4217001319921505`, 0.03447210696714538`, 218.70480172461387`, 0.0713049851341147`, 6.944425315128572`,
131416.31876315633`, 0, 0, 0, 9.80458899415031`, 0.11303184374317105`, 0, 0, 34690.75749174926`, 0, 0,
4.646129428351463`, 96710.99642644738`, 6.657758326443616`, 1.0000000000000993`, 0, 0, 0.4330655310324824`},
{0.06305409129179324`, 2.891634576752894`, 4.887865973915014`, 1.4252999692587962`, 0.37958790888649707`,
0.5644849529569385`, 0.3706753829912659`, 1888.9507831786343`, 0.129560920022812`, 6.557119455026179`,
51.592938499520955`, 0, 0, 0, 8.817751936046072`, 1.0102899497160374`, 0, 0, 0.016184485675544348`, 0, 0,

41.734133587783475`, 0.01457854338989386`, 0.7805522866618696`, 0.2153460291608431`, 0, 0, 0.9132998021993964`},
{0.07134729442997761`, 1.925040188749243`, 1.1575324502817335`, 0.9987470940766925`, 0.48237853230783223`,
0.6023915002525595`, 0.11093155711801116`, 121.08543179928522`, 0.23351841944906743`, 5.375810156733433`,
29915.823911628922`, 0, 0, 0, 20.825810736934756`, 1.1151253504108087`, 0, 0, 14789.245254759875`, 0, 0,
30.666587357627026`, 15073.894794121446`, 10.049685362694934`, 1.0000000000000995`, 0, 0, 0.7896670057017313`},
{0.1360789534218197`, 0.4455509561284261`, 2.009215820725272`, 0.8936493225292423`, 0.96227260378033`,
0.5587588217121244`, 0.041826495849356216`, 90.5779416890061`, 0.15671264889903508`, 7.812316525319764`,
68111.03518287199`, 0, 0, 0, 80.49776763498981`, 6.411209898539434`, 0, 0, 23092.275201082408`, 0, 0,
40.80743857477536`, 44891.037735599086`, 18.771917155196025`, 1.0000000000001383`, 0, 0, 0.8026463515983912`},
{0.1584938652096898 3`, 3.640724074855333`, 1.8861164484754733`, 1.1569149894265711`, 0.5885808127893892`,
0.1748767871381529`, 0.46502697264632115`, 193.59851086045592`, 0.1503277806325949`, 4.212693145809347`,
26164.56895608105`, 0, 0, 0, 8.868097048884618`, 0.9868829398639059`, 0, 0, 7996.875568213752`, 0, 0,
51.328121117521896`, 18106.510262959047`, 4.479586067441102`, 1.0000000000141984`, 0, 0, 0.18132889431597687`},
{0.2646664636377358`, 0.7835752608143567`, 7.19251403558616`, 1.494052846937745`, 0.8510899770532778`,
0.5885640033353514`, 0.48048609718479995`, 154.5393756420333`, 0.18357560397946`, 4.027566835631127`,
8249.340663343237`, 0, 0, 0, 36.74587992586362`, 17.922420843259424`, 0, 0, 1672.0632647379477`, 0, 0,
200.62236552402294`, 6321.986732239416`, 6.1604443985714115`, 1.0000000000304075`, 0, 0, 0.7960580692854555`},
{0.12495672263621771`, 3.9102582538632182`, 1.1196234634441904`, 1.2648993449094799`, 0.05034343178950351`,
0.5637162084123208`, 0.0377865951253564`, 87.92444212100203`, 0.05653305553347371`, 1.253921938823833`,
264111.2768128192`, 0, 0, 0, 7.534933223237838`, 0.07017288636376118`, 0, 0, 94826.08228671992`, 0, 0,
3.919915830018606`, 169273.6637568688`, 3.1324026814835038`, 1.000000000000137`, 0, 0, 0.1415128597843697`},
{0.22032142146595135`, 2.68397072068061`, 2.6883455170430324`, 1.1162234719994681`, 0.40997309115380154`,
0.5558969738438672`, 0.02165064984593792`, 1526.6454168541877`, 0.24054798278325668`, 4.966530798126495`,
49.03536488580379`, 0, 0, 0, 37.49712154398171`, 0.28928407597280986`, 0, 0, 0.03505329999561856`, 0, 0,
11.091856998145253`, 0.11032846974436966`, 0.7311122386497694`, 0.48362504035322307`, 0, 0, 0.688624887804471`},
{0.10964967041383433`, 0.7460930180313401`, 6.531951119775776`, 1.1455194328360876`, 0.1524629724176152`,
0.28792352560938195`, 0.09608079533773954`, 1051.3203547256705`, 0.17626541392472717`, 8.294356744338359`,
70.12448646374816`, 0, 0, 0, 30.49912016656039`, 3.396595300529472`, 0, 0, 0.010221911641800628`, 0, 0,
36.20251484004426`, 0.016011846321707466`, 1.7312349446859612`, 0.17459715725262526`, 0, 0, 0.927394773954033`},
{0.07988018646299105`, 2.6019950195572488`, 9.606629629213668`, 1.0662798233306474`, 0.7693895670648874`,
0.32124913017390155`, 0.04062772179058327`, 1822.0454711227774`, 0.21556365885863799`, 7.4522296585239225`,

77.67789447755827`, 0, 0, 0, 49.44846180450603`, 0.7390172740517948`, 0, 0, 0.007849358449318507`, 0, 0,
27.470275234993483`, 0.008957260236362246`, 0.8507303189082256`, 0.14648180752682116`, 0, 0, 0.40145492105319636`},
{0.1682679068810577`, 1.5454861727740674`, 1.469740171862469`, 0.9427424173065856`, 0.7350490973518766`,
0.3805201167376455`, 0.012952191039290932`, 4650.232844583341`, 0.019462289964433777`, 1.394906478790226`,
5.782774890399873`, 0, 0, 0, 4.829188176841602`, 0.038207958454593106`, 0, 0, 0.020206689360485416`, 0, 0,
0.8435695925928528`, 0.048573390338350474`, 0.06410459965505273`, 0.3578734872640926`, 0, 0, 0.11547351729422783`},
{0.1878353522665755`, 2.6490780379134238`, 9.484290061448991`, 1.1885927795104771`, 0.9576534469556572`,
0.2804046795618802`, 0.13562182147346188`, 68.8621810881709`, 0.06230339869012863`, 8.081807561887658`,
129865.38196915698`, 0, 0, 0, 213.9739256536155`, 10.15012215735319`, 0, 0, 35092.160591420135`, 0, 0,
384.12093841689665`, 94164.97637835183`, 24.91420723963223`, 1.000000000000055`, 0, 0, 0.6108021355796563`},
{0.195165600718623`, 2.3826254740878197`, 7.662410402528582`, 0.9519993324167042`, 0.5246436284800724`,
0.22761517290878341`, 0.02889008666683709`, 101.1296763305189`, 0.18152629399038878`, 2.6385617746928904`,
8075.8965636638795`, 0, 0, 0, 116.36193838402997`, 1.3223111672194472`, 0, 0, 2088.9695264407487`, 0, 0,
45.00817530982654`, 5824.214178724375`, 5.623471124146033`, 0.999999999999984`, 0, 0, 0.2428506212918482`},
{0.21914596002979703`, 3.4188550030486784`, 4.710500615641845`, 1.4527326168664971`, 0.03624911304432854`,
0.28384065291200533`, 0.4254237737178384`, 3505.5630456480862`, 0.12312118964896007`, 9.273808474100267`,
21.994509170033602`, 0, 0, 0, 3.5797079400817586`, 0.36907910089500506`, 0, 0, 0.00474714065346806`, 0, 0,
18.026113294508555`, 0.014861667084298997`, 0.5843420313366317`, 0.06781182687726517`, 0, 0, 1.104993871951961`},
{0.1814951436259516`, 1.11858432682377`, 5.7029405020905966`, 0.816783193035002`, 0.6766309251156815`,
0.4321734260024682`, 0.2931263656368919`, 87.29527865588257`, 0.044724422492498916`, 2.4174522941648533`,
160375.63566669732`, 0, 0, 0, 22.610745555794903`, 5.208739455075934`, 0, 0, 44607.30555428446`, 0, 0,
83.23449024223619`, 115657.27611916566`, 5.967103799448903`, 1.000000000000898`, 0, 0, 0.2217743181979818`},
{0.22203693974513244`, 0.9431525918127432`, 4.196712511517475`, 1.3261224346879132`, 0.8036004422541301`,
0.6271444136150295`, 0.10733581071086257`, 369.9451385562586`, 0.16273465181192748`, 5.731309489564488`,
225.90071966344982`, 0, 0, 0, 85.73344751490892`, 8.697178506715423`, 0, 0, 3.424698367719071`, 0, 0,
117.18237785809609`, 10.86299350169288`, 3.657985047874977`, 0.9999999961675067`, 0, 0, 1.1251763612012213`},
{0.18924733350115397`, 2.8150904404250037`, 2.4574844917932026`, 0.8737719488383665`, 0.6977173227044986`,
0.3482841124888072`, 0.48079795163551986`, 94.66261771766344`, 0.16471072416530214`, 1.3078470560939532`,
57313.14013302701`, 0, 0, 0, 3.472129594665993`, 0.5283172748743727`, 0, 0, 15468.44267495709`, 0, 0,
21.246584428717583`, 41819.45042358689`, 2.9016667944648593`, 1.000000000002547`, 0, 0, 0.08386177806463371`},
{0.07907817004881895`, 1.0481324229512046`, 3.8137728667258273`, 1.2920968808020492`, 0.04181350128653394`,

```
  0.5803710922764521`, 0.02851300126208627`, 266.99553906648964`, 0.22548933562284668`, 2.0140176417924156`,
  2720.1059443241925`, 0, 0, 0, 49.37930452604416`, 1.2218238658840577`, 0, 0, 1244.8759718356946`, 0, 0,
  18.294760128123777`, 1406.3216255787383`, 1.8166676355169908`, 0.9999999999999765`, 0, 0, 0.44352927612095394`},
 {0.22302087067747528`, 1.820151162075242`, 1.875184623833773`, 0.7813352529024251`, 0.8705057770695381`,
  0.22074020090969038`, 0.1367588752741694`, 187.45561858905666`, 0.07793294287132913`, 8.923384483271061`,
  51526.866471983536`, 0, 0, 0, 44.52287542734099`, 3.109676081754148`, 0, 0, 12278.600788756861`, 0, 0,
  80.85829334117707`, 39119.774837281315`, 9.770238060739464`, 1.0000000000008369`, 0, 0, 0.3561446829275311`},
 {0.21429795289589076`, 2.896671823601787`, 8.636702505614757`, 0.9811380648364925`, 0.20610104158853804`,
  0.1778381348410949`, 0.007936414694970003`, 185.13169573811163`, 0.014685687390926405`, 3.2353159752463547`,
  132.5848029001317`, 0, 0, 0, 119.18200137508019`, 0.3091914306230036`, 0, 0, 0.046544218469149856`, 0, 0,
  12.794658645496884`, 0.14249043910110062`, 3.7182116233445175`, 0.637771220332924`, 0, 0, 0.2523687303786486`},
 {0.17507336986882482`, 0.7983306290353371`, 2.750450064120228`, 0.8050075785267179`, 0.7818959542274213`,
  0.6526925477724106`, 0.09653256947066893`, 72.78219945862553`, 0.07700161447215975`, 3.655530763820291`,
  178673.84361342076`, 0, 0, 0, 34.94098801481757`, 3.792530410456041`, 0, 0, 51010.968739295444`, 0,
  0, 43.25275983164292`, 127580.88853516757`, 10.953387185997304`, 1.`, 0, 0, 0.38367480950420674`},
 {0.1470766374450453`, 1.2533511161361544`, 4.593526904612924`, 1.479552314271993`, 0.7400477950348978`,
  0.5768087033937906`, 0.10619660427114413`, 242.62879808709684`, 0.045931800134058565`, 3.771378090721337`,
  10744.69302168057`, 0, 0, 0, 63.5715783349201`, 4.838154824713873`, 0, 0, 3414.812063289552`, 0, 0,
  86.62723927992367`, 7174.843939648633`, 3.734095484894038`, 1.000000000000872`, 0, 0, 0.8190173474536607`},
 {0.0788585810797619`, 2.548378832414274`, 3.111482597871378`, 0.9346713900878518`, 0.19963135196405957`,
  0.5058944800586684`, 0.03749384602431879`, 1118.4164778142415`, 0.22872744797764172`, 1.4303214452036404`,
  14.58640832427198`, 0, 0, 0, 9.560662618618812`, 0.13292731229722263`, 0, 0, 0.024786684078228007`, 0, 0,
  4.839273555828064`, 0.027923467658307412`, 0.2979931039025121`, 0.4229115418020146`, 0, 0, 0.25829923738104343`},
 {0.24182502946725698`, 1.8945200327623395`, 6.300131593939071`, 0.7669603268824658`, 0.6959163653444247`,
  0.5105300710554361`, 0.2329066367569749`, 178.13280663193103`, 0.21420184435482204`, 3.5903109736592462`,
  684.3658492283569`, 0, 0, 0, 49.95781557132246`, 5.375957412063507`, 0, 0, 108.54608553203948`, 0, 0,
  145.49798589187859`, 374.9880047477282`, 4.804847584065826`, 0.9999999999402017`, 0, 0, 0.7462540209891684`},
 {0.05758087297506348`, 2.9919516996565125`, 9.945704556467732`, 1.1770757830874041`, 0.6136122924727272`,
  0.31603398268032157`, 0.21009959511709828`, 118.22678602178958`, 0.2258513747270965`, 6.274152149731609`,
  518.6124611616934`, 0, 0, 0, 137.30444178212255`, 8.62054807823717`, 0, 0, 2.318991480583124`, 0, 0,
  368.46090678074773`, 1.9075650553384622`, 11.732110604964895`, 0.9999946045542002`, 0, 0, 0.7535664838355827`},
```

```
{0.12816326660379745`, 2.7095777054297967`, 7.985014815061977`, 0.8582103259977079`, 0.28527001685481057`,
  0.4561351167228034`, 0.41006088059385215`, 4928.696897400591`, 0.12257221770331383`, 5.545857987270676`,
  13.653307289162441`, 0, 0, 0, 2.194945443809597`, 0.2884429110447941`, 0, 0, 0.001694785956147883`, 0, 0,
  11.16512115794637`, 0.00310299006191987`, 0.2520163123611485`, 0.041556989900579766`, 0, 0, 0.7475095657139902`}};
```

In[ ]:=

```
PatientsTrainingSet = result[[All, 1 ;; 10]];
```

In[ ]:=

```
(* Test with fully occupied receptors *)
OccRecCureTest[] := (tMax = 365; tEnd = tMax; tEndInit = tMax;
    NotebookDelete[prMDopt];
    prMDopt = PrintTemporary["Solving approximation with fully occupied receptors"];
    SolutionOccRec[0, 1];

    (* let's estimate how many active molecules got on cancer cells *)
    (* but there is no explicit binding in this version of the model, so let's consider their fate and apply conservation law then *)
    (* how many active receptors became inert and are left on cancer cells at the moment of cure *)
    If[tCure ≤ tMax,
      ActMolEst = NIntegrate[lambda * gamma * (fAN[t] * NN[t] + dA[t]), {t, 0, tCure}] +
          gamma * (fAN[tCure] * NN[tCure] + dA[tCure]) + NIntegrate[omega * gamma * dA[t], {t, 0, tCure}],
      ActMolEst =
        NIntegrate[lambda * gamma * (fAN[t] * NN[t] + dA[t]), {t, 0, tEndInit}] + NIntegrate[omega * gamma * dA[t], {t, 0, tEndInit}]];
    (* and how many active nuclides were released back in blood *)

    (* Now let's pick aconst based on that *)
    aconst0 = ActMolEst / Min[tCure, tMax];
    (* volume of drug distribution is of the order of 1, and since we perform estimation of aconst0, there is no need to divide by it *)

    (* Is it curative within a year?*)
    If[tCure > tMax, CurFlgOR = 0, CurFlgOR = 1];
    NotebookDelete[prMDopt];
    Return[CurFlgOR]);
```

In[ ]:=

```
(* script for mulidose optimization in curative case *)

MDoptCURE[] :=(


   (* Find the case with const a (concentration of active antibodies in blood), which is curative within a year and least toxic *)
```

```
NotebookDelete[prMDopt];
prMDopt = PrintTemporary["Solving approximation with constant a"];

ConstaRes = Array[f, {2, 3}];
ConstaRes[[1, 1]] = "ka";
ConstaRes[[1, 2]] = "CureFlg";
ConstaRes[[1, 3]] = "Tox";

ka = 1;

Stop = 0;
ijk = 2;
dk = 1;

While[Stop == 0,

  NotebookDelete[prca];
  prca = PrintTemporary["ka=" <> ToString[N[ka]]];

  ka = ka * (1 + RandomReal[0.00001]);
  aconst = ka * aconst0;
  tEndInit = Min[tMax, 2 * tCure - Log[0.0017] / lambda];
  tEnd = tEndInit;
  SolutionConsta[0, 1];

  t = tEnd; If[NN[t] == 0 && tEnd < tMax, CureFlg = 1, CureFlg = 0];
  Clear[t];

  ConstaRes[[ijk, 1]] = N[ka];
  ConstaRes[[ijk, 2]] = CureFlg;
  ConstaRes[[ijk, 3]] = If[NumberQ[ActBlood[tEndInit] / nCpm], ActBlood[tEndInit] / nCpm, ActBlood2[tEndInit] / nCpm];
```

```
If[Abs[ConstaRes[[ijk, 3]] - ConstaRes[[ijk - 1, 3]]] / ConstaRes[[ijk, 3]] < 0.001 && ijk > 2 && ConstaRes[[ijk, 2]] == 1 && ConstaRes[[ijk - 1, 2]] == 1, Stop = 1,

  If[ConstaRes[[ijk, 2]] == 0 || (ConstaRes[[ijk - 1, 2]] == 0 || ijk == 2), (*not curative or previous not curative *)
    ConstaRes = Join[ConstaRes, {{"", "", ""}}]; ijk++; ka = ka * (1 + Abs[dk]),
    (* curative, and toxicity decreased compared to previous curative *)
    If[ConstaRes[[ijk, 2]] == 1 && ConstaRes[[ijk, 3]] < ConstaRes[[ijk - 1, 3]],
      ConstaRes = Join[ConstaRes, {{"", "", ""}}]; ijk++; ka = ka * (1 + dk),
      (* curative, but toxicity increased compared to previous curative *)
      ConstaRes = Join[ConstaRes, {{"", "", ""}}]; ijk++; dk = -dk / 2.; ka = ka * (1 + dk)
     ];];];
];

NotebookDelete[prca];

tEndOpt = tEnd;

fFNac[t] = fFN[t];
NNac[t] = NN[t];
dFac[t] = dF[t];

(*so, the last run was with optimal ka *)

(* Discretize injections -- no more than 10 injections, no more frequently than once in a day *)
NotebookDelete[prMDopt];
prMDopt = PrintTemporary["Discretizing injections"];

InjMax = 10;
```

```
InterMin = Max[1, Ceiling[N[Round[tEndOpt] / InjMax]]];
InterMax = Min[Round[tMax / 2], Floor[N[Round[tEndOpt] / 2]]];

DiscrArray = Array[f, {InterMax - InterMin + 1 + 1, 3}];

DiscrArray[[1, 1]] = "Interval";
DiscrArray[[1, 2]] = "Nmin";
DiscrArray[[1, 3]] = "Ainj";

ii = 2;
Inter = InterMin;
Stop = 0;

kkmin = 1;

While[Stop == 0,

  NotebookDelete[prdis];
  prdis = PrintTemporary["Inter=" <> ToString[Inter] <> ", max is " <> ToString[InterMax]];

  InjDoses =
   Table[Max[0, NIntegrate[(lambda * aconst (*decay*) + kon * gamma/V * (fFNac[t] * NNac[t] + dFac[t]) * aconst (*binding*) +
          kappac * aconst (*clearance*)), {t, dd, Min[dd + Inter, tEndOpt]}]] / nCpm, {dd, 0, Round[tEndOpt], Inter}];

  Ainj = Table[{Inter * (dd - 1), kkmin * InjDoses[[dd]] * nCpm, kkmin * eta * InjDoses[[dd]] * nCpm}, {dd, 1, Length[InjDoses]}];

  FullSystemSolutionMD[];
```

```
    DiscrArray[[ii, 1]] = Inter;
    DiscrArray[[ii, 2]] = If[Nn < 0 || ! NumberQ[Nn], Cd, Nn];
    DiscrArray[[ii, 3]] = Ainj;

    If[tEnd < Ainj[[2, 1]], (* tumor hits the ceiling in between the second dose *)
      Stop = 1, ii++; Inter++];

    If[Inter - 1 == InterMax, Stop = 1];
  ];
  DiscrArray = DiscrArray[[1 ;; ii - 1]];

  NotebookDelete[prdis];

  If[Length[DiscrArray] == 1, (* Multidose optimization yields one injection, so single-dose setting is already better *)
    CurFlgOR = 0,
     (* Select best of the discretized schedules *)
     (* choose best interval, in terms of remaining viable cell number,
    normalize it to achieve Nm=Ncur, but then also check other schedules by increasing the interval *)
    Inter = DiscrArray[[Position[DiscrArray[[All, 2]], Min[DiscrArray[[2 ;;, 2]]]][[1, 1]], 1]];
    prInt = PrintTemporary["Optimal interval is " <> ToString[Inter]];

    InterArray = Array[f, {1, 3}];
    InterArray[[1, 1]] = "Interval";
    InterArray[[1, 2]] = "Tox";
    InterArray[[1, 3]] = "Schedule";

    Inter--;

    StopInt = 0;
    kji = 1;
```

```
kk = 1;

While[StopInt == 0,
  InterArray = Join[InterArray, {{"", "", ""}}];

  kji++;
  Inter++;

  NotebookDelete[prInt];
  prInt = PrintTemporary["Checking interval " <> ToString[Inter]];

  InjDoses =
    Table[Max[0, NIntegrate[(lambda * aconst (*decay*) + kon * gamma/V * (fFNac[t] * NNac[t] + dFac[t]) * aconst (*binding*) +
            kappac * aconst (*clearance*)), {t, dd, Min[dd + Inter, tEndOpt]}]]/nCpm, {dd, 0, Round[tEndOpt], Inter}];

  Ainj = Table[{Inter * (dd - 1), InjDoses[[dd]] * nCpm, eta * InjDoses[[dd]] * nCpm}, {dd, 1, Length[InjDoses]}];

  FullSystemSolutionMD[];

  (* If it is still not curative, adjust common multiplier to achieve Nm=Ncur... *)
  If[If[Nn < 0 || ! NumberQ[Nn], Cd, Nn] > Ncur, (*DiscrArray[[Position[DiscrArray[[All,2]],Min[DiscrArray[[2;;,2]]]][[1,1]],2]]*)

    NotebookDelete[prMDopt];
    prMDopt = PrintTemporary["Still not curative, adjusting multiplier"];

    kkadjRes = Array[f, {2, 2}];
    kkadjRes[[1, 1]] = "kk";
    kkadjRes[[1, 2]] = "Nmin";
```

```
Stop = 0;
ijk = 2;
dk = 0.05;

While[Stop == 0,

  NotebookDelete[prkk];
  prkk = PrintTemporary["kk=" <> ToString[N[kk]]];

  Ainj = Table[{Inter * (dd - 1), kk * InjDoses[[dd]] * nCpm, kk * eta * InjDoses[[dd]] * nCpm}, {dd, 1, Length[InjDoses]}];

  Quiet[kkadjRes[[ijk, 1]] = kk;
    Nmin = FullSystemSolutionMD[];
    kkadjRes[[ijk, 2]] = If[NumberQ[Nmin], Nmin, Cd]];


  If[(Abs[kkadjRes[[ijk, 2]] - kkadjRes[[ijk - 1, 2]]] / kkadjRes[[ijk, 2]] < 0.001 && ijk > 2 && kkadjRes[[ijk, 2]] < Cd) || kkadjRes[[ijk, 2]] < Ncur, Stop = 1,

    (* Nmin decreased compared to previous one or tumor burden is achieved *)
    If[(kkadjRes[[ijk, 2]] < kkadjRes[[ijk - 1, 2]] || kkadjRes[[ijk, 2]] == Cd) || ijk == 2,
        kkadjRes = Join[kkadjRes, {{"", ""}}]; ijk++; kk = kk * (1 + dk),
        (* Nmin increased compared to previous one *)
        kkadjRes = Join[kkadjRes, {{"", ""}}]; ijk++; dk = -dk / 2.; kk = kk * (1 + dk)
      ];];
];

NotebookDelete[prkk];
```

```
(* ...and adjust last dose for it to become curative, if not... *)
If[kkadjRes[[Length[kkadjRes], 2]] > Ncur,
  NotebookDelete[prMDopt];
  prMDopt = PrintTemporary["Still not curative, adjusting last dose"];

  Stop = 0;
  cou = 0;
  While[Stop == 0,

    (* delete last dose and replace current last by curative one *)
    LastDay = Ainj[[Length[Ainj] - cou, 1]];
    If[tEnd > LastDay, Ainj = Ainj[[1 ;; Length[Ainj] - cou - 1]];
      CurLast = FindCurativeSecondDose[LastDay];
      Ainj = Join[Ainj, {{LastDay, CurLast, eta * CurLast}}]; Stop = 1, cou++];
  ];];];
(* now Ainj contains best schedule *)

(* ...and also do this *)
(* now we have definitely curative schedule *)
(* but discretized schedule can be excessively curative, i.e. lead to Nmin<<Ncur, in this case adjust it to become less toxic, but yet curative *)

If[Nn < Ncur,
  NotebookDelete[prMDopt];
  prMDopt = PrintTemporary["Adjusting multiplier for toxicity"];

  InjDoses = Ainj[[All, 2]] / nCpm;

  kkadjRes = Array[f, {1, 3}];
  kkadjRes[[1, 1]] = "kk";
  kkadjRes[[1, 2]] = "Nmin";
```

```
kkadjRes[[1, 3]] = "Tox";
kk = 1;

kkold = kk;
Stop = 0;
ijk = 1;
dk = 0.05;
UncurCnt = 0;

kk = kk / (1 - dk);
While[Stop == 0,

  kk = kk * (1 - dk);
  NotebookDelete[prkk];
  prkk = PrintTemporary["kk=" <> ToString[N[kk]]];

  ijk++;
  Ainj = Table[{Inter * (dd - 1), kk * InjDoses[[dd]] * nCpm, kk * eta * InjDoses[[dd]] * nCpm}, {dd, 1, Length[InjDoses]}];

  kkadjRes = Join[kkadjRes, {{"", "", ""}}];
  Quiet[kkadjRes[[ijk, 1]] = kk;
    kkadjRes[[ijk, 2]] = FullSystemSolutionMD[];
    kkadjRes[[ijk, 3]] = If[tEnd < Ainj[[Length[Ainj], 1]],
        If[ActBloodpw[[Position[Sort[Join[{tEnd}, Ainj[[All, 1]]]], tEnd][[1, 1]] - 1]][365] / nCpm < 0,
          1000, ActBloodpw[[Position[Sort[Join[{tEnd}, Ainj[[All, 1]]]], tEnd][[1, 1]] - 1]][365] / nCpm],
        If[ActBloodpw[[npw]][tEnd] / nCpm < 0, 1000, ActBloodpw[[npw]][tEnd] / nCpm]];

    If[kkadjRes[[ijk, 2]] > Ncur && kkadjRes[[ijk - 1, 2]] < Ncur, dk = -dk / 2];
    If[kkadjRes[[ijk, 2]] < Ncur && kkadjRes[[ijk - 1, 2]] > Ncur, dk = -dk / 2];
    If[kkadjRes[[ijk, 2]] < Ncur && kkadjRes[[ijk, 2]] > 0.99 * Ncur, Stop = 1];
```

```
    If[kk < 10^(-20), Stop = 1];
    If[dk < 10^(-8), Stop = 1]];
  NotebookDelete[prkk];

  Ainj = Table[{Inter * (dd - 1), kk * InjDoses[[dd]] * nCpm, kk * eta * InjDoses[[dd]] * nCpm}, {dd, 1, Length[InjDoses]}];
  AinjBestYet = Ainj;

  (* is it curative without last doses *)
  NotebookDelete[prMDopt];
  prMDopt = PrintTemporary["Checking is it curative without last doses"];
  Stop = 0;
  While[Stop == 0 && Length[AinjBestYet] ≥ 2,
    Ainj = AinjBestYet[[1 ;; Length[AinjBestYet] - 1]];
    If[FullSystemSolutionMD[] < Ncur, AinjBestYet = Ainj, Stop = 1]];

  If[AinjBestYet[[Length[AinjBestYet], 2]] == 0, AinjBestYet = AinjBestYet[[1 ;; Length[AinjBestYet] - 1]]];

  (* make last dose a curative one *)
  NotebookDelete[prMDopt];
  prMDopt = PrintTemporary["Making last dose a curative one"];
  If[Length[AinjBestYet] ≥ 2,
    Ainj = AinjBestYet[[1 ;; Length[AinjBestYet] - 1]];
    (*If[tEnd>AinjBestYet[[Length[AinjBestYet],1]],*)

    Dmin = 0; Dmax = AinjBestYet[[Length[AinjBestYet], 2]] (*If[!NumberQ[ASC],Ainj[[1,2]],ASC]*);
    IC2 = FullSystemSolutionGetValues[AinjBestYet[[Length[AinjBestYet], 1]]][[2]];
    While[FullSystemSolutionIC[Join[IC2, {Dmax, eta * Dmax}]][[2, 1]] > Ncur, Dmax = 2 * Dmax];

    While[Abs[Dmin - Dmax] / Dmax > 0.000001,
      DA2 = (Dmin + Dmax) / 2; (*Ainj={{0,f1*ASC}};*)
      If[FullSystemSolutionIC[Join[IC2, {DA2, eta * DA2}]][[2, 1]] > Ncur, Dmin = DA2, Dmax = DA2]];
```

```
   CurLast = Dmax;
   AinjBestYet = Join[Ainj, {{AinjBestYet[[Length[AinjBestYet], 1]], CurLast, eta * CurLast}}] (*] *);

Ainj = AinjBestYet;
FullSystemSolutionToxicity[];
ToxLeastYet = If[ActBloodpw[[npw]][tEnd] / nCpm < 0, 1000, ActBloodpw[[npw]][tEnd] / nCpm];

If[ToxLeastYet < 0, ToxLeastYet = 10^23; StopInt = 1;
   InterArray[[kji, 1]] = Inter;
   InterArray[[kji, 2]] = ToxLeastYet;
   InterArray[[kji, 3]] = AinjBestYet,

   (* FIX PRE-LAST DOSE -- change to curative, if resulting schedule is less toxic *)
   If[Length[AinjBestYet] ≥ 3,

     NotebookDelete[prMDopt];
     prMDopt = PrintTemporary["Fixing pre-last dose"];

     (* delete last dose and replace current last by curative one *)
     Quiet[Stop = 0;
       cou = 1;
       While[Stop == 0,

         NotebookDelete[pr];
         pr = PrintTemporary[ToString[cou] <> " -- " <> ToString[Length[AinjBestYet]] <> " doses"];

         Ainj = AinjBestYet[[1 ;; Length[AinjBestYet] - 2]];
         FullSystemSolutionMD[];
         If[tEnd > AinjBestYet[[Length[AinjBestYet] - 1, 1]],
```

```
            Dmin = 0;
            Dmax = AinjBestYet[[Length[AinjBestYet] - 1, 2]] (*If[ ! NumberQ[ASC],Ainj[[1,2]],ASC] *);
            IC2 = FullSystemSolutionGetValues[AinjBestYet[[Length[AinjBestYet] - 1, 1]]][[2]];
            While[FullSystemSolutionIC[Join[IC2, {Dmax, eta * Dmax}]][[2, 1]] > Ncur, Dmax = 2 * Dmax];

            While[Abs[Dmin - Dmax] / Dmax > 0.000001,
              DA2 = (Dmin + Dmax) / 2; (*Ainj= {{0,f1*ASC}};*)
              If[FullSystemSolutionIC[Join[IC2, {DA2, eta * DA2}]][[2, 1]] > Ncur, Dmin = DA2, Dmax = DA2]];
            CurLast = Dmax;

            Ainj = Join[Ainj, {{AinjBestYet[[Length[AinjBestYet] - 1, 1]], CurLast, eta * CurLast}}];

            FullSystemSolutionToxicity[];
            If[ActBloodpw[[npw]][tEnd] / nCpm < ToxLeastYet && ActBloodpw[[npw]][tEnd] / nCpm > 0, AinjBestYet = Ainj;
              ToxLeastYet = ActBloodpw[[npw]][tEnd] / nCpm, Stop = 1];
            If[Length[AinjBestYet] == 2, Stop = 1];
            cou++, Stop = 1; Ainj = AinjBestYet]
        ]];
      NotebookDelete[pr];
      pr = PrintTemporary["Optimal toxicity after fixing pre-last dose is " <> ToString[ToxLeastYet]]];

    InterArray[[kji, 1]] = Inter;
    InterArray[[kji, 2]] = ToxLeastYet;
    InterArray[[kji, 3]] = AinjBestYet;

    If[kji ≥ 3 && InterArray[[kji, 2]] > InterArray[[kji - 1, 2]], StopInt = 1;
      ToxLeastYet = InterArray[[kji - 1, 2]];
      AinjBestYet = InterArray[[kji - 1, 3]],

      If[kji == 2 && InterArray[[kji, 1]] == InterMax, StopInt = 1;
```

```
        ToxLeastYet = InterArray[[kji, 2]];
        AinjBestYet = InterArray[[kji, 3]]
    ];];

If[kji > 10, StopInt = 1;  (* that's enough of increasing interval, as such cases show *)
   ToxLeastYet = InterArray[[kji, 2]];
   AinjBestYet = InterArray[[kji, 3]]];

(* if only one dose remains, do nothing, it's already optimized *)
If[Length[AinjBestYet] ≠ 1,

   NotebookDelete[prInt];
   prInt = PrintTemporary["Optimal toxicity now is " <> ToString[ToxLeastYet]];

   (* FIX FIRST DOSE -- is it profitable to increase or decrease it a bit *)
   NotebookDelete[prMDopt];
   prMDopt = PrintTemporary["Fixing first dose"];

   Quiet[ddinit = -0.04;
      dd = ddinit;
      ddmin = dd / 32;
      Stop = 0;
      ChngSgn = 0;
      ImprFlg = 0;

      While[Stop == 0,

         NotebookDelete[prFFD];
         prFFD = PrintTemporary["dd=" <> ToString[dd]];

         LastDay = AinjBestYet[[Length[AinjBestYet], 1]];
```

```
Ainj = AinjBestYet[[1 ;; Length[AinjBestYet] - 1]];
Ainj[[1, 2]] = (1 + dd) * AinjBestYet[[1, 2]];
Ainj[[1, 3]] = (1 + dd) * AinjBestYet[[1, 3]];

While[FullSystemSolutionMD[] < Ncur,
  Ainj = Ainj[[1 ;; Length[Ainj] - 1]];
  LastDay = Ainj[[Length[Ainj], 1]];
];

If[tEnd > LastDay, Stop = 1,
  If[tEnd < Ainj[[Length[Ainj], 1]] + (-Log[0.0017] / lambda), (* too much of a decrease of first dose *)

    If[Abs[dd] > Abs[ddmin], dd = dd / 2, Stop = 1]
    ,

    Dmin = 0; Dmax = Ainj[[Length[Ainj], 2]] (*If[ !NumberQ[ASC],Ainj[[1,2]],ASC]*);
    IC2 = FullSystemSolutionGetValues[Ainj[[Length[Ainj], 1]]][[2]];
    While[FullSystemSolutionIC[Join[IC2, {Dmax, eta * Dmax}]][[2, 1]] > Ncur, Dmax = 2 * Dmax];

    While[Abs[Dmin - Dmax] / Dmax > 0.000001,
      DA2 = (Dmin + Dmax) / 2; (*Ainj={{0,f1*ASC}};*)
      If[FullSystemSolutionIC[Join[IC2, {DA2, eta * DA2}]][[2, 1]] > Ncur, Dmin = DA2, Dmax = DA2]];
    CurDoseAlt = Dmax;

    Ainj = Join[Ainj, {{LastDay, CurDoseAlt, eta * CurDoseAlt}}];

    FullSystemSolutionToxicity[];
    ToxAlt = If[ActBloodpw[[npw]][tEnd] / nCpm < 0, 1000, ActBloodpw[[npw]][tEnd] / nCpm];

    NotebookDelete[prToxAlt];
```

```
                prToxAlt = PrintTemporary["ToxAlt=" <> ToString[ToxAlt]];

                If[ToxAlt < ToxLeastYet, AinjBestYet = Ainj; ToxLeastYet = ToxAlt; ImprFlg = 1,
                 If[Abs[dd] > Abs[ddmin], dd = dd / 2, If[ChngSgn == 0 && ImprFlg == 0, dd = -1 * ddinit;
                    ChngSgn = 1, Stop = 1]]];
             ]]]];
          NotebookDelete[prFFD];
          NotebookDelete[prToxAlt];];];
       NotebookDelete[prInt];]])
```

In[∘]:=

```
(* script for mulidose optimization in non-curative case *)
MDoptOS[] := (

   (* find the case with const a (concentration of active antibodies in blood) which provides greater OS under the same toxicity *)

   NotebookDelete[prMDopt];
   prMDopt = PrintTemporary["Solving approximation with constant a"];

   ConstaRes = Array[f, {2, 2}];
   ConstaRes⟦1, 1⟧ = "ka";
   ConstaRes⟦1, 2⟧ = "~OS";

   ka = 1;

   Stop = 0;
   ijk = 2;
   dk = 1;
```

```
While[Stop == 0,

  NotebookDelete[prca];
  prca = PrintTemporary["ka=" <> ToString[N[ka]]];

  Quiet[aconst = ka * aconst0;
   tEndInit = tMax;
   tEnd = tEndInit;
   SolutionConsta[0, 1];

   AB = ActBlood[tEndInit];
   ABm1 = ActBlood[tEndInit - 1];
   While[ (*still alive at 365 days *) AB < Abld,

     tAdd = (Abld - AB)/(AB - ABm1); tEndInit = tEndInit + 2 * tAdd; tEnd = tEndInit;
     SolutionConsta[0, 1]; AB = ActBlood[tEndInit]; ABm1 = ActBlood[tEndInit - 1];];

    (*find moment in time where ActBlood[t] =Abld*)
   tTOX = t /. NMinimize[{Abs[ActBlood[t] - Abld], t > 0, t < tEnd}, t][[2]];
    (*to save computational time, estimate OS roughly*)

   ConstaRes[[ijk, 1]] = N[ka];
   ConstaRes[[ijk, 2]] = tTOX + 1. * Log[Cd/(If[! NumberQ[NN[tEnd]], NN1[tEnd], NN[tEnd]] * Nnor)] / rho];

  If[(Abs[ConstaRes[[ijk, 2]] - ConstaRes[[ijk - 1, 2]]]/ConstaRes[[ijk, 2]] < 0.0001 && ijk > 2) || ConstaRes[[ijk, 2]] > tMax, Stop = 1,
```

```
      (* OS increased compared to previous case *)
      If[ConstaRes[[ijk, 2]] > ConstaRes[[ijk - 1, 2]] || ijk == 2,
        ConstaRes = Join[ConstaRes, {{"", ""}}]; ijk++; ka = ka * (1 + dk),
        (* OS decreased compared to previous case *)
        ConstaRes = Join[ConstaRes, {{"", ""}}]; ijk++; dk = -dk / 2.; ka = ka * (1 + dk)
      ]];
];

NotebookDelete[prca];

tEndOpt = tTOX;
fFNac[t] = fFN[t];
NNac[t] = NN[t];
dFac[t] = dF[t];

(*so, the last run was with optimal ka *)

(* Discretize injections -- no more than 10 injections, no more frequently than once in a day *)
If[tTOX < 1,

  NotebookDelete[prMDopt];
  prMDopt = PrintTemporary["Single-dose is better"];
  ATox = FindToxDose[];

  Ainj = {{0, ATox * nCpm, eta * ATox * nCpm}};
  Quiet[FullSystemSolutionMD[]];
  ToxTot = ActBloodpw[[1]][tEnd];

  DiscrArrayOpt = Array[f, 5];
```

```
DiscrArrayOpt[[1]] = 0;
DiscrArrayOpt[[2]] = 1;
DiscrArrayOpt[[3]] = tEnd;
DiscrArrayOpt[[4]] = ToxTot;
DiscrArrayOpt[[5]] = Select[Ainj, #[[1]] < Floor[tEnd] &]

, (* multi-dose is better *)
NotebookDelete[prMDopt];
prMDopt = PrintTemporary["Discretizing injections"];

InterMin = 1;
InterMax = Min[Round[tMax / 2], Ceiling[N[Ceiling[tEndOpt] / 2]]];

DiscrArray = Array[f, {InterMax - InterMin + 1 + 1, 5}];

DiscrArray[[1, 1]] = "Interval";
DiscrArray[[1, 2]] = "#Inj";
DiscrArray[[1, 3]] = "~OS";
DiscrArray[[1, 4]] = "ToxOS";
DiscrArray[[1, 5]] = "Schedule";

ii = 2;
InjMax = 10;

StopInt = 0;
Inter = InterMin;

While[StopInt == 0,

  NotebookDelete[prdis];
  prdis = PrintTemporary["InjMax=" <> ToString[InjMax] <> ", Inter=" <> ToString[Inter] <> ", max is " <> ToString[InterMax]];
```

```
InjDoses =
  Table[Max[0, NIntegrate[(lambda * aconst (*decay*) + kon * gamma/V * (fFNac[t] * NNac[t] + dFac[t]) * aconst (*binding*) +
          kappac * aconst (*clearance*)), {t, dd, dd + Inter}]]/nCpm, {dd, 0, Min[Round[tEndOpt], (InjMax - 1) * Inter], Inter}];

If[Length[InjDoses] == InjMax,
  InjDoses[[InjMax]] = NIntegrate[(lambda * aconst (*decay*) + kon * gamma/V * (fFNac[t] * NNac[t] + dFac[t]) * aconst
          (*binding*) + kappac * aconst (*clearance*)), {t, (InjMax - 2) * Inter, Round[tEndOpt]}]/nCpm];

kkmin = 1;
dk = 0.5;

Stop = 0;
ToxTotPrev = Abld;
ToxTot = Abld;

(* normalize toxic levels close to lethal level *)
While[Stop == 0,

  NotebookDelete[pr];
  pr = PrintTemporary["kkmin=" <> ToString[N[kkmin]]];

  Ainj = Table[{Inter * (dd - 1), kkmin * InjDoses[[dd]] * nCpm, kkmin * eta * InjDoses[[dd]] * nCpm}, {dd, 1, Length[InjDoses]}];

  Quiet[FullSystemSolutionMD[]];
  ToxTotPrev = ToxTot;
  ToxTot = ActBloodpw[[Min[Length[Ainj], Ceiling[tEnd / Inter]]]][tEnd];
```

```
        If[ (ToxTot / Abld - 1) * (ToxTotPrev / Abld - 1) < 0, dk = dk / 2]; (*changed sign*)

        If[ToxTot < 0.99 * Abld,
          kkmin = kkmin * (1 + dk); If[dk < 10^(-7), Stop = 1],
          If[ToxTot > Abld, kkmin = kkmin * (1 - dk), Stop = 1] ;];

      NotebookDelete[pr];

      DiscrArray[[ii, 1]] = Inter;
      DiscrArray[[ii, 2]] = InjMax;
      DiscrArray[[ii, 3]] = tEnd;
      DiscrArray[[ii, 4]] = ToxTot;
      DiscrArray[[ii, 5]] = Select[Ainj, #[[1]] < Floor[tEnd] &];

      NotebookDelete[prr];
      prr = PrintTemporary[MatrixForm[DiscrArray[[1 ;; ii]]]];

      If[DiscrArray[[ii, 3]] < Ainj[[2, 1]], StopInt = 1]; (* OS before second dose *)
      If[Inter > InterMin && DiscrArray[[ii, 3]] < DiscrArray[[ii - 1, 3]], StopInt = 1];
      If[Inter + 1 > tTOX, StopInt = 1];
      If[DiscrArray[[ii, 3]] > tMax, StopInt = 1]; (* OS is more than a year *)

      ii++;
      Inter++;];
    NotebookDelete[prr];

    (* select the best case with greater OS *)
    DiscrArrayOpt = DiscrArray[[Position[DiscrArray[[1 ;; ii - 1, 3]], Max[DiscrArray[[2 ;; ii - 1, 3]]]][[1, 1]]]];
  NotebookDelete[prMDopt];
```

```
(* check whether the chosen treatment is curative -- this can happen *)
prMDopt = PrintTemporary["Optimiation by OS provided a curative schedule"];
Ainj = DiscrArrayOpt[[5]];
Curative = 0;
If[FullSystemSolutionMD[] < Ncur,
  Print[""];
  Curative = 1;

   (* now we have curative schedule *)
   (* but it is excessively curative, i.e. leads to Nmin<<Ncur, so adjust it to become less toxic, but yet curative *)

  NotebookDelete[prMDopt];
  prMDopt = PrintTemporary["Adjusting multiplier for toxicity"];

  InjDoses = Ainj[[All, 2]] / nCpm;
  Inter = Ainj[[2, 1]];
  AinjBestYet = Ainj;

  kkadjRes = Array[f, {1, 3}];
  kkadjRes[[1, 1]] = "kk";
  kkadjRes[[1, 2]] = "Nmin";
  kkadjRes[[1, 3]] = "Tox";
  kk = 1;

  kkold = kk;
  Stop = 0;
  ijk = 1;
  dk = 0.05;
  UncurCnt = 0;

  kk = kk / (1 - dk);
```

```
While[Stop == 0,

  kk = kk * (1 - dk);
  NotebookDelete[prkk];
  prkk = PrintTemporary["kk=" <> ToString[N[kk]]];

  ijk++;
  Ainj = Table[{Inter * (dd - 1), kk * InjDoses[[dd]] * nCpm, kk * eta * InjDoses[[dd]] * nCpm}, {dd, 1, Length[InjDoses]}];

  kkadjRes = Join[kkadjRes, {{"", "", ""}}];
  Quiet[kkadjRes[[ijk, 1]] = kk;
    kkadjRes[[ijk, 2]] = FullSystemSolutionMD[];
    kkadjRes[[ijk, 3]] = If[tEnd < Ainj[[Length[Ainj], 1]],
        ActBloodpw[[Position[Sort[Join[{tEnd}, Ainj[[All, 1]]]], tEnd][[1, 1]] - 1]][365] / nCpm, ActBloodpw[[npw]][tEnd] / nCpm]];

  If[kkadjRes[[ijk, 2]] > Ncur && kkadjRes[[ijk - 1, 2]] < Ncur, dk = -dk / 2];
  If[kkadjRes[[ijk, 2]] < Ncur && kkadjRes[[ijk - 1, 2]] > Ncur, dk = -dk / 2];
  If[kkadjRes[[ijk, 2]] < Ncur && kkadjRes[[ijk, 2]] > 0.99 * Ncur, Stop = 1];
  If[kk < 10^(-20), Stop = 1]];
NotebookDelete[prkk];

Ainj = Table[{Inter * (dd - 1), kk * InjDoses[[dd]] * nCpm, kk * eta * InjDoses[[dd]] * nCpm}, {dd, 1, Length[InjDoses]}];
AinjBestYet = Ainj;

(* is it curative without last doses *)
NotebookDelete[prMDopt];
prMDopt = PrintTemporary["Checking is it curative without last doses"];
Stop = 0;
While[Stop == 0 && Length[AinjBestYet] ≥ 2,
  Ainj = AinjBestYet[[1 ;; Length[AinjBestYet] - 1]];
```

```
  If[FullSystemSolutionMD[] < Ncur, AinjBestYet = Ainj, Stop = 1]];

 (* make last dose a curative one *)
NotebookDelete[prMDopt];
prMDopt = PrintTemporary["Making last dose a curative one"];
If[Length[AinjBestYet] ≥ 2,
  Ainj = AinjBestYet[[1 ;; Length[AinjBestYet] - 1]];
  (*If[tEnd>AinjBestYet[[Length[AinjBestYet],1]],*)

  Dmin = 0; Dmax = AinjBestYet[[Length[AinjBestYet], 2]] (*If[!NumberQ[ASC],Ainj[[1,2]],ASC]*);
  IC2 = FullSystemSolutionGetValues[AinjBestYet[[Length[AinjBestYet], 1]]][[2]];
  While[FullSystemSolutionIC[Join[IC2, {Dmax, eta * Dmax}]][[2, 1]] > Ncur, Dmax = 2 * Dmax];

  While[Abs[Dmin - Dmax] / Dmax > 0.000001,
    DA2 = (Dmin + Dmax) / 2; (*Ainj={{0,f1*ASC}};*)
    If[FullSystemSolutionIC[Join[IC2, {DA2, eta * DA2}]][[2, 1]] > Ncur, Dmin = DA2, Dmax = DA2]];
  CurLast = Dmax;
  AinjBestYet = Join[Ainj, {{AinjBestYet[[Length[AinjBestYet], 1]], CurLast, eta * CurLast}}]] (*]*);

Ainj = AinjBestYet;
FullSystemSolutionToxicity[];
ToxLeastYet = ActBloodpw[[npw]][tEnd] / nCpm;

If[ToxLeastYet < 0, ToxLeastYet = 10^23; StopInt = 1;
  InterArray[[kji, 1]] = Inter;
  InterArray[[kji, 2]] = ToxLeastYet;
  InterArray[[kji, 3]] = AinjBestYet,

  (* FIX PRE-LAST DOSE -- change to curative, if resulting schedule is less toxic *)
  If[Length[AinjBestYet] ≥ 3,
```

```
NotebookDelete[prMDopt];
prMDopt = PrintTemporary["Fixing pre-last dose"];

(* delete last dose and replace current last by curative one *)
Quiet[Stop = 0;
  cou = 1;
  While[Stop == 0,

    NotebookDelete[pr];
    pr = PrintTemporary[ToString[cou] <> " -- " <> ToString[Length[AinjBestYet]] <> " doses"];

    Ainj = AinjBestYet[[1 ;; Length[AinjBestYet] - 2]];
    FullSystemSolutionMD[];
    If[tEnd > AinjBestYet[[Length[AinjBestYet] - 1, 1]],

      Dmin = 0; Dmax = AinjBestYet[[Length[AinjBestYet] - 1, 2]] (*If[!NumberQ[ASC],Ainj[[1,2]],ASC]*);
      IC2 = FullSystemSolutionGetValues[AinjBestYet[[Length[AinjBestYet] - 1, 1]]][[2]];
      While[FullSystemSolutionIC[Join[IC2, {Dmax, eta * Dmax}]][[2, 1]] > Ncur, Dmax = 2 * Dmax];

      While[Abs[Dmin - Dmax] / Dmax > 0.000001,
        DA2 = (Dmin + Dmax) / 2; (*Ainj={{0,f1*ASC}};*)
        If[FullSystemSolutionIC[Join[IC2, {DA2, eta * DA2}]][[2, 1]] > Ncur, Dmin = DA2, Dmax = DA2]];
      CurLast = Dmax;

      Ainj = Join[Ainj, {{AinjBestYet[[Length[AinjBestYet] - 1, 1]], CurLast, eta * CurLast}}];

      FullSystemSolutionToxicity[];
      If[ActBloodpw[[npw]][tEnd] / nCpm < ToxLeastYet, AinjBestYet = Ainj;
        ToxLeastYet = ActBloodpw[[npw]][tEnd] / nCpm, Stop = 1];
      If[Length[AinjBestYet] == 2, Stop = 1];
```

```
            cou ++, Stop = 1; Ainj = AinjBestYet]
          ]];
        NotebookDelete[pr];
        pr = PrintTemporary["Optimal toxicity after fixing pre-last dose is " <> ToString[ToxLeastYet]]];

      InterArray[[kji, 1]] = Inter;
      InterArray[[kji, 2]] = ToxLeastYet;
      InterArray[[kji, 3]] = AinjBestYet;

      If[kji ≥ 3 && InterArray[[kji, 2]] > InterArray[[kji - 1, 2]], StopInt = 1;
        ToxLeastYet = InterArray[[kji - 1, 2]];
        AinjBestYet = InterArray[[kji - 1, 3]],

        If[kji == 2 && InterArray[[kji, 1]] == InterMax, StopInt = 1;
          ToxLeastYet = InterArray[[kji, 2]];
          AinjBestYet = InterArray[[kji, 3]]]
      ];];

    If[kji > 10, StopInt = 1; (* that's enough of increasing interval, as such cases show *)
      ToxLeastYet = InterArray[[kji, 2]];
      AinjBestYet = InterArray[[kji, 3]]];

    (* if only one dose remains, do nothing, it's already optimized *)
    If[Length[AinjBestYet] ≠ 1,

      NotebookDelete[prInt];
      prInt = PrintTemporary["Optimal toxicity now is " <> ToString[ToxLeastYet]];

       (* FIX FIRST DOSE -- is it profitable to increase or decrease it a bit *)
      NotebookDelete[prMDopt];
```

```
prMDopt = PrintTemporary["Fixing first dose"];

Quiet[ddinit = -0.04;
  dd = ddinit;
  ddmin = dd / 32;
  Stop = 0;
  ChngSgn = 0;
  ImprFlg = 0;

  While[Stop == 0,

    NotebookDelete[prFFD];
    prFFD = PrintTemporary["dd=" <> ToString[dd] ];

    LastDay = AinjBestYet[[Length[AinjBestYet], 1]];

    Ainj = AinjBestYet[[1 ;; Length[AinjBestYet] - 1]];
    Ainj[[1, 2]] = (1 + dd) * AinjBestYet[[1, 2]];
    Ainj[[1, 3]] = (1 + dd) * AinjBestYet[[1, 3]];

    While[FullSystemSolutionMD[] < Ncur,
      Ainj = Ainj[[1 ;; Length[Ainj] - 1]];
      LastDay = Ainj[[Length[Ainj], 1]];
    ];

    If[tEnd > LastDay, Stop = 1,
      If[tEnd < Ainj[[Length[Ainj], 1]] + (-Log[0.0017] / lambda), (* too much of a decrease of first dose *)

          If[Abs[dd] > Abs[ddmin], dd = dd / 2, Stop = 1]
          ,
```

```
            Dmin = 0; Dmax = Ainj⟦Length[Ainj], 2⟧(*If[!NumberQ[ASC],Ainj⟦1,2⟧,ASC]*);
            IC2 = FullSystemSolutionGetValues[Ainj⟦Length[Ainj], 1⟧]⟦2⟧;
            While[FullSystemSolutionIC[Join[IC2, {Dmax, eta * Dmax}]]⟦2, 1⟧ > Ncur, Dmax = 2 * Dmax];

            While[Abs[Dmin - Dmax] / Dmax > 0.000001,
              DA2 = (Dmin + Dmax) / 2; (*Ainj={{0,f1*ASC}};*)
              If[FullSystemSolutionIC[Join[IC2, {DA2, eta * DA2}]]⟦2, 1⟧ > Ncur, Dmin = DA2, Dmax = DA2]];
            CurDoseAlt = Dmax;

            Ainj = Join[Ainj, {{LastDay, CurDoseAlt, eta * CurDoseAlt}}];

            FullSystemSolutionToxicity[];
            ToxAlt = ActBloodpw⟦npw⟧[tEnd] / nCpm;

            NotebookDelete[prToxAlt];
            prToxAlt = PrintTemporary["ToxAlt=" <> ToString[ToxAlt]];

            If[ToxAlt < ToxLeastYet, AinjBestYet = Ainj; ToxLeastYet = ToxAlt; ImprFlg = 1,
              If[Abs[dd] > Abs[ddmin], dd = dd / 2, If[ChngSgn == 0 && ImprFlg == 0, dd = -1 * ddinit;
                  ChngSgn = 1, Stop = 1]]]];
        ]]]];
    NotebookDelete[prFFD];
    NotebookDelete[prToxAlt];
    NotebookDelete[prMDopt];];

)
```

```
(* The outcome of multidose optimization for the training set was obtained by very long-running code hidden here,
it takes about a week for 1000 parameter sets.
   In the end of this code there is a script for exporting intermediate results. *)

cc0 = 2; Nlast = Length[PatientsTrainingSet];
SetBasicParameterValues[];

resultMD = Array[f, {Length[PatientsTrainingSet] + 1, 16}];

resultMD[[1, 1]] = "κc";
resultMD[[1, 2]] = "κp";
resultMD[[1, 3]] = "γ";
resultMD[[1, 4]] = "V";
resultMD[[1, 5]] = "ks";
resultMD[[1, 6]] = "ρ";
resultMD[[1, 7]] = "ω";
resultMD[[1, 8]] = "α";
resultMD[[1, 9]] = "kf";
resultMD[[1, 10]] = "N0";
resultMD[[1, 11]] = "SD-Acur";
resultMD[[1, 12]] = "SD-Tox";

resultMD[[1, 13]] = "MD-TotalTox";
resultMD[[1, 14]] = "MD-TotalDose";
resultMD[[1, 15]] = "CurFlg";
resultMD[[1, 16]] = "MD-Schedule";

Quiet[
  For[cc = cc0, cc ≤ Nlast, cc++,

    NotebookDelete[prcc];
```

```
prcc = PrintTemporary["cc=" <> ToString[cc]];

kappac = PatientsTrainingSet⟦cc, 1⟧;
kappap = PatientsTrainingSet⟦cc, 2⟧;
gamma = PatientsTrainingSet⟦cc, 3⟧ * Nnor / 10^7;
V = PatientsTrainingSet⟦cc, 4⟧;
ks = PatientsTrainingSet⟦cc, 5⟧;
rho = PatientsTrainingSet⟦cc, 6⟧;
omega = PatientsTrainingSet⟦cc, 7⟧;
alpha = PatientsTrainingSet⟦cc, 8⟧;
kf = PatientsTrainingSet⟦cc, 9⟧;
N0 = PatientsTrainingSet⟦cc, 10⟧ * 10^7 / Nnor;

resultMD⟦cc, 1⟧ = kappac;
resultMD⟦cc, 2⟧ = kappap;
resultMD⟦cc, 3⟧ = gamma * 10^7 / Nnor;
resultMD⟦cc, 4⟧ = V;
resultMD⟦cc, 5⟧ = ks;
resultMD⟦cc, 6⟧ = rho;
resultMD⟦cc, 7⟧ = omega;
resultMD⟦cc, 8⟧ = alpha;
resultMD⟦cc, 9⟧ = kf;
resultMD⟦cc, 10⟧ = N0 * Nnor / 10^7;

(* STEP 1. We define whether a virtual mouse can be potentially cured
  These are mice, for which cure is achieved within a year in approximation with fully occupied receptors and eta kept to initial value,
and mice that were cured in single-dose setting without achieveing lethal toxicity *)

OccRecCureTest[]; (* if cured within a year, CurFlgOR=1 *)

(* In training set, numerically is mouse cured by single dose without lethal toxicity? *)
```

```
ASC = result[[cc, 11]];
Tox = result[[cc, 16]] + result[[cc, 19]];
resultMD[[cc, 11]] = ASC;
resultMD[[cc, 12]] = Tox;

If[Tox < Abld / nCpm, NotebookDelete[progress];
  progress = PrintTemporary["Cured by single dose without exceeding toxicity, which is " <> ToString[result[[cc, 16]] + result[[cc, 19]]]];
  CurFlgOR = 1,
  NotebookDelete[progress];
  progress = PrintTemporary["Not cured by single dose without exceeding toxicity"];
];

(* After step 1. If a mouse is potential be potentially cured,
then we perform optimization with the goal of cure at the cost of least toxic decays level *)
If[CurFlgOR == 1,
  NotebookDelete[progress];
  progress = PrintTemporary["Cured in the setting when all the receptors are occupied"];

  If[tCure > 1, (* otherwise the cure is achieved within 1 day, and multidosing algorithm will not work *)

    (* 1→Step 2. Perform optimization of multidose schedule with curative intent *)
    MDoptCURE[];

    If[CurFlgOR == 0, (* it is set to zero if optimization of multidose setting yields one-dose schedule *)
      progress = PrintTemporary["Optization of multidose setting yields one-dose schedule"]];

    (* 2→Step 3. Compare it with minimal single curative dose setting... *)
    If[result[[cc, 16]] + result[[cc, 19]] < ToxLeastYet, (* single dose is better *)

      resultMD[[cc, 13]] = result[[cc, 16]] + result[[cc, 19]];
      resultMD[[cc, 14]] = result[[cc, 11]];
```

```
      resultMD[[cc, 15]] = 1;
      resultMD[[cc, 16]] = {{0, result[[cc, 11]] * nCpm, eta * result[[cc, 11]] * nCpm}},

      resultMD[[cc, 13]] = ToxLeastYet;
      resultMD[[cc, 14]] = Total[AinjBestYet[[All, 2]]] / nCpm;
      resultMD[[cc, 15]] = 1;
      resultMD[[cc, 16]] = AinjBestYet],

    (* the cure is achieved within 1 day, single-dosing is already optimal *)
   resultMD[[cc, 13]] = result[[cc, 16]] + result[[cc, 19]];
   resultMD[[cc, 14]] = result[[cc, 11]];
   resultMD[[cc, 15]] = 1;
   resultMD[[cc, 16]] = {{0, result[[cc, 11]] * nCpm, eta * result[[cc, 11]] * nCpm}};];

(* .. and compare to optimal double-dose setting *)
(* Compare the optimal multi- or single-dose schedule to double-dose schedule *)
NotebookDelete[progress];
progress = PrintTemporary["Comparing to double dose"];
ASC = FindCurDose[] * nCpm; (* single curative dose *)
Quiet[OptimizeTwoDose[]];
resultDDOpt = resultDD[[Length[resultDD]]];
If[resultDDOpt[[5]] < resultMD[[cc, 13]], (*Double dose is better*)
   resultMD[[cc, 13]] = resultDDOpt[[5]];
   resultMD[[cc, 14]] = (resultDDOpt[[1]] * ASC + resultDDOpt[[4]] * nCpm) / nCpm;
   resultMD[[cc, 15]] = 1;
   resultMD[[cc, 16]] =
     {{0, resultDDOpt[[1]] * ASC, eta * resultDDOpt[[1]] * ASC}, {resultDDOpt[[2]], resultDDOpt[[4]] * nCpm, eta * resultDDOpt[[4]] * nCpm}};
];

, NotebookDelete[progress];
```

```
  progress = PrintTemporary["Not cured in the setting when all the receptors are occupied"]];

 (* if the optimal discretized schedule is still curative but lethally toxic, move to optimization of OS *)
 If[CurFlgOR == 1 && resultMD[[cc, 13]] > Abld / nCpm, CurFlgOR = 0; NotebookDelete[progress];
  progress = PrintTemporary["Optimal discretized schedule is curative but lethally toxic"]];

 (* 1→ or 3→ if mouse cannot be potentially cured without lethal toxicity, or the optimized schedule is lethally toxic,
 then we perform optimization with the goal of increase of overall survival at the cost of non-lethal toxic decays level *)
 If[CurFlgOR == 0,

  (* Step 4. Perform optimization of multidose schedule with intent to prolong OS *)
  MDoptOS[];

  (* 4 → Step 5. Compare with single-dose non-lethally-toxic schedule *)
  If[Curative == 0,
   DTox = FindToxDose[];
   Ainj = {{0, DTox * nCpm, eta * DTox * nCpm}};
   FullSystemSolutionMD[];
   If[tEnd > DiscrArrayOpt[[3]],
     (* single-dose is best case *)
    resultMD[[cc, 13]] = ActBloodpw[[npw]][tEnd] / nCpm;
    resultMD[[cc, 14]] = DTox;
    resultMD[[cc, 15]] = 0;
    resultMD[[cc, 16]] = Ainj
    , (* multi-dose is better *)
    resultMD[[cc, 13]] = DiscrArrayOpt[[4]] / nCpm;
    resultMD[[cc, 14]] = Total[DiscrArrayOpt[[5, All, 2]]] / nCpm;
    resultMD[[cc, 15]] = 0;
    resultMD[[cc, 16]] = DiscrArrayOpt[[5]];],
   (* multi-dose OS optimization provided a curative schedule *)
```

```
                resultMD[[cc, 13]] = ToxLeastYet;
                resultMD[[cc, 14]] = Total[AinjBestYet[[All, 2]]] / nCpm;
                resultMD[[cc, 15]] = 1;
                resultMD[[cc, 16]] = AinjBestYet
              ];
            ];
            NotebookDelete[ResPrint];
            ResPrint = PrintTemporary[MatrixForm[Join[{resultMD[[1]]}, resultMD[[cc0 ;; cc]]]]];

            (* save intermediate results -- provide correct path for it *)
            (*If[Mod[cc,5]==1,Export["C:\\"<>ToString[cc]<>".xlsx",resultMD[[1;;cc]]]]*)];
      ];

(* with this script you can import the results *)
resultMD = {};
AppendTo[resultMD, Import[FileNames["C:\\MD-Train-results.xlsx"][[1]], "Data"]];
resultMD = resultMD[[1, 1]];

For[j = 2, j ≤ Length[resultMD], j++,
   x = ToExpression[StringSplit[resultMD[[j, 16]], {",", "{", "}"}]];
   y = 1 + (Length[x] - 3) / 5;
   ar = Array[f, {y, 3}];
   For[i = 1, i ≤ y, i++,
     ar[[i, 1]] = x[[5 * (i - 1) + 1]];
     ar[[i, 2]] = x[[5 * (i - 1) + 2]];
     ar[[i, 3]] = x[[5 * (i - 1) + 3]];
     resultMD[[j, 16]] = ar;
   ];];
```

In[ ]:=

```
(* a sample of results *)
MatrixForm[resultMD[[1 ;; 5]]]
```

Out[∘]//MatrixForm=

κ_c	κ_p	γ	V	k_s	ρ	ω	α	k_f	N_0	SD-Acur	SD-Tox	MD-TotalTox	MD-TotalDose	CurFlg
0.172487	1.2766	0.23246	0.933163	0.559533	0.182532	0.221539	78.8061	0.1919	1.54844	67743.1	19555.	230.263	819.218	0.
0.140906	0.941194	3.80523	1.32623	0.616836	0.48176	0.0830614	874.074	0.124911	5.51167	116.47	4.39936	2.97205	79.2084	1.
0.198005	1.47677	1.68458	1.08295	0.693023	0.274938	0.0668003	3207.85	0.188185	6.6557	31.8873	0.717332	0.567791	25.1273	1.
0.271193	3.00215	4.9147	1.4646	0.847118	0.282579	0.0495319	1933.86	0.197735	9.99299	105.136	1.00476	0.647205	66.4218	1.

{ {

In[∘]:=

```
(* check that single-dose curative-no-lethal-tox cases are cured in multidose setting *)
Select[Select[resultMD[[2 ;;]], #[[12]] < Abld / nCpm &], #[[15]] == 0 &]
```

Out[∘]=

{ }

In[∘]:=

```
(* check that in curative cases tox decays do not exceed Abld *)
Select[Select[resultMD[[2 ;;]], #[[13]] > Abld / nCpm &], #[[15]] == 1 &]
```

Out[∘]=

{ }

In[∘]:=

```
(* check that in non-curative cases tox decays do not exceed Abld *)
ExcAbld = Select[Select[resultMD[[2 ;;]], #[[13]] > 1.000001 * Abld / nCpm &], #[[15]] == 0 &];
Table[Position[resultMD, ExcAbld[[i]]][[1, 1]], {i, 1, Length[ExcAbld]}]
```

Out[∘]=

{ }

In[∘]:=

```
(* The results of in silico trial for training set are saved here *)
```

In[∘]:=

```
TestFree = {{"Free Res", "Free OS", "Free Tox", "Free Ainj"},
    {"TBUR", 48.06321698556508`, 0.`, {{0, 0, 0}}}, {"TBUR", 15.575130215566503`, 0.`, {{0, 0, 0}}},
    {"TBUR", 26.60547217417775`, 0.`, {{0, 0, 0}}}, {"TBUR", 24.44784088281995`, 0.`, {{0, 0, 0}}},
    {"TBUR", 15.383899746209982`, 0.`, {{0, 0, 0}}}, {"TBUR", 13.64451096950105`, 0.`, {{0, 0, 0}}},
    {"TBUR", 10.354551181237175`, 0.`, {{0, 0, 0}}}, {"TBUR", 18.61808950072029`, 0.`, {{0, 0, 0}}},
    {"TBUR", 16.87160707282588`, 0.`, {{0, 0, 0}}}, {"TBUR", 16.7024006736695`, 0.`, {{0, 0, 0}}},
    {"TBUR", 14.76129886240343`, 0.`, {{0, 0, 0}}}, {"TBUR", 16.45913983028539`, 0.`, {{0, 0, 0}}},
```

```
{"TBUR", 19.072226715622133`, 0.`, {{0, 0, 0}}}, {"TBUR", 21.498657494149153`, 0.`, {{0, 0, 0}}},
{"TBUR", 23.130710926246902`, 0.`, {{0, 0, 0}}}, {"TBUR", 51.28852143243468`, 0.`, {{0, 0, 0}}},
{"TBUR", 16.803404011783382`, 0.`, {{0, 0, 0}}}, {"TBUR", 11.961881390994156`, 0.`, {{0, 0, 0}}},
{"TBUR", 15.030895934839746`, 0.`, {{0, 0, 0}}}, {"TBUR", 16.4107171709515`, 0.`, {{0, 0, 0}}},
{"TBUR", 12.407051965482758`, 0.`, {{0, 0, 0}}}, {"TBUR", 10.694374054329307`, 0.`, {{0, 0, 0}}},
{"TBUR", 26.297965698699212`, 0.`, {{0, 0, 0}}}, {"TBUR", 11.629560459124086`, 0.`, {{0, 0, 0}}},
{"TBUR", 13.682048235673088`, 0.`, {{0, 0, 0}}}, {"TBUR", 30.082132991441014`, 0.`, {{0, 0, 0}}},
{"TBUR", 15.642538742641415`, 0.`, {{0, 0, 0}}}, {"TBUR", 12.81812608950302`, 0.`, {{0, 0, 0}}},
{"TBUR", 14.67798705595138`, 0.`, {{0, 0, 0}}}, {"TBUR", 37.407258251363146`, 0.`, {{0, 0, 0}}},
{"TBUR", 13.656950944149916`, 0.`, {{0, 0, 0}}}, {"TBUR", 48.677475515241795`, 0.`, {{0, 0, 0}}},
{"TBUR", 12.842375120012745`, 0.`, {{0, 0, 0}}}, {"TBUR", 21.69822735174708`, 0.`, {{0, 0, 0}}},
{"TBUR", 42.16863071467491`, 0.`, {{0, 0, 0}}}, {"TBUR", 14.371295273001591`, 0.`, {{0, 0, 0}}},
{"TBUR", 23.30887761580469`, 0.`, {{0, 0, 0}}}, {"TBUR", 21.43604397092713`, 0.`, {{0, 0, 0}}},
{"TBUR", 47.17459733382263`, 0.`, {{0, 0, 0}}}, {"TBUR", 21.85548740031712`, 0.`, {{0, 0, 0}}},
{"TBUR", 19.112567644189006`, 0.`, {{0, 0, 0}}}, {"TBUR", 12.261347315584116`, 0.`, {{0, 0, 0}}},
{"TBUR", 10.508746099757701`, 0.`, {{0, 0, 0}}}, {"TBUR", 15.046899406351711`, 0.`, {{0, 0, 0}}},
{"TBUR", 19.34357211720329`, 0.`, {{0, 0, 0}}}, {"TBUR", 29.95388478105533`, 0.`, {{0, 0, 0}}},
{"TBUR", 13.635007124121469`, 0.`, {{0, 0, 0}}}, {"TBUR", 20.579693328727803`, 0.`, {{0, 0, 0}}},
{"TBUR", 10.54219414285359`, 0.`, {{0, 0, 0}}}, {"TBUR", 17.5379944910435`, 0.`, {{0, 0, 0}}},
{"TBUR", 20.001140120624736`, 0.`, {{0, 0, 0}}}, {"TBUR", 22.89887806953096`, 0.`, {{0, 0, 0}}},
{"TBUR", 11.637404973993398`, 0.`, {{0, 0, 0}}}, {"TBUR", 13.74452207226901`, 0.`, {{0, 0, 0}}},
{"TBUR", 23.88716206270776`, 0.`, {{0, 0, 0}}}, {"TBUR", 18.361729457117654`, 0.`, {{0, 0, 0}}},
{"TBUR", 16.404323668936502`, 0.`, {{0, 0, 0}}}, {"TBUR", 18.185576113034166`, 0.`, {{0, 0, 0}}},
{"TBUR", 10.819642805077812`, 0.`, {{0, 0, 0}}}, {"TBUR", 20.38997938796948`, 0.`, {{0, 0, 0}}},
{"TBUR", 11.747202026018059`, 0.`, {{0, 0, 0}}}, {"TBUR", 21.523026146786393`, 0.`, {{0, 0, 0}}},
{"TBUR", 17.23647633271953`, 0.`, {{0, 0, 0}}}, {"TBUR", 42.76573431368189`, 0.`, {{0, 0, 0}}},
{"TBUR", 14.716355469103101`, 0.`, {{0, 0, 0}}}, {"TBUR", 29.30138042897503`, 0.`, {{0, 0, 0}}},
{"TBUR", 16.161444866426372`, 0.`, {{0, 0, 0}}}, {"TBUR", 29.996820078499233`, 0.`, {{0, 0, 0}}},
{"TBUR", 54.672608932807464`, 0.`, {{0, 0, 0}}}, {"TBUR", 39.19188557884543`, 0.`, {{0, 0, 0}}},
{"TBUR", 31.67532848676339`, 0.`, {{0, 0, 0}}}, {"TBUR", 48.366066772832035`, 0.`, {{0, 0, 0}}},
{"TBUR", 33.04001811126531`, 0.`, {{0, 0, 0}}}, {"TBUR", 23.87564369414348`, 0.`, {{0, 0, 0}}},
```

{"TBUR", 20.547830734412734`, 0.`, {{0, 0, 0}}}, {"TBUR", 17.511216603241273`, 0.`, {{0, 0, 0}}},
{"TBUR", 11.64028129031276`, 0.`, {{0, 0, 0}}}, {"TBUR", 13.885091738895186`, 0.`, {{0, 0, 0}}},
{"TBUR", 13.738222291629702`, 0.`, {{0, 0, 0}}}, {"TBUR", 12.004449072857657`, 0.`, {{0, 0, 0}}},
{"TBUR", 11.425097687240626`, 0.`, {{0, 0, 0}}}, {"TBUR", 27.34200641048181`, 0.`, {{0, 0, 0}}},
{"TBUR", 12.302192853509302`, 0.`, {{0, 0, 0}}}, {"TBUR", 15.907135879012184`, 0.`, {{0, 0, 0}}},
{"TBUR", 18.306927330659434`, 0.`, {{0, 0, 0}}}, {"TBUR", 11.358706653177666`, 0.`, {{0, 0, 0}}},
{"TBUR", 11.020248706408784`, 0.`, {{0, 0, 0}}}, {"TBUR", 44.83626616452527`, 0.`, {{0, 0, 0}}},
{"TBUR", 10.802824310073426`, 0.`, {{0, 0, 0}}}, {"TBUR", 11.517255189009635`, 0.`, {{0, 0, 0}}},
{"TBUR", 17.345523187868487`, 0.`, {{0, 0, 0}}}, {"TBUR", 11.240826551763899`, 0.`, {{0, 0, 0}}},
{"TBUR", 10.709763362995483`, 0.`, {{0, 0, 0}}}, {"TBUR", 11.920906714267707`, 0.`, {{0, 0, 0}}},
{"TBUR", 21.423014145639723`, 0.`, {{0, 0, 0}}}, {"TBUR", 24.905996250719024`, 0.`, {{0, 0, 0}}},
{"TBUR", 16.40161712230158`, 0.`, {{0, 0, 0}}}, {"TBUR", 23.12193862742964`, 0.`, {{0, 0, 0}}},
{"TBUR", 24.306985981260446`, 0.`, {{0, 0, 0}}}, {"TBUR", 44.07418038467887`, 0.`, {{0, 0, 0}}},
{"TBUR", 14.28103955663911`, 0.`, {{0, 0, 0}}}, {"TBUR", 47.84818641157776`, 0.`, {{0, 0, 0}}},
{"TBUR", 23.732375316273686`, 0.`, {{0, 0, 0}}}, {"TBUR", 11.3941049576263`, 0.`, {{0, 0, 0}}},
{"TBUR", 23.447884626047955`, 0.`, {{0, 0, 0}}}, {"TBUR", 22.096399595497132`, 0.`, {{0, 0, 0}}},
{"TBUR", 27.960412541439663`, 0.`, {{0, 0, 0}}}, {"TBUR", 15.07988067663409`, 0.`, {{0, 0, 0}}},
{"TBUR", 17.435841327368554`, 0.`, {{0, 0, 0}}}, {"TBUR", 23.872322241120195`, 0.`, {{0, 0, 0}}},
{"TBUR", 36.3589324678276`, 0.`, {{0, 0, 0}}}, {"TBUR", 15.231202146169432`, 0.`, {{0, 0, 0}}},
{"TBUR", 19.34791475646695`, 0.`, {{0, 0, 0}}}, {"TBUR", 40.830952293043836`, 0.`, {{0, 0, 0}}},
{"TBUR", 13.645792703229258`, 0.`, {{0, 0, 0}}}, {"TBUR", 17.09976247952999`, 0.`, {{0, 0, 0}}},
{"TBUR", 17.824997958353663`, 0.`, {{0, 0, 0}}}, {"TBUR", 13.848272441480516`, 0.`, {{0, 0, 0}}},
{"TBUR", 12.607477549288278`, 0.`, {{0, 0, 0}}}, {"TBUR", 24.6394080062515`, 0.`, {{0, 0, 0}}},
{"TBUR", 11.59039309382808`, 0.`, {{0, 0, 0}}}, {"TBUR", 23.7877522755938`, 0.`, {{0, 0, 0}}},
{"TBUR", 13.256863839126101`, 0.`, {{0, 0, 0}}}, {"TBUR", 14.073979950287825`, 0.`, {{0, 0, 0}}},
{"TBUR", 13.381222634327882`, 0.`, {{0, 0, 0}}}, {"TBUR", 12.149834372409941`, 0.`, {{0, 0, 0}}},
{"TBUR", 13.490987076934319`, 0.`, {{0, 0, 0}}}, {"TBUR", 23.397004045062662`, 0.`, {{0, 0, 0}}},
{"TBUR", 35.87321459080231`, 0.`, {{0, 0, 0}}}, {"TBUR", 29.89026513690699`, 0.`, {{0, 0, 0}}},
{"TBUR", 12.587536994821075`, 0.`, {{0, 0, 0}}}, {"TBUR", 17.03660121561207`, 0.`, {{0, 0, 0}}},
{"TBUR", 19.78877467830126`, 0.`, {{0, 0, 0}}}, {"TBUR", 17.198749282217157`, 0.`, {{0, 0, 0}}},
{"TBUR", 30.71492987525766`, 0.`, {{0, 0, 0}}}, {"TBUR", 25.510169087962797`, 0.`, {{0, 0, 0}}},

{"TBUR", 16.82517589641759`, 0.`, {{0, 0, 0}}}, {"TBUR", 28.137034475334016`, 0.`, {{0, 0, 0}}},
{"TBUR", 46.471328099444946`, 0.`, {{0, 0, 0}}}, {"TBUR", 23.944348494248327`, 0.`, {{0, 0, 0}}},
{"TBUR", 41.69784564674073`, 0.`, {{0, 0, 0}}}, {"TBUR", 20.4120799988928`, 0.`, {{0, 0, 0}}},
{"TBUR", 14.208743991315854`, 0.`, {{0, 0, 0}}}, {"TBUR", 13.053802901768826`, 0.`, {{0, 0, 0}}},
{"TBUR", 33.96893366945481`, 0.`, {{0, 0, 0}}}, {"TBUR", 21.317080212939118`, 0.`, {{0, 0, 0}}},
{"TBUR", 11.146905041125304`, 0.`, {{0, 0, 0}}}, {"TBUR", 15.994913594145412`, 0.`, {{0, 0, 0}}},
{"TBUR", 24.688300718209515`, 0.`, {{0, 0, 0}}}, {"TBUR", 10.892786751981323`, 0.`, {{0, 0, 0}}},
{"TBUR", 20.496793654502735`, 0.`, {{0, 0, 0}}}, {"TBUR", 22.43774678715645`, 0.`, {{0, 0, 0}}},
{"TBUR", 36.43593960552747`, 0.`, {{0, 0, 0}}}, {"TBUR", 16.9845410577661`, 0.`, {{0, 0, 0}}},
{"TBUR", 14.123071492394308`, 0.`, {{0, 0, 0}}}, {"TBUR", 12.390233675476065`, 0.`, {{0, 0, 0}}},
{"TBUR", 11.550260352656654`, 0.`, {{0, 0, 0}}}, {"TBUR", 44.08533096783269`, 0.`, {{0, 0, 0}}},
{"TBUR", 40.047711238975175`, 0.`, {{0, 0, 0}}}, {"TBUR", 29.036657897346217`, 0.`, {{0, 0, 0}}},
{"TBUR", 16.338609136167218`, 0.`, {{0, 0, 0}}}, {"TBUR", 10.584983103425673`, 0.`, {{0, 0, 0}}},
{"TBUR", 14.10125943463997`, 0.`, {{0, 0, 0}}}, {"TBUR", 17.55410239135919`, 0.`, {{0, 0, 0}}},
{"TBUR", 10.8247563261621`, 0.`, {{0, 0, 0}}}, {"TBUR", 17.271608840211048`, 0.`, {{0, 0, 0}}},
{"TBUR", 31.802899800811794`, 0.`, {{0, 0, 0}}}, {"TBUR", 15.380266389727884`, 0.`, {{0, 0, 0}}},
{"TBUR", 14.254464079345416`, 0.`, {{0, 0, 0}}}, {"TBUR", 21.807348781141854`, 0.`, {{0, 0, 0}}},
{"TBUR", 13.206078899498864`, 0.`, {{0, 0, 0}}}, {"TBUR", 11.549622447325776`, 0.`, {{0, 0, 0}}},
{"TBUR", 13.149739398480804`, 0.`, {{0, 0, 0}}}, {"TBUR", 18.795380654586523`, 0.`, {{0, 0, 0}}},
{"TBUR", 12.525724338844558`, 0.`, {{0, 0, 0}}}, {"TBUR", 23.62310726468693`, 0.`, {{0, 0, 0}}},
{"TBUR", 12.838392514120502`, 0.`, {{0, 0, 0}}}, {"TBUR", 21.65003556833198`, 0.`, {{0, 0, 0}}},
{"TBUR", 37.40512110669287`, 0.`, {{0, 0, 0}}}, {"TBUR", 11.293855584050153`, 0.`, {{0, 0, 0}}},
{"TBUR", 16.576247260386637`, 0.`, {{0, 0, 0}}}, {"TBUR", 18.767972495353188`, 0.`, {{0, 0, 0}}},
{"TBUR", 22.386252916375827`, 0.`, {{0, 0, 0}}}, {"TBUR", 25.12012831297297`, 0.`, {{0, 0, 0}}},
{"TBUR", 35.03273455459831`, 0.`, {{0, 0, 0}}}, {"TBUR", 14.61300821745166`, 0.`, {{0, 0, 0}}},
{"TBUR", 13.355353784754694`, 0.`, {{0, 0, 0}}}, {"TBUR", 20.183847393495498`, 0.`, {{0, 0, 0}}},
{"TBUR", 13.029323240322714`, 0.`, {{0, 0, 0}}}, {"TBUR", 11.609921353900138`, 0.`, {{0, 0, 0}}},
{"TBUR", 25.658713408967643`, 0.`, {{0, 0, 0}}}, {"TBUR", 20.475809116689145`, 0.`, {{0, 0, 0}}},
{"TBUR", 16.218321054277663`, 0.`, {{0, 0, 0}}}, {"TBUR", 11.226066360664612`, 0.`, {{0, 0, 0}}},
{"TBUR", 42.050751469583176`, 0.`, {{0, 0, 0}}}, {"TBUR", 12.882858778725932`, 0.`, {{0, 0, 0}}},
{"TBUR", 12.56907106523217`, 0.`, {{0, 0, 0}}}, {"TBUR", 17.43987909102979`, 0.`, {{0, 0, 0}}},

{"TBUR", 10.626991556496021`, 0.`, {{0, 0, 0}}}, {"TBUR", 14.598979323366477`, 0.`, {{0, 0, 0}}},
{"TBUR", 15.244734193272944`, 0.`, {{0, 0, 0}}}, {"TBUR", 11.395560828819518`, 0.`, {{0, 0, 0}}},
{"TBUR", 13.590009999366108`, 0.`, {{0, 0, 0}}}, {"TBUR", 39.23269715140823`, 0.`, {{0, 0, 0}}},
{"TBUR", 17.224499146221422`, 0.`, {{0, 0, 0}}}, {"TBUR", 17.45602569193859`, 0.`, {{0, 0, 0}}},
{"TBUR", 18.015823058083402`, 0.`, {{0, 0, 0}}}, {"TBUR", 29.430103293218373`, 0.`, {{0, 0, 0}}},
{"TBUR", 14.979005917346413`, 0.`, {{0, 0, 0}}}, {"TBUR", 11.603876384522977`, 0.`, {{0, 0, 0}}},
{"TBUR", 18.587587941749895`, 0.`, {{0, 0, 0}}}, {"TBUR", 10.536282122525705`, 0.`, {{0, 0, 0}}},
{"TBUR", 12.953013806535582`, 0.`, {{0, 0, 0}}}, {"TBUR", 14.456364027054`, 0.`, {{0, 0, 0}}},
{"TBUR", 18.230612977369624`, 0.`, {{0, 0, 0}}}, {"TBUR", 12.57129389661115`, 0.`, {{0, 0, 0}}},
{"TBUR", 12.620514020508201`, 0.`, {{0, 0, 0}}}, {"TBUR", 19.858695362958805`, 0.`, {{0, 0, 0}}},
{"TBUR", 16.28908274843925`, 0.`, {{0, 0, 0}}}, {"TBUR", 20.101890618070072`, 0.`, {{0, 0, 0}}},
{"TBUR", 35.72443638615082`, 0.`, {{0, 0, 0}}}, {"TBUR", 11.83015992867704`, 0.`, {{0, 0, 0}}},
{"TBUR", 19.29290057795537`, 0.`, {{0, 0, 0}}}, {"TBUR", 42.71998515200271`, 0.`, {{0, 0, 0}}},
{"TBUR", 20.928583225483383`, 0.`, {{0, 0, 0}}}, {"TBUR", 10.89830408413526`, 0.`, {{0, 0, 0}}},
{"TBUR", 17.94939601972694`, 0.`, {{0, 0, 0}}}, {"TBUR", 24.602244581625538`, 0.`, {{0, 0, 0}}},
{"TBUR", 12.883722294463105`, 0.`, {{0, 0, 0}}}, {"TBUR", 16.76374277375378`, 0.`, {{0, 0, 0}}},
{"TBUR", 10.546295619914138`, 0.`, {{0, 0, 0}}}, {"TBUR", 34.99510422227028`, 0.`, {{0, 0, 0}}},
{"TBUR", 38.29438130913484`, 0.`, {{0, 0, 0}}}, {"TBUR", 31.26958807241479`, 0.`, {{0, 0, 0}}},
{"TBUR", 21.780744507142266`, 0.`, {{0, 0, 0}}}, {"TBUR", 21.7364390563216`, 0.`, {{0, 0, 0}}},
{"TBUR", 15.37231865422585`, 0.`, {{0, 0, 0}}}, {"TBUR", 46.273727412489826`, 0.`, {{0, 0, 0}}},
{"TBUR", 13.266934620204339`, 0.`, {{0, 0, 0}}}, {"TBUR", 29.23441846082125`, 0.`, {{0, 0, 0}}},
{"TBUR", 17.452689728631984`, 0.`, {{0, 0, 0}}}, {"TBUR", 40.68063330048052`, 0.`, {{0, 0, 0}}},
{"TBUR", 22.85835031379234`, 0.`, {{0, 0, 0}}}, {"TBUR", 19.192963162202336`, 0.`, {{0, 0, 0}}},
{"TBUR", 32.64399325374426`, 0.`, {{0, 0, 0}}}, {"TBUR", 17.71116540129279`, 0.`, {{0, 0, 0}}},
{"TBUR", 11.11857324401638`, 0.`, {{0, 0, 0}}}, {"TBUR", 12.032935880955796`, 0.`, {{0, 0, 0}}},
{"TBUR", 23.27963038106922`, 0.`, {{0, 0, 0}}}, {"TBUR", 17.0494369686943`, 0.`, {{0, 0, 0}}},
{"TBUR", 26.51040638591145`, 0.`, {{0, 0, 0}}}, {"TBUR", 11.496692974718867`, 0.`, {{0, 0, 0}}},
{"TBUR", 13.061441886646385`, 0.`, {{0, 0, 0}}}, {"TBUR", 28.52378677441194`, 0.`, {{0, 0, 0}}},
{"TBUR", 40.37922044672485`, 0.`, {{0, 0, 0}}}, {"TBUR", 18.87702828832063`, 0.`, {{0, 0, 0}}},
{"TBUR", 54.439063728632036`, 0.`, {{0, 0, 0}}}, {"TBUR", 10.572571928422771`, 0.`, {{0, 0, 0}}},
{"TBUR", 18.07922033253259`, 0.`, {{0, 0, 0}}}, {"TBUR", 12.995071989913987`, 0.`, {{0, 0, 0}}},

{"TBUR", 30.38062898800741`, 0.`, {{0, 0, 0}}}, {"TBUR", 19.5862110604554`, 0.`, {{0, 0, 0}}},
{"TBUR", 18.576447322399627`, 0.`, {{0, 0, 0}}}, {"TBUR", 25.46575319636527`, 0.`, {{0, 0, 0}}},
{"TBUR", 19.352126390566543`, 0.`, {{0, 0, 0}}}, {"TBUR", 11.487111552159256`, 0.`, {{0, 0, 0}}},
{"TBUR", 23.153524175524996`, 0.`, {{0, 0, 0}}}, {"TBUR", 15.442879217168061`, 0.`, {{0, 0, 0}}},
{"TBUR", 14.525397013955203`, 0.`, {{0, 0, 0}}}, {"TBUR", 22.574796580513425`, 0.`, {{0, 0, 0}}},
{"TBUR", 38.40798867055705`, 0.`, {{0, 0, 0}}}, {"TBUR", 16.061410427082304`, 0.`, {{0, 0, 0}}},
{"TBUR", 10.652969027184504`, 0.`, {{0, 0, 0}}}, {"TBUR", 29.90505407546619`, 0.`, {{0, 0, 0}}},
{"TBUR", 31.285727379054855`, 0.`, {{0, 0, 0}}}, {"TBUR", 13.90456685634851`, 0.`, {{0, 0, 0}}},
{"TBUR", 39.287151124062554`, 0.`, {{0, 0, 0}}}, {"TBUR", 13.563323961296609`, 0.`, {{0, 0, 0}}},
{"TBUR", 42.9857012893227`, 0.`, {{0, 0, 0}}}, {"TBUR", 11.137761252946236`, 0.`, {{0, 0, 0}}},
{"TBUR", 20.239097755312994`, 0.`, {{0, 0, 0}}}, {"TBUR", 12.236200537483858`, 0.`, {{0, 0, 0}}},
{"TBUR", 19.56943109047689`, 0.`, {{0, 0, 0}}}, {"TBUR", 31.981570257288393`, 0.`, {{0, 0, 0}}},
{"TBUR", 35.244066083911136`, 0.`, {{0, 0, 0}}}, {"TBUR", 13.441081713614194`, 0.`, {{0, 0, 0}}},
{"TBUR", 17.309786312330917`, 0.`, {{0, 0, 0}}}, {"TBUR", 12.428284672299903`, 0.`, {{0, 0, 0}}},
{"TBUR", 56.279913937048825`, 0.`, {{0, 0, 0}}}, {"TBUR", 33.47088293523859`, 0.`, {{0, 0, 0}}},
{"TBUR", 28.836737899706538`, 0.`, {{0, 0, 0}}}, {"TBUR", 14.540192086637909`, 0.`, {{0, 0, 0}}},
{"TBUR", 17.491071983270462`, 0.`, {{0, 0, 0}}}, {"TBUR", 22.706536153209754`, 0.`, {{0, 0, 0}}},
{"TBUR", 30.957488578951097`, 0.`, {{0, 0, 0}}}, {"TBUR", 19.694050647736177`, 0.`, {{0, 0, 0}}},
{"TBUR", 13.974713660986392`, 0.`, {{0, 0, 0}}}, {"TBUR", 16.253620656208074`, 0.`, {{0, 0, 0}}},
{"TBUR", 12.815643729288379`, 0.`, {{0, 0, 0}}}, {"TBUR", 32.935566706435544`, 0.`, {{0, 0, 0}}},
{"TBUR", 33.12433904607939`, 0.`, {{0, 0, 0}}}, {"TBUR", 14.867252134165485`, 0.`, {{0, 0, 0}}},
{"TBUR", 17.203521845311187`, 0.`, {{0, 0, 0}}}, {"TBUR", 23.528295807245243`, 0.`, {{0, 0, 0}}},
{"TBUR", 10.965005447589007`, 0.`, {{0, 0, 0}}}, {"TBUR", 22.153151316676144`, 0.`, {{0, 0, 0}}},
{"TBUR", 13.242338620995016`, 0.`, {{0, 0, 0}}}, {"TBUR", 25.01301511349265`, 0.`, {{0, 0, 0}}},
{"TBUR", 50.41151119071046`, 0.`, {{0, 0, 0}}}, {"TBUR", 30.96153358752434`, 0.`, {{0, 0, 0}}},
{"TBUR", 10.988165293179636`, 0.`, {{0, 0, 0}}}, {"TBUR", 19.871751575962456`, 0.`, {{0, 0, 0}}},
{"TBUR", 25.78858617855647`, 0.`, {{0, 0, 0}}}, {"TBUR", 12.862401258594799`, 0.`, {{0, 0, 0}}},
{"TBUR", 10.58663094735088`, 0.`, {{0, 0, 0}}}, {"TBUR", 33.57476290606553`, 0.`, {{0, 0, 0}}},
{"TBUR", 43.39907856294462`, 0.`, {{0, 0, 0}}}, {"TBUR", 42.39302081238804`, 0.`, {{0, 0, 0}}},
{"TBUR", 16.998850562641785`, 0.`, {{0, 0, 0}}}, {"TBUR", 27.85728862645862`, 0.`, {{0, 0, 0}}},
{"TBUR", 19.859837197016`, 0.`, {{0, 0, 0}}}, {"TBUR", 32.12418558754242`, 0.`, {{0, 0, 0}}},

{"TBUR", 14.50289654691129`, 0.`, {{0, 0, 0}}}, {"TBUR", 11.834130368388898`, 0.`, {{0, 0, 0}}},
{"TBUR", 19.67306437125372`, 0.`, {{0, 0, 0}}}, {"TBUR", 41.36787774346368`, 0.`, {{0, 0, 0}}},
{"TBUR", 19.18675370433281`, 0.`, {{0, 0, 0}}}, {"TBUR", 18.998643619309416`, 0.`, {{0, 0, 0}}},
{"TBUR", 17.325547274462096`, 0.`, {{0, 0, 0}}}, {"TBUR", 16.50322190462333`, 0.`, {{0, 0, 0}}},
{"TBUR", 14.512009559542578`, 0.`, {{0, 0, 0}}}, {"TBUR", 40.966660415271946`, 0.`, {{0, 0, 0}}},
{"TBUR", 18.757283070546645`, 0.`, {{0, 0, 0}}}, {"TBUR", 15.167083158870538`, 0.`, {{0, 0, 0}}},
{"TBUR", 25.57566774347472`, 0.`, {{0, 0, 0}}}, {"TBUR", 32.73078058973471`, 0.`, {{0, 0, 0}}},
{"TBUR", 17.274493608682985`, 0.`, {{0, 0, 0}}}, {"TBUR", 16.84000975506643`, 0.`, {{0, 0, 0}}},
{"TBUR", 12.608548972771938`, 0.`, {{0, 0, 0}}}, {"TBUR", 29.29951733479661`, 0.`, {{0, 0, 0}}},
{"TBUR", 15.712265223118067`, 0.`, {{0, 0, 0}}}, {"TBUR", 48.806149665246934`, 0.`, {{0, 0, 0}}},
{"TBUR", 13.903682688484166`, 0.`, {{0, 0, 0}}}, {"TBUR", 35.42806657135003`, 0.`, {{0, 0, 0}}},
{"TBUR", 21.825574014005397`, 0.`, {{0, 0, 0}}}, {"TBUR", 16.185264009304028`, 0.`, {{0, 0, 0}}},
{"TBUR", 54.480194552847756`, 0.`, {{0, 0, 0}}}, {"TBUR", 12.322236193133987`, 0.`, {{0, 0, 0}}},
{"TBUR", 34.91607596998899`, 0.`, {{0, 0, 0}}}, {"TBUR", 14.07473634860874`, 0.`, {{0, 0, 0}}},
{"TBUR", 20.529360845012214`, 0.`, {{0, 0, 0}}}, {"TBUR", 13.23989378141205`, 0.`, {{0, 0, 0}}},
{"TBUR", 26.45575899471731`, 0.`, {{0, 0, 0}}}, {"TBUR", 12.843601060773778`, 0.`, {{0, 0, 0}}},
{"TBUR", 19.037709057692695`, 0.`, {{0, 0, 0}}}, {"TBUR", 11.348187010327562`, 0.`, {{0, 0, 0}}},
{"TBUR", 42.18173362553312`, 0.`, {{0, 0, 0}}}, {"TBUR", 13.146818961863138`, 0.`, {{0, 0, 0}}},
{"TBUR", 12.288437910609112`, 0.`, {{0, 0, 0}}}, {"TBUR", 11.862891599017601`, 0.`, {{0, 0, 0}}},
{"TBUR", 12.876118427299852`, 0.`, {{0, 0, 0}}}, {"TBUR", 11.521618318207226`, 0.`, {{0, 0, 0}}},
{"TBUR", 36.98994122688238`, 0.`, {{0, 0, 0}}}, {"TBUR", 11.654106408184136`, 0.`, {{0, 0, 0}}},
{"TBUR", 25.396770783891675`, 0.`, {{0, 0, 0}}}, {"TBUR", 29.147603110234375`, 0.`, {{0, 0, 0}}},
{"TBUR", 11.45125760989693`, 0.`, {{0, 0, 0}}}, {"TBUR", 42.524040543098636`, 0.`, {{0, 0, 0}}},
{"TBUR", 13.380291211910784`, 0.`, {{0, 0, 0}}}, {"TBUR", 13.565955238417244`, 0.`, {{0, 0, 0}}},
{"TBUR", 13.736230830883128`, 0.`, {{0, 0, 0}}}, {"TBUR", 19.99608584926742`, 0.`, {{0, 0, 0}}},
{"TBUR", 15.285682958458526`, 0.`, {{0, 0, 0}}}, {"TBUR", 31.358980806400933`, 0.`, {{0, 0, 0}}},
{"TBUR", 44.93420998914805`, 0.`, {{0, 0, 0}}}, {"TBUR", 32.34495327102383`, 0.`, {{0, 0, 0}}},
{"TBUR", 19.957236379071126`, 0.`, {{0, 0, 0}}}, {"TBUR", 14.63915779287526`, 0.`, {{0, 0, 0}}},
{"TBUR", 26.696455874395106`, 0.`, {{0, 0, 0}}}, {"TBUR", 15.047277513423579`, 0.`, {{0, 0, 0}}},
{"TBUR", 47.22196849178534`, 0.`, {{0, 0, 0}}}, {"TBUR", 27.15780456440386`, 0.`, {{0, 0, 0}}},
{"TBUR", 13.13159888705372`, 0.`, {{0, 0, 0}}}, {"TBUR", 24.695227866829743`, 0.`, {{0, 0, 0}}},

{"TBUR", 15.214403932907855`, 0.`, {{0, 0, 0}}}, {"TBUR", 16.977825998875804`, 0.`, {{0, 0, 0}}},
{"TBUR", 16.353281796102976`, 0.`, {{0, 0, 0}}}, {"TBUR", 16.945328729127105`, 0.`, {{0, 0, 0}}},
{"TBUR", 13.977842444746697`, 0.`, {{0, 0, 0}}}, {"TBUR", 36.97642396258682`, 0.`, {{0, 0, 0}}},
{"TBUR", 30.275346194222085`, 0.`, {{0, 0, 0}}}, {"TBUR", 12.44368447057198`, 0.`, {{0, 0, 0}}},
{"TBUR", 18.148273432437723`, 0.`, {{0, 0, 0}}}, {"TBUR", 11.687752648539746`, 0.`, {{0, 0, 0}}},
{"TBUR", 19.856325510863208`, 0.`, {{0, 0, 0}}}, {"TBUR", 10.043047806181987`, 0.`, {{0, 0, 0}}},
{"TBUR", 14.933278828168039`, 0.`, {{0, 0, 0}}}, {"TBUR", 17.59529410895807`, 0.`, {{0, 0, 0}}},
{"TBUR", 23.5438265762416`, 0.`, {{0, 0, 0}}}, {"TBUR", 23.616890996989824`, 0.`, {{0, 0, 0}}},
{"TBUR", 28.232703514568744`, 0.`, {{0, 0, 0}}}, {"TBUR", 38.914556259326346`, 0.`, {{0, 0, 0}}},
{"TBUR", 13.584800463923047`, 0.`, {{0, 0, 0}}}, {"TBUR", 20.62650836214822`, 0.`, {{0, 0, 0}}},
{"TBUR", 13.058562245890082`, 0.`, {{0, 0, 0}}}, {"TBUR", 11.918693859938937`, 0.`, {{0, 0, 0}}},
{"TBUR", 37.25922726693147`, 0.`, {{0, 0, 0}}}, {"TBUR", 11.798410384777355`, 0.`, {{0, 0, 0}}},
{"TBUR", 16.74750925926284`, 0.`, {{0, 0, 0}}}, {"TBUR", 11.501476631179864`, 0.`, {{0, 0, 0}}},
{"TBUR", 13.335240098112425`, 0.`, {{0, 0, 0}}}, {"TBUR", 18.516803585619495`, 0.`, {{0, 0, 0}}},
{"TBUR", 38.30133916634852`, 0.`, {{0, 0, 0}}}, {"TBUR", 22.210454147138304`, 0.`, {{0, 0, 0}}},
{"TBUR", 13.596131054330051`, 0.`, {{0, 0, 0}}}, {"TBUR", 22.89525627820263`, 0.`, {{0, 0, 0}}},
{"TBUR", 16.997747002488452`, 0.`, {{0, 0, 0}}}, {"TBUR", 50.41687880715714`, 0.`, {{0, 0, 0}}},
{"TBUR", 14.88126077539598`, 0.`, {{0, 0, 0}}}, {"TBUR", 23.724589563385628`, 0.`, {{0, 0, 0}}},
{"TBUR", 11.434506711311286`, 0.`, {{0, 0, 0}}}, {"TBUR", 36.68521417748782`, 0.`, {{0, 0, 0}}},
{"TBUR", 24.39612982945171`, 0.`, {{0, 0, 0}}}, {"TBUR", 55.778384804220494`, 0.`, {{0, 0, 0}}},
{"TBUR", 16.664386931152496`, 0.`, {{0, 0, 0}}}, {"TBUR", 14.329313212066275`, 0.`, {{0, 0, 0}}},
{"TBUR", 11.672087521939748`, 0.`, {{0, 0, 0}}}, {"TBUR", 54.01179027734922`, 0.`, {{0, 0, 0}}},
{"TBUR", 11.405247664970775`, 0.`, {{0, 0, 0}}}, {"TBUR", 25.34313301072408`, 0.`, {{0, 0, 0}}},
{"TBUR", 12.4808803217875`, 0.`, {{0, 0, 0}}}, {"TBUR", 37.41658185946944`, 0.`, {{0, 0, 0}}},
{"TBUR", 12.13577814510028`, 0.`, {{0, 0, 0}}}, {"TBUR", 48.99782417282634`, 0.`, {{0, 0, 0}}},
{"TBUR", 14.897553883151275`, 0.`, {{0, 0, 0}}}, {"TBUR", 10.804751397655568`, 0.`, {{0, 0, 0}}},
{"TBUR", 19.258401517776825`, 0.`, {{0, 0, 0}}}, {"TBUR", 11.44217468838711`, 0.`, {{0, 0, 0}}},
{"TBUR", 12.681675865218939`, 0.`, {{0, 0, 0}}}, {"TBUR", 20.940691654374874`, 0.`, {{0, 0, 0}}},
{"TBUR", 32.344052499241215`, 0.`, {{0, 0, 0}}}, {"TBUR", 12.570734772825132`, 0.`, {{0, 0, 0}}},
{"TBUR", 15.778985756942813`, 0.`, {{0, 0, 0}}}, {"TBUR", 12.33251768651675`, 0.`, {{0, 0, 0}}},
{"TBUR", 20.145972625709376`, 0.`, {{0, 0, 0}}}, {"TBUR", 31.31497512489917`, 0.`, {{0, 0, 0}}},

```
{"TBUR", 15.42334754350149`, 0.`, {{0, 0, 0}}}, {"TBUR", 10.939076856535786`, 0.`, {{0, 0, 0}}},
{"TBUR", 12.163904391800871`, 0.`, {{0, 0, 0}}}, {"TBUR", 23.016256180685332`, 0.`, {{0, 0, 0}}},
{"TBUR", 13.465559307822373`, 0.`, {{0, 0, 0}}}, {"TBUR", 16.938436116048713`, 0.`, {{0, 0, 0}}},
{"TBUR", 22.75496037581735`, 0.`, {{0, 0, 0}}}, {"TBUR", 31.98541612279679`, 0.`, {{0, 0, 0}}},
{"TBUR", 11.553484962581928`, 0.`, {{0, 0, 0}}}, {"TBUR", 12.668723899766286`, 0.`, {{0, 0, 0}}},
{"TBUR", 11.592799947685451`, 0.`, {{0, 0, 0}}}, {"TBUR", 11.397768046038085`, 0.`, {{0, 0, 0}}},
{"TBUR", 15.64953731758805`, 0.`, {{0, 0, 0}}}, {"TBUR", 37.61272990848145`, 0.`, {{0, 0, 0}}},
{"TBUR", 12.429747064742099`, 0.`, {{0, 0, 0}}}, {"TBUR", 16.020526869596214`, 0.`, {{0, 0, 0}}},
{"TBUR", 27.535683542489448`, 0.`, {{0, 0, 0}}}, {"TBUR", 14.866082346469833`, 0.`, {{0, 0, 0}}},
{"TBUR", 22.679929972937018`, 0.`, {{0, 0, 0}}}, {"TBUR", 24.640942814427707`, 0.`, {{0, 0, 0}}},
{"TBUR", 14.52864740194873`, 0.`, {{0, 0, 0}}}, {"TBUR", 14.468672192230859`, 0.`, {{0, 0, 0}}},
{"TBUR", 33.862599357409046`, 0.`, {{0, 0, 0}}}, {"TBUR", 18.13460289942999`, 0.`, {{0, 0, 0}}},
{"TBUR", 11.06860771521896`, 0.`, {{0, 0, 0}}}, {"TBUR", 14.695115025459263`, 0.`, {{0, 0, 0}}},
{"TBUR", 42.889333170982965`, 0.`, {{0, 0, 0}}}, {"TBUR", 18.32810270873234`, 0.`, {{0, 0, 0}}},
{"TBUR", 22.55042670715202`, 0.`, {{0, 0, 0}}}, {"TBUR", 20.819452761599024`, 0.`, {{0, 0, 0}}},
{"TBUR", 14.57502587384403`, 0.`, {{0, 0, 0}}}, {"TBUR", 16.870533647882578`, 0.`, {{0, 0, 0}}},
{"TBUR", 39.20446087246648`, 0.`, {{0, 0, 0}}}, {"TBUR", 16.02374795337168`, 0.`, {{0, 0, 0}}},
{"TBUR", 10.976746930863678`, 0.`, {{0, 0, 0}}}, {"TBUR", 39.88908495707163`, 0.`, {{0, 0, 0}}},
{"TBUR", 46.761009093754055`, 0.`, {{0, 0, 0}}}, {"TBUR", 15.030036299995903`, 0.`, {{0, 0, 0}}},
{"TBUR", 24.037263208192336`, 0.`, {{0, 0, 0}}}, {"TBUR", 29.400451669012387`, 0.`, {{0, 0, 0}}},
{"TBUR", 11.366636226483088`, 0.`, {{0, 0, 0}}}, {"TBUR", 13.498042485355949`, 0.`, {{0, 0, 0}}},
{"TBUR", 11.84669856650416`, 0.`, {{0, 0, 0}}}, {"TBUR", 24.382826944478264`, 0.`, {{0, 0, 0}}},
{"TBUR", 14.07918404802905`, 0.`, {{0, 0, 0}}}, {"TBUR", 13.410400016968563`, 0.`, {{0, 0, 0}}},
{"TBUR", 15.460927904001359`, 0.`, {{0, 0, 0}}}, {"TBUR", 13.26329234783915`, 0.`, {{0, 0, 0}}},
{"TBUR", 26.06104702095423`, 0.`, {{0, 0, 0}}}, {"TBUR", 10.411702967140554`, 0.`, {{0, 0, 0}}},
{"TBUR", 21.692286236340202`, 0.`, {{0, 0, 0}}}, {"TBUR", 14.077611414701128`, 0.`, {{0, 0, 0}}},
{"TBUR", 35.424907201842935`, 0.`, {{0, 0, 0}}}, {"TBUR", 12.756704090277578`, 0.`, {{0, 0, 0}}},
{"TBUR", 40.73725575537088`, 0.`, {{0, 0, 0}}}, {"TBUR", 24.742428262143257`, 0.`, {{0, 0, 0}}},
{"TBUR", 32.037639324317674`, 0.`, {{0, 0, 0}}}, {"TBUR", 12.877263243469512`, 0.`, {{0, 0, 0}}},
{"TBUR", 46.901118039879286`, 0.`, {{0, 0, 0}}}, {"TBUR", 12.22802986438405`, 0.`, {{0, 0, 0}}},
{"TBUR", 15.208786736663965`, 0.`, {{0, 0, 0}}}, {"TBUR", 41.14711213136567`, 0.`, {{0, 0, 0}}},
```

{"TBUR", 47.133377464371605`, 0.`, {{0, 0, 0}}}, {"TBUR", 14.29269281472364`, 0.`, {{0, 0, 0}}},
{"TBUR", 23.931105233664926`, 0.`, {{0, 0, 0}}}, {"TBUR", 21.841644424427226`, 0.`, {{0, 0, 0}}},
{"TBUR", 20.65377863194958`, 0.`, {{0, 0, 0}}}, {"TBUR", 44.53156059185303`, 0.`, {{0, 0, 0}}},
{"TBUR", 10.327095355615894`, 0.`, {{0, 0, 0}}}, {"TBUR", 12.710658236065246`, 0.`, {{0, 0, 0}}},
{"TBUR", 11.85073903088075`, 0.`, {{0, 0, 0}}}, {"TBUR", 40.22272644747394`, 0.`, {{0, 0, 0}}},
{"TBUR", 10.89534108750867`, 0.`, {{0, 0, 0}}}, {"TBUR", 32.25792330283363`, 0.`, {{0, 0, 0}}},
{"TBUR", 16.87709332476219`, 0.`, {{0, 0, 0}}}, {"TBUR", 33.286978451403115`, 0.`, {{0, 0, 0}}},
{"TBUR", 14.770455880866443`, 0.`, {{0, 0, 0}}}, {"TBUR", 45.07168787301143`, 0.`, {{0, 0, 0}}},
{"TBUR", 13.075067689650412`, 0.`, {{0, 0, 0}}}, {"TBUR", 11.363200443003201`, 0.`, {{0, 0, 0}}},
{"TBUR", 15.26142420633897`, 0.`, {{0, 0, 0}}}, {"TBUR", 15.941411939520073`, 0.`, {{0, 0, 0}}},
{"TBUR", 24.2160740318937`, 0.`, {{0, 0, 0}}}, {"TBUR", 15.09620667432739`, 0.`, {{0, 0, 0}}},
{"TBUR", 25.43371546298902`, 0.`, {{0, 0, 0}}}, {"TBUR", 19.405205756365106`, 0.`, {{0, 0, 0}}},
{"TBUR", 13.582663883745493`, 0.`, {{0, 0, 0}}}, {"TBUR", 10.91425602289378`, 0.`, {{0, 0, 0}}},
{"TBUR", 17.122917427875063`, 0.`, {{0, 0, 0}}}, {"TBUR", 16.489600048802778`, 0.`, {{0, 0, 0}}},
{"TBUR", 19.86192035390935`, 0.`, {{0, 0, 0}}}, {"TBUR", 27.22809658725469`, 0.`, {{0, 0, 0}}},
{"TBUR", 12.806494759536642`, 0.`, {{0, 0, 0}}}, {"TBUR", 13.611080159107692`, 0.`, {{0, 0, 0}}},
{"TBUR", 10.680871620195436`, 0.`, {{0, 0, 0}}}, {"TBUR", 31.149342552228052`, 0.`, {{0, 0, 0}}},
{"TBUR", 27.184047146656294`, 0.`, {{0, 0, 0}}}, {"TBUR", 12.703002766207302`, 0.`, {{0, 0, 0}}},
{"TBUR", 11.601292806475385`, 0.`, {{0, 0, 0}}}, {"TBUR", 12.933961553825862`, 0.`, {{0, 0, 0}}},
{"TBUR", 12.149872000439936`, 0.`, {{0, 0, 0}}}, {"TBUR", 28.91137474548539`, 0.`, {{0, 0, 0}}},
{"TBUR", 17.234568307510532`, 0.`, {{0, 0, 0}}}, {"TBUR", 11.601805233137206`, 0.`, {{0, 0, 0}}},
{"TBUR", 16.090776886690243`, 0.`, {{0, 0, 0}}}, {"TBUR", 12.677162792289378`, 0.`, {{0, 0, 0}}},
{"TBUR", 16.837148046668666`, 0.`, {{0, 0, 0}}}, {"TBUR", 11.187267040109601`, 0.`, {{0, 0, 0}}},
{"TBUR", 17.71215115096002`, 0.`, {{0, 0, 0}}}, {"TBUR", 13.105938841797277`, 0.`, {{0, 0, 0}}},
{"TBUR", 11.710366551024203`, 0.`, {{0, 0, 0}}}, {"TBUR", 15.09695814550823`, 0.`, {{0, 0, 0}}},
{"TBUR", 34.36946050120434`, 0.`, {{0, 0, 0}}}, {"TBUR", 10.87839009718447`, 0.`, {{0, 0, 0}}},
{"TBUR", 10.758772866747803`, 0.`, {{0, 0, 0}}}, {"TBUR", 26.333174380246106`, 0.`, {{0, 0, 0}}},
{"TBUR", 14.905034757077233`, 0.`, {{0, 0, 0}}}, {"TBUR", 21.480264546752586`, 0.`, {{0, 0, 0}}},
{"TBUR", 16.15484013376126`, 0.`, {{0, 0, 0}}}, {"TBUR", 16.026523347266657`, 0.`, {{0, 0, 0}}},
{"TBUR", 22.707528723878596`, 0.`, {{0, 0, 0}}}, {"TBUR", 45.54214963986977`, 0.`, {{0, 0, 0}}},
{"TBUR", 13.178649933245952`, 0.`, {{0, 0, 0}}}, {"TBUR", 21.33066825655316`, 0.`, {{0, 0, 0}}},

{"TBUR", 10.693335442166907`, 0.`, {{0, 0, 0}}}, {"TBUR", 24.140058957435954`, 0.`, {{0, 0, 0}}},
{"TBUR", 35.65468213963233`, 0.`, {{0, 0, 0}}}, {"TBUR", 15.408174964779144`, 0.`, {{0, 0, 0}}},
{"TBUR", 16.683697299628477`, 0.`, {{0, 0, 0}}}, {"TBUR", 11.683361847180175`, 0.`, {{0, 0, 0}}},
{"TBUR", 14.969235486279725`, 0.`, {{0, 0, 0}}}, {"TBUR", 12.860040934979654`, 0.`, {{0, 0, 0}}},
{"TBUR", 11.577785277027207`, 0.`, {{0, 0, 0}}}, {"TBUR", 32.92695357149144`, 0.`, {{0, 0, 0}}},
{"TBUR", 35.561653529886115`, 0.`, {{0, 0, 0}}}, {"TBUR", 36.98880609290846`, 0.`, {{0, 0, 0}}},
{"TBUR", 13.120459399874273`, 0.`, {{0, 0, 0}}}, {"TBUR", 18.760217294712056`, 0.`, {{0, 0, 0}}},
{"TBUR", 13.564820532196274`, 0.`, {{0, 0, 0}}}, {"TBUR", 15.302053695280298`, 0.`, {{0, 0, 0}}},
{"TBUR", 23.361624683765644`, 0.`, {{0, 0, 0}}}, {"TBUR", 14.490761015014344`, 0.`, {{0, 0, 0}}},
{"TBUR", 12.923774798432067`, 0.`, {{0, 0, 0}}}, {"TBUR", 42.42186597812847`, 0.`, {{0, 0, 0}}},
{"TBUR", 12.86249383834128`, 0.`, {{0, 0, 0}}}, {"TBUR", 13.316371878062519`, 0.`, {{0, 0, 0}}},
{"TBUR", 33.23512704372612`, 0.`, {{0, 0, 0}}}, {"TBUR", 16.71702059279312`, 0.`, {{0, 0, 0}}},
{"TBUR", 14.112740659617106`, 0.`, {{0, 0, 0}}}, {"TBUR", 10.846205559969924`, 0.`, {{0, 0, 0}}},
{"TBUR", 18.819846637549176`, 0.`, {{0, 0, 0}}}, {"TBUR", 21.778143035105746`, 0.`, {{0, 0, 0}}},
{"TBUR", 32.48765733501698`, 0.`, {{0, 0, 0}}}, {"TBUR", 23.8218783460135`, 0.`, {{0, 0, 0}}},
{"TBUR", 33.78567650573366`, 0.`, {{0, 0, 0}}}, {"TBUR", 11.826887858241546`, 0.`, {{0, 0, 0}}},
{"TBUR", 18.316335347925527`, 0.`, {{0, 0, 0}}}, {"TBUR", 36.464439212486084`, 0.`, {{0, 0, 0}}},
{"TBUR", 33.58843125815591`, 0.`, {{0, 0, 0}}}, {"TBUR", 11.90854724020301`, 0.`, {{0, 0, 0}}},
{"TBUR", 48.89190899453351`, 0.`, {{0, 0, 0}}}, {"TBUR", 19.71445978128771`, 0.`, {{0, 0, 0}}},
{"TBUR", 38.529907228233114`, 0.`, {{0, 0, 0}}}, {"TBUR", 25.793828311277053`, 0.`, {{0, 0, 0}}},
{"TBUR", 32.481984334256374`, 0.`, {{0, 0, 0}}}, {"TBUR", 45.97742122357253`, 0.`, {{0, 0, 0}}},
{"TBUR", 10.935529622937922`, 0.`, {{0, 0, 0}}}, {"TBUR", 25.88181048362016`, 0.`, {{0, 0, 0}}},
{"TBUR", 13.227039026649944`, 0.`, {{0, 0, 0}}}, {"TBUR", 22.45913154375276`, 0.`, {{0, 0, 0}}},
{"TBUR", 43.60640713465268`, 0.`, {{0, 0, 0}}}, {"TBUR", 42.30778031691784`, 0.`, {{0, 0, 0}}},
{"TBUR", 13.728194642400016`, 0.`, {{0, 0, 0}}}, {"TBUR", 41.35255911860099`, 0.`, {{0, 0, 0}}},
{"TBUR", 19.669940266058806`, 0.`, {{0, 0, 0}}}, {"TBUR", 12.729702000894928`, 0.`, {{0, 0, 0}}},
{"TBUR", 12.492633443146062`, 0.`, {{0, 0, 0}}}, {"TBUR", 16.705896697294722`, 0.`, {{0, 0, 0}}},
{"TBUR", 50.86128212278701`, 0.`, {{0, 0, 0}}}, {"TBUR", 12.215685804130477`, 0.`, {{0, 0, 0}}},
{"TBUR", 39.719620837830966`, 0.`, {{0, 0, 0}}}, {"TBUR", 15.811787424048298`, 0.`, {{0, 0, 0}}},
{"TBUR", 48.94807040017172`, 0.`, {{0, 0, 0}}}, {"TBUR", 18.41467986481607`, 0.`, {{0, 0, 0}}},
{"TBUR", 24.19385418644527`, 0.`, {{0, 0, 0}}}, {"TBUR", 40.584125901509545`, 0.`, {{0, 0, 0}}},

{"TBUR", 38.8958279388521`, 0.`, {{0, 0, 0}}}, {"TBUR", 28.55862349673592`, 0.`, {{0, 0, 0}}},
{"TBUR", 39.05560974035394`, 0.`, {{0, 0, 0}}}, {"TBUR", 11.677279664383269`, 0.`, {{0, 0, 0}}},
{"TBUR", 10.583748812811224`, 0.`, {{0, 0, 0}}}, {"TBUR", 47.26197853172193`, 0.`, {{0, 0, 0}}},
{"TBUR", 33.299350612789425`, 0.`, {{0, 0, 0}}}, {"TBUR", 12.51036116447944`, 0.`, {{0, 0, 0}}},
{"TBUR", 25.783663527587347`, 0.`, {{0, 0, 0}}}, {"TBUR", 21.41788797066659`, 0.`, {{0, 0, 0}}},
{"TBUR", 11.834044526773832`, 0.`, {{0, 0, 0}}}, {"TBUR", 15.832435477294512`, 0.`, {{0, 0, 0}}},
{"TBUR", 34.28723040620821`, 0.`, {{0, 0, 0}}}, {"TBUR", 34.99171293996418`, 0.`, {{0, 0, 0}}},
{"TBUR", 24.63580509663567`, 0.`, {{0, 0, 0}}}, {"TBUR", 34.988416969001996`, 0.`, {{0, 0, 0}}},
{"TBUR", 22.660804912069214`, 0.`, {{0, 0, 0}}}, {"TBUR", 10.458712340755353`, 0.`, {{0, 0, 0}}},
{"TBUR", 12.718593169435986`, 0.`, {{0, 0, 0}}}, {"TBUR", 21.65652654457781`, 0.`, {{0, 0, 0}}},
{"TBUR", 49.29545521631802`, 0.`, {{0, 0, 0}}}, {"TBUR", 20.033611728277037`, 0.`, {{0, 0, 0}}},
{"TBUR", 42.364423604933606`, 0.`, {{0, 0, 0}}}, {"TBUR", 12.477427574761816`, 0.`, {{0, 0, 0}}},
{"TBUR", 18.70532604277567`, 0.`, {{0, 0, 0}}}, {"TBUR", 13.125948084301214`, 0.`, {{0, 0, 0}}},
{"TBUR", 20.389233599068458`, 0.`, {{0, 0, 0}}}, {"TBUR", 12.165981740902751`, 0.`, {{0, 0, 0}}},
{"TBUR", 43.80392774889126`, 0.`, {{0, 0, 0}}}, {"TBUR", 13.609486675287132`, 0.`, {{0, 0, 0}}},
{"TBUR", 32.251203947356395`, 0.`, {{0, 0, 0}}}, {"TBUR", 27.029041297992062`, 0.`, {{0, 0, 0}}},
{"TBUR", 13.473149196109574`, 0.`, {{0, 0, 0}}}, {"TBUR", 17.53822259214256`, 0.`, {{0, 0, 0}}},
{"TBUR", 26.769352897221136`, 0.`, {{0, 0, 0}}}, {"TBUR", 28.570839519912493`, 0.`, {{0, 0, 0}}},
{"TBUR", 20.874362732849594`, 0.`, {{0, 0, 0}}}, {"TBUR", 48.32856800988171`, 0.`, {{0, 0, 0}}},
{"TBUR", 18.940540591145837`, 0.`, {{0, 0, 0}}}, {"TBUR", 16.80676190112244`, 0.`, {{0, 0, 0}}},
{"TBUR", 14.26087763878497`, 0.`, {{0, 0, 0}}}, {"TBUR", 20.420961183003847`, 0.`, {{0, 0, 0}}},
{"TBUR", 23.058095270394798`, 0.`, {{0, 0, 0}}}, {"TBUR", 28.3747233160563`, 0.`, {{0, 0, 0}}},
{"TBUR", 20.395716474849156`, 0.`, {{0, 0, 0}}}, {"TBUR", 16.764737617105876`, 0.`, {{0, 0, 0}}},
{"TBUR", 22.83371667079513`, 0.`, {{0, 0, 0}}}, {"TBUR", 28.029880525283286`, 0.`, {{0, 0, 0}}},
{"TBUR", 18.087678887282106`, 0.`, {{0, 0, 0}}}, {"TBUR", 12.94896603642323`, 0.`, {{0, 0, 0}}},
{"TBUR", 12.675770112102208`, 0.`, {{0, 0, 0}}}, {"TBUR", 13.253070300792047`, 0.`, {{0, 0, 0}}},
{"TBUR", 11.961408794472247`, 0.`, {{0, 0, 0}}}, {"TBUR", 22.21530008278033`, 0.`, {{0, 0, 0}}},
{"TBUR", 13.640589243352995`, 0.`, {{0, 0, 0}}}, {"TBUR", 29.6721269613534`, 0.`, {{0, 0, 0}}},
{"TBUR", 11.830063989758504`, 0.`, {{0, 0, 0}}}, {"TBUR", 47.359372067815094`, 0.`, {{0, 0, 0}}},
{"TBUR", 13.160977100773914`, 0.`, {{0, 0, 0}}}, {"TBUR", 28.033087091834208`, 0.`, {{0, 0, 0}}},
{"TBUR", 13.336639485549425`, 0.`, {{0, 0, 0}}}, {"TBUR", 13.988928045831925`, 0.`, {{0, 0, 0}}},

```
{"TBUR", 33.0256582965419`, 0.`, {{0, 0, 0}}}, {"TBUR", 10.515077905276268`, 0.`, {{0, 0, 0}}},
{"TBUR", 19.450361721900418`, 0.`, {{0, 0, 0}}}, {"TBUR", 10.762939404756512`, 0.`, {{0, 0, 0}}},
{"TBUR", 31.556638040557175`, 0.`, {{0, 0, 0}}}, {"TBUR", 17.099219378997855`, 0.`, {{0, 0, 0}}},
{"TBUR", 25.302447331903913`, 0.`, {{0, 0, 0}}}, {"TBUR", 43.81339351584165`, 0.`, {{0, 0, 0}}},
{"TBUR", 30.18552258874591`, 0.`, {{0, 0, 0}}}, {"TBUR", 12.908407879395686`, 0.`, {{0, 0, 0}}},
{"TBUR", 10.365796563981997`, 0.`, {{0, 0, 0}}}, {"TBUR", 13.226144107436296`, 0.`, {{0, 0, 0}}},
{"TBUR", 15.919419490197924`, 0.`, {{0, 0, 0}}}, {"TBUR", 13.856570499735756`, 0.`, {{0, 0, 0}}},
{"TBUR", 24.712056049212627`, 0.`, {{0, 0, 0}}}, {"TBUR", 17.106300959102477`, 0.`, {{0, 0, 0}}},
{"TBUR", 17.322459119645117`, 0.`, {{0, 0, 0}}}, {"TBUR", 15.46670909556155`, 0.`, {{0, 0, 0}}},
{"TBUR", 40.33007504928803`, 0.`, {{0, 0, 0}}}, {"TBUR", 21.619326991526847`, 0.`, {{0, 0, 0}}},
{"TBUR", 16.43689417140755`, 0.`, {{0, 0, 0}}}, {"TBUR", 14.598405973620398`, 0.`, {{0, 0, 0}}},
{"TBUR", 14.98137651070417`, 0.`, {{0, 0, 0}}}, {"TBUR", 22.717023415542723`, 0.`, {{0, 0, 0}}},
{"TBUR", 23.672646615201977`, 0.`, {{0, 0, 0}}}, {"TBUR", 16.382200598877855`, 0.`, {{0, 0, 0}}},
{"TBUR", 28.467533075986136`, 0.`, {{0, 0, 0}}}, {"TBUR", 21.66600625864843`, 0.`, {{0, 0, 0}}},
{"TBUR", 11.042244448974591`, 0.`, {{0, 0, 0}}}, {"TBUR", 47.741898661433325`, 0.`, {{0, 0, 0}}},
{"TBUR", 12.76637618750352`, 0.`, {{0, 0, 0}}}, {"TBUR", 12.750403449540226`, 0.`, {{0, 0, 0}}},
{"TBUR", 11.767046882255508`, 0.`, {{0, 0, 0}}}, {"TBUR", 14.082448830451854`, 0.`, {{0, 0, 0}}},
{"TBUR", 40.84614841103317`, 0.`, {{0, 0, 0}}}, {"TBUR", 46.96536329632068`, 0.`, {{0, 0, 0}}},
{"TBUR", 20.76956772782752`, 0.`, {{0, 0, 0}}}, {"TBUR", 11.970597875012833`, 0.`, {{0, 0, 0}}},
{"TBUR", 15.005006240553472`, 0.`, {{0, 0, 0}}}, {"TBUR", 13.076754953824741`, 0.`, {{0, 0, 0}}},
{"TBUR", 10.687138230978139`, 0.`, {{0, 0, 0}}}, {"TBUR", 34.48859972112343`, 0.`, {{0, 0, 0}}},
{"TBUR", 43.08190765276775`, 0.`, {{0, 0, 0}}}, {"TBUR", 16.366087201283104`, 0.`, {{0, 0, 0}}},
{"TBUR", 11.262212370511055`, 0.`, {{0, 0, 0}}}, {"TBUR", 13.009725623716392`, 0.`, {{0, 0, 0}}},
{"TBUR", 15.608821245745215`, 0.`, {{0, 0, 0}}}, {"TBUR", 21.0461271445896`, 0.`, {{0, 0, 0}}},
{"TBUR", 32.26810348417811`, 0.`, {{0, 0, 0}}}, {"TBUR", 28.82114794346613`, 0.`, {{0, 0, 0}}},
{"TBUR", 12.329574622712771`, 0.`, {{0, 0, 0}}}, {"TBUR", 22.178412626368406`, 0.`, {{0, 0, 0}}},
{"TBUR", 16.232830318421655`, 0.`, {{0, 0, 0}}}, {"TBUR", 16.238393976237294`, 0.`, {{0, 0, 0}}},
{"TBUR", 23.231285968436552`, 0.`, {{0, 0, 0}}}, {"TBUR", 13.659481116902418`, 0.`, {{0, 0, 0}}},
{"TBUR", 18.582688656218153`, 0.`, {{0, 0, 0}}}, {"TBUR", 23.05970948100761`, 0.`, {{0, 0, 0}}},
{"TBUR", 12.052661400237728`, 0.`, {{0, 0, 0}}}, {"TBUR", 35.35386450014168`, 0.`, {{0, 0, 0}}},
{"TBUR", 18.275119948611998`, 0.`, {{0, 0, 0}}}, {"TBUR", 16.212210303924188`, 0.`, {{0, 0, 0}}},
```

```
{"TBUR", 19.866543382633253`, 0.`, {{0, 0, 0}}}, {"TBUR", 11.909070906746388`, 0.`, {{0, 0, 0}}},
{"TBUR", 10.50167529694731`, 0.`, {{0, 0, 0}}}, {"TBUR", 22.30854453140307`, 0.`, {{0, 0, 0}}},
{"TBUR", 17.49793318949586`, 0.`, {{0, 0, 0}}}, {"TBUR", 27.84536437214363`, 0.`, {{0, 0, 0}}},
{"TBUR", 17.55037519571739`, 0.`, {{0, 0, 0}}}, {"TBUR", 12.265824143100668`, 0.`, {{0, 0, 0}}},
{"TBUR", 10.97552191447832`, 0.`, {{0, 0, 0}}}, {"TBUR", 12.855135991524493`, 0.`, {{0, 0, 0}}},
{"TBUR", 19.457194308298153`, 0.`, {{0, 0, 0}}}, {"TBUR", 12.854184679765657`, 0.`, {{0, 0, 0}}},
{"TBUR", 15.342948084104734`, 0.`, {{0, 0, 0}}}, {"TBUR", 20.313144895556743`, 0.`, {{0, 0, 0}}},
{"TBUR", 17.91258222508193`, 0.`, {{0, 0, 0}}}, {"TBUR", 41.693063461502966`, 0.`, {{0, 0, 0}}},
{"TBUR", 42.471523291586976`, 0.`, {{0, 0, 0}}}, {"TBUR", 21.82331413072227`, 0.`, {{0, 0, 0}}},
{"TBUR", 15.334853012393816`, 0.`, {{0, 0, 0}}}, {"TBUR", 15.795564746878552`, 0.`, {{0, 0, 0}}},
{"TBUR", 11.943477223716226`, 0.`, {{0, 0, 0}}}, {"TBUR", 14.148436620073113`, 0.`, {{0, 0, 0}}},
{"TBUR", 50.0590504269086`, 0.`, {{0, 0, 0}}}, {"TBUR", 17.672412334099075`, 0.`, {{0, 0, 0}}},
{"TBUR", 25.592591763981485`, 0.`, {{0, 0, 0}}}, {"TBUR", 11.270739509486726`, 0.`, {{0, 0, 0}}},
{"TBUR", 13.749102982524867`, 0.`, {{0, 0, 0}}}, {"TBUR", 19.607393384003693`, 0.`, {{0, 0, 0}}},
{"TBUR", 13.89750429302042`, 0.`, {{0, 0, 0}}}, {"TBUR", 16.39365561253116`, 0.`, {{0, 0, 0}}},
{"TBUR", 27.395334147795012`, 0.`, {{0, 0, 0}}}, {"TBUR", 13.012952377781055`, 0.`, {{0, 0, 0}}},
{"TBUR", 12.436668319625735`, 0.`, {{0, 0, 0}}}, {"TBUR", 49.49274320303074`, 0.`, {{0, 0, 0}}},
{"TBUR", 24.791977957221413`, 0.`, {{0, 0, 0}}}, {"TBUR", 15.17416648677446`, 0.`, {{0, 0, 0}}},
{"TBUR", 17.220696864583296`, 0.`, {{0, 0, 0}}}, {"TBUR", 21.219966432811695`, 0.`, {{0, 0, 0}}},
{"TBUR", 13.77838678908476`, 0.`, {{0, 0, 0}}}, {"TBUR", 15.713259732640687`, 0.`, {{0, 0, 0}}},
{"TBUR", 36.71715452834798`, 0.`, {{0, 0, 0}}}, {"TBUR", 19.53059523651704`, 0.`, {{0, 0, 0}}},
{"TBUR", 24.175697096773543`, 0.`, {{0, 0, 0}}}, {"TBUR", 33.28252895621355`, 0.`, {{0, 0, 0}}},
{"TBUR", 13.910987968398889`, 0.`, {{0, 0, 0}}}, {"TBUR", 13.362136785180317`, 0.`, {{0, 0, 0}}},
{"TBUR", 15.815101615612848`, 0.`, {{0, 0, 0}}}, {"TBUR", 17.903242159133516`, 0.`, {{0, 0, 0}}},
{"TBUR", 24.64383494366226`, 0.`, {{0, 0, 0}}}, {"TBUR", 42.80841006522777`, 0.`, {{0, 0, 0}}},
{"TBUR", 13.865780998272808`, 0.`, {{0, 0, 0}}}, {"TBUR", 21.063687749194674`, 0.`, {{0, 0, 0}}},
{"TBUR", 15.494700214198755`, 0.`, {{0, 0, 0}}}, {"TBUR", 15.966939742837381`, 0.`, {{0, 0, 0}}},
{"TBUR", 33.65046516337769`, 0.`, {{0, 0, 0}}}, {"TBUR", 24.87481384140964`, 0.`, {{0, 0, 0}}},
{"TBUR", 41.24418926681968`, 0.`, {{0, 0, 0}}}, {"TBUR", 19.20478053486381`, 0.`, {{0, 0, 0}}},
{"TBUR", 14.945931820728953`, 0.`, {{0, 0, 0}}}, {"TBUR", 11.013321563899583`, 0.`, {{0, 0, 0}}},
{"TBUR", 36.28678457103674`, 0.`, {{0, 0, 0}}}, {"TBUR", 17.05296327960182`, 0.`, {{0, 0, 0}}},
```

{"TBUR", 19.25971658066236`, 0.`, {{0, 0, 0}}}, {"TBUR", 15.950572817516548`, 0.`, {{0, 0, 0}}},
{"TBUR", 18.06766481816991`, 0.`, {{0, 0, 0}}}, {"TBUR", 18.200054830100743`, 0.`, {{0, 0, 0}}},
{"TBUR", 23.411457421147087`, 0.`, {{0, 0, 0}}}, {"TBUR", 32.31252368319767`, 0.`, {{0, 0, 0}}},
{"TBUR", 14.929279851706433`, 0.`, {{0, 0, 0}}}, {"TBUR", 11.040979085202878`, 0.`, {{0, 0, 0}}},
{"TBUR", 19.717473244185054`, 0.`, {{0, 0, 0}}}, {"TBUR", 13.296007125282928`, 0.`, {{0, 0, 0}}},
{"TBUR", 20.815440879862997`, 0.`, {{0, 0, 0}}}, {"TBUR", 18.66983351486171`, 0.`, {{0, 0, 0}}},
{"TBUR", 22.18917365370357`, 0.`, {{0, 0, 0}}}, {"TBUR", 24.332815444056717`, 0.`, {{0, 0, 0}}},
{"TBUR", 24.208123369247495`, 0.`, {{0, 0, 0}}}, {"TBUR", 32.944836325446786`, 0.`, {{0, 0, 0}}},
{"TBUR", 36.92748331324047`, 0.`, {{0, 0, 0}}}, {"TBUR", 14.041798403768025`, 0.`, {{0, 0, 0}}},
{"TBUR", 28.09699216470916`, 0.`, {{0, 0, 0}}}, {"TBUR", 16.170250411588558`, 0.`, {{0, 0, 0}}},
{"TBUR", 23.657075326477326`, 0.`, {{0, 0, 0}}}, {"TBUR", 14.89776836913991`, 0.`, {{0, 0, 0}}},
{"TBUR", 18.667826603545613`, 0.`, {{0, 0, 0}}}, {"TBUR", 21.61595836406774`, 0.`, {{0, 0, 0}}},
{"TBUR", 16.13780376577376`, 0.`, {{0, 0, 0}}}, {"TBUR", 18.7642249152172`, 0.`, {{0, 0, 0}}},
{"TBUR", 22.555268217069766`, 0.`, {{0, 0, 0}}}, {"TBUR", 18.026917716145988`, 0.`, {{0, 0, 0}}},
{"TBUR", 18.599074809035223`, 0.`, {{0, 0, 0}}}, {"TBUR", 12.877903825820145`, 0.`, {{0, 0, 0}}},
{"TBUR", 15.079362752028729`, 0.`, {{0, 0, 0}}}, {"TBUR", 17.811073047994938`, 0.`, {{0, 0, 0}}},
{"TBUR", 17.990752325334856`, 0.`, {{0, 0, 0}}}, {"TBUR", 45.893635119059375`, 0.`, {{0, 0, 0}}},
{"TBUR", 44.43788375634744`, 0.`, {{0, 0, 0}}}, {"TBUR", 15.115190269209219`, 0.`, {{0, 0, 0}}},
{"TBUR", 25.443603831280072`, 0.`, {{0, 0, 0}}}, {"TBUR", 24.782770817843314`, 0.`, {{0, 0, 0}}},
{"TBUR", 19.797682112824013`, 0.`, {{0, 0, 0}}}, {"TBUR", 14.561232531845715`, 0.`, {{0, 0, 0}}},
{"TBUR", 23.543415049287574`, 0.`, {{0, 0, 0}}}, {"TBUR", 13.740989361384829`, 0.`, {{0, 0, 0}}},
{"TBUR", 38.51721976591389`, 0.`, {{0, 0, 0}}}, {"TBUR", 12.2499581884403`, 0.`, {{0, 0, 0}}},
{"TBUR", 33.16596487989694`, 0.`, {{0, 0, 0}}}, {"TBUR", 9.940073006557478`, 0.`, {{0, 0, 0}}},
{"TBUR", 23.397182987274583`, 0.`, {{0, 0, 0}}}, {"TBUR", 11.853555656211329`, 0.`, {{0, 0, 0}}},
{"TBUR", 11.235916079052062`, 0.`, {{0, 0, 0}}}, {"TBUR", 49.686409683914164`, 0.`, {{0, 0, 0}}},
{"TBUR", 35.35214868052249`, 0.`, {{0, 0, 0}}}, {"TBUR", 16.432505258854217`, 0.`, {{0, 0, 0}}},
{"TBUR", 11.582609853078674`, 0.`, {{0, 0, 0}}}, {"TBUR", 22.635752916284545`, 0.`, {{0, 0, 0}}},
{"TBUR", 28.64782756782896`, 0.`, {{0, 0, 0}}}, {"TBUR", 20.28342745759608`, 0.`, {{0, 0, 0}}},
{"TBUR", 26.564990285776915`, 0.`, {{0, 0, 0}}}, {"TBUR", 28.053185379993153`, 0.`, {{0, 0, 0}}},
{"TBUR", 13.667131981393798`, 0.`, {{0, 0, 0}}}, {"TBUR", 28.687811042664265`, 0.`, {{0, 0, 0}}},
{"TBUR", 14.694512870599208`, 0.`, {{0, 0, 0}}}, {"TBUR", 38.534663297901815`, 0.`, {{0, 0, 0}}},

{"TBUR", 16.682198516464616`, 0.`, {{0, 0, 0}}}, {"TBUR", 28.44084598126558`, 0.`, {{0, 0, 0}}},
{"TBUR", 15.854857422442613`, 0.`, {{0, 0, 0}}}, {"TBUR", 19.890581969950656`, 0.`, {{0, 0, 0}}},
{"TBUR", 18.68544354217343`, 0.`, {{0, 0, 0}}}, {"TBUR", 11.345741507410272`, 0.`, {{0, 0, 0}}},
{"TBUR", 14.295716546322787`, 0.`, {{0, 0, 0}}}, {"TBUR", 14.790159408592888`, 0.`, {{0, 0, 0}}},
{"TBUR", 12.07491795117946`, 0.`, {{0, 0, 0}}}, {"TBUR", 10.57403520760616`, 0.`, {{0, 0, 0}}},
{"TBUR", 20.859837828167283`, 0.`, {{0, 0, 0}}}, {"TBUR", 13.650091657561859`, 0.`, {{0, 0, 0}}},
{"TBUR", 14.789285047940135`, 0.`, {{0, 0, 0}}}, {"TBUR", 17.539783694108117`, 0.`, {{0, 0, 0}}},
{"TBUR", 15.576781991119198`, 0.`, {{0, 0, 0}}}, {"TBUR", 19.403840862370068`, 0.`, {{0, 0, 0}}},
{"TBUR", 16.511432802125757`, 0.`, {{0, 0, 0}}}, {"TBUR", 42.98897502423996`, 0.`, {{0, 0, 0}}},
{"TBUR", 17.30215306322323`, 0.`, {{0, 0, 0}}}, {"TBUR", 16.230184219724393`, 0.`, {{0, 0, 0}}},
{"TBUR", 40.076633226951195`, 0.`, {{0, 0, 0}}}, {"TBUR", 12.657997113314245`, 0.`, {{0, 0, 0}}},
{"TBUR", 10.923875760479062`, 0.`, {{0, 0, 0}}}, {"TBUR", 11.027643230525713`, 0.`, {{0, 0, 0}}},
{"TBUR", 15.982775945738316`, 0.`, {{0, 0, 0}}}, {"TBUR", 13.663841772323265`, 0.`, {{0, 0, 0}}},
{"TBUR", 12.496661505845097`, 0.`, {{0, 0, 0}}}, {"TBUR", 33.33702978272119`, 0.`, {{0, 0, 0}}},
{"TBUR", 32.50776581843792`, 0.`, {{0, 0, 0}}}, {"TBUR", 25.75900761651251`, 0.`, {{0, 0, 0}}},
{"TBUR", 24.13819572326012`, 0.`, {{0, 0, 0}}}, {"TBUR", 23.831295367256025`, 0.`, {{0, 0, 0}}},
{"TBUR", 17.418668588351647`, 0.`, {{0, 0, 0}}}, {"TBUR", 14.690590247826242`, 0.`, {{0, 0, 0}}},
{"TBUR", 14.58668631579079`, 0.`, {{0, 0, 0}}}, {"TBUR", 23.110508159017936`, 0.`, {{0, 0, 0}}},
{"TBUR", 27.649404787663176`, 0.`, {{0, 0, 0}}}, {"TBUR", 20.402971985023456`, 0.`, {{0, 0, 0}}},
{"TBUR", 14.451154672093`, 0.`, {{0, 0, 0}}}, {"TBUR", 23.638157328748637`, 0.`, {{0, 0, 0}}},
{"TBUR", 27.342515021299192`, 0.`, {{0, 0, 0}}}, {"TBUR", 20.044327124870254`, 0.`, {{0, 0, 0}}},
{"TBUR", 21.140372706628398`, 0.`, {{0, 0, 0}}}, {"TBUR", 19.378044081491225`, 0.`, {{0, 0, 0}}},
{"TBUR", 12.969880857037644`, 0.`, {{0, 0, 0}}}, {"TBUR", 43.98177375655846`, 0.`, {{0, 0, 0}}},
{"TBUR", 27.942403387575297`, 0.`, {{0, 0, 0}}}, {"TBUR", 15.065055530242297`, 0.`, {{0, 0, 0}}},
{"TBUR", 13.133447583113266`, 0.`, {{0, 0, 0}}}, {"TBUR", 17.442301351839788`, 0.`, {{0, 0, 0}}},
{"TBUR", 17.787722967798597`, 0.`, {{0, 0, 0}}}, {"TBUR", 15.923430414857627`, 0.`, {{0, 0, 0}}},
{"TBUR", 20.90517785145848`, 0.`, {{0, 0, 0}}}, {"TBUR", 20.214542972695146`, 0.`, {{0, 0, 0}}},
{"TBUR", 17.109287733607584`, 0.`, {{0, 0, 0}}}, {"TBUR", 37.62769485234447`, 0.`, {{0, 0, 0}}},
{"TBUR", 38.945054261646746`, 0.`, {{0, 0, 0}}}, {"TBUR", 11.620434482343382`, 0.`, {{0, 0, 0}}},
{"TBUR", 14.293481981916766`, 0.`, {{0, 0, 0}}}, {"TBUR", 18.198643117092846`, 0.`, {{0, 0, 0}}},
{"TBUR", 23.66393678534455`, 0.`, {{0, 0, 0}}}, {"TBUR", 18.609790652838026`, 0.`, {{0, 0, 0}}},

```
{"TBUR", 11.394820503277003`, 0.`, {{0, 0, 0}}}, {"TBUR", 11.534546538960255`, 0.`, {{0, 0, 0}}},
{"TBUR", 26.96018029734404`, 0.`, {{0, 0, 0}}}, {"TBUR", 10.76444233468533`, 0.`, {{0, 0, 0}}},
{"TBUR", 13.671221192230108`, 0.`, {{0, 0, 0}}}, {"TBUR", 10.658407142409843`, 0.`, {{0, 0, 0}}},
{"TBUR", 23.74555431787879`, 0.`, {{0, 0, 0}}}, {"TBUR", 31.43889555794629`, 0.`, {{0, 0, 0}}},
{"TBUR", 18.276420134215673`, 0.`, {{0, 0, 0}}}, {"TBUR", 28.32246125888158`, 0.`, {{0, 0, 0}}},
{"TBUR", 28.564626492356293`, 0.`, {{0, 0, 0}}}, {"TBUR", 34.397867467439596`, 0.`, {{0, 0, 0}}},
{"TBUR", 10.954693744482926`, 0.`, {{0, 0, 0}}}, {"TBUR", 39.216732847528576`, 0.`, {{0, 0, 0}}},
{"TBUR", 13.896513744191326`, 0.`, {{0, 0, 0}}}, {"TBUR", 33.33667182162505`, 0.`, {{0, 0, 0}}},
{"TBUR", 16.862678234295064`, 0.`, {{0, 0, 0}}}, {"TBUR", 16.520726336783653`, 0.`, {{0, 0, 0}}},
{"TBUR", 12.974342545148046`, 0.`, {{0, 0, 0}}}, {"TBUR", 24.759588022772974`, 0.`, {{0, 0, 0}}},
{"TBUR", 16.400599932128966`, 0.`, {{0, 0, 0}}}, {"TBUR", 17.685156341198912`, 0.`, {{0, 0, 0}}},
{"TBUR", 11.76636594738288`, 0.`, {{0, 0, 0}}}, {"TBUR", 16.988665912763345`, 0.`, {{0, 0, 0}}},
{"TBUR", 15.523706784939291`, 0.`, {{0, 0, 0}}}, {"TBUR", 12.935376707680263`, 0.`, {{0, 0, 0}}},
{"TBUR", 13.537419881640139`, 0.`, {{0, 0, 0}}}, {"TBUR", 28.217054159784027`, 0.`, {{0, 0, 0}}},
{"TBUR", 22.589648813110042`, 0.`, {{0, 0, 0}}}, {"TBUR", 17.67766166842277`, 0.`, {{0, 0, 0}}},
{"TBUR", 11.963323326797754`, 0.`, {{0, 0, 0}}}, {"TBUR", 14.602361348174588`, 0.`, {{0, 0, 0}}},
{"TBUR", 17.245432460662414`, 0.`, {{0, 0, 0}}}, {"TBUR", 12.984914712426743`, 0.`, {{0, 0, 0}}},
{"TBUR", 12.49757188965857`, 0.`, {{0, 0, 0}}}, {"TBUR", 12.80452059403376`, 0.`, {{0, 0, 0}}},
{"TBUR", 44.444081752976636`, 0.`, {{0, 0, 0}}}, {"TBUR", 13.28178056163008`, 0.`, {{0, 0, 0}}},
{"TBUR", 15.937211037384804`, 0.`, {{0, 0, 0}}}, {"TBUR", 13.685303499684323`, 0.`, {{0, 0, 0}}},
{"TBUR", 24.641143795071706`, 0.`, {{0, 0, 0}}}, {"TBUR", 22.418199529996542`, 0.`, {{0, 0, 0}}},
{"TBUR", 23.329943952750835`, 0.`, {{0, 0, 0}}}, {"TBUR", 25.394457842914825`, 0.`, {{0, 0, 0}}},
{"TBUR", 36.20192045098103`, 0.`, {{0, 0, 0}}}, {"TBUR", 24.602347001256383`, 0.`, {{0, 0, 0}}},
{"TBUR", 19.269176801792575`, 0.`, {{0, 0, 0}}}, {"TBUR", 11.902196959379282`, 0.`, {{0, 0, 0}}},
{"TBUR", 25.674320854541833`, 0.`, {{0, 0, 0}}}, {"TBUR", 14.663391972224385`, 0.`, {{0, 0, 0}}},
{"TBUR", 31.809634311632383`, 0.`, {{0, 0, 0}}}, {"TBUR", 45.188360637440184`, 0.`, {{0, 0, 0}}},
{"TBUR", 12.125309379042935`, 0.`, {{0, 0, 0}}}, {"TBUR", 13.66640247818617`, 0.`, {{0, 0, 0}}},
{"TBUR", 17.49859212154337`, 0.`, {{0, 0, 0}}}, {"TBUR", 15.536991841188716`, 0.`, {{0, 0, 0}}},
{"TBUR", 23.33262374747416`, 0.`, {{0, 0, 0}}}, {"TBUR", 16.4365530173755`, 0.`, {{0, 0, 0}}}};
```

In[ ]:=

```
TestSDNumOpt = {{"NumOpt Res", "NumOpt OS", "NumOpt Tox", "NumOpt A", "Nn", "FrAcViable"},
```

```
{"TBUR", 52.70140532274077`, 229.79192370772853`, 817.579158858249`, 1.548441048845115`*^7, 0.023605648062942707`},
{"CURE", 365, 4.403889329888315`, 116.58616666375578`, 0.00965137530790896`, 0.011667469698721658`},
{"CURE", 365, 0.717339590508007`, 31.887673993937216`, 0.009996636165184182`, 0.013614993264863906`},
{"CURE", 365, 1.005771612378581`, 105.24013383778038`, 0.009716195079197335`, 0.010116922460558715`},
{"CURE", 365, 0.6810975489350403`, 42.72744090081215`, 0.009999996009535883`, 0.005658610854076266`},
{"CURE", 365, 0.5084770782075301`, 73.5538837498121`, 0.009999854636959288`, 0.006847603511379174`},
{"TBUR", 20.702583360415186`, 220.20594826765057`, 1694.2263532939717`, 1.9321391417179417`*^7, 0.07712191798713938`},
{"TBUR", 100.62474859522568`, 229.4369623187892`, 847.0163093115158`, 0.6025449003826571`, 0.013277946670364202`},
{"CURE", 365, 0.034966630181657216`, 8.432685624423241`, 0.009999248897619637`, 0.023977555223643844`},
{"CURE", 365, 1.118619320017856`, 79.62939990698891`, 0.009998867472208567`, 0.0181814939474261 6`},
{"CURE", 365, 19.308281293850833`, 443.4586119189351`, 0.00999996544653303`, 0.019580929014306827`},
{"CURE", 365, 2.093464534915794`, 61.19254417120219`, 0.009999864985196661`, 0.0156734263293163`},
{"CURE", 365, 0.9172198372566348`, 37.41763042879539`, 0.009999880859550486`, 0.01622048627305621`},
{"TBUR", 101.96731076006198`, 228.99712816105747`, 1202.7372724131537`, 121.98801053865385`, 0.025390334662442 73`},
{"CURE", 365, 0.7850521633543617`, 67.32535673836472`, 0.009998544049808487`, 0.008827814850296817`},
{"TBUR", 143.92267826241422`, 229.79428287896357`, 952.8283131750011`, 4514.676539601415`, 0.0013344500475485526`},
{"CURE", 365, 1.3426070157837735`, 31.968091081284168`, 0.009999799229689908`, 0.01973269815896927`},
{"TBUR", 43.72746691688882`, 229.63635066015075`, 629.1388795250341`, 385700.59985863266`, 0.01969760949668126`},
{"TBUR", 49.08360172971043`, 229.6221064388585`, 856.9594563935934`, 1.1137581392138442`*^6, 0.02949781499802577`},
{"CURE", 365, 1.2434322269956208`, 158.2169054432892`, 0.009999668016774174`, 0.008058877845867096`},
{"CURE", 365, 0.9757846152827412`, 51.485966539917634`, 0.009999858987792352`, 0.005451051909187211`},
{"TBUR", 16.963658064880615`, 229.00084864878002`, 1645.0168702476906`, 8.111100890263619`*^7, 0.0994944543954468 7`},
{"TBUR", 46.39193275103984`, 229.75485965396334`, 1255.7784941321927`, 2.947651237401796`*^7, 0.019406241825178683`},
{"TBUR", 64.31951637005825`, 229.7854048917598`, 541.2981771167957`, 101.94201132808575`, 0.001365985439455924`},
{"CURE", 365, 0.20530427642538057`, 117.49689259637626`, 0.009999517341141479`, 0.01137976449951292`},
{"CURE", 365, 0.2432306009666031`, 27.5081359835505`, 0.009996100288136892`, 0.023318390655123457`},
{"CURE", 365, 2.0755887750516266`, 76.7071727563354`, 0.009999696296132178`, 0.006423152632025295 5`},
{"TBUR", 51.13515097658142`, 229.39937077909124`, 810.2843033640007`, 26289.32839707288`, 0.022831398553319653`},
{"CURE", 365, 7.654069190981112`, 627.8231099891796`, 0.00999916260436892`, 0.018865684576878972`},
{"CURE", 365, 0.1574145113176128`, 16.257799625822074`, 0.00999985017655025`, 0.021017584812931592`},
{"TBUR", 15.675523732928712`, 229.5732500814992`, 1451.8765527323671`, 4.0707470595068745`*^7, 0.10021447417637622`},
```

```
{"TBUR", 75.44529976815448`, 229.77944178122098`, 921.6308947613368`, 1.6947791682515074`*^7, 0.01428361340523722`},
{"TBUR", 17.836433841970873`, 229.50949186269045`, 1102.3029397663317`, 4.656274919049821`*^7, 0.12451900095036282`},
{"CURE", 365, 3.005130534865098`, 193.33284423321186`, 0.009999802106915135`, 0.018909409947972418`},
{"CURE", 365, 1.707997372513549`, 79.86269582720229`, 0.009999899019907195`, 0.012109632825237974`},
{"TBUR", 49.273159603793395`, 229.09976674183838`, 1214.9048617011624`, 383516.26062419167`, 0.03775266219389413`},
{"CURE", 365, 0.2453935248513836`, 18.55455042687172`, 0.009998832929376556`, 0.020642621865579913`},
{"CURE", 365, 3.800156189242196`, 466.6170722198455`, 0.009999239700515165`, 0.02159638257665042`},
{"CURE", 365, 0.8180110212411604`, 36.91912649473084`, 0.00999999790188494`, 0.024676258805580827`},
{"CURE", 365, 0.6025355392470273`, 91.99317695156148`, 0.009992616332095226`, 0.011552573883788228`},
{"TBUR", 29.222748389160657`, 229.75709415354513`, 1115.4085204475807`, 1.1599347066236524`*^7, 0.016628948875044753`},
{"CURE", 365, 0.6574838479794635`, 48.70295628040397`, 0.009998197611885818`, 0.013890478664227258`},
{"TBUR", 39.2674290552072`, 229.54516250879752`, 1232.94760829524`, 500889.2285213694`, 0.019239579775954453`},
{"CURE", 365, 2.594443949994454`, 170.70234058150197`, 0.009999937620068644`, 0.009450530884881377`},
{"TBUR", 37.29914492510725`, 229.71299470850175`, 853.0225224243968`, 1.5212579972220052`*^7, 0.03271869784102541`},
{"CURE", 365, 1.3970469931430523`, 169.6571910562468`, 0.009999876978005235`, 0.023705146222626847`},
{"CURE", 365, 181.83594580178863`, 991.1699656363973`, 0.00999998356519429`, 0.008627261046367476`},
{"CURE", 365, 0.4187830536369054`, 60.89648851999438`, 0.009999795604995927`, 0.022910115534222484`},
{"CURE", 365, 0.6936281603518767`, 122.34983651782183`, 0.00999915324466878`, 0.022449933041310304`},
{"TBUR", 22.56728074215603`, 229.67994371101705`, 1320.2228859851234`, 6.1478795622477025`*^7, 0.11001018650879298`},
{"TBUR", 63.527433757334094`, 229.79525044946047`, 872.8487676068357`, 210290.90052023402`, 0.0012242540992553582`},
{"CURE", 365, 0.6805118720913651`, 48.022836368382244`, 0.009999153634262996`, 0.012875855732122791`},
{"CURE", 365, 6.433957950579239`, 311.0022794486728`, 0.00999996913911215`, 0.007644675868576964`},
{"CURE", 365, 4.001880234928654`, 42.60912893663879`, 0.009999928587012286`, 0.009486871881176657`},
{"CURE", 365, 2.5407787078406945`, 106.92504849220919`, 0.009999168868177548`, 0.02274219489036883`},
{"CURE", 365, 1.8222958700804774`, 47.1254888546048`, 0.009999999028040387`, 0.01104376363834214`},
{"CURE", 365, 170.53252350172707`, 961.2728551058846`, 0.009998544012636256`, 0.01353971705664239`},
{"CURE", 365, 154.33327122128404`, 698.8628526840386`, 0.00999997525354631`, 0.001405950392996475`},
{"CURE", 365, 0.4901959201535579`, 27.694641136356577`, 0.009999620528257435`, 0.0203279004643896`},
{"TBUR", 38.987550081970845`, 229.69011555046498`, 531.5329854111923`, 2.83339265497387`*^7, 0.04168310040063307`},
{"CURE", 365, 0.6414592346579371`, 142.9497814785936`, 0.009999778581170949`, 0.0157982686137941`},
{"TBUR", 102.1871059874951`, 229.67978809582132`, 504.34657598796656`, 67.88276116843674`, 0.008776422571840256`},
```

{"CURE", 365, 1.1911567761773192`, 93.72952985424547`, 0.009999739716403205`, 0.013914446187342902`},
{"CURE", 365, 1.9036334733383191`, 69.68107013653777`, 0.009740566216430014`, 0.013137999395560217`},
{"TBUR", 95.15788352654593`, 229.5676581783385`, 928.5588816592569`, 0.10639254553442275`, 0.009026734492513263`},
{"CURE", 365, 0.43554264978495427`, 19.903874308664403`, 0.009999966832277551`, 0.013515135821735344`},
{"TBUR", 64.90857878654226`, 229.54030715565145`, 745.2004117714732`, 20265.231282517325`, 0.020016949869253085`},
{"CURE", 365, 1.4880821235350323`, 153.60024905823985`, 0.009704185647723875`, 0.014857851359190677`},
{"TBUR", 174.22229556235118`, 229.7371237966175`, 1194.0770696338857`, 169.08354337894684`, 0.007217932478668606`},
{"TBUR", 126.79941285020074`, 229.69804618173956`, 520.9630496878374`, 7416.37031309048`, 0.012242693824727228`},
{"CURE", 365, 1.4284903825926982`, 215.44744072320046`, 0.009999234065936943`, 0.023941623765780462`},
{"CURE", 365, 0.046106363992441696`, 5.396126526524246`, 0.009998826673947659`, 0.018603407090519636`},
{"CURE", 365, 0.33515637503891693`, 14.044370964429136`, 0.009999959311411784`, 0.013933259817362025`},
{"CURE", 365, 1.7415281549737605`, 28.236675077117283`, 0.009999854748814499`, 0.023057233255779897`},
{"CURE", 365, 0.8811333882534299`, 41.06101881145546`, 0.009999899441408165`, 0.008517805451478596`},
{"TBUR", 73.51290343437738`, 229.02417004201095`, 1181.9235063854019`, 8352.880387239586`, 0.029694507183069277`},
{"CURE", 365, 1.1788659170145654`, 220.78734004938778`, 0.009998386709740247`, 0.02281657938694594`},
{"CURE", 365, 1.918090969668336`, 162.60729167463066`, 0.009997527870615091`, 0.013057299039569526`},
{"CURE", 365, 0.2464473150569143`, 20.557693603006435`, 0.009999231718831717`, 0.01342906225289222`},
{"TBUR", 27.800991498711152`, 229.6228977444067`, 1351.0977054897107`, 2.1476189577846814`*^7, 0.024963987249442102`},
{"CURE", 365, 1.1979362602873749`, 62.21070420490669`, 0.009999774082846098`, 0.015206058324212264`},
{"CURE", 365, 0.1328318599603014`, 7.598542349284617`, 0.00999990416417731`, 0.02064603655564474`},
{"CURE", 365, 1.3466451665287344`, 44.28802888995205`, 0.009999680325832769`, 0.021338337157014865`},
{"TBUR", 18.243355718253092`, 229.65287533098518`, 774.4499639460915`, 3.951771093200938`*^7, 0.22912009745860604`},
{"CURE", 365, 0.19294000039973994`, 52.126916644003934`, 0.009999990217678757`, 0.017496141749842188`},
{"CURE", 365, 31.889088600786337`, 215.94950444357002`, 0.009999920531565464`, 0.010043547217169277`},
{"CURE", 365, 0.6020828695324756`, 227.4729240865987`, 0.009656295220303314`, 0.007105384996025115`},
{"CURE", 365, 0.945319961133685`, 63.86234003993293`, 0.009999689507681233`, 0.030163029376595353`},
{"TBUR", 65.05615597985508`, 229.69278857572226`, 1177.7051749982334`, 3.1349294140571318`, 0.005458930057259543`},
{"TBUR", 14.314841387678593`, 229.41089600708347`, 1717.8654896585567`, 9.980846637487562`*^7, 0.170938285797302`},
{"TBUR", 55.96058620683505`, 229.78511126657665`, 954.6701074901382`, 98278.82609004166`, 0.0026637675226668773`},
{"TBUR", 77.74695750589652`, 229.6078325571305`, 952.7152282313297`, 0.07278417759963234`, 0.007052032118192185`},
{"CURE", 365, 3.515177532156977`, 502.3896632498788`, 0.009999994256014203`, 0.007818545336791853`},

```
{"CURE", 365, 0.12290328880042899`, 30.85928421832529`, 0.009989118115922797`, 0.01775609433826727`},
{"CURE", 365, 0.5268312642766899`, 19.868016888064`, 0.009998853565860999`, 0.01284721661308411`},
{"TBUR", 110.32312946896674`, 229.75718395232516`, 1006.2122962349339`, 34.328849057883325`, 0.003646694050902847 7`},
{"CURE", 365, 1.155110947044526 7`, 166.86334630161292`, 0.009598058570621229`, 0.02447231521445445 2`},
{"CURE", 365, 0.30578635772197726`, 100.97298265246533`, 0.009998281227438505`, 0.012911393782797079`},
{"TBUR", 75.19651550049991`, 229.6744590155511`, 963.1479717555803`, 47740.25217567358`, 0.015051159725730718`},
{"CURE", 365, 0.19793511400287495`, 5.165644693434317`, 0.009999773976808983`, 0.02537276150159499`},
{"TBUR", 77.17089864670282`, 229.77095941378332`, 1195.6909408067402`, 0.7690322184386328`, 0.0019368555499669057`},
{"CURE", 365, 0.12071297801849783`, 5.333962480338528`, 0.009999873268932341`, 0.01581099384567838`},
{"CURE", 365, 0.2231677672092708`, 36.915241604738156`, 0.00965528294718142`, 0.023302442253810295`},
{"TBUR", 25.387989742534906`, 229.46489788051596`, 1293.3884424912303`, 1.2622036823968204`*^7, 0.041228685370046446`},
{"CURE", 365, 0.5508566558346543`, 52.13482080576142`, 0.009668803002312506`, 0.01817384574002696`},
{"TBUR", 46.06712119074343 5`, 229.76212554603708`, 527.4715703412107`, 2.8229037825988317`*^6, 0.020400082626279053`},
{"TBUR", 128.13426571641023`, 229.79768452045207`, 541.1907902516816`, 0.08001526263125992`, 0.0003678657221797806`},
{"CURE", 365, 5.127348501317175`, 218.39636757188694`, 0.00999985772572401`, 0.00845007116007947 2`},
{"CURE", 365, 0.09501526994825371`, 13.596905130737142`, 0.00999912229273153`, 0.008421900214600769`},
{"CURE", 365, 0.08001804376402233`, 41.867519799280146`, 0.009998951927292525`, 0.011112282568936416`},
{"CURE", 365, 0.18184115387206282`, 10.347676473329471`, 0.009998784433746416`, 0.01574706798125372`},
{"CURE", 365, 0.12941275457541873`, 6.91814469911552`, 0.009999779575819318`, 0.010197870950727921`},
{"TBUR", 47.959690157994814`, 229.5215544251325`, 1401.4301483254687`, 1.9920643355392953`*^6, 0.040008936900411025`},
{"CURE", 365, 0.26852704764920293`, 27.45774275295392`, 0.009999848060097209`, 0.025239481896642685`},
{"TBUR", 30.69348952617905 7`, 228.85924856389633`, 1192.1747880232963`, 2.027844202297487`*^7, 0.07079047413750557`},
{"TBUR", 45.96997685106568`, 229.7417215469557`, 1200.1713838075336`, 2.6387862168016317`*^6, 0.010021464294089157`},
{"CURE", 365, 0.2284520662453947`, 52.340131348885414`, 0.009999967797570114`, 0.024708212459274233`},
{"TBUR", 20.464387169169285`, 229.44489476215642`, 1132.9515893835774`, 3.0938079790542003`*^7, 0.09726332453719524`},
{"CURE", 365, 0.9617987188932369`, 45.47805849369191`, 0.009999629399308494`, 0.020541770273108505`},
{"TBUR", 43.8486311657397`, 229.71839584764044`, 678.7134779127022`, 1.0054922503033573`*^7, 0.04100287305780426`},
{"CURE", 365, 1.365697121351972`, 41.1816320073827`, 0.009999152034772642`, 0.020780665786382463`},
{"CURE", 365, 7.427734912224884`, 242.41768828662686`, 0.009999898044798176`, 0.016209149064753264`},
{"CURE", 365, 4.561525038205796`, 195.42326157533014`, 0.009999667675896039`, 0.010539910666230926`},
{"TBUR", 22.60379272204638 6`, 228.99246004626977`, 1758.9389931528187`, 4.710838195465512`*^7, 0.10840868923422911`},
```

```
{"CURE", 365, 0.03619696391658695`, 3.3042053582531965`, 0.009982966416974541`, 0.021002459048884536`},
{"CURE", 365, 5.396531533875047`, 189.14425352938247`, 0.009999933425202933`, 0.017054477302977555`},
{"CURE", 365, 0.423603758419866`, 18.954063379694603`, 0.009999842456579828`, 0.01918651831655969`},
{"CURE", 365, 5.3224097548178095`, 80.42073080000324`, 0.009997674357704179`, 0.01959222219961226`},
{"TBUR", 96.96945081529154`, 229.78252732550322`, 927.0063744093244`, 130929.2151787454`, 0.003848350534769275`},
{"CURE", 365, 3.4105952601457092`, 373.10158364208615`, 0.009999191687502433`, 0.018648602412310557`},
{"CURE", 365, 0.18225166698962225`, 14.839800505204645`, 0.009999585584436628`, 0.013943872739823492`},
{"CURE", 365, 0.7008229957884909`, 42.357170080788926`, 0.009999867738577325`, 0.012632005098556148`},
{"CURE", 365, 7.784723280710702`, 588.9517568599194`, 0.009999461531909257`, 0.02159406973771451`},
{"TBUR", 48.89016469170332`, 229.5457788248055`, 1170.9025458285685`, 1.5359461545943774`*^6, 0.02723011398442866`},
{"CURE", 365, 1.29983407289497`, 91.11293450438286`, 0.00999994155392189`, 0.015759240004456946`},
{"CURE", 365, 1.3887411688996352`, 168.49706269000316`, 0.009994304854055306`, 0.024245941796870753`},
{"CURE", 365, 0.2127224939140294`, 21.908871508238615`, 0.00999992926877114`, 0.02268769229464067`},
{"CURE", 365, 0.35526018703009016`, 15.635946848883803`, 0.009997104136179968`, 0.021823974697810236`},
{"CURE", 365, 0.07854008901059034`, 8.535152158193894`, 0.009988367849179539`, 0.014352330993410807`},
{"CURE", 365, 0.26658498977768647`, 72.48321661807263`, 0.009998979026985981`, 0.015190910654022996`},
{"TBUR", 66.58762371393307`, 229.68352543379896`, 767.9050632527002`, 1.4561808629395634`*^7, 0.057871037392724936`},
{"CURE", 365, 7.146755396625121`, 102.5520117068653`, 0.009999908029257501`, 0.013560943149031512`},
{"CURE", 365, 0.5569511432631485`, 21.437253089908197`, 0.009999894682499332`, 0.01894044096654872`},
{"CURE", 365, 6.754106593949964`, 330.62533642346443`, 0.009999766053810798`, 0.021412353693660863`},
{"CURE", 365, 48.3445426002658`, 418.00907573062307`, 0.009989040997826175`, 0.019443020934966967`},
{"CURE", 365, 1.1045608968451983`, 54.674618615562295`, 0.009999836354116616`, 0.008778672146444021`},
{"CURE", 365, 1.8209257782154897`, 120.57841915503047`, 0.009998203861803195`, 0.01956719109676588`},
{"CURE", 365, 2.698498841451828`, 263.5464120483333`, 0.009997475105936132`, 0.018631407963267954`},
{"CURE", 365, 0.2134590959666292`, 37.80436767377949`, 0.00966524402239812`, 0.02106784767714309`},
{"CURE", 365, 1.739475113341851`, 49.19052016198898`, 0.009993911976109127`, 0.008538603252998085`},
{"CURE", 365, 0.46008845102711887`, 29.572061228824285`, 0.009998395915230942`, 0.007531675398599421`},
{"TBUR", 65.34520398663517`, 229.7220268591953`, 1115.4102737025214`, 21798.7625962284`, 0.007984247008743848`},
{"CURE", 365, 0.37069497621075453`, 23.69707063940198`, 0.009999804737150461`, 0.019807976609280725`},
{"CURE", 365, 0.6673355648074047`, 46.485132894905966`, 0.009997798407773716`, 0.01144369645034393`},
{"TBUR", 30.245928761052618`, 229.77882429087575`, 1147.906854027196`, 6.257243755037331`*^6, 0.004668583656259683`},
```

```
{"TBUR", 20.142071548690826`, 229.70131433130592`, 1048.486779363532`, 4.699759298331439`*^7, 0.043630986683239864`},
{"TBUR", 29.973260958201358`, 228.69466981770788`, 1249.678043566252`, 8.01142611671648`*^6, 0.10021643292783476`},
{"CURE", 365, 3.452619949336431`, 151.93962730497594`, 0.009726201668015876`, 0.016353450643679483`},
{"CURE", 365, 3.57850252249936`, 183.26145098268896`, 0.009999334135787762`, 0.01834843966967872`},
{"CURE", 365, 0.3116839531683893`, 34.57495920559338`, 0.009999819386906559`, 0.022424645172407658`},
{"CURE", 365, 4.611061354924938`, 180.13245235576252`, 0.009999337720963836`, 0.017219209485506162`},
{"CURE", 365, 0.1989851278061841`, 105.78904799789814`, 0.009999289513047146`, 0.017676343941969777`},
{"CURE", 365, 0.8129972138066086`, 49.9720334097109`, 0.009999892756214556`, 0.018079106367464425`},
{"CURE", 365, 2.864829473655663`, 20.734364749356565`, 0.009999932354634101`, 0.012907526719983634`},
{"CURE", 365, 16.946721888515643`, 758.8741694907665`, 0.00998006588328593`, 0.011716628307213815`},
{"TBUR", 22.1859253880336`, 229.4751503818593`, 887.808853701541`, 7.348654585309644`*^7, 0.1848300536729545`},
{"CURE", 365, 0.8552134385074094`, 27.123682688549014`, 0.009999914066527511`, 0.022083895403132433`},
{"CURE", 365, 0.5980736079280584`, 109.91231010367247`, 0.009999820126259305`, 0.01179719249783872`},
{"CURE", 365, 2.3890893828922444`, 129.16337723285324`, 0.009999826430741276`, 0.01878575129035609`},
{"CURE", 365, 0.3456169689149329`, 36.47192351981279`, 0.009999980810434793`, 0.01742328615466202`},
{"CURE", 365, 3.6632462410826254`, 96.42445373362199`, 0.009999283571254885`, 0.02276947625212802`},
{"CURE", 365, 6.665850912534509`, 535.7932929165146`, 0.009992026001301332`, 0.014389886099234256`},
{"TBUR", 23.946748154339954`, 229.70371980325092`, 896.1289250223261`, 6.186836781574085`*^6, 0.01487768343128957`},
{"CURE", 365, 3.2001003511697026`, 171.93411868014448`, 0.009999880635721936`, 0.012234054616280831`},
{"TBUR", 22.718661636167138`, 228.7359903774966`, 1261.3117667248373`, 5.362400051743099`*^7, 0.07729148475151168`},
{"CURE", 365, 0.4918149021093473`, 20.978380011876826`, 0.009999761970554648`, 0.025466881039940846`},
{"CURE", 365, 1.386957813604475`, 72.92025086916618`, 0.009999962806065738`, 0.006317351834932512`},
{"CURE", 365, 0.4409875400957414`, 117.10549371503804`, 0.00999958192304449`, 0.01679565893200257`},
{"TBUR", 150.96202977489796`, 229.4333538301771`, 1769.4024186385304`, 191.17382909206674`, 0.021872658541848035`},
{"TBUR", 12.523061373602996`, 229.43570633839786`, 827.4806661354867`, 5.398273141094878`*^7, 0.19447380846282677`},
{"CURE", 365, 1.1101994171669363`, 93.88058510840187`, 0.009995253570601787`, 0.011282551778035995`},
{"CURE", 365, 1.1038957098555053`, 47.082190932009254`, 0.009999995373945642`, 0.006384501646374082`},
{"CURE", 365, 2.228900302075953`, 96.11744605206538`, 0.00999977190148623`, 0.010508340231303924`},
{"CURE", 365, 0.5942924607187303`, 62.5786513647811`, 0.009998532070806929`, 0.02368493809526141`},
{"CURE", 365, 1.8327711411442182`, 192.28914874592638`, 0.009999424048962657`, 0.013475401133869188`},
{"TBUR", 81.69110076605341`, 229.7860349857919`, 954.269488736203`, 16.28362160377262`, 0.001218663331952846`},
```

```
{"CURE", 365, 3.676150615082786`, 538.4986335885135`, 0.009999802480212238`, 0.017377173132881677`},
{"TBUR", 22.095556937017744`, 229.54760596218662`, 818.8555284550315`, 8.411462154648635`*^7, 0.18442095334732944`},
{"TBUR", 73.14744487404978`, 229.70764148927242`, 847.6111010501259`, 2.4448893304385004`, 0.005649701347940945`},
{"TBUR", 34.90580653688788`, 229.73773583696104`, 930.6803201374256`, 2.671854831282897`*^6, 0.009288785014998285`},
{"TBUR", 133.06217937979656`, 229.5972852768662`, 1178.5441074873272`, 0.6870442079152662`, 0.010417738718584651`},
{"TBUR", 30.10966789999773`, 229.44493235969847`, 707.826714515686`, 6.385828434268044`*^7, 0.10700231870595016`},
{"CURE", 365, 0.34093621304634175`, 24.3257910858765`, 0.009592153343398396`, 0.02399494661451732`},
{"TBUR", 13.419682189884178`, 228.9056897664444`, 1603.7347294154924`, 9.368280708056207`*^7, 0.13270749605803694`},
{"TBUR", 72.53431691686565`, 229.74792311738054`, 461.5711002600821`, 1.758101511511735`*^7, 0.03510302189136765`},
{"TBUR", 56.198452685568434`, 229.7336041019841`, 1219.692124316567`, 184.34135159153604`, 0.004894464210557644`},
{"CURE", 365, 2.02021493378022`, 257.8486229059581`, 0.00999906321415619`, 0.016884011313328888`},
{"CURE", 365, 0.23748077590298397`, 8.360888711248338`, 0.0099999664622722`, 0.012920126373425371`},
{"TBUR", 13.78821797411644`, 229.55596238656332`, 1350.8978344264785`, 7.877832682736377`*^7, 0.12867340743680045`},
{"TBUR", 35.36869351871867`, 229.5791032998571`, 1251.7267219643845`, 2.8691383956244905`*^6, 0.029792129532614536`},
{"TBUR", 27.923012251357747`, 229.6475673116757`, 722.6092835476525`, 3.945507542936359`*^7, 0.050444220760243505`},
{"CURE", 365, 2.6911583475303384`, 146.73979239044206`, 0.009995166553135851`, 0.00622271804691055`},
{"CURE", 365, 0.21913292812817536`, 27.6386617247663`, 0.009999867392176348`, 0.00977170345290480 2`},
{"TBUR", 181.09514037421673`, 229.68457297596706`, 1050.403963641117`, 1.573679190065731`, 0.008278559377679379`},
{"CURE", 365, 4.250536770020683`, 52.90970703095785`, 0.009999848903533608`, 0.008293765923218302`},
{"CURE", 365, 1.4235720289938951`, 68.1150077938996`, 0.00964108472136539`, 0.01767945874120946`},
{"TBUR", 34.13769433861112`, 229.6140629436865`, 1265.51431881754`, 1.1006922639585307`*^7, 0.045801476027682614`},
{"CURE", 365, 32.70884756353449`, 450.4945143645525`, 0.009999566330998933`, 0.02370217991931104`},
{"TBUR", 44.09949466233615`, 229.49093220609694`, 1586.854390847056`, 1.099260837649892`*^6, 0.026424091083281354`},
{"TBUR", 23.00481071494442`, 229.24564564533912`, 1015.6114959716799`, 2.9363583112466767`*^7, 0.04550453162631275`},
{"CURE", 365, 0.19430376768756885`, 12.277538758420013`, 0.009999830273179208`, 0.017657617445490936`},
{"TBUR", 20.458610614468206`, 228.4698483787658`, 1831.0223167820984`, 4.035057547637437`*^7, 0.11583628753602726`},
{"CURE", 365, 0.2905462895821216`, 15.063997144246477`, 0.009999582040557089`, 0.01605163801215893`},
{"TBUR", 20.438962194389624`, 229.6036096181078`, 1288.7414402710765`, 2.979677734696839`*^7, 0.0683353778943017`},
{"CURE", 365, 1.8288944249273194`, 108.5283627292384`, 0.009999149205609119`, 0.006482727361381489`},
{"TBUR", 31.943513843203263`, 229.30741913275062`, 948.5380983352662`, 8.603940363916334`*^6, 0.046246441439642814`},
{"CURE", 365, 6.7441222305821995`, 365.6552875579414`, 0.00999977816567976`, 0.017759028395490904`},
```

```
{"CURE", 365, 0.1378768010994316`, 16.978374148148582`, 0.009636500196860886`, 0.021193481284866544`},
{"CURE", 365, 0.6849214518571913`, 62.54918880880595`, 0.009999891450746885`, 0.006696930007116847`},
{"TBUR", 56.389199960273785`, 229.77873472842947`, 907.8336550060071`, 461967.92312837276`, 0.004287319600478848`},
{"TBUR", 153.30098559473782`, 229.59705415037476`, 1452.6506147886578`, 3.901133992918239`, 0.01282647971106265`},
{"CURE", 365, 0.15515014528117346`, 16.216648297894316`, 0.009576805496979673`, 0.01723347146506736`},
{"CURE", 365, 0.08569638578848326`, 12.076418089918576`, 0.009666518524191982`, 0.014727552168912736`},
{"TBUR", 48.52151886924714`, 229.7592187778826`, 485.0945217985858`, 3.178863308772511`*^7, 0.09871335364860628`},
{"TBUR", 34.09791950136657`, 229.5215848860736`, 1172.913529245477`, 5.90084364296337`*^7, 0.0972177964958956`},
{"TBUR", 19.78937604944560`, 228.77916651434526`, 1462.7116682654932`, 3.263772018884812`*^7, 0.06937679343413922`},
{"CURE", 365, 0.6594125157148889`, 15.16630347509383`, 0.00999987464204714`, 0.005571716541719994`},
{"CURE", 365, 4.185935327628897`, 162.61032326867013`, 0.009998933719349627`, 0.019719072230560183`},
{"CURE", 365, 1.2830167558689156`, 277.6695965195157`, 0.009999600940042165`, 0.017926494296611643`},
{"CURE", 365, 1.0028852131582333`, 72.21087842829046`, 0.009999784705065743`, 0.0153292617778320`},
{"CURE", 365, 10.41409381391447`, 504.61550895525374`, 0.009999501127465981`, 0.013994246946914455`},
{"CURE", 365, 0.7937095566679112`, 38.02556792435533`, 0.009998873638615815`, 0.012160160984805983`},
{"TBUR", 117.84345389391454`, 229.6350085114155`, 1468.8489371851872`, 22440.9522770323`, 0.019322054854237346`},
{"CURE", 365, 1.21793166303902`, 17.406482651061882`, 0.00999979354600695`, 0.025254716002364137`},
{"CURE", 365, 7.242680398246301`, 205.02679150902674`, 0.009999855790607255`, 0.011856857995244883`},
{"CURE", 365, 0.17119421958943926`, 15.113372553329318`, 0.00999879267674309`, 0.021040251113046853`},
{"CURE", 365, 0.2582727858427214`, 30.670421570942512`, 0.00999987763069694`, 0.013645213986146598`},
{"TBUR", 61.63206579147233`, 229.7850106925153`, 1170.0671198493558`, 2.1344247301621333`*^7, 0.016521841091451137`},
{"CURE", 365, 0.7142040903767056`, 372.50120912152545`, 0.009999776463382067`, 0.014807927096160297`},
{"TBUR", 104.7530468668919`, 229.72683800895854`, 1236.9643153642355`, 462.94846966531566`, 0.008592674492929761`},
{"CURE", 365, 2.9369516037811576`, 72.31350020142574`, 0.009997246922110606`, 0.015738435817626444`},
{"TBUR", 120.85303372037666`, 229.5987088118307`, 595.2936078372755`, 15758.332755515254`, 0.021372294421430692`},
{"TBUR", 24.008440108759817`, 229.7570164069571`, 847.4883732042815`, 1.378327695847128`*^7, 0.06815310425482989`},
{"CURE", 365, 1.7379801731842468`, 55.67353769564302`, 0.009999759203504896`, 0.021450516612895265`},
{"TBUR", 48.34059736160332`, 229.72450649579957`, 1141.5513048673936`, 2.5635598213416934`*^7, 0.04793775426804615`},
{"CURE", 365, 0.41551443400057764`, 41.81085187038952`, 0.009999434320720894`, 0.013999646073508556`},
{"TBUR", 27.408422870785206`, 227.59837086817384`, 1588.4182942541024`, 9.16797989146587`*^6, 0.06886862879376646`},
{"CURE", 365, 6.808412257171393`, 298.7116344805929`, 0.009977438223474373`, 0.007484605431288873`},
```

```
{"TBUR", 105.6885385687728`, 229.47760513414522`, 768.034365804572`, 111.81499380599719`, 0.0168219194602816 5`},
{"CURE", 365, 1.3171720572970094`, 61.677990166611195`, 0.009999943055422011`, 0.005979749959946787`},
{"CURE", 365, 0.7024093581371824`, 106.83260721287002`, 0.009680893246426327`, 0.018596171593358503`},
{"CURE", 365, 2.5061959620923364`, 94.40185835612182`, 0.00999931447000758`, 0.015098214529472201`},
{"CURE", 365, 0.17494417520692682`, 40.133996026354865`, 0.009998931850790071`, 0.022043375136747465`},
{"CURE", 365, 16.873526292916132`, 600.1267934047381`, 0.009998293742503104`, 0.016169038405361016`},
{"CURE", 365, 0.36982517669576853`, 168.19567161638844`, 0.00999679098127055`, 0.025481295195320004`},
{"CURE", 365, 4.443087011405831`, 50.27416847857208`, 0.009999969990116394`, 0.016700642350464534`},
{"TBUR", 102.9254717071595`, 229.7947171020637 1`, 823.7821748382167`, 845642.0382213292`, 0.0026607512356275577`},
{"CURE", 365, 1.703843865524397`, 50.06693349660645`, 0.00999978169810746`, 0.018787280261923486`},
{"CURE", 365, 0.07247947355992108`, 15.861036877465137`, 0.009642090538401605`, 0.01673767571856705`},
{"TBUR", 18.8822320188327`, 227.1761782194638`, 987.1737008345755`, 1.2341700274455292`*^7, 0.09527262843136929`},
{"TBUR", 155.3805391780838`, 229.67300279598462`, 1095.710701189543`, 0.3428135191601346`, 0.008264370421133553`},
{"CURE", 365, 2.5519743085570252`, 183.6778998687646`, 0.009624452576473244`, 0.025030505253306804`},
{"CURE", 365, 0.05197032716656968 5`, 6.640827167530567`, 0.009614627783676619`, 0.02329682903421762`},
{"TBUR", 110.17504923425562`, 229.7734419370688`, 704.9084216669987`, 47.77086259247433`, 0.0028571977943170554`},
{"CURE", 365, 4.596816533449376`, 208.95024151267833`, 0.009998758996940253`, 0.020118219364317205`},
{"CURE", 365, 0.10144183098280886`, 26.11497989119209`, 0.009999753600435594`, 0.020383214463203077`},
{"CURE", 365, 2.442256603420054`, 347.8214828663897`, 0.009997461433631014`, 0.02435872691210825`},
{"CURE", 365, 5.25856606214456`, 519.8715635304371`, 0.009971813538345784`, 0.01660171260276786`},
{"CURE", 365, 1.176340607325032`, 95.69020912502161`, 0.009996103266680798`, 0.013148619726024297`},
{"TBUR", 95.5179554097782 1`, 229.7284781808126`, 982.0533197804501`, 529.1623290114989`, 0.006834768416057311`},
{"TBUR", 59.1656385510538`, 229.73025647812815`, 1213.901123247649`, 1.947717244141315`*^7, 0.039463318799844715`},
{"CURE", 365, 1.8220682359305336`, 25.7303654317282`, 0.009999757092424438`, 0.0184183506667035`},
{"CURE", 365, 0.17690219137868876`, 47.06528080193095`, 0.009985183909937677`, 0.01781284047046381`},
{"TBUR", 128.39497012748026`, 229.4788055380522`, 1184.3631606352958`, 103.12671919101898`, 0.020971431982977722`},
{"CURE", 365, 4.03378981381895`, 65.07313091022633`, 0.00999979190381963`, 0.028072690256827428`},
{"TBUR", 61.16899684493345`, 229.33332707825383`, 760.6864743483694`, 2458.8497862129852`, 0.028596516783064838`},
{"TBUR", 80.11697712206684`, 229.7557818301099`, 961.6314062319308`, 3.3561034705148563`*^6, 0.016100971777634778`},
{"CURE", 365, 6.4932084698544825`, 253.64987294087226`, 0.00999949240417488`, 0.0117011171687103`},
{"TBUR", 178.12085942862902`, 229.67586173148842`, 1207.9383031945483`, 2.0634825491054745`, 0.0087634780152609`},
```

```
{"TBUR", 29.288126673722896`, 229.14995407769075`, 2055.5914823632493`, 3.12166525277943`*^6, 0.03493661176524097`},
{"CURE", 365, 1.5117644447325538`, 130.8590519813411`, 0.00999992066657804`, 0.009870201868912307`},
{"CURE", 365, 2.4703423607938784`, 207.3438160555331`, 0.009999968025753557`, 0.004177749092278042 5`},
{"TBUR", 111.04843307827619`, 229.75409502141952`, 611.8373214571102`, 0.612415411569521`, 0.0031149029669420686`},
{"CURE", 365, 0.5836512308863021`, 68.6074382162557`, 0.009999764263713112`, 0.016156839446203036`},
{"CURE", 365, 0.32507395718288135`, 17.752111843390306`, 0.00999977631804025`, 0.029022374793292637`},
{"CURE", 365, 5.684399089245398`, 201.7568730799413`, 0.009993465894987593`, 0.017873736608610618`},
{"TBUR", 47.1005946036295`, 229.5888795397428`, 556.2727275647617`, 771497.4362830601`, 0.0368883293288922`},
{"CURE", 365, 4.024509558680923`, 209.76124287865005`, 0.00998588147382291`, 0.009270830334132014`},
{"CURE", 365, 0.3385040232024598`, 7.774766593901272`, 0.009999882208914372`, 0.026888652708428978`},
{"CURE", 365, 0.21349295719639302`, 24.129307474002978`, 0.009999929889335181`, 0.029506236399768286`},
{"CURE", 365, 0.41684114168513103`, 54.56590427972123`, 0.009999981433082226`, 0.02695898802124803`},
{"CURE", 365, 0.13810997417213372`, 49.28848275932714`, 0.009605395334459808`, 0.01897717884753653`},
{"CURE", 365, 0.23980151135832709`, 76.29057737519767`, 0.009999802829787139`, 0.01911626802519151`},
{"CURE", 365, 5.635386631636808`, 293.69162176858265`, 0.009999850679055376`, 0.01465110681999023`},
{"TBUR", 63.816706761776345`, 229.76971769766166`, 607.2266992769744`, 827019.9684456901`, 0.012529410516750664`},
{"TBUR", 48.87876855552933`, 229.58210404712892`, 742.6647668135795`, 1.0510450968017415`*^6, 0.04735562756442318 6`},
{"CURE", 365, 5.652423746221615`, 87.38956499837494`, 0.009999963702771163`, 0.005324985594868886`},
{"CURE", 365, 2.4798199642183993`, 27.788414184089152`, 0.009999845903338671`, 0.019242132643249494`},
{"TBUR", 18.64546888088687`, 229.0967820030005`, 1015.2047408254526`, 4.7148191409051225`*^7, 0.13077873163724335`},
{"TBUR", 133.0471916419186`, 229.72654031662654`, 615.3311202400612`, 21.79668036578473`, 0.006206560805209287`},
{"CURE", 365, 15.755940616532584`, 448.8377200496436`, 0.009998569302718906`, 0.024014891655696084`},
{"TBUR", 23.857512808625092`, 227.14065956849643`, 1487.6508431685597`, 7.177036179616737`*^7, 0.11290271661824174`},
{"CURE", 365, 1.426731643682769`, 68.83636700398544`, 0.009999903065223192`, 0.00980163268859966`},
{"CURE", 365, 3.747638540572233`, 459.3327568939671`, 0.009988972986464766`, 0.01492334608335502`},
{"TBUR", 70.35368401844887`, 229.5502727811527`, 1755.6744324533563`, 0.10655673862746409`, 0.008268482197782135`},
{"CURE", 365, 0.578956759453394`, 112.67208838189302`, 0.009998716927682153`, 0.008196686035516095`},
{"TBUR", 15.452638363759943`, 229.4381577776998`, 1211.1713053050794`, 9.842771813142875`*^7, 0.1891404749629692`},
{"CURE", 365, 2.5268871890324713`, 88.42715985029211`, 0.009999184319460045`, 0.009322265902345332`},
{"TBUR", 219.23025030742596`, 229.77157977650106`, 695.8379572316221`, 0.11337402613011832`, 0.0025335967043231358`},
{"CURE", 365, 0.8565974964087554`, 27.885893531933927`, 0.0099968053932 2841`, 0.02641458459594712`},
```

```
{"CURE", 365, 0.2462857025336534`, 108.01719309187555`, 0.009562182458016705`, 0.019586240112291292`},
{"CURE", 365, 1.8522399466004877`, 196.1649781609609 7`, 0.009999113283084745`, 0.009943246257230445`},
{"CURE", 365, 82.4729203826393`, 571.552348817696`, 0.009999937053066902`, 0.012165532783008267`},
{"TBUR", 16.140136586031453`, 229.00857826713673`, 1422.621740792927`, 4.1290128285309464`*^7, 0.14889440263357034`},
{"CURE", 365, 0.6651872092092497`, 127.34895612255886`, 0.009999660155662051`, 0.010912288370829196`},
{"TBUR", 112.70273826656809`, 229.79550604813005`, 747.5984262165272`, 457.26334518907413`, 0.0008101213415923132`},
{"CURE", 365, 0.10838779888109933`, 6.286148542103592`, 0.00999983293165955`, 0.012313696838806138`},
{"CURE", 365, 0.19377060079337968`, 38.109576359613634`, 0.009712962613313673`, 0.021603865593593796`},
{"TBUR", 61.45924862936793`, 229.7505715627171`, 756.3221844873931`, 72091.77998072238`, 0.00590139093648662`},
{"CURE", 365, 1.9786411600361111`, 203.2683286381046`, 0.009673302442172103`, 0.0227349062072616`},
{"TBUR", 34.62089802328402`, 229.52400191961218`, 918.783608737745`, 3.433838390798457`*^7, 0.07560250681897016`},
{"TBUR", 125.04245980251774`, 229.628082517832`, 1509.2658467041822`, 172.71925309102457`, 0.01293770598206867`},
{"CURE", 365, 0.3381905776832934`, 29.08684470151638`, 0.0099998541997975`, 0.02153994058255524`},
{"TBUR", 17.446507009707574`, 228.73209973881163`, 1635.4353319971183`, 7.448076551427086`*^7, 0.09554526786203324`},
{"TBUR", 51.84606267889583`, 229.60984904302188`, 1088.345277183934`, 1.0169626050961163`*^6, 0.023045749823428788`},
{"CURE", 365, 0.2293261908976757`, 100.64965936506871`, 0.009999275373816086`, 0.029028183124481535`},
{"CURE", 365, 2.101083136805176`, 58.15450284057323`, 0.009999959183175254`, 0.011139770680425654`},
{"TBUR", 23.61912291684355`, 229.7016721987418`, 1054.6617432644496`, 4.461346915500395`*^7, 0.0947823984104729`},
{"CURE", 365, 0.3192779421392667`, 8.715454955813225`, 0.009999854672954502`, 0.016618092201923975`},
{"TBUR", 20.122506458656822`, 229.64202542440995`, 995.0221465763295`, 5.424569346408827`*^7, 0.11059007793933626`},
{"CURE", 365, 0.7899421028664199`, 45.403466767600946`, 0.0099998339102086`, 0.017481575053530644`},
{"CURE", 365, 2.72551090866 2687`, 97.80445747204139`, 0.009999931625834941`, 0.014686412638475792`},
{"CURE", 365, 0.3661133157908036`, 49.79224490766081`, 0.009998428552309063`, 0.025230738766003763`},
{"CURE", 365, 0.8099458013176162`, 177.74348955008023`, 0.009999974363773796`, 0.013133322386806014`},
{"TBUR", 84.74362723522542`, 229.60328905331596`, 812.453956353037`, 75094.84453023993`, 0.019677953756311074`},
{"TBUR", 83.11919052328749`, 229.7266599291585`, 788.1669922878868`, 264820.37883216713`, 0.012476970726293718`},
{"CURE", 365, 10.886820848226298`, 541.0361530421275`, 0.009999162492070298`, 0.021287508674797204`},
{"TBUR", 77.50911305560749`, 229.5296593752051`, 1143.8699845263836`, 1067.3020585980348`, 0.015282040180004446`},
{"CURE", 365, 7.9711319668713525`, 435.50906213200847`, 0.009997565244399586`, 0.006513778621334096`},
{"CURE", 365, 8.913076227441225`, 532.8192404319075`, 0.009997013361134333`, 0.02624467615071882`},
{"CURE", 365, 0.43278390901600944`, 21.3356358177959`, 0.009999794006130108`, 0.015054605553125907`},
```

```
{"TBUR", 154.29312135307416`, 229.64873694345386`, 892.4015050185354`, 1126.6501969127744`, 0.01544057078794247`},
{"CURE", 365, 3.583448883919988`, 95.97039892414129`, 0.009999188497379361`, 0.019707966652369846`},
{"TBUR", 192.60033196051427`, 229.3264560941518`, 1342.7758809139855`, 0.025880569265499608`, 0.016242771167524376`},
{"CURE", 365, 0.41283642987957375`, 27.876679747166055`, 0.00999991738669643`, 0.006528690118033236`},
{"CURE", 365, 7.9949860794309355`, 102.54714731530905`, 0.00999973923284564`, 0.02262968400682272`},
{"CURE", 365, 0.06091289368395384`, 12.909614435422016`, 0.009999145384968851`, 0.024828503734742245`},
{"TBUR", 31.232545697045257`, 229.57020438580088`, 1285.3436321961256`, 5.724289318051632`*^6, 0.02423283641447614`},
{"CURE", 365, 0.28531601178551697`, 34.1277416366056`, 0.009999880895847328`, 0.018279937609436806`},
{"CURE", 365, 5.333202717676054`, 287.5148889343771`, 0.00999909237736091`, 0.0164127180245623`},
{"CURE", 365, 5.2617376749825455`, 231.5525188932019`, 0.009999688432603564`, 0.0226838371109391`},
{"CURE", 365, 6.384875210256725`, 311.86385086651535`, 0.009996044735335718`, 0.00834402481926364`},
{"CURE", 365, 0.8507628135604157`, 70.80597486853279`, 0.009999848798977815`, 0.021656079344611192`},
{"TBUR", 57.0334983374229`, 229.53550228650835`, 1786.1687956358262`, 5019.61185550928`, 0.014896052838206698`},
{"TBUR", 59.326233105880014`, 229.67490179404643`, 1074.952162692421`, 48175.403586358145`, 0.012788097808372012`},
{"CURE", 365, 0.1151262315034552`, 23.535255124818885`, 0.009995825129435792`, 0.014763728297202629`},
{"CURE", 365, 3.4545420813712537`, 33.47456732719112`, 0.00999996004439415`, 0.015997715248036774`},
{"CURE", 365, 3.0255236260831544`, 190.56980437391263`, 0.009999909255167045`, 0.021139216433114755`},
{"CURE", 365, 1.2213596709077643`, 246.37888386494564`, 0.009994666306666294`, 0.013389646895436641`},
{"TBUR", 60.884918549725036`, 229.6189690948649`, 1377.9856232592938`, 126.19791166109033`, 0.009011841365792595`},
{"CURE", 365, 0.7816347788717722`, 32.27115414900167`, 0.009998899149260066`, 0.01401874823156647`},
{"TBUR", 25.84028185639385`, 229.538545666217`, 1168.302468751606`, 1.8715941731713913`*^7, 0.025516777813644694`},
{"CURE", 365, 2.0697000385421616`, 324.1043451466409`, 0.009997755346016005`, 0.01459265838170032`},
{"TBUR", 75.59008946051149`, 229.75938663822794`, 1090.916425554376`, 0.011115875449822525`, 0.0020389936681497315`},
{"CURE", 365, 0.17367992081856914`, 11.653608065793993`, 0.00999985027790335`, 0.021538753541013957`},
{"CURE", 365, 1.3343700948998998`, 295.60118363409237`, 0.009999986287442808`, 0.016035360000815107`},
{"CURE", 365, 0.570387097954444`, 14.584070353552667`, 0.009999828958581782`, 0.020410716868221847`},
{"TBUR", 137.84264532305411`, 229.6211921097459`, 585.3666783633985`, 13343.708272220652`, 0.019253789133970103`},
{"CURE", 365, 3.7783422791502486`, 156.24143653760956`, 0.009999519398830037`, 0.011303034932613674`},
{"CURE", 365, 133.94424858241086`, 330.98693558447894`, 0.009996690088719127`, 0.0016600867251494228`},
{"TBUR", 92.5087915406586`, 229.76234615920467`, 582.5303501831856`, 0.01852298246234162`, 0.002264968339553062`},
{"TBUR", 83.56672358911081`, 229.76849547025458`, 600.2180626517847`, 556.5531358456166`, 0.002978616545733837`},
```

```
{"CURE", 365, 0.8865354123028163`, 20.341328638627232`, 0.009999981849951453`, 0.019783517462529574`},
{"CURE", 365, 0.5090076494932007`, 11.869506184524745`, 0.009999854564040294`, 0.023211136321396326`},
{"TBUR", 122.6310814362678`, 229.6892919249087`, 1197.7746843036853`, 80977.07876818153`, 0.016370384935830085`},
{"CURE", 365, 2.655632721780921`, 36.58172241256094`, 0.009999829452509556`, 0.028685905725888576`},
{"TBUR", 93.37091230084832`, 229.42541010626348`, 715.501149704582`, 267.4154758750325`, 0.020121634828535604`},
{"TBUR", 32.53350536458907`, 229.52988625546286`, 1336.3484483016164`, 1.0833443952969892`*^7, 0.03940254297834358 5`},
{"CURE", 365, 0.26250997360283884`, 24.422369880412926`, 0.009999816477520209`, 0.012933523886268802`},
{"TBUR", 49.714099183811385`, 229.77234152677784`, 1175.717860523023`, 60894.35219117039`, 0.003430897607171022`},
{"CURE", 365, 0.11406568684602326`, 15.36264928445481`, 0.009999560592563968`, 0.024529260284443064`},
{"TBUR", 42.59288047274977`, 229.68998223218983`, 573.6584418698361`, 1.0307304506298423`*^7, 0.0829330918219664`},
{"TBUR", 64.97989851804316`, 229.74157247800363`, 1035.2339252672698`, 3.4515988820325645`, 0.00324925879346344`},
{"CURE", 365, 5.198175644880513`, 471.9168424274748`, 0.009999914675351954`, 0.015334768720921401`},
{"TBUR", 22.228361312829435`, 229.49358812228166`, 1266.8625718668889`, 2.1289279591692727`*^7, 0.05800889305518085`},
{"CURE", 365, 0.15975461376561895`, 74.49571331753731`, 0.009634955450848757`, 0.01771724463295752`},
{"TBUR", 92.19541703840848`, 229.62596104541268`, 525.2953426461471`, 5.420103865704857`, 0.00923928391050494`},
{"CURE", 365, 0.8020625918175788`, 44.78462043962761`, 0.00999642671935808`, 0.021009357455569068`},
{"CURE", 365, 2.0742621593773323`, 272.62830709199153`, 0.009999899998463256`, 0.020049889262832615`},
{"TBUR", 132.97615935869513`, 229.76838038780753`, 814.6240476558083`, 1143.607934650941`, 0.003813029915867813 8`},
{"CURE", 365, 3.593291984562134`, 108.87180702768802`, 0.00999973371341754`, 0.013865979879974094`},
{"CURE", 365, 3.4435946958328154`, 297.6542560735927`, 0.009999913543027792`, 0.010666710471001112`},
{"TBUR", 89.41989401489626`, 229.6434947348093`, 757.3241696859662`, 2.780117353014707`, 0.00809944729038786`},
{"TBUR", 13.79008807073441`, 229.38164472307673`, 1290.93125569193`, 3.259097863295619`*^7, 0.13376463649559717`},
{"CURE", 365, 0.8292371836752066`, 28.821929012338842`, 0.009997679626209127`, 0.021146770113480033`},
{"TBUR", 27.52964845796373`, 229.05296194258915`, 1641.8575048446655`, 4.808882514209867`*^6, 0.0435374030683853`},
{"CURE", 365, 0.15607927660291862`, 9.202196006629729`, 0.009999912615132146`, 0.009122374365746304`},
{"TBUR", 41.29792085164803`, 229.77053875497614`, 890.302858854595`, 1.234503836294538`*^6, 0.00688415161588498`},
{"CURE", 365, 0.5982487320840485`, 194.00170856991676`, 0.009999421255928762`, 0.02368952993324324`},
{"CURE", 365, 0.40738066232825243`, 18.095042927230782`, 0.009999517722393518`, 0.015955014184899488`},
{"CURE", 365, 5.075303308724657`, 498.79621464256434`, 0.00999997799687013`, 0.021312860720571123`},
{"CURE", 365, 0.24218983130463753`, 11.585305945624066`, 0.00999987892562039`, 0.02382558250555501 2`},
{"TBUR", 76.00010636889593`, 229.64728830138384`, 1061.4906712582238`, 10.898873041862352`, 0.007591793074565418`},
```

```
{"TBUR", 82.53673966981046`, 229.75505319453148`, 822.7420563446846`, 224.4589169750807`, 0.003831750286696218 5`},
{"TBUR", 50.14572223115015`, 229.7409516462298`, 507.2885377783525`, 7486.612698615132`, 0.00816167375629020 9`},
{"CURE", 365, 0.1602805256206483 5`, 8.59241659450946`, 0.00999976538883657 5`, 0.02080921730501598 6`},
{"TBUR", 43.408951103587775`, 229.72068241010632`, 550.5316942616514`, 9.47070292130638 7`*^7, 0.14806660357558976`},
{"CURE", 365, 3.550256178306406`, 334.8707652815217`, 0.00999771011191017`, 0.017497739837385704`},
{"TBUR", 19.99295364509191`, 229.63430099350876`, 842.44645030875`, 3.581478588882678`*^7, 0.13084264458293648`},
{"CURE", 365, 8.583553501440294`, 486.63926866596387`, 0.009998380559620 36`, 0.00981852870688503 1`},
{"CURE", 365, 3.278978732311173`, 62.83765074284079`, 0.009999919688312 51`, 0.006351212259364607`},
{"TBUR", 95.45563866514314`, 229.633729040277`, 617.105852553719`, 41.381788860927166`, 0.009677251683712623`},
{"TBUR", 112.07114439975321`, 229.67138963724932`, 1185.3090416757682`, 36954.51135866464`, 0.017766125274719286`},
{"CURE", 365, 0.43195378713328275`, 172.77686939360316`, 0.009672792847595906`, 0.01612246311207321`},
{"CURE", 365, 2.1358765143810046`, 35.94675333808501`, 0.009999855769796446`, 0.014525045626622777`},
{"CURE", 365, 0.321554152664810 7`, 54.830134781323416`, 0.009997594885627285`, 0.026088207593453653`},
{"CURE", 365, 4.4467967984599825`, 453.3734756525281`, 0.009999405162607793`, 0.011837892270485944`},
{"CURE", 365, 0.08235174689005031`, 26.42383639075827`, 0.009996326892585058`, 0.0279321476239204`},
{"TBUR", 53.799154991424444`, 228.93569996460081`, 1155.1688359913073`, 146957.30489138525`, 0.03776013901734165`},
{"CURE", 365, 0.0948164771628869 1`, 21.043013518612817`, 0.009999942508327484`, 0.015800065650295462`},
{"CURE", 365, 0.1604280566466299`, 14.840275049261608`, 0.009998831325355 83`, 0.021131454077397468`},
{"CURE", 365, 2.7987064975133786`, 161.5061716782205`, 0.009999562657912148`, 0.026476392576773398`},
{"CURE", 365, 1.653773470208331`, 57.79248624348925`, 0.00999999727159927`, 0.007784031667889068`},
{"TBUR", 171.07123590779725`, 229.76806596553396`, 1056.2984067515326`, 334.07280306011967`, 0.004091321093280081`},
{"TBUR", 97.24252214831314`, 229.72805508924515`, 764.5629210221141`, 0.09805825427831799`, 0.003978262192372461`},
{"CURE", 365, 0.24742651348216801`, 26.706628602275785`, 0.009999843465536945`, 0.02228837569728467`},
{"TBUR", 15.943066717934325`, 228.1006760666324`, 1362.209835303457`, 6.093120594420567`*^7, 0.18516980495040952`},
{"CURE", 365, 2.856659142013854`, 113.43524078037935`, 0.00999993921018542`, 0.023428644569996833`},
{"TBUR", 17.943022027035557`, 228.7104034296614`, 1256.2772951628033`, 4.5584512065957315`*^7, 0.11633610021693291`},
{"CURE", 365, 0.13582103871292925`, 35.703354315599334`, 0.009997831423519133`, 0.02770378924797765`},
{"TBUR", 73.72678259577975`, 229.53856361555603`, 979.7749649850946`, 1.9184599871064363`, 0.010079015617104831`},
{"TBUR", 53.36605548133027`, 229.64787709691277`, 638.7313756189849`, 3.583163782496636`*^7, 0.1297744637605052`},
{"TBUR", 20.718732373668654`, 228.22970669365714`, 1539.2359866594015`, 6.887542862502483`*^7, 0.18751149449154753`},
{"CURE", 365, 1.7033658198849713`, 69.35120937765485`, 0.009998638147975625`, 0.025119431704752795`},
```

```
{"CURE", 365, 1.3297535995475673`, 139.97969240219317`, 0.009998289576599552`, 0.014628677068676342`},
{"CURE", 365, 0.5407916071325568`, 33.32127763618644`, 0.009986612542104733`, 0.016813274449988654`},
{"TBUR", 66.36147715791877`, 229.6522553790365`, 709.8153439321017`, 7823.741031184832`, 0.012700587962495997`},
{"TBUR", 46.61715082424158`, 229.530826306158`, 647.245619924445`, 43295.745724439286`, 0.02018891332960405`},
{"TBUR", 16.237629570110666`, 228.61533037866366`, 1275.1493313438014`, 8.659052494024475`*^7, 0.2710641844729076`},
{"CURE", 365, 3.9830196961190483`, 224.38258565618338`, 0.009999866758802742`, 0.022533639045061584`},
{"CURE", 365, 0.9574525391101876`, 55.910157178437345`, 0.009999730254270514`, 0.010813284913166727`},
{"CURE", 365, 2.102704352255025`, 70.01346568370124`, 0.009999900292342975`, 0.014570124599996102`},
{"CURE", 365, 1.1954315890695029`, 18.442743194672495`, 0.009999873009577801`, 0.015427175383929237`},
{"TBUR", 20.624899381074847`, 229.6089383497546`, 1224.4522114803917`, 2.125434673297826`*^7, 0.024568051009242756`},
{"TBUR", 99.60262282527778`, 229.52893170450656`, 751.687455051824`, 10.789018648253554`, 0.013227330221979345`},
{"CURE", 365, 1.308744825568288`, 83.94591063898233`, 0.009999810411954331`, 0.018744327704524763`},
{"TBUR", 33.31059551738588`, 229.44141018823183`, 1029.4622100026986`, 1.4261566356112922`*^7, 0.05922375612877704`},
{"CURE", 365, 1.6913659531504788`, 151.31905742874383`, 0.009997287170242224`, 0.013936665478095038`},
{"CURE", 365, 3.0126367735457653`, 117.04285959818942`, 0.009999926211297546`, 0.00725559950934784`},
{"CURE", 365, 0.2738991311902238`, 43.75467617528231`, 0.009999456987958706`, 0.010887225374734707`},
{"CURE", 365, 1.4099249179299536`, 41.634601827593656`, 0.009999737994915684`, 0.012256933425663532`},
{"CURE", 365, 0.053021768011500915`, 4.9331985797699875`, 0.009999789912597172`, 0.01915421097548001`},
{"CURE", 365, 2.8180574530167406`, 116.74440667269357`, 0.00999973730721281`, 0.010882220572724758`},
{"CURE", 365, 0.47109266083696494`, 65.05636070606424`, 0.009999923877078595`, 0.028475660303777867`},
{"CURE", 365, 3.154232423842538`, 107.47952608572385`, 0.00999985901376113`, 0.004248667376263893`},
{"CURE", 365, 1.2192708900908125`, 104.59493045461626`, 0.00999970307728711`, 0.021366789211253606`},
{"CURE", 365, 0.11032252058946779`, 48.384717313888196`, 0.009999890847939502`, 0.007906689820975137`},
{"CURE", 365, 14.74777831916519`, 248.66221239847926`, 0.009999729012512575`, 0.01814555309173153`},
{"TBUR", 76.65017808727696`, 229.76391540104686`, 1149.3585491180422`, 16.57053761638259`, 0.002786013683306274`},
{"TBUR", 111.77310608486046`, 229.752549656339`, 1246.3749112580952`, 4611.110177856232`, 0.006225249436855353`},
{"CURE", 365, 0.2627899464924906`, 40.02606038490514`, 0.009999616562426024`, 0.006389446868231402`},
{"TBUR", 29.112954362725663`, 229.6356121438989`, 1044.4954944911763`, 1.1110790752592713`*^7, 0.04881531093087641`},
{"TBUR", 140.66673488952017`, 229.70490003707582`, 1245.0503271504454`, 0.030789057778268476`, 0.005419225147944351`},
{"TBUR", 26.507837734512137`, 229.0129543638542`, 1289.173617613943`, 4.556656925924955`*^7, 0.09753255892017805`},
{"TBUR", 83.50832241853723`, 229.5971169579591`, 1291.8043766523665`, 9242.82437755005`, 0.018589600590963422`},
```

```
{"CURE", 365, 0.21891573764544553`, 14.752938785843687`, 0.009999865464351955`, 0.024867665220752986`},
{"CURE", 365, 1.9635263144849253`, 188.00505626100662`, 0.00998964218637123`, 0.01419225868062162`},
{"CURE", 365, 0.5006746437214933`, 206.52399032339508`, 0.00999992671299151`, 0.01046111102899206`},
{"CURE", 365, 0.4872536301443029`, 36.50532871029123`, 0.009682892370314323`, 0.024073134971039755`},
{"TBUR", 54.75498698066986`, 229.43472489165237`, 1094.0863104870446`, 691432.2420126521`, 0.03232578619031034`},
{"CURE", 365, 2.745433600586913`, 163.20466763004757`, 0.009999842616026223`, 0.009988995543427798`},
{"CURE", 365, 1.4492037363736505`, 136.4722068404403`, 0.009999947430343813`, 0.006848877128068537`},
{"CURE", 365, 36.07882617876973`, 347.25247238788506`, 0.009999861762447795`, 0.014736526839585788`},
{"CURE", 365, 0.6594085220182293`, 38.931695876075345`, 0.009997019745983904`, 0.010797428405047911`},
{"CURE", 365, 0.7052821591959295`, 177.85374445566404`, 0.009691505106248709`, 0.012182369351637927`},
{"TBUR", 58.708791267513426`, 229.47472336040897`, 714.3163876784478`, 941562.9277742625`, 0.0409328607846261`},
{"CURE", 365, 4.420324720541935`, 258.95940846809134`, 0.009995987707339102`, 0.014141404714362713`},
{"CURE", 365, 0.34614738248491117`, 140.41914947768063`, 0.009999948044707607`, 0.015323694313818706`},
{"CURE", 365, 1.1713317762882403`, 106.79076546752066`, 0.009999726740002742`, 0.017433595717579117`},
{"CURE", 365, 6.927032163936434`, 233.98556808449982`, 0.009999970284235191`, 0.019287488272968802`},
{"CURE", 365, 0.08483391113560994`, 19.849510202600893`, 0.009987032529243229`, 0.019026062733696846`},
{"CURE", 365, 1.2395553430369879`, 160.52257831090384`, 0.009998558020479564`, 0.03123197750744166`},
{"CURE", 365, 0.17685340215725295`, 19.17459101517087`, 0.00972709742727546`, 0.01837659388703453`},
{"TBUR", 36.96981255806451`, 229.06309725728403`, 1208.848242508738`, 7.047002620841318`*^6, 0.06567093665550049`},
{"CURE", 365, 1.255472309094088`, 39.68475428019911`, 0.009999725834474085`, 0.024470206527042813`},
{"TBUR", 31.9243164692905`, 229.77076299217882`, 1018.6963480397275`, 3.4610065999125615`*^7, 0.060557304048158796`},
{"CURE", 365, 1.4565437051331966`, 321.8021879790802`, 0.0099999146782745`, 0.01414602826272523 5`},
{"TBUR", 57.062123766009016`, 229.7652226066227`, 1163.1093276174445`, 12507.196089124256`, 0.003962103607824653`},
{"TBUR", 20.067862951040105`, 228.8378282162604 8`, 1398.6915641081962`, 7.396831999010141`*^7, 0.08838012355215773`},
{"CURE", 365, 13.345217559782268`, 306.5003267307166`, 0.009993828586831378`, 0.014641229502698097`},
{"CURE", 365, 0.22789953333221202`, 44.23103384013264`, 0.009998792987903883`, 0.022511473199632082`},
{"CURE", 365, 0.576166585440161`, 62.540795989142175`, 0.009999952184310752`, 0.013793303968641498`},
{"TBUR", 27.346858418724693`, 229.13932334915182`, 1116.9382353832848`, 2.2907286854466986`*^7, 0.07590450394613184`},
{"CURE", 365, 0.9269701786009211`, 120.81425546502963`, 0.009997427485985321`, 0.020489248170878365`},
{"CURE", 365, 12.75671109531688`, 713.5640315405354`, 0.009999991506412667`, 0.01525457272499272`},
{"CURE", 365, 1.7211063854190833`, 136.47780498199245`, 0.009999310900380783`, 0.019807750838271965`},
```

```
{"CURE", 365, 0.09921538163345217`, 26.590883846479766`, 0.009995453440666357`, 0.026363905559462553`},
{"CURE", 365, 7.575397924847024`, 379.8040324354292`, 0.009995293027660977`, 0.01690943341913055`},
{"CURE", 365, 2.670914337720122`, 65.84103880262477`, 0.009999938482806217`, 0.015722739159265656`},
{"TBUR", 20.646372954013014`, 229.31114548279535`, 1161.5953919762062`, 2.861394334334613`*^7, 0.041434214041861275`},
{"TBUR", 84.57990852236694`, 229.7870055067977`, 653.0068156593726`, 393704.6802519324`, 0.004385377760657541 5`},
{"CURE", 365, 0.7654975542340251`, 24.203504798549968`, 0.009999758966191465`, 0.02016861018003922`},
{"TBUR", 81.43996540557748`, 229.4756189239324`, 628.2420896228991`, 492803.54067603254`, 0.04323899305888695`},
{"CURE", 365, 1.5278525954073718`, 33.04091384895502`, 0.009999853315822939`, 0.011240949878764814`},
{"CURE", 365, 0.1952414397637435`, 8.713366945968357`, 0.009999948176119985`, 0.02093883829329674`},
{"CURE", 365, 8.808344561684406`, 121.81514655115433`, 0.0099999404756 5569`, 0.013398494131847634`},
{"CURE", 365, 0.3934421315725003`, 134.37381845496233`, 0.00964474435245102`, 0.012681164017230325`},
{"TBUR", 94.41060551714803`, 229.72389540021464`, 1300.4426637448764`, 586567.6261604251`, 0.015436359499357476`},
{"CURE", 365, 1.1234505299592077`, 20.98426597777796`, 0.009999915169429606`, 0.02383032973476207`},
{"CURE", 365, 3.8392380126697314`, 110.97245524147648`, 0.00999951085521148`, 0.01404371559844459`},
{"CURE", 365, 2.6141008637728502`, 116.95358294760247`, 0.009999851812724099`, 0.010106641838104679`},
{"CURE", 365, 0.4326055102324096`, 22.460995404880858`, 0.009999911353959631`, 0.007259008861010352`},
{"TBUR", 90.48734136258498`, 229.6438559946277`, 1227.8736884970417`, 75.49098418704189`, 0.010172455726527977`},
{"CURE", 365, 0.3013379541916594`, 14.062228725472254`, 0.009999954507903794`, 0.026484024001026892`},
{"TBUR", 44.902782817165786`, 229.57695734838165`, 1516.4831206673073`, 23843.5664431646`, 0.012199817596047284`},
{"TBUR", 16.09771012876661 4`, 229.15671033250084`, 1299.7685372202022`, 3.546682887992379`*^7, 0.16312595686596162`},
{"TBUR", 60.06317787870501`, 229.38062087217924`, 1090.6885024120932`, 759.6588980169176`, 0.018486375866877906`},
{"TBUR", 176.08930069125944`, 229.71754156804843`, 683.9083723018043`, 0.05855317261085476`, 0.005418352884761505`},
{"CURE", 365, 5.445113838719442`, 130.76848642538334`, 0.009999930987 26709`, 0.016451860566797122`},
{"CURE", 365, 0.049433070116838985`, 16.705163746750795`, 0.009998051576049981`, 0.02652273800625311`},
{"CURE", 365, 0.19029525336518657`, 10.745436419399514`, 0.009999916444918537`, 0.021864491384442523`},
{"TBUR", 152.37065494935047`, 229.66965587547347`, 1400.006505313673`, 0.7186546115151224`, 0.008765678419284044`},
{"TBUR", 43.441923440287276`, 229.5972947106412`, 744.2203422596582`, 437520.9586802615`, 0.024056229068615667`},
{"TBUR", 214.25969107123012`, 229.67443888598842`, 1264.4702555003919`, 0.19624264500644292`, 0.00809362368721133`},
{"CURE", 365, 2.5225448738471843`, 149.7916679552216`, 0.009999857227222781`, 0.01957618043936284`},
{"TBUR", 44.61212174572604`, 229.52867704205786`, 1663.003512683668`, 146117.199984999`, 0.019078190182265656`},
{"CURE", 365, 3.762125041572507`, 127.86282731973519`, 0.009999724185003014`, 0.006071394609395375`},
```

```
{"TBUR", 52.80048697798943`, 229.7783336913585`, 907.275243307415`, 25196.2028538957`, 0.003089120885724739`},
{"CURE", 365, 0.1533204965174365 7`, 18.240718957465933`, 0.009990799586 18656`, 0.01775204790971 9713`},
```

```
{"TBUR", 52.80048697798943`, 229.7783336913585`, 907.275243307415`, 25196.2028538957`, 0.003089120885724739`},
{"CURE", 365, 0.15332049651743657`, 18.240718957465933`, 0.009990799586618656`, 0.017752047909719713`},
{"CURE", 365, 0.9443883674603839`, 44.96271263595521`, 0.009999757305951996`, 0.01920416194195333`},
{"CURE", 365, 1.1080742443578457`, 104.47558398889062`, 0.009657467726365335`, 0.025371189725697935`},
{"CURE", 365, 0.581120927492058`, 92.5995936572873`, 0.009673976169463194`, 0.0123513506916452 7`},
{"CURE", 365, 0.6124022138188215`, 289.9522427049994`, 0.00999995488796847`, 0.01012321545224424`},
{"CURE", 365, 0.8438091397954443`, 189.5667435854124`, 0.009999881317524094`, 0.013637606596543313`},
{"TBUR", 21.441596494987664`, 229.6753805730573`, 1226.5342017223961`, 7.450517421109603`*^7, 0.04921078765324544`},
{"TBUR", 73.63979090365123`, 229.59579185925952`, 1113.121399377522`, 357.09283103485177`, 0.012210786824411544`},
{"CURE", 365, 3.917267507409427`, 162.9147677912991`, 0.009999511232206892`, 0.020800730302799202`},
{"TBUR", 86.88302577635307`, 229.5946009196477`, 1087.894690664191`, 14986.955255102677`, 0.01862339439196576`},
{"TBUR", 15.31352521331403`, 229.42892265185847`, 1467.4857814688435`, 4.122343563126204`*^7, 0.09127693320777912`},
{"TBUR", 26.031106796184574`, 229.37502361940687`, 1165.299143038298`, 9.901172079369856`*^6, 0.02890078451763095`},
{"CURE", 365, 10.304965088111683`, 386.1274581380708`, 0.009999875941067209`, 0.012951081164744027`},
{"CURE", 365, 0.7669666525430928`, 134.57575238565775`, 0.009999930452680486`, 0.017174520989337198`},
{"CURE", 365, 2.384163945701003`, 363.0224104595148`, 0.009996657883789601`, 0.010030518660911938`},
{"CURE", 365, 0.045455068454475306`, 25.076597395367305`, 0.009999178638310296`, 0.010933338020469871`},
{"TBUR", 18.350971295790625`, 229.14211814762`, 1138.8118440226503`, 7.039558454460138`*^7, 0.11094117236229376`},
{"TBUR", 32.016373988305816`, 228.904399759165`, 1313.4351594824543`, 6.06441762817642`*^6, 0.05407101275520678`},
{"CURE", 365, 1.0151134004559743`, 44.453145244668015`, 0.00999934272425189`, 0.014766350305042206`},
{"CURE", 365, 0.07786306399969233`, 5.0477584053114795`, 0.00999985922189412`, 0.011180209853234308`},
{"TBUR", 42.518772094581024`, 229.68172162261934`, 775.4883291846828`, 9.48294897523894`*^6, 0.03367338873385901`},
{"TBUR", 54.31491872264501`, 229.2618087994201`, 1466.0910671635677`, 6558.1091011352355`, 0.021524547235998608`},
{"TBUR", 23.51077242016955`, 229.3533053050158`, 1535.5296057148987`, 4.3025576111317664`*^7, 0.1237771872753335`},
{"TBUR", 24.725134059276947`, 228.8479670614112`, 1337.1435494171944`, 3.1428786749072764`*^7, 0.06177097851179374`},
{"CURE", 365, 4.134527531660482`, 142.88412248938323`, 0.009999912240970948`, 0.02034182949096167`},
{"CURE", 365, 4.699286822809973`, 387.74559281158906`, 0.009997155997252748`, 0.017911637745110294`},
{"TBUR", 35.20138160340028`, 229.5013964054368`, 1066.247251912167`, 2.4292983997957066`*^7, 0.06444389942475993`},
{"TBUR", 39.18189235038725`, 229.66265389755733`, 612.7985434783133`, 4.250865043129077`*^6, 0.0266766268513228`},
{"CURE", 365, 2.231464660136193`, 35.95835439216669`, 0.00999997809084319`, 0.01364016327406866 6`},
{"CURE", 365, 0.6632224833797844`, 93.02075134430964`, 0.009999897944227116`, 0.015396477124936445`},
```

```
{"TBUR", 176.0167357226551`, 229.7850798096233`, 630.0501337804292`, 0.13853909686505175`, 0.0016129512924819348`},
{"CURE", 365, 1.631566251865803`, 180.07811675585023`, 0.009999724068697001`, 0.015450987552639508`},
{"CURE", 365, 7.291305360011944`, 297.28738888341553`, 0.00999978581227838`, 0.018365635235227936`},
{"CURE", 365, 5.356575406785117`, 236.0704884405618`, 0.009999889220735579`, 0.008707043470686668`},
{"CURE", 365, 2.0011645846473693`, 89.48837779980384`, 0.009999938674715025`, 0.021424351368296943`},
{"TBUR", 95.412818604117`, 229.72612049170087`, 637.9117289342379`, 18.559931045773045`, 0.005563300575314096`},
{"CURE", 365, 1.5691305101716184`, 117.89197024119771`, 0.009999881537106051`, 0.020935810908123336`},
{"CURE", 365, 0.45800696445623806`, 66.0353740781253`, 0.009999866974285914`, 0.00694255125688498`},
{"TBUR", 118.8243564905838`, 229.7596541005801`, 996.2310158578971`, 0.06886920134259927`, 0.0026564710736143534`},
{"CURE", 365, 0.530698750291567`, 76.8889017516358`, 0.00971204011060419 8`, 0.019399790544459877`},
{"TBUR", 85.04675831732308`, 229.7104841968323`, 1217.0306833166826`, 0.15492912845217172`, 0.004457719632127511`},
{"CURE", 365, 2.9947573096112987`, 225.71962026078097`, 0.009999276372296166`, 0.020545304587827228`},
{"CURE", 365, 0.13785762921431494`, 38.136323178273756`, 0.0099998466901004`, 0.014023392608356349`},
{"CURE", 365, 0.6783957313416907`, 28.18705203004953`, 0.009999241511784085`, 0.01435871936305822`},
{"CURE", 365, 0.5367465692415506`, 43.98131087185115`, 0.009692451073680886`, 0.024748251981645795`},
{"TBUR", 21.97646992855342`, 229.40047761284052`, 968.1465015913313`, 3.4664980752399296`*^7, 0.1594094945657252`},
{"TBUR", 23.745816971983118`, 229.53931334179262`, 984.6674228969372`, 6.881869706233187`*^7, 0.09540808068911848`},
{"TBUR", 76.73134173736968`, 229.37959866437737`, 897.4219505410446`, 0.45587154345008274`, 0.012128045375722752`},
{"TBUR", 75.48783859293465`, 229.6209853915854`, 1247.9843992935982`, 63.59931720484274`, 0.00977898475328899`},
{"TBUR", 46.43506461713933`, 229.68845354906261`, 687.2868945724088`, 611253.5390014511`, 0.01546604086836162`},
{"CURE", 365, 17.287186762380944`, 493.9480866335014`, 0.009999881202611414`, 0.013895292061908988`},
{"TBUR", 43.43676109611835`, 229.68026378735928`, 856.6977830937037`, 3.192323714009316`*^7, 0.09020922813517634`},
{"CURE", 365, 0.7783001587367261`, 35.42941104619079`, 0.00999970536599397`, 0.02887243801618389`},
{"CURE", 365, 0.41873395023533694`, 36.8320246521671`, 0.009999630755705712`, 0.027534040555770793`},
{"TBUR", 17.60225185499527`, 229.45422157616048`, 1418.7593201587079`, 9.133686457942215`*^7, 0.16215290847514072`},
{"CURE", 365, 6.32003635107973`, 735.0843025192282`, 0.009998060240702586`, 0.019509854596512794`},
{"CURE", 365, 5.142852457783996`, 285.30518005767885`, 0.009998770929616319`, 0.021973101584352842`},
{"CURE", 365, 0.28446135325805166`, 80.58261104969404`, 0.009999183413222227`, 0.009815032749507431`},
{"TBUR", 61.61529150075207`, 229.74197571370226`, 940.7396203593205`, 224898.22940622678`, 0.008889985364726237`},
{"CURE", 365, 0.7048582686320558`, 12.850238991474182`, 0.009999439180825952`, 0.01917471024161002`},
{"CURE", 365, 103.36311376139692`, 607.7228818417328`, 0.00999973947778128`, 0.010665761565002744`},
```

```
{"CURE", 365, 0.7252342536459632`, 55.847077142041094`, 0.00999983187664587`, 0.0168821111014591`},
{"TBUR", 58.61431408685387`, 229.46588305270052`, 946.6726350784304`, 4138.66755298144`, 0.02100478633143965`},
{"CURE", 365, 1.3558443526918929`, 48.720120276570526`, 0.009999902782248472`, 0.004566137379171605`},
{"CURE", 365, 0.5727818107655294`, 29.475117122782912`, 0.009999664186630841`, 0.02065071751122532`},
{"CURE", 365, 8.86781574199241`, 159.61464999274136`, 0.009999992108959839`, 0.019134859070410345`},
{"TBUR", 54.16045601700556`, 229.59476761418793`, 1649.8541006289036`, 20378.485685159274`, 0.016315048969241838`},
{"TBUR", 16.607775224830444`, 228.48632453310887`, 1805.4318076685854`, 9.660042085393697`*^7, 0.09928114783631427`},
{"TBUR", 61.82043116133504`, 229.4376768048852`, 1037.1835447612564`, 273736.2436168704`, 0.030352225195066085`},
{"CURE", 365, 1.4635205387842096`, 272.5455876101427`, 0.009998763124254077`, 0.01886331418144582`},
{"TBUR", 89.89634795884076`, 229.64152686012662`, 629.343133725618`, 89880.72622388335`, 0.021625285014345286`},
{"CURE", 365, 4.17599003579503`, 184.15305318940673`, 0.009999935825073664`, 0.01848937936678474`},
{"CURE", 365, 5.687599321005833`, 119.28328582921795`, 0.009999912130188283`, 0.023427840186464793`},
{"TBUR", 33.24437699238964`, 229.69172979481374`, 557.0612539743122`, 8.356990380735083`*^6, 0.028221824242846607`},
{"CURE", 365, 0.1599692578538922`, 13.174796973675276`, 0.009997311531454384`, 0.006265686147337565`},
{"CURE", 365, 0.20245785871624689`, 6.289641794800871`, 0.00999979776316258`, 0.02345072195407261 7`},
{"CURE", 365, 0.23289303283586762`, 31.377351537482884`, 0.009996156327643039`, 0.016764960141555783`},
{"TBUR", 16.285682961236155`, 229.02169826252774`, 1460.579710257681`, 4.023445632685 6665`*^7, 0.0965007971 4986329`},
{"CURE", 365, 0.042285148246927355`, 8.53311633304141`, 0.00999824605967133`, 0.01742197190632 3764`},
{"TBUR", 28.521929604347115`, 229.57052264952895`, 1457.7990479218333`, 7.377305493119007`*^7, 0.09399280468498084`},
{"CURE", 365, 3.1157133592343262`, 55.00754443080858`, 0.009999068020181691`, 0.01777576916143 8967`},
{"CURE", 365, 6.6314130779958145`, 191.9126899677546`, 0.009999792152255197`, 0.020593209122869566`},
{"TBUR", 88.39051109371347`, 229.7269857895096`, 981.7657859701857`, 47602.17465662184`, 0.011655160701417306`},
{"CURE", 365, 1.3009523485810353`, 66.02876569518462`, 0.009999572998602666`, 0.02804318086312575`},
{"TBUR", 14.951099879532178`, 226.94646053075`, 1055.6418127762647`, 9.51311671217389`*^7, 0.20232879549370186`},
{"CURE", 365, 0.33760639050316066`, 35.514879351048855`, 0.009999902317390116`, 0.023966597079587496`},
{"CURE", 365, 0.4879188344905859`, 206.33727841349278`, 0.009999718047639312`, 0.017338533164297707`},
{"CURE", 365, 0.05477927675398563`, 15.129004524290249`, 0.009998596242916567`, 0.026256299120095545`},
{"CURE", 365, 0.789156652345387`, 15.983229436267044`, 0.009998070004944098`, 0.030274178684084073`},
{"CURE", 365, 1.1209548730467735`, 225.3227379760394`, 0.00999955276597476`, 0.018402946620047517`},
{"TBUR", 16.143643487826655`, 229.55459646886885`, 1053.9858634848345`, 3.2052596491028216`*^7, 0.1856520307376752`},
{"CURE", 365, 0.5042957512378021`, 36.34624023357966`, 0.0099999636325935`, 0.025390400170703972`},
```

```
{"TBUR", 25.464461810768682`, 229.62545887633908`, 1239.833517074585`, 6.3609508913283244`*^7, 0.09494505381165809`},
{"TBUR", 59.59796168165531`, 229.74996628505284`, 1129.9693027295568`, 60.25406066275024`, 0.003918031466944146`},
{"TBUR", 42.77712689552655`, 229.37923687120795`, 1795.6696841591283`, 271469.37784283754`, 0.03166483764862433`},
{"CURE", 365, 0.12425964973938107`, 18.942661150942655`, 0.009596993201678583`, 0.024662497952172634`},
{"TBUR", 109.94027142645443`, 229.77539743940457`, 479.9719491757846`, 758543.3557507405`, 0.007862188789789708`},
{"CURE", 365, 7.780129849043117`, 381.0998124931715`, 0.009995331571947026`, 0.016873183682816956`},
{"TBUR", 146.3591933749227`, 229.52042618938736`, 1167.3574643386037`, 896.2155279139027`, 0.021046445468154623`},
{"CURE", 365, 0.8807319097410684`, 78.43101454624538`, 0.009999221801523834`, 0.0210155651943865 8`},
{"CURE", 365, 0.5421825488771733`, 35.30084013643003`, 0.009999731860197545`, 0.030616473671534666`},
{"CURE", 365, 0.10573126848709145`, 13.305681530937902`, 0.009999987941296485`, 0.015865384192445535`},
{"TBUR", 115.45454468564924`, 229.66190437016212`, 1301.1106538772585`, 0.36026432098773853`, 0.007549765085767337`},
{"CURE", 365, 0.1426503215818051`, 8.036719930292259`, 0.009999845430569808`, 0.0243352890217882`},
{"CURE", 365, 4.667857295719127`, 191.70206783032378`, 0.009999657958002124`, 0.012611766388984319`},
{"CURE", 365, 0.9792727550554428`, 27.969346016513246`, 0.009989679017300579`, 0.02808909442355648`},
{"TBUR", 153.44807577117842`, 229.74166369720683`, 863.466662105761`, 1.7226216786908832`, 0.004850738844072217`},
{"CURE", 365, 11.023861832999566`, 332.08041316909754`, 0.009999246022890823`, 0.01789647604522198`},
{"CURE", 365, 0.23574954587950206`, 66.2469755897497`, 0.009567221375295785`, 0.01737752595056148`},
{"CURE", 365, 0.05851713476120625`, 9.787954834477132`, 0.009999814959411995`, 0.022853726418006006`},
{"CURE", 365, 0.33738489631428015`, 24.80572464520403`, 0.009999720928313109`, 0.01179311178375209`},
{"TBUR", 71.69442416506885`, 229.7294114552799`, 1174.0294760151914`, 0.42246758605715645`, 0.0035417468516584887`},
{"CURE", 365, 0.6848339834901203`, 23.229787236539575`, 0.009999592381403844`, 0.016807128422434932`},
{"CURE", 365, 2.089443793278995`, 69.83564733257448`, 0.009999811923245776`, 0.00986546222421508`},
{"TBUR", 12.777783212969021`, 228.77080260398796`, 971.5855111573875`, 7.41363923833616`*^7, 0.20571784033978197`},
{"CURE", 365, 0.18640911869878715`, 91.90357716595113`, 0.00999981454085699`, 0.021467907266266028`},
{"TBUR", 58.79113254387577`, 229.56868431111786`, 1256.0783007270413`, 2.814019308719552`*^7, 0.08369264770228813`},
{"TBUR", 47.93527424572178`, 229.77724992701766`, 1132.6202241997973`, 4.946774459682585`*^7, 0.025215645788823318`},
{"CURE", 365, 5.08344895625907`, 147.1358006477116`, 0.009999819401992105`, 0.010409754449345582`},
{"CURE", 365, 1.0482805656851273`, 372.32325648190437`, 0.009999897906609793`, 0.01980160369502742`},
{"CURE", 365, 6.870571233103954`, 347.75214754555213`, 0.009999836103541609`, 0.020917774151039813`},
{"TBUR", 24.689282588651942`, 229.59072488615897`, 1049.8218830008257`, 2.1922266859101966`*^7, 0.03367742095869785`},
{"TBUR", 18.60039153536929`, 228.93483142582375`, 1279.2124996687235`, 4.721491520685097`*^7, 0.09435304589168303`},
```

```
{"TBUR", 60.17020380379656`, 229.77344211574584`, 1057.4187366585984`, 547903.8261425204`, 0.004685281220600954`},
{"CURE", 365, 0.2989107917639120`, 137.06184934205177`, 0.009736975602182106`, 0.017423241861043843`},
{"TBUR", 24.95201333346673`, 229.44999430654983`, 902.1723948026956`, 8.557828905511698`*^7, 0.26904139803263555`},
{"CURE", 365, 0.24125298662151545`, 32.761434871367484`, 0.009999984443899313`, 0.021584463377517484`},
{"CURE", 365, 0.217047760694615`, 12.4900777710721`, 0.009993518959655699`, 0.021994897569161828`},
{"TBUR", 40.28668172426557`, 229.72143951045794`, 729.3913119717648`, 1.6138335116361887`*^6, 0.023619822768064774`},
{"TBUR", 42.55968322541777`, 229.61816127973435`, 670.285143099333`, 485388.34293490095`, 0.02254184383154467`},
{"CURE", 365, 1.2829680995333121`, 306.2258567897296`, 0.009999690561550569`, 0.022233233526066733`},
{"TBUR", 22.972078032842138`, 229.22069476032146`, 1053.6904400273374`, 2.116263899161003`*^7, 0.12047643026963437`},
{"CURE", 365, 0.20407961859103602`, 31.237567445426265`, 0.009999972870564174`, 0.029016894642174957`},
{"TBUR", 21.085899108584904`, 228.65999769392036`, 1583.7634023867156`, 5.6312664511737764`*^7, 0.10800544794520246`},
{"CURE", 365, 0.6022954214297019`, 47.256021494061535`, 0.00999968111198273`, 0.010324344235749112`},
{"CURE", 365, 0.0682374617102988`, 13.69939056811259`, 0.009999960129015514`, 0.008644780132227685`},
{"CURE", 365, 1.4132790067895338`, 107.9941435773733`, 0.009998955332516879`, 0.013975723256217825`},
{"CURE", 365, 10.149605374816419`, 539.5371385844079`, 0.009985905116698486`, 0.008328411408056404`},
{"CURE", 365, 0.07599277766737358`, 23.30814069408579`, 0.009999517467049517`, 0.02543973662591289`},
{"CURE", 365, 0.43494094241376435`, 87.5717986444274`, 0.009988721794254947`, 0.00955417938296096`},
{"CURE", 365, 0.8896139057716261`, 170.5842132882581`, 0.009999660364214588`, 0.013464387240902405`},
{"CURE", 365, 1.0289762740117407`, 144.37253300308473`, 0.009999881669504366`, 0.01595184477099988`},
{"TBUR", 96.56513156124043`, 229.70852800596768`, 1056.1800620430397`, 0.2896089235042601`, 0.005202522407327387`},
{"TBUR", 36.50290869499375`, 229.7706324481959`, 901.234403409456`, 2.747416228964452`*^7, 0.012032860407687437`},
{"CURE", 365, 4.848718471403237`, 311.939335601995`, 0.00999812345668301`, 0.013781910380366197`},
{"CURE", 365, 0.7206374564807154`, 8.083261825734397`, 0.009999550742425806`, 0.013582783040537902`},
{"TBUR", 57.976588984410576`, 229.67182465841913`, 1046.2557624515734`, 1.8317045486884972`*^6, 0.020568247275467456`},
{"CURE", 365, 0.649950614410015`, 129.45212941581747`, 0.009714904576037997`, 0.011602341223893854`},
{"CURE", 365, 0.5730735283991223`, 122.90853249850572`, 0.009998737176421319`, 0.009209931726198063`},
{"CURE", 365, 161.53393486911102`, 327.10615362086565`, 0.009995065126536312`, 0.004306287294406303`},
{"CURE", 365, 0.032967182707480114`, 12.368189152136694`, 0.009997253570452974`, 0.016788382447937296`},
{"CURE", 365, 3.6009823001832286`, 150.30150054856108`, 0.009665044225464213`, 0.024702630258149297`},
{"TBUR", 61.891145096201505`, 229.65026148378968`, 1277.642459869385`, 50795.4516067389`, 0.013088697681936998`},
{"TBUR", 19.134783034342963`, 229.37969694418229`, 1058.910756613079`, 2.055442432220964`*^7, 0.037547241041149544`},
```

```
{"CURE", 365, 1.4850503001389923`, 92.52148855861351`, 0.009999935833531677`, 0.008069435539143376`},
{"TBUR", 15.571824327048066`, 229.4608466427711`, 1010.0449115351627`, 7.23494261394152`*^7, 0.1632042961897719`},
{"CURE", 365, 1.640212636830736`, 21.06740151660475`, 0.009999645417711638`, 0.01895898565809314`},
{"CURE", 365, 5.059638463948856`, 159.10785815806506`, 0.009999895413420432`, 0.022415668591725356`},
{"CURE", 365, 1.1883181254248723`, 30.30422039783605`, 0.009999929283503656`, 0.014373611086358075`},
{"CURE", 365, 13.655235674476394`, 188.19238040048012`, 0.009998168764128651`, 0.016067631288777258`},
{"TBUR", 48.77830888650257`, 229.46424086547006`, 1271.4411971443578`, 62501.166411852115`, 0.018494797068021512`},
{"CURE", 365, 4.433077230047953`, 508.265102830343`, 0.009999573198326138`, 0.027974412100339817`},
{"CURE", 365, 27.40523311025159`, 350.12876229800474`, 0.009999667986325915`, 0.008087040599694955`},
{"CURE", 365, 0.4904604017837187`, 18.58642503441135`, 0.009999770006857117`, 0.018242312675017015`},
{"TBUR", 17.044994372193774`, 229.6345340019842`, 891.2531230324194`, 6.483734227937327`*^7, 0.10671558601928363`},
{"CURE", 365, 0.72886312227051`, 53.13239805444406`, 0.009999911058237757`, 0.016512589585839867`},
{"CURE", 365, 0.292480349451233`, 15.757236619172923`, 0.009999973173069051`, 0.028314022824623513`},
{"TBUR", 73.2597954961464`, 229.6823639747851`, 707.0281068902269`, 0.306644962842791`, 0.0055221735445182794`},
{"TBUR", 49.53942657194034`, 229.54956834641126`, 869.0455193268622`, 1.3688221571929974`*^6, 0.027546378796733013`},
{"CURE", 365, 0.2976512679629559`, 128.33945020210692`, 0.009741585867579049`, 0.004021095361204997`},
{"CURE", 365, 6.227637871361216`, 110.81064441548416`, 0.009999691897393833`, 0.028800778601320604`},
{"CURE", 365, 0.1053425126823813`, 12.709097137450808`, 0.009999570024162 64`, 0.017966514520991107`},
{"TBUR", 46.097698556368115`, 229.56256423802097`, 579.5686642747177`, 1.8416000063781843`*^7, 0.10490813961871315`},
{"CURE", 365, 0.32471450089710746`, 78.94411649272473`, 0.009998399455159979`, 0.02394076722376911`},
{"TBUR", 103.23170984970939`, 229.53935609643938`, 1461.9454958564356`, 9017.239575918431`, 0.022534698460464728`},
{"CURE", 365, 7.281122280254655`, 270.0642203506971`, 0.00999987843765867`, 0.004928429705312454`},
{"TBUR", 15.543185339142287`, 227.89281615833033`, 1754.0421521036246`, 8.920933051147842`*^7, 0.18494217217968595`},
{"CURE", 365, 1.786293384722261`, 95.38033516732825`, 0.009999915135080986`, 0.019830735457726224`},
{"TBUR", 99.94375847417845`, 229.7708955928454`, 1013.7600587543687`, 0.013222268308078653`, 0.0017790789655571881`},
{"TBUR", 33.00781759989928`, 229.46314702623116`, 1758.0097680342824`, 6.369051649058398`*^6, 0.041972693013073555`},
{"CURE", 365, 1.7107472352645983`, 229.73740150281287`, 0.009998257535171442`, 0.010671517435459455`},
{"TBUR", 18.58313638134368`, 229.69049895723066`, 674.267223383251`, 2.597638512857568`*^7, 0.1764687333226281`},
{"TBUR", 95.38034622535297`, 229.78768968608813`, 930.3042469526594`, 2.2387127738907884`, 0.0011557774240688315`},
{"TBUR", 86.90562470830251`, 229.79618752568516`, 861.5827896720483`, 0.09454227595596774`, 0.0005335670114193213`},
{"TBUR", 191.8949750485541`, 229.72444938252866`, 792.1898357491744`, 0.5119197039808487`, 0.005661510127802185`},
```

```
{"CURE", 365, 0.3782172933585036`, 58.43849020191097`, 0.009999571169885145`, 0.012104597128219407`},
{"TBUR", 81.81599547806525`, 229.4447522488278`, 1461.5518901222633`, 58.085461852577446`, 0.015446314109624354`},
{"CURE", 365, 0.3784432037897473`, 28.74979306476609`, 0.009999850099475105`, 0.011437887315498354`},
{"CURE", 365, 0.5634185385188327`, 39.23762997286449`, 0.009635896897474266`, 0.015630783959479835`},
{"CURE", 365, 27.335894825975767`, 554.8342331937914`, 0.009997242144351146`, 0.02541866889988218`},
{"TBUR", 33.13676151131448`, 229.6062419109467`, 904.043994451824`, 8.356461348797534`*^7, 0.12848668760669846`},
{"CURE", 365, 0.2744534050269108`, 22.673453305500082`, 0.009999851095480065`, 0.016100913148848973`},
{"CURE", 365, 0.6770847388233867`, 10.318782692596496`, 0.00999973697852625`, 0.014009868335349345`},
{"CURE", 365, 0.10064643926988041`, 22.321621559454748`, 0.009756678872899324`, 0.006153063224460015`},
{"CURE", 365, 0.6975876061041552`, 28.50614203268516`, 0.009999695969760086`, 0.018565405674190558`},
{"CURE", 365, 1.0240216205771564`, 187.91672270144437`, 0.00999841566023497`, 0.024702648723149825`},
{"CURE", 365, 2.1582687213280924`, 35.869077295722946`, 0.009999695296878741`, 0.018155684829707197`},
{"CURE", 365, 0.29356054044981117`, 28.317909301051248`, 0.009999947223059835`, 0.006827827880333166`},
{"TBUR", 51.78179165511722`, 229.72516256002936`, 1315.1112712057013`, 2206.06404632272`, 0.009772906948022641`},
{"TBUR", 68.5357378037342`, 229.72819742087086`, 973.6569818697478`, 158.6472363371425`, 0.005249904341536014`},
{"CURE", 365, 0.1984389349867106`, 25.03494676965739`, 0.009996908178019353`, 0.01225166711114145`},
{"CURE", 365, 1.0659771389984947`, 184.69310736291442`, 0.009999915576103165`, 0.029706635696976843`},
{"CURE", 365, 0.2449657058086599`, 49.605824109425214`, 0.009999887679224637`, 0.009591634122134606`},
{"CURE", 365, 8.27516735139247`, 500.38135516329106`, 0.009999024506522649`, 0.01931155990809243`},
{"TBUR", 18.657208296010666`, 229.6635256381769`, 1154.2702928342317`, 8.874326085906798`*^7, 0.1031742208792454`},
{"CURE", 365, 4.407784184388581`, 155.34491521969727`, 0.009999959418370405`, 0.004670505350379037 6`},
{"CURE", 365, 5.109838464869132`, 45.85025593334029`, 0.009999777488218606`, 0.01796026693633011 3`},
{"TBUR", 159.26158344310593`, 229.70238620850682`, 1235.7440499255536`, 0.061391459031746365`, 0.007299335878322651`},
{"CURE", 365, 7.436354285508701`, 225.6289499353664`, 0.009999945995152184`, 0.020731040230000063`},
{"CURE", 365, 0.07585912456254405`, 17.658829837225817`, 0.009999599810593418`, 0.01747454955613812`},
{"TBUR", 16.818852890452227`, 228.13703859645116`, 1367.3363527498748`, 8.616308543170305`*^7, 0.2266706525350374`},
{"CURE", 365, 2.4543190165817768`, 112.51914831072534`, 0.009999726596053053`, 0.012623220296834605`},
{"CURE", 365, 8.311715262608105`, 264.76692705552733`, 0.009998863593326706`, 0.01726330130906508`},
{"CURE", 365, 1.3913035479628768`, 36.43377781583799`, 0.009999978829548301`, 0.009011596314129821`},
{"TBUR", 62.61266968541777`, 229.74255309358273`, 1155.6606240021554`, 8.154318190322855`*^6, 0.018226581640438527`},
{"CURE", 365, 1.0658671532428978`, 130.46835172270556`, 0.00999983495094769`, 0.009284314885981875`},
```

```
{"CURE", 365, 2.1126412985185437`, 33.48885238320409`, 0.009999867415025981`, 0.011419714885198969`},
{"CURE", 365, 0.9172665682216932`, 37.42981781319722`, 0.009999823331420445`, 0.008577797077136027`},
{"TBUR", 53.12917867636318`, 229.41859939349757`, 1629.2305627622106`, 170228.9387570907`, 0.024312049819348293`},
{"CURE", 365, 5.164651671428515`, 90.45830726666428`, 0.009999999269759358`, 0.020754596420037737`},
{"TBUR", 58.32790349281815`, 229.74062060832208`, 744.9830081588344`, 460585.5175198899`, 0.011373914010395133`},
{"CURE", 365, 11.79335699841111`, 188.09881390089404`, 0.00999971228077926`, 0.01605789395956766`},
{"TBUR", 83.08420951950171`, 229.7648752657414`, 953.6339338202228`, 4.202532395820601`, 0.002394196541520006`},
{"CURE", 365, 4.834213893658332`, 255.0347756254378`, 0.009999608575913668`, 0.021961169962681985`},
{"CURE", 365, 1.4334833633189945`, 159.7442535845741`, 0.00999954309028409`, 0.013771104584455447`},
{"CURE", 365, 0.12573267817443062`, 8.932753323106047`, 0.009999974174640611`, 0.020786508949461862`},
{"TBUR", 84.87189436495302`, 229.69776360180438`, 1381.9137909537872`, 5.154174005569877`, 0.006398069993710632`},
{"CURE", 365, 1.450571304032044`, 69.05943910168973`, 0.009999409232810817`, 0.006108993676531758`},
{"CURE", 365, 0.017759893981965234`, 4.720696383150441`, 0.009999946093193296`, 0.01938836297203155`},
{"CURE", 365, 1.1257779412850182`, 125.06594326384041`, 0.009999215709196168`, 0.01018404063213855`},
{"CURE", 365, 1.0286611066982478`, 69.7747361024778`, 0.009997798916666667`, 0.0170025972437948`},
{"CURE", 365, 1.176172309186346`, 36.755817191611776`, 0.009999305625763994`, 0.01973360843582584`},
{"CURE", 365, 4.001268982031855`, 443.1341398516781`, 0.009998501107085778`, 0.016841918910353956`},
{"TBUR", 71.88012764711878`, 229.73726028302744`, 722.1153039681284`, 601149.1411314489`, 0.012454933475025161`},
{"CURE", 365, 2.8523821355028094`, 153.20805321325332`, 0.009999720744823809`, 0.01901758107486054`},
{"TBUR", 16.530819725655558`, 229.67642722536561`, 1105.364122892681`, 2.9941173167216185`*^7, 0.04605102570986204`},
{"CURE", 365, 4.474545825449263`, 70.28300333809248`, 0.00999983190027349`, 0.01806754729627012`},
{"CURE", 365, 1.5754049594969983`, 320.8341710834453`, 0.009999692066596613`, 0.015043512691637471`},
{"CURE", 365, 0.49184979560389047`, 70.03383979116278`, 0.009630072357256048`, 0.023118307126838922`},
{"TBUR", 44.29761290230306`, 229.66798136679773`, 1294.6779615000676`, 591928.3068419263`, 0.019630715645636093`},
{"TBUR", 24.386806142061037`, 229.48974169420794`, 1436.4724548239458`, 4.829474782317017`*^7, 0.08411719692223954`},
{"TBUR", 61.18314093562741`, 229.7576962345752`, 976.6138463271293`, 63698.06956015773`, 0.00579937129017101`},
{"CURE", 365, 1.219499270841244`, 68.15267049586495`, 0.009999940074731482`, 0.010230261836497468`},
{"TBUR", 198.18369512751508`, 229.63505804013224`, 1033.1834936141972`, 0.20433444278638557`, 0.01084679396368698`},
{"TBUR", 139.87544387210755`, 229.76463618993176`, 1000.9656808250833`, 586.7735208354746`, 0.004662894083013455`},
{"CURE", 365, 0.2469055032689859`, 106.72017302968764`, 0.009997937748283383`, 0.01919725759416184`},
{"CURE", 365, 8.92555724868028`, 145.99406673211902`, 0.009998197585490545`, 0.009622527383315366`},
```

```
{"CURE", 365, 1.5086050560747963`, 88.98448939654105`, 0.009999946178261446`, 0.019988050579312005`},
{"CURE", 365, 0.6894544913769298`, 68.97758421673204`, 0.009998892842351894`, 0.015379945578393306`},
{"CURE", 365, 0.444835347715188`, 24.793236957216784`, 0.009999506732723606`, 0.010953336669714582`},
{"CURE", 365, 0.2833318476274697`, 43.22783933354197`, 0.009999491982209478`, 0.0313540733304811`},
{"CURE", 365, 4.996723359766419`, 381.22075548073616`, 0.009999846263250085`, 0.011884079086814636`},
{"CURE", 365, 2.116775985383466`, 78.29105120738927`, 0.00999960978755013`, 0.02105891080121465`},
{"CURE", 365, 14.421459412626966`, 393.88261969853096`, 0.009999924436253225`, 0.012665251180967353`},
{"TBUR", 67.39735268910034`, 229.44777524410395`, 1946.9966245952412`, 703.0526315710903`, 0.01741449249139932`},
{"TBUR", 46.55002073213759`, 229.55600406717207`, 858.4155346217912`, 2.269626771896927`*^6, 0.041309764993916025`},
{"TBUR", 49.02325002605657`, 229.4753405927738`, 781.0426408366154`, 680968.8933595939`, 0.04236557689183867`},
{"CURE", 365, 6.813603503080188`, 110.15769510973314`, 0.009999971733981945`, 0.020248899485782592`},
{"CURE", 365, 0.8126467796088896`, 32.47123239745076`, 0.009999981816692606`, 0.015816271487980305`},
{"CURE", 365, 0.7767020858870675`, 77.90011260853517`, 0.00999976132954926`, 0.022058028869133165`},
{"TBUR", 59.83084437945171`, 229.06214456451903`, 1031.1453347457084`, 2469.7233075940585`, 0.024991224107593916`},
{"CURE", 365, 0.13451343746961827`, 8.678562167180337`, 0.009999871213135506`, 0.023919060944956563`},
{"CURE", 365, 0.021188437329575097`, 3.2964441533703983`, 0.009999861458537113`, 0.020220648514987425`},
{"CURE", 365, 0.09916094803464254`, 11.611985157740964`, 0.00999176482723247`, 0.015513156403762884`},
{"CURE", 365, 14.263268725393116`, 130.2181598697714`, 0.009999884908492044`, 0.00644668209731934`},
{"TBUR", 113.30343221926812`, 229.655538590626`, 679.6786447575219`, 1.1854781298906583`, 0.007867717837677413`},
{"TBUR", 18.828383790789037`, 228.70556909792458`, 1269.0436210130388`, 3.867096162578347`*^7, 0.13214646149032183`},
{"CURE", 365, 0.5873980415219014`, 132.7422998053295`, 0.009999742169254427`, 0.007887747264857687`},
{"CURE", 365, 1.0703306788947502`, 31.87064015250537`, 0.009999778964079071`, 0.01390398164170123`},
{"CURE", 365, 0.44880981792347796`, 40.267101369925875`, 0.009999910696143921`, 0.02413492929066661`},
{"CURE", 365, 7.410249035417187`, 341.203036878694`, 0.009960338840855667`, 0.015768193159148017`},
{"TBUR", 145.89409245306794`, 229.77449772686919`, 663.547822676207`, 2.168427245840071`, 0.002230724481799269`},
{"CURE", 365, 52.97597085323322`, 436.4318016393896`, 0.009999994010403584`, 0.01036065380343412`},
{"TBUR", 15.56676884299709`, 229.4536256093489`, 1325.288916136089`, 2.002632914376134`*^7, 0.06383970319253504`},
{"CURE", 365, 0.7606274464781632`, 31.70594602583216`, 0.009999819642692939`, 0.012456632814634638`},
{"CURE", 365, 0.39429075132433816`, 20.68819482025977`, 0.00999999684242594`, 0.02054416068091479`},
{"TBUR", 93.3537985992926`, 229.7474641555391`, 1055.8004823483923`, 1951.351947957114`, 0.005357483105672757 6`},
{"CURE", 365, 0.25452186708801194`, 48.51088981804953`, 0.009999162788302887`, 0.01274506590877596 2`},
```

```
{"CURE", 365, 146.552678995562`, 873.2251285057775`, 0.009999983641433455`, 0.012566318581112252`},
{"TBUR", 88.63819744947982`, 229.4704728650499`, 1559.8279659371628`, 1417.018607120011`, 0.02023844627239932`},
{"TBUR", 99.51598480550166`, 229.37395182261352`, 1100.2034169749209`, 0.05712154797569246`, 0.012699378672134033`},
{"CURE", 365, 0.18917180874036107`, 63.624178216266685`, 0.009634068064195093`, 0.015413901197990871`},
{"CURE", 365, 1.10179760787051`, 42.13889668435912`, 0.009999815787144244`, 0.024685661350688077`},
{"TBUR", 55.40636188689949`, 229.58520565238112`, 911.8643881145276`, 2.9148857507360233`*^6, 0.0410754581051509`},
{"CURE", 365, 3.6664121786631525`, 209.9312233399246`, 0.009999770420302511`, 0.028534467940543137`},
{"TBUR", 64.42734344213997`, 229.69498527994136`, 1040.786483664262`, 112328.88393780467`, 0.011259646097300683`},
{"TBUR", 75.24842745990948`, 229.51839158608666`, 1188.8225645768018`, 285.68194725026217`, 0.01461297185584549`},
{"CURE", 365, 5.068845755516543`, 176.24747708579977`, 0.00999897609968619`, 0.016042367168513955`},
{"TBUR", 94.61468185139343`, 229.7820107276657`, 1120.659518994783`, 111978.74989015484`, 0.0036489777586357275`},
{"CURE", 365, 2.956706404757465`, 174.65795317022784`, 0.009999826673939444`, 0.017547866887057475`},
{"TBUR", 70.50417176204205`, 229.55104619692597`, 830.861818288502`, 30858.229249116626`, 0.01959406718928355`},
{"CURE", 365, 3.1751723461148313`, 160.50041936004226`, 0.009999804433305899`, 0.019818347442889996`},
{"CURE", 365, 0.4617484696599675`, 66.65512883821334`, 0.009999193385589902`, 0.014412090967218632`},
{"CURE", 365, 3.2837715400736744`, 37.991009131486834`, 0.009999977790369426`, 0.01406125899419`},
{"TBUR", 91.41792801500685`, 229.71670700854943`, 1205.1927059575135`, 1353.2924835846177`, 0.007562478721931283`},
{"CURE", 365, 0.04068753087358899`, 3.797934238837696`, 0.009998257942671816`, 0.024404014823352527`},
{"CURE", 365, 0.3341762729858494`, 122.94605067174925`, 0.009999991762134897`, 0.0231000982585398`},
{"TBUR", 14.914687190519906`, 228.2427857208865`, 1659.326060445685`, 4.737681248427658`*^7, 0.1262035277092501`},
{"CURE", 365, 6.055275846631403`, 531.2201385231588`, 0.009983477881971877`, 0.014810895895078618`},
{"CURE", 365, 1.7800370001739974`, 38.24450640376617`, 0.009999942237554503`, 0.0188182553754588`},
{"CURE", 365, 1.368096495205839`, 156.18017362184656`, 0.009999926778156882`, 0.010594032824104056`},
{"CURE", 365, 0.586690977328830 5`, 35.03721407090137`, 0.009612620275565774`, 0.0246164325917753`},
{"CURE", 365, 0.1340256633444807`, 10.528257762891618`, 0.009647823516972963`, 0.021932613207714394`},
{"CURE", 365, 8.277305211623947`, 184.09040356774736`, 0.009999999042395458`, 0.02511598904640341`},
{"TBUR", 36.71383326039693`, 229.7054834863811`, 789.1492533683779`, 1.6719349451557292`*^7, 0.05589014299821541`},
{"CURE", 365, 2.406273389407619`, 76.16163462993998`, 0.009999889461076012`, 0.02856087827180902`},
{"TBUR", 180.42560183097595`, 229.6940427099333`, 1075.0652476360922`, 0.09413309565241333`, 0.006951766114520264`},
{"CURE", 365, 4.838481004847429`, 53.55956788253574`, 0.0099999480834819`, 0.013613974250939288`},
{"CURE", 365, 0.07072641210551932`, 32.670020485190406`, 0.009999413292861801`, 0.0251655662173408 7`},
```

```
{"CURE", 365, 7.034176245566717`, 218.95352476804396`, 0.009999984821598657`, 0.022962362413791588`},
{"TBUR", 68.97949025913316`, 229.73007129879971`, 751.8531376437138`, 23068.365664733454`, 0.008537134645819048`},
{"TBUR", 41.90258209331105`, 229.7224007952343`, 637.4983990819832`, 3.713870684575756`*^6, 0.016483154191601183`},
{"CURE", 365, 0.1744757767652571`, 12.38560377634291`, 0.009995991541039263`, 0.02225772477415476`},
{"CURE", 365, 0.5406776310928884`, 51.23772297917848`, 0.009998874020170835`, 0.016316499648204207`},
{"TBUR", 42.14582261715636`, 229.5398779466169`, 1614.5777345958509`, 1.6689562301181897`*^6, 0.022496698026126687`},
{"TBUR", 63.4186880605331`, 229.79526975467442`, 1006.1465491746602`, 19600.918324178998`, 0.0008925945936169171`},
{"CURE", 365, 0.06833792878011587`, 36.0779165848758`, 0.009999371360698376`, 0.007241205551996665`},
{"TBUR", 102.42741984096735`, 229.79528707414326`, 705.4010863053171`, 0.013235739213819047`, 0.0005026193170618784`},
{"CURE", 365, 1.542846722948319`, 95.98577788114977`, 0.009999880609341994`, 0.01701174327082215`},
{"CURE", 365, 0.07620842098083569`, 22.943747342738593`, 0.00999972131641958`, 0.01417280976386614`},
{"CURE", 365, 0.14462604274173183`, 12.326674416443408`, 0.009999874474471527`, 0.017303611805121043`},
{"CURE", 365, 2.2475641242808124`, 205.63270743866093`, 0.009996972999245813`, 0.01776063268530834`},
{"TBUR", 99.53286675294879`, 229.38579598953555`, 1393.532611445377`, 3.8749455412052733`, 0.015591051426856734`},
{"CURE", 365, 1.7686224163575195`, 96.45344085697309`, 0.009720075372022458`, 0.019959196296650005`},
{"TBUR", 160.0203859297691`, 229.65777995497038`, 1107.1015985388503`, 632.453802165287`, 0.013123314116103076`},
{"TBUR", 27.386045421611453`, 229.56734963640642`, 1342.9836416244507`, 3.61629659415634`*^7, 0.036275796898553855`},
{"CURE", 365, 2.099593901482453`, 194.43303920199304`, 0.009999713997254054`, 0.015641989159843283`},
{"TBUR", 110.0626009494386`, 229.4221413876846`, 1376.270063299882`, 211.739503066525`, 0.019678689264921174`},
{"TBUR", 32.91819417433076`, 229.3988139133487`, 1187.6075589029413`, 4.941292476418828`*^7, 0.09405658810565759`},
{"TBUR", 63.79811149670853`, 229.47436152845745`, 1631.339728455794`, 8396.063029056535`, 0.019986522290439367`},
{"TBUR", 40.20112134117334`, 229.6118833831943`, 966.1512974688883`, 4.208017131153068`*^7, 0.059814304683869945`},
{"TBUR", 25.874920399961436`, 228.1675757676982`, 1599.4734432822781`, 3.3088428282177612`*^7, 0.09109377843232824`},
{"TBUR", 143.52045189548073`, 229.60642089268762`, 1076.3907083712127`, 319.62211902884724`, 0.016196361141685423`},
{"CURE", 365, 4.125864148070426`, 208.89895703278532`, 0.009988387449051247`, 0.009882383108663838`},
{"CURE", 365, 0.4640831589972832`, 30.40234603191725`, 0.009999907182850655`, 0.01597524397772435`},
{"CURE", 365, 1.5314266325218449`, 166.12597787567233`, 0.009999837026315765`, 0.011304818775033042`},
{"CURE", 365, 0.274290504573376`, 45.05577847769551`, 0.009999910122388963`, 0.019204978490788994`},
{"CURE", 365, 0.9043448696506137`, 53.950218583880485`, 0.009999735612749187`, 0.01720632243159995`},
{"CURE", 365, 2.50617881344381`, 341.0711915753612`, 0.009999836463198 64`, 0.010541466816340725`},
{"CURE", 365, 0.19328697864335198`, 21.32934294253058`, 0.009999790888457464`, 0.024834693599918637`},
```

{"TBUR", 119.44787067601314`, 229.72867271142292`, 998.9669701927589`, 696.6024119868772`, 0.007283425540913256`5`},
{"TBUR", 73.53584616320154`, 229.5567524286718`, 1434.038936966344`, 361.4297527968302`, 0.01358518771605084`},
{"TBUR", 12.23172205598565`, 229.72822406833922`, 1401.3801805596604`, 8.952098728322259`*^7, 0.0823521974656481`3`},
{"CURE", 365, 1.0275825486080519`, 20.30259699812229`, 0.00999972057403334`, 0.015297385539019572`},
{"TBUR", 52.63226442623845`, 229.6694711918392`, 488.252572260405`, 7.476521607461454`*^6, 0.0837914130805186`},
{"CURE", 365, 0.924377957955534`, 26.531164195912513`, 0.009999762741784043`, 0.021113475993167635`},
{"CURE", 365, 7.431171248399493`, 74.07811321882703`, 0.00999983163785913`, 0.016035141520208577`},
{"TBUR", 63.99503948093398`, 229.75642499432047`, 978.4196989159838`, 525924.5206415585`, 0.0084840129955202`5`},
{"CURE", 365, 0.03690861771930858`, 7.718156198707505`, 0.009973463366190267`, 0.02391206807338941`8`},
{"CURE", 365, 0.07123486321789267`, 18.721372338783812`, 0.009999463136565426`, 0.026334446059325268`},
{"TBUR", 19.73249502165981`, 229.13075823856093`, 959.2057780215614`, 3.9080801156438336`*^7, 0.14720939965003094`},
{"CURE", 365, 2.4498897299221776`, 68.38848784609428`, 0.009999886634934087`, 0.017495285986905872`},
{"TBUR", 34.64860656097315`, 229.5446295948899`, 1340.914800794501`, 2.0379602253181167`*^7, 0.09473042364563045`},
{"CURE", 365, 5.586012279848347`, 347.4635941997093`, 0.009999930147074947`, 0.016843413715562666`},
{"TBUR", 62.07479165154697`, 229.7397884113187`, 1181.8682788547715`, 3324.07974025308`, 0.006081275676396176`},
{"TBUR", 34.6894024182568`, 229.5084790608652`, 1288.4758221475704`, 2.8129727580868844`*^7, 0.07299702343047333`},
{"CURE", 365, 0.5010618416636816`, 31.78867092489188`, 0.009999832770683087`, 0.0157123106580708`},
{"CURE", 365, 2.5210515382369163`, 148.09564192717437`, 0.009667338754031328`, 0.00611906512723841`},
{"TBUR", 50.80467659508667`, 229.6080256156286`, 1343.7813726224397`, 181812.45937876593`, 0.01701460264875576`},
{"CURE", 365, 3.517190316147665`, 202.74030240746063`, 0.009598098257059186`, 0.017771181239711824`},
{"TBUR", 13.807953047005945`, 228.91794740549756`, 1557.2831163908309`, 6.991109163451484`*^7, 0.13210933142430695`},
{"CURE", 365, 2.5746019547732923`, 57.227831520030755`, 0.009999913472823217`, 0.010173011520782595`},
{"CURE", 365, 0.8310665291796261`, 64.7196238663545`, 0.009999853392125703`, 0.015560787476347494`},
{"TBUR", 16.074316820154277`, 229.50337828224772`, 750.2094611368684`, 3.968142168145161`*^7, 0.19170584093560664`},
{"CURE", 365, 3.250945041097175`, 208.85857687098226`, 0.009996977331975533`, 0.014347323424589746`},
{"CURE", 365, 0.07940986917405722`, 8.123765716353835`, 0.009998663494878724`, 0.023548629198553817`},
{"TBUR", 27.33319539793822`, 229.65326465704047`, 927.3193304162276`, 1.6769233307174902`*^7, 0.06369583598914823`},
{"CURE", 365, 0.06774895085233082`, 21.144238445330227`, 0.009629163410371327`, 0.02604988365411036`},
{"CURE", 365, 0.2972804407784868`, 40.5841633506734`, 0.00962981967297916`, 0.017085705055030473`},
{"CURE", 365, 0.3918591708095973`, 27.669133878553346`, 0.009699156389295053`, 0.02921535970018582`},
{"TBUR", 98.14380514479039`, 229.74025539032522`, 624.7745896640577`, 0.018388713794645326`, 0.0031143268685310125`},

```
{"TBUR", 35.18679462596534`, 229.49529534157583`, 781.5519613968698`, 2.5397196437110823`*^7, 0.09131985754561138`},
{"CURE", 365, 0.2738197882754762`, 45.78954544358616`, 0.009997849479568947`, 0.012319522508306228`},
{"CURE", 365, 0.27561360407457064`, 31.848854223790493`, 0.009999855009841158`, 0.013003706029987376`},
{"TBUR", 17.14350587994442`, 227.436553554007`, 1634.7445495505078`, 5.2146563089949675`*^7, 0.10973499136132799`},
{"CURE", 365, 1.8984520089894443`, 517.041951798736`, 0.009999326141321008`, 0.020939189337271544`},
{"CURE", 365, 2.8046326199936096`, 207.5350901772567`, 0.009998722253280199`, 0.017877407555964377`},
{"TBUR", 80.73469140353815`, 229.73339378537702`, 828.5475217668636`, 0.018759725396029773`, 0.0034173387334250666`},
{"TBUR", 32.15175157918659 5`, 229.27656401800064`, 1014.3605485715365`, 1.1690239731986092`*^7, 0.05994833077619726`},
{"CURE", 365, 0.3195317894276555`, 17.21115518641997`, 0.009999779102214433`, 0.022337712429316335`},
{"CURE", 365, 21.3615402294722`, 334.81045967698157`, 0.009999999572135928`, 0.021493439738719547`},
{"TBUR", 52.385350633717785`, 229.62981497314942`, 1036.6181200429014`, 1.043263907608314`*^7, 0.04536769479714176`},
{"CURE", 365, 0.20713561051695648`, 41.420569550042316`, 0.009997659259572774`, 0.018234302547175823`},
{"CURE", 365, 0.875464455903919`, 90.28688251238714`, 0.009999959116155142`, 0.009678818683475912`},
{"TBUR", 18.84610710964836`, 229.635682360344`, 986.0104161814642`, 9.014567082907547`*^7, 0.14673670934044333`},
{"CURE", 365, 1.9045903618542233`, 162.5120557819706`, 0.009968751275301124`, 0.010771837397689311`},
{"TBUR", 40.69294535391018`, 229.72467454648816`, 717.604617319609`, 1.0591058680506488`*^6, 0.015213646383789045`},
{"CURE", 365, 0.17558706241312216`, 24.953240884319843`, 0.009999976307454889`, 0.00976935033209478`},
{"CURE", 365, 5.054303455786547`, 121.32090235225469`, 0.009999794098959547`, 0.02139701659728863`},
{"CURE", 365, 0.32971899286692097`, 61.40263902667965`, 0.009996535626691975`, 0.007477978692692318`},
{"TBUR", 15.833420859786894`, 229.67068184363043`, 1193.7001198216487`, 2.3238471342304833`*^7, 0.0999639929272128`},
{"CURE", 365, 4.16982669842086`, 177.58052723272377`, 0.009999799811943318`, 0.01854810139675303`},
{"CURE", 365, 0.42893703994599985`, 106.74852079349704`, 0.00999878329798318`, 0.01142603192326281`},
{"TBUR", 41.75730580685035`, 229.56477856414836`, 1300.6662037498074`, 1.1866128197998634`*^7, 0.042833988948924234`},
{"TBUR", 99.26820667184707`, 229.49391166301103`, 1526.4871933585719`, 66.25787914768254`, 0.015843522538948995`},
{"TBUR", 55.91227925938318`, 229.6175069598605`, 1306.9744150262134`, 481812.44337301183`, 0.020567269935947343`},
{"TBUR", 30.034783288335237`, 229.63286178975622`, 1137.1541414762803`, 3.4047733169518984`*^6, 0.024425310514982843`},
{"CURE", 365, 0.13347254352248084`, 20.28808565564795`, 0.009994172838292258`, 0.02397486548106004`},
{"CURE", 365, 0.12870452510929603`, 5.154319429378407`, 0.00999984123247369`, 0.024162620929714163`},
{"TBUR", 67.64682253172563`, 229.65063352980872`, 1207.4833335374528`, 1009.878083929633`, 0.010336833402853235`},
{"CURE", 365, 1.1507412235358288`, 136.43802240094422`, 0.009998531647504421`, 0.0048365214802104884`},
{"CURE", 365, 7.725986471861457`, 538.8634860071157`, 0.00999919647894687`, 0.023087199686997552`},
```

```
{"CURE", 365, 0.04791236313010207`, 20.774233113708775`, 0.009648331363578427`, 0.015047334140741435`},
{"TBUR", 85.25680852538876`, 229.6974408137708`, 788.2222198185169`, 1.2520883274988572`, 0.005459952485763423`},
{"CURE", 365, 0.25670759445169944`, 22.602990992500054`, 0.009678602214926781`, 0.012857285446483237`},
{"CURE", 365, 0.13332503872021678`, 50.097824448037514`, 0.009995158686002 72`, 0.010569624966566446`},
{"CURE", 365, 4.244777719627667`, 357.8270076957162`, 0.00999909227958637`, 0.018613971745796244`},
{"CURE", 365, 0.014073486052691075`, 4.8883092601608595`, 0.009999845746078208`, 0.02512379638317749`},
{"CURE", 365, 0.3520751990252756`, 24.460899315767374`, 0.00999984650599141`, 0.019308532716339817`},
{"TBUR", 13.20212585869900 8`, 229.2689375528951 2`, 1186.4021961312546`, 7.528063016828725`*^7, 0.13951227490746973`},
{"TBUR", 65.18815525062955`, 229.63821251813818`, 1100.8617642051295`, 1564.7989923194148`, 0.010513437929963834`},
{"CURE", 365, 1.4034762517150097`, 343.6934176837445`, 0.0099995231728698`, 0.010528735294835074`},
{"TBUR", 106.62570447050734`, 229.49266976109604`, 1298.398368484096`, 93.2355591817667`, 0.01651565401174185`},
{"CURE", 365, 2.1957623395266532`, 307.91258958654197`, 0.009995266993390475`, 0.016859678092863244`},
{"TBUR", 39.36368480901633`, 229.5097474419792`, 1593.9577032390398`, 435548.76272731804`, 0.021011396998608666`},
{"CURE", 365, 3.9653442139784763`, 225.63181267065062`, 0.00958510295085305`, 0.013952663930158926`},
{"TBUR", 79.67678845881093`, 229.63425539879566`, 1139.494736822028`, 228718.4362503434`, 0.02018088209914454`},
{"TBUR", 18.75182196683196`, 228.68536654797745`, 1476.5378367273431`, 4.489137931085856`*^7, 0.06774459843189558`},
{"CURE", 365, 0.3795220334267991`, 15.074599800220147`, 0.009999832236512695`, 0.011888539996249104`},
{"TBUR", 69.57090024568947`, 229.7834205382241`, 739.2831763468291`, 0.15695226939366927`, 0.0011249153019279793`},
{"CURE", 365, 0.054903395435211944`, 15.357154736152749`, 0.009999504547427342`, 0.024479763286254445`},
{"CURE", 365, 0.5016839949190386`, 24.91636778725762`, 0.009999771018109777`, 0.024838030031020114`},
{"CURE", 365, 0.47095309816824166`, 56.11645803531293`, 0.009999681303105521`, 0.012186795144665218`},
{"CURE", 365, 0.0641839302886854 1`, 23.875324337773478`, 0.009999816227494716`, 0.016193158374792602`},
{"TBUR", 80.84949111708892`, 229.72209515839464`, 462.5165429868198`, 412812.67166631046`, 0.015286754944810056`},
{"TBUR", 90.46574217710452`, 229.7921570739821`, 884.5513060218407`, 121362.9468663302`, 0.00238231080406854 7`},
{"TBUR", 18.62568075867038`, 229.10285619967615`, 1419.4395830756744`, 5.606626367509145`*^7, 0.04762217394845703`},
{"CURE", 365, 0.8570528304032118`, 77.48405353224275`, 0.009704935535291422`, 0.02187245595133051`},
{"CURE", 365, 1.4035985989435757`, 34.09535408888692`, 0.009999711735021692`, 0.006697388051323795`},
{"CURE", 365, 3.376012046397105`, 98.52363481904182`, 0.009999762171449158`, 0.021179056955757607`},
{"TBUR", 30.56319740923042`, 229.51575402803297`, 1088.7704415070382`, 3.2735847771910567`*^7, 0.07906346772999774`},
{"CURE", 365, 0.4409935988416167`, 198.6376115111413`, 0.009999434686325935`, 0.012216953436596598`},
{"CURE", 365, 1.485134761330885`, 122.9444348501155`, 0.009999996078581189`, 0.005938484704643241`},
```

```
{"CURE", 365, 0.037887965466482515`, 7.919495495049151`, 0.009999246252558752`, 0.011013568942268348`},
{"TBUR", 46.385588647625774`, 229.731114320614`, 1241.6709282523707`, 154850.66562304186`, 0.008664074394039517`},
{"CURE", 365, 0.23349379143830487`, 7.237248414886909`, 0.0096142242201719`, 0.021417461839124848`},
{"TBUR", 66.80749997298717`, 229.71681586114482`, 1219.1714075991983`, 27.938303597287423`, 0.004650199769142143`},
{"CURE", 365, 2.1319832873082745`, 234.98172850215303`, 0.00999996738487558`, 0.015012654107576571`},
{"TBUR", 69.58404692149618`, 229.7301480382095`, 552.6759250540484`, 2852.352727369173`, 0.006510541307028965`},
{"TBUR", 21.90311168663427`, 229.34701908804314`, 1071.9619863911678`, 4.27772561837068`*^7, 0.03573764110030299`},
{"CURE", 365, 11.102903078594853`, 268.9484004777007`, 0.009999924643806031`, 0.006565961027418166`},
{"CURE", 365, 0.06765410098771288`, 6.788829340514491`, 0.009999666814680216`, 0.016569619116144417`},
{"TBUR", 31.147391056621217`, 229.12565406974403`, 1213.747713440343`, 6.937291721698093`*^7, 0.09323596772092424`},
{"CURE", 365, 0.39247249997215405`, 23.57517737254557`, 0.00999997486062471`, 0.00802695680955981`},
{"TBUR", 37.33235650882394`, 229.43876111558652`, 1363.3555874071626`, 601592.3645465076`, 0.025437479163253368`},
{"CURE", 365, 18.810682493831713`, 672.8632756257077`, 0.009999431449832369`, 0.02077204508029378`},
{"TBUR", 20.58565929608093`, 229.62390279923864`, 1046.5397897519567`, 6.944425315128572`*^7, 0.0896977109125906`},
{"CURE", 365, 1.0264744757531021`, 51.59294049266626`, 0.009999916904054494`, 0.015129052483499893`},
{"TBUR", 17.56910907398438`, 228.80544496824487`, 912.0300707064177`, 5.3758101567334324`*^7, 0.175182739115241`},
{"TBUR", 18.691757069902472`, 224.1761238364697`, 1207.2519038852893`, 7.807151324060234`*^7, 0.1439093724731041`},
{"TBUR", 122.49723533947788`, 229.7605893225748`, 817.3236219506515`, 27064.6677070629`, 0.006487556710201737`},
{"TBUR", 74.59334799675523`, 229.72761654192666`, 1275.83573065306`, 0.1225810535936468`, 0.005054416013204946`},
{"TBUR", 19.548901161152735`, 229.5560749724095`, 883.4388657620076`, 1.253921938823833`*^7, 0.13069231617879984`},
{"CURE", 365, 0.3246812579711938`, 49.08448924969286`, 0.009611683605397891`, 0.015308901667494513`},
{"CURE", 365, 3.406816211038532`, 70.12446633580986`, 0.00999998920638468`, 0.024688062639219775`},
{"CURE", 365, 0.7468723607447225`, 77.67848270587022`, 0.00999932332346296`, 0.010952574624211791`},
{"CURE", 365, 0.058414647679717185`, 5.782774878552444`, 0.009999865573104973`, 0.011139657603748866`},
{"TBUR", 107.9833780983641`, 229.45590546821728`, 1481.9965959850117`, 152.2040428746339`, 0.018871667864178064`},
{"TBUR", 161.75593599129311`, 229.71665936314892`, 1032.5943999541437`, 0.16184866523479893`, 0.005618349877361380 5`},
{"CURE", 365, 0.37382607393689327`, 21.9944995731149`, 0.009999240393967 95`, 0.026567645411348793`},
{"TBUR", 57.72924640760163`, 229.72974610794984`, 955.6212482954328`, 80058.52099820181`, 0.009439498123726377`},
{"CURE", 365, 12.121894209566868`, 225.9007900496694`, 0.009999400684036272`, 0.016202413571388845`},
{"TBUR", 46.06977035279166`, 229.76991248577002`, 911.3340284949853`, 3.8375914456068804`*^6, 0.013492407766529893`},
{"TBUR", 80.99139600350333`, 229.73649366065692`, 558.5076893003364`, 0.32219803932788443`, 0.003613355109317311`},
```

```
{"TBUR", 87.63379153865736`, 229.7313370182687`, 1106.967474535892`, 267287.62207168335`, 0.01146449377150544`},
{"CURE", 365, 0.3557338179813116`, 132.58419412111527`, 0.009998334924554033`, 0.0280441658005276`},
{"TBUR", 17.18987798863921`, 227.11788278980205`, 1261.0777071902626`, 3.655530763820291`*^7, 0.10854427159189402`},
{"TBUR", 83.28299080428316`, 229.70942028873145`, 856.464161872864`, 0.06828460874802425`, 0.004495156656521723`},
{"CURE", 365, 0.15771399769302968`, 14.58640843371478`, 0.009998254005416577`, 0.02058924703309752`},
{"CURE", 365, 113.92199938292532`, 684.3656557660996`, 0.009999995935136542`, 0.0070208866363758605`},
{"CURE", 365, 10.939570415583406`, 518.6125233682752`, 0.009996781473601637`, 0.02262213296674285`},
{"CURE", 365, 0.2901375985244421`, 13.653302744491517`, 0.009999983709698627`, 0.018458268386862424`}};
```

In[∘]:=

```
TestMDNumOpt = {{"NumOpt Res", "NumOpt OS", "NumOpt Tox", "NumOpt Ainj", "Nn", "FrAcViable", "RecAvgOcc", "MaxEtaInj"},
  {"TBUR", 52.70140532274077`, 229.79192370772853`, {{0, 0.06213601607322693`, 110.60210861034392`}}, 1.548441048845115`*^7,
   0.023605648062942707`, 0.48490055603872556`, 2404.719457090413`}, {"CURE", 365, 3.010242035822389`,
   {{0, 0.004248814545348463 5`, 7.562889890720266`}, {20, 0.0017710265430701839`, 3.1524272466649275`}},
   0.00999998383584689`, 0.020356161199290106`, 0.5259898652197571`, 1788.2050996803816`},
  {"CURE", 365, 0.5678391662874382`, {{0, 0.001148133851407738`, 2.0436782555057733`},
     {9, 0.0006134100101931511`, 1.0918698181438087`}, {18, 0.00014827630933890603`, 0.2639318306232527`}},
   0.009947488281047086`, 0.01873105240010708`, 0.29697684061221513`, 1782.3246646249913`}, {"CURE", 365,
   0.6445836263243523`, {{0, 0.003675519559409394`, 6.5424248157487215`}, {35, 0.0013739087107577305`, 2.4455575051487606`}},
   0.00976270777770234`, 0.01739032617688768`, 0.18810325030930086`, 1781.524606769224`},
  {"CURE", 365, 0.6810979755469122`, {{0, 0.0032472874954616333`, 5.780171741921707`}}, 0.009999825152713384`,
   0.005658607173970566`, 0.48261742244029165`, 1781.4534360198604`}, {"CURE", 365, 0.5014254277384449`,
   {{0, 0.004527977083638434`, 8.059799208876413`}, {4, 0.0009886158599958412`, 1.7597362307925974`}},
   0.009939615034304478`, 0.007032244129735178`, 0.3650495403939277`, 1780.5528721072276`}, {"TBUR", 50.70007528482451`,
   228.3955842770731`, {{0, 0.07478081190509338`, 133.10984519106623`}, {3, 0.02420447841686193`, 43.083971582014236`},
     {6, 0.012612915599626136`, 22.450989767334523`}, {9, 0.00682037091778674`, 12.140260233660399`},
     {12, 0.003843764418599704`, 6.841900665107474`}, {15, 0.0022827236554921475`, 4.063248106776022`},
     {18, 0.001447650922358986`, 2.576818641798995`}, {21, 0.0009917147311051856`, 1.7652522213672306`},
     {24, 0.0007370580126330141`, 1.311963262486765`}, {27, 0.03862141783524298`, 68.74612374673251`}},
   1.7885224114786475`*^6, 0.05847921079652532`, 0.7952138356553052`, 1969.5288377694853`}, {"CURE", 365,
   70.53063167990467`, {{0, 0.03835099860951311`, 68.26477752493334`}, {34, 0.0012858001244048563`, 2.2887242214406442`}},
   0.009958745551522947`, 0.02156801113644875`, 0.9999741808479062`, 3258.594850297307`},
```

```
{"CURE", 365, 0.034966551681460394`, {{0, 0.0006408827083114687`, 1.1407712207944143`}},
  0.009998268974311901`, 0.023977788707759382`, 0.0881214476230777`, 1781.2059515169244`},
{"CURE", 365, 1.1186179181760687`, {{0, 0.006051826995240729`, 10.772252051528497`}},
  0.009999958109352345`, 0.01818153840326934`, 0.35573736206777135`, 1780.640181066733`},
{"CURE", 365, 6.0762351380075525`, {{0, 0.027317048236165088`, 48.62434586037386`},
    {2, 0.0032553301132664863`, 5.794487601614345`}, {4, 0.00026661012661018287`, 0.4745660253661254`},
    {6, 0.00005084671352405546`, 0.09050715007281872`}, {8, 0.00003887407647026247`, 0.06919585611706719`},
    {10, 0.000038352355305037285`, 0.06826719244296635`}, {12, 0.00003833215148585499`, 0.06823122964482188`},
    {14, 0.000038331279800146115`, 0.06822967804426008`}, {16, 7.737099545555471`*^-6, 0.013772037191088738`}},
  0.009999985470628461`, 0.021915544304684703`, 0.9923370873282571`, 2026.0868059620634`}, {"CURE", 365,
  1.6594727309505646`, {{0, 0.0033484560170481837`, 5.960251710345767`}, {47, 0.000303016698694427`, 0.53936972367608`}},
  0.009999802092248065`, 0.021230919710246612`, 0.4583037429522768`, 1799.179121868684`},
{"CURE", 365, 0.5659070376660903`, {{0, 0.002003853940810287`, 3.566860014642311`},
    {5, 0.000678900071338035`, 1.2084421269817023`}, {10, 0.00015789269193489016`, 0.2810489916441045`},
    {15, 0.000041004643669296884`, 0.07298826573134845`}, {20, 9.17868209959381`*^-6, 0.016338054137276983`}},
  0.009999993103208897`, 0.0169375977238952`, 0.9273678882766383`, 1971.1762438759456`}, {"TBUR", 187.61104105006842`,
  227.96229816038266`, {{0, 0.05805689208543195`, 103.34126791206887`}, {11, 0.00041937435116902493`, 0.7464863450808644`},
    {22, 0.0004188052245751153`, 0.7454732997437052`}, {33, 0.0004188581975321326`, 0.745567591607196`},
    {44, 0.00041942716598504043`, 0.746580355453372`}, {55, 0.0004257509262112402`, 0.7578366486560074`},
    {66, 0.0005121390692787315`, 0.911607543316142`}, {77, 0.0019203212710493416`, 3.418171862467828`},
    {88, 0.005351620584583996`, 9.525884640559513`}, {99, 0.16835676758953336`, 299.6750463093693`}},
  619.0155898659677`, 0.035445377542452694`, 0.8427226438218788`, 3355.6511422536787`},
{"CURE", 365, 0.7037221812111484`, {{0, 0.002591359278219956`, 4.612619515231521`},
    {5, 0.0016461169540192089`, 2.9300881781541914`}, {10, 0.0003453341580002149`, 0.6146948012403824`}},
  0.00996752247020752`, 0.010439499488415986`, 0.38000027239517914`, 1781.406517373055`},
{"CURE", 365, 3.2888101245915378`, {{0, 0.00192738361647891`, 3.4307428373324598`},
    {29, 0.00012829422911458198`, 0.22836372782395592`}, {58, 0.000038446745527670887`, 0.06843520703925417`},
    {87, 0.00003497361527688665`, 0.06225303519285823`}, {116, 0.00003430954842300174`, 0.061070996192943094`},
    {145, 0.00003423026592671799`, 0.06092987334955802`}, {174, 0.00003422010068971649`, 0.06091177922769536`},
    {203, 0.000034218768413197936`, 0.060909407775492315`}, {232, 0.00006126422364899329`, 0.10905031809520806`}},
  0.00997824500491207`, 0.0430126500623433`, 0.6931210382155257`, 2267.876878948134`}, {"CURE", 365, 0.8566049213801836`,
```

```
{{0, 0.0015549279501936623`, 2.767771751344719`}, {20, 0.00010037390856143756`, 0.17866555723935887`}},
 0.00999990339507628`, 0.03306937501475631`, 0.7054741893318833`, 2269.418137574579`}, {"TBUR", 111.02815096076179`,
 228.38268790053846`, {{0, 0.012031044591275023`, 21.415259372469542`}, {9, 0.0017535353859072257`, 3.1212929869148613`},
   {18, 0.0006793650895686698`, 1.2092698594322322`}, {27, 0.001319350980203518`, 2.3484447447622623`},
   {36, 0.0027231901812622295`, 4.847278522646769`}, {45, 0.002801797130132536`, 4.987198891635914`},
   {54, 0.002154576190903892`, 3.8351456198089275`}, {63, 0.0020073876366172995`, 3.5731499931787924`},
   {72, 0.002164021510218747`, 3.8519582881893695`}, {81, 0.3476386329021076`, 618.7967665657516`}},
 930418.6193935758`, 0.062408016297356934`, 0.2249500551085974`, 1780.1392179387585`},
{"TBUR", 49.08517943450247`, 230.2448124105461`, {{0, 0.06525943756103517`, 116.16179885864261`}},
 1.113482819571788`*^6, 0.02944014695024829`, 0.7069894621317676`, 2631.5975651821577`},
{"CURE", 365, 1.2434347006227626`, {{0, 0.01202450847016938`, 21.4036250769015`}},
 0.009998448776535036`, 0.008058861529873198`, 0.34544849253806204`, 1780.296436433956`},
{"CURE", 365, 0.5660213707327385`, {{0, 0.00148047650131468`, 2.63524817234013`}, {4, 0.000563553233111273`, 1.0031247549380657`},
   {8, 0.00014252043683027357`, 0.253686377557887`}, {12, 0.000018604314410515655`, 0.033115679650717864`}},
 0.009999974228953934`, 0.01096523259726005`, 0.567789785022085`, 1806.0828664310695`},
{"TBUR", 16.966620161097595`, 229.62121753048058`, {{0, 0.1252718257904053`, 222.98384990692142`}},
 8.110907066136248`*^7, 0.09936637111432783`, 0.5243213018568377`, 2010.7478279991726`}, {"TBUR", 109.57942425537146`,
 227.64650890329142`, {{0, 0.025477958308998464`, 45.35076579001727`}, {11, 0.015888177374833612`, 28.28095572720383`},
   {22, 0.013997156666115111`, 24.9149388656849`}, {33, 0.012708823338629131`, 22.62170554275985`},
   {44, 0.011594006888857906`, 20.637332262167075`}, {55, 0.01059471335062146`, 18.8585897641062`},
   {66, 0.009697638039628169`, 17.261795710538138`}, {77, 0.00889246983395413`, 15.82859630443835`},
   {88, 0.008169485660248063`, 14.541684475241553`}, {99, 0.4913848813548331`, 874.6650888116029`}},
 3.9224986532165095`*^7, 0.30685503148729065`, 0.7045119182607307`, 2029.1087808998607`}, {"TBUR", 365,
 229.68103905162425`, {{0, 0.016904408506187817`, 30.08984714101431`}, {35, 0.0027953668272540093`, 4.975752952512136`},
   {70, 0.0027953234810351745`, 4.975675796242611`}, {105, 0.0027953150986639638`, 4.975660875621855`},
   {140, 0.0027953122824852863`, 4.9756558628238094`}, {175, 0.0027953112957081606`, 4.975654106360526`},
   {210, 0.0027953109487773463`, 4.975653488823676`}, {245, 0.0027953108267734766`, 4.975653271656789`},
   {280, 0.002795310783869965`, 4.975653195288538`}, {315, 0.0034342389601654546`, 6.112945349094509`}},
 639.9204425813534`, 0.003313537412005891`, 0.10021802868718883`, 1780.0441994720177`}, {"CURE", 365,
 0.20458133919968244`, {{0, 0.008126085091965381`, 14.464431463698379`}, {3, 0.0008053947580637102`, 1.4336026693534043`}},
 0.009999807187669406`, 0.011489557749382564`, 0.5117802101269089`, 1780.645233782277`},
```

{"CURE", 365, 0.24323118782870964`, {{0, 0.0020906232721839813`, 3.721309424487487`}},
0.009999929466496402`, 0.023318330422353723`, 0.05728047082364014`, 1780.215708529951`},
{"CURE", 365, 2.0776807981856584`, {{0, 0.005835574997878423`, 10.387323496223592`}}, 0.009710594141353467`,
0.006416406415621021 5`, 0.29746699878731014`, 1780.3331222249772`}, {"TBUR", 90.23581281748854`,
229.02911182341705`, {{0, 0.033043982300796304`, 58.81828849541741`}, {4, 0.004270394677615477`, 7.601302526155549`},
{8, 0.0005746948021371916`, 1.022956747804201`}, {12, 0.00011340062716394836`, 0.20185311635182807`},
{16, 0.000045033043888157 82`, 0.08015881812092092`}, {20, 0.00003261957856176928`, 0.05806284983994932`},
{24, 0.000029610254574828756`, 0.05270625314319518 6`}, {28, 0.000028596220527084043`, 0.0509012725382096`},
{32, 0.000028139930198497 58`, 0.05008907575332569`}, {36, 0.023823847565958692`, 42.40644866740647`}},
4364.568788538207`, 0.0220016106416546`, 0.18060911793456427`, 1780.0431722837138`}, {"CURE", 365, 3.585396202664531`,
{{0, 0.035627614034272344`, 63.417152981004776`}, {2, 0.010499432277189491`, 18.688989453397298`},
{4, 0.0020662299452216767`, 3.677889302494585`}, {6, 0.0003892474099078845`, 0.6928603896360345`},
{8, 0.00008484836703367901`, 0.15103009331994866`}, {10, 0.00003143714723591271`, 0.05595812207992462`},
{12, 0.000022663021677909 16`, 0.04034017858667831`}, {14, 0.000016807627248878512`, 0.02991757650300375`}},
0.009999999210131122`, 0.01957114284806292`, 0.9721163059990122`, 1901.63753535236`}, {"CURE", 365, 0.10526478152559618`,
{{0, 0.0006301523134968638`, 1.1216711180244174`}, {45, 0.00016059140628444773`, 0.28585270318631695`}},
0.009999835849825915`, 0.03688057380005944`, 0.11536147233457353`, 1786.6032968364025`},
{"TBUR", 15.676204431546905`, 230.1022913757328`, {{0, 0.11056374549865722`, 196.80346698760985`}},
4.0707470595068745`*^7, 0.10011201734110423`, 0.5200648837680323`, 2027.7129445665419`},
{"TBUR", 75.45914109182651`, 230.26292382988603`, {{0, 0.07018431663513186`, 124.9280836105347`}},
1.692168650239558`*^7, 0.01424903010379206`, 0.4452319769526781`, 2359.077631404743`},
{"TBUR", 17.838894666589656`, 230.13687255024288`, {{0, 0.08394290924072267`, 149.41837844848635`}},
4.656274919049821`*^7, 0.12442749228025818`, 0.49607837989589776`, 2087.883896980782`}, {"CURE", 365,
1.5980886198649993`, {{0, 0.010746193496689548`, 19.128224424107394`}, {3, 0.0032163868960182044`, 5.725168674912404`},
{6, 0.0006954570706542276`, 1.2379135857645251`}, {9, 0.00015715631349416545`, 0.27973823801961456`},
{12, 0.000046738011095316186`, 0.08319365974966281`}, {15, 0.000015346309584245838`, 0.02731643105995759`}},
0.009999981626816428`, 0.01969799388186445`, 0.9538726300184939`, 1874.7616888572552`}, {"CURE", 365,
0.45247700829903703`, {{0, 0.0010707349791007664`, 1.9059082627993642`}, {8, 0.0004171230815626617`, 0.7424790851815378`},
{16, 0.000059783903872871975`, 0.1064153488937121`}, {24, 0.000013180698947801 66`, 0.023461644127086956`}},
0.00999997070332326`, 0.06511821414524971`, 0.35763331964299194`, 2173.7017245892384`}, {"TBUR", 85.98237701437289`,
229.54957444995813`, {{0, 0.07289310954689024`, 129.74973499346464`}, {4, 0.0027097651981073576`, 4.823382052631096`},

```
{8, 0.0004960032198322301`, 0.8828857313013695`}, {12, 0.0002930622586983115`, 0.5216508204829944`},
{16, 0.0002664227925798093`, 0.4742325707920605`}, {20, 0.0002609407558429203`, 0.46447454540039806`},
{24, 0.0002593105109517466`, 0.46157270949412327`}, {28, 0.000258679970034066`, 0.460450346660637 5`},
{32, 0.0002583859711785202`, 0.45992702869776597`}, {36, 0.018633962790358043`, 33.16845376683732`}},
121962.27659314705`, 0.03522529221298324`, 0.1900318523215928`, 1780.0181964146154`},
{"CURE", 365, 0.24539479086801122`, {{0, 0.0014101529351823305`, 2.5100722246245484`}}, 0.009998673154598679`,
0.020642505320522992`, 0.13500810341296374`, 1781.0894871865428`}, {"CURE", 365, 2.580245950253217`,
{{0, 0.01950459361878954`, 34.71817664144538`}, {39, 0.0044262708688378605`, 7.8787621465313915`}},
0.009998548222766536`, 0.03677716166009913`, 0.4655051925265284`, 1782.7256991050622`}, {"CURE", 365,
0.27568593598066854`, {{0, 0.0006651506077833032`, 1.18396808185428`}, {8, 0.0002851585570330557 3`, 0.5075822315188392`},
{16, 0.0000373731899122874`, 0.06652427804387159`}, {24, 5.622290662846925`*^-6, 0.010007677379867526`}},
0.009999873781132554`, 0.10285206603351303`, 0.08430386609540683`, 1873.3217507403226`}, {"CURE", 365,
0.34687449479368904`, {{0, 0.0038077763657982347`, 6.777841931120858`}, {4, 0.0018535340738794343`, 3.299290651505393`},
{8, 0.0007634272500983332`, 1.358900505175033`}, {12, 0.00022070378926982543`, 0.39285274490028926`}},
0.009999969189987837`, 0.01283396548411052`, 0.8041654387608378`, 1784.9830664527578`},
{"TBUR", 29.227480494602833`, 230.2402459783765`, {{0, 0.08494092941284181`, 151.19485435485845`}},
1.1591709949496733`*^7, 0.016598723801484007`, 0.5370914122215195`, 2189.1758891464847`}, {"CURE", 365,
0.6208637061036848`, {{0, 0.002903532131703635`, 5.1682871944324695`}, {5, 0.0005840049890956372`, 1.039528880590234`}},
0.00999992393603809`, 0.015499634323819515`, 0.37813861691749073`, 1781.6404275351742`}, {"TBUR", 88.7570889245065`,
230.0872592935243`, {{0, 0.07013092813738786`, 124.83305208455039`}, {6, 0.0024280205613064643`, 4.321876599125507`},
{12, 0.0009277338341657874`, 1.6513662248151015`}, {18, 0.0013685847224634243`, 2.4360808059848953`},
{24, 0.0030501711705164694`, 5.429304683519316`}, {30, 0.003170963051620341`, 5.644314231884207`},
{36, 0.0018154617237414966`, 3.2315218682598643`}, {42, 0.0012367617538607543`, 2.2014359218721427`},
{48, 0.0010822956692640496`, 1.9264862912900083`}, {54, 0.08654438266550503`, 154.04900114459898`}},
530679.7198090092`, 0.03591606194597795`, 0.7404398860876164`, 2222.2274682455163`}, {"CURE", 365,
1.1980749561253758`, {{0, 0.004423110346586026`, 7.873136416923127`}, {5, 0.0013101931956396948`, 2.332143888238657`},
{10, 0.0001536696072348360 3`, 0.27353190087800816`}, {15, 0.0000191576509602814 36`, 0.03410061870930096`}},
0.009999932174318583`, 0.02665051965008184`, 0.43811513797903734`, 1842.1748442974024`}, {"TBUR", 79.85391134439388`,
229.487154687409`, {{0, 0.009010023154098771`, 16.037841214295813`}, {6, 0.0052932580224703905`, 9.421999279997296`},
{12, 0.004202940373138667`, 7.481233864186826`}, {18, 0.0035321523963320767`, 6.287231265471096`},
{24, 0.002995939208331463`, 5.332771790830004`}, {30, 0.0025466902372256357`, 4.533108622261631`},
```

```
{36, 0.00217117605308422б`, 3.864693374489922`}, {42, 0.0018580332866064307`, 3.3072992501594465`},
  {48, 0.0015969624775966367`, 2.842593210122013`}, {54, 0.13279045738120654`, 236.36701413854766`}},
 1.094261539471857`*^7, 0.10025438695934143`, 0.6366130075139214`, 2014.5479385337758`},
{"CURE", 365, 0.9434029961723961`, {{0, 0.010412789016913233`, 18.534764450105556`},
  {9, 0.0016362360747052237`, 2.912500212975298`}, {18, 0.00018844531360707448`, 0.3354326582205926`}},
 0.009988917087036487`, 0.02544993046869049`, 0.9100587198853697`, 1787.090704778027`},
{"CURE", 365, 12.766470599946821`, {{0, 0.027124541008529707`, 48.28168299518288`},
  {2, 0.0010955293257127361`, 1.9500421997686699`}, {4, 0.00007091605251815847`, 0.12623057348232206`},
  {6, 0.000046692228987539027`, 0.08311216759781948`}, {8, 0.00004611600576922433`, 0.0820864902692193`},
  {10, 0.0000460922479420568`, 0.0820442013368611`}, {12, 0.000046090397149747165`, 0.08204090692654996`},
  {14, 0.000046090169630703555`, 0.08204050194265232`}, {16, 0.00004609013017789982`, 0.08204043171666167`},
  {18, 0.0000460901211546045`, 0.082040415655196`}, {20, 6.257537208541714`*^-6, 0.011138416231204252`}},
 0.009999999950687012`, 0.02310028857592997`, 0.9967084402268105`, 2002.9164350661297`}, {"CURE", 365,
 0.39668123658908416`, {{0, 0.003702506502015658`, 6.590461573587872`}, {32, 0.0006763398639018597`, 1.2038849577453101`}},
 0.009879535275428833`, 0.025122466701892308`, 0.09097607166186411`, 1780.209686863143`},
{"CURE", 365, 0.6943331238183913`, {{0, 0.00930783079044062`, 16.56793880698431`}},
 0.009536767652508804`, 0.022410868278461067`, 0.18286382617215308`, 1780.1525345828554`},
{"TBUR", 22.570504753906494`, 230.25954608749007`, {{0, 0.10053801536560059`, 178.95766735076904`}},
 6.1478795622477025`*^7, 0.10986869624141496`, 0.43425928423307625`, 2066.978368019087`}, {"TBUR", 255.5842487875131`,
 229.2239932299592`, {{0, 0.011292096713756371`, 20.09993215048634`}, {25, 0.0025228075540412735`, 4.490597446193466`},
  {50, 0.0026940950948014394`, 4.795489268746561`}, {75, 0.001822629487605105`, 3.244280487937087`},
  {100, 0.001415587562836463`, 2.5197458618489037`}, {125, 0.0011203535165675803`, 1.9942292594902928`},
  {150, 0.0009245450540672426`, 1.6456901962396917`}, {175, 0.0007916608536446208`, 1.409156319487425`},
  {200, 0.0006998582275618997`, 1.2457476450601814`}, {225, 0.25684671119199054`, 457.18714592174314`}},
 994422.9702934751`, 0.08128395446504116`, 0.8055582909817752`, 2132.9507895685997`}, {"CURE", 365, 0.678943595920611`,
 {{0, 0.003284762007597346`, 5.846876373523276`}, {7, 0.0003563722923486282`, 0.6343426803805582`}},
 0.009968297268850422`, 0.013023865440278039`, 0.31583583352313505`, 1780.7259886777792`},
{"CURE", 365, 3.4234899996586172`, {{0, 0.009503469681226971`, 16.91617603258401`}, {5, 0.00265749201775924`, 4.730335791611447`},
  {10, 0.0004018004236601599`, 0.7152047541150846`}, {15, 0.000024154424587501255`, 0.042994875765752234`}},
 0.009974399728586348`, 0.016857439915066764`, 0.4921039124893289`, 1786.7228632064569`}, {"CURE", 365,
 2.6026540823008895`, {{0, 0.0014069763087879302`, 2.5044178296425157`}, {3, 0.00055061663295263З`, 0.9800976066556867`},
```

{6, 0.0001232447129145821`, 0.21937558898795614`}, {9, 0.000026175670837253656`, 0.046592694090311505`}},
0.009999958707614434`, 0.01742583013264786`, 0.6314981206353135`, 1826.7682210499727`}, {"CURE", 365,
1.2856439157463735`, {{0, 0.004388203990120266`, 7.811003102414074`}, {18, 0.0002774793815502433`, 0.4939132991594331`}},
0.009999926469761614`, 0.04630237463454228`, 0.726809148878093`, 2340.9863900459013`},
{"CURE", 365, 1.822296633221726`, {{0, 0.0035815386352702388`, 6.375138770781025`}},
0.009999862266391001`, 0.011043758752741745`, 0.11040406437167896`, 1780.2675581372037`},
{"CURE", 365, 5.580713994849188`, {{0, 0.03759806033277844`, 66.92454739234562`}, {3, 0.0025940299999038375`, 4.617373399828831`},
{6, 0.00011847376631999542`, 0.21088330404959185`}, {9, 0.000026984284337738363`, 0.0480320261211742 8`},
{12, 0.000024506901039656 44`, 0.04362228385058846`}, {15, 0.000024462579430162 09`, 0.04354339138568852`},
{18, 0.000024461919159737494`, 0.04354221610433274`}, {21, 0.000043931169719117 58`, 0.07819748210002929`}},
0.00999999968798946`, 0.02491814031411584`, 0.9941807494005628`, 2073.2100202642523`},
{"CURE", 365, 1.0606237333126909`, {{0, 0.002680107359293269`, 4.7705910995420195`},
{4, 0.0006922070657577445`, 1.2321285770487853`}, {8, 0.00010642392367718474`, 0.18943458414538883`},
{12, 0.0000258906120413403`, 0.046085289433585724`}, {16, 0.000017155913671833887`, 0.03053752633586432`},
{20, 0.000016404053104805066`, 0.029199214526553018`}, {24, 0.000032814016184678 83`, 0.05840894880872831 6`}},
0.009999982544140959`, 0.02376582195184622`, 0.9779883484825671`, 2116.6489305354658`},
{"CURE", 365, 0.49019559268481333`, {{0, 0.0021047913488543013`, 3.7465286009606564`}},
0.009999953899924294`, 0.02032791711507987`, 0.049089645526329426`, 1780.2241355909302`},
{"TBUR", 39.00567394411929`, 230.22458806213734`, {{0, 0.04047746181488038`, 72.04988203048707`}},
2.824649273079598`*^7, 0.041573535722278177`, 0.6978785655392818`, 2823.96255144484`},
{"CURE", 365, 0.6414584563230179`, {{0, 0.010864170529524056`, 19.33822354255282`}}, 0.009999181086712243`,
0.015798430431166022`, 0.3173045123595449`, 1780.35600475309`}, {"TBUR", 108.22701910311268`, 229.19647688399834`,
{{0, 0.007283845530641766`, 12.965245044542343`}, {2, 0.0084738850056049`, 15.083515309976724`},
{4, 0.007640726090945576`, 13.600492441883127`}, {6, 0.005657945454899781`, 10.07114290972161`},
{8, 0.0036508177905380523`, 6.498455667157733`}, {10, 0.002147388158930296`, 3.822350922895927`},
{12, 0.0011911066146388992`, 2.1201697740572403`}, {14, 0.000638293663900969`, 1.136162721743725`},
{16, 0.0003359245848222787`, 0.5979457609836559`}, {18, 0.00147150927294566`, 2.6192865058432746`}},
25.1312433498418`, 0.00915076371590218`, 0.7532633733067069`, 4084.569679294991`}, {"CURE", 365, 1.1362873122075134`,
{{0, 0.005443834702396229`, 9.690025770265285`}, {7, 0.001349140238519135`, 2.40146962456406`}}, 0.009914257255184118`,
0.015056234654657928`, 0.37553375994506966`, 1780.7816796841091`}, {"CURE", 365, 0.7094138598342137`,
{{0, 0.001640045966450 3806`, 2.9192818202816775`}, {28, 0.0003769008923450949`, 0.670883588374269`}},

```
0.009782696898525163`, 0.039023924462227945`, 0.06092269802738394`, 1781.0970589579176`},
{"CURE", 365, 4.3952717707139675`, {{0, 0.023207082842327094`, 41.308607459342234`},
  {3, 0.002065033102448729`, 3.675758922358738`}, {6, 0.00011518333547179144`, 0.20502633713978877`},
  {9, 0.000022989207070118318`, 0.04092078858481061`}, {12, 0.0000198900918910789`, 0.03540436356612045`},
  {15, 0.000019812641755206356`, 0.03526650232426732`}, {18, 0.00001981033974219117`, 0.03526240474110028`},
  {21, 0.000019810201598136034`, 0.035262158844682145`}, {24, 6.775951523920837`*^-6, 0.01206119371257909`}},
 0.009999997255460176`, 0.02605470630802215`, 0.9945473644964072`, 2124.347683972345`},
{"CURE", 365, 0.43554262740599037`, {{0, 0.0015126943709650566`, 2.6925959803178006`}}, 0.00999939408248491`,
 0.01351513654928443`, 0.17634555134007054`, 1780.812872210605`}, {"TBUR", 112.2553259141986`, 228.84446846033006`,
 {{0, 0.021230974327562935`, 37.79113430306202`}, {6, 0.00036453291462096294`, 0.648868588025314`},
  {12, 0.00006284666867112769`, 0.1118670702346073`}, {18, 0.000051613951596299035`, 0.09187283384141227`},
  {24, 0.00005164257630112814`, 0.09192378581600809`}, {30, 0.00005688483256558713`, 0.1012550019667451`},
  {36, 0.0000793992209600094`, 0.14133061330881672`}, {42, 0.00018541989275726762`, 0.33004740910793634`},
  {48, 0.0005526550029531773`, 0.9837259052566556`}, {54, 0.138681271970818`, 246.85266410805602`}},
 51402.042693081785`, 0.04262457450064976`, 0.8249640400220284`, 2896.0052724901984`}, {"CURE", 365,
 0.9200868444092943`, {{0, 0.005131261067440104`, 9.133644700043385`}, {35, 0.002036090281928644`, 3.6242407018329867`}},
 0.009763709419448466`, 0.02722826554946175`, 0.28604446452246174`, 1781.3702667781083`},
{"TBUR", 365, 228.51226092801406`, {{0, 0.036002747925741`, 64.08489130781898`}, {31, 0.0007336458558119779`, 1.305889623345321`},
  {62, 0.0008278846203003793`, 1.473634624134675`}, {93, 0.0022208806107071317`, 3.9531674870586944`},
  {124, 0.0016374034342814736`, 2.914578113021023`}, {155, 0.0014442143736141794`, 2.5707015850332393`},
  {186, 0.0012500674468259255`, 2.225120055350147`}, {217, 0.0011197976128063162`, 1.9932397507952426`},
  {248, 0.0010225444097523581`, 1.8201290493591975`}, {279, 0.04687095077596408`, 83.43029238121606`}},
 179.39090331850696`, 0.008998395554879295`, 0.8675287087617305`, 2271.003764070008`}, {"TBUR", 215.015428701527`,
 229.23578246881863`, {{0, 0.026866379232427684`, 47.82215503372128`}, {11, 0.009534237887929732`, 16.970943440514922`},
  {22, 0.001970154980282215`, 3.5068758649023426`}, {33, 0.00040090083307668705`, 0.7136034828765028`},
  {44, 0.00009838162369649086`, 0.17511929017975375`}, {55, 0.00003886433308908785`, 0.06917851289857636`},
  {66, 0.000027044491743140918`, 0.04813919530279084`}, {77, 0.00002451059803514703`, 0.04362886450256171`},
  {88, 0.000023801766382700988`, 0.04236714416120776`}, {99, 0.0007177157397274711`, 1.2775340167148985`}},
 892.4600956885622`, 0.012788366402422334`, 0.7843585362332756`, 3824.754597129778`},
{"CURE", 365, 1.4299637511852352`, {{0, 0.01639044584895568`, 29.17499361114111`}}, 0.009680264269543637`,
 0.023915497500474753`, 0.2799031578545323`, 1780.1680086737206`}, {"CURE", 365, 0.044226139232106575`,
```

```
{{0, 0.00037729716673457533`, 0.6715889567875442`}, {26, 0.000015030156097398459`, 0.026753677853369254`}},
0.009973718019268864`, 0.01954027469587128`, 0.0420590733328188`, 1780.7497515890368`},
{"CURE", 365, 0.33515630479236597`, {{0, 0.0010673719730117512`, 1.899922111960917`}},
0.009999465840535437`, 0.013933262858233236`, 0.12040848820023464`, 1780.7079511414433`},
{"CURE", 365, 1.1210576477066025`, {{0, 0.0012074472637196455`, 2.149256129420969`},
{8, 0.00016315584266418602`, 0.29041739994225113`}, {16, 8.582571282994045`*^-6, 0.0152769768837294`}},
0.009999969063372994`, 0.041832639838478305`, 0.49694092692048875`, 1889.248624688573`}, {"CURE", 365,
0.8758775119725929`, {{0, 0.002652541815220023`, 4.721524431091641`}, {5, 0.0004495787408997594`, 0.8002501588015717`}},
0.00995699158774767`, 0.008676671950742464`, 0.25794905405161855`, 1780.9231697654616`},
{"CURE", 365, 146.5275151563389`, {{0, 0.05796960905840l844`, 103.18590412395528`},
{7, 0.0034911293658283537`, 6.214210271174469`}, {14, 0.003466994083950115`, 6.171249469431204`},
{21, 0.00346656432286579`, 6.170484494701107`}, {28, 0.0034665395580506333`, 6.170440413330128`},
{35, 0.0034665369882142566`, 6.170435839021377`}, {42, 0.003466536617796391`, 6.170435179677575`},
{49, 0.003466536552895103`, 6.170435064153283`}, {56, 0.00345599169774452`, 6.151665221985246`}},
0.009987165301791084`, 0.035663964610191765`, 0.9752312618018634`, 3001.979513089626`},
{"CURE", 365, 1.1788606077163708`, {{0, 0.016779774188528156`, 29.867998055580117`}},
0.009999909388126078`, 0.022816884122441553`, 0.7251753734571513`, 1781.4369476075103`},
{"CURE", 365, 1.4123267890693663`, {{0, 0.007749403470126346`, 13.793938176824897`},
{2, 0.003814673446341092`, 6.790118734487143`}, {4, 0.0012922501663736228`, 2.3002052961450485`},
{6, 0.00042060522977515686`, 0.7486773089997791`}, {8, 0.0001378076761371829`, 0.24529766352418556`},
{10, 0.000046828546954777733`, 0.08335481357950363`}, {12, 0.000013941766339093848`, 0.024816344083587048`}},
0.009999995839136602`, 0.013417612152475403`, 0.9287198968458427`, 1861.7639952723762`},
{"CURE", 365, 0.246445475728762`, {{0, 0.0015623732343105754`, 2.781024357072824`}}, 0.009999717756978959`,
0.013429175353107442`, 0.11231181448178919`, 1780.6358672372062`}, {"TBUR", 54.39346638027193`,
179.38661664412024`, {{0, 0.046282516340458`, 82.38287908601524`}, {6, 0.012703238832022927`, 22.611765121000808`},
{12, 0.009693655186639808`, 17.254706232218858`}, {18, 0.010470936392888703`, 18.638266779341894`},
{24, 0.01318743325856878`, 23.47363120025243`}, {30, 0.016795377403096924`, 29.895771777512525`},
{36, 0.020037480972849225`, 35.66671613167162`}, {42, 0.022429742317210972`, 39.92494132463553`},
{48, 0.024385299856115837`, 43.405833743886184`}, {54, 5.237375669262508`, 9322.528691287262`}},
1.979350251963227`*^7, 0.09618016054429665`, 0.8136743998294336`, 2027.8796649623562`},
{"CURE", 365, 1.1979364634740284`, {{0, 0.004728014309108413`, 8.415865470212976`}},
```

```
0.009999627108885622`, 0.015206056036136414`, 0.10660687887969691`, 1780.1885554901492`},
{"CURE", 365, 0.1328316761718486`, {{0, 0.000577488437641308`, 1.0279294190015282`}}, 0.009999572823003663`,
  0.02064606739467465`, 0.08937899016643976`, 1781.5048908291105`}, {"CURE", 365, 0.8889361458343319`,
  {{0, 0.0019858752154254507`, 3.5348578834573026`}, {21, 0.00021969486355383541`, 0.3910568571258271`}},
  0.009815732922538376`, 0.041488362322269316`, 0.677781361631167`, 1974.5464746177117`},
{"TBUR", 18.244658804610708`, 230.2440001496436`, {{0, 0.05897614955902101`, 104.97754621505739`}},
  3.951771093200938`*^7, 0.22897888331257366`, 0.5692031443194675`, 2202.128467421238`},
{"CURE", 365, 0.19294049815787173`, {{0, 0.003961655022627672`, 7.051745940277256`}},
  0.00999928897896748`, 0.017496096453344493`, 0.4721999391136825`, 1781.325071634099`},
{"CURE", 365, 3.1912484882104755`, {{0, 0.007538371507208598`, 13.418301282831305`},
    {3, 0.00025575387770884734`, 0.45524190232174827`}, {6, 0.00006483590481723973`, 0.11540791057468673`},
    {9, 0.0000631942908323835`, 0.11248583768164262`}, {12, 0.00006318489058357535`, 0.11246910523876412`},
    {15, 0.00006318477819319671`, 0.11246890518389016`}, {18, 0.00012002217583853444`, 0.2136394729925913`}},
  0.009999999649756673`, 0.02050633270233852`, 0.9966330729953594`, 2127.3664037239137`},
{"CURE", 365, 0.5834821315926492`, {{0, 0.00976503525729211`, 17.381762757979953`},
    {2, 0.005382657504200114`, 9.581130357476201`}, {4, 0.001886330810624697`, 3.3576688429119605`}},
  0.0099553172748074`, 0.007635636308035909`, 0.5813104529849474`, 1780.6135388841594`},
{"CURE", 365, 0.40390047528236006`, {{0, 0.0017756338415112728`, 3.1606282378900654`},
    {13, 0.0003098478677418893`, 0.5515292045805629`}, {26, 0.000014405650997183134`, 0.025642058774985977`}},
  0.009999990683424715`, 0.08989987807122661`, 0.17101221353252985`, 1851.2693509121916`},
{"CURE", 365, 107.8024523052649`, {{0, 0.045984200091459815`, 81.85187616279848`},
    {21, 0.0024239970266149895`, 4.314714707374681`}, {42, 0.002772927039294201`, 4.935810129943677`}},
  0.009992923308538317`, 0.00954988669525999`, 0.9999526314288625`, 2323.3234412928027`},
{"TBUR", 14.316722686291174`, 230.07950852072432`, {{0, 0.1308194160461426`, 232.85856056213385`}},
  9.980846637487562`*^7, 0.17078660400893578`, 0.44713843112647866`, 1974.6911892154603`},
{"TBUR", 55.98296115851492`, 230.2617489832525`, {{0, 0.07270032882690432`, 129.40658531188967`}},
  97526.2300335666`, 0.002656275525085459`, 0.6903194992433329`, 2361.346542167314`},
{"CURE", 365, 7.226867528161666`, {{0, 0.021731965263457626`, 38.68289816895459`},
    {3, 0.0011913691924984383`, 2.12063716264722`}, {6, 0.000057580209456250915`, 0.10249277283212664`},
    {9, 0.000032558204496244024`, 0.05795360400331436`}, {12, 0.00003206657010837536`, 0.05707849479290814`},
    {15, 0.000032049921059147054`, 0.05704885948528175`}, {18, 0.0000320487747671368`, 0.057046819085503506`},
```

```
{21, 0.00003204864942152745`, 0.05704659597031886`}, {24, 0.00002199980396296587`, 0.03915965105407924`}},
0.00999999785472652`, 0.02274228138975349`, 0.9919822416610519`, 2195.200926475176`}, {"CURE", 365,
3.374318950098466`, {{0, 0.025581681652683836`, 45.53539334177723`}, {7, 0.011151676452657099`, 19.849984085729638`}},
0.009999955990647116`, 0.008476136252402372`, 0.6097098595014456`, 1780.5260216851766`},
{"CURE", 365, 0.12290367608986404`, {{0, 0.002345312433306137`, 4.174656131284924`}}, 0.00999798551513393`,
0.017756079353623794`, 0.06589646418453877`, 1780.239749752353`}, {"CURE", 365, 0.35694914595790095`,
{{0, 0.0008747250272112735`, 1.557010548436067`}, {6, 0.00035048131218503795`, 0.6238567356893676`},
{12, 0.00010904599360745416`, 0.19410186862126844`}, {18, 0.000023861024874668333`, 0.04247262427690963`}},
0.0099996621990481`, 0.015202130869854957`, 0.821702864953383`, 1856.378221565742`}, {"CURE", 365,
41.109802611579326`, {{0, 0.018186397693354495`, 32.371787894171`}, {9, 0.00031268096193115064`, 0.5565721122374481`},
{18, 0.0001240146045812662`, 0.22074599615465382`}, {27, 0.00012343580718134612`, 0.21971573678279607`},
{36, 0.00012342798656135906`, 0.21970181607921913`}, {45, 0.000123427037362214`, 0.2197001265047409`},
{54, 0.00012342683264687635`, 0.2196997621114399`}, {63, 0.00012342677534367324`, 0.2196996601117384`},
{72, 0.00012342675690574906`, 0.21969962729223336`}, {81, 0.000047477426638055335`, 0.0845098194157385`}},
0.00999999758726582`, 0.014663641105422913`, 0.9999897196101301`, 2360.9591758724764`},
{"CURE", 365, 1.1538454173105601`, {{0, 0.012668941003333174`, 22.55071498593305`}}, 0.009999371363624549`,
0.024503951525521345`, 0.625646931945289`, 1781.2679381633009`}, {"CURE", 365, 0.3054641354232927`,
{{0, 0.006983291480244503`, 12.430258834835215`}, {7, 0.0006815348908037837`, 1.2131321056307351`}},
0.00997133724807188`, 0.013015254475200513`, 0.24292804028610412`, 1780.284466336552`}, {"TBUR", 192.50272161794672`,
229.28395726837064`, {{0, 0.052104050823616445`, 92.74521046603726`}, {16, 0.0007077381809547466`, 1.259773962099449`},
{32, 0.0009625377060465823`, 1.7133171167629162`}, {48, 0.003889322989808828`, 6.922994921859714`},
{64, 0.00662692199364081`, 11.795921148680641`}, {80, 0.004241390014649394`, 7.549674226075922`},
{96, 0.003792393441132731`, 6.7504603252162605`}, {112, 0.0038717491206253386`, 6.891713434713102`},
{128, 0.003547449544960772`, 6.314460190030174`}, {144, 0.23378208241913842`, 416.13210670606634`}},
47042.344483283734`, 0.08386130062230907`, 0.7796786526062754`, 2391.402718926102`},
{"CURE", 365, 0.08842386868484241`, {{0, 0.00013104866085033134`, 0.23326661631358978`},
{11, 0.00003358077673912248`, 0.05977378259563801`}, {22, 2.7708963967742615`*^-6, 0.004932195586258185 6`}},
0.009823821955027323`, 0.08174686571383781`, 0.12215898761567158`, 2197.3076156663737`},
{"CURE", 365, 26.450515349267985`, {{0, 0.013690719659223475`, 24.369480993417785`},
{4, 0.0002856557691008459 5`, 0.5084672689995058`}, {8, 0.0002447456282172967`, 0.43564721822678815`},
{12, 0.0002445501818268 6146`, 0.4352993236518134`}, {16, 0.0002445449305120609`, 0.43528997631146843`},
```

```
{20, 0.00024454452733174454`, 0.4352892586505052`}, {24, 0.0002445444663307172`, 0.4352891500686765`},
{28, 0.0002445444516964449`, 0.43528912401967196`}, {32, 0.0001742002510739813`, 0.3100764469116867`}},
0.009997075404657024`, 0.010331233605763218`, 0.9998399621778372`, 2220.098732394038`},
{"CURE", 365, 0.1207129846232886`, {{0, 0.00040538117028732245`, 0.7215784831114339`}},
0.00999986002684584`, 0.015810992954201696`, 0.09487285718173151`, 1781.915375507749`},
{"CURE", 365, 0.22294149158743984`, {{0, 0.0028027813913168004`, 4.988950876543905`}}, 0.009996280663303297`,
0.023328312025482502`, 0.0637470382766467`, 3.865704031853719`*^8}, {"TBUR", 47.21487504921707`, 228.36279034176877`,
{{0, 0.029468521872069695`, 52.45396893228405`}, {2, 0.009632312477724862`, 17.145516210350255`},
{4, 0.006012015053149862`, 10.701386794606755`}, {6, 0.003937706105929049`, 7.009116868553709`},
{8, 0.002688173087279081`, 4.784948095356764`}, {10, 0.0019098696095417203`, 3.3995679049842624`},
{12, 0.0014115231540437114`, 2.512511214197806`}, {14, 0.001084452767155327`, 1.9303259255364822`},
{16, 0.000864814352021594`, 1.5393695465984374`}, {18, 0.06009956392243344`, 106.97722378193151`}},
3.6774396012734934`*^6, 0.04213030608987968`, 0.7114841090375015`, 2243.2539782004665`},
{"CURE", 365, 0.5502911275457596`, {{0, 0.003958298084808277`, 7.045770590958734`}},
0.009999518096037895`, 0.018194171625189718`, 0.355288748305624`, 1780.8937950164009`},
{"TBUR", 46.071344205658725`, 230.25427792360412`, {{0, 0.040168175697326676`, 71.49935274124148`}},
2.821036959146886`*^6, 0.0203729827776689`, 0.6470583518807256`, 2908.1278092676066`}, {"CURE", 365, 1.4991208823109305`,
{{0, 0.0005223241866042924`, 0.9297370521556404`}, {8, 0.00010869930381973231`, 0.19348476079912352`},
{16, 0.000024244607039312203`, 0.043155400529975727`}, {24, 0.00001749483693011191`, 0.031140809735599198`},
{32, 0.000017178795839737312`, 0.030578256594732418`}, {40, 0.00004741717588695626`, 0.08440257307878261`}},
0.009999985093454365`, 0.023230034874105507`, 0.9864542040783902`, 2724.4726476015585`}, {"CURE", 365,
3.6545947557383824`, {{0, 0.007137193292249268`, 12.704204060203697`}, {17, 0.0047016303013351236`, 8.368901936376519`}},
0.009748104977634852`, 0.013976533275180177`, 0.4305340497104015`, 1780.882902037025`},
{"CURE", 365, 0.09501555351361736`, {{0, 0.0010333678185536887`, 1.8393947170255658`}}, 0.009998649358690402`,
0.008421837704973777`, 0.15485099665398724`, 1781.5341077505118`}, {"CURE", 365, 0.07986580634893985`,
{{0, 0.0028637383542707623`, 5.097454270601957`}, {4, 0.000316914440916651`, 0.564107704831639`}}, 0.009968538992764408`,
0.01119723338291308`, 0.27124290849488597`, 1780.8784476890628`}, {"CURE", 365, 0.1203129580093548`,
{{0, 0.0004325328765851717`, 0.7699085203216056`}, {27, 0.00008275336790341135`, 0.14730099486807222`}},
0.009837056536435181`, 0.025325004701136928`, 0.07255149231700675`, 1784.9535561849184`},
{"CURE", 365, 0.12941274763074379`, {{0, 0.0005257789693042072`, 0.9358865653614887`}}, 0.009999798260716373`,
0.01019787147666962`, 0.10285512960346034`, 1782.0452206990474`}, {"TBUR", 192.8001733187005`, 227.96074339789993`,
```

```
  {{0, 0.07300701547022401`, 129.95248753699875`}, {14, 0.02126593948111036`, 37.85337227637644`},
    {28, 0.006394557235312674`, 11.382311878856559`}, {42, 0.001993842864555619`, 3.5490402989090017`},
    {56, 0.0006642626583511386`, 1.1823875318650268`}, {70, 0.00026015545788190945`, 0.4630767150297988`},
    {84, 0.0001361898531896717`, 0.24241793867761566`}, {98, 0.00009624213300148852`, 0.17131099674264957`},
    {112, 0.00008151648470767784`, 0.14509934277966655`}, {126, 0.005601367348455971`, 9.970433880251628`}},
  3260.1673712046927`, 0.04094539748594965`, 0.1901582584976571`, 1780.0279663078593`},
{"CURE", 365, 0.2688020472152992`, {{0, 0.002088868507621947`, 3.718185943567065`}}, 0.009702100845649644`,
  0.025212251796741438`, 0.11207800453840307`, 1780.4665285946915`}, {"TBUR", 48.05599166651512`, 229.85105160821485`,
  {{0, 0.053141838477027664`, 94.59247248910926`}, {5, 0.013836455198493232`, 24.62889025331795`},
    {10, 0.010488131309552864`, 18.668873731004098`}, {15, 0.011361519295547708`, 20.223504346074918`},
    {20, 0.013624853003788498`, 24.252238346743525`}, {25, 0.0155394407144941`, 27.6602044717995`},
    {30, 0.01651624838069539`, 29.398922117637788`}, {35, 0.01700201405526311`, 30.263585018368335`},
    {40, 0.017476631813916427`, 31.108404628771236`}, {45, 0.2037592703382946`, 362.6915012021644`}},
  2.2274776820327573`*^7, 0.20673376415167233`, 0.6759369150467642`, 1995.620312037538`}, {"TBUR", 161.12954833505697`,
  228.68559806398747`, {{0, 0.036304699168364964`, 64.62236451968964`}, {16, 0.006774150196656145`, 12.057987350047938`},
    {32, 0.00679810004151662`, 12.100618073899582`}, {48, 0.008585083517577637`, 15.281448661288193`},
    {64, 0.007836868935265591`, 13.949626704772752`}, {80, 0.00688317270674158`, 12.252047418000013`},
    {96, 0.006378883912538749 5`, 11.354413364318974`}, {112, 0.005928184472664673`, 10.55216836134312`},
    {128, 0.005471153517302044`, 9.738653260797639`}, {144, 0.382544030629935`, 680.9283745212841`}},
  2.2742168928297465`*^6, 0.12806778383209524`, 0.21833301227590066`, 1780.0591697152151`},
{"CURE", 365, 0.22845214757177276`, {{0, 0.003977851323332922`, 7.080575355532602`}},
  0.009999833983536942`, 0.024708197825540738`, 0.3620957898212465`, 1780.9168319979324`},
{"TBUR", 20.46852092525105`, 230.07047954804915`, {{0, 0.08627687454223637`, 153.57283668518073`}},
  3.0936990017047305`*^7, 0.09716346195147801`, 0.4909317574927164`, 2106.5093765645256`},
{"CURE", 365, 0.9617980250909907`, {{0, 0.0034563300048445437`, 6.152267408623287`}},
  0.009999983029247452`, 0.020541775521954363`, 0.10905531758179472`, 1780.257193309988`},
{"TBUR", 43.85618510482511`, 230.26285247486277`, {{0, 0.051685595512390145`, 92.00036001205446`}},
  1.0045541196252914`*^7, 0.04093398150760795`, 0.532856649986227`, 2559.5517174283023`}, {"CURE", 365,
  1.31708869349862`, {{0, 0.0021908628227927 6`, 3.8997358245711133`}, {29, 0.0008227433600250819`, 1.464483180844646`}},
  0.009738739003554694`, 0.024494818004926762`, 0.1436221502969663`, 1781.402608911194`},
{"CURE", 365, 5.057315150242534`, {{0, 0.008642977852784388`, 15.384500577956212`},
```

```
{10, 0.0033796945663087034`, 6.015856328029492`}, {20, 0.0006147847326111647`, 1.0943168240478731`}},
0.009935889766189253`, 0.02645804820343211`, 0.30163182762686147`, 1780.6229307047413`},
{"CURE", 365, 2.930176391526962`, {{0, 0.006846873407944163`, 12.18743466614061`},
{5, 0.0023102921686644507`, 4.112320060222722`}, {10, 0.0004119336618866424`, 0.7332419181582235`}},
0.009999945296364238`, 0.019325574765754628`, 0.3563744004343975`, 1781.4044150495545`},
{"TBUR", 22.608375066483774`, 229.64935182862703`, {{0, 0.13394725799560545`, 238.4261192321777`}},
4.709913009715836`*^7, 0.10823951600434038`, 0.4729871609936208`, 2013.6412545947273`},
{"CURE", 365, 0.03619751654436967`, {{0, 0.00025112336963110715`, 0.44699959794337074`}}, 0.00998662025124769`,
0.02100175418931788`, 0.03215682759086765`, 1781.417340393436`}, {"CURE", 365, 3.397605851215074`,
{{0, 0.008337478695575181`, 14.840712078123822`}, {19, 0.0006822466456583762`, 1.2143990292719096`}},
0.009999936501124091`, 0.03336340229487511`, 0.6216351632726157`, 1817.0770510290706`}, {"CURE", 365, 0.402905376371641`,
{{0, 0.0011679109987897342`, 2.078881577845727`}, {6, 0.00020060428335695187`, 0.3570756243753743`}},
0.009913320493313227`, 0.021454102761564287`, 0.16152377945174212`, 1781.4929679328136`}, {"CURE", 365,
2.9558864203601227`, {{0, 0.0029948680149921216`, 5.330865066685976`}, {13, 0.00047635085799829107`, 0.8479045272369581`}},
0.009999462270262015`, 0.04153979259580732`, 0.7693534214628364`, 2453.0054556663817`},
{"TBUR", 96.96945081529154`, 229.78252732550322`, {{0, 0.07045248445510865`, 125.40542233009342`}},
130929.2151787454`, 0.003848350534769275`, 0.6481210914495674`, 2395.5738046085016`},
{"CURE", 365, 2.8059892014978685`, {{0, 0.016054244037098257`, 28.576554386034896`},
{9, 0.006078276057409472`, 10.819331382188858`}, {18, 0.0012526533352532176`, 2.2297229367507274`}},
0.009960345413470104`, 0.024111571906122668`, 0.5836135462803432`, 1780.6834871402666`}, {"CURE", 365,
0.1548012138295399`, {{0, 0.0007105296481891985`, 1.2647427737767736`}, {20, 0.00021220417106304602`, 0.3777234244922219`}},
0.009768400310926444`, 0.019406619185635344`, 0.16959852735053155`, 1784.2492965461645`},
{"CURE", 365, 0.7008229699069691`, {{0, 0.0032191448096120876`, 5.7300777611095155`}},
0.009999849679931894`, 0.012632005529736458`, 0.18610967950890356`, 1780.4183085816899`},
{"CURE", 365, 5.398856023004571`, {{0, 0.03826530553712193`, 68.11224385607703`}, {2, 0.0055483592043746475`, 9.876079383786873`},
{4, 0.0005805289881916317`, 1.0333415989811046`}, {6, 0.00007780365093157309`, 0.13849049865820012`},
{8, 0.00003208004514311679`, 0.05710248035474789`}, {10, 0.00002844909778420388`, 0.0506393940558829`},
{12, 0.000028204557117958655`, 0.050204111669966404`}, {14, 7.783943474163172`*^-6, 0.013855419384010446`}},
0.00999999412332922`, 0.022094852049100965`, 0.9875701108551284`, 1820.9730673532822`}, {"TBUR", 218.40890960224726`,
228.89197279813436`, {{0, 0.04149459160048902`, 73.86037304887046`}, {17, 0.01717942879184894`, 30.579383249491112`},
{34, 0.006966232674964839`, 12.399894161437413`}, {51, 0.0028623826778786696`, 5.095041166624031`},
```

{68, 0.0011995809209107543`, 2.135254039221143`}, {85, 0.0005238205300125356`, 0.9324005434223134`},
{102, 0.0002486623591853209`, 0.44261899934987115`}, {119, 0.00013613156429087483`, 0.2423141844377572`},
{136, 0.00008941056179677804`, 0.1591507999982649`}, {153, 0.026294681686773846`, 46.80453340245745`}},
4201.31768082325`, 0.03016880180111845`, 0.1964095575923364`, 1780.0428086984218`}, {"CURE", 365, 1.0202419814790948`,
{{0, 0.004293241473846522`, 7.641969823446808`}, {22, 0.0014592902500261086`, 2.5975366450464734`}},
0.009999996800099465`, 0.019698436721932087`, 0.7289510128575217`, 1791.8715283659697`}, {"CURE", 365,
0.5814558657567421`, {{0, 0.008522845650213228`, 15.170665257379545`}, {5, 0.003557540944483986`, 6.332422881181495`},
{10, 0.0010290685679888753`, 1.831742051020198`}, {15, 0.00029555422614747`, 0.5260886522542496`},
{20, 0.00008619869236974135`, 0.15343367241813963`}, {25, 0.000019644832738344688`, 0.03496780227425354`}},
0.009999995155592098`, 0.024858763998773858`, 0.917689804101315`, 1818.53953036184`},
{"CURE", 365, 0.21272269444043831`, {{0, 0.001665075424107715`, 2.9638342549117325`}}, 0.00999967510971836`,
0.022687655579820245`, 0.6219955362449184`, 1788.1228962594782`}, {"CURE", 365, 0.26275987743422213`,
{{0, 0.0006416992586781915`, 1.1422246804471807`}, {48, 0.00023468294963238531`, 0.41773565034564586`}},
0.009752044310737867`, 0.033223979712677675`, 0.05726702412048269`, 1782.720874915346`}, {"CURE", 365, 0.05157174509286368`,
{{0, 0.0002919022038102313`, 0.5195859227822117`}, {41, 0.00010426945839486552`, 0.1855996359428606`}},
0.009998264214051672`, 0.02547711257839649`, 0.06617052363361885`, 1787.151194096195`},
{"CURE", 365, 0.26658516533122406`, {{0, 0.0055087280184902`, 9.805535872912555`}}, 0.009998893263440235`,
0.01519090008195615`, 0.1667405677081614`, 1780.3219246724605`}, {"TBUR", 115.13083032465657`, 228.3606987060051`,
{{0, 0.010483949731169218`, 18.661430521481208`}, {6, 0.0035090403035628473`, 6.2460917403418685`},
{12, 0.002430051444645526`, 4.325491571469037`}, {18, 0.0017851325927563761`, 3.1775360151063494`},
{24, 0.0013312541909950883`, 2.3696324599712573`}, {30, 0.0010013269855785926`, 1.7823620343298947`},
{36, 0.0007602412839726924`, 1.3532294854713927`}, {42, 0.0005838970206370874`, 1.0393366967340159`},
{48, 0.0004548610582850612`, 0.809652683747409`}, {54, 0.06473697850324558`, 115.23182173577716`}},
7.618080475290346`*^6, 0.11452449188343827`, 0.5120704144527096`, 2356.608383619752`}, {"CURE", 365,
3.780644150344162`, {{0, 0.0029991600191231734`, 5.338504834039249`}, {8, 0.0009824354000310606`, 1.748735012055288`},
{16, 0.00017207419770102087`, 0.3062920719078172`}, {24, 0.00001289882680270442`, 0.022959911708813868`}},
0.00999988939620186`, 0.030368624662705124`, 0.3690180827051889`, 1791.5353003669895`}, {"CURE", 365,
0.3719106760574928`, {{0, 0.0008960771791581628`, 1.5950173789015298`}, {14, 0.0000902312518454244`, 0.16061162828485542`}},
0.00999989670586323`, 0.03903866434004902`, 0.6128379907486854`, 1928.5876211925797`}, {"CURE", 365, 4.202907246258883`,
{{0, 0.014825240085228147`, 26.388927351706098`}, {14, 0.000924940096004621`, 1.6463933708882252`}},
0.0099980128125251`, 0.042909932641726226`, 0.5282896978312048`, 1786.965353077895`}, {"CURE", 365,

19.107566876488953`, {{0, 0.010067235607514897`, 17.919679381376515`}, {7, 0.0028248320993941463`, 5.028201136921581`},
{14, 0.00029945733052751615`, 0.5330340483389787`}, {21, 0.000026814740030727965`, 0.04773023725469579`},
{28, 4.701588215842848`*^-6, 0.008368827024200269`}, {35, 0.000011483726525961034`, 0.02044103321621064`}},
0.009999992689268206`, 0.0617048474216196`, 0.6675185643688185`, 2261.190786976846`}, {"CURE", 365,
0.4827688332305949`, {{0, 0.0011919575471130567`, 2.121684433861241`}, {5, 0.00041732765840687728`, 0.7428432319642336`},
{10, 0.00006520330750088782`, 0.11606188735158032`}, {15, 0.000015971616803229555`, 0.0284294779097486`}},
0.009999967564327118`, 0.027352571615028125`, 0.5829141396725028`, 2022.8377302138695`},
{"CURE", 365, 1.8209203750972458`, {{0, 0.009163933658202117`, 16.31180191159977`}}, 0.009998998438617613`,
0.019567330038344405`, 0.5553909521835197`, 1780.935981360384`}, {"CURE", 365, 1.713813451931022`,
{{0, 0.016424212398852136`, 29.2350980699568`}, {6, 0.0035178590822504984`, 6.261789166405888`}}, 0.00999981159027937`,
0.019304886118356637`, 0.9539477853069631`, 1912.505370861501`}, {"CURE", 365, 0.18797482606624547`,
{{0, 0.001750859625730687`, 3.116530133800623`}, {48, 0.0007360774990753981`, 1.3102179483542087`}},
0.009998575131071968`, 0.0267619539244985`, 0.11246377188704822`, 1781.1724305718003`},
{"CURE", 365, 1.5641819614526165`, {{0, 0.0023973608821059333`, 4.2673023701485615`},
{2, 0.000959802687854463`, 1.7084487843809442`}, {4, 0.0003002287851417602`, 0.5344072375523331`},
{6, 0.00009498229320131352`, 0.1690684818983381`}, {8, 0.000014649975974977364`, 0.02607695723545971`}},
0.009999998921289366`, 0.009138339408535725`, 0.8907718431347801`, 1819.4448449291951`},
{"CURE", 365, 0.46008960719728653`, {{0, 0.0022474822147737646`, 4.000518342297301`}}, 0.009993548593916 07`,
0.007531655906278498`, 0.2534993827606503`, 1780.8961964282032`}, {"TBUR", 348.4698169840748`, 228.86390457383175`,
{{0, 0.03196751095447501`, 56.90216949896552`}, {32, 0.009073138603663202`, 16.1501867145205`},
{64, 0.0052867615586971554`, 9.410435574480937`}, {96, 0.004020259272258902`, 7.156061504620847`},
{128, 0.003143565376569 4107`, 5.595546370293551`}, {160, 0.002453676461136773`, 4.367544100823456`},
{192, 0.0019168910164923626`, 3.4120660093564057`}, {224, 0.0015052815939966657`, 2.679401237314065`},
{256, 0.00119148692552 4047`, 2.120846727432804`}, {288, 0.04652913575281219`, 82.82186164000571`}},
1465.095496219109`, 0.019902430925621217`, 0.1541900711194978`, 1780.047528498597`},
{"CURE", 365, 0.25541401861250307`, {{0, 0.0007138507581050385`, 1.2706543494269686`},
{8, 0.00037939659462648867`, 0.6753259384351499`}, {16, 0.00013688395752858634`, 0.24365344440088368`},
{24, 0.00005001426180211209`, 0.08902538600775954`}, {32, 0.000013052146031224881`, 0.02323281993558029`}},
0.009981849886145215`, 0.031542680795137445`, 0.6914266253260797`, 1818.1374007215718`},
{"CURE", 365, 0.48402245470410493`, {{0, 0.00196722877342876`, 3.5016672167031926`},
{3, 0.0009242017638126424`, 1.6450791395865032`}, {6, 0.0003269647187289486`, 0.5819971993375286`},

{9, 0.00011658885292733067`, 0.20752815821064857`}, {12, 7.760121346454478`*^-6, 0.01381301599668897`}},
0.00999596212264164`, 0.013099860496036713`, 0.8409700944203435`, 1795.5123396178271`}, {"TBUR", 72.13008692410035`,
162.4475884269784`, {{0, 0.014707864064423587`, 26.179998034673986`}, {8, 0.00920292217341309`, 16.3812014686753`},
{16, 0.009187803000214252`, 16.354289340381364`}, {24, 0.010010559104224439`, 17.818795205519503`},
{32, 0.010873188218360863`, 19.354275028682338`}, {40, 0.011638807891306687`, 20.717078046525902`},
{48, 0.012360420492486717`, 22.001548476626354`}, {56, 0.013074030721287143`, 23.27177468389111`},
{64, 0.013785129264545318`, 24.537530090890662`}, {72, 2.5894563816588487`, 4609.23235935275`}},
9.121158768363455`*^6, 0.09049147742145154`, 0.8381064474886702`, 2061.9714674436173`},
{"TBUR", 20.149096245622005`, 230.23029746687965`, {{0, 0.0798446846008301`, 142.12353858947756`}},
4.694826441411909`*^7, 0.04354850958293336`, 0.5859044687563336`, 2165.8435109464326`},
{"TBUR", 29.984644331768664`, 229.89679002615227`, {{0, 0.09516586303710936`, 169.39523620605468`}},
8.008595668207758`*^6, 0.10007397939809905`, 0.6192493979722495`, 2299.003777786342`}, {"CURE", 365, 2.6656367888221673`,
{{0, 0.005883296657683187`, 10.472268050676073`}, {69, 0.003063673651193004`, 5.4533390991235455`}},
0.009772451441397618`, 0.022510239050389284`, 0.17864522555063259`, 5.039825634511669`*^9}, {"CURE", 365,
1.8735610140071741`, {{0, 0.005710426812620591`, 10.164559726464653`}, {42, 0.0016090132964287885`, 2.8640436676432435`}},
0.009999922650717549`, 0.03909709899198647`, 0.3798682151034777`, 1783.5234609413226`}, {"CURE", 365, 0.23706564595999383`,
{{0, 0.0013926793568013018`, 2.478969255106317`}, {44, 0.0005816993764652762`, 1.0354248901081917`}},
0.009758105244591923`, 0.03368270755007892`, 0.09272604711021754`, 1781.7681258417044`}, {"CURE", 365,
2.7488977844609477`, {{0, 0.010151762434587507`, 18.070137133565762`}, {4, 0.0012629821591135814`, 2.2481082432221746`},
{8, 0.00008161133482738616`, 0.14526817599274736`}, {12, 0.000021230263472805183`, 0.03778986898159323`},
{16, 0.000018807386859258388`, 0.03347714860947993`}, {20, 0.000010265230375622292`, 0.018272110068608797`}},
0.009999992458332291`, 0.022002833481671355`, 0.9459509686752333`, 2042.2170490929223`},
{"CURE", 365, 0.19898590736770677`, {{0, 0.008039998608685078`, 14.31119752345944`}}, 0.00999940173598099`,
0.017676095978983817`, 0.19800825257113747`, 1780.1860515544727`}, {"CURE", 365, 0.6709830796191399`,
{{0, 0.00262053343200524`, 4.664549508969327`}, {27, 0.00038285661406463415`, 0.6814847730350487`}},
0.009999794819035516`, 0.026012499567645612`, 0.47588023775387456`, 1792.543466516886`}, {"CURE", 365,
0.7584070203143282`, {{0, 0.0006402206460263543`, 1.1395927499269105`}, {3, 0.00034601866063501953`, 0.6159132159303347`},
{6, 0.00011753106708965125`, 0.20920529941957922`}, {9, 0.00004017832682983254`, 0.07151742175710193`},
{12, 0.0000175876161654072`, 0.03130595677442482`}, {15, 0.000011459727115112691`, 0.020398314264900592`},
{18, 9.949987984325547`*^-6, 0.017710978612099477`}, {21, 0.000027416383945254228`, 0.04880116342255252`}},
0.009999997064052094`, 0.01960099872716057`, 0.9459058724683191`, 2211.363619115534`}, {"CURE", 365,

```
14.358161705730723`, {{0, 0.043832572029786666`, 78.02197821302026`}, {11, 0.00829931646354562`, 14.772783305111204`}},
  0.009998564778473185`, 0.014008724412892035`, 0.8743370421844568`, 2061.7860513678093`},
{"TBUR", 22.189838712331376`, 230.1166373568551`, {{0, 0.0676086902618408`, 120.34346866607663`}}, 7.348654585309644`*^7,
  0.1846632817993948`, 0.5431195466485371`, 2194.5542546779534`}, {"CURE", 365, 0.6191676646276854`,
  {{0, 0.0008657879514184848`, 1.541102553524903`}, {26, 0.00039345976123614874`, 0.7003583750003447`}},
  0.00963241961274836`, 0.04346772718819333`, 0.6633953456606182`, 2090.987042377581`}, {"CURE", 365, 0.5959646577524085`,
  {{0, 0.007518002011091198`, 13.382043579742332`}, {6, 0.0008093308410764037`, 1.4406088971159987`}},
  0.009964532013955833`, 0.012002186701664214`, 0.45094274288839753`, 1780.4854119770052`},
{"CURE", 365, 1.6454161729404486`, {{0, 0.005498963137868487`, 9.788154385405907`},
    {6, 0.0012303670168746605`, 2.190053290036896`}, {12, 0.0000648446550988288`, 0.11542348607591527`}},
  0.009999945123058256`, 0.03312877846971578`, 0.23424083794688655`, 1781.8839985719198`}, {"CURE", 365,
  0.258993702595483`, {{0, 0.0015245264031281752`, 2.7136569975681515`}, {36, 0.0004975402280831075`, 0.8856216059879313`}},
  0.009748262795071431`, 0.026983659114461475`, 0.20009809353451444`, 1783.5578679115454`},
{"CURE", 365, 3.6632448163427918`, {{0, 0.007328255703589278`, 13.044295152388914`}}, 0.009999877530354548`,
  0.022769470136192665`, 0.3411990832736804`, 1780.6355298293226`}, {"CURE", 365, 5.3610625298216`,
  {{0, 0.03298343511194064`, 58.71051449925435`}, {14, 0.006423211761204773`, 11.433316934944495`}},
  0.00999976413928141`, 0.015320090212514426`, 0.8868297937228057`, 1913.2369609135515`},
{"TBUR", 23.95602741735963`, 230.18973084006973`, {{0, 0.06824228286743166`, 121.47126350402836`}},
  6.170492770719757`*^6, 0.01483934171123977`, 0.69127969960146`, 2306.679392265782`}, {"CURE", 365,
  1.407719831744281`, {{0, 0.004491774644868488`, 7.9953588678659075`}, {7, 0.001154770654809748`, 2.055491765561351`},
    {14, 0.00010733361400390818`, 0.19105383292695655`}, {21, 9.48792965074156`*^-6, 0.016888514778319975`}},
  0.009999950114412196`, 0.03566457733138875`, 0.3408494830175806`, 1840.9765055926562`}, {"TBUR", 34.238070137602506`,
  228.90796548333864`, {{0, 0.03133410905838044`, 55.77471412391718`}, {3, 0.015693325917600934`, 27.93412013332966`},
    {6, 0.014386467895325444`, 25.60791285367929`}, {9, 0.014172545696825484`, 25.227131340349363`},
    {12, 0.014547904099677303`, 25.8952692974256`}, {15, 0.015234455829310097`, 27.117331376171975`},
    {18, 0.01606609934639256`, 28.59765683657876`}, {21, 0.016951771683875162`, 30.17415359729779`},
    {24, 0.01785105188436268`, 31.774872354165566`}, {27, 0.1362601290434926`, 242.54302969741684`}},
  6.219700978123265`*^7, 0.30322183088200305`, 0.6442402469972331`, 1874.4914297237196`},
{"CURE", 365, 0.4918148177546451`, {{0, 0.0015943566149625513`, 2.837954774633341`}}, 0.009999827847038293`,
  0.025466885952154787`, 0.09949686604563819`, 1780.603174390758`}, {"CURE", 365, 1.0649567441911656`,
  {{0, 0.002992647095670581`, 5.326911830293634`}, {26, 0.0012381444417255006`, 2.2038971062713912`}},
```

0.009755013068956434`, 0.008853214610197377`, 0.20630950934063394`, 1781.6085192781466`}, {"CURE", 365,
0.43941124934323034`, {{0, 0.00809901594533203`, 14.416248382691014`}, {7, 0.0007923714927329932`, 1.410421257064728`}},
0.009969461301297363`, 0.01697770091358036`, 0.5643872235496327`, 1781.0893938925867`}, {"CURE", 365,
22.72631248585618`, {{0, 0.05134308966980217`, 91.39069961224787`}, {43, 0.0011096321101495697`, 1.9751451560662343`},
{86, 0.0011141410523395315`, 1.9831710731643661`}, {129, 0.0011120986134426584`, 1.979535531927932`},
{172, 0.0011095281692678916`, 1.9749601412968474`}, {215, 0.0011093165646480678`, 1.9745834850735609`},
{258, 0.00110929348985853 7`, 1.974542411948196`}, {301, 0.0010807145011426363`, 1.9236718120338925`}},
0.00999756737737057`, 0.052896024525920335`, 0.8703739593036496`, 2306.493828399106`},
{"TBUR", 12.523971701171433`, 230.00462219777356`, {{0, 0.06301455974578857`, 112.16591634750365`}},
5.398273141094878`*^7, 0.19436084611386184`, 0.6841292600347594`, 2147.856661405851`}, {"CURE", 365,
0.8040479535450753`, {{0, 0.005438215495939791 6`, 9.68002358277283`}, {4, 0.0013997250115395027`, 2.4915105205403147`},
{8, 0.0002929887872215583`, 0.5215200412543738`}, {12, 0.000015710232310295523`, 0.02796421351232603`}},
0.009999995659359042`, 0.011630165107294511`, 0.9194289027978957`, 1823.844034982128`}, {"CURE", 365,
0.3912072331212439`, {{0, 0.000797618432694015`, 1.4197608101953467`}, {6, 0.00030551189861908323`, 0.5438111795419681`},
{12, 0.000055398210964647804`, 0.0986088155170731`}, {18, 0.000016307053528962192`, 0.029026555281552704`}},
0.00999995614758029`, 0.02449158888001225`, 0.3391351976046906`, 1920.6754840396193`}, {"CURE", 365,
0.9887235012615642`, {{0, 0.0023286849422660045`, 4.145059197233488`}, {9, 0.0007742962626064108`, 1.3782473474394115`},
{18, 0.00013809989360681702`, 0.2458178106201343`}, {27, 0.00001609226975854247`, 0.028644240170205598`}},
0.009999991192368402`, 0.02824233473228301`, 0.2149057231336129`, 1788.0033750875277`}, {"CURE", 365,
0.4386532307201965`, {{0, 0.002710907177122318`, 4.825414775277726`}, {41, 0.0007525642560184463`, 1.3395643757128346`}},
0.009747587892892089`, 0.037198776916083434`, 0.17403278126898336`, 1782.714532873887`}, {"CURE", 365, 1.0292059994535716`,
{{0, 0.007160847899298300 5`, 12.746309260750975`}, {37, 0.0007792170763751984`, 1.3870063959478534`}},
0.00978554492577612`, 0.026401823946180437`, 0.5496217458000259`, 1796.8526773157346`}, {"CURE", 365, 30.694931868746924`,
{{0, 0.010954776227929251`, 19.499501685714073`}, {57, 0.00030353420066861047`, 0.5402908771901267`},
{114, 0.0002356579586453065`, 0.4194711663886456`}, {171, 0.00023488478238432392`, 0.41809491264409654`},
{228, 0.00023486779187318854`, 0.4180646695342756`}, {285, 0.00009848658427417812`, 0.17530612000803705`}},
0.009983865003309179`, 0.007891950922631724`, 0.8898735961784954`, 2362.8530949980336`}, {"CURE", 365,
3.675758391057579`, {{0, 0.04002523974725114`, 71.24492675010704`}, {3, 0.0009242165839587945`, 1.6451055194466542`}},
0.009991834154785524`, 0.01750001356456878`, 0.7597727125912849`, 1780.5532037859502`},
{"TBUR", 22.096644934161382`, 230.0947773526568`, {{0, 0.062357735633850095`, 110.99676942825317`}},
8.411462154648635`*^7, 0.18424775174309282`, 0.5820169140718918`, 2234.974396866577`},

{"CURE", 365, 13.602918170928321`, {{0, 0.013186734834077929`, 23.47238800465871`},
{21, 0.00013515146210750083`, 0.24056960255135146`}, {42, 0.00013535702434077406`, 0.24093550332657782`},
{63, 0.0001350854984840202`, 0.2404521873015559`}, {84, 0.00009281615401472768`, 0.16521275414621525`}},
0.009963608467453575`, 0.027374211070872616`, 0.882632727272723`, 2341.4736557240735`}, {"TBUR", 82.82733104925046`,
228.75573457861995`, {{0, 0.011303718270332023`, 20.120618521191002`}, {5, 0.0029468204019667543`, 5.2453403155008225`},
{10, 0.0012247698016205732`, 2.18009024688462`}, {15, 0.0006425749540027806`, 1.143783418124949`},
{20, 0.00041349316121182126`, 0.7360178269570418`}, {25, 0.00031434288606588663`, 0.5595303371972782`},
{30, 0.0002677603534522039`, 0.4766134291449229`}, {35, 0.00024383048205599042`, 0.43401825805966293`},
{40, 0.0002303390771561127`, 0.41000355733788063`}, {45, 0.05772645354492437`, 102.75308730996538`}},
722653.8280532808`, 0.012819203809511803`, 0.7576650383948679`, 2373.108223711211`}, {"CURE", 365, 191.7499890296858 5`,
{{0, 0.07469675943089307`, 132.96023178698965`}, {26, 0.0046819422815707785`, 8.333857261195986`}},
0.009993036868633206`, 0.01176384152848869`, 0.9999739408043614`, 2464.7995921485494`},
{"TBUR", 30.113952690600364`, 229.9987633451909`, {{0, 0.05390263557434082`, 95.94669132232667`}},
6.385312141663634`*^7, 0.10686878653247375`, 0.5694520919968891`, 2379.0483938951384`},
{"CURE", 365, 0.34058352131272396`, {{0, 0.0018468931093662206`, 3.2874697346718724`}},
0.009998844298041517`, 0.024025353386486217`, 0.03575931019900817`, 1780.1838960527125`},
{"TBUR", 13.420966537628798`, 229.47856882033847`, {{0, 0.12212809562683109`, 217.38801021575932`}},
9.368280708056207`*^7, 0.13257081835626208`, 0.521154229805927`, 1993.2919947944054`}, {"TBUR", 134.7915227994572`,
229.53278769941275`, {{0, 0.0030214022619649396`, 5.378096026297593`}, {10, 0.0016472402572258159`, 2.932087657861952`},
{20, 0.0014099625540889608`, 2.50973334627835`}, {30, 0.0012327708281690746`, 2.194332074140952`},
{40, 0.0010837512681670359`, 1.9290772573373238`}, {50, 0.0009569255512916432`, 1.7033274812991248`},
{60, 0.0008488223820174923`, 1.5109038399911363`}, {70, 0.0007566312839542233`, 1.3468036854385175`},
{80, 0.0006779826302615332`, 1.2068090818655293`}, {90, 0.11349599855854363`, 202.0228774342077`}},
2.399620409959235`*^7, 0.19105164285375947`, 0.5459508952175889`, 2207.214763171631`}, {"TBUR", 193.9591198893562`,
229.36903782970256`, {{0, 0.04996198626132382`, 88.93233554515639`}, {17, 0.003271353700108422`, 5.823009586192992`},
{34, 0.00327118604055733 5`, 5.822711152192056`}, {51, 0.003271180459273232`, 5.822701217506353`},
{68, 0.003271180542029397`, 5.822701364812326`}, {85, 0.003271181993745757`, 5.8227039488674475`},
{102, 0.0032711842098830544`, 5.822707893591837`}, {119, 0.0032711871074530083`, 5.822713051266356`},
{136, 0.00327119075834845`, 5.822719549860241`}, {153, 0.22168800839066957`, 394.60465493539186`}},
29.122431344947884`, 0.034436900719935444`, 0.7281827904016829`, 2746.9486811678494`}, {"CURE", 365,
1.3492548809684315`, {{0, 0.015388386416181118`, 27.391327820802392`}, {3, 0.0037391959495610308`, 6.6557687902186355`},

```
    {6, 0.0006085012339640471`, 1.083132196456004`}, {9, 0.0001046703777537917`, 0.1863132724017492 5`}},
  0.009994613131610246`, 0.017399173042370143`, 0.9403093653051673`, 1783.5908469612168`},
 {"CURE", 365, 0.23748082266088094`, {{0, 0.0006354276652796127`, 1.1310612441977106`}},
  0.009999896601357848`, 0.012920123683266992`, 0.11558758046155605`, 1781.5457429331136`},
 {"TBUR", 13.790174254331742`, 230.17233629387425`, {{0, 0.10287398338317873`, 183.11569042205812`}},
  7.877832682736377`*^7, 0.12856650273881612`, 0.5176572471432562`, 2024.4318066143278`}, {"TBUR", 68.08383457654784`,
  229.8471105142363`, {{0, 0.046438679341417544`, 82.66084922772323`}, {3, 0.008635625275900066`, 15.371412991102117`},
    {6, 0.003519494409045170 7`, 6.264700048100403`}, {9, 0.001771794485501084`, 3.1537941841919297`},
    {12, 0.0010844759646673 1`, 1.9303672171078115`}, {15, 0.0007808463425595391`, 1.3899064897559794`},
    {18, 0.0006323922626570956`, 1.1256582275296303`}, {21, 0.0005524465583517095`, 0.983354873866043`},
    {24, 0.0005044915553541964`, 0.8979949685304695`}, {27, 0.036228985253654346`, 64.48759375150473`}},
  339493.3969616597`, 0.029445799256621445`, 0.7076687845362059`, 2251.227116374861`},
 {"TBUR", 27.92779041366026`, 230.17462612516275`, {{0, 0.05502836227416993`, 97.95048484802246`}},
  3.9433622751290895`*^7, 0.050370933613543514`, 0.6467091705318018`, 2413.435560454463`},
 {"CURE", 365, 1.2099304822271597`, {{0, 0.0033930524742646715`, 6.039633404191115`},
    {3, 0.001384104347927493`, 2.463705739310937`}, {6, 0.00027469607536898693`, 0.4889590141567968`},
    {9, 0.000051535858475628476`, 0.09173382808661867`}, {12, 0.000013282428427951389`, 0.02364272260175347`}},
  0.009999934106196703`, 0.01714393059426183`, 0.6863474606117442`, 1992.1203234932902`}, {"CURE", 365,
  0.19046188783224563`, {{0, 0.001281328357560166`, 2.2807644764570956`}, {32, 0.000503342861604978`, 0.8959502936568609`}},
  0.00973799546570727`, 0.012392055326593848`, 0.1442585229992739`, 1781.9792210922994`},
 {"CURE", 365, 3.2804814705333047`, {{0, 0.015159367329571447`, 26.983673846637174`},
    {6, 0.002323124845740629`, 4.135162225418319`}, {12, 0.0002741062994289688 5`, 0.48790921298356454`},
    {18, 0.000050818557471057015`, 0.0904570322984815`}, {24, 0.000029232261385200777`, 0.05203342526565738`},
    {30, 0.000027623367983032127`, 0.04916959500979719`}, {36, 3.4477792655412328`*^-6, 0.006137047092663394`}},
  0.009999997854294144`, 0.03813323869578901`, 0.9797613917192697`, 2067.979961980297`},
 {"CURE", 365, 4.2505364419913825`, {{0, 0.004021137426731079`, 7.157624619581321`}}, 0.009999872753878054`,
  0.008293766592439276`, 0.10601463697326226`, 1780.2232679167726`}, {"CURE", 365, 1.417245531564513`,
  {{0, 0.004861278282517745 4`, 8.653075342881586`}, {5, 0.0003099686371125429 4`, 0.5517441740603265`}},
  0.009999475195589674`, 0.0179882258592361`, 0.7497400809990531`, 1783.782641316472`}, {"TBUR", 55.787712294614686`,
  191.95756498545813`, {{0, 0.04918077355949568`, 87.54177693590232`}, {6, 0.014767376541570069`, 26.28593024399472`},
    {12, 0.010991577050898515`, 19.565007150599357`}, {18, 0.010800994324082725`, 19.22576989686725`},
```

```
{24, 0.01240001927300131`, 22.072034305942335`}, {30, 0.01527450590684335`, 27.18862051418116`},
  {36, 0.019017357309548552`, 33.85089601099642`}, {42, 0.023154411040239315`, 41.21485165162597`},
  {48, 0.027335786794164887`, 48.65770049361349`}, {54, 9.914336739013605`, 17647.519395444215`}},
 1.0472180012081653`*^7, 0.14281279200985594`, 0.7265578871073073`, 2061.309446469213`}, {"CURE", 365,
 14.438929888420962`, {{0, 0.012669339222792423`, 22.551423816570512`}, {7, 0.0030769821293362043`, 5.477028190218444`},
  {14, 0.00019103599779952252`, 0.3400440760831501`}, {21, 0.0000251366153587609`, 0.0447431753385944`}},
 0.009962555543844641`, 0.06681612997118798`, 0.7227914400591218`, 2007.8093558993794`}, {"TBUR", 200.37177518713025`,
 229.64400363383493`, {{0, 0.06942741146452247`, 123.58079240685001`}, {16, 0.021028526942020644`, 37.43077956796746`},
  {32, 0.008131334301119734`, 14.473775055993128`}, {48, 0.0034712834052209505`, 6.178884461293292`},
  {64, 0.0014744936596087347`, 2.6245987141035476`}, {80, 0.0006405604895361905`, 1.1401976713744193`},
  {96, 0.0002932846244871928`, 0.5220466315872031`}, {112, 0.00014823449432322265`, 0.2638573998953363`},
  {128, 0.00008721209459706894`, 0.1552375283827827`}, {144, 0.024712961070737227`, 43.98907070591226`}},
 6704.9589023714325`, 0.02866480367832167`, 0.1642610093443026`, 1780.0128860912891`},
{"TBUR", 23.008263952481347`, 229.77602265421322`, {{0, 0.07734115600585939`, 137.66725769042972`}},
 2.935323250280968`*^7, 0.04543454452455778`, 0.6317289288440249`, 2206.6334021920156`}, {"CURE", 365, 0.19231580078881752`,
 {{0, 0.0008204519757556778`, 1.4604045168451065`}, {7, 0.0001015261498539193`, 0.18071654673997636`}},
 0.00999979561610195`, 0.018267360076361972`, 0.3660625796003572`, 1783.7171624853888`},
{"TBUR", 20.468973277961815`, 229.42724737443947`, {{0, 0.1394365692138672`, 248.19709320068364`}},
 4.0328854346616745`*^7, 0.11565479858827202`, 0.4840346131237827`, 2013.555631162851`},
{"CURE", 365, 0.2905464098798346`, {{0, 0.0011448642538847125`, 2.0378583719147882`}},
 0.009999884934634887`, 0.01605163067815484`, 0.0358834958859133`, -3.218950891846049`*^8},
{"TBUR", 20.441475919222622`, 230.14708162140283`, {{0, 0.0981406307220459`, 174.6903226852417`}},
 2.979677734696839`*^7, 0.06825121192969062`, 0.4913201373204419`, 2077.9166473062114`}, {"CURE", 365,
 1.310250039237746`, {{0, 0.0028881852707802196`, 5.140969781988791`}, {5, 0.0018654308387871625`, 3.3204668930411496`},
  {10, 0.0009687228157811465`, 1.724326612090441`}, {15, 0.00017852713295943312`, 0.31777829666779095`}},
 0.00997490607798071`, 0.009921286532222457`, 0.3398271825824106`, 1780.9698954479393`}, {"TBUR", 58.71194677141193`,
 228.9602415854664`, {{0, 0.0251436845383972`, 44.75575847834701`}, {3, 0.005826464041932692`, 10.371105994640192`},
  {6, 0.0026525789957100167`, 4.72159061236383`}, {9, 0.0013552595955140803`, 2.4123620800150625`},
  {12, 0.0007940640084414896`, 1.4134339350258516`}, {15, 0.0005396960254721605`, 0.9606589253404457`},
  {18, 0.0004184169946625629 4`, 0.744782250499362`}, {21, 0.0003574566213234996`, 0.6362727859558294`},
  {24, 0.0003254469626309157`, 0.5792955934830298`}, {27, 0.057415895402471384`, 102.20029381639907`}},
```

```
2.069066051291831`*^6, 0.050943864048794016`, 0.8157581632973171`, 2372.0021710324822`}, {"CURE", 365,
 5.874946438390239`, {{0, 0.02167604544643477`, 38.583360894653886`}, {15, 0.004594662986954339`, 8.178500116778723`}},
 0.009999756247953722`, 0.019671689604929952`, 0.8653504053530461`, 1929.1546645209066`}, {"CURE", 365,
 0.13440653597145336`, {{0, 0.0008121092028287607`, 1.445554381035194`}, {40, 0.000375201335353466`, 0.6678583769291695`}},
 0.009997744570779976`, 0.02562324180175685`, 0.21463693607350096`, 1785.3496199583421`}, {"CURE", 365,
 0.6347098626695713`, {{0, 0.0025670172033936765`, 4.569290622040745`}, {25, 0.001782467772333666`, 3.1727926347539257`}},
 0.009997361999350612`, 0.007661981333570263`, 0.27852613399100534`, 1781.3408906822026`},
{"TBUR", 56.41388064186295`, 230.2600323162284`, {{0, 0.06913362503051758`, 123.0578525543213`}},
 458742.1010706369`, 0.004274648180566665`, 0.63937961239124`, 2400.7332464263814`},
{"CURE", 365, 7.8685074120583325`, {{0, 0.03233152378118332`, 57.55011233050631`}, {3, 0.004661555957664181`, 8.297569604642243`},
  {6, 0.0005113797120467348`, 0.9102558874431878`}, {9, 0.00007433472417652274`, 0.13231580903421047`},
  {12, 0.00003503712612986061`, 0.06236608451115189`}, {15, 0.000032128393322824554`, 0.057188540114627706`},
  {18, 0.00003194825669590047`, 0.05686789691870284`}, {21, 0.000031937538660918824`, 0.0568488188164355`},
  {24, 0.000031936810118059286`, 0.05684752201014553`}, {27, 0.000019293823455753885`, 0.03434300575124191`}},
 0.009999989414198576`, 0.0386415521951121`, 0.9897396494483801`, 2006.5805159931967`},
{"CURE", 365, 0.15515021107449212`, {{0, 0.001232465768674373`, 2.193789068240384`}}, 0.009576635352906671`,
 0.01723346385846862`, 0.3276904923669384`, 1782.3684812133881`}, {"CURE", 365, 0.08183169479849337`,
 {{0, 0.000678499636256515`, 1.2077293525365966`}, {3, 0.0002566047167795973`, 0.4567563958676832`}},
 0.009999943190344675`, 0.015150336037116484`, 0.6571301225672205`, 1790.3575528233453`},
{"TBUR", 48.52543356042437`, 230.26249641629488`, {{0, 0.036941065788269056`, 65.75509710311893`}},
 3.178863308772511`*^7, 0.09858884099447884`, 0.5813391988621223`, 2853.1739973695203`},
{"TBUR", 34.10461872434427`, 230.1391036529168`, {{0, 0.08932006835937502`, 158.98972167968753`}},
 5.897120547502396`*^7, 0.0970371002571751`, 0.4708119248440114`, 2164.872251647911`}, {"TBUR", 41.49257971833915`,
 229.36747248912536`, {{0, 0.05397993644980394`, 96.08428688065102`}, {3, 0.02543094277941889`, 45.267078147365616`},
  {6, 0.01853048888834956`, 32.984270221262214`}, {9, 0.013633517224681318`, 24.267660659932748`},
  {12, 0.010097411454449434`, 17.97339238891999`}, {15, 0.007530041172763439`, 13.403473287518922`},
  {18, 0.005658004499842187`, 10.071248009719092`}, {21, 0.004287855753396232`, 7.632383241045294`},
  {24, 0.00328186857491323`, 5.841726063345549`}, {27, 0.06923389346215618`, 123.236330362638`}},
 9.165523587051028`*^6, 0.08731269834839163`, 0.7100311580818666`, 1876.227731779159`},
{"CURE", 365, 0.6594122709751479`, {{0, 0.0011526386403235816`, 2.0516967797759755`}},
 0.009999965804059296`, 0.005571718591067788`, 0.2773646052516447`, 1782.6703321175148`},
```

```
{"CURE", 365, 2.627471026346351`, {{0, 0.007879750978269573`, 14.025956741319838`},
    {7, 0.0013187384300306837`, 2.347354405454617`}, {14, 0.00012174469616236494`, 0.21670555916900963`},
    {21, 0.000017911888766929188`, 0.031883162005133955`}, {28, 7.848600919575249`*^-6, 0.013970509636843943`}},
  0.009999986040746481`, 0.028303025000716003`, 0.8946703268570718`, 2011.989573469426`}, {"CURE", 365,
  1.268084386470455`, {{0, 0.019203614426943162`, 34.18243367995883`}, {5, 0.001990933318053979`, 3.543861306136083`}},
  0.00996259123797137`, 0.018162840342623724`, 0.7883946200149696`, 1781.4242283019985`},
{"CURE", 365, 1.002884840452645`, {{0, 0.005488024754699122`, 9.768684063364438`}},
  0.009999919759430072`, 0.015329268034230265`, 0.13210165761894574`, 1780.2206672779346`},
{"CURE", 365, 7.968418834012323`, {{0, 0.031088817315007337`, 55.33809482071306`},
    {2, 0.004394321529393307 5`, 7.821892322320086`}, {4, 0.0003342938652267579`, 0.595043080103629`},
    {6, 0.000042999067866157`, 0.07653983408017595`}, {8, 0.000025680763923600 99`, 0.04571175978400 9764`},
    {10, 0.000024806821264443227`, 0.04415614185070894 4`}, {12, 7.770815478485108`*^-6, 0.013832051551703493`}},
  0.0099999647419318`, 0.015793909536423876`, 0.9719167907643134`, 1982.4313216331755`},
{"CURE", 365, 0.7937102497622507`, {{0, 0.002889945651877305`, 5.144103260341603`}}, 0.009999234864769318`,
  0.012160150105609495`, 0.07600673087484831`, 1780.222293807568`}, {"CURE", 365, 24.570072153460202`,
  {{0, 0.03720669841011636`, 66.22792317000713`}, {14, 0.0010358375428599167`, 1.8437908262906513`},
    {28, 0.00007886487467289201`, 0.14037947691774777`}, {42, 0.00005762296441210828`, 0.10256887665355273`},
    {56, 0.00005520411527239711`, 0.09826332518486687`}, {70, 0.00005477006245136794`, 0.09750307111634349`},
    {84, 0.00005472657809070396`, 0.09741330900145304`}, {98, 0.00005472134430193981 6`, 0.09740399285745288`},
    {112, 0.00005472078051486128`, 0.09740298931645307`}, {126, 0.00002656881722297795`, 0.047292494656900746`}},
  0.009999989101796357`, 0.056685702927047596`, 0.8773216977352912`, 2039.488701238278`}, {"CURE", 365,
  0.7834102060883597`, {{0, 0.0005423859994070884`, 0.9654470789446175`}, {23, 0.00031355966427286 2`, 0.5581362024056944`}},
  0.009999528117563532`, 0.05185253577424495`, 0.26946112026228725`, 1789.0462627957497`}, {"CURE", 365,
  2.7911316319048733`, {{0, 0.005278149556190699`, 9.395106210019444`}, {8, 0.0007694244676976506`, 1.369575552501818`},
    {16, 0.000035164767001393216`, 0.06259328526247991`}, {24, 2.409407597059366`*^-6, 0.004288745522765672`}},
  0.009999986387567937`, 0.03738027619447775`, 0.33994916759215876`, 1889.6415432656615`}, {"CURE", 365,
  0.11134587183308992`, {{0, 0.0005743081570265141`, 1.0222685195071952`}, {18, 0.0001773672982303913`, 0.3157137908500966`}},
  0.009999695988748168`, 0.03861987659565844`, 0.03264161875006072`, 1780.5912053529635`}, {"CURE", 365,
  0.25817296790538785`, {{0, 0.002214404437422049`, 3.941639898611248`}, {7, 0.00011525564706677164`, 0.20515505177885354`}},
  0.009999933158460718`, 0.013750211004577989`, 0.40194805733809735`, 1781.8433211605661`},
{"TBUR", 61.63206579147233`, 229.7850106925153`, {{0, 0.0889251011085510 5`, 158.28667997322086`}},
```

2.1344247301621333`*^7, 0.016521841091451137`, 0.36770782854737577`, 2193.2526185978063`},
{"CURE", 365, 0.7149505943063293`, {{0, 0.028338399576150707`, 50.44235124554826`}}, 0.009616137735756832`,
0.01478494531826042`, 0.5328292857240242`, 1780.2209582739388`}, {"CURE", 365, 26.791704377833856`,
{{0, 0.02186353438300982`, 38.91709120175748`}, {27, 0.00013068830534873995`, 0.2326251835207571`},
{54, 0.00014889421882786037`, 0.26503170951359145`}, {81, 0.00012739949861045158`, 0.22677110752660382`},
{108, 0.0001260857084485755`, 0.22443256103846443`}, {135, 0.00012587550123225601`, 0.2240583921934157`},
{162, 0.0001258566726737801`, 0.22402487735932858`}, {189, 0.00012521321020970305`, 0.22287951417327143`}},
0.009999986794582966`, 0.03578716243531738`, 0.9062848139676041`, 2173.1139194896605`}, {"CURE", 365,
1.7957006281142072`, {{0, 0.0030227043084195947`, 5.380413668986878`}, {8, 0.00037670370575696`, 0.6705325962473887`}},
0.009999946721314405`, 0.030435234748552767`, 0.8078251330894958`, 2349.399314790712`}, {"TBUR", 258.77343224176514`,
229.05556006669283`, {{0, 0.023709494376988043`, 42.20289999103871`}, {20, 0.0006367426550514127`, 1.1334019259915147`},
{40, 0.0006855242138426032`, 1.2202331006398337`}, {60, 0.0018395641886163152`, 3.274424255737041`},
{80, 0.005618750353206335`, 10.001375628707278`}, {100, 0.004453377115761492`, 7.927011266055455`},
{120, 0.0044086806229492745`, 7.8474515088497085`}, {140, 0.005119913988476543`, 9.113446899488247`},
{160, 0.005453513343850435`, 9.707253752053775`}, {180, 1.7193717297641946`, 3060.4816789802667`}},
16075.9639675044`, 0.08300203269006902`, 0.7620473948878758`, 3453.7804727390117`},
{"TBUR", 24.008987784981617`, 230.24386979551662`, {{0, 0.06453819274902345`, 114.87798309326175`}},
1.378327695847128`*^7, 0.0680849456700261`, 0.6106270186242605`, 2277.536334728725`}, {"CURE", 365,
1.6896160221995922`, {{0, 0.003596510535138539`, 6.4017887525466`}, {10, 0.000586202018491214`, 1.043439592914361`}},
0.009999991947622605`, 0.02235189154680646`, 0.8364508961121792`, 1791.8181483952428`}, {"TBUR", 81.81112103708753`,
229.0844229329865`, {{0, 0.023787364561494353`, 42.341508919459955`}, {7, 0.011293646994874658`, 20.10269165087689`},
{14, 0.011000731960508233`, 19.581302889704656`}, {21, 0.011195032363140029`, 19.92715760638925`},
{28, 0.011414588052293855`, 20.317966733083065`}, {35, 0.011608005121340182`, 20.662249115985528`},
{42, 0.011788098302164074`, 20.98281497785205`}, {49, 0.011962965293325654`, 21.294078222119666`},
{56, 0.012134704074934504`, 21.599773253383418`}, {63, 0.8518269922671444`, 1516.2520462355171`}},
2.6026708788657483`*^7, 0.30854169552814836`, 0.5999086936107884`, 1957.3067903976746`}, {"CURE", 365,
0.3374728057570491`, {{0, 0.0019383510927112586`, 3.4502649450260403`}, {34, 0.0006140108392358904`, 1.092939293839885`}},
0.009749275276505563`, 0.019188108572905167`, 0.16306000240488858`, 1781.6922257531612`}, {"TBUR", 49.41064067227592`,
228.84360323867543`, {{0, 0.06860048553567122`, 122.10886425349477`}, {2, 0.016007539685984503`, 28.493420641052417`},
{4, 0.007825252724237784`, 13.928949849143255`}, {6, 0.004086926549769404`, 7.274729258589539`},
{8, 0.0022711490000238746`, 4.0426452200424965`}, {10, 0.0013458807816141979`, 2.395667791273272`},

```
{12, 0.0008540352549003795`, 1.5201827537226755`}, {14, 0.0005823868858070561`, 1.03664865673656`},
{16, 0.0004270357762214547`, 0.7601236816741894`}, {18, 0.04088447013384182`, 72.77435683823843`}},
2.5769516122620674`*^6, 0.05626037642909041`, 0.7457517595326102`, 2307.341653889572`},
{"CURE", 365, 6.577080780878027`, {{0, 0.01848042385239934`, 32.89515445727083`}, {1, 0.003525079887267345`, 6.274642199335874`},
{2, 0.0005742889902319206`, 1.0222344026128187`}, {3, 0.00008839818726747159`, 0.15734877333609942`}},
0.009999993423357355`, 0.007720941797173735`, 0.9530646873955705`, 1784.7206890694556`},
{"TBUR", 164.49086979225737`, 229.51517423349395`, {{0, 0.03529830355846217`, 62.83098033406266`},
{5, 0.002910280254448271`, 5.180298852917923`}, {10, 0.002893737884433627`, 5.150853434291855`},
{15, 0.002893467315759983`, 5.150371822052770`}, {20, 0.002893454468793314`, 5.150348954452101`},
{25, 0.002893453312459768`, 5.150346896178388`}, {30, 0.002893453156451445`, 5.150346618483572`},
{35, 0.00289345312861708`, 5.150346568938402`}, {40, 0.002893453121512149`, 5.150346556291626`}},
0.010273291594114744`, 0.016802733956558645`, 0.07931174419214934`, 1780.0224531990334`},
{"CURE", 365, 1.317171958587842`, {{0, 0.004687526904438497`, 8.343797889900525`}},
0.009999962754382816`, 0.005979750028874671`, 0.26058620243710817`, 1780.6684206326129`},
{"CURE", 365, 0.7016849040133043`, {{0, 0.008111174986720003`, 14.437891476361607`}}, 0.009998728605601567`,
0.018616459600116864`, 0.38005213295262424`, 1780.6419769337933`}, {"CURE", 365, 1.7366684724165085`,
{{0, 0.004735197215143071`, 8.428651042954666`}, {8, 0.0004863030370743587`, 0.8656194059923584`}},
0.00999987675355246`, 0.024723881964194085`, 0.8347791722077852`, 2537.9354644412006`},
{"CURE", 365, 0.17494325898743882`, {{0, 0.003050168604859626 6`, 5.429300116650135`}},
0.009997243771691242`, 0.02204336749960574`, 0.30123899746699273`, 1781.4010144017905`},
{"CURE", 365, 6.079018531258666`, {{0, 0.013904793853794882`, 24.75053305975489`}, {8, 0.003339914129563598`, 5.945047150623204`},
{16, 0.0002471210317930380 3`, 0.43987543659160766`}, {24, 0.00002400806549880885`, 0.04273435658787975`}},
0.009999971519752356`, 0.05412974387480806`, 0.6432693870771192`, 2153.881434629738`},
{"CURE", 365, 0.37020885364198247`, {{0, 0.012795674046096344`, 22.776299802051494`}}, 0.009693767648290426`,
0.0254539400642366 8`, 0.29293767318345576`, 1780.2160110398352`}, {"CURE", 365, 1.5630771815262927`,
{{0, 0.0022518296095206626`, 4.0082567049467785`}, {4, 0.0003852960616851482`, 0.6858269897995637`},
{8, 0.00004468285964076598 4`, 0.07953549016056345`}, {12, 0.00001929407258420723 2`, 0.03434344919988887`},
{16, 0.00001779850869040158`, 0.031681345468914816`}, {20, 0.00002640458270339898 7`, 0.0470001572120502`}},
0.009999989768465703`, 0.024871688320441877`, 0.9570683123602038`, 2104.1586739505774`}, {"TBUR", 341.4841856756524`,
229.23933820060924`, {{0, 0.017820689091049297`, 31.720826582067748`}, {34, 0.007113207114374583`, 12.661508663586755`},
{68, 0.00377729863109521`, 6.723591563349473`}, {102, 0.0022409766092961535`, 3.9889383645471526`},
```

```
    {136, 0.0014720829689434112`, 2.620307684719272`}, {170, 0.0010815534460017 63`, 1.9251651338831381`},
    {204, 0.0008767149013172967`, 1.5605525243447878`}, {238, 0.0007635110643328163`, 1.3590496945124129`},
    {272, 0.0006969205423635266`, 1.2405185654070772`}, {306, 0.41724456483307726`, 742.6953254028774`}},
  829785.4849291958`, 0.185009533798382`, 0.743685087078986`, 2423.697819388714`},
 {"CURE", 365, 1.355706814521042`, {{0, 0.0027299293587394716`, 4.85927425855626`},
    {3, 0.0006718627401268078`, 1.1959156774257178`}, {6, 0.00007427018283377204`, 0.13220092544411421`},
    {9, 0.000011541977347773 07`, 0.020544719679036063`}, {12, 0.000016898733328505735`, 0.03007974532474021`}},
  0.009999999127780657`, 0.0222259312697165 3`, 0.9486043304435292`, 2373.03614379878 8`},
 {"CURE", 365, 0.07240475580707215`, {{0, 0.0012042391925768947`, 2.143545762786873`}},
  0.00999966629092199`, 0.016760277880216508`, 0.1831294138587254`, 1781.513338952081`},
 {"TBUR", 18.8841149148648`, 227.70725327314202`, {{0, 0.07517555236816406`, 133.81248321533204`}},
  1.2341700274455292`*^7, 0.09518739644979725`, 0.5329798427071838`, 2128.1240388394813`},
 {"CURE", 365, 5.284214671371257`, {{0, 0.015081334494880127`, 26.84477540088663`},
    {34, 0.000031296485663479 85`, 0.05570774448099413`}, {68, 0.000026183390704933402`, 0.04660643545478146`}},
  0.009999981593967401`, 0.04818725962788048`, 0.7551508612444043`, 2395.4308294433354`}, {"CURE", 365,
  2.297387820172124`, {{0, 0.010738092607712394`, 19.11380484172806`}, {26, 0.001818690856410418`, 3.237269724410544`}},
  0.009999875619593528`, 0.029362935292641`, 0.45104772195126425`, 1780.7846953750666`},
 {"CURE", 365, 0.051917072380796195`, {{0, 0.0005041991896833952`, 0.8974745576364435`}},
  0.009996271438067188`, 0.02333077821190177`, 0.08562641575263048`, 1781.5202241329478`},
 {"CURE", 365, 6.020269543566309`, {{0, 0.004176029722701665`, 7.433332906408965`},
    {8, 0.0001349666055466363`, 0.2402405578730126`}, {16, 0.00004787345453639547`, 0.08521474907478394`},
    {24, 0.00004733289980469778`, 0.08425256165236204`}, {32, 0.00004732765257074106`, 0.08424322157591908`},
    {40, 0.00004732741053220338`, 0.08424279074732204`}, {48, 0.00006604666773697122`, 0.11756306857180877`}},
  0.009999984704272455`, 0.0347413859803547`, 0.9987774978572553`, 2441.4886616993203`}, {"CURE", 365,
  2.9162391905243124`, {{0, 0.012836537566148899`, 22.849036867745042`}, {2, 0.0025794230304602658`, 4.591372994219274`},
    {4, 0.0003569271602458058`, 0.6353303452375344`}, {6, 0.000056106260566478544`, 0.09986914380833181`},
    {8, 0.000020427230868765606`, 0.03636047094640278`}, {10, 0.00001671959767914007`, 0.029760883868869326`},
    {12, 0.000016392878266618338`, 0.02917932331458064`}, {14, 3.7242654913814303`*^-6, 0.006629192574658946`}},
  0.009999996261301549`, 0.02089974355248173`, 0.9820839847241207`, 1970.7878074380667`},
 {"CURE", 365, 0.10144177256721937`, {{0, 0.0019847374215682815`, 3.5328326103915413`}},
  0.009999997970373806`, 0.020383231393294014`, 0.3669817296472343`, 1781.7388036115567`},
```

```
{"CURE", 365, 1.0687823445244802`, {{0, 0.020304080757489334`, 36.141263748331006`},
    {5, 0.005294625066240246`, 9.424432617907637`}, {10, 0.0009926712515202613`, 1.7669548277060652`},
    {15, 0.00018912759131367596`, 0.3366471125383432`}, {20, 0.000035033383300991824`, 0.06235942227576543 6`}},
  0.0099966795289007`, 0.024944607725288153`, 0.9453966246861208`, 1792.2827997735644`}, {"CURE", 365,
  3.9763947844414353`, {{0, 0.033579640933371555`, 59.77176086140136`}, {2, 0.005739806340086514`, 10.216855285353995`},
    {4, 0.0006984176969133092`, 1.2431835005056902`}, {6, 0.00009090581965427589`, 0.1618123589846111`},
    {8, 0.000022290452089253188`, 0.03967700471887067`}, {10, 8.36386742081908`*^-6, 0.014887684009057961`}},
  0.009999997145162361`, 0.016838560876472845`, 0.9811303788724319`, 1856.1750182504302`},
{"CURE", 365, 0.9001329258657192`, {{0, 0.005258880856252013`, 9.360807924128585`},
    {3, 0.0017035524306541646`, 3.032323326564413`}, {6, 0.0004144506127333533`, 0.7377220906653689`},
    {9, 0.00010273279373261257`, 0.18286437284405035`}, {12, 5.5330404143133154`*^-6, 0.009848811937477702`}},
  0.00999999736181789`, 0.013557350682734113`, 0.9230264904937747`, 1812.6533446709811`}, {"TBUR", 358.94906053140267`,
  227.72102939994207`, {{0, 0.04503909123050394`, 80.16958239029701`}, {30, 0.0016182957007790358`, 2.880566347386684`},
    {60, 0.0016210719132527523`, 2.885508005589899`}, {90, 0.0016639778090198562`, 2.9618805000553445`},
    {120, 0.001901538203941076`, 3.3847380030151153`}, {150, 0.0018357373657143304`, 3.2676125109715084`},
    {180, 0.0017377120555645408`, 3.093127458904883`}, {210, 0.0016972609151584533`, 3.0211244289820467`},
    {240, 0.0016674820213499432`, 2.9681179980028984`}, {270, 0.05136917457827097`, 91.43713074932232`}},
  654.3490767423301`, 0.02142554584146257`, 0.8258276741420197`, 2412.3871247895277`},
{"TBUR", 59.17180849670849`, 230.25739483669386`, {{0, 0.09244136810302737`, 164.5456352233887`}},
  1.946788557720336`*^7, 0.03938341640669892`, 0.40743815628408264`, 2185.253624044241`},
{"CURE", 365, 1.8220680468959316`, {{0, 0.0019555075738313496`, 3.480803481419802`}},
  0.009999796654654452`, 0.01841835281806582`, 0.13329737078534953`, 1780.6552823778939`},
{"CURE", 365, 0.1769016575510806`, {{0, 0.003576950761246986`, 6.3669723550196355`}},
  0.009993159377568214`, 0.01781307437224037`, 0.08495146407630558`, 1780.2355538196284`},
{"CURE", 365, 33.45155133176841`, {{0, 0.04407788323418956`, 78.45863215685742`},
    {4, 0.0007014798273642567`, 1.248634092708377`}, {8, 0.00006794232985802522`, 0.1209373471472849`},
    {12, 0.0000552103975260135`, 0.09827450759630404`}, {16, 0.000054832397895752804`, 0.09760166825444`},
    {20, 0.0000548127492307598`, 0.09756669363075245`}, {24, 0.000054811255594829916`, 0.09756403495879726`},
    {28, 0.0000548111149640093 2`, 0.09756378463593661`}, {32, 0.000011118982242760097`, 0.01979178839211297`}},
  0.009994727625089435`, 0.042287689569619126`, 0.9979918356989668`, 1978.0315350066844`}, {"CURE", 365,
  1.8645456220382217`, {{0, 0.001825440695403661`, 3.249284437818517`}, {10, 0.0005137109341366556`, 0.9144054627632472`},
```

```
  {20, 0.0000374392619817333`, 0.06664188632748529`}, {30, 1.582913096831887`*^-6, 0.0028175853123607584`}},
0.009934440599688888`, 0.0799017540023893`, 0.08986180386276431`, 1834.742956019734`}, {"TBUR", 103.20678090502192`,
228.3555879932151`, {{0, 0.02952804506911629`, 52.559920223027`}, {8, 0.00017576171505064145`, 0.3128558527901418`},
  {16, 0.00016268648836381021`, 0.2895819492875822`}, {24, 0.0001683825739712292`, 0.29972098166878797`},
  {32, 0.00027918096479739483`, 0.4969421173393628`}, {40, 0.0039025202344710025`, 6.946486017358384`},
  {48, 0.014100081113628772`, 25.098144382259214`}, {56, 0.008375071941194702`, 14.907628055326567`},
  {64, 0.006039804663320913`, 10.750852300711225`}, {72, 6.01120803256152`, 10699.950297959505`}},
5414.9713162603075`, 0.08506880885646813`, 0.8205810075265192`, 1909.9112615027154`}, {"TBUR", 318.6543089159828`,
229.33682920034195`, {{0, 0.05567535092314153`, 99.10212464319193`}, {24, 0.002826253463342751`, 5.030731164750096`},
  {48, 0.002440759829338247`, 4.34455249622208`}, {72, 0.002411966209543928`, 4.293299852988191`},
  {96, 0.002408786093501572`, 4.287639246432798`}, {120, 0.0024084196511301686`, 4.2869869790117`},
  {144, 0.0024083772167659907`, 4.286911445843463`}, {168, 0.0024083722999105207`, 4.286902693840727`},
  {192, 0.002408371730187603`, 4.286901679733933`}, {216, 0.0028097670087013533`, 5.001385275488409`}},
40095.11686906748`, 0.02758886226627752`, 0.6777484927081228`, 2335.5341154733856`}, {"CURE", 365, 5.057069017765882`,
{{0, 0.01387972104732453`, 24.705903464237664`}, {15, 0.001150031396470979`, 2.0470558857183425`}},
0.009999916372501656`, 0.015728123164846033`, 0.6328283856157078`, 1784.5219699836011`}, {"CURE", 365,
2.368795116894621`, {{0, 0.017095706169832123`, 30.43035698230118`}, {7, 0.0033528538395496497`, 5.968079834398376`},
  {14, 0.00047992993019099447`, 0.8542752757399703`}, {21, 0.00008584302040504901`, 0.15280057632098723`},
  {28, 0.0000355592349013476`, 0.06329543812439874`}, {35, 0.000022675638188052692`, 0.040362635974733795`}},
0.009999989311061832`, 0.039819800135633764`, 0.9678296029169579`, 2008.890539077545`}, {"TBUR", 115.09471121006274`,
229.3012431117721`, {{0, 0.11632072285187818`, 207.05088667634317`}, {8, 0.026843834893863095`, 47.782026111076306`},
  {16, 0.007806854315277539`, 13.896200681194019`}, {24, 0.002839086602169125`, 5.053574151861043`},
  {32, 0.0012207846208074646`, 2.1729966250372867`}, {40, 0.000601976105899569`, 1.0715174685012328`},
  {48, 0.00033899582171494904`, 0.6034125626526093`}, {56, 0.00021896436500327423`, 0.3897565697058281`},
  {64, 0.00016068890480800137`, 0.28602625055824243`}, {72, 0.006581890534523704`, 11.715765151452194`}},
9397.727476778831`, 0.03285214872804212`, 0.24702617532486773`, 1780.0155389429667`}, {"CURE", 365,
1.1217611070017817`, {{0, 0.005271002613808421`, 9.38238465257899`}, {34, 0.0020186435257508887`, 3.5931854758365818`}},
0.009761742210439028`, 0.014587303363145338`, 0.26633588698965455`, 1782.0375914531908`}, {"CURE", 365,
1.9512118637164488`, {{0, 0.005717763532513398`, 10.177619087873849`}, {2, 0.003933562194243109`, 7.001740705752733`},
  {4, 0.002322578855974899`, 4.13419036363532`}, {6, 0.000591331288317491`, 1.052569693205134`}},
0.0099999884178024`, 0.0056362971494696 8`, 0.5194927806465368`, 1780.9888618246373`},
```

```
{"CURE", 365, 2.8865088004626043`, {{0, 0.004145579021250463`, 7.379130657825824`},
    {5, 0.00046453121542095426`, 0.8268655634492987`}, {10, 0.000031934508708707655`, 0.05684342550149963`},
    {15, 0.000012569257992967916`, 0.02237327922748289`}, {20, 0.000011940352325707501`, 0.02125382713975935`},
    {25, 0.000011918289282577964`, 0.021214554922988776`}, {30, 0.00001191689607219872`, 0.02121207500851372`},
    {35, 0.0000119167480969281`, 0.021211811612532015`}, {40, 0.000017977089278232648`, 0.03199921891525411`}},
  0.009999986089347677`, 0.033523517784649924`, 0.9730011439017188`, 2555.608716335905`},
{"CURE", 365, 0.4948998427712954`, {{0, 0.00313169373218742`, 5.574414843293607`},
    {6, 0.001276571863018305`, 2.2722979161725836`}, {12, 0.00038305054536385484`, 0.6818299707476616`}},
  0.009963589222379745`, 0.018289302177513306`, 0.7521670931220457`, 1783.37528116362`},
{"CURE", 365, 0.16444893936744712`, {{0, 0.0005946744356589176`, 1.0585204954728733`},
    {10, 0.00008288051996961495`, 0.1475273255459146`}, {20, 2.494605427512035`*^-6, 0.004440397660971422`}},
  0.009999930486443405`, 0.07360275385074645`, 0.1074574586548887`, 1950.231643175513`}, {"CURE", 365,
  4.892849651943322`, {{0, 0.012113482659719677`, 21.561999134301026`}, {11, 0.002494772017162101`, 4.440694190548539`}},
  0.009999430479120759`, 0.019692697941656342`, 0.7244412994430836`, 2399.028399701968`}, {"TBUR", 77.17975805333374`,
  229.64044236196733`, {{0, 0.011551206916195166`, 20.561148310827395`}, {3, 0.0017044193466978883`, 3.0338664371222417`},
    {6, 0.0005821120985781354`, 1.036159535469081`}, {9, 0.0002677894039563092`, 0.4766651390422304`},
    {12, 0.00015846176226445272`, 0.28206193683072583`}, {15, 0.00011339039724891694`, 0.20183490710307214`},
    {18, 0.00009242554909555856`, 0.16451747739009423`}, {21, 0.00008188440790339528`, 0.1457542460680436`},
    {24, 0.0000763103722606162`, 0.13583246262389684`}, {27, 0.032201518368481`, 57.31870269589618`}},
  253679.46054685433`, 0.03749825919446025`, 0.6639881846406256`, 3400.911583412979`},
{"CURE", 365, 3.455695687662219`, {{0, 0.011170781985086906`, 19.88399193345469`},
    {2, 0.0031434927442246597`, 5.5954170847198945`}, {4, 0.0006551264008510233`, 1.1661249935148217`},
    {6, 0.00014260803128300113`, 0.253842295683742`}, {8, 7.3008765947735785`*^-6, 0.012995560338696697`}},
  0.009999994874802473`, 0.010248116526678102`, 0.9152823724191917`, 1805.208267735229`},
{"CURE", 365, 0.33850393032671655`, {{0, 0.0005908821040211769`, 1.051770145157695`}},
  0.0099999540004627`, 0.026888660370233287`, 0.0987417983640507`, 1781.3160038187032`},
{"CURE", 365, 0.21349349730037567`, {{0, 0.0018338315554705907`, 3.2642201687376513`}}, 0.009999792403284842`,
  0.02950616001973457`, 0.4383754646313938`, 1783.879816777316`}, {"CURE", 365, 0.38207772980840576`,
  {{0, 0.0031517266311966987`, 5.610073403530124`}, {3, 0.0011239165392331269`, 2.000571439834966`}},
  0.009919999557157893`, 0.02710175804014197`, 0.8751424562136281`, 1789.727747974569`},
{"CURE", 365, 0.13795230764885918`, {{0, 0.003742195804245038`, 6.6611085315561676`}},
```

```
0.009999595744971362`, 0.019008074076396306`, 0.4345585258455725`, 1781.856711771362`},
{"CURE", 365, 0.24009014583565316`, {{0, 0.005803879923232556`, 10.330906263353949`}}, 0.009630649829526354`,
 0.0190896424415146`, 0.631510324859885`, 1781.5874527907067`}, {"CURE", 365, 3.8336091497998663`,
 {{0, 0.01160669289229439`, 20.659913348284014`}, {37, 0.003578520392158659`, 6.369766298042413`}},
 0.009754252379832025`, 0.023919200097387402`, 0.4383430254706106`, 1782.375422092853`},
{"TBUR", 63.81876204557676`, 230.2622802081095`, {{0, 0.04624171257019044`, 82.31024837493898`}},
 826881.2321774792`, 0.01250846332320709`, 0.5849935436116913`, 2831.044511542389`},
{"TBUR", 48.88245435305306`, 230.2294258679703`, {{0, 0.056555633544921886`, 100.66902770996096`}},
 1.0505287624993033`*^6, 0.04726838819236934`, 0.6334874776379188`, 2725.5492300093215`}, {"CURE", 365,
 2.8473220617698534`, {{0, 0.002523810637153069`, 4.492382934132463`}, {15, 0.000838403401789414`, 1.492358055185157`}},
 0.009996933376352617`, 0.011786217050682736`, 0.10252533400333042`, 1781.1973066883352`}, {"CURE", 365, 1.7794559718024403`,
 {{0, 0.0011193173233351114`, 1.9923848355364981`}, {14, 0.00040946538363089124`, 0.7288483828629865`}},
 0.009999278642035736`, 0.032182211950488294`, 0.044560212001930735`, 1780.2290189943924`},
{"TBUR", 18.648218283104594`, 229.7222999759216`, {{0, 0.07731018066406252`, 137.6121215820313`}},
 4.7148191409051225`*^7, 0.130671002464314`, 0.5536948018811867`, 2132.0805202214597`},
{"CURE", 365, 2.533750349986619`, {{0, 0.006224007571590293 5`, 11.078733477430722`},
   {15, 0.00008179364564230211`, 0.14559268924329774`}, {30, 0.000023794163220990268`, 0.04235361053336268`},
   {45, 0.000023657713129489963`, 0.04211072937049213`}, {60, 0.00003498468121634436`, 0.06227273256509297`}},
 0.009999997539379902`, 0.04739803663283277`, 0.7473253792191534`, 2665.182868666714`},
{"CURE", 365, 8.482462496442245`, {{0, 0.014479554780982685`, 25.77360751014918`}, {7, 0.005026325004077259`, 8.946858507257524`},
   {14, 0.0008015835752991465`, 1.426818764032481`}, {21, 0.00011034865751678448`, 0.19642061037987635`},
   {28, 0.000017065596561677875`, 0.030376761879786617`}, {35, 0.000013361268286051 13`, 0.02378305754917101`}},
 0.009999993582067272`, 0.048995679326592795`, 0.8208531095336423`, 2334.446426602261`},
{"TBUR", 23.86251223469357`, 227.80181826272715`, {{0, 0.11328804016113282`, 201.6527114868164`}}, 7.176379664460865`*^7,
 0.1127425254757519`, 0.4771147828364258`, 2044.2127164017536`}, {"CURE", 365, 1.2951319761519218`,
 {{0, 0.0031389383353817367`, 5.587310236979492`}, {36, 0.0016023447628018953`, 2.852173677787374`}},
 0.009752723430129782`, 0.011537379339895683`, 0.16554663140523618`, 1780.624015702365`},
{"CURE", 365, 2.952660575760677`, {{0, 0.02815521113818526`, 50.116275825969765`}, {3, 0.005519332419364366`, 9.824411706468572`},
   {6, 0.0009188038437505351`, 1.6354708418759523`}, {9, 0.0001238192525198311`, 0.22039826948529936`}},
 0.009999976390719355`, 0.015283665506356063`, 0.9504628405119728`, 1786.7899687861657`}, {"CURE", 365,
 5.7750732045442925`, {{0, 0.04641725709366048`, 82.62271762671564`}, {3, 0.0035833594081637627`, 6.378379746531497`},
```

{6, 0.000179693253032076`, 0.3198539903970953`}, {9, 0.0000574490004960071`, 0.10225922088289265`},
{12, 0.00005428322622888177`, 0.09662414268740954`}, {15, 0.00005420889681182255`, 0.09649183632504414`},
{18, 0.00005420628300657704`, 0.09648718375170713`}, {21, 0.000028141592229566735`, 0.050092034168628785`}},
0.009999994263725527`, 0.022388128739335263`, 0.9952982896873188`, 1930.9487179814`}, {"CURE", 365,
0.48602908545262175`, {{0, 0.004452800932852413`, 7.925985660477296`}, {36, 0.002674975436042587`, 4.761456276155805`}},
0.009991083313662665`, 0.010397135884393414`, 0.1670586090527126`, 1780.4820869805735`},
{"TBUR", 15.454529898587198`, 230.03913996949228`, {{0, 0.0922334861755371`, 164.17560539245605`}},
9.842771813142875`*^7, 0.18896648410253647`, 0.5498628558012285`, 2062.103935222582`},
{"CURE", 365, 0.9939696265319411`, {{0, 0.0016793034745330852`, 2.9891601846688918`},
{8, 0.0007434659295958465`, 1.3233693546806067`}, {16, 0.00016749479733548723`, 0.2981407392571673`},
{24, 0.00003672091373232875`, 0.06536322644354517`}, {32, 7.784538550563225`*^-6, 0.01385647862000254`}},
0.009999986426234307`, 0.030016149538941453`, 0.3026479582664826`, 1822.9946599828763`}, {"TBUR", 365,
229.52646700495174`, {{0, 0.04036877149291295`, 71.85641325738507`}, {31, 0.0008047285908657821`, 1.432416891741092`},
{62, 0.0007915186225938364`, 1.4089031482170289`}, {93, 0.0007924384336118413`, 1.4105404118290774`},
{124, 0.0008047678543642436`, 1.4324867807683535`}, {155, 0.0007965437265711527`, 1.4178478332966518`},
{186, 0.0007929089460209584`, 1.411377923917306`}, {217, 0.0007919776323699056`, 1.409720185618432`},
{248, 0.0007916430971938942`, 1.4091247130051316`}, {279, 0.006102394764863069`, 10.862262681456262`}},
0.12415848132852074`, 0.002542465448356687`, 0.013826416105981447`, 1780.003113693492`}, {"CURE", 365,
0.5745276824362303`, {{0, 0.001102050512382029`, 1.9616499120400117`}, {44, 0.00032074610832403333`, 0.5709280728167793`}},
0.009751987763765863`, 0.04551456019081689`, 0.13571091838788354`, 1783.4051161145742`},
{"CURE", 365, 0.246029813914844`, {{0, 0.008201124258235425`, 14.598001179659056`}}, 0.009997217337802603`,
0.019618793800853206`, 0.33644405328884475`, 1780.3956114349091`}, {"CURE", 365, 1.2688880755928333`,
{{0, 0.007454269170116519`, 13.268599122807405`}, {20, 0.00262908663682335`, 4.679774213545562`}},
0.009799988465526299`, 0.015883987808155687`, 0.4927815898876682`, 1782.038582951803`}, {"CURE", 365,
4.569793745643918`, {{0, 0.016049588284497887`, 28.568267146406242`}, {3, 0.0028379040633541974`, 5.051469232770471`},
{6, 0.0003538021433467008`, 0.6297678151571274`}, {9, 0.00006195213600290194`, 0.11027480208516545`},
{12, 0.000031439828589867456`, 0.055962894889964074`}, {15, 0.000028731929439448016`, 0.051142834402217466`},
{18, 0.000028531795716938044`, 0.05078659637614972`}, {21, 0.00001714411852900979`, 0.030516530981637424`}},
0.009992058135795297`, 0.028081223080926927`, 0.9810404836790125`, 1984.850282911748`},
{"TBUR", 16.142567163123566`, 229.60489976743398`, {{0, 0.10833592414855958`, 192.83794498443606`}},
4.1290128285309464`*^7, 0.14873216783824622`, 0.4884443586307996`, 2024.6720124217475`},

```
{"CURE", 365, 0.6651868390948152`, {{0, 0.009678515387985802`, 17.227757390614727`}}, 0.009999261313598375`,
  0.010912312873876262`, 0.36165269711600845`, 1780.353572399142`}, {"TBUR", 365, 230.17782456740758`,
  {{0, 0.004054676381144608`, 7.217323958437403`}, {31, 0.00026336919479576064`, 0.46879716673645394`},
   {62, 0.00026659338858051 5`, 0.47453623167331677`}, {93, 0.00029152688914711077`, 0.5189178626818571`},
   {124, 0.0003879261844354418`, 0.6905086082950864`}, {155, 0.00044089299738474805`, 0.7847895353448516`},
   {186, 0.000403143306910424`, 0.7175950863005548`}, {217, 0.0003906066704336048`, 0.6952798733718164`},
   {248, 0.00038334424036626675`, 0.6823527478519549`}, {279, 0.14545205034664974`, 258.9046496170365`}},
  1578.2168461317635`, 0.03433133738130732`, 0.8755976340703795`, 2532.8775644595516`}, {"CURE", 365, 0.0780369955114573`,
  {{0, 0.00015155462031396096`, 0.26976722415885046`}, {11, 0.00008778979693207564`, 0.15626583853909462`},
   {22, 0.000037116535607038276`, 0.06606743338052813`}, {33, 0.000012659388968632945`, 0.022533712364166646`}},
  0.009948042180131667`, 0.022964694068684587`, 0.2819159252552321`, 1811.4305590008564`}, {"CURE", 365,
  0.18312318095537036`, {{0, 0.0018877419791271043`, 3.3601807228462457`}, {15, 0.0008384795981241675`, 1.4924936846610182`}},
  0.00989357217315269`, 0.023861257925014086`, 0.15760184528714977`, 1780.597551667767`}, {"TBUR", 251.90376218061664`,
  219.30251769059177`, {{0, 0.04237803479960282`, 75.43290194329302`}, {22, 0.0018118702540804387`, 3.225129052263181`},
   {44, 0.00155265137698008`, 2.763719451024542`}, {66, 0.0008589937571674401`, 1.5290088877580432`},
   {88, 0.0007298188044954466`, 1.299077472001895`}, {110, 0.0006687573344066075`, 1.1903880552437613`},
   {132, 0.0006417498021517183`, 1.1423146478300585`}, {154, 0.0006273293861453739`, 1.1166463073387656`},
   {176, 0.0006190680590307984`, 1.101941145074821`}, {198, 0.02138417780527148`, 38.06383649338323`}},
  117400.74563279406`, 0.01942297459661906`, 0.051827437902854705`, 1780.0112018827872`}, {"CURE", 365,
  1.4623284578076328`, {{0, 0.007099161607580559`, 12.636507661493397`}, {27, 0.0035300654483617264`, 6.2835164980838725`}},
  0.009999376233653 27`, 0.038756660863808`, 0.67270365963192`, 1819.982093357942`}, {"TBUR", 62.82150048745036`,
  228.27465794273988`, {{0, 0.019121536550371975`, 34.036335059662115`}, {4, 0.008730504347578407`, 15.540297738689567`},
   {8, 0.006895348940194253`, 12.273721113545772`}, {12, 0.00568442182605632`, 10.118270850380249`},
   {16, 0.0047970765790974505`, 8.538796310793463`}, {20, 0.004109414882889025`, 7.314758491542466`},
   {24, 0.0035563644007195073`, 6.330328633280724`}, {28, 0.0031000457593192334`, 5.518081451588235`},
   {32, 0.0027170561196373385`, 4.836359892954462`}, {36, 0.08353442860163256`, 148.69128291090598`}},
  2.0317113851850774`*^7, 0.12583743139149725`, 0.6583622990852324`, 1971.7708494410854`}, {"TBUR", 365,
  229.08372612690516`, {{0, 0.11119746697015095`, 197.93149120686869`}, {31, 0.00048134015187573884`, 0.8567854703388151`},
   {62, 0.0002600712965188516`, 0.46292690780355583`}, {93, 0.00025963451525744404`, 0.46214943715825046`},
   {124, 0.0002594960697621533`, 0.46190300417663294`}, {155, 0.00025942514248368444`, 0.46177675362095827`},
   {186, 0.00025938731766701403`, 0.461709425447285`}, {217, 0.0002593668859508322`, 0.46167305699248135`},
```

```
{248, 0.0002593561452296394`, 0.46165393850875813`}, {279, 0.001020674042129838`, 1.8167997949911119`}},
0.082123384834992`, 0.012954873543836485`, 0.05971240426716833`, 1780.00514710428`}, {"CURE", 365, 0.32978103185379065`,
{{0, 0.0017905861598253485`, 3.1872433644891207`}, {9, 0.0004007718617203816`, 0.7133739138622793`}},
0.009917150050024241`, 0.022781173966564067`, 0.7399891118159442`, 1789.1001069545616`},
{"TBUR", 17.45022862370758`, 229.37916327425972`, {{0, 0.12454216957092284`, 221.68506183624265`}},
7.447743136137328`*^7, 0.09542683295033709`, 0.5014901240004698`, 2010.6523554591654`}, {"TBUR", 98.03430436467411`,
230.12632242154405`, {{0, 0.05973433248898331`, 106.32711183039031`}, {5, 0.006636869298343455`, 11.81362735105135`},
{10, 0.001542970495256489 5`, 2.7464874815565516`}, {15, 0.0006077392704536171`, 1.0817759014074386`},
{20, 0.00040059218271207487`, 0.7130540852274933`}, {25, 0.00034402969336425065`, 0.6123728541883661`},
{30, 0.00032429667752835125`, 0.5772480860004653`}, {35, 0.0003156988674882852`, 0.5619439841291476`},
{40, 0.0003112545473045357`, 0.5540330942020736`}, {45, 0.015117965026779557`, 26.90997774766761`}},
235385.6550942043`, 0.0215546079067745`, 0.1987936506043982`, 1780.0289848875855`},
{"CURE", 365, 0.22932538399742824`, {{0, 0.007649348213776369`, 13.615839820521938`}}, 0.009998969390307798`,
0.02902828968770319`, 0.23519824240608853`, 1780.3073335329454`}, {"CURE", 365, 1.3539905681808475`,
{{0, 0.00203308141930644`, 3.618884926365464`}, {23, 0.0007747096068139703`, 1.3789831001288673`}},
0.009758839252050903`, 0.019620324777599334`, 0.5226134544763047`, 1790.1071341546144`},
{"TBUR", 23.620451549361434`, 230.228731900187`, {{0, 0.0803149223327637`, 142.9605617523194`}}, 4.461346915500395`*^7,
0.0946781322560968`, 0.5040332519308252`, 2150.3545489373455`}, {"CURE", 365, 0.3180030843646453`,
{{0, 0.0005828555552762622`, 1.0374828883917468`}, {7, 0.00007752078867732 62`, 0.13798700384564064`}},
0.00999984243253698`, 0.01700605529408631`, 0.5654094446824109`, 1794.535790000521`},
{"TBUR", 20.123937490339728`, 230.16714475011057`, {{0, 0.07577322959899904`, 134.8763486862183`}},
5.424569346408827`*^7, 0.11046730768503034`, 0.5842700309188491`, 2165.0863217061533`},
{"CURE", 365, 0.7899421396441835`, {{0, 0.0034506636320177416`, 6.142181264991581`}}, 0.009999814806909372`,
0.017481573768144364`, 0.10038752317841731`, 1780.3299485945604`}, {"CURE", 365, 0.7648580759452949`,
{{0, 0.0017782801105232534`, 3.165338596731391`}, {9, 0.0002977952202567255`, 0.5300754920569715`},
{18, 0.0000153722179954912`, 0.027362548031974337`}, {27, 8.202716311479155`*^-6, 0.014600835034432897`}},
0.009999900742112683`, 0.06737641189838693`, 0.43662284663703077`, 2051.487340263756`},
{"CURE", 365, 0.3661138398684648`, {{0, 0.0037842158060601523`, 6.735904134787071`}},
0.009997253682331309`, 0.0252306693784043`, 0.3228536724948263`, 1781.0488225891427`},
{"CURE", 365, 0.7887593137832396`, {{0, 0.010987149426767607`, 19.557125979646344`},
{3, 0.002219496630587888`, 3.950704002446441`}, {6, 0.00020625774837133523`, 0.3671387921009767`}},
```

0.009999835419060715`, 0.013602259377881664`, 0.7265704778634278`, 1781.5149225043317`}, {"TBUR", 179.22252175632826`,
230.0332554549538`, {{0, 0.05440706390356071`, 96.84457374833806`}, {11, 0.0005096170178920219`, 0.907118291847799`},
{22, 0.0003642742974182395`, 0.6484082494044664`}, {33, 0.0003838364490157074`, 0.6832288792479592`},
{44, 0.0005102027865325368`, 0.9081609600279156`}, {55, 0.000903586057051836`, 1.6083831815522678`},
{66, 0.000982960342727959`, 1.7496694100557673`}, {77, 0.0006597803292627511`, 1.174408986087697`},
{88, 0.0005229368056848457`, 0.9308275141190254`}, {99, 0.03792762370183939`, 67.51117018927413`}},
74097.50506016317`, 0.037437294786096`, 0.7026001842567299`, 2848.3485851354526`}, {"TBUR", 346.7535842582502`,
228.63502149519562`, {{0, 0.04943271163130551`, 87.99022670372382`}, {27, 0.005548478597937392`, 9.876291904328559`},
{54, 0.0007250813323008997`, 1.2906447714956015`}, {81, 0.0002350018539420009`, 0.41830330001676164`},
{108, 0.00017812869223446048`, 0.3170690721773397`}, {135, 0.00016615930175284264`, 0.2957635571200599`},
{162, 0.00016176649467435398`, 0.28794436052035005`}, {189, 0.00015971330615542034`, 0.28428968495664825`},
{216, 0.0001586506757576973`, 0.2823982028487012`}, {243, 0.00334183455444675`, 5.948465506915214`}},
1423.8796429106817`, 0.013039327495229076`, 0.7295413476862123`, 2652.936085173487`}, {"CURE", 365,
5.369020390657864`, {{0, 0.022615311197160936`, 40.255253930946466`}, {18, 0.001284018075693991`, 2.285552174735304`}},
0.009999815100124768`, 0.04455641755828381`, 0.7231611268012755`, 1959.7932162294862`}, {"CURE", 365,
49.80234949015177`, {{0, 0.03092376838271807`, 55.04430772123816`}, {4, 0.0017669723866614174`, 3.1452108482573227`},
{8, 0.0017009157116310476`, 3.0276299667032647`}, {12, 0.0016990897308024784`, 3.0243797208284118`},
{16, 0.0016989837806786724`, 3.024191129608037`}, {20, 0.0016989741583688172`, 3.0241740018964944`},
{24, 0.0016989729992304622`, 3.0241719386302224`}, {28, 0.0016989728308513 74`, 3.0241716389154454`},
{32, 0.0016989728029785925`, 3.0241715893018943`}, {36, 0.0016948313607190583`, 3.0167998220799235`}},
0.009990671878332157`, 0.02916461403029871`, 0.9882240268246633`, 2488.8559224552423`},
{"CURE", 365, 7.492084529856005`, {{0, 0.025701940411583434`, 45.74945393261851`}, {1, 0.005406062922530087`, 9.622792002103552`},
{2, 0.0009916420283376335`, 1.7651228104409875`}, {3, 0.00016894989772551691`, 0.3007308179514201`}},
0.009999963886107027`, 0.006790890920328969`, 0.9366348284623026`, 1782.5459704004188`},
{"CURE", 365, 6.004322363627177`, {{0, 0.033656853374940655`, 59.90919900739437`},
{3, 0.005907288780433 5955`, 10.514974029171801`}, {6, 0.0007525952601612414`, 1.3396195630870098`},
{9, 0.0000971316194594542`, 0.1728942826378285`}, {12, 0.000019634593452840088`, 0.034949576346055354`},
{15, 0.000011615519160437284`, 0.02067562410557837`}, {18, 4.149222483357872`*^-6, 0.007385616020377012`}},
0.009999983879162844`, 0.026979700474669646`, 0.9815784403042146`, 2046.4174745974753`}, {"CURE", 365,
0.39442697348304784`, {{0, 0.0010539804093991178`, 1.8760851287304297`}, {36, 0.0004189438132788448`, 0.7457199876363436`}},
0.00973635202746143`, 0.018134304942497362`, 0.06885755378759655`, 1781.2228058851904`}, {"CURE", 365,

```
4.269266440981306`, {{0, 0.014980091839188048`, 26.664563473754722`}, {12, 0.0011257087275317154`, 2.0037615350064533`},
  {24, 0.00007303758995833576`, 0.13000691012583765`}, {36, 0.000019850773226311282`, 0.03533437634283408`},
  {48, 0.000016941129691949257`, 0.030155210851669677`}, {60, 0.000016727122404498586`, 0.029774277880007485`},
  {72, 0.000016702825417360767`, 0.029731029242902163`}, {84, 0.000016699445613635205`, 0.029725013192270666`},
  {96, 0.000016698947174059056`, 0.029724125969825113`}, {108, 0.000016006396493979 24`, 0.028491385759283047`}},
 0.009999998071039426`, 0.07128123699331214`, 0.6883569973042509`, 2338.2509745108064`},
{"CURE", 365, 1.3948645503460495`, {{0, 0.005686332223867728`, 10.121671358484557`},
  {4, 0.0012300891466233243`, 2.1895586809895176`}, {8, 0.00015474140029725928`, 0.27543969252912154`},
  {12, 0.000032061857478536435`, 0.05707010631179485`}, {16, 0.000040578417990917544`, 0.07222958402383323`}},
 0.009999997262168716`, 0.020817670715573208`, 0.9711281596747467`, 2172.830784801878`},
{"CURE", 365, 1.623752587868971`, {{0, 0.03735210746725839`, 66.48675129171993`}, {5, 0.012846770937024292`, 22.867252267903236`},
  {10, 0.003332852019580206`, 5.9324765948527665`}, {15, 0.0008545583223247068`, 1.521113813737978`},
  {20, 0.00022306434855218378`, 0.3970545404228871`}, {25, 0.00006265653748634461`, 0.11152863672569341`},
  {30, 0.000017587385828007994`, 0.03130554677385423`}}, 0.009982863555092333`,
 0.03193722167177613`, 0.9470131622741603`, 1853.4552611684906`}, {"CURE", 365, 0.38324117165150157`,
 {{0, 0.0010593138303923103`, 1.8855786180983125`}, {22, 0.0008885589581958935`, 1.5816349455886904`}},
 0.009996039837421972`, 0.007457947864217251`, 0.18584373497162782`, 1781.3411996774673`},
{"CURE", 365, 5.157281683784892`, {{0, 0.004549115967612908`, 8.097426422350976`},
  {7, 0.0005354832731855023`, 0.953160226270194`}, {14, 8.695423728804904`*^-6, 0.01547785423727273`}},
 0.009999993131482983`, 0.04213934889489012`, 0.22462188366799543`, 1791.0376133092905`}, {"CURE", 365,
 0.06086343419595992`, {{0, 0.0008732063204119452`, 1.5543072503332624`}, {10, 0.00010459914643671149`, 0.18618648065734641`}},
 0.009970593198847863`, 0.025132711927886586`, 0.22677810780838073`, 1782.889902869146`},
{"TBUR", 31.238302127985463`, 230.1096549308479`, {{0, 0.09788187980651858`, 174.22974605560307`}}, 5.71663847397466`*^6,
 0.024174528462415176`, 0.6231619611142271`, 2167.4268583348567`}, {"CURE", 365, 0.2201733691250701`,
 {{0, 0.001193105847615732`, 2.123728408756003`}, {35, 0.0005045174813190338`, 0.8980411167478801`}},
 0.009999747528470408`, 0.030538518008810914`, 0.4677033240668431`, 1795.836766706381`}, {"CURE", 365,
 3.8822772851109733`, {{0, 0.01880939126860364`, 33.48071645811448`}, {2, 0.002345544461649496`, 4.1750691417361026`},
  {4, 0.00020725549427270918`, 0.36891477980542225`}, {6, 0.000040787100667608 32`, 0.07260103918834279`},
  {8, 0.000029627313228211095`, 0.052736617546215746`}, {10, 0.000024248935545658977`, 0.043163105271272977`}},
 0.009999994286930967`, 0.017124138643484964`, 0.9847501201044914`, 1907.511961308649`}, {"CURE", 365,
 2.8085841609171633`, {{0, 0.00843946069398716`, 15.022240035297147`}, {7, 0.0010880551670456589`, 1.936738197341273`},
```

{14, 0.00003087146802415642`, 0.05495121308299843`}, {21, 4.954527635777885`*^-7, 0.0008819059191684637`}},
0.009989898416800206`, 0.05215730975039604`, 0.35902607885481613`, 1828.0185275786218`}, {"CURE", 365,
3.366890718026392`, {{0, 0.010532173746005178`, 18.747269267889216`}, {4, 0.0022870173704072636`, 4.0708909193249285`},
{8, 0.00025119629819210764`, 0.44712941078195156`}, {12, 0.00003165601024690232`, 0.05634769823948613 4`}},
0.00999954400749238`, 0.017341409246392093`, 0.7409605860000641`, 1843.7992976652026`}, {"CURE", 365,
0.6203773835571167`, {{0, 0.0027444395859043316`, 4.88510246290971`}, {36, 0.001108694823816436`, 1.9734767863932563`}},
0.009999412618088462`, 0.03547399000194234`, 0.2560386330196102`, 1783.6263239338539`}, {"TBUR", 191.76571467575477`,
228.3234745296366`, {{0, 0.06871234370238855`, 122.30797179025161`}, {14, 0.04797596762088632`, 85.39722236517765`},
{28, 0.010342128198791501`, 18.408988193848874`}, {42, 0.0025042612590056444`, 4.457585041030047`},
{56, 0.0008304982522162406`, 1.4782868889449081`}, {70, 0.00027669646955081863`, 0.49251971580045717`},
{84, 0.00009184044744512169`, 0.1634759964523166`}, {98, 0.00003679054078925521`, 0.06548716260487428`},
{112, 0.00002057461684422863`, 0.036622817982726964`}, {126, 0.005368898334028418`, 9.556639034570583`}},
12.68352008415853`, 0.015118112991911575`, 0.1326055717707714`, 1780.0175014471918`}, {"TBUR", 174.568060140346`,
228.25125165353285`, {{0, 0.06507896596734344`, 115.84055942187133`}, {13, 0.0008608149485598354`, 1.5322506084365068`},
{26, 0.0009208580245265443`, 1.6391272836572488`}, {39, 0.002108217231352264 5`, 3.752626671807031`},
{52, 0.0018667718109013388`, 3.3228538234043823`}, {65, 0.0010664360115396389`, 1.8982561005405574`},
{78, 0.0008973461654022675`, 1.5972761744160362`}, {91, 0.0007928267140494653`, 1.4112315510080482`},
{104, 0.000695022996187218`, 1.237140933213248`}, {117, 0.02853925142466002`, 50.79986753589484`}},
46935.09522921381`, 0.022463534476357495`, 0.6363159319409244`, 2370.8728503574093`},
{"CURE", 365, 0.11524332983473881`, {{0, 0.0017904633043253199`, 3.1870246816990697`}}, 0.009589089223979093`,
0.014743424786855487`, 0.11786034792618905`, 1780.713102734724`}, {"CURE", 365, 0.42358166036449174`,
{{0, 0.0009340881623308515`, 1.6626769289489156`}, {9, 0.0004653359948626457`, 0.8282980708555092`},
{18, 0.00017058432262545502`, 0.3036400942733099`}, {27, 0.0000658599826586238`, 0.11723076913235035`},
{36, 0.00002968416413894393`, 0.05283781216732019`}, {45, 0.000011701115235367918`, 0.020827985118954895`}},
0.009999989411546248`, 0.026883875507685062`, 0.8893883139776043`, 1948.603534830299`}, {"CURE", 365,
1.9514864458316772`, {{0, 0.008834816130774591`, 15.725972712778772`}, {15, 0.0004436579848767394`, 0.7897112130805961`}},
0.009999897575945925`, 0.04072448152811877`, 0.44971790839567255`, 1801.2545426724516`}, {"CURE", 365,
0.7997289655982532`, {{0, 0.01572882794593813`, 27.997313743769872`}, {7, 0.0032473756783406196`, 5.780328707446303`}},
0.009999319893950488`, 0.013530119329430568`, 0.9341046212695355`, 1849.9997343806938`},
{"CURE", 365, 84.1917087100026`, {{0, 0.03891048398155535`, 69.26066148716852`}, {16, 0.014657287495846653`, 26.08997174260704`},
{32, 0.002991798420536178`, 5.325401188554397`}, {48, 0.0008634714742483397`, 1.5369792241620446`}},

```
0.009999999650247804`, 0.016476428923053812`, 0.9999020821078556`, 2279.771094968188`}, {"CURE", 365,
0.6404745008234457`, {{0, 0.0015451428606542`, 2.7503542919644763`}, {26, 0.0004483263192256104`, 0.7980208482215865`}},
0.009741346666965172`, 0.019550837226116696`, 0.18044945523713388`, 1783.843780187963`}, {"TBUR", 46.043820171482075`,
211.6114841473689`, {{0, 0.035463875243398064`, 63.12569793324855`}, {5, 0.017717071031703877`, 31.5363864364329`},
  {10, 0.01433337871260535`, 25.513414108437527`}, {15, 0.01288224063115974`, 22.93038832346434`},
  {20, 0.01246532212782791`, 22.18827338753368`}, {25, 0.012680341601811725`, 22.571008051224872`},
  {30, 0.01332173156223584`, 23.712682180779797`}, {35, 0.014270524755569723`, 25.40153406491411`},
  {40, 0.015449456435887042`, 27.500032455878934`}, {45, 3.887057444021695`, 6918.962250358618`}},
3.357650555195935`*^7, 0.10520511808982314`, 0.8275174711057021`, 2032.3004083796507`}, {"CURE", 365,
1.8389207070595721`, {{0, 0.014532838836375381`, 25.868453128748175`}, {26, 0.007398741158612995`, 13.16975926233113`}},
0.009862226504698264`, 0.016970392058579895`, 0.5569647825565025`, 1780.464110361996`}, {"CURE", 365,
4.085008359747398`, {{0, 0.007760464836841428`, 13.813627409577741`}, {3, 0.00045955084312543934`, 0.818000500763282`},
  {6, 0.00005077861227676967`, 0.09038592985265002`}, {9, 0.00004349662552851103`, 0.07742399344074963`},
  {12, 0.000043386613586881216`, 0.07722817218464857`}, {15, 0.00004338326948475712`, 0.07722221968286769`},
  {18, 0.00004338301212494317`, 0.07722176158239885`}, {21, 0.00008300835367870175`, 0.1477548695480891`}},
0.009999990673090589`, 0.021071309389643484`, 0.9924335719515888`, 2114.3608636099407`},
{"CURE", 365, 0.17367995126506364`, {{0, 0.0008856743647988029`, 1.576500369341869`}}, 0.009999785394512599`,
0.021538749416913704`, 0.06276273934402579`, 1780.7583849675236`}, {"CURE", 365, 1.1723486021286786`,
 {{0, 0.01662461056758136`, 29.59180681029482`}, {13, 0.004026653378974955`, 7.16744301457542`}},
0.009999591492539575`, 0.017863295982299358`, 0.7769996422280855`, 1781.0239051392593`},
{"CURE", 365, 0.5709732505546623`, {{0, 0.0011094877103031552`, 1.9748881243396164`}}, 0.009547864986542845`,
0.02038008922625644`, 0.41925685811802865`, 1784.2283478584743`}, {"TBUR", 365, 230.11910813713678`,
 {{0, 0.025610719360597903`, 45.58708046186426`}, {26, 0.0020396734655307542`, 3.630618768644742`},
  {52, 0.0020394621917419866`, 3.630242701300736`}, {78, 0.00203944341932315`, 3.630209286395207`},
  {104, 0.0020394390669620567`, 3.6302015391924605`}, {130, 0.0020394378652230966`, 3.630199400097112`},
  {156, 0.0020394375219903874`, 3.6301987891428893`}, {182, 0.002039437423297973`, 3.630198613470392`},
  {208, 0.0020394373948813735`, 3.630198562888845`}, {234, 0.002666956591249042`, 4.747182732423294`}},
210.90854769235415`, 0.019236771252198293`, 0.0706370720937552`, 1780.0259675750506`},
{"CURE", 365, 2.68790905803938`, {{0, 0.006319907164820471`, 11.249434753380438`},
  {5, 0.0018792019445971722`, 3.344979461382966`}, {10, 0.0002617307591578729`, 0.46588075130101375`}},
0.0099995945772958`, 0.01785190682731983`, 0.44673833969873467`, 1781.5815618569459`}, {"CURE", 365,
```

48.637314463341724`, {{0, 0.0051182351714727375`, 9.110458605221474`}, {8, 0.0013094918477720555`, 2.330895489034259`},
{16, 0.001309479041611848`, 2.3308726940690896`}, {24, 0.0024119341891674076`, 4.293242856717986`}},
0.00999972516181686`, 0.004327948283431003`, 0.9996120322671125`, 2770.2319147576013`}, {"CURE", 365,
6.231384719329837`, {{0, 0.004408299946054957`, 7.846773903977823`}, {20, 0.0003789341822447049`, 0.6745028443955746`}},
0.009999990899215917`, 0.022702519725361768`, 0.5835929910724424`, -473.1646228169885`}, {"TBUR", 361.64140719606445`,
229.86652020510581`, {{0, 0.033056614413829156`, 58.8407736566159`}, {31, 0.0004513053962542659`, 0.8033236053325933`},
{62, 0.00044418419260236025`, 0.7906478628322012`}, {93, 0.00044361699113351475`, 0.7896382442176564`},
{124, 0.0004432807697835402`, 0.7890397702147016`}, {155, 0.0004430104633845512`, 0.7885586248245011`},
{186, 0.00044278299270755967`, 0.7881537270194564`}, {217, 0.000442588194434448`, 0.7878069860933175`},
{248, 0.00044241859761094834`, 0.7875051037474881`}, {279, 0.010558399036167994`, 18.79395028437903`}},
156.71517014163084`, 0.004366859058164194`, 0.889394695634117`, 2997.8897481305125`}, {"CURE", 365, 0.7361257715970096`,
{{0, 0.0011088870009500346`, 1.973818861691061`}, {6, 0.00017507826004397312`, 0.3116393028782722`}},
0.009999949347063093`, 0.026522713171049844`, 0.21205243285010758`, 1782.8485035644387`},
{"CURE", 365, 0.360164608177346`, {{0, 0.0004401757057245553`, 0.7835127561897085`},
{10, 0.00017889949673849457`, 0.3184411041945204`}, {20, 0.000019814100626385463`, 0.035269099114966125`}},
0.009999971273000001`, 0.03859104779261103`, 0.13151541890248544`, 1784.7812334828216`}, {"TBUR", 231.0299137173057`,
227.95662728226617`, {{0, 0.038122411160817875`, 67.85789186625583`}, {19, 0.0008070604565450507`, 1.4365676126501905`},
{38, 0.00082775653113722`, 1.4734066254242515`}, {57, 0.001030045225147157`, 1.8334805007619395`},
{76, 0.001870613249406775`, 3.3296915839440597`}, {95, 0.003665696851941795`, 6.524940396456395`},
{114, 0.004813419607449154`, 8.567886901259493`}, {133, 0.0047138853165290115`, 8.390715863421642`},
{152, 0.004566400999790864`, 8.128193779627738`}, {171, 8.089489388401047`, 14399.291111353865`}},
76800.87842909343`, 0.10990602112938981`, 0.711594459202182`, 2266.0025986132737`},
{"CURE", 365, 2.6556328223972594`, {{0, 0.0027802110056610978`, 4.948775590076754`}}, 0.009999816965765186`,
0.028685904555854742`, 0.08254415169899677`, 1780.17123692515`}, {"TBUR", 131.93812193977462`, 229.50548375298874`,
{{0, 0.05169677728719346`, 92.02026357120437`}, {7, 0.00022809560906709062`, 0.4060101841394213`},
{14, 0.00006916509211758054`, 0.12311386396929337`}, {21, 0.00006831452283325233`, 0.12159985064318915`},
{28, 0.00006826642333233274`, 0.12151423353155229`}, {35, 0.00006825870355423345`, 0.12150049232653555`},
{42, 0.00006825826166614502`, 0.12149970576573815`}, {49, 0.00006826143865390593`, 0.12150536080395255`},
{56, 0.00006826875128129787`, 0.12151837728071022`}, {63, 0.002321948114472626`, 4.133067643761275`}},
269.36021638188544`, 0.020801566814746385`, 0.7733537462715906`, 4035.9194073541566`}, {"TBUR", 63.51010939547635`,
228.5350544921238`, {{0, 0.03795264670948308`, 67.5557111428799`}, {3, 0.008630121887994344`, 15.361616960629933`},

```
{6, 0.004509951011968413`, 8.027712801303775`}, {9, 0.00273949037411507`, 4.876292865924825`},
   {12, 0.0018270437788200783`, 3.25213792629974`}, {15, 0.0012935193763776466`, 2.302464489952211`},
   {18, 0.0009603770021077172`, 1.7094710637517367`}, {21, 0.0007475555683082734`, 1.3306489115887268`},
   {24, 0.000610784728309644`, 1.0871968163911663`}, {27, 0.06296294451269177`, 112.07404123259136`}},
  3.6279901159594837`*^6, 0.04538310333684311`, 0.2915237553467313`, 1780.0674409374624`},
 {"CURE", 365, 0.21925029225516193`, {{0, 0.000856677614464915`, 1.5248861537475487`},
   {9, 0.0004969357299616261`, 0.8845455993316945`}, {18, 0.0001564923453679858`, 0.2785563747550147`}},
  0.009939730700546722`, 0.017221078942077122`, 0.33982864117263667`, 1784.0578517081592`},
 {"TBUR", 49.717948585858046`, 230.25394512818357`, {{0, 0.08953362464904785`, 159.36985187530516`}},
  60818.93567321338`, 0.003423230260019565`, 0.6916048702200108`, 2230.404673230863`}, {"CURE", 365, 0.10174600155152001`,
  {{0, 0.0006538343535463969`, 1.1638251493125864`}, {70, 0.0003803695780588162`, 0.6770578489446928`}},
  0.009772132472179732`, 0.02984006709702793`, 0.0431174419776249`, 1780.2756308295109`},
 {"TBUR", 42.597855663876445`, 230.25333584089364`, {{0, 0.04368541240692139`, 77.76003408432008`}},
  1.0304250526882214`*^7, 0.08283887945032145`, 0.52507791676163`, 2636.2761406080303`},
 {"CURE", 365, 38.662639097611816`, {{0, 0.01903927448969614`, 33.88990859165912`},
   {4, 0.0008927126487111918`, 1.5890285147059213`}, {8, 0.00013585242980441545`, 0.24181732505185946`},
   {12, 0.00012190884841783799`, 0.21699775018375161`}, {16, 0.00012153277995539729`, 0.21632834832060718`},
   {20, 0.00012150778671460236`, 0.2162838603519922`}, {24, 0.00012150496877847046`, 0.2162788444256774`},
   {28, 0.00012150454330939297`, 0.21627808709071947`}, {32, 0.00007107058337943732`, 0.12650563841539844`}},
  0.009999998715415096`, 0.011941677567690006`, 0.9998283024482785`, 2348.6409532442267`}, {"CURE", 365,
  3.6064968443604233`, {{0, 0.020443437613958212`, 36.38931895284562`}, {34, 0.004011051469862145`, 7.139671616354618`}},
  0.009999052887264452`, 0.024008716546621848`, 0.6787667667451434`, 1840.8811502557728`},
 {"TBUR", 22.23114628568708`, 230.02322076899216`, {{0, 0.0964745044708252`, 171.72461795806885`}},
  2.128888058773611`*^7, 0.057929380760670855`, 0.5093293307778972`, 2096.3572784253806`},
 {"CURE", 365, 0.15959185022720176`, {{0, 0.005656033868166442`, 10.067740285336267`}}, 0.009998832149396904`,
  0.017738509986188335`, 0.12965787721346575`, 1780.2560754235687`}, {"TBUR", 213.7387164263371`, 229.0135517246524`,
  {{0, 0.024176980615619324`, 43.0350254958024`}, {15, 0.0018844967982371934`, 3.354404300862204`},
   {30, 0.0018844942862212993`, 3.354399829473913`}, {45, 0.0018844952039971782`, 3.3544014631149777`},
   {60, 0.001884504977989145`, 3.354418860820678`}, {75, 0.0018846234742296633`, 3.354629784128801`},
   {90, 0.0018861989982098789`, 3.357434216813584`}, {105, 0.0019078737364938956`, 3.396015250959134`},
   {120, 0.0021838727208455553`, 3.8872934431050847`}, {135, 0.05656389475344904`, 100.68373266113929`}},
```

```
  4.748111923598696`, 0.024419694311392084`, 0.9125636911722755`, 4042.763502633539`}, {"CURE", 365, 0.6808991040170471`,
  {{0, 0.0020421786920470195`, 3.635078071843695`}, {38, 0.0008405632472146404`, 1.4962025800420597`}},
  0.009722656976730455`, 0.028802395272751996`, 0.1687512653494331`, 1781.007051865547`},
{"CURE", 365, 2.0742704274293358`, {{0, 0.02071982633092144`, 36.881290869040164`}}, 0.00999968191953034`,
  0.020049810656451554`, 0.7327495162591562`, 1781.0773118731622`}, {"CURE", 365, 101.87319683403977`,
  {{0, 0.0259209149083661 9`, 46.13922853689182`}, {31, 0.0005519655500605033`, 0.9824986791076958`},
   {62, 0.00024036392303221335`, 0.4278477829973397`}, {93, 0.0002387603668659475`, 0.4249934530213865`},
   {124, 0.00023847887340977273`, 0.4244923946693955`}, {155, 0.00023837116213214113`, 0.42430066859521115`},
   {186, 0.0002383287271773691`, 0.42422513437571696`}, {217, 0.000238311941076 4463`, 0.42419525511607437`},
   {248, 0.00023830529438616875`, 0.42418342400738035`}, {279, 0.0017135792505759532`, 3.0501710660251966`}},
  0.009999990359677541`, 0.00800410434158267`, 0.9999837615200177`, 2542.396310764679`},
{"CURE", 365, 1.1004808967284638`, {{0, 0.0020230943636887207`, 3.6011079673659223`},
   {8, 0.0005606623384577634`, 0.9979789624548188`}, {16, 0.00004258596659740502`, 0.07580302054338094`},
   {24, 2.70649312663511`*^-6, 0.004817557765410496`}, {32, 6.084741156718943`*^-7, 0.0010830839258959717`}},
  0.009999917656668775`, 0.059005709089608366`, 0.1475856948486117`, 1914.4565133992687`}, {"CURE", 365,
  3.22540648116331`, {{0, 0.016740075361578857`, 29.79733414361037`}, {15, 0.004516966359672732`, 8.040200120217463`}},
  0.009999938716535116`, 0.011756562899403774`, 0.7192805181188322`, 1781.5431194099767`}, {"CURE", 365,
  128.10667502319828`, {{0, 0.032898596865815415`, 58.55950242115144`}, {25, 0.005779395469248605`, 10.287323935262517`}},
  0.009999398031707431`, 0.01187659136183409`, 0.9999410412419509`, 2874.624687926338`},
{"TBUR", 13.791354148483354`, 229.93969393903643`, {{0, 0.09830739021301271`, 174.98715457916265`}},
  3.259097863295619`*^7, 0.1336443328154008`, 0.5506998838613032`, 2040.4345246091304`},
{"CURE", 365, 0.8292396317599798`, {{0, 0.0021904729584312477`, 3.899041866007621`}}, 0.009999968067402823`,
  0.02114670077644765`, 0.0631457279732782`, 1780.1764360968057`}, {"TBUR", 63.75404772006436`, 229.82747889842318`,
  {{0, 0.08157838144377229`, 145.20951896991468`}, {3, 0.013879048124225577`, 24.704705661121526`},
   {6, 0.004503554239647066`, 8.016326546571777`}, {9, 0.0017828953242725214`, 3.173553677205088`},
   {12, 0.000867456612176433`, 1.5440727696740506`}, {15, 0.0005180240429751678`, 0.9220827964957987`},
   {18, 0.00036857621353954164`, 0.6560656601003843`}, {21, 0.00029700231626366786`, 0.5286641229493287`},
   {24, 0.0002583085422078689`, 0.4597892051300066`}, {27, 0.03011599438897749`, 53.60647001237994`}},
  248772.4664399888`, 0.0414220035364936`, 0.27644984883747026`, 1780.036115423547`},
{"CURE", 365, 0.15607929227696446`, {{0, 0.0006993669663006331`, 1.244873200015127`}}, 0.009999872648502377`,
  0.00912237180029802 3`, 0.1831373438936696`, 1782.16959748918`}, {"TBUR", 119.73767314461234`, 228.82152604122706`,
```

```
{{0, 0.004915672824935782`, 8.749897628385693`}, {8, 0.0022266122306458104`, 3.9633697705495425`},
  {16, 0.0012369473570921974`, 2.2017662956241115`}, {24, 0.0007713149268560854`, 1.372940569803832`},
  {32, 0.0005232602321286967`, 0.9314032131890801`}, {40, 0.00037867834910616576`, 0.6740474614089751`},
  {48, 0.0002893861952608089`, 0.5151074275642399`}, {56, 0.00023225500958603913`, 0.4134139170631496`},
  {64, 0.00019483114188711037`, 0.34679943255905643`}, {72, 0.058589655182130954`, 104.28958622419309`}},
 98314.5270403848`, 0.007928349798511586`, 0.8183322288626614`, 2402.143370984786`}, {"CURE", 365, 0.5903587490167899`,
 {{0, 0.012974823465895891`, 23.095185769294687`}, {6, 0.0018575664435892308`, 3.3064682695888314`}},
 0.009957878971088347`, 0.023938118221300 94`, 0.7621013771456777`, 1781.5819104603202`},
{"CURE", 365, 0.4073811993374568`, {{0, 0.001375224700674704`, 2.447899967200973`}}, 0.009999843865109864`,
 0.015954996022004776`, 0.0799512449048806`, 1780.5685850219895`}, {"CURE", 365, 3.621484451163788`,
 {{0, 0.020091569586623136`, 35.762993864189184`}, {30, 0.005939818774801632`, 10.572877419146904`}},
 0.00999996553229443`, 0.03421175048454697`, 0.6859545088911574`, 1817.0711564100343`},
{"CURE", 365, 0.24218990208502136`, {{0, 0.0008804835042594188`, 1.5672606375817655`}}, 0.009999805387170968`,
 0.023825575140045328`, 0.044248132269644344`, 1780.3724752827898`}, {"TBUR", 319.4515085192676`, 228.65985600693918`,
 {{0, 0.07861749346555294`, 139.93913836868424`}, {27, 0.0006800990147546101`, 1.2105762462632057`},
  {54, 0.00035914287443262913`, 0.6392743164900798`}, {81, 0.00009317607480107482`, 0.16585341314591318`},
  {108, 0.00007625909015420806`, 0.13574118047449035`}, {135, 0.00007201455436366682`, 0.12818590676732694`},
  {162, 0.00007122154823480898`, 0.12677435585795999`}, {189, 0.00007104440357954007`, 0.12645903837158134`},
  {216, 0.00007100588924872833`, 0.12639048286273644`}, {243, 0.000328694582642413`, 0.5850763571034951`}},
 0.058211628856356765`, 0.007620483812134885`, 0.7299148948025032`, 2496.8967625240944`},
{"CURE", 365, 172.315382211405`, {{0, 0.04101014683099802`, 72.99806135917648`}, {31, 0.003059440194853924`, 5.445803546839984`},
  {62, 0.0006214255142462348`, 1.106137415358298`}, {93, 0.0001531401858173825`, 0.27258953075494086`},
  {124, 0.00009219863600462298`, 0.16411357208822888`}, {155, 0.00007915230265019042`, 0.14089109871733893`},
  {186, 0.00007460261826752016`, 0.13279266051618588`}, {217, 0.00007249394715170316`, 0.12903922593003161`},
  {248, 0.0000713168211930756`, 0.12694394172367454`}, {279, 0.003153569984670142`, 5.613354572712852`}},
 0.009996209800420456`, 0.004985413641819962`, 0.8522586685575354`, 2559.7028386558122`}, {"TBUR", 89.1414786312633`,
 229.7574959150538`, {{0, 0.017212874070146808`, 30.638915844861323`}, {5, 0.0009460294433935883`, 1.683932409240587`},
  {10, 0.0001879793817231475`, 0.3346032994672025`}, {15, 0.0001448658129369067`, 0.2578611470276939`},
  {20, 0.00014061221234089815`, 0.25028973796679865`}, {25, 0.00014399719269137713`, 0.2563150029906513`},
  {30, 0.00015291399607975228`, 0.2721869130219591`}, {35, 0.0001602542849759128`, 0.28525262725712475`},
  {40, 0.0001583761427043158 2`, 0.28190953401368213`}, {45, 0.08280252532191638`, 147.38849507301114`}},
```

```
7134.29300312851`, 0.028239863488180854`, 0.8916298034150671`, 3285.021293141486`}, {"CURE", 365, 0.15022896654037152`,
  {{0, 0.0005550701120053112`, 0.988024799369454`}, {10, 0.00005620091103988064`, 0.10003762165098753`}},
  0.009990837684621 57`, 0.0234398481977693`, 0.04610237933257725`, 1781.0520732329353`},
{"TBUR", 43.413686068331764`, 230.25665308484014`, {{0, 0.041924257278442394`, 74.62517795562746`}},
  9.470702921306387`*^7, 0.1478790583239159`, 0.5598647515101602`, 2647.5302232083354`}, {"CURE", 365,
  1.8723089353128761`, {{0, 0.02167589060428523`, 38.58308527562771`}, {3, 0.0037575041260732773`, 6.688357344410433`},
    {6, 0.0004106740790294589`, 0.7309998606724368`}, {9, 0.00005689307864168057`, 0.10126967998219141`},
    {12, 0.000022588975783934 63`, 0.04020837689540364`}, {15, 5.942000556248625`*^-6, 0.010576760990122553`}},
  0.009999993187646869`, 0.01768796034998372`, 0.9782508833032159`, 1993.3126641529857`},
{"TBUR", 19.995516170120933`, 230.17840171628478`, {{0, 0.06415423870086673`, 114.19454488754279`}},
  3.581478588882678`*^7, 0.13071561305348542`, 0.5891551561050153`, 2218.038509731538`}, {"CURE", 365,
  5.446008440964269`, {{0, 0.02440982571628475`, 43.44948977498685`}, {12, 0.0005606563753064381`, 0.9979683480454599`}},
  0.00999991119922978`, 0.015532903216679094`, 0.7942514208801192`, 1940.4206249341346`},
{"CURE", 365, 2.4607964387782895`, {{0, 0.002221020748957598`, 3.9534169331445246`},
    {4, 0.0010591929133892863`, 1.8853633858329297`}, {8, 0.0003061042267026202`, 0.544865523530664`}}, 0.009999852275656267`,
  0.009162133911482545`, 0.4926545618642712`, 1782.7063953598713`}, {"TBUR", 175.02093280164482`, 229.11295511776368`,
  {{0, 0.025311044037172385`, 45.053658386166845`}, {11, 0.0001314461649274001`, 0.23397417357077216`},
    {22, 0.00010931324346555348`, 0.1945775733686852`}, {33, 0.00010930143049135693`, 0.19455654627461533`},
    {44, 0.00010930058193984194`, 0.19455503585291864`}, {55, 0.00010930043286475884`, 0.19455477049927072`},
    {66, 0.0001093004433605998`, 0.1945547891818676`}, {77, 0.0001093005249464851`, 0.19455493440474347`},
    {88, 0.00010930065625066004`, 0.1945551681261749`}, {99, 0.02308041118567682`, 41.08313191050473`}},
  45.11436696440336`, 0.011439295961934187`, 0.8797665548459559`, 3501.6371932770594`}, {"TBUR", 354.30873848946084`,
  228.55183215574442`, {{0, 0.07881992125150342`, 140.2994598276761`}, {28, 0.001303221451346696`, 2.319734183397119`},
    {56, 0.00199118644248467`, 3.544311867622712`}, {84, 0.0025572858894926413`, 4.551968883296901`},
    {112, 0.0017770361995033171`, 3.163124435115905`}, {140, 0.0015442136393237847`, 2.7487002779963365`},
    {168, 0.0014007380488122846`, 2.4933137268858667`}, {196, 0.0013177959955833088`, 2.3456768721382897`},
    {224, 0.0012703280141114863`, 2.2611838651184457`}, {252, 0.034407438808679934`, 61.24524107945029`}},
  37648.35128111331`, 0.03829135642324496`, 0.6972010686587684`, 2321.7455879690524`}, {"CURE", 365, 0.4223977169333269`,
  {{0, 0.010887890065931053`, 19.380444317357274`}, {7, 0.0020884653230682664`, 3.7174682750615142`}},
  0.009942758328759396`, 0.016611942983570608`, 0.6291019399303657`, 1781.0189064710594`}, {"CURE", 365,
  1.0209119273096585`, {{0, 0.001806955161001833`, 3.2163801865832626`}, {3, 0.000493019415906506`, 0.8775745603135806`},
```

{6, 0.00008173992124738679`, 0.14549705982034847`}, {9, 0.00002447651100633778`, 0.043568189591281246`},
{12, 0.000017741024709180735`, 0.031579023982341706`}, {15, 0.000027463906355731167`, 0.048885753313201476`}},
0.009999994049206923`, 0.01757775547459423`, 0.9606921527953396`, 2075.339481686715`}, {"CURE", 365,
0.28218144092791136`, {{0, 0.0026669303821566796`, 4.74713608023889`}, {49, 0.0009003582210693933`, 1.60263763350352`}},
0.009999886982616052`, 0.034062108413650045`, 0.22600083523493347`, 1782.0004376787588`},
{"CURE", 365, 4.318195434379393`, {{0, 0.029368067868941614`, 52.275160806716066`},
{2, 0.003802435948593307`, 6.768335988496087`}, {4, 0.0003519951934562689`, 0.6265514443521585`}},
0.009994719513065287`, 0.012349963963143726`, 0.7189158216548763`, 1780.4256246039802`},
{"CURE", 365, 0.08235183277513594`, {{0, 0.0020082136038214573`, 3.5746202148021937`}}, 0.009993621570497956`,
0.027932117236846726`, 0.10510628155046543`, 1780.5477012407412`}, {"TBUR", 137.71369120763381`, 229.29206804902748`,
{{0, 0.06366596356755791`, 113.3254151502531`}, {7, 0.0005538920085467948`, 0.9859277752132948`},
{14, 0.00035644099664873703`, 0.6344649740347519`}, {21, 0.00035000724708947534`, 0.6230128998192661`},
{28, 0.0003491808213486847`, 0.6215418620006588`}, {35, 0.0003489790957103199`, 0.6211827903643693`},
{42, 0.0003489158904609367`, 0.6210702850205686`}, {49, 0.0003488767305020218`, 0.6210005802935988`},
{56, 0.00034882300424914905`, 0.6209049475634854`}, {63, 0.022807676935898972`, 40.59766494590017`}},
13.44962691276699`, 0.03567765262736589`, 0.172003875198208`, 1780.0192301078914`}, {"CURE", 365, 0.09446900422758027`,
{{0, 0.0014073567441248254`, 2.505095004542189`}, {5, 0.0001931314526105746`, 0.34377398564682277`}},
0.009998419518611785`, 0.015980792731295268`, 0.4032792864755621`, 1783.5828728342185`}, {"CURE", 365, 0.1498481131574041`,
{{0, 0.0008233447718388603`, 1.4655536938731712`}, {11, 0.00023029643877718584`, 0.4099276610233908`}},
0.009848730787840248`, 0.025458668254739455`, 0.040740432263917915`, 1780.3023970623362`}, {"CURE", 365,
1.4546602800233017`, {{0, 0.005400779803841752`, 9.613388050838319`}, {37, 0.0010809089853070447`, 1.9240179938465396`}},
0.009749006786403166`, 0.06020226082482937`, 0.2451140267579303`, 1783.236620346858`},
{"CURE", 365, 1.6537748454051746`, {{0, 0.004392232576147072`, 7.818173985541788`}}, 0.009997836144267 65`,
0.007784025025657857`, 0.1426331198340918`, 1780.271022335815`}, {"TBUR", 365, 229.9008982485788`,
{{0, 0.015256620131665125`, 27.15678383436392`}, {31, 0.0004196935505295989`, 0.7470545199426861`},
{62, 0.0004209746620227895`, 0.7493348984005653`}, {93, 0.000436571040645359`, 0.7770964523487391`},
{124, 0.0005543530824097727`, 0.9867484866893954`}, {155, 0.0009295472378593043`, 1.654594083389562`},
{186, 0.0009552812908212064`, 1.7004006976617474`}, {217, 0.000821262396622874`, 1.4618470659887155`},
{248, 0.0007941434726532388`, 1.413575381322765`}, {279, 0.11407921316330676`, 203.06099943068605`}},
339.0143867177117`, 0.026593924320985986`, 0.8697525631741547`, 2304.460752246641`}, {"CURE", 365,
4.076322692130266`, {{0, 0.00836450903233895`, 14.88882607756333`}, {3, 0.0007145751020562052`, 1.2719436816600451`},

```
    {6, 0.00005149130224085021`, 0.09165451798871337`}, {9, 0.000022742490331178 23`, 0.04048163278949725`},
    {12, 0.000021894599460804527`, 0.03897238704023206`}, {15, 0.000021873219636261058`, 0.038934330952544685`},
    {18, 0.000021872431297599214`, 0.038932927709726596`}, {21, 0.000019460927476214607`, 0.034640450907662004`}},
  0.009999996333013924`, 0.02601906305631053`, 0.993453091117767`, 2187.685367742925`}, {"CURE", 365,
  0.24457291533270362`, {{0, 0.001745545245444746`, 3.107070536891648`}, {1, 0.00034154898052460574`, 0.6079571853337982`}},
  0.00999991565972793`, 0.022314512327365775`, 0.7110197585023966`, 1788.8349330671958`},
 {"TBUR", 15.944896763905072`, 228.7284031657029`, {{0, 0.10373541831970215`, 184.64904460906985`}},
  6.093120594420567`*^7, 0.18499469406819186`, 0.5089653426503726`, 2030.8471151720046`}, {"CURE", 365,
  2.368047857645819`, {{0, 0.004569180762733 7485`, 8.133141757666072`}, {69, 0.002542997280820281`, 4.5265351598601`}},
  0.009993668461468 15`, 0.03053446101507106`, 0.4452770259004471`, 1782.6805272017941`},
 {"TBUR", 17.947045991462232`, 229.3692762725821`, {{0, 0.09566841125488282`, 170.2897720336914`}}, 4.558160028266961`*^7,
  0.11621094115192389`, 0.5216702641213732`, 2070.5467010748143`}, {"CURE", 365, 0.13544712613408702`,
  {{0, 0.0024149748859071396`, 4.298655296914708`}, {1, 0.00033900041604346314`, 0.6034207405573644`}},
  0.009997802406371326`, 0.027699355316967177`, 0.5873099132001962`, 1784.0435014044137`}, {"TBUR", 250.7096395186635`,
  131.680312828188`, {{0, 0.04179083501572555`, 74.38768632799147`}, {22, 0.003572906433942544`, 6.359773452417729`},
    {44, 0.00357291901554233`, 6.359795847665347`}, {66, 0.003574459932959975`, 6.362538680668755`},
    {88, 0.00370158292265462 7`, 6.588817602325235`}, {110, 0.00377093883852704 6`, 6.712271132578142`},
    {132, 0.0036538695218247407`, 6.503887748848038`}, {154, 0.003633911824108049`, 6.4683630469123266`},
    {176, 0.003609558573043412`, 6.425014260017272`}, {198, 0.032256299681803484`, 57.41621343361021`}},
  1.3826593293128007`, 0.023450105894567697`, 0.11090266684529822`, 1780.0176779542353`},
 {"TBUR", 53.37044561778921`, 230.25723623578259`, {{0, 0.04864086627960206`, 86.58074197769167`}},
  3.5827698242220104`*^7, 0.12960248460650828`, 0.4564206802264399`, 2577.0975444491123`},
 {"TBUR", 20.725963637807325`, 229.08688390640998`, {{0, 0.11721636772155765`, 208.6451345443726`}},
  6.885205442921187`*^7, 0.18727126722255336`, 0.5100389565340083`, 2047.0486147695503`},
 {"CURE", 365, 0.6411904755918663`, {{0, 0.0015539684312676119`, 2.766063807656349`},
    {10, 0.0005276924946412216`, 0.9392926404613745`}, {20, 0.00006476715273412251`, 0.11528553186673808`},
    {30, 7.058231528471488`*^-6, 0.012563652120679248`}, {40, 9.117094190461817`*^-6, 0.016228427659022035`}},
  0.009999970420676995`, 0.08409684622530986`, 0.6785189072713843`, 2353.5309573220793`},
 {"CURE", 365, 1.015624493004501`, {{0, 0.00938729070463417`, 16.709377454248823`},
    {5, 0.00104662590432925`, 1.862994109706065`}, {10, 0.00005589394876202498`, 0.09949122879640446`}},
  0.009999977846058742`, 0.0150799670213587 3`, 0.9494988949714749`, 1791.0469371723307`},
```

```
{"CURE", 365, 0.5407800124378717`, {{0, 0.0025323637822517783`, 4.507607532408166`}}, 0.009999786621997164`,
  0.016813738470346357`, 0.09166589631881794`, 1780.3039793331004`}, {"TBUR", 115.39518521501685`,
  229.80372778815007`, {{0, 0.040545638872487584`, 72.1712371930279`}, {6, 0.007874142035548912`, 14.015972823277062`},
    {12, 0.0010505776143507144`, 1.8700281535442718`}, {18, 0.00019928397757074356`, 0.35472548007592347`},
    {24, 0.00008324237207279701`, 0.14817142228957866`}, {30, 0.0000643711958709558`, 0.11458072865030132`},
    {36, 0.00006016977185872394`, 0.10710219390852861`}, {42, 0.00005881591534965492`, 0.10469232932238576`},
    {48, 0.00005823929820472589`, 0.10366595080441209`}, {54, 0.008932386749085072`, 15.89964841337143`}},
  2451.663031695299`, 0.015579430435717573`, 0.8127698012122064`, 3004.0358136859404`}, {"TBUR", 200.47889017037699`,
  228.29075666062224`, {{0, 0.01890324523122268`, 33.64777651157637`}, {17, 0.001318163176511957`, 2.3463304541912833`},
    {34, 0.0013173267944813541`, 2.34484169417681`}, {51, 0.001318111430402654`, 2.3462383461167238`},
    {68, 0.00132120922900474 3`, 2.3517524276284423`}, {85, 0.0013321048627861144`, 2.3711466557592837`},
    {102, 0.0013665595957037839`, 2.432476080352735`}, {119, 0.0014431441659523857`, 2.568796615395246`},
    {136, 0.0015082398448268552`, 2.684666923791802`}, {153, 0.02211986133347472`, 39.373353173584995`}},
  5504.277264310644`, 0.019522297355719698`, 0.1987175701238049`, 1780.0813633376974`},
{"TBUR", 16.239537108011593`, 229.330242327327`, {{0, 0.09710556030273437`, 172.8478973388672`}}, 8.659052494024475`*^7,
  0.2708749252405819`, 0.49915448012743013`, 2042.2862392493473`}, {"CURE", 365, 2.981395197643459`,
  {{0, 0.010061306829231231`, 17.90912615603159`}, {42, 0.0027418669098920267`, 4.8805230996078075`}},
  0.009740857073440287`, 0.033941202884615315`, 0.3089816164263805`, 1781.4024341964096`}, {"CURE", 365, 0.5936091541134879`,
  {{0, 0.0017846495889077385`, 3.1766762682557745`}, {33, 0.0007917866121303879`, 1.4093801695920904`}},
  0.009783627698783152`, 0.01946180927134679`, 0.23318611164417857`, 1784.9781502045103`}, {"CURE", 365,
  1.9902240621163603`, {{0, 0.004217839415474184`, 7.507754159544047`}, {4, 0.0008201721975341313`, 1.4599065116107537`}},
  0.009999915279103738`, 0.016220157736482967`, 0.4306163974271295`, 1781.39615190245`},
{"CURE", 365, 1.1966444801373206`, {{0, 0.0014030477184632008`, 2.497424938864497`}},
  0.009629699003284345`, 0.015409770710514473`, 0.040847929614956965`, 1780.1928638417448`},
{"TBUR", 20.62939301791234`, 230.10987338824134`, {{0, 0.09324485778808596`, 165.975846862793`}},
  2.1240904190349128`*^7, 0.024527386637739453`, 0.5617053767200433`, 2116.5447660023415`},
{"CURE", 365, 32.57191242719888`, {{0, 0.014239999793477355`, 25.347199632389692`},
    {3, 0.001981714501135058`, 3.5274518120204035`}, {6, 0.0019577839529364067`, 3.4848554362268045`},
    {9, 0.001957146891679881`, 3.483721467190188`}, {12, 0.001957115803158658`, 3.483666129622411`},
    {15, 0.0019571135289657383`, 3.4836620815590145`}, {18, 0.001957113308661122`, 3.483661689416798`},
    {21, 0.001957113282675028`, 3.48366164316155`}, {24, 0.0019587565149108716`, 3.4865865965413514`}},
```

```
0.009990441334205279`, 0.04243265492899171`, 0.8366714512643829`, 2964.0708074489435`}, {"CURE", 365,
0.5573834677268151`, {{0, 0.002064480256171733`, 3.674774855985685`}, {12, 0.0006112186064926608`, 1.0879691195569363`},
  {24, 0.00007456661524767509`, 0.13272857514086164`}, {36, 6.436063021798843`*^-6, 0.01145619217880194`}},
0.00999999490070948`, 0.05499705681253219`, 0.15054531406494312`, 1796.7695804490656`}, {"TBUR", 61.16095125876474`,
229.1128270018262`, {{0, 0.028927746069262655`, 51.491388003287526`}, {3, 0.00824479695228216`, 14.675738575062244`},
  {6, 0.004843924147894142`, 8.622184983251572`}, {9, 0.003217252181543743`, 5.726708883147862`},
  {12, 0.002315424376630646`, 4.12145539040255`}, {15, 0.0017480478284185746`, 3.111525134585063`},
  {18, 0.0013554028924468102`, 2.412617148555322`}, {21, 0.0010683207936848568`, 1.9016110127590449`},
  {24, 0.0008540070441782879`, 1.5201325386373523`}, {27, 0.06075142124029466`, 108.1375298077245`}},
6.60662349256681`*^6, 0.06817872075046405`, 0.6495604621937905`, 2284.4067848685004`}, {"CURE", 365,
1.6720274433256042`, {{0, 0.009315194939024359`, 16.58104699146336`}, {20, 0.0020110534587034377`, 3.579675156492119`}},
0.009999953376220075`, 0.014677440138183449`, 0.5486649900806273`, 1780.8630223847692`},
{"CURE", 365, 3.0156739977817812`, {{0, 0.00890415334348995`, 15.849392951412112`}}, 0.009658459182054017`,
0.007247829700819508`, 0.2066442063513949`, 1780.161577561703`}, {"CURE", 365, 0.2578451463437831`,
 {{0, 0.0024275074256297287`, 4.320963217620917`}, {8, 0.0007411475274690425`, 1.3192425988948957`}},
0.0099994950959626`, 0.011700631116710632`, 0.5672482577268989`, 1782.424498393112`},
{"CURE", 365, 1.0419861160124364`, {{0, 0.002488565993080148`, 4.429647467682663`},
  {3, 0.00047076706166219507`, 0.8379653697587073`}, {6, 0.00007283347614659932`, 0.12964358754094676`},
  {9, 0.000027631536319875687`, 0.04918413464937872`}, {12, 4.1023726064619055`*^-6, 0.0073022232395021916`}},
0.009999996630629096`, 0.013166316506325422`, 0.9595063768203443`, 1956.4508427852713`},
{"CURE", 365, 0.05302179011578308`, {{0, 0.00037492324486723997`, 0.6673633758636872`}},
0.009999629308232132`, 0.019154201162910865`, 0.0809360580181738`, 1781.9206284958134`},
{"CURE", 365, 1.1497489168256945`, {{0, 0.003279474350029803`, 5.837464343053049`},
  {8, 0.0002956143625498418`, 0.5261935653387184`}, {16, 0.000010670121713796583`, 0.018992816650557916`}},
0.009999982911167602`, 0.03193135255036664`, 0.5956090552482426`, 2250.07203200702`}, {"CURE", 365,
0.3668552471696376`, {{0, 0.002669910608553349`, 4.752440883224962`}, {52, 0.0009467183476855679`, 1.685158658880311`}},
0.009999865781914098`, 0.04384603709024823`, 0.3683467507902321`, 1786.5689999342294`},
{"CURE", 365, 3.1542315207694673`, {{0, 0.008168441690769312`, 14.539826209569377`}}, 0.009999934785458901`,
0.004248668564549102`, 0.6680190393459822`, 1781.5256701945934`}, {"CURE", 365, 0.9949536884528708`,
 {{0, 0.005484958153040078`, 9.763225512411339`}, {29, 0.0008679919802476933`, 1.545025724840894`}},
0.009724288566933505`, 0.03124819220392439`, 0.4065199466906466`, 1784.3183704227206`}, {"CURE", 365,
```

0.10966785510382938`, {{0, 0.003199197508794287`, 5.694571565653831`}, {3, 0.0004727621146581975`, 0.8415165640915915`}},
0.009954080479639568`, 0.00800697071931754`, 0.3932260464018066`, 1781.1798331224452`}, {"CURE", 365, 11.19892883569196`,
{{0, 0.014362729388136162`, 25.565658310882366`}, {20, 0.000020302653420931308`, 0.03613872308925773`}},
0.009999585877175851`, 0.02575683344580499`, 0.601757026891448`, 2142.3087660536057`}, {"TBUR", 77.00790650899185`,
230.1116510763281 7`, {{0, 0.00018275892603032706`, 0.3253108883339822`}, {1, 0.0002082416908595283`, 0.3706702097299603`},
{2, 0.0002109876315831826`, 0.375557984218065`}, {3, 0.00018940096539455298`, 0.33713371840230427`},
{4, 0.00015475725560600 7`, 0.2754679149786925`}, {5, 0.00011826671887055866`, 0.21051475958959437`},
{6, 0.00008643292592885644`, 0.15385060815336446`}, {7, 0.00006141664550356895`, 0.10932162899635274`},
{8, 0.00004291853240268130 6`, 0.07639498767677272`}, {9, 0.5570549486675435`, 991.5578086282273`}},
1249.977327960725`, 0.03031398454901036`, 0.7831777396391927`, 2153.63887664886`}, {"CURE", 365, 12.443017455671539`,
{{0, 0.013384387642623058`, 23.824210003869045`}, {23, 0.0002753515935351431`, 0.49012583649255465`},
{46, 0.00005818805145245417 4`, 0.10357473158536844`}, {69, 0.00005014737697092772`, 0.08926233100825133`},
{92, 0.00004885623833030644`, 0.08696410422794545`}, {115, 0.00004878311536757511 6`, 0.08683394535428371`},
{138, 0.00004877683622375439 4`, 0.08682276847828284`}, {161, 0.00004585599036753388`, 0.0816236628542103`}},
0.00999998786756934 1`, 0.0425594796771692 7`, 0.7626511675873454`, 2137.2730965994556`},
{"CURE", 365, 0.26278971017147323`, {{0, 0.003041977895332576`, 5.414720653691985`}}, 0.009999502016548685`,
0.006389535012539652`, 0.18104742259670875`, 1780.4999103794548`}, {"TBUR", 52.96372128216135`,
228.99432157082688`, {{0, 0.02825574321537451`, 50.29522292336663`}, {5, 0.015836359499888878`, 28.1887199098022`},
{10, 0.013414588566460436`, 23.877967648299574`}, {15, 0.0123542384590942`, 21.990544457187678`},
{20, 0.011634549951619697`, 20.70949891388306`}, {25, 0.010976154999287568`, 19.537555898731874`},
{30, 0.010343104843637228`, 18.410726621674268`}, {35, 0.009744804834129935`, 17.345752604751286`},
{40, 0.009186709291091342`, 16.352342538142587`}, {45, 0.22249661050538969`, 396.0439666995936`}},
1.0720870342769023`*^7, 0.27440908384766916`, 0.6736014814750575`, 1991.7833036426523`}, {"CURE", 365,
2.5337642139232734`, {{0, 0.013143388221913594`, 23.395231035006198`}, {4, 0.0036090385518991477`, 6.424088622380484`},
{8, 0.0006587635148902966`, 1.172599056504728`}, {12, 0.0001289850423083005`, 0.22959337530877488`},
{16, 0.00003945085544436917 3`, 0.07022252269097713`}, {20, 0.00002529377706714337 2`, 0.04502292317951521`},
{24, 0.00002332243697225631 7`, 0.04151393781061625`}, {28, 0.00002927059262097916`, 0.05210165486534290 6`}},
0.009999998895938235`, 0.030810935047462806`, 0.9638257771793688`, 2027.8093979379325`}, {"TBUR", 46.9402299888178`,
230.08431035988485`, {{0, 0.04255110933156526 5`, 75.74097461018617`}, {3, 0.017643675804597907`, 31.405742932184275`},
{6, 0.013490827762774705`, 24.013673417738975`}, {9, 0.010588503955255181`, 18.847537040354222`},
{12, 0.008448211537248403`, 15.037816536302156`}, {15, 0.006835608825192275`, 12.16738370884225`},

{18, 0.005603763885367132`, 9.974699715953495`}, {21, 0.004652064747267413`, 8.280675250135994`},
{24, 0.003909400839001749`, 6.958733493423113`}, {27, 0.15862622867219486`, 282.3546870365069`}},
2.394990673308113`*^7, 0.11820052311330242`, 0.7609104042451785`, 1925.2532550838`}, {"CURE", 365,
24.102748307073757`, {{0, 0.0351837583067579`, 62.62708978602907`}, {7, 0.0006936402827531319`, 1.2346797033005747`},
{14, 0.00006882990165702412`, 0.12251722494950293`}, {21, 0.00005608835630206144`, 0.09983727421766934`},
{28, 0.00005484129181649414 5`, 0.09761749943335958`}, {35, 0.00005459380986677609`, 0.09717698156286143`},
{42, 0.00005455760254599213`, 0.097112532531866`}, {49, 0.00005455320132081695 5`, 0.09710469835105419`},
{56, 0.00005455266207397035`, 0.09710373849166723`}, {63, 0.00004081933540089356`, 0.07265841701359053`}},
0.009999991850733553`, 0.05161718086775122`, 0.8351797921233193`, 1964.285706194949`},
{"CURE", 365, 0.21891564594983798`, {{0, 0.0011212228905185656`, 1.995776745123047`}},
0.009999926308482239`, 0.024867676648271393`, 0.11016488789735532`, 1780.7078226555875`},
{"CURE", 365, 1.3301792890982012`, {{0, 0.010584079433975538`, 18.839661392476458`},
{3, 0.0032004982408462947`, 5.696886868706405`}, {6, 0.000718630509604796`, 1.2791623070965368`},
{9, 0.00016558260142101482`, 0.29473703052940636`}, {12, 0.00003951447127118738 6`, 0.07033575886271355`}},
0.009999950214314266`, 0.01449677277306178`, 0.9419939967447647`, 1845.9784191019435`}, {"CURE", 365,
0.4988841399089817`, {{0, 0.01412622810227922`, 25.14468602205701`}, {4, 0.0015276771750035668`, 2.719265371506349`}},
0.009965004848726776`, 0.010657104312547629`, 0.4638833698961766`, 1780.2961827181698`}, {"CURE", 365, 0.389004157293689`,
{{0, 0.0015521062768652052`, 2.7627491728200653`}, {63, 0.0006624087301681652`, 1.1790875396993339`}},
0.009754250997982907`, 0.033049637995949066`, 0.0809638582577495`, 1780.69085430791`}, {"TBUR", 117.4421245988279`,
229.38412787596206`, {{0, 0.03307699644502299`, 58.87705367214092`}, {6, 0.01783317280279292`, 31.743047588971397`},
{12, 0.006414837091049752`, 11.418410022068558`}, {18, 0.002265048556890014`, 4.0317864312642255`},
{24, 0.0008323965830994209`, 1.481665917916969`}, {30, 0.0003182523897003692 4`, 0.5664892536666574`},
{36, 0.0001291341070058844`, 0.22985871047047426`}, {42, 0.00005915822834232301 5`, 0.10530164644933497`},
{48, 0.00003327836587970948`, 0.05923549126588288`}, {54, 0.025082499254264687`, 44.64684867259113`}},
43453.27048387717`, 0.03462767961034044`, 0.7364544123799394`, 2730.4553057839767`}, {"CURE", 365, 2.5752088680828353`,
{{0, 0.009787873502352272`, 17.42241483418704`}, {4, 0.0018501481780329413`, 3.2932637568986354`}},
0.009915534709580858`, 0.010989719336739543`, 0.5005069348353788`, 1780.7256045979393`},
{"CURE", 365, 1.2913125391413178`, {{0, 0.005254567604438628`, 9.353130335900758`},
{3, 0.0030390864812201964`, 5.409573936571949`}, {6, 0.000966045623521716`, 1.7195612098686546`}},
0.009999961141636642`, 0.008183867552247936`, 0.5062199602470125`, 1780.544611666054`},
{"CURE", 365, 8.307571863275443`, {{0, 0.004414480186172575`, 7.857774731387183`},

{9, 0.001780699563168618`, 3.1696452224401406`}, {18, 0.0002635061096501167`, 0.4690408751772077`},
{27, 0.000028166921976397054`, 0.05013712111798676`}, {36, 9.265055561965836`*^-6, 0.016491798900299188`}},
0.009999933728280539`, 0.0861047799291407`, 0.4240761949293936`, 2181.263679913462`}, {"CURE", 365,
0.4915489081982489`, {{0, 0.0017553777765816042`, 3.124572442315255`}, {3, 0.000746282252919083`, 1.3283824101959678`},
{6, 0.0002484141122049255`, 0.4421771197247674`}, {9, 0.00008143527695998696`, 0.14495479298877678`}},
0.009999957618739057`, 0.011996485122258165`, 0.84633050576025`, 1803.8234187222506`}, {"CURE", 365,
0.6915259444806819`, {{0, 0.010397593936602239`, 18.507717207151988`}, {10, 0.002858759361928513`, 5.088591664232752`}},
0.009925429941300138`, 0.012675007201245586`, 0.39504481141864123`, 1780.3464378195881`}, {"TBUR", 98.22800608026213`,
228.9500485183779`, {{0, 0.022248152418298514`, 39.60171130457135`}, {5, 0.0011172699481005746`, 1.9887405076190225`},
{10, 0.0003325810662538301 5`, 0.5919942979318178`}, {15, 0.00022416967848745315`, 0.3990220277076666`},
{20, 0.0002015940616519574`, 0.35883742974048416`}, {25, 0.00019525678607295148`, 0.3475570792098537`},
{30, 0.00019361074905734906`, 0.34462713332208134`}, {35, 0.00019412043936342988`, 0.3455343820669052`},
{40, 0.00019626347037141176`, 0.34934897726111297`}, {45, 0.04128145693737635`, 73.48099334852992`}},
491271.0959507047`, 0.048685746886068595`, 0.19167653754949396`, 1780.0707733897398`}, {"CURE", 365,
3.1633567468795816`, {{0, 0.013383022229630961`, 23.82177956874311`}, {10, 0.002424444017004029`, 4.3155103502671714`}},
0.009999556693476828`, 0.019727780998752466`, 0.8342516049181727`, 2040.2702808251631`},
{"CURE", 365, 0.3461477987750599`, {{0, 0.010671867804569038`, 18.99592469213289`}}, 0.00999951506214088`,
0.015323670282110407`, 0.35275436472808813`, 1780.2658958255045`}, {"CURE", 365, 0.6789064351954621`,
{{0, 0.004970307100399716`, 8.847146638711495`}, {6, 0.0018278988757316308`, 3.2536599988023025`},
{12, 0.0005638818346041301`, 1.0037096655953515`}, {18, 0.0000895272383082983`, 0.159358484188771`}},
0.009978049961903207`, 0.019800315879192076`, 0.8439746759405939`, 1790.7417462410187`},
{"CURE", 365, 1.9257157610652336`, {{0, 0.012143414684134754`, 21.61527813775986`},
{2, 0.003775010493457009`, 6.719518678353475`}, {4, 0.000805550791995962`, 1.4338804097528124`},
{6, 0.0001659066365407871`, 0.295313813042601`}, {8, 0.000043049005775335 95`, 0.07662723028009799`},
{10, 0.000020745567261836573`, 0.0369271097260691`}, {12, 0.000017024012959895843`, 0.030302743068614604`},
{14, 0.000016464204966554617`, 0.029306284840467215`}, {16, 0.000014273707559376788`, 0.025407199455690685`}},
0.009999989861544404`, 0.021561032828229944`, 0.9731850449754562`, 1963.8860101680646`},
{"CURE", 365, 0.08483376151337257`, {{0, 0.001508560167522239`, 2.6852370981895857`}},
0.009971751507767383`, 0.01902571727145754`, 0.055590624885041025`, 1780.319103684354`},
{"CURE", 365, 1.2395514494111146`, {{0, 0.012199679057460928`, 21.715428722280453`}},
0.009998347813885972`, 0.031232080738174815`, 0.35166264943937203`, 1780.4160749616901`},

```
{"CURE", 365, 0.1766727615370314`, {{0, 0.00145580913096526`, 2.591340253118163`}}, 0.009998536133525486`,
  0.01839593567413445`, 0.06313655727453828`, 1780.4424574298355`}, {"TBUR", 66.19476115559169`, 229.2006850156225`,
  {{0, 0.05158412898187777`, 91.81974958774245`}, {3, 0.009335408981297902`, 16.61702798671027`},
    {6, 0.004115004231003997`, 7.324707531187116`}, {9, 0.002298794403904644`, 4.091854038950268`},
    {12, 0.001557019910867752`, 2.7714954413446`}, {15, 0.001218393173617545`, 2.16873984903923`},
    {18, 0.001048271262030774`, 1.8659228464147777`}, {21, 0.0009454813241910144`, 1.6829567570600057`},
    {24, 0.0008585043155479605`, 1.5281376816753698`}, {27, 0.050575181213593966`, 90.02382256019726`}},
  3.1929777162245917`*^6, 0.0657493454130685`, 0.6814741621368369`, 2439.911276082972`},
{"CURE", 365, 0.7534089240588607`, {{0, 0.0015337913973563233`, 2.7301486872942555`},
    {9, 0.0002905051663439081`, 0.5170991960921564`}, {18, 0.000011054354064721369`, 0.019676750235204037`}},
  0.009999995853461908`, 0.04937023897335587`, 0.13881754594237286`, 1784.382149970579`},
{"TBUR", 31.925358330968926`, 230.25960527075372`, {{0, 0.07757607460021973`, 138.08541278839112`}},
  3.4610065999125615`*^7, 0.060478624050297844`, 0.4952351940530084`, 2208.7386236389343`},
{"CURE", 365, 1.4565434429639201`, {{0, 0.024456962023137123`, 43.53339240118409`}}, 0.009999977606007883`,
  0.014145997577823703`, 0.486404132074001`, 1780.37041693074`}, {"TBUR", 152.13802993645`, 227.02513926790584`,
  {{0, 0.01700052532327559`, 30.260935075430552`}, {14, 0.004431257537773516`, 7.887638417236858`},
    {28, 0.00550187791028876 8`, 9.793342680314007`}, {42, 0.011188482189628021`, 19.91549829753788`},
    {56, 0.009052789143537408`, 16.113964675496586`}, {70, 0.01122323532400561`, 19.977358876729983`},
    {84, 0.01295147853992011`, 23.053631801057797`}, {98, 0.01443027530534967`, 25.685890043522413`},
    {112, 0.016815889681097936`, 29.932283632354327`}, {126, 1.783154168257748`, 3174.0144194987915`}},
  63566.54240487005`, 0.08754601648613246`, 0.8768418493978484`, 2097.679829787099`}, {"TBUR", 31.273444915447033`,
  222.80607549713508`, {{0, 0.04175847199729758`, 74.33008015518969`}, {3, 0.025492831622241814`, 45.37724028759044`},
    {6, 0.02549976529661426`, 45.389582227973385`}, {9, 0.025933529197155376`, 46.16168197093657`},
    {12, 0.0266402510676405`, 47.41964690040009`}, {15, 0.027578695310666392`, 49.090077652986174`},
    {18, 0.028730305978843216`, 51.13994464234093`}, {21, 0.03008449290321733`, 53.550397367726845`},
    {24, 0.03163523606035405`, 56.310720187430206`}, {27, 3.4564595079817297`, 6152.497924207479`}},
  8.41776847383769`*^7, 0.13894020425385187`, 0.7584629190587053`, 1854.8175513765336`}, {"CURE", 365,
  7.334198601231345`, {{0, 0.010948191670821197`, 19.48778117406173`}, {18, 0.002169272362629212`, 3.8613048054799974`}},
  0.009999755585481867`, 0.029943034110809504`, 0.6996943364872497`, 1957.729906437746`},
{"CURE", 365, 0.2278991060042154`, {{0, 0.003361553193050999`, 5.983564683630778`}}, 0.009999428092123659`,
  0.022511406728372665`, 0.5415341372942659`, 1782.644585811527`}, {"CURE", 365, 0.5428214554133469`,
```

```
{{0, 0.0030895153218636236`, 5.499337272917249`}, {20, 0.0011871541808530187`, 2.1131344419183735`}},
0.009800861634288121`, 0.01673735886681994`, 0.6428462078446369`, 1787.4977689234563`}, {"TBUR", 53.449541218062286`,
229.29916851832718`, {{0, 0.03207261833450716`, 57.08926063542275`}, {4, 0.015958276034880387`, 28.405731342087083`},
  {8, 0.012512013308336848`, 22.271383688839588`}, {12, 0.0101785518245383`, 18.117822247678173`},
  {16, 0.00849082408148491`, 15.113666865043138`}, {20, 0.007218449894244028`, 12.84884081175437`},
  {24, 0.006225843614930039`, 11.08200163457547`}, {28, 0.005428889023433914`, 9.663422461712367`},
  {32, 0.004773798470573067`, 8.49736127762006`}, {36, 0.14492816428257665`, 257.9721324229865`}},
1.2061228925735828`*^7, 0.13027902181864426`, 0.7204007759539979`, 1896.009129492166`}, {"CURE", 365,
0.860548417883519`, {{0, 0.005968218997364339`, 10.623429815308523`}, {29, 0.002532390469725926`, 4.507655036112148`}},
0.00999967525057186`, 0.025362253330691347`, 0.18534135215091455`, 1780.485862872074`}, {"CURE", 365,
8.015745837503044`, {{0, 0.029826976518394385`, 53.09201820274201`}, {36, 0.005073717258103342`, 9.031216719423949`}},
0.009998301206116722`, 0.025235033590386338`, 0.6745897596555153`, 1838.6690225482291`}, {"CURE", 365,
1.6794545839161499`, {{0, 0.009156571827119587`, 16.298697852272863`}, {5, 0.0009689368681636567`, 1.7247076253313092`}},
0.009942062198540703`, 0.02104311848132337`, 0.24769819293031092`, 1780.2270725606936`},
{"CURE", 365, 0.09931738827142979`, {{0, 0.0020229289703774292`, 3.6008135672718247`}}, 0.009631551173390391`,
0.026333450622917934`, 0.07108660864610111`, 1780.2995179551774`}, {"CURE", 365, 5.9382791057049324`,
{{0, 0.02222613197812132`, 39.56251492105595`}, {9, 0.0042781780697626`, 7.615156964177427 5`}}, 0.00888082925601106 1`,
0.01988859501696818`, 0.9032087947361904`, 2208.138088373683`}, {"CURE", 365, 1.5531900775783984`,
{{0, 0.0023518419060297576`, 4.186278592732969`}, {31, 0.00047133826344263 72`, 0.8389821089278942`}},
0.009999817788100213`, 0.030341821338021557`, 0.6053942843208758`, 1855.1241883562493`},
{"TBUR", 20.650189440547635`, 229.82847427758165`, {{0, 0.08845816612243655`, 157.45553569793705`}},
2.8604757470278826`*^7, 0.041371452670487315`, 0.5685697160070564`, 2129.074955789649`}, {"TBUR", 187.22145890011927`,
229.48670249637996`, {{0, 0.0020698040790678588`, 3.6842512607407882`}, {12, 0.000657985576549847`, 1.1712143262587276`},
  {24, 0.00024904204301823626`, 0.44329483657246055`}, {36, 0.00013121668197753656`, 0.2335656939200151`},
  {48, 0.00008776431403992442`, 0.15622047899106548`}, {60, 0.00006860493150095518`, 0.12211677807170022`},
  {72, 0.00005906975318980896`, 0.10514416067785995`}, {84, 0.00005388641373881676`, 0.09591781645509383`},
  {96, 0.00005086790317968 3155`, 0.090544867659836`}, {108, 0.049587866775729926`, 88.26640286079927`}},
157603.16911329646`, 0.012406889595796295`, 0.7918152381743335`, 2718.7345468705357`},
{"CURE", 365, 0.4421918129049762`, {{0, 0.0008600236037000025`, 1.5308420145860044`},
  {9, 0.00016470787139730945`, 0.2931800110872108`}, {18, 0.0000142651797661025`, 0.02539201998366245`}},
0.00999919018855153`, 0.04244992754867237`, 0.43093116078196636`, 1850.9288671226775`}, {"TBUR", 128.9990081986891`,
```

```
230.15558523616625`, {{0, 0.020024942260906307`, 35.644397224413225`}, {6, 0.0009978437587015196`, 1.7761618904887047`},
    {12, 0.00023981383520405968`, 0.4268686266632262`}, {18, 0.00013043573405570813`, 0.23217560661916045`},
    {24, 0.00010654680010556148`, 0.1896533041878994`}, {30, 0.00010109054674354431`, 0.17994117320350886`},
    {36, 0.00010262014215797353`, 0.18266385304119287`}, {42, 0.00010953419431528523`, 0.1949708658812077`},
    {48, 0.00012327527806542835`, 0.21942999495646245`}, {54, 0.03971395070549117`, 70.69083225577427`}},
  316114.54843140685`, 0.056088783902579493`, 0.7345081230572931`, 3437.571167817211`}, {"CURE", 365, 0.9294995597750549`,
  {{0, 0.0012304442776345417`, 2.190190814189484`}, {15, 0.00030707325021729387`, 0.5465903853867831`}},
  0.00973904853743346`, 0.02195808151440193`, 0.051094447880530566`, 1781.056884842847`},
{"CURE", 365, 0.19524061833380654`, {{0, 0.0006622131636799535`, 1.1787394313503172`}}, 0.009998925501832106`,
  0.02093892975092179`, 0.08325147633947173`, 1781.1546349771506`}, {"CURE", 365, 7.049996005262779`,
  {{0, 0.005554770682732638`, 9.887491815264097`}, {29, 0.0018821291970151403`, 3.35018997068695`}},
  0.009670330506433046`, 0.02107775094812013`, 0.4068997951678737`, 1782.8232425650272`},
{"CURE", 365, 0.39304120504089496`, {{0, 0.01020221909932953`, 18.15994999680656`}}, 0.00999946292181278`,
  0.012698616923534408`, 0.2909022505124309`, 1780.2246288517567`}, {"TBUR", 365, 229.3776243204121`,
  {{0, 0.09253241191166466`, 164.7076932027631`}, {23, 0.0024175452019817717`, 4.303230459527554`},
    {46, 0.0005439882272169962`, 0.9682990444462533`}, {69, 0.00048606715944772647`, 0.8651995438169532`},
    {92, 0.0004815889109726255`, 0.8572282615312734`}, {115, 0.0004810922900579046`, 0.8563442763030702`},
    {138, 0.00048103461185235505`, 0.8562416090971919`}, {161, 0.00048102787296841547`, 0.8562296138837796`},
    {184, 0.00048102708492270236`, 0.8562282111624103`}, {207, 0.0005019413026997409`, 0.8934555188055389`}},
  0.35965177542295707`, 0.015490487734370078`, 0.11104714276229553`, 1780.009389010183`}, {"CURE", 365,
  0.30613688314672904`, {{0, 0.0004852722169705667`, 0.8637845462076088`}, {6, 0.00025472941613062066`, 0.4534183607125048`},
    {12, 0.00007618245531627462`, 0.13560477046296882`}, {18, 0.000022266995162479207`, 0.03963525138921299`},
    {24, 8.52993353910533`*^-6, 0.015183281699607488`}, {30, 5.17106669589546`*^-6, 0.009204498718693919`},
    {36, 4.383866948142577`*^-6, 0.007803283167693789`}, {42, 0.000011500877574492425`, 0.020471562082596515`}},
  0.00999998777857782`, 0.052665221495137325`, 0.8147607575425471`, 2115.433638141848`},
{"CURE", 365, 3.493336728790061`, {{0, 0.006292821493189316`, 11.201222257876982`},
    {3, 0.001260807485687092`, 2.2442373245230236`}, {6, 0.00013304225694893245`, 0.23681521736909975`}},
  0.009999995593160551`, 0.016299787852276378`, 0.6173236305775464`, 1781.2968857669387`},
{"CURE", 365, 2.3559305602285834`, {{0, 0.006703734332826475`, 11.932647112431125`},
    {2, 0.0012350265934189322`, 2.1983473362857`}, {4, 0.0001809448971689992`, 0.32208191696081856`}},
  0.009999959168879378`, 0.011273839731974326`, 0.8185916646128246`, 1781.8526088320916`}, {"CURE", 365,
```

```
0.3163549252069758`, {{0, 0.0008535173289495435`, 1.5192608455301875`}, {27, 0.0003840591184027411`, 0.6836252307568792`}},
0.00979093454826865`, 0.010726886487370883`, 0.0891098537679161`, 1782.1072736333256`}, {"TBUR", 217.59335998640515`,
228.14200080371668`, {{0, 0.0649917172021832`, 115.6852566198861`}, {15, 0.0012991403487436926`, 2.3124698207637726`},
  {30, 0.001299000696077915`, 2.312221239018689`}, {45, 0.0012990258280533083`, 2.3122659739348888`},
  {60, 0.0012991761948125333`, 2.3125336267663092`}, {75, 0.001299974663990539`, 2.313954901903159`},
  {90, 0.0013043309800921672`, 2.3217091445640574`}, {105, 0.001328141403360043`, 2.3640916979808764`},
  {120, 0.0014493691954885239`, 2.5798771679695727`}, {135, 0.15950132968367906`, 283.9123668369487`}},
44.85646238767835`, 0.028280156449634785`, 0.8374250788552444`, 6344.233017577855`},
{"CURE", 365, 0.16237136950934303`, {{0, 0.0004403790419583306`, 0.7838746946858283`},
  {16, 0.00011325685077337115`, 0.20159719437660062`}, {32, 0.000011511773136874358`, 0.020490956183636357`}},
0.009999948464821131`, 0.05960400492824641`, 0.133552074924821`, 1797.9742228657508`}, {"TBUR", 141.22977388107986`,
229.63407199343737`, {{0, 0.10271241250759498`, 182.82809426351906`}, {11, 0.0029797788005178263`, 5.304006264921731`},
  {22, 0.000452432414832878`, 0.8053296984025229`}, {33, 0.0003039072174133876 6`, 0.5409548469958301`},
  {44, 0.0003094909402720857`, 0.5508938736843125`}, {55, 0.0003555372383850203 4`, 0.6328562843253362`},
  {66, 0.0004124617659238569`, 0.7341819433444653`}, {77, 0.0004483115247725059 5`, 0.7979945140950606`},
  {88, 0.0004377965361116435`, 0.7792778342787254`}, {99, 0.01357139282671691`, 24.1570792315561`}},
7323.663247196291`, 0.012917863186289314`, 0.7017986962293234`, 5442.830603434135`},
{"TBUR", 16.09991866016497`, 229.75112514760932`, {{0, 0.09898036956787112`, 176.1850578308106`}},
3.546682887992379`*^7, 0.1629588152528585`, 0.5109079444205135`, 2045.7485599795268`}, {"TBUR", 106.55912486168423`,
228.73428316908928`, {{0, 0.03997774492659391`, 71.16038596933716`}, {7, 0.0005058385837636261`, 0.9003926790992545`},
  {14, 0.0004992922571985587`, 0.8887402178134346`}, {21, 0.00049916328776129`, 0.8885106522150963`},
  {28, 0.0004991725458195254`, 0.8885271315587552`}, {35, 0.00049926462813081 4`, 0.8886910380728489`},
  {42, 0.00049965609008568 1`, 0.8893878403525121`}, {49, 0.0005014730349325142`, 0.8926220021798754`},
  {56, 0.000510704322024058`, 0.9090536932028233`}, {63, 0.05515019911501342`, 98.1673544247239`}},
41580.58921109704`, 0.0245448614078943`, 0.15400431713504392`, 1780.0262176787594`},
{"CURE", 365, 1.3083674719472285`, {{0, 0.005837288854658519`, 10.390374161292165`},
  {6, 0.0020400101382465033`, 3.6312180460787755`}, {12, 0.0004880740019865646 3`, 0.868771723536085`},
  {18, 0.00011743152029820209`, 0.20902810613079967`}, {24, 0.000033052895822710 47`, 0.058834154564424625`},
  {30, 0.000014683154090189345`, 0.026136014280537037`}, {36, 0.000012600866287959494`, 0.0224295419925679`}},
0.00999999311016379`, 0.03569430926821475`, 0.943336541551741`, 2156.926879068339`},
{"CURE", 365, 5.445117313374496`, {{0, 0.009938411193603136`, 17.690371924613583`}},
```

```
0.009999993398089583`, 0.016451853001422528`, 0.33327117814131124`, 1780.4863777840687`},
{"CURE", 365, 0.04943310738935026`, {{0, 0.0012695933599207025`, 2.2598761806588503`}}, 0.009997625235053029`,
 0.026522716583517084`, 0.18658254517338616`, 1781.0697572699805`}, {"CURE", 365, 0.14522706734379298`,
 {{0, 0.00048182536904587433`, 0.8576491569016562`}, {14, 0.000050664963904379`, 0.0901836357497946`}},
 0.00999985385024322`, 0.041342204913385146`, 0.4621092098797298`, 1874.761266424623`}, {"CURE", 365,
 8.093778200442454`, {{0, 0.025944168309507982`, 46.18061959092421`}, {4, 0.0010518846652108169`, 1.872354704075254`},
  {8, 0.0000742226823123579`, 0.13211637451599706`}, {12, 0.0000526465391596112`, 0.09371083970410794`},
  {16, 0.0000523165930185082l`, 0.09312353557294463`}, {20, 0.0000523104684025082 26`, 0.09311263375646464`},
  {24, 0.0000523100693173012 26`, 0.09311192338479618`}, {28, 0.00005231000117210908`, 0.09311180208635417`},
  {32, 0.00005230998356590480 5`, 0.09311177074731056`}, {36, 0.00004427795964384088`, 0.07881476816603677`}},
 0.009999997352354363`, 0.03416438777755674`, 0.997478843134907`, 2045.423727460403`}, {"TBUR", 72.1184092087263`,
 230.08379007328995`, {{0, 0.030349502647287942`, 54.02211471217254`}, {3, 0.0037843817314938476`, 6.736199482059048`},
  {6, 0.0010397774690226519`, 1.8508038948603203`}, {9, 0.00041761220447337 52`, 0.7433497239626078`},
  {12, 0.00026656274004023 7`, 0.4744816772716219`}, {15, 0.00022495102055654682`, 0.4004128165906533`},
  {18, 0.00021122967018539644`, 0.37598881293000563`}, {21, 0.0002057994901616406`, 0.36632309248772027`},
  {24, 0.00020328881989558763`, 0.36185409941414604`}, {27, 0.021014948498753405`, 37.406608327781065`}},
 153155.82673908933`, 0.023179857444678348`, 0.7656706458712454`, 2857.2877004173356`},
{"CURE", 365, 1.429388341888615`, {{0, 0.014926618429645872`, 26.569380804769654`}, {5, 0.00526189744107993`, 9.366177445122277`},
  {10, 0.0014324520325700212`, 2.549764617974638`}, {15, 0.00038912545383766264`, 0.6926433078310396`},
  {20, 0.00011147412327743021`, 0.1984239394338258`}, {25, 0.00003806607304605811`, 0.06775761002198344`},
  {30, 0.000019184313820698314`, 0.034148078600843`}, {35, 0.000014671356924883401`, 0.026115015326292453`},
  {40, 0.000013700729211818161`, 0.024387297997036327`}, {45, 0.000013706738505282783`, 0.024397994539403355`}},
 0.009999997066168607`, 0.03686672088837038`, 0.9581113095588643`, 2013.012309273665`},
{"CURE", 365, 1.8862590232840932`, {{0, 0.006434950484543647`, 11.454211862487693`},
  {5, 0.0019406093605597995`, 3.4542846617964433`}, {10, 0.00018177943531299 46`, 0.3235673948571304`}},
 0.009999971089525354`, 0.032017343993259444`, 0.23099991653189553`, 1780.766123329344`}, {"TBUR", 81.73680636815219`,
 230.08724160683525`, {{0, 0.05236231151904259 5`, 93.20491450389582`}, {5, 0.0016614832670845707`, 2.9574402154105353`},
  {10, 0.0004004831602978107`, 0.7128600253301031`}, {15, 0.00030184113833517 93`, 0.5372772262366192`},
  {20, 0.00029098038798731825`, 0.5179450906174264`}, {25, 0.0003049166933984467 5`, 0.5427517142492352`},
  {30, 0.0003530115588087958`, 0.6283605746796564`}, {35, 0.00048441166084140506`, 0.862252756297701`},
  {40, 0.0007980941472166765`, 1.4206075820456843`}, {45, 0.08513031053990355`, 151.53195276102832`}},
```

```
81238.0765571852`, 0.022137430337686115`, 0.8197321090131533`, 2159.3146886347013`},
{"CURE", 365, 3.762126473904325`, {{0, 0.009717578539540535`, 17.297289800382153`}},
  0.009999610875223917`, 0.006071390777662093`, 0.47490389113501413`, 1780.614171853847`},
{"TBUR", 52.80214150297674`, 230.2601168481161`, {{0, 0.06909110069274904`, 122.9821592330933`}}, 25188.395466078324`,
  0.0030829304285765794`, 0.6977169311444136`, 2410.7414129827366`}, {"CURE", 365, 0.13294500048002442`,
  {{0, 0.0010397229803466395`, 1.8507069050170182`}, {20, 0.00015795046048205908`, 0.2811518196580652`}},
  0.009999787128175262`, 0.021242213269667753`, 0.0627113938435829`, 1781.224667881797`}, {"CURE", 365,
  0.6355170684629339`, {{0, 0.0026131634387261972`, 4.651430920932631`}, {6, 0.0006202348986491448`, 1.104018119595477`},
    {12, 0.00010102802763995095`, 0.17982988919911266`}, {18, 0.000029815330490971123`, 0.053071288273928596`}},
  0.009999993588828608`, 0.020352993574724216`, 0.9354807439192228`, 1895.0167219760908`}, {"CURE", 365,
  0.924706854397969`, {{0, 0.004600675044050828`, 8.189201578410474`}, {50, 0.0020178012295615933`, 3.5916861886196365`}},
  0.009743969373683003`, 0.03424806477412374`, 0.13321122878092653`, 1780.227293867808`}, {"CURE", 365,
  0.4906933560453421`, {{0, 0.004077716420335571`, 7.258335228197316`}, {36, 0.0017036206956214947`, 3.0324448382062603`}},
  0.009758538599750096`, 0.0162635521454633`, 0.32591461034900665`, 1782.1541557599726`}, {"CURE", 365,
  0.5866409749731667`, {{0, 0.01696802035886134`, 30.20307623877319`}, {5, 0.004478123571711384`, 7.971059957646263`}},
  0.009917544833206761`, 0.010678478279367897`, 0.6897952869409755`, 1780.5406827818713`},
{"CURE", 365, 0.843809180361248`, {{0, 0.01440707317121391`, 25.64459024476076`}},
  0.009999953896049107`, 0.013637640479163448`, 0.5549295930414538`, 1780.5175686972239`},
{"TBUR", 21.44496191008965`, 230.1749992710648`, {{0, 0.0934034061431885`, 166.2580629348755`}}, 7.450517421109603`*^7,
  0.04913346461776874`, 0.5704759336625295`, 2119.3562074885367`}, {"TBUR", 217.1463148550105`, 228.7873905492961`,
  {{0, 0.07852961095535806`, 139.78270750053733`}, {16, 0.00025949969651916597`, 0.4619094598041154`},
    {32, 0.0002506548202853745`, 0.44616558010796664`}, {48, 0.0002505187273661910`7`, 0.44592333471182005`},
    {64, 0.0002505225704400546`6`, 0.4459301753832972`}, {80, 0.00025056171057759324`, 0.445999844828116`},
    {96, 0.0002506224835066749`, 0.44610802064188126`}, {112, 0.0002507030213232532`, 0.4462513779553906`},
    {128, 0.0002508031954407959`4`, 0.44642968788461673`}, {144, 0.005135713502995852`, 9.141570035332617`}},
  72.26749741632634`, 0.012495855211698603`, 0.8383468442382156`, 2513.887490176772`}, {"CURE", 365, 3.721633469348757`,
  {{0, 0.007552728634804629`, 13.44385696995224`}, {33, 0.003906123539772467`, 6.952899900794991`}},
  0.009999920536486438`, 0.02479007804912594`, 0.6691008309146286`, 1806.8204807860363`}, {"TBUR", 318.4671276860406`,
  228.9199849102732`, {{0, 0.07813989830173135`, 139.08901897708182`}, {23, 0.0035040794202391384`, 6.237261368025665`},
    {46, 0.00027750721965883775`, 0.49396285099273113`}, {69, 0.00009232165066851001`, 0.1643325381899478`},
    {92, 0.00007144910322251059`, 0.12717940373606884`}, {115, 0.00006879826770065968`, 0.12246091650717422`},
```

```
    {138, 0.00006820740903051683`, 0.12140918807431995`}, {161, 0.00006806532419421761`, 0.12115627706570735`},
    {184, 0.00006803034059670318`, 0.12109400626213165`}, {207, 0.0003312373046134263`, 0.5896024022118989`}},
  1.5212057012576632`, 0.018770219012927074`, 0.6040051842734971`, 2534.196684957089`},
 {"TBUR", 15.314963758499578`, 229.96078737966823`, {{0, 0.11175242424011234`, 198.91931514739997`}},
  4.122343563126204`*^7, 0.09117534467045268`, 0.5199705521504011`, 2025.190011363844`}, {"TBUR", 46.64453268416391`,
  225.20650587324903`, {{0, 0.03822140263259417`, 68.03409668601763`}, {5, 0.017449480733084603`, 31.060075704890593`},
    {10, 0.014095438260600876`, 25.089880103869557`}, {15, 0.013271185134148494`, 23.62270953878432`},
    {20, 0.013714268247218183`, 24.411397480048365`}, {25, 0.014908718464894622`, 26.53751886751243`},
    {30, 0.01655625757439023`, 29.47013848241461`}, {35, 0.018440995353699967`, 32.82497172958595`},
    {40, 0.0204135411421291`, 36.3361032329898`}, {45, 2.5949568468952204`, 4619.023187473493`}},
  9.828801974287048`*^6, 0.10868421939234632`, 0.7805361728500919`, 2072.7689958929973`}, {"CURE", 365,
  8.375539029742216`, {{0, 0.02142235137750017`, 38.1317854519503`}, {19, 0.0035227611789336935`, 6.2705148985019745`}},
  0.009999586080375796`, 0.016208729215337076`, 0.8192187505781805`, 2110.2235855692093`}, {"CURE", 365,
  0.5334098857419258`, {{0, 0.006221134651771846`, 11.073619680153886`}, {5, 0.0023699078893841072`, 4.218436043103711`},
    {10, 0.0007517670046252754`, 1.3381452682329904`}, {15, 0.00009097199884997514`, 0.16193015795295576`}},
  0.009993252360358525`, 0.019561459000301002`, 0.8188099008343404`, 1782.6135417276937`}, {"CURE", 365,
  1.6974942677147042`, {{0, 0.016277924885004645`, 28.97470629530827`}, {22, 0.005691330408033655`, 10.130568126299906`}},
  0.009999944744008315`, 0.013024993883744545`, 0.6895684282214538`, 1785.808974461571`},
 {"CURE", 365, 0.04545490155754764`, {{0, 0.0019058146218992442`, 3.3923500269806546`}},
  0.009998613758844999`, 0.010933300441962819`, 0.1470769481307068`, 1780.8925839106787`},
 {"TBUR", 18.357004473237595`, 229.78128305372076`, {{0, 0.08672314643859863`, 154.36720066070555`}},
  7.036674316248423`*^7, 0.11078015707423626`, 0.55887625406725`, 2111.047837648635`}, {"TBUR", 63.09304255150685`,
  229.44534747946213`, {{0, 0.05345564646260932`, 95.1510507034446`}, {3, 0.009866536315142848`, 17.56243464095427`},
    {6, 0.0037803092852819083`, 6.728950527801796`}, {9, 0.0016409202230207682`, 2.9208379969769678`},
    {12, 0.0008317629352156704`, 1.4805380246838933`}, {15, 0.0005067324963468355`, 0.9019838434973672`},
    {18, 0.0003675857051333633`, 0.6543025551373867`}, {21, 0.0003036309998986804`, 0.5404631798196511`},
    {24, 0.0002721054846649122`, 0.48434776270354374`}, {27, 0.04309067414632943`, 76.7013999804664`}},
  834485.5652296057`, 0.04923169554329694`, 0.27791523588392214`, 1780.0420223052643`},
 {"CURE", 365, 0.7451010722817135`, {{0, 0.002824397944976279`, 5.027428342057776`},
    {6, 0.0003824957176574l034`, 0.6808423774301904`}, {12, 0.000050764486380185046`, 0.09036078575672939`}},
  0.009999995401734577`, 0.015678229942920077`, 0.950310352763764`, 1896.9268452176104`}, {"CURE", 365, 0.07365371831602986`,
```

{{0, 0.00019948741217790973`, 0.35508759367667936`}, {30, 0.00013616326295738017`, 0.2423706080641367`}},
0.009999527343855345`, 0.01365358605986204`, 0.15061278718680648`, 1789.8567771346402`},
{"TBUR", 42.523236305316125`, 230.23437581936543`, {{0, 0.059055223464965834`, 105.11829776763918`}},
9.475781821707174`*^6, 0.03360615533961062`, 0.6472190288312025`, 2528.741768107302`}, {"TBUR", 175.17273959892674`,
228.46632742878012`, {{0, 0.06737189362231324`, 119.92197064771757`}, {14, 0.0015234559857578308`, 2.711751654648939`},
{28, 0.001522423368617232`, 2.7099135961386733`}, {42, 0.0015235331255738346`, 2.7118889635214254`},
{56, 0.0015305512122674868`, 2.724381157836126`}, {70, 0.0015672604603934573`, 2.789723619500354`},
{84, 0.0016146985382595395`, 2.87416339810198`}, {98, 0.001571954332025753`, 2.7980787110058403`},
{112, 0.0015442829031186063`, 2.748823567551119`}, {126, 0.034948650542867735`, 62.20859796630457`}},
8600.023006039064`, 0.02152376728902805`, 0.7233072244415357`, 18139.711242377954`},
{"TBUR", 23.51397724041276`, 229.9381257749695`, {{0, 0.11693411827087406`, 208.1427305221558`}},
4.302122468758077`*^7, 0.12358795187957115`, 0.46503258104619555`, 2040.2122942237054`}, {"TBUR", 46.88180161322569`,
230.18296014144607`, {{0, 0.03747811903048946`, 66.71105187427125`}, {3, 0.015556703814273615`, 27.69093278940704`},
{6, 0.011122265340141992`, 19.797632305452744`}, {9, 0.008136862481602673`, 14.48361521725276`},
{12, 0.00605182518671535`, 10.772248832353322`}, {15, 0.00457277085385737`, 8.139532119863214`},
{18, 0.003511380792185058`, 6.250257810089403`}, {21, 0.0027418674811998897`, 4.880524116535804`},
{24, 0.002178719132236025`, 3.878120055375812`}, {27, 0.12594863827710145`, 224.1885761332406`}},
1.418313320574332`*^7, 0.08627407208173678`, 0.7954626234085048`, 1957.202153198838`}, {"CURE", 365,
2.221413463251318`, {{0, 0.005755372453872358`, 10.244562967892797`}, {18, 0.0003821817245131801`, 0.6802834696334605`}},
0.009999953980380167`, 0.04419759872933312`, 0.6946215941797206`, 1928.4562289594012`},
{"CURE", 365, 2.458502032361327`, {{0, 0.025261490707080683`, 44.96545345860361`}, {2, 0.004181035963636641`, 7.442244015273221`},
{4, 0.0004211491081526329`, 0.7496454125116867`}, {6, 0.00005283615136235458`, 0.09404834942499116`},
{8, 0.000021789820991855646`, 0.03878588136550305`}, {10, 0.00001958408153481695`, 0.03485966513197418`},
{12, 0.00001787892339868917`, 0.03182448364966724`}}, 0.009999999979821147`, 0.018125359805559134`,
0.9867982440361103`, 1982.7174583158774`}, {"TBUR", 61.3432944807755`, 230.12125007498335`,
{{0, 0.038533010093216954`, 68.58875796592618`}, {4, 0.012699193643410217`, 22.604564685270187`},
{8, 0.008515934193136879`, 15.158362863783646`}, {12, 0.006451501174244872`, 11.483672090155872`},
{16, 0.005114715689655442`, 9.104193927586687`}, {20, 0.004097439663563887 5`, 7.29344260114372`},
{24, 0.003289088926366 3646`, 5.854578288932129`}, {28, 0.002650008276179687`, 4.717014731599843`},
{32, 0.002150119507314529`, 3.827212723019862`}, {36, 0.1117510185869938`, 198.91681308484894`}},
2.218716714128696`*^7, 0.1112160547728924 1`, 0.60459008206845`, 2015.7525591509038`}, {"TBUR", 81.71409714859696`,

```
  228.34576883706578`, {{0, 0.010309885011171364`, 18.35159531988503`}, {4, 0.0015863641204007838`, 2.8237281343133946`},
    {8, 0.0005119489168594062`, 0.9112690720097432`}, {12, 0.00024635985298866763`, 0.4385205383198284`},
    {16, 0.0001718128949258949`, 0.30582695296809287`}, {20, 0.00014713965556921384`, 0.2619085869132006`},
    {24, 0.00013739938766795998`, 0.24457091004896875`}, {28, 0.00013299780678600702`, 0.23673609607909252`},
    {32, 0.0001308925513995346`, 0.23298874149117163`}, {36, 0.03413445415880403`, 60.759328402671166`}},
  312923.71629013395`, 0.023211921834982042`, 0.7599838284551262`, 3087.9622487397064`}, {"CURE", 365,
  0.7679445589409145`, {{0, 0.001398624046374028`, 2.4895508025457698`}, {2, 0.0007130612903649202`, 1.2692490968495578`},
    {4, 0.0002249680900119271 4`, 0.4004432002212303`}, {6, 0.00006832167280661728`, 0.12161257759577876`},
    {8, 0.000025387975784831398`, 0.04519059689699988 4`}, {10, 0.000014243370948877 33`, 0.025353200289001645`},
    {12, 0.00001152185009457674`, 0.020508893168346594`}, {14, 0.000010903264331680471`, 0.01940781051039123 7`},
    {16, 0.000010771357474542102`, 0.019173016304684938`}, {18, 0.000010221844843834195`, 0.018194883822024867`}},
  0.00999015177111753`, 0.01718565616016629`, 0.9537885949728503`, 2031.6861737012605`},
 {"CURE", 365, 0.6392009958448261`, {{0, 0.005967776731409778`, 10.622642581909405`},
    {4, 0.0009920971414236522`, 1.7659329117341012`}, {8, 0.00007446604818201356`, 0.13254956576398416`}},
  0.00999529243432298`, 0.015907651445860763`, 0.7733804673170754`, 1783.8200253812697`},
 {"CURE", 365, 1.8029725440219122`, {{0, 0.0016644928179858714`, 2.9627972160148506`},
    {10, 0.00023250064883094753`, 0.41385115491908664`}, {20, 0.00002702037664430977`, 0.048096270426871396`},
    {30, 0.000016237836449761`, 0.028903348880574584`}, {40, 0.000015860555807649 42`, 0.028231789337615967`},
    {50, 0.000015847056150131866`, 0.02820775994723472`}, {60, 0.000015846087160760 18`, 0.028206035146153115`},
    {70, 0.0000158459607935609`, 0.028205810212538398`}, {80, 0.000029366361402529942`, 0.0522721232965033`}},
  0.009999989960829939`, 0.043451781629558636`, 0.9741315084531911`, 2617.7521012920124`},
 {"CURE", 365, 1.6315660214204644`, {{0, 0.013685934990740223`, 24.360964283517596`}},
  0.009999783185618364`, 0.015450998714801937`, 0.39912164307619574`, 1780.4026710871258`},
 {"CURE", 365, 2.927088566885267`, {{0, 0.01848653404786665`, 32.90603060520264`},
    {2, 0.003293252645000096`, 5.861989708100171`}, {4, 0.00033748701519934285`, 0.6007268870548302`},
    {6, 0.000048521846183422396`, 0.08636888620649187`}, {8, 0.000025588847966886205`, 0.045548149381057446`},
    {10, 0.00002409899075285957`, 0.04289620354009003`}, {12, 0.000024019404875962717`, 0.042754540679213644`},
    {14, 0.000024015677327279136`, 0.042747905642556865`}, {16, 4.5332506273581535`*^-6, 0.008069186116697514`}},
  0.009999999744586048`, 0.019302942827646802`, 0.9872869647809054`, 2041.0348889411484`},
 {"CURE", 365, 1.7809833299648445`, {{0, 0.004082483294189026`, 7.266820263656467`},
    {9, 0.0015645174459230824`, 2.784841053743087`}, {18, 0.00030568981807719685`, 0.5441278761774104`},
```

```
{27, 0.000057104003340748096`, 0.10164512594653162`}, {36, 0.000014161337279919024`, 0.025207180358255865`}},
0.009913068301367876`, 0.032092027197401514`, 0.4028069574643461`, 1827.7066945552538`},
{"CURE", 365, 1.0993326533535377`, {{0, 0.005260079195208545 5`, 9.36294096747121`},
{4, 0.0013313073595440138`, 2.369727099988345`}, {8, 0.00019817236477009 15`, 0.35274680929076285`},
{12, 0.0000402364791869711`, 0.07162093295280857`}, {16, 0.0000281008166091851`, 0.050019453564349475`}},
0.00999999739481734`, 0.022149087510692885`, 0.9592278020961372`, 2007.824421741609`}, {"TBUR", 217.2909493869432`,
229.8269203656705`, {{0, 0.01866964126987034`, 33.2319614603692`}, {14, 0.013077118239698603`, 23.277270466663516`},
{28, 0.003141045570305957 7`, 5.591061115144605`}, {42, 0.0005476581557449048`, 0.9748315172259306`},
{56, 0.0001007910796410416 6`, 0.17940812176105414`}, {70, 0.000027666893605942788`, 0.049247070618578165`},
{84, 0.000014782502524470487`, 0.026312854493557466`}, {98, 0.000012276033839650294`, 0.021851340234577524`},
{112, 0.000011713151669812924`, 0.020849409972267006`}, {126, 0.034954138806913664`, 62.218367076306315`}},
1.0964486318615265`, 0.016035996088764777`, 0.7066876998688684`, 3040.551439251019`}, {"CURE", 365,
1.4914956751347797`, {{0, 0.006182280936849803 5`, 11.00446006759265`}, {40, 0.0022551728187776474`, 4.014207617424213`}},
0.00974307444920794`, 0.024408762795320872`, 0.336326599657448`, 1781.8771718424234`},
{"CURE", 365, 0.4580067248761056`, {{0, 0.005018685827547737`, 8.933260773034972`}},
0.009999562812959367`, 0.006942527346749422`, 0.13982819461002072`, 1780.1607035239642`},
{"CURE", 365, 1.5969382942942512`, {{0, 0.00574534286304715`, 10.226710296223928`},
{4, 0.0012918870260470625`, 2.2995589063637714`}, {8, 0.0001864121192948937`, 0.33181357234491077`},
{12, 0.000036727868792738376`, 0.0653756064510743`}, {16, 0.000019276386271926645`, 0.034311967564029426`},
{20, 0.000017591188915061408`, 0.03131231626880931`}, {24, 0.000017457967032256635`, 0.031075181317416815`},
{28, 0.000017448654836230677`, 0.031058605608490608`}, {32, 0.000010351471423761413`, 0.018425619134295315`}},
0.009999997011161853`, 0.029902784945994545`, 0.9796412187419804`, 2152.538800380843`},
{"CURE", 365, 0.5301581162707819`, {{0, 0.005837725636868262`, 10.391151633625508`}}, 0.009988109727699554`,
0.019420116219341445`, 0.12849431217345236`, 1780.151961405619`}, {"TBUR", 87.03800090478263`, 229.85376634103062`,
{{0, 0.010286044205442019`, 18.309158685686793`}, {1, 0.0010161057448829544`, 1.8086682258916587`},
{2, 0.00017206089552454233`, 0.3062683940336854`}, {3, 0.000065564546064424 48`, 0.11670489199467558`},
{4, 0.0000507574296083814`, 0.0903482247029189`}, {5, 0.000048403445268610 25`, 0.08615813257812624`},
{6, 0.000047966094552870675`, 0.08537964830410981`}, {7, 0.000047871674623993024`, 0.08521158083070758`},
{8, 0.000047848326324793386`, 0.08517002085813223`}, {9, 0.08282924788014115`, 147.43606122665125`}},
0.7207125659276865`, 0.00508544202528791`, 0.6990290421774435`, 2130.3296190840856`},
{"CURE", 365, 0.8780063625628581`, {{0, 0.010377948287346443`, 18.47274795147667`},
```

```
{4, 0.0053657276064697`, 9.550995139516191`}, {8, 0.0018723105140790964`, 3.3327127150607923`},
{12, 0.0006307214494778799`, 1.1226841800706262`}, {16, 0.00021531574544292998`, 0.3832620268884154`},
{20, 0.00007723640975527041`, 0.13748080936438134`}, {24, 0.00003541355368626165`, 0.06303612556154574`}},
0.00999998765386682`, 0.021487990666602837`, 0.9205122737782411`, 1883.4691220387563`},
{"CURE", 365, 0.13785903851001918`, {{0, 0.0028983897731922207`, 5.159133796282152`}}, 0.00999491186480714`,
0.014022777933982579`, 0.06650145502763012`, 1780.175242305726`}, {"CURE", 365, 0.35426790425404453`,
{{0, 0.0008354646616606067`, 1.48712709775588`}, {20, 0.0002943104370598895`, 0.5238725779666034`}}, 0.009999387769029826`,
0.033048396349820215`, 0.04941039890374316`, 1781.1062310932336`}, {"CURE", 365, 0.34313424675509296`,
{{0, 0.0014692669208862734`, 2.6152951191775666`}, {38, 0.0006583420282128602`, 1.171848810218891`}},
0.009758068761256483`, 0.04515751357780836`, 0.16570715739913977`, 1782.2558105097248`},
{"TBUR", 21.97929755126802`, 230.00138029810816`, {{0, 0.07372658729553225`, 131.23332538604743`}},
3.4664980752399296`*^7, 0.15924009738000147`, 0.5342210641918455`, 2167.508556474439`},
{"TBUR", 23.750782478483007`, 230.12895617479228`, {{0, 0.07498469352722167`, 133.4727544784546`}},
6.881481431149216`*^7, 0.09528618125706972`, 0.5530665008801158`, 2186.228189207325`}, {"CURE", 365, 38.7074387047483`,
{{0, 0.037653905121362054`, 67.02395111602446`}, {21, 0.0010760080403198606`, 1.9152943117693517`}},
0.009965164177100264`, 0.021274122273894688`, 0.9999221769717055`, 2955.1669727907192`}, {"TBUR", 283.2412782226097`,
223.01749513918347`, {{0, 0.06215275342889786`, 110.6319011034382`}, {24, 0.006430135579506069`, 11.445641331520802`},
{48, 0.006430137499095197`, 11.44564474838945`}, {72, 0.00643017075617713`, 11.445703945995291`},
{96, 0.006430407772505147`, 11.446125835059162`}, {120, 0.006432090783514113`, 11.449121594655121`},
{144, 0.006443072815241331`, 11.46866961112957`}, {168, 0.006477188246634259`, 11.529395079008983`},
{192, 0.006473047845512551`, 11.522025165012339`}, {216, 0.07699206645733123`, 137.04587829404957`}},
36.22778077257219`, 0.023304272317763077`, 0.8689999756463249`, 2324.1968570100453`},
{"TBUR", 46.437760938989825`, 230.23917689211746`, {{0, 0.052338480949401876`, 93.16249608993535`}},
610856.2835259815`, 0.015436795118640802`, 0.747577161154734`, 2803.711877423674`}, {"CURE", 365, 10.944379996053057`,
{{0, 0.02102244439504822`, 37.41995102318583`}, {20, 0.002835593892163409`, 5.0473571280508684`}},
0.009999769408686592`, 0.02640026219845098`, 0.6635853197155483`, 1792.4897852660856`},
{"TBUR", 43.43942517770038`, 230.22010500764532`, {{0, 0.06523951053619387`, 116.12632875442509`}},
3.192291366254209`*^7, 0.09008855185172464`, 0.4318695773267847`, 2300.8609300271232`},
{"CURE", 365, 0.36518758695888454`, {{0, 0.0011348826564351428`, 2.0200911284545535`},
{14, 0.00014248249157002593`, 0.25361883499464616`}, {28, 4.315758621355583`*^-6, 0.007682050346012937`}},
0.00999997324847296`, 0.076501485958357`, 0.14422488949954282`, 1868.8214384492144`},
```

```
{"CURE", 365, 0.41873358916091824`, {{0, 0.0027992315664304604`, 4.98263218824622`}},
  0.009999917216576344`, 0.02753406526293212`, 0.0840270760713405`, 1780.3135917960797`},
{"TBUR", 17.60564788500462`, 230.16731055025468`, {{0, 0.10804179191589358`, 192.3143896102906`}},
  9.133686457942215`*^7, 0.16202251225163913`, 0.42547122011387806`, 2012.037682231093`}, {"CURE", 365,
  4.188531867553629`, {{0, 0.039665148963937566`, 70.60396515580887`}, {19, 0.010716928024672195`, 19.07613188391651`}},
  0.00999731704524962`, 0.02277824362552568`, 0.8341582032950198`, 2001.8483777396787`}, {"CURE", 365,
  3.5823856685054674`, {{0, 0.013660437356669943`, 24.3155784948725`}, {28, 0.0014955734991382442`, 2.6621208284660747`}},
  0.009999956787499825`, 0.037556615588240506`, 0.4513778966526159`, 1784.1829737827163`},
{"CURE", 365, 0.2844609068053049`, {{0, 0.00612426895045205`, 10.90119873180465`}}, 0.009999950899839466`,
  0.009815061089628728`, 0.16840994976735416`, 1780.1765279399408`}, {"TBUR", 253.98965879013355`, 228.4973735347345`,
  {{0, 0.053088975666602124`, 94.49837668655177`}, {20, 0.001827105844705189`, 3.2522484035752366`},
    {40, 0.0016028051780965584`, 2.852993217011874`}, {60, 0.0013645286271160143`, 2.4288609562665058`},
    {80, 0.0012958940667284911`, 2.3066914387767143`}, {100, 0.0012767157701460805`, 2.2725540708600236`},
    {120, 0.001270733670710373`, 2.261905933864464`}, {140, 0.0012688735422092415`, 2.25859490513245`},
    {160, 0.0012682924267186002`, 2.2575605195591084`}, {180, 0.009954403573480083`, 17.718838360794546`}},
  2002.2194657833475`, 0.011620661659630178`, 0.7863845061248451`, 2415.345363222235`},
{"CURE", 365, 0.7048581298629535`, {{0, 0.0009766179749377618`, 1.738379995389216`}},
  0.009997110448219989`, 0.01917471159677694`, 0.10934645593154539`, 1781.3536626528958`},
{"CURE", 365, 34.192279490969035`, {{0, 0.023571592645368192`, 41.95743490875538`},
    {6, 0.0004873121653970479`, 0.8674156544067452`}, {12, 0.00009731798918251566`, 0.17322602074487786`}},
  0.009999999259240729`, 0.020625495674427875`, 0.9984775295991395`, 2020.9882336841981`}, {"CURE", 365,
  0.2856371699018831 5`, {{0, 0.0013157571374664888`, 2.34204770469035`}, {22, 0.000365985014409922`, 0.6514533256496612`}},
  0.009768412755906593`, 0.05158100409753778`, 0.09206796153955404`, 1782.6834744206424`}, {"TBUR", 104.91865796124311`,
  228.1327856161775`, {{0, 0.030406984126999707`, 54.124431746059486`}, {7, 0.00041739161424129094`, 0.7429570733494979`},
    {14, 0.000394414669232754`, 0.7020581112343021`}, {21, 0.00039519641991658843`, 0.7034496274515274`},
    {28, 0.00040823209831820527`, 0.7266531350064054`}, {35, 0.0005513084870399891`, 0.9813291069311805`},
    {42, 0.00252975219476351 1`, 4.50295890667905`}, {49, 0.007625785186211364`, 13.573897631456227`},
    {56, 0.005810833343620945`, 10.343283351645281`}, {63, 1.3511540693749866`, 2405.054243487476`}},
  16516.2370510113`, 0.058804033994678526`, 0.8146107686650905`, 1837.6716467029964`}, {"CURE", 365,
  0.7944985802690219`, {{0, 0.0012745431316942`, 2.268686774415676`}, {4, 0.0006291433888890883`, 1.1198752322225771`},
    {8, 0.00022911392297173637`, 0.4078227828896907`}, {12, 0.0000595551647690091`, 0.1060081932888362`}},
```

```
0.009999948787816416`, 0.008545274648540416`, 0.5598163429232912`, 1789.3221790166845`},
{"CURE", 365, 0.5727814963459201`, {{0, 0.002240176995512068`, 3.987391705201148`}}, 0.009999834164218447`,
 0.02065072946664916`, 0.13714003096441002`, 1780.6007153791797`}, {"CURE", 365, 6.907337856781433`,
 {{0, 0.010186627816268544`, 18.13219751295801`}, {2, 0.001421647650272847 1`, 2.5305328174856676`},
  {4, 0.0001487019806708158`, 0.26468952559405207`}, {6, 0.000036968518075913 07`, 0.06580396217512527`},
  {8, 0.00002915771649937735`, 0.05190073536889168 4`}, {10, 0.00002872616059267474 7`, 0.05113256585496104 4`},
  {12, 0.00002870674848869406`, 0.05109801230987543`}, {14, 2.8903161703388137`*^-6, 0.005144762783203088`}},
 0.009996771307991934`, 0.020101694324519234`, 0.986952764923642`, 2063.2658365506736`}, {"TBUR", 182.0927405695317`,
 229.30810967025002`, {{0, 0.0955822576850984`, 170.13641867947516`}, {14, 0.02492420219361248 3`, 44.36507990463022`},
  {28, 0.003633827995940699`, 6.468213832774444`}, {42, 0.0005699127065444043`, 1.0144446176490396`},
  {56, 0.00011040855288673024`, 0.19652722413837984`}, {70, 0.000040275521359029045`, 0.0716904280190717`},
  {84, 0.00002864699488115522`, 0.05099165088845629`}, {98, 0.0000259424744431638`, 0.04617760450883156`},
  {112, 0.000024988569679475138`, 0.04447965402946574`}, {126, 0.000444857025111922`, 0.7918455046992212`}},
 4.4310141164527215`, 0.016322833563712436`, 0.15859812190501713`, 1780.0089670989378`},
{"TBUR", 16.61171672612266`, 229.16286839620315`, {{0, 0.13748779296875`, 244.72827148437497`}}, 9.659100111161293`*^7,
 0.09915533147048138`, 0.4940545995064747`, 1989.3827067661864`}, {"TBUR", 88.24107648368496`, 229.22902328352595`,
 {{0, 0.05632384303549764`, 100.25644060318578`}, {4, 0.015453722415530413`, 27.50762589964414`},
  {8, 0.0035113214504152177`, 6.250152181739087`}, {12, 0.0009304324874904726`, 1.6561698277330412`},
  {16, 0.0003227731142703451`, 0.5745361434012142`}, {20, 0.00014936897938722316`, 0.26587678330925724`},
  {24, 0.00008995787383678942`, 0.16012501542948515`}, {28, 0.0000664043786543072`, 0.1181997940046668`},
  {32, 0.00005594452980863292`, 0.09958126305936658`}, {36, 0.0024172338232601003`, 4.302676205402977`}},
 98184.8018863977`, 0.03014998940641252`, 0.6616900905174526`, 2790.5360268834747`}, {"CURE", 365, 1.3498803304449258`,
 {{0, 0.01180665028361052`, 21.015837504826727`}, {34, 0.006443937076531014 4`, 11.470207996225206`}},
 0.009999865087757558`, 0.023515756761254294`, 0.6101578130414015`, 1784.9426465784802`}, {"TBUR", 147.44547977961167`,
 229.0501423181017`, {{0, 0.01276661549458061`, 22.724575580353488`}, {8, 0.00047022757367856134`, 0.8370050811478393`},
  {16, 0.00007565229579291868`, 0.13466108651139527`}, {24, 0.00005146882217838196`, 0.0916145034775199`},
  {32, 0.00005295076577947148 4`, 0.09425236308745923`}, {40, 0.00006849234834296071`, 0.12191638005047008`},
  {48, 0.00011277762238035261`, 0.20074416783702767`}, {56, 0.0002093921417297349`, 0.3727180122789282`},
  {64, 0.0003658886761089286 4`, 0.6512818434738931`}, {72, 0.2702692190962858`, 481.0792099913888`}},
 113285.24131752233`, 0.07168961682103317`, 0.7514581987479191`, 2460.1400748864576`}, {"CURE", 365,
 2.1621955837139284`, {{0, 0.005495015849434104`, 9.781128211992703`}, {8, 0.0016301822585066848`, 2.901724420141899`},
```

```
{16, 0.00020857778461868835`, 0.37126845662126523`}, {24, 0.000018702903399984985`, 0.033291168051973276`}},
0.009999969169988066`, 0.04390082952778009`, 0.398983782187224`, 1789.4553811362443`}, {"CURE", 365,
1.645956016665143`, {{0, 0.003341499059865987 5`, 5.947868326561458`}, {6, 0.001104977793934444`, 1.9668604732033101`},
{12, 0.00014465362351130776`, 0.2574834498501278`}, {18, 0.000018671987559865147`, 0.03323613785655996`},
{24, 7.331384293540206`*^-6, 0.013049864042501566`}, {30, 6.478223518894448`*^-6, 0.011531237863632117`},
{36, 6.416472797250729`*^-6, 0.011421321579106298`}, {42, 0.000016389984887325343`, 0.02917417309943911`}},
0.009999998988183022`, 0.060931108821882816`, 0.7570620736548809`, 2084.635768896988`}, {"TBUR", 70.86480719850037`,
229.96910777503177`, {{0, 0.005436617222960317`, 9.677178656869364`}, {5, 0.003473902058335157`, 6.18354566383658`},
{10, 0.002651237659971113`, 4.719203034748582`}, {15, 0.0020604823544800727`, 3.6676585909745296`},
{20, 0.0016144808084247093`, 2.8737758389959827`}, {25, 0.001274426864787327`, 2.2684798193214424`},
{30, 0.0010143008463436062`, 1.8054555064916191`}, {35, 0.0008149011460753945`, 1.450524040014202`},
{40, 0.0006618122331283112`, 1.1780257749683938`}, {45, 0.08724929981748936`, 155.30375367513105`}},
1.1653918800153762`*^7, 0.06214045476176766`, 0.7433344414088408`, 1898.9705161038655`},
{"CURE", 365, 0.15997113266586874`, {{0, 0.0010012961967500846`, 1.7823072302151506`}},
0.009999865191037002`, 0.006265608320545507`, 0.12286399979238359`, 1780.9590936933157`},
{"CURE", 365, 0.10763593678116633`, {{0, 0.0001752448911724092`, 0.31193590628688833`},
{10, 0.00004704246623770215`, 0.08373558990310982`}, {20, 6.400895620950948`*^-6, 0.011393594205292689`}},
0.00999992755551806`, 0.06454548361200237`, 0.3325206643185744`, 1950.6736391077327`},
{"CURE", 365, 0.23289239032055117`, {{0, 0.0023846722448976738`, 4.244716595917859`}},
0.00999717713268228 8`, 0.016765008380204047`, 0.06872097682185282`, 1780.151678149341`},
{"TBUR", 16.287984099154556`, 229.56927890818739`, {{0, 0.11122651100158694`, 197.98318958282474`}},
4.0234456326856665`*^7, 0.09637593820005645`, 0.5295715968744136`, 2032.3126210349694`}, {"CURE", 365, 0.04185118210068640 4`,
{{0, 0.0005058423508015835`, 0.9003993844268187`}, {7, 0.00013958271165206136`, 0.2484572267406692`}},
0.009999929341373073`, 0.017759148224652122`, 0.30085294416373`, 1785.1340709789526`},
{"TBUR", 28.524948006322006`, 230.14868866220965`, {{0, 0.11101475715637207`, 197.6062677383423`}},
7.377008703092396`*^7, 0.09385077073649839`, 0.4588195446670277`, 2068.165317029916`}, {"CURE", 365,
2.3758168562500783`, {{0, 0.002591955493579701`, 4.613680778571867`}, {29, 0.0006614933793633045`, 1.177458215266682`}},
0.009999961480741672`, 0.02395170912071806`, 0.7460986964162525`, 1988.8998136689772`}, {"CURE", 365, 4.190660196433903`,
{{0, 0.008459511373778624`, 15.057930245325949`}, {19, 0.0008462231169741801`, 1.5062771482140405`}},
0.0099999049985946`, 0.03522751904253925`, 0.3986074258497466`, 1782.5456762139413`}, {"TBUR", 335.16222764352887`,
229.4177625562105`, {{0, 0.07094289314728525`, 126.27834980216772`}, {24, 0.0007435775066460891`, 1.3235679618300387`},
```

```
{48, 0.0002663047800438859`, 0.47402250847811694`}, {72, 0.0002474944973589269`, 0.4405402052988899`},
{96, 0.00024335301575067035`, 0.43316836803619324`}, {120, 0.00024198677397759837`, 0.4307364576801251`},
{144, 0.00024150064789217067`, 0.4298711532480638`}, {168, 0.00024132670412231987`, 0.4295615333377294`},
{192, 0.00024126571234364666`, 0.4294529679716911`}, {216, 0.0013670449230575757`, 2.4333399630424846`}},
3.9836575883055345`, 0.011830674575221137`, 0.8160570324370602`, 2427.196766343451`}, {"CURE", 365, 0.9211348449882711`,
{{0, 0.0025592749583453565`, 4.555509425854734`}, {68, 0.0010316529540447272`, 1.8363422581996147`}},
0.00976643417435635`, 0.043204217028435465`, 0.07386938159015902`, 1780.4998259197678`},
{"TBUR", 14.95374628830667`, 227.58719225217703`, {{0, 0.08038955688476565`, 143.0934112548828 5`}}, 9.51311671217389`*^7,
0.20215758946157375`, 0.5883574242806581`, 2090.9442016157386`}, {"CURE", 365, 0.30995078654436037`,
{{0, 0.0019222351731649557`, 3.421578608233621`}, {10, 0.0005454956858887013`, 0.9709823208818883`}},
0.00989451042712882`, 0.02784181812365005`, 0.32323304485073484`, 1781.297267347237`},
{"CURE", 365, 0.488428696713223`, {{0, 0.015697272715251183`, 27.94114543314711`}},
0.009603384234277288`, 0.01730939138283185`, 0.460977619556992`, 1780.3413813273323`},
{"CURE", 365, 0.05477911476488951`, {{0, 0.0011498010526293104`, 2.0466458736801725`}}, 0.009998551211049655`,
0.02625638738922478`, 0.12526058937407805`, 1780.7049395830522`}, {"CURE", 365, 0.49261655587498415`,
{{0, 0.0005952154642065848`, 1.059483526287721`}, {50, 0.00017463383401129478`, 0.3108482245401047`}},
0.00999975303590112`, 0.05439448137407066 5`, 0.08450312808721319`, 1782.2134829320266`}, {"CURE", 365,
0.8625095595216368`, {{0, 0.008562264043089499`, 15.24082999669931`}, {56, 0.004200998794971893`, 7.47777785504997`}},
0.009995971190546123`, 0.02653843811866488`, 0.4201517843467208`, 1782.3010496778734`},
{"TBUR", 16.145083191520648`, 230.14415476015847`, {{0, 0.08026345252990724`, 142.8689455032349`}},
3.2052596491028216`*^7, 0.18551775461506487`, 0.5176817786284302`, 2087.910269569733`}, {"CURE", 365, 0.23961237221808168`,
{{0, 0.0009551902277133453`, 1.700238605329755`}, {14, 0.00031310251787777403`, 0.5573224818224378`},
{28, 0.000043728609824319 27`, 0.07783692548728831`}, {42, 3.3128265324548973`*^-6, 0.005896831227769717`}},
0.009962046593398853`, 0.06761048616454801`, 0.16045341325000886`, 1797.5227059803337`},
{"TBUR", 25.468078147920135`, 230.1693137794198`, {{0, 0.09441617965698243`, 168.06079978942873`}},
6.3609508913283244`*^7, 0.09480073108733292`, 0.4745206712613276`, 2106.1922430815916`},
{"TBUR", 260.61272039799616`, 230.015666803193`, {{0, 0.08010477230319338`, 142.5864946996842`},
{22, 0.0010328137893678973`, 1.8384085450748573`}, {44, 0.0018775375103363768`, 3.3420167683987505`},
{66, 0.00031341233301684316`, 0.5578739527699809`}, {88, 0.00026013875894002295`, 0.46304699091324086`},
{110, 0.00020093417270313857`, 0.3576628274115866`}, {132, 0.00019158888628872966`, 0.34102821759393875`},
{154, 0.0001874595645038794`, 0.3336780248169053`}, {176, 0.0001861754151769544`, 0.33139223901497883`},
```

{198, 0.00170414843995786`, 3.033384223124991`}}, 0.5485452668228927`, 0.003935815081979674`,
0.12422942946029868`, 1780.0135505963344`}, {"TBUR", 121.7286743403362`, 228.1665987293114`,
{{0, 0.07508029586778023`, 133.64292664464878`}, {10, 0.0018270129145260847`, 3.2520829878564306`},
{20, 0.0017138849145644144`, 3.0507151479246573`}, {30, 0.0017059332836671037`, 3.0365612449274444`},
{40, 0.0017042672561605428`, 3.033595715965766`}, {50, 0.0017038851536702567`, 3.032915573533056`},
{60, 0.0017039606947451319`, 3.033050036646334`}, {70, 0.0017042668838367742`, 3.0335950532294578`},
{80, 0.0017047227081534338`, 3.034406420513112`}, {90, 0.07596408814159086`, 135.2160768920317`}},
34525.22077009691`, 0.03642427840849541`, 0.21096696636476314`, 1780.023219548391`},
{"CURE", 365, 0.12425884002674975`, {{0, 0.0014396339873473684`, 2.562548497478316`}}, 0.00959627684812173`,
0.024663205880202734`, 0.4400926479041348`, 1783.6362285560124`}, {"TBUR", 354.369217333277`, 228.05776033231743`,
{{0, 0.02813926047315891`, 50.08788364222287`}, {29, 0.0015095052199391578`, 2.686919291491701`},
{58, 0.001393225643328633`, 2.479941645124967`}, {87, 0.001380237669353972`, 2.456823051450071`},
{116, 0.0013762784427656296`, 2.449775628122821`}, {145, 0.001374773937670009`, 2.447097609052616`},
{174, 0.0013741701977635276`, 2.4460229520190793`}, {203, 0.001373923910939139`, 2.4455845614716676`},
{232, 0.0013738228566389683`, 2.445404684817364`}, {261, 0.014495574530567224`, 25.802122664409662`}},
784175.3884999485`, 0.051692665329612156`, 0.5405712203662989`, 3343.7044074299774`}, {"CURE", 365,
5.270975258251704`, {{0, 0.019984874167141918`, 35.57307601751261`}, {9, 0.002512508391636755`, 4.472264937113423`}},
0.00999994487523094`, 0.02514509196148782`, 0.8541360203940564`, 2274.7153289712223`}, {"TBUR", 365,
228.01740034888795`, {{0, 0.06738882026922198`, 119.95210007921511`}, {31, 0.0016421427024683481`, 2.92301401039366`},
{62, 0.0020509205541598776`, 3.6506385864045825`}, {93, 0.0058648568182219285`, 10.439445136435033`},
{124, 0.004090210365545525`, 7.280574450671034`}, {155, 0.0038538594190279715`, 6.859869765869789`},
{186, 0.0033865903332601036`, 6.028130793202985`}, {217, 0.003084621356251764`, 5.49062601412814`},
{248, 0.0028318715393031635`, 5.040731339959631`}, {279, 0.08901950422808683`, 158.45471752599457`}},
988.1468977595223`, 0.041366554320394426`, 0.7864502222015289`, 2540.215437298817`}, {"CURE", 365,
0.8216714630270862`, {{0, 0.00405331830919025`, 7.214906590358645`}, {34, 0.0012532248713631807`, 2.230740271026462`}},
0.00974599744278639`, 0.026550349350235466`, 0.47756518231126893`, 1789.0010536554669`},
{"CURE", 365, 0.5421824785473142`, {{0, 0.0026828635131487966`, 4.775497053404858`}},
0.00999977076970309`, 0.03061647781744066`, 0.08412018632415527`, 1780.2315876522712`},
{"CURE", 365, 0.10573195824906438`, {{0, 0.0010112382750812037`, 1.8000041296445426`}},
0.009995294566590084`, 0.015865278987909584`, 0.08210813394629578`, 1780.6735160781845`},
{"CURE", 365, 3.4655936816029214`, {{0, 0.01939407953216679`, 34.521461567256885`}, {3, 0.004939459788992219`, 8.79223842440615`},

```
{6, 0.0008282659122013632`, 1.4743133237184263`}, {9, 0.00013981766960407754`, 0.24887545189525803`},
  {12, 0.000040912078129942576`, 0.0728234990712978`}, {15, 0.000028354533161357464`, 0.05047106902721628`},
  {18, 0.000026999280803648773`, 0.04805871983049481`}, {21, 0.00002687216358151966`, 0.04783245117510499`},
  {24, 0.000026861180139096154`, 0.04781290064759115`}, {27, 6.369068386293852`*^-6, 0.011336941727603056`}},
 0.009999996742705235`, 0.031512804483007265`, 0.9777229331098607`, 2039.7842682633964`},
{"CURE", 365, 0.07668847700034159`, {{0, 0.0002313204178001266`, 0.41175034368422536`},
  {11, 0.00005548198816768757 6`, 0.09875793893848389`}, {22, 5.798229601640785`*^-6, 0.010320848690920597`}},
 0.009999914762354022`, 0.0645627356802572`, 0.26696895364812184`, 1922.5472821091414`},
{"CURE", 365, 1.9315954569396097`, {{0, 0.003713718541803317`, 6.610419004409904`},
  {14, 0.0017366804757006182`, 3.0912912467471`}, {28, 0.000488998879885539`, 0.8704180061962594`},
  {42, 0.00013557774398364084`, 0.24132838429088066`}, {56, 0.00003538913393215834`, 0.06299265839924185`}},
 0.009999992983029548`, 0.03574043087139338`, 0.31209073261867515`, 1786.835529119555`}, {"CURE", 365,
 0.6577639606663762`, {{0, 0.0010628361345187685`, 1.891848319443408`}, {38, 0.0003853490497161367`, 0.6859213084947232`}},
 0.009752082793846368`, 0.0492795988825123`, 0.035135013856568705`, 1780.418866696183`},
{"CURE", 365, 2.930867549605359`, {{0, 0.007694564501101273`, 13.696324811960267`},
  {28, 0.00014141638509719906`, 0.2517211654730143`}, {56, 0.000019421316503170012`, 0.03456994337564262`},
  {84, 0.000020319713738135 5`, 0.03616909045388119`}, {112, 0.000018639620719933338`, 0.03317852488148134`},
  {140, 0.00001838702273670474`, 0.03272890047133443 5`}, {168, 0.000018370277328468413`, 0.03269909364467377`},
  {196, 0.0000183662330392494`, 0.03269189480986393`}, {224, 0.000039361533140188536`, 0.07006352898953559`}},
 0.009999961060642801`, 0.04072415575548314`, 0.764816842906784`, 2318.009100463783`},
{"CURE", 365, 8.985729975123862`, {{0, 0.023870566005741993`, 42.48960749022075`},
  {2, 0.001194372968955112`, 2.125983884740099`}, {4, 0.00005268910439677329`, 0.09378660582625646`},
  {6, 0.00002312794658729144`, 0.04116774492537876`}, {8, 0.000022591662639461003`, 0.040213159498240586`},
  {10, 0.000022582384945178013`, 0.04019664520241686`}, {12, 0.000015482587673205 78`, 0.027559006058306284`}},
 0.009981873249283272`, 0.01820917071932163`, 0.9961113450331366`, 2149.9128228157497`},
{"CURE", 365, 0.23575063107558114`, {{0, 0.005034792862820658`, 8.96193129582077`}}, 0.009563406982293406`,
 0.017377340884659306`, 0.12597031437633166`, 1780.3054991482513`}, {"CURE", 365, 0.058304459185504046`,
 {{0, 0.0006323018823072228`, 1.1254973505068566`}, {6, 0.00011263095545628192`, 0.20048310071218184`}},
 0.009961852611178643`, 0.02309092073038309`, 0.37172550115137487`, 1786.6531388018172`}, {"CURE", 365,
 0.21228535933265202`, {{0, 0.0007448148621614686`, 1.3257704546474143`}, {7, 0.000417895341359561`, 0.7438537076200186`},
  {14, 0.0001902637560257418`, 0.3386694857258204`}, {21, 0.000049519797577127354`, 0.0881452396872867`}},
```

0.009969093380545909`, 0.01716785319827763`, 0.6318304354766975`, 1793.865928108083`},
{"CURE", 365, 18.06829010272497`, {{0, 0.017803501126458864`, 31.690232005096778`},
{17, 0.00009760127292248695`, 0.1737302658020268`}, {34, 0.00009752472692863406`, 0.17359401393296864`},
{51, 0.00010175441680213114`, 0.18112286190779342`}, {68, 0.00009614838078133828`, 0.17114411779078215`},
{85, 0.00009609836198823447`, 0.17105508433905736`}, {102, 0.00011261652527683145`, 0.20045741499275999`}},
0.00997560529179709`, 0.017413716365393444`, 0.9232733092821136`, 2251.2432878624236`},
{"CURE", 365, 0.6848353652622163`, {{0, 0.0017654674346579428`, 3.142532033691138`}},
0.00999986415141674`, 0.016807091478539156`, 0.03816272704883995`, 1780.1763108151933`},
{"CURE", 365, 2.0894427933295194`, {{0, 0.005307506684223886`, 9.447361897918517`}},
0.009999966265373564`, 0.009865467148751127`, 0.15217475097480412`, 1780.241770440198`},
{"TBUR", 12.778613670244727`, 229.3429909164419`, {{0, 0.07398847579956057`, 131.69948692321782`}},
7.41363923833616`*^7, 0.20558627074696156`, 0.6407165114364337`, 2101.6437508413237`},
{"CURE", 365, 0.18640916932644572`, {{0, 0.006984673574797295`, 12.432718963139186`}}, 0.00999964238072884`,
0.021467895991646852`, 0.44888524296641646`, 1780.8716561379772`}, {"TBUR", 93.70484036557116`,
228.17351798592026`, {{0, 0.05265796921094479`, 93.7311851954817`}, {8, 0.011506318689670603`, 20.48124726761367`},
{16, 0.008628826369937342`, 15.359310938488466`}, {24, 0.008340557455945776`, 14.84619227158348`},
{32, 0.008962375214496414`, 15.953027881803614`}, {40, 0.00984345594994084`, 17.521351590894696`},
{48, 0.010617396744709313`, 18.89896620558258`}, {56, 0.011150802800188632`, 19.848428984335765`},
{64, 0.011483124889571532`, 20.439962303437323`}, {72, 0.8187439419452588`, 1457.3642166625605`}},
2.6540327920924112`*^7, 0.388622271478678`, 0.604864315658915`, 2051.829555804161`},
{"TBUR", 47.941112059445466`, 230.26306607683202`, {{0, 0.08625164031982424`, 153.52791976928717`}},
4.944039012802179`*^7, 0.025165775025228373`, 0.41473992152758565`, 2198.1364246113135`},
{"CURE", 365, 5.0834473138024325`, {{0, 0.011182317277260007`, 19.904524753522814`}}, 0.009999912810889142`,
0.010409757935652573`, 0.2798047774734428`, 1780.2302313091486`}, {"CURE", 365, 1.0220970074859808`,
{{0, 0.02207139316380066`, 39.28707983156517`}, {13, 0.005563329746542018`, 9.902726948844792`}},
0.009999777223455917`, 0.020674172606991278`, 0.5729024807187939`, 1780.266948822991`}, {"CURE", 365,
5.017574072999525`, {{0, 0.015857497928077 18`, 28.22634631197738`}, {30, 0.0032875552784428536`, 5.851848395628279`}},
0.009999611566849295`, 0.03167963164898379`, 0.728082827820229`, 2271.897548275794`},
{"TBUR", 24.69704044358323`, 230.13076295066693`, {{0, 0.07994635581970216`, 142.30451335906986`}},
2.1875555226345807`*^7, 0.03359215905617098`, 0.6634585893327088`, 2226.6233044987084`},
{"TBUR", 18.602722997501694`, 229.4867051344844`, {{0, 0.09741497993469236`, 173.3986642837524`}},

4.721491520685097`*^7, 0.09423436767494735`, 0.5440997531119642`, 2075.541927507532`}, {"TBUR", 200.3334797336206`,
229.6571927546577`, {{0, 0.05182517002340216`, 92.24880264165583`}, {19, 0.002824633415357201`, 5.027847479335817`},
{38, 0.002155937505661658`, 3.837568760077751`}, {57, 0.002139374267040848`, 3.8080861953327103`},
{76, 0.002159163862118293 4`, 3.843311674570562`}, {95, 0.002126788004719308`, 3.7856826484003676`},
{114, 0.0020466520593871488`, 3.6430406657091243`}, {133, 0.0019573484224218746`, 3.4840801919109365`},
{152, 0.0018819790173200456`, 3.349922650829681`}, {171, 0.11736553419635455`, 208.9106508695111`}},
852613.5520460242`, 0.0806732706619129`, 0.839127703360341`, 2201.0642236596673`}, {"CURE", 365, 0.2793808793350692`,
{{0, 0.007284459109006131 5`, 12.966337214030915`}, {13, 0.002560927160222986`, 4.558450345196916`}},
0.009918450769453776`, 0.018818672014891475`, 0.541042513913243`, 1780.798920336537`},
{"TBUR", 24.955318795006523`, 230.1516137164932`, {{0, 0.06870250701904296`, 122.29046249389646`}},
8.557828905511698`*^7, 0.268838956865232`, 0.47144859987912646`, 2183.660728058419`},
{"CURE", 365, 0.22500135985689593`, {{0, 0.0015008942278776256`, 2.6715917256221737`},
{10, 0.0007116042788050374`, 1.2666556162729667`}, {20, 0.00016641429730588326`, 0.29621744920447224`}},
0.00997223852624014`, 0.023892133697456512`, 0.6297657191428593`, 1787.1923450093223`},
{"CURE", 365, 0.21704625368068625`, {{0, 0.0009492394564092738`, 1.6896462324085073`}}, 0.009983474665152493`,
0.02199500560789419`, 0.04915205939022248`, 1780.3567879990503`}, {"TBUR", 83.93331719686603`, 228.71462654666283`,
{{0, 0.007125422685199724`, 12.683252379655508`}, {4, 0.002267070395982922`, 4.035385304849601`},
{8, 0.0011252112162892156`, 2.002875964994804`}, {12, 0.0006061197438918068`, 1.078893144127416`},
{16, 0.00034972863007187407`, 0.6225169615279359`}, {20, 0.00021908528152311745`, 0.38997180111114904`},
{24, 0.00015157632065114696`, 0.2698058507590416`}, {28, 0.00011612586267675675`, 0.20670403556462705`},
{32, 0.00009701485301067501`, 0.17268643835900152`}, {36, 0.044498814841466665`, 79.20789041781066`}},
109147.95485101339`, 0.019789079471311796`, 0.7135804643309508`, 2685.0827379087527`}, {"TBUR", 70.22173123358239`,
229.53161761833053`, {{0, 0.012774628015125326`, 22.738837866923078`}, {3, 0.002914960371394225`, 5.188629461081721`},
{6, 0.0008256456071577609`, 1.4696491807408143`}, {9, 0.00032648223079719414`, 0.5811383708190055`},
{12, 0.00018280259972845424`, 0.3253886275166485`}, {15, 0.00013531601152105428`, 0.24086250050747662`},
{18, 0.00012186678623411159`, 0.21692287949671862`}, {21, 0.0001237999233511914`, 0.2203638635651207`},
{24, 0.00013133687759355`, 0.23377964211651903`}, {27, 0.03532744945101763`, 62.882860022811386`}},
232885.57575284483`, 0.023076885749753022`, 0.8061739702228559`, 2943.7978271352536`}, {"CURE", 365,
1.272974839932943`, {{0, 0.021178597656832724`, 37.69790382916225`}, {7, 0.002150369097943988 5`, 3.8276569943402996`}},
0.009967125683362112`, 0.022482268572909445`, 0.7764918345832857`, 1781.217193684466`},
{"TBUR", 22.977930156559896`, 229.9323467244304`, {{0, 0.08024095535278322`, 142.82890052795412`}},

```
2.1155527739015408`*^7, 0.12031881663395683`, 0.5777975000027958`, 2182.0558988818743`},
{"CURE", 365, 0.2040793015407386`, {{0, 0.0023740515724111175`, 4.225811798891789`}},
 0.00999943660133056`, 0.029016941804310973`, 0.21680560164355517`, 1780.8652566211942`},
{"TBUR", 21.090848168532954`, 229.37238539316962`, {{0, 0.12060723304748536`, 214.68087482452395`}},
 5.630732993897926`*^7, 0.10787163055448795`, 0.4806903289617826`, 2032.4228751869334`},
{"CURE", 365, 0.6022989788991627`, {{0, 0.0035914785612970405`, 6.392831839108733`}}, 0.009989218133328068`,
 0.010324281747851667`, 0.18283093948018386`, 1780.4815354085622`}, {"CURE", 365, 0.06600790993207763`,
 {{0, 0.0007392191150553554`, 1.3158100247985325`}, {5, 0.00027706211968677905`, 0.4931705730424667`}},
 0.009999641878909324`, 0.00903905178657893`, 0.3331769253106393`, 1783.4597122623281`},
{"CURE", 365, 1.1442344571017535`, {{0, 0.007247700476677486`, 12.900906848485926`},
  {4, 0.0008193589764479941`, 1.4584589780774295`}, {8, 0.00006513713849150037`, 0.11594410651487065`}},
 0.009999992427246224`, 0.014343116113161615`, 0.96311297129011`, 1798.7667326125368`},
{"CURE", 365, 5.357775797692294`, {{0, 0.014175440018657292`, 25.232283233209984`},
  {5, 0.006203576339154303`, 11.04236588369466`}, {10, 0.001695054462864361`, 3.0171969438985626`},
  {15, 0.0004564998226676282`, 0.8125696843483782`}, {20, 0.0001104258530994039`, 0.19655801851693894`}},
 0.009999976727246847`, 0.017525165786778787`, 0.657995703615002`, 1789.2165330857326`},
{"CURE", 365, 0.07599399799190173`, {{0, 0.0017714464247158908`, 3.1531746359942856`}}, 0.009997438609889994`,
 0.02543929276367669 7`, 0.05380511975613086 5`, 1780.275631429616`}, {"CURE", 365, 0.3651535296317894`,
 {{0, 0.0033942920982232673`, 6.041839934837416`}, {41, 0.0021485398411911594`, 3.8244009173202635`}},
 0.009802942821987867`, 0.012118317442231599`, 0.10610572748116381`, 1780.434873441735`},
{"CURE", 365, 0.6405211277291181`, {{0, 0.00938359101459877`, 16.70279200598581`},
  {4, 0.0023491811466503742`, 4.181542441037666`}, {8, 0.00042441154308833057`, 0.7554525466972284`}},
 0.009999930602789088`, 0.014863485420542714`, 0.8577553513424405`, 1782.906372540217`}, {"CURE", 365,
 0.9987780115381529`, {{0, 0.007900065005928799`, 14.062115710553261`}, {12, 0.002756012661470084`, 4.905702537416749`}},
 0.009922550941515028`, 0.016694806806120625`, 0.375024439609324`, 1780.3573613358406`}, {"CURE", 365,
 201.2138802218061`, {{0, 0.07020676164344959`, 124.96803572534027`}, {30, 0.0005452538960244157`, 0.9705519349234597`},
  {60, 0.0006978363092650211`, 1.2421486304917375`}, {90, 0.0001137480145618899`, 0.20247146592016402`},
  {120, 0.00007316081462766992`, 0.13022625003725244`}, {150, 0.000057065748391193774`, 0.10157703213632492`},
  {180, 0.00005545040113692313`, 0.09870171402372317`}, {210, 0.00005459872625335214`, 0.09718573273096683`},
  {240, 0.00005451957438621824`, 0.09704484240746847`}, {270, 0.00025190720354934134`, 0.4483948223178277`}},
 0.009991115740485132`, 0.005851464139333795`, 0.8915180645974066`, 2406.3248572715233`}, {"TBUR", 94.39120746329122`,
```

```
228.47483458765882`, {{0, 0.00511642755743482`, 9.10724105223398`}, {8, 0.0030382962306124196`, 5.408167290490107`},
   {16, 0.0023248932866942133`, 4.1383100503157`}, {24, 0.0018747968252934588`, 3.3371383490223567`},
   {32, 0.0015262455098429973`, 2.716717007520536`}, {40, 0.0012528940757847561`, 2.230151454896866`},
   {48, 0.0010394280981062514`, 1.8501820146291275`}, {56, 0.0008727578257888746`, 1.5535089299041969`},
   {64, 0.0007424996612588665`, 1.3216493970407823`}, {72, 0.13692870885117128`, 243.7331017550849`}},
 1.635396720703695`*^7, 0.0794615852358029`, 0.7032218925381963`, 2024.7405413674126`},
{"CURE", 365, 4.2763469718483496`, {{0, 0.021108215570966662`, 37.572623716320656`}, {3, 0.0024234215319828`, 4.313690326929384`},
   {6, 0.00021907184553991992`, 0.3899478850610575`}, {9, 0.000013795794979544982`, 0.024556515063590068`}},
 0.009999989568812533`, 0.013984177729115334`, 0.974911584369535`, 1808.1583966884107`},
{"CURE", 365, 0.7206383792464857`, {{0, 0.0006143286740556054`, 1.0935050398189776`}}, 0.009999966644881418`,
 0.013582764252416559`, 0.08378878818981825`, 1781.215769395031`}, {"TBUR", 159.98426111353933`, 228.78729573051808`,
 {{0, 0.060517688830850014`, 107.72148611891302`}, {9, 0.010656128289324026`, 18.967908354996766`},
   {18, 0.002392740038850336`, 4.259077269153598`}, {27, 0.0007239484681777303`, 1.28862827335636`},
   {36, 0.0003356862928618933 4`, 0.5975216012941701`}, {45, 0.0002411858759499370 9`, 0.429310859190888`},
   {54, 0.0002144025533523823 2`, 0.38163654496724053`}, {63, 0.0002047270806232971 6`, 0.36441420350946896`},
   {72, 0.0002004574983688544`, 0.35681434709656085`}, {81, 0.004731788793411597`, 8.422584052272642`}},
 2950.296156272829`, 0.02077099768938029`, 0.6010116607029751`, 2400.5447118481707`}, {"CURE", 365, 0.6190605737611479`,
 {{0, 0.005209139784236734`, 9.272268815941386`}, {38, 0.004023825547529854`, 7.16240947460314`}}, 0.009793679538525073`,
 0.013058474252786277`, 0.29262953067876457`, 1780.9212963088828`}, {"CURE", 365, 0.5508356060330517`,
 {{0, 0.006538719579616430 5`, 11.638920851717247`}, {10, 0.002438721622822977`, 4.340924488624899`}},
 0.009904389460836886`, 0.009824809001612474`, 0.3711688466193911`, 1780.6332237072038`}, {"CURE", 365,
 161.34160453763883`, {{0, 0.015415379035824765`, 27.439374683768083`}, {1, 0.009455646864416295`, 16.83105141866101`}},
 0.00999008811323772`, 0.004348083574753761`, 0.9997639255962711`, 3253.162667766932`},
{"CURE", 365, 0.0330007316357834`, {{0, 0.0009409204318877552`, 1.674838368760204`}}, 0.009670229732014287`,
 0.016769840791425464`, 0.06459602738097149`, 1780.4992224859252`}, {"CURE", 365, 1.9103464632270453`,
 {{0, 0.005135176142618174`, 9.14061353386035`}, {20, 0.00104665219912663`, 1.8630409144454019`}},
 0.009762749368601692`, 0.056099027527872956`, 0.2617237263446142`, 1781.2844656418097`}, {"TBUR", 356.6303229320038`,
 228.62862944702366`, {{0, 0.04573634801077244`, 81.41069945917495`}, {33, 0.01053103119048332`, 18.74523551906031`},
   {66, 0.00647028195866262 2`, 11.517101886419468`}, {99, 0.005021438286268526`, 8.938160149557975`},
   {132, 0.003801418287030407`, 6.766524550914124`}, {165, 0.0028186482871015678`, 5.017193951040791`},
   {198, 0.002085821861123733`, 3.7127629128002444`}, {231, 0.0015508549617734642`, 2.7605218319567664`},
```

```
{264, 0.001160931979988692`, 2.0664589243798717`}, {297, 0.04076315524545908`, 72.55841633691715`}},
3035.327362887097`, 0.02081372404045802`, 0.15007707098528136`, 1780.026220522695`},
{"TBUR", 19.13852711595611`, 229.885730093951`, {{0, 0.08063849449157717`, 143.53652019500737`}}, 2.055442432220964`*^7,
0.037494278890396075`, 0.6192277284538117`, 2163.6892589315776`}, {"CURE", 365, 1.4538223657841673`,
{{0, 0.005713811374015267`, 10.170584245747175`}, {4, 0.001172720786414034`, 2.0874429998169806`}},
0.00999977583707516`, 0.00840080333475866`, 0.42907522899307765`, 1780.609310131594`},
{"TBUR", 15.573788009876782`, 230.02747756607437`, {{0, 0.0769172477722168`, 136.9127010345459`}},
7.23494261394152`*^7, 0.1630534574772148`, 0.6079357468278915`, 2121.3585091877044`},
{"CURE", 365, 1.2828146444794823`, {{0, 0.00100869839061457`, 1.7954831352939347`},
{4, 0.0002274960744786865`, 0.4049430125720619`}, {8, 0.000018754252510802436`, 0.03338256946922834`}},
0.00999994111594489`, 0.02873107943968316`, 0.3580595058092362`, 1789.7677405702266`}, {"CURE", 365,
1.6427567562120153`, {{0, 0.008345745434273927`, 14.85542687300759`}, {3, 0.0024367432009112316`, 4.337402897621992`},
{6, 0.00047050846287965335`, 0.8375050639257828`}, {9, 0.00009589660229905653`, 0.17069595209232058`},
{12, 0.000029366303861718 54`, 0.052272020873859`}, {15, 0.000018304359311783827`, 0.03258175957497521`},
{18, 0.00001667657262515396`, 0.029684299272774047`}, {21, 5.137636025966445`*^-6, 0.009144992126220273`}},
0.009999999258902678`, 0.0251000511851493`, 0.9650634442149845`, 1950.9833583595835`},
{"CURE", 365, 0.8288892494970419`, {{0, 0.0012188364953248318`, 2.1695289616782003`},
{4, 0.0003516112030858578`, 0.6258679414928269`}, {8, 0.000039179608566004094`, 0.06973970324748728`}},
0.009999921776692823`, 0.025794223724091316`, 0.2431905493024117`, 1784.514702889057`},
{"CURE", 365, 6.283718339367014`, {{0, 0.004631584288609813`, 8.244220033725467`}, {6, 0.001848061413838219`, 3.28954931663203`},
{12, 0.0003174474350597064`, 0.5650564344062773`}, {18, 0.000047061080589181354`, 0.08376872344874281`},
{24, 9.640641161024232`*^-6, 0.017160341266623132`}, {30, 0.000011307513840341 83`, 0.020127374635808458`}},
0.009999983449496644`, 0.0431999564893843`, 0.7231805790757752`, 2045.5610899387582`}, {"TBUR", 89.13943130602564`,
228.97362861107501`, {{0, 0.0752309678681851`, 133.91112280536947`}, {5, 0.015461932162498455`, 27.52223924924725`},
{10, 0.0025948694288307613`, 4.618867583318755`}, {15, 0.0005582303183360467`, 0.9936499666381631`},
{20, 0.00017446089301016967`, 0.31054038955810204`}, {25, 0.00008750655151018631`, 0.15576166168813163`},
{30, 0.0000643607544780255`, 0.1145621429708854`}, {35, 0.00005689278740805139`, 0.10126916158633147`},
{40, 0.000053904095498014984`, 0.09594928998646667`}, {45, 0.0043331442934957335`, 7.712996842422404`}},
9079.847406530313`, 0.018195990613129782`, 0.14411311578710737`, 1780.0121267256586`},
{"CURE", 365, 2.233222034928952`, {{0, 0.030317538636829275`, 53.9652187735561`}, {8, 0.005793040561701206`, 10.311612199828145`},
{16, 0.0009505304513447579`, 1.6919442033936691`}, {24, 0.00010673090568937173`, 0.18998101212708168`}},
```

0.009999838353994202`, 0.02958920305380679`, 0.936948963971377`, 1786.4944822132823`}, {"CURE", 365,
19.014808089806632`, {{0, 0.011974403670591768`, 21.314438533653348`}, {14, 0.006589601440119365`, 11.729490563412469`}},
0.009999395752474832`, 0.013460528977963512`, 0.6907529726945315`, 1799.7515384389644`}, {"CURE", 365, 0.3785598539752888`,
{{0, 0.0008051685200521667`, 1.433199965692857`}, {45, 0.00028071526846622555`, 0.49967317786988147`}},
0.009999902099315627`, 0.026133379381600035`, 0.11113997972349546`, 1783.1893476490654`},
{"TBUR", 17.049281076901508`, 230.18673320667966`, {{0, 0.06787097930908204`, 120.81034317016602`}},
6.483734227937327`*^7, 0.10658970201778624`, 0.6504338929745312`, 2195.9780277326063`},
{"CURE", 365, 0.7288635482778612`, {{0, 0.004038064550448804`, 7.187754899798871`}}, 0.009999245659966365`,
0.016512559317071646`, 0.3519677457380313`, 1781.0485332655146`}, {"CURE", 365, 0.2489341579089408`,
{{0, 0.0006945840058040657`, 1.236359530331237`}, {55, 0.0003256898765138705`, 0.5797279801946894`}},
0.009766148771014397`, 0.036626055897224785`, 0.04259222004900425`, 1780.8904503809895`}, {"CURE", 365,
186.76529190838718`, {{0, 0.014386699357584562`, 25.60832485650052`}, {12, 0.034694776538432684`, 61.75670223841018`}},
0.009983869389438535`, 0.005932040838821674`, 0.9996418889967243`, 2553.7216245315108`}, {"TBUR", 77.18179197979966`,
228.05419222919616`, {{0, 0.04880652066398899`, 86.87560678190039`}, {4, 0.009225327370831185`, 16.42108272007951`},
{8, 0.0028754614664453144`, 5.118321410272659`}, {12, 0.001006018785673867 3`, 1.7907134384994838`},
{16, 0.00042642740773437393`, 0.7590407857671856`}, {20, 0.0002427983644442941`, 0.43218108871084343`},
{24, 0.00018187426219365204`, 0.32373618670470067`}, {28, 0.00015954243532193 9`, 0.2839855348730514`},
{32, 0.00015016074924895128`, 0.2672861336631333`}, {36, 0.010135919657973542`, 18.041936991192905`}},
743700.0339701164`, 0.0315207495378477`, 0.7612273633112175`, 2592.514575344362`}, {"CURE", 365, 0.292116942047819`,
{{0, 0.007307938657919575`, 13.008130811096844`}, {2, 0.0022748018023244507`, 4.049147208137523`}},
0.009932881061709154`, 0.004136519349913272`, 0.2646633101248961`, 1780.2769583757856`}, {"CURE", 365,
2.895586443107779`, {{0, 0.0034028073216222383`, 6.056997032487584`}, {9, 0.0006044353995498025`, 1.0758950111986485`},
{18, 0.000022122388733399843`, 0.03937785194545172`}, {27, 2.118179477311618`*^-6, 0.0037703594696146796`}},
0.009959450242782941`, 0.07756219543640813`, 0.5205058032657618`, 2258.2611653648537`},
{"CURE", 365, 0.10534248119954921`, {{0, 0.0009658910999464645`, 1.7192861579047067`}},
0.009998748782895103`, 0.01796652278047311`, 0.11194971346777241`, 1781.1143016030012`},
{"TBUR", 46.10521733675777`, 230.2445223701893`, {{0, 0.04413548946380616`, 78.56117124557497`}},
1.8406053530654456`*^7, 0.10477244563603587`, 0.5628117143598024`, 2771.745705864704`}, {"CURE", 365,
0.317559683546119`, {{0, 0.004739787665975871`, 8.43682204543705`}, {12, 0.0011606778888043922`, 2.066006642071818`}},
0.00999820967306249`, 0.02486548868831468`, 0.6044056503192714`, 1781.759204352431`}, {"TBUR", 365,
229.1553650378025`, {{0, 0.070409439598714 1`, 125.32880248571108`}, {31, 0.0022141359995364934`, 3.9411620791749584`},

```
{62, 0.003174018363861818`, 5.649752687674037`}, {93, 0.002323505078682653`, 4.135839040055123`},
{124, 0.002139932007570633`, 3.809078973475727`}, {155, 0.002054818884818950`, 3.657577614977732`},
{186, 0.002021950656579726`, 3.599072168711912`}, {217, 0.002008767836910112`, 3.575606749699999`},
{248, 0.002003471258913313`, 3.566178840865697`}, {279, 0.025875696993745`, 46.05874064886695`}},
279.8879795832612`, 0.023728358575648444`, 0.8326778543109259`, 2322.2501465125556`},
{"CURE", 365, 4.538897833097549`, {{0, 0.005929132865799`, 10.553856501122667`},
{3, 0.003906119719176237`, 6.952893100133703`}, {6, 0.001914106418538538`, 3.407109424998599`},
{9, 0.000933148694881081`, 1.661004676888324`}, {12, 0.000182828416950444`, 0.3254345821717919`}},
0.009999910593512`, 0.009010356779734595`, 0.5279726812498997`, 1780.9262472331138`},
{"TBUR", 15.548490946126897`, 228.76366290935334`, {{0, 0.13357435226440428`, 237.76234703063963`}},
8.920586728506228`*^7, 0.1847810522629491`, 0.46979356756327456`, 1986.0051205330499`}, {"CURE", 365,
1.5439682649346316`, {{0, 0.005352240774422926`, 9.526988578472809`}, {7, 0.0009267754707420849`, 1.649660337920911`}},
0.00987695116314701`, 0.024842695832552032`, 0.18859705618069733`, 1780.6975127014366`},
{"CURE", 365, 52.39008305491437`, {{0, 0.019189550013929167`, 34.15739902479391`},
{4, 0.0003189451321822512`, 0.5677223352844071`}, {8, 0.0002783131464702631`, 0.4953974007170683`},
{12, 0.0002781553650505548`, 0.4951165497899876`}, {16, 0.0002781516554150722`, 0.4951099466388285`},
{20, 0.0002781513928157084`, 0.4951094792119609`}, {24, 0.0002781513548281701`, 0.495109411594142`},
{28, 0.0002781513459294294`, 0.4951093957543843`}, {32, 0.000325308737127154`, 0.5790495520863354`}},
0.00999723353430831`, 0.006707674020362`, 0.9999843924265246`, 2333.638060726628`}, {"TBUR", 64.65880757648914`,
228.46302716160943`, {{0, 0.07849768226398733`, 139.72587442989743`}, {4, 0.012788448774234056`, 22.763438818136617`},
{8, 0.005563354319591639`, 9.902770688873117`}, {12, 0.003738334052523072`, 6.654234613491069`},
{16, 0.003321453975377314`, 5.91218807617162`}, {20, 0.003359000045197576`, 5.979020080451685`},
{24, 0.003397629898996801`, 6.047781220214306`}, {28, 0.003189875474064926`, 5.677978343835568`},
{32, 0.002777324405612606`, 4.943637441990439`}, {36, 0.10285816471871383`, 183.0875331993106`}},
4.195876889970613`*^6, 0.06198039227260211`, 0.6624511642360496`, 1967.0064357998826`}, {"CURE", 365,
1.4176771202417109`, {{0, 0.009079214967785682`, 16.161002642658513`}, {38, 0.00512626755482221`, 9.124756247583534`}},
0.009999789815251754`, 0.01390519185830331`, 0.4576404855374086`, 1781.6634166608856`},
{"TBUR", 18.58528870031779`, 230.22262463957637`, {{0, 0.05134700298309326`, 91.397665309906`}}, 2.597638512857568`*^7,
0.17636102966901634`, 0.6903248799640455`, 2321.0731874582034`}, {"CURE", 365, 32.953621199910636`,
{{0, 0.007614555962308703`, 13.55390961290949`}, {9, 0.000513063667293717`, 0.9132533277828173`},
{18, 0.000512090723204769`, 0.911521487304490`}, {27, 0.000512082975897335`, 0.911507697097256`},
```

```
{36, 0.0005120824574619592`, 0.9115067742822874`}, {45, 0.0005120823696355041`, 0.9115066179511974`},
  {54, 0.0005120823454075576`, 0.9115065748254526`}, {63, 0.0005120823366061809`, 0.911506559159002`},
  {72, 0.0005120823328646198`, 0.9115065524990232`}, {81, 0.0004427332688148476`, 0.7880652184904288`}},
 0.009994525752274035`, 0.007382151492638201`, 0.9998637286833575`, 2368.5123948400114`}, {"CURE", 365,
 143.78888355702736`, {{0, 0.032291797376458216`, 57.47939933009563`}, {33, 0.0016183906632301764`, 2.880735380549714`},
  {66, 0.0014649299428551104`, 2.6075752982820966`}, {99, 0.0014539239491057204`, 2.587984629408182`},
  {132, 0.001453186709962209`, 2.586672343732732`}, {165, 0.001453141636503787`, 2.5865921129767413`},
  {198, 0.0014531388778867324`, 2.5865872026383836`}, {231, 0.0001673931874260762`, 0.29795987361841564`}},
 0.00998624638153798`, 0.0009181887945730282`, 0.9194605419110384`, 2438.3732023639923`}, {"CURE", 365,
 1.6400011729985593`, {{0, 0.004901040466287979`, 8.723852029992601`}, {11, 0.0008215405127818032`, 1.4623421127516096`},
  {22, 0.00004546939210548665`, 0.08093551794776623`}, {33, 6.531390735664555`*^-6, 0.011625875509482908`},
  {44, 5.210569607747678`*^-6, 0.009274813901790867`}, {55, 5.16538162476507`*^-6, 0.009194379292081823`},
  {66, 5.1622496223051226`*^-6, 0.00918880432770312`}, {77, 9.310894152786219`*^-6, 0.016573391591959467`}},
 0.009961372320187938`, 0.06950363059226418`, 0.7899484321047908`, 2413.243925154943`},
{"CURE", 365, 0.22322685984249543`, {{0, 0.003177614112104377`, 5.656153119545791`},
  {6, 0.0009191125278347919`, 1.6360202995459294`}, {12, 0.00010205648493381157`, 0.1816605431821846`}},
 0.009976833772708237`, 0.013313802366286343`, 0.8524139712276719`, 1787.3198734728817`}, {"CURE", 365,
 60.685950981557504`, {{0, 0.04355997372459042`, 77.53675322977094`}, {3, 0.0028924776640809123`, 5.148610242064024`},
  {6, 0.0027782983209139332`, 4.945371011226801`}, {9, 0.002774877098935716`, 4.939281236105575`},
  {12, 0.002774687159571302`, 4.938943144036918`}, {15, 0.002774671135462876`, 4.93891462112392`},
  {18, 0.002774669323854189`, 4.9389113964604565`}, {21, 0.002774669070399758`, 4.93891094531157`},
  {24, 0.0027746690286993363`, 4.938910871084818`}, {27, 0.002747813449349562`, 4.89110793984222`}},
 0.00999995389809607`, 0.02564717902139023`, 0.9784373628720977`, 2399.5729372805317`}, {"CURE", 365,
 0.2748909227611292`, {{0, 0.0011798871205515068`, 2.100199074581682`}, {17, 0.0003689333007197237`, 0.6567012752811081`}},
 0.009999818701983408`, 0.019061719228001248`, 0.21869130518613564`, 1783.2316206555279`},
{"CURE", 365, 0.5404928077367063`, {{0, 0.0022859141264512336`, 4.068927145083196`},
  {5, 0.0005847794737392971`, 1.0409074632559487`}, {10, 0.00007876874616034778`, 0.14020836816541907`}},
 0.009999945070644528`, 0.016469239911709427`, 0.759135747297076`, 1788.313641187337`},
{"CURE", 365, 15.266582234101907`, {{0, 0.038215814193509064`, 68.02414926444614`},
  {2, 0.0020074715673172004`, 3.573299389824617`}, {4, 0.00009873163427321258`, 0.17574230900631838`},
  {6, 0.000022771510514872803`, 0.04053328871647359`}, {8, 0.00002021715829418401`, 0.035986541763647545`},
```

```
{10, 0.000020128460786832316`, 0.035828660200561524`}, {12, 0.000020124008454387336`, 0.035820735048809456`},
{14, 0.000020123669799663216`, 0.035820132243400526`}, {16, 0.000013903453745512137`, 0.024748147667011604`}},
0.009999993728990178`, 0.026728291963074892`, 0.9972442213408431`, 1962.9699199489762`},
{"TBUR", 33.14252113786084`, 230.19694174731248`, {{0, 0.06884503364562988`, 122.54415988922119`}}, 8.354415727121226`*^7,
0.12828885465966752`, 0.5076340993597268`, 2261.5597436174908`}, {"CURE", 365, 0.22209911308201927`,
{{0, 0.001051141295242984`, 1.8710315055325115`}, {30, 0.0002095363114855012`, 0.37297463444419215`}},
0.00975510021747927`, 0.024623145384721608`, 0.4434247255031338`, 1797.4748344431366`}, {"CURE", 365, 0.42139096627148565`,
{{0, 0.0003921156806431666`, 0.6979659115448366`}, {32, 0.00009635464959656016`, 0.17151127628187707`}},
0.009999264866024643`, 0.02479628338366427`, 0.09954126808658333`, 1788.1087006263267`}, {"CURE", 365,
0.0981250206588834`, {{0, 0.0011863239430199726`, 2.1116566185755516`}, {3, 0.0004719507400080915 4`, 0.840072317214403`}},
0.00999999913682737`, 0.0064025995419260745`, 0.2569613525456227`, 1781.8731430872763`}, {"CURE", 365,
0.6537411095717834`, {{0, 0.001646514763807895`, 2.930796279578053`}, {8, 0.00042141520453366227`, 0.7501190640699189`}},
0.009999913814108375`, 0.02078668562840782`, 0.8380536429228961`, 1808.657306831357`}, {"CURE", 365,
0.548023378087629`, {{0, 0.008546336009828312`, 15.212478097494392`}, {8, 0.0035762366865662687`, 6.365701302087958`},
{16, 0.0012564471721619595`, 2.2364759664482876`}, {24, 0.00031656674739379703`, 0.5634888103609587`}},
0.009985496136471771`, 0.026994477908338727`, 0.830402019718501`, 1783.0850597280682`},
{"CURE", 365, 2.1582675565782643`, {{0, 0.0027260484306559345`, 4.852366206567563`}}, 0.009999922076293659`,
0.0181556961897077`, 0.04298291383342194`, 1780.1618168611442`}, {"CURE", 365, 0.2922455340679021`,
{{0, 0.0018508585519167095`, 3.294528222411743`}, {5, 0.000290822762095439`, 0.5176645165298814`}},
0.009999923032085271`, 0.006935362061229899`, 0.23944788607122014`, 1781.422775578008`},
{"CURE", 365, 82.8679010158424`, {{0, 0.03759001841982912`, 66.91023278729584`}, {18, 0.006425189015942819`, 11.43683644837822`},
{36, 0.001100286077482822`, 1.9585092179194232`}, {54, 0.00022082434493928433`, 0.3930673339919261`}},
0.009999959377136153`, 0.021805924679705487`, 0.9438426922969437`, 2161.50773557332`},
{"CURE", 365, 68.47328995038426`, {{0, 0.024722839062550354`, 44.00665353133963`},
{25, 0.003968629551376031`, 7.064160601449336`}, {50, 0.0006343718839962624`, 1.129181953513347`}},
0.009999672662969175`, 0.013284566965462225`, 0.9999650698628625`, 2425.476672599237`}, {"CURE", 365,
0.19809666144841784`, {{0, 0.0016362815704771003`, 2.9125811954492384`}, {6, 0.0002627632763249772`, 0.4677186318584594`}},
0.00996408507617601`, 0.01235364727775617`, 0.13922433805804163`, 1780.4936631050784`}, {"CURE", 365,
0.7315014546836218`, {{0, 0.006597237795003305`, 11.743083275105882`}, {56, 0.002610789831211803`, 4.647205899557009`}},
0.009768209732472976`, 0.05081352341780216`, 0.4295890069001722`, 1783.4395832116438`}, {"CURE", 365,
0.24065653566292614`, {{0, 0.002902932826883564`, 5.167220431852744`}, {7, 0.0008016705688200159`, 1.4269736124996282`}},
```

0.009928660312542538`, 0.009964308461057485`, 0.3330068231980925`, 1781.25023835736`}, {"CURE", 365,
5.965612992031404`, {{0, 0.029662606734079902`, 52.79943998666223`}, {8, 0.005619878632797431`, 10.003383966379426`}},
0.00999973765114507`, 0.021871507512810084`, 0.922858937310032`, 2076.100425954591`},
{"TBUR", 18.660572747504208`, 230.21127166174142`, {{0, 0.08790034294128418`, 156.46261043548583`}},
8.874326085906798`*^7, 0.10303876539548956`, 0.5735295261420265`, 2110.8104986626986`},
{"CURE", 365, 4.407784490035505`, {{0, 0.011806214367528007`, 21.01506157419985`}},
0.009999923401377071`, 0.004670505039107311`, 0.5536950715501437`, 1780.6071358998124`},
{"CURE", 365, 5.109838378322492`, {{0, 0.003484619392973603З`, 6.202622519493014`}},
0.0099997756737547`, 0.017960267631014954`, 0.07398866699685105`, 1780.2160970468785`},
{"CURE", 365, 9.068372100728512`, {{0, 0.0122797975752165`, 21.858039683885373`}, {8, 0.0017535919237167438`, 3.121393624215804`},
{16, 0.00012018434363164611`, 0.21392813166433008`}, {24, 0.000021004057555722413`, 0.0373872224491859`},
{32, 0.00001548449236834536`, 0.02756239641565474`}, {40, 0.000015160395599050014`, 0.026985504166309026`},
{48, 0.000015137205829013755`, 0.02694422637564448`}, {56, 0.000025953501374131808`, 0.04619723244595462`}},
0.009999991711147406`, 0.05828717891414183`, 0.6095360311561328`, 2099.4714523998587`}, {"CURE", 365,
5.12824097666857 4`, {{0, 0.009945724113150952`, 17.703388921408695`}, {73, 0.001889064513054436`, 3.362534833236896`}},
0.009999855218684519`, 0.03166906042921503`, 0.5322977136981933`, 1788.7672483016022`},
{"CURE", 365, 0.07585909388138315`, {{0, 0.0013420705904605107`, 2.388885651019709`}},
0.009999932677944317`, 0.01747461690057782`, 0.3883555413475168`, 1783.3587732482915`},
{"TBUR", 16.823675403542563`, 228.97983448337155`, {{0, 0.10412581443786623`, 185.3439496994019`}},
8.616308543170305`*^7, 0.2265070165222851`, 0.48610173133280465`, 2035.8278601610127`}, {"CURE", 365,
1.6170970539614393`, {{0, 0.005130873162969076`, 9.132954230084955`}, {14, 0.0006645717889238957`, 1.1829377842845343`}},
0.009999858157199604`, 0.021852429269065745`, 0.75689788776549`, 2063.519356343115`}, {"CURE", 365,
6.849231671086752`, {{0, 0.0182583450849103`, 32.499854251140334`}, {2, 0.0012824419272668853`, 2.2827466305350557`},
{4, 0.00009645904994275542`, 0.17169710889810463`}, {6, 0.00004768047655771356`, 0.08487124827273014`},
{8, 0.00004615986610259153`, 0.08216456166261293`}, {10, 0.000027827452794827003`, 0.04953286597479206`}},
0.00999999296194717`, 0.017806834862738832`, 0.9912423084286637`, 1912.0468822897415`}, {"CURE", 365,
1.0298230945031612`, {{0, 0.0016613802684022126`, 2.9572568777559387`}, {23, 0.00037193445376988563`, 0.6620433277103964`}},
0.009999953757637433`, 0.012785932885446922`, 0.6531162610124921`, 1816.89908715492`},
{"TBUR", 62.617188466955746`, 230.25210983076022`, {{0, 0.0880062198638916`, 156.65107135772703`}},
8.152237440349103`*^6, 0.01818952222185812`, 0.5169971063468749`, 2233.115515032087`}, {"CURE", 365,
1.0364465546952355`, {{0, 0.006742617667892332`, 12.001859448848352`}, {7, 0.002902016180883719`, 5.165588801973019`}},

0.009912072435918616`, 0.009713689483510032`, 0.2591915204667393`, 1780.2948675092364`}, {"CURE", 365, 2.066251373684524`,
{{0, 0.0023669920864448656`, 4.2132459138718605`}, {23, 0.00011514167085681219`, 0.20495217412512567`}},
0.009999425426902974`, 0.01179663663256981`, 0.4948035589549776`, 1785.8827705079163`}, {"CURE", 365,
0.6765817636334884`, {{0, 0.0015076730615155842`, 2.68365804949774`}, {30, 0.000565103851086577`, 1.005884854934107`}},
0.009999554626753624`, 0.012657717720458287`, 0.2310434200909179`, 1784.28044698645`}, {"TBUR", 276.3049551429505`,
229.52831807629158`, {{0, 0.08507927211237143`, 151.44110436002111`}, {24, 0.01885857722200225`, 33.5682674551640 1`},
{48, 0.006387539464215356 5`, 11.369820246303336`}, {72, 0.0029643317567697824`, 5.276510527050213`},
{96, 0.0015206302629758746`, 2.706721868097057`}, {120, 0.000816834177071285`, 1.4539648351868875`},
{144, 0.00045813721267552493`, 0.8154842385624345`}, {168, 0.00027247304074934126`, 0.4850020125338274`},
{192, 0.00017536645917547527`, 0.31215229733234595`}, {216, 0.009345439641802764`, 16.634882562408922`}},
55.96896621814019`, 0.0239394524103772`, 0.1602480180880105`, 1780.0146899689848`}, {"CURE", 365, 3.1436600594694375`,
{{0, 0.0036031887559494933`, 6.413675985590099`}, {5, 0.0005817384071960853`, 1.0354943648090318`},
{10, 0.000030866544359089 53`, 0.054942448959179366`}, {15, 1.6649986063428471`*^-6, 0.002963697519290268`}},
0.009999995228369123`, 0.041637109913935336`, 0.5150888566548992`, 1946.4394568050886`}, {"TBUR", 108.66860103948653`,
228.53791023509595`, {{0, 0.006211326701280833`, 11.056161528279883`}, {6, 0.0011001655836825144`, 1.9582947389548753`},
{12, 0.0003169355468639554`, 0.5641452734178406`}, {18, 0.00015862576971061392`, 0.2823538700848928`},
{24, 0.00011595314643038119`, 0.20639660064607848`}, {30, 0.0001039378913775418`, 0.1850094466520244`},
{36, 0.00010366066635095974`, 0.18451598610470835`}, {42, 0.00010976153518043349`, 0.19537553262117158`},
{48, 0.00011995210930775418`, 0.21351475456780242`}, {54, 0.05322393822404827`, 94.73861003880593`}},
209952.61957467327`, 0.018244784182055437`, 0.7758368233663747`, 2586.8664815557786`}, {"CURE", 365,
8.234146630765053`, {{0, 0.00843435081531609`, 15.01314445126264`}, {20, 0.0014013008076735557`, 2.4943154376589294`}},
0.009999805493172864`, 0.027065944005173617`, 0.725304983116898`, 2051.988983756177`}, {"CURE", 365,
4.5363651039017965`, {{0, 0.00595630797687311`, 10.602228198834137`}, {6, 0.0007445945093200385`, 1.3253782265896688`},
{12, 0.00007554440167755028`, 0.13446903498603952`}, {18, 0.00004124341662286516`, 0.07341328158869999`},
{24, 0.000040124161838184974`, 0.07142100807196926`}, {30, 0.00010395028813466934`, 0.18503151287971142`}},
0.009999952159931042`, 0.02562158947011371`, 0.997252321298549`, 2240.3595522096607`}, {"CURE", 365,
2.6394297404875195`, {{0, 0.008025860391286672`, 14.286031496490276`}, {8, 0.0024261901991117023`, 4.31861855441883`},
{16, 0.00027444414708292753`, 0.488510581807611`}, {24, 0.000017347288875906013`, 0.030878174199112703`}},
0.009999943108527696`, 0.05138023133041479`, 0.3360822545394121`, 1789.60743410765`},
{"CURE", 365, 0.838710823231548`, {{0, 0.009167955667267401`, 16.31896108773598`}, {3, 0.002670148680788578`, 4.75286465180367`},
{6, 0.0005266483924996159`, 0.9374341386493164`}, {9, 0.00011202385538140971`, 0.1994024625789093`},

```
{12, 0.00001829822135975621`, 0.03257083402036605`}}, 0.009999985685700024`,
0.014073701115814808`, 0.9432950557097314`, 1840.9521247513615`}, {"CURE", 365, 0.12285003536715865`,
{{0, 0.000515955831942605 4`, 0.9184013808578376`}, {11, 0.0001476266037408928`, 0.26277535465878915`}},
0.0099998821971916 6`, 0.021819262661867404`, 0.5355089817126556`, 1789.3519423444097`}, {"CURE", 365,
8.383013405056795`, {{0, 0.02098185496365314`, 37.347701835302594`}, {3, 0.0015779246789807667`, 2.808705928585765`},
{6, 0.00011894021349026604`, 0.21171358001267357`}, {9, 0.00005707232318597605`, 0.10158873527103737`},
{12, 0.000054774920285720806`, 0.09749935810858303`}, {15, 0.00005465934777052087`, 0.09729363903152716`},
{18, 0.0000546492934398109 1`, 0.0972757423228634`}, {21, 0.00005464801588551554`, 0.09727346827621765`},
{24, 0.00005464781295305703`, 0.09727310705644152`}, {27, 0.00003833963250071612`, 0.0682445458512747`}},
0.009999993434522708`, 0.0297618806025045`, 0.9960342731747833`, 1996.2200211243228`},
{"CURE", 365, 1.4505708335981222`, {{0, 0.005248515681582511`, 9.34235791321687`}},
0.009999926910196594`, 0.006108995939481725`, 0.18788604512690818`, 1780.2301277430447`},
{"CURE", 365, 0.017760086262557696`, {{0, 0.00035877662188784563`, 0.6386223869603652`}}, 0.009997540235211154`,
0.019387986412957546`, 0.13404288026351402`, 1783.5659823312133`}, {"CURE", 365, 0.9898359570889238`,
{{0, 0.005988145644013343`, 10.658899246343752`}, {32, 0.002277205597350983`, 4.0534259632847505`}},
0.00972742315056259`, 0.012622608753432038`, 0.24900360121281645`, 1781.2956651532377`},
{"CURE", 365, 0.7147405889053394`, {{0, 0.004164534189943776`, 7.412870858099922`},
{3, 0.0010350963020609736`, 1.8424714176685328`}, {6, 0.00016062701937730794`, 0.2859160944916081`},
{9, 0.000031273889157101175`, 0.05566752269964009`}, {12, 0.000012718998113236715`, 0.022639816641561353`}},
0.009999999639321048`, 0.017474623737824536`, 0.9586628363860065`, 1838.0069819431953`},
{"CURE", 365, 1.0152255541187147`, {{0, 0.0015134455965173804`, 2.693933161800937`},
{7, 0.0007572607640285794`, 1.3479241599708713`}, {14, 0.00014146804838399934`, 0.2518131261235188`}},
0.009957318746661496`, 0.025864641143541457`, 0.31906173039723495`, 1782.0584068258797`},
{"CURE", 365, 2.926592732693426`, {{0, 0.02723144197071806`, 48.471966707878146`}, {3, 0.005091350774097999`, 9.06260437789444`},
{6, 0.0007360945672869921`, 1.310248329770846`}, {9, 0.00012229486843845454`, 0.2176848658204491`},
{12, 3.0142970118326007`*^-6, 0.005365448681062029`}}, 0.009999979343290924`, 0.01758575274755263`,
0.9525749253270994`, 1783.2840616305218`}, {"TBUR", 205.77973899658244`, 228.3494893624436`,
{{0, 0.028467204189925453`, 50.67162345806731`}, {19, 0.0018315556410310446`, 3.2601690410352595`},
{38, 0.0033160711098204915`, 5.902606575480474`}, {57, 0.00602197842391446`, 10.71912159456774`},
{76, 0.004904161920839694`, 8.729408219094656`}, {95, 0.004252547249482463`, 7.569534104078784`},
{114, 0.004214613938685208`, 7.502012810859672`}, {133, 0.003971407141555028`, 7.069104711967951`},
```

```
    {152, 0.003703611309387587`, 6.592428130709905`}, {171, 0.41973824575818947`, 747.1340774495773`}},
  672387.76055662`, 0.14207781116013318`, 0.7873860601969421`, 2407.677169109959`}, {"CURE", 365, 1.9422326791599263`,
  {{0, 0.006171220383429845`, 10.984772282505125`}, {24, 0.0017967984481779419`, 3.1983012377567364`}},
  0.009748831512333023`, 0.033200318968231037`, 0.22842354440262416`, 1780.9068839095858`},
 {"TBUR", 16.535305804542197`, 230.1889731493486`, {{0, 0.08417602539062502`, 149.83332519531254`}},
  2.9941173167216185`*^7, 0.045978034937596356`, 0.6580261195371838`, 2141.09994890 6728`}, {"CURE", 365,
  1.7022653030955286`, {{0, 0.004121997861506609`, 7.337156193481762`}, {3, 0.0009757731768622059`, 1.7368762548147263`},
    {6, 0.00012287723885 3223`, 0.21872148515873693`}, {9, 0.000019933362933261007`, 0.03548138602120459`},
    {12, 9.876593417034333`*^-6, 0.01758033628232111`}, {15, 0.000032088662221233 04`, 0.057117818753794805`}},
  0.00998124337025869`, 0.019473996378120723`, 0.9787202225087372`, 2502.08224793627`}, {"CURE", 365,
  1.0810110933524057`, {{0, 0.019994385541920314`, 35.59000626461816`}, {5, 0.00415202670729961 7`, 7.390607538993319`}},
  0.009922446549574625`, 0.01562807541818052`, 0.9218400529735077`, 1783.5645815147643`}, {"CURE", 365,
  0.41096070395052664`, {{0, 0.0032435141043262923`, 5.7734551057008`}, {42, 0.001003495861584834`, 1.7862226336210043`}},
  0.009735483403477942`, 0.03280021316554442`, 0.3318482718465363`, 1784.7402531616235`},
 {"TBUR", 44.30027252617322`, 230.24083894454986`, {{0, 0.09859271049499513`, 175.4950246810913`}},
  591444.2315741914`, 0.01958782407838304`, 0.7036979181332945`, 2239.267707271272`},
 {"TBUR", 24.390459589884795`, 230.0671506625649`, {{0, 0.10939068794250491`, 194.71542453765872`}},
  4.828497383584037`*^7, 0.0839835978205076`, 0.4981058253064984`, 2068.143414665361`}, {"TBUR", 263.2390321731134`,
  230.18682040147434`, {{0, 0.04953781587387166`, 88.17731225549156`}, {24, 0.002787351331169623`, 4.961485369481928`},
    {48, 0.0029074140199954034`, 5.175196955591818`}, {72, 0.0030469122964951714`, 5.423503887761404`},
    {96, 0.0028790718131973493`, 5.124747827491281`}, {120, 0.002765580419863198`, 4.922733147356492`},
    {144, 0.002717730167869411`, 4.83755969880755`}, {168, 0.002692939591049738`, 4.793432472068533`},
    {192, 0.002680163635824442`, 4.7706912717675065`}, {216, 0.04936631276948819`, 87.87203672968897`}},
  78302.11079249499`, 0.03304528368762984`, 0.12679772457992003`, 1780.0191938226135`}, {"CURE", 365, 0.820102094202406`,
  {{0, 0.002745189567573441`, 4.886437430280725`}, {29, 0.0007305694321375407`, 1.3004135892048225`}},
  0.009756677908733568`, 0.01673442123863003`, 0.18153066463736547`, 1781.7872488196476`},
 {"CURE", 365, 7.47487776283584`, {{0, 0.014110713236449504`, 25.11706956088012`},
    {14, 0.001147647172072024`, 2.0428119662882027`}, {28, 0.000031934304224387 41`, 0.05684306151940959`},
    {42, 9.125901746256528`*^-6, 0.01624410510833662`}, {56, 8.783707407065752`*^-6, 0.015634999184577038`},
    {70, 8.7767473368866`*^-6, 0.01562261025965815`}, {84, 0.000017412377393346 38`, 0.030994031760156558`}},
  0.00999966046726969`, 0.06187987809011663`, 0.8626863707399429`, 2266.5840229701516`}, {"CURE", 365,
```

26.353717636452078`, {{0, 0.012728576192256884`, 22.656865622217254`}, {12, 0.0003361152313957123`, 0.598285111884368`},
{24, 0.00011303083344145004`, 0.2011948835257811`}, {36, 0.00011158250290748608`, 0.19861685517532524`},
{48, 0.00011154921049009732`, 0.19855759467237324`}, {60, 0.00011154451526734125`, 0.19854923717586745`},
{72, 0.00011154352720124991`, 0.19854747841822487`}, {84, 0.00011154329088938307`, 0.1985470577831019`},
{96, 0.0001115432312839896`, 0.19854695168550146`}, {108, 0.00005972030686994`, 0.1063021462284932`}},
0.009999997373889166`, 0.0258776628482984`, 0.9999220229484647`, 2342.207569861176`}, {"CURE", 365, 0.24691081076339497`,
{{0, 0.007705201941103779`, 13.715259455164729`}, {5, 0.00042266458214294263`, 0.7523429562144378`}},
0.009983671757507263`, 0.019268390832133903`, 0.5625090090594531`, 1780.8641261314058`}, {"CURE", 365,
6.274801721375873`, {{0, 0.00687924042441745`, 12.245047955463061`}, {23, 0.0009761556258342382`, 1.7375570139849439`}},
0.00999164770796525`, 0.01451670137978792`, 0.7150798805148458`, 1891.0289427468615`},
{"CURE", 365, 1.1350065918427164`, {{0, 0.00408264709754465`, 7.267111833629477`},
{5, 0.0009045891349940053`, 1.6101686602893295`}, {10, 0.00008891207559880749`, 0.15826349456587735`}},
0.00999995744871133`, 0.031150211863156644`, 0.4824411709068644`, 1784.154051398322`},
{"CURE", 365, 0.6901547167130785`, {{0, 0.005247524492042928`, 9.340593595836411`}},
0.00959326774154621`, 0.015359463955483373`, 0.18468975880761848`, 1780.4419590695784`},
{"CURE", 365, 0.4448355542784745`, {{0, 0.001884286874090113`, 3.354030635880401`}}, 0.00999392551537305`,
0.010953331125338367`, 0.0531524109658091`, 1780.2498651891303`}, {"CURE", 365, 0.23609243828460463`,
{{0, 0.0017083626612874082`, 3.0408855370915866`}, {69, 0.0010023029713989305`, 1.7840992890900962`}},
0.009770921379880407`, 0.04212609427464854`, 0.11382218002956773`, 1048.0955565416027`},
{"CURE", 365, 4.4522998774793425`, {{0, 0.02212065657607823`, 39.37476870541925`},
{3, 0.0037194746593741685`, 6.62066489368602`}, {6, 0.0004440474449134032`, 0.7904044519458577`}},
0.00998619328971682`, 0.013425676285263307`, 0.829023744311087`, 1781.2993958330924`},
{"CURE", 365, 1.5979758663996095`, {{0, 0.002971472120563716`, 5.289220374603413`},
{8, 0.0012984183842752323`, 2.3111847240099133`}, {16, 0.0002419990065381007`, 0.43075823163781923`}},
0.009999923943531695`, 0.032367443161955854`, 0.2776840280071991`, 1781.3017928523218`}, {"CURE", 365,
10.299995145880809`, {{0, 0.019158450622136548`, 34.10204210740306`}, {20, 0.002551696453695908`, 4.542019687578716`}},
0.009999311745302138`, 0.019384939257419673`, 0.7508671979751674`, 2070.048051368675`},
{"TBUR", 179.155138415111`, 230.2174994338959`, {{0, 0.10741490266179524`, 191.19852673799554`},
{12, 0.036983008250876914`, 65.8297546865609`}, {24, 0.0023521465739343756`, 4.1868209016031885`},
{36, 0.0003960071537188958`, 0.7048927336196347`}, {48, 0.0007102630554041556`, 1.264268238619397`},
{60, 0.0004592509917512606`, 0.8174667653172439`}, {72, 0.00008081213001077252`, 0.14384559141917508`},

{84, 0.00002169148856733715 3`, 0.03861084964986013 4`}, {96, 0.00001891404935627179 5`, 0.03366700785416379 4`},
{108, 0.00020274254750317 2`, 0.3608817345556462`}}, 11.978839248228557`, 0.017430365740177235`,
0.11085491338537573`, 1780.0060536636324`}, {"TBUR", 106.29838801250044`, 228.33182977644188`,
{{0, 0.02967389375055995`, 52.81953087599671`}, {6, 0.0025198095291294395`, 4.485260961850402`},
{12, 0.0009759872776760629`, 1.7372573542633918`}, {18, 0.0006323642694475729`, 1.12560839961668`},
{24, 0.0005198028355368523`, 0.925249047255597`}, {30, 0.0004753542518183628`, 0.8461305682366856`},
{36, 0.00045531717783153493`, 0.8104645765401322`}, {42, 0.00044502520033663854`, 0.7921448565992166`},
{48, 0.0004390726461214289`, 0.7815493100961435`}, {54, 0.0359494479294466 7`, 63.99001731441507`}},
459381.61471384665`, 0.04476836456596378`, 0.6239342280526907`, 2641.123616217593`},
{"TBUR", 49.02568526863226`, 230.24033138814144`, {{0, 0.05947819709777833`, 105.87119083404544`}},
680625.5102557082`, 0.042286099086467056`, 0.737182659949755`, 2999.7094017272266`},
{"CURE", 365, 4.835366601065029`, {{0, 0.006248747828554546`, 11.122771134827094`},
{2, 0.0013524257211833066`, 2.4073177837062865`}, {4, 0.0001748164397245708`, 0.3111732627097361`},
{6, 0.000033367138161559966`, 0.05939350592757674`}, {8, 0.00001989720771477169`, 0.03541702973229361`},
{10, 0.000018832776233444846`, 0.03352234169553183`}, {12, 0.00001876185760530162`, 0.03339610653743688`},
{14, 0.000018757656546542715`, 0.03338862865284603`}, {16, 0.000014544580055301905`, 0.025889352498437393`}},
0.009999997696424033`, 0.02264231652265036`, 0.9761987945252331`, 2121.7353939806585`}, {"CURE", 365,
0.39512981234367456`, {{0, 0.000831659605815592`, 1.4803540983517538`}, {10, 0.0003096739933837671`, 0.5512197082231055`},
{20, 0.0000576249726122206`, 0.10257245124975269`}, {30, 5.865206243326704`*^-6, 0.010440067113121534`}},
0.009958043805428765`, 0.04032912838230459`, 0.1600535291492213`, 1790.9978818182014`},
{"CURE", 365, 0.7767020926201219`, {{0, 0.005920408608870827`, 10.538327323790073`}}, 0.009999260698594243`,
0.022058040705343757`, 0.329080319652518`, 1780.531079541004`}, {"TBUR", 106.35262706962288`, 228.89453185935315`,
{{0, 0.054112267158050245`, 96.31983554132943`}, {6, 0.0002490442058838842`, 0.4432986864733139`},
{12, 0.0001941082568318016`, 0.34551269716060695`}, {18, 0.00019289501915188955`, 0.3433531340903634`},
{24, 0.00019643776907292694`, 0.34965922894980994`}, {30, 0.00022505739994220354`, 0.40060217189712233`},
{36, 0.000403536023247243`, 0.7182941213800926`}, {42, 0.0004991763950136842`, 0.8885339831243579`},
{48, 0.00030606967401340326`, 0.5448040197438578`}, {54, 0.023283279064808017`, 41.44423673535827`}}, 2633.415235037467`,
0.026724623999242282`, 0.8528821086251052`, 3351.2290270069398`}, {"CURE", 365, 0.05310064620489042 5`,
{{0, 0.00014974237673022912`, 0.2665414305798078`}, {9, 0.00006479097603851468`, 0.11532793734855616`},
{18, 9.193593994518662`*^-6, 0.016364597310243218`}, {27, 2.749760564816654`*^-6, 0.004894573805373645`}},
0.009999224925570 95`, 0.10043855398066212`, 0.16222219975188001`, 1985.5752735553658`},

```
{"CURE", 365, 0.02118843164008544`, {{0, 0.00025052969095111104`, 0.44594284989297767`}}, 0.009999949037298232`,
  0.020220654645380833`, 0.1074465670998172`, 1784.8829292308594`}, {"CURE", 365, 0.09298567311928951`,
  {{0, 0.0006177506053328196`, 1.099596077492419`}, {26, 0.00020515677150187013`, 0.3651790532733288`}},
  0.009813988983887637`, 0.01780329931392621`, 0.0341531963258324`, 1780.5446975617178`},
{"CURE", 365, 4.120135249186183`, {{0, 0.002134577612450266`, 3.799548150161474`},
   {5, 0.0006882273029936867`, 1.2250445993287622`}, {10, 0.00007838711356508868`, 0.13952906214585786`},
   {15, 7.888168389275749`*^-6, 0.014040939732910832`}, {20, 1.7336795646745978`*^-6, 0.003085949625120784`}},
  0.009936808369011039`, 0.02937893192361655`, 0.3237587770707608`, 2042.0938554433692`}, {"TBUR", 365,
  229.12745081651698`, {{0, 0.014904708654750264`, 26.53038140545547`}, {32, 0.002135224794993405`, 3.800700135088261`},
   {64, 0.002135991378915372`, 3.802064654469362`}, {96, 0.002178908997124253 6`, 3.8784580148811716`},
   {128, 0.002158009672661260 3`, 3.841257217337044`}, {160, 0.002146927939786283 4`, 3.821531732819585`},
   {192, 0.002140720059708021 5`, 3.8104817062802785`}, {224, 0.002137884974090466 8`, 3.805435253881031`},
   {256, 0.002136505029256926 2`, 3.8029789520773285`}, {288, 0.020020184366468196`, 35.635928172313385`}},
  2.251052485943967`, 0.008526520519875097`, 0.8958528093981016`, 3144.4357716123654`},
{"TBUR", 18.831408698971078`, 229.34676224896702`, {{0, 0.0966405963897705`, 172.0202615737915`}},
  3.867096162578347`*^7, 0.1320197666647114`, 0.49686555598167637`, 2063.9155627920204`}, {"CURE", 365,
  0.5694572412140817`, {{0, 0.007667216316926176`, 13.647645044128593`}, {6, 0.002126643184841346`, 3.785424869017596`}},
  0.009922610557622003`, 0.008311737846104523`, 0.44784655391544037`, 1780.392837867394`},
{"CURE", 365, 1.0703325436193671`, {{0, 0.0024221728064597768`, 4.311467595498403`}}, 0.009999518110634707`,
  0.013903956732205729`, 0.09585776966923518`, 1780.301631833525`}, {"CURE", 365, 0.3643235856371008`,
  {{0, 0.0019279888135920512`, 3.4318200881938514`}, {37, 0.0005127044284142228`, 0.9126138825773167`}},
  0.0099968538958389`, 0.03492992768081792`, 0.17076032459677426`, 1783.5375054481033`}, {"CURE", 365,
  6.768737520227029`, {{0, 0.019448573102085556`, 34.61846012171229`}, {14, 0.005807213286374578`, 10.33683964974675`}},
  0.009994623855012891`, 0.016732528567761046`, 0.8694826721985172`, 1941.224429715231`}, {"TBUR", 365,
  228.64750310661503`, {{0, 0.003875710998051851`, 6.898765576532295`}, {31, 0.00059626985338694`, 1.061360339028753`},
   {62, 0.0005962706714028766`, 1.0613617950971204`}, {93, 0.0005962780625927655`, 1.0613749514151227`},
   {124, 0.0005963382653466341`, 1.0614821123170086`}, {155, 0.0005968259577159068`, 1.0623502047343143`},
   {186, 0.0006006137794340964`, 1.0690925273926917`}, {217, 0.0006213405955681616`, 1.1059862601113275`},
   {248, 0.000647604450145371`, 1.1527359212587605`}, {279, 0.04265846057735534`, 75.9320598276925`}},
  5.089740311642557`, 0.003545539979513862`, 0.8863973810832269`, 2777.4163850529512`}, {"CURE", 365,
  3.99433124690871`, {{0, 0.013725332340101004`, 24.431091565379788`}, {3, 0.0018657320396385903`, 3.321003030556691`},
```

```
{6, 0.000147545371134556`, 0.2626307606195097`}, {9, 0.00004411118517860679`, 0.07851790961792009`},
{12, 0.000039382372219642434`, 0.07010062255096354`}, {15, 0.000039209906994596267`, 0.06979363445038136`},
{18, 0.00003920422398845635`, 0.0697835186994523`}, {21, 0.000031345226606706644`, 0.055794503359937825`}},
0.009999993478930517`, 0.022581462863479708`, 0.9827655638191872`, 2052.1842026582713`},
{"TBUR", 15.56745995879745`, 229.95475540676358`, {{0, 0.1009238052368164`, 179.6443733215332`}}, 2.002632914376134`*^7,
0.06377562399281211`, 0.5706169884328031`, 2061.8713728087187`}, {"CURE", 365, 0.6065259118710473`,
{{0, 0.0013734979064129657`, 2.444826273415079`}, {32, 0.000544642174352768`, 0.9694630703479271`}},
0.009999881476747956`, 0.017488857553224806`, 0.08709859458920123`, 1781.3996162909486`},
{"CURE", 365, 0.39429098911709287`, {{0, 0.0015723037349138936`, 2.7987006481467303`}}, 0.00999976607513007`,
0.020544146737513307`, 0.04417493537521626`, 1780.1682294083746`}, {"TBUR", 355.2202493867274`, 227.96806229354723`,
{{0, 0.04835840615074192`, 86.07796294832062`}, {29, 0.0011677929401067795`, 2.0786714333900678`},
{58, 0.003221733564620741`, 5.734685745024919`}, {87, 0.0015387891340398552`, 2.7390446585909425`},
{116, 0.0012889667994816915`, 2.294360903077411`}, {145, 0.0011073255398717677`, 1.9710394609717465`},
{174, 0.0010145866950250545`, 1.8059643171445972`}, {203, 0.0009695364950691508`, 1.7257749612230886`},
{232, 0.0009449360838314177`, 1.6819862292199232`}, {261, 0.02178681121848683`, 38.78052396890656`}},
511.7468549506541`, 0.006443243273228058`, 0.8298334903688207`, 2340.163828954891`}, {"CURE", 365, 0.22441982696324744`,
{{0, 0.002285836776526309`, 4.06878946221683`}, {13, 0.0010152494494998397`, 1.8071440201097146`}},
0.009895507131477944`, 0.014603589073761553`, 0.5247926136477075`, 1781.8865635961622`}, {"CURE", 365,
11.409650494652277`, {{0, 0.03414461516810293`, 60.77741499922321`}, {2, 0.002049893559786353`, 3.6488105364197074`},
{4, 0.00010309081146209179`, 0.1835016444025234`}, {6, 0.00003374270029659962`, 0.060062006527947326`},
{8, 0.00003166067199968545`, 0.056355996159440105`}, {10, 0.000031586601568506515`, 0.0562241507919416`},
{12, 0.000031582300351749 77`, 0.05621649462611457 6`}, {14, 0.00003158191022137675`, 0.056215800194050605`},
{16, 0.00003158186193781099`, 0.05621571424930355`}, {18, 1.6938405758597443`*^-6, 0.0030150362250303446`}},
0.009999283790549634`, 0.023229901107260018`, 0.5294065937270893`, 2127.9542024367647`}, {"TBUR", 181.16679664492935`,
228.49351805599812`, {{0, 0.07885369462135297`, 140.3595764260083`}, {13, 0.002056310873037148`, 3.6602333540061243`},
{26, 0.002054764067576131`, 3.657480040285513`}, {39, 0.002064099797965822`, 3.674097640379163`},
{52, 0.002196017894873954 6`, 3.9089118528756392`}, {65, 0.004344437525001576`, 7.733098794502804`},
{78, 0.014834511551156592`, 26.405430561058733`}, {91, 0.014256214643924238`, 25.376062066185142`},
{104, 0.010543757382835979`, 18.767888141448044`}, {117, 1.6764754454467738`, 2984.126292895258`}},
1156.000304932031`, 0.055505073318037376`, 0.7115082709760175`, 1813.2122649519654`}, {"CURE", 365,
230.18050048892442`, {{0, 0.050461411892601636`, 89.82131316883091`}, {3, 0.003723646699938822`, 6.628091125891103`},
```

{6, 0.0037030362315527685`, 6.591404492163929`}, {9, 0.0037028269893382617`, 6.591032041022106`},
{12, 0.0037028205995135698`, 6.591020667134154`}, {15, 0.003702820146327859`, 6.5910198604635895`},
{18, 0.003702820085446949`, 6.591019752095568`}, {21, 0.0037028200719691606`, 6.591019728105106`},
{24, 0.003702820068502558`, 6.591019721934554`}, {27, 0.003702820068502558`, 6.591019721934554`}},
0.009608543780062914`, 0.012687552429726896`, 0.7605335476686528`, 2960.7370447031744`},
{"CURE", 365, 0.18917215265585036`, {{0, 0.004835446180787339`, 8.607094201801464`}},
0.00963449245040926`, 0.015413849496808692`, 0.1440778502864592`, 1780.1956112433063`},
{"CURE", 365, 1.1017974153113728`, {{0, 0.0032025556027971444`, 5.7005489729789165`}},
0.00999988941339648`, 0.02468566591943332`, 0.06978763423142441`, 1780.2147489479119`},
{"TBUR", 55.41163411953648`, 230.21430361960483`, {{0, 0.0694405746459961`, 123.60422286987306`}},
2.913885889858133`*^6, 0.04099498339110434`, 0.5797843571745386`, 2484.884570833023`}, {"CURE", 365,
1.555759304220796`, {{0, 0.00504454118633353`, 8.979283311673685`}, {10, 0.0016238369041234291`, 2.890429689339704`},
{20, 0.00015250952793418866`, 0.27146695972285584`}, {30, 0.000016698404004816724`, 0.02972315912857377`}},
0.009999971665222889`, 0.0889012355504328`, 0.5706818900849004`, 2265.217256877464`}, {"TBUR", 289.7034149002831`,
229.61498993679515`, {{0, 0.058665253406223625`, 104.42415106307804`}, {24, 0.009928881268360671`, 17.673408657681993`},
{48, 0.0027424135121416073`, 4.881496051612061`}, {72, 0.0010149894118311271`, 1.806681153059406`},
{96, 0.0004012687398041141`, 0.7142583568513232`}, {120, 0.00019157923358750003`, 0.3410110357857501`},
{144, 0.00012061234476688047`, 0.21468997368504725`}, {168, 0.0000941151374964672`, 0.16752494474371166`},
{192, 0.00008244687281612538`, 0.14675543361270318`}, {216, 0.006205660297466212`, 11.046075329489858`}},
1028.8697670492604`, 0.01134699823502972`, 0.7967691023933493`, 2419.0044186533946`}, {"TBUR", 190.47617766234734`,
230.1114675108354`, {{0, 0.07583801393144507`, 134.99166479797225`}, {13, 0.0005944217132373082`, 1.0580706495624086`},
{26, 0.0005939891802653417`, 1.057300740872308`}, {39, 0.0005994830513285364`, 1.067079831364795`},
{52, 0.0007676572973663787`, 1.3664299893121543`}, {65, 0.0019244499637874884`, 3.425520935541729`},
{78, 0.001093767619221925`, 1.9469063622150267`}, {91, 0.000861345584880459`, 1.5331951410872169`},
{104, 0.0008890453525622961`, 1.5825007275608873`}, {117, 0.03814893861640904`, 67.9051107372081`}},
270.23148740168006`, 0.021055649465313395`, 0.11349825871551922`, 1780.0123543048096`}, {"CURE", 365,
3.949409246278487`, {{0, 0.011048031056845806`, 19.665495281185535`}, {2, 0.0018952893182531483`, 3.373614986490604`},
{4, 0.00018765437589510876`, 0.33402478909329353`}, {6, 0.00002710437704889792`, 0.048245791147038294`},
{8, 0.000014741483814303063`, 0.02623984118945945`}, {10, 0.000013964377778426236`, 0.024856592445598697`},
{12, 0.000013924929211031339`, 0.024786373995635783`}, {14, 1.244301210436377`*^-6, 0.002214856154576751`}},
0.009998231637710407`, 0.017101366714300043`, 0.9806955721850076`, 2169.2195837033187`}, {"CURE", 365,

118.3188828143135`, {{0, 0.040009777402441195`, 71.21740377634532`}, {30, 0.001429867349314271`, 2.545163881779402`},
{60, 0.0006208710270057392`, 1.1051504280702156`}, {90, 0.0005537641593045922`, 0.985700203562174`},
{120, 0.0005465483298887554`, 0.9728560272019846`}, {150, 0.0005456478144013652`, 0.9712531096344299`},
{180, 0.0005455339669299941`, 0.9710504611353896`}, {210, 0.000545519542366269`, 0.9710247854119588`},
{240, 0.0005455177142993709`, 0.9710215314528801`}, {270, 0.0004177556383249607`, 0.7436050362184301`}},
0.009995763394976823`, 0.0073465595083500555`, 0.839044938742902`, 2254.637541397437`}, {"CURE", 365,
2.7982065786555617`, {{0, 0.011681123908024841`, 20.792400556284218`}, {6, 0.0014090956716399897`, 2.5081902955191815`}},
0.009999735908160888`, 0.018245750311741568`, 0.8939293070288458`, 1783.6962287613974`}, {"TBUR", 131.6212859013011`,
229.2088954275003`, {{0, 0.03177322817114974`, 56.55634614464653`}, {7, 0.019538465073865318`, 34.77846783148026`},
{14, 0.005768278387543847`, 10.267535529828045`}, {21, 0.0015342381132915305`, 2.7309438416589242`},
{28, 0.0005243049395816157`, 0.9332627924552759`}, {35, 0.0002588627962871193`, 0.46077577739107234`},
{42, 0.00016140536912151796`, 0.2873015570363019`}, {49, 0.00010469749679246664`, 0.1863615442905906`},
{56, 0.0000648932311039429`, 0.11550995136501838`}, {63, 0.004014417704894606`, 7.145663514712399`}},
4744.046227631977`, 0.01990681893961804`, 0.6427220647271572`, 3010.5955544011376`},
{"CURE", 365, 2.1420027926982153`, {{0, 0.006627314330817879`, 11.796619508855825`},
{7, 0.0015503428005845183`, 2.7596101850404424`}, {14, 0.00010400645207541082`, 0.18513148469423127`}},
0.009999981236835242`, 0.035088775112130904`, 0.28593917125946083`, 1781.876071534133`}, {"CURE", 365,
0.3658149326272356`, {{0, 0.0029381597824250463`, 5.229924412716582`}, {38, 0.0010323269876984754`, 1.837542038103286`}},
0.009737687258604783`, 0.020316254892722763`, 0.10743966646113294`, 1781.0798690610372`}, {"CURE", 365,
0.650546389845122`, {{0, 0.0014117655414822299`, 2.512942663838369`}, {5, 0.000508163637374582`, 0.9045312745267561`},
{10, 0.00012384591805110218`, 0.22044573413096186`}, {15, 0.00003806189470029551`, 0.06775017256652602`},
{20, 0.00002004908499865520 3`, 0.035687371297606255`}, {25, 0.000018809707050818666`, 0.033481278550457225`}},
0.009999995388431689`, 0.02081692772283467`, 0.9451044587976738`, 2037.2219643906894`}, {"TBUR", 365,
228.14063715299335`, {{0, 0.04451617572946601`, 79.23879279844948`}, {31, 0.005053071405495053 5`, 8.994467101781195`},
{62, 0.005053038199285431`, 8.994407994728068`}, {93, 0.005053075039803358`, 8.994473570849978`},
{124, 0.0050530946728456335`, 8.994508517665228`}, {155, 0.005053004225910421`, 8.99434752212055`},
{186, 0.005052946397954006`, 8.994244588358129`}, {217, 0.0050529381741913956`, 8.994229950060685`},
{248, 0.005052937084682047`, 8.994228010734044`}, {279, 0.00651991877840894`, 11.60545542556791`}},
0.48334701825320275`, 0.007789899401090183`, 0.8340677417203343`, 2290.555095920506`},
{"CURE", 365, 0.04068727743337053`, {{0, 0.0002886412487960461`, 0.5137814228569622`}},
0.009998818918244669`, 0.024404179495325615`, 0.03508621092392826`, 1780.726952380901`},

{"CURE", 365, 0.3345606099675976`, {{0, 0.00935319437270869`, 16.648685983421473`}},
0.00959246215309545`, 0.023059456881764594`, 0.5373419272802269`, 1781.2647661991787`},
{"TBUR", 14.91688719241972`, 228.86426662254763`, {{0, 0.1263615036010742`, 224.9234764099121`}},
4.737681248427658`*^7, 0.1260712291625983`, 0.48964071543409926`, 1990.2769451082036`},
{"CURE", 365, 5.156892468244217`, {{0, 0.03645943621947393`, 64.8977964706636`}, {3, 0.0036341790745496806`, 6.4688387526984314`},
{6, 0.00030163596807152433`, 0.5369120231673132`}, {9, 0.000020755166157211003`, 0.03694419575983559`}},
0.009999983474627345`, 0.014968575886256065`, 0.9780673940989938`, 1816.0521262512555`},
{"CURE", 365, 1.7800376668669033`, {{0, 0.0029065835544715944`, 5.173718726959438`}}, 0.009999631020087135`,
0.01881824746894679`, 0.11316653847909847`, 1780.2782912028777`}, {"CURE", 365, 1.3327510754348095`,
{{0, 0.009139663760350462`, 16.268601493423823`}, {8, 0.0024325001100851808`, 4.329850195951622`}},
0.009926923271485833`, 0.011063370627551477`, 0.43932045974775535`, 1780.5314206925202`},
{"CURE", 365, 0.5860831831646826`, {{0, 0.0026601684788248032`, 4.73509989230815`}},
0.009993477655443 12`, 0.024648218806295613`, 0.37036496197821633`, 1781.406929426221`},
{"CURE", 365, 0.1338890242959437`, {{0, 0.0007993509243474735`, 1.4228446453385026`}}, 0.00999887365570532`,
0.021960025021170722`, 0.08702566128849291`, 1780.908562754213`}, {"CURE", 365, 4.6307613006601445`,
{{0, 0.0069954353355744`, 12.451874897322432`}, {32, 0.0009551813110971778`, 1.7002227337529767`}},
0.009999773823709854`, 0.053026075213262504`, 0.46815717051280403`, 1786.2958157466485`}, {"TBUR", 37.20247823326694`,
229.25108822050183`, {{0, 0.030191577833979195`, 53.74100854448296`}, {1, 0.030082226479609512`, 53.54636313370492`}},
1.6777361299614392`*^7, 0.056921964996287835`, 0.5183821740685085`, 2363.9430458246384`}, {"CURE", 365,
1.062069014984234`, {{0, 0.0020269361214898547`, 3.607946296251942`}, {9, 0.0005866140864673607`, 1.044173073911902`},
{18, 0.000036464295208657 36`, 0.0649064454714101`}, {27, 2.722952078579142`*^-6, 0.0048468546998708725`}},
0.009999992665997628`, 0.08632521469450266`, 0.15702748226541713`, 1879.4139275418115`},
{"CURE", 365, 3.5308852500213876`, {{0, 0.012688052394346006`, 22.584733261935888`},
{6, 0.0025254967059872447`, 4.495384136657295`}, {12, 0.00032190705216495743`, 0.5729945528536242`},
{18, 0.000048752273897635 36`, 0.08677904753779093`}, {24, 0.000017797582446054385`, 0.031679696753976805`},
{30, 0.000014731899842859061`, 0.026222781720289128`}, {36, 3.7994021866586038`*^-6, 0.006762935892252315`}},
0.009999994013463484`, 0.0389373177007514`, 0.9534213402756783`, 2034.8455659699698`}, {"CURE", 365,
1.3717182160315586`, {{0, 0.0024496550352835146`, 4.360385962804656`}, {4, 0.00043797252896185 39`, 0.7795911015520999`},
{8, 0.00006111434880112314`, 0.1087835408659992`}, {12, 0.000027581692240251096`, 0.04909541218764696`},
{16, 0.00002518287896951767`, 0.04482552456574145`}, {20, 0.000025757324643749377`, 0.045848037865873895`}},
0.00999998668979486`, 0.01938975724873452`, 0.9721587619051232`, 2109.816506835408`},

{"CURE", 365, 0.07072668341941975`, {{0, 0.002482930771518407`, 4.419616773302764`}}, 0.009998205994216436`,
0.025165462879508292`, 0.13588732137633516`, 1780.5122212531114`}, {"CURE", 365, 2.295704360828526`,
{{0, 0.014890719489711837`, 26.50548069168707`}, {5, 0.0010051549926547517`, 1.7891758869254577`},
{10, 0.0000566356875939476`, 0.10081152391722674`}, {15, 0.000050130072622582694`, 0.08923152926819719`}},
0.009999994821027178`, 0.024348102601986315`, 0.9892292115585186`, 2243.726704310872`}, {"TBUR", 128.44122585471123`,
228.5858389364445`, {{0, 0.04417701838821738`, 78.63509273102693`}, {7, 0.008452395145033431`, 15.04526335815951`},
{14, 0.0013228365752142063`, 2.3546491038812873`}, {21, 0.00030850795089925297`, 0.5491441526006703`},
{28, 0.00014592649331174188`, 0.25974915809490057`}, {35, 0.00011541084952451563`, 0.20543131215363783`},
{42, 0.00010775563442732146`, 0.19180502928063223`}, {49, 0.00010503344103276334`, 0.18695952503831875`},
{56, 0.00010376729839839934`, 0.18470579114915087`}, {63, 0.003479270794950563 3`, 6.193102015012004`}},
7600.912843990756`, 0.009356414684318295`, 0.8097058045144581`, 2715.873810116199`}, {"TBUR", 80.40925195068886`,
228.1340065657729`, {{0, 0.005489833942820321`, 9.77190441822017`}, {4, 0.0020707586166405817`, 3.685950337620235`},
{8, 0.00110499749742536`, 1.9668955454171404`}, {12, 0.0006285841518479559`, 1.1188797902893617`},
{16, 0.00038015026324345156`, 0.6766674685733437`}, {20, 0.00024652550040086743`, 0.43881539071354403`},
{24, 0.00017281607674607435`, 0.3076126166080123`}, {28, 0.00013116122928918153`, 0.23346698813474315`},
{32, 0.00010699971562473818`, 0.19045949381203395`}, {36, 0.04318936366039482`, 76.87706731550277`}},
1.4665655945252085`*^6, 0.021753396986808303`, 0.7723068785596315`, 2797.034611666154`}, {"CURE", 365,
0.15672232258518345`, {{0, 0.0005365379466043371`, 0.95503754495572`}, {32, 0.0003076312284482207`, 0.5475835866378328`}},
0.009732693044029215`, 0.030034542254056933`, 0.027934348451528382`, 1780.5859773000673`}, {"CURE", 365,
0.4033697918336215`, {{0, 0.002336443067159685`, 4.15886865954424`}, {27, 0.0005176676211571601`, 0.921448365659745`}},
0.009999984357930689`, 0.024208311852570844`, 0.20980410256183693`, 1783.7566710523517`}, {"TBUR", 200.20287137968091`,
228.98819793816665`, {{0, 0.0552318205931489`, 98.31264065580505`}, {15, 0.021350710199598888`, 38.00426415528602`},
{30, 0.009351039557578513`, 16.644850412489753`}, {45, 0.004894844990873027 5`, 8.71282408375399`},
{60, 0.0028334117155466346`, 5.04347285367301`}, {75, 0.0017404964426739127`, 3.0980836679595645`},
{90, 0.0011140656121124924`, 1.983036789560237`}, {105, 0.000738533347737097`, 1.3145893589720326`},
{120, 0.0005071986230961664`, 0.9028135491111763`}, {135, 0.044067037985661604`, 78.43932761447766`}},
305.7488818122696`, 0.026816834453084734`, 0.2114207327750321`, 1780.0259850379653`},
{"TBUR", 260.98262923649673`, 218.3676228216844`, {{0, 0.03357476867204392`, 59.76308823623818`},
{29, 0.0028953599396275456`, 5.15374069253703`}, {58, 0.005902293915227264`, 10.50608316910453`},
{87, 0.008325352075161562`, 14.81912669378758`}, {116, 0.007422577433499373`, 13.212187831628883`},
{145, 0.0075308456068109 1`, 13.40490518012342`}, {174, 0.00762309295979217`, 13.569105468430061`},

{203, 0.00762755841737468`, 13.577053982926932`}, {232, 0.007660813817566687`, 13.636248595268702`}},
24226.78958331858`, 0.016913296057260052`, 0.7401340142489298`, 2281.2458053919154`}, {"CURE", 365,
0.0678781145131004`, {{0, 0.002220951539899406`, 3.953293741020943`}, {3, 0.0005064429754018378`, 0.9014684962152713`}},
0.009998045114990865`, 0.007351654486059575`, 0.20271641390045567`, 1780.600972230818`}, {"CURE", 365, 3.1923764811572286`,
{{0, 0.0015124714396190485`, 2.692199162521906`}, {22, 0.000037734727191102999`, 0.06716781440016333`},
{44, 0.0000372051163905047`, 0.06622510717509834`}, {66, 0.00003716611640622048`, 0.06615568720307245`}},
0.009999943741010885`, 0.018456917469545465`, 0.8216330112305719`, 2593.665847024346`},
{"CURE", 365, 0.7963745662391666`, {{0, 0.003380384709916461`, 6.017084783651301`},
{7, 0.0003921885157512023`, 0.6980955580371401`}, {14, 0.000012196571182295726`, 0.021709896704486392`}},
0.009999897019110995`, 0.0405550957483588`, 0.27684469574738674`, 1840.9707012452086`},
{"CURE", 365, 0.07620868530989264`, {{0, 0.0017437306295444016`, 3.1038405205890345`}},
0.009999793173837649`, 0.014172916104372534`, 0.20828338399946011`, 1781.145053258669`},
{"CURE", 365, 0.14462607194806434`, {{0, 0.0009368274415644433`, 1.667552845984709`}},
0.009999767699640333`, 0.017303608001352503`, 0.0844253417584689`, 1780.8409296436912`},
{"CURE", 365, 1.34566837163679`, {{0, 0.01095315151887485`, 19.496609703597237`}, {3, 0.003858116657399794`, 6.867447650171633`},
{6, 0.000990006126961436`, 1.7622109059913562`}, {9, 0.0002565862609022334`, 0.45672354440597546`},
{12, 0.00007342517286713454`, 0.1306968077034995`}, {15, 0.000016081481804438662`, 0.028625037611900817`}},
0.009999990368369347`, 0.018374384908899543`, 0.9399017493264068`, 1820.371346525042`},
{"TBUR", 365, 224.15015231113216`, {{0, 0.0824819144375462`, 146.81780769883224`}, {31, 0.005113242205854775`, 9.1015711264215`},
{62, 0.005113242759690078`, 9.101572112248338`}, {93, 0.005113247570293487`, 9.101580675122408`},
{124, 0.005113283080871238`, 9.101643883950803`}, {155, 0.005113543131271097`, 9.102106773662554`},
{186, 0.0051152732732367735`, 9.105186426361456`}, {217, 0.005119492327578435`, 9.112696343089615`},
{248, 0.005117038209934505`, 9.108328013683417`}, {279, 0.03530438840361416`, 62.841811358433205`}},
4.504429668664868`, 0.02333287674326099`, 0.7931315920967372`, 2539.8760815511223`}, {"CURE", 365, 1.462847103006295`,
{{0, 0.003881268023817715`, 6.908657082395532`}, {68, 0.002117727157420723`, 3.769554340208887`}},
0.00977452832103147`, 0.02605774757027965`, 0.3446234152692685`, 1783.2114671343759`},
{"CURE", 365, 39.47406098413505`, {{0, 0.026884350390667226`, 47.85414369538766`},
{12, 0.003808289009050265`, 6.778754436109472`}, {24, 0.0003618576452118732`, 0.6441066084771342`},
{36, 0.00005207859300969077`, 0.09269989555724957`}, {48, 0.00002766131535053029`, 0.04923714132394392`},
{60, 0.00002629237918813558`, 0.04680043495488134`}, {72, 0.000026235260984978524`, 0.04669876455326177`},
{84, 0.000026232813630248l6`, 0.04669440826184172`}, {96, 0.000034673625757517334`, 0.061719053848380855`}},

0.009999992555338679`, 0.035697125275112186`, 0.9999656174879986`, 2403.6109507487804`}, {"TBUR", 49.92813947734132`,
222.13095114814203`, {{0, 0.03969888075884036`, 70.66400775073583`}, {5, 0.02148802500430348`, 38.248684507660194`},
{10, 0.018636625134674054`, 33.17319273971981`}, {15, 0.017043174512184768`, 30.336850631688883`},
{20, 0.016170947288501887`, 28.78428617353336`}, {25, 0.015754032354111935`, 28.04217759031924`},
{30, 0.01563630079877569`, 27.832615421820726`}, {35, 0.01571938833835843`, 27.98051124227801`},
{40, 0.015938312455798388`, 28.370196171321133`}, {45, 2.0301584240698882`, 3613.6819948444013`}},
4.6714861496101275`*^7, 0.16694499278887062`, 0.7805777909531152`, 1984.9798254832854`}, {"CURE", 365,
1.4073392796946185`, {{0, 0.007240734844120121`, 12.888508022533815`}, {41, 0.002705025267663728`, 4.814944976441436`}},
0.009747672510736686`, 0.026035001611815576`, 0.18979148785163946`, 1780.574282088639`}, {"TBUR", 365,
228.06586982681938`, {{0, 0.06090280033814885`, 108.40698460190495`}, {31, 0.004389480515377529`, 7.813275317372002`},
{62, 0.004389491655338623`, 7.81329514650275`}, {93, 0.0043895531141322445`, 7.813404543155396`},
{124, 0.004389697198094012`, 7.8136610126073425`}, {155, 0.004389599057791495`, 7.813486322868862`},
{186, 0.004389481949891766`, 7.813277870807343`}, {217, 0.004389467076528161`, 7.813251396220127`},
{248, 0.004389464795599096`, 7.813247336166391`}, {279, 0.008354142353217117`, 14.870373388726469`}},
1.2451055272455436`, 0.0197762392546857`, 0.08520256659875415`, 1780.0141835297643`}, {"TBUR", 52.50804047888889`,
228.0325504778472`, {{0, 0.050631528758883375`, 90.12412119081239`}, {5, 0.023890637066419335`, 42.525333978226406`},
{10, 0.019657871543048884`, 34.991011346627005`}, {15, 0.01681905930008699`, 29.937925554154837`},
{20, 0.014767380409930246`, 26.285937129675833`}, {25, 0.013230646918645244`, 23.55055151518853`},
{30, 0.012048154735507847`, 21.44571542920397`}, {35, 0.011116918191546012`, 19.7881143809519`},
{40, 0.010368385008343542`, 18.455725314851506`}, {45, 0.913206287848843`, 1625.5071923709402`}},
5.0798808006146334`*^7, 0.2659531698997752`, 0.7127883621949196`, 2050.1934245742277`}, {"TBUR", 128.48282839886897`,
229.24097009044357`, {{0, 0.07891510023659665`, 140.46887842114202`}, {8, 0.0006527601046834504`, 1.1619129863365416`},
{16, 0.0006042121189877227`, 1.0754975717981463`}, {24, 0.0006048128075744377`, 1.076566797482499`},
{32, 0.0006197517719221338`, 1.103158154021398`}, {40, 0.0007266210770189874`, 1.2933855170937973`},
{48, 0.0016002087899550052`, 2.8483716461199093`}, {56, 0.005277204693742763`, 9.393424354862116`},
{64, 0.0068881296428239674`, 12.260870764226661`}, {72, 0.23172750130183423`, 412.47495231726487`}},
7134.413714076967`, 0.03943291533572157`, 0.8414215735725151`, 2127.298745967474`},
{"TBUR", 40.21207565091959`, 230.20315067201346`, {{0, 0.0735746479034424`, 130.96287326812748`}},
4.2026490519880265`*^7, 0.05968953148188366`, 0.5281697171154044`, 2293.6528891640933`}, {"TBUR", 45.260050859739366`,
229.77988421467936`, {{0, 0.056794508463013`, 101.09422506416315`}, {2, 0.017858919559733847`, 31.788876816326248`},
{4, 0.012592365775286031`, 22.414411080009135`}, {6, 0.009101512443053033`, 16.200692148634396`},

```
    {8, 0.0066772145955320454`, 11.885441980047041`}, {10, 0.004959783535563328`, 8.828414693302724`},
    {12, 0.0037299913975895097`, 6.6393846877093265`}, {14, 0.002842738382170457`, 5.060074320263414`},
    {16, 0.0021985902099491217`, 3.9134905737094368`}, {18, 0.07903499471490978`, 140.6822905925394`}},
  1.5988583516172867`*^7, 0.08737002258689329`, 0.7438126365739384`, 2074.9693752171042`}, {"TBUR", 365,
  229.15682642538474`, {{0, 0.07891495421098581`, 140.4686184955547`}, {31, 0.001175490630640689`, 2.0923733225404257`},
    {62, 0.00011284740959540026`, 0.20086838907981658`}, {93, 0.00010774269969671884`, 0.19178200546015953`},
    {124, 0.0001082262891299038`, 0.19264279465122874`}, {155, 0.00010815967423258203`, 0.192524220133996`},
    {186, 0.0001067151775580088`, 0.18995301605325562`}, {217, 0.00010515671989444338`, 0.1871789614121092`},
    {248, 0.00010452505963570766`, 0.1860546061515596`}, {279, 0.0008580396691993716`, 1.5273106111748813`}},
  0.4497811776198051`, 0.0162182975595764 9`, 0.049876034814541496`, 1780.0059434520253`}, {"CURE", 365,
  3.6862179139612983`, {{0, 0.01301858300228318`, 23.173077744064063`}, {6, 0.0023133996518475333`, 4.117851380288609`}},
  0.009998455706499787`, 0.010607045772154438`, 0.925495368922066`, 1954.8878058430778`},
 {"CURE", 365, 0.3327179791950079`, {{0, 0.0010747211241657372`, 1.913003601015012`},
    {11, 0.0004446265279928348`, 0.791435219827246`}, {22, 0.0000972109958389657`, 0.17303557259335894`}},
  0.00999974182794738`, 0.025283075982719484`, 0.3718065577481536`, 1785.6085097580715`},
 {"CURE", 365, 1.531426412642617`, {{0, 0.012625572525945195`, 22.473519096182446`}},
  0.009999394980899783`, 0.01130484588411255`, 0.2314559293998201`, 1780.2045274221075`},
 {"CURE", 365, 0.2742904914518179`, {{0, 0.0034242390135363556`, 6.0951454440947135`}}, 0.009997839007358369`,
  0.01920497910069501`, 0.5135048077096853`, 1781.749482013125`}, {"CURE", 365, 0.6839086457026424`,
  {{0, 0.002542134299672449`, 4.524999053416958`}, {19, 0.0003290901021385906 4`, 0.5857803818066913`}},
  0.00999981803472412`, 0.029959921997688085`, 0.5600845846551559`, 1816.0708328651747`}, {"CURE", 365,
  2.3837612488415405`, {{0, 0.016589719744507042`, 29.52970114522254`}, {30, 0.007705485739802832`, 13.71576461684904`}},
  0.009738093134373765`, 0.012051155399774032`, 0.486569071447759`, 1781.533784362839`}, {"CURE", 365, 0.13511744690196267`,
  {{0, 0.0007943064570525613`, 1.413865493553559`}, {52, 0.00028752372912081123`, 0.5117922378350441`}},
  0.009784917969338018`, 0.04150290213544477`, 0.19469731659865777`, 1785.645256375038`}, {"CURE", 365,
  22.99547409882237`, {{0, 0.01415485334503047`, 25.195638954154234`}, {7, 0.0025193124008551873`, 4.484376073522234`},
    {14, 0.0002806409496618591`, 0.4995408903981092`}, {21, 0.00004736045664021979`, 0.08430161281959124`},
    {28, 0.00002834991741486950 8`, 0.05046285299846773`}, {35, 0.00002723317979013379 5`, 0.04847506002643816`},
    {42, 0.00002717829042037964`, 0.04837735694827577`}, {49, 0.00002717503573714382 5`, 0.0483715636121160 1`},
    {56, 0.00002717475109141728 3`, 0.04837105694272277`}, {63, 5.735558112778346`*^-6, 0.010209293440745455`}},
  0.00999996713979673`, 0.032599962252495916`, 0.999894916587619`, 2375.474591138653`}, {"TBUR", 218.70518016224509`,
```

```
228.91430172772834`, {{0, 0.09908017333065368`, 176.36270852856356`}, {17, 0.002811991880025248`, 5.005345546444942`},
   {34, 0.0001620158687764667`, 0.2883882464221107`}, {51, 0.00011586190903652008`, 0.20623419808500576`},
   {68, 0.00011826855944828888`, 0.21051803581795422`}, {85, 0.0001269002558171151`, 0.2258824553544649`},
   {102, 0.00013702751131110813`, 0.2439089701337725`}, {119, 0.00014609819486858058`, 0.2600547868660734`},
   {136, 0.00015224972320488018`, 0.27100450730468667`}, {153, 0.006116032697071938`, 10.886538200788051`}},
  14.569506333462037`, 0.013208034130212225`, 0.11414622544785398`, 1780.0105516352023`},
 {"TBUR", 12.232345924131293`, 230.23715579919903`, {{0, 0.1067183303833008`, 189.9586280822754`}}, 8.952098728322259`*^7,
  0.08227250501257079`, 0.6519486036521821`, 2038.7941770612524`}, {"CURE", 365, 0.6043041236325875`,
  {{0, 0.0010342211194418424`, 1.8409135926064792`}, {7, 0.00032411526929323255`, 0.576925179341954`},
   {14, 0.00008270535531416076`, 0.14721553245920616`}, {21, 0.000026463666434090625`, 0.047105326252681316`}},
  0.00999997628673259`, 0.01690081361977004`, 0.9166603777382143`, 2000.7689017069035`},
 {"TBUR", 52.63584791596752`, 230.25323850019618`, {{0, 0.0371815586090088`, 66.18317432403566`}},
  7.474879438321969`*^6, 0.0836788610864367`, 0.598026981991002`, 3045.8207375277952`},
 {"CURE", 365, 0.924377690150668`, {{0, 0.002016367907063495`, 3.589134874573021`}}, 0.00999979789928558`,
  0.021113482773358422`, 0.04778255626380203`, 1780.2448797835152`}, {"CURE", 365, 4.13138502455838`,
  {{0, 0.0022539459981439935`, 4.012023876696308`}, {7, 0.0007681696791911457`, 1.3673420289602392`},
   {14, 0.0001316129155731354`, 0.234270989720181`}, {21, 0.000017371566396267487`, 0.030921388185356127`}},
  0.009999956430691827`, 0.034591402460024415`, 0.4922594277298461`, 1801.151613725029`}, {"TBUR", 249.4304833342768`,
  229.4562603119112`, {{0, 0.016074318617529088`, 28.612287139201776`}, {19, 0.005753022225101857`, 10.240379560681305`},
   {38, 0.002043517450639547`, 3.6374610621383936`}, {57, 0.0008069952086048266`, 1.4364514713165915`},
   {76, 0.0003564008869646012`, 0.6343935787969901`}, {95, 0.00018399126427743654`, 0.3275044504138371`},
   {114, 0.00011582969934290312`, 0.20617686483036754`}, {133, 0.00008754060194488146`, 0.1558222714618898`},
   {152, 0.00007469101880403947`, 0.13295001347119026`}, {171, 0.049222545234883765`, 87.6161305180931`}},
  3288.104134046685`, 0.008540987547424963`, 0.8005517312543124`, 2369.0718359290595`},
 {"CURE", 365, 0.03690915613945711`, {{0, 0.0005865882441875597`, 1.0441270746538562`}},
  0.009985897358891925`, 0.023912112389551227`, 0.031710603279362364`, 1780.4719539332455`},
 {"CURE", 365, 0.07130918411611463`, {{0, 0.0014242473702044643`, 2.5351603189639462`}},
  0.009668208837547385`, 0.0263044926267825`, 0.20219011717016913`, 1781.0408305766496`},
 {"TBUR", 19.736033227383984`, 229.75375689032478`, {{0, 0.07304573059082031`, 130.02140045166016`}},
  3.9080801156438336`*^7, 0.14708073909138103`, 0.5331768341396009`, 2149.7433704371897`}, {"CURE", 365,
  1.6660145219897151`, {{0, 0.0024948120366255202`, 4.440765425193426`}, {50, 0.0009617546843920333`, 1.7119233382178194`}},
```

```
0.009999863414996502`, 0.02840301789695553`, 0.5159530724560792`, 1803.104093556403`},
{"TBUR", 34.653079157301505`, 230.20974540577282`, {{0, 0.1021137523651123`, 181.76247920989988`}},
2.0374113034368224`*^7, 0.09455204733247873`, 0.5495819626610543`, 2149.2419738057583`}, {"CURE", 365,
2.836320600129074`, {{0, 0.010298844751638132`, 18.331943657915875`}, {25, 0.003025693739245612`, 5.385734855857189`}},
0.00999970781008137`, 0.039582334334909756`, 0.6360567132200535`, 1803.7722806310016`},
{"CURE", 365, 50.39420712867294`, {{0, 0.026430835982641263`, 47.04688804910145`},
{8, 0.00040140494096845355`, 0.7145007949238473`}, {16, 0.00019702660640654148`, 0.35070735940364384`},
{24, 0.00019366768530226748`, 0.3447284798380361`}, {32, 0.0001925991736918387`, 0.3428265291714729`},
{40, 0.0001923294627799054`, 0.3423464437482316`}, {48, 0.00019230349112419226`, 0.3423002142010622`},
{56, 0.00019230101780311028`, 0.3422958116895363`}, {64, 0.0001815858396227369`, 0.3232227945284717`}},
0.00999999292912654`, 0.019593331087707052`, 0.9462741560668017`, 2241.6096020721316`}, {"TBUR", 62.33862579430315`,
228.239928381665`, {{0, 0.035893760360742565`, 63.890893442121765`}, {3, 0.01124501700754251`, 20.01613027342567`},
{6, 0.00779392412795059`, 13.873184947752051`}, {9, 0.005662414666495034`, 10.079098106361158`},
{12, 0.004218585857552782`, 7.509082826443952`}, {15, 0.0031962080811243146`, 5.68925038440128`},
{18, 0.0024571910337039863`, 4.373800039993096`}, {21, 0.0019180696454226002`, 3.414163968852229`},
{24, 0.0015231296647517498`, 2.7111708032581148`}, {27, 0.07174747481151482`, 127.71050516449638`}},
1.2711181529541625`*^7, 0.0897161533033746`, 0.5844348963332495`, 2101.277735203461`}, {"CURE", 365,
0.3491729029169801`, {{0, 0.0011354913254371381`, 2.021174559278106`}, {21, 0.00021419623927112583`, 0.381269305902604`}},
0.009999974822350854`, 0.03425520968097717`, 0.6135015572262145`, 1913.7701515225835`},
{"CURE", 365, 1.9414993657836097`, {{0, 0.005360474603638795`, 9.541644794477055`},
{4, 0.0025980548477380918`, 4.624537628973803`}, {8, 0.0007065955638703985`, 1.2577401036893092`}},
0.009999919201799348`, 0.00859360430821851`, 0.47270716004172225`, 1780.8631845201346`}, {"TBUR", 159.8123628577545`,
229.13680642061374`, {{0, 0.05596952971090401`, 99.62576288540915`}, {13, 0.003198825411072998`, 5.693909231709936`},
{26, 0.003166965163711924 5`, 5.637197991407226`}, {39, 0.003164785830151558`, 5.63331877769773`},
{52, 0.003165270189849801`, 5.634180937932647`}, {65, 0.003166916897018944`, 5.637112076693721`},
{78, 0.003169649479161922`, 5.641976072908221`}, {91, 0.0031736765297707584`, 5.64914422299195`},
{104, 0.003179288673219763`, 5.659133838331179`}, {117, 0.10016197899240674`, 178.288322606484`}},
80784.50416420275`, 0.03839762037298747`, 0.8773875712917976`, 2158.9198860353376`},
{"CURE", 365, 2.491364958419851`, {{0, 0.009238407263779785`, 16.444364929528017`},
{6, 0.001628756603336371`, 2.89918675393874`}, {12, 0.00007645896383192896`, 0.13609695562083354`}},
0.009999969480810222`, 0.028797426640760307`, 0.3962415029460738`, 1782.3908202434425`},
```

{"TBUR", 13.809153816632454`, 229.48297477761201`, {{0, 0.11859069824218751`, 211.0914428710938`}},
6.991109163451484`*^7, 0.1319774363075776`, 0.517856448259022`, 2000.2673156269464`}, {"CURE", 365, 1.595514851484176`,
{{0, 0.002305137053626838 7`, 4.103143955455773`}, {16, 0.0003095449925469934`, 0.5509900867336482`}},
0.009999708540460433`, 0.01981428193984074`, 0.693314561772077`, 1956.7788473499734`},
{"CURE", 365, 0.8319139296747491`, {{0, 0.004923622494406418`, 8.764048040043424`}},
0.009665137983209768`, 0.015543756418939571`, 0.15223234780742936`, 1780.1972175639085`},
{"TBUR", 16.076114613733125`, 230.06878078112638`, {{0, 0.05713017940521243`, 101.69171934127812`}},
3.968142168145161`*^7, 0.19156430140512162`, 0.6589782216515118`, 2222.80310222042`}, {"CURE", 365,
2.6196722100108984`, {{0, 0.014033074517729564`, 24.978872641558628`}, {3, 0.0015407086565632719`, 2.742461408682624`},
{6, 0.00013973614804208042`, 0.24873034351490309`}, {9, 0.000025162112236733 68`, 0.044788559781385955`}},
0.009999996243946123`, 0.014718045377691356`, 0.9771754292887644`, 1882.9163163978158`},
{"CURE", 365, 0.07941103568938597`, {{0, 0.0006174149562679532`, 1.0989986221569568`}},
0.009998511005124371`, 0.02354831391017197`, 0.15964480673667278`, 1782.5774173597574`},
{"TBUR", 27.337010974666224`, 230.2035256743096`, {{0, 0.07061750411987304`, 125.69915733337402`}},
1.6764407675161561`*^7, 0.06360309336979088`, 0.5781974799273685`, 2253.841553904803 7`},
{"CURE", 365, 0.06767989957860635`, {{0, 0.0016053675023967092`, 2.8575541542661425`}},
0.009999364273013735`, 0.02608024714290631`, 0.06539425979674586`, 1780.2593834333095`},
{"CURE", 365, 0.2969762356955536`, {{0, 0.003081347222749006`, 5.484798056493231`}},
0.009999008660889715`, 0.017107095932454554`, 0.2854782248861779`, 1781.257632747709`},
{"CURE", 365, 0.26347552849969263`, {{0, 0.000947120125981874`, 1.6858738242477356`},
{16, 0.00038860726176927847`, 0.6917209259493157`}, {32, 0.00006711363305393327`, 0.11946226683600122`}},
0.009999907983357275`, 0.05044020161409782 5`, 0.18617951504031652`, 1783.258408198171`}, {"CURE", 365,
2.550732902539532`, {{0, 0.005484757970750338 4`, 9.762869187935603`}, {4, 0.0006986750975407447`, 1.2436416736225255`},
{8, 0.00006262002781872705`, 0.11146364951733416`}, {12, 0.000022795740785641075`, 0.04057641859844112`},
{16, 0.000021053858671838924`, 0.037475868435873284`}, {20, 0.00005102763422903949`, 0.09082918892769029`}},
0.00999999725638435`, 0.02426990932962118`, 0.9895067850538114`, 2536.2870669708223`},
{"TBUR", 35.19388043303459`, 230.19249919812233`, {{0, 0.05951698303222656 4`, 105.94022979736329`}},
2.538053803371155`*^7, 0.09116947076953202`, 0.626106012535309`, 2474.264644431496`}, {"CURE", 365,
0.2729201630169085`, {{0, 0.0028187889018887`, 5.017444245361886`}, {7, 0.0006491615489532108`, 1.155507557136715`}},
0.009949541013640097`, 0.012470510219938245`, 0.12978856500843516`, 1780.2386342076547`},
{"CURE", 365, 0.23959418779043665`, {{0, 0.0016917640657313473`, 3.0113400370017986`},

```
{4, 0.0006036336309349599`, 1.0744678630642286`}, {8, 0.00014126687642097502`, 0.2514550400293355`}},
0.009973163076133487`, 0.013679978624216526`, 0.8057329589582602`, 1790.4468708811974`},
{"TBUR", 17.14724261571764`, 228.0829543295679`, {{0, 0.12448956489562987`, 221.59142551422119`}},
5.2141479200841635`*^7, 0.1095947979819098`, 0.4919559082173027`, 2003.422339960824`}, {"CURE", 365,
1.6900230453213487`, {{0, 0.03457991936187761`, 61.552256464142154`}, {1, 0.005614223303326913`, 9.993317479921904`}},
0.009946806565268895`, 0.020976611173720176`, 0.8890642283468599`, 1781.3491849820355`}, {"CURE", 365,
2.5561613273533452`, {{0, 0.010252248440542754`, 18.249002224166098`}, {40, 0.003909076079905533`, 6.958155422231849`}},
0.00973517049586041`, 0.02190279541751606`, 0.525792911887182`, 1782.6711836849581`}, {"CURE", 365,
6.530235410272422`, {{0, 0.009352773053202725`, 16.64793603470085`}, {3, 0.000304445537300237`, 0.541913056394421 8`},
  {6, 0.000035833324957611594`, 0.06378331842454864`}, {9, 0.000032884779229489 07`, 0.058534907028490554`},
  {12, 0.00003284500357645957`, 0.05846410636609804`}, {15, 0.00003284346748403794`, 0.05846137212158754`},
  {18, 0.000032843334137813254`, 0.0584611347653076`}, {21, 0.000018611871381562943`, 0.03312913105918204`}},
0.00999999763062965`, 0.022447309719863518`, 0.9952381319327396`, 2162.4859449497685`}, {"TBUR", 54.843490340803626`,
229.16715724275247`, {{0, 0.03159712353729983`, 56.24287989639369`}, {3, 0.008222452133920241`, 14.635964798378028`},
  {6, 0.004214759244062585`, 7.5022714544314`}, {9, 0.002816481106445567 2`, 5.01333636947311`},
  {12, 0.00233240219373979`, 4.151675904856827`}, {15, 0.0022386328737808714`, 3.984766515329951`},
  {18, 0.00231925430558856 8`, 4.128272663947651`}, {21, 0.00243143487264874`, 4.327954073314757`},
  {24, 0.002470215760345 7607`, 4.396984053415453`}, {27, 0.10349221289687216`, 184.21613895643245`}},
9.568848622804614`*^6, 0.07785549560271317`, 0.6742807658565474`, 2088.5169310045712`},
{"CURE", 365, 0.22587741241369896`, {{0, 0.0005855043934895961`, 1.042197820411481`},
  {12, 0.00025523249517648904`, 0.4543138414141505`}, {24, 0.00005554798291769654`, 0.09887540959349983`}},
0.009927314640572255`, 0.03756823870290324`, 0.24301017287714846`, 1787.6815702084796`}, {"CURE", 365,
9.161325134624152`, {{0, 0.008977802866480802`, 15.980489102335827`}, {9, 0.0021097397645640013`, 3.7553367809239226`},
  {18, 0.00015294275106520405`, 0.2722380968960632`}, {27, 0.000018372202616903635`, 0.03270252065808847`}},
0.009999994183594785`, 0.061587720380254764`, 0.5570903399917133`, 1900.5148150357584`}, {"TBUR", 100.41139347171874`,
228.5366934173556`, {{0, 0.02495761937611151`, 44.42456248947849`}, {5, 0.005383038599249481`, 9.581808706664077`},
  {10, 0.0027309532573092524`, 4.861096798010469`}, {15, 0.0017952869421678803`, 3.1956107570588275`},
  {20, 0.0013796781403169792`, 2.455827089764223`}, {25, 0.0011421692996349242`, 2.033061353350165`},
  {30, 0.0009645317867677677`, 1.7168665804466268`}, {35, 0.0008099843822916475`, 1.4417722004791325`},
  {40, 0.000674586181248381`, 1.2007634026221181`}, {45, 0.05464415897664525`, 97.26660297842854`}},
5.246121145742527`*^6, 0.05917588257192026`, 0.6304281599751758`, 2321.586290533585`},
```

```
{"CURE", 365, 0.2071352990894908`, {{0, 0.0031479586475605284`, 5.603366392657741`}}, 0.009996317529283408`,
  0.01823433178839978`, 0.09781480835690358`, 1780.323844255942`}, {"CURE", 365, 0.812464278863128`,
  {{0, 0.004713658687435209`, 8.390312463634674`}, {5, 0.0016858784636162943`, 3.000863665237004`}},
  0.009999975261653993`, 0.010643590617094153`, 0.5716752199578525`, 1781.0521346371681`},
 {"TBUR", 18.84729322707904`, 230.16976223296712`, {{0, 0.0750869655609131`, 133.6547986984253`}},
  9.014567082907547`*^7, 0.14659424865340226`, 0.5918346827784692`, 2156.007984405044`},
 {"CURE", 365, 1.6137857069379242`, {{0, 0.009081559815249992`, 16.16517647114499`}, {2, 0.00269483952590699`, 4.796814356114442`},
    {4, 0.0006323916258003054`, 1.1256570939245436`}, {6, 0.00015066724130763135`, 0.26818768952758376`},
    {8, 1.4721047523603493`*^-6, 0.0026203464592014216`}}, 0.009999999538448568`, 0.011169350282386747`,
  0.927111835008304`, 1794.7466992703874`}, {"TBUR", 80.25728556072299`, 229.86061644081823`,
  {{0, 0.018081685429519965`, 32.18540006454554`}, {4, 0.002875376675347078`, 5.118170482117799`},
    {8, 0.0009708313921341475`, 1.7280798779987825`}, {12, 0.0005170448520252693`, 0.9203398366049793`},
    {16, 0.00039154105583079444`, 0.6969430793788141`}, {20, 0.0003495238487525192`, 0.6221524507794841`},
    {24, 0.0003323493150277505`, 0.5915817807493958`}, {28, 0.0003239071187387976`, 0.5765546713550597`},
    {32, 0.0003189569861615784`, 0.5677434353676095`}, {36, 0.03230373555951538`, 57.50064929593737`}},
  219610.66439640775`, 0.01612758160182246`, 0.783109300606468`, 2630.252814304548`}, {"CURE", 365, 0.14817839326350093`,
  {{0, 0.0009671883380661217`, 1.7215952417576965`}, {32, 0.00052139710067302`, 0.9280868391979756`}},
  0.009999934641569145`, 0.013318436583196333`, 0.2253056591175488`, 1785.1748327569278`}, {"CURE", 365,
  3.53836596983165`, {{0, 0.00497900619284827`, 8.862631023269921`}, {47, 0.0015317276384940069`, 2.726475196519332`}},
  0.009745319781396764`, 0.034096545400064925`, 0.20550879156577273`, 1780.804850305876`}, {"CURE", 365,
  0.3279195198271717`, {{0, 0.003733313764110284`, 6.645298500116305`}, {5, 0.0009074423367114358`, 1.6152473593463559`}},
  0.00994428940522556`, 0.007603004607822313`, 0.15587307803642883`, 1780.322099992854`},
 {"TBUR", 15.834379050314354`, 230.1868416225504`, {{0, 0.09090301513671875`, 161.80736694335937`}},
  2.3238471342304833`*^7, 0.09986782009756522`, 0.5695009474128031`, 2083.8194648310664`}, {"CURE", 365,
  3.2255868263631466`, {{0, 0.009312316637554344`, 16.575923614846733`}, {56, 0.0008701023124375233`, 1.5487821161387914`}},
  0.009999899305238424`, 0.02574366669811815`, 0.638429482060078`, 1857.2587561393434`}, {"CURE", 365,
  0.24677700156858773`, {{0, 0.003674131169609434`, 6.539953481904792`}, {4, 0.00213762126672947`, 3.8049658547784566`},
    {8, 0.0010616432859983733`, 1.8897250490771045`}, {12, 0.0003715142428380613`, 0.6612953522517492`}},
  0.009999625947491646`, 0.013694641578878173`, 0.7287010711823099`, 1781.8251560142605`}, {"TBUR", 102.5128382075052`,
  229.26554922583048`, {{0, 0.05549198487515628`, 98.77573307777817`}, {5, 0.017864450071331247`, 31.798721126969618`},
    {10, 0.008514881698308273`, 15.156489422988722`}, {15, 0.004190202879828544`, 7.458561126094809`},
```

{20, 0.0021073337729443026`, 3.751054115840858`}, {25, 0.0010986713941203634`, 1.9556350815342467`},
{30, 0.0006099351041347777`, 1.0856844853599041`}, {35, 0.0003729491872387465`, 0.6638495532849689`},
{40, 0.0002573606502112678`, 0.4581019573760567`}, {45, 0.011911140172379861`, 21.20182950683615`}},
190351.66609670536`, 0.04147952633867849`, 0.6683693744115276`, 2256.2268539623874`}, {"TBUR", 306.24208652824063`,
229.2573201509441`, {{0, 0.11442641647379825`, 203.67902132336087`}, {24, 0.00022347368512743023`, 0.3977831595268258`},
{48, 0.00018115895618638433`, 0.32246294201176406`}, {72, 0.00018111775766483062`, 0.32238960864339855`},
{96, 0.00018110503507388136`, 0.32236696243150886`}, {120, 0.0001810985929533474`, 0.3223554954569584`},
{144, 0.00018109500660648783`, 0.32234911175954833`}, {168, 0.00018109297344169728`, 0.32234549272622115`},
{192, 0.00018109181684285166`, 0.322343433980276`}, {216, 0.0012827263586104019`, 2.283252918326516`}},
43.99984750720154`, 0.01608365127643695`, 0.8192393209565417`, 2359.8872942463513`}, {"TBUR", 202.03101551007694`,
229.7985515088447`, {{0, 0.07977749032472932`, 142.0039327780182`}, {16, 0.003113020279977139`, 5.541176098359307`},
{32, 0.0033953320732841544`, 6.043691090445795`}, {48, 0.001825096258797347`, 3.248671340659278`},
{64, 0.0014653296849185618`, 2.60828683915504`}, {80, 0.0013010253634143268`, 2.3158251468775015`},
{96, 0.0012293695216058058`, 2.188277748458334`}, {112, 0.0011956751325776227`, 2.128301735988168`},
{128, 0.001179181035019342`, 2.0989422423344286`}, {144, 0.02321146788160708`, 41.3164128292605 9`}},
165782.19650640633`, 0.029567829622408292`, 0.1635114778230083`, 1780.0151139216869`}, {"TBUR", 62.98809271172485`,
229.58553123483526`, {{0, 0.04223324868369197`, 75.17518265697171`}, {6, 0.011943156737520378`, 21.258818992786274`},
{12, 0.007125481305119273`, 12.683356723112308`}, {18, 0.006553521679783765`, 11.665268590015103`},
{24, 0.007271729878170264`, 12.943679183143072`}, {30, 0.008088781600021428`, 14.398031248038142`},
{36, 0.008366038521530552`, 14.891548568324382`}, {42, 0.008168095629657765`, 14.539210220790823`},
{48, 0.007828525721341434`, 13.934775783987751`}, {54, 0.7826718222849122`, 1393.1558436671437`}},
2.7978487223155824`*^6, 0.17065675950757705`, 0.7720677829658921`, 2121.0445549333695`}, {"CURE", 365,
0.12564204596632125`, {{0, 0.0011718661844692235`, 2.085921808355218`}, {19, 0.0002803189936634302`, 0.49896780872090574`}},
0.00992195968699511 6`, 0.026008083277360677`, 0.034186047278801944`, 1780.1539979497425`},
{"CURE", 365, 0.0882466778452384`, {{0, 0.00018476650597000477`, 0.32888438062660846`},
{9, 0.00004563475533387816`, 0.08122986449430311`}, {18, 5.611667804100467`*^-6, 0.009988768691298831`}},
0.00999936802788052`, 0.04935834016006562`, 0.37546655489866504`, 1890.841588846692`}, {"TBUR", 212.2441139029631 4`,
229.45683728154836`, {{0, 0.08802713597834848`, 156.6883020414603`}, {16, 0.00030634138717018254`, 0.5452876691629249`},
{32, 0.00022507039825583284`, 0.40062530889538245`}, {48, 0.00028195423358615726`, 0.5018785357833598`},
{64, 0.00041748218456401766`, 0.7431182885239515`}, {80, 0.00029515507962736304`, 0.5253760417367063`},
{96, 0.00023412263273720418`, 0.41673828627222337`}, {112, 0.00022283813556696526`, 0.39665188130919815`},

{128, 0.0002155175213674575`, 0.3836211880340744`}, {144, 0.0032525970932947294`, 5.789622826064619`}},
112.85591874023147`, 0.010961526518364321`, 0.12807070799663434`, 1780.0107068922075`}, {"CURE", 365,
1.1048519612329641`, {{0, 0.007154841575360873`, 12.735618004142353`}, {3, 0.002807143795789583`, 4.996715956505457`}},
0.009999357782778513`, 0.005092808011355147`, 0.3417510846189834`, 1780.2662143327082`}, {"CURE", 365,
4.707384576486673`, {{0, 0.03481058119605968`, 61.962834528986235`}, {4, 0.006486465867467034`, 11.54590924409132`}},
0.009999964095316855`, 0.023543066920828734`, 0.97825516832219`, 2613.4319675057895`},
{"CURE", 365, 0.04786342906746407`, {{0, 0.0015772625679368487`, 2.8075273709275907`}}, 0.009999912394567032`,
0.015069119701376763`, 0.11041574702260236`, 1780.4478668483036`}, {"CURE", 365, 185.65730684902644`,
{{0, 0.04564336780948647`, 81.24519470088592`}, {26, 0.004924131942175874`, 8.764954857073056`}}, 0.009978765828067438`,
0.006470646368895295`, 0.9999484975269104`, 2722.091332738785`}, {"CURE", 365, 0.23175618280133728`,
{{0, 0.001029664142968337`, 1.83280217448364`}, {42, 0.0005122014756775054`, 0.9117186267059597`}}, 0.009755714800921243`,
0.015376374431604608`, 0.054662556325443146`, 1780.829551523255`}, {"CURE", 365, 0.13316171382499742`,
{{0, 0.0035028309911201093`, 6.235039164193795`}, {4, 0.0003028156885028508`, 0.5390119255350745`}},
0.009974745085183727`, 0.01067815507022298`, 0.29872250339836115`, 1780.7756507316756`}, {"CURE", 365,
2.959217143000278`, {{0, 0.022843676171294524`, 40.66174358490425`}, {7, 0.00423888589261248`, 7.545216888850216`}},
0.009999185673083439`, 0.01920399971082365`, 0.9514115057238542`, 2016.2428016125664`},
{"CURE", 365, 0.014073954933837741`, {{0, 0.000371524144728274`, 0.661312977616327 6`}},
0.009999160919501218`, 0.025122862285534193`, 0.028724702449275143`, 1780.6995103455076`},
{"CURE", 365, 0.3520750523606531`, {{0, 0.0018590275894632211`, 3.3090691092445335`}},
0.009999967717083247`, 0.019308541098289434`, 0.11595660674726889`, 1780.3581645908314`},
{"TBUR", 13.203385179956994`, 229.82963194160774`, {{0, 0.09034726142883301`, 160.81812534332275`}},
7.528063016828725`*^7, 0.13939405527045606`, 0.6019371187644598`, 2061.242092433003`}, {"TBUR", 314.05415423807744`,
228.46072771950594`, {{0, 0.06994607664384397`, 124.50401642604228`}, {27, 0.0012695015805902665`, 2.2597128134506743`},
{54, 0.0012857196034869937`, 2.288580894206849`}, {81, 0.0013013668773571006`, 2.316433041695639`},
{108, 0.0012726037391476105`, 2.265234655682747`}, {135, 0.0012687783628048197`, 2.2584254857925794`},
{162, 0.0012679352693054215`, 2.25692477936365`}, {189, 0.0012677713725901339`, 2.2566330432104382`},
{216, 0.001267738917365845`, 2.256575272911204`}, {243, 0.004413591661647413`, 7.8561931577323945`}},
7.143951578471868`, 0.010888745889379567`, 0.8547941719836779`, 2460.8071087606822`}, {"CURE", 365,
1.386735464241201`, {{0, 0.021941425527126048`, 39.05573743828436`}, {6, 0.003879583696789937`, 6.905658980286088`}},
0.009947383567559802`, 0.010826159165950226`, 0.4705755433043258`, 1780.2067857025395`}, {"CURE", 365,
24.60141713409871`, {{0, 0.03728646099388444`, 66.36990056911431`}, {4, 0.0016810034326064267`, 2.9921861100394396`},

{8, 0.001636811376944175`, 2.9135242509606316`}, {12, 0.0016359059538773137`, 2.9119125979016185`},
{16, 0.0016358692581128621`, 2.9118472794408947`}, {20, 0.001635867071356439`, 2.9118433870144615`},
{24, 0.0016358669082143238`, 2.9118430966214963`}, {28, 0.0016301663452869806`, 2.9016960946108257`}},
0.009999877870870375`, 0.035700715361966215`, 0.9750722713198253`, 2452.2710936433914`},
{"CURE", 365, 1.537855748945488`, {{0, 0.021445350557369156`, 38.1727239921171`}, {6, 0.0017437174138672758`, 3.103816996683751`},
{12, 0.00007377488444318238`, 0.13131929430886463`}}, 0.009999991632542258`, 0.017210720759985393`,
0.9712546638311421`, 1792.3409222903208`}, {"TBUR", 73.73451941584086`, 228.0044404208945`,
{{0, 0.07256427276569793`, 129.16440552294233`}, {4, 0.003992265398090591`, 7.106232408601252`},
{8, 0.0009648006044439511`, 1.717345075910233`}, {12, 0.0005880393361226486`, 1.0467100182983144`},
{16, 0.0005150741681007913`, 0.9168320192194086`}, {20, 0.0005068045804136042`, 0.9021121531362157`},
{24, 0.0005308143730017773`, 0.9448495839431635`}, {28, 0.000591172391505519`, 1.052286856879824`},
{32, 0.0006961779275018094`, 1.239196710953221`}, {36, 0.04424796097825656`, 78.76137054129669`}},
260107.14814382835`, 0.020846401353785623`, 0.7705833965386216`, 2206.779923196704`},
{"CURE", 365, 3.9127623490399595`, {{0, 0.014883244828406632`, 26.492175794563803`},
{2, 0.001902283865690872`, 3.386065280929752`}, {4, 0.00015149548617656054`, 0.2696619653942778`}},
0.009994917399925399`, 0.014512021235637157`, 0.5863001957949895`, 1780.7512863105587`}, {"TBUR", 365,
229.25250149186945`, {{0, 0.06761246026805334`, 120.35017927713494`}, {31, 0.003025158224899525`, 5.384781640321153`},
{62, 0.0029807123125388164`, 5.305667916319092`}, {93, 0.002927227599535608`, 5.210465127173381`},
{124, 0.0029209953740170903`, 5.199371765750421`}, {155, 0.002920241056041624`, 5.1980290797540905`},
{186, 0.0029201491270727208`, 5.197865446189443`}, {217, 0.0029201379654774226`, 5.197845578549812`},
{248, 0.0029201366092188917`, 5.197843164409627`}, {279, 0.0038621161128728435`, 6.874566680913661`}},
714.5101346029156`, 0.025804129931992027`, 0.7418025375118898`, 2391.8735128954336`},
{"TBUR", 18.756802951860973`, 229.29789174659575`, {{0, 0.11244175910949708`, 200.1463312149048`}},
4.487093710650328`*^7, 0.06763869917573773`, 0.5285088444224926`, 2044.6895379632676`},
{"CURE", 365, 0.25669843747573484`, {{0, 0.0005663580390931807`, 1.0081173095858618`},
{5, 0.00015965649012067567`, 0.2841885524148028`}, {10, 0.000017941524321050014`, 0.031935913291469026`}},
0.009999931910428162`, 0.02206089267248591`, 0.2810453255742326`, 1804.4555140873704`}, {"CURE", 365,
23.67749741509654`, {{0, 0.006362729754854313`, 11.325658963640677`}, {6, 0.00020674382327362135`, 0.36800400542704603`},
{12, 0.00017517561248183255`, 0.31181259021766194`}, {18, 0.00017482127919151748`, 0.31118187696090116`},
{24, 0.00017480210719747054`, 0.31114775081149765`}, {30, 0.00017479992205280098`, 0.3111438612539858`},
{36, 0.00017479955231192027`, 0.3111432031152181`}, {42, 0.00017479947345611392`, 0.3111430627518828`},

```
{48, 0.00017479945406428027`, 0.3111430282344189`}, {54, 0.00013386487363845734`, 0.23827947507645408`}},
0.009999997641872569`, 0.008741030564766892`, 0.9997695602666634`, 2588.722439125104`},
{"CURE", 365, 0.05490306989305068`, {{0, 0.0011671370312508378`, 2.077503915626491`}},
0.009998005884027494`, 0.024479922653360485`, 0.10136676952364862`, 1780.5096232520311`},
{"CURE", 365, 0.23265443329636093`, {{0, 0.0007084641147620543`, 1.2610661242764565`},
{10, 0.0001351347993130553`, 0.24053994277723847`}, {20, 8.130305787900784`*^-6, 0.014471944302463395`}},
0.0099999465179507`, 0.07214496731469767`, 0.2282692203740646`, 1957.3587558473487`}, {"CURE", 365,
0.40703065874644784`, {{0, 0.0025589078990216904`, 4.55485606025861`}, {33, 0.0009976204961710858`, 1.775764483184533`}},
0.00999976158206616`, 0.015860745297629326`, 0.288288268904343`, 1783.6453825854644`}, {"CURE", 365, 0.06340243650942158`,
{{0, 0.001469764143591412`, 2.616180175592713`}, {6, 0.00034021273900218253`, 0.6055786754238849`}},
0.00994088770681641`, 0.01651069614600244`, 0.4493297132766388`, 1782.2064234475095`},
{"TBUR", 136.65460711672995`, 230.04276809036523`, {{0, 0.025506383770070855`, 45.40136311072612`},
{7, 0.0008841849257814014`, 1.5738491678908944`}, {14, 0.00027231618405207733`, 0.4847228076126977`},
{21, 0.00021083053269002456`, 0.37527834818824374`}, {28, 0.00019605526611452315`, 0.34897837368385126`},
{35, 0.0001906860844374233`, 0.3394212302986135`}, {42, 0.00018848210071634744`, 0.3354981392750984`},
{49, 0.00018745576431940638`, 0.33367126048854334`}, {56, 0.00018691022126579308`, 0.33270019385311167`},
{63, 0.0189140607699302 8`, 33.667028170475895`}}, 397688.0351712972`, 0.03632348780257241`,
0.7229383853068183`, 3675.581779595992`}, {"TBUR", 336.70788381160077`, 228.77371207705778`,
{{0, 0.010890033938492728`, 19.384260410517054`}, {31, 0.005341222298576901 6`, 9.507375691466883`},
{62, 0.0033143840477690384`, 5.899603605028888`}, {93, 0.00210225733473619`, 3.742018055830418`},
{124, 0.001362069325351616`, 2.424483399125876`}, {155, 0.0009113206597615843`, 1.6221507743756203`},
{186, 0.0006356205508566069`, 1.1314045805247603`}, {217, 0.0004662421893216262`, 0.8299110969924947`},
{248, 0.00036146917018978447`, 0.6434151229378163`}, {279, 0.18386930619350803`, 327.2873650244443`}},
568116.8661869386`, 0.08367360578214389`, 0.6557524120982847`, 1690.614254921185`},
{"TBUR", 18.63154962069527`, 229.67647110008107`, {{0, 0.10809359550476078`, 192.40659999847418`}},
5.601806352112307`*^7, 0.047536497044212325`, 0.5624762427771646`, 2063.37789154796`}, {"CURE", 365,
0.5825315339907032`, {{0, 0.002349068345507347`, 4.181341655003077`}, {12, 0.0011709244820895758`, 2.084245578119445`},
{24, 0.0003853458640056788`, 0.6859156379301082`}, {36, 0.00007045141101104979`, 0.12540351159966862`}},
0.00997117892389836`, 0.03729461450001689`, 0.42497926689740284`, 1782.7405652112047`}, {"CURE", 365,
0.9404214788057618`, {{0, 0.0013474483935928116`, 2.3984581405952046`}, {24, 0.0003905760828587649`, 0.6952254274886015`}},
0.00975111655343773`, 0.010848595229859061`, 0.07198139824147123`, 1781.3582594833194`}, {"CURE", 365,
```

1.6366818543012238`, {{0, 0.002838241769628316 4`, 5.052070349938403`}, {13, 0.0007743642965200791`, 1.378368447805741`},
{26, 0.00009800626227397612`, 0.1744511468476775`}, {39, 4.4004475536430776`*^-7, 0.0007832796645484678`}},
0.009999940381199255`, 0.0516701033051022`, 0.2075125274445983`, 1783.6902207226885`}, {"TBUR", 31.255607420310913`,
228.0365839097122`, {{0, 0.04237879742503249`, 75.43425941655785`}, {1, 0.04128427378281614`, 73.48600733341273`}},
3.158852447480009`*^7, 0.079844753580026`, 0.563135124247471`, 2214.3513077826924`}, {"CURE", 365, 0.4379415353849823`,
{{0, 0.013284876675153535`, 23.647080481773294`}, {5, 0.0017388099293012273`, 3.095081674156185`}},
0.009958283144321036`, 0.012494972511851584`, 0.531549466834371`, 1780.3820689937193`},
{"CURE", 365, 1.4851355653844576`, {{0, 0.00934378203159932`, 16.63193201624679`}},
0.00999979244430632`, 0.005938481339421845 5`, 0.4390725560732446`, 1780.6411881123586`},
{"CURE", 365, 0.037888008794779285`, {{0, 0.0006018823311469592`, 1.0713505494415876`}}, 0.009999310438637422`,
0.011013549876071636`, 0.13628781765521877`, 1781.3354243793162`}, {"TBUR", 179.89413565212718`, 230.08015686437037`,
{{0, 0.06628313488775199`, 117.98398010019852`}, {15, 0.00361201270938453`, 6.4293826227044635`},
{30, 0.005372797480801107`, 9.56357951582597`}, {45, 0.002385064407541456`, 4.245414645423791`},
{60, 0.0016712336701663183`, 2.974795932896046`}, {75, 0.0012370881940936653`, 2.202016985486724`},
{90, 0.001028515547709069`, 1.8307576749221428`}, {105, 0.0009161564068534283`, 1.6307584041991023`},
{120, 0.0008511245919865262`, 1.5150017737360166`}, {135, 0.031351760879797505`, 55.806134366039565`}},
106402.81028566152`, 0.018307892245838217`, 0.7363434051905084`, 2190.84190107526`},
{"CURE", 365, 0.23325550069165035`, {{0, 0.0005494823520775273`, 0.9780785866979986`}}, 0.009999354286708057`,
0.021445712172768007`, 0.10240385084454783`, 1782.0925922225808`}, {"TBUR", 214.8886445171056`, 229.07530859434766`,
{{0, 0.08006857264480424`, 142.52205930775156`}, {16, 0.007940888155415266`, 14.134780916639174`},
{32, 0.00037426999825486266`, 0.6662005968936555`}, {48, 0.002303642322915345`, 4.100483334789314`},
{64, 0.00083423925476626 7`, 1.4849458734839551`}, {80, 0.0000791738339744187`, 0.14092942447446527`},
{96, 0.00014367257757302054`, 0.2557371880799766`}, {112, 0.00007439628163125812`, 0.13242538130363943`},
{128, 0.000033617785332890576`, 0.05983965789254522`}, {144, 0.0006266274534353754`, 1.1153968671149683`}},
0.10596605109719855`, 0.004661966392472072`, 0.11077610759097263`, 1780.0071720185983`}, {"CURE", 365,
2.0587432012322733`, {{0, 0.015001233547577451`, 26.70219571468786`}, {8, 0.0024702601395067457`, 4.397063048322008`}},
0.009999860781214188`, 0.01575170891448625`, 0.7807405355404068`, 1781.700748378492`}, {"TBUR", 107.63556964234982`,
229.05649462554373`, {{0, 0.03382596151987901`, 60.21021150538463`}, {6, 0.0037723658608185225`, 6.71481123225697`},
{12, 0.0002521993223274497`, 0.4489147937428605`}, {18, 0.00011052396259859596`, 0.1967326534255008`},
{24, 0.00010436460998142283`, 0.18576900576693264`}, {30, 0.00011139847354020521`, 0.19828928290156528`},
{36, 0.00012037892542110477`, 0.2142744872495665`}, {42, 0.00011403649853245934`, 0.2029849673877776`},

```
      {48, 0.00010341121864961701`, 0.18407196919631827`}, {54, 0.00433893605931791`, 7.723306185585879`}},
    3361.620390111932`, 0.008324075748317636`, 0.5929090446324413`, 3285.1462889885097`},
  {"TBUR", 21.913977042321566`, 229.87403581540894`, {{0, 0.08163237571716309`, 145.3056287765503`}},
    4.26714637984647`*^7, 0.03564040883667103`, 0.6255578636129872`, 2185.894961196052`}, {"CURE", 365,
    8.871210043239412`, {{0, 0.009584128249882572`, 17.059748284790977`}, {2, 0.004640330451346838`, 8.259788203397372`},
      {4, 0.0018460774044764365`, 3.286017779968057`}, {6, 0.0004431501672855294`, 0.7888072977682423`}},
    0.009999933898201313`, 0.008739453882373401`, 0.7304093395097047`, 1781.4587309615297`},
  {"CURE", 365, 0.0676539480102549`, {{0, 0.0005159498866717123`, 0.918390798275648`}},
    0.009999071996407339`, 0.016569659047392545`, 0.10048646727260613`, 1781.3426166049449`},
  {"TBUR", 31.14968285047954`, 229.6606111942693`, {{0, 0.09242968559265138`, 164.52484035491946`}}, 6.937277630710958`*^7,
    0.0930990300422366`, 0.45322344792121066`, 2118.332599284839`}, {"CURE", 365, 0.19614618135754114`,
    {{0, 0.0005912654485034429`, 1.0524524983361285`}, {10, 0.0002689153554919673`, 0.47866933277570184`}},
    0.009999784818184053`, 0.021382540992517705`, 0.1584753316670985`, 1785.5121706962773`}, {"TBUR", 75.07969097281601`,
    228.2126864074949`, {{0, 0.06588514594118686`, 117.2755597753126`}, {4, 0.022474277322836304`, 40.00421363464863`},
      {8, 0.0074531640832760345`, 13.266632068231342`}, {12, 0.002729271509511436`, 4.858103286930357`},
      {16, 0.001098982996532879`, 1.9561897338285248`}, {20, 0.0004834003906112014`, 0.8604526952879386`},
      {24, 0.00023820841288479358`, 0.4240109749349326`}, {28, 0.00013823978659090717`, 0.24606682013181475`},
      {32, 0.00009675697178207352`, 0.1722274097720909`}, {36, 0.004648596528673346`, 8.274501821038555`}},
    52598.75684119272`, 0.024751709788263975`, 0.7080967392158584`, 2390.657389429022`}, {"CURE", 365,
    12.198558946914309`, {{0, 0.037330454531714276`, 66.4482090664514`}, {7, 0.00498105610360693`, 8.866279864420335`}},
    0.009999660012248154`, 0.028505816975501124`, 0.9021054069142024`, 2378.801636945893`},
  {"TBUR", 20.58733243693395`, 230.13768410761827`, {{0, 0.07969641685485843`, 141.859622001648`}},
    6.944425315128572`*^7, 0.089589324779531`, 0.5858994766353509`, 2157.2976335945227`},
  {"CURE", 365, 1.0264744353916906`, {{0, 0.003921063325963593`, 6.9794927202151955`}},
    0.009999933012588648`, 0.01512905316526403`, 0.18869081371555146`, 1780.5935326849988`},
  {"TBUR", 17.573787831078796`, 229.4614545419986`, {{0, 0.06945319175720216`, 123.62668132781985`}},
    5.3758101567334324`*^7, 0.17503019561334648`, 0.5663148974982807`, 2152.4921884831283`},
  {"TBUR", 18.69669426202294`, 224.8310782969542`, {{0, 0.09193501472473146`, 163.644326210022`}}, 7.806865256570081`*^7,
    0.1437549774878748`, 0.5115455896906891`, 2064.809117759628`}, {"CURE", 365, 14.354764035111074`,
    {{0, 0.008044397619308225`, 14.319027762368641`}, {26, 0.0002908024691234178`, 0.5176283950396837`},
      {52, 0.00006247948949775323`, 0.11121349130600075`}, {78, 0.000054744728822787186`, 0.0974456173045612`},
```

```
{104, 0.00005417180742478065`, 0.09642581721610957`}, {130, 0.0000541079999423220`, 0.0963122398973333`},
{156, 0.00005410044570491884`, 0.09629879335475554`}, {182, 0.00005409954494584818`, 0.09629719000360978`},
{208, 0.00005409943744676603`, 0.09629699865524353`}, {234, 0.00006226336019668534`, 0.1108287811500999`}},
0.009999994815869799`, 0.046952971787944844`, 0.6436357060490739`, 2454.1501570910777`}, {"CURE", 365,
21.682731417305654`, {{0, 0.019687938158513746`, 35.04452992215447`}, {2, 0.0012656088417068736`, 2.2527837382382345`},
{4, 0.0001388928674937568`, 0.2472293041388871`}, {6, 0.00008651894566870003`, 0.15400372329028605`},
{8, 0.00008392527142048142`, 0.1493869831284569`}, {10, 0.00008375867606406887`, 0.14909044339404257`},
{12, 0.00008374459076835808`, 0.14906537156767738`}, {14, 0.00008374313988575817`, 0.14906278899664951`},
{16, 0.00008374296904674508`, 0.14906248490320623`}, {18, 0.000024103929020197295`, 0.04290499365595185`}},
0.009999996218372458`, 0.023624155931818356`, 0.990507522310849`, 2015.7977830352452`},
{"TBUR", 19.550021232763275`, 230.08437666035115`, {{0, 0.06727590560913084`, 119.7511119842529`}},
1.253921938823833`*^7, 0.13057902380708605`, 0.5672033236164874`, 2190.8747176890115`},
{"CURE", 365, 0.32433737619559816`, {{0, 0.003726687731321088`, 6.633504161751537`}}, 0.00999077359818696`,
0.01532929122365393`, 0.4357200567065646`, 1781.5165287412526`}, {"CURE", 365, 1.9473288123785741`,
{{0, 0.0025048480841775595`, 4.458629589836056`}, {28, 0.0005993257055992607`, 1.0667997559666842`}},
0.009735362362291682`, 0.05333710895074256`, 0.1161131919483985`, 1781.327530242758`}, {"CURE", 365, 0.5441141405307915`,
{{0, 0.003187930248167922`, 5.674515841738901`}, {41, 0.0010956237643295098`, 1.9502103005065277`}}, 0.009997409025221296`,
0.016234853756169725`, 0.10681178745762757`, 1780.8479802978347`}, {"CURE", 365, 0.058024329815242116`,
{{0, 0.0003691723482467881`, 0.6571267798792828`}, {5, 0.00006861529186167817`, 0.12213521951378717`}},
0.009952146829062631`, 0.011366429726301629`, 0.333358603846433`, 1786.6521375437883`},
{"CURE", 365, 15.28375761331063`, {{0, 0.050029352732192`, 89.05224786330176`}, {4, 0.002932182921314458`, 5.219285599939735`},
{8, 0.0001623568238866624`, 0.288995146518259`}, {12, 0.000052059870500807855`, 0.09266656949143798`},
{16, 0.000048106480220114720`, 0.08562953479180421`}, {20, 0.00004793224562669648`, 0.08531939721551975`},
{24, 0.00004791989267053275`, 0.08529740895354829`}, {28, 0.000047918690831047964`, 0.08529526967926536`},
{32, 0.00004783181838913873`, 0.08514063673266693`}}, 0.009999996264482792`,
0.04061753559390791`, 0.9956289874334937`, 1961.773613135305`}, {"CURE", 365, 2.0070309716074552`,
{{0, 0.009439305525466396`, 16.801963835330184`}, {4, 0.002987906251950709`, 5.318473128472261`},
{8, 0.0006611092451633885`, 1.1767744563908316`}, {12, 0.00014656242983786166`, 0.2608811251113937`},
{16, 0.000040021363474916074`, 0.07123802698535062`}, {20, 0.000018875651755036206`, 0.033598660123964445`},
{24, 0.000015078174727839543`, 0.026839151015554383`}, {28, 0.000014484350221602289`, 0.025782143394452076`},
{32, 0.000014401489045747117`, 0.025634650501429868`}, {36, 6.23293651466816`*^-6, 0.011094626996109325`}},
```

0.009999997988969772`, 0.03521385422860075`, 0.9653771028885936`, 2080.543890315715`},
{"CURE", 365, 0.37382624154849015`, {{0, 0.0016715826969225538`, 2.975417200522146`}}, 0.00999975913720591`,
0.0265676322863124`, 0.05935656879203995`, 1780.3910199577974`}, {"TBUR", 245.80288797473085`, 230.17034559622093`,
{{0, 0.05585074463898289`, 99.41432545738954`}, {20, 0.008638964266920486`, 15.377356395118467`},
{40, 0.001292166746911452`, 2.3000568095023843`}, {60, 0.00027841708026535083`, 0.4955824028723245`},
{80, 0.00013311809035574792`, 0.23695020083323132`}, {100, 0.00010686250869304748`, 0.19021526547362452`},
{120, 0.00009898871649783746`, 0.17619991536615065`}, {140, 0.00009542199186654153`, 0.16985114552244396`},
{160, 0.00009340603198474442`, 0.16626273693284505`}, {180, 0.006424274372750444`, 11.435208383495791`}},
1044.6559866235505`, 0.009366098205173081`, 0.1700810334091074`, 1780.017832965185`}, {"CURE", 365,
9.99957849391229`, {{0, 0.015665018696391354`, 27.88373327957661`}, {2, 0.0006713976559359684`, 1.1950878275660237`},
{4, 0.000074687934202293`, 0.13294452288008152`}, {6, 0.00006360428035002288`, 0.11321561902304073`},
{8, 0.0000634644676954835`, 0.11296675249796062`}, {10, 0.00004506836118911974`, 0.08022168291663315`}},
0.009976988722491215`, 0.016993710361059327`, 0.993571198869675`, 1962.2052178468284`},
{"TBUR", 46.06977035279166`, 229.76991248577002`, {{0, 0.06926138616561889`, 123.28526737480162`}},
3.8375914456068804`*^6, 0.013492407766529893`, 0.4791699961381854`, 2343.714050343499`}, {"TBUR", 84.84952466336571`,
228.80795004177764`, {{0, 0.001975239872374726`, 3.5159269728270126`}, {2, 0.001960015258677809`, 3.4888271604465007`},
{4, 0.001173059304543805 5`, 2.0880455620879737`}, {6, 0.0005551708624719077`, 0.9882041351999957`},
{8, 0.0002459671990444626`, 0.4378216142991434`}, {10, 0.00010803512876786698`, 0.19230252920680324`},
{12, 0.0000480575056685467`, 0.08554236009001312`}, {14, 0.000022200703828874808`, 0.039517252815397155`},
{16, 0.000011174568115659744`, 0.019890731245874343`}, {18, 0.04099930938080665`, 72.97877069783584`}},
50.985062787499736`, 0.007832543148500975`, 0.711959760631892`, 3278.3369954516565`}, {"TBUR", 365,
228.96169006369522`, {{0, 0.07138067003642234`, 127.05759266483179`}, {31, 0.00935713315535197`, 16.65569701652650 6`},
{62, 0.001268539768643422`, 2.2580007881852913`}, {93, 0.00026992443284677573`, 0.4804654904672607`},
{124, 0.00014015925467587454`, 0.2494834733230567`}, {155, 0.00011916788451322887`, 0.2121188344335474`},
{186, 0.00011400642773114805`, 0.20293144136144353`}, {217, 0.00011240383691980339`, 0.20007882971725`},
{248, 0.00011186432333964842`, 0.19911849554457417`}, {279, 0.0011450073066997966`, 2.038113005925638`}},
11.66994511981349`, 0.01163146111124433`, 0.07202338361134586`, 1780.0052192783905`}, {"CURE", 365,
0.3557345872737103`, {{0, 0.009572586779519795`, 17.039204467545236`}, {8, 0.0005135321908341936`, 0.9140872996848648`}},
0.009986246093869488`, 0.028141333852399513`, 0.5879962556292545`, 1780.7982228988312`},
{"TBUR", 17.192670569410637`, 227.70834015931527`, {{0, 0.09603397369384765`, 170.94047317504882`}},
3.655530763820291`*^7, 0.10843774209718872`, 0.4995007371332774`, 2047.2109486322922`}, {"CURE", 365,

```
7.743102987363201`, {{0, 0.01197149887482627`, 21.30926799719076`}, {3, 0.0008027456320706625`, 1.4288872250857794`},
   {6, 0.00006571135006985667`, 0.11696620312434486`}, {9, 0.000044987006564165825`, 0.08007687168421516`},
   {12, 0.000044573931556905753`, 0.07934159817129224`}, {15, 0.00004456339310492418`, 0.07932283972676504`},
   {18, 0.000044562818447271684`, 0.0793218168361436`}, {21, 0.00003883204132455347`, 0.06912103355770517`}},
  0.009999988324321536`, 0.023147008785264045`, 0.994704818291562`, 2208.550525278121`},
 {"CURE", 365, 0.15771399662008465`, {{0, 0.0011085670326446706`, 1.9732493181075135`}},
  0.009998160767966951`, 0.020589118275272395`, 0.3747989299026255`, 1783.4659812988575`},
 {"CURE", 365, 6.5114968404986575`, {{0, 0.014424733835485098`, 25.67602622716347`},
   {2, 0.0011596158303861684`, 2.0641161780873793`}, {4, 0.00008628981093153648`, 0.15359586345813492`},
   {6, 0.00003536878038240698`, 0.06295642908068443`}, {8, 0.000033362399061284163`, 0.05938507032908581`},
   {10, 0.0000332759762627805`, 0.05923123774749285`}, {12, 0.000033270665668451623`, 0.05922178488984388`},
   {14, 0.00003327019775420278`, 0.05922095200248095`}, {16, 0.000027759554293365123`, 0.04941200664218992`}},
  0.009999994729049179`, 0.023799660215281397`, 0.9920844605480665`, 2002.9999932375194`}, {"CURE", 365,
  6.26703314963031`, {{0, 0.022466294512313686`, 39.99000423191836`}, {15, 0.003036452618745075`, 5.404885661366234`}},
  0.009999716019707372`, 0.04147853633918916`, 0.7901290646836072`, 2578.17492761391`},
 {"CURE", 365, 0.290137697000936`, {{0, 0.0010376513539763455`, 1.8470194100778952`}},
  0.009999849125651055`, 0.018458261920669992`, 0.0361093192056095`, 1780.2234263753148`}};
```

```
(* The trial was performed via these long-running codes *)
```

```
(* Make Kaplan-Meier curve for FREE GROWTH (training set) *)
```

```
Npar = 1000; (*how many sets will be tested*)
SetBasicParameterValues[];
TestFree = Array[f, {Npar + 1, 4}];

TestFree[[1, 1]] = "Free Res";
TestFree[[1, 2]] = "Free OS";
TestFree[[1, 3]] = "Free Tox";
TestFree[[1, 4]] = "Free Ainj";
```

```
Quiet[For[ij = 2, ij ≤ Npar + 1, ij++,

    NotebookDelete[pr]; (* To see the code running *)
    pr = PrintTemporary["Set " <> ToString[ij - 1] <> " of " <> ToString[Npar]];

    kappac = PatientsTrainingSet[[ij, 1]];
    kappap = PatientsTrainingSet[[ij, 2]];
    gamma = PatientsTrainingSet[[ij, 3]] * Nnor / 10^7;
    V = PatientsTrainingSet[[ij, 4]];
    ks = PatientsTrainingSet[[ij, 5]];
    rho = PatientsTrainingSet[[ij, 6]];
    omega = PatientsTrainingSet[[ij, 7]];
    alpha = PatientsTrainingSet[[ij, 8]];
    kf = PatientsTrainingSet[[ij, 9]];
    N0 = PatientsTrainingSet[[ij, 10]] * 10^7 / Nnor;

    Ainj = {{0, 0, 0}};

    res = PatientTEST[];

    TestFree[[ij, 1]] = res[[1]];
    TestFree[[ij, 2]] = res[[2]];
    TestFree[[ij, 3]] = res[[3]];
    TestFree[[ij, 4]] = Ainj;
  ]];

(* TEST FOR NUMERICARILLY OPTIMAL SINGLE DOSES *)
Npar = 1000; (*how many sets will be tested*)
SetBasicParameterValues[];
TestSDNumOpt = Array[f, {Npar + 1, 6}];
```

```
TestSDNumOpt[[1, 1]] = "NumOpt Res";
TestSDNumOpt[[1, 2]] = "NumOpt OS";
TestSDNumOpt[[1, 3]] = "NumOpt Tox";
TestSDNumOpt[[1, 4]] = "NumOpt A";
TestSDNumOpt[[1, 5]] = "Nn";
TestSDNumOpt[[1, 6]] = "FrAcViable";

Quiet[For[ij = 2, ij ≤ Npar + 1, ij++,

    NotebookDelete[pr]; (* To see the code running *)
    pr = PrintTemporary["Set " <> ToString[ij - 1] <> " of " <> ToString[Npar]];

    kappac = PatientsTrainingSet[[ij, 1]];
    kappap = PatientsTrainingSet[[ij, 2]];
    gamma = PatientsTrainingSet[[ij, 3]] * Nnor / 10^7;
    V = PatientsTrainingSet[[ij, 4]];
    ks = PatientsTrainingSet[[ij, 5]];
    rho = PatientsTrainingSet[[ij, 6]];
    omega = PatientsTrainingSet[[ij, 7]];
    alpha = PatientsTrainingSet[[ij, 8]];
    kf = PatientsTrainingSet[[ij, 9]];
    N0 = PatientsTrainingSet[[ij, 10]] * 10^7 / Nnor;

    AA = FindToxDose[];
    AA = AA * 0.998;
    Ainj = {{0, AA * nCpm, eta * AA * nCpm}};
    res = PatientTEST[];

    (* optimal treament cannot be lethally toxic -- it is either cure or death due to tumor burden *)
    While[res[[1]] == "TOX",
```

```
      AA = AA * 0.999;
      Ainj = {{0, AA * nCpm, eta * AA * nCpm}};
      res = PatientTEST[];];

    (* If it is not curative, then it's the best treatment *)
    (* But if it is curative, descalate dose *)
    If[res[[1]] == "CURE",
      AA = FindCurDose[];
      Ainj = {{0, AA * nCpm, eta * AA * nCpm}};
      res = PatientTEST[];

      (* To avoid errors, TBUR cannot be achieved *)
      While[res[[1]] == "TBUR",
        AA = AA * 1.001;
        Ainj = {{0, AA * nCpm, eta * AA * nCpm}};
        res = PatientTEST[];
      ];
    ];

    TestSDNumOpt[[ij, 1]] = res[[1]];
    TestSDNumOpt[[ij, 2]] = res[[2]];
    TestSDNumOpt[[ij, 3]] = res[[3]];
    TestSDNumOpt[[ij, 4]] = AA;

    (* run the simulation and write down interesting measures *)
    TestSDNumOpt[[ij, 5]] = FullSystemSolutionMD[];
    TestSDNumOpt[[ij, 6]] = (SDpw[[npw]][tEnd] + CFNpw[[npw]][tEnd] + CFDpw[[npw]][tEnd] + UNpw[[npw]][tEnd]) / Total[Ainj[[All, 2]]];

  ]];

(* Make Kaplan-Meier curve for these optimal MD schedules (training set) *)
```

```
Npar = Length[resultMD] - 1; (*how many sets will be tested*)
SetBasicParameterValues[];
TestMDNumOpt = Array[f, {Npar + 1, 8}];

TestMDNumOpt[[1, 1]] = "NumOpt Res";
TestMDNumOpt[[1, 2]] = "NumOpt OS";
TestMDNumOpt[[1, 3]] = "NumOpt Tox";
TestMDNumOpt[[1, 4]] = "NumOpt Ainj";
TestMDNumOpt[[1, 5]] = "Nn";
TestMDNumOpt[[1, 6]] = "FrAcViable";
TestMDNumOpt[[1, 7]] = "RecAvgOcc";
(*TestMDNumOpt[[1,8]]="MaxEtaObs";*)
TestMDNumOpt[[1, 8]] = "MaxEtaInj";

Quiet[For[ij = 2, ij ≤ Npar + 1, ij++,

    NotebookDelete[pr]; (* To see the code running *)
    pr = PrintTemporary["Set " <> ToString[ij] <> " of " <> ToString[Npar + 1]];

    kappac = resultMD[[ij, 1]];
    kappap = resultMD[[ij, 2]];
    gamma = resultMD[[ij, 3]] * Nnor / 10^7;
    V = resultMD[[ij, 4]];
    ks = resultMD[[ij, 5]];
    rho = resultMD[[ij, 6]];
    omega = resultMD[[ij, 7]];
    alpha = resultMD[[ij, 8]];
    kf = resultMD[[ij, 9]];
    N0 = resultMD[[ij, 10]] * 10^7 / Nnor;

    If[resultMD[[ij, 12]] < resultMD[[ij, 13]], (* additional check whether SD is better, should not happen *)
```

```
    Ainj = {{0, resultMD[[ij, 11]] * nCpm, eta * resultMD[[ij, 11]] * nCpm}}
    , Ainj = resultMD[[ij, 16]]];

  res = PatientTEST[];

  (* cure, which was defined previously, should be achieved *)
  While[resultMD[[ij, 15]] == 1 && res[[1]] ≠ "CURE",
    Ainj[[Length[Ainj], 2]] = Ainj[[Length[Ainj], 2]] * 1.001;
    Ainj[[Length[Ainj], 3]] = Ainj[[Length[Ainj], 3]] * 1.001;
    res = PatientTEST[];
  ];

  (* optimal treament cannot be lethally toxic -- it is either cure or death due to tumor burden *)
  While[res[[1]] == "TOX",
    Ainj[[Length[Ainj], 2]] = Ainj[[Length[Ainj], 2]] * 0.99;
    Ainj[[Length[Ainj], 3]] = Ainj[[Length[Ainj], 3]] * 0.99;
    res = PatientTEST[];];

  TestMDNumOpt[[ij, 1]] = res[[1]];
  TestMDNumOpt[[ij, 2]] = res[[2]];
  TestMDNumOpt[[ij, 3]] = res[[3]];
  TestMDNumOpt[[ij, 4]] = Ainj;
  (* It cannot be worse that a single-dose result
     although due to the numerical errors it can be
     so replace it if so *)

  If[TestMDNumOpt[[ij, 2]] < TestSDNumOpt[[ij, 2]],
    TestMDNumOpt[[ij, 1]] = TestSDNumOpt[[ij, 1]];
    TestMDNumOpt[[ij, 2]] = TestSDNumOpt[[ij, 2]];
    TestMDNumOpt[[ij, 3]] = TestSDNumOpt[[ij, 3]];
    Ainj = {{0, TestSDNumOpt[[ij, 4]] * nCpm, eta * TestSDNumOpt[[ij, 4]] * nCpm}};
```

```
    TestMDNumOpt[[ij, 4]] = Ainj;
   ];

   (* run the simulation and write down interesting measures*)
   TestMDNumOpt[[ij, 5]] = FullSystemSolutionMD[];
   TestMDNumOpt[[ij, 6]] = (SDpw[[npw]][tEnd] + CFNpw[[npw]][tEnd] + CFDpw[[npw]][tEnd] + UNpw[[npw]][tEnd]) / Total[Ainj[[All, 2]]];

   Quiet[If[resultMD[[ij, 15]] == 1,
      tt = t /. NMinimize[{Piecewise[Table[{NNpw[[nn]][t], tB[[nn]] ≤ t < tE[[nn]]}, {nn, 1, npw}]], t > 0, t < tE[[npw]]}, t][[2]],

      tt = t /.
        NMinimize[{Piecewise[Table[{Abs[Cd - Nnor * NNpw[[nn]][t]], tB[[nn]] ≤ t < tE[[nn]]}, {nn, 1, npw}]], t > 0, t < tE[[npw]]}, t][[2]]];

   TestMDNumOpt[[ij, 7]] =
    NIntegrate[Piecewise[Table[{(NNpw[[nn]][t] + DDpw[[nn]][t] - fFNpw[[nn]][t] * NNpw[[nn]][t] - dFpw[[nn]][t]) / (NNpw[[nn]][t] + DDpw[[nn]][t]), tB[[nn]] ≤ t < tE[[nn]]},
          {nn, 1, npw}]], {t, 0, tt}] / tt;

   TestMDNumOpt[[ij, 8]] = NIntegrate[Piecewise[Table[{bpw[[nn]][t], tB[[nn]] ≤ t < tE[[nn]]}, {nn, 1, npw}]], {t, 0, tt}] /
      NIntegrate[Piecewise[Table[{apw[[nn]][t], tB[[nn]] ≤ t < tE[[nn]]}, {nn, 1, npw}]], {t, 0, tt}];
 ]];
```

```
(* Figure S.29, left and middle *)

TestMDNumOptSM = SurvivalModelFit[TestMDNumOpt⟦2 ;; Length[TestMDNumOpt], 2⟧];
TestSDNumOptSM = SurvivalModelFit[TestSDNumOpt⟦2 ;; Length[TestSDNumOpt], 2⟧];

GraphicsGrid[{{Plot[{TestSDNumOptSM[t], TestMDNumOptSM[t]}, {t, 0, 365},
      PlotStyle → {
        Darker[Green] (*Single dose*), Darker[Gray] (*Multi-dose*)}, Exclusions → None, PlotRange → {0.55, 1.01}]
    ,
    Show[
      ListPlot[Join[{{0, 1}}, Thread[
          {Sort[TestSDNumOpt⟦2 ;;, 3⟧], Table[N[1 - (i - 1) / (Length[TestSDNumOpt] - 1)], {i, 1, Length[TestSDNumOpt] - 1}]}]],
        Joined → True, PlotStyle → Darker[Green], PlotRange → {{0, 280}, {0, 1}}] (* Single dose *),
      ListPlot[Join[{{0, 1}}, Thread[
          {Sort[TestMDNumOpt⟦2 ;;, 3⟧], Table[N[1 - (i - 1) / (Length[TestMDNumOpt] - 1)], {i, 1, Length[TestMDNumOpt] - 1}]}]],
        Joined → True, PlotStyle → Darker[Gray], PlotRange → {{0, 280}, {0, 1}}] (* Multi-dose *),
      Plot[1000 * (x - Abld / nCpm), {x, 0, 400}, PlotStyle → Directive[Gray, Dashed]]]}}]
```

Out[•]=

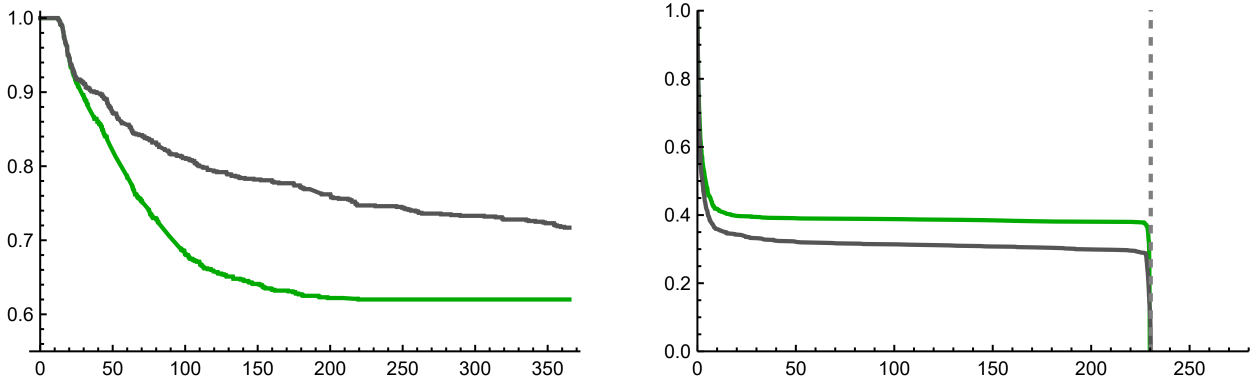

In[ ]:=
```
(* survival rates *)
PercentForm[TestSDNumOptSM[364]]
PercentForm[Total[resultMD[[2 ;;, 15]]] / (Length[resultMD] - 1)]
PercentForm[TestMDNumOptSM[364]]
```
Out[ ]//PercentForm=

62%

Out[ ]//PercentForm=

69.8%

Out[ ]//PercentForm=

71.7%

In[ ]:=
```
(* toxicity-related deaths *)
PercentForm[N[Length[Select[TestSDNumOpt[[2 ;;, 3]], # > Abld / nCpm &]] / Length[TestSDNumOpt]]]
PercentForm[N[Length[Select[TestMDNumOpt[[2 ;;, 3]], # > Abld / nCpm &]] / Length[TestMDNumOpt]]]
```
Out[ ]//PercentForm=

0%

Out[ ]//PercentForm=

0%

In[ ]:=

```
(* Figure S.29, right *)
(* fraction of activity spent on viable cells in curative setting *)
Histogram[{Select[TestSDNumOpt⟦2 ;;⟧, #⟦1⟧ == "CURE" &]⟦All, 6⟧ (* MD*),
    Select[TestMDNumOpt⟦2 ;;⟧, #⟦1⟧ == "CURE" &]⟦All, 6⟧ (* SD*)},
  PlotRange → {{0, Max[Select[TestMDNumOpt⟦2 ;;⟧, #⟦1⟧ == "CURE" &]⟦All, 6⟧]}, Automatic},
  Ticks → {Table[{i, PercentForm[i]}, {i, 0.02, 0.10, 0.02}], Automatic}, ChartStyle → {Darker[Green], Darker[Gray]}, ImageSize → 300]
```

Out[ ]=

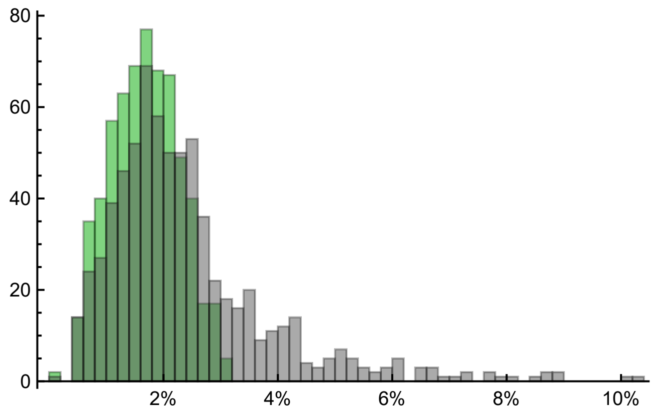

In[ ]:=

```
(* "The window in the parameter space for which the use of multi-dose schedules can be beneficial is defined
by the ratio of the maximal possible rate of cancer cell damage due to irradiation from nuclides, anchored to
them, and their proliferation rate, αλγ / (ν [η+1]) / ρ. This measure lies within the range ≈1.2-11.9 for the parameter
sets, for which optimized multi-dose schedule yields an increase in overall survival of at least one day compared
to the optimal single doses." *)
Min[Select[Thread[{TestMDNumOpt⟦All, 2⟧⟦2 ;;⟧ - TestSDNumOpt⟦All, 2⟧⟦2 ;;⟧,
        resultMD⟦2 ;;, 8⟧ * lambda * resultMD⟦2 ;;, 3⟧ * Nnor / 10^7 / (nu * (eta + 1)) / resultMD⟦2 ;;, 6⟧}], #⟦1⟧ ≥ 1 &]⟦All, 2⟧]
Max[Select[Thread[{TestMDNumOpt⟦All, 2⟧⟦2 ;;⟧ - TestSDNumOpt⟦All, 2⟧⟦2 ;;⟧,
        resultMD⟦2 ;;, 8⟧ * lambda * resultMD⟦2 ;;, 3⟧ * Nnor / 10^7 / (nu * (eta + 1)) / resultMD⟦2 ;;, 6⟧}], #⟦1⟧ ≥ 1 &]⟦All, 2⟧]
```

Out[ ]=

1.16973

Out[ ]=

11.9306

```
(* "Out of all the mice having C_SD > 0 and C_LT > 0,
[illegible]
```

```
are not found during numerical optimization." *)

(* calculate CSD and CLT and C0 for all cases *)
CSD = Array[f, Length[resultMD]];
CSD[[1]] = "CSD";
For[i = 2, i ≤ Length[resultMD], i++,
  CSD[[i]] = (resultMD[[i, 8]] * resultMD[[i, 5]] * lambda * resultMD[[i, 3]] * Nnor / 10^7) /
      (nu * (eta * (lambda + resultMD[[i,1]]) / resultMD[[i,1]] + 1)) - lambda - resultMD[[i, 6]];
  If[CSD[[i]] > 0, CSD[[i]] = 1, CSD[[i]] = 0]];

CLT = Array[f, Length[resultMD]];
CLT[[1]] = "CLT";
For[i = 2, i ≤ Length[resultMD], i++,

  chi = resultMD[[i, 8]] * lambda * resultMD[[i, 3]] * Nnor / 10^7 / nu;
  FANC = 1 / (eta * (lambda + resultMD[[i,1]]) / resultMD[[i,1]] + 1) * resultMD[[i, 6]] / (lambda + resultMD[[i, 6]]);
  Aa = resultMD[[i, 6]] + resultMD[[i, 7]] + lambda;
  Bb = (resultMD[[i, 6]] + resultMD[[i, 7]] + lambda * (1 + resultMD[[i, 5]])) -
    (resultMD[[i, 6]] + resultMD[[i, 7]]) / (chi * FANC) * (resultMD[[i, 6]] + resultMD[[i, 7]] + lambda);
  Cc = lambda * resultMD[[i, 5]] - (resultMD[[i, 6]] + resultMD[[i, 7]]) / (chi * FANC) * lambda;
  Quiet[kDD = y /. Solve[{Aa * y^2 + Bb * y + Cc == 0, y > 0}, y][[1]]];
  CLT[[i]] = If[NumberQ[kDD], resultMD[[i, 7]] / resultMD[[i, 6]] - kDD, 10];
  If[CLT[[i]] > 0, CLT[[i]] = 1, CLT[[i]] = 0]];

(* out of CSD>0, how many are not cured without lethal toxicity *)
```

```
x = Select[Thread[{CSD⟦2 ;;⟧, resultMD⟦2 ;;, 15⟧, TestMDNumOpt⟦2 ;;, 2⟧}], #⟦1⟧ + #⟦2⟧ == 2 &];
PercentForm[1 - N[Length[x] / Total[CSD⟦2 ;;⟧]]]

(* out of CLT>0, how many are not cured without lethal toxicity *)
x = Select[Thread[{CLT⟦2 ;;⟧, resultMD⟦2 ;;, 15⟧, TestMDNumOpt⟦2 ;;, 2⟧}], #⟦1⟧ + #⟦2⟧ == 2 &];
PercentForm[1 - N[Length[x] / Total[CLT⟦2 ;;⟧]]]
```

Out[ ]//PercentForm=

7.132%

Out[ ]//PercentForm=

14.27%

In[ ]:=

```
(* "Only seven of the cured mice correspond to both C_SD<0 and C_LT<0." *)
Length[Select[Select[Thread[{CLT⟦2 ;;⟧, resultMD⟦2 ;;⟧}], #⟦2, 15⟧ == 1 &], #⟦1⟧ == 0 &]⟦All, 2, 12⟧] (* If CLT<0 then CSD<0 as well *)
```

Out[ ]=

7

In[ ]:=

```
(* "for some multi-dose treatments this measure
[fraction of injected activity spent on viable cancer cells]
exceeds the corresponding theoretical threshold for a single-dose setting,
estimated to be <5%" *)
(* how many of cases exceed 5% of this measure *)
Length[Select[Select[TestMDNumOpt⟦2 ;;⟧, #⟦1⟧ == "CURE" &]⟦All, 6⟧, # > 0.05 &]]
(* there is not a single case like this for single-dose treatment *)
Length[Select[Select[TestSDNumOpt⟦2 ;;⟧, #⟦1⟧ == "CURE" &]⟦All, 6⟧, # > 0.05 &]]
```

Out[ ]=

46

Out[ ]=

0

In[∘]:=

```
(* "The average fraction of occupied receptors [in the simulations with optimized multi-dose schedules]
for [virtual mice, which cannot be cured by a single dose without exceeding lethal toxicity,]
up to the moments of cure is ≈86% at kS < 1/3; ≈94% at 1/3 < kS < 2/3 and ≈95% at kS > 2/3" *)
CureMdNotSd =
   Select[Select[Thread[{resultMD[[2 ;;, 5]], TestMDNumOpt[[2 ;;, 7]], resultMD[[2 ;;, 15]], resultMD[[2 ;;, 12]]}], #[[3]] == 1 &], #[[4]] > Abld / nCpm &];
PercentForm[Mean[Select[CureMdNotSd, #[[1]] ≤ 1 / 3 &][[All, 2]]]]
PercentForm[Mean[Select[Select[CureMdNotSd, #[[1]] > 1 / 3 &], #[[1]] ≤ 2 / 3 &][[All, 2]]]]
PercentForm[Mean[Select[CureMdNotSd, #[[1]] > 2 / 3 &][[All, 2]]]]
```

Out[∘]//PercentForm=

86.26%

Out[∘]//PercentForm=

94.25%

Out[∘]//PercentForm=

95.45%

(* S.5.3.2 Cancer binding capacity−based multi−dose optimization *)

In[∘]:=

```
(* We need at first to stratify the training set by ks, it is perfomed with this script, with output being the size of groups *)
ksb1 = 1 / 3; ksb2 = 2 / 3;

TestMDNumOptKs = Thread[{TestMDNumOpt[[All, 1]], TestMDNumOpt[[All, 2]], TestMDNumOpt[[All, 3]], TestMDNumOpt[[All, 4]], resultMD[[All, 5]]}];
TestMDNumOptKsLow = Select[TestMDNumOptKs[[2 ;;]], #[[5]] ≤ ksb1 &][[All, 1 ;; 4]];
resultMDKsLow = Select[resultMD[[2 ;;]], #[[5]] ≤ ksb1 &];
Length[resultMDKsLow]

TestSDNumOptKs = Thread[{TestSDNumOpt[[All, 1]], TestSDNumOpt[[All, 2]], TestSDNumOpt[[All, 3]], TestSDNumOpt[[All, 4]], resultMD[[All, 5]]}];
TestSDNumOptKsLow = Select[TestSDNumOptKs[[2 ;;]], #[[5]] ≤ ksb1 &][[All, 1 ;; 4]];

TestMDNumOptKs = Thread[{TestMDNumOpt[[All, 1]], TestMDNumOpt[[All, 2]], TestMDNumOpt[[All, 3]], TestMDNumOpt[[All, 4]], resultMD[[All, 5]]}];
TestMDNumOptKsMid = Select[Select[TestMDNumOptKs[[2 ;;]], #[[5]] > ksb1 &], #[[5]] ≤ ksb2 &][[All, 1 ;; 4]];
resultMDKsMid = Select[Select[resultMD[[2 ;;]], #[[5]] > ksb1 &], #[[5]] ≤ ksb2 &];
```

```
Length[resultMDKsMid]

TestSDNumOptKs = Thread[{TestSDNumOpt[[All, 1]], TestSDNumOpt[[All, 2]], TestSDNumOpt[[All, 3]], TestSDNumOpt[[All, 4]], resultMD[[All, 5]]}];
TestSDNumOptKsMid = Select[Select[TestSDNumOptKs[[2 ;;]], #[[5]] > ksb1 &], #[[5]] ≤ ksb2 &][[All, 1 ;; 4]];

TestMDNumOptKs = Thread[{TestMDNumOpt[[All, 1]], TestMDNumOpt[[All, 2]], TestMDNumOpt[[All, 3]], TestMDNumOpt[[All, 4]], resultMD[[All, 5]]}];
TestMDNumOptKsHigh = Select[TestMDNumOptKs[[2 ;;]], #[[5]] > ksb2 &][[All, 1 ;; 4]];
resultMDKsHigh = Select[resultMD[[2 ;;]], #[[5]] > ksb2 &];
Length[resultMDKsHigh]

TestSDNumOptKs = Thread[{TestSDNumOpt[[All, 1]], TestSDNumOpt[[All, 2]], TestSDNumOpt[[All, 3]], TestSDNumOpt[[All, 4]], resultMD[[All, 5]]}];
TestSDNumOptKsHigh = Select[TestSDNumOptKs[[2 ;;]], #[[5]] > ksb2 &][[All, 1 ;; 4]];

TestMDNumOptSMKsLow = SurvivalModelFit[TestMDNumOptKsLow[[2 ;; Length[TestMDNumOptKsLow], 2]]];
TestSDNumOptSMKsLow = SurvivalModelFit[TestSDNumOptKsLow[[2 ;; Length[TestSDNumOptKsLow], 2]]];
TestMDNumOptSMKsMid = SurvivalModelFit[TestMDNumOptKsMid[[2 ;; Length[TestMDNumOptKsMid], 2]]];
TestSDNumOptSMKsMid = SurvivalModelFit[TestSDNumOptKsMid[[2 ;; Length[TestSDNumOptKsMid], 2]]];
TestMDNumOptSMKsHigh = SurvivalModelFit[TestMDNumOptKsHigh[[2 ;; Length[TestMDNumOptKsHigh], 2]]];
TestSDNumOptSMKsHigh = SurvivalModelFit[TestSDNumOptKsHigh[[2 ;; Length[TestSDNumOptKsHigh], 2]]];
```

Out[∘]=
339

Out[∘]=
322

Out[∘]=
339

(* Figure S.30: Dependence of one−year overall survival on the ratio of first dose to initial cancer binding capacity *)

In[∘]:=
(* The outcomes of universal schedules with varied X, k, T for the training set are saved here in a closed cell *)

In[∘]:=
```
(* results of capacity−based schedules for Low ks *)
OutcomeKsLow = {{"X", "T", "kM", "OS", "AUC", "ToxDeath", "ToxAvg"},
```

```
{2.6`, 28.`, 0.66`, 0.6047197640117987`, 0.6855539371519964`, 0.`, 81.64430771980723`},
{2.5`, 19.`, 0.04`, 0.6106194690265481`, 0.6900576693241259`, 0.`, 66.7817777217914`},
{2.8000000000000003`, 11.`, 1.49`, 0.601769911504424`, 0.6743100441728646`, 0.`, 87.02150977117006`},
{0.7000000000000001`, 5.`, 0.59`, 0.6076696165191734`, 0.6706474247364671`, 0.`, 66.9289258360851`},
{2.3000000000000003`, 28.`, 0.12`, 0.6194690265486721`, 0.6931478589765028`, 0.`, 68.81924829370159`},
{2.9000000000000004`, 25.`, 1.45`, 0.5988200589970495`, 0.6773390198048426`, 0.0029498525073746312`, 86.1404686359212`},
{2.1`, 23.`, 0.15`, 0.6194690265486721`, 0.6996490136245975`, 0.`, 68.84487435530056`},
{1.5`, 17.`, 0.92`, 0.6047197640117987`, 0.6842161014021801`, 0.`, 80.4417209100409`},
{1.3`, 13.`, 0.89`, 0.6106194690265481`, 0.6853472644829826`, 0.`, 80.3734711201532`},
{1.3`, 39.`, 0.61`, 0.6253687315634214`, 0.6922288643133061`, 0.`, 71.88009378436941`},
{2.7`, 16.`, 1.28`, 0.5958702064896748`, 0.6730173354770242`, 0.`, 85.78297980301078`},
{1.2000000000000002`, 6.`, 0.19`, 0.6165191740412974`, 0.6882395389213106`, 0.`, 64.09223104557606`},
{2.6`, 37.`, 0.56`, 0.6076696165191734`, 0.6809275737198868`, 0.`, 79.02378523933648`},
{2.3000000000000003`, 6.`, 0.39`, 0.6047197640117987`, 0.6781209119844278`, 0.`, 80.21309282800064`},
{2.2`, 39.`, 0.3`, 0.6135693215339227`, 0.6855713832161491`, 0.`, 73.4305609243804`},
{2.5`, 24.`, 0.34`, 0.6047197640117987`, 0.6869433675465921`, 0.`, 77.4429141136851`},
{1.6`, 3.`, 1.47`, 0.601769911504424`, 0.6727518891912914`, 0.`, 87.45230939583577`},
{0.7000000000000001`, 15.`, 1.2`, 0.5899705014749256`, 0.6572234634459622`, 0.`, 71.85063390617634`},
{2.6`, 39.`, 0.77`, 0.6076696165191734`, 0.6795357154595792`, 0.`, 80.91996492350574`},
{0.7000000000000001`, 8.`, 1.3800000000000001`, 0.5929203539823001`, 0.6580278278596037`, 0.0029498525073746312`,
  72.18314207567198`}, {0.8`, 37.`, 1.2`, 0.5870206489675509`, 0.6514509246390197`, 0.`, 71.06608131559796`},
{2.4000000000000004`, 18.`, 1.45`, 0.5958702064896748`, 0.6738356942726652`, 0.`, 85.17517669222912`},
{2.`, 35.`, 0.5700000000000001`, 0.6165191740412974`, 0.6891265067463661`, 0.`, 76.4489695400791`},
{2.7`, 23.`, 1.36`, 0.601769911504424`, 0.6794528739007502`, 0.`, 86.01622471989329`},
{1.`, 7.`, 0.09`, 0.6165191740412974`, 0.6865797017750512`, 0.`, 45.210320365512274`},
{1.5`, 13.`, 0.48`, 0.6076696165191734`, 0.6894678560455006`, 0.`, 76.38212512604441`},
{0.7000000000000001`, 10.`, 1.3800000000000001`, 0.5870206489675509`, 0.6533256028249791`, 0.0029498525073746312`,
  72.10759594070755`}, {1.`, 13.`, 1.32`, 0.5988200589970495`, 0.67145247719354`, 0.`, 79.7156134760456`},
{0.7000000000000001`, 20.`, 0.5700000000000001`, 0.5870206489675509`, 0.656198257828072`, 0.`, 65.56692287951314`},
{1.6`, 16.`, 1.1500000000000001`, 0.5988200589970495`, 0.6787291701519889`, 0.0029498525073746312`, 82.78318398434341`},
{1.3`, 10.`, 1.31`, 0.6076696165191734`, 0.6799532761080546`, 0.0029498525073746312`, 82.00350802660445`},
```

```
{1.5`, 38.`, 0.36`, 0.6253687315634214`, 0.6950385154116235`, 0.`, 68.89118299348199`},
{1.5`, 40.`, 0.76`, 0.6106194690265481`, 0.6864575410569631`, 0.`, 75.10707187700287`},
{0.9`, 33.`, 1.05`, 0.5811209439528016`, 0.655482241303204`, 0.`, 72.85577706269471`},
{2.8000000000000003`, 36.`, 0.52`, 0.6076696165191734`, 0.6802683662691915`, 0.`, 80.25342727868959`},
{2.`, 21.`, 0.78`, 0.5988200589970495`, 0.6829974599280243`, 0.`, 80.95675156524842`},
{2.`, 7.`, 1.1500000000000001`, 0.601769911504424`, 0.6750554575252692`, 0.`, 85.28390206759263`},
{1.2000000000000002`, 34.`, 1.12`, 0.601769911504424`, 0.6764910948816891`, 0.0029498525073746312`, 75.81955072740989`},
{1.9000000000000001`, 37.`, 0.53`, 0.6194690265486721`, 0.689544659224207`, 0.`, 75.91557449320231`},
{2.1`, 30.`, 0.8`, 0.601769911504424`, 0.6854555272460138`, 0.`, 80.67791989740132`},
{0.8`, 23.`, 0.76`, 0.5870206489675509`, 0.6591722305768659`, 0.0029498525073746312`, 70.71281062329597`},
{3.`, 31.`, 0.56`, 0.6076696165191734`, 0.6849351809281624`, 0.`, 81.95866770003808`},
{2.6`, 7.`, 0.8`, 0.601769911504424`, 0.6747194646664078`, 0.`, 85.17438835821784`},
{1.8`, 19.`, 0.01`, 0.6076696165191734`, 0.6785472917218498`, 0.`, 49.76233077719453`},
{2.4000000000000004`, 39.`, 0.37`, 0.6165191740412974`, 0.6865782718174138`, 0.`, 63.606801127571316`},
{0.7000000000000001`, 24.`, 1.04`, 0.5840707964601762`, 0.6516679791096619`, 0.`, 69.75261038471565`},
{1.7000000000000002`, 4.`, 0.92`, 0.601769911504424`, 0.6745186849750544`, 0.`, 85.09170669582716`},
{2.3000000000000003`, 12.`, 1.12`, 0.601769911504424`, 0.6768643799855233`, 0.`, 85.29546051833407`},
{2.5`, 18.`, 1.23`, 0.5988200589970495`, 0.6773166894254035`, 0.`, 85.88780494183678`},
{2.8000000000000003`, 39.`, 0.19`, 0.6194690265486721`, 0.6854060490238887`, 0.`, 75.01006776745241`},
{2.1`, 4.`, 0.77`, 0.5988200589970495`, 0.6719609385704118`, 0.`, 85.05667549301495`},
{2.6`, 14.`, 0.64`, 0.5988200589970495`, 0.6782372165674511`, 0.`, 83.45527988974243`},
{2.5`, 18.`, 0.18`, 0.6076696165191734`, 0.6885963879602878`, 0.`, 74.02739821777179`},
{0.9`, 20.`, 0.12`, 0.5958702064896748`, 0.6739091863921776`, 0.`, -137.8740198243919`},
{3.`, 21.`, 0.02`, 0.6076696165191734`, 0.6766699880285983`, 0.`, 70.78519263614835`},
{1.5`, 15.`, 1.3`, 0.6076696165191734`, 0.6827635410952039`, 0.`, 82.0991419379636`},
{1.`, 28.`, 0.25`, 0.6194690265486721`, 0.6927072598231706`, 0.`, 62.550877088208075`},
{3.`, 33.`, 0.5`, 0.6106194690265481`, 0.6885574952880841`, 0.`, 80.95925798961979`},
{1.9000000000000001`, 36.`, 0.67`, 0.6106194690265481`, 0.6856711493505079`, 0.`, 77.39220198382802`},
{1.1`, 38.`, 0.75`, 0.6106194690265481`, 0.679843839430644`, 0.`, 71.51697166722015`},
{1.1`, 21.`, 0.54`, 0.5958702064896748`, 0.6802645011041565`, 0.0029498525073746312`, 72.94232277286683`},
{1.`, 33.`, 0.63`, 0.6047197640117987`, 0.6757990152291855`, 0.`, 70.94154864789469`},
```

```
{2.6`, 5.`, 0.3`, 0.601769911504424`, 0.674506488984589`, 0.`, 79.66784409982118`},
{2.4000000000000004`, 34.`, 0.29`, 0.6165191740412974`, 0.6868250110115038`, 0.`, -892.6148694504403`},
{1.2000000000000002`, 8.`, 0.8200000000000001`, 0.6135693215339227`, 0.6850452613080914`, 0.`, 80.40670106114153`},
{1.6`, 21.`, 1.07`, 0.601769911504424`, 0.6831215933195339`, 0.`, 81.07341259574055`},
{2.8000000000000003`, 26.`, 0.3`, 0.6076696165191734`, 0.6860984027023413`, 0.`, 78.26855432167483`},
{2.2`, 40.`, 0.17`, 0.6165191740412974`, 0.6830269527852653`, 0.`, 69.27927529981642`},
{2.1`, 38.`, 0.9`, 0.601769911504424`, 0.6793921559728215`, 0.`, 79.7727191054227`},
{2.7`, 18.`, 0.68`, 0.5988200589970495`, 0.6794036568907178`, 0.`, 83.4445065205006`},
{2.3000000000000003`, 40.`, 0.55`, 0.6076696165191734`, 0.6791637826124257`, 0.`, 77.44927848651781`},
{1.6`, 38.`, 0.91`, 0.601769911504424`, 0.6799681175265767`, 0.0029498525073746312`, 76.43043072684276`},
{2.9000000000000004`, 14.`, 0.68`, 0.5988200589970495`, 0.6765272119886055`, 0.`, 84.48849704041729`},
{2.1`, 3.`, 0.35000000000000003`, 0.5988200589970495`, 0.6712473471188161`, 0.`, 78.98997480518662`},
{2.5`, 36.`, 0.65`, 0.6047197640117987`, 0.678257026382896`, 0.0029498525073746312`, 3.7529359055399354`*^17},
{1.1`, 22.`, 1.27`, 0.5958702064896748`, 0.6739715835796077`, 0.`, 78.63311947787155`},
{2.2`, 19.`, 0.24`, 0.6076696165191734`, 0.6892430782586598`, 0.`, 73.3506351560342`},
{2.`, 13.`, 1.18`, 0.6047197640117987`, 0.6800033487579152`, 0.`, 84.81670357331373`},
{3.`, 11.`, 0.3`, 0.601769911504424`, 0.6793864688612385`, 0.`, 80.37284775640624`},
{2.4000000000000004`, 31.`, 0.79`, 0.601769911504424`, 0.6853380926352161`, 0.`, 81.56524048772425`},
{2.`, 16.`, 1.12`, 0.5988200589970495`, 0.6783027542593758`, 0.`, 84.21740415675966`},
{1.4000000000000001`, 8.`, 1.36`, 0.6076696165191734`, 0.6800042227097332`, 0.`, 83.79441794843159`},
{0.7000000000000001`, 38.`, 1.09`, 0.5870206489675509`, 0.6438203131455459`, 0.`, 67.12272733270291`},
{0.7000000000000001`, 11.`, 0.16`, 0.5870206489675509`, 0.6548915621196423`, 0.`, 46.935434226292436`},
{1.2000000000000002`, 27.`, 0.62`, 0.6106194690265481`, 0.6912557374214333`, 0.`, 73.28235928060369`},
{1.6`, 29.`, 0.31`, 0.628318584070796`, 0.7077725932335246`, 0.`, 70.18780754963784`},
{2.5`, 26.`, 1.41`, 0.601769911504424`, 0.6801863316695385`, 0.`, 84.44717588620156`},
{2.6`, 10.`, 1.01`, 0.6047197640117987`, 0.6783589810630314`, 0.`, 85.72009424262431`},
{1.1`, 27.`, 0.41000000000000003`, 0.6194690265486721`, 0.6960017899681036`, 0.`, 68.98457973615749`},
{0.7000000000000001`, 25.`, 1.16`, 0.5840707964601762`, 0.6512181349015973`, 0.`, 70.21143423365848`},
{1.7000000000000002`, 40.`, 0.04`, 0.6135693215339227`, 0.679662512485261`, 0.`, 51.746556222284795`},
{2.2`, 17.`, 0.15`, 0.6106194690265481`, 0.692217055102666`, 0.`, 70.37134616314219`},
{1.2000000000000002`, 5.`, 0.59`, 0.6076696165191734`, 0.6807520234702675`, 0.`, 78.63970673486156`},
```

```
{1.9000000000000001`, 27.`, 1.09`, 0.5988200589970495`, 0.6810253428991638`, 0.`, 81.14852775599839`},
{1.7000000000000002`, 25.`, 0.84`, 0.5988200589970495`, 0.683872280960527`, 0.0029498525073746312`, 79.13488148980471`},
{2.6`, 28.`, 1.21`, 0.601769911504424`, 0.680444847500521`, 0.`, 84.50122201343707`},
{2.3000000000000003`, 30.`, 1.29`, 0.5988200589970495`, 0.6787979950794908`, 0.`, 82.96373101619902`},
{1.4000000000000001`, 31.`, 0.08`, 0.6312684365781706`, 0.6956456552749541`, 0.`, 50.94318004679779`},
{2.8000000000000003`, 31.`, 1.28`, 0.601769911504424`, 0.6799565060296751`, 0.`, 84.66624461124795`},
{2.6`, 29.`, 0.37`, 0.6106194690265481`, 0.6894769783482836`, 0.`, 78.57825253019485`},
{3.`, 4.`, 0.47000000000000003`, 0.5988200589970495`, 0.6710416591978715`, 0.`, 83.91851173057769`},
{2.5`, 7.`, 0.14`, 0.6047197640117987`, 0.6804092198003932`, 0.`, 73.35377955520623`},
{2.4000000000000004`, 34.`, 0.93`, 0.6047197640117987`, 0.6809859290258998`, 0.`, 81.78983757765315`},
{1.7000000000000002`, 18.`, 1.48`, 0.601769911504424`, 0.6792376439931711`, 0.`, 83.03513255278543`},
{1.4000000000000001`, 32.`, 0.73`, 0.6076696165191734`, 0.6934301033452538`, 0.`, 75.54383949103273`},
{2.3000000000000003`, 14.`, 0.47000000000000003`, 0.5988200589970495`, 0.682574303379694`, 0.`, 80.71983255318978`},
{2.9000000000000004`, 22.`, 0.67`, 0.601769911504424`, 0.6813988611424417`, 0.`, 83.34914023649904`},
{1.2000000000000002`, 5.`, 1.3800000000000001`, 0.601769911504424`, 0.6744776647658186`, 0.0029498525073746312`,
  84.47511461333978`}, {2.7`, 20.`, 0.43`, 0.5988200589970495`, 0.6827431908330408`, 0.`, 80.67177283414878`},
{2.1`, 22.`, 0.87`, 0.5988200589970495`, 0.6820827278429794`, 0.`, 82.35131590746865`},
{2.7`, 10.`, 1.43`, 0.601769911504424`, 0.6739609746953509`, 0.`, 86.6585356754637`},
{1.8`, 16.`, 1.1`, 0.601769911504424`, 0.6816919141287832`, 0.`, 83.7309765616358`},
{0.9`, 24.`, 1.35`, 0.5870206489675509`, 0.6625744383127681`, 0.0029498525073746312`, -3715.6309627690243`},
{1.4000000000000001`, 17.`, 0.02`, 0.6194690265486721`, 0.6932571610677001`, 0.`, 38.12519072285082`},
{2.`, 16.`, 0.7000000000000001`, 0.5988200589970495`, 0.6814562317684006`, 0.`, 81.51850481954386`},
{2.1`, 4.`, 0.08`, 0.601769911504424`, 0.674769950933775`, 0.`, 66.05101588191283`},
{1.7000000000000002`, 30.`, 1.08`, 0.601769911504424`, 0.6849240076482694`, 0.`, 80.00664405739307`},
{0.9`, 31.`, 0.42`, 0.5988200589970495`, 0.6724862733038228`, 0.`, 66.88080771980306`},
{2.2`, 34.`, 0.9400000000000001`, 0.6047197640117987`, 0.6819105993465227`, 0.`, 80.58623067061008`},
{2.9000000000000004`, 4.`, 0.45`, 0.5988200589970495`, 0.6710952891560281`, 0.`, 83.43702217394976`},
{2.2`, 35.`, 1.01`, 0.6076696165191734`, 0.6831126484664536`, 0.`, 81.09487715108756`},
{2.7`, 17.`, 0.98`, 0.5988200589970495`, 0.6780908841876695`, 0.`, 85.72904105460988`},
{2.`, 9.`, 0.67`, 0.6047197640117987`, 0.679993134668827`, 0.`, 82.55788779296638`},
{2.3000000000000003`, 5.`, 0.01`, 0.5929203539823001`, 0.6665575239211246`, 0.`, 61.20317178171784`},
```

```
{1.4000000000000001`, 15.`, 1.28`, 0.601769911504424`, 0.67892776803987`, 0.0029498525073746312`, 81.84096707194342`},
{1.6`, 32.`, 1.04`, 0.601769911504424`, 0.683563580960986`, 0.`, 79.24878779370195`},
{1.1`, 6.`, 0.32`, 0.6165191740412974`, 0.6864749655022466`, 0.`, 70.74084844490208`},
{1.1`, 33.`, 0.04`, 0.6194690265486721`, 0.6789196521356092`, 0.0029498525073746312`, 1.2754556582406343`*^21},
{1.`, 18.`, 1.06`, 0.5929203539823001`, 0.6718896137181347`, 0.`, 78.32581891997583`},
{1.2000000000000002`, 12.`, 0.63`, 0.6106194690265481`, 0.6880727736060902`, 0.`, 77.164636591293`},
{2.4000000000000004`, 4.`, 0.71`, 0.5988200589970495`, 0.6716133920482079`, 0.`, 85.25703089294475`},
{1.4000000000000001`, 8.`, 0.36`, 0.6135693215339227`, 0.68866729285152`, 0.`, 73.85620887602485`},
{0.8`, 13.`, 1.29`, 0.5899705014749256`, 0.6604752562284176`, 0.0029498525073746312`, 75.70417476492696`},
{1.9000000000000001`, 17.`, 1.09`, 0.601769911504424`, 0.6820787765138304`, 0.`, 83.7851480535014`},
{1.7000000000000002`, 14.`, 0.77`, 0.6047197640117987`, 0.6849172841526806`, 0.`, 81.6032484460229`},
{3.`, 28.`, 0.87`, 0.601769911504424`, 0.6802517562330704`, 0.`, 83.88454013473097`},
{2.3000000000000003`, 40.`, 0.55`, 0.6076696165191734`, 0.6791637826124257`, 0.`, 77.44927848651781`},
{1.6`, 38.`, 0.91`, 0.601769911504424`, 0.6799681175265767`, 0.0029498525073746312`, 76.43043072684276`},
{2.9000000000000004`, 14.`, 0.68`, 0.5988200589970495`, 0.6765272119886055`, 0.`, 84.48849704041729`},
{2.1`, 3.`, 0.35000000000000003`, 0.5988200589970495`, 0.6712473471188161`, 0.`, 78.98997480518662`},
{2.5`, 36.`, 0.65`, 0.6047197640117987`, 0.678257026382896`, 0.0029498525073746312`, 3.7529359055399354`*^17},
{1.1`, 22.`, 1.27`, 0.5958702064896748`, 0.6739715835796077`, 0.`, 78.63311947787155`},
{2.2`, 19.`, 0.24`, 0.6076696165191734`, 0.6892430782586598`, 0.`, 73.3506351560342`},
{2.`, 13.`, 1.18`, 0.6047197640117987`, 0.6800033487579152`, 0.`, 84.81670357331373`},
{3.`, 11.`, 0.3`, 0.601769911504424`, 0.6793864688612385`, 0.`, 80.37284775640624`},
{2.4000000000000004`, 31.`, 0.79`, 0.601769911504424`, 0.6853380926352161`, 0.`, 81.56524048772425`},
{2.`, 16.`, 1.12`, 0.5988200589970495`, 0.6783027542593758`, 0.`, 84.21740415675966`},
{1.4000000000000001`, 8.`, 1.36`, 0.6076696165191734`, 0.6800042227097332`, 0.`, 83.79441794843159`},
{0.7000000000000001`, 38.`, 1.09`, 0.5870206489675509`, 0.6438203131455459`, 0.`, 67.12272733270291`},
{0.7000000000000001`, 11.`, 0.16`, 0.5870206489675509`, 0.6548915621196423`, 0.`, 46.935434226292436`},
{1.2000000000000002`, 27.`, 0.62`, 0.6106194690265481`, 0.6912557374214333`, 0.`, 73.28235928060369`},
{1.6`, 29.`, 0.31`, 0.628318584070796`, 0.7077725932335246`, 0.`, 70.18780754963784`},
{2.5`, 26.`, 1.41`, 0.601769911504424`, 0.6801863316695385`, 0.`, 84.44717588620156`},
{2.6`, 10.`, 1.01`, 0.6047197640117987`, 0.6783589810630314`, 0.`, 85.72009424262431`},
{1.1`, 27.`, 0.41000000000000003`, 0.6194690265486721`, 0.6960017899681036`, 0.`, 68.98457973615749`},
```

{0.7000000000000001`, 25.`, 1.16`, 0.5840707964601762`, 0.6512181349015973`, 0.`, 70.21143423365848`},
{1.7000000000000002`, 40.`, 0.04`, 0.6135693215339227`, 0.679662512485261`, 0.`, 51.746556222284795`},
{2.2`, 17.`, 0.15`, 0.6106194690265481`, 0.692217055102666`, 0.`, 70.37134616314219`},
{1.2000000000000002`, 5.`, 0.59`, 0.6076696165191734`, 0.6807520234702675`, 0.`, 78.63970673486156`},
{1.9000000000000001`, 27.`, 1.09`, 0.5988200589970495`, 0.6810253428991638`, 0.`, 81.14852775599839`},
{1.7000000000000002`, 25.`, 0.84`, 0.5988200589970495`, 0.683872280960527`, 0.0029498525073746312`, 79.13488148980471`},
{2.6`, 28.`, 1.21`, 0.601769911504424`, 0.680444847500521`, 0.`, 84.50122201343707`},
{2.3000000000000003`, 30.`, 1.29`, 0.5988200589970495`, 0.6787979950794908`, 0.`, 82.96373101619902`},
{1.4000000000000001`, 31.`, 0.08`, 0.6312684365781706`, 0.6956456552749541`, 0.`, 50.94318004679779`},
{2.8000000000000003`, 31.`, 1.28`, 0.601769911504424`, 0.6799565060296751`, 0.`, 84.66624461124795`},
{2.6`, 29.`, 0.37`, 0.6106194690265481`, 0.6894769783482836`, 0.`, 78.57825253019485`},
{3.`, 4.`, 0.47000000000000003`, 0.5988200589970495`, 0.6710416591978715`, 0.`, 83.91851173057769`},
{2.5`, 7.`, 0.14`, 0.6047197640117987`, 0.6804092198003932`, 0.`, 73.35377955520623`},
{2.4000000000000004`, 34.`, 0.93`, 0.6047197640117987`, 0.6809859290258998`, 0.`, 81.78983757765315`},
{1.7000000000000002`, 18.`, 1.48`, 0.601769911504424`, 0.6792376439931711`, 0.`, 83.03513255278543`},
{1.4000000000000001`, 32.`, 0.73`, 0.6076696165191734`, 0.6934301033452538`, 0.`, 75.54383949103273`},
{2.3000000000000003`, 14.`, 0.47000000000000003`, 0.5988200589970495`, 0.682574303379694`, 0.`, 80.71983255318978`},
{2.9000000000000004`, 22.`, 0.67`, 0.601769911504424`, 0.6813988611424417`, 0.`, 83.34914023649904`},
{1.2000000000000002`, 5.`, 1.3800000000000001`, 0.601769911504424`, 0.6744776647658186`, 0.0029498525073746312`,
84.47511461333978`}, {2.7`, 20.`, 0.43`, 0.5988200589970495`, 0.6827431908330408`, 0.`, 80.67177283414878`},
{2.1`, 22.`, 0.87`, 0.5988200589970495`, 0.6820827278429794`, 0.`, 82.35131590746865`},
{2.7`, 10.`, 1.43`, 0.601769911504424`, 0.6739609746953509`, 0.`, 86.6585356754637`},
{1.8`, 16.`, 1.1`, 0.601769911504424`, 0.6816919141287832`, 0.`, 83.7309765616358`},
{0.9`, 24.`, 1.35`, 0.5870206489675509`, 0.6625744383127681`, 0.0029498525073746312`, -3715.6309627690243`},
{1.4000000000000001`, 17.`, 0.02`, 0.6194690265486721`, 0.6932571610677001`, 0.`, 38.12519072285082`},
{2.`, 16.`, 0.7000000000000001`, 0.5988200589970495`, 0.6814562317684006`, 0.`, 81.51850481954386`},
{2.1`, 4.`, 0.08`, 0.601769911504424`, 0.674769950933775`, 0.`, 66.05101588191283`},
{1.7000000000000002`, 30.`, 1.08`, 0.601769911504424`, 0.6849240076482694`, 0.`, 80.00664405739307`},
{0.9`, 31.`, 0.42`, 0.5988200589970495`, 0.6724862733038228`, 0.`, 66.88080771980306`},
{2.2`, 34.`, 0.9400000000000001`, 0.6047197640117987`, 0.6819105993465227`, 0.`, 80.58623067061008`},
{2.9000000000000004`, 4.`, 0.45`, 0.5988200589970495`, 0.6710952891560281`, 0.`, 83.43702217394976`},

```
{2.2`, 35.`, 1.01`, 0.6076696165191734`, 0.6831126484664536`, 0.`, 81.09487715108756`},
{2.7`, 17.`, 0.98`, 0.5988200589970495`, 0.6780908841876695`, 0.`, 85.72904105460988`},
{2.`, 9.`, 0.67`, 0.6047197640117987`, 0.679993134668827`, 0.`, 82.5578879296638`},
{2.3000000000000003`, 5.`, 0.01`, 0.5929203539823001`, 0.6665575239211246`, 0.`, 61.20317178171784`},
{1.4000000000000001`, 15.`, 1.28`, 0.601769911504424`, 0.67892776803987`, 0.0029498525073746312`, 81.84096707194342`},
{1.6`, 32.`, 1.04`, 0.601769911504424`, 0.683563580960986`, 0.`, 79.24878779370195`},
{1.1`, 6.`, 0.32`, 0.6165191740412974`, 0.6864749655022466`, 0.`, 70.74084844490208`},
{1.1`, 33.`, 0.04`, 0.6194690265486721`, 0.6789196521356092`, 0.0029498525073746312`, 1.2754556582406343`*^21},
{1.`, 18.`, 1.06`, 0.5929203539823001`, 0.6718896137181347`, 0.`, 78.32581891997583`},
{1.2000000000000002`, 12.`, 0.63`, 0.6106194690265481`, 0.6880727736060902`, 0.`, 77.164636591293`},
{2.4000000000000004`, 4.`, 0.71`, 0.5988200589970495`, 0.6716133920482079`, 0.`, 85.25703089294475`},
{1.4000000000000001`, 8.`, 0.36`, 0.6135693215339227`, 0.68866729285152`, 0.`, 73.85620887602485`},
{0.8`, 13.`, 1.29`, 0.5899705014749256`, 0.6604752562284176`, 0.0029498525073746312`, 75.70417476492696`},
{1.9000000000000001`, 17.`, 1.09`, 0.601769911504424`, 0.6820787765138304`, 0.`, 83.7851480535014`}};
```

In[ ]:=

```
(* results of capacity-based schedules for Mid ks *)
OutcomeKsMid = {{"X", "T", "kM", "OS", "AUC", "ToxDeath", "ToxAvg"}, {2.5`, 23.`, 0.87`, 0.6708074534161501`, 0.7366590516095083`,
    0.`, 83.13836680971666`}, {0.8`, 22.`, 0.59`, 0.6739130434782619`, 0.7115163836942003`, 0.`, 69.7261341694818`},
   {1.`, 34.`, 0.9400000000000001`, 0.6521739130434793`, 0.7029533126964742`, 0.006211180124223602`, 73.64939763599446`},
   {2.8000000000000003`, 37.`, 0.9400000000000001`, 0.6645962732919265`,
    0.7257783100352794`, 0.003105590062111801`, 83.22436549088543`},
   {2.4000000000000004`, 25.`, 0.51`, 0.6863354037267089`, 0.7417839779331444`, 0.003105590062111801`, 80.50403529023484`},
   {1.7000000000000002`, 4.`, 0.86`, 0.6708074534161501`, 0.7296341662310092`, 0.`, 83.63446732913359`},
   {2.3000000000000003`, 24.`, 1.12`, 0.6708074534161501`, 0.7341114684851374`, 0.006211180124223602`, 83.71685616312249`},
   {1.3`, 30.`, 0.47000000000000003`, 0.7049689440993798`, 0.7459409513809202`, 0.`, 72.19237640244097`},
   {0.9`, 10.`, 0.72`, 0.6956521739130442`, 0.7394659639582503`, 0.`, 76.26917426764999`},
   {2.7`, 4.`, 0.72`, 0.658385093167703`, 0.7210053990324662`, 0.`, 85.02188779031124`},
   {1.7000000000000002`, 24.`, 0.38`, 0.7049689440993798`, 0.754921072266055`, 0.`, 74.863423870843`},
   {2.7`, 38.`, 1.01`, 0.6677018633540382`, 0.7271573571883586`, 0.`, 82.94042972499244`},
   {2.`, 36.`, 0.41000000000000003`, 0.6925465838509325`, 0.7419190257944998`, 0.`, 76.47470194459528`},
   {2.3000000000000003`, 38.`, 0.97`, 0.6770186335403736`, 0.7343789752504442`, 0.`, 81.545598995081`},
```

```
{1.4000000000000001`, 19.`, 0.03`, 0.701863354037268`, 0.755892809414532`, 0.`, 39.757880779995766`},
{3.`, 14.`, 1.11`, 0.6614906832298147`, 0.7284031397289761`, 0.`, 85.94851439700248`},
{1.7000000000000002`, 20.`, 0.25`, 0.7049689440993798`, 0.7572571363259291`, 0.`, 70.33420182349865`},
{0.9`, 36.`, 0.9400000000000001`, 0.6335403726708084`, 0.6894119475433934`, 0.015527950310559006`, 74.03709283442689`},
{1.3`, 34.`, 0.98`, 0.6956521739130442`, 0.7392935083515881`, 0.006211180124223602`, 76.8459753230947`},
{2.`, 17.`, 1.24`, 0.6708074534161501`, 0.7373923429455947`, 0.003105590062111801`, 84.56896089324853`},
{2.`, 19.`, 1.4000000000000001`, 0.6739130434782619`, 0.7395636417214626`, 0.`, 84.31044016066704`},
{1.6`, 10.`, 1.1`, 0.6801242236024854`, 0.740831984107058`, 0.003105590062111801`, 83.79884995888443`},
{2.9000000000000004`, 14.`, 0.92`, 0.6614906832298147`, 0.7282661378167878`, 0.003105590062111801`, 85.47760233414316`},
{1.1`, 35.`, 0.33`, 0.6925465838509325`, 0.7295941674143405`, 0.`, -6.163528757947376`*^18},
{2.3000000000000003`, 34.`, 0.17`, 0.6863354037267089`, 0.7369531386352579`, 0.`, 70.65407201858179`},
{1.8`, 23.`, 0.52`, 0.6925465838509325`, 0.7527631726112407`, 0.003105590062111801`, 78.65679758572426`},
{2.8000000000000003`, 39.`, 0.56`, 0.6770186335403736`, 0.7325710472715474`, 0.`, 81.35530701993719`},
{2.2`, 22.`, 1.23`, 0.6739130434782619`, 0.7392611067197288`, 0.`, 83.81034104781206`},
{2.2`, 9.`, 1.32`, 0.6739130434782619`, 0.7338111293595138`, 0.`, 86.01916634659722`},
{1.6`, 36.`, 0.98`, 0.6863354037267089`, 0.7394476139891176`, 0.009316770186335404`, 79.85720641820866`},
{1.5`, 39.`, 0.08`, 0.6956521739130442`, 0.7414094902842916`, 0.`, 52.36887866714998`},
{1.4000000000000001`, 19.`, 0.44`, 0.7080745341614917`, 0.7585336987676625`, 0.`, 74.84294153260875`},
{2.2`, 27.`, 0.72`, 0.6832298136645971`, 0.7425698114627801`, 0.003105590062111801`, 81.59415375126704`},
{1.1`, 35.`, 1.46`, 0.6739130434782619`, 0.720125665949517`, 0.006211180124223602`, 76.45272271022557`},
{3.`, 21.`, 1.34`, 0.6614906832298147`, 0.729962930950077`, 0.003105590062111801`, 86.12149757863729`},
{2.5`, 34.`, 1.46`, 0.6677018633540382`, 0.7257431488714465`, 0.003105590062111801`, 83.55966327606204`},
{2.8000000000000003`, 9.`, 0.19`, 0.6739130434782619`, 0.7356136898876922`, 0.`, 76.34117441933985`},
{1.9000000000000001`, 27.`, 0.11`, 0.6894409937888206`, 0.7456156872089871`, 0.`, 63.42344918192101`},
{1.5`, 29.`, 0.36`, 0.701863354037268`, 0.7517215226405012`, 0.`, 72.39019386442382`},
{1.9000000000000001`, 35.`, 0.6`, 0.6925465838509325`, 0.7433814846190782`, 0.`, 78.64569955731554`},
{1.4000000000000001`, 13.`, 0.54`, 0.7049689440993798`, 0.7585303967413229`, 0.`, 78.1782039114437`},
{1.6`, 26.`, 0.36`, 0.701863354037268`, 0.7532814753941636`, 0.`, 73.1599960378128`},
{0.7000000000000001`, 11.`, 1.3800000000000001`, 0.6677018633540382`, 0.7114617493958716`, 0.003105590062111801`,
  71.26206259656445`}, {1.5`, 6.`, 0.19`, 0.6925465838509325`, 0.7473578744323247`, 0.`, 66.67698862282737`},
{1.1`, 5.`, 0.07`, 0.6987577639751561`, 0.7414783937159423`, 0.`, 40.261975577672814`},
```

{1.3`, 32.`, 0.99`, 0.6925465838509325`, 0.7388250251645404`, 0.006211180124223602`, 77.05863793604556`},
{1.5`, 14.`, 0.28`, 0.701863354037268`, 0.7579322971430246`, 0.`, 71.01057178590403`},
{2.1`, 28.`, 1.28`, 0.6770186335403736`, 0.7405955158974774`, 0.`, 83.09688819568531`},
{1.9000000000000001`, 27.`, 0.03`, 0.6832298136645971`, 0.7328543672051434`, 0.`, -1.116237846607126`*^17},
{1.5`, 31.`, 1.25`, 0.6894409937888206`, 0.7415327952477178`, 0.003105590062111801`, 79.96194957398322`},
{1.4000000000000001`, 6.`, 1.1400000000000001`, 0.6770186335403736`, 0.7342327333758455`, 0.003105590062111801`,
83.50711014787525`}, {0.9`, 35.`, 0.01`, 0.5714285714285722`, 0.6280078364888632`, 0.`, -35.73971828337178`},
{2.7`, 31.`, 0.78`, 0.6708074534161501`, 0.731176766012621`, 0.003105590062111801`, 82.5025867827026`},
{2.`, 4.`, 1.17`, 0.6614906832298147`, 0.7222666574350785`, 0.003105590062111801`, 86.16530373056105`},
{1.`, 7.`, 0.77`, 0.6894409937888206`, 0.7395638831356615`, 0.`, 79.3102354199048`},
{1.6`, 16.`, 0.37`, 0.701863354037268`, 0.7588256265808547`, 0.`, 74.92521410606892`},
{1.7000000000000002`, 31.`, 1.31`, 0.6770186335403736`, 0.734300714142005`, 0.009316770186335404`, 81.36547603281683`},
{0.7000000000000001`, 29.`, 1.11`, 0.6428571428571439`, 0.6859901256606679`, 0.`, 68.0131918111589`},
{2.1`, 20.`, 1.29`, 0.6739130434782619`, 0.7401756129849723`, 0.`, 84.11217578942718`},
{1.3`, 22.`, 0.77`, 0.7049689440993798`, 0.7554374633507686`, 0.`, 78.4185901280553`},
{2.7`, 39.`, 0.36`, 0.6770186335403736`, 0.7322365444494546`, 0.`, 78.60089608957351`},
{0.7000000000000001`, 9.`, 0.52`, 0.6894409937888206`, 0.727674719564280 9`, 0.`, 65.86953320003714`},
{2.8000000000000003`, 26.`, 1.4000000000000001`, 0.658385093167703`, 0.7278824078824997`, 0.006211180124223602`,
-6.08058834166655`*^33}, {1.8`, 32.`, 1.46`, 0.6801242236024854`, 0.7350927637604435`, 0.`, 80.99215732460573`},
{1.4000000000000001`, 39.`, 0.78`, 0.6925465838509325`, 0.7435995669085533`, 0.`, 77.0467192989504`},
{2.3000000000000003`, 28.`, 0.93`, 0.6801242236024854`, 0.7414072230406068`, 0.`, 82.49244371558298`},
{1.1`, 33.`, 0.5`, 0.6956521739130442`, 0.7338646302265969`, 0.`, 71.46026175705227`},
{2.2`, 31.`, 0.72`, 0.6832298136645971`, 0.7389599597404489`, 0.003105590062111801`, 80.91275636911807`},
{1.`, 12.`, 0.34`, 0.701863354037268`, 0.743839664099079`, 0.`, 68.35659916480485`},
{2.2`, 21.`, 1.09`, 0.6708074534161501`, 0.7369196569490869`, 0.003105590062111801`, 83.61694776327552`},
{2.5`, 40.`, 0.34`, 0.6832298136645971`, 0.7340087350816948`, 0.`, 77.3652847727262`},
{2.4000000000000004`, 27.`, 0.09`, 0.6801242236024854`, 0.7368561359023621`, 0.`, 67.96352676905498`},
{1.9000000000000001`, 34.`, 1.24`, 0.6801242236024854`, 0.7360209937243154`, 0.003105590062111801`, 81.30887975091224`},
{1.`, 28.`, 1.45`, 0.6490683229813675`, 0.7052698021644199`, 0.009316770186335404`, 75.66232316795087`},
{2.9000000000000004`, 11.`, 0.98`, 0.658385093167703`, 0.7260934728040349`, 0.`, 85.55795924932035`},
{1.5`, 36.`, 0.6900000000000001`, 0.6956521739130442`, 0.7465662824086391`, 0.`, 77.34597373722833`},

```
{0.9`, 39.`, 0.9500000000000001`, 0.6242236024844731`, 0.6823927251410017`, 0.012422360248447204`, 73.15427565459811`},
{2.5`, 30.`, 0.45`, 0.6832298136645971`, 0.7374105297544434`, 0.`, 79.53106393806326`},
{1.1`, 8.`, 0.3`, 0.7080745341614917`, 0.7534117099812871`, 0.`, 68.78779664561478`},
{2.5`, 16.`, 1.22`, 0.6677018633540382`, 0.7333622762349269`, 0.`, 85.24518236568777`},
{1.2000000000000002`, 8.`, 0.91`, 0.6863354037267089`, 0.7416479598626348`, 0.003105590062111801`, 80.82350491175443`},
{2.8000000000000003`, 12.`, 0.64`, 0.6708074534161501`, 0.7351639328022207`, 0.`, 83.951018300017`},
{2.1`, 5.`, 1.47`, 0.6614906832298147`, 0.7231437231906866`, 0.003105590062111801`, 87.21645331375566`},
{2.9000000000000004`, 39.`, 0.62`, 0.6739130434782619`, 0.7318189667223138`, 0.`, 81.992524365468`},
{1.3`, 7.`, 0.35000000000000003`, 0.7111801242236035`, 0.7601831434289865`, 0.`, 73.22898810518451`},
{2.9000000000000004`, 29.`, 1.06`, 0.6708074534161501`, 0.7321772998698588`, 0.`, 83.97154376431658`},
{2.3000000000000003`, 10.`, 0.87`, 0.6739130434782619`, 0.7351019196739876`, 0.003105590062111801`, 84.3947112302837 5`},
{1.`, 27.`, 0.96`, 0.6770186335403736`, 0.7247672533681274`, 0.`, 75.30696024721942`},
{2.7`, 12.`, 1.31`, 0.658385093167703`, 0.7250101586967115`, 0.003105590062111801`, 86.10638655805862`},
{2.7`, 21.`, 1.44`, 0.6614906832298147`, 0.7304597633452821`, 0.003105590062111801`, 85.79738908514413`},
{1.1`, 11.`, 1.18`, 0.6770186335403736`, 0.732358708782037`, 0.006211180124223602`, 80.84147263725393`},
{0.9`, 33.`, 1.3`, 0.639751552795032`, 0.6876623952156355`, 0.006211180124223602`, 73.11042845259263`},
{2.2`, 17.`, 0.68`, 0.6801242236024854`, 0.7460638373605123`, 0.`, 82.3939246188333`},
{3.`, 28.`, 1.03`, 0.6708074534161501`, 0.7335756453507063`, 0.`, 84.25521859900056`},
{1.6`, 28.`, 1.19`, 0.6956521739130442`, 0.7512999069254139`, 0.`, 80.949398834841`},
{1.2000000000000002`, 27.`, 1.32`, 0.6894409937888206`, 0.7372734407797229`, 0.006211180124223602`, 77.40923270169638`},
{1.3`, 7.`, 0.86`, 0.6894409937888206`, 0.7445415402553239`, 0.003105590062111801`, 81.95094973343502`},
{0.8`, 39.`, 0.08`, 0.5807453416149075`, 0.6337163336533734`, 0.`, 30.5508700805139`},
{3.`, 39.`, 0.42`, 0.6739130434782619`, 0.7298237807324921`, 0.`, 80.40997410097971`},
{1.6`, 19.`, 0.92`, 0.6925465838509325`, 0.7521492495515091`, 0.`, 81.4975744550504`},
{2.`, 8.`, 1.49`, 0.6677018633540382`, 0.7290520833680089`, 0.003105590062111801`, 86.24381245903398`},
{0.9`, 17.`, 0.75`, 0.6925465838509325`, 0.7367674468509032`, 0.`, 75.4108848334395`},
{1.7000000000000002`, 19.`, 1.09`, 0.6832298136645971`, 0.7462925073528295`, 0.`, 82.58190461704388`},
{2.`, 8.`, 0.81`, 0.6770186335403736`, 0.7367151557426271`, 0.`, 83.5889516219732`},
{2.7`, 39.`, 1.01`, 0.6645962732919265`, 0.7251989540810923`, 0.`, 82.89849467553562`},
{2.3000000000000003`, 7.`, 1.2`, 0.6677018633540382`, 0.7296541347943498`, 0.`, 86.36867658600589`},
{1.9000000000000001`, 40.`, 1.1500000000000001`, 0.6708074534161501`, 0.7311748680722969`, 0.003105590062111801`,
```

80.64404830870716`}, {2.6`, 38.`, 0.76`, 0.6739130434782619`, 0.7313423198441446`, 0.`, 81.82661732462032`},
{0.7000000000000001`, 11.`, 0.25`, 0.6863354037267089`, 0.7211993358759445`, 0.`, 54.49909710542681`},
{1.5`, 10.`, 0.91`, 0.6832298136645971`, 0.7439251550989061`, 0.003105590062111801`, 82.24846584922304`},
{0.7000000000000001`, 23.`, 0.99`, 0.6521739130434793`, 0.6969151430046997`, 0.012422360248447204`, 71.1292524473986`},
{2.2`, 3.`, 0.18`, 0.6645962732919265`, 0.7228033446929063`, 0.`, 72.39567513795716`},
{1.5`, 35.`, 1.3800000000000001`, 0.6801242236024854`, 0.733947071167675`, 0.006211180124223602`, 80.05852661890349`},
{3.`, 25.`, 1.25`, 0.6645962732919265`, 0.7292923192465551`, 0.003105590062111801`, 85.46420041337689`},
{1.2000000000000002`, 39.`, 1.22`, 0.6894409937888206`, 0.7356516943218416`, 0.003105590062111801`, 76.5569143024417`},
{1.2000000000000002`, 25.`, 0.05`, 0.7049689440993798`, 0.7470105806318251`, 0.`, 37.73497088173173`},
{2.2`, 26.`, 0.87`, 0.6801242236024854`, 0.7428060683510281`, 0.003105590062111801`, 82.61739947818296`},
{1.4000000000000001`, 12.`, 1.49`, 0.6801242236024854`, 0.7396049695759049`, 0.006211180124223602`, 83.0323763851061`},
{0.7000000000000001`, 16.`, 0.19`, 0.6708074534161501`, 0.7068263594950746`, 0.`, 49.25298227622709`},
{2.7`, 39.`, 0.79`, 0.6708074534161501`, 0.7301239921041262`, 0.`, 82.26250909773347`},
{2.9000000000000004`, 40.`, 0.76`, 0.6677018633540382`, 0.7276922043340978`, 0.`, 82.45640473144805`},
{1.7000000000000002`, 27.`, 1.23`, 0.6832298136645971`, 0.7442336764969737`, 0.003105590062111801`, 82.1275733686024`},
{2.9000000000000004`, 14.`, 1.47`, 0.6552795031055911`, 0.7234486733333422`, 0.003105590062111801`, 86.49272184032088`},
{1.9000000000000001`, 37.`, 1.16`, 0.6739130434782619`, 0.7314420376898605`, 0.006211180124223602`, 80.98514005276597`},
{2.5`, 30.`, 1.08`, 0.6708074534161501`, 0.7318387077665974`, 0.`, 83.30362268241802`},
{1.2000000000000002`, 9.`, 0.24`, 0.7111801242236035`, 0.7572813682920365`, 0.`, 66.13151182808919`},
{1.9000000000000001`, 38.`, 1.07`, 0.6801242236024854`, 0.7379486342193812`, 0.`, 80.74070269874468`},
{2.7`, 30.`, 0.42`, 0.6832298136645971`, 0.7363777824653671`, 0.`, 80.03059697562823`},
{1.5`, 13.`, 0.84`, 0.6863354037267089`, 0.7460665150013208`, 0.006211180124223602`, 81.64810719284922`},
{2.1`, 15.`, 0.17`, 0.6894409937888206`, 0.7497992289181195`, 0.`, 70.37993800345333`},
{0.9`, 6.`, 0.09`, 0.6894409937888206`, 0.7272118538935753`, 0.`, 38.58340833791254`},
{1.7000000000000002`, 23.`, 0.32`, 0.7049689440993798`, 0.7564903481131541`, 0.`, 72.96069065881146`},
{2.7`, 37.`, 1.32`, 0.6677018633540382`, 0.7245789992983306`, 0.003105590062111801`, 83.58161199728103`},
{1.6`, 6.`, 1.3800000000000001`, 0.6677018633540382`, 0.726081644335004`, 0.009316770186335404`, 85.04396749264285`},
{2.8000000000000003`, 10.`, 0.5`, 0.6708074534161501`, 0.7342227998221592`, 0.`, 82.67485807323058`},
{1.1`, 5.`, 0.84`, 0.6708074534161501`, 0.7256374125686654`, 0.009316770186335404`, 80.8131299987543`},
{1.2000000000000002`, 37.`, 1.18`, 0.6956521739130442`, 0.7373736713012495`, 0.003105590062111801`, 76.67728705952116`},
{0.8`, 22.`, 0.6900000000000001`, 0.6708074534161501`, 0.7108184492525624`, 0.`, 70.34839448956394`},

```
{2.8000000000000003`, 3.`, 0.02`, 0.658385093167703`, 0.7162550038884132`, 0.`, 67.03650230969988`},
{1.3`, 38.`, 1.08`, 0.6832298136645971`, 0.734447685016281`, 0.006211180124223602`, 76.66594838436257`},
{2.2`, 16.`, 0.2`, 0.6863354037267089`, 0.7468418371649042`, 0.`, 72.49597906184616`},
{0.9`, 24.`, 0.51`, 0.6801242236024854`, 0.716868755577829`, 0.`, 69.63829987274255`},
{2.3000000000000003`, 18.`, 0.77`, 0.6770186335403736`, 0.7437994941195514`, 0.`, 83.43300663358343`},
{2.4000000000000004`, 20.`, 1.21`, 0.6708074534161501`, 0.7374772959694476`, 0.`, 84.75713993337858`},
{2.9000000000000004`, 4.`, 0.77`, 0.658385093167703`, 0.7207221042042896`, 0.`, 85.90639014004203`},
{2.7`, 35.`, 0.93`, 0.6770186335403736`, 0.7319099049290699`, 0.`, 83.22872649337852`},
{1.2000000000000002`, 9.`, 1.49`, 0.6801242236024854`, 0.7365373406129275`, 0.003105590062111801`, 82.13393036853219`},
{2.5`, 36.`, 1.44`, 0.6645962732919265`, 0.7245911338911032`, 0.006211180124223602`, 83.41573812849522`},
{1.2000000000000002`, 13.`, 0.63`, 0.6987577639751561`, 0.7508942737019669`, 0.`, 78.07607172082992`},
{2.`, 36.`, 0.15`, 0.6894409937888206`, 0.7389877157166217`, 0.`, 67.22807243459327`},
{2.9000000000000004`, 36.`, 0.74`, 0.6739130434782619`, 0.7315919023953277`, 0.`, 82.62650798353192`},
{0.8`, 16.`, 1.17`, 0.6552795031055911`, 0.70568098425518`, 0.009316770186335404`, 73.42361875186053`},
{2.3000000000000003`, 22.`, 0.18`, 0.6894409937888206`, 0.7427683129652893`, 0.`, 71.57401130570008`},
{2.2`, 38.`, 1.07`, 0.6708074534161501`, 0.7309265851947871`, 0.003105590062111801`, 81.39129554279448`},
{2.9000000000000004`, 33.`, 0.29`, 0.6801242236024854`, 0.7331703943001795`, 0.`, 78.47836221949926`},
{1.3`, 39.`, 0.36`, 0.7049689440993798`, 0.7479993073933684`, 0.`, 69.30125584718081`},
{2.2`, 23.`, 0.45`, 0.6894409937888206`, 0.7479181288840709`, 0.`, 79.5410228806899`},
{1.3`, 23.`, 0.5700000000000001`, 0.7080745341614917`, 0.7568922976362148`, 0.`, 76.58422592400679`},
{0.7000000000000001`, 27.`, 1.37`, 0.6366459627329203`, 0.6810866490292269`, 0.003105590062111801`, 68.45931951326965`},
{2.3000000000000003`, 33.`, 1.48`, 0.6770186335403736`, 0.7323299957562848`, 0.003105590062111801`, 83.00903694686961`},
{3.`, 24.`, 0.54`, 0.6832298136645971`, 0.7402811842787047`, 0.`, 82.46238943133586`},
{1.7000000000000002`, 38.`, 1.16`, 0.6832298136645971`, 0.7378305953895222`, 0.`, 80.29549142558255`},
{1.5`, 3.`, 0.75`, 0.6677018633540382`, 0.7273312681534185`, 0.`, 82.67700798027008`},
{0.9`, 34.`, 1.07`, 0.6552795031055911`, 0.6999655674222484`, 0.`, 72.78929840016288`},
{0.7000000000000001`, 30.`, 1.27`, 0.6242236024844731`, 0.6705804595736696`, 0.006211180124223602`, 68.24016372758616`},
{3.`, 12.`, 0.99`, 0.658385093167703`, 0.7258551680757019`, 0.`, 85.31268774903067`},
{0.9`, 25.`, 0.6900000000000001`, 0.6708074534161501`, 0.7165023294155569`, 0.`, 73.05598333017214`},
{0.7000000000000001`, 27.`, 0.68`, 0.6552795031055911`, 0.6948406478293172`, 0.`, 66.11855820279983`},
{2.9000000000000004`, 32.`, 0.65`, 0.6801242236024854`, 0.7349789835111902`, 0.`, 82.28781397022401`},
```

```
{3.`, 5.`, 0.58`, 0.658385093167703`, 0.7218030851610554`, 0.003105590062111801`, 84.4598240218978`},
{0.9`, 39.`, 0.6`, 0.6490683229813675`, 0.6952680763544977`, 0.`, 69.74980413174698`},
{1.2000000000000002`, 15.`, 1.16`, 0.6925465838509325`, 0.7456967994059446`, 0.`, 80.49939937539645`},
{1.2000000000000002`, 22.`, 0.72`, 0.701863354037268`, 0.7512775740309068`, 0.`, 77.10164646383416`},
{3.`, 27.`, 1.3900000000000001`, 0.6645962732919265`, 0.7295730067707377`, 0.003105590062111801`, 85.52394645520852`},
{1.2000000000000002`, 38.`, 0.68`, 0.6956521739130442`, 0.7387128765460859`, 0.`, 74.0496330769935`},
{1.7000000000000002`, 30.`, 0.81`, 0.6925465838509325`, 0.7448627452346448`, 0.003105590062111801`, 79.38752458802834`},
{1.2000000000000002`, 20.`, 1.49`, 0.6863354037267089`, 0.7402319320691311`, 0.003105590062111801`, 79.54713380493604`},
{3.`, 32.`, 0.34`, 0.6863354037267089`, 0.7385534516687455`, 0.`, 79.88900225300046`},
{1.6`, 13.`, 0.45`, 0.701863354037268`, 0.7589806397174904`, 0.`, 77.80312999285253`},
{1.5`, 18.`, 0.7000000000000001`, 0.6987577639751561`, 0.7569841649539187`, 0.`, 79.53776983935106`},
{2.5`, 39.`, 0.6`, 0.6770186335403736`, 0.7336195310520423`, 0.`, 80.63583001748485`},
{0.7000000000000001`, 40.`, 0.53`, 0.6024844720496904`, 0.6523628592324321`, 0.`, 61.28999531374361`},
{0.7000000000000001`, 29.`, 0.42`, 0.6490683229813675`, 0.6881060840284497`, 0.`, 61.751748660492716`},
{0.9`, 37.`, 0.4`, 0.6552795031055911`, 0.7024607457717134`, 0.`, 65.93252366845337`},
{1.7000000000000002`, 20.`, 0.68`, 0.6925465838509325`, 0.7528698131172252`, 0.003105590062111801`, 80.88089395586807`},
{2.`, 14.`, 0.27`, 0.6894409937888206`, 0.7513942802189548`, 0.`, 74.65334212788282`},
{1.3`, 28.`, 1.4000000000000001`, 0.6894409937888206`, 0.7432178431387724`, 0.003105590062111801`, 79.37789513407061`},
{2.`, 14.`, 0.65`, 0.6801242236024854`, 0.7437765124160934`, 0.003105590062111801`, 82.11062696762356`},
{2.9000000000000004`, 7.`, 0.8`, 0.658385093167703`, 0.7239842435378555`, 0.`, 85.52893564053339`},
{1.7000000000000002`, 37.`, 0.43`, 0.6987577639751561`, 0.7481453426243635`, 0.`, 75.41795709352859`},
{2.8000000000000003`, 38.`, 1.`, 0.6677018633540382`, 0.7270714828065189`, 0.`, 82.98602424904095`},
{2.3000000000000003`, 20.`, 1.2`, 0.6739130434782619`, 0.7396365185531136`, 0.`, 84.42502663515079`},
{1.9000000000000001`, 13.`, 1.36`, 0.6739130434782619`, 0.7370052358896181`, 0.`, 84.81961412286664`},
{2.9000000000000004`, 7.`, 1.34`, 0.658385093167703`, 0.7221383186926194`, 0.003105590062111801`, 87.9885970169024`},
{2.3000000000000003`, 19.`, 1.24`, 0.6739130434782619`, 0.7391328304064678`, 0.`, 84.84207287733138`},
{2.7`, 39.`, 1.3`, 0.6614906832298147`, 0.7230345914300959`, 0.003105590062111801`, 83.52364935840392`},
{2.`, 39.`, 1.1400000000000001`, 0.6677018633540382`, 0.729570042723378`, 0.003105590062111801`, 80.58174623475163`},
{1.4000000000000001`, 30.`, 0.01`, 0.6832298136645971`, 0.7285206194165329`, 0.`, -9.884783098960197`},
{1.4000000000000001`, 29.`, 0.2`, 0.7111801242236035`, 0.7555279392038438`, 0.`, 63.91771113724006`},
{3.`, 4.`, 1.05`, 0.6614906832298147`, 0.7223767752260473`, 0.`, 87.5734993106031`}};
```

In[ ]:=

```
OutcomeKsHigh = {{"X", "T", "kM", "OS", "AUC", "ToxDeath", "ToxAvg"},
    {1.1`, 32.`, 0.18`, 0.6401179941002945`, 0.6706554989092429`, 0.`, 53.15910193617849`},
    {2.4000000000000004`, 39.`, 0.9400000000000001`, 0.6460176991150436`, 0.7021841044092255`, 0.`, 81.38871824507048`},
    {0.7000000000000001`, 25.`, 0.86`, 0.5250737463126836`, 0.5715744458324908`, 0.`, 56.62508188833331`},
    {1.4000000000000001`, 34.`, 1.48`, 0.6784660766961647`, 0.7245979698415758`, 0.0029498525073746312`, 77.88341255304819`},
    {2.1`, 5.`, 0.75`, 0.6460176991150436`, 0.7065283801504376`, 0.`, 83.27282985272258`},
    {1.1`, 30.`, 0.42`, 0.6371681415929199`, 0.669639395877392`, 0.`, 64.6465201024285`},
    {2.9000000000000004`, 39.`, 0.05`, 0.6224188790560468`, 0.6835913417076437`, 0.`, 69.93892720568978`},
    {0.8`, 30.`, 1.46`, 0.5162241887905596`, 0.5659872472185565`, 0.008849557522123894`, 59.88040068597158`},
    {3.`, 40.`, 0.37`, 0.6342182890855452`, 0.6926887282415561`, 0.`, 78.57290308918351`},
    {2.8000000000000003`, 31.`, 1.04`, 0.6401179941002945`, 0.7039897196397574`, 0.`, 83.65742112400945`},
    {1.1`, 13.`, 0.42`, 0.6755162241887901`, 0.7095837958044281`, 0.`, 68.06604308909964`},
    {0.7000000000000001`, 18.`, 0.98`, 0.5693215339233032`, 0.6092612884284997`, 0.`, 59.67255020691748`},
    {1.2000000000000002`, 23.`, 0.74`, 0.6784660766961647`, 0.7194032906756649`, 0.`, 73.1723881584215`},
    {0.7000000000000001`, 19.`, 0.32`, 0.5457227138643059`, 0.5857002614431703`, 0.`, 49.16302671451609`},
    {1.5`, 12.`, 0.27`, 0.7109144542772852`, 0.749804769558999`, 0.`, 69.72891228566876`},
    {1.8`, 22.`, 1.47`, 0.6843657817109138`, 0.7366826368407994`, 0.`, 82.6481421656849`},
    {2.`, 20.`, 1.05`, 0.6814159292035392`, 0.7355310828172477`, 0.`, 82.21280160551093`},
    {1.4000000000000001`, 17.`, 1.18`, 0.6961651917404122`, 0.7390383300018695`, 0.`, 78.85011116930451`},
    {1.5`, 17.`, 1.12`, 0.6932153392330376`, 0.7387521669970545`, 0.`, 79.64328812778815`},
    {1.2000000000000002`, 10.`, 0.6900000000000001`, 0.6932153392330376`, 0.7358935381762419`, 0.`, 75.45861752215717`},
    {1.9000000000000001`, 19.`, 0.86`, 0.6814159292035392`, 0.7364217249349145`, 0.`, 81.348155782693`},
    {2.2`, 10.`, 0.22`, 0.6696165191740407`, 0.7265620759240473`, 0.`, 73.6305027127524`},
    {2.`, 40.`, 0.8`, 0.6666666666666661`, 0.7162656092232573`, 0.`, 78.79608118579674`},
    {1.3`, 14.`, 1.37`, 0.6961651917404122`, 0.7373918842419793`, 0.`, 78.95444160583635`},
    {0.8`, 7.`, 1.23`, 0.6548672566371674`, 0.6991813456011575`, 0.`, 72.79642318672248`},
    {2.1`, 38.`, 0.84`, 0.6637168141592914`, 0.7153042082794994`, 0.`, 79.79082626705976`},
    {2.`, 30.`, 1.49`, 0.6755162241887901`, 0.728268068820355`, 0.`, 82.60935803436162`},
    {1.9000000000000001`, 22.`, 1.02`, 0.6814159292035392`, 0.73558596419`, 0.`, 81.48968154879468`},
    {0.9`, 27.`, 1.26`, 0.5604719764011793`, 0.6069419564256426`, 0.0029498525073746312`, 63.996985869969905`},
    {1.5`, 39.`, 0.51`, 0.6902654867256629`, 0.7284056209164339`, 0.`, 73.36612392173895`},
```

{2.`, 38.`, 1.31`, 0.6666666666666661`, 0.7174863990332039`, 0.0029498525073746312`, 80.97062974747378`},
{2.8000000000000003`, 20.`, 0.36`, 0.6489675516224181`, 0.7131653467149706`, 0.`, 79.10116157641221`},
{1.2000000000000002`, 34.`, 1.2`, 0.6725663716814154`, 0.7077852591250084`, 0.`, 74.4756386446432`},
{2.6`, 32.`, 1.32`, 0.6371681415929199`, 0.7029111301952182`, 0.`, 83.42779806706223`},
{1.1`, 34.`, 0.05`, 0.6106194690265481`, 0.6492286398919811`, 0.`, 31.0007045268281`},
{1.1`, 23.`, 1.09`, 0.6401179941002945`, 0.6830304814337919`, 0.`, 71.3170255003077`},
{1.1`, 21.`, 0.3`, 0.6578171091445421`, 0.692155494419323`, 0.`, 62.262594798817254`},
{1.`, 28.`, 0.84`, 0.5870206489675509`, 0.6294184134516758`, 0.`, 65.15890398970878`},
{2.5`, 7.`, 0.7000000000000001`, 0.643067846607669`, 0.7050703744140578`, 0.`, 83.5136866260659`},
{1.6`, 31.`, 1.31`, 0.6843657817109138`, 0.7332131987229975`, 0.`, 79.91045105064981`},
{1.9000000000000001`, 34.`, 1.`, 0.6725663716814154`, 0.724493677088881`, 0.`, 80.04140407407225`},
{0.7000000000000001`, 32.`, 0.76`, 0.5014749262536865`, 0.5477660325502695`, 0.`, 53.41994891838377`},
{1.8`, 28.`, 0.64`, 0.6814159292035392`, 0.7348943753964104`, 0.`, 78.34663715605151`},
{0.9`, 11.`, 1.37`, 0.6401179941002945`, 0.68293102635938`, 0.0058997050147492625`, 74.26806128400798`},
{1.7000000000000002`, 12.`, 0.75`, 0.6843657817109138`, 0.7351836443582086`, 0.`, 79.9722338435381`},
{1.`, 23.`, 0.9`, 0.6135693215339227`, 0.6564121014568769`, 0.`, 67.05362463625363`},
{1.6`, 31.`, 1.21`, 0.6843657817109138`, 0.7330619631285649`, 0.`, 79.7432933189236`},
{1.`, 19.`, 0.97`, 0.6342182890855452`, 0.6728603614483332`, 0.`, 68.81805224698658`},
{1.`, 33.`, 0.63`, 0.5811209439528016`, 0.6187045589626942`, 0.`, 62.34263583560435`},
{1.2000000000000002`, 13.`, 0.77`, 0.6932153392330376`, 0.7330192937534266`, 0.`, 75.25949218721112`},
{2.1`, 17.`, 0.14`, 0.6932153392330376`, 0.7373961848215224`, 0.`, 68.62410666475961`},
{2.8000000000000003`, 37.`, 0.67`, 0.6401179941002945`, 0.6997325422962376`, 0.`, 81.36210639094071`},
{1.6`, 20.`, 0.05`, 0.6991150442477868`, 0.7428468019234704`, 0.`, 51.640562104139306`},
{3.`, 6.`, 0.27`, 0.6342182890855452`, 0.6975357868327582`, 0.`, 79.07445265704033`},
{1.8`, 4.`, 0.06`, 0.6755162241887901`, 0.7268372602991294`, 0.`, 57.65948807463491`},
{0.8`, 34.`, 0.5700000000000001`, 0.5162241887905596`, 0.5616261073159959`, 0.`, 54.529533592716824`},
{1.6`, 28.`, 1.05`, 0.6873156342182883`, 0.7367357069903376`, 0.`, 79.51175139083796`},
{0.7000000000000001`, 12.`, 0.01`, 0.466076696165191`, 0.5195219919280689`, 0.`, 5.3404153962878524`},
{2.6`, 15.`, 0.17`, 0.6607669616519167`, 0.71947865244871`, 0.`, 73.83168563562927`},
{1.3`, 13.`, 0.52`, 0.7020648967551615`, 0.7413456040363262`, 0.`, 73.83400217656886`},
{1.2000000000000002`, 4.`, 1.32`, 0.6666666666666661`, 0.7190021675376279`, 0.`, 83.27054906770964`},

```
{2.`, 40.`, 0.71`, 0.6755162241887901`, 0.722839263611574`, 0.`, 78.30828178001182`},
{0.9`, 40.`, 0.12`, 0.4955752212389727`, 0.5494352991580576`, 0.`, 35.23119806184972`},
{2.7`, 10.`, 0.9`, 0.643067846607669`, 0.705772867062792`, 0.`, 84.66326031882274`},
{2.3000000000000003`, 13.`, 0.19`, 0.6666666666666661`, 0.7243353686512303`, 0.`, 72.84008254445256`},
{2.3000000000000003`, 30.`, 0.99`, 0.6578171091445421`, 0.7159905324119409`, 0.`, 82.34728954353764`},
{2.8000000000000003`, 24.`, 1.03`, 0.643067846607669`, 0.7096690122124135`, 0.`, 84.25325217666443`},
{2.9000000000000004`, 28.`, 1.03`, 0.6401179941002945`, 0.7042462562903216`, 0.`, 83.67464621880195`},
{3.`, 4.`, 0.64`, 0.628318584070796`, 0.6904878506736353`, 0.`, 84.63584832505339`},
{1.5`, 15.`, 0.79`, 0.6932153392330376`, 0.7421598096099921`, 0.`, 78.64106611587982`},
{2.4000000000000004`, 40.`, 0.8300000000000001`, 0.6460176991150436`, 0.7017246289173388`, 0.`, 80.51111045534122`},
{1.6`, 4.`, 0.35000000000000003`, 0.6725663716814154`, 0.7264349874127445`, 0.`, 75.51892910322042`},
{0.9`, 25.`, 1.3`, 0.5693215339233032`, 0.6155844671437458`, 0.`, 64.76014033560006`},
{2.7`, 8.`, 0.97`, 0.643067846607669`, 0.7044794422634665`, 0.`, 85.29474456906156`},
{2.8000000000000003`, 38.`, 1.28`, 0.6371681415929199`, 0.6983312661440227`, 0.`, 83.33763517465613`},
{2.4000000000000004`, 13.`, 0.15`, 0.6666666666666661`, 0.723602744292254`, 0.`, 71.76182267237857`},
{2.9000000000000004`, 36.`, 1.1500000000000001`, 0.6342182890855452`, 0.6940208499029993`, 0.`, 83.41767150183958`},
{1.6`, 9.`, 1.46`, 0.6784660766961647`, 0.7289505009684232`, 0.0029498525073746312`, 82.90138454014159`},
{1.6`, 21.`, 1.1`, 0.6902654867256629`, 0.7410099811497127`, 0.`, 80.48171590068156`},
{2.1`, 29.`, 1.31`, 0.6755162241887901`, 0.7276799754240756`, 0.`, 82.65622392948849`},
{2.9000000000000004`, 20.`, 0.51`, 0.6460176991150436`, 0.7121248871469034`, 0.`, 81.61498473317974`},
{2.`, 23.`, 0.06`, 0.6843657817109138`, 0.7323914051580137`, 0.`, 60.27968513431957`},
{0.9`, 3.`, 1.23`, 0.6401179941002945`, 0.6934400746090513`, 0.0029498525073746312`, 80.8884228816312`},
{2.3000000000000003`, 27.`, 0.44`, 0.6637168141592914`, 0.7208045807525696`, 0.`, 78.58659444487319`},
{1.3`, 31.`, 0.65`, 0.6902654867256629`, 0.727478499008149`, 0.`, 73.88950103631929`},
{1.7000000000000002`, 9.`, 0.88`, 0.6725663716814154`, 0.7274804632439104`, 0.`, 81.40787128871875`},
{2.5`, 17.`, 1.28`, 0.643067846607669`, 0.7071605544513851`, 0.`, 84.53871759078233`},
{2.6`, 21.`, 0.72`, 0.6489675516224181`, 0.7144899393247213`, 0.`, 82.42188121235745`},
{2.4000000000000004`, 14.`, 1.02`, 0.6519174041297928`, 0.7121497639583949`, 0.`, 84.05520141101833`},
{1.4000000000000001`, 40.`, 0.53`, 0.6932153392330376`, 0.7273395089967382`, 0.`, 73.11864498495054`},
{3.`, 11.`, 0.78`, 0.6371681415929199`, 0.7008202651752734`, 0.`, 84.34191261550775`},
{2.6`, 17.`, 0.03`, 0.643067846607669`, 0.7011582393882883`, 0.`, 65.67752495272195`},
```

```
{0.8`, 22.`, 0.8200000000000001`, 0.5663716814159285`, 0.6086205907702187`, 0.`, 61.268182020829016`},
{1.5`, 14.`, 0.9400000000000001`, 0.6961651917404122`, 0.741907734250081`, 0.`, 79.88933933317871`},
{2.4000000000000004`, 32.`, 0.02`, 0.6401179941002945`, 0.6927459683966055`, 0.`, 61.87649665045021`},
{2.8000000000000003`, 21.`, 0.65`, 0.6489675516224181`, 0.7141769637930752`, 0.`, 82.4689277342947`},
{1.2000000000000002`, 34.`, 1.24`, 0.6666666666666661`, 0.7035371641069255`, 0.0058997050147492625`, 74.38341113299681`},
{2.1`, 37.`, 0.23`, 0.6725663716814154`, 0.7162608604935371`, 0.`, 71.34738459767246`},
{1.2000000000000002`, 20.`, 0.29`, 0.6873156342182883`, 0.7208918999373515`, 0.`, 65.2074650286764`},
{1.7000000000000002`, 8.`, 0.54`, 0.6755162241887901`, 0.7302160315652079`, 0.`, 78.4988037233318`},
{1.7000000000000002`, 20.`, 1.24`, 0.6873156342182883`, 0.7368505817438896`, 0.`, 81.49639716100559`},
{2.8000000000000003`, 23.`, 0.4`, 0.6460176991150436`, 0.7111224594138726`, 0.`, 79.56871154706973`},
{2.2`, 7.`, 0.47000000000000003`, 0.6578171091445421`, 0.7175896834970306`, 0.`, 80.17377636696244`},
{2.5`, 3.`, 0.73`, 0.6342182890855452`, 0.6944199554694579`, 0.`, 84.767986043416`},
{0.8`, 38.`, 0.64`, 0.5073746312684357`, 0.5525572443743851`, 0.0029498525073746312`, 54.1239167319018`},
{1.2000000000000002`, 31.`, 0.79`, 0.6725663716814154`, 0.7084729694126187`, 0.`, 72.68023428211531`},
{2.1`, 30.`, 0.98`, 0.6696165191740407`, 0.7248124348411482`, 0.`, 81.46491843050252`},
{0.7000000000000001`, 38.`, 1.01`, 0.4837758112094387`, 0.529325121390359`, 0.`, 51.965022595001614`},
{3.`, 7.`, 1.34`, 0.6401179941002945`, 0.70131167582857`, 0.`, 87.56408100662861`},
{2.3000000000000003`, 24.`, 1.25`, 0.6607669616519167`, 0.720724576174495`, 0.`, 83.55405618474704`},
{2.2`, 10.`, 0.08`, 0.6607669616519167`, 0.7185824457574663`, 0.`, 65.3045179369445`},
{0.9`, 3.`, 0.35000000000000003`, 0.6873156342182883`, 0.7277158718897496`, 0.`, 68.46994465809159`},
{1.9000000000000001`, 24.`, 1.17`, 0.6784660766961647`, 0.7335520793605319`, 0.`, 81.98436312416653`},
{1.2000000000000002`, 5.`, 1.07`, 0.6725663716814154`, 0.7228414216665017`, 0.`, 79.93863283910545`},
{0.9`, 31.`, 0.27`, 0.5486725663716806`, 0.5892343949799882`, 0.`, 49.64810417499576`},
{2.4000000000000004`, 5.`, 1.`, 0.6401179941002945`, 0.7009208647798209`, 0.`, 85.5367806238846`},
{2.5`, 23.`, 0.49`, 0.6519174041297928`, 0.7164347444177218`, 0.`, 79.87232334317208`},
{2.9000000000000004`, 3.`, 1.43`, 0.6194690265486721`, 0.6831957872606945`, 0.`, 88.37107318151422`},
{0.7000000000000001`, 6.`, 1.05`, 0.6489675516224181`, 0.6922840510266092`, 0.`, 68.02135246818409`},
{1.3`, 21.`, 1.32`, 0.6932153392330376`, 0.7345067799987882`, 0.`, 77.41808016201244`},
{2.3000000000000003`, 22.`, 1.25`, 0.6578171091445421`, 0.7181827137136676`, 0.`, 83.56931379066971`},
{1.2000000000000002`, 38.`, 0.59`, 0.6784660766961647`, 0.7088113616327052`, 0.`, 71.12630674933699`},
{1.7000000000000002`, 13.`, 0.3`, 0.7020648967551615`, 0.7476401938973833`, 0.`, 72.77328128845465`},
```

```
{1.`, 24.`, 0.11`, 0.5870206489675509`, 0.6280892646628238`, 0.`, 41.16059941479885`},
{2.8000000000000003`, 26.`, 0.08`, 0.643067846607669`, 0.70299270531328`, 0.`, 70.91521137345403`},
{1.7000000000000002`, 15.`, 0.46`, 0.6961651917404122`, 0.7462250621854801`, 0.`, 76.64855038637405`},
{0.7000000000000001`, 10.`, 1.`, 0.6312684365781706`, 0.6711872760374092`, 0.`, 65.36166398527818`},
{2.8000000000000003`, 34.`, 0.92`, 0.6371681415929199`, 0.6971542502300977`, 0.`, 82.67494588281575`},
{1.8`, 21.`, 1.33`, 0.6843657817109138`, 0.737224310214056`, 0.`, 82.49016379379177`},
{2.7`, 15.`, 0.44`, 0.6489675516224181`, 0.7151158443944273`, 0.`, 80.79670407356325`},
{1.7000000000000002`, 9.`, 0.45`, 0.6843657817109138`, 0.7352669812440047`, 0.`, 76.45144292837762`},
{2.3000000000000003`, 33.`, 0.2`, 0.6607669616519167`, 0.7114710303271014`, 0.`, 72.09127668101641`},
{0.8`, 40.`, 0.46`, 0.489675516224188`, 0.5371691720846108`, 0.`, 50.00302263652054`},
{3.`, 24.`, 0.9500000000000001`, 0.6401179941002945`, 0.70614138090335`, 0.`, 84.03502634805326`},
{1.1`, 14.`, 0.78`, 0.6637168141592914`, 0.7046099340709495`, 0.`, 72.46075163066837`},
{1.8`, 21.`, 0.15`, 0.6991150442477868`, 0.7447707502293507`, 0.`, 66.19513939286058`},
{2.9000000000000004`, 36.`, 0.8200000000000001`, 0.6342182890855452`, 0.694189347078344`, 0.`, 82.38657380258412`},
{1.4000000000000001`, 23.`, 0.31`, 0.7020648967551615`, 0.7417311659252657`, 0.`, 69.38115392645669`},
{1.8`, 36.`, 1.23`, 0.6725663716814154`, 0.7219090450458204`, 0.`, 79.93870042176563`},
{2.7`, 28.`, 0.32`, 0.6519174041297928`, 0.7105895752591256`, 0.`, 78.00627765112439`},
{0.9`, 21.`, 0.91`, 0.5929203539823001`, 0.6335677544192837`, 0.`, 64.7654722335573`},
{2.1`, 31.`, 1.04`, 0.6696165191740407`, 0.7246201781448581`, 0.`, 81.71436851142667`},
{0.7000000000000001`, 40.`, 0.78`, 0.47492625368731484`, 0.5224019319016674`, 0.`, 50.963050268705715`},
{3.`, 4.`, 1.29`, 0.6253687315634214`, 0.6884388455279847`, 0.`, 87.83063513746866`},
{1.`, 5.`, 0.34`, 0.6932153392330376`, 0.731896133023877`, 0.`, 68.24524824156752`},
{2.1`, 11.`, 1.07`, 0.6637168141592914`, 0.7210581731172787`, 0.`, 83.89904672480266`},
{1.9000000000000001`, 5.`, 0.76`, 0.6578171091445421`, 0.7151851881313365`, 0.`, 82.93504502839455`},
{2.5`, 26.`, 0.8`, 0.6519174041297928`, 0.7153320871353824`, 0.`, 82.11729916589019`},
{2.`, 14.`, 0.84`, 0.6755162241887901`, 0.7302415383718874`, 0.`, 81.9325897724062`},
{2.7`, 9.`, 0.43`, 0.6460176991150436`, 0.7087328271857396`, 0.`, 80.77179105354313`},
{2.8000000000000003`, 27.`, 1.4000000000000001`, 0.6460176991150436`, 0.7110147119219729`, 0.`, 84.68619053976754`},
{2.6`, 19.`, 0.47000000000000003`, 0.6489675516224181`, 0.7159578270961194`, 0.`, 80.42322488983004`},
{1.7000000000000002`, 27.`, 0.33`, 0.6932153392330376`, 0.7391070635599956`, 0.`, 72.6922580896972`},
{1.6`, 22.`, 0.13`, 0.7079646017699106`, 0.7490507231418275`, 0.`, 61.75895996192324`},
```

```
{1.5`, 10.`, 0.19`, 0.7138643067846598`, 0.7519669195073905`, 0.`, 65.32482541840602`},
{2.1`, 14.`, 0.89`, 0.6696165191740407`, 0.7255862255183393`, 0.`, 82.67147030529905`},
{0.7000000000000001`, 20.`, 1.1300000000000001`, 0.5516224188790553`, 0.5942247934233782`, 0.`, 58.96074616473307`},
{1.6`, 23.`, 0.52`, 0.6902654867256629`, 0.7415078076445628`, 0.`, 75.66607772884481`},
{1.5`, 28.`, 0.37`, 0.6991150442477868`, 0.7375167669941706`, 0.`, 71.90472421494543`},
{3.`, 19.`, 0.16`, 0.6460176991150436`, 0.7081887819338886`, 0.`, 75.7375090028133`},
{0.8`, 28.`, 0.72`, 0.5309734513274328`, 0.5773054400117467`, 0.`, 57.315816894491455`},
{1.`, 11.`, 0.27`, 0.6696165191740407`, 0.7019614149357772`, 0.`, 61.05873815507349`},
{3.`, 5.`, 1.04`, 0.6312684365781706`, 0.6929214086299853`, 0.`, 86.48903913284191`},
{1.5`, 32.`, 0.13`, 0.6961651917404122`, 0.7340463891241581`, 0.`, 59.49287337321649`},
{1.`, 6.`, 0.61`, 0.6814159292035392`, 0.7240793707830064`, 0.`, 74.03355959712093`},
{2.9000000000000004`, 35.`, 1.46`, 0.6312684365781706`, 0.6939472986459849`, 0.0029498525073746312`, 84.24733175967587`},
{1.5`, 29.`, 0.21`, 0.6991150442477868`, 0.739786959834164`, 0.`, 66.11636544658909`},
{1.3`, 21.`, 1.47`, 0.6932153392330376`, 0.7343544682504625`, 0.`, 77.76611598969632`},
{0.7000000000000001`, 27.`, 1.08`, 0.5191740412979343`, 0.5671288528866663`, 0.`, 56.40383326303431`},
{2.7`, 34.`, 0.14`, 0.6460176991150436`, 0.7014375981588421`, 0.`, 72.7520106342402`},
{1.2000000000000002`, 4.`, 1.19`, 0.6666666666666661`, 0.7192006722471486`, 0.`, 83.08186915335641`},
{1.7000000000000002`, 11.`, 0.51`, 0.6843657817109138`, 0.7357002603600066`, 0.`, 76.9328575339271`},
{2.7`, 9.`, 0.04`, 0.6460176991150436`, 0.7060422578076196`, 0.`, 67.84961595661517`},
{2.1`, 38.`, 0.19`, 0.6725663716814154`, 0.7159546466322918`, 0.`, 69.92788838975366`},
{0.8`, 27.`, 0.62`, 0.5339233038348075`, 0.5790301733279896`, 0.0058997050147492625`, 57.75426775134211`},
{2.9000000000000004`, 22.`, 0.59`, 0.6460176991150436`, 0.7113806173408996`, 0.`, 82.14217537062537`},
{2.2`, 13.`, 1.23`, 0.6548672566371674`, 0.7141455682059241`, 0.0029498525073746312`, 84.33432728592403`},
{2.8000000000000003`, 36.`, 1.03`, 0.6401179941002945`, 0.7002312248078112`, 0.`, 83.22541428710495`},
{1.4000000000000001`, 13.`, 0.68`, 0.6961651917404122`, 0.742872398654658`, 0.`, 76.85648643339299`},
{1.4000000000000001`, 12.`, 0.29`, 0.7138643067846598`, 0.7497820446739563`, 0.`, 69.54435230151962`},
{2.3000000000000003`, 33.`, 1.11`, 0.6519174041297928`, 0.7101504924243152`, 0.`, 82.12226514518042`},
{1.4000000000000001`, 7.`, 1.22`, 0.6814159292035392`, 0.7319623160945814`, 0.`, 81.14959173473487`},
{1.7000000000000002`, 8.`, 1.1500000000000001`, 0.6725663716814154`, 0.7280789526211235`, 0.`, 83.64396374257367`},
{1.5`, 8.`, 0.18`, 0.705014749262536`, 0.7462171918033413`, 0.`, 64.81577548333286`},
{1.1`, 24.`, 0.35000000000000003`, 0.643067846607669`, 0.6798745762280377`, 0.`, 63.155983112187435`},
```

```
{2.1`, 9.`, 0.9`, 0.6607669616519167`, 0.7196254157606431`, 0.`, 83.6469333120327`},
{1.3`, 13.`, 0.6900000000000001`, 0.6991150442477868`, 0.7405602538443704`, 0.`, 75.91137405172458`},
{1.2000000000000002`, 36.`, 1.31`, 0.6725663716814154`, 0.7056389341399577`, 0.0029498525073746312`, 74.55402103824154`},
{2.5`, 8.`, 0.67`, 0.643067846607669`, 0.7060482090149258`, 0.`, 83.27453561952602`},
{1.2000000000000002`, 26.`, 0.54`, 0.6784660766961647`, 0.713535282517965`, 0.`, 71.4867795694154`},
{1.2000000000000002`, 36.`, 0.53`, 0.6784660766961647`, 0.7086426158503905`, 0.`, 70.62943192490087`},
{1.1`, 29.`, 0.49`, 0.628318584070796`, 0.6639549392390146`, 0.0058997050147492625`, 66.17545649512529`},
{1.8`, 23.`, 1.06`, 0.6843657817109138`, 0.7383038764524646`, 0.`, 81.32976097153406`},
{2.8000000000000003`, 5.`, 1.27`, 0.6312684365781706`, 0.6934918230347846`, 0.`, 87.18266201610514`},
{1.`, 31.`, 0.73`, 0.5781710914454269`, 0.6194703985816635`, 0.`, 63.34059700310237`},
{0.8`, 26.`, 1.06`, 0.5486725663716806`, 0.5940542035642861`, 0.`, 59.917052798355`},
{2.4000000000000004`, 18.`, 0.55`, 0.6548672566371674`, 0.718141802483834`, 0.`, 80.72135257404867`},
{1.6`, 25.`, 1.18`, 0.6873156342182883`, 0.7386710482061319`, 0.`, 80.22659294597075`},
{0.9`, 18.`, 1.47`, 0.6047197640117987`, 0.6453385834790017`, 0.0058997050147492625`, 68.13968280035235`},
{0.7000000000000001`, 4.`, 0.71`, 0.6578171091445421`, 0.7010291175018353`, 0.`, 66.90736178620604`}}
```

Out[ ]=

```
{{X, T, kM, OS, AUC, ToxDeath, ToxAvg}, {1.1, 32., 0.18, 0.640118, 0.670655, 0., 53.1591}, {2.4, 39., 0.94, 0.646018, 0.702184, 0., 81.3887},
{0.7, 25., 0.86, 0.525074, 0.571574, 0., 56.6251}, {1.4, 34., 1.48, 0.678466, 0.724598, 0.00294985, 77.8834}, {2.1, 5., 0.75, 0.646018, 0.706528, 0., 83.2728},
{1.1, 30., 0.42, 0.637168, 0.669639, 0., 64.6465}, {2.9, 39., 0.05, 0.622419, 0.683591, 0., 69.9389}, {0.8, 30., 1.46, 0.516224, 0.565987, 0.00884956, 59.8804},
{3., 40., 0.37, 0.634218, 0.692689, 0., 78.5729}, {2.8, 31., 1.04, 0.640118, 0.70399, 0., 83.6574}, {1.1, 13., 0.42, 0.675516, 0.709584, 0., 68.066},
{0.7, 18., 0.98, 0.569322, 0.609261, 0., 59.6726}, {1.2, 23., 0.74, 0.678466, 0.719403, 0., 73.1724}, {0.7, 19., 0.32, 0.545723, 0.5857, 0., 49.163},
{1.5, 12., 0.27, 0.710914, 0.749805, 0., 69.7289}, {1.8, 22., 1.47, 0.684366, 0.736683, 0., 82.6481}, {2., 20., 1.05, 0.681416, 0.735531, 0., 82.2128},
{1.4, 17., 1.18, 0.696165, 0.739038, 0., 78.8501}, {1.5, 17., 1.12, 0.693215, 0.738752, 0., 79.6433}, {1.2, 10., 0.69, 0.693215, 0.735894, 0., 75.4586},
{1.9, 19., 0.86, 0.681416, 0.736422, 0., 81.3482}, {2.2, 10., 0.22, 0.669617, 0.726562, 0., 73.6305}, {2., 40., 0.8, 0.666667, 0.716266, 0., 78.7961},
{1.3, 14., 1.37, 0.696165, 0.737392, 0., 78.9544}, {0.8, 7., 1.23, 0.654867, 0.699181, 0., 72.7964}, {2.1, 38., 0.84, 0.663717, 0.715304, 0., 79.7908},
{2., 30., 1.49, 0.675516, 0.728268, 0., 82.6094}, {1.9, 22., 1.02, 0.681416, 0.735586, 0., 81.4897}, {0.9, 27., 1.26, 0.560472, 0.606942, 0.00294985, 63.997},
{1.5, 39., 0.51, 0.690265, 0.728406, 0., 73.3661}, {2., 38., 1.31, 0.666667, 0.717486, 0.00294985, 80.9706}, {2.8, 20., 0.36, 0.648968, 0.713165, 0., 79.1012},
{1.2, 34., 1.2, 0.672566, 0.707785, 0., 74.4756}, {2.6, 32., 1.32, 0.637168, 0.702911, 0., 83.4278}, {1.1, 34., 0.05, 0.610619, 0.649229, 0., 31.0007},
{1.1, 23., 1.09, 0.640118, 0.68303, 0., 71.317}, {1.1, 21., 0.3, 0.657817, 0.692155, 0., 62.2626}, {1., 28., 0.84, 0.587021, 0.629418, 0., 65.1589},
{2.5, 7., 0.7, 0.643068, 0.70507, 0., 83.5137}, {1.6, 31., 1.31, 0.684366, 0.733213, 0., 79.9105}, {1.9, 34., 1., 0.672566, 0.724494, 0., 80.0414},
{0.7, 32., 0.76, 0.501475, 0.547766, 0., 53.4199}, {1.8, 28., 0.64, 0.681416, 0.734894, 0., 78.3466}, {0.9, 11., 1.37, 0.640118, 0.682931, 0.00589971, 74.2681},
{1.7, 12., 0.75, 0.684366, 0.735184, 0., 79.9722}, {1., 23., 0.9, 0.613569, 0.656412, 0., 67.0536}, {1.6, 31., 1.21, 0.684366, 0.733062, 0., 79.7433},
```

{1., 19., 0.97, 0.634218, 0.67286, 0., 68.8181}, {1., 33., 0.63, 0.581121, 0.618705, 0., 62.3426}, {1.2, 13., 0.77, 0.693215, 0.733019, 0., 75.2595},
{2.1, 17., 0.14, 0.693215, 0.737396, 0., 68.6241}, {2.8, 37., 0.67, 0.640118, 0.699733, 0., 81.3621}, {1.6, 20., 0.05, 0.699115, 0.742847, 0., 51.6406},
{3., 6., 0.27, 0.634218, 0.697536, 0., 79.0745}, {1.8, 4., 0.06, 0.675516, 0.726837, 0., 57.6595}, {0.8, 34., 0.57, 0.516224, 0.561626, 0., 54.5295},
{1.6, 28., 1.05, 0.687316, 0.736736, 0., 79.5118}, {0.7, 12., 0.01, 0.466077, 0.519522, 0., 5.34042}, {2.6, 15., 0.17, 0.660767, 0.719479, 0., 73.8317},
{1.3, 13., 0.52, 0.702065, 0.741346, 0., 73.834}, {1.2, 4., 1.32, 0.666667, 0.719002, 0., 83.2705}, {2., 40., 0.71, 0.675516, 0.722839, 0., 78.3083},
{0.9, 40., 0.12, 0.495575, 0.549435, 0., 35.2312}, {2.7, 10., 0.9, 0.643068, 0.705773, 0., 84.6633}, {2.3, 13., 0.19, 0.666667, 0.724335, 0., 72.8401},
{2.3, 30., 0.99, 0.657817, 0.715991, 0., 82.3473}, {2.8, 24., 1.03, 0.643068, 0.709669, 0., 84.2533}, {2.9, 28., 1.03, 0.640118, 0.704246, 0., 83.6746},
{3., 4., 0.64, 0.628319, 0.690488, 0., 84.6358}, {1.5, 15., 0.79, 0.693215, 0.74216, 0., 78.6411}, {2.4, 40., 0.83, 0.646018, 0.701725, 0., 80.5111},
{1.6, 4., 0.35, 0.672566, 0.726435, 0., 75.5189}, {0.9, 25., 1.3, 0.569322, 0.615584, 0., 64.7601}, {2.7, 8., 0.97, 0.643068, 0.704479, 0., 85.2947},
{2.8, 38., 1.28, 0.637168, 0.698331, 0., 83.3376}, {2.4, 13., 0.15, 0.666667, 0.723603, 0., 71.7618}, {2.9, 36., 1.15, 0.634218, 0.694021, 0., 83.4177},
{1.6, 9., 1.46, 0.678466, 0.728951, 0.00294985, 82.9014}, {1.6, 21., 1.1, 0.690265, 0.74101, 0., 80.4817}, {2.1, 29., 1.31, 0.675516, 0.72768, 0., 82.6562},
{2.9, 20., 0.51, 0.646018, 0.712125, 0., 81.615}, {2., 23., 0.06, 0.684366, 0.732391, 0., 60.2797}, {0.9, 3., 1.23, 0.640118, 0.69344, 0.00294985, 80.8884},
{2.3, 27., 0.44, 0.663717, 0.720805, 0., 78.5866}, {1.3, 31., 0.65, 0.690265, 0.727478, 0., 73.8895}, {1.7, 9., 0.88, 0.672566, 0.72748, 0., 81.4079},
{2.5, 17., 1.28, 0.643068, 0.707161, 0., 84.5387}, {2.6, 21., 0.72, 0.648968, 0.71449, 0., 82.4219}, {2.4, 14., 1.02, 0.651917, 0.71215, 0., 84.0552},
{1.4, 40., 0.53, 0.693215, 0.72734, 0., 73.1186}, {3., 11., 0.78, 0.637168, 0.70082, 0., 84.3419}, {2.6, 17., 0.03, 0.643068, 0.701158, 0., 65.6775},
{0.8, 22., 0.82, 0.566372, 0.608621, 0., 61.2682}, {1.5, 14., 0.94, 0.696165, 0.741908, 0., 79.8893}, {2.4, 32., 0.02, 0.640118, 0.692746, 0., 61.8765},
{2.8, 21., 0.65, 0.648968, 0.714177, 0., 82.4689}, {1.2, 34., 1.24, 0.666667, 0.703537, 0.00589971, 74.3834}, {2.1, 37., 0.23, 0.672566, 0.716261, 0., 71.3474},
{1.2, 20., 0.29, 0.687316, 0.720892, 0., 65.2075}, {1.7, 8., 0.54, 0.675516, 0.730216, 0., 78.4988}, {1.7, 20., 1.24, 0.687316, 0.736851, 0., 81.4964},
{2.8, 23., 0.4, 0.646018, 0.711122, 0., 79.5687}, {2.2, 7., 0.47, 0.657817, 0.71759, 0., 80.1738}, {2.5, 3., 0.73, 0.634218, 0.69442, 0., 84.768},
{0.8, 38., 0.64, 0.507375, 0.552557, 0.00294985, 54.1239}, {1.2, 31., 0.79, 0.672566, 0.708473, 0., 72.6802}, {2.1, 30., 0.98, 0.669617, 0.724812, 0., 81.4649},
{0.7, 38., 1.01, 0.483776, 0.529325, 0., 51.965}, {3., 7., 1.34, 0.640118, 0.701312, 0., 87.5641}, {2.3, 24., 1.25, 0.660767, 0.720725, 0., 83.5541},
{2.2, 10., 0.08, 0.660767, 0.718582, 0., 65.3045}, {0.9, 3., 0.35, 0.687316, 0.727716, 0., 68.4699}, {1.9, 24., 1.17, 0.678466, 0.733552, 0., 81.9844},
{1.2, 5., 1.07, 0.672566, 0.722841, 0., 79.9386}, {0.9, 31., 0.27, 0.548673, 0.589234, 0., 49.6481}, {2.4, 5., 1., 0.640118, 0.700921, 0., 85.5368},
{2.5, 23., 0.49, 0.651917, 0.716435, 0., 79.8723}, {2.9, 3., 1.43, 0.619469, 0.683196, 0., 88.3711}, {0.7, 6., 1.05, 0.648968, 0.692284, 0., 68.0214},
{1.3, 21., 1.32, 0.693215, 0.734507, 0., 77.4181}, {2.3, 22., 1.25, 0.657817, 0.718183, 0., 83.5693}, {1.2, 38., 0.59, 0.678466, 0.708811, 0., 71.1263},
{1.7, 13., 0.3, 0.702065, 0.74764, 0., 72.7733}, {1., 24., 0.11, 0.587021, 0.628089, 0., 41.1606}, {2.8, 26., 0.08, 0.643068, 0.702993, 0., 70.9152},
{1.7, 15., 0.46, 0.696165, 0.746225, 0., 76.6486}, {0.7, 10., 1., 0.631268, 0.671187, 0., 65.3617}, {2.8, 34., 0.92, 0.637168, 0.697154, 0., 82.6749},
{1.8, 21., 1.33, 0.684366, 0.737224, 0., 82.4902}, {2.7, 15., 0.44, 0.648968, 0.715116, 0., 80.7967}, {1.7, 9., 0.45, 0.684366, 0.735267, 0., 76.4514},
{2.3, 33., 0.2, 0.660767, 0.711471, 0., 72.0913}, {0.8, 40., 0.46, 0.489676, 0.537169, 0., 50.003}, {3., 24., 0.95, 0.640118, 0.706141, 0., 84.035},
{1.1, 14., 0.78, 0.663717, 0.70461, 0., 72.4608}, {1.8, 21., 0.15, 0.699115, 0.744771, 0., 66.1951}, {2.9, 36., 0.82, 0.634218, 0.694189, 0., 82.3866},
{1.4, 23., 0.31, 0.702065, 0.741731, 0., 69.3812}, {1.8, 36., 1.23, 0.672566, 0.721909, 0., 79.9387}, {2.7, 28., 0.32, 0.651917, 0.71059, 0., 78.0063},
{0.9, 21., 0.91, 0.59292, 0.633568, 0., 64.7655}, {2.1, 31., 1.04, 0.669617, 0.72462, 0., 81.7144}, {0.7, 40., 0.78, 0.474926, 0.522402, 0., 50.9631},
{3., 4., 1.29, 0.625369, 0.688439, 0., 87.8306}, {1., 5., 0.34, 0.693215, 0.731896, 0., 68.2452}, {2.1, 11., 1.07, 0.663717, 0.721058, 0., 83.899},

```
{1.9, 5., 0.76, 0.657817, 0.715185, 0., 82.935}, {2.5, 26., 0.8, 0.651917, 0.715332, 0., 82.1173}, {2., 14., 0.84, 0.675516, 0.730242, 0., 81.9326},
{2.7, 9., 0.43, 0.646018, 0.708733, 0., 80.7718}, {2.8, 27., 1.4, 0.646018, 0.711015, 0., 84.6862}, {2.6, 19., 0.47, 0.648968, 0.715958, 0., 80.4232},
{1.7, 27., 0.33, 0.693215, 0.739107, 0., 72.6923}, {1.6, 22., 0.13, 0.707965, 0.749051, 0., 61.759}, {1.5, 10., 0.19, 0.713864, 0.751967, 0., 65.3248},
{2.1, 14., 0.89, 0.669617, 0.725586, 0., 82.6715}, {0.7, 20., 1.13, 0.551622, 0.594225, 0., 58.9607}, {1.6, 23., 0.52, 0.690265, 0.741508, 0., 75.6661},
{1.5, 28., 0.37, 0.699115, 0.737517, 0., 71.9047}, {3., 19., 0.16, 0.646018, 0.708189, 0., 75.7375}, {0.8, 28., 0.72, 0.530973, 0.577305, 0., 57.3158},
{1., 11., 0.27, 0.669617, 0.701961, 0., 61.0587}, {3., 5., 1.04, 0.631268, 0.692921, 0., 86.489}, {1.5, 32., 0.13, 0.696165, 0.734046, 0., 59.4929},
{1., 6., 0.61, 0.681416, 0.724079, 0., 74.0336}, {2.9, 35., 1.46, 0.631268, 0.693947, 0.00294985, 84.2473}, {1.5, 29., 0.21, 0.699115, 0.739787, 0., 66.1164},
{1.3, 21., 1.47, 0.693215, 0.734354, 0., 77.7661}, {0.7, 27., 1.08, 0.519174, 0.567129, 0., 56.4038}, {2.7, 34., 0.14, 0.646018, 0.701438, 0., 72.752},
{1.2, 4., 1.19, 0.666667, 0.719201, 0., 83.0819}, {1.7, 11., 0.51, 0.684366, 0.7357, 0., 76.9329}, {2.7, 9., 0.04, 0.646018, 0.706042, 0., 67.8496},
{2.1, 38., 0.19, 0.672566, 0.715955, 0., 69.9279}, {0.8, 27., 0.62, 0.533923, 0.57903, 0.00589971, 57.7543}, {2.9, 22., 0.59, 0.646018, 0.711381, 0., 82.1422},
{2.2, 13., 1.23, 0.654867, 0.714146, 0.00294985, 84.3343}, {2.8, 36., 1.03, 0.640118, 0.700231, 0., 83.2254}, {1.4, 13., 0.68, 0.696165, 0.742872, 0., 76.8565},
{1.4, 12., 0.29, 0.713864, 0.749782, 0., 69.5444}, {2.3, 33., 1.11, 0.651917, 0.71015, 0., 82.1223}, {1.4, 7., 1.22, 0.681416, 0.731962, 0., 81.1496},
{1.7, 8., 1.15, 0.672566, 0.728079, 0., 83.644}, {1.5, 8., 0.18, 0.705015, 0.746217, 0., 64.8158}, {1.1, 24., 0.35, 0.643068, 0.679875, 0., 63.156},
{2.1, 9., 0.9, 0.660767, 0.719625, 0., 83.6469}, {1.3, 13., 0.69, 0.699115, 0.74056, 0., 75.9114}, {1.2, 36., 1.31, 0.672566, 0.705639, 0.00294985, 74.554},
{2.5, 8., 0.67, 0.643068, 0.706048, 0., 83.2745}, {1.2, 26., 0.54, 0.678466, 0.713535, 0., 71.4868}, {1.2, 36., 0.53, 0.678466, 0.708643, 0., 70.6294},
{1.1, 29., 0.49, 0.628319, 0.663955, 0.00589971, 66.1755}, {1.8, 23., 1.06, 0.684366, 0.738304, 0., 81.3298}, {2.8, 5., 1.27, 0.631268, 0.693492, 0., 87.1827},
{1., 31., 0.73, 0.578171, 0.61947, 0., 63.3406}, {0.8, 26., 1.06, 0.548673, 0.594054, 0., 59.9171}, {2.4, 18., 0.55, 0.654867, 0.718142, 0., 80.7214},
{1.6, 25., 1.18, 0.687316, 0.738671, 0., 80.2266}, {0.9, 18., 1.47, 0.60472, 0.645339, 0.00589971, 68.1397}, {0.7, 4., 0.71, 0.657817, 0.701029, 0., 66.9074}}
```

```
(* They were obtained by these long-running codes, they use randomization and the results will differ from the provided ones *)
```

```
(* FINDING OPTIMAL UNIVERSAL PROTOCOL FOR LOW ks *)
Npar = Length[resultMDKsLow];
SetBasicParameterValues[];

Nnn = 200;

TESTTABLE = Array[f, {Nnn + 1, 7}];
TESTTABLE[[1, 1]] = "X";
TESTTABLE[[1, 2]] = "T";
TESTTABLE[[1, 3]] = "kM";
TESTTABLE[[1, 4]] = "OS";
```

```
TESTTABLE[[1, 5]] = "AUC";
TESTTABLE[[1, 6]] = "ToxDeath";
TESTTABLE[[1, 7]] = "ToxAvg";

BestSchedules = Array[f, {Npar + 1, 7}];
BestSchedules[[1, 1]] = "X";
BestSchedules[[1, 2]] = "T";
BestSchedules[[1, 3]] = "kM";
BestSchedules[[1, 4]] = "KsLow Res";
BestSchedules[[1, 5]] = "KsLow OS";
BestSchedules[[1, 6]] = "KsLow Tox";
BestSchedules[[1, 7]] = "Ainj";

TestMDKsLow = Array[f, {Nnn + 1, Npar + 1, 5}];

For[ii = 2, ii ≤ Nnn + 1, ii++,

  X = Floor[RandomReal[{0.7, 3}], 0.1];
  T = RandomInteger[{3, 40}];
  kM = Floor[RandomReal[{0.01, 1.5}], 0.01];

  While[Length[Select[Select[Select[TESTTABLE[[2 ;; ii]], #[[1]] == X &], #[[2]] == T &], #[[3]] == kM &]] > 1,
    X = Floor[RandomReal[{0.7, 3}], 0.1];
    T = RandomInteger[{3, 40}];
    kM = Floor[RandomReal[{0.01, 1.5}], 0.01]];

  TESTTABLE[[ii, 1]] = X;
  TESTTABLE[[ii, 2]] = T;
  TESTTABLE[[ii, 3]] = kM;

  NotebookDelete[pr];
```

```
pr = PrintTemporary["ii=" <> ToString[ii] <> "; X=" <> ToString[X] <> "; T=" <> ToString[T] <> "; kM=" <> ToString[kM]];

TestMDKsLow[[ii, 1, 1]] = "KsLow Res";
TestMDKsLow[[ii, 1, 2]] = "KsLow OS";
TestMDKsLow[[ii, 1, 3]] = "KsLow Tox";
TestMDKsLow[[ii, 1, 4]] = "Ainj";
TestMDKsLow[[ii, 1, 5]] = "DoseMaxTox";

(* for-cycle begins *)
Quiet[For[ij = 1, ij ≤ Npar, ij++,

   NotebookDelete[prr]; (* To see the code running *)
   prr = PrintTemporary["Set " <> ToString[ij] <> " of " <> ToString[Npar]];

   kappac = resultMDKsLow[[ij, 1]];
   kappap = resultMDKsLow[[ij, 2]];
   gamma = resultMDKsLow[[ij, 3]] * Nnor / 10^7;
   V = resultMDKsLow[[ij, 4]];
   ks = resultMDKsLow[[ij, 5]];
   rho = resultMDKsLow[[ij, 6]];
   omega = resultMDKsLow[[ij, 7]];
   alpha = resultMDKsLow[[ij, 8]];
   kf = resultMDKsLow[[ij, 9]];
   N0 = resultMDKsLow[[ij, 10]] * 10^7 / Nnor;

   Dose = Min[(X * gamma * N0 + 3)/(eta + 1), Min[
      (Abld - (gamma * N0)/(eta + 1) * (lambda * 0.5)/((lambda + 0.5) * (lambda + 0.4))) * (lambda + 0.04)/lambda + (gamma * N0)/(eta + 1), ((lambda + 0.5) * (lambda + 0.4))/(lambda * 0.5) * Abld]];
```

```
(* overall level of toxic decays due to this dose cannot exceed *)
DoseMaxTox = Max[lambda/(lambda + 0.04) * (Dose - (gamma * N0)/(eta + 1)) + (lambda * 0.5)/((lambda + 0.5) * (lambda + 0.4)) * (gamma * N0)/(eta + 1),
    lambda/(lambda + 0.04 + kon * gamma * N0 / V) * Dose + (lambda * 0.5)/((lambda + 0.5) * (lambda + 0.4)) * Dose];

(* remaining safe dose *)
Asfd = Max[(Abld - DoseMaxTox) * (lambda + 0.04)/lambda, 0];

Ainj = {{0, Dose, eta * Dose}};

(* next doses -- estimation *)
NextDoses = Min[Asfd, kM * (gamma * N0)/(eta + 1)];
If[NextDoses ≠ 0, (* how many of them are ok t0 inject *)
  nn = Round[Asfd / NextDoses];
  (* a remainder, last dose *)
  DoseLast = Asfd - (nn * NextDoses);

  Ainj = Join[Ainj, Table[{i * T, NextDoses, eta * NextDoses}, {i, 1, nn}]];
  If[DoseLast > 0, Ainj = Join[Ainj, {{(nn + 1) * T, DoseLast, eta * DoseLast}}]];
  Ainj = Select[Ainj, #[[1]] < 365 &];
  If[Length[Ainj] > 10, Ainj = Ainj[[1 ;; 10]]];

res = PatientTEST2[];
If[! NumberQ[res[[2]]], res = PatientTEST2[]];
If[res[[3]] < 0, res = PatientTEST2[]];

TestMDKsLow[[ii, 1 + ij, 1]] = res[[1]]; (*Res*)
```

```
      TestMDKsLow[[ii, 1 + ij, 2]] = res[[2]]; (*OS*)
      TestMDKsLow[[ii, 1 + ij, 3]] = res[[3]]; (*TOX*)
      TestMDKsLow[[ii, 1 + ij, 4]] = Ainj;
      TestMDKsLow[[ii, 1 + ij, 5]] = DoseMaxTox;

      change = 0;

      If[ii == 2,
        change = 1,

        If[res[[1]] == "CURE" && BestSchedules[[1 + ij, 4]] == "CURE" && res[[3]] < BestSchedules[[1 + ij, 6]], (*cure and less toxic*)
          change = 1,

          If[res[[1]] == "CURE" && BestSchedules[[1 + ij, 4]] == "TBUR",
            change = 1,

            If[res[[1]] == "TBUR" && BestSchedules[[1 + ij, 4]] == "TBUR" && res[[2]] > BestSchedules[[1 + ij, 5]], (*not cure, but greater OS*)
              change = 1]]]];

      If[BestSchedules[[1 + ij, 4]] == "TOX", change = 1];

      If[change == 1,
        BestSchedules[[1 + ij, 1]] = X;
        BestSchedules[[1 + ij, 2]] = T;
        BestSchedules[[1 + ij, 3]] = kM;
        BestSchedules[[1 + ij, 4]] = res[[1]];
        BestSchedules[[1 + ij, 5]] = res[[2]];
        BestSchedules[[1 + ij, 6]] = res[[3]];
        BestSchedules[[1 + ij, 7]] = Ainj]
    ]];
  (* for-cycle ends *)
```

```
  TestMDKsLowSM = SurvivalModelFit[TestMDKsLow[[ii, 2 ;;, 2]]];
  TESTTABLE[[ii, 4]] = TestMDKsLowSM[364]; (* OS *)
  TESTTABLE[[ii, 5]] = Total[TestMDKsLow[[ii, 2 ;;, 2]]] / 365 / (Npar - 1); (* AUC *)
  TESTTABLE[[ii, 6]] = N[Length[Select[TestMDKsLow[[ii, 2 ;;, 3]], # > Abld / nCpm &]] / Npar]; (* Tox Deaths *)
  TESTTABLE[[ii, 7]] = Mean[TestMDKsLow[[ii, 2 ;;, 3]]]; (* "ToxAvg" *)

  NotebookDelete[prTab];
  prTab = PrintTemporary[MatrixForm[TESTTABLE[[1 ;; ii]]]];
]
OutcomeKsLow = TESTTABLE;

(* FINDING OPTIMAL UNIVERSAL PROTOCOL FOR MID ks *)

Npar = Length[resultMDKsMid];
SetBasicParameterValues[];

Nnn = 200;

TESTTABLE = Array[f, {Nnn + 1, 7}];
TESTTABLE[[1, 1]] = "X";
TESTTABLE[[1, 2]] = "T";
TESTTABLE[[1, 3]] = "kM";
TESTTABLE[[1, 4]] = "OS";
TESTTABLE[[1, 5]] = "AUC";
TESTTABLE[[1, 6]] = "ToxDeath";
TESTTABLE[[1, 7]] = "ToxAvg";

BestSchedules = Array[f, {Npar + 1, 7}];
BestSchedules[[1, 1]] = "X";
BestSchedules[[1, 2]] = "T";
```

```
BestSchedules[[1, 3]] = "kM";
BestSchedules[[1, 4]] = "KsMid Res";
BestSchedules[[1, 5]] = "KsMid OS";
BestSchedules[[1, 6]] = "KsMid Tox";
BestSchedules[[1, 7]] = "Ainj";

TestMDKsMid = Array[f, {Nnn + 1, Npar + 1, 5}];

For[ii = 2, ii ≤ Nnn + 1, ii++,

  X = Floor[RandomReal[{0.7, 3}], 0.1];
  T = RandomInteger[{3, 40}];
  kM = Floor[RandomReal[{0.01, 1.5}], 0.01];

  While[Length[Select[Select[Select[TESTTABLE[[2 ;; ii]], #[[1]] == X &], #[[2]] == T &], #[[3]] == kM &]] > 1,
    X = Floor[RandomReal[{0.7, 3}], 0.1];
    T = RandomInteger[{3, 40}];
    kM = Floor[RandomReal[{0.01, 1.5}], 0.01]];

  TESTTABLE[[ii, 1]] = X;
  TESTTABLE[[ii, 2]] = T;
  TESTTABLE[[ii, 3]] = kM;

  NotebookDelete[pr];
  pr = PrintTemporary["ii=" <> ToString[ii] <> "; X=" <> ToString[X] <> "; T=" <> ToString[T] <> "; kM=" <> ToString[kM]];

  TestMDKsMid[[ii, 1, 1]] = "KsMid Res";
  TestMDKsMid[[ii, 1, 2]] = "KsMid OS";
  TestMDKsMid[[ii, 1, 3]] = "KsMid Tox";
  TestMDKsMid[[ii, 1, 4]] = "Ainj";
  TestMDKsMid[[ii, 1, 5]] = "DoseMaxTox";
```

```
(* for begins *)
Quiet[For[ij = 1, ij ≤ Npar, ij++,

  NotebookDelete[prr]; (* To see the code running *)
  prr = PrintTemporary["Set " <> ToString[ij] <> " of " <> ToString[Npar]];

  kappac = resultMDKsMid[[ij, 1]];
  kappap = resultMDKsMid[[ij, 2]];
  gamma = resultMDKsMid[[ij, 3]] * Nnor / 10^7;
  V = resultMDKsMid[[ij, 4]];
  ks = resultMDKsMid[[ij, 5]];
  rho = resultMDKsMid[[ij, 6]];
  omega = resultMDKsMid[[ij, 7]];
  alpha = resultMDKsMid[[ij, 8]];
  kf = resultMDKsMid[[ij, 9]];
  N0 = resultMDKsMid[[ij, 10]] * 10^7 / Nnor;

  Dose = Min[(X * gamma * N0 + 3)/(eta + 1), Min[
     (Abld - (gamma * N0)/(eta + 1) * (lambda * 0.5)/((lambda + 0.5) * (lambda + 0.4))) * (lambda + 0.04)/lambda + (gamma * N0)/(eta + 1), ((lambda + 0.5) * (lambda + 0.4))/(lambda * 0.5) * Abld]];

  (* overall level of toxic decays due to this dose cannot exceed *)
  DoseMaxTox = Max[lambda/(lambda + 0.04) * (Dose - (gamma * N0)/(eta + 1)) + (lambda * 0.5)/((lambda + 0.5) * (lambda + 0.4)) * (gamma * N0)/(eta + 1),
     lambda/(lambda + 0.04 + kon * gamma * N0 / V) * Dose + (lambda * 0.5)/((lambda + 0.5) * (lambda + 0.4)) * Dose];

  (* remaining safe dose *)
```

```
Asfd = Max[ (Abld - DoseMaxTox) * (lambda + 0.04)/lambda, 0];

Ainj = {{0, Dose, eta * Dose}};

(* next doses -- estimation *)
NextDoses = Min[Asfd, kM * (gamma * N0)/(eta + 1)];
If[NextDoses ≠ 0, (* how many of them are ok t0 inject *)
  nn = Round[Asfd / NextDoses];
  (* a remainder, last dose *)
  DoseLast = Asfd - (nn * NextDoses);

  Ainj = Join[Ainj, Table[{i * T, NextDoses, eta * NextDoses}, {i, 1, nn}]];
  If[DoseLast > 0, Ainj = Join[Ainj, {{(nn + 1) * T, DoseLast, eta * DoseLast}}]];
  Ainj = Select[Ainj, #[[1]] < 365 &];
  If[Length[Ainj] > 10, Ainj = Ainj[[1 ;; 10]]];

res = PatientTEST2[];
If[! NumberQ[res[[2]]], res = PatientTEST2[]];
If[res[[3]] < 0, res = PatientTEST2[]];

TestMDKsMid[[ii, 1 + ij, 1]] = res[[1]]; (*Res*)
TestMDKsMid[[ii, 1 + ij, 2]] = res[[2]]; (*OS*)
TestMDKsMid[[ii, 1 + ij, 3]] = res[[3]]; (*TOX*)
TestMDKsMid[[ii, 1 + ij, 4]] = Ainj;
TestMDKsMid[[ii, 1 + ij, 5]] = DoseMaxTox;

change = 0;

If[ii == 2,
```

```
change = 1,

If [res[[1]] == "CURE" && BestSchedules[[1 + ij, 4]] == "CURE" && res[[3]] < BestSchedules[[1 + ij, 6]], (*cure and less toxic*)
  change = 1,

  If [res[[1]] == "CURE" && BestSchedules[[1 + ij, 4]] == "TBUR",
    change = 1,

    If [res[[1]] == "TBUR" && BestSchedules[[1 + ij, 4]] == "TBUR" && res[[2]] > BestSchedules[[1 + ij, 5]], (*not cure, but greater OS*)
      change = 1] ] ] ];

If [BestSchedules[[1 + ij, 4]] == "TOX", change = 1] ;

If [change == 1,
  BestSchedules[[1 + ij, 1]] = X;
  BestSchedules[[1 + ij, 2]] = T;
  BestSchedules[[1 + ij, 3]] = kM;
  BestSchedules[[1 + ij, 4]] = res[[1]];
  BestSchedules[[1 + ij, 5]] = res[[2]];
  BestSchedules[[1 + ij, 6]] = res[[3]];
  BestSchedules[[1 + ij, 7]] = Ainj ]
]];
(* for−cycle ends *)

TestMDKsMidSM = SurvivalModelFit [TestMDKsMid[[ii, 2 ;;, 2]]] ;
TESTTABLE[[ii, 4]] = TestMDKsMidSM [364] ; (*OS*)
TESTTABLE[[ii, 5]] = Total [TestMDKsMid[[ii, 2 ;;, 2]]] /365/ (Npar − 1) ; (* AUC *)
TESTTABLE[[ii, 6]] = N[Length [Select [TestMDKsMid[[ii, 2 ;;, 3]], # > Abld / nCpm &] ] /Npar]; (* Tox Deaths *)
TESTTABLE[[ii, 7]] = Mean [TestMDKsMid[[ii, 2 ;;, 3]]] ; (* "ToxAvg" *)
```

```
  NotebookDelete[prTab];
  prTab = PrintTemporary[MatrixForm[TESTTABLE⟦1 ;; ii⟧]];
]
OutcomeKsMid = TESTTABLE;

(* FINDING OPTIMAL UNIVERSAL PROTOCOL FOR HIGH ks *)

Npar = Length[resultMDKsHigh];
SetBasicParameterValues[];

Nnn = 200;

TESTTABLE = Array[f, {Nnn + 1, 7}];
TESTTABLE⟦1, 1⟧ = "X";
TESTTABLE⟦1, 2⟧ = "T";
TESTTABLE⟦1, 3⟧ = "kM";
TESTTABLE⟦1, 4⟧ = "OS";
TESTTABLE⟦1, 5⟧ = "AUC";
TESTTABLE⟦1, 6⟧ = "ToxDeath";
TESTTABLE⟦1, 7⟧ = "ToxAvg";

BestSchedules = Array[f, {Npar + 1, 7}];
BestSchedules⟦1, 1⟧ = "X";
BestSchedules⟦1, 2⟧ = "T";
BestSchedules⟦1, 3⟧ = "kM";
BestSchedules⟦1, 4⟧ = "KsHigh Res";
BestSchedules⟦1, 5⟧ = "KsHigh OS";
BestSchedules⟦1, 6⟧ = "KsHigh Tox";
BestSchedules⟦1, 7⟧ = "Ainj";

TestMDKsHigh = Array[f, {Nnn + 1, Npar + 1, 5}];
```

```
For[ii = 2, ii ≤ Nnn + 1, ii++,

  X = Floor[RandomReal[{0.7, 3}], 0.1];
  T = RandomInteger[{3, 40}];
  kM = Floor[RandomReal[{0.01, 1.5}], 0.01];

  While[Length[Select[Select[Select[TESTTABLE⟦2 ;; ii⟧, #⟦1⟧ == X &], #⟦2⟧ == T &], #⟦3⟧ == kM &]] > 1,
   X = Floor[RandomReal[{0.7, 3}], 0.1];
   T = RandomInteger[{3, 40}];
   kM = Floor[RandomReal[{0.01, 1.5}], 0.01]];

  TESTTABLE⟦ii, 1⟧ = X;
  TESTTABLE⟦ii, 2⟧ = T;
  TESTTABLE⟦ii, 3⟧ = kM;

  NotebookDelete[pr];
  pr = PrintTemporary["ii=" <> ToString[ii] <> "; X=" <> ToString[X] <> "; T=" <> ToString[T] <> "; kM=" <> ToString[kM]];

  TestMDKsHigh⟦ii, 1, 1⟧ = "KsHigh Res";
  TestMDKsHigh⟦ii, 1, 2⟧ = "KsHigh OS";
  TestMDKsHigh⟦ii, 1, 3⟧ = "KsHigh Tox";
  TestMDKsHigh⟦ii, 1, 4⟧ = "Ainj";
  TestMDKsHigh⟦ii, 1, 5⟧ = "DoseMaxTox";

  (* for begins *)
  Quiet[For[ij = 1, ij ≤ Npar, ij++,

     NotebookDelete[prr]; (* To see the code running *)
     prr = PrintTemporary["Set " <> ToString[ij] <> " of " <> ToString[Npar]];
```

```
kappac = resultMDKsHigh[[ij, 1]];
kappap = resultMDKsHigh[[ij, 2]];
gamma = resultMDKsHigh[[ij, 3]] * Nnor / 10^7;
V = resultMDKsHigh[[ij, 4]];
ks = resultMDKsHigh[[ij, 5]];
rho = resultMDKsHigh[[ij, 6]];
omega = resultMDKsHigh[[ij, 7]];
alpha = resultMDKsHigh[[ij, 8]];
kf = resultMDKsHigh[[ij, 9]];
N0 = resultMDKsHigh[[ij, 10]] * 10^7 / Nnor;
```

$$\text{Dose} = \text{Min}\left[\frac{X * \text{gamma} * \text{N0} + 3}{\text{eta} + 1}, \text{Min}\left[\left(\text{Abld} - \frac{\text{gamma} * \text{N0}}{\text{eta} + 1} * \frac{\text{lambda} * 0.5}{(\text{lambda} + 0.5) * (\text{lambda} + 0.4)}\right) * \frac{\text{lambda} + 0.04}{\text{lambda}} + \frac{\text{gamma} * \text{N0}}{\text{eta} + 1}, \frac{(\text{lambda} + 0.5) * (\text{lambda} + 0.4)}{\text{lambda} * 0.5} * \text{Abld}\right]\right];$$

(* overall level of toxic decays due to this dose cannot exceed *)

$$\text{DoseMaxTox} = \text{Max}\left[\frac{\text{lambda}}{\text{lambda} + 0.04} * \left(\text{Dose} - \frac{\text{gamma} * \text{N0}}{\text{eta} + 1}\right) + \frac{\text{lambda} * 0.5}{(\text{lambda} + 0.5) * (\text{lambda} + 0.4)} * \frac{\text{gamma} * \text{N0}}{\text{eta} + 1}, \frac{\text{lambda}}{\text{lambda} + 0.04 + \text{kon} * \text{gamma} * \text{N0} / \text{V}} * \text{Dose} + \frac{\text{lambda} * 0.5}{(\text{lambda} + 0.5) * (\text{lambda} + 0.4)} * \text{Dose}\right];$$

(* remaining safe dose *)

$$\text{Asfd} = \text{Max}\left[(\text{Abld} - \text{DoseMaxTox}) * \frac{\text{lambda} + 0.04}{\text{lambda}}, 0\right];$$

```
Ainj = {{0, Dose, eta * Dose}};
```

(* next doses -- estimation *)

```
NextDoses = Min[Asfd, kM * (gamma * N0)/(eta + 1)];

If[NextDoses ≠ 0, (* how many of them are ok t0 inject *)
  nn = Round[Asfd / NextDoses];
   (* a remainder, last dose *)
  DoseLast = Asfd - (nn * NextDoses);

  Ainj = Join[Ainj, Table[{i * T, NextDoses, eta * NextDoses}, {i, 1, nn}]];
  If[DoseLast > 0, Ainj = Join[Ainj, {{(nn + 1) * T, DoseLast, eta * DoseLast}}]];
  Ainj = Select[Ainj, #[[1]] < 365 &];
  If[Length[Ainj] > 10, Ainj = Ainj[[1 ;; 10]]];

res = PatientTEST2[];
If[! NumberQ[res[[2]]], res = PatientTEST2[]];
If[res[[3]] < 0, res = PatientTEST2[]];

TestMDKsHigh[[ii, 1 + ij, 1]] = res[[1]]; (*Res*)
TestMDKsHigh[[ii, 1 + ij, 2]] = res[[2]]; (*OS*)
TestMDKsHigh[[ii, 1 + ij, 3]] = res[[3]]; (*TOX*)
TestMDKsHigh[[ii, 1 + ij, 4]] = Ainj;
TestMDKsHigh[[ii, 1 + ij, 5]] = DoseMaxTox;

change = 0;

If[ii == 2,
  change = 1,

  If[res[[1]] == "CURE" && BestSchedules[[1 + ij, 4]] == "CURE" && res[[3]] < BestSchedules[[1 + ij, 6]], (*cure and less toxic*)
    change = 1,

    If[res[[1]] == "CURE" && BestSchedules[[1 + ij, 4]] == "TBUR",
```

```
            change = 1,

            If[res[[1]] == "TBUR" && BestSchedules[[1 + ij, 4]] == "TBUR" && res[[2]] > BestSchedules[[1 + ij, 5]], (*not cure, but greater OS*)
              change = 1]]]];

        If[BestSchedules[[1 + ij, 4]] == "TOX", change = 1];

        If[change == 1,
          BestSchedules[[1 + ij, 1]] = X;
          BestSchedules[[1 + ij, 2]] = T;
          BestSchedules[[1 + ij, 3]] = kM;
          BestSchedules[[1 + ij, 4]] = res[[1]];
          BestSchedules[[1 + ij, 5]] = res[[2]];
          BestSchedules[[1 + ij, 6]] = res[[3]];
          BestSchedules[[1 + ij, 7]] = Ainj]
      ]];
    (* for-cycle ends *)

    TestMDKsHighSM = SurvivalModelFit[TestMDKsHigh[[ii, 2 ;;, 2]]];
    TESTTABLE[[ii, 4]] = TestMDKsHighSM[364]; (*OS*)
    TESTTABLE[[ii, 5]] = Total[TestMDKsHigh[[ii, 2 ;;, 2]]] / 365 / (Npar - 1); (* AUC *)
    TESTTABLE[[ii, 6]] = N[Length[Select[TestMDKsHigh[[ii, 2 ;;, 3]], # > Abld / nCpm &]] / Npar]; (* Tox Deaths *)
    TESTTABLE[[ii, 7]] = Mean[TestMDKsHigh[[ii, 2 ;;, 3]]]; (* "ToxAvg" *)

    NotebookDelete[prTab];
    prTab = PrintTemporary[MatrixForm[TESTTABLE[[1 ;; ii]]]];
  ]
  OutcomeKsHigh = TESTTABLE;
```

```
(* Figure S.30 itself *)
GraphicsGrid[{{ListPlot[OutcomeKsLow⟦2 ;;, {1, 4}⟧, AxesLabel → {"X", "1-year OS"}, PlotRange → {0.99 * Min[OutcomeKsLow⟦2 ;;, 4⟧],
      1.01 * Max[OutcomeKsLow⟦2 ;;, 4⟧]}, Ticks → {Automatic, Table[{i, PercentForm[i]}, {i, 0.58, 0.63, 0.01}]}],
   ListPlot[OutcomeKsMid⟦2 ;;, {1, 4}⟧, AxesLabel → {"X", "1-year OS"},
    PlotRange → {0.99 * Min[OutcomeKsMid⟦2 ;;, 4⟧], 1.01 * Max[OutcomeKsMid⟦2 ;;, 4⟧]},
    Ticks → {Automatic, Table[{i, PercentForm[i]}, {i, 0.58, 0.70, 0.02}]}],
   ListPlot[OutcomeKsHigh⟦2 ;;, {1, 4}⟧, AxesLabel → {"X", "1-year OS"},
    PlotRange → {0.99 * Min[OutcomeKsHigh⟦2 ;;, 4⟧], 1.01 * Max[OutcomeKsHigh⟦2 ;;, 4⟧]},
    Ticks → {Automatic, Table[{i, PercentForm[i]}, {i, 0.50, 0.70, 0.05}]}]}}, ImageSize → 1000]
```

Out[∘]=

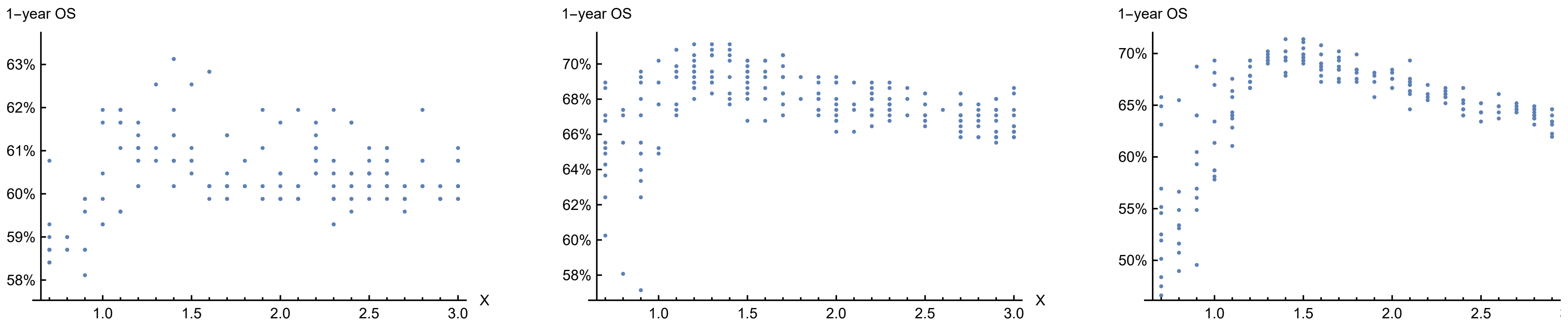


```
(* Figure S.31: Dependence of one-year overall survival on inter-dose interval T and the ratio of second and subsequent doses to initial cancer binding capacity k under fixed ratio of first dose to it X=1.4 *)
```

In[∘]:=

```
(* The outcomes of universal schedules with X=1.4 and varied k, T for the training set are saved here in a closed cell *)
```

In[∘]:=

```
OutcomeKsHighX = {{"X", "T", "kM", "OS", "AUC", "ToxDeath", "ToxAvg"},
   {1.4`, 3.`, 0.05`, 0.6784660766961647`, 0.7248482900165698`, 0.`, 46.01409450338261`},
   {1.4`, 10.`, 0.05`, 0.7079646017699106`, 0.7413859100982002`, 0.`, -14.098909347251078`},
   {1.4`, 17.`, 0.05`, 0.7109144542772852`, 0.7443305923507942`, 0.`, 46.10630950350258`},
   {1.4`, 24.`, 0.05`, 0.705014749262536`, 0.7417180383740529`, 0.`, 46.1591456532844`},
   {1.4`, 31.`, 0.05`, 0.6932153392330376`, 0.7339913452829742`, 0.`, 46.011676849438054`},
```

```
{1.4`, 38.`, 0.05`, 0.6902654867256629`, 0.7240994476977215`, 0.`, -4.37114744165144`*^18},
{1.4`, 45.`, 0.05`, 0.6666666666666661`, 0.7071262713363441`, 0.`, 43.758040792093695`},
{1.4`, 7.`, 0.05`, 0.7020648967551615`, 0.7393980685961132`, 0.`, 46.096872962806465`},
{1.4`, 14.`, 0.05`, 0.7168141592920344`, 0.7479022669419106`, 0.`, 46.13290763166162`},
{1.4`, 21.`, 0.05`, 0.7079646017699106`, 0.7419086026538954`, 0.`, 46.06311485332422`},
{1.4`, 28.`, 0.05`, 0.7020648967551615`, 0.7356769725397224`, 0.`, 46.03366886654738`},
{1.4`, 35.`, 0.05`, 0.6932153392330376`, 0.7266747125260817`, 0.`, 45.9079580475595`},
{1.4`, 42.`, 0.05`, 0.6725663716814154`, 0.7111302120134985`, 0.`, 43.87374638432661`},
{1.4`, 49.`, 0.05`, 0.6548672566371674`, 0.7003099912087908`, 0.`, 42.040586124813935`},
{1.4`, 3.`, 0.1`, 0.6843657817109138`, 0.7296994806530532`, 0.`, 55.33017354876644`},
{1.4`, 10.`, 0.1`, 0.719764011799409`, 0.7488384478720457`, 0.`, 54.826817932705275`},
{1.4`, 17.`, 0.1`, 0.7286135693215329`, 0.7557217735870206`, 0.`, 55.13868573071142`},
{1.4`, 24.`, 0.1`, 0.705014749262536`, 0.7406177101225359`, 0.`, 54.84643625468161`},
{1.4`, 31.`, 0.1`, 0.7020648967551615`, 0.73609425648886`, 0.`, 54.64952014973411`},
{1.4`, 38.`, 0.1`, 0.6902654867256629`, 0.7261059297787142`, 0.`, -4.2736041140822134`*^20},
{1.4`, 45.`, 0.1`, 0.6696165191740407`, 0.7097233373356409`, 0.`, 52.100686864973596`},
{1.4`, 7.`, 0.1`, 0.7109144542772852`, 0.7466940611203055`, 0.`, 55.11053958328414`},
{1.4`, 14.`, 0.1`, 0.7286135693215329`, 0.7557073181662357`, 0.`, 55.15390727815931`},
{1.4`, 21.`, 0.1`, 0.7109144542772852`, 0.7444005201746812`, 0.`, 54.87783776367667`},
{1.4`, 28.`, 0.1`, 0.7020648967551615`, 0.7404750349348578`, 0.`, 54.861321307552736`},
{1.4`, 35.`, 0.1`, 0.6961651917404122`, 0.7319082116997263`, 0.`, -3.406843600331924`*^18},
{1.4`, 42.`, 0.1`, 0.6814159292035392`, 0.7189044578792996`, 0.`, 52.64884091749347`},
{1.4`, 49.`, 0.1`, 0.6666666666666661`, 0.7080865720740112`, 0.`, 50.433789128984046`},
{1.4`, 3.`, 0.15`, 0.6843657817109138`, 0.7310933124125734`, 0.`, 61.71070821834994`},
{1.4`, 10.`, 0.15`, 0.719764011799409`, 0.7515927464639818`, 0.`, 60.4946141302529`},
{1.4`, 17.`, 0.15`, 0.7227138643067836`, 0.7526201381288228`, 0.`, 60.61507163543391`},
{1.4`, 24.`, 0.15`, 0.705014749262536`, 0.742439526454534`, 0.`, 60.22248616459923`},
{1.4`, 31.`, 0.15`, 0.6991150442477868`, 0.7344833988327069`, 0.`, 59.81238467108587`},
{1.4`, 38.`, 0.15`, 0.6873156342182883`, 0.725304677235462`, 0.`, 59.28338308676024`},
{1.4`, 45.`, 0.15`, 0.6696165191740407`, 0.7138572660952911`, 0.`, 57.535724630998764`},
{1.4`, 7.`, 0.15`, 0.7079646017699106`, 0.746002573653284`, 0.`, 60.493352442838315`},
```

```
{1.4`, 14.`, 0.15`, 0.7256637168141582`, 0.7542892321794992`, 0.`, 60.64061550556058`},
{1.4`, 21.`, 0.15`, 0.7109144542772852`, 0.746368758034702`, 0.`, 60.34957378366103`},
{1.4`, 28.`, 0.15`, 0.6991150442477868`, 0.7380465392549286`, 0.`, 60.074362621140146`},
{1.4`, 35.`, 0.15`, 0.6932153392330376`, 0.7292358579520615`, 0.`, 59.52077654967581`},
{1.4`, 42.`, 0.15`, 0.6873156342182883`, 0.724336916151435`, 0.`, 57.859022793262504`},
{1.4`, 49.`, 0.15`, 0.6725663716814154`, 0.7147578749161766`, 0.`, 55.70723748048927`},
{1.4`, 3.`, 0.2`, 0.6843657817109138`, 0.7321724851829878`, 0.`, 66.48980607801026`},
{1.4`, 10.`, 0.2`, 0.7138643067846598`, 0.7488657490032998`, 0.`, 64.36080352223027`},
{1.4`, 17.`, 0.2`, 0.719764011799409`, 0.7513524336379281`, 0.`, 64.70959556321735`},
{1.4`, 24.`, 0.2`, 0.7079646017699106`, 0.7445347387531349`, 0.`, 64.31000892890447`},
{1.4`, 31.`, 0.2`, 0.705014749262536`, 0.7357337812140347`, 0.`, 63.65019060517266`},
{1.4`, 38.`, 0.2`, 0.6902654867256629`, 0.7266395416645085`, 0.`, 63.12963897383025`},
{1.4`, 45.`, 0.2`, 0.6755162241887901`, 0.7161913266724161`, 0.`, 61.212272205893335`},
{1.4`, 7.`, 0.2`, 0.7020648967551615`, 0.7425177379353399`, 0.`, 64.4566051124547`},
{1.4`, 14.`, 0.2`, 0.7227138643067836`, 0.753969016371637`, 0.`, 64.74685159980662`},
{1.4`, 21.`, 0.2`, 0.7168141592920344`, 0.7487273862722144`, 0.`, 64.4911852570685`},
{1.4`, 28.`, 0.2`, 0.7020648967551615`, 0.7393991887205681`, 0.`, 64.02532737895609`},
{1.4`, 35.`, 0.2`, 0.6932153392330376`, 0.7283662245419087`, 0.`, 63.20978712996527`},
{1.4`, 42.`, 0.2`, 0.6843657817109138`, 0.7233908402157404`, 0.`, 61.67466302660453`},
{1.4`, 49.`, 0.2`, 0.6755162241887901`, 0.7153243008588267`, 0.`, 59.46631383478288`},
{1.4`, 3.`, 0.25`, 0.6843657817109138`, 0.7336471864512687`, 0.`, 70.37425424869488`},
{1.4`, 10.`, 0.25`, 0.7109144542772852`, 0.7487157073299684`, 0.`, 67.53594127833037`},
{1.4`, 17.`, 0.25`, 0.7227138643067836`, 0.7527705853455708`, 0.`, 67.88360207562208`},
{1.4`, 24.`, 0.25`, 0.7079646017699106`, 0.744734936814908`, 0.`, 67.40018203875267`},
{1.4`, 31.`, 0.25`, 0.7020648967551615`, 0.7372182004242052`, 0.`, 66.66625282902193`},
{1.4`, 38.`, 0.25`, 0.6932153392330376`, 0.7275033993686354`, 0.`, 66.14553770242972`},
{1.4`, 45.`, 0.25`, 0.6755162241887901`, 0.7159476675370351`, 0.`, 64.2868260419904`},
{1.4`, 7.`, 0.25`, 0.6991150442477868`, 0.7410222312178659`, 0.`, 67.38887191053969`},
{1.4`, 14.`, 0.25`, 0.7227138643067836`, 0.7540381404479762`, 0.`, 67.9372677512227`},
{1.4`, 21.`, 0.25`, 0.7168141592920344`, 0.7498264535961905`, 0.`, 67.64140925363277`},
{1.4`, 28.`, 0.25`, 0.705014749262536`, 0.7393289637141678`, 0.`, 67.02951792310076`},
```

```
{1.4`, 35.`, 0.25`, 0.6932153392330376`, 0.7279774306533738`, 0.`, 66.18479362659383`},
{1.4`, 42.`, 0.25`, 0.6873156342182883`, 0.7246918656501313`, 0.`, 64.84974702990989`},
{1.4`, 49.`, 0.25`, 0.6755162241887901`, 0.7152726373498793`, 0.`, 62.792141506521354`},
{1.4`, 3.`, 0.3`, 0.6784660766961647`, 0.728717022959857`, 0.`, 72.49745739807145`},
{1.4`, 10.`, 0.3`, 0.7109144542772852`, 0.7492025741431826`, 0.`, 70.03655779165929`},
{1.4`, 17.`, 0.3`, 0.7138643067846598`, 0.7485573841806069`, 0.`, 69.46488294519294`},
{1.4`, 24.`, 0.3`, 0.705014749262536`, 0.7465017633644891`, 0.`, 69.54825495377865`},
{1.4`, 31.`, 0.3`, 0.7020648967551615`, 0.7393309910605979`, 0.`, 69.04106973303081`},
{1.4`, 38.`, 0.3`, 0.6932153392330376`, 0.7284800440192535`, 0.`, 68.541991725`},
{1.4`, 45.`, 0.3`, 0.6755162241887901`, 0.7161655668737945`, 0.`, 66.28415349596777`},
{1.4`, 7.`, 0.3`, 0.6961651917404122`, 0.7410378294005225`, 0.`, 70.23702601470265`},
{1.4`, 14.`, 0.3`, 0.7109144542772852`, 0.7456396289902448`, 0.`, 69.31049869428901`},
{1.4`, 21.`, 0.3`, 0.7109144542772852`, 0.7466433024601402`, 0.`, 69.5679862042126`},
{1.4`, 28.`, 0.3`, 0.7020648967551615`, 0.7393632834978925`, 0.`, 69.09612890084054`},
{1.4`, 35.`, 0.3`, 0.6961651917404122`, 0.7302875439309484`, 0.`, 68.64805395913544`},
{1.4`, 42.`, 0.3`, 0.6843657817109138`, 0.7221567836403968`, 0.`, 66.87959504563992`},
{1.4`, 49.`, 0.3`, 0.6696165191740407`, 0.7116597526293149`, 0.`, 64.69555938819927`},
{1.4`, 3.`, 0.35`, 0.6666666666666661`, 0.7214885515835952`, 0.`, 73.8756514401308`},
{1.4`, 10.`, 0.35`, 0.7020648967551615`, 0.7450696059712105`, 0.`, 71.38901207479641`},
{1.4`, 17.`, 0.35`, 0.7079646017699106`, 0.7448980212613842`, 0.`, 70.60021158240507`},
{1.4`, 24.`, 0.35`, 0.6991150442477868`, 0.7418843208409518`, 0.`, 70.47943101220558`},
{1.4`, 31.`, 0.35`, 0.7020648967551615`, 0.7398741370649315`, 0.`, 70.12710292943387`},
{1.4`, 38.`, 0.35`, 0.6932153392330376`, 0.7273975380883608`, 0.`, 69.35821295632509`},
{1.4`, 45.`, 0.35`, 0.6755162241887901`, 0.7168727517218045`, 0.`, 67.56096034220687`},
{1.4`, 7.`, 0.35`, 0.6961651917404122`, 0.7404759408106555`, 0.`, 71.30228898759263`},
{1.4`, 14.`, 0.35`, 0.7109144542772852`, 0.7466509893724241`, 0.`, 70.79437742738949`},
{1.4`, 21.`, 0.35`, 0.705014749262536`, 0.7441099773049099`, 0.`, 70.73669681049995`},
{1.4`, 28.`, 0.35`, 0.6991150442477868`, 0.737553471753443`, 0.`, 70.15745716394022`},
{1.4`, 35.`, 0.35`, 0.6932153392330376`, 0.7277837141264062`, 0.`, 69.4079494505298`},
{1.4`, 42.`, 0.35`, 0.6843657817109138`, 0.7219507837276378`, 0.`, 68.19380992102968`},
{1.4`, 49.`, 0.35`, 0.6725663716814154`, 0.7151794767374511`, 0.`, 66.33654204974427`},
```

{1.4`, 3.`, 0.4`, 0.6666666666666661`, 0.7212822813708301`, 0.`, 75.84595399470666`},
{1.4`, 10.`, 0.4`, 0.7020648967551615`, 0.7455611544184821`, 0.`, 73.23686113849163`},
{1.4`, 17.`, 0.4`, 0.7079646017699106`, 0.744789558225596`, 0.`, 72.3315455025828`},
{1.4`, 24.`, 0.4`, 0.6991150442477868`, 0.7417120610452437`, 0.`, 72.1662863490468`},
{1.4`, 31.`, 0.4`, 0.6991150442477868`, 0.7365834692313055`, 0.`, 71.73439385848982`},
{1.4`, 38.`, 0.4`, 0.6932153392330376`, 0.7274151076115682`, 0.`, 70.99210293205358`},
{1.4`, 45.`, 0.4`, 0.6814159292035392`, 0.7189499654246744`, 0.`, 69.27823194789296`},
{1.4`, 7.`, 0.4`, 0.6961651917404122`, 0.7413523386422546`, 0.`, 73.24272648468538`},
{1.4`, 14.`, 0.4`, 0.7079646017699106`, 0.7462636539846798`, 0.`, 72.68875295012745`},
{1.4`, 21.`, 0.4`, 0.7079646017699106`, 0.7472969085694195`, 0.`, 72.42271175381507`},
{1.4`, 28.`, 0.4`, 0.7020648967551615`, 0.7398180829354535`, 0.`, 72.01756622541015`},
{1.4`, 35.`, 0.4`, 0.6932153392330376`, 0.7295015342598042`, 0.`, 71.19163017410868`},
{1.4`, 42.`, 0.4`, 0.6873156342182883`, 0.7232248210155927`, 0.`, 69.88986452555561`},
{1.4`, 49.`, 0.4`, 0.6725663716814154`, 0.7145625810712765`, 0.`, 68.04686134262083`},
{1.4`, 3.`, 0.45`, 0.6666666666666661`, 0.7210922346275911`, 0.`, 77.13655154454763`},
{1.4`, 10.`, 0.45`, 0.6991150442477868`, 0.7432342076758296`, 0.`, 73.80528972809932`},
{1.4`, 17.`, 0.45`, 0.7079646017699106`, 0.7457557528884946`, 0.`, 73.50209790012349`},
{1.4`, 24.`, 0.45`, 0.6991150442477868`, 0.7421761015274599`, 0.`, 73.18233574801206`},
{1.4`, 31.`, 0.45`, 0.6991150442477868`, 0.735779267738695`, 0.`, 72.63625579866417`},
{1.4`, 38.`, 0.45`, 0.6932153392330376`, 0.7277127962354211`, 0.`, 72.13955361110696`},
{1.4`, 45.`, 0.45`, 0.6784660766961647`, 0.7180223278262311`, 0.`, 70.2063580207736`},
{1.4`, 7.`, 0.45`, 0.6932153392330376`, 0.739630056127254`, 0.`, 74.67497901887052`},
{1.4`, 14.`, 0.45`, 0.705014749262536`, 0.7455667541613638`, 0.`, 73.7497357785274`},
{1.4`, 21.`, 0.45`, 0.705014749262536`, 0.7455770834926477`, 0.`, 73.30676636375848`},
{1.4`, 28.`, 0.45`, 0.7020648967551615`, 0.7393572347543071`, 0.`, 72.96443348352028`},
{1.4`, 35.`, 0.45`, 0.6932153392330376`, 0.7290159532578925`, 0.`, 72.1101054117634`},
{1.4`, 42.`, 0.45`, 0.6873156342182883`, 0.7226879776275567`, 0.`, 70.87820550156694`},
{1.4`, 49.`, 0.45`, 0.6725663716814154`, 0.7143946224119374`, 0.`, 68.88769193607199`},
{1.4`, 3.`, 0.5`, 0.6666666666666661`, 0.7208537864173843`, 0.`, 78.15252602819048`},
{1.4`, 10.`, 0.5`, 0.6961651917404122`, 0.7412172203772796`, 0.`, 74.79416530896388`},
{1.4`, 17.`, 0.5`, 0.705014749262536`, 0.7443027320071386`, 0.`, 74.28031272318786`},

```
{1.4`, 24.`, 0.5`, 0.6991150442477868`, 0.742373407612726`, 0.`, 74.12374805197449`},
{1.4`, 31.`, 0.5`, 0.6991150442477868`, 0.7358894615385527`, 0.`, 73.51161349971846`},
{1.4`, 38.`, 0.5`, 0.6932153392330376`, 0.7277727060892943`, 0.`, 73.03836848056915`},
{1.4`, 45.`, 0.5`, 0.6784660766961647`, 0.7181421415916367`, 0.`, 71.04680894456517`},
{1.4`, 7.`, 0.5`, 0.6932153392330376`, 0.7404879014589268`, 0.`, 75.88467152952316`},
{1.4`, 14.`, 0.5`, 0.7020648967551615`, 0.7444759553732417`, 0.`, 74.62844626319716`},
{1.4`, 21.`, 0.5`, 0.705014749262536`, 0.7455055514641058`, 0.`, 74.23948383359036`},
{1.4`, 28.`, 0.5`, 0.7020648967551615`, 0.7409951810822991`, 0.`, 73.93536628824742`},
{1.4`, 35.`, 0.5`, 0.6932153392330376`, 0.7302244783426118`, 0.`, 73.07367146361025`},
{1.4`, 42.`, 0.5`, 0.6873156342182883`, 0.7226959217864463`, 0.`, 71.90422566168014`},
{1.4`, 49.`, 0.5`, 0.6696165191740407`, 0.7116279007903945`, 0.`, 69.78137400025643`}};
```

In[ ]:=

```
OutcomeKsMidX = {{"X", "T", "kM", "OS", "AUC", "ToxDeath", "ToxAvg"},
{1.4`, 10.`, 0.05`, 0.7142857142857153`, 0.7591729736204582`, 0.`, 45.247433978119716`},
{1.4`, 17.`, 0.05`, 0.7142857142857153`, 0.762023498906282`, 0.`, 45.041703316683005`},
{1.4`, 24.`, 0.05`, 0.7142857142857153`, 0.765254093777405`, 0.`, 45.19890858844046`},
{1.4`, 14.`, 0.05`, 0.7142857142857153`, 0.7619583838290835`, 0.`, 45.1123682181885`},
{1.4`, 24.`, 0.1`, 0.7142857142857153`, 0.7631075543742905`, 0.`, 54.72541630285257`},
{1.4`, 14.`, 0.1`, 0.7142857142857153`, 0.7640007492150527`, 0.`, 54.74453408551143`},
{1.4`, 21.`, 0.1`, 0.7142857142857153`, 0.7660260183441574`, 0.`, 54.37883458384397`},
{1.4`, 21.`, 0.05`, 0.7111801242236035`, 0.7630614790275554`, 0.`, -3.3556213068229073`},
{1.4`, 17.`, 0.1`, 0.7111801242236035`, 0.763354282986669`, 0.`, -7.016356658390817`*^32},
{1.4`, 21.`, 0.2`, 0.7111801242236035`, 0.7610255058355826`, 0.`, 64.38273128479216`},
{1.4`, 21.`, 0.25`, 0.7111801242236035`, 0.7601645995238392`, 0.`, 67.45052117111666`},
{1.4`, 21.`, 0.3`, 0.7111801242236035`, 0.7614203906077874`, 0.`, 69.98292669565639`},
{1.4`, 10.`, 0.4`, 0.7111801242236035`, 0.7621038346130069`, 0.`, 75.28561826112986`},
{1.4`, 10.`, 0.1`, 0.7080745341614917`, 0.757833352482827`, 0.`, 54.76379678862979`},
{1.4`, 31.`, 0.1`, 0.7080745341614917`, 0.7548249164248642`, 0.`, 53.48690224401046`},
{1.4`, 28.`, 0.1`, 0.7080745341614917`, 0.7588754007395649`, 0.`, 54.07961788379429`},
{1.4`, 10.`, 0.15`, 0.7080745341614917`, 0.759393960792547`, 0.`, 61.38752915203793`},
{1.4`, 31.`, 0.15`, 0.7080745341614917`, 0.7521503530592277`, 0.`, 59.73594412625307`},
```

```
{1.4`, 28.`, 0.15`, 0.7080745341614917`, 0.7543778719943111`, 0.`, -195.93975183546672`},
{1.4`, 10.`, 0.2`, 0.7080745341614917`, 0.7590867634545193`, 0.`, 65.60136110643177`},
{1.4`, 31.`, 0.2`, 0.7080745341614917`, 0.752384211671686 7`, 0.`, 63.36918590581414`},
{1.4`, 14.`, 0.2`, 0.7080745341614917`, 0.7607597015116649`, 0.`, 65.18298828155213`},
{1.4`, 35.`, 0.2`, 0.7080745341614917`, 0.7495274387370326`, 0.`, 63.538114582950264`},
{1.4`, 10.`, 0.25`, 0.7080745341614917`, 0.7592565404571282`, 0.`, 68.9139685013169`},
{1.4`, 31.`, 0.25`, 0.7080745341614917`, 0.7515775689561967`, 0.`, 66.29503366356553`},
{1.4`, 10.`, 0.3`, 0.7080745341614917`, 0.75977665861779`, 0.`, 71.4401964116387`},
{1.4`, 10.`, 0.35`, 0.7080745341614917`, 0.7603161139978317`, 0.`, 73.73428372680482`},
{1.4`, 21.`, 0.35`, 0.7080745341614917`, 0.7591613772897995`, 0.`, 72.20857654538563`},
{1.4`, 24.`, 0.4`, 0.7080745341614917`, 0.7581696817596506`, 0.`, 73.39167304460528`},
{1.4`, 21.`, 0.4`, 0.7080745341614917`, 0.7589318837237213`, 0.`, 73.72264479874403`},
{1.4`, 24.`, 0.45`, 0.7080745341614917`, 0.7574074812707816`, 0.`, 74.53202514393207`},
{1.4`, 21.`, 0.45`, 0.7080745341614917`, 0.7591333148307589`, 0.`, 74.86453969256343`},
{1.4`, 24.`, 0.5`, 0.7080745341614917`, 0.7571647578440222`, 0.`, 75.85970332306323`},
{1.4`, 21.`, 0.5`, 0.7080745341614917`, 0.7589018590209969`, 0.`, 76.2121988554172`},
{1.4`, 7.`, 0.05`, 0.7049689440993798`, 0.7513392372028258`, 0.`, 45.3019499842705`},
{1.4`, 24.`, 0.15`, 0.7049689440993798`, 0.7590810033648255`, 0.`, 60.172218817683635`},
{1.4`, 14.`, 0.15`, 0.7049689440993798`, 0.7575179001734254`, 0.`, 60.8535298394285`},
{1.4`, 21.`, 0.15`, 0.7049689440993798`, 0.7584742455745331`, 0.`, 60.603406880212326`},
{1.4`, 35.`, 0.15`, 0.7049689440993798`, 0.7498215991507658`, 0.`, -2.0425250838144848`*^19},
{1.4`, 38.`, 0.2`, 0.7049689440993798`, 0.746362407009282 8`, 0.`, 62.83782938979863`},
{1.4`, 7.`, 0.2`, 0.7049689440993798`, 0.7564045525025772`, 0.`, 66.10691654670866`},
{1.4`, 17.`, 0.25`, 0.7049689440993798`, 0.7591288483506281`, 0.`, 67.98130394317889`},
{1.4`, 38.`, 0.25`, 0.7049689440993798`, 0.7487179946866841`, 0.`, 65.96942654512502`},
{1.4`, 7.`, 0.25`, 0.7049689440993798`, 0.7559294322029111`, 0.`, 69.45076981327566`},
{1.4`, 14.`, 0.25`, 0.7049689440993798`, 0.7584717667379118`, 0.`, 68.42014658577283`},
{1.4`, 35.`, 0.25`, 0.7049689440993798`, 0.7492791643191004`, 0.`, 66.221509414666`},
{1.4`, 17.`, 0.3`, 0.7049689440993798`, 0.7583558363435785`, 0.`, 70.50897798777483`},
{1.4`, 38.`, 0.3`, 0.7049689440993798`, 0.7502488048068369`, 0.`, 68.52682864993253`},
{1.4`, 35.`, 0.3`, 0.7049689440993798`, 0.7508097263812247`, 0.`, 68.5818985283574`},
```

```
{1.4`, 17.`, 0.35`, 0.7049689440993798`, 0.757836478811787`, 0.`, 72.69727874096742`},
{1.4`, 24.`, 0.35`, 0.7049689440993798`, 0.75763284145798`, 0.`, 71.90032969719043`},
{1.4`, 28.`, 0.35`, 0.7049689440993798`, 0.7539182816870026`, 0.`, 71.27667272060874`},
{1.4`, 17.`, 0.4`, 0.7049689440993798`, 0.758371925577107`, 0.`, 74.22595878279508`},
{1.4`, 38.`, 0.4`, 0.7049689440993798`, 0.7493679170780831`, 0.`, 71.66730281808324`},
{1.4`, 28.`, 0.4`, 0.7049689440993798`, 0.7546117898790923`, 0.`, 72.75227886431948`},
{1.4`, 35.`, 0.4`, 0.7049689440993798`, 0.7510746022345333`, 0.`, 72.03718109395886`},
{1.4`, 17.`, 0.45`, 0.7049689440993798`, 0.7579998678641128`, 0.`, 75.3051845372276`},
{1.4`, 31.`, 0.45`, 0.7049689440993798`, 0.750223681023016`, 0.`, 73.21417932116992`},
{1.4`, 28.`, 0.45`, 0.7049689440993798`, 0.7543219751493753`, 0.`, 73.85861741737543`},
{1.4`, 17.`, 0.5`, 0.7049689440993798`, 0.758029271450135`, 0.`, 76.67243421561051`},
{1.4`, 28.`, 0.5`, 0.7049689440993798`, 0.7541097065825728`, 0.`, 75.20404910838032`},
{1.4`, 31.`, 0.05`, 0.701863354037268`, 0.7448556950800123`, 0.`, 44.22564823364016`},
{1.4`, 28.`, 0.05`, 0.701863354037268`, 0.7556910057446785`, 0.`, 44.79247167217296`},
{1.4`, 35.`, 0.1`, 0.701863354037268`, 0.7487491046623214`, 0.`, -144.20574040916517`},
{1.4`, 17.`, 0.15`, 0.701863354037268`, 0.7569552213290031`, 0.`, 60.61108536244444`},
{1.4`, 17.`, 0.2`, 0.701863354037268`, 0.7568528880189616`, 0.`, 64.69817415078678`},
{1.4`, 24.`, 0.2`, 0.701863354037268`, 0.7567649402576866`, 0.`, 64.08768239797485`},
{1.4`, 28.`, 0.2`, 0.701863354037268`, 0.7501042560301314`, 0.`, 63.477167915136974`},
{1.4`, 24.`, 0.25`, 0.701863354037268`, 0.7576983993118521`, 0.`, 67.22843706462893`},
{1.4`, 28.`, 0.25`, 0.701863354037268`, 0.7514049614747687`, 0.`, 66.6961141902899`},
{1.4`, 24.`, 0.3`, 0.701863354037268`, 0.7570063537065588`, 0.`, 69.96584902287826`},
{1.4`, 31.`, 0.3`, 0.701863354037268`, 0.7485454494263611`, 0.`, 68.87981328371416`},
{1.4`, 7.`, 0.3`, 0.701863354037268`, 0.754467223264745`, 0.`, 72.07346104752287`},
{1.4`, 14.`, 0.3`, 0.701863354037268`, 0.7579203472601319`, 0.`, 71.04184318628559`},
{1.4`, 28.`, 0.3`, 0.701863354037268`, 0.7500937364553446`, 0.`, 69.0270634844442`},
{1.4`, 31.`, 0.35`, 0.701863354037268`, 0.7477668808809473`, 0.`, 70.67062618559729`},
{1.4`, 38.`, 0.35`, 0.701863354037268`, 0.7479020396042962`, 0.`, 70.28675346281658`},
{1.4`, 14.`, 0.35`, 0.701863354037268`, 0.757715709103588`, 0.`, 73.20556908149646`},
{1.4`, 35.`, 0.35`, 0.701863354037268`, 0.7498253565000329`, 0.`, 70.60389728622317`},
{1.4`, 31.`, 0.4`, 0.701863354037268`, 0.7484642101749418`, 0.`, 72.13090293753538`},
```

{1.4`, 14.`, 0.4`, 0.701863354037268`, 0.7578369375286143`, 0.`, 74.6470743794467`},
{1.4`, 10.`, 0.45`, 0.701863354037268`, 0.7565090854736061`, 0.`, 76.42625073946581`},
{1.4`, 38.`, 0.45`, 0.701863354037268`, 0.7467892025761969`, 0.`, 72.75660917654956`},
{1.4`, 14.`, 0.45`, 0.701863354037268`, 0.7573715904109938`, 0.`, 75.71968694595829`},
{1.4`, 35.`, 0.45`, 0.701863354037268`, 0.748889314599124`, 0.`, 72.90266598103018`},
{1.4`, 10.`, 0.5`, 0.701863354037268`, 0.756398340908456`, 0.`, 77.87787300701943`},
{1.4`, 31.`, 0.5`, 0.701863354037268`, 0.748279752458227`, 0.`, 74.21822651837263`},
{1.4`, 38.`, 0.5`, 0.701863354037268`, 0.747719921677235`, 0.`, 74.21057786472855`},
{1.4`, 14.`, 0.5`, 0.701863354037268`, 0.7571516958834157`, 0.`, 77.06144040918007`},
{1.4`, 35.`, 0.5`, 0.701863354037268`, 0.7497540676190523`, 0.`, 74.514770323531`},
{1.4`, 38.`, 0.05`, 0.6987577639751561`, 0.7392075469104772`, 0.`, 44.26633791751621`},
{1.4`, 35.`, 0.05`, 0.6987577639751561`, 0.739978945881899`, 0.`, 44.286645524638146`},
{1.4`, 7.`, 0.1`, 0.6987577639751561`, 0.7506049480238237`, 0.`, 55.02088806903586`},
{1.4`, 7.`, 0.15`, 0.6987577639751561`, 0.7515157585565311`, 0.`, 61.588165738017`},
{1.4`, 7.`, 0.35`, 0.6987577639751561`, 0.7528742400158073`, 0.`, 74.30812408914716`},
{1.4`, 7.`, 0.4`, 0.6987577639751561`, 0.7525963001162916`, 0.`, 75.79515797080231`},
{1.4`, 7.`, 0.45`, 0.6987577639751561`, 0.752455473917723‍9`, 0.`, 77.04932053360439`},
{1.4`, 38.`, 0.15`, 0.6956521739130442`, 0.7413935758869388`, 0.`, 59.13414138170837`},
{1.4`, 7.`, 0.5`, 0.6956521739130442`, 0.7520675607941048`, 0.`, 78.71055611597473`},
{1.4`, 38.`, 0.1`, 0.6925465838509325`, 0.7395593217246007`, 0.`, 52.78783106704791`},
{1.4`, 42.`, 0.15`, 0.6925465838509325`, 0.7382777565418794`, 0.`, 56.81602166799536`},
{1.4`, 45.`, 0.1`, 0.689441`, 0.73424`, 0.003105590062111801`, 7.827650144098218`*^15},
{1.4`, 3.`, 0.05`, 0.6894409937888206`, 0.7382597114750328`, 0.`, 45.40187581199986`},
{1.4`, 3.`, 0.1`, 0.6894409937888206`, 0.7401939952930505`, 0.`, 55.60073564186628`},
{1.4`, 45.`, 0.15`, 0.6894409937888206`, 0.7354154890771519`, 0.`, -2.919732660944252`*^19},
{1.4`, 45.`, 0.2`, 0.6894409937888206`, 0.7349480077409738`, 0.`, 60.38944787370869`},
{1.4`, 42.`, 0.2`, 0.6894409937888206`, 0.7369740632525423`, 0.`, 60.64387489418652`},
{1.4`, 42.`, 0.25`, 0.6894409937888206`, 0.7371564724761758`, 0.`, 63.848863888727514`},
{1.4`, 42.`, 0.35`, 0.6894409937888206`, 0.7381198683639618`, 0.`, 68.00824059595352`},
{1.4`, 42.`, 0.4`, 0.6894409937888206`, 0.7394130599681255`, 0.`, 69.87825784078353`},
{1.4`, 42.`, 0.45`, 0.6894409937888206`, 0.7378372005819748`, 0.`, 70.53103981843834`},

```
{1.4`, 45.`, 0.5`, 0.6894409937888206`, 0.7371698837698758`, 0.`, 71.8099176413778`},
{1.4`, 42.`, 0.5`, 0.6894409937888206`, 0.7389754161604114`, 0.`, 72.154001281106`},
{1.4`, 42.`, 0.05`, 0.6863354037267089`, 0.7300117125370367`, 0.`, 42.667785327726946`},
{1.4`, 42.`, 0.1`, 0.6863354037267089`, 0.7345842747394163`, 0.`, 51.077064748556126`},
{1.4`, 45.`, 0.25`, 0.6863354037267089`, 0.7349404836067445`, 0.003105590062111801`, 1.0269126011325853`*^20},
{1.4`, 49.`, 0.25`, 0.6863354037267089`, 0.734696998625918`, 0.`, 61.545386782980245`},
{1.4`, 45.`, 0.3`, 0.6863354037267089`, 0.7341714362035148`, 0.`, 65.81309692987965`},
{1.4`, 42.`, 0.3`, 0.6863354037267089`, 0.7379224798157952`, 0.`, 66.22500032160875`},
{1.4`, 45.`, 0.35`, 0.6863354037267089`, 0.7339765102258974`, 0.`, 67.5565807429624`},
{1.4`, 49.`, 0.35`, 0.6863354037267089`, 0.7330281588300889`, 0.`, 66.01659164631104`},
{1.4`, 45.`, 0.4`, 0.6863354037267089`, 0.7370118473274113`, 0.`, 69.48430261220288`},
{1.4`, 49.`, 0.4`, 0.6863354037267089`, 0.7343022751089231`, 0.`, 67.63619736707962`},
{1.4`, 45.`, 0.45`, 0.6863354037267089`, 0.7365487729974397`, 0.`, 70.57147930016126`},
{1.4`, 49.`, 0.45`, 0.6863354037267089`, 0.7358457591180978`, 0.`, 68.80341260351346`},
{1.4`, 49.`, 0.15`, 0.68323`, 0.730124`, 0.003105590062111801`, 3.3222047550469445`*^32},
{1.4`, 49.`, 0.1`, 0.6832298136645971`, 0.7302235648143267`, 0.`, 48.73714279277443`},
{1.4`, 3.`, 0.15`, 0.6832298136645971`, 0.7368370969461795`, 0.`, 62.13432449742792`},
{1.4`, 49.`, 0.2`, 0.6832298136645971`, 0.7327022811325392`, 0.`, 58.536206578352036`},
{1.4`, 49.`, 0.3`, 0.6832298136645971`, 0.7312085013823899`, 0.`, 63.585156753042035`},
{1.4`, 49.`, 0.5`, 0.6832298136645971`, 0.7351121656108788`, 0.`, 70.23112474451241`},
{1.4`, 3.`, 0.2`, 0.6801242236024854`, 0.7354444504431028`, 0.`, 66.28926161193732`},
{1.4`, 3.`, 0.45`, 0.6801242236024854`, 0.7352396502778535`, 0.`, 77.1412955743286`},
{1.4`, 3.`, 0.25`, 0.6770186335403736`, 0.733041496112589`, 0.`, 69.61217383436984`},
{1.4`, 3.`, 0.3`, 0.6770186335403736`, 0.7331069569363062`, 0.`, 72.25079995244857`},
{1.4`, 3.`, 0.35`, 0.6770186335403736`, 0.7331950910049915`, 0.`, 74.49117936321778`},
{1.4`, 3.`, 0.4`, 0.6770186335403736`, 0.7328566694219181`, 0.`, 75.98222348684031`},
{1.4`, 3.`, 0.5`, 0.6770186335403736`, 0.7330187362966457`, 0.`, 78.81318357659644`},
{1.4`, 45.`, 0.05`, 0.6739130434782619`, 0.7235902448943331`, 0.`, 42.66959849540814`},
{1.4`, 49.`, 0.05`, 0.6708074534161501`, 0.7205450504816749`, 0.`, 40.81053461072823`}};
```

In[ ]:=

```
OutcomeKsLowX = {{"X", "T", "kM", "OS", "AUC", "ToxDeath", "ToxAvg"},
```

```
{1.4`, 28.`, 0.1`, 0.6401179941002945`, 0.7106528557193353`, 0.`, 54.342829940256784`},
{1.4`, 28.`, 0.15`, 0.6401179941002945`, 0.7149675428234289`, 0.`, 60.16980226875079`},
{1.4`, 28.`, 0.2`, 0.6342182890855452`, 0.7126992311044694`, 0.`, 63.62278864826384`},
{1.4`, 35.`, 0.2`, 0.6342182890855452`, 0.6980453216165673`, 0.`, -128.4776528907778`},
{1.4`, 24.`, 0.05`, 0.6312684365781706`, 0.6996218231085881`, 0.`, 45.77661306833761`},
{1.4`, 31.`, 0.05`, 0.6312684365781706`, 0.6929784467783688`, 0.`, -1.368446332511419`*^18},
{1.4`, 28.`, 0.3`, 0.6312684365781706`, 0.7113639252450835`, 0.`, 68.51179737135486`},
{1.4`, 28.`, 0.35`, 0.6312684365781706`, 0.7092293200485463`, 0.`, 70.21897984938894`},
{1.4`, 28.`, 0.05`, 0.628318584070796`, 0.6988476051048341`, 0.`, 45.54701478821774`},
{1.4`, 35.`, 0.05`, 0.628318584070796`, 0.6883217275158818`, 0.`, 45.17895788608434`},
{1.4`, 31.`, 0.1`, 0.628318584070796`, 0.7006551367072249`, 0.`, 53.789666670334114`},
{1.4`, 35.`, 0.1`, 0.628318584070796`, 0.6902225741103085`, 0.`, -5.041902545172894`},
{1.4`, 24.`, 0.15`, 0.628318584070796`, 0.7093126473988438`, 0.`, 60.189406974860304`},
{1.4`, 31.`, 0.15`, 0.628318584070796`, 0.7011661606517264`, 0.`, 59.14941041919297`},
{1.4`, 35.`, 0.15`, 0.628318584070796`, 0.6915637926867452`, 0.0029498525073746312`, 8.768301262676186`*^20},
{1.4`, 31.`, 0.2`, 0.628318584070796`, 0.7069028753051221`, 0.`, 62.398866680418905`},
{1.4`, 38.`, 0.2`, 0.628318584070796`, 0.6922380849002806`, 0.`, 61.08021395400519`},
{1.4`, 31.`, 0.25`, 0.628318584070796`, 0.7105897093593575`, 0.`, 65.60047377502467`},
{1.4`, 38.`, 0.25`, 0.628318584070796`, 0.6955090499967955`, 0.`, 63.93670271280086`},
{1.4`, 28.`, 0.25`, 0.628318584070796`, 0.7137624778392263`, 0.`, 66.75339518337013`},
{1.4`, 35.`, 0.25`, 0.628318584070796`, 0.6969921780039996`, 0.`, 64.35956487460372`},
{1.4`, 31.`, 0.3`, 0.628318584070796`, 0.710840679302165`, 0.`, 67.60202706839158`},
{1.4`, 35.`, 0.3`, 0.628318584070796`, 0.6959670798111294`, 0.`, 66.03979443385987`},
{1.4`, 31.`, 0.35`, 0.628318584070796`, 0.7087545122205305`, 0.`, 69.49393002612555`},
{1.4`, 42.`, 0.35`, 0.628318584070796`, 0.6931745192868448`, 0.`, 66.02246678402769`},
{1.4`, 28.`, 0.4`, 0.628318584070796`, 0.7072115373418546`, 0.`, 71.83745329140085`},
{1.4`, 42.`, 0.4`, 0.628318584070796`, 0.6942080404502358`, 0.`, 67.87112798043003`},
{1.4`, 38.`, 0.45`, 0.628318584070796`, 0.6949397999540026`, 0.`, 70.06373393595757`},
{1.4`, 42.`, 0.45`, 0.628318584070796`, 0.6934500288193362`, 0.`, 68.77194314963101`},
{1.4`, 17.`, 0.05`, 0.6253687315634214`, 0.7040099578148687`, 0.`, 46.51677781017657`},
{1.4`, 21.`, 0.05`, 0.6253687315634214`, 0.7018989469917344`, 0.`, 46.072433622811154`},
```

```
{1.4`, 17.`, 0.1`, 0.6253687315634214`, 0.707450186764154`, 0.`, 55.43194749368504`},
{1.4`, 24.`, 0.1`, 0.6253687315634214`, 0.7070779996100954`, 0.0029498525073746312`, 5.292248890894531`*^19},
{1.4`, 21.`, 0.1`, 0.6253687315634214`, 0.707151791279356`, 0.`, 54.61280514449666`},
{1.4`, 21.`, 0.15`, 0.6253687315634214`, 0.7099953474651859`, 0.`, 60.77707957420597`},
{1.4`, 24.`, 0.2`, 0.6253687315634214`, 0.7111976477123243`, 0.`, 63.902393213770665`},
{1.4`, 45.`, 0.2`, 0.6253687315634214`, 0.6883640169818793`, 0.`, 59.61084166173547`},
{1.4`, 21.`, 0.2`, 0.6253687315634214`, 0.7082750067008239`, 0.`, 64.2256369077348`},
{1.4`, 24.`, 0.25`, 0.6253687315634214`, 0.708941607662316`, 0.`, 66.74601523897817`},
{1.4`, 21.`, 0.25`, 0.6253687315634214`, 0.7076287227267878`, 0.`, 67.12479696025463`},
{1.4`, 42.`, 0.25`, 0.6253687315634214`, 0.691696247132594`, 0.`, 62.80911031602544`},
{1.4`, 24.`, 0.3`, 0.6253687315634214`, 0.7069150904241736`, 0.`, 68.82993036500855`},
{1.4`, 38.`, 0.3`, 0.6253687315634214`, 0.6953113959673303`, 0.`, 65.49840544238545`},
{1.4`, 24.`, 0.35`, 0.6253687315634214`, 0.7067100517022143`, 0.`, 70.68343766629921`},
{1.4`, 38.`, 0.35`, 0.6253687315634214`, 0.6930116647338873`, 0.`, 66.84783471288445`},
{1.4`, 35.`, 0.35`, 0.6253687315634214`, 0.6926154276538069`, 0.`, 67.46203697034557`},
{1.4`, 31.`, 0.4`, 0.6253687315634214`, 0.7057302081467864`, 0.`, -3.668552762640826`*^16},
{1.4`, 38.`, 0.4`, 0.6253687315634214`, 0.692414234792228`, 0.`, 68.59506948658321`},
{1.4`, 35.`, 0.4`, 0.6253687315634214`, 0.6914650716074482`, 0.`, 69.42636790565868`},
{1.4`, 35.`, 0.45`, 0.6253687315634214`, 0.6917305083342556`, 0.`, 70.45570772359332`},
{1.4`, 38.`, 0.5`, 0.6253687315634214`, 0.6946099315180195`, 0.`, 71.09598577212232`},
{1.4`, 35.`, 0.5`, 0.6253687315634214`, 0.6937932283788661`, 0.`, 72.06682833497548`},
{1.4`, 42.`, 0.5`, 0.6253687315634214`, 0.6934062797573262`, 0.`, 69.76119052426179`},
{1.4`, 14.`, 0.05`, 0.6224188790560468`, 0.7021764341088976`, 0.`, 46.80720322806668`},
{1.4`, 38.`, 0.1`, 0.6224188790560468`, 0.6866491531307533`, 0.`, 53.00552015818567`},
{1.4`, 42.`, 0.1`, 0.6224188790560468`, 0.6869675197659579`, 0.`, -629.2031881314289`},
{1.4`, 49.`, 0.1`, 0.6224188790560468`, 0.6837334979108739`, 0.`, 49.87626501947149`},
{1.4`, 17.`, 0.15`, 0.6224188790560468`, 0.7058206696323455`, 0.`, 61.39041971127582`},
{1.4`, 38.`, 0.15`, 0.6224188790560468`, 0.6863614739277136`, 0.`, 57.96737040412442`},
{1.4`, 10.`, 0.2`, 0.6224188790560468`, 0.6983763068546002`, 0.`, 66.1701542753862`},
{1.4`, 17.`, 0.2`, 0.6224188790560468`, 0.7046525206026988`, 0.`, 64.96558402444259`},
{1.4`, 49.`, 0.2`, 0.6224188790560468`, 0.6836587243140605`, 0.`, 57.962193996550475`},
```

{1.4`, 42.`, 0.3`, 0.6224188790560468`, 0.6928554731444966`, 0.`, 64.46770878098252`},
{1.4`, 49.`, 0.3`, 0.6224188790560468`, 0.687411623365323`, 0.`, 62.57810754765612`},
{1.4`, 10.`, 0.05`, 0.6194690265486721`, 0.6954478628272119`, 0.`, 47.245875824783674`},
{1.4`, 38.`, 0.05`, 0.6194690265486721`, 0.6852803229225801`, 0.`, 45.11740560176284`},
{1.4`, 10.`, 0.1`, 0.6194690265486721`, 0.6962897339949015`, 0.`, 56.16322764996007`},
{1.4`, 7.`, 0.1`, 0.6194690265486721`, 0.693157844282061`, 0.`, 56.69383127885759`},
{1.4`, 14.`, 0.1`, 0.6194690265486721`, 0.7013018969813454`, 0.`, 55.71894995483987`},
{1.4`, 45.`, 0.15`, 0.6194690265486721`, 0.6851383541960684`, 0.`, 56.57294555160374`},
{1.4`, 7.`, 0.15`, 0.6194690265486721`, 0.6930606497065926`, 0.`, 62.97857386232641`},
{1.4`, 14.`, 0.15`, 0.6194690265486721`, 0.7006970143977224`, 0.`, 61.689419910488574`},
{1.4`, 42.`, 0.15`, 0.6194690265486721`, 0.6847082655244734`, 0.`, -48.60907744375631`},
{1.4`, 49.`, 0.15`, 0.6194690265486721`, 0.6827309217933795`, 0.`, 54.80028547508715`},
{1.4`, 42.`, 0.2`, 0.6194690265486721`, 0.6872164722284502`, 0.0029498525073746312`, 1.2124674456345398`*^20},
{1.4`, 17.`, 0.25`, 0.6194690265486721`, 0.7027536694404456`, 0.`, 67.64533812940778`},
{1.4`, 45.`, 0.25`, 0.6194690265486721`, 0.6870298727978256`, 0.`, 62.36506664840421`},
{1.4`, 49.`, 0.25`, 0.6194690265486721`, 0.683901220949673`, 0.`, 60.683134845113706`},
{1.4`, 21.`, 0.3`, 0.6194690265486721`, 0.7024892648387584`, 0.`, 68.76969334178267`},
{1.4`, 49.`, 0.35`, 0.6194690265486721`, 0.6840121048705686`, 0.`, 64.10926370652322`},
{1.4`, 24.`, 0.4`, 0.6194690265486721`, 0.7015923446981908`, 0.`, 71.67591211063436`},
{1.4`, 49.`, 0.4`, 0.6194690265486721`, 0.6866180857897002`, 0.`, 65.45980228483756`},
{1.4`, 31.`, 0.45`, 0.6194690265486721`, 0.70059290481135`, 0.`, 71.69239859676799`},
{1.4`, 28.`, 0.45`, 0.6194690265486721`, 0.702245015606366`, 0.`, 72.09185937454765`},
{1.4`, 49.`, 0.45`, 0.6194690265486721`, 0.686134039028626`, 0.`, 66.75565448238768`},
{1.4`, 31.`, 0.5`, 0.6194690265486721`, 0.7049798543762867`, 0.`, 73.24541107643202`},
{1.4`, 10.`, 0.15`, 0.6165191740412974`, 0.6955143756583692`, 0.`, 62.35462762727378`},
{1.4`, 7.`, 0.2`, 0.6165191740412974`, 0.691060457394202`, 0.`, 66.73073198990166`},
{1.4`, 14.`, 0.2`, 0.6165191740412974`, 0.699257492167016`, 0.`, 65.41935585610169`},
{1.4`, 10.`, 0.25`, 0.6165191740412974`, 0.693908687380036`, 0.`, 69.07457377379164`},
{1.4`, 7.`, 0.25`, 0.6165191740412974`, 0.6907923448493637`, 0.`, 69.95693076511287`},
{1.4`, 10.`, 0.3`, 0.6165191740412974`, 0.6928042615501876`, 0.`, 70.82022234368527`},
{1.4`, 10.`, 0.35`, 0.6165191740412974`, 0.6931423198382007`, 0.`, 72.88282123826188`},

```
{1.4`, 45.`, 0.35`, 0.6165191740412974`, 0.6875429758930146`, 0.`, 65.52949949849074`},
{1.4`, 7.`, 0.35`, 0.6165191740412974`, 0.6901698459581058`, 0.`, 74.30200186232327`},
{1.4`, 21.`, 0.35`, 0.6165191740412974`, 0.6998559018686491`, 0.`, 70.54504529798695`},
{1.4`, 45.`, 0.4`, 0.6165191740412974`, 0.6882502737679755`, 0.`, 67.09526979509175`},
{1.4`, 45.`, 0.45`, 0.6165191740412974`, 0.6869520542474596`, 0.`, 68.00357538649405`},
{1.4`, 45.`, 0.5`, 0.6165191740412974`, 0.6874388386398659`, 0.`, 68.84624280546703`},
{1.4`, 28.`, 0.5`, 0.6165191740412974`, 0.7018774582698021`, 0.`, 73.24536914808166`},
{1.4`, 49.`, 0.5`, 0.6165191740412974`, 0.6850134302226977`, 0.`, 67.44122830752774`},
{1.4`, 45.`, 0.05`, 0.6135693215339227`, 0.676573855709`, 0.`, 43.20002403482492`},
{1.4`, 7.`, 0.05`, 0.6135693215339227`, 0.6883327013729323`, 0.`, 47.47216398552406`},
{1.4`, 42.`, 0.05`, 0.6135693215339227`, 0.6789185763377402`, 0.`, -153.50238861884085`},
{1.4`, 49.`, 0.05`, 0.6135693215339227`, 0.6763384591203232`, 0.`, 42.64566451987915`},
{1.4`, 45.`, 0.1`, 0.6135693215339227`, 0.6796244268744465`, 0.`, -14.321561818770498`},
{1.4`, 14.`, 0.25`, 0.6135693215339227`, 0.6966754189924445`, 0.`, 68.35724874883778`},
{1.4`, 17.`, 0.3`, 0.6135693215339227`, 0.6985309478290725`, 0.`, 69.77573797188253`},
{1.4`, 45.`, 0.3`, 0.6135693215339227`, 0.6872035325965619`, 0.`, 64.00620686894209`},
{1.4`, 7.`, 0.3`, 0.6135693215339227`, 0.6883546175301987`, 0.`, 71.7385054296376`},
{1.4`, 14.`, 0.3`, 0.6135693215339227`, 0.695087222347307`, 0.`, 70.14025392949394`},
{1.4`, 10.`, 0.4`, 0.6135693215339227`, 0.6908810477858699`, 0.`, 74.8529528390647`},
{1.4`, 7.`, 0.4`, 0.6135693215339227`, 0.6874939253710438`, 0.`, 75.79764225953991`},
{1.4`, 10.`, 0.45`, 0.6135693215339227`, 0.6902493772066776`, 0.`, 75.85306022215755`},
{1.4`, 24.`, 0.45`, 0.6135693215339227`, 0.6967239735848509`, 0.`, 71.75856993950774`},
{1.4`, 7.`, 0.45`, 0.6135693215339227`, 0.6876966892504416`, 0.`, 77.02965238719946`},
{1.4`, 10.`, 0.5`, 0.6135693215339227`, 0.6896759458359021`, 0.`, 76.71043756837987`},
{1.4`, 7.`, 0.5`, 0.6135693215339227`, 0.6875432739094167`, 0.`, 77.82458767470621`},
{1.4`, 3.`, 0.05`, 0.6106194690265481`, 0.6785757869418998`, 0.`, 47.76730867453385`},
{1.4`, 3.`, 0.1`, 0.6106194690265481`, 0.6800296555927718`, 0.`, 57.0848661935312`},
{1.4`, 3.`, 0.15`, 0.6106194690265481`, 0.6807333017532471`, 0.`, 63.75035237624216`},
{1.4`, 3.`, 0.2`, 0.6106194690265481`, 0.6811900339750935`, 0.`, 68.0023971377489`},
{1.4`, 17.`, 0.35`, 0.6106194690265481`, 0.6953077511670203`, 0.`, 71.48106498282628`},
{1.4`, 14.`, 0.35`, 0.6106194690265481`, 0.6927123378569604`, 0.`, 72.01401112463398`},
```

```
{1.4`, 17.`, 0.4`, 0.6106194690265481`, 0.6940028245033488`, 0.`, 73.30126466003648`},
{1.4`, 14.`, 0.4`, 0.6106194690265481`, 0.6922027694590542`, 0.`, 73.8848894676654`},
{1.4`, 21.`, 0.4`, 0.6106194690265481`, 0.6951814510249057`, 0.`, 71.89762844088976`},
{1.4`, 14.`, 0.45`, 0.6106194690265481`, 0.6915558975293224`, 0.`, 74.87979820416942`},
{1.4`, 21.`, 0.45`, 0.6106194690265481`, 0.6955168719988096`, 0.`, 73.32683877328265`},
{1.4`, 24.`, 0.5`, 0.6106194690265481`, 0.6960817091744159`, 0.`, 73.66538900541867`},
{1.4`, 14.`, 0.5`, 0.6106194690265481`, 0.6919188576576352`, 0.`, 75.92649498212393`},
{1.4`, 21.`, 0.5`, 0.6106194690265481`, 0.6951038209961384`, 0.`, 74.29278326615722`},
{1.4`, 3.`, 0.25`, 0.6076696165191734`, 0.6788447333973928`, 0.`, 71.34944089744182`},
{1.4`, 3.`, 0.3`, 0.6076696165191734`, 0.6786947565362739`, 0.`, 73.26217044640451`},
{1.4`, 17.`, 0.45`, 0.6076696165191734`, 0.6917204659515392`, 0.`, 74.2158153570603`},
{1.4`, 17.`, 0.5`, 0.6076696165191734`, 0.6913463167471221`, 0.`, 75.10299495754737`},
{1.4`, 3.`, 0.35`, 0.6047197640117987`, 0.6765943756102468`, 0.`, 75.28300079437209`},
{1.4`, 3.`, 0.4`, 0.6047197640117987`, 0.6766175753280046`, 0.`, 77.1161191341626`},
{1.4`, 3.`, 0.45`, 0.6047197640117987`, 0.6766206955797325`, 0.`, 78.27064933146033`},
{1.4`, 3.`, 0.5`, 0.6047197640117987`, 0.6767066327906881`, 0.`, 79.15978971660073`}};
```

```
(* They were obtained by these long-running codes, they use randomization and the results will differ from the provided ones *)
```

```
(* FINDING OPTIMAL UNIVERSAL PROTOCOL FOR LOW ks WITH FIXED X *)
Npar = Length[resultMDKsLow];
SetBasicParameterValues[];

X = 1.4;
Nnn = 140;

TESTTABLE = Array[f, {Nnn + 1, 7}];
TESTTABLE[[1, 1]] = "X";
TESTTABLE[[1, 2]] = "T";
TESTTABLE[[1, 3]] = "kM";
TESTTABLE[[1, 4]] = "OS";
```

```
TESTTABLE[[1, 5]] = "AUC";
TESTTABLE[[1, 6]] = "ToxDeath";
TESTTABLE[[1, 7]] = "ToxAvg";

BestSchedules = Array[f, {Npar + 1, 7}];
BestSchedules[[1, 1]] = "X";
BestSchedules[[1, 2]] = "T";
BestSchedules[[1, 3]] = "kM";
BestSchedules[[1, 4]] = "KsLow Res";
BestSchedules[[1, 5]] = "KsLow OS";
BestSchedules[[1, 6]] = "KsLow Tox";
BestSchedules[[1, 7]] = "Ainj";

TestMDKsLow = Array[f, {Nnn + 1, Npar + 1, 5}];

ii = 1;

Do[Do[
    ii++;

    TESTTABLE[[ii, 1]] = X;
    TESTTABLE[[ii, 2]] = T;
    TESTTABLE[[ii, 3]] = kM;

    NotebookDelete[pr];
    pr = PrintTemporary["ii=" <> ToString[ii] <> "; X=" <> ToString[X] <> "; T=" <> ToString[T] <> "; kM=" <> ToString[kM]];

    TestMDKsLow[[ii, 1, 1]] = "KsLow Res";
    TestMDKsLow[[ii, 1, 2]] = "KsLow OS";
    TestMDKsLow[[ii, 1, 3]] = "KsLow Tox";
    TestMDKsLow[[ii, 1, 4]] = "Ainj";
```

```
TestMDKsLow[[ii, 1, 5]] = "DoseMaxTox";

(* for begins *)
Quiet[For[ij = 1, ij ≤ Npar, ij++,

    NotebookDelete[prr]; (* To see the code running *)
    prr = PrintTemporary["Set " <> ToString[ij] <> " of " <> ToString[Npar]];

    kappac = resultMDKsLow[[ij, 1]];
    kappap = resultMDKsLow[[ij, 2]];
    gamma = resultMDKsLow[[ij, 3]] * Nnor / 10^7;
    V = resultMDKsLow[[ij, 4]];
    ks = resultMDKsLow[[ij, 5]];
    rho = resultMDKsLow[[ij, 6]];
    omega = resultMDKsLow[[ij, 7]];
    alpha = resultMDKsLow[[ij, 8]];
    kf = resultMDKsLow[[ij, 9]];
    N0 = resultMDKsLow[[ij, 10]] * 10^7 / Nnor;
```

$$\text{Dose} = \text{Min}\Big[\frac{X*\text{gamma}*N0+3}{\text{eta}+1}, \text{Min}\Big[\Big(\text{Abld} - \frac{\text{gamma}*N0}{\text{eta}+1} * \frac{\text{lambda}*0.5}{(\text{lambda}+0.5)*(\text{lambda}+0.4)}\Big) * \frac{\text{lambda}+0.04}{\text{lambda}} + \frac{\text{gamma}*N0}{\text{eta}+1}, \frac{(\text{lambda}+0.5)*(\text{lambda}+0.4)}{\text{lambda}*0.5} * \text{Abld}\Big]\Big];$$

```
    (* overall level of toxic decays due to this dose cannot exceed *)
```

$$\text{DoseMaxTox} = \text{Max}\Big[\frac{\text{lambda}}{\text{lambda}+0.04} * \Big(\text{Dose} - \frac{\text{gamma}*N0}{\text{eta}+1}\Big) + \frac{\text{lambda}*0.5}{(\text{lambda}+0.5)*(\text{lambda}+0.4)} * \frac{\text{gamma}*N0}{\text{eta}+1}, \frac{\text{lambda}}{\text{lambda}+0.04+\text{kon}*\text{gamma}*N0/V} * \text{Dose} + \frac{\text{lambda}*0.5}{(\text{lambda}+0.5)*(\text{lambda}+0.4)} * \text{Dose}\Big];$$

```
(* remaining safe dose *)
Asfd = Max[(Abld - DoseMaxTox) * (lambda + 0.04)/lambda, 0];

Ainj = {{0, Dose, eta * Dose}};

(* next doses -- estimation *)
NextDoses = Min[Asfd, kM * (gamma * N0)/(eta + 1)];
If[NextDoses ≠ 0, (* how many of them are ok t0 inject *)
  nn = Round[Asfd / NextDoses];
  (* a remainder, last dose *)
  DoseLast = Asfd - (nn * NextDoses);

  Ainj = Join[Ainj, Table[{i * T, NextDoses, eta * NextDoses}, {i, 1, nn}]];
  If[DoseLast > 0, Ainj = Join[Ainj, {{(nn + 1) * T, DoseLast, eta * DoseLast}}]];
  Ainj = Select[Ainj, #[[1]] < 365 &];
  If[Length[Ainj] > 10, Ainj = Ainj[[1 ;; 10]]];

res = PatientTEST2[];
If[! NumberQ[res[[2]]], res = PatientTEST2[]];
If[res[[3]] < 0, res = PatientTEST2[]];

TestMDKsLow[[ii, 1 + ij, 1]] = res[[1]]; (*Res*)
TestMDKsLow[[ii, 1 + ij, 2]] = res[[2]]; (*OS*)
TestMDKsLow[[ii, 1 + ij, 3]] = res[[3]]; (*TOX*)
TestMDKsLow[[ii, 1 + ij, 4]] = Ainj;
TestMDKsLow[[ii, 1 + ij, 5]] = DoseMaxTox;

change = 0;
```

```
        If [ii == 2,
          change = 1,

          If [res⟦1⟧ == "CURE" && BestSchedules⟦1 + ij, 4⟧ == "CURE" && res⟦3⟧ < BestSchedules⟦1 + ij, 6⟧, (*cure and less toxic*)
            change = 1,

            If [res⟦1⟧ == "CURE" && BestSchedules⟦1 + ij, 4⟧ == "TBUR",
              change = 1,

              If [res⟦1⟧ == "TBUR" && BestSchedules⟦1 + ij, 4⟧ == "TBUR" && res⟦2⟧ > BestSchedules⟦1 + ij, 5⟧, (*not cure, but greater OS*)
                change = 1] ] ] ];

        If [BestSchedules⟦1 + ij, 4⟧ == "TOX", change = 1];

        If [change == 1,
          BestSchedules⟦1 + ij, 1⟧ = X;
          BestSchedules⟦1 + ij, 2⟧ = T;
          BestSchedules⟦1 + ij, 3⟧ = kM;
          BestSchedules⟦1 + ij, 4⟧ = res⟦1⟧;
          BestSchedules⟦1 + ij, 5⟧ = res⟦2⟧;
          BestSchedules⟦1 + ij, 6⟧ = res⟦3⟧;
          BestSchedules⟦1 + ij, 7⟧ = Ainj]
      ]];
    (* for-cycle ends *)

    TestMDKsLowSM = SurvivalModelFit [TestMDKsLow⟦ii, 2 ;;, 2⟧];
    TESTTABLE⟦ii, 4⟧ = TestMDKsLowSM [364]; (*OS*)
    TESTTABLE⟦ii, 5⟧ = Total [TestMDKsLow⟦ii, 2 ;;, 2⟧] / 365 / (Npar - 1); (* AUC *)
    TESTTABLE⟦ii, 6⟧ = N[Length [Select [TestMDKsLow⟦ii, 2 ;;, 3⟧, # > Abld / nCpm &] ] / Npar]; (* Tox Deaths *)
    TESTTABLE⟦ii, 7⟧ = Mean [TestMDKsLow⟦ii, 2 ;;, 3⟧]; (* "ToxAvg" *)
```

```
        NotebookDelete[prTab];
        prTab = PrintTemporary[MatrixForm[TESTTABLE[[1 ;; ii]]]];

        , {T, Join[Table[3 + (j - 1) * 7, {j, 1, 7}], Table[7 + (j - 1) * 7, {j, 1, 7}]]}],
      {kM, Table[N[i / 20], {i, 1, 10}]}];
OutcomeKsLowX = TESTTABLE;
(* FINDING OPTIMAL UNIVERSAL PROTOCOL FOR MID ks WITH FIXED X *)
Npar = Length[resultMDKsMid];
SetBasicParameterValues[];

X = 1.4;
Nnn = 140;

TESTTABLE = Array[f, {Nnn + 1, 7}];
TESTTABLE[[1, 1]] = "X";
TESTTABLE[[1, 2]] = "T";
TESTTABLE[[1, 3]] = "kM";
TESTTABLE[[1, 4]] = "OS";
TESTTABLE[[1, 5]] = "AUC";
TESTTABLE[[1, 6]] = "ToxDeath";
TESTTABLE[[1, 7]] = "ToxAvg";

BestSchedules = Array[f, {Npar + 1, 7}];
BestSchedules[[1, 1]] = "X";
BestSchedules[[1, 2]] = "T";
BestSchedules[[1, 3]] = "kM";
BestSchedules[[1, 4]] = "KsMid Res";
BestSchedules[[1, 5]] = "KsMid OS";
BestSchedules[[1, 6]] = "KsMid Tox";
```

```
BestSchedules[[1, 7]] = "Ainj";

TestMDKsMid = Array[f, {Nnn + 1, Npar + 1, 5}];

ii = 1;

Do[Do[
   ii++;

   TESTTABLE[[ii, 1]] = X;
   TESTTABLE[[ii, 2]] = T;
   TESTTABLE[[ii, 3]] = kM;

   NotebookDelete[pr];
   pr = PrintTemporary["ii=" <> ToString[ii] <> "; X=" <> ToString[X] <> "; T=" <> ToString[T] <> "; kM=" <> ToString[kM]];

   TestMDKsMid[[ii, 1, 1]] = "KsMid Res";
   TestMDKsMid[[ii, 1, 2]] = "KsMid OS";
   TestMDKsMid[[ii, 1, 3]] = "KsMid Tox";
   TestMDKsMid[[ii, 1, 4]] = "Ainj";
   TestMDKsMid[[ii, 1, 5]] = "DoseMaxTox";

   (* for begins *)
   Quiet[For[ij = 1, ij ≤ Npar, ij++,

      NotebookDelete[prr]; (* To see the code running *)
      prr = PrintTemporary["Set " <> ToString[ij] <> " of " <> ToString[Npar]];

      kappac = resultMDKsMid[[ij, 1]];
      kappap = resultMDKsMid[[ij, 2]];
```

```
gamma = resultMDKsMid[[ij, 3]] * Nnor / 10^7;
V = resultMDKsMid[[ij, 4]];
ks = resultMDKsMid[[ij, 5]];
rho = resultMDKsMid[[ij, 6]];
omega = resultMDKsMid[[ij, 7]];
alpha = resultMDKsMid[[ij, 8]];
kf = resultMDKsMid[[ij, 9]];
N0 = resultMDKsMid[[ij, 10]] * 10^7 / Nnor;

Dose = Min[(X * gamma * N0 + 3)/(eta + 1), Min[(Abld - (gamma * N0)/(eta + 1) * (lambda * 0.5)/((lambda + 0.5) * (lambda + 0.4))) * (lambda + 0.04)/lambda + (gamma * N0)/(eta + 1),
    ((lambda + 0.5) * (lambda + 0.4))/(lambda * 0.5) * Abld]];

(* overall level of toxic decays due to this dose cannot exceed *)
DoseMaxTox = Max[lambda/(lambda + 0.04) * (Dose - (gamma * N0)/(eta + 1)) + (lambda * 0.5)/((lambda + 0.5) * (lambda + 0.4)) * (gamma * N0)/(eta + 1),
   lambda/(lambda + 0.04 + kon * gamma * N0 / V) * Dose + (lambda * 0.5)/((lambda + 0.5) * (lambda + 0.4)) * Dose];

(* remaining safe dose *)
Asfd = Max[(Abld - DoseMaxTox) * (lambda + 0.04)/lambda, 0];

Ainj = {{0, Dose, eta * Dose}};

(* next doses -- estimation *)
NextDoses = Min[Asfd, kM * (gamma * N0)/(eta + 1)];

If[NextDoses ≠ 0, (* how many of them are ok t0 inject *)
```

```
    nn = Round[Asfd / NextDoses];
    (* a remainder, last dose *)
    DoseLast = Asfd - (nn * NextDoses);

    Ainj = Join[Ainj, Table[{i * T, NextDoses, eta * NextDoses}, {i, 1, nn}]];
    If[DoseLast > 0, Ainj = Join[Ainj, {{(nn + 1) * T, DoseLast, eta * DoseLast}}]];
    Ainj = Select[Ainj, #[[1]] < 365 &];
    If[Length[Ainj] > 10, Ainj = Ainj[[1 ;; 10]]];

  res = PatientTEST2[];
  If[! NumberQ[res[[2]]], res = PatientTEST2[]];
  If[res[[3]] < 0, res = PatientTEST2[]];

  TestMDKsMid[[ii, 1 + ij, 1]] = res[[1]]; (*Res*)
  TestMDKsMid[[ii, 1 + ij, 2]] = res[[2]]; (*OS*)
  TestMDKsMid[[ii, 1 + ij, 3]] = res[[3]]; (*TOX*)
  TestMDKsMid[[ii, 1 + ij, 4]] = Ainj;
  TestMDKsMid[[ii, 1 + ij, 5]] = DoseMaxTox;

  change = 0;

  If[ii == 2,
    change = 1,

    If[res[[1]] == "CURE" && BestSchedules[[1 + ij, 4]] == "CURE" && res[[3]] < BestSchedules[[1 + ij, 6]], (*cure and less toxic*)
      change = 1,

      If[res[[1]] == "CURE" && BestSchedules[[1 + ij, 4]] == "TBUR",
        change = 1,

        If[res[[1]] == "TBUR" && BestSchedules[[1 + ij, 4]] == "TBUR" && res[[2]] > BestSchedules[[1 + ij, 5]], (*not cure, but greater OS*)
```

```
                    change = 1] ] ] ];

        If[BestSchedules[[1 + ij, 4]] == "TOX", change = 1];

        If[change == 1,
          BestSchedules[[1 + ij, 1]] = X;
          BestSchedules[[1 + ij, 2]] = T;
          BestSchedules[[1 + ij, 3]] = kM;
          BestSchedules[[1 + ij, 4]] = res[[1]];
          BestSchedules[[1 + ij, 5]] = res[[2]];
          BestSchedules[[1 + ij, 6]] = res[[3]];
          BestSchedules[[1 + ij, 7]] = Ainj]
      ]];
    (* for-cycle ends *)

    TestMDKsMidSM = SurvivalModelFit[TestMDKsMid[[ii, 2 ;;, 2]]];
    TESTTABLE[[ii, 4]] = TestMDKsMidSM[364]; (*OS*)
    TESTTABLE[[ii, 5]] = Total[TestMDKsMid[[ii, 2 ;;, 2]]] / 365 / (Npar - 1); (* AUC *)
    TESTTABLE[[ii, 6]] = N[Length[Select[TestMDKsMid[[ii, 2 ;;, 3]], # > Abld / nCpm &]] / Npar]; (* Tox Deaths *)
    TESTTABLE[[ii, 7]] = Mean[TestMDKsMid[[ii, 2 ;;, 3]]]; (* "ToxAvg" *)

    NotebookDelete[prTab];
    prTab = PrintTemporary[MatrixForm[TESTTABLE[[1 ;; ii]]]];

    , {T, Join[Table[3 + (j - 1) * 7, {j, 1, 7}], Table[7 + (j - 1) * 7, {j, 1, 7}]]}],
  {kM, Table[N[i / 20], {i, 1, 10}]}];
OutcomeKsMidX = TESTTABLE;

(* FINDING OPTIMAL UNIVERSAL PROTOCOL FOR HIGH ks WITH FIXED X *)
Npar = Length[resultMDKsHigh];
```

```
SetBasicParameterValues[ ];

X = 1.4;
Nnn = 140;

TESTTABLE = Array[f, {Nnn + 1, 7}];
TESTTABLE⟦1, 1⟧ = "X";
TESTTABLE⟦1, 2⟧ = "T";
TESTTABLE⟦1, 3⟧ = "kM";
TESTTABLE⟦1, 4⟧ = "OS";
TESTTABLE⟦1, 5⟧ = "AUC";
TESTTABLE⟦1, 6⟧ = "ToxDeath";
TESTTABLE⟦1, 7⟧ = "ToxAvg";

BestSchedules = Array[f, {Npar + 1, 7}];
BestSchedules⟦1, 1⟧ = "X";
BestSchedules⟦1, 2⟧ = "T";
BestSchedules⟦1, 3⟧ = "kM";
BestSchedules⟦1, 4⟧ = "KsHigh Res";
BestSchedules⟦1, 5⟧ = "KsHigh OS";
BestSchedules⟦1, 6⟧ = "KsHigh Tox";
BestSchedules⟦1, 7⟧ = "Ainj";

TestMDKsHigh = Array[f, {Nnn + 1, Npar + 1, 5}];

ii = 1;

Do[Do[
    ii++;

    TESTTABLE⟦ii, 1⟧ = X;
```

```
TESTTABLE[[ii, 2]] = T;
TESTTABLE[[ii, 3]] = kM;

NotebookDelete[pr];
pr = PrintTemporary["ii=" <> ToString[ii] <> "; X=" <> ToString[X] <> "; T=" <> ToString[T] <> "; kM=" <> ToString[kM]];

TestMDKsHigh[[ii, 1, 1]] = "KsHigh Res";
TestMDKsHigh[[ii, 1, 2]] = "KsHigh OS";
TestMDKsHigh[[ii, 1, 3]] = "KsHigh Tox";
TestMDKsHigh[[ii, 1, 4]] = "Ainj";
TestMDKsHigh[[ii, 1, 5]] = "DoseMaxTox";

(* for begins *)
Quiet[For[ij = 1, ij ≤ Npar, ij++,

  NotebookDelete[prr]; (* To see the code running *)
  prr = PrintTemporary["Set " <> ToString[ij] <> " of " <> ToString[Npar]];

  kappac = resultMDKsHigh[[ij, 1]];
  kappap = resultMDKsHigh[[ij, 2]];
  gamma = resultMDKsHigh[[ij, 3]] * Nnor / 10^7;
  V = resultMDKsHigh[[ij, 4]];
  ks = resultMDKsHigh[[ij, 5]];
  rho = resultMDKsHigh[[ij, 6]];
  omega = resultMDKsHigh[[ij, 7]];
  alpha = resultMDKsHigh[[ij, 8]];
  kf = resultMDKsHigh[[ij, 9]];
  N0 = resultMDKsHigh[[ij, 10]] * 10^7 / Nnor;
```

```
Dose = Min[(X * gamma * N0 + 3)/(eta + 1), Min[(Abld - (gamma * N0)/(eta + 1) * (lambda * 0.5)/((lambda + 0.5) * (lambda + 0.4))) * (lambda + 0.04)/lambda + (gamma * N0)/(eta + 1),
    ((lambda + 0.5) * (lambda + 0.4))/(lambda * 0.5) * Abld]];

(* overall level of toxic decays due to this dose cannot exceed *)
DoseMaxTox = Max[lambda/(lambda + 0.04) * (Dose - (gamma * N0)/(eta + 1)) + (lambda * 0.5)/((lambda + 0.5) * (lambda + 0.4)) * (gamma * N0)/(eta + 1),
   lambda/(lambda + 0.04 + kon * gamma * N0 / V) * Dose + (lambda * 0.5)/((lambda + 0.5) * (lambda + 0.4)) * Dose];

(* remaining safe dose *)
Asfd = Max[(Abld - DoseMaxTox) * (lambda + 0.04)/lambda, 0];


Ainj = {{0, Dose, eta * Dose}};

(* next doses -- estimation *)
NextDoses = Min[Asfd, kM * (gamma * N0)/(eta + 1)];
If[NextDoses ≠ 0, (* how many of them are ok t0 inject *)
  nn = Round[Asfd / NextDoses];
  (* a remainder, last dose *)
  DoseLast = Asfd - (nn * NextDoses);

  Ainj = Join[Ainj, Table[{i * T, NextDoses, eta * NextDoses}, {i, 1, nn}]];
  If[DoseLast > 0, Ainj = Join[Ainj, {{(nn + 1) * T, DoseLast, eta * DoseLast}}]];
  Ainj = Select[Ainj, #[[1]] < 365 &];
  If[Length[Ainj] > 10, Ainj = Ainj[[1 ;; 10]]];
```

```
res = PatientTEST2[];
If[! NumberQ[res[[2]]], res = PatientTEST2[]];
If[res[[3]] < 0, res = PatientTEST2[]];

TestMDKsHigh[[ii, 1 + ij, 1]] = res[[1]]; (*Res*)
TestMDKsHigh[[ii, 1 + ij, 2]] = res[[2]]; (*OS*)
TestMDKsHigh[[ii, 1 + ij, 3]] = res[[3]]; (*TOX*)
TestMDKsHigh[[ii, 1 + ij, 4]] = Ainj;
TestMDKsHigh[[ii, 1 + ij, 5]] = DoseMaxTox;

change = 0;

If[ii == 2,
  change = 1,

  If[res[[1]] == "CURE" && BestSchedules[[1 + ij, 4]] == "CURE" && res[[3]] < BestSchedules[[1 + ij, 6]], (*cure and less toxic*)
    change = 1,

    If[res[[1]] == "CURE" && BestSchedules[[1 + ij, 4]] == "TBUR",
      change = 1,

      If[res[[1]] == "TBUR" && BestSchedules[[1 + ij, 4]] == "TBUR" && res[[2]] > BestSchedules[[1 + ij, 5]], (*not cure, but greater OS*)
        change = 1]]]];

If[BestSchedules[[1 + ij, 4]] == "TOX", change = 1];

If[change == 1,
  BestSchedules[[1 + ij, 1]] = X;
  BestSchedules[[1 + ij, 2]] = T;
  BestSchedules[[1 + ij, 3]] = kM;
  BestSchedules[[1 + ij, 4]] = res[[1]];
```

```
BestSchedules[[1 + ij, 5]] = res[[2]];
BestSchedules[[1 + ij, 6]] = res[[3]];
BestSchedules[[1 + ij, 7]] = Ainj]
]];
(* for-cycle ends *)

TestMDKsHighSM = SurvivalModelFit[TestMDKsHigh[[ii, 2 ;;, 2]]];
TESTTABLE[[ii, 4]] = TestMDKsHighSM[364]; (*OS*)
TESTTABLE[[ii, 5]] = Total[TestMDKsHigh[[ii, 2 ;;, 2]]] /365/ (Npar - 1); (* AUC *)
TESTTABLE[[ii, 6]] = N[Length[Select[TestMDKsHigh[[ii, 2 ;;, 3]], # > Abld / nCpm &]] /Npar]; (* Tox Deaths *)
TESTTABLE[[ii, 7]] = Mean[TestMDKsHigh[[ii, 2 ;;, 3]]]; (* "ToxAvg" *)

NotebookDelete[prTab];
prTab = PrintTemporary[MatrixForm[TESTTABLE[[1 ;; ii]]]];

, {T, Join[Table[3 + (j - 1) * 7, {j, 1, 7}], Table[7 + (j - 1) * 7, {j, 1, 7}]]}],
{kM, Table[N[i / 20], {i, 1, 10}]}];
OutcomeKsHighX = TESTTABLE;
```

In[ ]:=

```
(* Figure S.31 itself *) GraphicsGrid[{{ListPlot[Table[Style[{OutcomeKsLowX[[t, {2, 3}]]},
    ColorData["SandyTerrain"][(OutcomeKsLowX[[t, 4]] - Min[OutcomeKsLowX[[2 ;;, 4]]]) / (Max[OutcomeKsLowX[[2 ;;, 4]]] - Min[OutcomeKsLowX[[2 ;;, 4]]])]], {t, 2, Length[OutcomeKsLowX]}],
   AxesLabel → {"T", "k"}, PlotStyle → PointSize[Large], ImageSize → 300, PlotRange → {{0, 51}, Automatic}],

  ListPlot[Table[Style[{OutcomeKsMidX[[t, {2, 3}]]},
    ColorData["SandyTerrain"][(OutcomeKsMidX[[t, 4]] - Min[OutcomeKsMidX[[2 ;;, 4]]]) / (Max[OutcomeKsMidX[[2 ;;, 4]]] - Min[OutcomeKsMidX[[2 ;;, 4]]])]], {t, 2, Length[OutcomeKsMidX]}],
   AxesLabel → {"T", "k"}, PlotStyle → PointSize[Large], ImageSize → 300, PlotRange → {{0, 51}, Automatic}],

  ListPlot[Table[Style[{OutcomeKsHighX[[t, {2, 3}]]},
    ColorData["SandyTerrain"][(OutcomeKsHighX[[t, 4]] - Min[OutcomeKsHighX[[2 ;;, 4]]]) / (Max[OutcomeKsHighX[[2 ;;, 4]]] - Min[OutcomeKsHighX[[2 ;;, 4]]])]], {t, 2, Length[OutcomeKsHighX]}],
   AxesLabel → {"T", "k"}, PlotStyle → PointSize[Large], ImageSize → 300, PlotRange → {{0, 51}, Automatic}]}}, ImageSize → 900]
```

Out[ ]=

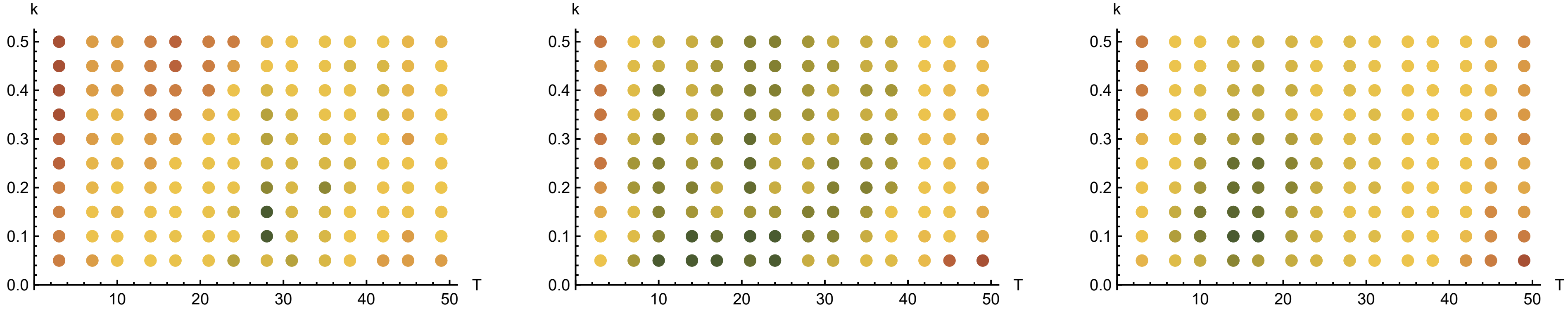


```
(* maximal survival rates and what are the (T,k) sets corresponding to them *)
```

In[∘]:=

```
(* Low ks *)
PercentForm[Max[OutcomeKsLowX[[2 ;;, 4]]]]
pos = Position[OutcomeKsLowX[[2 ;;, 4]], Max[OutcomeKsLowX[[2 ;;, 4]]]];
Length[pos];
For[j = 1, j ≤ Length[pos], j++,
  Print[OutcomeKsLowX[[2 ;;]][[Position[OutcomeKsLowX[[2 ;;, 4]], Max[OutcomeKsLowX[[2 ;;, 4]]]][[j, 1]]]][[2 ;; 3]]]
]
```

Out[∘]//PercentForm=

64.01%

{28., 0.1}

{28., 0.15}

In[∘]:=

```
(* Mid ks *)
PercentForm[Max[OutcomeKsMidX[[2 ;;, 4]]]]
pos = Position[OutcomeKsMidX[[2 ;;, 4]], Max[OutcomeKsMidX[[2 ;;, 4]]]];
Length[pos];
For[j = 1, j ≤ Length[pos], j++,
  Print[OutcomeKsMidX[[2 ;;]][[Position[OutcomeKsMidX[[2 ;;, 4]], Max[OutcomeKsMidX[[2 ;;, 4]]]][[j, 1]]]][[2 ;; 3]]]
]
```

Out[∘]//PercentForm=

71.43%

{10., 0.05}

{17., 0.05}

{24., 0.05}

{14., 0.05}

{24., 0.1}

{14., 0.1}

{21., 0.1}

In[∘]:=

```
(* High ks *)
PercentForm[Max[OutcomeKsHighX[[2 ;;, 4]]]]
pos = Position[OutcomeKsHighX[[2 ;;, 4]], Max[OutcomeKsHighX[[2 ;;, 4]]]];
Length[pos];
For[j = 1, j ≤ Length[pos], j++,
  Print[OutcomeKsHighX[[2 ;;]][[Position[OutcomeKsHighX[[2 ;;, 4]], Max[OutcomeKsHighX[[2 ;;, 4]]]][[j, 1]]]][[2 ;; 3]]]
]
```

Out[∘]//PercentForm=

72.86%

{17., 0.1}

{14., 0.1}

```
(* values of survival rate from upper left corner of parametric plane, T=3, k=0.5 *)
PercentForm[Select[Select[OutcomeKsLowX, #[[2]] == 3 &], #[[3]] == 0.5 &][[1, 4]]]
PercentForm[Select[Select[OutcomeKsMidX, #[[2]] == 3 &], #[[3]] == 0.5 &][[1, 4]]]
PercentForm[Select[Select[OutcomeKsHighX, #[[2]] == 3 &], #[[3]] == 0.5 &][[1, 4]]]
```

Out[∘]//PercentForm=

60.47%

Out[∘]//PercentForm=

67.7%

Out[∘]//PercentForm=

66.67%

```
In[ ]:=
(* values of survival rate from lower right corner of parametric plane, T=49, k=0.05 *)
PercentForm[Select[Select[OutcomeKsLowX, #[[2]] == 49 &], #[[3]] == 0.05 &][[1, 4]]]
PercentForm[Select[Select[OutcomeKsMidX, #[[2]] == 49 &], #[[3]] == 0.05 &][[1, 4]]]
PercentForm[Select[Select[OutcomeKsHighX, #[[2]] == 49 &], #[[3]] == 0.05 &][[1, 4]]]
Out[ ]//PercentForm=
61.36%
Out[ ]//PercentForm=
67.08%
Out[ ]//PercentForm=
65.49%

(* Figure S.32: Survival curves and toxicity curves for personalized numerically optimized single-dose
    and multi-dose schedules and optimized universal schedules for three groups of virtual mice from the training set *)

In[ ]:=
(* The outcomes of applying optimized universal schedules are saved here in a closed cell *)

In[ ]:=
TestMDKsLow = {{"KsLow Res", "KsLow OS", "KsLow Tox", "Ainj", "DoseMaxTox"},
    {"CURE", 365, 37.85811424082171`, {{0, 0.010210340227471118`, 18.174405604898592`},
        {28, 0.0006089923776821234`, 1.0840064322741796`}, {56, 0.0006089923776821234`, 1.0840064322741796`},
        {84, 0.0006089923776821234`, 1.0840064322741796`}, {112, 0.0006089923776821234`, 1.0840064322741796`},
        {140, 0.0006089923776821234`, 1.0840064322741796`}, {168, 0.0006089923776821234`, 1.0840064322741796`},
        {196, 0.0006089923776821234`, 1.0840064322741796`}, {224, 0.0006089923776821234`, 1.0840064322741796`},
        {252, 0.0006089923776821234`, 1.0840064322741796`}}, 0.003417705936400931 7`},
    {"CURE", 365, 121.70638575375398`, {{0, 0.022722775577214457`, 40.446540527441734`},
        {28, 0.001502737759806647 8`, 2.674873212455833`}, {56, 0.0015027377598066478`, 2.674873212455833`},
        {84, 0.0015027377598066478`, 2.674873212455833`}, {112, 0.0015027377598066478`, 2.674873212455833`},
        {140, 0.0015027377598066478`, 2.674873212455833`}, {168, 0.0015027377598066478`, 2.674873212455833`},
        {196, 0.0015027377598066478`, 2.674873212455833`}, {224, 0.0015027377598066478`, 2.674873212455833`},
        {252, 0.0015027377598066478`, 2.674873212455833`}}, 0.006860334658641895`},
    {"TBUR", 101.68328251983546`, 51.07066127059965`, {{0, 0.06568603011405001`, 116.92113360300903`}}, 0.0175`},
    {"CURE", 365, 10.236590242054982`, {{0, 0.003383155900875551 8`, 6.022017503558482`},
```

{28, 0.00012133635435386853`, 0.21597871074988598`}, {56, 0.00012133635435386853`, 0.21597871074988598`},
{84, 0.00012133635435386853`, 0.21597871074988598`}, {112, 0.00012133635435386853`, 0.21597871074988598`},
{140, 0.00012133635435386853`, 0.21597871074988598`}, {168, 0.00012133635435386853`, 0.21597871074988598`},
{196, 0.00012133635435386853`, 0.21597871074988598`}, {224, 0.00012133635435386853`, 0.21597871074988598`},
{252, 0.00012133635435386853`, 0.21597871074988598`}}, 0.0015392977610289523`}, {"CURE", 365, 16.032821559846468`,
{{0, 0.004354732418849423`, 7.751423705551973`}, {28, 0.0001907346770662879`, 0.3395077251779925`},
{56, 0.0001907346770662879`, 0.3395077251779925`}, {84, 0.0001907346770662879`, 0.3395077251779925`},
{112, 0.0001907346770662879`, 0.3395077251779925`}, {140, 0.0001907346770662879`, 0.3395077251779925`},
{168, 0.0001907346770662879`, 0.3395077251779925`}, {196, 0.0001907346770662879`, 0.3395077251779925`},
{224, 0.0001907346770662879`, 0.3395077251779925`}, {252, 0.0001907346770662879`, 0.3395077251779925`}},
0.0018066140054801435`}, {"TBUR", 39.83063484053233`, 4.754750025861075`,
{{0, 0.0158046590425311 9`, 28.132293095705517`}, {28, 0.0010085865787578426`, 1.7952841101889598`}}, 0.004956907706142255`},
{"CURE", 365, 97.77897444806229`, {{0, 0.04870154613466617`, 86.68875211970578`},
{28, 0.0033583642281960558`, 5.977888326188979`}, {56, 0.0033583642281960558`, 5.977888326188979`}}, 0.014008044851719996`},
{"CURE", 365, 42.86748862475913`, {{0, 0.014796063111466381`, 26.33699233841016`},
{28, 0.0009365440122532133`, 1.6670483418107196`}, {56, 0.0009365440122532133`, 1.6670483418107196`},
{84, 0.0009365440122532133`, 1.6670483418107196`}, {112, 0.0009365440122532133`, 1.6670483418107196`},
{140, 0.0009365440122532133`, 1.6670483418107196`}, {168, 0.0009365440122532133`, 1.6670483418107196`},
{196, 0.0009365440122532133`, 1.6670483418107196`}, {224, 0.0009365440122532133`, 1.6670483418107196`},
{252, 0.0009365440122532133`, 1.6670483418107196`}}, 0.004679406066823872`},
{"CURE", 365, 56.673789067660856`, {{0, 0.07056243453891543`, 125.60113347926948`}}, 0.0175`},
{"TBUR", 24.842295098809224`, 9.795206857030692`, {{0, 0.02891380659532062`, 51.4665757396707`}}, 0.008563713785684034`},
{"TBUR", 26.972047784333824`, 4.199847868455267`, {{0, 0.007135891793292206`, 12.701887392060128`}}, 0.002571812695899977`},
{"CURE", 365, 44.241494835346806`, {{0, 0.018054187692372128`, 32.13645409242239`},
{28, 0.001169267196603624`, 2.081295609954451`}, {56, 0.001169267196603624`, 2.081295609954451`},
{84, 0.001169267196603624`, 2.081295609954451`}, {112, 0.001169267196603624`, 2.081295609954451`},
{140, 0.001169267196603624`, 2.081295609954451`}, {168, 0.001169267196603624`, 2.081295609954451`},
{196, 0.001169267196603624`, 2.081295609954451`}, {224, 0.001169267196603624`, 2.081295609954451`},
{252, 0.001169267196603624`, 2.081295609954451`}}, 0.0055758353349355735`},
{"TBUR", 223.1147844312819`, 74.02481461714565`, {{0, 0.033829790500790276`, 60.21702709140669`},
{28, 0.002296095968633492`, 4.0870508241676164`}, {56, 0.002296095968633492`, 4.0870508241676164`},

```
{84, 0.002296095968633492`, 4.0870508241676164`}, {112, 0.002296095968633492`, 4.0870508241676164`},
{140, 0.002296095968633492`, 4.0870508241676164`}, {168, 0.0004367931768362379`, 0.7774918547685035`}},
0.009916280805452194`}, {"CURE", 365, 119.84256358713938`, {{0, 0.06076536786174534`, 108.16235479390672`},
{28, 0.0002714744148405887`, 0.4832244584162479`}}, 0.017327243554192354`}, {"CURE", 365, 90.18661112394149`,
{{0, 0.060200576530459626`, 107.15702622421813`}, {28, 0.0005156661769087758`, 0.917885794897621`}}, 0.0171718487965126`},
{"TBUR", 117.6196216472981`, 81.01108664156139`, {{0, 0.04856667125499047`, 86.44867483388305`},
{28, 0.0033487303082192204`, 5.960739948630212`}, {56, 0.0033487303082192204`, 5.960739948630212`}}, 0.013970935838030675`},
{"CURE", 365, 65.54040982350666`, {{0, 0.04920985438476635`, 87.5935408048841`}, {28, 0.003394671960346068‌4`, 6.042516089416002`},
{56, 0.0033946719603460684`, 6.042516089416002`}}, 0.014147899047376861`},
{"CURE", 365, 75.52104760690248`, {{0, 0.01165281518644063`, 20.742011031864322`},
{28, 0.0007120263033228029`, 1.2674068199145891`}, {56, 0.0007120263033228029`, 1.2674068199145891`},
{84, 0.0007120263033228029`, 1.2674068199145891`}, {112, 0.0007120263033228029`, 1.2674068199145891`},
{140, 0.0007120263033228029`, 1.2674068199145891`}, {168, 0.0007120263033228029`, 1.2674068199145891`},
{196, 0.0007120263033228029`, 1.2674068199145891`}, {224, 0.0007120263033228029`, 1.2674068199145891`},
{252, 0.0007120263033228029`, 1.2674068199145891`}}, 0.0038145835694161265`},
{"TBUR", 69.92638777049167`, 18.396786278146944`, {{0, 0.022040661497943034`, 39.2323774663386`},
{28, 0.0014540153255729743`, 2.588147279519894`}, {56, 0.0014540153255729743`, 2.588147279519894`}}, 0.006672660120831563`},
{"TBUR", 365, 36.34592411484939`, {{0, 0.016550633155242866`, 29.4601270163323`},
{28, 0.0010618704439515339`, 1.8901293902337304`}, {56, 0.0010618704439515339`, 1.8901293902337304`},
{84, 0.0010618704439515339`, 1.8901293902337304`}, {112, 0.0010618704439515339`, 1.8901293902337304`},
{140, 0.0010618704439515339`, 1.8901293902337304`}, {168, 0.0010618704439515339`, 1.8901293902337304`},
{196, 0.0010618704439515339`, 1.8901293902337304`}, {224, 0.0010618704439515339`, 1.8901293902337304`},
{252, 0.0010618704439515339`, 1.8901293902337304`}}, 0.005162152475420692`},
{"CURE", 365, 58.089692983405705`, {{0, 0.07057577289923456`, 125.62487576063751`}}, 0.0175`},
{"CURE", 365, 29.914341955709975`, {{0, 0.006292168257002894‌6`, 11.200059497465153`},
{28, 0.0003291229512201073`, 0.585838853171791`}, {56, 0.0003291229512201073`, 0.585838853171791`},
{84, 0.0003291229512201073`, 0.585838853171791`}, {112, 0.0003291229512201073`, 0.585838853171791`},
{140, 0.0003291229512201073`, 0.585838853171791`}, {168, 0.0003291229512201073`, 0.585838853171791`},
{196, 0.0003291229512201073`, 0.585838853171791`}, {224, 0.0003291229512201073`, 0.585838853171791`},
{252, 0.0003291229512201073`, 0.585838853171791`}}, 0.002339673484110173`},
{"CURE", 365, 125.46645032772514`, {{0, 0.02822324551772677`, 50.23737702155365`},
```

```
{28, 0.0018956284698432415`, 3.37421867632097`}, {56, 0.0018956284698432415`, 3.37421867632097`},
{84, 0.0018956284698432415`, 3.37421867632097`}, {112, 0.0018956284698432415`, 3.37421867632097`},
{140, 0.0018956284698432415`, 3.37421867632097`}, {168, 0.0018956284698432415`, 3.37421867632097`},
{196, 0.0018956284698432415`, 3.37421867632097`}, {224, 0.0018956284698432415`, 3.37421867632097`}}, 0.008373715169608465`},
{"CURE", 365, 17.424600639362822`, {{0, 0.005656603910875465`, 10.068754961358328`},
{28, 0.00028372549792529087`, 0.5050313863070177`}, {56, 0.00028372549792529087`, 0.5050313863070177`},
{84, 0.00028372549792529087`, 0.5050313863070177`}, {112, 0.00028372549792529087`, 0.5050313863070177`},
{140, 0.00028372549792529087`, 0.5050313863070177`}, {168, 0.00028372549792529087`, 0.5050313863070177`},
{196, 0.00028372549792529087`, 0.5050313863070177`}, {224, 0.00028372549792529087`, 0.5050313863070177`},
{252, 0.00028372549792529087`, 0.5050313863070177`}}, 0.002164806480872832 5`},
{"TBUR", 89.27116133955128`, 13.668829929745636`, {{0, 0.009991191346496944`, 17.78432059676456`},
{28, 0.000593338886183968`, 1.056143217407463`}, {56, 0.000593338886183968`, 1.056143217407463`},
{84, 0.000593338886183968`, 1.056143217407463`}}, 0.0033574100618568815`}, {"CURE", 365, 139.04517813502375`,
{{0, 0.02837034310214003`, 50.49921072180925`}, {28, 0.001906135440158474 1`, 3.3929210834820838`},
{56, 0.0019061354401584741`, 3.3929210834820838`}, {84, 0.0019061354401584741`, 3.3929210834820838`},
{112, 0.0019061354401584741`, 3.3929210834820838`}, {140, 0.0019061354401584741`, 3.3929210834820838`},
{168, 0.0019061354401584741`, 3.3929210834820838`}, {196, 0.0019061354401584741`, 3.3929210834820838`},
{224, 0.0009347579100593209`, 1.6638690799055913`}}, 0.00841418709652905`}, {"CURE", 365, 87.6532567321043`,
{{0, 0.044396616376779365`, 79.02597715066727`}, {28, 0.0030508692454898553`, 5.430547256971942`},
{56, 0.0030508692454898553`, 5.430547256971942`}, {84, 0.0012468882216321283`, 2.2194610345051884`}},
0.012823601182883377`}, {"CURE", 365, 130.68670919240978`, {{0, 0.0255527661288482`, 45.483923709349796`},
{28, 0.0017048799420662005`, 3.0346862968778368`}, {56, 0.0017048799420662005`, 3.0346862968778368`},
{84, 0.0017048799420662005`, 3.0346862968778368`}, {112, 0.0017048799420662005`, 3.0346862968778368`},
{140, 0.0017048799420662005`, 3.0346862968778368`}, {168, 0.0017048799420662005`, 3.0346862968778368`},
{196, 0.0017048799420662005`, 3.0346862968778368`}, {224, 0.0017048799420662005`, 3.0346862968778368`},
{252, 0.0017048799420662005`, 3.0346862968778368`}}, 0.007638968592356864`},
{"TBUR", 77.42587480923144`, 12.614722739124714`, {{0, 0.018307207535812338`, 32.58682941374596`},
{28, 0.001187340042563639`, 2.1134652757632777`}, {56, 0.001187340042563639`, 2.1134652757632777`}}, 0.005645450350396337`},
{"CURE", 365, 33.37372815740596`, {{0, 0.004918732727414227`, 8.755344254797324`},
{28, 0.0002310204133923454`, 0.4112163358383748`}, {56, 0.0002310204133923454`, 0.4112163358383748`},
{84, 0.0002310204133923454`, 0.4112163358383748`}, {112, 0.0002310204133923454`, 0.4112163358383748`},
```

```
{140, 0.0002310204133923454`, 0.4112163358383748`}, {168, 0.0002310204133923454`, 0.4112163358383748`},
  {196, 0.0002310204133923454`, 0.4112163358383748`}, {224, 0.0002310204133923454`, 0.4112163358383748`},
  {252, 0.0002310204133923454`, 0.4112163358383748`}}, 0.001961791123870432`},
{"TBUR", 92.80139148733957`, 8.511900451153286`, {{0, 0.008102075193317146`, 14.421693844104519`},
  {28, 0.0004584020180996967`, 0.8159555922174602`}, {56, 0.0004584020180996967`, 0.8159555922174602`},
  {84, 0.0004584020180996967`, 0.8159555922174602`}}, 0.0028376450963004726`}, {"CURE", 365, 86.20215222518262`,
 {{0, 0.05719278640658695`, 101.80315980372477`}, {28, 0.0018161067637140693`, 3.2326700394110435`}}, 0.01634429569581832`},
{"TBUR", 38.289513182207784`, 6.711018329042206`, {{0, 0.002927216944551707`, 5.2104461613020385`},
  {28, 0.0000887692860450224 6`, 0.15800932916013996`}}, 0.0014138522739790447`},
{"CURE", 365, 98.65823491577686`, {{0, 0.02590554227635936`, 46.11186525191966`},
  {28, 0.0017300782383169976`, 3.079539264204256`}, {56, 0.0017300782383169976`, 3.079539264204256`},
  {84, 0.0017300782383169976`, 3.079539264204256`}, {112, 0.0017300782383169976`, 3.079539264204256`},
  {140, 0.0017300782383169976`, 3.079539264204256`}, {168, 0.0017300782383169976`, 3.079539264204256`},
  {196, 0.0017300782383169976`, 3.079539264204256`}, {224, 0.0017300782383169976`, 3.079539264204256`},
  {252, 0.0017300782383169976`, 3.079539264204256`}}, 0.007736030216572836`},
{"TBUR", 20.809265560934843`, 22.955907699487955`, {{0, 0.023779620748492644`, 42.327724932316904`}}, 0.007151111429103178`},
{"CURE", 365, 76.05409188908583`, {{0, 0.014640520126688593`, 26.060125825505693`}, {28, 0.0009254337990548`, 1.647272162317544`},
  {56, 0.0009254337990548`, 1.647272162317544`}, {84, 0.0009254337990548`, 1.647272162317544`},
  {112, 0.0009254337990548`, 1.647272162317544`}, {140, 0.0009254337990548`, 1.647272162317544`},
  {168, 0.0009254337990548`, 1.647272162317544`}, {196, 0.0009254337990548`, 1.647272162317544`},
  {224, 0.0009254337990548`, 1.647272162317544`}, {252, 0.0009254337990548`, 1.647272162317544`}}, 0.004636610501294728`},
{"CURE", 365, 103.24449223336974`, {{0, 0.038335784499285284`, 68.2376964087278`},
  {28, 0.0026179526828117063`, 4.659955775404837`}, {56, 0.0026179526828117063`, 4.659955775404837`},
  {84, 0.0026179526828117063`, 4.659955775404837`}, {112, 0.0026179526828117063`, 4.659955775404837`}},
 0.011156044602655955`}, {"TBUR", 33.56432738549344`, 4.269941521542843`,
 {{0, 0.005253162649080185`, 9.35062951536273`}, {28, 0.0002549082649399138`, 0.45373671159304657`}}, 0.002053805030174263`},
{"CURE", 365, 132.28386382609926`, {{0, 0.024669252547336026`, 43.911269534258125`}, {28, 0.001641771829101045`,
   2.92235385579986`}, {56, 0.001641771829101045`, 2.92235385579986`}, {84, 0.001641771829101045`, 2.92235385579986`},
  {112, 0.001641771829101045`, 2.92235385579986`}, {140, 0.001641771829101045`, 2.92235385579986`},
  {168, 0.001641771829101045`, 2.92235385579986`}, {196, 0.001641771829101045`, 2.92235385579986`},
  {224, 0.001641771829101045`, 2.92235385579986`}, {252, 0.001641771829101045`, 2.92235385579986`}}, 0.007395881683438996`},
```

```
{"CURE", 365, 45.98610002697291`, {{0, 0.010707788234918419`, 19.059863058154786`},
    {28, 0.0006445243782140736`, 1.1472533932210511`}, {56, 0.0006445243782140736`, 1.1472533932210511`},
    {84, 0.0006445243782140736`, 1.1472533932210511`}, {112, 0.0006445243782140736`, 1.1472533932210511`},
    {140, 0.0006445243782140736`, 1.1472533932210511`}, {168, 0.0006445243782140736`, 1.1472533932210511`},
    {196, 0.0006445243782140736`, 1.1472533932210511`}, {224, 0.0006445243782140736`, 1.1472533932210511`},
    {252, 0.0006445243782140736`, 1.1472533932210511`}}, 0.003554572081990625`}, {"CURE", 365, 58.31693949680892`,
  {{0, 0.02312437250406222`, 41.16138305723075`}, {28, 0.001531423254581488`, 2.7259333931550485`},
    {56, 0.001531423254581488`, 2.7259333931550485`}, {84, 0.001531423254581488`, 2.7259333931550485`},
    {112, 0.001531423254581488`, 2.7259333931550485`}, {140, 0.001531423254581488`, 2.7259333931550485`},
    {168, 0.001531423254581488`, 2.7259333931550485`}, {196, 0.001531423254581488`, 2.7259333931550485`},
    {224, 0.001531423254581488`, 2.7259333931550485`}, {252, 0.001531423254581488`, 2.7259333931550485`}},
  0.006970828665322871`}, {"CURE", 365, 79.79241268776074`, {{0, 0.011590853968742487`, 20.631720064361627`},
    {28, 0.0007076005020586497`, 1.2595288936643965`}, {56, 0.0007076005020586497`, 1.2595288936643965`},
    {84, 0.0007076005020586497`, 1.2595288936643965`}, {112, 0.0007076005020586497`, 1.2595288936643965`},
    {140, 0.0007076005020586497`, 1.2595288936643965`}, {168, 0.0007076005020586497`, 1.2595288936643965`},
    {196, 0.0007076005020586497`, 1.2595288936643965`}, {224, 0.0007076005020586497`, 1.2595288936643965`},
    {252, 0.0007076005020586497`, 1.2595288936643965`}}, 0.0037975357716253455`},
{"TBUR", 94.10105085577638`, 6.408036165545593`, {{0, 0.004897864817975589`, 8.718199375996548`},
    {28, 0.0002295298484324426`, 0.4085631302097478`}, {56, 0.0002295298484324426`, 0.4085631302097478`},
    {84, 0.0002295298484324426`, 0.4085631302097478`}}, 0.001956049598547942`},
{"CURE", 365, 12.48419169848081`, {{0, 0.0047542606506942265`, 8.462583958235724`},
    {28, 0.00021927240791234532`, 0.3903048860839747`}, {56, 0.00021927240791234532`, 0.3903048860839747`},
    {84, 0.00021927240791234532`, 0.3903048860839747`}, {112, 0.00021927240791234532`, 0.3903048860839747`},
    {140, 0.00021927240791234532`, 0.3903048860839747`}, {168, 0.00021927240791234532`, 0.3903048860839747`},
    {196, 0.00021927240791234532`, 0.3903048860839747`}, {224, 0.00021927240791234532`, 0.3903048860839747`},
    {252, 0.00021927240791234532`, 0.3903048860839747`}}, 0.001916538838484238`}, {"CURE", 365, 118.06129354316239`,
  {{0, 0.022703561819780598`, 40.41234003920947`}, {28, 0.0015013653485613718`, 2.6724303204392417`},
    {56, 0.0015013653485613718`, 2.6724303204392417`}, {84, 0.0015013653485613718`, 2.6724303204392417`},
    {112, 0.0015013653485613718`, 2.6724303204392417`}, {140, 0.0015013653485613718`, 2.6724303204392417`},
    {168, 0.0015013653485613718`, 2.6724303204392417`}, {196, 0.0015013653485613718`, 2.6724303204392417`},
    {224, 0.0015013653485613718`, 2.6724303204392417`}, {252, 0.0015013653485613718`, 2.6724303204392417`}},
```

```
0.00685504825105176`}, {"TBUR", 55.5946698828071`, 7.763385703077804`,
  {{0, 0.01047986680730434`, 18.654162917001727`}, {28, 0.0006282442762416393`, 1.118274811710118`}}, 0.003491862558929275 3`},
{"CURE", 365, 11.86418662847841`, {{0, 0.004121795489164946 5`, 7.336795970713605`},
   {28, 0.00017409632494596816`, 0.3098914584038233`}, {56, 0.00017409632494596816`, 0.3098914584038233`},
   {84, 0.00017409632494596816`, 0.3098914584038233`}, {112, 0.00017409632494596816`, 0.3098914584038233`},
   {140, 0.00017409632494596816`, 0.3098914584038233`}, {168, 0.00017409632494596816`, 0.3098914584038233`},
   {196, 0.00017409632494596816`, 0.3098914584038233`}, {224, 0.00017409632494596816`, 0.3098914584038233`},
   {252, 0.00017409632494596816`, 0.3098914584038233`}}, 0.0017425245343110594`},
{"CURE", 365, 82.22318266541394`, {{0, 0.04457455955471176`, 79.34271600738694`}, {28, 0.003063579472485027`, 5.453171461023349`},
   {56, 0.003063579472485027`, 5.453171461023349`}, {84, 0.0011445327019629133`, 2.037268209493986`}}, 0.01287255986104266`},
{"CURE", 365, 105.29106803279451`, {{0, 0.020961535283230522`, 37.31153280415033`},
   {28, 0.001376934881664938 1`, 2.4509440893635897`}, {56, 0.0013769348816649381`, 2.4509440893635897`},
   {84, 0.0013769348816649381`, 2.4509440893635897`}, {112, 0.0013769348816649381`, 2.4509440893635897`},
   {140, 0.0013769348816649381`, 2.4509440893635897`}, {168, 0.0013769348816649381`, 2.4509440893635897`},
   {196, 0.0013769348816649381`, 2.4509440893635897`}, {224, 0.0013769348816649381`, 2.4509440893635897`},
   {252, 0.0013769348816649381`, 2.4509440893635897`}}, 0.006375753020187189`}, {"CURE", 365, 108.28739397127454`,
  {{0, 0.029076312167280346`, 51.755835657759015`}, {28, 0.00195656180195421 1`, 3.4826800074784954`},
   {56, 0.00195656180195421 1`, 3.4826800074784954`}, {84, 0.00195656180195421 1`, 3.4826800074784954`},
   {112, 0.00195656180195421 1`, 3.4826800074784954`}, {140, 0.00195656180195421 1`, 3.4826800074784954`},
   {168, 0.00195656180195421 1`, 3.4826800074784954`}, {196, 0.00195656180195421 1`, 3.4826800074784954`},
   {224, 0.00027654236484277693`, 0.49224540942014294`}}, 0.00860842501366766`},
{"TBUR", 14.646454799924522`, 3.428738967715794`, {{0, 0.003806149967655205`, 6.774946942426265`}}, 0.0016556789037025898`},
{"TBUR", 18.433809835948463`, 7.388400309464117`, {{0, 0.03727282411773203`, 66.34562692956301`}}, 0.010863585314127882`},
{"CURE", 365, 75.33528200807787`, {{0, 0.0303473848114552`, 54.01834496439025`}, {28, 0.002047352705109558`, 3.6442878150950135`},
   {56, 0.002047352705109558`, 3.6442878150950135`}, {84, 0.002047352705109558`, 3.6442878150950135`},
   {112, 0.002047352705109558`, 3.6442878150950135`}, {140, 0.002047352705109558`, 3.6442878150950135`},
   {168, 0.002047352705109558`, 3.6442878150950135`}, {196, 0.002047352705109558`, 3.6442878150950135`}},
  0.008958143599265056`}, {"CURE", 365, 67.18690141937809`, {{0, 0.05028856172066351`, 89.51363986278105`},
   {28, 0.0034717224843387228`, 6.179666022122927`}, {56, 0.0013294775313066201`, 2.366470005725784`}}, 0.014444690899134784`},
{"CURE", 365, 61.6255724744214`, {{0, 0.01003325299947483`, 17.859190339065197`},
   {28, 0.0005963432899681026`, 1.0614910561432227`}, {56, 0.0005963432899681026`, 1.0614910561432227`},
```

```
{84, 0.0005963432899681026`, 1.0614910561432227`}, {112, 0.0005963432899681026`, 1.0614910561432227`},
{140, 0.0005963432899681026`, 1.0614910561432227`}, {168, 0.0005963432899681026`, 1.0614910561432227`},
{196, 0.0005963432899681026`, 1.0614910561432227`}, {224, 0.0005963432899681026`, 1.0614910561432227`},
{252, 0.0005963432899681026`, 1.0614910561432227`}}, 0.003368982761383323`},
{"CURE", 365, 84.41429102343794`, {{0, 0.04613573172184507`, 82.12160246488422`}, {28, 0.003175091770137406`, 5.651663350844583`},
{56, 0.003175091770137406`, 5.651663350844583`}, {84, 0.00024652362784853294`, 0.43881205757038866`}},
0.013302095438466964`}, {"CURE", 365, 155.45924403549736`, {{0, 0.026590127530873166`, 47.33042700495424`},
{28, 0.0017789771850679839`, 3.1665793894210115`}, {56, 0.0017789771850679839`, 3.1665793894210115`},
{84, 0.0017789771850679839`, 3.1665793894210115`}, {112, 0.0017789771850679839`, 3.1665793894210115`},
{140, 0.0017789771850679839`, 3.1665793894210115`}, {168, 0.0017789771850679839`, 3.1665793894210115`},
{196, 0.0017789771850679839`, 3.1665793894210115`}, {224, 0.0017789771850679839`, 3.1665793894210115`},
{252, 0.0008155780457301774`, 1.4517289213997158`}}, 0.007924384665098335`},
{"TBUR", 17.092601054042667`, 3.557659772023006`, {{0, 0.0019269097330628924`, 3.4298993248519483`}}, 0.0011386311646522 5`},
{"TBUR", 16.345081264948103`, 17.22745465065279`, {{0, 0.017573122333286274`, 31.28015775324957`}}, 0.005443476655256313`},
{"CURE", 365, 71.95776673030812`, {{0, 0.021080630047211106`, 37.52352148403577`},
{28, 0.001385441650520694`, 2.4660861379268355`}, {56, 0.001385441650520694`, 2.4660861379268355`},
{84, 0.001385441650520694`, 2.4660861379268355`}, {112, 0.001385441650520694`, 2.4660861379268355`},
{140, 0.001385441650520694`, 2.4660861379268355`}, {168, 0.001385441650520694`, 2.4660861379268355`},
{196, 0.001385441650520694`, 2.4660861379268355`}, {224, 0.001385441650520694`, 2.4660861379268355`},
{252, 0.001385441650520694`, 2.4660861379268355`}}, 0.0064085203467457615`},
{"TBUR", 149.10527294626402`, 32.791730050684336`, {{0, 0.07062711489865942`, 125.71626451961376`}}, 0.017500000000000005`},
{"CURE", 365, 66.35304504909621`,
{{0, 0.029476173749488625`, 52.467589274089754`}, {28, 0.001985123343540517`, 3.53351955150212`},
{56, 0.001985123343540517`, 3.53351955150212`}, {84, 0.001985123343540517`, 3.53351955150212`},
{112, 0.001985123343540517`, 3.53351955150212`}, {140, 0.001985123343540517`, 3.53351955150212`},
{168, 0.001985123343540517`, 3.53351955150212`}, {196, 0.001985123343540517`, 3.53351955150212`}}, 0.008718441563552105`},
{"CURE", 365, 14.21095565729069`, {{0, 0.004877124040646364`, 8.681280792350528`},
{28, 0.00022804836433749795`, 0.40592608852074635`}, {56, 0.00022804836433749795`, 0.40592608852074635`},
{84, 0.00022804836433749795`, 0.40592608852074635`}, {112, 0.00022804836433749795`, 0.40592608852074635`},
{140, 0.00022804836433749795`, 0.40592608852074635`}, {168, 0.00022804836433749795`, 0.40592608852074635`},
{196, 0.00022804836433749795`, 0.40592608852074635`}, {224, 0.00022804836433749795`, 0.40592608852074635`},
```

```
{252, 0.00022804836433749795`, 0.40592608852074635`}}, 0.0019503430519197782`},
{"CURE", 365, 48.44938101455724`, {{0, 0.06480294631670447`, 115.34924444373395`}}, 0.017500000000000005`},
{"TBUR", 21.040658017853342`, 2.9740311929089955`, {{0, 0.003721870682174089`, 6.624929814269878`}}, 0.001632490588975898`},
{"CURE", 365, 62.43423252948516`, {{0, 0.016256945595200026`, 28.937363159456048`},
  {28, 0.001040892761091331`, 1.8527891147425692`}, {56, 0.001040892761091331`, 1.8527891147425692`},
  {84, 0.001040892761091331`, 1.8527891147425692`}, {112, 0.001040892761091331`, 1.8527891147425692`},
  {140, 0.001040892761091331`, 1.8527891147425692`}, {168, 0.001040892761091331`, 1.8527891147425692`},
  {196, 0.001040892761091331`, 1.8527891147425692`}, {224, 0.001040892761091331`, 1.8527891147425692`},
  {252, 0.001040892761091331`, 1.8527891147425692`}}, 0.005081348283326378`},
{"TBUR", 24.938539784578825`, 4.523312955341252`, {{0, 0.004446890288689887`, 7.915464713867999`}}, 0.0018319700069958542`},
{"CURE", 365, 132.99088319257925`, {{0, 0.06263917041791892`, 111.49772334389567`}}, 0.017500000000000005`},
{"TBUR", 21.207287953928113`, 3.178416966585667`, {{0, 0.0033693216788103367`, 5.997392588282399`}}, 0.0015354914604247664`},
{"CURE", 365, 87.98729631296342`, {{0, 0.04523374584177651`, 80.51606759836218`}, {28, 0.003110664207275366`, 5.536982288950152`},
  {56, 0.003110664207275366`, 5.536982288950152`}, {84, 0.0007653591042149598`, 1.3623392055026284`}}, 0.013053926124421834`},
{"TBUR", 15.989897809039267`, 2.2875143201027344`, {{0, 0.00473906976417775`, 8.435544180236395`}}, 0.0019123592698570165`},
{"TBUR", 14.493356948953418`, 11.649137374915322`, {{0, 0.04531787255092028`, 80.6658131406381`}}, 0.013077072459816243`},
{"CURE", 365, 141.16660601015974`, {{0, 0.0402949938408028`, 71.72508903662897`}, {28, 0.002757896207205815`, 4.909055248826351`},
  {56, 0.002757896207205815`, 4.909055248826351`}, {84, 0.002757896207205815`, 4.909055248826351`},
  {112, 0.0008483053133960371`, 1.509983457844946`}}, 0.011695094768627786`}, {"CURE", 365, 87.32740904090387`,
 {{0, 0.03495840461222227`, 62.22596020975564`}, {28, 0.0023767112623072056`, 4.230546046906826`},
  {56, 0.0023767112623072056`, 4.230546046906826`}, {84, 0.0023767112623072056`, 4.230546046906826`},
  {112, 0.0023767112623072056`, 4.230546046906826`}, {140, 0.0023767112623072056`, 4.230546046906826`}},
 0.010226803836959564`}, {"CURE", 365, 23.17099180745805`, {{0, 0.008093338995549966`, 14.406143412078938`},
  {28, 0.0004577780039734696`, 0.8148448470727759`}, {56, 0.0004577780039734696`, 0.8148448470727759`},
  {84, 0.0004577780039734696`, 0.8148448470727759`}, {112, 0.0004577780039734696`, 0.8148448470727759`},
  {140, 0.0004577780039734696`, 0.8148448470727759`}, {168, 0.0004577780039734696`, 0.8148448470727759`},
  {196, 0.0004577780039734696`, 0.8148448470727759`}, {224, 0.0004577780039734696`, 0.8148448470727759`},
  {252, 0.0004577780039734696`, 0.8148448470727759`}}, 0.002835241448687853`},
{"CURE", 365, 39.325875672836666`, {{0, 0.006105532702864413`, 10.867848211098655`},
  {28, 0.0003157918402102158`, 0.5621094755741841`}, {56, 0.0003157918402102158`, 0.5621094755741841`},
  {84, 0.0003157918402102158`, 0.5621094755741841`}, {112, 0.0003157918402102158`, 0.5621094755741841`},
```

```
{140, 0.0003157918402102158`, 0.5621094755741841`}, {168, 0.0003157918402102158`, 0.5621094755741841`},
{196, 0.0003157918402102158`, 0.5621094755741841`}, {224, 0.0003157918402102158`, 0.5621094755741841`},
{252, 0.0003157918402102158`, 0.5621094755741841`}}, 0.0022883232152529054`},
{"TBUR", 27.23914035559213`, 5.4685299582192215`, {{0, 0.014100162462441022`, 25.09828918314502`}}, 0.004487938339303506`},
{"CURE", 365, 75.64661102391673`, {{0, 0.033171787056311415`, 59.04578096023432`}, {28, 0.002249095722599287`, 4.00339038622673`},
{56, 0.002249095722599287`, 4.00339038622673`}, {84, 0.002249095722599287`, 4.00339038622673`},
{112, 0.002249095722599287`, 4.00339038622673`}, {140, 0.002249095722599287`, 4.00339038622673`},
{168, 0.0009562871243236643`, 1.7021910812961225`}}, 0.009735239985341758`}, {"CURE", 365, 18.6235125119903`,
{{0, 0.006272095560096733`, 11.164330096972185`}, {28, 0.00032768918715538156`, 0.5832867531365792`},
{56, 0.00032768918715538156`, 0.5832867531365792`}, {84, 0.00032768918715538156`, 0.5832867531365792`},
{112, 0.00032768918715538156`, 0.5832867531365792`}, {140, 0.00032768918715538156`, 0.5832867531365792`},
{168, 0.00032768918715538156`, 0.5832867531365792`}, {196, 0.00032768918715538156`, 0.5832867531365792`},
{224, 0.00032768918715538156`, 0.5832867531365792`}, {252, 0.00032768918715538156`, 0.5832867531365792`}},
0.002334150750847586 5`}, {"CURE", 365, 65.11998913923324`, {{0, 0.037994632319794774`, 67.63044552923469`},
{28, 0.002593584669990956`, 4.616580712583901`}, {56, 0.002593584669990956`, 4.616580712583901`},
{84, 0.002593584669990956`, 4.616580712583901`}, {112, 0.002593584669990956`, 4.616580712583901`}}, 0.011062181157296118`},
{"CURE", 365, 59.20178337514752`, {{0, 0.02145112739556279`, 38.18300676410177`},
{28, 0.0014119057468315287`, 2.513192229360121`}, {56, 0.0014119057468315287`, 2.513192229360121`},
{84, 0.0014119057468315287`, 2.513192229360121`}, {112, 0.0014119057468315287`, 2.513192229360121`},
{140, 0.0014119057468315287`, 2.513192229360121`}, {168, 0.0014119057468315287`, 2.513192229360121`},
{196, 0.0014119057468315287`, 2.513192229360121`}, {224, 0.0014119057468315287`, 2.513192229360121`},
{252, 0.0014119057468315287`, 2.513192229360121`}}, 0.006510457721629045`},
{"TBUR", 130.8641218064927`, 42.2807614358704`, {{0, 0.01778860643425368`, 31.663719452971552`},
{28, 0.0011502971067380208`, 2.047528849993677`}, {56, 0.0011502971067380208`, 2.047528849993677`},
{84, 0.0011502971067380208`, 2.047528849993677`}, {112, 0.0011502971067380208`, 2.047528849993677`}},
0.005502764214747432`}, {"CURE", 365, 28.772063688440454`, {{0, 0.007278532228209645`, 12.955787366213167`},
{28, 0.0003995775205920180 3`, 0.7112479866537921`}, {56, 0.00039957752059201803`, 0.7112479866537921`},
{84, 0.00039957752059201803`, 0.7112479866537921`}, {112, 0.00039957752059201803`, 0.7112479866537921`},
{140, 0.00039957752059201803`, 0.7112479866537921`}, {168, 0.00039957752059201803`, 0.7112479866537921`},
{196, 0.00039957752059201803`, 0.7112479866537921`}, {224, 0.00039957752059201803`, 0.7112479866537921`},
{252, 0.00039957752059201803`, 0.7112479866537921`}}, 0.002611058297932981`},
```

```
{"TBUR", 41.43241746348001`, 5.082277498557025`, {{0, 0.009756556945543948`, 17.366671363068228`},
  {28, 0.0005765792861158968`, 1.0263111292862963`}}, 0.0032928535542411878`},
{"CURE", 365, 53.493604881910514`, {{0, 0.013299874531987728`, 23.673776666938156`},
  {28, 0.0008296733994333096`, 1.476818650991291`}, {56, 0.0008296733994333096`, 1.476818650991291`},
  {84, 0.0008296733994333096`, 1.476818650991291`}, {112, 0.0008296733994333096`, 1.476818650991291`},
  {140, 0.0008296733994333096`, 1.476818650991291`}, {168, 0.0008296733994333096`, 1.476818650991291`},
  {196, 0.0008296733994333096`, 1.476818650991291`}, {224, 0.0008296733994333096`, 1.476818650991291`},
  {252, 0.0008296733994333096`, 1.476818650991291`}}, 0.004267749851736392`}, {"CURE", 365, 62.27402795153233`,
 {{0, 0.05897472725049537`, 104.97501450588176`}, {28, 0.0010456712940607015`, 1.8612949034280488`}}, 0.016834572812870464`},
{"CURE", 365, 40.469555252797015`, {{0, 0.005858656768362874 5`, 10.428409047685916`},
  {28, 0.00029815784488867737`, 0.5307209639018458`}, {56, 0.00029815784488867737`, 0.5307209639018458`},
  {84, 0.00029815784488867737`, 0.5307209639018458`}, {112, 0.00029815784488867737`, 0.5307209639018458`},
  {140, 0.00029815784488867737`, 0.5307209639018458`}, {168, 0.00029815784488867737`, 0.5307209639018458`},
  {196, 0.00029815784488867737`, 0.5307209639018458`}, {224, 0.00029815784488867737`, 0.5307209639018458`},
  {252, 0.00029815784488867737`, 0.5307209639018458`}}, 0.002220398613911208 5`},
{"TBUR", 15.343406771302448`, 4.031474685652979`, {{0, 0.00441740748467172`, 7.862985322715662`}}, 0.0018238582090054475`},
{"CURE", 365, 82.72033300475852`, {{0, 0.026833523309074767`, 47.76367149015309`},
  {28, 0.00179636259779667`, 3.1975254240780724`}, {56, 0.00179636259779667`, 3.1975254240780724`},
  {84, 0.00179636259779667`, 3.1975254240780724`}, {112, 0.00179636259779667`, 3.1975254240780724`},
  {140, 0.00179636259779667`, 3.1975254240780724`}, {168, 0.00179636259779667`, 3.1975254240780724`},
  {196, 0.00179636259779667`, 3.1975254240780724`}, {224, 0.00179636259779667`, 3.1975254240780724`},
  {252, 0.0005712607562902514`, 1.0168441461966475`}}, 0.00799135174812316`},
{"CURE", 365, 144.32356714201327`, {{0, 0.036244619722233924`, 64.51542310557639`},
  {28, 0.002468583770165181 5`, 4.394079110894023`}, {56, 0.0024685837701651815`, 4.394079110894023`},
  {84, 0.0024685837701651815`, 4.394079110894023`}, {112, 0.0024685837701651815`, 4.394079110894023`},
  {140, 0.0009988684396647803`, 1.777985822603309`}}, 0.01058068866888377`},
{"CURE", 365, 25.508537741671862`, {{0, 0.008727716477871206`, 15.535335330610748`},
  {28, 0.0005030906812821295`, 0.8955014126821906`}, {56, 0.0005030906812821295`, 0.8955014126821906`},
  {84, 0.0005030906812821295`, 0.8955014126821906`}, {112, 0.0005030906812821295`, 0.8955014126821906`},
  {140, 0.0005030906812821295`, 0.8955014126821906`}, {168, 0.0005030906812821295`, 0.8955014126821906`},
  {196, 0.0005030906812821295`, 0.8955014126821906`}, {224, 0.0005030906812821295`, 0.8955014126821906`},
```

```
{252, 0.0005030906812821295`, 0.8955014126821906`}}, 0.0030097819022716398`},
{"CURE", 365, 20.720572020426072`, {{0, 0.007926977832016024`, 14.110020540988522`},
  {28, 0.0004458950637210451`, 0.7936932134234603`}, {56, 0.0004458950637210451`, 0.7936932134234603`},
  {84, 0.0004458950637210451`, 0.7936932134234603`}, {112, 0.0004458950637210451`, 0.7936932134234603`},
  {140, 0.0004458950637210451`, 0.7936932134234603`}, {168, 0.0004458950637210451`, 0.7936932134234603`},
  {196, 0.0004458950637210451`, 0.7936932134234603`}, {224, 0.0004458950637210451`, 0.7936932134234603`},
  {252, 0.0004458950637210451`, 0.7936932134234603`}}, 0.0027894694064084`}, {"CURE", 365, 117.7751410094465`,
 {{0, 0.03407282940750926`, 60.64963634536649`}, {28, 0.0023134558905419907`, 4.117951485164744`},
  {56, 0.0023134558905419907`, 4.117951485164744`}, {84, 0.0023134558905419907`, 4.117951485164744`},
  {112, 0.0023134558905419907`, 4.117951485164744`}, {140, 0.0023134558905419907`, 4.117951485164744`},
  {168, 0.0002449138757415897`, 0.43594669882002973`}}, 0.009983149700076294`},
{"TBUR", 143.31045434637042`, 24.48763561396859`, {{0, 0.014899313648137118`, 26.52077829368407`},
  {28, 0.000943919050586837`, 1.6801759100445703`}, {56, 0.000943919050586837`, 1.6801759100445703`},
  {84, 0.000943919050586837`, 1.6801759100445703`}, {112, 0.000943919050586837`, 1.6801759100445703`},
  {140, 0.000943919050586837`, 1.6801759100445703`}}, 0.004707814066801011`},
{"CURE", 365, 131.71850310157635`, {{0, 0.019726495251762795`, 35.113161548137775`},
  {28, 0.0012887177365601002`, 2.2939175710769786`}, {56, 0.0012887177365601002`, 2.2939175710769786`},
  {84, 0.0012887177365601002`, 2.2939175710769786`}, {112, 0.0012887177365601002`, 2.2939175710769786`},
  {140, 0.0012887177365601002`, 2.2939175710769786`}, {168, 0.0012887177365601002`, 2.2939175710769786`},
  {196, 0.0012887177365601002`, 2.2939175710769786`}, {224, 0.0012887177365601002`, 2.2939175710769786`},
  {252, 0.0012887177365601002`, 2.2939175710769786`}}, 0.006035948324570089`},
{"CURE", 365, 88.01776284143315`, {{0, 0.05181936386987191`, 92.238467688372`}, {28, 0.003581065494996466`, 6.374296581093709`},
  {56, 0.0005582807465443779`, 0.9937397288489928`}}, 0.01486587057356492`}, {"CURE", 365, 166.3222224339134`,
 {{0, 0.05495167082402091`, 97.81397406675721`}, {28, 0.002785069870600729`, 4.9574243696692974`}}, 0.01572768280961772`},
{"TBUR", 62.414770671391395`, 21.37071840700219`, {{0, 0.030152561215753406`, 53.67155896404106`},
  {28, 0.002033436733988001`, 3.6195173864986416`}, {56, 0.002033436733988001`, 3.6195173864986416`}}, 0.008904540500620716`},
{"CURE", 365, 62.688914578436105`, {{0, 0.01394329877924922`, 24.819071827063613`},
  {28, 0.000875632274237702`, 1.5586254481431094`}, {56, 0.000875632274237702`, 1.5586254481431094`},
  {84, 0.000875632274237702`, 1.5586254481431094`}, {112, 0.000875632274237702`, 1.5586254481431094`},
  {140, 0.000875632274237702`, 1.5586254481431094`}, {168, 0.000875632274237702`, 1.5586254481431094`},
  {196, 0.000875632274237702`, 1.5586254481431094`}, {224, 0.000875632274237702`, 1.5586254481431094`},
```

```
{252, 0.000875632274237702`, 1.5586254481431094`}}, 0.004444779401323965`},
{"TBUR", 28.642219182947287`, 6.098621096596466`, {{0, 0.011635078716246966`, 20.7104401149196`},
  {28, 0.0007107594125946839`, 1.2651517544185373`}}, 0.0038097036175911508`},
{"CURE", 365, 33.92282146351274`, {{0, 0.00943564028236479`, 16.795439702609325`},
  {28, 0.0005536566673173857`, 0.9855088678249466`}, {56, 0.0005536566673173857`, 0.9855088678249466`},
  {84, 0.0005536566673173857`, 0.9855088678249466`}, {112, 0.0005536566673173857`, 0.9855088678249466`},
  {140, 0.0005536566673173857`, 0.9855088678249466`}, {168, 0.0005536566673173857`, 0.9855088678249466`},
  {196, 0.0005536566673173857`, 0.9855088678249466`}, {224, 0.0005536566673173857`, 0.9855088678249466`},
  {252, 0.0005536566673173857`, 0.9855088678249466`}}, 0.003204557639718922`},
{"CURE", 365, 27.54262520114216`, {{0, 0.006849674217333698`, 12.192420106853982`},
  {28, 0.0003689448055294505`, 0.6567217538424219`}, {56, 0.0003689448055294505`, 0.6567217538424219`},
  {84, 0.0003689448055294505`, 0.6567217538424219`}, {112, 0.0003689448055294505`, 0.6567217538424219`},
  {140, 0.0003689448055294505`, 0.6567217538424219`}, {168, 0.0003689448055294505`, 0.6567217538424219`},
  {196, 0.0003689448055294505`, 0.6567217538424219`}, {224, 0.0003689448055294505`, 0.6567217538424219`},
  {252, 0.0003689448055294505`, 0.6567217538424219`}}, 0.002493063769710707`},
{"CURE", 365, 40.27089891643015`, {{0, 0.0074094888443141885`, 13.188890142879256`},
  {28, 0.00040893156459948556`, 0.7278981849870843`}, {56, 0.00040893156459948556`, 0.7278981849870843`},
  {84, 0.00040893156459948556`, 0.7278981849870843`}, {112, 0.00040893156459948556`, 0.7278981849870843`},
  {140, 0.00040893156459948556`, 0.7278981849870843`}, {168, 0.00040893156459948556`, 0.7278981849870843`},
  {196, 0.00040893156459948556`, 0.7278981849870843`}, {224, 0.00040893156459948556`, 0.7278981849870843`},
  {252, 0.00040893156459948556`, 0.7278981849870843`}}, 0.00264708925396731`},
{"CURE", 365, 44.26589774192142`, {{0, 0.011976882738517682`, 21.318851274561474`},
  {28, 0.0007351739856140206`, 1.3086096943929566`}, {56, 0.0007351739856140206`, 1.3086096943929566`},
  {84, 0.0007351739856140206`, 1.3086096943929566`}, {112, 0.0007351739856140206`, 1.3086096943929566`},
  {140, 0.0007351739856140206`, 1.3086096943929566`}, {168, 0.0007351739856140206`, 1.3086096943929566`},
  {196, 0.0007351739856140206`, 1.3086096943929566`}, {224, 0.0007351739856140206`, 1.3086096943929566`},
  {252, 0.0007351739856140206`, 1.3086096943929566`}}, 0.003903746408746975`}, {"CURE", 365, 53.6904691335086`,
 {{0, 0.012986598290009511`, 23.11614495621693`}, {28, 0.0008072965250062943`, 1.4369878145112038`},
  {56, 0.0008072965250062943`, 1.4369878145112038`}, {84, 0.0008072965250062943`, 1.4369878145112038`},
  {112, 0.0008072965250062943`, 1.4369878145112038`}, {140, 0.0008072965250062943`, 1.4369878145112038`},
  {168, 0.0008072965250062943`, 1.4369878145112038`}, {196, 0.0008072965250062943`, 1.4369878145112038`},
```

{224, 0.0008072965250062943`, 1.4369878145112038`}, {252, 0.0008072965250062943`, 1.4369878145112038`}},
0.00418155609660525 8`}, {"TBUR", 35.949839862437024`, 4.769811729523964`,
{{0, 0.013559091612255979`, 24.135183069815643`}, {28, 0.0008481889051667563`, 1.5097762511968262`}}, 0.004339069953769544`},
{"CURE", 365, 133.09805130546349`, {{0, 0.05152577980639263 4`, 91.71588805537888`},
{28, 0.0035600952047479466`, 6.336969464451345`}, {56, 0.0007061843054267168`, 1.257008063659556`}}, 0.01478509485716158`},
{"CURE", 365, 49.28021790930065`, {{0, 0.009956103726349537`, 17.721864632902175`},
{28, 0.0005908326276020103`, 1.0516820771315782`}, {56, 0.0005908326276020103`, 1.0516820771315782`},
{84, 0.0005908326276020103`, 1.0516820771315782`}, {112, 0.0005908326276020103`, 1.0516820771315782`},
{140, 0.0005908326276020103`, 1.0516820771315782`}, {168, 0.0005908326276020103`, 1.0516820771315782`},
{196, 0.0005908326276020103`, 1.0516820771315782`}, {224, 0.0005908326276020103`, 1.0516820771315782`},
{252, 0.0005908326276020103`, 1.0516820771315782`}}, 0.003347756173901567`},
{"TBUR", 214.381096541578`, 13.410663615852997`, {{0, 0.0062571764833729785`, 11.137774140403902`}}, 0.0023300459670382886`},
{"CURE", 365, 116.80323989021011`, {{0, 0.016972819850769375`, 30.211619334369487`},
{28, 0.001092026636489141 8`, 1.9438074129506724`}, {56, 0.0010920266364891418`, 1.9438074129506724`},
{84, 0.0010920266364891418`, 1.9438074129506724`}, {112, 0.0010920266364891418`, 1.9438074129506724`},
{140, 0.0010920266364891418`, 1.9438074129506724`}, {168, 0.0010920266364891418`, 1.9438074129506724`},
{196, 0.0010920266364891418`, 1.9438074129506724`}, {224, 0.0010920266364891418`, 1.9438074129506724`},
{252, 0.0010920266364891418`, 1.9438074129506724`}}, 0.005278311480725552`},
{"CURE", 365, 72.13466316159605`, {{0, 0.03049817150990257`, 54.28674528762657`}, {28, 0.0020581231835700843`, 3.66345926675475`},
{56, 0.0020581231835700843`, 3.66345926675475`}, {84, 0.0020581231835700843`, 3.66345926675475`},
{112, 0.0020581231835700843`, 3.66345926675475`}, {140, 0.0020581231835700843`, 3.66345926675475`},
{168, 0.0020581231835700843`, 3.66345926675475`}, {196, 0.0010089843413484817`, 1.7959921276002975`}},
0.008999630536419738`}, {"TBUR", 51.59002899964628`, 6.131581152734481`,
{{0, 0.007343242897182699`, 13.070972356985203`}, {28, 0.00040419971123295046`, 0.7194754859946518`}},
0.002628862570355985 6`}, {"CURE", 365, 137.35243785337906`,
{{0, 0.023843260297045112`, 42.4410033287403`}, {28, 0.0015827723826516945`, 2.817334841120016`},
{56, 0.0015827723826516945`, 2.817334841120016`}, {84, 0.0015827723826516945`, 2.817334841120016`},
{112, 0.0015827723826516945`, 2.817334841120016`}, {140, 0.0015827723826516945`, 2.817334841120016`},
{168, 0.0015827723826516945`, 2.817334841120016`}, {196, 0.0015827723826516945`, 2.817334841120016`},
{224, 0.0015827723826516945`, 2.817334841120016`}, {252, 0.0015827723826516945`, 2.817334841120016`}},
0.007168620997112388`}, {"CURE", 365, 14.480526829909442`, {{0, 0.004940838189645928`, 8.794691977569752`},

```
    {28, 0.00023259937498032392`, 0.41402688746497657`}, {56, 0.00023259937498032392`, 0.41402688746497657`},
    {84, 0.00023259937498032392`, 0.41402688746497657`}, {112, 0.00023259937498032392`, 0.41402688746497657`},
    {140, 0.00023259937498032392`, 0.41402688746497657`}, {168, 0.00023259937498032392`, 0.41402688746497657`},
    {196, 0.00023259937498032392`, 0.41402688746497657`}, {224, 0.00023259937498032392`, 0.41402688746497657`},
    {252, 0.00023259937498032392`, 0.41402688746497657`}}, 0.0019678731452411826`},
  {"CURE", 365, 139.63755060453482`, {{0, 0.04394792882169804`, 78.2273133026225`}, {28, 0.0030188201344126184`, 5.37349983925446`},
    {56, 0.0030188201344126184`, 5.37349983925446`}, {84, 0.0015049798686572555`, 2.678864166209915`}}, 0.012700150821602052`},
  {"TBUR", 21.21486307399575`, 2.782149094590236`, {{0, 0.00439166248150519`, 7.817159217079238`}}, 0.0018167748167738063`},
  {"TBUR", 18.181035675673378`, 2.3824017503958634`, {{0, 0.0033490222038675462`, 5.961259522884232`}}, 0.0015299063322278083`},
  {"CURE", 365, 149.01208488935453`, {{0, 0.0429333926141746`, 76.4214388532308`}, {28, 0.002946353262446658`, 5.244508807155051`},
    {56, 0.002946353262446658`, 5.244508807155051`}, {84, 0.002946353262446658`, 5.244508807155051`}}, 0.012421014794909596`},
  {"", 365, 149.01208488935453`, {{0, 0.015635247810469446`, 27.830741102635614`},
    {28, 0.0009964857764677181`, 1.7737446821125382`}, {56, 0.0009964857764677181`, 1.7737446821125382`},
    {84, 0.0009964857764677181`, 1.7737446821125382`}, {112, 0.0009964857764677181`, 1.7737446821125382`},
    {140, 0.0009964857764677181`, 1.7737446821125382`}, {168, 0.0009964857764677181`, 1.7737446821125382`},
    {196, 0.0009964857764677181`, 1.7737446821125382`}, {224, 0.0009964857764677181`, 1.7737446821125382`},
    {252, 0.0009964857764677181`, 1.7737446821125382`}}, 0.004910296478426465`},
  {"TBUR", 117.80530153066995`, 25.592740860988446`, {{0, 0.02192885802239696`, 39.03336727986659`},
    {28, 0.001446029363033969`, 2.573932266200465`}, {56, 0.001446029363033969`, 2.573932266200465`},
    {84, 0.001446029363033969`, 2.573932266200465`}, {112, 0.001446029363033969`, 2.573932266200465`}}, 0.00664189889446733`},
  {"CURE", 365, 78.24560846369847`, {{0, 0.051228516630498065`, 91.18675960228656`},
    {28, 0.003538862120755477`, 6.299174574944749`}, {56, 0.0008559413498603471`, 1.5235756027514178`}}, 0.014703306882335386`},
  {"CURE", 365, 88.87622918772699`, {{0, 0.03688099001319412`, 65.64816222348554`},
    {28, 0.002514038790948053`, 4.474989047887534`}, {56, 0.002514038790948053`, 4.474989047887534`},
    {84, 0.002514038790948053`, 4.474989047887534`}, {112, 0.002514038790948053`, 4.474989047887534`},
    {140, 0.0005419088951615029`, 0.9645978333874752`}}, 0.010755777417029457`},
  {"TBUR", 19.500644658049744`, 2.9930870557168925`, {{0, 0.0096933513419075`, 17.25416538859535`}}, 0.0032754633803539048`},
  {"CURE", 365, 74.22311506976256`, {{0, 0.05022713202114949`, 89.40429499764609`},
    {28, 0.0034673346486591497`, 6.171855674613287`}, {56, 0.0013604249576468662`, 2.421556424611422`}}, 0.014427789341441628`},
  {"CURE", 365, 70.44378193717394`, {{0, 0.022370814090448173`, 39.82004908099775`},
    {28, 0.001475976536090557`, 2.6301238234241193`}, {56, 0.001475976536090557`, 2.6301238234241193`},
```

{84, 0.0014775976536090557`, 2.6301238234241193`}, {112, 0.0014775976536090557`, 2.6301238234241193`},
{140, 0.0014775976536090557`, 2.6301238234241193`}, {168, 0.0014775976536090557`, 2.6301238234241193`},
{196, 0.0014775976536090557`, 2.6301238234241193`}, {224, 0.0014775976536090557`, 2.6301238234241193`},
{252, 0.0014775976536090557`, 2.6301238234241193`}}, 0.006763497177400556`}, {"TBUR", 37.87993038890295`,
20.620875229755622`, {{0, 0.05583948266993663`, 99.3942791524872`}, {28, 0.00240121776978034`, 4.274167630209005`}},
0.015971952328321604`}, {"TBUR", 45.36191238502929`, 7.086878495172183`,
{{0, 0.018693755176637773`, 33.27488421441524`}, {28, 0.0012149505883368847`, 2.1626120472396546`}}, 0.005751803747926372`},
{"CURE", 365, 101.16157470913976`, {{0, 0.03138126916509062`, 55.8586591138613`},
{28, 0.0021212015875120877`, 3.775738825771516`}, {56, 0.0021212015875120877`, 3.775738825771516`},
{84, 0.0021212015875120877`, 3.775738825771516`}, {112, 0.0021212015875120877`, 3.775738825771516`},
{140, 0.0021212015875120877`, 3.775738825771516`}, {168, 0.0021212015875120877`, 3.775738825771516`},
{196, 0.0002487000325439685`, 0.442686057928264`}}, 0.009242603008789506`},
{"CURE", 365, 50.09987600881457`, {{0, 0.019211862087196756`, 34.197114515210224`},
{28, 0.001251958224805383`6`, 2.2284856401535826`}, {56, 0.0012519582248053836`, 2.2284856401535826`},
{84, 0.0012519582248053836`, 2.2284856401535826`}, {112, 0.0012519582248053836`, 2.2284856401535826`},
{140, 0.0012519582248053836`, 2.2284856401535826`}, {168, 0.0012519582248053836`, 2.2284856401535826`},
{196, 0.0012519582248053836`, 2.2284856401535826`}, {224, 0.0012519582248053836`, 2.2284856401535826`},
{252, 0.0012519582248053836`, 2.2284856401535826`}}, 0.00589435391355168`},
{"CURE", 365, 12.93121451993907`, {{0, 0.004633646464663738`, 8.247890707101453`},
{28, 0.0002106571089101675`, 0.3749696538600982`}, {56, 0.0002106571089101675`, 0.3749696538600982`},
{84, 0.0002106571089101675`, 0.3749696538600982`}, {112, 0.0002106571089101675`, 0.3749696538600982`},
{140, 0.0002106571089101675`, 0.3749696538600982`}, {168, 0.0002106571089101675`, 0.3749696538600982`},
{196, 0.0002106571089101675`, 0.3749696538600982`}, {224, 0.0002106571089101675`, 0.3749696538600982`},
{252, 0.0002106571089101675`, 0.3749696538600982`}}, 0.0018833534633328859`},
{"CURE", 365, 36.88637708265995`, {{0, 0.009544130504725532`, 16.988552298411445`},
{28, 0.0005614059689145813`, 0.9993026246679548`}, {56, 0.0005614059689145813`, 0.9993026246679548`},
{84, 0.0005614059689145813`, 0.9993026246679548`}, {112, 0.0005614059689145813`, 0.9993026246679548`},
{140, 0.0005614059689145813`, 0.9993026246679548`}, {168, 0.0005614059689145813`, 0.9993026246679548`},
{196, 0.0005614059689145813`, 0.9993026246679548`}, {224, 0.0005614059689145813`, 0.9993026246679548`},
{252, 0.0005614059689145813`, 0.9993026246679548`}}, 0.00323440726891913`},
{"CURE", 365, 46.32385336144564`, {{0, 0.009105819761262722`, 16.208359175047647`},

{28, 0.00053009805866723 8`, 0.9435745444276835`}, {56, 0.000530098058667238`, 0.9435745444276835`},
{84, 0.000530098058667238`, 0.9435745444276835`}, {112, 0.000530098058667238`, 0.9435745444276835`},
{140, 0.000530098058667238`, 0.9435745444276835`}, {168, 0.000530098058667238`, 0.9435745444276835`},
{196, 0.000530098058667238`, 0.9435745444276835`}, {224, 0.000530098058667238`, 0.9435745444276835`},
{252, 0.000530098058667238`, 0.9435745444276835`}}, 0.003113811948141483`},
{"TBUR", 143.87114046896022`, 21.219421723495408`, {{0, 0.022355141422196582`, 39.79215173150992`},
{28, 0.0014764781773053707`, 2.62813115560356`}, {56, 0.0014764781773053707`, 2.62813115560356`},
{84, 0.0014764781773053707`, 2.62813115560356`}, {112, 0.0014764781773053707`, 2.62813115560356`},
{140, 0.0014764781773053707`, 2.62813115560356`}}, 0.006759185052992402`}, {"CURE", 365, 135.55124964551658`,
{{0, 0.04415587425330569`, 78.59745617088413`}, {28, 0.0030336733795274496`, 5.3999386155588605`},
{56, 0.0030336733795274496`, 5.3999386155588605`}, {84, 0.001385366613671295`, 2.4659525723349054`}}, 0.01275736421735606`},
{"TBUR", 21.20656243273727`, 9.023739089152265`, {{0, 0.026854854091485515`, 47.80164028284422`}}, 0.007997220626734119`},
{"TBUR", 39.71522696547551`, 3.4262478261181886`,
{{0, 0.005182482236099832`, 9.2248183802577`}, {28, 0.0002498596640127457`, 0.44475020194268733`}}, 0.002034358262776 4993`},
{"CURE", 365, 88.00960766655827`, {{0, 0.02254573821837179`, 40.13141402870178`},
{28, 0.001490092234175 0285`, 2.6523641768315507`}, {56, 0.0014900922341750285`, 2.6523641768315507`},
{84, 0.0014900922341750285`, 2.6523641768315507`}, {112, 0.0014900922341750285`, 2.6523641768315507`},
{140, 0.0014900922341750285`, 2.6523641768315507`}, {168, 0.0014900922341750285`, 2.6523641768315507`},
{196, 0.0014900922341750285`, 2.6523641768315507`}, {224, 0.0014900922341750285`, 2.6523641768315507`},
{252, 0.0014900922341750285`, 2.6523641768315507`}}, 0.006811625204452871`},
{"CURE", 365, 71.11872731458023`, {{0, 0.035353050378032234`, 62.92842967289738`},
{28, 0.0024049002455793465`, 4.280722437131237`}, {56, 0.0024049002455793465`, 4.280722437131237`},
{84, 0.0024049002455793465`, 4.280722437131237`}, {112, 0.0024049002455793465`, 4.280722437131237`},
{140, 0.0024049002455793465`, 4.280722437131237`}}, 0.010335385324936323`}, {"CURE", 365, 99.81709277179151`,
{{0, 0.044786533338108024`, 79.72002934183229`}, {28, 0.003078720457013331`, 5.480122413483729`},
{56, 0.003078720457013331`, 5.480122413483729`}, {84, 0.0010226022800839856`, 1.8202320585494944`}}, 0.012930881603747772`},
{"CURE", 365, 107.88637110160394`, {{0, 0.025355698390438774`, 45.13314313498102`}, {28, 0.001690803675036956`,
3.009630541565782`}, {56, 0.001690803675036956`, 3.009630541565782`}, {84, 0.001690803675036956`, 3.009630541565782`},
{112, 0.001690803675036956`, 3.009630541565782`}, {140, 0.001690803675036956`, 3.009630541565782`},
{168, 0.001690803675036956`, 3.009630541565782`}, {196, 0.001690803675036956`, 3.009630541565782`},
{224, 0.001690803675036956`, 3.009630541565782`}, {252, 0.001690803675036956`, 3.009630541565782`}}, 0.0075847480479534 7`},

```
{"CURE", 365, 107.79368915821084`, {{0, 0.016926812918083362`, 30.129726994188385`},
   {28, 0.0010887404270115695`, 1.9379579600805936`}, {56, 0.0010887404270115695`, 1.9379579600805936`},
   {84, 0.0010887404270115695`, 1.9379579600805936`}, {112, 0.0010887404270115695`, 1.9379579600805936`},
   {140, 0.0010887404270115695`, 1.9379579600805936`}, {168, 0.0010887404270115695`, 1.9379579600805936`},
   {196, 0.0010887404270115695`, 1.9379579600805936`}, {224, 0.0010887404270115695`, 1.9379579600805936`},
   {252, 0.0010887404270115695`, 1.9379579600805936`}}, 0.005265653290416474`},
{"TBUR", 159.97321671459682`, 35.45153932649181`, {{0, 0.02730657849862815`, 48.60570972755811`},
   {28, 0.0018301522541933402`, 3.2576710124641455`}, {56, 0.0018301522541933402`, 3.2576710124641455`},
   {84, 0.0018301522541933402`, 3.2576710124641455`}, {112, 0.0018301522541933402`, 3.2576710124641455`},
   {140, 0.0018301522541933402`, 3.2576710124641455`}}, 0.008121506537118336`}, {"CURE", 365, 84.8403508299355`,
  {{0, 0.021287530721164655`, 37.89180468367309`}, {28, 0.0014002202700888044`, 2.492392080758072`},
   {56, 0.0014002202700888044`, 2.492392080758072`}, {84, 0.0014002202700888044`, 2.492392080758072`},
   {112, 0.0014002202700888044`, 2.492392080758072`}, {140, 0.0014002202700888044`, 2.492392080758072`},
   {168, 0.0014002202700888044`, 2.492392080758072`}, {196, 0.0014002202700888044`, 2.492392080758072`},
   {224, 0.0014002202700888044`, 2.492392080758072`}, {252, 0.0014002202700888044`, 2.492392080758072`}},
  0.006465446291447496`}, {"CURE", 365, 65.15542133493142`, {{0, 0.026735236862776696`, 47.58872161574252`},
   {28, 0.0017893421373468077`, 3.1850290044773177`}, {56, 0.0017893421373468077`, 3.1850290044773177`},
   {84, 0.0017893421373468077`, 3.1850290044773177`}, {112, 0.0017893421373468077`, 3.1850290044773177`},
   {140, 0.0017893421373468077`, 3.1850290044773177`}, {168, 0.0017893421373468077`, 3.1850290044773177`},
   {196, 0.0017893421373468077`, 3.1850290044773177`}, {224, 0.0017893421373468077`, 3.1850290044773177`},
   {252, 0.0006699193210593472`, 1.192456391485638`}}, 0.007964309551014851`},
{"CURE", 365, 188.563932158575`, {{0, 0.030791982713711117`, 54.809729230405786`},
   {28, 0.002079109698127837`, 3.7008152626675503`}, {56, 0.002079109698127837`, 3.7008152626675503`},
   {84, 0.002079109698127837`, 3.7008152626675503`}, {112, 0.002079109698127837`, 3.7008152626675503`},
   {140, 0.002079109698127837`, 3.7008152626675503`}, {168, 0.002079109698127837`, 3.7008152626675503`},
   {196, 0.0007560337795456257`, 1.3457401275912138`}}, 0.009080468747437408`},
{"CURE", 365, 92.96831245435109`, {{0, 0.04686037351858056`, 83.41146486307339`}, {28, 0.003226851898475655`, 5.743796379286667`},
   {56, 0.003226851898475655`, 5.743796379286667`}}, 0.013501470907194488`},
{"TBUR", 264.8395657888087`, 16.175204511086555`, {{0, 0.008712681677458464`, 15.508573385876065`},
   {28, 0.0005020167669669337`, 0.893589845201142`}, {56, 0.0005020167669669337`, 0.893589845201142`},
   {84, 0.0005020167669669337`, 0.893589845201142`}, {112, 0.0005020167669669337`, 0.893589845201142`},
```

{140, 0.000502016766966933 7`, 0.893589845201142`}, {168, 0.0005020167669669337`, 0.893589845201142`},
{196, 0.0005020167669669337`, 0.893589845201142`}, {224, 0.0005020167669669337`, 0.893589845201142`},
{252, 0.0005020167669669337`, 0.893589845201142`}}, 0.003005645278641842`},
{"CURE", 365, 16.945297913692723`, {{0, 0.004876202411328774`, 8.679640292165217`},
{28, 0.00022798253367195578`, 0.40580890993608126`}, {56, 0.00022798253367195578`, 0.40580890993608126`},
{84, 0.00022798253367195578`, 0.40580890993608126`}, {112, 0.00022798253367195578`, 0.40580890993608126`},
{140, 0.00022798253367195578`, 0.40580890993608126`}, {168, 0.00022798253367195578`, 0.40580890993608126`},
{196, 0.00022798253367195578`, 0.40580890993608126`}, {224, 0.00022798253367195578`, 0.40580890993608126`},
{252, 0.00022798253367195578`, 0.40580890993608126`}}, 0.0019500894779774312`},
{"TBUR", 13.450688612391271`, 2.594267902753244`, {{0, 0.011249818453519711`, 20.024676847265088`}}, 0.003703704424873631`},
{"CURE", 365, 25.132445073026368`, {{0, 0.007311275644423858`, 13.014070647074467`},
{28, 0.00040191633603589046`, 0.715411078143885`}, {56, 0.00040191633603589046`, 0.715411078143885`},
{84, 0.00040191633603589046`, 0.715411078143885`}, {112, 0.00040191633603589046`, 0.715411078143885`},
{140, 0.00040191633603589046`, 0.715411078143885`}, {168, 0.00040191633603589046`, 0.715411078143885`},
{196, 0.00040191633603589046`, 0.715411078143885`}, {224, 0.00040191633603589046`, 0.715411078143885`},
{252, 0.00040191633603589046`, 0.715411078143885`}}, 0.002620067209625432`},
{"TBUR", 94.34552882040872`, 41.96344873644497`, {{0, 0.03168769639900743`, 56.40409959023323`},
{28, 0.002143089247077574 5`, 3.8146988597980824`}, {56, 0.0021430892470775745`, 3.8146988597980824`},
{84, 0.0021430892470775745`, 3.8146988597980824`}}, 0.009326912351237417`},
{"TBUR", 46.853949028488614`, 6.760176155624078`, {{0, 0.008168110227547284`, 14.539236205034166`},
{28, 0.0004631188062589923`, 0.8243514751410063`}}, 0.002855813750056554`},
{"TBUR", 15.603072483399645`, 4.9226773866659705`, {{0, 0.029843667940537864`, 53.121728934157396`}}, 0.008819552659993356`},
{"CURE", 365, 104.80602853536497`, {{0, 0.034619855055155226`, 61.6233419981763`},
{28, 0.0023525291510881306`, 4.187501888936873`}, {56, 0.0023525291510881306`, 4.187501888936873`},
{84, 0.0023525291510881306`, 4.187501888936873`}, {112, 0.0023525291510881306`, 4.187501888936873`},
{140, 0.0023525291510881306`, 4.187501888936873`}}, 0.010133656468243301`},
{"TBUR", 14.229170335498118`, 2.8637373315784638`, {{0, 0.015105633792968325`, 26.888028151483617`}}, 0.0047645802867029895`},
{"CURE", 365, 134.99156062540163`,
{{0, 0.05882267653683224`, 104.70436423556139`}, {28, 0.0011114115588919677`, 1.9783125748277026`}}, 0.01679273809888693`},
{"TBUR", 21.836941727937358`, 8.28624216310501`, {{0, 0.02264848228337014`, 40.31429846439885`}}, 0.006839893855544074`},
{"CURE", 365, 21.11852373060223`, {{0, 0.007174992839428131`, 12.771487254182073`},

```
{28, 0.0003921818499647671`, 0.6980836929372854`}, {56, 0.0003921818499647671`, 0.6980836929372854`},
{84, 0.0003921818499647671`, 0.6980836929372854`}, {112, 0.0003921818499647671`, 0.6980836929372854`},
{140, 0.0003921818499647671`, 0.6980836929372854`}, {168, 0.0003921818499647671`, 0.6980836929372854`},
{196, 0.0003921818499647671`, 0.6980836929372854`}, {224, 0.0003921818499647671`, 0.6980836929372854`},
{252, 0.0003921818499647671`, 0.6980836929372854`}}, 0.0025825708241727406`},
{"CURE", 365, 59.43806827500103`, {{0, 0.015090357461105748`, 26.860836280768233`},
{28, 0.0009575650372274541`, 1.7044657662648683`}, {56, 0.0009575650372274541`, 1.7044657662648683`},
{84, 0.0009575650372274541`, 1.7044657662648683`}, {112, 0.0009575650372274541`, 1.7044657662648683`},
{140, 0.0009575650372274541`, 1.7044657662648683`}, {168, 0.0009575650372274541`, 1.7044657662648683`},
{196, 0.0009575650372274541`, 1.7044657662648683`}, {224, 0.0009575650372274541`, 1.7044657662648683`},
{252, 0.0009575650372274541`, 1.7044657662648683`}}, 0.004760377208935105`},
{"CURE", 365, 64.2000720306495`, {{0, 0.02160209450484372`, 38.451728218621824`},
{28, 0.001422689111780166З`, 2.532386618968696`}, {56, 0.0014226891117801663`, 2.532386618968696`},
{84, 0.0014226891117801663`, 2.532386618968696`}, {112, 0.0014226891117801663`, 2.532386618968696`},
{140, 0.0014226891117801663`, 2.532386618968696`}, {168, 0.0014226891117801663`, 2.532386618968696`},
{196, 0.0014226891117801663`, 2.532386618968696`}, {224, 0.0014226891117801663`, 2.532386618968696`},
{252, 0.0014226891117801663`, 2.532386618968696`}}, 0.006551994296404225`},
{"CURE", 365, 98.12220922611985`, {{0, 0.034518390471728`, 61.44273503967584`}, {28, 0.0023452816808433295`, 4.174601391901127`},
{56, 0.0023452816808433295`, 4.174601391901127`}, {84, 0.0023452816808433295`, 4.174601391901127`},
{112, 0.0023452816808433295`, 4.174601391901127`}, {140, 0.0023452816808433295`, 4.174601391901127`}},
0.010105739849341131`}, {"TBUR", 84.50431332847087`, 8.428304628476125`,
{{0, 0.00901883145274338`, 16.053519985883216`}, {28, 0.0005238846080587135`, 0.93251460234451`},
{56, 0.0005238846080587135`, 0.93251460234451`}, {84, 0.0005238846080587135`, 0.93251460234451`}}, 0.0030898782820695156`},
{"CURE", 365, 29.32850077585337`, {{0, 0.012983434669359438`, 23.1105137114598`},
{28, 0.0008070705521027177`, 1.4365855827428375`}, {56, 0.0008070705521027177`, 1.4365855827428375`},
{84, 0.0008070705521027177`, 1.4365855827428375`}, {112, 0.0008070705521027177`, 1.4365855827428375`},
{140, 0.0008070705521027177`, 1.4365855827428375`}, {168, 0.0008070705521027177`, 1.4365855827428375`},
{196, 0.0008070705521027177`, 1.4365855827428375`}, {224, 0.0008070705521027177`, 1.4365855827428375`},
{252, 0.0008070705521027177`, 1.4365855827428375`}}, 0.004180685668825869`},
{"TBUR", 20.91065070832618`, 9.182294436035223`, {{0, 0.011739381509425615`, 20.896099086777593`}}, 0.003838401131829587`},
{"CURE", 365, 65.9562970112469`, {{0, 0.041189930746600496`, 73.31807672894888`}, {28, 0.002821820271905651`, 5.022840083992058`},
```

{56, 0.002821820271905651`, 5.022840083992058`}, {84, 0.002821820271905651`, 5.022840083992058`},
{112, 0.000269600445473259`, 0.47988879294240105`}}, 0.011941324651969867`},
{"CURE", 365, 63.50424013201218`, {{0, 0.01428953504429177`, 25.43537237883935`},
{28, 0.0009003634360264557`, 1.602646916127091`}, {56, 0.0009003634360264557`, 1.602646916127091`},
{84, 0.0009003634360264557`, 1.602646916127091`}, {112, 0.0009003634360264557`, 1.602646916127091`},
{140, 0.0009003634360264557`, 1.602646916127091`}, {168, 0.0009003634360264557`, 1.602646916127091`},
{196, 0.0009003634360264557`, 1.602646916127091`}, {224, 0.0009003634360264557`, 1.602646916127091`},
{252, 0.0009003634360264557`, 1.602646916127091`}}, 0.004540041664616407`}, {"CURE", 365, 67.90148969839713`,
{{0, 0.03677117566961318`, 65.45269269191147`}, {28, 0.002506194909263699 7`, 4.461026938489385`},
{56, 0.0025061949092636997`, 4.461026938489385`}, {84, 0.0025061949092636997`, 4.461026938489385`},
{112, 0.0025061949092636997`, 4.461026938489385`}, {140, 0.0006207634757971862`, 1.1049589869189915`}},
0.010725563473639649`}, {"TBUR", 33.6513260963426`, 18.63377268012092`,
{{0, 0.03657554499534806`, 65.10447009171955`}, {28, 0.002492221289673334`, 4.436153895618535`}}, 0.010671738318156206`},
{"CURE", 365, 186.21225943563158`, {{0, 0.033754842608928394`, 60.08361984389254`},
{28, 0.0022907425477862145`, 4.077521735059462`}, {56, 0.0022907425477862145`, 4.077521735059462`},
{84, 0.0022907425477862145`, 4.077521735059462`}, {112, 0.0022907425477862145`, 4.077521735059462`},
{140, 0.0022907425477862145`, 4.077521735059462`}, {168, 0.0004959645634389723`, 0.8828169229213708`}},
0.009895659898491792`}, {"CURE", 365, 88.20182970430947`, {{0, 0.04642806551539979`, 82.64195661741162`},
{28, 0.003195972755391314 7`, 5.68883150459654`}, {56, 0.0031959727553913147`, 5.68883150459654`},
{84, 0.00007836895227263541`, 0.13949673504529103`}}, 0.013382527159873925`},
{"TBUR", 23.787155993436016`, 16.26039451510626`, {{0, 0.04207280696481568`, 74.88959639737192`}}, 0.012184236198927303`},
{"CURE", 365, 173.82417353525025`, {{0, 0.0242240191956636 3`, 43.118754168281264`},
{28, 0.0016099694468387313`, 2.865745615372942`}, {56, 0.0016099694468387313`, 2.865745615372942`},
{84, 0.0016099694468387313`, 2.865745615372942`}, {112, 0.0016099694468387313`, 2.865745615372942`},
{140, 0.0016099694468387313`, 2.865745615372942`}, {168, 0.0016099694468387313`, 2.865745615372942`},
{196, 0.0016099694468387313`, 2.865745615372942`}, {224, 0.0016099694468387313`, 2.865745615372942`},
{252, 0.0016099694468387313`, 2.865745615372942`}}, 0.007273381699884758`},
{"TBUR", 50.827502547390424`, 3.6616311925310785`, {{0, 0.007807061127068996`, 13.896568806182811`},
{28, 0.0004373295847962574`, 0.7784466609373382`}}, 0.0027564759338199003`},
{"CURE", 365, 21.016534160358283`, {{0, 0.008757675332539915`, 15.58866209192105`},
{28, 0.0005052305994727516`, 0.8993104670614978`}, {56, 0.0005052305994727516`, 0.8993104670614978`},

```
{84, 0.0005052305994727516`, 0.8993104670614978`}, {112, 0.0005052305994727516`, 0.8993104670614978`},
  {140, 0.0005052305994727516`, 0.8993104670614978`}, {168, 0.0005052305994727516`, 0.8993104670614978`},
  {196, 0.0005052305994727516`, 0.8993104670614978`}, {224, 0.0005052305994727516`, 0.8993104670614978`},
  {252, 0.0005052305994727516`, 0.8993104670614978`}}, 0.0030180246792119473`}, {"CURE", 365, 55.183627605697495`,
 {{0, 0.05385133120892107`, 95.8553695518795`}, {28, 0.0032608099441478627`, 5.804241700583195`}}, 0.015424939126451362`},
{"CURE", 365, 78.07345393753316`, {{0, 0.048434791894528435`, 86.21392957226061`},
  {28, 0.003339310353900504`, 5.943972429942897`}, {56, 0.003339310353900504`, 5.943972429942897`}}, 0.013934651001265726`},
{"CURE", 365, 69.669342195943`, {{0, 0.03668697781778949`, 65.30282051566529`}, {28, 0.002500180776990579`, 4.450321783043231`},
  {56, 0.002500180776990579`, 4.450321783043231`}, {84, 0.002500180776990579`, 4.450321783043231`},
  {112, 0.002500180776990579`, 4.450321783043231`}, {140, 0.0006812235767227823`, 1.2125779665665524`}},
 0.010702397564291304`}, {"TBUR", 120.97041139941145`, 29.320307802533257`,
 {{0, 0.02884265939024597`, 51.33993371463783`}, {28, 0.0019398723178803267`, 3.4529727258269816`},
  {56, 0.0019398723178803267`, 3.4529727258269816`}, {84, 0.0019398723178803267`, 3.4529727258269816`},
  {112, 0.0019398723178803267`, 3.4529727258269816`}}, 0.008544138586703913`},
{"CURE", 365, 42.87289509704602`, {{0, 0.009873140299328825`, 17.57418973280531`},
  {28, 0.0005849066685291022`, 1.041133869981802`}, {56, 0.0005849066685291022`, 1.041133869981802`},
  {84, 0.0005849066685291022`, 1.041133869981802`}, {112, 0.0005849066685291022`, 1.041133869981802`},
  {140, 0.0005849066685291022`, 1.041133869981802`}, {168, 0.0005849066685291022`, 1.041133869981802`},
  {196, 0.0005849066685291022`, 1.041133869981802`}, {224, 0.0005849066685291022`, 1.041133869981802`},
  {252, 0.0005849066685291022`, 1.041133869981802`}}, 0.0033249298999769705`},
{"TBUR", 13.955657153570622`, 1.7936733705880161`, {{0, 0.007201635860953359`, 12.818911832496978`}}, 0.0025899012941081488`},
{"TBUR", 56.34305477975944`, 13.51639026025193`, {{0, 0.04105399098980746`, 73.07610396185727`},
  {28, 0.0028121102892775763`, 5.005556314914086`}, {56, 0.0028121102892775763`, 5.005556314914086`}}, 0.011903922651627883`},
{"CURE", 365, 78.59782017753659`, {{0, 0.036949554720485084`, 65.77020740246346`},
  {28, 0.0025189362700402643`, 4.483706560671671`}, {56, 0.0025189362700402643`, 4.483706560671671`},
  {84, 0.0025189362700402643`, 4.483706560671671`}, {112, 0.0025189362700402643`, 4.483706560671671`},
  {140, 0.0004926745140822024`, 0.8769606350663203`}}, 0.010774642076390655`},
{"CURE", 365, 32.20712275262948`, {{0, 0.010251619093731922`, 18.24788198684282`},
  {28, 0.0006119408681293234`, 1.0892547452701955`}, {56, 0.0006119408681293234`, 1.0892547452701955`},
  {84, 0.0006119408681293234`, 1.0892547452701955`}, {112, 0.0006119408681293234`, 1.0892547452701955`},
  {140, 0.0006119408681293234`, 1.0892547452701955`}, {168, 0.0006119408681293234`, 1.0892547452701955`},
```

```
{196, 0.0006119408681293234`, 1.0892547452701955`}, {224, 0.0006119408681293234`, 1.0892547452701955`},
{252, 0.0006119408681293234`, 1.0892547452701955`}}, 0.003429063262663873`}, {"CURE", 365, 16.5729690255237`,
{{0, 0.003392697599037470 3`, 6.039001726286697`}, {28, 0.00012201790422257699`, 0.21719186951618705`},
{56, 0.00012201790422257699`, 0.21719186951618705`}, {84, 0.00012201790422257699`, 0.21719186951618705`},
{112, 0.00012201790422257699`, 0.21719186951618705`}, {140, 0.00012201790422257699`, 0.21719186951618705`},
{168, 0.00012201790422257699`, 0.21719186951618705`}, {196, 0.00012201790422257699`, 0.21719186951618705`},
{224, 0.00012201790422257699`, 0.21719186951618705`}, {252, 0.00012201790422257699`, 0.21719186951618705`}},
0.0015419230312687581`}, {"TBUR", 278.8154591512636`, 60.49701978911725`,
{{0, 0.03430509335452594`, 61.06306617105617`}, {28, 0.002330046172471754`, 4.1474821869997225`},
{56, 0.002330046172471754`, 4.1474821869997225`}, {84, 0.002330046172471754`, 4.1474821869997225`},
{112, 0.002330046172471754`, 4.1474821869997225`}, {140, 0.002330046172471754`, 4.1474821869997225`},
{168, 0.0000615414090666365`, 0.10954370813861296`}}, 0.010047054009092926`}, {"CURE", 365, 63.59916386624027`,
{{0, 0.0543649559660543`, 96.76962161957665`}, {28, 0.0030387404336249574`, 5.408957971852424`}}, 0.015566256087693211`},
{"TBUR", 25.908668621923876`, 8.240780509387898`, {{0, 0.04080995595272677`, 72.64172159585365`}}, 0.01183677968509864`},
{"CURE", 365, 13.393444393928775`, {{0, 0.0053125038970803186`, 9.456256936802967`},
{28, 0.00025914692551135183`, 0.4612815274102063`}, {56, 0.00025914692551135183`, 0.4612815274102063`},
{84, 0.00025914692551135183`, 0.4612815274102063`}, {112, 0.00025914692551135183`, 0.4612815274102063`},
{140, 0.00025914692551135183`, 0.4612815274102063`}, {168, 0.00025914692551135183`, 0.4612815274102063`},
{196, 0.00025914692551135183`, 0.4612815274102063`}, {224, 0.00025914692551135183`, 0.4612815274102063`},
{252, 0.00025914692551135183`, 0.4612815274102063`}}, 0.0020701319784516086`},
{"CURE", 365, 130.78774162770972`, {{0, 0.06246291701852984`, 111.1839922929831`}}, 0.0175`},
{"CURE", 365, 57.65171316334956`, {{0, 0.020885125848273053`, 37.175524009926036`},
{28, 0.0013714770648822615`, 2.4412291754904256`}, {56, 0.0013714770648822615`, 2.4412291754904256`},
{84, 0.0013714770648822615`, 2.4412291754904256`}, {112, 0.0013714770648822615`, 2.4412291754904256`},
{140, 0.0013714770648822615`, 2.4412291754904256`}, {168, 0.0013714770648822615`, 2.4412291754904256`},
{196, 0.0013714770648822615`, 2.4412291754904256`}, {224, 0.0013714770648822615`, 2.4412291754904256`},
{252, 0.0013714770648822615`, 2.4412291754904256`}}, 0.006354729989251794`},
{"CURE", 365, 73.0763764019854`, {{0, 0.03077119707254162`, 54.77273078912408`},
{28, 0.002077625009472874`, 3.6981725168617157`}, {56, 0.002077625009472874`, 3.6981725168617157`},
{84, 0.002077625009472874`, 3.6981725168617157`}, {112, 0.002077625009472874`, 3.6981725168617157`},
{140, 0.002077625009472874`, 3.6981725168617157`}, {168, 0.002077625009472874`, 3.6981725168617157`},
```

{196, 0.000773928739108443`, 1.3775931556130285`}}, 0.009074749857125475`},
{"TBUR", 13.743950194613186`, 2.4703547854279524`, {{0, 0.008557736943093754`, 15.232771758706882`}},
0.002963014313687854 3`}, {"TBUR", 31.84773234462368`, 3.7452834289837793`,
{{0, 0.007787623129702206`, 13.861969170869926`}, {28, 0.0004359411564129152`, 0.775975258414989`}}, 0.0027511278296205744`},
{"TBUR", 19.38027790096907`, 2.7474968034704648`, {{0, 0.012311598783524747`, 21.91464583467405`}}, 0.0039958390380495385`},
{"CURE", 365, 54.49592908602367`, {{0, 0.01513724391287323`, 26.94429416491435`},
{28, 0.0009609140694965602`, 1.710427043703877`}, {56, 0.0009609140694965602`, 1.710427043703877`},
{84, 0.0009609140694965602`, 1.710427043703877`}, {112, 0.0009609140694965602`, 1.710427043703877`},
{140, 0.0009609140694965602`, 1.710427043703877`}, {168, 0.0009609140694965602`, 1.710427043703877`},
{196, 0.0009609140694965602`, 1.710427043703877`}, {224, 0.0009609140694965602`, 1.710427043703877`},
{252, 0.0009609140694965602`, 1.710427043703877`}}, 0.004773277387120002`},
{"TBUR", 24.926319974607555`, 7.879669675000838`, {{0, 0.02137287629119078`, 38.043719798319586`}}, 0.006488927980061694`},
{"CURE", 365, 29.263035921860546`, {{0, 0.008919870707611239`, 15.877369859548004`},
{28, 0.0005168159834064177`, 0.9199324504634235`}, {56, 0.0005168159834064177`, 0.9199324504634235`},
{84, 0.0005168159834064177`, 0.9199324504634235`}, {112, 0.0005168159834064177`, 0.9199324504634235`},
{140, 0.0005168159834064177`, 0.9199324504634235`}, {168, 0.0005168159834064177`, 0.9199324504634235`},
{196, 0.0005168159834064177`, 0.9199324504634235`}, {224, 0.0005168159834064177`, 0.9199324504634235`},
{252, 0.0005168159834064177`, 0.9199324504634235`}}, 0.0030626505606832647`},
{"CURE", 365, 130.76760230689132`, {{0, 0.03105656462596284`, 55.28068503421385`},
{28, 0.0020980084061458177`, 3.7344549629395556`}, {56, 0.0020980084061458177`, 3.7344549629395556`},
{84, 0.0020980084061458177`, 3.7344549629395556`}, {112, 0.0020980084061458177`, 3.7344549629395556`},
{140, 0.0020980084061458177`, 3.7344549629395556`}, {168, 0.0020980084061458177`, 3.7344549629395556`},
{196, 0.0005282475600978508`, 0.9402806569741745`}}, 0.00915326491101734`},
{"TBUR", 96.6006576171223`, 18.619242998323234`, {{0, 0.019223503275967228`, 34.21783583122166`},
{28, 0.0012527897382889884`, 2.2299657341543995`}, {56, 0.0012527897382889884`, 2.2299657341543995`},
{84, 0.0012527897382889884`, 2.2299657341543995`}}, 0.005897556830466099 5`},
{"CURE", 365, 118.19163661706632`, {{0, 0.021054176025726023`, 37.47643332579232`},
{28, 0.0013835520775574736`, 2.462722698052303`}, {56, 0.0013835520775574736`, 2.462722698052303`},
{84, 0.0013835520775574736`, 2.462722698052303`}, {112, 0.0013835520775574736`, 2.462722698052303`},
{140, 0.0013835520775574736`, 2.462722698052303`}, {168, 0.0013835520775574736`, 2.462722698052303`},
{196, 0.0013835520775574736`, 2.462722698052303`}, {224, 0.0013835520775574736`, 2.462722698052303`},

```
{252, 0.0013835520775574736`, 2.462722698052303`}}, 0.006401241877635815`},
{"TBUR", 18.144168570368823`, 2.3263004268407883`, {{0, 0.006637630753570811`, 11.814982741356044`}}, 0.0024347228553875 38`},
{"TBUR", 98.18247616463732`, 28.676539712054392`,
  {{0, 0.03121280859284861`, 55.558799295270525`}, {28, 0.002109168689494801`, 3.7543202673007454`},
   {56, 0.002109168689494801`, 3.7543202673007454`}, {84, 0.002109168689494801`, 3.7543202673007454`}}, 0.009196253342369261`},
{"TBUR", 33.134645767427806`, 4.075766306654412`, {{0, 0.008720952738741334`, 15.523295874959574`},
   {28, 0.0005026075570585672`, 0.8946414515642497`}}, 0.0030079209501909796`},
{"CURE", 365, 76.06137560037028`, {{0, 0.019693642174343613`, 35.054683070331635`},
   {28, 0.001286371088173016`, 2.2897405369479684`}, {56, 0.001286371088173016`, 2.2897405369479684`},
   {84, 0.001286371088173016`, 2.2897405369479684`}, {112, 0.001286371088173016`, 2.2897405369479684`},
   {140, 0.001286371088173016`, 2.2897405369479684`}, {168, 0.001286371088173016`, 2.2897405369479684`},
   {196, 0.001286371088173016`, 2.2897405369479684`}, {224, 0.001286371088173016`, 2.2897405369479684`},
   {252, 0.001286371088173016`, 2.2897405369479684`}}, 0.006026909241068283`}, {"CURE", 365, 91.68564596510055`,
  {{0, 0.04467777579209855`, 79.52644090993542`}, {28, 0.003070952060869797`, 5.4662946683482385`},
   {56, 0.003070952060869797`, 5.4662946683482385`}, {84, 0.0010851612119244514`, 1.9315869572255235`}},
  0.012900958424031973`}, {"CURE", 365, 103.21851582302267`, {{0, 0.04902990331967669`, 87.2732279090245`},
   {28, 0.0033818183128396643`, 6.019636596854602`}, {56, 0.0033818183128396643`, 6.019636596854602`}}, 0.014098387926003656`},
{"CURE", 365, 36.019296492092174`, {{0, 0.012179934976089456`, 21.68028425743923`},
   {28, 0.0007496777168691475`, 1.3344263360270825`}, {56, 0.0007496777168691475`, 1.3344263360270825`},
   {84, 0.0007496777168691475`, 1.3344263360270825`}, {112, 0.0007496777168691475`, 1.3344263360270825`},
   {140, 0.0007496777168691475`, 1.3344263360270825`}, {168, 0.0007496777168691475`, 1.3344263360270825`},
   {196, 0.0007496777168691475`, 1.3344263360270825`}, {224, 0.0007496777168691475`, 1.3344263360270825`},
   {252, 0.0007496777168691475`, 1.3344263360270825`}}, 0.003959613507808087`},
{"TBUR", 56.67741793728194`, 11.765984458811072`, {{0, 0.03049990023587604`, 54.289822419859355`},
   {28, 0.002058246663996761`, 3.6636790619142343`}, {56, 0.002058246663996761`, 3.6636790619142343`}}, 0.009000106172179109`},
{"TBUR", 100.63558739430233`, 19.66100690443528`, {{0, 0.014679076664390884`, 26.128756462615772`},
   {28, 0.0009281878374621067`, 1.65217435068255`}, {56, 0.0009281878374621067`, 1.65217435068255`},
   {84, 0.0009281878374621067`, 1.65217435068255`}}, 0.004647218815376989`},
{"CURE", 365, 9.391001750799493`, {{0, 0.0030908529939281675`, 5.501718329192138`},
   {28, 0.00010045757528619821`, 0.1788144840094328`}, {56, 0.00010045757528619821`, 0.1788144840094328`},
   {84, 0.00010045757528619821`, 0.1788144840094328`}, {112, 0.00010045757528619821`, 0.1788144840094328`},
```

```
{140, 0.00010045757528619821`, 0.1788144840094328`}, {168, 0.00010045757528619821`, 0.1788144840094328`},
{196, 0.00010045757528619821`, 0.1788144840094328`}, {224, 0.00010045757528619821`, 0.1788144840094328`},
{252, 0.00010045757528619821`, 0.1788144840094328`}}, 0.0014588745376576337`},
{"TBUR", 13.54556527169727`, 1.7708312267955064`, {{0, 0.005935872424689241`, 10.565852915946849`}}, 0.0022416434658401577`},
{"CURE", 365, 73.63063644358728`, {{0, 0.06506476798443651`, 115.81528701229699`}}, 0.0175`},
{"TBUR", 12.406820362508068`, 2.172575677856114`, {{0, 0.007594251059293447`, 13.517766885542335`}}, 0.0026979240986872467`},
{"CURE", 365, 30.692014240735865`, {{0, 0.0113075919103696`, 20.12751360045789`},
{28, 0.0006873674978891576`, 1.2235141462427004`}, {56, 0.0006873674978891576`, 1.2235141462427004`},
{84, 0.0006873674978891576`, 1.2235141462427004`}, {112, 0.0006873674978891576`, 1.2235141462427004`},
{140, 0.0006873674978891576`, 1.2235141462427004`}, {168, 0.0006873674978891576`, 1.2235141462427004`},
{196, 0.0006873674978891576`, 1.2235141462427004`}, {224, 0.0006873674978891576`, 1.2235141462427004`},
{252, 0.0006873674978891576`, 1.2235141462427004`}}, 0.0037196000164491485`}, {"CURE", 365, 106.2683996944215`,
{{0, 0.043539840710103674`, 77.50091646398454`}, {28, 0.0029896709835844485`, 5.321614350780318`},
{56, 0.0029896709835844485`, 5.321614350780318`}, {84, 0.0029896709835844485`, 5.321614350780318`}}, 0.01258787085252244`},
{"CURE", 365, 48.1641823446958`, {{0, 0.012866848787137215`, 22.90299084110424`},
{28, 0.0007987429890868446`, 1.4217625205745834`}, {56, 0.0007987429890868446`, 1.4217625205745834`},
{84, 0.0007987429890868446`, 1.4217625205745834`}, {112, 0.0007987429890868446`, 1.4217625205745834`},
{140, 0.0007987429890868446`, 1.4217625205745834`}, {168, 0.0007987429890868446`, 1.4217625205745834`},
{196, 0.0007987429890868446`, 1.4217625205745834`}, {224, 0.0007987429890868446`, 1.4217625205745834`},
{252, 0.0007987429890868446`, 1.4217625205745834`}}, 0.0041486086274244716`},
{"CURE", 365, 54.78904550885328`, {{0, 0.015534807695847204`, 27.651957698608022`},
{28, 0.0009893114825661297`, 1.7609744389677109`}, {56, 0.0009893114825661297`, 1.7609744389677109`},
{84, 0.0009893114825661297`, 1.7609744389677109`}, {112, 0.0009893114825661297`, 1.7609744389677109`},
{140, 0.0009893114825661297`, 1.7609744389677109`}, {168, 0.0009893114825661297`, 1.7609744389677109`},
{196, 0.0009893114825661297`, 1.7609744389677109`}, {224, 0.0009893114825661297`, 1.7609744389677109`},
{252, 0.0009893114825661297`, 1.7609744389677109`}}, 0.004882661728371927`},
{"CURE", 365, 53.29157895419131`, {{0, 0.01879228163111211`, 33.450261303379555`},
{28, 0.0012219881922279085`, 2.1751389821656772`}, {56, 0.0012219881922279085`, 2.1751389821656772`},
{84, 0.0012219881922279085`, 2.1751389821656772`}, {112, 0.0012219881922279085`, 2.1751389821656772`},
{140, 0.0012219881922279085`, 2.1751389821656772`}, {168, 0.0012219881922279085`, 2.1751389821656772`},
{196, 0.0012219881922279085`, 2.1751389821656772`}, {224, 0.0012219881922279085`, 2.1751389821656772`},
```

{252, 0.0012219881922279085`, 2.1751389821656772`}}, 0.005778911980064482`},
{"TBUR", 22.077902622814793`, 2.716429362744646`, {{0, 0.006557007591055358`, 11.671473512078538`}}, 0.002412540473845059`},
{"CURE", 365, 19.872964982613993`, {{0, 0.005422206180405974`, 9.651527001122632`},
{28, 0.00026698280289175577`, 0.47522938914732527`}, {56, 0.00026698280289175577`, 0.47522938914732527`},
{84, 0.00026698280289175577`, 0.47522938914732527`}, {112, 0.00026698280289175577`, 0.47522938914732527`},
{140, 0.00026698280289175577`, 0.47522938914732527`}, {168, 0.00026698280289175577`, 0.47522938914732527`},
{196, 0.00026698280289175577`, 0.47522938914732527`}, {224, 0.00026698280289175577`, 0.47522938914732527`},
{252, 0.00026698280289175577`, 0.47522938914732527`}}, 0.0021003150899655525`},
{"TBUR", 159.4806131073347`, 71.42149668851201`, {{0, 0.04402052164737105`, 78.35652853232047`},
{28, 0.003024005336246405`, 5.382729498518601`}, {56, 0.003024005336246405`, 5.382729498518601`},
{84, 0.0014632234131282847`, 2.6045376753683467`}}, 0.012720123763695669`},
{"CURE", 365, 39.65898452797232`, {{0, 0.014952963574056004`, 26.616275161819686`},
{28, 0.0009477511881524725`, 1.686997114911401`}, {56, 0.0009477511881524725`, 1.686997114911401`},
{84, 0.0009477511881524725`, 1.686997114911401`}, {112, 0.0009477511881524725`, 1.686997114911401`},
{140, 0.0009477511881524725`, 1.686997114911401`}, {168, 0.0009477511881524725`, 1.686997114911401`},
{196, 0.0009477511881524725`, 1.686997114911401`}, {224, 0.0009477511881524725`, 1.686997114911401`},
{252, 0.0009477511881524725`, 1.686997114911401`}}, 0.004722575124161297`}, {"CURE", 365, 102.17396757738416`,
{{0, 0.042758072554347604`, 76.10936914673873`}, {28, 0.0029338304010304438`, 5.22221811383419`},
{56, 0.0029338304010304438`, 5.22221811383419`}, {84, 0.0029338304010304438`, 5.22221811383419`}}, 0.012372777832505805`},
{"CURE", 365, 19.072752219168372`, {{0, 0.0039839827405920795`, 7.091489278253902`},
{28, 0.0001642525571907634`, 0.29236955179955887`}, {56, 0.0001642525571907634`, 0.29236955179955887`},
{84, 0.0001642525571907634`, 0.29236955179955887`}, {112, 0.0001642525571907634`, 0.29236955179955887`},
{140, 0.0001642525571907634`, 0.29236955179955887`}, {168, 0.0001642525571907634`, 0.29236955179955887`},
{196, 0.0001642525571907634`, 0.29236955179955887`}, {224, 0.0001642525571907634`, 0.29236955179955887`},
{252, 0.0001642525571907634`, 0.29236955179955887`}}, 0.0017046072054085243`}, {"CURE", 365, 154.063481738107`,
{{0, 0.060338331650895596`, 107.40223033859417`}, {28, 0.00045610671887566 72`, 0.8118699595986876`}}, 0.017209750269806395`},
{"CURE", 365, 41.00983577715125`, {{0, 0.011645680320525955`, 20.7293109705362`},
{28, 0.0007115166700431831`, 1.2664996726768658`}, {56, 0.0007115166700431831`, 1.2664996726768658`},
{84, 0.0007115166700431831`, 1.2664996726768658`}, {112, 0.0007115166700431831`, 1.2664996726768658`},
{140, 0.0007115166700431831`, 1.2664996726768658`}, {168, 0.0007115166700431831`, 1.2664996726768658`},
{196, 0.0007115166700431831`, 1.2664996726768658`}, {224, 0.0007115166700431831`, 1.2664996726768658`},

```
{252, 0.0007115166700431831`, 1.2664996726768658`}}, 0.0038126205067795876`},
{"CURE", 365, 122.12469276346116`, {{0, 0.049342514070383224`, 87.82967504528214`},
  {28, 0.003404147652175846`, 6.059382820873006`}, {56, 0.003404147652175846`, 6.059382820873006`}}, 0.014184398580139479`},
{"CURE", 365, 35.66068682650014`, {{0, 0.0155982286250729`, 27.764846952629764`},
  {28, 0.0009938415489393934`, 1.7690379571121202`}, {56, 0.0009938415489393934`, 1.7690379571121202`},
  {84, 0.0009938415489393934`, 1.7690379571121202`}, {112, 0.0009938415489393934`, 1.7690379571121202`},
  {140, 0.0009938415489393934`, 1.7690379571121202`}, {168, 0.0009938415489393934`, 1.7690379571121202`},
  {196, 0.0009938415489393934`, 1.7690379571121202`}, {224, 0.0009938415489393934`, 1.7690379571121202`},
  {252, 0.0009938415489393934`, 1.7690379571121202`}}, 0.00490011146206329`},
{"CURE", 365, 116.54940841224978`, {{0, 0.02056067670821067`, 36.598004540614994`},
  {28, 0.0013483021263063773`, 2.3999777848253516`}, {56, 0.0013483021263063773`, 2.3999777848253516`},
  {84, 0.0013483021263063773`, 2.3999777848253516`}, {112, 0.0013483021263063773`, 2.3999777848253516`},
  {140, 0.0013483021263063773`, 2.3999777848253516`}, {168, 0.0013483021263063773`, 2.3999777848253516`},
  {196, 0.0013483021263063773`, 2.3999777848253516`}, {224, 0.0013483021263063773`, 2.3999777848253516`},
  {252, 0.0013483021263063773`, 2.3999777848253516`}}, 0.006265462161106089`},
{"CURE", 365, 114.70881874988531`, {{0, 0.03622016396541323`, 64.47189185843554`},
  {28, 0.0024668369303922745`, 4.390969736098248`}, {56, 0.0024668369303922745`, 4.390969736098248`},
  {84, 0.0024668369303922745`, 4.390969736098248`}, {112, 0.0024668369303922745`, 4.390969736098248`},
  {140, 0.001016429428378457`, 1.8092443825136533`}}, 0.010573959995487922`},
{"TBUR", 15.429325212813996`, 1.9591959826742817`, {{0, 0.005446635908710595`, 9.695011917504859`}}, 0.0021070366019643938`},
{"TBUR", 15.074657971659535`, 2.0674942453865786`, {{0, 0.00619662467709595`, 11.02999192523079`}}, 0.002313385949891655`},
{"CURE", 365, 77.32413065563344`, {{0, 0.024021045138654402`, 42.757460346804834`},
  {28, 0.0015954712999095009`, 2.8399389138389117`}, {56, 0.0015954712999095009`, 2.8399389138389117`},
  {84, 0.0015954712999095009`, 2.8399389138389117`}, {112, 0.0015954712999095009`, 2.8399389138389117`},
  {140, 0.0015954712999095009`, 2.8399389138389117`}, {168, 0.0015954712999095009`, 2.8399389138389117`},
  {196, 0.0015954712999095009`, 2.8399389138389117`}, {224, 0.0015954712999095009`, 2.8399389138389117`},
  {252, 0.0015954712999095009`, 2.8399389138389117`}}, 0.0072175361111565745`},
{"TBUR", 24.982080489662852`, 6.003967567031226`, {{0, 0.012706434362964831`, 22.6174531660774`}}, 0.0041044727507001276`},
{"CURE", 365, 54.23534913689628`, {{0, 0.03800277728002203`, 67.64494355843921`},
  {28, 0.0025941664528643313`, 4.617616286098509`}, {56, 0.0025941664528643313`, 4.617616286098509`},
  {84, 0.0025941664528643313`, 4.617616286098509`}, {112, 0.0025941664528643313`, 4.617616286098509`}},
```

```
0.011064422133831547`}, {"CURE", 365, 85.45250993925498`, {{0, 0.015874975166058986`, 28.257455795584995`},
  {28, 0.001013609159009828`, 1.8042243030374938`}, {56, 0.001013609159009828`, 1.8042243030374938`},
  {84, 0.001013609159009828`, 1.8042243030374938`}, {112, 0.001013609159009828`, 1.8042243030374938`},
  {140, 0.001013609159009828`, 1.8042243030374938`}, {168, 0.001013609159009828`, 1.8042243030374938`},
  {196, 0.001013609159009828`, 1.8042243030374938`}, {224, 0.001013609159009828`, 1.8042243030374938`},
  {252, 0.001013609159009828`, 1.8042243030374938`}}, 0.00497625424418438`},
{"CURE", 365, 144.4842211268541`, {{0, 0.04345746530412278`, 77.35428824133855`}, {28, 0.002983787026014385`, 5.311140906305606`},
  {56, 0.002983787026014385`, 5.311140906305606`}, {84, 0.002983787026014385`, 5.311140906305606`}}, 0.012565206364698184`},
{"CURE", 365, 53.43760689078042`, {{0, 0.018432965455096213`, 32.81067851007126`},
  {28, 0.001196322751083916`, 2.1294544969293705`}, {56, 0.001196322751083916`, 2.1294544969293705`},
  {84, 0.001196322751083916`, 2.1294544969293705`}, {112, 0.001196322751083916`, 2.1294544969293705`},
  {140, 0.001196322751083916`, 2.1294544969293705`}, {168, 0.001196322751083916`, 2.1294544969293705`},
  {196, 0.001196322751083916`, 2.1294544969293705`}, {224, 0.001196322751083916`, 2.1294544969293705`},
  {252, 0.001196322751083916`, 2.1294544969293705`}}, 0.005680050954745098`},
{"TBUR", 26.487676090048804`, 3.6286380153007167`, {{0, 0.00394501144181744`, 7.022120366435043`}}, 0.0016938847753815542`},
{"TBUR", 108.76889797269538`, 23.69958302539122`, {{0, 0.006430523419158289`, 11.446331686101756`},
  {28, 0.0003390054628026354`, 0.6034297237886913`}, {56, 0.0003390054628026354`, 0.6034297237886913`},
  {84, 0.0003390054628026354`, 0.6034297237886913`}}, 0.0023777400508330324`}, {"CURE", 365, 167.42299529483176`,
 {{0, 0.044444371015458034`, 79.1109804075153`}, {28, 0.003054280291109761`, 5.436618918175374`},
  {56, 0.003054280291109761`, 5.436618918175374`}, {84, 0.00121941905470274`, 2.1705659173708773`}}, 0.012836740231049472`},
{"TBUR", 14.0556458555863`, 1.9718420300344235`, {{0, 0.003988382266033368`, 7.099320433539394`}}, 0.0017058176758105723`},
{"CURE", 365, 23.25265498355992`, {{0, 0.006370071872853977`, 11.33872793368008`},
  {28, 0.0003346874952094704`, 0.5957437414728574`}, {56, 0.0003346874952094704`, 0.5957437414728574`},
  {84, 0.0003346874952094704`, 0.5957437414728574`}, {112, 0.0003346874952094704`, 0.5957437414728574`},
  {140, 0.0003346874952094704`, 0.5957437414728574`}, {168, 0.0003346874952094704`, 0.5957437414728574`},
  {196, 0.0003346874952094704`, 0.5957437414728574`}, {224, 0.0003346874952094704`, 0.5957437414728574`},
  {252, 0.0003346874952094704`, 0.5957437414728574`}}, 0.0023611076188728244`},
{"TBUR", 14.382774648216751`, 2.4167274880589997`, {{0, 0.0028707196503808103`, 5.109880977677842`}}, 0.0013983078014439183`},
{"CURE", 365, 110.48916922572616`, {{0, 0.03094359449311555`, 55.07959819774568`},
  {28, 0.00208993911094244`, 3.7200916174775434`}, {56, 0.00208993911094244`, 3.7200916174775434`},
  {84, 0.00208993911094244`, 3.7200916174775434`}, {112, 0.00208993911094244`, 3.7200916174775434`},
```

```
{140, 0.00208993911094244`, 3.7200916174775434`}, {168, 0.00208993911094244`, 3.7200916174775434`},
{196, 0.0006255068143408808`, 1.1134021295267678`}}, 0.009122182694548307`}, {"CURE", 365, 133.4031100819212`,
{{0, 0.01832870414461087`, 32.625093377407346`}, {28, 0.001188875514620677`, 2.116198416024805`},
{56, 0.001188875514620677`, 2.116198416024805`}, {84, 0.001188875514620677`, 2.116198416024805`},
{112, 0.001188875514620677`, 2.116198416024805`}, {140, 0.001188875514620677`, 2.116198416024805`},
{168, 0.001188875514620677`, 2.116198416024805`}, {196, 0.001188875514620677`, 2.116198416024805`},
{224, 0.001188875514620677`, 2.116198416024805`}, {252, 0.001188875514620677`, 2.116198416024805`}}, 0.005651364853913202`},
{"TBUR", 35.565463573000955`, 5.1553222037208215`, {{0, 0.009262644546597781`, 16.487507292944052`},
{28, 0.0005412998290483135`, 0.963513695705998`}}, 0.0031569601838975885`},
{"TBUR", 117.86891436008933`, 47.71108324973211`, {{0, 0.05136440348562454`, 91.42863820441167`},
{28, 0.003548568324693083`, 6.316451617953688`}, {56, 0.0007874834463885145`, 1.401720534571556`}}, 0.014740694327493531`},
{"CURE", 365, 94.53072578780979`, {{0, 0.044615361957336966`, 79.4153442840598`},
{28, 0.0030664939298153983`, 5.4583591950714085`}, {56, 0.0030664939298153983`, 5.4583591950714085`},
{84, 0.001121062562938856`, 1.9954913620311638`}}, 0.012883786094728405`},
{"CURE", 365, 20.648021611139644`, {{0, 0.007732063917674915`, 13.763073773461349`},
{28, 0.0004319726412681088`, 0.7689113014572336`}, {56, 0.0004319726412681088`, 0.7689113014572336`},
{84, 0.0004319726412681088`, 0.7689113014572336`}, {112, 0.0004319726412681088`, 0.7689113014572336`},
{140, 0.0004319726412681088`, 0.7689113014572336`}, {168, 0.0004319726412681088`, 0.7689113014572336`},
{196, 0.0004319726412681088`, 0.7689113014572336`}, {224, 0.0004319726412681088`, 0.7689113014572336`},
{252, 0.0004319726412681088`, 0.7689113014572336`}}, 0.0027358414578021457`},
{"TBUR", 22.141487838028315`, 13.400594898110713`, {{0, 0.04139458806865542`, 73.68236676220664`}}, 0.011997633368493037`},
{"TBUR", 17.47247869988918`, 3.6129169895267923`, {{0, 0.0020731569591066297`, 3.6902193872098006`}}, 0.0011788691268756922`},
{"CURE", 365, 15.712456874997827`, {{0, 0.004367259378307236`, 7.7737216933868805`},
{28, 0.00019162945988470315`, 0.34110043859477157`}, {56, 0.00019162945988470315`, 0.34110043859477157`},
{84, 0.00019162945988470315`, 0.34110043859477157`}, {112, 0.00019162945988470315`, 0.34110043859477157`},
{140, 0.00019162945988470315`, 0.34110043859477157`}, {168, 0.00019162945988470315`, 0.34110043859477157`},
{196, 0.00019162945988470315`, 0.34110043859477157`}, {224, 0.00019162945988470315`, 0.34110043859477157`},
{252, 0.00019162945988470315`, 0.34110043859477157`}}, 0.0018100606303158673`},
{"CURE", 365, 86.56423337617822`, {{0, 0.07062426371925833`, 125.71118942027982`}}, 0.017500000000000005`},
{"CURE", 365, 153.1526847079538`, {{0, 0.03270517879364019`, 58.215218252679534`},
{28, 0.002215766560979914`, 3.944064478544247`}, {56, 0.002215766560979914`, 3.944064478544247`},
```

{84, 0.002215766560979914`, 3.944064478544247`}, {112, 0.002215766560979914`, 3.944064478544247`},
{140, 0.002215766560979914`, 3.944064478544247`}, {168, 0.002215766560979914`, 3.944064478544247`}}, 0.009606858981787569`},
{"CURE", 365, 78.85256032951503`, {{0, 0.04502946163191177`, 80.15244170480295`}, {28, 0.003096072477999314`, 5.511009010838778`},
{56, 0.003096072477999314`, 5.511009010838778`}, {84, 0.0008828663708822073`, 1.5715021401703289`}}, 0.012997720064712198`},
{"CURE", 365, 127.63728274072514`, {{0, 0.05616004829189834`, 99.96488595957905`},
{28, 0.0022626188222591094`, 4.027461503621215`}}, 0.016060151658562387`},
{"TBUR", 13.500771029288856`, 5.382674519758018`, {{0, 0.023336096318683778`, 41.538251447257124`}}, 0.007029081632473152`},
{"CURE", 365, 85.50892628034237`, {{0, 0.015718837307520876`, 27.97953040738716`},
{28, 0.0010024564548285345`, 1.7843724895947914`}, {56, 0.0010024564548285345`, 1.7843724895947914`},
{84, 0.0010024564548285345`, 1.7843724895947914`}, {112, 0.0010024564548285345`, 1.7843724895947914`},
{140, 0.0010024564548285345`, 1.7843724895947914`}, {168, 0.0010024564548285345`, 1.7843724895947914`},
{196, 0.0010024564548285345`, 1.7843724895947914`}, {224, 0.0010024564548285345`, 1.7843724895947914`},
{252, 0.0010024564548285345`, 1.7843724895947914`}}, 0.004933295007120787`}, {"CURE", 365, 78.38504901788846`,
{{0, 0.06054625071127344`, 107.77232626606673`}, {28, 0.0003662113559180795`, 0.6518562135341814`}}, 0.017266956409870315`},
{"CURE", 365, 12.595368189648322`, {{0, 0.004914085230200164`, 8.747071709756291`},
{28, 0.00023068844930562656`, 0.4106254397640153`}, {56, 0.00023068844930562656`, 0.4106254397640153`},
{84, 0.00023068844930562656`, 0.4106254397640153`}, {112, 0.00023068844930562656`, 0.4106254397640153`},
{140, 0.00023068844930562656`, 0.4106254397640153`}, {168, 0.00023068844930562656`, 0.4106254397640153`},
{196, 0.00023068844930562656`, 0.4106254397640153`}, {224, 0.00023068844930562656`, 0.4106254397640153`},
{252, 0.00023068844930562656`, 0.4106254397640153`}}, 0.001960512427361843`},
{"TBUR", 66.07255286624404`, 7.023448082841398`, {{0, 0.008105890138589483`, 14.42848444668928`},
{28, 0.00045867451419057803`, 0.8164406352592289`}, {56, 0.00045867451419057803`, 0.8164406352592289`}},
0.002838694727311640 3`}, {"CURE", 365, 90.88417084078279`, {{0, 0.05329043946377009`, 94.85698224551075`},
{28, 0.0035033156909448668`, 6.235901929881863`}}, 0.015270617287580541`}, {"CURE", 365, 77.93641651704655`,
{{0, 0.018395683583023827`, 32.74431677778241`}, {28, 0.0011936597602216024`, 2.124714373194452`},
{56, 0.0011936597602216024`, 2.124714373194452`}, {84, 0.0011936597602216024`, 2.124714373194452`},
{112, 0.0011936597602216024`, 2.124714373194452`}, {140, 0.0011936597602216024`, 2.124714373194452`},
{168, 0.0011936597602216024`, 2.124714373194452`}, {196, 0.0011936597602216024`, 2.124714373194452`},
{224, 0.0011936597602216024`, 2.124714373194452`}, {252, 0.0011936597602216024`, 2.124714373194452`}},
0.005669793347810256`}, {"TBUR", 32.56808456828738`, 3.6986417405412655`,
{{0, 0.01734870849488019`, 30.880701120886737`}, {28, 0.0011188758253541998`, 1.9915989691304756`}}, 0.005381732198308452`},

```
{"TBUR", 19.19635599338616`, 2.6111823582491733`, {{0, 0.011243213514005404`, 20.01292005492962`}}, 0.0037018871643767 7`},
{"CURE", 365, 53.49757497489765`, {{0, 0.01679127231446345`, 29.888464719744942`},
  {28, 0.0010790589553244328`, 1.9207249404774904`}, {56, 0.0010790589553244328`, 1.9207249404774904`},
  {84, 0.0010790589553244328`, 1.9207249404774904`}, {112, 0.0010790589553244328`, 1.9207249404774904`},
  {140, 0.0010790589553244328`, 1.9207249404774904`}, {168, 0.0010790589553244328`, 1.9207249404774904`},
  {196, 0.0010790589553244328`, 1.9207249404774904`}, {224, 0.0010790589553244328`, 1.9207249404774904`},
  {252, 0.0010790589553244328`, 1.9207249404774904`}}, 0.005228361111715121`}, {"CURE", 365, 86.23889612339876`,
 {{0, 0.030061205427922812`, 53.508945661702604`}, {28, 0.00202691132057153`, 3.6079021506173232`},
  {56, 0.00202691132057153`, 3.6079021506173232`}, {84, 0.00202691132057153`, 3.6079021506173232`},
  {112, 0.00202691132057153`, 3.6079021506173232`}, {140, 0.00202691132057153`, 3.6079021506173232`},
  {168, 0.00202691132057153`, 3.6079021506173232`}, {196, 0.00202691132057153`, 3.6079021506173232`}}, 0.008879405181209454`},
{"CURE", 365, 91.33737499027916`, {{0, 0.025229929992288378`, 44.909275386273315`},
  {28, 0.0016818202180262137`, 2.9936399880866604`}, {56, 0.0016818202180262137`, 2.9936399880866604`},
  {84, 0.0016818202180262137`, 2.9936399880866604`}, {112, 0.0016818202180262137`, 2.9936399880866604`},
  {140, 0.0016818202180262137`, 2.9936399880866604`}, {168, 0.0016818202180262137`, 2.9936399880866604`},
  {196, 0.0016818202180262137`, 2.9936399880866604`}, {224, 0.0016818202180262137`, 2.9936399880866604`},
  {252, 0.0016818202180262137`, 2.9936399880866604`}}, 0.00755014456048516 5`},
{"TBUR", 49.45350856158591`, 29.936577759191117`, {{0, 0.07151666116089621`, 127.29965686639525`}}, 0.0175`},
{"TBUR", 30.065307615052856`, 21.11021272407089`,
 {{0, 0.021381341995514714`, 38.05878875201619`}, {28, 0.0014069210753995232`, 2.504319514211151`}}, 0.006491257205032288`},
{"TBUR", 40.06381071226334`, 5.457139554633498`, {{0, 0.021596353303723975`, 38.441508880628675`},
  {28, 0.0014222790259858988`, 2.5316566662549`}}, 0.006550414681938892`}, {"CURE", 365, 87.76918810695699`,
 {{0, 0.022436446469630358`, 39.93687471594204`}, {28, 0.0014822856806934977`, 2.638468511634426`},
  {56, 0.0014822856806934977`, 2.638468511634426`}, {84, 0.0014822856806934977`, 2.638468511634426`},
  {112, 0.0014822856806934977`, 2.638468511634426`}, {140, 0.0014822856806934977`, 2.638468511634426`},
  {168, 0.0014822856806934977`, 2.638468511634426`}, {196, 0.0014822856806934977`, 2.638468511634426`},
  {224, 0.0014822856806934977`, 2.638468511634426`}, {252, 0.0014822856806934977`, 2.638468511634426`}},
 0.006781555046022129 6`}, {"TBUR", 71.21664889229635`, 62.518315357736924`,
 {{0, 0.05337229449505667`, 95.00268420120088`}, {28, 0.0034679250551174403`, 6.172906598109043`}}, 0.015293138601288904`},
{"TBUR", 329.06360578072247`, 78.28760561155545`, {{0, 0.01669273107516727`, 29.71306131379774`},
  {28, 0.0010720202953747057`, 1.9081961257669762`}, {56, 0.0010720202953747057`, 1.9081961257669762`},
```

```
{84, 0.0010720202953747057`, 1.9081961257669762`}, {112, 0.0010720202953747057`, 1.9081961257669762`},
{140, 0.0010720202953747057`, 1.9081961257669762`}, {168, 0.0010720202953747057`, 1.9081961257669762`},
{196, 0.0010720202953747057`, 1.9081961257669762`}, {224, 0.0010720202953747057`, 1.9081961257669762`},
{252, 0.0010720202953747057`, 1.9081961257669762`}}, 0.005201248811731631`},
{"CURE", 365, 61.08227030574554`, {{0, 0.020186102259447898`, 35.93126202181726`},
{28, 0.0013215468085376076`, 2.3523533191969417`}, {56, 0.0013215468085376076`, 2.3523533191969417`},
{84, 0.0013215468085376076`, 2.3523533191969417`}, {112, 0.0013215468085376076`, 2.3523533191969417`},
{140, 0.0013215468085376076`, 2.3523533191969417`}, {168, 0.0013215468085376076`, 2.3523533191969417`},
{196, 0.0013215468085376076`, 2.3523533191969417`}, {224, 0.0013215468085376076`, 2.3523533191969417`},
{252, 0.0013215468085376076`, 2.3523533191969417`}}, 0.00616240302674223`},
{"TBUR", 215.16414785553968`, 16.766250841104455`, {{0, 0.005247505549801886`, 9.340559878647356`},
{28, 0.0002545041864200353`, 0.45301745182766284`}, {56, 0.0002545041864200353`, 0.45301745182766284`},
{84, 0.0002545041864200353`, 0.45301745182766284`}, {112, 0.0002545041864200353`, 0.45301745182766284`},
{140, 0.0002545041864200353`, 0.45301745182766284`}, {168, 0.0002545041864200353`, 0.45301745182766284`},
{196, 0.0002545041864200353`, 0.45301745182766284`}}, 0.002052248555202311`},
{"CURE", 365, 175.34044874068232`, {{0, 0.044870957941106085`, 79.87030513516883`},
{28, 0.0030847507857989067`, 5.4908563987220536`}, {56, 0.0030847507857989067`, 5.4908563987220536`},
{84, 0.0009740400131112298`, 1.733791223337989`}}, 0.012954109900639702`},
{"TBUR", 118.45450671137722`, 12.493459016051306`, {{0, 0.011496313302837553`, 20.463437679050845`},
{28, 0.0007008475973511543`, 1.2475087232850548`}, {56, 0.0007008475973511543`, 1.2475087232850548`},
{84, 0.0007008475973511543`, 1.2475087232850548`}, {112, 0.0007008475973511543`, 1.2475087232850548`}},
0.003771524175739594`}, {"TBUR", 92.45600422044123`, 30.187148268283284`,
{{0, 0.056927997391562`, 101.33183535698036`}, {28, 0.0019305902774890522`, 3.436450693930513`}}, 0.016271442550688787`},
{"TBUR", 113.56378602595807`, 77.10449478997067`, {{0, 0.050052060913755025`, 89.09266842648394`},
{28, 0.0034548295695595458`, 6.1495966338159915`}, {56, 0.001448623341217991`, 2.578549547368024`}}, 0.014379620874959751`},
{"CURE", 365, 67.65348667487072`, {{0, 0.06066342795991235`, 107.98090176864399`},
{28, 0.0003155488948737528`, 0.56167703287528`}}, 0.017299196157807614`}, {"CURE", 365, 171.46056372558047`,
{{0, 0.02948068085628742`, 52.47561192419161`}, {28, 0.0019854452797404307`, 3.5340925979379665`},
{56, 0.0019854452797404307`, 3.5340925979379665`}, {84, 0.0019854452797404307`, 3.5340925979379665`},
{112, 0.0019854452797404307`, 3.5340925979379665`}, {140, 0.0019854452797404307`, 3.5340925979379665`},
{168, 0.0019854452797404307`, 3.5340925979379665`}, {196, 0.0019854452797404307`, 3.5340925979379665`}},
```

0.008719681633521385`}, {"TBUR", 97.08531053611158`, 48.62652410010246`,
{{0, 0.041595707838062525`, 74.0403599517513`}, {28, 0.0028508043498672244`, 5.074431742763659`},
{56, 0.0028508043498672244`, 5.074431742763659`}, {84, 0.0028508043498672244`, 5.074431742763659`}}, 0.012052968774864234`},
{"TBUR", 80.46757827895654`, 5.982677729654816`, {{0, 0.0058441559635560745`, 10.402597615129812`}}, 0.0022164089120084 11`},
{"CURE", 365, 63.18686742695123`, {{0, 0.047393179274489466`, 84.35985910859125`},
{28, 0.0032649094524691484`, 5.811538825395084`}, {56, 0.0032649094524691484`, 5.811538825395084`}}, 0.013648065262921165`},
{"TBUR", 355.5393552137795`, 43.20720433735056`, {{0, 0.018509360555097096`, 32.94666178807283`},
{28, 0.0012017795439411216`, 2.1391675882151966`}, {56, 0.0012017795439411216`, 2.1391675882151966`},
{84, 0.0012017795439411216`, 2.1391675882151966`}, {112, 0.0012017795439411216`, 2.1391675882151966`},
{140, 0.0012017795439411216`, 2.1391675882151966`}, {168, 0.0012017795439411216`, 2.1391675882151966`},
{196, 0.0012017795439411216`, 2.1391675882151966`}, {224, 0.0012017795439411216`, 2.1391675882151966`},
{252, 0.0012017795439411216`, 2.1391675882151966`}}, 0.005701070041609505`},
{"CURE", 365, 63.476049815327045`, {{0, 0.012123275957973459`, 21.579431205192755`},
{28, 0.0007456306441465762`, 1.3272225465809055`}, {56, 0.0007456306441465762`, 1.3272225465809055`},
{84, 0.0007456306441465762`, 1.3272225465809055`}, {112, 0.0007456306441465762`, 1.3272225465809055`},
{140, 0.0007456306441465762`, 1.3272225465809055`}, {168, 0.0007456306441465762`, 1.3272225465809055`},
{196, 0.0007456306441465762`, 1.3272225465809055`}, {224, 0.0007456306441465762`, 1.3272225465809055`},
{252, 0.0007456306441465762`, 1.3272225465809055`}}, 0.003944024539099122`},
{"CURE", 365, 83.76258306079075`, {{0, 0.022188377976555096`, 39.495312798268074`},
{28, 0.0014645665026166933`, 2.606928374657714`}, {56, 0.0014645665026166933`, 2.606928374657714`},
{84, 0.0014645665026166933`, 2.606928374657714`}, {112, 0.0014645665026166933`, 2.606928374657714`},
{140, 0.0014645665026166933`, 2.606928374657714`}, {168, 0.0014645665026166933`, 2.606928374657714`},
{196, 0.0014645665026166933`, 2.606928374657714`}, {224, 0.0014645665026166933`, 2.606928374657714`},
{252, 0.0014645665026166933`, 2.606928374657714`}}, 0.0067133023281879915`}, {"CURE", 365, 82.17199343179315`,
{{0, 0.03250840404973661`, 57.86495920853116`}, {28, 0.0022017112221296583`, 3.919045975390792`},
{56, 0.0022017112221296583`, 3.919045975390792`}, {84, 0.0022017112221296583`, 3.919045975390792`},
{112, 0.0022017112221296583`, 3.919045975390792`}, {140, 0.0022017112221296583`, 3.919045975390792`},
{168, 0.0022017112221296583`, 3.919045975390792`}}, 0.009552719050891705`},
{"CURE", 365, 36.555482807875485`, {{0, 0.010151157900934257`, 18.06906106366298`},
{28, 0.0006047650686437762`, 1.0764818221859216`}, {56, 0.0006047650686437762`, 1.0764818221859216`},
{84, 0.0006047650686437762`, 1.0764818221859216`}, {112, 0.0006047650686437762`, 1.0764818221859216`},

```
    {140, 0.0006047650686437762`, 1.0764818221859216`}, {168, 0.0006047650686437762`, 1.0764818221859216`},
    {196, 0.0006047650686437762`, 1.0764818221859216`}, {224, 0.0006047650686437762`, 1.0764818221859216`},
    {252, 0.0006047650686437762`, 1.0764818221859216`}}, 0.003401422713232148`},
{"CURE", 365, 40.306244632428346`, {{0, 0.012953384851178777`, 23.057025035098224`},
    {28, 0.0008049241365183848`, 1.4327649630027248`}, {56, 0.0008049241365183848`, 1.4327649630027248`},
    {84, 0.0008049241365183848`, 1.4327649630027248`}, {112, 0.0008049241365183848`, 1.4327649630027248`},
    {140, 0.0008049241365183848`, 1.4327649630027248`}, {168, 0.0008049241365183848`, 1.4327649630027248`},
    {196, 0.0008049241365183848`, 1.4327649630027248`}, {224, 0.0008049241365183848`, 1.4327649630027248`},
    {252, 0.0008049241365183848`, 1.4327649630027248`}}, 0.004172417864495596`}, {"CURE", 365, 161.9815508192740 2`,
  {{0, 0.02977701546300575`, 53.00308752415023`}, {28, 0.0020066120373631686`, 3.57176942650644`},
    {56, 0.0020066120373631686`, 3.57176942650644`}, {84, 0.0020066120373631686`, 3.57176942650644`},
    {112, 0.0020066120373631686`, 3.57176942650644`}, {140, 0.0020066120373631686`, 3.57176942650644`},
    {168, 0.0020066120373631686`, 3.57176942650644`}, {196, 0.0020066120373631686`, 3.57176942650644`}}, 0.008801214124996227`},
{"CURE", 365, 73.3106307326864`, {{0, 0.03657949860314831`, 65.111507513604`}, {28, 0.002492503690230495`, 4.436656568610281`},
    {56, 0.002492503690230495`, 4.436656568610281`}, {84, 0.002492503690230495`, 4.436656568610281`},
    {112, 0.002492503690230495`, 4.436656568610281`}, {140, 0.0007584013671754139`, 1.3499544335722367`}},
  0.010672826100301661`}, {"CURE", 365, 97.53511410017056`, {{0, 0.024992721522386668`, 44.487044309848265`},
    {28, 0.0016648767558903772`, 2.9634806254848716`}, {56, 0.0016648767558903772`, 2.9634806254848716`},
    {84, 0.0016648767558903772`, 2.9634806254848716`}, {112, 0.0016648767558903772`, 2.9634806254848716`},
    {140, 0.0016648767558903772`, 2.9634806254848716`}, {168, 0.0016648767558903772`, 2.9634806254848716`},
    {196, 0.0016648767558903772`, 2.9634806254848716`}, {224, 0.0016648767558903772`, 2.9634806254848716`},
    {252, 0.0016648767558903772`, 2.9634806254848716`}}, 0.0074848798323314085`},
{"CURE", 365, 101.91544235811533`, {{0, 0.019925784029660797`, 35.467895572796216`},
    {28, 0.0013029526492671006`, 2.319255715695439`}, {56, 0.0013029526492671006`, 2.319255715695439`},
    {84, 0.0013029526492671006`, 2.319255715695439`}, {112, 0.0013029526492671006`, 2.319255715695439`},
    {140, 0.0013029526492671006`, 2.319255715695439`}, {168, 0.0013029526492671006`, 2.319255715695439`},
    {196, 0.0013029526492671006`, 2.319255715695439`}, {224, 0.0013029526492671006`, 2.319255715695439`},
    {252, 0.0013029526492671006`, 2.319255715695439`}}, 0.006090779958191759`},
{"CURE", 365, 137.950103490169`, {{0, 0.03573197102601518`, 63.60290842630702`}, {28, 0.002431966006149556 4`, 4.328899490946211`},
    {56, 0.0024319660061495564`, 4.328899490946211`}, {84, 0.0024319660061495564`, 4.328899490946211`},
    {112, 0.0024319660061495564`, 4.328899490946211`}, {140, 0.0024319660061495564`, 4.328899490946211`}},
```

```
0.010439640257707905`}, {"CURE", 365, 11.232141726337657`, {{0, 0.002765486272294865`, 4.92256556468486`},
  {28, 0.0000772170951695338`, 0.13744642940177015`}, {56, 0.0000772170951695338`, 0.13744642940177015`},
  {84, 0.0000772170951695338`, 0.13744642940177015`}, {112, 0.0000772170951695338`, 0.13744642940177015`},
  {140, 0.0000772170951695338`, 0.13744642940177015`}, {168, 0.0000772170951695338`, 0.13744642940177015`},
  {196, 0.0000772170951695338`, 0.13744642940177015`}, {224, 0.0000772170951695338`, 0.13744642940177015`},
  {252, 0.0000772170951695338`, 0.13744642940177015`}}, 0.0013693542492527735`},
{"CURE", 365, 46.01568496390412`, {{0, 0.015147279313478272`, 26.962157177991326`},
  {28, 0.0009616308838254913`, 1.7117029732093745`}, {56, 0.0009616308838254913`, 1.7117029732093745`},
  {84, 0.0009616308838254913`, 1.7117029732093745`}, {112, 0.0009616308838254913`, 1.7117029732093745`},
  {140, 0.0009616308838254913`, 1.7117029732093745`}, {168, 0.0009616308838254913`, 1.7117029732093745`},
  {196, 0.0009616308838254913`, 1.7117029732093745`}, {224, 0.0009616308838254913`, 1.7117029732093745`},
  {252, 0.0009616308838254913`, 1.7117029732093745`}}, 0.004776038492963626`},
{"TBUR", 97.75963388777471`, 46.87228362699658`, {{0, 0.061807146078827445`, 110.01672002031285`}}, 0.0175`},
{"TBUR", 120.64926001903947`, 51.33502994818917`,
 {{0, 0.05934321095126112`, 105.63091549324479`}, {28, 0.0008863546062876173`, 1.5777111991919588`}},
 0.016935956159635154`}, {"TBUR", 62.289559823156814`, 20.462771925908932`,
 {{0, 0.053947034147911674`, 96.02572078328278`}, {28, 0.0032194320617111483`, 5.730589069845844`}}, 0.015451270506183817`},
{"", 365, 20.462771925908932`, {{0, 0.008791899765396596`, 15.64958158240594`},
  {28, 0.0005076752018196574`, 0.9036618592389902`}, {56, 0.0005076752018196574`, 0.9036618592389902`},
  {84, 0.0005076752018196574`, 0.9036618592389902`}, {112, 0.0005076752018196574`, 0.9036618592389902`},
  {140, 0.0005076752018196574`, 0.9036618592389902`}, {168, 0.0005076752018196574`, 0.9036618592389902`},
  {196, 0.0005076752018196574`, 0.9036618592389902`}, {224, 0.0005076752018196574`, 0.9036618592389902`},
  {252, 0.0005076752018196574`, 0.9036618592389902`}}, 0.0030274410727645596`},
{"TBUR", 11.78356544694685`, 1.7742654389284604`, {{0, 0.0028060077030688454`, 4.994693711462545`}}, 0.0013805031773031125`},
{"TBUR", 43.608370952540625`, 5.043000878935599`,
 {{0, 0.005908211341627828`, 10.516616188097533`}, {28, 0.00030169745726474535`, 0.5370214739312468`}},
 0.0022340328899311704`}, {"CURE", 365, 74.09368793068404`,
 {{0, 0.05410982873386315`, 96.3154951462764`}, {28, 0.003149046603230118`, 5.60530295374961`}}, 0.015496061252489927`},
{"CURE", 365, 116.2502101877864`, {{0, 0.02293212595874503`, 40.81918420656615`},
  {28, 0.0015176913584874028`, 2.701490618107577`}, {56, 0.0015176913584874028`, 2.701490618107577`},
  {84, 0.0015176913584874028`, 2.701490618107577`}, {112, 0.0015176913584874028`, 2.701490618107577`},
```

```
    {140, 0.0015176913584874028`, 2.701490618107577`}, {168, 0.0015176913584874028`, 2.701490618107577`},
    {196, 0.0015176913584874028`, 2.701490618107577`}, {224, 0.0015176913584874028`, 2.701490618107577`},
    {252, 0.0015176913584874028`, 2.701490618107577`}}, 0.006917934607518678`},
  {"CURE", 365, 31.710823077784507`, {{0, 0.010438826120344278`, 18.581110494212815`},
    {28, 0.0006253127986016347`, 1.1130567815109098`}, {56, 0.0006253127986016347`, 1.1130567815109098`},
    {84, 0.0006253127986016347`, 1.1130567815109098`}, {112, 0.0006253127986016347`, 1.1130567815109098`},
    {140, 0.0006253127986016347`, 1.1130567815109098`}, {168, 0.0006253127986016347`, 1.1130567815109098`},
    {196, 0.0006253127986016347`, 1.1130567815109098`}, {224, 0.0006253127986016347`, 1.1130567815109098`},
    {252, 0.0006253127986016347`, 1.1130567815109098`}}, 0.003480570764505569`},
  {"TBUR", 25.710332223466885`, 4.0306579543657834`, {{0, 0.020507344351171335`, 36.50307294508497`}}, 0.006250788478562571`},
  {"CURE", 365, 37.60353722068754`, {{0, 0.015448931948043039`, 27.499098867516608`},
    {28, 0.0009831775005801177`, 1.7500559510326095`}, {56, 0.0009831775005801177`, 1.7500559510326095`},
    {84, 0.0009831775005801177`, 1.7500559510326095`}, {112, 0.0009831775005801177`, 1.7500559510326095`},
    {140, 0.0009831775005801177`, 1.7500559510326095`}, {168, 0.0009831775005801177`, 1.7500559510326095`},
    {196, 0.0009831775005801177`, 1.7500559510326095`}, {224, 0.0009831775005801177`, 1.7500559510326095`},
    {252, 0.0009831775005801177`, 1.7500559510326095`}}, 0.004859034168454857`}, {"CURE", 365, 80.09097689884221`,
   {{0, 0.044094195793351436`, 78.48766851216556`}, {28, 0.003029267775245004`, 5.392096639936107`},
    {56, 0.003029267775245004`, 5.392096639936107`}, {84, 0.0014208449663362863`, 2.5291040400785896`}}, 0.012740394216565087`},
  {"TBUR", 14.598063529832238`, 2.4851279191744458`, {{0, 0.002745451596752228`, 4.886903842218966`}}, 0.0013638419770582743`},
  {"CURE", 365, 16.461935374029895`, {{0, 0.0061676043536750946`, 10.97833574954167`},
    {28, 0.00032022552955383594`, 0.570001442605828`}, {56, 0.00032022552955383594`, 0.570001442605828`},
    {84, 0.00032022552955383594`, 0.570001442605828`}, {112, 0.00032022552955383594`, 0.570001442605828`},
    {140, 0.00032022552955383594`, 0.570001442605828`}, {168, 0.00032022552955383594`, 0.570001442605828`},
    {196, 0.00032022552955383594`, 0.570001442605828`}, {224, 0.00032022552955383594`, 0.570001442605828`},
    {252, 0.00032022552955383594`, 0.570001442605828`}}, 0.002305401397233054`},
  {"CURE", 365, 100.23614387702867`, {{0, 0.031564921177470076`, 56.185559695896735`},
    {28, 0.002134319588396335`, 3.7990888673454766`}, {56, 0.002134319588396335`, 3.7990888673454766`},
    {84, 0.002134319588396335`, 3.7990888673454766`}, {112, 0.002134319588396335`, 3.7990888673454766`},
    {140, 0.002134319588396335`, 3.7990888673454766`}, {168, 0.002134319588396335`, 3.7990888673454766`},
    {196, 0.00009058870423029296`, 0.16124789352992147`}}, 0.009293132396158355`},
  {"CURE", 365, 47.059192703776816`, {{0, 0.013663697925587616`, 24.321382307545957`},
```

```
{28, 0.0008556607846904447`, 1.5230761967489916`}, {56, 0.0008556607846904447`, 1.5230761967489916`},
{84, 0.0008556607846904447`, 1.5230761967489916`}, {112, 0.0008556607846904447`, 1.5230761967489916`},
{140, 0.0008556607846904447`, 1.5230761967489916`}, {168, 0.0008556607846904447`, 1.5230761967489916`},
{196, 0.0008556607846904447`, 1.5230761967489916`}, {224, 0.0008556607846904447`, 1.5230761967489916`},
{252, 0.0008556607846904447`, 1.5230761967489916`}}, 0.004367850977506111`},
{"TBUR", 24.228584312348108`, 3.612852594031461`, {{0, 0.012137867355766366`, 21.605403893264132`}}, 0.00394803916644667`},
{"CURE", 365, 99.61762616754555`,
{{0, 0.05264855746126654`, 93.71443228105444`}, {28, 0.0036402936086675102`, 6.479722623428168`},
{56, 0.00014054457341470183`, 0.25016934067816926`}}, 0.015094012065947685`}, {"CURE", 365, 93.9807071816734`,
{{0, 0.034202619179897926`, 60.88066214021831`}, {28, 0.0023227265885697526`, 4.1344533276541595`},
{56, 0.0023227265885697526`, 4.1344533276541595`}, {84, 0.0023227265885697526`, 4.1344533276541595`},
{112, 0.0023227265885697526`, 4.1344533276541595`}, {140, 0.0023227265885697526`, 4.1344533276541595`},
{168, 0.00014244480545414807`, 0.25355175370838356`}}, 0.010018859614716331`},
{"TBUR", 20.041011416027903`, 12.43203815431727`, {{0, 0.02542923355320966`, 45.264035724713196`}}, 0.007604980261457367`},
{"TBUR", 39.355083512962665`, 16.453833704561855`,
{{0, 0.020348566258137704`, 36.22044793948511`}, {28, 0.0013331513798725938`, 2.3730094561732167`}},
0.006207102816398376`}, {"TBUR", 44.78708842797186`, 10.240855430677678`,
{{0, 0.028427387850220302`, 50.60075037339214`}, {28, 0.0019102100650213506`, 3.400173915738004`}}, 0.008429882193662815`},
{"CURE", 365, 8.99958968562135`, {{0, 0.0027609302349881214`, 4.9144558182788565`},
{28, 0.00007689166393333777`, 0.13686716180134123`}, {56, 0.00007689166393333777`, 0.13686716180134123`},
{84, 0.00007689166393333777`, 0.13686716180134123`}, {112, 0.00007689166393333777`, 0.13686716180134123`},
{140, 0.00007689166393333777`, 0.13686716180134123`}, {168, 0.00007689166393333777`, 0.13686716180134123`},
{196, 0.00007689166393333777`, 0.13686716180134123`}, {224, 0.00007689166393333777`, 0.13686716180134123`},
{252, 0.00007689166393333777`, 0.13686716180134123`}}, 0.0013681007167106762`},
{"CURE", 365, 93.75090666351471`, {{0, 0.018362847659094666`, 32.68586883318851`},
{28, 0.0011913143370838055`, 2.120539520009174`}, {56, 0.0011913143370838055`, 2.120539520009174`},
{84, 0.0011913143370838055`, 2.120539520009174`}, {112, 0.0011913143370838055`, 2.120539520009174`},
{140, 0.0011913143370838055`, 2.120539520009174`}, {168, 0.0011913143370838055`, 2.120539520009174`},
{196, 0.0011913143370838055`, 2.120539520009174`}, {224, 0.0011913143370838055`, 2.120539520009174`},
{252, 0.0011913143370838055`, 2.120539520009174`}}, 0.0056607589838610995`},
{"CURE", 365, 53.13492092778356`, {{0, 0.01611336018407209`, 28.68178112764832`},
```

```
    {28, 0.0010306366602964784`, 1.8345332553277316`}, {56, 0.0010306366602964784`, 1.8345332553277316`},
    {84, 0.0010306366602964784`, 1.8345332553277316`}, {112, 0.0010306366602964784`, 1.8345332553277316`},
    {140, 0.0010306366602964784`, 1.8345332553277316`}, {168, 0.0010306366602964784`, 1.8345332553277316`},
    {196, 0.0010306366602964784`, 1.8345332553277316`}, {224, 0.0010306366602964784`, 1.8345332553277316`},
    {252, 0.0010306366602964784`, 1.8345332553277316`}}, 0.005041842683766662`},
  {"CURE", 365, 41.31234734434712`, {{0, 0.009905537018514684`, 17.631855892956136`},
    {28, 0.0005872207198995209`, 1.0452528814211473`}, {56, 0.0005872207198995209`, 1.0452528814211473`},
    {84, 0.0005872207198995209`, 1.0452528814211473`}, {112, 0.0005872207198995209`, 1.0452528814211473`},
    {140, 0.0005872207198995209`, 1.0452528814211473`}, {168, 0.0005872207198995209`, 1.0452528814211473`},
    {196, 0.0005872207198995209`, 1.0452528814211473`}, {224, 0.0005872207198995209`, 1.0452528814211473`},
    {252, 0.0005872207198995209`, 1.0452528814211473`}}, 0.0033338434226332847`},
  {"TBUR", 335.56383946815134`, 42.006553825477205`, {{0, 0.0073920597907089205`, 13.157866427461878`},
    {28, 0.00040768663219910924`, 0.7256822053144144`}, {56, 0.00040768663219910924`, 0.7256822053144144`},
    {84, 0.00040768663219910924`, 0.7256822053144144`}, {112, 0.00040768663219910924`, 0.7256822053144144`},
    {140, 0.00040768663219910924`, 0.7256822053144144`}, {168, 0.00040768663219910924`, 0.7256822053144144`},
    {196, 0.00040768663219910924`, 0.7256822053144144`}, {224, 0.00040768663219910924`, 0.7256822053144144`},
    {252, 0.00040768663219910924`, 0.7256822053144144`}}, 0.002642293883692393`},
  {"TBUR", 33.51470737816053`, 4.793276556037628`, {{0, 0.01244176807368608`, 22.146347171161224`},
    {28, 0.000768380080983192`, 1.3677165441500818`}}, 0.004031653371913196`},
  {"CURE", 365, 39.74711855159691`, {{0, 0.008018502414193248`, 14.272934297263982`},
    {28, 0.00045243253387656113`, 0.8053299103002788`}, {56, 0.00045243253387656113`, 0.8053299103002788`},
    {84, 0.00045243253387656113`, 0.8053299103002788`}, {112, 0.00045243253387656113`, 0.8053299103002788`},
    {140, 0.00045243253387656113`, 0.8053299103002788`}, {168, 0.00045243253387656113`, 0.8053299103002788`},
    {196, 0.00045243253387656113`, 0.8053299103002788`}, {224, 0.00045243253387656113`, 0.8053299103002788`},
    {252, 0.00045243253387656113`, 0.8053299103002788`}}, 0.0028146511673196265`},
  {"TBUR", 311.0106831338574`, 38.31278889917992`, {{0, 0.017770287505006206`, 31.631111758911047`},
    {28, 0.0011489886117917723`, 2.045199728989355`}, {56, 0.0011489886117917723`, 2.045199728989355`},
    {84, 0.0011489886117917723`, 2.045199728989355`}, {112, 0.0011489886117917723`, 2.045199728989355`},
    {140, 0.0011489886117917723`, 2.045199728989355`}, {168, 0.0011489886117917723`, 2.045199728989355`},
    {196, 0.0011489886117917723`, 2.045199728989355`}, {224, 0.0011489886117917723`, 2.045199728989355`},
    {252, 0.0011489886117917723`, 2.045199728989355`}}, 0.00549772400712795`},
```

```
{"TBUR", 321.57322014710786`, 36.24246174917051`, {{0, 0.007768064383688333`, 13.827154602965232`},
    {28, 0.0004345441031262101`, 0.7734885035646539`}, {56, 0.0004345441031262101`, 0.7734885035646539`},
    {84, 0.0004345441031262101`, 0.7734885035646539`}, {112, 0.0004345441031262101`, 0.7734885035646539`},
    {140, 0.0004345441031262101`, 0.7734885035646539`}, {168, 0.0004345441031262101`, 0.7734885035646539`},
    {196, 0.0004345441031262101`, 0.7734885035646539`}, {224, 0.0004345441031262101`, 0.7734885035646539`},
    {252, 0.0004345441031262101`, 0.7734885035646539`}}, 0.002745746503050942`},
{"TBUR", 27.096104606036857`, 5.48555568993516`, {{0, 0.008521955446411131`, 15.169080694611814`}}, 0.002953169514912779`},
{"TBUR", 18.738092790728334`, 2.516459001654221`, {{0, 0.0031440965161270537`, 5.596491798706156`}}, 0.001473523778486591`},
{"TBUR", 17.656105337072837`, 2.470555111877694`, {{0, 0.0027880340222618933`, 4.96270055962617`}}, 0.001375557960160258`},
{"CURE", 365, 126.50047763632118`,
  {{0, 0.044272692843122394`, 78.80539326075787`}, {28, 0.0030420175645143577`, 5.414791264835557`},
    {56, 0.0030420175645143577`, 5.414791264835557`}, {84, 0.0013181708514810988`, 2.346344115636356`}}, 0.01278950528513012`},
{"CURE", 365, 76.6007697241491`, {{0, 0.036023578179242795`, 64.12196915905217`},
    {28, 0.0024527950885229574`, 4.365975257570864`}, {56, 0.0024527950885229574`, 4.365975257570864`},
    {84, 0.0024527950885229574`, 4.365975257570864`}, {112, 0.0024527950885229574`, 4.365975257570864`},
    {140, 0.0011575921328295293`, 2.060513996436562`}}, 0.010519872053777319`},
{"TBUR", 85.4852331565927`, 18.650057965420213`, {{0, 0.007722295434882128`, 13.745685874090189`},
    {28, 0.0004312748924971955`, 0.7676693086450079`}, {56, 0.0004312748924971955`, 0.7676693086450079`},
    {84, 0.0004312748924971955`, 0.7676693086450079`}}, 0.002733153790813652`},
{"CURE", 365, 69.76985576596748`, {{0, 0.023649324102084167`, 42.09579690170982`},
    {28, 0.0015689197972973412`, 2.7926772391892674`}, {56, 0.0015689197972973412`, 2.7926772391892674`},
    {84, 0.0015689197972973412`, 2.7926772391892674`}, {112, 0.0015689197972973412`, 2.7926772391892674`},
    {140, 0.0015689197972973412`, 2.7926772391892674`}, {168, 0.0015689197972973412`, 2.7926772391892674`},
    {196, 0.0015689197972973412`, 2.7926772391892674`}, {224, 0.0015689197972973412`, 2.7926772391892674`},
    {252, 0.0015689197972973412`, 2.7926772391892674`}}, 0.007115262054876705`},
{"CURE", 365, 29.858064353759403`, {{0, 0.005182812128331675`, 9.225405588430382`},
    {28, 0.0002498832277435916`, 0.4447921453835931`}, {56, 0.0002498832277435916`, 0.4447921453835931`},
    {84, 0.0002498832277435916`, 0.4447921453835931`}, {112, 0.0002498832277435916`, 0.4447921453835931`},
    {140, 0.0002498832277435916`, 0.4447921453835931`}, {168, 0.0002498832277435916`, 0.4447921453835931`},
    {196, 0.0002498832277435916`, 0.4447921453835931`}, {224, 0.0002498832277435916`, 0.4447921453835931`},
    {252, 0.0002498832277435916`, 0.4447921453835931`}}, 0.0020344490281983254`},
```

```
{"CURE", 365, 110.59024749772466`, {{0, 0.03649481272701886`, 64.96076665409358`},
    {28, 0.002486454699078391`, 4.425889364359536`}, {56, 0.002486454699078391`, 4.425889364359536`},
    {84, 0.002486454699078391`, 4.425889364359536`}, {112, 0.002486454699078391`, 4.425889364359536`},
    {140, 0.0008192119045805808`, 1.4581971901534339`}}, 0.010649525917612819`}};
```

In[ ]:=

```
TestMDKsMid = {{"KsMid Res", "KsMid OS", "KsMid Tox", "Ainj", "DoseMaxTox"},
  {"TBUR", 50.35160533188224`, 5.0939892311922375`, {{0, 0.0019673956729458256`, 3.5019642978435694`},
    {14, 0.00002021062378745951`, 0.03597491034167793`}, {28, 0.00002021062378745951`, 0.03597491034167793`},
    {42, 0.00002021062378745951`, 0.03597491034167793`}}, 0.0011497703278600556`}, {"CURE", 365, 77.20451184626056`,
   {{0, 0.018170919210108587`, 32.34423619399328`}, {14, 0.001177605162156228`, 2.096137188638086`},
    {28, 0.001177605162156228`, 2.096137188638086`}, {42, 0.001177605162156228`, 2.096137188638086`},
    {56, 0.001177605162156228`, 2.096137188638086`}, {70, 0.001177605162156228`, 2.096137188638086`},
    {84, 0.001177605162156228`, 2.096137188638086`}, {98, 0.001177605162156228`, 2.096137188638086`},
    {112, 0.001177605162156228`, 2.096137188638086`}, {126, 0.001177605162156228`, 2.096137188638086`}}, 0.005607952445994898`},
  {"CURE", 365, 37.66321133620305`, {{0, 0.01318080588196551`, 23.46183446989861`}, {14, 0.0008211684958602941`,
      1.4616799226313235`}, {28, 0.0008211684958602941`, 1.4616799226313235`}, {42, 0.0008211684958602941`, 1.4616799226313235`},
    {56, 0.0008211684958602941`, 1.4616799226313235`}, {70, 0.0008211684958602941`, 1.4616799226313235`},
    {84, 0.0008211684958602941`, 1.4616799226313235`}, {98, 0.0008211684958602941`, 1.4616799226313235`},
    {112, 0.0008211684958602941`, 1.4616799226313235`}, {126, 0.0008211684958602941`, 1.4616799226313235`}},
   0.004234989710083124`}, {"TBUR", 26.65590674198072`, 4.274691861357993`,
   {{0, 0.01100341075945254`, 19.58607115182552`}, {14, 0.0006656402728236536`, 1.1848396856261034`}}, 0.0036359086536056334`},
  {"TBUR", 13.088967062813461`, 2.4275529076815228`, {{0, 0.014645035888898731`, 26.06816388223974`}}, 0.0046378529526843165`},
  {"CURE", 365, 113.58471212523746`, {{0, 0.023565669637190027`, 41.94689195419825`},
    {14, 0.001562944478376331`, 2.7820411715098694`}, {28, 0.001562944478376331`, 2.7820411715098694`},
    {42, 0.001562944478376331`, 2.7820411715098694`}, {56, 0.001562944478376331`, 2.7820411715098694`},
    {70, 0.001562944478376331`, 2.7820411715098694`}, {84, 0.001562944478376331`, 2.7820411715098694`},
    {98, 0.001562944478376331`, 2.7820411715098694`}, {112, 0.001562944478376331`, 2.7820411715098694`},
    {126, 0.001562944478376331`, 2.7820411715098694`}}, 0.007092245651152057`}, {"CURE", 365, 35.45202986661635`,
   {{0, 0.012127445577528876`, 21.586853128001398`}, {14, 0.0007459284741148204`, 1.3277526839243803`},
    {28, 0.0007459284741148204`, 1.3277526839243803`}, {42, 0.0007459284741148204`, 1.3277526839243803`},
    {56, 0.0007459284741148204`, 1.3277526839243803`}, {70, 0.0007459284741148204`, 1.3277526839243803`},
```

{84, 0.0007459284741148204`, 1.3277526839243803`}, {98, 0.0007459284741148204`, 1.3277526839243803`},
{112, 0.0007459284741148204`, 1.3277526839243803`}, {126, 0.0007459284741148204`, 1.3277526839243803`}},
0.0039451717539810425`}, {"CURE", 365, 77.94224159781614`, {{0, 0.029442582277799518`, 52.40779645448314`},
{14, 0.0019827239527055807`, 3.5292486358159336`}, {28, 0.0019827239527055807`, 3.5292486358159336`},
{42, 0.0019827239527055807`, 3.5292486358159336`}, {56, 0.0019827239527055807`, 3.5292486358159336`},
{70, 0.0019827239527055807`, 3.5292486358159336`}, {84, 0.0019827239527055807`, 3.5292486358159336`},
{98, 0.0019827239527055807`, 3.5292486358159336`}}, 0.008709199320773072`}, {"CURE", 365, 34.48481725813796`,
{{0, 0.013007104321995849`, 23.15264569315261`}, {14, 0.0008087612415767469`, 1.4395950100066095`},
{28, 0.0008087612415767469`, 1.4395950100066095`}, {42, 0.0008087612415767469`, 1.4395950100066095`},
{56, 0.0008087612415767469`, 1.4395950100066095`}, {70, 0.0008087612415767469`, 1.4395950100066095`},
{84, 0.0008087612415767469`, 1.4395950100066095`}, {98, 0.0008087612415767469`, 1.4395950100066095`},
{112, 0.0008087612415767469`, 1.4395950100066095`}, {126, 0.0008087612415767469`, 1.4395950100066095`}},
0.004187198056201621`}, {"TBUR", 19.565148224060053`, 3.845998044756764`,
{{0, 0.009854052246805389`, 17.540212999313592`}, {14, 0.0005835432362059998`, 1.0387069604466797`}}, 0.0033196780784065056`},
{"TBUR", 59.83390190943187`, 6.2470891651997675`, {{0, 0.0027722317970938853`, 4.934572598827116`},
{14, 0.00007769891836946377`, 0.1383040746976455`}, {28, 0.00007769891836946377`, 0.1383040746976455`},
{42, 0.00007769891836946377`, 0.1383040746976455`}, {56, 0.00007769891836946377`, 0.1383040746976455`}},
0.0013712101899046605`}, {"CURE", 365, 51.6638816541804`, {{0, 0.011706123103434838`, 20.836899124114012`},
{14, 0.0007158340116795318`, 1.2741845407895667`}, {28, 0.0007158340116795318`, 1.2741845407895667`},
{42, 0.0007158340116795318`, 1.2741845407895667`}, {56, 0.0007158340116795318`, 1.2741845407895667`},
{70, 0.0007158340116795318`, 1.2741845407895667`}, {84, 0.0007158340116795318`, 1.2741845407895667`},
{98, 0.0007158340116795318`, 1.2741845407895667`}, {112, 0.0007158340116795318`, 1.2741845407895667`},
{126, 0.0007158340116795318`, 1.2741845407895667`}}, 0.0038292505276091107`},
{"CURE", 365, 76.4183439341573`, {{0, 0.0436351022194491`, 77.6704819506194`}, {14, 0.0029964753771091223`, 5.333726171254238`},
{28, 0.0029964753771091223`, 5.333726171254238`}, {42, 0.0029964753771091223`, 5.333726171254238`}}, 0.012614080778810155`},
{"CURE", 365, 175.92961932029027`, {{0, 0.024538799134554975`, 43.679062459507854`},
{14, 0.001632453728188113`, 2.9057676361748412`}, {28, 0.001632453728188113`, 2.9057676361748412`},
{42, 0.001632453728188113`, 2.9057676361748412`}, {56, 0.001632453728188113`, 2.9057676361748412`},
{70, 0.001632453728188113`, 2.9057676361748412`}, {84, 0.001632453728188113`, 2.9057676361748412`},
{98, 0.001632453728188113`, 2.9057676361748412`}, {112, 0.001632453728188113`, 2.9057676361748412`},
{126, 0.001632453728188113`, 2.9057676361748412`}}, 0.007359989177048254`}, {"CURE", 365, 101.62663447253188`,

{{0, 0.01410880950798533`, 25.113680924213888`}, {14, 0.0008874544691474242`, 1.5796689550824152`},
{28, 0.0008874544691474242`, 1.5796689550824152`}, {42, 0.0008874544691474242`, 1.5796689550824152`},
{56, 0.0008874544691474242`, 1.5796689550824152`}, {70, 0.0008874544691474242`, 1.5796689550824152`},
{84, 0.0008874544691474242`, 1.5796689550824152`}, {98, 0.0008874544691474242`, 1.5796689550824152`},
{112, 0.0008874544691474242`, 1.5796689550824152`}, {126, 0.0008874544691474242`, 1.5796689550824152`}},
0.00449031745787805`}, {"CURE", 365, 142.46528510828142`, {{0, 0.018983410380582613`, 33.790470477437054`},
{14, 0.0012356402457615158`, 2.199439637455498`}, {28, 0.0012356402457615158`, 2.199439637455498`},
{42, 0.0012356402457615158`, 2.199439637455498`}, {56, 0.0012356402457615158`, 2.199439637455498`},
{70, 0.0012356402457615158`, 2.199439637455498`}, {84, 0.0012356402457615158`, 2.199439637455498`},
{98, 0.0012356402457615158`, 2.199439637455498`}, {112, 0.0012356402457615158`, 2.199439637455498`},
{126, 0.0012356402457615158`, 2.199439637455498`}}, 0.0058314984913375775`},
{"TBUR", 17.797283959987887`, 2.6111524739740584`, {{0, 0.009612216957627779`, 17.109746184577446`},
{14, 0.0005662692869790277`, 1.0079593308226693`}}, 0.0032531403430014335`}, {"CURE", 365, 117.56940211711563`,
{{0, 0.05501666174137658`, 97.92965789965031`}, {14, 0.0027569705607345864`, 4.9074075981075636`}}, 0.015745564188623447`},
{"CURE", 365, 66.5633066097822`, {{0, 0.009870904652652173`, 17.57021028172087`},
{14, 0.0005847469794807701`, 1.0408496234757707`}, {28, 0.0005847469794807701`, 1.0408496234757707`},
{42, 0.0005847469794807701`, 1.0408496234757707`}, {56, 0.0005847469794807701`, 1.0408496234757707`},
{70, 0.0005847469794807701`, 1.0408496234757707`}, {84, 0.0005847469794807701`, 1.0408496234757707`},
{98, 0.0005847469794807701`, 1.0408496234757707`}, {112, 0.0005847469794807701`, 1.0408496234757707`},
{126, 0.0005847469794807701`, 1.0408496234757707`}}, 0.0033243147917868623`},
{"CURE", 365, 60.26868049832492`, {{0, 0.012265889311843452`, 21.833282975081346`},
{14, 0.0007558173122801473`, 1.3453548158586621`}, {28, 0.0007558173122801473`, 1.3453548158586621`},
{42, 0.0007558173122801473`, 1.3453548158586621`}, {56, 0.0007558173122801473`, 1.3453548158586621`},
{70, 0.0007558173122801473`, 1.3453548158586621`}, {84, 0.0007558173122801473`, 1.3453548158586621`},
{98, 0.0007558173122801473`, 1.3453548158586621`}, {112, 0.0007558173122801473`, 1.3453548158586621`},
{126, 0.0007558173122801473`, 1.3453548158586621`}}, 0.003983262690145235`},
{"CURE", 365, 58.52039933767902`, {{0, 0.0224247021050622`, 39.91596974701071`}, {14, 0.0014814467975100578`, 2.636975299567903`},
{28, 0.0014814467975100578`, 2.636975299567903`}, {42, 0.0014814467975100578`, 2.636975299567903`},
{56, 0.0014814467975100578`, 2.636975299567903`}, {70, 0.0014814467975100578`, 2.636975299567903`},
{84, 0.0014814467975100578`, 2.636975299567903`}, {98, 0.0014814467975100578`, 2.636975299567903`}, {112,
0.0014814467975100578`, 2.636975299567903`}, {126, 0.0014814467975100578`, 2.636975299567903`}}, 0.006778323741671165`},

```
{"TBUR", 12.684101146705471`, 4.253714830397434`, {{0, 0.019133701048777357`, 34.057987866823694`}}, 0.005872848952422886`},
{"TBUR", 35.21118086462591`, 3.734934116838327`, {{0, 0.003937904455963025`, 7.009469931614184`},
    {14, 0.00016096125114583093`, 0.28651102703957904`}, {28, 0.00016096125114583093`, 0.28651102703957904`}},
  0.0016919293835695562`}, {"CURE", 365, 26.791573404469837`, {{0, 0.009854396283699097`, 17.54082538498439`},
    {14, 0.000583567810269836`, 1.0387507022803082`}, {28, 0.000583567810269836`, 1.0387507022803082`},
    {42, 0.000583567810269836`, 1.0387507022803082`}, {56, 0.000583567810269836`, 1.0387507022803082`},
    {70, 0.000583567810269836`, 1.0387507022803082`}, {84, 0.000583567810269836`, 1.0387507022803082`},
    {98, 0.000583567810269836`, 1.0387507022803082`}, {112, 0.000583567810269836`, 1.0387507022803082`},
    {126, 0.000583567810269836`, 1.0387507022803082`}}, 0.003319772735542815`}, {"CURE", 365, 79.74552044654558`,
  {{0, 0.015820805110021666`, 28.161033095838565`}, {14, 0.0010097398692928768`, 1.7973369673413206`},
    {28, 0.0010097398692928768`, 1.7973369673413206`}, {42, 0.0010097398692928768`, 1.7973369673413206`},
    {56, 0.0010097398692928768`, 1.7973369673413206`}, {70, 0.0010097398692928768`, 1.7973369673413206`},
    {84, 0.0010097398692928768`, 1.7973369673413206`}, {98, 0.0010097398692928768`, 1.7973369673413206`},
    {112, 0.0010097398692928768`, 1.7973369673413206`}, {126, 0.0010097398692928768`, 1.7973369673413206`}},
  0.004961350079999962`}, {"CURE", 365, 54.42111599266809`, {{0, 0.008131341460470084`, 14.47378779963675`},
    {14, 0.0004604924657534781`, 0.819676589041191`}, {28, 0.0004604924657534781`, 0.819676589041191`},
    {42, 0.0004604924657534781`, 0.819676589041191`}, {56, 0.0004604924657534781`, 0.819676589041191`},
    {70, 0.0004604924657534781`, 0.819676589041191`}, {84, 0.0004604924657534781`, 0.819676589041191`},
    {98, 0.0004604924657534781`, 0.819676589041191`}, {112, 0.0004604924657534781`, 0.819676589041191`},
    {126, 0.0004604924657534781`, 0.819676589041191`}}, 0.0028456973170774254`},
{"TBUR", 30.808714533807155`, 4.013087666275063`, {{0, 0.009402300739024795`, 16.736095315464134`},
    {14, 0.0005512752713645289`, 0.9812699830288614`}, {28, 0.0005512752713645289`, 0.9812699830288614`}},
  0.003195384711645307`}, {"CURE", 365, 68.49195246466198`, {{0, 0.06502285606095995`, 115.7406837885087`}}, 0.0175`},
{"CURE", 365, 127.73632648179536`, {{0, 0.047315215064738105`, 84.22108281523383`},
    {14, 0.003259340580344052`, 5.801626233012412`}, {28, 0.003259340580344052`, 5.801626233012412`}}, 0.013626614456559764`},
{"TBUR", 17.673539002653513`, 4.0964537568800905`, {{0, 0.022787428045890977`, 40.56162192168594`},
    {14, 0.001507355793283542`, 2.683093312044705`}}, 0.006878122918034111`}, {"CURE", 365, 148.05343618339248`,
  {{0, 0.05510552147507449`, 98.08782822563259`}, {14, 0.0027185513893581204`, 4.839021473057454`}}, 0.015770012752226652`},
{"CURE", 365, 31.317410415655605`, {{0, 0.008757101977013636`, 15.587641519084272`},
    {14, 0.0005051896455065888`, 0.899237569001728`}, {28, 0.0005051896455065888`, 0.899237569001728`},
    {42, 0.0005051896455065888`, 0.899237569001728`}, {56, 0.0005051896455065888`, 0.899237569001728`},
```

```
{70, 0.0005051896455065888`, 0.899237569001728`}, {84, 0.0005051896455065888`, 0.899237569001728`},
{98, 0.0005051896455065888`, 0.899237569001728`}, {112, 0.0005051896455065888`, 0.899237569001728`},
{126, 0.0005051896455065888`, 0.899237569001728`}}, 0.00301786692813091`},
{"CURE", 365, 178.4556132614792`, {{0, 0.02384498900592984`, 42.444080430555125`},
{14, 0.0015828958618577468`, 2.817554634106789`}, {28, 0.0015828958618577468`, 2.817554634106789`},
{42, 0.0015828958618577468`, 2.817554634106789`}, {56, 0.0015828958618577468`, 2.817554634106789`},
{70, 0.0015828958618577468`, 2.817554634106789`}, {84, 0.0015828958618577468`, 2.817554634106789`},
{98, 0.0015828958618577468`, 2.817554634106789`}, {112, 0.0015828958618577468`, 2.817554634106789`},
{126, 0.0015828958618577468`, 2.817554634106789`}}, 0.007169096628170023`}, {"CURE", 365, 48.09963343177620`,
{{0, 0.05611663241988438`, 99.88760570739419`}, {14, 0.0022813899996861494`, 4.060874199441346`}}, 0.01604820636383609`},
{"CURE", 365, 34.480983832114056`, {{0, 0.011017886303311767`, 19.611837619894946`},
{14, 0.0006666742402421698`, 1.1866801476310622`}, {28, 0.0006666742402421698`, 1.1866801476310622`},
{42, 0.0006666742402421698`, 1.1866801476310622`}, {56, 0.0006666742402421698`, 1.1866801476310622`},
{70, 0.0006666742402421698`, 1.1866801476310622`}, {84, 0.0006666742402421698`, 1.1866801476310622`},
{98, 0.0006666742402421698`, 1.1866801476310622`}, {112, 0.0006666742402421698`, 1.1866801476310622`},
{126, 0.0006666742402421698`, 1.1866801476310622`}}, 0.0036398914052976016`},
{"CURE", 365, 94.95704296369348`, {{0, 0.017323997203885944`, 30.83671502291698`},
{14, 0.0011171107331403253`, 1.988457104989779`}, {28, 0.0011171107331403253`, 1.988457104989779`},
{42, 0.0011171107331403253`, 1.988457104989779`}, {56, 0.0011171107331403253`, 1.988457104989779`},
{70, 0.0011171107331403253`, 1.988457104989779`}, {84, 0.0011171107331403253`, 1.988457104989779`},
{98, 0.0011171107331403253`, 1.988457104989779`}, {112, 0.0011171107331403253`, 1.988457104989779`},
{126, 0.0011171107331403253`, 1.988457104989779`}}, 0.005374933218112946`}, {"CURE", 365, 99.03990120824635`,
{{0, 0.044800498538159525`, 79.74488739792396`}, {14, 0.003079717971302724`, 5.481897988918849`},
{28, 0.003079717971302724`, 5.481897988918849`}, {42, 0.0010145692926710114`, 1.8059333409544003`}}, 0.01293472394118771`},
{"CURE", 365, 82.47241965754456`, {{0, 0.023200964140067507`, 41.29771616932016`},
{14, 0.001536894085724723`, 2.7356714725900066`}, {28, 0.001536894085724723`, 2.7356714725900066`},
{42, 0.001536894085724723`, 2.7356714725900066`}, {56, 0.001536894085724723`, 2.7356714725900066`},
{70, 0.001536894085724723`, 2.7356714725900066`}, {84, 0.001536894085724723`, 2.7356714725900066`},
{98, 0.001536894085724723`, 2.7356714725900066`}, {112, 0.001536894085724723`, 2.7356714725900066`},
{126, 0.001536894085724723`, 2.7356714725900066`}}, 0.006991901826432200 5`},
{"CURE", 365, 57.70391554330885`, {{0, 0.025168055557306404`, 44.7991388920054`}, {14, 0.001677400615527501`, 2.985773095638952`},
```

```
{28, 0.001677400615527501`, 2.985773095638952`}, {42, 0.001677400615527501`, 2.985773095638952`},
{56, 0.001677400615527501`, 2.985773095638952`}, {70, 0.001677400615527501`, 2.985773095638952`},
{84, 0.001677400615527501`, 2.985773095638952`}, {98, 0.001677400615527501`, 2.985773095638952`},
{112, 0.001677400615527501`, 2.985773095638952`}, {126, 0.001677400615527501`, 2.985773095638952`}},
0.007533120639794795`}, {"CURE", 365, 17.295690177495274`, {{0, 0.00414001590761786`, 7.36922831555979`},
{14, 0.00017539778340689056`, 0.3122080544642652`}, {28, 0.00017539778340689056`, 0.3122080544642652`},
{42, 0.00017539778340689056`, 0.3122080544642652`}, {56, 0.00017539778340689056`, 0.3122080544642652`},
{70, 0.00017539778340689056`, 0.3122080544642652`}, {84, 0.00017539778340689056`, 0.3122080544642652`},
{98, 0.00017539778340689056`, 0.3122080544642652`}, {112, 0.00017539778340689056`, 0.3122080544642652`},
{126, 0.00017539778340689056`, 0.3122080544642652`}}, 0.0017475376380070185`},
{"TBUR", 21.687847252747044`, 3.0200494453514115`, {{0, 0.002551198687380343`, 4.5411336635370105`},
{14, 0.00006191083910421077`, 0.11020129360549516`}}, 0.001310395895101396`},
{"CURE", 365, 67.67486617274798`, {{0, 0.03382481033816414`, 60.20816240193217`}, {14, 0.002295740242731625`, 4.086417632062292`},
{28, 0.002295740242731625`, 4.086417632062292`}, {42, 0.002295740242731625`, 4.086417632062292`},
{56, 0.002295740242731625`, 4.086417632062292`}, {70, 0.002295740242731625`, 4.086417632062292`},
{84, 0.00044072501695575396`, 0.784490530181242`}}, 0.009914910580518445`}, {"CURE", 365, 24.07574592231725`,
{{0, 0.004446076281074058`, 7.914015780311824`}, {14, 0.00019725923865376188`, 0.3511214448036961`},
{28, 0.00019725923865376188`, 0.3511214448036961`}, {42, 0.00019725923865376188`, 0.3511214448036961`},
{56, 0.00019725923865376188`, 0.3511214448036961`}, {70, 0.00019725923865376188`, 0.3511214448036961`},
{84, 0.00019725923865376188`, 0.3511214448036961`}, {98, 0.00019725923865376188`, 0.3511214448036961`},
{112, 0.00019725923865376188`, 0.3511214448036961`}, {126, 0.00019725923865376188`, 0.3511214448036961`}},
0.0018317460437209162`}, {"CURE", 365, 62.84437782610733`, {{0, 0.02483573334885825`, 44.20760536096768`},
{14, 0.0016536633149240615`, 2.9435207005648296`}, {28, 0.0016536633149240615`, 2.9435207005648296`},
{42, 0.0016536633149240615`, 2.9435207005648296`}, {56, 0.0016536633149240615`, 2.9435207005648296`},
{70, 0.0016536633149240615`, 2.9435207005648296`}, {84, 0.0016536633149240615`, 2.9435207005648296`},
{98, 0.0016536633149240615`, 2.9435207005648296`}, {112, 0.0016536633149240615`, 2.9435207005648296`},
{126, 0.0016536633149240615`, 2.9435207005648296`}}, 0.007441686642505884`}, {"CURE", 365, 113.82650232180359`,
{{0, 0.015923302646537223`, 28.34347871083626`}, {14, 0.0010170611219011308`, 1.8103687969840128`},
{28, 0.0010170611219011308`, 1.8103687969840128`}, {42, 0.0010170611219011308`, 1.8103687969840128`},
{56, 0.0010170611219011308`, 1.8103687969840128`}, {70, 0.0010170611219011308`, 1.8103687969840128`},
{84, 0.0010170611219011308`, 1.8103687969840128`}, {98, 0.0010170611219011308`, 1.8103687969840128`},
```

{112, 0.0010170611219011308`, 1.8103687969840128`}, {126, 0.0010170611219011308`, 1.8103687969840128`}},
0.004989550902086499`}, {"CURE", 365, 48.36242717261314`, {{0, 0.05046868654379278`, 89.83426204795114`},
{14, 0.0034845885431336703`, 6.202567606777933`}, {28, 0.0012387331561925664`, 2.204945018022768`}}, 0.014494249827701488`},
{"CURE", 365, 73.97789812206722`, {{0, 0.03895156670660635`, 69.3337887377593`}, {14, 0.0026619371261917832`, 4.738248084621374`},
{28, 0.0026619371261917832`, 4.738248084621374`}, {42, 0.0026619371261917832`, 4.738248084621374`},
{56, 0.0026619371261917832`, 4.738248084621374`}}, 0.011325468815793799`},
{"CURE", 365, 55.00462197741542`, {{0, 0.021304678554241608`, 37.92232782655006`},
{14, 0.00140144511530858 7`, 2.4945723052492847`}, {28, 0.00140144511530858 7`, 2.4945723052492847`},
{42, 0.00140144511530858 7`, 2.4945723052492847`}, {56, 0.00140144511530858 7`, 2.4945723052492847`},
{70, 0.00140144511530858 7`, 2.4945723052492847`}, {84, 0.00140144511530858 7`, 2.4945723052492847`},
{98, 0.00140144511530858 7`, 2.4945723052492847`}, {112, 0.00140144511530858 7`, 2.4945723052492847`},
{126, 0.00140144511530858 7`, 2.4945723052492847`}}, 0.006470164287666842`},
{"TBUR", 11.746669091038006`, 2.202459968011037`, {{0, 0.0028252518837551324`, 5.028948353084136`}}, 0.0013857979554430023`},
{"TBUR", 17.676921163485666`, 3.5755751107844675`,
{{0, 0.00716944399075671 4`, 12.76161030354695`}, {14, 0.0003917855036310945`, 0.6973781964633482`}}, 0.002581044132903005`},
{"CURE", 365, 71.90716696157334`, {{0, 0.03623029656388118`, 64.4899278837085`}, {14, 0.0024675606874256994`, 4.392258023617745`},
{28, 0.0024675606874256994`, 4.392258023617745`}, {42, 0.0024675606874256994`, 4.392258023617745`},
{56, 0.0024675606874256994`, 4.392258023617745`}, {70, 0.0010091534954093581`, 1.7962932218286574`}}, 0.01057674784401954`},
{"TBUR", 12.075782078119252`, 1.9210343217865828`, {{0, 0.008414090938710208`, 14.97708187090417`}}, 0.002923492042706763`},
{"TBUR", 84.83536870162162`, 11.783959203547335`, {{0, 0.012925980798723806`, 23.008245821728373`},
{14, 0.0008029667042001723`, 1.4292807334763067`}, {28, 0.0008029667042001723`, 1.4292807334763067`},
{42, 0.0008029667042001723`, 1.4292807334763067`}, {56, 0.0008029667042001723`, 1.4292807334763067`},
{70, 0.0008029667042001723`, 1.4292807334763067`}, {84, 0.0008029667042001723`, 1.4292807334763067`}},
0.004164878007109704`}, {"CURE", 365, 119.5885901052497`, {{0, 0.02515103223612912`, 44.76883738030983`},
{14, 0.0016761846640148377`, 2.983608701946411`}, {28, 0.0016761846640148377`, 2.983608701946411`},
{42, 0.0016761846640148377`, 2.983608701946411`}, {56, 0.0016761846640148377`, 2.983608701946411`},
{70, 0.0016761846640148377`, 2.983608701946411`}, {84, 0.0016761846640148377`, 2.983608701946411`},
{98, 0.0016761846640148377`, 2.983608701946411`}, {112, 0.0016761846640148377`, 2.983608701946411`},
{126, 0.0016761846640148377`, 2.983608701946411`}}, 0.0075284369013539024`}, {"CURE", 365, 26.70172614692699`,
{{0, 0.008545905551020817`, 15.211711880817054`}, {14, 0.0004901041865071018`, 0.8723854519826412`},
{28, 0.0004901041865071018`, 0.8723854519826412`}, {42, 0.0004901041865071018`, 0.8723854519826412`},

```
{56, 0.0004901041865071018`, 0.8723854519826412`}, {70, 0.0004901041865071018`, 0.8723854519826412`},
{84, 0.0004901041865071018`, 0.8723854519826412`}, {98, 0.0004901041865071018`, 0.8723854519826412`},
{112, 0.0004901041865071018`, 0.8723854519826412`}, {126, 0.0004901041865071018`, 0.8723854519826412`}},
0.0029597590648864913`}, {"TBUR", 21.233540062525794`, 13.612839016023415`,
{{0, 0.024698834897352338`, 43.96392611728716`}, {14, 0.0016438848541022106`, 2.926115040301935`}}, 0.007404020870175301 5`},
{"CURE", 365, 24.78536897717058`, {{0, 0.009867765766761797`, 17.564623064836`}, {14, 0.0005845227733457433`, 1.040450536555423`},
{28, 0.0005845227733457433`, 1.040450536555423`}, {42, 0.0005845227733457433`, 1.040450536555423`},
{56, 0.0005845227733457433`, 1.040450536555423`}, {70, 0.0005845227733457433`, 1.040450536555423`},
{84, 0.0005845227733457433`, 1.040450536555423`}, {98, 0.0005845227733457433`, 1.040450536555423`},
{112, 0.0005845227733457433`, 1.040450536555423`}, {126, 0.0005845227733457433`, 1.040450536555423`}},
0.003323451169444768`}, {"TBUR", 24.699247685858843`, 3.236232613527706`,
{{0, 0.01212630800444053`, 21.584828247904145`}, {14, 0.0007458472188942241`, 1.327608049631719`}},
0.00394485876600723`}, {"TBUR", 24.38126413540278`, 13.438520003614292`,
{{0, 0.04410725894658433`, 78.5109209249201`}, {14, 0.003030200857618782`, 5.393757526561432`}}, 0.01274398836792456`},
{"CURE", 365, 13.838052140928394`, {{0, 0.004673847049778564`, 8.319447748605844`},
{14, 0.00021352857927551232`, 0.38008087111041194`}, {28, 0.00021352857927551232`, 0.38008087111041194`},
{42, 0.00021352857927551232`, 0.38008087111041194`}, {56, 0.00021352857927551232`, 0.38008087111041194`},
{70, 0.00021352857927551232`, 0.38008087111041194`}, {84, 0.00021352857927551232`, 0.38008087111041194`},
{98, 0.00021352857927551232`, 0.38008087111041194`}, {112, 0.00021352857927551232`, 0.38008087111041194`},
{126, 0.00021352857927551232`, 0.38008087111041194`}}, 0.0018944141150045069`},
{"TBUR", 16.806300810389097`, 2.5315866037859025`, {{0, 0.006136123862120172`, 10.922300474573907`},
{14, 0.00031797692301419863`, 0.5659989229652735`}}, 0.0022967399623174717`},
{"CURE", 365, 11.07958496428256`, {{0, 0.0032865718119366332`, 5.850097825247207`},
{14, 0.00011443749085823148`, 0.20369873372765204`}, {28, 0.00011443749085823148`, 0.20369873372765204`},
{42, 0.00011443749085823148`, 0.20369873372765204`}, {56, 0.00011443749085823148`, 0.20369873372765204`},
{70, 0.00011443749085823148`, 0.20369873372765204`}, {84, 0.00011443749085823148`, 0.20369873372765204`},
{98, 0.00011443749085823148`, 0.20369873372765204`}, {112, 0.00011443749085823148`, 0.20369873372765204`},
{126, 0.00011443749085823148`, 0.20369873372765204`}}, 0.0015127239447095403`}, {"CURE", 365, 87.74674916943229`,
{{0, 0.05406815117295691`, 96.2413090878633`}, {14, 0.003167066208830706`, 5.637377851718656`}}, 0.015484594230744098`},
{"TBUR", 108.89875272804511`, 27.962331050263803`, {{0, 0.03984602484766603`, 70.92592422884553`}}, 0.011571566973441218`},
{"CURE", 365, 22.17241739883439`, {{0, 0.008720712657340953`, 15.522868530066896`},
```

```
    {14, 0.0005025904083871116`, 0.8946109269290586`}, {28, 0.0005025904083871116`, 0.8946109269290586`},
    {42, 0.0005025904083871116`, 0.8946109269290586`}, {56, 0.0005025904083871116`, 0.8946109269290586`},
    {70, 0.0005025904083871116`, 0.8946109269290586`}, {84, 0.0005025904083871116`, 0.8946109269290586`},
    {98, 0.0005025904083871116`, 0.8946109269290586`}, {112, 0.0005025904083871116`, 0.8946109269290586`},
    {126, 0.0005025904083871116`, 0.8946109269290586`}}, 0.003007854895014546`},
{"CURE", 365, 65.05789607052641`, {{0, 0.06531535045604005`, 116.26132381175128`}}, 0.0175`},
{"CURE", 365, 47.461962609417874`, {{0, 0.00841431466562127`, 14.977480104805862`},
    {14, 0.0004807048375499913`, 0.8556546108389845`}, {28, 0.0004807048375499913`, 0.8556546108389845`},
    {42, 0.0004807048375499913`, 0.8556546108389845`}, {56, 0.0004807048375499913`, 0.8556546108389845`},
    {70, 0.0004807048375499913`, 0.8556546108389845`}, {84, 0.0004807048375499913`, 0.8556546108389845`},
    {98, 0.0004807048375499913`, 0.8556546108389845`}, {112, 0.0004807048375499913`, 0.8556546108389845`},
    {126, 0.0004807048375499913`, 0.8556546108389845`}}, 0.0029235535981648676`},
{"CURE", 365, 123.27587727079808`, {{0, 0.022847339494862316`, 40.66826430085492`},
    {14, 0.0015116351824957805`, 2.6907106248424895`}, {28, 0.0015116351824957805`, 2.6907106248424895`},
    {42, 0.0015116351824957805`, 2.6907106248424895`}, {56, 0.0015116351824957805`, 2.6907106248424895`},
    {70, 0.0015116351824957805`, 2.6907106248424895`}, {84, 0.0015116351824957805`, 2.6907106248424895`},
    {98, 0.0015116351824957805`, 2.6907106248424895`}, {112, 0.0015116351824957805`, 2.6907106248424895`},
    {126, 0.0015116351824957805`, 2.6907106248424895`}}, 0.006894606749458985`},
{"CURE", 365, 84.11501205728523`, {{0, 0.029204007864372855`, 51.98313399858368`},
    {14, 0.00196568292317510`, 3.4989156032516866`}, {28, 0.00196568292317510`, 3.4989156032516866`},
    {42, 0.00196568292317510`, 3.4989156032516866`}, {56, 0.00196568292317510`, 3.4989156032516866`},
    {70, 0.00196568292317510`, 3.4989156032516866`}, {84, 0.00196568292317510`, 3.4989156032516866`},
    {98, 0.00196568292317510`, 3.4989156032516866`}, {112, 0.00015748432528569535`, 0.2803220990085377`}},
  0.00864355877158364`}, {"CURE", 365, 70.99576750326418`, {{0, 0.010061148087485308`, 17.908843595723848`},
    {14, 0.0005983357962545654`, 1.0650377173331265`}, {28, 0.0005983357962545654`, 1.0650377173331265`},
    {42, 0.0005983357962545654`, 1.0650377173331265`}, {56, 0.0005983357962545654`, 1.0650377173331265`},
    {70, 0.0005983357962545654`, 1.0650377173331265`}, {84, 0.0005983357962545654`, 1.0650377173331265`},
    {98, 0.0005983357962545654`, 1.0650377173331265`}, {112, 0.0005983357962545654`, 1.0650377173331265`},
    {126, 0.0005983357962545654`, 1.0650377173331265`}}, 0.003376657720614683`},
{"CURE", 365, 113.41391142257046`, {{0, 0.061621963837140706`, 109.68709563011046`}}, 0.017500000000000005`},
{"CURE", 365, 15.152537258986941`, {{0, 0.004990418176356901`, 8.882944353915285`},
```

```
{14, 0.00023614080260253635`, 0.4203306286325147`}, {28, 0.00023614080260253635`, 0.4203306286325147`},
{42, 0.00023614080260253635`, 0.4203306286325147`}, {56, 0.00023614080260253635`, 0.4203306286325147`},
{70, 0.00023614080260253635`, 0.4203306286325147`}, {84, 0.00023614080260253635`, 0.4203306286325147`},
{98, 0.00023614080260253635`, 0.4203306286325147`}, {112, 0.00023614080260253635`, 0.4203306286325147`},
{126, 0.00023614080260253635`, 0.4203306286325147`}}, 0.0019815144134298747`},
{"CURE", 365, 53.3297503912962`, {{0, 0.009783978667444288`, 17.415482028050832`},
{14, 0.0005785379805373496`, 1.0297976053564823`}, {28, 0.0005785379805373496`, 1.0297976053564823`},
{42, 0.0005785379805373496`, 1.0297976053564823`}, {56, 0.0005785379805373496`, 1.0297976053564823`},
{70, 0.0005785379805373496`, 1.0297976053564823`}, {84, 0.0005785379805373496`, 1.0297976053564823`},
{98, 0.0005785379805373496`, 1.0297976053564823`}, {112, 0.0005785379805373496`, 1.0297976053564823`},
{126, 0.0005785379805373496`, 1.0297976053564823`}}, 0.003300398273137925`},
{"TBUR", 50.581689541851254`, 5.17488974766839`, {{0, 0.010057615542210074`, 17.90255566513393`},
{14, 0.0005980834715920488`, 1.064588579433847`}, {28, 0.0005980834715920488`, 1.064588579433847`},
{42, 0.0005980834715920488`, 1.064588579433847`}}, 0.003375685788174098`}, {"CURE", 365, 87.95133352118785`,
{{0, 0.035135437051326206`, 62.54107795136065`}, {14, 0.002389356436528915 5`, 4.253054457021469`},
{28, 0.0023893564365289155`, 4.253054457021469`}, {42, 0.0023893564365289155`, 4.253054457021469`},
{56, 0.0023893564365289155`, 4.253054457021469`}, {70, 0.0023893564365289155`, 4.253054457021469`}}, 0.010275511937548477`},
{"CURE", 365, 26.254413299126057`, {{0, 0.009136232232853616`, 16.262493374479437`},
{14, 0.0005322703780665875`, 0.9474412729585258`}, {28, 0.0005322703780665875`, 0.9474412729585258`},
{42, 0.0005322703780665875`, 0.9474412729585258`}, {56, 0.0005322703780665875`, 0.9474412729585258`},
{70, 0.0005322703780665875`, 0.9474412729585258`}, {84, 0.0005322703780665875`, 0.9474412729585258`},
{98, 0.0005322703780665875`, 0.9474412729585258`}, {112, 0.0005322703780665875`, 0.9474412729585258`},
{126, 0.0005322703780665875`, 0.9474412729585258`}}, 0.003122179531692298`},
{"CURE", 365, 86.15086712570385`, {{0, 0.01987141368336661`, 35.37111635639257`}, {14, 0.00129906905310323`, 2.3123429145237493`},
{28, 0.00129906905310323`, 2.3123429145237493`}, {42, 0.00129906905310323`, 2.3123429145237493`},
{56, 0.00129906905310323`, 2.3123429145237493`}, {70, 0.00129906905310323`, 2.3123429145237493`},
{84, 0.00129906905310323`, 2.3123429145237493`}, {98, 0.00129906905310323`, 2.3123429145237493`},
{112, 0.00129906905310323`, 2.3123429145237493`}, {126, 0.00129906905310323`, 2.3123429145237493`}}, 0.006075820686830217`},
{"CURE", 365, 86.89669987222895`, {{0, 0.05177478711370247`, 92.1591210623904`}, {14, 0.0035778814409843625`,
6.3686289649521655`}, {28, 0.0005807378949425398`, 1.0337134529977208`}}, 0.014853605877137428`},
{"TBUR", 60.399902137982956`, 6.272432213601204`, {{0, 0.003903047904307495`, 6.947425269667341`},
```

{14, 0.0001584714974561502`, 0.28207926547194734`}, {28, 0.0001584714974561502`, 0.28207926547194734`},
{42, 0.0001584714974561502`, 0.28207926547194734`}, {56, 0.0001584714974561502`, 0.28207926547194734`}},
0.0016823390710098201`}, {"TBUR", 18.79090462873622`, 9.145453375800422`,
{{0, 0.022813036399302506`, 40.60720479075846`}, {14, 0.0015091849613843653`, 2.6863492312641704`}}, 0.006885168712918929`},
{"TBUR", 14.020197642524783`, 1.9124311780624503`, {{0, 0.00707995856169511`, 12.602326239817295`},
{14, 0.00038539368726955126`, 0.6860007633398012`}}, 0.0025564234176146854`}, {"CURE", 365, 71.11158121669163`,
{{0, 0.02495783445800318`, 44.42494533524566`}, {14, 0.001662384822720128`, 2.9590449844418276`},
{28, 0.001662384822720128`, 2.9590449844418276`}, {42, 0.001662384822720128`, 2.9590449844418276`},
{56, 0.001662384822720128`, 2.9590449844418276`}, {70, 0.001662384822720128`, 2.9590449844418276`},
{84, 0.001662384822720128`, 2.9590449844418276`}, {98, 0.001662384822720128`, 2.9590449844418276`},
{112, 0.001662384822720128`, 2.9590449844418276`}, {126, 0.001662384822720128`, 2.9590449844418276`}},
0.007475281124603921`}, {"TBUR", 14.03400525607571`, 2.0915975522406876`,
{{0, 0.0086121086984189`, 15.329553483185641`}, {14, 0.000494832982749822`, 0.8808027092946832`}}, 0.0029779739727253604`},
{"CURE", 365, 100.03337683226`, {{0, 0.03545218435602371`, 63.1048881537222`}, {14, 0.002411981244007309`, 4.29332661433301`},
{28, 0.002411981244007309`, 4.29332661433301`}, {42, 0.002411981244007309`, 4.29332661433301`},
{56, 0.002411981244007309`, 4.29332661433301`}, {70, 0.002411981244007309`, 4.29332661433301`}}, 0.010362660709019762`},
{"TBUR", 192.83641847725218`, 9.982319172028987`, {{0, 0.002689756032523986`, 4.787765737892695`},
{14, 0.00007180779232875667`, 0.12781787034518688`}, {28, 0.00007180779232875667`, 0.12781787034518688`},
{42, 0.00007180779232875667`, 0.12781787034518688`}, {56, 0.00007180779232875667`, 0.12781787034518688`},
{70, 0.00007180779232875667`, 0.12781787034518688`}, {84, 0.00007180779232875667`, 0.12781787034518688`},
{98, 0.00007180779232875667`, 0.12781787034518688`}, {112, 0.00007180779232875667`, 0.12781787034518688`},
{126, 0.00007180779232875667`, 0.12781787034518688`}}, 0.001348518089761027`},
{"CURE", 365, 57.72798448043272`, {{0, 0.023099066831555836`, 41.11633896016939`},
{14, 0.0015296157065453175`, 2.722715957650665`}, {28, 0.0015296157065453175`, 2.722715957650665`},
{42, 0.0015296157065453175`, 2.722715957650665`}, {56, 0.0015296157065453175`, 2.722715957650665`},
{70, 0.0015296157065453175`, 2.722715957650665`}, {84, 0.0015296157065453175`, 2.722715957650665`},
{98, 0.0015296157065453175`, 2.722715957650665`}, {112, 0.0015296157065453175`, 2.722715957650665`},
{126, 0.0015296157065453175`, 2.722715957650665`}}, 0.006963866149028401`}, {"CURE", 365, 50.590192451483865`,
{{0, 0.04121345722596484`, 73.35995386221741`}, {14, 0.002823500734717389`, 5.025831307796952 5`},
{28, 0.002823500734717389`, 5.0258313077969525`}, {42, 0.002823500734717389`, 5.0258313077969525`},
{56, 0.00025438720748404905`, 0.45280922932160733`}}, 0.011947797647140592`},

{"CURE", 365, 14.258838196269368`, {{0, 0.0030198083887158374`, 5.375258931914191`},
{14, 0.00009538296062817465`, 0.16978166991815088`}, {28, 0.00009538296062817465`, 0.16978166991815088`},
{42, 0.00009538296062817465`, 0.16978166991815088`}, {56, 0.00009538296062817465`, 0.16978166991815088`},
{70, 0.00009538296062817465`, 0.16978166991815088`}, {84, 0.00009538296062817465`, 0.16978166991815088`},
{98, 0.00009538296062817465`, 0.16978166991815088`}, {112, 0.00009538296062817465`, 0.16978166991815088`},
{126, 0.00009538296062817465`, 0.16978166991815088`}}, 0.0014393275676531688`}, {"CURE", 365, 150.1985777401109`,
{{0, 0.02383480028512669`, 42.42594450752551`}, {14, 0.0015821680960860932`, 2.816259211033246`},
{28, 0.0015821680960860932`, 2.816259211033246`}, {42, 0.0015821680960860932`, 2.816259211033246`},
{56, 0.0015821680960860932`, 2.816259211033246`}, {70, 0.0015821680960860932`, 2.816259211033246`},
{84, 0.0015821680960860932`, 2.816259211033246`}, {98, 0.0015821680960860932`, 2.816259211033246`},
{112, 0.0015821680960860932`, 2.816259211033246`}, {126, 0.0015821680960860932`, 2.816259211033246`}}, 0.0071662933383308`},
{"CURE", 365, 83.79652911287062`, {{0, 0.027940413517425655`, 49.733936061017666`},
{14, 0.0018754261841074475`, 3.3382586077112566`}, {28, 0.0018754261841074475`, 3.3382586077112566`},
{42, 0.0018754261841074475`, 3.3382586077112566`}, {56, 0.0018754261841074475`, 3.3382586077112566`},
{70, 0.0018754261841074475`, 3.3382586077112566`}, {84, 0.0018754261841074475`, 3.3382586077112566`},
{98, 0.0018754261841074475`, 3.3382586077112566`}, {112, 0.0018754261841074475`, 3.3382586077112566`}},
0.008295897739141144`}, {"CURE", 365, 32.48812308474696`, {{0, 0.009063981240207384`, 16.133886607569142`},
{14, 0.0005271095928775708`, 0.9382550753220761`}, {28, 0.0005271095928775708`, 0.9382550753220761`},
{42, 0.0005271095928775708`, 0.9382550753220761`}, {56, 0.0005271095928775708`, 0.9382550753220761`},
{70, 0.0005271095928775708`, 0.9382550753220761`}, {84, 0.0005271095928775708`, 0.9382550753220761`},
{98, 0.0005271095928775708`, 0.9382550753220761`}, {112, 0.0005271095928775708`, 0.9382550753220761`},
{126, 0.0005271095928775708`, 0.9382550753220761`}}, 0.0031023006403700392`},
{"TBUR", 190.66669120203238`, 60.774143506940554`, {{0, 0.06529932521698123`, 116.23279888622659`}}, 0.0175`},
{"CURE", 365, 31.403416519698084`, {{0, 0.00716612071953343`, 12.755694880769505`},
{14, 0.0003915481271151456`, 0.6969556662649592`}, {28, 0.0003915481271151456`, 0.6969556662649592`},
{42, 0.0003915481271151456`, 0.6969556662649592`}, {56, 0.0003915481271151456`, 0.6969556662649592`},
{70, 0.0003915481271151456`, 0.6969556662649592`}, {84, 0.0003915481271151456`, 0.6969556662649592`},
{98, 0.0003915481271151456`, 0.6969556662649592`}, {112, 0.0003915481271151456`, 0.6969556662649592`},
{126, 0.0003915481271151456`, 0.6969556662649592`}}, 0.002580129779410237`},
{"CURE", 365, 46.43644239406646`, {{0, 0.006911836804616949`, 12.303069512218169`},
{14, 0.0003733849903353969`, 0.6646252827970065`}, {28, 0.0003733849903353969`, 0.6646252827970065`},

```
{42, 0.0003733849903353969`, 0.6646252827970065`}, {56, 0.0003733849903353969`, 0.6646252827970065`},
{70, 0.0003733849903353969`, 0.6646252827970065`}, {84, 0.0003733849903353969`, 0.6646252827970065`},
{98, 0.0003733849903353969`, 0.6646252827970065`}, {112, 0.0003733849903353969`, 0.6646252827970065`},
{126, 0.0003733849903353969`, 0.6646252827970065`}}, 0.00251016697164l299`},
{"CURE", 365, 118.26378243178394`, {{0, 0.020176963121525796`, 35.914994356315916`},
{14, 0.0013208940129717432`, 2.351191343089703`}, {28, 0.0013208940129717432`, 2.351191343089703`},
{42, 0.0013208940129717432`, 2.351191343089703`}, {56, 0.0013208940129717432`, 2.351191343089703`},
{70, 0.0013208940129717432`, 2.351191343089703`}, {84, 0.0013208940129717432`, 2.351191343089703`},
{98, 0.0013208940129717432`, 2.351191343089703`}, {112, 0.0013208940129717432`, 2.351191343089703`},
{126, 0.0013208940129717432`, 2.351191343089703`}}, 0.006159888515551744`},
{"CURE", 365, 48.96546045636961`, {{0, 0.0343128364153922`, 61.076848819398116`}, {14, 0.002330599248247915`, 4.148466661881288`},
{28, 0.002330599248247915`, 4.148466661881288`}, {42, 0.002330599248247915`, 4.148466661881288`},
{56, 0.002330599248247915`, 4.148466661881288`}, {70, 0.002330599248247915`, 4.148466661881288`},
{84, 0.00005542825982889077`, 0.09866230249542557`}}, 0.01004918440841098`},
{"CURE", 365, 56.25271766359014`, {{0, 0.01625662117201454`, 28.936785686185882`},
{14, 0.0010408695880066534`, 1.852747866651843`}, {28, 0.0010408695880066534`, 1.852747866651843`},
{42, 0.0010408695880066534`, 1.852747866651843`}, {56, 0.0010408695880066534`, 1.852747866651843`},
{70, 0.0010408695880066534`, 1.852747866651843`}, {84, 0.0010408695880066534`, 1.852747866651843`},
{98, 0.0010408695880066534`, 1.852747866651843`}, {112, 0.0010408695880066534`, 1.852747866651843`},
{126, 0.0010408695880066534`, 1.852747866651843`}}, 0.005081259022639286`},
{"CURE", 365, 26.06937456586712`, {{0, 0.010295303371576803`, 18.325640001406708`},
{14, 0.0006150611736896724`, 1.0948088891676169`}, {28, 0.0006150611736896724`, 1.0948088891676169`},
{42, 0.0006150611736896724`, 1.0948088891676169`}, {56, 0.0006150611736896724`, 1.0948088891676169`},
{70, 0.0006150611736896724`, 1.0948088891676169`}, {84, 0.0006150611736896724`, 1.0948088891676169`},
{98, 0.0006150611736896724`, 1.0948088891676169`}, {112, 0.0006150611736896724`, 1.0948088891676169`},
{126, 0.0006150611736896724`, 1.0948088891676169`}}, 0.003441082405653667`}, {"CURE", 365, 77.17878692685647`,
{{0, 0.03352475534585738`, 59.67406451562613`}, {14, 0.0022743077432811425`, 4.048267783040433`},
{28, 0.0022743077432811425`, 4.048267783040433`}, {42, 0.0022743077432811425`, 4.048267783040433`},
{56, 0.0022743077432811425`, 4.048267783040433`}, {70, 0.0022743077432811425`, 4.048267783040433`},
{84, 0.0006776185373843623`, 1.2061609965441649`}}, 0.009832354474860864`},
{"TBUR", 136.13641543697497`, 32.76761062182046`, {{0, 0.01717017767617121`, 30.562916263584757`}}, 0.005332611838673161`},
```

```
{"CURE", 365, 83.54770936611733`, {{0, 0.031290870027548545`, 55.69774864903641`},
    {14, 0.002114744506259083`, 3.7642452211411674`}, {28, 0.002114744506259083`, 3.7642452211411674`},
    {42, 0.002114744506259083`, 3.7642452211411674`}, {56, 0.002114744506259083`, 3.7642452211411674`},
    {70, 0.002114744506259083`, 3.7642452211411674`}, {84, 0.002114744506259083`, 3.7642452211411674`},
    {98, 0.00032652726421979715`, 0.5812185303112389`}}, 0.009217730898870907`},
{"TBUR", 139.67072139481283`, 15.53212316691974`, {{0, 0.003439032828783894`, 6.121478435235331`},
    {14, 0.00012532756349017867`, 0.22308306301251804`}, {28, 0.00012532756349017867`, 0.22308306301251804`},
    {42, 0.00012532756349017867`, 0.22308306301251804`}, {56, 0.00012532756349017867`, 0.22308306301251804`},
    {70, 0.00012532756349017867`, 0.22308306301251804`}, {84, 0.00012532756349017867`, 0.22308306301251804`},
    {98, 0.00012532756349017867`, 0.22308306301251804`}, {112, 0.00012532756349017867`, 0.22308306301251804`},
    {126, 0.00012532756349017867`, 0.22308306301251804`}}, 0.001554671548109642`},
{"TBUR", 164.50564465720888`, 158.1513944951196`, {{0, 0.032178242244311595`, 57.277271194874636`},
    {14, 0.002178128236027872`, 3.877068260129612`}, {28, 0.002178128236027872`, 3.877068260129612`},
    {42, 0.002178128236027872`, 3.877068260129612`}, {56, 0.002178128236027872`, 3.877068260129612`},
    {70, 0.002178128236027872`, 3.877068260129612`}, {84, 0.002178128236027872`, 3.877068260129612`}}, 0.009461879459511404`},
{"CURE", 365, 106.99499410963611`, {{0, 0.02954427474805083`, 52.588809051530475`}, {14, 0.001989987700580674`, 3.5421781070336`},
    {28, 0.001989987700580674`, 3.5421781070336`}, {42, 0.001989987700580674`, 3.5421781070336`},
    {56, 0.001989987700580674`, 3.5421781070336`}, {70, 0.001989987700580674`, 3.5421781070336`},
    {84, 0.001989987700580674`, 3.5421781070336`}, {98, 0.001989987700580674`, 3.5421781070336`}}, 0.008737178639677604`},
{"TBUR", 34.636148412132414`, 5.1233813138714375`, {{0, 0.004743358380346655`, 8.443177917017046`},
    {14, 0.0002184936743160902`, 0.38891874028264056`}, {28, 0.0002184936743160902`, 0.38891874028264056`}},
  0.0019135392250607044`}, {"CURE", 365, 88.24632548334847`, {{0, 0.050836228688422996`, 90.48848706539293`},
    {14, 0.003510841553464401`, 6.249297965166633`}, {28, 0.0010535705469357105`, 1.8753555735455647`}}, 0.014595374117927204`},
{"TBUR", 17.94724290066911`, 2.213572433127671`, {{0, 0.005246106934356579`, 9.33807034315471`},
    {14, 0.000254404285316799`, 0.45283962786390225`}}, 0.00205186374492607`}, {"CURE", 365, 73.2246269433121`,
  {{0, 0.056404938879055296`, 100.40079120471843`}, {14, 0.0021567385428015026`, 3.8389946061866747`}}, 0.016127530018217227`},
{"TBUR", 147.77465243427005`, 74.94233565679625`, {{0, 0.014257512652194859`, 25.378372520906847`},
    {14, 0.0008980761223052475`, 1.5985754977033404`}, {28, 0.0008980761223052475`, 1.5985754977033404`},
    {42, 0.0008980761223052475`, 1.5985754977033404`}, {56, 0.0008980761223052475`, 1.5985754977033404`},
    {70, 0.0008980761223052475`, 1.5985754977033404`}, {84, 0.0008980761223052475`, 1.5985754977033404`},
    {98, 0.0008980761223052475`, 1.5985754977033404`}, {112, 0.0008980761223052475`, 1.5985754977033404`},
```

```
{126, 0.0008980761223052475`, 1.5985754977033404`}}, 0.0045312311330367215`},
{"CURE", 365, 23.472671157825918`, {{0, 0.006877095324485355`, 12.241229677583933`},
  {14, 0.0003709034560402831`, 0.6602081517517039`}, {28, 0.0003709034560402831`, 0.6602081517517039`},
  {42, 0.0003709034560402831`, 0.6602081517517039`}, {56, 0.0003709034560402831`, 0.6602081517517039`},
  {70, 0.0003709034560402831`, 0.6602081517517039`}, {84, 0.0003709034560402831`, 0.6602081517517039`},
  {98, 0.0003709034560402831`, 0.6602081517517039`}, {112, 0.0003709034560402831`, 0.6602081517517039`},
  {126, 0.0003709034560402831`, 0.6602081517517039`}}, 0.0025006083194675922`}, {"CURE", 365, 71.06304967693458`,
 {{0, 0.03585136140819831`, 63.81542330659298`}, {14, 0.0024404938905912086`, 4.344079125252351`},
  {28, 0.0024404938905912086`, 4.344079125252351`}, {42, 0.0024404938905912086`, 4.344079125252351`},
  {56, 0.0024404938905912086`, 4.344079125252351`}, {70, 0.0024404938905912086`, 4.344079125252351`}}, 0.010472488919648956`},
{"TBUR", 12.447824695838039`, 1.5637501397356315`, {{0, 0.004578773562390334`, 8.150216941054795`}}, 0.0018682559204358496`},
{"TBUR", 14.735038565033873`, 32.92602317478095`,
 {{0, 0.05910257756485907`, 105.20258806544915`}, {14, 0.0009903942531146739`, 1.7629017705441195`}}, 0.0168697491116543`},
{"CURE", 365, 111.970655734828`, {{0, 0.023839240404272995`, 42.43384791960593`},
  {14, 0.0015824852474536862`, 2.8168237404675613`}, {28, 0.0015824852474536862`, 2.8168237404675613`},
  {42, 0.0015824852474536862`, 2.8168237404675613`}, {56, 0.0015824852474536862`, 2.8168237404675613`},
  {70, 0.0015824852474536862`, 2.8168237404675613`}, {84, 0.0015824852474536862`, 2.8168237404675613`},
  {98, 0.0015824852474536862`, 2.8168237404675613`}, {112, 0.0015824852474536862`, 2.8168237404675613`},
  {126, 0.0015824852474536862`, 2.8168237404675613`}}, 0.007167514977546188`}, {"CURE", 365, 136.5729181786075`,
 {{0, 0.05612078570292702`, 99.8949985512101`}, {14, 0.002279594296656779`, 4.0576778480490665`}}, 0.016049349083945688`},
{"CURE", 365, 89.08095191457147`, {{0, 0.027547204951393945`, 49.034024813481224`},
  {14, 0.001847339857962326`, 3.28826494717294`}, {28, 0.001847339857962326`, 3.28826494717294`},
  {42, 0.001847339857962326`, 3.28826494717294`}, {56, 0.001847339857962326`, 3.28826494717294`},
  {70, 0.001847339857962326`, 3.28826494717294`}, {84, 0.001847339857962326`, 3.28826494717294`},
  {98, 0.001847339857962326`, 3.28826494717294`}, {112, 0.001847339857962326`, 3.28826494717294`}}, 0.008187711677402194`},
{"CURE", 365, 73.41374655863406`, {{0, 0.02483626257166646`, 44.2085473775663`},
  {14, 0.0016537011165532194`, 2.9435879874647304`}, {28, 0.0016537011165532194`, 2.9435879874647304`},
  {42, 0.0016537011165532194`, 2.9435879874647304`}, {56, 0.0016537011165532194`, 2.9435879874647304`},
  {70, 0.0016537011165532194`, 2.9435879874647304`}, {84, 0.0016537011165532194`, 2.9435879874647304`},
  {98, 0.0016537011165532194`, 2.9435879874647304`}, {112, 0.0016537011165532194`, 2.9435879874647304`},
  {126, 0.0016537011165532194`, 2.9435879874647304`}}, 0.007441832251061618`},
```

```
{"TBUR", 213.74525546169093`, 26.55364244095081`, {{0, 0.008246599612581711`, 14.678947310395445`},
   {14, 0.00046872519090430866`, 0.8343308398096694`}, {28, 0.00046872519090430866`, 0.8343308398096694`},
   {42, 0.00046872519090430866`, 0.8343308398096694`}, {56, 0.00046872519090430866`, 0.8343308398096694`},
   {70, 0.00046872519090430866`, 0.8343308398096694`}, {84, 0.00046872519090430866`, 0.8343308398096694`},
   {98, 0.00046872519090430866`, 0.8343308398096694`}, {112, 0.00046872519090430866`, 0.8343308398096694`},
   {126, 0.00046872519090430866`, 0.8343308398096694`}}, 0.002877409051351444`},
{"TBUR", 167.66924914116066`, 23.823617631108984`, {{0, 0.005723508598113519`, 10.187845304642064`},
   {14, 0.0002885044041565805`, 0.5135378393987133`}, {28, 0.0002885044041565805`, 0.5135378393987133`},
   {42, 0.0002885044041565805`, 0.5135378393987133`}, {56, 0.0002885044041565805`, 0.5135378393987133`},
   {70, 0.0002885044041565805`, 0.5135378393987133`}, {84, 0.0002885044041565805`, 0.5135378393987133`},
   {98, 0.0002885044041565805`, 0.5135378393987133`}, {112, 0.0002885044041565805`, 0.5135378393987133`},
   {126, 0.0002885044041565805`, 0.5135378393987133`}}, 0.002183214407986957`},
{"TBUR", 223.5647771391596`, 85.1646762770885`, {{0, 0.016103428383253245`, 28.664102522190774`},
   {14, 0.0010299272459522752`, 1.8332704977950498`}, {28, 0.0010299272459522752`, 1.8332704977950498`},
   {42, 0.0010299272459522752`, 1.8332704977950498`}, {56, 0.0010299272459522752`, 1.8332704977950498`},
   {70, 0.0010299272459522752`, 1.8332704977950498`}, {84, 0.0010299272459522752`, 1.8332704977950498`},
   {98, 0.0010299272459522752`, 1.8332704977950498`}, {112, 0.0010299272459522752`, 1.8332704977950498`},
   {126, 0.0010299272459522752`, 1.8332704977950498`}}, 0.005039110082014338`},
{"CURE", 365, 105.50579177242362`, {{0, 0.02705204260217158`, 48.15263583186541`},
   {14, 0.0018119711187321566`, 3.225308591343239`}, {28, 0.0018119711187321566`, 3.225308591343239`},
   {42, 0.0018119711187321566`, 3.225308591343239`}, {56, 0.0018119711187321566`, 3.225308591343239`},
   {70, 0.0018119711187321566`, 3.225308591343239`}, {84, 0.0018119711187321566`, 3.225308591343239`},
   {98, 0.0018119711187321566`, 3.225308591343239`}, {112, 0.0018119711187321566`, 3.225308591343239`},
   {126, 0.00035191413584262365`, 0.6264071617998701`}}, 0.008051474400009173`},
{"CURE", 365, 12.195295990895348`, {{0, 0.004185850126066275`, 7.45081322439797`},
   {14, 0.0001786716561532059`, 0.3180355479527065`}, {28, 0.0001786716561532059`, 0.3180355479527065`},
   {42, 0.0001786716561532059`, 0.3180355479527065`}, {56, 0.0001786716561532059`, 0.3180355479527065`},
   {70, 0.0001786716561532059`, 0.3180355479527065`}, {84, 0.0001786716561532059`, 0.3180355479527065`},
   {98, 0.0001786716561532059`, 0.3180355479527065`}, {112, 0.0001786716561532059`, 0.3180355479527065`},
   {126, 0.0001786716561532059`, 0.3180355479527065`}}, 0.0017601483083107194`}, {"CURE", 365, 105.53684139768633`,
  {{0, 0.0613536410867627`, 109.20948113443761`}, {14, 0.00001713008015063427`, 0.030491542668128997`}}, 0.017489099039904143`},
```

```
{"CURE", 365, 35.13873281148228`, {{0, 0.006630290263163903`, 11.801916668431746`},
  {14, 0.00035327452308875075`, 0.6288286510979764`}, {28, 0.00035327452308875075`, 0.6288286510979764`},
  {42, 0.00035327452308875075`, 0.6288286510979764`}, {56, 0.00035327452308875075`, 0.6288286510979764`},
  {70, 0.00035327452308875075`, 0.6288286510979764`}, {84, 0.00035327452308875075`, 0.6288286510979764`},
  {98, 0.00035327452308875075`, 0.6288286510979764`}, {112, 0.00035327452308875075`, 0.6288286510979764`},
  {126, 0.00035327452308875075`, 0.6288286510979764`}}, 0.002432703217930576`},
{"CURE", 365, 75.50392752915013`, {{0, 0.020267654117974508`, 36.07642432999462`},
  {14, 0.0013273719412895083`, 2.3627220554953245`}, {28, 0.0013273719412895083`, 2.3627220554953245`},
  {42, 0.0013273719412895083`, 2.3627220554953245`}, {56, 0.0013273719412895083`, 2.3627220554953245`},
  {70, 0.0013273719412895083`, 2.3627220554953245`}, {84, 0.0013273719412895083`, 2.3627220554953245`},
  {98, 0.0013273719412895083`, 2.3627220554953245`}, {112, 0.0013273719412895083`, 2.3627220554953245`},
  {126, 0.0013273719412895083`, 2.3627220554953245`}}, 0.006184840926532984`},
{"TBUR", 13.708773916222343`, 3.106942068212827`, {{0, 0.01203020573419859`, 21.41376620687349`}}, 0.003918417515638311`},
{"CURE", 365, 43.28482487475215`, {{0, 0.008504230495431564`, 15.137530281868184`},
  {14, 0.0004871273968221552`, 0.8670867663434363`}, {28, 0.0004871273968221552`, 0.8670867663434363`},
  {42, 0.0004871273968221552`, 0.8670867663434363`}, {56, 0.0004871273968221552`, 0.8670867663434363`},
  {70, 0.0004871273968221552`, 0.8670867663434363`}, {84, 0.0004871273968221552`, 0.8670867663434363`},
  {98, 0.0004871273968221552`, 0.8670867663434363`}, {112, 0.0004871273968221552`, 0.8670867663434363`},
  {126, 0.0004871273968221552`, 0.8670867663434363`}}, 0.002948292732445021`},
{"CURE", 365, 82.69516400025365`, {{0, 0.01485430251407242`, 26.440658475048906`},
  {14, 0.0009407039695822161`, 1.6744530658563448`}, {28, 0.0009407039695822161`, 1.6744530658563448`},
  {42, 0.0009407039695822161`, 1.6744530658563448`}, {56, 0.0009407039695822161`, 1.6744530658563448`},
  {70, 0.0009407039695822161`, 1.6744530658563448`}, {84, 0.0009407039695822161`, 1.6744530658563448`},
  {98, 0.0009407039695822161`, 1.6744530658563448`}, {112, 0.0009407039695822161`, 1.6744530658563448`},
  {126, 0.0009407039695822161`, 1.6744530658563448`}}, 0.004695429857123161`},
{"CURE", 365, 50.323619238327176`, {{0, 0.015906487816184502`, 28.313548312808415`},
  {14, 0.0010158600625902224`, 1.8082309114105959`}, {28, 0.0010158600625902224`, 1.8082309114105959`},
  {42, 0.0010158600625902224`, 1.8082309114105959`}, {56, 0.0010158600625902224`, 1.8082309114105959`},
  {70, 0.0010158600625902224`, 1.8082309114105959`}, {84, 0.0010158600625902224`, 1.8082309114105959`},
  {98, 0.0010158600625902224`, 1.8082309114105959`}, {112, 0.0010158600625902224`, 1.8082309114105959`},
  {126, 0.0010158600625902224`, 1.8082309114105959`}}, 0.004984924527099227`}, {"CURE", 365, 130.4217696072433`,
```

```
{{0, 0.044672559561133016`, 79.51715601881676`}, {14, 0.003070579472943688`, 5.465631461839765`},
  {28, 0.003070579472943688`, 5.465631461839765`}, {42, 0.0010881616643014132`, 1.9369277624565155`}}, 0.012899523248061682`},
{"CURE", 365, 77.91945645651958`, {{0, 0.04310462044792056`, 76.7262243972986`}, {14, 0.002958583821999941`, 5.266279203159895`},
  {28, 0.002958583821999941`, 5.266279203159895`}, {42, 0.002958583821999941`, 5.266279203159895`}}, 0.012468125836207641`},
{"CURE", 365, 163.99220808489045`, {{0, 0.025365161662147816`, 45.14998775862311`},
  {14, 0.0016914796230161734`, 3.0108337289687888`}, {28, 0.0016914796230161734`, 3.0108337289687888`},
  {42, 0.0016914796230161734`, 3.0108337289687888`}, {56, 0.0016914796230161734`, 3.0108337289687888`},
  {70, 0.0016914796230161734`, 3.0108337289687888`}, {84, 0.0016914796230161734`, 3.0108337289687888`},
  {98, 0.0016914796230161734`, 3.0108337289687888`}, {112, 0.0016914796230161734`, 3.0108337289687888`},
  {126, 0.0016914796230161734`, 3.0108337289687888`}}, 0.007587351740206919`},
{"CURE", 365, 58.167358840543955`, {{0, 0.02281513943500205`, 40.610948194303646`},
  {14, 0.0015093351782200473`, 2.6866166172316843`}, {28, 0.0015093351782200473`, 2.6866166172316843`},
  {42, 0.0015093351782200473`, 2.6866166172316843`}, {56, 0.0015093351782200473`, 2.6866166172316843`},
  {70, 0.0015093351782200473`, 2.6866166172316843`}, {84, 0.0015093351782200473`, 2.6866166172316843`},
  {98, 0.0015093351782200473`, 2.6866166172316843`}, {112, 0.0015093351782200473`, 2.6866166172316843`},
  {126, 0.0015093351782200473`, 2.6866166172316843`}}, 0.006885747334977766`},
{"CURE", 365, 92.90365788311222`, {{0, 0.03149484089030112`, 56.060816784736`}, {14, 0.002129313853598552`, 3.7901786594054223`},
  {28, 0.002129313853598552`, 3.7901786594054223`}, {42, 0.002129313853598552`, 3.7901786594054223`},
  {56, 0.002129313853598552`, 3.7901786594054223`}, {70, 0.002129313853598552`, 3.7901786594054223`},
  {84, 0.002129313853598552`, 3.7901786594054223`}, {98, 0.0001509228500385807`, 0.2686426730686737`}},
 0.009273850745326434`}, {"TBUR", 39.02379642905142`, 8.144415763594708`, {{0, 0.020858465988658124`, 37.128069459811464`},
  {14, 0.001369572789195481`, 2.4378395647679563`}, {28, 0.001369572789195481`, 2.4378395647679563`}}, 0.006347394886541901`},
{"CURE", 365, 83.42900501840758`, {{0, 0.04013691261759356`, 71.44370445931655`}, {14, 0.002746604691262299`, 4.888956350446892`},
  {28, 0.002746604691262299`, 4.888956350446892`}, {42, 0.002746604691262299`, 4.888956350446892`},
  {56, 0.0009505274620254175`, 1.6919388824052433`}}, 0.011651600840846712`},
{"CURE", 365, 85.48648580033532`, {{0, 0.032487428988973756`, 57.827623600373286`},
  {14, 0.00220021300350374`, 3.9163791462366575`}, {28, 0.00220021300350374`, 3.9163791462366575`},
  {42, 0.00220021300350374`, 3.9163791462366575`}, {56, 0.00220021300350374`, 3.9163791462366575`},
  {70, 0.00220021300350374`, 3.9163791462366575`}, {84, 0.00220021300350374`, 3.9163791462366575`}}, 0.009546948044319877`},
{"CURE", 365, 51.69370730250739`, {{0, 0.0626875584741077`, 111.5838540839117`}}, 0.0175`},
{"CURE", 365, 50.78057251047113`, {{0, 0.009483882824429213`, 16.881311427484`},
```

```
{14, 0.0005571025631791301`, 0.9916425624588516`}, {28, 0.0005571025631791301`, 0.9916425624588516`},
{42, 0.0005571025631791301`, 0.9916425624588516`}, {56, 0.0005571025631791301`, 0.9916425624588516`},
{70, 0.0005571025631791301`, 0.9916425624588516`}, {84, 0.0005571025631791301`, 0.9916425624588516`},
{98, 0.0005571025631791301`, 0.9916425624588516`}, {112, 0.0005571025631791301`, 0.9916425624588516`},
{126, 0.0005571025631791301`, 0.9916425624588516`}}, 0.0032178309279559973`},
{"CURE", 365, 67.88468788810387`, {{0, 0.06846437002428478`, 121.8665786432269`}}, 0.0175`},
{"TBUR", 15.60925161276212`, 2.721388614923584`,
{{0, 0.004039228872090284`, 7.189827392320706`}, {14, 0.00016819870944063516`, 0.2993937028043306`}}, 0.0017198074373195983`},
{"CURE", 365, 26.862809278636686`, {{0, 0.006161146072359546`, 10.966840008799991`},
{14, 0.00031976422374558233`, 0.5691803182671366`}, {28, 0.00031976422374558233`, 0.5691803182671366`},
{42, 0.00031976422374558233`, 0.5691803182671366`}, {56, 0.00031976422374558233`, 0.5691803182671366`},
{70, 0.00031976422374558233`, 0.5691803182671366`}, {84, 0.00031976422374558233`, 0.5691803182671366`},
{98, 0.00031976422374558233`, 0.5691803182671366`}, {112, 0.00031976422374558233`, 0.5691803182671366`},
{126, 0.00031976422374558233`, 0.5691803182671366`}}, 0.002303624487772046`},
{"TBUR", 138.1398289193181`, 52.26819711914506`, {{0, 0.021163197812971223`, 37.670492107088776`},
{14, 0.001391339348074988`, 2.4765840395734786`}, {28, 0.001391339348074988`, 2.4765840395734786`},
{42, 0.001391339348074988`, 2.4765840395734786`}, {56, 0.001391339348074988`, 2.4765840395734786`},
{70, 0.001391339348074988`, 2.4765840395734786`}, {84, 0.001391339348074988`, 2.4765840395734786`},
{98, 0.001391339348074988`, 2.4765840395734786`}, {112, 0.001391339348074988`, 2.4765840395734786`},
{126, 0.001391339348074988`, 2.4765840395734786`}}, 0.006431237759782615`}, {"CURE", 365, 95.1443864429122`,
{{0, 0.05283973133277016`, 94.05472177233088`}, {14, 0.0036539488852034836`, 6.5040290156622005`},
{28, 0.000044233841743476 86`, 0.07873623830338881`}}, 0.015146610991942845`},
{"CURE", 365, 37.944303135384665`, {{0, 0.012158627183409827`, 21.642356386469494`},
{14, 0.0007481557316777454`, 1.3317172023863868`}, {28, 0.0007481557316777454`, 1.3317172023863868`},
{42, 0.0007481557316777454`, 1.3317172023863868`}, {56, 0.0007481557316777454`, 1.3317172023863868`},
{70, 0.0007481557316777454`, 1.3317172023863868`}, {84, 0.0007481557316777454`, 1.3317172023863868`},
{98, 0.0007481557316777454`, 1.3317172023863868`}, {112, 0.0007481557316777454`, 1.3317172023863868`},
{126, 0.0007481557316777454`, 1.3317172023863868`}}, 0.003953750954513221`},
{"CURE", 365, 96.0261024973797`, {{0, 0.023134170878899667`, 41.17882416444141`},
{14, 0.001532123138498 4486`, 2.7271791865272386`}, {28, 0.0015321231384984486`, 2.7271791865272386`},
{42, 0.0015321231384984486`, 2.7271791865272386`}, {56, 0.0015321231384984486`, 2.7271791865272386`},
```

```
{70, 0.0015321231384984486`, 2.7271791865272386`}, {84, 0.0015321231384984486`, 2.7271791865272386`},
  {98, 0.0015321231384984486`, 2.7271791865272386`}, {112, 0.0015321231384984486`, 2.7271791865272386`},
  {126, 0.0015321231384984486`, 2.7271791865272386`}}, 0.006973524556706427`},
{"CURE", 365, 98.9404640997319`, {{0, 0.04676533444007161`, 83.24229530332747`}, {14, 0.0032200633928678732`, 5.731712839304814`},
  {28, 0.0032200633928678732`, 5.731712839304814`}}, 0.013475322179767341`},
{"CURE", 365, 11.448406852026986`, {{0, 0.003423997130210264`, 6.094714891774269`},
  {14, 0.00012425358502063364`, 0.22117138133672787`}, {28, 0.00012425358502063364`, 0.22117138133672787`},
  {42, 0.00012425358502063364`, 0.22117138133672787`}, {56, 0.00012425358502063364`, 0.22117138133672787`},
  {70, 0.00012425358502063364`, 0.22117138133672787`}, {84, 0.00012425358502063364`, 0.22117138133672787`},
  {98, 0.00012425358502063364`, 0.22117138133672787`}, {112, 0.00012425358502063364`, 0.22117138133672787`},
  {126, 0.00012425358502063364`, 0.22117138133672787`}}, 0.0015505346773629254`},
{"CURE", 365, 116.37163205405669`, {{0, 0.017552604037204787`, 31.24363518622452`},
  {14, 0.0011334397926630998`, 2.017522830940318`}, {28, 0.0011334397926630998`, 2.017522830940318`},
  {42, 0.0011334397926630998`, 2.017522830940318`}, {56, 0.0011334397926630998`, 2.017522830940318`},
  {70, 0.0011334397926630998`, 2.017522830940318`}, {84, 0.0011334397926630998`, 2.017522830940318`},
  {98, 0.0011334397926630998`, 2.017522830940318`}, {112, 0.0011334397926630998`, 2.017522830940318`},
  {126, 0.0011334397926630998`, 2.017522830940318`}}, 0.005437831321358703`}, {"CURE", 365, 67.18772270646657`,
 {{0, 0.029067872411461342`, 51.74081289240119`}, {14, 0.001955958962252854`, 3.4816069528100804`},
  {28, 0.001955958962252854`, 3.4816069528100804`}, {42, 0.001955958962252854`, 3.4816069528100804`},
  {56, 0.001955958962252854`, 3.4816069528100804`}, {70, 0.001955958962252854`, 3.4816069528100804`},
  {84, 0.001955958962252854`, 3.4816069528100804`}, {98, 0.001955958962252854`, 3.4816069528100804`},
  {112, 0.00028441123438990856`, 0.5062519972140372`}}, 0.008606102928080075`},
{"TBUR", 55.5464108120954`, 5.487126062005967`, {{0, 0.00442798896265064`, 7.881820353518139`},
  {14, 0.00019596728733780342`, 0.34882177146129006`}, {28, 0.00019596728733780342`, 0.34882177146129006`},
  {42, 0.00019596728733780342`, 0.34882177146129006`}}, 0.0018267695607124303`},
{"CURE", 365, 81.25493319940026`, {{0, 0.02746825034688609`, 48.89348561745724`},
  {14, 0.001841700243354621`, 3.2782264331712256`}, {28, 0.001841700243354621`, 3.2782264331712256`},
  {42, 0.001841700243354621`, 3.2782264331712256`}, {56, 0.001841700243354621`, 3.2782264331712256`},
  {70, 0.001841700243354621`, 3.2782264331712256`}, {84, 0.001841700243354621`, 3.2782264331712256`},
  {98, 0.001841700243354621`, 3.2782264331712256`}, {112, 0.001841700243354621`, 3.2782264331712256`}},
 0.00816598837721048`}, {"CURE", 365, 64.6705573448618`, {{0, 0.024417690248963696`, 43.46348864315538`},
```

```
{14, 0.0016238030935030218`, 2.8903695064353787`}, {28, 0.0016238030935030218`, 2.8903695064353787`},
{42, 0.0016238030935030218`, 2.8903695064353787`}, {56, 0.0016238030935030218`, 2.8903695064353787`},
{70, 0.0016238030935030218`, 2.8903695064353787`}, {84, 0.0016238030935030218`, 2.8903695064353787`},
{98, 0.0016238030935030218`, 2.8903695064353787`}, {112, 0.0016238030935030218`, 2.8903695064353787`},
{126, 0.0016238030935030218`, 2.8903695064353787`}}, 0.007326667691949535`},
{"CURE", 365, 68.71852704432007`, {{0, 0.03356944205068881`, 59.753606850226085`},
{14, 0.002277499650769102`, 4.053949378369001`}, {28, 0.002277499650769102`, 4.053949378369001`},
{42, 0.002277499650769102`, 4.053949378369001`}, {56, 0.002277499650769102`, 4.053949378369001`},
{70, 0.002277499650769102`, 4.053949378369001`}, {84, 0.0006423383684310332`, 1.1433622958072391`}}, 0.009844649422187657`},
{"TBUR", 18.71657102066627`, 2.4121768248409574`, {{0, 0.00297882728837028`, 5.3023125732990986`},
{14, 0.00009245573917492054`, 0.16457121573135855`}}, 0.0014280521676870424`}, {"CURE", 365, 71.16578704829334`,
{{0, 0.03134769062726605`, 55.79888931653357`}, {14, 0.0021188031205246183`, 3.771469554533821`},
{28, 0.0021188031205246183`, 3.771469554533821`}, {42, 0.0021188031205246183`, 3.771469554533821`},
{56, 0.0021188031205246183`, 3.771469554533821`}, {70, 0.0021188031205246183`, 3.771469554533821`},
{84, 0.0021188031205246183`, 3.771469554533821`}, {98, 0.000277608766782636`, 0.4941436048730921`}}, 0.009233364324589783`},
{"TBUR", 13.74017351381077`, 1.8555681044118768`, {{0, 0.004745486599403267`, 8.446966146937815`}}, 0.0019141247759823852`},
{"CURE", 365, 81.75283366766742`, {{0, 0.03692799977697408`, 65.73183960301385`}, {14, 0.002517396631218049`, 4.480966003568128`},
{28, 0.002517396631218049`, 4.480966003568128`}, {42, 0.002517396631218049`, 4.480966003568128`},
{56, 0.002517396631218049`, 4.480966003568128`}, {70, 0.00050815251063311`, 0.9045114689269358`}}, 0.010768711522860262`},
{"CURE", 365, 50.25961115380377`, {{0, 0.015159723974705734`, 26.98430867497621`},
{14, 0.0009625197881988816`, 1.7132852229940092`}, {28, 0.0009625197881988816`, 1.7132852229940092`},
{42, 0.0009625197881988816`, 1.7132852229940092`}, {56, 0.0009625197881988816`, 1.7132852229940092`},
{70, 0.0009625197881988816`, 1.7132852229940092`}, {84, 0.0009625197881988816`, 1.7132852229940092`},
{98, 0.0009625197881988816`, 1.7132852229940092`}, {112, 0.0009625197881988816`, 1.7132852229940092`},
{126, 0.0009625197881988816`, 1.7132852229940092`}}, 0.004779462474545364`},
{"CURE", 365, 48.56590876622347`, {{0, 0.018191525523039476`, 32.380915431010266`},
{14, 0.0011790770416512917`, 2.098757134139299`}, {28, 0.0011790770416512917`, 2.098757134139299`},
{42, 0.0011790770416512917`, 2.098757134139299`}, {56, 0.0011790770416512917`, 2.098757134139299`},
{70, 0.0011790770416512917`, 2.098757134139299`}, {84, 0.0011790770416512917`, 2.098757134139299`},
{98, 0.0011790770416512917`, 2.098757134139299`}, {112, 0.0011790770416512917`, 2.098757134139299`},
{126, 0.0011790770416512917`, 2.098757134139299`}}, 0.005613621996547806`},
```

```
{"CURE", 365, 14.88376048923372`, {{0, 0.005412697255753424`, 9.634601115241095`},
   {14, 0.00026630359398800225`, 0.474020397298644`}, {28, 0.00026630359398800225`, 0.474020397298644`},
   {42, 0.00026630359398800225`, 0.474020397298644`}, {56, 0.00026630359398800225`, 0.474020397298644`},
   {70, 0.00026630359398800225`, 0.474020397298644`}, {84, 0.00026630359398800225`, 0.474020397298644`},
   {98, 0.00026630359398800225`, 0.474020397298644`}, {112, 0.00026630359398800225`, 0.474020397298644`},
   {126, 0.00026630359398800225`, 0.474020397298644`}}, 0.002097698836917167`},
{"TBUR", 32.64210981128178`, 24.317191978990575`, {{0, 0.06177507185817554`, 109.95962790755246`}}, 0.0175`},
{"CURE", 365, 33.517421571640945`, {{0, 0.012236803201132834`, 21.781509698016443`},
   {14, 0.0007537397329436744`, 1.3416567246397404`}, {28, 0.0007537397329436744`, 1.3416567246397404`},
   {42, 0.0007537397329436744`, 1.3416567246397404`}, {56, 0.0007537397329436744`, 1.3416567246397404`},
   {70, 0.0007537397329436744`, 1.3416567246397404`}, {84, 0.0007537397329436744`, 1.3416567246397404`},
   {98, 0.0007537397329436744`, 1.3416567246397404`}, {112, 0.0007537397329436744`, 1.3416567246397404`},
   {126, 0.0007537397329436744`, 1.3416567246397404`}}, 0.003975260036996445`}, {"CURE", 365, 60.38062749148225`,
  {{0, 0.043067565122702434`, 76.66026591841033`}, {14, 0.0029559370130557896`, 5.261567883239305`},
   {28, 0.0029559370130557896`, 5.261567883239305`}, {42, 0.0029559370130557896`, 5.261567883239305`}}, 0.012457930560600444`},
{"CURE", 365, 72.26509846683622`, {{0, 0.025531728481556278`, 45.446476697170176`},
   {14, 0.001703377252973920 7`, 3.0320115102935787`}, {28, 0.0017033772529739207`, 3.0320115102935787`},
   {42, 0.0017033772529739207`, 3.0320115102935787`}, {56, 0.0017033772529739207`, 3.0320115102935787`},
   {70, 0.0017033772529739207`, 3.0320115102935787`}, {84, 0.0017033772529739207`, 3.0320115102935787`},
   {98, 0.0017033772529739207`, 3.0320115102935787`}, {112, 0.0017033772529739207`, 3.0320115102935787`},
   {126, 0.0017033772529739207`, 3.0320115102935787`}}, 0.007633180365941208`},
{"CURE", 365, 49.11429712597165`, {{0, 0.01029806818749142`, 18.330561373734728`},
   {14, 0.0006152586605407165`, 1.0951604157624752`}, {28, 0.0006152586605407165`, 1.0951604157624752`},
   {42, 0.0006152586605407165`, 1.0951604157624752`}, {56, 0.0006152586605407165`, 1.0951604157624752`},
   {70, 0.0006152586605407165`, 1.0951604157624752`}, {84, 0.0006152586605407165`, 1.0951604157624752`},
   {98, 0.0006152586605407165`, 1.0951604157624752`}, {112, 0.0006152586605407165`, 1.0951604157624752`},
   {126, 0.0006152586605407165`, 1.0951604157624752`}}, 0.0034418431076603767`},
{"CURE", 365, 59.5188871564592`, {{0, 0.06199629702565542`, 110.35340870566665`}}, 0.0175`},
{"CURE", 365, 53.77067602781945`,
  {{0, 0.05545944888974011`, 98.71781902373739`}, {14, 0.0025655282208992772`, 4.566640233200713`}}, 0.015867391132155007`},
{"CURE", 365, 77.16067264551775`, {{0, 0.04351413285811783`, 77.45515648744974`}, {14, 0.002987834708442603`, 5.318345781027833`},
```

```
{28, 0.002987834708442603`, 5.318345781027833`}, {42, 0.002987834708442603`, 5.318345781027833`}}, 0.012580797681939752`},
{"CURE", 365, 68.01768603082772`, {{0, 0.05621275359608591`, 100.05870140103292`},
{14, 0.002239831289258908`, 3.986899694880856`}}, 0.01607465281592615`},
{"CURE", 365, 40.645336939876636`, {{0, 0.06672056652053904`, 118.76260840655948`}}, 0.017500000000000001`},
{"TBUR", 48.45963858567536`, 5.704436854817407`, {{0, 0.009439114850657604`, 16.801624434170535`},
{14, 0.0005539048507668725`, 0.9859506343650329`}, {28, 0.0005539048507668725`, 0.9859506343650329`},
{42, 0.0005539048507668725`, 0.9859506343650329`}}, 0.003205513620570601`},
{"CURE", 365, 77.622641298203`, {{0, 0.022841598897085837`, 40.65804603681279`}, {14, 0.0015112251397974607`, 2.68998074883948`},
{28, 0.0015112251397974607`, 2.68998074883948`}, {42, 0.0015112251397974607`, 2.68998074883948`},
{56, 0.0015112251397974607`, 2.68998074883948`}, {70, 0.0015112251397974607`, 2.68998074883948`},
{84, 0.0015112251397974607`, 2.68998074883948`}, {98, 0.0015112251397974607`, 2.68998074883948`},
{112, 0.0015112251397974607`, 2.68998074883948`}, {126, 0.0015112251397974607`, 2.68998074883948`}}, 0.006893027300995458`},
{"TBUR", 31.742797307745956`, 32.29875278039446`, {{0, 0.04858782283433005`, 86.48632464510749`},
{14, 0.003350241135314905`, 5.963429220860531`}, {28, 0.003350241135314905`, 5.963429220860531`}}, 0.01397675541132075`},
{"TBUR", 41.11763611550462`, 28.77416432654785`, {{0, 0.038369088150131014`, 68.2969769072332`},
{14, 0.0026203315150149734`, 4.664190096726653`}, {28, 0.0026203315150149734`, 4.664190096726653`}}, 0.011165207655391272`},
{"CURE", 365, 63.90227215005293`, {{0, 0.036479530822040286`, 64.93356486323171`},
{14, 0.0024853631344370635`, 4.4239463792979725`}, {28, 0.0024853631344370635`, 4.4239463792979725`},
{42, 0.0024853631344370635`, 4.4239463792979725`}, {56, 0.0024853631344370635`, 4.4239463792979725`},
{70, 0.0008301854092167184`, 1.4777300284057588`}}, 0.010645321306476838`}, {"CURE", 365, 80.79437809128548`,
{{0, 0.037388027028500845`, 66.5506881107315`}, {14, 0.002550255720612818 5`, 4.539455182690817`},
{28, 0.0025502557206128185`, 4.539455182690817`}, {42, 0.0025502557206128185`, 4.539455182690817`},
{56, 0.0025502557206128185`, 4.539455182690817`}, {70, 0.00017781992549689707`, 0.3165194673844768`}},
0.01089528184948753`}, {"TBUR", 17.776167900893515`, 3.7272816222760663`,
{{0, 0.005122577800296626`, 9.118188484527995`}, {14, 0.0002455807757410882`, 0.43713378081913695`}}, 0.0020178763609307497`},
{"CURE", 365, 98.90873365379954`, {{0, 0.04766527701351741`, 84.84419308406099`},
{14, 0.0032843450052568585`, 5.846134109357208`}, {28, 0.0032843450052568585`, 5.846134109357208`}}, 0.013722929305407795`},
{"TBUR", 23.99145415067894`, 3.3620007584127403`, {{0, 0.013263793060405534`, 23.60955164752185`},
{14, 0.000827096151463153`, 1.4722311496044125`}}, 0.004257822518892099`},
{"CURE", 365, 69.79838656905744`, {{0, 0.040974669729416414`, 72.93491211836121`}, {14, 0.00280644448496393`, 4.995471183235796`},
{28, 0.00280644448496393`, 4.995471183235796`}, {42, 0.00280644448496393`, 4.995471183235796`},
```

```
{56, 0.00040879751926853336`, 0.7276595842979894`}}, 0.011882098470988887`},
{"CURE", 365, 94.77798445986025`, {{0, 0.03961303971802943`, 70.51121069809238`},
  {14, 0.0027091851984362885`, 4.822349653216594`}, {28, 0.0027091851984362885`, 4.822349653216594`},
  {42, 0.0027091851984362885`, 4.822349653216594`}, {56, 0.0012892863121594968`, 2.2949296356439044`}},
 0.011507464240701953`}, {"TBUR", 14.641226202897524`, 2.333708546672937`,
 {{0, 0.005436618971708473`, 9.677181769641082`}, {14, 0.00026801228798479154`, 0.47706187261292893`}}, 0.002104280576133412`},
{"TBUR", 178.95605480530125`, 34.64768860584625`, {{0, 0.013161576522177013`, 23.427606209475083`},
  {14, 0.000819794970161159`, 1.4592350468867863`}, {28, 0.000819794970161159`, 1.4592350468867863`},
  {42, 0.000819794970161159`, 1.4592350468867863`}, {56, 0.000819794970161159`, 1.4592350468867863`},
  {70, 0.000819794970161159`, 1.4592350468867863`}, {84, 0.000819794970161159`, 1.4592350468867863`},
  {98, 0.000819794970161159`, 1.4592350468867863`}, {112, 0.000819794970161159`, 1.4592350468867863`},
  {126, 0.000819794970161159`, 1.4592350468867863`}}, 0.004229699009714425`},
{"TBUR", 22.737683661792715`, 7.488201430194446`, {{0, 0.05082065071962163`, 90.4607582809265`},
  {14, 0.0035097288414071594`, 6.247317337704744`}}, 0.014591088048802315`},
{"CURE", 365, 87.08170430269914`, {{0, 0.03588534500384879`, 63.87591410685084`}, {14, 0.002442921290280528`, 4.34839989669934`},
  {28, 0.002442921290280528`, 4.34839989669934`}, {42, 0.002442921290280528`, 4.34839989669934`},
  {56, 0.002442921290280528`, 4.34839989669934`}, {70, 0.002442921290280528`, 4.34839989669934`}}, 0.010481839050075306`},
{"CURE", 365, 124.62765277550345`, {{0, 0.017215848144351693`, 30.644209696946014`},
  {14, 0.0011093858003164499`, 1.9747067245632808`}, {28, 0.0011093858003164499`, 1.9747067245632808`},
  {42, 0.0011093858003164499`, 1.9747067245632808`}, {56, 0.0011093858003164499`, 1.9747067245632808`},
  {70, 0.0011093858003164499`, 1.9747067245632808`}, {84, 0.0011093858003164499`, 1.9747067245632808`},
  {98, 0.0011093858003164499`, 1.9747067245632808`}, {112, 0.0011093858003164499`, 1.9747067245632808`},
  {126, 0.0011093858003164499`, 1.9747067245632808`}}, 0.005345177455287481`},
{"CURE", 365, 11.879225717446761`, {{0, 0.004191990817065171`, 7.461743654376005`},
  {14, 0.00017911027693884136`, 0.3188162929511376`}, {28, 0.00017911027693884136`, 0.3188162929511376`},
  {42, 0.00017911027693884136`, 0.3188162929511376`}, {56, 0.00017911027693884136`, 0.3188162929511376`},
  {70, 0.00017911027693884136`, 0.3188162929511376`}, {84, 0.00017911027693884136`, 0.3188162929511376`},
  {98, 0.00017911027693884136`, 0.3188162929511376`}, {112, 0.00017911027693884136`, 0.3188162929511376`},
  {126, 0.00017911027693884136`, 0.3188162929511376`}}, 0.0017618378370568269`},
{"CURE", 365, 139.12824535187252`, {{0, 0.061770964138018254`, 109.9523161656725`}}, 0.0175`},
{"TBUR", 12.149819729383534`, 1.9394456863043361`, {{0, 0.0036933078874932025`, 6.574088039737901`}}, 0.0016246319192119892`},
```

{"TBUR", 49.96548421190675`, 12.696066365657185`,
{{0, 0.026922122662323186`, 47.92137833893527`}, {14, 0.0018026911230286998`, 3.2087901989910854`},
{28, 0.0018026911230286998`, 3.2087901989910854`}, {42, 0.0018026911230286998`, 3.2087901989910854`}},
0.00801572867153889`}, {"TBUR", 41.72153142217946`, 4.24999782110559`,
{{0, 0.004048946710594861`, 7.2071251448588525`}, {14, 0.00016889284076239067`, 0.30062925655705536`},
{28, 0.00016889284076239067`, 0.30062925655705536`}}, 0.001722481170211624`}, {"CURE", 365, 54.6479664104486`,
{{0, 0.06046769306130407`, 107.63249364912124`}, {14, 0.00040017634423737187`, 0.7123138927425219`}}, 0.0172453423263944`},
{"TBUR", 42.53163481618853`, 4.575729746366986`, {{0, 0.005845181152972383 5`, 10.404422452290843`},
{14, 0.0002971953009322137`, 0.5290076356593404`}, {28, 0.0002971953009322137`, 0.5290076356593404`},
{42, 0.0002971953009322137`, 0.5290076356593404`}}, 0.0022166909791225798`},
{"TBUR", 22.22307076420394`, 4.911501283511918`, {{0, 0.011537070554771822`, 20.535985587493844`},
{14, 0.0007037588296321736`, 1.252690716745269`}}, 0.003782737986818459`}, {"CURE", 365, 24.348269629825435`,
{{0, 0.005974429718327161`, 10.634484898622345`}, {14, 0.0003064273413146977`, 0.545440667540162`},
{28, 0.0003064273413146977`, 0.545440667540162`}, {42, 0.0003064273413146977`, 0.545440667540162`},
{56, 0.0003064273413146977`, 0.545440667540162`}, {70, 0.0003064273413146977`, 0.545440667540162`},
{84, 0.0003064273413146977`, 0.545440667540162`}, {98, 0.0003064273413146977`, 0.545440667540162`},
{112, 0.0003064273413146977`, 0.545440667540162`}, {126, 0.0003064273413146977`, 0.545440667540162`}},
0.0022522519879079658`}, {"TBUR", 26.21485376393986`, 5.6825094535733705`,
{{0, 0.0067668650095189424`, 12.045019716943717`}, {14, 0.00036302986211411074`, 0.6461931545631171`}},
0.00247027992713165 7`}, {"TBUR", 15.830556425363437`, 2.4184258147165894`,
{{0, 0.012814100602445195`, 22.809099072352446`}, {14, 0.0007949752616088432`, 1.415055965663741`}}, 0.004134095672065182`},
{"CURE", 365, 52.27057101619141`, {{0, 0.011546774929125755`, 20.553259373843844`},
{14, 0.0007044519992288829`, 1.2539245586274117`}, {28, 0.0007044519992288829`, 1.2539245586274117`},
{42, 0.0007044519992288829`, 1.2539245586274117`}, {56, 0.0007044519992288829`, 1.2539245586274117`},
{70, 0.0007044519992288829`, 1.2539245586274117`}, {84, 0.0007044519992288829`, 1.2539245586274117`},
{98, 0.0007044519992288829`, 1.2539245586274117`}, {112, 0.0007044519992288829`, 1.2539245586274117`},
{126, 0.0007044519992288829`, 1.2539245586274117`}}, 0.0037854080152300677`},
{"CURE", 365, 128.2178119617213`, {{0, 0.01873488956787081`, 33.34810343081004`},
{14, 0.0012178887591392444`, 2.167841991267855`}, {28, 0.0012178887591392444`, 2.167841991267855`},
{42, 0.0012178887591392444`, 2.167841991267855`}, {56, 0.0012178887591392444`, 2.167841991267855`},
{70, 0.0012178887591392444`, 2.167841991267855`}, {84, 0.0012178887591392444`, 2.167841991267855`},

{98, 0.0012178887591392444`, 2.167841991267855`}, {112, 0.0012178887591392444`, 2.167841991267855`},
{126, 0.0012178887591392444`, 2.167841991267855`}}, 0.00576312132382367`}, {"CURE", 365, 47.0644455485403`,
{{0, 0.011044550481195846`, 19.659299856528605`}, {14, 0.0006685788243767466`, 1.190070307390609`},
{28, 0.0006685788243767466`, 1.190070307390609`}, {42, 0.0006685788243767466`, 1.190070307390609`},
{56, 0.0006685788243767466`, 1.190070307390609`}, {70, 0.0006685788243767466`, 1.190070307390609`},
{84, 0.0006685788243767466`, 1.190070307390609`}, {98, 0.0006685788243767466`, 1.190070307390609`},
{112, 0.0006685788243767466`, 1.190070307390609`}, {126, 0.0006685788243767466`, 1.190070307390609`}},
0.003647227696121319`}, {"TBUR", 39.39204136880601`, 8.54269001621025`,
{{0, 0.0176583019387295`, 31.43177745093851`}, {14, 0.0011409896427720082`, 2.0309615641341745`},
{28, 0.0011409896427720082`, 2.0309615641341745`}}, 0.005466912680942073 5`}, {"CURE", 365, 87.05910842593168`,
{{0, 0.020349127660856142`, 36.22144723632393`}, {14, 0.001333191480066768`, 2.373080834518847`},
{28, 0.001333191480066768`, 2.373080834518847`}, {42, 0.001333191480066768`, 2.373080834518847`},
{56, 0.001333191480066768`, 2.373080834518847`}, {70, 0.001333191480066768`, 2.373080834518847`},
{84, 0.001333191480066768`, 2.373080834518847`}, {98, 0.001333191480066768`, 2.373080834518847`},
{112, 0.001333191480066768`, 2.373080834518847`}, {126, 0.001333191480066768`, 2.373080834518847`}}, 0.006207257278824691`},
{"CURE", 365, 41.26394783084124`, {{0, 0.014825431691670277`, 26.38926841117309`},
{14, 0.0009386417679820634`, 1.670782347008073`}, {28, 0.0009386417679820634`, 1.670782347008073`},
{42, 0.0009386417679820634`, 1.670782347008073`}, {56, 0.0009386417679820634`, 1.670782347008073`},
{70, 0.0009386417679820634`, 1.670782347008073`}, {84, 0.0009386417679820634`, 1.670782347008073`},
{98, 0.0009386417679820634`, 1.670782347008073`}, {112, 0.0009386417679820634`, 1.670782347008073`},
{126, 0.0009386417679820634`, 1.670782347008073`}}, 0.004687486437664183`},
{"CURE", 365, 41.80350626921954`, {{0, 0.011774081518238756`, 20.957865102464986`},
{14, 0.000720688184165526`, 1.2828249678146362`}, {28, 0.000720688184165526`, 1.2828249678146362`},
{42, 0.000720688184165526`, 1.2828249678146362`}, {56, 0.000720688184165526`, 1.2828249678146362`},
{70, 0.000720688184165526`, 1.2828249678146362`}, {84, 0.000720688184165526`, 1.2828249678146362`},
{98, 0.000720688184165526`, 1.2828249678146362`}, {112, 0.000720688184165526`, 1.2828249678146362`},
{126, 0.000720688184165526`, 1.2828249678146362`}}, 0.0038479483737263926`},
{"CURE", 365, 50.01844329188789`, {{0, 0.051097788740260115`, 90.95406395766301`},
{14, 0.0035295244143099093`, 6.282553457471638`}, {28, 0.000921800238732692`, 1.6408044249441918`}}, 0.014667338857154 71`},
{"CURE", 365, 42.24013658098119`, {{0, 0.014951251898180992`, 26.613228378762166`},
{14, 0.0009476289255899716`, 1.6867794875501494`}, {28, 0.0009476289255899716`, 1.6867794875501494`},

```
{42, 0.0009476289255899716`, 1.6867794875501494`}, {56, 0.0009476289255899716`, 1.6867794875501494`},
{70, 0.0009476289255899716`, 1.6867794875501494`}, {84, 0.0009476289255899716`, 1.6867794875501494`},
{98, 0.0009476289255899716`, 1.6867794875501494`}, {112, 0.0009476289255899716`, 1.6867794875501494`},
{126, 0.0009476289255899716`, 1.6867794875501494`}}, 0.004722104179507777`}, {"CURE", 365, 70.47120985513155`,
{{0, 0.01567842113396629`, 27.907589618459998`}, {14, 0.0009995695852889213`, 1.77923386181428`},
{28, 0.0009995695852889213`, 1.77923386181428`}, {42, 0.0009995695852889213`, 1.77923386181428`},
{56, 0.0009995695852889213`, 1.77923386181428`}, {70, 0.0009995695852889213`, 1.77923386181428`},
{84, 0.0009995695852889213`, 1.77923386181428`}, {98, 0.0009995695852889213`, 1.77923386181428`},
{112, 0.0009995695852889213`, 1.77923386181428`}, {126, 0.0009995695852889213`, 1.77923386181428`}}, 0.004922175039182256`},
{"TBUR", 155.10590368723922`, 52.61454700639731`, {{0, 0.013619049410802464`, 24.241907951228388`},
{14, 0.0008524716050629338`, 1.5173994570120222`}, {28, 0.0008524716050629338`, 1.5173994570120222`},
{42, 0.0008524716050629338`, 1.5173994570120222`}, {56, 0.0008524716050629338`, 1.5173994570120222`},
{70, 0.0008524716050629338`, 1.5173994570120222`}, {84, 0.0008524716050629338`, 1.5173994570120222`},
{98, 0.0008524716050629338`, 1.5173994570120222`}, {112, 0.0008524716050629338`, 1.5173994570120222`},
{126, 0.0008524716050629338`, 1.5173994570120222`}}, 0.004355566537658203`},
{"CURE", 365, 31.273822279669222`, {{0, 0.01232231411712181`, 21.93371912847682`},
{14, 0.0007598476555143156`, 1.3525288268154818`}, {28, 0.0007598476555143156`, 1.3525288268154818`},
{42, 0.0007598476555143156`, 1.3525288268154818`}, {56, 0.0007598476555143156`, 1.3525288268154818`},
{70, 0.0007598476555143156`, 1.3525288268154818`}, {84, 0.0007598476555143156`, 1.3525288268154818`},
{98, 0.0007598476555143156`, 1.3525288268154818`}, {112, 0.0007598476555143156`, 1.3525288268154818`},
{126, 0.0007598476555143156`, 1.3525288268154818`}}, 0.003998787218334075`},
{"CURE", 365, 43.09076704956969`, {{0, 0.013564271896602831`, 24.14440397595304`},
{14, 0.0008485589254772457`, 1.5104348873494975`}, {28, 0.0008485589254772457`, 1.5104348873494975`},
{42, 0.0008485589254772457`, 1.5104348873494975`}, {56, 0.0008485589254772457`, 1.5104348873494975`},
{70, 0.0008485589254772457`, 1.5104348873494975`}, {84, 0.0008485589254772457`, 1.5104348873494975`},
{98, 0.0008485589254772457`, 1.5104348873494975`}, {112, 0.0008485589254772457`, 1.5104348873494975`},
{126, 0.0008485589254772457`, 1.5104348873494975`}}, 0.004340495239509585`},
{"TBUR", 12.410470746085103`, 5.9990774312822674`, {{0, 0.03354644687486813`, 59.71267543726527`}}, 0.00983832260805911`},
{"TBUR", 198.73682396323176`, 12.974534381466498`, {{0, 0.004443457112156315`, 7.909353659638241`},
{14, 0.00019707215515963732`, 0.35078843618415445`}, {28, 0.00019707215515963732`, 0.35078843618415445`},
{42, 0.00019707215515963732`, 0.35078843618415445`}, {56, 0.00019707215515963732`, 0.35078843618415445`},
```

```
{70, 0.00019707215515963732`, 0.35078843618415445`}, {84, 0.00019707215515963732`, 0.35078843618415445`},
{98, 0.00019707215515963732`, 0.35078843618415445`}, {112, 0.00019707215515963732`, 0.35078843618415445`},
{126, 0.00019707215515963732`, 0.35078843618415445`}}, 0.0018310254145314272`},
{"TBUR", 189.80085440697817`, 8.790448590116341`, {{0, 0.0027966931193702466`, 4.978113752479039`},
{14, 0.00007944615567491814`, 0.1414141571013543`}, {28, 0.00007944615567491814`, 0.1414141571013543`},
{42, 0.00007944615567491814`, 0.1414141571013543`}, {56, 0.00007944615567491814`, 0.1414141571013543`},
{70, 0.00007944615567491814`, 0.1414141571013543`}, {84, 0.00007944615567491814`, 0.1414141571013543`},
{98, 0.00007944615567491814`, 0.1414141571013543`}, {112, 0.00007944615567491814`, 0.1414141571013543`},
{126, 0.00007944615567491814`, 0.1414141571013543`}}, 0.0013779403945609688`}, {"CURE", 365, 54.01254147275854`,
{{0, 0.013363226575735635`, 23.78654330480943`}, {14, 0.0008341985454153032`, 1.4848734108392396`},
{28, 0.0008341985454153032`, 1.4848734108392396`}, {42, 0.0008341985454153032`, 1.4848734108392396`},
{56, 0.0008341985454153032`, 1.4848734108392396`}, {70, 0.0008341985454153032`, 1.4848734108392396`},
{84, 0.0008341985454153032`, 1.4848734108392396`}, {98, 0.0008341985454153032`, 1.4848734108392396`},
{112, 0.0008341985454153032`, 1.4848734108392396`}, {126, 0.0008341985454153032`, 1.4848734108392396`}},
0.0042851803166557316`}, {"TBUR", 96.4718976609383`, 35.37041781081608`,
{{0, 0.0558461903845914`, 99.40621888457268`}, {14, 0.002398317639112129`, 4.26900539761959`}}, 0.015973797866019556`},
{"CURE", 365, 42.83824878937363`, {{0, 0.012048575780429237`, 21.446464889164044`},
{14, 0.000740294917179132`, 1.3177249525788548`}, {28, 0.000740294917179132`, 1.3177249525788548`},
{42, 0.000740294917179132`, 1.3177249525788548`}, {56, 0.000740294917179132`, 1.3177249525788548`},
{70, 0.000740294917179132`, 1.3177249525788548`}, {84, 0.000740294917179132`, 1.3177249525788548`},
{98, 0.000740294917179132`, 1.3177249525788548`}, {112, 0.000740294917179132`, 1.3177249525788548`},
{126, 0.000740294917179132`, 1.3177249525788548`}}, 0.003923471787409938`}, {"CURE", 365, 17.831202377482136`,
{{0, 0.006112326587531304`, 10.879941325805722`}, {14, 0.0003162771176864223`, 0.5629732694818317`},
{28, 0.0003162771176864223`, 0.5629732694818317`}, {42, 0.0003162771176864223`, 0.5629732694818317`},
{56, 0.0003162771176864223`, 0.5629732694818317`}, {70, 0.0003162771176864223`, 0.5629732694818317`},
{84, 0.0003162771176864223`, 0.5629732694818317`}, {98, 0.0003162771176864223`, 0.5629732694818317`},
{112, 0.0003162771176864223`, 0.5629732694818317`}, {126, 0.0003162771176864223`, 0.5629732694818317`}},
0.0022901924614736513`}, {"TBUR", 27.876292988780705`, 3.5319598174277265`,
{{0, 0.007732049185597614`, 13.763047550363753`}, {14, 0.00043197158897687304`, 0.768909428378834`}}, 0.0027358374044687186`},
{"CURE", 365, 18.693914910955534`, {{0, 0.00573447752302286`, 10.207369990980691`},
{14, 0.00028928789879296 2`, 0.5149324598514723`}, {28, 0.000289287898792962`, 0.5149324598514723`},
```

```
{42, 0.00028928789879296`, 0.5149324598514723`}, {56, 0.00028928789879296`, 0.5149324598514723`},
{70, 0.00028928789879296`, 0.5149324598514723`}, {84, 0.00028928789879296`, 0.5149324598514723`},
{98, 0.00028928789879296`, 0.5149324598514723`}, {112, 0.00028928789879296`, 0.5149324598514723`},
{126, 0.00028928789879296`, 0.5149324598514723`}}, 0.002186232360518938`},
{"CURE", 365, 141.70519840986827`, {{0, 0.020444467081428712`, 36.39115140494311`},
{14, 0.0013400014386790943`, 2.385202560848788`}, {28, 0.0013400014386790943`, 2.385202560848788`},
{42, 0.0013400014386790943`, 2.385202560848788`}, {56, 0.0013400014386790943`, 2.385202560848788`},
{70, 0.0013400014386790943`, 2.385202560848788`}, {84, 0.0013400014386790943`, 2.385202560848788`},
{98, 0.0013400014386790943`, 2.385202560848788`}, {112, 0.0013400014386790943`, 2.385202560848788`},
{126, 0.0013400014386790943`, 2.385202560848788`}}, 0.006233488641341315`},
{"TBUR", 16.204917691723377`, 1.9876773125241252`, {{0, 0.004512176772592811 5`, 8.031674655215204`},
{14, 0.0002019807023336728`, 0.35952565015393756`}}, 0.0018499327071717989`},
{"CURE", 365, 128.42978053688037`, {{0, 0.03330243230917634`, 59.27832951033388`},
{14, 0.0022584275263753534`, 4.020000996948129`}, {28, 0.0022584275263753534`, 4.020000996948129`},
{42, 0.0022584275263753534`, 4.020000996948129`}, {56, 0.0022584275263753534`, 4.020000996948129`},
{70, 0.0022584275263753534`, 4.020000996948129`}, {84, 0.0008531426519037023`, 1.51859392038859`}}, 0.009771185273957886`},
{"CURE", 365, 15.543854982420708`, {{0, 0.005879729925188143`, 10.465919266834895`},
{14, 0.0002996630703761965`, 0.5334002652696298`}, {28, 0.0002996630703761965`, 0.5334002652696298`},
{42, 0.0002996630703761965`, 0.5334002652696298`}, {56, 0.0002996630703761965`, 0.5334002652696298`},
{70, 0.0002996630703761965`, 0.5334002652696298`}, {84, 0.0002996630703761965`, 0.5334002652696298`},
{98, 0.0002996630703761965`, 0.5334002652696298`}, {112, 0.0002996630703761965`, 0.5334002652696298`},
{126, 0.0002996630703761965`, 0.5334002652696298`}}, 0.002226196610298574 4`}, {"CURE", 365, 38.9004067782163`,
{{0, 0.011983924132124596`, 21.331384955181782`}, {14, 0.0007356769423002288`, 1.3095049572944073`},
{28, 0.0007356769423002288`, 1.3095049572944073`}, {42, 0.0007356769423002288`, 1.3095049572944073`},
{56, 0.0007356769423002288`, 1.3095049572944073`}, {70, 0.0007356769423002288`, 1.3095049572944073`},
{84, 0.0007356769423002288`, 1.3095049572944073`}, {98, 0.0007356769423002288`, 1.3095049572944073`},
{112, 0.0007356769423002288`, 1.3095049572944073`}, {126, 0.0007356769423002288`, 1.3095049572944073`}},
0.0039056837537320387`}, {"TBUR", 50.86633710443653`, 5.285277455079956`,
{{0, 0.008809762735968714`, 15.68137767002431`}, {14, 0.0005089511282890944`, 0.905933008354588`},
{28, 0.0005089511282890944`, 0.905933008354588`}, {42, 0.0005089511282890944`, 0.905933008354588`}}, 0.003032355829471565`},
{"CURE", 365, 38.59186260346032`, {{0, 0.007857147783085692`, 13.985723053892531`},
```

```
    {14, 0.0004409072030831642`, 0.7848148214880323`}, {28, 0.0004409072030831642`, 0.7848148214880323`},
    {42, 0.0004409072030831642`, 0.7848148214880323`}, {56, 0.0004409072030831642`, 0.7848148214880323`},
    {70, 0.0004409072030831642`, 0.7848148214880323`}, {84, 0.0004409072030831642`, 0.7848148214880323`},
    {98, 0.0004409072030831642`, 0.7848148214880323`}, {112, 0.0004409072030831642`, 0.7848148214880323`},
    {126, 0.0004409072030831642`, 0.7848148214880323`}}, 0.0027702566052706917`},
{"TBUR", 44.60952393524888`, 5.669258675001827`, {{0, 0.006817621063973111`, 12.135365493872138`},
    {14, 0.0003666552945751227`, 0.6526464243437184`}, {28, 0.0003666552945751227`, 0.6526464243437184`},
    {42, 0.0003666552945751227`, 0.6526464243437184`}}, 0.0024842447745820084`},
{"CURE", 365, 57.056894324550456`, {{0, 0.019673323212630683`, 35.01851531848261`},
    {14, 0.001284919733764949 7`, 2.2871571261016106`}, {28, 0.0012849197337649497`, 2.2871571261016106`},
    {42, 0.0012849197337649497`, 2.2871571261016106`}, {56, 0.0012849197337649497`, 2.2871571261016106`},
    {70, 0.0012849197337649497`, 2.2871571261016106`}, {84, 0.0012849197337649497`, 2.2871571261016106`},
    {98, 0.0012849197337649497`, 2.2871571261016106`}, {112, 0.0012849197337649497`, 2.2871571261016106`},
    {126, 0.0012849197337649497`, 2.2871571261016106`}}, 0.006021318751347951`},
{"CURE", 365, 11.52769025171978`, {{0, 0.0031597983350784126`, 5.624441036439574`},
    {14, 0.00010538224251121571`, 0.18758039166996396`}, {28, 0.00010538224251121571`, 0.18758039166996396`},
    {42, 0.00010538224251121571`, 0.18758039166996396`}, {56, 0.00010538224251121571`, 0.18758039166996396`},
    {70, 0.00010538224251121571`, 0.18758039166996396`}, {84, 0.00010538224251121571`, 0.18758039166996396`},
    {98, 0.00010538224251121571`, 0.18758039166996396`}, {112, 0.00010538224251121571`, 0.18758039166996396`},
    {126, 0.00010538224251121571`, 0.18758039166996396`}}, 0.0014778439233187077`},
{"CURE", 365, 80.0594483468875`, {{0, 0.044236965673289`, 78.74179889845442`}, {14, 0.003039465623811972`, 5.41024881038531`},
    {28, 0.003039465623811972`, 5.41024881038531`}, {42, 0.0013387216423408422`, 2.382924523366699`}}, 0.012779675433658775`},
{"CURE", 365, 25.948024466164103`, {{0, 0.007672704592223135`, 13.65741417415718`},
    {14, 0.0004277326894501245`, 0.7613641872212216`}, {28, 0.0004277326894501245`, 0.7613641872212216`},
    {42, 0.0004277326894501245`, 0.7613641872212216`}, {56, 0.0004277326894501245`, 0.7613641872212216`},
    {70, 0.0004277326894501245`, 0.7613641872212216`}, {84, 0.0004277326894501245`, 0.7613641872212216`},
    {98, 0.0004277326894501245`, 0.7613641872212216`}, {112, 0.0004277326894501245`, 0.7613641872212216`},
    {126, 0.0004277326894501245`, 0.7613641872212216`}}, 0.0027195095357565036`},
{"CURE", 365, 34.265416453984706`, {{0, 0.011511957057527219`, 20.49128356239845`},
    {14, 0.0007019650084004164`, 1.249497714952741`}, {28, 0.0007019650084004164`, 1.249497714952741`},
    {42, 0.0007019650084004164`, 1.249497714952741`}, {56, 0.0007019650084004164`, 1.249497714952741`},
```

```
{70, 0.0007019650084004164`, 1.249497714952741`}, {84, 0.0007019650084004164`, 1.249497714952741`},
   {98, 0.0007019650084004164`, 1.249497714952741`}, {112, 0.0007019650084004164`, 1.249497714952741`},
   {126, 0.0007019650084004164`, 1.249497714952741`}}, 0.003775828344969082`}, {"CURE", 365, 64.46924697620673`,
  {{0, 0.043697728636582195`, 77.78195697311631`}, {14, 0.003000948692618629`, 5.341688672861159`},
   {28, 0.003000948692618629`, 5.341688672861159`}, {42, 0.003000948692618629`, 5.341688672861159`}}, 0.012631311597301288`},
 {"CURE", 365, 103.24481495057704`, {{0, 0.03215753709117714`, 57.240416022295314`},
   {14, 0.002176649296518268`, 3.874435747802517`}, {28, 0.002176649296518268`, 3.874435747802517`},
   {42, 0.002176649296518268`, 3.874435747802517`}, {56, 0.002176649296518268`, 3.874435747802517`},
   {70, 0.002176649296518268`, 3.874435747802517`}, {84, 0.002176649296518268`, 3.874435747802517`}}, 0.009456182714402506`},
 {"TBUR", 51.38617963968121`, 6.701691831176162`, {{0, 0.008153874637187135`, 14.5138968541931`},
   {14, 0.0004621019783761244`, 0.8225415215095014`}, {28, 0.0004621019783761244`, 0.8225415215095014`},
   {42, 0.0004621019783761244`, 0.8225415215095014`}}, 0.002851897018350691 6`}, {"CURE", 365, 95.36395582116369`,
  {{0, 0.031938236163852114`, 56.85006037165677`}, {14, 0.002160984944566479 6`, 3.8465532013283337`},
   {28, 0.0021609849445664796`, 3.8465532013283337`}, {42, 0.0021609849445664796`, 3.8465532013283337`},
   {56, 0.0021609849445664796`, 3.8465532013283337`}, {70, 0.0021609849445664796`, 3.8465532013283337`},
   {84, 0.0021609849445664796`, 3.8465532013283337`}}, 0.009395845006344845`},
 {"CURE", 365, 28.499435156774997`, {{0, 0.009528281969357535`, 16.960341905456414`},
   {14, 0.0005602739306740101`, 0.997287596599738`}, {28, 0.0005602739306740101`, 0.997287596599738`},
   {42, 0.0005602739306740101`, 0.997287596599738`}, {56, 0.0005602739306740101`, 0.997287596599738`},
   {70, 0.0005602739306740101`, 0.997287596599738`}, {84, 0.0005602739306740101`, 0.997287596599738`},
   {98, 0.0005602739306740101`, 0.997287596599738`}, {112, 0.0005602739306740101`, 0.997287596599738`},
   {126, 0.0005602739306740101`, 0.997287596599738`}}, 0.003230046757033293 6`},
 {"CURE", 365, 72.20857864440913`, {{0, 0.019808982794828348`, 35.25998937479446`},
   {14, 0.0012946097039219255`, 2.304405272981027`}, {28, 0.0012946097039219255`, 2.304405272981027`},
   {42, 0.0012946097039219255`, 2.304405272981027`}, {56, 0.0012946097039219255`, 2.304405272981027`},
   {70, 0.0012946097039219255`, 2.304405272981027`}, {84, 0.0012946097039219255`, 2.304405272981027`},
   {98, 0.0012946097039219255`, 2.304405272981027`}, {112, 0.0012946097039219255`, 2.304405272981027`},
   {126, 0.0012946097039219255`, 2.304405272981027`}}, 0.006058643665408784`}, {"CURE", 365, 89.80908136194608`,
  {{0, 0.03630984187215959`, 64.63151853244408`}, {14, 0.0024732424951598724`, 4.402371641384573`},
   {28, 0.0024732424951598724`, 4.402371641384573`}, {42, 0.0024732424951598724`, 4.402371641384573`},
   {56, 0.0024732424951598724`, 4.402371641384573`}, {70, 0.000952034254686436`, 1.6946209733418562`}}, 0.01059863366842896`},
```

```
{"CURE", 365, 106.99976330638678`, {{0, 0.04710616042780696`, 83.84896556149639`},
  {14, 0.003244081062775413`, 5.775046429174023`}, {28, 0.003244081062775413`, 5.775046429174023`}}, 0.013569095877841865`},
{"CURE", 365, 102.66975680777298`, {{0, 0.04243718955519192`, 75.53819740824161`},
  {14, 0.002910910186805037 6`, 5.181420132512967`}, {28, 0.0029109101868050376`, 5.181420132512967`},
  {42, 0.0029109101868050376`, 5.181420132512967`}}, 0.01228449118018797`},
{"CURE", 365, 66.49584525085629`, {{0, 0.03799497431477604`, 67.63105428030136`}, {14, 0.002593609098203904`, 4.616624194802949`},
  {28, 0.002593609098203904`, 4.616624194802949`}, {42, 0.002593609098203904`, 4.616624194802949`},
  {56, 0.002593609098203904`, 4.616624194802949`}}, 0.011062275252627075`},
{"CURE", 365, 41.23217125217675`, {{0, 0.01061733857732739`, 18.898862667642753`},
  {14, 0.0006380636883861427`, 1.135753365327334`}, {28, 0.0006380636883861427`, 1.135753365327334`},
  {42, 0.0006380636883861427`, 1.135753365327334`}, {56, 0.0006380636883861427`, 1.135753365327334`},
  {70, 0.0006380636883861427`, 1.135753365327334`}, {84, 0.0006380636883861427`, 1.135753365327334`},
  {98, 0.0006380636883861427`, 1.135753365327334`}, {112, 0.0006380636883861427`, 1.135753365327334`},
  {126, 0.0006380636883861427`, 1.135753365327334`}}, 0.003529686072158325`},
{"CURE", 365, 46.442656683060186`, {{0, 0.01720851511311245`, 30.631156901340162`},
  {14, 0.0011088620123707897`, 1.9737743820200055`}, {28, 0.0011088620123707897`, 1.9737743820200055`},
  {42, 0.0011088620123707897`, 1.9737743820200055`}, {56, 0.0011088620123707897`, 1.9737743820200055`},
  {70, 0.0011088620123707897`, 1.9737743820200055`}, {84, 0.0011088620123707897`, 1.9737743820200055`},
  {98, 0.0011088620123707897`, 1.9737743820200055`}, {112, 0.0011088620123707897`, 1.9737743820200055`},
  {126, 0.0011088620123707897`, 1.9737743820200055`}}, 0.005343159870120431 5`},
{"CURE", 365, 15.642454347168183`, {{0, 0.005332209294777788`, 9.491332544704463`},
  {14, 0.00026055445391831 4`, 0.4637869279745989`}, {28, 0.000260554453918314`, 0.4637869279745989`},
  {42, 0.000260554453918314`, 0.4637869279745989`}, {56, 0.000260554453918314`, 0.4637869279745989`},
  {70, 0.000260554453918314`, 0.4637869279745989`}, {84, 0.000260554453918314`, 0.4637869279745989`},
  {98, 0.000260554453918314`, 0.4637869279745989`}, {112, 0.000260554453918314`, 0.4637869279745989`},
  {126, 0.000260554453918314`, 0.4637869279745989`}}, 0.002075553654264533`},
{"CURE", 365, 35.35549333211515`, {{0, 0.004559346379382283`, 8.115636555300464`},
  {14, 0.00020534995996149223`, 0.36552292873145614`}, {28, 0.00020534995996149223`, 0.36552292873145614`},
  {42, 0.00020534995996149223`, 0.36552292873145614`}, {56, 0.00020534995996149223`, 0.36552292873145614`},
  {70, 0.00020534995996149223`, 0.36552292873145614`}, {84, 0.00020534995996149223`, 0.36552292873145614`},
  {98, 0.00020534995996149223`, 0.36552292873145614`}, {112, 0.00020534995996149223`, 0.36552292873145614`},
```

```
{126, 0.00020534995996149223`, 0.36552292873145614`}}, 0.0018629107916622472`},
{"CURE", 365, 100.47695899716514`, {{0, 0.03075299155971836`, 54.74032497629868`},
  {14, 0.0020763246156997833`, 3.6958578159456144`}, {28, 0.0020763246156997833`, 3.6958578159456144`},
  {42, 0.0020763246156997833`, 3.6958578159456144`}, {56, 0.0020763246156997833`, 3.6958578159456144`},
  {70, 0.0020763246156997833`, 3.6958578159456144`}, {84, 0.0020763246156997833`, 3.6958578159456144`},
  {98, 0.0007896023915657351`, 1.4054922569870083`}}, 0.009069740854513545`},
{"CURE", 365, 45.5579297315062`, {{0, 0.01929036583072185`, 34.33685117868489`},
  {14, 0.001257565635057175 7`, 2.2384668304017725`}, {28, 0.0012575656350571757`, 2.2384668304017725`},
  {42, 0.0012575656350571757`, 2.2384668304017725`}, {56, 0.0012575656350571757`, 2.2384668304017725`},
  {70, 0.0012575656350571757`, 2.2384668304017725`}, {84, 0.0012575656350571757`, 2.2384668304017725`},
  {98, 0.0012575656350571757`, 2.2384668304017725`}, {112, 0.0012575656350571757`, 2.2384668304017725`},
  {126, 0.0012575656350571757`, 2.2384668304017725`}}, 0.005915953165392647`},
{"CURE", 365, 147.34308577229072`, {{0, 0.031190118075598603`, 55.51841017456552`},
  {14, 0.002107547938262657 7`, 3.7514353301075305`}, {28, 0.0021075479382626577`, 3.7514353301075305`},
  {42, 0.0021075479382626577`, 3.7514353301075305`}, {56, 0.0021075479382626577`, 3.7514353301075305`},
  {70, 0.0021075479382626577`, 3.7514353301075305`}, {84, 0.0021075479382626577`, 3.7514353301075305`},
  {98, 0.00041326753320245803`, 0.7356162091003753`}}, 0.009190010350959201`},
{"CURE", 365, 82.33589825695073`, {{0, 0.043223881445865524`, 76.93850897364064`},
  {14, 0.0029671024647102955`, 5.281442387184326`}, {28, 0.0029671024647102955`, 5.281442387184326`},
  {42, 0.0029671024647102955`, 5.281442387184326`}}, 0.012500938899811354`}, {"CURE", 365, 99.61391929557708`,
 {{0, 0.04400076091895824`, 78.32135443574568`}, {14, 0.0030225938556454894`, 5.380217063048971`},
  {28, 0.0030225938556454894`, 5.380217063048971`}, {42, 0.0014745900732567414`, 2.624770330397`}}, 0.012714686864378726`},
{"TBUR", 40.38277771249627`, 7.65158694098007`, {{0, 0.016617473260082304`, 29.5791024029465`},
  {14, 0.0010666447371543506`, 1.8986276321347442`}, {28, 0.0010666447371543506`, 1.8986276321347442`}},
 0.005180542633554263`}, {"CURE", 365, 97.07931905854441`, {{0, 0.01898044157309571`, 33.785186000110365`},
  {14, 0.0012354281880838799`, 2.199062174789306`}, {28, 0.0012354281880838799`, 2.199062174789306`},
  {42, 0.0012354281880838799`, 2.199062174789306`}, {56, 0.0012354281880838799`, 2.199062174789306`},
  {70, 0.0012354281880838799`, 2.199062174789306`}, {84, 0.0012354281880838799`, 2.199062174789306`},
  {98, 0.0012354281880838799`, 2.199062174789306`}, {112, 0.0012354281880838799`, 2.199062174789306`},
  {126, 0.0012354281880838799`, 2.199062174789306`}}, 0.005830681663786462`},
{"TBUR", 217.8663156437204`, 105.0211758113723`, {{0, 0.025673846218382385`, 45.69944626872065`},
```

```
{14, 0.0017135285198900713`, 3.050080765404327`}, {28, 0.0017135285198900713`, 3.050080765404327`},
{42, 0.0017135285198900713`, 3.050080765404327`}, {56, 0.0017135285198900713`, 3.050080765404327`},
{70, 0.0017135285198900713`, 3.050080765404327`}, {84, 0.0017135285198900713`, 3.050080765404327`},
{98, 0.0017135285198900713`, 3.050080765404327`}, {112, 0.0017135285198900713`, 3.050080765404327`},
{126, 0.0017135285198900713`, 3.050080765404327`}}, 0.007672282154606792`},
{"CURE", 365, 135.84367274006013`, {{0, 0.052014515156524256`, 92.58583697861317`},
{14, 0.003595004872614491`, 6.399108673253794`}, {28, 0.0004599662485628283`, 0.8187399224418345`}}, 0.014919563831978072`},
{"CURE", 365, 10.151570561834733`, {{0, 0.003833621764228307`, 6.8238467403263865`},
{14, 0.0001535124874504939`, 0.2732522276618792`}, {28, 0.0001535124874504939`, 0.2732522276618792`},
{42, 0.0001535124874504939`, 0.2732522276618792`}, {56, 0.0001535124874504939`, 0.2732522276618792`},
{70, 0.0001535124874504939`, 0.2732522276618792`}, {84, 0.0001535124874504939`, 0.2732522276618792`},
{98, 0.0001535124874504939`, 0.2732522276618792`}, {112, 0.0001535124874504939`, 0.2732522276618792`},
{126, 0.0001535124874504939`, 0.2732522276618792`}}, 0.0016632373999737463`},
{"TBUR", 12.491734522914562`, 2.239837475891243`, {{0, 0.013176716877328474`, 23.454556041644683`}}, 0.004233864675314482`},
{"CURE", 365, 85.02825526545944`, {{0, 0.02164109727436291`, 38.521153148365975`},
{14, 0.00142547502388868`, 2.5373455425218503`}, {28, 0.00142547502388868`, 2.5373455425218503`},
{42, 0.00142547502388868`, 2.5373455425218503`}, {56, 0.00142547502388868`, 2.5373455425218503`},
{70, 0.00142547502388868`, 2.5373455425218503`}, {84, 0.00142547502388868`, 2.5373455425218503`},
{98, 0.00142547502388868`, 2.5373455425218503`}, {112, 0.00142547502388868`, 2.5373455425218503`},
{126, 0.00142547502388868`, 2.5373455425218503`}}, 0.0065627253851843525`},
{"TBUR", 74.37783082216477`, 5.619592388448245`, {{0, 0.002809907886260692`, 5.001636037544031`},
{14, 0.00008039006759566425`, 0.14309432032028235`}, {28, 0.00008039006759566425`, 0.14309432032028235`},
{42, 0.00008039006759566425`, 0.14309432032028235`}, {56, 0.00008039006759566425`, 0.14309432032028235`},
{70, 0.00008039006759566425`, 0.14309432032028235`}}, 0.0013815762603842992`},
{"CURE", 365, 106.7132958895514`, {{0, 0.02824346981571764`, 50.2733762719774`}, {14, 0.0018970730625568752`, 3.376790051351238`},
{28, 0.0018970730625568752`, 3.376790051351238`}, {42, 0.0018970730625568752`, 3.376790051351238`},
{56, 0.0018970730625568752`, 3.376790051351238`}, {70, 0.0018970730625568752`, 3.376790051351238`},
{84, 0.0018970730625568752`, 3.376790051351238`}, {98, 0.0018970730625568752`, 3.376790051351238`},
{112, 0.0018970730625568752`, 3.376790051351238`}}, 0.008379279613875659`},
{"CURE", 365, 63.25819258595731`, {{0, 0.011990801976579885`, 21.343627518312196`},
{14, 0.0007361682169041783`, 1.3103794260894372`}, {28, 0.0007361682169041783`, 1.3103794260894372`},
```

{42, 0.0007361682169041783`, 1.3103794260894372`}, {56, 0.0007361682169041783`, 1.3103794260894372`},
{70, 0.0007361682169041783`, 1.3103794260894372`}, {84, 0.0007361682169041783`, 1.3103794260894372`},
{98, 0.0007361682169041783`, 1.3103794260894372`}, {112, 0.0007361682169041783`, 1.3103794260894372`},
{126, 0.0007361682169041783`, 1.3103794260894372`}}, 0.003907576100362175`},
{"CURE", 365, 62.170294534444125`, {{0, 0.02212988359265986`, 39.391192794934554`},
{14, 0.0014603883323384623`, 2.599491231562463`}, {28, 0.0014603883323384623`, 2.599491231562463`},
{42, 0.0014603883323384623`, 2.599491231562463`}, {56, 0.0014603883323384623`, 2.599491231562463`},
{70, 0.0014603883323384623`, 2.599491231562463`}, {84, 0.0014603883323384623`, 2.599491231562463`},
{98, 0.0014603883323384623`, 2.599491231562463`}, {112, 0.0014603883323384623`, 2.599491231562463`},
{126, 0.0014603883323384623`, 2.599491231562463`}}, 0.006697208383207755`},
{"CURE", 365, 55.76320438742923`, {{0, 0.0706639041885576`, 125.78174945563252`}}, 0.0175`},
{"CURE", 365, 85.31365016223236`, {{0, 0.03399775717305141`, 60.51600776803151`},
{14, 0.002308093588080715`, 4.108406586783673`}, {28, 0.002308093588080715`, 4.108406586783673`},
{42, 0.002308093588080715`, 4.108406586783673`}, {56, 0.002308093588080715`, 4.108406586783673`},
{70, 0.002308093588080715`, 4.108406586783673`}, {84, 0.0003041834308669406`, 0.5414465069431543`}}, 0.009962494581918765`},
{"CURE", 365, 23.087016975742188`, {{0, 0.008115676491962764`, 14.445904155693722`},
{14, 0.00045937353943152653`, 0.8176849001881172`}, {28, 0.00045937353943152653`, 0.8176849001881172`},
{42, 0.00045937353943152653`, 0.8176849001881172`}, {56, 0.00045937353943152653`, 0.8176849001881172`},
{70, 0.00045937353943152653`, 0.8176849001881172`}, {84, 0.00045937353943152653`, 0.8176849001881172`},
{98, 0.00045937353943152653`, 0.8176849001881172`}, {112, 0.00045937353943152653`, 0.8176849001881172`},
{126, 0.00045937353943152653`, 0.8176849001881172`}}, 0.00284138731115061`},
{"CURE", 365, 60.80628614291213`, {{0, 0.06650372818883074`, 118.37663617611872`}}, 0.017500000000000001`},
{"CURE", 365, 33.57644071960616`, {{0, 0.010126975509821159`, 18.026016407481663`},
{14, 0.0006030377549928407`, 1.0734072038872564`}, {28, 0.0006030377549928407`, 1.0734072038872564`},
{42, 0.0006030377549928407`, 1.0734072038872564`}, {56, 0.0006030377549928407`, 1.0734072038872564`},
{70, 0.0006030377549928407`, 1.0734072038872564`}, {84, 0.0006030377549928407`, 1.0734072038872564`},
{98, 0.0006030377549928407`, 1.0734072038872564`}, {112, 0.0006030377549928407`, 1.0734072038872564`},
{126, 0.0006030377549928407`, 1.0734072038872564`}}, 0.003394769252743329 5`}, {"CURE", 365, 61.56100053214176`,
{{0, 0.060104065534129066`, 106.98523665074974`}, {14, 0.0005573934289883452`, 0.9921603035992544`}}, 0.017145295090643782`},
{"CURE", 365, 25.48359611954478`, {{0, 0.009680651636760301`, 17.231559913433337`},
{14, 0.0005711574783456363`, 1.0166603114552326`}, {28, 0.0005711574783456363`, 1.0166603114552326`},

```
{42, 0.0005711574783456363`, 1.0166603114552326`}, {56, 0.0005711574783456363`, 1.0166603114552326`},
{70, 0.0005711574783456363`, 1.0166603114552326`}, {84, 0.0005711574783456363`, 1.0166603114552326`},
{98, 0.0005711574783456363`, 1.0166603114552326`}, {112, 0.0005711574783456363`, 1.0166603114552326`},
{126, 0.0005711574783456363`, 1.0166603114552326`}}, 0.0032719692268592675`},
{"CURE", 365, 50.96429609804262`, {{0, 0.013140687693499435`, 23.390424094428994`},
{14, 0.0008183029109698601`, 1.456579181526351`}, {28, 0.0008183029109698601`, 1.456579181526351`},
{42, 0.0008183029109698601`, 1.456579181526351`}, {56, 0.0008183029109698601`, 1.456579181526351`},
{70, 0.0008183029109698601`, 1.456579181526351`}, {84, 0.0008183029109698601`, 1.456579181526351`},
{98, 0.0008183029109698601`, 1.456579181526351`}, {112, 0.0008183029109698601`, 1.456579181526351`},
{126, 0.0008183029109698601`, 1.456579181526351`}}, 0.004223951728743991`}, {"CURE", 365, 30.87570849834189`,
{{0, 0.010676982097280277`, 19.005028133158895`}, {14, 0.000642323939811349`, 1.143336612864201`},
{28, 0.000642323939811349`, 1.143336612864201`}, {42, 0.000642323939811349`, 1.143336612864201`},
{56, 0.000642323939811349`, 1.143336612864201`}, {70, 0.000642323939811349`, 1.143336612864201`},
{84, 0.000642323939811349`, 1.143336612864201`}, {98, 0.000642323939811349`, 1.143336612864201`},
{112, 0.000642323939811349`, 1.143336612864201`}, {126, 0.000642323939811349`, 1.143336612864201`}}, 0.003546096186508253`},
{"CURE", 365, 91.83725482571577`, {{0, 0.038473663831104905`, 68.48312161936673`},
{14, 0.002627801206513107 8`, 4.677486147593331`}, {28, 0.0026278012065131078`, 4.677486147593331`},
{42, 0.0026278012065131078`, 4.677486147593331`}, {56, 0.0026278012065131078`, 4.677486147593331`}}, 0.011193980251045567`},
{"TBUR", 26.069523794256472`, 10.042654131508034`, {{0, 0.027373843127476333`, 48.72544076690787`},
{14, 0.0018349568705396384`, 3.2662232295605564`}}, 0.008140013497337588`},
{"CURE", 365, 16.34847014389053`, {{0, 0.005169072539755111`, 9.200949120764097`},
{14, 0.0002489018285595513`, 0.4430452548360013`}, {28, 0.0002489018285595513`, 0.4430452548360013`},
{42, 0.0002489018285595513`, 0.4430452548360013`}, {56, 0.0002489018285595513`, 0.4430452548360013`},
{70, 0.0002489018285595513`, 0.4430452548360013`}, {84, 0.0002489018285595513`, 0.4430452548360013`},
{98, 0.0002489018285595513`, 0.4430452548360013`}, {112, 0.0002489018285595513`, 0.4430452548360013`},
{126, 0.0002489018285595513`, 0.4430452548360013`}}, 0.002030668764728958`},
{"TBUR", 30.762896827666346`, 7.298003544155818`, {{0, 0.0270575022350957`, 48.16235397847035`},
{14, 0.0018123610925124508`, 3.2260027446721624`}, {28, 0.0018123610925124508`, 3.2260027446721624`}},
0.008052976544762943`}, {"CURE", 365, 22.31487248746473`, {{0, 0.007225965654909531`, 12.862218865738965`},
{14, 0.0003958227653562956 4`, 0.7045645223342062`}, {28, 0.00039582276535629564`, 0.7045645223342062`},
{42, 0.00039582276535629564`, 0.7045645223342062`}, {56, 0.00039582276535629564`, 0.7045645223342062`},
```

```
{70, 0.00039582276535629564`, 0.7045645223342062`}, {84, 0.00039582276535629564`, 0.7045645223342062`},
  {98, 0.00039582276535629564`, 0.7045645223342062`}, {112, 0.00039582276535629564`, 0.7045645223342062`},
  {126, 0.00039582276535629564`, 0.7045645223342062`}}, 0.0025965953105117137`},
{"CURE", 365, 174.26635357902765`, {{0, 0.04334986111383023`, 77.16275278261782`}, {14, 0.00297610101242206`, 5.297459802111267`},
  {28, 0.00297610101242206`, 5.297459802111267`}, {42, 0.00297610101242206`, 5.297459802111267`}}, 0.012535600515334717`},
{"TBUR", 20.268735499737183`, 2.298076997262595`, {{0, 0.005377992123209006`, 9.57282597931203`},
  {14, 0.0002638246559491153`, 0.46960788758942523`}}, 0.0020881501852944395`},
{"CURE", 365, 71.5325678491759`, {{0, 0.015019424513120985`, 26.73457563335535`}, {14, 0.0009524983980856854`, 1.69544714859252`},
  {28, 0.0009524983980856854`, 1.69544714859252`}, {42, 0.0009524983980856854`, 1.69544714859252`},
  {56, 0.0009524983980856854`, 1.69544714859252`}, {70, 0.0009524983980856854`, 1.69544714859252`},
  {84, 0.0009524983980856854`, 1.69544714859252`}, {98, 0.0009524983980856854`, 1.69544714859252`},
  {112, 0.0009524983980856854`, 1.69544714859252`}, {126, 0.0009524983980856854`, 1.69544714859252`}}, 0.004740860959918835`},
{"CURE", 365, 37.13081439866168`, {{0, 0.008255308348363325`, 14.69444886008672`},
  {14, 0.00046934724346013807`, 0.8354380933590457`}, {28, 0.00046934724346013807`, 0.8354380933590457`},
  {42, 0.00046934724346013807`, 0.8354380933590457`}, {56, 0.00046934724346013807`, 0.8354380933590457`},
  {70, 0.00046934724346013807`, 0.8354380933590457`}, {84, 0.00046934724346013807`, 0.8354380933590457`},
  {98, 0.00046934724346013807`, 0.8354380933590457`}, {112, 0.00046934724346013807`, 0.8354380933590457`},
  {126, 0.00046934724346013807`, 0.8354380933590457`}}, 0.002879805143167161`},
{"CURE", 365, 12.152609216456971`, {{0, 0.004861001544375775`, 8.652582748988879`},
  {14, 0.00022689675746102728`, 0.4038762282806286`}, {28, 0.00022689675746102728`, 0.4038762282806286`},
  {42, 0.00022689675746102728`, 0.4038762282806286`}, {56, 0.00022689675746102728`, 0.4038762282806286`},
  {70, 0.00022689675746102728`, 0.4038762282806286`}, {84, 0.00022689675746102728`, 0.4038762282806286`},
  {98, 0.00022689675746102728`, 0.4038762282806286`}, {112, 0.00022689675746102728`, 0.4038762282806286`},
  {126, 0.00022689675746102728`, 0.4038762282806286`}}, 0.0019459071633669961`},
{"TBUR", 13.251015378548768`, 4.463628216199999`, {{0, 0.01774854848671649`, 31.59241630635535`}}, 0.0054917428078917`},
{"CURE", 365, 43.668665963213236`, {{0, 0.012041861612066539`, 21.434513669478438`},
  {14, 0.0007398153337246534`, 1.316871294029883`}, {28, 0.0007398153337246534`, 1.316871294029883`},
  {42, 0.0007398153337246534`, 1.316871294029883`}, {56, 0.0007398153337246534`, 1.316871294029883`},
  {70, 0.0007398153337246534`, 1.316871294029883`}, {84, 0.0007398153337246534`, 1.316871294029883`},
  {98, 0.0007398153337246534`, 1.316871294029883`}, {112, 0.0007398153337246534`, 1.316871294029883`},
  {126, 0.0007398153337246534`, 1.316871294029883`}}, 0.003921624474060834`},
```

{"CURE", 365, 29.0631429207621`, {{0, 0.0074112014117598`, 13.191938512932444`},
{14, 0.0004090538908456006`, 0.7281159257051691`}, {28, 0.0004090538908456006`, 0.7281159257051691`},
{42, 0.0004090538908456006`, 0.7281159257051691`}, {56, 0.0004090538908456006`, 0.7281159257051691`},
{70, 0.0004090538908456006`, 0.7281159257051691`}, {84, 0.0004090538908456006`, 0.7281159257051691`},
{98, 0.0004090538908456006`, 0.7281159257051691`}, {112, 0.0004090538908456006`, 0.7281159257051691`},
{126, 0.0004090538908456006`, 0.7281159257051691`}}, 0.0026475604439245197`},
{"CURE", 365, 84.31707200586311`, {{0, 0.045070691151492044`, 80.22583024965584`}, {14, 0.003099017443683618`, 5.51625104975684`},
{28, 0.003099017443683618`, 5.51625104975684`}, {42, 0.0008591505479358793`, 1.5292879753258652`}}, 0.01300906381389802`},
{"CURE", 365, 62.689241158646105`, {{0, 0.04333301189101397`, 77.13276116600487`},
{14, 0.00297489749650613`, 5.295317543781771`}, {28, 0.00297489749650613`, 5.295317543781771`},
{42, 0.00297489749650613`, 5.295317543781771`}}, 0.012530964677722503`},
{"TBUR", 17.94536238498276`, 2.479993514458287`, {{0, 0.0035418133014880763`, 6.304427676648776`},
{14, 0.00013266902582619172`, 0.23615086597062127`}}, 0.0015829502162926648`},
{"CURE", 365, 149.76107506802106`, {{0, 0.046718994517465064`, 83.15981024108781`},
{14, 0.0032167533983959774`, 5.72582104914484`}, {28, 0.0032167533983959774`, 5.72582104914484`}}, 0.013462572371748952`},
{"CURE", 365, 175.74745419221614`, {{0, 0.024515891754953965`, 43.638287323818055`},
{14, 0.00163081748678804l`, 2.902855126482713`}, {28, 0.00163081748678804l`, 2.902855126482713`},
{42, 0.00163081748678804l`, 2.902855126482713`}, {56, 0.00163081748678804l`, 2.902855126482713`},
{70, 0.00163081748678804l`, 2.902855126482713`}, {84, 0.00163081748678804l`, 2.902855126482713`},
{98, 0.00163081748678804l`, 2.902855126482713`}, {112, 0.00163081748678804l`, 2.902855126482713`},
{126, 0.00163081748678804l`, 2.902855126482713`}}, 0.007353686518871695 5`},
{"TBUR", 110.15706052756907`, 43.062880602942066`, {{0, 0.058449531020566506`, 104.04016521660839`},
{14, 0.0012727438175109923`, 2.2654839951695664`}}, 0.01669007211612937`},
{"TBUR", 18.487438550355304`, 3.138542172863064`, {{0, 0.010588672407730405`, 18.84783688576012`},
{14, 0.000636016104843501`, 1.1321086666214317`}}, 0.003521798960173109`}, {"CURE", 365, 92.47083720017284`,
{{0, 0.05610827118288319`, 99.87272270553207`}, {14, 0.00228500504312483`, 4.067308976762198`}}, 0.016045905881647837`},
{"TBUR", 171.90630664187717`, 68.65799558539592`, {{0, 0.028273003418042276`, 50.32594608411525`},
{14, 0.0018991826055800637`, 3.3805450379325133`}, {28, 0.0018991826055800637`, 3.3805450379325133`},
{42, 0.0018991826055800637`, 3.3805450379325133`}, {56, 0.0018991826055800637`, 3.3805450379325133`},
{70, 0.0018991826055800637`, 3.3805450379325133`}, {84, 0.0018991826055800637`, 3.3805450379325133`},
{98, 0.0018991826055800637`, 3.3805450379325133`}, {112, 0.0018991826055800637`, 3.3805450379325133`}},

```
0.008387405388338591`}, {"CURE", 365, 105.96277065354252`, {{0, 0.050020759942737796`, 89.03695269807328`},
    {14, 0.003452593785915458`, 6.145616938929515`}, {28, 0.0014643923341090123`, 2.606618354714042`}}, 0.014371008832711703`},
  {"CURE", 365, 143.60729870063201`, {{0, 0.01972613420362249`, 35.11251888244803`},
    {14, 0.0012886919474072215`, 2.293871666384854`}, {28, 0.0012886919474072215`, 2.293871666384854`},
    {42, 0.0012886919474072215`, 2.293871666384854`}, {56, 0.0012886919474072215`, 2.293871666384854`},
    {70, 0.0012886919474072215`, 2.293871666384854`}, {84, 0.0012886919474072215`, 2.293871666384854`},
    {98, 0.0012886919474072215`, 2.293871666384854`}, {112, 0.0012886919474072215`, 2.293871666384854`},
    {126, 0.0012886919474072215`, 2.293871666384854`}}, 0.006035848987018031`},
  {"CURE", 365, 80.71010719545387`, {{0, 0.036176098978268005`, 64.39345618131705`},
    {14, 0.002463689431310473`, 4.3853671877326414`}, {28, 0.002463689431310473`, 4.3853671877326414`},
    {42, 0.002463689431310473`, 4.3853671877326414`}, {56, 0.002463689431310473`, 4.3853671877326414`},
    {70, 0.0010480712519212228`, 1.8655668284197766`}}, 0.010561836105441657`},
  {"CURE", 365, 64.08686356218911`, {{0, 0.034350565984542614`, 61.14400745248585`},
    {14, 0.0023332942174729447`, 4.153263707101842`}, {28, 0.0023332942174729447`, 4.153263707101842`},
    {42, 0.0023332942174729447`, 4.153263707101842`}, {56, 0.0023332942174729447`, 4.153263707101842`},
    {70, 0.0023332942174729447`, 4.153263707101842`}, {84, 0.00002564075190714457`, 0.04564053839471734`}},
  0.010059565193190631`}, {"CURE", 365, 72.7626414845259`, {{0, 0.022708508258128198`, 40.42114469946819`},
    {14, 0.0015017186655862007`, 2.6730592247434375`}, {28, 0.0015017186655862007`, 2.6730592247434375`},
    {42, 0.0015017186655862007`, 2.6730592247434375`}, {56, 0.0015017186655862007`, 2.6730592247434375`},
    {70, 0.0015017186655862007`, 2.6730592247434375`}, {84, 0.0015017186655862007`, 2.6730592247434375`},
    {98, 0.0015017186655862007`, 2.6730592247434375`}, {112, 0.0015017186655862007`, 2.6730592247434375`},
    {126, 0.0015017186655862007`, 2.6730592247434375`}}, 0.006856409197202708`},
  {"TBUR", 184.76593532055102`, 71.9420197154919`, {{0, 0.025895654182466876`, 46.09426444479104`},
    {14, 0.0017293719458961064`, 3.0782820636950694`}, {28, 0.0017293719458961064`, 3.0782820636950694`},
    {42, 0.0017293719458961064`, 3.0782820636950694`}, {56, 0.0017293719458961064`, 3.0782820636950694`},
    {70, 0.0017293719458961064`, 3.0782820636950694`}, {84, 0.0017293719458961064`, 3.0782820636950694`},
    {98, 0.0017293719458961064`, 3.0782820636950694`}, {112, 0.0017293719458961064`, 3.0782820636950694`},
    {126, 0.0017293719458961064`, 3.0782820636950694`}}, 0.007733309640194937`},
  {"CURE", 365, 114.4810630198188`, {{0, 0.031102344495806764`, 55.36217320253604`}, {14, 0.002101278396848955`, 3.74027554639114`},
    {28, 0.002101278396848955`, 3.74027554639114`}, {42, 0.002101278396848955`, 3.74027554639114`},
    {56, 0.002101278396848955`, 3.74027554639114`}, {70, 0.002101278396848955`, 3.74027554639114`},
```

```
{84, 0.00210127839684895`, 3.74027554639114`}, {98, 0.000488834346285119`, 0.870125136387512`}}, 0.00916586062803164`},
{"TBUR", 21.55073375303804`, 11.095640927920833`, {{0, 0.0403054232340138`, 71.74365335654457`},
  {14, 0.002758641163863744`, 4.910381271677464`}}, 0.011697964276251216`},
{"CURE", 365, 158.32083647609957`, {{0, 0.03638088288651024`, 64.75797153798823`}, {14, 0.002478316853327775`, 4.41140399892344`},
  {28, 0.002478316853327775`, 4.41140399892344`}, {42, 0.002478316853327775`, 4.41140399892344`},
  {56, 0.002478316853327775`, 4.41140399892344`}, {70, 0.0009010217074008862`, 1.6038186391735776`}}, 0.01061817965045601`},
{"TBUR", 214.20169868373696`, 64.36401633957058`, {{0, 0.026919215916618562`, 47.91620433158104`},
  {14, 0.0018024834983355123`, 3.2084206270372118`}, {28, 0.0018024834983355123`, 3.2084206270372118`},
  {42, 0.0018024834983355123`, 3.2084206270372118`}, {56, 0.0018024834983355123`, 3.2084206270372118`},
  {70, 0.0018024834983355123`, 3.2084206270372118`}, {84, 0.0018024834983355123`, 3.2084206270372118`},
  {98, 0.0018024834983355123`, 3.2084206270372118`}, {112, 0.0018024834983355123`, 3.2084206270372118`},
  {126, 0.00048524371131587925`, 0.863733806142265`}}, 0.008014928919454562`},
{"TBUR", 13.426189579957155`, 3.8894890517042167`, {{0, 0.013154947797435262`, 23.415807079434767`}}, 0.004227875205031333`},
{"CURE", 365, 31.57858332610333`, {{0, 0.0060882501736438405`, 10.837085309086037`},
  {14, 0.00031455737383731774`, 0.5599121254304256`}, {28, 0.00031455737383731774`, 0.5599121254304256`},
  {42, 0.00031455737383731774`, 0.5599121254304256`}, {56, 0.00031455737383731774`, 0.5599121254304256`},
  {70, 0.00031455737383731774`, 0.5599121254304256`}, {84, 0.00031455737383731774`, 0.5599121254304256`},
  {98, 0.00031455737383731774`, 0.5599121254304256`}, {112, 0.00031455737383731774`, 0.5599121254304256`},
  {126, 0.00031455737383731774`, 0.5599121254304256`}}, 0.0022835681591966976`},
{"CURE", 365, 69.02499304132947`, {{0, 0.01758009594162013`, 31.29257077608383`},
  {14, 0.0011354035001213385`, 2.0210182302159825`}, {28, 0.0011354035001213385`, 2.0210182302159825`},
  {42, 0.0011354035001213385`, 2.0210182302159825`}, {56, 0.0011354035001213385`, 2.0210182302159825`},
  {70, 0.0011354035001213385`, 2.0210182302159825`}, {84, 0.0011354035001213385`, 2.0210182302159825`},
  {98, 0.0011354035001213385`, 2.0210182302159825`}, {112, 0.0011354035001213385`, 2.0210182302159825`},
  {126, 0.0011354035001213385`, 2.0210182302159825`}}, 0.005445395350032885`},
{"CURE", 365, 43.41126097234357`, {{0, 0.006689501267022066`, 11.907312255299278`},
  {14, 0.00035750388050719104`, 0.6363569073028`}, {28, 0.00035750388050719104`, 0.6363569073028`},
  {42, 0.00035750388050719104`, 0.6363569073028`}, {56, 0.00035750388050719104`, 0.6363569073028`},
  {70, 0.00035750388050719104`, 0.6363569073028`}, {84, 0.00035750388050719104`, 0.6363569073028`},
  {98, 0.00035750388050719104`, 0.6363569073028`}, {112, 0.00035750388050719104`, 0.6363569073028`},
  {126, 0.00035750388050719104`, 0.6363569073028`}}, 0.002448994331279586`},
```

```
{"TBUR", 80.18394341934523`, 6.207067749704881`, {{0, 0.0033946773149359207`, 6.042525620585939`},
    {14, 0.00012215931250103776`, 0.2174435762518472`}, {28, 0.00012215931250103776`, 0.2174435762518472`},
    {42, 0.00012215931250103776`, 0.2174435762518472`}, {56, 0.00012215931250103776`, 0.2174435762518472`},
    {70, 0.00012215931250103776`, 0.2174435762518472`}}, 0.0015424677235387583`},
{"CURE", 365, 64.25786381747083`, {{0, 0.015853683086390926`, 28.219555893775848`},
    {14, 0.0010120882961763954`, 1.8015171671939838`}, {28, 0.0010120882961763954`, 1.8015171671939838`},
    {42, 0.0010120882961763954`, 1.8015171671939838`}, {56, 0.0010120882961763954`, 1.8015171671939838`},
    {70, 0.0010120882961763954`, 1.8015171671939838`}, {84, 0.0010120882961763954`, 1.8015171671939838`},
    {98, 0.0010120882961763954`, 1.8015171671939838`}, {112, 0.0010120882961763954`, 1.8015171671939838`},
    {126, 0.0010120882961763954`, 1.8015171671939838`}}, 0.004970396014113842`},
{"CURE", 365, 83.48213689490763`, {{0, 0.038202297296997055`, 68.00008918865475`},
    {14, 0.002608417882648262`, 4.642983831113906`}, {28, 0.002608417882648262`, 4.642983831113906`},
    {42, 0.002608417882648262`, 4.642983831113906`}, {56, 0.002608417882648262`, 4.642983831113906`}}, 0.011119317389783003`},
{"TBUR", 21.885158782330205`, 3.399041981307574`, {{0, 0.014351386237921617`, 25.54546750350048`},
    {14, 0.0009047813784285876`, 1.610510853602886`}}, 0.004557059190760856`},
{"CURE", 365, 69.85925478168873`, {{0, 0.037056245436819936`, 65.96011687753949`},
    {14, 0.0025265570354927533`, 4.497271523177101`}, {28, 0.0025265570354927533`, 4.497271523177101`},
    {42, 0.0025265570354927533`, 4.497271523177101`}, {56, 0.0025265570354927533`, 4.497271523177101`},
    {70, 0.00041606292200812786`, 0.7405920011744676`}}, 0.01080399659564964`}, {"CURE", 365, 66.83725891301297`,
  {{0, 0.028378450728777187`, 50.51364229722339`}, {14, 0.0019067145563468422`, 3.393951910297379`},
    {28, 0.0019067145563468422`, 3.393951910297379`}, {42, 0.0019067145563468422`, 3.393951910297379`},
    {56, 0.0019067145563468422`, 3.393951910297379`}, {70, 0.0019067145563468422`, 3.393951910297379`},
    {84, 0.0019067145563468422`, 3.393951910297379`}, {98, 0.0019067145563468422`, 3.393951910297379`},
    {112, 0.0009271987036423516`, 1.6504136924833859`}}, 0.008416417801228027`}, {"CURE", 365, 65.83875001311614`,
  {{0, 0.008337147986592056`, 14.84012341613386`}, {14, 0.0004751929319050475`, 0.8458434187909845`},
    {28, 0.0004751929319050475`, 0.8458434187909845`}, {42, 0.0004751929319050475`, 0.8458434187909845`},
    {56, 0.0004751929319050475`, 0.8458434187909845`}, {70, 0.0004751929319050475`, 0.8458434187909845`},
    {84, 0.0004751929319050475`, 0.8458434187909845`}, {98, 0.0004751929319050475`, 0.8458434187909845`},
    {112, 0.0004751929319050475`, 0.8458434187909845`}, {126, 0.0004751929319050475`, 0.8458434187909845`}},
  0.002902322221682161`}, {"TBUR", 18.127131535600668`, 15.30202230883204`,
  {{0, 0.03888439589658998`, 69.21422469593016`}, {14, 0.002657139211190614`, 4.729707795919293`}}, 0.011306987668567468`},
```

```
{"CURE", 365, 166.40418346020442`, {{0, 0.02687841072406854`, 47.843571088842`}, {14, 0.0017995688417247963`, 3.203232538270137`},
   {28, 0.0017995688417247963`, 3.203232538270137`}, {42, 0.0017995688417247963`, 3.203232538270137`},
   {56, 0.0017995688417247963`, 3.203232538270137`}, {70, 0.0017995688417247963`, 3.203232538270137`},
   {84, 0.0017995688417247963`, 3.203232538270137`}, {98, 0.0017995688417247963`, 3.203232538270137`},
   {112, 0.0017995688417247963`, 3.203232538270137`}, {126, 0.0005262033948098058`, 0.9366420427614542`}},
  0.008003701918158437`}, {"TBUR", 14.117363163440183`, 2.6956827219265977`,
  {{0, 0.006575937441977716`, 11.705168646720335`}, {14, 0.00034939217871830883`, 0.6219180781185897`}}, 0.002417748768367388`},
{"CURE", 365, 27.10399867989483`, {{0, 0.00793030980829155`, 14.115951458758959`},
   {14, 0.00044613306202643984`, 0.7941168504070629`}, {28, 0.00044613306202643984`, 0.7941168504070629`},
   {42, 0.00044613306202643984`, 0.7941168504070629`}, {56, 0.00044613306202643984`, 0.7941168504070629`},
   {70, 0.00044613306202643984`, 0.7941168504070629`}, {84, 0.00044613306202643984`, 0.7941168504070629`},
   {98, 0.00044613306202643984`, 0.7941168504070629`}, {112, 0.00044613306202643984`, 0.7941168504070629`},
   {126, 0.00044613306202643984`, 0.7941168504070629`}}, 0.0027903861549795077`},
{"CURE", 365, 35.516054153041274`, {{0, 0.012179927641189964`, 21.680271201318135`},
   {14, 0.0007496771929477551`, 1.3344254034470042`}, {28, 0.0007496771929477551`, 1.3344254034470042`},
   {42, 0.0007496771929477551`, 1.3344254034470042`}, {56, 0.0007496771929477551`, 1.3344254034470042`},
   {70, 0.0007496771929477551`, 1.3344254034470042`}, {84, 0.0007496771929477551`, 1.3344254034470042`},
   {98, 0.0007496771929477551`, 1.3344254034470042`}, {112, 0.0007496771929477551`, 1.3344254034470042`},
   {126, 0.0007496771929477551`, 1.3344254034470042`}}, 0.003959611489708897`},
{"CURE", 365, 53.44254345105494`, {{0, 0.01757711424265554`, 31.28726335192686`},
   {14, 0.001135190521623867 5`, 2.020639128490484`}, {28, 0.0011351905216238675`, 2.020639128490484`},
   {42, 0.0011351905216238675`, 2.020639128490484`}, {56, 0.0011351905216238675`, 2.020639128490484`},
   {70, 0.0011351905216238675`, 2.020639128490484`}, {84, 0.0011351905216238675`, 2.020639128490484`},
   {98, 0.0011351905216238675`, 2.020639128490484`}, {112, 0.0011351905216238675`, 2.020639128490484`},
   {126, 0.0011351905216238675`, 2.020639128490484`}}, 0.005444574975564639`},
{"CURE", 365, 126.2843030238856`, {{0, 0.05073622070352637`, 90.31047285227693`},
   {14, 0.003503698125971784 6`, 6.236582664229776`}, {28, 0.001103953175705023`, 1.965036652754941`}}, 0.01456785826256930 6`}};
```

In[∘]:=

```
TestMDKsHigh = {{"KsHigh Res", "KsHigh OS", "KsHigh Tox", "Ainj", "DoseMaxTox"},
  {"CURE", 365, 35.32032337958981`, {{0, 0.010497997888133983`, 18.68643624087849`},
    {14, 0.0006295393534437566`, 1.1205800491298867`}, {28, 0.0006295393534437566`, 1.1205800491298867`},
```

```
{42, 0.0006295393534437566`, 1.1205800491298867`}, {56, 0.0006295393534437566`, 1.1205800491298867`},
{70, 0.0006295393534437566`, 1.1205800491298867`}, {84, 0.0006295393534437566`, 1.1205800491298867`},
{98, 0.0006295393534437566`, 1.1205800491298867`}, {112, 0.0006295393534437566`, 1.1205800491298867`},
{126, 0.0006295393534437566`, 1.1205800491298867`}}, 0.0034968510825767267`},
{"CURE", 365, 61.52054069421517`, {{0, 0.04029059463672674`, 71.7172584533736`}, {14, 0.002757581978343239`, 4.908495921450966`},
{28, 0.002757581978343239`, 4.908495921450966`}, {42, 0.002757581978343239`, 4.908495921450966`},
{56, 0.0008511500288137415`, 1.51504705128846`}}, 0.011693884386645073`}, {"CURE", 365, 27.015390578499158`,
{{0, 0.010566375195517511`, 18.80814784802117`}, {14, 0.0006344234468282943`, 1.1292737353543638`},
{28, 0.0006344234468282943`, 1.1292737353543638`}, {42, 0.0006344234468282943`, 1.1292737353543638`},
{56, 0.0006344234468282943`, 1.1292737353543638`}, {70, 0.0006344234468282943`, 1.1292737353543638`},
{84, 0.0006344234468282943`, 1.1292737353543638`}, {98, 0.0006344234468282943`, 1.1292737353543638`},
{112, 0.0006344234468282943`, 1.1292737353543638`}, {126, 0.0006344234468282943`, 1.1292737353543638`}},
0.0035156641813675117`}, {"CURE", 365, 94.42852388078904`, {{0, 0.020614848489708983`, 36.69443031168199`},
{14, 0.0013521715392705424`, 2.4068653399015654`}, {28, 0.0013521715392705424`, 2.4068653399015654`},
{42, 0.0013521715392705424`, 2.4068653399015654`}, {56, 0.0013521715392705424`, 2.4068653399015654`},
{70, 0.0013521715392705424`, 2.4068653399015654`}, {84, 0.0013521715392705424`, 2.4068653399015654`},
{98, 0.0013521715392705424`, 2.4068653399015654`}, {112, 0.0013521715392705424`, 2.4068653399015654`},
{126, 0.0013521715392705424`, 2.4068653399015654`}}, 0.00628036680002795`},
{"TBUR", 13.656238784712723`, 23.234794194123126`, {{0, 0.04679417886133694`, 83.29363837317975`}}, 0.013483258335307717`},
{"CURE", 365, 119.59493054100281`, {{0, 0.03530682796717578`, 62.846153781572895`},
{14, 0.002401598644803885`, 4.2748455877509155`}, {28, 0.002401598644803885`, 4.2748455877509155`},
{42, 0.002401598644803885`, 4.2748455877509155`}, {56, 0.002401598644803885`, 4.2748455877509155`},
{70, 0.002401598644803885`, 4.2748455877509155`}}, 0.010322667848699462`}, {"CURE", 365, 70.43039477256137`,
{{0, 0.036459879754539105`, 64.89858596307961`}, {14, 0.0024839594867584087`, 4.421447886429967`},
{28, 0.0024839594867584087`, 4.421447886429967`}, {42, 0.0024839594867584087`, 4.421447886429967`},
{56, 0.0024839594867584087`, 4.421447886429967`}, {70, 0.0008442962861415285`, 1.5028473893319207`}},
0.010639914578888535`}, {"CURE", 365, 20.368973433786195`, {{0, 0.004931702911533762`, 8.778431182530097`},
{14, 0.00023194685511516934`, 0.41286540210500144`}, {28, 0.00023194685511516934`, 0.41286540210500144`},
{42, 0.00023194685511516934`, 0.41286540210500144`}, {56, 0.00023194685511516934`, 0.41286540210500144`},
{70, 0.00023194685511516934`, 0.41286540210500144`}, {84, 0.00023194685511516934`, 0.41286540210500144`},
{98, 0.00023194685511516934`, 0.41286540210500144`}, {112, 0.00023194685511516934`, 0.41286540210500144`},
```

```
{126, 0.00023194685511516934`, 0.41286540210500144`}}, 0.0019653596960256185`}, {"CURE", 365, 77.9571509219465`,
  {{0, 0.016226135760666185`, 28.882521653985812`}, {14, 0.001038692058624628`, 1.848871864351838`},
   {28, 0.001038692058624628`, 1.848871864351838`}, {42, 0.001038692058624628`, 1.848871864351838`},
   {56, 0.001038692058624628`, 1.848871864351838`}, {70, 0.001038692058624628`, 1.848871864351838`},
   {84, 0.001038692058624628`, 1.848871864351838`}, {98, 0.001038692058624628`, 1.848871864351838`},
   {112, 0.001038692058624628`, 1.848871864351838`}, {126, 0.001038692058624628`, 1.848871864351838`}}, 0.00507287137069275`},
 {"CURE", 365, 61.51556337179143`, {{0, 0.04783721578729754`, 85.15024410138963`}, {14, 0.003296626346241153`, 5.867994896309252`},
   {28, 0.003296626346241153`, 5.867994896309252`}}, 0.013770235952318428`}, {"CURE", 365, 21.539549323072592`,
  {{0, 0.007747160241411203`, 13.789945229711941`}, {14, 0.000433050950106415`, 0.7708306911894187`},
   {28, 0.000433050950106415`, 0.7708306911894187`}, {42, 0.000433050950106415`, 0.7708306911894187`},
   {56, 0.000433050950106415`, 0.7708306911894187`}, {70, 0.000433050950106415`, 0.7708306911894187`},
   {84, 0.000433050950106415`, 0.7708306911894187`}, {98, 0.000433050950106415`, 0.7708306911894187`},
   {112, 0.000433050950106415`, 0.7708306911894187`}, {126, 0.000433050950106415`, 0.7708306911894187`}},
  0.0027399950087490335`}, {"TBUR", 35.48311369578471`, 4.771973007664696`,
  {{0, 0.007815225623432986`, 13.911101609710716`}, {14, 0.00043791276310797096`, 0.7794847183321884`},
   {28, 0.00043791276310797096`, 0.7794847183321884`}}, 0.00275872228546126`},
 {"TBUR", 90.31136572917089`, 12.600309039207312`, {{0, 0.0031880388475619284`, 5.674709148660233`},
   {14, 0.00010739942197432397`, 0.19117097111429665`}, {28, 0.00010739942197432397`, 0.19117097111429665`},
   {42, 0.00010739942197432397`, 0.19117097111429665`}, {56, 0.00010739942197432397`, 0.19117097111429665`},
   {70, 0.00010739942197432397`, 0.19117097111429665`}, {84, 0.00010739942197432397`, 0.19117097111429665`}},
  0.0014856139214596811`}, {"CURE", 365, 92.13004406147806`, {{0, 0.027867297259822753`, 49.6037891224845`},
   {14, 0.0018702035942786688`, 3.3289623978160305`}, {28, 0.0018702035942786688`, 3.3289623978160305`},
   {42, 0.0018702035942786688`, 3.3289623978160305`}, {56, 0.0018702035942786688`, 3.3289623978160305`},
   {70, 0.0018702035942786688`, 3.3289623978160305`}, {84, 0.0018702035942786688`, 3.3289623978160305`},
   {98, 0.0018702035942786688`, 3.3289623978160305`}, {112, 0.0018702035942786688`, 3.3289623978160305`}},
  0.008275780781774274`}, {"TBUR", 22.613612550820843`, 13.111011552178118`,
  {{0, 0.028584148985258948`, 50.879785193760924`}, {14, 0.0019214072889526827`, 3.4201049743357754`}}, 0.008473012916893778`},
 {"CURE", 365, 39.85837959530844`, {{0, 0.05729541135276459`, 101.98583220792098`},
   {14, 0.0017717360996278`, 3.1536902573376264`}}, 0.016372531572964078`}, {"TBUR", 14.527746279307427`, 1.6621291891185157`,
  {{0, 0.004691880981300117`, 8.351548146714208`}, {14, 0.00021481671724133747`, 0.3823737566895807`}}, 0.0018993759093231728`},
 {"TBUR", 63.47885099892364`, 4.800370137673951`, {{0, 0.003928878821798185`, 6.99340430280077`},
```

```
{14, 0.00016031656299119953`, 0.28536348212433515`}, {28, 0.00016031656299119953`, 0.28536348212433515`},
{42, 0.00016031656299119953`, 0.28536348212433515`}, {56, 0.00016031656299119953`, 0.28536348212433515`}},
0.0016894461014151448`}, {"TBUR", 14.688931880414026`, 3.9261344239792653`,
{{0, 0.009347675557834705`, 16.638862492945776`}, {14, 0.0005473734727080938`, 0.9743247814204069`}}, 0.0031803553258809942`},
{"CURE", 365, 54.20198071163997`, {{0, 0.019209578099920237`, 34.19304901785802`},
{14, 0.0012517950828570604`, 2.2281952474855675`}, {28, 0.0012517950828570604`, 2.2281952474855675`},
{42, 0.0012517950828570604`, 2.2281952474855675`}, {56, 0.0012517950828570604`, 2.2281952474855675`},
{70, 0.0012517950828570604`, 2.2281952474855675`}, {84, 0.0012517950828570604`, 2.2281952474855675`},
{98, 0.0012517950828570604`, 2.2281952474855675`}, {112, 0.0012517950828570604`, 2.2281952474855675`},
{126, 0.0012517950828570604`, 2.2281952474855675`}}, 0.005893725505094049`}, {"CURE", 365, 85.25298483834199`,
{{0, 0.014019352014866336`, 24.95444658646208`}, {14, 0.0008810646482103531`, 1.5682950738144286`},
{28, 0.0008810646482103531`, 1.5682950738144286`}, {42, 0.0008810646482103531`, 1.5682950738144286`},
{56, 0.0008810646482103531`, 1.5682950738144286`}, {70, 0.0008810646482103531`, 1.5682950738144286`},
{84, 0.0008810646482103531`, 1.5682950738144286`}, {98, 0.0008810646482103531`, 1.5682950738144286`},
{112, 0.0008810646482103531`, 1.5682950738144286`}, {126, 0.0008810646482103531`, 1.5682950738144286`}},
0.004465704428789558`}, {"TBUR", 23.961012911946536`, 9.512935818629131`,
{{0, 0.049056659910195845`, 87.32085464014861`}, {14, 0.003383729497876747`, 6.02303850622061`}}, 0.014105749642924128`},
{"CURE", 365, 29.787217819108754`, {{0, 0.010762898682979395`, 19.157959655703323`},
{14, 0.0006484608387898576`, 1.1542602930459465`}, {28, 0.0006484608387898576`, 1.1542602930459465`},
{42, 0.0006484608387898576`, 1.1542602930459465`}, {56, 0.0006484608387898576`, 1.1542602930459465`},
{70, 0.0006484608387898576`, 1.1542602930459465`}, {84, 0.0006484608387898576`, 1.1542602930459465`},
{98, 0.0006484608387898576`, 1.1542602930459465`}, {112, 0.0006484608387898576`, 1.1542602930459465`},
{126, 0.0006484608387898576`, 1.1542602930459465`}}, 0.0035697349824242467`},
{"TBUR", 23.473511907329367`, 3.0294159634068607`, {{0, 0.0025690324658656405`, 4.57287778924084`},
{14, 0.00006318468042458915`, 0.11246873115576868`}}, 0.0013153026199973477`}, {"CURE", 365, 70.88054699366477`,
{{0, 0.030246916394120353`, 53.83951118153423`}, {14, 0.0020401763895856405`, 3.63151397346244`},
{28, 0.0020401763895856405`, 3.63151397346244`}, {42, 0.0020401763895856405`, 3.63151397346244`},
{56, 0.0020401763895856405`, 3.63151397346244`}, {70, 0.0020401763895856405`, 3.63151397346244`},
{84, 0.0020401763895856405`, 3.63151397346244`}, {98, 0.0020401763895856405`, 3.63151397346244`}}, 0.00893050106209885`},
{"CURE", 365, 109.08564012667915`, {{0, 0.016330189746902545`, 29.06773774948653`},
{14, 0.0010461244862129393`, 1.862101585459032`}, {28, 0.0010461244862129393`, 1.862101585459032`},
```

```
{42, 0.0010461244862129393`, 1.862101585459032`}, {56, 0.0010461244862129393`, 1.862101585459032`},
{70, 0.0010461244862129393`, 1.862101585459032`}, {84, 0.0010461244862129393`, 1.862101585459032`},
{98, 0.0010461244862129393`, 1.862101585459032`}, {112, 0.0010461244862129393`, 1.862101585459032`},
{126, 0.0010461244862129393`, 1.862101585459032`}}, 0.005101500429038959`},
{"CURE", 365, 59.981174473288384`, {{0, 0.049929757696445795`, 88.87496869967352`},
{14, 0.003446093625466029`, 6.1340466533295315`}, {28, 0.0015102379973012284`, 2.6882236351961866`}},
0.014345970785511747`}, {"CURE", 365, 38.94944174104419`, {{0, 0.006921994711706745`, 12.321150586838005`},
{14, 0.0003741105551275253`, 0.6659167881269951`}, {28, 0.0003741105551275253`, 0.6659167881269951`},
{42, 0.0003741105551275253`, 0.6659167881269951`}, {56, 0.0003741105551275253`, 0.6659167881269951`},
{70, 0.0003741105551275253`, 0.6659167881269951`}, {84, 0.0003741105551275253`, 0.6659167881269951`},
{98, 0.0003741105551275253`, 0.6659167881269951`}, {112, 0.0003741105551275253`, 0.6659167881269951`},
{126, 0.0003741105551275253`, 0.6659167881269951`}}, 0.0025129617835006786`},
{"CURE", 365, 89.21510340732364`, {{0, 0.04421937723302881`, 78.71049147479128`}, {14, 0.00303820930665053`, 5.408012565837944`},
{28, 0.00303820930665053`, 5.408012565837944`}, {42, 0.001348838770538829`, 2.4009330115581564`}}, 0.012774836210284052`},
{"CURE", 365, 13.388065439976568`, {{0, 0.005101184362499228`, 9.080108165248625`},
{14, 0.00024405267304127392`, 0.4344137580134676`}, {28, 0.00024405267304127392`, 0.4344137580134676`},
{42, 0.00024405267304127392`, 0.4344137580134676`}, {56, 0.00024405267304127392`, 0.4344137580134676`},
{70, 0.00024405267304127392`, 0.4344137580134676`}, {84, 0.00024405267304127392`, 0.4344137580134676`},
{98, 0.00024405267304127392`, 0.4344137580134676`}, {112, 0.00024405267304127392`, 0.4344137580134676`},
{126, 0.00024405267304127392`, 0.4344137580134676`}}, 0.002011990243530726`},
{"TBUR", 26.54570386518139`, 4.3121807727612085`, {{0, 0.0029980146323928644`, 5.336466045659298`},
{14, 0.00009382626374796226`, 0.16701074947137284`}}, 0.001433313079814235`},
{"CURE", 365, 65.09952834135368`, {{0, 0.06558575234857306`, 116.74263918046005`}}, 0.0175`}, {"CURE", 365,
97.04258248337662`, {{0, 0.02974008099047888`, 52.93734416305241`}, {14, 0.002003973860754107`, 3.5670734721423103`},
{28, 0.002003973860754107`, 3.5670734721423103`}, {42, 0.002003973860754107`, 3.5670734721423103`},
{56, 0.002003973860754107`, 3.5670734721423103`}, {70, 0.002003973860754107`, 3.5670734721423103`},
{84, 0.002003973860754107`, 3.5670734721423103`}, {98, 0.002003973860754107`, 3.5670734721423103`}}, 0.008791052100385692`},
{"CURE", 365, 51.91560984401012`, {{0, 0.012941722325384576`, 23.036265739184547`},
{14, 0.0008040910989616561`, 1.431282156151748`}, {28, 0.0008040910989616561`, 1.431282156151748`},
{42, 0.0008040910989616561`, 1.431282156151748`}, {56, 0.0008040910989616561`, 1.431282156151748`},
{70, 0.0008040910989616561`, 1.431282156151748`}, {84, 0.0008040910989616561`, 1.431282156151748`},
```

```
{98, 0.0008040910989616561`, 1.431282156151748`}, {112, 0.0008040910989616561`, 1.431282156151748`},
    {126, 0.0008040910989616561`, 1.431282156151748`}}, 0.004169209076985352`},
 {"CURE", 365, 77.13691313412222`, {{0, 0.03964049523608987 5`, 70.56008152023998`},
    {14, 0.0027111463068691774`, 4.825840426227136`}, {28, 0.0027111463068691774`, 4.825840426227136`},
    {42, 0.0027111463068691774`, 4.825840426227136`}, {56, 0.0012715323880001673`, 2.263327650640298`}}, 0.01151501825815874`},
 {"CURE", 365, 62.84420113025962`, {{0, 0.010520918183476329`, 18.727234366587865`},
    {14, 0.0006311765173967812`, 1.1234942009662705`}, {28, 0.0006311765173967812`, 1.1234942009662705`},
    {42, 0.0006311765173967812`, 1.1234942009662705`}, {56, 0.0006311765173967812`, 1.1234942009662705`},
    {70, 0.0006311765173967812`, 1.1234942009662705`}, {84, 0.0006311765173967812`, 1.1234942009662705`},
    {98, 0.0006311765173967812`, 1.1234942009662705`}, {112, 0.0006311765173967812`, 1.1234942009662705`},
    {126, 0.0006311765173967812`, 1.1234942009662705`}}, 0.0035031572943462377`},
 {"CURE", 365, 50.09132096419581`, {{0, 0.013396449244495486`, 23.845679655201966`},
    {14, 0.0008365715931838639`, 1.4890974358672777`}, {28, 0.0008365715931838639`, 1.4890974358672777`},
    {42, 0.0008365715931838639`, 1.4890974358672777`}, {56, 0.0008365715931838639`, 1.4890974358672777`},
    {70, 0.0008365715931838639`, 1.4890974358672777`}, {84, 0.0008365715931838639`, 1.4890974358672777`},
    {98, 0.0008365715931838639`, 1.4890974358672777`}, {112, 0.0008365715931838639`, 1.4890974358672777`},
    {126, 0.0008365715931838639`, 1.4890974358672777`}}, 0.004294321088256562`},
 {"CURE", 365, 119.49568866187433`, {{0, 0.016918007742299047`, 30.114053781292306`},
    {14, 0.0010881114858841184`, 1.9368384448737308`}, {28, 0.0010881114858841184`, 1.9368384448737308`},
    {42, 0.0010881114858841184`, 1.9368384448737308`}, {56, 0.0010881114858841184`, 1.9368384448737308`},
    {70, 0.0010881114858841184`, 1.9368384448737308`}, {84, 0.0010881114858841184`, 1.9368384448737308`},
    {98, 0.0010881114858841184`, 1.9368384448737308`}, {112, 0.0010881114858841184`, 1.9368384448737308`},
    {126, 0.0010881114858841184`, 1.9368384448737308`}}, 0.005263230664427835 5`},
 {"TBUR", 69.26766922660613`, 40.92561127938669`, {{0, 0.06253676287697073`, 111.3154379210079`}}, 0.0175`},
 {"CURE", 365, 104.78561702704243`, {{0, 0.016706591644129857`, 29.737733126551145`},
    {14, 0.0010730103360148903`, 1.9099583981065047`}, {28, 0.0010730103360148903`, 1.9099583981065047`},
    {42, 0.0010730103360148903`, 1.9099583981065047`}, {56, 0.0010730103360148903`, 1.9099583981065047`},
    {70, 0.0010730103360148903`, 1.9099583981065047`}, {84, 0.0010730103360148903`, 1.9099583981065047`},
    {98, 0.0010730103360148903`, 1.9099583981065047`}, {112, 0.0010730103360148903`, 1.9099583981065047`},
    {126, 0.0010730103360148903`, 1.9099583981065047`}}, 0.005205062361331165`},
 {"TBUR", 16.85809076939111`, 2.544360822813359`, {{0, 0.003989658970533901`, 7.101592967550343`},
```

```
      {14, 0.0001646580021866077`, 0.29309124389216173`}}, 0.0017061689439258915`},
   {"CURE", 365, 77.15462019669664`, {{0, 0.03597226539779448`, 64.03063240807418`}, {14, 0.0024491298898480784`, 4.35945120392958`},
      {28, 0.0024491298898480784`, 4.35945120392958`}, {42, 0.0024491298898480784`, 4.35945120392958`},
      {56, 0.0024491298898480784`, 4.35945120392958`}, {70, 0.0011944383928936802`, 2.1261003393507507`}}, 0.01050575403036346`},
   {"CURE", 365, 90.47689786309905`, {{0, 0.026638922372808578`, 47.41728182359927`},
      {14, 0.0017824625309205136`, 3.1727833050385144`}, {28, 0.0017824625309205136`, 3.1727833050385144`},
      {42, 0.0017824625309205136`, 3.1727833050385144`}, {56, 0.0017824625309205136`, 3.1727833050385144`},
      {70, 0.0017824625309205136`, 3.1727833050385144`}, {84, 0.0017824625309205136`, 3.1727833050385144`},
      {98, 0.0017824625309205136`, 3.1727833050385144`}, {112, 0.0017824625309205136`, 3.1727833050385144`},
      {126, 0.0007665984635517424`, 1.3645452651221015`}}, 0.00793780991123537`},
   {"CURE", 365, 76.88178540261305`, {{0, 0.06739261216381552`, 119.95884965159163`}}, 0.0175`},
   {"CURE", 365, 19.27110007427119`, {{0, 0.003546353421650795 4`, 6.312509090538415`},
      {14, 0.0001329933201235288`, 0.23672810981988124`}, {28, 0.0001329933201235288`, 0.23672810981988124`},
      {42, 0.0001329933201235288`, 0.23672810981988124`}, {56, 0.0001329933201235288`, 0.23672810981988124`},
      {70, 0.0001329933201235288`, 0.23672810981988124`}, {84, 0.0001329933201235288`, 0.23672810981988124`},
      {98, 0.0001329933201235288`, 0.23672810981988124`}, {112, 0.0001329933201235288`, 0.23672810981988124`},
      {126, 0.0001329933201235288`, 0.23672810981988124`}}, 0.0015841993694461253`},
   {"CURE", 365, 57.805711279609426`, {{0, 0.04219247617832135`, 75.102607597412`}, {14, 0.0028934306598857114`, 5.150306574596566`},
      {28, 0.0028934306598857114`, 5.150306574596566`}, {42, 0.0028934306598857114`, 5.150306574596566`}}, 0.01221716157756601`},
   {"CURE", 365, 43.53699821283776`, {{0, 0.007356175398897839`, 13.093992210038152`},
      {14, 0.00040512346135546047`, 0.7211197612127196`}, {28, 0.00040512346135546047`, 0.7211197612127196`},
      {42, 0.00040512346135546047`, 0.7211197612127196`}, {56, 0.00040512346135546047`, 0.7211197612127196`},
      {70, 0.00040512346135546047`, 0.7211197612127196`}, {84, 0.00040512346135546047`, 0.7211197612127196`},
      {98, 0.00040512346135546047`, 0.7211197612127196`}, {112, 0.00040512346135546047`, 0.7211197612127196`},
      {126, 0.00040512346135546047`, 0.7211197612127196`}}, 0.002632420774703142`},
   {"TBUR", 15.22575081575173`, 8.841138478054615`, {{0, 0.023385239171043806`, 41.62572572445797`},
      {14, 0.0015500565879373153`, 2.759100726528421`}}, 0.007042602629009685`},
   {"CURE", 365, 12.068472838635353`, {{0, 0.0022349112613695354`, 3.978142045237773`},
      {14, 0.00003931888010343877`, 0.06998760658412101`}, {28, 0.00003931888010343877`, 0.06998760658412101`},
      {42, 0.00003931888010343877`, 0.06998760658412101`}, {56, 0.00003931888010343877`, 0.06998760658412101`},
      {70, 0.00003931888010343877`, 0.06998760658412101`}, {84, 0.00003931888010343877`, 0.06998760658412101`},
```

{98, 0.00003931888010343877`, 0.06998760658412101`}, {112, 0.00003931888010343877`, 0.06998760658412101`},
{126, 0.00003931888010343877`, 0.06998760658412101`}}, 0.0012233736530811164`},
{"CURE", 365, 82.76525053688651`, {{0, 0.039018610480258`, 69.45312665485925`}, {14, 0.002666725967166901`, 4.746772221557084`},
{28, 0.002666725967166901`, 4.746772221557084`}, {42, 0.002666725967166901`, 4.746772221557084`},
{56, 0.002666725967166901`, 4.746772221557084`}}, 0.011343915010668672`}, {"CURE", 365, 30.37703952177452`,
{{0, 0.010391952802363782`, 18.497675988207533`}, {14, 0.0006219647044601708`, 1.107097173939104`},
{28, 0.0006219647044601708`, 1.107097173939104`}, {42, 0.0006219647044601708`, 1.107097173939104`},
{56, 0.0006219647044601708`, 1.107097173939104`}, {70, 0.0006219647044601708`, 1.107097173939104`},
{84, 0.0006219647044601708`, 1.107097173939104`}, {98, 0.0006219647044601708`, 1.107097173939104`},
{112, 0.0006219647044601708`, 1.107097173939104`}, {126, 0.0006219647044601708`, 1.107097173939104`}},
0.003467674199905961`}, {"CURE", 365, 66.18219999323013`, {{0, 0.016821022939961027`, 29.94142083313063`},
{14, 0.0010811840000028309`, 1.924507520005039`}, {28, 0.0010811840000028309`, 1.924507520005039`},
{42, 0.0010811840000028309`, 1.924507520005039`}, {56, 0.0010811840000028309`, 1.924507520005039`},
{70, 0.0010811840000028309`, 1.924507520005039`}, {84, 0.0010811840000028309`, 1.924507520005039`},
{98, 0.0010811840000028309`, 1.924507520005039`}, {112, 0.0010811840000028309`, 1.924507520005039`},
{126, 0.0010811840000028309`, 1.924507520005039`}}, 0.005236546597192550`},
{"CURE", 365, 48.17256551547474`, {{0, 0.013161938335499486`, 23.428250237189083`},
{14, 0.0008198208139698639`, 1.4592810488663577`}, {28, 0.0008198208139698639`, 1.4592810488663577`},
{42, 0.0008198208139698639`, 1.4592810488663577`}, {56, 0.0008198208139698639`, 1.4592810488663577`},
{70, 0.0008198208139698639`, 1.4592810488663577`}, {84, 0.0008198208139698639`, 1.4592810488663577`},
{98, 0.0008198208139698639`, 1.4592810488663577`}, {112, 0.0008198208139698639`, 1.4592810488663577`},
{126, 0.0008198208139698639`, 1.4592810488663577`}}, 0.004229798557796092`},
{"TBUR", 29.253693518228925`, 49.108086459727936`, {{0, 0.04824966070825117`, 85.88439606068708`},
{14, 0.0033260866977378417`, 5.920434321973358`}, {28, 0.0033260866977378417`, 5.920434321973358`}}, 0.013883714639043188`},
{"TBUR", 16.12161535836143`, 3.5283303069574043`, {{0, 0.009607573119237305`, 17.101480152242402`},
{14, 0.0005659375842368509`, 1.0073688999415946`}}, 0.003251862653169069 5`}, {"CURE", 365, 61.881414477200636`,
{{0, 0.009412264065659152`, 16.75383003687329`}, {14, 0.0005519869375526972`, 0.982536748843801`},
{28, 0.0005519869375526972`, 0.982536748843801`}, {42, 0.0005519869375526972`, 0.982536748843801`},
{56, 0.0005519869375526972`, 0.982536748843801`}, {70, 0.0005519869375526972`, 0.982536748843801`},
{84, 0.0005519869375526972`, 0.982536748843801`}, {98, 0.0005519869375526972`, 0.982536748843801`},
{112, 0.0005519869375526972`, 0.982536748843801`}, {126, 0.0005519869375526972`, 0.982536748843801`}},

```
0.0031981259873028476`}, {"TBUR", 27.249278971374114`, 11.737776446380721`,
  {{0, 0.029811383487502213`, 53.06426260775394`}, {14, 0.0020090668962557726`, 3.5761390753352753`}}, 0.00881067002586213 1`},
 {"CURE", 365, 35.20257369543556`, {{0, 0.010555638978223782`, 18.789037381238334`},
   {14, 0.0006336565741644565`, 1.1279087020127325`}, {28, 0.0006336565741644565`, 1.1279087020127325`},
   {42, 0.0006336565741644565`, 1.1279087020127325`}, {56, 0.0006336565741644565`, 1.1279087020127325`},
   {70, 0.0006336565741644565`, 1.1279087020127325`}, {84, 0.0006336565741644565`, 1.1279087020127325`},
   {98, 0.0006336565741644565`, 1.1279087020127325`}, {112, 0.0006336565741644565`, 1.1279087020127325`},
   {126, 0.0006336565741644565`, 1.1279087020127325`}}, 0.00351271025521401`}, {"CURE", 365, 148.14841667643336`,
  {{0, 0.043113776221021374`, 76.74252167341804`}, {14, 0.002959237805792856`, 5.267443294311284`},
   {28, 0.002959237805792856`, 5.267443294311284`}, {42, 0.002959237805792856`, 5.267443294311284`}}, 0.012470644924344378`},
 {"CURE", 365, 35.89496708429509`, {{0, 0.007597063089938404`, 13.52277230009036`},
   {14, 0.0004223297250012152`, 0.751746910502163`}, {28, 0.0004223297250012152`, 0.751746910502163`},
   {42, 0.0004223297250012152`, 0.751746910502163`}, {56, 0.0004223297250012152`, 0.751746910502163`},
   {70, 0.0004223297250012152`, 0.751746910502163`}, {84, 0.0004223297250012152`, 0.751746910502163`},
   {98, 0.0004223297250012152`, 0.751746910502163`}, {112, 0.0004223297250012152`, 0.751746910502163`},
   {126, 0.0004223297250012152`, 0.751746910502163`}}, 0.0026986977911935862`}, {"CURE", 365, 46.0776785707896`,
  {{0, 0.006357148015366821`, 11.31572346735294`}, {14, 0.0003337643625318164`, 0.5941005653066331`},
   {28, 0.0003337643625318164`, 0.5941005653066331`}, {42, 0.0003337643625318164`, 0.5941005653066331`},
   {56, 0.0003337643625318164`, 0.5941005653066331`}, {70, 0.0003337643625318164`, 0.5941005653066331`},
   {84, 0.0003337643625318164`, 0.5941005653066331`}, {98, 0.0003337643625318164`, 0.5941005653066331`},
   {112, 0.0003337643625318164`, 0.5941005653066331`}, {126, 0.0003337643625318164`, 0.5941005653066331`}},
  0.002357551792869 0285`}, {"CURE", 365, 51.49423745929725`, {{0, 0.013538145928461157`, 24.097899752660858`},
   {14, 0.0008466927848956975`, 1.5071131571143415`}, {28, 0.0008466927848956975`, 1.5071131571143415`},
   {42, 0.0008466927848956975`, 1.5071131571143415`}, {56, 0.0008466927848956975`, 1.5071131571143415`},
   {70, 0.0008466927848956975`, 1.5071131571143415`}, {84, 0.0008466927848956975`, 1.5071131571143415`},
   {98, 0.0008466927848956975`, 1.5071131571143415`}, {112, 0.0008466927848956975`, 1.5071131571143415`},
   {126, 0.0008466927848956975`, 1.5071131571143415`}}, 0.004333307029876354`},
 {"TBUR", 43.1443882867023`, 9.182199776419747`, {{0, 0.013192100187207815`, 23.481938333229913`},
   {14, 0.0008219752319490301`, 1.4631159128692737`}, {28, 0.0008219752319490301`, 1.4631159128692737`},
   {42, 0.0008219752319490301`, 1.4631159128692737`}}, 0.0042380971866484846`},
 {"CURE", 365, 31.66094668663461`, {{0, 0.006137546086605192`, 10.924832034157241`},
```

{14, 0.0003180785104774142`, 0.5661797486497973`}, {28, 0.0003180785104774142`, 0.5661797486497973`},
{42, 0.0003180785104774142`, 0.5661797486497973`}, {56, 0.0003180785104774142`, 0.5661797486497973`},
{70, 0.0003180785104774142`, 0.5661797486497973`}, {84, 0.0003180785104774142`, 0.5661797486497973`},
{98, 0.0003180785104774142`, 0.5661797486497973`}, {112, 0.0003180785104774142`, 0.5661797486497973`},
{126, 0.0003180785104774142`, 0.5661797486497973`}}, 0.002297131268304256`},
{"CURE", 365, 115.38004816288098`, {{0, 0.06174541814203848`, 109.9068442928285`}}, 0.0175`},
{"CURE", 365, 48.16869654624766`, {{0, 0.017502486833471494`, 31.15442656357926`},
{14, 0.001129859992396436`, 2.0111507864656564`}, {28, 0.001129859992396436`, 2.0111507864656564`},
{42, 0.001129859992396436`, 2.0111507864656564`}, {56, 0.001129859992396436`, 2.0111507864656564`},
{70, 0.001129859992396436`, 2.0111507864656564`}, {84, 0.001129859992396436`, 2.0111507864656564`},
{98, 0.001129859992396436`, 2.0111507864656564`}, {112, 0.001129859992396436`, 2.0111507864656564`},
{126, 0.001129859992396436`, 2.0111507864656564`}}, 0.005424042245113515`},
{"CURE", 365, 10.448896055221754`, {{0, 0.0027328715252717`, 4.864511314983626`},
{14, 0.00007488747038216481`, 0.13329969728025337`}, {28, 0.00007488747038216481`, 0.13329969728025337`},
{42, 0.00007488747038216481`, 0.13329969728025337`}, {56, 0.00007488747038216481`, 0.13329969728025337`},
{70, 0.00007488747038216481`, 0.13329969728025337`}, {84, 0.00007488747038216481`, 0.13329969728025337`},
{98, 0.00007488747038216481`, 0.13329969728025337`}, {112, 0.00007488747038216481`, 0.13329969728025337`},
{126, 0.00007488747038216481`, 0.13329969728025337`}}, 0.0013603807391620402`},
{"CURE", 365, 62.96973807213786`, {{0, 0.06793327747371673`, 120.92123390321578`}}, 0.0175`},
{"TBUR", 19.078218818124018`, 2.6238676394088545`,
{{0, 0.007254719712366681`, 12.913401088012693`}, {14, 0.0003978766266032349`, 0.7082203953537581`}}, 0.00260450660366257`},
{"CURE", 365, 88.47719982243069`, {{0, 0.028933682506103345`, 51.501954860863954`},
{14, 0.001946373969012996`, 3.4645456648431336`}, {28, 0.001946373969012996`, 3.4645456648431336`},
{42, 0.001946373969012996`, 3.4645456648431336`}, {56, 0.001946373969012996`, 3.4645456648431336`},
{70, 0.001946373969012996`, 3.4645456648431336`}, {84, 0.001946373969012996`, 3.4645456648431336`},
{98, 0.001946373969012996`, 3.4645456648431336`}, {112, 0.00040952419766148405`, 0.7289530718374416`}},
0.0085691823758848`}, {"CURE", 365, 37.34712298829652`, {{0, 0.010544980494285684`, 18.77006527982852`},
{14, 0.0006328952538831637`, 1.1265535519120313`}, {28, 0.0006328952538831637`, 1.1265535519120313`},
{42, 0.0006328952538831637`, 1.1265535519120313`}, {56, 0.0006328952538831637`, 1.1265535519120313`},
{70, 0.0006328952538831637`, 1.1265535519120313`}, {84, 0.0006328952538831637`, 1.1265535519120313`},
{98, 0.0006328952538831637`, 1.1265535519120313`}, {112, 0.0006328952538831637`, 1.1265535519120313`},

```
{126, 0.0006328952538831637`, 1.1265535519120313`}}, 0.0035097777163504546`},
{"CURE", 365, 44.272200733659766`, {{0, 0.009935231307220141`, 17.68471172685185`},
  {14, 0.0005893417405213393`, 1.049028298127984`}, {28, 0.0005893417405213393`, 1.049028298127984`},
  {42, 0.0005893417405213393`, 1.049028298127984`}, {56, 0.0005893417405213393`, 1.049028298127984`},
  {70, 0.0005893417405213393`, 1.049028298127984`}, {84, 0.0005893417405213393`, 1.049028298127984`},
  {98, 0.0005893417405213393`, 1.049028298127984`}, {112, 0.0005893417405213393`, 1.049028298127984`},
  {126, 0.0005893417405213393`, 1.049028298127984`}}, 0.003342013407798164`}, {"CURE", 365, 143.32895538413925`,
 {{0, 0.03443922719507041`, 61.30182440722534`}, {14, 0.002339627161082073`, 4.16453634672609`},
  {28, 0.002339627161082073`, 4.16453634672609`}, {42, 0.002339627161082073`, 4.16453634672609`},
  {56, 0.002339627161082073`, 4.16453634672609`}, {70, 0.002339627161082073`, 4.16453634672609`}}, 0.010083959135806921`},
{"CURE", 365, 72.21139172294333`, {{0, 0.024507220443333883`, 43.62285238913431`},
  {14, 0.0016301981073866068`, 2.90175263114816`}, {28, 0.0016301981073866068`, 2.90175263114816`},
  {42, 0.0016301981073866068`, 2.90175263114816`}, {56, 0.0016301981073866068`, 2.90175263114816`},
  {70, 0.0016301981073866068`, 2.90175263114816`}, {84, 0.0016301981073866068`, 2.90175263114816`},
  {98, 0.0016301981073866068`, 2.90175263114816`}, {112, 0.0016301981073866068`, 2.90175263114816`},
  {126, 0.0016301981073866068`, 2.90175263114816`}}, 0.0073513007238119495`}, {"TBUR", 61.47255462613012`,
 8.453474481472194`, {{0, 0.0039678518473779635`, 7.062776288332775`}, {14, 0.00016310035053261228`, 0.29031862394804986`},
  {28, 0.00016310035053261228`, 0.29031862394804986`}, {42, 0.00016310035053261228`, 0.29031862394804986`},
  {56, 0.00016310035053261228`, 0.29031862394804986`}}, 0.00170016900654938 2`},
```
[truncated: 2,163,598 more chars]
